# MRP1-CD28 bi-specific oligonucleotide aptamers: target costimulation to drug-resistant melanoma cancer stem cells

**DOI:** 10.18632/oncotarget.8095

**Published:** 2016-03-15

**Authors:** Mario Martínez Soldevilla, Helena Villanueva, Noelia Casares, Juan Jose Lasarte, Maurizio Bendandi, Susana Inoges, Ascensión López-Díaz de Cerio, Fernando Pastor

**Affiliations:** ^1^ Program of Molecular Therapies, Aptamer Unit, Centro de Investigación Médica Aplicada (CIMA), Pamplona, Spain; ^2^ Instituto de Investigación Sanitaria de Navarra (IDISNA), Recinto de Complejo Hospitalario de Navarra, Irunlarrea, Pamplona, Spain; ^3^ Program Immunology and Immunotherapy, Centro de Investigación Médica Aplicada (CIMA), Pamplona, Spain; ^4^ Ross University School of Medicine, Roseau, Portsmouth, Commonwealth of Dominica; ^5^ Clínica Universidad de Navarra, Pamplona, Spain

**Keywords:** aptamer, cancer immunotherapy, costimutaltion, targeting

## Abstract

In this work we show a clinically feasible strategy to convert in situ the own tumor into an endogenous vaccine by coating the melanoma cancerous cells with CD28 costimulatory ligands. This therapeutic approach is aimed at targeting T-cell costimulation to chemotherapy-resistant tumors which are refractory and been considered as untreatable cancers. These tumors are usually defined by an enrichment of cancer stem cells and characterized by the higher expression of chemotherapy-resistant proteins. In this work we develop the first aptamer that targets chemotherapy-resistant tumors expressing MRP1 through a novel combinatorial peptide-cell SELEX. With the use of the MRP1 aptamer we engineer a MRP1-CD28 bivalent aptamer that is able to bind MRP1-expressing tumors and deliver the CD28 costimulatory signal to tumor-infiltrating lymphocytes. The bi-specific aptamer is able to enhance costimulation in chemotherapy-resistant tumors. Melanoma-bearing mice systemically treated with MRP1-CD28 bivalent aptamer show reduced growth, thus proving an improved mice survival.

Besides, we have designed a technically feasible and translational whole-cell vaccine (Aptvax). Disaggregated cells from tumors can be directly decorated with costimulatory ligand aptamers to generate the vaccine Aptvax. CD28Aptvax made of irradiated tumor cells coated with the CD28-agonistic aptamer attached to MRP1 elicits a strong tumor- cell immune response against melanoma tumors reducing tumor growth.

## INTRODUCTION

Aptamers are defined as single-stranded oligonucleotide ligands that are selected through a complex combinatorial technique known as Systematic Evolution of Ligands by Exponential Enrichment (SELEX). Aptamers display high affinity and specificity for their targets, and represent a new therapeutic platform in life science. There are several aptamers in clinical trials; one of them (Macugen anti VEGF aptamer) has already been approved by the FDA for the treatment of macular degeneration [1]. Aptamers can be chemically synthesized, they are less immunogenic compounds than protein-based products, and they show higher plasticity. Considering all these advantages, it is easy to envision that in the near future aptamers will gain a significant niche in the clinic [2].

Chemotherapy is still one of the first-line treatments for oncologic patients. However, resistance to chemotherapy drugs is quite common among patients undergoing different chemotherapy regimens [3]. Lack of therapeutic approaches to tackle residual chemotherapy-resistant tumor cells puts those patients at high risk of disease recurrence.

Chemotherapy treatment will act as a positive selection pressure on the tumors, favoring the survival of those cells with higher expression of chemotherapy-resistant mechanisms, such as MRP1, ABCC2, etc. [4–6]. Multidrug Resistant-associated Protein 1 (MRP1) has been directly correlated with chemotherapy drug resistance in several types of tumors [4, 7–10], and, along with other chemotherapy-resistant proteins, it is highly expressed in cancer stem cells [11–13]. It has been evidenced that tumor stem cells are the main responsible cells for chemotherapy drug resistance in the long term. Cancer stem cells are significantly enriched in chemotherapy-resistant tumor lesions [14, 15]. They have also been underscored as key players in tumor progression and metastasis spreading [16]. For this reason, cancer stem cells are in the spotlight for the development of novel targeting oncologic-therapeutic approaches.

New therapeutic approaches are desired to eradicate chemotherapy-resistant tumors. Cancer immunotherapy has emerged with strength within the arsenal of therapeutic strategies in oncology upon the success of recent clinical trials. Tumor immunity can be promoted by either counteracting tumor immunosuppression (releasing the brakes) or by triggering immune activation (pushing the gas pedal). T-cell activation is provided by TCR and costimulatory ligands' engagement. CD28 has been underscored as one of the major costimulatory receptors on T lymphocytes, promoting proliferation and cell differentiation into effector T cells [17]. The artificial expression of CD28 ligand (B7.2) on melanoma tumors has been shown to favor tumor immune rejection and to work as a tumor vaccine with irradiated tumor cells [18]. This sort of vaccines based on tumor-irradiated cells that are transfected with immune-modulatory genes (GVAX, FVAX, IVAX, etc.) has shown limited success in clinical trials, basically due to the cumbersome technological challenge of generating autologous tumor-transfected cells, which implies the generation of a stable transfected self-tumor cell line. In order to circumvent this caveat, in clinical trials allogeneic tumor cell lines were used instead of autologous tumors, limiting the tumor-specific antigen repertoire and, therefore, their efficacy [4, 19]. A plausible, clinically feasible approach is to use as tumor vaccine self-tumor irradiated cells decorated with immune-potentiated aptamers, obtained directly from primary tumor disaggregation.

We have recently generated a CD28 agonist aptamer that was proved to potentiate the antitumor immune response induced in tumor-specific antigen vaccination, enhancing tumor-bearing mice survival [20]. We had also previously shown for the first time that bi-specific PSMA-4-1BB aptamer could be used to target 4-1BB costimulation to PSMA-expressing tumors eliciting a potent antitumor immune response and displaying reduced toxicity with a wider therapeutic index than conventional 4-1BB agonist [21]. That work was extended by Schrand et al., 2014, to target 4-1BB costimulation by using a bi-specific aptamer to VEGF as a tumor stroma marker, showing again a better therapeutic index than regular 4-1BB agonist [22].

In a quest to find a target which is broadly expressed among most of the tumors, especially the most aggressive ones, and which may target key cells that are critical for chemotherapy resistance and tumor progression (cancer stem-like cells), we selected through a combinatorial peptide-Cell High Throughput (HT) SELEX an aptamer against the extracellular exposed motif of MRP1. This aptamer was used to isolate from melanoma B16/F10 cells a cancer stem cell subpopulation that shows a more aggressive tumor phenotype and higher chemotherapy resistance profile. The MRP1 aptamer was used to generate a bi-specific MRP1-CD28 aptamer, which was used to create a CD28Aptvax (irradiated tumor cells coated with CD28 agonistic aptamer).

## RESULTS

### MRP1 aptamer selection by peptide-cell HT-SELEX

**Figure 1:**
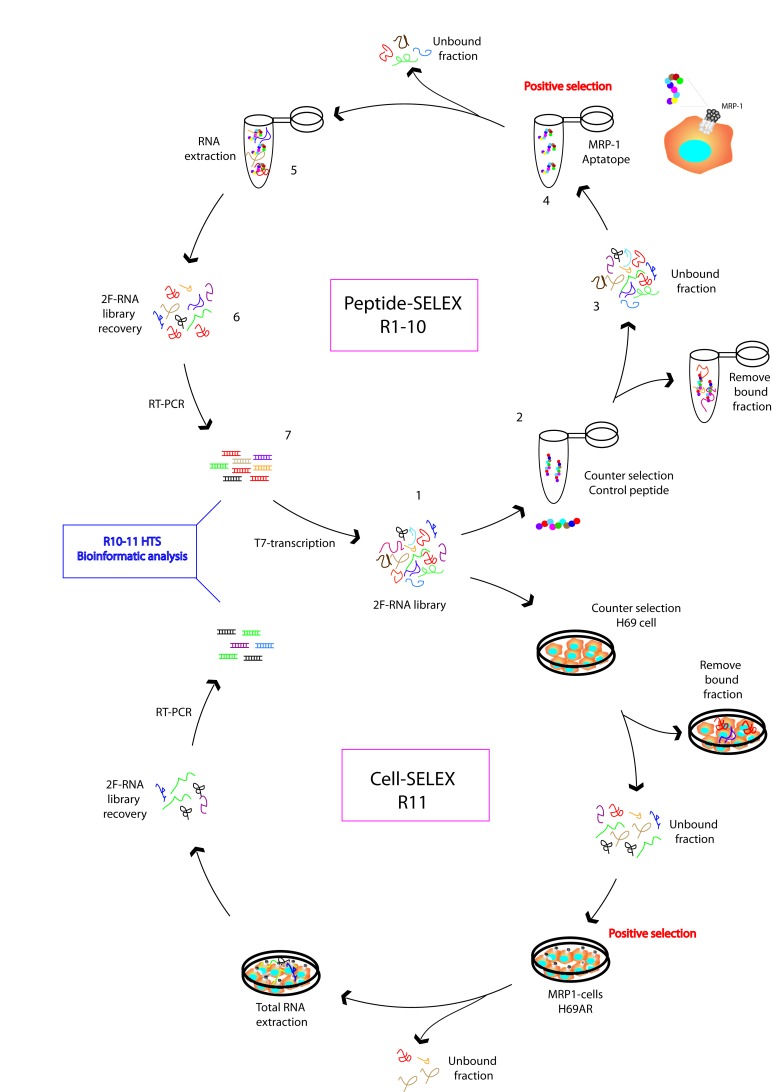
MRP1 aptamer selection strategy **A.** SELEX procedure as described in materials and methods was used for the selection of aptamers against MRP1 aptatope peptide and a control peptide was used for counter-selection. The library obtained after round 10 was allocated into an additional selection step. Cell SELEX was performed in the last round, using H69AR cells as positive selection and H69 as counter selection.

Since MRP1 is 17 trans-membrane protein with very few exposed extracellular amino-acids, it is not feasible to obtain the recombinant soluble protein to perform regular SELEX. Therefore, we performed a combined aptamer selection using Peptide-SELEX and Cell-SELEX as shown in Figure 1. Studying the MRP1 structure [23, 24], its pattern of glycosylation [25] and the homology of most conserved regions among different species, we identified one peptide from extracellular domain to perform the SELEX (RLSVYGALG). The peptide was synthesized with a biotin group at the N terminal; as control for counter-selection we used a scrambled peptide. We started the selection with a 25N-randomized DNA library flanked at 5' and 3' for constant region; the DNA library was transcribed with MutT7, which allows for the incorporation of 2'-Fluoro-pirimidin, conferring stability and RNAses resistance. We performed 10 rounds of selection against MRP1-peptide; the selection restriction was increased in each round as described in Table S1. During each SELEX round we performed counter-selection against the scrambled peptide. As the binding of aptamer to a protein peptide does not assure the binding of the aptamer to the native protein exposed in the cell-membrane surface, we performed the last round of selection (R11) by Cell-SELEX by using the chemotherapy-resistant tumor cell line that has high MRP1 expression (H69AR) [10] and as counter selection the parental cell line H69.

**Figure 2:**
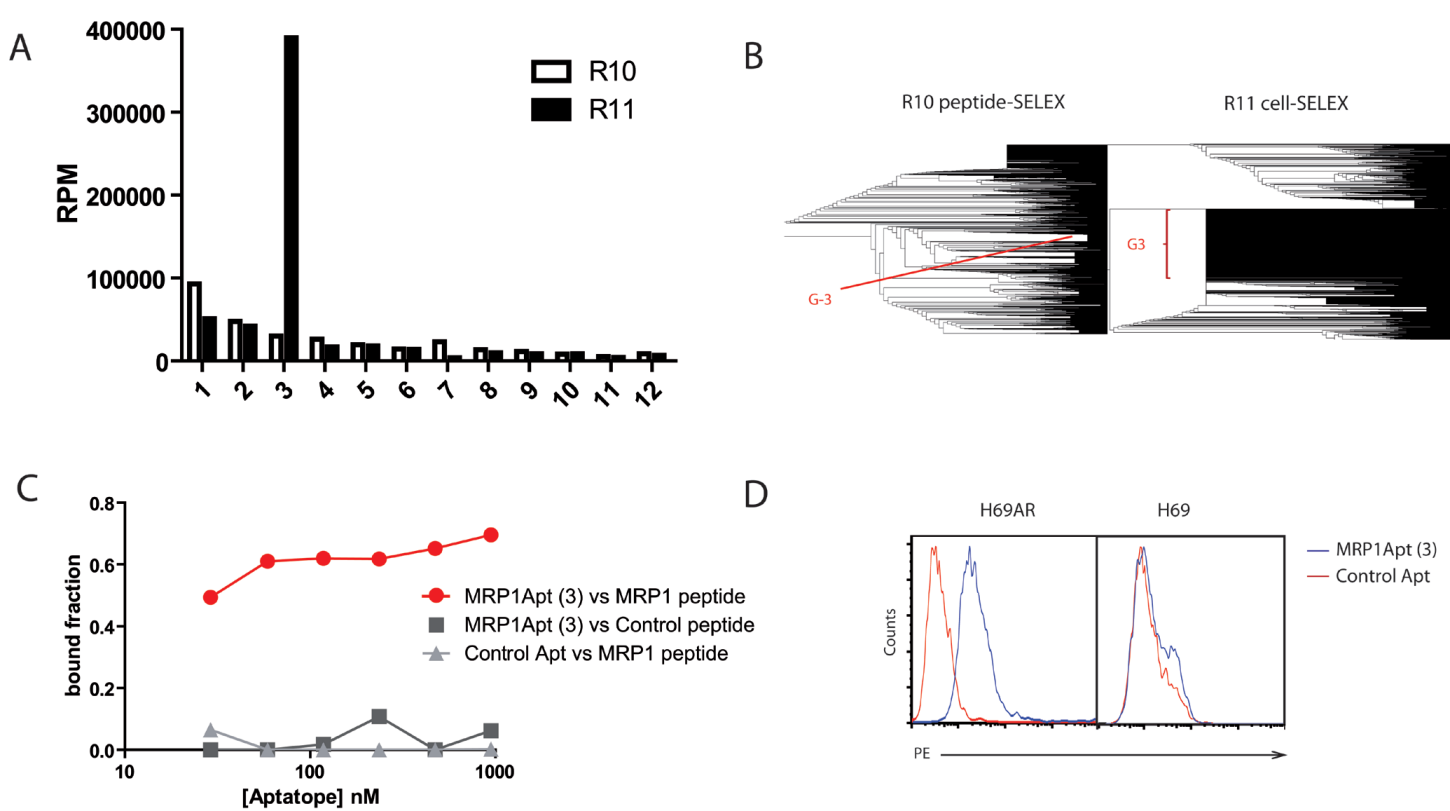
Selection and characterization of MRP1 aptamers **A.** Frequencies of species obtained from the deep sequencing analysis from selection rounds R10 and R11. **B.** Phylogenetic representation of every main sequence at rounds R10 and R11 from alignment using Clustal W and FASTAptamer. **C.** Nitrocellulose filter binding assay of MRP1Apt to the MRP1-peptide; the control peptide and a control aptamer were used to ensure any unspecificity. **D.** Binding of the MRP1Apt aptamer to MRP1 expressing cells by flow cytometry. MRP1Apt biotin-labeled and PE-labeled streptavidin were used to determine the binding to H69AR cells, and H69 cells were used as control. (C and D are representative data of two independent experiments).

The aptamer libraries obtained at rounds R10 and R11, before and after Cell-SELEX respectively, were HT-sequenced by Ion Torrent. The sequences obtained from the deep sequencing analysis from each round of selection (R10 and R11) (Supplementary Data 1, 2, 3, 4) were aligned using Clustal W and FASTAptamer for R10 and for R11 [26]. The phylogenetic representation of the sequences at rounds R10 and R11 is shown in Figure 2. 12 major sequences were identified after Cell-SELEX at R11 that were also present at R10 (Supplementary Table 2). Frequencies changed dramatically for aptamer 3 (Figure 2A, 2B), clearly indicating that not all the aptamers that bind to the peptide recognized the native MRP1 protein with the same affinity. The aptamer 3 that was most abundant in R11 was used for further experiments and would be named MRP1Apt.

To determine the affinity of the MRP1Apt (aptamer 3) to its target we performed a nitrocellulose filter-binding assay to the MRP1-peptide. The aptamer showed a high affinity to MRP1-peptide with a Kd of 50 nM (Figure 2C), but not to the scrambled peptide. The peptide to which MRP1Apt binds is quite conserved among species (same in human and mouse). Therefore, the aptamer will likely bind to all the MRP1 homologues within all the species that have this preserved peptide. In order to ensure the binding of the MRP1Apt aptamer to MRP1-expressing cells, we performed a flow cytometry assay with biotin-labeled MRP1Apt and PE-streptavidin. We utilized the high MRP1-expressing human cell line H69AR, which has been selected from H69 (lung cancer human cell line) after several passes with anthracycline drugs [10]. As seen in Figure 2D the MRP1Apt binds to H69AR but not to H69, proving that the aptamer binds to MRP1 on the cell. We evaluated whether the aptamer would be able to reverse chemotherapy resistance to doxorubicin and etoposide in H69AR by MTT, but it did not have any inhibition (Supplementary Figure 1), indicating that the aptamer binds to regions of MRP1 proteins that might not be important for the expulsion of chemotherapy drugs. Most of the MRP1 protein is exposed within the cell, and the pore function is regulated from cytosolic domain [24], indicating that its function might be easily modulated by targeting the cytoplasmic domain of MRP1.

As drug resistance has been associated with cancer stem cells [13], we reasoned that the MRP1Apt could be used to enrich a subpopulation of cancer stem cells. So we stained melanoma B16/F10 cells MRP1Apt that bind to a highly preserved peptide in MRP1 (identical sequence in mouse), and we performed sorting of those cells that have a higher expression of MRP1. These mouse B16 isolated cells mirror the chemotherapy-resistant tumor cells enriched in cancer patients that are refractory to chemotherapy treatments [10]. The sorted cells were expanded for a few days and analyzed for MRP1 expression by flow cytometry (Supplementary Figure 2A). To prove whether those cells have a stronger cancer stem phenotype, we performed an analysis on a set of known genes that are up-regulated on cancer stem cells (TERT, CD44, Aldh, ABCG2, notch1 and β-catenin) by qRT-PCR (Supplementary Figure 2B). As we can see in Supplementary Figure 1B, these melanoma cells have a higher expression of CD44 30-fold change and Aldh 15-fold change, and the other markers are up-regulated 5 to 3 times. These results indicate that these selected cells show phenotype-like cancer stem cells. Another characteristic of cancer stem cells is their capacity to grow forming spheres; indeed, we observed that these cells could grow forming spheres while the parental B16/F10 cell line did not (Supplementary Figure 2C). Finally, to study whether this subpopulation of cells were able to tolerate higher doses of chemotherapy (another characteristic of cancer stem cells), we performed an MTT assay in the presence of the chemotherapy drugs doxorubicin and etoposide (chemotherapy drugs that have been associated with drug resistance through MRP1 overexpression) [10]. The B16/F10 cancer stem cells displayed a much higher resistance to chemotherapy drugs than the parental B16/F10 cell line (Supplementary Figure 2D, 2E).

### Functional characterization of bi-specific MRP1-CD28 aptamer conjugates

**Figure 3:**
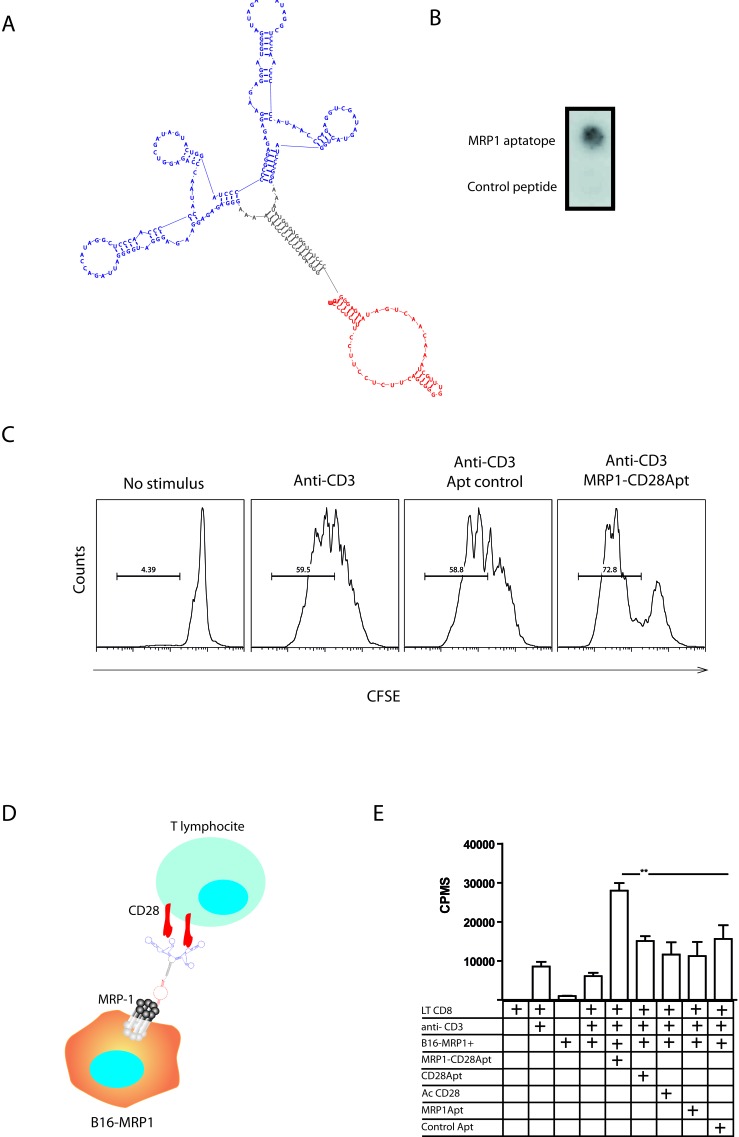
Characterization of MRP1-CD28 bi-specific aptamer **A.** Bi-specific aptamer construct secondary structure predicted, in blue the CD28 aptamer, in red the MRP1 aptamer and in grey the linker. **B.** Nitrocellulose binding assay of MRP1-CD28 bi-specific aptamer to MRP1 aptatope (respresentative data of two independent experiments). **C.** CFSE dilution assay on isolated CD4 T cells suboptimally activated with anti-CD3 and the MRP1-CD28 bi-specific aptamer or the control aptamer (respresentative data of two independent experiments). **D.** Mode of action of the bi-specific aptamer. Two CD28 aptamer units allow for costimulation by CD28 crosslinking and MRP1Apt unit, thereby generating a trivalent aptamer. **E.** ^3^H-thymidine proliferation assay of CD8 T cells suboptimally activated with anti-CD3 antibody and co-cultured with B16-MRP1 cells or B16-MRP1 coated with the bi-specific aptamer MRP1-CD28, MRP1Apt or CD28Apt-dimer or control aptamer or in the anti-CD28 antibody 37.51 (Data are shown as mean ± SEM of cuatriplicates, the experiment was repeated twice).

Based on the premise that lack of costimulation at the tumor site might hamper the ability of tumor-specific T cells to eliminate the tumor cells, we developed a bi-specific aptamer that is aimed at targeting CD28 costimulation to chemotherapy-resistant MRP1 cancer cells. We have previously shown that CD28 agonistic aptamers are able to costimulate T lymphocytes and promote tumor immunity when used as adjuvants in tumor-antigen vaccination. MRP1-CD28 bi-specific aptamer will provide to the infiltrating lymphocytes the CD28 costimulus; concurrently, the tumor cell will elicit the TCR signal through MHC class I. The bi-specific aptamer construct was engineered based on secondary predicted structures using the algorithm software RNAstructure. The sequence and structure of the MRP1-CD28 aptamer conjugate is specified in Figure 3A; the bi-specific aptamer is made of a single 2-Fluoro pyrimidine RNA oligonucleotide and not two independent aptamer conjugated by hybridization [21, 22]. The bi-specific aptamer contains two CD28 aptamer motifs to allow for the costimulation by CD28 crosslinking and the MRP1 aptamer motif. First, we characterized *in vitro* whether the MRP1-CD28 conjugated aptamer maintains its binding and costimulation capacity. In order to do that, we performed a nitrocellulose filter-binding assay to MRP1 peptide; the MRP1-CD28 bi-specific aptamer is capable of binding to MRP1 aptatope (Figure 3B). We further assayed the binding of the bi-specific aptamer to MRP1-B16 cells and to the B16/F10 (Supplementary Figure 3). To test if the CD28 maintains the costimulation capacity, we performed a CFSE dilution assay on isolated CD4 T cells suboptimally activated with anti-CD3 and the MRP1-CD28 bi-specific aptamer; the MRP1-CD28 aptamer construct induces a potent proliferation signal on T cells (Figure 3C). The final *in vitro* characterization experiment consisted in coating irradiated B16/F10 like cancer stem cells with the MRP1-CD28 bi-specific aptamer or the non-targeting CD28 agonistic aptamer and, after cell-washing, culture with isolated CD4 lymphocytes activated with a suboptimal dose of anti-CD3 (Figure 3D). Proliferation was measured by ^3^H thymidine incorporation. As it is shown in Figure 3E, only the cancer-like stem cells that were pre-incubated with MRP1-CD28 aptamer are able to trigger the CD28 costimulatory signal.

Specific *in vivo* targeting of MRP1-CD28 conjugates was tested in mice co-implanted with B16-MRP1 and the parental cell line contralaterally in opposite flanks. When tumors reached 10 mm of diameter, mice were injected intravenously with 250 pmols of bi-specific aptamer MRP1-CD28 (Supplementary Figure 4A). Mice were sacrificed 24 hours later, and tumors were excised and disaggregated to measure by qRT-PCR the accumulation of the aptamer in each tumor. As we observed in Supplementary Figure 4B, the bi-specific aptamer concentration was 3-fold higher in B16-MRP1hi tumors compared with the parental B16 tumors.

**Figure 4:**
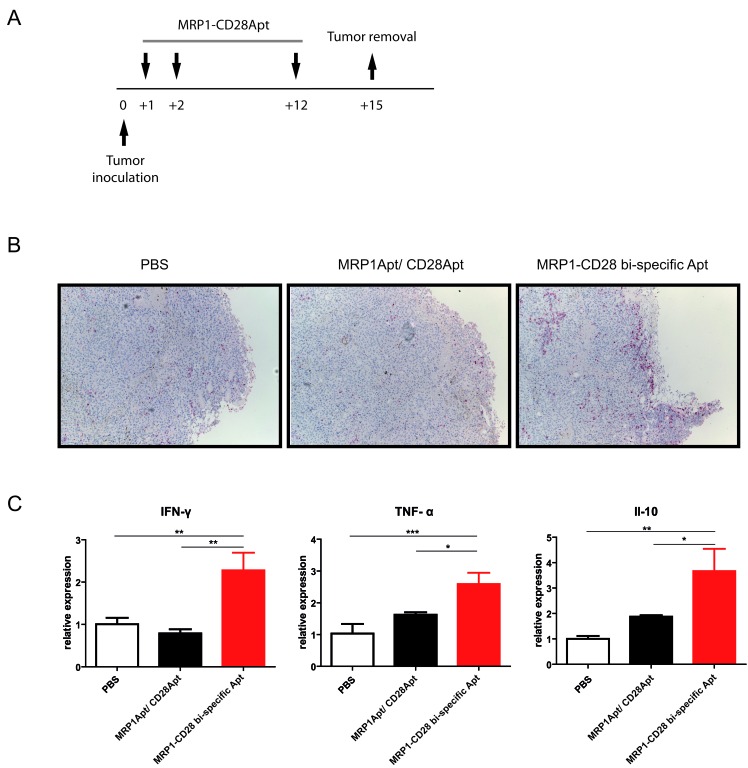
MRP1-CD28 bi-specific aptamer-elicited *in vivo* immune response **A.** Evaluation of lymphocyte infiltration by immunohistochemistry using anti-CD3. Tumor bearing mice treated intravenously with MRP1-CD28 bi-specific aptamer, the unconjugated MRP1 and CD28 aptamers and the PBS as control were excised and stained with anti-CD3 or **B.** disaggregated to determine by qRT-PCR the relative expression of IFN-γ, TNF-α and Il-10 (mean ± SEM of three tumor bearing mice per group).

To evaluate *in vivo* the immune response elicited by the treatment of MRP1-CD28 bi-specific aptamer, we treated B16-MRP1 tumor mice with the bi-specific aptamer as indicated in Figure 4A, and at day 15 mice were sacrificed to excise the tumor and assess T-lymphocyte infiltration by anti-CD3 immunohistochemistry (Figure 4B) and by qRT-PCR for the production of immuno-cytokines. We observed a significant increase of IFN-γ, TNF-α and IL-10 cytokines on the group of mice treated with the bi-specific aptamer versus the control groups (Figure 4C).

### Tumor inhibition by MRP1 targeting CD28 costimulation in combination with Gvax and Treg blockade

**Figure 5:**
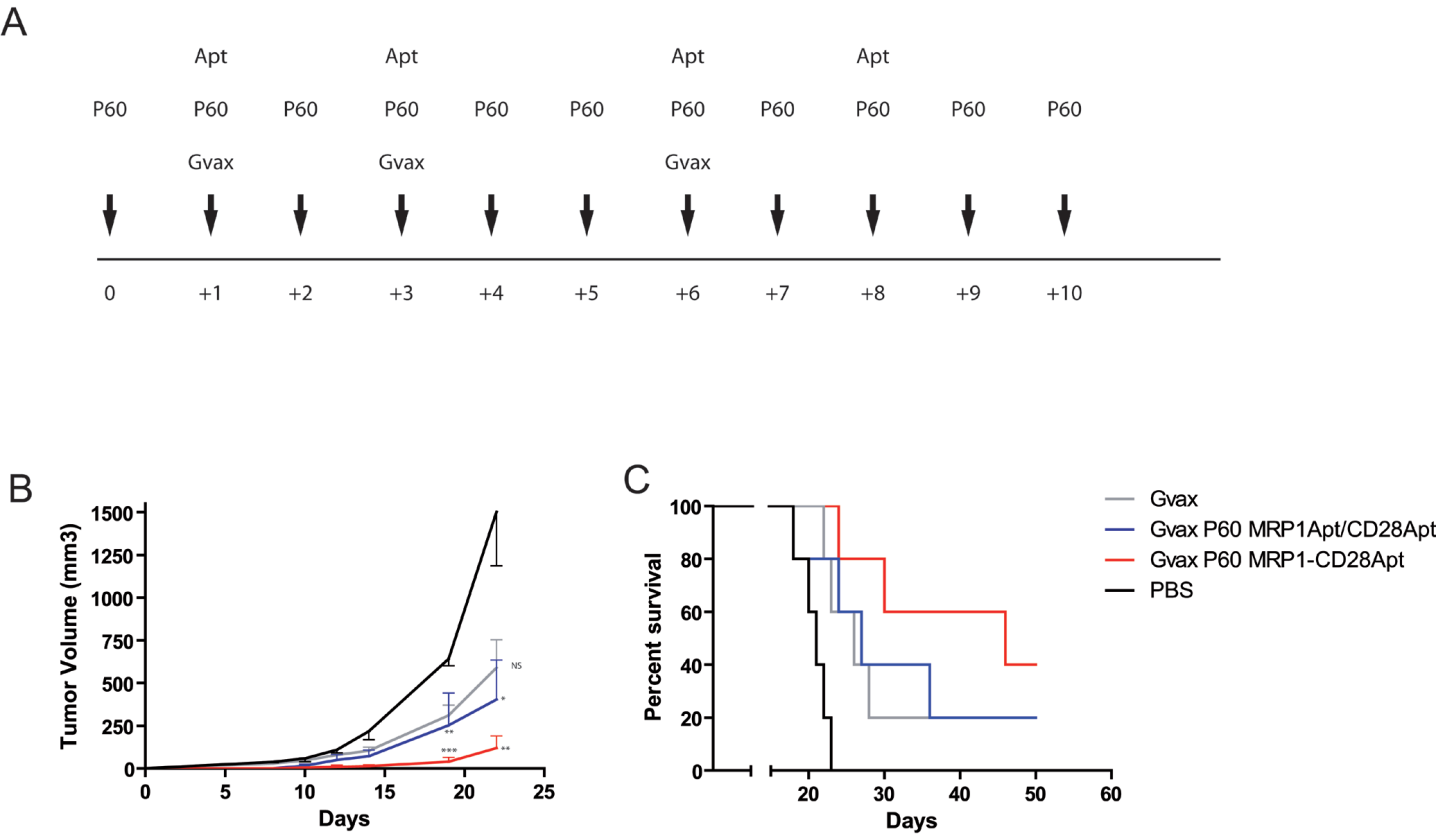
Tumor inhibition by CD28-MRP1 bi-specific aptamer in combination with GVAX and transient Foxp3 blockade **A.** Vaccination calendar. Mice injected with B16-MRP1 cells were treated for 10 days with P60; on days 1, 3, 6, they were treated with Gvax and the 200 pmol/injection of MRP1-CD28 bi-specific aptamer or the unconjugated aptamers intravenously at 1, 3, 6 and 8. **B.** Tumor growth of B16-MRP1 tumors treated with P60 plus Gvax and MRP1-CD28 bi-specific aptamer or the unconjugated aptamers (five mice per group). **C.** Survival of B16-MRP1 -bearing mice treated with P60 plus Gvax and MRP1-CD28 bi-specific aptamer or the unconjugated aptamers (five mice per group).

As we have shown, the bi-specific aptamer is enriched in tumors with higher concentration of MRP1, which indicates that the bi-specific MRP1-CD28 aptamer could be used to elicit targeting tumor immunity in a much more efficient fashion than the CD28 agonist aptamers. However, as the stimulus induced by CD28 tumor-targeting promotes Th1 cytokines (IFN-γ, TNF-α) but it also promotes the immunosuppressive cytokines (IL-10), we reasoned that, in order to have an antitumor effect, the immunosuppressive environment should be diminished. In order to do that, we treated the mice with Foxp3 blockade peptide (P60) [27] that inhibited Treg function transiently and we boosted the tumor immune response with Gvax (GM-CSF producing B16-F10 irradiated cells) immunization. B16-MRP1-bearing mice were treated as shown in the immunization calendar (Figure 5A). Mice that were treated either with Gvax or with Gvax plus P60 displayed a slight inhibition in tumor growth, while those mice that were treated with the whole combination (Gvax, P60 and MRP1-CD28 bi-specific aptamer) had a significant inhibition in tumor growth (Figure 5B) and around 50% survival rate in 50 days of follow-up (Figure 5C).

### CD28Aptvax improves tumor mice survival

**Figure 6:**
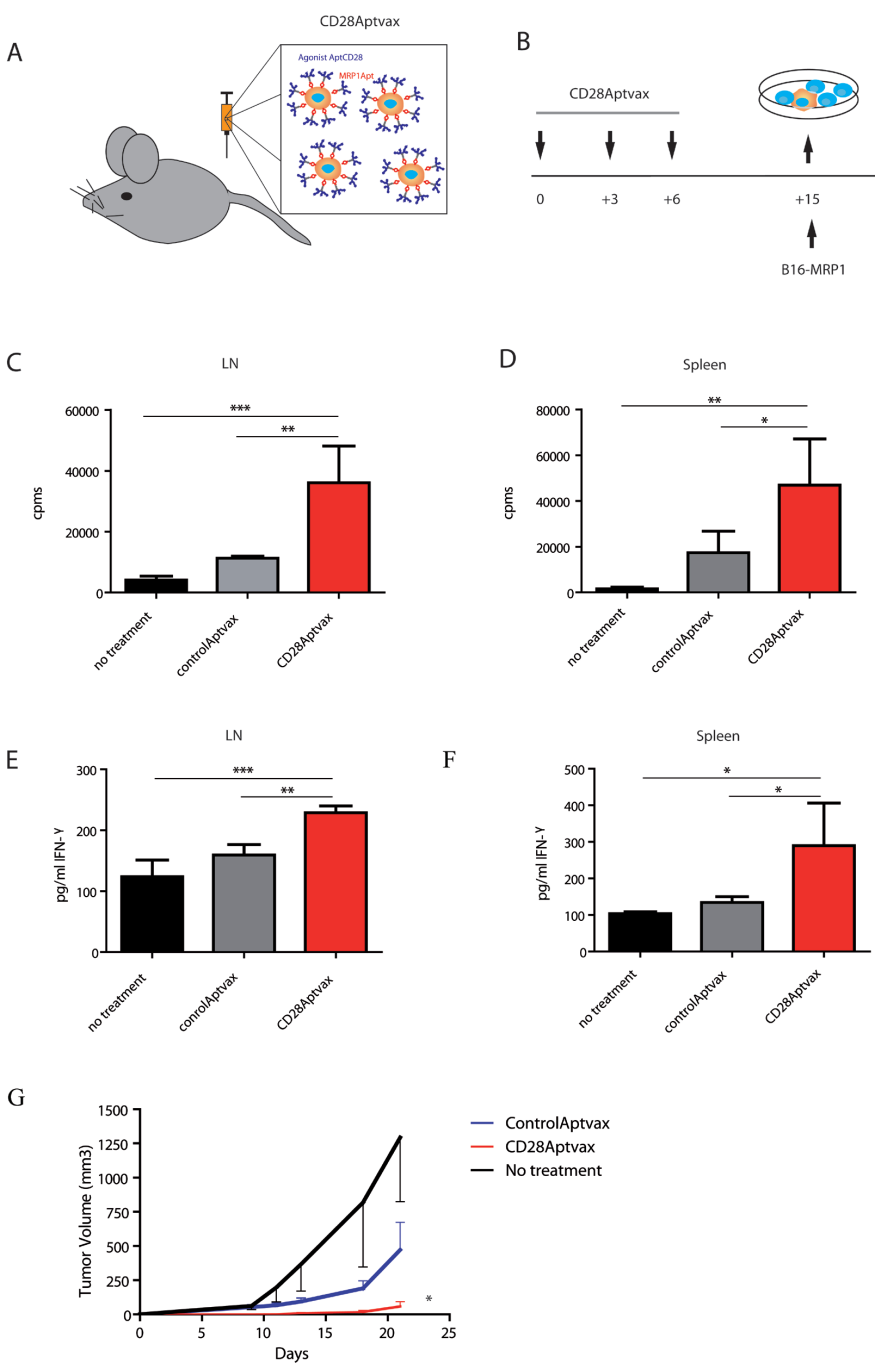
CD28Aptvax elicits cellular antitumor immunity **A.** Irradiated B16-MRP1 cells were ex-vivo coated with a control aptamer or with MRP1-CD28 bi-specific aptamer. **B.** The CD28Aptvax was injected at days +1, +3 and +6; at day +15 mice were sacrificed to assess the antitumor immune response against irradiated B16 tumor cells. The immune response elicited by the CD28Aptvax was measured by ^3^H thymidine proliferation on T cells derived from the lymph node **C.** or the proximal spleen **D.** the immune response was also measured by IFN-γ production by ELISA **E.** and **F.** (mean ± SEM of four treated mice per group). The overall antitumor effect of the vaccine on mice vaccinated as in B) and challenge at day +15 was evaluated by tumor measurement **G.** (5 mice per group).

It is usually technically cumbersome to generate a stable tumor cell line from primary tumors. This is a major technical limitation for the use of self-gene modified irradiated cell-based vaccines in the clinic (e.g. GVAX, IVAX or FLVAX, among others), which have shown very promising results in preclinical models. An alternative to this approach is to artificially link to the cell membrane an immune-modulatory ligand on disaggregated tumor cells obtained directly from the primary tumor samples. To that end, we generated CD28Aptvax, consisting of irradiated tumor cells coated *ex vivo* with bi-specific aptamer that binds to cell-surface protein on tumor cells (MRP1) while the other part of the aptamer provides the costimulatory receptor stimulus (CD28 agonist) (Figure 6A). We used the B16-MRP1 irradiated cells, and we coated them with the bi-specific aptamer MRP1-CD28. The CD28 agonistic aptamer-coated tumor cells were injected subcutaneously in C57BL6 mice as shown in the vaccine calendar (Figure 6B), and the tumor growth was monitored by caliper measure. We observed a statistically significant delay in the tumor growth of the mice treated with CD28Aptvax versus the control vaccine (Figure 6G).

The immune response elicited by this new formulation of vaccine CD28Aptvax was determined in mice immunized as in Figure 6B, by measuring the proliferation of T lymphocytes and the production of IFN-γ by ELISA on the supernatant of cultured isolated T cells from proximal lymph node or spleen from immunized mice. As can be seen in Figure 6C-6F, LN and spleen lymphocytes from the mice that have been vaccinated with CD28Aptvax have a higher T-cell proliferation and production of IFN-γ than the control groups.

## DISCUSSION

Here we described a new aptamer to target MRP1-expressing cells; we used a novel SELEX combinatorial approach to identify an aptamer that binds to a preserved aptatope on MRP1. This approach could be used for aptamer selections in which it is impossible to obtain a recombinant soluble protein as it occurs with multiple trans-membrane proteins. Thanks to the progress in the last few years in the field of HT-SELEX and the existence of new valuable software packages to perform the analysis, we were able to follow the evolution of the aptamer library from the peptide-SELEX (R10) to the CELL-SELEX (R11), which allowed us to identify the aptamer that is able to bind to the MRP1 aptatope and to the MRP1-expressing cell.

MRP1 is a 190 KDa transmembrane protein which functions as a transporter of various molecules across extra- and intra-cellular membranes, and is involved in multi-drug resistance [10, 28]. MRP1 is associated with chemotherapy resistance in several types of tumors (lung, melanoma, etc.) [4, 8, 10], being highly up-regulated in tumor patients that relapse upon chemotherapy treatment [29]. But, at the same time, it is an up-regulated marker on cancer stem cells, which are precisely the cells that are more resistant to chemotherapy and radiotherapy and, therefore, the ones responsible for tumor relapse in the long term [15]. Here we were able to isolate a subpopulation of chemotherapy-resistant cells with a cancer stem cell-like phenotype by using the MRP1 aptamer. It has previously been confirmed by other groups that chemotherapy resistance is associated with the enrichment of cancer stem cells [14, 15]. Here we proposed a clinically feasible approach that will target chemotherapy-resistant tumor cells with cancer stem cell-like phenotype. This approach could be a desirable therapeutic tool for patients that, though apparently responsive to conventional chemotherapy treatment, still maintain residual disease, possibly enriched in chemotherapy-resistant cells, and who, in the end, are bound to relapse.

CD28 is a receptor expressed in T lymphocytes which engulfs different types of subpopulation. In order to elucidate the immunological nature of the infiltrating T lymphocytes upon treatment with MRP1-CD28Apt, we assessed the expression of TNF-α, IFN-γ and IL-10. TNF-α and INF-γ are key cytokines of a Th1 polarized T-cell response, while IL-10 is characteristic of Th2 polarized T-cell response and immunosuppressive cytokines. This indicates that CD28 might also be triggering immunosuppression [30]. Following this line of thought, we performed an *in vivo* experiment to treat B16-MRP1 tumors with the bi-specific aptamer or its controls in the presence of Gvax so as to boost the efficacy of the treatment and P60 peptide to abrogate Treg function [27]. Based on the results, immunization with Gvax provided the boosting of T-cell response elicited by the bi-specific aptamer and the P60 peptide transiently blocked FOXP3 activity [27], favoring the efficacy of the treatment.

This approach would provide two possible major advantages versus systemic costimulation with conventional agonist: 1- the targeting, concentrating the costimulation signal at the tumor and reducing side effects on other tissues [21, 22]. 2- The immobilization of the costimulatory aptamer to the tumor might provide a more efficient crosslink of the CD28 receptors, delivering a more potent costimulatory signaling.

Previous bi-specific aptamers were designed to enhance 4-1BB costimulation at the tumor site [21, 22]. Here we described the use of a new bi-specific aptamer to target CD28 costimulation to cancer stem cells, which are the key players in chemotherapy resistance. The generation of this type of constructs reflects the aptamer plasticity to generate hetero-dimers which is more complicated to achieve with antibodies. The same approach to target costimulation chemotherapy-resistant tumors with overexpression of MRP1 could be used with other stimulatory aptamers such as 4-1BB, OX-40 or CD40 [31–33]. The reduced toxicity associated with targeting approaches would ideally allow for the combination of several of these target stimulatory approaches in the future. This is highly unlikely to happen with non-targeting approaches such as antibodies, which have already shown some toxicity by themselves in clinical trials [34]. Their combination, even if it could favor the antitumor response, would inevitably exacerbate the systemic auto-reactive immunity [35].

In order to bypass the technical limitations of using genetically modified irradiated cell-based vaccines, we attempted to generate a new type of vaccines that consists in artificially linking costimulatory ligands to the irradiated tumor-cell membrane “Aptvax”. The use of genetically modified vaccines has shown limited success in clinical trials due to the problem of generating a stably transfected self-tumor cell line. We coated irradiated B16-MRP1 cells with the bi-specific aptamer and subcutaneously injected them in mice (CD28AptVax). The tumors expressing the CD28 costimulatory aptamer were significantly smaller than those of control (Figure 6G), which was associated with a stronger antitumor immune response (Figure 6C-6F). These results reinforce the idea of anchoring the CD28Apt-dimer aptamer to the cell surface so as to better deliver the costimulatory signal, which must therefore be further investigated. The irradiated cells already induce an immune response due to the antigen input, which recruits the immune system cells. The bi-specific aptamer provides proper CD28 co-stimulatory ligands to boost that pre-existing immune response. The results provide the proof of concept that the CD28AptVax is effective, thus delaying tumor growth and suggesting that the CD28AptVax could be used in the future as a new type of vaccine, bypassing the technical problems of genetically modified patient-specific irradiated tumor cell-based vaccines.

## MATERIALS AND METHODS

### MRP1-Aptamer selection by peptide-cell HT SELEX

The 25N randomized RNA library consisting of two constant flanking sequences of 22 nucleotides and 46 nucleotides at the 5′ and 3′ end respectively was transcribed from partially randomized DNA sequences: Forward primer 5′- GGGGAAT TCTAATACGACTCACTATA GGGAGAGGGAGAATAG -3′ and Reverse primer 5′- GGGAGAAGGGAGAAAGGAAG -3′. This sort of 25N randomized libraries is known to present about 4^11^ different starting sequences. The selection was carried out in the presence of 150 mmol/l NaCl, 2 mmol/l CaCl_2_, 20 mmol/l HEPES (pH 7.4), 0,01% BSA binding buffer. Before each round of selection, in order to eliminate those specific RNAs that bind to biotinylated MRP1 peptide (RLSVYGALG) and sepharose streptavidin (Strp) beads (Amersha GE Healthcare Bio-science), the RNA library was subject to counter-selection with a scrambled peptide (DEGTHQ). During the counter-selection step, the library is incubated with the scrambled peptide and sepharose Strp beads (Amersha GE Healthcare Bio-science) at 37°C for 30 minutes. The pre-cleared RNA pool present in the supernatant obtained after bead-pelleting by centrifugation was subsequently used for the selection step.

The pre-cleared RNA pool was incubated with the bead-coupled MRP1 peptide and washed three times with washing buffer that contained 150 mmol/l NaCl, 2 mmol/l CaCl_2_, 20 mmol/l HEPES (pH 7.4), so as to remove the non-interacting RNA species. The MRP-1 peptide-binding RNA species were extracted after 30 minutes of incubation through phenol:chloroform:isoamyl alcohol (25:24:1), followed by reverse transcription and PCR amplification. The PCR product was transcribed in the presence of 2′-F-pirimidines and T7 polymerase using Durascribe T7 transcription Kit (Epicentre, Madison, WI) and used as library for the next round of selection.

The selection was carried out in ten rounds while increasing the stringency throughout the rounds of selection. The RNA-protein ratio was increased in each round as well as the final volume of binding reaction. RNA species from round 10 were analyzed with Clustal W and FastaAptamer software.

The last round of selection (R11) was performed by Cell-SELEX, using the chemotherapy-resistant tumor cell line that has the highest MRP1 expression (H69AR) [10] and the parental cell line H69 as counter selection. The selection was carried out against 500,000 cells in binding buffer containing 150 mmol/l NaCl, 2 mmol/l CaCl_2_, 20 mmol/l HEPES (pH 7.4) at 37°C for 30 minutes. RNA extraction was done with TRIzol, followed by reverse transcription and PCR amplification with the specific primers. R11 was deep-sequenced with Ion Torrent and analyzed with Clustal W and FASTAptamer software.

### Measurement of MRP1Apt aptamer affinity to its target by filter-binding assay

α-ATP P32 radiolabelled MRP1Apt was tested for binding to its target by nitrocellulose binding assay as described [32]. The aptamer transcription was carried out with α-ATP P32 and PAGE purified; we used 100000 cpm labeled aptamer for the binding assay. The aptamer was re-folded by heating at 65°C and progressive cooling until 37°C in binding buffer containing 150 mmol/l NaCl, 2 mmol/l CaCl2, 20 mmol/l HEPES (pH 7.4). The labeled aptamer was incubated with the protein at different concentrations in binding buffer for 30 minutes at 37°C and then added into the dot blot machine including a nitrocellulose filter. The aptamer bound to the protein is identified in a film. As binding control we used the IgG1 protein and randomized 2′F- RNA library (control Apt).

### Measurement of MRP1Apt aptamer affinity to its target by flow cytometry

10^5^ H69 cells were centrifuged and subsequently washed with PBS and stained with 50 pmol of the biotin-MRP1Apt and 0.05 μg/μl of streptavidin-PE for 30 minutes at 37 ^°^C. Afterwards, cells were washed twice with PBS and analyzed by flow cytometry (FacsCalibur).

### Isolation of melanoma cancer stem cells

10^6^ melanoma B16/F10 cells were stained with PE-labelled MRP1Apt and the sorting of those cells that have the highest expression of MRP1 (less than 1%) was carried out. The sorted cells were expanded for a few days and analyzed for MRP1 expression assessed by flow cytometry.

### Assessment of melanoma cancer stem-cell phenotype by qPCR

2 μg of RNA extracted from B16/F10 cells or B16MRP1 cells cultured in the cell culture media for the same period of time, were retro-transcribed and analyzed in a Fast Real Time PCR system (Applied biosystems, 7900 HT, Foster city, CA, USA) for stem-cell genes (TERT, CD44, Aldh, ABCG2, notch1 and β-catenin) using the following primers: TERT Fwd 5′- ACTCAGCAACCTCCAGCCTA -3′; TERT Rev 5′- CATATTGGCACTCTGCATGG -3′; CD44 Fw 5′- AAGGTGGAGCAAACACAACC -3′; CD44 ReV 5′- AGCTTTTTCTTCTGCCCACA -3′; ABCG2 Fwd 5′- CACCTTATTGGCCTCAGGAA -3′; ABCG2 Rev 5′- CCTGCTTGGAAGGCTCTATG -3′; Notch1 Fwd 5′- CTGGACCCCATGGACATC -3′; Notch1 Rev 5′- AGGATGACTGCACACATTGC -3′; β-catenin Fwd 5′- ACAAACTGTTTTGAAAATCCA -3′; and β-catenin Rev 5′-CGAGTCATTGCATACTGTCC-3′.

### Culture of melanoma sphere stem cells

B16/F10 and B16MRP1 cells were grown in a serum-free culture medium DMEM/F12+GlutMAX^TM^-I (Gibco, Grand Island, NY) containing 1% N2 supplement (Gibco, Grand Island, NY), 2% B27 supplement (Gibco, Grand Island, NY), 20 ng/ml murine FGF-2 (Sigma, St. Louis, MO, USA), and 50 ng/ml EGF (Sigma-Aldrich, St. Louis, MO, USA). 2·10^4^ cells were plated in a 6-well ultra-low attachment plate (Corning, Lowell, MA, USA) and supplemented with medium after 3 days. After 7 days cells were analyzed for melanoma sphere formation by light microscopy.

### MTT assay

A total of 15,000 B16/F10 or B16-MRP1 cells were plated in 0.1 ml in 96-well flat bottom plates and then exposed to doxorubicin and etoposide (final volume of 0.2 ml per well). 48h afterwards, 20 μl of 5 mg/ml MTT solution in PBS were added to each well for 4h. After removal of the medium, 170 μl of DMSO were added to each well to dissolve the formazan crystals. The absorbance at 540 nm was determined by using a plate reader.

### Generation of MRP1-CD28 Bi-specific aptamer

For the construction of the MRP1-CD28 Bi-specific aptamer, the CD28Apt7-dimer was used as template for a first PCR with the following primers: Fwd 5′- GGGAG ACCC ACCCATAAAAGGGAGAGAGGAAGAGG-3′ and Rev 5′ - GACTATTCTCCCGGGAG ACCC ACCCATATTTCCCGGGATCCAGTACTATC-3′. This first PCR product was used in a second PCR with the primers FwD 5′-GGGAGACCCACCCATAAAAG-3′ and Rev5′-GGGAGAAAGGAAGGAGAAGTCGCCCCAAACGATTTGTTGACTATTCTCCCGGG-3′. Finally, this product was used as template for the final PCR with the following primers: Fwd 5′-GGAATTCTAATACGACTCACTATAGGG-3′ and Rev 5′-GGGAGAAAGGAAGGAGAAGTC-3′. In order to generate the MRP1-CD28 bi-specific aptamer with 2′-fluoro-RNA pyrimidines, *in vitro* transcription of PCR-DNA template with the kit Durascribe (Epicentre, Madison, WI) was performed.

### MRP1-CD28 bi-specific aptamer proliferation assays

To evaluate the costimulatory capacity of each aptamer construct, a CD4 polyclonal proliferation assay by CFSE dilution was performed. CD4 T lymphocytes purified from the spleen of C57B6 mice were sub-optimally activated with an anti-CD3 antibody. CD4+ lymphocytes were isolated from the spleen using the Miltenyi negative selection kit (Miltenyi Biotec, Auburn, CA). 10^5^ purified CD4+ lymphocytes were stained with 2 μmol/l of CFSE. CFSE-labeled cells were incubated with 1 μg/ml of plate coated with antibody anti-CD3 145-2C11 (BD Biosciences, San Jose, CA) and 150 pmol of MRP1-CD28 bi-specific aptamer or the unconjugated aptamer, and randomized 2′F-RNA oligo as Apt control.

To evaluate the dual function of MRP1-CD28 bi-specific aptamer we performed H^3^ thymidine proliferation assay. 10,000 B16MRP1-irradiated tumor cells were incubated with 50 pmol of MRP1-CD28 bi-specific aptamer, or MRP1/CD28 unconjugated aptamer or CD28 monoclonal antibody (37.51) or control aptamer. After 30 minutes of incubation at 37°C in PBS, cells were washed twice to culture them with 100,000 isolated CD4 T cell/well, activated in 96-well CD3-coated plates (1 μg/ml). T-cell proliferation was assessed at day 4 upon the addition of H^3^.

### Measurement of *in vivo* targeting of melanoma cancer stem cells by qPCR

B16/F10 or B16MRP1 tumor were implanted in opposite flanks of CD57BL6 mice. At day 15, when tumors reached 10 mm of diameter each, mice were intravenously injected with 250 pmol of MPR1-CD28Apt. We assured that the size of the tumors was equal. Cells isolated from tumors placed in opposite flanks of C57BL6 mice were processed and RNA-extracted with TRIzol and 2μg of RNA was retro-transcribed (Applied biosystems, Foster city, CA, USA). qRT-PCR was performed with SYBR green (Applied biosystems, Foster city, CA, USA) and the following primers: Fwd 5′- GGAATTCTAATACGACTCACTATAGGG -3′ and Rev 5′- GGGAGAAAGGAAGGAGAAGGTC -3′ in the Fast RT-PCR system (7900 HT, Applied biosystems, Foster city, CA, USA).

### Assessment of *in vivo* MRP1-CD28 bi-specific aptamer-mediated immune response by immunohistochemistry

6-8 week-old female C57BL6 mice were subcutaneously implanted with 10^5^ B16-MRP1 cells. At days 1 and 2 after tumor implantation, mice were injected intravenously with 200 pmol of bi-specific aptamer or the aptamers separately at the same concentration with three mice per group. When tumors reached around 5 mm of diameter, they were injected again with 200 pmol of bi-specific aptamer or the aptamers separately at the same concentration. 3 days later, mice were sacrificed and the tumor was surgically removed. The tumors that were selected have a similar size of 10 mm of diameter and no sign of necrosis. Tumors were paraffin-embedded and stained with anti-CD3 SP7 clone and developed with EnVision system (Dako, CA, USA).

### Assessment of *in vivo* MRP1-CD28 bi-specific aptamer-mediated immune response by qRT-PCR

6-8 week-old female C57BL6 mice were subcutaneously implanted with 10^5^ B16-MRP1 cells. At days 1 and 2 after tumor implantation, mice were injected intravenously with 200 pmol of bi-specific aptamer or the aptamers separately at the same concentration with three mice per group. When tumors reached around 5 mm of diameter at day 12, they were injected again with 200 pmol of bi-specific aptamer or the aptamers separately at the same concentration. 3 days later, mice were sacrificed and the tumor was surgically removed. Total RNA from tumor tissue was extracted with RNAse kit (Qiagen GmbH, Hilden, Germany). 2 μg of total RNA was retro-transcribed using the retro-transcriptase (Applied biosystems, Foster city, CA, USA), and qRT-PCR was performed using the following primers: TNF-α Fwd 5′- CCTCACACACTCAGATCATCT -3′; and Rev 5′- GCTACGACGTGGGCTACAG -3′; IFN-γ Fwd 5′- GCCACGGCACAGTCATTGA -3′; Rev 5′- TGCTGATGGCCTGATTGTCTT -3′; IL-10 Fwd 5′- TGAATTCCCTGGGTGAGAAG -3′; and Rev 5′- TCTTCACCTGCTCCACTGC -3′.

### Assessment of antitumor effect of MRP1-CD28 bi-specific aptamer in combination with GVAX and Treg ablation

10^5^ B16-MRP1 cells were injected in C57B6 mice in the right flank. Mice were treated for 10 days with P60 (100 μg/mouse); at days 1, 3 and 6, they were treated with B16 Gvax (10^6^ cells) subcutaneously or PBS (Gibco, Grand Island, NY), and 200 pmol/injection of the MRP1-CD28 bi-specific aptamer or the unconjugated aptamers intravenously at days 1, 3, 6 and 8. B16 Gvax cells are irradiated B16 transfected cells that secrete GM-CSF; these cells were previously used in some of our previous studies [21] and have been kindly provided by Dr G. Dranoff. B16 Gvax cells were irradiated at 5,000 rads and injected subcutaneously in inguinal area 500,000 cell/leg. Tumor size was calculated by measuring the long (D) and short diameter (d) of the tumor with a caliper following the formula Dxd^2^/2. Mice were sacrificed if they showed signs of discomfort (loss of weight and mobility), and when the tumor reached 1,500 mm^3^.

### CD28Aptvax studies

B16-MRP1 cells were irradiated at 5,000 rad. The cells were incubated at 40,000 cell/pmol of aptamer in PBS at 20,000 cell/μl at 37°C sacking for 30 minutes; afterwards, the cells were washed twice with PBS. The vaccine was performed subcutaneously in the inguinal area, 500,000 cells per leg. As control Aptvax we used B16-MRP1 irradiated cells incubated with unconjugated MRP1/CD28 aptamer and washed as with CD28Aptvax. Mice were vaccinated three times, three days apart, and at day 15 after the first vaccine the mice were inoculated with B16-MRP1 tumor to follow tumor growth and survival. For immunological studies mice were vaccinated with CD28Aptvax three times, three days apart, and at day 15 mice were sacrificed to extract the spleen and the inguinal lymph nodes. The lymphocytes from the spleen and lymph nodes were incubated with B16-MRP1 irradiated cells in a 1:10 ratio. INF-γ production was measured by ELISA and proliferation after 72 hours was assessed by H^3^ thymidine incorporation.

### Statistics

Student's two-tailed t test was used for paired comparisons. Log-Rank test was used for the survival studies. Data are presented as mean ± SEM. * p value < 0.05, ** p value < 0.01.

## SUPPLEMENTARY MATERIALS FIGURES AND TABLES, DATAS










