# Supplementary material for: MRP1-CD28 bi-specific oligonucleotide aptamers: target costimulation to drug-resistant melanoma cancer stem cells
Source: Oncotarget. 2016 Mar 15;7(17):23182–96. doi: 10.18632/oncotarget.8095 (PMC5029619; doi:10.18632/oncotarget.8095)
Supplement: Supplementary file 1 [file oncotarget-07-23182-s001.pdf]

MRP1-CD28 Bi-specific oligonucleotide aptamers: target costimulation to drug-resistant melanoma cancer stem cells

Supplementary Material

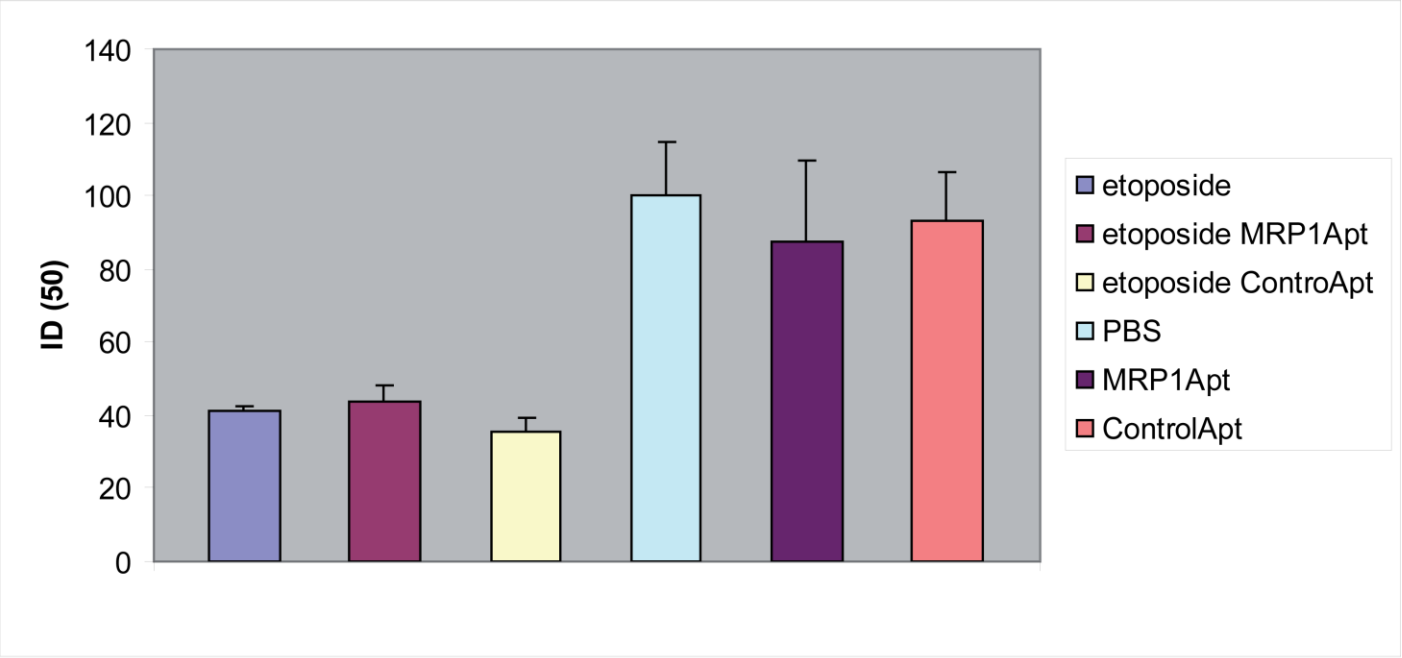

Supplementary Figure 1: MRP1Apt (3) does not reverse chemotherapy resistance in H69AR cells treated with etoposide.

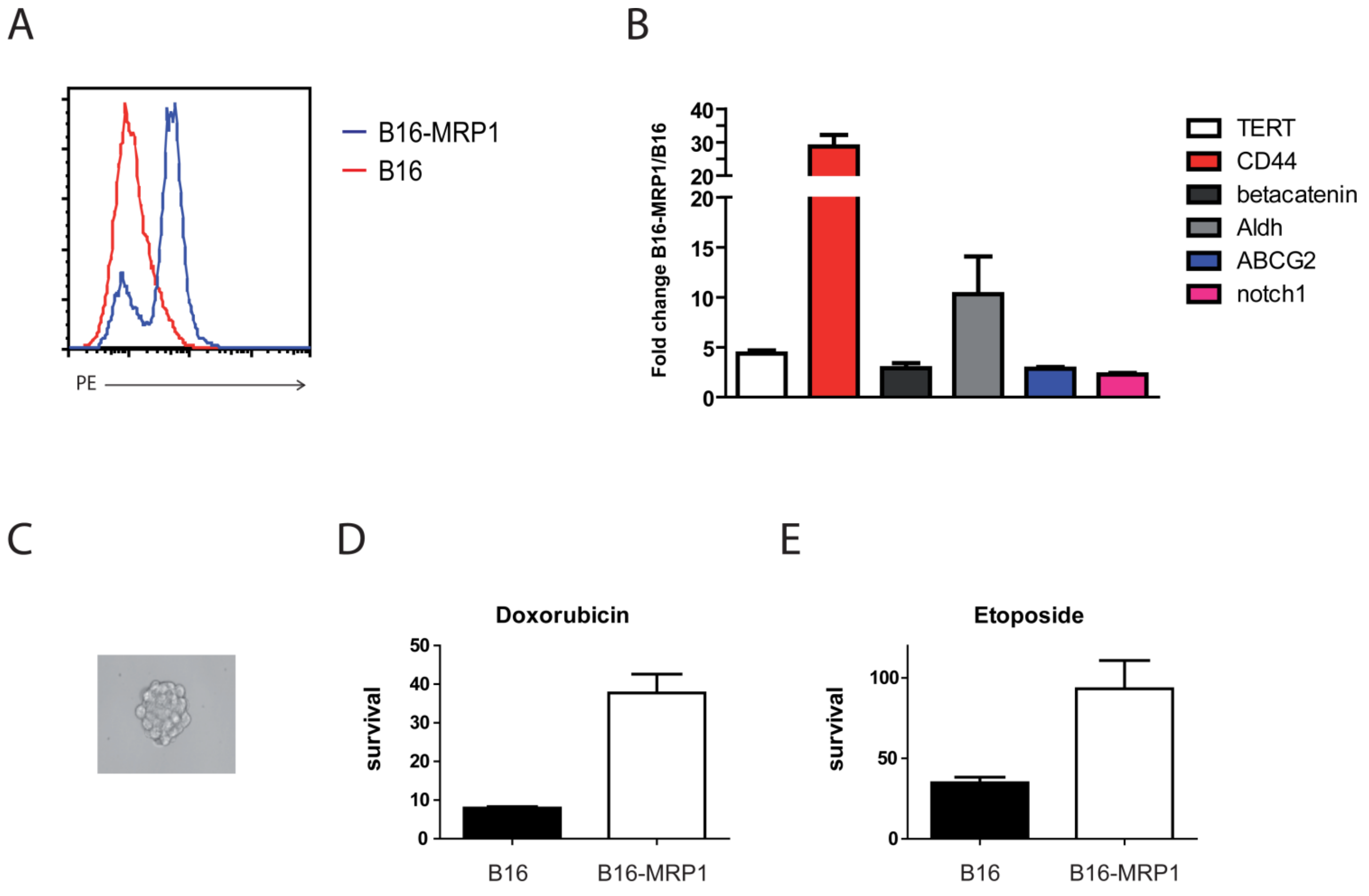

Supplementary figure 2. Characterization of melanoma cancer stem cells isolated with MRP1Apt. A) B16/F10 cells were sorted on the basis of their MRP1 expression. Afterwards, culture and expansion, cells were analyzed for MRP1 expression by flow cytometry. B) GAPDH-normalized qRT-PCR of TERT, CD44, Beta-catenin, Aldh, ABCG2 and Notch1 genes in B16-MRP1 versus B16/F10 cells. The results are shown in fold change of B16-MRP1 cells over B16/F10. C) Picture of B16-MRP1 cells growing in spheres. D) MTT assay performed as described in materials and methods of B16-MRP1 cells and its parental B16/F10 in the presence of doxorubicin. E) MTT assay carried out as described in materials and methods of B16-MRP1 cells and its parental B16/F10 in presence of etoposide.

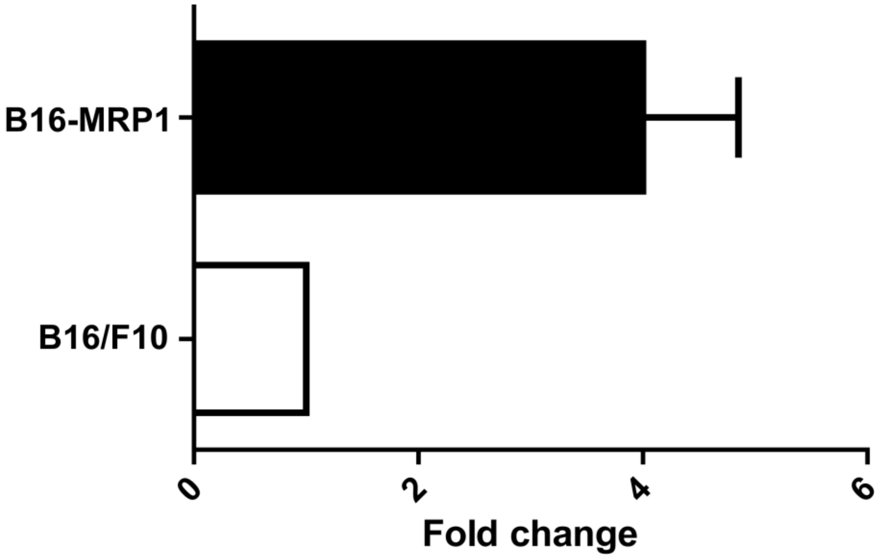

Supplementary Figure 3: MRP1-CD28 bi-specific aptamer binds to B16-MRP1 expressing cells and not to the parental cell line. B16-MRP1 cancer stem cell and the parental B16/F10 were incubated with the same amount of α-ATP P32-labeled MRP1-CD28 bi-specific aptamer.

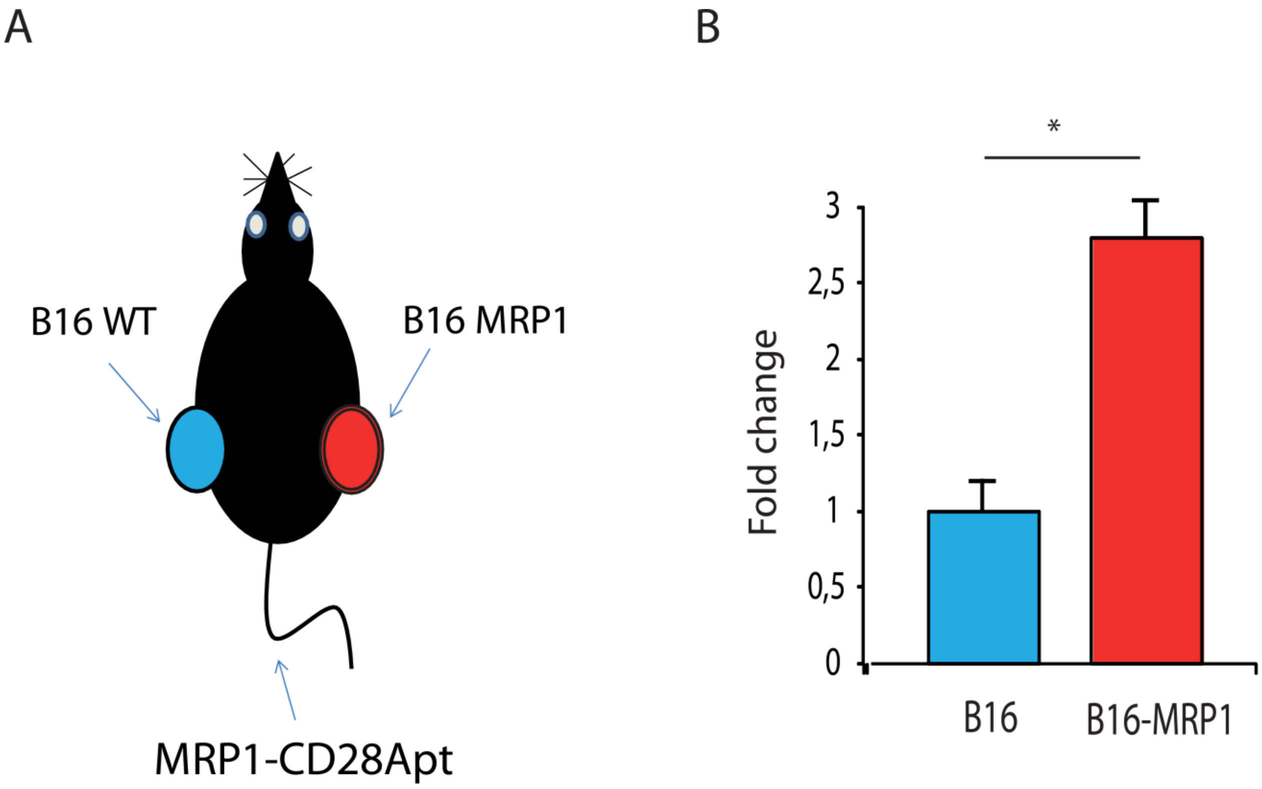

Supplementary figure 4. MRP1-CD28 bi-specific aptamer-mediated in vivo targeting of melanoma cancer stem cells. A) B16-MRP1 cancer cells and the parental cell line contralaterally co-implanted in opposite flanks. Mice were injected intravenously with 250 pmols of MRP1-CD28 bi-specific aptamer when tumors reached 10 mm of diameter and sacrificed 24 hours later. Tumors were excised and disaggregated (mean ± SEM of three tumor bearing mice per group). B) MRP1-CD28 bi-specific aptamer concentration determined by qRT-PCR.

Table S1. SELEX conditions

Round	**RNA (µM)**	**Peptide (nM)**
1	3	2
2	2	2
3	1	1
4	0.5	1
5	0.25	0.5
6	0.15	0.25
7	0.07	0.25
8	0.05	0.15
9	0.02	0.15
10	0.01	0.075

Round	**RNA (µM)**	**H69AR**
11	1	500.000 cells

Table S2:

	Randon-Sequence	RPM R10	RPM R11
1	TTTTTCCCTTCACTAACCGCGAACTGCA	92891.26	51312.97
2	ATCACCGTTGGTTCGGGTGCGCGTTCTC	47691.57	42120.15
3	TCAACAAATCGTTTGGGGCGACTTCTC	30179.27	389894.89
4	CTACGTTTAACCGATCGTGTTGTTCTC	26026.18	16741.54
5	TGACGTGTTGTGCGGCGCTCACTGTCTC	19588.85	18177.19
6	AGACGTATTCGTTTGCGCTCTGTTCAC	14535.90	13893.39
7	ATCACGACCCTTCTACCACGATTGAG	23118.99	3890.15
8	CAAACGTTTCCCCGACGTCGCTTTCTC	13428.40	9818.01
9	TCACATTCATGTTTGTGACCGTGTCTC	11559.48	8706.54
10	CCACCAACTATTGTTCGTCGCCGTTCT	8098.57	8984.39
11	TTTGCGCATTCGGTGCACTGTTATTCT	5537.48	4283.81
12	TCTTGCAATTTCCTGCGGTGCATTCTC	8998.40	6993.01
