# Supplementary material for: MRP1-CD28 bi-specific oligonucleotide aptamers: target costimulation to drug-resistant melanoma cancer stem cells
Source: Oncotarget. 2016 Mar 15;7(17):23182–96. doi: 10.18632/oncotarget.8095 (PMC5029619; doi:10.18632/oncotarget.8095)
Supplement: Supplementary file 2 [file oncotarget-07-23182-s002.pdf]

CLUSTAL 2.1 multiple sequence alignment

```
FC88X_00969_00285      -------------------CGCACCATCAATTTTGTCGTGCCGTTCTC------
-----
FC88X_00979_01201      -------------------CGCACCATCAATCTTGTCGTGCCGTTCTCC-----
-----
FC88X_01044_01235      ---------------------CCATTCTCAATTTGTCTTCCCCGTCCT------
-----
FC88X_01056_00382      --------------------TCAATGCTTGGCTTGCC-----------------
-----
FC88X_01004_00449      ------------------CAAGACCAATCAATTCGTCGAGCCTCTC--------
-----
FC88X_01080_01089      ---------------------CACAAACTGAATTCG------------------
-----
FC88X_01122_00750      ------------------CTTCACAATTTGATTTCCCGGATCTTT---------
-----
FC88X_00998_00903      --------------------ACAGCAAT-CGCACAGTCTTCTCTTCCT------
-----
FC88X_01037_00311      -------------------CTCAGTAATTCGCTCAGCTTCAATCTC--------
-----
FC88X_00957_01210      -----------------TCAACAA---TCGTTTGG-GGGCGAC-TTCTC-----
-----
FC88X_01029_00361      ----------------GTCAACAAAA-TCGTTTGG-GGGCGAC-TTCTC-----
-----
FC88X_00992_01217      -----------------TCAACAAA--TCGTTTGG-GGGCGAC-TTCTC-----
-----
FC88X_01013_00304      -----------------TCAACAAA--TCGTTTGG-GGGCGAC-TTCTC-----
-----
FC88X_01093_00528      -----------------TCAACAAA--TCGTTTGG-GGGCGAC-TTCTC-----
-----
FC88X_01030_00924      -----------------TCAACAAA--TCGTTTGG-GGGCGAC-TTCTC-----
-----
FC88X_00969_00272      -----------------TCAACAAA--TCGTTTGG-GG-CGAC-TTCTC-----
-----
FC88X_01127_00643      -----------------TCAACAAA--TCGTTTGG-GG-CGAC-TTCTC-----
-----
FC88X_01111_00674      -----------------TCAACAAA--TCGTTTGG-GG-CGAC-TTCTC-----
-----
FC88X_01124_00723      -----------------TCAACAAA--TCGTTTGG-GG-CGAC-TTCTC-----
-----
FC88X_01118_00799      -----------------TCAACAAA--TCGTTTGG-GG-CGAC-TTCTC-----
-----
FC88X_01142_00774      -----------------TCAACAAA--TCGTTTGG-GG-CGAC-TTCTC-----
-----
FC88X_01130_00817      -----------------TCAACAAA--TCGTTTGG-GG-CGAC-TTCTC-----
-----
FC88X_01106_00887      -----------------TCAACAAA--TCGTTTGG-GG-CGAC-TTCTC-----
-----
FC88X_01129_00877      -----------------TCAACAAA--TCGTTTGG-GG-CGAC-TTCTC-----
-----
FC88X_01131_00943      -----------------TCAACAAA--TCGTTTGG-GG-CGAC-TTCTC-----
-----
FC88X_01144_00958      -----------------TCAACAAA--TCGTTTGG-GG-CGAC-TTCTC-----
-----
FC88X_01145_01104      -----------------TCAACAAA--TCGTTTGG-GG-CGAC-TTCTC-----
-----
```

FC88X_01135_01154 -----------------TCAACAAA--TCGTTTGG-GG-CGAC-TTCTC-----
-----
FC88X_00988_00362 -----------------TCAACAAA--TCGTTTGG-GG-CGAC-TTCTC-----
-----
FC88X_00957_00353 -----------------TCAACAAA--TCGTTTGG-GG-CGAC-TTCTC-----
-----
FC88X_00989_00591 -----------------TCAACAAA--TCGTTTGG-GG-CGAC-TTCTC-----
-----
FC88X_00975_00543 -----------------TCAACAAA--TCGTTTGG-GG-CGAC-TTCTC-----
-----
FC88X_00951_00525 -----------------TCAACAAA--TCGTTTGG-GG-CGAC-TTCTC-----
-----
FC88X_00988_00649 -----------------TCAACAAA--TCGTTTGG-GG-CGAC-TTCTC-----
-----
FC88X_00994_00507 -----------------TCAACAAA--TCGTTTGG-GG-CGAC-TTCTC-----
-----
FC88X_00964_00723 -----------------TCAACAAA--TCGTTTGG-GG-CGAC-TTCTC-----
-----
FC88X_00968_00768 -----------------TCAACAAA--TCGTTTGG-GG-CGAC-TTCTC-----
-----
FC88X_01053_00521 -----------------TCAACAAA--TCGTTTGG-GG-CGAC-TTCTC-----
-----
FC88X_01051_00584 -----------------TCAACAAA--TCGTTTGG-GG-CGAC-TTCTC-----
-----
FC88X_01093_00592 -----------------TCAACAAA--TCGTTTGG-GG-CGAC-TTCTC-----
-----
FC88X_01060_00636 -----------------TCAACAAA--TCGTTTGG-GG-CGAC-TTCTC-----
-----
FC88X_00975_00268 -----------------TCAACAAA--TCGTTTGG--CGCGAC-TTCTC-----
-----
FC88X_01009_01018 -----------------TCAACAAA--TCGTTTGG---GCGAC-TTCTC-----
-----
FC88X_01055_00417 -----------------TCAACAAA--TCGTTTGG---GCGAC-TTCTC-----
-----
FC88X_01096_00470 -----------------TCAACAAA--TCGTTTGG--TGCGAC-TTCTC-----
-----
FC88X_01008_01134 -----------------TCAACAAA--TCGTTTGC--GGCGAC-TTCTC-----
-----
FC88X_01067_00405 -----------------TCAACAAA--TCGTTTG---GGCGAC-----------
-----
FC88X_01077_00936 -----------------TCAACAAA--TCGTTTG---GGCGAC-TTCTC-----
-----
FC88X_00959_00325 ----------------TACAACAAA--TCGTTTGG---GCGAC-TTCTCC----
-----
FC88X_00996_00859 ----------------TTCAACAAA--TCGTT-GG---GCGAC-TTCTC-----
-----
FC88X_00953_00966 -----------------TCAACAAA--TCGTT-GG-TGGCGAC-TTCTC-----
-----
FC88X_00950_01026 -----------------TCAACAAA--TCGTTCGG-TGGCGAC-TTCTC-----
-----
FC88X_01126_00850 -----------------TCAACAAA--TCGTTCGG--GGCGAC-TTCTC-----
-----
FC88X_01144_00914 -----------------TCAACAAA--TCGTTCGG--GGCGAC-TTCTC-----
-----
FC88X_01004_00401 -----------------TCAACAAA--TCGTTGGG--TGCGAC-TTCTC-----
-----

```
FC88X_01035_00554      -----------------TCAACAAA--TCGTTGGG---GCGAC-
TTCTCCTTCC-----
FC88X_01107_00475      -----------------TCAACAAA--TCGTTTGG---GCGAC-TTCTC-----
-----
FC88X_01106_01238      -----------------TCAACAAA--TCGTTTGG---GCGAC-TTCTC-----
-----
FC88X_01001_00838      -----------------TCAACAAA--TCGTTTGG-G-ACGAC-TTCTC-----
-----
FC88X_01124_00657      -----------------TCAACAAA--TCGTTTGG-GGACGAC-TTCTC-----
-----
FC88X_01081_00585      -----------------TCAACAAA--TCGTTTGG-G--CGAC-TTCTC-----
-----
FC88X_01096_00683      -----------------TCAACAAA--TCGTTTGG--AGCGAC-TTCTC-----
-----
FC88X_01054_00812      -----------------TCAACAAA--TCGTTTGG-GAGCGAC-TTCTC-----
-----
FC88X_01130_00751      -----------------TCAACAAA--TCGTTTGG-GAGCGAC-TTCTC-----
-----
FC88X_01124_00614      -----------------TCAACAAA--TCGTTTGG--GGCGAC-TTCTC-----
-----
FC88X_01104_00655      -----------------TCAACAAA--TCGTTTGG--GGCGAC-TTCTC-----
-----
FC88X_01114_00737      -----------------TCAACAAA--TCGTTTGG--GGCGAC-TTCTC-----
-----
FC88X_01111_00758      -----------------TCAACAAA--TCGTTTGG--GGCGAC-TTCTC-----
-----
FC88X_01136_00798      -----------------TCAACAAA--TCGTTTGG--GGCGAC-TTCTC-----
-----
FC88X_01124_00828      -----------------TCAACAAA--TCGTTTGG--GGCGAC-TTCTC-----
-----
FC88X_01100_00882      -----------------TCAACAAA--TCGTTTGG--GGCGAC-TTCTC-----
-----
FC88X_01125_00858      -----------------TCAACAAA--TCGTTTGG--GGCGAC-TTCTC-----
-----
FC88X_01119_00912      -----------------TCAACAAA--TCGTTTGG--GGCGAC-TTCTC-----
-----
FC88X_01138_00967      -----------------TCAACAAA--TCGTTTGG--GGCGAC-TTCTC-----
-----
FC88X_01124_01114      -----------------TCAACAAA--TCGTTTGG--GGCGAC-TTCTC-----
-----
FC88X_01129_01197      -----------------TCAACAAA--TCGTTTGG--GGCGAC-TTCTC-----
-----
FC88X_01129_01230      -----------------TCAACAAA--TCGTTTGG--GGCGAC-TTCTC-----
-----
FC88X_00976_00397      -----------------TCAACAAA--TCGTTTGG--GGCGAC-TTCTC-----
-----
FC88X_00979_00594      -----------------TCAACAAA--TCGTTTGG--GGCGAC-TTCTC-----
-----
FC88X_00967_00528      -----------------TCAACAAA--TCGTTTGG--GGCGAC-TTCTC-----
-----
FC88X_00995_00467      -----------------TCAACAAA--TCGTTTGG--GGCGAC-TTCTC-----
-----
FC88X_00975_00615      -----------------TCAACAAA--TCGTTTGG--GGCGAC-TTCTC-----
-----
FC88X_00967_00695      -----------------TCAACAAA--TCGTTTGG--GGCGAC-TTCTC-----
-----
```

```
FC88X_00983_00651        -----------------TCAACAAA--TCGTTTGG--GGCGAC-TTCTC-----
-----
FC88X_00995_00725        -----------------TCAACAAA--TCGTTTGG--GGCGAC-TTCTC-----
-----
FC88X_01094_00494        -----------------TCAACAAA--TCGTTTGG--GGCGAC-TTCTC-----
-----
FC88X_01090_00543        -----------------TCAACAAA--TCGTTTGG--GGCGAC-TTCTC-----
-----
FC88X_01076_00565        -----------------TCAACAAA--TCGTTTGG--GGCGAC-TTCTC-----
-----
FC88X_01056_00619        -----------------TCAACAAA--TCGTTTGG--GGCGAC-TTCTC-----
-----
FC88X_00999_00337        -----------------TCAACAAAA-TCGTTTGG--GGCGAC-TTCTC-----
-----
FC88X_01124_00725        -----------------TCAACAAAA-TCGTTTGG--GGCGAC-TTCTC-----
-----
FC88X_00999_00803        -----------------TCAACAAAA-TCGTTTGG--GGCGAC-TTCTC-----
-----
FC88X_00996_00999        -----------------TCAACAAAA-TCGTTTGG--GGCGAC-TTCTC-----
-----
FC88X_01007_00699        -----------------TCAACAAAA-TCGTTTGG--GGCGAC-TTCTC-----
-----
FC88X_01017_00696        -----------------TCAACAAAA-TCGTTTGG--GGCGAC-TTCTC-----
-----
FC88X_01025_01031        -----------------TCAACAAAA-TCGTTTGG--GGCGAC-TTCTC-----
-----
FC88X_01054_00432        -----------------TCAACAAAA-TCGTTTGG--GGCGAC-TTCTC-----
-----
FC88X_01089_00508        -----------------TCAACAAAA-TCGTTTGG--GGCGAC-TTCTC-----
-----
FC88X_01116_00517        -----------------TCAACAAAA-TCGTTTGG--GGCGAC-TTCTC-----
-----
FC88X_01104_01105        -----------------TCAACAAAA-TCGTTTGG--GGCGAC-TTCTC-----
-----
FC88X_00958_01116        -----------------TCAACAAA--TCATTTGG--GGCGAC-TTCTC-----
-----
FC88X_01005_00897        -----------------TCAACAAA--TCATTTGG--GGCGAC-TTCTC-----
-----
FC88X_00988_00263        -----------------TCAACAAA--TCGTTTGG--GGCGAC-TTCTC-----
-----
FC88X_01134_00609        -----------------TCAACAAA--TCGTTTGG--GGCGAC-TTCTC-----
-----
FC88X_01137_00689        -----------------TCAACAAA--TCGTTTGG--GGCGAC-TTCTC-----
-----
FC88X_01139_00741        -----------------TCAACAAA--TCGTTTGG--GGCGAC-TTCTC-----
-----
FC88X_01132_00793        -----------------TCAACAAA--TCGTTTGG--GGCGAC-TTCTC-----
-----
FC88X_01119_00827        -----------------TCAACAAA--TCGTTTGG--GGCGAC-TTCTC-----
-----
FC88X_01139_00815        -----------------TCAACAAA--TCGTTTGG--GGCGAC-TTCTC-----
-----
FC88X_01120_00882        -----------------TCAACAAA--TCGTTTGG--GGCGAC-TTCTC-----
-----
FC88X_01104_00930        -----------------TCAACAAA--TCGTTTGG--GGCGAC-TTCTC-----
-----
```

```
FC88X_01119_00994      -----------------TCAACAAA--TCGTTTGG--GGCGAC-TTCTC-----
-----
FC88X_01120_01120      -----------------TCAACAAA--TCGTTTGG--GGCGAC-TTCTC-----
-----
FC88X_01116_01161      -----------------TCAACAAA--TCGTTTGG--GGCGAC-TTCTC-----
-----
FC88X_01118_01207      -----------------TCAACAAA--TCGTTTGG--GGCGAC-TTCTC-----
-----
FC88X_00998_00282      -----------------TCAACAAA--TCGTTTGG--GGCGAC-TTCTC-----
-----
FC88X_00967_00340      -----------------TCAACAAA--TCGTTTGG--GGCGAC-TTCTC-----
-----
FC88X_00996_00325      -----------------TCAACAAA--TCGTTTGG--GGCGAC-TTCTC-----
-----
FC88X_00962_00619      -----------------TCAACAAA--TCGTTTGG--GGCGAC-TTCTC-----
-----
FC88X_00972_00601      -----------------TCAACAAA--TCGTTTGG--GGCGAC-TTCTC-----
-----
FC88X_00957_00688      -----------------TCAACAAA--TCGTTTGG--GGCGAC-TTCTC-----
-----
FC88X_00997_00675      -----------------TCAACAAA--TCGTTTGG--GGCGAC-TTCTC-----
-----
FC88X_00992_00709      -----------------TCAACAAA--TCGTTTGG--GGCGAC-TTCTC-----
-----
FC88X_00999_00787      -----------------TCAACAAA--TCGTTTGG--GGCGAC-TTCTC-----
-----
FC88X_01080_00535      -----------------TCAACAAA--TCGTTTGG--GGCGAC-TTCTC-----
-----
FC88X_01074_00563      -----------------TCAACAAA--TCGTTTGG--GGCGAC-TTCTC-----
-----
FC88X_01099_00557      -----------------TCAACAAA--TCGTTTGG--GGCGAC-TTCTC-----
-----
FC88X_01020_00394      -----------------TCAACAA-A-TCGTTTGG-AGGCGAC-TTCTC-----
-----
FC88X_01080_00677      -----------------TCAACAA-A-TCGTTTG--AGGCGAC-TTCTC-----
-----
FC88X_00994_00316      -----------------TCAACAA-A-TCGTTTGG-AGGCGAC-TTCTC-----
-----
FC88X_00991_00262      -----------------TCAACAA-A-TCGTTTG--GGGCGAC-TTCTC-----
-----
FC88X_01138_00640      -----------------TCAACAA-A-TCGTTTG--GGGCGAC-TTCTC-----
-----
FC88X_01143_00662      -----------------TCAACAA-A-TCGTTTG--GGGCGAC-TTCTC-----
-----
FC88X_01142_00738      -----------------TCAACAA-A-TCGTTTG--GGGCGAC-TTCTC-----
-----
FC88X_01134_00779      -----------------TCAACAA-A-TCGTTTG--GGGCGAC-TTCTC-----
-----
FC88X_01120_00849      -----------------TCAACAA-A-TCGTTTG--GGGCGAC-TTCTC-----
-----
FC88X_01143_00844      -----------------TCAACAA-A-TCGTTTG--GGGCGAC-TTCTC-----
-----
FC88X_01122_00850      -----------------TCAACAA-A-TCGTTTG--GGGCGAC-TTCTC-----
-----
FC88X_01107_00921      -----------------TCAACAA-A-TCGTTTG--GGGCGAC-TTCTC-----
-----
```

```
FC88X_01134_00992      -----------------TCAACAA-A-TCGTTTG--GGGCGAC-TTCTC-----
-----
FC88X_01121_01103      -----------------TCAACAA-A-TCGTTTG--GGGCGAC-TTCTC-----
-----
FC88X_01125_01153      -----------------TCAACAA-A-TCGTTTG--GGGCGAC-TTCTC-----
-----
FC88X_01125_01206      -----------------TCAACAA-A-TCGTTTG--GGGCGAC-TTCTC-----
-----
FC88X_00950_00325      -----------------TCAACAA-A-TCGTTTG--GGGCGAC-TTCTC-----
-----
FC88X_00969_00335      -----------------TCAACAA-A-TCGTTTG--GGGCGAC-TTCTC-----
-----
FC88X_00965_00352      -----------------TCAACAA-A-TCGTTTG--GGGCGAC-TTCTC-----
-----
FC88X_00964_00602      -----------------TCAACAA-A-TCGTTTG--GGGCGAC-TTCTC-----
-----
FC88X_00973_00636      -----------------TCAACAA-A-TCGTTTG--GGGCGAC-TTCTC-----
-----
FC88X_00962_00654      -----------------TCAACAA-A-TCGTTTG--GGGCGAC-TTCTC-----
-----
FC88X_00999_00362      -----------------TCAACAA-A-TCGTTTG--GGGCGAC-TTCTC-----
-----
FC88X_00992_00734      -----------------TCAACAA-A-TCGTTTG--GGGCGAC-TTCTC-----
-----
FC88X_00957_00821      -----------------TCAACAA-A-TCGTTTG--GGGCGAC-TTCTC-----
-----
FC88X_01084_00520      -----------------TCAACAA-A-TCGTTTG--GGGCGAC-TTCTC-----
-----
FC88X_01074_00580      -----------------TCAACAA-A-TCGTTTG--GGGCGAC-TTCTC-----
-----
FC88X_01052_00624      -----------------TCAACAA-A-TCGTTTG--GGGCGAC-TTCTC-----
-----
FC88X_00965_00587      -----------------TCAACAA-G-TCGTTTG--GGGCGAC-TTCTC-----
-----
FC88X_01016_00844      -----------------TCAACAA-G-TCGTTTG--GGGCGAC-TTCTC-----
-----
FC88X_01099_00570      -----------------TCAACAA-G-TCGTTTG--GGGCGAC-TTCTC-----
-----
FC88X_01073_00560      -----------------TCAACAAAG-TCGTTT---------------------
-----
FC88X_01139_00681      -----------------TCAACAAAG-TCGTTTG--GGGCGAC-TTCTC-----
-----
FC88X_01138_00634      -----------------TCAACAA-A-TCGTTTG--GGGCGAC-TTCTC-----
-----
FC88X_01140_00687      -----------------TCAACAA-A-TCGTTTG--GGGCGAC-TTCTC-----
-----
FC88X_01142_00716      -----------------TCAACAA-A-TCGTTTG--GGGCGAC-TTCTC-----
-----
FC88X_01133_00788      -----------------TCAACAA-A-TCGTTTG--GGGCGAC-TTCTC-----
-----
FC88X_01120_00820      -----------------TCAACAA-A-TCGTTTG--GGGCGAC-TTCTC-----
-----
FC88X_01139_00828      -----------------TCAACAA-A-TCGTTTG--GGGCGAC-TTCTC-----
-----
FC88X_01121_00868      -----------------TCAACAA-A-TCGTTTG--GGGCGAC-TTCTC-----
-----
```

```
FC88X_01106_00944      -----------------TCAACAA-A-TCGTTTG--GGGCGAC-TTCTC-----
-----
FC88X_01134_00985      -----------------TCAACAA-A-TCGTTTG--GGGCGAC-TTCTC-----
-----
FC88X_01120_01128      -----------------TCAACAA-A-TCGTTTG--GGGCGAC-TTCTC-----
-----
FC88X_01121_01185      -----------------TCAACAA-A-TCGTTTG--GGGCGAC-TTCTC-----
-----
FC88X_01119_01210      -----------------TCAACAA-A-TCGTTTG--GGGCGAC-TTCTC-----
-----
FC88X_00999_00256      -----------------TCAACAA-A-TCGTTTG--GGGCGAC-TTCTC-----
-----
FC88X_00969_00315      -----------------TCAACAA-A-TCGTTTG--GGGCGAC-TTCTC-----
-----
FC88X_00997_00334      -----------------TCAACAA-A-TCGTTTG--GGGCGAC-TTCTC-----
-----
FC88X_00963_00612      -----------------TCAACAA-A-TCGTTTG--GGGCGAC-TTCTC-----
-----
FC88X_00973_00627      -----------------TCAACAA-A-TCGTTTG--GGGCGAC-TTCTC-----
-----
FC88X_00961_00699      -----------------TCAACAA-A-TCGTTTG--GGGCGAC-TTCTC-----
-----
FC88X_00998_00399      -----------------TCAACAA-A-TCGTTTG--GGGCGAC-TTCTC-----
-----
FC88X_00992_00722      -----------------TCAACAA-A-TCGTTTG--GGGCGAC-TTCTC-----
-----
FC88X_00951_00847      -----------------TCAACAA-A-TCGTTTG--GGGCGAC-TTCTC-----
-----
FC88X_01084_00505      -----------------TCAACAA-A-TCGTTTG--GGGCGAC-TTCTC-----
-----
FC88X_01074_00571      -----------------TCAACAA-A-TCGTTTG--GGGCGAC-TTCTC-----
-----
FC88X_01044_01187      -----------------TCAACAG-A-TCGTTTG--GGGCGAC-TTCTC-----
-----
FC88X_01129_00925      -----------------TCAACA--A-TCGTTTG--GGGCGAC-TTCTC-----
-----
FC88X_01050_00622      -----------------TCAACAAAC-TCGTTTG--GGGCGAC-TTCTC-----
-----
FC88X_01143_00935      -----------------TCAACAA-C-TCGTTTG--GGGCGAC-TTCTC-----
-----
FC88X_01129_00643      -----------------TCAACAA-A-TCGTTTG--GGGCGAC-TTCTC-----
-----
FC88X_01115_00698      -----------------TCAACAA-A-TCGTTTG--GGGCGAC-TTCTC-----
-----
FC88X_01128_00704      -----------------TCAACAA-A-TCGTTTG--GGGCGAC-TTCTC-----
-----
FC88X_01122_00797      -----------------TCAACAA-A-TCGTTTG--GGGCGAC-TTCTC-----
-----
FC88X_01144_00765      -----------------TCAACAA-A-TCGTTTG--GGGCGAC-TTCTC-----
-----
FC88X_01131_00810      -----------------TCAACAA-A-TCGTTTG--GGGCGAC-TTCTC-----
-----
FC88X_01108_00862      -----------------TCAACAA-A-TCGTTTG--GGGCGAC-TTCTC-----
-----
FC88X_01130_00872      -----------------TCAACAA-A-TCGTTTG--GGGCGAC-TTCTC-----
-----
```

```
FC88X_01134_00901       -----------------TCAACAA-A-TCGTTTG--GGGCGAC-TTCTC-----
-----
FC88X_01107_01149       -----------------TCAACAA-A-TCGTTTG--GGGCGAC-TTCTC-----
-----
FC88X_01103_01187       -----------------TCAACAA-A-TCGTTTG--GGGCGAC-TTCTC-----
-----
FC88X_01137_01154       -----------------TCAACAA-A-TCGTTTG--GGGCGAC-TTCTC-----
-----
FC88X_00988_00367       -----------------TCAACAA-A-TCGTTTG--GGGCGAC-TTCTC-----
-----
FC88X_00989_00330       -----------------TCAACAA-A-TCGTTTG--GGGCGAC-TTCTC-----
-----
FC88X_00951_00633       -----------------TCAACAA-A-TCGTTTG--GGGCGAC-TTCTC-----
-----
FC88X_00977_00533       -----------------TCAACAA-A-TCGTTTG--GGGCGAC-TTCTC-----
-----
FC88X_00961_00501       -----------------TCAACAA-A-TCGTTTG--GGGCGAC-TTCTC-----
-----
FC88X_00994_00647       -----------------TCAACAA-A-TCGTTTG--GGGCGAC-TTCTC-----
-----
FC88X_00950_00597       -----------------TCAACAA-A-TCGTTTG--GGGCGAC-TTCTC-----
-----
FC88X_00967_00703       -----------------TCAACAA-A-TCGTTTG--GGGCGAC-TTCTC-----
-----
FC88X_00969_00779       -----------------TCAACAA-A-TCGTTTG--GGGCGAC-TTCTC-----
-----
FC88X_01055_00535       -----------------TCAACAA-A-TCGTTTG--GGGCGAC-TTCTC-----
-----
FC88X_01057_00599       -----------------TCAACAA-A-TCGTTTG--GGGCGAC-TTCTC-----
-----
FC88X_01094_00591       -----------------TCAACAA-A-TCGTTTG--GGGCGAC-TTCTC-----
-----
FC88X_01064_00615       -----------------TCAACAAA--TCGTTTG--GGGCGAC-TTCTC-----
-----
FC88X_00985_00958       -----------------TCAACAAA--TCGTTTG--GGGCGAC-TTCTC-----
-----
FC88X_00985_00974       -----------------TCAACAAA--TCGTTTG--GGGCGAC-TTCTC-----
-----
FC88X_01131_00963       -----------------TCAACAAA--TCGTTTG--GGGCGAC-TTCTC-----
-----
FC88X_01129_00985       -----------------TCAACAAA--TCGTTTG--GGGCGAC-TTCTC-----
-----
FC88X_01124_00969       -----------------TCAACAAA--TCGTTTG--GGGCGAC-TTCTC-----
-----
FC88X_01146_00933       -----------------TCAACAAA--TCGTTTG--GGGCGAC-TTCTC-----
-----
FC88X_01143_00940       -----------------TCAACAAA--TCGTTTG--GGGCGAC-TTCTC-----
-----
FC88X_01141_00943       -----------------TCAACAAA--TCGTTTG--GGGCGAC-TTCTC-----
-----
FC88X_01049_00766       -----------------TCAACAAA--TCGTTTG--GGGCGAC-TTCTC-----
-----
FC88X_01016_00827       -----------------TCAACAAA--TCGTTTG--GGGCGAC-TTCTC-----
-----
FC88X_01016_00803       -----------------TCAACAAA--TCGTTTG--GGGCGAC-TTCTC-----
-----
```

FC88X_01012_00842 -----------------TCAACAAA--TCGTTTG--GGGCGAC-TTCTC-----
-----
FC88X_01007_00806 -----------------TCAACAAA--TCGTTTG--GGGCGAC-TTCTC-----
-----
FC88X_01006_00840 -----------------TCAACAAA--TCGTTTG--GGGCGAC-TTCTC-----
-----
FC88X_01006_00828 -----------------TCAACAAA--TCGTTTG--GGGCGAC-TTCTC-----
-----
FC88X_01005_00828 -----------------TCAACAAA--TCGTTTG--GGGCGAC-TTCTC-----
-----
FC88X_01004_00817 -----------------TCAACAAA--TCGTTTG--GGGCGAC-TTCTC-----
-----
FC88X_01003_00826 -----------------TCAACAAA--TCGTTTG--GGGCGAC-TTCTC-----
-----
FC88X_01020_01204 -----------------TCAACAAA--TCGTTTG--GGGCGAC-TTCTC-----
-----
FC88X_01019_01222 -----------------TCAACAAA--TCGTTTG--GGGCGAC-TTCTC-----
-----
FC88X_01017_01229 -----------------TCAACAAA--TCGTTTG--GGGCGAC-TTCTC-----
-----
FC88X_01016_01216 -----------------TCAACAAA--TCGTTTG--GGGCGAC-TTCTC-----
-----
FC88X_01007_01200 -----------------TCAACAAA--TCGTTTG--GGGCGAC-TTCTC-----
-----
FC88X_01004_01226 -----------------TCAACAAA--TCGTTTG--GGGCGAC-TTCTC-----
-----
FC88X_01048_01171 -----------------TCAACAAA--TCGTTTG--GGGCGAC-TTCTC-----
-----
FC88X_01046_01156 -----------------TCAACAAA--TCGTTTG--GGGCGAC-TTCTC-----
-----
FC88X_01044_01155 -----------------TCAACAAA--TCGTTTG--GGGCGAC-TTCTC-----
-----
FC88X_01042_01159 -----------------TCAACAAA--TCGTTTG--GGGCGAC-TTCTC-----
-----
FC88X_01037_01165 -----------------TCAACAAA--TCGTTTG--GGGCGAC-TTCTC-----
-----
FC88X_01029_01173 -----------------TCAACAAA--TCGTTTG--GGGCGAC-TTCTC-----
-----
FC88X_01024_01160 -----------------TCAACAAA--TCGTTTG--GGGCGAC-TTCTC-----
-----
FC88X_01018_01195 -----------------TCAACAAA--TCGTTTG--GGGCGAC-TTCTC-----
-----
FC88X_01006_01192 -----------------TCAACAAA--TCGTTTG--GGGCGAC-TTCTC-----
-----
FC88X_01003_01163 -----------------TCAACAAA--TCGTTTG--GGGCGAC-TTCTC-----
-----
FC88X_00950_00989 -----------------TCAACAAA--TCGTTTG--GGGCGAC-TTCTC-----
-----
FC88X_00997_00920 -----------------TCAACAAA--TCGTTTG--GGGCGAC-TTCTC-----
-----
FC88X_00996_00947 -----------------TCAACAAA--TCGTTTG--GGGCGAC-TTCTC-----
-----
FC88X_00994_00907 -----------------TCAACAAA--TCGTTTG--GGGCGAC-TTCTC-----
-----
FC88X_00991_00936 -----------------TCAACAAA--TCGTTTG--GGGCGAC-TTCTC-----
-----

```
FC88X_01084_00497       -----------------TCAACAAA--TCGTTTG--GGGCGAC-TTCTC-----
-----
FC88X_01084_00486       -----------------TCAACAAA--TCGTTTG--GGGCGAC-TTCTC-----
-----
FC88X_01080_00451       -----------------TCAACAAA--TCGTTTG--GGGCGAC-TTCTC-----
-----
FC88X_01078_00465       -----------------TCAACAAA--TCGTTTG--GGGCGAC-TTCTC-----
-----
FC88X_01077_00454       -----------------TCAACAAA--TCGTTTG--GGGCGAC-TTCTC-----
-----
FC88X_01075_00499       -----------------TCAACAAA--TCGTTTG--GGGCGAC-TTCTC-----
-----
FC88X_01075_00493       -----------------TCAACAAA--TCGTTTG--GGGCGAC-TTCTC-----
-----
FC88X_01071_00473       -----------------TCAACAAA--TCGTTTG--GGGCGAC-TTCTC-----
-----
FC88X_01068_00457       -----------------TCAACAAA--TCGTTTG--GGGCGAC-TTCTC-----
-----
FC88X_01066_00498       -----------------TCAACAAA--TCGTTTG--GGGCGAC-TTCTC-----
-----
FC88X_01065_00498       -----------------TCAACAAA--TCGTTTG--GGGCGAC-TTCTC-----
-----
FC88X_01063_00492       -----------------TCAACAAA--TCGTTTG--GGGCGAC-TTCTC-----
-----
FC88X_01061_00465       -----------------TCAACAAA--TCGTTTG--GGGCGAC-TTCTC-----
-----
FC88X_01060_00481       -----------------TCAACAAA--TCGTTTG--GGGCGAC-TTCTC-----
-----
FC88X_01058_00465       -----------------TCAACAAA--TCGTTTG--GGGCGAC-TTCTC-----
-----
FC88X_01056_00482       -----------------TCAACAAA--TCGTTTG--GGGCGAC-TTCTC-----
-----
FC88X_01055_00489       -----------------TCAACAAA--TCGTTTG--GGGCGAC-TTCTC-----
-----
FC88X_01054_00479       -----------------TCAACAAA--TCGTTTG--GGGCGAC-TTCTC-----
-----
FC88X_01053_00479       -----------------TCAACAAA--TCGTTTG--GGGCGAC-TTCTC-----
-----
FC88X_01050_00477       -----------------TCAACAAA--TCGTTTG--GGGCGAC-TTCTC-----
-----
FC88X_01007_00312       -----------------TCAACAAA--TCGTTTG--GGGCGAC-TTCTC-----
-----
FC88X_01006_00331       -----------------TCAACAAA--TCGTTTG--GGGCGAC-TTCTC-----
-----
FC88X_01006_00330       -----------------TCAACAAA--TCGTTTG--GGGCGAC-TTCTC-----
-----
FC88X_01005_00322       -----------------TCAACAAA--TCGTTTG--GGGCGAC-TTCTC-----
-----
FC88X_01000_00333       -----------------TCAACAAA--TCGTTTG--GGGCGAC-TTCTC-----
-----
FC88X_01028_00297       -----------------TCAACAAA--TCGTTTG--GGGCGAC-TTCTC-----
-----
FC88X_01020_00289       -----------------TCAACAAA--TCGTTTG--GGGCGAC-TTCTC-----
-----
FC88X_01011_00297       -----------------TCAACAAA--TCGTTTG--GGGCGAC-TTCTC-----
-----
```

```
FC88X_01009_00298      -----------------TCAACAAA--TCGTTTG--GGGCGAC-TTCTC-----
-----
FC88X_01004_00270      -----------------TCAACAAA--TCGTTTG--GGGCGAC-TTCTC-----
-----
FC88X_01001_00267      -----------------TCAACAAA--TCGTTTG--GGGCGAC-TTCTC-----
-----
FC88X_00998_01269      -----------------TCAACAAA--TCGTTTG--GGGCGAC-TTCTC-----
-----
FC88X_00987_01270      -----------------TCAACAAA--TCGTTTG--GGGCGAC-TTCTC-----
-----
FC88X_00987_01255      -----------------TCAACAAA--TCGTTTG--GGGCGAC-TTCTC-----
-----
FC88X_00985_01275      -----------------TCAACAAA--TCGTTTG--GGGCGAC-TTCTC-----
-----
FC88X_00977_01265      -----------------TCAACAAA--TCGTTTG--GGGCGAC-TTCTC-----
-----
FC88X_00973_01258      -----------------TCAACAAA--TCGTTTG--GGGCGAC-TTCTC-----
-----
FC88X_00972_01265      -----------------TCAACAAA--TCGTTTG--GGGCGAC-TTCTC-----
-----
FC88X_00970_01271      -----------------TCAACAAA--TCGTTTG--GGGCGAC-TTCTC-----
-----
FC88X_00967_01263      -----------------TCAACAAA--TCGTTTG--GGGCGAC-TTCTC-----
-----
FC88X_00965_01253      -----------------TCAACAAA--TCGTTTG--GGGCGAC-TTCTC-----
-----
FC88X_00961_01250      -----------------TCAACAAA--TCGTTTG--GGGCGAC-TTCTC-----
-----
FC88X_00951_01273      -----------------TCAACAAA--TCGTTTG--GGGCGAC-TTCTC-----
-----
FC88X_00950_01255      -----------------TCAACAAA--TCGTTTG--GGGCGAC-TTCTC-----
-----
FC88X_00998_01237      -----------------TCAACAAA--TCGTTTG--GGGCGAC-TTCTC-----
-----
FC88X_00997_01224      -----------------TCAACAAA--TCGTTTG--GGGCGAC-TTCTC-----
-----
FC88X_00997_01202      -----------------TCAACAAA--TCGTTTG--GGGCGAC-TTCTC-----
-----
FC88X_00996_01225      -----------------TCAACAAA--TCGTTTG--GGGCGAC-TTCTC-----
-----
FC88X_00991_01228      -----------------TCAACAAA--TCGTTTG--GGGCGAC-TTCTC-----
-----
FC88X_00982_01216      -----------------TCAACAAA--TCGTTTG--GGGCGAC-TTCTC-----
-----
FC88X_00977_01211      -----------------TCAACAAA--TCGTTTG--GGGCGAC-TTCTC-----
-----
FC88X_00974_01217      -----------------TCAACAAA--TCGTTTG--GGGCGAC-TTCTC-----
-----
FC88X_00973_01212      -----------------TCAACAAA--TCGTTTG--GGGCGAC-TTCTC-----
-----
FC88X_00960_01206      -----------------TCAACAAA--TCGTTTG--GGGCGAC-TTCTC-----
-----
FC88X_00960_01205      -----------------TCAACAAA--TCGTTTG--GGGCGAC-TTCTC-----
-----
FC88X_00955_01216      -----------------TCAACAAA--TCGTTTG--GGGCGAC-TTCTC-----
-----
```

FC88X_00952_01247 -----------------TCAACAAA--TCGTTTG--GGGCGAC-TTCTC-----
-----
FC88X_00951_01214 -----------------TCAACAAA--TCGTTTG--GGGCGAC-TTCTC-----
-----
FC88X_00992_01105 -----------------TCAACAAA--TCGTTTG--GGGCGAC-TTCTC-----
-----
FC88X_00986_01136 -----------------TCAACAAA--TCGTTTG--GGGCGAC-TTCTC-----
-----
FC88X_00984_01114 -----------------TCAACAAA--TCGTTTG--GGGCGAC-TTCTC-----
-----
FC88X_00979_01111 -----------------TCAACAAA--TCGTTTG--GGGCGAC-TTCTC-----
-----
FC88X_00967_01115 -----------------TCAACAAA--TCGTTTG--GGGCGAC-TTCTC-----
-----
FC88X_00957_01127 -----------------TCAACAAA--TCGTTTG--GGGCGAC-TTCTC-----
-----
FC88X_00998_01031 -----------------TCAACAAA--TCGTTTG--GGGCGAC-TTCTC-----
-----
FC88X_00997_01024 -----------------TCAACAAA--TCGTTTG--GGGCGAC-TTCTC-----
-----
FC88X_00960_00995 -----------------TCAACAAA--TCGTTTG--GGGCGAC-TTCTC-----
-----
FC88X_00960_00959 -----------------TCAACAAA--TCGTTTG--GGGCGAC-TTCTC-----
-----
FC88X_00959_00997 -----------------TCAACAAA--TCGTTTG--GGGCGAC-TTCTC-----
-----
FC88X_00959_00981 -----------------TCAACAAA--TCGTTTG--GGGCGAC-TTCTC-----
-----
FC88X_00957_00954 -----------------TCAACAAA--TCGTTTG--GGGCGAC-TTCTC-----
-----
FC88X_00954_00981 -----------------TCAACAAA--TCGTTTG--GGGCGAC-TTCTC-----
-----
FC88X_01021_00336 -----------------TCAACAAA--TCGTTTG--GGGCGAC-TTCTC-----
-----
FC88X_01021_00313 -----------------TCAACAAA--TCGTTTG--GGGCGAC-TTCTC-----
-----
FC88X_01015_00326 -----------------TCAACAAA--TCGTTTG--GGGCGAC-TTCTC-----
-----
FC88X_01014_00303 -----------------TCAACAAA--TCGTTTG--GGGCGAC-TTCTC-----
-----
FC88X_01012_00304 -----------------TCAACAAA--TCGTTTG--GGGCGAC-TTCTC-----
-----
FC88X_01010_00312 -----------------TCAACAAA--TCGTTTG--GGGCGAC-TTCTC-----
-----
FC88X_01009_01147 -----------------TCAACAAA--TCGTTTG--GGGCGAC-TTCTC-----
-----
FC88X_01005_01139 -----------------TCAACAAA--TCGTTTG--GGGCGAC-TTCTC-----
-----
FC88X_01035_00447 -----------------TCAACAAA--TCGTTTG--GGGCGAC-TTCTC-----
-----
FC88X_01035_00403 -----------------TCAACAAA--TCGTTTG--GGGCGAC-TTCTC-----
-----
FC88X_01034_00438 -----------------TCAACAAA--TCGTTTG--GGGCGAC-TTCTC-----
-----
FC88X_01033_00402 -----------------TCAACAAA--TCGTTTG--GGGCGAC-TTCTC-----
-----

FC88X_01031_00438 -----------------TCAACAAA--TCGTTTG--GGGCGAC-TTCTC-----
-----
FC88X_01027_00430 -----------------TCAACAAA--TCGTTTG--GGGCGAC-TTCTC-----
-----
FC88X_01021_00400 -----------------TCAACAAA--TCGTTTG--GGGCGAC-TTCTC-----
-----
FC88X_01012_00439 -----------------TCAACAAA--TCGTTTG--GGGCGAC-TTCTC-----
-----
FC88X_01004_00445 -----------------TCAACAAA--TCGTTTG--GGGCGAC-TTCTC-----
-----
FC88X_01047_00370 -----------------TCAACAAA--TCGTTTG--GGGCGAC-TTCTC-----
-----
FC88X_01046_00353 -----------------TCAACAAA--TCGTTTG--GGGCGAC-TTCTC-----
-----
FC88X_00995_01017 -----------------TCAACAAA--TCGTTTG--GGGCGAC-TTCTC-----
-----
FC88X_00994_01048 -----------------TCAACAAA--TCGTTTG--GGGCGAC-TTCTC-----
-----
FC88X_00990_01005 -----------------TCAACAAA--TCGTTTG--GGGCGAC-TTCTC-----
-----
FC88X_00987_01049 -----------------TCAACAAA--TCGTTTG--GGGCGAC-TTCTC-----
-----
FC88X_00982_01044 -----------------TCAACAAA--TCGTTTG--GGGCGAC-TTCTC-----
-----
FC88X_00980_01048 -----------------TCAACAAA--TCGTTTG--GGGCGAC-TTCTC-----
-----
FC88X_00960_01047 -----------------TCAACAAA--TCGTTTG--GGGCGAC-TTCTC-----
-----
FC88X_00957_01037 -----------------TCAACAAA--TCGTTTG--GGGCGAC-TTCTC-----
-----
FC88X_00954_01011 -----------------TCAACAAA--TCGTTTG--GGGCGAC-TTCTC-----
-----
FC88X_00996_00975 -----------------TCAACAAA--TCGTTTG--GGGCGAC-TTCTC-----
-----
FC88X_00987_00986 -----------------TCAACAAA--TCGTTTG--GGGCGAC-TTCTC-----
-----
FC88X_00980_00981 -----------------TCAACAAA--TCGTTTG--GGGCGAC-TTCTC-----
-----
FC88X_00976_00961 -----------------TCAACAAA--TCGTTTG--GGGCGAC-TTCTC-----
-----
FC88X_00975_00997 -----------------TCAACAAA--TCGTTTG--GGGCGAC-TTCTC-----
-----
FC88X_00975_00962 -----------------TCAACAAA--TCGTTTG--GGGCGAC-TTCTC-----
-----
FC88X_00971_00986 -----------------TCAACAAA--TCGTTTG--GGGCGAC-TTCTC-----
-----
FC88X_00966_00957 -----------------TCAACAAA--TCGTTTG--GGGCGAC-TTCTC-----
-----
FC88X_01040_00385 -----------------TCAACAAA--TCGTTTG--GGGCGAC-TTCTC-----
-----
FC88X_01040_00374 -----------------TCAACAAA--TCGTTTG--GGGCGAC-TTCTC-----
-----
FC88X_01040_00350 -----------------TCAACAAA--TCGTTTG--GGGCGAC-TTCTC-----
-----
FC88X_01038_00375 -----------------TCAACAAA--TCGTTTG--GGGCGAC-TTCTC-----
-----

FC88X_01036_00387 -----------------TCAACAAA--TCGTTTG--GGGCGAC-TTCTC-----
-----
FC88X_01034_00353 -----------------TCAACAAA--TCGTTTG--GGGCGAC-TTCTC-----
-----
FC88X_01033_00366 -----------------TCAACAAA--TCGTTTG--GGGCGAC-TTCTC-----
-----
FC88X_01031_00387 -----------------TCAACAAA--TCGTTTG--GGGCGAC-TTCTC-----
-----
FC88X_01031_00384 -----------------TCAACAAA--TCGTTTG--GGGCGAC-TTCTC-----
-----
FC88X_01031_00376 -----------------TCAACAAA--TCGTTTG--GGGCGAC-TTCTC-----
-----
FC88X_01030_00365 -----------------TCAACAAA--TCGTTTG--GGGCGAC-TTCTC-----
-----
FC88X_01029_00396 -----------------TCAACAAA--TCGTTTG--GGGCGAC-TTCTC-----
-----
FC88X_01029_00389 -----------------TCAACAAA--TCGTTTG--GGGCGAC-TTCTC-----
-----
FC88X_01029_00358 -----------------TCAACAAA--TCGTTTG--GGGCGAC-TTCTC-----
-----
FC88X_01028_00353 -----------------TCAACAAA--TCGTTTG--GGGCGAC-TTCTC-----
-----
FC88X_01027_00391 -----------------TCAACAAA--TCGTTTG--GGGCGAC-TTCTC-----
-----
FC88X_01027_00369 -----------------TCAACAAA--TCGTTTG--GGGCGAC-TTCTC-----
-----
FC88X_01026_00392 -----------------TCAACAAA--TCGTTTG--GGGCGAC-TTCTC-----
-----
FC88X_01026_00374 -----------------TCAACAAA--TCGTTTG--GGGCGAC-TTCTC-----
-----
FC88X_01023_00392 -----------------TCAACAAA--TCGTTTG--GGGCGAC-TTCTC-----
-----
FC88X_01023_00389 -----------------TCAACAAA--TCGTTTG--GGGCGAC-TTCTC-----
-----
FC88X_01023_00354 -----------------TCAACAAA--TCGTTTG--GGGCGAC-TTCTC-----
-----
FC88X_01022_00389 -----------------TCAACAAA--TCGTTTG--GGGCGAC-TTCTC-----
-----
FC88X_01021_00375 -----------------TCAACAAA--TCGTTTG--GGGCGAC-TTCTC-----
-----
FC88X_01019_00372 -----------------TCAACAAA--TCGTTTG--GGGCGAC-TTCTC-----
-----
FC88X_01019_00362 -----------------TCAACAAA--TCGTTTG--GGGCGAC-TTCTC-----
-----
FC88X_01018_00357 -----------------TCAACAAA--TCGTTTG--GGGCGAC-TTCTC-----
-----
FC88X_01015_00377 -----------------TCAACAAA--TCGTTTG--GGGCGAC-TTCTC-----
-----
FC88X_01014_00386 -----------------TCAACAAA--TCGTTTG--GGGCGAC-TTCTC-----
-----
FC88X_01014_00380 -----------------TCAACAAA--TCGTTTG--GGGCGAC-TTCTC-----
-----
FC88X_01006_00382 -----------------TCAACAAA--TCGTTTG--GGGCGAC-TTCTC-----
-----
FC88X_01001_00361 -----------------TCAACAAA--TCGTTTG--GGGCGAC-TTCTC-----
-----

FC88X_01000_00359 -----------------TCAACAAA--TCGTTTG--GGGCGAC-TTCTC-----
-----
FC88X_01045_00322 -----------------TCAACAAA--TCGTTTG--GGGCGAC-TTCTC-----
-----
FC88X_01033_00331 -----------------TCAACAAA--TCGTTTG--GGGCGAC-TTCTC-----
-----
FC88X_01026_00338 -----------------TCAACAAA--TCGTTTG--GGGCGAC-TTCTC-----
-----
FC88X_01046_00573 -----------------TCAACAAA--TCGTTTG--GGGCGAC-TTCTC-----
-----
FC88X_01044_00591 -----------------TCAACAAA--TCGTTTG--GGGCGAC-TTCTC-----
-----
FC88X_01043_00587 -----------------TCAACAAA--TCGTTTG--GGGCGAC-TTCTC-----
-----
FC88X_01028_00789 -----------------TCAACAAA--TCGTTTG--GGGCGAC-TTCTC-----
-----
FC88X_01028_00766 -----------------TCAACAAA--TCGTTTG--GGGCGAC-TTCTC-----
-----
FC88X_01027_00771 -----------------TCAACAAA--TCGTTTG--GGGCGAC-TTCTC-----
-----
FC88X_01025_00756 -----------------TCAACAAA--TCGTTTG--GGGCGAC-TTCTC-----
-----
FC88X_01024_00793 -----------------TCAACAAA--TCGTTTG--GGGCGAC-TTCTC-----
-----
FC88X_01021_00798 -----------------TCAACAAA--TCGTTTG--GGGCGAC-TTCTC-----
-----
FC88X_01020_00784 -----------------TCAACAAA--TCGTTTG--GGGCGAC-TTCTC-----
-----
FC88X_01016_00792 -----------------TCAACAAA--TCGTTTG--GGGCGAC-TTCTC-----
-----
FC88X_01016_00762 -----------------TCAACAAA--TCGTTTG--GGGCGAC-TTCTC-----
-----
FC88X_01015_00776 -----------------TCAACAAA--TCGTTTG--GGGCGAC-TTCTC-----
-----
FC88X_01002_00635 -----------------TCAACAAA--TCGTTTG--GGGCGAC-TTCTC-----
-----
FC88X_01001_00609 -----------------TCAACAAA--TCGTTTG--GGGCGAC-TTCTC-----
-----
FC88X_01000_00649 -----------------TCAACAAA--TCGTTTG--GGGCGAC-TTCTC-----
-----
FC88X_01000_00609 -----------------TCAACAAA--TCGTTTG--GGGCGAC-TTCTC-----
-----
FC88X_01029_00486 -----------------TCAACAAA--TCGTTTG--GGGCGAC-TTCTC-----
-----
FC88X_01028_00489 -----------------TCAACAAA--TCGTTTG--GGGCGAC-TTCTC-----
-----
FC88X_01028_00457 -----------------TCAACAAA--TCGTTTG--GGGCGAC-TTCTC-----
-----
FC88X_01026_00470 -----------------TCAACAAA--TCGTTTG--GGGCGAC-TTCTC-----
-----
FC88X_01023_00452 -----------------TCAACAAA--TCGTTTG--GGGCGAC-TTCTC-----
-----
FC88X_01022_00491 -----------------TCAACAAA--TCGTTTG--GGGCGAC-TTCTC-----
-----
FC88X_01021_00480 -----------------TCAACAAA--TCGTTTG--GGGCGAC-TTCTC-----
-----

FC88X_01020_00479 -----------------TCAACAAA--TCGTTTG--GGGCGAC-TTCTC-----
-----
FC88X_01017_00485 -----------------TCAACAAA--TCGTTTG--GGGCGAC-TTCTC-----
-----
FC88X_01011_00476 -----------------TCAACAAA--TCGTTTG--GGGCGAC-TTCTC-----
-----
FC88X_01010_00485 -----------------TCAACAAA--TCGTTTG--GGGCGAC-TTCTC-----
-----
FC88X_01000_00498 -----------------TCAACAAA--TCGTTTG--GGGCGAC-TTCTC-----
-----
FC88X_01045_00446 -----------------TCAACAAA--TCGTTTG--GGGCGAC-TTCTC-----
-----
FC88X_01044_00431 -----------------TCAACAAA--TCGTTTG--GGGCGAC-TTCTC-----
-----
FC88X_01043_00404 -----------------TCAACAAA--TCGTTTG--GGGCGAC-TTCTC-----
-----
FC88X_01008_00770 -----------------TCAACAAA--TCGTTTG--GGGCGAC-TTCTC-----
-----
FC88X_01007_00797 -----------------TCAACAAA--TCGTTTG--GGGCGAC-TTCTC-----
-----
FC88X_01005_00761 -----------------TCAACAAA--TCGTTTG--GGGCGAC-TTCTC-----
-----
FC88X_01002_00780 -----------------TCAACAAA--TCGTTTG--GGGCGAC-TTCTC-----
-----
FC88X_01001_00770 -----------------TCAACAAA--TCGTTTG--GGGCGAC-TTCTC-----
-----
FC88X_01041_00656 -----------------TCAACAAA--TCGTTTG--GGGCGAC-TTCTC-----
-----
FC88X_01040_00668 -----------------TCAACAAA--TCGTTTG--GGGCGAC-TTCTC-----
-----
FC88X_01039_00699 -----------------TCAACAAA--TCGTTTG--GGGCGAC-TTCTC-----
-----
FC88X_01038_00657 -----------------TCAACAAA--TCGTTTG--GGGCGAC-TTCTC-----
-----
FC88X_01034_00691 -----------------TCAACAAA--TCGTTTG--GGGCGAC-TTCTC-----
-----
FC88X_01032_00661 -----------------TCAACAAA--TCGTTTG--GGGCGAC-TTCTC-----
-----
FC88X_01029_00698 -----------------TCAACAAA--TCGTTTG--GGGCGAC-TTCTC-----
-----
FC88X_01025_00688 -----------------TCAACAAA--TCGTTTG--GGGCGAC-TTCTC-----
-----
FC88X_01023_00683 -----------------TCAACAAA--TCGTTTG--GGGCGAC-TTCTC-----
-----
FC88X_01023_00654 -----------------TCAACAAA--TCGTTTG--GGGCGAC-TTCTC-----
-----
FC88X_01022_00652 -----------------TCAACAAA--TCGTTTG--GGGCGAC-TTCTC-----
-----
FC88X_01019_00674 -----------------TCAACAAA--TCGTTTG--GGGCGAC-TTCTC-----
-----
FC88X_01019_00671 -----------------TCAACAAA--TCGTTTG--GGGCGAC-TTCTC-----
-----
FC88X_01018_00698 -----------------TCAACAAA--TCGTTTG--GGGCGAC-TTCTC-----
-----
FC88X_01013_00666 -----------------TCAACAAA--TCGTTTG--GGGCGAC-TTCTC-----
-----

```
FC88X_01013_00658      -----------------TCAACAAA--TCGTTTG--GGGCGAC-TTCTC-----
-----
FC88X_01003_00650      -----------------TCAACAAA--TCGTTTG--GGGCGAC-TTCTC-----
-----
FC88X_01002_00665      -----------------TCAACAAA--TCGTTTG--GGGCGAC-TTCTC-----
-----
FC88X_01049_00628      -----------------TCAACAAA--TCGTTTG--GGGCGAC-TTCTC-----
-----
FC88X_01048_00625      -----------------TCAACAAA--TCGTTTG--GGGCGAC-TTCTC-----
-----
FC88X_01043_00612      -----------------TCAACAAA--TCGTTTG--GGGCGAC-TTCTC-----
-----
FC88X_01036_00610      -----------------TCAACAAA--TCGTTTG--GGGCGAC-TTCTC-----
-----
FC88X_01036_00600      -----------------TCAACAAA--TCGTTTG--GGGCGAC-TTCTC-----
-----
FC88X_01034_00627      -----------------TCAACAAA--TCGTTTG--GGGCGAC-TTCTC-----
-----
FC88X_01030_00620      -----------------TCAACAAA--TCGTTTG--GGGCGAC-TTCTC-----
-----
FC88X_01027_00600      -----------------TCAACAAA--TCGTTTG--GGGCGAC-TTCTC-----
-----
FC88X_01023_00602      -----------------TCAACAAA--TCGTTTG--GGGCGAC-TTCTC-----
-----
FC88X_01022_00603      -----------------TCAACAAA--TCGTTTG--GGGCGAC-TTCTC-----
-----
FC88X_01021_00648      -----------------TCAACAAA--TCGTTTG--GGGCGAC-TTCTC-----
-----
FC88X_01020_00611      -----------------TCAACAAA--TCGTTTG--GGGCGAC-TTCTC-----
-----
FC88X_01019_00640      -----------------TCAACAAA--TCGTTTG--GGGCGAC-TTCTC-----
-----
FC88X_01016_00621      -----------------TCAACAAA--TCGTTTG--GGGCGAC-TTCTC-----
-----
FC88X_01016_00616      -----------------TCAACAAA--TCGTTTG--GGGCGAC-TTCTC-----
-----
FC88X_01012_00638      -----------------TCAACAAA--TCGTTTG--GGGCGAC-TTCTC-----
-----
FC88X_01011_00619      -----------------TCAACAAA--TCGTTTG--GGGCGAC-TTCTC-----
-----
FC88X_01010_00639      -----------------TCAACAAA--TCGTTTG--GGGCGAC-TTCTC-----
-----
FC88X_01009_00647      -----------------TCAACAAA--TCGTTTG--GGGCGAC-TTCTC-----
-----
FC88X_01005_00636      -----------------TCAACAAA--TCGTTTG--GGGCGAC-TTCTC-----
-----
FC88X_01028_00542      -----------------TCAACAAA--TCGTTTG--GGGCGAC-TTCTC-----
-----
FC88X_01027_00532      -----------------TCAACAAA--TCGTTTG--GGGCGAC-TTCTC-----
-----
FC88X_01025_00527      -----------------TCAACAAA--TCGTTTG--GGGCGAC-TTCTC-----
-----
FC88X_01023_00534      -----------------TCAACAAA--TCGTTTG--GGGCGAC-TTCTC-----
-----
FC88X_01022_00547      -----------------TCAACAAA--TCGTTTG--GGGCGAC-TTCTC-----
-----
```

FC88X_01021_00513 -----------------TCAACAAA--TCGTTTG--GGGCGAC-TTCTC-----
-----
FC88X_01020_00529 -----------------TCAACAAA--TCGTTTG--GGGCGAC-TTCTC-----
-----
FC88X_01015_00527 -----------------TCAACAAA--TCGTTTG--GGGCGAC-TTCTC-----
-----
FC88X_01014_00520 -----------------TCAACAAA--TCGTTTG--GGGCGAC-TTCTC-----
-----
FC88X_01010_00540 -----------------TCAACAAA--TCGTTTG--GGGCGAC-TTCTC-----
-----
FC88X_01009_00530 -----------------TCAACAAA--TCGTTTG--GGGCGAC-TTCTC-----
-----
FC88X_01009_00523 -----------------TCAACAAA--TCGTTTG--GGGCGAC-TTCTC-----
-----
FC88X_01002_00512 -----------------TCAACAAA--TCGTTTG--GGGCGAC-TTCTC-----
-----
FC88X_01000_00547 -----------------TCAACAAA--TCGTTTG--GGGCGAC-TTCTC-----
-----
FC88X_01049_00455 -----------------TCAACAAA--TCGTTTG--GGGCGAC-TTCTC-----
-----
FC88X_01044_00471 -----------------TCAACAAA--TCGTTTG--GGGCGAC-TTCTC-----
-----
FC88X_01042_00487 -----------------TCAACAAA--TCGTTTG--GGGCGAC-TTCTC-----
-----
FC88X_01041_00458 -----------------TCAACAAA--TCGTTTG--GGGCGAC-TTCTC-----
-----
FC88X_01037_00497 -----------------TCAACAAA--TCGTTTG--GGGCGAC-TTCTC-----
-----
FC88X_01037_00482 -----------------TCAACAAA--TCGTTTG--GGGCGAC-TTCTC-----
-----
FC88X_01037_00481 -----------------TCAACAAA--TCGTTTG--GGGCGAC-TTCTC-----
-----
FC88X_00972_00554 -----------------TCAACAAA--TCGTTTG--GGGCGAC-TTCTC-----
-----
FC88X_00972_00551 -----------------TCAACAAA--TCGTTTG--GGGCGAC-TTCTC-----
-----
FC88X_00971_00550 -----------------TCAACAAA--TCGTTTG--GGGCGAC-TTCTC-----
-----
FC88X_00969_00561 -----------------TCAACAAA--TCGTTTG--GGGCGAC-TTCTC-----
-----
FC88X_01079_00839 -----------------TCAACAAA--TCGTTTG--GGGCGAC-TTCTC-----
-----
FC88X_01078_00827 -----------------TCAACAAA--TCGTTTG--GGGCGAC-TTCTC-----
-----
FC88X_01071_00821 -----------------TCAACAAA--TCGTTTG--GGGCGAC-TTCTC-----
-----
FC88X_01068_00822 -----------------TCAACAAA--TCGTTTG--GGGCGAC-TTCTC-----
-----
FC88X_01063_00822 -----------------TCAACAAA--TCGTTTG--GGGCGAC-TTCTC-----
-----
FC88X_01054_00849 -----------------TCAACAAA--TCGTTTG--GGGCGAC-TTCTC-----
-----
FC88X_01097_00799 -----------------TCAACAAA--TCGTTTG--GGGCGAC-TTCTC-----
-----
FC88X_01042_00568 -----------------TCAACAAA--TCGTTTG--GGGCGAC-TTCTC-----
-----

FC88X_01039_00560 -----------------TCAACAAA--TCGTTTG--GGGCGAC-TTCTC-----
-----
FC88X_01039_00559 -----------------TCAACAAA--TCGTTTG--GGGCGAC-TTCTC-----
-----
FC88X_01038_00570 -----------------TCAACAAA--TCGTTTG--GGGCGAC-TTCTC-----
-----
FC88X_01027_00594 -----------------TCAACAAA--TCGTTTG--GGGCGAC-TTCTC-----
-----
FC88X_01025_00550 -----------------TCAACAAA--TCGTTTG--GGGCGAC-TTCTC-----
-----
FC88X_01024_00565 -----------------TCAACAAA--TCGTTTG--GGGCGAC-TTCTC-----
-----
FC88X_01024_00562 -----------------TCAACAAA--TCGTTTG--GGGCGAC-TTCTC-----
-----
FC88X_01022_00566 -----------------TCAACAAA--TCGTTTG--GGGCGAC-TTCTC-----
-----
FC88X_01018_00590 -----------------TCAACAAA--TCGTTTG--GGGCGAC-TTCTC-----
-----
FC88X_01017_00596 -----------------TCAACAAA--TCGTTTG--GGGCGAC-TTCTC-----
-----
FC88X_01016_00586 -----------------TCAACAAA--TCGTTTG--GGGCGAC-TTCTC-----
-----
FC88X_01013_00567 -----------------TCAACAAA--TCGTTTG--GGGCGAC-TTCTC-----
-----
FC88X_01007_00559 -----------------TCAACAAA--TCGTTTG--GGGCGAC-TTCTC-----
-----
FC88X_01001_00563 -----------------TCAACAAA--TCGTTTG--GGGCGAC-TTCTC-----
-----
FC88X_01049_00501 -----------------TCAACAAA--TCGTTTG--GGGCGAC-TTCTC-----
-----
FC88X_01048_00535 -----------------TCAACAAA--TCGTTTG--GGGCGAC-TTCTC-----
-----
FC88X_01047_00528 -----------------TCAACAAA--TCGTTTG--GGGCGAC-TTCTC-----
-----
FC88X_01041_00518 -----------------TCAACAAA--TCGTTTG--GGGCGAC-TTCTC-----
-----
FC88X_01040_00545 -----------------TCAACAAA--TCGTTTG--GGGCGAC-TTCTC-----
-----
FC88X_01039_00506 -----------------TCAACAAA--TCGTTTG--GGGCGAC-TTCTC-----
-----
FC88X_01110_00508 -----------------TCAACAAA--TCGTTTG--GGGCGAC-TTCTC-----
-----
FC88X_01110_00503 -----------------TCAACAAA--TCGTTTG--GGGCGAC-TTCTC-----
-----
FC88X_01106_00545 -----------------TCAACAAA--TCGTTTG--GGGCGAC-TTCTC-----
-----
FC88X_01103_00504 -----------------TCAACAAA--TCGTTTG--GGGCGAC-TTCTC-----
-----
FC88X_01102_00546 -----------------TCAACAAA--TCGTTTG--GGGCGAC-TTCTC-----
-----
FC88X_01102_00508 -----------------TCAACAAA--TCGTTTG--GGGCGAC-TTCTC-----
-----
FC88X_01101_00528 -----------------TCAACAAA--TCGTTTG--GGGCGAC-TTCTC-----
-----
FC88X_01113_00471 -----------------TCAACAAA--TCGTTTG--GGGCGAC-TTCTC-----
-----

```
FC88X_01111_00456      -----------------TCAACAAA--TCGTTTG--GGGCGAC-TTCTC-----
-----
FC88X_01105_00450      -----------------TCAACAAA--TCGTTTG--GGGCGAC-TTCTC-----
-----
FC88X_01100_00493      -----------------TCAACAAA--TCGTTTG--GGGCGAC-TTCTC-----
-----
FC88X_01066_01265      -----------------TCAACAAA--TCGTTTG--GGGCGAC-TTCTC-----
-----
FC88X_01066_01252      -----------------TCAACAAA--TCGTTTG--GGGCGAC-TTCTC-----
-----
FC88X_01064_01252      -----------------TCAACAAA--TCGTTTG--GGGCGAC-TTCTC-----
-----
FC88X_01096_01216      -----------------TCAACAAA--TCGTTTG--GGGCGAC-TTCTC-----
-----
FC88X_01088_01224      -----------------TCAACAAA--TCGTTTG--GGGCGAC-TTCTC-----
-----
FC88X_01088_01212      -----------------TCAACAAA--TCGTTTG--GGGCGAC-TTCTC-----
-----
FC88X_01083_01224      -----------------TCAACAAA--TCGTTTG--GGGCGAC-TTCTC-----
-----
FC88X_01079_01230      -----------------TCAACAAA--TCGTTTG--GGGCGAC-TTCTC-----
-----
FC88X_01078_01227      -----------------TCAACAAA--TCGTTTG--GGGCGAC-TTCTC-----
-----
FC88X_01078_01204      -----------------TCAACAAA--TCGTTTG--GGGCGAC-TTCTC-----
-----
FC88X_01076_01200      -----------------TCAACAAA--TCGTTTG--GGGCGAC-TTCTC-----
-----
FC88X_01075_01228      -----------------TCAACAAA--TCGTTTG--GGGCGAC-TTCTC-----
-----
FC88X_01071_01202      -----------------TCAACAAA--TCGTTTG--GGGCGAC-TTCTC-----
-----
FC88X_01070_01201      -----------------TCAACAAA--TCGTTTG--GGGCGAC-TTCTC-----
-----
FC88X_01069_01242      -----------------TCAACAAA--TCGTTTG--GGGCGAC-TTCTC-----
-----
FC88X_01066_01201      -----------------TCAACAAA--TCGTTTG--GGGCGAC-TTCTC-----
-----
FC88X_01063_01245      -----------------TCAACAAA--TCGTTTG--GGGCGAC-TTCTC-----
-----
FC88X_01062_01231      -----------------TCAACAAA--TCGTTTG--GGGCGAC-TTCTC-----
-----
FC88X_01059_01222      -----------------TCAACAAA--TCGTTTG--GGGCGAC-TTCTC-----
-----
FC88X_01052_01228      -----------------TCAACAAA--TCGTTTG--GGGCGAC-TTCTC-----
-----
FC88X_01050_01248      -----------------TCAACAAA--TCGTTTG--GGGCGAC-TTCTC-----
-----
FC88X_01050_01218      -----------------TCAACAAA--TCGTTTG--GGGCGAC-TTCTC-----
-----
FC88X_01099_01186      -----------------TCAACAAA--TCGTTTG--GGGCGAC-TTCTC-----
-----
FC88X_01097_01165      -----------------TCAACAAA--TCGTTTG--GGGCGAC-TTCTC-----
-----
FC88X_01096_01176      -----------------TCAACAAA--TCGTTTG--GGGCGAC-TTCTC-----
-----
```

```
FC88X_01096_01170       -----------------TCAACAAA--TCGTTTG--GGGCGAC-TTCTC-----
-----
FC88X_01096_01167       -----------------TCAACAAA--TCGTTTG--GGGCGAC-TTCTC-----
-----
FC88X_01091_01164       -----------------TCAACAAA--TCGTTTG--GGGCGAC-TTCTC-----
-----
FC88X_01084_01186       -----------------TCAACAAA--TCGTTTG--GGGCGAC-TTCTC-----
-----
FC88X_01082_01153       -----------------TCAACAAA--TCGTTTG--GGGCGAC-TTCTC-----
-----
FC88X_01074_01183       -----------------TCAACAAA--TCGTTTG--GGGCGAC-TTCTC-----
-----
FC88X_01067_01158       -----------------TCAACAAA--TCGTTTG--GGGCGAC-TTCTC-----
-----
FC88X_01057_01168       -----------------TCAACAAA--TCGTTTG--GGGCGAC-TTCTC-----
-----
FC88X_01056_01158       -----------------TCAACAAA--TCGTTTG--GGGCGAC-TTCTC-----
-----
FC88X_01055_01178       -----------------TCAACAAA--TCGTTTG--GGGCGAC-TTCTC-----
-----
FC88X_01054_01152       -----------------TCAACAAA--TCGTTTG--GGGCGAC-TTCTC-----
-----
FC88X_01052_01199       -----------------TCAACAAA--TCGTTTG--GGGCGAC-TTCTC-----
-----
FC88X_01052_01170       -----------------TCAACAAA--TCGTTTG--GGGCGAC-TTCTC-----
-----
FC88X_01099_01118       -----------------TCAACAAA--TCGTTTG--GGGCGAC-TTCTC-----
-----
FC88X_01099_01114       -----------------TCAACAAA--TCGTTTG--GGGCGAC-TTCTC-----
-----
FC88X_01093_01133       -----------------TCAACAAA--TCGTTTG--GGGCGAC-TTCTC-----
-----
FC88X_01073_01134       -----------------TCAACAAA--TCGTTTG--GGGCGAC-TTCTC-----
-----
FC88X_01072_01146       -----------------TCAACAAA--TCGTTTG--GGGCGAC-TTCTC-----
-----
FC88X_01072_01144       -----------------TCAACAAA--TCGTTTG--GGGCGAC-TTCTC-----
-----
FC88X_01071_01127       -----------------TCAACAAA--TCGTTTG--GGGCGAC-TTCTC-----
-----
FC88X_01068_01106       -----------------TCAACAAA--TCGTTTG--GGGCGAC-TTCTC-----
-----
FC88X_01098_01090       -----------------TCAACAAA--TCGTTTG--GGGCGAC-TTCTC-----
-----
FC88X_01099_00955       -----------------TCAACAAA--TCGTTTG--GGGCGAC-TTCTC-----
-----
FC88X_01099_00954       -----------------TCAACAAA--TCGTTTG--GGGCGAC-TTCTC-----
-----
FC88X_01095_00990       -----------------TCAACAAA--TCGTTTG--GGGCGAC-TTCTC-----
-----
FC88X_01078_00958       -----------------TCAACAAA--TCGTTTG--GGGCGAC-TTCTC-----
-----
FC88X_01074_00978       -----------------TCAACAAA--TCGTTTG--GGGCGAC-TTCTC-----
-----
FC88X_01058_00987       -----------------TCAACAAA--TCGTTTG--GGGCGAC-TTCTC-----
-----
```

FC88X_01098_00917 -----------------TCAACAAA--TCGTTTG--GGGCGAC-TTCTC-----
-----
FC88X_01097_00900 -----------------TCAACAAA--TCGTTTG--GGGCGAC-TTCTC-----
-----
FC88X_01096_00910 -----------------TCAACAAA--TCGTTTG--GGGCGAC-TTCTC-----
-----
FC88X_01091_00947 -----------------TCAACAAA--TCGTTTG--GGGCGAC-TTCTC-----
-----
FC88X_01087_00949 -----------------TCAACAAA--TCGTTTG--GGGCGAC-TTCTC-----
-----
FC88X_01077_00946 -----------------TCAACAAA--TCGTTTG--GGGCGAC-TTCTC-----
-----
FC88X_01077_00917 -----------------TCAACAAA--TCGTTTG--GGGCGAC-TTCTC-----
-----
FC88X_01075_00907 -----------------TCAACAAA--TCGTTTG--GGGCGAC-TTCTC-----
-----
FC88X_01096_00786 -----------------TCAACAAA--TCGTTTG--GGGCGAC-TTCTC-----
-----
FC88X_01095_00750 -----------------TCAACAAA--TCGTTTG--GGGCGAC-TTCTC-----
-----
FC88X_01091_00781 -----------------TCAACAAA--TCGTTTG--GGGCGAC-TTCTC-----
-----
FC88X_01085_00797 -----------------TCAACAAA--TCGTTTG--GGGCGAC-TTCTC-----
-----
FC88X_01083_00776 -----------------TCAACAAA--TCGTTTG--GGGCGAC-TTCTC-----
-----
FC88X_01082_00784 -----------------TCAACAAA--TCGTTTG--GGGCGAC-TTCTC-----
-----
FC88X_01080_00785 -----------------TCAACAAA--TCGTTTG--GGGCGAC-TTCTC-----
-----
FC88X_01077_00786 -----------------TCAACAAA--TCGTTTG--GGGCGAC-TTCTC-----
-----
FC88X_01077_00758 -----------------TCAACAAA--TCGTTTG--GGGCGAC-TTCTC-----
-----
FC88X_01067_00795 -----------------TCAACAAA--TCGTTTG--GGGCGAC-TTCTC-----
-----
FC88X_01063_00786 -----------------TCAACAAA--TCGTTTG--GGGCGAC-TTCTC-----
-----
FC88X_01055_00769 -----------------TCAACAAA--TCGTTTG--GGGCGAC-TTCTC-----
-----
FC88X_01053_00771 -----------------TCAACAAA--TCGTTTG--GGGCGAC-TTCTC-----
-----
FC88X_01051_00771 -----------------TCAACAAA--TCGTTTG--GGGCGAC-TTCTC-----
-----
FC88X_01095_00748 -----------------TCAACAAA--TCGTTTG--GGGCGAC-TTCTC-----
-----
FC88X_01092_00715 -----------------TCAACAAA--TCGTTTG--GGGCGAC-TTCTC-----
-----
FC88X_01089_00733 -----------------TCAACAAA--TCGTTTG--GGGCGAC-TTCTC-----
-----
FC88X_01088_00730 -----------------TCAACAAA--TCGTTTG--GGGCGAC-TTCTC-----
-----
FC88X_01075_00747 -----------------TCAACAAA--TCGTTTG--GGGCGAC-TTCTC-----
-----
FC88X_01075_00729 -----------------TCAACAAA--TCGTTTG--GGGCGAC-TTCTC-----
-----

FC88X_01073_00701 -----------------TCAACAAA--TCGTTTG--GGGCGAC-TTCTC-----
-----
FC88X_01071_00710 -----------------TCAACAAA--TCGTTTG--GGGCGAC-TTCTC-----
-----
FC88X_01064_00743 -----------------TCAACAAA--TCGTTTG--GGGCGAC-TTCTC-----
-----
FC88X_01059_00746 -----------------TCAACAAA--TCGTTTG--GGGCGAC-TTCTC-----
-----
FC88X_01054_00726 -----------------TCAACAAA--TCGTTTG--GGGCGAC-TTCTC-----
-----
FC88X_01053_00703 -----------------TCAACAAA--TCGTTTG--GGGCGAC-TTCTC-----
-----
FC88X_01073_00914 -----------------TCAACAAA--TCGTTTG--GGGCGAC-TTCTC-----
-----
FC88X_01069_00905 -----------------TCAACAAA--TCGTTTG--GGGCGAC-TTCTC-----
-----
FC88X_01066_00912 -----------------TCAACAAA--TCGTTTG--GGGCGAC-TTCTC-----
-----
FC88X_01063_00936 -----------------TCAACAAA--TCGTTTG--GGGCGAC-TTCTC-----
-----
FC88X_01062_00904 -----------------TCAACAAA--TCGTTTG--GGGCGAC-TTCTC-----
-----
FC88X_01056_00949 -----------------TCAACAAA--TCGTTTG--GGGCGAC-TTCTC-----
-----
FC88X_01045_00754 -----------------TCAACAAA--TCGTTTG--GGGCGAC-TTCTC-----
-----
FC88X_01035_00792 -----------------TCAACAAA--TCGTTTG--GGGCGAC-TTCTC-----
-----
FC88X_01029_00803 -----------------TCAACAAA--TCGTTTG--GGGCGAC-TTCTC-----
-----
FC88X_01020_00831 -----------------TCAACAAA--TCGTTTG--GGGCGAC-TTCTC-----
-----
FC88X_01009_00844 -----------------TCAACAAA--TCGTTTG--GGGCGAC-TTCTC-----
-----
FC88X_01009_00824 -----------------TCAACAAA--TCGTTTG--GGGCGAC-TTCTC-----
-----
FC88X_01008_00841 -----------------TCAACAAA--TCGTTTG--GGGCGAC-TTCTC-----
-----
FC88X_01127_00593 -----------------TCAACAAA--TCGTTTG--GGGCGAC-TTCTC-----
-----
FC88X_01125_00595 -----------------TCAACAAA--TCGTTTG--GGGCGAC-TTCTC-----
-----
FC88X_01118_00587 -----------------TCAACAAA--TCGTTTG--GGGCGAC-TTCTC-----
-----
FC88X_01117_00592 -----------------TCAACAAA--TCGTTTG--GGGCGAC-TTCTC-----
-----
FC88X_01114_00596 -----------------TCAACAAA--TCGTTTG--GGGCGAC-TTCTC-----
-----
FC88X_01108_00571 -----------------TCAACAAA--TCGTTTG--GGGCGAC-TTCTC-----
-----
FC88X_01103_00563 -----------------TCAACAAA--TCGTTTG--GGGCGAC-TTCTC-----
-----
FC88X_01100_00593 -----------------TCAACAAA--TCGTTTG--GGGCGAC-TTCTC-----
-----
FC88X_01132_00537 -----------------TCAACAAA--TCGTTTG--GGGCGAC-TTCTC-----
-----

```
FC88X_01125_00516      -----------------TCAACAAA--TCGTTTG--GGGCGAC-TTCTC-----
-----
FC88X_01123_00501      -----------------TCAACAAA--TCGTTTG--GGGCGAC-TTCTC-----
-----
FC88X_01121_00503      -----------------TCAACAAA--TCGTTTG--GGGCGAC-TTCTC-----
-----
FC88X_01114_00539      -----------------TCAACAAA--TCGTTTG--GGGCGAC-TTCTC-----
-----
FC88X_01114_00533      -----------------TCAACAAA--TCGTTTG--GGGCGAC-TTCTC-----
-----
FC88X_01112_00517      -----------------TCAACAAA--TCGTTTG--GGGCGAC-TTCTC-----
-----
FC88X_01055_00906      -----------------TCAACAAA--TCGTTTG--GGGCGAC-TTCTC-----
-----
FC88X_01052_00937      -----------------TCAACAAA--TCGTTTG--GGGCGAC-TTCTC-----
-----
FC88X_01096_00869      -----------------TCAACAAA--TCGTTTG--GGGCGAC-TTCTC-----
-----
FC88X_01096_00866      -----------------TCAACAAA--TCGTTTG--GGGCGAC-TTCTC-----
-----
FC88X_01093_00895      -----------------TCAACAAA--TCGTTTG--GGGCGAC-TTCTC-----
-----
FC88X_01088_00865      -----------------TCAACAAA--TCGTTTG--GGGCGAC-TTCTC-----
-----
FC88X_01083_00883      -----------------TCAACAAA--TCGTTTG--GGGCGAC-TTCTC-----
-----
FC88X_01080_00866      -----------------TCAACAAA--TCGTTTG--GGGCGAC-TTCTC-----
-----
FC88X_01077_00884      -----------------TCAACAAA--TCGTTTG--GGGCGAC-TTCTC-----
-----
FC88X_01074_00887      -----------------TCAACAAA--TCGTTTG--GGGCGAC-TTCTC-----
-----
FC88X_01073_00858      -----------------TCAACAAA--TCGTTTG--GGGCGAC-TTCTC-----
-----
FC88X_01069_00859      -----------------TCAACAAA--TCGTTTG--GGGCGAC-TTCTC-----
-----
FC88X_01067_00858      -----------------TCAACAAA--TCGTTTG--GGGCGAC-TTCTC-----
-----
FC88X_01066_00863      -----------------TCAACAAA--TCGTTTG--GGGCGAC-TTCTC-----
-----
FC88X_01064_00862      -----------------TCAACAAA--TCGTTTG--GGGCGAC-TTCTC-----
-----
FC88X_01062_00884      -----------------TCAACAAA--TCGTTTG--GGGCGAC-TTCTC-----
-----
FC88X_01058_00893      -----------------TCAACAAA--TCGTTTG--GGGCGAC-TTCTC-----
-----
FC88X_01053_00868      -----------------TCAACAAA--TCGTTTG--GGGCGAC-TTCTC-----
-----
FC88X_01099_00833      -----------------TCAACAAA--TCGTTTG--GGGCGAC-TTCTC-----
-----
FC88X_01092_00801      -----------------TCAACAAA--TCGTTTG--GGGCGAC-TTCTC-----
-----
FC88X_01089_00816      -----------------TCAACAAA--TCGTTTG--GGGCGAC-TTCTC-----
-----
FC88X_01087_00802      -----------------TCAACAAA--TCGTTTG--GGGCGAC-TTCTC-----
-----
```

```
FC88X_01085_00838      -----------------TCAACAAA--TCGTTTG--GGGCGAC-TTCTC-----
-----
FC88X_01085_00813      -----------------TCAACAAA--TCGTTTG--GGGCGAC-TTCTC-----
-----
FC88X_01112_00619      -----------------TCAACAAA--TCGTTTG--GGGCGAC-TTCTC-----
-----
FC88X_01143_00582      -----------------TCAACAAA--TCGTTTG--GGGCGAC-TTCTC-----
-----
FC88X_01131_00580      -----------------TCAACAAA--TCGTTTG--GGGCGAC-TTCTC-----
-----
FC88X_01130_00566      -----------------TCAACAAA--TCGTTTG--GGGCGAC-TTCTC-----
-----
FC88X_00974_00443      -----------------TCAACAAA--TCGTTTG--GGGCGAC-TTCTC-----
-----
FC88X_00972_00416      -----------------TCAACAAA--TCGTTTG--GGGCGAC-TTCTC-----
-----
FC88X_00969_00401      -----------------TCAACAAA--TCGTTTG--GGGCGAC-TTCTC-----
-----
FC88X_00968_00426      -----------------TCAACAAA--TCGTTTG--GGGCGAC-TTCTC-----
-----
FC88X_00968_00416      -----------------TCAACAAA--TCGTTTG--GGGCGAC-TTCTC-----
-----
FC88X_00965_00436      -----------------TCAACAAA--TCGTTTG--GGGCGAC-TTCTC-----
-----
FC88X_00965_00428      -----------------TCAACAAA--TCGTTTG--GGGCGAC-TTCTC-----
-----
FC88X_00964_00422      -----------------TCAACAAA--TCGTTTG--GGGCGAC-TTCTC-----
-----
FC88X_00961_00401      -----------------TCAACAAA--TCGTTTG--GGGCGAC-TTCTC-----
-----
FC88X_00957_00415      -----------------TCAACAAA--TCGTTTG--GGGCGAC-TTCTC-----
-----
FC88X_00991_00481      -----------------TCAACAAA--TCGTTTG--GGGCGAC-TTCTC-----
-----
FC88X_00979_00481      -----------------TCAACAAA--TCGTTTG--GGGCGAC-TTCTC-----
-----
FC88X_00979_00477      -----------------TCAACAAA--TCGTTTG--GGGCGAC-TTCTC-----
-----
FC88X_00977_00477      -----------------TCAACAAA--TCGTTTG--GGGCGAC-TTCTC-----
-----
FC88X_00975_00489      -----------------TCAACAAA--TCGTTTG--GGGCGAC-TTCTC-----
-----
FC88X_00972_00494      -----------------TCAACAAA--TCGTTTG--GGGCGAC-TTCTC-----
-----
FC88X_00971_00495      -----------------TCAACAAA--TCGTTTG--GGGCGAC-TTCTC-----
-----
FC88X_00971_00472      -----------------TCAACAAA--TCGTTTG--GGGCGAC-TTCTC-----
-----
FC88X_00968_00462      -----------------TCAACAAA--TCGTTTG--GGGCGAC-TTCTC-----
-----
FC88X_00967_00470      -----------------TCAACAAA--TCGTTTG--GGGCGAC-TTCTC-----
-----
FC88X_00961_00484      -----------------TCAACAAA--TCGTTTG--GGGCGAC-TTCTC-----
-----
FC88X_00960_00496      -----------------TCAACAAA--TCGTTTG--GGGCGAC-TTCTC-----
-----
```

FC88X_00959_00475 -----------------TCAACAAA--TCGTTTG--GGGCGAC-TTCTC-----
-----
FC88X_00958_00499 -----------------TCAACAAA--TCGTTTG--GGGCGAC-TTCTC-----
-----
FC88X_01004_01269 -----------------TCAACAAA--TCGTTTG--GGGCGAC-TTCTC-----
-----
FC88X_01000_01262 -----------------TCAACAAA--TCGTTTG--GGGCGAC-TTCTC-----
-----
FC88X_01047_01238 -----------------TCAACAAA--TCGTTTG--GGGCGAC-TTCTC-----
-----
FC88X_01047_01228 -----------------TCAACAAA--TCGTTTG--GGGCGAC-TTCTC-----
-----
FC88X_01036_01217 -----------------TCAACAAA--TCGTTTG--GGGCGAC-TTCTC-----
-----
FC88X_01028_01233 -----------------TCAACAAA--TCGTTTG--GGGCGAC-TTCTC-----
-----
FC88X_01046_01142 -----------------TCAACAAA--TCGTTTG--GGGCGAC-TTCTC-----
-----
FC88X_01041_01142 -----------------TCAACAAA--TCGTTTG--GGGCGAC-TTCTC-----
-----
FC88X_01031_01147 -----------------TCAACAAA--TCGTTTG--GGGCGAC-TTCTC-----
-----
FC88X_01020_01123 -----------------TCAACAAA--TCGTTTG--GGGCGAC-TTCTC-----
-----
FC88X_01048_01028 -----------------TCAACAAA--TCGTTTG--GGGCGAC-TTCTC-----
-----
FC88X_01048_01021 -----------------TCAACAAA--TCGTTTG--GGGCGAC-TTCTC-----
-----
FC88X_01048_01006 -----------------TCAACAAA--TCGTTTG--GGGCGAC-TTCTC-----
-----
FC88X_01045_01014 -----------------TCAACAAA--TCGTTTG--GGGCGAC-TTCTC-----
-----
FC88X_01044_01001 -----------------TCAACAAA--TCGTTTG--GGGCGAC-TTCTC-----
-----
FC88X_01042_01005 -----------------TCAACAAA--TCGTTTG--GGGCGAC-TTCTC-----
-----
FC88X_01037_01045 -----------------TCAACAAA--TCGTTTG--GGGCGAC-TTCTC-----
-----
FC88X_01036_01043 -----------------TCAACAAA--TCGTTTG--GGGCGAC-TTCTC-----
-----
FC88X_01033_01032 -----------------TCAACAAA--TCGTTTG--GGGCGAC-TTCTC-----
-----
FC88X_01031_01027 -----------------TCAACAAA--TCGTTTG--GGGCGAC-TTCTC-----
-----
FC88X_01029_01039 -----------------TCAACAAA--TCGTTTG--GGGCGAC-TTCTC-----
-----
FC88X_01028_01015 -----------------TCAACAAA--TCGTTTG--GGGCGAC-TTCTC-----
-----
FC88X_01027_01013 -----------------TCAACAAA--TCGTTTG--GGGCGAC-TTCTC-----
-----
FC88X_01025_01036 -----------------TCAACAAA--TCGTTTG--GGGCGAC-TTCTC-----
-----
FC88X_01025_01026 -----------------TCAACAAA--TCGTTTG--GGGCGAC-TTCTC-----
-----
FC88X_01023_01048 -----------------TCAACAAA--TCGTTTG--GGGCGAC-TTCTC-----
-----

```
FC88X_01023_01009      -----------------TCAACAAA--TCGTTTG--GGGCGAC-TTCTC-----
-----
FC88X_01017_01022      -----------------TCAACAAA--TCGTTTG--GGGCGAC-TTCTC-----
-----
FC88X_01017_01008      -----------------TCAACAAA--TCGTTTG--GGGCGAC-TTCTC-----
-----
FC88X_01015_01002      -----------------TCAACAAA--TCGTTTG--GGGCGAC-TTCTC-----
-----
FC88X_01012_01038      -----------------TCAACAAA--TCGTTTG--GGGCGAC-TTCTC-----
-----
FC88X_01012_01006      -----------------TCAACAAA--TCGTTTG--GGGCGAC-TTCTC-----
-----
FC88X_01011_01026      -----------------TCAACAAA--TCGTTTG--GGGCGAC-TTCTC-----
-----
FC88X_01010_01012      -----------------TCAACAAA--TCGTTTG--GGGCGAC-TTCTC-----
-----
FC88X_01008_01022      -----------------TCAACAAA--TCGTTTG--GGGCGAC-TTCTC-----
-----
FC88X_01008_01001      -----------------TCAACAAA--TCGTTTG--GGGCGAC-TTCTC-----
-----
FC88X_01006_01026      -----------------TCAACAAA--TCGTTTG--GGGCGAC-TTCTC-----
-----
FC88X_01006_01025      -----------------TCAACAAA--TCGTTTG--GGGCGAC-TTCTC-----
-----
FC88X_01003_01009      -----------------TCAACAAA--TCGTTTG--GGGCGAC-TTCTC-----
-----
FC88X_01001_01001      -----------------TCAACAAA--TCGTTTG--GGGCGAC-TTCTC-----
-----
FC88X_01000_01046      -----------------TCAACAAA--TCGTTTG--GGGCGAC-TTCTC-----
-----
FC88X_01000_01024      -----------------TCAACAAA--TCGTTTG--GGGCGAC-TTCTC-----
-----
FC88X_01048_00989      -----------------TCAACAAA--TCGTTTG--GGGCGAC-TTCTC-----
-----
FC88X_01048_00979      -----------------TCAACAAA--TCGTTTG--GGGCGAC-TTCTC-----
-----
FC88X_01048_00970      -----------------TCAACAAA--TCGTTTG--GGGCGAC-TTCTC-----
-----
FC88X_01044_00978      -----------------TCAACAAA--TCGTTTG--GGGCGAC-TTCTC-----
-----
FC88X_01044_00965      -----------------TCAACAAA--TCGTTTG--GGGCGAC-TTCTC-----
-----
FC88X_01044_00955      -----------------TCAACAAA--TCGTTTG--GGGCGAC-TTCTC-----
-----
FC88X_01042_00991      -----------------TCAACAAA--TCGTTTG--GGGCGAC-TTCTC-----
-----
FC88X_01039_00982      -----------------TCAACAAA--TCGTTTG--GGGCGAC-TTCTC-----
-----
FC88X_01035_00959      -----------------TCAACAAA--TCGTTTG--GGGCGAC-TTCTC-----
-----
FC88X_01033_00978      -----------------TCAACAAA--TCGTTTG--GGGCGAC-TTCTC-----
-----
FC88X_01028_00954      -----------------TCAACAAA--TCGTTTG--GGGCGAC-TTCTC-----
-----
FC88X_01026_00981      -----------------TCAACAAA--TCGTTTG--GGGCGAC-TTCTC-----
-----
```

FC88X_01025_00977 -----------------TCAACAAA--TCGTTTG--GGGCGAC-TTCTC-----
-----
FC88X_01025_00974 -----------------TCAACAAA--TCGTTTG--GGGCGAC-TTCTC-----
-----
FC88X_01011_00990 -----------------TCAACAAA--TCGTTTG--GGGCGAC-TTCTC-----
-----
FC88X_01006_00982 -----------------TCAACAAA--TCGTTTG--GGGCGAC-TTCTC-----
-----
FC88X_01046_00918 -----------------TCAACAAA--TCGTTTG--GGGCGAC-TTCTC-----
-----
FC88X_01045_00943 -----------------TCAACAAA--TCGTTTG--GGGCGAC-TTCTC-----
-----
FC88X_01045_00919 -----------------TCAACAAA--TCGTTTG--GGGCGAC-TTCTC-----
-----
FC88X_01045_00909 -----------------TCAACAAA--TCGTTTG--GGGCGAC-TTCTC-----
-----
FC88X_01042_00936 -----------------TCAACAAA--TCGTTTG--GGGCGAC-TTCTC-----
-----
FC88X_01040_00941 -----------------TCAACAAA--TCGTTTG--GGGCGAC-TTCTC-----
-----
FC88X_01040_00939 -----------------TCAACAAA--TCGTTTG--GGGCGAC-TTCTC-----
-----
FC88X_01040_00912 -----------------TCAACAAA--TCGTTTG--GGGCGAC-TTCTC-----
-----
FC88X_01039_00921 -----------------TCAACAAA--TCGTTTG--GGGCGAC-TTCTC-----
-----
FC88X_01036_00903 -----------------TCAACAAA--TCGTTTG--GGGCGAC-TTCTC-----
-----
FC88X_01034_00946 -----------------TCAACAAA--TCGTTTG--GGGCGAC-TTCTC-----
-----
FC88X_01032_00902 -----------------TCAACAAA--TCGTTTG--GGGCGAC-TTCTC-----
-----
FC88X_01031_00927 -----------------TCAACAAA--TCGTTTG--GGGCGAC-TTCTC-----
-----
FC88X_01029_00915 -----------------TCAACAAA--TCGTTTG--GGGCGAC-TTCTC-----
-----
FC88X_01028_00937 -----------------TCAACAAA--TCGTTTG--GGGCGAC-TTCTC-----
-----
FC88X_01028_00922 -----------------TCAACAAA--TCGTTTG--GGGCGAC-TTCTC-----
-----
FC88X_01027_00929 -----------------TCAACAAA--TCGTTTG--GGGCGAC-TTCTC-----
-----
FC88X_01027_00921 -----------------TCAACAAA--TCGTTTG--GGGCGAC-TTCTC-----
-----
FC88X_01023_00936 -----------------TCAACAAA--TCGTTTG--GGGCGAC-TTCTC-----
-----
FC88X_01021_00933 -----------------TCAACAAA--TCGTTTG--GGGCGAC-TTCTC-----
-----
FC88X_01019_00937 -----------------TCAACAAA--TCGTTTG--GGGCGAC-TTCTC-----
-----
FC88X_01019_00916 -----------------TCAACAAA--TCGTTTG--GGGCGAC-TTCTC-----
-----
FC88X_01017_00900 -----------------TCAACAAA--TCGTTTG--GGGCGAC-TTCTC-----
-----
FC88X_01011_00923 -----------------TCAACAAA--TCGTTTG--GGGCGAC-TTCTC-----
-----

```
FC88X_01008_00927      -----------------TCAACAAA--TCGTTTG--GGGCGAC-TTCTC-----
-----
FC88X_01008_00911      -----------------TCAACAAA--TCGTTTG--GGGCGAC-TTCTC-----
-----
FC88X_01005_00927      -----------------TCAACAAA--TCGTTTG--GGGCGAC-TTCTC-----
-----
FC88X_01004_00942      -----------------TCAACAAA--TCGTTTG--GGGCGAC-TTCTC-----
-----
FC88X_01004_00926      -----------------TCAACAAA--TCGTTTG--GGGCGAC-TTCTC-----
-----
FC88X_01004_00912      -----------------TCAACAAA--TCGTTTG--GGGCGAC-TTCTC-----
-----
FC88X_01047_00871      -----------------TCAACAAA--TCGTTTG--GGGCGAC-TTCTC-----
-----
FC88X_01046_00895      -----------------TCAACAAA--TCGTTTG--GGGCGAC-TTCTC-----
-----
FC88X_01046_00871      -----------------TCAACAAA--TCGTTTG--GGGCGAC-TTCTC-----
-----
FC88X_01045_00878      -----------------TCAACAAA--TCGTTTG--GGGCGAC-TTCTC-----
-----
FC88X_01044_00857      -----------------TCAACAAA--TCGTTTG--GGGCGAC-TTCTC-----
-----
FC88X_01041_00896      -----------------TCAACAAA--TCGTTTG--GGGCGAC-TTCTC-----
-----
FC88X_01034_00861      -----------------TCAACAAA--TCGTTTG--GGGCGAC-TTCTC-----
-----
FC88X_01033_00862      -----------------TCAACAAA--TCGTTTG--GGGCGAC-TTCTC-----
-----
FC88X_01032_00884      -----------------TCAACAAA--TCGTTTG--GGGCGAC-TTCTC-----
-----
FC88X_01032_00859      -----------------TCAACAAA--TCGTTTG--GGGCGAC-TTCTC-----
-----
FC88X_01031_00893      -----------------TCAACAAA--TCGTTTG--GGGCGAC-TTCTC-----
-----
FC88X_01025_00884      -----------------TCAACAAA--TCGTTTG--GGGCGAC-TTCTC-----
-----
FC88X_01023_00893      -----------------TCAACAAA--TCGTTTG--GGGCGAC-TTCTC-----
-----
FC88X_01020_00896      -----------------TCAACAAA--TCGTTTG--GGGCGAC-TTCTC-----
-----
FC88X_01020_00893      -----------------TCAACAAA--TCGTTTG--GGGCGAC-TTCTC-----
-----
FC88X_01017_00878      -----------------TCAACAAA--TCGTTTG--GGGCGAC-TTCTC-----
-----
FC88X_01016_00894      -----------------TCAACAAA--TCGTTTG--GGGCGAC-TTCTC-----
-----
FC88X_01012_00861      -----------------TCAACAAA--TCGTTTG--GGGCGAC-TTCTC-----
-----
FC88X_01006_00897      -----------------TCAACAAA--TCGTTTG--GGGCGAC-TTCTC-----
-----
FC88X_01006_00886      -----------------TCAACAAA--TCGTTTG--GGGCGAC-TTCTC-----
-----
FC88X_01006_00871      -----------------TCAACAAA--TCGTTTG--GGGCGAC-TTCTC-----
-----
FC88X_01004_00873      -----------------TCAACAAA--TCGTTTG--GGGCGAC-TTCTC-----
-----
```

```
FC88X_01003_00891      -----------------TCAACAAA--TCGTTTG--GGGCGAC-TTCTC-----
-----
FC88X_01003_00862      -----------------TCAACAAA--TCGTTTG--GGGCGAC-TTCTC-----
-----
FC88X_01001_00858      -----------------TCAACAAA--TCGTTTG--GGGCGAC-TTCTC-----
-----
FC88X_01047_00813      -----------------TCAACAAA--TCGTTTG--GGGCGAC-TTCTC-----
-----
FC88X_01046_00818      -----------------TCAACAAA--TCGTTTG--GGGCGAC-TTCTC-----
-----
FC88X_01043_00841      -----------------TCAACAAA--TCGTTTG--GGGCGAC-TTCTC-----
-----
FC88X_01043_00833      -----------------TCAACAAA--TCGTTTG--GGGCGAC-TTCTC-----
-----
FC88X_01042_00830      -----------------TCAACAAA--TCGTTTG--GGGCGAC-TTCTC-----
-----
FC88X_01041_00823      -----------------TCAACAAA--TCGTTTG--GGGCGAC-TTCTC-----
-----
FC88X_01040_00816      -----------------TCAACAAA--TCGTTTG--GGGCGAC-TTCTC-----
-----
FC88X_01040_00803      -----------------TCAACAAA--TCGTTTG--GGGCGAC-TTCTC-----
-----
FC88X_01035_00841      -----------------TCAACAAA--TCGTTTG--GGGCGAC-TTCTC-----
-----
FC88X_01033_00834      -----------------TCAACAAA--TCGTTTG--GGGCGAC-TTCTC-----
-----
FC88X_01033_00816      -----------------TCAACAAA--TCGTTTG--GGGCGAC-TTCTC-----
-----
FC88X_01033_00807      -----------------TCAACAAA--TCGTTTG--GGGCGAC-TTCTC-----
-----
FC88X_01030_00832      -----------------TCAACAAA--TCGTTTG--GGGCGAC-TTCTC-----
-----
FC88X_00983_00436      -----------------TCAACAAA--TCGTTTG--GGGCGAC-TTCTC-----
-----
FC88X_00975_00439      -----------------TCAACAAA--TCGTTTG--GGGCGAC-TTCTC-----
-----
FC88X_00952_00415      -----------------TCAACAAA--TCGTTTG--GGGCGAC-TTCTC-----
-----
FC88X_00965_00578      -----------------TCAACAAA--TCGTTTG--GGGCGAC-TTCTC-----
-----
FC88X_00988_00928      -----------------TCAACAAA--TCGTTTG--GGGCGAC-TTCTC-----
-----
FC88X_00986_00929      -----------------TCAACAAA--TCGTTTG--GGGCGAC-TTCTC-----
-----
FC88X_00985_00945      -----------------TCAACAAA--TCGTTTG--GGGCGAC-TTCTC-----
-----
FC88X_00984_00938      -----------------TCAACAAA--TCGTTTG--GGGCGAC-TTCTC-----
-----
FC88X_00979_00942      -----------------TCAACAAA--TCGTTTG--GGGCGAC-TTCTC-----
-----
FC88X_00975_00933      -----------------TCAACAAA--TCGTTTG--GGGCGAC-TTCTC-----
-----
FC88X_00972_00906      -----------------TCAACAAA--TCGTTTG--GGGCGAC-TTCTC-----
-----
FC88X_00969_00928      -----------------TCAACAAA--TCGTTTG--GGGCGAC-TTCTC-----
-----
```

FC88X_00967_00944 -----------------TCAACAAA--TCGTTTG--GGGCGAC-TTCTC-----
-----
FC88X_00966_00902 -----------------TCAACAAA--TCGTTTG--GGGCGAC-TTCTC-----
-----
FC88X_00965_00921 -----------------TCAACAAA--TCGTTTG--GGGCGAC-TTCTC-----
-----
FC88X_00964_00936 -----------------TCAACAAA--TCGTTTG--GGGCGAC-TTCTC-----
-----
FC88X_01071_00383 -----------------TCAACAAA--TCGTTTG--GGGCGAC-TTCTC-----
-----
FC88X_01063_00351 -----------------TCAACAAA--TCGTTTG--GGGCGAC-TTCTC-----
-----
FC88X_01060_00359 -----------------TCAACAAA--TCGTTTG--GGGCGAC-TTCTC-----
-----
FC88X_01057_00381 -----------------TCAACAAA--TCGTTTG--GGGCGAC-TTCTC-----
-----
FC88X_01040_01275 -----------------TCAACAAA--TCGTTTG--GGGCGAC-TTCTC-----
-----
FC88X_01037_01255 -----------------TCAACAAA--TCGTTTG--GGGCGAC-TTCTC-----
-----
FC88X_00955_00463 -----------------TCAACAAA--TCGTTTG--GGGCGAC-TTCTC-----
-----
FC88X_00952_00463 -----------------TCAACAAA--TCGTTTG--GGGCGAC-TTCTC-----
-----
FC88X_00999_00405 -----------------TCAACAAA--TCGTTTG--GGGCGAC-TTCTC-----
-----
FC88X_00997_00423 -----------------TCAACAAA--TCGTTTG--GGGCGAC-TTCTC-----
-----
FC88X_00993_00418 -----------------TCAACAAA--TCGTTTG--GGGCGAC-TTCTC-----
-----
FC88X_00993_00403 -----------------TCAACAAA--TCGTTTG--GGGCGAC-TTCTC-----
-----
FC88X_00988_00442 -----------------TCAACAAA--TCGTTTG--GGGCGAC-TTCTC-----
-----
FC88X_00986_00410 -----------------TCAACAAA--TCGTTTG--GGGCGAC-TTCTC-----
-----
FC88X_00980_00855 -----------------TCAACAAA--TCGTTTG--GGGCGAC-TTCTC-----
-----
FC88X_00977_00865 -----------------TCAACAAA--TCGTTTG--GGGCGAC-TTCTC-----
-----
FC88X_00977_00863 -----------------TCAACAAA--TCGTTTG--GGGCGAC-TTCTC-----
-----
FC88X_00973_00886 -----------------TCAACAAA--TCGTTTG--GGGCGAC-TTCTC-----
-----
FC88X_00970_00877 -----------------TCAACAAA--TCGTTTG--GGGCGAC-TTCTC-----
-----
FC88X_00970_00870 -----------------TCAACAAA--TCGTTTG--GGGCGAC-TTCTC-----
-----
FC88X_00966_00868 -----------------TCAACAAA--TCGTTTG--GGGCGAC-TTCTC-----
-----
FC88X_00966_00865 -----------------TCAACAAA--TCGTTTG--GGGCGAC-TTCTC-----
-----
FC88X_00958_00851 -----------------TCAACAAA--TCGTTTG--GGGCGAC-TTCTC-----
-----
FC88X_00955_00861 -----------------TCAACAAA--TCGTTTG--GGGCGAC-TTCTC-----
-----

FC88X_00951_00891 -----------------TCAACAAA--TCGTTTG--GGGCGAC-TTCTC-----
-----
FC88X_00998_00846 -----------------TCAACAAA--TCGTTTG--GGGCGAC-TTCTC-----
-----
FC88X_00998_00827 -----------------TCAACAAA--TCGTTTG--GGGCGAC-TTCTC-----
-----
FC88X_00996_00833 -----------------TCAACAAA--TCGTTTG--GGGCGAC-TTCTC-----
-----
FC88X_00994_00832 -----------------TCAACAAA--TCGTTTG--GGGCGAC-TTCTC-----
-----
FC88X_00993_00833 -----------------TCAACAAA--TCGTTTG--GGGCGAC-TTCTC-----
-----
FC88X_00993_00816 -----------------TCAACAAA--TCGTTTG--GGGCGAC-TTCTC-----
-----
FC88X_00992_00805 -----------------TCAACAAA--TCGTTTG--GGGCGAC-TTCTC-----
-----
FC88X_00992_00802 -----------------TCAACAAA--TCGTTTG--GGGCGAC-TTCTC-----
-----
FC88X_00991_00847 -----------------TCAACAAA--TCGTTTG--GGGCGAC-TTCTC-----
-----
FC88X_00991_00834 -----------------TCAACAAA--TCGTTTG--GGGCGAC-TTCTC-----
-----
FC88X_00990_00828 -----------------TCAACAAA--TCGTTTG--GGGCGAC-TTCTC-----
-----
FC88X_00990_00827 -----------------TCAACAAA--TCGTTTG--GGGCGAC-TTCTC-----
-----
FC88X_00989_00803 -----------------TCAACAAA--TCGTTTG--GGGCGAC-TTCTC-----
-----
FC88X_00987_00841 -----------------TCAACAAA--TCGTTTG--GGGCGAC-TTCTC-----
-----
FC88X_00962_00947 -----------------TCAACAAA--TCGTTTG--GGGCGAC-TTCTC-----
-----
FC88X_00956_00913 -----------------TCAACAAA--TCGTTTG--GGGCGAC-TTCTC-----
-----
FC88X_00955_00938 -----------------TCAACAAA--TCGTTTG--GGGCGAC-TTCTC-----
-----
FC88X_00952_00937 -----------------TCAACAAA--TCGTTTG--GGGCGAC-TTCTC-----
-----
FC88X_00950_00934 -----------------TCAACAAA--TCGTTTG--GGGCGAC-TTCTC-----
-----
FC88X_00998_00856 -----------------TCAACAAA--TCGTTTG--GGGCGAC-TTCTC-----
-----
FC88X_00995_00898 -----------------TCAACAAA--TCGTTTG--GGGCGAC-TTCTC-----
-----
FC88X_00995_00887 -----------------TCAACAAA--TCGTTTG--GGGCGAC-TTCTC-----
-----
FC88X_00995_00863 -----------------TCAACAAA--TCGTTTG--GGGCGAC-TTCTC-----
-----
FC88X_00993_00862 -----------------TCAACAAA--TCGTTTG--GGGCGAC-TTCTC-----
-----
FC88X_00993_00858 -----------------TCAACAAA--TCGTTTG--GGGCGAC-TTCTC-----
-----
FC88X_00989_00893 -----------------TCAACAAA--TCGTTTG--GGGCGAC-TTCTC-----
-----
FC88X_00986_00857 -----------------TCAACAAA--TCGTTTG--GGGCGAC-TTCTC-----
-----

```
FC88X_00985_00881      -----------------TCAACAAA--TCGTTTG--GGGCGAC-TTCTC-----
-----
FC88X_00982_00858      -----------------TCAACAAA--TCGTTTG--GGGCGAC-TTCTC-----
-----
FC88X_00981_00897      -----------------TCAACAAA--TCGTTTG--GGGCGAC-TTCTC-----
-----
FC88X_01088_00492      -----------------TCAACAAA--TCGTTTG--GGGCGAC-TTCTC-----
-----
FC88X_01082_00465      -----------------TCAACAAA--TCGTTTG--GGGCGAC-TTCTC-----
-----
FC88X_01082_00460      -----------------TCAACAAA--TCGTTTG--GGGCGAC-TTCTC-----
-----
FC88X_01082_00456      -----------------TCAACAAA--TCGTTTG--GGGCGAC-TTCTC-----
-----
FC88X_01073_00450      -----------------TCAACAAA--TCGTTTG--GGGCGAC-TTCTC-----
-----
FC88X_01090_00419      -----------------TCAACAAA--TCGTTTG--GGGCGAC-TTCTC-----
-----
FC88X_01089_00444      -----------------TCAACAAA--TCGTTTG--GGGCGAC-TTCTC-----
-----
FC88X_01088_00425      -----------------TCAACAAA--TCGTTTG--GGGCGAC-TTCTC-----
-----
FC88X_01087_00415      -----------------TCAACAAA--TCGTTTG--GGGCGAC-TTCTC-----
-----
FC88X_01085_00444      -----------------TCAACAAA--TCGTTTG--GGGCGAC-TTCTC-----
-----
FC88X_01083_00426      -----------------TCAACAAA--TCGTTTG--GGGCGAC-TTCTC-----
-----
FC88X_01082_00416      -----------------TCAACAAA--TCGTTTG--GGGCGAC-TTCTC-----
-----
FC88X_01080_00432      -----------------TCAACAAA--TCGTTTG--GGGCGAC-TTCTC-----
-----
FC88X_01078_00429      -----------------TCAACAAA--TCGTTTG--GGGCGAC-TTCTC-----
-----
FC88X_01078_00402      -----------------TCAACAAA--TCGTTTG--GGGCGAC-TTCTC-----
-----
FC88X_01077_00431      -----------------TCAACAAA--TCGTTTG--GGGCGAC-TTCTC-----
-----
FC88X_01075_00442      -----------------TCAACAAA--TCGTTTG--GGGCGAC-TTCTC-----
-----
FC88X_01070_00419      -----------------TCAACAAA--TCGTTTG--GGGCGAC-TTCTC-----
-----
FC88X_01070_00418      -----------------TCAACAAA--TCGTTTG--GGGCGAC-TTCTC-----
-----
FC88X_01070_00409      -----------------TCAACAAA--TCGTTTG--GGGCGAC-TTCTC-----
-----
FC88X_01064_00448      -----------------TCAACAAA--TCGTTTG--GGGCGAC-TTCTC-----
-----
FC88X_01064_00447      -----------------TCAACAAA--TCGTTTG--GGGCGAC-TTCTC-----
-----
FC88X_01060_00432      -----------------TCAACAAA--TCGTTTG--GGGCGAC-TTCTC-----
-----
FC88X_01060_00401      -----------------TCAACAAA--TCGTTTG--GGGCGAC-TTCTC-----
-----
FC88X_01059_00447      -----------------TCAACAAA--TCGTTTG--GGGCGAC-TTCTC-----
-----
```

```
FC88X_01058_00437       -----------------TCAACAAA--TCGTTTG--GGGCGAC-TTCTC-----
-----
FC88X_01056_00404       -----------------TCAACAAA--TCGTTTG--GGGCGAC-TTCTC-----
-----
FC88X_01056_00403       -----------------TCAACAAA--TCGTTTG--GGGCGAC-TTCTC-----
-----
FC88X_01053_00420       -----------------TCAACAAA--TCGTTTG--GGGCGAC-TTCTC-----
-----
FC88X_01050_00434       -----------------TCAACAAA--TCGTTTG--GGGCGAC-TTCTC-----
-----
FC88X_01087_00397       -----------------TCAACAAA--TCGTTTG--GGGCGAC-TTCTC-----
-----
FC88X_01076_00390       -----------------TCAACAAA--TCGTTTG--GGGCGAC-TTCTC-----
-----
FC88X_01087_00670       -----------------TCAACAAA--TCGTTTG--GGGCGAC-TTCTC-----
-----
FC88X_01084_00673       -----------------TCAACAAA--TCGTTTG--GGGCGAC-TTCTC-----
-----
FC88X_01082_00667       -----------------TCAACAAA--TCGTTTG--GGGCGAC-TTCTC-----
-----
FC88X_01070_00678       -----------------TCAACAAA--TCGTTTG--GGGCGAC-TTCTC-----
-----
FC88X_01068_00654       -----------------TCAACAAA--TCGTTTG--GGGCGAC-TTCTC-----
-----
FC88X_01067_00683       -----------------TCAACAAA--TCGTTTG--GGGCGAC-TTCTC-----
-----
FC88X_01065_00655       -----------------TCAACAAA--TCGTTTG--GGGCGAC-TTCTC-----
-----
FC88X_01056_00682       -----------------TCAACAAA--TCGTTTG--GGGCGAC-TTCTC-----
-----
FC88X_01051_00668       -----------------TCAACAAA--TCGTTTG--GGGCGAC-TTCTC-----
-----
FC88X_01093_00615       -----------------TCAACAAA--TCGTTTG--GGGCGAC-TTCTC-----
-----
FC88X_01091_00618       -----------------TCAACAAA--TCGTTTG--GGGCGAC-TTCTC-----
-----
FC88X_01090_00634       -----------------TCAACAAA--TCGTTTG--GGGCGAC-TTCTC-----
-----
FC88X_01084_00628       -----------------TCAACAAA--TCGTTTG--GGGCGAC-TTCTC-----
-----
FC88X_01082_00636       -----------------TCAACAAA--TCGTTTG--GGGCGAC-TTCTC-----
-----
FC88X_01081_00628       -----------------TCAACAAA--TCGTTTG--GGGCGAC-TTCTC-----
-----
FC88X_01080_00625       -----------------TCAACAAA--TCGTTTG--GGGCGAC-TTCTC-----
-----
FC88X_01079_00611       -----------------TCAACAAA--TCGTTTG--GGGCGAC-TTCTC-----
-----
FC88X_01078_00625       -----------------TCAACAAA--TCGTTTG--GGGCGAC-TTCTC-----
-----
FC88X_01076_00621       -----------------TCAACAAA--TCGTTTG--GGGCGAC-TTCTC-----
-----
FC88X_01075_00623       -----------------TCAACAAA--TCGTTTG--GGGCGAC-TTCTC-----
-----
FC88X_01073_00614       -----------------TCAACAAA--TCGTTTG--GGGCGAC-TTCTC-----
-----
```

```
FC88X_01071_00649      -----------------TCAACAAA--TCGTTTG--GGGCGAC-TTCTC-----
-----
FC88X_00952_00499      -----------------TCGACAAA--TCGTTTG--GGGCGAC-TTCTC-----
-----
FC88X_00959_00615      -----------------TCGACAAA--TCGTTTG--GGGCGAC-TTCTC-----
-----
FC88X_00969_00739      -----------------TCGACAAA--TCGTTTG--GGGCGAC-TTCTC-----
-----
FC88X_01117_00797      -----------------TCGACAAA--TCGTTTG--GGGCGAC-TTCTC-----
-----
FC88X_01018_00549      -----------------TCAACAAA--CCGTTTG--GGGCGAC-TTCTC-----
-----
FC88X_01138_00724      ------------------CTACTAT--CCGTTAGATGGGCATT-TTCTC-----
-----
FC88X_01113_00731      -----------------TCAACAAA--CCGTTTG--GGGCGAC-TTCTC-----
-----
FC88X_00971_00253      -----------------TCAACAAA--TCGTTTG--GGGCGAC-TTCTC-----
-----
FC88X_01127_00601      -----------------TCAACAAA--TCGTTTG--GGGCGAC-TTCTC-----
-----
FC88X_01107_00672      -----------------TCAACAAA--TCGTTTG--GGGCGAC-TTCTC-----
-----
FC88X_01119_00742      -----------------TCAACAAA--TCGTTTG--GGGCGAC-TTCTC-----
-----
FC88X_01116_00798      -----------------TCAACAAA--TCGTTTG--GGGCGAC-TTCTC-----
-----
FC88X_01141_00778      -----------------TCAACAAA--TCGTTTG--GGGCGAC-TTCTC-----
-----
FC88X_01126_00814      -----------------TCAACAAA--TCGTTTG--GGGCGAC-TTCTC-----
-----
FC88X_01104_00855      -----------------TCAACAAA--TCGTTTG--GGGCGAC-TTCTC-----
-----
FC88X_01127_00881      -----------------TCAACAAA--TCGTTTG--GGGCGAC-TTCTC-----
-----
FC88X_01124_00940      -----------------TCAACAAA--TCGTTTG--GGGCGAC-TTCTC-----
-----
FC88X_01143_00963      -----------------TCAACAAA--TCGTTTG--GGGCGAC-TTCTC-----
-----
FC88X_01135_01140      -----------------TCAACAAA--TCGTTTG--GGGCGAC-TTCTC-----
-----
FC88X_01133_01177      -----------------TCAACAAA--TCGTTTG--GGGCGAC-TTCTC-----
-----
FC88X_01133_01212      -----------------TCAACAAA--TCGTTTG--GGGCGAC-TTCTC-----
-----
FC88X_00997_00283      -----------------TCAACAAA--TCGTTTG--GGGCGAC-TTCTC-----
-----
FC88X_00983_00564      -----------------TCAACAAA--TCGTTTG--GGGCGAC-TTCTC-----
-----
FC88X_00969_00545      -----------------TCAACAAA--TCGTTTG--GGGCGAC-TTCTC-----
-----
FC88X_00951_00514      -----------------TCAACAAA--TCGTTTG--GGGCGAC-TTCTC-----
-----
FC88X_00988_00634      -----------------TCAACAAA--TCGTTTG--GGGCGAC-TTCTC-----
-----
FC88X_00985_00502      -----------------TCAACAAA--TCGTTTG--GGGCGAC-TTCTC-----
-----
```

```
FC88X_00955_00729      -----------------TCAACAAA--TCGTTTG--GGGCGAC-TTCTC-----
-----
FC88X_00999_00744      -----------------TCAACAAA--TCGTTTG--GGGCGAC-TTCTC-----
-----
FC88X_01096_00488      -----------------TCAACAAA--TCGTTTG--GGGCGAC-TTCTC-----
-----
FC88X_01093_00546      -----------------TCAACAAA--TCGTTTG--GGGCGAC-TTCTC-----
-----
FC88X_00958_00381      ----------------GTCAACAAA--TCGTTTG--GGGCGAC-TTCTC-----
-----
FC88X_01115_01236      ----------------GTCAACAAA--TCGTTTG--GG-CGAC-TTCTC-----
-----
FC88X_00972_00375      ----------------GTCAACAAA--TCGTTTG--GGGCGAC-TTCTC-----
-----
FC88X_00972_00882      ----------------GTCAACAAA--TCGTTTG--GGGCGAC-TTCTC-----
-----
FC88X_00969_00640      ----------------GTCAACAAA--TCGTTTG--GGGCGAC-TTCTC-----
-----
FC88X_00965_00713      ----------------GTCAACAAA--TCGTTTG--GGGCGAC-TTCTC-----
-----
FC88X_00969_00713      ----------------GTCAACAAA--TCGTTTG--GGGCGAC-TTCTC-----
-----
FC88X_01026_00610      ----------------GTCAACAAA--TCGTTTG--GGGCGAC-TTCTC-----
-----
FC88X_01009_00820      ----------------GTCAACAAA--TCGTTTG--GGGCGAC-TTCTC-----
-----
FC88X_01072_00404      ----------------GTCAACAAA--TCGTTTG--GGGCGAC-TTCTC-----
-----
FC88X_01124_00642      ----------------GTCAACAAA--TCGTTTG--GGGCGAC-TTCTC-----
-----
FC88X_01117_00705      ----------------GTCAACAAA--TCGTTTG--GGGCGAC-TTCTC-----
-----
FC88X_01062_00565      ----------------GTCAACAAA--TCGTTTG--GGGCGAC-TTCTC-----
-----
FC88X_01065_00567      ----------------GTCAACAAA--TCGTTTG--GGGCGAC-TTCTC-----
-----
FC88X_01107_01177      ----------------GTCAACAAA--TCGTTTG--GGGCGAC-TTCTC-----
-----
FC88X_01111_01213      ----------------GTCAACAAA--TCGTTTG--GGGCGAC-TTCTC-----
-----
FC88X_00972_00283      -----------------TCAACAAA--TCGTTTG--GGGCGAC-TTCTC-----
-----
FC88X_01131_00611      -----------------TCAACAAA--TCGTTTG--GGGCGAC-TTCTC-----
-----
FC88X_01118_00654      -----------------TCAACAAA--TCGTTTG--GGGCGAC-TTCTC-----
-----
FC88X_01130_00726      -----------------TCAACAAA--TCGTTTG--GGGCGAC-TTCTC-----
-----
FC88X_01125_00774      -----------------TCAACAAA--TCGTTTG--GGGCGAC-TTCTC-----
-----
FC88X_01146_00759      -----------------TCAACAAA--TCGTTTG--GGGCGAC-TTCTC-----
-----
FC88X_01131_00826      -----------------TCAACAAA--TCGTTTG--GGGCGAC-TTCTC-----
-----
FC88X_01114_00879      -----------------TCAACAAA--TCGTTTG--GGGCGAC-TTCTC-----
-----
```

FC88X_01130_00881 -----------------TCAACAAA--TCGTTTG--GGGCGAC-TTCTC-----
-----
FC88X_01137_00921 -----------------TCAACAAA--TCGTTTG--GGGCGAC-TTCTC-----
-----
FC88X_01108_01131 -----------------TCAACAAA--TCGTTTG--GGGCGAC-TTCTC-----
-----
FC88X_01104_01160 -----------------TCAACAAA--TCGTTTG--GGGCGAC-TTCTC-----
-----
FC88X_01142_01173 -----------------TCAACAAA--TCGTTTG--GGGCGAC-TTCTC-----
-----
FC88X_00989_00375 -----------------TCAACAAA--TCGTTTG--GGGCGAC-TTCTC-----
-----
FC88X_00960_00340 -----------------TCAACAAA--TCGTTTG--GGGCGAC-TTCTC-----
-----
FC88X_00951_00636 -----------------TCAACAAA--TCGTTTG--GGGCGAC-TTCTC-----
-----
FC88X_00979_00529 -----------------TCAACAAA--TCGTTTG--GGGCGAC-TTCTC-----
-----
FC88X_00977_00671 -----------------TCAACAAA--TCGTTTG--GGGCGAC-TTCTC-----
-----
FC88X_00999_00630 -----------------TCAACAAA--TCGTTTG--GGGCGAC-TTCTC-----
-----
FC88X_00955_00560 -----------------TCAACAAA--TCGTTTG--GGGCGAC-TTCTC-----
-----
FC88X_00973_00718 -----------------TCAACAAA--TCGTTTG--GGGCGAC-TTCTC-----
-----
FC88X_00984_00755 -----------------TCAACAAA--TCGTTTG--GGGCGAC-TTCTC-----
-----
FC88X_01057_00504 -----------------TCAACAAA--TCGTTTG--GGGCGAC-TTCTC-----
-----
FC88X_01058_00552 -----------------TCAACAAA--TCGTTTG--GGGCGAC-TTCTC-----
-----
FC88X_01086_00584 -----------------TCAACAAA--TCGTTTG--GGGCGAC-TTCTC-----
-----
FC88X_01059_00620 -----------------TCAACAAA--TCGTTTG--GGGCGAC-TTCTC-----
-----
FC88X_00963_00332 -----------------TCAACAAA--TCGTTTG--GGGCGAC-TCCTC-----
-----
FC88X_01075_00838 -----------------TCAACAAA--TCGTTTG--GGGCGAC-TCCTC-----
-----
FC88X_01019_01184 -----------------TCAACAAA--TCGTTTG--GGGCGAC-----------
-----
FC88X_01046_00653 -----------------TCAACAAA--TCGTTTG--GG-CGAC-TCTCC-----
-----
FC88X_01004_00940 -----------------TCAACAAA--TCGTTTG--GG-CGAC-TCTCCTTCC-
-----
FC88X_00990_00782 -----------------TCAACAAA--TCGTTTG--GGGCGAC-TCTCCC----
-----
FC88X_00961_00926 -----------------TCAACAAA--TCGTTTG--GGGCGAC-TCTC------
-----
FC88X_01072_00366 -----------------TCAACAAA--TCGTTTG--GGGCGGC-TCTC------
-----
FC88X_01129_00589 -----------------TCAACAAAAATCGTTTG--GGGCGAC-TCTC------
-----
FC88X_01073_00381 -----------------TCAACAAAATCGTTTGG--GGGCGAC-TCTC------
-----

```
FC88X_00981_00878       -----------------TCAACAAA--TCGTTTG--GGGCGAC-TCTC------
-----
FC88X_01023_00453       -----------------TCAACAAA--TCGTTTG--GGGCGAC-TCTC------
-----
FC88X_01056_01252       -----------------TCAACAAA--TCGTTTG--GGGCGAC-TCTC------
-----
FC88X_00959_00360       -----------------TCAACAAA--TCGTTTG--GGGCGAC-TCT-------
-----
FC88X_01082_01256       -----------------TCAACAAA--TCGTTTG--GGGCGAC-TCT-------
-----
FC88X_01129_00670       -----------------TCAACAAA--TCGTTTG--GGGCGAC-TCT-------
-----
FC88X_00964_00773       --------------CCGTCAACAAA----TCTGG--GGTCTAT-TCTG------
-----
FC88X_01021_00465       --------------CCGTCAACAAA----TCTGG--GGTCTAT-TCTG------
-----
FC88X_01018_00754       --------------CCGTCAACAAA----TCTGG--GGTCTAT-TCTG------
-----
FC88X_01067_00918       --------------CCGTCAACAAA----TCTGG--GGTCTAT-TCTG------
-----
FC88X_01138_00985       --------------CCGTCAACAAA----TCTGG--GGTCTAT-TCTG------
-----
FC88X_00962_00439       --------------CCGTCAACGAA----TCTGG--GGTCTAT-TCTG------
-----
FC88X_01049_01034       --------------TCGTCAACAAA----TCTGG--GGTCTAT-TCTG------
-----
FC88X_01137_00919       ----------------TTCAACTAA----T------------------------
-----
FC88X_01034_00403       -----------------TCAACAAA----T------------------------
-----
FC88X_01036_00533       ----------------TTCAACAAA----TCGT---------------------
-----
FC88X_01004_00687       -----------------TCAACAAA----TC-----------------------
-----
FC88X_01125_01229       -----------------TCAACAAA----TCGTT--TGGGGTC-GACGTTC---
-----
FC88X_01107_01133       ----------------ATCAACAAA--TCGTTTG--G-----------------
-----
FC88X_01071_00570       -----------------TCAACAAA--TCGTT----------------------
-----
FC88X_01019_01149       -----------------TCAACAAA--TCGTTTG--GGGCGAC-TTTTC-----
-----
FC88X_01009_00753       -----------------TCAACAAA--TCGTTTG--------------------
-----
FC88X_01007_00506       -----------------TCAACAAA--TCGTTTG--G-----------------
-----
FC88X_00987_00376       -----------------TCAACAAA--TCGTTTG--GGGCGAC-TTCTT-----
-----
FC88X_00961_00758       -----------------TCAACAAA--TCGTTTG--GGGCGAC-TTCTT-----
-----
FC88X_01133_00947       -----------------TCAACAAA--TCGTTTG--GGGCGAC-TTCTT-----
-----
FC88X_01033_00401       -----------------TCAACAAA--TCGTTTG--GGGCGAC-TTC-------
-----
FC88X_01025_01112       -----------------TCAACAAA--TCGTTTG--GGGCGTA-CTTCCTC---
-----
```

FC88X_01090_00741 -----------------TCAACAAAA-TCGTTTG--GGGCG-A-CTTCCTC---
-----
FC88X_01010_00760 -----------------TCAACAAA--TCGTT-G--GGGCG-------------
-----
FC88X_01014_00668 -----------------TCAACAAA--TCGTTTG--GGGCGAC-TTCCC-----
-----
FC88X_00972_00721 -----------------TCAACAAA--TCGTTTG--GGGCGAC-TT--------
-----
FC88X_00957_00649 -----------------TCAACAAA--TCGTTTG--GG----------------
-----
FC88X_01146_00772 -----------------TCAACAAA--TCGTTTG--GGCCGAC-TTCTC-----
-----
FC88X_00974_00677 --------------TACAACTGAAA--TCGTTTG--GGGCG----ACTTCC---
-----
FC88X_01098_00799 --------------TACAAC-AAAT--CCGTTTG--GGGTCCG-TACTTCC---
-----
FC88X_01125_01241 --------------TACGAC--AAA--TCGTTT---------------------
-----
FC88X_00999_00686 -------------CTACTAC--CAA--TCGTTGG--GTCGCTA-CTTCTC----
-----
FC88X_01025_00426 -----CCACGTAATCCTTTACGCCT--ACGTTCT--C-----------------
-----
FC88X_01003_00643 ----------------TTCACGCAT--TCGAT----------------------
-----
FC88X_01098_00951 ---------------GATCACGCAA--TCGTTT---------------------
-----
FC88X_01091_00805 ----TTCACCAATCGATTGGGCCGT--ACCTTCT--C-----------------
-----
FC88X_00974_00999 ----------------------CAA--TCGTTT---------------------
-----
FC88X_01139_00947 -----------------TCAACAAA--TCGTTTG--GGGCTAC-TTCTC-----
-----
FC88X_01044_00407 -----------------TCAACAAA--TCGTTTG--G-----------------
-----
FC88X_00974_00643 -----------------TCAACAAA--TCGTTTG--GGGTGCT-TCTCCT----
-----
FC88X_01048_00587 -----------------TCAACAA---TCGTTTG--G-----------------
-----
FC88X_00955_00838 -----------------TCAACAAA--TCGTTTG--GGGTGAC-TTCTC-----
-----
FC88X_00952_00842 -----------------TCAACAAA--TCGTTTG--GGG---------------
-----
FC88X_00992_00801 -----------------TCAACAAA--TCGTTTG--G-----------------
-----
FC88X_00988_00900 -----------------TCAACAAA--TCGTTTG--GGGCGGC-TTCTC-----
-----
FC88X_01030_00795 -----------------TCAACAAA--TCGTTTG--GGGCGGC-TTCTC-----
-----
FC88X_01110_00532 -----------------TCAACAAA--TCGTTTG--GGGC--------------
-----
FC88X_01104_00789 -----------------TCAACAAA--TCGTTTG--GGGCAAC-TTCTC-----
-----
FC88X_01115_01005 -----------------TCAACAAA--TCGTTTG--GGGC--------------
-----
FC88X_01048_00387 -----------------TCAACAAA--TCGTTTG--G-----------------
-----

```
FC88X_01097_00691      -----------------TCAACAAA--TCGTTTG--GGGCGAC-TTCT------
-----
FC88X_01114_00811      -----------------TCAACAAA--TCGTTTG--GGGCGAC-TTCT------
-----
FC88X_01012_01175      -----------------TCAACAAA--TCGTTTG--GGGCGAC-TTCT------
-----
FC88X_00978_00637      -----------------TCAACAAA--TCGTTTG--GGGCGAC-TTCTCT----
-----
FC88X_01000_01174      -----------------TCAACAAA--TCGTTTG--GGGCGAC-TTCTCTACC-
-----
FC88X_01020_00353      -----------------TCAACAAA--TCGTTTG--GGGCGAC-TTCT------
-----
FC88X_01141_00619      -----------------TCAACAAA--TCGTTTG--GGGCGAC-TTCTC-----
-----
FC88X_01108_00732      -----------------TCAACAAA--TCGTTTG--GGGCGAC-TTCTC-----
-----
FC88X_01146_00713      -----------------TCAACAAA--TCGTTTG--GGGCGAC-TTCTC-----
-----
FC88X_01135_00767      -----------------TCAACAAA--TCGTTTG--GGGCGAC-TTCTC-----
-----
FC88X_01121_00807      -----------------TCAACAAA--TCGTTTG--GGGCGAC-TTCTC-----
-----
FC88X_01144_00811      -----------------TCAACAAA--TCGTTTG--GGGCGAC-TTCTC-----
-----
FC88X_01122_00851      -----------------TCAACAAA--TCGTTTG--GGGCGAC-TTCTC-----
-----
FC88X_01115_00936      -----------------TCAACAAA--TCGTTTG--GGGCGAC-TTCTC-----
-----
FC88X_01135_00989      -----------------TCAACAAA--TCGTTTG--GGGCGAC-TTCTC-----
-----
FC88X_01121_01141      -----------------TCAACAAA--TCGTTTG--GGGCGAC-TTCTC-----
-----
FC88X_01126_01173      -----------------TCAACAAA--TCGTTTG--GGGCGAC-TTCTC-----
-----
FC88X_01125_01217      -----------------TCAACAAA--TCGTTTG--GGGCGAC-TTCTC-----
-----
FC88X_00953_00323      -----------------TCAACAAA--TCGTTTG--GGGCGAC-TTCTC-----
-----
FC88X_00972_00306      -----------------TCAACAAA--TCGTTTG--GGGCGAC-TTCTC-----
-----
FC88X_00970_00375      -----------------TCAACAAA--TCGTTTG--GGGCGAC-TTCTC-----
-----
FC88X_00964_00614      -----------------TCAACAAA--TCGTTTG--GGGCGAC-TTCTC-----
-----
FC88X_00974_00621      -----------------TCAACAAA--TCGTTTG--GGGCGAC-TTCTC-----
-----
FC88X_00965_00655      -----------------TCAACAAA--TCGTTTG--GGGCGAC-TTCTC-----
-----
FC88X_00963_00808      -----------------TCAACAAA--TCGTTTG--GGGCGAC-TTCTC-----
-----
FC88X_00992_00743      -----------------TCAACAAA--TCGTTTG--GGGCGAC-TTCTC-----
-----
FC88X_00958_00809      -----------------TCAACAAA--TCGTTTG--GGGCGAC-TTCTC-----
-----
FC88X_01084_00524      -----------------TCAACAAA--TCGTTTG--GGGCGAC-TTCTC-----
-----
```

```
FC88X_01075_00598      -----------------TCAACAAA--TCGTTTG--GGGCGAC-TTCTC-----
-----
FC88X_01056_00621      -----------------TCAACAAA--TCGTTTG--GGGCGAC-TTCTC-----
-----
FC88X_00955_00841      -----------------TCAACAAA--TCGTTTG--GGGCGAC-TTCTCTCC--
-----
FC88X_01052_01159      -----------------TCAACAAA--TCGTTTG--GGGCGAC-TTCT------
-----
FC88X_01127_00610      -----------------TCAACAAA--TCGTTTG--GGGCGAC-TTCTC-----
-----
FC88X_01109_00664      -----------------TCAACAAA--TCGTTTG--GGGCGAC-TTCTC-----
-----
FC88X_01122_00718      -----------------TCAACAAA--TCGTTTG--GGGCGAC-TTCTC-----
-----
FC88X_01117_00790      -----------------TCAACAAA--TCGTTTG--GGGCGAC-TTCTC-----
-----
FC88X_01142_00755      -----------------TCAACAAA--TCGTTTG--GGGCGAC-TTCTC-----
-----
FC88X_01130_00802      -----------------TCAACAAA--TCGTTTG--GGGCGAC-TTCTC-----
-----
FC88X_01104_00880      -----------------TCAACAAA--TCGTTTG--GGGCGAC-TTCTC-----
-----
FC88X_01128_00898      -----------------TCAACAAA--TCGTTTG--GGGCGAC-TTCTC-----
-----
FC88X_01125_00922      -----------------TCAACAAA--TCGTTTG--GGGCGAC-TTCTC-----
-----
FC88X_01144_00950      -----------------TCAACAAA--TCGTTTG--GGGCGAC-TTCTC-----
-----
FC88X_01142_01122      -----------------TCAACAAA--TCGTTTG--GGGCGAC-TTCTC-----
-----
FC88X_01134_01178      -----------------TCAACAAA--TCGTTTG--GGGCGAC-TTCTC-----
-----
FC88X_01136_01226      -----------------TCAACAAA--TCGTTTG--GGGCGAC-TTCTC-----
-----
FC88X_00950_00375      -----------------TCAACAAA--TCGTTTG--GGGCGAC-TTCTC-----
-----
FC88X_00986_00585      -----------------TCAACAAA--TCGTTTG--GGGCGAC-TTCTC-----
-----
FC88X_00973_00520      -----------------TCAACAAA--TCGTTTG--GGGCGAC-TTCTC-----
-----
FC88X_00951_00516      -----------------TCAACAAA--TCGTTTG--GGGCGAC-TTCTC-----
-----
FC88X_00988_00645      -----------------TCAACAAA--TCGTTTG--GGGCGAC-TTCTC-----
-----
FC88X_00992_00526      -----------------TCAACAAA--TCGTTTG--GGGCGAC-TTCTC-----
-----
FC88X_00963_00729      -----------------TCAACAAA--TCGTTTG--GGGCGAC-TTCTC-----
-----
FC88X_00960_00774      -----------------TCAACAAA--TCGTTTG--GGGCGAC-TTCTC-----
-----
FC88X_01099_00478      -----------------TCAACAAA--TCGTTTG--GGGCGAC-TTCTC-----
-----
FC88X_01098_00545      -----------------TCAACAAA--TCGTTTG--GGGCGAC-TTCTC-----
-----
FC88X_01080_00579      -----------------TCAACAAA--TCGTTTG--GGGCGAC-TTCTC-----
-----
```

```
FC88X_00962_00354       ------------------TCAGCAAA--TCGTTT--GGGGCGAC-TTCTC-----
-----
FC88X_01130_00868       ------------------TCAGCAAA--TCGTTT--GGGGCGAC-TTCTC-----
-----
FC88X_00972_00842       ------------------TCAACAAA--TCGTTTT-GGGGCGAC-TTCTC-----
-----
FC88X_00972_00277       ------------------TCAACAAA--TCGTTT--GGGGCGAC-TTCTC-----
-----
FC88X_01129_00603       ------------------TCAACAAA--TCGTTT--GGGGCGAC-TTCTC-----
-----
FC88X_01111_00697       ------------------TCAACAAA--TCGTTT--GGGGCGAC-TTCTC-----
-----
FC88X_01126_00730       ------------------TCAACAAA--TCGTTT--GGGGCGAC-TTCTC-----
-----
FC88X_01119_00763       ------------------TCAACAAA--TCGTTT--GGGGCGAC-TTCTC-----
-----
FC88X_01142_00797       ------------------TCAACAAA--TCGTTT--GGGGCGAC-TTCTC-----
-----
FC88X_01130_00833       ------------------TCAACAAA--TCGTTT--GGGGCGAC-TTCTC-----
-----
FC88X_01107_00886       ------------------TCAACAAA--TCGTTT--GGGGCGAC-TTCTC-----
-----
FC88X_01129_00898       ------------------TCAACAAA--TCGTTT--GGGGCGAC-TTCTC-----
-----
FC88X_01132_00907       ------------------TCAACAAA--TCGTTT--GGGGCGAC-TTCTC-----
-----
FC88X_01144_01047       ------------------TCAACAAA--TCGTTT--GGGGCGAC-TTCTC-----
-----
FC88X_01100_01162       ------------------TCAACAAA--TCGTTT--GGGGCGAC-TTCTC-----
-----
FC88X_01136_01191       ------------------TCAACAAA--TCGTTT--GGGGCGAC-TTCTC-----
-----
FC88X_00988_00364       ------------------TCAACAAA--TCGTTT--GGGGCGAC-TTCTC-----
-----
FC88X_00960_00359       ------------------TCAACAAA--TCGTTT--GGGGCGAC-TTCTC-----
-----
FC88X_00990_00587       ------------------TCAACAAA--TCGTTT--GGGGCGAC-TTCTC-----
-----
FC88X_00977_00524       ------------------TCAACAAA--TCGTTT--GGGGCGAC-TTCTC-----
-----
FC88X_00958_00521       ------------------TCAACAAA--TCGTTT--GGGGCGAC-TTCTC-----
-----
FC88X_00993_00648       ------------------TCAACAAA--TCGTTT--GGGGCGAC-TTCTC-----
-----
FC88X_00998_00521       ------------------TCAACAAA--TCGTTT--GGGGCGAC-TTCTC-----
-----
FC88X_00964_00736       ------------------TCAACAAA--TCGTTT--GGGGCGAC-TTCTC-----
-----
FC88X_00968_00775       ------------------TCAACAAA--TCGTTT--GGGGCGAC-TTCTC-----
-----
FC88X_01054_00541       ------------------TCAACAAA--TCGTTT--GGGGCGAC-TTCTC-----
-----
FC88X_01056_00583       ------------------TCAACAAA--TCGTTT--GGGGCGAC-TTCTC-----
-----
FC88X_01089_00572       ------------------TCAACAAA--TCGTTT--GGGGCGAC-TTCTC-----
-----
```

FC88X_00989_00476 ------------GGGAATCAACAAA--TCGTTT--GGGGCGAC-TTCTC-----
-----
FC88X_01015_00661 ------------GGGAATCAACAAA--TCGTTT--GGGGCGAC-TTCTC-----
-----
FC88X_01049_00457 -----------------TCAATAAA--TCGTTT--GGGGCGAC-TTCTC-----
-----
FC88X_01031_00530 -----------------TCAATAAA--TCGTTT--GGGGCGAC-TTCTC-----
-----
FC88X_01071_00560 -----------------TCAATAAA--TCGTTT--GGGGCGAC-TTCTC-----
-----
FC88X_01065_00748 -----------------TCAATAAA--TCGTTT--GGGGCGAC-TTCTC-----
-----
FC88X_01141_01199 ----------------ATCAACAAA--TCGTTT--GGGGCGAC-TTCTC-----
-----
FC88X_00982_00296 -----------------TCAACAAA--TCGTTT--GGGGCGAC-TTCTC-----
-----
FC88X_00990_00691 -----------------TCAACAAA--TCGTTT--GGGGCGAC-TTCTC-----
-----
FC88X_01122_00695 -----------------TCAACAAA--TCGTTT--GGGGCGAC-TTCTC-----
-----
FC88X_01133_00738 -----------------TCAACAAA--TCGTTT--GGGGCGAC-TTCTC-----
-----
FC88X_01126_00756 -----------------TCAACAAA--TCGTTT--GGGGCGAC-TTCTC-----
-----
FC88X_01108_00802 -----------------TCAACAAA--TCGTTT--GGGGCGAC-TTCTC-----
-----
FC88X_01134_00847 -----------------TCAACAAA--TCGTTT--GGGGCGAC-TTCTC-----
-----
FC88X_01114_00893 -----------------TCAACAAA--TCGTTT--GGGGCGAC-TTCTC-----
-----
FC88X_01131_00860 -----------------TCAACAAA--TCGTTT--GGGGCGAC-TTCTC-----
-----
FC88X_01104_00986 -----------------TCAACAAA--TCGTTT--GGGGCGAC-TTCTC-----
-----
FC88X_01111_01131 -----------------TCAACAAA--TCGTTT--GGGGCGAC-TTCTC-----
-----
FC88X_01104_01172 -----------------TCAACAAA--TCGTTT--GGGGCGAC-TTCTC-----
-----
FC88X_01101_01217 -----------------TCAACAAA--TCGTTT--GGGGCGAC-TTCTC-----
-----
FC88X_00989_00389 -----------------TCAACAAA--TCGTTT--GGGGCGAC-TTCTC-----
-----
FC88X_00961_00301 -----------------TCAACAAA--TCGTTT--GGGGCGAC-TTCTC-----
-----
FC88X_00981_00300 -----------------TCAACAAA--TCGTTT--GGGGCGAC-TTCTC-----
-----
FC88X_00972_00656 -----------------TCAACAAA--TCGTTT--GGGGCGAC-TTCTC-----
-----
FC88X_00980_00661 -----------------TCAACAAA--TCGTTT--GGGGCGAC-TTCTC-----
-----
FC88X_00950_00675 -----------------TCAACAAA--TCGTTT--GGGGCGAC-TTCTC-----
-----
FC88X_00959_00584 -----------------TCAACAAA--TCGTTT--GGGGCGAC-TTCTC-----
-----
FC88X_00975_00713 -----------------TCAACAAA--TCGTTT--GGGGCGAC-TTCTC-----
-----

FC88X_00989_00781 -----------------TCAACAAA--TCGTTT--GGGGCGAC-TTCTC-----
-----
FC88X_01068_00547 -----------------TCAACAAA--TCGTTT--GGGGCGAC-TTCTC-----
-----
FC88X_01063_00582 -----------------TCAACAAA--TCGTTT--GGGGCGAC-TTCTC-----
-----
FC88X_00959_00400 ----------------ATCAACAAA--TCGTTT--GGGGCGAC-TTCTC-----
-----
FC88X_01054_00934 -------------GGGGATCAACAAA--TCGTTT--GGGGCGAC-TTCTC-----
-----
FC88X_01063_00598 -----------------TCAACAAA--TCGCTT--GGGGCGAC-TTCTC-----
-----
FC88X_01132_00825 -----------------TCAACAAA--TCGCTT--GGGGCGAC-TTCTC-----
-----
FC88X_01126_00600 -----------------TCAACAAA--TCGTTT--GGGGCGAC-TTCTC-----
-----
FC88X_01104_00696 -----------------TCAACAAA--TCGTTT--GGGGCGAC-TTCTC-----
-----
FC88X_01119_00700 -----------------TCAACAAA--TCGTTT--GGGGCGAC-TTCTC-----
-----
FC88X_01114_00781 -----------------TCAACAAA--TCGTTT--GGGGCGAC-TTCTC-----
-----
FC88X_01137_00759 -----------------TCAACAAA--TCGTTT--GGGGCGAC-TTCTC-----
-----
FC88X_01125_00840 -----------------TCAACAAA--TCGTTT--GGGGCGAC-TTCTC-----
-----
FC88X_01103_00864 -----------------TCAACAAA--TCGTTT--GGGGCGAC-TTCTC-----
-----
FC88X_01127_00864 -----------------TCAACAAA--TCGTTT--GGGGCGAC-TTCTC-----
-----
FC88X_01123_00904 -----------------TCAACAAA--TCGTTT--GGGGCGAC-TTCTC-----
-----
FC88X_01140_00973 -----------------TCAACAAA--TCGTTT--GGGGCGAC-TTCTC-----
-----
FC88X_01126_01111 -----------------TCAACAAA--TCGTTT--GGGGCGAC-TTCTC-----
-----
FC88X_01132_01198 -----------------TCAACAAA--TCGTTT--GGGGCGAC-TTCTC-----
-----
FC88X_01131_01206 -----------------TCAACAAA--TCGTTT--GGGGCGAC-TTCTC-----
-----
FC88X_00982_00364 -----------------TCAACAAA--TCGTTT--GGGGCGAC-TTCTC-----
-----
FC88X_00982_00575 -----------------TCAACAAA--TCGTTT--GGGGCGAC-TTCTC-----
-----
FC88X_00968_00538 -----------------TCAACAAA--TCGTTT--GGGGCGAC-TTCTC-----
-----
FC88X_00997_00489 -----------------TCAACAAA--TCGTTT--GGGGCGAC-TTCTC-----
-----
FC88X_00984_00610 -----------------TCAACAAA--TCGTTT--GGGGCGAC-TTCTC-----
-----
FC88X_00974_00582 -----------------TCAACAAA--TCGTTT--GGGGCGAC-TTCTC-----
-----
FC88X_00953_00740 -----------------TCAACAAA--TCGTTT--GGGGCGAC-TTCTC-----
-----
FC88X_00997_00720 -----------------TCAACAAA--TCGTTT--GGGGCGAC-TTCTC-----
-----

```
FC88X_01095_00498       -----------------TCAACAAA--TCGTTT--GGGGCGAC-TTCTC-----
-----
FC88X_01091_00516       -----------------TCAACAAA--TCGTTT--GGGGCGAC-TTCTC-----
-----
FC88X_01085_00572       -----------------TCAACAAA--TCGTTT--GGGGCGAC-TTCTC-----
-----
FC88X_01055_00646       -----------------TCAACAAA--TCGTTT--GGGGCGAC-TTCTC-----
-----
FC88X_01060_00627       -----------------TCAACAAA--TCGTTT--GGGGCGAC-TTCTC-----
-----
FC88X_01027_00643       -----------------TCAACG-AA-TCGTTTG--GGGCAAC-TTCTCCC---
-----
FC88X_01010_00765       -----------------TCAACG-AA-TCGTT----------------------
-----
FC88X_01112_00618       -----------------TCAAC--AA-TCGTTTG--GGCGACT-TCTCCC----
-----
FC88X_00973_00716       -----------------TCAACAAAA-TCGTTTG--GGGCGAC-TTCTCCC---
-----
FC88X_01044_00339       -----------------TCAACAAAA-TCGTTTG--GGGCGAC-TTCTCCC---
-----
FC88X_00966_00608       -----------------TCAACA-AA-TCGTTTG--GGGCGAC-TTCTCCC---
-----
FC88X_00973_00618       -----------------TCAACA-AA-TCGTTTG--GGGCGAC-TTCTCCC---
-----
FC88X_00967_00752       -----------------TCAACA-AA-TCGTTTG--GGGCGAC-TTCTCCC---
-----
FC88X_00970_00883       -----------------TCAACA-AA-TCGTTTG--GGGCGAC-TTCTCCC---
-----
FC88X_01051_00548       -----------------TCAACA-AA-TCGTTTG--GGGCGAC-TTCTCCC---
-----
FC88X_01050_01051       -----------------TCAACA-AA-TCGTTTG--GGGCGAC-TTCTCCC---
-----
FC88X_01119_00497       -----------------TCAACA-AA-TCGTTTG--GGGCGAC-TTCTCCC---
-----
FC88X_01123_00498       -----------------TCAACA-AA-TCGTTTG--GGGCGAC-TTCTCCC---
-----
FC88X_01012_00373       -----------------TCAACA-AA-TCGTTTG--GGGCGAC-TTCTCCC---
-----
FC88X_01017_00282       -----------------TCAACA-AA-TCGTTTG--GGGCGAC-TTCTCCC---
-----
FC88X_01072_01112       -----------------TCAACA-AA-TCGTTTG--GGGCGAC-TTCTCCCA--
-----
FC88X_01023_00762       -----------------TCAACA-AA-TCGTTTG--GGGCGAC-TTCTCCC---
-----
FC88X_01005_00832       -----------------TCAACA-AA-TCGTTTG--GGGCGAC-TTCTCCC---
-----
FC88X_01033_00874       -----------------TCAACA-AA-TCGTTTG--GGGCGAC-TTCTCCC---
-----
FC88X_00985_00264       -----------------TCAACA-AA-TCGTTTG--GGGCGAC-TTCTC-----
-----
FC88X_01133_00619       -----------------TCAACA-AA-TCGTTTG--GGGCGAC-TTCTC-----
-----
FC88X_01137_00654       -----------------TCAACA-AA-TCGTTTG--GGGCGAC-TTCTC-----
-----
FC88X_01135_00718       -----------------TCAACA-AA-TCGTTTG--GGGCGAC-TTCTC-----
-----
```

FC88X_01132_00778 -----------------TCAACA-AA-TCGTTTG--GGGCGAC-TTCTC-----
-----
FC88X_01116_00835 -----------------TCAACA-AA-TCGTTTG--GGGCGAC-TTCTC-----
-----
FC88X_01137_00820 -----------------TCAACA-AA-TCGTTTG--GGGCGAC-TTCTC-----
-----
FC88X_01120_00856 -----------------TCAACA-AA-TCGTTTG--GGGCGAC-TTCTC-----
-----
FC88X_01146_00892 -----------------TCAACA-AA-TCGTTTG--GGGCGAC-TTCTC-----
-----
FC88X_01118_00990 -----------------TCAACA-AA-TCGTTTG--GGGCGAC-TTCTC-----
-----
FC88X_01116_01141 -----------------TCAACA-AA-TCGTTTG--GGGCGAC-TTCTC-----
-----
FC88X_01110_01182 -----------------TCAACA-AA-TCGTTTG--GGGCGAC-TTCTC-----
-----
FC88X_01111_01201 -----------------TCAACA-AA-TCGTTTG--GGGCGAC-TTCTC-----
-----
FC88X_00993_00382 -----------------TCAACA-AA-TCGTTTG--GGGCGAC-TTCTC-----
-----
FC88X_00965_00333 -----------------TCAACA-AA-TCGTTTG--GGGCGAC-TTCTC-----
-----
FC88X_00996_00301 -----------------TCAACA-AA-TCGTTTG--GGGCGAC-TTCTC-----
-----
FC88X_00954_00633 -----------------TCAACA-AA-TCGTTTG--GGGCGAC-TTCTC-----
-----
FC88X_00971_00630 -----------------TCAACA-AA-TCGTTTG--GGGCGAC-TTCTC-----
-----
FC88X_00954_00654 -----------------TCAACA-AA-TCGTTTG--GGGCGAC-TTCTC-----
-----
FC88X_00994_00699 -----------------TCAACA-AA-TCGTTTG--GGGCGAC-TTCTC-----
-----
FC88X_00988_00732 -----------------TCAACA-AA-TCGTTTG--GGGCGAC-TTCTC-----
-----
FC88X_00999_00778 -----------------TCAACA-AA-TCGTTTG--GGGCGAC-TTCTC-----
-----
FC88X_01079_00529 -----------------TCAACA-AA-TCGTTTG--GGGCGAC-TTCTC-----
-----
FC88X_01069_00560 -----------------TCAACA-AA-TCGTTTG--GGGCGAC-TTCTC-----
-----
FC88X_01098_00576 -----------------TCAACA-AA-TCGTTTG--GGGCGAC-TTCTC-----
-----
FC88X_01058_00636 -----------------TCAACAAA--TCGTTTG--GGGCGAC-TTCTC-----
-----
FC88X_00959_00411 -----------------TCAACAAA--TCGTTTG--GGGCGAC-TTCTCCT---
-----
FC88X_01007_00322 -----------------TCAACAAA--TCGTTTG--GGGCGACCTTCTCCT---
-----
FC88X_01140_00929 -----------------TCAACAAA--TCGTTTG--GGGCGAC-TTCTCC----
-----
FC88X_00983_00297 -----------------TCAACAAA--TCGTTTG--GGGCGAC-TTCTC-----
-----
FC88X_00991_00659 -----------------TCAACAAA--TCGTTTG--GGGCGAC-TTCTC-----
-----
FC88X_01125_00680 -----------------TCAACAAA--TCGTTTG--GGGCGAC-TTCTC-----
-----

```
FC88X_01134_00747       -----------------TCAACAAA--TCGTTTG--GGGCGAC-TTCTC-----
-----
FC88X_01129_00795       -----------------TCAACAAA--TCGTTTG--GGGCGAC-TTCTC-----
-----
FC88X_01110_00838       -----------------TCAACAAA--TCGTTTG--GGGCGAC-TTCTC-----
-----
FC88X_01135_00842       -----------------TCAACAAA--TCGTTTG--GGGCGAC-TTCTC-----
-----
FC88X_01119_00867       -----------------TCAACAAA--TCGTTTG--GGGCGAC-TTCTC-----
-----
FC88X_01138_00862       -----------------TCAACAAA--TCGTTTG--GGGCGAC-TTCTC-----
-----
FC88X_01117_00967       -----------------TCAACAAA--TCGTTTG--GGGCGAC-TTCTC-----
-----
FC88X_01115_01123       -----------------TCAACAAA--TCGTTTG--GGGCGAC-TTCTC-----
-----
FC88X_01107_01197       -----------------TCAACAAA--TCGTTTG--GGGCGAC-TTCTC-----
-----
FC88X_01107_01213       -----------------TCAACAAA--TCGTTTG--GGGCGAC-TTCTC-----
-----
FC88X_00990_00389       -----------------TCAACAAA--TCGTTTG--GGGCGAC-TTCTC-----
-----
FC88X_00962_00325       -----------------TCAACAAA--TCGTTTG--GGGCGAC-TTCTC-----
-----
FC88X_00995_00301       -----------------TCAACAAA--TCGTTTG--GGGCGAC-TTCTC-----
-----
FC88X_00975_00573       -----------------TCAACAAA--TCGTTTG--GGGCGAC-TTCTC-----
-----
FC88X_00980_00698       -----------------TCAACAAA--TCGTTTG--GGGCGAC-TTCTC-----
-----
FC88X_00950_00688       -----------------TCAACAAA--TCGTTTG--GGGCGAC-TTCTC-----
-----
FC88X_00993_00693       -----------------TCAACAAA--TCGTTTG--GGGCGAC-TTCTC-----
-----
FC88X_00977_00737       -----------------TCAACAAA--TCGTTTG--GGGCGAC-TTCTC-----
-----
FC88X_00991_00761       -----------------TCAACAAA--TCGTTTG--GGGCGAC-TTCTC-----
-----
FC88X_01075_00522       -----------------TCAACAAA--TCGTTTG--GGGCGAC-TTCTC-----
-----
FC88X_01064_00560       -----------------TCAACAAA--TCGTTTG--GGGCGAC-TTCTC-----
-----
FC88X_01049_00394       -----------------TCAACAAA--TCGTTTG--GGGCGAC-
TTCTCCTTCC-----
FC88X_01104_00516       -----------------TCAACAAA--TCGTTTG--GGGCGAC-
TTCTCCTTCC-----
FC88X_01120_01158       -----------------TCAACAAA--TCGTTTG--GGGCGAC-
TTCTCCTTCC-----
FC88X_01051_01225       -----------------TCAACAAA--TCGTTTG--GGGCGAC-TTCTCCT---
-----
FC88X_01101_01146       -----------------TCAACAAA--TCGTTTG--GGGCGAC-TTCTCC----
-----
FC88X_00984_00299       -----------------TCAACAAA--TCGTTTG--GGGCGAC-TTCTC-----
-----
FC88X_01133_00616       -----------------TCAACAAA--TCGTTTG--GGGCGAC-TTCTC-----
-----
```

FC88X_01131_00699 -----------------TCAACAAA--TCGTTTG--GGGCGAC-TTCTC-----
-----
FC88X_01135_00710 -----------------TCAACAAA--TCGTTTG--GGGCGAC-TTCTC-----
-----
FC88X_01132_00772 -----------------TCAACAAA--TCGTTTG--GGGCGAC-TTCTC-----
-----
FC88X_01115_00808 -----------------TCAACAAA--TCGTTTG--GGGCGAC-TTCTC-----
-----
FC88X_01137_00806 -----------------TCAACAAA--TCGTTTG--GGGCGAC-TTCTC-----
-----
FC88X_01120_00854 -----------------TCAACAAA--TCGTTTG--GGGCGAC-TTCTC-----
-----
FC88X_01144_00863 -----------------TCAACAAA--TCGTTTG--GGGCGAC-TTCTC-----
-----
FC88X_01118_00971 -----------------TCAACAAA--TCGTTTG--GGGCGAC-TTCTC-----
-----
FC88X_01116_01126 -----------------TCAACAAA--TCGTTTG--GGGCGAC-TTCTC-----
-----
FC88X_01108_01169 -----------------TCAACAAA--TCGTTTG--GGGCGAC-TTCTC-----
-----
FC88X_01111_01200 -----------------TCAACAAA--TCGTTTG--GGGCGAC-TTCTC-----
-----
FC88X_00991_00358 -----------------TCAACAAA--TCGTTTG--GGGCGAC-TTCTC-----
-----
FC88X_00964_00342 -----------------TCAACAAA--TCGTTTG--GGGCGAC-TTCTC-----
-----
FC88X_00992_00304 -----------------TCAACAAA--TCGTTTG--GGGCGAC-TTCTC-----
-----
FC88X_00976_00594 -----------------TCAACAAA--TCGTTTG--GGGCGAC-TTCTC-----
-----
FC88X_00968_00641 -----------------TCAACAAA--TCGTTTG--GGGCGAC-TTCTC-----
-----
FC88X_00951_00654 -----------------TCAACAAA--TCGTTTG--GGGCGAC-TTCTC-----
-----
FC88X_00994_00690 -----------------TCAACAAA--TCGTTTG--GGGCGAC-TTCTC-----
-----
FC88X_00986_00725 -----------------TCAACAAA--TCGTTTG--GGGCGAC-TTCTC-----
-----
FC88X_00992_00757 -----------------TCAACAAA--TCGTTTG--GGGCGAC-TTCTC-----
-----
FC88X_01076_00529 -----------------TCAACAAA--TCGTTTG--GGGCGAC-TTCTC-----
-----
FC88X_01067_00556 -----------------TCAACAAA--TCGTTTG--GGGCGAC-TTCTC-----
-----
FC88X_01097_00559 -----------------TCAACAAA--TCGTTTG--GGGCGAC-TTCTC-----
-----
FC88X_01077_00588 -----------------TCAACAAA--TCGTTTG--GGGCGAC-TTCTC-----
-----
FC88X_01085_00513 -----------------TCAACAAA--TCGTTTG--GGGCGAC-TTCTC-----
-----
FC88X_01094_00451 -----------------TCAACAAA--TCGTTTG--GGGCGAC-TTCTC-----
-----
FC88X_00993_00725 -----------------TCAACAAA--TCGTTTG--GGGCGAC-TTCTC-----
-----
FC88X_00967_00800 -----------------TCAACAAA--TCGTTTG--GGGCGAC-TTCTC-----
-----

FC88X_00967_00654 -----------------TCAACAAA--TCGTTTG--GGGCGAC-TTCTC-----
-----
FC88X_00975_00605 -----------------TCAACAAA--TCGTTTG--GGGCGAC-TTCTC-----
-----
FC88X_00995_00454 -----------------TCAACAAA--TCGTTTG--GGGCGAC-TTCTC-----
-----
FC88X_00971_00352 -----------------TCAACAAA--TCGTTTG--GGGCGAC-TTCTC-----
-----
FC88X_00955_00318 -----------------TCAACAAA--TCGTTTG--GGGCGAC-TTCTC-----
-----
FC88X_00975_00384 -----------------TCAACAAA--TCGTTTG--GGGCGAC-TTCTC-----
-----
FC88X_01128_01237 -----------------TCAACAAA--TCGTTTG--GGGCGAC-TTCTC-----
-----
FC88X_01126_01197 -----------------TCAACAAA--TCGTTTG--GGGCGAC-TTCTC-----
-----
FC88X_01122_01108 -----------------TCAACAAA--TCGTTTG--GGGCGAC-TTCTC-----
-----
FC88X_01137_00997 -----------------TCAACAAA--TCGTTTG--GGGCGAC-TTCTC-----
-----
FC88X_01118_00949 -----------------TCAACAAA--TCGTTTG--GGGCGAC-TTCTC-----
-----
FC88X_01123_00894 -----------------TCAACAAA--TCGTTTG--GGGCGAC-TTCTC-----
-----
FC88X_01146_00809 -----------------TCAACAAA--TCGTTTG--GGGCGAC-TTCTC-----
-----
FC88X_01124_00824 -----------------TCAACAAA--TCGTTTG--GGGCGAC-TTCTC-----
-----
FC88X_01136_00773 -----------------TCAACAAA--TCGTTTG--GGGCGAC-TTCTC-----
-----
FC88X_01100_00775 -----------------TCAACAAA--TCGTTTG--GGGCGAC-TTCTC-----
-----
FC88X_01109_00702 -----------------TCAACAAA--TCGTTTG--GGGCGAC-TTCTC-----
-----
FC88X_01142_00607 -----------------TCAACAAA--TCGTTTG--GGGCGAC-TTCTC-----
-----
FC88X_01092_00628 -----------------TCAACAAA--TCGTCTG--GGGCGAC-TTCTC-----
-----
FC88X_00995_00938 -----------------ACAACAAA--TCGTTTG--GGGCGAC-TTCTC-----
-----
FC88X_01038_00858 -----------------ACAACAAA--TCGTTTG--GGGCGAC-TTCTC-----
-----
FC88X_00971_01124 -----------------CCAACAAA--TCGTTTG--GGGCGAC-TTCTC-----
-----
FC88X_01034_00913 -----------------CCAACAAA--TCGTTTG--GGGCGAC-TTCTC-----
-----
FC88X_01014_00378 -----------------CTGACAAG--TCGATG---------------------
-----
FC88X_01134_00553 ----------------TCTAACAAA--TCGTTGG--GTCGACTTCCT-------
-----
FC88X_01037_00557 ------------------TAACAAA--TCG------------------------
-----
FC88X_01096_00788 ------------------TAACAAA--TC-------------------------
-----
FC88X_00969_00662 -----------------TTAACAAA--TCGTTTG--GGGCGACTTCTC------
-----

FC88X_00977_01006 -----------------TTAACAAA--TCGTTTG--GGGCGACTTCTC------
-----

FC88X_01048_00592 -----------------TTAACAAA--TCGTTTG--GGGCGACTTCTC------
-----

FC88X_01004_00554 -----------------TCGAACAAA-TCGTTTGG-G--CGAC-TTCT------
-----

FC88X_01131_00539 -----------------TCAAACAAA-TCGTTTGG-GGGCGAC-TTCTCCT---
-----

FC88X_01123_00540 -----------------TCAAACAAA-TCGTTTGG-GG-CGAC-TTCTC-----
-----

FC88X_01146_00807 -----------------TCAAACAAA-TCGTTTGG-GG-CGAC-TTCTC-----
-----

FC88X_01000_01175 -----------------TCAACCAAA----------------------------
-----

FC88X_01130_00929 -----------------TCAACCAAA-TCGTTTGG-GG-CGGC-TTCTC-----
-----

FC88X_01101_00732 -------------TCAACAAAAAAAA-TCGTTTGG-GG-CGAC-TTCTC-----
-----

FC88X_00984_00368 -----------------CTCCCAAATCACGTTACGTGCGACCCA----------
-----

FC88X_01073_00638 -----------------TACACAAATCGT-------------------------
-----

FC88X_00980_00429 -----------------TCCCCAAATAGCTACCGACGACAGCGTC---------
-----

FC88X_01095_00881 -----------------TCCCCAAATAGCTACCGACGACAGCGTC---------
-----

FC88X_01009_00981 ------------------GGCCGAACGTCG-CATGC--TCCCGGCCGCCACT--
-----

FC88X_01114_00588 -------------------GCCGA-CGTCG-CATGCG-TCCCGGC---------
-----

FC88X_01056_01154 ------------------GGCCGA-CGTCG-CATGC--TCCCGCCGCAT-----
-----

FC88X_01096_01161 ------------------GGCCCGACGTCG-CATGC--TCCCGGCGC-------
-----

FC88X_00982_01003 ------------------GGCCGA-CGTCG-CAT--------------------
-----

FC88X_01023_00994 ------------------GGCCGAACGTCGACATGTCGTCCCGGCCG-------
-----

FC88X_01118_00875 ------------------GGCCGA-CGTCGACATGTCGTCCCGGCCAGT-----
-----

FC88X_00955_00884 -------------------GCCGA-CGTCAC-----------------------
-----

FC88X_00977_00271 -------------------ATCACCGTTGGTTCC-GGGTGCGCGACTCTTCC--
-----

FC88X_01038_00602 -------------------ATCACCGTTGGTTC--GGGTGCGCG-CTCTT----
-----

FC88X_01081_00842 -----------------TACTCACCGTTGGTTC--GGGTGCGCA----------
-----

FC88X_01021_00341 -------------------ATCACC-TTGGTTC--GG-----------------
-----

FC88X_01002_00813 -------------------ATCACCGTTGGTTC--GGCTGCGCGTTCTC-----
-----

FC88X_00999_00279 -------------------ATCACCGTCGGTTC--GGGTGCGCGTTCGTCC---
-----

FC88X_00964_00769 -------------------ATCACCGTTGGCTC--GGGTGCGCGTTCTCCC---
-----

FC88X_01005_00884 -------------------ATCACCGTTGGCTC--GGGTGCGCGTTCTC-----
-----
FC88X_01098_00774 -------------------ATCACCGTTGGTC---GGGTGCGCGTTCTC-----
-----
FC88X_01095_00806 -------------------ATCACCGTTGGTCC--GGGTGCGCGTTCTC-----
-----
FC88X_01024_00975 -------------------ATCACCGTTGGTTC--AGGTGCGCGTTCTC-----
-----
FC88X_01085_00461 -------------------ATCACCGTTGGTTC---GGTGCGCGTTCTC-----
-----
FC88X_01083_00810 -------------------ATCACCGTTCGGTTC-GGGTGCGCGTTCTC-----
-----
FC88X_01113_00779 -------------------ATCACCGTTCGGTTC-GGGTGCGCGTTCTC-----
-----
FC88X_01078_00774 -------------------ATCACCGTCGGTTC--GGGTGCGCGTTCTC-----
-----
FC88X_00996_00288 -------------------ATCACCGTTGGTTC--GGGTGCGCGTTCTC-----
-----
FC88X_00998_00298 -------------------ATCACCGTTGGTTC--GGGTGCGCGTTCTC-----
-----
FC88X_00956_00324 -------------------ATCACCGTTGGTTC--GGGTGCGCGTTCTC-----
-----
FC88X_00961_00322 -------------------ATCACCGTTGGTTC--GGGTGCGCGTTCTC-----
-----
FC88X_00966_00348 -------------------ATCACCGTTGGTTCG-GG-TGCGCGTTCTC-----
-----
FC88X_01021_00929 -------------------ATCACCGTTGGTTCG-GGCTGCGCGTTCTC-----
-----
FC88X_00956_00390 -------------------ATCACCGTTGGTTC--GGGTGCGCGTTCTC-----
-----
FC88X_00968_00352 -------------------ATCACCGTTGGTTC--GGGTGCGCGTTCTC-----
-----
FC88X_00965_00405 -------------------ATCACCGTTGGTTC--GGGTGCGCGTTCTC-----
-----
FC88X_00999_00438 -------------------ATCACCGTTGGTTC--GGGTGCGCGTTCTC-----
-----
FC88X_00973_00590 -------------------ATCACCGTTGGTTC--GGGTGCGCGTTCTC-----
-----
FC88X_00999_00618 -------------------ATCACCGTTGGTTC--GGGTGCGCGTTCTC-----
-----
FC88X_00960_00414 -------------------ATCACCGTTGGTTC--GGGTGCGCGTTCTC-----
-----
FC88X_00990_00402 ------------------GATCACCGTTGGTTC--GGGTGCGCGTTCTC-----
-----
FC88X_00994_00424 -------------------ATCACCGTTGGTTC--GGGTGCGCGTTCTC-----
-----
FC88X_00951_00475 -------------------ATCACCGTTGGTTC--GGGTGCGCGTTCTC-----
-----
FC88X_00959_00649 -------------------ATCACCGTTGGTTC--GGGTGCGCGTTCTC-----
-----
FC88X_00954_00692 -------------------ATCACCGTTGGTTC--GGGTGCGCGTTCTC-----
-----
FC88X_00980_00675 -------------------ATCACCGTTGGTTC--GGGTGCGCGTTCTC-----
-----
FC88X_00981_00696 -------------------ATCACCGTTGGTTC--GGGTGCGCGTTCTC-----
-----

```
FC88X_00993_00669      -------------------ATCACCGTTGGTTC--GGGTGCGCGTTCTC-----
-----
FC88X_00966_00705      -------------------ATCACCGTTGGTTC--GGGTGCGCGTTCTC-----
-----
FC88X_00973_00741      -------------------ATCACCGTTGGTTC--GGGTGCGCGTTCTC-----
-----
FC88X_00954_00783      -------------------ATCACCGTTGGTTC--GGGTGCGCGTTCTC-----
-----
FC88X_00991_00755      -------------------ATCACCGTTGGTTC--GGGTGCGCGTTCTC-----
-----
FC88X_00979_00834      -------------------ATCACCGTTGGTTC--GGGTGCGCGTTCTC-----
-----
FC88X_00990_00838      -------------------ATCACCGTTGGTTC--GGGTGCGCGTTCTC-----
-----
FC88X_00979_00877      -------------------ATCACCGTTGGTTC--GGGTGCGCGTTCTC-----
-----
FC88X_00994_00898      -------------------ATCACCGTTGGTTC--GGGTGCGCGTTCTC-----
-----
FC88X_00961_00937      -------------------ATCACCGTTGGTTC--GGGTGCGCGTTCTC-----
-----
FC88X_00989_00910      -------------------ATCACCGTTGGTTC--GGGTGCGCGTTCTC-----
-----
FC88X_00963_00975      -------------------ATCACCGTTGGTTC--GGGTGCGCGTTCTC-----
-----
FC88X_00981_00985      -------------------ATCACCGTTGGTTC--GGGTGCGCGTTCTC-----
-----
FC88X_00989_00977      -------------------ATCACCGTTGGTTC--GGGTGCGCGTTCTC-----
-----
FC88X_00990_00975      -------------------ATCACCGTTGGTTC--GGGTGCGCGTTCTC-----
-----
FC88X_00994_00999      -------------------ATCACCGTTGGTTC--GGGTGCGCGTTCTC-----
-----
FC88X_00952_01029      -------------------ATCACCGTTGGTTC--GGGTGCGCGTTCTC-----
-----
FC88X_00979_01048      -------------------ATCACCGTTGGTTC--GGGTGCGCGTTCTC-----
-----
FC88X_00996_01003      -------------------ATCACCGTTGGTTC--GGGTGCGCGTTCTC-----
-----
FC88X_00970_01109      -------------------ATCACCGTTGGTTC--GGGTGCGCGTTCTC-----
-----
FC88X_00981_01126      -------------------ATCACCGTTGGTTC--GGGTGCGCGTTCTC-----
-----
FC88X_01003_00269      -------------------ATCACCGTTGGTTC--GGGTGCGCGTTCTC-----
-----
FC88X_01020_00346      -------------------ATCACCGTTGGTTC--GGGTGCGCGTTCTC-----
-----
FC88X_01026_00337      -------------------ATCACCGTTGGTTC--GGGTGCGCGTTCTC-----
-----
FC88X_01028_00379      -------------------ATCACCGTTGGTTC--GGGTGCGCGTTCTC-----
-----
FC88X_01046_00396      -------------------ATCACCGTTGGTTC--GGGTGCGCGTTCTC-----
-----
FC88X_01000_00433      -------------------ATCACCGTTGGTTC--GGGTGCGCGTTCTC-----
-----
FC88X_01025_00418      -------------------ATCACCGTTGGTTC--GGGTGCGCGTTCTC-----
-----
```

FC88X_01034_00416   -------------------ATCACCGTTGGTTC--GGGTGCGCGTTCTC-----
-----
FC88X_01046_00448   -------------------ATCACCGTTGGTTC--GGGTGCGCGTTCTC-----
-----
FC88X_01030_00520   -------------------ATCACCGTTGGTTC--GGGTGCGCGTTCTC-----
-----
FC88X_01006_00572   -------------------ATCACCGTTGGTTC--GGGTGCGCGTTCTC-----
-----
FC88X_01031_00592   -------------------ATCACCGTTGGTTC--GGGTGCGCGTTCTC-----
-----
FC88X_01046_00576   -------------------ATCACCGTTGGTTC--GGGTGCGCGTTCTC-----
-----
FC88X_01003_00608   -------------------ATCACCGTTGGTTC--GGGTGCGCGTTCTC-----
-----
FC88X_01020_00608   -------------------ATCACCGTTGGTTC--GGGTGCGCGTTCTC-----
-----
FC88X_01048_00636   -------------------ATCACCGTTGGTTC--GGGTGCGCGTTCTC-----
-----
FC88X_01014_00658   -------------------ATCACCGTTGGTTC--GGGTGCGCGTTCTC-----
-----
FC88X_01034_00698   -------------------ATCACCGTTGGTTC--GGGTGCGCGTTCTC-----
-----
FC88X_01023_00770   -------------------ATCACCGTTGGTTC--GGGTGCGCGTTCTC-----
-----
FC88X_01007_00833   -------------------ATCACCGTTGGTTC--GGGTGCGCGTTCTC-----
-----
FC88X_01025_00833   -------------------ATCACCGTTGGTTC--GGGTGCGCGTTCTC-----
-----
FC88X_01033_00822   -------------------ATCACCGTTGGTTC--GGGTGCGCGTTCTC-----
-----
FC88X_01034_00820   -------------------ATCACCGTTGGTTC--GGGTGCGCGTTCTC-----
-----
FC88X_01047_00845   -------------------ATCACCGTTGGTTC--GGGTGCGCGTTCTC-----
-----
FC88X_01021_00872   -------------------ATCACCGTTGGTTC--GGGTGCGCGTTCTC-----
-----
FC88X_01043_00867   -------------------ATCACCGTTGGTTC--GGGTGCGCGTTCTC-----
-----
FC88X_01044_00850   -------------------ATCACCGTTGGTTC--GGGTGCGCGTTCTC-----
-----
FC88X_01003_00966   -------------------ATCACCGTTGGTTC--GGGTGCGCGTTCTC-----
-----
FC88X_01009_01037   -------------------ATCACCGTTGGTTC--GGGTGCGCGTTCTC-----
-----
FC88X_01045_01027   -------------------ATCACCGTTGGTTC--GGGTGCGCGTTCTC-----
-----
FC88X_01002_01129   -------------------ATCACCGTTGGTTC--GGGTGCGCGTTCTC-----
-----
FC88X_01017_01113   -------------------ATCACCGTTGGTTC--GGGTGCGCGTTCTC-----
-----
FC88X_01019_01213   -------------------ATCACCGTTGGTTC--GGGTGCGCGTTCTC-----
-----
FC88X_01026_01214   -------------------ATCACCGTTGGTTC--GGGTGCGCGTTCTC-----
-----
FC88X_01039_01253   -------------------ATCACCGTTGGTTC--GGGTGCGCGTTCTC-----
-----

```
FC88X_01041_01275       -------------------ATCACCGTTGGTTC--GGGTGCGCGTTCTC-----
-----
FC88X_01042_01256       -------------------ATCACCGTTGGTTC--GGGTGCGCGTTCTC-----
-----
FC88X_01050_00337       -------------------ATCACCGTTGGTTC--GGGTGCGCGTTCTC-----
-----
FC88X_01068_00379       -------------------ATCACCGTTGGTTC--GGGTGCGCGTTCTC-----
-----
FC88X_01077_00382       -------------------ATCACCGTTGGTTC--GGGTGCGCGTTCTC-----
-----
FC88X_01067_00439       -------------------ATCACCGTTGGTTC--GGGTGCGCGTTCTC-----
-----
FC88X_01073_00443       -------------------ATCACCGTTGGTTC--GGGTGCGCGTTCTC-----
-----
FC88X_01092_00444       -------------------ATCACCGTTGGTTC--GGGTGCGCGTTCTC-----
-----
FC88X_01076_00462       -------------------ATCACCGTTGGTTC--GGGTGCGCGTTCTC-----
-----
FC88X_01069_00545       -------------------ATCACCGTTGGTTC--GGGTGCGCGTTCTC-----
-----
FC88X_01083_00517       -------------------ATCACCGTTGGTTC--GGGTGCGCGTTCTC-----
-----
FC88X_01094_00521       -------------------ATCACCGTTGGTTC--GGGTGCGCGTTCTC-----
-----
FC88X_01052_00582       -------------------ATCACCGTTGGTTC--GGGTGCGCGTTCTC-----
-----
FC88X_01053_00565       -------------------ATCACCGTTGGTTC--GGGTGCGCGTTCTC-----
-----
FC88X_01057_00580       -------------------ATCACCGTTGGTTC--GGGTGCGCGTTCTC-----
-----
FC88X_01079_00594       -------------------ATCACCGTTGGTTC--GGGTGCGCGTTCTC-----
-----
FC88X_01098_00598       -------------------ATCACCGTTGGTTC--GGGTGCGCGTTCTC-----
-----
FC88X_01056_00610       -------------------ATCACCGTTGGTTC--GGGTGCGCGTTCTC-----
-----
FC88X_01094_00609       -------------------ATCACCGTTGGTTC--GGGTGCGCGTTCTC-----
-----
FC88X_01088_00666       -------------------ATCACCGTTGGTTC--GGGTGCGCGTTCTC-----
-----
FC88X_01056_00753       -------------------ATCACCGTTGGTTC--GGGTGCGCGTTCTC-----
-----
FC88X_01082_00887       -------------------ATCACCGTTGGTTC--GGGTGCGCGTTCTC-----
-----
FC88X_01082_00982       -------------------ATCACCGTTGGTTC--GGGTGCGCGTTCTC-----
-----
FC88X_01099_01142       -------------------ATCACCGTTGGTTC--GGGTGCGCGTTCTC-----
-----
FC88X_01072_01184       -------------------ATCACCGTTGGTTC--GGGTGCGCGTTCTC-----
-----
FC88X_01106_00488       -------------------ATCACCGTTGGTTC--GGGTGCGCGTTCTC-----
-----
FC88X_01121_00531       -------------------ATCACCGTTGGTTC--GGGTGCGCGTTCTC-----
-----
FC88X_01129_00527       -------------------ATCACCGTTGGTTC--GGGTGCGCGTTCTC-----
-----
```

FC88X_01130_00600 -------------------ATCACCGTTGGTTC--GGGTGCGCGTTCTC-----
-----
FC88X_01122_00667 -------------------ATCACCGTTGGTTC--GGGTGCGCGTTCTC-----
-----
FC88X_01105_00732 -------------------ATCACCGTTGGTTC--GGGTGCGCGTTCTC-----
-----
FC88X_01127_00715 -------------------ATCACCGTTGGTTC--GGGTGCGCGTTCTC-----
-----
FC88X_01132_00717 -------------------ATCACCGTTGGTTC--GGGTGCGCGTTCTC-----
-----
FC88X_01137_00708 -------------------ATCACCGTTGGTTC--GGGTGCGCGTTCTC-----
-----
FC88X_01136_00791 -------------------ATCACCGTTGGTTC--GGGTGCGCGTTCTC-----
-----
FC88X_01144_00776 -------------------ATCACCGTTGGTTC--GGGTGCGCGTTCTC-----
-----
FC88X_01122_00804 -------------------ATCACCGTTGGTTC--GGGTGCGCGTTCTC-----
-----
FC88X_01144_00832 -------------------ATCACCGTTGGTTC--GGGTGCGCGTTCTC-----
-----
FC88X_01111_00853 -------------------ATCACCGTTGGTTC--GGGTGCGCGTTCTC-----
-----
FC88X_01111_00869 -------------------ATCACCGTTGGTTC--GGGTGCGCGTTCTC-----
-----
FC88X_01133_00858 -------------------ATCACCGTTGGTTC--GGGTGCGCGTTCTC-----
-----
FC88X_01124_00941 -------------------ATCACCGTTGGTTC--GGGTGCGCGTTCTC-----
-----
FC88X_01134_00933 -------------------ATCACCGTTGGTTC--GGGTGCGCGTTCTC-----
-----
FC88X_01132_00970 -------------------ATCACCGTTGGTTC--GGGTGCGCGTTCTC-----
-----
FC88X_01110_01149 -------------------ATCACCGTTGGTTC--GGGTGCGCGTTCTC-----
-----
FC88X_01128_01132 -------------------ATCACCGTTGGTTC--GGGTGCGCGTTCTC-----
-----
FC88X_01103_01164 -------------------ATCACCGTTGGTTC--GGGTGCGCGTTCTC-----
-----
FC88X_00960_01251 -------------------ATCACCGTTGGTTC--GGGTGCGCGTTCTCT----
-----
FC88X_01030_00685 -------------------ATCACCGTTGGTTC--GGGTGCGCGTCCTC-----
-----
FC88X_00964_00659 -------------------ATCACCGTTGGTTC--GGGTGTGCGTTCTC-----
-----
FC88X_00966_00856 -------------------ATCACCGTTGGTTC--GGGTGTGCGTTCTC-----
-----
FC88X_01041_00336 ------------------AATCACCGTTGGTTC--GGGTGCGCGTTCTC-----
-----
FC88X_01061_00687 -------------------ATCACCGTTGGTTC--GGGTGCGCGTTCTCCC---
-----
FC88X_01047_00915 -------------------ATCACCGCTGGTTC--GGGTGCGCGTTCTC-----
-----
FC88X_00962_00485 -------------------ATCACCGTTGGTTC--GGGTGCGTGTTCTC-----
-----
FC88X_01138_00588 -------------------ATCACCGTTGGTTC--GGGTGCGTGTTCTC-----
-----

```
FC88X_01142_00743       -------------------ATCACCGTTAGTTC--GGGTGCGCGTTCTC-----
-----
FC88X_01095_00865       -------------------ACCACCGTTGGTTC--GGGTGCGCGTTCTC-----
-----
FC88X_01034_00481       -------------------ATCACCATTGGTTC--GGGTGCGCGTTCTC-----
-----
FC88X_01144_00854       -------------------ATCACCATTGGTTC--GGGTGCGCGTTCTC-----
-----
FC88X_01018_00984       -------------------GACACCAATCGCGCC-GGGTC-ATGTTCTC-----
-----
FC88X_01056_00994       --------------------ACACCAATCGCGCC-GGGTC-ATGTTCTC-----
-----
FC88X_01099_01164       --------------------ACACCAATCGCGCC-GGGTC-ATGTTCTC-----
-----
FC88X_00970_00661       --------------------ACACCAATCGTGCC-GGGTC-ATGTTCTC-----
-----
FC88X_00959_01262       --------------------ACACCAATCGTGCC-GGGTCGATGTTCTCC----
-----
FC88X_00958_00746       --------------------ACACCAATCGTGCC-GGGTC-ATGTTCTC-----
-----
FC88X_00960_00752       --------------------ACACCAATCGTGCC-GGGTC-ATGTTCTC-----
-----
FC88X_01004_00522       --------------------ACACCAATCGTGCC-GGGTC-ATGTTCTC-----
-----
FC88X_01030_01175       --------------------ACACCAATCGTGCC-GGGTC-ATGTTCTC-----
-----
FC88X_01068_00610       --------------------ACACCAATCGTGCC-GGGTC-ATGTTCTC-----
-----
FC88X_01069_00936       --------------------ACACCAATCGTGCC-GGGTC-ATGTTCTC-----
-----
FC88X_01109_00486       --------------------ACACCAATCGTGCC-GGGTC-ATGTTCTC-----
-----
FC88X_01130_00863       --------------------ACACCAATCGTGCC-GGGTC-ATGTTCTC-----
-----
FC88X_01098_00449       --------------------ACACCAATCGTGCC-GGGTC-ATGTTCTCC----
-----
FC88X_01090_00761       --------------------ACACCAATCGTGCC-GGGTC-ATGTTCTCCC---
-----
FC88X_01070_00582       --------------------ACACCAATCGTACC-GGGTC-ATGTTCTC-----
-----
FC88X_01007_00505       -------------------GACACCAATCGTGCC-GGGTC-ATGTTCAC-----
-----
FC88X_01040_00396       --------------------ACACCAATCGCGC--GGGTC-ATGTTCTC-----
-----
FC88X_00959_00836       -------------------TTCGCCAAT-ATCCC-GCGGC-GTGTTCTC-----
-----
FC88X_01032_00437       -------------------TTCGCCAAT-ATCCC-GCGGC-GTGTTCTC-----
-----
FC88X_01078_00534       -------------------TTCGCCAAT-ATCCC-GCGGC-GTGTTCTC-----
-----
FC88X_01071_00893       -------------------TTCGCCAAT-ATCCC-GCGGC-GTGTTCTC-----
-----
FC88X_01091_00864       -------------------TTCGCCAAT-ATCCC-GCGGC-GTGTTCTC-----
-----
FC88X_01033_00789       -------------------TTCGCCAGT-ATCCC-GCGGC-GTGTTCTC-----
-----
```

```
FC88X_00983_00896       -------------------TTCGCCAAT-ACCCC-GCGGC-GTGTTCTC-----
-----
FC88X_01013_00898       -------------------TTCGCCAAT-ATCTC-GCGGC-GTGTTCTC-----
-----
FC88X_01033_00473       ------------------TCGTCGGATCGAGTCT-GGCGCCCTGCT--------
-----
FC88X_01005_00609       ------------------CCGTGATATCTTCTCC-GGCACGTTCTCCT------
-----
FC88X_01011_00833       ---------------------CTGTATCGCGTCC-AACACAGCGTTCTC-----
-----
FC88X_01090_00511       ---------------------CTGTATCGCGTCC-AACACAGCGTTCTC-----
-----
FC88X_01137_00833       ---------------------CCATGTCGCGTCT-GGCCCCACGTTCTC-----
-----
FC88X_01011_00772       ---------------------CCGCGTCGTTTCT-GGATTCGCATTCTC-----
-----
FC88X_00964_00922       --------------------CCGCCAGTAGTAT--TGGCCCACTATCTC-----
-----
FC88X_00992_00340       --------------------CTAAAAACTCCCCT-GCGTCGACTTTAC------
-----
FC88X_01009_00338       --------------------TCACAAACTCG-----------------------
-----
FC88X_01081_00814       -----------------TCAACAAT--TTATCTG--CGTCGGCTTTCT------
-----
FC88X_01059_00943       -----------------TCAACAGT--TTATCTGG-CGTCGGCTTTCT------
-----
FC88X_01079_00479       ----------------CTCAATAA------------------------------
-----
FC88X_01006_00782       --------------CCCTTGACTAT--TCATTTT--GCGATACTGCC-------
-----
FC88X_01085_00748       ------------------TCACAAT--TCATT----------------------
-----
FC88X_01056_00853       -----------------TTAGACAA--TCATTGG--GCGTAACTACTTC-----
-----
FC88X_00988_00311       ----------------------CTACACGATACCTTGAAGCTGCGTCTC-----
-----
FC88X_00950_00448       ----------------------TTAACCCATCCCATGAGTCGCTGTCTT-----
-----
FC88X_01034_00364       ------------------------------TCCAATGAATCGTTGG--------
-----
FC88X_00980_00511       ------------------CTACGAG--TCTTTTGTTT-----------------
-----
FC88X_00973_00788       ------------------CTACGAA--T--------------------------
-----
FC88X_01044_00998       --------------TTTCCTACAAA--TCGATTC--------------------
-----
FC88X_01113_00560       ---------------TTCCTACAA---CTGATTCG-------------------
-----
FC88X_01073_00923       TACAGCATTTGCGGGTACCTATAAC--TC-------------------------
-----
FC88X_00968_00628       ---------------CTGTTTCTTCCTTCGTCCCGAATTTTC------------
-----
FC88X_00979_00922       --------------TCGTAGAATTCCATCGGCAAGACTTTCT------------
-----
FC88X_00957_01010       ----------------------TCCCTTCGTCTTGAGTGTCACTTTTCT-----
-----
```

FC88X_01026_00414 ----------------------TCCCTTCGTCTTGAGTGTCACTTTTCT-----
-----
FC88X_01005_00833 ----------------------TCCCTTCGTCTTGAGTGTCACTTTTCT-----
-----
FC88X_01132_00580 ----------------------TCCCTTCGTCTTGAGTGTCACTTTTCT-----
-----
FC88X_01074_00545 -------------------TTCGAATTTTGTTTC--GTACGACTCCTC------
-----
FC88X_01112_00507 -----------CCGTGTGCGCTAATTTCTCTTTC--TTGC--------------
-----
FC88X_01020_00798 ------------------ACAACAAACCTTGTGCACTCCCGTCGC---------
-----
FC88X_01090_00650 --------------------TTCAGAACTGTTGGACTC----------------
-----
FC88X_01093_00488 --------------------CCAACAACTCTTTGGCCCGTGTTTCTC-------
-----
FC88X_01123_00746 --------------------CCAATAACTCTTTGGCCCGTGTTTCTC-------
-----
FC88X_01063_00594 --------------------TCTACAACTCTTCGGC------------------
-----
FC88X_00957_00665 ---------------------TCTTGTATTTT-GTGCATCACTTTTCTC-----
-----
FC88X_01064_00449 ---------------------TCTTGTATTTT-GTGCATCACTTTTCTC-----
-----
FC88X_01111_00742 ---------------------TCTTGTATTTT-GTGCATCACTTTTCTC-----
-----
FC88X_01141_01139 ---------------------TCTTGTATTTT-GTGCATCACTTTTCTC-----
-----
FC88X_00992_00294 ---------------------TCTTGTATTTT-GTGCATCGCTTTTCT------
-----
FC88X_00960_00914 ---------------------TCTTGTATTTTTGTGCATCACTTTCT-------
-----
FC88X_00991_01103 ---------------------TCTTGTATTTT-GTGCGTCACTTTTCTC-----
-----
FC88X_01121_00600 ---------------------TCACACAGTCTTTTGTGCTCCCTTTTG------
-----
FC88X_00962_00465 ---------------------TCACATTGTTT-GTGCCTCCCTTACACACC---
-----
FC88X_01001_00375 ----------------------CAGGTCTTTTGTTGCCTCTGTTGTTCT-----
-----
FC88X_00995_00330 ----------------------CCAGACCATTTGTGTTTGAGGCATCTC-----
-----
FC88X_01047_00433 ----------------------CCAGACCATTTGTGTTTGAGGCATCTC-----
-----
FC88X_01031_00552 ----------------------CCAGACCATTTGTGTTTGAGGCATCTC-----
-----
FC88X_00998_00699 ---------------------CCACAATCGTGTTTGCGTT-GGCATCTC-----
-----
FC88X_01046_00509 ---------------------CCACAATCGTGTTTGCGTT-GGCATCTC-----
-----
FC88X_01018_00878 ---------------------CCACAATCGTGTTTGCGTT-GGCATCTC-----
-----
FC88X_01068_00443 ---------------------CCACAATCGTGTTTGCGTT-GGCATCTC-----
-----
FC88X_01094_00646 ---------------------CCACAATCGTGTTTGCGTT-GGCATCTC-----
-----

```
FC88X_01117_00585      ---------------------CCACAATCGTGTTTGCGTT-GGCATCTC-----
-----
FC88X_01044_00825      ---------------------CCACGATCGTGTTTGCGTT-GGCATCTC-----
-----
FC88X_00963_00436      ---------------------TCACATTCATGTTT-GTGACCGTGTCTC-----
-----
FC88X_00973_00591      ---------------------TCACATTCATGTTT-GTGACCGTGTCTC-----
-----
FC88X_00951_00857      ---------------------TCACATTCATGTTT-GTGACCGTGTCTC-----
-----
FC88X_00982_00966      ---------------------TCACATTCATGTTT-GTGACCGTGTCTC-----
-----
FC88X_01002_00407      ---------------------TCACATTCATGTTT-GTGACCGTGTCTC-----
-----
FC88X_01003_00460      ---------------------TCACATTCATGTTT-GTGACCGTGTCTC-----
-----
FC88X_01034_00514      ---------------------TCACATTCATGTTT-GTGACCGTGTCTC-----
-----
FC88X_01017_01131      ---------------------TCACATTCATGTTT-GTGACCGTGTCTC-----
-----
FC88X_01089_00881      ---------------------TCACATTCATGTTT-GTGACCGTGTCTC-----
-----
FC88X_01065_01160      ---------------------TCACATTCATGTTT-GTGACCGTGTCTC-----
-----
FC88X_01103_00445      ---------------------TCACATTCATGTTT-GTGACCGTGTCTC-----
-----
FC88X_01106_00502      ---------------------TCACATTCATGTTT-GTGACCGTGTCTC-----
-----
FC88X_01101_00660      ---------------------TCACATTCATGTTT-GTGACCGTGTCTC-----
-----
FC88X_01124_00930      ---------------------TCACATTCATGTTT-GTGACCGTGTCTC-----
-----
FC88X_01108_01140      ---------------------TCACATTCATGTTT-GTGACCGTGTCTC-----
-----
FC88X_01124_01157      ---------------------TCACATTCATGTTT-GTGACCGTGTCTC-----
-----
FC88X_01130_01150      ---------------------TCACATTCATGTTT-GTGACCGTGTCTC-----
-----
FC88X_01029_01236      ---------------------TCACATTCATGTTT-GTGACCGTGTCTCCC---
-----
FC88X_01000_00293      ---------------------TCACATTCATATTT-GTGACCGTGTCTC-----
-----
FC88X_00957_00578      ---------------------TCACATTCATGTTTTGTGACCGTGTCTC-----
-----
FC88X_00976_00561      ---------------------TCACATTTATGTTT-GTGACCGTGTCTC-----
-----
FC88X_01141_00590      ---------------------TCACATTCGTGTTT-GTGACCGTGTCTC-----
-----
FC88X_01078_00859      ---------------------TCACACTCATGTTT-GTGACCGTGTCTC-----
-----
FC88X_00965_00562      ---------------------TTACATTCATGTTT-GTGACCGTGTCTCCC---
-----
FC88X_00955_00844      ---------------------TCACATTCATGCTT-GTGACGGTGTCTC-----
-----
FC88X_01051_00558      ---------------------TCACATTCATGTTT-GTGACCGCGTCTC-----
-----
```

FC88X_00974_00395 --------------------ATCACGTAG-CGTTCGTGTGTCGATTCTC-----
-----
FC88X_01003_00387 --------------------ATCACGTAGGCGTTCGCGTGTCGATTCTC-----
-----
FC88X_01089_00928 --------------------ATCACGTAG-CGTTCGTGTGTCGATTCTC-----
-----
FC88X_01057_01180 --------------------ATCACGTAG-CGTTCGTGTGTCGATTCTC-----
-----
FC88X_01126_00806 --------------------ATCACGTAG-CGTTCGTGTGTCGATTCTC-----
-----
FC88X_01115_00888 --------------------ATCACGTAG-CGTTCGTGTGTCGATTCTC-----
-----
FC88X_01089_00429 --------------------ATCACGTAG-CGTTCGTGTGTCGATTCTCCC---
-----
FC88X_00994_00681 --------------------------CTCTCGTTTGTGCGGCGCCT-
CTGTGTT-----
FC88X_01014_00437 --------------------------
CTGCCGATAGTGTCGCGTCCGCTGTGCC-----
FC88X_01069_00568 -----------------------ACGAAA-CCTCTGTGT---------------
-----
FC88X_01039_00400 ----------------------TTCCTCTGTGTCAAGAGCGAGTGTCTC-----
-----
FC88X_01034_00797 ------------------------TTTTCTCCTAGTTTGCTGCGTCTTTGC---
-----
FC88X_01111_00788 ------------------------TTTTCTCCTAGTTTGCTGCGTCTTTGC---
-----
FC88X_01097_00796 ----------------------TTTTTTGTACTATTTCTCTGCGTTGTTG----
-----
FC88X_01114_00475 --------------------------TTCTACGAA-------------------
-----
FC88X_00999_00292 -----------------CCACCAACTATTGTTCG----TCGCCGTTCT------
-----
FC88X_00991_00400 -----------------CCACCAACTATTGTTCG----TCGCCGTTCT------
-----
FC88X_00986_00491 -----------------CCACCAACTATTGTTCG----TCGCCGTTCT------
-----
FC88X_00970_00586 -----------------CCACCAACTATTGTTCG----TCGCCGTTCT------
-----
FC88X_00956_00665 -----------------CCACCAACTATTGTTCG----TCGCCGTTCT------
-----
FC88X_01040_00772 -----------------CCACCAACTATTGTTCG----TCGCCGTTCT------
-----
FC88X_01005_00931 -----------------CCACCAACTATTGTTCG----TCGCCGTTCT------
-----
FC88X_01057_00820 -----------------CCACCAACTATTGTTCG----TCGCCGTTCT------
-----
FC88X_01134_00589 -----------------CCACCAACTATTGTTCG----TCGCCGTTCT------
-----
FC88X_01147_00701 -----------------CCACCAACTATTGTTCG----TCGCCGTTCT------
-----
FC88X_01123_00871 -----------------CCACCAACTATTGTTCG----TCGCCGTTCT------
-----
FC88X_01133_01214 -----------------CCACCAACTATTGTTCG----TCGCCGTTCT------
-----
FC88X_00990_00821 -----------------CCACCAACTATTGGTCG----TCGCCGTTCT------
-----

```
FC88X_01103_00562      -----------------CCACCAACTATTGCTCG----TCGCCGTTCT------
-----
FC88X_01065_00586      -----------------CCTCCAACTATTGTTCG----TCGCCGTTCT------
-----
FC88X_01097_00425      -----------------CCACCAACTATTATTCG----TCGCCGTTCT------
-----
FC88X_00952_00386      -----------------CCACCAACTACTGTTCG----TCGCCGTTCT------
-----
FC88X_01034_00307      -----------------TTACTAACTACTAGGCG----CAACTGTTCT------
-----
FC88X_01044_00437      -----------------TTACTAACTACTAGGCG----CAACTGTTCTCC----
-----
FC88X_01130_01205      -----------------TCACTAACTACTAGGCG----CAACTGTTCT------
-----
FC88X_00987_00842      -----------------TTACTAACTACTAGGCG----CGACTGTTCT------
-----
FC88X_01072_00549      -------------------TGTCTCGGTTTCGCG----CAGAACTTTCTC----
-----
FC88X_01099_00829      ------------------CTGTCTCTGAGTCGGG----CCAAATTATTCT----
-----
FC88X_00974_00369      ------------------TTCCCTCAGTGTGGGA----CGGACCTGTTCTT---
-----
FC88X_00998_00725      --------------------------------GG----CCGAACGT--------
-----
FC88X_00966_00997      ------------------CTCCCCACAACTAAAG----CCGATCCTTCGC----
-----
FC88X_01055_00937      -------------------TCCCATCGTTTGAGG----ACAGCTCCTTATC---
-----
FC88X_01124_00654      ----------------CTTTACTAACGTTAGAAG----CCGGTTCTC-------
-----
FC88X_01029_00428      --------------------CCTCACATTAGTTG----ACGCGCTCCCCAT---
-----
FC88X_01004_01267      --------------------CCTCACATTAGTTG----ACGCGCTCCCCAT---
-----
FC88X_01081_00480      -------------------------TACTAACAA----TCGT------------
-----
FC88X_01033_00829      ---------------------------------T----TCATATGC--------
-----
FC88X_01057_00502      --------------------------ACAAAATG----
TCATATTCTCGACGGGCTCGA
FC88X_01086_00904      -------------------------TACAAATCC----
CTTAGTTCCCGATCACCT---
FC88X_00958_00465      -----------------TCTCCCTAT----TTTGCGCGACAAGTTGCA------
-----
FC88X_01010_01028      -----------------TCTCCCTAT----TTTGCGCGACAAGTTGCA------
-----
FC88X_01064_00597      -----------------TCTCCCTAT----TTTGCGCGACAAGTTGCA------
-----
FC88X_01100_00709      -----------------TCTCCCTAT----TTTGCGCGACAAGTTGCA------
-----
FC88X_01111_00818      -----------------TCTCCCTAT----TTTGCGCGACAAGTTGCA------
-----
FC88X_01085_01173      -----------------TCTCCCTAT----TTTGCGCGACAAGTTGCACC----
-----
FC88X_01027_00888      -----------------TCTCCCTAT----TCTGCGCGACAAGTTGCA------
-----
```

```
FC88X_00986_01025       -----------------TCTCCCTAT----TTTGCGCGACAAGCTGCA------
-----
FC88X_01026_01259       ------------------CTCCCCAT----CGTGCGCCATTAGAGTGGC-----
-----
FC88X_00988_00852       -----------------TCCGCCAAT----TCTGCGCGTTAGCATTCTC-----
-----
FC88X_01033_01205       -----------------TCCGCCAAT----TCTGCGCGTTAGCATTCTC-----
-----
FC88X_01047_01255       -----------------TCCGCCAAT----TCTGCGCGTTAGCATTCTC-----
-----
FC88X_01092_00582       -----------------TCCGCCAAT----TCTGCGCGTTAGCATTCTC-----
-----
FC88X_01054_00810       -----------------TCCGCCAAT----TCTGCGCGTTAGCATTCTC-----
-----
FC88X_01052_00947       -----------------TCCGCCAAT----TCTGCGCGTTAGCATTCTC-----
-----
FC88X_01139_00890       -----------------TCCGCCAAT----TCTGCGCGTTAGCATTCTC-----
-----
FC88X_01126_00910       -----------------TCCGCCAAT----TCTGCGCGTTAGCATTCTC-----
-----
FC88X_01048_00872       -----------------TCCGCCAAT----TCTGCGCGTTAGCATTCTCAT---
-----
FC88X_01098_01260       ------------------TCGCCAAT----TCTGCGCGTTAGCATTCTC-----
-----
FC88X_01083_00577       -----------------TCCGCCAAT----TCTGCGCGT---------------
-----
FC88X_01008_00372       -----------------TCTCCCAAT----CATCTGCGCGCGCATGAT------
-----
FC88X_01034_00751       -----------------TCTCCCAAT----CGTTCGCGTCAGCCCGCG------
-----
FC88X_01006_00318       ------------------TCCCCAAC----TTTCCCTTTTGCGCTTCACG----
-----
FC88X_01012_01158       ---------------------CAGCTGTTTTTACGCGCCTACTATTCC------
-----
FC88X_01145_00749       ---------------------CAGCTGTTTTTACGCGCCTACTATTCC------
-----
FC88X_01128_01136       ---------------------CAGCTGTTTTTACGCGCCTACTATTCC------
-----
FC88X_00990_00280       --------------------TCAGCTATTTA-ACCTTTCAACTATTCT------
-----
FC88X_01082_00643       --------------------TCGGCTATTAC-ACGGTGCCACTGTCTCCC----
-----
FC88X_01065_00408       ----------------------TTCAGCCTTATAGGTCGTCCTGTTCTA-----
-----
FC88X_01138_00874       ----------------------TTCAGCCTTATAGGTCGTCCTGTTCTA-----
-----
FC88X_00984_00409       ---------------------GTTCAGCCTTATAGGTCGTCCTGTTCCA-----
-----
FC88X_00970_00706       ----------------------ACCAGCACTCCATGTTGTCATGTTCTC-----
-----
FC88X_01000_00276       ----------------------ACCAGCACTCCATGTTGTC-------------
-----
FC88X_01069_01174       --------------------TCTTCAGCCCAACAT-TTGTCCTATTCT------
-----
FC88X_01111_00712       --------------------TCTTCAGCCCAACAT-TTGTCCTATTCT------
-----
```

```
FC88X_01003_00518       --------------------TCACCAAACCTCCCA-TGGTGCTATTGC------
-----
FC88X_01018_00627       --------------------TCACCAAACCTCCCA-TGGTGCTATTGC------
-----
FC88X_01061_00489       --------------------TCACCAAACCTCCCA-TGGTGCTATTGC------
-----
FC88X_01011_00780       --------------------TCACCAAGCCTCCCA-TGGTGCTATTGC------
-----
FC88X_00951_00964       --------------------TCACCAAACCTCTCA-TGGTGCTATTGC------
-----
FC88X_00992_00746       --------------------TCACCACTCCCTTTG-AACTATTCTTGT------
-----
FC88X_00996_00759       ---------------------CTAAGGCGTCTCTCTCATTGCTATTCT------
-----
FC88X_01133_00563       ---------------------CTAAGGCGTCTCTCTCATTGCTATTCT------
-----
FC88X_00965_00451       -----------------------ATAGCGTC-CTCCCAAAACCAGTCTGTT---
-----
FC88X_01056_00875       ---------------------------AACTACTCCA-----------------
-----
FC88X_01112_00990       ---------------------TAACGGCACCACTCCACGCTCTATATT------
-----
FC88X_00954_00370       ----------------------TTTCCTACAACTCATC----------------
-----
FC88X_00975_00733       ----------------------TTTCCTACAACTCATCGG--------------
-----
FC88X_00985_00845       ----------------------TTTCCTACAACTATCCGGGCGA----------
-----
FC88X_01026_00637       ---------------------TTCCCTGACAACAATTACCGCGCGCCT------
-----
FC88X_01140_00719       --------------------TTTCCCTTACAACTA-------------------
-----
FC88X_01051_00739       --------------------------CTACAACAAT------------------
-----
FC88X_00969_00421       --------------------------CTACAACAA-------------------
-----
FC88X_00986_00422       -------------TTAACTCTTTCCTCTACGCAACTATTCTTC-----------
-----
FC88X_01095_01153       --------------------------CTAC-CAACT------------------
-----
FC88X_01050_00698       -------------------------TCTACGAAATTT-----------------
-----
FC88X_01049_00805       ----------------------CCTTCTCCAGAACCAAGTCGTGTGAC------
-----
FC88X_01032_00960       -------------------------TCTACAAAACTCAAGATTGG---------
-----
FC88X_01045_00352       ------------------GACACTCCCTA--GAAACATGCGCCATTTG------
-----
FC88X_01023_00646       -------------------ACACTCCCTA--GAAACATGCGCCATTTG------
-----
FC88X_01030_00797       -------------------ACACTCCCTA--GAAACATGCGCCATTTG------
-----
FC88X_01058_00372       -------------------ACACTCCCTA--GAAACATGCGCCATTTG------
-----
FC88X_01074_00725       -------------------ACACTCCCTA--GAAACATGCGCCATTTG------
-----
```

```
FC88X_01122_00704       -------------------ACACTCCCTA--GAAACATGCGCCATTTG------
-----
FC88X_01140_00619       -------------------ACACTCCCTTACGAACCACGCGCGAAT--------
-----
FC88X_01036_00583       --------------------------CTA--CAAACAA----------------
-----
FC88X_01018_00401       ---------------ACTAACAACAACC--------------------------
-----
FC88X_01032_00772       -------------------ACAACAACCAGTCACCTTCGCACTCTC--------
-----
FC88X_00992_00697       -------------------------CCGATAAAACTTCGCAGCCGTCCCATT--
-----
FC88X_01047_00605       ----------------------CCGATAAAAAAACTTCGCAGTCGTCCCATT--
-----
FC88X_01076_01079       ----------------------CATGCAACAAAACTGCGTCGCTGTCCC-----
-----
FC88X_01091_01096       -------------------------ACAAGAACAGTTCGAG-------------
-----
FC88X_01028_00305       -------------------GTGCAAAAAAAAAAACCTCTCGAGC----------
-----
FC88X_00959_00519       ------------------------------TCATCGTAACCGTATGAGC-
CCCATTCA-
FC88X_01039_00543       ------------------------------
TCATCGTAACCGTATGAGCTCCCATTTA-
FC88X_01043_00583       ---------------------TTCAGTTCCTCCACTCCACACCGTATT------
-----
FC88X_01114_01174       -------------------CCAACCGTTCCCCGACCTCACCGTATA--------
-----
FC88X_00995_00877       -------------------CCTGGGACCCTCCGCCTTCAACGTATT--------
-----
FC88X_00999_00522       -------------------------------------CACAACGTAAT------
-----
FC88X_00979_00739       -------------------------TCCCGACATCTTCAACGTGCTATTCTA--
-----
FC88X_00986_00846       -------------------------TCCCGACATCTTCAACGTGCTATTCTA--
-----
FC88X_01071_00453       -------------------------TCCCGACATCTTCAACGTGCTATTCTA--
-----
FC88X_01062_00761       ------------------------------------TCAACGTG----------
-----
FC88X_00972_00648       -----------------TTGTAACCATTTCCCTG-CCAACG-TATCT-------
-----
FC88X_01008_00399       -------------------TTAACTAATCCCTTGTCCCACG-TGTC--------
-----
FC88X_00964_01259       -------------------TCCCCCACTAAATCGTCTGCAGATGTCC-------
-----
FC88X_01023_00832       -------------------TTACCAACTAACCTGTCTGCCG-TGTC--------
-----
FC88X_01081_00923       ------------------TTTGTACA-TCGCCCG-TGCAACATGCTT-------
-----
FC88X_01089_01093       ------------------TTTGTACA-TCGCCCG-TGCAACATGCTT-------
-----
FC88X_01104_00736       ------------------TTTGTACA-TCGCCCG-TGCAACATGCTT-------
-----
FC88X_01125_00919       ------------------TTTGTACAATCGCCCGGTGCAACATGCTT-------
-----
```

FC88X_01113_00715 ---------------CCCCCAATGAGCTTGCCCCGTCCTGTA------------
-----
FC88X_01133_00965 ------------------GCAATAAGC---------------------------
-----
FC88X_00983_00632 --------------------CGCAATTTGGAC-ATGTGGCCGCTTCTC------
-----
FC88X_00967_00852 --------------------CGCAATTTGGAC-ATGTGGCCGCTTCTC------
-----
FC88X_01045_00497 --------------------CGCAATTTGGAC-ATGTGGCCGCTTCTC------
-----
FC88X_01065_00803 --------------------CGCAATTTGGAC-ATGTGGCCGCTTCTC------
-----
FC88X_01128_00850 --------------------CGCAATTTGGAC-GTGTGGCCGCTTCTC------
-----
FC88X_00995_00608 ------------------GTCGCACTGTCGTCGATGTGTGCCTGTC--------
-----
FC88X_01104_00501 -------------------TCGCACTGTCGTCGATGTGTGCCTGTC--------
-----
FC88X_01089_00723 --------------------CGCAATTTGGAC-ATGTGGGCCGGCT--------
-----
FC88X_01038_00824 ---------------CGTGGTACTTTGTCGCC-ACCTCATCTCCC---------
-----
FC88X_01140_01144 ---------------TGTGGTGCAATTGTGCC-GCCT-ATCTGC----------
-----
FC88X_00993_00830 ------------------GGCGCAACTTTGTG-ACGTCCACTTCTC--------
-----
FC88X_00966_00346 -----------------CCAACATCTCTTCCACG--CGGCGATATT--------
-----
FC88X_01030_00784 -----------------CCAAGTTCACTCCC-TG--CGGCGACATCTT------
-----
FC88X_00965_00597 -------------------------TCTCCCTGG--
GAACGACATGGCGCACAA-----
FC88X_00951_00408 -------------------CGTGCAGGTATATCTGGTCGTTATGCTC-------
-----
FC88X_00994_00765 -------------------CGTGCAGGTATATCTGGTCGTTATGCTC-------
-----
FC88X_00994_00826 -------------------CGTGCAGGTATATCTGGTCGTTATGCTC-------
-----
FC88X_01049_00370 -------------------CGTGCAGGTATATCTGGTCGTTATGCTC-------
-----
FC88X_01003_00817 -------------------CGTGCAGGTATATCTGGTCGTTATGCTC-------
-----
FC88X_01026_00841 -------------------CGTGCAGGTATATCTGGTCGTTATGCTC-------
-----
FC88X_01071_00515 -------------------CGTGCAGGTATATCTGGTCGTTATGCTC-------
-----
FC88X_01114_00828 -------------------CGTGCAGGTATATCTGGTCGTTATGCTC-------
-----
FC88X_00986_00836 -------------------CGTGCAGGTATATCCGGTCGTTATGCTC-------
-----
FC88X_00975_01203 --------------------------------CCAGTCGATAT-----------
-----
FC88X_01147_00833 --------------------------------CCAGTCGATAT-----------
-----
FC88X_01101_00878 --------------------------------CCAGTCGATAT-----------
-----

FC88X_00991_00743   ----------------------------
CCATCCAGTTGTGACTCGCGCTATTGTC---
FC88X_01004_00585   ------------------------CTTCCCTTCCAGTTGTAAC--GCGATATT-
-----
FC88X_00952_00544   ------------------------CTACTCTTCAAGCCGTGACT-GCTATA---
-----
FC88X_00987_00630   --------------------------------CCAGTCGATAC-----------
-----
FC88X_00967_00643   ------------------------CCACTTGTCATGC---TATGTCCCTTCCT-
-----
FC88X_01137_01136   --------------------CTCCCCACGTGTAAGGCACATATGTCCA------
-----
FC88X_01010_00519   -------------------ACCACCCATCTGTGGCACC--AATGTTC-------
-----
FC88X_01046_00623   ----------------TTGCTTGCTATTTTCCTTCGGACGGAC-----------
-----
FC88X_01114_01219   --------------------CCGGTAATTTCGATCGGCCCCACACGT-------
-----
FC88X_01014_00849   -------------------ATTCCAATTCGCTTTGGCTCCCATGTTCCGT----
-----
FC88X_01009_00830   -------------------CTCCCAATTGGATGACTCGCGCGTGCC--------
-----
FC88X_01039_01043   -------------------CTCCCAATTGGATGACTCGCGCGTGCC--------
-----
FC88X_01066_00862   -------------------CTCCCAATTGAATGAC-CGCGCGTGCC--------
-----
FC88X_01070_00998   ---------------------CGCAATTGG------------------------
-----
FC88X_01086_00445   -------------------CTCCCTAGTCAAGTAGCCGCTCGTGGC--------
-----
FC88X_01079_00892   -------------------TTCCCTGGTGCTTGAGCCGCGATCTTTCC------
-----
FC88X_00978_00335   -----------------TCTTCCAATTTT-G--G--GCGGTACTGTCTC-----
-----
FC88X_00995_00328   -----------------TCTTCCAATTTT-G--G--GCGGTACTGTCTC-----
-----
FC88X_00993_00944   -----------------TCTTCCAATTTT-G--G--GCGGTACTGTCTC-----
-----
FC88X_01032_00468   -----------------TCTTCCAATTTT-G--G--GCGGTACTGTCTC-----
-----
FC88X_01048_00506   -----------------TCTTCCAATTTT-G--G--GCGGTACTGTCTC-----
-----
FC88X_01141_00850   -----------------TCTTCCAATTTT-G--G--GCGGTACTGTCTC-----
-----
FC88X_00991_00394   -----------------TCTTCCAATTTTTG--G--GCGGTACTGTCTC-----
-----
FC88X_01027_00933   -------------------AACCAATTCGCGC-G--GTAGGTCTATCTC-----
-----
FC88X_00974_00404   -----------------TCTTGCAATTTCCT-----GCGGTGCATTCTC-----
-----
FC88X_01050_00415   -----------------TCTTGCAATTTCCT-----GCG-TGCATTCTC-----
-----
FC88X_00998_00411   -----------------TCTTGCAATTTCCT-----GCGGTGCATTCTC-----
-----
FC88X_00980_00471   -----------------TCTTGCAATTTCCT-----GCGGTGCATTCTC-----
-----

FC88X_00978_01275 -----------------TCTTGCAATTTCCT-----GCGGTGCATTCTC-----
-----
FC88X_01025_00380 -----------------TCTTGCAATTTCCT-----GCGGTGCATTCTC-----
-----
FC88X_01036_00378 -----------------TCTTGCAATTTCCT-----GCGGTGCATTCTC-----
-----
FC88X_01038_00435 -----------------TCTTGCAATTTCCT-----GCGGTGCATTCTC-----
-----
FC88X_01041_00912 -----------------TCTTGCAATTTCCT-----GCGGTGCATTCTC-----
-----
FC88X_01031_01001 -----------------TCTTGCAATTTCCT-----GCGGTGCATTCTC-----
-----
FC88X_01073_00404 -----------------TCTTGCAATTTCCT-----GCGGTGCATTCTC-----
-----
FC88X_01086_00494 -----------------TCTTGCAATTTCCT-----GCGGTGCATTCTC-----
-----
FC88X_01082_00864 -----------------TCTTGCAATTTCCT-----GCGGTGCATTCTC-----
-----
FC88X_01102_00719 -----------------TCTTGCAATTTCCT-----GCGGTGCATTCTC-----
-----
FC88X_01102_00807 -----------------TCTTGCAATTTCCT-----GCGGTGCATTCTC-----
-----
FC88X_01136_01000 -----------------TCTTGCAATTTCCT-----GCGGTGCATTCTC-----
-----
FC88X_01103_00482 -----------------TCTTGCAATTTCCT-----GCGGTGCATTCTCCC---
-----
FC88X_01136_01104 -----------------TCTTGCAATTTCCT-----GCGGTGCATTCTCCC---
-----
FC88X_01001_00983 -----------------TCTTGCGATTTCCT-----GCGGTGCATTCTC-----
-----
FC88X_01089_01060 -----------------TCCTGCAATTTCCT-----GCGGTGCATTCTC-----
-----
FC88X_01057_00506 -----------------TCTTGCAATTTCCT-----GCGGTGCATTCGTC----
-----
FC88X_01062_00621 -----------------TCTTGCAATTTCCT-----GCGGTGCATTTCTC----
-----
FC88X_00980_01225 -----------------TTTTGCAGTTCCCT-----GCGGTGCATTCTC-----
-----
FC88X_01024_01267 ------------------CTGACAATTCCGCCCT--GCGCTACTTCTC------
-----
FC88X_01072_00632 ------------------CTGACAATTCCCCCCT--GCGCTACTTCTC------
-----
FC88X_01024_00307 -----------AATCAGGCCTGCGTGGACT--------GGTGCGTTCTC-----
-----
FC88X_01130_00708 -----------AATCAGGCCTGCGTGGACT--------GGTGCGTTCTC-----
-----
FC88X_01117_01210 -----------------CCGTGCGTTGATTC-----ACGGTGCGTCCTC-----
-----
FC88X_01031_01151 ------------------AGACGTA-TTCGTTG-TCGCTCTGT-TCAC------
-----
FC88X_01069_01216 ------------------AGACGTA-TTCGTTG--CGCTCTGT-TCAC------
-----
FC88X_01145_00634 ------------------AGACGTA-TCCGTTT-GCGCTCTGT-TCAC------
-----
FC88X_00991_00292 ------------------AGACGTA-TTCGTTT-GCGCTCTGT-TCAC------
-----

FC88X_00997_00282 ------------------AGACGTA-TTCGTTT-GCGCTCTGT-TCAC------
-----
FC88X_00991_00339 ------------------AGACGTA-TTCGTTT-GCGCTCTGT-TCAC------
-----
FC88X_00973_00433 ------------------AGACGTA-TTCGTTT-GCGCTCTGT-TCAC------
-----
FC88X_00968_00459 ------------------AGACGTA-TTCGTTT-GCGCTCTGT-TCAC------
-----
FC88X_00961_00540 ------------------AGACGTA-TTCGTTT-GCGCTCTGT-TCAC------
-----
FC88X_00964_00809 ------------------AGACGTA-TTCGTTT-GCGCTCTGT-TCAC------
-----
FC88X_00975_00842 ------------------AGACGTA-TTCGTTT-GCGCTCTGT-TCAC------
-----
FC88X_00962_00867 ------------------AGACGTA-TTCGTTT-GCGCTCTGT-TCAC------
-----
FC88X_00983_00852 ------------------AGACGTA-TTCGTTT-GCGCTCTGT-TCAC------
-----
FC88X_00987_00851 ------------------AGACGTA-TTCGTTT-GCGCTCTGT-TCAC------
-----
FC88X_00978_00919 ------------------AGACGTA-TTCGTTT-GCGCTCTGT-TCAC------
-----
FC88X_00959_00994 ------------------AGACGTA-TTCGTTT-GCGCTCTGT-TCAC------
-----
FC88X_00971_00967 ------------------AGACGTA-TTCGTTT-GCGCTCTGT-TCAC------
-----
FC88X_00982_01214 ------------------AGACGTA-TTCGTTT-GCGCTCTGT-TCAC------
-----
FC88X_00991_01214 ------------------AGACGTA-TTCGTTT-GCGCTCTGT-TCAC------
-----
FC88X_00982_01260 ------------------AGACGTA-TTCGTTT-GCGCTCTGT-TCAC------
-----
FC88X_01018_00290 ------------------AGACGTA-TTCGTTT-GCGCTCTGT-TCAC------
-----
FC88X_01049_00328 ------------------AGACGTA-TTCGTTT-GCGCTCTGT-TCAC------
-----
FC88X_01021_00415 ------------------AGACGTA-TTCGTTT-GCGCTCTGT-TCAC------
-----
FC88X_01025_00470 ------------------AGACGTA-TTCGTTT-GCGCTCTGT-TCAC------
-----
FC88X_01030_00549 ------------------AGACGTA-TTCGTTT-GCGCTCTGT-TCAC------
-----
FC88X_01049_00601 ------------------AGACGTA-TTCGTTT-GCGCTCTGT-TCAC------
-----
FC88X_01035_00879 ------------------AGACGTA-TTCGTTT-GCGCTCTGT-TCAC------
-----
FC88X_01016_00930 ------------------AGACGTA-TTCGTTT-GCGCTCTGT-TCAC------
-----
FC88X_01044_00979 ------------------AGACGTA-TTCGTTT-GCGCTCTGT-TCAC------
-----
FC88X_01000_01037 ------------------AGACGTA-TTCGTTT-GCGCTCTGT-TCAC------
-----
FC88X_01011_01042 ------------------AGACGTA-TTCGTTT-GCGCTCTGT-TCAC------
-----
FC88X_01043_01004 ------------------AGACGTA-TTCGTTT-GCGCTCTGT-TCAC------
-----

FC88X_01047_01036 ------------------AGACGTA-TTCGTTT-GCGCTCTGT-TCAC------
-----
FC88X_01017_01202 ------------------AGACGTA-TTCGTTT-GCGCTCTGT-TCAC------
-----
FC88X_01090_00593 ------------------AGACGTA-TTCGTTT-GCGCTCTGT-TCAC------
-----
FC88X_01050_00771 ------------------AGACGTA-TTCGTTT-GCGCTCTGT-TCAC------
-----
FC88X_01055_00898 ------------------AGACGTA-TTCGTTT-GCGCTCTGT-TCAC------
-----
FC88X_01075_00853 ------------------AGACGTA-TTCGTTT-GCGCTCTGT-TCAC------
-----
FC88X_01058_00946 ------------------AGACGTA-TTCGTTT-GCGCTCTGT-TCAC------
-----
FC88X_01087_00936 ------------------AGACGTA-TTCGTTT-GCGCTCTGT-TCAC------
-----
FC88X_01094_00939 ------------------AGACGTA-TTCGTTT-GCGCTCTGT-TCAC------
-----
FC88X_01093_01159 ------------------AGACGTA-TTCGTTT-GCGCTCTGT-TCAC------
-----
FC88X_01104_00561 ------------------AGACGTA-TTCGTTT-GCGCTCTGT-TCAC------
-----
FC88X_01119_00599 ------------------AGACGTA-TTCGTTT-GCGCTCTGT-TCAC------
-----
FC88X_01131_00594 ------------------AGACGTA-TTCGTTT-GCGCTCTGT-TCAC------
-----
FC88X_01113_00733 ------------------AGACGTA-TTCGTTT-GCGCTCTGT-TCAC------
-----
FC88X_01139_00906 ------------------AGACGTA-TTCGTTT-GCGCTCTGT-TCAC------
-----
FC88X_01103_00978 ------------------AGACGTA-TTCGTTT-GCGCTCTGT-TCAC------
-----
FC88X_01133_01001 ------------------AGACGTA-TTCGTTT-GCGCTCTGT-TCAC------
-----
FC88X_00984_00488 ------------------AGACGTA-TTCGTTT-GCGCTCTGT-TCACCC----
-----
FC88X_01036_00451 ------------------AGACGTA-TTGGTTT-GCGCTCTGT-TCAC------
-----
FC88X_00972_00704 ------------------AGACGTA-TTCGTCT-GCGCTCTGT-TCAC------
-----
FC88X_01030_00347 ------------------AGACGTA-TTCGTCT-GCGCTCTGT-TCAC------
-----
FC88X_00965_00832 ------------------AGACGTA-TTCGTTT-GCGCTCTGC-TCACCC----
-----
FC88X_01106_00675 ------------------AGACGTA-TTCGTTT-GCGCTCTGC-TCAC------
-----
FC88X_01049_00466 ------------------AGACGCA-TTCGTTT-GCGCTCTGT-TCAC------
-----
FC88X_01119_00591 ------------------AGACGCA-TTCGTTTTGCGCTCTGT-TCAC------
-----
FC88X_01103_00879 ------------------AGACGGA-TTCGTTT-GCGCTCTGT-TCAC------
-----
FC88X_01081_01076 ------------------AGACGTA-TTCGTTT-GCGCTCTGT-TCGC------
-----
FC88X_01142_00920 ------------------AGACGTA-TTCGTTT-GCGCTCTGT-TCGC------
-----

```
FC88X_01079_01258       -----------------GAGACGTAATTCGTTT-GCGCTCTGT-TCGC------
-----
FC88X_01068_00918       ------------------AGACGTATTTCGTTT-GCGCTCTG------------
-----
FC88X_00980_00863       ------------------AGACGTA-TTCGTTTTGCGCTCTGT-T---------
-----
FC88X_00960_00500       ------------------AGACGTA-TTCGTTT-GCGCTCTGT-TCTC------
-----
FC88X_01000_01009       ------------------AGACGTA-TTCGTTT-GCGCTCTGT-TCTC------
-----
FC88X_01031_00340       ------------------AGACGTA-TTCGTTT-GCGCTCTTCGTCAC------
-----
FC88X_00984_00732       -----------------CCGACATACTTAGTTTGCCGCATTCTC----------
-----
FC88X_00994_00997       -----------------CCGACATACTTAGTTTGCCGCATTCTC----------
-----
FC88X_01043_00405       -----------------CCGACGTACTTAGTTTGCCGCATTCTC----------
-----
FC88X_01056_00470       -----------------CCGACGTGTTCATTTCGCTGTATTCTC----------
-----
FC88X_01077_00943       -----------------CCGACGTGTTCATTTCGCTGTATTCTC----------
-----
FC88X_01015_01023       -----------------CCGACGCGTTCATTTCGCTGTATTCTC----------
-----
FC88X_01108_01115       -----------------CCGACGTGTTCACTTCGCTGTATTCTC----------
-----
FC88X_00990_00793       -----------------CCAGACGACGGACATCGGAGGCATTCT----------
-----
FC88X_01115_00558       --------------CTCCCTGACGACTAAC-TCGATGACATT------------
-----
FC88X_01020_00812       --------------------AACGACGTACTTCGT-------------------
-----
FC88X_00986_00740       -------------------CTCTGCTCTCTCCAACGTCGTGGTGTT--------
-----
FC88X_01072_00462       ------------------TCTCCGTTGTCTCCTGAGTCGGTGTCGCCC------
-----
FC88X_00991_00340       --------------------------TTCACGTAAGTCGTT-------------
-----
FC88X_00963_00364       ---------------CCAACAGGTATCTGATTGCGCGTTCTC------------
-----
FC88X_00982_00850       ---------------CCAACAGGTATCTGATTGCGCGTTCTC------------
-----
FC88X_00981_01033       ---------------CCAACAGGTATCTGATTGCGCGTTCTC------------
-----
FC88X_01047_00408       ---------------CCAACAGGTATCTGATTGCGCGTTCTC------------
-----
FC88X_01085_00779       ---------------CCAACAGGTATCTGATTGCGCGTTCTC------------
-----
FC88X_01104_01170       ---------------CCAACAGGTATCTGATCGCGCGTTCCC------------
-----
FC88X_00972_00418       ---------------CCAACAGGTATCTAATTGCGCGTTCTC------------
-----
FC88X_01006_00386       ---------------CCAAGACGTAACCGGTACCGCATTCTC------------
-----
FC88X_00970_01014       -----------------AATACGTAC----------------------------
-----
```

```
FC88X_01043_00584      --------------CTTGGGTCAAGCCTAAGACTATTTCTT-------------
-----
FC88X_01025_00970      --------------CTTGGGCCAAGCCTAAGACTATTTCTT-------------
-----
FC88X_01124_00587      ----------------------AGACGTATTAGAATTCCCC-------------
-----
FC88X_01132_01168      ----------------------AGACGTATT-----------------------
-----
FC88X_01087_00491      ---------------ATCAACCCAAGGCAATAGTTATCTCTT------------
-----
FC88X_01056_01213      -------------TAACCAATCCGACGTGGTACCAATCTC--------------
-----
FC88X_00950_00433      ------------------CGCGTTT-CTATTGGCGTGTCTATATCTC-------
-----
FC88X_00950_00820      ------------------CGCGTTT-CTATTGGCGTGTCTATATCTC-------
-----
FC88X_00988_00916      ------------------CGCGTTT-CTATTGGCGTGTCTATATCTC-------
-----
FC88X_01027_00617      ------------------CGCGTTT-CTATTGGCGTGTCTATATCTC-------
-----
FC88X_01009_00442      -----------------CTTCGTTC-TTACTGGCGGGCTTATCTC---------
-----
FC88X_01079_00714      -----------------CTTCGTTC-TTACTGGCGGGCTTATCTC---------
-----
FC88X_01057_00849      -----------------CTTCGTCC-TTACTGGCGGGCTTACCTCTCC------
-----
FC88X_00999_00841      -----------------CTTCGTAT-TGTGGTTGATGCC---------------
-----
FC88X_01032_00352      -----------------CTTCGTCT-TGCTGGTGATGCCTATTG----------
-----
FC88X_00993_00483      ------------------TGACGTG-TTGT-GCGGCGCTCACT-GTCTC-----
-----
FC88X_01144_00589      ------------------TGACGTG-TTGT-GCAGCGCTCACT-GTCTC-----
-----
FC88X_00964_00814      ------------------TGACGTG-TTGT-GCGGCGCTCACT-GTCTC-----
-----
FC88X_00967_00885      ------------------TGACGTG-TTGT-GCGGCGCTCACT-GTCTC-----
-----
FC88X_00957_00917      ------------------TGACGTG-TTGT-GCGGCGCTCACT-GTCTC-----
-----
FC88X_00984_00986      ------------------TGACGTG-TTGT-GCGGCGCTCACT-GTCTC-----
-----
FC88X_00990_00998      ------------------TGACGTG-TTGT-GCGGCGCTCACT-GTCTC-----
-----
FC88X_01023_00416      ------------------TGACGTG-TTGT-GCGGCGCTCACT-GTCTC-----
-----
FC88X_01026_00538      ------------------TGACGTG-TTGT-GCGGCGCTCACT-GTCTC-----
-----
FC88X_01024_00639      ------------------TGACGTG-TTGT-GCGGCGCTCACT-GTCTC-----
-----
FC88X_01045_00627      ------------------TGACGTG-TTGT-GCGGCGCTCACT-GTCTC-----
-----
FC88X_01020_00678      ------------------TGACGTG-TTGT-GCGGCGCTCACT-GTCTC-----
-----
FC88X_01031_00987      ------------------TGACGTG-TTGT-GCGGCGCTCACT-GTCTC-----
-----
```

```
FC88X_01029_01007      ------------------TGACGTG-TTGT-GCGGCGCTCACT-GTCTC-----
-----
FC88X_01039_01120      ------------------TGACGTG-TTGT-GCGGCGCTCACT-GTCTC-----
-----
FC88X_01049_01186      ------------------TGACGTG-TTGT-GCGGCGCTCACT-GTCTC-----
-----
FC88X_01083_00393      ------------------TGACGTG-TTGT-GCGGCGCTCACT-GTCTC-----
-----
FC88X_01089_00432      ------------------TGACGTG-TTGT-GCGGCGCTCACT-GTCTC-----
-----
FC88X_01056_00461      ------------------TGACGTG-TTGT-GCGGCGCTCACT-GTCTC-----
-----
FC88X_01069_00452      ------------------TGACGTG-TTGT-GCGGCGCTCACT-GTCTC-----
-----
FC88X_01056_00526      ------------------TGACGTG-TTGT-GCGGCGCTCACT-GTCTC-----
-----
FC88X_01092_00506      ------------------TGACGTG-TTGT-GCGGCGCTCACT-GTCTC-----
-----
FC88X_01092_00518      ------------------TGACGTG-TTGT-GCGGCGCTCACT-GTCTC-----
-----
FC88X_01086_00837      ------------------TGACGTG-TTGT-GCGGCGCTCACT-GTCTC-----
-----
FC88X_01056_01082      ------------------TGACGTG-TTGT-GCGGCGCTCACT-GTCTC-----
-----
FC88X_01050_01197      ------------------TGACGTG-TTGT-GCGGCGCTCACT-GTCTC-----
-----
FC88X_01080_01174      ------------------TGACGTG-TTGT-GCGGCGCTCACT-GTCTC-----
-----
FC88X_01076_01204      ------------------TGACGTG-TTGT-GCGGCGCTCACT-GTCTC-----
-----
FC88X_01108_00501      ------------------TGACGTG-TTGT-GCGGCGCTCACT-GTCTC-----
-----
FC88X_01117_00765      ------------------TGACGTG-TTGT-GCGGCGCTCACT-GTCTC-----
-----
FC88X_01135_00988      ------------------TGACGTG-TTGT-GCGGCGCTCACT-GTCTC-----
-----
FC88X_01143_01250      ------------------TGACGTG-TTGT-GCGGCGCTCACT-GTCTC-----
-----
FC88X_01095_00467      ------------------TGACGTG-TTGT-GCGGCGCTCACT-GTCTCC----
-----
FC88X_00980_00913      ------------------TGACGTG-TTGT-GCGGCGCTCACT-GTCTCCC---
-----
FC88X_00970_01134      ------------------TGACGTG-TTGT-GCGGCGCTCACT-GTCTCCC---
-----
FC88X_00974_00933      ------------------TGACGTG-TTGT-GTGGCGCTCACT-GTCTC-----
-----
FC88X_00987_00870      ------------------TGACGTG-TTGT-GCGGCGCTCACT-ATCTC-----
-----
FC88X_01080_00408      ------------------TGACGTG-TTGT-GCGGCGCTCACTGATCTC-----
-----
FC88X_01089_01132      ------------------TGACGTA-TTGT-GCGGCGCTCACT-GTCTC-----
-----
FC88X_00972_00555      ------------------TGACGTG-TTGTCGCGGCGCT---------------
-----
FC88X_01049_01122      ------------------TGACGTG-TTGT-GCGGCGCTCACTGTCCTC-----
-----
```

FC88X_01007_00294 ------------------TGACGTG-TTGT-GCGGCG-TCACT-GTCCC-----
-----
FC88X_01014_00487 ------------------TGACGTG-TTGT-GC---------------------
-----
FC88X_00984_00564 ---------------------------TGACGTGATT-----------------
-----
FC88X_00971_00796 ---------------TTCTGACGGA-TTGCTCTGACTATTCAC-----------
-----
FC88X_01060_00351 -----------------TGGACGTG-TTTGTGTGGCGCTCCACCTGTC-------
-----
FC88X_00961_00544 ------------------CTACGTTTAAACGATCGTGT---TGTTCTC------
-----
FC88X_01011_00551 ------------------CTACGTTTAACCGA-CGTGT---TGTTCTC------
-----
FC88X_01006_00940 ------------------CTACGTTTAATCGATCGTGT---TGTTCTC------
-----
FC88X_01052_00402 ------------------CTACGTTTAATCGATCGTGT---TGTTCTC------
-----
FC88X_00990_00704 ------------------CGACGTTTAACCGATCATGT---TGTTCTC------
-----
FC88X_01050_01195 ------------------CTACGTTTAACCGATCGCGT---TGTTCTC------
-----
FC88X_01029_01034 -----------------GCTACGTTTAACCGATCGTGT---TGTTCTC------
-----
FC88X_00964_00371 ------------------CTACGTTTAACCGATCGTGT---TGTTCTC------
-----
FC88X_00991_00900 ------------------CTACGTCTAACCGATCGTGT---TGTTCTC------
-----
FC88X_01022_00379 ------------------CTACGT-TATCCGATCGTGT---TGTTCTC------
-----
FC88X_00999_00365 ------------------CTACGTTTAACCGATCGTGT---TGTTCTC------
-----
FC88X_00952_00489 ------------------CTACGTTTAACCGATCGTGT---TGTTCTC------
-----
FC88X_00973_00530 ------------------CTACGTTTAACCGATCGTGT---TGTTCTC------
-----
FC88X_00968_00605 ------------------CTACGTTTAACCGATCGTGT---TGTTCTC------
-----
FC88X_00989_00726 ------------------CTACGTTTAACCGATCGTGT---TGTTCTC------
-----
FC88X_01045_00676 ------------------CTACGTTTAACCGATCGTGT---TGTTCTCC-----
-----
FC88X_00974_00794 ------------------CTACGTTTAACCGATCGTGT---TGTTCTC------
-----
FC88X_00988_00765 ------------------CTACGTTTAACCGATCGTGT---TGTTCTC------
-----
FC88X_00981_00833 ------------------CTACGTTTAACCGATCGTGT---TGTTCTC------
-----
FC88X_00956_00881 ------------------CTACGTTTAACCGATCGTGT---TGTTCTC------
-----
FC88X_00974_00959 ------------------CTACGTTTAACCGATCGTGT---TGTTCTC------
-----
FC88X_01005_00274 ------------------CTACGTTTAACCGATCGTGT---TGTTCTC------
-----
FC88X_01008_00310 ------------------CTACGTTTAACCGATCGTGT---TGTTCTC------
-----

FC88X_01046_00337 ------------------CTACGTTTAACCGATCGTGT---TGTTCTC------
-----
FC88X_01004_00466 ------------------CTACGTTTAACCGATCGTGT---TGTTCTC------
-----
FC88X_01009_00488 ------------------CTACGTTTAACCGATCGTGT---TGTTCTC------
-----
FC88X_01023_00464 ------------------CTACGTTTAACCGATCGTGT---TGTTCTC------
-----
FC88X_01028_00458 ------------------CTACGTTTAACCGATCGTGT---TGTTCTC------
-----
FC88X_01029_00473 ------------------CTACGTTTAACCGATCGTGT---TGTTCTC------
-----
FC88X_01002_00638 ------------------CTACGTTTAACCGATCGTGT---TGTTCTC------
-----
FC88X_01043_00799 ------------------CTACGTTTAACCGATCGTGT---TGTTCTC------
-----
FC88X_01015_00903 ------------------CTACGTTTAACCGATCGTGT---TGTTCTC------
-----
FC88X_01020_00920 ------------------CTACGTTTAACCGATCGTGT---TGTTCTC------
-----
FC88X_01016_01171 ------------------CTACGTTTAACCGATCGTGT---TGTTCTC------
-----
FC88X_01074_00400 ------------------CTACGTTTAACCGATCGTGT---TGTTCTC------
-----
FC88X_01084_00443 ------------------CTACGTTTAACCGATCGTGT---TGTTCTC------
-----
FC88X_01064_00490 ------------------CTACGTTTAACCGATCGTGT---TGTTCTC------
-----
FC88X_01085_00605 ------------------CTACGTTTAACCGATCGTGT---TGTTCTC------
-----
FC88X_01075_00693 ------------------CTACGTTTAACCGATCGTGT---TGTTCTC------
-----
FC88X_01064_00721 ------------------CTACGTTTAACCGATCGTGT---TGTTCTC------
-----
FC88X_01081_00749 ------------------CTACGTTTAACCGATCGTGT---TGTTCTC------
-----
FC88X_01092_00732 ------------------CTACGTTTAACCGATCGTGT---TGTTCTC------
-----
FC88X_01089_00815 ------------------CTACGTTTAACCGATCGTGT---TGTTCTC------
-----
FC88X_01088_00877 ------------------CTACGTTTAACCGATCGTGT---TGTTCTC------
-----
FC88X_01096_00860 ------------------CTACGTTTAACCGATCGTGT---TGTTCTC------
-----
FC88X_01059_00933 ------------------CTACGTTTAACCGATCGTGT---TGTTCTC------
-----
FC88X_01098_00937 ------------------CTACGTTTAACCGATCGTGT---TGTTCTC------
-----
FC88X_01143_00584 ------------------CTACGTTTAACCGATCGTGT---TGTTCTC------
-----
FC88X_01146_00611 ------------------CTACGTTTAACCGATCGTGT---TGTTCTC------
-----
FC88X_01115_00787 ------------------CTACGTTTAACCGATCGTGT---TGTTCTC------
-----
FC88X_01122_00774 ------------------CTACGTTTAACCGATCGTGT---TGTTCTC------
-----

FC88X_01100_00890 ------------------CTACGTTTAACCGATCGTGT---TGTTCTC------
-----
FC88X_01114_00887 ------------------CTACGTTTAACCGATCGTGT---TGTTCTC------
-----
FC88X_01133_00923 ------------------CTACGTTTAACCGATCGTGT---TGTTCTC------
-----
FC88X_01133_00979 ------------------CTACGTTTAACCGATCGTGT---TGTTCTC------
-----
FC88X_01104_01159 ------------------CTACGTTTAACCGATCGTGT---TGTTCTC------
-----
FC88X_01142_01273 ------------------CTACGTTTAACCGATCGTGT---TGTTCTC------
-----
FC88X_01052_00733 -----------------GCTACGTTTAACCGATCGTGT---TGTTCTCC-----
-----
FC88X_00997_00347 ------------------CTACGTTTAACCGATCGTGT---TGTTCT-------
-----
FC88X_01123_01128 ------------------CTACGTTTAACCGATCGTGT---TGTTCTCCC----
-----
FC88X_01039_01107 ------------------CTACGTTTAACCGATCGTGT---TGTTCTCACA---
-----
FC88X_01097_00570 ------------------CTACGTTTAACCGATCGTGT---TATTCTC------
-----
FC88X_01123_00581 ------------------CTGCGTTTAACCGATCGTGT---TGTTCTC------
-----
FC88X_01061_01270 ------------------CAACGTTTAACCGATCGTGT---TGTTCTC------
-----
FC88X_01111_00924 ------------------CCACGTTTAACCGATCGTGT---TGTTCTC------
-----
FC88X_01043_00328 ------------------CTACGTTCAACCGATCGTGT---TGTTCTC------
-----
FC88X_01136_00894 ------------------CTACGTTCAACCGATCGTGT---TGTTCTC------
-----
FC88X_01031_00949 ------------------CTACGTTTAACCGAT-GTGT---TGTCTC-------
-----
FC88X_01082_00409 ------------------CTACGTTTAAC-------------------------
-----
FC88X_00955_00577 --------------------ACATATAACCGATCATGTGTCTGATCTC------
-----
FC88X_00996_00673 --------------------ACATATAACCGATCATGTGTCTGATCTC------
-----
FC88X_01043_00533 --------------------ACATATAACCGATCATGTGTCTGATCTC------
-----
FC88X_00994_00541 ---------------CCTGTACTTGTGAATGGCCACGTCTC-------------
-----
FC88X_01093_01120 ---------------CCTGTACTTGTGAATGGCCACGTCTCCC-----------
-----
FC88X_00987_00845 ---------------------CTTGTAAA-------------------------
-----
FC88X_01060_01225 -------------CTTGTCATTTGATCAATGTCTACATCTC-------------
-----
FC88X_00959_00399 ------------------CGCTACAATTTGTAATCCGT--TTATTCT-------
-----
FC88X_01042_00661 ------------------CGCTACAATTTGTAATCCGT--TTATTCT-------
-----
FC88X_01018_00826 ------------------CGCTACAATTTGTAATCCGT--TTATTCT-------
-----

```
FC88X_01030_00909      ------------------CGCTACAATTTGTAATCCGT--TTATTCT-------
-----
FC88X_01038_00923      ------------------CGCTACAATTTGTAATCCGT--TTATTCT-------
-----
FC88X_01087_00850      ------------------CGCTACAATTTGTAATCCGT--TTATTCT-------
-----
FC88X_01104_00795      ----------------------------------CCGT--TTACTC--------
-----
FC88X_01105_00648      --------------TCAATCTCTTGCTATATC--CCGT--TTATTCC-------
-----
FC88X_01112_00600      ---------------CAATCTAGTGCTCGATCG-TTAT--TCATG---------
-----
FC88X_01147_00979      ------------------CTTCATTCTAGCCCGTTTGA--TCATTTT-------
-----
FC88X_00963_00525      ---------------TACAACCACTTTCA-GTTTCCG---TTATTCT-------
-----
FC88X_00989_00599      -----------------TCAACACTTTCA-GTTTCCGG--TTATTCT-------
-----
FC88X_01010_00771      ----------------CCAAGTACTTTGACGCTCCCGG--TTACTC--------
-----
FC88X_00982_00523      -----------------TTACCACTTT-ACATGCGTGC--CTATTCT-------
-----
FC88X_01092_01066      -----------------TTACCA-TTT-ACATGCGTGT--CTATTCT-------
-----
FC88X_00963_00686      -----------------TTACCATTTT-ACATGCGTGC--CTATTCT-------
-----
FC88X_01024_00531      -----------------TTACCATTTT-ACATGCGTGC--CTATTCT-------
-----
FC88X_01063_00498      -----------------TTACCATTTT-ACATGCGTGC--CTATTCT-------
-----
FC88X_01060_00735      -----------------TTACCATTTT-ACATGCGTGC--CTATTCT-------
-----
FC88X_01063_00737      -----------------TTACCATTTT-ACATGCGTGC--CTATTCT-------
-----
FC88X_01088_00829      -----------------TTACCATTTT-ACATGCGTGC--CTATTCT-------
-----
FC88X_01111_00735      -----------------TTACCATTTT-ACATGCGTGC--CTATTCT-------
-----
FC88X_01139_00742      -----------------TTACCATTTT-ACATGCGTGC--CTATTCT-------
-----
FC88X_00994_00523      -----------------TTACCATTTT-ATATGCGTGC--CTATTCT-------
-----
FC88X_00992_00721      -----------------TTACCATTTT-ACACGCGTGC--CTATTCT-------
-----
FC88X_01030_00435      -----------------CTACCATTTTTACATGCGTGC--CTATTCT-------
-----
FC88X_01004_00822      -----------------TTACCATTTT-ACATGCGCGC--CTATTCT-------
-----
FC88X_00966_00746      ---------------TCGCACCATCTTTAGAT---CGC--CTTTTCTCC-----
-----
FC88X_00954_00704      -----------------CCACCTGTTTTGTGGTTGAGC--TATCTC--------
-----
FC88X_01098_00751      -----------------CCACCTGTTTTGTGGTTGAGC--TATCTC--------
-----
FC88X_01005_01116      -----------------CCACCTGTTTTGTGGTTGAGC--AATCTC--------
-----
```

FC88X_01080_00469 -----------------CCACCTGTTTTGTGGTCGAGC--TATCTC--------
-----
FC88X_01066_00854 -----------------CCACCTGTTTTGTGGT-GAGC--TATCTC--------
-----
FC88X_01006_00555 ----------------GCCACC-ATTTAGTTGATGCGT--ATTCTC--------
-----
FC88X_01113_00885 ----------------ACCCTCATTTGTGCTGTGCTGC--TCAC----------
-----
FC88X_01129_00859 -----------------CCGCCATTTGTTCATTGGTGC----------------
-----
FC88X_01145_00614 -----------------CCTCCTTTGATGATGGGGCAG--CTCCCC--------
-----
FC88X_01093_00949 -------------------ACCGTGATTTGTCAGTCCCGGGCTCTC--------
-----
FC88X_01057_01097 --------------------CCGTATTTA-------------------------
-----
FC88X_00951_00411 --------------------TGGTGCCTTATTTACCA-----------------
-----
FC88X_00975_00602 ------------------TTTCTTCCCTGATTAAAGGCGGCGTGTCC-------
-----
FC88X_01094_01131 -------------------ACCTTTCCCCATTGACTGCTACGTGTC--------
-----
FC88X_01037_00796 ----------------------TTCACGAATT----------------------
-----
FC88X_01131_00662 ----------------------TTGCCGAATTGACTCCCCGACAAAACT-----
-----
FC88X_01134_00941 -------------------------------------TCCGGGACT--------
-----
FC88X_01143_00968 --------------------CCGTCAAATTTCCCACGTCCGGTACTC-------
-----
FC88X_01029_00825 --------------------CCGCGACTTGTCTCCCGTCGGGTAACC-------
-----
FC88X_00955_00789 -----------------------TCCCGAATTTAT-CCGCGCGTCTGTCTC---
-----
FC88X_01082_00922 -----------------------TCCCGAATTTAT-CCGCGCGTCTGTCTC---
-----
FC88X_00974_00446 ----------------------CTCCCGGATATTTGTCGCGCATTTGAC-----
-----
FC88X_00983_00835 --------------------CCTGACCTTTCCCAGTGCGCGCTGTTTT------
-----
FC88X_01140_00862 --------------------CCTGACCTTTCC-AGTGCGCGCTGTTTT------
-----
FC88X_01091_00431 --------------------TCTGACTGTTTCCAAAGCA-GCTGTTCT------
-----
FC88X_01115_00611 -------------------GCTTACTTTTTCCGGTTGCCTGTTTTC--------
-----
FC88X_01131_00698 -------------------CTTCACGTTTTCCTGCGGTGCCCTGTT--------
-----
FC88X_01067_01269 ------------------CTGTCTGATTATCCAAAGGCGCCTTCTT--------
-----
FC88X_01122_00737 ---------------------CTCCTGATTAACACAGCGCTTTGTCCC------
-----
FC88X_00956_00458 ----------------------TTTGCGCATTCGGTGCACTGTTATTCT-----
-----
FC88X_01118_00992 ----------------------TTTGCGCATTCGGTGCACTGTTATTCTCC---
-----

```
FC88X_00966_00502      ----------------------TTTGCGCATTCGGTGCACTGTTATTCT-----
-----
FC88X_00952_00843      ----------------------TTTGCGCATTCGGTGCACTGTTATTCT-----
-----
FC88X_00979_00848      ----------------------TTTGCGCATTCGGTGCACTGTTATTCT-----
-----
FC88X_01011_00432      ----------------------TTTGCGCATTCGGTGCACTGTTATTCT-----
-----
FC88X_00991_00859      ----------------------TTTGCGCATTCGGTGCACTGTTATTCTT----
-----
FC88X_01075_00484      ----------------------TTTGCGCATTCGGTGCACTGTTATTCT-----
-----
FC88X_01089_00829      ----------------------TTTGCGCATTCGGTGCACTGTTATTCT-----
-----
FC88X_01067_01146      ----------------------TTTGCGCATTCGGTGCACTGTTATTCT-----
-----
FC88X_01113_00713      ----------------------TTTGCGCATTCGGTGCACTGTTATTCT-----
-----
FC88X_01126_01211      ----------------------TTTGCGCATTCGGTGCACTGTTATTCT-----
-----
FC88X_00967_00434      ----------------------TTTGCGCATTCAGTGCACTGTTATTCT-----
-----
FC88X_00982_00317      ----------------------TTTGCGCATTCGGTGCACAGTTATTCT-----
-----
FC88X_00999_00903      ----------------------TTTGCGCATTCGGTGCACTGTTATTCC-----
-----
FC88X_01122_00555      ----------------------TCTGCGCATTCGGTGCACTGTTATTCT-----
-----
FC88X_00952_00967      -----------------------TTTCTCGTTAGGCGCCCTGTGACTGTT----
-----
FC88X_01026_00422      -----------------------TTTCTCGTTAGGCGCCCTGTGACTGTT----
-----
FC88X_01109_00508      -----------------------TTTCTCGTTAGGCGCCCTGTGACTGTT----
-----
FC88X_01122_00711      -----------------------TTTCTCGTTAGGCGCCCTGTGACTGTT----
-----
FC88X_01016_01267      -----------------------TTCCTCGTTAGGCGCCCTGTGACTGTT----
-----
FC88X_01047_00379      -----------------------CCGCGAAATTTGTGCCCTCTTACTATTCC--
-----
FC88X_01048_01048      -----------------------CCGCGAAATTTGTGCCCTCTTACTATT----
-----
FC88X_00968_00497      ------------------------------ACTTGTGC----------------
-----
FC88X_01035_00783      ---------------------TCCTGCGCTA-TGCGGCCACTTGTTCTC-----
-----
FC88X_01067_00655      ---------------------TCCTGTGTTAGCGCTGTCCCTTGTTTTCC----
-----
FC88X_00953_00434      ---------------------ACCTGCTCTA--GGTGCCCCCTCTATTCT----
-----
FC88X_00991_00346      --------------------TCTTGCCCCGTCTAT---GCGACTTGTTCC----
-----
FC88X_00966_01041      ------------------------------TCTA----GCGATTTT--------
-----
FC88X_01045_01248      ------------------------------TCTA-----CGATTTCTT------
-----
```

FC88X_01034_00452 ------------------CGCATACGTTGGCCCGC---GTGATTTCT-------
-----
FC88X_01079_00847 -----------------------ACGCTGACCC-C---
TCGATTTCTCCATTTC-----
FC88X_00981_00332 ---------------------CCTCTGGCCGTACTGCTCCCGTTTCT-------
-----
FC88X_01036_00961 ---------------------CTCAGGACTACTCAGCCCCGACTTTCT------
-----
FC88X_00988_00567 --------------------CCGGGGCATTATT--GCACGAGTTATCTC-----
-----
FC88X_01087_00524 ---------------------CGCGACATTTTTCGGCCCCGGTTTTCT------
-----
FC88X_00964_00541 --------------------CCGTTGCATCTTTT-GCCCCCGCTTTCT------
-----
FC88X_01009_00433 ----------------------CCCTTTGATCAGGTCGACGGCTTTCTT-----
-----
FC88X_01130_00789 ----------------------CCCTTTGATCAGGTCAACGGCTTTCTC-----
-----
FC88X_01023_01049 ---------------------------------------CGGCGTTCTTG----
-----
FC88X_01025_00638 ---------------------TTCCCGTGTTCCTGACACCGACATTCT------
-----
FC88X_01027_01214 ----------------------CACGACTTTTTCGGCATCGCTATAATCTC---
-----
FC88X_01040_01207 ----------------------CACGACTTTTTCGGCATCGCTATAACC-----
-----
FC88X_01037_00502 ----------------------CACGACTTTTTCGGGATCGCTATAATCTC---
-----
FC88X_01097_00508 ----------------------CACGACTTTTTCG-------------------
-----
FC88X_00952_00426 ----------------------------
TTTTTCGCTCCCCGACAACCGACGTATT---
FC88X_01061_00484 ----------------------------
TTTTTCGCTTCCCGACAACCGACGTATT---
FC88X_01035_00951 --------------------------AGCTCTTCTCCTGCCGGCTAC--
ACGTA-----
FC88X_01037_00339 ---------------------CCAAACTTTTCCTGGTCCCCGGCTAC-------
-----
FC88X_00981_00776 --------------------CTAACAGTTCTTTTGCTCCACCGCTAT-------
-----
FC88X_01080_00609 --------------------CTCACGATTTTTCTGAGCCTCCCCGGG-------
-----
FC88X_01105_00531 --------------------CTCACGATTTTTCTGAGCCTCCCTGGG-------
-----
FC88X_00963_00459 ---------------------CCACGGGATTTATGGTCTTGGCATTCT------
-----
FC88X_01001_00951 --------------------CTACCACGGTTTTTGCTATTCGCGACC-------
-----
FC88X_01047_00611 -------------------CACAACAGCATGTTCTACTCCCATTTA--------
-----
FC88X_01078_00917 ------------------AGACAACAGCAT------------------------
-----
FC88X_01098_00804 ---------------------CAACAGTATTTTTTCACACGTGCGTCTC-----
-----
FC88X_01103_01158 ---------------------CAACAGTATTTTTT-ACACGTGCGTCTC-----
-----

```
FC88X_01109_00860      ---------------------CAACAGTATTTTTTTACACGTGCGTCTC-----
-----
FC88X_01063_00811      ---------------------CAACAGTATTTTTT-------------------
-----
FC88X_01133_00876      ----------------------ACCTGACCCTGCATCTT-CGGCATCTC-----
-----
FC88X_01142_00889      ----------------------ACCTGACCCTTCTCCGCGCATCATATC-----
-----
FC88X_01097_00820      ---------------------CCCCAGCATTTTCTGCGACTGTTATCGC-----
-----
FC88X_00969_00290      ------------------------CCTCCCGTTAGTTA----------------
-----
FC88X_00957_00915      -------------------TCTCTCCTCCGGCTACTAAATTCTGCC--------
-----
FC88X_01085_01071      ----------------CCATCTGTAGTTCGGCCTCCCATTAGT-----------
-----
FC88X_01129_00595      --------------------CTGTAGTCTATCCGCAGCCCAGTCTC--------
-----
FC88X_01132_00894      -------------------CCGATAGTCCGACGGCTCCCGTATCTC--------
-----
FC88X_01134_01101      ----------------TCCCCCACATTCCGGCCGCGACGTGCC-----------
-----
FC88X_01119_01173      ---------------TCCCCTCATATCCGGCTCGC-ACGTGCC-----------
-----
FC88X_01061_00802      --------------TCTTCCCTATATCCCA-CGGCCACGTCTG-----------
-----
FC88X_00964_00868      -----------------TCCCGATATA-CGTCGTGGGCTCTTCGC---------
-----
FC88X_01074_00913      -----------------TCCCGATATA-CGTCGTGGGCTCTTCGC---------
-----
FC88X_01087_00693      ---------------TGTACCGTTATCTCGTCTTTGCCTGTT------------
-----
FC88X_00977_00932      -------------------CCGATAACTCCCCGGCGCCTTCGCGGT--------
-----
FC88X_01003_00428      ------------------ACTGTTGT---GCCGTCGCC----------------
-----
FC88X_01144_01205      ------------TCAATGACTATTGTCC-GCCGGCGCCAC--------------
-----
FC88X_01108_00563      ------------------TCTGTCGTTCGGTCGACGCTGCCT------------
-----
FC88X_00964_00369      ----------------CTTGATGTCCTGAGGACCTTGCTTCTC-----------
-----
FC88X_01048_00408      ------------------CCTTGCCCTGGGCCCCCTGCCTGTTGT---------
-----
FC88X_00975_00397      ---------------CGCCTTTGTGGCCGGCTGCGCGTTCTT------------
-----
FC88X_00950_01008      ---------------TCTTGCCTTCTATGGCCCCGCGCTTCT------------
-----
FC88X_00997_00425      ---------------CTTTTATGCCACTGACCCCAC-GCTGTT-----------
-----
FC88X_01090_00864      ---------------CCTTTATGCCACTGACCCCAC-GCTGTT-----------
-----
FC88X_01058_00518      -----------------TTTATGTGTCAGGAGGCAC-GCTATTCT---------
-----
FC88X_00992_00621      -----------------CTTACGACCTCA-CCGCAGGGCTGTTCT---------
-----
```

FC88X_00995_00715 ------------------CTACAACCTTGGCCGCACTGCTATTAT---------
-----
FC88X_00957_00458 ----------------CCAATAGTCATGAGACCGTCGGCTGCTC----------
-----
FC88X_01052_00501 ----------------CCAATAGTCATGAGACCGCCGGCTGCTCCC--------
-----
FC88X_01015_00424 ----------------CTAAAAACATTATTCCCGTCG-CTGTTCT---------
-----
FC88X_01026_00996 ----------------CTAAAAACATTATTCCCGTCG-CTGTTCT---------
-----
FC88X_00973_00543 -----------------CTAAAACATTATTCCCGTCG-CTGTTCT---------
-----
FC88X_01117_01269 ----------------CCAAAAATTATTGACCCGGCC-GCATTCT---------
-----
FC88X_00960_00828 -------------------ACTATGCTCGGTGGCCTAT-TATTCTC--------
-----
FC88X_01047_00673 -------------------ACTATGCTCGGTGGCCTAT-TATTCTC--------
-----
FC88X_00967_00366 -------------------CCAATACACGGAAGCTTGTCTGTTCTC--------
-----
FC88X_01102_00452 ------------------TCGAACACTCGGCGACGTCACTATTCT---------
-----
FC88X_01019_00673 ------------------GATGACACGCGGCCGCATCTCTATTCTT--------
-----
FC88X_01131_01143 -------------------ATGACACGCGGCCGCATCTCTATTCTT--------
-----
FC88X_01021_00409 -------------------ATGACACGCGGCCGCATCTCTATTCTT--------
-----
FC88X_00951_00846 ------------------CCACGTCCTCGGCAGCACCGCAATATT---------
-----
FC88X_01013_00294 ------------------CCACGTCCTCGGCAGCACCGCAATATT---------
-----
FC88X_01056_00352 ------------------CCACGTCCTCGGCAGCACCGCAATATT---------
-----
FC88X_01030_00525 ------------------TTCCACGTTCGTCTGCACCTCTATTTT---------
-----
FC88X_01083_00480 -------------------TACACGTCC--------------------------
-----
FC88X_00970_00280 -----------------CTTCTAGCATTGTCAGAGCTGTCCTC-----------
-----
FC88X_01043_00411 -----------------CTTCTAGCATTGTCAGAGCTGTCCTC-----------
-----
FC88X_01118_00491 ------------------CTCTAACTTTTTAGGCCCTGACATCTC---------
-----
FC88X_00993_00477 ------------------CTCAGCTTTTGTTGCATGTGATTTCTC---------
-----
FC88X_00964_00627 ------------------CTCAGCTTTTGTTGCATGTGATTTCTC---------
-----
FC88X_01000_00761 ------------------CTCAGCTTTTGTTGCACGTGATTTCTC---------
-----
FC88X_01048_00774 -------------------TTAGCGTTTTTTGACCCCGACATTTCTC-------
-----
FC88X_00973_00563 -----------------GCTGCAAGT-TAGTCCCAGTCTACTCTC---------
-----
FC88X_00985_00726 -----------------CCTGCAACCATATTGCCGGTCACGTCTC---------
-----

```
FC88X_01052_00363      -------------------TCTGCAGATCTGA-CATGACTTTTTCTC-------
-----
FC88X_01086_00597      -------------------TCTGCAGATCTGA-CATGACTTTTTCTC-------
-----
FC88X_01113_01202      -------------------TCTGCAGATCTGA-CATGACTTTTTCTC-------
-----
FC88X_00972_00251      -------------------TTTGCCTGTCGGC-CATCTAGTTTTCTC-------
-----
FC88X_01025_00909      -----------------CTCCTGCGCGTTTGA-CGCCTCCTTTT----------
-----
FC88X_01046_00400      --------------------CTGCGTGTCCGATCGCGTCTGTATCTC-------
-----
FC88X_01025_00499      --------------------CTGCGTGTCCGATCGCGTCTGTATCTC-------
-----
FC88X_01000_00688      --------------------CTGCGTGTCCGATCGCGTCTGTATCTCCC-----
-----
FC88X_01001_00483      ----------------CACTTTACTTGTCC-ATCGCGTCTGTT-----------
-----
FC88X_01073_00712      --------------------------------TCGAGT-CTGA-----------
-----
FC88X_01092_01209      -----------------TTTCCTCCCTACTTGTCGCGTACTGAC----------
-----
FC88X_00996_00398      -------------------CTTGCAGCTCGAGCCTGTGACTGTTCT--------
-----
FC88X_00987_00849      -------------------CTTGCAGCTCGAGCCTGTGACTGTTCT--------
-----
FC88X_00967_00923      -------------------CTTGCAGCTCGAGCCTGTGACTGTTCT--------
-----
FC88X_00981_00916      -------------------CTTGCAGCTCGAGCCTGTGACTGTTCT--------
-----
FC88X_01002_01017      -------------------CTTGCAGCTCGAGCCTGTGACTGTTCT--------
-----
FC88X_01087_00494      -------------------CTTGCAGCTCGAGCCTGTGACTGTTCT--------
-----
FC88X_01080_00574      -------------------CTTGCAGCTCGAGCCTGTGACTGTTCT--------
-----
FC88X_01060_00704      -------------------CTTGCAGCTCGAGCCTGTGACTGTTCT--------
-----
FC88X_01142_00972      -------------------CTTGCAGCTCGAGCCTGTGACTGTTCT--------
-----
FC88X_01031_00941      -------------------CTTGCAGCTCGAGCCTGTTACG-------------
-----
FC88X_00975_00548      -------------------CTACCGGTTTGGGCCTGTG-CCGTTCT--------
-----
FC88X_00951_01256      ---------------------ATCTAGCC-CGACTGGCCCGTCTGTTCT-----
-----
FC88X_01015_00341      -----------------------CTAGTCGCAACTGGACC--------------
-----
FC88X_01039_01266      -----------------------CTAGTC--GACTCGACATCTGTGGCTTCA--
-----
FC88X_01126_01204      -----------------------CTAGTC--GACTCGACCAT------------
-----
FC88X_01009_00765      ------------------------CCGTCTAATCCAGACCGCTTGTCCCGT---
-----
FC88X_00997_00319      -----------------TCGAGCGTTTTTTCTCCCACGGCGTATA---------
-----
```

FC88X_00952_01111 -----------------TAGAGCGTTTTT-CTCCCACGACGTATA---------
-----
FC88X_00965_00964 ----------------------CTTTTTCCTGCCTGGATTGCGTATTCT-----
-----
FC88X_01066_00980 ----------------------CTTTTTCCTGCCTGGACTGCGTATTCT-----
-----
FC88X_00992_00749 ----------------------CTTTCCCCCACCGAGATCG-GTATTATT----
-----
FC88X_01019_00504 ------------------------CTTTCCTCC-CGGTGA--------------
-----
FC88X_01106_01257 --------------------CCACTTTTCCTCCACAGTGACTTATTC-------
-----
FC88X_01096_00733 ------------------------CTTTCCTACA--------------------
-----
FC88X_01140_00931 ----------------------GCCTTTCGTACGGGCTTCGACTATTCT-----
-----
FC88X_00954_00483 -----------------CTCGGAAACCTCCCAC-CGGGCATTGT----------
-----
FC88X_01092_00414 ----------------GCTCGGAAACCTCCCAC-CGGGCATTGT----------
-----
FC88X_00967_01042 -----------------CTCGGAAACCTCCCAC-CGGGCATTGT----------
-----
FC88X_01024_00308 -----------------CTCGGAAACCTCCCAC-CGGGCATTGTCC--------
-----
FC88X_01090_00802 ----------------CTATCGTAAACTCTGAC-CCAGCATTCT----------
-----
FC88X_01094_01122 -----------------CTAGTTCTCCTCCGACACAGGCATCTG----------
-----
FC88X_01130_00674 ----------------TCTCGTCTTC-TCTCACCCAGTGTGTG-----------
-----
FC88X_01138_01207 ----------------TCTCGTCTTCCTCTCA----------------------
-----
FC88X_01049_00415 --------------TCTGTCGTTTTACTCCCACAGCACACT-------------
-----
FC88X_01012_00637 -----------------CTCCTTTGTCTCCCACATCGCGATTTC----------
-----
FC88X_01023_00824 -------------------CCTCTTTTCCTCCGATCGCGATTTCT---------
-----
FC88X_01084_00405 -----------------TCCACGACTATACCAAATCCCGATTGCTCC-------
-----
FC88X_01133_00676 ------------------ACACGCCTTTACC---TCCCGCGTTCTCCT------
-----
FC88X_00978_00729 -------------------------CTTCCGACCCGGTTATCGCAAATTGTT--
-----
FC88X_01108_00504 -------------------------CTTCCGACCCGGTTATCGCAAATTGTT--
-----
FC88X_00956_00491 -------------------------CTTCCGACCCGGTTATCGCAAATTGTC--
-----
FC88X_01070_00589 ------------------------CCGTTCGCCCCTTTGACTGCAGATTATT--
-----
FC88X_01107_01239 ---------------------------------------ACTGCCGATT-----
-----
FC88X_00962_00814 -----------------CAGCAGTTTATCCCAGCTCGTCGCGTCC---------
-----
FC88X_01066_00756 -------------------ATATTTCATCCCT-TTCGCCGCGTTATA-------
-----

FC88X_00999_00313 -----------------CAACGTTTGCTTCCAGATCCCCGTGTA----------
-----
FC88X_01036_00787 -----------CCACCAACCGGACGCCTCCCAGATATA----------------
-----
FC88X_01113_00698 ---------------------------TCCCAGATC------------------
-----
FC88X_00970_00419 -------------------CTTACGAATTCCCAACGTCCCGCGCGC--------
-----
FC88X_00997_00415 -------------------CTCCCGAATCGCCTACTCCCAGCTAGC--------
-----
FC88X_01035_00526 -----------------------CCAATCCCTATGTCGAT--------------
-----
FC88X_01016_00671 -------------------TCTCCGAATGCCCCATTCGATGGTTAT--------
-----
FC88X_01036_00402 -------------------TTAACTAATCTCCCATTGGAAGCCAGT--------
-----
FC88X_01079_00694 -----------------------TTACTCCCT--GATTGCATTTAACCA-----
-----
FC88X_01078_00826 ------------------CGCGATTGCTCCCATGGCTTGCATGTT---------
-----
FC88X_01030_00675 -------------------GCCTCCATTGCACTGGAACGCCTGTTCTG------
-----
FC88X_00981_00272 ------------------ATCACGA-CCCTTCTACCAC-GATTGAG--------
-----
FC88X_00979_00887 ------------------ATCACGA-CCCTTCTACCAC-GATTGAG--------
-----
FC88X_00960_00971 ------------------ATCACGA-CCCTTCTACCAC-GATTGAG--------
-----
FC88X_00992_01031 ------------------ATCACGA-CCCTTCTACCAC-GATTGAG--------
-----
FC88X_01044_00498 ------------------ATCACGA-CCCTTCTACCAC-GATTGAG--------
-----
FC88X_01041_00795 ------------------ATCACGA-CCCTTCTACCAC-GATTGAG--------
-----
FC88X_01075_00411 ------------------ATCACGA-CCCTTCTACCAC-GATTGAG--------
-----
FC88X_01088_00494 ------------------ATCACGA-CCCTTCTACCAC-GATTGAG--------
-----
FC88X_01105_00835 ------------------ATCACGA-CCCTTCTACCAC-GATTGAG--------
-----
FC88X_01050_00538 ------------------ATCACGA-CCCTTCTACCAT-GATTGAG--------
-----
FC88X_00971_00284 ------------------ATCACGA-CCCTCCTACCACCGATTGAG--------
-----
FC88X_00965_01049 -----------------AGTCACGA-CC-TTCTACCAC-GACTGAGC-------
-----
FC88X_00985_00356 ------------------ATCACGAACCCTTCT---------------------
-----
FC88X_01077_00611 ------------------ATCAGAA-CCTTTCTGCATCCCACTATT--------
-----
FC88X_01099_01188 ------------------ATCAGAA-CCTTTCTGCATCCCACTATT--------
-----
FC88X_01113_00883 ------------------ATCAGAA-CCTTTCTGCATCCCACTATT--------
-----
FC88X_01126_00619 ------------------ATCAGAA-CCTTTCTGCATCCCACTATTCC------
-----

```
FC88X_00961_00968      ------------------ATCAGAA-CCTTTCTGTATCCCACTATT--------
-----
FC88X_00957_01146      ----------------------CGACATTTTCTGACCCATTCTACTATT-----
-----
FC88X_01066_01156      -------------------TTACGGCACATTCTG---CAGTCTCCTGTT-----
-----
FC88X_00992_00866      ----------------TCTCCCCGTTAGCCTCCGCGCGATGTG-----------
-----
FC88X_00969_00925      ----------------TCTCCCCGTTAGCCTCCGCGCGATGTG-----------
-----
FC88X_00996_01030      ----------------TCTCCCCGTTAGCCTCCGCGCGATGTG-----------
-----
FC88X_01042_00795      ----------------TCTCCCCGTTAGCCTCCGCGCGATGTG-----------
-----
FC88X_01060_00369      ----------------TCTCCCCGTTAGCCTCCGCGCGATGTG-----------
-----
FC88X_01133_00805      ----------------TCTCCCCGTTAGCCTCCGCGCGATGTG-----------
-----
FC88X_00989_00695      ----------------CCTCCCCGTTAGCCTCCGCGCGATGTG-----------
-----
FC88X_01115_00535      ----------------TCTCCCCGTTAGCCTCC---------------------
-----
FC88X_00992_00842      -------------------CTGCAAAACCTCCCGCGCTAGCATAAC--------
-----
FC88X_00950_00399      --------------------CTTGGGTATATCCTCACCGTTTGCATT-------
-----
FC88X_00999_00389      --------------------AGCAAACCATACCGCCACATTT------------
-----
FC88X_01120_00904      ----------------------CAAAACGTTCTTCCCCATTTGCCTACT-----
-----
FC88X_00985_00347      -------------------CTATCGTCACTT-GACATGGTGC-TTCTC------
-----
FC88X_00970_00804      -------------------CTATCGTCACTT-GACATGGTGC-TTCTC------
-----
FC88X_01020_00785      -------------------CTATCGTCACTT-GACATGGTGC-TTCTC------
-----
FC88X_01095_00538      -------------------CTATCGTCACTT-GACATGGTGC-TTCTC------
-----
FC88X_01083_01181      -------------------CTATCGTCACTT-GACATGGTGC-TTCTC------
-----
FC88X_01139_00694      -------------------CTATCGTCACTT-GACATGGTGC-TTCTC------
-----
FC88X_00950_00540      -------------------CTATCGTCACTT-GACATGGTGCATTCTC------
-----
FC88X_01123_00948      -------------------CTACCGTCACTT-GACATGGTCC-TTCTC------
-----
FC88X_01061_00937      -------------------CTATCGTCACTTTGACA------------------
-----
FC88X_01012_00483      -------------------CTACGGTCCCTC-TATCTGGCACGTTCTC------
-----
FC88X_00990_00655      ------------------TTTCTCATTCTG-CCGAACCTCTATTTCT-------
-----
FC88X_01068_00475      ------------------TTTCTCTCTGTA-CCGCTTCTGTTTACC--------
-----
FC88X_00964_00950      -------------------------CTGTG-CCGTATCGGTGTGTCT-------
-----
```

FC88X_01011_01017 ------------------CGTGCCTGTATA-CGGTACC-TGTTCGC--------
-----
FC88X_01127_00955 ------------------CGTGCCTGTATA-CGGTACCCTGTTCGC--------
-----
FC88X_01060_00693 ------------------CGTGCCTGTATA-CGGTACC-TGTTCGC--------
-----
FC88X_01119_00499 ------------------CGTGCCTGTATA-CGGTACC-TGTTCGC--------
-----
FC88X_01119_00510 ------------------CGTGCCTGTATA-CGGTACC-TGTTCG---------
-----
FC88X_00968_00723 -----------------ACATGCCTGC-TG-CGGTCTCTGACTATC--------
-----
FC88X_01070_00751 ----------------------CCAT-GTGCCGGTACCTGTCGCGTTCTC----
-----
FC88X_01136_01267 ---------------------CCCATAGTACCAGAACCTGGGTCGTTCT-----
-----
FC88X_01030_01223 ----------------------CCGGCGTATCTGTACCTGGTCTGTTCA-----
-----
FC88X_00972_00369 ---------------------GCCTGACTCCCGGCACTTT-TCGACCACA----
-----
FC88X_01020_00622 ----------------------------TCTTGGCTCTTTCTCCACCATT----
-----
FC88X_01027_00972 -----------------TTCAACCGTCTCTGGCGCAACCATATT----------
-----
FC88X_01069_00468 -------------------TTCCCGTCTCTGTTGCGCCACCATTGGCTTC----
-----
FC88X_01040_00448 --------------------TACCGTCT--------------------------
-----
FC88X_01064_01236 ------------------TTTTTCCCTTCACTAACT-G-CGAACTGCA------
-----
FC88X_01096_01214 ------------------TTTTTCCCTTCACTAACT-G-CGAACTGCA------
-----
FC88X_00978_00561 ------------------TTTTTCCCTTCACTAAC--G-CGAACTGCA------
-----
FC88X_00974_00295 ------------------TTTTTCCCTTCACTAACC-G-CGAACTGCA------
-----
FC88X_00985_00279 ------------------TTTTTCCCTTCACTAACC-G-CGAACTGCA------
-----
FC88X_00986_00255 ------------------TTTTTCCCTTCACTAACC-G-CGAACTGCA------
-----
FC88X_00995_00255 ------------------TTTTTCCCTTCACTAACC-G-CGAACTGCA------
-----
FC88X_00998_00260 ------------------TTTTTCCCTTCACTAACC-G-CGAACTGCA------
-----
FC88X_00978_00331 ------------------TTTTTCCCTTCACTAACC-G-CGAACTGCA------
-----
FC88X_00978_00488 ------------------TTTTTCCCTTCACTAACC-G-CGAACTGCA------
-----
FC88X_00979_00557 ------------------TTTTTCCCTTCACTAACC-G-CGAACTGCA------
-----
FC88X_00956_00347 -------------------TTTTCCCTTCACTAACC-G-CGAACTGCA------
-----
FC88X_01120_00784 -------------------TTTTCCCTTCACTAACC-G-CGAACTGCACC----
-----
FC88X_00986_00488 -------------------TTTTCCCTTCACTAACC-G-CGAACTGCA------
-----

```
FC88X_00974_00777       -------------------TTTTCCCTTCACTAACC-G-CGAACTGCA------
-----
FC88X_00951_00919       -------------------TTTTCCCTTCACTAACC-G-CGAACTGCA------
-----
FC88X_00999_01132       -------------------TTTTCCCTTCACTAACC-G-CGAACTGCA------
-----
FC88X_00952_00493       -------------------TTTTCCCTTCACTAACC-G-CGAACTGCA------
-----
FC88X_01105_01127       ------------------CTTTTCCCTTCACTAACC-G-CGAACTGCA------
-----
FC88X_00990_00708       -------------------TTTTCCCTTCACTAACC-G-CGAACTGCA------
-----
FC88X_00992_00878       -------------------TTTTCCCTTCACTAACC-G-CGAACTGCA------
-----
FC88X_00997_00994       -------------------TTTTCCCTTCACTAACC-G-CGAACTGCA------
-----
FC88X_01006_00347       -------------------TTTTCCCTTCACTAACC-G-CGAACTGCA------
-----
FC88X_01025_00429       -------------------TTTTCCCTTCACTAACC-G-CGAACTGCA------
-----
FC88X_01014_00528       -------------------TTTTCCCTTCACTAACC-G-CGAACTGCA------
-----
FC88X_00953_00867       -------------------TTTTCCCTTCACTAACC-G-CGAACTGCA------
-----
FC88X_01041_00800       -------------------TTTTCCCTTCACTAACC-G-CGAACTGCACTT---
-----
FC88X_00957_00981       -------------------TTTTCCCTTCACTAACC-G-CGAACTGCA------
-----
FC88X_01000_00321       -------------------TTTTCCCTTCACTAACC-G-CGAACTGCA------
-----
FC88X_01009_00416       -------------------TTTTCCCTTCACTAACC-G-CGAACTGCA------
-----
FC88X_01006_00515       -------------------TTTTCCCTTCACTAACC-G-CGAACTGCA------
-----
FC88X_01005_00559       -------------------TTTTCCCTTCACTAACC-G-CGAACTGCA------
-----
FC88X_01033_00695       -------------------TTTTCCCTTCACTAACC-G-CGAACTGCA------
-----
FC88X_01021_00847       -------------------TTTTCCCTTCACTAACC-G-CGAACTGCA------
-----
FC88X_01040_00812       -------------------TTTTCCCTTCACTAACC-G-CGAACTGCA------
-----
FC88X_01018_01031       -------------------TTTTCCCTTCACTAACC-G-CGAACTGCA------
-----
FC88X_01014_01143       -------------------TTTTCCCTTCACTAACC-G-CGAACTGCA------
-----
FC88X_01013_01204       -------------------TTTTCCCTTCACTAACC-G-CGAACTGCA------
-----
FC88X_01040_01252       -------------------TTTTCCCTTCACTAACC-G-CGAACTGCA------
-----
FC88X_01092_00533       -------------------TTTTCCCTTCACTAACC-G-CGAACTGCA------
-----
FC88X_01078_00716       -------------------TTTTCCCTTCACTAACC-G-CGAACTGCA------
-----
FC88X_01058_00786       -------------------TTTTCCCTTCACTAACC-G-CGAACTGCA------
-----
```

FC88X_01081_00775 -------------------TTTTCCCTTCACTAACC-G-CGAACTGCA------
-----
FC88X_01087_00793 -------------------TTTTCCCTTCACTAACC-G-CGAACTGCA------
-----
FC88X_01099_00843 -------------------TTTTCCCTTCACTAACC-G-CGAACTGCA------
-----
FC88X_01085_00906 -------------------TTTTCCCTTCACTAACC-G-CGAACTGCA------
-----
FC88X_01083_00979 -------------------TTTTCCCTTCACTAACC-G-CGAACTGCA------
-----
FC88X_01057_01232 -------------------TTTTCCCTTCACTAACC-G-CGAACTGCA------
-----
FC88X_01110_00483 -------------------TTTTCCCTTCACTAACC-G-CGAACTGCA------
-----
FC88X_01117_00523 -------------------TTTTCCCTTCACTAACC-G-CGAACTGCA------
-----
FC88X_01123_00510 -------------------TTTTCCCTTCACTAACC-G-CGAACTGCA------
-----
FC88X_01135_00684 -------------------TTTTCCCTTCACTAACC-G-CGAACTGCA------
-----
FC88X_01132_00776 -------------------TTTTCCCTTCACTAACC-G-CGAACTGCA------
-----
FC88X_01102_00801 -------------------TTTTCCCTTCACTAACC-G-CGAACTGCA------
-----
FC88X_01139_00852 -------------------TTTTCCCTTCACTAACC-G-CGAACTGCA------
-----
FC88X_01131_00951 -------------------TTTTCCCTTCACTAACC-G-CGAACTGCA------
-----
FC88X_01130_01174 -------------------TTTTCCCTTCACTAACC-G-CGAACTGCA------
-----
FC88X_01134_01171 -------------------TTTTCCCTTCACTAACC-G-CGAACTGCA------
-----
FC88X_00964_00302 ------------------TTTTTCCCTTCACTAACC-G-CGAACTGCA------
-----
FC88X_00997_00376 ------------------TTTTTCCCTTCACTAACC-G-CGAACTGCA------
-----
FC88X_00958_00514 ------------------TTTTTCCCTTCACTAACC-G-CGAACTGCA------
-----
FC88X_00986_00623 ------------------TTTTTCCCTTCACTAACC-G-CGAACTGCA------
-----
FC88X_00981_00718 ------------------TTTTTCCCTTCACTAACC-G-CGAACTGCA------
-----
FC88X_00984_00709 ------------------TTTTTCCCTTCACTAACC-G-CGAACTGCA------
-----
FC88X_00965_00799 ------------------TTTTTCCCTTCACTAACC-G-CGAACTGCA------
-----
FC88X_00984_00883 ------------------TTTTTCCCTTCACTAACC-G-CGAACTGCA------
-----
FC88X_00960_00939 ------------------TTTTTCCCTTCACTAACC-G-CGAACTGCA------
-----
FC88X_00974_00931 ------------------TTTTTCCCTTCACTAACC-G-CGAACTGCA------
-----
FC88X_00979_00928 ------------------TTTTTCCCTTCACTAACC-G-CGAACTGCA------
-----
FC88X_00968_00957 ------------------TTTTTCCCTTCACTAACC-G-CGAACTGCA------
-----

FC88X_00972_00995 ------------------TTTTTCCCTTCACTAACC-G-CGAACTGCA-----------
FC88X_00993_00985 ------------------TTTTTCCCTTCACTAACC-G-CGAACTGCA-----------
FC88X_00998_01047 ------------------TTTTTCCCTTCACTAACC-G-CGAACTGCA-----------
FC88X_00983_01221 ------------------TTTTTCCCTTCACTAACC-G-CGAACTGCA-----------
FC88X_00980_01267 ------------------TTTTTCCCTTCACTAACC-G-CGAACTGCA-----------
FC88X_01013_00291 ------------------TTTTTCCCTTCACTAACC-G-CGAACTGCA-----------
FC88X_01041_00322 ------------------TTTTTCCCTTCACTAACC-G-CGAACTGCA-----------
FC88X_01015_00387 ------------------TTTTTCCCTTCACTAACC-G-CGAACTGCA-----------
FC88X_01025_00351 ------------------TTTTTCCCTTCACTAACC-G-CGAACTGCA-----------
FC88X_01047_00383 ------------------TTTTTCCCTTCACTAACC-G-CGAACTGCA-----------
FC88X_01015_00406 ------------------TTTTTCCCTTCACTAACC-G-CGAACTGCA-----------
FC88X_01024_00407 ------------------TTTTTCCCTTCACTAACC-G-CGAACTGCA-----------
FC88X_01035_00433 ------------------TTTTTCCCTTCACTAACC-G-CGAACTGCA-----------
FC88X_01047_00437 ------------------TTTTTCCCTTCACTAACC-G-CGAACTGCA-----------
FC88X_01022_00478 ------------------TTTTTCCCTTCACTAACC-G-CGAACTGCA-----------
FC88X_01026_00494 ------------------TTTTTCCCTTCACTAACC-G-CGAACTGCA-----------
FC88X_01008_00530 ------------------TTTTTCCCTTCACTAACC-G-CGAACTGCA-----------
FC88X_01019_00529 ------------------TTTTTCCCTTCACTAACC-G-CGAACTGCA-----------
FC88X_01034_00614 ------------------TTTTTCCCTTCACTAACC-G-CGAACTGCA-----------
FC88X_01027_00650 ------------------TTTTTCCCTTCACTAACC-G-CGAACTGCA-----------
FC88X_01002_00799 ------------------TTTTTCCCTTCACTAACC-G-CGAACTGCA-----------
FC88X_01015_00758 ------------------TTTTTCCCTTCACTAACC-G-CGAACTGCA-----------
FC88X_01025_00765 ------------------TTTTTCCCTTCACTAACC-G-CGAACTGCA-----------
FC88X_01043_00777 ------------------TTTTTCCCTTCACTAACC-G-CGAACTGCA-----------
FC88X_01001_00832 ------------------TTTTTCCCTTCACTAACC-G-CGAACTGCA-----------
FC88X_01007_00811 ------------------TTTTTCCCTTCACTAACC-G-CGAACTGCA-----------
FC88X_01027_00842 ------------------TTTTTCCCTTCACTAACC-G-CGAACTGCA-----------
FC88X_01044_00815 ------------------TTTTTCCCTTCACTAACC-G-CGAACTGCA-----------

```
FC88X_01046_00810       ------------------TTTTTCCCTTCACTAACC-G-CGAACTGCA------
-----
FC88X_01005_00891       ------------------TTTTTCCCTTCACTAACC-G-CGAACTGCA------
-----
FC88X_01031_00851       ------------------TTTTTCCCTTCACTAACC-G-CGAACTGCA------
-----
FC88X_01000_00905       ------------------TTTTTCCCTTCACTAACC-G-CGAACTGCA------
-----
FC88X_01003_00939       ------------------TTTTTCCCTTCACTAACC-G-CGAACTGCA------
-----
FC88X_01005_00928       ------------------TTTTTCCCTTCACTAACC-G-CGAACTGCA------
-----
FC88X_01043_00923       ------------------TTTTTCCCTTCACTAACC-G-CGAACTGCA------
-----
FC88X_01035_01040       ------------------TTTTTCCCTTCACTAACC-G-CGAACTGCA------
-----
FC88X_01042_01038       ------------------TTTTTCCCTTCACTAACC-G-CGAACTGCA------
-----
FC88X_01007_01124       ------------------TTTTTCCCTTCACTAACC-G-CGAACTGCA------
-----
FC88X_01021_01119       ------------------TTTTTCCCTTCACTAACC-G-CGAACTGCA------
-----
FC88X_01000_01178       ------------------TTTTTCCCTTCACTAACC-G-CGAACTGCA------
-----
FC88X_01007_01229       ------------------TTTTTCCCTTCACTAACC-G-CGAACTGCA------
-----
FC88X_01017_01233       ------------------TTTTTCCCTTCACTAACC-G-CGAACTGCA------
-----
FC88X_01038_01214       ------------------TTTTTCCCTTCACTAACC-G-CGAACTGCA------
-----
FC88X_01049_01200       ------------------TTTTTCCCTTCACTAACC-G-CGAACTGCA------
-----
FC88X_01019_01261       ------------------TTTTTCCCTTCACTAACC-G-CGAACTGCA------
-----
FC88X_01050_00388       ------------------TTTTTCCCTTCACTAACC-G-CGAACTGCA------
-----
FC88X_01060_00490       ------------------TTTTTCCCTTCACTAACC-G-CGAACTGCA------
-----
FC88X_01072_00472       ------------------TTTTTCCCTTCACTAACC-G-CGAACTGCA------
-----
FC88X_01091_00548       ------------------TTTTTCCCTTCACTAACC-G-CGAACTGCA------
-----
FC88X_01080_00568       ------------------TTTTTCCCTTCACTAACC-G-CGAACTGCA------
-----
FC88X_01087_00596       ------------------TTTTTCCCTTCACTAACC-G-CGAACTGCA------
-----
FC88X_01094_00583       ------------------TTTTTCCCTTCACTAACC-G-CGAACTGCA------
-----
FC88X_01058_00607       ------------------TTTTTCCCTTCACTAACC-G-CGAACTGCA------
-----
FC88X_01080_00627       ------------------TTTTTCCCTTCACTAACC-G-CGAACTGCA------
-----
FC88X_01097_00717       ------------------TTTTTCCCTTCACTAACC-G-CGAACTGCA------
-----
FC88X_01099_00772       ------------------TTTTTCCCTTCACTAACC-G-CGAACTGCA------
-----
```

```
FC88X_01092_00802      ------------------TTTTTCCCTTCACTAACC-G-CGAACTGCA------
-----
FC88X_01059_00893      ------------------TTTTTCCCTTCACTAACC-G-CGAACTGCA------
-----
FC88X_01072_00972      ------------------TTTTTCCCTTCACTAACC-G-CGAACTGCA------
-----
FC88X_01076_00984      ------------------TTTTTCCCTTCACTAACC-G-CGAACTGCA------
-----
FC88X_01087_00994      ------------------TTTTTCCCTTCACTAACC-G-CGAACTGCA------
-----
FC88X_01089_00961      ------------------TTTTTCCCTTCACTAACC-G-CGAACTGCA------
-----
FC88X_01086_01239      ------------------TTTTTCCCTTCACTAACC-G-CGAACTGCA------
-----
FC88X_01104_00487      ------------------TTTTTCCCTTCACTAACC-G-CGAACTGCA------
-----
FC88X_01100_00510      ------------------TTTTTCCCTTCACTAACC-G-CGAACTGCA------
-----
FC88X_01110_00541      ------------------TTTTTCCCTTCACTAACC-G-CGAACTGCA------
-----
FC88X_01111_00532      ------------------TTTTTCCCTTCACTAACC-G-CGAACTGCA------
-----
FC88X_01131_00527      ------------------TTTTTCCCTTCACTAACC-G-CGAACTGCA------
-----
FC88X_01126_00579      ------------------TTTTTCCCTTCACTAACC-G-CGAACTGCA------
-----
FC88X_01106_00617      ------------------TTTTTCCCTTCACTAACC-G-CGAACTGCA------
-----
FC88X_01127_00614      ------------------TTTTTCCCTTCACTAACC-G-CGAACTGCA------
-----
FC88X_01100_00686      ------------------TTTTTCCCTTCACTAACC-G-CGAACTGCA------
-----
FC88X_01141_00674      ------------------TTTTTCCCTTCACTAACC-G-CGAACTGCA------
-----
FC88X_01106_00709      ------------------TTTTTCCCTTCACTAACC-G-CGAACTGCA------
-----
FC88X_01138_00824      ------------------TTTTTCCCTTCACTAACC-G-CGAACTGCA------
-----
FC88X_01107_00889      ------------------TTTTTCCCTTCACTAACC-G-CGAACTGCA------
-----
FC88X_01112_00867      ------------------TTTTTCCCTTCACTAACC-G-CGAACTGCA------
-----
FC88X_01115_00887      ------------------TTTTTCCCTTCACTAACC-G-CGAACTGCA------
-----
FC88X_01130_00862      ------------------TTTTTCCCTTCACTAACC-G-CGAACTGCA------
-----
FC88X_01135_00884      ------------------TTTTTCCCTTCACTAACC-G-CGAACTGCA------
-----
FC88X_01109_00906      ------------------TTTTTCCCTTCACTAACC-G-CGAACTGCA------
-----
FC88X_01138_00916      ------------------TTTTTCCCTTCACTAACC-G-CGAACTGCA------
-----
FC88X_01100_00992      ------------------TTTTTCCCTTCACTAACC-G-CGAACTGCA------
-----
FC88X_01120_00954      ------------------TTTTTCCCTTCACTAACC-G-CGAACTGCA------
-----
```

FC88X_01124_01119 ------------------TTTTTCCCTTCACTAACC-G-CGAACTGCA------
-----
FC88X_01145_01124 ------------------TTTTTCCCTTCACTAACC-G-CGAACTGCA------
-----
FC88X_01135_01156 ------------------TTTTTCCCTTCACTAACC-G-CGAACTGCA------
-----
FC88X_01102_01233 ------------------TTTTTCCCTTCACTAACC-G-CGAACTGCA------
-----
FC88X_00952_00350 ------------------TTTTTCCCTTCACTAACC-G-CGAACTGCAAT----
-----
FC88X_00997_00316 -------------------TTTTCCCTTCATTAACC-G-CGAACTGCA------
-----
FC88X_01116_00737 ------------------TTTTTCCCTTCACTAACC-G-CGAACTGCAC-----
-----
FC88X_01074_00642 -------------------TTCTCCCTTCACTAACC-G-CGAACTGCA------
-----
FC88X_00951_00430 -----------------TTTTTTCCCTTCACTAACC-G-CGAACTGCA------
-----
FC88X_00954_00921 -----------------TTTTTTCCCTTCACTAACC-G-CGAA-TGCA------
-----
FC88X_00965_00512 -----------------TTTTTTCCCTTCACTAACC-G-CGAACTGCA------
-----
FC88X_00977_00601 -----------------TTTTTTCCCTTCACTAACC-G-CGAACTGCA------
-----
FC88X_00998_00825 -----------------TTTTTTCCCTTCACTAACC-G-CGAACTGCA------
-----
FC88X_01078_00379 -----------------TTTTTTCCCTTCACTAACC-G-CGAACTGCA------
-----
FC88X_01094_00450 -----------------TTTTTTCCCTTCACTAACC-G-CGAACTGCA------
-----
FC88X_01050_00634 -----------------TTTTTTCCCTTCACTAACC-G-CGAACTGCA------
-----
FC88X_01092_00784 -----------------TTTTTTCCCTTCACTAACC-G-CGAACTGCA------
-----
FC88X_01080_00842 -----------------TTTTTTCCCTTCACTAACC-G-CGAACTGCA------
-----
FC88X_01065_00980 -----------------TTTTTTCCCTTCACTAACC-G-CGAACTGCA------
-----
FC88X_01118_00905 -----------------TTTTTTCCCTTCACTAACC-G-CGAACTGCA------
-----
FC88X_01111_01010 -----------------TTTTTTCCCTTCACTAACC-G-CGAACTGCA------
-----
FC88X_01137_01013 -----------------TTTTTTCCCTTCACTAACC-G-CGAACTGCA------
-----
FC88X_01125_00820 ----------------TTTTTTTCCCTTCACTAACC-G-CGAACTGCA------
-----
FC88X_01066_00422 ------------------TTTTTCCCTTCACTGACC-G-CGAACTGCA------
-----
FC88X_01105_00485 ----------------GTTTTTTCCCTTCACTAACC-G-CGAACTGCA------
-----
FC88X_01011_00947 ------------------TTTTTCCCTTCACTAGCC-G-CGAACTGCA------
-----
FC88X_01090_00919 ------------------TTTTTCCCTCCACTAGCC-G-CGAACTGCA------
-----
FC88X_00992_00642 ------------------TTTTTCCCTACACTAACC-G-CGAACTGCA------
-----

```
FC88X_00989_00968      ------------------TTTTTCCCTTCACTAACC-G-CGAACCGCA------
-----
FC88X_01086_00946      -------------------TTTTCCCTTCACTAACC-G-CGAACAGCA------
-----
FC88X_01007_01208      ------------------TTTTTCCCTTCACTAACC-G-CGGACTGCA------
-----
FC88X_01005_00694      ------------------TTCTTCCCTTCACTAACC-G-CGAACTGCA------
-----
FC88X_01063_00801      ------------------TTCTTCCCTTCACTAACC-G-CGAACTGCA------
-----
FC88X_01025_00533      ------------------TTTTTCCCCTCACTAACC-G-CGAACTGCA------
-----
FC88X_01069_00450      ------------------TTTTTCCCCTCACTAACC-G-CGAACTGCA------
-----
FC88X_01119_00628      ------------------TTTTTCCCCTCACTAACC-G-CGAACTGCA------
-----
FC88X_00994_00715      -------------------TTTTCCCCTCACTAACC-G-CGAACTGCA------
-----
FC88X_01023_00929      -------------------TTTTCCCCTCACTAACC-G-CGAACTGCA------
-----
FC88X_01030_01138      -------------------TTTTCCCCTCACTAACC-G-CGAACTGCA------
-----
FC88X_01056_01255      -----------------TTTTTCCCCTTCACTAACC-G-CGAACTGCA------
-----
FC88X_01020_01211      ------------------TTTTCCCCTTCACTAACC-G-CGAACTGCA------
-----
FC88X_01124_00594      -------------------TTTCCCCTTCACTAACC-G-CGAACTGCA------
-----
FC88X_01092_00438      ------------------TTTTCCCCTTCACTAACC-G-CGAACTGCA------
-----
FC88X_01140_00698      ------------------TTTTCCCCTTCACTAACC-G-CGAACTGCA------
-----
FC88X_01142_01188      ----------------TTTTTTCCCCTTCACTAACC-G-CGAACTGCA------
-----
FC88X_00994_00803      -------------------CTTTCCCTTCACTAACC-G-CGAACTGCA------
-----
FC88X_01071_00858      -------------------CTTTCCCTTCACTAACC-G-CGAACTGCA------
-----
FC88X_01014_00920      -------------------TTTTCCCTTCACTAACC-G-CGAGCTGCA------
-----
FC88X_01143_00688      -------------------TTTTCCCTTCACTAACC-GTCGAACTGCA------
-----
FC88X_01011_00584      ------------------TTTTTCCCTTCAC-GAAC-CGCGAACTGCA------
-----
FC88X_01094_01155      -------------------TTTTCCCTTCAC-GAAC-CGCGAACTGCA------
-----
FC88X_01100_00597      ------------------TTTTTCCCTTCAC-GAAC-CGCGAACTGCA------
-----
FC88X_00996_00450      -------------------TTTTCCCTTCAC-AAAC-CGCGAACTGCA------
-----
FC88X_00985_00693      -----------------TTTTTTCCCTTCACTAAAC-CGCGAACTGCA------
-----
FC88X_00964_00448      ------------------TTTTTCCCTCCACTAACC-G-CGAACTGCA------
-----
FC88X_01029_00869      -------------------TTTTCCCTCCACTAACC-G-CGAACTGCA------
-----
```

FC88X_01035_00937 ------------------TTTTTCCCTTCACTAACC-A-CGAACCGCA------
-----
FC88X_01092_00462 ------------------TTTTTCCCTTCACTAACC-G-CGAACTCGC------
-----
FC88X_01114_00651 ------------------TTTTTCCCTTCACTAACC-G-CGAACGCA-------
-----
FC88X_01076_00607 ------------------TTTTTCCCTTCACTAACG-C-CG-------------
-----
FC88X_01114_00545 -------------------TCTTCCCTTCACTAAC-------------------
-----
FC88X_01113_00787 ------------------TTTTTCCCGTCACTAGC-------------------
-----
FC88X_01075_00657 -------------------TTTCCTTCCTACTAACC--GCGAACTGCA------
-----
FC88X_01139_01030 -------------------TTTCCTCCCTACTAACC--GCGAACTGCA------
-----
FC88X_01088_00446 -------------------TTTTCCTCCTACTAACC--GCGAACTGCA------
-----
FC88X_01018_00988 --------------------TTTTCCTTCACTAAC-------------------
-----
FC88X_01068_01270 -------------------TTTTCCCTACACTAATCCGGCGAACTGC-------
-----
FC88X_01051_00470 -----------TCCCCCCATTTTTCCCACACTCCTTGT----------------
-----
FC88X_01094_00592 -------------------TTTCCCTTACACT----------------------
-----
FC88X_01041_00422 -----------CCCAAA--TTTTCCCGTCACCACTTTACT--------------
-----
FC88X_01114_01191 ----------CCCCAAAATTTTTCCCGTCAC-----------------------
-----
FC88X_00961_00312 -----------------CGTTGA-ACTTTCCTGTCCCCACGTATT---------
-----
FC88X_01098_00941 -------------------TTGACACCTAGCTCCCCTCACGTTTTAC-------
-----
FC88X_00999_00593 --------------------CTTCACTTTTCCCT---CATTTTGCCTGCT----
-----
FC88X_01117_01105 ----------------------TTACTTTTCCCTTCGCATCTGGCATTCT----
-----
FC88X_01032_00836 -----------------------TACTTTAC-----------------------
-----
FC88X_00996_00342 ------ACGTATTTACTGTGTTCAGTTTTACCTATTC-----------------
-----
FC88X_01070_00779 ------ACGTATTTACTGTGTTCAGTTTTACCTATTC-----------------
-----
FC88X_00969_00442 -------------------------TCTTACCTAA-------------------
-----
FC88X_01055_01252 ----------TTCGCCTTTATGCCGTGTGACCCATT------------------
-----
FC88X_01029_00323 ----------------TGCGTGCGTTTTCCCCCAGCCGTGTGTCCC--------
-----
FC88X_01006_01034 ----------------TGCGTGCGTTTTTCCCCAGCCGTGTGTC----------
-----
FC88X_01074_00689 ------------------------TTTTCCCCT---------------------
-----
FC88X_00981_00904 --------------------TCTCTTTCTTCCCCACTGTGTTGTGTG-------
-----

```
FC88X_00975_00545      --------------TCAACTTACA-TCACGCTTGCCTGTTTTCCC---------
-----
FC88X_01050_00727      -----------------CCTTACTCTCCTGCATGCCCCATTTTCCC--------
-----
FC88X_00972_00892      ---------------CTCCTTACTCCTTCATGCCTATGGCAAC-----------
-----
FC88X_01109_00574      --------------TCTCTTTACCTTCTGCCATGGCTTGTT-------------
-----
FC88X_00993_00481      -----------CTAATTCCTCGCTGTCTTTCACGTTTC----------------
-----
FC88X_00989_00956      -----------CAACTTCTTCTGCTGCTTTCCCATGTT----------------
-----
FC88X_00981_00632      -----------ATAACCCTTCGCCAACTGTCCCGTACG----------------
-----
FC88X_00976_00639      ---------------------TTTCCTTCACCTA--------------------
-----
FC88X_01077_00708      -----------------CTCCTTTCCCTCCGCTACTGCATTCCT----------
-----
FC88X_01046_00526      --------------CCTATGCCTCCTTCTCCTCACGGTACT-------------
-----
FC88X_01010_00811      -------------CGCGATGCCTCCCTTCCTTCCCGACACA-------------
-----
FC88X_01024_00926      ----------------TTAACCTCCCTTTGCCAGCGTG-CTATC----------
-----
FC88X_01052_00871      ----------------TTAACCTCCCTTTGCCAGCGTG-CTATC----------
-----
FC88X_00963_00801      --------------------CCTCCTTTTCCTCGAATGTCTATCACC-------
-----
FC88X_01016_00857      ------------------------TTTACCTCA---------------------
-----
FC88X_01060_00410      ------------------CGCATGGTTAACTCAACCTTGCCTCCCCC-------
-----
FC88X_00959_00336      ----------------------ACCTGATCTGTACTCTCCGGGCGTGTA-----
-----
FC88X_00974_01202      ----------------------GCCTGATCTGTACTCCCCGGGCGTGTACC---
-----
FC88X_01008_00532      ----------------------------CTCATCGTCCTCA-------------
-----
FC88X_01002_00307      --------------------ACTCCTGATCT-TCCACGCCGGCCTAAC------
-----
FC88X_01079_00777      -----------------------CCTGACCT-TGGCCTCC--CCTAACACTG--
-----
FC88X_00996_00832      ----------------------CCTAGGCCTAT--CCGCCGCGTTGTTCT----
-----
FC88X_01144_01227      -----------------CTAATCCCTGACCTATG-TCGCCGCGTT---------
-----
FC88X_01099_00717      ------------------------------------CACCTTGTCC--------
-----
FC88X_00962_00894      ------------------CACTCCTTGCCCTGTAGCTGCCGCAGC---------
-----
FC88X_01012_00758      --------------------CTCCCTGGTCTCTA-CTCCCGCCATG--------
-----
FC88X_01096_00471      ---------------------TCAATCGTCCTCCCACGTTGCACTGTG------
-----
FC88X_01121_00671      ---------------------TCAATGATCCTCC-ACGTTGCACTTTG------
-----
```

FC88X_01142_00858 ----------------------TAACCTCATCTCTCCACGGCACTTATC-----
-----
FC88X_00993_00836 -----------------------CCATTCACTCCTACAATGCACTTTGCT----
-----
FC88X_01140_00859 ----------------------TTAATTCTCTGCTCC--CTGGCTTTGCTA---
-----
FC88X_01023_00687 ----------------------CTGACACTTTCCACCGCCAGCATTAC------
-----
FC88X_01059_00482 ------------------------CATACTTTCCCACG----------------
-----
FC88X_01079_00593 ----------------ATTCAATCTACACTTATCCCTGCTAAC-----------
-----
FC88X_01009_01042 ----------------------TCTATCCATTTTTCGCTCGGACCTTTT-----
-----
FC88X_01052_00512 -----------------------CCATCCTATTCTTCACCGCGCCTTTAT----
-----
FC88X_01062_00553 ---------------------TTATGCTCTATCTCGCTGCGAACCTTT------
-----
FC88X_01129_00794 ----------------------TCTGTTCT-TCTC-CCACGCACCTTGGT----
-----
FC88X_00994_00489 --------------------------------------ATACACCT--------
-----
FC88X_01129_00541 ----------------------TTCGCTGTTCCTATCAATCCACCTGTCTTC--
-----
FC88X_00961_00688 ----------------------TTGACCAATCTTCTATCTCCACCTGC------
-----
FC88X_01106_00543 ----------------------TTCTCCAATCTTCTTATTGCGCCTGTT-----
-----
FC88X_00978_00843 --------------------------------------CTCCGCCGGTC-----
-----
FC88X_01040_00860 -----------------------CCCACACTTTTGCGACTCGGCCGTTCA----
-----
FC88X_01015_00332 --------------------CTTCGTCTTATTCGCCAGTCGCTGCGTTCC----
-----
FC88X_01074_00597 --------------------TTTCTCCTTATTCGCC---TCCTACGTTCG----
-----
FC88X_00965_00874 ------------------------TACTAAACTCCCTAGCGCTTCATTTCA---
-----
FC88X_01050_00665 ------------------------TCTTATACTCCC---CGCTTCAGTTTGTT-
-----
FC88X_01026_00913 -------------------CTTTTCACTAATATCCCTGCCGCTGTT--------
-----
FC88X_01070_00931 ----------------------TTTTCTAAT-TCCCTGCCTCTGTATGCT----
-----
FC88X_01053_00576 --------------------TCTCCAATTCTCTTCCT-CCGTTGTGTG------
-----
FC88X_01101_00672 ----------------------TCCATTACTATTCCT-CCGTCTTGCTTTG---
-----
FC88X_00957_00451 -----------------------CCGTTTAATCTCCCACTGCGGCATATC----
-----
FC88X_00970_00901 -----------------------CCGTTTAATCTCCCACTGCGGCATATC----
-----
FC88X_01096_00644 -----------------------CCGTTTAATCTCCCACTGCGGCATATC----
-----
FC88X_01106_00447 -----------------------CCGTTTAATCTCCCACTGCGGCATATC----
-----

FC88X_01138_00594 -----------------------CCGTTTAATCTCCCACTGCGGCATATC----
-----

FC88X_01140_00877 -----------------------CCGTTTAATCTCCCACTGCGGCATATC----
-----

FC88X_01105_01113 -----------------------CCGTTTAATCTCC-ACTGCGGCATAT-----
-----

FC88X_01097_00447 -----------------------CCATTTAATC---------------------
-----

FC88X_01083_00645 -----------------------CCGTTCTATCTCCCGTGGCGGCGTGCCCC--
-----

FC88X_01043_00476 -----------------------CCTTTTGCTCTCCTCATGCTACA--------
-----

FC88X_01066_00408 ----------------CTCTTTACCGTTTGATCTCC-CGTGCTAT---------
-----

FC88X_01133_00540 -----------------------CCTTTATATCTCCCCTTGCGCGCTACA----
-----

FC88X_01121_01146 -----------------------CCATTCAATCTTCTCCTGCCAGCTACT----
-----

FC88X_01144_00888 -----------------------TCATGCGATCTCCTTCTCCGACCTATG----
-----

FC88X_00969_00657 -------------------TGTCACAATTCTACTCCCGGATACTAAT-------
-----

FC88X_01118_00571 -------------------------------------GGAT-CTAA--------
-----

FC88X_01133_00910 -----------------------TCACGTATTCCGCTGCAT-CGAACTTTT---
-----

FC88X_01147_01043 ----------------------CTCAC-TATCCCGC-GTAT-CTAATTTCTC--
-----

FC88X_00977_00939 -----------------------TTTTAAACTCCCCGGCAGTCGCATTTT----
-----

FC88X_01070_00462 ---------------------AGGTTCAGTCTCCCGGG--GTCTCATTTCT---
-----

FC88X_00959_00869 ------------------TTCTACCTATTCGGCTCCGCAATATCA---------
-----

FC88X_01042_00505 ------------------TTCTACCTAA--------------------------
-----

FC88X_00971_00446 ---------ACTACTCGCCTCCACCTATCACTTTTG------------------
-----

FC88X_00987_00950 -------------------TTCGCGCTCCCCTTACCTCAACATATT--------
-----

FC88X_01011_01231 -------------------TTCGCGCTCCTCTTA--------------------
-----

FC88X_01013_00550 -----------------ACTTCACCCTTCTCACGACACAAACCT----------
-----

FC88X_01121_01110 -------------------TTTACGCCTC-CATGACAATAACTATTA-------
-----

FC88X_00953_00458 -------------------CTATCAGCTCAATTATCCCACTTTTCT--------
-----

FC88X_01049_00412 -------------------TTATCGCATCAACGGTCCCGCTATTCC--------
-----

FC88X_01086_00634 ----------------CTCGTTATTCTTCTCCTCTTGGACTGTC----------
-----

FC88X_01059_00910 -------------------TTCAATCATCGCCGCTTTGACTGTTCT--------
-----

FC88X_01035_00313 ----------------CTTTCCGCGGGTTATCCCTT--TTCTGTT---------
-----

FC88X_01079_00843 ------------------TTACATGCGTTGCCCCTCAGTTCTGTT---------
-----
FC88X_01016_00638 ------------------------ACGTTTCCTCTGTTTATTGCTCTTCCT---
-----
FC88X_01109_00743 ---------------------------TTTCCACT-------------------
-----
FC88X_00959_00387 -------------------TTTAGCAC-GTTACCTCGGC-CTATTTTC------
-----
FC88X_01037_00928 -------------------TTTAGCAC-GTTACCTCGGC-CTATTTTC------
-----
FC88X_01005_00299 -------------------CTTCGCACTATGACCTTTGCACTATTC--------
-----
FC88X_01052_00771 -------------------TTTCGCACTATGACCTT-GCACTATTC--------
-----
FC88X_01063_00583 -----------------TTCACCGATGTGTGACCTTCGC-CTGTT---------
-----
FC88X_01130_00563 -----------------TTCACCGATGTGTGACCTTCGC-CTGTT---------
-----
FC88X_01134_01173 -----------------TTCACCGATGTGTGACCTTCGC-CTGTT---------
-----
FC88X_00973_00692 -----------------TTAACCCATCACCGACCTACGC-CTATT---------
-----
FC88X_00999_01268 --------------CCTAGCTTTGTACTAACGGCTGTTCGC-------------
-----
FC88X_01092_00629 -------------------CTACGTACTAACGA---------------------
-----
FC88X_01084_01096 ---------GGGTCCTCCCCTACGTACTGTCATGTCTA----------------
-----
FC88X_01120_00957 ------------------CCTACGTAC---------------------------
-----
FC88X_01022_00927 --------------------TACGTACTC-------------------------
-----
FC88X_01097_00772 --------CTATCACGCGATTACGTTCTCTACT---------------------
-----
FC88X_01047_00420 -------------------CAACGTACTCCA-----------------------
-----
FC88X_01040_01011 ------------------CCAACGTATCCACAGCGCTCCCGTCAC---------
-----
FC88X_01072_00928 --------------------CTCGTACTCCAGAGACCTGCCCATATT-------
-----
FC88X_01118_01139 --------------------CTCGTACTCCAGAGACCTGCCCATATT-------
-----
FC88X_00989_00694 ---------------------CGATCGTGCCACATCCGTCCTATTCTT------
-----
FC88X_01119_00755 ---------------------CGATCGTGCCACATCCGTCCTATTCTT------
-----
FC88X_00984_00383 ----------------GTCCACGATCCCGGCA-ATTTTACCTATT---------
-----
FC88X_00972_00435 ------------------TTATCTGCACGCCCAACAACCGCTATT---------
-----
FC88X_01147_00610 ------------------TTATCTGCACGCCCAACAACCGCTATT---------
-----
FC88X_01041_00479 ------------------TTACCTCCTCTGCCTACGAACGCTGTT---------
-----
FC88X_00998_00698 ------------------ACATGTGATCTCCGCACTTGCGCTCTT---------
-----

```
FC88X_01136_00920       ------------------ACATGTGATCTCCGCACTTGCGCTCTT---------
-----
FC88X_01055_00474       --------------------ATCTGAACTCCAAGTTCCGCTATTTTC-------
-----
FC88X_01057_00525       --------------------ATCTGAGCTCAA----------------------
-----
FC88X_00991_00489       ------------------CCAGGTAGTTAACGCAATCGGCTTTTTCT-------
-----
FC88X_00990_00750       ------------------CCAGGTAGTTAACGCAATCGGCTTTTTCT-------
-----
FC88X_01042_00539       ------------------CCAGGTAGTTAACGCGATCGGCTTTTTCT-------
-----
FC88X_01043_01241       ----------------TCCCAGCTATATTGCGCG-TAGGCTCTT----------
-----
FC88X_00956_00937       -------------------AATCTAACGCTTACGCCTGGTTTATTCT-------
-----
FC88X_01115_00938       --------------------ATCTAACGTTTACGCCTGGTTTATTCT-------
-----
FC88X_01139_00907       -------------------GATCTAACGTTTACGCCCGGTTTATTCC-------
-----
FC88X_01024_00545       -------------------CTCCCAACTCTAACGCCTTGTTCAGGC--------
-----
FC88X_00970_00851       --------------------CTGTGCACT-CACATCCGGCTTTGTCTC------
-----
FC88X_01074_00873       --------------------CTGTGCACT-CACATCCGGCTTTGTCTC------
-----
FC88X_01133_00610       --------------------CTGTGCACT-CACATCCGGCTTTGTCTC------
-----
FC88X_01130_01204       --------------------CTGTGCACT-CACATCCGGCTTTGTCTC------
-----
FC88X_01082_00929       ---------------------CGTGGACTACGCGCCCGGTTTATTCTT------
-----
FC88X_01032_01155       ------------------------TTAACGCACCCTCGTTACCTGTTCAC----
-----
FC88X_01070_00447       ------------------------TTAACGCACCCTCGTTACCTGTTCAC----
-----
FC88X_01097_00606       ---------------------TACACTTAGCGCTCTCGTTAC-TATTCAC----
-----
FC88X_01023_00642       -----------------------CGAAACACTCACTCGCTGTTTTTTCTC----
-----
FC88X_00975_00281       -------------------ACGGCTCTTGTCCGGCTCCCTACTATT--------
-----
FC88X_01016_00610       -------------------ACGGCTCTTGTCCGGCTCCCTACTATT--------
-----
FC88X_01118_00501       ----------------ATTACCGCTCATGTCC---CCTGTACTATT--------
-----
FC88X_01044_00505       --------------------CCTGTGTATTGCAGCTCCCAGCTTTTTT------
-----
FC88X_01087_00875       ---------------------------------GTTCCCACCT-----------
-----
FC88X_01002_00854       ----------------------GCTGTTGTGTTCCAGCTGCCTGTTCTG-----
-----
FC88X_01062_00595       ----------------------TCTGTTGATTGTCTCCCGCCGCTGTTA-----
-----
FC88X_00960_00321       -----------------------CAAGTTCCCGACTGTCG--------------
-----
```

FC88X_01014_00783 -------------------CCCCCATGTACCTGACAGTTGGCTCCT--------
-----
FC88X_00973_00860 -------------------CATGCGATTG-TTACCGCCCGTTTTCTC-------
-----
FC88X_01046_00518 -------------------CATGCAATTACTTGCCGTCTGTCCTC---------
-----
FC88X_00960_00345 --------------------TTTCACGTTGCCGACTGCTGCTATTCC-------
-----
FC88X_00985_00308 --------------------TTTCACGTTGCCGACTGCTGCTATTCC-------
-----
FC88X_00966_00696 --------------------TTTCACGTTGCCGACTGCTGCTATTCC-------
-----
FC88X_00974_00815 --------------------TTTCACGTTGCCGACTGCTGCTATTCC-------
-----
FC88X_00957_01204 --------------------TTTCACGTTGCCGACTGCTGCTATTCC-------
-----
FC88X_01026_00438 --------------------TTTCACGTTGCCGACTGCTGCTATTCC-------
-----
FC88X_01020_00815 --------------------TTTCACGTTGCCGACTGCTGCTATTCC-------
-----
FC88X_01073_00537 --------------------TTTCACGTTGCCGACTGCTGCTATTCC-------
-----
FC88X_01074_00596 --------------------TTTCACGTTGCCGACTGCTGCTATTCC-------
-----
FC88X_01059_00710 --------------------TTTCACGTTGCCGACTGCTGCTATTCC-------
-----
FC88X_01078_00851 --------------------TTTCACGTTGCCGACTGCTGCTATTCC-------
-----
FC88X_01127_00618 --------------------TTTCACGTTGCCGACTGCTGCTATTCC-------
-----
FC88X_01116_00806 --------------------TTTCACGTTGCCGACTGCTGCTATTCC-------
-----
FC88X_00952_01008 --------------------TTTCACGTTGCCGACTGCTGCTATTCCCC-----
-----
FC88X_00956_00981 --------------------TTTCACGTTGTCGACTGCTGCTATTCC-------
-----
FC88X_01047_00846 --------------------TTTCACGTTGCCGACTGCTGTCCTCATTC-----
-----
FC88X_00981_00464 -------------CTCCCATTTTCACGTTGCTCGATGCC---------------
-----
FC88X_00989_00851 --------------------TTTCACGTTGC-----------------------
-----
FC88X_00999_00343 --------------------TCCTCGTTGTCCGAGTGTTGCTAAGCTC------
-----
FC88X_00955_00480 -------------------TTCGTACTTGTCCGGTCGCTATC------------
-----
FC88X_01059_00838 -------------------CAAACGTTTCCCCGACGTCGCTTTCTCC-------
-----
FC88X_01126_00671 -------------------CAAACGTTTCCCCGACGTCGCTTTCTCC-------
-----
FC88X_00951_00322 -------------------CAAACGTTTCCCCGACGTCGCTTTCTC--------
-----
FC88X_00997_00580 -------------------CAAACGTTTCCCCGACGTCGCTTTCTC--------
-----
FC88X_00994_00938 -------------------CAAACGTTTCCCCGACGTCGCTTTCTC--------
-----

```
FC88X_00997_01120      -------------------CAAACGTTTCCCCGACGTCGCTTTCTC--------
-----
FC88X_01047_00376      -------------------CAAACGTTTCCCCGACGTCGCTTTCTC--------
-----
FC88X_01027_00638      -------------------CAAACGTTTCCCCGACGTCGCTTTCTC--------
-----
FC88X_01003_00665      -------------------CAAACGTTTCCCCGACGTCGCTTTCTC--------
-----
FC88X_01001_00753      -------------------CAAACGTTTCCCCGACGTCGCTTTCTC--------
-----
FC88X_01022_00851      -------------------CAAACGTTTCCCCGACGTCGCTTTCTC--------
-----
FC88X_01031_00932      -------------------CAAACGTTTCCCCGACGTCGCTTTCTC--------
-----
FC88X_01063_00608      -------------------CAAACGTTTCCCCGACGTCGCTTTCTC--------
-----
FC88X_01095_00799      -------------------CAAACGTTTCCCCGACGTCGCTTTCTC--------
-----
FC88X_01138_00841      -------------------CAAACGTTTCCCCGACGTCGCTTTCTC--------
-----
FC88X_01144_00808      -------------------CAAACGTTTCCCCGACGTCGCTTTCTC--------
-----
FC88X_01125_00859      -------------------CAAACGTTTCCCCGACGTCGCTTTCTC--------
-----
FC88X_01104_00916      -------------------CAAACGTTTCCCCGACGTCGCTTTCTC--------
-----
FC88X_01130_00997      -------------------CAAACGTTTCCCCGACGTCGCTTTCTC--------
-----
FC88X_01021_00675      ------------------GCAAACGTTTCCCCGACGTCGCTTTCTCCC------
-----
FC88X_00961_00647      -------------------CAAACGTTTCCCTGACGTCGCTTTCTC--------
-----
FC88X_01020_00591      -------------------CAAACGTTTCCC-GACGTCGCTTTCTC--------
-----
FC88X_00976_00847      -------------------CAAACGTTTCCCCGACGTCGCTCTCTC--------
-----
FC88X_00995_00630      -------------------CAAACGTTTCCCCGACGTC----------------
-----
FC88X_01022_00939      -----------------CAAAAACGTTCCCCCGACGTCGCTTTCTC--------
-----
FC88X_01048_01221      -------------------CAAACGTTCCCCCGACGTCGCTTTCTC--------
-----
FC88X_01076_00632      ------------------CAAAACGTTTCCCCGACGTCGCTTTCTC--------
-----
FC88X_01132_00887      ------------------CAAAACGCTTCCCCGACGTCGCTTTCTC--------
-----
FC88X_01147_01128      ------------------CAAAACGTTTCCCCGACGTCGCTTTCCC--------
-----
FC88X_01137_01182      -------------------CAAACGTT-CCCCGACGTT----------------
-----
FC88X_00970_00616      --------------------CAACGTTTACCCGACTGTC---------------
-----
FC88X_01083_00668      --------------------CAACGTT-ACCCGAT-------------------
-----
FC88X_00998_00442      ---------------TCTCGCGATGGTCCGCTACTTGTTCTC------------
-----
```

FC88X_01025_00290 ---------------TCTCGCGATGGTCCGCTACTTGTTCTC------------
-----
FC88X_01134_00563 ---------------TCTCGCGATGGTCCGCTACCTGTTCTC------------
-----
FC88X_01144_01202 -----------------TCCCAATGTTCCGACACG-GTTCTGTG----------
-----
FC88X_01058_00441 -----------------CGCAAATGTCTCCCAAACTGTC---------------
-----
FC88X_01096_00982 ----------------TCGCTAACTATTCCTCCACTGTACATGT----------
-----
FC88X_00975_00343 --------------CCTGTACGTTTGTACACGTTATTCGCA-------------
-----
FC88X_01144_00906 --------------CCTGTACGTTTGTACACGTTATTCGCA-------------
-----
FC88X_01020_00897 -------------TCCAGCGCCCGTTGAAACGTAATTATT--------------
-----
FC88X_01072_00615 -------------TCCAGCGCCCGTTGAAACGTAATTATT--------------
-----
FC88X_00969_00851 -------------TCCAGCGCCCGTTGAAACGTAATTATTCC------------
-----
FC88X_01098_00786 --------------------------TATACGTAA-------------------
-----
FC88X_00972_00461 --------------------CCAGACTATACCTGGTAATCAGTTCTC-------
-----
FC88X_01005_00793 -----------------GTACCTCAGTAAACGAAGT--TCTATTCT--------
-----
FC88X_00959_00357 --------------------TCTTGTTTTATGTAACTGGCTGTATCTC------
-----
FC88X_00991_00442 ------------------CGTTATGTTTTATG-AACTG--CGTATCTC------
-----
FC88X_01075_00601 --------------------ACATGCTTTACGAGATTGCCTATGCTC-------
-----
FC88X_01127_00633 --------------------ACATATTTTACGA---------------------
-----
FC88X_00996_00667 ----------------------CTACTGCATGTACATCAGTAACGTCCC-----
-----
FC88X_00993_00841 ----------------------CTACTGCATGTACATCAGTAACGTCTC-----
-----
FC88X_01069_00362 --------------------GGGGAATGCGTGTCCATC----------------
-----
FC88X_00970_00467 --------------ATCGTAGCTGTTTCTATGTGTCCATTCCCC----------
-----
FC88X_01043_00608 ---------------------------CTAAGT-TCCACT--------------
-----
FC88X_01091_01177 ---------------CTGCGCGCTGTTCTACTTCTTGATTCC------------
-----
FC88X_00953_00981 ----------------CGTGCGCGAGTCG---TGCCCTGTTCCCGT--------
-----
FC88X_01057_00872 ------------------CACGCTAGTCGAGGTGCCCCGTTGTTTT--------
-----
FC88X_01048_01124 ---------------------ACGAGGTGT------------------------
-----
FC88X_01132_00962 ---------------------ACGAGGTGTCCTCCCCACCTTGTACTA------
-----
FC88X_00997_00359 ------------------TCGCTGGCTTGGTTGTCCGT-TATTCTC--------
-----

FC88X_01109_00559 ------------------TCGCTGGCTTGGTTGTCCGT-TATTCTC--------
-----
FC88X_01049_00533 ------------------TCACTGGCTTGGTTGTCCGT-TATTCTC--------
-----
FC88X_01023_00838 ------------------TCACTGGCTTGGTTGTCCGT-TATTCTC--------
-----
FC88X_01061_01231 ------------------TCACTGGCTTGGTTGTCCGT-TATTCTC--------
-----
FC88X_01054_00564 ------------------TCACTGGCTTGGTTGTCCAT-TATTCTCCC------
-----
FC88X_00976_00695 ------------------TCGGTTGCTCGATTAACGGTCTATTCT---------
-----
FC88X_00953_00648 ------------------TCGTTCGCTTTGCTGCCGCGCTATTGT---------
-----
FC88X_01048_00862 ------------------TCGTTTGCTTTGCTGCCGCGCTATTGT---------
-----
FC88X_00999_00901 ---------------CACACCTTTGAGTTGTTCCCGCG-TATT-----------
-----
FC88X_01009_00512 --------------CGACTCGTTCAGTTGCCTCCCTTTTGCT------------
-----
FC88X_01040_00576 --------------CGACTCGTTCAGTTGCCTCCCTTTTGCT------------
-----
FC88X_01034_00780 -------------------CTCTCGGTGATTCCCTCG-----------------
-----
FC88X_01069_00779 ------------------CTACACCCTGGCCTCCCCGTAGATTCC---------
-----
FC88X_01030_01153 --------------------------------TTCCGTTGATT-----------
-----
FC88X_01078_00604 ------------------TTTTTCGCTATCTGTCCCGTTGGTTCTC--------
-----
FC88X_00981_00693 -----------------TCTAGTTGGTTTTATTGCTGCTATTCTC---------
-----
FC88X_00964_00902 -----------------TCTAGTTGGTTTTATTGCTGCTATTCTC---------
-----
FC88X_00971_00828 ---------------TCCCTGATTGCTTCATTTTCTATTCTCC-----------
-----
FC88X_00957_00512 -----------------TCCAGTGTTTAATTTCTTGGCTTTTCT----------
-----
FC88X_01039_00355 ------------------CCACGAGTTTGCTTGCGTGGTTTACTC---------
-----
FC88X_00962_01209 -------------------TTCGCATTTGACTTTCGCCTACTTTCT--------
-----
FC88X_01137_01031 -------------------------TTTGACTTTCGA-----------------
-----
FC88X_01104_01019 -------------------CGTAACGTTTGCTTACGCCTTTTCTC---------
-----
FC88X_01008_00875 ------------------TCTGTCCCTTCTTTGTTTGCGCGTTTT---------
-----
FC88X_01036_01150 ------------------CTATTCCCTGGTTTGCAAACGCGTGTT---------
-----
FC88X_00965_00616 -------------------TTTTCTGTCATACGCAGGGCTATTCTT--------
-----
FC88X_00972_00889 -------------------TTCTCTGTCATACGCAGGGCTATTCTT--------
-----
FC88X_01077_00780 -------------------TTTTCGGTCGTACGCAGGGCTATTCTT--------
-----

```
FC88X_01005_00604      -----------------GTCCTTTTTTCGTCCGCTGTACTATTGT---------
-----
FC88X_01052_00882      --------------------------TAGTACGCT-------------------
-----
FC88X_01025_01203      -------------------TTCTAGTTCCTCGTCTAGTATATTCGC--------
-----
FC88X_01134_00559      -------------------TTCTAGTTCCTCGTCTAGTATATTCGC--------
-----
FC88X_01037_00607      -------------------TTCTAGTTCCTGGTCTAGTATATTCGC--------
-----
FC88X_00987_01233      ------------------TCCGCGATGTCGTTCCTGGACCATTTTC--------
-----
FC88X_00999_00689      -------------------TCACGTGTTTTCCGTCTAGACAATGTT--------
-----
FC88X_01132_00848      ---------------------------TTTCCTTGTA-----------------
-----
FC88X_00983_00879      -------------------TGACATGCTTT--TCGTATTCACTTTTC-------
-----
FC88X_01105_01179      ---------------------ACATAGTTTCGTCGTAGTCATGTTCTC------
-----
FC88X_00958_00905      --------------------TTATTCTCTCTTGTAAGGGCATTGTTG-------
-----
FC88X_01099_00659      --------------------TTATTCTCTCTTGTAAGGGCATTGTTG-------
-----
FC88X_01034_00825      -------------------TTTACTTTTTGTTGTATGCACATTCTC--------
-----
FC88X_01038_00769      --------------------------------------CCATTGTCG-------
-----
FC88X_01041_00876      -------------------CCGATTAGTCTCCGTCGGGCCATTGTC--------
-----
FC88X_00953_00985      -----------TTCGGCATCGTTCGTCTCCTGCATATTCC--------------
-----
FC88X_01124_01144      -----------TTCGGCGCCGTTCGTCTCCTGCATATT----------------
-----
FC88X_01092_00609      ------------CCAGCG-CGTTTGCGTTATGCGTATTCC--------------
-----
FC88X_01080_00724      ----------------CG-CGTATGCCTCCTACGTA------------------
-----
FC88X_01036_00500      -----------------ACGTTTTCGTTTTACGGCATCCTATTCTT--------
-----
FC88X_01137_00881      -----------------ACGTTTCG--TTTACGGCATCCTATTCTT--------
-----
FC88X_01094_00676      ----------------------------TTACGGTCATC---------------
-----
FC88X_01039_00630      -------------------CTTATGTTTCTCGCGTGCC-TATTCGT--------
-----
FC88X_01063_01220      -------------------CTTATGTTTCTCGCGTGCC-TATTCGT--------
-----
FC88X_01076_00928      -------------------CTTACGTTTCTCGCGTGCC-TATTCGC--------
-----
FC88X_01074_00379      ----------------TTGCACGTTTGTCTCCCGTGTCGTATT-----------
-----
FC88X_00997_00539      --------------------CTACGTTTTAGTTACATTGCATTTCTC-------
-----
FC88X_01011_01002      --------------------CTACGTTTTAGTTACATTGCATTTCTC-------
-----
```

FC88X_01143_00830 -------------------GCTACGTTT-AGTTACATTGCATTTCTCCC-----
-----
FC88X_01034_00575 --------------------CTACGTTTTAGTTACA------------------
-----
FC88X_00953_00589 --------------------CTACGTTTA-------------------------
-----
FC88X_00967_00804 --------------------CTACGTTA--------------------------
-----
FC88X_01036_00314 --------------TTCCGTCTCCGTTAGCATTGCAGTAC--------------
-----
FC88X_00955_00641 ------------------CCCAACATTCGCTCCTCAGGTTGCTATCC-------
-----
FC88X_01137_01123 ------------------CCTAACGTTCCCTCG-CAGTATGGTATT--------
-----
FC88X_01012_00645 ------------------CCTAACGTTTTGCAC---AGTTGGTATCTC------
-----
FC88X_01079_00653 ---------------CCACCTTACGTTATGGTT---------------------
-----
FC88X_01038_00810 -------------------CTTCCGTTACCCCCGCGGCGGCATGTG--------
-----
FC88X_01126_00570 -------------------CTTCCGTTACAC-----------------------
-----
FC88X_01062_00666 -----------------TCTTACAGTTTT--CTAA-------------------
-----
FC88X_01085_00689 -------------TCCTCTCTTCTGTTTC--CCAGCCTAGT-------------
-----
FC88X_01069_00570 ---------------TCCCCTACCGTTTCAGCCAAGCTGCT-------------
-----
FC88X_01065_00527 -------------------CTCCCTTTTCAACGACGTTGGCCATTA--------
-----
FC88X_01143_00975 -------------------CTTCGTTGTCAACGGT-TCGGCTATTCC-------
-----
FC88X_00955_00304 --------------------CTAGACGTTCCCACATCTTGG-------------
-----
FC88X_00987_00422 ----------------TTTTCTAGCTATTCGC-CACCTTGGAGGT---------
-----
FC88X_01103_00810 -----------------TTTCTACCTATTCGTCACGCGTGTTTC----------
-----
FC88X_01131_00859 -----------------CTTCCAACCTCTCGTCA-GCGTGCTGCT---------
-----
FC88X_00995_00365 ----------------ATCTCTCATCTTTCTCCTCGGACCCATCC---------
-----
FC88X_01091_01265 -------------------ACTCCCTTCTAACAGCTGGCCCATGCG--------
-----
FC88X_01022_00567 --------------------CTCTTCTTA-------------------------
-----
FC88X_01107_00836 --------------------CCCTTCTTCCTCTTCACGATGATGCCA-------
-----
FC88X_01128_00807 ----------------CTCACTTCTTTTCTCTCTCGGGATGA------------
-----
FC88X_00995_00850 ------------------ATCTTCTCGTCCG-AGCTTGCTATTCAC--------
-----
FC88X_01047_01127 ------------------ATCTTCTCGTCCG-AGCTTGCTATTCAC--------
-----
FC88X_00966_00826 -------------------TCTCGTCTTCCCCATCGCGCTATTAAC--------
-----

FC88X_01043_00508 ------------------GCCAAGTATTCCGTGACATGCTATTCGT--------
-----
FC88X_01102_00599 -------------------CCCAGTATTCCGTGACATGCTATTCGT--------
-----
FC88X_01078_00511 -------------------ACATCTATTCAGT----------------------
-----
FC88X_00951_00465 ----------------------CCAATTTCGC-ATCTC----------------
-----
FC88X_01076_01180 --------------------CTCCAACTTCTCTATCTCCGAACATAC-------
-----
FC88X_01017_00368 -----------------TTTCTCCAGTTTCCCTCTCGCCAAGGCGC--------
-----
FC88X_01132_00861 ----------------------CCAAGTTCCCT-TCTCCATGGCAATTAC----
-----
FC88X_00996_00348 --------------------------GTTTACCTTCTCA---------------
-----
FC88X_00983_00475 -------------------TCACGCATTTGCCCGTTTGACTTTCAC--------
-----
FC88X_01021_00958 -------------------TCACGCATTTGCCCGTTTGACTTTCAC--------
-----
FC88X_01142_01221 -------------------TCACGCATTTGCCCGCTTGACTTTCAC--------
-----
FC88X_01099_00477 ------------------TTCCCCTATCTGAGCGTTGGACATTCC---------
-----
FC88X_01045_00572 -------------------TCACACATTTACCCAAAT---TTCTATTCT-----
-----
FC88X_01020_00822 -------------------TCACACATTTACCCAAAT---TTCTATTCT-----
-----
FC88X_01104_00673 -------------------TCACACATCTACCCAAAT---TTCTATTCT-----
-----
FC88X_01013_01164 --------------------CCCTCATTTGCGCACATGAATTCTATT-------
-----
FC88X_00952_00458 ------------------CTCAGTGGGTCCTCTCTGTGTGTTCTC---------
-----
FC88X_00951_00752 --------------------------TCCTACTCTGCG----------------
-----
FC88X_01146_00684 -----------------------GGTTCTCCCAGTTTGAGTTGTCAACGCGT--
-----
FC88X_01143_00960 --------------------------TCCCTGTGTGTAAGTGGTCT--------
-----
FC88X_01010_00305 -------------------CCATTGCTTTGTATAA-GCGGA-CTTCTC------
-----
FC88X_01103_00836 -------------------CCATTGCATAATGTGT-GCAGCTCTTCTC------
-----
FC88X_01032_00990 --------------------TTGTACATTGTCTGTTAGAGCTATTCT-------
-----
FC88X_01077_00703 --------------------TTGTGCATTGTCTGTTAGAACTATTCT-------
-----
FC88X_01044_00948 -------------------CTTGTGCGG-GTGTGT-AAGGCAATTCTC------
-----
FC88X_00961_00504 -------------------ACCACCCTTGTTC----------------------
-----
FC88X_01090_00808 -------------------TCCACCCTTCTTCCACAACGCCATGTT--------
-----
FC88X_01031_00954 -------------------CCCACTCCTCTCCAGCCTTGGTCGTCTC-------
-----

```
FC88X_00991_00685       -------------------ATCACCGTTGGTCGGTGGTGCGCTGTT--------
-----
FC88X_01009_00814       -------------------ATCACCGTTGGTCGGTGGTGCGCT-----------
-----
FC88X_00996_00634       -------------------ATCACCGTTGGTCGG--------------------
-----
FC88X_01120_00515       -------------------ATCACCGTTGGTCGGTCGG----------------
-----
FC88X_01036_00634       -------------------CACATTTCTGTTTCGTCGCGCCG-TATT-------
-----
FC88X_01097_00567       -------------------CACATTTCTGTTTCGTCGCGCCG-TATT-------
-----
FC88X_01046_00678       -------------------TCCATTCCTGTT-CGTCACTTCGCTATT-------
-----
FC88X_01093_00595       -------------------TCCATTCCTGTT-CGTCACTTCGCTATT-------
-----
FC88X_00977_00651       ------------------ACATGCACTTGTT-CGTCTATTCC-TATTCC-----
-----
FC88X_00957_00879       --------------------CCACGTAATCCACATTTGTGTCTTCTC-------
-----
FC88X_01006_01130       --------------------TCACATAATCCACATTTGTGTCTTCTC-------
-----
FC88X_01065_00483       --------------------TCAATTCATCCTCATTGCTATCGCCTC-------
-----
FC88X_00975_00327       ------------------TTCTGGACTGTACTCTGGGGCTGTCTC---------
-----
FC88X_00957_00357       -----------------GTTCTGGACTGTACTCTGGGGCTGTCTC---------
-----
FC88X_00966_00811       ------------------TTCTGGACTGTACTCTGGGGCTGTCTC---------
-----
FC88X_00973_00811       ------------------TTCTGGACTGTACTCTGGGGCTGTCTC---------
-----
FC88X_00999_00813       ------------------TTCTGGACTGTACTCTGGGGCTGTCTC---------
-----
FC88X_00959_01273       ------------------TTCTGGACTGTACTCTGGGGCTGTCTC---------
-----
FC88X_01017_00583       ------------------TTCTGGACTGTACTCTGGGGCTGTCTC---------
-----
FC88X_01022_00635       ------------------TTCTGGACTGTACTCTGGGGCTGTCTC---------
-----
FC88X_01010_00651       ------------------TTCTGGACTGTACTCTGGGGCTGTCTC---------
-----
FC88X_01043_00663       ------------------TTCTGGACTGTACTCTGGGGCTGTCTC---------
-----
FC88X_01038_00763       ------------------TTCTGGACTGTACTCTGGGGCTGTCTC---------
-----
FC88X_01017_01104       ------------------TTCTGGACTGTACTCTGGGGCTGTCTC---------
-----
FC88X_01061_00631       ------------------TTCTGGACTGTACTCTGGGGCTGTCTC---------
-----
FC88X_01098_00899       ------------------TTCTGGACTGTACTCTGGGGCTGTCTC---------
-----
FC88X_01052_00934       ------------------TTCTGGACTGTACTCTGGGGCTGTCTC---------
-----
FC88X_01145_00738       ------------------TTCTGGACTGTACTCTGGGGCTGTCTC---------
-----
```

FC88X_01121_01125 ------------------TTCTGGACTGTACTCTGGGGCTGTCTC---------
-----
FC88X_00981_00315 ----------------CCTTCCTGTCTTTCCCCTGGGGGCTTCT----------
-----
FC88X_01093_00495 ------------------CCGAATATTTCTCCCTGGGATCGTCTC---------
-----
FC88X_01135_00941 ------------------CCTGATGCTTGGCCTTGGGTGCTTCTC---------
-----
FC88X_01018_00935 -------------------CTTAGACCTTGGTGGCATTCTGTTCTC--------
-----
FC88X_01092_00541 -------------------CTTAGACCTTGGTGGCATTCTGTTCTC--------
-----
FC88X_01080_00942 -------------------CTTAGACCTTGGTGGCATTCTGTTCTC--------
-----
FC88X_01139_00888 -------------------CTTAGACCTTGGTGGCATTCTGTTCTC--------
-----
FC88X_01089_00407 -------------------TTTAGACCTTGGTGGCATTCTGTTCTC--------
-----
FC88X_01142_00593 -------------------CTTAGACCTCGGTGGCATTCTGT------------
-----
FC88X_00973_00867 ------------CCAATAGGTTAGAC-TTGGTGGCTTTCT--------------
-----
FC88X_00963_00855 --------------------------AGTA-CTTCCATGTA-------------
-----
FC88X_01079_00738 --------------------------AGTA-CTTCCATGTA-------------
-----
FC88X_01040_00330 ------------------------ATAGTAGCTTCCCCTTGCTATCTTCAT---
-----
FC88X_00970_01205 ----------------TTTGACAACGTGT---GTCCTGCTGTTCTC--------
-----
FC88X_01053_00758 --------------------CCAGCATCT---GTCCTGTGCTCTTCTTCG----
-----
FC88X_01040_00802 ------------------TTGTGACATGTAACGTCCTTCTATCTT---------
-----
FC88X_01133_00758 ------------------TTGTGACATGTAACGTCCTTCTATCTT---------
-----
FC88X_01046_00612 ---------------GCCAACCGACATGT---GTTCTTCTATTCTT--------
-----
FC88X_00956_00920 ------------------ATGTCTGACCATTGCTGCTTCTATCTC---------
-----
FC88X_01104_00830 ------------------TCCTCTGTCGACCATGGCGTC-ATCCC---------
-----
FC88X_00986_00292 ----------------AGCTTAGATCTTGGCTCACGTTTTCTC-----------
-----
FC88X_01033_01110 ----------------AGCTTAGATCTTGGCTCACGTTTT--------------
-----
FC88X_00991_00578 ----------------AGCTTAGATCTTGGCTCACGTTTTCTC-----------
-----
FC88X_00989_00747 --------------CGAGCCTA-ATCGTTGCTCCCGCATAT-------------
-----
FC88X_01012_00886 -----------------CCCTAGATTGATCTCCCCGCTACTACA----------
-----
FC88X_01075_00458 ------------------CCTAG-TAGTTCTCACCCGCATTT------------
-----
FC88X_01058_00494 -------------------CTTGATTGTCCGTCCTGCTACGTCTCCTCC-----
-----

```
FC88X_00972_00769      -----------------CCGACAA-TTCGACTCCCCGGCAATGTT---------
-----
FC88X_01065_00642      -----------------CCGACAAATTCGACTCCCCGGCAATGTT---------
-----
FC88X_01042_00323      -----------------CCGACAA-TTCGACTCCCCGGCAATGTT---------
-----
FC88X_01045_00838      -----------------CCGACAA-TTCGACTCCCCGGCAATGTT---------
-----
FC88X_01025_01130      -----------------CCGACAA-TTCGACTCCCCGGCAATGTT---------
-----
FC88X_01070_01137      -----------------CCGACAA-TTCGACTCCCCGGCAATGTT---------
-----
FC88X_01119_00689      -----------------CCGACAA-TTCGACTCCCCGGCAATGTT---------
-----
FC88X_01133_01123      -----------------CCGACAA-TTCGACTCCCCGGCAATGTT---------
-----
FC88X_01036_00656      -----------------CTGACAA-TTCGACTCCCCGGCAGTGTT---------
-----
FC88X_00992_00671      -----------------CCGACAA-TTCGACTCCCCGGCACT------------
-----
FC88X_01142_00865      --------------CCTGTTGCAA-CACGACTCCCCGGCACT------------
-----
FC88X_01049_00626      -------------------------CTAGAGTCAC-------------------
-----
FC88X_01142_00847      ----------------CTCCCCCGCGTAGAGTGACACAGACTC-----------
-----
FC88X_01058_00872      -----------------------CACTGCAGTTACAAAGCGCCTCCCGGA----
-----
FC88X_01034_00515      ---------------CCACCACAACTCCGACACTGCTATTGC------------
-----
FC88X_01110_00596      ------------------CCACGACTCCGA------------------------
-----
FC88X_01085_00637      ---------------TCTCGACCGCCGATGCCTCCC------------------
-----
FC88X_01093_00801      ---------TTACCTTCTCCACTACGCCTATTGCCT------------------
-----
FC88X_01087_00401      ------------CTATCTCCACATCACCGAGGGCCTC-----------------
-----
FC88X_00986_00840      -------------------TTCAACGTTTATCCGCATGAACATCTC--------
-----
FC88X_00962_01241      -------------------TTCAACGTTTATCCGCATGAACATCTC--------
-----
FC88X_01071_01072      -------------------TTCAACGTTTATCCGCATGAACATCTC--------
-----
FC88X_01092_00468      --------------------CCAACGCCTCTTCG---GCACATC----------
-----
FC88X_01060_00628      ------------------CCGAAAACTGTTCTGACATGATGCTCTT--------
-----
FC88X_01123_00664      --------------------CAAACACGTTACGACCTGCGCCTCTT--------
-----
FC88X_01113_01172      ------------TCTCGTATAAACACCGCTGCGACCTGTC--------------
-----
FC88X_00985_00278      -----------------------TCCCTGATTCCCAGCAAACGGCTCGAC----
-----
FC88X_01033_01016      -----------------------TCCCTGATTCCCAGCAAACGGCTCGAC----
-----
```

FC88X_01121_00497 -------------------CTAATCCCTCCTTCACTGCA-ACCTGCA-------
-----
FC88X_01121_00862 ----------------------ATCCCTTCTTTCCTGCATACCT----------
-----
FC88X_01081_00525 -------------------------CTCCTG-
CATGCGACACTGCTGTACCACT-----
FC88X_01133_00681 ------------------------TCTCCTGACCTGGCTAACTGCTCTGC----
-----
FC88X_00979_00990 ----------------CTAACCACTGTCCGGTCCCAGCATGTT-----------
-----
FC88X_01121_00731 ---------------TCTTACCGTCCTCCGCACCTAGC-TGTT-----------
-----
FC88X_01108_00870 ---------------ATTAGGTATTTTCAAGTCCTAGCCTGCTCCC--------
-----
FC88X_01032_01033 -----------------ATTCCAAATCCCTAGACCACCGTGCC-----------
-----
FC88X_01059_00902 --------------------------------ACTACCGTGC------------
-----
FC88X_01001_00553 ---------------------CCATCACGGATTCAGCCTCGCTATACT------
-----
FC88X_01126_00617 --------------------TCCATCCAGCATACTGCCCTGCCTTATT------
-----
FC88X_01007_00986 ------------------TTCGCGATCCGAATCCCAGCTACTGTT---------
-----
FC88X_01001_01048 ------------------CTCGCGATCCGAGTCCCAGCTACTGTT---------
-----
FC88X_01132_01176 ---------------------ACGTTACCAATCCCGAATACTGTCGTT------
-----
FC88X_00989_00360 -------------------ACTGTTGCTAAA--CTTCAGTCTCGTTCT------
-----
FC88X_01052_00833 -------------------ACTGTTGCTAAAAACTTCAGTCTCGTTCT------
-----
FC88X_00985_00938 -------------------ACTGTTGCTAAA--CTTCAGTCTCGTTCT------
-----
FC88X_00989_00923 -------------------ACTGTTGCTAAA--CTTCAGTCTCGTTCT------
-----
FC88X_01128_01023 -------------------ACTGTTGCTAAA--CTTCAGTCTCGTTCT------
-----
FC88X_01055_00770 -------------------ACTGTTGCTAAA--CTTCA----------------
-----
FC88X_01077_00500 ----------------------CCTATAACT--CCCGAACACTGGCCTTGA---
-----
FC88X_01130_01151 -------------------------ATTATT--CCTAACCACAGCCC-------
-----
FC88X_01001_00859 --------------------CTGATGATAAA--CTCACGCCCTGGCTATG----
-----
FC88X_01015_00674 -----------CTGCTAAAAATGAGGCTGCACACTTCTC---------------
-----
FC88X_01068_00829 -----------CTGCTAAAA-TGAGGCTGCACACTTCTC---------------
-----
FC88X_01076_00926 ----------GAACCGTTCCCCTTTGCTGCACACCTTAT---------------
-----
FC88X_01082_01056 ------------------------TGCTGCACACT-------------------
-----
FC88X_00993_00392 --------------TCTGCCCATCTCCTG-GCCTTTCATATT------------
-----

```
FC88X_01129_01229      --------------TCTTCTTCCATTCTACGCTTTTCTTGC-------------
-----
FC88X_00997_00749      ----------CGTTTATGCTTCATCTCCACAACTTTCA----------------
-----
FC88X_01022_00625      -------------------------TCCA-AACT--------------------
-----
FC88X_00990_00334      ---------------TTCTGTGCATTTCTCCCAGCACACCGTC-----------
-----
FC88X_01008_00662      ---------------TTCTGTGCATTTCTCCCAGCACACCGTC-----------
-----
FC88X_01132_01130      ---------------TTCTGTGCATTTCTCCCAGCACACCGTC-----------
-----
FC88X_01013_01211      ---------------TTCTGTGCATTTCCCCCAGCACACCGTC-----------
-----
FC88X_00980_00614      ---------------TTCTGTGCATTTCTCCCAGCACATCGTC-----------
-----
FC88X_01049_00473      ---------------TCCTGTGCATTTCTCCCAGCACACCGTT-----------
-----
FC88X_01059_00799      --------------TTCGTGTGCATTTCTCCCAGCACACCGTC-----------
-----
FC88X_01046_01020      ------------------TCCCTGTGTTCTCCC-CACAGCGGTTAA--------
-----
FC88X_01094_01130      ------------------TCCCTGTGTTCTCCC-CACAGCGGTTAA--------
-----
FC88X_01121_01192      ---------------CTTTCCCTTCGTCCCCGCGCACACTGG------------
-----
FC88X_00976_00422      -----------------CTGTAATTTCCCCAGACAGCGCCGTAGT---------
-----
FC88X_00999_00479      -----------------CTGTAATTTCCCCAGACAGCGCCGTAGT---------
-----
FC88X_00954_00731      -----------------CTGTAATTTCCCCAGACAGCGCCGTAGT---------
-----
FC88X_00964_00827      -----------------CTGTAATTTCCCCAGACAGCGCCGTAGT---------
-----
FC88X_00967_00803      -----------------CTGTAATTTCCCCAGACAGCGCCGTAGT---------
-----
FC88X_01012_00919      -----------------CTGTAATTTCCCCAGACAGCGCCGTAGT---------
-----
FC88X_01091_00740      -----------------CTGTAATTTCCCCAGACAGCGCCGTAGT---------
-----
FC88X_01033_00940      --------------------CCTGTATTCCGCACACCGACGTATT---------
-----
FC88X_01018_00520      ----------------ATATCAAATCTCCCAAACCACGCTCGC-----------
-----
FC88X_01112_00949      ------------------TCGCAACCTCCCAGACACCGCTAGTCC---------
-----
FC88X_00967_00887      ------------------------TCTCCCAATCAACGGCACGCCGGTAAC---
-----
FC88X_01090_00551      ------------------------TCTCCCAGTATCTAGCACGACGCTAAC---
-----
FC88X_01014_00554      --------------------------CCTCGAGCTCTCTCACTCCACTGACAT-
-----
FC88X_01035_00892      -----------------------------------CTATCACACCACTCA----
-----
FC88X_01027_00461      ----------------TTTGGACGTCTCCCAGTCCTCGTTACGCC---------
-----
```

FC88X_01024_00847 ----------------TTTGGACGTCTCCCAGTCCTCGTTACG-----------
-----
FC88X_01049_01141 ----------------TTTGGACGTCTCCCAGTCCTCGTTACG-----------
-----
FC88X_01142_00921 ----------------TTTGGACGTCTCCCAGTCCTCGTTACG-----------
-----
FC88X_01062_00561 ----------------------------CCCATCCCCGTGCCG-----------
-----
FC88X_01008_00780 ----------------CCACAAACTCTGGCACTTCTCCTTGCG-----------
-----
FC88X_00996_00356 ------------------CTATCCCTCTGAGAACGTGCGTGTCTC---------
-----
FC88X_01028_00408 -------------------TAACACTCTGA------------------------
-----
FC88X_00968_00959 ------------------TCTTTCTCCCGCTGAACGGTGCTTCTC---------
-----
FC88X_01146_00718 ---------------CTAGCATTATTCCGCTA---GGTGCTTCTC---------
-----
FC88X_00950_00467 -----------------CTGTCTCCTTTACCGCA-CCAACATGTT---------
-----
FC88X_01005_00500 ---------------TTCTCTCATTTCAACCTCATCCAACAC------------
-----
FC88X_01067_00594 ---------------CTATTTCACGCTGACCGCCCACATTTT------------
-----
FC88X_01024_00425 ----------------CTTCCTTCTCTCCACATGC--AACGTGCA---------
-----
FC88X_01025_00620 ----------------CTTCCTTCTCTTCACATGC--AACGTGCA---------
-----
FC88X_00979_00814 ----------------TTCCCCTTTCTTGACGCGCT-AACGTGTT---------
-----
FC88X_01016_00587 -----------------CTCCCTGCTTTCTCGCACTGAACGTCA----------
-----
FC88X_00982_00710 ---------------TTTGGCTTTGTCCCACAACATGTTCTC------------
-----
FC88X_01088_00553 -----------------CCTTTCTCCCCGCAAACAGGATCTCTG----------
-----
FC88X_01048_00403 -------------------TTCTCGCCGCACAAA-CGAACATTGTT--------
-----
FC88X_01016_01140 -------------------TTCTCGCCGCACAAA-CGAACATTGTT--------
-----
FC88X_01010_00831 -------------------TTCTCGCCGCACAAAACGAACATTGTT--------
-----
FC88X_00960_00469 ------------------------CCGCACCAGCGTTGACAATTTATTCTC---
-----
FC88X_01032_00535 ------------------------CCGCACCAGCGTTGACAATTTATTCTC---
-----
FC88X_01015_01271 ---------------------TTATGCCACCAGCCCAGTCTATTTCTC------
-----
FC88X_01059_00798 ------------------CCAGTATCCCTCCAGCCCTGATTTATT---------
-----
FC88X_01017_00667 ----------------TCACTGGATTCTCCCGCAGTACGTTTAT----------
-----
FC88X_01075_01198 -----------------CCCTGGACTACACCGCTGCAGATTATT----------
-----
FC88X_00974_00491 ------------------------CTACACCA----------------------
-----

```
FC88X_00967_00724       ---------------CTACTACATCTCCACCACCGCACGAACCCT---------
-----
FC88X_01073_00516       ------------------------CTACACCATGA-------------------
-----
FC88X_01102_00764       ------------------------CCACAGCATGACTCTGGCTCTCCTACG---
-----
FC88X_00990_00518       -------------------------TACACCAGC--------------------
-----
FC88X_01106_00577       -------------CCAATTACTCCCTACAGCAGCGACATT--------------
-----
FC88X_01128_00876       --------------TTCATCCTTCCCCAGCCAGCGCGGTTA-------------
-----
FC88X_01110_01126       ---------------CCATCCTTCTGCAAC-AGCACAGTCCT------------
-----
FC88X_00988_00605       -------------CGACACCCTGCTCCCGACAGCCACGAG--------------
-----
FC88X_00999_00296       ------------CTGCACATTATCTCGCTCCCGACGGACC--------------
-----
FC88X_01098_00438       --------GCGCCTGCGC-TTATCTT--TCCCAACGAT----------------
-----
FC88X_00967_00666       ---------------CACACGGTTTT-CTCCCACCGGCTGGCC-----------
-----
FC88X_00966_00784       ---------------CACACGGTTTTTCTCCCACCGGCTGGCC-----------
-----
FC88X_00962_00968       ---------------CACACGGTTTC--TCCCACCGGCTGGCC-----------
-----
FC88X_01074_01245       ---------------CACACGGTTTC--TCCCACCGGCTGGCCCC---------
-----
FC88X_00994_00363       -------------------TCATGTGCCTCCCGACGTGACATTCGC--------
-----
FC88X_00979_00825       -------------------TCATGTGCCTCCCGACGTGACATTCGC--------
-----
FC88X_00996_00907       -------------------TCATGTGCCTCCCGACGTGACATTCGC--------
-----
FC88X_01027_00663       -------------------TCATGTGCCTCCCGACGTGACATTCGC--------
-----
FC88X_01082_00589       -------------------TCATGTGCCTCCCGACGTGACATTCGC--------
-----
FC88X_01131_00741       -------------------TCATGTGCCTCCCGACGTGACATTCGC--------
-----
FC88X_01117_00946       -------------------TCATGTGCCTCCCGACGTGACATTCGC--------
-----
FC88X_01051_00359       ------------------TCCATGTGCCTCCCGACGTGACATTCGC--------
-----
FC88X_01147_01198       -------------------------ACCTCCCGGCGCGA---------------
-----
FC88X_01048_00583       ----------------CCAGAATTTGTCTCCCGCTGGCGTGAT-----------
-----
FC88X_01010_00875       ----------------CCAGAATTTGTCTCCCGCCGGCGTGAT-----------
-----
FC88X_01023_00579       ---------------GCCAGCAATAAACTCCC-CCGGCGCGAA-----------
-----
FC88X_00979_00566       ------------------ACCACAT-TTCTCTCCCTGCACAAT-ACG-------
-----
FC88X_01048_00846       ------------------ACCAATT-TTCTATTCCCGCACAGTTACAG------
-----
```

```
FC88X_00970_00708      ----------------ACACCACATATCCCGTCCCAGCGCATT-----------
-----
FC88X_00979_00737      -------------------CCACGTATCTTGCCCCTGGCTGTTCGC--------
-----
FC88X_01095_00518      ----------------TCTTCCCATATTTTGCACC--GCTATTCG---------
-----
FC88X_00966_00939      ----------------TCACTTCCTATCTGGTCGC--GATATTCT---------
-----
FC88X_00955_01021      -----------------------CTATCTCG-CGC--CAT--------------
-----
FC88X_01011_00934      ----------------ACGACCACTATCTCGCCCC--ACTATTCTA--------
-----
FC88X_00960_00988      -------------------CCAAATTCTCTCCGCCGGCAAGTTGTT--------
-----
FC88X_01127_00664      -------------------CCAAATTCTCTCCGCCGGCAAGTTGTT--------
-----
FC88X_01027_01152      -------------------CCAAATTCTCTCCGCCGGCAAGTTGTTCTTC----
-----
FC88X_00991_01272      -----------------CCAAAAAACTCTTCCCCCAGCAGGCTGAT--------
-----
FC88X_00985_00940      -------------------CCAATTCTCTGTTTTCCCCCGGCAGCC--------
-----
FC88X_01044_00859      -------------------ACAACTACTCTCCGCGGACCAGCATAT--------
-----
FC88X_01106_00588      ------------TAGTTAAATTACTGCTCCCCACCTGCA---------------
-----
FC88X_01013_00412      ---------------------------TTTAGACCT------------------
-----
FC88X_01118_00824      -----------ACCACACGTCCGTCTCTTTAGCCCTCA----------------
-----
FC88X_00963_00314      -----------TCTACACCTCTCGTGTCCAAAGCTCCC----------------
-----
FC88X_00967_00524      ----------------CTCCCAACGATTCAACACCAC-CAGTGGT---------
-----
FC88X_01015_00369      ----------------CTCCCAACGATTCAACACCAC-CAGTGGT---------
-----
FC88X_01008_00510      -------------------CTATTGAAACCACAAC-------------------
-----
FC88X_00972_00368      -------------------CCATCGGTTTCAAACCTCGCAGTTGTT--------
-----
FC88X_00951_00747      -----------------CTATAACCCCGTAGTTG--CAATGTTCTC--------
-----
FC88X_00976_00845      -----------------CTATAACCCCGTAGTTG--CAATGTTCTC--------
-----
FC88X_00967_00660      -----------------CTATAACCCCGTAGCTG--------------------
-----
FC88X_00967_00697      -------------------TTGACGCCTTTGGTGACCACTGATCTC--------
-----
FC88X_00959_00966      -------------------TTGACGCCTTTGGTGACCACTGATCTC--------
-----
FC88X_01141_00728      -------------------TTGACGCCTTTGGTGACCACTGATCTC--------
-----
FC88X_01077_00555      -------------------CTGACGCCTTTGGTGACCACTGATCTC--------
-----
FC88X_01029_00492      ------------------TACAGCATCTCGCCCTAGGTACCATTTT--------
-----
```

```
FC88X_01126_00916      -----------------CCACCACGAGCGCTCCCTTTGACCACTT---------
-----
FC88X_00994_00343      ----------------------TCGGTTACTCACCGCGCTATCCTCGT------
-----
FC88X_00989_00921      -------------------CTCTCGGATACACACGGGGCGCTCCCTA-------
-----
FC88X_01010_00571      -----------------------CGATTACAGACGTCGCCCACCGA--------
-----
FC88X_01035_00480      ---------------CCGCGCATTTCCCACACCGCT------------------
-----
FC88X_01088_01213      ---------------CCGCGCATTTTCCACACCGCTC-----------------
-----
FC88X_00984_00301      --------------ACCGGCGATTTTCCACAGCACTCTTTTT------------
-----
FC88X_00983_00408      -------------TCCTGTCA-TTGACCACGCCACTATTCT-------------
-----
FC88X_01108_00916      -------------TCCCGTCAATTGACCACGCCACTATTCT-------------
-----
FC88X_01010_00773      --------------CTAGCGT-TTCCCCC-GCAATCTATTTGTC----------
-----
FC88X_01134_00923      --------------CTAGCGT-TTCCCCC-GCAATCTATTTGTC----------
-----
FC88X_00986_00844      --------------CTAGCGT-TTCCCCCCGCAATCTATTTGTCC---------
-----
FC88X_01135_00857      --------------TTAGCGT-TTCCCCC-GCAATCTATTTGTC----------
-----
FC88X_00989_00594      --------------CCATCGTGTTCGCCCCGCGATGAATCT-------------
-----
FC88X_00996_00360      ----------------------TCCTCCCAACGATTTAGCTGTTTTGCA-----
-----
FC88X_00954_00691      ----------------------TCCTCCCAACGATTTAGCTGTTTTGCA-----
-----
FC88X_01004_00829      ----------------------TCCTCCCAACGATTTAGCTGTTTTGCA-----
-----
FC88X_01060_00635      ----------------------TCCTCCCAACGATTTAGCTGTTTTGCA-----
-----
FC88X_01092_00945      ----------------------TCCTCCCAACGATTTAGCTGTTTTGCA-----
-----
FC88X_01040_00361      --------------------ATGATCCTCGCCGGATGATCTGTTCC--------
-----
FC88X_01046_00624      --------------------ATGATCCTCGCCGGATGATCTGTTCCCC------
-----
FC88X_00964_00738      -----------------TTGCACCCTCCGGACGATTGACTAGTT----------
-----
FC88X_01087_00852      -----------------TTGCACCCTCCGGACGATTGACTAGTT----------
-----
FC88X_00998_00904      ---------------------------CCGATGATTG-----------------
-----
FC88X_00987_00276      -------------ACCACTTCCAACGGATCA-CGTATTATTCC-----------
-----
FC88X_00970_00458      -------------ACCACTTCCAACGGATCA-CGTATTATTCC-----------
-----
FC88X_01061_00398      ----------------TCTCCCAATCTATCAGCGTATGATC-------------
-----
FC88X_01102_00467      ----------------TCTCCCAATCTATCAGCGTATGATC-------------
-----
```

```
FC88X_01045_01017      --------------------TTACCTTCTCACG-TATGTTGGCATTTT------
-----
FC88X_01112_00816      --------------------TTACCTTCTCACG-TATGTTGGCATTTT------
-----
FC88X_01032_00409      ----------------------------TCACGCTATATTG-------------
-----
FC88X_01078_00629      ----------------------------TCACGCAAGAT---------------
-----
FC88X_01039_00951      -------------TTCCCTTCTAAC-CCGTACGCAACAATG-------------
-----
FC88X_01066_00807      -------------TTCCCTTCTAAC-CCGTACGCAACAATG-------------
-----
FC88X_01108_00764      -----------CTTTCTATTCTATTACCGCACGAGACA----------------
-----
FC88X_00986_00465      ------------------------------TCTCC-AATAT-------------
-----
FC88X_01140_00857      ---------------CCTGACAACGACAGCTCTCCGAATATT------------
-----
FC88X_00990_00783      -----------------TCCTCAACCTTACACTGCAGCGCGTGC----------
-----
FC88X_00996_01019      -------------------------TTTACACT---------------------
-----
FC88X_01009_00464      --------------TTCTCTCAACTCTTACACGCTAGCTGT-------------
-----
FC88X_01133_00967      --------------TTCTCTCAACT-TTACACGCTAGCTGTCC-----------
-----
FC88X_01025_01273      ----------------TCTATCAATACTGGCCGGTAGCTTTCT-----------
-----
FC88X_01068_00760      ----------------TCTATCAATATTGGCCGGTAGCTTTCT-----------
-----
FC88X_00986_00832      ----------------TCTCTCAAT-TTGCAGTTCAGCTTTCTC----------
-----
FC88X_00970_00300      ------------------CCGCAAT-TGACGTCATCCCTGGTACTC--------
-----
FC88X_01057_01217      ---------------------CAATCTGAC------------------------
-----
FC88X_01069_00599      --------------------TTAATCTGACCCGACAGCCTACTTGTT-------
-----
FC88X_01082_01064      --------------------TTAATCTGACCCGACAGCCTACTTGTT-------
-----
FC88X_00978_00964      ------------------CTCTAATCTGTACGATATAACTATTCC---------
-----
FC88X_01060_01145      ----------------------AATCCGTA------------------------
-----
FC88X_01116_00516      ------------------CTCCCATCCGTGATATGAGCGGACACAT--------
-----
FC88X_00965_00327      ----------------CACTTGCATGTGAAGACTCGCTGTTCT-----------
-----
FC88X_01112_01112      ----------------CACTTACATGTGAAGATTCGCTGTTCT-----------
-----
FC88X_01030_00532      ---------------TTTCCCCGTTATCGTAGATCGCTGTTCTCC---------
-----
FC88X_01143_01145      ---------------TCTCTCCGTTATCGTAGATCGCTGTCCT-----------
-----
FC88X_01033_01007      --------------------------------ATCACTGT--------------
-----
```

```
FC88X_01035_01150      ---------------TCTCACCGGTGACGGTATACGCTTTCTC-----------
-----
FC88X_01008_01025      ---------------CAGTCCCAGTTGAGT-CTTCGCG-TTCTC----------
-----
FC88X_01090_00452      -----------------CCCACGGTTGTGTGCATCCCGATTCTCCC--------
-----
FC88X_00974_00622      -----------------CCCAAGCATCAGTGCACCGTTGTTCTC----------
-----
FC88X_00983_00789      -----------------CCCAAGCATCAGTGCACGGTTGCTCTCCC--------
-----
FC88X_01038_00504      ------------------CCGTGAATTAGTTCACGATGTGCTTCT---------
-----
FC88X_01009_00652      -----------------CTCCCAAATGAGTCTACGGTCACTCAC----------
-----
FC88X_00977_00335      -----------------CTTGGCTATCTGCTAAATGCGTATTCT----------
-----
FC88X_01002_00487      -----------------CTTGGCTATCTGCTAAATGCGTATTCT----------
-----
FC88X_01099_00473      -----------------CTTGGCTATTTGCTAAATGCGTATTCT----------
-----
FC88X_00987_00912      ---------------ACCTCTATTCGCTGC-AAATGGCCATTGC----------
-----
FC88X_00959_00854      ----------------CCAATCTCTCGTGGTCATTGCGCTTCT-----------
-----
FC88X_01048_00602      ----------------CCAATCTCTCGTGGTCATTGCGCTTCT-----------
-----
FC88X_01094_01153      ----------------------TCTCCTCTGCATT-CGCT--------------
-----
FC88X_01002_00502      ------------------CCATTCATTCGATGAATTTGCCGTTCT---------
-----
FC88X_01132_00815      -----------------CTCAAACGTGCGATGAATGAGCCTTCT----------
-----
FC88X_01146_00931      -----------------TCCTCACCCGTGCAAATTGAGCTTTT-----------
-----
FC88X_00978_00734      ------------------TCAGTGCATT-CACAG-GGCTTGGTTCTT-------
-----
FC88X_01136_00736      -----------------CTCATTGCAATACGCAG-TGCGTTGTTC---------
-----
FC88X_01078_00603      ----------------ACCCTGGATACGAAACAG-TGCATTCTC----------
-----
FC88X_01145_00837      ----------------ACCCTGGATACGAAACAG-TACATTCTC----------
-----
FC88X_01019_00384      -----------------ACCTGGACACGAAACAG-TGCATTCTCC---------
-----
FC88X_01066_00601      ----------------------ATTGCAAACCAAATGCATCCTC----------
-----
FC88X_00976_01215      ---------------TGTGATGAGTACAACTCACCTCGATTCTC----------
-----
FC88X_01071_00377      ---------------TGTGATGAGTACAACTCACCTCGATTCTC----------
-----
FC88X_01071_00669      ---------------TGTGATGAGTACAACTCACCTCGATTCTC----------
-----
FC88X_01065_00811      ---------------TGTGATGAGTACAACTCACCTCGATTCTC----------
-----
FC88X_01141_01176      ---------------TGTGATGAGTACAACTCACCTCGATTCTC----------
-----
```

FC88X_01024_00599 ---------------CGTGACGAGTACAACTCACCTCGATTCTC----------
-----
FC88X_01080_01171 ---------------------CAGTACAAC------------------------
-----
FC88X_01133_00939 ------------------CCTCTGCACAACGCAACTTGCGTCT-----------
-----
FC88X_00980_00336 ------------------CCATCACCCCGCTAAACGCGCAA-TATT--------
-----
FC88X_01005_01016 ------------------CCAACAGCATGCCAAACTCGTAAATATT--------
-----
FC88X_00976_00721 --------------------ACCGTACTTTCAACCTCGAACACTATT-------
-----
FC88X_01141_00811 ----------------------------CACAAACTCGAA--------------
-----
FC88X_00994_00587 ----------------CCCAAACAGCCCCAGCACCTACATCGC-----------
-----
FC88X_01012_00374 --------------------CACATCCCTCATACCTATCACGCAA---------
-----
FC88X_01130_00779 -----------------ACGAACATCCTCCTGAGCTGTCTATCT----------
-----
FC88X_01138_00792 ---------------ACACGAA-ATCCCCGGTACCCGTCTAACT----------
-----
FC88X_01027_01207 ----------------TACGTACAACTTC-------------------------
-----
FC88X_00964_00386 --------------ACGATCTATCTCCCG-CCGTCAGCACGC------------
-----
FC88X_01042_00882 ------------TCACGATC-ATCTCCAA-TCTCCAGCACGCTTCT--------
-----
FC88X_01038_00342 ------------------TCGATCCCCCG-TCGACAGCTCA-------------
-----
FC88X_00984_00360 ---------------------CCAAACCT-TCCCCACTATTGAATTTAT-----
-----
FC88X_01130_01232 ---------------------CCAACACC-TTCT--------------------
-----
FC88X_01071_00490 -------------------CTATCAACCG-TCGCCATTACGTTATT--------
-----
FC88X_01143_01201 -------------------CTATCAACCG-TCGCCATTACGTTATTCC------
-----
FC88X_01036_01012 -----------------CATCATCAGCCC-CACTCTTTATTCTC----------
-----
FC88X_00968_00551 ------------------TCTCGAACTCAGCGATCAACAGGCTACTCCTC----
-----
FC88X_01043_00752 ------------------CCTCCTGCTCA-CGTGCAACAGTCTTTT--------
-----
FC88X_01099_00802 ------------------CCTCCCACTCG-CAAGCACCATATTTGC--------
-----
FC88X_01068_01240 ------------------TCTCTCATCCA-CGAGTTCAGCATATT---------
-----
FC88X_00955_00347 ---------------------CTTTGTTAATTCCCGCCAATCTATTCAC-----
-----
FC88X_01071_00498 ----------------------ATTGTTA--TCCCGCAGTTCTATTCCCC----
-----
FC88X_01013_00942 -------------------CACGTTGATAACAAGCGCGACTATTCC--------
-----
FC88X_01118_00845 -------------------CACGTTGATAACAAGCGCGACTATTCC--------
-----

FC88X_01087_00671 -------------------CACGTTGATAACAAGCGCGACTATTTCCCTC----
-----
FC88X_01070_01203 --------------------------CTAACTACC-------------------
-----
FC88X_01082_01268 ----------------------GTTACTAACTACCTATG---------------
-----
FC88X_00953_00426 -----------------TCGTAAATTCTAACCACCCCTGCTGTT----------
-----
FC88X_00981_01211 -----------------CCGTTATTTTAAACTCCCGCGGCCGGTT---------
-----
FC88X_01075_00399 -----------------CCGTTATTTTAA-CTCCCGCGGCCGGTT---------
-----
FC88X_01075_00483 -----------------CCGTTATTTTAA-CTCCCGCGGCCGGTT---------
-----
FC88X_01125_00802 -----------------CCGTTATTTTAA-CTCCCGCGGCCGGTT---------
-----
FC88X_01130_00906 -----------------CCGTTATTTTAA-CTCCCGCGGCCGGTT---------
-----
FC88X_00966_00591 -----------------CCGTCAGTGTGACAACCCGCG-CCATTT---------
-----
FC88X_00964_00443 -----------------TTCTCGTCAGTTGTTGCTAA-CTATTCC---------
-----
FC88X_01141_01166 -----------------TTCTCGTCAGTTGTTGCTAA-CTATTCC---------
-----
FC88X_01000_00312 ------------------------------CTGCTAA-CTAT------------
-----
FC88X_00995_00361 ------------------------------CTGCTAA-CTATTCTCAA------
-----
FC88X_00991_00447 ------------------CTTACTAACCTCCTGCAAATCTATTTT---------
-----
FC88X_01048_01019 ------------------CCTCGCTGTAAACTGTTTTGCCATATT---------
-----
FC88X_01030_01270 ------------------CCTAGTTGTTAACTACTTTCGCATGTT---------
-----
FC88X_01049_01039 ------------------CCTCCTG-TTATCTAGTGTCCATGTGAC--------
-----
FC88X_01083_00612 ------------------CCTCCTGGCTATGTAGCTGCCCCTGATC--------
-----
FC88X_00967_00828 ------------------------------TTGACAACTGT-------------
-----
FC88X_01105_00538 ---------------TTACCCACTTTCTATTTTGAAACTATC------------
-----
FC88X_01025_01047 --------------CTATAATCTCGTCGATTGTCTATCTCTT------------
-----
FC88X_01058_00579 --------------CTAATAACGCTTCGAATTCCCAAACTCT------------
-----
FC88X_00965_00331 -----------------ACTGCTAATATCCCACCAACTGCTATT----------
-----
FC88X_00982_00892 -----------------TCTACTATTATCCCAACTGACATTGTC----------
-----
FC88X_00975_00614 -------------------TACTAGTAACA------------------------
-----
FC88X_01133_00737 -----------------TCCACTATTACCACGAATCCCACTCTT----------
-----
FC88X_01043_00372 -------------------TTGTCAAATCCCGCGCATAACTATTCC--------
-----

```
FC88X_01027_00879      -------------------TTGTCAAATCCCGCGCATAACTATTCC--------
-----
FC88X_01138_00592      -------------------TTGTCAAATCCCGCGCATAACTATTCC--------
-----
FC88X_00961_01014      -------------------TTGTCGAATCCCGCGCATAACTATTCC--------
-----
FC88X_01032_00599      ----------------------------------CACAACTATT----------
-----
FC88X_01061_00476      -----------------ACACCAGTCCCATCAGCCACAATTATT----------
-----
FC88X_01061_00767      ------------------CCGTTTCCCAGCCGGCCGCAACTAATA---------
-----
FC88X_00978_00352      ----------------TCCCGAACTTAGCGACGGACCTCCCGG-----------
-----
FC88X_01021_00407      -----------------CCGAGACTCTGCGTAGGTGCTCCCATT----------
-----
FC88X_00960_00534      -----------------------GTCTGCGCAAACACTCCTACA----------
-----
FC88X_01041_00523      --------------ACTACGAACCTCTGTTCTAGCGCTCCT-------------
-----
FC88X_01112_00935      ------------------TTGCCTGCTGCCCCACGCCGCTGTTCTC--------
-----
FC88X_01117_01100      ------------------TTACCAGCTTGCACGAGCCTCCCTGTTCT-------
-----
FC88X_00992_00640      -------------------------TTGCAACAACCCTCTCATC----------
-----
FC88X_00985_00466      ----------------GTGCAAAAACCTCTCGACGATACCTGTT----------
-----
FC88X_01053_00423      -------------------CACAAACATCT------------------------
-----
FC88X_01058_00907      ----------------CATCGTAACCAACCCGAACAAACCTGTT----------
-----
FC88X_00990_00393      ----------------------CTGTTAACTAAGCAGCCCCACCGTATT-----
-----
FC88X_00991_00827      ----------------------ATATTCGATAAGCACCCCGATCATATT-----
-----
FC88X_01066_00372      --------------------CAAAAC-ATCCCATCACGTCTACTAT--------
-----
FC88X_01075_00443      ------------------ATCAACACTATTCGATCCCGCCAAATA---------
-----
FC88X_00977_00316      -----------------------CCTCTA-CCGTCTGATTTT------------
-----
FC88X_01041_00371      ----------------ACGTGAACCTTTATCCGTCCGTTTCTT-----------
-----
FC88X_01033_01207      -------------------ACATCTCTCAGCTTCACCGTCTATTTT--------
-----
FC88X_01052_00476      ----------------CTTAGATCTTTCAACCGCCCGGCTTTTT----------
-----
FC88X_01026_00381      -------------------ACATCTGCCAATCGCACGTCTGTTCAC--------
-----
FC88X_01128_00741      -------------------ACATCTACCAATCGCACGTCTGTTCAC--------
-----
FC88X_00996_00397      -------------------ACATCTACCA-------------------------
-----
FC88X_00999_00425      ---------------------CACCTCCCGGCATATCATACGCACACT------
-----
```

FC88X_01070_00622 -----------------CCCGCACGTTTCGGCAT-TCCTAGTCACCC-------
-----
FC88X_01069_00649 ---------------------CGCTGTCT---TAGCCACA--------------
-----
FC88X_01120_00684 ---------------------TGCTGTCT---TAGCCAC---------------
-----
FC88X_01074_00543 -----------------CTAACGCCGTCTGTATAGCCCCCATTTT---------
-----
FC88X_00981_00720 -----------------TTGAGGCATCTCTCAAACCCACTGTTTT---------
-----
FC88X_00975_00572 ----------------TCCACTACTGATCTCCTATTCACTATT-----------
-----
FC88X_01036_01129 -------------CCATCCACTCCCTATCTCCGATCTATT--------------
-----
FC88X_01133_01165 -----------------------------ACCAACTCACT--------------
-----
FC88X_01060_00422 ----------------CCACTATCTCCCTGGCAACCAGTGTTGT----------
-----
FC88X_01128_01215 ----------------CCAATAGCTCCCGGGCCAACTGTT--------------
-----
FC88X_01140_00772 ----------------TCTCAAGCTCCCTGGCCACTTATTGAC-----------
-----
FC88X_01135_00901 ----------------CCTACAGCTACCCTGTCCCTTTGTGTA-----------
-----
FC88X_01003_01157 ----------------CTGACGAATCTCCCGAGTCAGACTGGG-----------
-----
FC88X_01052_00576 -------------------------CTCCCTAGTCA------------------
-----
FC88X_01015_00942 ----------------CTTACTTCTCCCAATAGTCCGACTGTG-----------
-----
FC88X_00974_00431 ----------------TCAATTGCCCTGCCGAGTCAAACTATC-----------
-----
FC88X_01070_00585 ------------------CTCCCAACAGATTCGACGAACCGTCC----------
-----
FC88X_01143_00925 -------------------CTCCCACTTATGAGTCGTACTGACCGT--------
-----
FC88X_00998_00254 ---------------ATTGGCGAGAAA-TGC-CGTCTAGTGCTC----------
-----
FC88X_01146_00701 ---------------ATTGGCGAGAAAATGC-CGTCTAGTGCTC----------
-----
FC88X_00956_00825 ---------------ATTGGCGAGAAA-TGC-CGTCTAGTGCTC----------
-----
FC88X_01002_00483 ---------------ATTGGCGAGAAA-TGC-CGTCTAGTGCTC----------
-----
FC88X_01042_01029 ---------------ATTGGCGAGAAA-TGC-CGTCTAGTGCTC----------
-----
FC88X_01043_01186 ---------------ATTGGCGAGAAA-TGC-CGTCTAGTGCTC----------
-----
FC88X_01052_00543 ---------------ATTGGCGAGAAA-TGC-CGTCTAGTGCTC----------
-----
FC88X_01071_00525 ---------------ATTGGCGAGAAA-TGC-CGTCTAGTGCTC----------
-----
FC88X_01094_00659 ---------------ATTGGCGAGAAA-TGC-CGTCTAGTGCTC----------
-----
FC88X_01095_01185 ---------------ATTGGCGAGAAA-TGC-CGTCTAGTGCTC----------
-----

```
FC88X_01126_00938      ---------------ATTGGCGAGAAA-TGC-CGTCTAGTGCTC----------
-----
FC88X_01110_01040      ---------------ATTGGCGAGAAA-TGC-CGTCTAGTGCTC----------
-----
FC88X_01062_00741      ---------------CACGGCTCGAAAGTGC-AGTC-AGTTCTC----------
-----
FC88X_01071_00726      ---------------CACGGCTCGAAAGTGC-AGTC-AGTTCTC----------
-----
FC88X_01109_00457      ---------------CACGGCTCGAAAGTGC-AGTC-AGTTCTC----------
-----
FC88X_01140_01041      ---------------CACGGCTCGAAAGTGC-AGTC-AGTTCTC----------
-----
FC88X_00955_00330      ----------CTGCCACGTTTGGACAATTGC-A-----GTTCTC----------
-----
FC88X_01120_01119      --------------CAGGTAAAGTCAAATGC-ACGC--GTTCTC----------
-----
FC88X_00959_00599      ----------------TTCACACACATTTACAGGGGCTGTTCT-----------
-----
FC88X_00978_00758      ----------------TTCACACACATTTACAGGGGCTGTTCT-----------
-----
FC88X_01037_01257      ----------------TTCACACACATTTACAGGGGCTGTTCT-----------
-----
FC88X_01069_00429      ----------------TTCACACACATTTACAGGGGCTGTTCT-----------
-----
FC88X_01124_00591      ----------------TTCACACACATTTACAGGGGCTGTTCT-----------
-----
FC88X_00998_00685      ---------------TCCTACACACAACAGC-GGGCTTGTTCT-----------
-----
FC88X_01010_00757      ---------------TCCTACACACAACAGC-GGGCTTGTTCT-----------
-----
FC88X_01053_00694      ---------------TCCTACACACAACAGC-GGGCTTGTTCT-----------
-----
FC88X_01132_00873      ----------------CTACTACTCAAGTGCGCGTTTCGTTCTC----------
-----
FC88X_01144_00868      ----------------CTACTACTCAAGTGCGCGTTTCGTTCTC----------
-----
FC88X_01000_00516      ----------------CTACTACTCAAGTGCGCGTTTTGTTCTC----------
-----
FC88X_00990_00808      ----------------CTAC-ACTCAACT-------------------------
-----
FC88X_00997_00558      ---------------TCTCCCTGCGTATGGA-ACTGTCATCTC-----------
-----
FC88X_01112_00660      ---------------------------TGGACACTATCATCC------------
-----
FC88X_01019_00959      ---------------------------GGGGAACTGTCGTACTT----------
-----
FC88X_00978_00802      ----------------CTGGCACACACACGTCAGTATCTTCTCCC---------
-----
FC88X_01053_00554      ----------------CTGGCACACACACGTCAGTATCTTCTC-----------
-----
FC88X_01070_00563      ----------------TGGCTAAACCCCCGTACGTCACTTCTC-----------
-----
FC88X_01137_01159      --------------CAGTGCAAAAGATCCGTCTGTCTACTCTC-----------
-----
FC88X_01075_00827      ----------------TCCCCACAGACATGCAACACTATTCTT-----------
-----
```

```
FC88X_01078_00843      ----------------------CAGACGTGC-----------------------
-----
FC88X_00957_00443      ---------------CATACCACACATTTGCTACTCCTTTAA------------
-----
FC88X_00985_00433      ---------------TCTCATGAAACAA-ACATGGCTATACCTC----------
-----
FC88X_01015_00452      ------------------CACGAAACGATATTTTGCTATTTTCAC---------
-----
FC88X_00993_00835      ------------------TCGACAAATTAACCACGGCTCCCTTTT---------
-----
FC88X_01060_00571      ------------------TCGACAAATTAACCACGGCTCCCTTTT---------
-----
FC88X_01004_00923      --------------------GCCAAGTTTCCCACGCTTCTCCATGTG-------
-----
FC88X_01059_00961      --------------------GCCAAGTTTCCCACGCTTCGCCCTGTG-------
-----
FC88X_01053_00628      ----------------------------AACACT-GCTC---------------
-----
FC88X_01136_00857      -------------------CTGTAAGCTAACACTTGCTCTATTCGC--------
-----
FC88X_01002_00792      -----------------TTAGCTAACACGACGATACTCATTCTC----------
-----
FC88X_01132_00930      -----------------TTAGCTAACACGACGATACTCATTCTCCTC-------
-----
FC88X_01076_00553      -----------------TTAGCAATCATGGCGTAAACAATTCTC----------
-----
FC88X_01127_00617      -----------------TGGGTCACTTGTA-GGTGCACTGTTTTC---------
-----
FC88X_01106_00835      ------------------CTATCACTACTGTAGTGAGCACTTCTC---------
-----
FC88X_01146_01172      ------------TACCCCCAACCAAAACTGCAATGCTTT---------------
-----
FC88X_01111_01219      -------------------------TACTACAATCTTT----------------
-----
```
