# Supplementary material for: MRP1-CD28 bi-specific oligonucleotide aptamers: target costimulation to drug-resistant melanoma cancer stem cells
Source: Oncotarget. 2016 Mar 15;7(17):23182–96. doi: 10.18632/oncotarget.8095 (PMC5029619; doi:10.18632/oncotarget.8095)
Supplement: Supplementary file 5 [file oncotarget-07-23182-s005.pdf]

CLUSTAL 2.1 multiple sequence alignment

```
FC88X_00969_00285      -------------------CGCACCATCAATTTTGTCGTGCCGTTCTC------
-----
FC88X_00979_01201      -------------------CGCACCATCAATCTTGTCGTGCCGTTCTCC-----
-----
FC88X_01044_01235      ---------------------CCATTCTCAATTTGTCTTCCCCGTCCT------
-----
FC88X_01056_00382      --------------------TCAATGCTTGGCTTGCC-----------------
-----
FC88X_01004_00449      ------------------CAAGACCAATCAATTCGTCGAGCCTCTC--------
-----
FC88X_01080_01089      ---------------------CACAAACTGAATTCG------------------
-----
FC88X_01122_00750      ------------------CTTCACAATTTGATTTCCCGGATCTTT---------
-----
FC88X_00998_00903      --------------------ACAGCAAT-CGCACAGTCTTCTCTTCCT------
-----
FC88X_01037_00311      -------------------CTCAGTAATTCGCTCAGCTTCAATCTC--------
-----
FC88X_00957_01210      -----------------TCAACAA---TCGTTTGG-GGGCGAC-TTCTC-----
-----
FC88X_01029_00361      ----------------GTCAACAAAA-TCGTTTGG-GGGCGAC-TTCTC-----
-----
FC88X_00992_01217      -----------------TCAACAAA--TCGTTTGG-GGGCGAC-TTCTC-----
-----
FC88X_01013_00304      -----------------TCAACAAA--TCGTTTGG-GGGCGAC-TTCTC-----
-----
FC88X_01093_00528      -----------------TCAACAAA--TCGTTTGG-GGGCGAC-TTCTC-----
-----
FC88X_01030_00924      -----------------TCAACAAA--TCGTTTGG-GGGCGAC-TTCTC-----
-----
FC88X_00969_00272      -----------------TCAACAAA--TCGTTTGG-GG-CGAC-TTCTC-----
-----
FC88X_01127_00643      -----------------TCAACAAA--TCGTTTGG-GG-CGAC-TTCTC-----
-----
FC88X_01111_00674      -----------------TCAACAAA--TCGTTTGG-GG-CGAC-TTCTC-----
-----
FC88X_01124_00723      -----------------TCAACAAA--TCGTTTGG-GG-CGAC-TTCTC-----
-----
FC88X_01118_00799      -----------------TCAACAAA--TCGTTTGG-GG-CGAC-TTCTC-----
-----
FC88X_01142_00774      -----------------TCAACAAA--TCGTTTGG-GG-CGAC-TTCTC-----
-----
FC88X_01130_00817      -----------------TCAACAAA--TCGTTTGG-GG-CGAC-TTCTC-----
-----
FC88X_01106_00887      -----------------TCAACAAA--TCGTTTGG-GG-CGAC-TTCTC-----
-----
FC88X_01129_00877      -----------------TCAACAAA--TCGTTTGG-GG-CGAC-TTCTC-----
-----
FC88X_01131_00943      -----------------TCAACAAA--TCGTTTGG-GG-CGAC-TTCTC-----
-----
FC88X_01144_00958      -----------------TCAACAAA--TCGTTTGG-GG-CGAC-TTCTC-----
-----
FC88X_01145_01104      -----------------TCAACAAA--TCGTTTGG-GG-CGAC-TTCTC-----
-----
```

```
FC88X_01135_01154       -----------------TCAACAAA--TCGTTTGG-GG-CGAC-TTCTC-----
-----
FC88X_00988_00362       -----------------TCAACAAA--TCGTTTGG-GG-CGAC-TTCTC-----
-----
FC88X_00957_00353       -----------------TCAACAAA--TCGTTTGG-GG-CGAC-TTCTC-----
-----
FC88X_00989_00591       -----------------TCAACAAA--TCGTTTGG-GG-CGAC-TTCTC-----
-----
FC88X_00975_00543       -----------------TCAACAAA--TCGTTTGG-GG-CGAC-TTCTC-----
-----
FC88X_00951_00525       -----------------TCAACAAA--TCGTTTGG-GG-CGAC-TTCTC-----
-----
FC88X_00988_00649       -----------------TCAACAAA--TCGTTTGG-GG-CGAC-TTCTC-----
-----
FC88X_00994_00507       -----------------TCAACAAA--TCGTTTGG-GG-CGAC-TTCTC-----
-----
FC88X_00964_00723       -----------------TCAACAAA--TCGTTTGG-GG-CGAC-TTCTC-----
-----
FC88X_00968_00768       -----------------TCAACAAA--TCGTTTGG-GG-CGAC-TTCTC-----
-----
FC88X_01053_00521       -----------------TCAACAAA--TCGTTTGG-GG-CGAC-TTCTC-----
-----
FC88X_01051_00584       -----------------TCAACAAA--TCGTTTGG-GG-CGAC-TTCTC-----
-----
FC88X_01093_00592       -----------------TCAACAAA--TCGTTTGG-GG-CGAC-TTCTC-----
-----
FC88X_01060_00636       -----------------TCAACAAA--TCGTTTGG-GG-CGAC-TTCTC-----
-----
FC88X_00975_00268       -----------------TCAACAAA--TCGTTTGG--CGCGAC-TTCTC-----
-----
FC88X_01009_01018       -----------------TCAACAAA--TCGTTTGG---GCGAC-TTCTC-----
-----
FC88X_01055_00417       -----------------TCAACAAA--TCGTTTGG---GCGAC-TTCTC-----
-----
FC88X_01096_00470       -----------------TCAACAAA--TCGTTTGG--TGCGAC-TTCTC-----
-----
FC88X_01008_01134       -----------------TCAACAAA--TCGTTTGC--GGCGAC-TTCTC-----
-----
FC88X_01067_00405       -----------------TCAACAAA--TCGTTTG---GGCGAC-----------
-----
FC88X_01077_00936       -----------------TCAACAAA--TCGTTTG---GGCGAC-TTCTC-----
-----
FC88X_00959_00325       ----------------TACAACAAA--TCGTTTGG---GCGAC-TTCTCC----
-----
FC88X_00996_00859       ----------------TTCAACAAA--TCGTT-GG---GCGAC-TTCTC-----
-----
FC88X_00953_00966       -----------------TCAACAAA--TCGTT-GG-TGGCGAC-TTCTC-----
-----
FC88X_00950_01026       -----------------TCAACAAA--TCGTTCGG-TGGCGAC-TTCTC-----
-----
FC88X_01126_00850       -----------------TCAACAAA--TCGTTCGG--GGCGAC-TTCTC-----
-----
FC88X_01144_00914       -----------------TCAACAAA--TCGTTCGG--GGCGAC-TTCTC-----
-----
FC88X_01004_00401       -----------------TCAACAAA--TCGTTGGG--TGCGAC-TTCTC-----
-----
```

FC88X_01035_00554 ------------------TCAACAAA--TCGTTGGG---GCGAC-
TTCTCCTTCC-----
FC88X_01107_00475 ------------------TCAACAAA--TCGTTTGG---GCGAC-TTCTC-----
-----
FC88X_01106_01238 ------------------TCAACAAA--TCGTTTGG---GCGAC-TTCTC-----
-----
FC88X_01001_00838 ------------------TCAACAAA--TCGTTTGG-G-ACGAC-TTCTC-----
-----
FC88X_01124_00657 ------------------TCAACAAA--TCGTTTGG-GGACGAC-TTCTC-----
-----
FC88X_01081_00585 ------------------TCAACAAA--TCGTTTGG-G--CGAC-TTCTC-----
-----
FC88X_01096_00683 ------------------TCAACAAA--TCGTTTGG--AGCGAC-TTCTC-----
-----
FC88X_01054_00812 ------------------TCAACAAA--TCGTTTGG-GAGCGAC-TTCTC-----
-----
FC88X_01130_00751 ------------------TCAACAAA--TCGTTTGG-GAGCGAC-TTCTC-----
-----
FC88X_01124_00614 ------------------TCAACAAA--TCGTTTGG--GGCGAC-TTCTC-----
-----
FC88X_01104_00655 ------------------TCAACAAA--TCGTTTGG--GGCGAC-TTCTC-----
-----
FC88X_01114_00737 ------------------TCAACAAA--TCGTTTGG--GGCGAC-TTCTC-----
-----
FC88X_01111_00758 ------------------TCAACAAA--TCGTTTGG--GGCGAC-TTCTC-----
-----
FC88X_01136_00798 ------------------TCAACAAA--TCGTTTGG--GGCGAC-TTCTC-----
-----
FC88X_01124_00828 ------------------TCAACAAA--TCGTTTGG--GGCGAC-TTCTC-----
-----
FC88X_01100_00882 ------------------TCAACAAA--TCGTTTGG--GGCGAC-TTCTC-----
-----
FC88X_01125_00858 ------------------TCAACAAA--TCGTTTGG--GGCGAC-TTCTC-----
-----
FC88X_01119_00912 ------------------TCAACAAA--TCGTTTGG--GGCGAC-TTCTC-----
-----
FC88X_01138_00967 ------------------TCAACAAA--TCGTTTGG--GGCGAC-TTCTC-----
-----
FC88X_01124_01114 ------------------TCAACAAA--TCGTTTGG--GGCGAC-TTCTC-----
-----
FC88X_01129_01197 ------------------TCAACAAA--TCGTTTGG--GGCGAC-TTCTC-----
-----
FC88X_01129_01230 ------------------TCAACAAA--TCGTTTGG--GGCGAC-TTCTC-----
-----
FC88X_00976_00397 ------------------TCAACAAA--TCGTTTGG--GGCGAC-TTCTC-----
-----
FC88X_00979_00594 ------------------TCAACAAA--TCGTTTGG--GGCGAC-TTCTC-----
-----
FC88X_00967_00528 ------------------TCAACAAA--TCGTTTGG--GGCGAC-TTCTC-----
-----
FC88X_00995_00467 ------------------TCAACAAA--TCGTTTGG--GGCGAC-TTCTC-----
-----
FC88X_00975_00615 ------------------TCAACAAA--TCGTTTGG--GGCGAC-TTCTC-----
-----
FC88X_00967_00695 ------------------TCAACAAA--TCGTTTGG--GGCGAC-TTCTC-----
-----

FC88X_00983_00651 -----------------TCAACAAA--TCGTTTGG--GGCGAC-TTCTC-----
-----
FC88X_00995_00725 -----------------TCAACAAA--TCGTTTGG--GGCGAC-TTCTC-----
-----
FC88X_01094_00494 -----------------TCAACAAA--TCGTTTGG--GGCGAC-TTCTC-----
-----
FC88X_01090_00543 -----------------TCAACAAA--TCGTTTGG--GGCGAC-TTCTC-----
-----
FC88X_01076_00565 -----------------TCAACAAA--TCGTTTGG--GGCGAC-TTCTC-----
-----
FC88X_01056_00619 -----------------TCAACAAA--TCGTTTGG--GGCGAC-TTCTC-----
-----
FC88X_00999_00337 -----------------TCAACAAAA-TCGTTTGG--GGCGAC-TTCTC-----
-----
FC88X_01124_00725 -----------------TCAACAAAA-TCGTTTGG--GGCGAC-TTCTC-----
-----
FC88X_00999_00803 -----------------TCAACAAAA-TCGTTTGG--GGCGAC-TTCTC-----
-----
FC88X_00996_00999 -----------------TCAACAAAA-TCGTTTGG--GGCGAC-TTCTC-----
-----
FC88X_01007_00699 -----------------TCAACAAAA-TCGTTTGG--GGCGAC-TTCTC-----
-----
FC88X_01017_00696 -----------------TCAACAAAA-TCGTTTGG--GGCGAC-TTCTC-----
-----
FC88X_01025_01031 -----------------TCAACAAAA-TCGTTTGG--GGCGAC-TTCTC-----
-----
FC88X_01054_00432 -----------------TCAACAAAA-TCGTTTGG--GGCGAC-TTCTC-----
-----
FC88X_01089_00508 -----------------TCAACAAAA-TCGTTTGG--GGCGAC-TTCTC-----
-----
FC88X_01116_00517 -----------------TCAACAAAA-TCGTTTGG--GGCGAC-TTCTC-----
-----
FC88X_01104_01105 -----------------TCAACAAAA-TCGTTTGG--GGCGAC-TTCTC-----
-----
FC88X_00958_01116 -----------------TCAACAAA--TCATTTGG--GGCGAC-TTCTC-----
-----
FC88X_01005_00897 -----------------TCAACAAA--TCATTTGG--GGCGAC-TTCTC-----
-----
FC88X_00988_00263 -----------------TCAACAAA--TCGTTTGG--GGCGAC-TTCTC-----
-----
FC88X_01134_00609 -----------------TCAACAAA--TCGTTTGG--GGCGAC-TTCTC-----
-----
FC88X_01137_00689 -----------------TCAACAAA--TCGTTTGG--GGCGAC-TTCTC-----
-----
FC88X_01139_00741 -----------------TCAACAAA--TCGTTTGG--GGCGAC-TTCTC-----
-----
FC88X_01132_00793 -----------------TCAACAAA--TCGTTTGG--GGCGAC-TTCTC-----
-----
FC88X_01119_00827 -----------------TCAACAAA--TCGTTTGG--GGCGAC-TTCTC-----
-----
FC88X_01139_00815 -----------------TCAACAAA--TCGTTTGG--GGCGAC-TTCTC-----
-----
FC88X_01120_00882 -----------------TCAACAAA--TCGTTTGG--GGCGAC-TTCTC-----
-----
FC88X_01104_00930 -----------------TCAACAAA--TCGTTTGG--GGCGAC-TTCTC-----
-----

FC88X_01119_00994 -----------------TCAACAAA--TCGTTTGG--GGCGAC-TTCTC-----
-----
FC88X_01120_01120 -----------------TCAACAAA--TCGTTTGG--GGCGAC-TTCTC-----
-----
FC88X_01116_01161 -----------------TCAACAAA--TCGTTTGG--GGCGAC-TTCTC-----
-----
FC88X_01118_01207 -----------------TCAACAAA--TCGTTTGG--GGCGAC-TTCTC-----
-----
FC88X_00998_00282 -----------------TCAACAAA--TCGTTTGG--GGCGAC-TTCTC-----
-----
FC88X_00967_00340 -----------------TCAACAAA--TCGTTTGG--GGCGAC-TTCTC-----
-----
FC88X_00996_00325 -----------------TCAACAAA--TCGTTTGG--GGCGAC-TTCTC-----
-----
FC88X_00962_00619 -----------------TCAACAAA--TCGTTTGG--GGCGAC-TTCTC-----
-----
FC88X_00972_00601 -----------------TCAACAAA--TCGTTTGG--GGCGAC-TTCTC-----
-----
FC88X_00957_00688 -----------------TCAACAAA--TCGTTTGG--GGCGAC-TTCTC-----
-----
FC88X_00997_00675 -----------------TCAACAAA--TCGTTTGG--GGCGAC-TTCTC-----
-----
FC88X_00992_00709 -----------------TCAACAAA--TCGTTTGG--GGCGAC-TTCTC-----
-----
FC88X_00999_00787 -----------------TCAACAAA--TCGTTTGG--GGCGAC-TTCTC-----
-----
FC88X_01080_00535 -----------------TCAACAAA--TCGTTTGG--GGCGAC-TTCTC-----
-----
FC88X_01074_00563 -----------------TCAACAAA--TCGTTTGG--GGCGAC-TTCTC-----
-----
FC88X_01099_00557 -----------------TCAACAAA--TCGTTTGG--GGCGAC-TTCTC-----
-----
FC88X_01020_00394 -----------------TCAACAA-A-TCGTTTGG-AGGCGAC-TTCTC-----
-----
FC88X_01080_00677 -----------------TCAACAA-A-TCGTTTG--AGGCGAC-TTCTC-----
-----
FC88X_00994_00316 -----------------TCAACAA-A-TCGTTTGG-AGGCGAC-TTCTC-----
-----
FC88X_00991_00262 -----------------TCAACAA-A-TCGTTTG--GGGCGAC-TTCTC-----
-----
FC88X_01138_00640 -----------------TCAACAA-A-TCGTTTG--GGGCGAC-TTCTC-----
-----
FC88X_01143_00662 -----------------TCAACAA-A-TCGTTTG--GGGCGAC-TTCTC-----
-----
FC88X_01142_00738 -----------------TCAACAA-A-TCGTTTG--GGGCGAC-TTCTC-----
-----
FC88X_01134_00779 -----------------TCAACAA-A-TCGTTTG--GGGCGAC-TTCTC-----
-----
FC88X_01120_00849 -----------------TCAACAA-A-TCGTTTG--GGGCGAC-TTCTC-----
-----
FC88X_01143_00844 -----------------TCAACAA-A-TCGTTTG--GGGCGAC-TTCTC-----
-----
FC88X_01122_00850 -----------------TCAACAA-A-TCGTTTG--GGGCGAC-TTCTC-----
-----
FC88X_01107_00921 -----------------TCAACAA-A-TCGTTTG--GGGCGAC-TTCTC-----
-----

```
FC88X_01134_00992      -----------------TCAACAA-A-TCGTTTG--GGGCGAC-TTCTC-----
-----
FC88X_01121_01103      -----------------TCAACAA-A-TCGTTTG--GGGCGAC-TTCTC-----
-----
FC88X_01125_01153      -----------------TCAACAA-A-TCGTTTG--GGGCGAC-TTCTC-----
-----
FC88X_01125_01206      -----------------TCAACAA-A-TCGTTTG--GGGCGAC-TTCTC-----
-----
FC88X_00950_00325      -----------------TCAACAA-A-TCGTTTG--GGGCGAC-TTCTC-----
-----
FC88X_00969_00335      -----------------TCAACAA-A-TCGTTTG--GGGCGAC-TTCTC-----
-----
FC88X_00965_00352      -----------------TCAACAA-A-TCGTTTG--GGGCGAC-TTCTC-----
-----
FC88X_00964_00602      -----------------TCAACAA-A-TCGTTTG--GGGCGAC-TTCTC-----
-----
FC88X_00973_00636      -----------------TCAACAA-A-TCGTTTG--GGGCGAC-TTCTC-----
-----
FC88X_00962_00654      -----------------TCAACAA-A-TCGTTTG--GGGCGAC-TTCTC-----
-----
FC88X_00999_00362      -----------------TCAACAA-A-TCGTTTG--GGGCGAC-TTCTC-----
-----
FC88X_00992_00734      -----------------TCAACAA-A-TCGTTTG--GGGCGAC-TTCTC-----
-----
FC88X_00957_00821      -----------------TCAACAA-A-TCGTTTG--GGGCGAC-TTCTC-----
-----
FC88X_01084_00520      -----------------TCAACAA-A-TCGTTTG--GGGCGAC-TTCTC-----
-----
FC88X_01074_00580      -----------------TCAACAA-A-TCGTTTG--GGGCGAC-TTCTC-----
-----
FC88X_01052_00624      -----------------TCAACAA-A-TCGTTTG--GGGCGAC-TTCTC-----
-----
FC88X_00965_00587      -----------------TCAACAA-G-TCGTTTG--GGGCGAC-TTCTC-----
-----
FC88X_01016_00844      -----------------TCAACAA-G-TCGTTTG--GGGCGAC-TTCTC-----
-----
FC88X_01099_00570      -----------------TCAACAA-G-TCGTTTG--GGGCGAC-TTCTC-----
-----
FC88X_01073_00560      -----------------TCAACAAAG-TCGTTT---------------------
-----
FC88X_01139_00681      -----------------TCAACAAAG-TCGTTTG--GGGCGAC-TTCTC-----
-----
FC88X_01138_00634      -----------------TCAACAA-A-TCGTTTG--GGGCGAC-TTCTC-----
-----
FC88X_01140_00687      -----------------TCAACAA-A-TCGTTTG--GGGCGAC-TTCTC-----
-----
FC88X_01142_00716      -----------------TCAACAA-A-TCGTTTG--GGGCGAC-TTCTC-----
-----
FC88X_01133_00788      -----------------TCAACAA-A-TCGTTTG--GGGCGAC-TTCTC-----
-----
FC88X_01120_00820      -----------------TCAACAA-A-TCGTTTG--GGGCGAC-TTCTC-----
-----
FC88X_01139_00828      -----------------TCAACAA-A-TCGTTTG--GGGCGAC-TTCTC-----
-----
FC88X_01121_00868      -----------------TCAACAA-A-TCGTTTG--GGGCGAC-TTCTC-----
-----
```

FC88X_01106_00944 -----------------TCAACAA-A-TCGTTTG--GGGCGAC-TTCTC-----
-----
FC88X_01134_00985 -----------------TCAACAA-A-TCGTTTG--GGGCGAC-TTCTC-----
-----
FC88X_01120_01128 -----------------TCAACAA-A-TCGTTTG--GGGCGAC-TTCTC-----
-----
FC88X_01121_01185 -----------------TCAACAA-A-TCGTTTG--GGGCGAC-TTCTC-----
-----
FC88X_01119_01210 -----------------TCAACAA-A-TCGTTTG--GGGCGAC-TTCTC-----
-----
FC88X_00999_00256 -----------------TCAACAA-A-TCGTTTG--GGGCGAC-TTCTC-----
-----
FC88X_00969_00315 -----------------TCAACAA-A-TCGTTTG--GGGCGAC-TTCTC-----
-----
FC88X_00997_00334 -----------------TCAACAA-A-TCGTTTG--GGGCGAC-TTCTC-----
-----
FC88X_00963_00612 -----------------TCAACAA-A-TCGTTTG--GGGCGAC-TTCTC-----
-----
FC88X_00973_00627 -----------------TCAACAA-A-TCGTTTG--GGGCGAC-TTCTC-----
-----
FC88X_00961_00699 -----------------TCAACAA-A-TCGTTTG--GGGCGAC-TTCTC-----
-----
FC88X_00998_00399 -----------------TCAACAA-A-TCGTTTG--GGGCGAC-TTCTC-----
-----
FC88X_00992_00722 -----------------TCAACAA-A-TCGTTTG--GGGCGAC-TTCTC-----
-----
FC88X_00951_00847 -----------------TCAACAA-A-TCGTTTG--GGGCGAC-TTCTC-----
-----
FC88X_01084_00505 -----------------TCAACAA-A-TCGTTTG--GGGCGAC-TTCTC-----
-----
FC88X_01074_00571 -----------------TCAACAA-A-TCGTTTG--GGGCGAC-TTCTC-----
-----
FC88X_01044_01187 -----------------TCAACAG-A-TCGTTTG--GGGCGAC-TTCTC-----
-----
FC88X_01129_00925 -----------------TCAACA--A-TCGTTTG--GGGCGAC-TTCTC-----
-----
FC88X_01050_00622 -----------------TCAACAAAC-TCGTTTG--GGGCGAC-TTCTC-----
-----
FC88X_01143_00935 -----------------TCAACAA-C-TCGTTTG--GGGCGAC-TTCTC-----
-----
FC88X_01129_00643 -----------------TCAACAA-A-TCGTTTG--GGGCGAC-TTCTC-----
-----
FC88X_01115_00698 -----------------TCAACAA-A-TCGTTTG--GGGCGAC-TTCTC-----
-----
FC88X_01128_00704 -----------------TCAACAA-A-TCGTTTG--GGGCGAC-TTCTC-----
-----
FC88X_01122_00797 -----------------TCAACAA-A-TCGTTTG--GGGCGAC-TTCTC-----
-----
FC88X_01144_00765 -----------------TCAACAA-A-TCGTTTG--GGGCGAC-TTCTC-----
-----
FC88X_01131_00810 -----------------TCAACAA-A-TCGTTTG--GGGCGAC-TTCTC-----
-----
FC88X_01108_00862 -----------------TCAACAA-A-TCGTTTG--GGGCGAC-TTCTC-----
-----
FC88X_01130_00872 -----------------TCAACAA-A-TCGTTTG--GGGCGAC-TTCTC-----
-----

```
FC88X_01134_00901       -----------------TCAACAA-A-TCGTTTG--GGGCGAC-TTCTC-----
-----
FC88X_01107_01149       -----------------TCAACAA-A-TCGTTTG--GGGCGAC-TTCTC-----
-----
FC88X_01103_01187       -----------------TCAACAA-A-TCGTTTG--GGGCGAC-TTCTC-----
-----
FC88X_01137_01154       -----------------TCAACAA-A-TCGTTTG--GGGCGAC-TTCTC-----
-----
FC88X_00988_00367       -----------------TCAACAA-A-TCGTTTG--GGGCGAC-TTCTC-----
-----
FC88X_00989_00330       -----------------TCAACAA-A-TCGTTTG--GGGCGAC-TTCTC-----
-----
FC88X_00951_00633       -----------------TCAACAA-A-TCGTTTG--GGGCGAC-TTCTC-----
-----
FC88X_00977_00533       -----------------TCAACAA-A-TCGTTTG--GGGCGAC-TTCTC-----
-----
FC88X_00961_00501       -----------------TCAACAA-A-TCGTTTG--GGGCGAC-TTCTC-----
-----
FC88X_00994_00647       -----------------TCAACAA-A-TCGTTTG--GGGCGAC-TTCTC-----
-----
FC88X_00950_00597       -----------------TCAACAA-A-TCGTTTG--GGGCGAC-TTCTC-----
-----
FC88X_00967_00703       -----------------TCAACAA-A-TCGTTTG--GGGCGAC-TTCTC-----
-----
FC88X_00969_00779       -----------------TCAACAA-A-TCGTTTG--GGGCGAC-TTCTC-----
-----
FC88X_01055_00535       -----------------TCAACAA-A-TCGTTTG--GGGCGAC-TTCTC-----
-----
FC88X_01057_00599       -----------------TCAACAA-A-TCGTTTG--GGGCGAC-TTCTC-----
-----
FC88X_01094_00591       -----------------TCAACAA-A-TCGTTTG--GGGCGAC-TTCTC-----
-----
FC88X_01064_00615       -----------------TCAACAAA--TCGTTTG--GGGCGAC-TTCTC-----
-----
FC88X_00985_00958       -----------------TCAACAAA--TCGTTTG--GGGCGAC-TTCTC-----
-----
FC88X_00985_00974       -----------------TCAACAAA--TCGTTTG--GGGCGAC-TTCTC-----
-----
FC88X_01131_00963       -----------------TCAACAAA--TCGTTTG--GGGCGAC-TTCTC-----
-----
FC88X_01129_00985       -----------------TCAACAAA--TCGTTTG--GGGCGAC-TTCTC-----
-----
FC88X_01124_00969       -----------------TCAACAAA--TCGTTTG--GGGCGAC-TTCTC-----
-----
FC88X_01146_00933       -----------------TCAACAAA--TCGTTTG--GGGCGAC-TTCTC-----
-----
FC88X_01143_00940       -----------------TCAACAAA--TCGTTTG--GGGCGAC-TTCTC-----
-----
FC88X_01141_00943       -----------------TCAACAAA--TCGTTTG--GGGCGAC-TTCTC-----
-----
FC88X_01049_00766       -----------------TCAACAAA--TCGTTTG--GGGCGAC-TTCTC-----
-----
FC88X_01016_00827       -----------------TCAACAAA--TCGTTTG--GGGCGAC-TTCTC-----
-----
FC88X_01016_00803       -----------------TCAACAAA--TCGTTTG--GGGCGAC-TTCTC-----
-----
```

FC88X_01012_00842 -----------------TCAACAAA--TCGTTTG--GGGCGAC-TTCTC-----
-----
FC88X_01007_00806 -----------------TCAACAAA--TCGTTTG--GGGCGAC-TTCTC-----
-----
FC88X_01006_00840 -----------------TCAACAAA--TCGTTTG--GGGCGAC-TTCTC-----
-----
FC88X_01006_00828 -----------------TCAACAAA--TCGTTTG--GGGCGAC-TTCTC-----
-----
FC88X_01005_00828 -----------------TCAACAAA--TCGTTTG--GGGCGAC-TTCTC-----
-----
FC88X_01004_00817 -----------------TCAACAAA--TCGTTTG--GGGCGAC-TTCTC-----
-----
FC88X_01003_00826 -----------------TCAACAAA--TCGTTTG--GGGCGAC-TTCTC-----
-----
FC88X_01020_01204 -----------------TCAACAAA--TCGTTTG--GGGCGAC-TTCTC-----
-----
FC88X_01019_01222 -----------------TCAACAAA--TCGTTTG--GGGCGAC-TTCTC-----
-----
FC88X_01017_01229 -----------------TCAACAAA--TCGTTTG--GGGCGAC-TTCTC-----
-----
FC88X_01016_01216 -----------------TCAACAAA--TCGTTTG--GGGCGAC-TTCTC-----
-----
FC88X_01007_01200 -----------------TCAACAAA--TCGTTTG--GGGCGAC-TTCTC-----
-----
FC88X_01004_01226 -----------------TCAACAAA--TCGTTTG--GGGCGAC-TTCTC-----
-----
FC88X_01048_01171 -----------------TCAACAAA--TCGTTTG--GGGCGAC-TTCTC-----
-----
FC88X_01046_01156 -----------------TCAACAAA--TCGTTTG--GGGCGAC-TTCTC-----
-----
FC88X_01044_01155 -----------------TCAACAAA--TCGTTTG--GGGCGAC-TTCTC-----
-----
FC88X_01042_01159 -----------------TCAACAAA--TCGTTTG--GGGCGAC-TTCTC-----
-----
FC88X_01037_01165 -----------------TCAACAAA--TCGTTTG--GGGCGAC-TTCTC-----
-----
FC88X_01029_01173 -----------------TCAACAAA--TCGTTTG--GGGCGAC-TTCTC-----
-----
FC88X_01024_01160 -----------------TCAACAAA--TCGTTTG--GGGCGAC-TTCTC-----
-----
FC88X_01018_01195 -----------------TCAACAAA--TCGTTTG--GGGCGAC-TTCTC-----
-----
FC88X_01006_01192 -----------------TCAACAAA--TCGTTTG--GGGCGAC-TTCTC-----
-----
FC88X_01003_01163 -----------------TCAACAAA--TCGTTTG--GGGCGAC-TTCTC-----
-----
FC88X_00950_00989 -----------------TCAACAAA--TCGTTTG--GGGCGAC-TTCTC-----
-----
FC88X_00997_00920 -----------------TCAACAAA--TCGTTTG--GGGCGAC-TTCTC-----
-----
FC88X_00996_00947 -----------------TCAACAAA--TCGTTTG--GGGCGAC-TTCTC-----
-----
FC88X_00994_00907 -----------------TCAACAAA--TCGTTTG--GGGCGAC-TTCTC-----
-----
FC88X_00991_00936 -----------------TCAACAAA--TCGTTTG--GGGCGAC-TTCTC-----
-----

FC88X_01084_00497 -----------------TCAACAAA--TCGTTTG--GGGCGAC-TTCTC-----
-----
FC88X_01084_00486 -----------------TCAACAAA--TCGTTTG--GGGCGAC-TTCTC-----
-----
FC88X_01080_00451 -----------------TCAACAAA--TCGTTTG--GGGCGAC-TTCTC-----
-----
FC88X_01078_00465 -----------------TCAACAAA--TCGTTTG--GGGCGAC-TTCTC-----
-----
FC88X_01077_00454 -----------------TCAACAAA--TCGTTTG--GGGCGAC-TTCTC-----
-----
FC88X_01075_00499 -----------------TCAACAAA--TCGTTTG--GGGCGAC-TTCTC-----
-----
FC88X_01075_00493 -----------------TCAACAAA--TCGTTTG--GGGCGAC-TTCTC-----
-----
FC88X_01071_00473 -----------------TCAACAAA--TCGTTTG--GGGCGAC-TTCTC-----
-----
FC88X_01068_00457 -----------------TCAACAAA--TCGTTTG--GGGCGAC-TTCTC-----
-----
FC88X_01066_00498 -----------------TCAACAAA--TCGTTTG--GGGCGAC-TTCTC-----
-----
FC88X_01065_00498 -----------------TCAACAAA--TCGTTTG--GGGCGAC-TTCTC-----
-----
FC88X_01063_00492 -----------------TCAACAAA--TCGTTTG--GGGCGAC-TTCTC-----
-----
FC88X_01061_00465 -----------------TCAACAAA--TCGTTTG--GGGCGAC-TTCTC-----
-----
FC88X_01060_00481 -----------------TCAACAAA--TCGTTTG--GGGCGAC-TTCTC-----
-----
FC88X_01058_00465 -----------------TCAACAAA--TCGTTTG--GGGCGAC-TTCTC-----
-----
FC88X_01056_00482 -----------------TCAACAAA--TCGTTTG--GGGCGAC-TTCTC-----
-----
FC88X_01055_00489 -----------------TCAACAAA--TCGTTTG--GGGCGAC-TTCTC-----
-----
FC88X_01054_00479 -----------------TCAACAAA--TCGTTTG--GGGCGAC-TTCTC-----
-----
FC88X_01053_00479 -----------------TCAACAAA--TCGTTTG--GGGCGAC-TTCTC-----
-----
FC88X_01050_00477 -----------------TCAACAAA--TCGTTTG--GGGCGAC-TTCTC-----
-----
FC88X_01007_00312 -----------------TCAACAAA--TCGTTTG--GGGCGAC-TTCTC-----
-----
FC88X_01006_00331 -----------------TCAACAAA--TCGTTTG--GGGCGAC-TTCTC-----
-----
FC88X_01006_00330 -----------------TCAACAAA--TCGTTTG--GGGCGAC-TTCTC-----
-----
FC88X_01005_00322 -----------------TCAACAAA--TCGTTTG--GGGCGAC-TTCTC-----
-----
FC88X_01000_00333 -----------------TCAACAAA--TCGTTTG--GGGCGAC-TTCTC-----
-----
FC88X_01028_00297 -----------------TCAACAAA--TCGTTTG--GGGCGAC-TTCTC-----
-----
FC88X_01020_00289 -----------------TCAACAAA--TCGTTTG--GGGCGAC-TTCTC-----
-----
FC88X_01011_00297 -----------------TCAACAAA--TCGTTTG--GGGCGAC-TTCTC-----
-----

FC88X_01009_00298 -----------------TCAACAAA--TCGTTTG--GGGCGAC-TTCTC-----
-----
FC88X_01004_00270 -----------------TCAACAAA--TCGTTTG--GGGCGAC-TTCTC-----
-----
FC88X_01001_00267 -----------------TCAACAAA--TCGTTTG--GGGCGAC-TTCTC-----
-----
FC88X_00998_01269 -----------------TCAACAAA--TCGTTTG--GGGCGAC-TTCTC-----
-----
FC88X_00987_01270 -----------------TCAACAAA--TCGTTTG--GGGCGAC-TTCTC-----
-----
FC88X_00987_01255 -----------------TCAACAAA--TCGTTTG--GGGCGAC-TTCTC-----
-----
FC88X_00985_01275 -----------------TCAACAAA--TCGTTTG--GGGCGAC-TTCTC-----
-----
FC88X_00977_01265 -----------------TCAACAAA--TCGTTTG--GGGCGAC-TTCTC-----
-----
FC88X_00973_01258 -----------------TCAACAAA--TCGTTTG--GGGCGAC-TTCTC-----
-----
FC88X_00972_01265 -----------------TCAACAAA--TCGTTTG--GGGCGAC-TTCTC-----
-----
FC88X_00970_01271 -----------------TCAACAAA--TCGTTTG--GGGCGAC-TTCTC-----
-----
FC88X_00967_01263 -----------------TCAACAAA--TCGTTTG--GGGCGAC-TTCTC-----
-----
FC88X_00965_01253 -----------------TCAACAAA--TCGTTTG--GGGCGAC-TTCTC-----
-----
FC88X_00961_01250 -----------------TCAACAAA--TCGTTTG--GGGCGAC-TTCTC-----
-----
FC88X_00951_01273 -----------------TCAACAAA--TCGTTTG--GGGCGAC-TTCTC-----
-----
FC88X_00950_01255 -----------------TCAACAAA--TCGTTTG--GGGCGAC-TTCTC-----
-----
FC88X_00998_01237 -----------------TCAACAAA--TCGTTTG--GGGCGAC-TTCTC-----
-----
FC88X_00997_01224 -----------------TCAACAAA--TCGTTTG--GGGCGAC-TTCTC-----
-----
FC88X_00997_01202 -----------------TCAACAAA--TCGTTTG--GGGCGAC-TTCTC-----
-----
FC88X_00996_01225 -----------------TCAACAAA--TCGTTTG--GGGCGAC-TTCTC-----
-----
FC88X_00991_01228 -----------------TCAACAAA--TCGTTTG--GGGCGAC-TTCTC-----
-----
FC88X_00982_01216 -----------------TCAACAAA--TCGTTTG--GGGCGAC-TTCTC-----
-----
FC88X_00977_01211 -----------------TCAACAAA--TCGTTTG--GGGCGAC-TTCTC-----
-----
FC88X_00974_01217 -----------------TCAACAAA--TCGTTTG--GGGCGAC-TTCTC-----
-----
FC88X_00973_01212 -----------------TCAACAAA--TCGTTTG--GGGCGAC-TTCTC-----
-----
FC88X_00960_01206 -----------------TCAACAAA--TCGTTTG--GGGCGAC-TTCTC-----
-----
FC88X_00960_01205 -----------------TCAACAAA--TCGTTTG--GGGCGAC-TTCTC-----
-----
FC88X_00955_01216 -----------------TCAACAAA--TCGTTTG--GGGCGAC-TTCTC-----
-----

```
FC88X_00952_01247      ------------------TCAACAAA--TCGTTTG--GGGCGAC-TTCTC-----
-----
FC88X_00951_01214      ------------------TCAACAAA--TCGTTTG--GGGCGAC-TTCTC-----
-----
FC88X_00992_01105      ------------------TCAACAAA--TCGTTTG--GGGCGAC-TTCTC-----
-----
FC88X_00986_01136      ------------------TCAACAAA--TCGTTTG--GGGCGAC-TTCTC-----
-----
FC88X_00984_01114      ------------------TCAACAAA--TCGTTTG--GGGCGAC-TTCTC-----
-----
FC88X_00979_01111      ------------------TCAACAAA--TCGTTTG--GGGCGAC-TTCTC-----
-----
FC88X_00967_01115      ------------------TCAACAAA--TCGTTTG--GGGCGAC-TTCTC-----
-----
FC88X_00957_01127      ------------------TCAACAAA--TCGTTTG--GGGCGAC-TTCTC-----
-----
FC88X_00998_01031      ------------------TCAACAAA--TCGTTTG--GGGCGAC-TTCTC-----
-----
FC88X_00997_01024      ------------------TCAACAAA--TCGTTTG--GGGCGAC-TTCTC-----
-----
FC88X_00960_00995      ------------------TCAACAAA--TCGTTTG--GGGCGAC-TTCTC-----
-----
FC88X_00960_00959      ------------------TCAACAAA--TCGTTTG--GGGCGAC-TTCTC-----
-----
FC88X_00959_00997      ------------------TCAACAAA--TCGTTTG--GGGCGAC-TTCTC-----
-----
FC88X_00959_00981      ------------------TCAACAAA--TCGTTTG--GGGCGAC-TTCTC-----
-----
FC88X_00957_00954      ------------------TCAACAAA--TCGTTTG--GGGCGAC-TTCTC-----
-----
FC88X_00954_00981      ------------------TCAACAAA--TCGTTTG--GGGCGAC-TTCTC-----
-----
FC88X_01021_00336      ------------------TCAACAAA--TCGTTTG--GGGCGAC-TTCTC-----
-----
FC88X_01021_00313      ------------------TCAACAAA--TCGTTTG--GGGCGAC-TTCTC-----
-----
FC88X_01015_00326      ------------------TCAACAAA--TCGTTTG--GGGCGAC-TTCTC-----
-----
FC88X_01014_00303      ------------------TCAACAAA--TCGTTTG--GGGCGAC-TTCTC-----
-----
FC88X_01012_00304      ------------------TCAACAAA--TCGTTTG--GGGCGAC-TTCTC-----
-----
FC88X_01010_00312      ------------------TCAACAAA--TCGTTTG--GGGCGAC-TTCTC-----
-----
FC88X_01009_01147      ------------------TCAACAAA--TCGTTTG--GGGCGAC-TTCTC-----
-----
FC88X_01005_01139      ------------------TCAACAAA--TCGTTTG--GGGCGAC-TTCTC-----
-----
FC88X_01035_00447      ------------------TCAACAAA--TCGTTTG--GGGCGAC-TTCTC-----
-----
FC88X_01035_00403      ------------------TCAACAAA--TCGTTTG--GGGCGAC-TTCTC-----
-----
FC88X_01034_00438      ------------------TCAACAAA--TCGTTTG--GGGCGAC-TTCTC-----
-----
FC88X_01033_00402      ------------------TCAACAAA--TCGTTTG--GGGCGAC-TTCTC-----
-----
```

FC88X_01031_00438 -----------------TCAACAAA--TCGTTTG--GGGCGAC-TTCTC-----
-----

FC88X_01027_00430 -----------------TCAACAAA--TCGTTTG--GGGCGAC-TTCTC-----
-----

FC88X_01021_00400 -----------------TCAACAAA--TCGTTTG--GGGCGAC-TTCTC-----
-----

FC88X_01012_00439 -----------------TCAACAAA--TCGTTTG--GGGCGAC-TTCTC-----
-----

FC88X_01004_00445 -----------------TCAACAAA--TCGTTTG--GGGCGAC-TTCTC-----
-----

FC88X_01047_00370 -----------------TCAACAAA--TCGTTTG--GGGCGAC-TTCTC-----
-----

FC88X_01046_00353 -----------------TCAACAAA--TCGTTTG--GGGCGAC-TTCTC-----
-----

FC88X_00995_01017 -----------------TCAACAAA--TCGTTTG--GGGCGAC-TTCTC-----
-----

FC88X_00994_01048 -----------------TCAACAAA--TCGTTTG--GGGCGAC-TTCTC-----
-----

FC88X_00990_01005 -----------------TCAACAAA--TCGTTTG--GGGCGAC-TTCTC-----
-----

FC88X_00987_01049 -----------------TCAACAAA--TCGTTTG--GGGCGAC-TTCTC-----
-----

FC88X_00982_01044 -----------------TCAACAAA--TCGTTTG--GGGCGAC-TTCTC-----
-----

FC88X_00980_01048 -----------------TCAACAAA--TCGTTTG--GGGCGAC-TTCTC-----
-----

FC88X_00960_01047 -----------------TCAACAAA--TCGTTTG--GGGCGAC-TTCTC-----
-----

FC88X_00957_01037 -----------------TCAACAAA--TCGTTTG--GGGCGAC-TTCTC-----
-----

FC88X_00954_01011 -----------------TCAACAAA--TCGTTTG--GGGCGAC-TTCTC-----
-----

FC88X_00996_00975 -----------------TCAACAAA--TCGTTTG--GGGCGAC-TTCTC-----
-----

FC88X_00987_00986 -----------------TCAACAAA--TCGTTTG--GGGCGAC-TTCTC-----
-----

FC88X_00980_00981 -----------------TCAACAAA--TCGTTTG--GGGCGAC-TTCTC-----
-----

FC88X_00976_00961 -----------------TCAACAAA--TCGTTTG--GGGCGAC-TTCTC-----
-----

FC88X_00975_00997 -----------------TCAACAAA--TCGTTTG--GGGCGAC-TTCTC-----
-----

FC88X_00975_00962 -----------------TCAACAAA--TCGTTTG--GGGCGAC-TTCTC-----
-----

FC88X_00971_00986 -----------------TCAACAAA--TCGTTTG--GGGCGAC-TTCTC-----
-----

FC88X_00966_00957 -----------------TCAACAAA--TCGTTTG--GGGCGAC-TTCTC-----
-----

FC88X_01040_00385 -----------------TCAACAAA--TCGTTTG--GGGCGAC-TTCTC-----
-----

FC88X_01040_00374 -----------------TCAACAAA--TCGTTTG--GGGCGAC-TTCTC-----
-----

FC88X_01040_00350 -----------------TCAACAAA--TCGTTTG--GGGCGAC-TTCTC-----
-----

FC88X_01038_00375 -----------------TCAACAAA--TCGTTTG--GGGCGAC-TTCTC-----
-----

FC88X_01036_00387 -----------------TCAACAAA--TCGTTTG--GGGCGAC-TTCTC-----
-----
FC88X_01034_00353 -----------------TCAACAAA--TCGTTTG--GGGCGAC-TTCTC-----
-----
FC88X_01033_00366 -----------------TCAACAAA--TCGTTTG--GGGCGAC-TTCTC-----
-----
FC88X_01031_00387 -----------------TCAACAAA--TCGTTTG--GGGCGAC-TTCTC-----
-----
FC88X_01031_00384 -----------------TCAACAAA--TCGTTTG--GGGCGAC-TTCTC-----
-----
FC88X_01031_00376 -----------------TCAACAAA--TCGTTTG--GGGCGAC-TTCTC-----
-----
FC88X_01030_00365 -----------------TCAACAAA--TCGTTTG--GGGCGAC-TTCTC-----
-----
FC88X_01029_00396 -----------------TCAACAAA--TCGTTTG--GGGCGAC-TTCTC-----
-----
FC88X_01029_00389 -----------------TCAACAAA--TCGTTTG--GGGCGAC-TTCTC-----
-----
FC88X_01029_00358 -----------------TCAACAAA--TCGTTTG--GGGCGAC-TTCTC-----
-----
FC88X_01028_00353 -----------------TCAACAAA--TCGTTTG--GGGCGAC-TTCTC-----
-----
FC88X_01027_00391 -----------------TCAACAAA--TCGTTTG--GGGCGAC-TTCTC-----
-----
FC88X_01027_00369 -----------------TCAACAAA--TCGTTTG--GGGCGAC-TTCTC-----
-----
FC88X_01026_00392 -----------------TCAACAAA--TCGTTTG--GGGCGAC-TTCTC-----
-----
FC88X_01026_00374 -----------------TCAACAAA--TCGTTTG--GGGCGAC-TTCTC-----
-----
FC88X_01023_00392 -----------------TCAACAAA--TCGTTTG--GGGCGAC-TTCTC-----
-----
FC88X_01023_00389 -----------------TCAACAAA--TCGTTTG--GGGCGAC-TTCTC-----
-----
FC88X_01023_00354 -----------------TCAACAAA--TCGTTTG--GGGCGAC-TTCTC-----
-----
FC88X_01022_00389 -----------------TCAACAAA--TCGTTTG--GGGCGAC-TTCTC-----
-----
FC88X_01021_00375 -----------------TCAACAAA--TCGTTTG--GGGCGAC-TTCTC-----
-----
FC88X_01019_00372 -----------------TCAACAAA--TCGTTTG--GGGCGAC-TTCTC-----
-----
FC88X_01019_00362 -----------------TCAACAAA--TCGTTTG--GGGCGAC-TTCTC-----
-----
FC88X_01018_00357 -----------------TCAACAAA--TCGTTTG--GGGCGAC-TTCTC-----
-----
FC88X_01015_00377 -----------------TCAACAAA--TCGTTTG--GGGCGAC-TTCTC-----
-----
FC88X_01014_00386 -----------------TCAACAAA--TCGTTTG--GGGCGAC-TTCTC-----
-----
FC88X_01014_00380 -----------------TCAACAAA--TCGTTTG--GGGCGAC-TTCTC-----
-----
FC88X_01006_00382 -----------------TCAACAAA--TCGTTTG--GGGCGAC-TTCTC-----
-----
FC88X_01001_00361 -----------------TCAACAAA--TCGTTTG--GGGCGAC-TTCTC-----
-----

FC88X_01000_00359 -----------------TCAACAAA--TCGTTTG--GGGCGAC-TTCTC-----
-----
FC88X_01045_00322 -----------------TCAACAAA--TCGTTTG--GGGCGAC-TTCTC-----
-----
FC88X_01033_00331 -----------------TCAACAAA--TCGTTTG--GGGCGAC-TTCTC-----
-----
FC88X_01026_00338 -----------------TCAACAAA--TCGTTTG--GGGCGAC-TTCTC-----
-----
FC88X_01046_00573 -----------------TCAACAAA--TCGTTTG--GGGCGAC-TTCTC-----
-----
FC88X_01044_00591 -----------------TCAACAAA--TCGTTTG--GGGCGAC-TTCTC-----
-----
FC88X_01043_00587 -----------------TCAACAAA--TCGTTTG--GGGCGAC-TTCTC-----
-----
FC88X_01028_00789 -----------------TCAACAAA--TCGTTTG--GGGCGAC-TTCTC-----
-----
FC88X_01028_00766 -----------------TCAACAAA--TCGTTTG--GGGCGAC-TTCTC-----
-----
FC88X_01027_00771 -----------------TCAACAAA--TCGTTTG--GGGCGAC-TTCTC-----
-----
FC88X_01025_00756 -----------------TCAACAAA--TCGTTTG--GGGCGAC-TTCTC-----
-----
FC88X_01024_00793 -----------------TCAACAAA--TCGTTTG--GGGCGAC-TTCTC-----
-----
FC88X_01021_00798 -----------------TCAACAAA--TCGTTTG--GGGCGAC-TTCTC-----
-----
FC88X_01020_00784 -----------------TCAACAAA--TCGTTTG--GGGCGAC-TTCTC-----
-----
FC88X_01016_00792 -----------------TCAACAAA--TCGTTTG--GGGCGAC-TTCTC-----
-----
FC88X_01016_00762 -----------------TCAACAAA--TCGTTTG--GGGCGAC-TTCTC-----
-----
FC88X_01015_00776 -----------------TCAACAAA--TCGTTTG--GGGCGAC-TTCTC-----
-----
FC88X_01002_00635 -----------------TCAACAAA--TCGTTTG--GGGCGAC-TTCTC-----
-----
FC88X_01001_00609 -----------------TCAACAAA--TCGTTTG--GGGCGAC-TTCTC-----
-----
FC88X_01000_00649 -----------------TCAACAAA--TCGTTTG--GGGCGAC-TTCTC-----
-----
FC88X_01000_00609 -----------------TCAACAAA--TCGTTTG--GGGCGAC-TTCTC-----
-----
FC88X_01029_00486 -----------------TCAACAAA--TCGTTTG--GGGCGAC-TTCTC-----
-----
FC88X_01028_00489 -----------------TCAACAAA--TCGTTTG--GGGCGAC-TTCTC-----
-----
FC88X_01028_00457 -----------------TCAACAAA--TCGTTTG--GGGCGAC-TTCTC-----
-----
FC88X_01026_00470 -----------------TCAACAAA--TCGTTTG--GGGCGAC-TTCTC-----
-----
FC88X_01023_00452 -----------------TCAACAAA--TCGTTTG--GGGCGAC-TTCTC-----
-----
FC88X_01022_00491 -----------------TCAACAAA--TCGTTTG--GGGCGAC-TTCTC-----
-----
FC88X_01021_00480 -----------------TCAACAAA--TCGTTTG--GGGCGAC-TTCTC-----
-----

FC88X_01020_00479 -----------------TCAACAAA--TCGTTTG--GGGCGAC-TTCTC-----
-----
FC88X_01017_00485 -----------------TCAACAAA--TCGTTTG--GGGCGAC-TTCTC-----
-----
FC88X_01011_00476 -----------------TCAACAAA--TCGTTTG--GGGCGAC-TTCTC-----
-----
FC88X_01010_00485 -----------------TCAACAAA--TCGTTTG--GGGCGAC-TTCTC-----
-----
FC88X_01000_00498 -----------------TCAACAAA--TCGTTTG--GGGCGAC-TTCTC-----
-----
FC88X_01045_00446 -----------------TCAACAAA--TCGTTTG--GGGCGAC-TTCTC-----
-----
FC88X_01044_00431 -----------------TCAACAAA--TCGTTTG--GGGCGAC-TTCTC-----
-----
FC88X_01043_00404 -----------------TCAACAAA--TCGTTTG--GGGCGAC-TTCTC-----
-----
FC88X_01008_00770 -----------------TCAACAAA--TCGTTTG--GGGCGAC-TTCTC-----
-----
FC88X_01007_00797 -----------------TCAACAAA--TCGTTTG--GGGCGAC-TTCTC-----
-----
FC88X_01005_00761 -----------------TCAACAAA--TCGTTTG--GGGCGAC-TTCTC-----
-----
FC88X_01002_00780 -----------------TCAACAAA--TCGTTTG--GGGCGAC-TTCTC-----
-----
FC88X_01001_00770 -----------------TCAACAAA--TCGTTTG--GGGCGAC-TTCTC-----
-----
FC88X_01041_00656 -----------------TCAACAAA--TCGTTTG--GGGCGAC-TTCTC-----
-----
FC88X_01040_00668 -----------------TCAACAAA--TCGTTTG--GGGCGAC-TTCTC-----
-----
FC88X_01039_00699 -----------------TCAACAAA--TCGTTTG--GGGCGAC-TTCTC-----
-----
FC88X_01038_00657 -----------------TCAACAAA--TCGTTTG--GGGCGAC-TTCTC-----
-----
FC88X_01034_00691 -----------------TCAACAAA--TCGTTTG--GGGCGAC-TTCTC-----
-----
FC88X_01032_00661 -----------------TCAACAAA--TCGTTTG--GGGCGAC-TTCTC-----
-----
FC88X_01029_00698 -----------------TCAACAAA--TCGTTTG--GGGCGAC-TTCTC-----
-----
FC88X_01025_00688 -----------------TCAACAAA--TCGTTTG--GGGCGAC-TTCTC-----
-----
FC88X_01023_00683 -----------------TCAACAAA--TCGTTTG--GGGCGAC-TTCTC-----
-----
FC88X_01023_00654 -----------------TCAACAAA--TCGTTTG--GGGCGAC-TTCTC-----
-----
FC88X_01022_00652 -----------------TCAACAAA--TCGTTTG--GGGCGAC-TTCTC-----
-----
FC88X_01019_00674 -----------------TCAACAAA--TCGTTTG--GGGCGAC-TTCTC-----
-----
FC88X_01019_00671 -----------------TCAACAAA--TCGTTTG--GGGCGAC-TTCTC-----
-----
FC88X_01018_00698 -----------------TCAACAAA--TCGTTTG--GGGCGAC-TTCTC-----
-----
FC88X_01013_00666 -----------------TCAACAAA--TCGTTTG--GGGCGAC-TTCTC-----
-----

FC88X_01013_00658 -----------------TCAACAAA--TCGTTTG--GGGCGAC-TTCTC-----
-----
FC88X_01003_00650 -----------------TCAACAAA--TCGTTTG--GGGCGAC-TTCTC-----
-----
FC88X_01002_00665 -----------------TCAACAAA--TCGTTTG--GGGCGAC-TTCTC-----
-----
FC88X_01049_00628 -----------------TCAACAAA--TCGTTTG--GGGCGAC-TTCTC-----
-----
FC88X_01048_00625 -----------------TCAACAAA--TCGTTTG--GGGCGAC-TTCTC-----
-----
FC88X_01043_00612 -----------------TCAACAAA--TCGTTTG--GGGCGAC-TTCTC-----
-----
FC88X_01036_00610 -----------------TCAACAAA--TCGTTTG--GGGCGAC-TTCTC-----
-----
FC88X_01036_00600 -----------------TCAACAAA--TCGTTTG--GGGCGAC-TTCTC-----
-----
FC88X_01034_00627 -----------------TCAACAAA--TCGTTTG--GGGCGAC-TTCTC-----
-----
FC88X_01030_00620 -----------------TCAACAAA--TCGTTTG--GGGCGAC-TTCTC-----
-----
FC88X_01027_00600 -----------------TCAACAAA--TCGTTTG--GGGCGAC-TTCTC-----
-----
FC88X_01023_00602 -----------------TCAACAAA--TCGTTTG--GGGCGAC-TTCTC-----
-----
FC88X_01022_00603 -----------------TCAACAAA--TCGTTTG--GGGCGAC-TTCTC-----
-----
FC88X_01021_00648 -----------------TCAACAAA--TCGTTTG--GGGCGAC-TTCTC-----
-----
FC88X_01020_00611 -----------------TCAACAAA--TCGTTTG--GGGCGAC-TTCTC-----
-----
FC88X_01019_00640 -----------------TCAACAAA--TCGTTTG--GGGCGAC-TTCTC-----
-----
FC88X_01016_00621 -----------------TCAACAAA--TCGTTTG--GGGCGAC-TTCTC-----
-----
FC88X_01016_00616 -----------------TCAACAAA--TCGTTTG--GGGCGAC-TTCTC-----
-----
FC88X_01012_00638 -----------------TCAACAAA--TCGTTTG--GGGCGAC-TTCTC-----
-----
FC88X_01011_00619 -----------------TCAACAAA--TCGTTTG--GGGCGAC-TTCTC-----
-----
FC88X_01010_00639 -----------------TCAACAAA--TCGTTTG--GGGCGAC-TTCTC-----
-----
FC88X_01009_00647 -----------------TCAACAAA--TCGTTTG--GGGCGAC-TTCTC-----
-----
FC88X_01005_00636 -----------------TCAACAAA--TCGTTTG--GGGCGAC-TTCTC-----
-----
FC88X_01028_00542 -----------------TCAACAAA--TCGTTTG--GGGCGAC-TTCTC-----
-----
FC88X_01027_00532 -----------------TCAACAAA--TCGTTTG--GGGCGAC-TTCTC-----
-----
FC88X_01025_00527 -----------------TCAACAAA--TCGTTTG--GGGCGAC-TTCTC-----
-----
FC88X_01023_00534 -----------------TCAACAAA--TCGTTTG--GGGCGAC-TTCTC-----
-----
FC88X_01022_00547 -----------------TCAACAAA--TCGTTTG--GGGCGAC-TTCTC-----
-----

FC88X_01021_00513 -----------------TCAACAAA--TCGTTTG--GGGCGAC-TTCTC-----
-----
FC88X_01020_00529 -----------------TCAACAAA--TCGTTTG--GGGCGAC-TTCTC-----
-----
FC88X_01015_00527 -----------------TCAACAAA--TCGTTTG--GGGCGAC-TTCTC-----
-----
FC88X_01014_00520 -----------------TCAACAAA--TCGTTTG--GGGCGAC-TTCTC-----
-----
FC88X_01010_00540 -----------------TCAACAAA--TCGTTTG--GGGCGAC-TTCTC-----
-----
FC88X_01009_00530 -----------------TCAACAAA--TCGTTTG--GGGCGAC-TTCTC-----
-----
FC88X_01009_00523 -----------------TCAACAAA--TCGTTTG--GGGCGAC-TTCTC-----
-----
FC88X_01002_00512 -----------------TCAACAAA--TCGTTTG--GGGCGAC-TTCTC-----
-----
FC88X_01000_00547 -----------------TCAACAAA--TCGTTTG--GGGCGAC-TTCTC-----
-----
FC88X_01049_00455 -----------------TCAACAAA--TCGTTTG--GGGCGAC-TTCTC-----
-----
FC88X_01044_00471 -----------------TCAACAAA--TCGTTTG--GGGCGAC-TTCTC-----
-----
FC88X_01042_00487 -----------------TCAACAAA--TCGTTTG--GGGCGAC-TTCTC-----
-----
FC88X_01041_00458 -----------------TCAACAAA--TCGTTTG--GGGCGAC-TTCTC-----
-----
FC88X_01037_00497 -----------------TCAACAAA--TCGTTTG--GGGCGAC-TTCTC-----
-----
FC88X_01037_00482 -----------------TCAACAAA--TCGTTTG--GGGCGAC-TTCTC-----
-----
FC88X_01037_00481 -----------------TCAACAAA--TCGTTTG--GGGCGAC-TTCTC-----
-----
FC88X_00972_00554 -----------------TCAACAAA--TCGTTTG--GGGCGAC-TTCTC-----
-----
FC88X_00972_00551 -----------------TCAACAAA--TCGTTTG--GGGCGAC-TTCTC-----
-----
FC88X_00971_00550 -----------------TCAACAAA--TCGTTTG--GGGCGAC-TTCTC-----
-----
FC88X_00969_00561 -----------------TCAACAAA--TCGTTTG--GGGCGAC-TTCTC-----
-----
FC88X_01079_00839 -----------------TCAACAAA--TCGTTTG--GGGCGAC-TTCTC-----
-----
FC88X_01078_00827 -----------------TCAACAAA--TCGTTTG--GGGCGAC-TTCTC-----
-----
FC88X_01071_00821 -----------------TCAACAAA--TCGTTTG--GGGCGAC-TTCTC-----
-----
FC88X_01068_00822 -----------------TCAACAAA--TCGTTTG--GGGCGAC-TTCTC-----
-----
FC88X_01063_00822 -----------------TCAACAAA--TCGTTTG--GGGCGAC-TTCTC-----
-----
FC88X_01054_00849 -----------------TCAACAAA--TCGTTTG--GGGCGAC-TTCTC-----
-----
FC88X_01097_00799 -----------------TCAACAAA--TCGTTTG--GGGCGAC-TTCTC-----
-----
FC88X_01042_00568 -----------------TCAACAAA--TCGTTTG--GGGCGAC-TTCTC-----
-----

```
FC88X_01039_00560      -----------------TCAACAAA--TCGTTTG--GGGCGAC-TTCTC-----
-----
FC88X_01039_00559      -----------------TCAACAAA--TCGTTTG--GGGCGAC-TTCTC-----
-----
FC88X_01038_00570      -----------------TCAACAAA--TCGTTTG--GGGCGAC-TTCTC-----
-----
FC88X_01027_00594      -----------------TCAACAAA--TCGTTTG--GGGCGAC-TTCTC-----
-----
FC88X_01025_00550      -----------------TCAACAAA--TCGTTTG--GGGCGAC-TTCTC-----
-----
FC88X_01024_00565      -----------------TCAACAAA--TCGTTTG--GGGCGAC-TTCTC-----
-----
FC88X_01024_00562      -----------------TCAACAAA--TCGTTTG--GGGCGAC-TTCTC-----
-----
FC88X_01022_00566      -----------------TCAACAAA--TCGTTTG--GGGCGAC-TTCTC-----
-----
FC88X_01018_00590      -----------------TCAACAAA--TCGTTTG--GGGCGAC-TTCTC-----
-----
FC88X_01017_00596      -----------------TCAACAAA--TCGTTTG--GGGCGAC-TTCTC-----
-----
FC88X_01016_00586      -----------------TCAACAAA--TCGTTTG--GGGCGAC-TTCTC-----
-----
FC88X_01013_00567      -----------------TCAACAAA--TCGTTTG--GGGCGAC-TTCTC-----
-----
FC88X_01007_00559      -----------------TCAACAAA--TCGTTTG--GGGCGAC-TTCTC-----
-----
FC88X_01001_00563      -----------------TCAACAAA--TCGTTTG--GGGCGAC-TTCTC-----
-----
FC88X_01049_00501      -----------------TCAACAAA--TCGTTTG--GGGCGAC-TTCTC-----
-----
FC88X_01048_00535      -----------------TCAACAAA--TCGTTTG--GGGCGAC-TTCTC-----
-----
FC88X_01047_00528      -----------------TCAACAAA--TCGTTTG--GGGCGAC-TTCTC-----
-----
FC88X_01041_00518      -----------------TCAACAAA--TCGTTTG--GGGCGAC-TTCTC-----
-----
FC88X_01040_00545      -----------------TCAACAAA--TCGTTTG--GGGCGAC-TTCTC-----
-----
FC88X_01039_00506      -----------------TCAACAAA--TCGTTTG--GGGCGAC-TTCTC-----
-----
FC88X_01110_00508      -----------------TCAACAAA--TCGTTTG--GGGCGAC-TTCTC-----
-----
FC88X_01110_00503      -----------------TCAACAAA--TCGTTTG--GGGCGAC-TTCTC-----
-----
FC88X_01106_00545      -----------------TCAACAAA--TCGTTTG--GGGCGAC-TTCTC-----
-----
FC88X_01103_00504      -----------------TCAACAAA--TCGTTTG--GGGCGAC-TTCTC-----
-----
FC88X_01102_00546      -----------------TCAACAAA--TCGTTTG--GGGCGAC-TTCTC-----
-----
FC88X_01102_00508      -----------------TCAACAAA--TCGTTTG--GGGCGAC-TTCTC-----
-----
FC88X_01101_00528      -----------------TCAACAAA--TCGTTTG--GGGCGAC-TTCTC-----
-----
FC88X_01113_00471      -----------------TCAACAAA--TCGTTTG--GGGCGAC-TTCTC-----
-----
```

```
FC88X_01111_00456      -----------------TCAACAAA--TCGTTTG--GGGCGAC-TTCTC-----
-----
FC88X_01105_00450      -----------------TCAACAAA--TCGTTTG--GGGCGAC-TTCTC-----
-----
FC88X_01100_00493      -----------------TCAACAAA--TCGTTTG--GGGCGAC-TTCTC-----
-----
FC88X_01066_01265      -----------------TCAACAAA--TCGTTTG--GGGCGAC-TTCTC-----
-----
FC88X_01066_01252      -----------------TCAACAAA--TCGTTTG--GGGCGAC-TTCTC-----
-----
FC88X_01064_01252      -----------------TCAACAAA--TCGTTTG--GGGCGAC-TTCTC-----
-----
FC88X_01096_01216      -----------------TCAACAAA--TCGTTTG--GGGCGAC-TTCTC-----
-----
FC88X_01088_01224      -----------------TCAACAAA--TCGTTTG--GGGCGAC-TTCTC-----
-----
FC88X_01088_01212      -----------------TCAACAAA--TCGTTTG--GGGCGAC-TTCTC-----
-----
FC88X_01083_01224      -----------------TCAACAAA--TCGTTTG--GGGCGAC-TTCTC-----
-----
FC88X_01079_01230      -----------------TCAACAAA--TCGTTTG--GGGCGAC-TTCTC-----
-----
FC88X_01078_01227      -----------------TCAACAAA--TCGTTTG--GGGCGAC-TTCTC-----
-----
FC88X_01078_01204      -----------------TCAACAAA--TCGTTTG--GGGCGAC-TTCTC-----
-----
FC88X_01076_01200      -----------------TCAACAAA--TCGTTTG--GGGCGAC-TTCTC-----
-----
FC88X_01075_01228      -----------------TCAACAAA--TCGTTTG--GGGCGAC-TTCTC-----
-----
FC88X_01071_01202      -----------------TCAACAAA--TCGTTTG--GGGCGAC-TTCTC-----
-----
FC88X_01070_01201      -----------------TCAACAAA--TCGTTTG--GGGCGAC-TTCTC-----
-----
FC88X_01069_01242      -----------------TCAACAAA--TCGTTTG--GGGCGAC-TTCTC-----
-----
FC88X_01066_01201      -----------------TCAACAAA--TCGTTTG--GGGCGAC-TTCTC-----
-----
FC88X_01063_01245      -----------------TCAACAAA--TCGTTTG--GGGCGAC-TTCTC-----
-----
FC88X_01062_01231      -----------------TCAACAAA--TCGTTTG--GGGCGAC-TTCTC-----
-----
FC88X_01059_01222      -----------------TCAACAAA--TCGTTTG--GGGCGAC-TTCTC-----
-----
FC88X_01052_01228      -----------------TCAACAAA--TCGTTTG--GGGCGAC-TTCTC-----
-----
FC88X_01050_01248      -----------------TCAACAAA--TCGTTTG--GGGCGAC-TTCTC-----
-----
FC88X_01050_01218      -----------------TCAACAAA--TCGTTTG--GGGCGAC-TTCTC-----
-----
FC88X_01099_01186      -----------------TCAACAAA--TCGTTTG--GGGCGAC-TTCTC-----
-----
FC88X_01097_01165      -----------------TCAACAAA--TCGTTTG--GGGCGAC-TTCTC-----
-----
FC88X_01096_01176      -----------------TCAACAAA--TCGTTTG--GGGCGAC-TTCTC-----
-----
```

FC88X_01096_01170 ------------------TCAACAAA--TCGTTTG--GGGCGAC-TTCTC-----
-----
FC88X_01096_01167 ------------------TCAACAAA--TCGTTTG--GGGCGAC-TTCTC-----
-----
FC88X_01091_01164 ------------------TCAACAAA--TCGTTTG--GGGCGAC-TTCTC-----
-----
FC88X_01084_01186 ------------------TCAACAAA--TCGTTTG--GGGCGAC-TTCTC-----
-----
FC88X_01082_01153 ------------------TCAACAAA--TCGTTTG--GGGCGAC-TTCTC-----
-----
FC88X_01074_01183 ------------------TCAACAAA--TCGTTTG--GGGCGAC-TTCTC-----
-----
FC88X_01067_01158 ------------------TCAACAAA--TCGTTTG--GGGCGAC-TTCTC-----
-----
FC88X_01057_01168 ------------------TCAACAAA--TCGTTTG--GGGCGAC-TTCTC-----
-----
FC88X_01056_01158 ------------------TCAACAAA--TCGTTTG--GGGCGAC-TTCTC-----
-----
FC88X_01055_01178 ------------------TCAACAAA--TCGTTTG--GGGCGAC-TTCTC-----
-----
FC88X_01054_01152 ------------------TCAACAAA--TCGTTTG--GGGCGAC-TTCTC-----
-----
FC88X_01052_01199 ------------------TCAACAAA--TCGTTTG--GGGCGAC-TTCTC-----
-----
FC88X_01052_01170 ------------------TCAACAAA--TCGTTTG--GGGCGAC-TTCTC-----
-----
FC88X_01099_01118 ------------------TCAACAAA--TCGTTTG--GGGCGAC-TTCTC-----
-----
FC88X_01099_01114 ------------------TCAACAAA--TCGTTTG--GGGCGAC-TTCTC-----
-----
FC88X_01093_01133 ------------------TCAACAAA--TCGTTTG--GGGCGAC-TTCTC-----
-----
FC88X_01073_01134 ------------------TCAACAAA--TCGTTTG--GGGCGAC-TTCTC-----
-----
FC88X_01072_01146 ------------------TCAACAAA--TCGTTTG--GGGCGAC-TTCTC-----
-----
FC88X_01072_01144 ------------------TCAACAAA--TCGTTTG--GGGCGAC-TTCTC-----
-----
FC88X_01071_01127 ------------------TCAACAAA--TCGTTTG--GGGCGAC-TTCTC-----
-----
FC88X_01068_01106 ------------------TCAACAAA--TCGTTTG--GGGCGAC-TTCTC-----
-----
FC88X_01098_01090 ------------------TCAACAAA--TCGTTTG--GGGCGAC-TTCTC-----
-----
FC88X_01099_00955 ------------------TCAACAAA--TCGTTTG--GGGCGAC-TTCTC-----
-----
FC88X_01099_00954 ------------------TCAACAAA--TCGTTTG--GGGCGAC-TTCTC-----
-----
FC88X_01095_00990 ------------------TCAACAAA--TCGTTTG--GGGCGAC-TTCTC-----
-----
FC88X_01078_00958 ------------------TCAACAAA--TCGTTTG--GGGCGAC-TTCTC-----
-----
FC88X_01074_00978 ------------------TCAACAAA--TCGTTTG--GGGCGAC-TTCTC-----
-----
FC88X_01058_00987 ------------------TCAACAAA--TCGTTTG--GGGCGAC-TTCTC-----
-----

FC88X_01098_00917 -----------------TCAACAAA--TCGTTTG--GGGCGAC-TTCTC-----
-----
FC88X_01097_00900 -----------------TCAACAAA--TCGTTTG--GGGCGAC-TTCTC-----
-----
FC88X_01096_00910 -----------------TCAACAAA--TCGTTTG--GGGCGAC-TTCTC-----
-----
FC88X_01091_00947 -----------------TCAACAAA--TCGTTTG--GGGCGAC-TTCTC-----
-----
FC88X_01087_00949 -----------------TCAACAAA--TCGTTTG--GGGCGAC-TTCTC-----
-----
FC88X_01077_00946 -----------------TCAACAAA--TCGTTTG--GGGCGAC-TTCTC-----
-----
FC88X_01077_00917 -----------------TCAACAAA--TCGTTTG--GGGCGAC-TTCTC-----
-----
FC88X_01075_00907 -----------------TCAACAAA--TCGTTTG--GGGCGAC-TTCTC-----
-----
FC88X_01096_00786 -----------------TCAACAAA--TCGTTTG--GGGCGAC-TTCTC-----
-----
FC88X_01095_00750 -----------------TCAACAAA--TCGTTTG--GGGCGAC-TTCTC-----
-----
FC88X_01091_00781 -----------------TCAACAAA--TCGTTTG--GGGCGAC-TTCTC-----
-----
FC88X_01085_00797 -----------------TCAACAAA--TCGTTTG--GGGCGAC-TTCTC-----
-----
FC88X_01083_00776 -----------------TCAACAAA--TCGTTTG--GGGCGAC-TTCTC-----
-----
FC88X_01082_00784 -----------------TCAACAAA--TCGTTTG--GGGCGAC-TTCTC-----
-----
FC88X_01080_00785 -----------------TCAACAAA--TCGTTTG--GGGCGAC-TTCTC-----
-----
FC88X_01077_00786 -----------------TCAACAAA--TCGTTTG--GGGCGAC-TTCTC-----
-----
FC88X_01077_00758 -----------------TCAACAAA--TCGTTTG--GGGCGAC-TTCTC-----
-----
FC88X_01067_00795 -----------------TCAACAAA--TCGTTTG--GGGCGAC-TTCTC-----
-----
FC88X_01063_00786 -----------------TCAACAAA--TCGTTTG--GGGCGAC-TTCTC-----
-----
FC88X_01055_00769 -----------------TCAACAAA--TCGTTTG--GGGCGAC-TTCTC-----
-----
FC88X_01053_00771 -----------------TCAACAAA--TCGTTTG--GGGCGAC-TTCTC-----
-----
FC88X_01051_00771 -----------------TCAACAAA--TCGTTTG--GGGCGAC-TTCTC-----
-----
FC88X_01095_00748 -----------------TCAACAAA--TCGTTTG--GGGCGAC-TTCTC-----
-----
FC88X_01092_00715 -----------------TCAACAAA--TCGTTTG--GGGCGAC-TTCTC-----
-----
FC88X_01089_00733 -----------------TCAACAAA--TCGTTTG--GGGCGAC-TTCTC-----
-----
FC88X_01088_00730 -----------------TCAACAAA--TCGTTTG--GGGCGAC-TTCTC-----
-----
FC88X_01075_00747 -----------------TCAACAAA--TCGTTTG--GGGCGAC-TTCTC-----
-----
FC88X_01075_00729 -----------------TCAACAAA--TCGTTTG--GGGCGAC-TTCTC-----
-----

FC88X_01073_00701 -----------------TCAACAAA--TCGTTTG--GGGCGAC-TTCTC----------
FC88X_01071_00710 -----------------TCAACAAA--TCGTTTG--GGGCGAC-TTCTC----------
FC88X_01064_00743 -----------------TCAACAAA--TCGTTTG--GGGCGAC-TTCTC----------
FC88X_01059_00746 -----------------TCAACAAA--TCGTTTG--GGGCGAC-TTCTC----------
FC88X_01054_00726 -----------------TCAACAAA--TCGTTTG--GGGCGAC-TTCTC----------
FC88X_01053_00703 -----------------TCAACAAA--TCGTTTG--GGGCGAC-TTCTC----------
FC88X_01073_00914 -----------------TCAACAAA--TCGTTTG--GGGCGAC-TTCTC----------
FC88X_01069_00905 -----------------TCAACAAA--TCGTTTG--GGGCGAC-TTCTC----------
FC88X_01066_00912 -----------------TCAACAAA--TCGTTTG--GGGCGAC-TTCTC----------
FC88X_01063_00936 -----------------TCAACAAA--TCGTTTG--GGGCGAC-TTCTC----------
FC88X_01062_00904 -----------------TCAACAAA--TCGTTTG--GGGCGAC-TTCTC----------
FC88X_01056_00949 -----------------TCAACAAA--TCGTTTG--GGGCGAC-TTCTC----------
FC88X_01045_00754 -----------------TCAACAAA--TCGTTTG--GGGCGAC-TTCTC----------
FC88X_01035_00792 -----------------TCAACAAA--TCGTTTG--GGGCGAC-TTCTC----------
FC88X_01029_00803 -----------------TCAACAAA--TCGTTTG--GGGCGAC-TTCTC----------
FC88X_01020_00831 -----------------TCAACAAA--TCGTTTG--GGGCGAC-TTCTC----------
FC88X_01009_00844 -----------------TCAACAAA--TCGTTTG--GGGCGAC-TTCTC----------
FC88X_01009_00824 -----------------TCAACAAA--TCGTTTG--GGGCGAC-TTCTC----------
FC88X_01008_00841 -----------------TCAACAAA--TCGTTTG--GGGCGAC-TTCTC----------
FC88X_01127_00593 -----------------TCAACAAA--TCGTTTG--GGGCGAC-TTCTC----------
FC88X_01125_00595 -----------------TCAACAAA--TCGTTTG--GGGCGAC-TTCTC----------
FC88X_01118_00587 -----------------TCAACAAA--TCGTTTG--GGGCGAC-TTCTC----------
FC88X_01117_00592 -----------------TCAACAAA--TCGTTTG--GGGCGAC-TTCTC----------
FC88X_01114_00596 -----------------TCAACAAA--TCGTTTG--GGGCGAC-TTCTC----------
FC88X_01108_00571 -----------------TCAACAAA--TCGTTTG--GGGCGAC-TTCTC----------
FC88X_01103_00563 -----------------TCAACAAA--TCGTTTG--GGGCGAC-TTCTC----------
FC88X_01100_00593 -----------------TCAACAAA--TCGTTTG--GGGCGAC-TTCTC----------
FC88X_01132_00537 -----------------TCAACAAA--TCGTTTG--GGGCGAC-TTCTC----------

FC88X_01125_00516   -----------------TCAACAAA--TCGTTTG--GGGCGAC-TTCTC-----
-----
FC88X_01123_00501   -----------------TCAACAAA--TCGTTTG--GGGCGAC-TTCTC-----
-----
FC88X_01121_00503   -----------------TCAACAAA--TCGTTTG--GGGCGAC-TTCTC-----
-----
FC88X_01114_00539   -----------------TCAACAAA--TCGTTTG--GGGCGAC-TTCTC-----
-----
FC88X_01114_00533   -----------------TCAACAAA--TCGTTTG--GGGCGAC-TTCTC-----
-----
FC88X_01112_00517   -----------------TCAACAAA--TCGTTTG--GGGCGAC-TTCTC-----
-----
FC88X_01055_00906   -----------------TCAACAAA--TCGTTTG--GGGCGAC-TTCTC-----
-----
FC88X_01052_00937   -----------------TCAACAAA--TCGTTTG--GGGCGAC-TTCTC-----
-----
FC88X_01096_00869   -----------------TCAACAAA--TCGTTTG--GGGCGAC-TTCTC-----
-----
FC88X_01096_00866   -----------------TCAACAAA--TCGTTTG--GGGCGAC-TTCTC-----
-----
FC88X_01093_00895   -----------------TCAACAAA--TCGTTTG--GGGCGAC-TTCTC-----
-----
FC88X_01088_00865   -----------------TCAACAAA--TCGTTTG--GGGCGAC-TTCTC-----
-----
FC88X_01083_00883   -----------------TCAACAAA--TCGTTTG--GGGCGAC-TTCTC-----
-----
FC88X_01080_00866   -----------------TCAACAAA--TCGTTTG--GGGCGAC-TTCTC-----
-----
FC88X_01077_00884   -----------------TCAACAAA--TCGTTTG--GGGCGAC-TTCTC-----
-----
FC88X_01074_00887   -----------------TCAACAAA--TCGTTTG--GGGCGAC-TTCTC-----
-----
FC88X_01073_00858   -----------------TCAACAAA--TCGTTTG--GGGCGAC-TTCTC-----
-----
FC88X_01069_00859   -----------------TCAACAAA--TCGTTTG--GGGCGAC-TTCTC-----
-----
FC88X_01067_00858   -----------------TCAACAAA--TCGTTTG--GGGCGAC-TTCTC-----
-----
FC88X_01066_00863   -----------------TCAACAAA--TCGTTTG--GGGCGAC-TTCTC-----
-----
FC88X_01064_00862   -----------------TCAACAAA--TCGTTTG--GGGCGAC-TTCTC-----
-----
FC88X_01062_00884   -----------------TCAACAAA--TCGTTTG--GGGCGAC-TTCTC-----
-----
FC88X_01058_00893   -----------------TCAACAAA--TCGTTTG--GGGCGAC-TTCTC-----
-----
FC88X_01053_00868   -----------------TCAACAAA--TCGTTTG--GGGCGAC-TTCTC-----
-----
FC88X_01099_00833   -----------------TCAACAAA--TCGTTTG--GGGCGAC-TTCTC-----
-----
FC88X_01092_00801   -----------------TCAACAAA--TCGTTTG--GGGCGAC-TTCTC-----
-----
FC88X_01089_00816   -----------------TCAACAAA--TCGTTTG--GGGCGAC-TTCTC-----
-----
FC88X_01087_00802   -----------------TCAACAAA--TCGTTTG--GGGCGAC-TTCTC-----
-----

```
FC88X_01085_00838      -----------------TCAACAAA--TCGTTTG--GGGCGAC-TTCTC-----
-----
FC88X_01085_00813      -----------------TCAACAAA--TCGTTTG--GGGCGAC-TTCTC-----
-----
FC88X_01112_00619      -----------------TCAACAAA--TCGTTTG--GGGCGAC-TTCTC-----
-----
FC88X_01143_00582      -----------------TCAACAAA--TCGTTTG--GGGCGAC-TTCTC-----
-----
FC88X_01131_00580      -----------------TCAACAAA--TCGTTTG--GGGCGAC-TTCTC-----
-----
FC88X_01130_00566      -----------------TCAACAAA--TCGTTTG--GGGCGAC-TTCTC-----
-----
FC88X_00974_00443      -----------------TCAACAAA--TCGTTTG--GGGCGAC-TTCTC-----
-----
FC88X_00972_00416      -----------------TCAACAAA--TCGTTTG--GGGCGAC-TTCTC-----
-----
FC88X_00969_00401      -----------------TCAACAAA--TCGTTTG--GGGCGAC-TTCTC-----
-----
FC88X_00968_00426      -----------------TCAACAAA--TCGTTTG--GGGCGAC-TTCTC-----
-----
FC88X_00968_00416      -----------------TCAACAAA--TCGTTTG--GGGCGAC-TTCTC-----
-----
FC88X_00965_00436      -----------------TCAACAAA--TCGTTTG--GGGCGAC-TTCTC-----
-----
FC88X_00965_00428      -----------------TCAACAAA--TCGTTTG--GGGCGAC-TTCTC-----
-----
FC88X_00964_00422      -----------------TCAACAAA--TCGTTTG--GGGCGAC-TTCTC-----
-----
FC88X_00961_00401      -----------------TCAACAAA--TCGTTTG--GGGCGAC-TTCTC-----
-----
FC88X_00957_00415      -----------------TCAACAAA--TCGTTTG--GGGCGAC-TTCTC-----
-----
FC88X_00991_00481      -----------------TCAACAAA--TCGTTTG--GGGCGAC-TTCTC-----
-----
FC88X_00979_00481      -----------------TCAACAAA--TCGTTTG--GGGCGAC-TTCTC-----
-----
FC88X_00979_00477      -----------------TCAACAAA--TCGTTTG--GGGCGAC-TTCTC-----
-----
FC88X_00977_00477      -----------------TCAACAAA--TCGTTTG--GGGCGAC-TTCTC-----
-----
FC88X_00975_00489      -----------------TCAACAAA--TCGTTTG--GGGCGAC-TTCTC-----
-----
FC88X_00972_00494      -----------------TCAACAAA--TCGTTTG--GGGCGAC-TTCTC-----
-----
FC88X_00971_00495      -----------------TCAACAAA--TCGTTTG--GGGCGAC-TTCTC-----
-----
FC88X_00971_00472      -----------------TCAACAAA--TCGTTTG--GGGCGAC-TTCTC-----
-----
FC88X_00968_00462      -----------------TCAACAAA--TCGTTTG--GGGCGAC-TTCTC-----
-----
FC88X_00967_00470      -----------------TCAACAAA--TCGTTTG--GGGCGAC-TTCTC-----
-----
FC88X_00961_00484      -----------------TCAACAAA--TCGTTTG--GGGCGAC-TTCTC-----
-----
FC88X_00960_00496      -----------------TCAACAAA--TCGTTTG--GGGCGAC-TTCTC-----
-----
```

FC88X_00959_00475 -----------------TCAACAAA--TCGTTTG--GGGCGAC-TTCTC-----
-----
FC88X_00958_00499 -----------------TCAACAAA--TCGTTTG--GGGCGAC-TTCTC-----
-----
FC88X_01004_01269 -----------------TCAACAAA--TCGTTTG--GGGCGAC-TTCTC-----
-----
FC88X_01000_01262 -----------------TCAACAAA--TCGTTTG--GGGCGAC-TTCTC-----
-----
FC88X_01047_01238 -----------------TCAACAAA--TCGTTTG--GGGCGAC-TTCTC-----
-----
FC88X_01047_01228 -----------------TCAACAAA--TCGTTTG--GGGCGAC-TTCTC-----
-----
FC88X_01036_01217 -----------------TCAACAAA--TCGTTTG--GGGCGAC-TTCTC-----
-----
FC88X_01028_01233 -----------------TCAACAAA--TCGTTTG--GGGCGAC-TTCTC-----
-----
FC88X_01046_01142 -----------------TCAACAAA--TCGTTTG--GGGCGAC-TTCTC-----
-----
FC88X_01041_01142 -----------------TCAACAAA--TCGTTTG--GGGCGAC-TTCTC-----
-----
FC88X_01031_01147 -----------------TCAACAAA--TCGTTTG--GGGCGAC-TTCTC-----
-----
FC88X_01020_01123 -----------------TCAACAAA--TCGTTTG--GGGCGAC-TTCTC-----
-----
FC88X_01048_01028 -----------------TCAACAAA--TCGTTTG--GGGCGAC-TTCTC-----
-----
FC88X_01048_01021 -----------------TCAACAAA--TCGTTTG--GGGCGAC-TTCTC-----
-----
FC88X_01048_01006 -----------------TCAACAAA--TCGTTTG--GGGCGAC-TTCTC-----
-----
FC88X_01045_01014 -----------------TCAACAAA--TCGTTTG--GGGCGAC-TTCTC-----
-----
FC88X_01044_01001 -----------------TCAACAAA--TCGTTTG--GGGCGAC-TTCTC-----
-----
FC88X_01042_01005 -----------------TCAACAAA--TCGTTTG--GGGCGAC-TTCTC-----
-----
FC88X_01037_01045 -----------------TCAACAAA--TCGTTTG--GGGCGAC-TTCTC-----
-----
FC88X_01036_01043 -----------------TCAACAAA--TCGTTTG--GGGCGAC-TTCTC-----
-----
FC88X_01033_01032 -----------------TCAACAAA--TCGTTTG--GGGCGAC-TTCTC-----
-----
FC88X_01031_01027 -----------------TCAACAAA--TCGTTTG--GGGCGAC-TTCTC-----
-----
FC88X_01029_01039 -----------------TCAACAAA--TCGTTTG--GGGCGAC-TTCTC-----
-----
FC88X_01028_01015 -----------------TCAACAAA--TCGTTTG--GGGCGAC-TTCTC-----
-----
FC88X_01027_01013 -----------------TCAACAAA--TCGTTTG--GGGCGAC-TTCTC-----
-----
FC88X_01025_01036 -----------------TCAACAAA--TCGTTTG--GGGCGAC-TTCTC-----
-----
FC88X_01025_01026 -----------------TCAACAAA--TCGTTTG--GGGCGAC-TTCTC-----
-----
FC88X_01023_01048 -----------------TCAACAAA--TCGTTTG--GGGCGAC-TTCTC-----
-----

FC88X_01023_01009 -----------------TCAACAAA--TCGTTTG--GGGCGAC-TTCTC-----
-----
FC88X_01017_01022 -----------------TCAACAAA--TCGTTTG--GGGCGAC-TTCTC-----
-----
FC88X_01017_01008 -----------------TCAACAAA--TCGTTTG--GGGCGAC-TTCTC-----
-----
FC88X_01015_01002 -----------------TCAACAAA--TCGTTTG--GGGCGAC-TTCTC-----
-----
FC88X_01012_01038 -----------------TCAACAAA--TCGTTTG--GGGCGAC-TTCTC-----
-----
FC88X_01012_01006 -----------------TCAACAAA--TCGTTTG--GGGCGAC-TTCTC-----
-----
FC88X_01011_01026 -----------------TCAACAAA--TCGTTTG--GGGCGAC-TTCTC-----
-----
FC88X_01010_01012 -----------------TCAACAAA--TCGTTTG--GGGCGAC-TTCTC-----
-----
FC88X_01008_01022 -----------------TCAACAAA--TCGTTTG--GGGCGAC-TTCTC-----
-----
FC88X_01008_01001 -----------------TCAACAAA--TCGTTTG--GGGCGAC-TTCTC-----
-----
FC88X_01006_01026 -----------------TCAACAAA--TCGTTTG--GGGCGAC-TTCTC-----
-----
FC88X_01006_01025 -----------------TCAACAAA--TCGTTTG--GGGCGAC-TTCTC-----
-----
FC88X_01003_01009 -----------------TCAACAAA--TCGTTTG--GGGCGAC-TTCTC-----
-----
FC88X_01001_01001 -----------------TCAACAAA--TCGTTTG--GGGCGAC-TTCTC-----
-----
FC88X_01000_01046 -----------------TCAACAAA--TCGTTTG--GGGCGAC-TTCTC-----
-----
FC88X_01000_01024 -----------------TCAACAAA--TCGTTTG--GGGCGAC-TTCTC-----
-----
FC88X_01048_00989 -----------------TCAACAAA--TCGTTTG--GGGCGAC-TTCTC-----
-----
FC88X_01048_00979 -----------------TCAACAAA--TCGTTTG--GGGCGAC-TTCTC-----
-----
FC88X_01048_00970 -----------------TCAACAAA--TCGTTTG--GGGCGAC-TTCTC-----
-----
FC88X_01044_00978 -----------------TCAACAAA--TCGTTTG--GGGCGAC-TTCTC-----
-----
FC88X_01044_00965 -----------------TCAACAAA--TCGTTTG--GGGCGAC-TTCTC-----
-----
FC88X_01044_00955 -----------------TCAACAAA--TCGTTTG--GGGCGAC-TTCTC-----
-----
FC88X_01042_00991 -----------------TCAACAAA--TCGTTTG--GGGCGAC-TTCTC-----
-----
FC88X_01039_00982 -----------------TCAACAAA--TCGTTTG--GGGCGAC-TTCTC-----
-----
FC88X_01035_00959 -----------------TCAACAAA--TCGTTTG--GGGCGAC-TTCTC-----
-----
FC88X_01033_00978 -----------------TCAACAAA--TCGTTTG--GGGCGAC-TTCTC-----
-----
FC88X_01028_00954 -----------------TCAACAAA--TCGTTTG--GGGCGAC-TTCTC-----
-----
FC88X_01026_00981 -----------------TCAACAAA--TCGTTTG--GGGCGAC-TTCTC-----
-----

FC88X_01025_00977 ------------------TCAACAAA--TCGTTTG--GGGCGAC-TTCTC-----
-----
FC88X_01025_00974 ------------------TCAACAAA--TCGTTTG--GGGCGAC-TTCTC-----
-----
FC88X_01011_00990 ------------------TCAACAAA--TCGTTTG--GGGCGAC-TTCTC-----
-----
FC88X_01006_00982 ------------------TCAACAAA--TCGTTTG--GGGCGAC-TTCTC-----
-----
FC88X_01046_00918 ------------------TCAACAAA--TCGTTTG--GGGCGAC-TTCTC-----
-----
FC88X_01045_00943 ------------------TCAACAAA--TCGTTTG--GGGCGAC-TTCTC-----
-----
FC88X_01045_00919 ------------------TCAACAAA--TCGTTTG--GGGCGAC-TTCTC-----
-----
FC88X_01045_00909 ------------------TCAACAAA--TCGTTTG--GGGCGAC-TTCTC-----
-----
FC88X_01042_00936 ------------------TCAACAAA--TCGTTTG--GGGCGAC-TTCTC-----
-----
FC88X_01040_00941 ------------------TCAACAAA--TCGTTTG--GGGCGAC-TTCTC-----
-----
FC88X_01040_00939 ------------------TCAACAAA--TCGTTTG--GGGCGAC-TTCTC-----
-----
FC88X_01040_00912 ------------------TCAACAAA--TCGTTTG--GGGCGAC-TTCTC-----
-----
FC88X_01039_00921 ------------------TCAACAAA--TCGTTTG--GGGCGAC-TTCTC-----
-----
FC88X_01036_00903 ------------------TCAACAAA--TCGTTTG--GGGCGAC-TTCTC-----
-----
FC88X_01034_00946 ------------------TCAACAAA--TCGTTTG--GGGCGAC-TTCTC-----
-----
FC88X_01032_00902 ------------------TCAACAAA--TCGTTTG--GGGCGAC-TTCTC-----
-----
FC88X_01031_00927 ------------------TCAACAAA--TCGTTTG--GGGCGAC-TTCTC-----
-----
FC88X_01029_00915 ------------------TCAACAAA--TCGTTTG--GGGCGAC-TTCTC-----
-----
FC88X_01028_00937 ------------------TCAACAAA--TCGTTTG--GGGCGAC-TTCTC-----
-----
FC88X_01028_00922 ------------------TCAACAAA--TCGTTTG--GGGCGAC-TTCTC-----
-----
FC88X_01027_00929 ------------------TCAACAAA--TCGTTTG--GGGCGAC-TTCTC-----
-----
FC88X_01027_00921 ------------------TCAACAAA--TCGTTTG--GGGCGAC-TTCTC-----
-----
FC88X_01023_00936 ------------------TCAACAAA--TCGTTTG--GGGCGAC-TTCTC-----
-----
FC88X_01021_00933 ------------------TCAACAAA--TCGTTTG--GGGCGAC-TTCTC-----
-----
FC88X_01019_00937 ------------------TCAACAAA--TCGTTTG--GGGCGAC-TTCTC-----
-----
FC88X_01019_00916 ------------------TCAACAAA--TCGTTTG--GGGCGAC-TTCTC-----
-----
FC88X_01017_00900 ------------------TCAACAAA--TCGTTTG--GGGCGAC-TTCTC-----
-----
FC88X_01011_00923 ------------------TCAACAAA--TCGTTTG--GGGCGAC-TTCTC-----
-----

FC88X_01008_00927 -----------------TCAACAAA--TCGTTTG--GGGCGAC-TTCTC-----
-----
FC88X_01008_00911 -----------------TCAACAAA--TCGTTTG--GGGCGAC-TTCTC-----
-----
FC88X_01005_00927 -----------------TCAACAAA--TCGTTTG--GGGCGAC-TTCTC-----
-----
FC88X_01004_00942 -----------------TCAACAAA--TCGTTTG--GGGCGAC-TTCTC-----
-----
FC88X_01004_00926 -----------------TCAACAAA--TCGTTTG--GGGCGAC-TTCTC-----
-----
FC88X_01004_00912 -----------------TCAACAAA--TCGTTTG--GGGCGAC-TTCTC-----
-----
FC88X_01047_00871 -----------------TCAACAAA--TCGTTTG--GGGCGAC-TTCTC-----
-----
FC88X_01046_00895 -----------------TCAACAAA--TCGTTTG--GGGCGAC-TTCTC-----
-----
FC88X_01046_00871 -----------------TCAACAAA--TCGTTTG--GGGCGAC-TTCTC-----
-----
FC88X_01045_00878 -----------------TCAACAAA--TCGTTTG--GGGCGAC-TTCTC-----
-----
FC88X_01044_00857 -----------------TCAACAAA--TCGTTTG--GGGCGAC-TTCTC-----
-----
FC88X_01041_00896 -----------------TCAACAAA--TCGTTTG--GGGCGAC-TTCTC-----
-----
FC88X_01034_00861 -----------------TCAACAAA--TCGTTTG--GGGCGAC-TTCTC-----
-----
FC88X_01033_00862 -----------------TCAACAAA--TCGTTTG--GGGCGAC-TTCTC-----
-----
FC88X_01032_00884 -----------------TCAACAAA--TCGTTTG--GGGCGAC-TTCTC-----
-----
FC88X_01032_00859 -----------------TCAACAAA--TCGTTTG--GGGCGAC-TTCTC-----
-----
FC88X_01031_00893 -----------------TCAACAAA--TCGTTTG--GGGCGAC-TTCTC-----
-----
FC88X_01025_00884 -----------------TCAACAAA--TCGTTTG--GGGCGAC-TTCTC-----
-----
FC88X_01023_00893 -----------------TCAACAAA--TCGTTTG--GGGCGAC-TTCTC-----
-----
FC88X_01020_00896 -----------------TCAACAAA--TCGTTTG--GGGCGAC-TTCTC-----
-----
FC88X_01020_00893 -----------------TCAACAAA--TCGTTTG--GGGCGAC-TTCTC-----
-----
FC88X_01017_00878 -----------------TCAACAAA--TCGTTTG--GGGCGAC-TTCTC-----
-----
FC88X_01016_00894 -----------------TCAACAAA--TCGTTTG--GGGCGAC-TTCTC-----
-----
FC88X_01012_00861 -----------------TCAACAAA--TCGTTTG--GGGCGAC-TTCTC-----
-----
FC88X_01006_00897 -----------------TCAACAAA--TCGTTTG--GGGCGAC-TTCTC-----
-----
FC88X_01006_00886 -----------------TCAACAAA--TCGTTTG--GGGCGAC-TTCTC-----
-----
FC88X_01006_00871 -----------------TCAACAAA--TCGTTTG--GGGCGAC-TTCTC-----
-----
FC88X_01004_00873 -----------------TCAACAAA--TCGTTTG--GGGCGAC-TTCTC-----
-----

```
FC88X_01003_00891      -----------------TCAACAAA--TCGTTTG--GGGCGAC-TTCTC-----
-----
FC88X_01003_00862      -----------------TCAACAAA--TCGTTTG--GGGCGAC-TTCTC-----
-----
FC88X_01001_00858      -----------------TCAACAAA--TCGTTTG--GGGCGAC-TTCTC-----
-----
FC88X_01047_00813      -----------------TCAACAAA--TCGTTTG--GGGCGAC-TTCTC-----
-----
FC88X_01046_00818      -----------------TCAACAAA--TCGTTTG--GGGCGAC-TTCTC-----
-----
FC88X_01043_00841      -----------------TCAACAAA--TCGTTTG--GGGCGAC-TTCTC-----
-----
FC88X_01043_00833      -----------------TCAACAAA--TCGTTTG--GGGCGAC-TTCTC-----
-----
FC88X_01042_00830      -----------------TCAACAAA--TCGTTTG--GGGCGAC-TTCTC-----
-----
FC88X_01041_00823      -----------------TCAACAAA--TCGTTTG--GGGCGAC-TTCTC-----
-----
FC88X_01040_00816      -----------------TCAACAAA--TCGTTTG--GGGCGAC-TTCTC-----
-----
FC88X_01040_00803      -----------------TCAACAAA--TCGTTTG--GGGCGAC-TTCTC-----
-----
FC88X_01035_00841      -----------------TCAACAAA--TCGTTTG--GGGCGAC-TTCTC-----
-----
FC88X_01033_00834      -----------------TCAACAAA--TCGTTTG--GGGCGAC-TTCTC-----
-----
FC88X_01033_00816      -----------------TCAACAAA--TCGTTTG--GGGCGAC-TTCTC-----
-----
FC88X_01033_00807      -----------------TCAACAAA--TCGTTTG--GGGCGAC-TTCTC-----
-----
FC88X_01030_00832      -----------------TCAACAAA--TCGTTTG--GGGCGAC-TTCTC-----
-----
FC88X_00983_00436      -----------------TCAACAAA--TCGTTTG--GGGCGAC-TTCTC-----
-----
FC88X_00975_00439      -----------------TCAACAAA--TCGTTTG--GGGCGAC-TTCTC-----
-----
FC88X_00952_00415      -----------------TCAACAAA--TCGTTTG--GGGCGAC-TTCTC-----
-----
FC88X_00965_00578      -----------------TCAACAAA--TCGTTTG--GGGCGAC-TTCTC-----
-----
FC88X_00988_00928      -----------------TCAACAAA--TCGTTTG--GGGCGAC-TTCTC-----
-----
FC88X_00986_00929      -----------------TCAACAAA--TCGTTTG--GGGCGAC-TTCTC-----
-----
FC88X_00985_00945      -----------------TCAACAAA--TCGTTTG--GGGCGAC-TTCTC-----
-----
FC88X_00984_00938      -----------------TCAACAAA--TCGTTTG--GGGCGAC-TTCTC-----
-----
FC88X_00979_00942      -----------------TCAACAAA--TCGTTTG--GGGCGAC-TTCTC-----
-----
FC88X_00975_00933      -----------------TCAACAAA--TCGTTTG--GGGCGAC-TTCTC-----
-----
FC88X_00972_00906      -----------------TCAACAAA--TCGTTTG--GGGCGAC-TTCTC-----
-----
FC88X_00969_00928      -----------------TCAACAAA--TCGTTTG--GGGCGAC-TTCTC-----
-----
```

```
FC88X_00967_00944      -----------------TCAACAAA--TCGTTTG--GGGCGAC-TTCTC-----
-----
FC88X_00966_00902      -----------------TCAACAAA--TCGTTTG--GGGCGAC-TTCTC-----
-----
FC88X_00965_00921      -----------------TCAACAAA--TCGTTTG--GGGCGAC-TTCTC-----
-----
FC88X_00964_00936      -----------------TCAACAAA--TCGTTTG--GGGCGAC-TTCTC-----
-----
FC88X_01071_00383      -----------------TCAACAAA--TCGTTTG--GGGCGAC-TTCTC-----
-----
FC88X_01063_00351      -----------------TCAACAAA--TCGTTTG--GGGCGAC-TTCTC-----
-----
FC88X_01060_00359      -----------------TCAACAAA--TCGTTTG--GGGCGAC-TTCTC-----
-----
FC88X_01057_00381      -----------------TCAACAAA--TCGTTTG--GGGCGAC-TTCTC-----
-----
FC88X_01040_01275      -----------------TCAACAAA--TCGTTTG--GGGCGAC-TTCTC-----
-----
FC88X_01037_01255      -----------------TCAACAAA--TCGTTTG--GGGCGAC-TTCTC-----
-----
FC88X_00955_00463      -----------------TCAACAAA--TCGTTTG--GGGCGAC-TTCTC-----
-----
FC88X_00952_00463      -----------------TCAACAAA--TCGTTTG--GGGCGAC-TTCTC-----
-----
FC88X_00999_00405      -----------------TCAACAAA--TCGTTTG--GGGCGAC-TTCTC-----
-----
FC88X_00997_00423      -----------------TCAACAAA--TCGTTTG--GGGCGAC-TTCTC-----
-----
FC88X_00993_00418      -----------------TCAACAAA--TCGTTTG--GGGCGAC-TTCTC-----
-----
FC88X_00993_00403      -----------------TCAACAAA--TCGTTTG--GGGCGAC-TTCTC-----
-----
FC88X_00988_00442      -----------------TCAACAAA--TCGTTTG--GGGCGAC-TTCTC-----
-----
FC88X_00986_00410      -----------------TCAACAAA--TCGTTTG--GGGCGAC-TTCTC-----
-----
FC88X_00980_00855      -----------------TCAACAAA--TCGTTTG--GGGCGAC-TTCTC-----
-----
FC88X_00977_00865      -----------------TCAACAAA--TCGTTTG--GGGCGAC-TTCTC-----
-----
FC88X_00977_00863      -----------------TCAACAAA--TCGTTTG--GGGCGAC-TTCTC-----
-----
FC88X_00973_00886      -----------------TCAACAAA--TCGTTTG--GGGCGAC-TTCTC-----
-----
FC88X_00970_00877      -----------------TCAACAAA--TCGTTTG--GGGCGAC-TTCTC-----
-----
FC88X_00970_00870      -----------------TCAACAAA--TCGTTTG--GGGCGAC-TTCTC-----
-----
FC88X_00966_00868      -----------------TCAACAAA--TCGTTTG--GGGCGAC-TTCTC-----
-----
FC88X_00966_00865      -----------------TCAACAAA--TCGTTTG--GGGCGAC-TTCTC-----
-----
FC88X_00958_00851      -----------------TCAACAAA--TCGTTTG--GGGCGAC-TTCTC-----
-----
FC88X_00955_00861      -----------------TCAACAAA--TCGTTTG--GGGCGAC-TTCTC-----
-----
```

FC88X_00951_00891 -----------------TCAACAAA--TCGTTTG--GGGCGAC-TTCTC-----
-----
FC88X_00998_00846 -----------------TCAACAAA--TCGTTTG--GGGCGAC-TTCTC-----
-----
FC88X_00998_00827 -----------------TCAACAAA--TCGTTTG--GGGCGAC-TTCTC-----
-----
FC88X_00996_00833 -----------------TCAACAAA--TCGTTTG--GGGCGAC-TTCTC-----
-----
FC88X_00994_00832 -----------------TCAACAAA--TCGTTTG--GGGCGAC-TTCTC-----
-----
FC88X_00993_00833 -----------------TCAACAAA--TCGTTTG--GGGCGAC-TTCTC-----
-----
FC88X_00993_00816 -----------------TCAACAAA--TCGTTTG--GGGCGAC-TTCTC-----
-----
FC88X_00992_00805 -----------------TCAACAAA--TCGTTTG--GGGCGAC-TTCTC-----
-----
FC88X_00992_00802 -----------------TCAACAAA--TCGTTTG--GGGCGAC-TTCTC-----
-----
FC88X_00991_00847 -----------------TCAACAAA--TCGTTTG--GGGCGAC-TTCTC-----
-----
FC88X_00991_00834 -----------------TCAACAAA--TCGTTTG--GGGCGAC-TTCTC-----
-----
FC88X_00990_00828 -----------------TCAACAAA--TCGTTTG--GGGCGAC-TTCTC-----
-----
FC88X_00990_00827 -----------------TCAACAAA--TCGTTTG--GGGCGAC-TTCTC-----
-----
FC88X_00989_00803 -----------------TCAACAAA--TCGTTTG--GGGCGAC-TTCTC-----
-----
FC88X_00987_00841 -----------------TCAACAAA--TCGTTTG--GGGCGAC-TTCTC-----
-----
FC88X_00962_00947 -----------------TCAACAAA--TCGTTTG--GGGCGAC-TTCTC-----
-----
FC88X_00956_00913 -----------------TCAACAAA--TCGTTTG--GGGCGAC-TTCTC-----
-----
FC88X_00955_00938 -----------------TCAACAAA--TCGTTTG--GGGCGAC-TTCTC-----
-----
FC88X_00952_00937 -----------------TCAACAAA--TCGTTTG--GGGCGAC-TTCTC-----
-----
FC88X_00950_00934 -----------------TCAACAAA--TCGTTTG--GGGCGAC-TTCTC-----
-----
FC88X_00998_00856 -----------------TCAACAAA--TCGTTTG--GGGCGAC-TTCTC-----
-----
FC88X_00995_00898 -----------------TCAACAAA--TCGTTTG--GGGCGAC-TTCTC-----
-----
FC88X_00995_00887 -----------------TCAACAAA--TCGTTTG--GGGCGAC-TTCTC-----
-----
FC88X_00995_00863 -----------------TCAACAAA--TCGTTTG--GGGCGAC-TTCTC-----
-----
FC88X_00993_00862 -----------------TCAACAAA--TCGTTTG--GGGCGAC-TTCTC-----
-----
FC88X_00993_00858 -----------------TCAACAAA--TCGTTTG--GGGCGAC-TTCTC-----
-----
FC88X_00989_00893 -----------------TCAACAAA--TCGTTTG--GGGCGAC-TTCTC-----
-----
FC88X_00986_00857 -----------------TCAACAAA--TCGTTTG--GGGCGAC-TTCTC-----
-----

FC88X_00985_00881 -----------------TCAACAAA--TCGTTTG--GGGCGAC-TTCTC-----
-----
FC88X_00982_00858 -----------------TCAACAAA--TCGTTTG--GGGCGAC-TTCTC-----
-----
FC88X_00981_00897 -----------------TCAACAAA--TCGTTTG--GGGCGAC-TTCTC-----
-----
FC88X_01088_00492 -----------------TCAACAAA--TCGTTTG--GGGCGAC-TTCTC-----
-----
FC88X_01082_00465 -----------------TCAACAAA--TCGTTTG--GGGCGAC-TTCTC-----
-----
FC88X_01082_00460 -----------------TCAACAAA--TCGTTTG--GGGCGAC-TTCTC-----
-----
FC88X_01082_00456 -----------------TCAACAAA--TCGTTTG--GGGCGAC-TTCTC-----
-----
FC88X_01073_00450 -----------------TCAACAAA--TCGTTTG--GGGCGAC-TTCTC-----
-----
FC88X_01090_00419 -----------------TCAACAAA--TCGTTTG--GGGCGAC-TTCTC-----
-----
FC88X_01089_00444 -----------------TCAACAAA--TCGTTTG--GGGCGAC-TTCTC-----
-----
FC88X_01088_00425 -----------------TCAACAAA--TCGTTTG--GGGCGAC-TTCTC-----
-----
FC88X_01087_00415 -----------------TCAACAAA--TCGTTTG--GGGCGAC-TTCTC-----
-----
FC88X_01085_00444 -----------------TCAACAAA--TCGTTTG--GGGCGAC-TTCTC-----
-----
FC88X_01083_00426 -----------------TCAACAAA--TCGTTTG--GGGCGAC-TTCTC-----
-----
FC88X_01082_00416 -----------------TCAACAAA--TCGTTTG--GGGCGAC-TTCTC-----
-----
FC88X_01080_00432 -----------------TCAACAAA--TCGTTTG--GGGCGAC-TTCTC-----
-----
FC88X_01078_00429 -----------------TCAACAAA--TCGTTTG--GGGCGAC-TTCTC-----
-----
FC88X_01078_00402 -----------------TCAACAAA--TCGTTTG--GGGCGAC-TTCTC-----
-----
FC88X_01077_00431 -----------------TCAACAAA--TCGTTTG--GGGCGAC-TTCTC-----
-----
FC88X_01075_00442 -----------------TCAACAAA--TCGTTTG--GGGCGAC-TTCTC-----
-----
FC88X_01070_00419 -----------------TCAACAAA--TCGTTTG--GGGCGAC-TTCTC-----
-----
FC88X_01070_00418 -----------------TCAACAAA--TCGTTTG--GGGCGAC-TTCTC-----
-----
FC88X_01070_00409 -----------------TCAACAAA--TCGTTTG--GGGCGAC-TTCTC-----
-----
FC88X_01064_00448 -----------------TCAACAAA--TCGTTTG--GGGCGAC-TTCTC-----
-----
FC88X_01064_00447 -----------------TCAACAAA--TCGTTTG--GGGCGAC-TTCTC-----
-----
FC88X_01060_00432 -----------------TCAACAAA--TCGTTTG--GGGCGAC-TTCTC-----
-----
FC88X_01060_00401 -----------------TCAACAAA--TCGTTTG--GGGCGAC-TTCTC-----
-----
FC88X_01059_00447 -----------------TCAACAAA--TCGTTTG--GGGCGAC-TTCTC-----
-----

FC88X_01058_00437 -----------------TCAACAAA--TCGTTTG--GGGCGAC-TTCTC-----
-----
FC88X_01056_00404 -----------------TCAACAAA--TCGTTTG--GGGCGAC-TTCTC-----
-----
FC88X_01056_00403 -----------------TCAACAAA--TCGTTTG--GGGCGAC-TTCTC-----
-----
FC88X_01053_00420 -----------------TCAACAAA--TCGTTTG--GGGCGAC-TTCTC-----
-----
FC88X_01050_00434 -----------------TCAACAAA--TCGTTTG--GGGCGAC-TTCTC-----
-----
FC88X_01087_00397 -----------------TCAACAAA--TCGTTTG--GGGCGAC-TTCTC-----
-----
FC88X_01076_00390 -----------------TCAACAAA--TCGTTTG--GGGCGAC-TTCTC-----
-----
FC88X_01087_00670 -----------------TCAACAAA--TCGTTTG--GGGCGAC-TTCTC-----
-----
FC88X_01084_00673 -----------------TCAACAAA--TCGTTTG--GGGCGAC-TTCTC-----
-----
FC88X_01082_00667 -----------------TCAACAAA--TCGTTTG--GGGCGAC-TTCTC-----
-----
FC88X_01070_00678 -----------------TCAACAAA--TCGTTTG--GGGCGAC-TTCTC-----
-----
FC88X_01068_00654 -----------------TCAACAAA--TCGTTTG--GGGCGAC-TTCTC-----
-----
FC88X_01067_00683 -----------------TCAACAAA--TCGTTTG--GGGCGAC-TTCTC-----
-----
FC88X_01065_00655 -----------------TCAACAAA--TCGTTTG--GGGCGAC-TTCTC-----
-----
FC88X_01056_00682 -----------------TCAACAAA--TCGTTTG--GGGCGAC-TTCTC-----
-----
FC88X_01051_00668 -----------------TCAACAAA--TCGTTTG--GGGCGAC-TTCTC-----
-----
FC88X_01093_00615 -----------------TCAACAAA--TCGTTTG--GGGCGAC-TTCTC-----
-----
FC88X_01091_00618 -----------------TCAACAAA--TCGTTTG--GGGCGAC-TTCTC-----
-----
FC88X_01090_00634 -----------------TCAACAAA--TCGTTTG--GGGCGAC-TTCTC-----
-----
FC88X_01084_00628 -----------------TCAACAAA--TCGTTTG--GGGCGAC-TTCTC-----
-----
FC88X_01082_00636 -----------------TCAACAAA--TCGTTTG--GGGCGAC-TTCTC-----
-----
FC88X_01081_00628 -----------------TCAACAAA--TCGTTTG--GGGCGAC-TTCTC-----
-----
FC88X_01080_00625 -----------------TCAACAAA--TCGTTTG--GGGCGAC-TTCTC-----
-----
FC88X_01079_00611 -----------------TCAACAAA--TCGTTTG--GGGCGAC-TTCTC-----
-----
FC88X_01078_00625 -----------------TCAACAAA--TCGTTTG--GGGCGAC-TTCTC-----
-----
FC88X_01076_00621 -----------------TCAACAAA--TCGTTTG--GGGCGAC-TTCTC-----
-----
FC88X_01075_00623 -----------------TCAACAAA--TCGTTTG--GGGCGAC-TTCTC-----
-----
FC88X_01073_00614 -----------------TCAACAAA--TCGTTTG--GGGCGAC-TTCTC-----
-----

FC88X_01071_00649 -----------------TCAACAAA--TCGTTTG--GGGCGAC-TTCTC-----
-----
FC88X_00952_00499 -----------------TCGACAAA--TCGTTTG--GGGCGAC-TTCTC-----
-----
FC88X_00959_00615 -----------------TCGACAAA--TCGTTTG--GGGCGAC-TTCTC-----
-----
FC88X_00969_00739 -----------------TCGACAAA--TCGTTTG--GGGCGAC-TTCTC-----
-----
FC88X_01117_00797 -----------------TCGACAAA--TCGTTTG--GGGCGAC-TTCTC-----
-----
FC88X_01018_00549 -----------------TCAACAAA--CCGTTTG--GGGCGAC-TTCTC-----
-----
FC88X_01138_00724 ------------------CTACTAT--CCGTTAGATGGGCATT-TTCTC-----
-----
FC88X_01113_00731 -----------------TCAACAAA--CCGTTTG--GGGCGAC-TTCTC-----
-----
FC88X_00971_00253 -----------------TCAACAAA--TCGTTTG--GGGCGAC-TTCTC-----
-----
FC88X_01127_00601 -----------------TCAACAAA--TCGTTTG--GGGCGAC-TTCTC-----
-----
FC88X_01107_00672 -----------------TCAACAAA--TCGTTTG--GGGCGAC-TTCTC-----
-----
FC88X_01119_00742 -----------------TCAACAAA--TCGTTTG--GGGCGAC-TTCTC-----
-----
FC88X_01116_00798 -----------------TCAACAAA--TCGTTTG--GGGCGAC-TTCTC-----
-----
FC88X_01141_00778 -----------------TCAACAAA--TCGTTTG--GGGCGAC-TTCTC-----
-----
FC88X_01126_00814 -----------------TCAACAAA--TCGTTTG--GGGCGAC-TTCTC-----
-----
FC88X_01104_00855 -----------------TCAACAAA--TCGTTTG--GGGCGAC-TTCTC-----
-----
FC88X_01127_00881 -----------------TCAACAAA--TCGTTTG--GGGCGAC-TTCTC-----
-----
FC88X_01124_00940 -----------------TCAACAAA--TCGTTTG--GGGCGAC-TTCTC-----
-----
FC88X_01143_00963 -----------------TCAACAAA--TCGTTTG--GGGCGAC-TTCTC-----
-----
FC88X_01135_01140 -----------------TCAACAAA--TCGTTTG--GGGCGAC-TTCTC-----
-----
FC88X_01133_01177 -----------------TCAACAAA--TCGTTTG--GGGCGAC-TTCTC-----
-----
FC88X_01133_01212 -----------------TCAACAAA--TCGTTTG--GGGCGAC-TTCTC-----
-----
FC88X_00997_00283 -----------------TCAACAAA--TCGTTTG--GGGCGAC-TTCTC-----
-----
FC88X_00983_00564 -----------------TCAACAAA--TCGTTTG--GGGCGAC-TTCTC-----
-----
FC88X_00969_00545 -----------------TCAACAAA--TCGTTTG--GGGCGAC-TTCTC-----
-----
FC88X_00951_00514 -----------------TCAACAAA--TCGTTTG--GGGCGAC-TTCTC-----
-----
FC88X_00988_00634 -----------------TCAACAAA--TCGTTTG--GGGCGAC-TTCTC-----
-----
FC88X_00985_00502 -----------------TCAACAAA--TCGTTTG--GGGCGAC-TTCTC-----
-----

```
FC88X_00955_00729      -----------------TCAACAAA--TCGTTTG--GGGCGAC-TTCTC-----
-----
FC88X_00999_00744      -----------------TCAACAAA--TCGTTTG--GGGCGAC-TTCTC-----
-----
FC88X_01096_00488      -----------------TCAACAAA--TCGTTTG--GGGCGAC-TTCTC-----
-----
FC88X_01093_00546      -----------------TCAACAAA--TCGTTTG--GGGCGAC-TTCTC-----
-----
FC88X_00958_00381      ----------------GTCAACAAA--TCGTTTG--GGGCGAC-TTCTC-----
-----
FC88X_01115_01236      ----------------GTCAACAAA--TCGTTTG--GG-CGAC-TTCTC-----
-----
FC88X_00972_00375      ----------------GTCAACAAA--TCGTTTG--GGGCGAC-TTCTC-----
-----
FC88X_00972_00882      ----------------GTCAACAAA--TCGTTTG--GGGCGAC-TTCTC-----
-----
FC88X_00969_00640      ----------------GTCAACAAA--TCGTTTG--GGGCGAC-TTCTC-----
-----
FC88X_00965_00713      ----------------GTCAACAAA--TCGTTTG--GGGCGAC-TTCTC-----
-----
FC88X_00969_00713      ----------------GTCAACAAA--TCGTTTG--GGGCGAC-TTCTC-----
-----
FC88X_01026_00610      ----------------GTCAACAAA--TCGTTTG--GGGCGAC-TTCTC-----
-----
FC88X_01009_00820      ----------------GTCAACAAA--TCGTTTG--GGGCGAC-TTCTC-----
-----
FC88X_01072_00404      ----------------GTCAACAAA--TCGTTTG--GGGCGAC-TTCTC-----
-----
FC88X_01124_00642      ----------------GTCAACAAA--TCGTTTG--GGGCGAC-TTCTC-----
-----
FC88X_01117_00705      ----------------GTCAACAAA--TCGTTTG--GGGCGAC-TTCTC-----
-----
FC88X_01062_00565      ----------------GTCAACAAA--TCGTTTG--GGGCGAC-TTCTC-----
-----
FC88X_01065_00567      ----------------GTCAACAAA--TCGTTTG--GGGCGAC-TTCTC-----
-----
FC88X_01107_01177      ----------------GTCAACAAA--TCGTTTG--GGGCGAC-TTCTC-----
-----
FC88X_01111_01213      ----------------GTCAACAAA--TCGTTTG--GGGCGAC-TTCTC-----
-----
FC88X_00972_00283      -----------------TCAACAAA--TCGTTTG--GGGCGAC-TTCTC-----
-----
FC88X_01131_00611      -----------------TCAACAAA--TCGTTTG--GGGCGAC-TTCTC-----
-----
FC88X_01118_00654      -----------------TCAACAAA--TCGTTTG--GGGCGAC-TTCTC-----
-----
FC88X_01130_00726      -----------------TCAACAAA--TCGTTTG--GGGCGAC-TTCTC-----
-----
FC88X_01125_00774      -----------------TCAACAAA--TCGTTTG--GGGCGAC-TTCTC-----
-----
FC88X_01146_00759      -----------------TCAACAAA--TCGTTTG--GGGCGAC-TTCTC-----
-----
FC88X_01131_00826      -----------------TCAACAAA--TCGTTTG--GGGCGAC-TTCTC-----
-----
FC88X_01114_00879      -----------------TCAACAAA--TCGTTTG--GGGCGAC-TTCTC-----
-----
```

FC88X_01130_00881 -----------------TCAACAAA--TCGTTTG--GGGCGAC-TTCTC-----
-----
FC88X_01137_00921 -----------------TCAACAAA--TCGTTTG--GGGCGAC-TTCTC-----
-----
FC88X_01108_01131 -----------------TCAACAAA--TCGTTTG--GGGCGAC-TTCTC-----
-----
FC88X_01104_01160 -----------------TCAACAAA--TCGTTTG--GGGCGAC-TTCTC-----
-----
FC88X_01142_01173 -----------------TCAACAAA--TCGTTTG--GGGCGAC-TTCTC-----
-----
FC88X_00989_00375 -----------------TCAACAAA--TCGTTTG--GGGCGAC-TTCTC-----
-----
FC88X_00960_00340 -----------------TCAACAAA--TCGTTTG--GGGCGAC-TTCTC-----
-----
FC88X_00951_00636 -----------------TCAACAAA--TCGTTTG--GGGCGAC-TTCTC-----
-----
FC88X_00979_00529 -----------------TCAACAAA--TCGTTTG--GGGCGAC-TTCTC-----
-----
FC88X_00977_00671 -----------------TCAACAAA--TCGTTTG--GGGCGAC-TTCTC-----
-----
FC88X_00999_00630 -----------------TCAACAAA--TCGTTTG--GGGCGAC-TTCTC-----
-----
FC88X_00955_00560 -----------------TCAACAAA--TCGTTTG--GGGCGAC-TTCTC-----
-----
FC88X_00973_00718 -----------------TCAACAAA--TCGTTTG--GGGCGAC-TTCTC-----
-----
FC88X_00984_00755 -----------------TCAACAAA--TCGTTTG--GGGCGAC-TTCTC-----
-----
FC88X_01057_00504 -----------------TCAACAAA--TCGTTTG--GGGCGAC-TTCTC-----
-----
FC88X_01058_00552 -----------------TCAACAAA--TCGTTTG--GGGCGAC-TTCTC-----
-----
FC88X_01086_00584 -----------------TCAACAAA--TCGTTTG--GGGCGAC-TTCTC-----
-----
FC88X_01059_00620 -----------------TCAACAAA--TCGTTTG--GGGCGAC-TTCTC-----
-----
FC88X_00963_00332 -----------------TCAACAAA--TCGTTTG--GGGCGAC-TCCTC-----
-----
FC88X_01075_00838 -----------------TCAACAAA--TCGTTTG--GGGCGAC-TCCTC-----
-----
FC88X_01019_01184 -----------------TCAACAAA--TCGTTTG--GGGCGAC-----------
-----
FC88X_01046_00653 -----------------TCAACAAA--TCGTTTG--GG-CGAC-TCTCC-----
-----
FC88X_01004_00940 -----------------TCAACAAA--TCGTTTG--GG-CGAC-TCTCCTTCC-
-----
FC88X_00990_00782 -----------------TCAACAAA--TCGTTTG--GGGCGAC-TCTCCC----
-----
FC88X_00961_00926 -----------------TCAACAAA--TCGTTTG--GGGCGAC-TCTC------
-----
FC88X_01072_00366 -----------------TCAACAAA--TCGTTTG--GGGCGGC-TCTC------
-----
FC88X_01129_00589 -----------------TCAACAAAAATCGTTTG--GGGCGAC-TCTC------
-----
FC88X_01073_00381 -----------------TCAACAAAATCGTTTGG--GGGCGAC-TCTC------
-----

```
FC88X_00981_00878      -----------------TCAACAAA--TCGTTTG--GGGCGAC-TCTC------
-----
FC88X_01023_00453      -----------------TCAACAAA--TCGTTTG--GGGCGAC-TCTC------
-----
FC88X_01056_01252      -----------------TCAACAAA--TCGTTTG--GGGCGAC-TCTC------
-----
FC88X_00959_00360      -----------------TCAACAAA--TCGTTTG--GGGCGAC-TCT-------
-----
FC88X_01082_01256      -----------------TCAACAAA--TCGTTTG--GGGCGAC-TCT-------
-----
FC88X_01129_00670      -----------------TCAACAAA--TCGTTTG--GGGCGAC-TCT-------
-----
FC88X_00964_00773      --------------CCGTCAACAAA----TCTGG--GGTCTAT-TCTG------
-----
FC88X_01021_00465      --------------CCGTCAACAAA----TCTGG--GGTCTAT-TCTG------
-----
FC88X_01018_00754      --------------CCGTCAACAAA----TCTGG--GGTCTAT-TCTG------
-----
FC88X_01067_00918      --------------CCGTCAACAAA----TCTGG--GGTCTAT-TCTG------
-----
FC88X_01138_00985      --------------CCGTCAACAAA----TCTGG--GGTCTAT-TCTG------
-----
FC88X_00962_00439      --------------CCGTCAACGAA----TCTGG--GGTCTAT-TCTG------
-----
FC88X_01049_01034      --------------TCGTCAACAAA----TCTGG--GGTCTAT-TCTG------
-----
FC88X_01137_00919      ----------------TTCAACTAA----T------------------------
-----
FC88X_01034_00403      -----------------TCAACAAA----T------------------------
-----
FC88X_01036_00533      ----------------TTCAACAAA----TCGT---------------------
-----
FC88X_01004_00687      -----------------TCAACAAA----TC-----------------------
-----
FC88X_01125_01229      -----------------TCAACAAA----TCGTT--TGGGGTC-GACGTTC---
-----
FC88X_01107_01133      ----------------ATCAACAAA--TCGTTTG--G-----------------
-----
FC88X_01071_00570      -----------------TCAACAAA--TCGTT----------------------
-----
FC88X_01019_01149      -----------------TCAACAAA--TCGTTTG--GGGCGAC-TTTTC-----
-----
FC88X_01009_00753      -----------------TCAACAAA--TCGTTTG--------------------
-----
FC88X_01007_00506      -----------------TCAACAAA--TCGTTTG--G-----------------
-----
FC88X_00987_00376      -----------------TCAACAAA--TCGTTTG--GGGCGAC-TTCTT-----
-----
FC88X_00961_00758      -----------------TCAACAAA--TCGTTTG--GGGCGAC-TTCTT-----
-----
FC88X_01133_00947      -----------------TCAACAAA--TCGTTTG--GGGCGAC-TTCTT-----
-----
FC88X_01033_00401      -----------------TCAACAAA--TCGTTTG--GGGCGAC-TTC-------
-----
FC88X_01025_01112      -----------------TCAACAAA--TCGTTTG--GGGCGTA-CTTCCTC---
-----
```

```
FC88X_01090_00741      -----------------TCAACAAAA-TCGTTTG--GGGCG-A-CTTCCTC---
-----
FC88X_01010_00760      -----------------TCAACAAA--TCGTT-G--GGGCG-------------
-----
FC88X_01014_00668      -----------------TCAACAAA--TCGTTTG--GGGCGAC-TTCCC-----
-----
FC88X_00972_00721      -----------------TCAACAAA--TCGTTTG--GGGCGAC-TT--------
-----
FC88X_00957_00649      -----------------TCAACAAA--TCGTTTG--GG----------------
-----
FC88X_01146_00772      -----------------TCAACAAA--TCGTTTG--GGCCGAC-TTCTC-----
-----
FC88X_00974_00677      --------------TACAACTGAAA--TCGTTTG--GGGCG----ACTTCC---
-----
FC88X_01098_00799      --------------TACAAC-AAAT--CCGTTTG--GGGTCCG-TACTTCC---
-----
FC88X_01125_01241      --------------TACGAC--AAA--TCGTTT---------------------
-----
FC88X_00999_00686      -------------CTACTAC--CAA--TCGTTGG--GTCGCTA-CTTCTC----
-----
FC88X_01025_00426      -----CCACGTAATCCTTTACGCCT--ACGTTCT--C-----------------
-----
FC88X_01003_00643      ----------------TTCACGCAT--TCGAT----------------------
-----
FC88X_01098_00951      ---------------GATCACGCAA--TCGTTT---------------------
-----
FC88X_01091_00805      ----TTCACCAATCGATTGGGCCGT--ACCTTCT--C-----------------
-----
FC88X_00974_00999      ----------------------CAA--TCGTTT---------------------
-----
FC88X_01139_00947      -----------------TCAACAAA--TCGTTTG--GGGCTAC-TTCTC-----
-----
FC88X_01044_00407      -----------------TCAACAAA--TCGTTTG--G-----------------
-----
FC88X_00974_00643      -----------------TCAACAAA--TCGTTTG--GGGTGCT-TCTCCT----
-----
FC88X_01048_00587      -----------------TCAACAA---TCGTTTG--G-----------------
-----
FC88X_00955_00838      -----------------TCAACAAA--TCGTTTG--GGGTGAC-TTCTC-----
-----
FC88X_00952_00842      -----------------TCAACAAA--TCGTTTG--GGG---------------
-----
FC88X_00992_00801      -----------------TCAACAAA--TCGTTTG--G-----------------
-----
FC88X_00988_00900      -----------------TCAACAAA--TCGTTTG--GGGCGGC-TTCTC-----
-----
FC88X_01030_00795      -----------------TCAACAAA--TCGTTTG--GGGCGGC-TTCTC-----
-----
FC88X_01110_00532      -----------------TCAACAAA--TCGTTTG--GGGC--------------
-----
FC88X_01104_00789      -----------------TCAACAAA--TCGTTTG--GGGCAAC-TTCTC-----
-----
FC88X_01115_01005      -----------------TCAACAAA--TCGTTTG--GGGC--------------
-----
FC88X_01048_00387      -----------------TCAACAAA--TCGTTTG--G-----------------
-----
```

FC88X_01097_00691 -----------------TCAACAAA--TCGTTTG--GGGCGAC-TTCT------
-----
FC88X_01114_00811 -----------------TCAACAAA--TCGTTTG--GGGCGAC-TTCT------
-----
FC88X_01012_01175 -----------------TCAACAAA--TCGTTTG--GGGCGAC-TTCT------
-----
FC88X_00978_00637 -----------------TCAACAAA--TCGTTTG--GGGCGAC-TTCTCT----
-----
FC88X_01000_01174 -----------------TCAACAAA--TCGTTTG--GGGCGAC-TTCTCTACC-
-----
FC88X_01020_00353 -----------------TCAACAAA--TCGTTTG--GGGCGAC-TTCT------
-----
FC88X_01141_00619 -----------------TCAACAAA--TCGTTTG--GGGCGAC-TTCTC-----
-----
FC88X_01108_00732 -----------------TCAACAAA--TCGTTTG--GGGCGAC-TTCTC-----
-----
FC88X_01146_00713 -----------------TCAACAAA--TCGTTTG--GGGCGAC-TTCTC-----
-----
FC88X_01135_00767 -----------------TCAACAAA--TCGTTTG--GGGCGAC-TTCTC-----
-----
FC88X_01121_00807 -----------------TCAACAAA--TCGTTTG--GGGCGAC-TTCTC-----
-----
FC88X_01144_00811 -----------------TCAACAAA--TCGTTTG--GGGCGAC-TTCTC-----
-----
FC88X_01122_00851 -----------------TCAACAAA--TCGTTTG--GGGCGAC-TTCTC-----
-----
FC88X_01115_00936 -----------------TCAACAAA--TCGTTTG--GGGCGAC-TTCTC-----
-----
FC88X_01135_00989 -----------------TCAACAAA--TCGTTTG--GGGCGAC-TTCTC-----
-----
FC88X_01121_01141 -----------------TCAACAAA--TCGTTTG--GGGCGAC-TTCTC-----
-----
FC88X_01126_01173 -----------------TCAACAAA--TCGTTTG--GGGCGAC-TTCTC-----
-----
FC88X_01125_01217 -----------------TCAACAAA--TCGTTTG--GGGCGAC-TTCTC-----
-----
FC88X_00953_00323 -----------------TCAACAAA--TCGTTTG--GGGCGAC-TTCTC-----
-----
FC88X_00972_00306 -----------------TCAACAAA--TCGTTTG--GGGCGAC-TTCTC-----
-----
FC88X_00970_00375 -----------------TCAACAAA--TCGTTTG--GGGCGAC-TTCTC-----
-----
FC88X_00964_00614 -----------------TCAACAAA--TCGTTTG--GGGCGAC-TTCTC-----
-----
FC88X_00974_00621 -----------------TCAACAAA--TCGTTTG--GGGCGAC-TTCTC-----
-----
FC88X_00965_00655 -----------------TCAACAAA--TCGTTTG--GGGCGAC-TTCTC-----
-----
FC88X_00963_00808 -----------------TCAACAAA--TCGTTTG--GGGCGAC-TTCTC-----
-----
FC88X_00992_00743 -----------------TCAACAAA--TCGTTTG--GGGCGAC-TTCTC-----
-----
FC88X_00958_00809 -----------------TCAACAAA--TCGTTTG--GGGCGAC-TTCTC-----
-----
FC88X_01084_00524 -----------------TCAACAAA--TCGTTTG--GGGCGAC-TTCTC-----
-----

FC88X_01075_00598	-----------------TCAACAAA--TCGTTTG--GGGCGAC-TTCTC-----
-----
FC88X_01056_00621	-----------------TCAACAAA--TCGTTTG--GGGCGAC-TTCTC-----
-----
FC88X_00955_00841	-----------------TCAACAAA--TCGTTTG--GGGCGAC-TTCTCTCC--
-----
FC88X_01052_01159	-----------------TCAACAAA--TCGTTTG--GGGCGAC-TTCT------
-----
FC88X_01127_00610	-----------------TCAACAAA--TCGTTTG--GGGCGAC-TTCTC-----
-----
FC88X_01109_00664	-----------------TCAACAAA--TCGTTTG--GGGCGAC-TTCTC-----
-----
FC88X_01122_00718	-----------------TCAACAAA--TCGTTTG--GGGCGAC-TTCTC-----
-----
FC88X_01117_00790	-----------------TCAACAAA--TCGTTTG--GGGCGAC-TTCTC-----
-----
FC88X_01142_00755	-----------------TCAACAAA--TCGTTTG--GGGCGAC-TTCTC-----
-----
FC88X_01130_00802	-----------------TCAACAAA--TCGTTTG--GGGCGAC-TTCTC-----
-----
FC88X_01104_00880	-----------------TCAACAAA--TCGTTTG--GGGCGAC-TTCTC-----
-----
FC88X_01128_00898	-----------------TCAACAAA--TCGTTTG--GGGCGAC-TTCTC-----
-----
FC88X_01125_00922	-----------------TCAACAAA--TCGTTTG--GGGCGAC-TTCTC-----
-----
FC88X_01144_00950	-----------------TCAACAAA--TCGTTTG--GGGCGAC-TTCTC-----
-----
FC88X_01142_01122	-----------------TCAACAAA--TCGTTTG--GGGCGAC-TTCTC-----
-----
FC88X_01134_01178	-----------------TCAACAAA--TCGTTTG--GGGCGAC-TTCTC-----
-----
FC88X_01136_01226	-----------------TCAACAAA--TCGTTTG--GGGCGAC-TTCTC-----
-----
FC88X_00950_00375	-----------------TCAACAAA--TCGTTTG--GGGCGAC-TTCTC-----
-----
FC88X_00986_00585	-----------------TCAACAAA--TCGTTTG--GGGCGAC-TTCTC-----
-----
FC88X_00973_00520	-----------------TCAACAAA--TCGTTTG--GGGCGAC-TTCTC-----
-----
FC88X_00951_00516	-----------------TCAACAAA--TCGTTTG--GGGCGAC-TTCTC-----
-----
FC88X_00988_00645	-----------------TCAACAAA--TCGTTTG--GGGCGAC-TTCTC-----
-----
FC88X_00992_00526	-----------------TCAACAAA--TCGTTTG--GGGCGAC-TTCTC-----
-----
FC88X_00963_00729	-----------------TCAACAAA--TCGTTTG--GGGCGAC-TTCTC-----
-----
FC88X_00960_00774	-----------------TCAACAAA--TCGTTTG--GGGCGAC-TTCTC-----
-----
FC88X_01099_00478	-----------------TCAACAAA--TCGTTTG--GGGCGAC-TTCTC-----
-----
FC88X_01098_00545	-----------------TCAACAAA--TCGTTTG--GGGCGAC-TTCTC-----
-----
FC88X_01080_00579	-----------------TCAACAAA--TCGTTTG--GGGCGAC-TTCTC-----
-----

```
FC88X_00962_00354      -----------------TCAGCAAA--TCGTTT--GGGGCGAC-TTCTC-----
-----
FC88X_01130_00868      -----------------TCAGCAAA--TCGTTT--GGGGCGAC-TTCTC-----
-----
FC88X_00972_00842      -----------------TCAACAAA--TCGTTTT-GGGGCGAC-TTCTC-----
-----
FC88X_00972_00277      -----------------TCAACAAA--TCGTTT--GGGGCGAC-TTCTC-----
-----
FC88X_01129_00603      -----------------TCAACAAA--TCGTTT--GGGGCGAC-TTCTC-----
-----
FC88X_01111_00697      -----------------TCAACAAA--TCGTTT--GGGGCGAC-TTCTC-----
-----
FC88X_01126_00730      -----------------TCAACAAA--TCGTTT--GGGGCGAC-TTCTC-----
-----
FC88X_01119_00763      -----------------TCAACAAA--TCGTTT--GGGGCGAC-TTCTC-----
-----
FC88X_01142_00797      -----------------TCAACAAA--TCGTTT--GGGGCGAC-TTCTC-----
-----
FC88X_01130_00833      -----------------TCAACAAA--TCGTTT--GGGGCGAC-TTCTC-----
-----
FC88X_01107_00886      -----------------TCAACAAA--TCGTTT--GGGGCGAC-TTCTC-----
-----
FC88X_01129_00898      -----------------TCAACAAA--TCGTTT--GGGGCGAC-TTCTC-----
-----
FC88X_01132_00907      -----------------TCAACAAA--TCGTTT--GGGGCGAC-TTCTC-----
-----
FC88X_01144_01047      -----------------TCAACAAA--TCGTTT--GGGGCGAC-TTCTC-----
-----
FC88X_01100_01162      -----------------TCAACAAA--TCGTTT--GGGGCGAC-TTCTC-----
-----
FC88X_01136_01191      -----------------TCAACAAA--TCGTTT--GGGGCGAC-TTCTC-----
-----
FC88X_00988_00364      -----------------TCAACAAA--TCGTTT--GGGGCGAC-TTCTC-----
-----
FC88X_00960_00359      -----------------TCAACAAA--TCGTTT--GGGGCGAC-TTCTC-----
-----
FC88X_00990_00587      -----------------TCAACAAA--TCGTTT--GGGGCGAC-TTCTC-----
-----
FC88X_00977_00524      -----------------TCAACAAA--TCGTTT--GGGGCGAC-TTCTC-----
-----
FC88X_00958_00521      -----------------TCAACAAA--TCGTTT--GGGGCGAC-TTCTC-----
-----
FC88X_00993_00648      -----------------TCAACAAA--TCGTTT--GGGGCGAC-TTCTC-----
-----
FC88X_00998_00521      -----------------TCAACAAA--TCGTTT--GGGGCGAC-TTCTC-----
-----
FC88X_00964_00736      -----------------TCAACAAA--TCGTTT--GGGGCGAC-TTCTC-----
-----
FC88X_00968_00775      -----------------TCAACAAA--TCGTTT--GGGGCGAC-TTCTC-----
-----
FC88X_01054_00541      -----------------TCAACAAA--TCGTTT--GGGGCGAC-TTCTC-----
-----
FC88X_01056_00583      -----------------TCAACAAA--TCGTTT--GGGGCGAC-TTCTC-----
-----
FC88X_01089_00572      -----------------TCAACAAA--TCGTTT--GGGGCGAC-TTCTC-----
-----
```

FC88X_00989_00476 ------------GGGAATCAACAAA--TCGTTT--GGGGCGAC-TTCTC----------
FC88X_01015_00661 ------------GGGAATCAACAAA--TCGTTT--GGGGCGAC-TTCTC----------
FC88X_01049_00457 -----------------TCAATAAA--TCGTTT--GGGGCGAC-TTCTC----------
FC88X_01031_00530 -----------------TCAATAAA--TCGTTT--GGGGCGAC-TTCTC----------
FC88X_01071_00560 -----------------TCAATAAA--TCGTTT--GGGGCGAC-TTCTC----------
FC88X_01065_00748 -----------------TCAATAAA--TCGTTT--GGGGCGAC-TTCTC----------
FC88X_01141_01199 ----------------ATCAACAAA--TCGTTT--GGGGCGAC-TTCTC----------
FC88X_00982_00296 -----------------TCAACAAA--TCGTTT--GGGGCGAC-TTCTC----------
FC88X_00990_00691 -----------------TCAACAAA--TCGTTT--GGGGCGAC-TTCTC----------
FC88X_01122_00695 -----------------TCAACAAA--TCGTTT--GGGGCGAC-TTCTC----------
FC88X_01133_00738 -----------------TCAACAAA--TCGTTT--GGGGCGAC-TTCTC----------
FC88X_01126_00756 -----------------TCAACAAA--TCGTTT--GGGGCGAC-TTCTC----------
FC88X_01108_00802 -----------------TCAACAAA--TCGTTT--GGGGCGAC-TTCTC----------
FC88X_01134_00847 -----------------TCAACAAA--TCGTTT--GGGGCGAC-TTCTC----------
FC88X_01114_00893 -----------------TCAACAAA--TCGTTT--GGGGCGAC-TTCTC----------
FC88X_01131_00860 -----------------TCAACAAA--TCGTTT--GGGGCGAC-TTCTC----------
FC88X_01104_00986 -----------------TCAACAAA--TCGTTT--GGGGCGAC-TTCTC----------
FC88X_01111_01131 -----------------TCAACAAA--TCGTTT--GGGGCGAC-TTCTC----------
FC88X_01104_01172 -----------------TCAACAAA--TCGTTT--GGGGCGAC-TTCTC----------
FC88X_01101_01217 -----------------TCAACAAA--TCGTTT--GGGGCGAC-TTCTC----------
FC88X_00989_00389 -----------------TCAACAAA--TCGTTT--GGGGCGAC-TTCTC----------
FC88X_00961_00301 -----------------TCAACAAA--TCGTTT--GGGGCGAC-TTCTC----------
FC88X_00981_00300 -----------------TCAACAAA--TCGTTT--GGGGCGAC-TTCTC----------
FC88X_00972_00656 -----------------TCAACAAA--TCGTTT--GGGGCGAC-TTCTC----------
FC88X_00980_00661 -----------------TCAACAAA--TCGTTT--GGGGCGAC-TTCTC----------
FC88X_00950_00675 -----------------TCAACAAA--TCGTTT--GGGGCGAC-TTCTC----------
FC88X_00959_00584 -----------------TCAACAAA--TCGTTT--GGGGCGAC-TTCTC----------
FC88X_00975_00713 -----------------TCAACAAA--TCGTTT--GGGGCGAC-TTCTC----------

```
FC88X_00989_00781      -----------------TCAACAAA--TCGTTT--GGGGCGAC-TTCTC-----
-----
FC88X_01068_00547      -----------------TCAACAAA--TCGTTT--GGGGCGAC-TTCTC-----
-----
FC88X_01063_00582      -----------------TCAACAAA--TCGTTT--GGGGCGAC-TTCTC-----
-----
FC88X_00959_00400      ----------------ATCAACAAA--TCGTTT--GGGGCGAC-TTCTC-----
-----
FC88X_01054_00934      ------------GGGGATCAACAAA--TCGTTT--GGGGCGAC-TTCTC-----
-----
FC88X_01063_00598      -----------------TCAACAAA--TCGCTT--GGGGCGAC-TTCTC-----
-----
FC88X_01132_00825      -----------------TCAACAAA--TCGCTT--GGGGCGAC-TTCTC-----
-----
FC88X_01126_00600      -----------------TCAACAAA--TCGTTT--GGGGCGAC-TTCTC-----
-----
FC88X_01104_00696      -----------------TCAACAAA--TCGTTT--GGGGCGAC-TTCTC-----
-----
FC88X_01119_00700      -----------------TCAACAAA--TCGTTT--GGGGCGAC-TTCTC-----
-----
FC88X_01114_00781      -----------------TCAACAAA--TCGTTT--GGGGCGAC-TTCTC-----
-----
FC88X_01137_00759      -----------------TCAACAAA--TCGTTT--GGGGCGAC-TTCTC-----
-----
FC88X_01125_00840      -----------------TCAACAAA--TCGTTT--GGGGCGAC-TTCTC-----
-----
FC88X_01103_00864      -----------------TCAACAAA--TCGTTT--GGGGCGAC-TTCTC-----
-----
FC88X_01127_00864      -----------------TCAACAAA--TCGTTT--GGGGCGAC-TTCTC-----
-----
FC88X_01123_00904      -----------------TCAACAAA--TCGTTT--GGGGCGAC-TTCTC-----
-----
FC88X_01140_00973      -----------------TCAACAAA--TCGTTT--GGGGCGAC-TTCTC-----
-----
FC88X_01126_01111      -----------------TCAACAAA--TCGTTT--GGGGCGAC-TTCTC-----
-----
FC88X_01132_01198      -----------------TCAACAAA--TCGTTT--GGGGCGAC-TTCTC-----
-----
FC88X_01131_01206      -----------------TCAACAAA--TCGTTT--GGGGCGAC-TTCTC-----
-----
FC88X_00982_00364      -----------------TCAACAAA--TCGTTT--GGGGCGAC-TTCTC-----
-----
FC88X_00982_00575      -----------------TCAACAAA--TCGTTT--GGGGCGAC-TTCTC-----
-----
FC88X_00968_00538      -----------------TCAACAAA--TCGTTT--GGGGCGAC-TTCTC-----
-----
FC88X_00997_00489      -----------------TCAACAAA--TCGTTT--GGGGCGAC-TTCTC-----
-----
FC88X_00984_00610      -----------------TCAACAAA--TCGTTT--GGGGCGAC-TTCTC-----
-----
FC88X_00974_00582      -----------------TCAACAAA--TCGTTT--GGGGCGAC-TTCTC-----
-----
FC88X_00953_00740      -----------------TCAACAAA--TCGTTT--GGGGCGAC-TTCTC-----
-----
FC88X_00997_00720      -----------------TCAACAAA--TCGTTT--GGGGCGAC-TTCTC-----
-----
```

```
FC88X_01095_00498       -----------------TCAACAAA--TCGTTT--GGGGCGAC-TTCTC-----
-----
FC88X_01091_00516       -----------------TCAACAAA--TCGTTT--GGGGCGAC-TTCTC-----
-----
FC88X_01085_00572       -----------------TCAACAAA--TCGTTT--GGGGCGAC-TTCTC-----
-----
FC88X_01055_00646       -----------------TCAACAAA--TCGTTT--GGGGCGAC-TTCTC-----
-----
FC88X_01060_00627       -----------------TCAACAAA--TCGTTT--GGGGCGAC-TTCTC-----
-----
FC88X_01027_00643       -----------------TCAACG-AA-TCGTTTG--GGGCAAC-TTCTCCC---
-----
FC88X_01010_00765       -----------------TCAACG-AA-TCGTT----------------------
-----
FC88X_01112_00618       -----------------TCAAC--AA-TCGTTTG--GGCGACT-TCTCCC----
-----
FC88X_00973_00716       -----------------TCAACAAAA-TCGTTTG--GGGCGAC-TTCTCCC---
-----
FC88X_01044_00339       -----------------TCAACAAAA-TCGTTTG--GGGCGAC-TTCTCCC---
-----
FC88X_00966_00608       -----------------TCAACA-AA-TCGTTTG--GGGCGAC-TTCTCCC---
-----
FC88X_00973_00618       -----------------TCAACA-AA-TCGTTTG--GGGCGAC-TTCTCCC---
-----
FC88X_00967_00752       -----------------TCAACA-AA-TCGTTTG--GGGCGAC-TTCTCCC---
-----
FC88X_00970_00883       -----------------TCAACA-AA-TCGTTTG--GGGCGAC-TTCTCCC---
-----
FC88X_01051_00548       -----------------TCAACA-AA-TCGTTTG--GGGCGAC-TTCTCCC---
-----
FC88X_01050_01051       -----------------TCAACA-AA-TCGTTTG--GGGCGAC-TTCTCCC---
-----
FC88X_01119_00497       -----------------TCAACA-AA-TCGTTTG--GGGCGAC-TTCTCCC---
-----
FC88X_01123_00498       -----------------TCAACA-AA-TCGTTTG--GGGCGAC-TTCTCCC---
-----
FC88X_01012_00373       -----------------TCAACA-AA-TCGTTTG--GGGCGAC-TTCTCCC---
-----
FC88X_01017_00282       -----------------TCAACA-AA-TCGTTTG--GGGCGAC-TTCTCCC---
-----
FC88X_01072_01112       -----------------TCAACA-AA-TCGTTTG--GGGCGAC-TTCTCCCA--
-----
FC88X_01023_00762       -----------------TCAACA-AA-TCGTTTG--GGGCGAC-TTCTCCC---
-----
FC88X_01005_00832       -----------------TCAACA-AA-TCGTTTG--GGGCGAC-TTCTCCC---
-----
FC88X_01033_00874       -----------------TCAACA-AA-TCGTTTG--GGGCGAC-TTCTCCC---
-----
FC88X_00985_00264       -----------------TCAACA-AA-TCGTTTG--GGGCGAC-TTCTC-----
-----
FC88X_01133_00619       -----------------TCAACA-AA-TCGTTTG--GGGCGAC-TTCTC-----
-----
FC88X_01137_00654       -----------------TCAACA-AA-TCGTTTG--GGGCGAC-TTCTC-----
-----
FC88X_01135_00718       -----------------TCAACA-AA-TCGTTTG--GGGCGAC-TTCTC-----
-----
```

```
FC88X_01132_00778      -----------------TCAACA-AA-TCGTTTG--GGGCGAC-TTCTC-----
-----
FC88X_01116_00835      -----------------TCAACA-AA-TCGTTTG--GGGCGAC-TTCTC-----
-----
FC88X_01137_00820      -----------------TCAACA-AA-TCGTTTG--GGGCGAC-TTCTC-----
-----
FC88X_01120_00856      -----------------TCAACA-AA-TCGTTTG--GGGCGAC-TTCTC-----
-----
FC88X_01146_00892      -----------------TCAACA-AA-TCGTTTG--GGGCGAC-TTCTC-----
-----
FC88X_01118_00990      -----------------TCAACA-AA-TCGTTTG--GGGCGAC-TTCTC-----
-----
FC88X_01116_01141      -----------------TCAACA-AA-TCGTTTG--GGGCGAC-TTCTC-----
-----
FC88X_01110_01182      -----------------TCAACA-AA-TCGTTTG--GGGCGAC-TTCTC-----
-----
FC88X_01111_01201      -----------------TCAACA-AA-TCGTTTG--GGGCGAC-TTCTC-----
-----
FC88X_00993_00382      -----------------TCAACA-AA-TCGTTTG--GGGCGAC-TTCTC-----
-----
FC88X_00965_00333      -----------------TCAACA-AA-TCGTTTG--GGGCGAC-TTCTC-----
-----
FC88X_00996_00301      -----------------TCAACA-AA-TCGTTTG--GGGCGAC-TTCTC-----
-----
FC88X_00954_00633      -----------------TCAACA-AA-TCGTTTG--GGGCGAC-TTCTC-----
-----
FC88X_00971_00630      -----------------TCAACA-AA-TCGTTTG--GGGCGAC-TTCTC-----
-----
FC88X_00954_00654      -----------------TCAACA-AA-TCGTTTG--GGGCGAC-TTCTC-----
-----
FC88X_00994_00699      -----------------TCAACA-AA-TCGTTTG--GGGCGAC-TTCTC-----
-----
FC88X_00988_00732      -----------------TCAACA-AA-TCGTTTG--GGGCGAC-TTCTC-----
-----
FC88X_00999_00778      -----------------TCAACA-AA-TCGTTTG--GGGCGAC-TTCTC-----
-----
FC88X_01079_00529      -----------------TCAACA-AA-TCGTTTG--GGGCGAC-TTCTC-----
-----
FC88X_01069_00560      -----------------TCAACA-AA-TCGTTTG--GGGCGAC-TTCTC-----
-----
FC88X_01098_00576      -----------------TCAACA-AA-TCGTTTG--GGGCGAC-TTCTC-----
-----
FC88X_01058_00636      -----------------TCAACAAA--TCGTTTG--GGGCGAC-TTCTC-----
-----
FC88X_00959_00411      -----------------TCAACAAA--TCGTTTG--GGGCGAC-TTCTCCT---
-----
FC88X_01007_00322      -----------------TCAACAAA--TCGTTTG--GGGCGACCTTCTCCT---
-----
FC88X_01140_00929      -----------------TCAACAAA--TCGTTTG--GGGCGAC-TTCTCC----
-----
FC88X_00983_00297      -----------------TCAACAAA--TCGTTTG--GGGCGAC-TTCTC-----
-----
FC88X_00991_00659      -----------------TCAACAAA--TCGTTTG--GGGCGAC-TTCTC-----
-----
FC88X_01125_00680      -----------------TCAACAAA--TCGTTTG--GGGCGAC-TTCTC-----
-----
```

```
FC88X_01134_00747      -----------------TCAACAAA--TCGTTTG--GGGCGAC-TTCTC-----
-----
FC88X_01129_00795      -----------------TCAACAAA--TCGTTTG--GGGCGAC-TTCTC-----
-----
FC88X_01110_00838      -----------------TCAACAAA--TCGTTTG--GGGCGAC-TTCTC-----
-----
FC88X_01135_00842      -----------------TCAACAAA--TCGTTTG--GGGCGAC-TTCTC-----
-----
FC88X_01119_00867      -----------------TCAACAAA--TCGTTTG--GGGCGAC-TTCTC-----
-----
FC88X_01138_00862      -----------------TCAACAAA--TCGTTTG--GGGCGAC-TTCTC-----
-----
FC88X_01117_00967      -----------------TCAACAAA--TCGTTTG--GGGCGAC-TTCTC-----
-----
FC88X_01115_01123      -----------------TCAACAAA--TCGTTTG--GGGCGAC-TTCTC-----
-----
FC88X_01107_01197      -----------------TCAACAAA--TCGTTTG--GGGCGAC-TTCTC-----
-----
FC88X_01107_01213      -----------------TCAACAAA--TCGTTTG--GGGCGAC-TTCTC-----
-----
FC88X_00990_00389      -----------------TCAACAAA--TCGTTTG--GGGCGAC-TTCTC-----
-----
FC88X_00962_00325      -----------------TCAACAAA--TCGTTTG--GGGCGAC-TTCTC-----
-----
FC88X_00995_00301      -----------------TCAACAAA--TCGTTTG--GGGCGAC-TTCTC-----
-----
FC88X_00975_00573      -----------------TCAACAAA--TCGTTTG--GGGCGAC-TTCTC-----
-----
FC88X_00980_00698      -----------------TCAACAAA--TCGTTTG--GGGCGAC-TTCTC-----
-----
FC88X_00950_00688      -----------------TCAACAAA--TCGTTTG--GGGCGAC-TTCTC-----
-----
FC88X_00993_00693      -----------------TCAACAAA--TCGTTTG--GGGCGAC-TTCTC-----
-----
FC88X_00977_00737      -----------------TCAACAAA--TCGTTTG--GGGCGAC-TTCTC-----
-----
FC88X_00991_00761      -----------------TCAACAAA--TCGTTTG--GGGCGAC-TTCTC-----
-----
FC88X_01075_00522      -----------------TCAACAAA--TCGTTTG--GGGCGAC-TTCTC-----
-----
FC88X_01064_00560      -----------------TCAACAAA--TCGTTTG--GGGCGAC-TTCTC-----
-----
FC88X_01049_00394      -----------------TCAACAAA--TCGTTTG--GGGCGAC-
TTCTCCTTCC-----
FC88X_01104_00516      -----------------TCAACAAA--TCGTTTG--GGGCGAC-
TTCTCCTTCC-----
FC88X_01120_01158      -----------------TCAACAAA--TCGTTTG--GGGCGAC-
TTCTCCTTCC-----
FC88X_01051_01225      -----------------TCAACAAA--TCGTTTG--GGGCGAC-TTCTCCT---
-----
FC88X_01101_01146      -----------------TCAACAAA--TCGTTTG--GGGCGAC-TTCTCC----
-----
FC88X_00984_00299      -----------------TCAACAAA--TCGTTTG--GGGCGAC-TTCTC-----
-----
FC88X_01133_00616      -----------------TCAACAAA--TCGTTTG--GGGCGAC-TTCTC-----
-----
```

FC88X_01131_00699 -----------------TCAACAAA--TCGTTTG--GGGCGAC-TTCTC-----
-----
FC88X_01135_00710 -----------------TCAACAAA--TCGTTTG--GGGCGAC-TTCTC-----
-----
FC88X_01132_00772 -----------------TCAACAAA--TCGTTTG--GGGCGAC-TTCTC-----
-----
FC88X_01115_00808 -----------------TCAACAAA--TCGTTTG--GGGCGAC-TTCTC-----
-----
FC88X_01137_00806 -----------------TCAACAAA--TCGTTTG--GGGCGAC-TTCTC-----
-----
FC88X_01120_00854 -----------------TCAACAAA--TCGTTTG--GGGCGAC-TTCTC-----
-----
FC88X_01144_00863 -----------------TCAACAAA--TCGTTTG--GGGCGAC-TTCTC-----
-----
FC88X_01118_00971 -----------------TCAACAAA--TCGTTTG--GGGCGAC-TTCTC-----
-----
FC88X_01116_01126 -----------------TCAACAAA--TCGTTTG--GGGCGAC-TTCTC-----
-----
FC88X_01108_01169 -----------------TCAACAAA--TCGTTTG--GGGCGAC-TTCTC-----
-----
FC88X_01111_01200 -----------------TCAACAAA--TCGTTTG--GGGCGAC-TTCTC-----
-----
FC88X_00991_00358 -----------------TCAACAAA--TCGTTTG--GGGCGAC-TTCTC-----
-----
FC88X_00964_00342 -----------------TCAACAAA--TCGTTTG--GGGCGAC-TTCTC-----
-----
FC88X_00992_00304 -----------------TCAACAAA--TCGTTTG--GGGCGAC-TTCTC-----
-----
FC88X_00976_00594 -----------------TCAACAAA--TCGTTTG--GGGCGAC-TTCTC-----
-----
FC88X_00968_00641 -----------------TCAACAAA--TCGTTTG--GGGCGAC-TTCTC-----
-----
FC88X_00951_00654 -----------------TCAACAAA--TCGTTTG--GGGCGAC-TTCTC-----
-----
FC88X_00994_00690 -----------------TCAACAAA--TCGTTTG--GGGCGAC-TTCTC-----
-----
FC88X_00986_00725 -----------------TCAACAAA--TCGTTTG--GGGCGAC-TTCTC-----
-----
FC88X_00992_00757 -----------------TCAACAAA--TCGTTTG--GGGCGAC-TTCTC-----
-----
FC88X_01076_00529 -----------------TCAACAAA--TCGTTTG--GGGCGAC-TTCTC-----
-----
FC88X_01067_00556 -----------------TCAACAAA--TCGTTTG--GGGCGAC-TTCTC-----
-----
FC88X_01097_00559 -----------------TCAACAAA--TCGTTTG--GGGCGAC-TTCTC-----
-----
FC88X_01077_00588 -----------------TCAACAAA--TCGTTTG--GGGCGAC-TTCTC-----
-----
FC88X_01085_00513 -----------------TCAACAAA--TCGTTTG--GGGCGAC-TTCTC-----
-----
FC88X_01094_00451 -----------------TCAACAAA--TCGTTTG--GGGCGAC-TTCTC-----
-----
FC88X_00993_00725 -----------------TCAACAAA--TCGTTTG--GGGCGAC-TTCTC-----
-----
FC88X_00967_00800 -----------------TCAACAAA--TCGTTTG--GGGCGAC-TTCTC-----
-----

```
FC88X_00967_00654       -----------------TCAACAAA--TCGTTTG--GGGCGAC-TTCTC-----
-----
FC88X_00975_00605       -----------------TCAACAAA--TCGTTTG--GGGCGAC-TTCTC-----
-----
FC88X_00995_00454       -----------------TCAACAAA--TCGTTTG--GGGCGAC-TTCTC-----
-----
FC88X_00971_00352       -----------------TCAACAAA--TCGTTTG--GGGCGAC-TTCTC-----
-----
FC88X_00955_00318       -----------------TCAACAAA--TCGTTTG--GGGCGAC-TTCTC-----
-----
FC88X_00975_00384       -----------------TCAACAAA--TCGTTTG--GGGCGAC-TTCTC-----
-----
FC88X_01128_01237       -----------------TCAACAAA--TCGTTTG--GGGCGAC-TTCTC-----
-----
FC88X_01126_01197       -----------------TCAACAAA--TCGTTTG--GGGCGAC-TTCTC-----
-----
FC88X_01122_01108       -----------------TCAACAAA--TCGTTTG--GGGCGAC-TTCTC-----
-----
FC88X_01137_00997       -----------------TCAACAAA--TCGTTTG--GGGCGAC-TTCTC-----
-----
FC88X_01118_00949       -----------------TCAACAAA--TCGTTTG--GGGCGAC-TTCTC-----
-----
FC88X_01123_00894       -----------------TCAACAAA--TCGTTTG--GGGCGAC-TTCTC-----
-----
FC88X_01146_00809       -----------------TCAACAAA--TCGTTTG--GGGCGAC-TTCTC-----
-----
FC88X_01124_00824       -----------------TCAACAAA--TCGTTTG--GGGCGAC-TTCTC-----
-----
FC88X_01136_00773       -----------------TCAACAAA--TCGTTTG--GGGCGAC-TTCTC-----
-----
FC88X_01100_00775       -----------------TCAACAAA--TCGTTTG--GGGCGAC-TTCTC-----
-----
FC88X_01109_00702       -----------------TCAACAAA--TCGTTTG--GGGCGAC-TTCTC-----
-----
FC88X_01142_00607       -----------------TCAACAAA--TCGTTTG--GGGCGAC-TTCTC-----
-----
FC88X_01092_00628       -----------------TCAACAAA--TCGTCTG--GGGCGAC-TTCTC-----
-----
FC88X_00995_00938       -----------------ACAACAAA--TCGTTTG--GGGCGAC-TTCTC-----
-----
FC88X_01038_00858       -----------------ACAACAAA--TCGTTTG--GGGCGAC-TTCTC-----
-----
FC88X_00971_01124       -----------------CCAACAAA--TCGTTTG--GGGCGAC-TTCTC-----
-----
FC88X_01034_00913       -----------------CCAACAAA--TCGTTTG--GGGCGAC-TTCTC-----
-----
FC88X_01014_00378       -----------------CTGACAAG--TCGATG---------------------
-----
FC88X_01134_00553       ----------------TCTAACAAA--TCGTTGG--GTCGACTTCCT-------
-----
FC88X_01037_00557       ------------------TAACAAA--TCG------------------------
-----
FC88X_01096_00788       ------------------TAACAAA--TC-------------------------
-----
FC88X_00969_00662       -----------------TTAACAAA--TCGTTTG--GGGCGACTTCTC------
-----
```

FC88X_00977_01006 -----------------TTAACAAA--TCGTTTG--GGGCGACTTCTC------
-----
FC88X_01048_00592 -----------------TTAACAAA--TCGTTTG--GGGCGACTTCTC------
-----
FC88X_01004_00554 -----------------TCGAACAAA-TCGTTTGG-G--CGAC-TTCT------
-----
FC88X_01131_00539 -----------------TCAAACAAA-TCGTTTGG-GGGCGAC-TTCTCCT---
-----
FC88X_01123_00540 -----------------TCAAACAAA-TCGTTTGG-GG-CGAC-TTCTC-----
-----
FC88X_01146_00807 -----------------TCAAACAAA-TCGTTTGG-GG-CGAC-TTCTC-----
-----
FC88X_01000_01175 -----------------TCAACCAAA----------------------------
-----
FC88X_01130_00929 -----------------TCAACCAAA-TCGTTTGG-GG-CGGC-TTCTC-----
-----
FC88X_01101_00732 -------------TCAACAAAAAAAA-TCGTTTGG-GG-CGAC-TTCTC-----
-----
FC88X_00984_00368 -----------------CTCCCAAATCACGTTACGTGCGACCCA----------
-----
FC88X_01073_00638 -----------------TACACAAATCGT-------------------------
-----
FC88X_00980_00429 -----------------TCCCCAAATAGCTACCGACGACAGCGTC---------
-----
FC88X_01095_00881 -----------------TCCCCAAATAGCTACCGACGACAGCGTC---------
-----
FC88X_01009_00981 ------------------GGCCGAACGTCG-CATGC--TCCCGGCCGCCACT--
-----
FC88X_01114_00588 -------------------GCCGA-CGTCG-CATGCG-TCCCGGC---------
-----
FC88X_01056_01154 ------------------GGCCGA-CGTCG-CATGC--TCCCGCCGCAT-----
-----
FC88X_01096_01161 ------------------GGCCCGACGTCG-CATGC--TCCCGGCGC-------
-----
FC88X_00982_01003 ------------------GGCCGA-CGTCG-CAT--------------------
-----
FC88X_01023_00994 ------------------GGCCGAACGTCGACATGTCGTCCCGGCCG-------
-----
FC88X_01118_00875 ------------------GGCCGA-CGTCGACATGTCGTCCCGGCCAGT-----
-----
FC88X_00955_00884 -------------------GCCGA-CGTCAC-----------------------
-----
FC88X_00977_00271 -------------------ATCACCGTTGGTTCC-GGGTGCGCGACTCTTCC--
-----
FC88X_01038_00602 -------------------ATCACCGTTGGTTC--GGGTGCGCG-CTCTT----
-----
FC88X_01081_00842 -----------------TACTCACCGTTGGTTC--GGGTGCGCA----------
-----
FC88X_01021_00341 -------------------ATCACC-TTGGTTC--GG-----------------
-----
FC88X_01002_00813 -------------------ATCACCGTTGGTTC--GGCTGCGCGTTCTC-----
-----
FC88X_00999_00279 -------------------ATCACCGTCGGTTC--GGGTGCGCGTTCGTCC---
-----
FC88X_00964_00769 -------------------ATCACCGTTGGCTC--GGGTGCGCGTTCTCCC---
-----

```
FC88X_01005_00884       -------------------ATCACCGTTGGCTC--GGGTGCGCGTTCTC-----
-----
FC88X_01098_00774       -------------------ATCACCGTTGGTC---GGGTGCGCGTTCTC-----
-----
FC88X_01095_00806       -------------------ATCACCGTTGGTCC--GGGTGCGCGTTCTC-----
-----
FC88X_01024_00975       -------------------ATCACCGTTGGTTC--AGGTGCGCGTTCTC-----
-----
FC88X_01085_00461       -------------------ATCACCGTTGGTTC---GGTGCGCGTTCTC-----
-----
FC88X_01083_00810       -------------------ATCACCGTTCGGTTC-GGGTGCGCGTTCTC-----
-----
FC88X_01113_00779       -------------------ATCACCGTTCGGTTC-GGGTGCGCGTTCTC-----
-----
FC88X_01078_00774       -------------------ATCACCGTCGGTTC--GGGTGCGCGTTCTC-----
-----
FC88X_00996_00288       -------------------ATCACCGTTGGTTC--GGGTGCGCGTTCTC-----
-----
FC88X_00998_00298       -------------------ATCACCGTTGGTTC--GGGTGCGCGTTCTC-----
-----
FC88X_00956_00324       -------------------ATCACCGTTGGTTC--GGGTGCGCGTTCTC-----
-----
FC88X_00961_00322       -------------------ATCACCGTTGGTTC--GGGTGCGCGTTCTC-----
-----
FC88X_00966_00348       -------------------ATCACCGTTGGTTCG-GG-TGCGCGTTCTC-----
-----
FC88X_01021_00929       -------------------ATCACCGTTGGTTCG-GGCTGCGCGTTCTC-----
-----
FC88X_00956_00390       -------------------ATCACCGTTGGTTC--GGGTGCGCGTTCTC-----
-----
FC88X_00968_00352       -------------------ATCACCGTTGGTTC--GGGTGCGCGTTCTC-----
-----
FC88X_00965_00405       -------------------ATCACCGTTGGTTC--GGGTGCGCGTTCTC-----
-----
FC88X_00999_00438       -------------------ATCACCGTTGGTTC--GGGTGCGCGTTCTC-----
-----
FC88X_00973_00590       -------------------ATCACCGTTGGTTC--GGGTGCGCGTTCTC-----
-----
FC88X_00999_00618       -------------------ATCACCGTTGGTTC--GGGTGCGCGTTCTC-----
-----
FC88X_00960_00414       -------------------ATCACCGTTGGTTC--GGGTGCGCGTTCTC-----
-----
FC88X_00990_00402       ------------------GATCACCGTTGGTTC--GGGTGCGCGTTCTC-----
-----
FC88X_00994_00424       -------------------ATCACCGTTGGTTC--GGGTGCGCGTTCTC-----
-----
FC88X_00951_00475       -------------------ATCACCGTTGGTTC--GGGTGCGCGTTCTC-----
-----
FC88X_00959_00649       -------------------ATCACCGTTGGTTC--GGGTGCGCGTTCTC-----
-----
FC88X_00954_00692       -------------------ATCACCGTTGGTTC--GGGTGCGCGTTCTC-----
-----
FC88X_00980_00675       -------------------ATCACCGTTGGTTC--GGGTGCGCGTTCTC-----
-----
FC88X_00981_00696       -------------------ATCACCGTTGGTTC--GGGTGCGCGTTCTC-----
-----
```

FC88X_00993_00669 -------------------ATCACCGTTGGTTC--GGGTGCGCGTTCTC-----
-----
FC88X_00966_00705 -------------------ATCACCGTTGGTTC--GGGTGCGCGTTCTC-----
-----
FC88X_00973_00741 -------------------ATCACCGTTGGTTC--GGGTGCGCGTTCTC-----
-----
FC88X_00954_00783 -------------------ATCACCGTTGGTTC--GGGTGCGCGTTCTC-----
-----
FC88X_00991_00755 -------------------ATCACCGTTGGTTC--GGGTGCGCGTTCTC-----
-----
FC88X_00979_00834 -------------------ATCACCGTTGGTTC--GGGTGCGCGTTCTC-----
-----
FC88X_00990_00838 -------------------ATCACCGTTGGTTC--GGGTGCGCGTTCTC-----
-----
FC88X_00979_00877 -------------------ATCACCGTTGGTTC--GGGTGCGCGTTCTC-----
-----
FC88X_00994_00898 -------------------ATCACCGTTGGTTC--GGGTGCGCGTTCTC-----
-----
FC88X_00961_00937 -------------------ATCACCGTTGGTTC--GGGTGCGCGTTCTC-----
-----
FC88X_00989_00910 -------------------ATCACCGTTGGTTC--GGGTGCGCGTTCTC-----
-----
FC88X_00963_00975 -------------------ATCACCGTTGGTTC--GGGTGCGCGTTCTC-----
-----
FC88X_00981_00985 -------------------ATCACCGTTGGTTC--GGGTGCGCGTTCTC-----
-----
FC88X_00989_00977 -------------------ATCACCGTTGGTTC--GGGTGCGCGTTCTC-----
-----
FC88X_00990_00975 -------------------ATCACCGTTGGTTC--GGGTGCGCGTTCTC-----
-----
FC88X_00994_00999 -------------------ATCACCGTTGGTTC--GGGTGCGCGTTCTC-----
-----
FC88X_00952_01029 -------------------ATCACCGTTGGTTC--GGGTGCGCGTTCTC-----
-----
FC88X_00979_01048 -------------------ATCACCGTTGGTTC--GGGTGCGCGTTCTC-----
-----
FC88X_00996_01003 -------------------ATCACCGTTGGTTC--GGGTGCGCGTTCTC-----
-----
FC88X_00970_01109 -------------------ATCACCGTTGGTTC--GGGTGCGCGTTCTC-----
-----
FC88X_00981_01126 -------------------ATCACCGTTGGTTC--GGGTGCGCGTTCTC-----
-----
FC88X_01003_00269 -------------------ATCACCGTTGGTTC--GGGTGCGCGTTCTC-----
-----
FC88X_01020_00346 -------------------ATCACCGTTGGTTC--GGGTGCGCGTTCTC-----
-----
FC88X_01026_00337 -------------------ATCACCGTTGGTTC--GGGTGCGCGTTCTC-----
-----
FC88X_01028_00379 -------------------ATCACCGTTGGTTC--GGGTGCGCGTTCTC-----
-----
FC88X_01046_00396 -------------------ATCACCGTTGGTTC--GGGTGCGCGTTCTC-----
-----
FC88X_01000_00433 -------------------ATCACCGTTGGTTC--GGGTGCGCGTTCTC-----
-----
FC88X_01025_00418 -------------------ATCACCGTTGGTTC--GGGTGCGCGTTCTC-----
-----

FC88X_01034_00416 -------------------ATCACCGTTGGTTC--GGGTGCGCGTTCTC-----
-----
FC88X_01046_00448 -------------------ATCACCGTTGGTTC--GGGTGCGCGTTCTC-----
-----
FC88X_01030_00520 -------------------ATCACCGTTGGTTC--GGGTGCGCGTTCTC-----
-----
FC88X_01006_00572 -------------------ATCACCGTTGGTTC--GGGTGCGCGTTCTC-----
-----
FC88X_01031_00592 -------------------ATCACCGTTGGTTC--GGGTGCGCGTTCTC-----
-----
FC88X_01046_00576 -------------------ATCACCGTTGGTTC--GGGTGCGCGTTCTC-----
-----
FC88X_01003_00608 -------------------ATCACCGTTGGTTC--GGGTGCGCGTTCTC-----
-----
FC88X_01020_00608 -------------------ATCACCGTTGGTTC--GGGTGCGCGTTCTC-----
-----
FC88X_01048_00636 -------------------ATCACCGTTGGTTC--GGGTGCGCGTTCTC-----
-----
FC88X_01014_00658 -------------------ATCACCGTTGGTTC--GGGTGCGCGTTCTC-----
-----
FC88X_01034_00698 -------------------ATCACCGTTGGTTC--GGGTGCGCGTTCTC-----
-----
FC88X_01023_00770 -------------------ATCACCGTTGGTTC--GGGTGCGCGTTCTC-----
-----
FC88X_01007_00833 -------------------ATCACCGTTGGTTC--GGGTGCGCGTTCTC-----
-----
FC88X_01025_00833 -------------------ATCACCGTTGGTTC--GGGTGCGCGTTCTC-----
-----
FC88X_01033_00822 -------------------ATCACCGTTGGTTC--GGGTGCGCGTTCTC-----
-----
FC88X_01034_00820 -------------------ATCACCGTTGGTTC--GGGTGCGCGTTCTC-----
-----
FC88X_01047_00845 -------------------ATCACCGTTGGTTC--GGGTGCGCGTTCTC-----
-----
FC88X_01021_00872 -------------------ATCACCGTTGGTTC--GGGTGCGCGTTCTC-----
-----
FC88X_01043_00867 -------------------ATCACCGTTGGTTC--GGGTGCGCGTTCTC-----
-----
FC88X_01044_00850 -------------------ATCACCGTTGGTTC--GGGTGCGCGTTCTC-----
-----
FC88X_01003_00966 -------------------ATCACCGTTGGTTC--GGGTGCGCGTTCTC-----
-----
FC88X_01009_01037 -------------------ATCACCGTTGGTTC--GGGTGCGCGTTCTC-----
-----
FC88X_01045_01027 -------------------ATCACCGTTGGTTC--GGGTGCGCGTTCTC-----
-----
FC88X_01002_01129 -------------------ATCACCGTTGGTTC--GGGTGCGCGTTCTC-----
-----
FC88X_01017_01113 -------------------ATCACCGTTGGTTC--GGGTGCGCGTTCTC-----
-----
FC88X_01019_01213 -------------------ATCACCGTTGGTTC--GGGTGCGCGTTCTC-----
-----
FC88X_01026_01214 -------------------ATCACCGTTGGTTC--GGGTGCGCGTTCTC-----
-----
FC88X_01039_01253 -------------------ATCACCGTTGGTTC--GGGTGCGCGTTCTC-----
-----

FC88X_01041_01275 -------------------ATCACCGTTGGTTC--GGGTGCGCGTTCTC-----
-----
FC88X_01042_01256 -------------------ATCACCGTTGGTTC--GGGTGCGCGTTCTC-----
-----
FC88X_01050_00337 -------------------ATCACCGTTGGTTC--GGGTGCGCGTTCTC-----
-----
FC88X_01068_00379 -------------------ATCACCGTTGGTTC--GGGTGCGCGTTCTC-----
-----
FC88X_01077_00382 -------------------ATCACCGTTGGTTC--GGGTGCGCGTTCTC-----
-----
FC88X_01067_00439 -------------------ATCACCGTTGGTTC--GGGTGCGCGTTCTC-----
-----
FC88X_01073_00443 -------------------ATCACCGTTGGTTC--GGGTGCGCGTTCTC-----
-----
FC88X_01092_00444 -------------------ATCACCGTTGGTTC--GGGTGCGCGTTCTC-----
-----
FC88X_01076_00462 -------------------ATCACCGTTGGTTC--GGGTGCGCGTTCTC-----
-----
FC88X_01069_00545 -------------------ATCACCGTTGGTTC--GGGTGCGCGTTCTC-----
-----
FC88X_01083_00517 -------------------ATCACCGTTGGTTC--GGGTGCGCGTTCTC-----
-----
FC88X_01094_00521 -------------------ATCACCGTTGGTTC--GGGTGCGCGTTCTC-----
-----
FC88X_01052_00582 -------------------ATCACCGTTGGTTC--GGGTGCGCGTTCTC-----
-----
FC88X_01053_00565 -------------------ATCACCGTTGGTTC--GGGTGCGCGTTCTC-----
-----
FC88X_01057_00580 -------------------ATCACCGTTGGTTC--GGGTGCGCGTTCTC-----
-----
FC88X_01079_00594 -------------------ATCACCGTTGGTTC--GGGTGCGCGTTCTC-----
-----
FC88X_01098_00598 -------------------ATCACCGTTGGTTC--GGGTGCGCGTTCTC-----
-----
FC88X_01056_00610 -------------------ATCACCGTTGGTTC--GGGTGCGCGTTCTC-----
-----
FC88X_01094_00609 -------------------ATCACCGTTGGTTC--GGGTGCGCGTTCTC-----
-----
FC88X_01088_00666 -------------------ATCACCGTTGGTTC--GGGTGCGCGTTCTC-----
-----
FC88X_01056_00753 -------------------ATCACCGTTGGTTC--GGGTGCGCGTTCTC-----
-----
FC88X_01082_00887 -------------------ATCACCGTTGGTTC--GGGTGCGCGTTCTC-----
-----
FC88X_01082_00982 -------------------ATCACCGTTGGTTC--GGGTGCGCGTTCTC-----
-----
FC88X_01099_01142 -------------------ATCACCGTTGGTTC--GGGTGCGCGTTCTC-----
-----
FC88X_01072_01184 -------------------ATCACCGTTGGTTC--GGGTGCGCGTTCTC-----
-----
FC88X_01106_00488 -------------------ATCACCGTTGGTTC--GGGTGCGCGTTCTC-----
-----
FC88X_01121_00531 -------------------ATCACCGTTGGTTC--GGGTGCGCGTTCTC-----
-----
FC88X_01129_00527 -------------------ATCACCGTTGGTTC--GGGTGCGCGTTCTC-----
-----

```
FC88X_01130_00600      -------------------ATCACCGTTGGTTC--GGGTGCGCGTTCTC-----
-----
FC88X_01122_00667      -------------------ATCACCGTTGGTTC--GGGTGCGCGTTCTC-----
-----
FC88X_01105_00732      -------------------ATCACCGTTGGTTC--GGGTGCGCGTTCTC-----
-----
FC88X_01127_00715      -------------------ATCACCGTTGGTTC--GGGTGCGCGTTCTC-----
-----
FC88X_01132_00717      -------------------ATCACCGTTGGTTC--GGGTGCGCGTTCTC-----
-----
FC88X_01137_00708      -------------------ATCACCGTTGGTTC--GGGTGCGCGTTCTC-----
-----
FC88X_01136_00791      -------------------ATCACCGTTGGTTC--GGGTGCGCGTTCTC-----
-----
FC88X_01144_00776      -------------------ATCACCGTTGGTTC--GGGTGCGCGTTCTC-----
-----
FC88X_01122_00804      -------------------ATCACCGTTGGTTC--GGGTGCGCGTTCTC-----
-----
FC88X_01144_00832      -------------------ATCACCGTTGGTTC--GGGTGCGCGTTCTC-----
-----
FC88X_01111_00853      -------------------ATCACCGTTGGTTC--GGGTGCGCGTTCTC-----
-----
FC88X_01111_00869      -------------------ATCACCGTTGGTTC--GGGTGCGCGTTCTC-----
-----
FC88X_01133_00858      -------------------ATCACCGTTGGTTC--GGGTGCGCGTTCTC-----
-----
FC88X_01124_00941      -------------------ATCACCGTTGGTTC--GGGTGCGCGTTCTC-----
-----
FC88X_01134_00933      -------------------ATCACCGTTGGTTC--GGGTGCGCGTTCTC-----
-----
FC88X_01132_00970      -------------------ATCACCGTTGGTTC--GGGTGCGCGTTCTC-----
-----
FC88X_01110_01149      -------------------ATCACCGTTGGTTC--GGGTGCGCGTTCTC-----
-----
FC88X_01128_01132      -------------------ATCACCGTTGGTTC--GGGTGCGCGTTCTC-----
-----
FC88X_01103_01164      -------------------ATCACCGTTGGTTC--GGGTGCGCGTTCTC-----
-----
FC88X_00960_01251      -------------------ATCACCGTTGGTTC--GGGTGCGCGTTCTCT----
-----
FC88X_01030_00685      -------------------ATCACCGTTGGTTC--GGGTGCGCGTCCTC-----
-----
FC88X_00964_00659      -------------------ATCACCGTTGGTTC--GGGTGTGCGTTCTC-----
-----
FC88X_00966_00856      -------------------ATCACCGTTGGTTC--GGGTGTGCGTTCTC-----
-----
FC88X_01041_00336      ------------------AATCACCGTTGGTTC--GGGTGCGCGTTCTC-----
-----
FC88X_01061_00687      -------------------ATCACCGTTGGTTC--GGGTGCGCGTTCTCCC---
-----
FC88X_01047_00915      -------------------ATCACCGCTGGTTC--GGGTGCGCGTTCTC-----
-----
FC88X_00962_00485      -------------------ATCACCGTTGGTTC--GGGTGCGTGTTCTC-----
-----
FC88X_01138_00588      -------------------ATCACCGTTGGTTC--GGGTGCGTGTTCTC-----
-----
```

```
FC88X_01142_00743       -------------------ATCACCGTTAGTTC--GGGTGCGCGTTCTC-----
-----
FC88X_01095_00865       -------------------ACCACCGTTGGTTC--GGGTGCGCGTTCTC-----
-----
FC88X_01034_00481       -------------------ATCACCATTGGTTC--GGGTGCGCGTTCTC-----
-----
FC88X_01144_00854       -------------------ATCACCATTGGTTC--GGGTGCGCGTTCTC-----
-----
FC88X_01018_00984       -------------------GACACCAATCGCGCC-GGGTC-ATGTTCTC-----
-----
FC88X_01056_00994       --------------------ACACCAATCGCGCC-GGGTC-ATGTTCTC-----
-----
FC88X_01099_01164       --------------------ACACCAATCGCGCC-GGGTC-ATGTTCTC-----
-----
FC88X_00970_00661       --------------------ACACCAATCGTGCC-GGGTC-ATGTTCTC-----
-----
FC88X_00959_01262       --------------------ACACCAATCGTGCC-GGGTCGATGTTCTCC----
-----
FC88X_00958_00746       --------------------ACACCAATCGTGCC-GGGTC-ATGTTCTC-----
-----
FC88X_00960_00752       --------------------ACACCAATCGTGCC-GGGTC-ATGTTCTC-----
-----
FC88X_01004_00522       --------------------ACACCAATCGTGCC-GGGTC-ATGTTCTC-----
-----
FC88X_01030_01175       --------------------ACACCAATCGTGCC-GGGTC-ATGTTCTC-----
-----
FC88X_01068_00610       --------------------ACACCAATCGTGCC-GGGTC-ATGTTCTC-----
-----
FC88X_01069_00936       --------------------ACACCAATCGTGCC-GGGTC-ATGTTCTC-----
-----
FC88X_01109_00486       --------------------ACACCAATCGTGCC-GGGTC-ATGTTCTC-----
-----
FC88X_01130_00863       --------------------ACACCAATCGTGCC-GGGTC-ATGTTCTC-----
-----
FC88X_01098_00449       --------------------ACACCAATCGTGCC-GGGTC-ATGTTCTCC----
-----
FC88X_01090_00761       --------------------ACACCAATCGTGCC-GGGTC-ATGTTCTCCC---
-----
FC88X_01070_00582       --------------------ACACCAATCGTACC-GGGTC-ATGTTCTC-----
-----
FC88X_01007_00505       -------------------GACACCAATCGTGCC-GGGTC-ATGTTCAC-----
-----
FC88X_01040_00396       --------------------ACACCAATCGCGC--GGGTC-ATGTTCTC-----
-----
FC88X_00959_00836       -------------------TTCGCCAAT-ATCCC-GCGGC-GTGTTCTC-----
-----
FC88X_01032_00437       -------------------TTCGCCAAT-ATCCC-GCGGC-GTGTTCTC-----
-----
FC88X_01078_00534       -------------------TTCGCCAAT-ATCCC-GCGGC-GTGTTCTC-----
-----
FC88X_01071_00893       -------------------TTCGCCAAT-ATCCC-GCGGC-GTGTTCTC-----
-----
FC88X_01091_00864       -------------------TTCGCCAAT-ATCCC-GCGGC-GTGTTCTC-----
-----
FC88X_01033_00789       -------------------TTCGCCAGT-ATCCC-GCGGC-GTGTTCTC-----
-----
```

```
FC88X_00983_00896      -------------------TTCGCCAAT-ACCCC-GCGGC-GTGTTCTC-----
-----
FC88X_01013_00898      -------------------TTCGCCAAT-ATCTC-GCGGC-GTGTTCTC-----
-----
FC88X_01033_00473      ------------------TCGTCGGATCGAGTCT-GGCGCCCTGCT--------
-----
FC88X_01005_00609      ------------------CCGTGATATCTTCTCC-GGCACGTTCTCCT------
-----
FC88X_01011_00833      ---------------------CTGTATCGCGTCC-AACACAGCGTTCTC-----
-----
FC88X_01090_00511      ---------------------CTGTATCGCGTCC-AACACAGCGTTCTC-----
-----
FC88X_01137_00833      ---------------------CCATGTCGCGTCT-GGCCCCACGTTCTC-----
-----
FC88X_01011_00772      ---------------------CCGCGTCGTTTCT-GGATTCGCATTCTC-----
-----
FC88X_00964_00922      --------------------CCGCCAGTAGTAT--TGGCCCACTATCTC-----
-----
FC88X_00992_00340      --------------------CTAAAAACTCCCCT-GCGTCGACTTTAC------
-----
FC88X_01009_00338      --------------------TCACAAACTCG-----------------------
-----
FC88X_01081_00814      -----------------TCAACAAT--TTATCTG--CGTCGGCTTTCT------
-----
FC88X_01059_00943      -----------------TCAACAGT--TTATCTGG-CGTCGGCTTTCT------
-----
FC88X_01079_00479      ----------------CTCAATAA------------------------------
-----
FC88X_01006_00782      --------------CCCTTGACTAT--TCATTTT--GCGATACTGCC-------
-----
FC88X_01085_00748      ------------------TCACAAT--TCATT----------------------
-----
FC88X_01056_00853      -----------------TTAGACAA--TCATTGG--GCGTAACTACTTC-----
-----
FC88X_00988_00311      ----------------------CTACACGATACCTTGAAGCTGCGTCTC-----
-----
FC88X_00950_00448      ----------------------TTAACCCATCCCATGAGTCGCTGTCTT-----
-----
FC88X_01034_00364      ------------------------------TCCAATGAATCGTTGG--------
-----
FC88X_00980_00511      ------------------CTACGAG--TCTTTTGTTT-----------------
-----
FC88X_00973_00788      ------------------CTACGAA--T--------------------------
-----
FC88X_01044_00998      --------------TTTCCTACAAA--TCGATTC--------------------
-----
FC88X_01113_00560      ---------------TTCCTACAA---CTGATTCG-------------------
-----
FC88X_01073_00923      TACAGCATTTGCGGGTACCTATAAC--TC-------------------------
-----
FC88X_00968_00628      ---------------CTGTTTCTTCCTTCGTCCCGAATTTTC------------
-----
FC88X_00979_00922      --------------TCGTAGAATTCCATCGGCAAGACTTTCT------------
-----
FC88X_00957_01010      ----------------------TCCCTTCGTCTTGAGTGTCACTTTTCT-----
-----
```

FC88X_01026_00414 ----------------------TCCCTTCGTCTTGAGTGTCACTTTTCT-----
-----
FC88X_01005_00833 ----------------------TCCCTTCGTCTTGAGTGTCACTTTTCT-----
-----
FC88X_01132_00580 ----------------------TCCCTTCGTCTTGAGTGTCACTTTTCT-----
-----
FC88X_01074_00545 -------------------TTCGAATTTTGTTTC--GTACGACTCCTC------
-----
FC88X_01112_00507 -----------CCGTGTGCGCTAATTTCTCTTTC--TTGC--------------
-----
FC88X_01020_00798 ------------------ACAACAAACCTTGTGCACTCCCGTCGC---------
-----
FC88X_01090_00650 --------------------TTCAGAACTGTTGGACTC----------------
-----
FC88X_01093_00488 --------------------CCAACAACTCTTTGGCCCGTGTTTCTC-------
-----
FC88X_01123_00746 --------------------CCAATAACTCTTTGGCCCGTGTTTCTC-------
-----
FC88X_01063_00594 --------------------TCTACAACTCTTCGGC------------------
-----
FC88X_00957_00665 ---------------------TCTTGTATTTT-GTGCATCACTTTTCTC-----
-----
FC88X_01064_00449 ---------------------TCTTGTATTTT-GTGCATCACTTTTCTC-----
-----
FC88X_01111_00742 ---------------------TCTTGTATTTT-GTGCATCACTTTTCTC-----
-----
FC88X_01141_01139 ---------------------TCTTGTATTTT-GTGCATCACTTTTCTC-----
-----
FC88X_00992_00294 ---------------------TCTTGTATTTT-GTGCATCGCTTTTCT------
-----
FC88X_00960_00914 ---------------------TCTTGTATTTTTGTGCATCACTTTCT-------
-----
FC88X_00991_01103 ---------------------TCTTGTATTTT-GTGCGTCACTTTTCTC-----
-----
FC88X_01121_00600 ---------------------TCACACAGTCTTTTGTGCTCCCTTTTG------
-----
FC88X_00962_00465 ---------------------TCACATTGTTT-GTGCCTCCCTTACACACC---
-----
FC88X_01001_00375 ----------------------CAGGTCTTTTGTTGCCTCTGTTGTTCT-----
-----
FC88X_00995_00330 ----------------------CCAGACCATTTGTGTTTGAGGCATCTC-----
-----
FC88X_01047_00433 ----------------------CCAGACCATTTGTGTTTGAGGCATCTC-----
-----
FC88X_01031_00552 ----------------------CCAGACCATTTGTGTTTGAGGCATCTC-----
-----
FC88X_00998_00699 ---------------------CCACAATCGTGTTTGCGTT-GGCATCTC-----
-----
FC88X_01046_00509 ---------------------CCACAATCGTGTTTGCGTT-GGCATCTC-----
-----
FC88X_01018_00878 ---------------------CCACAATCGTGTTTGCGTT-GGCATCTC-----
-----
FC88X_01068_00443 ---------------------CCACAATCGTGTTTGCGTT-GGCATCTC-----
-----
FC88X_01094_00646 ---------------------CCACAATCGTGTTTGCGTT-GGCATCTC-----
-----

```
FC88X_01117_00585      ---------------------CCACAATCGTGTTTGCGTT-GGCATCTC-----
-----
FC88X_01044_00825      ---------------------CCACGATCGTGTTTGCGTT-GGCATCTC-----
-----
FC88X_00963_00436      ---------------------TCACATTCATGTTT-GTGACCGTGTCTC-----
-----
FC88X_00973_00591      ---------------------TCACATTCATGTTT-GTGACCGTGTCTC-----
-----
FC88X_00951_00857      ---------------------TCACATTCATGTTT-GTGACCGTGTCTC-----
-----
FC88X_00982_00966      ---------------------TCACATTCATGTTT-GTGACCGTGTCTC-----
-----
FC88X_01002_00407      ---------------------TCACATTCATGTTT-GTGACCGTGTCTC-----
-----
FC88X_01003_00460      ---------------------TCACATTCATGTTT-GTGACCGTGTCTC-----
-----
FC88X_01034_00514      ---------------------TCACATTCATGTTT-GTGACCGTGTCTC-----
-----
FC88X_01017_01131      ---------------------TCACATTCATGTTT-GTGACCGTGTCTC-----
-----
FC88X_01089_00881      ---------------------TCACATTCATGTTT-GTGACCGTGTCTC-----
-----
FC88X_01065_01160      ---------------------TCACATTCATGTTT-GTGACCGTGTCTC-----
-----
FC88X_01103_00445      ---------------------TCACATTCATGTTT-GTGACCGTGTCTC-----
-----
FC88X_01106_00502      ---------------------TCACATTCATGTTT-GTGACCGTGTCTC-----
-----
FC88X_01101_00660      ---------------------TCACATTCATGTTT-GTGACCGTGTCTC-----
-----
FC88X_01124_00930      ---------------------TCACATTCATGTTT-GTGACCGTGTCTC-----
-----
FC88X_01108_01140      ---------------------TCACATTCATGTTT-GTGACCGTGTCTC-----
-----
FC88X_01124_01157      ---------------------TCACATTCATGTTT-GTGACCGTGTCTC-----
-----
FC88X_01130_01150      ---------------------TCACATTCATGTTT-GTGACCGTGTCTC-----
-----
FC88X_01029_01236      ---------------------TCACATTCATGTTT-GTGACCGTGTCTCCC---
-----
FC88X_01000_00293      ---------------------TCACATTCATATTT-GTGACCGTGTCTC-----
-----
FC88X_00957_00578      ---------------------TCACATTCATGTTTTGTGACCGTGTCTC-----
-----
FC88X_00976_00561      ---------------------TCACATTTATGTTT-GTGACCGTGTCTC-----
-----
FC88X_01141_00590      ---------------------TCACATTCGTGTTT-GTGACCGTGTCTC-----
-----
FC88X_01078_00859      ---------------------TCACACTCATGTTT-GTGACCGTGTCTC-----
-----
FC88X_00965_00562      ---------------------TTACATTCATGTTT-GTGACCGTGTCTCCC---
-----
FC88X_00955_00844      ---------------------TCACATTCATGCTT-GTGACGGTGTCTC-----
-----
FC88X_01051_00558      ---------------------TCACATTCATGTTT-GTGACCGCGTCTC-----
-----
```

FC88X_00974_00395 --------------------ATCACGTAG-CGTTCGTGTGTCGATTCTC-----
-----
FC88X_01003_00387 --------------------ATCACGTAGGCGTTCGCGTGTCGATTCTC-----
-----
FC88X_01089_00928 --------------------ATCACGTAG-CGTTCGTGTGTCGATTCTC-----
-----
FC88X_01057_01180 --------------------ATCACGTAG-CGTTCGTGTGTCGATTCTC-----
-----
FC88X_01126_00806 --------------------ATCACGTAG-CGTTCGTGTGTCGATTCTC-----
-----
FC88X_01115_00888 --------------------ATCACGTAG-CGTTCGTGTGTCGATTCTC-----
-----
FC88X_01089_00429 --------------------ATCACGTAG-CGTTCGTGTGTCGATTCTCCC---
-----
FC88X_00994_00681 --------------------------CTCTCGTTTGTGCGGCGCCT-
CTGTGTT-----
FC88X_01014_00437 --------------------------
CTGCCGATAGTGTCGCGTCCGCTGTGCC-----
FC88X_01069_00568 -----------------------ACGAAA-CCTCTGTGT---------------
-----
FC88X_01039_00400 ----------------------TTCCTCTGTGTCAAGAGCGAGTGTCTC-----
-----
FC88X_01034_00797 ------------------------TTTTCTCCTAGTTTGCTGCGTCTTTGC---
-----
FC88X_01111_00788 ------------------------TTTTCTCCTAGTTTGCTGCGTCTTTGC---
-----
FC88X_01097_00796 ----------------------TTTTTTGTACTATTTCTCTGCGTTGTTG----
-----
FC88X_01114_00475 --------------------------TTCTACGAA-------------------
-----
FC88X_00999_00292 -----------------CCACCAACTATTGTTCG----TCGCCGTTCT------
-----
FC88X_00991_00400 -----------------CCACCAACTATTGTTCG----TCGCCGTTCT------
-----
FC88X_00986_00491 -----------------CCACCAACTATTGTTCG----TCGCCGTTCT------
-----
FC88X_00970_00586 -----------------CCACCAACTATTGTTCG----TCGCCGTTCT------
-----
FC88X_00956_00665 -----------------CCACCAACTATTGTTCG----TCGCCGTTCT------
-----
FC88X_01040_00772 -----------------CCACCAACTATTGTTCG----TCGCCGTTCT------
-----
FC88X_01005_00931 -----------------CCACCAACTATTGTTCG----TCGCCGTTCT------
-----
FC88X_01057_00820 -----------------CCACCAACTATTGTTCG----TCGCCGTTCT------
-----
FC88X_01134_00589 -----------------CCACCAACTATTGTTCG----TCGCCGTTCT------
-----
FC88X_01147_00701 -----------------CCACCAACTATTGTTCG----TCGCCGTTCT------
-----
FC88X_01123_00871 -----------------CCACCAACTATTGTTCG----TCGCCGTTCT------
-----
FC88X_01133_01214 -----------------CCACCAACTATTGTTCG----TCGCCGTTCT------
-----
FC88X_00990_00821 -----------------CCACCAACTATTGGTCG----TCGCCGTTCT------
-----

```
FC88X_01103_00562      -----------------CCACCAACTATTGCTCG----TCGCCGTTCT------
-----
FC88X_01065_00586      -----------------CCTCCAACTATTGTTCG----TCGCCGTTCT------
-----
FC88X_01097_00425      -----------------CCACCAACTATTATTCG----TCGCCGTTCT------
-----
FC88X_00952_00386      -----------------CCACCAACTACTGTTCG----TCGCCGTTCT------
-----
FC88X_01034_00307      -----------------TTACTAACTACTAGGCG----CAACTGTTCT------
-----
FC88X_01044_00437      -----------------TTACTAACTACTAGGCG----CAACTGTTCTCC----
-----
FC88X_01130_01205      -----------------TCACTAACTACTAGGCG----CAACTGTTCT------
-----
FC88X_00987_00842      -----------------TTACTAACTACTAGGCG----CGACTGTTCT------
-----
FC88X_01072_00549      -------------------TGTCTCGGTTTCGCG----CAGAACTTTCTC----
-----
FC88X_01099_00829      ------------------CTGTCTCTGAGTCGGG----CCAAATTATTCT----
-----
FC88X_00974_00369      ------------------TTCCCTCAGTGTGGGA----CGGACCTGTTCTT---
-----
FC88X_00998_00725      --------------------------------GG----CCGAACGT--------
-----
FC88X_00966_00997      ------------------CTCCCCACAACTAAAG----CCGATCCTTCGC----
-----
FC88X_01055_00937      -------------------TCCCATCGTTTGAGG----ACAGCTCCTTATC---
-----
FC88X_01124_00654      ----------------CTTTACTAACGTTAGAAG----CCGGTTCTC-------
-----
FC88X_01029_00428      --------------------CCTCACATTAGTTG----ACGCGCTCCCCAT---
-----
FC88X_01004_01267      --------------------CCTCACATTAGTTG----ACGCGCTCCCCAT---
-----
FC88X_01081_00480      -------------------------TACTAACAA----TCGT------------
-----
FC88X_01033_00829      ---------------------------------T----TCATATGC--------
-----
FC88X_01057_00502      --------------------------ACAAAATG----
TCATATTCTCGACGGGCTCGA
FC88X_01086_00904      -------------------------TACAAATCC----
CTTAGTTCCCGATCACCT---
FC88X_00958_00465      -----------------TCTCCCTAT----TTTGCGCGACAAGTTGCA------
-----
FC88X_01010_01028      -----------------TCTCCCTAT----TTTGCGCGACAAGTTGCA------
-----
FC88X_01064_00597      -----------------TCTCCCTAT----TTTGCGCGACAAGTTGCA------
-----
FC88X_01100_00709      -----------------TCTCCCTAT----TTTGCGCGACAAGTTGCA------
-----
FC88X_01111_00818      -----------------TCTCCCTAT----TTTGCGCGACAAGTTGCA------
-----
FC88X_01085_01173      -----------------TCTCCCTAT----TTTGCGCGACAAGTTGCACC----
-----
FC88X_01027_00888      -----------------TCTCCCTAT----TCTGCGCGACAAGTTGCA------
-----
```

```
FC88X_00986_01025       -----------------TCTCCCTAT----TTTGCGCGACAAGCTGCA------
-----
FC88X_01026_01259       ------------------CTCCCCAT----CGTGCGCCATTAGAGTGGC-----
-----
FC88X_00988_00852       -----------------TCCGCCAAT----TCTGCGCGTTAGCATTCTC-----
-----
FC88X_01033_01205       -----------------TCCGCCAAT----TCTGCGCGTTAGCATTCTC-----
-----
FC88X_01047_01255       -----------------TCCGCCAAT----TCTGCGCGTTAGCATTCTC-----
-----
FC88X_01092_00582       -----------------TCCGCCAAT----TCTGCGCGTTAGCATTCTC-----
-----
FC88X_01054_00810       -----------------TCCGCCAAT----TCTGCGCGTTAGCATTCTC-----
-----
FC88X_01052_00947       -----------------TCCGCCAAT----TCTGCGCGTTAGCATTCTC-----
-----
FC88X_01139_00890       -----------------TCCGCCAAT----TCTGCGCGTTAGCATTCTC-----
-----
FC88X_01126_00910       -----------------TCCGCCAAT----TCTGCGCGTTAGCATTCTC-----
-----
FC88X_01048_00872       -----------------TCCGCCAAT----TCTGCGCGTTAGCATTCTCAT---
-----
FC88X_01098_01260       ------------------TCGCCAAT----TCTGCGCGTTAGCATTCTC-----
-----
FC88X_01083_00577       -----------------TCCGCCAAT----TCTGCGCGT---------------
-----
FC88X_01008_00372       -----------------TCTCCCAAT----CATCTGCGCGCGCATGAT------
-----
FC88X_01034_00751       -----------------TCTCCCAAT----CGTTCGCGTCAGCCCGCG------
-----
FC88X_01006_00318       ------------------TCCCCAAC----TTTCCCTTTTGCGCTTCACG----
-----
FC88X_01012_01158       ---------------------CAGCTGTTTTTACGCGCCTACTATTCC------
-----
FC88X_01145_00749       ---------------------CAGCTGTTTTTACGCGCCTACTATTCC------
-----
FC88X_01128_01136       ---------------------CAGCTGTTTTTACGCGCCTACTATTCC------
-----
FC88X_00990_00280       --------------------TCAGCTATTTA-ACCTTTCAACTATTCT------
-----
FC88X_01082_00643       --------------------TCGGCTATTAC-ACGGTGCCACTGTCTCCC----
-----
FC88X_01065_00408       ----------------------TTCAGCCTTATAGGTCGTCCTGTTCTA-----
-----
FC88X_01138_00874       ----------------------TTCAGCCTTATAGGTCGTCCTGTTCTA-----
-----
FC88X_00984_00409       ---------------------GTTCAGCCTTATAGGTCGTCCTGTTCCA-----
-----
FC88X_00970_00706       ----------------------ACCAGCACTCCATGTTGTCATGTTCTC-----
-----
FC88X_01000_00276       ----------------------ACCAGCACTCCATGTTGTC-------------
-----
FC88X_01069_01174       --------------------TCTTCAGCCCAACAT-TTGTCCTATTCT------
-----
FC88X_01111_00712       --------------------TCTTCAGCCCAACAT-TTGTCCTATTCT------
-----
```

FC88X_01003_00518 --------------------TCACCAAACCTCCCA-TGGTGCTATTGC------
-----
FC88X_01018_00627 --------------------TCACCAAACCTCCCA-TGGTGCTATTGC------
-----
FC88X_01061_00489 --------------------TCACCAAACCTCCCA-TGGTGCTATTGC------
-----
FC88X_01011_00780 --------------------TCACCAAGCCTCCCA-TGGTGCTATTGC------
-----
FC88X_00951_00964 --------------------TCACCAAACCTCTCA-TGGTGCTATTGC------
-----
FC88X_00992_00746 --------------------TCACCACTCCCTTTG-AACTATTCTTGT------
-----
FC88X_00996_00759 ---------------------CTAAGGCGTCTCTCTCATTGCTATTCT------
-----
FC88X_01133_00563 ---------------------CTAAGGCGTCTCTCTCATTGCTATTCT------
-----
FC88X_00965_00451 -----------------------ATAGCGTC-CTCCCAAAACCAGTCTGTT---
-----
FC88X_01056_00875 ---------------------------AACTACTCCA-----------------
-----
FC88X_01112_00990 ---------------------TAACGGCACCACTCCACGCTCTATATT------
-----
FC88X_00954_00370 ----------------------TTTCCTACAACTCATC----------------
-----
FC88X_00975_00733 ----------------------TTTCCTACAACTCATCGG--------------
-----
FC88X_00985_00845 ----------------------TTTCCTACAACTATCCGGGCGA----------
-----
FC88X_01026_00637 ---------------------TTCCCTGACAACAATTACCGCGCGCCT------
-----
FC88X_01140_00719 --------------------TTTCCCTTACAACTA-------------------
-----
FC88X_01051_00739 --------------------------CTACAACAAT------------------
-----
FC88X_00969_00421 --------------------------CTACAACAA-------------------
-----
FC88X_00986_00422 -------------TTAACTCTTTCCTCTACGCAACTATTCTTC-----------
-----
FC88X_01095_01153 --------------------------CTAC-CAACT------------------
-----
FC88X_01050_00698 -------------------------TCTACGAAATTT-----------------
-----
FC88X_01049_00805 ----------------------CCTTCTCCAGAACCAAGTCGTGTGAC------
-----
FC88X_01032_00960 -------------------------TCTACAAAACTCAAGATTGG---------
-----
FC88X_01045_00352 ------------------GACACTCCCTA--GAAACATGCGCCATTTG------
-----
FC88X_01023_00646 -------------------ACACTCCCTA--GAAACATGCGCCATTTG------
-----
FC88X_01030_00797 -------------------ACACTCCCTA--GAAACATGCGCCATTTG------
-----
FC88X_01058_00372 -------------------ACACTCCCTA--GAAACATGCGCCATTTG------
-----
FC88X_01074_00725 -------------------ACACTCCCTA--GAAACATGCGCCATTTG------
-----

FC88X_01122_00704 -------------------ACACTCCCTA--GAAACATGCGCCATTTG------
-----
FC88X_01140_00619 -------------------ACACTCCCTTACGAACCACGCGCGAAT--------
-----
FC88X_01036_00583 --------------------------CTA--CAAACAA----------------
-----
FC88X_01018_00401 ---------------ACTAACAACAACC--------------------------
-----
FC88X_01032_00772 -------------------ACAACAACCAGTCACCTTCGCACTCTC--------
-----
FC88X_00992_00697 -------------------------CCGATAAAACTTCGCAGCCGTCCCATT--
-----
FC88X_01047_00605 ----------------------CCGATAAAAAAACTTCGCAGTCGTCCCATT--
-----
FC88X_01076_01079 ----------------------CATGCAACAAAACTGCGTCGCTGTCCC-----
-----
FC88X_01091_01096 -------------------------ACAAGAACAGTTCGAG-------------
-----
FC88X_01028_00305 -------------------GTGCAAAAAAAAAAACCTCTCGAGC----------
-----
FC88X_00959_00519 ------------------------------TCATCGTAACCGTATGAGC-
CCCATTCA-
FC88X_01039_00543 ------------------------------
TCATCGTAACCGTATGAGCTCCCATTTA-
FC88X_01043_00583 ---------------------TTCAGTTCCTCCACTCCACACCGTATT------
-----
FC88X_01114_01174 -------------------CCAACCGTTCCCCGACCTCACCGTATA--------
-----
FC88X_00995_00877 -------------------CCTGGGACCCTCCGCCTTCAACGTATT--------
-----
FC88X_00999_00522 -------------------------------------CACAACGTAAT------
-----
FC88X_00979_00739 -------------------------TCCCGACATCTTCAACGTGCTATTCTA--
-----
FC88X_00986_00846 -------------------------TCCCGACATCTTCAACGTGCTATTCTA--
-----
FC88X_01071_00453 -------------------------TCCCGACATCTTCAACGTGCTATTCTA--
-----
FC88X_01062_00761 ------------------------------------TCAACGTG----------
-----
FC88X_00972_00648 -----------------TTGTAACCATTTCCCTG-CCAACG-TATCT-------
-----
FC88X_01008_00399 -------------------TTAACTAATCCCTTGTCCCACG-TGTC--------
-----
FC88X_00964_01259 -------------------TCCCCCACTAAATCGTCTGCAGATGTCC-------
-----
FC88X_01023_00832 -------------------TTACCAACTAACCTGTCTGCCG-TGTC--------
-----
FC88X_01081_00923 ------------------TTTGTACA-TCGCCCG-TGCAACATGCTT-------
-----
FC88X_01089_01093 ------------------TTTGTACA-TCGCCCG-TGCAACATGCTT-------
-----
FC88X_01104_00736 ------------------TTTGTACA-TCGCCCG-TGCAACATGCTT-------
-----
FC88X_01125_00919 ------------------TTTGTACAATCGCCCGGTGCAACATGCTT-------
-----

FC88X_01113_00715 ---------------CCCCCAATGAGCTTGCCCCGTCCTGTA------------
-----
FC88X_01133_00965 ------------------GCAATAAGC---------------------------
-----
FC88X_00983_00632 --------------------CGCAATTTGGAC-ATGTGGCCGCTTCTC------
-----
FC88X_00967_00852 --------------------CGCAATTTGGAC-ATGTGGCCGCTTCTC------
-----
FC88X_01045_00497 --------------------CGCAATTTGGAC-ATGTGGCCGCTTCTC------
-----
FC88X_01065_00803 --------------------CGCAATTTGGAC-ATGTGGCCGCTTCTC------
-----
FC88X_01128_00850 --------------------CGCAATTTGGAC-GTGTGGCCGCTTCTC------
-----
FC88X_00995_00608 ------------------GTCGCACTGTCGTCGATGTGTGCCTGTC--------
-----
FC88X_01104_00501 -------------------TCGCACTGTCGTCGATGTGTGCCTGTC--------
-----
FC88X_01089_00723 --------------------CGCAATTTGGAC-ATGTGGGCCGGCT--------
-----
FC88X_01038_00824 ---------------CGTGGTACTTTGTCGCC-ACCTCATCTCCC---------
-----
FC88X_01140_01144 ---------------TGTGGTGCAATTGTGCC-GCCT-ATCTGC----------
-----
FC88X_00993_00830 ------------------GGCGCAACTTTGTG-ACGTCCACTTCTC--------
-----
FC88X_00966_00346 -----------------CCAACATCTCTTCCACG--CGGCGATATT--------
-----
FC88X_01030_00784 -----------------CCAAGTTCACTCCC-TG--CGGCGACATCTT------
-----
FC88X_00965_00597 -------------------------TCTCCCTGG--
GAACGACATGGCGCACAA-----
FC88X_00951_00408 -------------------CGTGCAGGTATATCTGGTCGTTATGCTC-------
-----
FC88X_00994_00765 -------------------CGTGCAGGTATATCTGGTCGTTATGCTC-------
-----
FC88X_00994_00826 -------------------CGTGCAGGTATATCTGGTCGTTATGCTC-------
-----
FC88X_01049_00370 -------------------CGTGCAGGTATATCTGGTCGTTATGCTC-------
-----
FC88X_01003_00817 -------------------CGTGCAGGTATATCTGGTCGTTATGCTC-------
-----
FC88X_01026_00841 -------------------CGTGCAGGTATATCTGGTCGTTATGCTC-------
-----
FC88X_01071_00515 -------------------CGTGCAGGTATATCTGGTCGTTATGCTC-------
-----
FC88X_01114_00828 -------------------CGTGCAGGTATATCTGGTCGTTATGCTC-------
-----
FC88X_00986_00836 -------------------CGTGCAGGTATATCCGGTCGTTATGCTC-------
-----
FC88X_00975_01203 --------------------------------CCAGTCGATAT-----------
-----
FC88X_01147_00833 --------------------------------CCAGTCGATAT-----------
-----
FC88X_01101_00878 --------------------------------CCAGTCGATAT-----------
-----

FC88X_00991_00743   ----------------------------
CCATCCAGTTGTGACTCGCGCTATTGTC---
FC88X_01004_00585   ------------------------CTTCCCTTCCAGTTGTAAC--GCGATATT-
-----
FC88X_00952_00544   ------------------------CTACTCTTCAAGCCGTGACT-GCTATA---
-----
FC88X_00987_00630   --------------------------------CCAGTCGATAC-----------
-----
FC88X_00967_00643   ------------------------CCACTTGTCATGC---TATGTCCCTTCCT-
-----
FC88X_01137_01136   --------------------CTCCCCACGTGTAAGGCACATATGTCCA------
-----
FC88X_01010_00519   -------------------ACCACCCATCTGTGGCACC--AATGTTC-------
-----
FC88X_01046_00623   ----------------TTGCTTGCTATTTTCCTTCGGACGGAC-----------
-----
FC88X_01114_01219   --------------------CCGGTAATTTCGATCGGCCCCACACGT-------
-----
FC88X_01014_00849   -------------------ATTCCAATTCGCTTTGGCTCCCATGTTCCGT----
-----
FC88X_01009_00830   -------------------CTCCCAATTGGATGACTCGCGCGTGCC--------
-----
FC88X_01039_01043   -------------------CTCCCAATTGGATGACTCGCGCGTGCC--------
-----
FC88X_01066_00862   -------------------CTCCCAATTGAATGAC-CGCGCGTGCC--------
-----
FC88X_01070_00998   ---------------------CGCAATTGG------------------------
-----
FC88X_01086_00445   -------------------CTCCCTAGTCAAGTAGCCGCTCGTGGC--------
-----
FC88X_01079_00892   -------------------TTCCCTGGTGCTTGAGCCGCGATCTTTCC------
-----
FC88X_00978_00335   -----------------TCTTCCAATTTT-G--G--GCGGTACTGTCTC-----
-----
FC88X_00995_00328   -----------------TCTTCCAATTTT-G--G--GCGGTACTGTCTC-----
-----
FC88X_00993_00944   -----------------TCTTCCAATTTT-G--G--GCGGTACTGTCTC-----
-----
FC88X_01032_00468   -----------------TCTTCCAATTTT-G--G--GCGGTACTGTCTC-----
-----
FC88X_01048_00506   -----------------TCTTCCAATTTT-G--G--GCGGTACTGTCTC-----
-----
FC88X_01141_00850   -----------------TCTTCCAATTTT-G--G--GCGGTACTGTCTC-----
-----
FC88X_00991_00394   -----------------TCTTCCAATTTTTG--G--GCGGTACTGTCTC-----
-----
FC88X_01027_00933   -------------------AACCAATTCGCGC-G--GTAGGTCTATCTC-----
-----
FC88X_00974_00404   -----------------TCTTGCAATTTCCT-----GCGGTGCATTCTC-----
-----
FC88X_01050_00415   -----------------TCTTGCAATTTCCT-----GCG-TGCATTCTC-----
-----
FC88X_00998_00411   -----------------TCTTGCAATTTCCT-----GCGGTGCATTCTC-----
-----
FC88X_00980_00471   -----------------TCTTGCAATTTCCT-----GCGGTGCATTCTC-----
-----

```
FC88X_00978_01275       -----------------TCTTGCAATTTCCT-----GCGGTGCATTCTC-----
-----
FC88X_01025_00380       -----------------TCTTGCAATTTCCT-----GCGGTGCATTCTC-----
-----
FC88X_01036_00378       -----------------TCTTGCAATTTCCT-----GCGGTGCATTCTC-----
-----
FC88X_01038_00435       -----------------TCTTGCAATTTCCT-----GCGGTGCATTCTC-----
-----
FC88X_01041_00912       -----------------TCTTGCAATTTCCT-----GCGGTGCATTCTC-----
-----
FC88X_01031_01001       -----------------TCTTGCAATTTCCT-----GCGGTGCATTCTC-----
-----
FC88X_01073_00404       -----------------TCTTGCAATTTCCT-----GCGGTGCATTCTC-----
-----
FC88X_01086_00494       -----------------TCTTGCAATTTCCT-----GCGGTGCATTCTC-----
-----
FC88X_01082_00864       -----------------TCTTGCAATTTCCT-----GCGGTGCATTCTC-----
-----
FC88X_01102_00719       -----------------TCTTGCAATTTCCT-----GCGGTGCATTCTC-----
-----
FC88X_01102_00807       -----------------TCTTGCAATTTCCT-----GCGGTGCATTCTC-----
-----
FC88X_01136_01000       -----------------TCTTGCAATTTCCT-----GCGGTGCATTCTC-----
-----
FC88X_01103_00482       -----------------TCTTGCAATTTCCT-----GCGGTGCATTCTCCC---
-----
FC88X_01136_01104       -----------------TCTTGCAATTTCCT-----GCGGTGCATTCTCCC---
-----
FC88X_01001_00983       -----------------TCTTGCGATTTCCT-----GCGGTGCATTCTC-----
-----
FC88X_01089_01060       -----------------TCCTGCAATTTCCT-----GCGGTGCATTCTC-----
-----
FC88X_01057_00506       -----------------TCTTGCAATTTCCT-----GCGGTGCATTCGTC----
-----
FC88X_01062_00621       -----------------TCTTGCAATTTCCT-----GCGGTGCATTTCTC----
-----
FC88X_00980_01225       -----------------TTTTGCAGTTCCCT-----GCGGTGCATTCTC-----
-----
FC88X_01024_01267       ------------------CTGACAATTCCGCCCT--GCGCTACTTCTC------
-----
FC88X_01072_00632       ------------------CTGACAATTCCCCCCT--GCGCTACTTCTC------
-----
FC88X_01024_00307       -----------AATCAGGCCTGCGTGGACT--------GGTGCGTTCTC-----
-----
FC88X_01130_00708       -----------AATCAGGCCTGCGTGGACT--------GGTGCGTTCTC-----
-----
FC88X_01117_01210       -----------------CCGTGCGTTGATTC-----ACGGTGCGTCCTC-----
-----
FC88X_01031_01151       ------------------AGACGTA-TTCGTTG-TCGCTCTGT-TCAC------
-----
FC88X_01069_01216       ------------------AGACGTA-TTCGTTG--CGCTCTGT-TCAC------
-----
FC88X_01145_00634       ------------------AGACGTA-TCCGTTT-GCGCTCTGT-TCAC------
-----
FC88X_00991_00292       ------------------AGACGTA-TTCGTTT-GCGCTCTGT-TCAC------
-----
```

FC88X_00997_00282 ------------------AGACGTA-TTCGTTT-GCGCTCTGT-TCAC------
-----
FC88X_00991_00339 ------------------AGACGTA-TTCGTTT-GCGCTCTGT-TCAC------
-----
FC88X_00973_00433 ------------------AGACGTA-TTCGTTT-GCGCTCTGT-TCAC------
-----
FC88X_00968_00459 ------------------AGACGTA-TTCGTTT-GCGCTCTGT-TCAC------
-----
FC88X_00961_00540 ------------------AGACGTA-TTCGTTT-GCGCTCTGT-TCAC------
-----
FC88X_00964_00809 ------------------AGACGTA-TTCGTTT-GCGCTCTGT-TCAC------
-----
FC88X_00975_00842 ------------------AGACGTA-TTCGTTT-GCGCTCTGT-TCAC------
-----
FC88X_00962_00867 ------------------AGACGTA-TTCGTTT-GCGCTCTGT-TCAC------
-----
FC88X_00983_00852 ------------------AGACGTA-TTCGTTT-GCGCTCTGT-TCAC------
-----
FC88X_00987_00851 ------------------AGACGTA-TTCGTTT-GCGCTCTGT-TCAC------
-----
FC88X_00978_00919 ------------------AGACGTA-TTCGTTT-GCGCTCTGT-TCAC------
-----
FC88X_00959_00994 ------------------AGACGTA-TTCGTTT-GCGCTCTGT-TCAC------
-----
FC88X_00971_00967 ------------------AGACGTA-TTCGTTT-GCGCTCTGT-TCAC------
-----
FC88X_00982_01214 ------------------AGACGTA-TTCGTTT-GCGCTCTGT-TCAC------
-----
FC88X_00991_01214 ------------------AGACGTA-TTCGTTT-GCGCTCTGT-TCAC------
-----
FC88X_00982_01260 ------------------AGACGTA-TTCGTTT-GCGCTCTGT-TCAC------
-----
FC88X_01018_00290 ------------------AGACGTA-TTCGTTT-GCGCTCTGT-TCAC------
-----
FC88X_01049_00328 ------------------AGACGTA-TTCGTTT-GCGCTCTGT-TCAC------
-----
FC88X_01021_00415 ------------------AGACGTA-TTCGTTT-GCGCTCTGT-TCAC------
-----
FC88X_01025_00470 ------------------AGACGTA-TTCGTTT-GCGCTCTGT-TCAC------
-----
FC88X_01030_00549 ------------------AGACGTA-TTCGTTT-GCGCTCTGT-TCAC------
-----
FC88X_01049_00601 ------------------AGACGTA-TTCGTTT-GCGCTCTGT-TCAC------
-----
FC88X_01035_00879 ------------------AGACGTA-TTCGTTT-GCGCTCTGT-TCAC------
-----
FC88X_01016_00930 ------------------AGACGTA-TTCGTTT-GCGCTCTGT-TCAC------
-----
FC88X_01044_00979 ------------------AGACGTA-TTCGTTT-GCGCTCTGT-TCAC------
-----
FC88X_01000_01037 ------------------AGACGTA-TTCGTTT-GCGCTCTGT-TCAC------
-----
FC88X_01011_01042 ------------------AGACGTA-TTCGTTT-GCGCTCTGT-TCAC------
-----
FC88X_01043_01004 ------------------AGACGTA-TTCGTTT-GCGCTCTGT-TCAC------
-----

```
FC88X_01047_01036      ------------------AGACGTA-TTCGTTT-GCGCTCTGT-TCAC------
-----
FC88X_01017_01202      ------------------AGACGTA-TTCGTTT-GCGCTCTGT-TCAC------
-----
FC88X_01090_00593      ------------------AGACGTA-TTCGTTT-GCGCTCTGT-TCAC------
-----
FC88X_01050_00771      ------------------AGACGTA-TTCGTTT-GCGCTCTGT-TCAC------
-----
FC88X_01055_00898      ------------------AGACGTA-TTCGTTT-GCGCTCTGT-TCAC------
-----
FC88X_01075_00853      ------------------AGACGTA-TTCGTTT-GCGCTCTGT-TCAC------
-----
FC88X_01058_00946      ------------------AGACGTA-TTCGTTT-GCGCTCTGT-TCAC------
-----
FC88X_01087_00936      ------------------AGACGTA-TTCGTTT-GCGCTCTGT-TCAC------
-----
FC88X_01094_00939      ------------------AGACGTA-TTCGTTT-GCGCTCTGT-TCAC------
-----
FC88X_01093_01159      ------------------AGACGTA-TTCGTTT-GCGCTCTGT-TCAC------
-----
FC88X_01104_00561      ------------------AGACGTA-TTCGTTT-GCGCTCTGT-TCAC------
-----
FC88X_01119_00599      ------------------AGACGTA-TTCGTTT-GCGCTCTGT-TCAC------
-----
FC88X_01131_00594      ------------------AGACGTA-TTCGTTT-GCGCTCTGT-TCAC------
-----
FC88X_01113_00733      ------------------AGACGTA-TTCGTTT-GCGCTCTGT-TCAC------
-----
FC88X_01139_00906      ------------------AGACGTA-TTCGTTT-GCGCTCTGT-TCAC------
-----
FC88X_01103_00978      ------------------AGACGTA-TTCGTTT-GCGCTCTGT-TCAC------
-----
FC88X_01133_01001      ------------------AGACGTA-TTCGTTT-GCGCTCTGT-TCAC------
-----
FC88X_00984_00488      ------------------AGACGTA-TTCGTTT-GCGCTCTGT-TCACCC----
-----
FC88X_01036_00451      ------------------AGACGTA-TTGGTTT-GCGCTCTGT-TCAC------
-----
FC88X_00972_00704      ------------------AGACGTA-TTCGTCT-GCGCTCTGT-TCAC------
-----
FC88X_01030_00347      ------------------AGACGTA-TTCGTCT-GCGCTCTGT-TCAC------
-----
FC88X_00965_00832      ------------------AGACGTA-TTCGTTT-GCGCTCTGC-TCACCC----
-----
FC88X_01106_00675      ------------------AGACGTA-TTCGTTT-GCGCTCTGC-TCAC------
-----
FC88X_01049_00466      ------------------AGACGCA-TTCGTTT-GCGCTCTGT-TCAC------
-----
FC88X_01119_00591      ------------------AGACGCA-TTCGTTTTGCGCTCTGT-TCAC------
-----
FC88X_01103_00879      ------------------AGACGGA-TTCGTTT-GCGCTCTGT-TCAC------
-----
FC88X_01081_01076      ------------------AGACGTA-TTCGTTT-GCGCTCTGT-TCGC------
-----
FC88X_01142_00920      ------------------AGACGTA-TTCGTTT-GCGCTCTGT-TCGC------
-----
```

```
FC88X_01079_01258      -----------------GAGACGTAATTCGTTT-GCGCTCTGT-TCGC------
-----
FC88X_01068_00918      ------------------AGACGTATTTCGTTT-GCGCTCTG------------
-----
FC88X_00980_00863      ------------------AGACGTA-TTCGTTTTGCGCTCTGT-T---------
-----
FC88X_00960_00500      ------------------AGACGTA-TTCGTTT-GCGCTCTGT-TCTC------
-----
FC88X_01000_01009      ------------------AGACGTA-TTCGTTT-GCGCTCTGT-TCTC------
-----
FC88X_01031_00340      ------------------AGACGTA-TTCGTTT-GCGCTCTTCGTCAC------
-----
FC88X_00984_00732      -----------------CCGACATACTTAGTTTGCCGCATTCTC----------
-----
FC88X_00994_00997      -----------------CCGACATACTTAGTTTGCCGCATTCTC----------
-----
FC88X_01043_00405      -----------------CCGACGTACTTAGTTTGCCGCATTCTC----------
-----
FC88X_01056_00470      -----------------CCGACGTGTTCATTTCGCTGTATTCTC----------
-----
FC88X_01077_00943      -----------------CCGACGTGTTCATTTCGCTGTATTCTC----------
-----
FC88X_01015_01023      -----------------CCGACGCGTTCATTTCGCTGTATTCTC----------
-----
FC88X_01108_01115      -----------------CCGACGTGTTCACTTCGCTGTATTCTC----------
-----
FC88X_00990_00793      -----------------CCAGACGACGGACATCGGAGGCATTCT----------
-----
FC88X_01115_00558      --------------CTCCCTGACGACTAAC-TCGATGACATT------------
-----
FC88X_01020_00812      --------------------AACGACGTACTTCGT-------------------
-----
FC88X_00986_00740      -------------------CTCTGCTCTCTCCAACGTCGTGGTGTT--------
-----
FC88X_01072_00462      ------------------TCTCCGTTGTCTCCTGAGTCGGTGTCGCCC------
-----
FC88X_00991_00340      --------------------------TTCACGTAAGTCGTT-------------
-----
FC88X_00963_00364      ---------------CCAACAGGTATCTGATTGCGCGTTCTC------------
-----
FC88X_00982_00850      ---------------CCAACAGGTATCTGATTGCGCGTTCTC------------
-----
FC88X_00981_01033      ---------------CCAACAGGTATCTGATTGCGCGTTCTC------------
-----
FC88X_01047_00408      ---------------CCAACAGGTATCTGATTGCGCGTTCTC------------
-----
FC88X_01085_00779      ---------------CCAACAGGTATCTGATTGCGCGTTCTC------------
-----
FC88X_01104_01170      ---------------CCAACAGGTATCTGATCGCGCGTTCCC------------
-----
FC88X_00972_00418      ---------------CCAACAGGTATCTAATTGCGCGTTCTC------------
-----
FC88X_01006_00386      ---------------CCAAGACGTAACCGGTACCGCATTCTC------------
-----
FC88X_00970_01014      -----------------AATACGTAC----------------------------
-----
```

FC88X_01043_00584 ---------------CTTGGGTCAAGCCTAAGACTATTTCTT--------------
FC88X_01025_00970 ---------------CTTGGGCCAAGCCTAAGACTATTTCTT--------------
FC88X_01124_00587 ------------------------AGACGTATTAGAATTCCCC--------------
FC88X_01132_01168 ------------------------AGACGTATT----------------------------
FC88X_01087_00491 ----------------ATCAACCCAAGGCAATAGTTATCTCTT-------------
FC88X_01056_01213 --------------TAACCAATCCGACGTGGTACCAATCTC---------------
FC88X_00950_00433 -------------------CGCGTTT-CTATTGGCGTGTCTATATCTC--------
FC88X_00950_00820 -------------------CGCGTTT-CTATTGGCGTGTCTATATCTC--------
FC88X_00988_00916 -------------------CGCGTTT-CTATTGGCGTGTCTATATCTC--------
FC88X_01027_00617 -------------------CGCGTTT-CTATTGGCGTGTCTATATCTC--------
FC88X_01009_00442 ------------------CTTCGTTC-TTACTGGCGGGCTTATCTC----------
FC88X_01079_00714 ------------------CTTCGTTC-TTACTGGCGGGCTTATCTC----------
FC88X_01057_00849 ------------------CTTCGTCC-TTACTGGCGGGCTTACCTCTCC-------
FC88X_00999_00841 ------------------CTTCGTAT-TGTGGTTGATGCC--------------------
FC88X_01032_00352 ------------------CTTCGTCT-TGCTGGTGATGCCTATTG---------------
FC88X_00993_00483 -------------------TGACGTG-TTGT-GCGGCGCTCACT-GTCTC----------
FC88X_01144_00589 -------------------TGACGTG-TTGT-GCAGCGCTCACT-GTCTC----------
FC88X_00964_00814 -------------------TGACGTG-TTGT-GCGGCGCTCACT-GTCTC----------
FC88X_00967_00885 -------------------TGACGTG-TTGT-GCGGCGCTCACT-GTCTC----------
FC88X_00957_00917 -------------------TGACGTG-TTGT-GCGGCGCTCACT-GTCTC----------
FC88X_00984_00986 -------------------TGACGTG-TTGT-GCGGCGCTCACT-GTCTC----------
FC88X_00990_00998 -------------------TGACGTG-TTGT-GCGGCGCTCACT-GTCTC----------
FC88X_01023_00416 -------------------TGACGTG-TTGT-GCGGCGCTCACT-GTCTC----------
FC88X_01026_00538 -------------------TGACGTG-TTGT-GCGGCGCTCACT-GTCTC----------
FC88X_01024_00639 -------------------TGACGTG-TTGT-GCGGCGCTCACT-GTCTC----------
FC88X_01045_00627 -------------------TGACGTG-TTGT-GCGGCGCTCACT-GTCTC----------
FC88X_01020_00678 -------------------TGACGTG-TTGT-GCGGCGCTCACT-GTCTC----------
FC88X_01031_00987 -------------------TGACGTG-TTGT-GCGGCGCTCACT-GTCTC----------

```
FC88X_01029_01007      ------------------TGACGTG-TTGT-GCGGCGCTCACT-GTCTC-----
-----
FC88X_01039_01120      ------------------TGACGTG-TTGT-GCGGCGCTCACT-GTCTC-----
-----
FC88X_01049_01186      ------------------TGACGTG-TTGT-GCGGCGCTCACT-GTCTC-----
-----
FC88X_01083_00393      ------------------TGACGTG-TTGT-GCGGCGCTCACT-GTCTC-----
-----
FC88X_01089_00432      ------------------TGACGTG-TTGT-GCGGCGCTCACT-GTCTC-----
-----
FC88X_01056_00461      ------------------TGACGTG-TTGT-GCGGCGCTCACT-GTCTC-----
-----
FC88X_01069_00452      ------------------TGACGTG-TTGT-GCGGCGCTCACT-GTCTC-----
-----
FC88X_01056_00526      ------------------TGACGTG-TTGT-GCGGCGCTCACT-GTCTC-----
-----
FC88X_01092_00506      ------------------TGACGTG-TTGT-GCGGCGCTCACT-GTCTC-----
-----
FC88X_01092_00518      ------------------TGACGTG-TTGT-GCGGCGCTCACT-GTCTC-----
-----
FC88X_01086_00837      ------------------TGACGTG-TTGT-GCGGCGCTCACT-GTCTC-----
-----
FC88X_01056_01082      ------------------TGACGTG-TTGT-GCGGCGCTCACT-GTCTC-----
-----
FC88X_01050_01197      ------------------TGACGTG-TTGT-GCGGCGCTCACT-GTCTC-----
-----
FC88X_01080_01174      ------------------TGACGTG-TTGT-GCGGCGCTCACT-GTCTC-----
-----
FC88X_01076_01204      ------------------TGACGTG-TTGT-GCGGCGCTCACT-GTCTC-----
-----
FC88X_01108_00501      ------------------TGACGTG-TTGT-GCGGCGCTCACT-GTCTC-----
-----
FC88X_01117_00765      ------------------TGACGTG-TTGT-GCGGCGCTCACT-GTCTC-----
-----
FC88X_01135_00988      ------------------TGACGTG-TTGT-GCGGCGCTCACT-GTCTC-----
-----
FC88X_01143_01250      ------------------TGACGTG-TTGT-GCGGCGCTCACT-GTCTC-----
-----
FC88X_01095_00467      ------------------TGACGTG-TTGT-GCGGCGCTCACT-GTCTCC----
-----
FC88X_00980_00913      ------------------TGACGTG-TTGT-GCGGCGCTCACT-GTCTCCC---
-----
FC88X_00970_01134      ------------------TGACGTG-TTGT-GCGGCGCTCACT-GTCTCCC---
-----
FC88X_00974_00933      ------------------TGACGTG-TTGT-GTGGCGCTCACT-GTCTC-----
-----
FC88X_00987_00870      ------------------TGACGTG-TTGT-GCGGCGCTCACT-ATCTC-----
-----
FC88X_01080_00408      ------------------TGACGTG-TTGT-GCGGCGCTCACTGATCTC-----
-----
FC88X_01089_01132      ------------------TGACGTA-TTGT-GCGGCGCTCACT-GTCTC-----
-----
FC88X_00972_00555      ------------------TGACGTG-TTGTCGCGGCGCT---------------
-----
FC88X_01049_01122      ------------------TGACGTG-TTGT-GCGGCGCTCACTGTCCTC-----
-----
```

```
FC88X_01007_00294       ------------------TGACGTG-TTGT-GCGGCG-TCACT-GTCCC-----
-----
FC88X_01014_00487       ------------------TGACGTG-TTGT-GC---------------------
-----
FC88X_00984_00564       ---------------------------TGACGTGATT-----------------
-----
FC88X_00971_00796       ---------------TTCTGACGGA-TTGCTCTGACTATTCAC-----------
-----
FC88X_01060_00351       -----------------TGGACGTG-TTTGTGTGGCGCTCCACCTGTC------
-----
FC88X_00961_00544       ------------------CTACGTTTAAACGATCGTGT---TGTTCTC------
-----
FC88X_01011_00551       ------------------CTACGTTTAACCGA-CGTGT---TGTTCTC------
-----
FC88X_01006_00940       ------------------CTACGTTTAATCGATCGTGT---TGTTCTC------
-----
FC88X_01052_00402       ------------------CTACGTTTAATCGATCGTGT---TGTTCTC------
-----
FC88X_00990_00704       ------------------CGACGTTTAACCGATCATGT---TGTTCTC------
-----
FC88X_01050_01195       ------------------CTACGTTTAACCGATCGCGT---TGTTCTC------
-----
FC88X_01029_01034       -----------------GCTACGTTTAACCGATCGTGT---TGTTCTC------
-----
FC88X_00964_00371       ------------------CTACGTTTAACCGATCGTGT---TGTTCTC------
-----
FC88X_00991_00900       ------------------CTACGTCTAACCGATCGTGT---TGTTCTC------
-----
FC88X_01022_00379       ------------------CTACGT-TATCCGATCGTGT---TGTTCTC------
-----
FC88X_00999_00365       ------------------CTACGTTTAACCGATCGTGT---TGTTCTC------
-----
FC88X_00952_00489       ------------------CTACGTTTAACCGATCGTGT---TGTTCTC------
-----
FC88X_00973_00530       ------------------CTACGTTTAACCGATCGTGT---TGTTCTC------
-----
FC88X_00968_00605       ------------------CTACGTTTAACCGATCGTGT---TGTTCTC------
-----
FC88X_00989_00726       ------------------CTACGTTTAACCGATCGTGT---TGTTCTC------
-----
FC88X_01045_00676       ------------------CTACGTTTAACCGATCGTGT---TGTTCTCC-----
-----
FC88X_00974_00794       ------------------CTACGTTTAACCGATCGTGT---TGTTCTC------
-----
FC88X_00988_00765       ------------------CTACGTTTAACCGATCGTGT---TGTTCTC------
-----
FC88X_00981_00833       ------------------CTACGTTTAACCGATCGTGT---TGTTCTC------
-----
FC88X_00956_00881       ------------------CTACGTTTAACCGATCGTGT---TGTTCTC------
-----
FC88X_00974_00959       ------------------CTACGTTTAACCGATCGTGT---TGTTCTC------
-----
FC88X_01005_00274       ------------------CTACGTTTAACCGATCGTGT---TGTTCTC------
-----
FC88X_01008_00310       ------------------CTACGTTTAACCGATCGTGT---TGTTCTC------
-----
```

```
FC88X_01046_00337       ------------------CTACGTTTAACCGATCGTGT---TGTTCTC------
-----
FC88X_01004_00466       ------------------CTACGTTTAACCGATCGTGT---TGTTCTC------
-----
FC88X_01009_00488       ------------------CTACGTTTAACCGATCGTGT---TGTTCTC------
-----
FC88X_01023_00464       ------------------CTACGTTTAACCGATCGTGT---TGTTCTC------
-----
FC88X_01028_00458       ------------------CTACGTTTAACCGATCGTGT---TGTTCTC------
-----
FC88X_01029_00473       ------------------CTACGTTTAACCGATCGTGT---TGTTCTC------
-----
FC88X_01002_00638       ------------------CTACGTTTAACCGATCGTGT---TGTTCTC------
-----
FC88X_01043_00799       ------------------CTACGTTTAACCGATCGTGT---TGTTCTC------
-----
FC88X_01015_00903       ------------------CTACGTTTAACCGATCGTGT---TGTTCTC------
-----
FC88X_01020_00920       ------------------CTACGTTTAACCGATCGTGT---TGTTCTC------
-----
FC88X_01016_01171       ------------------CTACGTTTAACCGATCGTGT---TGTTCTC------
-----
FC88X_01074_00400       ------------------CTACGTTTAACCGATCGTGT---TGTTCTC------
-----
FC88X_01084_00443       ------------------CTACGTTTAACCGATCGTGT---TGTTCTC------
-----
FC88X_01064_00490       ------------------CTACGTTTAACCGATCGTGT---TGTTCTC------
-----
FC88X_01085_00605       ------------------CTACGTTTAACCGATCGTGT---TGTTCTC------
-----
FC88X_01075_00693       ------------------CTACGTTTAACCGATCGTGT---TGTTCTC------
-----
FC88X_01064_00721       ------------------CTACGTTTAACCGATCGTGT---TGTTCTC------
-----
FC88X_01081_00749       ------------------CTACGTTTAACCGATCGTGT---TGTTCTC------
-----
FC88X_01092_00732       ------------------CTACGTTTAACCGATCGTGT---TGTTCTC------
-----
FC88X_01089_00815       ------------------CTACGTTTAACCGATCGTGT---TGTTCTC------
-----
FC88X_01088_00877       ------------------CTACGTTTAACCGATCGTGT---TGTTCTC------
-----
FC88X_01096_00860       ------------------CTACGTTTAACCGATCGTGT---TGTTCTC------
-----
FC88X_01059_00933       ------------------CTACGTTTAACCGATCGTGT---TGTTCTC------
-----
FC88X_01098_00937       ------------------CTACGTTTAACCGATCGTGT---TGTTCTC------
-----
FC88X_01143_00584       ------------------CTACGTTTAACCGATCGTGT---TGTTCTC------
-----
FC88X_01146_00611       ------------------CTACGTTTAACCGATCGTGT---TGTTCTC------
-----
FC88X_01115_00787       ------------------CTACGTTTAACCGATCGTGT---TGTTCTC------
-----
FC88X_01122_00774       ------------------CTACGTTTAACCGATCGTGT---TGTTCTC------
-----
```

FC88X_01100_00890 ------------------CTACGTTTAACCGATCGTGT---TGTTCTC------
-----
FC88X_01114_00887 ------------------CTACGTTTAACCGATCGTGT---TGTTCTC------
-----
FC88X_01133_00923 ------------------CTACGTTTAACCGATCGTGT---TGTTCTC------
-----
FC88X_01133_00979 ------------------CTACGTTTAACCGATCGTGT---TGTTCTC------
-----
FC88X_01104_01159 ------------------CTACGTTTAACCGATCGTGT---TGTTCTC------
-----
FC88X_01142_01273 ------------------CTACGTTTAACCGATCGTGT---TGTTCTC------
-----
FC88X_01052_00733 -----------------GCTACGTTTAACCGATCGTGT---TGTTCTCC-----
-----
FC88X_00997_00347 ------------------CTACGTTTAACCGATCGTGT---TGTTCT-------
-----
FC88X_01123_01128 ------------------CTACGTTTAACCGATCGTGT---TGTTCTCCC----
-----
FC88X_01039_01107 ------------------CTACGTTTAACCGATCGTGT---TGTTCTCACA---
-----
FC88X_01097_00570 ------------------CTACGTTTAACCGATCGTGT---TATTCTC------
-----
FC88X_01123_00581 ------------------CTGCGTTTAACCGATCGTGT---TGTTCTC------
-----
FC88X_01061_01270 ------------------CAACGTTTAACCGATCGTGT---TGTTCTC------
-----
FC88X_01111_00924 ------------------CCACGTTTAACCGATCGTGT---TGTTCTC------
-----
FC88X_01043_00328 ------------------CTACGTTCAACCGATCGTGT---TGTTCTC------
-----
FC88X_01136_00894 ------------------CTACGTTCAACCGATCGTGT---TGTTCTC------
-----
FC88X_01031_00949 ------------------CTACGTTTAACCGAT-GTGT---TGTCTC-------
-----
FC88X_01082_00409 ------------------CTACGTTTAAC-------------------------
-----
FC88X_00955_00577 --------------------ACATATAACCGATCATGTGTCTGATCTC------
-----
FC88X_00996_00673 --------------------ACATATAACCGATCATGTGTCTGATCTC------
-----
FC88X_01043_00533 --------------------ACATATAACCGATCATGTGTCTGATCTC------
-----
FC88X_00994_00541 ---------------CCTGTACTTGTGAATGGCCACGTCTC-------------
-----
FC88X_01093_01120 ---------------CCTGTACTTGTGAATGGCCACGTCTCCC-----------
-----
FC88X_00987_00845 ---------------------CTTGTAAA-------------------------
-----
FC88X_01060_01225 -------------CTTGTCATTTGATCAATGTCTACATCTC-------------
-----
FC88X_00959_00399 ------------------CGCTACAATTTGTAATCCGT--TTATTCT-------
-----
FC88X_01042_00661 ------------------CGCTACAATTTGTAATCCGT--TTATTCT-------
-----
FC88X_01018_00826 ------------------CGCTACAATTTGTAATCCGT--TTATTCT-------
-----

FC88X_01030_00909 -------------------CGCTACAATTTGTAATCCGT--TTATTCT-------
-----
FC88X_01038_00923 -------------------CGCTACAATTTGTAATCCGT--TTATTCT-------
-----
FC88X_01087_00850 -------------------CGCTACAATTTGTAATCCGT--TTATTCT-------
-----
FC88X_01104_00795 ----------------------------------CCGT--TTACTC--------
-----
FC88X_01105_00648 --------------TCAATCTCTTGCTATATC--CCGT--TTATTCC-------
-----
FC88X_01112_00600 ---------------CAATCTAGTGCTCGATCG-TTAT--TCATG---------
-----
FC88X_01147_00979 ------------------CTTCATTCTAGCCCGTTTGA--TCATTTT-------
-----
FC88X_00963_00525 ---------------TACAACCACTTTCA-GTTTCCG---TTATTCT-------
-----
FC88X_00989_00599 -----------------TCAACACTTTCA-GTTTCCGG--TTATTCT-------
-----
FC88X_01010_00771 ----------------CCAAGTACTTTGACGCTCCCGG--TTACTC--------
-----
FC88X_00982_00523 -----------------TTACCACTTT-ACATGCGTGC--CTATTCT-------
-----
FC88X_01092_01066 -----------------TTACCA-TTT-ACATGCGTGT--CTATTCT-------
-----
FC88X_00963_00686 -----------------TTACCATTTT-ACATGCGTGC--CTATTCT-------
-----
FC88X_01024_00531 -----------------TTACCATTTT-ACATGCGTGC--CTATTCT-------
-----
FC88X_01063_00498 -----------------TTACCATTTT-ACATGCGTGC--CTATTCT-------
-----
FC88X_01060_00735 -----------------TTACCATTTT-ACATGCGTGC--CTATTCT-------
-----
FC88X_01063_00737 -----------------TTACCATTTT-ACATGCGTGC--CTATTCT-------
-----
FC88X_01088_00829 -----------------TTACCATTTT-ACATGCGTGC--CTATTCT-------
-----
FC88X_01111_00735 -----------------TTACCATTTT-ACATGCGTGC--CTATTCT-------
-----
FC88X_01139_00742 -----------------TTACCATTTT-ACATGCGTGC--CTATTCT-------
-----
FC88X_00994_00523 -----------------TTACCATTTT-ATATGCGTGC--CTATTCT-------
-----
FC88X_00992_00721 -----------------TTACCATTTT-ACACGCGTGC--CTATTCT-------
-----
FC88X_01030_00435 -----------------CTACCATTTTTACATGCGTGC--CTATTCT-------
-----
FC88X_01004_00822 -----------------TTACCATTTT-ACATGCGCGC--CTATTCT-------
-----
FC88X_00966_00746 ---------------TCGCACCATCTTTAGAT---CGC--CTTTTCTCC-----
-----
FC88X_00954_00704 -----------------CCACCTGTTTTGTGGTTGAGC--TATCTC--------
-----
FC88X_01098_00751 -----------------CCACCTGTTTTGTGGTTGAGC--TATCTC--------
-----
FC88X_01005_01116 -----------------CCACCTGTTTTGTGGTTGAGC--AATCTC--------
-----

```
FC88X_01080_00469      -----------------CCACCTGTTTTGTGGTCGAGC--TATCTC--------
-----
FC88X_01066_00854      -----------------CCACCTGTTTTGTGGT-GAGC--TATCTC--------
-----
FC88X_01006_00555      ----------------GCCACC-ATTTAGTTGATGCGT--ATTCTC--------
-----
FC88X_01113_00885      ----------------ACCCTCATTTGTGCTGTGCTGC--TCAC----------
-----
FC88X_01129_00859      -----------------CCGCCATTTGTTCATTGGTGC----------------
-----
FC88X_01145_00614      -----------------CCTCCTTTGATGATGGGGCAG--CTCCCC--------
-----
FC88X_01093_00949      -------------------ACCGTGATTTGTCAGTCCCGGGCTCTC--------
-----
FC88X_01057_01097      --------------------CCGTATTTA-------------------------
-----
FC88X_00951_00411      --------------------TGGTGCCTTATTTACCA-----------------
-----
FC88X_00975_00602      ------------------TTTCTTCCCTGATTAAAGGCGGCGTGTCC-------
-----
FC88X_01094_01131      -------------------ACCTTTCCCCATTGACTGCTACGTGTC--------
-----
FC88X_01037_00796      ----------------------TTCACGAATT----------------------
-----
FC88X_01131_00662      ----------------------TTGCCGAATTGACTCCCCGACAAAACT-----
-----
FC88X_01134_00941      -------------------------------------TCCGGGACT--------
-----
FC88X_01143_00968      --------------------CCGTCAAATTTCCCACGTCCGGTACTC-------
-----
FC88X_01029_00825      --------------------CCGCGACTTGTCTCCCGTCGGGTAACC-------
-----
FC88X_00955_00789      -----------------------TCCCGAATTTAT-CCGCGCGTCTGTCTC---
-----
FC88X_01082_00922      -----------------------TCCCGAATTTAT-CCGCGCGTCTGTCTC---
-----
FC88X_00974_00446      ----------------------CTCCCGGATATTTGTCGCGCATTTGAC-----
-----
FC88X_00983_00835      --------------------CCTGACCTTTCCCAGTGCGCGCTGTTTT------
-----
FC88X_01140_00862      --------------------CCTGACCTTTCC-AGTGCGCGCTGTTTT------
-----
FC88X_01091_00431      --------------------TCTGACTGTTTCCAAAGCA-GCTGTTCT------
-----
FC88X_01115_00611      -------------------GCTTACTTTTTCCGGTTGCCTGTTTTC--------
-----
FC88X_01131_00698      -------------------CTTCACGTTTTCCTGCGGTGCCCTGTT--------
-----
FC88X_01067_01269      ------------------CTGTCTGATTATCCAAAGGCGCCTTCTT--------
-----
FC88X_01122_00737      ---------------------CTCCTGATTAACACAGCGCTTTGTCCC------
-----
FC88X_00956_00458      ----------------------TTTGCGCATTCGGTGCACTGTTATTCT-----
-----
FC88X_01118_00992      ----------------------TTTGCGCATTCGGTGCACTGTTATTCTCC---
-----
```

FC88X_00966_00502 ----------------------TTTGCGCATTCGGTGCACTGTTATTCT-----
-----
FC88X_00952_00843 ----------------------TTTGCGCATTCGGTGCACTGTTATTCT-----
-----
FC88X_00979_00848 ----------------------TTTGCGCATTCGGTGCACTGTTATTCT-----
-----
FC88X_01011_00432 ----------------------TTTGCGCATTCGGTGCACTGTTATTCT-----
-----
FC88X_00991_00859 ----------------------TTTGCGCATTCGGTGCACTGTTATTCTT----
-----
FC88X_01075_00484 ----------------------TTTGCGCATTCGGTGCACTGTTATTCT-----
-----
FC88X_01089_00829 ----------------------TTTGCGCATTCGGTGCACTGTTATTCT-----
-----
FC88X_01067_01146 ----------------------TTTGCGCATTCGGTGCACTGTTATTCT-----
-----
FC88X_01113_00713 ----------------------TTTGCGCATTCGGTGCACTGTTATTCT-----
-----
FC88X_01126_01211 ----------------------TTTGCGCATTCGGTGCACTGTTATTCT-----
-----
FC88X_00967_00434 ----------------------TTTGCGCATTCAGTGCACTGTTATTCT-----
-----
FC88X_00982_00317 ----------------------TTTGCGCATTCGGTGCACAGTTATTCT-----
-----
FC88X_00999_00903 ----------------------TTTGCGCATTCGGTGCACTGTTATTCC-----
-----
FC88X_01122_00555 ----------------------TCTGCGCATTCGGTGCACTGTTATTCT-----
-----
FC88X_00952_00967 -----------------------TTTCTCGTTAGGCGCCCTGTGACTGTT----
-----
FC88X_01026_00422 -----------------------TTTCTCGTTAGGCGCCCTGTGACTGTT----
-----
FC88X_01109_00508 -----------------------TTTCTCGTTAGGCGCCCTGTGACTGTT----
-----
FC88X_01122_00711 -----------------------TTTCTCGTTAGGCGCCCTGTGACTGTT----
-----
FC88X_01016_01267 -----------------------TTCCTCGTTAGGCGCCCTGTGACTGTT----
-----
FC88X_01047_00379 -----------------------CCGCGAAATTTGTGCCCTCTTACTATTCC--
-----
FC88X_01048_01048 -----------------------CCGCGAAATTTGTGCCCTCTTACTATT----
-----
FC88X_00968_00497 ------------------------------ACTTGTGC----------------
-----
FC88X_01035_00783 ---------------------TCCTGCGCTA-TGCGGCCACTTGTTCTC-----
-----
FC88X_01067_00655 ---------------------TCCTGTGTTAGCGCTGTCCCTTGTTTTCC----
-----
FC88X_00953_00434 ---------------------ACCTGCTCTA--GGTGCCCCCTCTATTCT----
-----
FC88X_00991_00346 --------------------TCTTGCCCCGTCTAT---GCGACTTGTTCC----
-----
FC88X_00966_01041 ------------------------------TCTA----GCGATTTT--------
-----
FC88X_01045_01248 ------------------------------TCTA-----CGATTTCTT------
-----

FC88X_01034_00452 ------------------CGCATACGTTGGCCCGC---GTGATTTCT-------
-----
FC88X_01079_00847 -----------------------ACGCTGACCC-C---
TCGATTTCTCCATTTC-----
FC88X_00981_00332 ---------------------CCTCTGGCCGTACTGCTCCCGTTTTCT------
-----
FC88X_01036_00961 ---------------------CTCAGGACTACTCAGCCCCGACTTTCT------
-----
FC88X_00988_00567 --------------------CCGGGGCATTATT--GCACGAGTTATCTC-----
-----
FC88X_01087_00524 ---------------------CGCGACATTTTTCGGCCCCGGTTTTCT------
-----
FC88X_00964_00541 --------------------CCGTTGCATCTTTT-GCCCCCGCTTTCT------
-----
FC88X_01009_00433 ----------------------CCCTTTGATCAGGTCGACGGCTTTCTT-----
-----
FC88X_01130_00789 ----------------------CCCTTTGATCAGGTCAACGGCTTTCTC-----
-----
FC88X_01023_01049 ---------------------------------------CGGCGTTCTTG----
-----
FC88X_01025_00638 ---------------------TTCCCGTGTTCCTGACACCGACATTCT------
-----
FC88X_01027_01214 ----------------------CACGACTTTTTCGGCATCGCTATAATCTC---
-----
FC88X_01040_01207 ----------------------CACGACTTTTTCGGCATCGCTATAACC-----
-----
FC88X_01037_00502 ----------------------CACGACTTTTTCGGGATCGCTATAATCTC---
-----
FC88X_01097_00508 ----------------------CACGACTTTTTCG-------------------
-----
FC88X_00952_00426 ----------------------------
TTTTTCGCTCCCCGACAACCGACGTATT---
FC88X_01061_00484 ----------------------------
TTTTTCGCTTCCCGACAACCGACGTATT---
FC88X_01035_00951 --------------------------AGCTCTTCTCCTGCCGGCTAC--
ACGTA-----
FC88X_01037_00339 ---------------------CCAAACTTTTCCTGGTCCCCGGCTAC-------
-----
FC88X_00981_00776 --------------------CTAACAGTTCTTTTGCTCCACCGCTAT-------
-----
FC88X_01080_00609 --------------------CTCACGATTTTTCTGAGCCTCCCCGGG-------
-----
FC88X_01105_00531 --------------------CTCACGATTTTTCTGAGCCTCCCTGGG-------
-----
FC88X_00963_00459 ---------------------CCACGGGATTTATGGTCTTGGCATTCT------
-----
FC88X_01001_00951 --------------------CTACCACGGTTTTTGCTATTCGCGACC-------
-----
FC88X_01047_00611 -------------------CACAACAGCATGTTCTACTCCCATTTA--------
-----
FC88X_01078_00917 ------------------AGACAACAGCAT------------------------
-----
FC88X_01098_00804 ---------------------CAACAGTATTTTTTCACACGTGCGTCTC-----
-----
FC88X_01103_01158 ---------------------CAACAGTATTTTTT-ACACGTGCGTCTC-----
-----

```
FC88X_01109_00860      ---------------------CAACAGTATTTTTTTACACGTGCGTCTC-----
-----
FC88X_01063_00811      ---------------------CAACAGTATTTTTT-------------------
-----
FC88X_01133_00876      ----------------------ACCTGACCCTGCATCTT-CGGCATCTC-----
-----
FC88X_01142_00889      ----------------------ACCTGACCCTTCTCCGCGCATCATATC-----
-----
FC88X_01097_00820      ---------------------CCCCAGCATTTTCTGCGACTGTTATCGC-----
-----
FC88X_00969_00290      ------------------------CCTCCCGTTAGTTA----------------
-----
FC88X_00957_00915      -------------------TCTCTCCTCCGGCTACTAAATTCTGCC--------
-----
FC88X_01085_01071      ----------------CCATCTGTAGTTCGGCCTCCCATTAGT-----------
-----
FC88X_01129_00595      --------------------CTGTAGTCTATCCGCAGCCCAGTCTC--------
-----
FC88X_01132_00894      -------------------CCGATAGTCCGACGGCTCCCGTATCTC--------
-----
FC88X_01134_01101      ----------------TCCCCCACATTCCGGCCGCGACGTGCC-----------
-----
FC88X_01119_01173      ---------------TCCCCTCATATCCGGCTCGC-ACGTGCC-----------
-----
FC88X_01061_00802      --------------TCTTCCCTATATCCCA-CGGCCACGTCTG-----------
-----
FC88X_00964_00868      -----------------TCCCGATATA-CGTCGTGGGCTCTTCGC---------
-----
FC88X_01074_00913      -----------------TCCCGATATA-CGTCGTGGGCTCTTCGC---------
-----
FC88X_01087_00693      ---------------TGTACCGTTATCTCGTCTTTGCCTGTT------------
-----
FC88X_00977_00932      -------------------CCGATAACTCCCCGGCGCCTTCGCGGT--------
-----
FC88X_01003_00428      ------------------ACTGTTGT---GCCGTCGCC----------------
-----
FC88X_01144_01205      ------------TCAATGACTATTGTCC-GCCGGCGCCAC--------------
-----
FC88X_01108_00563      ------------------TCTGTCGTTCGGTCGACGCTGCCT------------
-----
FC88X_00964_00369      ----------------CTTGATGTCCTGAGGACCTTGCTTCTC-----------
-----
FC88X_01048_00408      ------------------CCTTGCCCTGGGCCCCCTGCCTGTTGT---------
-----
FC88X_00975_00397      ---------------CGCCTTTGTGGCCGGCTGCGCGTTCTT------------
-----
FC88X_00950_01008      ---------------TCTTGCCTTCTATGGCCCCGCGCTTCT------------
-----
FC88X_00997_00425      ---------------CTTTTATGCCACTGACCCCAC-GCTGTT-----------
-----
FC88X_01090_00864      ---------------CCTTTATGCCACTGACCCCAC-GCTGTT-----------
-----
FC88X_01058_00518      -----------------TTTATGTGTCAGGAGGCAC-GCTATTCT---------
-----
FC88X_00992_00621      -----------------CTTACGACCTCA-CCGCAGGGCTGTTCT---------
-----
```

FC88X_00995_00715 ------------------CTACAACCTTGGCCGCACTGCTATTAT---------
-----
FC88X_00957_00458 ----------------CCAATAGTCATGAGACCGTCGGCTGCTC----------
-----
FC88X_01052_00501 ----------------CCAATAGTCATGAGACCGCCGGCTGCTCCC--------
-----
FC88X_01015_00424 ----------------CTAAAAACATTATTCCCGTCG-CTGTTCT---------
-----
FC88X_01026_00996 ----------------CTAAAAACATTATTCCCGTCG-CTGTTCT---------
-----
FC88X_00973_00543 -----------------CTAAAACATTATTCCCGTCG-CTGTTCT---------
-----
FC88X_01117_01269 ----------------CCAAAAATTATTGACCCGGCC-GCATTCT---------
-----
FC88X_00960_00828 -------------------ACTATGCTCGGTGGCCTAT-TATTCTC--------
-----
FC88X_01047_00673 -------------------ACTATGCTCGGTGGCCTAT-TATTCTC--------
-----
FC88X_00967_00366 -------------------CCAATACACGGAAGCTTGTCTGTTCTC--------
-----
FC88X_01102_00452 ------------------TCGAACACTCGGCGACGTCACTATTCT---------
-----
FC88X_01019_00673 ------------------GATGACACGCGGCCGCATCTCTATTCTT--------
-----
FC88X_01131_01143 -------------------ATGACACGCGGCCGCATCTCTATTCTT--------
-----
FC88X_01021_00409 -------------------ATGACACGCGGCCGCATCTCTATTCTT--------
-----
FC88X_00951_00846 ------------------CCACGTCCTCGGCAGCACCGCAATATT---------
-----
FC88X_01013_00294 ------------------CCACGTCCTCGGCAGCACCGCAATATT---------
-----
FC88X_01056_00352 ------------------CCACGTCCTCGGCAGCACCGCAATATT---------
-----
FC88X_01030_00525 ------------------TTCCACGTTCGTCTGCACCTCTATTTT---------
-----
FC88X_01083_00480 -------------------TACACGTCC--------------------------
-----
FC88X_00970_00280 -----------------CTTCTAGCATTGTCAGAGCTGTCCTC-----------
-----
FC88X_01043_00411 -----------------CTTCTAGCATTGTCAGAGCTGTCCTC-----------
-----
FC88X_01118_00491 ------------------CTCTAACTTTTTAGGCCCTGACATCTC---------
-----
FC88X_00993_00477 ------------------CTCAGCTTTTGTTGCATGTGATTTCTC---------
-----
FC88X_00964_00627 ------------------CTCAGCTTTTGTTGCATGTGATTTCTC---------
-----
FC88X_01000_00761 ------------------CTCAGCTTTTGTTGCACGTGATTTCTC---------
-----
FC88X_01048_00774 -------------------TTAGCGTTTTTTGACCCCGACATTTCTC-------
-----
FC88X_00973_00563 -----------------GCTGCAAGT-TAGTCCCAGTCTACTCTC---------
-----
FC88X_00985_00726 -----------------CCTGCAACCATATTGCCGGTCACGTCTC---------
-----

```
FC88X_01052_00363       -------------------TCTGCAGATCTGA-CATGACTTTTTCTC-------
-----
FC88X_01086_00597       -------------------TCTGCAGATCTGA-CATGACTTTTTCTC-------
-----
FC88X_01113_01202       -------------------TCTGCAGATCTGA-CATGACTTTTTCTC-------
-----
FC88X_00972_00251       -------------------TTTGCCTGTCGGC-CATCTAGTTTTCTC-------
-----
FC88X_01025_00909       -----------------CTCCTGCGCGTTTGA-CGCCTCCTTTT----------
-----
FC88X_01046_00400       --------------------CTGCGTGTCCGATCGCGTCTGTATCTC-------
-----
FC88X_01025_00499       --------------------CTGCGTGTCCGATCGCGTCTGTATCTC-------
-----
FC88X_01000_00688       --------------------CTGCGTGTCCGATCGCGTCTGTATCTCCC-----
-----
FC88X_01001_00483       ----------------CACTTTACTTGTCC-ATCGCGTCTGTT-----------
-----
FC88X_01073_00712       --------------------------------TCGAGT-CTGA-----------
-----
FC88X_01092_01209       -----------------TTTCCTCCCTACTTGTCGCGTACTGAC----------
-----
FC88X_00996_00398       -------------------CTTGCAGCTCGAGCCTGTGACTGTTCT--------
-----
FC88X_00987_00849       -------------------CTTGCAGCTCGAGCCTGTGACTGTTCT--------
-----
FC88X_00967_00923       -------------------CTTGCAGCTCGAGCCTGTGACTGTTCT--------
-----
FC88X_00981_00916       -------------------CTTGCAGCTCGAGCCTGTGACTGTTCT--------
-----
FC88X_01002_01017       -------------------CTTGCAGCTCGAGCCTGTGACTGTTCT--------
-----
FC88X_01087_00494       -------------------CTTGCAGCTCGAGCCTGTGACTGTTCT--------
-----
FC88X_01080_00574       -------------------CTTGCAGCTCGAGCCTGTGACTGTTCT--------
-----
FC88X_01060_00704       -------------------CTTGCAGCTCGAGCCTGTGACTGTTCT--------
-----
FC88X_01142_00972       -------------------CTTGCAGCTCGAGCCTGTGACTGTTCT--------
-----
FC88X_01031_00941       -------------------CTTGCAGCTCGAGCCTGTTACG-------------
-----
FC88X_00975_00548       -------------------CTACCGGTTTGGGCCTGTG-CCGTTCT--------
-----
FC88X_00951_01256       ---------------------ATCTAGCC-CGACTGGCCCGTCTGTTCT-----
-----
FC88X_01015_00341       -----------------------CTAGTCGCAACTGGACC--------------
-----
FC88X_01039_01266       -----------------------CTAGTC--GACTCGACATCTGTGGCTTCA--
-----
FC88X_01126_01204       -----------------------CTAGTC--GACTCGACCAT------------
-----
FC88X_01009_00765       ------------------------CCGTCTAATCCAGACCGCTTGTCCCGT---
-----
FC88X_00997_00319       -----------------TCGAGCGTTTTTTCTCCCACGGCGTATA---------
-----
```

```
FC88X_00952_01111      -----------------TAGAGCGTTTTT-CTCCCACGACGTATA---------
-----
FC88X_00965_00964      ----------------------CTTTTTCCTGCCTGGATTGCGTATTCT-----
-----
FC88X_01066_00980      ----------------------CTTTTTCCTGCCTGGACTGCGTATTCT-----
-----
FC88X_00992_00749      ----------------------CTTTCCCCCACCGAGATCG-GTATTATT----
-----
FC88X_01019_00504      ------------------------CTTTCCTCC-CGGTGA--------------
-----
FC88X_01106_01257      --------------------CCACTTTTCCTCCACAGTGACTTATTC-------
-----
FC88X_01096_00733      ------------------------CTTTCCTACA--------------------
-----
FC88X_01140_00931      ----------------------GCCTTTCGTACGGGCTTCGACTATTCT-----
-----
FC88X_00954_00483      -----------------CTCGGAAACCTCCCAC-CGGGCATTGT----------
-----
FC88X_01092_00414      ----------------GCTCGGAAACCTCCCAC-CGGGCATTGT----------
-----
FC88X_00967_01042      -----------------CTCGGAAACCTCCCAC-CGGGCATTGT----------
-----
FC88X_01024_00308      -----------------CTCGGAAACCTCCCAC-CGGGCATTGTCC--------
-----
FC88X_01090_00802      ----------------CTATCGTAAACTCTGAC-CCAGCATTCT----------
-----
FC88X_01094_01122      -----------------CTAGTTCTCCTCCGACACAGGCATCTG----------
-----
FC88X_01130_00674      ----------------TCTCGTCTTC-TCTCACCCAGTGTGTG-----------
-----
FC88X_01138_01207      ----------------TCTCGTCTTCCTCTCA----------------------
-----
FC88X_01049_00415      --------------TCTGTCGTTTTACTCCCACAGCACACT-------------
-----
FC88X_01012_00637      -----------------CTCCTTTGTCTCCCACATCGCGATTTC----------
-----
FC88X_01023_00824      -------------------CCTCTTTTCCTCCGATCGCGATTTCT---------
-----
FC88X_01084_00405      -----------------TCCACGACTATACCAAATCCCGATTGCTCC-------
-----
FC88X_01133_00676      ------------------ACACGCCTTTACC---TCCCGCGTTCTCCT------
-----
FC88X_00978_00729      -------------------------CTTCCGACCCGGTTATCGCAAATTGTT--
-----
FC88X_01108_00504      -------------------------CTTCCGACCCGGTTATCGCAAATTGTT--
-----
FC88X_00956_00491      -------------------------CTTCCGACCCGGTTATCGCAAATTGTC--
-----
FC88X_01070_00589      ------------------------CCGTTCGCCCCTTTGACTGCAGATTATT--
-----
FC88X_01107_01239      ---------------------------------------ACTGCCGATT-----
-----
FC88X_00962_00814      -----------------CAGCAGTTTATCCCAGCTCGTCGCGTCC---------
-----
FC88X_01066_00756      -------------------ATATTTCATCCCT-TTCGCCGCGTTATA-------
-----
```

```
FC88X_00999_00313      -----------------CAACGTTTGCTTCCAGATCCCCGTGTA----------
-----
FC88X_01036_00787      -----------CCACCAACCGGACGCCTCCCAGATATA----------------
-----
FC88X_01113_00698      ---------------------------TCCCAGATC------------------
-----
FC88X_00970_00419      -------------------CTTACGAATTCCCAACGTCCCGCGCGC--------
-----
FC88X_00997_00415      -------------------CTCCCGAATCGCCTACTCCCAGCTAGC--------
-----
FC88X_01035_00526      -----------------------CCAATCCCTATGTCGAT--------------
-----
FC88X_01016_00671      -------------------TCTCCGAATGCCCCATTCGATGGTTAT--------
-----
FC88X_01036_00402      -------------------TTAACTAATCTCCCATTGGAAGCCAGT--------
-----
FC88X_01079_00694      -----------------------TTACTCCCT--GATTGCATTTAACCA-----
-----
FC88X_01078_00826      ------------------CGCGATTGCTCCCATGGCTTGCATGTT---------
-----
FC88X_01030_00675      -------------------GCCTCCATTGCACTGGAACGCCTGTTCTG------
-----
FC88X_00981_00272      ------------------ATCACGA-CCCTTCTACCAC-GATTGAG--------
-----
FC88X_00979_00887      ------------------ATCACGA-CCCTTCTACCAC-GATTGAG--------
-----
FC88X_00960_00971      ------------------ATCACGA-CCCTTCTACCAC-GATTGAG--------
-----
FC88X_00992_01031      ------------------ATCACGA-CCCTTCTACCAC-GATTGAG--------
-----
FC88X_01044_00498      ------------------ATCACGA-CCCTTCTACCAC-GATTGAG--------
-----
FC88X_01041_00795      ------------------ATCACGA-CCCTTCTACCAC-GATTGAG--------
-----
FC88X_01075_00411      ------------------ATCACGA-CCCTTCTACCAC-GATTGAG--------
-----
FC88X_01088_00494      ------------------ATCACGA-CCCTTCTACCAC-GATTGAG--------
-----
FC88X_01105_00835      ------------------ATCACGA-CCCTTCTACCAC-GATTGAG--------
-----
FC88X_01050_00538      ------------------ATCACGA-CCCTTCTACCAT-GATTGAG--------
-----
FC88X_00971_00284      ------------------ATCACGA-CCCTCCTACCACCGATTGAG--------
-----
FC88X_00965_01049      -----------------AGTCACGA-CC-TTCTACCAC-GACTGAGC-------
-----
FC88X_00985_00356      ------------------ATCACGAACCCTTCT---------------------
-----
FC88X_01077_00611      ------------------ATCAGAA-CCTTTCTGCATCCCACTATT--------
-----
FC88X_01099_01188      ------------------ATCAGAA-CCTTTCTGCATCCCACTATT--------
-----
FC88X_01113_00883      ------------------ATCAGAA-CCTTTCTGCATCCCACTATT--------
-----
FC88X_01126_00619      ------------------ATCAGAA-CCTTTCTGCATCCCACTATTCC------
-----
```

```
FC88X_00961_00968      ------------------ATCAGAA-CCTTTCTGTATCCCACTATT--------
-----
FC88X_00957_01146      ----------------------CGACATTTTCTGACCCATTCTACTATT-----
-----
FC88X_01066_01156      -------------------TTACGGCACATTCTG---CAGTCTCCTGTT-----
-----
FC88X_00992_00866      ----------------TCTCCCCGTTAGCCTCCGCGCGATGTG-----------
-----
FC88X_00969_00925      ----------------TCTCCCCGTTAGCCTCCGCGCGATGTG-----------
-----
FC88X_00996_01030      ----------------TCTCCCCGTTAGCCTCCGCGCGATGTG-----------
-----
FC88X_01042_00795      ----------------TCTCCCCGTTAGCCTCCGCGCGATGTG-----------
-----
FC88X_01060_00369      ----------------TCTCCCCGTTAGCCTCCGCGCGATGTG-----------
-----
FC88X_01133_00805      ----------------TCTCCCCGTTAGCCTCCGCGCGATGTG-----------
-----
FC88X_00989_00695      ----------------CCTCCCCGTTAGCCTCCGCGCGATGTG-----------
-----
FC88X_01115_00535      ----------------TCTCCCCGTTAGCCTCC---------------------
-----
FC88X_00992_00842      -------------------CTGCAAAACCTCCCGCGCTAGCATAAC--------
-----
FC88X_00950_00399      --------------------CTTGGGTATATCCTCACCGTTTGCATT-------
-----
FC88X_00999_00389      --------------------AGCAAACCATACCGCCACATTT------------
-----
FC88X_01120_00904      ----------------------CAAAACGTTCTTCCCCATTTGCCTACT-----
-----
FC88X_00985_00347      -------------------CTATCGTCACTT-GACATGGTGC-TTCTC------
-----
FC88X_00970_00804      -------------------CTATCGTCACTT-GACATGGTGC-TTCTC------
-----
FC88X_01020_00785      -------------------CTATCGTCACTT-GACATGGTGC-TTCTC------
-----
FC88X_01095_00538      -------------------CTATCGTCACTT-GACATGGTGC-TTCTC------
-----
FC88X_01083_01181      -------------------CTATCGTCACTT-GACATGGTGC-TTCTC------
-----
FC88X_01139_00694      -------------------CTATCGTCACTT-GACATGGTGC-TTCTC------
-----
FC88X_00950_00540      -------------------CTATCGTCACTT-GACATGGTGCATTCTC------
-----
FC88X_01123_00948      -------------------CTACCGTCACTT-GACATGGTCC-TTCTC------
-----
FC88X_01061_00937      -------------------CTATCGTCACTTTGACA------------------
-----
FC88X_01012_00483      -------------------CTACGGTCCCTC-TATCTGGCACGTTCTC------
-----
FC88X_00990_00655      ------------------TTTCTCATTCTG-CCGAACCTCTATTTCT-------
-----
FC88X_01068_00475      ------------------TTTCTCTCTGTA-CCGCTTCTGTTTACC--------
-----
FC88X_00964_00950      -------------------------CTGTG-CCGTATCGGTGTGTCT-------
-----
```

```
FC88X_01011_01017      ------------------CGTGCCTGTATA-CGGTACC-TGTTCGC--------
-----
FC88X_01127_00955      ------------------CGTGCCTGTATA-CGGTACCCTGTTCGC--------
-----
FC88X_01060_00693      ------------------CGTGCCTGTATA-CGGTACC-TGTTCGC--------
-----
FC88X_01119_00499      ------------------CGTGCCTGTATA-CGGTACC-TGTTCGC--------
-----
FC88X_01119_00510      ------------------CGTGCCTGTATA-CGGTACC-TGTTCG---------
-----
FC88X_00968_00723      -----------------ACATGCCTGC-TG-CGGTCTCTGACTATC--------
-----
FC88X_01070_00751      ----------------------CCAT-GTGCCGGTACCTGTCGCGTTCTC----
-----
FC88X_01136_01267      ---------------------CCCATAGTACCAGAACCTGGGTCGTTCT-----
-----
FC88X_01030_01223      ----------------------CCGGCGTATCTGTACCTGGTCTGTTCA-----
-----
FC88X_00972_00369      ---------------------GCCTGACTCCCGGCACTTT-TCGACCACA----
-----
FC88X_01020_00622      ----------------------------TCTTGGCTCTTTCTCCACCATT----
-----
FC88X_01027_00972      -----------------TTCAACCGTCTCTGGCGCAACCATATT----------
-----
FC88X_01069_00468      -------------------TTCCCGTCTCTGTTGCGCCACCATTGGCTTC----
-----
FC88X_01040_00448      --------------------TACCGTCT--------------------------
-----
FC88X_01064_01236      ------------------TTTTTCCCTTCACTAACT-G-CGAACTGCA------
-----
FC88X_01096_01214      ------------------TTTTTCCCTTCACTAACT-G-CGAACTGCA------
-----
FC88X_00978_00561      ------------------TTTTTCCCTTCACTAAC--G-CGAACTGCA------
-----
FC88X_00974_00295      ------------------TTTTTCCCTTCACTAACC-G-CGAACTGCA------
-----
FC88X_00985_00279      ------------------TTTTTCCCTTCACTAACC-G-CGAACTGCA------
-----
FC88X_00986_00255      ------------------TTTTTCCCTTCACTAACC-G-CGAACTGCA------
-----
FC88X_00995_00255      ------------------TTTTTCCCTTCACTAACC-G-CGAACTGCA------
-----
FC88X_00998_00260      ------------------TTTTTCCCTTCACTAACC-G-CGAACTGCA------
-----
FC88X_00978_00331      ------------------TTTTTCCCTTCACTAACC-G-CGAACTGCA------
-----
FC88X_00978_00488      ------------------TTTTTCCCTTCACTAACC-G-CGAACTGCA------
-----
FC88X_00979_00557      ------------------TTTTTCCCTTCACTAACC-G-CGAACTGCA------
-----
FC88X_00956_00347      -------------------TTTTCCCTTCACTAACC-G-CGAACTGCA------
-----
FC88X_01120_00784      -------------------TTTTCCCTTCACTAACC-G-CGAACTGCACC----
-----
FC88X_00986_00488      -------------------TTTTCCCTTCACTAACC-G-CGAACTGCA------
-----
```

FC88X_00974_00777 -------------------TTTTCCCTTCACTAACC-G-CGAACTGCA------
-----
FC88X_00951_00919 -------------------TTTTCCCTTCACTAACC-G-CGAACTGCA------
-----
FC88X_00999_01132 -------------------TTTTCCCTTCACTAACC-G-CGAACTGCA------
-----
FC88X_00952_00493 -------------------TTTTCCCTTCACTAACC-G-CGAACTGCA------
-----
FC88X_01105_01127 ------------------CTTTTCCCTTCACTAACC-G-CGAACTGCA------
-----
FC88X_00990_00708 -------------------TTTTCCCTTCACTAACC-G-CGAACTGCA------
-----
FC88X_00992_00878 -------------------TTTTCCCTTCACTAACC-G-CGAACTGCA------
-----
FC88X_00997_00994 -------------------TTTTCCCTTCACTAACC-G-CGAACTGCA------
-----
FC88X_01006_00347 -------------------TTTTCCCTTCACTAACC-G-CGAACTGCA------
-----
FC88X_01025_00429 -------------------TTTTCCCTTCACTAACC-G-CGAACTGCA------
-----
FC88X_01014_00528 -------------------TTTTCCCTTCACTAACC-G-CGAACTGCA------
-----
FC88X_00953_00867 -------------------TTTTCCCTTCACTAACC-G-CGAACTGCA------
-----
FC88X_01041_00800 -------------------TTTTCCCTTCACTAACC-G-CGAACTGCACTT---
-----
FC88X_00957_00981 -------------------TTTTCCCTTCACTAACC-G-CGAACTGCA------
-----
FC88X_01000_00321 -------------------TTTTCCCTTCACTAACC-G-CGAACTGCA------
-----
FC88X_01009_00416 -------------------TTTTCCCTTCACTAACC-G-CGAACTGCA------
-----
FC88X_01006_00515 -------------------TTTTCCCTTCACTAACC-G-CGAACTGCA------
-----
FC88X_01005_00559 -------------------TTTTCCCTTCACTAACC-G-CGAACTGCA------
-----
FC88X_01033_00695 -------------------TTTTCCCTTCACTAACC-G-CGAACTGCA------
-----
FC88X_01021_00847 -------------------TTTTCCCTTCACTAACC-G-CGAACTGCA------
-----
FC88X_01040_00812 -------------------TTTTCCCTTCACTAACC-G-CGAACTGCA------
-----
FC88X_01018_01031 -------------------TTTTCCCTTCACTAACC-G-CGAACTGCA------
-----
FC88X_01014_01143 -------------------TTTTCCCTTCACTAACC-G-CGAACTGCA------
-----
FC88X_01013_01204 -------------------TTTTCCCTTCACTAACC-G-CGAACTGCA------
-----
FC88X_01040_01252 -------------------TTTTCCCTTCACTAACC-G-CGAACTGCA------
-----
FC88X_01092_00533 -------------------TTTTCCCTTCACTAACC-G-CGAACTGCA------
-----
FC88X_01078_00716 -------------------TTTTCCCTTCACTAACC-G-CGAACTGCA------
-----
FC88X_01058_00786 -------------------TTTTCCCTTCACTAACC-G-CGAACTGCA------
-----

```
FC88X_01081_00775       -------------------TTTTCCCTTCACTAACC-G-CGAACTGCA------
-----
FC88X_01087_00793       -------------------TTTTCCCTTCACTAACC-G-CGAACTGCA------
-----
FC88X_01099_00843       -------------------TTTTCCCTTCACTAACC-G-CGAACTGCA------
-----
FC88X_01085_00906       -------------------TTTTCCCTTCACTAACC-G-CGAACTGCA------
-----
FC88X_01083_00979       -------------------TTTTCCCTTCACTAACC-G-CGAACTGCA------
-----
FC88X_01057_01232       -------------------TTTTCCCTTCACTAACC-G-CGAACTGCA------
-----
FC88X_01110_00483       -------------------TTTTCCCTTCACTAACC-G-CGAACTGCA------
-----
FC88X_01117_00523       -------------------TTTTCCCTTCACTAACC-G-CGAACTGCA------
-----
FC88X_01123_00510       -------------------TTTTCCCTTCACTAACC-G-CGAACTGCA------
-----
FC88X_01135_00684       -------------------TTTTCCCTTCACTAACC-G-CGAACTGCA------
-----
FC88X_01132_00776       -------------------TTTTCCCTTCACTAACC-G-CGAACTGCA------
-----
FC88X_01102_00801       -------------------TTTTCCCTTCACTAACC-G-CGAACTGCA------
-----
FC88X_01139_00852       -------------------TTTTCCCTTCACTAACC-G-CGAACTGCA------
-----
FC88X_01131_00951       -------------------TTTTCCCTTCACTAACC-G-CGAACTGCA------
-----
FC88X_01130_01174       -------------------TTTTCCCTTCACTAACC-G-CGAACTGCA------
-----
FC88X_01134_01171       -------------------TTTTCCCTTCACTAACC-G-CGAACTGCA------
-----
FC88X_00964_00302       ------------------TTTTTCCCTTCACTAACC-G-CGAACTGCA------
-----
FC88X_00997_00376       ------------------TTTTTCCCTTCACTAACC-G-CGAACTGCA------
-----
FC88X_00958_00514       ------------------TTTTTCCCTTCACTAACC-G-CGAACTGCA------
-----
FC88X_00986_00623       ------------------TTTTTCCCTTCACTAACC-G-CGAACTGCA------
-----
FC88X_00981_00718       ------------------TTTTTCCCTTCACTAACC-G-CGAACTGCA------
-----
FC88X_00984_00709       ------------------TTTTTCCCTTCACTAACC-G-CGAACTGCA------
-----
FC88X_00965_00799       ------------------TTTTTCCCTTCACTAACC-G-CGAACTGCA------
-----
FC88X_00984_00883       ------------------TTTTTCCCTTCACTAACC-G-CGAACTGCA------
-----
FC88X_00960_00939       ------------------TTTTTCCCTTCACTAACC-G-CGAACTGCA------
-----
FC88X_00974_00931       ------------------TTTTTCCCTTCACTAACC-G-CGAACTGCA------
-----
FC88X_00979_00928       ------------------TTTTTCCCTTCACTAACC-G-CGAACTGCA------
-----
FC88X_00968_00957       ------------------TTTTTCCCTTCACTAACC-G-CGAACTGCA------
-----
```

FC88X_00972_00995 ------------------TTTTTCCCTTCACTAACC-G-CGAACTGCA------
-----
FC88X_00993_00985 ------------------TTTTTCCCTTCACTAACC-G-CGAACTGCA------
-----
FC88X_00998_01047 ------------------TTTTTCCCTTCACTAACC-G-CGAACTGCA------
-----
FC88X_00983_01221 ------------------TTTTTCCCTTCACTAACC-G-CGAACTGCA------
-----
FC88X_00980_01267 ------------------TTTTTCCCTTCACTAACC-G-CGAACTGCA------
-----
FC88X_01013_00291 ------------------TTTTTCCCTTCACTAACC-G-CGAACTGCA------
-----
FC88X_01041_00322 ------------------TTTTTCCCTTCACTAACC-G-CGAACTGCA------
-----
FC88X_01015_00387 ------------------TTTTTCCCTTCACTAACC-G-CGAACTGCA------
-----
FC88X_01025_00351 ------------------TTTTTCCCTTCACTAACC-G-CGAACTGCA------
-----
FC88X_01047_00383 ------------------TTTTTCCCTTCACTAACC-G-CGAACTGCA------
-----
FC88X_01015_00406 ------------------TTTTTCCCTTCACTAACC-G-CGAACTGCA------
-----
FC88X_01024_00407 ------------------TTTTTCCCTTCACTAACC-G-CGAACTGCA------
-----
FC88X_01035_00433 ------------------TTTTTCCCTTCACTAACC-G-CGAACTGCA------
-----
FC88X_01047_00437 ------------------TTTTTCCCTTCACTAACC-G-CGAACTGCA------
-----
FC88X_01022_00478 ------------------TTTTTCCCTTCACTAACC-G-CGAACTGCA------
-----
FC88X_01026_00494 ------------------TTTTTCCCTTCACTAACC-G-CGAACTGCA------
-----
FC88X_01008_00530 ------------------TTTTTCCCTTCACTAACC-G-CGAACTGCA------
-----
FC88X_01019_00529 ------------------TTTTTCCCTTCACTAACC-G-CGAACTGCA------
-----
FC88X_01034_00614 ------------------TTTTTCCCTTCACTAACC-G-CGAACTGCA------
-----
FC88X_01027_00650 ------------------TTTTTCCCTTCACTAACC-G-CGAACTGCA------
-----
FC88X_01002_00799 ------------------TTTTTCCCTTCACTAACC-G-CGAACTGCA------
-----
FC88X_01015_00758 ------------------TTTTTCCCTTCACTAACC-G-CGAACTGCA------
-----
FC88X_01025_00765 ------------------TTTTTCCCTTCACTAACC-G-CGAACTGCA------
-----
FC88X_01043_00777 ------------------TTTTTCCCTTCACTAACC-G-CGAACTGCA------
-----
FC88X_01001_00832 ------------------TTTTTCCCTTCACTAACC-G-CGAACTGCA------
-----
FC88X_01007_00811 ------------------TTTTTCCCTTCACTAACC-G-CGAACTGCA------
-----
FC88X_01027_00842 ------------------TTTTTCCCTTCACTAACC-G-CGAACTGCA------
-----
FC88X_01044_00815 ------------------TTTTTCCCTTCACTAACC-G-CGAACTGCA------
-----

FC88X_01046_00810 ------------------TTTTTCCCTTCACTAACC-G-CGAACTGCA------
-----
FC88X_01005_00891 ------------------TTTTTCCCTTCACTAACC-G-CGAACTGCA------
-----
FC88X_01031_00851 ------------------TTTTTCCCTTCACTAACC-G-CGAACTGCA------
-----
FC88X_01000_00905 ------------------TTTTTCCCTTCACTAACC-G-CGAACTGCA------
-----
FC88X_01003_00939 ------------------TTTTTCCCTTCACTAACC-G-CGAACTGCA------
-----
FC88X_01005_00928 ------------------TTTTTCCCTTCACTAACC-G-CGAACTGCA------
-----
FC88X_01043_00923 ------------------TTTTTCCCTTCACTAACC-G-CGAACTGCA------
-----
FC88X_01035_01040 ------------------TTTTTCCCTTCACTAACC-G-CGAACTGCA------
-----
FC88X_01042_01038 ------------------TTTTTCCCTTCACTAACC-G-CGAACTGCA------
-----
FC88X_01007_01124 ------------------TTTTTCCCTTCACTAACC-G-CGAACTGCA------
-----
FC88X_01021_01119 ------------------TTTTTCCCTTCACTAACC-G-CGAACTGCA------
-----
FC88X_01000_01178 ------------------TTTTTCCCTTCACTAACC-G-CGAACTGCA------
-----
FC88X_01007_01229 ------------------TTTTTCCCTTCACTAACC-G-CGAACTGCA------
-----
FC88X_01017_01233 ------------------TTTTTCCCTTCACTAACC-G-CGAACTGCA------
-----
FC88X_01038_01214 ------------------TTTTTCCCTTCACTAACC-G-CGAACTGCA------
-----
FC88X_01049_01200 ------------------TTTTTCCCTTCACTAACC-G-CGAACTGCA------
-----
FC88X_01019_01261 ------------------TTTTTCCCTTCACTAACC-G-CGAACTGCA------
-----
FC88X_01050_00388 ------------------TTTTTCCCTTCACTAACC-G-CGAACTGCA------
-----
FC88X_01060_00490 ------------------TTTTTCCCTTCACTAACC-G-CGAACTGCA------
-----
FC88X_01072_00472 ------------------TTTTTCCCTTCACTAACC-G-CGAACTGCA------
-----
FC88X_01091_00548 ------------------TTTTTCCCTTCACTAACC-G-CGAACTGCA------
-----
FC88X_01080_00568 ------------------TTTTTCCCTTCACTAACC-G-CGAACTGCA------
-----
FC88X_01087_00596 ------------------TTTTTCCCTTCACTAACC-G-CGAACTGCA------
-----
FC88X_01094_00583 ------------------TTTTTCCCTTCACTAACC-G-CGAACTGCA------
-----
FC88X_01058_00607 ------------------TTTTTCCCTTCACTAACC-G-CGAACTGCA------
-----
FC88X_01080_00627 ------------------TTTTTCCCTTCACTAACC-G-CGAACTGCA------
-----
FC88X_01097_00717 ------------------TTTTTCCCTTCACTAACC-G-CGAACTGCA------
-----
FC88X_01099_00772 ------------------TTTTTCCCTTCACTAACC-G-CGAACTGCA------
-----

FC88X_01092_00802 ------------------TTTTTCCCTTCACTAACC-G-CGAACTGCA------
-----
FC88X_01059_00893 ------------------TTTTTCCCTTCACTAACC-G-CGAACTGCA------
-----
FC88X_01072_00972 ------------------TTTTTCCCTTCACTAACC-G-CGAACTGCA------
-----
FC88X_01076_00984 ------------------TTTTTCCCTTCACTAACC-G-CGAACTGCA------
-----
FC88X_01087_00994 ------------------TTTTTCCCTTCACTAACC-G-CGAACTGCA------
-----
FC88X_01089_00961 ------------------TTTTTCCCTTCACTAACC-G-CGAACTGCA------
-----
FC88X_01086_01239 ------------------TTTTTCCCTTCACTAACC-G-CGAACTGCA------
-----
FC88X_01104_00487 ------------------TTTTTCCCTTCACTAACC-G-CGAACTGCA------
-----
FC88X_01100_00510 ------------------TTTTTCCCTTCACTAACC-G-CGAACTGCA------
-----
FC88X_01110_00541 ------------------TTTTTCCCTTCACTAACC-G-CGAACTGCA------
-----
FC88X_01111_00532 ------------------TTTTTCCCTTCACTAACC-G-CGAACTGCA------
-----
FC88X_01131_00527 ------------------TTTTTCCCTTCACTAACC-G-CGAACTGCA------
-----
FC88X_01126_00579 ------------------TTTTTCCCTTCACTAACC-G-CGAACTGCA------
-----
FC88X_01106_00617 ------------------TTTTTCCCTTCACTAACC-G-CGAACTGCA------
-----
FC88X_01127_00614 ------------------TTTTTCCCTTCACTAACC-G-CGAACTGCA------
-----
FC88X_01100_00686 ------------------TTTTTCCCTTCACTAACC-G-CGAACTGCA------
-----
FC88X_01141_00674 ------------------TTTTTCCCTTCACTAACC-G-CGAACTGCA------
-----
FC88X_01106_00709 ------------------TTTTTCCCTTCACTAACC-G-CGAACTGCA------
-----
FC88X_01138_00824 ------------------TTTTTCCCTTCACTAACC-G-CGAACTGCA------
-----
FC88X_01107_00889 ------------------TTTTTCCCTTCACTAACC-G-CGAACTGCA------
-----
FC88X_01112_00867 ------------------TTTTTCCCTTCACTAACC-G-CGAACTGCA------
-----
FC88X_01115_00887 ------------------TTTTTCCCTTCACTAACC-G-CGAACTGCA------
-----
FC88X_01130_00862 ------------------TTTTTCCCTTCACTAACC-G-CGAACTGCA------
-----
FC88X_01135_00884 ------------------TTTTTCCCTTCACTAACC-G-CGAACTGCA------
-----
FC88X_01109_00906 ------------------TTTTTCCCTTCACTAACC-G-CGAACTGCA------
-----
FC88X_01138_00916 ------------------TTTTTCCCTTCACTAACC-G-CGAACTGCA------
-----
FC88X_01100_00992 ------------------TTTTTCCCTTCACTAACC-G-CGAACTGCA------
-----
FC88X_01120_00954 ------------------TTTTTCCCTTCACTAACC-G-CGAACTGCA------
-----

FC88X_01124_01119   ------------------TTTTTCCCTTCACTAACC-G-CGAACTGCA------
-----
FC88X_01145_01124   ------------------TTTTTCCCTTCACTAACC-G-CGAACTGCA------
-----
FC88X_01135_01156   ------------------TTTTTCCCTTCACTAACC-G-CGAACTGCA------
-----
FC88X_01102_01233   ------------------TTTTTCCCTTCACTAACC-G-CGAACTGCA------
-----
FC88X_00952_00350   ------------------TTTTTCCCTTCACTAACC-G-CGAACTGCAAT----
-----
FC88X_00997_00316   -------------------TTTTCCCTTCATTAACC-G-CGAACTGCA------
-----
FC88X_01116_00737   ------------------TTTTTCCCTTCACTAACC-G-CGAACTGCAC-----
-----
FC88X_01074_00642   -------------------TTCTCCCTTCACTAACC-G-CGAACTGCA------
-----
FC88X_00951_00430   -----------------TTTTTTCCCTTCACTAACC-G-CGAACTGCA------
-----
FC88X_00954_00921   -----------------TTTTTTCCCTTCACTAACC-G-CGAA-TGCA------
-----
FC88X_00965_00512   -----------------TTTTTTCCCTTCACTAACC-G-CGAACTGCA------
-----
FC88X_00977_00601   -----------------TTTTTTCCCTTCACTAACC-G-CGAACTGCA------
-----
FC88X_00998_00825   -----------------TTTTTTCCCTTCACTAACC-G-CGAACTGCA------
-----
FC88X_01078_00379   -----------------TTTTTTCCCTTCACTAACC-G-CGAACTGCA------
-----
FC88X_01094_00450   -----------------TTTTTTCCCTTCACTAACC-G-CGAACTGCA------
-----
FC88X_01050_00634   -----------------TTTTTTCCCTTCACTAACC-G-CGAACTGCA------
-----
FC88X_01092_00784   -----------------TTTTTTCCCTTCACTAACC-G-CGAACTGCA------
-----
FC88X_01080_00842   -----------------TTTTTTCCCTTCACTAACC-G-CGAACTGCA------
-----
FC88X_01065_00980   -----------------TTTTTTCCCTTCACTAACC-G-CGAACTGCA------
-----
FC88X_01118_00905   -----------------TTTTTTCCCTTCACTAACC-G-CGAACTGCA------
-----
FC88X_01111_01010   -----------------TTTTTTCCCTTCACTAACC-G-CGAACTGCA------
-----
FC88X_01137_01013   -----------------TTTTTTCCCTTCACTAACC-G-CGAACTGCA------
-----
FC88X_01125_00820   ----------------TTTTTTTCCCTTCACTAACC-G-CGAACTGCA------
-----
FC88X_01066_00422   ------------------TTTTTCCCTTCACTGACC-G-CGAACTGCA------
-----
FC88X_01105_00485   ----------------GTTTTTTCCCTTCACTAACC-G-CGAACTGCA------
-----
FC88X_01011_00947   ------------------TTTTTCCCTTCACTAGCC-G-CGAACTGCA------
-----
FC88X_01090_00919   ------------------TTTTTCCCTCCACTAGCC-G-CGAACTGCA------
-----
FC88X_00992_00642   ------------------TTTTTCCCTACACTAACC-G-CGAACTGCA------
-----

FC88X_00989_00968 ------------------TTTTTCCCTTCACTAACC-G-CGAACCGCA------
-----
FC88X_01086_00946 -------------------TTTTCCCTTCACTAACC-G-CGAACAGCA------
-----
FC88X_01007_01208 ------------------TTTTTCCCTTCACTAACC-G-CGGACTGCA------
-----
FC88X_01005_00694 ------------------TTCTTCCCTTCACTAACC-G-CGAACTGCA------
-----
FC88X_01063_00801 ------------------TTCTTCCCTTCACTAACC-G-CGAACTGCA------
-----
FC88X_01025_00533 ------------------TTTTTCCCCTCACTAACC-G-CGAACTGCA------
-----
FC88X_01069_00450 ------------------TTTTTCCCCTCACTAACC-G-CGAACTGCA------
-----
FC88X_01119_00628 ------------------TTTTTCCCCTCACTAACC-G-CGAACTGCA------
-----
FC88X_00994_00715 -------------------TTTTCCCCTCACTAACC-G-CGAACTGCA------
-----
FC88X_01023_00929 -------------------TTTTCCCCTCACTAACC-G-CGAACTGCA------
-----
FC88X_01030_01138 -------------------TTTTCCCCTCACTAACC-G-CGAACTGCA------
-----
FC88X_01056_01255 -----------------TTTTTCCCCTTCACTAACC-G-CGAACTGCA------
-----
FC88X_01020_01211 ------------------TTTTCCCCTTCACTAACC-G-CGAACTGCA------
-----
FC88X_01124_00594 -------------------TTTCCCCTTCACTAACC-G-CGAACTGCA------
-----
FC88X_01092_00438 ------------------TTTTCCCCTTCACTAACC-G-CGAACTGCA------
-----
FC88X_01140_00698 ------------------TTTTCCCCTTCACTAACC-G-CGAACTGCA------
-----
FC88X_01142_01188 ----------------TTTTTTCCCCTTCACTAACC-G-CGAACTGCA------
-----
FC88X_00994_00803 -------------------CTTTCCCTTCACTAACC-G-CGAACTGCA------
-----
FC88X_01071_00858 -------------------CTTTCCCTTCACTAACC-G-CGAACTGCA------
-----
FC88X_01014_00920 -------------------TTTTCCCTTCACTAACC-G-CGAGCTGCA------
-----
FC88X_01143_00688 -------------------TTTTCCCTTCACTAACC-GTCGAACTGCA------
-----
FC88X_01011_00584 ------------------TTTTTCCCTTCAC-GAAC-CGCGAACTGCA------
-----
FC88X_01094_01155 -------------------TTTTCCCTTCAC-GAAC-CGCGAACTGCA------
-----
FC88X_01100_00597 ------------------TTTTTCCCTTCAC-GAAC-CGCGAACTGCA------
-----
FC88X_00996_00450 -------------------TTTTCCCTTCAC-AAAC-CGCGAACTGCA------
-----
FC88X_00985_00693 -----------------TTTTTTCCCTTCACTAAAC-CGCGAACTGCA------
-----
FC88X_00964_00448 ------------------TTTTTCCCTCCACTAACC-G-CGAACTGCA------
-----
FC88X_01029_00869 -------------------TTTTCCCTCCACTAACC-G-CGAACTGCA------
-----

FC88X_01035_00937 ------------------TTTTTCCCTTCACTAACC-A-CGAACCGCA------
-----
FC88X_01092_00462 ------------------TTTTTCCCTTCACTAACC-G-CGAACTCGC------
-----
FC88X_01114_00651 ------------------TTTTTCCCTTCACTAACC-G-CGAACGCA-------
-----
FC88X_01076_00607 ------------------TTTTTCCCTTCACTAACG-C-CG-------------
-----
FC88X_01114_00545 -------------------TCTTCCCTTCACTAAC-------------------
-----
FC88X_01113_00787 ------------------TTTTTCCCGTCACTAGC-------------------
-----
FC88X_01075_00657 -------------------TTTCCTTCCTACTAACC--GCGAACTGCA------
-----
FC88X_01139_01030 -------------------TTTCCTCCCTACTAACC--GCGAACTGCA------
-----
FC88X_01088_00446 -------------------TTTTCCTCCTACTAACC--GCGAACTGCA------
-----
FC88X_01018_00988 --------------------TTTTCCTTCACTAAC-------------------
-----
FC88X_01068_01270 -------------------TTTTCCCTACACTAATCCGGCGAACTGC-------
-----
FC88X_01051_00470 -----------TCCCCCCATTTTTCCCACACTCCTTGT----------------
-----
FC88X_01094_00592 -------------------TTTCCCTTACACT----------------------
-----
FC88X_01041_00422 -----------CCCAAA--TTTTCCCGTCACCACTTTACT--------------
-----
FC88X_01114_01191 ----------CCCCAAAATTTTTCCCGTCAC-----------------------
-----
FC88X_00961_00312 -----------------CGTTGA-ACTTTCCTGTCCCCACGTATT---------
-----
FC88X_01098_00941 -------------------TTGACACCTAGCTCCCCTCACGTTTTAC-------
-----
FC88X_00999_00593 --------------------CTTCACTTTTCCCT---CATTTTGCCTGCT----
-----
FC88X_01117_01105 ----------------------TTACTTTTCCCTTCGCATCTGGCATTCT----
-----
FC88X_01032_00836 -----------------------TACTTTAC-----------------------
-----
FC88X_00996_00342 ------ACGTATTTACTGTGTTCAGTTTTACCTATTC-----------------
-----
FC88X_01070_00779 ------ACGTATTTACTGTGTTCAGTTTTACCTATTC-----------------
-----
FC88X_00969_00442 -------------------------TCTTACCTAA-------------------
-----
FC88X_01055_01252 ----------TTCGCCTTTATGCCGTGTGACCCATT------------------
-----
FC88X_01029_00323 ----------------TGCGTGCGTTTTCCCCCAGCCGTGTGTCCC--------
-----
FC88X_01006_01034 ----------------TGCGTGCGTTTTTCCCCAGCCGTGTGTC----------
-----
FC88X_01074_00689 ------------------------TTTTCCCCT---------------------
-----
FC88X_00981_00904 --------------------TCTCTTTCTTCCCCACTGTGTTGTGTG-------
-----

```
FC88X_00975_00545      --------------TCAACTTACA-TCACGCTTGCCTGTTTTCCC---------
-----
FC88X_01050_00727      -----------------CCTTACTCTCCTGCATGCCCCATTTTCCC--------
-----
FC88X_00972_00892      ---------------CTCCTTACTCCTTCATGCCTATGGCAAC-----------
-----
FC88X_01109_00574      --------------TCTCTTTACCTTCTGCCATGGCTTGTT-------------
-----
FC88X_00993_00481      -----------CTAATTCCTCGCTGTCTTTCACGTTTC----------------
-----
FC88X_00989_00956      -----------CAACTTCTTCTGCTGCTTTCCCATGTT----------------
-----
FC88X_00981_00632      -----------ATAACCCTTCGCCAACTGTCCCGTACG----------------
-----
FC88X_00976_00639      ---------------------TTTCCTTCACCTA--------------------
-----
FC88X_01077_00708      -----------------CTCCTTTCCCTCCGCTACTGCATTCCT----------
-----
FC88X_01046_00526      --------------CCTATGCCTCCTTCTCCTCACGGTACT-------------
-----
FC88X_01010_00811      -------------CGCGATGCCTCCCTTCCTTCCCGACACA-------------
-----
FC88X_01024_00926      ----------------TTAACCTCCCTTTGCCAGCGTG-CTATC----------
-----
FC88X_01052_00871      ----------------TTAACCTCCCTTTGCCAGCGTG-CTATC----------
-----
FC88X_00963_00801      --------------------CCTCCTTTTCCTCGAATGTCTATCACC-------
-----
FC88X_01016_00857      ------------------------TTTACCTCA---------------------
-----
FC88X_01060_00410      ------------------CGCATGGTTAACTCAACCTTGCCTCCCCC-------
-----
FC88X_00959_00336      ----------------------ACCTGATCTGTACTCTCCGGGCGTGTA-----
-----
FC88X_00974_01202      ----------------------GCCTGATCTGTACTCCCCGGGCGTGTACC---
-----
FC88X_01008_00532      ----------------------------CTCATCGTCCTCA-------------
-----
FC88X_01002_00307      --------------------ACTCCTGATCT-TCCACGCCGGCCTAAC------
-----
FC88X_01079_00777      -----------------------CCTGACCT-TGGCCTCC--CCTAACACTG--
-----
FC88X_00996_00832      ----------------------CCTAGGCCTAT--CCGCCGCGTTGTTCT----
-----
FC88X_01144_01227      -----------------CTAATCCCTGACCTATG-TCGCCGCGTT---------
-----
FC88X_01099_00717      ------------------------------------CACCTTGTCC--------
-----
FC88X_00962_00894      ------------------CACTCCTTGCCCTGTAGCTGCCGCAGC---------
-----
FC88X_01012_00758      --------------------CTCCCTGGTCTCTA-CTCCCGCCATG--------
-----
FC88X_01096_00471      ---------------------TCAATCGTCCTCCCACGTTGCACTGTG------
-----
FC88X_01121_00671      ---------------------TCAATGATCCTCC-ACGTTGCACTTTG------
-----
```

```
FC88X_01142_00858       ----------------------TAACCTCATCTCTCCACGGCACTTATC-----
-----
FC88X_00993_00836       -----------------------CCATTCACTCCTACAATGCACTTTGCT----
-----
FC88X_01140_00859       ----------------------TTAATTCTCTGCTCC--CTGGCTTTGCTA---
-----
FC88X_01023_00687       ----------------------CTGACACTTTCCACCGCCAGCATTAC------
-----
FC88X_01059_00482       ------------------------CATACTTTCCCACG----------------
-----
FC88X_01079_00593       ----------------ATTCAATCTACACTTATCCCTGCTAAC-----------
-----
FC88X_01009_01042       ----------------------TCTATCCATTTTTCGCTCGGACCTTTT-----
-----
FC88X_01052_00512       -----------------------CCATCCTATTCTTCACCGCGCCTTTAT----
-----
FC88X_01062_00553       ---------------------TTATGCTCTATCTCGCTGCGAACCTTT------
-----
FC88X_01129_00794       ----------------------TCTGTTCT-TCTC-CCACGCACCTTGGT----
-----
FC88X_00994_00489       --------------------------------------ATACACCT--------
-----
FC88X_01129_00541       ----------------------TTCGCTGTTCCTATCAATCCACCTGTCTTC--
-----
FC88X_00961_00688       ----------------------TTGACCAATCTTCTATCTCCACCTGC------
-----
FC88X_01106_00543       ----------------------TTCTCCAATCTTCTTATTGCGCCTGTT-----
-----
FC88X_00978_00843       --------------------------------------CTCCGCCGGTC-----
-----
FC88X_01040_00860       -----------------------CCCACACTTTTGCGACTCGGCCGTTCA----
-----
FC88X_01015_00332       --------------------CTTCGTCTTATTCGCCAGTCGCTGCGTTCC----
-----
FC88X_01074_00597       --------------------TTTCTCCTTATTCGCC---TCCTACGTTCG----
-----
FC88X_00965_00874       ------------------------TACTAAACTCCCTAGCGCTTCATTTCA---
-----
FC88X_01050_00665       ------------------------TCTTATACTCCC---CGCTTCAGTTTGTT-
-----
FC88X_01026_00913       -------------------CTTTTCACTAATATCCCTGCCGCTGTT--------
-----
FC88X_01070_00931       ----------------------TTTTCTAAT-TCCCTGCCTCTGTATGCT----
-----
FC88X_01053_00576       --------------------TCTCCAATTCTCTTCCT-CCGTTGTGTG------
-----
FC88X_01101_00672       ----------------------TCCATTACTATTCCT-CCGTCTTGCTTTG---
-----
FC88X_00957_00451       -----------------------CCGTTTAATCTCCCACTGCGGCATATC----
-----
FC88X_00970_00901       -----------------------CCGTTTAATCTCCCACTGCGGCATATC----
-----
FC88X_01096_00644       -----------------------CCGTTTAATCTCCCACTGCGGCATATC----
-----
FC88X_01106_00447       -----------------------CCGTTTAATCTCCCACTGCGGCATATC----
-----
```

```
FC88X_01138_00594      -----------------------CCGTTTAATCTCCCACTGCGGCATATC----
-----
FC88X_01140_00877      -----------------------CCGTTTAATCTCCCACTGCGGCATATC----
-----
FC88X_01105_01113      -----------------------CCGTTTAATCTCC-ACTGCGGCATAT-----
-----
FC88X_01097_00447      -----------------------CCATTTAATC---------------------
-----
FC88X_01083_00645      -----------------------CCGTTCTATCTCCCGTGGCGGCGTGCCCC--
-----
FC88X_01043_00476      -----------------------CCTTTTGCTCTCCTCATGCTACA--------
-----
FC88X_01066_00408      ----------------CTCTTTACCGTTTGATCTCC-CGTGCTAT---------
-----
FC88X_01133_00540      -----------------------CCTTTATATCTCCCCTTGCGCGCTACA----
-----
FC88X_01121_01146      -----------------------CCATTCAATCTTCTCCTGCCAGCTACT----
-----
FC88X_01144_00888      -----------------------TCATGCGATCTCCTTCTCCGACCTATG----
-----
FC88X_00969_00657      -------------------TGTCACAATTCTACTCCCGGATACTAAT-------
-----
FC88X_01118_00571      -------------------------------------GGAT-CTAA--------
-----
FC88X_01133_00910      -----------------------TCACGTATTCCGCTGCAT-CGAACTTTT---
-----
FC88X_01147_01043      ----------------------CTCAC-TATCCCGC-GTAT-CTAATTTCTC--
-----
FC88X_00977_00939      -----------------------TTTTAAACTCCCCGGCAGTCGCATTTT----
-----
FC88X_01070_00462      ---------------------AGGTTCAGTCTCCCGGG--GTCTCATTTCT---
-----
FC88X_00959_00869      ------------------TTCTACCTATTCGGCTCCGCAATATCA---------
-----
FC88X_01042_00505      ------------------TTCTACCTAA--------------------------
-----
FC88X_00971_00446      ---------ACTACTCGCCTCCACCTATCACTTTTG------------------
-----
FC88X_00987_00950      -------------------TTCGCGCTCCCCTTACCTCAACATATT--------
-----
FC88X_01011_01231      -------------------TTCGCGCTCCTCTTA--------------------
-----
FC88X_01013_00550      -----------------ACTTCACCCTTCTCACGACACAAACCT----------
-----
FC88X_01121_01110      -------------------TTTACGCCTC-CATGACAATAACTATTA-------
-----
FC88X_00953_00458      -------------------CTATCAGCTCAATTATCCCACTTTTCT--------
-----
FC88X_01049_00412      -------------------TTATCGCATCAACGGTCCCGCTATTCC--------
-----
FC88X_01086_00634      ----------------CTCGTTATTCTTCTCCTCTTGGACTGTC----------
-----
FC88X_01059_00910      -------------------TTCAATCATCGCCGCTTTGACTGTTCT--------
-----
FC88X_01035_00313      ----------------CTTTCCGCGGGTTATCCCTT--TTCTGTT---------
-----
```

```
FC88X_01079_00843      ------------------TTACATGCGTTGCCCCTCAGTTCTGTT---------
-----
FC88X_01016_00638      ------------------------ACGTTTCCTCTGTTTATTGCTCTTCCT---
-----
FC88X_01109_00743      ---------------------------TTTCCACT-------------------
-----
FC88X_00959_00387      -------------------TTTAGCAC-GTTACCTCGGC-CTATTTTC------
-----
FC88X_01037_00928      -------------------TTTAGCAC-GTTACCTCGGC-CTATTTTC------
-----
FC88X_01005_00299      -------------------CTTCGCACTATGACCTTTGCACTATTC--------
-----
FC88X_01052_00771      -------------------TTTCGCACTATGACCTT-GCACTATTC--------
-----
FC88X_01063_00583      -----------------TTCACCGATGTGTGACCTTCGC-CTGTT---------
-----
FC88X_01130_00563      -----------------TTCACCGATGTGTGACCTTCGC-CTGTT---------
-----
FC88X_01134_01173      -----------------TTCACCGATGTGTGACCTTCGC-CTGTT---------
-----
FC88X_00973_00692      -----------------TTAACCCATCACCGACCTACGC-CTATT---------
-----
FC88X_00999_01268      --------------CCTAGCTTTGTACTAACGGCTGTTCGC-------------
-----
FC88X_01092_00629      -------------------CTACGTACTAACGA---------------------
-----
FC88X_01084_01096      ---------GGGTCCTCCCCTACGTACTGTCATGTCTA----------------
-----
FC88X_01120_00957      ------------------CCTACGTAC---------------------------
-----
FC88X_01022_00927      --------------------TACGTACTC-------------------------
-----
FC88X_01097_00772      --------CTATCACGCGATTACGTTCTCTACT---------------------
-----
FC88X_01047_00420      -------------------CAACGTACTCCA-----------------------
-----
FC88X_01040_01011      ------------------CCAACGTATCCACAGCGCTCCCGTCAC---------
-----
FC88X_01072_00928      --------------------CTCGTACTCCAGAGACCTGCCCATATT-------
-----
FC88X_01118_01139      --------------------CTCGTACTCCAGAGACCTGCCCATATT-------
-----
FC88X_00989_00694      ---------------------CGATCGTGCCACATCCGTCCTATTCTT------
-----
FC88X_01119_00755      ---------------------CGATCGTGCCACATCCGTCCTATTCTT------
-----
FC88X_00984_00383      ----------------GTCCACGATCCCGGCA-ATTTTACCTATT---------
-----
FC88X_00972_00435      ------------------TTATCTGCACGCCCAACAACCGCTATT---------
-----
FC88X_01147_00610      ------------------TTATCTGCACGCCCAACAACCGCTATT---------
-----
FC88X_01041_00479      ------------------TTACCTCCTCTGCCTACGAACGCTGTT---------
-----
FC88X_00998_00698      ------------------ACATGTGATCTCCGCACTTGCGCTCTT---------
-----
```

```
FC88X_01136_00920       ------------------ACATGTGATCTCCGCACTTGCGCTCTT---------
-----
FC88X_01055_00474       --------------------ATCTGAACTCCAAGTTCCGCTATTTTC-------
-----
FC88X_01057_00525       --------------------ATCTGAGCTCAA----------------------
-----
FC88X_00991_00489       ------------------CCAGGTAGTTAACGCAATCGGCTTTTTCT-------
-----
FC88X_00990_00750       ------------------CCAGGTAGTTAACGCAATCGGCTTTTTCT-------
-----
FC88X_01042_00539       ------------------CCAGGTAGTTAACGCGATCGGCTTTTTCT-------
-----
FC88X_01043_01241       ----------------TCCCAGCTATATTGCGCG-TAGGCTCTT----------
-----
FC88X_00956_00937       -------------------AATCTAACGCTTACGCCTGGTTTATTCT-------
-----
FC88X_01115_00938       --------------------ATCTAACGTTTACGCCTGGTTTATTCT-------
-----
FC88X_01139_00907       -------------------GATCTAACGTTTACGCCCGGTTTATTCC-------
-----
FC88X_01024_00545       -------------------CTCCCAACTCTAACGCCTTGTTCAGGC--------
-----
FC88X_00970_00851       --------------------CTGTGCACT-CACATCCGGCTTTGTCTC------
-----
FC88X_01074_00873       --------------------CTGTGCACT-CACATCCGGCTTTGTCTC------
-----
FC88X_01133_00610       --------------------CTGTGCACT-CACATCCGGCTTTGTCTC------
-----
FC88X_01130_01204       --------------------CTGTGCACT-CACATCCGGCTTTGTCTC------
-----
FC88X_01082_00929       ---------------------CGTGGACTACGCGCCCGGTTTATTCTT------
-----
FC88X_01032_01155       ------------------------TTAACGCACCCTCGTTACCTGTTCAC----
-----
FC88X_01070_00447       ------------------------TTAACGCACCCTCGTTACCTGTTCAC----
-----
FC88X_01097_00606       ---------------------TACACTTAGCGCTCTCGTTAC-TATTCAC----
-----
FC88X_01023_00642       -----------------------CGAAACACTCACTCGCTGTTTTTTCTC----
-----
FC88X_00975_00281       -------------------ACGGCTCTTGTCCGGCTCCCTACTATT--------
-----
FC88X_01016_00610       -------------------ACGGCTCTTGTCCGGCTCCCTACTATT--------
-----
FC88X_01118_00501       ----------------ATTACCGCTCATGTCC---CCTGTACTATT--------
-----
FC88X_01044_00505       --------------------CCTGTGTATTGCAGCTCCCAGCTTTTTT------
-----
FC88X_01087_00875       ---------------------------------GTTCCCACCT-----------
-----
FC88X_01002_00854       ----------------------GCTGTTGTGTTCCAGCTGCCTGTTCTG-----
-----
FC88X_01062_00595       ----------------------TCTGTTGATTGTCTCCCGCCGCTGTTA-----
-----
FC88X_00960_00321       -----------------------CAAGTTCCCGACTGTCG--------------
-----
```

```
FC88X_01014_00783      -------------------CCCCCATGTACCTGACAGTTGGCTCCT--------
-----
FC88X_00973_00860      -------------------CATGCGATTG-TTACCGCCCGTTTTCTC-------
-----
FC88X_01046_00518      -------------------CATGCAATTACTTGCCGTCTGTCCTC---------
-----
FC88X_00960_00345      --------------------TTTCACGTTGCCGACTGCTGCTATTCC-------
-----
FC88X_00985_00308      --------------------TTTCACGTTGCCGACTGCTGCTATTCC-------
-----
FC88X_00966_00696      --------------------TTTCACGTTGCCGACTGCTGCTATTCC-------
-----
FC88X_00974_00815      --------------------TTTCACGTTGCCGACTGCTGCTATTCC-------
-----
FC88X_00957_01204      --------------------TTTCACGTTGCCGACTGCTGCTATTCC-------
-----
FC88X_01026_00438      --------------------TTTCACGTTGCCGACTGCTGCTATTCC-------
-----
FC88X_01020_00815      --------------------TTTCACGTTGCCGACTGCTGCTATTCC-------
-----
FC88X_01073_00537      --------------------TTTCACGTTGCCGACTGCTGCTATTCC-------
-----
FC88X_01074_00596      --------------------TTTCACGTTGCCGACTGCTGCTATTCC-------
-----
FC88X_01059_00710      --------------------TTTCACGTTGCCGACTGCTGCTATTCC-------
-----
FC88X_01078_00851      --------------------TTTCACGTTGCCGACTGCTGCTATTCC-------
-----
FC88X_01127_00618      --------------------TTTCACGTTGCCGACTGCTGCTATTCC-------
-----
FC88X_01116_00806      --------------------TTTCACGTTGCCGACTGCTGCTATTCC-------
-----
FC88X_00952_01008      --------------------TTTCACGTTGCCGACTGCTGCTATTCCCC-----
-----
FC88X_00956_00981      --------------------TTTCACGTTGTCGACTGCTGCTATTCC-------
-----
FC88X_01047_00846      --------------------TTTCACGTTGCCGACTGCTGTCCTCATTC-----
-----
FC88X_00981_00464      -------------CTCCCATTTTCACGTTGCTCGATGCC---------------
-----
FC88X_00989_00851      --------------------TTTCACGTTGC-----------------------
-----
FC88X_00999_00343      --------------------TCCTCGTTGTCCGAGTGTTGCTAAGCTC------
-----
FC88X_00955_00480      -------------------TTCGTACTTGTCCGGTCGCTATC------------
-----
FC88X_01059_00838      -------------------CAAACGTTTCCCCGACGTCGCTTTCTCC-------
-----
FC88X_01126_00671      -------------------CAAACGTTTCCCCGACGTCGCTTTCTCC-------
-----
FC88X_00951_00322      -------------------CAAACGTTTCCCCGACGTCGCTTTCTC--------
-----
FC88X_00997_00580      -------------------CAAACGTTTCCCCGACGTCGCTTTCTC--------
-----
FC88X_00994_00938      -------------------CAAACGTTTCCCCGACGTCGCTTTCTC--------
-----
```

FC88X_00997_01120 -------------------CAAACGTTTCCCCGACGTCGCTTTCTC--------
-----
FC88X_01047_00376 -------------------CAAACGTTTCCCCGACGTCGCTTTCTC--------
-----
FC88X_01027_00638 -------------------CAAACGTTTCCCCGACGTCGCTTTCTC--------
-----
FC88X_01003_00665 -------------------CAAACGTTTCCCCGACGTCGCTTTCTC--------
-----
FC88X_01001_00753 -------------------CAAACGTTTCCCCGACGTCGCTTTCTC--------
-----
FC88X_01022_00851 -------------------CAAACGTTTCCCCGACGTCGCTTTCTC--------
-----
FC88X_01031_00932 -------------------CAAACGTTTCCCCGACGTCGCTTTCTC--------
-----
FC88X_01063_00608 -------------------CAAACGTTTCCCCGACGTCGCTTTCTC--------
-----
FC88X_01095_00799 -------------------CAAACGTTTCCCCGACGTCGCTTTCTC--------
-----
FC88X_01138_00841 -------------------CAAACGTTTCCCCGACGTCGCTTTCTC--------
-----
FC88X_01144_00808 -------------------CAAACGTTTCCCCGACGTCGCTTTCTC--------
-----
FC88X_01125_00859 -------------------CAAACGTTTCCCCGACGTCGCTTTCTC--------
-----
FC88X_01104_00916 -------------------CAAACGTTTCCCCGACGTCGCTTTCTC--------
-----
FC88X_01130_00997 -------------------CAAACGTTTCCCCGACGTCGCTTTCTC--------
-----
FC88X_01021_00675 ------------------GCAAACGTTTCCCCGACGTCGCTTTCTCCC------
-----
FC88X_00961_00647 -------------------CAAACGTTTCCCTGACGTCGCTTTCTC--------
-----
FC88X_01020_00591 -------------------CAAACGTTTCCC-GACGTCGCTTTCTC--------
-----
FC88X_00976_00847 -------------------CAAACGTTTCCCCGACGTCGCTCTCTC--------
-----
FC88X_00995_00630 -------------------CAAACGTTTCCCCGACGTC----------------
-----
FC88X_01022_00939 -----------------CAAAAACGTTCCCCCGACGTCGCTTTCTC--------
-----
FC88X_01048_01221 -------------------CAAACGTTCCCCCGACGTCGCTTTCTC--------
-----
FC88X_01076_00632 ------------------CAAAACGTTTCCCCGACGTCGCTTTCTC--------
-----
FC88X_01132_00887 ------------------CAAAACGCTTCCCCGACGTCGCTTTCTC--------
-----
FC88X_01147_01128 ------------------CAAAACGTTTCCCCGACGTCGCTTTCCC--------
-----
FC88X_01137_01182 -------------------CAAACGTT-CCCCGACGTT----------------
-----
FC88X_00970_00616 --------------------CAACGTTTACCCGACTGTC---------------
-----
FC88X_01083_00668 --------------------CAACGTT-ACCCGAT-------------------
-----
FC88X_00998_00442 ---------------TCTCGCGATGGTCCGCTACTTGTTCTC------------
-----

```
FC88X_01025_00290      ---------------TCTCGCGATGGTCCGCTACTTGTTCTC------------
-----
FC88X_01134_00563      ---------------TCTCGCGATGGTCCGCTACCTGTTCTC------------
-----
FC88X_01144_01202      -----------------TCCCAATGTTCCGACACG-GTTCTGTG----------
-----
FC88X_01058_00441      -----------------CGCAAATGTCTCCCAAACTGTC---------------
-----
FC88X_01096_00982      ----------------TCGCTAACTATTCCTCCACTGTACATGT----------
-----
FC88X_00975_00343      --------------CCTGTACGTTTGTACACGTTATTCGCA-------------
-----
FC88X_01144_00906      --------------CCTGTACGTTTGTACACGTTATTCGCA-------------
-----
FC88X_01020_00897      -------------TCCAGCGCCCGTTGAAACGTAATTATT--------------
-----
FC88X_01072_00615      -------------TCCAGCGCCCGTTGAAACGTAATTATT--------------
-----
FC88X_00969_00851      -------------TCCAGCGCCCGTTGAAACGTAATTATTCC------------
-----
FC88X_01098_00786      --------------------------TATACGTAA-------------------
-----
FC88X_00972_00461      --------------------CCAGACTATACCTGGTAATCAGTTCTC-------
-----
FC88X_01005_00793      -----------------GTACCTCAGTAAACGAAGT--TCTATTCT--------
-----
FC88X_00959_00357      --------------------TCTTGTTTTATGTAACTGGCTGTATCTC------
-----
FC88X_00991_00442      ------------------CGTTATGTTTTATG-AACTG--CGTATCTC------
-----
FC88X_01075_00601      --------------------ACATGCTTTACGAGATTGCCTATGCTC-------
-----
FC88X_01127_00633      --------------------ACATATTTTACGA---------------------
-----
FC88X_00996_00667      ----------------------CTACTGCATGTACATCAGTAACGTCCC-----
-----
FC88X_00993_00841      ----------------------CTACTGCATGTACATCAGTAACGTCTC-----
-----
FC88X_01069_00362      --------------------GGGGAATGCGTGTCCATC----------------
-----
FC88X_00970_00467      --------------ATCGTAGCTGTTTCTATGTGTCCATTCCCC----------
-----
FC88X_01043_00608      ---------------------------CTAAGT-TCCACT--------------
-----
FC88X_01091_01177      ---------------CTGCGCGCTGTTCTACTTCTTGATTCC------------
-----
FC88X_00953_00981      ----------------CGTGCGCGAGTCG---TGCCCTGTTCCCGT--------
-----
FC88X_01057_00872      ------------------CACGCTAGTCGAGGTGCCCCGTTGTTTT--------
-----
FC88X_01048_01124      ---------------------ACGAGGTGT------------------------
-----
FC88X_01132_00962      ---------------------ACGAGGTGTCCTCCCCACCTTGTACTA------
-----
FC88X_00997_00359      ------------------TCGCTGGCTTGGTTGTCCGT-TATTCTC--------
-----
```

```
FC88X_01109_00559       ------------------TCGCTGGCTTGGTTGTCCGT-TATTCTC--------
-----
FC88X_01049_00533       ------------------TCACTGGCTTGGTTGTCCGT-TATTCTC--------
-----
FC88X_01023_00838       ------------------TCACTGGCTTGGTTGTCCGT-TATTCTC--------
-----
FC88X_01061_01231       ------------------TCACTGGCTTGGTTGTCCGT-TATTCTC--------
-----
FC88X_01054_00564       ------------------TCACTGGCTTGGTTGTCCAT-TATTCTCCC------
-----
FC88X_00976_00695       ------------------TCGGTTGCTCGATTAACGGTCTATTCT---------
-----
FC88X_00953_00648       ------------------TCGTTCGCTTTGCTGCCGCGCTATTGT---------
-----
FC88X_01048_00862       ------------------TCGTTTGCTTTGCTGCCGCGCTATTGT---------
-----
FC88X_00999_00901       ---------------CACACCTTTGAGTTGTTCCCGCG-TATT-----------
-----
FC88X_01009_00512       --------------CGACTCGTTCAGTTGCCTCCCTTTTGCT------------
-----
FC88X_01040_00576       --------------CGACTCGTTCAGTTGCCTCCCTTTTGCT------------
-----
FC88X_01034_00780       -------------------CTCTCGGTGATTCCCTCG-----------------
-----
FC88X_01069_00779       ------------------CTACACCCTGGCCTCCCCGTAGATTCC---------
-----
FC88X_01030_01153       --------------------------------TTCCGTTGATT-----------
-----
FC88X_01078_00604       ------------------TTTTTCGCTATCTGTCCCGTTGGTTCTC--------
-----
FC88X_00981_00693       -----------------TCTAGTTGGTTTTATTGCTGCTATTCTC---------
-----
FC88X_00964_00902       -----------------TCTAGTTGGTTTTATTGCTGCTATTCTC---------
-----
FC88X_00971_00828       ---------------TCCCTGATTGCTTCATTTTCTATTCTCC-----------
-----
FC88X_00957_00512       -----------------TCCAGTGTTTAATTTCTTGGCTTTTCT----------
-----
FC88X_01039_00355       ------------------CCACGAGTTTGCTTGCGTGGTTTACTC---------
-----
FC88X_00962_01209       -------------------TTCGCATTTGACTTTCGCCTACTTTCT--------
-----
FC88X_01137_01031       -------------------------TTTGACTTTCGA-----------------
-----
FC88X_01104_01019       -------------------CGTAACGTTTGCTTACGCCTTTTCTC---------
-----
FC88X_01008_00875       ------------------TCTGTCCCTTCTTTGTTTGCGCGTTTT---------
-----
FC88X_01036_01150       ------------------CTATTCCCTGGTTTGCAAACGCGTGTT---------
-----
FC88X_00965_00616       -------------------TTTTCTGTCATACGCAGGGCTATTCTT--------
-----
FC88X_00972_00889       -------------------TTCTCTGTCATACGCAGGGCTATTCTT--------
-----
FC88X_01077_00780       -------------------TTTTCGGTCGTACGCAGGGCTATTCTT--------
-----
```

```
FC88X_01005_00604       -----------------GTCCTTTTTTCGTCCGCTGTACTATTGT---------
-----
FC88X_01052_00882       --------------------------TAGTACGCT-------------------
-----
FC88X_01025_01203       -------------------TTCTAGTTCCTCGTCTAGTATATTCGC--------
-----
FC88X_01134_00559       -------------------TTCTAGTTCCTCGTCTAGTATATTCGC--------
-----
FC88X_01037_00607       -------------------TTCTAGTTCCTGGTCTAGTATATTCGC--------
-----
FC88X_00987_01233       ------------------TCCGCGATGTCGTTCCTGGACCATTTTC--------
-----
FC88X_00999_00689       -------------------TCACGTGTTTTCCGTCTAGACAATGTT--------
-----
FC88X_01132_00848       ---------------------------TTTCCTTGTA-----------------
-----
FC88X_00983_00879       -------------------TGACATGCTTT--TCGTATTCACTTTTC-------
-----
FC88X_01105_01179       ---------------------ACATAGTTTCGTCGTAGTCATGTTCTC------
-----
FC88X_00958_00905       --------------------TTATTCTCTCTTGTAAGGGCATTGTTG-------
-----
FC88X_01099_00659       --------------------TTATTCTCTCTTGTAAGGGCATTGTTG-------
-----
FC88X_01034_00825       -------------------TTTACTTTTTGTTGTATGCACATTCTC--------
-----
FC88X_01038_00769       --------------------------------------CCATTGTCG-------
-----
FC88X_01041_00876       -------------------CCGATTAGTCTCCGTCGGGCCATTGTC--------
-----
FC88X_00953_00985       -----------TTCGGCATCGTTCGTCTCCTGCATATTCC--------------
-----
FC88X_01124_01144       -----------TTCGGCGCCGTTCGTCTCCTGCATATT----------------
-----
FC88X_01092_00609       ------------CCAGCG-CGTTTGCGTTATGCGTATTCC--------------
-----
FC88X_01080_00724       ----------------CG-CGTATGCCTCCTACGTA------------------
-----
FC88X_01036_00500       -----------------ACGTTTTCGTTTTACGGCATCCTATTCTT--------
-----
FC88X_01137_00881       -----------------ACGTTTCG--TTTACGGCATCCTATTCTT--------
-----
FC88X_01094_00676       ----------------------------TTACGGTCATC---------------
-----
FC88X_01039_00630       -------------------CTTATGTTTCTCGCGTGCC-TATTCGT--------
-----
FC88X_01063_01220       -------------------CTTATGTTTCTCGCGTGCC-TATTCGT--------
-----
FC88X_01076_00928       -------------------CTTACGTTTCTCGCGTGCC-TATTCGC--------
-----
FC88X_01074_00379       ----------------TTGCACGTTTGTCTCCCGTGTCGTATT-----------
-----
FC88X_00997_00539       --------------------CTACGTTTTAGTTACATTGCATTTCTC-------
-----
FC88X_01011_01002       --------------------CTACGTTTTAGTTACATTGCATTTCTC-------
-----
```

```
FC88X_01143_00830      -------------------GCTACGTTT-AGTTACATTGCATTTCTCCC-----
-----
FC88X_01034_00575      --------------------CTACGTTTTAGTTACA------------------
-----
FC88X_00953_00589      --------------------CTACGTTTA-------------------------
-----
FC88X_00967_00804      --------------------CTACGTTA--------------------------
-----
FC88X_01036_00314      --------------TTCCGTCTCCGTTAGCATTGCAGTAC--------------
-----
FC88X_00955_00641      ------------------CCCAACATTCGCTCCTCAGGTTGCTATCC-------
-----
FC88X_01137_01123      ------------------CCTAACGTTCCCTCG-CAGTATGGTATT--------
-----
FC88X_01012_00645      ------------------CCTAACGTTTTGCAC---AGTTGGTATCTC------
-----
FC88X_01079_00653      ---------------CCACCTTACGTTATGGTT---------------------
-----
FC88X_01038_00810      -------------------CTTCCGTTACCCCCGCGGCGGCATGTG--------
-----
FC88X_01126_00570      -------------------CTTCCGTTACAC-----------------------
-----
FC88X_01062_00666      -----------------TCTTACAGTTTT--CTAA-------------------
-----
FC88X_01085_00689      -------------TCCTCTCTTCTGTTTC--CCAGCCTAGT-------------
-----
FC88X_01069_00570      ---------------TCCCCTACCGTTTCAGCCAAGCTGCT-------------
-----
FC88X_01065_00527      -------------------CTCCCTTTTCAACGACGTTGGCCATTA--------
-----
FC88X_01143_00975      -------------------CTTCGTTGTCAACGGT-TCGGCTATTCC-------
-----
FC88X_00955_00304      --------------------CTAGACGTTCCCACATCTTGG-------------
-----
FC88X_00987_00422      ----------------TTTTCTAGCTATTCGC-CACCTTGGAGGT---------
-----
FC88X_01103_00810      -----------------TTTCTACCTATTCGTCACGCGTGTTTC----------
-----
FC88X_01131_00859      -----------------CTTCCAACCTCTCGTCA-GCGTGCTGCT---------
-----
FC88X_00995_00365      ----------------ATCTCTCATCTTTCTCCTCGGACCCATCC---------
-----
FC88X_01091_01265      -------------------ACTCCCTTCTAACAGCTGGCCCATGCG--------
-----
FC88X_01022_00567      --------------------CTCTTCTTA-------------------------
-----
FC88X_01107_00836      --------------------CCCTTCTTCCTCTTCACGATGATGCCA-------
-----
FC88X_01128_00807      ----------------CTCACTTCTTTTCTCTCTCGGGATGA------------
-----
FC88X_00995_00850      ------------------ATCTTCTCGTCCG-AGCTTGCTATTCAC--------
-----
FC88X_01047_01127      ------------------ATCTTCTCGTCCG-AGCTTGCTATTCAC--------
-----
FC88X_00966_00826      -------------------TCTCGTCTTCCCCATCGCGCTATTAAC--------
-----
```

```
FC88X_01043_00508      ------------------GCCAAGTATTCCGTGACATGCTATTCGT--------
-----
FC88X_01102_00599      -------------------CCCAGTATTCCGTGACATGCTATTCGT--------
-----
FC88X_01078_00511      -------------------ACATCTATTCAGT----------------------
-----
FC88X_00951_00465      ----------------------CCAATTTCGC-ATCTC----------------
-----
FC88X_01076_01180      --------------------CTCCAACTTCTCTATCTCCGAACATAC-------
-----
FC88X_01017_00368      -----------------TTTCTCCAGTTTCCCTCTCGCCAAGGCGC--------
-----
FC88X_01132_00861      ----------------------CCAAGTTCCCT-TCTCCATGGCAATTAC----
-----
FC88X_00996_00348      --------------------------GTTTACCTTCTCA---------------
-----
FC88X_00983_00475      -------------------TCACGCATTTGCCCGTTTGACTTTCAC--------
-----
FC88X_01021_00958      -------------------TCACGCATTTGCCCGTTTGACTTTCAC--------
-----
FC88X_01142_01221      -------------------TCACGCATTTGCCCGCTTGACTTTCAC--------
-----
FC88X_01099_00477      ------------------TTCCCCTATCTGAGCGTTGGACATTCC---------
-----
FC88X_01045_00572      -------------------TCACACATTTACCCAAAT---TTCTATTCT-----
-----
FC88X_01020_00822      -------------------TCACACATTTACCCAAAT---TTCTATTCT-----
-----
FC88X_01104_00673      -------------------TCACACATCTACCCAAAT---TTCTATTCT-----
-----
FC88X_01013_01164      --------------------CCCTCATTTGCGCACATGAATTCTATT-------
-----
FC88X_00952_00458      ------------------CTCAGTGGGTCCTCTCTGTGTGTTCTC---------
-----
FC88X_00951_00752      --------------------------TCCTACTCTGCG----------------
-----
FC88X_01146_00684      -----------------------GGTTCTCCCAGTTTGAGTTGTCAACGCGT--
-----
FC88X_01143_00960      --------------------------TCCCTGTGTGTAAGTGGTCT--------
-----
FC88X_01010_00305      -------------------CCATTGCTTTGTATAA-GCGGA-CTTCTC------
-----
FC88X_01103_00836      -------------------CCATTGCATAATGTGT-GCAGCTCTTCTC------
-----
FC88X_01032_00990      --------------------TTGTACATTGTCTGTTAGAGCTATTCT-------
-----
FC88X_01077_00703      --------------------TTGTGCATTGTCTGTTAGAACTATTCT-------
-----
FC88X_01044_00948      -------------------CTTGTGCGG-GTGTGT-AAGGCAATTCTC------
-----
FC88X_00961_00504      -------------------ACCACCCTTGTTC----------------------
-----
FC88X_01090_00808      -------------------TCCACCCTTCTTCCACAACGCCATGTT--------
-----
FC88X_01031_00954      -------------------CCCACTCCTCTCCAGCCTTGGTCGTCTC-------
-----
```

```
FC88X_00991_00685       -------------------ATCACCGTTGGTCGGTGGTGCGCTGTT--------
-----
FC88X_01009_00814       -------------------ATCACCGTTGGTCGGTGGTGCGCT-----------
-----
FC88X_00996_00634       -------------------ATCACCGTTGGTCGG--------------------
-----
FC88X_01120_00515       -------------------ATCACCGTTGGTCGGTCGG----------------
-----
FC88X_01036_00634       -------------------CACATTTCTGTTTCGTCGCGCCG-TATT-------
-----
FC88X_01097_00567       -------------------CACATTTCTGTTTCGTCGCGCCG-TATT-------
-----
FC88X_01046_00678       -------------------TCCATTCCTGTT-CGTCACTTCGCTATT-------
-----
FC88X_01093_00595       -------------------TCCATTCCTGTT-CGTCACTTCGCTATT-------
-----
FC88X_00977_00651       ------------------ACATGCACTTGTT-CGTCTATTCC-TATTCC-----
-----
FC88X_00957_00879       --------------------CCACGTAATCCACATTTGTGTCTTCTC-------
-----
FC88X_01006_01130       --------------------TCACATAATCCACATTTGTGTCTTCTC-------
-----
FC88X_01065_00483       --------------------TCAATTCATCCTCATTGCTATCGCCTC-------
-----
FC88X_00975_00327       ------------------TTCTGGACTGTACTCTGGGGCTGTCTC---------
-----
FC88X_00957_00357       -----------------GTTCTGGACTGTACTCTGGGGCTGTCTC---------
-----
FC88X_00966_00811       ------------------TTCTGGACTGTACTCTGGGGCTGTCTC---------
-----
FC88X_00973_00811       ------------------TTCTGGACTGTACTCTGGGGCTGTCTC---------
-----
FC88X_00999_00813       ------------------TTCTGGACTGTACTCTGGGGCTGTCTC---------
-----
FC88X_00959_01273       ------------------TTCTGGACTGTACTCTGGGGCTGTCTC---------
-----
FC88X_01017_00583       ------------------TTCTGGACTGTACTCTGGGGCTGTCTC---------
-----
FC88X_01022_00635       ------------------TTCTGGACTGTACTCTGGGGCTGTCTC---------
-----
FC88X_01010_00651       ------------------TTCTGGACTGTACTCTGGGGCTGTCTC---------
-----
FC88X_01043_00663       ------------------TTCTGGACTGTACTCTGGGGCTGTCTC---------
-----
FC88X_01038_00763       ------------------TTCTGGACTGTACTCTGGGGCTGTCTC---------
-----
FC88X_01017_01104       ------------------TTCTGGACTGTACTCTGGGGCTGTCTC---------
-----
FC88X_01061_00631       ------------------TTCTGGACTGTACTCTGGGGCTGTCTC---------
-----
FC88X_01098_00899       ------------------TTCTGGACTGTACTCTGGGGCTGTCTC---------
-----
FC88X_01052_00934       ------------------TTCTGGACTGTACTCTGGGGCTGTCTC---------
-----
FC88X_01145_00738       ------------------TTCTGGACTGTACTCTGGGGCTGTCTC---------
-----
```

```
FC88X_01121_01125      ------------------TTCTGGACTGTACTCTGGGGCTGTCTC---------
-----
FC88X_00981_00315      ----------------CCTTCCTGTCTTTCCCCTGGGGGCTTCT----------
-----
FC88X_01093_00495      ------------------CCGAATATTTCTCCCTGGGATCGTCTC---------
-----
FC88X_01135_00941      ------------------CCTGATGCTTGGCCTTGGGTGCTTCTC---------
-----
FC88X_01018_00935      -------------------CTTAGACCTTGGTGGCATTCTGTTCTC--------
-----
FC88X_01092_00541      -------------------CTTAGACCTTGGTGGCATTCTGTTCTC--------
-----
FC88X_01080_00942      -------------------CTTAGACCTTGGTGGCATTCTGTTCTC--------
-----
FC88X_01139_00888      -------------------CTTAGACCTTGGTGGCATTCTGTTCTC--------
-----
FC88X_01089_00407      -------------------TTTAGACCTTGGTGGCATTCTGTTCTC--------
-----
FC88X_01142_00593      -------------------CTTAGACCTCGGTGGCATTCTGT------------
-----
FC88X_00973_00867      ------------CCAATAGGTTAGAC-TTGGTGGCTTTCT--------------
-----
FC88X_00963_00855      --------------------------AGTA-CTTCCATGTA-------------
-----
FC88X_01079_00738      --------------------------AGTA-CTTCCATGTA-------------
-----
FC88X_01040_00330      ------------------------ATAGTAGCTTCCCCTTGCTATCTTCAT---
-----
FC88X_00970_01205      ----------------TTTGACAACGTGT---GTCCTGCTGTTCTC--------
-----
FC88X_01053_00758      --------------------CCAGCATCT---GTCCTGTGCTCTTCTTCG----
-----
FC88X_01040_00802      ------------------TTGTGACATGTAACGTCCTTCTATCTT---------
-----
FC88X_01133_00758      ------------------TTGTGACATGTAACGTCCTTCTATCTT---------
-----
FC88X_01046_00612      ---------------GCCAACCGACATGT---GTTCTTCTATTCTT--------
-----
FC88X_00956_00920      ------------------ATGTCTGACCATTGCTGCTTCTATCTC---------
-----
FC88X_01104_00830      ------------------TCCTCTGTCGACCATGGCGTC-ATCCC---------
-----
FC88X_00986_00292      ----------------AGCTTAGATCTTGGCTCACGTTTTCTC-----------
-----
FC88X_01033_01110      ----------------AGCTTAGATCTTGGCTCACGTTTT--------------
-----
FC88X_00991_00578      ----------------AGCTTAGATCTTGGCTCACGTTTTCTC-----------
-----
FC88X_00989_00747      --------------CGAGCCTA-ATCGTTGCTCCCGCATAT-------------
-----
FC88X_01012_00886      -----------------CCCTAGATTGATCTCCCCGCTACTACA----------
-----
FC88X_01075_00458      ------------------CCTAG-TAGTTCTCACCCGCATTT------------
-----
FC88X_01058_00494      -------------------CTTGATTGTCCGTCCTGCTACGTCTCCTCC-----
-----
```

```
FC88X_00972_00769      -----------------CCGACAA-TTCGACTCCCCGGCAATGTT---------
-----
FC88X_01065_00642      -----------------CCGACAAATTCGACTCCCCGGCAATGTT---------
-----
FC88X_01042_00323      -----------------CCGACAA-TTCGACTCCCCGGCAATGTT---------
-----
FC88X_01045_00838      -----------------CCGACAA-TTCGACTCCCCGGCAATGTT---------
-----
FC88X_01025_01130      -----------------CCGACAA-TTCGACTCCCCGGCAATGTT---------
-----
FC88X_01070_01137      -----------------CCGACAA-TTCGACTCCCCGGCAATGTT---------
-----
FC88X_01119_00689      -----------------CCGACAA-TTCGACTCCCCGGCAATGTT---------
-----
FC88X_01133_01123      -----------------CCGACAA-TTCGACTCCCCGGCAATGTT---------
-----
FC88X_01036_00656      -----------------CTGACAA-TTCGACTCCCCGGCAGTGTT---------
-----
FC88X_00992_00671      -----------------CCGACAA-TTCGACTCCCCGGCACT------------
-----
FC88X_01142_00865      --------------CCTGTTGCAA-CACGACTCCCCGGCACT------------
-----
FC88X_01049_00626      -------------------------CTAGAGTCAC-------------------
-----
FC88X_01142_00847      ----------------CTCCCCCGCGTAGAGTGACACAGACTC-----------
-----
FC88X_01058_00872      -----------------------CACTGCAGTTACAAAGCGCCTCCCGGA----
-----
FC88X_01034_00515      ---------------CCACCACAACTCCGACACTGCTATTGC------------
-----
FC88X_01110_00596      ------------------CCACGACTCCGA------------------------
-----
FC88X_01085_00637      ---------------TCTCGACCGCCGATGCCTCCC------------------
-----
FC88X_01093_00801      ---------TTACCTTCTCCACTACGCCTATTGCCT------------------
-----
FC88X_01087_00401      ------------CTATCTCCACATCACCGAGGGCCTC-----------------
-----
FC88X_00986_00840      -------------------TTCAACGTTTATCCGCATGAACATCTC--------
-----
FC88X_00962_01241      -------------------TTCAACGTTTATCCGCATGAACATCTC--------
-----
FC88X_01071_01072      -------------------TTCAACGTTTATCCGCATGAACATCTC--------
-----
FC88X_01092_00468      --------------------CCAACGCCTCTTCG---GCACATC----------
-----
FC88X_01060_00628      ------------------CCGAAAACTGTTCTGACATGATGCTCTT--------
-----
FC88X_01123_00664      --------------------CAAACACGTTACGACCTGCGCCTCTT--------
-----
FC88X_01113_01172      ------------TCTCGTATAAACACCGCTGCGACCTGTC--------------
-----
FC88X_00985_00278      -----------------------TCCCTGATTCCCAGCAAACGGCTCGAC----
-----
FC88X_01033_01016      -----------------------TCCCTGATTCCCAGCAAACGGCTCGAC----
-----
```

FC88X_01121_00497 -------------------CTAATCCCTCCTTCACTGCA-ACCTGCA-------
-----
FC88X_01121_00862 ----------------------ATCCCTTCTTTCCTGCATACCT----------
-----
FC88X_01081_00525 -------------------------CTCCTG-
CATGCGACACTGCTGTACCACT-----
FC88X_01133_00681 ------------------------TCTCCTGACCTGGCTAACTGCTCTGC----
-----
FC88X_00979_00990 ----------------CTAACCACTGTCCGGTCCCAGCATGTT-----------
-----
FC88X_01121_00731 ---------------TCTTACCGTCCTCCGCACCTAGC-TGTT-----------
-----
FC88X_01108_00870 ---------------ATTAGGTATTTTCAAGTCCTAGCCTGCTCCC--------
-----
FC88X_01032_01033 -----------------ATTCCAAATCCCTAGACCACCGTGCC-----------
-----
FC88X_01059_00902 --------------------------------ACTACCGTGC------------
-----
FC88X_01001_00553 ---------------------CCATCACGGATTCAGCCTCGCTATACT------
-----
FC88X_01126_00617 --------------------TCCATCCAGCATACTGCCCTGCCTTATT------
-----
FC88X_01007_00986 ------------------TTCGCGATCCGAATCCCAGCTACTGTT---------
-----
FC88X_01001_01048 ------------------CTCGCGATCCGAGTCCCAGCTACTGTT---------
-----
FC88X_01132_01176 ---------------------ACGTTACCAATCCCGAATACTGTCGTT------
-----
FC88X_00989_00360 -------------------ACTGTTGCTAAA--CTTCAGTCTCGTTCT------
-----
FC88X_01052_00833 -------------------ACTGTTGCTAAAAACTTCAGTCTCGTTCT------
-----
FC88X_00985_00938 -------------------ACTGTTGCTAAA--CTTCAGTCTCGTTCT------
-----
FC88X_00989_00923 -------------------ACTGTTGCTAAA--CTTCAGTCTCGTTCT------
-----
FC88X_01128_01023 -------------------ACTGTTGCTAAA--CTTCAGTCTCGTTCT------
-----
FC88X_01055_00770 -------------------ACTGTTGCTAAA--CTTCA----------------
-----
FC88X_01077_00500 ----------------------CCTATAACT--CCCGAACACTGGCCTTGA---
-----
FC88X_01130_01151 -------------------------ATTATT--CCTAACCACAGCCC-------
-----
FC88X_01001_00859 --------------------CTGATGATAAA--CTCACGCCCTGGCTATG----
-----
FC88X_01015_00674 -----------CTGCTAAAAATGAGGCTGCACACTTCTC---------------
-----
FC88X_01068_00829 -----------CTGCTAAAA-TGAGGCTGCACACTTCTC---------------
-----
FC88X_01076_00926 ----------GAACCGTTCCCCTTTGCTGCACACCTTAT---------------
-----
FC88X_01082_01056 ------------------------TGCTGCACACT-------------------
-----
FC88X_00993_00392 --------------TCTGCCCATCTCCTG-GCCTTTCATATT------------
-----

```
FC88X_01129_01229      --------------TCTTCTTCCATTCTACGCTTTTCTTGC-------------
-----
FC88X_00997_00749      ----------CGTTTATGCTTCATCTCCACAACTTTCA----------------
-----
FC88X_01022_00625      -------------------------TCCA-AACT--------------------
-----
FC88X_00990_00334      ---------------TTCTGTGCATTTCTCCCAGCACACCGTC-----------
-----
FC88X_01008_00662      ---------------TTCTGTGCATTTCTCCCAGCACACCGTC-----------
-----
FC88X_01132_01130      ---------------TTCTGTGCATTTCTCCCAGCACACCGTC-----------
-----
FC88X_01013_01211      ---------------TTCTGTGCATTTCCCCCAGCACACCGTC-----------
-----
FC88X_00980_00614      ---------------TTCTGTGCATTTCTCCCAGCACATCGTC-----------
-----
FC88X_01049_00473      ---------------TCCTGTGCATTTCTCCCAGCACACCGTT-----------
-----
FC88X_01059_00799      --------------TTCGTGTGCATTTCTCCCAGCACACCGTC-----------
-----
FC88X_01046_01020      ------------------TCCCTGTGTTCTCCC-CACAGCGGTTAA--------
-----
FC88X_01094_01130      ------------------TCCCTGTGTTCTCCC-CACAGCGGTTAA--------
-----
FC88X_01121_01192      ---------------CTTTCCCTTCGTCCCCGCGCACACTGG------------
-----
FC88X_00976_00422      -----------------CTGTAATTTCCCCAGACAGCGCCGTAGT---------
-----
FC88X_00999_00479      -----------------CTGTAATTTCCCCAGACAGCGCCGTAGT---------
-----
FC88X_00954_00731      -----------------CTGTAATTTCCCCAGACAGCGCCGTAGT---------
-----
FC88X_00964_00827      -----------------CTGTAATTTCCCCAGACAGCGCCGTAGT---------
-----
FC88X_00967_00803      -----------------CTGTAATTTCCCCAGACAGCGCCGTAGT---------
-----
FC88X_01012_00919      -----------------CTGTAATTTCCCCAGACAGCGCCGTAGT---------
-----
FC88X_01091_00740      -----------------CTGTAATTTCCCCAGACAGCGCCGTAGT---------
-----
FC88X_01033_00940      --------------------CCTGTATTCCGCACACCGACGTATT---------
-----
FC88X_01018_00520      ----------------ATATCAAATCTCCCAAACCACGCTCGC-----------
-----
FC88X_01112_00949      ------------------TCGCAACCTCCCAGACACCGCTAGTCC---------
-----
FC88X_00967_00887      ------------------------TCTCCCAATCAACGGCACGCCGGTAAC---
-----
FC88X_01090_00551      ------------------------TCTCCCAGTATCTAGCACGACGCTAAC---
-----
FC88X_01014_00554      --------------------------CCTCGAGCTCTCTCACTCCACTGACAT-
-----
FC88X_01035_00892      -----------------------------------CTATCACACCACTCA----
-----
FC88X_01027_00461      ----------------TTTGGACGTCTCCCAGTCCTCGTTACGCC---------
-----
```

FC88X_01024_00847 ----------------TTTGGACGTCTCCCAGTCCTCGTTACG-----------
-----
FC88X_01049_01141 ----------------TTTGGACGTCTCCCAGTCCTCGTTACG-----------
-----
FC88X_01142_00921 ----------------TTTGGACGTCTCCCAGTCCTCGTTACG-----------
-----
FC88X_01062_00561 ----------------------------CCCATCCCCGTGCCG-----------
-----
FC88X_01008_00780 ----------------CCACAAACTCTGGCACTTCTCCTTGCG-----------
-----
FC88X_00996_00356 ------------------CTATCCCTCTGAGAACGTGCGTGTCTC---------
-----
FC88X_01028_00408 -------------------TAACACTCTGA------------------------
-----
FC88X_00968_00959 ------------------TCTTTCTCCCGCTGAACGGTGCTTCTC---------
-----
FC88X_01146_00718 ---------------CTAGCATTATTCCGCTA---GGTGCTTCTC---------
-----
FC88X_00950_00467 -----------------CTGTCTCCTTTACCGCA-CCAACATGTT---------
-----
FC88X_01005_00500 ---------------TTCTCTCATTTCAACCTCATCCAACAC------------
-----
FC88X_01067_00594 ---------------CTATTTCACGCTGACCGCCCACATTTT------------
-----
FC88X_01024_00425 ----------------CTTCCTTCTCTCCACATGC--AACGTGCA---------
-----
FC88X_01025_00620 ----------------CTTCCTTCTCTTCACATGC--AACGTGCA---------
-----
FC88X_00979_00814 ----------------TTCCCCTTTCTTGACGCGCT-AACGTGTT---------
-----
FC88X_01016_00587 -----------------CTCCCTGCTTTCTCGCACTGAACGTCA----------
-----
FC88X_00982_00710 ---------------TTTGGCTTTGTCCCACAACATGTTCTC------------
-----
FC88X_01088_00553 -----------------CCTTTCTCCCCGCAAACAGGATCTCTG----------
-----
FC88X_01048_00403 -------------------TTCTCGCCGCACAAA-CGAACATTGTT--------
-----
FC88X_01016_01140 -------------------TTCTCGCCGCACAAA-CGAACATTGTT--------
-----
FC88X_01010_00831 -------------------TTCTCGCCGCACAAAACGAACATTGTT--------
-----
FC88X_00960_00469 ------------------------CCGCACCAGCGTTGACAATTTATTCTC---
-----
FC88X_01032_00535 ------------------------CCGCACCAGCGTTGACAATTTATTCTC---
-----
FC88X_01015_01271 ---------------------TTATGCCACCAGCCCAGTCTATTTCTC------
-----
FC88X_01059_00798 ------------------CCAGTATCCCTCCAGCCCTGATTTATT---------
-----
FC88X_01017_00667 ----------------TCACTGGATTCTCCCGCAGTACGTTTAT----------
-----
FC88X_01075_01198 -----------------CCCTGGACTACACCGCTGCAGATTATT----------
-----
FC88X_00974_00491 ------------------------CTACACCA----------------------
-----

```
FC88X_00967_00724      ---------------CTACTACATCTCCACCACCGCACGAACCCT---------
-----
FC88X_01073_00516      ------------------------CTACACCATGA-------------------
-----
FC88X_01102_00764      ------------------------CCACAGCATGACTCTGGCTCTCCTACG---
-----
FC88X_00990_00518      -------------------------TACACCAGC--------------------
-----
FC88X_01106_00577      -------------CCAATTACTCCCTACAGCAGCGACATT--------------
-----
FC88X_01128_00876      --------------TTCATCCTTCCCCAGCCAGCGCGGTTA-------------
-----
FC88X_01110_01126      ---------------CCATCCTTCTGCAAC-AGCACAGTCCT------------
-----
FC88X_00988_00605      -------------CGACACCCTGCTCCCGACAGCCACGAG--------------
-----
FC88X_00999_00296      ------------CTGCACATTATCTCGCTCCCGACGGACC--------------
-----
FC88X_01098_00438      --------GCGCCTGCGC-TTATCTT--TCCCAACGAT----------------
-----
FC88X_00967_00666      ---------------CACACGGTTTT-CTCCCACCGGCTGGCC-----------
-----
FC88X_00966_00784      ---------------CACACGGTTTTTCTCCCACCGGCTGGCC-----------
-----
FC88X_00962_00968      ---------------CACACGGTTTC--TCCCACCGGCTGGCC-----------
-----
FC88X_01074_01245      ---------------CACACGGTTTC--TCCCACCGGCTGGCCCC---------
-----
FC88X_00994_00363      -------------------TCATGTGCCTCCCGACGTGACATTCGC--------
-----
FC88X_00979_00825      -------------------TCATGTGCCTCCCGACGTGACATTCGC--------
-----
FC88X_00996_00907      -------------------TCATGTGCCTCCCGACGTGACATTCGC--------
-----
FC88X_01027_00663      -------------------TCATGTGCCTCCCGACGTGACATTCGC--------
-----
FC88X_01082_00589      -------------------TCATGTGCCTCCCGACGTGACATTCGC--------
-----
FC88X_01131_00741      -------------------TCATGTGCCTCCCGACGTGACATTCGC--------
-----
FC88X_01117_00946      -------------------TCATGTGCCTCCCGACGTGACATTCGC--------
-----
FC88X_01051_00359      ------------------TCCATGTGCCTCCCGACGTGACATTCGC--------
-----
FC88X_01147_01198      -------------------------ACCTCCCGGCGCGA---------------
-----
FC88X_01048_00583      ----------------CCAGAATTTGTCTCCCGCTGGCGTGAT-----------
-----
FC88X_01010_00875      ----------------CCAGAATTTGTCTCCCGCCGGCGTGAT-----------
-----
FC88X_01023_00579      ---------------GCCAGCAATAAACTCCC-CCGGCGCGAA-----------
-----
FC88X_00979_00566      ------------------ACCACAT-TTCTCTCCCTGCACAAT-ACG-------
-----
FC88X_01048_00846      ------------------ACCAATT-TTCTATTCCCGCACAGTTACAG------
-----
```

```
FC88X_00970_00708      ----------------ACACCACATATCCCGTCCCAGCGCATT-----------
-----
FC88X_00979_00737      -------------------CCACGTATCTTGCCCCTGGCTGTTCGC--------
-----
FC88X_01095_00518      ----------------TCTTCCCATATTTTGCACC--GCTATTCG---------
-----
FC88X_00966_00939      ----------------TCACTTCCTATCTGGTCGC--GATATTCT---------
-----
FC88X_00955_01021      -----------------------CTATCTCG-CGC--CAT--------------
-----
FC88X_01011_00934      ----------------ACGACCACTATCTCGCCCC--ACTATTCTA--------
-----
FC88X_00960_00988      -------------------CCAAATTCTCTCCGCCGGCAAGTTGTT--------
-----
FC88X_01127_00664      -------------------CCAAATTCTCTCCGCCGGCAAGTTGTT--------
-----
FC88X_01027_01152      -------------------CCAAATTCTCTCCGCCGGCAAGTTGTTCTTC----
-----
FC88X_00991_01272      -----------------CCAAAAAACTCTTCCCCCAGCAGGCTGAT--------
-----
FC88X_00985_00940      -------------------CCAATTCTCTGTTTTCCCCCGGCAGCC--------
-----
FC88X_01044_00859      -------------------ACAACTACTCTCCGCGGACCAGCATAT--------
-----
FC88X_01106_00588      ------------TAGTTAAATTACTGCTCCCCACCTGCA---------------
-----
FC88X_01013_00412      ---------------------------TTTAGACCT------------------
-----
FC88X_01118_00824      -----------ACCACACGTCCGTCTCTTTAGCCCTCA----------------
-----
FC88X_00963_00314      -----------TCTACACCTCTCGTGTCCAAAGCTCCC----------------
-----
FC88X_00967_00524      ----------------CTCCCAACGATTCAACACCAC-CAGTGGT---------
-----
FC88X_01015_00369      ----------------CTCCCAACGATTCAACACCAC-CAGTGGT---------
-----
FC88X_01008_00510      -------------------CTATTGAAACCACAAC-------------------
-----
FC88X_00972_00368      -------------------CCATCGGTTTCAAACCTCGCAGTTGTT--------
-----
FC88X_00951_00747      -----------------CTATAACCCCGTAGTTG--CAATGTTCTC--------
-----
FC88X_00976_00845      -----------------CTATAACCCCGTAGTTG--CAATGTTCTC--------
-----
FC88X_00967_00660      -----------------CTATAACCCCGTAGCTG--------------------
-----
FC88X_00967_00697      -------------------TTGACGCCTTTGGTGACCACTGATCTC--------
-----
FC88X_00959_00966      -------------------TTGACGCCTTTGGTGACCACTGATCTC--------
-----
FC88X_01141_00728      -------------------TTGACGCCTTTGGTGACCACTGATCTC--------
-----
FC88X_01077_00555      -------------------CTGACGCCTTTGGTGACCACTGATCTC--------
-----
FC88X_01029_00492      ------------------TACAGCATCTCGCCCTAGGTACCATTTT--------
-----
```

```
FC88X_01126_00916      -----------------CCACCACGAGCGCTCCCTTTGACCACTT---------
-----
FC88X_00994_00343      ----------------------TCGGTTACTCACCGCGCTATCCTCGT------
-----
FC88X_00989_00921      -------------------CTCTCGGATACACACGGGGCGCTCCCTA-------
-----
FC88X_01010_00571      -----------------------CGATTACAGACGTCGCCCACCGA--------
-----
FC88X_01035_00480      ---------------CCGCGCATTTCCCACACCGCT------------------
-----
FC88X_01088_01213      ---------------CCGCGCATTTTCCACACCGCTC-----------------
-----
FC88X_00984_00301      --------------ACCGGCGATTTTCCACAGCACTCTTTTT------------
-----
FC88X_00983_00408      -------------TCCTGTCA-TTGACCACGCCACTATTCT-------------
-----
FC88X_01108_00916      -------------TCCCGTCAATTGACCACGCCACTATTCT-------------
-----
FC88X_01010_00773      --------------CTAGCGT-TTCCCCC-GCAATCTATTTGTC----------
-----
FC88X_01134_00923      --------------CTAGCGT-TTCCCCC-GCAATCTATTTGTC----------
-----
FC88X_00986_00844      --------------CTAGCGT-TTCCCCCCGCAATCTATTTGTCC---------
-----
FC88X_01135_00857      --------------TTAGCGT-TTCCCCC-GCAATCTATTTGTC----------
-----
FC88X_00989_00594      --------------CCATCGTGTTCGCCCCGCGATGAATCT-------------
-----
FC88X_00996_00360      ----------------------TCCTCCCAACGATTTAGCTGTTTTGCA-----
-----
FC88X_00954_00691      ----------------------TCCTCCCAACGATTTAGCTGTTTTGCA-----
-----
FC88X_01004_00829      ----------------------TCCTCCCAACGATTTAGCTGTTTTGCA-----
-----
FC88X_01060_00635      ----------------------TCCTCCCAACGATTTAGCTGTTTTGCA-----
-----
FC88X_01092_00945      ----------------------TCCTCCCAACGATTTAGCTGTTTTGCA-----
-----
FC88X_01040_00361      --------------------ATGATCCTCGCCGGATGATCTGTTCC--------
-----
FC88X_01046_00624      --------------------ATGATCCTCGCCGGATGATCTGTTCCCC------
-----
FC88X_00964_00738      -----------------TTGCACCCTCCGGACGATTGACTAGTT----------
-----
FC88X_01087_00852      -----------------TTGCACCCTCCGGACGATTGACTAGTT----------
-----
FC88X_00998_00904      ---------------------------CCGATGATTG-----------------
-----
FC88X_00987_00276      -------------ACCACTTCCAACGGATCA-CGTATTATTCC-----------
-----
FC88X_00970_00458      -------------ACCACTTCCAACGGATCA-CGTATTATTCC-----------
-----
FC88X_01061_00398      ----------------TCTCCCAATCTATCAGCGTATGATC-------------
-----
FC88X_01102_00467      ----------------TCTCCCAATCTATCAGCGTATGATC-------------
-----
```

```
FC88X_01045_01017       --------------------TTACCTTCTCACG-TATGTTGGCATTTT------
-----
FC88X_01112_00816       --------------------TTACCTTCTCACG-TATGTTGGCATTTT------
-----
FC88X_01032_00409       ----------------------------TCACGCTATATTG-------------
-----
FC88X_01078_00629       ----------------------------TCACGCAAGAT---------------
-----
FC88X_01039_00951       -------------TTCCCTTCTAAC-CCGTACGCAACAATG-------------
-----
FC88X_01066_00807       -------------TTCCCTTCTAAC-CCGTACGCAACAATG-------------
-----
FC88X_01108_00764       -----------CTTTCTATTCTATTACCGCACGAGACA----------------
-----
FC88X_00986_00465       ------------------------------TCTCC-AATAT-------------
-----
FC88X_01140_00857       ---------------CCTGACAACGACAGCTCTCCGAATATT------------
-----
FC88X_00990_00783       -----------------TCCTCAACCTTACACTGCAGCGCGTGC----------
-----
FC88X_00996_01019       -------------------------TTTACACT---------------------
-----
FC88X_01009_00464       --------------TTCTCTCAACTCTTACACGCTAGCTGT-------------
-----
FC88X_01133_00967       --------------TTCTCTCAACT-TTACACGCTAGCTGTCC-----------
-----
FC88X_01025_01273       ----------------TCTATCAATACTGGCCGGTAGCTTTCT-----------
-----
FC88X_01068_00760       ----------------TCTATCAATATTGGCCGGTAGCTTTCT-----------
-----
FC88X_00986_00832       ----------------TCTCTCAAT-TTGCAGTTCAGCTTTCTC----------
-----
FC88X_00970_00300       ------------------CCGCAAT-TGACGTCATCCCTGGTACTC--------
-----
FC88X_01057_01217       ---------------------CAATCTGAC------------------------
-----
FC88X_01069_00599       --------------------TTAATCTGACCCGACAGCCTACTTGTT-------
-----
FC88X_01082_01064       --------------------TTAATCTGACCCGACAGCCTACTTGTT-------
-----
FC88X_00978_00964       ------------------CTCTAATCTGTACGATATAACTATTCC---------
-----
FC88X_01060_01145       ----------------------AATCCGTA------------------------
-----
FC88X_01116_00516       ------------------CTCCCATCCGTGATATGAGCGGACACAT--------
-----
FC88X_00965_00327       ----------------CACTTGCATGTGAAGACTCGCTGTTCT-----------
-----
FC88X_01112_01112       ----------------CACTTACATGTGAAGATTCGCTGTTCT-----------
-----
FC88X_01030_00532       ---------------TTTCCCCGTTATCGTAGATCGCTGTTCTCC---------
-----
FC88X_01143_01145       ---------------TCTCTCCGTTATCGTAGATCGCTGTCCT-----------
-----
FC88X_01033_01007       --------------------------------ATCACTGT--------------
-----
```

```
FC88X_01035_01150       ---------------TCTCACCGGTGACGGTATACGCTTTCTC-----------
-----
FC88X_01008_01025       ---------------CAGTCCCAGTTGAGT-CTTCGCG-TTCTC----------
-----
FC88X_01090_00452       -----------------CCCACGGTTGTGTGCATCCCGATTCTCCC--------
-----
FC88X_00974_00622       -----------------CCCAAGCATCAGTGCACCGTTGTTCTC----------
-----
FC88X_00983_00789       -----------------CCCAAGCATCAGTGCACGGTTGCTCTCCC--------
-----
FC88X_01038_00504       ------------------CCGTGAATTAGTTCACGATGTGCTTCT---------
-----
FC88X_01009_00652       -----------------CTCCCAAATGAGTCTACGGTCACTCAC----------
-----
FC88X_00977_00335       -----------------CTTGGCTATCTGCTAAATGCGTATTCT----------
-----
FC88X_01002_00487       -----------------CTTGGCTATCTGCTAAATGCGTATTCT----------
-----
FC88X_01099_00473       -----------------CTTGGCTATTTGCTAAATGCGTATTCT----------
-----
FC88X_00987_00912       ---------------ACCTCTATTCGCTGC-AAATGGCCATTGC----------
-----
FC88X_00959_00854       ----------------CCAATCTCTCGTGGTCATTGCGCTTCT-----------
-----
FC88X_01048_00602       ----------------CCAATCTCTCGTGGTCATTGCGCTTCT-----------
-----
FC88X_01094_01153       ----------------------TCTCCTCTGCATT-CGCT--------------
-----
FC88X_01002_00502       ------------------CCATTCATTCGATGAATTTGCCGTTCT---------
-----
FC88X_01132_00815       -----------------CTCAAACGTGCGATGAATGAGCCTTCT----------
-----
FC88X_01146_00931       -----------------TCCTCACCCGTGCAAATTGAGCTTTT-----------
-----
FC88X_00978_00734       ------------------TCAGTGCATT-CACAG-GGCTTGGTTCTT-------
-----
FC88X_01136_00736       -----------------CTCATTGCAATACGCAG-TGCGTTGTTC---------
-----
FC88X_01078_00603       ----------------ACCCTGGATACGAAACAG-TGCATTCTC----------
-----
FC88X_01145_00837       ----------------ACCCTGGATACGAAACAG-TACATTCTC----------
-----
FC88X_01019_00384       -----------------ACCTGGACACGAAACAG-TGCATTCTCC---------
-----
FC88X_01066_00601       ----------------------ATTGCAAACCAAATGCATCCTC----------
-----
FC88X_00976_01215       ---------------TGTGATGAGTACAACTCACCTCGATTCTC----------
-----
FC88X_01071_00377       ---------------TGTGATGAGTACAACTCACCTCGATTCTC----------
-----
FC88X_01071_00669       ---------------TGTGATGAGTACAACTCACCTCGATTCTC----------
-----
FC88X_01065_00811       ---------------TGTGATGAGTACAACTCACCTCGATTCTC----------
-----
FC88X_01141_01176       ---------------TGTGATGAGTACAACTCACCTCGATTCTC----------
-----
```

```
FC88X_01024_00599       ---------------CGTGACGAGTACAACTCACCTCGATTCTC----------
-----
FC88X_01080_01171       ---------------------CAGTACAAC------------------------
-----
FC88X_01133_00939       ------------------CCTCTGCACAACGCAACTTGCGTCT-----------
-----
FC88X_00980_00336       ------------------CCATCACCCCGCTAAACGCGCAA-TATT--------
-----
FC88X_01005_01016       ------------------CCAACAGCATGCCAAACTCGTAAATATT--------
-----
FC88X_00976_00721       --------------------ACCGTACTTTCAACCTCGAACACTATT-------
-----
FC88X_01141_00811       ----------------------------CACAAACTCGAA--------------
-----
FC88X_00994_00587       ----------------CCCAAACAGCCCCAGCACCTACATCGC-----------
-----
FC88X_01012_00374       --------------------CACATCCCTCATACCTATCACGCAA---------
-----
FC88X_01130_00779       -----------------ACGAACATCCTCCTGAGCTGTCTATCT----------
-----
FC88X_01138_00792       ---------------ACACGAA-ATCCCCGGTACCCGTCTAACT----------
-----
FC88X_01027_01207       ----------------TACGTACAACTTC-------------------------
-----
FC88X_00964_00386       --------------ACGATCTATCTCCCG-CCGTCAGCACGC------------
-----
FC88X_01042_00882       ------------TCACGATC-ATCTCCAA-TCTCCAGCACGCTTCT--------
-----
FC88X_01038_00342       ------------------TCGATCCCCCG-TCGACAGCTCA-------------
-----
FC88X_00984_00360       ---------------------CCAAACCT-TCCCCACTATTGAATTTAT-----
-----
FC88X_01130_01232       ---------------------CCAACACC-TTCT--------------------
-----
FC88X_01071_00490       -------------------CTATCAACCG-TCGCCATTACGTTATT--------
-----
FC88X_01143_01201       -------------------CTATCAACCG-TCGCCATTACGTTATTCC------
-----
FC88X_01036_01012       -----------------CATCATCAGCCC-CACTCTTTATTCTC----------
-----
FC88X_00968_00551       ------------------TCTCGAACTCAGCGATCAACAGGCTACTCCTC----
-----
FC88X_01043_00752       ------------------CCTCCTGCTCA-CGTGCAACAGTCTTTT--------
-----
FC88X_01099_00802       ------------------CCTCCCACTCG-CAAGCACCATATTTGC--------
-----
FC88X_01068_01240       ------------------TCTCTCATCCA-CGAGTTCAGCATATT---------
-----
FC88X_00955_00347       ---------------------CTTTGTTAATTCCCGCCAATCTATTCAC-----
-----
FC88X_01071_00498       ----------------------ATTGTTA--TCCCGCAGTTCTATTCCCC----
-----
FC88X_01013_00942       -------------------CACGTTGATAACAAGCGCGACTATTCC--------
-----
FC88X_01118_00845       -------------------CACGTTGATAACAAGCGCGACTATTCC--------
-----
```

```
FC88X_01087_00671       -------------------CACGTTGATAACAAGCGCGACTATTTCCCTC-----
-----
FC88X_01070_01203       --------------------------CTAACTACC--------------------
-----
FC88X_01082_01268       ----------------------GTTACTAACTACCTATG----------------
-----
FC88X_00953_00426       -----------------TCGTAAATTCTAACCACCCCTGCTGTT-----------
-----
FC88X_00981_01211       -----------------CCGTTATTTTAAACTCCCGCGGCCGGTT----------
-----
FC88X_01075_00399       -----------------CCGTTATTTTAA-CTCCCGCGGCCGGTT----------
-----
FC88X_01075_00483       -----------------CCGTTATTTTAA-CTCCCGCGGCCGGTT----------
-----
FC88X_01125_00802       -----------------CCGTTATTTTAA-CTCCCGCGGCCGGTT----------
-----
FC88X_01130_00906       -----------------CCGTTATTTTAA-CTCCCGCGGCCGGTT----------
-----
FC88X_00966_00591       -----------------CCGTCAGTGTGACAACCCGCG-CCATTT----------
-----
FC88X_00964_00443       -----------------TTCTCGTCAGTTGTTGCTAA-CTATTCC----------
-----
FC88X_01141_01166       -----------------TTCTCGTCAGTTGTTGCTAA-CTATTCC----------
-----
FC88X_01000_00312       ------------------------------CTGCTAA-CTAT-------------
-----
FC88X_00995_00361       ------------------------------CTGCTAA-CTATTCTCAA-------
-----
FC88X_00991_00447       ------------------CTTACTAACCTCCTGCAAATCTATTTT----------
-----
FC88X_01048_01019       ------------------CCTCGCTGTAAACTGTTTTGCCATATT----------
-----
FC88X_01030_01270       ------------------CCTAGTTGTTAACTACTTTCGCATGTT----------
-----
FC88X_01049_01039       ------------------CCTCCTG-TTATCTAGTGTCCATGTGAC---------
-----
FC88X_01083_00612       ------------------CCTCCTGGCTATGTAGCTGCCCCTGATC---------
-----
FC88X_00967_00828       ------------------------------TTGACAACTGT--------------
-----
FC88X_01105_00538       ---------------TTACCCACTTTCTATTTTGAAACTATC-------------
-----
FC88X_01025_01047       --------------CTATAATCTCGTCGATTGTCTATCTCTT-------------
-----
FC88X_01058_00579       --------------CTAATAACGCTTCGAATTCCCAAACTCT-------------
-----
FC88X_00965_00331       -----------------ACTGCTAATATCCCACCAACTGCTATT-----------
-----
FC88X_00982_00892       -----------------TCTACTATTATCCCAACTGACATTGTC-----------
-----
FC88X_00975_00614       -------------------TACTAGTAACA-------------------------
-----
FC88X_01133_00737       -----------------TCCACTATTACCACGAATCCCACTCTT-----------
-----
FC88X_01043_00372       -------------------TTGTCAAATCCCGCGCATAACTATTCC---------
-----
```

```
FC88X_01027_00879      -------------------TTGTCAAATCCCGCGCATAACTATTCC--------
-----
FC88X_01138_00592      -------------------TTGTCAAATCCCGCGCATAACTATTCC--------
-----
FC88X_00961_01014      -------------------TTGTCGAATCCCGCGCATAACTATTCC--------
-----
FC88X_01032_00599      ----------------------------------CACAACTATT----------
-----
FC88X_01061_00476      -----------------ACACCAGTCCCATCAGCCACAATTATT----------
-----
FC88X_01061_00767      ------------------CCGTTTCCCAGCCGGCCGCAACTAATA---------
-----
FC88X_00978_00352      ----------------TCCCGAACTTAGCGACGGACCTCCCGG-----------
-----
FC88X_01021_00407      -----------------CCGAGACTCTGCGTAGGTGCTCCCATT----------
-----
FC88X_00960_00534      -----------------------GTCTGCGCAAACACTCCTACA----------
-----
FC88X_01041_00523      --------------ACTACGAACCTCTGTTCTAGCGCTCCT-------------
-----
FC88X_01112_00935      ------------------TTGCCTGCTGCCCCACGCCGCTGTTCTC--------
-----
FC88X_01117_01100      ------------------TTACCAGCTTGCACGAGCCTCCCTGTTCT-------
-----
FC88X_00992_00640      -------------------------TTGCAACAACCCTCTCATC----------
-----
FC88X_00985_00466      ----------------GTGCAAAAACCTCTCGACGATACCTGTT----------
-----
FC88X_01053_00423      -------------------CACAAACATCT------------------------
-----
FC88X_01058_00907      ----------------CATCGTAACCAACCCGAACAAACCTGTT----------
-----
FC88X_00990_00393      ----------------------CTGTTAACTAAGCAGCCCCACCGTATT-----
-----
FC88X_00991_00827      ----------------------ATATTCGATAAGCACCCCGATCATATT-----
-----
FC88X_01066_00372      --------------------CAAAAC-ATCCCATCACGTCTACTAT--------
-----
FC88X_01075_00443      ------------------ATCAACACTATTCGATCCCGCCAAATA---------
-----
FC88X_00977_00316      -----------------------CCTCTA-CCGTCTGATTTT------------
-----
FC88X_01041_00371      ----------------ACGTGAACCTTTATCCGTCCGTTTCTT-----------
-----
FC88X_01033_01207      -------------------ACATCTCTCAGCTTCACCGTCTATTTT--------
-----
FC88X_01052_00476      ----------------CTTAGATCTTTCAACCGCCCGGCTTTTT----------
-----
FC88X_01026_00381      -------------------ACATCTGCCAATCGCACGTCTGTTCAC--------
-----
FC88X_01128_00741      -------------------ACATCTACCAATCGCACGTCTGTTCAC--------
-----
FC88X_00996_00397      -------------------ACATCTACCA-------------------------
-----
FC88X_00999_00425      ---------------------CACCTCCCGGCATATCATACGCACACT------
-----
```

```
FC88X_01070_00622      -----------------CCCGCACGTTTCGGCAT-TCCTAGTCACCC-------
-----
FC88X_01069_00649      ---------------------CGCTGTCT---TAGCCACA--------------
-----
FC88X_01120_00684      ---------------------TGCTGTCT---TAGCCAC---------------
-----
FC88X_01074_00543      -----------------CTAACGCCGTCTGTATAGCCCCCATTTT---------
-----
FC88X_00981_00720      -----------------TTGAGGCATCTCTCAAACCCACTGTTTT---------
-----
FC88X_00975_00572      ----------------TCCACTACTGATCTCCTATTCACTATT-----------
-----
FC88X_01036_01129      -------------CCATCCACTCCCTATCTCCGATCTATT--------------
-----
FC88X_01133_01165      -----------------------------ACCAACTCACT--------------
-----
FC88X_01060_00422      ----------------CCACTATCTCCCTGGCAACCAGTGTTGT----------
-----
FC88X_01128_01215      ----------------CCAATAGCTCCCGGGCCAACTGTT--------------
-----
FC88X_01140_00772      ----------------TCTCAAGCTCCCTGGCCACTTATTGAC-----------
-----
FC88X_01135_00901      ----------------CCTACAGCTACCCTGTCCCTTTGTGTA-----------
-----
FC88X_01003_01157      ----------------CTGACGAATCTCCCGAGTCAGACTGGG-----------
-----
FC88X_01052_00576      -------------------------CTCCCTAGTCA------------------
-----
FC88X_01015_00942      ----------------CTTACTTCTCCCAATAGTCCGACTGTG-----------
-----
FC88X_00974_00431      ----------------TCAATTGCCCTGCCGAGTCAAACTATC-----------
-----
FC88X_01070_00585      ------------------CTCCCAACAGATTCGACGAACCGTCC----------
-----
FC88X_01143_00925      -------------------CTCCCACTTATGAGTCGTACTGACCGT--------
-----
FC88X_00998_00254      ---------------ATTGGCGAGAAA-TGC-CGTCTAGTGCTC----------
-----
FC88X_01146_00701      ---------------ATTGGCGAGAAAATGC-CGTCTAGTGCTC----------
-----
FC88X_00956_00825      ---------------ATTGGCGAGAAA-TGC-CGTCTAGTGCTC----------
-----
FC88X_01002_00483      ---------------ATTGGCGAGAAA-TGC-CGTCTAGTGCTC----------
-----
FC88X_01042_01029      ---------------ATTGGCGAGAAA-TGC-CGTCTAGTGCTC----------
-----
FC88X_01043_01186      ---------------ATTGGCGAGAAA-TGC-CGTCTAGTGCTC----------
-----
FC88X_01052_00543      ---------------ATTGGCGAGAAA-TGC-CGTCTAGTGCTC----------
-----
FC88X_01071_00525      ---------------ATTGGCGAGAAA-TGC-CGTCTAGTGCTC----------
-----
FC88X_01094_00659      ---------------ATTGGCGAGAAA-TGC-CGTCTAGTGCTC----------
-----
FC88X_01095_01185      ---------------ATTGGCGAGAAA-TGC-CGTCTAGTGCTC----------
-----
```

FC88X_01126_00938 ---------------ATTGGCGAGAAA-TGC-CGTCTAGTGCTC----------
-----
FC88X_01110_01040 ---------------ATTGGCGAGAAA-TGC-CGTCTAGTGCTC----------
-----
FC88X_01062_00741 ---------------CACGGCTCGAAAGTGC-AGTC-AGTTCTC----------
-----
FC88X_01071_00726 ---------------CACGGCTCGAAAGTGC-AGTC-AGTTCTC----------
-----
FC88X_01109_00457 ---------------CACGGCTCGAAAGTGC-AGTC-AGTTCTC----------
-----
FC88X_01140_01041 ---------------CACGGCTCGAAAGTGC-AGTC-AGTTCTC----------
-----
FC88X_00955_00330 ----------CTGCCACGTTTGGACAATTGC-A-----GTTCTC----------
-----
FC88X_01120_01119 --------------CAGGTAAAGTCAAATGC-ACGC--GTTCTC----------
-----
FC88X_00959_00599 ----------------TTCACACACATTTACAGGGGCTGTTCT-----------
-----
FC88X_00978_00758 ----------------TTCACACACATTTACAGGGGCTGTTCT-----------
-----
FC88X_01037_01257 ----------------TTCACACACATTTACAGGGGCTGTTCT-----------
-----
FC88X_01069_00429 ----------------TTCACACACATTTACAGGGGCTGTTCT-----------
-----
FC88X_01124_00591 ----------------TTCACACACATTTACAGGGGCTGTTCT-----------
-----
FC88X_00998_00685 ---------------TCCTACACACAACAGC-GGGCTTGTTCT-----------
-----
FC88X_01010_00757 ---------------TCCTACACACAACAGC-GGGCTTGTTCT-----------
-----
FC88X_01053_00694 ---------------TCCTACACACAACAGC-GGGCTTGTTCT-----------
-----
FC88X_01132_00873 ----------------CTACTACTCAAGTGCGCGTTTCGTTCTC----------
-----
FC88X_01144_00868 ----------------CTACTACTCAAGTGCGCGTTTCGTTCTC----------
-----
FC88X_01000_00516 ----------------CTACTACTCAAGTGCGCGTTTTGTTCTC----------
-----
FC88X_00990_00808 ----------------CTAC-ACTCAACT-------------------------
-----
FC88X_00997_00558 ---------------TCTCCCTGCGTATGGA-ACTGTCATCTC-----------
-----
FC88X_01112_00660 ---------------------------TGGACACTATCATCC------------
-----
FC88X_01019_00959 ---------------------------GGGGAACTGTCGTACTT----------
-----
FC88X_00978_00802 ----------------CTGGCACACACACGTCAGTATCTTCTCCC---------
-----
FC88X_01053_00554 ----------------CTGGCACACACACGTCAGTATCTTCTC-----------
-----
FC88X_01070_00563 ----------------TGGCTAAACCCCCGTACGTCACTTCTC-----------
-----
FC88X_01137_01159 --------------CAGTGCAAAAGATCCGTCTGTCTACTCTC-----------
-----
FC88X_01075_00827 ----------------TCCCCACAGACATGCAACACTATTCTT-----------
-----

```
FC88X_01078_00843      ----------------------CAGACGTGC-----------------------
-----
FC88X_00957_00443      ---------------CATACCACACATTTGCTACTCCTTTAA------------
-----
FC88X_00985_00433      ---------------TCTCATGAAACAA-ACATGGCTATACCTC----------
-----
FC88X_01015_00452      ------------------CACGAAACGATATTTTGCTATTTTCAC---------
-----
FC88X_00993_00835      ------------------TCGACAAATTAACCACGGCTCCCTTTT---------
-----
FC88X_01060_00571      ------------------TCGACAAATTAACCACGGCTCCCTTTT---------
-----
FC88X_01004_00923      --------------------GCCAAGTTTCCCACGCTTCTCCATGTG-------
-----
FC88X_01059_00961      --------------------GCCAAGTTTCCCACGCTTCGCCCTGTG-------
-----
FC88X_01053_00628      ----------------------------AACACT-GCTC---------------
-----
FC88X_01136_00857      -------------------CTGTAAGCTAACACTTGCTCTATTCGC--------
-----
FC88X_01002_00792      -----------------TTAGCTAACACGACGATACTCATTCTC----------
-----
FC88X_01132_00930      -----------------TTAGCTAACACGACGATACTCATTCTCCTC-------
-----
FC88X_01076_00553      -----------------TTAGCAATCATGGCGTAAACAATTCTC----------
-----
FC88X_01127_00617      -----------------TGGGTCACTTGTA-GGTGCACTGTTTTC---------
-----
FC88X_01106_00835      ------------------CTATCACTACTGTAGTGAGCACTTCTC---------
-----
FC88X_01146_01172      ------------TACCCCCAACCAAAACTGCAATGCTTT---------------
-----
FC88X_01111_01219      -------------------------TACTACAATCTTT----------------
-----
```
