# Supplementary material for: MRP1-CD28 bi-specific oligonucleotide aptamers: target costimulation to drug-resistant melanoma cancer stem cells
Source: Oncotarget. 2016 Mar 15;7(17):23182–96. doi: 10.18632/oncotarget.8095 (PMC5029619; doi:10.18632/oncotarget.8095)
Supplement: Supplementary file 3 [file oncotarget-07-23182-s003.pdf]

```
@T6XXI:00014:00023
GGGGAATTCTAATACGACTCACTATAGGGAGAGGGAGAATAGTCCGATATACGTCGTGGCTCTCTGC
+
?AF4@69649999==;<;B@@:CB@??<.77777.716003;6559998:==<<<;73243433381
@T6XXI:00024:00017
GGGAGAAGGAGAAAGGAAGACATAGGTGCCGTGGAGAAAGGCATGGCTATT
+
8B88-1-1-////+/,424337733136601110033..+.4...3555.10
@T6XXI:00025:00009
GGGGAATCAATACGACTCACTATAGGGAGAGGG
+
,,,*,*//8:9;>=948::000..557765572
@T6XXI:00026:00011
GGGAGAAGGGAGAAAGGAAGGAGAACACGCACCCGGACCAACGGTGATCTATTCTCCCCTCTCCCTATAGTGAGTCG
TATTAGAATTCCCC
+
//+/;;249-48446<@BCE@BBB@CCBBCCDC>@=@A596;989BAB@??>==7777-
7====9??@@A@A>>;8////,/9>7A>@CC7
@T6XXI:00026:00012
GGGAGAAGGGAGAAAGGAAGAAAGATCGCGGCTCAAACACCAGGGAACTATTCTCCCTCTCCCTATAGTGAGTCGTA
TTAGAATTCCCC
+
<<8<CB@CE@BBAB?B@C@BBC>AABBBA<=:;:BC>BBB?A?@<@=BAAA>;88?8>>99?9??AAAC??@A>?>=
=9<@@<<;<AA6
@T6XXI:00029:00009
CTATCGACTCGAGATCTGCGGCTTTGGACAGTCCCTCTGTACTGTGAAGCCACAGATGTCGAGTCGACTAGCTTCCT
TTCTCCCCTTC
+
AAA@99:AB?=>CA?9999;7;CD>C@BB>?777.7>7777=:0//73.9:9;;:000000>==>A@?8787737:=
?97/000+,*+
@T6XXI:00029:00012
GGGAGAAGGGAAAGAAAGGAGAGAGCGCCCCAAACGATTTGTTGACTATTCTCCCCTC
+
<<19=<-11-11--**)*)******,899969989?CKC9BB@C;;8?>B6////+-.
@T6XXI:00031:00016
GGGGAATTCTAATACGACTCACTATAGGGAGAGGGAGAATAGACACGACT
+
999/42424@@@CCCCC@@>A<AABBAE@CBAAA<???>??///++++*,
@T6XXI:00032:00011
GGGGGAATTCTAATACGACTCACTATAGGGAGAGGGAGAATAGTCAACAAATCGTTTGGGGCGACTTCTCCTTCCTT
TCTCCCTTCTCCC
+
9994+424244488=>A@A???788::@@>BCDCC>CD>6:;.///78;=7???AC?@CC8BDBBD>::;4434244
-8=;;:=3835>:
@T6XXI:00037:00008
GGGGAATTCTAATACGACGTCACTATAGGGAGAGGGAGAATAGTACGAC
+
999-959594499:<111.0;4=8==;>;6<?777.7==4615....++
@T6XXI:00037:00015
GGGAGAAGGGAGAAAGGAAGGAGAAGTCGCCCCAAACGATTTGTTGA
+
//+/8B?CB?BBCC>B@C@DA@??=87439<95@<277111,16011
@T6XXI:00039:00012
GGGAGAAGGGAGAAAGGAAGGAGAATGCACCGCAGGAAATTACAAGACTATTCTCCCTCCTCC
+
:A>BKD@BA?AABB=B?C@C@@@A<;9BBC?BBCF9;:5;>@9<>=966110..35+.-,-13
@T6XXI:00048:00009
```

```
GGGAGAAGGGAGAAAGGAAGCTAGTCGACTCGAGCATCTGTGGCTTCACAGTACAGAGGGACTCGTCCAAAGCCGC
+
4:69BBADC>BA?@<A5;49888731711./5.......4444698611../.344:74442-----222+..466
@T6XXI:00014:00075
CTAGTCACTCGAGATCTGCGGCTTGGACAGTCCCTCTGTACTGATGAAGCCACA
+
8:111:1::CB@@>;99111,00,.346997>A;?76/<0/,,,,,00+42444
@T6XXI:00028:00084
GGGAGAAGGGAGAAAGGAAGGGGAGAAGGGAGAAAGGAAGCTAGTCGACTCGACATCTGTGGCTTCACAGTACAGAG
GGACTGTCCAAAGCCGCA
+
CD>BBA>AA:AAAB>B@CACCB9ABB?AC=?:99,7<>;@AA@?@BCCC?C@<<3788743<==<;??C==<?=;4/
/,/88///9:74447449
@T6XXI:00029:00088
CGACTCGAGATCTGCGGCTTTGGACAGTCCCTCTGTACTGTGAAGCCACAGATGTCGAGTCGACTAGCTTCCTTTCT
CCCTTCTCCCCTTCCTTTCTCCCTTCCTCCCTTCCTTCTCCCTTCCTCCC
+
NCCCCCBCAAABBBAB@AAA<BADCDCCBB9;;99>?9;;@@A@;;9;?>???AAACDBFBB@@?C@AB?@=@C9<;
@A7=071111,10177957644+0/0/000+04423/003303/0/000+
@T6XXI:00030:00092
GGGGAATTCTAATACGACTCACTATAGGGAGAGGGAGAA
+
A::292959989@@@A@B@@>??;;71766....+...,
@T6XXI:00030:00094
GGGAGAAGGGAGAAAGGAAGTGCAGTTCGCGGTTAGTGAAGGGAAAACTATTCTCCCTCTCCCTAT
+
DE@;<9;@@<ABBEBCAC@<78>=4424:;;23133337111,166,337953167,11111,100
@T6XXI:00031:00060
GGGGAATTCTAATACGACTCACTATAGGAGAGGGAGAATAGTCAACAAATCGTTTGGGCGACTTCTCCTTCCTTTCT
CCCTTCTCCC
+
7@@1<-9599888499;;<BC;;>;00,.;:=<9??898>@=868894261111,11,1757:7445,-
,/46938/88149;6775
@T6XXI:00032:00095
GGGGAATTCTAATACGACTCACTATAGGGAGAGGGAGAATAGTTTGTACATCGCCCGTGCAACATGCTTCCTTTCTC
CCTTCTCCC
+
?>>4@6969>@=ACCCC@@@B?>?>777.7>===9?A?<:9444-
4:;:=BBCBF>CCBBB?BBBAAA?=;;>6<<;;:;387<?:
@T6XXI:00037:00083
GGGAGAAGGGAGAAAGGAAGGAGAAGTCGCCCCAAACGATTTGTTGACTATTCTCCCTCTCCCTATAGTGAGTCGTA
TTAGAATTCCCC
+
BBACDC?A@:@@@@<@=@=A?B@A?A@A??@@7@I>BBCDB>CC@CBAAA?<=<;?:888=@:>??????AA????@
@?D??<?>@@@5
@T6XXI:00037:00090
CTAGTCGACTCGAGATCTGCGGCTTTGGACAGTCCCTCTGTACTGTGAAGCCACAGATGTCGAGTCGACTAGCTTCC
TTTCTCCCTTCCTCCC
+
AA-,,-
,,/1:>CCDCCBBBC@AAA=A?BBBCCCC>BAAC:::???=A<<@=AACBBBBAABB::<<;839311013662633
522,.,.-22
@T6XXI:00038:00089
CGACTCGAGATCTGCGGCTTTGGACAGTCCCTCTGTACTGTGAAGCCACAGATGTCGAGTCGACCTAGCTTCCTTTC
TCCCTTCTCCC
+
```

```
CBBBABCCDBBBCDB?94491959CGCECC@C?@@CCCCCCAB>BB<@@AA???@???@>>?00,0<07948;>?6;
;B?8=5:1=>:
@T6XXI:00039:00085
GGGAGAAGGGAGAAAGGAAGTGCAGTTCGCGGTTAGTGAAGGGAAAACTATTCTCCCTCTCCCTATAGTGAGTCGTA
TTAGAACTTC
+
44-4@@>AA:<;AB<@=@?B339:4429=99;<8;:99?7::7:BB8@@?;77666,111668<<;===;;734-1-
-,---,-3/0
@T6XXI:00039:00093
GGGGAATTCTAATACGACTCACTATAGGGAGAGGGAGAATAGTCAACAAATCGTTTGGGGCGACTTCTCCTTCCTTT
CTCCCTTCC
+
DDB6=;=6;99@BCDCBBCCC@B7;777.7==@B>AAA?@?888<=?@A<BB???9?BB4;7><<371130234463
6433+-,-,
@T6XXI:00039:00095
GGGGAATTCTAATACGACTCACTATAGGGAGAGGGAGAATAGTCAACAAATCGTTTGGGGCGACTTCTCCTTCCTTT
CTCCCTTCTCCC
+
@@?4>>@>@@@@BCCCA@@BA=;@@6995:@@BC>BBB@AAAADA@AAA<BBBBC?BBB8BBBBC?BBB>?<?>@A;
@@@D<@689A?:
@T6XXI:00040:00097
GGGAGAAGGGAGAAAGGAAGGAGAAGTCGCCCCCAAAACGATTTGTTG
+
99-4999AC?BBBH@C?B?A<<;;:;/103>>>/B@>*0..,,*,.,.
@T6XXI:00041:00090
GGGAGAAGGGAGAAAGGAAGCTAGTCACTCGACCATCTGTGGCTTCACAGTACAGAGGGACTGTCCAAAGCCGCAGA
TCTCGAGTCGACTAG
+
BDADDC@EE>BCBB>B>?=@AABBCC>;33:0/,....47989;9<CCCCBCB?9300,4::=@@>AA<@@>???=<
=<===AAAA@??=88
@T6XXI:00042:00079
GGGGAATTCTAATACGACTCACTATAGGGAGAGGGAGAATAGTCACATTCATGTTTGTGACCCGTGTCTAGC
+
A=;0;,/,///7699;;<BBBABBB::;7;CCDE>CAAA?@?=@B88:>ABABCC<>>=034,//----...
@T6XXI:00042:00083
TCGAGATCTGCGGCTTTGGACAGTCCCTCTGTACTGTGAAGCCACAGATGTCGAGTCGACTAGCTTCCTTTCTCCCT
TC
+
BD>>@BCCCDA@=ACC?C@CC;;;??<?CCB@@@D@@@C@@@=ACCCAA??BDCECCCAAAA894898996//88+/
,.
@T6XXI:00043:00083
GGGAGAAGGGAGAAAGGAAGGAGAATGCAGCGAAATGAACACGTCGGAGCCGC
+
::599>;>B?A@@A<A@C@D@CAA>@A;8837>>6??A5;:6117A26/7533
@T6XXI:00044:00076
GGGAGAAGGGAGAAAGGAAGGAGACACGGTCACAAACATGAATGCGACTATTCTCCCTCTCCCTATAGTGAGTCGTA
TTAGAACTTCCCC
+
@B>=><@BC@CCCC?C@C@B?ABCCCCC@BBBBBC@BBBCC@BBCCCCBCB?B@><<9961789:87A@?ABBBABA
A;;>9,//58974
@T6XXI:00044:00077
GGGGAATTCTAATACGACTCACTATAGGGAGAGGGAGAATAGTCAACAAATCGTTTGGGGCGACTACTCCTTA
+
EEC:D@C<;?C@CDCBBBBBA:;;CAAB@BBBCC>?@@CB???@@>@@B?CCCCC9AAA477?7717.5,-,,
@T6XXI:00044:00084
GGGGAATCTAATACGACTCACTATAGGAGAGGGA
+
```

111*/,///656=====;<<3644..,.0.16,1
@T6XXI:00044:00087
GGGGAATTCTAATACGACTCACTATAGGGAGAGGGAGAATAGCCGTAATGTTTGCTTACGCCTTTTCTCTTCCTTTC
TCCCTTCTCCC
+
CEECC@D@BBBDCCBAAABAA@BBBB@C>?;?>>=;?B@AA??;>=:8=;;;6;?>;==;979991;;;::;344-
44;91889??A<
@T6XXI:00044:00090
GGGAGAAGGGAGAAAGGAAGATCAGCCGGGAGTTGTTGAGATTACAGCTATTCTCCCCCTCCCTATAGTGAGTCGTA
TTAGAATTCCCC
+
DC@DCCACC>BBCC?C@C?BBCCCCC@CC>A@A??@<@@???<??B??ACC6949999/8@A=BBBBCCCCC????A
@<=;97859;;1
@T6XXI:00044:00098
GGGAGAAGGGAGAAAGGAAGGAGAAGTCGCCCCAAACGATTTGTTGACTATTCTCCCTCCTCCCTATAGTG
+
9=<AEFBCDACCCC<A=@7;9;BCACBDDDDC8BB>B?88996988111.+*++000++*+222244887/
@T6XXI:00046:00083
GGGAGAAGGGAGAAAGGAAGCTAGTCGACTCGACATCTGTGGCTTCACAGTACAGAGGGACTGTCCAAAGCCGCAGA
TCTCGAGTCGACTAGCTACGTACG
+
DD@CCB@BB>B9998;@B?<;;>?>011000<1;11116998<;<?AA???@>>;444-
8;<@HC>BD?==;=?>>?@<==<<<>?;444444//6660,+
@T6XXI:00046:00088
GGGAGAAGGGAGAAAGGAAGCTAGTCGACTCGACATCTGTGGCTTCACAGTACAGAGGGACTGTCCAAAGCCGCAGA
TCTCGAGTCGACAT
+
CCCBBB@CHBBBBC>B@B?BC?>>>111@>>>3;:::6::B:???A???BCC@@=744-
499;??=?A:????>===;;884999/,,55.
@T6XXI:00046:00097
GGGGAATTCTAATACGACTCACTATAGGGAGAGGGAGAATAGTACAACTGAAATC
+
999/98><@BB>BCBDBBBKDCB@?:77.7>>>>6;9?<==511-/,/---32--
@T6XXI:00049:00084
GGGAGAAGGGAGAAAGGAAGGAGAATGCACCGCAGGAAATTGCAAGACTATTCTCCCTCTCCCTATAGTGAGTCGTA
TTAGAATTCCCC
+
DICCCB@DC@CCCC>C;;:<CCBB?BBCCC@CCBB@BB>B?BAA=A???AA?ACCC=?=AAA<A???????A@?AA<
<8;==855<>@6
@T6XXI:00049:00097
GGGGAATTCTAATACGACTCACTATAGG
+
DDD9EAC9;;@?998>@@;4402,,,,*
@T6XXI:00004:00130
CTAGTCGACTCGAGATCTGCGGCTTTGGACAGTCCCTCTGTACGTGAGC
+
ECCDC:@@CCECCCCBBBBBB@BBB?C?BBBB:77-7==:>@777****
@T6XXI:00005:00133
GGGGAATTCTAATACGACTCACTATAGGGAGAGGGAGAATAGCCATGTCGCGTCTGGCCACACGTTCTCCTTCCTTT
CTCCCTTCTCCC
+
BCC8A@A@CCD@DCB<<<@BA>?A8777.7====9?<<=>8779;;;<<7879;;;8;;<<8@;;7830/323:574
7444+-,-+000
@T6XXI:00006:00122
CTAGTCACTCGAGATCTGCGGCTTTGGACAGTCCCTCTGTACTGTGAAGCCACAGATGTCGAGTCGACTAGCTTCCT
TTCTCCCTTCTCCC
+

```
KB<;;944:@999ABBBCCB@CCC>C=@@CB???;?BBBBBDC;88=<ADACCC@@@CBBBC:;<BAA?;;88998<
<;>CCC==<;;::1
@T6XXI:00006:00132
GGGGAATTCTAATACGACTCACTATAGGAGAGGGAGAATAGCTGTCCCT
+
@@A6@<@@B@A?BCCCC@@<B:66=./.0>>BC>BC>;;55,,,,,,**
@T6XXI:00006:00144
GGAGAAGG
+
92/**)*)
@T6XXI:00010:00117
GGGAGAAGGGAGAAAGGAAGGAGAAGTCGCCCCAAACGATTTGTTGACTATTCTCCCTCTCCCTATAGTGAGTCGTA
TTAGAATTCCCC
+
CDACDD@BA=BCCCAC@CBCA@@>@BBBCCCC9CC>CBCBC>AA?BCCCB>=>B==;888@C<A@@???AAAAAAAA
A=@@A>A=@@@5
@T6XXI:00012:00116
GGGGAATTCTAATACGACTCACTATAGGGAGAGGGAGAATAGACTGTTGCTAAACTTCAGTCTCGTTCTCTTCCTTT
CTCCCTTCCACC
+
;44,48?<?@@=@BBCCCBB@?>>?777.7>>>?:=77404444;:;7;;;5=:@A>@@A===@@@=@A@@><:=;7
94//,/77,--,
@T6XXI:00013:00148
GGGGAATTCTAATACGACTCACTATAGGAGAGGGAGAATAGCCAATTCATGTTGTACGCTGCAGTTCTCTTCCTTTC
TCCCTTCTCCC
+
=@?4?<?=AAADCCCCCBB@B:999//-
0;;BC>CBB@DA??;<;=568<><=@?BCDCCCCCBB?BCCB@DBCC>BBBC>C@C???9
@T6XXI:00015:00109
GGGGAATTCTAATACGACTCACTATAGGGAGAGGGAGAATAGTCCAACGAAATCG
+
999/48>8>@B?BCCCCCDCA?AA@9::7:@CCE?AABA=88//,/---472400
@T6XXI:00017:00120
GGGAGAAGGGAGAAAGGAAGCTAGTCACTCGACATCTGTGGCTTCACAGTACAGAGGGACTGTCCAAAGCCGCAGAT
CTCGAGTCGACTAG
+
DDACCB?AA=BB==:B=><@CB@AA????@>=??:;:CDE?AA@CB?;>?@CA=888466<;@??@A<AA=@@<<:<
=A>99=A@@@???C
@T6XXI:00018:00126
CTAGTCACTCGAGATCTGCGGCTTTGGACAGTCCCTCTGTACTGTGAAGCCACAGATGTCGAGTCGACTAGCTTCCT
TTCCTCCCTTCCTCCC
+
;@9444444;499?BBBCCC<<;C<@788><777.16<<?ABBBAA=<>B>BBCCCCBBC?ABBCB?A?A>66:755
7-/-/7700*/,.--+
@T6XXI:00019:00113
GGGAGAAGGGAGAAAGGAAGCTAGTCGACTCGACATCTGTGGCTTCACAGTACAGAGGGACTGTCCAAAGCCGCAGA
TCTCGAG
+
DDABAA@DD?BBBB:A=B6<;AABBBBBBBCC??@BCBBBCC@BB?ACC>AACB?9733-
4889=?=?@;AA>AA@@===@???A
@T6XXI:00019:00133
GGGAGAAGGGAGAAAGGAAGGAGAAGCGCGCTTAAGACTGCGTTAAGCTATTCTCCCTCTCCCTATAGTGAGTCGTA
TTAGAATTCCCC
+
AA<AAA@EC@BBBC<@=@AB@B@@=@DCCCAA>BECCCACCCD@C?AABBDBDDBC@=<<DCBCCC@?@CCCBB@@C
C@CCC@C@FCC:
@T6XXI:00023:00125
```

```
CTAGTCGACTCGAGATCTGCGGCTTTGGGACAGTCCCTCTGTACTGTGAAGCCACAGATGTCGAGTCGACTAGCTTC
CTTTCCTCC
+
-
,,,,,,/44;;B?AEC::9CAB??577,37777@@<AA<<9?788===<AEAA@@@?<;:?@=:9488733796645
55+.,,22
@T6XXI:00024:00109
GGGGAATTCTAATACGACTCACTATAGGGAGAGGGAGAATAGTCGTA
+
CCC:C@CACBB@CBBBBBBB@?>?A116.7==BCACA>;:4./-++*
@T6XXI:00024:00117
GGGGAATTCTAATACGACTCACTATAGGGAGAGGGAGAATAGTGACGTGTTGTGCGGCGCTCACTGCCTCCTTCCTT
CTCCCTTCTCCC
+
494,489244424444;?BBBCCCC???9???BC>BBB@@@@????=<<A:<<@@@;?????AA???=@;7877,/-
.31566../:86
@T6XXI:00024:00140
GGGTCAATACGACTCACTATAGGGAGAGGGAGAATAGCTATCGTCACTCGACATAGTGCTTCTCCTTCCTTTCTCCC
TTCTCCC
+
DC>BDDACB@@@BBBBCCKC:9:5;@@@CBCEC?C9;;CCCCBBBBBCCCCBCBBBBAA?>A::7;DB8;<7<@@@;
A?@@@A<
@T6XXI:00024:00149
GGGGAATTCTAATACGACTCACTATAGGGAGAGGGAGAATAGCCGCGTGATTACGAATTTAGCTGTTCTCTTCCTTT
CTCCCTTCTCCC
+
EEE:C@C@CCB@BCCCCBAAC9??7677-
777>B>??@?@@AA>A@?????;=?<<38818@@@@@?ABAA3738?:>@@A:=;=<994
@T6XXI:00025:00128
GGGAGAAGGGAGAAAGGAAGTGCAGTTCGCGGTTAGTGAAGGGAAAAACTATTCTCCCTCTCCCTATAGTGAGTCGT
ATTAGAATTCCCC
+
DDCCCC?BB?CCBB?B?B>ABBBBBB@BBBC@B@ABABB>9?-
6666,8AAB@@??B>??@CCCC@@@@AAAA@A@??><??=;=8<<<4
@T6XXI:00026:00136
GGGAGAAGGGAGAAAGGAAGGGAATAGCAGCAGTCGGCAACGTGAAACTATTCTCCCTCTCCCTATAGTGAGTCGTA
TTAGAATTCCCC
+
:@:=?=?BB<A@@B<A>@;>>0843?79:777??9@?=@=?GCDCB>CCCC@BCCD@<:;AB>B<999@A?@A==<<
;3444/02444.
@T6XXI:00027:00106
CTAGTCACTCGAGATCTGCGGCTTTGGACAGTCCCTCTGTACTGTGAAGCCACAGATGTCGAGTCGACTAGCTTCCT
TTCTCCCTTCTCCC
+
///////999499ABBAG<;9<@@<A9;<CCBBB<BB??@@CBBC;838B=<<=BB@@@A@CCABC@@?B??:;?>@
B<B???6979003,
@T6XXI:00027:00125
GGGAGAAGGGAGAAAGGAAGCTAGTCACTCGACATCTGTGGCTTCACAGTACAGAGGGACTGTCCAAAGCCGCAGAT
CTCGAGTCGACTAG
+
CF@AAA@CC>CCCD?C?B=@@B@BBB???DC::<;::BAA<;<9<BBBCBDCC=77717=>?@@>@C<A@>988888
=<=8==<=====A<
@T6XXI:00030:00141
GGGGAATTCTAATACGACTCACTATAGGAGAGGGAGAATAGTGACGTGTTGTGCGGCGCTCACTGTCTCCTTCCTTT
CTCCCTTCTCCC
+
```

```
BDHCC;:<<CCCCB@BBBBBA7444//,0;:=?=@ECCA@@???A<<<A?===AA?=????<==@<:??5:9;/33,
4::=7?=?@@A;
@T6XXI:00031:00108
GGGAGAAGGGAGAAAGGAAGGGGAGAAGGGAGAAAGGAAGCTAGTCACTCGA
+
CB?CDC@CC>BBBC>AC@@CCC:B@@=@C>B::7.73747A781?=111...
@T6XXI:00032:00116
GGGGAATTCTAATACGACTCACTATAGCGAGAGGGAGAATAGATCACCGTTGGTTCGGGTGCGCGTTCTCCTTCCTT
TCTCCCTTCTCCC
+
EDC:C@B?BBB?BBBBCCCCB@CAC===@CBBCC>BBB>AAC@??AC@CC595:;>?><B@???CC@ECB?A>A9==
9=@@C<A>===?:
@T6XXI:00033:00102
GGGAGAAGGGAGAAAGGAAGGAGACACGGTCACAAACATGAATGTGACTATTCTCCCTCTCCCTATAGTGAGTCGTA
TTAGAACTTCCCC
+
DC>CBC@CH>@ACC>B=@=@=@BCCCCC@C@=9997;CFCC@AAABBBBBE@CC@@<@AAA@;@@@@AAA@@@@A==
=<;;;.0/9@=54
@T6XXI:00033:00137
GGGGAATTCTAATACGACTCACTATAGGGAGAGGGAGAATAGTCTAAGTGA
+
CBB9B=?=ACB@BBBBB?>?B@CB@766-1;=??<AAA???87///,--0,
@T6XXI:00033:00139
GGGGAATTCTAATACGACTCACTATAGGGAGAGGGAGAATAGTCAACAAATCGTTTGGGGCGACTTCTCCTTCCTTT
CTCCCTTCTCCC
+
B?A4@<@=@CC@CC???BB?B@B?;111.7<=BC>ABB@CA??=<;:>::A???E>BBB7BCBBC@B?@==<@=@@;
@@@@;@<??AA<
@T6XXI:00034:00141
GGGAGAAGGGAGAAAGGAAGCTAGTCACTCGACATCTGTGGCTTCACAGTACAGAGGGACTGTCCAAAACCGCAGAT
CTCGAGTCGACTAG
+
DE>CCC?BA=ABBB>C@B@BCB>@@::;AAA@?@@@@CCC@BB?BC@@@?@@@<<<=;===<;:<===4::<=====
???ACCCBBAAABB
@T6XXI:00034:00148
GGGGAATTCTA
+
BDC9B><=11/
@T6XXI:00035:00118
GGGGAATTCTAATACGACTCACTATAGGGAGAGGGAGAATAGTCAACCAAATC
+
>DB1>,1,//77/444=9..........+111668<??984++++*+**..0.
@T6XXI:00035:00122
GGGGAATTCTAATACGACTCACTATAGGAGAGGGAGAATAGCTAAAAACATTATTCCCGTCGCTGTTCTCTTCCTTT
CTCCCTTCTCCC
+
C@@6@?A@CBB@BBBBCCCBC:999..,0;;>B?EDD@A??@B@@@@2BCC?@B?CC>?@A<=@CG@C;;<CC@CB8
;>CE=A?A??<9
@T6XXI:00036:00106
GGGAGAAGGGAGAAAGGAAGGAGACAGCCCCAGAGTACAGTCCAGAACTATCTCCCTCTCCCTATAGTGAGTCGTA
+
==:?CCCDCBFCBB>@7969<877A??@BB69989483377;:4722,,,,,22306/77@7>;30444034433+
@T6XXI:00036:00110
GGGGAATTCTAATACGACTCACTATAGGGAGAGGGAGAATAGCCAAACTTT
+
9>?/969;=@A799B??=<<>:@@CCCB>A@???9?=?83004/00+---+
@T6XXI:00036:00119
```

```
GGGGAATTCGACTCACTATAGGGAGAGGGAGAATAGCCGTGGTTTCTTCCATCGCGGTATTCTCTTCCTTTCTCCCT
TCTCCC
+
EEEBC@C?CCCCCCCC>;;78808@==?<?BA=BC<=<=CG?HC@BCBDCCBCCBB?BBBCBBBBBB9;<9;@@@:@
687??<
@T6XXI:00036:00134
GGGAGAAGGGAGAAAGGAAGCTAGTCGACTCGACATCTGTGGCTTCACAGTACAGAGGGACTGTCCAAAGCCGCAGA
TCTCGA
+
AA<ACC@CC@AA?B>B?B>BAC?@@???@@A@;;;AA@@BB=?A@CCCCAAADC:8:;:====;<9=A<AC>999AA
AA989@
@T6XXI:00036:00149
GGGAGAAGGGAGAAAGGAAGCTAGTCACTCGACCATCTGTGGCTTCACAGTACAGAGGGACTGTCCAAAGCCGCAGA
TCTCGAGTCGACTAG
+
GC?BA@=@@:?99909;>=ACAACC?100:>00,.333:;?=?@=@BBBBBCC>;333,4;;;<=>AA=BA@B@<?;
<=<<AAAA???ABAA
@T6XXI:00037:00135
GGGAGAAGGGAGAAAGGAAGCTAGTCGACTCGACATCTGTGGCTTCACAGTACAGAGGGACTGTCCAAAGCCGCAGA
TCTCGAG
+
AA>ABB@BC?BBDG>C@C=@@CAAACBCA@B>@ABBCCCCC@BB?BCC???@?;655555;<==@=@@<@A?AAAA?
??AA===
@T6XXI:00038:00101
GGGAGAAGGGAGAAAGGAAGCTAGTCGACTCGACATCTGTGGCTTCACAGTACAGAGGGACTGTCCAAAGCCGCAGA
TCTCGAG
+
DIDCCB>@=:=@@@:@@C;<;CCCCBBB@@@>99:???CCC?AB?ACAAC@@@>655555;<>??>AB=AB>AAA@<
@=A>A=@
@T6XXI:00038:00102
GGGAGAAGGGAGAAAGGAAGCTGCGTCGACACTCTGTGGCTTCACAGTACAGAGGGACTGTCCCAAAGCCGCAGATC
TCGAGTCGACT
+
GDAAAA@CC@CCBA=B=>;:;00..6336861111634/39<=ACCBCFBB@@AB=A>DD877.9?9AC?A@@@@DB
BBBCBBBAAAA
@T6XXI:00039:00121
GGGGAATTCTAATACGACTCACTATAGGGAGAGGGAGAATAGTCAACAAATCGTTTGGGGCGACTTCTCCTTCCTTT
CTCCCTTCTCCC
+
999/9<@?CDB@BA@@BABBBA@99111.7@ACC>CCC@A<<<A=<==<;>A@BC;???6?AABCACCC>?<??AB<
A@@@<@>@@AA<
@T6XXI:00040:00110
GGGAGAAGGGAGAAAGGAAGAGAAGCACACCGCTGAAGAATTTGTGGCTATTCTCCCTCTCCTATAGTGAGTCGTAT
TAGAACTTCCCC
+
BB=BAA@AC@DDDD>B@C@CA@@@GCA@@?=A;:=C?B@?B?69996:BBA<>944-
4444239??B@??661....,-222.10111,
@T6XXI:00040:00120
GGGAGAAGGGAGAAAGGAAGCTAGTCGACTCGACATCTGTGGCTTCACAGTACAGAGGGACTGTCAAGCCAGCAGAT
CTCGAGTCGACTAG
+
CD@CCC@CC>B@@=:>==<@AA;;;0.,0707-0/55556@?@BBJBBBCBB??<77718;88//,+++*+++-
1100030006446:8:<
@T6XXI:00040:00122
GGGAGAAGGGAGAAAGGAAGGGACACGCCGCCTTTAATCAGGGAAGAAACTATTCTCCCTCTCCCTATAGTGAGTCG
TATTAGAATTCCCC
+
```

```
@BACCC@DC>9<;B@B@C@CC>ABB???>>?>@@>C@CBA@BBB@?BB>CBCC@BCBBABBC@A;BBBABBCCBA@@
@@@>@AA?A=BBA6
@T6XXI:00040:00128
GGGAGAAGGGAGAAAGGAAGTGCAGCTCGCGGTTAGTGAAGGGAAAAACTATTCTCCCTCTCCCTATAGTGAGTCGT
ATTAGAATTCCCC
+
DDACBC@BB=??=A;B@B@BB;;;111/8888;;???AA>AB>BCCC2CC<<;<CCC?DC@??<?AAAACAA???A@
AA>@@@<::<??5
@T6XXI:00041:00109
GGGAGAAGGGAGAAAGGAAGCTAGTCACTCGACATCTGTGGCTTCACAGTACAGAGGGACTGTCCAAAGCCGCAGAT
CTCGAGTCGACTAG
+
DB@ECC@BC>DDBB=B?B?BDB>AA::;?@B:;:AAABCD=?@@CCB@BBCD@<88946;;@A@?=A<AA>@@???A
A@@?@CA===@@@?
@T6XXI:00041:00117
GGGGAATTCTAATACGACTCACTATAGGAGAGGGAGAATAGCCAGCAAGTCTCCCTTATCACCAGCCCTTCCTTCTC
CCTTCTCCC
+
CEH:C@CABCC@BBBCCBAAB>==9..,0;;>B?CCC@@<??8><<<9<AA@A@;@?@@@@A?>@>>5676,/,/16
71;8;;;;6
@T6XXI:00041:00147
GGGAGAAGGGAGAAAGGAAGCTAGTCGACTCGACATCTGTGGCTTCACAGTACAGAGGGACTGTCCAAAGCCGCAGA
TCTC
+
CE@CEB@BD>CAAA==<?6;;A@@;::?ABAB@@?BBBABC?AB?AC@CA@@>A<===;<<<<??8<;6<?<?@BA?
?888
@T6XXI:00042:00112
GGGAGAAGGGAGAAAGGAAGGATATGCCGCAGTGGGAGATTAAACGGCTATTCTCCCTCTCCCTATAGTGAGTCGTA
TTAGAATTCCCC
+
BDACECBCB>@??B>B@BAB=>@?A;;;<CBCBCC=??>?@BC<AA>BCCCAABAA?BBAAA@ABBBB@@@C==?@A
?<??=:95;;<4
@T6XXI:00043:00119
GGGAGAAGGGAGAAAGGAAGAGGAGTGACAGAGGCCAAGTTACTGGCTATTCTCCCTCTCCCTATAGTGAGTCGTAT
TACGAATTCCCC
+
CB=B@@=>?:?99919<?@BCD@CBAAA@??:858=A=<=9<@AC?BBA@?F99919>>BCCCDBD@B@CCCC>=00
,0//:::,111,
@T6XXI:00044:00110
GGGGAATTCTAATACGACTCACTATAGGGAGAGGGAGAATAGTGACGTGTTGTGCGGCGCTCACTGTCTCCTTCCTT
TCTCCCTTCTCCC
+
@F@,459=BBC<=<DCACB;;78??777,611676167;6<<887<<789;@?@@@=@@?A?=;=88877>;9>388
1878A<@:=004,
@T6XXI:00044:00122
GGGGAATTCTAATACGACTCACTATAGGGAGAGGGAGAATAGTTTTTCCCTTCACTAACCGCGAACTGCACTTCCTT
TCTCCCTTCTCCC
+
A@B8AAC@CBB@BA@@BBBBA?><;6695:@@@C>A?@>=AA@CAA1@@:@?@BB??>?>@@B@><;<=>?@@A;<=
=??BC8?;?;;;7
@T6XXI:00044:00127
GGGAGAAGGGAGAAAGGAAGTGCAGTTCGCGGTTAGTGAAGGGAAAAACTATTCTCCCTCTCCCTATAGTGAGTCGT
ATTAGAACTTCCC
+
AD@ABD@B=9A@@@<AACECCABBCC@CBAA=@@D@@@@677.7777+377?@CCCC>BBBCC?CA@@@@@@?=;44
44/40;/43/30+
@T6XXI:00045:00133
```

```
GGGAGAAGGGAGAAAGGAAGAATATTAGGAGGACAGTAGATGCGAGGCTATTCTCCCTCTCCCTATAGTGAGTCGTA
TTAGAATTCCCC
+
GD<==?AEDACBBB8;9;>BB@BCD@BB?::7:???AA<;;:66//,/8;>=@???:;;;=?:<><=;<<?>>?AAB
B?@=@=B7<<9/
@T6XXI:00045:00141
GGGGAATTCTAATACGACTCACTATAGGGAGAGGGAGAATAGTCAACAAATCGTTTGGGGCGACTTCTCCTTCCTTT
CTCCCTTCTCCC
+
EECCD@B@BCB@CBBBAABBB@>97111.7>>>@>CED?@?AAAA@@AA<C?86=-
6114:>?AC@;::;<7:;==7>@AA@A=??AA<
@T6XXI:00047:00131
GGGGAATTCTAATACGACTCACTATAGGGAGAGGGAGAATAGTCAACAAATCGTTTGGGGCGACTTCTCCTTCCTTT
CTCCCTTCTCCC
+
@@:2B6<8<CB?BDCCCDGBBBB??761.7=7776;??:<===AA>AAA<C;;;C>B?B9:::?B<A@@;;7:9;;7
====7;34444-
@T6XXI:00047:00133
GGGGAATTCTAATACGACTCACTATAGGGAGAGGGAGAATAGCTACGTTTAACCGATCGTG
+
@@;2B596<9><AA@ACC@@@@@@BBA@5:7===.7663088>A@@::7.74737:/,++0
@T6XXI:00047:00139
GGGAGAAGGGAGAAAGGAAGAGAAGGGAGAAAGGAAGCTAGTCGACTCGACATCTGTGGCTTCACAGTACAGAGGGA
CTGTCCAAAGCCGCAGATCTCGAGGGGAATTCTAATACGACTCACTATAGGAGAGGGAGAATAGATCACCGTTGTCG
GGTG
+
CD<AAA?AC@B;94-95949888>BB<@=DD>DAC?C@?@C????:70:7/--2223556=;>@AAAAA<:<874-
4B==?@>@>:==388;<878<==AAAA4@>@@:9:??==77//.1127393288444-
4;;:@@=<=@:55**)***...09
@T6XXI:00047:00143
GGGAGAAGGGAGAAAGAAAGCTAGTCACTCGAGATCTGCGGCTTTGGACAGTCCCTCTGTACTGTGAAGCCACAGAT
GTCGAGTCGACTAGCTTCCTTTCTCCCTTCTCCC
+
B:5@@@=@B?BA>>999909>>>?;:;?@@AA:::AABAB@ADE>C<<ACCBCB>B===A889@AAC@@@>@A@@@@
AAAA?@@@C???B@@@9;9;=69:;;4:7:;::7
@T6XXI:00047:00149
GGGAGAAGGGAGAAAGGAAGTGCAGTTCGCGGTTAGTGAAGGGAAAACTATTCTCCCTCTCCCTATAGTGAGTCGTA
TTAGAATTCCCC
+
ECBCCC@CC>DBBB=B@B?B?BBBCA@@@@A@B>BBBBB@??;?@C8B@;>@B?@@;CBCCC>B;?A===;A=<A@@
@>@@?9;3444-
@T6XXI:00048:00101
GGGAGAAGGGAGAAAGGAAGGAGAAAGCGACGTCGGGGAAACGTTTGCTATTCTCCCTCTCCCTATAGTGAGTCGTA
TTAGAATTCCCC
+
AD?CCB@BB@ABCC>CAC=@=?>@A6;<BCCBAACCB4<=9<9AB<@CABA?ACBC@BBBAA:?>?88:::9@@@@:
:58==::<<9<4
@T6XXI:00048:00103
GGGAGAAGGGAGAAAGGAAGCTAGTCGATCGACATCTGTGGCTTCACAGTACAGAGGGACTGTCCAAAGCCGCAGAT
CTCGAGTCGACTAG
+
CC@ECB@BBBDAA?=A>@=?A@>@@BCA?AB;:<?????C@AA?AAAABBAAA=<;<7<?=A@@>@@<@A?BA@=@>
588?AAA@??????
@T6XXI:00048:00108
GGGGAATTCTAATACGACTCACTATAGGGAGAGGGAGAATAGTGACGTGTTGTGCGGCGCTCACTGTCTCCTTCCTT
TCTCCCTTCTCCC
+
```

```
BDKDD<:9;A@>BCCCCA>@@?<>>777.7==?B@;;:@AAAAAAA???=;=<99=9==<<<<:=443/.,/,/568
2:::;687;444-
@T6XXI:00048:00113
GGGAGAAGGGAGAAAGGAAGGAGAAGTCGCCCCAAACGATTTGTTGACTATTCTCCCTCTCCCTATAGTGAGTCGTA
TTAGAATTCCCC
+
CEBCCB@BCACBCB<>;><@=@CC?BCBCCCC9CC>BAABD>CC@BABACC>A???<@@@@A;A@AA@@????<<<?
@=??A<=8<==1
@T6XXI:00048:00148
GGGGAATTCTAATACGACTCACTATAGG
+
999/4297;CCDCCCCCBBBCC@@A:63
@T6XXI:00049:00123
GGGGAATTCTAATACGACTCACTATAGGGAGAGGGAGAATAGGCAATTTAGCGATGCGGGC
+
FCD:C;;;<CB>BBBCC<<;CCC?@777-7>7775:??:://-/978;576;96:7.43+,
@T6XXI:00004:00198
GGGAGAAGGGAGAAAGGAAGAACAGGGCACCGCAGGAAAACGTGAAGCTATTCTCCCTCTCCCTATAGTGAGTCGTA
TTAGAATTCCCC
+
CB@CDD?BB?BBBB>C@C@CC@BCCC>BBB@DCCB>@@@8@BBBH@BBBCHBC;::8;BAAB>BABCCCDCDD:;:A
A?@@@=?8<;;1
@T6XXI:00005:00151
GGGGAATTCTAATACGACTCACTATAGGGAGAGGGAGAATAGTTCAACAAATCGTTTGGGGCGACTTCTCCTTCCTT
TCTCCCTTCCTCCC
+
944,9<@@BDB@BBBCBABBB@B@;996.7><<?<?@<:87//,-
.33;=9<<<<989??58886601770344:;;:999=5:;A6/.54
@T6XXI:00005:00162
GGGAGAAGGGAGAAAGGAAGCTAGTCGGACTCGACATCTGTGGCTTCACAGTACAGAGGACTGTCCAAAGCCGCA
+
AA?ACC@BC98999049977<7737:74:A=:<=>000.0..587::????BA:66//,/64/.78@;4:88>=?
@T6XXI:00005:00165
GGGAGAAGGGAGAAAGGAAGCTAGTCACTCGACATCTGTGGCTTCACAGTACAGAGGGACTGTCCAAAGCCGCAGAT
CTCGAGTCGACTAG
+
CC>B@>58909@@@<A=@=;;;AA@:7686=776666777589<>CDBBACCBA88858@@A@@>@@<@HAA999AA
===BA@@@??9:9@
@T6XXI:00006:00154
GGGGAATTCTAATACGACTCACTATAGGGAGAGGGAGAATAGTCAACAAATCGTTTGGGGCGACTTCTCCTTCCTTT
CTCCCTTCTCCC
+
A??4>;>=@BB@CBBBCBBB??;99111.7??CC>CBB>@9AABC<9<<7??AAA<AAA5@@AAB>@BB<<9<<@@<
@@@A?A8:8AA?
@T6XXI:00006:00184
GGGGAATTCTAATACGACTCACTATAGGGAGAGGGAGAATAGTTCGCCAATATCCCGCGGCGTGTTCTCCTTCCTTT
CTCCCTTCTCCC
+
B?:7A8;;<CC@CB??BBBB?AB97111.7>>>@>BCA>B=A>;@@@9?;?@??A>BCC@BAB@B?===9<;><?B<
AAAA=A=@<<;6
@T6XXI:00007:00180
GGGAGAAGGGAGAAAGGAAAGTGGCAGGT
+
??>?BA@CB;<<>@8<-11+...,///,/
@T6XXI:00007:00183
GGGGAATTCTAATACGACTCACTATAGGGAGAGGGAGAATAGTCAACAAAATCGTTTGGGGCGACTTCTCCTTCCTT
TCTCCCTTCTCCC
```

```
+
444,4295;;;?A;<<B?@@A?<?>777,6<=>@>BCA?AA==<;94444+1666>8<9<56699;8?6700/3/43
,04444::<98=:
@T6XXI:00008:00163
GGGAGAAGGGAGAAAGGAAGGGACAGTCCATCTGTGGCTTCACAGTACAGAGGGACTGTCCAAAGCCGCAGATCTCG
AGTCGACTAG
+
AA?AEC@EC;;<CC>@=@?@@<?>>?:;4;AB???B?BCACBBB@ACB@===C;??B@@B?AA;@@;<?????>?@?
??@CDCBBBE
@T6XXI:00010:00171
GGGAGAAGGGAGAAAGGAAGGTGTACGTGCGGTTGGGGAAGTCGCGACTATTCTCCCTCTCCCTATAGTGAGTCGTA
TTAGAACTTCCCC
+
94-4??<>>194@A>BCL@C@BBBCCGCA<=;9@CDL:D@B>9933739<>=@<BA;@66172600043--
+,++++3347<;44/344+
@T6XXI:00011:00170
GGGAGAAGGGAGAAAGGAAGGTGAACAGAGCGCAAACGAATACGTCTCTATTCTCCCTCTCCCTATAGTGAGTCGTA
TTAGAATTCCCC
+
HE@CAB>AA=BCBB=@?A@D?BAB@CBCCCCB>@@<@@A?AAADCCCCBEB@CBBB>ABAED@CCCB@@@BA@AAA;
:8:BD?CECCD8
@T6XXI:00011:00172
GGGGAATTCTAATACGACTCACTATAGGGAGAGGAGAATAGTAGGACGT
+
999/4:A@CBB?BCCCBBAA@??>9::?86=;;:?=97;784?;3//,+
@T6XXI:00012:00154
GCTATCACTCGAGATCTGCGGCTTTGGACAGTCCCTCTGTACTGTGAAGCCACAGATGTCGAGTCGACTAGCTTCCT
TTCT
+
4444444499=ABBBAB>>;888,77<736699916::::??A;535;@=@@ABBCC<::@;7993333110101
>,1.
@T6XXI:00013:00151
GGGAGAAGGGAGAAAGGAAGTGCAGTTCGCAGTTAGTGAAGGGAAAAACTATTCTCCCTCTCCCTATAGTGAGTCGT
ATTAGAACTTCCCC
+
EEACCB@BB>CCBB;@>@@@>>@BCCB?@@ABC?BBBCC@CD>CCCC2CCCD?BAA<;AAA?A<AAA=<<=A;77;;
;;76;;.00>;97.
@T6XXI:00013:00155
GGGAGAAGGGAGAAAGGAAGGAGAAGTCGCCCAAACCGATTGTTCG
+
DEADEC=@@9>>>>9>595948377768799,38,31375,..,..
@T6XXI:00013:00179
GGGGAATTCTAATACGACTCACTATAGGAGAGGGAGAATAGTCAACAAATCGTTTGGGGCGACTTCTCCTTCCTTTC
TTCCTTTCCATC
+
=99292924999=@BCBABBDA>>>77477111,1102356888<<<<;AAA??;@CA7??BAA>?AA5758>;;6<
732223+/,///
@T6XXI:00014:00154
GGGAGAAGGGAGAAAGGAAGGAGAAGTCGCCCCAAACGATTTGTTGACTATTCTCCCTCTCCCTATAGTGAGTCGTA
TTAGAATTCCCC
+
@B6;;B>BB@CCCN@H=A=@A@BC@CCDCCCC9CB<A?771,1;:6?BBB@@@@AC=988>@:>???@@0//;/..4
99<=77:9>=>4
@T6XXI:00014:00194
GGGAGAAGGGAGAAAGGAAGCTAGTCGACTCGACATCGTGTGCTTCA
+
CECCCB>==6>98809=B@ADAC@?:;;A@>>:77********2247
```

```
@T6XXI:00014:00195
GGGGAATTCTAATACGACTCACTATAGGGAGAGGGAGGATAGTCAACAAATCGTTTGGGGCGACTTCTCCTTCCTTT
CTCCCTTCTCCC
+
@A>6@=A>ABB@CCB?BBBBBAB97111.777?C@BA??AAAAAA?8884:????<A?A5?@@AA>A@A7869>BA=
@A??;?=@@BBA
@T6XXI:00016:00150
GGGAGAAGGGAGAAAGGAAGCAGTCCATCTGTGGCTTCACAGTACAGAGGGACTGTCCAAAGCCGCAGATCTCGAGT
CGACTAG
+
BD@BBA@CC>BCBC?C@B@B?>>675:???ABB?BC?CB?A==@@@@<<=<<==<<A?C=:?:;:======<<<:<<
BBBBAAB
@T6XXI:00016:00157
GGGAGAAGGGAGAAAGGAAGGAGATAGCTCAACCACAAAACAGGTGCTATTCTCCCTCTCCCTATAGTGAGTCGTAT
TAGAATTCCCC
+
DCAEEC@CC>CBBB?B@C@B@BBBCA?=ACCBDCC@@BB7>>::://1HEEBDCCA=BBBBB>ABBBCBDDCA???AA
??BA=;;?@@5
@T6XXI:00017:00180
GGGGAATTCTAATACGACTCACTATAGGGAGAGGGAGAATAGTCAACAAATCGTTTGGGGCGACTTCTCCTTCCTTT
CTCCCTTCTCCC
+
AA?6A=A>BCC@CCBAA???>@B?6111.677BE@A@=@AA?AAA?==<3866662;;;5?ABBCACCC;988>AA<
??>>7>599CC<
@T6XXI:00018:00163
GGGAGAAGGGAGAAAGGAAGTGCAGTTCGCGGTTAGTGAAGGGAAAGCTATTCTCCCTCCTCCCTATAGTGAGTCGT
ATTAGAACTTCCCC
+
CFBCCB@CEACA@A>A@CEDCAAA<958==<4717<;;>><@<?A<::;AB?>;:?60/,,2202/+/77@;<<:8?
<<56<9564/000+
@T6XXI:00019:00151
GGGAGAAGGGAGAAAGGAAGCTAGTCACTCGACATCTGTGGCTTCACAGTACAGAGGGACTGTCCAAAGCCGCAGAT
CTCGAGTCGACTAG
+
DDDBBB@CB>CCBB>C?A>@BA??@A:97?A???:88CCCA@@A@CCCBACCB;333,4::=@@>AA<@A?BA@@AA
???@?CB????@@A
@T6XXI:00020:00189
GGGAGAAGGGAGAAAGGAAGCTAGTCACTCGACATCTGTGGCTTCACAGTACAGAGGACTGTCCAAAGCCGCAGATC
TCGAGTCGACTAG
+
CD@BBB;;80999909;>=@BBABB?:::?>=<?:77BBC?ABDCBBB:::<=:4723;4000/00+0449<<<<@A
BA@@???@@AA@@
@T6XXI:00021:00163
CTATCGACTCGAGATCTGCGGCTTTGGACAGTCCCTCTGTACTGTGAAGCCACAGATGTCGAGTCGACTAGCTTCCT
TCTCCCTTCTCCC
+
>11///44499:4449499:?CCD>B@?@@BBBC>BBDCCB@@:630/3=;=?BB@?;;:>>::110;;;8..,.,.
,.55501,00030
@T6XXI:00021:00171
TTGACTAATACGACTCACTATAGGAGGAG
+
-*,,,,,*,,//44444;433771331..
@T6XXI:00021:00172
GGGAGAAGGGAGAAAGGAAGTGCAGTTCGCGGTTAGTGAAGGGAAAACTATTCTCCCTCTCCCTATAGTGAGTCGTA
TTAGAACTTCC
+
```

CCACEC=@@:>@>><A>B?@@@CC>>;<;10,.,.66;;<<@?BCC9<<;A@BAAC;?<?<?;?<:88470=65373
65000231,--
@T6XXI:00021:00184
CTAGTCGACTCGAGATCTGCGGCTTTGGACAGTCCCTCTGTACTGTGAAGCCACAGATGTCGAGTCGACTAGCTTCC
TTTCTCCCTTCCTCCC
+
A994494444::949>CDCCD@CAA<A@CCCCCCB<ABCCC@@@BDCA>AC?BBCCCCBBBBABAB@@@@@@@=DC?
CC@D>=<66,/,///,
@T6XXI:00021:00185
GGGAGAAGGGAGAAAGGAAGGAGAAATCACATGCAACAAAAGCTGAGCTATTCTCCCTCTCCCTATAGTGAGTCGTA
TTAGAATTCCCCC
+
HDADDJ?BB=>@@B=B?BBC?BBCC?CCBCCCCBB>9966-
:<??DCBA@>>BABB:>??AA;@@@A@AAAAA???A@<?>=88/4444,
@T6XXI:00022:00157
GGGAGAAGGGAGAAAGGAAGCTAGTCGACTCGACATCTGTGGCTTCACAGTACAGAGGGACTGTCCAAAGCCGCAGA
TCTCGAGTCGACTAG
+
ED>CBB>BA<@BBB>C@CACCCBBB:::=@@=;;::::CDJ?BC?A???AAC?==777-4::999244-
4;9?@<==@???@AA@888899<
@T6XXI:00022:00167
CTATCGACTCGAGATCTGCGGCTTTGGACAGTCCCTCTGTACTGTGAAGCCACAGATGTCGAGTCGACTAGCTTCCT
TTCTCCCTTCTCCC
+
C:771////8;>BBDBBBBA>ABC>C?BAA?9::5:BCDCBBB@B@@=@C@CCCDEC@;::?@@BBBBBCA@?2757
@9??BB>@;<;;;4
@T6XXI:00022:00186
CTAGTCACTCGAGATCTGCGGCTTTGGACAC
+
CB;;99=99BA>>?BA@;10,0.404,....
@T6XXI:00023:00176
GGGAGAAGGGAGAAAGGAAGAAAAGGAACGGAGCAGAACGGCCACGACTATTCTCCCTCTCCCTATAGTGAGTCGTA
TTAGAATTCCCC
+
::6<@>?AC>BBCC>@=@@??B@6?>B?BB?A@CBBB@BC@C>ABBBBCCB@???B>BBCEC>@@@@A@?A:;;BCA
A<?;<<=88664
@T6XXI:00023:00183
GGGAGAAGGGAGAAAGGAAGGAGACAGTGAGCGCCGCACAACACGTCACTATTCTCCCTCTCCCTATAGTGAGTCGT
ATTAGAATTCCCC
+
AA<ADC@EC=BBCB>A?@AB?>@>CBBBBC?;;;>??B>;;????>>ABBCBACBAA>ACCCC>BBCBBBBBCBBBB
CC?ABB>@<??@8
@T6XXI:00024:00162
GGGGAATTCTAATACGACTCACTATAGGAGAGGGAGAAATAGCTGGT
+
>>?4>6924988444:87373.445..,.22557//.442000,.,.
@T6XXI:00024:00178
GGGGAATTCTAAGTACGACTCACTATAGGGAGAGGGAGAATAGAGACGTAATTCGT
+
@::2:21,111,////49;;;;6>>=711,1766626638833.,+00++*.4445
@T6XXI:00024:00182
GGGGAATTCTAATACGACTCACTATAGGGAGAGGGAGAATAGCTACGTTTAACCGATCGTGTTGTTCTCCTTCCTTT
CTCCCTTCTCCC
+
CDG:E>?=?BB?BCBCBAAAA@BCB???9?AACC>CCA=A====9=?@C>B?B?@BB@@<<@?AA89:A>@=A=?@;
888A<A>@@AC?
@T6XXI:00025:00164

```
GGGAGAAGGGAGAAAGGAAGCTATCGACCATCTGTGGCTTCACAGTACAGAGGGACTGTCCAAAGCCGCAGATCTCG
AGTCGACTAG
+
@C<@CB@DC@CCCC@C@C@BCCC???7741444???=@C@BDDABCBBBC@@@<???@A@;@@<@C@CA@@DC??@C
CACB@??BB?
@T6XXI:00025:00167
GGGGAATTCTAATACGACTCACTATAGGGAGAGGGAGAATAGTTAGATCCTCGTCAATCAAGCTCCCTCCTTCCTTT
CTCCCTTCTCCC
+
DCC9B8;8;BB?BBCBBABAA97<;666.7???A>DB?=?AAA>===?A>@A@@@@=@AA=?6==A>AA=A>A88=7
<@>?;@9<<<>:
@T6XXI:00026:00157
GGGGAATTCTAATACGACTCACTATAGGGAGAGGGAGAATAGCTGGCACACACACGTCAGTATCTTCTCCTTCCTTT
CTCCCTTCTCCC
+
AAA9AADAC@@?@ABBBBBBB@<996665:BBCC>AAA?A<<:>>?@@@@>@@<<:<<=AA=@??;<=>:=;=:==7
???A<A;==AA<
@T6XXI:00027:00184
GGGAGAAGGGAGAAAGGAAGGGAATAGTCGCGCTTGTTATCAACGTGCTATTCTCCCTCTCCCTATAGTGAGTCGTA
TTAGAATTCCCC
+
9@5?99599-
48=?0828<>>7@=@BC?@@CCCC>BBDCCBB@BBAAA;;<@B>>@<@@BBG@DGFBAAABBBCCBCC?C;;;DDII
F9
@T6XXI:00028:00155
GGGAGAAGGGAGAAAGGAAGAGTCAGTTCGCGGTTAGTGAAGGGAAAAACTATTCTCCCTCTCCCTATAGTGAGTCG
TATTAGAATTCCCC
+
EDACBB=@>:>98809<;:10001::;;;@?=<973;8?@=??.7776+3BCC@@>>>=1//>>=>99???AAAA@@
@@A>@@@=?>?CC6
@T6XXI:00029:00151
GGGAGAAGGGAGAAAGGAAGAGTAGTCTGGGGCGGTCGGGGTGTGAACTATTCTCCCTCTCCCTATAGTGAGTCGTA
TTAGAACTTCCCC
+
991949598-449?096:;?BDCCCAA;899/7<<@>BBB7>A<>B?BBCGADCCB<?=777-
:@?=EDF@@?@@@@>;==;/./,///*
@T6XXI:00029:00171
CTATCACTCGAGATCTGCGGCTTTGGACAGTCCCTCTGTACTGTGAAGCCACAGATGTCGAGTCGACTAGCTTCCTT
TCTCCCTCTCCC
+
CA@?<::9=@BBBBBBCCE@;:::;@CCCB@@@<?BB????@:44;8;@@@C?@@@@?@A???@C?@>7311010..
+..-3-..4442
@T6XXI:00029:00191
GGGAGAAGGGAGAAAGGAAGGAGAAGTCGCCCCAAACGATTTGTTGACTATTCTCCCTCTCCT
+
94-449284-48>>987=<<<:<:@BBA@@@@5?B087777,663667=78588992///,++
@T6XXI:00030:00196
GGGAGAAGGGAGAAAGGAAGGAGAAGTCGCCCCAAACGATTTGTTGACTATTCTCCCTCTCCCTATAGTGAGTCGTA
TTAGAATTCCCC
+
CCACCC@AA=BCBC?CAB?A@BBB@B@BBBBB7BB>BDDDC<AA@AAACAA>@A==;@@@@A=A<<<===:;;=B<?
?>?;948/444-
@T6XXI:00031:00160
GGGAGAAGGGAGAAAGGAAGCTAGTCACTCGACATCTGTGGCTTCACAGTACAGAGGGACTGTCCAAAGCCGCAGAT
CTCGAGTCGACTAG
+
```

CC>??A@CC?BCBB>BAB?AAA<@?A@??CB:::???ABB@ABBCBBB?BC@?844317=?AA@=@CA@@>@@==>A
=A<?<@A<@;;AAA
@T6XXI:00031:00169
GGGGAATTCTAATACGACTCACTATAGGAGAGGGAGAATAGACACCAATCGTGCTGGGTCATGTTCTCCTTCCTTTC
TCCCTTCTCCC
+
??@7A@CACAA?@BBBAAAAB7;;://,0::?F6:;<9;<=A>??686:@??=B:::5:@@AAD@D:;:;?B>BC89
8A@<A>@DCC?
@T6XXI:00031:00173
GGGGAATTCTAATACGACTCACTATAGGGAGAGGGAGAATAGCCTGCAGGTCGAGTGTAGGACTGTTCCCTTCCTTT
CTCCCTTCTCCC
+
@AB9A@C@AABACCCCCBCA@?;>;166.777==9?CA=??AA>C@?@C?AAAB===AAA>AABAA:<:4:34344+
022248./.4;6
@T6XXI:00031:00174
GGGGAATTCTAATACGACTCACTATAGGGAGAGGGAGAATAGCCTCTAACCCTATAACGTCTCACTGTTCTTCCTTT
CTCCCTTCTCCC
+
@CA6@@ECCCC@CCBB?>BBB@<97111.7<<AD@CB@>@==99=C@C@CC?CAB?<?????<===AA?AA?B>AA6
89AA:>378;;6
@T6XXI:00031:00175
GGGGAATTCTAATACGACTCACTATAGGGAGAGGGAGGAATAGTTGCTCGCTAAGCACTCGTACCAAACTTCTTCCT
T
+
/88*/,,*,,,*//8::7B;87887711,1337:6/.,-3---446440----,-
1444;65;529:44420;48/00
@T6XXI:00031:00183
GGGGAATTCTAA
+
444,42424489
@T6XXI:00031:00194
GGGAGAAGGGAGAAAGGAAGCTAGTCACTCGACATCTGTGGCTTCACAGTACAGAGGGACTGTCCAAAGCCGCAGAT
CTCGAGTCGACTAG
+
@=:9@A=@@>ACCC>CAC?BCB:;:?@@?AA::7>AAAFC??@@CCDC@BBAA>;;;7;====>9;;7<?<@AA@@@
A?@99=@???@@<<
@T6XXI:00032:00151
GGGAGAAGGGAGAAAGGAAGCTAGTCGACTCGACATCTGTGGCTTCACAGTACAGAGGGACTGTCCAAAGCCGCAGA
TCTCGAGTCGACTAG
+
EEB<;;79919999142959>>::<0//9777-0.///5677@B?BBAACCBA?=733-
49;=>?>@@;@@=?>>??889@@AA@@@@@@A@
@T6XXI:00032:00154
GGGAGAAGGGAGAAAGGAAGGAGAAGCACCATGTCAAGTGACGATAGCTATTCTCCCTCTCCCTATAGTGAGTCGTA
TTAGAATTCCCC
+
BB>BBB=@@>ACCC>C=@=@@BBB?AB>96:0//7744;?:A<@BBB?@=A?>>?CA@@@@@<AAA@@@AABB===@
@;>?@=;;;=;4
@T6XXI:00032:00161
GGGGAATTCTAATACGACTCACTATAGGGAGAGGGAGAATAGCTTCGCACTATGACCTTTGCACTATTCCTTCCTTT
CTCCCTTCTCCC
+
999,9?C?BBC@CCCCCBCBC@<?????9???CCAFA?<?@AAD?BBAAA@CB@?B?BC@CCB>>::77;?<@>A@:
@AAC>B=@444-
@T6XXI:00032:00165
GGGAGAAGGGAGAAAGGAAGCTAGTCGACTCGACATCTGTGGCTCACAGT
+

```
99-4442495499A;A7654..,,0,.......116666333.....771
@T6XXI:00033:00153
GGGGAATTCTAATACGACTCACTATAGGAGAGGGAGAATAGTTACAACGCTCACACAGACTATTTCTC
+
999/9:B@CBB@BBBBBBB9::;;;A@=>>000,0//,---238:<88@884554433030000+011
@T6XXI:00033:00162
GGGAGAAGGGAGAAAGGAAGGGAACAGTCCTACACTCGACCTGCAGGCTATTCTCCCTCTCCCTATAGTGAGTCGTA
TTAGAATTCCCC
+
CC>BBB@BC?ABAB>B@@@??;?=?>>;?>>>>BBAA==B?BAAA@>BCCCBCCCC?B?BCC?B@B@BBACCBAAAA
A?@?B9;2444-
@T6XXI:00034:00152
GGGAGAAGGGAGAAAGGAAGGAAGAAAAGAGTGGTAATGGAGAGAATGCTGTCTATTCTCCCTCTCCCTATAGTGAG
TCGTATTAGAATTCCCC
+
DD>BBB@BC>BBCB>B?BDB>?97733,8>>BC??=;?C?BAA??;?@A::;BCCC>B:665:BCCE>@??@@AAA?
??@???>@AA?C@CCC:
@T6XXI:00034:00197
CTAGTCGACTCGAGATCTGCGGCTTTGGACAGTCCCTCTGTACTGTGAAGCCACAGATGGACTGTCTTCCTTTCTCC
CTTCTCCC
+
EEGCCCBBBCCCDCCC@A@B@?;<@<@>ABBAABB>BCCBBBB@BCCA=@CDCBBBB@@=@@BBAAA;?;??9?@AA
<A>@???9
@T6XXI:00035:00168
TGCGGCTTTGGACAGTCCCTCTGTACTGTGAAGCCACAGATGTCGAGTCGACTAGCTTCCTTTCTCCCTTCTCCC
+
7;<A?ABB:C@BBBC??@>@CCC?BBB?@?A@BB?BAABBB@@?@B@C<<==?C::;?B@CC>CBBB>B>?@CC>
@T6XXI:00035:00171
GGGAGAAGGGAGAAAGGAAGCTAGTCACTCGAGATCTGCGGCTTTGGACAGTCCCTCTGTACTGTGAAGCCACAGAT
GTCGAGTCGACTAGCTTCCTTTCTCCCTTCTCCC
+
A;:@<<:4=-
99>@<8;@<@AABAB??????774:16?@A>BCD>B>ACCCCBB>CCA@@@@??@@B@@@=@AA@AAA@===?A@AA
A???;?;?==;7:433-4:;444,
@T6XXI:00035:00172
GGGGAATTCTAATACGACTCACTATAGGAGAGGGAGAATAGCCATTCAATCTGTCACCGGTTATGTCTCCTTCCTTT
CTCCCTTCTCC
+
DDD:C?B?BFDBCBCBBCCCCCCCC::5:@??A>BC@>A===>@A>AA>A?<<<BBB?BCG@CBBBABCBB>F677.
7=?>.424443
@T6XXI:00035:00181
GGGAGAAGGGAGAAAGGAAGTGCAGTTCGCGGTTAGTGAAGGGAAAAACTATTCTCCCTCTCCCTATAGTGAGTCGT
ATTAGAACTTCCCC
+
4:6:;B=>@<@AAB<@<?;877;9AA;888713137777977,1111+3777:<<=>;>AAAC;?96473350001-
--,---2454442.
@T6XXI:00035:00193
GGGAGAAGGGAGAAAGGAAGCTAGTCGACTCGACATCTGTGGCTTCACAGTACAGAGGGACTGTCCAAAGCCGCAGA
TCTCGAGTCGACTAGCTTCCTTTCTCCCTTCTCC
+
CB<@@><?@199@@9=<>>@@80-
7,,,,,,,033.6676631711666686608 5;;3;8888:7<;6;D?A@@@>===8478<855;3333737>AB7<
353+.,--.,
@T6XXI:00035:00196
GCTAGTCGACTCGAGATCTGCGGCTTTGGACAGTCCCTCTGTACTGTGAAGCCACAGATGTCGAGTCCGACTAGCTT
CCTTTCTCCCTTCCTCCC
+
```

```
@A>>?94494:==?CBABBCBBB@??<?687339688????AC?<?:6440424444377;9311142443337688
<<<A>=66533,.-/772
@T6XXI:00036:00161
GGGGAATTCTAATACGAC
+
111+1,1,///,////43
@T6XXI:00036:00165
GGGAGAAGGGAGAAAGGAAGCTAGTCGACTCGACATCTGTGGCTTCACAGTACAGAGGGACTGTCCAAAGCCGCAGA
TCTCGAGTCG
+
CC?@@A@BC?CABA=B@A?AC;9=7/00<<><3::A><ACC<?@>AAABBBBC@AA==;<<<<==<=<;<<;@===<
<<@@==<<9=
@T6XXI:00036:00170
GGGAGAAGGGAGAAAGGAAGGAGAAGTCGCCCCAAACGATTCGTTGACTATTCTCCCTCTCCCTATAGTGAGTCGTA
TTAGAATTCCCC
+
CE@CBB>BB>BBBE?B?B@D@CBB?A?>88;8.8D:@>@C????>B??888<=;;;6887@A<@???@@@@@A@@;;
<30042356;:0
@T6XXI:00036:00181
GGGAGAAGGGAGAAAGGAAGCTAGTCACTCGACATCTGTGGCTTCACAGTACAGAGGGACTGTCCAAAGCCGCAGAT
GTCGAGTCGACTAGCTTCCTTTCTCCCTTCTCCC
+
BA?C@>><B>C;;909595:DB?AA/000/..33../99>:=@@BCBCCCCB@>744-
49999;8;;6;=<??778=788<<??@@A=<<;;:2424;:;:::4:387BA:
@T6XXI:00037:00166
GGGAGAAGGGAGAAAGGAAGCTAGCTCGACTCGACATCTGTGGCTTCACAGTACAGAGGGATCGCAAAGC
+
@@?:B@59409911-1,1,..,20,/...6699<<<76::AB=AB?<??BBAA?=?=:82/./----+-1
@T6XXI:00039:00169
GGGGAATTCTAATACGACTCACTATAGGGAGAGGAGAATAGCTACCGGCGTAAGATCC
+
BCC9CAD@BCB@B@@ABAAAA?<97111.7>775:@;9:;447==<>3..--,--12,
@T6XXI:00039:00172
GGGAGAAGGGAGAAAGGAAGGATAAAGCAAATACGGGAAAAGGTTAACTATTCTCCCTCTCCCTATAGTGAGTCGTA
TTAGAATTCCCC
+
DC?AAA@CC@CBBB?C@C@B@BAA><??AA<ABAAB>ACC9C>B>B>AAB@>@@@@>=9=AA;<>BBBBB>B@@@??
??@A@?A/733+
@T6XXI:00039:00192
GGGGAATTCTAATACGACTCACTATAGGAGAGGGAGAATAGTCAACAAATCGTTTGGGGCGACTCTCCTTCCTTTCT
CCCTTCTCCC
+
DEJ:FBCBAAAECCCCCCCCBB>997//,0@:==<?CA?ABB>?>====:>?@?>8??<2<=777:>@;==?;==183
885><??AA@
@T6XXI:00040:00165
GGGGAATTCTAATACGACTCACTATAGGGAGAGGGAGAATAGTTAGCGTTCTCTGACCCCGACATTTCTCCTTCCTT
TCTCCCTTCTCCC
+
499/9<@@CAB?BBBBBAA?@?@<9116.6>=@@>BB@=?AAA>??:?=;;;<68:;==5??@;<;6;:;9;7:244
+33344<;<<AA7
@T6XXI:00040:00180
GGGGAATTCTAATACGACTCACTATAGGAGAGGGAGAATAGATCACCGTTGGTTCGGGTGCGCGTTCTCCTTCCTTT
CTCCCTTCTCCC
+
@AC6@@B?BBB@C??=A?BB?ABB?6607><<?=@ED?A<?<<<?B>@@887:?C?C?C@@@BBB@B::5:?A?AL@
AB?@<@?AAAA=
@T6XXI:00040:00186
```

```
GGGAGAAGGGAGAAAGGAAGTCCACGGAGAATGCCTTAACGCAAAAGCTATTCTCCCCCTCCCTATAGTGAGTCGTA
TTAGAATTCCCC
+
CC?BBB@DCBBBAA=B?B>DAC@ECC@AA@?@@@>BAC=:?;;;;0;?AAA@CBBB?=/43718>>?@?C@@A==<8
88<@?<@>@?@5
@T6XXI:00041:00150
GGGAGAAGGGAGAAAGGAAGTGCAGTTCGCGGTTAGTGAAGGGAAAAACTATTCTCCCTCTCCCTATAGTGAGTCGT
ATTAGAATTCCCC
+
GC@CBB@BB>BBBB>B@BAB>>ACED@BDCC?A>AAA@@=@C>CCCC2BBCC@CC?C>DCBBA>B===@@@A@A@@@
@@=@AA?A?AAA6
@T6XXI:00041:00151
CTATCGACTCGAGATCTGCGGCTTTGGACAGTCCCTCTGTACTGTGAAGCCACAGATGTCGAGTCGACTAGCTTCCT
TTCTCCCTTCTCCC
+
C8661////8;BBBBBBBB??BDD<B>AABB@@@;@CA?>99>???;<8@?BBCBA@@B?@@@@@@@ACCCAA?B@B
B>BBBC;A=;?@@<
@T6XXI:00041:00164
TTACGACTCACTATAGGGAGAGGGAGAATAGCCACCTGCTTTGTGGTTGAGCTATCTCCTTCCTTTCTCCCTTCTCC
+
?<???C<<<CCCDB99919999B>A@C@DCBC@CC@CCAAA<@A@@CB=?DBACCAAA@D>@?A@199991959894
@T6XXI:00042:00175
GGGGAATTCTAATACGACTCACTATAGGGAGAGGAGAATATAGGAACAAG
+
8880>?C@CCD=AACBCAABCCBABABB>BB>?AC:437;933/-,-1,-
@T6XXI:00042:00187
GGGGAATTCTAATACGACTCACTATAGGAGAGGGAGAATAGATCACCGTTGGTTTCGGGTG
+
CCC9E@C?AAAA;;;@A@@A@;:@<00,.,,.53...--42;9:7747=:=477-999040
@T6XXI:00043:00150
CTAGTCGACTCGAGATCTGCGGCTTTGGACAGTCCCTCTGTACTGTGAAGCCACAGATGTCAGTCGACTAGCTTCCT
TTCTCCCTTCTCCC
+
CC;<<A=BCCBBBBBBBA@@C@BBB>B@CCCBBCC>BBBBCCCCCBBB?@B@BFB?@0111,2,//844471621>>
>78677,0/0+000
@T6XXI:00043:00154
GGGAATTCTAATACGACTCACTACTAGGAGAGGGAGAATAGTCAACAAATCGTTTGGGGCGACTTCTCCTTCCTTTC
TCCCTTCTCCC
+
11-/,/,///789@@@980;,--
,,,,*,4.11,177><<=?:=444=<CCC@C>CBB6:::@@=>?@5:58;=;4<<??:?9==<B:
@T6XXI:00043:00155
GGGAGAAGGGAGAAAGGAAGCTAGTCACTCGACATCTGTGGCTTCACAGTACAGAGGGACTGTCCAAAGCCGCAGAT
CTCGAAGATC
+
BA<?>><>@:@999095949@?>100//7::.//6668@>@DD?BBBBAAAAA>??@;@@@@AA<??7=?<>??A??
?A@/,+*(((
@T6XXI:00043:00157
GGGAGAAGGGAGAAAGGAAGGAGAAGTCGCCCCAAACGATTTGTTGACTATTCTCCCTCTCCCTATAGTGAGTCGTA
TTAGAATTCCCC
+
BEBECD?AA=ABAA>C?B?B@CBB@B?BBBBB8@?=?CCD@8??@CA>AA?>A<894<<<AA<A?@@AA=<<;;;::
?>AAA>@8;;;0
@T6XXI:00043:00188
GGGGAATTCTAATACGACTCACTATAGGGAGAGGGAGAATAGTCAACAAATCGTTTGGGGCGACTTCTCCTTCCTTT
CTCCCTTCTCCC
+
```

```
CCDAC>B>ACC@CCBBA???>@B?>111-
6<<<?=?CABA?>??A>@@?;BCCCC>B@@7BCCC@>@CC?A?A>A>;:?@@;@><@AA<
@T6XXI:00043:00197
GGGAGAAGGGAGAAAGGAAGGAGAAGTCGCCCCAAACGATTTGTTGACTATTCTCCCTCTCCCTATAGTGAGTCGTA
TTAGAATTCCCC
+
CB@AAA@BB>BBBB?B@B@B@BBB>?@BBBBB7@@=BCCCC=>?>@?<>?A?<<<=;88>A=;=:;<==A<==?AAA
A>A@?;=89990
@T6XXI:00044:00151
GGGAGAAGGGAGAAAGGAAGGAGAAGTCGCCCCAAACGATTTGTTGACTATC
+
99599=;=>:>>?@<@?@;>;88373143333,3<673311,167667110-
@T6XXI:00044:00157
GGGAGAAGGGAGAAAGGAAGCTAGTCACTCGACATCTGTGGCTTCACAGTACAGAGGGACTGTCCAAAGCCGCAGAT
CTCGAGTCGACTAGCTTCCTTTCTCCCTTCTCCT
+
CCAC@@@AC<@ABA<A8;8<B@>>>677616166678@CCA@@BBCB@@@BAA@AAC=????@@?;A=A=:??@@CC
888@@?=?@@===AAA>@=@B=AAAA<A>?:970
@T6XXI:00044:00174
CTATCACTCGAGATCTGCGGCTTTGGACAGTCCCTCTGTACTGTGAAGCCACAGATGTCGAGTCGACTAGCTTCCTT
TCTCCCTTCTCCCCTTCCTTTCTCCCTTCTCCC
+
ECCCBEGD@??CCBBBCBB@BBB=B?CCBBA?>;????@???899A>AC@CBBBBBCCDGBB?@@;;<?796<<>?C
>CA<@<?:;0044-4797:;6<>>A<A9<<;<4
@T6XXI:00044:00189
GGGGAATTCTAATACGACTCACTATAGGGAGAGGGAGAATAGTTAGCGTTTTCTGACCCCGACATTTCTCCTTCCTT
CTCCCTTCT
+
CKKBH;:<<AB?@AABCA@AA:88>777.7>>??>AA?<?AAA>@@@@@A@4====<CD5=<<ADC<@;9B999,/,
--.-+-338
@T6XXI:00045:00157
GGGGAATTCTAATACGACTCACTATAGGAGAGGGAGAATAGCTCCGTAACAGCTGGTAGCGGCTTCTCCTTCCTTTC
TCCCTTCTCCC
+
<<<4<>A?BBA?ABB@BBBCE>777//,0;0775:BB<<=??BBAAAB@CC<<<A>@;:<<?=A;=43/378244,4
9941748@A@<
@T6XXI:00045:00170
GGGGAATTCTAATACGACTCACTATAGGGAGAGGGAGAATAGAGACGTATTCGTTTGCGCTCTGTTCACCTTCCTTT
CTCCCTTCTCCC
+
499/99@>ABBACCCBBBAAA@997116.7==BC@BB@@AA@?<????<@>A===7<>=??==?==?8848=@8<<6
;???:?>>7771
@T6XXI:00045:00189
GGGAGAAGGGAGAAAGGAAGTGCAGTTCGCGGTTAGTGAAGGGAAAAACTATTCTCCCTCTCCCTATAGTGAGTCGT
ATTAGAATTCCCC
+
CC@BAA@CB>BBBB>B?B?BB@BBBB@BBBB?AABABCC@CC>BBBB2BCBC@BCCC>AAACC>B??A<<<AAA???
AA?A@A>A9<<=4
@T6XXI:00045:00198
GGGAGAAGGGAGAAAGGAAGGAGAAGAGTGATGCACAAAAATGCAAGACTATTCTCCCTCTCCCTATAGTGAGTCGT
ATTAGAATTCCCC
+
AA?ABBACDBBAAB>B@B@B@BB?>>>???????@CA===.<=C@>:6<CCC<===99=AAAA<AAA?::A@AA@@@
@@>AAA=A=@CC6
@T6XXI:00046:00152
GGGAGAAGGGAGAAAGGAAGCTAGTCACTCACTCTGTGGCTTCACAGTACAGAGGGACTGTCCAAAGCCGCAGATCT
CGAGTCGACTAGC
```

```
+
BC899@699-948=9=?C@CCAA@?;00...334988:;=@?@@ACCB;88//..++00.--
2222320153336664413-.-122-00
@T6XXI:00046:00158
GGGGAATTCTAATACGACTCACTATAGGGAGAGGGAGAATAGTCAACAAATCGTTTGGGGCGACTTCTCCTTCCTTT
CTCCCTTCTCCC
+
BBB9B@BBECB@CBAABGBCA<;@7177.7>@@B>BBB?@?@@@A>@@@;BBBCD<AAB7AACBB?BCC??=?>AA<
@@AA<A>@<<<5
@T6XXI:00046:00174
GGGGAATTCTAATACGACTCACTATAGGGAGAGGGAGAATAGCTAGCGTTTCCCCCGCAATCTATTTGTCCTTCCTT
TCTCCCTTCTCCC
+
CCC:CBC@BBB@CCCCCBBBBA>97111.7>>@K>CDA?B=?<AAAACCC<>==>1???AC==??=7=8828:;8<8
9=A?A9=;=ACCA
@T6XXI:00046:00175
GGGGAATTCTAATACGACTCACTATAGGGAGAGGGAGAATAGTCAACAAATCGTTTGGGGCGACTTCTCCTTCCTTT
CTCCCTTCTCCC
+
???4><@=@AC@BBDBBAABAA??=777.7>??B?BC@>AAABAA=@AA<C??=@;CCC8CCC@A=?BB?=;=>AB6
;88813284AB?
@T6XXI:00047:00150
GGGAGAAGGGAGAAAGGAAGCAGGTCGACATCTGTGGCTTCACAGTACAGAGGGACTGTCCAAAGCCGCAGATCTCG
AGTCGACTAG
+
BDACBA@AA??BBB?C?B?BCCCBBCC::::::@AB>A?<=<;<<=<DBA<==7<==@@@>@C?@A>???=>>><==
?===AAAAAA
@T6XXI:00047:00171
GGGAGAAGGGAGAAAGGAAGAGAAACGAAT
+
DEBECC@EB=?@@@:;:;:00,,2*,,/70
@T6XXI:00047:00177
GGGAGAAGGGAGAAAGGAAGAGAATAGGCACGCGTGTAAAATGGTAACTATTCTCCCTCTCCCTATAGTGAGTCGTA
TTAGAATTCCCC
+
CKBCCB<==<;<;B=B@BBBCBB?BC@>@BBBBCBCCCCC9BB?::8;CCG?AABDAE??AB@BCGADAAAAA?<;;
;8<@@?A?BAA6
@T6XXI:00047:00182
GGGGAATTCTAATACGACTCACTATAGGAGAGGGAGAATAGTCAACAAATCGTTTGGGGCGACTTCTCCTTCCTTTC
TCCCTTCTCC
+
DID:C;<;<@A@@@BCCBBBA=;;;00,0;;>>:A@><?898AA9;<=7>===<6<>>.77==?;:77:;8<9<A;A
<AA:;3/./,
@T6XXI:00048:00164
GGGGAATTCTAATACGACTCACTATAGGGAGAGGGAGAATAGTCCTACTATCTTTCGCTACATTTCGCCTTCCTTTC
TCCCTTCTCCC
+
EFF8BBBACCBBCCC;<;CCC?A><776.7==AB>CCA;==@@A>@@@??????:;<<B@A?DE>C@?;=<;244-
44;=4;8;;;;6
@T6XXI:00048:00167
GGAGAAGGGAGAAAGGAAGAACAAACGGTCGCTGGGAATCGAGACGCTATTCTCCCTCTCCTATAGTGAGTGTA
+
424;;8=?<?AAC@C@CB;;9;CC<CB@BCCB::858,///8//4=::4924444-4;4424/4.....44...
@T6XXI:00048:00176
GGGAGAAGGGAGAAAGGAAGGAGAAGTCGCCCCAAACGATTTGTTGACTATTCTCCCTCTCCCTATAGTGAGTCGTA
TTAGAATTCCC
+
```

```
CC>ABB@BB>BCBH@C>@>@@BCC@CDB?>;;5;;:@BAAA=AB>B@@@AA>AAAA<A==687=478C===@AAA==
<;=4/,-,++,
@T6XXI:00048:00178
GGGGAATACTAATACGACTCACTATAGGAGAGGGAGAATAGCTATAACTCCGTAGTTGCAATGTTCTCCTTCCTTTC
TCCCTTCTCCC
+
@==6@@ABCBB@B??@BACBB>;;4./,0;;AC>BB@?B<?<>@BC>@A=;=<588?@@@=AAA?AAB<?><;<@<5
88C;?;<7771
@T6XXI:00049:00151
GGGAGAAGGGAGAAAGGAAGGAGAAGTCGCCCCAAACGATTTGTTGACTATTCTCCCTCTCCCTATAGTGAGTCGTA
TTAGAATTCCCC
+
CCABBB@AA=AAAA?A@B?B@BBD@B?BBBBB8BB<BB??@;@?>@@?AA?=@AAA;???@A<A;;===<<::?AAA
A>C??::/444-
@T6XXI:00049:00163
GGGAGAAGGGAGAAAGGAAGAGAATGCTTGCGCGTAGGGAGGCTGTGGCTATTCTCCCCCTCCCTATAGTGAGTCGT
ATTAGAATTCCCC
+
==5>>>;>>:>>@C=BAC@AA@@8;:FCACA@@?<==<.7747??@@?A????BBBECC2BCB>BBB<=<<<88>AA
AA=AAA?A?<<<4
@T6XXI:00049:00165
GGGAGAAGGGAGAAAGGAAGTGCAGTTCGCGGTTAGTGAAGGGAAAAACTATTCTCCCTCTCCCTATAGTGAGTCGT
ATTATGAACTTC
+
BBA>==ACNBCBBC?CAE@:<;@B@B?BBBA>B=<88??<AB>BECC2E>?D31/>>=/1/@A=A:;A@@@@?@?81
00++++00++*.
@T6XXI:00049:00166
GGGAGAAGGGAGAAAGGAAGGAGACAGTGAGCGCCGCACAACACGTCACTATTC
+
@@@@AA>A:9899>9@=@@C?BBCCBA@@@;;<C@AABBCBCAAABBCCCC?=>
@T6XXI:00049:00167
CTAGTCACTCGAGATCTGCGGCTTTGGACAGTCCCTCTGTACTGTGAAGCCACAGATGTCGAGTCGACTAGCCTTTC
TCCCTTCTCCCCTTCCTTTCTCCCTTCTCCC
+
C=:<;<::=CBCCBBAABB?>???;?>>?A@@@@;@CA@?;;;>>:;?;A@AABBBB@A@@@A=?@CDDBBB?BC>C
DBCCC=?4444,348/00+0;;;-434444-
@T6XXI:00049:00195
GGGAGAAGGGAGAAAGGAAGCTAGTCACTCGAGATCTGCGGCTTTGGACAGTCCCTCTGTACTGTGAAGCCACAGAT
GTCGAGTCGACTAGCTTCCTTTCTCCCTTCTCCC
+
CHACCC@CC@CCCC@C@C>@@@B>??@:::@@?;?????@>??@<?@C:::?>>.774433338=9B=8838;:887
:??=883733?A889@>;;;936///,/,/---+
@T6XXI:00049:00198
GGGAGAAGGGAGAAAGGAAGCTAGGTCACTCGACCATCTGTGGCTTCACAGTACAGAGGGACTGTCCAAAGCCGCAG
ATCTCGAGTCGACTAG
+
CC?BA=<=@<BBBB>C?B?AB@;;:?600<<:00,/7735>B;??BCCCABBABA@?>=7===>?@>AA<AA>@@CB
AAA@@???@@???A@?
@T6XXI:00004:00211
GGGAGAAGGGAGAAAGGAAGGGGAGAAGGGAGAAAGGAAGCTAGTCGACTCGACATCTGATGGCGTTCA
+
CB>CCC?BD?@F@C>C@C?BBB7A;@?@=6=161-74747=711=711116103000453354---,--
@T6XXI:00004:00248
CTCGAGATCTGCGGCTTTGGACAGTCCCTCTGTACTGTGAAGCCACAGATGTCGAGTCGACTAGCTTCCTTTCTCCC
TTCTCCC
+
```

```
DCC<<?CCCBBBC@CCC@C@CCCCCECACCBBBBBBBBDDE@CCABBBBCC@@ACCBBBBDBAA@BD@C@CC?CCCC@
C>AABC=
@T6XXI:00005:00212
GGGGAATTCTAATACGACTCACTATAGGAGAGGGAGAATAGTTCCTCGACTTGATCGCGACGTGGCACTTCTTCCTT
CCCTTTCCTT
+
CDC9DBC@BBB@B@AABB@@A:995/.,0;;>@?ACA>?833/4740-3..,.......----
2327??876;70023422334444
@T6XXI:00006:00215
GGGGAATTCTAATACGACTCACTATAGGGAGAGGGAGAATAGTCAACAAATCGTTTGGGGCGACTTCTCCTTCCTTT
CTCCCTTCTCCC
+
999/4:BACCB@BBBBCCCBCECA?677.7>@CC@BB@?A8>?==>???;AA??A8?9<5?@BAA=@??5858?CD?
==?A=D<??@C<
@T6XXI:00006:00247
GGGAGAAGGGAGAAAGGAAGCTAGTCGACTCGACATCTGTGGCTTCACAGTACAGAGGGACTGTCCAAAGCCGCAGA
TCTCGAGTCGACTAG
+
CC@CCBAB@;998809<>=?AAAA@9:777778611118=><?B@BCCCCCB?=8733-4:;;;;244-
4434@A@=<<AABB@????@A@@
@T6XXI:00007:00201
GGGAGAAGGGAGAAAGGAAGCTAGTCGACTCGACATCTGTGGCTTCACAGTACAGAGGGACTGTCCAAAGCCGCAGA
TCTCGAGTCGACTAG
+
C@5>?>?AA>AA>>9?5948>?;;:0.....4.1198868;8:;8:CCCCBCCAA87718;::;;/44-
3825:???DA???@@<====<<@
@T6XXI:00007:00234
GGGAGAAGGGAGAAAGGAAGCGTAGTCGACTCG
+
4404;C;>@999:;:==?=;774.,,,,,,,,.
@T6XXI:00007:00237
GGGAGAAGGGAGAAAGGAAGGAGAAGTCGCCCCAAACGATTTGTTGACTATTCTCCCTCTCCCTATAGTGAGTCGTA
TTAGAATTCCCC
+
EKACCB===5=;446;>B?A@CCB?B@C><;<6;;:ACCCB>CC?BAAAAA=AACC<889?@;@???@@@AAA@>>>
??ACD>?3844,
@T6XXI:00008:00208
GGGGAATTCTAATACGACTCACTATAGGGAGAGGGAGAATAGACACCAATCGTGCCGGGTCATGTTCTCCTTCCTTT
CTCCCTTCTCCC
+
CCB9C@D@BBB@CB@@@????@B?>111-
6<<BCBCA?=?A<<<?@;=;?:77>=377.7?AAB@?AAA?A;===A;@@@@AA;?=AD;
@T6XXI:00008:00213
GGGAGAAGGGAGAAAGGAAGGAGAAGTCGCCCCAAACGATTTGTTGACTATTCTCCCTCTCCCTATAGTGAGTCGTA
TTAGAATTCCCC
+
CKADCC@CB>CBBC>B?B?B@BAA@CCBA?>;5@;<ACCEC>BA>ABCCAA>=9884=?@AB=A???@<<==<;?@A
;9;//,-,///.
@T6XXI:00008:00226
GGGGAATTCTAATACGACTCACTATAGGGAGAGGGAGAATAGACACTGAAATCGTTTGGGGCGACTTCT
+
999/9<ABCBB?BB??@BCCBBBBBBBB>BACCC77792400000003;;48999=;===58/,,,***
@T6XXI:00010:00202
GGGAGAAGGGAGAAAGGAAGCTAGTCGACTCGACATCTGTGGCTTCACAGTACAGAGGGACTGTCCAAAGCCGCAGA
TCTCGA
+
```

```
CC@CBB@CC?CAEB=B@B>ABB9;::;:B??=;::777???:?B@BCB@>@@=4:300,3::=@@>@A<@@>A@@@A
A@@???
@T6XXI:00010:00208
GCTAGTCGACTCGAGATCTGCGGCTTGGACAGTCCCTCTGTACTGTGAAGCCACAGATGTCGAGTCGGCTAGCTTCC
TTCTCCCTTCTCCC
+
44:99?:949::?;<;@AA@@@>885313399668816l;998??::68=B=>?@??777317644244666666,
.,.33303499999
@T6XXI:00010:00239
GGGGAATTCTAATACGACTCACTATAGGGAGAGGGAGAATAGTCAACAAATCGTTTGGGGCGACTTCTCCTTCCTTT
CTCCCTTCCTCCC
+
CDC9C9:<<DBBCC9;<DBA@???>7776:>@BD>BAB?AA?@@A=7771:@@ACCCCC9AABAB@DCD?@>@>AA<
@==969,/,/661
@T6XXI:00012:00219
GGGGAATTCTAATACGACTCACTATAGGGAGAGGGAGAATAGTTTTCCCTTCACTAACCGCGAACTGCACTTCCTTT
CTCCCTTCTCCC
+
499/95954999>:;<@@AACBA?=777-
7==BE>CAA>AA@@@@4@B=A??AABB@C@C@@=>@AA=@??:=477.7>??<C>@<::6
@T6XXI:00012:00221
GGGAGAAGGGAGAAAGGAAGCTAGTCGACTCGACATCTGTGGCTTCACAGTACAGAGGGACTGTCCAAAGCCGCAGA
TCTCGAGTCGACTAG
+
CEA@A?@CC=C@@=:@782:A>9::/00::9:0:6:@@BCC=@B??CBCBBBBAA??@;?@@@@A>@?<?C>@????
A=@@AA@@???AA@@
@T6XXI:00012:00231
CTATCGACTCGAGATCTGCGGCTTTGGACAGTCCCTCTGTACTGTGAAGCCACAGATGTCGAGTCGACTAGCTTCCT
TTCTCCCTTCTCCC
+
B=;8/////79@?BBBA@@C@BBB=B=??B@???<?BCDC@<>>A==<=C?BBCBBABBBBCC?@@BCCBA??<?;?
C@@??D>A>A???;
@T6XXI:00012:00234
GGGGAATTCTAATACGACTCACTATAGGGAGAGGG
+
CCH:C@B?@AACDBB?CBCBBA<97111,00000,
@T6XXI:00012:00242
GGGAGAAGGGAGAAAGGAAGGGGAGAAGGGAGAAAGGAAGCTAGTCGACTCGACATCTGTGGCTTCACAGTACAGAG
GGACTGTCCAAAGCCGCAGATCTCGAGTCGCTA
+
DECDAABDC@CCBC<@<?<;;B7BCC>AA8?;;66><@<ABCCCC:;;??BB889AA@@@A???>????@@AA?=<<
;7<@AAAA?AA:???AA@<<<@@@AA???@@@?
@T6XXI:00013:00210
GGGAGAAGGGAGAAAGGAAGGAGAAGTCGCCCCAAACGATTTGTTGACTATTCTCCCTCTCCCTATAGTGAGTCGTA
TTAGAATTCCCC
+
CF@CECBCC=ABBC@CAC@B=;<;@CBCCCCC8@@<@CCBB>B@:??99<=:>@AA?A@@@C<A<<<AAAABA@@88
9>BBD>B@ACD5
@T6XXI:00014:00202
CTAGTCGACTCGAGATCTGCGGCTTTGGACAGTCCCTCTGTACTGTGAAGCCACAGATGTCGAGTCGACTAGCTTCC
TTTCTCCCTTCTCCC
+
1--
,,,,,/1:@CCCCBCBCC@BBB>B?@ABB???8????AA:;;CCA>AC=AACGBBABCCCAABB@@@A8:8?A?CE=
@<BC>@383;<6
@T6XXI:00014:00206
```

```
GGGAGAAGGGAGAAAGGAAGTGCAGTTCGCGGTTAGTGAAGGGAAAAACTATTCTCCCTCTCCCTATAGTGAGTCGT
ATTAGAATTCCCC
+
9>:9995990948;9=;=>A79;CDC?BBBB=?=?B;<;@CC>CCCC2C@@@=???=6=>=<<8977>??@AA@===
AA>AABAA>?A?5
@T6XXI:00015:00213
GGGAGAAGGGAGAAAGGAAGGAGAAGTCGCCCCAAACGATTTGTTGACTATTCTCCCTCTCCCTATAGTGAGTCGTA
CATTCA
+
99049=59469=;C<A=@8843@@>BB>>=;;.00+.../766<>==9?;:::9::4;==>@;@A===AB=<<476,
+++*++
@T6XXI:00015:00233
GGGGAATTCTAATACGACTCACTATAGGGAGAGGGAGAATAGTCCCAGGGGCGAGGACACAATATTCTCCTTCCTTT
CTCCCTTCTCCC
+
ACB9B?B?@AA?BBCCC@@@C?>>>777.7=:??9>0/.-8856601111*----
0347==;?@@>@9:5749/481888=;=<=<<<;
@T6XXI:00015:00238
GGGAGAAGGGAGAAAGGAAGAAAATGCGACTGCCGGGGAGTTTAAAACTATTCTCCCTCTCCCTATAGTGAGTCGTA
TTAGAATTCCCC
+
CC@B@@@BB>ABBC>B@B@BBBB8BBBBBB??>@BBB8CCCC>BBB9BAAC@E???=?AAAC>CCC@@@AA===A9=
=>@@?:;3444-
@T6XXI:00016:00218
GGGAGAAGGGAGAAAGGAAGGAGAAGTCGCCCCAAACGATTTGTTGACTATTCTCCCTCTCCCTATAGTGAGTCGTA
TTAGAATTCCCC
+
CA:AB@<@@<@@@@<@=A=@BBCC::;AA???.886:?BDE>CB?C??@@@>A@AA<A???A<AA@=<=CAAA@A=<
<;?65568;;@5
@T6XXI:00016:00232
GCTAGTCACTCGAGATCTGCGGCTTTGGACAGTCCCTCTGTACTGTGAAGCCACAGATGTCGAGTCGACTAGCTTCC
TTTCTCCCTTCTCCC
+
444444444444::=?BCBBB=??>2<1:9ACDDB;BBBCCB???855239=?>BCC;;<????ABC??;7766010
17,616623/0000+
@T6XXI:00016:00236
GGGGAATTCTAATACGACTCACTATAGGAGAGGGAGAATAGTCAACAAATCGTTTGGGGCGACTTCTCCTTCCTTTC
TCCCTTCCTCC
+
@A?1;-1,1667:9<<A@?AB010000,.7/61167456;<;443444-:===F.761,163:=:933,-,-
0173./4;44/-,--,
@T6XXI:00017:00218
TCGAGATCTGCGGCTTTGGACAGTCCCTCTGTACTGTGAAGCCACAGATGTCGAGTCGACTAGCTTCCTTTCTCCCT
TCTCCC
+
ACAAABBBACBA=@>?;?;>ABB@>?;BBBBB??>>>>??A;<==><<?<<BBBBBBCEBAB@AA@ABEC@BBCC@D
;;<BB:
@T6XXI:00017:00245
GGGGAATTCTAATACGACTCACTATAGGGAGAGGGAGAATAGTCAACAAATCGTTTGGGGCGACTTCTCCTTCCTTT
CTCCCTTCTCCC
+
CCC9C===>BB?BBBBCBAAB?997116.7==?B?DB@>@@@A@@>@AA<B<<<=;?CC7BABBBACBB??<?=?A<
@@@A>A<>@AA;
@T6XXI:00018:00204
GGGGAATTCTAATACGACTCACTATAGGGAGAGGGAGAATAGTCAACAAATCGTTTGGGGCGACTTCTCCTTCCTTT
CTCCCT
+
```

```
944,48>?BAB?BCCCBAAA???@???C>CCCCC>BBA=AAA@?==8881:@>B?.777.7167>:<;633/4/044
933101
@T6XXI:00018:00241
GGGAGAAGGGAGAAAGGAAGCTAGTCACTCGACATCTGTGGCTTCACAGTACAGAGGGACTGTCCAAAGCCGCAGAT
CTCGAGTCGACTAG
+
CC>BCC?@=:=@==9>5948A;8;B???AA@:::<<;>:;3:A>BCBBBA@C?=43317>=?@@>AC<@@=@A<<<<
9====;;9=9A???
@T6XXI:00019:00209
GGGGAATTCTAATACGACTCACTATAGGAGAGGGAGAATAGTTTTTCCCTTCACTAACCGCGAACTGCACTTCCTT
CTCCCTTCTCC
+
==92>69694424888=?;;;BBB@??577===9?B?>@AAAAACC-=;-
4/0667;;?@><?>:;4337660101011.5033.--0
@T6XXI:00019:00226
GGGAGAAGGGAGAAAGGAAGAACGGTCAAACGCAGGAAAGCAATGGACTATTCTCCTCTCCCTATAGTGAGTCCGTA
+
DE?@?=59819>8;5=;=<77<??7;@@@<A??@A=@@>@CC@BC?AAA??:?A@:??9?@:=:94-+*****)*+0
@T6XXI:00020:00216
GGGAGAAGGGAGAAAGGAAGGAGAAGTCGCCCCAAACGATTTGTTGACTATTCTCCCTCTCCCTATAGTGAGTCGTA
TTAGAATTCCCC
+
BBBCDB@BA=BB@B=B?B?B@CBD@@AA?>;;6@@<ABABC?CC@CBBBAA>A@??;????A<A@@@@?A@@;<@:<
<?A==;=A@AA6
@T6XXI:00020:00239
GGGGAATTCTAATACGACTCACTATAGGGAGAGGGAGAATAGTCAACAAATCGTTTGGGGCGACTTCTCCTTCCTTT
CTCCCTTCTCCC
+
ECC7B7;:<AA@CCBBBBBCCCB>=777.7>?AC>BC@>A898=;9733,677=?.777-
71;=>3:6:;8;=>AA<A;;;684:8;<6
@T6XXI:00020:00243
GGGAGAAGGGAGAAAGGAAGGCATGAGTACGACGAC
+
?@<@;979@:@999049>9>43333634.......4
@T6XXI:00020:00249
CTAGTCACTCGAGATCTGCGGCTTTGGACAGTCCCTCTGTACTGTGAAGCCACAGATGTCGAGTCGACTAGCTTCCT
TTCTCCCTTCTCCC
+
C=1////44AAABBDCBABC@CCC>C?B@>>:??;?A@A?>>CAC@A;=AACDICCCBCBBBBBBBCCCCBBB=?=?
C>BCEC>@689@A;
@T6XXI:00021:00222
GGGAGAAGGGAGAAAGGAAGAGAACGGCGACGAACAATAGTTGGTGGCTATTCTCCCTCTCCCTGATAG
+
940449594-448;-429;777B=@A=>>>99;44;9499>?A?AB@CCAA?7616,1.335+./.433
@T6XXI:00021:00237
GGGAGAAGGGAGAAAGGAAGGATAGGGCACCAATGAACAAATGGCGGCTATTCTCCCTCTCCCTATAGTGAGTCGTA
TTAAGAATTCCCC
+
??:B<<8<C?CDDD>B@CDD8;;???9;;;<@=8BB<99919@>996;ABB@CBBA:@@99909>>=ACCCABA>00
.,.,..,.59997
@T6XXI:00022:00207
GGGGAATTCTAATACGACTCACTATAGGAGAGGGAGAATAGTCAACAAATCGTTTGGGGCGACTTCTCCTTCCTTTC
TCCCTTCTCCC
+
999/4:B@DBBA@>@BBBBBA>967./,0<<@B>BDC?A:::==;=A=;AAAAB>BCC9CCCBB?CCC=?<<;<=;=
???:@;??CC;
@T6XXI:00022:00217
```

```
CTAGTCACTCGAGATCTGCGGCTTTGGACAGTCCCTCTGTACTGTGAAGCCACAGATGTCGAGTCGACTAGCCTTCC
TTTCTCCCTTCTCCC
+
CC:<<@;:9>>@<<<BCB@A>ABA<B=@@BAABC>CCEB???A9:8@9<ACECBCCCBB;;;AABD@::7./,.,0,
/:,06>96420000+
@T6XXI:00022:00225
GGGGAATTCTAATACGACTCACTATAGGGAGAGGGAGAATAGTCAACAAATCGTTTGGGGCGACTTCTCCTTCCTTT
CTCCCTTCTCCC
+
BCD:C@B@BBB@BBBBBAAAB@>97116.7>>>@>BEA@@<>@==<;@@<BCBAC>B@@8@@C@@>@BC==99;A@<
@A@A<A;==AB>
@T6XXI:00023:00230
CTAGTCGACTCGAGATCTGCGGCTTTGGACAGTCCCTCTGTACTGTGAAGCCACAGATGTCGAGTCGACTAGCTTCC
TTTCTCCCTTCCTCCC
+
B<1///////89??DCCBBB@=@>@<@8;<CCDDC<AABBBCBBBBB@>?B?BCCA@@BCCCCBBBC@@?BA?8:?>
@F=B><<68,/,/771
@T6XXI:00023:00245
GGGGAATTCTAATACGACTCACTATAGGGAGAGGGAGAATAGTACACAACTCG
+
?=@6@>A@BCC@C??=A?B??AB>966659AACC?BBC?90+,,+**+*----
@T6XXI:00024:00230
GGGGAATCTAATACGACTCACTATAGGGAGAGGGAGAATAGTTTCACACACATTTACCAGGGGCTGTTCTCTTCCTT
TCTCCCTTCTCCC
+
88217+////,/48443;;;>><6616,16677888644333303403 4<<4392/0,0000+/:4499>?>?C@CB
>B???<?DB?==9
@T6XXI:00025:00212
CTAGTCGACTCGAGATCTGCGGCTTTGGACAGTCCCTCTGTACTGTGAAGCCACAGATGGAGTCGACTAGCTTCCTT
TCTCCCCTTCCTTTCTCCCTTCTCCC
+
D1111=::BEDCCCCCBAAAA??CD>B<??>>77:57??BBBBB@@B=;<C@CCDC>>>;103>>>>CB@CC;;;:C
=ABCCB7A??>@A;@ADC>D688<<7
@T6XXI:00025:00237
GGGAGAAGGGAGAAAGGAAGCTAGTCACTCGACATCTGTGGCTTCACAGTACAGAGGGACTGTCCAAAGCCGCAGAT
CTCGAGTCGACTAG
+
CCCAA?@BC<A@==5>5948A@>@B:???A@:::AA??@L?BB@CBCC??@A@<88848:<<AA<;:;?A>AA=;==
:::====<<<<<<<
@T6XXI:00025:00249
GGGAGAAGGGAGAAAGGAAGCTAGTCACTCGACATCTGTGGCTTCACAGTACAGAGGGACTGTCCAAAGCCGCAGAT
CTCGAGTCGACTAG
+
CCACCC@BC>B@==9>5948BB???@>76177116669:::?A=@@F???BBAA77717??@@A?CA:>@=A@@AA@
@888====<<<<==
@T6XXI:00027:00229
GGGAGAAGGGAGAAAGGAAGGAGAAGTCGCCCCAAACGATTTGTTGACTATTCTCCCTCTCCCTATAGTGAGTCGTA
TTAGAATTCCCC
+
CDACAA?BC>BBAB>B?B?A?ABC@ABBDCLN9CC?DDCCB>BC@CCCC??<?AAA<@?@@@<@AA=A:?;A=<??@
@?A@@<=38870
@T6XXI:00027:00242
GGGAGAAGGGAGAAAGGAAGGAGAAGTCGCCCCAAACGATTTGTTGACTATTCTCCCTCTCCCTATAGTGAGTCGTA
TTAGAAT
+
@@:@CA?CC@AAFC?C@C@C@BBB?@EBBBBB9BE>CB@?C>B>????B??<?A==;<<@==;=A==@?@@@A@@?>
>?AAA>=
```

```
@T6XXI:00027:00246
GGGGAATTCTAATACGACTCACTATAGGGAGAGGGAGAATAGACACCAATCGCGCCGGGTCATGTTCTCCTTCCTTT
CTCCCTTCTCCC
+
;;9/9:BBCCB@CCCBBBBBBA@<916659::DE>CCA>@??@@AA>A;B<=:>>2664:@=?@B@DBA>A5858>:
;=?>7==>>??<
@T6XXI:00028:00226
CTATCACTCGAGATCTGCGGCTTTGGACAGTCCCTCTGTACTGTGAAGCCACAGATGTCGAGTCGACTAGCTTCCTT
TCTCCCTTCCTCCC
+
DBC;1111:::;;;BCBCB>:::6;>ABCBBBC>CBBA????B<=>>BD@CCBCB;;;@@A??AA;;79A?;>A;==
;?77728,/,/661
@T6XXI:00028:00239
GGGGAATTCTAATACGACTCACTATAGGGAGAGGGAGAATAGCTTCGTCCGATCTCGAACCTATTCACCTTCCTTTC
TCCCTTCTCCC
+
999/4<B@CAA?BCCBB@?BB@>;?9::.7>@AB>CCA>@=A@B?BAAA@CDCBBAAA@::?/.-
/>>;C5868808??B<B=@@BB=
@T6XXI:00029:00222
GGGGAATTCTAATACG
+
444,424244424444
@T6XXI:00030:00204
GGGGAATTCTAATACGACTCACTATAGGGAGAGGGAGAATAGAGACGTATTCGTTTGCGCTCTGTTCACCTTCCTTT
CTCCCTTCTCC
+
999,4242444248@>@@@@AACBB@A@-
77==<6?9699:64333<668<==AA<AA==@?@AA>884;88=8==:8333032300/
@T6XXI:00030:00221
GGGAGAAGGGAGAAAGGAAGCTAGTCACTCGACATCTGTGGCTTCACAGTACAGAGGGACTGTCCAAAGCCGCAGAT
CTCGAGTCGACTAG
+
@C@BBB@DD>B@==:?=@?BCBBB@7::??=77::::@@B??@=@CB???CB@4300,4::=AB?AA<AA?AAA@@=
9==AAAA??AAAAA
@T6XXI:00030:00226
GGGAGAAGGGAGAAAGGAAGCTAGTCACTCGACATCTGTGGCTTCACAGTACAGAGGGACTGTCCAAAGCCGCAGAC
TCGAGTCGACTACGT
+
IDCC><<<B:@;;9095958@?>?A:66=?B?BB::8BCC>:;:;BACDBBBC<<<?7<=@@@B>AB<??>@88888
8;;848=:61.****
@T6XXI:00031:00228
GGGGAATTCTAATACGACCTCACTATAGGAGAGGGGAAGAATAGAC
+
444,48989A@<911110,.4437??7740;::::+0,0.,++---
@T6XXI:00031:00238
GGGGAATTCTAATACGACTCACTATAGGAGAGGGAGAATAGCCACACACTCCCTGCTTACGCATCCCTTCCTTCC
+
CKB8D695444999>@A8<;<:>?9:;:0/09303732555589999000000+0008<<::@;444,-,-,-,-,
@T6XXI:00031:00245
GGGAGAAGGGAGAAAGGAAGGAGAAGTCGCCCCAAACGATTTGTTGACTATTCTCCCTCTCCCTATAGTGAGTCGTA
TTAGAATTCCCC
+
EE@AA@>B@<BCCCAC@C=@>@BD@@;AAB??8??<BB>>B>BA@C???==:::==;<8>?C<@?@???A???@@@@
@?A@@<@=@CC6
@T6XXI:00032:00200
GGGAGAAGGGAGAAAGGAAGCTAGTCGACTCGACATCTGTGGCTTCACAGTACAGAGGACTGTCCAAAGCCGCAGAT
CTCGAGTCGACTAGCTTCCTTTCTCCCTTCTCCC
```

```
+
DB@B;;79>:>899042423993..0.......33111668>:><=@BAAACCAA7738>>=>?>@@;>>8;;?:==
888?@C@@@@?>>><<9=?@A<AAAA<?<?AAC=
@T6XXI:00032:00204
GGGAGAAGGGAGAAAGGAAGCTAGTCGACTCGAGATCTGCGGCTTTGGACAGTCCCTCTGTACTGTGAAGCCACAGA
TGTCGAGTCCGACTA
+
4<89A@ACF>ACAA<>6;6797771111666676>;;7;:A>CBD8:59;<<==?6=;:4373311-/,-
241./.<8559611-.,.277;
@T6XXI:00032:00210
GGGAGAAGGGAGAAAGGAAGTGCAGTTCGCGGTTAGTGAAGGGAAGAACTATTCTCCCTCTCCCTATAGTGAGTCGT
ATTAGAACTTCCCC
+
CC>BCC?BB=B>==:@>B>ABBCDDC@CCCC@C@CCCBB?BC>CABC@BBBB?BBBC>C===C<@=====B@@@???
??@@@@<==;>AA6
@T6XXI:00032:00237
GGGGAATTCTAATACGACTCACTATAAGGAGAGGGAGAATAGTCAACAATCGTTGGGCGACTCTC
+
CCC:C8;:<@@;>AA<<<D?>7=@,,+,+,00778::<<:==:44/04/776636>75;7...,,
@T6XXI:00033:00204
GGGAGAAGGGAGAAAGGAAGCTAGTCGACTCGACATCTGTGGCTTCACAGTACAGAGGGACTGTCCAAAGCCGCAGA
TCTCGCGA
+
CCACBA===5=944-
;?A=@@EABA@CB>A:?:9::::CCC?@B@CC>99<A?8833417?AA?<;?@:;<9<>:>@@?><==<;
@T6XXI:00034:00215
GGGAGAAGGGAGAAAGGAAGGGAATAGCAGCAGTCGGCAACGTGAAACTATTCTCCCTCTCCCTATAGTGAGTCGTA
TTAGAATTCCC
+
GC@BBB@CC>BAAC?B?B@BC>C@BBBBCDAACBAA?AB@BBBCC?>BBBA?@>@>99A???;=CA===A@@?<<<8
869AA@D@AC>
@T6XXI:00034:00222
GGGAGAAGGGAGAAAGGAAGGACAGCCCCCGGGGAGAGAGTAAACGGCTATTCTCCCTCTCCCTATAGTGAGTCGTA
TTAGAATTCCCC
+
?A<@CB?CD@DCCC>B?BDA?@?978888+8?A7BCCCCB?8?8995:CCC@BBBB@CDCBC?BBBBBBBCCA@@@@
A=?BC@C?CDC9
@T6XXI:00034:00243
GGGAGAAGGAGAAAGGAAGAATAACGTGGGGTTAGGGATCAAGTGGCTATTCTCCCTCTCCCTATAGTGAGTCGTAT
TAGAACTTCCCC
+
>=01//,/,///7+/,4239933148>>>>4>;984-
4999244999;942499969>77;2;;????A@??:69944403/160111,
@T6XXI:00035:00229
GGGGAATTCTAATACGACTCACTATAGGGAGAGGGAGAATAGCAGCCAACTCTCAAGTTGCCAAATTCCTCCTTCCT
TTCTCCCTTCTCCC
+
CDC:C@E=<9C;88=@;::AA@??>777.7>>>C>??;:7433000/08344433236<44733,-,-,-
301040110348848<=6884
@T6XXI:00035:00238
GGGGAATTCTAATACGACTCACTATAGGGAGAGGGAGAATAGTCAACAAATCGTTTGGGGCGACTTCTCCTTCCTTT
CTCCCTTCTCCC
+
BAA4@<@=@BBBCCBBBBBB@>;96116.6??DE>BB@?=9=?AA>@??<BCB@@;?@C9CBBAC?>A<<>:=;>?;
?@@A<A<??AA<
@T6XXI:00036:00214
```

```
GGGAGAAGGGAGAAAGGAAGCTAGTCGACTCGACATCTGTGGCTTCACAGTACAGAGGGACTGTCCAAAGCCGCAGA
TCTCGAGTCGACTAGCTTCCTTTCTCCCTTCTCCC
+
CC@C<;7<@:>99909<@<@@>::>000:0/.0616??;??8:<=?A@ADCBA?????<?CC@@@<==7;;8;>@@?
??@@???A===A?>===2424:4:;==7?A===D?
@T6XXI:00036:00232
GGGAGAAGGGAGAAAGGAAGCTAG
+
?C>ABDACB:A911-/,1,...22
@T6XXI:00036:00248
CTATCGACTCGAGATCTGCGGCTTTGGACAGTCCCTCTGTACTGTGAAGCCACAGATGTCGAGTCGACTAGCCCTTC
TCCCCTTCCTTTCTCCCTTCTCCC
+
C:7711188:BBCBBAABBB@?AA<A?AA@>>@?;???????>@CAA9<@ABBBBBBCCBBCC@AACCDCBBB:>:<
7666-636344-4:994848000+
@T6XXI:00037:00200
GGGAGAAGGGAGAAAGGAAGATAAACGTACTGCGGGAGAATCCAGTGACTATTCTCCCTCTCCCTATAGTGAGTCGT
ATTAGAATTCCCC
+
BC?BBB@BB?BBCB>B@B@BCAAA>BCAAABB?@A>BBE@AB>@BBB?<<B?@@??>9>?A>>;::;;688=@A@AA
==;=AA>A=AAA5
@T6XXI:00037:00225
GGGAGAAGGGAGAAAGGAAGCTAGTCACTCGACATCTGTGGCTTCACAGTACAGAGGGACTGTCCAAAGCCGCAGAT
CTCGAGTCGACTAG
+
CG;:=B@CC@DDBB?B@B?@@??BA?????=???:::F??=?A?B???????<=77717==???>AA<??<@AAA??
???=<=A@@A@@@A
@T6XXI:00037:00238
GGGAGAAGGGAGAAAGGAAGAGAACGGCGACGAACAATAGTTGGTGGCTATTCTCCCTCTCCCTATAGTGAGTCGTA
TTAGAATTCCCC
+
DC>@@B=@@<@BBC@C@C@BBBC@CC@BBEBCC@BB?BDNC@C?;;7;@CC@DCDC@BBCBB?A?@@AA988==887
34;8943/000+
@T6XXI:00038:00206
GGGGAATTCTAATACGACTCACTATAGGGAGAGGAGA
+
C8818+989>?;9989=<7;>?;?<777.77::,0.-
@T6XXI:00038:00209
CTAGTCGACTCGAGATCTGCGGCTTTGGACAGTCCCTCTGTACTGTGAAGCCACAGATGTCGAGTCGACTAGCTTCC
TTTCTCCCTTCTCCC
+
GA1//1////8;9499@AABD@CCC>C?CBCC???=?CCCBBBCBBBA>?B@BBBAAB;;:@@==991104...,.,
..+.666,0/0333+
@T6XXI:00038:00222
GGGGAATTCTAATACGACTCACTATAGGAGAGGGAGAATAGTTTCTCGTTAGGCGCCCTGTGACTGTTCTTCCTTTC
TCCCTTCCTCCC
+
CJEAC9;;;AA<ABBBCAAAB<@@?993:?>@A>BBD@A??;;:;==<<:<84400018@@AAABBB=BB?A>AA;8
///,/,-,.782
@T6XXI:00039:00205
GGGGAATTCTAATACGGACTCACTATAGGGAGAGGGAGAATAGATCACCGTTGGTTCGGGTGCGCGTTCTCCTTCCT
TTCTCCCTTCTCCC
+
CDCBH::>;CCACB@?@BBBB@AA>9611-
1;;?B>CCA?B<?=<=?<<AA5:59>???<@BCACBC@CCC?A=?;?B<AB@@<@>AAAB>
@T6XXI:00039:00235
```

```
GGGAGAAGGGAGAAAGGAAGGAGAAGTCGCCCCAAACGATTTGTTGACTATTCTCCCTCTCCCTATAGTGAGTCGTA
TTAGAATTCCCC
+
FK?BBB@BB>ABAA=B?B?B@CDC@B;BBBCC8@B<@B???;?B@CABB@@>@AAA<AA@@@;@==???@@AA???A
A=@@A>A8<;;0
@T6XXI:00039:00238
GGGGAATTCTAATACGACTCACTATAGGAGAGGGAGAATAGTGACTAGCTGGTCTACGTTGCTCTCTCCTTCCTTCT
CCTCTCC
+
:CC8A8<8;@@@CCDBBB<;;=?::00,//077-
76326@<==A==8554<;?<9=AA@?AB@@@A??3824797666,/-112
@T6XXI:00039:00244
CTATCGACTCGAGATCTGCGGCTTTGGACAGTCCCGCTGTACTGTGAAGCCACAGATGTCGAGTCGACTAGCTTCCT
TTCTCCCTTCTCCC
+
C866/1//789>@AACCCBA>A??<@?@ABA@@B>BCCCA@ABBCCC?AB@BCCCBAAB?BBCCBBBCCCCBD@CAD
C=@@?C=A><??>7
@T6XXI:00040:00213
GGGAGAAGGGAGAAAGGAAGTGCAGTTCGCGGTTAGTGAAGGGAAAAACTATTCTCCCTCTCCCTATAGTGAGTCGT
ATAGAACTTCCCC
+
CC>CAAA@B>@99909>@?CCCCCCCDCAA@@C?B@@@A?BC=BBCB2B;;<?ABDD>::;CC<B=<<??>>>;8//
//-22018/444+
@T6XXI:00040:00240
GGGAGAAAGGGAGAAAGGAAGGGGAGAAGGGAGAAAGGAAGCTAGTCGACTCGACATCTGTGGCTTCACAGTACAGG
+
??81///+/65,,/8+/,/,.695884-6116/?>9@?>,......5998??B887@<;8>;84838;@?;788/,*
@T6XXI:00040:00243
GGGAGAAGGGAGAAAGGAAGCTATCGACTCGACATCTGTGGCTTCACAGTACAGAGGGACTGTCCAAAGCCGCAGA
+
FDBAA@;=95=>>@<B?B@CCCBBBBCBBB??::::7?@A;@@@CCCCB?A>74000-4;;:>?>@@<@CB??:==
@T6XXI:00041:00221
GGGGAATTCTAATACGACTCACTATAGGGAGAGGGAGAATAGTCTCGCGATGGTCCGCTACTTGTTCTCCTTCCTTT
CTCCCTTCTCCC
+
=994>>A@CCCACA@ABBBBB@;97111.7>>BD=BB@>A9==??AAAAC@@@<<<=>CBBB@CC@B;:7;>A=AB=
AAGC=A>??;;6
@T6XXI:00041:00225
CTAGTCGACTCGAGATCTGCGGCTTTGGACAGTCCCTCTGTACTGTGAAGCCACAGATGTCGAGCGATGCTTCCTTT
CTCCCTTCTCCC
+
D?499<;<@CCECCCCCBABB@BBB@B>@><>777.7==AABBBABAB=?B@CBCBABBBCCCCCCCCBA?;?>=@?
CCCC==;=??C@
@T6XXI:00041:00241
GGGAGAAGGGAGAAAGGAAGCTAGTCACTCGACATCTGTGGCTTCACAGTACAGAGGGACTGTCCAAAGCCGCAGAT
GTCGAGTCGACTAGCTTCCTTTCTCCCTTCTCCC
+
>75<11-/656/44-99;9<><;;AB>@?<<<7769?BAB8:;?A??ABB@@:8444-
4<<;;=<??<?@<ACA??==<>?@?@@A???@@<;78/4418;;=1858?@?;
@T6XXI:00041:00247
GGGAGAAGGGAGAAAGGAAGGGAGAAGGGAGAAAGGAAGGGAGAAGGGAGAAAGGAAC
+
DG@CCB<==<499>0949273,333117;111=-707311,16603444300+0/0/1
@T6XXI:00042:00212
GGGGAATTCTAATACGACTCACTATAGGAGAGGGAGAATAGTTTGCGCATTCGGTGCACTGTTATTCTCTTCCTTTC
TCCCTTCTCCC
+
```

```
CGC:C@C@CCB@CBBBBBBBB@<<A6137>7776:B@=@@@@A=<<<>?B>AB?B@AACDCC@@@=@AAA@C?BB>B
BCC>C@A?;=;
@T6XXI:00042:00229
GGGAGAAGGGAGAAAGGAAGTGCAGTTCGCGGTTAGTGAAGGGAAAGCTATTCTCCCTCTCCCTATAGTGAGTCGTA
TTAGAATTCCCC
+
EF@BBA=>=:@@@@<@=@=@AABBBC@CCCC@C???>DD?BB>CC?CCCBB@>AB@;C@BBA;BA@@?AA<>@AAA8
888AACC;AAA6
@T6XXI:00043:00200
GGGGAATTCTAATACGACTCACTATAGGAGAGGGAGAATAGTCAACAAATCGTTTGGGGCGACTTCTCCTTCCTTTC
TCCCTTCTCCC
+
CCC:C@B@BBB@CCA@@>>><:997//,0::>@=BGH>A<AA@<:<@A;BA977,166-
7??=?;<==4438<@@<@ABA;@C@AAA<
@T6XXI:00043:00213
GGGGAATTCTAATACGACTCACTATAGGAGAGGGAGAATAGTTTAGCACGTTACCTCGGCCTATTTTCCTTCCTTTC
CTCCTTCCTCCC
+
A@@6?7<:<AA?BBBBCACCBBCB?7937>777.7?;9>@98858>=AABA?@?477747:<7611,17;:;::4+/
,/.,.,-,000+
@T6XXI:00043:00238
CTAGTCACTCGAGATCTGCGGCTTTGGACAGTCCCTCTGTACTGTGAAGCCACAGATGTCGAGTCGACTAGCTTCCT
TTCTCCCTTCTCCC
+
A<1/186==C@?ACCCCCCC@:73/348?AAABBCBBBCCCBAAAA@9=A>A@CBB;<;BA?AA@@????8:62656
@>B@@C>A>AAAB;
@T6XXI:00044:00211
GGGGAATTCTAATACGACTCACTATAGGGAGAGGGAGAATAGTTTTCCCTTCACTAACCGCGAACTGCACTTCCTTT
CTCCCTTCTCCC
+
FDC8A=A=@BC@CBAABBBBBB>97111.7>>?B=?@=>AAACAD08858<@BDBB?B?B@@<><A@@:::@C=??;
?BBC>B@C@@@;
@T6XXI:00044:00222
GGGGAATTCTAATACGACTCACTATAGGGAGAGGGAGAATAGTTTTTCCCTTCACTAACCGCGAACTGCACTTCCTT
TCTCCCTTCTCCC
+
BEDDE>@C>AA=@ABBCCCCB=996166.7>==?=@CA@@@@AAAA1AA<A?ACB>@@C@??A@>AAA<>:8:?:;?
8ABCC>B@<<<A>
@T6XXI:00045:00200
GGGAGAAGGGAGAAAGGAAGTGCAGTTCGCGGTTAGTGAAGGGAAAAACTATTCTCCCACTCCCTATAGTGAGTCGT
ATTAGAATTCCCC
+
CD>ABA@DC>BBBC>B?A@B@?ABBB>?ABB@B@BBBA<5675:CCC2CCAA>ACCC<@?B??;>>A?@@><<=<?<
@<:;A=<A:=984
@T6XXI:00045:00207
GGGAGAAGGGAGAAAGGAAGCTAGTCACTCGAGATCTGCGGCTTGGACAGTCCTCTGT
+
88+//4249599@C=@>97.....330000,,,+,,,,770173707111.3334.--
@T6XXI:00045:00227
GGGAGAAGGGAGAAAGGAAGCTAGTCACTCGACATCTGTGGCTTCACAGTACAGAGGGACTGTCCAAAGCCGCAGAT
CTCGAGTCGACTAG
+
FKCBBB=@@<@@@@<B=@=?B????::7>@>7::961=??;@BBCCCCCB>974000-
4;;>@B<>>6<<;<<=@AAA@@@@@AA@???=;
@T6XXI:00046:00228
GGGAGAAGGGAGAAAGGAAGCTAGTCACTCGACATCTGTGGCTTCACAGTACAGAGGGACTGTCCAAAGCCGCAGAT
CTCGAGTCGACTAG
```

```
+
CDACCBBCB=BB==:@696;CAAAC=@?ACC::;;::BBB<?@=@CBBCC@AB>98846<<<@@>==;?A?AA=::?
<@@@:@??@CA@@@
@T6XXI:00047:00206
CTATCACTCGAGATCTGCGGCTTTGGACAGTCCCTCTGTACTGTGAAGCCACAGATGTCGAGTCGACTAGCTTCCTT
TCTCCCTTCTCCC
+
CA@@<::;BBBCCCBBB@ABABB>B?AA??>?B>BCCA>=>@AAA@>@C@CCCBCCCBBBBCCBBBBBCDCB=?=?B
>?A>@5<484471
@T6XXI:00047:00207
GGGGAATTCTAATACGACTCACTATAGGGAGAGGGAGAATAGTCAACAAATCGTTTGGGGCGACTTCTCCTTCCTTT
CTCCCTTCTCCC
+
CBC7B=@=@CCDCCBBBB><BAB?;111.6==@C>BB??B<<=@@>??>5?=666-
777.7>BKE@BCC?@=?=@C<A???4=378@@<
@T6XXI:00047:00210
CTAGTCGACTCGAGATCTGCGGCTTTGGACAGTCCCTCTGTACTGTGAAGCCACAGATGTCGAGTCGACTAGCTTCC
TTTCTCCCTTCTCCCCTTCCTTTCTCCCTTCTC
+
C7-,,-
,,7::>DBBBCCBBB@CCC>C@CC@@9994:?BCCBBCA@?;:;B@BABBBAB@??A99:@A???BAA=A@CDABBB
CBA;;4444-4/4/30,3999-42499
@T6XXI:00047:00213
GGGGAATTCTAATACGACTCACTATAGGGAGAGGAGAATAGCTAGGCGCATATTCGTGCTCTGTCTCCTCTTCCTC
+
C>>5A=@=ABBBDBBBCCBBBCCB669;6;=7637:747//./..,..-14449000...5140000/000/0200
@T6XXI:00047:00218
CTATCACTCGAGATCTGCGGCTTTGGACAGTCCCTCTGTACTGTGAAGCCACAGATGTCGAGTCGTACTTAG
+
DCDB@::9?A?BCCCDB?>=@BC>B>@B??BCC>C@@@AA@>><;;<<@=@CCCBBBDBCA>;8----,*,*
@T6XXI:00047:00233
CTAGTCACTCGAGATCTGCGGCTTTGGACAGTCCCTCTGTACTGTGAAGCCACAGATGTCGAGTCGACTAGCTTCCT
TTCTCCCTTCCTCCC
+
94949944:=@A?BBBBDCB?BAA<A=@@AAAAA<?BB:::???@??8<A?BBBBB@@@AAA??ACCCEDDCC?C?C
F>B?@?69,/,///,
@T6XXI:00047:00237
GGGGAATTCTAATACGACTCACTATAGGGAGAGGGAGAATAGCCGCAACCCTGTCCTCTTGAATCTATTCTTCCTTT
CTCCCTTCTCCC
+
A=92?59:=CB@DBBBBBBBBB<97111.7==?@>BBA?@CCC?A<?=??9?=??A<;;@DA?A@888;;;8;344-
4;;;9;9;444-
@T6XXI:00047:00238
GGGGAATTCTAATACGACTCACTATAGGAGAGGGAGAATAGTTGCTTGCTATTTTCCTTCGGACGGACCTTCCTTTC
TCCCTTCTCCC
+
@@>4><@>@AAADDCCCBBBB:6;5/.,000==<?CA>A>??>A?:;ACB??::589B?AA@A@@>@A>@=@<?<;<
@AC=A85;AC>
@T6XXI:00048:00206
GGGGAATTCTAATACGACTCACTATAGGAGAGGGAGAATAGATCACGACCCTTCTACCACGATTGAGCTTCCTTTCT
CCCTTCTCCC
+
CEF9D@C@@@@?BCBBBCCBA7;;7//,0><?A>CC@<???@@???@::6-
6:;??C@B@A@@>@@@AA=A?DD?BEGE?B>@:88/
@T6XXI:00048:00217
GGGAGAAGGGAGAAAGGAAGCTAGTCACTCGACATCTGTGGCTTCACAGTACAGAGGGACTGTCCAAAGCCGCAGAT
CTCGAGTCGACTAG
```

```
+
DD@@@@?A@:@@885@496:AA@@@@?==??:::CCCBAA>?@=@CCBBBA?;93331388=@C>??<?A???;==@
<??@@@@@@AAAA@
@T6XXI:00048:00221
CTATCACTCGAGATCTGCGGCTTTGGACAGTCCCTCTGTACTGTGAAGCCACAGATGTCGAGTCGACTAGCTTCCTT
TCTCCCTTCTCCC
+
CBBC;::=994BB?<<<CC@CCC?C@CC?????9?@@AB@A@CAAA=@B?BBCBB:;:ABAAB<<;@@?::67706:
5:;:=-8343884
@T6XXI:00048:00232
CTAGTCACTCGAGATCTGCGGCTTTGGACAGTCCCTCTGTACTGTGAAGCCACAGATGTCGAGTCGACTAGCTTCCT
TTCTCCCTTCTCCC
+
B:///1/4:AACCBECBB@@?ABB<B@?<??AAA<ACCCCC@@@BAA;=ABCB@AA@@??@@AABBBC@@@??:?>@
>7>777-49>;;96
@T6XXI:00048:00237
GGGAGAAGGGAGAAAGGAAGCTAGTCACTCGACATCTGTGGCTTCACAGTACAGAGGACTGTCCAAAGCCGCAGATC
TCGAGTCGACTAG
+
44-49:249-
4489699@97<>8000.....455<==:BB<:;8;@>@B????;842494403/30+0444666440-----
32234665
@T6XXI:00049:00211
GGAGAAGGGAGAAAGAAGAGTAGCTGGGACGGAATTTTAACGCGAATGCTATTCTCCCTCTCCCTATAGTG
+
>,1//,//+,,//+//,.667=;;>8>6:1/,/,///*49944482337633133176111111,1667110
@T6XXI:00004:00273
GGGAGAAGGGAGAAAGGAAGCTAGTCGACTCGACATCTGGTGGCTTCACAGTACAGAGGGACTGTCC
+
44-4;;249199991829477?881<77611134.....,.444688867666631---+--22333
@T6XXI:00004:00288
GGGAGAAGGGAGAAAGGAAGGAGAAGTCGCCCCAAACGATTTGTTGACTATTCTCCCTCTCCCTATAGTGAGTCGTA
TTAGAATTCCCC
+
CCBCCB?BB=??>><@?B?B@CBB@B?CBBCC7BB<CB@@@;@@>@BCC?<<;===;88>?CAA?:888?====A6<
=>BA@=@9;;<4
@T6XXI:00005:00264
GGGGAATTCTAATACGACTCACTATAGGGAGAGGGAGAATAGCTCAACAATT
+
CCB9C>A?ABBBCBBBB?>>>?DCC=<<;=>>CC=AA@?C4++,,,*,,*42
@T6XXI:00005:00277
GGGGAATTCTAATACGACTCCTATAGGGAGAGGGAGAATAGCCAAAAA
+
444-4:@?BBB?BBBBBC@@=?>?777.7?777.7<<:81,,+,,,+)
@T6XXI:00005:00296
GGGAGAAGGGAGAAAGGAAGGAGACAGTGAGTGCCGCACAACACGTCACTATTCTCCCTCTCCCTATAGTGAGTCGT
ATTAGAATTCCCC
+
,-+,/9284-448;-
969997377@@@@ADDCCDBBC@@?<@771611///8699@A<?CBAA<BCCDCC@@@CCCC??;7:::@?BEE:
@T6XXI:00006:00280
GGGGAATTCTAATACGACTCACTATAGGGAGAGGGAGAATAGTCAACAAATCGTTTGGGGCGACTTCTCCTTCCTTT
CTCCCTTCTCCC
+
BD@4@5958989>?AADCC@@?BB=777-7/5::60.-,---
28<;>==386761,1662;8;663;11002878808000+0241110
@T6XXI:00006:00282
```

```
GGGAGAAGGGAGAAAGGAAGCTAGTCGATCGAGCATCTGCGTTTG
+
BA>AAABCGCDBBC?B=@=@BC?AB:;1===011/77555...+.
@T6XXI:00006:00287
GGGAGAAGGGAGAAAGGAAGTCGTAGAGATAGCTGGGAAAGGTGTTGCTATTCTCCCTCTCCCTATAGTGAGTCGTA
TTAGAATTCCCC
+
DC@CDE@BC>BBBC@C@C@CCBBBCC@><ABCCCCC<BC>A?@<<:;<BBB?AAAA;??@AB<@@@===AA===??>
=>@=<9<<???5
@T6XXI:00007:00261
GGGGAATTCTAATACGACTCACTATAGGGAGAGGGAGAATAGACACCAATCGCGCCGGGTCATGTTCTCCTTCCTTT
CTCCCTTCTCCC
+
CCC:D@C@BBB@B???BBBBB?;76166.7>>>A>BCA>A@?@@AC><:B@?;77311,7;?@@?@AAC?A;>;?C@
AC@@<@=@???:
@T6XXI:00007:00263
GGGGAATTCTAATACGACTCACTATAGGGAGAGGGAGAATAGTCAACAAATCGTTTCGGGGCGACTT
+
AAA9A@BABCC@CBB@BBCB@?BB=::<;<ABCC<??C?A<====:<>?6>;00/+....*..100/
@T6XXI:00007:00293
GGGAGAAGGGAGAAAGGAAGAACGGTGTGCTGGGAGAAATGCACAGAACTATTCTCCCTCTCCCTATAGTGAGTCTA
TTAGAATCGGGCGAATTCTAATACGACTCACTATAGGGAGAGGGAGAATAGTCAACAAATCGTTTGGGGCTACTTCT
CCTTCCTTTCTCCCTTCTCCC
+
CD@BBA7;;9<CBB?GAC@BB>>A?ABBBCCCC>CBCC>CFAAAB9959998<=@BE>CAABC>@@????>>=3/.4
43311000043568977100/00004495<8667166,1733346<=;@?>>BA;9:C;??9975399233316011
83677011,100426588:;4
@T6XXI:00008:00281
GGGGAATTCTAATACGACTCACTATAGGGAGAGGGAGAATAGTTTTTCCCTTCACTAACCGCGAACTGCACTTCCTT
TCTCCCTTCTCCC
+
CCC:C@C@BBB@BBBBBBBBB@>97116.7==>BCDED>A==<???1@@;A>@BBCC@C@<=@@>@@A=?>A?C@DC
>?BAB>BD???AA
@T6XXI:00008:00291
CGAGATCTGCGGCTTTGGACAGTCCCTCTGTACTGTGAAGCCACAGATGTCGAGCTTCCTTTCTCCCTTCTCCC
+
<<BBCBBBCCD>@@CCDACBBBBBD>BBBCCAA@:::A9==>CC?==@?>BBCCBB>A59908???:A=A@@@<
@T6XXI:00009:00270
GGGAGAAGGGAGAAAGGAAGCTAGTCGACTCGACATCTGTGGCTTCACAGTACAGAGGGACTGTCCAAAGCCGCAGA
TCTCGA
+
BB>BBA;=@<@A@@<B@B=@@CBBB:;;A??=:9:::7??A=AB?BBCCCCB?44000-
4:;;;=38858CAAA?BAA????>
@T6XXI:00009:00284
GGGAGAAGGGAGAAAGGAAGCTAGTCGACTCGAGATCTGCGGCTTTGGACAGTCCCTCTGTACTGTGAAGCCACAGA
TGTCGAGTCGACTAGCTTCCTTTCTCCCTTCTCCC
+
CHBB9969>:>999094948>>;;</0000==>>??CBBBC>BBC>C@CBCBBBC>@>>>@@@@8<<A?AA<==?<@
=<<======A===A???<?>AA<@@AC<A889AA=
@T6XXI:00010:00256
GGGGAATTCTAATACGACTCACTATAGGGAGAGGGAGAATAGTCTCCCTGCGTATGGAACTGTCATCTCCTTCCTTT
CTCCCTTCTCC
+
999/48=8=@A<@@<<<CCB;:;>./.3+.,,,20/,.-
..47<6;6431177///77798?;986...,.447>?;>977+.,...,
@T6XXI:00010:00282
```

```
GGGGAATTCTAATACGACTCACTATAGGGAGAGGGAGAATAGTCAACAAATCGTTTGGGGCGACTTCTCCTTCCTTT
CTCCCTTCTCCC
+
===6?>CADCC@DBBBCAAACB??7777.7==@C>CCA>AA<;44/333,11111,17>8?CC:?:::::6;@>AC?
988A<?8;;;;6
@T6XXI:00011:00287
GGGGAATTCTAATACGACTCACTATAGGGAGAGGAGAA
+
=885?<A>BBB@FCBAA?BA?@DB@<<6237//,0,,+
@T6XXI:00013:00291
CTAGTCGACTCGAGATCTGCGGCTTTGGACAGTCCCTCTGTACTGTGAAGCCACAGATGTCGAGTCGACTAGCTTCC
TTTCTCCCTTCTCCC
+
E11111///1:>B@@AC;<<BA@@G@C?B@?B???<?AACCCCBBAB??@EACCCGBBBBBBBCBBLC<<;<:6998
;?9?<@C>A?<8881
@T6XXI:00014:00253
GGGAGAAGGGAGAAAGGAAGGAGAAGTCGCCCCAAACGATTTGTTGACTATTCTCCCTCTCCCTATAGTGAGTCGTA
TTAGAATTCCCC
+
CC@CCC@CB>CB?B=B?A>A?ABB@CCAAACC9CC>CCBCC?BB@DAB?ABCAABC<><<<C<AAAA@@@@@A???@
@>A@>;;/444-
@T6XXI:00014:00261
GGGGAATTCTAATACGACTCACTATAGGGAGAGGGAGAATAGTCAACAAATCGTTTGGGGCGACTTCTCCTTCCTTT
CTCCCTTCTCCC
+
CCB9B?BABCD@CBBBCCCB@??<;669.7BBCC>DA?<A??@@@<??@;BCCCC>CCC8?@CBC8::A@C?A=BB7
>>AC<A=@@@AB
@T6XXI:00014:00278
GGGGAATTCTAATACGACTCACTATAGGGAGAGGGAGAATAGCCATTGTATTTCCTCTCGCTATGTATCCTTCCTTT
CTCCCTTCTCCC
+
444,9<@?CCB@B@???BB?BC>97111.7=<AD@CB@@A==998=:334>>7=;@@999AA>??=<;?;856566,
./8:6:7:;<=7
@T6XXI:00015:00254
GGGGAATTCTAATACGACTCACTATAGGGAGAGGGAGAATAGTTGTGTTGTCCCAGCATTGCCTATATTCTTCCTTT
CTCCCTTCTCCC
+
CDD9C@C@CCCBBBCCCCBBBCB<7111.7>>@E@CFA@AC@@?@<;;8;9996<?BBC@CC>BABBD=@@BDBBB=
A?==6=47:?C<
@T6XXI:00015:00267
CTAGTCGACTCGAGATCTGCGGCTTTGGACAGTCCCTCTGTACTGTGAAGCCACAGATGTCGAGTCGACTAGCTTCC
TTTCTCCCTTCTCCC
+
C8,-,-
,,/>;BCCBBBAA@C?BCB<B>AABB???=@CCCCCBB?BBA;=AADDBBBCBBBAAABBB:;;?@?63?:??9?@A
A>@9<<?@7
@T6XXI:00015:00287
GGGGAATTCTAATACGACTCACTATAGGGAGAGGGACGAATAGTCCTCCACATCTCCAACAACCCCTTACACTTCCT
TTCTCCCTCTCCC
+
111*/,/,//776:@;;;BBBBCB>A9?-161.5333.-,-----2-0/040167777-
0.44444799945;8:244+.287,/-1460
@T6XXI:00015:00297
GGGAGAAGGGAGAAAGGAAGCTAGTCGAACTCGACATCTGTGGCTCACAGTACAGAGGGACTGC
+
990499244-499;-469=<>@C;:>0/*,222,**,,,,*00,,.336788868888443---
@T6XXI:00016:00278
```

```
GGGAGAAGGGAGAAAGGAAGCTAGTCACTCGACATCTGTGGCTTCACAGTACAGAGGGACTGTCCAAAGCCGCAGAT
CTCGAGTCGACTAG
+
CCACCB@CC>CCBB=A>?>?C?BBB7;::@A:::ABACBC::;@CCCBBCCDC=44414?A?@??AA<@@=@@==;A
;AA@@???@@@???
@T6XXI:00016:00287
GGGGAATTCTAATACGACTCACTATAGGGAGAGGGAGAATAGTCAACAAATCGTTTGGGGCGACTTCTCCTTCCTTT
CTCCCTTCTCCC
+
===2>595444244448373;7976111,111168<9<<=@?@AA>@?@:B:77?.777,1669?566;20/348:4
:999-4/43880
@T6XXI:00017:00257
GGGGAATTCTAATACGACTCACTATAGGAGAGGGAGAATAGATCACCGTTGATTCGGGTGCGCGTTCTCCTTCCTTT
CTCCCTTCTCCC
+
CCB9B=A?@BB?@@@@A@@CCBAB@::5:::AA>D@===@@?@CA=9;:,0..5557-
/988403555?>@<@8;:4444:6<@9:9C?
@T6XXI:00017:00279
GGGGAATTCTAATACGACTCACTATAGGAGAGGAGAATAGTTTACGGACCCCTATTTTC
+
---*,2@<@AA@CCCCC>>>B:99;//,0>:=:=63/3:99;944727344000000+0
@T6XXI:00019:00254
GGGGAATTCTAATACGACTCACTATAGGGAGAGGGAGAATAGCAAACGTTTCCCCGACGTCGCTTTCTCCTTCCTTT
CTCCCTTCTCCC
+
@@>4@<@=@CC@<;<AACCCCCCCB96659??AD>CB?<?899CC<@BBB>CFD7AABB;;<CCB??::4734244+
38840;373884
@T6XXI:00019:00287
GGGAGAAGGGAGAAAGGAAGCTAGTCGACTCGAGATCTGCGGCTTTGGACAGTCCCTCTGTACTGTGAAGCCACAGA
TGTCAAGTCGACTAGCTTCCTTTCTCCCTTCTCCC
+
CB?C=<;<B>@9;909<B>ACC<;;:::@AB:<;BCCKBBC@BBB>B>???BBCC=A?C===AA@???9<<=@@@@A
@@A>><???@@@;;887-/-.5437A@<@9788?1
@T6XXI:00019:00291
GGGGAATTCTAATACGACTCACTATAGGGAGAGGGAGAATAGTCAACAAATCGTTTGGGGCGACTTCTCCTTCCTTT
CTCCCTTCTCCC
+
1880=-
1,///799;<CC??@:886;;68????C>CA?;==?BBC>???7=>7::5:BB5AAABC?BBD?@=@>@D<???A;?
==;<<6
@T6XXI:00021:00283
GGGGAATTCTAATACGACTCACTATAGGGGAGGAGGAAGATAGCTAACACTTCAAATTCCCAGCATTATTCTTCCTT
TCTCCCTTCTCCC
+
HCB7B79;<CC@CBABBBAAA@B>76166-67300,0,.-----
.47?A@@<<<B?C>AB=A<??C?BA??@=@=@@<@@@A=A??AAA;
@T6XXI:00021:00286
GGGGAATTCTAATACGACTCACTATAGGAGAGGGAGAATAGAGACGTATTCGTTTGCGCTCTGTTCACCTTCCTTTC
TCCCTTCTCCC
+
999/4295<CABBA@@CAAAC>=;://-
0==BC>CCA?AAAA878<>?@>ABAA:@?AAB<:??>@==:<?A9<<6;;;;6;9;4881
@T6XXI:00022:00267
GGGAGAAGGGAGAAAGGAAGAAC
+
44-444244-486858,/,.4,.
@T6XXI:00022:00288
```

```
GGGGATTAATACGACTCACTATAGGAGAGGGAGAATAGATAACACCGGTCGCCCTTGTCTATTCGCTTCCTTTCTCC
CTTCACCC
+
9<9/9B=@=@CA;<<AA??A?>??;?@BCB=>>=90//733377455../.3333.-032--,-----
.55757779533..-30
@T6XXI:00023:00258
GGGGAATTCTAATACGACTCACTATAGGGAGAGGGAGAATAGAGACGTATTCGTTTGCGCTCTGTTCACCTTCCTTT
CTCCCTTCTCCC
+
444,489899=9=>>?ABBBB:;8>776,77777-
7>;;<==@@@@@<<;8;;;=:==<;?;777588:8624/30+03340823000+
@T6XXI:00023:00265
GGGGAATTCTAATACGACTCACTATAGGGAGAGGGAGAATAGCAAATCGAACCGCCGAATTGCTGTCCCCTTCCTTT
CTCCCTTCTCCC
+
CDNCD@A>ADCBCC@?99<A@@BBA;;:59:9BB?CC@>@@@@<=;AA@@:<87738@=@>A@?;4444-
48:34418@AB<A:=9@@;
@T6XXI:00023:00269
GGGAGAAGGGAGAAAGGAAGGGGAGAAGGGAGAAAGGAAGCTAGTCACTCGACAG
+
BB>CBA@BB@ECBC>C@C@BBB8B>>566-7777.70737@::7CB=<00,**((
@T6XXI:00023:00273
CTAGTCGACTCGAGATCTGCGGCTTTGGACAGTCCCTCTGTACTGTGAAGCCACAGATGTCGAGTGACTACGTCTCC
T
+
B9994994?DCAAACCCCCCC@CBB>B=@@BBBBB>BCCCC?@?@@>::498<BBBDBBBD?==7766111111144
4
@T6XXI:00024:00251
GGGAGAAGGGAGAAAGGAAGGGAGAAG
+
CD@B?=684-999809494066;00,.
@T6XXI:00024:00258
GGGGAATTCTAATACGACTCACTATAGGAGAGGGAGAATAGCCAAACCATATTCGTCTGCCTATTTTCCTTC
+
>==2929594484999>AAB;:;><7747776999=;<<986:=;-43844;8;437004930000+04;56
@T6XXI:00025:00259
CTAGTCACTCGAGATCTGCGGCTTTGGACAGTCCCTCTGTACTGTGAAGCCACAGATGTCGAGTCGACTAGCTTCCT
TTCTCCCTTCTCCC
+
99944444:?=499@@@@BC@CDBCB?BCCBA??9?:::::CCC?==;=A@BA?@;8::999??>>88733110136
771666252338=4
@T6XXI:00025:00268
GGGGAATTCTAATACGACTCACTATAGGGAGAGGGAGAATAGTCAACAAATCGTTTGGGGCGACTTCTCCTTCCTTT
CTCCCTTCTCCC
+
CDD:E?A?AEE@CB??BBBA@A<97111.7====<?D@>@@>?>A>===;@@=9726685?@@AB?:::=?<?>AA<
A@AA<@<?>AA=
@T6XXI:00025:00272
CTATCACTCGAGATCTGCGGCTTTGGACAGTCCCTCTGTACTGTGAAGCCACAGATGTCGAGTCGACTAGCTTCCTT
TCTCCCATTCTCCC
+
ADC99444=999CCCBBBB?DEE>C@CBBAAAC>DECA@@BE?<<D38<:<<?BBCBAAA???@ACBBB888?::::
6>587,--,/*000
@T6XXI:00025:00286
CTCGAGATCTGCGGCTTTGGACAGTCCCTCTGTACTGTGAAGCCACAGATGTCGAGTCGACTAGCTTCCTTTCTCCC
TTCTCCC
+
```

```
BCCAAACDCCCCB@BBB=B?@CBACDD?CBBDCBDCBBCCBAA@@@BBBBA@BBBCBBBCCDCBBB@A@DC>BBCC?
B?BA@@<
@T6XXI:00025:00299
GGGAGAAGGGAGAAAGGAAGCTAGTCGACTCGACATCTGTGGCTTCACAGTACAGAGGGACTGTCCAAAGCCGCAGA
TCTCGCAGTCGACTA
+
CFADEC@CB?BAAA=B@B>?@C@?>0/.33338?8661>?<3:7<=????BA?;9343-
49:9;?8;;6<><@AAAA88:8//,500++-11
@T6XXI:00026:00252
GGGGAATTCTAATACGACTCACTATAGGGAGAGGGAGAATAGTTATACATTCGTCCCACAAGCCTTCTCTTCCTTCT
CCCTTCTCCC
+
ECB8C=@>@CCACCBBB?@@B@>97116.:@?CC>CCA=A<<<;<88=:>>7?776-
77736<:>7;;67470631=84+3/0000+
@T6XXI:00026:00261
CTAGTCGACTCGAGATCTGCGGCTTTGGACAGTCCCTCTGTACTGTGAAGCCACAGATGTCGGTCGGCTTCCTTTCT
CCCTTCTCCC
+
C?@AB<<@EICCCBBBBCCCB>??B>B@ACBC???;?AAABAAC@BBA=@C?BBCCC:;:?==?==:?@<@>BB@CB
CGAD?AFCCA
@T6XXI:00026:00290
GGGGAATTCTAATACGACTCACTATAGGGAGAGGGAGAATAGTCCGCCAATTCTGCGCGTTAGCATTCTCCTTCCTT
TCTCCCTTCTCCC
+
444,4999@CB89999983;77996667-
77>>@<BA@<?C?@??=<9?=@=?CBFBB??<?@A@A?BAA@DAD?BB<@????9?>;;;<6
@T6XXI:00027:00259
CTAGTCACTCGAGATCTGCGGCTTTGGACAGTCCCTCTGTACTGTGAAGCCACAGATGTCGAGTCGACTAGCTTCCT
TTCTCCCTTCTCCC
+
?99449444BAA;<>BBBCB?CBE>B>>>BCCCA;>>@@@@??@@@@>@CADBDBBB@@?AAAABB@@ADBCC>A?B
DBBBBB>A899AC?
@T6XXI:00027:00276
GGGAGAAGGGAGAAAGGAAGGAGAAGTCGCCCCAAACGATTTGTTGACTATTCTCCCTCTCCCTATAGTGAGTCGTA
TTAGAATTCCCC
+
CDACCC@CC@CBBB@C@C@B@CCC?BBCABBB5@@<@BCCC>CC>BAAAAA?AAAA=@>>>A=AAAA@@@@@@@@??
>?ABA?D58870
@T6XXI:00027:00279
GGGAGAAGGGAGAAAGGAAGCTAGTCACTCGACATCTGTGGCTTCACAGTACAGAGGGACTGTCCAAAGCCGCAGAT
CTCGAGTCGACTAG
+
ECAAA@@BB@?@@@9>;><AA@@??@=711111179678B@DCBBBBC@BBA=9334-
4;;ABA?AA<@@>@A@@@??@@@@@A@AA???@
@T6XXI:00027:00281
GGGGAATTCTAATACGACTCACTTATAGGGAGAGGG
+
499,42494449=;;;:8>>?13136111,11111,
@T6XXI:00028:00250
GGGGAATTCTAATACGACTCACTATAGGAGAGGGAGAATAGCCGCTACCTAAACGGTTTGTCTATTCCCTTCCTTTC
TCCCTTCTCCC
+
CCB9C=?=ACB@CCAAABCBB:997/.,0<@?@>BCC>AAAA>@@ACDBBBB>BC@@B=@58:B@8<<6<:>;>?<?
AAC:>;?AAA:
@T6XXI:00028:00259
GGGAGAAGGGAGAAAGGAAGAGAACAGCGCGCACTGGAGAGGTCAGGCTATTCTCCCTCTCCCTATAGTGAGTCGTA
TTAGAATTCCCC
```

```
+
CC@BBA@BCAAAAB>B@B@BBBB@BBBBBBBBBBBB@ABAB@BBBC?BBEC?@:7947>;=>;?<AA?;<=B@;@?@
@>=?=;?9<<<5
@T6XXI:00029:00276
GGGGAATTCTAATACGACTCACTAGTAGGAGGAGGAAGAAT
+
?CC7C>B>BCC?BBCBBBEC@?A:877>30.3..,.,.32-
@T6XXI:00030:00263
GGGAGAAGGGAGAAAGGAAGAATAACAGGCAATGGGGGATGATGTGGCTATTCTCCCTCTCCCTATAGTGAGTCGTA
TTAGAACTTCCCC
+
@?<ACEBCC@BBBC>C@C@AB?AC?BBC>@@=>>>>>/>@AA@@CB<>@AB=@=9;-44444-
4===AC@A=?:33777669:<<:>B?5
@T6XXI:00030:00274
GGGAGAAGGGAGAAAGGAAGGAGAAGTCGCCCCAAACGATTTGTTGACTATTCTCCCTCTCCCTATAGTGAGTCGTA
TTAGAATTCCCCAATC
+
FGCCCC@CB==;;96;?A@>=>@@=0/0.44459::::CBB@BB@CAA@BB??>@C:98434-
4;;;@@@====A???>@==7:3444,477,
@T6XXI:00030:00292
CTAGTCGACTCGAGATCTGCGGCTTTGGACAGTCCCTCTGTACTGTGAAGCCACAGATGGACTGCTTCCTTTCTCCC
TTCTCCC
+
IDADCCCECDD@@@BB;;<AF>AAA=A?BBACBCC>CCCBBBBBBBCA>AD>A@CAA@@=@@?>?@6:47@<???C=
BAABBEE
@T6XXI:00031:00250
GGGGAATTCTAATACGACTCACTATAGGAGAGGGAGAATAGATCACCGTTGGTTCGGGTGCGCGTTCTCCTTCCTTT
CTCCCTTCTCCC
+
@CC:C?B?B@@=A@@@E<<;CB>>:7747?>@A@BEC?A@A888CC=@B7:5:>@>@<A;;;BAA>B??=<>B@CC>
BBBC?C<<<<A?
@T6XXI:00031:00257
GGGGAATTCTAATACGACTCACTATAGGGAGAGGGAGAATAGATCACCGTTGGTTCGGGTGCGCGTTCTCCTTCCTT
TCTCCCTTCTCCC
+
CB@2B8;:<B@;@<<<@CDBBA>>7677.7==BC>CBA>@?<<==BB=AC=?=BABDECD;<<B@A>::::;:?;??
:>?;96;;889DA
@T6XXI:00031:00264
GGGAGAAGGGAGAAAGGAAGTAGTCGACTCGACATCTGTGGCTTCACAGTACAGAGGGACTGTCCAAAGCCGCAGAT
CTCGAGTCGACTAG
+
CDABBB@BB>BBBB>B?A>?777?;88@??=><<ABAAAC@CC@CEBBBBBBBB??@=?BCCA@>AA=AB@B@@@?@
@@@=AAABBBAAAB
@T6XXI:00031:00265
GGGAGAAGGAGAAAGG
+
991///,/,/,02*,*
@T6XXI:00032:00282
CTAGTCACTCGAGATCTGCGGCTTTGGACAGTCCCTCTGTACTGTGAAGCCACAGATGTCGAGTCGACTAGCTTCCT
TTCTCCCTTCCTCCC
+
B1111=:?BBBCHCDCCDDD@BB?<A@AB<7777.7>@CBBBB:;7778>>B?CCB@@@BCBBCB@;<;;7770631
1,645522,/,--33
@T6XXI:00032:00293
CTAGTCACTCGGAGATCTGCGGCTTTGGACAGTCCCTCTGTACTGTGAAGCCACAGATGTCGAGTCGACTAGCTTCC
TTTCTCCCTTCTCCC
+
```

```
CB<;<<4;B9989BBACCCBB?BCC?B>?@@??BB>BCCCA<AB?B@A=<B@BBBACBBCAABCCCCBAAB?7737:
=?:???B=@9<<?@:
@T6XXI:00032:00299
GGGGAATTCTAATACGACTCACTATAGGAGAGGAGATAGT
+
ABB9>8A?ACD@DCBB@?@BC;;=?//-0>7>:>/.+,,,
@T6XXI:00033:00251
GGGAGAAGGGAGAAAGGAAGCTAGTCACTCGACATCTGTGGCTTCACAGTACAGAGGGACTGTCCAAAGCCGCAGAT
CTCGAGTCGACTAG
+
CCCBBA@BA<BB===B=@=@CB>BB<:77>>771888??C=?@?BBBBBBBBB<37717>>@A@?AA<AA>??=<==
=???AAA@@@@A@@
@T6XXI:00033:00274
GGGGAATTCTAATACGACTCACTATAGGGAGAGGGAGAATAGTACGTCGAA
+
@=94>6969>>;>@A@BCDCCCCBB666.7>=?A>BCD>A=52/.,,**((
@T6XXI:00033:00291
GGGAGAAGGGAGAAAGGAAGCTAGTCACTCGACATCTGTGGCTTCACAGTACAGAGGGACTGTCCAAAGCCGCAGAT
CTCGAGTCGACTAG
+
:B:??><>>:>==C7;6:@B;>A@>@81111666?@?>::887779???BB@@>88818@?@AA9<<7<>;>?CAA@
A?><<778==?899
@T6XXI:00033:00292
GGGAGAAGGGAGAAAGGAAGGGAATAGCAGCAGTTGGCAACGTGAAACTATTCTCCCTCTCCCTATAGTGAGTCGTA
TTAGAATTCCCC
+
CK@CAA=@=<>@>@9@=@=?B>CBBBB>>===00,.44447;;>AB?CCCC@CBBC>CBA@@<<;<<?@AAA@@==7
838;?=<<=AA6
@T6XXI:00034:00266
GGGAGAAGGGAGAAAGGAAGCACACGGCACACCGGCAAACTGGGAGACTATTCTCCCTCTCCCTATAGTGAGTCGTA
TTAGAATTCCCC
+
FDACCC@CB>BBBC?B@B9::CCCCC@BBBCC@C@CED@BBBC@CBCCDCB>A@@@<@BCCD?ABDJEC@AA@@@A@
@=@?C=@5888.
@T6XXI:00034:00270
GGGAGAAGGGAGAAAGGAAGCTAGTCGACTCGACATCTGTGGCTTCACAGTACAGAGGGACTGTCCAAAGCCGCAGA
TCTCGAATCGACTAGCTTCCTTTCTCCCTTCTCCC
+
@@9999<>?194=>0424937888771661117@?99?????:785777>:;;A>;;;;7;==@??<?C<A@>@?@@B
B@@@@?=@888@><<<@;;57756769,/,/:8:6
@T6XXI:00034:00297
GGGGAATTCTAATACGACTCACTATAGGAGAGGGAGAATAGTCAACAAATCGTTTGGGGCGACTTCTCCTTCCTTTC
TCCCTTCTCCC
+
BBB9B@CBAAA@BBBBBBBBA:==5..,0<:=?=@DC>A>>>AB?@@@<B?8662;;;2?ABBD@CCC>=;=>AA<@
@@A;A<??AA=
@T6XXI:00035:00263
GGGAGAAGGGAGAAAGGAAGGAGATATAGACACGCCAGTAGAGACGCGCTATTCTCCCTCTCCCTATAGTGAGTCGT
ATTAGAATTCCCC
+
CCBBBB?AA=ABCC?B?A?A@CA?@=AACCC??>A@BB>@@@B>==???@C@@??==;<<??=;<???<<=;AA;=<
?@==@@;A?AA@7
@T6XXI:00035:00275
GGGAGAAGGGAGAAAGGAAGCTAGTCGACTCGACATCTGTGGCTTCACAGTACAGAGGGACTGTCCAAAGCCGCAGA
TCTCGAGTC
+
```

```
??199@599198@A9>=A>;;8>@>0//:///>??666?9:07?<=<=77:>744444-
4;;;;@38818=><==AAA@@@@@@@A
@T6XXI:00035:00289
GGGGAATTCTAATACGACTCACTATAGGGAGAGGGAGAATAGCTCCACTA
+
999,4298<CCBC@?=A?BB?AB>;666-9??AC>C>B9:5..00:0/,7
@T6XXI:00035:00290
GGGGAATTCTAATACGACTCACTATAGGGAGAGGAGAATAGTACGA
+
1//*/5>?CDCACCBBBBBCCBBBBBB;41700,0,,++*****++3
@T6XXI:00036:00262
GGGAGAAGGGAGAAAAGGAAGC
+
BCDB?=684-4/---*-+-*,*
@T6XXI:00036:00275
GGGGAATTCTAATACGACTCACTATAGGGAGAGGGAGAATAGACTTTGGTTTGCCTTAAGTACTATTCCCTTCCTTT
CTCCCTTCTCCCTAGTCGACTCGAGATCTGCGGCTTCCTTTCTCCCTTCCTCC
+
==96=@D?CCB@DCABBBCBC@?>?:7:59?AAE>BBC=@??@@DC=A>AA<??<?>A?A==<<@@>@A;<<=333+
-7686:.0/:;9@=====?BCC@@@AA@A@?=500/026606005+,*,*+++
@T6XXI:00036:00277
CTAGTCACTCGAGATCTGCGGCTTTGGACAGTCCCTCTGTACTGTGAAGCCACAGATGTCGAGTCGACTAGCTCCCT
TCTCCCCTTCCTTTCTCCCTTCTCCC
+
==1/1:8=?@@@CBCBDB@@=@BB>C@BCBBAAA=ACCCCC>>@@?A;8;;<CCCCBCCBBBB:9:??@@@A9@@8;
:61677.48;34817;;@7=:<@@@7
@T6XXI:00036:00278
GGGGAATTCTAATACGACTCACTATAGGGAGAGGGAGAATAGTTTTCCCTTCACTAACCGCGAACTGCACTTCCTTT
CTCCCTTCTCCC
+
CCC:D@D@CCC?BBCCCDBB?>;97166.7==BG>CDC@B43300+44-
48=ABBB?B>:::A==>??AAA@B:??9?<<<9??<==B<
@T6XXI:00036:00291
GGGAGAAGGGAGAAAGGAAGAATAATACGTGATCCGTTGGAAGTGGTCGT
+
6=8119599-/668+/,/,..6448<>A@@@CDC@;<9:,0,...,...-
@T6XXI:00036:00294
GGGAGAAGGGAGAAAGGAAGCTAGTCACTCGACATCTGTGGCTTCACAGTACAGAGGGACTGTCCAAAGCCGCAGAT
CTCGAGTCGACTAG
+
AA<ACB@CC=A944-
9<@;>?B??@B>9;==00////;7A>BB?BCCC@@B@;633317=>@A@?@@<@@?@@<;;@AAA===AAA@AAA@
@T6XXI:00036:00298
GGGAGAAGGGAGAAAGGAAGGAGACAGTGAGCGCCGCACAACACGTCACTATTCTCCCTCTCCCTATAGTGAGTCGT
ATTAGAATTCCCC
+
CEACCC@CB>BCDB=?=?@C@B>>ACBCDCC@@@@CCB985:BBAAACCCCC@BBBB=BBBAA=AABBBBBBBCCBA
AA?AAB>B?BCC:
@T6XXI:00037:00285
GGGGAATTCTAATACGACTCACTATAGGGAGAGGGAGAATAGTCAACAAATCGTTTGGGGCGACTTCTCCTTCCTTT
CTCCCTTCTCCC
+
@?=8A?CACCB?BABBBABB@<;97111.7>>?CBCDA>@<AAAA???::AA???8??A7ACCCC@AAA8868<AC>
@@@@<A>AAAA<
@T6XXI:00037:00293
GGGAGAAGGGAGAAAAGGAAGCTAGTCGACTCGACATCTGTGGCTTCACAGTACAGAGGGACTGTCCAAAGCCGCAG
ATCTCGAGTCGACTACGCTTCCTTTCTCCCTTCTCCC
```

```
+
CB>@99599084448,4242333...0..34...1111111138;<?ACCCCBB?AAAA69:==BA>A@;@A>AAA@
@@?>=;=8887;;;888::7:79;6;;@B1834:994
@T6XXI:00037:00297
GGGGAATTCTAATACGACTCACTATAGGGAGAGGGAGAATAGTCCACCGCTATTCCGCATAACATTCCCCTTCCTTT
CTCCCTTCTCCC
+
>>>4><>=BBBCEBA??>BBB@B97111.7=>>@>BCA?AAA<@=@@?A@=??@A?A@@@A;==A>@@@1888/44-
4;98;@;<A;82
@T6XXI:00038:00263
GGGAGAAGGGAGAAAGGAAGTGCAGTTCGCGGTTAGTGAAGGGAAAAAACTATTCTCCCTCTCCCTATAGTGAGTCG
TATTAGAATTCCCC
+
CECCA>>==6@@@@:@?B@BCCCBBB@BBBA@B@BBBBC<66,16666*8BCCBCBBB>B?CCC>@=>=AAA@@???
AAA>AA@>@=@?@5
@T6XXI:00038:00278
GGGAGAAGGGAGAAAGGAAGGAGAACAACACGATCGGTTAAACGTAGCTATTCTCCCTCTCCCTATAGTGAGTCGTA
TTAGAATTCCCC
+
BA<;<;<>A<<9;B<@@ABNBBBB?CC@BBCCCABB>A@NC>BBCCCCCBB?AABA9<<777.7?=?A??:?>;;;8
858?>>=;@@@5
@T6XXI:00039:00260
GGGAGAAGGGAGAAAGGAAGTGCAGTTCGCGGTTAGTGAAGGGAAAAACTATTCTCCCTCTCCCTATAGTGAGTCGT
ATTAGAACTTCCCC
+
AC>ADAACC@BBAB=B@B@AA?BCCC@BBBC?B?@?@BB?AC<AAAA2ACCC@CCCD>??@BC?B@?B??<==??;<
;;7;<@389>AAA6
@T6XXI:00039:00273
GGGGAATCTAATACGACGTCACTATAGGAGAGGGAGAATGAGTACAACAAATCGTTTGGGGCGACTTCTCCTTCCTT
TCTCCCTTCTCCC
+
?;;58,////,/4444333334664..,.4/..+.335../663199444-
6110;::;=4;771101.44474555-/.554449488-
@T6XXI:00039:00291
GGGGAATTCTAATACGACTCACTATAGGGAGAGGGAGAATAGTCAACAAATCGTTTGGGGCGACTTCTCCTTCCTTT
CTCCCTTCTCCC
+
ABC:C=?=@CD@CCCC@>BC@??B?:::7:??CD?CA?=?AA@A@>AAA<CABAC=AAA7ABBBB????;<9<=??<
?AAC=A<??AA<
@T6XXI:00040:00253
GGGAGAAGGGAGAAAGGAAGCGCGCGGTAGAGTCAAGAGAATGTTTAACTATTCTCCTCTCCCTATAGTGAGTCGTA
TTAGAATTCCCC
+
CC?AAB@CB>BCBB=A997:BCAA?>>>>>?????>BAB=:A@@A1838==;;@=<8;;77717??@ACA@???A==
>@A?>;?5888.
@T6XXI:00040:00257
GGGAGAAGGGAGAAAGGAAGCTAGTCACTCGACATCTGTGGCTTCACAGTACAGAGGGACTGTCCAAAGCCGCAGAT
CTCGAGTCGACTAG
+
CC@BBB?BB?B9996;==<@BBBBB@D?@@>1111//?@C?BB@CB???BC?>9330,38::44/44,34866==?>
@@@@@@===DAAAA
@T6XXI:00040:00258
GGGAGAAGGGAGAAAGGAAGCTAGTCGACTCGAGATCTGCGGCTTTGGACAGTCCCTCTGTACTGTGAAGCCACAGA
TGTCGAGTCGACTAGCTTCCTTTCTCCCTTCTCCC
+
CD@D<::<B:>999095968>>0000//./5:=>BBBBBBB?BBD>C=AACCA@A=@:??@???A@@@>@@=@==87
8??999AAA????@<;;:=:<>;?A@C=B:==AA;
```

```
@T6XXI:00040:00294
GGGGAATTCTAATACGACTCACTATAGGGAGAGGGAGAATAGTGTGCGCATTCGGTGCACTGTTATTCTCTTCCTTT
CTCCCTTCTCCC
+
CBB7B6;;<CCACBA??BBBBAB9;111.6==BC>BB@>A=<559?===8;:;A>AABCAC??<?A?A@?@>A>@@;
@AAC>=;==<<7
@T6XXI:00040:00295
GGGGAATTCTAATACGACTCACTATAGGAGAGGGAGAATAGTCACATTCATGTTGTGACCGTGTCTCCTTCTTCTCC
TCTCC
+
111+1-1,/,,2,/11:9>C@:>@@00,00061,1742350004449:;=<<<,0/.-2,--
33000/0/004311,--244
@T6XXI:00040:00299
GGGGAATTCTAATACGACTCACTATAGGAGAGGGAGAAT
+
/11+1-959489>;:<C@?@F>>;7//,00077-63320
@T6XXI:00041:00251
GGGAATTCTAATACGACTCACTATAGGGCTAGAGGGACGAATAGTCAACAAATCGTTTGGGGC
+
990424244424484437<>?00.555,..4333:77ABB7;:=:::8::8+333..+...*.
@T6XXI:00041:00261
GGGAGAAGGGAGAAAGGAAGGGGAGAAGGGAGAAAGGAAGCTAGTAGACTCGACATCTGTGGCTTCACAGTACAGAG
GGACTGTCCAAAGCCGCAGATCTCGAGT
+
CC>DCCACCACBDC>A494888.8>>577.7777-
:=A>BB???CCCC??:::==99994@5;;@CCCCCDBAAA788/8>>=>>48808>;>??@AB:;8?>>=
@T6XXI:00041:00271
GGGAGAAGGGAGAAAGGAAGGAGAAGTCGCCCCAAACGATTTGTTGACTATTCTCCCTCTCCCTATAGTGAGTCGTA
TTAGAATTCCCCCAATCGAATTCCCGCGGCCGCCATGGCGGCCGGAGCATGCGACGTCG
+
CCACDC@DD?BAAA=B?B?B@BBB@C@AABBB9BC>BCBBB<??>CB@B<@><A>>;@===A<@A@<?<A=???@A@
@?B<:78/4444,47444:73211+-++*+*++0224:44445444.//4877/44344
@T6XXI:00041:00280
GGGAGAAGGGAGAAAGGAAGCTAGTCGACTCGACATCTGTGGCTTCACAGTACAGAGGGACTGTCCAAAG
+
ECBBAB@CCAAAAB?B@B@BCCAAAACCCCC?@?BAABCCC?BB@CBBBBBA?99344-4;;=@@>AC=@
@T6XXI:00041:00292
GGGGAATTCTAATACGACTCACTATAGGAGAGGGAGAATAGATCACCGTTGGTTCGGGTGCGCGTTCTCCTTCCTTT
CTCCCTTCTCCC
+
944/9<>=ABB@CBBBBBBCB>;=://,000775:9A?AA????AA@CC:;8:>A@@=B@@@BDB@CCB?A7:7:B>
CB@A<A;=<?@7
@T6XXI:00042:00252
GGGGAATTCTAATACGACTCACTATAGGAGAGGGAGAATAGTCAACAAATCGTTTGGGGCGACTTCTCCTTCCTTTC
TCCCTTCTCCC
+
CCC:C@CABDB?AAAABBBBB:997//,0<<@C>CCA@B9=:<=<9983?=;;;6<<<2?@CCC?BAA>><?=@@:@
888188<<AB<
@T6XXI:00042:00258
GGGAGAAGGGAGAAAGGAAGCTAGTCACTCGACCATCTGTGGCTTCACAGTACAGAGGACTGTCCAAAGCCGCAGAT
CTCGAGTCGACTAG
+
99-499:;;:999909595333379657444..,....33638;:<:<???>00///,,/..--,-
3344748=@8884>>;7531-----
@T6XXI:00042:00264
GGGGAATTCTAATACGACTCACTATAGGAGAGGGAGAATAGTCCCAGCTATATTGCGCGTAGGCTCTTCTTCCTTTC
TCCCTTCTCCC
```

```
+
@@A6@@C@DBB@AAAAA???>:997//-
0::>A>ACA@?6=AACAA=8??AA=>==?BBBAA>AA>?=@?<?=?@<@ABA<A:==BA;
@T6XXI:00042:00265
GGGGAATTCTAATACGACTCACTATAGGGAGAGGGAGAATAGTTGCACGTTTGTCTCCCGTGTCGTATTCTTCCTTT
CTCCCTTCTCCC
+
CCG9E>B>BAA=@@@@CCBB@?>>>777-
7??>@=@<??@???>B@AB:::5;77:@C>A?????@;<9=C;>;??7==>>7=;=???:
@T6XXI:00042:00274
GGGAGAAGGAGAAAGGAAGCTATCGATCGAGCATCGTGTGGCTTCACAGTACAGAGGACTGTCCAAAGCGC
+
;?81//,/,,,//+/,/,.69A67771111666611116888?;6161633.--1101442--,--+----
@T6XXI:00042:00275
GGGAGAAGGGAGAAAGGAAGCTAAGT
+
;;89>><>>:>911,1,1-1...,31
@T6XXI:00042:00288
GGGGAATTCTAATACGACTCACTATAGGGAGAGGGAGAATAGTCAACAAATCGTTTGGGGCGACTTCTCCTTCCTTT
CTCCCTTCTCCC
+
499/9<@@BCC@DBBABBBBB@B>?996-6<<<?<?CA>AAAAAA<::;;A@996,111-
9??AC?BBB>=:=?CB=>@@C<A9<=BC<
@T6XXI:00042:00294
GGGGAATCTAATACGACTCACTATAGGAGAGGGAGAATAGTTACATT
+
/1/*/,1//7,/4;;A8:8?<78776016=>@9A9201+++*+++00
@T6XXI:00043:00264
GGGGAATTCTAATACGACTCACTATAGGAGAGGGAGAATAGTGACGTGTTGTGCGGCGCTCACTGTCTCCTTCCTTT
CTCCCTTCTCCC
+
<<<4<@B?<@<@CCCCCCC;<:BB?::5:?>?@<?=<344<;;;@@=<<9=888A388?873?A===@@@@?A9==9
<@;B1848=@A<
@T6XXI:00043:00274
GGGAGAAGGGAGAAAGGAAGCTAGTCACTCGACATCTGTGGCTTCACAGTACAGAGGGACTGTCCAAAGCCGCAGAT
CTCGAGTCGACTAG
+
CCABBB@CC@BCBB=A?A@AAAACAAB@???::6999??@=??<?AB?@@DB=;433-
4;;=??>AD?AA>===@AA@AA@===CAAA@@@
@T6XXI:00043:00279
GGGGAATTCTAATACGACTCACTATAGGAGAGGGAGAATAGTCAACAAATCGTTTGGGGCGACTTCTCCTTCCTTTC
TCCCATTCTCCC
+
DEC9B7;8;AA?CECBBAAAA7;;://,0><?B=BCD?CA===?;;;;-
:;??D>CCC9C??8?376734//4771687;-.-210165
@T6XXI:00044:00260
GGGGAATTCTAATACGACTCACTATAGGGAGAGGGAGAATGGACCTTTGACGCTGAAAAGGTGTGTTCTCTTCCTTT
CTCCCTTCTCCC
+
BC?9B=B>BCC@BBB?A?>B>C?97111.7>>?A>BBBEB:<01366.7?BAB@@@AA5@=@A===;<<<<>@>A@;
@??:7<<>;;;4
@T6XXI:00044:00269
GAATTCTAATACGACTCACTATAGGGAGAGGGAGAATAGTCAACAAATCGTTTGGGGCGACTTCTCCTTCCTTTCTC
CCTTCTCCC
+
A@=@>@@@?BBDCCBBBB@<97166.777>@=ADH?DA???A?@<=;???@C>BBB9BCCDD?BAA??@A?AA:>@A
A<A;==@@:
```

@T6XXI:00044:00281
GGGGAATTCTAATACGACTCACTATAGGAGAGGGAGAATAGTCAACAAATCGTTTGGTGCGACGTTCTCCTTCCTTT
CTCCCTTCTCCCGGGATTCTAACTACGACTCACTATAGGAGACGGGAGA
+
CCB8B8;8;BA@DBBBBBBBCBBBA9137>777.7?;=AA??<;8;<;6?CDCC>C@CA>>7//,55,,++*-,-
/+-1333<97799,//,/-2---,-044000-44/./9>4/7330042410
@T6XXI:00044:00282
GGGAGAAGGGAGAAAGGAAGGAGAAGTCGGGGATGAAGAGCGTGTGGCTATTCTCCCTCTCCCTATAGTGAGTCGTA
TTAGAATTCCCC
+
990444899-
44>@8;8;@C?CBB@BAAA>>2>879<>>ABCCCCD@@;;75:7>?:788?826?=?67384455877:=>@>=<=A
A6
@T6XXI:00044:00284
GGGGAATTCTAATACGACTCACTATAGGGAGAGGGAGAATAGTCCCCAAATAGCTACCGACGACAGCGTCCTTCCTT
TCTCCCTTCTCCC
+
GDE:CAC@ABA@BCCBB@@?><79=667.7==BC@CC@=@=B@@@@5@A<>???????@???????>>>?;=;=8;;
6<>>@;@>@@??:
@T6XXI:00044:00291
GGGGAATTCTAATACGACGTCACTATAGGGAGAGGGAGAATAGTACAACAAATCGTTTGGGGCCGTACTTC
+
BB>5>,1,/66,/4611...949;B888C=BBAA@:??=<>=66644<===;BBAAA:777,31....4,,
@T6XXI:00045:00258
GGGAATTCTAATACGACTCACTATAGGAGAGGGAGAATAGTCAACAAATCGTTTGGGGCGACTTCTCCTTCCTTTCT
CCCTTCTCCC
+
4914292444284=@@?ABC?>@>00,0::==<?A@?B@@@AB=7881:???C>CBC7AAECD;;;C??=?>DC;@@
AA:=:=8471
@T6XXI:00045:00266
GGGAGAAGGAGAAAGGAAGCGCAAGAGCATTGCAAAGAGTGAGAACTATTCGTCCCTCTCCTATAGTGAGTCGTATT
AGAATTCCCC
+
991///,/,////+/,42399<B;8;==9424444-
4;864000880555.....+,5./,.669944449887777331042333,
@T6XXI:00045:00278
GGGGAATTCTAATACGACTCACTATAGGGAGAGGAGAATAGTTACGGCCCTT
+
>AB9C?@=?CCACCB>>@BBB@@99111.7>77477:8:444244471101,
@T6XXI:00045:00282
GGGAGAAGGGAGAAAGGAAGGAGAATGCGGTACCGGTTACGTCTTGGCTATTCTCCCTCTCCCTATAGTGAGTCGTA
TAGATTCCCC
+
EK@CCC@CB:?844-
9<CACCCBA787>?<>@<7@;?5:671118661103/34>9:444==46644344003333003334<===5
@T6XXI:00045:00283
GGGGAATTCTAATACGACTCACTATAGGGAGAGGGAGAATAGATCACCGTTGGTTCGGGTGCGCGTTCTCCTTCCTT
TCTCCCTTCTCCC
+
<99/9696<BB?BDCBBBBBA>996177.7==?C?FB?>?A=<<A??:?B<?=@CCCC>B;;;AAA@BAA@B@C?BC
>BCAC<A?888>7
@T6XXI:00045:00295
GGGAGAAGGGAGAAAGGAAGACAGGTGCAGGGATTGTTGATTAGCAGCTATTCTCCCTCTCCCTATAGTGAGTCGTA
TTAGAATTCCCC
+

```
94-
9@>59919>AB?DBD?BAABB?BB???@5::<A><?@C<??BBBCAA?=@@AA<AA==<7<@?@A@@ACD???==;=
AA>@>AAA6
@T6XXI:00045:00299
GGGGAATTCTAATACGACTCACTATAGGGAGAGGGAGAATAGTCAACAAATCGTTTGGGGCGACTTCTCCTTCCTTT
CTCCCTTCTCCC
+
444-
96959>@;A@BBBBBCCEDC???B;?88:B>BCA?CC@???AAAD9@@BBC>CCC7??CCC?ABC<969>AA<=?@C
<A689@@<
@T6XXI:00046:00253
GGGAGAAGGGAGAAAGGAAGGAGAAGTCGCCCCA
+
4469?=@;;-94480478991333111.4466*.
@T6XXI:00046:00259
GGGAGAAGGGAGAAAGGAAGCTAGTCACTCGACATCTGTGGCTTCACAGTACAGAGGACTGTCCAAAGCCGCATATC
TCGAGTCGACTAG
+
BBABCB@BB==@885>784:C@@BBAA@A?>77616179>588@BCCBBABACA8868@@@@@<??;?@>@===AA?
?????@@@@A?89
@T6XXI:00046:00263
GGGAGAAGGGAGAAAGGAAGCTAGCGACACTC
+
CB?AAA=@@:>99909<><???>977,,,*(*
@T6XXI:00046:00275
GGGGAATTCTAATACGACTCACTATAGGAGAGGGAGAATAGCACTCACTCCTGAGTAATCTATTTCTTCTTCCTTTC
TCCCTTCTCCC
+
ECCCD?@=?BB@C@@@BCCB>>=97//,0<<?B?CB@>@<=@::;AA===<@@@???>@A@@?A;@@>@@>@>AA;@
AAA;?:6<;;7
@T6XXI:00046:00289
GGGAGAAGGGAGAAAGGAAGCTAGTCACTCGACATCTGTGGCTTCACAGTACAGAGGGACTGTCCAAAGCCGCAGAT
CTCGAGTCGACTAG
+
CC>AA?@BC=BBCG>@=A@CC>;@=6777677669>>AAC?AB@CBC999?@@=99878=<@A@>@=;==>?@===<
<<;<<=A???@@==
@T6XXI:00047:00253
CTAGTCACTCGAGATCTGCGGCTTTGGACAGTCCCCTCTGTACTGTGAAGCCACAGATGTCGAGTCGACTAGCTTCC
TTTCTCCCTTCTC
+
1994499444444448C99;=>??:?9;8?>7777-
7<<@@A;;;@9;<=B:99>93337176<688773971177011,1116-497--
@T6XXI:00047:00268
GGGAGAAGGGAGAAAGGAAGACTACGGCGCTGTCTGGGGAAATTACAGCTATTCTCCCTCTCCCTATAGTGAGTCGT
ATTAGAATTCCCC
+
DC@AAA@BA::8840959=BCCCCCA@????CCDCDDC9AB>B=AACDDCBDBCBAA=ACCEC<A9@??@@@=:?@@
@A=?@A=@38770
@T6XXI:00047:00277
GGGGAATTCTAATACGACTCACTATAGGGAGAGGGAGAATAGTTTGGCTTGTCCCACAACATGTTCTCCTTCCTTTC
TCCCTTCTCCC
+
@@=4>695<AA>BBBBBBCCCCA?=677.7>>>D?ECA>?:<68468:?9???C>BBB@CCCCC>??A>A?@>@A=A
@AA<A<;?@@<
@T6XXI:00048:00251
GGGAGAAGGGAGAAAGGAAGCTAGTCGACTCGGACATCT
+
```

```
99-444244-444;5=7=<777>7?11.5...,...33,
@T6XXI:00048:00285
GGGAGAAGGGAGAAAGGAAGCTAGTCGACTCGACATCTGTGGCTTCACAGTACAGAGGGACTGTCCAAAGCCGCAGA
TCTGAGTCGACTAG
+
CA>BA==ACABBBE<@=A?AAB@@@@@@CFCCC@B@AABBC?BB@CC>>>?@;48330-
4::;<=>AD<AA>===AAAAA9AABCCCFAAA
@T6XXI:00048:00292
GGGGAATTCTAATACGACTCACTATAGGGAGAGGGAGAATAGTTTTTCCCTTCACTAACCGCGAACTCGCACTTCCT
TCTCCCTTCTCCC
+
@@C6@>B@CCC@CCCCCBBBB?>@?96757>>@@>BB?<==;;;<<0=C?C<=?BC@@A><470++++++*0200*.
,.4457@?@@@@;
@T6XXI:00048:00295
GGGAGAAGGGAGAAAGGAAGACTACGGCGCTGTCTGGGGAAATTACAGCTATTCTCCCTCTCCCTATAGTGAGTCGT
ATTAGAATTCCCC
+
AC@CCCACB@===A?B@BA>>>ABCD@CB??CCCCECD:CC>B?BGCCCBBB>C;:;99B>CC>BBBBBB?CB=<@<
<<::?A>A<???5
@T6XXI:00048:00297
GGGAGAAGGGAGAAAGGAAGCTAGTCGACTCGACATCTGTGGCTTCACAGTACAGAGGGACTGTCCAAAGCCGCAGA
TCTCGAGTCGACTAG
+
CCAAACBCC==@@=9>;><>@801;0//777:076?86::A=AABCCCAABA@;937714;;???<?CA??=??>?@
????===@@@@AAA@
@T6XXI:00049:00250
CGAGATCTGCGGCTTTGGACAGTCCCTCTGTACTGTGAAGCCACAGATGTCGAGTCGACTAGCTTCCTTTCTCCCTT
CTCCC
+
DCBBBCCBBBB?BAC?C@CBBA@??6????CCCBBB?=:<@=@??=???ABCBCCDCBBBBBB?<=<=95944409<
?>?A:
@T6XXI:00049:00260
GGGGAATTCTAATACGACTCACTATAGGGAGAGGGAGAATAGTCAACAAATCGTTTGGGGCGACTTCTCCTTCCTTT
CTCCCTTCCTCCC
+
CCB9B6;;<BB@CCBBB:;:;78@>777-
777?B>BB@>@A?==:8;::4====>9?=?5?AAAC????=<9=?AB=@;@=6;././;;6
@T6XXI:00049:00261
GGGGAATTCTAATACGACTCACTATAGGAGAGGGAGAATAGTCAACAAATCGTTTGGGGCGACTTCTCCTTCCTTTC
TCCCTTCTCCC
+
BBB9BCC@CCCACBBBCAAA@>;>=//-
0;;>B@DCA>@??@@B<?>>7@>=<<6===2?@@BL@B@B?????AA<A;::6;00/>?9
@T6XXI:00049:00271
GGGGAATTCTAATACGACTCACTATAGGAGAGGGAGAATAGTCCTTTTCCGGACGTACCCCACATTTTCTTCCTTTC
TCCCTTCTCCC
+
CCC9C?B?BBB?BBB?@@CCCB@@?:737=777.7:7<@@@A@<???5?<@>BAAAFCCC7@BBBBC9AA=@:<<7=
ABC<A>A@@@:
@T6XXI:00049:00289
GGGAGAAGGGAGAAAGGAAGCTAGTCACTCGAGATCTGCGGCTTTGGACAGTCCCTCTGTACTGTGAAGCCACAGAT
GTCGAGTCGACTAGCTTCCTTTCTCCCTTCCTCCCC
+
>95?9964;:>944-
424239733111116>9<8887?BB>@?D<A7:7>>777.7744<<=;=:;=::924444@>738==;:;:::4444
4348;;-44994943/0000+
@T6XXI:00004:00318
```

```
GGGAGAAGGGAGAAAGGAAGAATGTGAAAACTGGGCAAGATGTTTAGCTATTCTCCCTCTC
+
:B?@;B8<;9<BBBBC>A>>?;?833111,3337679899;444-444444::///+/7//
@T6XXI:00004:00323
GGGAGAAGGGAGAAAGGAAGGAGAAGTCGCCCCAAACGATTTGTTGACTATTCTCCCTCTCCCTATAGTGAGTCGTA
TTAGAATTCCCC
+
BC@BBB@BD>BBBC>B>@=>@BBB=;?BBBBB8BB>BA?AAAAC@CB?@A=;<<==<<AA==;=::?=====:::9=
=;AAA=@=?CC6
@T6XXI:00004:00333
GGGGAGAAGGGAGAAAGGAAGGAGAAGTCGCCCCAAACGATTTGTTGACTATTCTCCCTCTCCCTATCAGTGA
+
499,444244-448858<999;>>?<???B??B797,3;666266388?<<A:@<=B;==7--+-----2--3
@T6XXI:00004:00340
GGGAGAAGGGAGAAAGGAAGGAGAAGTCGCCCCAAACGATTTGTTGACTATTCTCCCTCTCCCTATAGTGAGTCGTA
TTAGAATTCCCC
+
CKAAA@@CGCAA@C;;:<@C@CCC>?@>BBCD8@?<@@?AA?AA?AAD???>@@@A<@AAAA=A?<?@?<@AA????
?=??=9;2444-
@T6XXI:00005:00302
CTATCACTCGAGATCTGCGGCTTTGGACAGTCCCTCTGTACTGTGAAGCCACAGATGTCGAGTCGACTAGCTTCCTT
TCTCCCTTCTCCC
+
CA@?C:::DCCBBCBBB@?=@CEAC?BCCCCBC>CCC@?>?C@A??<?B@CCCCCBB@AA@ABBCCCCCDCD?B?A@
<@A??:?>@A@@<
@T6XXI:00005:00317
GGGAGAAGGGAGAAAGGAAGCTAGTCGACTCGACATCTGTGGCTTCACAGTACAGAGGGACTGTCCAAAGCCGCAGA
TCTCGAGTCGACTACG
+
491444244-4444-
4782837744........1;?@?<?C@?B???@><=:834000+099903/04200204434-----
13400000000
@T6XXI:00005:00319
GGGAGAAGGGAGAAAGGAAGGAGAAGTGCCCCAAACGATTTGTTGACTATTCTCCCTCTCCCTATAGTGAGTCGTAT
TAGAATTCCCC
+
BBABDBBCC@BBAC@C?C@>=>==9//.355*..+.9ABC>CC@C???CC?@?>=7:9:::4:::;<?@@@C===@@
B<<<;8565;1
@T6XXI:00005:00329
CGACTCACTATAGGAGAGGGAGAATAGTCAACAAATCGTTTGGGGCGACTTCTCCTTCCTTTCTCCCTTCTCCC
+
BBBBAAAB=<<11,1=;;=8BBCBCBBBCC@DHH?CCCCC?CCD9CDCBB?BBB?BCC@CB>BBBE>A?ADEC?
@T6XXI:00005:00341
GGGGAATTCTAATACGACTCACTATAGGGAGAGGGAGAATAGTCCGAGACTATAACCCACGTTGATCACCCTTCCTT
TCTCCCTTCTCCC
+
;<9/<<A>BCC@CBBB?<<BB@BA@77778@@A@;B?;:;9===<888;;==AA=@@;@AAA>C@BCBCC8:5::??
9?@==7?><==C;
@T6XXI:00006:00307
GGGGGAATTCTAAATACGACCTC
+
4:::/99924444-4////.,.6
@T6XXI:00006:00315
GGGGGAATTCTAATACGACTCACTATAGGGAGAGGGAGAATAGCCAAATCCAGCCTCACGCTCCCGTGTACTTCCTT
TCTCCCTTCTCCC
+
```

```
9999-
9=B@AA@=@A;;<FBBBBBBBCCC=@@CCC=AAA:==??>AH@AA<??A>AA>9:9044+44004==::<==999DC
;?44444,
@T6XXI:00006:00324
CTATCGACTCGAGATCTGCGGCTTTGGACAGTCCCTCTGTACTGTGAAGCCACAGATGTCGAGTCGACTAGCCTTCT
TCCTCCTTCTCCCTCTCC
+
CCCB<9:??BBBHDCBBC@@=@CC?B>AABB???;?BBB@@???AAB>?B@BCCCCBCCCCCCBA@@CA@@>:=477
374610024000+0339>
@T6XXI:00006:00327
GGGGAATTCTAATACGACTCACTATAGGGAGAGGGAGAATAGTCAACAAATCGTTTGGGGCGACTTCTCCTTCCTTT
CTCCCTTCTCCC
+
ABB9B@EAAA@@CDDBB?@ABAB?9166.7>?CD>B?@?@=@@@?@AAA>AABCC>???7<<AAA@BAB@C>A>@@;
@@AA<@>A@@B<
@T6XXI:00006:00348
GGGGAATTCTAATACGACTCACTATAGGAGAGGGAGAATAGTTTTTCCCTTCACTAACCGCGAACTGCACTTCCTTT
CTCCCTTCTCCC
+
A@92=;@?AAA@CBBBCCCBB:>9;//-
0<>@B>B@A>@@<<<<<0;=;?<?CCCC?C@@@@A>@@@=@?D?A;??9???D?C@?@@C@
@T6XXI:00007:00307
GGGGAATTCTAATACGACTCACTATAGGGAGAGGGAGAATAGTCCCGATATACGTCGTGGGCTCTTCGCCTTCCTTT
CTCCCTTCTCCC
+
499/9<@>BCCDCDBBA>@>CCBAA99959??BC>BBA;>8AA==:=<@<=??????@A@:@@<=;=CC@C>B@BB=
BBCC>C=BBCC>
@T6XXI:00007:00325
GGGGAATTCTAATACGACTCACTATAGGGAGAGGGAGAATGATCGTACTTATTG
+
CCC9C?C@DDB@CB@AABB@A?BEBAAA<A?ABB>B?=84//+++0+++*+.5<
@T6XXI:00007:00337
GACTCGAGATCTGCGGCTTTGGACAGTCCCTCTGTACTGTGAAGCCACAGATGTCGAGCTCTCTCA
+
CCCDBBBCCCCBFCC@CCC>CBAACCCDCBDBBCBBBCCCBB?BB@CAAABBBBCCB?@5.+*(((
@T6XXI:00008:00322
GGGAGAAGGGAGAAAGAAGAATTGCGCGTTATTGCTGAGGAGAGATCTATTCTCCCTCTCCCTAGTAGTGA
+
:@:>99584-/,00)**)*0)*)*/8999948233779B==776B7713373667777...+...----43
@T6XXI:00008:00337
GGGGAATTCTAATACGACTCACTATAGGGAGAGGGAGA
+
CCB-429598=9A@;<<B@@?BA@7:7=-71161,111
@T6XXI:00008:00347
GGGGAATTCTAATACGACTCACTATAGGGAGAGGGAGAATAGCTACGTTTAACCAATCGTGTTGTTCTCCTTCCTTT
CTCCCTTCTCCC
+
BA?7@5;:<BB?CD<;<BBBDFCC????9?@BCC>BAA=@?B=BCCBA?5:54;=:ACCCC@@AA<???=@=@=@A<
??@C?C>@???<
@T6XXI:00008:00348
GGGAGAAGGGAGAAAGGAAGCTAGTCGACTCGACATCTGGTGCTTCACTACAGAGGACGTGTCCAAAGCCGCAGATC
TCGAGTCGACTAG
+
CB?CDC>?>:=@996@7:6:B?==>/11????@@?A@>=5;::?@?<?:9966644-
.,,,,+*.4247:A@@C@A===9;;BCA>?===
@T6XXI:00009:00306
```

```
GGGGAATTCTAATACGACTCACTATAGGAGAGGGAGAATAGCTACAATTTTGTGTAGACCTTCAACCGCTTCCTTTC
TCCCTTCTCCC
+
DJA8;24544888=ABB;<<C>@>:0/,..577;885:4;;;11/:A::7699A:86<<96555+,++++*+00034
469368<8AA<
@T6XXI:00009:00314
GGGGAATTCTAATACGACTCACTATAGGGAGAGGGAGAATAGTCAACAAATCGTTTGGGGCGACTTCTCCTTCCTTT
CTCCCTTCTCCC
+
444,42424@@>@B?BBAABA@B<00/=.0/7;:6=/733<8>@A?@@@:BBBBD9??<2?@::6@>=9>8/0/00+
0---+-,--110
@T6XXI:00009:00333
GGGAGAAGGGAGAAAGGAAGCTAGTCGACTCGACATCTGTGGCTTCACAGTACAGAGGGACTGTCCAAAGCCGCAGA
TCTCGAGTCCGACTAG
+
CCACBB=>><@C@@>C>@=@I>>A@8;:B@BB::;;::@CE?BB?BCA@@?@<9<44317>><>?<??;?A>A@@@A
A@@@@@=::<===?9=
@T6XXI:00009:00343
GGGAGAAGGGAGAAAGGAAGCAGTCGACTCGACATCTGTGGCTTCACAGTACAGAGGGACTGTCCAAAGCCGCAGAT
CTCGAGTCGACTAG
+
BC>CBCADC@BBBC?C=@=@@@@@:;;CAFB=<=DBBBAA@<<;=CBCDCCCAB:7:8:??B??:>>:>>=B>@>@?
?:88@AAACAABCB
@T6XXI:00010:00305
CTATCACTCGAGATCTGCGGCTTTGGACAGTCCCTCTGTACTGTGAAGCCACAGATGTCGAGTCGACTAGCTTCCTT
TCTCCCTTCTCCC
+
CBCB:::ABB>?=CA@;;;?BCD?D?CBCC:::6:A>@@B???==A>@C@CCCCBC@@?BBBBBBBBCDBBB@CABB
?AC@@;@>=<=A<
@T6XXI:00010:00306
GGGAGAAGGGAGAAAGGAAGAGAACAGTCGGGCCAGTCGGGCTAGATCTATTCTCCCTCTCCCTATAGTGAGTCGTA
TTAGAATTCCCC
+
?A?A@A>AC@CCCC?B@DE@@@BACCCA>@@<@=@CDBDE@BCBCB?AA<;59@@@<<;<BCACCBBBCD@?:<<@@
?@CBB>B?BCC:
@T6XXI:00010:00317
GGGAGAAGGGAGAAAGGAAGCTAGTCGACTCGACATCTGTGGCTTCACAGTACAGAGGGACTGTCCAAAGCCGCAGA
TCTCGAGTCGACTAG
+
CD>A@>59919A>>9A895;B811>/00====3:;ABABCB<?@=?ABABBA@:;433-
4;:=?A?AA:@A@CA<<<<<?@??????AAAAA
@T6XXI:00010:00334
GGGAGAAGGAGAAAGGA
+
:A68-1-1-///7+/,4
@T6XXI:00010:00340
GGGAGAAGGGAGAAAGGAAGCAGTCCATCTGTGGCTTCACAGTACAGAGGGACTGTCCAAAGCCGCAGATCTCGAGT
CGACTAG
+
BB>BBC?BB>BB=>:A<@=?>>>7:6:AAACCC@CC>?:::<<@A==;;<;;=====<=<;;;;@@=AA=<AA@AAA
CDCCCCB
@T6XXI:00011:00304
GGGAGAAGGGAGAAAGGAAGCTAGTCACTCGACATCTGTGGCTTCACAGTACAGAGGGACTGTCCAAAGCCGCAGAT
CTCGAGTCGACTAG
+
CDACB@899-9@>><A8;:;BA@A?600777,,,///36:6<>?CCCCAAAB=9033-
4:;=>?BCC@@?<?@@?>?888?@@??@@@CCB
```

```
@T6XXI:00011:00341
GGGGAATTCTAATACGACTCACTATAGGAGAGGGAGAATAGCCACGTGCTCCTCGTGGCAGTCTTTC
+
CGE:C81-100*,,,,/6<99/664/0,.:9<=.74923300/001110004433--,...474333
@T6XXI:00011:00349
GGGAGAAGGGAGAAAGGAAGCTAGTCGACTCGAGATCTGCGGCTTTGGACAGTCCCTCTGTACTGTGAAGCCACAGA
TGTCGAGTCGACTAGCTTCCTTCTCCCTTCTCCC
+
99-
999:<B<A@@>9>596:B??AB;::BCCBCCB<;;AAABCEC?EB@@?ACBB<@=<<<<<;<>??=@A>AAAA@@??
?@@@AA????@<8678,/,./-305043440
@T6XXI:00012:00347
GGGAGAAGGGAGAAAGGAAGCTAGTCGACTCGACATCTGTGGCTTCACAGTACAGAGGGACTGTCCAAAGCCGCAGA
TCTCGAGTCGACTAGCTTCCTTTCTCCCTTCTCCC
+
??>>AA=@B:@99918482333330,,,,,,00..//67773:747=>?@AA>>@@??<?AAAAA=@@;@A>AAA==
<888>>?<==??@B88858=?@:;:;;58.0/::9
@T6XXI:00013:00302
GGGGAATTCTAATACGACTCACTATAGGAGAGGGAGAATAGTTTTTCCCTTCACTAACCGCG
+
999/42424/1,///6/08?>900//.,.55>?;==854844333+0303237...,.4.11
@T6XXI:00013:00314
GGGGAATTCTAATACGACTCACTATAGGGAGAGGGAGAATAGTTCCTAACTCTCCATCATTCTGCTATTCTTCCTTT
CTCCCTTCTCCC
+
DNE9B@BBCCCADC@AABCECCBCC???;??ABB>CCA>AAAA;=;=@=?AA??<?AA@A>???@AAA>A?=??A=;
A@@@;@<===A<
@T6XXI:00013:00327
GGGGAGAAAGGGGGAGAAAAAGGGAAAGCTAGTCACTCGACCATCCTGTGGCTTTCACAGTACAGAGGGACTGTCCA
AAAGCCGCAGATCTCGAGTCGACTAG
+
BCC9A?@@:>>>>/999@@A1BCACB<>;3337111111111136333...,*,,,*,.54697740334-
00035<;9==.40211//-17666>B?989A@88
@T6XXI:00013:00331
CTAGTCACTCGAGATCTGCGGCTTTGGACAGTCCCTCTGTACTGTGAAGCCACAGATGTCGAGTCGACTAGCTTCCT
TTCTCCCTTCTCCC
+
C:11188=?CCCCCCBBBCC@BBB>C?ABAB9994:ABCBAAC?@AA>@B@BCBBBBBB??@BBBBAAACABA?A?A
B>BECC>@??@?A<
@T6XXI:00013:00347
GGGGAATTCGTAATACGACTCACTATAGGGAGAGGGAGAATAG
+
C886>?::111/,/644433336771617661111,144424-
@T6XXI:00013:00348
GGGGAATTCTAATACGACTCACTATAGGGAGAGGAGAA
+
/11+/,42499584>9;9AACB?<=767,.7/0,.112
@T6XXI:00014:00307
GGGGAATTCTAATACGACTCACTATAGGGAGAGGAGAATAGTACGATGCTCT
+
8886@A@BC998<BBCCCCBB@BB<:??7;=7737=976///.-----3340
@T6XXI:00014:00342
GGGGTCAATACGACTCACTATAGGGAGAGGGAGAATAGTCAACAAATCGTTTGGGGCGACTTCTCCTTCCTTTCTCC
CTTCTCCC
+
CCB9DAA>ABDDCGCDBA;??777,6==AC=BCC@C>@@BBBBCD>CCCCB<???5?A@@A>?::8:@ECCC@B@@@
<@=@?>>7
```

```
@T6XXI:00014:00347
GGGAGAAGGGAGAAAGGAAGCTAGTCACTCGACCATCTGTGGCTTCACAGTACAGAGGACTGTCCAAAGCCGCAGAT
CTCGAGTCGACTAG
+
BB>@BA59809>>>9?6958>?==C?>;777,,*,,,,2375=?@CCCBBBB=77//,/99//8>AA<AC<???A@@
@@@?@@@?AAAA?A
@T6XXI:00015:00334
GGGAGAAGGGAGAAAGGAAGCTAGTCACTCGACATCTGTGGCTTCACAGTACAGAGGGACTGTCCAAAGCCGCAGAT
CTCGAGTCGACTAGCTTCCTTTCTCCCTTCTCCC
+
998;=B@CD=BA@@<A=@=?@??=>;77/......./5575?A?CB?@@CCCAA@@@<@A@@@?=A?:?A?AA????
@@BA===AA===AB@@;=;=C:@@A@<A89:AD@
@T6XXI:00015:00345
GGGGAATTCTAATACGACTCACTAATAGGGAGAGGGAAC
+
499/959284824/6//.0..79957?@@>66300)*)*
@T6XXI:00016:00328
GGGGAATTCTAATACGACTCACTATAGGGAGAGGAGAATAGTA
+
CCC9CACCBBB@C<<<CBCCCCC>?696-6=763:=:8;/,*+
@T6XXI:00016:00331
GGGAGAAGGGAGAAAGGAAGGAGAAGTCGCCCCAAACGACTTGTTGACTATTCTCCCTCTCCCTATAGTGAGTCGTA
TTAGAATTCCCC
+
DECCCB@BB>BCAA;?=?AB@B@A@>;93377-
8?8?BBEC?BB?BBBBAA>=?==;?9==@<@@@@A@@?A@<@:<@>A;;7:3444-
@T6XXI:00016:00334
GGGGAATTCTAATACGACTCACTATAGGGAGAGGGAGAATAGCCTCCCACAAAATTGTCGTCACGCGCTCTTCCTTT
CTCCCTTCTCCC
+
BBB7B?B?AAAACCA?BBBBDA@9>111.7>==A>B@A>A588=BBE>CB??@5B@CCBBBAACBBBBBBC?B?AA<
<<<?:?=@A@C?
@T6XXI:00016:00336
TGCGACTCGAGATCTGCGGCTTTGGACAGTCCCGTCATGTACTGTGAAGCCACAGATGTCGAGTCGACTAGCTTCCT
TTCTCCCTTCT
+
,///444;;?9=:9949A?999-499::4444-
444337333ADCB::>=>0.../3359B?68<:????;7746377.7===.737=
@T6XXI:00017:00342
GGGAGAAGGGAGAAAGGAAAGCTAGTCACTCGACATCCTGTGGCTTCACAGTACAGAGGGACTGTCCAAAGCCGCAG
ATCTCGAGTCGACTAG
+
@@@BBC=@?:>99804244-377633...,22,,,..,.555-
88@@CDBCBBA<<<@>:>>887;:=:7=<499::66///.//;85B77/.
@T6XXI:00017:00343
GGGAGAAGGGAGAAAGGAAGTTTTCGAAAATTAAACACTGTCTAATTTTCTGCAAAGTTTTTATTCATGAATTAAGA
GTATTTCCCTTTGTCCATTATTCCCAAGGCAAATATGGAAGTTTGATCATATACTAATCGTAATAAAGTTGGATTCT
CTTTAAGAGATTGATAAATTGTAAAGGCAAAAGCTGAATATATCATT
+
HCDCCB@DB:A99;7;>A?BECC9BAA@@260::5;CABABCCC@BCC9CBA@??:88888,1636??>=:;3747>
==9BB<=@7==7AAA=AD@CB?BC@C8;88@@78855;A@@BC>BA???<@A@C?:><==78484=4<=<<;??9<<
<<<7=>:::85234311012---334444774549;;888==74++*
@T6XXI:00018:00305
GGGAGAAGGGAGAAAGGAAGCTAGTCGACTCGACATCTGTGGCTTCACAGTACAGAGGGACTGTCCAAAGCCGCAGA
TCTCGAGTCGACTAG
+
```

```
;;6<CC@CC@CBBB>@<=;;;700:/0,,777-
0////;>C5;:@CBABABBCB@74717<=?AA=@?<AC?@@@AAA?989BAAAAA@A??
@T6XXI:00018:00312
GGGAGAAGGGAGAAAGGAAGGAGAATACAGCGAAATGAACACGTCGCTATTCTCCCTCTCCCTATAGTGAGTCGTAT
TAGAATTCCCC
+
@A?ABB@EC>CCBB>C?B?A@BBB@CACBCBBBB>BCC@BAECB;8;CCC>@???6==444+04458=C@@>>;<9;
7:365899==5
@T6XXI:00018:00314
GGGAGAAGGAGAAAGGAAGGAGAACAACACGATCGTTAAACGTAGCTATTCTCCCTA
+
94-///,/,/,,2*,*/,.6488:CC?AB;6<6///:::8:@?9444..,...5+..
@T6XXI:00018:00316
GGGGAATTCTAATACGACTCACTATAGGGAGAGGGAGAATAGTCAACAAATCGTTTGGGGCGACTTCTCCTTCCTTT
CTCCCTTCCTCCC
+
999/49@@CCBADCBBBBCDBA@?=661.7??BD>CCC;>>?@A=<8=65<>777.777.7>>>A@@@@A@>@?@@:
@@E?9;./-/872
@T6XXI:00018:00327
GGGAGAAGGGAGAAAGGAAGCTAGTCGACTCGACATCTGTGGCTTCACAGTACAGAGGGAC
+
/1,///,//+//44-4997338=6;<866666:5777///7544996617::6//---+--
@T6XXI:00018:00328
GGGGAATTCTAATACGACTCACTATAGGGAGAGGGAGAATAGTCAACAAATCGTTTGGGGCGACTTCTCCTTCCTTT
CTCCCTTCTCCC
+
CBB4<8<=@AB?CC;;;?;<:AAB>@BC>GBAACB@>A<??@AAA>@@?:??777.7==.7:B@B?;::>@;=<@@7
>?>@:>34-140
@T6XXI:00018:00333
GGGGAATTCTAATACGACTCACTATAGGGAGAGGGAGAATAGCTACGT
+
?==8@>B?BCCABB@ABCCBBBBB????9??ACC?C@@>@30.*)(((
@T6XXI:00019:00333
GGGGAATTCTAATACGACTCACTATAGGGAGAGGAGAATAGAG
+
///*/,4:A=ABGBDBCCBBBABC==??77://,/:7799<31
@T6XXI:00020:00314
GGGGAATTCTAATACGACTCACTATAGGGAGAGGGAGAATAGCAAACGTTTCCCCGACGTCGCTTTCTCCTTCCTTT
CTCCCTTCTCCC
+
BCC:CCCAACE@DCCC?>@CA<@?=676.7====9??;;=;<;>?:@ABC>DCC9@@@AAA???><>775:>@>AC@
>>?;6;.0.;;6
@T6XXI:00020:00340
GGGGAATTCTAATACGACTCACTATAGGGAGAGGGAGAAATAG
+
B@@9B=A>@BB@AA@BC;;;B=?>7716266776-144446@4
@T6XXI:00021:00329
GGGGAATTCTAATACGACTCACTATAGGGAGAGGGAGAATAGATCACCGTTGGTTCGGGTGCGCGTTCTCCTTCCTT
TCTCCCTTCTCCC
+
944,4<?>CCCACBBBB888A???=777.7???A>B??<?@=9><993:@>@?B?BBBCB@@@CFLA;<;@B<@6::
5;@@@;@>????:
@T6XXI:00021:00347
GGGGAATTCTAATACGACTCACTATAGGGAGAGGGAGAATAGTCCCACTATCCTCTCTACGTTAATTTACTTCCTTT
CTCCCTTCTCCC
+
```

```
@@A6@>A?BBE@CCHBC>@@A<@A?67:5:??@DAB??>@@@@CCA@@?<?<>@@===AA@@=?=AC>A@@>@?AA<
A@AA<A;==?A<
@T6XXI:00022:00321
GGGAGAAGGGAGAAAGGAAGCTAGTCACTCGACCATCTGTGGCTTCACAGTACAGAGGGACTGTCCAAAGCCGCAGA
TCTCGAGTCGACTAG
+
DG@BAB>@>6>998096958>>=?AA>=9:>00,/733:=A5::?CCBBBCBC@AA==99=@@@A>A@<A@>A@@AA
A=@@@=@A?@@@@@@
@T6XXI:00022:00334
GGGAGAAGGGAGAAAGGAAGCTAGTCACTCGACCATCTGTGGCTTCACAGTACAGAGGGACTGTCCAAAGCCGCAGA
TCTCGAGTCGACTAG
+
FDAAAA@CC@CB?=<B=@=?CCB@??>::::00,....7:B=?@@BBBACCCA=837717====@<??;?C>@@?A@
AAAA===@???????
@T6XXI:00023:00331
GGGGAATTCTAATACGACTCACTATAGGGAGAGGGAGAATAGCATCTCATC
+
=HH:C@CCCCC<<<DDCBDA@:88>777-7>>BB>B>@966,+++00074.
@T6XXI:00023:00334
CTATCACTCGAGATCTGCGGCTTTGGACAGTCCCTCTGTACTGTGAAGCCACAGATGTCGAGTCGACCT
+
==;;44:949;==9=:AA@?BBNAL<>;?A@@A=ABCEDCCA@?:655=<>BAACCB?96/../,,,*,
@T6XXI:00023:00340
GGGGAATTCTAATACGACTCACTATAGGGAGAGGGAGAATAGCTACACAATCGACTCCCGGCTTTTCTACCTTCCTT
TCTCCCTTCTCCC
+
CGD:D@B?BCC@CCBBB@@@AABB@<<A<??:CC>?==>@BA@BBCCB=;@7::?968,,+,111.,,++*+00*++
,+:AD<@<===C<
@T6XXI:00024:00303
GGGAGAAGGGAGAAAGGAAGGAGAAAGCGACGTCGGGGAAACGTTTGCTATTCTCCCTCTCCCTATAGTGAGTCGTA
TTAGAACTTCCCC
+
@C?>>><>>1949@0949<>;>7772;??>@AABCAA7CA6::==:=CBCC?CCBB<@@@@@<@AAA:;:B??:7:7
?:@=><AC>BBA5
@T6XXI:00024:00327
GGGGAATTCTAATACGACTCACTATAGGGAGAGGGAGAATAGTCAACAAATCGTTTGGGGCGACTTCTCCTTCCTTT
CTCCCTTCTCCC
+
@AA7A>B@CBB?CBBBB?>?BBBBC969.7<<=?<A@@>A@AAAA=??@;BABAE>BCB9BAA??=?AC<<9<9<;6
;=;;4<343;=:
@T6XXI:00024:00338
GGGGAATTCTAATACGACTCACTATAGGGAGAGGGAGAATAGCCCTTTTCCCCGTAGAACGCGAGTTTTATCTTCC
+
@:?6=<>@A@@@@B@@@CDCCBBBC???<@CDCC@C<:7::444+177,111,166688355.0----*.-44/0/
@T6XXI:00025:00311
GGGAGAAGGGAGAAAGGAAGGAGAACAACGGTGCACTGGTGCTTGGGCTATTCTCCCTCTCCCTATAGTGAGTCGTA
TTAGAATTCCCC
+
BBCAA@@CC>A@@B>B@CAC?BAC@CD?BB?B@BBBBCA<;;A=@@=ACBB?B99919>>>@<ACCCCCBCCABCBB
B?BBC?DDCCC9
@T6XXI:00025:00317
GGGGAATTCTAATACGACTCACTATAGGGAGAGGGAGAATAGCTACCATTTTACATGCGTGCCTACTCTCTTCCTTT
CTCCCTTCCTCCC
+
BCCAC;;==CC@CCBBBBBB@?>??777.7>>>@>BB?<>>?>?==>7777.7;:;<A@@@A>A@A@@?>;34244+
377327,/,/671
@T6XXI:00025:00325
```

```
GGGGAATTCTAATACGACCTCA
+
0880:;B>9998800,*(((((
@T6XXI:00026:00330
GGGGAATTCTAATACGACTCACTATAGGGAGAGGGGAG
+
?CC9B@C6999548998:A@@>>?>777.7?,,,,*,,
@T6XXI:00027:00314
CTATCGACTCGAGATCGTGCGGGCTTTGGACAGTCC
+
44494444444444444446666+..3+.,..3...7
@T6XXI:00028:00311
GGGAGAAGGGAGAAAGGAAGCTAGTCACTCGACATCTGTGGCTTCACAGTACAGAGGGACTGTCCAAAGCCGCAGAT
CTCGAGTCGACTAG
+
BD?DCB@BB=BB==:@696:CBBBC=????@:::@@?CCC?ABBBBCABABA?977717=>@AA?AA=AB<@@@A<<
<@CC@@???@@@@@
@T6XXI:00028:00335
GGGAGAAGGGAGAAAGGAAGCTAGTCACTCACATCTGTGGCTTCACAGTACAGAGGGACTGTCCAAAGCCGCAGATC
TCGAGTCGACTAGCTTCCTTTCTCCCTTCTCCC
+
?::=BC@CC?B<;<8;BB><<<B@>=;7111666889992677;::7<<=??B847,488;=>>BD:>>?@@A@@;/
//./.-445353306:99=?1888C>C<;;882
@T6XXI:00028:00336
GGGGAATTCTAATACGACTCACTATAGGGAGAGGGAGAATAGTTTTTCCCTTCACTAACCGCGAACTGCACTTCCTT
TCTCCCTTCTCCC
+
CHH:C@A>ABB>BBBBB@@@BA??=667.7>>?A>CAA=@??>?==1??:@>@BBBC?C?BCBA><??@BA?:=367
.7=:<6=./0@=9
@T6XXI:00028:00342
GGGAGAAGGGAGAAAGG
+
@;:911,/85/,,202*
@T6XXI:00028:00347
CTATCGACTCGAGATCTGCGGCTTTGGACAGTCCCTCTGTACTGTGAAGCCACAGATGTCGAGTCGACTAGCTTCCT
TTCTCCCTTCTCCC
+
C==81111;;ACBBBCCCCB?CCEAC@@><=777.7>>BBCCB@@@@>?BBCCCBBBCBBBCBB@@::;A8710631
=6<88=:?9=9?A=
@T6XXI:00029:00317
GGGGAATCTAATACGACTCACTATAGGAGAGGGAGAATAGTCCCGAATTTATTCGCGCGTCTGTCTCCTTCCTTCTC
CTTCTCCC
+
?=?1=-1/667/9<AA@?@?<?>777377611,110230035848;244-
4;3448983749<?8748;343498:;7:7:444,
@T6XXI:00029:00348
GGGAGAAGGGAGAAAGGAAGTGCAGTTCGCGGTAGTGAAGGGAAAAACTATTCTCCCCTACCTCCTGATAGT
+
DC?;995990948>9>>A=:779=99;888<579999911616>>?2CBBB><?@?>566686676111---
@T6XXI:00030:00310
GGGAGAAGGGAGAAAGGAAGAATATATGCGGTCGGGAGAGTACCAGTCTATTCTCCCTCTCCCTATAGTGAGTCGTA
TTAGAACTTCCCC
+
BBCBCC@BB>ABAB=BAC?AA@DBD=???97=6:7-
:?>;@=A?BCCCCBB<?17769?9@C=@>AAAA@AAA???<=:>>>388;@@@5
@T6XXI:00030:00333
```

```
GGGAGAAGGGAGAAAGGAAGCTAGTCACTCGAGATCTGCGGCTTTGGACAGTCCCTCTGTACTGTGAAGCCACAGAT
GTCGAGTCGACTAGCTTCCTTTCTCCCTTCTCC
+
CG@AA@?A@:@99909;>;888?>=?::66BB=;??<??B@???<????DKDDC>B@=?====@@@A>A@>?<<;=9
:887;9=87888??AA@C9<;-44::4:7444/
@T6XXI:00030:00347
GGGAGAAGGGAGAAAGGAAGCTAGTCACTCGACATCTGTGGCTTCACAGTACAGAGGGACTGTTCAAAGCCGCAGAT
CTCGAGTCGACTAGCTTCCTTTCTCCCTTCTCCC
+
9909>><>@:>99914242397;99=<;3./.....,,,,*..6444:::6=;;444-///.--
.4557455====<?=>@??<==:>><;;;5;5?BBAA@D9=:9::93
@T6XXI:00030:00348
GGGGAATTCTAATACGACTCACTATAGGAGAGGGAGAATAGTCAACAAATCGTTTGGGGCGACTTCTCCTTCCTTTC
TCCCTTCTCCC
+
DCB7B9=;=CDCCCB?BBBBA64;:/.-
0>>@C<BAC?@??AAC>???;AAAAC9?@@5<=>@@>???>@>@>AC<A98858387AA<
@T6XXI:00031:00331
GGGGAATTCTAATACGACTCACTATAGGGAGAGGGAGAATAGTCAACAAATCGTTTGGGGCGACTTCTCCTTCCTTT
CTCCCTTCTCCC
+
CCC9B?B?BCBEB???BBBBBCB<9666-
9=?CC>C@A?B;4;;;:88<;@AAAA>BCC9BBA??;??A;<;=>AC?@???<A>??DC<
@T6XXI:00031:00349
GGGAGAAGGGAGAAAGGAAGGAGAAGTCGCCCCAAACGATTTGTTGACTATTCTCCCTCTCCCTATAGTGAGTCGTA
TTAGAATTCCCC
+
CDBCCB@BC>BBBB>><B=@=@CC@BBB@@@@7@A<A@AAB>AAACCC@@A>@@AA<A===A<A@@@@@@??@788?
?<?A@?A9<==5
@T6XXI:00032:00326
GGGAGAAGGGAGAAAGGAAGAGAAGCGCC
+
GC>BCB@CC@AC;;;;;8*,---*,/.22
@T6XXI:00032:00327
GGGAGAAGGGAGAAAGGAAGCTAGTCACTCGAGATCTGCCTTTGGACAGTCCATCTGTGGCTTCACAGTACAGAGGG
ACTGTCCAAAGCCGCAGATCTCGAGTCGACTAG
+
DC@BBCACE>BBCB=@>B=?ABCCCBBB????A>A<??C@DE>B?AB????277<84488;==A@@?????;;344-
4::;=?>?C=BA>::8=@@=@?889@@@@@?>>
@T6XXI:00032:00336
GGGGAATTCTAATACGACTCACTATAGGAGAGGGAGAATAGTTTGTACATCGCCCGTGCAACATGCCTCTTCCTTTC
TCCCTTCTCCC
+
CCB7B7;:<AA;A@@:;;A?@:;?<00-0>>@B>C@@?@>>@=:?889@@@???9??CCC@CBBCE@?=<8:344-
4049499833@:
@T6XXI:00032:00337
GGGAGAAGGGAGAAAGGAAGCTAGTCGACTCGACATCTGTGGCTTCACAGTACAGAGGGACTGTCCAAAGCCGCAGA
TCTCGAGTCGACTA
+
CCBDDB?BB=?@@C=B?BACDC:>>/11>>@?1;;:::@AA?AAAC@??CCD@?@47317<=?@@>@C<??<???@B
B====>>@<<<???
@T6XXI:00033:00337
GGGGAATTCTAATACGACTCACTATAGGGAGAGGGAGAATAGTTTTT
+
AAA9A@A@B@@=@CCC<:<CBAAC::;@:>>@@@<@88348,,,,,)
@T6XXI:00035:00300
```

```
GGGGAATTCTAATACGACTCACTATAGGGAGAGGGAGAATAGCAAACGTTTCCCCGACGTCGCGTTCTCCTTCCTTT
CTCCCTTCTCCC
+
AA@4@<@=@CC@DBAABBBBBABB?66649??CC>CBA>AAA=999=CEE>BBB8CCCCAAACBA??77349;2481
8<DC@C????@?
@T6XXI:00035:00317
GGGAGAAGGGAGAAAGGAAGGGGAGAAGGGAGAAAGGAAGGGGAGAAGGGAGAAAGGAAGACAGTCCGACTCTGTGG
CTTCACAGTACAGAGGGACTGTCCAAAGCCGCAGATCTCGAAGTCGACTAG
+
EE?CCA<===9;;C>A>@@DDB8BCC@B?966698@DC@CBB9CCC>==;<<;<;=<>;8<8----,----
23<<<;8?9887;<<<@AB<<<7<<<9;=38814?=87844.../.+3474...--4
@T6XXI:00036:00300
GGGAGAAGGGAGAAAGGAAGGAGAAAGCGACGTCGGGGAAACGTTTGCTATTCTCCCTCTCCCTATAGTGAGTCGTA
TTAGAATTCCCC
+
CCCBCA>AA>@@@C?B?B@C@CCCC>?BBCCBCB???7AC?CBBBABDFCE@CCCGB@@@@@:BCBB@?ABBA@???
???CC@;:;AC4
@T6XXI:00036:00320
GGGGAATTCTAATACGACTCACTATAGGGAGAGGGAGAATAGCTCACACTCCGTCCAACACCTCTG
+
8C802*/,18;7>BBABBCCCCC;?977.6<667.7;;8:;77:679;<8823--,-,/--22344
@T6XXI:00037:00306
GGGGAATTCTAATACGACTCACTATAGGGAGAGGAGAATAGT
+
89=17+1,///7/99:@@A@@??>>777.7>??=>0/,./-+
@T6XXI:00037:00307
GGGGAATTCTAATACGACTCACTATAGGAGAGGGAGAATAGCACCGCTGTTGTTGCAGTTGACCTCTTCTTCCTTTC
TCCCTTCTCCC
+
AA?4=;@=@CC@CBBAA@BBB:997/.,0>:=?=?CAAA=A?;?<AAAAB?CC?@????>;<<;<<=;;84;/33-
7==C?A?BBBA=
@T6XXI:00037:00311
GGGGAATTCTAATACGACTCACTATAGGGAGAGGGAGAATAGTTTTCCCTCACTAACCGCGAACTGCACTTCCTTTC
TCCCTTCTCCC
+
C@;8B:<;<CC?DBB@@?BAB?99;677.7A777.7=;9;;3444,00+04=99>?BA???@>@???CB@37377.7
==>:>44444,
@T6XXI:00037:00331
CTAGTCACTCGAGATCTGCGGCTTTGGACAGTCCCTCTGTACTGTGACGACCACAG
+
=7///6699?=@>?ACBBBA>ABB>B@@@CCBBB>BBBCBBBCB366,,,)*,,,*
@T6XXI:00037:00349
GGGAGAAGGGAGAAAGGAAGCTAGTCGACTCGACATCTGTGGCTTCACAGTACAGAGGGACTGTCCAAAGCCAGATC
TCGAGTCGACTAG
+
CD@CCB@BC>BCCB=@>A>>A@9=>/10=@@;B@@@CBBBC@@@=?BB?@?CAAA<<<7<?@@AC=AA:>@?AA@@<
?=?A=AAAAAA??
@T6XXI:00038:00306
GGGAGAAGGGAGAAAGGAAGCTAGTCGACTCGACATCTGTGGCTTCACAGTACAGAGGGACTGTCCAAAGC
+
99199B6990989@9>;@>ABB;<;CBAAA??@???@::7A:???A:;<CCBB?;444,4334;=:=?:??
@T6XXI:00038:00343
GGGAGAAGGGAGAAAGGAAGCTAGTCACTCGAGATCTGCGGCTTTGGACAGTCCCTCTGTACTGTGAAGCCACAGAT
GTCGAGTCGACTAGCTTCCTTTCTCCCTCTCCC
+
```

44-
44489;589;919=@=@BBBBCA??889996?:8?@A?@AB5:6:?????B>;88?@===AABA<==:=@@@AA@:8
9=;;@?887@887=9468./.27,..8883
@T6XXI:00039:00307
GGGAGAAGGGAGAAAGGAAGGAGAAGCGGCCACATGTCCAAATTGCGCTATTCTCCCTCTCCCTATAGTGAGTCGTA
TTAGAATTCCCC
+
@A<@EC@CC>CBBC=@?@@B@BB?=;?<@B@B>>=BAA>BA<BAABBCCDCADAAA=BBBBA????>>;??@A=?=@
>??A@>@>@@A5
@T6XXI:00039:00308
CTAGTCACTCGAGATCTGCGGCTTTGGACAGTCCCTCTGTACTGTGAAGCCACAGATGTCGAGTCGACTAGCTTCCT
TTCTCCCTTCTCCC
+
B6///6699BBBBCCBB@BB@BBB>B>@?BCCCB;???????@AA=<=;B@BBABBCA@???BB@@@BBB@??>A?A
D?BBCCAA899AC:
@T6XXI:00040:00306
GGGGAATTCTAATACGACTCACTATAGGGAGAGGGAGAATAGTTACCAATGTTTGCTCCCTGTAACGCCTCCTTTCT
CCCTTCTCCC
+
444,42924449;8;110899=>?=777.0<;;;.1/;;;;@@=;;8<377;;498884-/311,---,--
22222000+044044,
@T6XXI:00040:00327
GGGAGAAGGGAGAAAGGAAGACTCGACATCTGTGGCTTCACAGTACAGAGGGACTGTCCAAAGCCGCAGATCTCGAG
TCGACTAG
+
CC@AAA@DB>B?==5>;=2<8@??7::@@AABBBAAA>@BAA@A@>;6000-
4::;<=?@@;@@>CB@@AA@@?????===DCC@
@T6XXI:00040:00329
GGGAGAAGGGAGAAAGGAAGCTAGTCGACTCGACATCTGTGGCTTCACAGTACAGAGGGACTGTCCAAAGCCGCAGA
TCTCGAG
+
DD??A@BCH>FBBB<@>B>ABE>BA@B@?AA???@@BBBCB?AA@CCC??@C=8:44317@>??C>@@<??>???AA
@@@@@@A
@T6XXI:00040:00332
GGGAGAAGGGAGAAAGGAAGGAGAAGTCGCCCCAAACGATTTGTTGACTATTCTCCCTCTCCCTATAGTGAGTCGTA
TTAGAATTCCCC
+
CH@CCA@BC@@A@C?C?B?B@BCC?@@BBBBB8>B>DA@BB>@?@CB@@@@=@@@C;<<<@C=AAA@@@@AA???@@
@>AAA>B>@@@5
@T6XXI:00041:00304
GGGGAATTCTAATACGACTCACTATAGGAGAGGGAGAATAGCTAAAAACATTATTCCCGTCGCTGTTCTCTTCCTTT
CTCCCTTCTCCC
+
DBB7@546<AA?CAAACBBBB:=97..,00077.7@??@@AABBA@?2>BB=AB@AA<@@@@@CDC?AAAA>A>AB>
BBBC>C>BBCC>
@T6XXI:00041:00313
GGGGAATTCTAATACGACTCACTATAGGGAGAGGGAGAATAGACATCTACCAATCGCACGTCTGTTCACCTTCCTTT
CTCCCTTCTCCC
+
EDC:C997;@@=@CCBCBBBA@;97611-6>==A>BB@AC<????>=;<<=?=?AA?===A@@@A>A??::48/44-
4;;=7=;====7
@T6XXI:00041:00341
GGGAGAAGGGAGAAAGGAAGCTAGTCACTCGACATCTGTGGCTTCACAGTACAGAGGGACTGTCCAAAGCCGCAGAT
CTCGAGTCGACTAGCTTCCTTTCTCCCTTCTCCC
+
B@>AAA?@@:>999094958BA?<<900....../??9>?5:;<???BBBBA;;444-
4;;;@A>BA:@@?C<<<<878>488===88888?A?B9<<7<??@;@>=<<B1

```
@T6XXI:00042:00311
GGGAGAAGGGAGAAAGGAAGGAGAAATGACACCATTCTGATTAGGGTCTATTCTCCCTCTCCCTATAGTGAGTCGTA
TTAGAATTCCCC
+
BB:@>>5991994:9=7=9>7<77>8>>7;9A<AA@B?CCC?ABB@CDCCNCBCBB>B::;C>DA::8?=>;;;787
=:==@9998AA5
@T6XXI:00042:00312
CTATCACTCGAGATCTGCGGCTTTGGACAGTCCCTCTGTACTGTGAAGCCACAGATGTCGAGTCGACTAGCTTCCTT
TCTCCCTTCCTCCC
+
B?=99::==BBAABCCC@@=@AA<A@BBCC???=@CCA@?<<>AAB>AB@CBBBCBBBCBBB::;@AAABA<<A>AA
=?>7916,/,///,
@T6XXI:00042:00325
GGGAGAAGGGAGAAAGGAAGCTAGTCACTCGAGATCTGCGGCTTTGGACAGTCCCTCTGTACCTGTGAAGCCACAGA
TGTCGAGTCGACTACGCTTCCTTTCTCCCTCTCCC
+
>>1944244-444:-424999@AAA@?<66676769>?@>;777-
7;==?BBBD@B?=:=7424:;;;8<;:>>;;68888=@=ABB=;;///7:76,//,////,./4245
@T6XXI:00042:00331
GGGAGAAGGGAGAAAGGAAGCTAGTCGACTCGACATCTGTGGCTTCACAGTACAGAGGGACTGTCCAAAGCCGCAGA
+
DD@AAA@CC>B???<@@D=@@CAAA>??@CB?<7:::7?>A;@@?BCDBBB@<::33317>?AAA=?A:>>>AA===
@T6XXI:00042:00334
GGGAGAAGGGAGAAAGGAAGCTAGTCACTCGACATCTGTGGCTTCACAGTACAGAGGGACTGTCCAAAGCCGCAGAT
CCACGAGTCGACCTAGCTTCCTTTCTCCCTTCTCCC
+
>>6;:?=>B<A@BA<@<@>AA@@@>0,,,,,,,,22,..601311111196633000+366:;<:?;45;5;8976,
,+++0013440--...-4444::5:=AA<A>;;;;6
@T6XXI:00042:00335
GGGAGAAGGGAGAAAGGAAGTGCAGTTCGCGGTTTGTGAAGGGAAAACTATTCTCCCTCTCCCTATAGTGAGTCGTA
TTAGAATTCCCC
+
CC?ACC@CC=ABBB>B@C@B>>BCCC@CCCB@CB<BBBB@??;???8B??B?BBCC>BBBCC>C@???>@?B@AA@@
@=@AC?D=???5
@T6XXI:00042:00340
CTCGAGATCTGCGGCTTTGGACAGTCCCTCTGTACTGTGAAGCCACAGATGTCGAGTCGACTAGCTTCCTTTCTCCC
TTCTCCC
+
CFCAAAAACCCCC@BCC?C?CCCCCDEBFCCBABBBA@@@=@B>A=888--,,,,,.3199973442454;-
88>>0968??A<
@T6XXI:00042:00349
GGGGAATTCTAATACGACTCACTATAGGGAGAGGGAGAATAGTTCATTTTTAACCTGTGCCGAGCTGCTCTTCCTTC
TCCCTTCTCC
+
DCB9B698;AA9<<CCCBBBB@<97111.7==BC>B@BAB@;82:44444,4:=<@A??><>>??;?=<00,/,/,.
//.+4/4882
@T6XXI:00043:00304
GGGAGAAGGGAGAAAGGAAGTGCAGTTCGCGGTTAGTGAAGGGAAAACTATTCTCCC
+
//+//17@C@<<<N@C?B?B889>9424443779777:8<>@-777-33331.5552
@T6XXI:00043:00313
GGGAGAAGGGAGAAAGGAAGGAGAAGTCGCCCCAAACGATTTGTTGACTATTCTCCCTCTCCCTATAGTGAGTCGTA
TTAGAATTCCCC
+
DIDAA?@DC@BBBC=B?B=>=@CC@BBBCCCC9CC>BB97:,676?BBCAA?@@=<;???@C<AA@???AAA===AA
@=@==:=>AAA5
@T6XXI:00043:00316
```

```
GGGAGAAGGGAGAAAGGAAGGAGAACGAAACGCGCACTTGAGTAGTAGCTATTCTCCCTCTCCCTATAGTGAGTCGT
ATTAGAATTCCCC
+
D@:999699098>>094;=@?CCC?CBB@<@@998@>>=@BBCCDEICAACCBCCCC>;;<BB<ABB9<;88<<>@@
@A>@<:>B>BAA7
@T6XXI:00043:00323
GCTAGTCACTCGAGATCTGCGGCTTTGGACAGTCCCTCTGTACTGTGAAGCCACAGATGTCGAGTCGACTAGCTTCC
TTTCCTCCCTTCTCCCCTTCCTT
+
/,//////////////868879388,39977:??626300./0/3>9;37<99?@>@??@AAFBCCCC@@ABDB=?4
77.747???@34.///+/55+,+
@T6XXI:00043:00327
GGGAGAAGGGAGAAAGGAAGGGGAGAAGGGGTA
+
EDADCC@EC?@AAB=@<>4---*-==+***)**
@T6XXI:00043:00331
GGGAGAAGGGAGAAAGGAAGCTAGTCACTCGACATCTGTGGCTTCACAGTACAGAGGGACTGTCCAAAGCCGCAGAT
CTCGAGTCGACTAG
+
CC@CCB=>><=@>@9@8;7;@@???BA@?A?;::???BDE?AA?ABBABAAB;8773-
49;=;<:<@<=><?<<;A????AA@@@@===??
@T6XXI:00043:00333
GGGGAATTCTAATACGACTCACTATAGGGAGAGGGAGAATAGTCAACAAATCGTTTGGGGCGACTTCTCCTTCCTTT
CTCCCTTCTCCC
+
<99/9<A?CCB@CB?=A>>>?AB?7111.7=<>A>BAA>A<@@AA>@@@<CBBBC>AC@7>ACCC@BBB7737;AA<
@@@A<A>AB@@<
@T6XXI:00043:00340
GGGGAATTCTAATACGACTCACTATAGGAGAGGGAGAATAGTACAACA
+
CDC:C9;9<BB?BBBBBABBE>=;<00,/:077,1444483334;480
@T6XXI:00044:00309
GGGAGAAGGGAGAAAGGAAGGGGAGAAGGGAGAAAGGAAGCTAGTCGACTCGACATCTGTGGCTTCACAGTACAGAG
GGACTGTCCAAAGCCGCGAGTCGACTACGCTTCCTTTCTCCCTTCTCCC
+
CD>@A>=@C>AAAB:;;<@CCC9CCD?BA>AB?B:B;?:?????BCAAABBB@?AA=<<<A>??<?AA=A=<<<<==
@<?>><<<9=A<AD>@@@?==?<888888??=@8;;699;;49243883
@T6XXI:00044:00337
ATACCGAGATCTGGCGGCTTTGGACAGTCCCTCTGTACTGTGAAGCCACAGATGTCGAGTCGACTGCTCCTTT
+
BECC=AAA@><12,1878;;;B=??CCCCC>CBC;:;AC@@?CBBB?BBBBDCC???@=777877711.422*
@T6XXI:00044:00340
GGGGAATTCTAATACGACTCACTATAGGGAGAGGGAGAATAGCTAAAACATTATTCCCGTCGCTGTTCTCTTCCTTT
CTCCCTTCTCCC
+
@AA6A@C@CCB@BBBBBBAAA9996167.7===><@@@AAAABB@@@7BDA?A=?A=;??<?ABCC@@@@B?B?CB9
????9?;??@A<
@T6XXI:00044:00343
GGGAGAAGGGAGAAAGGAAGCTAGTCGACTCGACATCTGTGGCTTCACAGTACAGAGGGACTGTCCAAAGCCGCAGA
TCTCGA
+
EEACCC@DC>B@??:@>A=@@BBBB?????==7777:7@BC8;;@CAAAAAC@@@88417B789?>BB=@@?@AA@?
@==<>?
@T6XXI:00044:00345
GGGGAATTCTAATACGACTCACTATAGGAGAGGGAGAATAGAGACGTATTCGTTTGCGCTCTGTTCACCTTCCTTTC
TCCCTTCTCCC
+
```

DED:D?<><CDBCCCBBBCCA5;=7//,0:0775:@B<@@@AA?8=?7737=??;@A@@B@;AA=?AA>B9<9<>:?
@@B>B?@???7
@T6XXI:00045:00340
GGGGAATTCTAATACGACTCACTATAGGGAGAGGGAGAATAGTTCGCCAATATCCCGCGGCGTGTTCTCCTTCCTTT
CTCCCTTCTCCC
+
?B?6B=A@@BBAAAACCCCBBA;<;677.777==8?AA?????>??AABABACCE>???=?AA@A?@@@>@>@?AA<
??@C<?<?<<<7
@T6XXI:00046:00327
GGGGAATTCTAATACGACTCACTATAGGAGAGGAGA
+
8>=17+1,/667688@@;3:C:00...,../47734
@T6XXI:00046:00329
GGGGAATTCTAATACGACTCACTATAGGAGAGGAGAATAGTCAACAATCGTTGGGCGACTTCTCCTTCC
+
CIC:B69894898@@AA@CCC=;;;00,,7,0,.2444;44-.,//,7777367779<8?:;;;33//4
@T6XXI:00046:00332
CGAGATCTGCGGCTTTGGACAGTCCCTCTGTACTGTGAAGCCACAGATGTCGAGCGACTTCTCGCCTTCCTTCCTCC
TCTCC
+
BBBBBBBABBB?@AB<B;>?BBB@@;BCCBB??@@BB@>@@>BB@<<><?4111/,***)*,,,/6;898888299<
;77;:
@T6XXI:00046:00347
GGGGAATTCTAATACGACTCACTATAGGGAGAGGGAGAATAGCTTGTCATTTGGTCAATGTCTACATCTCCTTCCTT
TCTCCCTTCTCCC
+
CGD:C>A>@BBBBBBBCCCB>=??7166.7==>@>BAA=A?@@C>BAAABB>B?BBA>A888<>==?<<<7:7:244
,48;99>;877;6
@T6XXI:00047:00312
CTATCGACTCGAGATCTGCGGCTTTGGACAGTCCCTCTGTACTGTGAAGCCACAGATGTCGATCGACTAGCTTCCTT
TCTCCCTTCTCCC
+
B>CBCBCDCDBBBABCCCA@>9775:8:;CCBBC>BBBBBBCC@CAA=?BABBA>>@BBBA@AAA:::DA@>;;344
-4;;;4<9<?==:
@T6XXI:00047:00327
GGGAGAAGGGAGAAAGGAAGCTAGTCACTCGACATCTGTGGCTTCACAGTACAGAGGGACTGTCCAAAGCCACAGAT
CTCG
+
DCABBB>BCAAAAE;<<<@EECCEE?A????@FBCCBDDC?BB?BCC???CB>634317==99:5842./,//.--
1446:
@T6XXI:00047:00329
GGGGAATTCTAATACGACTCACTATAGGGAGAGGAGAATAT
+
=8B9B+:<<CCBCCBB?>B>BCB?>661.7<773:88349.
@T6XXI:00048:00324
GGGGAATTCTA
+
DDD:CAA?@99
@T6XXI:00048:00326
GGGAGAAGGGAGAAAGGAAGTAGTCGACTCGACATCTGTGGCTTCACAGTACAGAGGGACTGTCCAAAGCCGCAGAT
CTCGAGTCGACTAGCTTCCTTTCTCCCTTCTCCC
+
44-
9@B5<=:;ACD?B>CADC@@??@@;<:=>999?>>4428@>ACAAABA9998880;?ABCCABD?CC?B;::>=777
33766666688;=;>:==.71==9;798994
@T6XXI:00048:00330

```
GGGGAATTCTAATACGACTCACTATAGGAGAGGGAGAATAGATCACGACCCTTCTACCACGATTGAGCTTCCTTTCT
CCCTTCTCCC
+
CDF:C?B?BCC@BBBBBBBA@799;//,0==@ABBAA>A?C@@@??@BBB<?:7:00,/866388839489244-
4:;;:>=?>BB>
@T6XXI:00048:00349
AGTCGACTCGAGATCTGCGGCTTTGGACAGTCCCTCTGTACTGTGAAGCCACAGATGTCGAGTCGACTAGCTTCCT
+
<@?1//8668;>CC?AAAA<>>?5>9>>>@@AA<AAA?@@CCDCCC@AB?BCAA?@@=<=@=@;;///9/40,,+,
@T6XXI:00049:00306
GGGAGAAGGGAGAAAGGAAGCTAGTCACTCGAGATCTGCGGCTTTGGACACCCTCTGTACTGTGAAGCCACAGATGT
CGAGTCGACTAGCTTCCTTTCTCCCTTCTCCC
+
94-449244-4444-
49;9777A;7=?866666;6111310111,1778?BC>CBDC?<<<BCBC<=<:;;;==;=///8///94447///5
6,//+/4;;59899885
@T6XXI:00049:00336
GGGAGAAGGGAGAAAGGAAGTGAAAGGGCGTTCGGAATAGTGGGTGGCTATTCTCCCTCTCCCTATAGTGAGTCGTA
TTAGATCCC
+
DC>CBA>@=<@>=>7:8;@DFBBB@BD>B>?@BB?A?<9????8?B;?ACC=A@AC?==<DGA@@@CDCCCCBB???
??@AABAC7
@T6XXI:00004:00354
GGGGAATTCTAATACGACTCACTATAGGGAGAGGGAGAATAGTTCCAATCCAAG
+
BCD:C::;;BB>BBBABBBBBBAA<;661-6==?A>AAD?D?@:9746,,,+*)*
@T6XXI:00004:00375
GGGAGAAGGGAGAAAGGAAGACAGAGGTCATTGAGAATAGCAGGGGACTATTCTCCCTCTCCCTATAGTGAGTCGTA
TTAGAATTCCCC
+
4<89=B<?>194>?<A?B@@@@B@??;??A?=ABBC???B?7777.7<=7;78ABA;@?@CC=@??@@AA@AA<<8=
9<AA=::5665.
@T6XXI:00004:00380
GGGGAATTCTAATACGACGTCACTATAGGGAGAGGGAGAATAGTCAACAAATCGTTTGGGGCGACTTCTCCTTCCTT
TC
+
499/42/7//1,//2//...4../;7333,38777-
:?@:??>?9;9430+3337926885;B95;7//.44,/,/42-
@T6XXI:00004:00392
GGGAGAAGGGAGAAAGGAAGGAGGAAGTCGCC
+
:?:?BB?AA9;<BB>B@B<?68.,.,......
@T6XXI:00004:00397
GGGAGAAGGGAGAAAG
+
995949599044//+,
@T6XXI:00005:00352
CGAGATCTGCGGCTTTGGACAGTCCCTCTGTACTGTGAAGCCACAGATGTCGAGTCGACTAGCTTCCTTTCTCCCTT
CTCCC
+
BBB?A@BBBBB?BCC>B?BCBBBDC>CAAAAAACBCC@>@A?FC@???@?EGCDBCDAA@@94499=@B=BCCC@C;
<;AB:
@T6XXI:00005:00381
GGGGAATTCTAATACGACTCACTATAGGGAGAGGGAGAATAGATGTCTGACCATTGCTGCTTCTATCTCCTTCCTTT
CTCCCTTCTCCC
+
```

```
AAA7A@B@CDC@?@?ABCCACAAA?88?8:ABCD>B@A>A====@?<<?==?@ABC@@???>AA@@@>>;=;=8;<7
<AAC?A=?AAA<
@T6XXI:00005:00382
GGGGAATTCTAATACGACTCACTATAGGGAGAGGGAGAATAGTCAACAAATCGTTTGGGGCGACTTCTCCTTCCTTT
CTCCCTTCTCCC
+
CIK:C::<;CC@CCBBBAAABAB>9666.7><<?<?AA@BAA@??<?AA=CCBBB>BCC7<?BAA=AAA;<9=>@@;
???A<A;==AD<
@T6XXI:00005:00384
GGGAGAAGGGAGAAAGGAAGCTAGTCGACTCGAGATCTGCGGCTTTGGACAGTCCCTCTGTACTGTGAAGCCACAGA
TGTCGAGTCGACTAGCTTCCTTTCTCCCTTCTCCC
+
DH?@@@=@@<ACCB=A8;7;:8>@A::;CAAACBCCCDCCC@B??=B@BCCAAACAA?????AA??>@@AA?@AA88
7<<<@?@@A??===<<<8<9<@<;>??6;00/559
@T6XXI:00005:00387
GGGAGAAGGGAGAAAGGAAGCTAGTCGACTCGAGATCTGCGGCTTTGGACAGTCCCTCTGTACTGTGAAGCCACAGA
TGTCGAGTCGACTAGCTTCCTTCTC
+
9959>;=AA:>99918;>;773..4......335688986;:::5+.,.43.67?;=3444488=<<@<58489;88
:/....135686000--,-,-,-33
@T6XXI:00005:00394
GGGAGAAGGGAGAAAGGAAGGGAGAGGACTAGTCGA
+
FEABBB@CC<@A??9><>58807731013..,,,,,
@T6XXI:00006:00353
GGGAGAAGGGAGAAAGGAAGGAGAAGTCGCCCCAAACGATTTGTTGACTATTCTCCCCTCTCCCTATAGTGAGTCGT
ATTCAGAATTCCCC
+
:F>@=C;=<<<CCDCC@C@D@CAB@CBCBBBB7A?:811662688?@A?44344444+000044334<;89=AAA?<
;;330-33344440
@T6XXI:00006:00378
GGGGAATTCTAATACGACTCACTATAGGGAGAGGAGAATAG
+
>=<0<,458989=BCBCCCGBBB@>:77.7>77474342,*
@T6XXI:00006:00379
GGGGAATTCTAATACGACTCACTATAGGGAGAGGGAGAATAGTGGGAATTCTAATACGACTCACTATAGGAGAGGGA
GA
+
CCB4@59544498499>@@AC@?>=777.7>;;;60--,-44,--+-,-,-1333003:;::<<<8300/030--+-
33
@T6XXI:00007:00395
GGGAGAAGGGAGAAAGGAAGCTAGTCGACTCGACATCTGTGGCTTCACAGTACAGA
+
A@:@EB?BB;<<CB@B8;58>833633,,,,,,,,,,/77?>=<<661/...--10
@T6XXI:00007:00398
CTATCGACTCGAGATCTGCGGCTTTGGACAGTCCCTCTGTACTGTGAAGCCACAGATGTCGAGTCGACTAGCTTCCT
TTCTCCCTTCTCCC
+
AA@=99:BCAAABCBBBBCC@CCCCC?BCCA???7;:CAA?@@?==A>ADBCCCB@@@A???BBCBBBA@@BE?B?B
C@BB?A7=8444:4
@T6XXI:00008:00374
GGGAGAAGGGAGAAAGGAAGCTAGTCACTCGACATCTGTGGCTTCACAGTACAGAGGGACTGTCCAAAGCCGCAGAT
CTCGAGTCGACT
+
GC>CCC@DD>B===:?<?<@CBBBC@CB??A::7787BCCCBBCCCBBCCCDAA==<7<@>??B>@@<@@>AAA@@A
@@@===A???A@
@T6XXI:00009:00378
```

```
GGGAGAAGGGAGAAAGGAAGGAAGAA
+
BB>CDC?BC:<9;B=>:=,-*-*-,*
@T6XXI:00009:00380
GGGAGAAGGGAGAAAGGAAGGAGAAGTCGCCCCAGAACG
+
EE=;<B>A@=BBBG@C@B@C@BCC@:797777*-,,*,,
@T6XXI:00009:00382
GGGGAATTCTAATACGACTCACTATAGGGAGAGGGAGAATAGTCAACAAATCGTTTGGGGCGACTTCTCCTTCCTTT
CTCCCTTCTCCC
+
CHC:C=<<<BABCC@@@CCCC@A@B991-
677=>:@<<8;@<<=@AA@C;BAAAC<A??49:???>@A@5838;=;9=<<<7<A@?@@<
@T6XXI:00009:00393
GGGGAATTCTAATACGACTCACTATAGGGAGAGGGAGAATAGTCAACAAATCGTTTGGGGCGACTTCTCCTTCCTTT
CTCCCTTCTCCC
+
?B@4@5969>>;@@AABACCCCCA@::7.:@@BC@C<<8;====??@@A<BBBBC>BBB7AABBB=??C@?=??BB=
@@@A<A>AAAA<
@T6XXI:00010:00378
GGGAGAAGGGAGAAAGGAAGGGGAGAAGGGAGAAAGGAAGCTAGTCGACTCGACATCTGTGGCTTCACAGTACAGAG
GGACTGTCCAAAGCCGCAGATCTCGAG
+
CF@CBB=@@<@@@C@C@C@CCC9CBA561,7777-
7;?6::AAACCECCE?=<=<==@;;;=7858?A=??>;::448-8;>@@@>@A889?AA;=;0/..444
@T6XXI:00010:00392
GGGAGAAGGGAGAAAGGAAGCTAGTCACTCGACATCTGTGGCTTCACAGTACAGAGGGACTGTCCAAAGCCGCAGAT
CTCGAGTCGACTAG
+
991444244-
444;6>?BABA@A:966611111179999996:59A?ABCEC?;<;96;;;<=A>AA=AA?@@A@>=<78848?=??
??>A
@T6XXI:00011:00365
GGGGAATTCTAATACGACTCACTATAGGGAGAGGGAGAATAGTCAACAAATCGTTTGGGGCGACTTCTCCTTCCTTT
CTCCCTTCTCCC
+
CCC9A=?=@BB@CBA@BAAAB@>97111.7>>>A?ACC>A:8?@A?AAA:>>?BB=B>>6?BAAB?<A>;<98;?@<
@A@A<@:==CC<
@T6XXI:00011:00391
GGGGAATTCTAATACGACTCACTATAGGGAGAGGGAGAATAGCTTTTCACTCCCCGACAACCGACGTATTCTTCCTT
TCTCCCTTCTCCC
+
CCB7B7;;<CB@CCBABCCCC>@>=666.7>>AC>B@A>A=@7999.7??=?@?1=>;;8<=@@A@@@A>@?8?9;<
7<<@A<A=@@@C?
@T6XXI:00012:00365
GGGGAATTCTAATACGACTCACTATAGGGAGAGGGAGAAT
+
499/959244424;@@;;:9B;<<>661,16677-79943
@T6XXI:00012:00369
GGGGAATTCTAATACGACTCACTATAGGGAGAGGGAGAATAGTCTTCCCTTATTCCAACTCC
+
DCB9B645944244:?>>?:;7?><777.7>===6=;;378<7702330400/,.3354.11
@T6XXI:00012:00375
GGGAGAAGGGAGAAAGGAAGCTAGTCGACTCGACATCTGTGGCTTCACAGTACAGAGGGACTGTCCAAAGCCGCAGA
TCTCGAGTCGACTAG
+
```

```
HDA;<;@CC<B@@@<>>?<>A800;0/,0307//.//.56>=>A?@BCFBBCAA@88817>A@A@=@A<@@?BBA??
<<A@89===<@===@
@T6XXI:00012:00381
GGGAGAAGGGAGAAAGGAAGCTAGTCGACTCGACATCTGTGGTCACAGTACAGAGGGACTGTCCAAAGCCGCAGATC
TCGAGTCGACTAG
+
BA>BCA@CC>BBBB>B<><@A@@AA=<==@ACCCBABCCCDCA??@?A<<:;;444+38:?@@;@@:@@889@@@@A
A@@?>784770:3
@T6XXI:00012:00386
GGGGAATTCTAATACGACTCACTATAGGGAGAGGGAGAATAGTCTAAACCTTTTCAATCTGTGACCATTCTTCCTTT
CTCCCTTCTCCC
+
CCC9C?B@BCC@CCBAAAB>>=B??777.7>>@A@BAA?AA?>???D<A>@@A5AA>CB@?@A8869A?@A;=:=B=
==<A=A?===CA
@T6XXI:00012:00388
GGGAGAAGGGAGAAAGGAAGGAGAACGCGCACCCGGAGCCAACGGTGATCTATTCTCCCTCTCCCTATAGTGAGTCG
TATTAGAATTCCCC
+
=C?@AA=@@:>>88589654-0009;;;AB:>>-1,--
,*/78:;;CC@??BC@CABB<A>>?A68;BB;;:@?C>><=<::74470666-
@T6XXI:00012:00391
GGGGAATTCTAATACGACTCACTATAGGGAGAGGGAGAATAGTGTCTCGATTCCCAGCGACCGTTTGTTCTTCCTTT
CTCCCTTCTCCC
+
CBB9B=A?ACBBDBBBBCCCCCCBB???;?AABD=@=@>@@@89:?A@A=@=;@:;3;7;@<@B@:??;??<?@CE>
B@AA<A>@@@=;
@T6XXI:00013:00354
GGGAGAAGGGAGAAAGGAAGGAGAAGTCGCCCCAAACGATTTGTTGACTATTCCTCCCTCTCCCTATAGTGAGTCGT
ATTCAGAAT
+
AB>BBB@DC?BBCC@D=A=?788857633333,33,316776??:897414,/,-
332343;403330308440303365533333
@T6XXI:00013:00355
GGGAGAAGGGAGAAAGGAAGCTAGTCGACTTGACATCTGTGGCTTCACAGTACAGAGGGACTGTCCAAAGCCGCAGA
TCTCGAGTCGACTAG
+
CD@CCC?AA=B@>>9@;?;>@>???:;:???A;;???AAA5:;?BBBCBFCBB<<<>7>>@A@@<??7==:>?<<=
@<==99:DACA@@@A
@T6XXI:00013:00358
GGGAGAAGGGAGAAAGGAAGCTAGTGACTCGACATCTGTGGCTTCACAGTACAGAGGGACTGTCCAAAGCCGCAGAT
CTCGAGTCGACTAG
+
;<6<BC=@@<@====B?B?BB?BBBBCBBBB::::;7>??8?@>ACDCBABB@?77714;;:99244-
4;:=?@@??989@@?>????@<<
@T6XXI:00013:00368
GGGGAATTCTAATACGACTCACTATAGGGAGAGGGAGAATAGTCAACAAATCGTTTGGGGCGACTTCTCCTTCCTTT
CTCCCTTCTCCC
+
CEF9A>A?BCD@CCBBAA?@A?<96166.7?>@B>BAA>@:?AAA?@@@:AB???<?AB6AABBB<??B=@=@=@A@
@A@C=B9<<AC<
@T6XXI:00013:00374
GGGAGAAGGGAGAAAGGAAGGAGAAGTCGCCCCAAACGATTTGTTGACTATTCTCCCTCTCCCTATAGTGAGTCGTA
TTAGAATTCCCC
+
BC@CCCACC@CBBA<>4848>>@A>A??8888.8?:BBBBB>BC@CBB@@A=?77718==>>:?<<<<@@@@AA@==
=?A?;9:7;;=4
@T6XXI:00013:00379
```

```
GGGAGAAGGGAGAAAGGAAGGTGAACAGAGCGCAAACGAATACGTCTCTATTCTCCCTCTCCCTATAGTGAGTCGTA
TTAGAATTCCCCC
+
@=<@@@?BC@CCCC@B?B>>=?787<AAACGCECA7;9959?BACA9999959984-
4:888:==;;B>;;98<9937771629<=ABC3
@T6XXI:00013:00380
GGGGAATTCTAATACGACTCACTATAGGGAGAGGGAGAATAGCCTGTAATTGCAGCCTGTTTTCTCCGCCTTCCTTC
TCCCTTCTCCC
+
?=9,9298@@@@CGE@AAAACBA??677.:::CD>CAA>A==<9?AAA?A@;8://,/////+/875999777-/--
223455<6;=:
@T6XXI:00013:00385
GGGAGAAGGGAGAAAGGAAGCTAGTC
+
AC<>@@<>>:>111-/,1,...7766
@T6XXI:00013:00396
GGGAGAAGGGAGAAAGGAAGCTAGTCGACTCGACATCTGTGGCTTCACAGTACAGAGGGACTGTCCAAAGCCGCAGA
TCTCGAGTCGACTAG
+
BDCCEGBCB=BCB@:?796:C:::A:::7//;00///.755-
::>ABAABBB;;;77717;;<??=?@<??>A989@?<<====A@@@@CAA
@T6XXI:00014:00351
CTATCGACTCGAGATCTGCGGCTTTGGACAGTCCCTCTGTACTGTGAAGCCACAGATGTCGAGTCGACTAGCTTCCT
TTCTCCCTTCTCCC
+
D:::1118::@BAABCCA?>>>><<@@CB@A:::5:@@ACCBBBA@@>@D?BCBBCBB@@@CCCCCCCBBGAA@C?C
C@AA@@1858?>A?
@T6XXI:00014:00363
GGGAGAAGGGAGAAAGGAAGCTAGTCGACTCGACATCTGTGGCTTCACAGTACAGAGGGACTGTCGAAAGCCGCAGA
TCTCGAGTCGACTAG
+
BB?BCC@CC>C?@=9>597;@?@?D/11K@77/0001/===5@@=BCCFCBBAAA<<<7<>>?A@@AA;@@>@@<77
7<<;@@@????AA@A
@T6XXI:00014:00369
GGGAGAAGGGAGAAAGGAAGGAGAAGTCGCCCCAAACGATTTGTTGACTATTCTCCCTCTCCCTATAGTGAGTCGTA
TTAGAATTCCCC
+
DC@BB@@BB=@@@B=B@B@B@BBB@ACC?<>>.880879BE<AA@CB@<A@=;730+4944418@@@@?@AA@???@
@>AAC?D;==<4
@T6XXI:00014:00378
GGGGAATTCTAATACGACTCACTATAGGGAGAGGGAGAATAGTCAACAAATCGTTTGGGGCGACTTCTCCT
+
:??299924442444983..0..43...+1677788;;88:44384044+1177=;@?>,..,,,*,,,00
@T6XXI:00014:00398
GGGGAATTCTAATACGACTCACTATAGGGAGAGGGAGAATAGCCAAGACGTAACCGGTACCGCATTCTCCTTCCTTT
CTCCCTTCTCCC
+
DDC:D<;<;CB@DBBBCBBB??;97116.7??BC>A???@>8738;=@@@@@BB>A=@==8:4487:;B3859>@>:
??>@;@699DH<
@T6XXI:00015:00383
GGGAGAAGGGAGAAAGGAAGAGAACGGCGACGAACAATAGTTGGTGGCTATTCTCCCTCCTCCCTATAGTGAGTCGT
+
44-4=><>B>999@19442377719;;@@@@?>==8444=878<;;;;;;;576677110111,1043336655583
@T6XXI:00016:00361
GGGAGAAGGGAGAAAGGAAGCCAGTCGACTCGACATCTGTGGCTTCACAGTACAGAGGGACTGTCCAAAGCCGCAGA
TCTCGAGTCGACTACG
+
```

:@:999244-444:-
42477?=A@A:::>>77<@=ABBBBEABB@BCBBCF@@>@888/8??BCB>AB=CD@BAAAA@>=;8;69;94;7//
.
@T6XXI:00016:00363
GGGAGAAGGGAGAAAGGAAGCTAGTCGACTCGACATCTGTGGCTTCACAGTACAGAGGGACTGTCCAAAGCCGCAGA
TCTCGAGTCGACTAG
+
CCACDB@BA=BCBB?B>?=AA@?@@:::????:::::ABD@BB@BCBAAAA@@>??@7>?????>AA;@@=<<;;<
;;<?>?<==@899?=
@T6XXI:00016:00380
GGGAGAAGGGAGAAAGGAAGGAGAAGTCGCCCCAAACGATTTGTTGACTATTCTCCCCTCTCCCTATAGTGAGTCGT
ATTCAG
+
9@694A>AB@CKCCAC@B=@=@@@D??@CBBB8BB:@8868266699<644430000+0444=;=9998<8?8883+
++*++.
@T6XXI:00017:00358
GGGGAATTCTAATACGACTCACTATAGGGAGAGGGAGAATAGTCCGCCAATTCTGCGCGTTAGCATTCTCCTTCCTT
TCTCCCTTCTCCC
+
BDC:C@B@C??=ECCCCCDAA7:7=6776:BBCE:A=B=@9@CC?884:=A7::BBBBBBACBB?@<;<<3737377
5:BBD@C9<844-
@T6XXI:00017:00362
GGGGAATTCTAATACGACTCACTATAGGGAGAGGGAGAATAGTCAACAAATCGTTTGGGGCGACTTCTCCTTCCTTT
CTCCCTTCTCCC
+
CCC9D@CACCB@BABBBBBBA@>>966959??BB>BAA>A?@@AA?===9?CCDE>BBB7AAAAA=A?A;=8:/44-
4;;=:@;<;<@:
@T6XXI:00017:00363
GGGGAATTCTAATACGACTCACTATAGGGAGAGGGAGAATAGTTTCGTAGCACTTGACCTACCCCGAACTT
+
FEC9B6;8<@@@CAAABCCBCAB?>777.7?==?=@?CCD?;;:27/-+++++-4544545333+00/044
@T6XXI:00017:00366
GGGGAATTCTAATACGACTCACTATAGGGAGGGGGAGAATAGTCACATTCATGTTTGTGACCGTGTCTCCTTCCTTT
CTCCCTTCTCCC
+
CDH:C=A=ACDACC@?ABBCB?AAB99946==?A2BCC@@<=@CCAA?;=?BA?A<AA???>@BA???@>A>A>@@<
@A<A76:<;;;6
@T6XXI:00017:00377
GGGAGAAGGGAGAAAGGAAGGAGAAGTCGCCCCCAAACGATTTGTTGACTATTCTCCTCTCCCTCATAGAGTGAGTC
GATTCATGAATAC
+
CE>AAA;==9>>>>9A>AACB?>>=@/000000*..+.447@;@CA@C@<@@8:992400944.------
1334444658/0----0.--
@T6XXI:00018:00350
CTAGTCACTCGAGATCTGCGGCTTTGGACAGTCCCTCTGTACTGTGAAGCCACAGATGTCGAGTCGACTAGCTTCCT
TTCTCCCTTCCTCCC
+
E>111;8=CECBBBC;<<CC@BCC?B@DB;::?=.77=?=?B>0./=7:CACCCC@@?@@@???@>>?;:7667701
61944533,/,+000
@T6XXI:00018:00354
CTAGTCACTCGAGATCTGCGGCTTTGGACAGTCCCTCTGTACTGTGAAGCCACAGATGTCGAGTCGACTAGCTTCCT
TTCTCCCTTCTCCC
+
B=111>>?BCCBBBBDBBCC=@@B=B>@@??77:5;BBABBAA?AAB9<@@CCCBAA@@?BBB@AABCCB<;:?C@C
D>DBCC>@><<<@;
@T6XXI:00018:00355

```
GGGAGAAGGGAGAAAGGAAGCTAGTCACTCGACATCTGTGGCTTCACATACAGAGGGACTGTCCAAAGCCGCAGATC
TCGAGTCGACTAGCTTCCTTTCTCCCTTCTCCC
+
:A:=?A=>A?>111-
/,/,...664<7,,,,,,,03533666847777>8666111,11005658>;585449445433663339:300444
/05344>865352336=;
@T6XXI:00018:00368
GGGGAATTCTAATACGACTCACTATAGGGAGAGGAGAATAGT
+
800*-+9249=@CDCCC?@@B???????<?BB?=A//,/,)(
@T6XXI:00018:00397
GGGGAATTCTAATACGACTCACTATAGGGAGAGGGAGAATAGTCAACAAATCGTTTGGGGCGACTTCTCCTTCCTTT
CTCCCCTTCTCCC
+
/1/*/,/,///,448>A;;;B:::A???:????766888;;99443433+67611,177,66=6627167423234+
00000+0438;@;
@T6XXI:00019:00379
GGGAGAAGGGAGAAAGGAAGCTAGTCGACTCGGACATCTGTGGCTTCACAGTACAGAGGGACTGTCCAAAGCCGCAG
ATCTCGAGTCGACTAG
+
CD@BBCEDDAABAH=B>@=:88:::0..744=;>?A???@::901,.79>=??99:444-
49944028846=8:<???C?999@?><<<?===
@T6XXI:00020:00357
GGGGAATTCTAATACGACTCACTATAGGGAGAGGGAGAATAGCTACGTTTAACCGATCGTGTTGTTCTCCTTCCTTT
CTCCCTTCTCCC
+
@?;6==A=ACC@CCB@;;<B?=??=77767?BCK@C??=??AAA@??BB6;632003;?@A@;=820032323/00+
000438563343
@T6XXI:00020:00360
GGGGAATTCTAATACGACTCACTATAGGGAGAGGGAGAATAGTTTGTACATCGCCCCGTGCAACATGCTTCTTCCTT
TCTCCCTTCTCCC
+
DEKAE?CA<<<@CBBAA@@@@???=776,16777;744999===:=<403<61116,1667>=911110/49445>:
::??A<A?===A?
@T6XXI:00020:00365
GGGAGAAGGGAGAAAGGAAGCTAGTCGACTCGACATCTGTGGCTTCACAGTACAGAGGGACTGTCCAAAGCCGCAGA
TCTCAGTCGACTAG
+
DG@ECB@BB>BBBB?D=?=>>@??????@CA?::;;;:@BC8<;@DCCBBACAAA<??:??A889?AA<@A@@@@A@
@@@@A@@???AA==
@T6XXI:00020:00367
GGGAGAAGGGAGAAAGGAAGCTAGTCGACTCGACATCTGTGGCTTCACAGTACAGAGGGACTGTCCAAAGCCGCAGA
TCTCGAGTCGACTAG
+
::;<BB?BB>BBBC=B?B>ACB////11<>>;1::::6>AC8:8@CCCCBBB?==43317<8<=?<??<?C>AAAA@
@@AA@?@B@@99:A@
@T6XXI:00020:00386
GGGAGAAGGGAGAAAGGAAGCTAGTCACTCGACATCTGTGGCTTCACAGTGGACTGTCCAAAGCCGCAGATCTCGAG
TCGACTAG
+
BE@CCA@BB>BBBB<?=?>??@AABC@>6>>11/:77>7B@A@??DCBB??;??;;<==@C<??>AAAAD?????AB
@@@CAA@=
@T6XXI:00021:00357
GGGAGAAGGGAGAAAGGAAGGAGAAGCGCCCCAAAGATTTGTGACTATT
+
44-44;249-4444-/68,.,...644488807963317,111113.-,
@T6XXI:00021:00361
```

```
GGGGAATTCTAATACGACTCACTATAGGGAGAGGGAGAATAGTCAACAAATCGTTTGGGGCGACTTCTCCTTCCTTT
CTCCCTTCCTCCC
+
CDD:D@B@BCC@CA@AB@@@BB??=777-
76=<<6?6<8<===>C:<=@7???BC<BCC7BAAAK@CBB@D>A>AA68/8828,/,/881
@T6XXI:00021:00378
GGGAGAAGGGAGAAAGGAAGCTAGTCGACTCGAGATCTGCGGCTTTGGACAGTCCCTCTGTACTGAAGCCACAGATG
TCGAGTCGACTAGCTTCCTTTCTCCCTTCTCCC
+
GDANAAAAB?>111-
1,/,.44...0...//7:>BC@BBBB?CCC>B?AAABBCC>@=<<><66;:::<454;6646344994//11///44
2424;6444>-424444-
@T6XXI:00021:00385
GGGAGAAGGGAGAAAGGAAGTGCAGTTCGCGGTTAGTGAAGGGAAAACTA
+
/8+//:799-8444-49;77746///,/668999?7339111,.550...
@T6XXI:00021:00395
GGGGAATTCTAATACGACTCACTATAGGGAGAGGGAGAATAGCTCCACAATTTCTAATGCCCGACCTGTTCTTCCTT
TCTCCCTTCTCCC
+
CCB7B8;;=BB@GCBBBBCCA??>>777.7==>A:@>=;????CEEBA?>CC@CCC@CAAA<A?>=@::7:A=@;>=
7;<::4:288>@:
@T6XXI:00021:00397
GGGGAATTCTAATACGACTCACTATAGGGAGAGGGAGAATAGTTGTCTCCTGATCTGTGGCGCACTATCCTTCCTTC
TCCCTTCTCC
+
ACC:CBC?A@B=@ABCA@@@;:<@?777.7777@9:4773337:==<@@>@?<?<?=>82848;@B@;75438,/,-
..20100002
@T6XXI:00022:00364
GGGAGAAGGGAGAAAGGAAGCTAGTCGACTCGACATCTGTGGCTTCACAGTACAGAGGGACTGTCCAAAGCCGCAGA
TCTCGAGTCGACTAG
+
AA:B<;7<B<ACCBAC:<:;A?<<>0//7///.1111168>279>9969961489;986;889A@>AC;??599CC8
99?<?=88836=6;;
@T6XXI:00022:00369
GGGAGAAGGGAGAAAGGAAGCTAGTCGACTCGAGATCTGCGGCTTTGGACAGTCCCTCTGTACTGTGAAGCCACAGA
TGTCGAGTCGACTAGCTTCCTTTCTCCCTC
+
CA:A;<;;B<BBCCAB=@=@@?;><0.....455688ACCC@CCC<A8:9@@>@B>884=?>>A@???A@@>?988<
<<<8?????<<<===9641+,,+,+00+,+
@T6XXI:00022:00372
GGGGAATTCTAATACGACTCACTATAGGGAGAGGGAGAATAGCTACATCCCTCCTGCAATGCACCTGTTCTTCCTTT
CTCCCTCTCCC
+
CFG:CCC@CCC@CCC@A@BBB@>>>777.777BC?A?@>AAA:;:C?<::444233=;8;;=;;8;=:8=>;>;=99
/.--+--1110
@T6XXI:00022:00379
GGGAGAAGGGAGAAAGGAAGCTAGTCACTCGACATCTGTGGCTTCACAGTACAGAGGGACTGTCCAAAGCCGCAGAT
CTCGAGTCGACTAG
+
HI@CCB@CB:=@@=9>=@=@CCBBC:;:::A????==BBCACC?BBC???A?>;;;;6;>>?@@?@C<AA????@@?
>???>>>?=;;;<;
@T6XXI:00022:00390
GGGGAATTCTAATACGACTCACTATAGGGAGAGGGAGAATAGCAAACGTTTCCCCGACGTCGCTTTCTCCTTCCTTC
CTCCTTC
+
```

```
>99,95924?A>A@@@@A@@@CCCCBAA=A???D:@>?577=<A@:?CC>;BBC7BBA???:::E=>>E=511+,+*
)**)*)+
@T6XXI:00022:00393
GGGGAATTCTAATACGACTCACTATAGGGAGAGGGAGAATAGCTCCGACGTTTTAGCCTTCGTGTTATTCCTTCCTT
TCTCCCTTCTCCC
+
=DJ9A>AIEED>BBCCCCCCC::8?777.7>>>@=A>><????BB>?==7444-449799;934-0-.,-,-,-
0110--04+32486=;
@T6XXI:00023:00352
GGGAGAAGGGAGAAAGGAAGCTAGTCACTCGACATCTGTGGCTTCACAGTACAGAGGGACTGTCCAAAGCCGCAGAT
CTCGAGTCGACTAG
+
DDA=<<:<<1;A@@:B=>;>>>=76:9677?6611111..5..,.447111104030+00000324=;<@<ABB?><
8878644...7748
@T6XXI:00023:00354
GGGAGAAGGGAGAAAGGAAGGGAGAAGTCGAGTCCGGAAAC
+
EDBB@@=@A<A@@>9>59499083977133333101049-4
@T6XXI:00023:00361
GGGAGAAGGGAGAAAGGAAGCTACGTCGACTCGACCATCCTGTGGCTTCACAGTACAGAGGACTGTCCAAAGCCGCC
AGA
+
99-4;?<AB=BBBC>C@C=?;=000=::7;/..00,...,..--
24@2666=9?;<>@<<9;?;4442//,/6,//,-77
@T6XXI:00023:00367
GGGGAATTCTAATACGACTCACTATAGGGAGAGGGAGAATAGCC
+
BFD:B8;:9?@5;9C<<<DGCC@@===?7=?777-77779+**.
@T6XXI:00023:00385
GGGGAATCTAATACGA
+
188*/,////,/4466
@T6XXI:00024:00371
GGGGAATTCTAATACGACTCACCTATAGGG
+
>>929899844988444?70..,.....5+
@T6XXI:00024:00374
GGGAGAAGGGAGAAAAGGGAAAGCTAGTCG
+
/857//,/85//444,44-44-33344...
@T6XXI:00024:00382
GGGAGAAGGGAGAAAGGAAGCAGAACAGGCAGCTGGAACACAACAGCCTATTCTCCCTCTCCCTATAGTGAGTCGTA
TTA
+
DH@ECC9;;8;AAB@B@CACCCB>>B?@?ACBBBA?BEECCC?A?8:4:B@:??@@<??FBB>?A=99=43.41-
+**)*
@T6XXI:00024:00392
GGGGAATTCTAATACGACTCACTATAGGGAGAGGGAGAATAGTTTTTCCCTTCACTAACCGCGAACTGCACTTCCTT
TCTCCCTTCTCCC
+
HKK9C@B?ACB@BBBABABBA@>?96695:>@CD>C@A?A;73774,39:?>@BCCCAB>?@@A?AA@??B??A:??
<?@@B<A=<<<<6
@T6XXI:00024:00394
GGGAGAAGGGAGAAAGGAAGCTAGTCGACTCGACC
+
==:?CDFGDABBBC?D@B@CF:83111..,,,,,*
@T6XXI:00025:00358
```

```
GGGAGAAGGGAGAAAGGAAGCTAGTCGACTCGAGATCTGCGGCTTTGGACAGTCCCTCTGTACTGTGAAGCCACAGA
TGTCGAGTCGACTAGCTTCCTTTCTCCCTTCTCCCCTTCCTTCTCCCTTCCT
+
EC@C<<9<B<ABBA>A9;68@>77:;::AAACEA@@?C?==;777.7@?B?@?CB>888>====;<<<<???A@@?@
@A@?>>8886<==>?872479:-44::4<9<9:?;1846,/,.,,,+/,/,-
@T6XXI:00025:00368
GGGAGAAGGGAGAAAGGAAGGAGAAGTCGCCCCAAACGATTTGTTGACTATTCTCCCTCTCCCTATAGTGAGTCGTA
TTAGAATTCCCCC
+
CEDCCC@BD>ABBCBB?B?B?BEC>@@CBBBB7BDACBBBD>CC?BBB?@:9:8885///88689<?D@?@A988=;
;896444/0034,
@T6XXI:00025:00375
CTATCGACTCGAGATCTGCGGCTTTGGACAGTCCCTCTGTACTGTAAAGCCACAGATGTCGAGTCGACTAGCTTCCT
TTCTCCCTTCTCCC
+
C==999:???@BFECCBBBA@CCC>CBA@@@:7:5:A@?AACCB@?A>AB@BCB;:;;::BB::=CCCCCBAA4858
>8>==A<B?BBCE<
@T6XXI:00025:00379
GGGAGAAGGGAGAAAGGAAGCTAGTCGACTCGAGACTGCGGCTTGGAC
+
9?><<;=>>:>999049;99;337176666>>@?87696601101016
@T6XXI:00025:00386
CTATCGACTCGAGATCTACGGCGTTCT
+
49944444:>A@@@@@-,,2*,,,*,,
@T6XXI:00025:00395
GGGGAATTCTAATACGACTCACTATAGGGAGAGGGAGAATAGTCAACAAATCGTTTGGGGCGACTTCTCCTTCCTTT
CTCCCTTCTCCC
+
CEC:C@A>ADBFBAA@@@AACBCDB:::5:@BCC<A>@=ABAA>><@AD<CABAB<@@@4::AAA?ABD?A<??A@7
=844-3284@D?
@T6XXI:00026:00353
GGGAGAAGGGAGAAAGGAAGATGGCGTTACGTAAAGGAGAATGATGCACTATTCTCCCTCTCCCTATAGTGAGTCGT
ATTAGAATTCCCCGGAGAAG
+
CC>BAA>BB>BCCC@C@C@DEBB@CCB=@>8;;@<?@B@@?@BAA>B@@ACC@DGBB=?>>@A<A?::CBBBBBBCA
===@;8,/,---*-,-33,-
@T6XXI:00026:00357
GGAGAAGGAGAAAGAAGCC
+
489442424444-442331
@T6XXI:00026:00366
GGGAGAAGGGAGAAAGGAAGCTAGTCGACTCGACATCTGTGGCTTCACAGTACAGAGGGACTGTCCAAAGCCGCAGA
TCTCGAGTCGACTAG
+
AA<ACC@DC?C>=85=4848>337@888ACCB:::???ACC=A@@BBCB@BBAA<;;;6;===AA<??<?@?@@@@@
@???@HAA@?==>C@
@T6XXI:00026:00373
GGGAGAAGGGAGAAAGGAAAGGAGAAGTCGCCCCAAACGATTTGTTGACTATTCTCCCTCCTCCCTATAGTGAGTCG
TATTCAGA
+
CD?A@@=@@9;;>?:@611+066.0,.......*.407167@=A@@@8100-,-2222--,-
2322465666:::95331,----
@T6XXI:00026:00377
GGGAGAAGGGAGAAAGGAAGCTAGTCACTCGACATCTGTGGCTTCACAGTACAGAGGGACTGTCCAAAGCCGCAGAT
CTCGAGTCGACTAG
+
```

```
CCACCC?B?:=?=?=B?B?BC@:?B://>>>00////;>B<AB@CFCCBBBCA@;<?7<<?===8;;6;?<@@@@@@
@@AA@@A@@@@@??
@T6XXI:00026:00394
GGGAGAAGGGAGAAAGGAAGCTAGTCGACTCGACATCTGTGGCTTCACAGTACAGAGGGACTGTCCAAAGCCGCAGA
TCTC
+
CC>AAA<>>:999>9@>B>AAACCBBC@??@@:::;;:???8:;8:BBABB@;:8433-
4;;?@A>BA=AD@===A?889@
@T6XXI:00027:00350
GGGGAATTCTAATACGACTCACTATAGGGAGAGGGAGAATAGAGACGTATTCGCTTGCGCTCTGTTCACCTTCCTTT
CTCCCTTCTCCC
+
CHH9B=A>@AA@FFCCB?@@@?@A<??@:>>???<?@@>@@@BC@@@@??=?AAA>A@A@@?8888<;;;>:<9;B9
;444-48<;<C<
@T6XXI:00027:00366
GGGAGAAGGGAGAAGGGAGAAGGGAGAAGGGAGAAGGGAGAAGGGAGAAGGGAGAAGGGAGAAGGGAGAAGGGAGAA
GGAAGAAAGGAAGGAGAACG
+
?A>BBB@CC@GDB@CC=<<<?BD?DBB:??9:99:AC>::8388-
4;;/4717;9;@@7==@7894487:=B:;40/023:=AA;A=;9;9,++*++
@T6XXI:00027:00383
CTATCGATCGAGATCTGCGGCTTTGGACAGTCCCTCTGTACTGTGAAGCCACAGATGTCGAGTCGACTAGCTTCCTT
TCTCCCTTCTCCC
+
BDAA:9:CDCBBBBBBAAA?CGG>C?CCCB???6777=>7700./578C@DBHAAABA@@BA@AA<<9BA@<<@<>>
/81666<:<116-
@T6XXI:00027:00392
GGGGAATCTAATACGACTCACTATAGGAGCAGGGAAGAATAGTCGCCGATATCCCAATGGCCA
+
8@818+944488:AAC;;;?733666366889;77663/0030003/0166644+0/00/101
@T6XXI:00028:00377
GGGGAATTCTAATACGACTCACTATAGGGAGAGGGAGAATAGATTCGCGATTCCCTGCACTGGACTATTCTTCCTTT
CTCCCTTCTCCC
+
ABC/969299954=>B@@@@B@??>777.777?A>A??<???@@=@CA?@@677.334377;;;;;;;:;6770191
:<=?6<37111,
@T6XXI:00028:00389
GGGAGAAGGGAGAAAGGAAGGGAATAGCAGCAGTCGGCAACGTGAAACTATTCTCCCTCTCCCTATAGTGAGTCGTA
TTAGAACTTCCCC
+
;C6444999-44990499>@?;?57767337>>>=A6;7478877;8@@CB=>767-
7>==?86?????AAA===AAD;<<A<AA2000+
@T6XXI:00028:00390
GGGAGAAGGGAGAAAGGAAGCTAGTCGACTCGACATCTGTGCG
+
99-9998=B?<;=CAEDCABCBBA??<BBA@::6...3,,,0*
@T6XXI:00029:00357
GGGGAATTCTAATACGACTCACTATAGGGAGAGGGAGAATAGTCTCCCCGTTAGCCCTC
+
AC@8@=?=>@?@?BBB@??A??>>=77:4:>?AB4:7837;;9:0034-0342---+-3
@T6XXI:00029:00381
GGGAGAAGGGAGAAAGGAAGCTAGTCGACTCGACCATCTGTGGCTTCACAGTACAGAGGGACTGTCCAAAGCCGCAG
ATCTCGAGTCGACTAG
+
BC<@A@=@@<@@==9>896;@>>?D:;1>>>;00,....?:B5:9C???AAA@>;;444-
4;;889>@AAAA>AAAA@@@@@=<<@@889@@@
@T6XXI:00029:00397
```

```
GGGGAATTCTAATACGACTCACTATAGGGAGAGGGAGAATAGTCAACAAATCGTTTGGGGCGACTTCTCCTTCCTTT
CTCCCTTCCTCCC
+
@?>2=296<@@@CDDDCCC@AA@?6677.7==@@<B<<9=@@@AC@@@@<BCBBC>CCB8BBBAA=??C7869>@A<
A;;828,/,/872
@T6XXI:00030:00367
GGGAGAAGGGAGAAAGGAAGCTAGTCGACTCGAGATCTGCGGCTTTGGACAGTCCCTCTGTACTGTGAAGCCACAGA
TGTCGAGTCGACTAGCTTCCTTTCTCCCTTCTCCC
+
CC:?@?<>@:?=>>9><@>?@?>@@:66:889????<<@>>=::9,169788BCD>A<<;8444403::==;=>>88
8=843>;7434433044/4/34,40330588:<>4
@T6XXI:00030:00370
CTAGTCACTCGAGATCTGCGGCTTTGGACAGTCCCT
+
///9>>6,,,00,**,,/6434498<53371111,.
@T6XXI:00030:00373
GGGGAATTCTAATACGACTCACTATAGGGAGAGGGAGAATAGATCACGACCCTTCTACCCACGA
+
9>?4?595944<=@BCB<;;B@A???@?9:::??:744/00000388=@??;;011111,---+
@T6XXI:00030:00392
GGGAGAAGGGAGAAAGGAAGCTAGTCGACTCGAGATCTGGCGGCTTTGGACAGTCCCTCTGTACTGTGAAGCCACAG
ATGTCGAGTCGACTAGCTTCCTTTCTCCCTTCTCCC
+
DC>B<;7<@:A@@>9>59588894:0..5565>>CCE@?=?A5::BBBBCBBAAAC<<;@788?=<<=A>AC>@@A@
???=<<<<<<AA@?@?<<8;99=6788@:@=@@@B;
@T6XXI:00030:00398
GGGAGAAGGAGAAAGGAAGGAGAAGTCAATGATACCGCA
+
<>,---*,**000*,*/54349::=<>>;>00.,*),,,
@T6XXI:00031:00351
GGGGAATTCTAATACGACTCACTATAGGAGAGGGAGAAA
+
GFC:C7;:<=@<@ABCA;;88788117366111,1333+
@T6XXI:00031:00367
GGGGAATTCTAATACGACTCACTATAGGGAGAGGGAGAATAGACACGCTCCCTTAATCACCTTCATTGCCTTCCTTT
CTCCCTTCTCCC
+
DHH:C>@AACC@CCBBAABBBBCB9161.7>=?A>AAA>@@@@;@?AA@@A=A=@>@?;;8;889;9::7:7:7::4
9<@A;@<@=<<7
@T6XXI:00031:00369
GGGGAATTCTAATACGACTCACTATAGGGAGAGGGAGAATAGTCTCCTTTCTCCATGTCTATCTACATTCTTCCTTT
CTCCCTTCTCCC
+
CDIBD::;;DC@CBAABABAA@ABB???=?CCCC;?>A=A=:=:??8;<7<?>?AA===?@A?==9ACC@@=?<?>/
:8>>:>=>777.
@T6XXI:00031:00370
GGGAGAAGGGAGAAAGGAAGGGGAGAAGGGAGAAAGGAAGGGGAGAAGGGAGAAAGGAAGGGGAGAAGGGAGAAAGG
AAGACTAGTCGACTCGACATCTGTGGCTTCACAGTACAGAGGGACTCGTCAAAGCCGCAGATCTCGAGTCGACTAG
+
AA?ACDACC@<<<B<@?@5888.888>??:::;7.7364777-7<879;:444:6;5?@DCD4=8834;-
480:67377/-
.44<71.////.,,,,,..4589@@?A@@@@@@@@?10.+..,,,,,,,003<<;<?;76766;;<:?B9<996
@T6XXI:00031:00372
GGGGAATTCTAATACGACTCACTATAGGGAGAGGGAGAATAGTGACGTGTTGTGCGGCGCTCACTGTCTCCTTCCTT
TCTCCCTTCTCCC
+
```

GCC9C@B>@BB@ACBB@@@CCC??;676.7==?B?C@A@AAA@@@@@???>@@?@@>A==@@777;7448349;7::
4:344-37;;?A:
@T6XXI:00031:00377
GGGGAATTCTAATACGACTCACTATAGGGAGAGGGAAGAATAGTTCT
+
:::5B1969=>;=BCCDDBBCCCB?@?C>=:::<,0,/97//.-,--
@T6XXI:00032:00359
CTAGTCGACTCGAGATCTGCGGCTTTGGACAGTCCCTCTGTACTGTCGAAGCCACAGATGTCGAGTCGA
+
//////69444449;;;8999==;>69,0......+.253...33100483464;88333773111//2
@T6XXI:00032:00360
GGGGAATTCTAATACGACTCACTATAGGGAGAGGGAGAATAGATCTCTCCTCAGACAATAACGCGCAATTCTTCCTT
TCTCCCTTCTCCC
+
EED:C<:<<CDADCBBBBCBBBBA?97757>@CC>CAA?@@?>>>>8809;98/-
0.,.5571007739=4713779,:7999A888@C;
@T6XXI:00032:00370
GGGAGAAGGGAGAAAGGAAGGAGAACGCACACCG
+
:C>B?A>BB=BCCC?B?B@BCCBB9<1<11,*)*
@T6XXI:00032:00394
GGGGAATTCTAATACGACTCACTATAGGAGAGGGAGAATAGTCAACAAATCGTTTGGGGCGACTTCTCCTT
+
J;A0?-1,///,/844477<>;771113;;7330.--,-..448<<>=;@8767,116-64557,..3,-,
@T6XXI:00033:00363
GGGAGAAGGGAGAAAGGAAGCTAGTCGACTCGACATCTGTGGCTTCACAGTACAGAGGGACTGTCCAAACCGCAGAT
CTCGAGT
+
ED?<<;=@@?@9;;09=@68;;8>=?:::?AA::::@==??5::?B??:;:74;;444-
4444>??@A7<9884;;488<:@@@
@T6XXI:00033:00371
GGGAGAAGGGAGAAAGGAAGGAAGGGAGAAAGGAAGCTAGTCGACTCGACATCTGTGGCTTCACAGTACAGAGGACT
GTCCAAAGCCGCAGATCTCGAGT
+
CG?ABB@CC@<=<C>B@C@B>C=@@=@DFC>C@C8;;CGCCBF@@BBA:::::7A@@:A?>@===AA@;77//./99
;899==;<A>@H@??878;=>??
@T6XXI:00033:00387
GGGGAATTCTAATACGACTCACTATAGGAGAGGAGA
+
BCA/<59?A=@=ABBBCA::;@>;;:740>0,+,..
@T6XXI:00034:00364
CTAGTCACTCGAGATCTGCGGCTTTGGACAGTCCCTCTGTACTGTGAAGCCACAGATGTCGAGTCCGACTAGCTTCC
T
+
111////////////68431333,3=<<@@=??=?C???DDB?<55:3;;9BCB;;;B>=::01,/...473333,
.
@T6XXI:00034:00394
GGGGAATTCTAATACGACTCACTATAGGAGAGGGAGAATAGTCAACAAACTC
+
A>B5>,/,/66,/4>;;7?>>5664..,.4.11,6668<A88744400*+++
@T6XXI:00035:00352
GGGGAATTCTAATACGACTCACTAATAGGAGAGGG
+
444,42424;;2499449933...,...,...6>>
@T6XXI:00035:00365
GGGGAATTCTAATACGACTCACTATAGGGAGAGGGAGAATAGTCAACAAATCGTTTGGGGCGACTTCTCCTTCCTTT
CTCCCTTCTCCC

+
DIFCC?A?ABB@CCA?@BBCGCA?>776.7==?A?C@@?DAAA??=AAC=CA?AC?BBB8CCC?B?>AA5838=@B<
???C?C8:5@A7
@T6XXI:00035:00387
GGGGAATTCTAATACGACTCACTATAGGGAGAGGGAGAATAGTCAACAAATCGTTTGGGGCGACTTCTCCTTCCTTT
CTCC
+
ACC5;8<@BCCBCDCC<;;AA7887777,7<>=79?;97;;:<=?485=;;A:::167=2@?;::9:00,-
01523+-000
@T6XXI:00035:00389
GGGGAATTCTAATACGACTCACTATAGGGAGAGGGAGAATAGTTTTTCCCTTCACTAACCGCGAACTGCACTTCCTT
TCTCCCTTCTCCC
+
CEK:C?A?BED@CBBBBBAAA;997166.7>==?=@HC@AA;;;;;0;A<A>>BCCE>?>=BAC???@=???=@377
.7==>:>:;<?@;
@T6XXI:00036:00375
GGGGAATTCTAATACGACTCACTATAGGGAGAGGGAGAATAGTCAACAAATCGTTTGGGCGACTTCTCCTCCTTTCT
CCCT
+
8?80?+1,///7849;<;???;9<;767,7<777-
:AA8:?>:00/000+11111,17288<@@:88>20020494222+.
@T6XXI:00036:00390
GGGGAATTCTAATACGACTCACTATAGGGAGAGGGAGAATAGATCACCGTTGGTTCGGGTGCGCGTTCTCCTTCCTT
TCC
+
DKK9B=A=@@@?BA@AA<;<CBCBA???9=?@@@<A@@>@@<;<=::3672627>@@B=@@;@@9=88..,.,,*,2
,
@T6XXI:00037:00387
GGGGAATTCTAATACGACTCACTATAGGGAGAGGGAGAATAGCCTGTCGCTTATGTGATTTGTTGTTCTCTTCCTTC
TCCCTTCTCCC
+
///*/,/,49;8=>C@A@B@@??><777-7=;=>9?1278/.-,.75>6@<=????@@AH<@@>@A=8://,/,/,-
2445;;AAAA=
@T6XXI:00037:00390
GGGAGAAGGGAGAAAGGAAGCTAGTCGACTCGAGATCTGCGGCTTTGGACAGTCCCTCTGTACTGTGAAGCCACAGA
TGTCGAGTCGACTAGCTTCCTTTCTCCCTCTCCC
+
BD>BAGBCB:@;9909;>;?@>;;:0.//..;=>AB==@CC@CBC>C?BBBBBCC>AA<88887==??=?A>@@@AA
A===?@888<844;44488,//,/-13,.----+
@T6XXI:00038:00369
TGGACAGTCCCTCTGTACTGTGAAGCCACAGATGTCGAGTCGACTAGCTTCCTTTCTCCCTTCTCCC
+
BF=<:CCBCC@CDCCAAAC@AAC@BB@@@CB@?@ABCCBCBDCCCB>?=@CADD;<<DC<@=?AAB:
@T6XXI:00038:00383
GGGAGAAGGGAGAAAGGAAGCTAGTCGACTCGACATCTGTGGCTTCACAGTACAGAGGGACTGTCCAAAGCCGCAGA
TCTCGAGTCGACTAG
+
CD@CCB@B?:=@>>:@>@>AA<;;;/00:;;@1::???BCCABC@CC????A==934417>>@@?=?B<@@<?@===
=9==???@???@@@@
@T6XXI:00039:00356
TCTAATACGACTCACTATAGGGAGAGGGAGAATAGTCAACAAATCGTTTGGGGCGACTTCTCCTTCCTTTCTCCCTT
CTCCC
+
?AA@CBCBBECBBAB99<88807>777,7==9>B==@CBBBC=@<;@@6;<<4<=>>B9<<<8;9;;=@7<<@A?B?
@;;;6
@T6XXI:00039:00360

```
GGGAGAAGGGAGAAAGGAAGAATATTACGTTTTCGGAGTGTACGGTGCTATTCTCCCTCTCCCTATAGTGAGTCGTA
TTAGAATTCCCC
+
=D>BBB?AA8;49@082897717@=77?8788,7A;??@BBBBC@CDDD??=??<B7=><<;36?>?@A<<=@:66/
/,.1646;==C5
@T6XXI:00039:00386
GGGAGAAGGGAGAAAGGAAGAGAACAGTTGCGCCTAGTAGTTAGTAACTATTCTCCCTCTCCCTATAGTGAGTCGTA
TTAGAACTTCCCC
+
DCACCCAAA=>@@C<@=@@CCCC@B??BACCDD@ABBBC;:5:?BB@CCBA=????<AB@@@>AAC=88=@@@B=9=
A:944--.,---*
@T6XXI:00039:00389
GGGGAATTCTAATACGACTCACTATAGGGAG
+
88802*/,/66789;;@>>>?<77;666,/,
@T6XXI:00040:00379
GGGGAATTCTAATACGACTCACTATAGGGAGAGGGAGAATAGTTTGCGCATTCGGTGCACAGTTATTCTCTTCCTTT
CTCCCTTCTCCC
+
BBA9>;==ACCBCBBABABBAA>?=777.7==BD>DDCB@?<<65;@@=AA@CCAC@?@ACEC@BB?ABAA=>4871
79;B6;8;<<<6
@T6XXI:00040:00383
GGGAGAAGGGAGAAAGGAAGCTAGTCACTCGACATCTGTGGCTTCACAGTACAGAGGACTGTCCAAAGCCGCAGATC
TCGAGTCGACTAG
+
FDABBC@EDABB=A647;;>?@AABCD:::@::6888>998:;7:DBBAAC=88..-
/8::;A@AA9====<<@A<==A===@@?>??>>
@T6XXI:00041:00383
GGGGAATTCTAATACGACTCACTATAGGGAGAGGGAGAATAGTCAACAAATCGTTTGGGCGACTTCTCCTTCCTTTC
TCCCTTCCTCCC
+
B?B7B698;AB@CBBBBBBBA<@<;677.7?===9???=@@CA@@<??A9??BBB<BC>BBBBB<??A<;8<;>>:>
///,.,/,/882
@T6XXI:00043:00380
GGGGAATTCTAATACGACTCACTATAGGGAGAGGGAGAATAGTTCGACCAA
+
CKH:D>;<<BB@BCCBBBCBBCBB=76757?BCE>B@A=>40334443/*.
@T6XXI:00043:00386
GGGAGAAGGGAGAAAGGAAGCTAGTCGACTCGACATCTGTGGCTTCACAGTACAGAGGACTGTCCCAAAGCCGCAGA
TCTCGAGTCGACTAG
+
CC@DBA?@B<>@==9>594883/.4/.,,,0,,.....4.1017761169?@====?<AA=99==<@@:<;55;844
48887?@@@=A@@;;
@T6XXI:00044:00351
GGGGAATTCTAATACGACTCACTATAGGGAGAGGGAAGAATAGTCAACAAATCGTTTGGGGCGACTTCTCCTTCCTT
TCTCCCTTCCTCCC
+
4::2:5959>>;@A@@@?A:;7@?>777-0<000,0,-
2222983323683::?>>88885?>>>?476<33/4:>>:A=<A<=9=:;=B4
@T6XXI:00044:00353
GGGGAATTCTAATACGACTCACTATAGGGAGAGGGAGAATAGCCCACACAGTTGAGGCGCACCTCCCGTCT
+
DHD:B898<AA?BCBCD?@@CA@<>777.77777.:>;9;:444-499@AAA<??@383448/0--+-3--
@T6XXI:00045:00351
GGGGAATTCTAATACGACTCACTATAGGAGAGGGAGAATAGTCAACAAATCGTTTGGGGCGACTTCTCCTTCCTTTC
TCCCTTCTCCC
+
```

```
DC@7@79:<=@?BBBBCCBCC>;;://,0<;>>=A@@>AAAAAA>@@B;BDCCC<???5?????;=?A6838=@@<@
AAC<A=@@@A;
@T6XXI:00046:00354
GGGAGAAGGGAGAAAGGAAGAAAATGTGCGAGTCGGAGAATTGGTGGCTATTCTCCCTCTCCCTATAGTGAGTCGTA
TTAGAACTTCCCC
+
DKBCCB===<@>>@9@;>;>AAA7AACCCCDBAAB@CCC@C@C@A@=AB@A?AADC<@@;990;B>?CCAAA@????
?:?=@39::??=5
@T6XXI:00046:00355
GGGAGAAGGGAGAAAGGAAGCTAGTCGACTGACATCTGTGGCTTCACAGTACAGAGGGACTGTCCAAAGCCGCAGAT
CTCGAGTCGACTAG
+
BGBCCC?BC>@@@B8;;<@DCA<<<BBFCBA@@@BBBBBC@BBBDBB@@?CBCA88868@@AAB=@@;@A???????
@@@A<<<;;;9996
@T6XXI:00046:00376
GGGAGAAGGGAGAAAGGAAGCTAGCTCGACATCTGTGGCTTCACAGTACAGACGGGACTGTCCAAAGCCGCAGATCT
CGAGTCGACTAG
+
KC@CCB@BD?CBBB=B>B??@@@DEECB@@@ABAAAB8<;=AAAAAA>A@?1111+18;=AB@BB=BB>AAB@AACB
?=>>88888611
@T6XXI:00047:00389
CTAGTCACTCGAGATCTGCGGCTTTGGACAGCCCTCTGTACTGTGAAGCCACAGATGTCGAGTCGACTAGCTTCCTT
TCTCCCTTCTCCC
+
C=1//669;AAABCCCCB@<<77708<?ABBCC>CCCDBBBBCA@?9=A?BBCDCCBBCCDD<>?CBABC<;:<@CC
@CDGD=?=??CB>
@T6XXI:00047:00390
GGGGAATCTAATGACGACTCACTATAGGGA
+
0220=+1///5/449@94444344....+.
@T6XXI:00049:00355
CTATCGACTCGAGATCTGCGGCTTTGGACAGTCCCTCTGTACTGTGAAGCCACAGATGTCGAGTCGACTAGCTTCCT
TTCCTCCC
+
418////678499?@CCFB?<88708488>@?B?;8:???AABA65550;79=@@D:::B?B?:<:<779;/.-
.444+.,.440
@T6XXI:00049:00363
GGGGAATTCTAATACGACTCACTATAGGGAGAGGGAGAATAGTTACACGTCTAACATCGTTCGGGTCGACTTCTCCT
TCCTTTCTCCCTTCTCCC
+
DDB6>6969@@>AA@ABCCCCCCBB991-
6<=;=6?<<88;6.)********+*++334::@<<36653004000/0/0/044344=;=555==;
@T6XXI:00049:00366
GGGAGAAGGAGAAAGAAGTGCAGTTCGCGGTTAGTGAAGGGAAAACTATTCTCCCCCTCCCTATAGTGAGTCGTATT
AGAATTCCCC
+
6<88-1-1-
,,,,*,/,.449=>A=:8:>4848A;;:@CC>BCD>C@?77611111+111,177;64449334988;=<;;;:;:?
5
@T6XXI:00004:00413
GGGGAATTCTAATACGACTCACTATAGGGAGGAGGAA
+
CEC9C?A=@AACCDCCA?@CB@BB>;::,0,+*0)*)
@T6XXI:00004:00422
GGGGAATTCTAATACGACTCACTATAGGGAGAGGGAGAATAGTCACATTCA
+
CCB9B=A?ACDBCBCB@@;;;AAA@ABA9==167-633/0000400-344.
```

```
@T6XXI:00005:00403
GGGAGAAGGGAGAAAGGAAGGAGCACTAGACGGCATTTCTCGCCAATCTATTCTCCCTCTCCCTATACGTGAGTCGT
ATTAGAACTTCCCC
+
DDBCDHANC?B<<<82A>:>=001:9:?BABB@D@BA<BB@BC?B?BCCBA>::7>7<<54424440335;8:=9><
<=<===;>>?AAB6
@T6XXI:00005:00427
CTAGTCGACTCGAGATCTGCGGCTTTGGACAGTCCCTCTGTACTGTGAAGCCACAGATGTCGAGTCGACTAGCTTCC
TTTCTCCCTTCTCCCCTTCCTTCTCCCTTCTCCC
+
CA;<<=9;CHDBB<<<@@@@B?BCC>C?CCAAABB>C@C@BC@@?A?A>AC@EDC@;@@:::?@@AB;;;A:71888
8?8?>BE>B:;////+/46,/,/-332221000,
@T6XXI:00005:00433
GGGAGAAGGGAGAAAGGAAGCTAGTCACTCGACATCTGTGGCTTCACAGTACAGAGGGACTGTCGAAAGCCGCAGAT
CTCGAGTCGACTAG
+
:C@CDBACC>BBBB?C@C@CCCC@@@B@BBCB@@@@@B>C?AA>AAABBBBB@><<<6<;;;44444-
44/04==?86566;7334424.-
@T6XXI:00005:00435
GGGAGAAGGGAGAAAGGAAGGAGAAGTCGCCCCAAACGATTTGTTGACTATTCTCCCTCTCCCTATAG
+
<;7;998@@6=@@@<@=@=?7;;>;=?:99BC6BB:87677,1.,...--023000+00000+03444
@T6XXI:00005:00437
GGGAGAGGAG
+
/1,1,,,*,,
@T6XXI:00005:00446
CAGTCGACTCGAGATCTGCGGCTTTGGACAGTCCCTCTGTACTGTGAAGCCACAGATGTCGAGTCGACTAGCTTCCT
TTCTCCCTTCTCCC
+
CCCBBCC;;<CCCCBC<>AB@>??>@@BBBCBBB>BBAABCCCCCCC@??>ACCBAA@@???A@@@?888@?A?B@C
CAA?AA<A>@@@@;
@T6XXI:00006:00404
GGGAGAAGGGAGAAAGGAAGGAGAATGACCGACAAAAACAGGTTGGTCTATTCTCCCTCTCCCTATAGTGACGT
+
9914;B>AB=CBAA<@=@?B@DCCIDBAC<?>77777,1;<:??C?BB@@@=B444-44044,3300------1
@T6XXI:00006:00424
GGGGAATTCTAATACGACTCACTATAGGGAGAGGGAGAATAGTCAACAAATCGTTTGGGGCGACTTCTCCTTCCTTT
CTCCCTTCCTCCC
+
999/99A=ACB@CDBB@?@?@?@DEDCB>B??BC>C<<9=AA@@;9444,7>?BB<@@@7?BBBF=?8;34/43344
<75444-.-.332
@T6XXI:00006:00434
GGGGAATTCTAATACGACTCACTATAGGGAGAGGGAGAATAGTCAACAAATCGTTGGGGCGACTC
+
ADF9B@C?<<<@BABDB;;;@@A?:::B>B?@@C:;8A?AA?A??9;76099//,...*,24...
@T6XXI:00007:00408
CTAGTCACTCGAGATCTGCGGCTTTGGACAGTCCCTCTGTACTGTGAAGCCACAGATGTCGAGTCGACTAGCTTCCT
TTCTCCCTTCTCCC
+
C:1111/4?BBBDBBAA@BB@BBB<B?AB@BBB@;@@@?@???>??A<?A@BBBBBCCCBBBBBCCCCCCCBA?ABC
F>BCBD>@=@@@@;
@T6XXI:00007:00410
GGGAGAAGGGAGAAAGGAAGGAGAATGCGGTACCGGTTACGTCTTGGCTATTCCTCCCTCTCCTATAGTGAGTCGTA
TTAGAATTCCCC
+
```

9@>49;<BC>CCDN?B@CBC?AAB>:78@7;;A@C>C;=;;8?B?B=868827234440004/0033343348===<
;988:5:8;:8.
@T6XXI:00007:00414
CTAGTCACTCGAGATCTGCGGCTTTGGACAGTCCCTCTGTACTGTGAAGCCACAGATGTCGAGTCGACTAGCTTCCT
TTCTCCCTTCTCCC
+
>=1////44BBBBBCBBBB>?9998;@CCCCBBB>BCCC?AAABACA>8;;<BCCCCAAABBB?@@?BBB;:65626
A;B@@C>?<??@A<
@T6XXI:00007:00415
GGGGAATTCTAATACGACTCACTATAGGGAGAGGGAGAATAGTCTGCAATTTCCTGCGGTGCATTCTCCTTCCTTTC
TCCCTTCTCCC
+
999/48=8=BB=@;;;B?B@@??@:::C=AAACC>A??;:688//-24,44-
4988=B@=>B;;6988;>;>=@@<?=>>.:4:66>;
@T6XXI:00007:00418
GGGAGAAGGGAGAAAGGAAGGAGAAGTCGCCCCAAACGATTTGTTGACTATTCTCCCTCTCCCTATAGTGAGTCGTA
TTAGAATTCCCC
+
BB>BA@=@H>AABC@C@B@C@CBB?@BBBBCC9CC>B???:8?@>BBBA@@=@@??:?988A<???==8<??>>?78
4;;:699/003+
@T6XXI:00007:00431
GGGAGAAGGGAGAAAGGAAGCTAGTCACTCGACCATCCTGTGGCTTCACAGTACAGAGGGACTGTCCAAAGCCGCAG
ATCTCGAGTCGACTAG
+
;;;>CDBHB=@@==9>595888?;=?A?77/..,...,.5357@A?@?BABAA=;9444+3::849233138565;<
737556462311---.
@T6XXI:00007:00433
GGGGAATTCTAATGACGACTCACTATAGGGAGAGGGAGAATAGTCGCG
+
:DE6?==86677///888;==8;=933;?5905772...,...33000
@T6XXI:00007:00441
CTCGAGATCTGCGGCTTTGGACAGTCCCTCTGTACTGTGAAGCCACAGATGTCGAGCGACTAGCTCCTTTCTCCCTT
+
76/////4488;;<==9-42499<<<DACCCCCBBBBCCEADCABA@@A;74980.07:8777.//,//+////+.3
@T6XXI:00008:00419
GGGGAATTCTAATACGACTCACTATAGGGAGAGGGAGAATAGCTACGTTTAACCGATCGTGTTGTTCTCCTTCCTTT
CTCCCTTCTCCC
+
@@@6@=@>ACCCCCBBB??>BACCC77659?@BC>B@@?A<@<>>>??@>@=><?@@AA;<@>@@69:A>A:=2441
8<;A=A>@<=A<
@T6XXI:00008:00427
GGGAGAAGGGAGAAAGGAAGGAGAATGCACCGCAGGAAATTGCAAGACTATTCTCCCTCTCCCTATAGTGAGTCGTA
TTAGAATTCCCC
+
BDACCB@BB;@944-
94948>B?@??BCCC@BCBB@??9>?BCC@B?ABBB>AAAB;<<@?@7<<@?@?AAAAAAA==:<;;;=:=>>4
@T6XXI:00008:00434
GGATTCTAATACGACTCACTATAGGGAGAGGGAGAATAGTTGCGTTCACC
+
/,//78:;?CCCAB@AABCBAA???9?ABCC>CA?772-/,----0-10/
@T6XXI:00009:00405
GGGGAATTCTAATACGACTCACTATAGGGAGAGGGAGAATAGTCAACAAATCGTTTGGGGCGACTTCTCCTTCCTTT
CTCCCTTCCTCCC
+
BFD:DACAAAA?AACCCBCC;:<B@@@LBCCFBCABAA>@===C=<;<=;:88A@;@?>8><?66011100/34443
444<;<:=<=<@1
@T6XXI:00009:00408

```
CTAGTCGACTCGAGATCTGCGGCTTTGGTCAGTCCCTCTGTACTGTGAAGCCACAGATGTCGAGTCGACTAGCTTCC
TTTCTCCCTTCTCCC
+
IA@?A94944:9949=?99:BABBB>B?AA961;;7;@ABCA@@BA@@?@?=BA?:::@@???A<AACCCD@??:?:
??9?@AA>@387;;4
@T6XXI:00009:00412
GGGGAATTCTAATACGACTCACTATAGGGAGAGGGAGAATAGCGACAACTGA
+
ABB8A=A@DBCADB@@?AABCDD@@?BB<ABBCC>CAB>>>/.---+**+++
@T6XXI:00009:00432
GGGAGAAGGGAGAAAGGAAGGAGAACGCGCACCCGAACCAACGGTGATCTATTCTCCCTCTCCCTATAGTGAGTCGT
ATTAGAATTCCCC
+
GH@BBB@DC>BBBB?C@C@B@B>B?BB@B@@@B@BC@C@CBCCBCCCDC@@@?A@?@<@@@CC?CDCBBBBBCCBBB
BB?BBE@D@DDC:
@T6XXI:00009:00436
GGGGAATTCTAATACGACTCACTATAGGAGAGGGAGAATAGCCGTATCCGCGTCTATGTTCCTCGTCTTCCTTTCCT
CCCTTCTCCC
+
==@6@ABDCBB@AA===?B?B:997..,0>0777:@@=A888>>?>??>@AAA@@@999-
/./9994/,/466,/,/660646:9;3
@T6XXI:00009:00444
GGGGGAATTCTAATACGACGTCACTATAGGGAGAGGAGAAT
+
1111*424249969/;10...446:99333,3111016771
@T6XXI:00010:00424
GGGGAATTCTAATACGACTCACTATAGGGAGAGGGAGAATAGTCAACAAATCGTTTGGGGCGACTTCTCCTTCCTTT
CTCCCTTCTCCC
+
@@@6@>A?BBCACCCBBBB@A<;9;67:.7?@AB>BAA>A=<5889;;=9<;166,111,6669A?BCBAA=?=?A:
?888188<<BA<
@T6XXI:00010:00428
GGGGAATTCTAATACGACTCACTATAGGGAGAGGGAGAATAGTCACTA
+
===4>696<AA@CCBBB?BB@?A>>611-6==AC>B@=780*****,*
@T6XXI:00010:00437
GGGGAATTCTAATACGACTCACTATAGGGAGAGGGAGAATAGCACATGTCTCT
+
ECC:C@A=<;96;;4==@;AAA881111,16779;?;:<<=4..---02----
@T6XXI:00010:00446
GGGGAATTCTAATACGACTCACTATAGGGAGAGGGAGAATAGTCCTCGGTTTTTCCACCATCACGATATTCTTCCTT
TCTCCCTTCTCCC
+
CCC9B>A>@@B>BBBBBBB@@?B?@766.7>==?<?AB>B@@=<:==AAACCC1@<???BAA@@@AABA>==;98<?
:==?@;@>???C>
@T6XXI:00011:00426
GGGAGAAGGGAGAAAGGAAGCTAGTCGACTCGACATCTGTGGCTTCACAGTACAGAGGGACTGTCCAAAGCCGCAGA
TCTCGAGTCGACT
+
CCAEDC@BB>BBBC>A>A@BBCB@B8;:AAA@::;:::@BB;?@?ABCCBACAAA<<<7<?>?@@>AA<@@=@AAC?
?:??@@CA??@>>
@T6XXI:00011:00439
GGGAGAAGGGAGAAAAG
+
CCABBD588091---**
@T6XXI:00011:00449
```

```
CTAGTCGACTCGAGATCTGCGGCTTTGGACAGTCCCTCTGTACTGTCGAAGCCACAGATGTCGAGTCGACCTAGCCT
TCCT
+
::94444444:99499@AA@@<8880848>@<A@@>?==>C6;3..--3,-8899@A@@@@@CCCB<<--+-
,,,*,2223
@T6XXI:00012:00416
CTAGTCGACTCGAGATCTGCGGCTTTGGACAGTCCCTCTGTACTGTGAAGCCACAGATGTCGAGTCGACTAGCTTCC
TTTCTCCCTTCTCCC
+
C7-,--
,,/C;BDBBBBBBBB@BBBAB@BBBCABB>BC@@A@C=<?=?<?C@CDCBAD@@?BBA?AAAAAD>;:?A=@A6:9=
@<=698AA=
@T6XXI:00012:00422
GGGAGAAGGGAGAAAGGAAGCTAGTCGACTCGACATCTGTGGCTTCACAGTACAGAGGGACTGTCCAAAGCCGCAGA
TCTCGAGTCGACTAG
+
CCACB@@BBBAABB>B696:A<;;=/00;==>0::::8BCL@CB>BB????><8:43317??@AA=A?<AA>??88=
==BA<<<??ABA@>?
@T6XXI:00012:00425
GGGGAATTCTAATACGACTCACTATAGGGAGAGGGAGAATAGTCAACAAATCGTTTGGGGCGACTTCTCCTTCCTTT
CTCCCTTCTCCC
+
AAA6@>A>BBB?BDCBCBCB?<??=6695:@?CC>C@@=@;A>@<<<<<:BBCCB>BBB8BCA@B?:::47338=;:
=>==7>9<<AA<
@T6XXI:00012:00449
GGGAGAAGGGAGAAAGGAAGGAGAACAGAATGCCACCAAGGTCTAAGCTATTCTCCCTCTCCCTATAGTGAGTCGTA
TTAGAATTCCCC
+
ID>CCC?BC?BBCC8:8:>A>CCCBEBBB@BBB?BB@C>@=@CBB?BBBBB@CCCC>CCCC@;C???A@@@@A@@?=
=9<@@;=5<;;4
@T6XXI:00013:00410
GGGAGAAGGGAGAAAGGAAGGAATAGGTAAAACTGAACGCAGTAAATACGTCTATTCTCCCTCTCCCTATAGTGAGT
CGTATTAGAACTTCCCC
+
@B>AADCCC>BBBC8:;=BCCE>CDC>:;88.8?8847:9BB9<<86677///::777==;555<;33...111355
8866888=:238/443+
@T6XXI:00013:00417
CTAGTCGACTCGAGATCTGCGGCTTTGGACAGTCCCCTCTGTACTGTGAAGCCACAGATGTCGAGTCCGACTAGCTT
CCTTTCTCCCTTCTCCC
+
BF<449:4449;44499::88100424,...5....*.766111...-
233@CB>?>979;7752,,*,,222088;<>>@;766>13232230
@T6XXI:00013:00419
GGGAGAAGGGAGAAAGGAAGCTAGTCGACTCGACATCTGTGGCTTCACAGTACAGAGGGACTGTCCAAAGCCGCAGA
TCTCGAGTCGACTAG
+
CBACEC@CC>CCBA;B@B@>;A:>>/11;;;:;??9768<@788@B@BD???;;;444-
4<<<A@@AC<8858>==??=>=@@@?????<==
@T6XXI:00013:00423
GGGAGAAGGGAGAAAGGAAGAGATAGACAGCTCAGGAGGATGTTCGTCTATTCTCCCTCTCCCTATAGTGAGTCGTA
TTAGAATTCCCC
+
BC@BBB@BB>BBBB>B?B?ABAACA88:BAA?@@@>@@>@AAA><@?:?AC=?=999=AAAC:B?::<<<@A@@@@@
@>AAA:;2444+
@T6XXI:00013:00426
GGGGAATTCTAATACGACTCACTATAGGGAGAGGGAGAATAGTTCGCCAATATCCCGCGGCGTGTTCTCCTTCCTTT
CTCCCTTCTCCC
```

```
+
CCC9CC?>ACCBCCBBBBAAA@>97116.7>>BF>C@A@A=999=@@>B@CB???;?BB?C===A>???=@;=8;<7
<==?:?<?AAA<
@T6XXI:00013:00442
GGGAGAAGGGAGAAAGGAAGGAGACGACCCCAGGGAGAAATATTCGGCTATTCTCCCTCCTCCCTATAGTGAGTCGT
ATTAGAACTTCCCC
+
?B@>>>5990948@9>4:6:@CCFCCBBAA7@@@<@B?B6;;:;@<<9@BB81/885/0,0..+.449:=?AACCB@
AB>B@=9>>3777,
@T6XXI:00013:00443
GGGGAATTCTAATACGACTCACTATAGGGAGAGGGAGAATAGCCTTCCACTTTATCATGCGCGTCTTCCTTCTTCCC
TT
+
@?=6@=@>@FD@CCCABB??B@BB?66646??CE>C@@>@=?:8<;?>BCAA<CCAA<=ABC@??6660101.,..+
.3
@T6XXI:00014:00409
CTAGTCGACCCGAGATCTGCGGCTTTGGACAGTCCCTCTGTACTGTGAAGCCACAGATGTCGAGTCGACTAGCTTCC
TTTCTCCCTTCTCCC
+
:11//686@C@AAAECCCCB>9433/;=@BCDBBB@CCCBBABBCCBB:9=@BBCDBCBBCCCCBCCCCCCC<<<<C
GE=@?CDCD;::CE?
@T6XXI:00014:00419
GGGGAATTCTAATACGACTCACTATAGGGAGAGGGAGAATAGAGACGTATTCGGTTGCGCTCTGTTCACCTTCCTTT
CTCCCTTCTCCC
+
DCB7B:;:<@>=BB<<=@@@CBBBC:9:.777=?9??B;==ADA@@=<<=;;;8:8::::<89;=:??@=@;>;>@7
====7=9<;;A1
@T6XXI:00014:00420
GGGAGAAGGGAGAAAGGAAGGAGACGATAGCTGAGTCAATGCCGGGGCTATTCTCCCTCCTCCCTATAGTGAGTCGT
ATTAGAATTCCCC
+
@;6@94244-
4;;A=B?B?ABCCCCCC@;;;>>>?996>@>A>BDD9ABCC<30760./,.440355AB?@@AA?>>@>384924/0
00+
@T6XXI:00014:00441
GGGGGAATTCCAATACGACTCACTATAGGGAGAGGGAGAATAGTTTTAAACTCCCCG
+
CBCC2@=AAC@A@C<:<;A?>@@>?>777.77777-600/043333+00+00000+0
@T6XXI:00015:00433
GGGAGAAGGGAGAAAGGAAGGGAGAAGGC
+
CC>BB=>==7;;><68800,,()((((((
@T6XXI:00016:00403
GGGGAATTCTAATACGACTCACTATAGGGAGAGGGAGAATAGTCTTGTATTTTGTGCATCACTTTTCTCCTTCCTTT
CTCCCTTCTCCC
+
CED:C?A?ACC@CCBBBBBDA>;96166.:@@BC>BBB=A=<=9855888;;0<AACCCCCBBCC9B??=?>@?CC<
@@@@;@>@??A<
@T6XXI:00016:00414
CTAGTCGACTCGAGATCTGCGGCTTTGGACAGTCCCTCTGTACTGTGAAGCCACAGATGTCGAGTCGACTAGCTTCC
TTTCTCCCTTCTCCC
+
B9999944==CCBBBBC??@@=@CC>D?CCBBAAA<ACBBBBBB>B@@<?B@CCCCBBBB?????ABACCC;;9273
7@9?::C<?:==?A<
@T6XXI:00016:00436
GGGAGAAGGGAGAAAGGAAGGAGAGGTC
+
```

```
44-44424A:ADGC?C:2--88--,*,0
@T6XXI:00016:00444
GGGGAATTCTAATACGACTCACTATAGGGAGAGGGAGAATAGTCAACAAATCGTTGGGGCGACTTCTCCTTCCTTCT
CCCTT
+
?=>4@6924448=>BBBABAA@???777.7>777.744/3>??==<@:9,../.,...*..111011100/044466
64+0/
@T6XXI:00016:00445
GGGGAATTCTAATACGACTCACTATAGGGAGAGGGAGAATAGTTTTTCCCTTCACTAACCGCGAACTGCACTTCCTT
TCTCCCTTCTCCC
+
999/4899:AA>@B;;;??;;;BBCCCF@BABCC>CA@:9;44444,33,023<<??=A>AAA>;>>=8:;B??:??
9=>@@<@=878@:
@T6XXI:00017:00406
GGGGGAATTCTAATACGACTCACTATAGGGAGAGGGAGA
+
////*/,/,/88,/444473337771111,17777,1--
@T6XXI:00017:00422
GGGGAATTCTAATACGACTCACTATAGGGAGAGGGAGAATAGTCAACAAATCGTTTGGGGCGACTTCTCCTTCCTTT
CTCCCTTCTCCC
+
==94>598<BBCCCBAA?<BBBB97111.7>?BC>CAA=@:?A???AAA:BC?BB>BBB8@===CDC@@>=>A?AA<
AAAA<A>@@AA<
@T6XXI:00018:00418
GGGAGAAGGGAGAAAGGAAGGGAC
+
<<:;@BAHCADD>>:<:<,**)**
@T6XXI:00018:00430
GGGAGAAGGGAGAAAGGAAGCTAGTCGACTCGACCATC
+
=@:@BB?CCABB?B==>B@;;8=;90../....101..
@T6XXI:00018:00434
CTAGTCGACTCGAGAATCTGC
+
<<@@?@>?///////,/8,//
@T6XXI:00018:00440
GGGAGAAGGGAGAAAGGAAGGGGAGAAGGGAGAAAGGAAGGGGAGAAGGGGGACAGTCCATCTGTGGCTTCACAGTA
CAGAGGGACTGTCCAAAGCCGCAGATCTCGAGTCGACTAG
+
DC?CCC@EC@ABBB=B>B>ABE8BCC@B?99111,7=>3777.77734;;;1>==:676://.4457=A;=<@@???
?????A;=9::A@>AA<??>@@@@===@@@?@>BAB;;;6
@T6XXI:00018:00449
CTAGTCGACTCGAGATCTGCGGCTTTGGACAGTCCCTCTGTACTGTGAAGCCACAGATGTCGAGTCGACTAGCTTCC
TTTCTCCCTTCTCCC
+
FD?B<;;;<BCBC=@=BCBBD@CBC>B?B@>=666-
7<=<>@????;;8<A@BBB@@@AA?@?===@101?00.57011,1117688=<==;
@T6XXI:00019:00408
GGGGAATTCTAATACGACTCACTATAGGGAGAGGAGAATAGC
+
DCB:B9;;;BB@BBBBBBBBBABB?776-/://,099781,,
@T6XXI:00019:00412
GGGAGAAGGGAGAAAGGAAGGAGAAGTCGCCCCAAACGATTTGTTGACTATTCTCCCTCTCCCTATAGTGAGTCGTA
TTAGAATTCCCC
+
CC?CBB@CH@B9996;>@>AACAB?ABBCCCC9CGACC:;6169;>BBAAA>@@AA<@???C<??AA@@AABA???@
@?C@@@@<>>?5
```

```
@T6XXI:00019:00432
GGGAGAAGGGAGAAAGGAAGTGCAGTTCGCGGTTAGTGAAGGGAAAAACTATTCTCCCTCTCCCTATAGTGAGTCGT
ATTAGAATTCCCC
+
CCACBB@BD>BBAA=B?B?ABBBBBB@BAAA=@=@CCCC@C?>??@B2CB@B@BCC?;???CC>CA???A99AAAA@
=<9=@@=@=?@?4
@T6XXI:00019:00433
GGGGAATCTAATACGACTCACTATAGGGAGAGGAGAATAGTCAACAAATCG
+
4992924444244866.,,,,,,,....+11699>==;:66834/000+111
@T6XXI:00019:00434
GGGAGAAGGGAGAAAGGAAGCTAGTCACTCGACATCTGTGGCTTCACAGTACAGAGGGACTGTCCAAAGCCGCAGAT
CTCGAGTCGACTAG
+
CCAAA?@CE>CCCC>C=@=@AA=ABB@>=>>01/>:>??C@BB?BCBCBCB?9=77717>>>??>AA<AA?A@<<<?
???AA@@???????
@T6XXI:00020:00406
GGGAGAGGAGA
+
94-4///5///
@T6XXI:00020:00408
GGGGAATTCTAATACGACTCACTATAGGGAGAGGGAGAATAGATCACCGTTGGTTCGGGTGCGCGTTCTCCTTCCTT
TCTCCCTTCTCCC
+
CCC9C@A>ACC@CBA@BCCBBBB?9666.6==?A>BAA?CA?@<@?=;?B?B?BADCC=BABCCCC@B??:=4747>
:>744-4::<<A<
@T6XXI:00020:00419
GGGAGAAGGGAGAAAGGAAGAATAAAGAGGGCTCGAACAAAGCCGGGCTATTCTCCCTCTCCCTATAGTGAGTCGTA
TTAGAATTCCCC
+
CHABBA>?=>BBBC>B@EBCC?BBC?B???;?CB??;777.1666;;BBCF@A773,44444-
4???@A@A@==>???@BAA>A9<<<4
@T6XXI:00020:00427
GGGAATTCTAATACGACCTCACTATAGGGGAAG
+
,,*,*,*,22*/444471337799611111,161
@T6XXI:00020:00446
GGGAGAAGGGAGAAAGGAAGGCAATGCACGCTTTAGGGAGGTTATAACTATTCTCCCTCTCCCTATAGTGAGTCGTA
TTAGAACTTCCCC
+
99049@@BB<BBBB>B@C@C@BC@CCCCCCBBB>BCC>CC?B?BB?>BBBB?BAAA<??@AB<@@@???AA<<<=A=
>;@<@>BA8<<<0
@T6XXI:00020:00448
GGGAGAAGGGAGAAAGGAAGGAGAAGTCGCCCCAAACGATTTGTTGACTATTCGTCCCTCTCCCTATAGTGAGTCGT
ATTAGAATTCCCCC
+
/1,//4244-44:;-42423777>@78:9???-
97,37111,1678<=<<9200000+0000303330336:;;==@@@=?<A<;/////+
@T6XXI:00021:00406
GGGAGAAGGGAGAAAGAAG
+
088819599-////+//54
@T6XXI:00021:00423
CTAGTCACTCGAGATCTGCGGCTTTGGACAGTCCCTCTGTACTGTGAAGCCACAGATGTCGAGTCGACTAGCTTCCT
TTCTCCCTTCTCCC
+
```

```
BA1/1::??AAABCCBCC@?<@?>8?9:<CDCBB>BCCCC@?B>?>A=8<<=DCCCCCCBCBBBBB;;<BA?8=?<>
??777=69493487
@T6XXI:00021:00440
GGGAGAAGGGAGAAAGGAAGCTAGTCGACTCGACATCTGTGGCTTCACAGTACAGAGGGACTGTCCAAAGCCGCAGA
TCTCGAGTCGACTAG
+
AA<@@==@@<@A@@<A=@<?A???A:;:ABC:::;AA;:;C=?@ABBBBB>?<==9987=<<@AB?AA:?A>A9<<=
<:;;<=<AAA????A
@T6XXI:00022:00437
GGGGAATTCTAATACGACTCACTATAGGGAGAGGGAGAATAGTCAACAAATCGTTTGGGGCGACTTCTCCTTCCTTT
CTCCCTTCTCCC
+
CCB8A=@>@@B@CCBBBBBBBA<97111.7>>>A>AAA>A=AA9969AA:BBCCCACCC7AA=;;9:;:,/,/5886
;;=?:?9<<?@;
@T6XXI:00023:00402
GGGAGAAGGGAGAAAGGAAGCTAGTCGACTCGACATCTGTGGCTTCACAGTACAGAGGGACTGTCCAAAGCCGCAGA
TCTCGAGTCGACTAG
+
GCACBB=?>:@B?==B=@=@B@;;;/00000;0:7:96<898<<?BCDCBBBAAA=997=<@@A@>@@<@A>A@@A=
=;<<<=???@?????
@T6XXI:00023:00404
GGGGAATTCTAATACGACTCACTATAGGGAGAGGGAGAATAGTCAACAAATCGTTTGGGGCGACTTCTACTTCCTTT
CTCCCTTCTCCC
+
?==,924:;@B;<<AA@@;:;7??>777,6777A>B@A?AB?AAC>@@>7:::AB<ADD7;:CBC@CC>?836017-
70444<565440
@T6XXI:00023:00420
GGGGAATTCTAATACGACTCACTATAGGAGAGGGAGAATAGCTCACGAACCTTTTGCGTGCTCTATCCTCCTTTCTC
CCTTCTCCC
+
=??4?6924889=?CDBAAAC>;;;00,/;;>><@?><>>;<?A::7B?A>@@A5??>A7449666623356443--
-+-,--222
@T6XXI:00023:00421
GGGAGAAGGGAGAAAGGAAGCGCGGC
+
CLAB>=5991991<8;:;,0,,*,*,
@T6XXI:00023:00427
GGGGAATTCTAATACGACTCACTATAGGGAGAGGGAGAATAGTCAACAAATCGTTTGGGGCGACTTCTCCTCCTTTC
TCCCTTCTCCC
+
499/429249999=@A@@FCCBBB@:::5:==BC:::A>@@AA??<?@@;B@@@C@CCC7?@@>>:;==.//9;;6;
7;;6;3736;4
@T6XXI:00024:00405
GGGAGAAGGGAGAAAGGAAGGGGAGAAGGAGAAAGG
+
DD@FCEEBA:@899049;9<@@5777113.../+.,
@T6XXI:00024:00434
GGGGAATTCTAATACGACTCACTATAGGGAGAGGGAGAATAGTGACGTGTTGTGCGGCGCTCACTGTCTCCTTCCTT
CTCCCTTCTCCC
+
CDD9B8;;<AB@BCBABBCBAAA>;111-
6<<?B>B@@>@@@@???@?<@>???CD>@AAAA888?;;<;5646,/,/666,/,/9994
@T6XXI:00024:00446
GGGAGAAGGAGAAAGGAAGCTAGTCACGTCGACATCGATGATGGCTTCACATACAGAGG
+
11-,,,*,*,,/8+/,47333731661111.45533......247?><:4361111...
@T6XXI:00025:00407
```

```
CTAATACGACTCACTATAGGGAGAGGGAGAATAGCCCCCCAGTGGCCGCTGTTGCCATGTCCTTCCTTTCTCCCTTC
TCCC
+
CCC@C>@@?@BBCB???888/7>777-7==:;?;A@@@B->::?,0,0/.24444228//-,-
447744<@@C9=;=@@@9
@T6XXI:00026:00408
CTAGTCACTCGAGATCTGCGGCTTGGACAGTCCCTCTGTACTGTGAAGCCACAGATGTCGAGTCGACTAGCTTCCTT
TCT
+
;9949=///8//////46666..,.6666//6826689999<:44;549;=997?><<>76114=333931188337
7..
@T6XXI:00026:00418
GGGGAATCTAATACGACTCACTATAGGGAG
+
08808+1///5/444433331331111,.5
@T6XXI:00026:00439
GGGAGAAG
+
491///,/
@T6XXI:00026:00446
GGGAGAAGGGAGAAAGGAAGGGGGAGAAGGGAGAAAGGAAGCTAGTCGACTCGAGATCTGCGGCTTTGGACAGTCCC
TCTGTACTGTGAAGCCACAGATGTCGAGTCGACTAGCTTCCTTCTCCC
+
AC@AA@?BB?BCCI>B?B=>>??2AAA?BD;??A@9@=?<?D???C:77.3/--8;;==<:94444-
4/330466;48666<:<<=>>=<?@?@?@?888;@@????BA9883,,*,*,*,222*
@T6XXI:00027:00405
GGGAGAAGGGAGAAAGGAAGAACAGTCAGTAGAAGCTGAGGGGAAGTCGTAT
+
:B;>>A;=9:=948-89?97957>>60066;.0,.44...440203000300
@T6XXI:00027:00444
GGGAGAAGGGAGAAAGGAAGGAGAATTGTAAACGGGCATATGCGCTGACTATTCTCCCTCTCCCTATAGTGAGTCGT
ATTAGAATTCCCC
+
CDACCC@CB>BBBBBB=B@B@>??@B@CCCC?CCC?DCCAAAB;:9A@@@@A>A???<???DC;@???@A@A@@@@@
@@<??A>=<<AA5
@T6XXI:00028:00403
GGGGAATTCTAATACGACTCACTATAGGAGAGGGAGAATAGATACCAC
+
@B@4@5929;?9@BBBBBCCCE@@?::8:BBBE>C@@48486///,+0
@T6XXI:00028:00415
GGGAGAAGGGAGAAAGGAAGTGCGAATAACGTGTACAAACGTACAGGCTATTCTCCCTCTCCCTATAGTGAGTCGTA
TTAGAATTCCCC
+
AA>ABB@CC>B@@@<??B@CBEBBDABB?BBBCCCAAA=BC@@?CC@A@@B?@???:888=>7=@AA===@@9:9@B
A@AAC@G@BBB8
@T6XXI:00028:00433
CTAGTCGACTCGAGATCTGCGGCTTGGACAGTCCCTCTGTACTGTGAAGCCACAGATGTCGAGTCGACCTAGCCTTC
CTTTCCTCCCTTCCTCCC
+
611/////44499:88=9999488437333667761611689888565 5=;898733376677666..,....,.36
666667111,02455584
@T6XXI:00028:00447
GGGGAATTCTAATACGACTCACTATAGGGAGAGGGAGAATGGCCAACAGGTATCTGATCGCACGTTCTCCTTCCTTC
TCCCTTCTCCC
+
C??7A=@=@BBBBBBBBBBBA@@<966649CCCD>C?@>A=9==??CCA>A??<@@@@C?>?@@=>/009:5:./..
;;;9;????C=
```

```
@T6XXI:00029:00400
GGGAGAAGGGAGAAAGGAAGGAGAAGTCGCCCCAAACGATTGTTGACTATTCTCCCCTCTCCCTATAGTGAGTCGTA
TTCAGA
+
49-9AA?BD>AACGBB?B@B8;9A:6133...*.404...,.3336944;7:4444-
4433?:;<<=<A;;5...333*..40
@T6XXI:00029:00408
GGGAGAAGGGAGAAAGGAAGGAGAAGTCGCCCCAAACGATTTGTTGACTATTCTCCCTCTCCCTATAGTGAGTCGTA
TTAGAATTCCCC
+
DC>BBC?AB>BBBB>A>B>A?@@@=>788>??5@C>CBBBB;BB?BBB@@@=@==9944444-
4:=AA@A@?0/00//794843/334,
@T6XXI:00029:00423
GGGGAATTCTAATACGACTCACTATAGGGAGAGGGAGAATAGTCAACAAATCGTTTGGGGCGACTTCTCCTTCCTTT
CTCCCTTCTCCC
+
DC@7@246<89<ABBBCABABCB>=6:959?BCC>CAA=>8>AAA>@@A<BCCBB<@@@5@>@@A;?=>;<8<9;A>
@???7=7:7881
@T6XXI:00029:00428
GGGGAATTCTAATACGACTCACTATAGGAGAGG
+
;;;,42424;;8=?AB@???0,--,,,*,,,66
@T6XXI:00029:00448
GGGAGAAGGGAGAAAGGAAGGGGAGAAGGGAGAAAGGAAGCTAGTCGACTCC
+
GCCAAABDC@BB>>;>;>9666+00.,..+.1779=>;>A:4:1111.2,,*
@T6XXI:00031:00404
GGGAGAAGGGAGAAAGGAAGGAGAAGTCGCCCCAAACGATTTGTTGACTATTCTCCCTCTCCCTATAGTGAGTCGTA
TTAGAATTCCCCC
+
CF<=>><>>:989B?B?B@D?BDDECAAADCC9CD?C??:9888<ABCAAAAA===7<<<@A<AC@@>==@?===AA
@>==:44/0034,
@T6XXI:00031:00412
GGGAGAAGGGAGAAAGGAAGTGCAGTTCGCGGTTAGTGAAGGGAAAACTATTCCTCCCTCTCCCTATAGTG
+
:B>BBB>BB=BBAC>B?B?CAAAA<;58@9843773337111,111,33331601>7,17111-1003396
@T6XXI:00031:00424
GGGGAATTCTAATACGACTCACTATA
+
///*/,/,44499444493.......
@T6XXI:00031:00438
GGGGAATTCTAATACGACTCACTATAGGGAGAGGAGAATAG
+
077*1,924>>BCBBBCBBBB>A@@777.7>>><>//,//-
@T6XXI:00032:00404
GGGAGAAGGGAGAAAGGAAGTGCAGTTCGCGGTTAGTGAAGGGAAAAACTATTCTCCCTCTCCCTATAGTGAGTCGT
TATTAGAACTTCCCC
+
>>:>>?<>>6>>AA<A@C?ABCCCCCAAA;;78<?B:;7:6626;;</>@@B?@@CD=??88626335658;400--
,--,-1223685990
@T6XXI:00032:00406
AGTCACTCGAGATCTGCGGCTTTGGACAGTCCCTCTGTACTGTGAAGCCACAGATGTCGAGTCGACTGCTTCCTTTC
TCCCTTCTCCC
+
CCCCBCCBBBBBCBBBBB>BBB>B@CCBCBCC>AAA???AAAAAA?AA@AAABB@A@@@@@@A?;;9@@?;?;>>7>
@@A7?<988@;
@T6XXI:00032:00422
```

```
GGGGAATTCTAATACGACTCACTATAGGAGAGGGAGAATAGTCACAATCGAT
+
DDC8A>A@BBCACBBBC@@@C>>;?/.,/:<>@>BBB?AA:;///5+0/.,,
@T6XXI:00032:00432
GGGAGAAGGGAGAAAGGAAGGAGAACGCGCACCCGAACCAACGGTGATCTATTCTCCCTCTCCCTATAGTGAGTCGT
ATTAGAATTCCCC
+
7?-119294-448;-
42477179C?BCCAA?@=694:;6:6:A@44;==999<@BCC>@@@??:>?@???ABA:::>:92:967;6444,
@T6XXI:00033:00401
GGGAGAAGGGAGAAAGGAAGCTAGTCGACTCGACATCTGTGGCTTCACAGTACAGAGGGACTGTCCAAAGCCGCAGA
TCTCGAGTCGACTAGCTTCCTT
+
AA<@@@>@@194>><>>A?;<;>=7,,,,,,,,4../:867>:<6:?=7::744;444,4::9;=?@A<AA>@@@<<
<<<;9;9440440000023/00
@T6XXI:00033:00403
GGGAGAAGGGAGAAAGGAAGGAGAAGTCGCCCCAAACGATTTGTTGACTATTCGTCCCTCTCCCTATAGTGAGTCGT
ATTAGAATTCCCCC
+
@B1949;>?<BBBL@BAB@C@CCC@????>>>2?A<A;BB>;BC=@>?4//,.----+---
0=;<8:?<<<?><<<@@@9854/0/0034+
@T6XXI:00033:00404
GGGGAATTCTAATACGACTCACTATAGGGAGAGGGAGAATAGTCAACAAATCGTTTGTGGCGACTTCTCCTTCCTTT
CTCCCTTCTCCC
+
CHD:C>AA@BBCBBBBBAAAB@<<9696.6=77:7:@@>A@@@@A;@?C?B???D>CBB?BBAAA?>??8978/44-
8==AAB9==?A<
@T6XXI:00033:00421
GGGGAATTCTAATACGACTCACTATAGGGAGAGGGAGAATAGCAAACGTTTCCCCGACGTCCGCTTTCTCCTTCCTT
CTCCCTTCTCCC
+
BDD/964248988=@AA??A878877/.+/./.5+.--,-
44444+016;6;??56:<;0/,..11,1773323/0/004444233330
@T6XXI:00033:00424
GGGGAATTCTAATACGACTCACTATAGGGAGAGGGAGAATAGTCAACAAATCGTTTGGGTGCGACTTCTCCTTCCTT
TCTCCCTTCTCCC
+
CCB9B=?=@BB@CBBBBBB?>:?9=666.7?==?<?@A>A@@???=@@B=CB?==.77.767669796442338;>;
:<6<?:=:>?>@<
@T6XXI:00033:00425
CTAGTCACTCGAGATCTGCGGCTTTGGACAGTCCCTCTGTACTGTGAAGCCACAGATGTCGAGTCGACTAGCTTCCT
TTCTCCCTTCCTCCC
+
==1/1::A?CCBCCBCBBBC@B@A>B@AAABA?A;AAA@??AAA@??<?B?BCCC@@@BBAAA:<<@@@B::7;::8
?8A67:67,/./;;6
@T6XXI:00033:00438
GGGGAATCTAATACGACTCACTATAGGGAGAGGGAGAATAGATCCACTTTGCGTTGAGTACTTCTCCTTCTCC
+
;;;5:,49=>;==>;;<BBFA??>777-7>777-68826>=400/....+....333565444000/02---0
@T6XXI:00034:00407
GGGGAATTCTAATACGACTCACTATAGGGAGAGGGAGAATAGTCAAACAAATCGTTTGGGGCGACTTCTCCCTTCCT
TTCTCCCTTCTCCC
+
6=:0<-
95888244;44322,,,,,,,,*11117,63684;44000+044+68:8>6669,1166601111,088:==:888:
469<;??:
@T6XXI:00035:00414
```

```
GGGAGAAGGGAGAAAGGAAGGAGAATGGTCTAGCCACAGATCGGACAACTATTCTCCCTCCTCCCTATAGTGAGTCC
GTATT
+
<;79>>;@C><<<E@CBB@C@CCC@>>???7>><380.0.,,2*,**)***,3667?>/42444+3222..+00++*
-003-
@T6XXI:00035:00423
GGGGAATTCTAATACGACTCACTATAGGAGAGGGAGAATAGTCACATTCATGTTTGTGACCGTGTCTCCTTCCTTTC
TCCCTTCTCCC
+
CB?7B69;<CC@CCCCBABAB7;=?//,0<0777;AA@A??@@@<<@>@@?A>A;::9@@=@@DC???>A>A?A@;@
???;>?@<;<7
@T6XXI:00035:00427
GGGAGAAGGGAGAAAGGAAGGAGAAGTCGCCCCAAACGATTTGTTGACTATTCGTCCCTCTCCCTATCAGTGAGTCG
TATTAGAATTCCCC
+
/1,//4244-448;-429<;579C@::;@CEI9CB<?6711,1636995557..-
133...55337;::866<;889<=<<<==;=5665.
@T6XXI:00035:00435
GGGAGAAGGGAGAAAGGAAGGAGAACGCGCACCGAACCAACGGTGATCTATTCTCCCTCTCCCTATAGTGAGTCGTA
CTTAGAATTCCCC
+
8A8119699-////+/,/,.,.443=9@?@11-
1;,1,/7//1:===9::AECACB<?A88;08@?000/34466611101667;;??=2
@T6XXI:00036:00401
GGGGAATCTAATACGACTCACTATAGGGAGAGGAG
+
88802*1//67/4448777;67><777,/3//,.1
@T6XXI:00036:00406
CAGTCCCTCTGTACTGTGAAGCCACAGAGGACTGTCCAAAGCCGCAGATCTCGAGTCGACTAGCTTCCTTTCTCCCT
TCTCCC
+
DCDDDCADCCDCCBBBBCC@CB>CCCC;:47>@9<<@EC?AC@@AABBBBAA@AAB?==?><;>><><?B>>>@?:<
5//456
@T6XXI:00036:00416
GGGGAATTCTAATACGACTCACTATAGGGAGAGGGAGAATAGTGACGTGTTGTGCGGCGCTCACTGTCTCCTTCCTT
TCTCCCTTCTCCC
+
CB@7B798<CCACCBBB>?AB?997116.7>>?D>C@@=@:9=9==;<::::<<==;<<<@A99=@<8:=/424/44
-4;;;-838<BA=
@T6XXI:00036:00417
GGGAGAAGGGAGAAAGGAAGCTAGTCGACTCGACATCTGTGGCTTCACAGTACAGAGGGACTGTCCAAAGCCGCAGA
TCTCGAGTCGACTAG
+
DDCBAA=@C@BABB799;>888>>;0/00//=10.75/0/775>;?CC?@@@;;9444-
4;;?CA=@@:@C?D@?888<<<AAAA==@@?@?
@T6XXI:00036:00445
GGGGAATTCTAATACGACTCACTATAGGGAGAGGGAGAATAGCCGATCTTTCTTTAGTTCCTCGCTGCTCTTCCTTT
CTCCCTTCTCCC
+
DCC:C@B?ADC@B@@@?<BB?@>;961959@@BD=A@A=@<<<<A<@=8<7<?@<@AA?A?A@=9=???@@;;3881
8=;B7?688@@;
@T6XXI:00037:00416
GGGGAATTCTAATACGACTCACTATAGGGAGAGGGAGAATAGTCAACAAATCGTTTGGGCGACTTCTC
+
@@>4>6959@@5:;;AA88;@788=777-777<;88:6;8=9@;=;===:;6661,11,111110197
@T6XXI:00038:00413
```

```
TCTGCGGCTTTGGACAGTCCCTCTGTACTGTGAAGCCACAGATGTCGAGTCGACTAGCTTCCTTTCTCCCTTCCTCC
C
+
AFFAA@?@@>9@>?>>@88808?@?8::B:77C@ADEH<<<CADCDJGBBBCCCA?@?;:=:=?9?><>6>,0,0//
,
@T6XXI:00038:00423
GGGGAATTCTAATACGACTCACTATAGGGAGAGGGAGAATAGCTCGTACAAC
+
@=92?<==@CCACB@@BBBB@A@?;66659@@CC>C@@>@47.00/..,+*+
@T6XXI:00038:00444
GGGGAATTCTAATACGACTCACTATAGGGAGAGGAGAATAGTACGA
+
C8708+969AA<ABBCCCCCC??<=777.7?<?::,,+,*()(***
@T6XXI:00038:00445
GGGAGAAGGGAGAAAGGAAGGTGAAAGTCAAACGGGCAAATGCGTGACTATTCTCCCTCTCCCTATAGTGAGTCGTA
TTCAGAACTTCCCC
+
44-
444899694CB8<;<ID@B>>;6/....+..4039A?>@A?;99?@992837727766>;>78:@>996633004/0
0388855442.
@T6XXI:00039:00407
GGGAGAAGGGAGAAAGGAAGCTAGTCGACTCGACATCTGTGGCTTCACAGTACAGAGGGACTGTCCAAAGCCGCAGA
TCTCGAGTC
+
9919@@<>@<@?>@<@>B?@@@AAA:;:B???:::??9:7?=>A@CAACAAB;;;444-
4;;>A@=@C;@@<@??AA?==<?>==>
@T6XXI:00039:00415
GGGAGAAGGGAGAAAGGAAGCTAGTCACTCGACATCTGTGGCTTCAGTACAGAGGACTGTCCAAAGCCGCAGATCTC
GAGTCGACTACG
+
8>;B?B@CC>A@BC>A?AACBB;<:9766111116669>>::;>?61111111924177<:=9?8:<;<88:@4338
366696333.--
@T6XXI:00039:00421
GGGGAATCTAATACGACGTCACTATAGGGAGAGGGAGAATAGCTCCGGATATTTGTCGCGCACCTCGACCTTCTTCT
CCCTTCTCC
+
88207+1///,/4844377;;7;;9378,3816689>;:8???::57;>3011,034335433:44400/0/00233
353553333
@T6XXI:00039:00442
GGGAGAAGGGAGAAAGGAAGGAGAAGTCGCCCCAAACGATTTGTTGACTATTCTCCCTCTCCCTATAGTGAGTCGTA
TTAGAATTC
+
CDBCBA>BC>AB=A=B@B@B@BBB@@@AABCC9CC>IC=??<?B@CCAA@<;<<==;==??A<@AA@@@@@@@AAA@
@=@AA?A<?
@T6XXI:00040:00402
GGGAGAAGGGAGAAAGGAAGTGCAGTTCGCGGTTAGTGAAGGGAAAAAACTATTCTCCCTCTCCCTATAGTGAGTCG
TATTAGAATTCCCC
+
CE>ABCAEE9;<CC>B?B?B;;<A@A=A;986;7;AB?B8:;7:CCCC-
CCBB?AACC>?????9?>>@@DAA@@?>>8838?A>>;@AC6
@T6XXI:00040:00421
GGGGAATTCTAATACGACTCACTATAGGGAGAGGGAGAATAGTTCCAACTGTTTCTCCCGGTGGATCCTTCCTTCTC
CTTCTCCC
+
;;@4@5969>>;@<;<CDGBDCCB;;:?6=>777.7448:888,/,-22--
00+0000+0200/0332424235555944834 4-
@T6XXI:00041:00423
```

```
GGGGAATTCTAATACGACTCACTATAGGGAGAGAGATATAAGGGACCCGTATCTCTCCTCGTCG
+
DFD4B6964449998;;;A@@?@CBAA@:;;,,,,,++22266438:49833430032000334
@T6XXI:00041:00445
GGGAGAAGGGAGAAAGGAAGGAGAAGTCGCCCCAAACGATTTGTTGACTATTCTCCCTCTCCCTATAGTGAGTCGTA
TTAGAATTCCCC
+
CCACCC@CE@BBAB=@<B7;7;BB>ABB@@@@7@?:??A>C>CC@B@@8:;7///71//-
79977;=;778=;;;=<<;=6553/000,
@T6XXI:00041:00447
CTAGTCACTCGAGATCTGCGGCTTTGGACAGTCCCTCTGTACTGTGAAGCCACAGATGTCGAGTCGACTAGCTTCCT
TCTCC
+
4494994444;44448>>>@<888/8488?7111,111111111133343454>988>:3199??A=;;;....,.,,
32555
@T6XXI:00042:00403
GGGGAATTCTAATACGACTCACTATAGGGAGAGGGAGAATAGTCAACAAATCGTTTGGGGCGACTTCTCCTTCCTTT
CTCCCT
+
/7707,924//,///8/.37367;688;85...4+.-2311665355;84;=77716772=7//.,.33,-,-
011+--3305
@T6XXI:00042:00447
GGGAGAAGGGAGAAAGGAAGCTAGCTCGACTCGAGATCTGCGGCTTTGGACAGTCCCTCTGTACTGTGAAGCCACAG
ATGTCGAGTCGACTACGCTTCC
+
9:594424;58444-
4242333711433373316111988<@??@C>?>??BCB@>7443333004<6=;?A;==@@???@@@@@?A;5,+*
**+-33,
@T6XXI:00043:00408
GGGGAATTCTAATACGACTCAGCTATAGGAGAGGAGAAT
+
78807,1,///,/449:@A@:31973331..,,*,,,*,
@T6XXI:00043:00439
GGGAGAAGGGAGAAAGGAAGGGGAGAAGGGAGAAAGGGAAAGC
+
CC>BBB?BC<@@@D<A<>4:88.8>>577.7<67,..+..+.4
@T6XXI:00044:00410
GGGAGAAGGGAGAAAGGAAGCTAGTCGACTCGACATCTGTGGCTTCACAGTACAGAGGACTGTCCAAAGCCGCAGAT
CTCGAGTGGACTAG
+
BC>;<;<>>:99;CABAC@A@@CBAAB@?BBC;;>CFBBBB?BB?BCC@@@A884..-
/;::;A?AA<@A=988;;9;<<80/=;9/0//.
@T6XXI:00044:00411
GGGGAATTCTAATACGACTCACTATAGGGAGAGGGAGAATAGTTTCACACTCATGCGCTCCGATTTATTCTTCCTTT
CTCCCTTCTCCC
+
CCB9B;:<<GGBCCBBBAAAB@;97116.7==<?<?AD@A>@@C>AA<<<AAA@@BBBCA=?8::08@=8737377-
:?AC<B>C;881
@T6XXI:00045:00406
GGGAGAAGGGAGAAAGGAAGAGAAGAGC
+
94-9?A?@@1989914242..446///2
@T6XXI:00045:00432
GGGGAATTCTAATACGACTCACTATAGGGAGAGGGAGAATAGCTACCAACTAAACTCACCGCAGCTTTCCTC
+
?BA7D8;;<BB@CCCBBABA?:??;677.7=<@C@B?C>@???BCC@B>A>AE>>==84/0668///+-,-0
@T6XXI:00045:00434
```

```
GGGGAATCTAATACGACT
+
8>K9@*//,,*,////.7
@T6XXI:00046:00415
GGGGAATTCTAATACGACTCACTATAGGAGAG
+
;8808,1,/6656:>>;:7=:7@8,,,+,,,0
@T6XXI:00046:00434
GGGGAATTCTAATACGACTCACTATAGGGAGAGGGAGAATAGCACGCGACTACAAAACGCTGGTATCTCCTTCCTTT
CTCCCTTCTCCC
+
DDC9?698<@@ACCA@@BBBB@<97111.7>>?B@C@@@@?AAB=???=>B@>:<<5:<==<;<=A=?<8<99/33-
4;;;:=;;<<<6
@T6XXI:00046:00439
GGGAGAAGGGAGAAAGGAAGGGAATAGCAGCAGTCGGCAACGTGAAACTATTCTCCCTCTCCCTATAGTGAGTCGTA
TTAGAACTTCCCC
+
DC>BA=;==:>>>><@;@>AB>B?BCCCBCCBBBFCE;;;<@@?CD>CBCC?CCCC>:::BA<@@@@@@@??@===?
A?AAA9;:3444,
@T6XXI:00046:00447
GGGAGAAGGGAGAAAGGAAGCTAGTCGACTCGACATCTGTGGCTTCACAGTACAGAGGGACTGTCCAAAGCCGCAGA
TCTCGAGTCGACTAG
+
DCCAA@@CC>BBCC>?=@;<>>777777>;;:;;;00/=5;5=??BAKBBBC?;9333-
49::;<?AA<AA<@@=<<>=>>===?AA<==@@
@T6XXI:00046:00448
GGGGAATTCTAATACGACTCACTATAGGGAAGAGGGAAGAATGAGTCAACAAATCGTTTGGGGCGACTTCTCCTTCC
TTTCTCCCTTCTCCC
+
CC@4A69699>59449937776;;6667,136666,6444/4.-1-
..54445696?<89CB5>>11/7777;99=>AC=>=?A<A>AA@>:
@T6XXI:00047:00410
CTATCGACTCGAGATCTGCGGCTTTGGACAGTCCCTCTGTACTGTGAAGCCACAGATGTCGAGTCGACTAGCTTCCT
TTCTCCCTTCTCCC
+
BB;:1111::=ABCB?@C@@=@CC>C?BBCCBCB>BBB::;BB?==A>AC@CCCCB??ACCCBA@@;;;BA@?8:7:
B>A?<<7<589@CA
@T6XXI:00047:00417
GGGGAATTCTAATACGACTCACTATAGGAGAGGAGAATAGTTGCGCATTCGTGCACTGTC
+
/1/*/,92498249@@BBFB:/107,,+***,*,00*--44283847;:11.40646...
@T6XXI:00047:00426
GGGAGAAGGGAGAAAGGAAGGAGAAGTCGCCCCAAACGATTTGTTGACTATTCTCCCTCTCCCTATAGTGAGTCGTA
TTAGAATTCCCCGGGAGAAGGGAGAAACGAAGCTAGTCGACTCGAGATCTGCGGCTTTGGACAGTCCCTCTGTACTG
ATGAAGCCACAGATGTCGGTCGA
+
CC>AA>59919>>B>A?AIE@AAB@BBBBCCC9BC@@@@BB>BD@C<<?AA?<<;;6;888A:=>?@=<<A@@===?
?=@@A>A>@AA5@@;@?;7:;6,*+.)***)*7=7377;@;@@@CAA@@?=774777.73778;??@;>>=74671-
..7757E?:88A?9=>74/440-
@T6XXI:00047:00436
GGGAGAAGGGAGAAAGGAAGGAGAAGTCGCCCCAAACGATTTGTTGACTATTCTCCCTCTCCCTATAGTGAGTCGTA
TTAGAATTCCCC
+
EDACCBBDD>ABAB>B@B@C@B>?>?CCCCCC9CC@C@@@BBBB?CAA?BC>A???:>??@A<@@A===?AAA@@>B
B?ACC@A9<<<0
@T6XXI:00047:00438
```

```
GGGAGAAGGGAGAAAGGAAGGAGAAGTGTCAGTACATCGCGGTAACACTATTCTCCCTCTCCCTATAGTGAGTCGTA
TTAGAATTCCCC
+
DC@@???AA<@@@A<?=@?C?BBC@BB>CBB@???AAA=@<<=><?>;;:?:?==>;?@@CC<@@@@<AAAA=<<@?
?<?AA>B3887,
@T6XXI:00048:00410
GGGGAATTCTAATACGACTCACTATAGGGAGAGGGAGAAATAGCCACC
+
9<;19?BACCC?BBC@@?A;000000.3+/.4=767444,36664//,
@T6XXI:00048:00424
GGGGAATTCTAATACGACTCACTATAGGGAGAGGGAGAATAGAGTA
+
:>>6>=?=@BB@CCBABABBA@AB?8??9;=;;?9?=:996/-+++
@T6XXI:00048:00427
GGGAGAAGGGAGAAAGGAAGGAGAAGTCGCCCCAAACGATTTGTTGACTATTCTCCCTCTCCCTATAGTGAGTCGTA
TTAGAATTCCCC
+
CDACCB@BB>BBBB>B@B>?>?AD@?>>>>?B8BB<ACBB@>@C@CB;?<C?:??A<@?@@A;A==A==A==::?==
=>@AA?@3884-
@T6XXI:00048:00431
GGGGAATTCTAATACGACTCACTATAGGAGAGGGAGAATAGTCAACAAATCGTTTGGGGCGACTTCCTCCTT
+
CDH:C@B>A=9599>>9888@<;::00,.:9<?9?=>:=:;0..55;;9B???B<@BC4;0777+,+,,++*
@T6XXI:00048:00432
GGGGAATTCAATACGACTCACTATA
+
:CA8A8/,/8,////4333>:;0,,
@T6XXI:00048:00439
GGGAGAAGGGAGAAAAGGAAGCTAGTCG
+
//+//424:-49888,49;233311...
@T6XXI:00049:00406
GGGAGAAGGGAGAAAGGAAGGAGAAGTCGCCCCAAACGATTTGTTGACTATTCTCCCTCTCCCTATAGTGAGTCGTA
TTAGAATTCCCC
+
?C>?ACCDD@CA@?=A@B@B@BCB@B?B<?BC9BD=AABBB>BD@CCB@@A>A?>>7;;<;>:>@@@899@@@@@@C
C<?@:9948881
@T6XXI:00049:00407
GGGAGAAGGGAGAAAGGAAGCTAGTCGACTCGAGATCTGCGGCTTTGGACAGTCCCTCTGTACTGTGAAGCCACAGA
TGTCGAGTCGACTAGCTTCCTTTCTCCCTTCCTCCC
+
AC>AAB?CB<A;<91;;>;?<::@@:::AA@??==??::6@B666,13;==?000,/.----
34<699=??>998?@@???D=9::4440-//-244660064406,-,-232
@T6XXI:00049:00420
GGGAGAAGGGAGAAAGGAAGCTAGTCACTCGAGCATCTGTGGCTTCACAGTACAGAGGGACTGTCCAAAGCCGCAGA
TCTCGAGTCGACTAG
+
CD@BB@@BB>@@BC@C?B?@>@????>@>>>>11/>==;>@<::6:BDCCBAA@?730,4;:99;;?=:?B;>?=<<
?@???B===CAAA@@
@T6XXI:00049:00441
GGGGAATTCTAATACGACTCACTATAGGGAGAGGGAGAATAGATC
+
0881?8B@BBD?BBCCC?>>A@BB???<6<>667.7::76///-0
@T6XXI:00004:00485
GGGGAATTCTAATACGACTCACTATAGGGAGAGGGAGAATAGCCGCCATTTGTTCATTGGTGCCCTATCCTTCCTTT
CTCCCTTCTCCC
+
```

```
@@@6@?CBCCC@CCBA@@@CCCBB@777.7>>>@>?=<>@@4424437??:AC=@??>=;:444-
7==?:?;?>AA>?@AC?A=???A<
@T6XXI:00004:00497
GGGGAATTCTAATACGACTCACTATAGGGAGAGGGAGAATAGTCAACAAATCGTTTGGGGCGACTTCTCCTTCCTTT
CTCCCTTCTCCC
+
===6@>A?BBB@CC??>BB?@@BA?=?B<:??BB>CAA?AAAA@@>@@A<CC99?6<??5?ACCC@CB@<?<?>AA<
??AA<A<??@A<
@T6XXI:00005:00484
GGGGAATTCTAATACGACTCACTATAGGGAGAGGGAGAATAGCTAAACAGCCTCCGCCCGCAACTCCTACTTCCTTT
CTCCCTTCTCCC
+
9994<@D?CBB?BB??<?BB??@?@@@>;<<<<>=>AA>@@AABBB>B???898>CBC?@AA689@=@@@@=@=A@:
?;=;1838;;B;
@T6XXI:00005:00491
GGGAGAAGGGAGAAAGGAAGAGAATAAACGGATTACAAATTGTAGCGCTATTCTCCCTCTCCCTATAGTGAGTCGTA
TTAGAATTCCCCC
+
DC>CCC@BB<@>??<@>A@BAAB?ACC8<<?BA@B?@B5<7;@@BCCCCCC=>88808=<79;BDC@CA@@=<;6?4
4/78823/0000+
@T6XXI:00006:00466
GGGGAATTCTAATACGACTCACTATAGGGAGAGGAGAATAGTA
+
---*-2@@CDD@CC@@@BBBBCB<?66649B?B>C/.-/,++*
@T6XXI:00006:00482
GGGGAATTCTAATACGACTCACTATAGGGAGAGGGAGAATAGTCAACAAATCGTTTGGGGCGACTTCCTCCTTG
+
9::29242444248883;BA::;76661,6117>8344:5;5900/000+1111;;?>?5961/.,.41101,/
@T6XXI:00006:00489
GGGGAATTCTAATACGACTCACTATAGGGAGAGGGAGAATAGTGACGTGTTGTCGCGGCGCTCACTGTCTCCTTCCT
TTCTCCCTTCTCCC
+
AKA1<,1,///564447<<??:BBBA@A=AAABC>C==;=@@@@?==?4438888AA>@A?>888;:=?8868?A>A
C<?;;<4:7:444-
@T6XXI:00006:00495
GGGGAATTCTAATACGACTCACTATAGGGAGAGGGAGAATAGTTTTCCCTTCACTAACCGCGAACTGCACTTCCTTT
CTCCCTTCTCCC
+
BAA6A===@CC@BBABBBBB?@B>;66646??CC?BAA?A=====5AA<@>=@@BB>?>@@;;<=>><>?B@C?@@>
A?<?:?8<<@@;
@T6XXI:00007:00467
GGGGAATTCTAATACGACTCAACTATAGGGAGAGGGAGAATAGCTTGCAG
+
///*/,/,///,44888::00,....47708666;-1088:444101...
@T6XXI:00007:00476
GGGAGAAGGGAGAAAGGAAGCTAGTCGACTCGACATCTGTGGCTTCACAGTACAGAGGGACTGTCCAAAGCCGCAGA
TCTCGAG
+
BB?BCB>@@:@===:@>C=@@CBBBCCCA@@A===BAACDC=?@@CCA@?>>;==<<<;<=====<><;<<;<=>>=
;8<<<<<
@T6XXI:00007:00482
GGGAGAAGGGAGAAAGGAAGCTAGTCGACTCGACATCGTGT
+
9;:448248-444;58;<<:;:DB@@1//.0,,,,,,,,0.
@T6XXI:00007:00483
GGGGAATTCTAATACGACTCACTATAGGGAGAGGGAGAATAGTCGTAACCAAT
+
```

499/4:A?BDB@CBCABABBCEDB?:::6:>@BB<><<9;897----,-,-,.
@T6XXI:00007:00494
GGGAGAAGGGAGAAAGGAAGCTAGTCACTCGACATCTGTGGCTTCACAGTACAGAGGACGTGTCC
+
44-44;24989AAB@C;<9;CB?AAA54..-..-..../989;>==88896003--,----3244
@T6XXI:00007:00498
GGGGAATTCTAATACGACTCACTATAGGGAGAGGGAGAATAGTTTCACGTTGCCGACTGCTGCTATTCCCTTCCTTT
CTCCCTTCCTCC
+
944,489;>@B@BBBBBDBB??876711,97;BC>A?A>AA@@A=AA@A=<;<:=;9447;;<69=;=<;;;:533+
3+++*+*++++*
@T6XXI:00008:00453
GGGGAATTCTAATACGACTCACTATAGGGAGAGGGAGAATAGCTAACGATTATTCTCCAGGCACGCACTCTTCCTTT
CTCCCTTCTCCC
+
EDD9B8<;;A@B<<=CCCDCCCB@<88?8?AACE??<<=@AAAAB@ACB@<??<=<=9<<=???CCC@A?=8:/44-
4:::4:34444,
@T6XXI:00008:00467
GGGAGAAGGGAGAAAGGAAGGAGACAAAGCCGGATGTGAGTGCACAGCTATTCTCCCTCTCCCTATAGTGAGTCGTA
TTACGAATTCCCC
+
9B<=<<@BC?A@HB9;9;=>;???99919>;>594;733B<<<ABDDCBCC=AAAC>::;;C=BBGCCA::::1::/
0,0//752,000+
@T6XXI:00008:00470
GGGGAATTCTAATACGACTCACTATAGGGAGAGGGAGAATAGTTTCACGTTGCCGACTGCTGCTATTCTCTTCCTTT
CTCCCTTCTCCC
+
BCD:B?A?BDB@BBBBBBBB@?@B<:8?8:ABAC>?>A>A=9==:??AAA<>><AAA?==:::<<=<AAAA>A;==9
=@CC9=9=AAA<
@T6XXI:00008:00478
CTAGTCACTCGAGATCTGCGGCTTTGGACAGTCCCTCTGTACTGTGAAGCCACAGATGTCGAGTCGACTAGCTTCCT
TTCTCCCTTCTCCC
+
C=:;;<::?DCCCBBBBAA@>BBB>B<ABCC::::CCCBBAA???C>AC@CCBBABABACC=?@CCBCCBBB?C<?
?<???C;=9=???:
@T6XXI:00008:00485
GGGGAATTCTAATACGACTCACTATAGGGAGAGGGAGAATAGCTACGCTTAACCGATCGTGTTGTTCTCCTTCCTTT
CTCCCTTCCTCCC
+
CCC8B8;:<9:9=AAA@C<;:CAA??@C>??????9?>=;>A??AAAA?@=B@B=@??44430/06<9<;:;7:344-
/22322,-,-133
@T6XXI:00008:00486
GGGGAATTCTAATACGACTCACTATAGGGAGAGGGAGAATAGTCAACAAATCGTTTGGGGCGACTTCTCCTTCCTTT
CTCCCTTCTCCC
+
@@>4?=@>ACC@CCA??BCB@AB>;661-
6=;?A>@AA>A??@BB>@@@;BBBBC=BBB8CC?@C@???8;8;56><A@@@<@>@???<
@T6XXI:00009:00461
GGGGAATTCTAATACGACTCACTATAGGGAGAGGGAGAATAGCTCCTTACTATTTAACTCCACTGTCCCCTTCCTTT
CTCCCTTCTCCC
+
@@?6@=@>ABC@CAAA?>>?>@BB?76641=;?A>AAA?@<@<BB@B=AA=@ACAA????>@@<???CC6?88/44-
4;;:7=:=>?A<
@T6XXI:00009:00473
GGGAGAAGGAGAAAGAAGCTAGTCGACTCGACATCGTGTGGCTTCA
+
44-//1,1,///7+/4277@AA>1111116111111166;;96,..

```
@T6XXI:00009:00485
GGGGAATTCTAATACGACTCACTATAGGGAGAGGGAGAATAGATCACCGTTGGTTCGGGTGCGCGTTCTCCTTCCTT
TCTCCCTTCTCCC
+
BCC:C>?=@AAADBBBBBBB@C?76111.7>>>>:=<<9=A889CCB?BB?B?A@BBB>BDDADCC@BBA?A?A?AK
>A===7=>===C?
@T6XXI:00010:00450
GGGAGAAGGGAGAAAGGAAGGGGAGAAGGGAGAAAGGAAGCTAGTCGACTCGAGATCTGCGGCTTTGGACAGTCCCT
CTGTACTGTGAAGCCACAGATGTCGAGTCGACTAGCTTCCTTTCTCCCTTCTCCC
+
DCDCCC?BB?BCBB>C?B?BEE9C?B?BC:=766,74747>:;;@1//65==??@@@???A<???:?<<==@AAA;@
=<<====>@??<?A@??>@AAAA=<<CCCCCBBB@;9585880888807;>666,
@T6XXI:00010:00463
GGGAGAAGGGAGAAAGGAAGCTAGTCGACTCGACATCTGTGGCTTCACAGTACAGAGGACTGTCCAAAGCCGCAGAT
CTCGAGTCGACCTAG
+
CGBBCC@CC?<;;A<@>B?CB@?=:.........577676999@<??<CCCCAA>8834::445<<=;==8874384
4448;;<<89A>;;<
@T6XXI:00010:00483
GGGGAATTCTAATACGACTCACTATAGGGAGAGGGAGAATAGTCAACACTTTTAGTTTCCGGTTATTCTCTTCCTTT
CTCCCTTCTCCC
+
BDC:C=?>ABBDCCCCBCCCC?><>777.777BC@GAA?B@@@@@@@@@?AA6AAAA<A=?=?=AA=;;8838;>A@
@@@C;A@A@@@;
@T6XXI:00010:00495
GGGGAATTCTAATACGACTCACTATAGGGAGAGGGAGAATAGTCTTGTATTTTGTGCATCACTTTTCTCCTTCCTTT
CTCCCTTCTCCC
+
BBA/969<@BC@ECCCC@A@BBA@@???9?@ABC>BAA?A@A@??;887==?5@@ACAAACC@@@5??@<=;>9<<7
=@@@<@@@@@@<
@T6XXI:00011:00455
GGGAGAAGGGAGAAAGGAAGGAGAAGTCGCCCCAAACGATTTGTTGACTATTCTCCCTCTCCCTATAGTGAGTCGTA
TTAGAATTCCCC
+
CB>BBBBEC@CBBB<@=@@C@ABCBBBCDCDC8BB?BDCEC>BBACCCA8847??=:@@@?>:?;788==>>?>=>>
?<??99;////+
@T6XXI:00011:00467
GGGAGAAGGGAGAAAGGAAGGAGAAGTCGCCCCAAACGATTTGTTGACTATTCTCCCTCTCCCTA
+
@C:?99598-499;:=<?=>?BBB@A:;9<<<5;9691111,16016>==7547743----.+-+
@T6XXI:00011:00469
GGGGAATCTAATAACGACTCACTATAGGGAGAAGGGAGAA
+
8A@10)-,**0*,*,//47>963?<777.111011,1332
@T6XXI:00011:00492
GGGAGAAGGGAGAAAGGAAGGGGAGAAGGGAGAAAGGAAGCTAGTCGACTCGACATCTGT
+
CD@CAA=>><@BBB=C<>4888.8>>:<=6=777-7074:@:;;>0..2++--2--3852
@T6XXI:00011:00494
GGGAGAAGGGAGAAAGGAAGGACGAAG
+
CCBB::244-9911-1-0,.,...3,.
@T6XXI:00012:00451
GGGAGAAGGGAGAAAGGAAGAGAACAACAGAGGCAACAAAAGACCTGCTATTCTCCCTCTCCCTATAGTGAGTCGTA
TTAGAATTCCCC
+
```

HC@CAA?BC>:<;C@C@B@CCCC?AC@CCBBB@CD@BBBB8BBC?BBCCBB?BAAA=BCCCE>B899A@@@ABAAA@
@=?@@>A;@@@5
@T6XXI:00012:00472
GGGGAATCTAATACGACGC
+
8>>0=+11//788611..,
@T6XXI:00012:00477
GGGAGAAGGGAGAAAGGAAGGAGAAGTCGCCCCAAACGATTTGTTGACTATTCTCCCTCTCCCTATAGTGAGTCGTA
TTAGAATTCCCCC
+
9<6;BBCCC>BBBB>B@C@C@BBA?AAA?@@@8@C?CCBBB?CD=A<;9???A???;@@@@@;@????AA@@AA@A?
@@=<553/---.+
@T6XXI:00012:00490
GGGGAATTCTAATACGACTCACTATAGGGAGAGGGAGAATAGCTAAAACATTATTCCCGTCGCTGTTCCTCTTCCTT
TCT
+
BAA9B=@=ACC?BBBBBCCCCCA??966.7><<<9:<;8<??;??@@5>::744/3998;;74/;;,0,..4,.-
/3043
@T6XXI:00013:00452
CTAGTCGACTCGAGATCTGCGGCTTTGGACAGTCCCTCTGTACTGTGAAGCCACAGATGTCGAGTCGACTAGCTTCC
TTTCTCCCTTCTCCC
+
::1///////8;99:ECBBBC@CCC>C?BBCCBCC>C?B=B@BBAA?A;=AACBBBCB@;::A??@CBACBCB?@C@
CD=@??B>@59/8;3
@T6XXI:00014:00474
TCTAATACGA
+
,///,////4
@T6XXI:00014:00481
GACTCGAGATCTGCGGCTTTGGACAGTCCCTCTGTACTGTGAAGCCACAGATGTCGAGTCGACTAGCTTCCTTTCTC
CCTTCTCCC
+
ABBCCBBBBBBECCD@CDD?CACBBBBBB>BBBBAAABBBBB?BC@BB?>????<<<B@@@B?A@:7;>@;??:?==
=-737>>@:
@T6XXI:00014:00496
GGGGAATTCTAATACGACTCACTATAGGGAGAGGAGAATAGA
+
,,,*-0>@CBB@CCBB>???>?B?C77679?:::9,,+,,++
@T6XXI:00015:00458
GGGAGAAGGGAGAAAGGAAGCTAGTCACTCGACATCTGTGGCTTCACAGTACAGAGGGACTGTCCAAAGCCGCAGAT
CTCGAGTCGACTAG
+
CC?CBB?AC:=;;;-
9>>>AD@??B8:7:7=776==<AAB?BB@CDCA??C@;;33317;;?A@>@C;@@?@@???@?@A???===AAA@@
@T6XXI:00015:00489
CTATCGCTCGAGATCTGCGGCTTTGGACAGTCCCTCTGTACTGTGAAGCCACAGATGTCGAGTCGACTAGCTTCCTT
TCTCCCTTCTCCC
+
B?82,,,,,,,4:?>:=885877.;>ABCBCCB>CCCC>>>@<AAA?AC>BBBCBBAABCCCBCCBBBCB;;<;@BB
<?@BCAA9<<;;6
@T6XXI:00016:00457
GGGGAATTCTAATACGACTCACTATAGGGAGAGGGAGAATAGACCCTTCACGACATCCCAGTCAGCTATTCTTCCTT
TCTCCCTTCTCCC
+
444,4242499548994AAA@>>?7777,76677164</038444+02367777../0,....47..3>83324;==
;=<84+0/00040
@T6XXI:00016:00470

```
GGGAGAGAATTTGGTAATGGGATATTGCAGTGACGTCGGAAGAAGATGGGGCCCTTATATCTCTCTCCCTATAGTGA
GTCGTATTCAGAACTTCCCC
+
CC>AAA@@=@A<A?CC=@@C=@BCCBCCCAB@CCBBDB@C@CC?BA@>>>/9909?C;;;@@A=???@<?;<@9:8A
A@;9664,.---,-0/000+
@T6XXI:00016:00495
GGGAGAAGGGAGAAAGGAAGGAGACACGGTCACAAACATGAATGTGACTATTCTCCCTCTCCCTATAGTGAGTCGTA
TTAGAATTCCCC
+
@A<ACCBCC@BBBB<=;;576;CCDCBC@CBDBAA=BBBCC@CCBBBBBBA::400,0;4:=;?@@AAAA@@@@AAA
A>A?85;34430
@T6XXI:00017:00480
GGGAGAAGGGAGAAAGGAAGGATGAATA
+
DCACCC@CC<@A>;;>;727+---,*,,
@T6XXI:00017:00495
CTAGTCGACTCGAGATCTGCGGCTTTGGACAGTCCCTCTGTACTGTGAAGCCACAGATGTCGAGTCGACTAGCTTCC
TTTCTCCCTTCTCCC
+
A@99994444:;@BBCBBBBB?BCC>C?BAA@???9?ABCCBB@@AAC>?B?BCCCBAAABBBCABBBBBBBBB?B>
BC=A?@B<@=?@@C<
@T6XXI:00018:00465
GGGGAATTCTAATACGACTCACTATAGGGAGAGGGAGAATAGTTGTCAAATCCCGCGCATAACTATTCCCTTCCTTT
CTCCCTTCTCCC
+
GCC:B>@>@CECCBBBAA@BB@CC==@B>BBBBB>B@A>@@@@>???BB>BCC>BBBBAAB?@??A??B18588<=9
@8<<7<9<5;;9
@T6XXI:00018:00466
GGGGAATTCTAATACGACTCACTATAGGGAGAGGGAGAATAGCTTCGACGCA
+
AA@7A898<CB@CBBACCBB<?BB@@@B>=B=BE>C::999+,,5,,,,2+-
@T6XXI:00018:00471
GGGGAATTCTAATACGACTCACTATAGGGAGAGGGAGAATAGACACCAATCGTGCCGGGTCATGTTCTCCTTCCTTT
CTCCCTTCTCCC
+
?=@6@>A>BCC@CA@ACCCCCAA?=666.7==>BBDBA?B=AA@@@>@>CC??77266-
7B??=A?AAA??=?=?@:@???;@>@@@@;
@T6XXI:00018:00475
GGGAGAAGGGAGAAAGGAAGCTAGTC
+
99-44424;:@111-/,/,...777>
@T6XXI:00018:00483
GGGGAATTCTAATACGACTCACTATAGGGAGAGGGAGAATAGTCAACAAATCGTTTGGGGCGACTTCTCCTTCCTTT
CTCCCTTCTCCC
+
@@=4><@=ACCCC@A@BBBB?AB79111-
6==AC>BAA?A;99<=;;@<;@CAAC>CCC9BBCCC@BA?;=:=?AC<@@@@<@<?@CA:
@T6XXI:00018:00484
GGGGAATTCTAATACGACTCACTATAGGGAGAGGGAGAATAGATTGGCGAGAAATGCCGTCTAGTGCTCCTTCCT
+
/<;0<-9244424433940,,,,,,00008<88?;458>A@AAA<?<?@>>@@<@=;99///88687.+*+*+*+
@T6XXI:00019:00458
CTAGTCACTCGAGATCTGCGGCTTTGGACAGTCCCTCTGTACTGTGAAGCCACAGATGTCGAGTCGACTAGCTTCCT
TTCCTCC
+
?:1////4444444;>@@986888/8<?><;666-
1=<??CCCBACC=@B>AABBAA;::?@>=>A88377//:7555+/,,22
```

```
@T6XXI:00019:00464
GGGGAATTCTAATACGACTCACTATAGGGAGAGGGAGAATAGCTCCACCGTCGA
+
DCC7B8;8<@@?@AACCCCBBBB?>777.7>@CE>C@A>@?/./75,,*++++0
@T6XXI:00019:00478
GGGAGAAGGGAGAAAGGAAGGAGAAGTCGCCCCAAACGATTTGTTGACTATTCTCCCTCTCCCTATAGTGAGTCGTA
TTAGAATTCCCC
+
GE@BBBACC@@@=C@C>@>A@BCC@C?@?@@>2??:?ABCC>BC@CAA?AA?A@AA<????B;@@AA?@@<A???==
=>@AA?;/444-
@T6XXI:00019:00493
GGGAGAAGGGAGAAAGGAAGTGCAGTTCGCGGTTAGTGAAGGGAAAAACTATTCTCCCTCTCCCTATAGTGA
+
9=599>599-4999:=<;;799@@@=<:6..,.,..43444406666/793101666211111,00044535
@T6XXI:00019:00495
CTAGTCGACTCGAGATCTGCGGCTTTGGACAGTCCCTCTGTACTGTGAAGCCACAGATGTCGAGTCGACTAGCTTCC
TTTCTCCC
+
<11/////:?@@CABACBAAA=??>8?588?6999:@A=;;;0//11556@?>@797711111668=7733166363
6661000+
@T6XXI:00020:00450
GGGGAATTCTAATACGACGTCACTATAGGAGAGGGAGAAT
+
:;;1<,959982446//...66226...,.4...+...5.
@T6XXI:00020:00471
CTAGTCGACTCGAGATCTGCGGCTTTGGACAGTCCCTCTGTACTGTGAAGCCACAGATGTCGAGTCGACTAGCTTCC
TTTCTCCCTTCTCCC
+
?11//////689??@CB@@9948880;?@ACCBBB@BBBCB@BBBB@C?AB?BBBBBBBBBC@??A?BCCCCAA?A?
BB>BBBC<?:<;;=:
@T6XXI:00020:00475
GGGGAATTCTAATACGACTCACTATAGGGAGAGGGAGAATAGTCAACAAATCGTTTGGGGCGACTTCTCCTTCCTTT
CTCCCTTCTCCC
+
GBC7B8;8<CC?BBBCCCCC@?>>>777.7==@C?C@@>A@@@==;=AA<CCA@=.777-
7=@AB@AAA=<9<=@@<@@@A<A689BD?
@T6XXI:00020:00478
GGGGAATTCTAATACGACTCACTATAGGAGAGGGAGAATAGTCAACAAATCGTTTGGGGCGACTTCTCCTTCCTTTC
CTCCCTTCCTCCC
+
EIC:A8;;<9;@DAA@CBAAA;;;9//,000>><A@A>@AA@@@=@?A=CBA?B>BCC8CCCAA7:1>:;79,//,/
,/6406,4//440
@T6XXI:00020:00481
GGGGAATTCTAATACGACTCACTATAGGGAGAGGGAGAATAGCCAGCCTCC
+
8970=+1,///568=@?@@BBBBAA:77.7777B<A;:779./,---,-+*
@T6XXI:00020:00495
GGGAGAAGGGAGAAAGGAAGCTATCGACATCTGTGGCTTCACAGTACAGAGGACTGTCCAAAGCCGCAGATCTCGAG
T
+
9A<@@B>BB@BDCH?C@B?BBBBDCC??844444424999FCEBBCA?;01,0000>C@CC>CC@CCBBAAAC??AB
?
@T6XXI:00021:00483
GGGAGAAGGGAGAAAGGAAGAATAGATGCGGGGGAGTTAATGTTAAACTATTCTCCCTCTCCCTATAGTGAGTCGTA
TTAGAATTCCCC
+
```

```
DC>DCB=>><@B@C=B?B@BB?BCBBBFBBBB?1>::67:;@A?BD@BBBC@CCBB>CCBBC>DBBBBABCCD??=9
958AA?A:ADC5
@T6XXI:00021:00490
GGGGAATCTAATACGACTCACTATAGGGAGAGGAAGA
+
8>A18+1///76449783311311111,..34,.,-8
@T6XXI:00022:00478
GGGGAATTCTAATACGACTCACTATAGGGAGAGGGAGAATAGTTATCTCCTCGGCTTCGCTAACCTGCGCTTCCTTT
CTCCCTTCTCCC
+
DDB9B=A?ADBCCCBABCCCCBA>=7776:@ABE>CAA=@@@?=?@<<<=@@B?CF@AAACB;?;788887?@=@@6
83?;-43838:7
@T6XXI:00022:00482
GGGGAATTCTAATACGACTCACTATAGGGAGAGGGAGAATAGTCAACAAATCGTTTGGGGCGACTTCTCCTTCCTTC
TCCCTTCTCCC
+
?CC9A?B@<==ABCD<<<DCCBB==777.7??CE>@@@?=<<?;:799:6???AB<BDB6??BBC<@88,/,/,/,-
---+-030031
@T6XXI:00023:00455
GGGAGAAGGGAGAAAGGAAGCTAGTCGACTCGAGATCTGCGGCTTTGGACAGTCCCTCTGTACTGTGAAGCCACAGA
TGTCGAGTCGACTAGCTTCCTTTCTCCCTTCCTCCC
+
CC<@@A@A@:A@@@<A>CC@@?>=</00;;;@D@BBCBBBBA:::7:5:;CBBCC?@@?999AA@@@A>@@>@@@AA
A???AAAA@==<888@@>@>AC799=928,/,//.+
@T6XXI:00023:00456
GGGGAATTCTAATACGACTCACTATAGGGAGAGGAGAATAGTCAACAAATCGTTTGGGGCGACTTCTCCTTCCTTTC
TCCCTCTCCCTATAGTTATAGAACTTCCCC
+
CCE:C?A@BCC@CBBBCBCBBC;7;111.0000,.3345;??@@>@@@;CCCBB>BEE7??CCE7::C7838<;;68
778./.3495;885,,*+++02324/000+
@T6XXI:00023:00471
GGGAGAAGGGAGAAAGGAAGGAGAAGTCGCCCCAAACGATTTGTTGACTATTCTCCCTCTCCCTATAGTGAGTCGTT
ATTC
+
/1+/6=;>=:;99B195951,079,/,000000000,,..3036889?@@:93344-
..44757=<>95300,++*)*.)+
@T6XXI:00023:00483
GGGGAATTCTAATACGACTCACTATAGGGAGAGGGAGAATAGCTGAGTGGCCAATGCGACCAACTATCTCCTCCTTT
+
///*/,/5611,447?<<?@>78777162;66;6600355;;=??7603/0/02403465:=;9944555...3110
@T6XXI:00023:00497
AGTCGACTCGAGATCTGCGGCTTTGGACAGTCCCTCTGTACTGTGAAGCCACAGATGTCGAGTCGACTAGCTTCCTT
TCTCCCTTCTCCC
+
<?<1//:999;>CCBBBAA>B@B=B@CCCCBBB>B@@@A??ABCB?>AE@B@?=<?@@@AA???AA@??@?A=A?BB
>B@AD<A9==@C<
@T6XXI:00024:00451
GGGGAATTCTAATACGACTCACTATAGGGAGAGGGAAGAATAGTTTCACGCTGCCGACTGCTGCAT
+
<A;6>:96444<4//:/0.84.3311171/.335+.,--,--8<<1448==58=97400033330/
@T6XXI:00024:00473
GGGGAATTCTAATACGACTCACTATAGGGAGAGGGAGAATAGCTTGCAGCTCGAGCCTGTGACTGTTCTCTTCCTTT
CTCCCTTCTCCC
+
CHN9B@B@CBB@CBBBBAAAA@<97116.777BC>B@@?A<??B=@@CCBBBCB=@?@??<A@@<<;;447;>:=?<
?@@C<A;==@@<
@T6XXI:00024:00486
```

```
GGGGAATTCTAATACGACTCACTATAGGGAGAGGGGAGAATAAGCCCGACAATTCGACTCCCCC
+
944,429244:944383.....67///7766..00+/..,--,-343833359<?966.,,,,)
@T6XXI:00025:00451
GGGGAATTCTAATACGACTCACTATAGGGAGAGGGAGAATAGTTTTCCCTTCACTAACCGCGAACTGCACTTCCTTT
CTCCCTTCTCCC
+
CB?7B===@CC@CB??BBBB@A@?7111-
6>>CC>B@A?C=@<<<5A=;A>@?CCCA?>>??<>=?<<@>A=?4995:>?B9?;<<<?;
@T6XXI:00025:00453
GGGGAATTCTAATACGACTCACTATAGGGAGAGGGAGAATAGCTCCCAAACCACGTTACGTGCGACCCACTTCCTTT
CTCCCTTCTCCC
+
DLK9C?A?ABB?BCBAB:;A>??=777-7<?A?<B8/,-
///..424455755??>>@@CACBCDKCHBB>@69919>>>8>;>788,
@T6XXI:00025:00457
CTAGTCGACTCGAGATCTGCGGCTTTGGACAGTCCCTCTGTACTGTGAAGCCACAGATGTCGAGTCGACTAGCTTCC
TTTCTCCCTTCTCCC
+
C99449449=@@CBBBBBBBC@CCC?C@B@:::::5:CCCBBAB@?;@<=A?@CC@@@CCBCCBABB;88:0/.,.,
.53576::77?7775
@T6XXI:00025:00459
GGGAGAAGGGAGAAAGGAAGCTAGTCACTCGACCATCTGTGGCTTCACAGTACAGAGGGACTGTCCAAAGCCGCAGA
TCTCGAGTCGACTAG
+
DC@CCC@BB>9;9C=B>B>A@@:;;@73337//,....73638;=?ACBECBA==444-
4;?<==?AC;??;>==;;<<<@@@A@AA===AD
@T6XXI:00025:00479
GGGGAATTCTAATACGACTCACTATAGGGAGAGGGAGAATAGCCATTCGATAAGCACCCCGATATATTCTTCCTTTC
TCCCTTCTCCC
+
999/98?<9<;;?@A@BADBBBCCBAAA<ABBBC>A??698??==@<??==@>9<<DCD9CDCA77747606366,9
7779?76144,
@T6XXI:00025:00482
CTAGTCGACTCGAGATCTGCGGCTTTGGACAGTCCCTCTGTACTGTGAAGCCACAGATGTCGAGTCGACTAGCCTTC
CTTTCTCCCTTCTCCC
+
;8,---
,,68:>CDBBBBBAA?ACC@C=ACAA:::5:@@ABCBA<??A>@B>BBCB?@?CBBABABBA@AB:;;<ADACB?BC
AC;@489BD;
@T6XXI:00026:00474
GGGAGAAGGAGAAGAAGA
+
BB>@11,1,1,,*,,*..
@T6XXI:00026:00478
GGGGAATTCTAATACGACTCACTATAGGAGAGGGAGAATAGTTAACGCACCCTCCGTTACCTGTTCACCTTCCTTTC
TCCCTTCTCCC
+
CGB8B7;8498949998>>@;/0:<00,/7/11,13325442224//-
/1626631101.53..,.455737477,1389;=555==4
@T6XXI:00026:00483
GGGAGAAGGGAGAAAGGAAGGCGTCGTTCGGGGAGTAGTATTGGTAACTATTCTCCCTCTCCCTATAGTGAGTCGTA
TTAGAACTTCCCC
+
@@:>@@=@@<AC>>:<:9,1,043;;:7:75:5/0.:::4837>./,/00/5575755:73=:?@@A??888><<;A
A8<8?8;32888.
@T6XXI:00027:00450
```

```
GGGAGAAGGGAGAAAGGAAGCTAGTCGACTCGACATCTGGTGGCTTCACAGTACAGAGGACTGTCCAAAGCCGCAGA
TCTCGAGTCGTAG
+
CA<@AB?AB<;<;B?CDC@CFC????:77==77/.43..,.54388:?=B@??;;;//./;>8:?<??7>B=@@@@A
???A@@=;;////
@T6XXI:00027:00460
GGGGAATTCTAATACGACTCACTATAGGGAGAGGGAGAATAGTGACATGTTGTGCGGCGCTCACTGTCTCCTTCCTT
TCTCCCTTCTCCC
+
999/954944888;@9488877331111,16637:7575.0.34899?6626?BCA@>=//...,,....,.534<<
;5334+0/--110
@T6XXI:00027:00487
GGGGAATTCTAATACGACTCACTATAGGGAGAGGGAGAATAGCTACCAGAACT
+
CCB9>;>=@BB@CCCCCAABBBB@????9????C>@@A??:8:/00:--+*++
@T6XXI:00027:00493
GGGAGAAGGGAGAAAGGAAGGCGTTAGCGCAATCAGGTATGTGGGAGCTATTCTCCCTAGAAACATGCGCCATTTGC
TTCCTTTCTCCCTTCTCCC
+
99594989C=CDCC=B8;67;?>B=@@BBCC@BBAB=999?@?>:>>?@995848>988999,33444442333,3<
7:A@CA;?@BD9?::74:7
@T6XXI:00027:00497
CTATCGACTCGAGATCTGCGGCTTTGGACAGTCCCTCTGTACTGTGAAGCCACAGATGTCGAGTCGACTAGCTTCCT
TTCTCCCTTCTCCC
+
C;::1111;;BCCBCBBBBB?A@A>B?>:?@?<?9??@BCB@@:??C=?B@BCCCCC?@?BCCBBBCDCB::9:?;?
C>E@@A=A@AAAA<
@T6XXI:00028:00453
GGGAGAAGGGAGAAAGGAAGGTGGGTGTGGAAGGTTAGATGCTGCATCTATTCTCCCTCTCCCTATAGTGAGTCGTA
TTAGAATTCCCC
+
CC?AA@=AC>BBCD@C@C?A=ABB<AA??;??A?B?BBBBBBBCCDBBBBBACCCB>AB<;;6;?=>?@??>C@@48
5>@AA<=<==A0
@T6XXI:00028:00494
GGGAGAAGGGAGAAAGGAAGCAGGCCATCTGTGGCTTCACAGTACAGAGGGACTGTCCAAAGCCGCAGATCTCGAGT
CGACTAG
+
4=5499<AA<@@@F<A?B=?>>@473::;:CED=@@<:9?@AABF=>88818B=899@CC<@@9<=A@A===A=999
BBC999A
@T6XXI:00028:00497
CAGTCCCTCTGTACTGTGAAGCCACAGATGTCGAGTCGACTAGCTTCCTTTCTCCCTTCCTCCC
+
@@@9991444BBHBBBB@@7::<ABB<;;AABIDCCCCCB9944424244-/66856,.,.462
@T6XXI:00029:00479
GGGAGAAGGGAGAAAGGAAGCTAGTCGACTCGACATCTGTGGCTTCACAGTACAGAGGGACTGTCCAAAGCCGCAGA
TCTCGAGTCGACTAG
+
:C?AAA@DG>A@AB9?=@8;;@=>@0.//....669?<<<?><@876????A;;;444-
4;;@A@>AA:==?A@@@@??<<<8661------
@T6XXI:00030:00456
GGGAGAAGGGAGAAAGGAAGAATGACGCCGGGAGCGATGATTACGTGGCTATTCTCCCTCTCCCTATAGTGAGTCGT
ATTACGAATTCCCC
+
CD>CCA>@@194888000)**)****//59:5====99=992844:A?==9;24994-44444-
44444833;;9944730../,.6@@@7
@T6XXI:00030:00462
GGGGAATTCTAATACGACTCACTATAGGGAGAGGGAGAATAGTACAA
```

```
+
22707+1,///7/444977;;>A@:::>.77777-7444484/-/.,
@T6XXI:00030:00471
GGGAGAAGGGAGAAAGGAAGCTAGTCGACTCGACATCTGTGGCTTCACAGTACAGAGGGACTGTCCAAAGCCGCAGA
TCTCGAGTCGACTAG
+
DDBCCC@BB=BB==:@696;C???C:;7?===??????AAL@BB?BBBCCCD:88000-
4;;;<=?@@;@A@AA<;;9==@889???@AA??
@T6XXI:00030:00494
GGGAGAAGGGAGAAAGGAAGGAGAAGTCGCCCCAAACGATTTGTTGACTATTCTCCCTCTCCCTAT
+
44-4?99@B:BBA@>>11,0900.,//99999-66-17779995,..----,-35;;==85;0-++
@T6XXI:00030:00498
GGGAGAAGGGAGAAAGGAAGCTAGTCACTCGACATCTGTGGCTTCACAGTACAGAGGGACTGTCCAAAGCCGCAGAT
CTCGAGTCGA
+
CE@ECC@BB=BAAB>B@B@BBAA===@>>B;00.///B@C=??@CA?>>A<=<<=<<;<===><;<<;<=>A<=>==
9==@?@A@@@
@T6XXI:00031:00451
GGGGAATTCTAATACGACTCACTATAGGGAGAGGGAGAATAGTTTTTTCCCTTCACTAACCGCGAACTGCACTTCCT
TTCTCCCTTCTCCC
+
CCB9C=@=@CB@CCBBBAAAB@;97111.7>>?C@C@B>@AA@AAAA-
AA<A=AAAAC@C?=?@@=?@B=???;=377.7>>@<@=><<<7
@T6XXI:00031:00473
GGGAGAAGGGAGAAAGGAAGCTAGTCGACTCGACATCTGTGGCTTCGACAGTACA
+
49-449244-444;6924237788>>7771111119868<@490;0../,,,.41
@T6XXI:00031:00477
GGGGAATTCTAATACGACTCACTATAGGGAGAGGGAGAATAGTCAACAAATCGTTTGGGGCGACTTCTCCTTCCTTT
CTCCCTTCTCCC
+
999/95<:<CC@DBBBB9;;A?>?>777-
7?===<?@@>A=AA@@>@AA<B???C>CCB8BBCCC@BBB57378??<?CDC?D688<<6
@T6XXI:00032:00469
GGGAGAAGGGAGAAAGGAAGGAGAAGTCGCCCCAAACGATTTGTTGACTATTCTCCCTCTCCCTATAGTGAGTCGTA
TTAGAATTCCCC
+
BCA;<;>AA<@@@B8;59>A>?@@A?=>8888.8>8>=761169;ABBA<;885534889996:==>@@@@A@@@A?
?<?AA>C>@@@5
@T6XXI:00032:00496
GGGAGAAGGGAGAAAGGAAGCTAGTCGACTCGACATCTGTGGCTTCACAGTACAGAGGGACTGTCCAAAGCCGCAGA
TCTCGAGTCGACTAGCTTCCTTTCTCCCTTCTCCC
+
EC>B9959>9><<<8;-
0,...4440///,,7,/....5310367=77:@;84;;44818>B<=<>DD?BA>A@@@?>888?@???@A@@?>>@
9<9=D<A@AC<A69:AA@
@T6XXI:00033:00479
GGGGAATTCTAATACGACTCACTATAGGGAGAGGGAGAATAGCTACCGTCCGTTCCCTGCTATGCTGTTCTTCCTTC
TCCTCTC
+
>:70;,1,/6676=@;;;BBCCBB?777.77<?B<?===?@:9=7:37??744/00+00044;5;;59498/0/043
16,+++,
@T6XXI:00034:00456
GGGGAATTCTAATACGACTCACTATAGGGAGAGGGAGAATAGTCAACAATTCGTTG
+
499/42924449999;9;8;??>>?77:5:??AB>A??>@>8803233/1011101
```

```
@T6XXI:00034:00485
GGGAGAAGGGAGAAAGGAAGGAGAACTCAACGGCAGACAACTGCTGCTATTCTCCCTCTCCCTATAGTGAGTCGTAT
TAGAATTCCCC
+
CC@CCBADD@BDCC@C?B?B>@;:7<CCG?CB=@BCCDC@@>8000...977;;;:9ABAB>B:::BBA<<<AA:77
:4332424;;2
@T6XXI:00034:00492
GGGAGAAGGGAGAAAGGAAGGAGAAGTCGCCCCAAACGATTTGTTGACTATTCTCCCTCTCCCTATAGTGAGTCGTA
TTAGAATTCCC
+
?B?;<=ADC@CDGC>B?@7;7;??8?=BBDDB7CC>B><A<;9A=>8549;89444-
388;B9=988DB=;;00/::;9;;7./-,51
@T6XXI:00034:00497
GGGGAATTCTAATACGACTCACTATAGGAGAGGGAGAATAGCAACGTTTGCTCCCAGATCCCCGTGTACTTCCTTTC
TCCCTTCTCCC
+
>:85812,/88,/498337767455/0,/9/11,100/08000/016885<6<;10000033.000000023;<=99
459-,*+++0*
@T6XXI:00035:00473
GGGGAATTCTAATACGACTCACTATAGGGAGAGGGAGAATAGTCAACAAATCGTTTGGGGCTACTTCTCCTTCCTTT
CTCCCTTCCTCCC
+
:??1=,458449:4;<@@CCEBB?=776.7<777.7=>;<<==99<<==;??A@?1116-617?>-//7-/-
.555557A?78/3/444,
@T6XXI:00035:00495
GGGGAATTCTAATACGACTCACTATAGGGAGAGGGAGAATAGTCCGCCAATTCTGCGCGTTAGCATTCTCCTTCCTT
TCTCCCTTCTCCC
+
?@@6@@A>BCCACCBAABBB@@>>966659ABCC>???>A<;?@=@?>B@B@@>@BAA===<B@AA?ACC=A>A;??
9?;;<7<=@@@A;
@T6XXI:00035:00499
AGTCACTCGAGATCTGCGGCTTTGGACAGTCCCTCTGTACTGTGAAGCCACAGATGTCGAGTCGACTAGCCTTCCTT
TCTCCCTTCTCCC
+
@@@A=?998B<>@<;;@@;>@@<A@BCBBDCC@AA@@?BBB@A@B?CB>???@@@@@@ABA888:40-
./,/,/,.52/636+0/-1440
@T6XXI:00036:00481
GGGAGAAGGGAGAAAGGAAGGAGAAGTCGCCCCAAACGATTTGTTGACTATTCTCCCTCTCCCTATAGTGAGTCGTA
TTAGAATTCCCC
+
BE>BBA?AA<;;;A<A?B?B@CDC?@=?@BBB7BB=BBBBB=BE@CBB@@@>?>?C=B===A<889@=<<@@===??
?@@A@=@9<<;1
@T6XXI:00037:00455
GGGGAATTCTAATACGACTCACTATAGGGAGAGGGAGAATAGTTTTTCCCTTCACTAACCGCGAACTGCACTTCCTT
TCTCCCTTCTCCC
+
C@@4><A>BBB?BBBBB@@@C@@?=666.7>>BC>B@@CA=;<<<;0;A7?=?BBB@>A?:::A<??A@BAB<>477
.7A@C@C@@@@@<
@T6XXI:00037:00476
GGGGAATTCTAATACGACTCACTATAGGGAGAGGGAGAATAGTTATAACCTCCCTAAAGCGTGCATTGCCTTCCTTC
TCCCTTCTCCC
+
?A?4@5959;@<BBBBCBBBCB@>;677.7??BF>CAA=@:@=>=A@;<:444-4=;:>@@@988?;//,/79,/,-
222479@<AA<
@T6XXI:00037:00481
GGGGAATCTAATACGACTCACTATAGGGAGAGGGAGAATAGCTAGCATATTGTT
+
```

```
;>>5>1118;::?A@@<;;C@A?>777.77777,03323344617318448002
@T6XXI:00037:00485
GGGGAATTCTAATA
+
4;;,42/,//7,//
@T6XXI:00037:00498
GGGAGAAGGGAGAAAGGAAGTGCAGCTCGCGGTTAGTGAAGGGAAAAACTATTCTCCCTCTCCCTATAGTGAGTCGT
ATTAGTTCCCCC
+
CD?BBC@DC:;;;A<A>@@?7999>>???CC=@@ACBBC?AA8?>>>/>@@@?BBCC?A?@?C>:;;@@@@?=888;
9:441101111.
@T6XXI:00038:00457
GGGGAATTCTAATACGACTCACTATAGGGAGAGGGAGAATAGTCAACAAATCGTTTGGGGCGACTTCTCCTTCCTTT
CTCCCTTCTCCC
+
07718+92444888@AAACC@?@A>===.77777.7;9=@:6886233:066666,111,1.022*,,2*+*+0003
446936230481
@T6XXI:00038:00462
GGGGAATTCTAATACGACTCACTATAGGGAGAGGGAGAATAGCTCGCTAACCGATTG
+
499/4292448888>998<>?;??7667-77777.:;;=B@8/0/....,.,.6701
@T6XXI:00039:00471
GGGGAATTCTAATACGACTCATTATAGGGAGAGGGAGAATAGTCAACAAATCGTTTGGGGCGACTTCTCCTTCCTTT
CTCCCTTCTCCC
+
DGD:C;:<<=A=;<<AAACCCC>==611-
6=7775:==<??DD@?<@AC<AA???6=>>5?A@??=<;;./,/566178??;@>???C;
@T6XXI:00039:00472
GGGAGAAGGGAGAAAGGAAGTGCAGTTCGCGGTTAGTGAAGGGAAAAACTATTCTCCCTCTCCCTATAGTGAGTCGT
ATTAGAATTCCCC
+
GH@BBB@DH>BBCC>B@B@CCCCBCC@CBCC?B?BCCCC@C?>??@@2B>>B@CBB@=BB???;??>?AA?:><=<<
??<?@A?HC@@A6
@T6XXI:00039:00473
GGGAGAAGGGAGAAAGGAAGGAGAACAACACGATCAGTTAAACGTAGCTATTCTCCCTCTCCCTATAGTGAGTCGTA
TTAGAATTCCCC
+
BBABDB@BB?B@BBBB?@?A?ABB@BC@B@AACBBB::;;C@C@@@BB@@?=?BBCBCCCCB>@@<<==<<<=<<<=
=:=AA>A?AAA5
@T6XXI:00039:00492
GGGAGAAGGGAGAAAGGAAGCTAGTCGACTCGACATCTGG
+
9=:AAC@AB8999919<><@A?=>>0//5;;;>7222,,*
@T6XXI:00040:00456
GGGAGAAGGGAGAAAGGAAGGAAGGGAGAAAGGAAG
+
8A-119599-4444-424231377963111,13676
@T6XXI:00040:00463
GGGAGAGGAGAAG
+
<;81--,*,,,*,
@T6XXI:00040:00471
GGGAGAAGGGAGAAAGGAAGCTAGTCACTCGAGATCTGCGGCTTTGGACAGTCCCTCTGTACTGTGAAGCCACAGAT
GTCGAGTCGACTAGCTTCCTTTCTCCCTTCTCCC
+
;;6=AA<?>:>>@A<C>B>;;;@>@??:76???@BBBC??:777.7=@ABBCCD>BAA898A@????>??>?AA@@@
@AA???<<<==:=7778;;>>188@@:=9;;<?4
```

```
@T6XXI:00040:00479
GGGGAATTCTAATACGACTCACTATAGGGAGAGGGAGAATAGGCCACTTCTTCCTAATCCTGTTCTATTCTTCCTTT
CTCCCTTCTCCC
+
944,48><@@@>BCBBC@@@AAB?>7775;@@BCAC==;=AA>A=AAA>AA6748@=@@>AAA;??AA774:;344+
0444+0/0000+
@T6XXI:00040:00491
GGGAGAAGGGAGAAAGGAAGGAGAAGTCGCCCCAAACGATTTGTTGACTATTCTCCCTCTCCCTATAGTGAGTCGTA
TTAGAATTCCCC
+
ED@CCC@CC>BBCC@B@C@C?@@?@CECCCBB8BBACCBB@;BG@BAA:==;=@AD?C=<=A<@@@@=<<<<<>>;<
;;865555===1
@T6XXI:00041:00455
GGGAGAAGGGAGAAAGGAAGCTAGTCACTCGACATCTGTGGCTTCACAGTACAGAGGGACTGTCCAAAGCCGCAGAT
CTCGAGTCGACTAG
+
BB?BCC@CC<@@885>;>;?BBBBDCC????:;:?????A>AC@ACDCBABBA=47848@AAAA=??:@C?AA===A
<?????B?????@?
@T6XXI:00041:00485
GGGAGAAGGGAGAAAGGAAGTGCAGTTCGCGGTTAGTGAAGGGAAAACTATTCTCCCTCTCCCTATAGTGAGTCGTA
TTAGAATTCCCC
+
CB@BBBACC=A@@B<B@>=>BBBBAB?BBAA@C@CCCCC?CA>AAA9CDCA?@CCC=CCCCC????@@@DAA@@@@@
@=@AA>A>A@@5
@T6XXI:00042:00454
GGGAGAAGGGAGAAAGGAAGCTCAATCGTGGGTAGAAGGGTCGTGATCTATTC
+
CC@CDC@B@199990949483999=?<<996617188:;983700-4---+*+
@T6XXI:00042:00460
GGGGAATTCTAATACGACTCACTATAGGGAGAGGGAGAATAGTCTGACGCTATACGCCACCTATTCATCTTCCTTTC
TCCCTTCTCCC
+
GFC9B798<@@?DBBBCABAA9<<=777.7==?@>BAA>A@AA???CBCCBBBCBB@<BB@C@?=?B::;>B677.7
777.434;:;9
@T6XXI:00042:00478
GGGGAATTCTAATACGACTCACTATAGGGAGAGGGAGAATAGTCAACAAATCGTTTGGGGCGACTTCTCCTTCCTTT
CTCCCTTCCTCCC
+
BEC9B;<;<AA7:;B@@@@B@CB?>777.777>>9@:::<AAAAA<??@<CAAAC@CCB7??F;;5:17:42:7880
;33337,/,/660
@T6XXI:00043:00473
GGGAGAAGGGAGAAAGGAAGGCTAGTCACTCGACATCTGTGGCTTCACAGTACAGAGGGACGTGTCCAAAGCCGCAG
ATCTCGAGTCGACTAGCTTCCTTTCTCCCTTCCTCCC
+
CC><=<>AC<@@@@<><>=@9;;BC?000....54458667017:8;;<=?CC@?:88,//----
334747=<=;<<<<455===<999<664;<3834;943330321,.-10
@T6XXI:00043:00475
GGGGAATTCTAATACGACTCACTATAGGGAGAGGGAGAATAGTTTCCTACTCCCTGGTATCACCATTCCACTTCCTT
TCTCCCTTCTCCC
+
CCC8B79:<@@=BCC<<<CCAAAA>776.6<<?AAAAA?@@<<;6;<?@@AAB<?@:===6@@=<<8;;::;8:244
+4::A;?<?CAC<
@T6XXI:00044:00451
GGGAGAAGGGAGAAAGGAAGGTGGTAGCATCACTGGCGAATGGGTGCTATTCTCCCTCTCCCTATAGTGAGTCGTAT
TAGAATTCCCC
+
```

```
DC>BCC@CCBCCBB>B?B@BABB?A;;<@@@CCBB;;<C@CCC:;<CB@@@EEDC?BBBBB>B@@?AAAAAAAA@?@
>@AA>A>@@@5
@T6XXI:00044:00465
GGGAGAAGGGAGAAAGGAAGGACAGTCCATCTGTGGCT
+
CB>AA@@CC>BB@=6><>275110...,..44611216
@T6XXI:00044:00467
GGGGAATTCTAATACGACTCACTATAGGGAGAGGGAGAATAGTCAACACTTTGGTTGCACCCCCACGTCTCCTCCTT
TCTCCCTTCCTCCC
+
B@;8@:;:<=>=@9;;;8>?A?@>?777.77A??6:8<;??@??@=?>8;;6:;=;>ABBCDE2?>>;000,0/784
47523+-,.,/66,
@T6XXI:00044:00490
GGGGAATTCTAATACGACTCACTATAGGGAGAGGGAGAATAGTTTTCCT
+
,--*-+1,/88569;;@@DFCBB>>777.77777-434444-/--*-,-
@T6XXI:00045:00451
CTAGTCGACTCGAGATCTGCGCTT
+
4444444444444;449/,,,,,*
@T6XXI:00045:00486
GGGGAATCTAATACGACTCACTATAAGG
+
8AA0?)1///5699=>880/,,,,,*,*
@T6XXI:00045:00487
GGGGAATTCTAATACGACTCACTATAGGGAGAGGGAGAATAGTCAACAAATCGTTTGGGGCGACTTCTCCTTCCTTT
CTCCCTTCTCCC
+
444,4595999<@C@@@@CCBBBA????9??@ACB@@A>@@?@@A<??@<@?@AL@BCC7@?BCB5:3=3738=?CA
@@CA=A588DC:
@T6XXI:00045:00493
GGGGAATCTAATACGACTCACTATAGGAGAGGG
+
=?<0<-1///78?@@@;:;?000/0.,..377;
@T6XXI:00045:00494
GGGAGAAGGGAGAAAGGAAGGAGAACATGACCCG
+
44-499244-44490823997731044//888+/
@T6XXI:00046:00454
GGGGAATTCTAATACGACTCACTATAGGGAGAGGGAGAATAGTCTCCCAGTCTGGATTCTCACATTTCCCTTCCTTT
CTCCCTTCTCC
+
CDC:C>@=@CC@CBABB>>>B@<97116.7>>@B>B@@?@<=<9==A<CCCCBB???:?A?895@A;CC9?37311,
7.--+-01--,
@T6XXI:00046:00456
GGGAGAAGGGAGAAAGGAAGGAGAAGTCGCCCCAAACGATTTGTTGACTATTCTCCCTCTCCCTATAGTGAGTCGTA
TTAGAATTCCCC
+
AC>BCA>BB=BBBB>D?B?A@CCB@?993377-
;B<BBCCC>CC@C??=AA?A7771748?@;@???AAA===;;88=>BDC?C3844+
@T6XXI:00046:00463
GGGAGAAGGGAGAAAGGAAGCTAGTCACTCGAGATCTGCGGCTTTGGACAGTCCCTCTGTACTGTGAAGCCACAGAT
GTCGAGTCGACTAGCTTCCTTTCTCCCTTCTCCC
+
99144=;=9-
84890499979;BCCAA::866149888;@59:?8679::@?CC>:88888<=AAAA>AA>???A><;<<9:::838
99=;9976,//,/666,/,.---+
```

```
@T6XXI:00046:00476
GGGAGAAGGGAGAAAGGAAGGAGATCAGACACGATGATCGGGTTAT
+
440449244-44:;-1,/,.,...443.6696667664000+-0--
@T6XXI:00046:00489
GGGAATCTAATACGACTCACTATAGGGAGAGGGAGAATAGTCCCACAATTC
+
1/+1,94449;44::;;9;6711111,11776,144256<:64240-,.,.
@T6XXI:00047:00483
GGGGAATTCTAATACGACTCACTATAGGGAGAGGGAGAATAGTGACGTGTTGTGCGGCGCTCACTGTCTCCTTCCTT
TCTCCCTTCTCCC
+
@@=4?595;@A?CCCAA@@@CCB?=677.777>@<???>?@AAA?>?@@<;:<@AA>@@@@@@<@<8;;=8876,//
,/668,/,/;;>9
@T6XXI:00047:00488
GGGGAATTCTAATACGACTCACTATAGGAGAGGA
+
88808,1,///76444883366711110110/,.
@T6XXI:00047:00489
GGGGAATTCTAATACGACTCACTATAGGGAGAGGGAGAATAGTCAACAAATCGTTTGGGGCGACTTCTCCTTCCTTT
CTCCCTTCTCCC
+
,-,*-+1,/66769;;;<CCCCB>>777-7====9<@@>@AA@??=A@D:A:;7?.777-
7;;666;44/4249;;9;444-48;:=A<
@T6XXI:00047:00493
GGGGAATTCTAATACGACTCACTATAGGGAGAGGAGAATAGTCAACAAATCGTTTGGGGCGACTTCTCCTTCCTTTC
TCCCTTCTCCC
+
@=94>6;8;BC@BEBABCCABCC@>::;699/0,.1101466649444-7===>-
777,667:>;;46,/,/,//,//44,44;6;=;
@T6XXI:00048:00467
GGGAGAAGGGAGAAAGGAAGCTAGTCGACTCGACATCTGTGGCTTCACAGTACAGAGGGACTGTCCAAAGCCGCAGA
TCTCGAG
+
DD@AA@@BA:@@==6;>@133>?BAABB?@@:::?@>>>7737847>@@BB?;:;444-
4:;:<<=?A<@@=?@==<<<<<?A@
@T6XXI:00048:00475
GGGGAATTCTAATACGACTCACTATAGGGAGAGGGAGAATAGTCAACAAATCGTTTGGGGCGACTTCTCCTTCCTTT
CTCCCTTCTCCC
+
DDC8A8;:<AB@CCCCCBCAA@A>@<:886=6997:CC>?=AAAA;??C<BAAAC9?AA7A@A@@>::;::7;;>=:
@A@@;@9<<=@7
@T6XXI:00049:00450
GGGAGAAGGGAGAAAGGAAGGAGGAAGTCGCCCCAAACGATTGTTGACTATCTCCCTCTCCCTATAGT
+
:D>?@AACC=;32@88+-+-*-,*,2,..89996BB:<@:;56=:?::74:::99-443./+-.--++
@T6XXI:00049:00481
GGGAGAAGGGAGAAAGGAAGCTAGTCGACTCGAACATCT
+
DDBCCC>@99=911,1-0,1...76661111110.3...
@T6XXI:00049:00488
GGGGAATTCTAATACGACTCACTATAGGAGAGGGAGAATAGTTTCTTCCCTGATTAAAGGCGGCGTGTCCCTTCCTT
TCTCCCTTCTCCC
+
@==4>695<ABBBBBCCCCBBBBBB::57>>?@=B@A>@@@@@7=<<?B;<?AD@CC@C?BB@CKIBBBC<?;?=@@
<AA=@:>384==7
@T6XXI:00004:00501
```

```
CTAGTCACTCGAGATCTGCGGCTTTGGACAGTCCCTCTGTACTGTGAAGCCACAGATGTCGAGTCGACTAGCTTCCT
TTCTCCCTTCTCCC
+
@<1////44A@@BBCBBBCC?B9?>C@CB?????<?BABBA?>?=<<9;A>CBADCBCDBBBCBBB@???9::;=@C
CCC@??7;24344,
@T6XXI:00004:00509
GGGGAATTCTAATACGACTCACTATAGGGAGAGGGAGAATAGTCAACTTCTTCACAATACCCGCTTATCCTTCCTTT
CTCCCTTCTCCC
+
BCC:C@C?BBBABA@@BBBB@A;66111.7??CC>CAA=A=<:???AB<;:<?AA=:A9;;:;==<<:6/334344+
4;9;4<8<===7
@T6XXI:00004:00513
GGGGAATTCTAATACGACTCACTATAGGGAGAGGGAGAATAGCCATCGCACCGGTGTCAGTCACATATTCTTCCTTT
CTCCCTTCTCCC
+
999-
424848924@AC;;<A???A@@>@:::::E?CBA>@??A>@?==:4427:=716A::<A;;=8424:7:7;<6<=;B
4:798894
@T6XXI:00004:00521
GGGAGAAGGGAGAAAGGAAGTGCAACTTGTCGCGCAAAATAGGGAGACTATTCTCCCTCTCCCTATAGTGAGTCGTA
TTAGAACTTC
+
990444244-
488@9?<@@BACHB=@A>BA@>>>9999,49?9:@797B><:<711,16100+0435585888334565464244/0
@T6XXI:00005:00529
GGGGAATTCTAATACGACTCACTATAGGGAGAGGGAGAATAGATCACCGTCGTGTCGGGTGCGCGTTCTCCTTCCTT
TCTCCCTTCTCCC
+
DDC9CBC@BBB@BBBDCAB@?CB>?66659???C>@AA?A=88;=?537,,,,,00035+54486686<6565?;?@
<@BAA<?<??@@;
@T6XXI:00005:00540
GGGGAATTCTAATACGACTCACTATAGGGAGAGGGAGAATAGTCAACAAACTCGTTTGGGGGCGACTTCTCCTTCCT
TTCTCCCTTCTCC
+
999/49@>AAA?BBBD@A>>000>>>>@=;;;CC>?<<598?@@9-
.+0*++++++*+005.@A@:6353323;=<=;:9><=;B;://.
@T6XXI:00005:00541
GGGAGAAGGGAGAAAGGAAGAGAACGGCGACGAACAATAGTTGGTGGCTATTCTCCCTCTCCCTATAGTGAGTCGTA
TTAGAATTCCCC
+
CD>C@@=@B=BCCE@D=@?@@@@@DC@CCBBBC?BB@CCCC@C@998:BBCBCBCC>BCCDC===AAAA@@??????
@>A@@;;24440
@T6XXI:00006:00510
GGGAGAAGGGAGAAAGGAAGTGTATGGCGCGAACGGAGAACTAAGGCTATCTCCCTCTCCCTATAGTGAGTCGTATT
AGAATTCCCC
+
BA?FCD@BB=ABBB=?=>@CCBBBBB@BBB>B@BA<<@BBBBB898<BCCGECCAAAABB@BCCCCBBCBBBCBBCA
CCC@C@CCC:
@T6XXI:00006:00511
GGGGAATTCTAATACGACTCACTATAGGGAGAGGGAGAATAGCCTTTCACCGTGTGTGTTTGATTATTCTTCCTTTC
TCCCTTCTCCC
+
@==6@>A>BBB@CC;9:CCCCCCB>6664:99CE>B=?=AA@@;AC=C@@=@AAAC=<==;@@@>@C???<?<>>:?
AAA<A>AAAA;
@T6XXI:00006:00534
GGGAGAAGGGAGAAAGGAAGACAGTCCATCTGTGGCTTCACAGTACAGAGGGACTGTCCAAAGCCGCAGATCTCGAG
TCGACTAG
```

```
+
DD@AA@@BC@CBBB>C>A7;:??>::7;BAACCC?@@??BA=AAA===9999=ACBA;:;=4;<;<998@@@@A?@@
@BAABCCB
@T6XXI:00006:00543
GGGAGAAGGGAGAAAGGAAGGAGAAGAGCTGCACACATTATGCAATGGCTATTCTCCCTCTCCCTATAGTGAGTCGT
ATTAGAATTCCCC
+
CB@B@@?@A=AAAA;@=>=>>??>>>>>;>>;>>>>?>;??@@C?>>=;<C@>???@?==<<<;<=<<<===<<<<:
;@?=@?=B>BCC8
@T6XXI:00007:00505
GGGAGAAGGGAGAAAGGAAGGAGAAAGTCGCCCCAAACGATTTGTTGACTATTCTCCCTCTCCCTATAGTGAGTCGT
ATTAGAATTCCCC
+
CD?ACCACC@B@BB@B>A:4640//,...446966666JCEC>>?>BB@@@A=AAAA;A>>?==@@@@@?@?AA??=
==>AAA>A:<;;4
@T6XXI:00007:00509
CTAGTCACTCGAGATCTGCGGCTTTGGACAGTCCCTCTGTACTGTGAAGCCACAGATGTCGAGTCGACTAGCTTCCT
TTCTCCCTTCTCCC
+
;8111=69?DAABBDCBBCB?BBC>C89:CBBBC>CCCBB>:::AAA=@B@B??ABC?===??@BBBABBCCC?B?B
C>CABA=A=???A;
@T6XXI:00007:00517
GGGAGAAGGGAGAAAGGAAGCTAGTCACTCGACATCTGTGGCTTCACAGTACAGAGGGACTGTCCAAAGCCGCAGAT
CTCGAGTCGACTAG
+
DC@BBAABB?BCBB:>=;<AABA@@@:7:97?85?A8:<C>AB>AAABBB?=99330-
4;;@AA?BA:@@;=?AAA::==ABB=8?>?C@@
@T6XXI:00007:00542
GGGAGAAGGGAGAAAGGAAGCTAGTCGACTCGACATCTGTGGCTTCACAGTACAGAGGACTGTCCAAAGCCGCAGAT
CTGGAGTCGACTAG
+
491999284-///6+/,.,.67>?@????:::??@B96113/07016668?;666./,-
4987>?AA=A;?===A8888>99<<@>B@@@A
@T6XXI:00007:00549
GGGGAATTCTAATACGACTCACTATAGGGAGAGGGAGAATAGTCAACAAATCGTTTGGGAGCGACTTCTCCTTCCTT
TCTCCCTTCTCCC
+
<99/95969@@69;<;BBDC@?@BAAAA>ACCCC>A6:8:===A=<4771/7777177+,,,001<999=;<>@?@@
<@AAC=A=<>?=;
@T6XXI:00008:00504
GGGGAATTCTAATACGACTCACTATAGGGAGAGGGAGAATAGATCACCGTTGGTTCGGGTGCGCGTTCTCCTTCCTT
TCTCCCTTCTCCC
+
AAA7A=@=@CC@CCBBBBBB?@B??66659;;?C>??A@A====>:88:D@C?B?CCB>B:;;CCC>AAC@C@E@CG
>@<==7<=A@@A;
@T6XXI:00008:00511
GGGAGAAGGGAGAAAGGAAGTAGTCGACTCGAGATCTGCGGCTTTGGACAGTCCCTCTGTACTGTGAAGCCACAGAT
GTCGAGTCGACTACTTCCTTTCTCCCTTCTCCC
+
DC@B:99;B?B@@=9>5848>>=?;;;AB@>@A=99949489F;=?@99;98??9>>?:;;@B???A?@C@CBBBBB
BBBCCACA==?B????@6::68??@<A:==?A<
@T6XXI:00008:00512
GGGAGAAGGGAGAAAGGAAGGAGAACAACACGATCGGTTAAACGTAGCTATTCTCCCTCTCCCTATAGTGAGTCGTA
TTAGAACTTCCCC
+
DC>BBBBCC<AACCAC=@=@@B<;>8859>=9484424;>>:@AACCDDCD<;:CC<???BA<=878<=<A@@??98
9:<<A>@@:@@>0
```

```
@T6XXI:00008:00515
CGACTCGAGATCTGCGGCTTTGGACAGTCCCTCTGTACTGTGAAGCCACAGATGTCGAGTTAGCT
+
BB@<;9=AABEFBBCDFBDC@GAE==@CCC>CBCB;;;;;77=<8:6:AAAB>?>7,*,*)*.**
@T6XXI:00008:00541
GGGGAATTCTAATACGACTCACTATAGGGAGAGGGAGAATAGTCTTGTCCTCTGTGCCTCCCGATTGTTCTTCCTTT
CTCCCTTCTCCC
+
BBH:C899;AAACBBABCBB@C@;;616.7>>AB>BAA@@8<<=A?@@=:<???@AA?BCC>CBB?B@=??<?;>>7
<??@;??B;;<7
@T6XXI:00008:00543
GGGAGAAGGGAGAAAGGAAGGGCACGCGCGAGTCATCCAATTGGGAGCTATTCTCCCTCTCCCTATAGTGAGTCGTA
TTAGAATTCCCC
+
CFABBBBDD>???C>B@B@BC>A??<@AA@AAACCCC@A@B@BB>BBBBBCACCCB>CBBCD>C>??BCCCBBBBBA
A?=A=>A:<<<4
@T6XXI:00009:00500
GGGGAATTCTAATACGACTCACTATAGGAGAGGGAGAATAGCCACCAACTATTGTTCGTCGCCGTTCTCTTCCTTTC
+
9;;1989>;??=@999>><>773;<??:?CABB>C>>;898@>AA>@;=555;==<=;<<<9200/0000/0/0444
@T6XXI:00009:00512
GGGAGAAGGGAGAAAGGAAGGAGAAGTCGCCCCAAACGATTTGTTGACTATTCTCCCTCTCCCTATAGTGAGTCGTA
TTAGAATTCCCC
+
BCBBB@@BD>CEBA?B@B@B@BA?>BABBBBC8AB>ACEDC?B?>@<<8?8<=;;;:;999;:;;;;;9<<=<<<<<
@>@AA=?3700+
@T6XXI:00009:00516
GGGAGAAGGGAGAAAGGAAGGAGAAGT
+
DCABBB@BC=C<;;09;:,0,000,,,
@T6XXI:00009:00519
GGGAGAAGGGAGAAAGGAAGGAGAGTCGCCCTAACGAGTTGATTGAG
+
;<14B@59919911,1;?,1,......777,31335333,...,.44
@T6XXI:00009:00545
GGGAGAAGGGAGAAAGGAAGGCGATGTGTGGCGGGGAATACTTGGTAGCTATTCTCCCTCTCCCTATAGTGAGTGTA
ATTAGAA
+
44-
4:C?BD8;8:;8;::,0,...447>>B@??CC6D@?<=@><88774<<<>@@C@853330135488443444403/04
400/
@T6XXI:00010:00507
GGGGAATTCTAATACGACTCACTATAGGGAGAGGGAGAATAGCCGACAGTTCGACTCCCCGGCAATGTTCTTCCTTT
TCTCCCTTCTCCC
+
CDBAD99:<BA?CCCBBBBCCB:67777.7>>BD?F<<9<777:<=<A@@;=889@AAA6A>??=@AA>?>:;/444
,49;A:?;<;994
@T6XXI:00010:00508
GGGGAATTCTAATACGACTCACTATAGGGAGAGGGAGAATAGCTAGCATTATTCCGCTAGGTGCTTCTCCTTCCTTT
CTCCTTCTCCC
+
999/92928482447<97;<@971>6611:??ADA===>?==?A:;7>?48:;?:;AFBB?BBAA;?7747::7::4
7//,-01---+
@T6XXI:00010:00519
GGGAGAAGGGAGAAAGGAAGGAGAAGTCGCCCCAAACGATTTGTTGACTATTCTCCCTCTCCCTATAGTGAGTCGTA
TTAGAATTCCCC
+
```

```
AA>BBB?BC?BBCC>A?@@C>CAB?BECCCCC7@@<?CC@A;?B@CBB@@D;==<?:??A?H@A@==<;;==788=A
A>A@@:;/444-
@T6XXI:00010:00544
GGGAGAAGGGAGAAAGGAAGCTAGTCACTCGACATCTGTGGCTTCACAGTACAGAGGGACTGTCCAAAGCCGCAGAT
CTCGAGTCGACTAG
+
CB>DCC@BB?BB?=<?797:CA?AB<???@;747::6?AC=@@@CCCB?@A=9833317>@BCC=@@<@@>@A<<<<
=<<:66AA==99>=
@T6XXI:00011:00502
GGGAGAAGGGAGAAAGGAAGCTAGTCGACTCGACATCTGTGGCTTCACAGTACAGAGGGACTTTCCAAAGCCGCAGA
TCTCG
+
CD@CCB?BB;@=>>:@?A5;;C?@ABBCC@@?77:CCCCCA=?A=A@AA@?:8:====466==;A?BB>BB@DCCBB
CBCCC
@T6XXI:00011:00518
CGAGATCTGCGGCTTTGGACAGTCCCTCTGTAACTGTGGAAGCCACAGATGTCGAGTCGACTAGCTTCCTTTCTCCC
TTCTCCC
+
;444444344=@BDC<??ACA888A><99B71011110/023;;C<;<=@<=BAECBBBCCCC<;;>:;9=:=9>>0
958>>>8
@T6XXI:00011:00525
GGGAGAAGGAGAAAG
+
6<8<11,/,,*,,*,
@T6XXI:00011:00529
GGGAGAAGGGAGAAAGGAAGCTAGTCACTCGACATCTGTGGCTTCACAGTACAGAGGGACTGTCCAAAGCCGCAGAT
CTCGAGTCGACTAG
+
CCBCBB=>=:@@>><@8:5;CA?AB:?=??:747::6ABC<:;@CDBAAC??<=33317<<?AA>AA<A=>AB;;;<
<==:;:<<<<8=;;
@T6XXI:00011:00531
GGGAGAAGGGAGAAAGGAAGCTAGTCACTCGACATCTGTGGCTTCACAGTACAGAGGGACTGTCCAAAGCCGCAGAT
CTCGAGTCGACTAGCTTCCTTTCTCCCTTCTCCC
+
CB>@AB>AB>B;99095858A>???6771101009?A?AA@AB>BBBBCB@@??;;;6;;;<@@?@A<AA?A>@@A=
588??@@@@AA????@=@?CC9==<<6;378?@:
@T6XXI:00011:00549
CTAGTCACTCGAGATCTGCGGCTTTGGACAGTCCCTCTGTACTGTGAAGCCACAGATGTCGAGTCGACTAGCCTTCC
TTTCTCCCTTCTCCC
+
<<1//6/44ABBBCCB?@@BBBBB>B?A@7::::6;A@AAB??89:A?AC=AA@C@???????CCC=<<>00,09:9
:;9>@AC;;:?<<=9
@T6XXI:00012:00503
GGGGAATTCTAATACGACTCACTATAGGGAGAGGGAGAATAGTCAACAAATCGTTTGGGGCGACTTCTCCTTCCTTT
CTCCCTTCTCCC
+
CJD:C>@>ABC@CBBAACBBBBBCBBCC>BB@CC>AA@>??878;8:9;6=??>>.777.7?77747<99>9;9<@;
@@@@<A>@@@C<
@T6XXI:00012:00510
GGGAGAAGGATGAAGAGACGATAGTGGAGATGAAAGAGACTCACGCATCTATTCTCCCTCTCCATAGTGAGTCGTAT
TAGAATTCCCC
+
>:5944696944/,///.......1?9=;;4449594444494339444433 13...+...4358BB>>>BB;;:??
;=>>4:5:AA5
@T6XXI:00012:00512
GGGAGAAGGGAGAAAGGAAGTGCAGTTCGCGGTTAGTGAAGGGAAAAAACTATTCTCCCTCTCCCTATAGTGAGTCG
TATTAGAACTTCCCC
```

```
+
CB>A@@=@@<@@AA=B@C@EECBBBD@@;331377>;?;777,17777(88717711616547>:00/;98<?:;;/
//6464:8882333.
@T6XXI:00012:00520
GGGGAATTCTAATACGACGTCACTATAGGAGAGGGAGAATAGTTTTCCGATTATACACGACCACTCTTCTCTTCCTT
TCTCCCTTCTCC
+
:>@0>-
1,///,/4333337;;7866..,.347>;@<=;=@7565*,*++003345<4235.//664689978/44-
49::6<;>@@9
@T6XXI:00012:00527
GGGAGAAGGGAGAAAGGAAGCTAGTCACTCGACATCTGTGGCTTCACAGTACAGAGGGACTGTCCAAAGCCGCAGAT
CTCGAGTCGACTAGCTACTTCT
+
CC@BA@=>@:>999042847??<?A<===7477011169@<?B@DAAACBCAAA???;?A@@@?>AA<AA>A?@?AC
899==@@@@@@;8///,,+*+0
@T6XXI:00012:00532
CTAGTCACTCGAGATCTGCGGCTTTGGACAGTCCCTCTGTACTGTGAAGCCACAGATGTCGAGTCGACTAGCTTCCT
TTCTCCCTTCTCCC
+
B?9<<<::=BBBBBCB@BBC@@??;@?@??A???;?CCB?9<<=C??<?C@CCAAACCB??CBABCCABA??<<A>A
C=?=@@;@>@??A<
@T6XXI:00012:00544
GGGAGAAGGGAGAAAGGAAGCTAGTCACTCGAGATCTGCGGCTTTGGACAGTCCCTCTGTACTGTGAAGCCACAGAT
GTCGAGTCGACTAGCTTCCTTTCTCCCTTCTCCC
+
CC>BA@=@C>A@@?<@=?=@CA@AB???BA?AAACCBB?@@BCA;@>>@>==??;=99999::;;;;:;=888====
9<9;9<<<===<==9888;<@<AA@@7<9<?@AB
@T6XXI:00013:00504
CTAGTCGACTCGAGATCTGCGGCTTTGGACAGTCCCTCTGTACTGTGAAGCCACAGATGTCGAGTCGACTAGCCTTC
CTT
+
>9944444449;=@?BB@>>?;88808;>876:8?;?BBA::7:4444/44988;@;:;@:;:@@<<8--
,,,*,*,*..
@T6XXI:00013:00510
CTCGAGATCTGCGGCTTTGGACAGTCCCTCTGTACTGTGAAGCCACAGATGTCGAGTCGACTAGCTTCCTTTCTCCC
TTCTCCC
+
CCBBABCCCCCAA@AAA;@@BBCAAACBDHCCBCCBBBBB?BDAC@A@CAAB<;;CCCABBCC@@A?BACE@CBBB=
B;?@AA<
@T6XXI:00013:00534
GGGAGAAGGGAGAAAGGAAGCTAGTCGACTCGACATCTGTGGCTTCACAGTACAGAGGGACTGTCCAAAGCCGCAGA
TCTCGAGTCGACTAG
+
AA?AAB?A=:@@==9>5848?700>///999907::96???39;@DCCCBB@?=9000,4::;=?<?@;?@=@@@AA
@@@@@@@@@AAAB??
@T6XXI:00013:00545
GGGGAATCTAATACGACTCACTATAGGAGAGGGAGAATAGATCACCGTTGGTTCGGGTGCGCGTTCTCCTTCCCTTT
CTCCCTTCTCCC
+
CB;58,1///784377878613161101<616,000/03444660,/4,/,.,/,2820005557=7766011,162
4575447.---+
@T6XXI:00013:00548
GGGGAATTCTAATACGACTCACTATAGGGAGAGGGAGAATAGACACAAC
+
@@@6@@E@<=<?A@AA@BDBA@>77777.7>>??5733/097.---+02
@T6XXI:00014:00509
```

```
GGGGAATTCTAATACGACTCACTATAGGGAGAGGGAGAATAGCTAA
+
AAA9A>A><<<ACCFCCBBACB@;;667.7==>B@E@;;:3.***)
@T6XXI:00014:00514
GGGGAATTCTAATACGACTCACTATAGGGAGAGGGAGAATAGTTTGACAACATGTGTCCTGCTGCCCTCCTTCCTTT
CTCCCTTCTCC
+
499/42924@@=BBBCCCD:::A@?=?@6=====6<;=<@CAAA69:C@=@??;;;8:989:39=B998@?99111,
16666601110
@T6XXI:00014:00527
GGGAGAAGGGAGAAAGGAAGGAGAAGTCGCCCCAAACGATTTGTTGACTATTCTCCCTCTCCCTATAGTGAGTCGTA
TTCAGAATTCCCCC
+
BB>BCC=@@8;9AB<@?@@C@CBB>>>?7788.33,1666?=CA@B::888<@ABA=@@884-
4444=<<<AAA<99=.////,/,4430+
@T6XXI:00014:00534
GGGGAATTCTAATACGACTCACTATAGGGAGAGGGAGAATAGTCAACAAATCGTTTGGGGCGACTTCCTCCT
+
499/49928;A899:;;AB>7787766261116,0044655688::<=;@>???;CAC5:/.33,,*+000
@T6XXI:00014:00537
GGGGAATTCTAATACGACTCACTATAGGGAGAG
+
7<<0<,1,///564447>7711366171,/700
@T6XXI:00014:00546
GGGGAATTCTAATACGACTCACTATAGGAGAGGGAGAATAGTCAACAAATCGTTTGGGGCGACTTCTCCTTCCTTTC
TCCCTTCTCCC
+
CCC:CC?=?BB@AA@CCCCBB>;54./,0<<BC>A@C=AA@@?=:7771:A@@C>AAA6A???B?C@A<?<?>@A;@
AAC=A<??AA<
@T6XXI:00015:00502
GGGGAATTCTAATACGACTCACTATAGGGAGAGGGAGAATAGTTCCCTGACAACAATCACCGCACGCCTCTTCCTTT
CTCCCTTCTCCC
+
AAA6@=@>BBB@CBBBBBBB?AB>8111.6<<?@>B@@>A===<AA<@?>>CBCA?A@877:>==??>@CC?@>?=9
;77718<?@AA<
@T6XXI:00015:00513
GGGAGAAGGGAGAAAGGAAGCTAGTCGACTCGACATCTGTGGCTTCACAGTACAGAGGGACTGTCCAAAGCCGCAGA
TCTCGACGAG
+
B@9@9859919>>>194313::BCBDDCCBBB:9::==>=:8;?:?ABBBA?@@A87858?;;:=344-
4?;@@@@A@???===@@@
@T6XXI:00015:00547
GGGAGAAGGGAGAAAGGAAGGAGAAAGTGAGCGCCGCACAACACGTCACTATTCTCCCTCTCCTATAGTGAGTCTAT
TAC
+
//+4496;B<BBDG?B>A5848877-1116@==@<>====8:811---
311340033+043424==;364+++++11,--
@T6XXI:00015:00548
GGGAGAAGGGAGAAAGGAAGCACTCGTCGAGTTTAGGGATGAAGGAACTATTCTCCCTCTCCCTATAGTGAGTCGTA
TTAGAACTTCCCC
+
<979BB@DDACCBB?B=@=><@BCCACBB<==C@EIHFDAACBCACC@AA@5;9<><A?@@D>BA??A?CCC=<<00
0,,70*+.3133.
@T6XXI:00016:00514
GGGGAATTCTAATACGACTCACTATAGGGAGAGGGAGAATAGTTTTTCCCTTCACTAACCGCGAACTGCACTTCCTT
TCTCCCTTCTCCC
+
```

```
=@@4A>A@CBB?CBBB?<<B@CB>8116.6??CD>ABB=@<@=<<?1=>7?:=@CCC@C???=A?AAA=@@A?C=AA
<?;;>7?<?AAC<
@T6XXI:00016:00521
GGGGAATTCTAATACGACTCACTATAGGGAGAGGGAGAATGACATACA
+
DCC9B?B?CCBEB???????>AB>9616.7>@BC>>;87//...,+++
@T6XXI:00016:00542
CTAGTCACTCGAGATCTGCGGCTTTGGACAGTCCCTCTGTACTGTGAAGCCACAGATGTCGAGTCGACTAGCTTCCT
TTCTCCCTTCTCCC
+
;?1////44=;;;9998937131727776661111,161169<@854=<<B>BABA?>::6===@@AAAB???E@:77
:6/--.,/7/47>7
@T6XXI:00016:00545
GGGAGAAGGGAGAAAGGAAGGGGAGAAGGGCTAGTCACTCGACATCTGTGGCTTCACAGTACAGAGGGACTGTCCAA
AGCCGCAGACTCTCGAGTCGACAAG
+
CCABBB@CC<;99?9>;<>BCI8@@A577.89944;444==98==@?@@?4831111111444444;69499;;966
2./,/668..33313311377;689
@T6XXI:00017:00521
GGGGAATTCTAATACGACTCACTATAGGGAGAGGGAGAATAGTCAACAAATCGTTTGGGGCGACTTCTCCTTCCTTT
CTCCCTTCTCCC
+
CCC:CBB?BBB@BBBB??BB??<77111.7?>@C@AAB?A<A@;:8:CC<BCCBA9??;2?=??@>@<<8<7<;=@<
?AAD?D9<<@C<
@T6XXI:00017:00532
CTATCGACTCGAGATCTGCGGCTTTGGACAGTCCCTCTGTACTGTGAAGCCACAGATGTCGAGTCGACTAGCTTCCC
TTTCTCCCTTCTCCC
+
D;::1111::BBCACBBBBB@A@?;A=<?;?9994:CCCCB?A<<<=8:A@B@AABB>AABBBBCBBCCCBA?477.
77.7:;;<A>@@@@<
@T6XXI:00017:00548
GGGAGAAGGGAGAAAGGAAGTGCAGTTTGCGGTTAGTGAAGGGAAAAACTATTCTCCCTCTCCCTATAGTGAGTCGT
ATTAGAACTTCCCC
+
/1,1/9598-444;-
427777<>>9997;BB@C?C<<<A>@?.7777,8>77;=8::86666680335:<>@A@888<@=?AC<>=3774-
@T6XXI:00018:00500
GGGAGAAGGGAGAAAGGAAGCGAAAAAAGGGGGATGTAAATAGCGATTGGCTATTCTCCCTCTCCCTATAGTGAGTC
GTATTAGAACTTCCCC
+
CDACCCABB=@@DD@C?C>??767666*7777+1=779@9:?599998;:=>@A60.;;3//---
+.94?@AAA???887?383;538/044+
@T6XXI:00018:00513
GGGAGAAGGGAGAAAGGAAGAGAACGATGACGGTAGGGAAGTTACCGCTATTCTCCCTCTCCCTATAGTGAGTCGTA
TTAGAATTCCCC
+
CCAEGC>A@199@@:>;>;?BBBBEDEB@@@B>@AAB=C?CCDDC@CBAGDBAAAE@BBBCC@BBBCCCCCDBBBCC
F7:792969AB8
@T6XXI:00018:00518
GGGGAATCTAATTA
+
;>>1<,-,,,*,*,
@T6XXI:00018:00539
GGGAGAAGGGAGAAAGGAAGCTAGTCACTCGACATCTGTGGCTTCACAGTACAGAGGGACTGTCCAAAGCCGCAGAT
CTCGAGTCGACTAGCTTCCTTTCTCCCTTCTCCC
+
```

CB>C==<;@>A999142847?>7//0,,,,0,,+///77@5:7?A?=?@??<<??AA<AAA@@@B@C9==?@A@?@@
??>@@===<<<<<???>A>@@;@AAD9=9<<<<7
@T6XXI:00019:00515
CTATCGACTCGAGATCTGCGGCTTTGGACAGTCCCTCTGTACTGTGAAGCCACAGATGTCGAGTCGACTAGCTTCCT
TTCTCCCTTCTCCC
+
B??984449::>BBB@??<><877.8>A@@B????9????BB@A==??>@C@BBAC@@?BAAACBA@@AA@7710101
99?6?B;@<@>??7
@T6XXI:00019:00518
CTATCACTCGAGATCTGCGGCTTTGGACAGTCCCTCTGTACTGTGAAGCCACAGATGTCGAGTCGACTAGCTTCCTT
TCTCCCTTCTCCC
+
B9949444;A;999@@@AB?BBB>B=?===777.7>>ABB===@@@>AC@CCCBBBBCCBB??A@@CBBBB?=?377
.7444-474444-
@T6XXI:00019:00532
GGGAGAAGGGAGAAAGGAAGAATGATGCGGGAGACGAACGACGCCGAACTATTCTCCCTCTCCCTATAGTGAGTCGT
ATTAGAATTCCC
+
9A:@@?59909444-
/,,*,00,,,,22288:BCCCC@C???@@:?;8:;99274084:<<<=;==>5487><=755<<>A@;94/34-
@T6XXI:00019:00543
TCGAGATCTGCGGCTTTGGACAGTCCCTCTGTACTGTGAAGCCACAGATGTCGAGTCGACTAGCTTCCTTTCTCCCT
TCTCCC
+
@CCBBBBB@@AE@CCC>C?BA@===?>BCCC????BBAA?@?<?@@@AA=ACBCCCCBCCCCBBCBGDCC?CCCC@B
@AAAB@
@T6XXI:00019:00547
GGGGAATTCTAATACGACTCACTATAGGGAGAGGGAGAATAGTCAACAAATCGTTTGGGGCGACTTCTCCTTCCTTT
CTCCCTTCTCCC
+
444,4865611,//6333>;669?>999266677-0887;<<9::9430+16111-
17728866101042323:=A<<:<<1337:?>;
@T6XXI:00020:00511
GGGGAATTCTAATACGACTCACTATAGGGAGAGGGAGAATAGTCAACAAATCGTTTGGGGCGACTTCTCCTTCCTTT
CTCCCTTCTCCC
+
88000)/,///568>988;766@>::<>86=;::.:B;;=@8848:8;=;==:87,111,1668@@<<95544;<>:
?<;<7<;>>A@<
@T6XXI:00020:00514
GGGAGAAGGGAGAAAGGAAGGAGAACAACACGATCGGTTAAACGTAGCTATTCTCCCTCTCCCTATAGTGAGTCGTA
TTAGAACTTCCCC
+
490444248-
44895877;;>AA@:@A69844444499233,3337;@?@>?><<@89999<88????====><<<AA>@@@?>;>=
=B0
@T6XXI:00020:00546
GGGAGAAGGGAGAAAGGAAGGAGAAGTCGCCCCAAACGATTTGTTGACTATTCTCCCTCTCCCTATAGTGAGTCGTA
TTAGATTCCCC
+
;C>CCCACH?BABBCBBC@@.000,0.6....*.30..6??;B@;==<;?@8:9@C:=?<;;4444<::886//..7
334///75554
@T6XXI:00021:00525
GGGGAATTCTAATACGACTCACTATAGGGAGAGGGAGAATAGTCAACAAATCGTTTGGGGCGACTTCTCCTTCCTTT
CTCCCTTCTCCC
+
CDC8A=@=@CC@CA>BBBB?=@@77111.7>>>@????@B@A@@A>AAA<CBBBC>B@@7BBBBC??<<8<:=>ACA
@???:>;??@@;

```
@T6XXI:00021:00538
GGGAGAAGGGAGAAAGGAAGCTAGTCACTCGACATCTGTGGCTTCACAGTACAGAGGGACTGTCCAAAGCCGCAGAT
CTCGAGTCGACTAG
+
HIAB?>=@@<@CAA>C;;8;BA?AB<;::B?;;4<<9@AB>AA?BBBBBBB==9033-
4::=@A<??<?@>@AAAAB899===<=?CAA@@
@T6XXI:00022:00500
GGGGAATTCTAATACGACTCACTATAGGGAGAGGGAGAATAGATCAAGTCTAAAGTACGCCCCGCTATTCTTCCTTT
CCTCCCTTCCTCCC
+
444,42424888=AAACC@@@<<;CCCD@CCBBBA?@?8880//7B:<??85335;;;;76616///44;765423+
-,---+-,-,034+
@T6XXI:00022:00527
CGAGATCTGCGGCTTTGGACAGTCCCTCTGTACTGTGAAGCCACAGATGTCGAGTCGACTAGCTTCCTTTCTCCCTT
CTCCC
+
@@@@@BCABBC@BBB>B?BA?B:7:.7==?CEDCA@=@>@@8<9ACAA@@BBB<<=CA@>@@9929599-
88=>0948377/
@T6XXI:00022:00528
GGGAGAAGGGAGAAAGGAAGCTAGTCACTCGACATCCTGTGGCTTCACAGTACAGAGGGACTGTCCAAAGCCGCAGA
TCTCGAGTCGACTA
+
99-99969>9;;<H>B?B?@?:777761.4131-
..,..33488889???A884849:6;>==;;27513993476>>7334.--.---++
@T6XXI:00022:00536
GGGAGAAGGGAGAAAGGAAGCGAATAGACAAGGGCACCGGGTGTTATCTATTCTCCCTCTCCCTATAGTGAGTCGTA
TTAGAATTCCCC
+
BD>BCB@BB>?>?B=A?B=@@BB?B???AA?ACAAAA?AC>CCB>BCCDCC@A??@;===A@<ABA@@@AA@@@@?@
@>AAA?@8;;;4
@T6XXI:00023:00525
GGGGAATTCTAATACGACTCACTATAGGGAGAGGGAGAATAGTCAACAAATCGTTTGGGGCGACTTCTCCTTCCTTT
CTCCCTTCTCCC
+
@>>4><A?BBC@BBBBBBCB868=<777.7?777.499:?@AA@@>@AA<ABBAC>CCB9CDCCC@A??9;8;8996
;99:7=8<;=@:
@T6XXI:00023:00529
GGGAGAAGGGAGAAAGGAAGCTAGTCACTCGACCATTCTGTGGCT
+
CNAA?>59919111,/,0,.647??A644.-.-,-.,....7011
@T6XXI:00024:00528
GGGAGAAGGGAGAAAGGAAGCTATCGACTCGAGATCTGTGGCTTCACAGTACAGAGGGACTGTCCAAAGCCGCAGAT
CTCGAGTCGACTAG
+
CC@A992880999909;>;:>00/>>07:@@?=;???@AB?BB@BBBB@?A=9400014;;>AA=AA<@@???<==<
<@?@@@A???@AAA
@T6XXI:00024:00532
GGGAGAAGGGAGAAAGGAAGGAGAAGTCGCCCCAAACGATTTGTTGACTATTCTCCCTCTCCCTATAGTGAGTCGTA
TTAGAATTCCCCC
+
CD@AAAACC@BBBCAC8:7;>ABB?BCC?>>>.77.7?;BC<CB@BB@AAA>???A?A===A<AAACC??======C
C>@>>:?>;?>>.
@T6XXI:00024:00540
GGGAGAAGGGAGAAAGGAAGGAGAACGCGCACCCGAACCAACGGTGATCTATTCTCCCTCTCCTATA
+
>>5?>>>>;6@999147<;@@BAA=BBCBBBCC@DC?DADBDDBA@>?999949:::60-,,*,03.
@T6XXI:00024:00546
```

GGGAGAAGGGAGAAAGGAAGCTAGTCACTCGACATCTGTGGCTTCACAGTACAGAGGGACTGTCCAAAGCCGCAGAT
CTCGAGTCGACTAGCTTCCTTTCTCCCTTCTCCC
+
99049@=@@9>99909484;BBBB<70/..-
.137:CA@@???;?:::BB@??>;;<7<?@CBA>BA:?@:==AA=A898A@@@==<=;;;843479:4:444-
4/3343+
@T6XXI:00025:00503
GGGAGAAGGGAGAAAGGAAGAACAGCTAGACACGCGACATCAAGGGGCTATTCTCCCTCTCCCTATAGTGAGTCGTA
TTAGAATTCC
+
;<8=BC@CC@@??B@BA?>AA?AA@ABBBBCCBCCCCCCCBB;>=>6>BBB@DBBB?BBCCC;A@@?@>BB??AC::
9?C??<B?CA
@T6XXI:00025:00520
GGGGAATTCTAATACGACTCACTATAGGAGAGG
+
>;;5BAB@BBBBBCC@@@A@?::<00,+,,,.,
@T6XXI:00025:00523
GGGAGAAGGGAGAAAGGAAGCTAGTCACTCGAGATCTGGCGGCTTTGGACAGTCCCTCTGTACTGTGAAGCCACAGA
TGTCGAGTCGACTAGCTTCCTTTCTCCCTTCTCCC
+
99594424858444-
4231.4.//777=??<?435:./,.3337::C@@@=@?BB<;<887;=?>??@588;?887B<56444<@??ADAA=
<<:;244-4;;;-424444+
@T6XXI:00025:00525
GGGGAATTCTAATACGACTCACTATAGGGAGAGGGAGAATAGTCTCCCCATTAATCGCGGCTTAATTGCCTTCCTTT
CTCCCTTCTCCC
+
ABB7A=>=@@@;?@@B?@@@:7877777,677==<>8737::440344-
7<:?;7717737?:<9????=<>??BB>AADB<A<????:
@T6XXI:00025:00531
GGGAGAAGGGAGAAAGGAAGCAGTCCATCTGTGGCTTCACAGTACAGAGGGACTGTCCAAAGCCGCAGATCTCGAGT
CGACTAG
+
DDBAA?@BB=BBBA>C8:8;BA??=;?A@?@?<9<?=?<==@@A=?<77718=?AA=>==7==?@@???@@==<@@?
<=<CCAA
@T6XXI:00025:00532
GGGAGAAGGGAGAAAGGAAGAACAAGTAGCTGTCGGGTCAGATTAACTATTCTCCCTCTCCCTATCAGTGAGTCGTA
TTCAGAATTCCC
+
;C:>>?5990949>09239;9?CB5:6?77731111,16673885588:?>?@?A<@887A17000000------
000*..8:9;9@?7
@T6XXI:00025:00540
CTAGTCACTCGAGATCTGCGGCTTTGGACAGTCCCTCTGTACTGTGAAGCCACAGATGTCGAGTCGACTAGCTTCCT
TTCTCCCTTCTCCC
+
DD<;;@9:99999999:F?@=@EC>C=@BCCAAA=ACCCCCA??@@A?AC@BCCCC@??CBBB:<;AABCAA?;@=?
C>?=<A:=384??:
@T6XXI:00025:00541
GGGAGAAGGGAGAAAGGAAGGAGAAGTCGCCCCAAACGATTTGTTGACTATTCTCCCTCTCCCTATAGTGAGTCGTA
TTAGAATTCCCC
+
49194=8>85449>9=7>;>;>?=:?AACCCC8AA>A??<A>??=BCBB@@?@AAAA@999D<@<===<<<??;<<?
@<>>@9;8:::1
@T6XXI:00026:00515
GGGAGAAGGGAGAAAGGAAGGAGAAGTCGCCCCAAACGATTTGTTGACTATCCTCCTCTCCCTATAGTGAGTCGTAT
+
@A>BCJDCCACBBB=BBC@E@CDB=>>?8888.33,177>C;BD?CA?<;::74434439?<@887;;66//--++*

```
@T6XXI:00026:00530
GGGGAATTCTAATACGACTCACTATAGGAGAGGGAGAATAGTCAACAAATCGTTTGGGGCGACTTCTCCTTCCTTTC
TCCCTTCCTCCC
+
EFC:CBC??AA?B@BB;;<<CBABC::5:??=><=4833:::44240308?<@A=AA@7@5;;:,/-101,.-
.44833101,.,-332
@T6XXI:00026:00533
GGGGAATTCTAATACGACTCACTATAGGGAGAGGGAGAATAGTCCAACAAATCGTTTGGGGCGACTTCTCCTTCCTT
TCTCCCTTCTCCC
+
999/4999=CC:<<BBA@A@CBA>=777.777>@<A@@:::;//,/76675?@@AA?ABC9CCDDD?AA?9=:=ABD
7?88813490371
@T6XXI:00026:00535
GGGGAATTCTAATACGACTCACTATAGGGAGAGGGAGAATAGTCTGTCGCTCGGTCGACCGCTGCTA
+
@@>4?<@=ABC@CCCDCC@??@@716999ACCBB>A//,////..35A@@@?=2/,0++*+++0023
@T6XXI:00026:00536
GGGGAATTCTAATACGACTCACTATAGGAGAGGGAGAATAGATCACCGTTGGTTCGGGTGCGCGTTCTCCTTCCTTT
CTCCCTTCTCCC
+
999/9<@>BBBEBBBBB@AA?:977..,0::=@?@BB>AA@A===:8??4:5:=BBCAC;;;BCCACCE?B?B?BC@
????<??@@?C<
@T6XXI:00028:00506
CTATCGACTCGAGATCTGCGGCTTTGGACAGTCCCTCTGTACTGTGAAGCCACAGATGTCGAGTCGACTAG
+
B@@949::?==C??@BBCDDAABB>B?AAAA???9??=AAA<====A>AC@A@;;:317761116......
@T6XXI:00028:00507
CTAGTCGACTCGAGATCTGCGGCTTTGGACAGTCCCTCTGTACTGTGAAGCCACAGATGTCGAGTCGACTAGCTTCC
TTTCTCCCTTCTCCC
+
B@999944999AB??ACFCCB?BCCA>;;:;00/76;@CCCB;99@?@<@C>ABBDBBBCCDC@@<<;B;:661770
11,67494;8;<==7
@T6XXI:00028:00509
GGGGAATTCTAATACGACTCACTATAGGGAGAGGGAGAATAGTCAACAAAATCGTTTGGGGCGACTTCTCCTTCCTT
CTCCCTTCTCC
+
DJB6@??3216766498880.345...403..176103556653440444+16161,17>-
78>>@>@><8624/4/03344440005
@T6XXI:00028:00524
GGGAGAAGGGAGAAAGGAAGCTAGTCACTCGACATCTGTGGCTTCACAGTACAGAGGGACTGTCCAAAGCCGCAGAT
CTCGAGTCGACTAGCTTCCTTTCTCCCTTCTCCC
+
FCAB;959@9>99914231387<6;>711101001667;@;=B?BBBCCC@=99033-
4::=?@>@A<@@>BAA@@????AAAAA@@@@@AAA>@>@A<AAAC;?<??CC<
@T6XXI:00029:00513
CTAGTCGACTCGAGATCTGCGGGCTTTGGACAGTCCCTCTGTACTGTGAAGCCACAGATGTCGAGTCGACTAGCTTC
CTTTCTCCCTTCTCCC
+
;999994444;;@?BB@>>888/8>?=C@B@><766-
1<??BDED999@>ADBBBBB@@???BAAACEGGCB?::5:@CKBBA@A7;387;;6
@T6XXI:00029:00517
GGGAGAAGGGAGAAAGGAAGATTCGCGCGTGGTTCGTAAGGGAGTGTCTATTCTCCCTCTCCTATAGTGAGTCGTAT
TAGAACTTCCC
+
@@9@9959904>@@9@=?=ABB@CCAAABAA@C@C???=??7?>><;<A<;8<<86168//,.1338<;;<??@C@;
75,,+,,*++*
@T6XXI:00030:00500
```

```
GGGAGAAGGGAGAAAGGAAGCTAGTCGTCGACATCTGTGGCTTCACAGTACAGAGGGACTGTCCAAAGCCGCAGATC
TCGA
+
CE>DCB@BB>@@;;5;:;,00<0::BA?BB;;<999B>>;>>;@@CCCCB@@>89919AF@CC@ECAGCADDCABC?
?@CC
@T6XXI:00030:00521
GGGAGAAGGGAGAAAGGAAGGAGAAGTCGCCCCAAACGATTTGTTGACTATTCTCCCTCTCCCTATAGTGAGTCGTA
TTCAGAATTCCCC
+
>?:@@A=A>:>9990958477<??;<CCDDCC8BA8=7711,11011144323.//,//-
2344.33665;<@A::==///;;46,///+
@T6XXI:00030:00537
GGGAGAAGGGAGAAAGGAAGGAGAAGTCGCCCCAAACGATTTGTTGACTAT
+
BB?BCC?DB<<9;B9@;>17137;:=6737772;7:96645+,,*,,,+-0
@T6XXI:00031:00506
CTAGTCGACTCGAGATCTGCGGCTTTGGACAGTCCCTCTGTACTGTGAAGCCACAGATGTCGAGTCGACTAGCTTCC
TTTCTCCCTTCTCCCCTTCCTTTCTCCCTTCTCCC
+
:--
,,,,,/1:@ABACBBBBB@BBC@A>?>;98??;?CCCC;A<==@@8=@=@CCBBBCCAC=?BBBBCCCDBC?B@CC>
BCHC;@<??BAA6A=?;==7<<@A:?8;;::4
@T6XXI:00031:00548
GGGAGAAGGGAGAAAGGAAGCTAGTCACTCGAGATCTGCGGCTTTGGACAGTCCCTCTGTACTGTGAAGCCACAGAT
GTCGAGTCGACTAGCTTCCTTTCCTCCTC
+
BC<@=;79B19999094847>77:BAAABCAC@?BABC??>???;?>?>>>>>>;<<<:<<=<<??=;<9::9=9;=
:<;==9==A@???>6<8599927,./,//
@T6XXI:00032:00503
GGGAGAAGGGAGAAAGGAAGAACAGACGGCTGTTGGGCAGAGCGTGGCTATTCTCCCTCTCCCTATAGTGAGTCGTA
TTAGAACTTCCCC
+
CB@@BA@AA=@@BB>B@B?AA@BB??BB?BCBC?CD<B??????AA<??@A??===9=@@AC;@???A@???AAA@9
:77::.//78884
@T6XXI:00032:00511
GGGGAATTCTAATACGACTCACTATAGCGAGAGGGAGAATAGTCACATTCATGTTTGTGACCGTGTCTCCTTCCTT
+
CDD:C@B@?AA=AAB@???<::;BAB???:;:CC>BBB=9://---3?<A?BCCD<AACCA>88655544222,--
@T6XXI:00032:00515
GGGAGAAGGGAGAAAGGAAGTGCAGTTCGCGGTTAGTGAAGGGAAAAAACTATTCTCCCTCTCCCTATAGTGAGTCG
TATTAGAATTCCCC
+
CC@BBB@AA:=@==:@=?=@ACDCCC@BBBB@B?AAA@@>>>9=??AB-
BBDC@C@@@=?BBCG>@;@@=====@9====:=AB?A<?@>1
@T6XXI:00032:00517
CTATCGACTCGAGATCTGCGGCTTTGGACAGTCCCTCTGTACTGTGAAGCCACAGATGTCGAGTCGACTAGCTTCCT
TTCTCCCTTCTCCC
+
D@?=99:====BCCBCC@??=>>>;B?A@?AABB>BCCCCB<<<@@C?@B?BBBD8::C?@@CCBAABBCCCC?CAB
CAB@@@7<9<<<@<
@T6XXI:00032:00522
GGGAGAAGGGAGAAAGGAAGGAGAAGTCGCCCCAAACGATTTGTTGACTATTCTCCCTCCTCCCTATAGTGAGTCGT
ATTCAGAATTCCCC
+
BB>BBB?BB>BCCC?C=@=@>@?A=7787833,11,1779D>DC@CB?<:777///,--,-.-+-
358A@AAAA@???@:<<C@99,,,,*
@T6XXI:00032:00538
```

```
GGGAGAAGGGAGAAAGGAAGCTAGTCACTCGACATCTGTGGCTTCACAGTACAGAGGGACTGTCCAAAGCCGCAGAT
CTCGAGTCGACTAG
+
DDBCCB@BB=??=8:@=?;@CBBBC@A????:89BBBBCC=??>ABBBCB?<8?700-
4;;=@?>BC?@@>:98AB@?@@===?@@@??@@
@T6XXI:00033:00510
GGGGAATTCTAATACGACTCACTATAGGGAGAGGGAGAATGGCCCTGAATTCATCGGC
+
AB@6A=@:<<<111;;>;AA?@>>6111,167779900/09277-111017777110-
@T6XXI:00033:00534
GGGAGAAGGGAGAAAAGGAAGCTAGTCGACTGACATCTGTGGCTTCACAGTACAGAGGGACTGTCCAAAGCCGCAGA
TCTCGAGTCGACTAGCTTCCTTCTCCCTC
+
99144;284-
44444,4999396???7::@@7976111111017966888866330000+044444233+03568<:<8==????A==
=@C;;;941+,+,...)**
@T6XXI:00033:00538
GGGGAATTCTAATACGACTCACTATAGGGAGAGGGAGAATAGTCAACAAATCGTTTGGGGCGACTTCTCCTTCCTTT
CTCCCTTCTCCC
+
?@@6@>B?BBBABB?@?<<<>AB;9111.6777:7:@@@@?@@AA>@@?;B?99988??6?BCCC@BB@;=;=?CC<
@@@A<@<?@@A<
@T6XXI:00033:00539
GGGAGAAGGGAGAAAGGAAGCTAGTCACTCGACATCTGTGGCTTCACAGTACAGAGGGACTGTCCAAAGCCGCAGAT
CTCGAGTCGACTAG
+
?=5449;@@<@>>?8>10,0;;>>@:7777474876169668?;?BBCCC<;8;444-
4;;=@@?BA=@@9=<AAAC?===@@@???AA@@
@T6XXI:00034:00508
GGGAGAAGGGAGAAAGGAAGCTAGTCACTCGACATCGTGGCTTCACAGTACAGAGGGACTGTCCAAAGCCGCAATCT
CGAGTCGACTAG
+
BH@BBB=@?>@C?@<B895;B?BBB=@@CB?A?=7767737737@CGCCA@>>33317??@@>?ACA@@>@?:::==
=@AAA==<<<<<
@T6XXI:00034:00513
GGGAGAAGGGAGAAAGGAAGAATAGGGGACGGTTGGTAACAGTGGGGCTATTCTCCCTCTCCCTATAGTGAGTCGTA
TTAGAATTCCCC
+
;B:?>>599-4448-
42377<>ACCCD9BBB>A=@>??<?BBBCCC9C@AA?B=<;6;888@;??AC@@@ACB???????6644/344-
@T6XXI:00034:00529
GGGGAATTCTAATACGAC
+
444,424244498/,,,,
@T6XXI:00034:00536
CTAGACGACTCGAGATCTGCGGCTTTGGACAGTCCCTCTGTACTGTGAAGCCACAGATGTCGAGTCGACTAGCTTCC
TTTCTCCCTTCTCCCTTCCTTCTCCCTTCT
+
<<<<<@@CCBBAA;;:::>>@=???6=47777777.08;;<==AA=4834868696::BAA??<=@@ADAC::32:/
4718AAC?A??;;=6858,/,/455+,+,.
@T6XXI:00034:00541
GGGAGAAGGGAGAAAGGAAGCTAGTCACTCGACATCTTGTGGCTTCACAGTACAGAGGGACTGTCCAAAGCCGCAGA
TCTCGAGTCGACTAGCTTCCCTTTCTCCCTTCTCCT
+
BB?BBB@BCAC@@=9>584887=?>=<7./5,,+,,,*,22*.31186?CC@==A@@?;@?<==AAAC:@@?AA???
A===C@<====@@A@@@38418@:?@DC<?<???<?
@T6XXI:00034:00542
```

```
CTAGTCACTCGAGATCTGCGGCTTTGGACAGTCCCTCTGTACTGTGAAGCCACAGATGTCGAGTCGACTAGCTTCCT
TTCTCCCTTCTCCC
+
F?4999444>>A@FABBB?B@AAA<A?AABA???9?@@B??=A=AAB>ACACCBC@@@BBCCA?@@@@@AAA<;?=@
E>C@@C<A>@@@A<
@T6XXI:00034:00543
GGGGAATTCTAATACGACTCACTATAGGAGAGGGAGAATAGCTATCCCCTCTTCTCCGGAATCAACACCTTCCTTTC
TCCCTTCTCCC
+
CDD9B@BACCC@CCCCCCC@@>;=<00,000>><A@@>A@@>A??5;;0;>><???<?<?:==@>@@@?@;@9<<7<
??@<@>@BBA:
@T6XXI:00035:00508
CTCGAGATCTGCGGCTTTGGACAGTCCCTCTGTACTGTGAAGCCACAGATGTCGAGTCGACTAGCTTCCTTTCTCCC
TTCTCCC
+
A<<<@ABB?=C@>699>9>;>99999919>99;<AABCAA?AC:;<CB8888888?<?@BA????><>5990;@>@=
B;;<DEB
@T6XXI:00035:00526
GGGAGAAGGGAGAAAGGAAGCTAGTCGACTCGACATCTGTGGCTTCACAGTACAGAGGACTGTCCAAAGCCGCAGAT
CTCGGC
+
CEACCC@C@<@A@96>474;@<<?C:;:ABAB:::AAAAA@=?A?ACD@@?<444..-
/999;@9<<7;?8????@:863.))
@T6XXI:00036:00504
GGGGAATTCTAATACGACTCACTATAGGAGAGGGAGAATAGCCTCCCAATTATCAAAAATGAACTGCTCTTCCTTTC
TCCCTTCTCCC
+
ACB9@=?====<99;@B>BBB>;<<00,.5479>88:;488?8?>A6924/4000333.300/49333;>0947>99
99;8601.110
@T6XXI:00036:00505
GGGGAATTCTAATACGACTCACTATAGGGAGAGGGAGAAATAGTCAACAAATCGTTT
+
9AA9AAA>>995488A?@@?000;00..+...1726000+039:0730044111111
@T6XXI:00036:00514
GGGGAATTCTAATACGACTCACTATAGGGAGAGGGAGAATAGACCACCGTTGGTTCGGGTGCGCGTTCTCCTTCCTT
TCTCCCTTCTCCC
+
DGD:C?C?;<<>BBCECCCA:78>>777,7>>>A>@BC>@6<=>9:8588-
/,/76886:8887;39:33/0/04484<:><5<5404>7
@T6XXI:00036:00522
GGGAGAAGGGAGAAAGGAAGCTAGTCGACTCGACATCTGTGGCTTCACAGTACAGAGGACTGTCCAAAGCCGCAG
+
EC@AA?@BB;A@>?:@@C8;;C@@@CCDCCCBA:::::ACA@AB?BB????=555.../998;=?AC=?><?A=A
@T6XXI:00036:00530
CAGGTCGGACTCGAGATCTGCGGCTTTGGACAGTCCCTCTGTACTGTGAAGCCCACATGATCGAGTCGACTAGCTTC
CTTTCTCCCTTCTCCC
+
B;;:<@@;?CBCBD99;BCCBB>BBB>C@DBBBAAA<ABAC?AABBC=;7.,,+,,,++++++.:=:?B???99:@;
;;>0//88,/,///7,
@T6XXI:00037:00501
GGGGAATTCTAATACGACTCACTATAGGGAGAGGGAGAATAGTACCTACAGTCCGTAACAACTAGTTCTCCTTCCTT
TCTCCCTTCTCC
+
?A@4@6958889=AACCDECCB@;=777.7>==?==CC?;58///.//46445688C>BA?BCCCC9;:B@A;=061
,134446234::
@T6XXI:00037:00509
```

```
GGGGAATTCTAATACGACTCACTATAGGGAGAGGGAGAATAGGCAACCGTCATGTCGCCTTGTTCTCTGCTTCCTTT
CTCCCTTCTCCC
+
99949@A@CBB@?=;>BBB?>CB77111.777?B?@@A?A@@=@=:::>>;@ACA855;CAED@CCBBBCC@C@CCB
C@>><@@@CCC>
@T6XXI:00037:00536
GGGGAATTCTAATACGACTCACTATAGGAGAGGGAGAATAGCACGACTTTTTCGGCATCGCTATAATCTCCTTCCTT
TCTCCCTTCTCCC
+
CKF:C>C>CBB?BABBCBDB?;999/0,/0077-
666;<AAAA@BA??@@@1AC>A??A@@@@@A>AA@@>@<;9:96///604065884
@T6XXI:00037:00541
GGGAGAAGGGAGAAAGGAAGGAGAAGTCGCCCCAAACGATTTGTTGACTATTCTCCCTCTCCCTATAGTGATCGTAT
TAGAATCCCC
+
4959=>;>?999;C@CBC@C9<;CCBAAABLD8C@;76111,17767895;79444,444484588837/:.--
1130334../774
@T6XXI:00037:00547
CTAGTCGACTCGAGATCTGCGGCTTTGGACAGTCCCTCTGTACTGTGAAGCCACAGATGTCGAGTCGACTAGCTTCC
TTTCTCCCTTCTCCC
+
B>9999444=A@BA@@CCCCC@CCC?C@A<;<777-
7??@AADA==<A>@C@CCBBBCCC@??B?;;;88?6765721771000,023000,
@T6XXI:00038:00510
GGGGAATTCTAATACGACTCACTATAGGGAGAGGAGAAT
+
?J;6:1<694424/.0.42700.3...538677;422,-
@T6XXI:00038:00517
GGGAGAAGGGAGAAAGGAAGAGCAATTAGTGCTAGTACGGCGGGAGACTATTCTCCCTCTCCCTATAGTGAGTCGTA
TTAGAATTCCCC
+
BB@BCBABA;@@@A;B@?><<<@A@B@B??>?ABCCCBB?BBB>@@@AA@@@@>??><<A==;==<<<<@;@@AA==
=;=@A>C@CDD:
@T6XXI:00039:00512
GGGAGAAGGGAGAAAGGAAGGAGAAGTCGCCCCAAACGATTTGTTGACTATTCTCCCTCTCCCTATAGTGAG
+
9;:4998>>:>988047777133631137788.33,17779;B>><<:=>=8;;:94:444:558333.++0
@T6XXI:00039:00514
CTAGTCGACTCGAGATCTGCGGCTTTGGACAGTCCCCTCTGTACTGTGAAGCCACAGATGTCGAGTCGACTAGCTTC
CTTTCTCCCTTCTCCCCTTCCT
+
B9999944?AB=BBAA@BB@@A@CDBL@C?==7777.7??ACAA?AA@@<<A=@BB@@@@A?ABB:<<??>>696::
>CC>AA?A:;9:3///+/46,,
@T6XXI:00039:00515
GGGAGAAGGGAGAAAGGAAGGAGAAGTCGCCCCAAACGATTTGTTGACTATTCTCCCTCTCCCTATAGTGAGTCGTA
TTCAGAATTCCCC
+
:AA99969>9944;0823137778AA:<;CCC9BC>CACA<8?@:::386<5>?>>:?@@@@688>63343333-
++6++.97465:990
@T6XXI:00039:00524
GGGAGAAGGAGAAA
+
4911//,/,,**0)
@T6XXI:00039:00528
GGGGAATCTAATACGACTCACTATAGGAG
+
8880=)////,/;99;;1007-,,,,*.3
```

```
@T6XXI:00039:00541
GGGGAATTCTAATACGACTCACTATAGGAGAGGGAGAATAGTCAACAAATCGTTTGGGGCGACTTCTCCTTCCTTTC
TCCCTTCTCCC
+
A@@4>6969999;CBBBCABBAACB9947===?<<BA@A999AA>@@A<CBBAB5:7:4::::CAC@A=?>?>AA<A
AA=;A;==??;
@T6XXI:00039:00544
GGGAGAAGGGAGAAAGGAAGCTAGTCACTCGACATCTGTGGCTTCACAGTACAGAGGGACTGTCCAAAGCCGCAGAT
CTCGAGTCGACTAG
+
CC@BB@===:@@==9@885:B???CCAAAA=::8???@BC=?@@ABBBCCCAA843317<<>@A>??;@A>A@=?<=
=@B8<=AAA???AA
@T6XXI:00039:00545
GGGAGAAGGGAGAAAGGAAGCTAGTCACTCGACATCGTGTGGCTTCACAGTACAGAGGACTGTCCAAAGCCGCAGAT
CCTCGAGTCGACTAG
+
991999599-848:-423999AA>>>761.1.--5...7//-
.B=;>@@=?@998//,/6668@<??;?A<A?>99//,/888;<8344385
@T6XXI:00039:00546
GCTAGTCACTCGAGATCTGCGGCTTGGACAGTCCCTCTGTACCTGTGAAGACCACAGATGTCGAGTCGACCTAGCTT
CCTTTCTCCCTTCCTCCCTTCCTTCT
+
<4444444444988=B=644,..,.,.654366266=<;7707;5335349?BCBBB;;77133.-
,,,*,04..,17999@@=944/0/033,02423235
@T6XXI:00040:00504
GGGAGAAGGGAGAAAGGAAGCAGAGTGCGTGATAACAGGGAGATAAACTATTCTCCCTCTCCCTATAGTGAGTCGTA
TTAGAATTCCCC
+
CD@DDC@BB>AA@B>B@B?>>?>??>????>;;;>>>==;<<<@=>;><==>A??@<===AC>CA???8>>AABC@?
?=?@C?B@BBB8
@T6XXI:00040:00513
CTAGTCACTCGAGATCTGCGGCTTTGGACAGTCCCTCTGTACTGTGATGCCACAGATGTCGAGTCGACTAGCTTCCT
TTCTCCCTTCTCCC
+
://////44BBBCBCBBB@>=@EC>B;@?@CBBB>BCCA@@<<>>>=;??@BCBBA??>>@BCCCBBBBCCCC@C?B
B>B@@@<@<??AA<
@T6XXI:00040:00520
GGGAGAAGGGAGAAAGGAAGCTAGTCGACTCGACATCTGTGGCCATC
+
CC?DDB@BB=BBBB=A=>=>BA7>>/11>=>:1;:96.>;858,-++
@T6XXI:00040:00528
GGGAGAAGGGAGAAAGGAAGGAGACAGTGAGCGCCGCACAACACGTCACTATTCTCCCTCTCCCTATAGTGAGTCGT
ATTAGAATTCCCC
+
44-
44988;588=A<@<@?B=@ADCDDCDBAA@><???8?>AAABCBCCB@@=@AAA=AA???9?A@GBBB?>>000;43
356371333,
@T6XXI:00040:00544
GGGAGAAGGGAGAAAGGAAGAGAACAGCCCCTGTAAATGTGTGTGAACTATTCTCCCTCTCCCTATAGTGAGTCGTA
TTAGAATTCCCC
+
BE?BAB=@B<@@@B<A=@@BABB@CDCCCC9BC@@@<ABABBBBBE?BBCC?BACC>CCBCCBA???BB@<<;=;;;
<;==?>=<ACC4
@T6XXI:00041:00507
GGGAGAGAATTTGGTAATGGGGATGTCTGAGGTGAGGAAGAGAGATAGTCCACCCTTATATCTCTCTCCCTATAGTG
AGTCGTATTAGAATTCCCC
+
```

```
/1,//4442485897<59:A?2>611379777661.,.,...----
0044/611,1778<587;;;444,4::;;98;5330>88588888:?AA6
@T6XXI:00041:00516
GGGAGAAGGGAGAAAGGAAGGGAATAGCAGCAGTCGGCAACGTGAAACTATTCTCCCTCTCCCTATAGTGAGTCGTA
TTAGAATTCCCC
+
CCBCBB@BC>BBBB>B@A@BB>B@B@>@@BCABBBCCBC@BA?@@?;AB@@A?@?@>?????;?AAAA@AAAC@???
?:==A>A?AAC4
@T6XXI:00041:00528
GGGGAATTCTAATACGACTCACTATAGGGAGAGGGAGAATAGCTTCCTTCTCCTCACATGCAACGTGCCTACTTCCT
TC
+
BBA7A:;;<BBACA@@A@ABB@C@?:8:8:??CC>CAA??;?=A?C?BB@AA>A<@@@@@B@>A@@;94///-,-
,+00
@T6XXI:00042:00509
GGGAGAAGGGAGAAAGGAAGTGAATGGGAGCTCATACGGTTACGATGACTATTCTCCCTCTCCCTATAGTGAGTCGT
ATTAGAATTCCCC
+
AA@@@@@AAB?BBC?CAC?ACAA?ACC>CB@C??:8?>>>A>AA@@@AABBCACBCC>BBAAB?BCCCCBCBEC@@@
@@<?@><>=@@@8
@T6XXI:00042:00531
GGGGAATTCTAATACGACTCACTATAGGGAGAGGGAGAATAGATTGGCGAGAAATGCCGTCTAGTGCTCCTTCCTTT
CTCCCTTCTCCC
+
BBA9B>B>BCD@CCBBACBB=?>>=777.7>==?>?HDBAAA8848=@=?@AA<???<???@@@@===A>A<@3781
8@@@<A>???A<
@T6XXI:00042:00534
GGGGAATTCTAATACGACTCACTATAGGAGAGGGAGAATAGTCAACAAATCGTTTGGGGCGACTTCTCCTTCCTTTC
TCCCTTCCTCCC
+
@<<1<-1,///5/437373363477/0,../11,34;<=7.../,/-
2241117699<7<@>>>=>;8,/,/7892;16616,/,,550
@T6XXI:00043:00514
GGGAGAAGGGAGAAAGGAAGGAGAAGTCGCCCCAAACGATTTGTTGACTATTCTCCCTCTCCCTATAGTGAGTCGTA
TTAGAATTCCCC
+
EC>AA@=@B>@@@A<B>A@CACCB?BBB?>;;5;;<?BBCC>BB?BBCCAA=::;=;<;?<;:<===;:::====:;
?>AAA>@38770
@T6XXI:00043:00515
GGGGAATTCTAATACGACTCACTATAGGAGAGGGAGAATAGTCAACAAATCGTTTGGGGCGACTTCTCCTTCCTTTC
TCCCTTCTCCC
+
ACC9B;;;<BB>BBBBB;<;B=<;;00,0:077.44==A??>AA?AAA<B???C>CCD9BA>>;,0./,/,/78828
555355<8?A;
@T6XXI:00043:00524
GGGAGAAGGGAGAAAGGAAGCTAGTCACTCGACATCTGTGGCTTCACAGTACAGAGGACTGTCCAAAGCCGCAGATC
TCGAGTCGACT
+
@A<@AA?B@:>999094858A?=?BC:;;CACCA>::;00,/7336788663344423366555440320099=??
?@?;;;8168/
@T6XXI:00043:00530
GGGAGAAGGGAGAAAGGAAGGAGAAGTCGCCCCAAACGATTTGTTGACTATTCTCCCTCTCCCTATAGTGAGTCGTA
TTAGAATTCCCC
+
>=99996990949@<@=@@B?BBC?A@@@CCC7@@<?B@??;=??AAACAA>@@@A<?===A;@@AA@@@@A@@@@@
?=@@A?A>BAA6
@T6XXI:00043:00537
```

```
GGGAGAAGGGAGAAAGGAAGGAGAAGTCGCCCCAAACGATTTGTTGACTATTCTCCCTCTCCCTATAGTGAGTCGTA
TTAGAATTCCCC
+
?CABBA9==7;9=?<@=@;>;771999988ED8@@=@>==>89<>::4=8?<===C@BA@?B;<<<<;9:8843355
5:=<<;=;<<</
@T6XXI:00043:00546
GGGAGAAGGGAGAAAGGAAGATGTCGCCGCAGGGAGTGAACTTGGCTATTCTCCCTCTCCCTATAGTGAGTCGTATT
AGAATTCCCC
+
AA<@>?;>>:99;BBB?BABBBBBCCBACCCCCB::;BC@CC?B?BCBB@CBBC>@@@CC>CBBBAAACDDCDCAA?
ACD@D@CCC:
@T6XXI:00044:00500
GGGGAATTCTAATACGACTCACTATAGGAGAGGGAGAATAGCCGTTCATTTTCTCCCGCGGCCGTGAGCTTCCTTTC
TCCCTTCTCCC
+
ABA9B>B?DCC@CB@<BBBB?:977//-
0>>BC>?@@>A<;:<==<<BBCB8?C>>899>>=<===BBAA?@>AA<?BAB=A>B===9
@T6XXI:00044:00502
GGGAGAAGGGAGAAAGGAAGGAGAAGTCGCCCCAAACGATTTGTTGACTATTCTCCCTCTCCCTATAGTGAGTCGTA
TTAGAATTCCCC
+
CC>AA@=ACCBBBD@CDC?B?ACC?<??>>>>.77.7==??;?A>A?=>AA<??A@;@@@@C:@@A=<<<<@@@@@A
A>A@?;>38770
@T6XXI:00044:00505
GGGGAATTCTAATACGACTCACTATAGGGAGAGGGAGAATAGTCAACAAATCGTTTGGGGCGACTTCTCCTTCCTTT
CTCCCTTCTCCC
+
944,49><@AA?GDFBCCCB8::?=777.7>>@AAAAA>??@@AB?9994:=9=?>BBB9???BB=??@<@<@=@@;
@@@@:?378=>:
@T6XXI:00044:00510
GGGGAATCTAATACGACTCACTATAGGGAGAGGGAGAATAGTCGGCGCGCCCTTT
+
8880=+1///56=7377;:61711111,1111>;889<==554:4330000+04+
@T6XXI:00044:00526
GGGAGAAGGGAGAAAGGAAGGAGAACGCGCACCGAACCAACGGTGATCATTCTCCCTCTCCCTATAGTGAGTCGTAC
T
+
2788-1,1/+/68;-4231313367==88;11-
/7,/,/744244448=A>BBA?793333,376111113.......
@T6XXI:00044:00545
CTATCACTCGAGATCTGCGGCTTTGGACAGTCCCTCTGTACTGTGAAGCCACAGATGTCGAGTCGACTAGCCTTCCT
TTCCTCCCTTCCT
+
B@948////:8111BCA??=877-
811111111,16;9=A@<<;;8288883333.....,,,/.222,//,.5555665-3364:8=;3
@T6XXI:00045:00523
GGGGAATTCTAATACGACTCACTATAGGAGAGGGAGAATAGTCAACAAATCGTTTGGGGCGACTTCTCCTTCCTTTC
TCCCTTCTCCC
+
BBB9B?C@CBBAB?>>BBBB>:977/.,0<:=?==DC?A8==?A:;;;;CAAAC>CCC9BBBCC?B@@9=99;@A;@
@@C<A?@@@@<
@T6XXI:00045:00539
GGGGAATTCTAATACGACTCACTATAGGGAGAGGGAGAATAGTCACATTCATGTTTGTGACCGTGTCTCCTTCCTTT
CTCCCTTCTCCC
+
:;:5;,459>?59:BAAACDBAA?>777.7>??B=?==:=AA@@@<<?BGCBBBC>;87;;9<?AAA==;=>A>@A<
AADC;@?A???;
```

```
@T6XXI:00045:00549
GGGGAATTCTAATACGACTCACTATAGGGAGAGGGAGAATAGTCGAACTAA
+
AA@4@6959998>=@@AAABCBCC@::7.777>@<??A:966,+++*+++0
@T6XXI:00046:00502
GGGAGAAGGGAGAAAGGAAGGCGAACAGCCGGTAGTACAAAGCTAGGCTATTCTCCCTCTCCCTATAGTGAGTCGTA
TTAGAACTTCCCC
+
;C@CCCBCB=?A>;86:7,0,0=:4>@???CCBBBCC???9;?<==>ACDD@????7:;77858AA@A?@@AA?==4
4344;79;=@@@1
@T6XXI:00046:00506
GGGAGAAGGGAGAAAGGAAGCTAGTCACTCGACATCTGTGGCTTCACAGTACAGAGGGACTGTCCAAAGCCGCAGAT
CTCGAGTCGACTAG
+
GC@@AB?BE?CCBB=BBA?B>>>AA?;::?<?;<???CBB?CC@CCC@BB<944000-
4;;=>?>@A<AC>?>?@A==<@?:?AAA@@@AA
@T6XXI:00046:00530
GGGGAATTCTAATACGACTCACTATAGGGAGAGGGAGAATAGTCAACAAATCGTTTGGGGCGACTTCTCCTTCCTTT
CTCCCTTCTCCC
+
CCB7B798;@@@DD@@B@AAA?>=;666.7==>B@A@A@A<??@?=?AC<B<?<@;@@?8CCB@B@B?A6988>AC>
BBBB=B>A@@@<
@T6XXI:00046:00549
GGGAGAAGGGAGAAAGGAAGGAGAACACGCCGCGGGATATTGGCGAACTATTCTCCCTCTCCCTATAGTGAGTCGTA
TTAGAACTTCCCC
+
CC:AA@59919?@A<A>B9;:>CE@CBBBB@CCBC<@A;;9;?ADCBAAAB>A>>>883377:8=CCDCCADDDA??
<7455.//77::6
@T6XXI:00047:00501
GGGAGAAGGGAGAAAGGAAGGAGAATCGACACACGAATGCCTACGTGATCTATTCTCCCTCTCCCTATAGTGAGTCG
TATTAGAATTCCC
+
CC>AAA=@C@<=<C@C@C=@=@CA@CBBAABCC=@@>@ABACBC?ACCCEBBB@BBCC@BBBBB?BCCCCAAAA???
BBC@BCC?E>@@=
@T6XXI:00047:00526
GGGAGAAGGGAGAAAGGAAGGAGAAGTCGCCCCAAACGATTTGTTGACTATTCTCCCTCTCCCTATAGTGAGTCGTA
TTAGAATTCCCC
+
491444244-
488;9=;<=A?CCCABFA>>??.8801668@;?A@A@@????BAAA<A@@@A;@@BBAA@@@===AAC=?=?:=5>>
?5
@T6XXI:00047:00530
GGGGAATTCTAATACGACTCACTATAGGGAGAGGAGAAT
+
8886;;C@BBC@ABBBBBBB@BCB@?@A9;;0,+,+00+
@T6XXI:00047:00549
GGGAGAAGGGAGAAAGGAAGAGAACAGCCCTGCGGTGAGG
+
4=59;;299-444;6?>@?A>BC5<9/685////,/,000
@T6XXI:00048:00514
GGGAGAAGGGAGAAAGGAAGCTAGTCGACTCGACATCTGTGGCTTCACAGTACAGAGGGACTGTCCAAAGCCGCAGA
TCTCGAGTCGACTAGCTTCCTTTCTCCCTTCTCCC
+
BEAAA@=@@9>99909;><@CB><</00/....668?<99<98:6:AAABC@@?????:@???AA9==9=B?AAA??
<==?80/;;;000;;=A<?=?A;@>@@1838>>>:
@T6XXI:00048:00521
```

```
GCTATCACTCGAGATCTGCGGCTTTGGACAGTCCCTCTGTACTGTGAAGCCACAGATGTCGAGTCGACTAGCTTCCT
TTCTCCCTTCTCCC
+
//4:44:?@A=@AD@>;A10,00/+0,..7??@@;:89:9:43<4423-
1,/46006?877?99;7777766677>>?;B84874/30487
@T6XXI:00048:00529
GGGAGAAGGGAGAAAGGAAGGAGAAGTCGCCCCAAACGATTTGTTGACTATTCTCCCTCTCCCTATAGTGAGTCGTA
TTAGAATTCCCC
+
BB?BB@@BCBB@BB>C@B>?=>A@?BCC??;;5?A<ABCCC=BB@CBB?@@?B88414;9;<7=AAA@@A???;@?@
@?ABD>B;==<4
@T6XXI:00048:00538
GGGGAATTCTAATACGACTCACTATAGGAGAGGGAGAATAGCCAAAAATCCACGTTCCGA
+
;BEA>1;699999@?;;<>B?796111019000,/.-0133449=;;.30/0000/0430
@T6XXI:00048:00544
GTCGACTCGAGATCTGCGGCTTTGGACAGTCCCTCTGTACTGTGAAGCCACAGATGTCGAGTCGACTAGCTTCCTTT
CTCCCTTCTCCC
+
CC<::CCCCBDECBCBBB:;<H@E?BBCCCBA?B????BBBBA>B?BB?BBBBCBBBBCBBAA????C8:;?B=??8
?<<<6<:<>@@<
@T6XXI:00049:00545
GGGAGAAGGGAGAAAGGAAGGGGAGAAGGGAGAAAGGAAGCTAG
+
=C>CBB=@=<111@-1-0,000*.677@C=>A@729,0,01,**
@T6XXI:00049:00548
GGAGAGAATTTGGTAATGGGCTGATCGGGTTCTGGAGTGTGGTAAGGGCTCCCCCTTATATCTCCTTCTCCCATAGT
GACTCGT
+
CAA@>>=248-
89931333,4444//6858,0089:::???>?@@=;2400000+044433300/023333004444444444/
@T6XXI:00004:00556
CTATCGACTCGAGATCTGCGGCTTTGGACAGTCCCTCTGTACTGTGAAGCCACAGATGTCGAGTCGACTAGCTTCCT
TTCTCCCTTCTCCC
+
C:::11111:@BCCCBBBCC@C@@=B@A96;6994:BBCCC@@=<?A>@B@BBBABBAA>@?@ABBAABCBBB?A?A
B>BBAA<A@@@@A<
@T6XXI:00004:00563
GGGGAATTCTAATACGACTCACTATAGGGAGAGGGAGAATAGTCAACAAATCGTTTGGGGCGACTTCTCCTTCCTTT
CTCCCTTCTCCC
+
BBA7A=@=ACC@DBAAADBB?@B@@<<=;=AC@?;>;;:;999==<;@@;A@977,111,6@ABC@CBA;=:=>AA<
AAAC=A=A@@@;
@T6XXI:00004:00564
GGGGAATTCTAATACGACTCACTATAGGAGAGGGAGAATAGCTAACC
+
2870?+9544888477>?A::6316110166<>74//,-/-...,.,
@T6XXI:00004:00568
GGGAGAAGGGAGAAAGGAAGCTAGTCACTCGACATCTGTGGCTTCACAGTACAGAGGGACTGTCCAAAGCCGCAGAT
CTCGAGTCGACTAG
+
@@<ABA@CD<@@@>9>;>488=?=0.....-
.++23///77776::<<::7>>==?=7=@===@@CC>AA<?<;88///;679<==77773
@T6XXI:00004:00593
GGGGAATTCTAATACGACTCACTATAGGGAGAGGAGAATAGTAC
+
111+/7@9>BB?BCGCCB<<??B@>@@B<<:86:?4424,,,,+
```

```
@T6XXI:00005:00563
GGGAGAAGGGAGAAAGGAAGCTAGTCACTCGACATCTGTGGCTTCACAGTACAGAGGGACTGTCCAAAGCCGCAGAT
CTCGAGTCGACTAGCTTCCTTTCTCCCTTCTCC
+
94-44959804444-4231.....0,,...2.--
56:=CA=>?@C????89::433+084==A>AA=??;;?>8888748===>>?@877<;8;;==78//706,.--,
@T6XXI:00005:00564
GGGAGAAGGGAGAAAGGAAGCTAGTCGACTCGAGATCTCGGCGTTTGGGACCAGTC
+
4<89=@@BC;;9:;-423133116?A:16;66666669?A>555=4444--,--+0
@T6XXI:00005:00590
GGGGAATTCTAATACGACTCACTATAGGGAGAGGGAGAATAGTCAACAAATCGTTTGGGGCGACTTCTCCTTCCTTT
CTCCCTTCTCCC
+
499/9;><>@@=@BCA@@CCB?>>=777,777>=89<:<>A<:9;8443+16111,111,1768<=><<<=:;238-
44:;4:79///,
@T6XXI:00006:00578
GGGAGAAGGGAGAAAGGAAGGAC
+
CDACCB@BB:=;11+-80)*)**
@T6XXI:00006:00585
GGGAGAAGGGAGAAAGGAAGGAGAAGTCGCCCCAAACGATTTGTTGACTATTCTCCCTCTCCCTATAGTGAGTCGTA
TTAGAATTCCCCC
+
<<6;ABACDAAAAB>BAC@C?B>C>@ACABBB9CGABA>@@;@C@CA@=@B?ABBD;@===@:@@??@>=>889=@A
@=<55/0/-146.
@T6XXI:00006:00591
GGGAGAAGGGAGAAAGGAAGCTAGTCGACTCGACATCTGTGGCTTCACAGTACAGAGGGACTGTCCAAAGCCGCAGA
TCTCGAGTCGACTA
+
DC@???AB@:=444-
9=?>BB@>>>0//=;;=1::::6?@@:<?=?CBBBBA@?A=<<7<?=<=>9;;6<=;??@@A@===AA@@@?<<<<
@T6XXI:00007:00571
GGGAGAAGGGAGAAAGGAAGAGAACAGTCACAGGCTCGAGCTGCAAGCTATTCTCCCTCTCCCTATAGTGAGTCGTA
TTAGAATTCCCC
+
BDACCB?B?:9:;B=BACBCDBB?<<;CDCCAA?BCBBBCBAABC?AACBB?BBBB>BA@<<:<CCDB??=B?<=><
<9<>;9;2444-
@T6XXI:00007:00574
GGGAGAAGGGAGAAAGGAAGGAGAAGTCGCCCCAAACGATTTGTTGACTATTCTCCCTCTCCCTATAGTGAGTCGTA
TTAGAATTCCCC
+
DC>BBB?@C:?988-
9484;@BBB@@CCCECC9DD@C::7?;@C@CBB=443488448??@=;=?@AB@B@AAAA999=;6424/000+
@T6XXI:00007:00575
GGGGAATTCTAATACGACTCACTATAGGGAGAGGGAGAATAGCTACTAC
+
944,48><>??5943333376136888>5779??8<;;;>-++0*****
@T6XXI:00007:00584
GGGGAATTCTAATACGACTCACTATAGGGAGAGGGAGAATAGTTGCTTGACTCC
+
/11+1,1,///,/44433>?@>D??7775:AABC>B@B>AA==98333..*000
@T6XXI:00008:00561
GGGAGAAGGGAGAAAGGAAGAATACCGTTGTGGAGAATATAAGCAGACTATTCTCCCTCTCCCTATAGTGAGTCGTA
TTAGAATTCCCC
+
```

```
9?:>996990944>0923779;>@B>@@=>;;8<CC@CCCC@BBB@@ACCCBCBAE>AABBB=AA?AAAABAAAA@?
=>BAA>;:BCD5
@T6XXI:00008:00568
GGGGAATTCTAATACGACTCACTATAGGGAGAGGGAGAATAGTCAACAAATCGTTTGGGGCGACTTCTCCTTCCTTT
CTCCCTTCTCCC
+
999/9<A@BBB@BBBBB?<@>AD?966646?BCC=ABB=B===@?>=<<7?BBBC>CCC9BBCBC?CAA<?<??AA:
>?@C<A>????;
@T6XXI:00008:00572
GGGAGAAGGGAGAAAGGAAGCTAGTCATCTGTGGCTTCACAGTACAGAGGGACTGTCCAAAGCCGCAGAT
+
6=6119699-4448-4231397;;:??:74@?;8<B:??@A@?>88444818==?AB>BC<??>???A@@
@T6XXI:00008:00574
GGGAATTCTAATACGACTCACTATAGGGAGAGGGAGAATAGCGCGTAACGTTGACGACGCTTCCTCACCTTCCTTTC
TCCCTTCTCCC
+
99195958449;9;<<<<CB@?>>776.7777A>@@A>@@@AAAAAA>?@?9<<?@BBBBB=?=?>>;792424;:?
@@A<@=;844-
@T6XXI:00008:00591
GGGGAATTCTAATACGACTCACTATAGGGAGAGGGAGAATAGTCAACAAATCGTTTGGGGCGACTTCTCCTCCTTTC
TCCCTTCTCCC
+
DCC4@6959449:==@@BBBBBCC;;:C???:::6:99=A?==<@=<:<1:777?6???2=>;00,0<7,/-
01222.00+0449;<7
@T6XXI:00009:00555
GGGAGAAGGGAGAAAGGAAGCTATCGACTCGACATCTGTGGCTTCACAGTACAGAGGGACTGTCCAAAGCCGCAGAT
CTCG
+
BB>BBB@CB=BBBB=B@C@CCDDC@==?BB????:::??C?BA?BBCB>>====44444::=<=;<<<AB>??:AA=
@@@@
@T6XXI:00009:00598
GGGGGAATTCTAATACGACTCACTATAGGGAGAGGGAGAATAGACGTGGCGCCACCGACTGCTCTATCTCCTTCCTT
TCTCCCTTCTCCC
+
4444+42489995:ABBCDGCABBB?@?C>C;;<E>B@@=??899@BB==8833=;=@@AAAAA@AA@@@=?;=;=;
-436606,/-000
@T6XXI:00010:00565
GGGAGAAGGGAGAAAGGAAGAGGCAATAGGCGTAGTGGAGAAGGTAACTATTCTCCCTCTCCCTATAGTGAGTCGTA
TTCAGAATTCCCC
+
94-444244-444;-42377;@=BAA@AA>88394..,...,.7777:B@@A=?>>8669;628;5;<?@;6//-
++0*++5424/000+
@T6XXI:00010:00569
GGGGAATTCTAATACGACTCACTATAGGGAGAGGGAGAATAGTCAAACCTCACCCCTTTCTCTTCTCTTCCTC
+
CBB7A=@=AA?=@=11.8447976676?8>>ACC>BAA>A998<:6465553344+00+00002311201,--
@T6XXI:00010:00580
GGGGAATTCTAATACGACTCACTATAGGGAGAGGGAGAATAGTTTTTCCCTTCACTAACCGCGAACTGCACTTCCTT
TCTCCCTTCCTCCC
+
444,4:?=@A@?BBB@@@CCBFCBB:77.7>;;@68//,-4-----
*00+04699?><?=:;8?<>?@<:::<?;?B;=;6606,/,.110
@T6XXI:00011:00556
GGGAGAAGGGAGAAAGGAAGCTAGTCGACTCGACATCTGTGGCTTCACAGTACAGAGGGACTGTCCAAAGCCGCAGA
TCTCGAGTCGACTAG
+
```

```
CD?;9928909@@@<CAB?DB6::@::::?A?:::::6:78;<A?BBEEBB??<==A@<AC???A<??:?@8<<??=
=><<<@@?????AA@
@T6XXI:00011:00575
GGGGAATTCTAATACGACTCACTATAGGGAGAGGGAGAATAGT
+
;;:1<,458892447783<>B@@77777.7=777,000433--
@T6XXI:00011:00577
GGGAGAAGGGAGAAAGGAAGGAGAAGTCGCCCCAAACGATTTGTTGACTATTCTCCCTCTCCCTATAGTGAGTCGTA
TTAGAATTCCCC
+
CC@CDC@BB=BBBB=B@C@C?BBC@???7777.77.7;?C@=CC;;;AA@@>A=<<7=?@?B?AA@???A===<<<8
8334423/444-
@T6XXI:00011:00585
GGGGAATTCTAATACGACTCACTATAGGGAGAGGGAGAATAGTCAACAAATCGTTTGGGGCGACTTCTCCTTCCTTT
CTCCCTTCTCCC
+
CCC:CAB?BBB@BBB<?>>>>?B@A@@@;=B?@C>;@@?A;99@=;::<;<>6662;;;5?CCCB@C@@=?=?<==:
;AAA:A??@@@;
@T6XXI:00011:00586
CTAGTCACTCGAGATCTGCGGCTTTGGACAGTCCCTCTGTACTGTGAAGCCACAGATGTCGAGTCGACTAGCTTCCT
TTCTCCCTTCTCCC
+
211//6/44==@?ACB@@<;:;BC<B;;;AABDC>CBCCJC==499D@AC=A@CBCCABACCAA@@;;;A??<=?@L
J<?<CC:;74034-
@T6XXI:00012:00553
GGGAGAAGGGAGAAAGGAAGGGAGAG
+
CC>B==;=>:>9990950,0:,0...
@T6XXI:00012:00554
GGGAGAAGGGAGAAAGGAAGGGGAGAAGGGCTAGTCACTCGACATCTGTGGCTTCACAGTACAGAGGGACTGTCCAA
AGCCGCAGATCTCGAGTCGACTAG
+
CB@BCC@A@=@CBB>B@B@BBB8BB@@@@>ABD@;4<99449==?BCCCC@B?=>>?>>?BCCB???=?BCCB<@??
;?B@CCCCCB8;;CCBBECCBBBC
@T6XXI:00012:00558
GGGAGAAGGGAGAAAGGAAGGAGAACAAGTTGCGGACCATCGCGAGACTATTCTCCCTCTCCCTATAGTGAGTCGTA
TTAGAATTCCCC
+
CE@CB?@BB>BBBB=B@D@>=>?>>AB?BB>??@=@C@BCCCBBCC?A?BB@BAAA@@@@@A>BA@<<===::<<==
=;<@@<?8<<<4
@T6XXI:00012:00585
GGGGAATTCTAATACGACTCACTATAGGGAGAGGGAGAATAGTCAACAAATCGTTTGGGGCGACTTCTCCTTCCTTT
CTCCCTTCTCCC
+
444,489@@AAACCDCCBCB?@B7=666.7>==?<?@@=@@AAA@>???<ACDDG>BBB9BBBBC;??>3434244-
4444-48:<=>7
@T6XXI:00012:00594
GGGGAATTCTAATACGACTCACTATAGGGGAGAGGGAGAATAGTCAACAAATCGTTTGGGGCGACTTCTCCTTCCTT
TCTCCCTTCTCCC
+
444,424:49;;=:9>>??>==>;=7777,16667;885;=@@@>>9444-
7==66,111,....45547777=<=@98;=@1858@BC<
@T6XXI:00013:00556
GGGAGAAGGGAGAAAGGAAGTGCATGCGGGTGGAGAAAATAGAGAGACTATTCTCCCTCTCCCTGATAGTGAGTCGT
ATTAGAATCCCC
+
```

```
990444244-4444-
4777797994?89903<77;;;>7BBBBBDC@@@CB?A>==;=;<<=;999=?=<A?=55,,,+**..)..550
@T6XXI:00013:00570
GGGGAATTCTAATACGACGTCACTATAGGAGAGGGAGAATAGTCACATTCATGTTTGTGACCGTGTCTCCTTCCTTT
CTCCCTTCTCCC
+
499/921,//6,/,,,,...099786;00,0:/76,16636?9666<6><??B9?C<966742134...53103-
00+0388099;003+
@T6XXI:00013:00577
CTAGTCGACTCGAGATCTGCGGCTTTGGACAGTCCCTCTGTACTGTGAAGCCACAGATGTCGACGTT
+
:<<BCCB=B>AAA;<<C><;C@BBB@BB<;;CA@A<???AAACB@@@A89<?BCBC@?:,,)((((
@T6XXI:00013:00581
CTAGTCGACTCGAGATC
+
,A1,,,,,/88771444
@T6XXI:00013:00593
GGGGAATTCTAATACGACTCACTATAGGGAGAGGGAGAATAGTCAACAAATCGTTTGGGGCGACTTCTCCTTCCTTT
CTCCCTTCTCCC
+
444/99@@CAB>BBBBB>BB>AA>96675:CCEC>BBB=?9=<@=<<=<;>?99=8???5?CCCE@?==:<9<:??<
?AAA<A?@@@@<
@T6XXI:00013:00599
GGGGAATTCTAATACGACTCACTATAGGGAGAGGGAGAATAGTCAACAAATCGTTTGGGGCGACTTCTCCTTCCTTT
CTCCCTTCTCCC
+
;;949<B?@AA@FCCCCAAABAB=7777.7>77768::<?89:?A>BDBACCBBA<AAA6ACCCF?@;;3838?BA<
ACAD5858<=@:
@T6XXI:00014:00557
GGGAGAAGGGAGAAAGGAAGAGCACGTTCAAGCTAGGGACAGATGAACTATTCTCCCTCTCCCTATAGTGAGTCGTA
TTAGAATTCCCC
+
94-9>>699-
889909487<ACDGCCB>;;9<BAACJ@;<;;;:?B?BBBC?BBBL?BBAAA=ACCCBB???@A@A?98<883:BCC
C8
@T6XXI:00014:00569
CTAGTCGACTCGAGATCTGCGGCTTTGGACAGTCCCTCTGTACTGTGAAGCCACAGATGTTGAGTCGACTAGCTTCC
TTTCTCCCTTCTCCC
+
>=111=8;ECCCBCCBB?A?B@BBB?B@BCBBAAA>AB@@@C@=:??A>ACACCCCCC@?=?BCBBBCBBB@::568
>A>CA@A<A>AAAA<
@T6XXI:00014:00589
GGGAGAAGGGAGAAAGGAAGTGCAGTTCGCGGTTAGTGAAGGGGAAAACTATTCTCCCTCTCCCTATAGTGAGTCGT
ATTAGAATTCCCC
+
BB@BCBBDB>BBBB?B?A@BBBBBBB@99:>486;;;CD@CCC7BBB7BCCB@CCCC8??CCC>???@AA=AAA@@@
@@>AAA;>8<;90
@T6XXI:00014:00594
GGGAGAAGGGAGAAAGGAAGCTAGTCGACTCGACATCTGTGGCTTCACAGTACAGAGGGACTGTCCAAAGCCGCAGA
TCTCGAGTCGACTA
+
;<:;AA@BB@@884-
9;?<??>;::0//<:::0:9977AA?>@B?BDCCC?;??AA@@<AA<@@@9<<5<=:<====AAAA@@@A@@??<;
@T6XXI:00014:00596
GGGGAATTCTAATACGACTCACTATAGGGAGAGGGAGAATAGTCGAACTAATCGTTGGGCGACTCTCTCC
+
AA@4?<=5999;=@@CBCCCBACCBAAD?BBBBD<@@A>@@@888:7:::2566444420,,,,2,,+55
```

```
@T6XXI:00014:00598
GGGGAATTCTAATACGACTCACTATAGGGAGAGGGAGAATAGCTCAACCCTGGACTTCGGCGCTGTCTCCTTCCTTT
CTCCCTTCTCCC
+
499/424:ACB?@ABBBBBB@ABA>?AB>>BCCB>@AC>@;@<@?@?@C;==:==A;??==<@=====A?A>A>@@:
@AA=;<;:::=;
@T6XXI:00015:00553
GGGAGAAGGGAGAAAGGAAGAATACGGCGCGACCAACGATCCGAGGGCTATTCTCCCTCTCCCTATAGTGAGTCGTA
TTAGAATTCCCC
+
DD?CCD?B@<>>>>9>>A?BB@CCCA>?BBBCB@C@BCCCB<?998-
9B>>>BCCG>CECBA<@B???BAAA@===<;383?<<<==C5
@T6XXI:00015:00554
GGGGAATTCTAATACGACTCACTATAGGGAGAG
+
888*1,/,///7;;?;883311311111,1611
@T6XXI:00015:00570
GGGAGAAGGGAGAAAGGAAGCTAGTCGACTCGACATCGTGTGGCTTCACAGTACAGAGGACTGTCCAAAGCCGCAGA
TCTCG
+
9909>>594-
4448587;=<<?BBBB10030,,,,2,,,0320033.../.?;44:3323333:<;<<488:7449647348
@T6XXI:00015:00576
GGGGAATTCTAATACGACTCACTATAGGGAGAGGGAGAATAGCAAAACGTTTCCCCGACGTCGCTTTCTCCTTCCTT
TCTCCCTTCTCCC
+
BCD:D@B?BBB@BABABBBB?@B;7111.6>>>B>@==>@==733708BCC>BBB6ABBBBBA=?;;?44279;7;<
7<@AC?A;==BC<
@T6XXI:00015:00585
GGGGAATTCTAATACGACTCACTATAGGGAGAGGGAGAATAGACCAGCACTCCATGTTGTCATGTTCTCCTTCCTTT
CTCCCTTCTCCC
+
994/9;>?BCC?BBCCCBBB?AB>;111-
676@B>A@@?CAAAA<??@A<<?>@A@@>==<<A??<??A9=;=>AD;@@AA7<9777=:
@T6XXI:00015:00587
GGGGAATTCTAATACGACTCACTATAGGGAGAGGAGAATAGCTAGGAC
+
===5=AB@DBCACBBBBBCBCCAAAAA?9??;;:;44244////.,.-
@T6XXI:00015:00591
CTAGTCGACTCGAGGATCTGCGGCTTTGGACAGTCCCTCTGTACTGTGAAGCCACAGATGTCGAGTCGACTAGCTTC
CTTTCTCCCTTCTCCC
+
44444444//////,/6666.0-
06>8;,0......+1......2245889B@CBACDBBBBBDCCCCB<<;B>>>A@CB6;7;949/433=4
@T6XXI:00015:00594
GGGGAATTCTAATACGACTCACTATAGGGAGAGGGAGAATAGCTCGATCTTCTCTCTTCAACTTGTACACTTCCTTT
CTCCCTTCTCCC
+
BA?4@5469>?;@@@@CCD@??B==700,000.77>5555=.//>;;7;@8<<?888@@@>AA=????A@?<??AB<
A???<?>A@@C:
@T6XXI:00016:00572
GGGGAATTCTAATACGACTCACTATAGGGAGAGGGAGAATAGCTTGCAGCTCGAGCCTGTGACTGTTCTCTTCCTTT
CTCCCTTCTCCC
+
BCC:C?B?BBB@BBBBBBBB?A<77111.7==BCAA@@=@@@ACA?AACC???@8:8;CAADAA??<?==<?A<??<
??AA:?;=;;;6
@T6XXI:00016:00574
```

```
GGGGAATTCTAATACGACTCACTATAGGGAGAGGGAGAATAGTACGACGAAATCG
+
8886?A@>BDDABABBBBB@?BCC::;E<>>777.44;:8///---1--0;1438
@T6XXI:00016:00584
GGGAGAAGGGAGAAAGGAAGCTAGTCACTCGACATCTGTGGCTTCACAGTACAGAGGGACTGTCCAAAGCCGCAGAT
CTCGAGTCGACTAG
+
44-4AA@DGAC;996;=>;@CAAAD?<8:8;93388671==::4:C???B::::444-
4;;;;@9<<7<?;??@@?@?>>;8;43333853
@T6XXI:00016:00585
GGGGAATTCTAATACGACTCACTATAGGGAGAGGGAGAATAGTCAACAAATCGTTTGGGGCGACTTCTCCTTCCTTT
CTCCCTTCTCCC
+
9<<49>B@AAA@CCCCCCC@?@B=?777.777@B?B@B<??@AAA>AAA7?????6?@@5@@@??=?A@>B>B>AB?
???@:?;?>@@:
@T6XXI:00016:00599
GGGAGAAGGGAGAAAGGAAGGAGAAGTCGCCCCAAACGATTTGTTGACTATTCTCCCTCTCCCTATAGTGAGTCGTA
TTAGAATTCCCC
+
FE@DBB@BE>A@@>9>;?=@BCCC;;;B@@BB5;:6688:@;CA>?>AA?;9;;;=600/:5589=8<8>>@@@=<8
1++**)*.,,,(
@T6XXI:00017:00569
GGGAGAAGGGAGAAAGGAAGCTAGTCATCGCATCGTGTGCTCACAGTGACAGACAGG
+
EE@DBB>??:@@?B?C@C@B>??@@@>B@=0011111111311676666--------
@T6XXI:00017:00570
CTAGTCGACTCGAGATCTGCGGCTTTGGACAGTCCCTCTGTACTGTGAAGCCACAGATGGACTGTCCCTTCCTTTCT
CCCTTCTCCC
+
CBBBBBBCCBBBCBCCBBABB@BBD>C=?@BB9::5:CABAAAAA>>>=AC@CCCCCCC@CC@@@CCBC?B=??9?A
@@9?<:88B:
@T6XXI:00017:00572
CTAGTCGACTCGAGATCTGCGGCTTTGGACAGTCCCTCTGTACTGTGAAGCCACAGATGTCGAGTCGACTAGCTTCC
TTTCTCCCTTCTCCC
+
C<1111////:=BAAACBBAB@BEC?C@CCA@@BB>C>>>@@@==AAA?@C@BCBBBBCBCCCDABBCCBCCBB?A@
AD>BB@@:=;????:
@T6XXI:00017:00573
GGGAGAAGGGAGAAAGGAAGGAGAAGTCGCCCCAAACGATTTGTTGACTATTCTCCCTCTCCCTATAGTGAGTCGTA
TTAGAATTCCCC
+
BCACBB?BB?BBBB>B???A@BB@>@CC?>>>6<<;ACCC>;>>>>9::==;:8:=;=:6:=;=:6:===<<<988?
A?A@@==8;;;0
@T6XXI:00017:00579
GGGGAGAAGGGAGAA
+
944,444888-//,2
@T6XXI:00017:00586
GGGGAATTCTAATACGACTCACTATAGGGAGAGGGAGAATAGTCAACAAATCGTTTGGGGCGACTTCTCCTTCCTTT
CTCCCTTCTCCC
+
ABA7A>B?BBB@BBB?BB???@B>911659??DE>BBB>B;;;==;<<<;A?>>98<9<7>BBBC?BB@=?=?<AC:
>>@C;B<??AA<
@T6XXI:00017:00588
GCTAGTCACTCGAGATCTGCGGCTTTGGACAGTCCCTCTGTACTGTGAAGCCACAGATGTCGAGCGACTAGCTTCCT
+
4444//////6668>8;86..,...+.34649997,1......-36=;;<C:99><<71116114333311113333
```

```
@T6XXI:00017:00596
GGGAGAAGGGAGAAAGGAAGCTAGTCGACTCGACATCTGTGGCTTCACAGTACAGAGGGACTGTCCAAAGCCGCAGA
TCTCGAGTCGACTAG
+
AA<>@>5990999918;<7?C@=>C/10>@@7,000/..3326==?BECCCAAAA?;:;??==<=8;;7;A>AAAAA
999AAA@A788;=;<
@T6XXI:00018:00566
GGGGAATTCTAATACGACTCACTATAGGGAGAGGGAGAATAGCACTCCCTACGAAAAACGCAGCGTATCCTTCCTTT
CTCCCTTCTCCC
+
?=@6@>A>BBBDCCBABCB?>?B??666-
6;;;?;<??>@@@@??@?CAAAA<<<66.<A==<<@A>A=44348?@<@@AC;@:==BA<
@T6XXI:00018:00577
GGGAGAAGGGAGAAAGGAAGGAGATAAGCCCGCCAGTAAGAACGAAGCTATTCTCCCTCTCCCTATAGTGAGTCGTA
CTTAGAATTCCCC
+
B:-9442495888;5=;;:;7371;;38==<=@?C@@<<<<599@<77;9924444-4=444-
44333779964.....,.33,42444,
@T6XXI:00018:00589
GGGGAATTCTAATACGACTCACTATAGGGAGAGGGAGAATAGTAGACC
+
88818+;:<AA<BBBCBELCCAA>>777.7>>AA@B@;;;:,++***)
@T6XXI:00018:00593
GGGAGAAGGGAGAAAGGAAGCTAGTCACTCGACATCTGTGGCTTCACAGTACAGAGGGACTGTCCAAAGCCGCAGAT
CTCGAGTCGACTAG
+
ABACCC>??<@CA@>C?@?AD@??A>:::@=::8?@?CCL=?@@CC@???@<=<44414>9@@A=??:?C?A>66;=
<<8<<<<==::::;
@T6XXI:00018:00595
GGGAGAAGGGAGAAAGGAAGAGAA
+
DK@C@?=@=9>911,1::,-,87*
@T6XXI:00019:00557
CTATCGACTCGAGATCTGCGGCTTTGGACAGTCCCTCTGTACTGTGAAGCCACAGATGTCGAGTCGACAGCTTCCTT
TCTCCCTTCTCCC
+
CCCB:::BBBB?AABCCCCC@BB@=B?A?:::??9=@???B@@?BAA68<@B@??A@??=<>=797996>?@;<;<>
88;?A782:344-
@T6XXI:00019:00573
GGGAGAAGGGAGAAAGGAAGGAGAAGTCGCCCCAAACGATTTGTTGACTATTCTCCCTCTCCCTATAGTGAGTCGTA
TTAGAATTCCCC
+
9?199959>5944?082:5;5;CC@BBBBBBB7AA<ABBDC>BB?BC@?CA:?87818<;AD;@@AA===AA889=<
<=A<;9;8>??4
@T6XXI:00019:00578
GGGAGAAGGGAGAAAGGAAGAATAAGTCCGAGCCGGGCGA
+
CF@CBCDDC>A@@D<@=@?BC@BB>AA7700//,.520,,
@T6XXI:00019:00590
GGGAGAAGGGAGAAAGGAAGTACACGAATAGCCTGGAGATGGTTTGGCTATTCTCCCTCTCCCTATAGTGAGTCGTA
TTAGAATTCCCC
+
BBBDCC@CC>BCBB=?=>>BCBCCCCB>B;;:=@B?BBB?>377.17@BCC@CB?A<AC???=@A??AAAAA???@A
A>A??<?>DHC4
@T6XXI:00019:00592
GGGAGAAGGGAGAAAGGAAGGAGAAAAGTGATGCACGAAATACAAGACTATTCTCCCTCTCCCTATAGTGAGTCGTA
TTAGAACTTCCCC
```

+
?A=@AA@CB:?9991427;>>ABC@?7ACF@?<@BFEDE<<=FCDKCCCCA<8888/3111626688968;?<@7:7
?6331,--,-22.
@T6XXI:00019:00598
GGGAGAAGGGAGAAAGGAAGCTAGTCGACTCGACATCTGTGGCTCCACAGTACAGAGGGACTGTCCAAAGCCGCAGA
TCTCGAGTCGACTAG
+
DB<:9928809>>>9>5857?700:/000//9/:6<<=@=55,,,*,7:@B@AAA88444???CC>@@;<;8<??@@
C@@@?@@@@@@@@>@
@T6XXI:00020:00563
GGGAGAAGGGAGAAAGGAAGAGAACGGCGACGAACAATAGTTGGTGGCTATTCTCCCTCTCCCTATAGTGAGTCGTA
TTAGAATTCCCC
+
CC?@@?@DDABDBC=B?C@CCBB@CCBBBBBBB?BC@CCCC@C@??@?CC?=B:::8:CCCC>@>>>AAAAAAA=8=
A::A=>A58770
@T6XXI:00020:00582
GGGGAATTCTAATACGACTCACTAGTAGGAGAGGGAGAATAGTCAACTAAATCGTTTGGGGCGACTTCTCCTTCCTT
TCTCCCTTCCTCC
+
///*/,1,4499=BD;:<B==78,,,,,*,037>868<684;4444//.44444530333.667660110235586;
4:74422,+*+++
@T6XXI:00020:00590
GGGGAATTCTAATACGACTCACTATAGGAGAGGGAGAATAGTCAACAAATCGTTTGGGGCGACTTCTCCTCCTTTCT
CCCTTCTCCC
+
999/42924=99=>@?@@@?@>?=<7736=777,0444699:66<877,1;;<>.111,167773854/00/00+43
44-49:033+
@T6XXI:00020:00595
CAGTCGACTCGAGATCTGCGGCTTTGGACAGTCCCTCTGTACTGTGAGCCACAGATGTCGAGTCGACTAGCTTCCTT
TCTCCCTTCTCCC
+
CCCBBBBBBBBBBBBBCBBA@CAA>B@CBBBCCC>@??B@BABAA9:<C@CBC=<=888==CBAA=8=?=<?=@547
-4:;=7>><==@<
@T6XXI:00021:00578
GGGAGAAGGGAGAAAGGAAGCTAGTCGACTCGACATCTGTGGCTTCACAGTACAGAGGGACTGTCCAAAG
+
CCCB@@694-9@ABAB@C@CC?:::??@?>>?????>@@@<<9==?CCCB>;===99998:=<==>>A<@
@T6XXI:00021:00581
GGGGAATTCTAATACGACTCACTATAGGGAGAGGAGAATAGTTTCC
+
EBC9H@C?A;96;;;ABCCCCB?;;667.77110044/0003444/
@T6XXI:00021:00593
GGGAGAAGGGAGAAAGGAAGGTGAACAGGTAACGGAGGATGCGTTAACTATTCTCCCTCTCCCTATAGTGAGTCGTA
TTAGAATTCCCC
+
CC@CCB@CC@BB>>696;<>>ABB?CBB@BBBBDBCC@CCCCCC@C@CCCGACBBB=;;;BA<???AABBBCCA??@
@597973/000+
@T6XXI:00021:00597
GGGGAATTCTAATACGACTCACTATAGGGAGAGGGAGAATAGTCAACAAATCGTTTGGGGCGACTTCTCCTTCCTTT
CTCCCTTCTCCC
+
944/99@AECC@CBBBBAAAACCCB?::8699BB>@AA>AA=<;;4;;;6??>>>.777-
7>?@B?B<=;=>A?CC>C???;@>AA@A;
@T6XXI:00022:00562
GGGAGAAGGGAGAAAGGAAGGAGAAGTCGCCCCAAACGATTTGTTGACTATTCTCCCTCTCCCTATAGTGAGTCGTA
TTAGAATTCCCC
+

CC@CCC@DB>??>=<@>A?AGBBB?BA@@<>>.77.:?BCC>CC@B@==AA=?=<<6<448=:?@?==A@@@A@?<<
<587977,,,,*
@T6XXI:00022:00563
AGGGAGAAAGGAAGGACAAATAGATTGCGGGGGAAACGCTAGCTATTCTCCCTCTCCCTATAGTGAGTCGTATTAGA
ACTTCCCC
+
DCC>CDCC>?=@==7=8776;A?@@=??====0@A>BCCCCAABCBABCBC???@A?9>444888=?B;=>8834;;
/77:=>>1
@T6XXI:00022:00567
GGGGAATTCTAATACGACTCACTATAGGGAGAGGGAGAATAGTTTTTCCCTTCACTAACCGCGAACTGCACTTCCTT
TCTCCCTTCTCCC
+
@@@6A?A@CBB@BAAABBBB?@B79161.6==AC>BAA=?9??AAC1@@;@=?@ECC>>>???=>==<8<?C@C=AA
=A>>A<@>???C=
@T6XXI:00022:00569
GGGGAATTCTAATACGACTCACTATAGGGAGAGGGAGAAT
+
444,424244494448777766311666267444+.--,-
@T6XXI:00022:00570
CTATCGACTCGAGATCTGCGGCTTTGGACAGTCCCTCTGTACTGTGAAGCCACAGATGTCGAGTCGTACCTTAC
+
4:@=99:@?=999C@?<<00,.5526.0/6<?@?;<6/1/>5:;A@A>?B?BABCB@@?A>>661,,,,*,*,,
@T6XXI:00022:00577
GGGGAATTCTAATACGACTCACTATAGGGAGAGGGAGAATAGTCCTGAGTCTTATCGAGCTGGCCTGTCCTTCCTTT
CTCCCTTCTCCC
+
CEE9BAB?BCC@CCBBBBBB@AB;?66659??AB?A??>@===A>AAAAAAB>ACC@A@@@@>>;9?:6488;9<C>
A@@@;@?AAAC<
@T6XXI:00022:00595
GGGAGAAGGGAGAAAGGAAGAAGAACAGGAGTACAAAAGTGTAAAGCCTATTCTCCCTCTCCCTATAGTGAGTCGTA
TTAGAATTCCCC
+
<<69DB>@@:?9990;=?=@C@CC?BBBCABB?;;;:48?A@AB?BBCDIECCBBB=@A@@A<A@@@BBBCCBBAB7
738;;:;:==>1
@T6XXI:00023:00580
GGGAGAAGGGAGAAAGGAAGGAGAAGTCGCCCCAAACGATTCGTT
+
44-4;;244-499804999<=;;?01133333,33,161.,...,
@T6XXI:00023:00584
GGGAGAAGGGAGAAAGGAAGCTAGTCGACTCGACATCTGTGGCTTCACAGTACAAGGACGGAC
+
CH@AA?@DD>CCBB>C?B7;:BBBBCBCBC@A88;BBBCDC<?A?BCA@@?<77400++,+**
@T6XXI:00023:00595
CTATCGACTCGAGATCTGCGGCTTTGGACAGTCCCTCTGTACTGTGAAGCCACAGATGTCGAGTCGACTAGCTTCCT
TTCTCCCTTCTCCC
+
B;;999::BCBDDCCBBBCC@CCD>C9<;CCAAB=ABBBBB???AAA?@C@CCCCCCAAACA@?@????=8?88<=A
CBB?@D<@9=<>=7
@T6XXI:00024:00560
GGGGAATTCTAATACGACTCACTATAGGGAGAGGGAGAATAGCCGACTGTC
+
=@=6A@B?BCC@CA<>BBBB>@A?=66659??CC>AAA>A9444,,+++*0
@T6XXI:00024:00564
GGGGAATTCTAATACGACTCACTATAGGGAGAGGAGAA
+
9@@4@6959//7/10888;<86966611,111100442
@T6XXI:00024:00574

```
GGGGAATTCTAATACGACTCACTATAGGGAGAGGGAGAATAGTCAACAAATCGTTTGGAGCGACTTCTCCTTCCTTT
CTCCCTTCTCCC
+
CCB7A=>=@BBACBAAB@@@>CCCC?<<;<?:AA>@@@>@<????>77811>>>@=>://1>=:B?8889<9<>AA?
@<<<7<=?@DA<
@T6XXI:00024:00583
GGGAGAAGGGAGAAAGGAAGCTAGTCACTCGACATCTGTGGCTTCACAGTACAGAGGGACTGTCCAAAGCCAGCAGA
TCTCGAGTCGACTAG
+
EECBB>644-
9>>>9>=?<@CCCCCB?>>>;00.666=>C>?@@BC@?@??<:;<==496=?773/9399.////.455?====A@@
@D???
@T6XXI:00024:00584
CTAGTCACTCGAGATCTGCGGCTTTGGACAGTTCCTCTGTACTGTGAAGCCAGATGTCGAGTCGACTAGCTTCCTTT
CTCCCTTCTCCC
+
AB111>>?BCBABBBBBB888:CC>C?A?=<?5:5:BCBCA@?=AA<;=@=@AAB@@BCCCCCCBB@??A@:?;?@<
@@AA>B@@AAB=
@T6XXI:00024:00595
GGGGAATTCTAATACGACTCACTATAGGGAGAGGGAGAATAGCACAACGTAA
+
449/9>A@CBB@BBBBB><<??DA@@@@>>?>@@>B@@?C=536,,+,,+**
@T6XXI:00024:00599
GGGAGAAGGGAGAAAGGAAGGAGAACGCGCACCCGAACCAACGGTGATCTATTCTCCCTCTCCCTATAGTGAGTCGT
ATTAGAATTCCCC
+
CD@CCB?@>9>99909<@;?=@BB?CAAAAAAC@CC@C@CADCAAAAAA@BBBA@@@<@FBCC>BEEBBBBB;8:??
?:@::8;@2444,
@T6XXI:00025:00556
GGGGAATTCTAATACGACTCACTATAGGGAGAGGGAGAATAGTCGACAACTCCCCAAACGACGCATGCACTTCCTTT
CTCCCTTCTCCC
+
CCB9B>B>BBB@CCCCA?AB@CA?@:8:8???BE>C@@=@@@@AA@@??AAA?A6AC<898<<<888=?<;8<:;;6
<<<A<A?AAAA=
@T6XXI:00025:00575
GGGGAATTCTAATACGACTCACTATAGGGAGAGGGAGAATAGCTGACAATTCCCCCCTGCGCTACTTCTCCTTCCTT
TCTCCCTTCTCCC
+
::80>-
1,1/1,//6077>>A?A?>A>D9??AAA:=??<??@A???:428;=8888+77==<>ABB>>>;2757?BC@CCCC>
?24444-
@T6XXI:00025:00589
GGGGAATTCTAATACGACTCACTATAGGGAGAGGGAGAATAGATCACCGTTGGTTCGGGTGCGCGTTCTCCTTCCTT
TCTCCCTTCTCCC
+
BCG9A>AAEBB??BACCCCB>A@>>66659==BC<>>A@A=<<<<BB>AB5:5:=@>@=B;;;CCC@CCF?A?ABEE
A@@@@<@>AAAB>
@T6XXI:00025:00599
GGGGAATTCTAATACGACTCACTATAGGGAGAGGGAGAATAGTCAACAATCGTTTGGGGCGACTTCTCCTTCCTTTC
TCCCCTT
+
>99,42488>@@@<<<@?BB?;;:::999@@?@>7:44/08;889/34/1;69>;?AC7>>B7:8;?=>A8883444
0000+02
@T6XXI:00026:00550
GGGAGAAGGGAGAAAGGAAGCTAGTCACTCGACATCTGT
+
CDBA@?@BA=@??=9>584;@???@?7...1.-+00,,,
```

```
@T6XXI:00026:00552
GGGAGAAGGGAGAAAGGAAAGCTAGTCGACTCGACATCTGTGGCTTCACAGTACAGAGGGACTGTCCAAAGCCGCAG
ATCTCGAGTCGACTAG
+
<<-
4@A=@@1999>9><>>6;:>;><0..777//11111686247>@CCCC?<;?4449499:44428817B9@898??B
=<AAA?????DA@
@T6XXI:00026:00554
GGGAGAAGGGAGAAAGGAAGCTAGTCACTCGACATCTGTGGCTTCACAGTACAGAGGGACTTGTCCAAAGCCGCAGA
TCTCGAGTCGACTAG
+
CCAA??@BB>BBBB?C?@?BA??BC@;:;B?:88???BBB?CC@CC????=:::6<=:<4547>?<@@;?A>AAB??
9999=<<<8==AA==
@T6XXI:00026:00564
GGGAGAAGGGAGAAAGGAAGCTAGTCGACTCGAGATCTGCGGCTTTGGACAGTCCCTCTGTACTGTGAAGCCACAGA
TGTCGAGTCGACTAGCTTCCTTTCTCCCTTCTCCC
+
CEAC<<:<DBCBBHAE?B?B?777<7::?AACBAABBBCBC?CCB>C@CBB?<>>;99=@@=<<<<<;9<A>AAAA@
?=<<<><<<?>>:=;742/46616///,/,/---+
@T6XXI:00026:00565
CTAGTCGACTCGAGATCTGCGGCTTGGGACAGTCCCCTCTGTACTGTGAAGCCACAGATGGACTGTCCTTCCTTTCT
CCCTTCTCCC
+
AAA@<<9<<<@@>@;;;@@BB?BC@CCAB>==7777-
7>776>><@@AA>?B@BBBBFCC@CCCA?97=37311,1116,10144;2
@T6XXI:00026:00577
GGGAGAAGGGAGAAAGGAAGGAGAAGTCGCCCCAAACGATTTGTTGACTATTCTCCCTCTCCCTATAGTGAGTCGTA
TTAGAATTCCCC
+
CDACAA?BC>BBBB>B?A?B?ADA@@@B;;772<@<ACEC@;@@>@>>?A=<=9999=AA<=:?=?A=<<<==::99
<<A@@=?3433+
@T6XXI:00026:00579
GGGGAATTCTAATACGACTCACTATAGGGAGAGGGAGAATAGTCAACAAATCGTTTGGGGCGACTTCTCCTTCCTTT
CTCCCTTCTCCC
+
@@@6@>B@CBB@BA??BBBB@CB?<6665:AACC>B@@?A<@@???<<<7?B=77,111-
7ABCC@CCA;?<>?AB=A88848=?@?@<
@T6XXI:00026:00581
AAAGGAAGCTAGTCACTCGACATCTGAGTGGCT
+
=95>69589494<9444943;...4...35447
@T6XXI:00026:00593
GGGGGAATTCTAATACGACTCACTATAGGAGAGGGAGAATAGTCAACAAATCGTTTGGGGCGACTTCTCCTTCCTTT
CTCCCTTCTCCC
+
BDBC3B?B?BDBCBBBCBB@@?;777//,000AA>A??>@CC@@@<<;8486;;<.777.7<>>@?CCA>@>@?@A<
A@A@;@689?A<
@T6XXI:00026:00598
GGGAATTCTAATACGACTCACTATAGGGAGAGGGAGAATAGTCAACAAATCGTTTGGGGCGACTTCTCCTTCCTTTC
TCCCTTCTCCC
+
BG<@=AA@AACBBBC;;<BBBB??7:6.7>==?<<??>AA@A@@>==<6=?777.777.7777<=:45<A9>:==9=
B@C?D383781
@T6XXI:00027:00558
GGGAGAAGGGAGAAAGGAAGACTCGACATCTGTGGCTTCACAGTACAGAGGGACTGTCCAAAGCCGCAGATCTCGAG
TCGACTAG
+
```

```
CNDCA@<@><9;;@9@9;7::?===:::::8CDB>A@;>>AB;=A@<?;?A<A@@@@A>AA;@@?AAA<=?<???A@
AA?@@AA?
@T6XXI:00027:00576
CTAGTCGACTCGAGATCTGCGGCTTTGGACAGTCCCTCTGTACTGTGAAGCCACAGATGTCGAGTCGACTAGCTTCC
TTTCTCCCTTCTCCC
+
;6/111//4=AABBABBBABB?BBC>B?BBAA???9?AACBBB@???A?BB@CCC???BB?B???BBCCCCA??;?:
?@<?@=CAC>????:
@T6XXI:00027:00585
GGGGAATTCTAATACGACTCACTATAGGGAGAGGGAGAATAGCTCAGCTTTTGTTGCATGTGATTTCTCCTTCCTTT
CTCCCTTCTCCC
+
499/9:B=A@ACADCCCCB?>@A>?66659==?C>@??<?AAACCA:737708@?AAAA=<<<BA<A??;?>A<==<
<@<=;:9/00;9
@T6XXI:00027:00586
GGGGAATTCTAATACGACTCACTATAGGGAGAGGGAGAATAGTCAACAAATCGTTTGGGGCGACTTCTCCTTCCTTT
CTCCCTTCTCCC
+
@@@6@=@@ADB@CC@@@CCCA>@==696.7>>?A>ADD?A5<<AA<??A:BBCCC<AAA7AABBC?BBA>@>@>AA<
===A<@;?@??7
@T6XXI:00028:00554
GGGAGAAGGGAGAAAGGAAGCTAGTCACTCGAGATCTGCGGCTTTGGACAGTCCCTCTGTACTGTGAAGCCACAGAT
GTCGAGTCGACTAGCTTCCTTTCTCCCTTCTCCC
+
@;-994244-4444-
4231....,0**,//3355::CA??=?CE>C@???B?E?9;0//88;;<=?@<@>499@A@@A???@=<<<<;:;;4
4348;;188@A:?<?@?D7
@T6XXI:00028:00588
GCTATCACTCGAGATCTGCGGCTTTGGACAGTCCCTCTGTACTGTGAAGCCACAGATGTCGAGTCGACTAGCTTCCT
TTCTCCCTTCTCCC
+
9:99::A?A@BBBBA?<=00,0;;6;9:9/:6=96166???<=AABA>?B@B:;8>;611133./..6956995721
776000+04438;6
@T6XXI:00028:00589
CTAGTCGACTCGA
+
4//////,,,,/7
@T6XXI:00029:00575
GGGAGAAGGGAGAAAGGAAGCTAGTCGACTCGACATCTGTGGCTTCACAGTACAGAGGGACTGTCCAAAGCCGCAGA
TCTCGAGTCGACTAG
+
CCBCCD?A=:@@>>9@8:6;C=::=/00;>>@1;::::ABA9=@=@CCCBBABA934417<??@@>@A<@@=@AAAA
A@@@???AAAA@AA@
@T6XXI:00029:00581
GGGAGAAGGGAGAAAGGAAGCTAGTCGACTCGACATCTGTGGCTTCACAGTACAGAGGGACTGTCCAAAGCCGCAGA
TCTCGAGTCGACTAG
+
AC@CCA89809999096868>>>>>77777<<==??<7::A?8:;;AAABBAA==77717;;;@A<??<?C>@@@@@
AAA@AAAA899AA?>
@T6XXI:00029:00590
GGGGAATTCTAATACGACTCACTATAGGGAGAGGGAGAATAGTCAACAAATCGTTTGGGGCGACTTCTCCTTCCTTT
CTCCCTTCTCCC
+
944,49@?BBB?BCBCCADCAABDCBBB>CCBBB=A<<9;;77-/,-
11067111,16626>>::7:?;@B8;8;=4934443283487
@T6XXI:00029:00591
```

```
GGGGAATTCTAATACGACTCACTATAGGGAGAGGGAGAATAGCTCCCAAATCACGTTACGTGCGACCCACTTCCTTT
CTCCCTTCTCCC
+
@==6@>B@CBB@AAAB@@AB?@>77111.7>>>A>AAAA@;??CCB=;;:;;999;:;BBBBB?A@D@CDB@B@BA;
AABB>B<>@CC>
@T6XXI:00030:00557
GGGGAATTCTAATACGACTCACTATAGGGAGAGGGAGAATAGTTCCCAAATCTCTCACCGTGGCGTGCCCTTCCTTT
CTCCCTTCTCCC
+
CDDDDCC@CBB@CCCBABBDD@@??9:959BBCC>B@@=@<AA<??:@C>A=<=@AAA?@@@;:=>777189;;@?<
?AAA7<9<>>>7
@T6XXI:00030:00560
CTAGTCACTCGAGATCTGCGGCTTTGGACAGTCCCTCTGTACTGTGAAGCCACAGATGTCGAGTCGACTAGCTTCCT
TTCTCCCTTCTCCC
+
B11/16699BB@;9<C@@@B?BCD>C?AA@@???<?CCCBB??<@@A?ACDBB@@@CCCBABBBCBB?BBCCC?A?A
B>B@AA<A>@@@B;
@T6XXI:00030:00592
GGGAGAAGGGAGAAAGGAAGAGAACAGCCCCCGTAAATGTGTGTGAACTATTCTCCCTCTCCCTATAGTGAGTCGTA
TTAGAATTCCCC
+
CCDCCB@CB>BBBB?C@B@BD@?>AC@@@==/=?==<@BACB@@@?9>?BD@BA??>A@CCC>CC=A=<<=><8<=@
@>@AB?A58770
@T6XXI:00031:00554
GGGGAATTCTAATACGACTCACTATAGGGAGAGGGAGAATAGTCAACAAAGTCGTTT
+
8>>6AAC?C??=AACCCCCAA@B?76:<6<>777.7;;4<=4400/3044440004+
@T6XXI:00031:00559
GGGGAATTCTAATACGACTCACTATAGGAGAGGGAGAATAGCAAACGTTCCCCGAC
+
8C>0=+1,1//568?;<;@BB@AB?:747=77768@@<>;9///,/00,---*-33
@T6XXI:00031:00583
GGGAGAAGGGAGAAAGGAAGAGA
+
CC@BBCBB;6998801:<:8--,
@T6XXI:00031:00585
GGGAGAAGGGAGAAAGGAAGCTAGTCGACTCGACATCTGTGGCTTCACAGTACAGAGGGACTGTCCAAAGCCGCAGA
TCTCGAGTCGACTAGCTTCCTTTCTCCCTTCTCCC
+
A>6=11,//+//44-4231396667771161116666887993:49>@@@A>=;;444-
49;447=@@;AA?999@AA<==@@@@@@@88/888,/,/725,,,,..+//775
@T6XXI:00031:00587
CTAGTCACTCGAGATCTGCGGCTTTGGACAGTCCCTCTGTACTGTGAAGCCACAGATGTCGAGTCGACTAGCTTCCT
TTCTCCCCTTCCTTTCTCCCTTCTCCC
+
>=//1=1:;CBBCCBBBBBC@BBB>B@B@@?>>>;>@@???===AAA68;?BBBCBB@@@@@@CCCDCBBBBB8:8:
C>B@@CH5=:=?AC:@@@A1838;<;7
@T6XXI:00031:00595
GGGGAATTCTAATACGACTCACTATAGGGAGAGGGAGAATAGTCAACAAATCGTTTGGGGCGACTTCTCCTTCCTTT
CTCCCTTCTCCC
+
499/4:CBDBB@BBAAAABA?@B?>161.7>>@B>@AB?@<?AAA>???<ABB??8???6?BBBC@?<<8<88;@@<
???A=B?@@@@<
@T6XXI:00032:00586
GGGGAATTCTAATACGACTCACTATAGGGAGAGGAGAATAGC
+
BAA:B@@@BBB@??@@@@@GCBBABCCB;::/,//,+,010
```

```
@T6XXI:00033:00551
GGGGAATTCTAATACGACTCACTATAGGGAGAGGGAGAATAGCTACGTTTAACCGATCGTGTTGTCTCCTTCCT
+
@@>4><><;;;>B<<<LCCCBCC==6:7.7>77778@@@CAA@BBCC=A<@?@?AA?>>@BB>761,,*+*+0.
@T6XXI:00033:00570
GGGGAATTCTAATACGACTCACTATAGGGAGAGGGAGAATAGTCTGCAGATCTGACATGACTTTTTCTCCTTCCTTT
CTCCCTTCTCCC
+
BCC9C@C@CBC@CBBBAAAA??<77111.7??AC>B@@A@589???AA@@=<@@?BCBAEEBCCC2BBC;?:<3::7
:==A1738===7
@T6XXI:00033:00578
GGGGGAATTCTAATACGACTCACTATAGGAGCAGGGAGAATAGCCCCTTCACCCCTAGCACTATGAACTAAC
+
4444+42489992449=7<<877816/.44...4755745455888+64:==AB6???<;6..***)**.)*
@T6XXI:00033:00582
GGGAGAAGGGAGAAAGGAAGCTAGTCGACTCGAGATCTGCGGCTTTGGACAGTCCCTCTGTACTGTGAAGCCACAGA
TGTCGAGTCGACTAGCTTCCTTTCTCCCTTCTCCC
+
DCBCCD?BA<=@AB=CAC@CCA@AB;;;BCDCCCCCCBB=99611.7<>@ABA?A@AAA???@@AA<=;=?>AAAA@
A<<<AA@@@==:;?@@A>A>A@;@@AC=A8;<@C>
@T6XXI:00034:00555
GGGAGAAGGGAGAAAGGAAGGAGAAGTCGCCCCAAACGATTTGTTGACTATTCTCCCTCTCCCTATAGTGAGTCGTA
TTAGAATTCCCC
+
AA<BCC@CC?BBAB=B?B>?>ABB?BBC@@@@5??:?AAAC<AA@BBBABA>@=<64;>>?@<@@@A@AAAA<;;;;
;8<?>>B38880
@T6XXI:00034:00593
GGGGAATTCTAATACGACTCACTATAGGGAGAGGGAGAATAGTCAACAAATCGTTTGGGGCGACTTCTCCTTCCTTT
CTCCCTTCTCCC
+
CDB:C@C@BBB@BBBCBBBB@@>9911159=?BD>B@@>@AAAAA>?@@;BCCBB5::7-
7>>@A>@@?9<9<=@@<@AAA<A<?@@C>
@T6XXI:00034:00597
GGGGAATTCTAATACGACTCACTATAGGGAGAGGGAGAATAGTTTTCCCTTCACTAACCGCGAACTGCACTTCCTTT
CTCCCTTCTCCC
+
CCEBCCCBDCC@CCBBBBBCBCCBBA<<<8???B>C@A=?94444-44-
4;?BBCC@C=A?<<>=@@=C><<9:666944?:?<@@@@:
@T6XXI:00035:00570
CTCGAGATCTGCGGCTTTGGACAGTCCCTCTGTACTGGAAGCCACAGATGTCGAGTCGACTAGCTTCCTTTCTCCCT
TCTCC
+
944;99<@A@@CC<=@G@CACAB?99919999>@;99@B@BB?>738>>718==99;;;?CA@9959=A@>BBDBB>
-111,
@T6XXI:00035:00574
GGGGAATTCTAATACGACTCACTATAGGGAGAGGGAGAATAGTTACTAACTACTAGGCGCAACTGTTCTCTT
+
;>;6:>B?BCD@B:9;>>>>==8::??C>CCA@>:7443444::64..9:00/7755==94944445//+00
@T6XXI:00035:00581
GGGAGAAGGGAGAAAGGAAGCAGTCGACTCGACATCTGTGGCTTCACAGTACAGAGGGACTGTCCAAAGCCGCAGA
+
BE><<<?A@:A;991;>A?BDBAAAACCB?=11111/67754424@BBHCGA@?88808>>===4<;6:77=????
@T6XXI:00035:00582
GGGAGAAGGGAGAAAGGAAGGGAATAGCAGCAGTCGGCAACGTGAAACTATTCTCCCTCTCCCTATAGTGAGTCGTA
TTAGAATTCCCC
+
```

```
>95>@@=AECCKCC>C@B@CC>C@BBBCCBBBBBBB=??@?@@EDDB@@A@?BBBD<???==7=887?@@@AA====
=>A@;:4/004-
@T6XXI:00035:00593
GGGGAATTCTAATACGACTCACTATAGGAGAGGGAGAATAGCTATTCACGCTGGACG
+
7>>5B@B@CBB@B@<<<B???:975..,0<077-43444878><<:77161--,---
@T6XXI:00035:00594
GGGGAATTCTAATACGACTCACTATAGGGAGAGGGAGAATAGTCAACAAATCGTTTGGGGCGACTTCTCCTTCCTTT
CTCCCTTCTCCC
+
499/4:A?GBB@CAB@BBBB@AB@>:::88??BC>CAA>@<?@@><773,7<777-
777.7??@B@BA@:=;=>BCA?@@A<AA===A;
@T6XXI:00036:00575
GGAGAAGGGAGAAAGGAAGGAGACATAGGCAATCTCGTAAAGTATGTCTAATTCTCCCTCTCCCTATAGTCGAGTCG
TATTAGAATTCCCC
+
/,/66,//+49885=;>17433148===?AB=A@A>>A@@<@BC>;;9/1,/7;4698773<B076:3....53-
67@@@;9=A>C@CCF7
@T6XXI:00036:00592
GGGGAATTCTAATACGACTCACTATAGGGAGAGGGAGAATAGTCAACAAATCGTTTGGGGCGACTTCTCCTTCCTTT
CTCCCTTCTCCC
+
944,49@=ABBCDC@?ABBBCAB>66::.:>?CC>???>@<@AAA>@@@:BCCCC>@A@7@?BBB?@?@:=7;8330
3???:?<??@@<
@T6XXI:00036:00596
GGGAGAAGGGAGAAAGGAAGGAGAAAGCGACGTCGGGGAAACGTTTGCTATTCTCCCTCTCCCTATAGTGAGTCGTA
TTAGAATTCCCC
+
@@<>@?>AA<BBBB=B@C@B>BCDD<?@BBBECBBBB7CB?CBCB@BDAA@ACCCCABABCC@CBAAACCCCBBB@?
?<?@?;65;<<1
@T6XXI:00036:00598
GGGGAATTCTAATACGACTCACTATAGGGAGAGGGAGAATAGTCAACAAATCGTTTGGGGCGACTTCTCCTTCCTTT
CTCCCTTCTCCC
+
BBA9C>A@BBBABBB>>>BB?CB;7111.6>>BC>@@@?A=???><=@@;B>?998?685????D@B@@>@=@>AA<
@AA=;A<>>AA<
@T6XXI:00037:00551
GCTAGTCACTCGAGATCTGCGGCTTTGGACAGTCCCTCTGTACTGTGAAGCCACAGATGTCGAGTCGACTAGCTTCC
TTTCTCCCTTCCTCCC
+
4/6///6144==@@ABCCB@@<?@@<A?AACDCCE?@@?BAA>>>:?>9=A?CBCCEBAABAA@:;;;8878::?A@
CC>A:8628,/,///,
@T6XXI:00037:00552
GGGGAATTCTAATACGACTCACTATAGGGAGAGGGAGAATAGTCAACAAATCGTTTGGGGCGACTTCTCCTTCCTTT
CTCCCTTCTCCC
+
?A@6?<>>AABBCCBBB@A@?@<77111.7>>>A>BAA>B<AAA=<9999?B777-111-
:@BBE@BB@>?;><?:::@?;;@>@@BC=
@T6XXI:00037:00565
GGGAGAAGGGAGAAAGGAAGGAGAAGTCGCCCCAAACGATTTGTTGACTATTCTCCCTCTCCCTATAGTGAGTCGTA
TTAGAATTCCCC
+
@@<ACCACG?@??A<@>B=@=@CC?CCC@@?@6?A>BCAAA@AE?AA???><@<<<7=<<AC<????AA@@@A?@=<
;;;44342444-
@T6XXI:00037:00566
GGGAGAAGGGAGAAAGGAAGGAGAACATGTTGTGGGACAAAGCCAAACTATTCTCCCTCTCCCTATAGTGAGTCGTA
TTAGAATTCCCC
```

```
+
CC>;@C5880999914777;?<?@:@;;;CCBCCCACAAA<>=:??9?BB@;>988/887<><A::;BB@B?AAAAA
A?@@@<?=?AA6
@T6XXI:00037:00569
GGGGAATTCTAATACGACTCACTATAGGAGAGGGAGAATAGTCAACAAATCGTTTGGGGCGACTTCTCCTTCCTTTC
TCCCTTCTCCC
+
CCC:C:<8<BB>ABCDBBBBA?>=77747=77757@@>CCB@==;=AC=CBBBC>CCB8@?:::90//9878.//,/
444-0/73:@:
@T6XXI:00037:00571
GGGAGAAGGGAGAAAGGAAGAGAACAACGCGGGCGGATAGGCCTAGCTATTCTCCCTCTCCCTATAGTGAGTCGTAT
TAGAATTCCCC
+
9919@@;>@9@AAB=B@C>AAB@;AA>BBBBCABB>BB@@=ACB<<<AAB@DDBC><<<AA<ABBBBBBAAAAAB??
;??B>C@CCEC
@T6XXI:00037:00580
GGGGAATTCTAATACGACTCACTATAGGAGAGGGAGAATAGTCAACAAATCGTTTGGGGCGACTTCTCCTTCCTTTC
TCCCTTCTCCC
+
CCC9C@C@CBB@CCB@<B<B?:977/.,0<<>B=CDD?B;:<<><=<;:<?9>>;>>@7@B@@A@?A?;=;9<AA<?
??B=B>@@@A<
@T6XXI:00037:00587
GGGAGAAGGGAGAAAGGAAGGAGAAGTCGCCCCAAACGATTTGTTGACTATTCTCCCTCTCCCTATAGTGAGTCGTA
TTAGAATTCCCC
+
BCBBBB=??>@AAB<@7;58<>??;?=>????.77.17>CE<BA@CB?@AA>???A<AAAAA;@AAA@=<==<?@@A
B@A@?;;2444-
@T6XXI:00037:00597
GGGGAATTCTAATACGACTCACTATAGGGAGAGGGA
+
///*/56,///,/679>>>877>71111,16111,0
@T6XXI:00038:00561
GGGAGAAGGGAGAAAGGAAGGAGAAGTCGCCCCAAACGATTGTT
+
44-4B?@CCACBCC@A<@6:7;>=;>=>8...*..+,0,,*,,*
@T6XXI:00038:00562
GGGAGAAGGGAGAAAGGAAGTGCAGCTTCGCGGTTAGTGAAGGGAAAACTATTCTCCCTCCTCCTATAGTGAGTCGT
A
+
44-
49@?AC:@99919;>;>884844424988887887777137,333,48891..660..,.333..33843330300
@T6XXI:00038:00583
CTAGTCACTCGAGATCTGCGGCTTTGGACAGTCCCTCTGTACTGTGAGCCACAGATGTCGAGTCGACTAGCTTCCTT
TCTCCCTTCTCCC
+
D?9;;A::9EBBDCCBCCAA?AAC<B?A??=77:57AABBA@A;ABBBB@BCC>>@ACBBBC;<;BBBI;>:>@>?C
>B<<<7<9<<AA@
@T6XXI:00038:00588
GGGAGAAGGGAGAAAGGAAGCTAGTCGACTCGACATCTGTGGCTTCACAGTACAGAGGACTGTCCAAAGCCGCAG
+
BB>A9929A?9;;@<@>C7:<C:::CCCCFCB:::???BDD89<@DC?A@B?>>?;<9<=@@@A>AA<AA?A@@=
@T6XXI:00038:00594
GGGAGAAGGGAGAAAGGAAGTGCAGTTCGCGGTTAGTGAAGGGAAAAACTATTCTCCCTCTCCCTATAGTGAGTCGT
ATTAGAATTCCCC
+
CCABBB@BB>BBBB>B>?>>BBBCBB@BBA??B?:;;BB?BC>CCCC2CCCB>?B>>;BBCCC>?<?<<<=BB?>>?
AA>=AA>@<?>>4
```

```
@T6XXI:00039:00559
GGGAGAAGGGAGAAAGGAAGTGCAGTTCGCGGTTAGTGAAGGGAAAAAACTATTCTCCCTCTCCCTATAGTGAGTCG
TATTAGAATTCCCC
+
DD@CBB?A?:BB?@=B@C?ABBCB9:;<CCBGC?@>>B@=9>-
67777*;CDE@CABB>BBBBC;;:??AB=AA===?>>;??>9;24440
@T6XXI:00039:00564
GGGGAATTCTAATACGACTCAGCTATAGGAGAGGGAGAATAGCTCCGCAAATCAACCTAACTAAACTGTTCTTCCTT
TCTCCT
+
499,459249988>@BB>>>1//;>?00,311668999<9111./,.-.57BBB@B@=<57799;?<:::8631,-
.+-++*+
@T6XXI:00039:00567
GGGAGAAGGGAGAAAGGAAGGAGAAGTCGCCCCAAACGATTTGTTGACTATTCTCCCTCTCCCCTATAGT
+
CDACCC@BB?CBBB:<9;>C6;:B:7788888.11,166;B8::><:;<6=8:444-090444+044443
@T6XXI:00039:00572
GGGAGAAAGGGAGAAAGGAAGCTAGTCACTCGAGATCTGCGGCTTTGGACAGTCCCTCTGTACTGTGAAGCCACAGA
TGCCGAGTCGACTAGCTTCCTTTCTCCCTTCTCC
+
AA:A499-
4=:;=?>:=<@57;B:;::768<<B<=C><ACC@???9==>7968>78;64444488///579789844:4424448
959;=:8997:8;:4444:7:8///5
@T6XXI:00039:00575
GGGAGAAGGGAGAAAGGAAGGAGAAGTCGCCCCAAACGATTTGTTGACTATTCTCCCTCTCCCTATAGTGAGTCGTA
TTAGAATTCCCCC
+
DC?DCB@BB=BBBC>B?B@B@?>?=?678888.77.179?D>B@>BB@@?@=??==;:<84717>>?@@AAA@AA98
8?A;;8326666.
@T6XXI:00039:00583
GGGAGAAGGGAGAAAGGAAGGAGAAGTCGCCCCAAACGATTTGTTGACTATTCTCCCTCTCCCTATAGTGAGTCGTA
TTAGAATTCCCCC
+
CG@CBA=@=9>99909<@>BADCB@B?93377-
77.7>@ED>CC?BB@@AA>@AAA<===AC<A===AAA???@@==>?@AA?B3////+
@T6XXI:00039:00584
GGGAGAAGGGAGAAAGGAAGCTAGTCGACTCGACATCTGGTGGCTTCACAGTACAGAGGGACTGTCCAAAGCCGCAG
ATCTCGAGTCGACTAG
+
99149>599-448;-
4679997697711116;?:459..,.221331....4734;333+7;;6<=?CC;8824339<888?>::///6777
<
@T6XXI:00039:00591
GGGGGATTCTAATACGACTCACTAATAGGGAG
+
9999/4-+---*,,269<9644..,.33536/
@T6XXI:00039:00597
GGGGAATTCTAATACGACTCACTATAGGGAGAGGGAGAATAGTCAACAAATCGTTTGGGGCAACTTCTCCTTCCTTT
CTCCCTTCTCCC
+
???8A@CCCBB@BB<<>BBB@AB;6166.7>@CC>A@A?A;9<<=<5441:A===-
777.7>DBB@C;;7:D@;<<6;=>@;@>@@A@<
@T6XXI:00040:00554
GGGAGAAGGGAGAAAGGAAGACGAATAGCATGTCACGGAATACTTGGCTATTCTCCCTCTCCCTATAGTGAGTCGTA
TTAGAATTCCCC
+
```

```
CE>BB?ABB?=?AC=BD@@BBCBB?AC@@B@8:8DGD@B?BB@B<<=?CBB?B?::8:7:CC>CCA??<@@@@@=<<
B?A@@?C@EEE:
@T6XXI:00040:00555
GGGAGAAGGGAGAAAGGAAGGAGAACGCGCACCCGAACCAACGGTGATCTATTCTCCCTCTCCCTATAGTGAGTCGT
ATTAGAATTCCCC
+
CDDBBB?AA=AA@C@D@B?B@CB@>AAABCCCC@CB@B=A>AC@BCCCB@@@?A@>?>@CCCC=CCCAA>B?BACBB
BB?AAC>C?BBB7
@T6XXI:00040:00557
GGGAGAAGGGAGAAAGGAAGCTAGTCACTCGAGATCTGCGGCTTTGGACAGTCCCTCTGTACTGTGAAGCCACAGAT
GTCGAGTCGACTAGCTTCCTTTCTCCCTTCTCCC
+
?A?=<<<<C?BDCB=B=?=@AA?@C???@@=@<=?CFCCB@CCC?B>BBBB>>>;=<<<<<==;<<=;==;=<<<==
?A:::.//:8/48<;;;=;==7????;@588=?:
@T6XXI:00040:00560
GGGAGAAGGGAGAAAGGAAGGAGAAGTCGCCCCAAACGATTTGTTGACTATTCTCCCGCGGCTTTGGACAGCCCTCT
TACTGTGAAGCCACAGATGTCGAGTCGACTAGCTTCCTTTCTCCCTTCTCCC
+
CB?C<<;<BAB<<;09;?5878?@=@A?>??>2=?:AABCB>BB=??<=???AAAC=?:?>AAA;<:=:>7771<CB
@CCCCCDCDDCADDCBBBAA@CDJLBBBBBAAA98::<>:767=6==?=;<6
@T6XXI:00040:00566
GGGAGAAGGGAGAAAGGAAGGAGAAGTCGCCCCAAACGATTGTTGA
+
;;;89>5990944;5877131331016A@:::675244..,..,.6
@T6XXI:00041:00576
GACTCGAGATCTGCGGCTTTGGACAGTCCCTCTGTACTGTGAAGCCACAGATGTCGAGTCGACTAGCTTCCTTTCTC
CCTTCTCCC
+
CCCECCCCDCCCICEDDDH?CCA@@@99909988>?@?A@;<98>;8;;@AC@???AA:;8BAB@::;;>;>?8?==
=6=:716;6
@T6XXI:00041:00578
GGGGGAATTCTAATACGACTCACTATAGGAGAGGGAGAATAGTCAACAAATCGTTTGGGGCGACTTCTCCTTCCTTT
CTCCCTTCTCCC
+
GDDD3C@C@CCCCBB?BDBBB>645:./-
0>>BE>DAA>A;??@<=@AC<CBBBC9???6?BBBBABB@=?;?<?=;=BBC<A689@>7
@T6XXI:00041:00579
GGGGAATTCTAATACGACTCACTATAGGGAGAGGGAGAATAGTCAACAAATCGTTTGGGGCGACTTCTCCTTCCTTT
CTCCCTTCTCCC
+
@>?4?>@>ABBBBCCCCBB@>@?><666-
6=677.4448=>8<<<544=388667,116,766<;:=84/3/478;:?@@@:;8:344-
@T6XXI:00041:00582
GGGGAATTCTAATACGACTCACTATAGGAGAGGGAGAATAGTTCTGTGCATTTTCTCCCAGCACACCGTCCTTCCTT
TCTCCCTTCTCCC
+
DCC9A>@@BBBBDCBABABB?:974..,0::BC>CBD>A999::;====8665.5;<A<ACAAA=<:;4448>A@AC
=@@<=;<:;???:
@T6XXI:00041:00589
GGGAGAAGGGAGAAAGGAAGGAGAAAAGGAAGGGGAGAAGGGAGAAAAGGAAGGAGAAATGCACTGTTTCGTATCCA
GGGTCTATTCTCCCTCTCCTAT
+
DC>999;>>099;;7?674.370000*/,.,...*.577??761044-
4792344000+0437>>17:,6661777100+04003:;000+02--,-13
@T6XXI:00041:00593
GGGAGAAGGGAGAAAGGAAGCTACTCGACTCGACATCTGTGGCTTCACAGTACAGAGGGACTGTCCAAAGCCGCAGA
TCTCGAGTCGACTAG
```

```
+
CC@BA=694-
9?>>9A8:4;B?78;:777:?>???AAADBA<=@@BBB;;?<>>;86575;9===8;;6;?<@???AAA@@A@AAAA
AAA@A
@T6XXI:00041:00598
GGGAGAAGGGAGAAAGGAAGCTAGTCACTCGACATCTGTGGCTTCACAGTACAGAGGGACTGTCCAAAGCCGCAGAT
CTCGAGTCGACTAG
+
CCACB?894-
999909;?<@@?CLC?@>>>600/0/.;>C;?@@CBC?BBA@>4300,3::=?A?@A;@@>@AAAA=8==@@???AA
???@
@T6XXI:00042:00573
GGGAGAAGGGAGAAAGGAAGCTAGTCGACTCGACATCTGTGGCTTCACAGTACAGAGGACTGTCCAAGCCGCAGATC
TCGAGTCCGACT
+
BB?BCB?BC7998=9=;?7776668898:?CB???????<A=7:26888:=:66=7837>879;././5329@998@
@@4744434:9;
@T6XXI:00042:00578
GGGGAATTCTAATACGACTCACTATAGGGAGAGGGAGAATAGTTCTTTAATTCCCCGCTGTTCGTTGTCCTTCCTTT
CTCCCTTCTCCC
+
999/98?=@CBBCB@@BBBB=@;77116.7?>>B>@@A?@AA@>ACC68388;9;4<?@@?9;:86://-
/;;9;>5==@@;?=?ACC=
@T6XXI:00042:00587
GAGGGAGGAAGGG
+
9111+//,/,//+
@T6XXI:00043:00578
GGGGAATTCTAATACGACTCACTATAGGAGAGGGAGAATAGTGCCGCGCGATCCCCTGTTGACAACTATCTTCCTTT
CTCCCTTCTCCC
+
ACD:C:;<<BB@GBAAB@@@?BB?9660777=?>AAB?BAAB9=9=<>A@8333708AA?BA??;?@?@??<?9<:4
9444-49;===7
@T6XXI:00043:00579
GGGAGAAGGGAGAAAAGGAAGCTAGTC
+
94-49;999684999,4231371..,,
@T6XXI:00043:00581
GGGGAATTCTAATACGACTCACTATAGGGAGAGGGAGAATAGTCAACAAATCGTTTGGGGCGACTTCTCCTTCCTTT
CTCCCTTCTCCC
+
999/49@>ABB?BCCCCBBBB@@=;666.777BC>@?@>A=>=?@?AAC;AAACC<AAA6A@A@@;?8858=@?AB=
???A=A>@@@@<
@T6XXI:00043:00599
GGGAGAAGGGAGAAAGGAAGCTAGTCACTCGACATCTGTGGCTTCACAGTACAGAGGGACTGTCCAAAGCCGCAGAT
CTCGAGTCGACT
+
AC?ECC@CB?:<<C>A@C?@@???BB??=A@:87966@AA>BC?CCC?@?AAAA???:<?=;8;78:7;;9<@@@@A
===AAAA===@@
@T6XXI:00044:00561
GGGGAATTCTAATACGACTCACTATAGGGAGAGGGAGAATAGTCAACAAATCGTTTGGGGCGACTTCTCCTTCCTTT
CTCCCTTCTCCC
+
AA@7A8;;<CFBCCBB?>@C?8<=7777.777BB>?==>?AAAAA;>>A=CBBBC>BBB7BCCCC??@=3837=@@<
???@<@<@<<<6
@T6XXI:00044:00581
```

```
GGGAGAAGGGAGAAAGGAAGCTAGTCGACTCGACATCTGTGGCTTCACAGTACAGAGGACTGTCCAAAGCCGCAGAT
CTCGAGTCGAC
+
CIDA??=@@>BCCD@C@C?CA????;9:@@@B;;;AAACA?;=@;7:7>>?::4444248?;=?=?=:::7;888<=
@888=;<;66<
@T6XXI:00044:00586
GTCGACTCGAGATCTGCGGCTTTGGACAGTCCCTCTGTACTGTGAAGCCACAGATGTCGAGTCGACTAGCTTCTTTC
TCCCTTCTCCCTTCCTTTCTCCCTTCTCC
+
JCA?@CCCBBBCDFBBCB@A?A<A@CCCCBBB=ACCCCBBBBBBC@BC@GD:;;ACCDDDCD??ABBB8:;?BBE:;
;CD>?:7777.3/3/44+314406,/--,
@T6XXI:00044:00587
GGGAGAAGGGAGAAAGGAAGGAGAAGTCGCCCCAAACGATTTGTTGACTATTCTCCCTCTCCCTATAGTGAGTCGTA
TTTCAGAAATTCCCCC
+
CC?BBC=@@<@BAA=B=@=@@CCC>??BBBJI7BB?BCB@@;@A@CCB@@;9979:9/0/755579==A<=8674+,
++*++0113457757.
@T6XXI:00044:00592
GGGGAATTCTAATACGACTCACTATAGGGAGAGGGAGAATAGTCAACAAATCGTTTGGGGCGACTTCTCCTTCCTTT
CTCCCTTCTCCC
+
CBCAB?@>@AB@B@>>BB?B?BB77111.6==@B>@@A@A<@>AA?AAA:BABBE>><>7<BBCC@CAA>@>@>AC=
@=<<3<388@A<
@T6XXI:00045:00576
CTAGGTCACTCGAGATCTGCGGCTTTGGACAGTCCCTCTGTACTGTGAAGCCACAGATGTCGAGTCGACTAGCTTCC
TTTCTCCCTTCTCCC
+
AA@B>A;<=BBCGDCDACCCC@@8?9?687>B::::;CCCCCA@8<<A9<@ADCCB@<;;BCFDBCBBBBCAAA?BA
DE@B@@@<A>AAAA<
@T6XXI:00045:00589
GGGAGAGAATTTGGTAATGGGGAAAGTGAGTCGGGGGGAGAAGAGAATATGACCCTTATATCTCTCTCCTATAGTGA
GTCGTATTAGAATTCCCC
+
99149>999<C=BAA>53377,83,199?C@<<<@A@-
B;;7;BAB@C=6;677,101611100090727>?99:33;;777;;887729@@@A7
@T6XXI:00046:00550
GGGGAATTCTA
+
FEE9C=>=>/1
@T6XXI:00046:00551
GGGAGAAGGGAGAAAGGAAGCCCAGTCTGACTCGGGAGATTCGTCAGCTATTCTCCCTCTCCCTATAGTGAGTCGTA
TTAGAATTCCCC
+
CCADCC@CC>BBBC?C@C@BBB=B@>>ACAAABBC>?>;>B??>CAAA@@@?@BCC>B??AA<A8<=AA=<<<<<<@
@>@AC=@3887,
@T6XXI:00046:00572
GGGAGAAGGGAGAAAGGAAGGAGAAGTGGACGTCACAAAGTTGCGCCCTATTCTCCCTCTCCCTATAGTGAGTCGTA
TTAGAATTCCCC
+
AA?>>>@ABB??AB@B?A?A@BB>;???>???==CBBB?AB@BCCBB<BCC@B??><@BBCCACCC<??CCCBB@@?
?=?CC>D>@@@5
@T6XXI:00046:00573
GGGGAATTCTAATACGACTCACTATAGGGAGAGGGAGAATAGCTTCTAGCATTGTCAGAGCTGTCCTCCTTCCTTTC
TCCCTTCTCCC
+
>>@0>-1,/777:BDA@ABBCCCC????9???AC>C??<?<<<@?AAC884B697<77771/...,..,.33,--+-
00406244==4
```

```
@T6XXI:00046:00577
GGGAGAAGGGAGAAAGGAAGGAGACAGTGAGCGCCGCACAACACGTCACTATTCTCCCTCTCCCTATAGTGA
+
:;131@<@?:=88;-82377777698;;8997<<131611011111699@==24377/83311,14443333
@T6XXI:00046:00589
GGGGAATTCTAATACGACTCACTATAGGGAGAGGGAGAAATAGCCTGTTGCAACACGACTCCCCGGCACTCTTCCTT
TCTCCCTTCTCCC
+
999/49@<>@@=BDCDCBBB::;?<7::7:AA??.7444+3;49?CBB???@>A@@=A;4444-
4349=<<<?A/381887=;A>@@@@:
@T6XXI:00047:00557
GGGGAATTCTAATAACGACTCACTATAGGGAGAGGAGATATAG
+
BBB4@5458667//,...47;763?>776,614555///:544
@T6XXI:00047:00562
GGGGAATTCTAATACGACTCACTATAGGAGAGGGAGAATAGTCAACAAATCGTTTGGGGCGACTTCTCCTTCCTTTC
TCCCTTCTCCC
+
CC>9B@A>ABBACCBCBBBB>:977/.,0::>@=BA@?@===@@>AAC9@@@?C>CCB9BCCCC@@@=473356<7?
@@@<A699<A:
@T6XXI:00048:00555
CTAGTCACTCGAGATCTGCGGCTTTGGACAGTC
+
494////,,,,,,/666;;;5716671311111
@T6XXI:00048:00580
GGGGAATTCTAATACGACTCACTATAGGAGAGGGAGAATAGCACACGGTTTTCTCCCACCGGCTGGCCCTTCCTTTC
TCCCTTCTCCC
+
CCB4@5958449=AC@@@AA:/1;:00,/:<BC>ADD=@?@@@=???@AAA5?<==7=C>A?A@@:<<7<<?=@B:?
??>:?<???C=
@T6XXI:00048:00594
GGGGAATTCTAATACGACTCACTATAGGAGAGGGAGAATAGTCAACAAATCGTTTGGGGCGACTTCTCCTTCCTTTC
TCCCTTCTCCC
+
///*1-95;AA@DCAABBBB@:977..,0<077.49948=;:83/4330<777=-111,11555,.-1,-,-
0172548=58588<<;
@T6XXI:00049:00550
GGGGAATTTAATACGAACTCACTATAGGAGAGGAGAATAGTTTGCGCACTCGTGCACTGTTATTCTCTTCCTTTCTC
CCTTCTCCC
+
BDD:C@CB<A5/////,/...6979674/3/0,.--,-1000+0004499/0.5.31;;;7444....33,--+-
24446440031
@T6XXI:00049:00555
GGGGGAATTCTAATACGACTCACTATAG
+
6<?<*1,424:;9:=7833336......
@T6XXI:00049:00558
GGGAGAAGGGAGAAAGGAAGGAGACACGGTCACAAACATGAATGTGACTATTCTCCCTCTCCCTATAGTGAGTCGTA
TTAGAATTCCCC
+
BB?BCCBCB@>@>B>B@B?A?BBABBAA@A?@@AB>AA@775;@??BCCBC?>;<54::444-
8>>?@?@@@AA@????A@@<?@DBC5
@T6XXI:00049:00569
GGGGAATTCTAATACGACTCACTATAGGGAGAGGAGAATATAGTACGAATCCGTT
+
---*/598=BBBCCA>ACBBBCCCAABB>BBBA>@883797...31---04/003
@T6XXI:00049:00570
```

```
GGGAGAAGGGAGAAAGGAAGGAGATACCAACGCAAACACGATTGTGGCTATTCTCCCTCTCCCTATAGTGAGTCGTA
TTAGAATTCCCC
+
CDABBBACC@@A@D>C?A@C@CCB::;=@<?CBBB>BCBBBB?A;:59ADC@CAAA>ACDGE>BBBACCCCCE@@??
@?????=8>??5
@T6XXI:00049:00571
GGGGAATCTAATACGACGTCACTATAGGAGAGGAG
+
88808*1///,/44.....4;//333313611011
@T6XXI:00049:00573
GGGAGAAGGGAGAAAGGAAGCTAGTCACTCGACATCTGTGGCTTCACAGTACAGAGGGACTGTCCAAAGCCGCAGAT
CTCGAGTCGACTAG
+
BBA@;<6;@>>@ABAB?A?AA:7:?BCBDDA:99@??BBB?AA?ABBB?@=A?<>??;>>A?7737;69;=@>???@
@@AAA@A???<<=?
@T6XXI:00049:00581
GGGGAATTCTAATACGACTCACTATAGGGAGAGGGAGAATAGTCAACAAATCGTTTGGGGCGACTTCTCCTTCCTTT
CTCCCTTCTCCC
+
BAA7A=@=@BB@BBAAEABA=<;76167.7>@CF>AAA??<?@@@>AAA<CB???9?@A7BCCCC=?<>9<9=?AC<
AAAA<A?@@@@;
@T6XXI:00004:00603
GGGGAATCTAATACGACTCACTATAGGGAGAGGGAGAATAGT
+
1:80>,1///,/4>988776>><8877,17111,104/0033
@T6XXI:00004:00623
GGGAGAAGGGAGAAAGGAAGGAGAAGTCGCCCCAAACGATTTGTTGACTATTCTCCCTCTCCCTATAGTGAGTCGTA
TTAGAATTCCCC
+
CC@CDC@CD>BBBB@B@B>A?ABC@AABBBBBB8AA@@AAAC>?@>@?@<AA>A@==;@@@;=<AA?:<<==99<@?<
;9<=;8:24470
@T6XXI:00004:00624
GGGAGAAGGGAGAAAGGAAGCTAGTCGACTCGACATCTGTGGCTTCACAGTACAGAGGGACTGTCCAAAGCCGCAAG
ATCTCGAGTCGACTAGCTTCCTTTCTCCCTTCTCCC
+
49-
44424@8;:9A7;6959;6777,,,,,003566A==AA>AE?AABBAB@DCA;;;6;<:;;B8<<7<@=@A@CC@A=
==888?<<;<<<<<?=@?A@<@>>>7=:;:;=6
@T6XXI:00004:00632
TTCTAATACGACTCACTATAGGGAGAGGGAGAATAGCTGCCACGTTTGGACAATTGCAGTTCTCCTTCCTTTCTCCC
TTCTCCC
+
HADCD?CBBCBBABBB>93377.7==?@=BCF@B@@CBCB@BAAAD?F@CBC@B@?==@A=@BC?B?B@CC>CCCC>
C>@::9-
@T6XXI:00004:00640
TGCGACTCGAGATCTGCGGCTTTGGACAGTCCCTCTGTATGTGAA
+
64494444494A444@44599909<>=@@@BB:933333766.**
@T6XXI:00005:00621
CTAGTCGACTCGAGATCTGCGGCTTTGGACAGTCCCTCTGTACTGTGAAGCCACAGATGTCGAGTCGACCTAGCTTC
CTTTCTCCCTTCTCCC
+
B9944444:??=BBGBBBBAA?ACC@C?BBBCBBB>BCCABBBAAA??<?B?BBAABCCDE<<;BCDFC@BBBAA?A
EFD>B@AA;@9<<==:
@T6XXI:00005:00625
GGGAGAAGGGAGAAAGGAAGCTAGTCGACTCACATCTGTGGCTTCACAGTACAGAGGGACTGTCCAAAGCCGCAGAT
GTCGAGTCGACTAGCTTCCTTTCTCCCTTCTCCC
```

+
A?:ABBBBB:?;9908484;C@@@>/00:0.....3111?779@C:;:B???;;>>>7?A@@@@?@@<@A>?????A
888>>>??@@@===AACB@AA?@@ABAB=@??@:
@T6XXI:00005:00630
GGGAGAAGGGAGAAAGGAAGCTAGTCACTCGACATCTGTGGCTTCACAGTACAGAGGGACTGTCCAAAGCCGCAGAT
CTCGAGTCGACTAG
+
DC?BBA@BC?BCBB>C?A>AA?9??8:77?9::4<=<BBC?BB@BA<A???=:====79=AA@@>AC<==;=??@A?
??AA@@?@AA@@@A
@T6XXI:00005:00641
CTAGTCACTCGAGATCTGCGGCTTTGGACAGTCCCTCTGTACTGTGAAGCCACAGATGTCGAGTCGACTAGCTTCCT
TTCTCCCTTCTCCC
+
D:49;<444B@@ABBCCCBB?BCC>C=CB@@???=?BBACBB@=@@@>=B?BCCCCBBBBBBAABC@@@CBDC@C@D
C>CBAA<A9<<@C<
@T6XXI:00005:00647
GGGGAATTCTAATACGACTCACTATAGGGAGAGGGAGAATAGTTCTACCTATTTCCCGCCAACGCTAGCCTTCCTTT
CTCCCTTCTCCC
+
CCC9B8959>>;>@@@@AACBBBAAAEE>AAAAB<A?AAC====;;;;79;;;4;7::79;=<<=????A:=8;94
99::-438@@C?
@T6XXI:00006:00602
GGGAGAAGGA
+
11-,,,*,**
@T6XXI:00006:00608
GGGGAATTCTAATACGACTCACTATAGGGAGAGGGAGAATAGATCCGTTATTCACCCCATTTTTCTATTCTTCCTTC
TTCCCTTCTCCC
+
BB?6?248;ABADDCBB??A?@B7;111.7>>AC>@AA=A=:::<:?=?AB>A@@@@6@@AAA1<:..,/5+,+,+,
+*+000347@C<
@T6XXI:00006:00642
GGGGAATTCTAATACGACTCACTATAGGGAGAGAGATATAAGGGAACAACGAAGAATGAGCGGACCCCACACTTGCC
CATTACCAAATTCTCTCCC
+
CDB9A89<<BB@CA??AA9::BA=B9995:??CC@@@<@=;=@7=9<@>?BB@CC?CB<>A@<773300>=?@>ACC
=A@?AA?CC9>;=???@A<
@T6XXI:00007:00602
GGGGAATTCTAATACGACTCACTATAGGGAGAGGGAGAATAGACCACTTCCAACGGATCACGTATTATTCTTCCTTT
CTCCCTTCTCCC
+
999/9<A?CBB@BA@@@BBB>AB77111.7>>BC>C@@>@@@AA>===?A?@>@@489A??>A==8<?<>><?=?@<
??AA<@=?@@C;
@T6XXI:00007:00610
GGGAGAAGGGAGAAAGGAAGGAGAACGCACCAGTCCACGCAGGCCTGATTCTATTCTCCCTCTCCCTATAGTGAGTC
GTATTAGAATTCCCC
+
BA>BCCBCH@CCBA>B@C>?=?CC@CCCGC@BAA@?BB@A@C=@=ACDDADDBB8;8?><;;;CC=??@B;7::7=@
ACCD@@@A3<;<CC7
@T6XXI:00007:00625
GGGAGAAGGGAGAAAGGAAGGAGAATGCACCGGAGGAAATTGCAAGACTATTCTCCCTCTCCCTATAGTGAGTCGTA
TTAGAATTCCCC
+
AAACDC=??<@CCE@B@B@C@CA>>=?CCC>C@CC?@A>A@EBB?CBCBBC?BCBE>?8==<7<AAAA==B@A????
?<?AA>C>@@@5
@T6XXI:00007:00634
GGGAGAAGGGAGAAAGGAAGGAG

```
+
49-449899-44/8+/,066,00
@T6XXI:00007:00635
GGGAGAAGGGAGAAAGGAAGGAGAATGCACCGCAGGAAATTGCGAGACTATTCTCCCTCTCCCTATAGTGAGTCGTA
TTAGAATTCCCC
+
@@<A@>=@@<@?==<@@B?C@CCC?CCCBB?BCCB>BB>B@BC?@?:::??>ABDD@DA@@@:??><==@?;;:///
6573A<><==A6
@T6XXI:00007:00643
CTAGTCGACTCGAGATCTGCGGCTTTGGACAGTCCCTCTGTACTGTGAAGCCACAGATGTCGAGTCGACTAGCTTCC
TTTCTCCCTTCTCCCCTTCCTT
+
A9949944=?BCCCAACCCBB@@@@>@?CCAAAAA=ACCCCBDBA???<?B>AB@@@@@??CCCBFCC@@@@@=@C@
FC?AA@>7:8:////+/78,/,
@T6XXI:00007:00646
GGGAGAAGGGAGAAAGGAAGGAGAAGTCGCCCCAAACGATTTGTTGACTATTCTCCCTCTCCCTATAGTGAGTCGTA
TTAGAATTCCCC
+
EDACBA@BD>BBBB?C@B@B@BBB@@@BBBBB8BB>@@@@?>??ABB>9==;<<=<;=<<<=;=;999===<<<??@
@?A@@=?37770
@T6XXI:00007:00649
GGGAGAAGGGAGAAAGGAAGAAGAGCGCACAGCGGGAGGACCATTGGCTATTCTCCCTCTCCCTATAGTGAGTCGTA
TTAGAATTCCCC
+
CC>=A:8@@<@AAB>B>A?BA?BC?@@BBDCCCC@>ACAB=?AA?A>@@BC?>@@?;????@;@A===A@??@???@
A>AAA=A?@AA6
@T6XXI:00008:00607
GGGAGAGAATTTGGTAATGGGTAAGCGC
+
BB>BBBBB>BB=B>>A9100,./,,2//
@T6XXI:00008:00608
GGGAGAAGGGAGAAAGGAAGGAGAAATGCAATGTAACTAAAACGTAGCTATTCTCCCTCTCCCTATAGTGAGTCGTA
TTAGAATTCCCC
+
BC@BBB@BB>BACE@C@B?B@CCC?8AACC@CAAA?BACCC8AAABBBBBB?BCCC>B?CB@>848?????AAA?>>
A>AAB=B?BBA6
@T6XXI:00008:00619
GGGGGAATTCTAATTACGACTCACTATAGGGAGAGGGGAGAATG
+
4444+48000,-+,*,2/33;;7697669>166//4:07++++0
@T6XXI:00008:00632
GGGGAATTCTAATACGACTCACTATAGGGAGAGGGAGAATAGTCAACAAATCGTTTGGGGCGACTTCTCCTTCCTTT
CTCCCTTCTCCC
+
@==2><>?@>@>@@?@A@@@CCC@?166.7<<>B<<=@=?AA?>>:;;;6?BBBC@BAB6@:::@>A>?;=;=8;<6
;94;18587@A<
@T6XXI:00008:00639
GGGAGAAGGGAGAAAGGAAGGAGAACAGTCGGGCAAGCACCAAAGGACTATTCTCCCTCTCCCTATAGTGAGTCGTA
TTAGAATTCCCC
+
AB>BEB?BB>9;;C=B>B@C@CCC>BBBBBBB<BB@B@??@BB>B@BBCAB?AABC?A9CBB;;;;:=AA@@AA???
@>AAC=@=@CC6
@T6XXI:00008:00649
GGGGAATTCTAATACGACTCACTATAGGGAGAGGGAGAATAGTTACATCTTTCCTGCAATGCACCTGTTCTTCCTTT
CTCCCTTCTCCC
+
```

```
;;9/9<A>BCC@CCBABBCCBBBCA996-
6=;;?9?AA>@AAA=A><?78858=?@A@=@@=<<<<=>88<:=8;;6;==?;@>AAA=;
@T6XXI:00009:00602
GGGGAATTCTAATACGACTCACTATAGGGAGAGGGAGAATAGTTTTCCCTTCACTAA
+
8C807+4544424444337;99>?5555+,,,,,*,+345:=BBB+00+0/0...,0
@T6XXI:00009:00607
GGGAGAAGGGAGAAAGGAAGGAGACAGCCCCAGAGTACGGTCCAGAACTATTCTCCCTCTCCCTATAGTGAGTCGTA
TTAGAATTCCCC
+
DFDCCB@BC=BBBD=?=??A?CBBBABBCB8BB?BBBBB@BB@???>??@@;=??@;B=AAA<@@@A===A??@A==
==@@C?D????5
@T6XXI:00009:00614
GGGAGAAGGGAGAAAGGAAGGAGATATAGCAATCAGCAAAGGTGTAACTATTCTCCCTCTCCCTATAGTGAGTCGTA
TTAGAATTCCC
+
AA@ADC@CD><<<D>B@B@C@;;;C???AAB>BBBBBBC>EACDBB@B@@@@CBBC>??:::5:BAB???A===@@@
A<??A>C@CC?
@T6XXI:00009:00647
GGGAGAAGGGAGAAAGGAAGGAATGAGC
+
CB>B9959>:>88:5657,0,.6,,,2,
@T6XXI:00010:00607
GGGAGAAGGGAGAAAGGAAGAAAGGAAGGGAATAGTCGCGCTTGGTTATCAACGTGCCTATTC
+
44-4;;9>A:;9@C<@481883,37666669211899622--,-,-3113=<422--,-2222
@T6XXI:00010:00608
GGGAGAAGGGAGAAAGGAAGGAGAAGTCGCCCCAAACGATTTGTTGACTATTCTCCCTCTCCCTATAGTGAGTCGTA
TTAGAATTCCCC
+
CG@CBB@BA?@@BB>B@B?AAAB@@@@C?>;>69A<AFCDD>C@8:<=HAA>@@<=:::<@A=A==A<<<AC<???@
@?A@@>A>AAA5
@T6XXI:00010:00621
GGGAGAAGGGAGAAAGGAAGAGAGAGCAGATATCGAGTGTGTATCAGCTATTCTCCCTCTCCCTATAGTGAGTCGTA
TTAGAATTCCCC
+
=?:999599-4444-
477:;BCCCCAC@>A???A;<;A888@<<<C???;848777.7>777.7@?AABCBBBBBB?=:<@A>C?BCC9
@T6XXI:00010:00631
GGGAGAAGGGAGAAAGGAAGCTAGTCGACTCGACATCTGTGGCTTCACAGTACAGAGGGACTGTCCAAAGCCGCAGA
TCTCGAGTCGAT
+
9<19>><AA<@@@B=BACBCCBCAA8::B@@@AA@BBAAA@AAB@C@@@CCAAAAA=@9==AAAB?AA<AA=@??@<
==<<<===;??A
@T6XXI:00010:00636
GGGAGAAGGGAGAAAGGAAGCATAGTCGTCGACATCTGTGGCTTCACAGTACCAGAGGACCTGTCCAAAGCCGCAGA
TCTCGAGTTCCA
+
49149>;>>9@@BC<A<@,0...34.1111144==9994424424496////,/633133111116;;:<?<:<<@>
90--+,,*)*00
@T6XXI:00010:00642
GGGGAATTCTAATACGACTCACTATAGGGAGAGGGAGAATAGTCCACTGAAA
+
499/49@9>@@=ABBBCCBB@@>76116.7>>@C>BAB?D==432448-++*
@T6XXI:00011:00617
GGGAGAAGGGAGAAAGGAAGCTAGTCGACTCGAGATCTGTGCGTTCACA
+
```

```
CCBDED@CH>B9996;?B?BCC:::8::AABBBAA@B>/0//--,--13
@T6XXI:00011:00622
GGGGAATTCTAATACGACTCACTATAGGGAGAGGGAGAATAGTCAACAAATCGTTTGGGGCGACTTCTCCTTCCTTT
CTCCCTTCTCCC
+
;<9/<<A@CBB@BBBBBBBB@A<76111.7>==?<?@@>AAAAAA<:<<;?@?>?;<8:6????C@AB??@?A?BC<
????<??CABA:
@T6XXI:00011:00626
GGGGAATTCTAATACGACTCACTATAAGGGAGAGGGAGAATAGTAGTTTAACCGATCGTGTG
+
444,429244924999?>;0..45..,..+.4477963323==;<===9998<300------
@T6XXI:00011:00645
GGGGAATTCTAATACGACTCACTATAGGAGAGGGAGAATAGTTTTCCCTTCACTAACCGCGAACTGCACTTCCTTTC
TCCCTTCTCCC
+
944-
4898=BIFCBB@?@BB?;:<:00,0>>BD>C@B?@??AAA4@A<@9<????@C@@?=A>BHA9::C=?;?B?:88=7
;9:444-
@T6XXI:00012:00608
GGGAGAAGGGAGAAAGGAAGAGAATAACAGTGCACCGAATGCGCAAACTATTCTCCCTCTCCCTATAGTGAGTCGTA
TTAGAATTCCCC
+
@@:=9969904///+/,.,.4..,.=:?;:7BBAA?@@=@<<<CCCADDDDBCBAC9::7>@;CDD==<9977/..4
4,.1424/000+
@T6XXI:00012:00629
GGGAGAAGGGAGAAAGGAAGCTAGTCACTCGACATCTGTGGCTTCACAGTACAGAGGGACTGTCCAAAGCCGCAGAT
CTCGAGTCGACTAGCTTCCTTTCTCCCTTCTCCC
+
94-
4=>@BB:>99919;>;8;:>@?=7611016379761766811111==777;99=>588@AAA>AD;=<9<><;;?;<;
888==>>?===@A>A>AA<@@AAAA:=9@;1
@T6XXI:00012:00635
GGGAGAAGGGAGAAAGGAAGCGTCGTTCGGGAGTAGTATTGGTGA
+
AA<AAC@BB:@;44-9<>;>887316269?;776111--,-2--+
@T6XXI:00012:00636
GGGAGAAGGGAGAAAGGAAGCTAGTCACTCGACATCTGTGGCTTCACAGTACAGAGGGACTGTCCAAAGCCGCAGAT
CTCGAGTCGACTAGCTTCCTTTCTCCCTTCTCCC
+
9;7<ABADC<A;;909685;B@>;0*******..,/8???@CB@CCCC::8A@@??@;@???AA>AA;@?<<<<?@@
@A@A=====@@A@@AA>A<??<?AAA<A????A<
@T6XXI:00012:00637
GGGGAATTCTAATACGACTCACTATAGGGAGAGGGAGAATAGCACAAACT
+
999/4295<BB?B=<=BBBCCBBAA??=6777;;9:;;6//.4330301+
@T6XXI:00013:00607
GGGAGAAGGGAGAAAGGAAGGCCGCGACGGCGAAAGAATATGGGGAACTATTCTCCCTCTCCCTATAGTGAGTCGTA
TTAGAATTCCCC
+
BB>BAA@BB>AABB=B?A?A@B@B???@C@@7998;@<@B@@@@6@>BBBB@CCCCACCCCC?CBBBB????@@B@C
C@A??<?=@@@5
@T6XXI:00013:00613
CTATCGACTCGAGATCTGCGGCTTTGGACAGTCCCTCTGTACTGTGAAGCCACAGATGTCGAGTCGACTAGCTTCCT
TTCCTCCCTTCTCCC
+
B;88/////6:=?BBBAC@@=@@ECE@CCAA:::5:CCBBAAA=A@A>@C@CCCCBBCCCCAAACCB@A@A>66;99
<,0,/9917.87;94
```

```
@T6XXI:00013:00628
GGGAGAAGGGAGAAAGGAAGCTAGTCACTCGACATCTGTGGCTTCACAGTACAGAGGGACTGTCCAAAGCCGCAGAT
CTCGAGTCGACTAG
+
;::313>>>;>>BC@C;<8:AAA@AA@:::<BBADCCAAA??@=?CACC??:;:444-
44;;;A9<<5;65:<AACAAA???@@AAAC:99
@T6XXI:00013:00634
GGGGAATTCTAATACGACTCACTATAGGGAGAGGGAGAATAGTCAACAAATCGTTTGGGGCGACTTCTCCTTCCTTT
CTCCCTTCTCCC
+
>>>2=;>=@@@DDBBBBCCCCCB=?666.7>>BB>B9858?????<<<<7?CBCC>BCC9BBBBCABBA;=;=>AD=
BBBA>C=?@@A<
@T6XXI:00014:00602
GGGAGAAGGGAGAAAGGAAGGGGAGAAGGGAGAAAGGAAGCTAGTCG
+
9909=><@A<@A@B<@=A@DCC9CCC@CC<?:896=:=37//0.:/.
@T6XXI:00014:00627
GGGGAATTCTAATACGACTCACTATAGGGAGAGGGAGAATAGTCAACAAATCGTTTGGGGCGACTTCTCCTTCCTTT
CTCCCTTCTCCC
+
999/98?<?A@?BCCCCB???@B;966659@@CC>B@@=@<@@AA????7AA???9?@>5?BBBB@BCA;=:=<CCA
A@@@:@?=AAA4
@T6XXI:00014:00630
GGGAGAAGGGAGAAAGGAAGGTAAGTGACGACCAGCCGCCCATGG
+
99-4999=A58;::09>A?C@CCDBBB@@>7430002000+0311
@T6XXI:00014:00639
GGGAGAAGGGAGAAAGGAAGGGACAGTCCATCTGTGGCTTCACAGTACAGAGGGACTGTCCAAAGCCGCAGATCTCG
AGTCGACTAG
+
BD>BBA@BC@DBBB=?=>@BB>>@@BA?=@@BBCCC>?A?BCCAAA;;:=9=9:9;====;<<<?<;=@=@AAA@<<
<?<BBBBCBB
@T6XXI:00014:00648
GGGAGAAGGGAGAAAGGAAGCTAGTCGACTCGACATCTGTGGTCCA
+
9<:;ABABB:@1//+1-1,0443450//53777;00/,7700+-,-
@T6XXI:00015:00600
GGGAGAAGGGAGAAAGGAAAGCTGAGTCCGACACATCTGTGGCTTCACAGTACAGAGGGACTGTCCAAACCGCAGAT
CTCGAGTCGACTAG
+
CC@@>92990999914233,33116111361...-
..,,,2*./::::BCDBBA@A@D?EBBCBBCDJ@CCBBACEDDDBABBCDCAAACB
@T6XXI:00015:00607
GGGAGAAGGGAGAAAGGAAGGGGGAGAAGGGAGAAAGGAAGCTAGTCACTC
+
CB:A==59919>??9><?48888+787016,1117.73<:7///./0.313
@T6XXI:00015:00609
GGGAGAAGGGAGAAAGGAAGCTAGTCACTCGAGATCTGCGGCTTTGGACAGTCCCTCTGTACTGTGAAGCCACAGAT
GTCGAGTCGACTAGCTTCCTTTCTCCCTTCTCCC
+
BA<A@@=@@:>99969>?>BBBA@AA??@A@B@@CBAAA@A>CB=B>9AB?@@@;99:::=;;<=<<;<=<<;<=AA
899????@@==<@@@@?@>AA:????<?>AAAA:
@T6XXI:00015:00630
CTAGTCACTCGAGATCTGCGGCTTTGGACAGTCCCTCTGTATTGTGAAGCCACAGATGTCGAGTCGACTAGCTTCCT
TTCTCCCTTCTCCC
+
```

```
CA999>444ABBAABBCC@A>@B@>B?BBCBCCB>BCCCA8989??A>@CDCCCFCAAABCBBAAAACBBG<<9<@C
C?@@@E=?=?BAA=
@T6XXI:00015:00633
GGGGAATTCTAATACGACTCACTATAGGAGAGGGAGAATAGTTTTTCCCTTCACTAACCGCGAACTGCACTTCCTTT
CTCCCTTCTCCC
+
;99/9;@=@CC?BDCCD@@@C000000,.39>?==CD>AA84444,4;:=<?ACCE@C>@@?@=???>AAA>A?BC>
A??@:?<?=;;6
@T6XXI:00015:00642
CTAGTCGACTCGAGATCTGCGGCTTTGGACAGTCCTCTGTACTGTGAGACCACAGATGTCGAGTCGACTAGCTTCCT
TTCTCCCTTCTCCC
+
B7----
,,68;BCCBBBBB@@>@CC>C89;CCCC@C7/,,,,,,04:<<C@BBBBCCA<::BB@@@CCCCCBC=?;?C>@@@D
>A<>>CC<
@T6XXI:00016:00611
GGGAGAAGGGAGAAAGGAAGTAGGCACGGTTGCAGGAGAACGGATAGCTATTCTCCCTCTCCCTATAGTGAGTCGTA
TTAGAATTCCCC
+
CC>BA=>@@<@B??=B?B?BCB@?BB?C@B?@@BA?BBB@AB?BBABB?B?@B<;>;?>CCC>A??@A@A@@@@@@@
@>@@A>A<???5
@T6XXI:00016:00613
GGGGAATTCTAATACGACTCACTATAGGGAGAGGGAGAATAGTTAACGCACCCTCCGTTACCTGTTCACCTTCCTTT
CTCCCTTCTCCC
+
BBB:B@B@BBB@?BBBBAA@?C?77111.7=<BC>B@A?A<==9=>AA@>==600,/:::=???=:??=:=474:?9
;@AC=A?A@@@<
@T6XXI:00016:00634
GGGAGAAGGGAGAAAGGAAGGAGAAGTCGCCCCAAACGATTTGTTGACTATTCTCCCTCTCCCTATAGTGAGTCGTA
TTAGAATTCCCC
+
CC@BBB@BB>:;;C=B?A@C@CBC?A?B;777,675:CCEE>BB?BB@?AA>A;:84<6;?@;@A@@AAA>===AA@
@>@A@>;3444-
@T6XXI:00016:00639
GGGAGAAGGGAGAAAGGAAGCTAGTCACTCGACATCTGTGGCTTCACAGTACAGAGGGACTGTCCAAAGCCGCAGAT
CTCGAGTCGACTAG
+
CCABC@==95=98809684;C???CAAA@@=;:7??9BCC@CC@CCC>???<<=<<<::====;==;=>><=<<<=
<<<:88=999;;;=
@T6XXI:00017:00611
GGGGAATTCTAATACGACTCACTATAGGAGAGGGAGAATAGTCCACACACATTTACAGGGGCTGTTCTCTTCCTTTC
TCCCTTCTCCC
+
AA?7A=@=@BCABBBBB?A>?:977//-
0==@C@@@?=@AAAA><?====<@@;@BBBCC8CCCC@CBABA?A?>;>>>?;=;:8887
@T6XXI:00017:00614
GGGAGAAGGGAGAAAGGAAGGAGAAGTCGCCCCAAACGATTTGTTGACTATTCTCCCTCTCCCTATAGTGAGTCGTA
TAGAATTCC
+
?C8::B@CB@B;;;149?<B?C?@>>::7777,7?;?@?9;;6601770--0----++0000*--
11138<897///--1103554
@T6XXI:00017:00618
GCTAGTACACTCGAGATCTGCGGCTTTGGACAGTCCCTCTGTACTGTGAAGCCACAGATGTCGAGTCGACTAGCTTC
CTTTCCTCCCTTCCTCC
+
4,,,,,,,,/48447>A?;?>?:777,10166689>;;;:?A@=;=63320;@=;><B667?BBABCE;;:@=66:7
447,0,/77,/,4/00/
```

```
@T6XXI:00017:00619
GGGGAATTCTAATACGACTCACTATAGGAGAGGGAGAATAGTCAACAAATCGTTTGGGGCGACTTCTCCTTCCTT
+
499/92959@A?CFDBBCAACBBBB:7377777.4;@;999DBA?BBA=CC@@?<?BB5A?77707++++**)*.
@T6XXI:00017:00622
GGGAGAAGGGAGAAAGGAAGACGATGAGCGCAAAGAACAGTTGAGGACTATTCTCCCTCTCCCTATAGTGAGTCGTA
TTAGTAACTTCCCC
+
9904;@<@A8;;;F?DBDBEDD@???=771111,110062339/.-
.479;>@@@A:883;;55:<<=;;==A=88//46/-/,/.2444.
@T6XXI:00018:00600
GGAGAAGGGAGAAAGGAAGCTAGTCACTCGACATCTGTGGCTTCACAGTACAGAGGGACTGTCCAAAGCCGCAGATC
TCGAGTCGACTAG
+
C?@??58809999084847<==?BABACC=:88;::??D@BB@CA<9>>====99898@?:@A>?@<@A>A???AA=
???=@A@???AAA
@T6XXI:00018:00604
GGGGAATTCTAATACGACTCACTATAGGGAGAGGGAGAATAGTCAACAAATGTTTGGGGCG
+
444,424:44488878387311311117,..333+---,--34444000+16772666266
@T6XXI:00018:00606
GGGGAATCTAATACGACTCACTATAGGGAGAGGGAGAATAGTCAACAAATCGTTTGGGGCGACTTCTCCTCCTTTCT
CCCTTCTCCC
+
8>A0@+1/665/4337:?AEDDCC:::67><=?<<??=@@@@?@<444-7=>>=-111,164577733-.-
22222/44-479333+
@T6XXI:00018:00608
GGGAGAAGGGAGAAAGGAAGCTAGTCGACTCGACATCTGTGGCTTCACAGTACAGAGGGACTGTCCAAAGCCGCAGA
TCTCGAGTCGACTAG
+
CB:A4859908888982713@777?7::?:::???:::@BA:?A>AA?ABB@@@AAAA69988;;344-
43/04468?==<@@A???@@===
@T6XXI:00018:00613
GGGAGAAGGGAGAAAGGAAGCTAGTCGACTCGACATCTGTGGCTTCACAGTACAGAGGGACTGTCCAAAGCCGCAGA
TCTCGAGTCGACT
+
BC?CBB@CB=;;<DBI?C?;96;69676999<:::??@@B@>=B=?BA???884;444-
4;448?>AA<A@:=<AACA??<888:9:=88
@T6XXI:00018:00615
GGGGAATTCTAATACGACTCACTA
+
CCB7B7;58;;8444973,,,,,,
@T6XXI:00018:00631
GGGAGAAGGGAGAAAGGAAGGAGAAGTCGCCCCAAACGATTTGTTGACTATTCTCCCTCTCCCTATAGTGAGTCGTA
TTAGAATTCCCC
+
GKCCCB?@=:BB?A=B@CAA?ABA?@@BBBBB7>>=@CEDC?B>A?::<A=;<<<=;=:?8=<AAA@@@@?@A???A
@>@<:7:3444-
@T6XXI:00018:00634
GGGAGAAGGGAGAAAGGAAGAACAGCATAGCAGGGAACGGACGGTAGCTATTCTCCCTCTCCCTATAGTGAGTCGTA
TTAGAATT
+
CC>ABA@CC>?A@B?B?B@BD@CB?BBB@>>CCC?A?BBABBB@DDDCBBB?C@@@<@B@AB>AAAAABBAAA@@A=
=9<??<?>
@T6XXI:00018:00638
GCTAGTCACTCGAGATCTGCGGCTTTGGACAGTCCCTCTGTACTGTGAAGCCACAGATGTCGAGTCGACTAGCTTCC
TTTCCTCCCTTCTCCC
```

```
+
4/6///7699999BBA@@@8858==6@598@CCCJ@B?/1/>3448964/1-
/6449><<9633./.....13375555+.,-3324/546;;
@T6XXI:00018:00644
AGAAAGGAAGAGAATAGTCAACAAATCGTTTGGGGCGACTTCTCCTTCCTTTCTCCCTTCTCCC
+
?9490499:ACCC@;<<BBBC;;919@9990999/8??7>;?;;7:@B=BB>BAAC<B>:98@<
@T6XXI:00019:00601
GGGAATTCTAATACGACTCACTATAGGGAGAGGGAGAATAGTTTTTCCCTTCACTAACCGCGAACTGCACTTCCTTT
CTCCCTTCTCCC
+
CD?C@C?BCC@CBBBA?A@@@>79161.777CE@@@A>;;44000+44,049>????F@B>??<????BBB?B?CC>
?<<?:=:?@@@;
@T6XXI:00019:00611
GGGAGAAGGGAGAAAGGAAGAACAGGAGACTGCAGAATGTGCCGTAACTATTCTCCCTCTCCCTATAGTGAGTCGTA
TTAGAATTCCCC
+
=?<ADHBCCABBBB=B=@=@C@CBB@CBBCCCAC??=BBBBCBBBADDCBA=@;;8039;?A;BCFCBCAAADBCB?
?:?::56==?@5
@T6XXI:00019:00616
GGGAGAAGGAGAAAGGAAGAACATGCTGGGACCGGACAGTGGTTAGCTATTCGTCCCTCCTC
+
49-9442424444-4231377331;8:39277777;;:@@@@A;>888345...44+.-,-3
@T6XXI:00019:00628
GGGAGAAGGGAGAAAGGAAGCTAGTCATCGACCATCTGTGGCTTCACAGTACAGAGGGACTGTCCAAAGCCGCAGAT
CTCGAGTCGACTAGCTTCCTTTCTCCCTTCTCCT
+
KC:@<<99@:@111-
/.,.6366>?0..1..,....54606756;BCDDCC???AA;@@@>=>9<=9=C>AA???A??????@A@AAAAA@
@;;244-4444-8=88:>A
@T6XXI:00021:00603
CTAGTCACTCGAGATCTGCGGCTTTGGACAGTCCCTCTGTACTGTGAAGCCACAGATGTCGAGTCGACTAGCTTCCT
TTCTCCCTTCTCCC
+
;11//6/444;;:999AA?@B@BE>C@A@=<9776///0:466=<=A>ADCCBBBCCBB::::BCAAA@::85621
7,138:7;3834;4
@T6XXI:00021:00617
GGGAGAAGGGAGAAAGGAAGCTAGTCACTCGACATCTGTGGCTTCACAGTACAGAGGGACTGTCCAAAGCCGCAGAT
CTCGAGTCGACTAG
+
BC@CB?899-
;@==<@=?<@C?8??:100;90/.///=>B?AA@CCBB>?<;;<<==;=<<:9>37717A?AA@@@CA@@:<?A@??
?CA<
@T6XXI:00021:00627
GGGAGAAGGGAGAAAGGAAGGAGAAGTCGCCCCAAACGATTTGTTGACTATTCTCCCTCTCCCTATAGTGAGTCGTA
TTAGAATTCCCC
+
CFDCCCACD?BFDG>B?A57488747=>>>>>2;99?A@CA;BBABBAA@@>AAAA<@@@@@7<<<<=@@C@A>@<;
741-+*+0000.
@T6XXI:00021:00630
GGGGAATTCTAATTGACGACGTCACTATAGGGAGAGGGAGAAATAGTAG
+
1/1*/,92449542446//////668<A@>?<A:66766111,166331
@T6XXI:00021:00633
GGGGAATCTAATACGACTCACTATAGGGAGAGGGAGAATAGTCAACAAATCGTTTGGGGCGACTTCTCCTTCCTTTC
TCCCTTCTCCC
+
```

```
???5>11111::@CC@@@@??<76661,1;>@@<@@@>@>==<@=<<<6=><??8?CC6??@8877A9/3/4244-
4=>A<=;@@@A<
@T6XXI:00021:00640
GGGAGAAGGGAGAAAAGGAAGCTAGTCACTCGACATCTGTGGCTTCACAGTACAGAGGGACTGTCCAAAGCCGCAGA
TCTCGAGTCGACTAGCTTCCTTTCTCCCTTCTCCC
+
DC@@99599199999,4231397667<7777777016667>5:;=?BBCCBB@A????5<<?788<?@<@A?AB@@A
A=<<=<?>>@@===>@@3848@;?AAD<@9<=CD<
@T6XXI:00022:00607
GGGGAATTCTAATACGACTCACTATAGGGAGAGGGAGAATAGCCAAGCCATTTGGGACTGCTGTTTACCCTTCCTTT
CTCCCTTCTCCC
+
9;<1;;@AJCC@CCBBBCCBA@<76166.7>>BCB;99>AAAA9=:<A>ACC7::7:BABBCBBA<@>=6824/44-
4444-48;:@@;
@T6XXI:00022:00644
GGGAGAAGGGAGAAAGGAAGCTAGTCACTCGAGATCTGCGGCTTTGGACAGTCCCTCTGTACTGTGAAGCCACAGAT
GTCGAGTCGACTAGCTTCCTTTCTCCCTTCTCCC
+
CC>AA@<@A<@@@@<A@B?CCB:?????@AAB@@CBBBACAC?>;?>??@@@@@==<<;<===99==>AA>@AA???
9==??AA@A=;<?@??<@9<<7<?>@7>:=7771
@T6XXI:00023:00608
GGGAGAAGGGAGAAAGGAAGCTAGTCGACTGACATCTGTGGCTTCACAGTACAGAGGGACTGTCCAAAGCCGCAGAT
CTCGAGTCGACTAG
+
BCABDD>??<@BAB>C?BAAC@>>@8::BBBBBB;;;BBC=@@B@EBBBA@C=<44414866=@9==9=A?C????@
@@@==<<<<<<=<=
@T6XXI:00023:00627
GGGAGAAGGGAGAAAGGAAGCTAGTCACTCGACATCTGTGGCTTCACAGTACAGAGGGACTGTCCAAAGCCGCAGAT
CTCGAGTCGACTAG
+
CC@BAA89919999042399?61/.0..030003///355,:7;??@B@?===<@DC9=990==97;6//,+0+--
.244655568;;;;9
@T6XXI:00023:00648
GGGAGAAGGGAGAAAGGAAGAAGATTAGAAGCGAACGTGTTTACGCTCTATTCTCCCTCTCCCTATAGTGAGTCGTA
TTAGAATTCCCC
+
8@1129;>>1948@0923977:<;B>@??:=<795998@BC>CCGCICCC@=@98908>><7<:99B???@BBBBBC
C?AAE?B?BCC8
@T6XXI:00024:00600
GGGAGAAGGGAGAAAGGAAGCTAGTCACTCGAGATCTGCGGCTTTGGACAGTCCCTCTGTACTGTGAACCACAGATG
TCGAGTCGACTAGCTTCCTTTCTCCCTTCTC
+
@959?><>@:999>9><A>BBA@AB:::?C?A?@BBCCBB>:::5:=@ACBBCC?AA@899===@@C?A>@???=78
8<<<?A@=<==?779<><>>:>9991;>ABC
@T6XXI:00024:00604
GGGAGAAGGGAGAAAGGAAGCTAGTCACTCGACATCTGTGGCTTCACAGTACAGAGGGACTGTCCAAAGCCGCAGAT
CTCGAGTCGACTAGCTTCCTTTCTCCCTTCTCCC
+
=95?4958>9>499042313?671=777610100116;@?:B@<;;BBBBA@@????:?>?==<>AC?@@>A@???@
@@???@@@@AAB@@@@>@=@CAA@AA<@8448;6
@T6XXI:00024:00608
GGGAGAAGGGAGAAAGGAAGCTAGTCGACTCGACATCTGTGGCTTCACAGTACAGAGGGACTGTCCAAAGCCGCAGA
TCTCGAGTCGAC
+
BD@A?C@EC@BDBB=B>A?CCA<<<0//,777,/....3.0/063666;:63337;<=9444300234+33;8<<=5
4333+++++...
@T6XXI:00024:00616
```

```
CTCGAGATCTGCGGCTTTGGACAGTCCCTCTGTACTGTGAAGCCACAGATGTCGAGTCGACTAGCTTCCTTTCTCCC
TTCTCCCCTTCCTTTCTCCCTTCTCCCTTCCTTCTCCTTCCTCC
+
C@@AAACCCBBBBABBA=CACCCCCCEBCCCCBABCBABB?BB@CBBBBBBCBBABB?ABBA@;9:5429909@@A>
E@A@CCC6?:?377.:BBB>B>>777.434347://,.,/,//,
@T6XXI:00024:00617
CTATCGACCGAGATCTGCGGCTTTGGACAGTCCCTCTGTACTGTGAAGCCACAGATGTCGAGTCGACTAGCTTCCTT
TCTCCCTTCTCCC
+
C@===4496:ABBBBBDBBACCB>B?BAAA:::5:?=?@A?@?BCA89=ACCCDDCB@@@AA@CBCBCB??;:<::B
<ABBC@CACBAA=
@T6XXI:00024:00643
GGGAGAAGGGAGAAAGGAAGCACACCAGTTTTAGGGATATCGACTGGCTATTCTCCCTCTCCCTATAGTGAGTCGTA
TTAGAATTCCCC
+
CKDCCC@CC>CCCCAC?B>BBBCCC@CB@AA7<<<8<B?@@CC@@@?ACDC@BCCD@BBBBB>BCCCCCC:;;CCCB
C?BBCAC@DCC:
@T6XXI:00025:00600
GGGGAATTCTAATACGACTCACTATAGGGAGAGGGAGAATAGTTACAACGT
+
,,,*,*424;;98=997@?A77861611,166678982220**)***)*..
@T6XXI:00025:00601
CTATCGACTCGAGATCTGCGGCTTTGGACAGTCCCTCTGTACTGTGAAGCCACAGATGTCGAGTCGACTAGCCTTCC
TTTCTCCCTTCTCCCCTTCCTTTCTCCC
+
?9944449===DCBCBCBA?<877.8<>=???BC>BBCB??;4444837C@CDDDA@???A770;1-
,,0,,*,25,./-/=:=;=:;:::8.68852221---+
@T6XXI:00025:00607
GGGGAATTCTAATACGACTCACTATAGGGAGAGGGAGAATAGTCTCCCGATGTTCCGCAGCCGCTTGGCCTCTTCCT
TTCTCCCTTCTCCC
+
=992>698;BBACA@>CACC@??=@76:5:@?CE>@???@AA==???:<CC::;:?CCBBB@AA@>C@C@BBB@C?A
C@CCCB<?:;??<6
@T6XXI:00025:00610
GGGAGAAGGGAGAAAGGAAGCTAGTCACTCGACATCTGTGGCTTCACAGTACAGAGGGACTGTCCAAAGCCGCAGAT
CTCGAGTCGACTAG
+
BC?CCB?A=:@@996>474;A@>>@9:::??777A<A?@F@?@>@CC@C>@:84000,3489;<=@@;@@>@989A@
@A@<=<AA@@>AAB
@T6XXI:00025:00613
GGGAGAAGGGAGAAAGGAAGGAGAAGTCGCCCCAAACGATTTGTTGACTATTCTCCCCTCTCCCTATAGTGAGTCGT
ATTAGAATTCCCC
+
991449244-444;-969>9,.07,0.44444*..+..16>;575//.449493333+40344458896313-/-
+0++*-33013555.
@T6XXI:00025:00615
GGGGAATTCTAATACGACTCACTATAGGGAGAGGGAGAATAGCTCAAAAAACCCAAGACTCCCCCGT
+
99929<=699><=@9;;C<8;78=>711,16111,0110--.-/757577+7334/00000000+00
@T6XXI:00025:00642
GGGAGAAGGGAGAAAGGAAGGGGAGAAGGGAGAAAGGAAGGGGAGAAGGGAGAAAGGAAGCTAGTCGACTCGACATC
TGTGGCTTCACAGTACAGAGGGACTGTCCAAAGCCGCAGATCTCG
+
CD@CCB@BB>@A?B=B<>>;@B7BBB<??777661:=B<@AA6??;=>>4<734-
48;388K:;8@@CCA==559?@@@A=>=A;==@@@AABA@<<;6;==>?A>AC<==;==@@@????@
@T6XXI:00025:00643
GGGGAATTCTAATACGACTCACTATAGGGAGAGGGAGAATAGTCAACAAATCGTTTGGGGGCGACTTCCT
```

```
+
444,42424449499A@?D@@@BC<<;D>B?:77.744238;;443444-16111,1111+1./.3,.,.
@T6XXI:00025:00649
GGGGAATTCTAATACGACTCACTATAGGGAGAGGGAGAATAGTCAACAAATCGTTTGGGGCGACTTCTCCTTCCTTT
CTCCCTTCTCCC
+
944,9>CACBB@BBBBBBBB?CB;9111-
6??CE>BBB>B::<?:87771:?=?B49994:ABCB@CC@=A>A;==99AA=;?=AAAA<
@T6XXI:00026:00614
GGGAGAAGGGAGAAAGGAAGGAGAAGTCGCCCCAAACGATTTGTTGACTATTCTCCCCTCTCCCTATAGTGAGTCTA
TTCAGAATACCCC
+
BC@DCC@CE?BB>>8;::,1,../,.797799,11,111;?;;@>885864430000+033330------000---
67,4444243444,
@T6XXI:00026:00623
GGGGAATTCTAATACGACTCACTATAGGGAGAGGGAGAATAGCGGCCAACTTGCCCTAACTGCTATAATCTTCCTTC
TCCCTTCTCCC
+
;;<4;@B?B<<:<@@@CDC@@?B;=777.777>?<B@@>@CCA@583:47@768?8;>;@@C88::7;>70,0,0,.
/796;9;5;84
@T6XXI:00027:00601
GGGAGAAGGGAGAAAGGAAGCTAGTCGACTCGACATCTGTGGCTTCACAGTACAGAGGGACTGTCCAAAGCCG
+
DKDCBB@BC@A@?B@BAB>@@@@@>ACCAB@BBBBBBBCB@<8:8:CCBAA?@9=33317???@@=??;??<?
@T6XXI:00027:00630
GGGAGAAGGGAGAAAGGAAGGAGAACAACACGATCGGTTAAACGTAGCTATTCTCCCTCTCCCTATAGTGAGTCGTA
TTAGAATTCCCC
+
DEDAAA@BB>CBBB=B=@=@@C@?=AB@@AAHECCC?B?BC>CBB:;;BBC?AACB>CBBC@>@@@AAAA?@@@A??
?9<=C?C:===4
@T6XXI:00027:00649
GGGGAATTCTAATACGACTCACTATAGGGAGAGGGAGAATAGTCAACAAATCGTTTGGGGCGACTTCTCCTTCCTTT
CTCCCTTCTCCC
+
9;9/9<A>BCCCDBA@ABBB@AB>>611.6<<@BCC@@?AAAA==<=??7?A???8?;?5??AAB?BAA<?=?>@D?
A@@@;@>@@AC>
@T6XXI:00028:00604
GGGGAATTCTAATACGACTCACTATAGGGAGAGGAGAATAGTACGAAC
+
8880=1;8;@@=ABB??@@@?AABDFGE>CBCCEB<<8:/++,+++*+
@T6XXI:00028:00608
GGGAGAAGGGAGAAAGGAAGCTAGTCGACTCGACATCTGTGGCTTCACAGTACAGAGGGACTGTCCAAAGCCGCAGA
TCTCGAGTCGACTAGCTTCCTTTCTCCCTTCTCCC
+
@>8<11,/757/44-
42313>:,,******,02../:@<9:?=?=BCBCCCAA@<<<>7<;?>>?<??;?@>AAAAAA???===@?BB@??<
<<9<;=C7==?A;@<????;
@T6XXI:00028:00616
GGGGAATTCTAATACGACTCACTATAGGGAGAGGGAGAATAGTCGACAAATCGTTTGGGGCGACTTCTCCTTCCTTT
CTCCCTTCTCCC
+
999/;<B@CBB@BBBBBBBBA@B;9116.7>?CD>AAC=@:??AA=@<=;@BA??<A9<6AA?AC????=?<?>AC=
AAAA<A>A???<
@T6XXI:00028:00639
GGGAGAAGGGAGAAAGGAAGCTAGTCACTCGATATCGTGTTCACTA
+
CCACBB894-9988095858B???B?AAEC?@0.------233133
```

```
@T6XXI:00028:00646
GGGAGAAGGGAGAAAGGAAGCTAGTCACTCGACATCTGTGGCTTCACAGTACAGAGGGACTGTCAAAGCCAGCAGAT
CTCGAGTCGACTAG
+
CD<DAB?A@:@111,1-0,0>679?;87<<>9<:888AAC=?@AABBBAA?;;;99941,,****....)**---
3548;::<?A???CC@
@T6XXI:00029:00603
GGGAGAAGGGAGAAAGGAAGGGAACAGTCCTACACTCGACCTGCAGGCTATTCTCCCTCTCCCTATAGTGAGTCGTA
TTAGAATTCCCC
+
BA=BDCCCD?BA@@>B@C@BC>C@B>ACC@CBBBABBA??;?@=7858BCCACBHE>CCCBA:??@??CCBBAA?67
4;=<;8558=?5
@T6XXI:00029:00607
GGGAGAAGGGAGAAAGGAAGCTAGTCACTCGACATCTGTGGCTTCACAGTACAGAGGACTGTCCAAAGCCGCAGATC
TCGAGTCGACTAG
+
CB:;9969909999194843961199916146331117..,..,...19?979///,/6/44328;56=;>??@888
;9440000566<<
@T6XXI:00029:00622
GCTTGGGACA
+
-,,***)**,
@T6XXI:00029:00623
GGGGAATTCTAATACGACTCACTATAGGGAGAGGGAGAATAGTTCTCGCCGCACAAACGAACATTGTTCTTCCTTTC
TCCCTTCTCCC
+
=>>4>59288=8@>AAAAAAA?>==777.77=AB>???>==<?>A@???<7773=?:@AA>@@A>@@688>@?C=<?
===6::;448-
@T6XXI:00029:00629
GGGGAATTCTAATACGACTCACTATAGGAGAGGGAGAATAGCTACGTTTAACCGATCGTGTTGTTCTCCTTCCTTTC
TCCCTTCTCCC
+
444/4:CACCC@CCC@AABB@7633//,0::AAAA@@>@A??ABBBAA;?<?>@AA@??>@?BB=??<=>9;/44,4
4;?<A<A@A@7
@T6XXI:00029:00635
GGGAGAAGGGAGAAAGGAAGGAGAACGCGCACCCGAACCAACGGTGATCTATTCTCCCTCTCCCTATAGTGAGTCGT
ATTAGAATTCCCC
+
CECCCBBBB>BBAA>B@C@B@BB@>BBBBB@BB>CD@CCAAACACCBBBBBB?C;::5;BBBC>C?CBBBC@<;B@>
?@?B@@>C@CCC:
@T6XXI:00029:00638
GGGGAATTCTAATACGACTCA
+
BBB8A?@<>;?24,,-,,*00
@T6XXI:00030:00613
CTAGTCGACTCGAGATCTGCGGCTTTGGACAGTCCCTCTGTACTGTGAAGCCACAGATGTCGAGTCGACTAGCTTCC
TTTCTCCCTTCTCCC
+
A>9449444:A>ECCDC@@@C@AAA<A?>><=777.7>>@@CB@788>;@BACCGDCCAAACC;:<BAA>?7713?:
<B<::8A;@<?>>?:
@T6XXI:00030:00630
GGGAGAAGGGAGAAAGGAAGGAGAAGTCGCCCCAAACGATTTGTTGACTATTCTCCCTCTCCCTATAGTGAGTCGTA
TTAGAATTCCCC
+
BA?CCC=?=:@?88:@=?=@@CCC>AA>BBBB8@B>AAADD@BA?BBA===>A@AA<===@A=AA?AAAA<<<=A<=
=>AAA>@9;;;0
@T6XXI:00030:00636
```

```
GGGGAATTCTAATACGACTCACTATAGGGAGAGGAGAATAGTAC
+
9@@9@8BABBB?@@@C@@@CCA===661.7>>;:A8834,,,,,
@T6XXI:00030:00639
GGGGAATTCTAATACGACTCACTATAGGGAGAGGGAGAATAGTCAACAAATCGTTTGGGGCGACTTCTCCTTCCTTT
CTCCCTTCTCCC
+
CCD:D@B?ACCBC@@@BBBB?@BA@<<<8899>?>AAA=?;AC@?=8771:?;==-777-
7?BBB?AA@>@>@?AA:>?CA>A8;<CC<
@T6XXI:00031:00607
GGGAGAAGGGAGAAAGGAAGGAGAAGTCGCCCCAAACGATTTGTCGACTATTCTCCCTCTCCCTATAGTGAGTCGTA
TTAGAATTCCCC
+
CC@BBB@BC@BABB=B=@<@@B@?=AB<<<<<2<?9?EE@?9////5224=99<?=:434:;6<=>?@@@A@@@@@@
@=?::79/444-
@T6XXI:00031:00630
GGGAGAAGGGAGAAAGGAAGAATAGATTTTGGCGGAGTAGCAGTTGACTATTCTCCCTCTCCCTATAGTGAGTCGTA
TTAGAATTCCCC
+
BCABBB@B@:;444-
;>?>BCBCCBBBBC9B?BC@BBA?BBBAA?ACBBB@>=6694:AACE>C:78AACAA@@???@>AAA>C@CCC:
@T6XXI:00031:00632
GGGAGAAGGGAGAAAGGGAAGGGAAGAAG
+
CC>B9928909999111-1;==00)**)*
@T6XXI:00031:00635
GGGAGAAGGGAGAAAGGAAGCTATCGACCGACCATCTGTGGCCTTCA
+
CB:;9959904;8:8>>@-00./97=997677366;6611,-,.,.5
@T6XXI:00031:00644
GGGAGAAGGGAGAAAGGAAGCTAGTCACTCGACATCTGTGGCTTCACAGTACAGAGGGACTGTCCAAAGCCGCAGAT
CTCGAGTCGACTAG
+
EE@@@=6440999909485;D???C7::??=7:8???ABC?AA@BC=99@@==<<<=;<<==?:8984<A>><<<<<
<<<<99=;;9999=
@T6XXI:00032:00603
GGGAGAAGGGAGAAAGGAAGGAGAGCCCCGATTTGTAATCTCGAGAGCTATTCTCCCTCTCCCTATAGTGAGTCGTA
TTAGAATTCCCC
+
CE@CCDBDD>BBCB?B?>@B@CA@?@@?6?>?@>=?=?@?A?BB@C<<?A=><9899==AAC:A=66;;===88<<<
<;<@A?C:===4
@T6XXI:00032:00612
GGGAGAAGGGAGAAAGGAAGTAGTCGACTCGACATCTGTGGCTTCACAGTACAGAGGGACTGTCCAAAGCCGCAGAT
CTCGAGTCGACTAG
+
BC>BBB@CD@CCBB>B631880000.0:777;<;=BAABC@AA@AFDCBBCCBB9775:@===@:??:?B=BBBBBB
?AA<B@@=??ACAA
@T6XXI:00032:00614
GGGAGAAGGGAGAAAGGAAGAGAAGTCGCCCCAAACGATTTGTTGACTATTCTCCCTCTCCCTATAGTGAGTCGTAT
+
:B:A<98;B894>>8;::,0.4577::CADD9CC>CBBBC>CB@B@44442405;7<;?<=;=558;;../553010
@T6XXI:00032:00615
GGGGAATTCTAATACGACTCACTATAGGGAGAGGGAGAATAGTTCCTCCGTCTGAGGATCTATCTACTCCTTCCTTC
TCCCTTCTCCC
+
BA@4@6969@A>A@@<;<BDB@@>7777-7>===5:7?>AA@@>A=@?>A@889==:=7336===;;;;9999-/,-
.//+/7;==>7
```

```
@T6XXI:00032:00622
GGGAGAAGGGAGAAAGGAAGCTAGTCGACTCGACATCTGTGGCTTCACAGTACAGAGGGACTGTCCAAAGCCGCAGA
TCTCGAGTCGACTACG
+
99044989C9@99904239331972,,,,,,,,22221110/0166661668343888144<8=A>@C<AA??@?;=
00.798=??8340-.+
@T6XXI:00032:00624
GGGGAATTCTAATACGACTCACTATAGGGAGAGGAGAATAGTAGAA
+
>?>6>?B@CBB@BBBBBBA@??AC??BC>???B?@7555./+...)
@T6XXI:00032:00632
GGGAGAAGGGAGAAAGGAAGGAGAATAAATTGTCAACGCTGGTGCGGCTATTCTCCCTCTCCCTATAGTGAGTCGTA
TTAGAATTCCCC
+
EHBCCBACB@CCBB>B@B@B@BB@@??=;??@C@B?ABBCB=B;;;>@@BB????@>CCBBC>CB???AAA@@@@??
A==AA>C=@@@8
@T6XXI:00033:00602
GGGAGAAGGGAGAAAGGAAGGAGAATCGACACACGAACGCCTACGTGATCTATTCTCCCTCTCCCTATAGTGAGTCG
TATTAGAATTCCCC
+
AA>AA@?CC?@@@?<ADC@C?BBC@CC?>AABBA996;B??CCDDA@@CEHCC?BCBB?CCCBB?@>>BBCCBB@BC
BBB@BB=9=3666-
@T6XXI:00033:00620
GGGAGAAGGGAGAAAGGAAGGAGAAGTCGCCCCAAACGATTTGTTGACTATTCTCCCTCTCCCTATAGTGAGTCGTA
TTAGAATTCCCC
+
CFADCB@DD>@@?B==<A=@<@CC?BABBCCC9CC>BBCCE=AA@C??=AA>AAAA<@???C=@<?<<<=<<<9==@
@>A@;;:56661
@T6XXI:00033:00622
GGGAGAAGGGAGAAAGGAAGTGCAGTTCGCGGTTAGTGAAGGGAAAAACTATTCTCCCTCTCCCTATAGTGAGTCGT
ATTAGAACTTCCCC
+
@A<ABB@DC@CBBC?B@BACABBBBB@BBAC?A=?>B?A>A?:?AAB2BCCB@BCCC>AA:::58>@?@A==>=<;=
<;8<;?8<<<?AA4
@T6XXI:00033:00626
GGGAGAAGGGAGAAAGGAAGGAATAGAAATCCGGCGCAGACCGCACGGCTATTCTCCCTCTCCCTATAGTGAGTCGT
ATTAGAATTCCCC
+
@A<?A?5999999@8<:;AC?ABBCBBB>BC@C?BB?CCCC=AAAAC@C@@@=AA==48>AAA<@@@@?@@A@@@@@
@@>@AA>A>@@@5
@T6XXI:00034:00607
CCTCGAGATCTGCGGCTTTGGACAGTCCCTCTGTACTGTGAAGCCACAGGATGTCG
+
1,,,,,,,8648895988-824444444-49///8///8692//566/.,.664..
@T6XXI:00034:00610
GGGGAATTCTAATACGACTCACTATAGGGAGAGGGAGAATAGTCAACAAATCGTTTGGGGCGACTTCTCCTTCCTTC
TCCCTTCTCCC
+
GBB9C@C?BFCBCCAAACCCC?BA@==9869:AA>AAA>@???>;9444-7717?=A@@8=????;?8:7778,/,-
222444<8;=<
@T6XXI:00034:00617
TTGGACAGTCCCTCTGTACTGTGAAGCCACAGATGTCGAGTCGACTAGCTTCCTTTCTCCCTTCTCCC
+
EAD?BCCCBCB@BDCBA@@@A>>>@C?A>>BB?????B?BBBBCCBCABB?A?ACACCCC=B?BBCC>
@T6XXI:00034:00619
CTATCACTCGAGATCTGCGGCTTTGCAGTCCCTCTGTACTGTGAAGCCACAGATGTCGAGTCGACTAGCTTCCTTTC
TCCCTTCTCC
```

```
+
C@@98///8///11CABCE<<:E=@?>>77;6:BA@AAA@<<<@<?BABBCC@@?ABBBBBBCB@;:97177016-
:8;@:;76**)
@T6XXI:00035:00605
GGGAGAAGGGAGAAAGGAAGGAGAAGTCGCCCCAAACGATTTGTTGACTATTCTCCCTCTCCCTATAGTGAGTCGTA
TTAGAATTCCCC
+
DCBBBB@BB>AAAB>CBB>>>?B@>?;<BBEC8?>;ACCC?;?;B?=6::;<<<<<;=<@<<;<=@@A@@@@AA@==
=>AAB=@?AAA6
@T6XXI:00035:00632
GGGAGAAGGGAGAAAGGAAGAGGATTGTAATGATGCATTAGGGAGTAGCTATTCTCCCCTCCTCCATAT
+
JE?ABB?BC@CCCC>C@C@BBA>AB?BBB?BBC?>B?>:666-7>;ACAAABBA@@@?176415*++**
@T6XXI:00035:00634
GGGGAATTCTAATACGACTCACTATAGGGAGAGGGAGAATAGTTTTCCCTTCACTAACCGCGAACTGCACTTCCTTT
CTCCCTTCTCCC
+
999/429=BBB?BCCBBBBB<=>?976956==?A>AAC?@<@A@@5@@<@?BCCCB?B?BBAA>A@?>?7747477.
7999499:430+
@T6XXI:00035:00639
GGGAGAAGGGAGAAAGGAAGCTAGCGACTCGACATCTGTGGCTTCACAGTACAGAGGGACTGTCCAAAGCCGCAGAT
CTCGAGTCGACTAG
+
EB>CCB?BC@AAAC>B?B@BBB?@A@:77>70/0010=>E=?@?BFCAAACAA;;;;6;9:;997994:;;=?@@@@
A@@AAAA===?@@@
@T6XXI:00035:00644
GGGAGAAGGGAGAAAGGAAGAACAGCATCGTCGAGGTAGAGACATTAGCTCATTCTCCCTCTCCCTTCTCCC
+
BB<AAB@CH@CCBB?B@B5784887>>>>>61144244448??=;:91000.8,/444-4@988,37777?;
@T6XXI:00036:00607
GGGAGAAGGGAGAAAGGAAGCTAGTCGACTCGACATCTGTGGCTTCACAGTACAGAGGGACTGTCCAAAGCCGCAGA
TCTC
+
CCABAA?BB>ABAC>C@I8<<BBBA@@@@CCA==?BAABBA>?B>ACCA@@<==>===:<=====;==;<<:????A
A@@?
@T6XXI:00036:00624
GGGAGAAGGGAGAAAGGAAGCTAGTCACTCGACATCTGTGGCTTCACAGTACAGAGGGACTGTCCAAAGCCGCAGAC
TCTCGAGTCGACTAG
+
9?0944244-444;-
423777;>6;>111106438<A9447.0,.77;<BA????@?7?@@:98@>>9987/,+0++.34443300000034
@T6XXI:00037:00600
GGGAGAAGGGAGAAAGGAAGCTAGTCACTCGACATCTGTGGCTTCACAGTACAGAGGGACTGTCCAAAGCCGCAGAT
CTCGAGTCGACTAG
+
BCACBB@BB;;444-
95848D:7;B????B?::8::7BBE@BB@BB>>99>===89948;<=AA;<=;=A?A?:<<<>=<;<?A<::>A==
@T6XXI:00037:00604
GGGAGAAGGGAGAAAGGAAGGAGAACATGTTGTGGGACAAAGCCAAAACTATTCTCCCTCTCCCTATAGTGAGTCGT
ATTAGAACTTCCCC
+
991999<@?5>8>@<?<@@C>;;:7<<CDC@CBBA:?>>?0873777,6@AC@BBAC>@@@CC<??A:::@?>;444
4:7:::244/444+
@T6XXI:00037:00607
GGGGAATTCTAATACGACTCACTATAGGAGAGGGAGAATAGTCAACAAATCGTTTGGGGCGACTTCTCCTTCCTTTC
TCCCTTCTCCC
+
```

```
9=?4><?@A@ACCCCBAABCD=;:000,.79@79<==;=@AB=:2444-
77;;E>DDCABAA:;67;;7:3434;4:330+35==AB;
@T6XXI:00037:00616
GGGAGAAGGGAGAAAGGAAGTGCAGTTCGCGGTTAGTGAAGGGAAAAACTATTCTCCCTCTCCCTATAGTGAGTCGT
ATTAGAATTCCCC
+
=AAACA?CC9;;=><@<@=A799BBA=>>884310=...,..+....*.379>AA@@;@B>BB>ACA===A@@@===
@A>A@C>;;>@@4
@T6XXI:00037:00647
GGGAGAAGGGAGAAAGGAAGAGAAG
+
HEBCBB=@=9>911,1-1,0..,*,
@T6XXI:00038:00625
CTCACTATAGGGAGAGGGAGAATAGTCAACAAATCGTTTGGGGCGACTTCTCCTTCCTTCTCCCTTCTCC
+
@AA@>:>9999098888,36;36?<AAB@B@?838444,334+38<<<;???;477,/,+055+,+,+-2
@T6XXI:00038:00631
GGGGAATTCTAATACGACTCACTATAGGGAGAGGGAGAATAGCCTTTCGTTTTCCGTTTCTGCGCGTTTCTTCCTTT
CTCCCTTCTCCC
+
CCC9B?B@DBB@BBBBABBA@AC77111.7=<BC@B@@@A<<<5?A<ABCDD9@=@AC<@?@A@=@<?;?=<=<==;
<:8836:<=>>;
@T6XXI:00038:00634
GGGGAATTCTAATACGACTCACTATAGGAGAGGGAGAATAGCGCACCATCAATTTCGTCGTGCCGTTCTCCTTCCTT
TCTCCCTTCTCCC
+
FBB7A8;8;AA?BBABBCABABA;?964:>?@A>@@@<??A@=<@B@@@@A@AA=CA=A@@@<;@C??==9=>B?BA
>BBBA=B>???@<
@T6XXI:00038:00644
GGGGAATTCTAATACGACTCACTATAGGGAGAGGGAGAATAGATCACCGTTGGTTCGGGTGCGCGTTCTCCTTCCTT
TCTCCCTTCTCCC
+
?>>,959=BDNACB@@????>AB>9661.6==ABBC@A>@<====A?>AC=?;?=@@@<B:;;CCC@A??=??A@CL
E@@@A<A?@@@B<
@T6XXI:00039:00616
GGGAGAAGGGAGAAAGGAAGACAGTCCATCTGTGGCTTCACAGTACAGAGGGACTGTCCAAAGCCGCAGATCTCGAG
TCGACTAG
+
CCBDDC@CC>??>C@B@B=@@B@?9:8;@AACCC>??>???;;9<<=;;<<;<<==A=<=A<A=;=<<<=;;98<@@
ACCCCB<B
@T6XXI:00039:00632
GGGGAATTCTAATACGACTCACTATAGGAGAGGGAGAATAGCTGATCCCGGTCCCTGCTATAGCGTTACTTCCTTTC
TCCCTTCTCCC
+
CBA4@5464489?:<<B@@@B><<<00,0;;?B>B@A?A???CCC?@>-
42444,4:>@ABBBBAB?ACC?BAAC>;;;8665////,
@T6XXI:00039:00643
GGGGAATTCTAATACGACTCACTATAGGAGAGGGAGAATAGCTTGGTTTTGTCCCGCCTCGCACTGTTCTTCCTTTC
TCCCTTCTCCC
+
==94?596<ABCCCB@????>:977//-
0::>C@DA@?AA=<?=>=?CC8@9884<?>@@AA=9=<<;=@?C;=99=AAA<@?AAA=;
@T6XXI:00040:00603
GGGAGAAGGGAGAAAGGAAGAAGAGTGAGTCATAATGCAGGGAAGAACTATTCTCCCTCTCCCTATAGTGAGTCGTA
TTACGAATTTCCCC
+
```

8A8119599-8448-
4237799<<::;BEE@DBB@AB:::D@D@CC:<;<;>A=BC?::977,3<1:6:>?BB@:;;;900/:,00,111,
@T6XXI:00040:00614
GGGAGAAGGGAGAAAGGAAGCTAGTCACTACATCTGCGGCTTTGGACAGTCCCTCTGTACTGTGAAGCCACAGATGC
GAGTCGACTA
+
>=0949888-
449914977;CB@@=;,,,,,,2,..7?<9<>572670;35;5599734>???@@>@@>@?===8878300350004
@T6XXI:00040:00615
GGGAGAAGGGAGAAAGGAAGCTAGTCGACTCGACATCTGTGGCTTCACAGTACAGAGGGACTGTCCAAAGCCGCAGA
TCTCGAGTCGAC
+
CDDECB@CC@AA@C=B?B?BC?/11/10>??>@??A@@@?;8;?;?BBABB@;88443-
4;9880;>;9>;.//;;:@888<;9:<::<
@T6XXI:00040:00630
GGGAGAAGGGAGAAAGGAAGGAGAAGTCGCCCCAAACGATTTGTTGACTATTCTCCCTCTCCCTATAGTGAGTCGTA
TTAGAATTCCCC
+
BCABBBBCB>?@>?=B@C@C@BB@@@@>7777-
:@;BDCCC<B>@CA::==<<<:::?@A@C>=::88<==<98<@AA>A@@;;3444+
@T6XXI:00041:00628
GGGAGAAGGGAGAAAGGAAGGAGAAGTCGCCCCAAACGATTGTTGACTAT
+
?::BBB>BC>>?@A195848133601173333,397778801.444/.-3
@T6XXI:00042:00612
GGGAGAAGGGAGAAAGGAAGCTAGTCACTCGAGATCTGCGGCTTTGGACAGTCCCTCTGTACTGTGAAGCCACAGAT
GTCGAGTCGACTAGCTTCCTTTCTCCCTTCTCCC
+
C@6<:BACD?@999047>;>A?=?AA:::B@B==@BBBCC@@@?;?=::;BBCDA@????A@@@?>>:>=;>????=
==@AA@A@@===A==<>@=?@:?=@@7>478AA:
@T6XXI:00042:00627
GGGGAATTCTAATACGACTCACTATAGGAGAGGGAGAATAGTTTTTCCCCTCACTAACCGCGAACTGCGCTTCCTTC
TCCCTTCTCCC
+
999292959@@@CCCCBCCCCBA@@:747=>>>:@;;9=@@@CCA0777+48<@7:5:<??>@=@???;:89;,/,/
6660:7:344-
@T6XXI:00042:00641
GGGGAATTCTAATACGACTCACTATAGGGAGAGGGAGAATAGTCAACAAATCGTTTGGGGCGACTTCTCCTTCCTTT
CTCCCTTCTCCC
+
BBC:C@B?BCC@CBAAB>??B?>97116.7==BCAC@@=@@?CBB;;;?<BCAA?8?865?:::CCCB@=@?A?AA<
A@A<;A;==@A=
@T6XXI:00043:00618
GGGAGAAGGGAGAAAGGAAGCTAGTCACTCGACATCTGTGGCTTCACAGTACAGAGGGACTGTCCAAAGCCGCAGAT
CTCGAGTCGACTACGCTTCCTTCTCCCTTCTCCC
+
B@:=?B?A@:>999194377AA@677,,,,+,,+003111013111116874440030+00004628;48456???AA
AB=<=@@AA::;...5555...,9772401000+
@T6XXI:00043:00633
CGAGATCTGCGGCTTTGGACAGTCCCTCTGTACTGTGAAGCCACAGATGTCGAGTCGACTAGCTTCCTTC
+
FCB?@@A:;;B>A>C@B@BCC88:D>@7:100//448556=;???@?A@8<;999>999944//,,*,*,
@T6XXI:00044:00605
GGGGAATTCTAATACGACTCACTATAGGGAGAGGAGAATAGTAGACAAATCGATTTGGGAGCGGTAATTCC
+
C>>6>;>=ABB@CCBA?>>>@ACCB?AB=??BCAC7737444;000++*,,,,,2022,///9216>?974
@T6XXI:00044:00625

```
GGGGAATTCTAATACGACTCACTATAGGAGAGGGAGAATAGCATGCAATTACTTGCCGTCTGTCCTCCTTCCTTTCT
CCCTTCTCCC
+
999/48>;9;<@BBBBCCCB?:977//-
0>>BL>BBB?A=?<<@@AA>C?BA@?7738;;>>==;<<8<=@;==49444-4=:==@;
@T6XXI:00044:00645
GGGAGAAGGGAGAAAGGAAGGAGACAGTGAGCGCCGCACAACACGTCACTATTCTCCCTCTCCCTATAGTGAGTCGT
ATTAGAATTCCCC
+
BC@CCB@BB=?AAC?C@C@C?BBBB@@BBCCDCC@CCCB@@@@A@@@BBBB@<=877,8<88807@F@CCB@@@B@?
?@=?=?<>4888.
@T6XXI:00045:00614
GGGGAATTCTAATACGACTCACTATAGGGAGAGGGAGAATAGCACCAACTA
+
@@@6@>A>BBB@DCCCCBBA@?977167.7>>?B?CAA>;;;///,/7-+*
@T6XXI:00046:00607
CTAGTCGACTCGAGATCTGCGGCTTTGGACAGTCCCTCTGTACTGTGAAGCCACAGATGGACTGTCTTCCTTTCTCC
CTTCTCCC
+
CCCCBBBCCCCCCBBBABBAA>ABB>C?CCEEAAA=ACCA@?B<=A@@>@C?BBCCEBD?ABA@@@@7;7:C<@AAB
=F@B@@C<
@T6XXI:00047:00604
GGGGAATTCTAATACGACTCACTATAGGAGAGGGAGAATAGTCAACAAATCGTTTGGGGCGACTTCTCCTTCCTTTC
TCCCTTCTCCC
+
CEC8B798<>@<AB@@?B>>0,-
77,,+.47;8;<;??A===@@=???:BBBBB>BCC9CAAAC>?@?3837<?CA@@A@<C6:8AA=
@T6XXI:00047:00605
GGGAGAAGGGAGAAAGGAAGGAGAAGTCCGCCCCAAACGATTTGTTGACTATTCTCCCTCTCCCTATAGTGAGTCGT
ATTAGAATTCCCC
+
HD>CDC?BB>BCBB>B?A@CBB;6317817733,11,166;C?CC?C@==A@>@?>?:?888A<A@@@A@@@A@@@=
==>AA?44/444-
@T6XXI:00047:00607
GGGGAATTCTAATACGACTCACTATAGGAGAGGGAGAATAGCGCACCATCAATTTTGTCGTGCCGTTCTCCTTCCTT
TCTCCCTTCTCCC
+
ACC:CAB@BFC?BBBCCCCCB?<?A6137==>ACA@AAB@@???B@>???B?@CE9;;:BBBC?CC@;:8<?>@>@A
<AAAA<A<???A=
@T6XXI:00047:00632
GGGGAATTCTAATACGACTCACTATAGGGAGAGGGAGAATAGATCTGAACTCCAAGTTCCGCTATTTTCCTTCCTTT
CTCCCTTCTCCC
+
9;9499A?GCCCCBCCECCBBA@=;677.7==BC>A??>??9:9AAECBC<<9<=@@>B?BCGCCCB4;6737361,
111769244=;;
@T6XXI:00047:00646
GGGGAATTCTAATACGACTCACTATAGGAGAGGGAGAATAGTCCATTCCTATCCCGATTAGCTATTCTCTTCCTTTC
TCCCTTCTCCC
+
DCE9C?B@BBBBCA@>BBB?B:993//-
0>;BE>B??=A==?<;<<:<;<==<=;=<?>A99?CA>A;@A>A>??:?=;=7=/78==7
@T6XXI:00047:00647
GGGGAATTCAATACGACTCACTATAGGAGAGGGAGAATAGTGACGTGTTGTGCGCGCTCACTGC
+
CCC:K<<;999944/4..,0,,,...,..111,104246;;844A5967//+++1234111--
@T6XXI:00048:00604
```

```
GGGAGAAGGGAGAAAGGAAGGAGAAGTCGCCCCAAACGATTTGTTGACTATTCTCCCTCTCCCTATAGTGAGTCGTA
TTAGAATTCCCC
+
CDACDC@CB>BBBB?BAB?A?ABC@@?BBBEC8@@>@BBCB;@@>@?<<==;<<>>;><<<=;===;<<<=A==@AA
A>@AA>A9<<<4
@T6XXI:00048:00626
GGGGAATTCTAATACGACTCACTATAGGGAGAGGGGA
+
944,98?<ABB?CCBABBBBCFDC:::>6==0,,,**
@T6XXI:00048:00633
GGGGAATTCTAATACGACTCACTATAGGGAGAGGGAGAATAGTTACCTTCTCGCGTATGTTGGCATTTTCTTCCTTT
CTCCCTTCTCCC
+
@>>4>697<9;@CCCABBBB?C?7=666.7AAAB>D@?=?8::7//./577B9AABD=<<9=:==ACC4@A=<3885
8::;6;=0//:9
@T6XXI:00048:00636
GGGAGAAGGGAGAAAGGAAGAGTAGCCGGGGACCAGGAAAAGTTTGGCTATTCTCCCTCTCCCTATAGTGAGTCGTA
TTAGAACC
+
BB>BCC@CC@BBBB>B@B@BBB@CBB>AAA9AB<=74333-
8>><A=AACD@CAA?>ACCCC<BBBBB>?CBCBBBBBC@A=;A?
@T6XXI:00048:00639
GGGGAATTCTAATACGACTCACTATAGGAGAGGGAGAATAGTTTTTTCCCTTCA
+
=A>6=5924442449@@@@@AC@??AA;>@????9?;:79;000000(00+0234
@T6XXI:00048:00643
GGGGAATTCTAATACGACTCACTATAGGGAGGGAGGAAGAATCGCTCTCTCATTCCCGACCGAACATCCTCTTCCTT
TCTCCCTTCTCCC
+
BBA9B>A?BHBAB???BBBBBAB>9111-
.77000)*)(((((***2534<@B>BB<A=:966,/.//,//39B56=7<??A:?<?@@@;
@T6XXI:00048:00645
GGGAGAAGGGAGAAAGGAAGCTAGTCGATCGACATCTGTGGCTTCACAGTACAGAGGACTGTCCAAAGCCGCAGATC
TCGAGTCGACTACG
+
AB<@@@<?@:@>>><A?B@CNCCA=;:00.7//.75988888?=?BCCDBB?==7738==;=@9<<7<=;@===AA@
@@????87/9;///
@T6XXI:00049:00613
GGGAGAAGGGAGAAAGGAAGTCAATACATGTGATGCAAATGTGGGAGCTATTCTCCCTCTCCCTATAGTGAGTCGTA
TTAGAATTCCCC
+
BD?CCC?BB=?@@B=B?B?BBCB@CC@@@CCAAABBBB>B;;;EABB@=<?=????;=@BAB=@@@???@?=<=??A
A>99:>HFCEC:
@T6XXI:00049:00638
GGGAGAAGGGAGAAAGGAAGGAGAAGTCGCCCCAAACGATTTGTTGACTATTCTCCCTCTCCCTATAGTGAGTCGTA
TTAGAATTCCCCC
+
DD@CBB?BA<ABBB=BBC@B??A@@A@BBBBB8CC>BDFCC=AB@B@@=CC@A@AA=A??<=<@9==AAA@?@===@
><;8837;:;;;.
@T6XXI:00049:00646
GGGGAATTCTAATACGACTCACTATAGGAGAGGGAGAATAGTCAACAAATCGTTTGGGGCGACTTCTCCTTCCTTTC
TCCCTTCCTCCC
+
BB@4@5959489B@@AAA@A?:::;00,///11,1668:?A?7838<:4=>>>>.777-
767BB8:76562424=;=47433,.,---+
@T6XXI:00004:00653
GGGAGAAGGGAGAAAGGAAGCTAGTCACTCGACATCTGTGGCTTCACAGTACAGAAGGGACT
```

```
+
EC>CCB@CC?BCBB?B>A@BCB977,,,,,,0036:CC@@@CB???==7774444/342---
@T6XXI:00005:00652
GGGAGAAGGGAGAAAGGAAGGAGAAGTCGCCCCCAAACGATTTGTTGACTATTCTCCCTCTCCCTATAGTGAGTCGT
ATTAGAATTC
+
CD@?A?@CC>BCBB>B?A>;;<775:BBACCCC2BC>CCCC@9??A?=B?AB>==<<;<AA<B=AA@???A:<<<@?
??><<=<@>=
@T6XXI:00005:00668
GGGAGAAGGGAGAAAGGAAGGAGAAGTCGCCCCAAACGATTTGTTGACTATTCTCCCCTCCTCCTAGATG
+
CC@BBC>@A=BBBB>B=?>@@CBB?;;??BBB6>><<@@?>999>>9=?A=<<<<<<4===874//+*((
@T6XXI:00005:00676
GGGGAATTCTAATACGACTCACTATAGGGAGAGGGAGAATAGTCCGTTC
+
?@@6@<@=@B@>BBBBCCCCB@CD><::8:?B?C>B==4900+,+**)*
@T6XXI:00006:00672
GGGGAATTCTAATACGACTCACTATAGGAGAGGGAGAATAGTCAACAAATCGTTTGGGGCGACTTCTCCTTCCTTTC
TCCCTTCTCCC
+
999/4295<AB@CBBBA@@@B;;:;//,0@:==9?==;>???=?>8881:AAAB9???5??@@A=AA?9<9=>AA<A
AAA<A8:8BB=
@T6XXI:00006:00681
GGGGAATTCTAATACGACTCACTATAGGGAGAGGGAGAATAGCCCAAGCATCACGTGCACCGTTGTTCTCCTTCCTT
CTCCCTTCTCC
+
@B@4@295444888=;;<B<?6331111,17111,144449003+09;3//.....33103397706653333,-
,--134=<=882
@T6XXI:00006:00682
GGGAGAAGGGAGAAAGGAAGTGCAGTTCGCGGTTAGTGAAGGGAAAACTATTCTCCCTACTC
+
49144424:58488-42313339BB;;>1/-+-*-,,,.77::::75;73.33...+.....
@T6XXI:00007:00659
GGGAGAAGGGAGAAAGGAAGAGA
+
CF?BBC@CB?BB888820)****
@T6XXI:00007:00689
GGGAGAAGGGAGAAAGGAAGAACTTGGGGAGTGGCACTGGTAACGCGCTATTCTCCCTCTCCCTATAGTGAGTCGTA
TTAGAATTCCCC
+
NHB@A?>??=BBDC@C@B@BB@C@?@@@7?<=C?CCB>>>>>>AABBBABB@BCCC@@@@CCBCCC>B=C@CCBCBB
B?CCC?CABCC:
@T6XXI:00008:00654
CTAGTCGACTCGAGATCTGCGGCTTTGGACAGTCCCTCTGTACTGTGAAGCCACAGATGTCGAGTCGACTAGCTTCC
TTTCTCCCTTCTCCC
+
@@1///////8:449@@;99?=378,39999?<??=@CDDC???===A>@D=AFCCCBAABBCEB9;<CCDCBB@C@
GC>CCCA<A>@@@@:
@T6XXI:00008:00663
GGGGAATTCTAATACGACTCACTATAGGGAGAGGGAGAATAGTCTTCCAATTTTGGGCGGTACTGTCTCCTTCCTTT
CTCCCTTCTCCC
+
;;9/9696<AA=@ABBCCCC?@CB?77757ABEC=B?A=@<<=8699<C;?<:589:=B?BBCCCCBBC895:=?@:
BAAA>B@DC@@<
@T6XXI:00008:00666
GGGAGAAGGGAGAAAGGAAGCTAGTCACTCGAGATCTGCGGCTTTGGACAGTCCCTCTGTACTGTGAAGCCACAGAT
GTCGAGTCGACTAGCTCCTTTCTCCCTTCTCC
```

```
+
CC>ABC?A@:A99909484;BBBBDCBA@?::4796?AAC@CCD@D@;::B??=7488<<<AA?@A@=@@;==ACBA
===???=???::;://-,--+----+-0000/
@T6XXI:00008:00669
GGGGAGAAGGGAGAAAGGAAGGAGAAGTCGCCCCAAA
+
///*///,//+/44;;B@A@:;=;;9/.,,222022*
@T6XXI:00008:00670
GGGAGAAGGGAGAAAGGAAGGGGAGAAGGGAGAAAGGAAGCTAGTCGACTCGAGATCTGCGGCTTTGGACCAGTCCC
TCTGTACTGTCGAAGCCACAGATGTCGAGTCGACTAGCTTCCTT
+
CC@CCB@CC>BBBDAC@C@CCB8?78477.7777.1010178;1::66?<=@@AA@@@@@A600;064//,-----
+-2448566683344-/-..44347<?B@:777;4A?38,**)*)
@T6XXI:00008:00671
GGGAGAAGGGAGAAAGGAAGGAGAAGTCGCCCCAAACGATTTGTTGACTATTCTCCCTCTCCCTATAGTGAGTCGTA
TTAGAATTCCCC
+
BB>BBC@ED@BB?B=B?<>?=??B@@>>>@CC9BB?AAAAA=AB?CB@A?<:?=CC>C??<<;@???@@BBA????A
A=ABC@D<???5
@T6XXI:00008:00685
CTAGTCGACTCGAGATCTGCGGCTTTGGACAGTCCCTCTGTACTGTGAAGCCACAGATGTCGGGTCGACTAGCTTCC
TTTCTCCCTTCTCCC
+
B=111;;ADCCCC??@CCBBB@BB?>>?@B>ABBC>GBBB@@@AA@@A<?BACBBBBBCBBBC>B;<;AAC::7>?;
<=9=ADC<@>AABA<
@T6XXI:00008:00698
GGGGAATTCTAATACGACTCACTATAGGAGAGGGAGAATAGTTTGGCTTTGTCCCACAACATGTTCTCCTTCCTTTC
TCCCTTCCTCCC
+
6<?1?-1,//67688=>??>7636611011117;656568<<;99/300+1111,1>>>9<<4434;;24/4244-
4444,0/0/0;;:
@T6XXI:00009:00673
GGGGAATTCTAATACGACTCACTATAGGGAGAGGGAGAATAGTCAACAAATCGTTTGGGGCGACTTCTCCTTCCTTT
CTCCCTTCTCCC
+
=6:1<-1,/78:><;;BBAAA?>=>777.7>>BG>CB?>=7//0;979;6????B<BBB7@B@::47=92424244-
4444-8=@DAA<
@T6XXI:00009:00685
GGGAGAAGGGAGAAAGGAAGAAGAACAGTACGCCATTGATTGACGAACTATTCTCCCTCTCCCTATAGTGAGTCGTA
TTAGAATTCCCC
+
EC@B@@@BBB??=B=B@>@DD@?@?BBB??ABA>BDACCC?AAABB@BAAB>@==<8?CAAA<ACCA?:;CBBBBBB
@>AAA?B@BAA1
@T6XXI:00009:00688
GGGGAATCTAATTACGACGTCACTATAGGGAGAGGGAGAATAGCCGACAATTCGACTCCCCGGCAATGTTCTTCCTT
TCTCCCTTCTCC
+
8BA1@+1///,/,///..6999@CB@@CB6:;B>>:>:747?7748>=;;B=;;;@>>>>.74::>;;<;9999<==
;<5441047./,
@T6XXI:00009:00697
CTATCGACTCGAGATCTGCGGCTTTGGACAGTCCCTCTGTACTGTGAAGCCACAGATGTCAGTCGACTAGCTTCCTT
TCTCCCTTCTCCC
+
ECCC:::CCCCABCCBCC??=@DC>C@CCBBBCC<AAA??AA?<AAA>?B?BBAAC:;;CCBACB@@@B><<=A>AA
>A?=?7>598AC;
@T6XXI:00009:00699
```

```
GGGGAATTCTAATACGACTCACTATAGGGAGAGGGAGAATAGACAGCCACATTGTCAAACTGTCCTTTTCTTCCTTT
CTCCCTTCTCCC
+
<;949<A>BBBACBB?CBBB?A;77111.7>>BC>CAA?A:<===658777=;=?<<<7=?@CCACDD9??=?@AB<
@@AA<A;==AC>
@T6XXI:00010:00677
GGGGAATTCTAATACGACTCACTATAGGAGAGGGAGAATAGTCAACAAATCGTTTGGGGCGACTTCTCCTTCCTTTC
TCCCTTCTCCC
+
999/9<>=BCC@CBA@ABBBB:964..,0::?@>BAA>A@@@AA>@@@<B@B?C>CCC6??BBB@??=68378;;7=
A@A;@;==DD;
@T6XXI:00010:00678
GGGGAATTCTAATACGACTCACTATAGGAGAGGGAGAATAGTCAACAAATCGTTTGGGGCGACTTCTCCTTCCTTTC
TCCCTTCTCCC
+
@@@6@>A>BBB@CBBBBBB?@:977//,0::>A=BAB>AA@@@@>AAA<CBBBC>CCC9CCBBB?CBA<?<?>AA<=
ABB;?=@?CC;
@T6XXI:00011:00667
GGGGAGAAGGGAAGAAA
+
444,4//,//+/,,000
@T6XXI:00011:00688
GGGAGAAGGGAGAAAGGAAGCTAGTCGACTCGACATCTGTGGCTTCACAGTACAGAGGGACTGTCCAAAGCCGCAGA
TCTCGAGTCGACTAG
+
CC>A::2480999909585;F?;;B/11>><<07::96??@=@B?BBCC>;<<==<==;<=<===<==;@A>A@<@@
@:>=AAAA?AAAA??
@T6XXI:00012:00655
GGGAGAAGGGAGAAAGG
+
99699424=-4///+/,
@T6XXI:00012:00669
GGGGAATTCTAATACGACTCACTATAGGGAGAGGGAGAATAGCCTGTCGCTTATCTGACTTGTTGTTCTCTTCCTTT
CTCCCTTCTCCC
+
@@=6><>=@BB@BBBBBBBCCCCBA111.6<;AC>BAC?A=?;:>>CC?=?:;=<??@BB?CCACA?A@AA>A>@@<
@@@@<A>AAA@<
@T6XXI:00012:00674
GGGGAATTCTAATACGACTCACTATAGGGAGAGGAGAATA
+
111+1,96<BA@CBBAACCB?@B@@776-1=113744344
@T6XXI:00012:00677
GGGAGAAGGGAGAAAGGGAGA
+
CE?@?@@A=981--+--*,,,
@T6XXI:00012:00679
GGGGAATTCTAATACGACTCACTATAGGAGAGGGAGAATAGTCAACCAAATCGTTTGG
+
444,424248898>>;:0..434461101177<6;46:::9766-/--3351616,11
@T6XXI:00012:00698
GGGAGAAGGGAGAAAGGAAGGAGAATGCACCGCAGGAAATTGCAAGACTATTCTCCCTCTCCCTATAGTGAGTCGTA
TTAAGAACTTCCCC
+
9@<??B@ECBDDBB=B8;9;?BBB?BB;;;@CEFK@CC>D=???:????>>:=744-49000+0=4==?984<<0--
3,-,-33337<::4
@T6XXI:00013:00668
```

```
GGGGAATTCTAATACGACTCACTATAGGGAGAGGGAGAATAGCATTATCTCCTGTACATTGCGCGTTGCCTTCCTTT
CTCCCTTCTCCC
+
999,9>A@CBB@CBBBBBBBB@;76111.777??>B@A>@AAA;;::::?<<::@=A?:<?A==AA=BB=B=B?AA=
AAA=;=;;@:=;
@T6XXI:00013:00678
GGGAGAAGGGAGAAAGGAAGGAGAAGTCGCCCCAAACGATTTGTTGACTATTCTCCCTCTCCCTATAGT
+
;A1999599-84>@<@<A@F<<>E?:788888-89666116,197668333544133----.+---0--
@T6XXI:00013:00679
GGGAGAAGGGAGAAAGGAAGGAGAAGTCGCCCCAAACGATTTGTTGACTATTCTCCCTCTCCCTATAGTGAGTCGTA
TTAGAATTCCCC
+
CB>CAA=@B<@@@@<@>?>A?;;;>>?@8888-
8801717747;66193=>;=@?@7<<<<?;@AADAA<<=C?=<883333:=:@@A5
@T6XXI:00013:00680
GGGAGAAGGGAGAAAGGAAGGAGAATGCACCGCAGGAAATGCAAGACTATTCTCCCTCTCCTATAGT
+
?C@CCCACC>BBCH?C?A?B@CCC@B:;;;374,,*,2*,,6667???6707740+0300/0000--
@T6XXI:00013:00681
CTAGTCGACTCGAGATCTGCGGCTTTGGACAGTCCCTCTGTACTGTGAAGCCACAGATGTCGAGTCGACTAGCTTCC
TTTCTCCTCTCC
+
A9999944:?@BCCCBB@=::8;CC>C>AADCBBB>BCCCBA<<@AAA>=@?ACCCCBCBBCAAABBBBC@A@B?B>
A@:@742/,)((
@T6XXI:00013:00682
GGGAGAAGGGAGAAAGGAAGGGGAGAAGGGAGAAAGGAAGGGGAGAAGGGAGAAAGGAAGCTAGTCACTCGACATCT
GTGGCTTCACAGTACAGAGGGACTGTCCAAAGCCGCAGATCTCGAGTCGACTAG
+
@@>ABC@BCA@>?B=B>;<>>?7ACC@@A;8:8@9?=?47:7-
7>?:==9;730+88:7;@:9:=;11688==>@AAA@@===:=@AAAAAA>>===<<:??AD@@@;@A>A===?A@@A
889CCCCBBBB
@T6XXI:00013:00691
GGGGAATTCTAATACGACTCACTATAGGGAGAGGGAGAATAGTTTTATTCTCACCGCCAACCCTCTAGTCTTCCTTT
CTCCCTTCTCCC
+
CCC:C99;<CCACCBBBBBB@CB==66659BABC<A?@?AAAAAA1>>:;:===;A>:?<??:@CCCCC??:?;>>:
ABCCA@>AAAA<
@T6XXI:00014:00652
GGGAGAAGGGAGAAAGGAAGCTAGTCGACTCGACATCTGTGGCTTCACAGTACAGAGGGACTGTCCAAAGCCGCAGA
TCTCGAGTCGACTAGCTTCCTTTCTCCCTTCTCCC
+
EC?BAA=@@:@99909485;C?;;>/0//....1169<;B@49;?ABBBBB@@?@??>;?AA@AA=AA<AA><<<<<
=<=??@?@@@<<;<=;;9==@@<@??A7;8<===7
@T6XXI:00014:00654
GGGAGAAGGGAGAAAGGAAGGAGAAGTCGCCCCAAACGATTTGTTGACTATTCTCCCTCTCCCTATAGTGAGTCGTA
TTAGAATTCCCC
+
CC?AAC@DC>BB?B@B@D=?>?BB?BCCCCCC8AB?BCCBC>CC>CAA>?A?ABAA4??A=C=????@AA@??=;;;
;8<>=4428880
@T6XXI:00014:00655
GGGAGAAGGGAGAAAGGAAGCTAGTCGACTCGACATCTGTGGCTTCACAGTACAGAGGACTGTCCAAAGCCGCAG
+
BB@CDC?BB=@@::5?<?<@@CCCB;;::??>7::796??@;@C?ABCAAACCCC8888AAAAA>?@78838>=>
@T6XXI:00014:00665
GGGAGAAGGGAGAAAGGAAGGAGAACAACACGATCGGTTAAACGTAGCTATTCTCCCTCTCCCTATAGTGAGTCGTA
TTAGAATTCCCC
```

```
+
CC@ABB?BB><<<C?B@A@CACBC@BB@BBCCDBCB?B@CD@CAAABCCCC;>AC>;;AACC>B@@@@@AA@@@???
@>@AD?C?@@@5
@T6XXI:00014:00673
ATACGACTCACTATAGGAGAGGGAGAATAGATCACCGTTGGTTCGGGTGCGCGTTCTCCTTCCTTTCTCCCTTCTCC
C
+
AABABBBBBBB===00,1<;;>9ADC@BBBCBB;:6;B=?=@ADBJ@CAAA@AC?A@@<>:=:==7>@@A<A>@@@C
;
@T6XXI:00014:00695
GGGAGAAGGGAGAAAGGAAGGAGAAGTCGCCCCAAACGATTTGTTGACTATTCTCCCTCTCCCTATAGTGAGTCGTA
TTAGAATTCCCC
+
AB?BBB@BD?CCBB>B>A?BAABC@ABBA???7?A=CCCA@;B@>C>@?@B>A<<<;A<@@A;>9<=??@<A??>@@
A?A??;=8;9;0
@T6XXI:00015:00670
GGGGAATTCTAATACGACTCACTATAGGGAGAGGGAGAATAGTCAACAAATCGTTTGGGGCGACTTCTCCTTCCTTT
CTCCCTTCTCCC
+
AAA7A6;8<A@ACBBBBBBABBA?7166.7><<?<?@@BDAA????<<<7?AAAC>CCC8B?A@C?AA?<?<?>BB=
B?@?:A?A@@@;
@T6XXI:00015:00674
GGGGAATTCTAATACGACTCACTATAGGGAGAGGGAGAAATAGTCAACAAATCGTTTGGGGCGACTTCTCCTTCCTT
TCTCCCTTCTCCC
+
AB@7C6959442489;:BCDBBBB777=.76;<626000+03444:533306;;?A<BCC7??ABD@CC@<@?A?AA
:@@AB>B388<;6
@T6XXI:00015:00686
GGGGAATTCTAATACGACTCACTATAGGAGAGGGAGAATAGTGACGTGTTGTGCGGCGCTCACTGTCTCCTTCCTTT
CTCCCTTCTCCC
+
DEC9B999<AC@C<:9BBCB<:>97//-
0=;?B>BAAB@A===9=AAA<;@AAA=88>AAA@@@B>:?<5843/00,3;;;1838???;
@T6XXI:00015:00687
GGGAGAAGGGAGAAAGGAAGGAGAATTGTTTACGCCATGATCGCTAACTATTCTCCCTCTCCCTATAGTGAGTCGTA
TTAGAATTCCCC
+
DC>?A@>=A<;<;CBC@D?B?BDC@?=??@;BBBBAC@@@<??C?@@@?B@>A=<@;@AAA@<A<A<<@@C@??===
=ADCD>A>@B@5
@T6XXI:00015:00689
GGGGAATTCTAATACGACTCACTATAGGGAGAGGGAGAATAGTTGCCCGTTCTAGCGGCCACTGGTCCCCTTCCTTT
CTCCCTTCTCCC
+
444,9;>=ABBCCCBBBBBB=@A>>616-6==?C>B@@?A=<@>@@A<AA>?<?@@A?A?@AAA<====4844/00-
49996<8;<@B<
@T6XXI:00015:00697
GGGAGAAGGGAGAAAGGAAGTGCAGTTCGCGGTTAGTGAAGGGAAAAACTATTCTCCCTCTCCCTATAGTGAGTCGT
ATTAGAATTCCC
+
BB>BBB@CC>AACC>C?BBACBBBCB@??>>=@=@@@EC@CC>BABB2BCAA>????;BCB@@;<<<<BAA???AA>
>=>ACC>A;>=7
@T6XXI:00016:00651
GGGAGAAGGGAGAAAGGAAGGAGAAGTCGCCCCAAACGATTTGTTGGACTATTCTC
+
490444244-4489-427;B?ABC@C>;<BBB.8807116;2660701100.7445
@T6XXI:00016:00660
```

GGGGAATTCTAATACGACTCACTATAGGGAGAGGGAGAATAGTCCCTGTTTAACGCAACCACCCCGAGTTCTTCCTT
TCTCCCTTCTCCC
+
CBB9B798<AAACA@@CCCCCCB7=666.7==?B?C@@>AAB=6548889498;><:4:24444,77668?;:8:<;
:?=<A<@9<8==9
@T6XXI:00016:00675
GGGGAATTCTAATACGACTCACTATAGGAGAGGGAGAATAGCTACGTTTAACCGATCGTGTGTCTCTTCCTT
+
CNK:E:<<=AB@CA@@BBCBB;977//,/;<?C=A>?>AAA=B?ABBB>@>A>??>;;74.---113/0/0/
@T6XXI:00016:00677
GGGAGAAGGGAGAAAGGAAGAAGAACCGCGGGACAGCTGCCATGTGGCTATTCTCCCTCTCCCTATAGTGAGTCGTA
TTAGAATTCCCC
+
CC>BB@@BB:?BBB=B>@@BB@BB?C@BBBB?AABBBCBA??@???>ACA?>?BBB<BBAAA<?AA@?<AAAA?@@@
@>@AA=A>AAA0
@T6XXI:00017:00658
GGGAGAAGGGAGAAAGGAAGCTAGTCGACTCGACATCTGTGGCTTCACAGTACAGAGGGACTGTCCAAAG
+
CDCCCC@BB?>===:@=@=AACBBB???@C@@??@AA@<<8=<?;?AABCCA?99333-4;;;@633331
@T6XXI:00017:00668
GGGGAATTCTAATACGACTCACTATAGGGAGAGGGAGAATAGTCTTGCAATTTCCTGCGGTGCATTCTCCTTCCTTT
CTCCCTTCTCCC
+
ECC9CAB?BCCACBBABCCCBBBA?961-
6<<<?=?AA>BA@@?@>?=A;@@>@?CC@@=@BB@@=@CB?C@C?BB59:AA<A@BBBD>
@T6XXI:00017:00669
GGGGAATTCTAATACGACTCACTATAGGGAGAGGGAGAATAGTCAGCAAATCGTTTGGGGCGACTTCTCCTTCCTTT
CTCCCTTCTCCC
+
@@@6@@B>BCB@BAB?<B>>?AB>;611.6>=@B>B@@?B888;;;ABB:A?BCC>???7AAA@>>@CA=9;9<AA<
@@@A;@=>?BA>
@T6XXI:00017:00670
GGGGAATTCTAATACGACTCACTATAGGGAGAGGGAGAATAGCCAAAAACTCTTCCCCAGCAGGCTGATCTTCCTTT
CTCCCTTCTCCC
+
;99/9=A?CBB@BAAA<<<??@A@?66659@@CC>?==?A:>;/433707ACC>@@@5@@@@@999;;:==<=9999
=BAC<@:=?A=;
@T6XXI:00017:00677
GGGAGAAGGGAGAAAGGAAGAGAACAGTCACAGGCTCGAGCTGCAAGCTATTCCTCCTCCTCCTATAGTGAGTCGTA
TAGATTCCCC
+
@@<ACA>BE;<=AEACDC@A@@@?BCCBBBCCC?C@A@BBCCCAB@BBBB@=@;7747438C@AC@>;0//.1-+--
-----01454
@T6XXI:00017:00683
GGGAGAAGGGAGAAAGGAAGCGATAG
+
9919>?>?F:@99901,1,0......
@T6XXI:00017:00686
GGGGAATTCTAATACGACTCACTATAGGGAGAGGGAGAATAGAGACGTATTCGTTTGCGCTCTGTTCACCTTCCTTT
CTCCCTTCTCCC
+
CCC9B>B>BCB@BBBBBBCCCCBBA69959??BC>B>@>A=8==AAA===?AAAB:@@@C::6<=<=<=;<558;;6
;@AC<A>AA@@<
@T6XXI:00017:00697
GGGAGAAGGGAGAAAGGAAGCTAGTCGACTCGACATCTGTGGCTTCACAGTACAGAGGACTGTCCAAAGCCGCAGAT
CTCG
+

```
DCACDB?BC<AABB<A>A7;;;:::A?CB??=?777??>>;:8;7:BBCFC@@A===<???@??=@B=AA>@@AAA?
??AA
@T6XXI:00017:00698
GGGAGAAGGGAGAAAGGAAGGAGAATGCTAACGCGCAGAATTGGCGGACTATTCTCCCTCTCCCTATAGTGAGTCGT
ATTAGAATTCCCC
+
BA>BA=<>B=BBBC@CDC@C@AAA8;:BCC@A@AAACCCAB>?>?@<<A?@@<<@AA;@<???<?@@???AA@@@AA
AA>AAA>@:???5
@T6XXI:00018:00655
GGGAGAAGGGAGAAAGGAAGGAGGATATACCAGCATGAATGCAATGGCTATTCTCCCTCTCCCTATAGTGAGTCGTA
TTAGAATTCCCC
+
A=:>AA>A>:>98809<>;?<886;>CCCC?BBABCDD;<;;:61/,../@><98D>ABBBB<?A::8CAA@8AABC
E?@@@=A=??@6
@T6XXI:00018:00662
GGGAGAAGGGAGAAAGGAAGCTAGTCGACTCGACATCTGTGGCTTCACATTACAGAGGGACTGTCCAAAGCCGCAGA
CTCGAGTCGACTAG
+
AA?BDB@B?=BA==:@6710>0,,7,,,;///;??::6BBD<?A?@A=6657>?>777.8===?H>@@;AA>AA<@<
<A@@@A@8888870
@T6XXI:00018:00663
GGGAGAAGGGAGAAAGGAAGGGGAGAAGGGAGAAAGGAAGCTAGTCGACTCGACATCTGTGGCTTCACAGTACAGAG
GGACTGTCCAAAGCCGCAGATCTCGAG
+
CD@CCC@BB>9;;B=@>A?BBB8BCE@BA;<9992?:?:??@BBBCCC@;=<==@@?@CA<=<=;<<;;99====43
317<<>??>AA<@@=@AAAA<9==A@@
@T6XXI:00018:00685
GGGGAATTCTAATACGACGTCACTATAGGGAGAGGGAGAATAGCCTTTTCCCACTGACCGAACTCTCGTCTTCCTTC
TCCCTTCTCCC
+
8?808+1,///,/4443333>788=;77?:?@A@><@::87??B4777,172333004/0025334;565:78464/
/.-+.31.33+
@T6XXI:00018:00695
GGGGAATTCTAATACGACTCACTATAGGAGAGGGAGAATAGATCACCGTTGGTTCGGGTGCGCGTTCTCCTTCCTTT
CTCCCTTCTCCC
+
BAA7A8;;<BB@CBAABAAAB:997..,0::>@>BHH>@??<==??<>B=?;??B@B>C;;;CCC@BBB?B?B@CC>
@<=<7<>???C<
@T6XXI:00019:00651
CTAGTCGACTCGAGATCTGCGGCTTTGGACAGTCCCTCTGTACTGTGAAGCCACAGATGTCGAGTGACTAGCTTCCT
TTCTCCCTTCTCCC
+
BA111>;>BDCBBACCCBCCB@BBC?CABA>>777.7=7::CEEA==A?BC?ABCCC:;:CCCCB@BBCC?A==?8;
<6;;9B5;8<>==:
@T6XXI:00019:00658
GGGAGAAGGGAGAAAGGAAGGAGAAGTCGCCCCAAACGATTTGTTGACTATTCTCCCTCTCCCTATAGTGAGTCGTA
TTAGAATTCCCC
+
DC@CCB@BB>?BAB?C@B?B@CBC@@@AAACC9CC>BB???<?D?CBB@AA?AAAA<A@A=<;==A@@@@A@@???@
C>@@A=A<A@@5
@T6XXI:00019:00683
GGGGAATTCTAATACGACTCACTATAGGAGAGGGAGAATAGTCAACAAATCGTTTGGGGCGACTTCTCCTTCCTTTC
TCCCTTCTCCC
+
DDB4@5458889499:CC@@@?:77//,0>077.7::79=888?<:;;777777.7<<2=?BCC?CCB<?;=:=B<A
AAD9<9<>>>:
@T6XXI:00019:00696
```

```
GGGGAATTCTAATACGACTCACTATAGGGAGAGGGAGAATAGCTCATGTACTTGTCACACGGGATTCTCCTTCCTTT
CTCCCTTCTCCC
+
CCB8B6959>>;@BBBCCBCCBBA?:::5:??AC>B@A>AAA@BB:889@@@A<=884?48817@999;2423/44,
7<?A<A=@=<<7
@T6XXI:00019:00699
GGGGAATTCTAATACGACTCACTATAGGGAGAGGGAGAATAGAATCAGGCCTGCGTGGACTGGTGCGTTCTCCTTCC
TTTCTCCCTTCTCC
+
994-
9=@?@@@@AAABCCAA=<;76166.777>@=AAAAAA??::<::=??BBBCAB>?AA?>ABBBB@C@@:=48/33,4
444-434302
@T6XXI:00020:00651
GGGGAATTCTAATACGACTCACTATAGGGAGAGGGAGAATAGCTTGGGCCAAGCCTAAGACTATTTCTCTTCCTTTC
TCCCTTCTCCC
+
ABC:C>?>ACC@BBA?@CDCCBB;B111-
677?C?A??@A@@@B@CC?C@C?AA>AA>?>?7+,++,,,+00*++,+889485<<<<7
@T6XXI:00020:00665
CTAGTCGACTCGAGATCTGCGGCTTTGGACAGTCCCTCTGTACTGTGAAGCCACAGATGTCGA
+
A11,,,,,,,,87/99A;;:;:A@@<<53311166289666=A:?544204244439<<8::6
@T6XXI:00020:00694
GGAATTCTAATACGACTCACTATAAGGGAGAGGGAGAATAGCTGGCATCGATGTGCCTTCGACTTCTCCTTCCTTCT
CCCTTCTCC
+
42424:;BCADCB@@@B?>>@<00,.5+./:@?;>5523000110188689:000:9:8;>?;83464,.,.,.,.5
55322,++*
@T6XXI:00020:00695
GGGGAATTCTAATACGACTCACTATAGGGAGAGGGAGAATAGTCTGCAGATCTGACATGACTTTTTCTCCTTCCTTT
CTCCCTTCTCCC
+
CCC9=6;:<>A?BBCBCBBB;;837111,116;62130203003333636853333//1009::::/0/797:<377,
1494+4/4000+
@T6XXI:00020:00696
CGAGATCTGCGGCTTTGGACAGTCCCTCTGTACTGTGAAGCCACAGATGTCGAGTCGACTAGCTTCCTTTCTCCCTT
CTCCC
+
EDBCDCCBBBB@BBB<B@BBAAACC>CBBBBCCCBBBA?@A=CAA>??AABBBCDCDBBBB<<;=ABCCBCBBB=B=
??A@:
@T6XXI:00021:00651
GGGAGAAGGGAGAAAGGAAGGGGAGAAGGCTAGTCACTCGACATCTGTGGCTTCACAGTACAGAGGGACTGTCCAAA
GCCGCAGATCTCGAGTCGACTAG
+
EDABAA@CDCBBBB>BCE@ABA8???@C?CCGBAA>999994449;AAC?BG?BCCCBCCECAAAGACCACAB@DD@
@BBCCBBB@@@C@@@CIGCFCD<
@T6XXI:00021:00652
GGGAGAAGGGAGAAAGGAAGCTAGTCACTCGACATCTGTGGCTTCACAGTACAGAGGGACTGTCCAAAGCCGCAGAT
CTCGAGTCGACTAG
+
CC@CCB>A=9>988-
9584;BA@@C<???@>7:8:::BDD@AA?ACAC><?==<34417>>>??>@@<AA????@AA===AA@@@@@@@@@
@T6XXI:00021:00664
GGGGAATTCTAATACGACTCACTATAGGGAGAGGAGAATAG
+
DDD9ECCACCC@CBBBBBBBA?ABCBBA:991137/,+,,+
@T6XXI:00021:00673
```

```
GGGAGAAGGGAGAAAGGAAGGGGAGAAGGGAGAAAGGAAGCTAGT
+
CC@@A@@BB>C@?A=@@B=>>>6?<<=?82<//.+0,0,07,,,:
@T6XXI:00021:00699
GGGAGAAGGGAGAAAGGAAGCTAGTCGACTCGACATCTGTGGCTTCACAGTACAGAGGACTGTCCAAAGCCGCAGAT
C
+
CC>AA@?AA:@@@>9><@=AAC??@CCBBCBBB???BBAA><?A<ABCBBBA7777734;<?>?=?B@AA>@@?@@A
A
@T6XXI:00022:00654
GGGGAATTCTAATACGACTCACTATAGGAGAGGGAGAATAGCTATCTCGCGCCATCCCTAACGTTGTTCTTCCTTCT
CCCTTCCTCCC
+
499/959249;2449=73336...../,../;688====877????<<@@??;?;::5:<;<@A<;99:;9;-/-
.33335/444<C;
@T6XXI:00022:00664
GGGAGAAGGGAGAAAGGAAGGAGAAGTCGCCCCAAACGATTTGTTGACTATTCTCCCTCTCCCTATAGTGAGTCGTA
TTAGAATTCCCC
+
?A69:A@BC>BBCC@C?B>B@BBB@BAABCBC.8807116@=CC@B@?=@@;==AD<?>;=@90///88;?@>=8<<
<<;8444/--.+
@T6XXI:00022:00665
GGGAGAAGGGAGAAAGGAAGCTAGTCACTACATCCTGTGGCTTCACAGTACAGAGGGACTGTCCAAAGCCGCAGATC
TACGAGTCGACTAGCTTCCTTTCTCCCTTCTCCC
+
CB@B==<<C>CBBA<@6858A877>:00......,0039:=A?=??C999AA@@@B<@@@@AA>AC?BA>AB@<<88
///865677/..954?:?>AC<???A;@;><<<6
@T6XXI:00022:00667
CTAGTCGACTCGAGATCTGCGGCTTTGGACAGTCCCTCTGTACTGTGAAGCCACAGATGTCGACGTCGTCC
+
444499449=@99>@99999848><,753977696,1116171110002399=9;:B?<7:00..5,,..,
@T6XXI:00022:00674
CTAGTCGACTCGAGATCTGCGGCTTTGGACAGTCCCTCTGTACTGTGAAGCCACAGATGTCGAGTCGACTAGCTTCC
TTTCTCCCTTCTCCC
+
B--,,-
,,/1:@BABACA@@C@@@@=@=?@CBBBB>BCCBBBB@>??A?AC@BBBAAAABBBCB?@@BBBCCBA?C?CE@AA?
A<A<??@@<
@T6XXI:00022:00677
GGGAGAAGGGAGAAAGGAAGTGCAGTTCGCGGTTAGTGAAGGGAAAACTATTCTCCCTCTCCCTATAGTGAGTCGTA
TTAGAATTCCCC
+
DC>???@CCBBCCC?B?A@BBBC=<<ACCCC@B?ABBBB@CB>CED7AABC?AACEABCBAA<?@@@@A@@@@@@@?
?>@AD>?=@BD6
@T6XXI:00022:00688
GGGAGAAGGGAGAAAGGAAGAGAACAGTCACAGGCTCGAGCTGCAAGCTATTCTCCCTCTCCCTATAGTGAGTCGTA
TTAGAATTCCCC
+
CE@BBC@DD@BB?BBB@?>????>AAABBBBAA?AB@BB?BBC?@??@@@@>@@>@=@==<<:;@AB??B>><@BB@
@>@AA?A9=984
@T6XXI:00023:00654
GGGAGAAGGGAGAAAAGGAAGCTAGTCGACTCACATCTGTGGCTTCACAGTACAGAGGGACTGTCCAAAGCCGCAGA
TCTCGAGTCGACTAGCTTCCTTTCTCCCTTCTCCC
+
>;:@4954=9=9111*/,.,...003.......1116699>8<<:;?89::9;=:444,4=849>5<<6;=8558<8
44<<<<54486003444/478844000+0/0000+
@T6XXI:00023:00675
```

```
GGGAGAAGGGAGAAAGGAAGGAGAAGTCGCCCCAAACGATTTGTTGACTATTCTCCCTCTCCCATAGTGAGTCGTAT
TAGAATTCCCC
+
DDACEC@BB>BBBB>A??>?>ABD@CBBCCBC8AC>BCCCC>B@@C?=<@A?=A=;:;888;::::::;<<<<<9==
<=5547;<=?5
@T6XXI:00024:00666
GGGAGAAGGGAGAAAGGAAGCTAGTCACTCGACATCTGTGGCTTCACAGTACAGAGGGACTGTCCAAAGCCGCAGAT
CTCGAGTCGACT
+
A?6@9959919>>>9@7;6:C;::BBCBBBA::79:6ABC>@A@ACCC@@B<<65555;=8=;<8;;:;;8<<???A
@@@887AABCAA
@T6XXI:00024:00668
GGGAGAAGGGAGAAAGGAAGGGGAGTAGGGAGAAAGGAAGCTAGTCGACTCGACATCCTGTGGCTTCACAGTACAGA
GGGACTGTCCAAAGCCGCAGATCTCGAGTCACTAG
+
ED@FAA=@@?99<A<@AD@CCC6@@BDCB?>9990929<?A>A430./00:911///,/644,798===97:???=7
77-7;<767377.6737==9;=@==<>:77,++++
@T6XXI:00024:00674
GGAGAAGGGAGAAAGGAAGGAGAAGTCGCCCCAAACGATTTGTTGACTATTCCTCCCTCTCCCTATAGTGAGTCGTA
+
-*,887;B;A>><8<-0,.3..064673733,33,16111,177711012,-,-333./.220------2356<;:=
@T6XXI:00024:00679
GGGGAATTCTAATACGACTCACTATAGGGAGAGGGAGAATAGTCAACAAATCGTTTGGGGCGACTTCTCCTTCCTTT
CTCCCTTCTCCC
+
C?:6=6=<=CC@CEAA@BBB@???=777.777AD?A==?B??=?>?@AC;@@BBB>BBC9CCB??:;?;/434244-
49;9,327444:
@T6XXI:00024:00683
GGGGAATTCTAATACGACTCACTATAGGGAGAGGGAGAATAGTCAACAAATCGTTTGGGGCGACTTCTCCTTCCTTT
CTCCCTTCTCCC
+
===4>69:<CC@DCCBBBBB>@>97111.7>>BC>@<=>@<@A<:8<133;/0/5+../-
/7>;;888558<?>AC;@@@A;@>@@CC<
@T6XXI:00025:00650
GGGAGAAGGGAGAAAGGAAGCTAGTCGACTCGACATCTGTGGCTTCACAGTACAGAGGACTGTCCAAAGCCGCAG
+
A@<@CC@A?:?998094848@@:67?8?=:969:66??==>98:6;A?77744444424;;:;89=C<AA=AA@=
@T6XXI:00025:00654
GGGAGAAGGGAGAAAGGAAGCTAGTCGACTCGACATCTGTGGCTTCACAGTACAGAGGGACTGTCCAAAGCCGCAGA
TCTCGAGTCGACTAG
+
<<9<C@>>>09999142713>;9>>:116611161796=:638:>AACACB@>;4030-
4::@AA>AA:@@?A@?=;;===<<<<<?@AAAA
@T6XXI:00025:00657
GGGGAATTCTAATACGACTCACTATAGGGAGAGGGAGAATAGTTTCCCTTCACTAACCGCGAACTGCACTTCCTTCT
CCCTTC
+
;>;5:,45989548>988>>?;767111,7;<BC>CACB??<;;4:;7;947:11772675546=;;:::,0,0,.2
222222
@T6XXI:00025:00681
GGGAGAAGGGAGAAAGGAAGCTAGTCACTCGACCATCTGTGGCTTCACAGTACAAAGGGACTGTCCAAAGCCGCAGA
TCTCGAGTCGACTAG
+
;C=>;B7991999919484337117?..,00,,*+,,,,375>AB?BBCB??=A@;AA@@@??>A9AA>ABABC?D@
CGCCAAACCBABB@>
@T6XXI:00026:00654
GGGAGAAGGGAGAAAGGAAAG
```

```
+
CC>B@?58809911-/,,,*,
@T6XXI:00026:00659
GGGGAATTCTAATACGACTCACTATAGGAGAGGGAGAATAGCCACGTAATCTCCCACTGCGCACTCACCTTCCTTTC
TCCCTTCTCCC
+
CCB7A>B>BCBBCC@@@BCBB:::7//.1>>CC>CAA>A<??;?ACCB@???:?99/0097AA@@A=<9<9<9=A<@
@AC;@>@@@@;
@T6XXI:00026:00662
CTAGTCACTCGAGATCTGCGGCTTTGGACAGTCCCTCTGTACTGTGAAGCCACAGATGTCGAGTCGACTAGCTTCCT
TTCTCCCTTCTCCC
+
B=/111/44@==BBBCCC>;>>DD=B<>@CCBBB>BB@@B@@<=AAA>?BBCCD@@ACCBBBBBBBAA@A@@A>A>C
C@B@ADAB9<<@H@
@T6XXI:00026:00678
GGGAGAAGGGAGAAAGGAAGCTAGTCGACTCGACATCTGTGGCTTCACAGTACAGAGGACTGTCCAAAGCCGCAGAT
CTCGAGTCGACTAG
+
DF@CCB>=96>998096858@800>/00><00076:67@?C?9;;;CCC@?=555..-.8777;244-
4;4?A@@@@C@@===AAAA===A
@T6XXI:00026:00689
GGGGAATTCTAATACGACTCACTATAGGGAGAGGAGAATA
+
/66065=8==888448333397997111,11110100/00
@T6XXI:00027:00652
GGGAGAAGGGAGAAAGGAAGGAGAAGTCGCCCCAAACGATTTGTTGACTATTCTCCCTCTCCCTATAGTGAGTCGTA
TTAGAATTCCCC
+
CD?BB=@BCABBBC?A@B>A?ACC@DBCCCCC9CC>CCCCC<B?B?>>=A=<=<<<;<;<;=;<<<;<===@<????
?>AAA>@>@??4
@T6XXI:00027:00669
GGGGAATTCTAATACGACTCACTATAGGGAGAGGGAGAATAGTTTTTTCCCTTCACTAACCGCGAACTGCACTTCCT
TTCTCCCTTCTCCC
+
GGC8B79:<=89?@ACCBCBCCB?=777.777==6<99240003333*340323;?6:8:?CCCCA@@;9100::9;
>6;3844=8<6>?5
@T6XXI:00027:00670
GGGAGAAGGGAGAAAGGAAGCTAGTCGACTCGACATCTGTGGCTTCACAGTACAGAGGGACTGTCCAAAGCCGCAGA
TCTCGAGTCGACTAG
+
CD?BAC=@@<@B=::@6:58>>=7:7;<<66::7?<<00.-,-
.,.548<=894:88818==?AA>AA=?@>@??==778;;=====<<<??
@T6XXI:00027:00675
GGGGAATTCTAATACGACTCACTATAGGGAGAGGGAGAATAGCCGGCAATTCGACTCCCCGGCAATGTTCTTCCTTC
TCCCTTCTCCC
+
BBA7A:<:<AA=999<;??@ACB??:::6:>@CJ=A??AC9:9>@=8:5:4777:==>>.7369546;7//,/,.,-
113477998=;
@T6XXI:00027:00697
TCGAGATCTGCGGCTTTGGACAGTCCCTCTGTACTGTGAAGCCACAGATGTCGAGTCGACTAGCTTCCTTTCTCCCT
TCTCCCCTTCCTTTCCTCCCTTCTCCC
+
ABBB<<<CCFCC>BCC>C?AABBBBB>BCCEAAAFBCB@>@@>BCC?==AABC@BCCBBBBCDDCAD@DCACBBBBB
>?8888.09;,00,0,000,0,0111,
@T6XXI:00028:00654
GGGAGAAGGGAGAAAGGAAGCTAGTCACTCGACATCTGTGGCTTCACAGTACAGAGGACTGTCCAACCGCAGATCTC
GAGTCGACTAG
```

```
+
CC@CCCAEE<<<CB=B;<9<CCBABB;:77BBB<CA=@@C=?@?BBBAAAA876//,/88669797848888=;;43
0@?9@;<;BCC
@T6XXI:00028:00674
GGGAGAAGGGGAGAAAAGGAAAGGAGAAGCGCGCCTTAAGACTGCGTTTAAGCTATTCTCCTCTCC
+
;;:9942444,/////*/,..+.,...,13>B7101042444444444-424334;244423...,
@T6XXI:00028:00676
GGGAGAAGGGAGAAAGGAAGCTAGTCGACTCGACATCTGTGGCTTCACAGTACAGAGGGACTGTCCAAAGCCGCAGA
TCTCGAGTCGACTAG
+
CCABBA>??>@CCC>B=?=@C>5;;/00:;;;1::ABAAAA;=??BB?;>>====99946:9:<@=?A<AC>BA===
=689====???@AA=
@T6XXI:00028:00688
GGGAGAAGGGAGAAAGGAAGGAGAAGTCGCCCCAAACGATTTGTTGACTATTCTCCCTCTCCCTATAGTGAGTCGTA
TTAGAATTCCCC
+
CCCBBB@CB>B@996>4748>A?@=?A;<<<<2>>:?CDD@;?>>??;8==<=9999=@ACCA?=?@@=<<<::?<<
A>@A@9;2444-
@T6XXI:00028:00691
CGAGATCTGCGGCTTTGGACAGTCCCTCTGTACTGTGAAGCCACAGATGTCGAGTCGACTAGCTTCCTTTCTCCCTT
CTCCC
+
168,,,,,,,/579;:;><:169::-1B@;;<CCCCC=;655600000--////777CCCBB=:8989958444-
4777;;6
@T6XXI:00028:00695
GGGGAATTCTAATACGACTCACTATAGGAGAGGGAGAATAGTCAACAAATCGTTTGGGGCGACTTCTCCTTCCTTTC
TCCCTTCTCCC
+
9?@4>;>>@@@@AACBBBBBC>===0/,0::>@>A@@@DDA@<<8;=:7>>777.7>>5BB???>@?=::8:344-
4::<6<:;4885
@T6XXI:00028:00696
GGGAGAAGGGAGAAAGGAAGGACAAATAGATGCGGGGGAAACGCTAGCTATTCTCCCTCTCCCTATAGTGAGTCGTA
TTAGAATTCCCC
+
@=:@CC@CF>BBCC>C@C@B@BCBB;95;/0/67557-//-
/88?CCBBCD@CAAA>?AABB=BB??@AAA@@@AAAA>@@?=;3444,
@T6XXI:00029:00655
GGGAGAAGGGAGAAAGGAAG
+
CC@B>9299199990420,0
@T6XXI:00029:00669
GGGAGAAGGGAGAAAGGAAGAGAAAGGTCGGGGTAGCAATGCCTCCAACTATTCTCCCTCTCCCTATAGTGAGTCGT
ATTAGAATTCCCC
+
CC>CB@@BB=@BBB=B@B@AA@@A=??CCCCC9BBB@D@CCD@BB?C@BCCBEBAAA=BBCBC;?>9>>?BBCCAA<
=?>@A=>AB@AA5
@T6XXI:00029:00673
GGGAGAAGGGAGAAAGGAAAGCTAGTCGACTCGACATCTGTGGCTTCACAGTACAGAGGGACTGTCCAAAGCCGCAG
ATCTCGAGTCGACTAG
+
EC@ECCB@=:@===9>588,7>>0..0....45/78766?:838?;????CB?998333-
4;;?@@?AA:@@@A???A==??@AC@889@@??
@T6XXI:00029:00679
GGGGAATTCTAATACGACTCACTATAGGAGAGGGAGAATAGTTTTCCCTTCACTAACCGCGAACTGCACTTCCTTTC
TCCCTTCTCCC
+
```

```
>@>4>;>=ABBCCBB@B@CA@70///0,0<;>><@;;9==<9==-44-
349=?BB?B?BA<<8;:::??AAB@CD>:89<7<>@@AA<
@T6XXI:00029:00684
GGGGAATTCTAATACGACTCACTATAGGAGAGGGAGAATAGTCAACAAATCGTTTGGGGCGACTTCTCCTTCCTTTC
TCCCTTCCTCCC
+
AA?2@2924999=?AACDDDB><;:/,+,,,/56>4774==AAA=??C1:7?==6?@@5BBAAA?BA?<?<?>AA<A
@@;6;./.,551
@T6XXI:00030:00651
GGGGAATTCTAATACGACTCACTATAGGGAGAGGGAGAATAGTCAACAAATCGTTTGGGGCGACTTCTCCTTCCTTT
CTCCCTTCTCCC
+
@@@6@@A?BCB@CCBBB????AB?>111.7==>@=ABH@B>>>AA<<<<;@?>>>;@>B6?????:?@@;?<??AA<
AAAA<A>@@@@;
@T6XXI:00030:00656
GGGAGAAGGGAGAAAGGAAGTGCAGTTCGCGGTTAGTGAAGGGAAAACTATTCTCCCTCTCCCTATAGTGAGTCGTA
TTAGAATTCCCC
+
BC>BBB@CC>BBBC>B@B@B??AABB@@>>@@C@A>ABD?@?8:??6@CBC?CCDD>???AB>?=?@@A=<<?<??@
@889A=;2884-
@T6XXI:00030:00657
GGGAGAAGGGAGAAAAGGAAGCTAGTCACTCACATCCTGTGGCTTCACAGTACAGAGGGACTGTCCAAAGCCGCAGA
TCTCGAGTCGACTAGCTTCCTTTCTCCCTTCTCCC
+
@A6;;@==@:@8999-847137;647</.......1016445..,0359?>?<A>788-4;;88:>@@6;;-/--
11300044303666688:<;<::4+0044,3=;?AC<
@T6XXI:00030:00658
GGGGAATTCTAATACGACTCACTATAGGGAGAGGGAGAATAGCCAACAGGTATCTGATTGCGCGTTCTCCTTCCTTT
CTCCCTTCTCCC
+
BBB7A=A>@BB>CBCBBCCB?A@77111.777BC@C@A>A?77378;;;9=AAA<8>;?;;88:=?@@?<?<><?A9
==AB<A>A=<<7
@T6XXI:00030:00661
GGGAGAAGGGAGAAAGGAAGTGCAGTTCGCGGTTAGTGAAGGGAAAAACTATTCTCCCTCTCCCTATAGTGAGTCGT
ATTAGAATTCCCC
+
CCBBBB?A@:BB===B>A?ADBCBBBEB:8:@C@BBBCE?B=8888?2@CDC@BBCB=BB???;::::====@@AA@
??>=AA>A>@@?5
@T6XXI:00030:00663
GGGGAATTCTAATACGACTCACTATAGGAGAGGGAGAATAGTCAACAAATCGTTTGGGGCGACTTCTCCTTCCTTTC
TCCCTTCTCCC
+
DKKAI?<<=CC@CCBBCAAACA???963777=?<?AA=?>@@AA?AAA;AA>??-777-
7???B@AA?<=:=8<<7=AAC<A>AA??<
@T6XXI:00030:00668
GGGAGAAGGGAGAAAGGAAGTGCAGTTCGCGGTTAGTGAAGGGAAAAAACTATTCTCCCTCTCCCTATAGTGAGTCG
TATTAGAATTCCCC
+
CDDCCC=@@<@@@@<@?B?BBBCBBCACCBAB@BBBBBB@CC<?????,BCCB@CBBB>CBCBC;=?=????=AA=>
=9=<?@@>A>AAA6
@T6XXI:00030:00677
GGGAGAAGGGAGAAAGGAAAGCTAGTCGACTCGACATCTGTGGCTTCACAGTACAGAGGGACTGTCCAAAGCCGCAG
ATCTCGAGTCGACCTAG
+
44-
4996991499914233,39766@:6611661166169;958<;?ACBBBADCA88818=;<=@?CA:BA>AAAA?98
8=4488874?<><=
```

```
@T6XXI:00030:00689
GGGAGAAGGGAGAAAGGAAGGAGAAGTCGCCCCAAACGTATTTGTGAT
+
BCADCD?A@=?B===B?A?A@BBB>B>99:>>6>;6/0..,,*,..00
@T6XXI:00031:00654
GGGGAATTCTAATACGACTCACTATAGGAGAGGGAGAATAGATCACCGTTGGTTCGGGTGCGCGTTCTCCTTCCTTT
CTCCCTTCTCCC
+
BBB9B?C@BCB@AAABBAAAA:695./-
0>>@L>CBBA@9:===:85:C=?=A?B@=<B:;:CBA>DA@>@7:7:B:;88858?AAAA?
@T6XXI:00031:00677
GGGAGAAGGGAGAAAGGAAGAATAGCTGACTGGGATGTCGTGAAGGGTCTATTCTCCCTCTCCCTATAGTGAGTCGT
ATTAGAATTCCCC
+
DC@BBBBDC>BCCB=A<;:?@<@<>@DFDCCCCBDCCAAACDC@CC@C@@@CBAACC>BBBBB>@=AA@@ACCCBB@
@@>@AD>A=@@@5
@T6XXI:00031:00680
GGGAGAAGGGAGAAAGGAAGCTAGTCACTCGACATCTGTGGCTTCACAGTACAGAGGGACTGTCCAAAGCCGCAGAT
CTCGAGTCGACTAG
+
EC>BBC59809>?>9>5848@@77:@0//9<00-///;;C>@@>@DDAAACAA9300-
49::=?>@A@BA>@A???@@@?@@@888@888@
@T6XXI:00031:00687
CTATCGACTCGAGATCTGCGGCTTTGGACAGTCCCTCTGTACTGTGAAGCCACAGATGTCGAGTCGACTAGCTTCCT
TTCTCCCTTCTCCC
+
D>:>311>;;BBCBABBBBB?BBB<B>AAB@@@@=@CAA@>><;;<=88>@BCCBAA?AAAAABBBCBCDBAA?BAC
C>A?=A>A=>?>A;
@T6XXI:00031:00689
GGGGAATTCTAATACGACTCACTATAGGGAGAGGGAGAATAGATCACACGACCCTTCCTACCAACGATTGATGCCTT
CCTTTCCTCCCTTCTCCC
+
DDE9B@B@BBB?BB;<<ECC;:;@@77:6:?=?C>@=<>@8444800..444403334444,.,.,,.,.333;242
489;-42444-424444-
@T6XXI:00032:00652
GGGAGAAGGGAGAAAGGAAGAACATACGTGGAAGGAAGACAACACGACTATTCTCCCTCTCCCTAGTGAGTCGTATT
AGAATTCCCC
+
BB?BCC@CC>BBBC@C?@>BB@BB?@BBBB@B><=<<AACC@BBB?<<<>=5644317>?@@<@???=@@????886
8AA?A>@@@5
@T6XXI:00032:00677
GGGAGAAGGGAGAAAGGAAGGAGAACGCGCACCCGAACCAACGGTGATCTATTCTCCCTCTCCCTATAGTGAGTCGT
ATTAGAATTCCCC
+
CKDCCC@BD>BBBC>B?B?@@BAA?BDBCCBCC@CB>B=A>ACBDFCDCA@@=@@?@<@@@CC>CAAABCDCB?BC?
@?@CAA<B>BBB8
@T6XXI:00032:00679
GGGGAATTCTAATACGACTCACTATAGGGAGAGAGATATAAGGGTGACTCGTCTCTTTCCCCACTGCGGCCGCTGCC
CATTACCAAATTCTCTCCC
+
;99/9<><@BBACCBBBBBBBAC?=?AB>ABABCA@A<@?>==;A;<ACCDC??8==.430-
44;;?@>A>???:::.7@<9968>99>BBBBCC=
@T6XXI:00032:00683
GGGAGAAGGGAGAAAGGAAGCTAGTCACTCGACATCTGTGGCTTCACAGTACAGAGGGACTGTCCCAAAGCCGCAGA
TCTCGAGTCGACTAG
+
```

```
CDDBB@894-999909<>;?B?<?B9:::@@:;9ABACCC=?@?ACBCBBB994000-
4:;B@AA;@@;@CBC@<;;@@AA@@?C@???AA=
@T6XXI:00033:00659
GGGGAATTCTAATACGACTCACTATAGGAGAGGGAGAATAGTCAACAAATCGTTTGGGGCGACTTCTCCTTCCTTCT
CCCTTCTCCC
+
CCB9C@HCDDDAAAACCA@@B>=::/0,/::<<8?5356A???A@A@A<BAABC>CCC9B??;:5:;77778./,-
222249733=:
@T6XXI:00033:00682
GGGAGAAGGGAGAAAGGAAGGGGAGAAGGGAGAAAGGAAGGGGAGAAGGGAGAAAGGAAGCTAGTCACTCGACATCT
GTGGCTTCACAGTACAGAGGGACTGTCCAAAGCCGCAGATCTCGAGTCGACTAG
+
CC@BBB@BC>ABBB>F@C?AA?6:::@CC>A@?C>B?B???@7??=<==;<?:::>;>:6???=:4616;=<>?@@?
AAC?875888<>==@@878?:??@@@A>AA<@C:====A@BDD==<?CCCCCCA
@T6XXI:00033:00687
GGGAGAAGGGAGAAAGGAAGGAGATAGACGTGGGGCAGAGCACGTATCTATTCTCCCTCTCCCTATAGTGAGTCGTA
TTAGAATTCCCC
+
??=ABCACC?CCCG@C?A?B>AABAAAA???@@A5@AAC@A???<<=<<@BACACA>99999088><;616117716
7=88879:9BB8
@T6XXI:00034:00651
CTATCACTCGAGATCTGCCGCTTTGGACAGTCCCTCTGTACTGTGAAGCCACAGATGTCGAGTCGACTAGCTTCCTT
TCTCCCTTCTCCC
+
CDB>9::?BBBCBD@@?><<<:?;@?AACCBBB<BCC?>>:::999:9@?@>>BBB@?@?ACB<<<BAA@:64:=>?
:@>==7=;>??A;
@T6XXI:00034:00652
GGGGAATTCTAATACGACTCACTATAGGGAGAGGGAGAATAGTCAACAAATCGTTTGGGGCGACTTCTCCTTCCTTT
CTCCCTTCTCCC
+
CCD9D=?>@BB?B@??CAAA@>;76166.7>>>B@BBA>B:?;@C<9=94?9???8???5??@CC>AA@>@>@?CC<
A?@@;?;??@A<
@T6XXI:00034:00662
GGGGAATTCTAATACGACTCACTATAGGAGAGGGAGAATAGTTTGGCTTTGTCCCACAACAT
+
@;;2;7<848;944;A883??9:99//,.4467,100/0300013:99947111,.55,//3
@T6XXI:00034:00681
GGGAGAAGGGAGAAAGGAAGCTAGTCGACTCGACATCTGTGGCTTCACAGTACAGAGGACTGTCCAAAGCCGCAGAT
CTCGGT
+
EDADCB?B?:?@??<@@C8;<B@@@CCCCCB@77::::CCC>?A?BBA@@@<444..-
/;;9BB=BB:BB?A@<<<<;;<=>A
@T6XXI:00034:00686
GGGAGAAGGGAGAAAGGAAGGAGAAGTCGCCCCAAACGATTTGTTGACTATTCTCCCTCTCCCTATAGTGAGTCGTA
TTAGAATTCCCC
+
CE?AA?BCDB;;;C@C@C@@>@CC@@BCCCCB8AA<AB???<?@=BBB@BC<??AA<@??AA;<;?999A???@@@@
@>AA@>@8;;;0
@T6XXI:00034:00693
CTAGTCACTCGAGATCTGCGGCTTTGGACAGTCCCTCTGTACTGTGAAGCCACAGATGTCGAGTCGACTAGCTTCCT
TTCTCCCTTCTCCC
+
DA9<;@::9CCBBBBBBBCC?ACC?B>>@??9::5:A@BAAAA<?<A>@B@BBBBCC===??C@BBBACBCCB?B@D
C>A@?C<A>@@@A<
@T6XXI:00035:00661
GGGAGAAGGGAGAAAGGAAGGAGAACGCGCACCCGAACCAACGGTGATCTATTCTCCCTCTCCCTATAGTGAGTCGT
ATTAGAATTCCCC
```

```
+
CDBBB@@CC>=@=ABC@?;;;>BB@AABBH@@?>>A<A>A@ADCCA?@BBBB?BBCC>CCBBB>BGDBB?BBCABBB
BB?@@@>CACCC:
@T6XXI:00035:00663
CTATCACTCGAGATCTGCGGCTTTGGACAGTCCCTCTGTACTGTGAAGCCACAGATGTCGAGTCGACTAGCTTCCTT
TCTCCCTTCTCCC
+
B>>99::4>>>>?ABBBBC@CCC>C@CBB@:::5:@@BBA>A;@@A<?B@CCBCCCCB??AAA@ABBBBBCC>B?AC
@C@AA;@8;<>?7
@T6XXI:00035:00667
GGGAGAAGGGAGAAAGGAAGCTAGTCACTCGAGATCTGCGGCTTTGGACAGTCCCTCTGTACTGTGAAGCCACAGAT
GTCGAGTCGACTAGCTTCCTTTCTCCCTTCTCCC
+
CC<AA@;>>:>99919584:CBCCCBC@@?BB??BDECB@=:::8:@CEDCCCCA===899A??@AA>@@>@@<<<<
@@@@=<=@@@@?==84347::-438848:==:<;
@T6XXI:00035:00677
GGGAGAAGGGAGAAAGGAAGGAGAAGTCGCCCCAAACGATTTGTTGACTATTCTCCCTCTCCCTATAGTGAGTCGTA
TTAGAATTCCCC
+
CC>BB>@BBBABBB?A?A>@?ACC@@?BBBBB8AA=ACCCC>@@?C@@<?<;=><<;<=ABA<@8=<@@@@@A<@:?
?>AAA?B<::8.
@T6XXI:00035:00687
GGGAGAAGGGAGAAAGGAAGGGGAGAAGGGAGAAAGGAAG
+
@@>@4954958;>>=B?B@BCI9:77161,.445+.,.,.
@T6XXI:00035:00695
CTAGCGACTCCGAGATCTGCGGCTTTGGACAGTCCCCTCTGTAACCTGTGGAACGCCCACAGG
+
6669>;77//,/666844433139967:77699;;?7717711013331101,-+000++.44
@T6XXI:00035:00696
GGGAGAAGGGAGAAAGGAAGCTAGTCACTCGACATCTGTGGCTTCACAGTACAGAGGGACTGTCCAAAGCCGCAGAT
CTCGAGTCGACTAGCTTCCTTTCTCCCTTCTCCC
+
>95=4958;:A@@A<>585<;;=???7766677361176606816;@ABBCB;:444-
4:;>A@=AC@@@=??A@@@=<;==8887===@@==;;7::466882:.//955
@T6XXI:00035:00697
GGGGAATTCTAATACGACTCACTATAGGGAGAGGGAGAATAGTCAACAAATCGTTTGGGGCGACTTCTCCTTCCTTT
CTCCCTTCTCCC
+
;>>1>,/,1//568=BBBBDCBD>=777.7=777-
7999=??@A@@8885:BBBE>BBB7;:BBB=??C6888>AC<@???7=;=>??;
@T6XXI:00036:00668
GTCGACTCGAGATCTGCGGCTTTGGACAGTCCCTCTGTACTGTGAAGCCACAGATGTCGAGTCGACTAGCTTCCTTT
CTCCCTTCTCCC
+
BBBCCCCCCDDBD@:<<BB@C@<@<@AA;<;C=A@>?999>><9B>@@=@???>::1....--
13369:::@B?BC7::??9?;?6??.
@T6XXI:00036:00669
GGGGAATTCTAATACGACTCACTATAGGAGAGGGAGAATAGCTTCTCCTATCG
+
?992=2928449499@?CCDB>;;000,000<7;98986722;-/.450,...
@T6XXI:00036:00683
GGGAGAAGGGAGAAAGGAAGCTAGTCGACTCGACATCTGTGGCTTCACAGTACAGAGGACTGTCCAAAGCCGCAGAT
CTCGAGTAG
+
HC8<;B?A><@@@@<@>B>@@?:0....34447888671..2-
/,/7?BBA@@===?;:;://.04403344<<===9==897=?6
```

```
@T6XXI:00036:00690
GGGAGAAGGGAGAAAGGAAGCTAGTCACTCGAGATCTGCGGCTTGGACAGTCCCTCTGTACTGTGAAGCCACAGATG
TCGAGTCGACTAGCTTCCTTCTCCCTT
+
@;:9<;79A<BBBB>B6:6:<;@?A?A:7:AA@CCCB@;9900,/445?=:::57=;==?888898<@@:==???B<
<<?????AC@@?=//,/,/,./.-+.4
@T6XXI:00037:00661
GGGGAATTCTAATACGACTCACTATAGGAGAGGAGAATAGTCAACAAATCGTTTGGGGCGACTTCTCCTT
+
999,49@<ACC@DCBBB:;:?;:<:00,,7,0,.2234:<<<=@@@?:A?<<=-777.7?>==:==:343
@T6XXI:00037:00683
GGGAGAAGGGAGAAAGGAAGGGGAGAAGGGAGAAAGGAAGCTAGT
+
AB?AA@@CB?BABB?B988:BB8B@@=??:>000,/,/,.4-..;
@T6XXI:00037:00684
GGGAGAAGGGAGAAAGGAAGGAGAAGTCGCCCCAAACGATTTGTTGACTA
+
@A69:A<9;:411:66,0,.3...658<?>DC8BC=:5557+..3...--
@T6XXI:00038:00672
GGGGAATTCTAATACGACTCACTATAGGAGAGGGAGAATAGTCTCCTTACTATCACGACCGACGTGCTCTTCCTTTC
TCCCTTCTCCC
+
BBB9A=@>ACB@CCB@>>??A:977..,0;:<?=?ABAA8==@@@>@>BCB@BBB>A?A;=<B@?===@?9;38818
;;A6;79444-
@T6XXI:00038:00677
GGGGAATTCTAATACGACTCACTATAGGGAGAGGGAGAATAGTTCAGCCTTATAGGTCGTCCTGTTCTACTTCCTTT
CTCCCTTCTCC
+
/11+1,928449@BBBCBCCCCCBB:77.7=777.733/0@885?8878=48;7834B@AA@CCC@?8:4:8;344-
486600+,++*
@T6XXI:00038:00693
GGGAGAAGGGAGAAAGGAAGGGACAGTCCATCTGTGGCTTCACAGTACAGAGGGACTGTCCAAAGCCGCAGATCTCG
AGTCGACTAG
+
CCB:<;?BA<AA@@>A8;5;B>>??A7848>=???B@AAABBB@A@@?>;30018>>@C=;==:<=;<<<<?AAA@@
@A?BBCBBBC
@T6XXI:00039:00666
GGGGAATTCTAATACGACTCACTATAGGAGAGGAGAATAGTCAACAAATCGTTTGG
+
?B@4@6954482449;??<;B9;;A?;:>777478;9>>=;555544476161,11
@T6XXI:00039:00671
GGGAGAAGGGAGAAAGGAAGGAGAAGTCGCCCCAAACGATTTGTTGACTATTCTCCCTCTCCCTATAGTGAGTCGTA
TTAGAATTCCCC
+
CD?@AC@CC>BA??=@@B@B@BBA?B???>>>.886;AAAE=B@?B??<AA=A?A=;AA==C<@@??@@@A@@@A@@
@<?@C@A58770
@T6XXI:00039:00680
GGGGAATTCTAATACGACTCACTATAGGGAGAGGGAGAATAGCCATTGTCTCCGCTCCGTTACGTTTGTCTTCCTTT
CTCCCTTCTCCC
+
CCB9B>A>BBB@C@@@BCBA?>@97111.7==BL>BAAB@8==;<><==<??>@A@A>@@?@AAAC;:837302<>:
@@@B=B>A???;
@T6XXI:00039:00682
GGGAGAAGGGAGAAAGGAAGGAGAAGTCGCCCCAAACGATTTGTTGACTATTCTCCCCCTCCCTATAGTGAGTCGTA
TTAGAATTCCCC
+
```

```
991444899544480497=ABCBC?>788833,33,1666;89?>:::<?@<@==;;;.444+003468==??===<
<8;;8482835.
@T6XXI:00039:00693
GGGAGAAGGGAGAAAGGAAGGAGAAAGCGACGTCGGGGAAACGTTTGACTATTCTCCCTCTCCCTCATAGTCGAGTC
GTAT
+
44-4::244-4499-84313;9>BB7:9>?B<8888629:6:999-44444989999-
444396773644...3...,.--
@T6XXI:00040:00650
GGGGAATTCTAATACGACTCACTATAGGAGAGGGAGAATAGTGACGTGTTGTGCGGCGCTCACTGTCTCCTCCTTTC
T
+
BBC9B?B?@@A@CCCCBCCCD;<:>;:900000-
/;;9@?@@@A@<<;?;<<<?A@AAAA@988<:::6,/,+,11,+
@T6XXI:00040:00659
GGGGAATTCTAATACGACTCACTATAGGAGAGGGAGAATAGTCAACAAATCGTTTGGGGCGACTTCTCCTTCCTTTC
TCCCTTCTCCC
+
8::0<-1,/67764:;AAAAC;;;:00,./:=<9?AA>A===?=9444-
777:B<B>@7@8//>;==;,/,/,//,08=A;@:<<ACA
@T6XXI:00040:00672
GGGGAATTCTAATACGACTCACTATAGGAGAGGGAGAATAGCTCCCACTGACTACACATGTCATTTCCCTTCCTTTC
TCCCTTCTCCC
+
CBC9B=?=@CC@CCCBBBCB@:977//-
0<;>A>A@@@C===87649848:@@@AAA@<<==@BB=BC<?<>;<=.77>@6=:=777.
@T6XXI:00040:00675
GGGAGAAGGGAGAAAGGAAGGAGAAGTCGCCCCAAACGATTTGTTGACTATTCTCCCCTCCTACTA
+
CD>BA==9=:>>>>7;8;>A?BBB?B?>@AAA5?@<?DBB@<@C@DAA>AA>A?::74679..,,.
@T6XXI:00040:00685
GGGGAATTCTAATACGACTCACTATAGGGAGAGGAGAATAGAGTAGC
+
CCCDC?A?BBB?BBCBBCCCCCCCA?<899888?8876///--+++
@T6XXI:00040:00690
GGAGAAGGGAGAAAGAAGAGCACTCGGTAGTTGGGAAATCTCAGCGGCTATTCTCCCTCTCCCTATAGTGAGTCGTA
TAGAATTCCCC
+
1,///,//+,,//+/.6699BBBCCC=@>9954858;:==977;;;>@??B:=::;,14030+0----
..55448337433/344==4
@T6XXI:00040:00691
GGGAGAAGGGAGAAAGGAAGCTAGTCGACTCGACATCTGTGGCTTCACAGATACAGGACGAGTCCAAAGCCGCAGAT
CTCG
+
DL@CBBACD@;<<D@B@C=@@B:::BCDB@@?AAA?????@<@A?@?@0***(((,****++++*49599899:=94
4=84
@T6XXI:00040:00692
GGGGAATTCTAATACGACTCACTATAGGGAGAGGGAGAATAGTCAACAAATCGTTTGGGGCGACTTCTCCTTCCTTT
CTCCCTTCCTCCC
+
944,42429CCCDBBBBBCEC?><=777.7>??B?@=<>@<<=9=;<@@;CAAAB>BCB8BBB@?;??<798;3441
886939,/,/972
@T6XXI:00041:00652
CTATCGACTCGAGATCTGCGGCTTTGGACAGTCCCTCTGTACTGTGAAGCCACAGATGTCGAGTCGACTAGCTTCCT
TTCTC
+
```

```
B<<7//////499BBDDECB?@?>8>488>>>>A6::BB??@@A@?@=ABACCCCCCCCCA@??A>88371110101
1-711
@T6XXI:00041:00666
GGGGAATTCTAATACGACTCACTATAGGGAGAGGGAGAATAGTCAACAAATCGTTTGGGGCGACTTCTCCTTCCTTT
CTCCCTTCTCCC
+
CBC7A6;8<949=@AACCCC;98?>777-
7==>@>BAA>AAAA@@>?@@<BCCCC<AAA688>??<7743424;>@<A@>>17/4-482
@T6XXI:00041:00668
GGGAGAAGGGAGAAAGGAAGTGCAGCTTACGCGGTTCGTGAAGGGAAAACTATTCTCCCTCTCCTATAGTGAGTCGT
ATTAGAACTTCCCC
+
=>:999>AA>DBCG@C@C?:88/6//,*,,22222*,,...467,161,499824/665....,.4449<9>==404
0423382310144.
@T6XXI:00041:00674
GGGAGAAGGGAGAAAGGAAGACAGGA
+
DE>CCB@BCBBBBC>@6350000.,.
@T6XXI:00041:00683
GGGAGAAGGGAGAAAGGAAGCTAGTCACTCGACATCTGTGGCTTCACAGTACAGAGGACTGTCCAAAGCCGCAGATC
TCGAGTCGACT
+
EC@==<@B@:>999142347>BC<=@A999111011111771757>BAAAB8////,/8::;A>AC<AA>===???A
AAA???@@@@@
@T6XXI:00041:00699
CTAGTCGACTCGAGATCTGCGGCTTTGGACAGTCCCTCTGTACTGTGAAGCCACAGATGTCGAGTCGTACTTAGC
+
:44444444:A=B?BCCA@@B@;<;5713366111,11616997/..44-46487377;AA?;6711,,,,*,,,
@T6XXI:00042:00653
GGGAGAAGGGAGAAAGGAAGAAGAGCGGCTCGATGGTCAGTCGAGAACTATTCTCCCTCTCCCTATAGTGAGTCGTA
CTTAGAATTCCCC
+
;;-94424:-4488-4237799<7;88483777770166;4:::442449;9;373,3>877-
6;6;6;;;00..5...4222,42444,
@T6XXI:00042:00671
GGGAGAAGGGAGAAAGGAAGGA
+
99-944244-444;-42.3434
@T6XXI:00042:00682
GGGAGAAGGGAGAAAGGAAGCTAGTCGACTCGACATCTGTGGCTCACAGTACAGAGGACTGTCCAAAGCCGCAGATC
TCGAGT
+
::59@@<>@199>>19479787711?=>:;;=00.//<:/7239?CABBB884::;3449856;AA;==;555<@@?
??@???
@T6XXI:00043:00657
AGGGAGAAGGGAGAAAGGAAGGAGAACGCGCACCCGAACCAACGGTGATCTATTCTCCCTCTCCCTATAGTGAGTCG
TAACTTAGAATTCCCC
+
444-49988>:9:;BBB?B?B?;88@CBABA???-
942424244244444449:;98=:994=A>BBBBDFCB8302,,*,,*,,.7>>?C@2
@T6XXI:00043:00674
GGGAGAAGGGAGAAAGGAAGGAGAAGTCGCCCCAAACGATTTGTTGACTATTCTCCCTCTCCCTATAGTGAGTCGTA
TTAGAATTCCCC
+
=?<AFCBEE@BBBB=B@B?CBCBB?B::;AAA5??:????>9AC?B@?ADB?A@AA<@888A588?A99:CC@A@==
=><:694/444-
@T6XXI:00043:00683
```

```
GGGGAATTCTAATACGACTCACTATAGGGAGAGGGAGAATAGTCACAATTCATGTTTGTGACCGTGTCTCCTTCCTT
+
:>?6?:@=@CC@CBABCCC?>@C>>111.7>7776:>B=@<;98300/17669@?C<??;6=89:146-+++00000
@T6XXI:00043:00693
GGGAGAAGGGAGAAAAGGAAAGG
+
CA6A99599184999/1,..+.,
@T6XXI:00043:00699
GGGAGAAGGGAGAAAGGAAGGAGAAGTCGCCCCAAACGATTTGTTGACTATTCTCC
+
44-4486;C<BCBB=A@B<@=?@@@@@???>?.880776662660/.3---2--03
@T6XXI:00044:00671
GGGAGAAGGGAGAAAGGAAGGGAAAAATAGCTGCCCCAGACTCAGGAACTATTCTCCCTCTCCTATAGTGAGTCGTA
TTAGAACTTCCCC
+
6:86:A<A@<>?>>9>=?>AB:@??>/>>789?>@@6ABD@AB;:6;>?AAB=@@BB>BB;:5;????@CCAA874:
9/404233/300+
@T6XXI:00044:00677
GGGAGAAGGGAGAAAGGAAGGGAGAGGAGAA
+
FC:;442991999919<>;>>0833.3334,
@T6XXI:00044:00683
GGGGAATTCTAATACGACTCACTATAGGGAGAGGGAGAATAGTCAACAAATCGTTTGGGGCGACTTCTCCTTCCTTT
CTCCCTTCTCCC
+
9>?2=6929>><@<;;ABA@@?>><777.7@BCD>?<=>@?@A@;:444-
7>>>><@@@6??AAC@??=>@>@>AA<AAAC7=3774:7
@T6XXI:00045:00673
GGGAGAAGGGAGAAAGGAAGGAGAAGTCGCCCCAAACGATTTGTTGACTATTCTCCCTCTCCCTATAGTGAGTCATA
TAAT
+
CD?B=<89@:8444-423139933516?8877-33,161::8:E@CCBBBC<>343-4;;;?7<<???88499/-
****)*
@T6XXI:00045:00682
GGGGAATCTAATACGACTCACTATAGGAGAGGGAGAATAGATCACCGTTGGTTCGGGTGCGCGTTCTCCTTCCTTTC
TCCCTTCTCCC
+
?@@6@@D@@A@CCBBBBBCB5447//,0;:==9?5??AA@AAABA=AB7;;<@CBC?C;;;?=<:899999A>A?:=
=>=7?>988C<
@T6XXI:00045:00689
GGGAGAAGGGAGAAAGGAAGGAGAAGTCGCCCCAAACGATTTGTTGACTATTCTCCCTCTCCCTATAGTGAGTCGTA
TTAGAATTCCCC
+
EC@CCC@BC>AA@B>B@B?A?ACC@CAA?>;>5@B>CCCBD>::57==6?A>878?:>=>@A7==A?@@B???@@@@
??AAA@A97661
@T6XXI:00046:00658
GGGAGAAGGGAGAAAGGAAGCTAGTCGACTCGACATCTGTGGCTTCACAGTACAGAGGGACTGTCCAAAGCCGCAGA
+
CDAAA?@CEBCA@?:@>@5;;C:;;CCCECC@77::::@A?<?B@CB????=<94000,4;;?@=?AA>@@>@@9=;
@T6XXI:00046:00663
GGGAGAAGGGAGAAAGGAAGCTAGTCACTCGACATCTGTGGCTTCACAGTACAGAGGGACTGTCCAAAGCCGCAGAT
CTCGAGTCGACTAG
+
CFABB@==>:>99909;>;>?@@AAB?;79777388:BCC?CCBBCCAAAC??>77718A@AA@?AA<??8::AAAA
AAA===A888?==;
@T6XXI:00046:00679
```

```
CGAGATCTGCGGCTTTGGACAGTCCCTCTGTACTGTGAAGCCACAGATGTCGAGTCGACTAGCTTCCTTTCTCCCTT
CTCCC
+
FGCBCCCCBBA>?AA<A>A@@A???;?BCCCAABBBCA>AA>C<<<<=AACDBECCCBBBC<=<>B@CC?DBCB<A=
???B8
@T6XXI:00047:00658
CTAGTCACTCGAGATCTGCGGCTTTGGACAGTCCCTCTGTACTGTGAAGCCACAGATGTCGAGTCGACTAGCTTCCT
TTCTCCCTTCTCCC
+
B@9;;A::9BBBCCE@@?BB@CCC@C?AA?=:7:5:BCDCCA?<???:<@@CCC@@@BCCBBBABBBBBC;;7=>>B
C>B@@@7>;>>@@:
@T6XXI:00047:00684
GGGAGAAGGGAGAAAGGAAGGAGAAGTCGCCCCAAACGATTTGTTGACTATTCTCCCTCTCCCTATAGTGAGTCGTA
TTAGAATTCCCC
+
9914498@C>BBCC<?=@=@=@CC@CAACCCC7@A<@9@A<;<?@FCB@@@<??BA=????C=AAA???@@@<<<<<
<<@?>;;/444-
@T6XXI:00047:00692
GGGAGAAGGGAGAAAGGAAGGGGAGAAGGGAGAAAGGAAGCTAGTCGACTCGACATCT
+
DD@BFB@BC>BA@@:><<;7??6?BC?A?9=661,73737>/0.@0..55/009----
@T6XXI:00047:00693
GGGAGAAGGGAGAAAGGAAGGAGAAGTCGCCCCAAACGATTTGTTGACTATTCTCCCTCTCCCTATAGTGAGTCGTA
TTCAGAATTCCCC
+
8>8119699-444;-
427;>9>??=?ACCCLG8CA<????<8@<@AAB@?=9;444,08394334?6;8<3730111,..4422,330-
@T6XXI:00048:00680
GGGGAATTCTAATACGACTCACTATAGGAGAGGGAGAATAGCCACCAACTATTGTTCGTCGCCGTTCTCTTCCTTTC
TCCCTTCTCCC
+
AA@7A=@=@CC@CCCAAABB@:975..-
0==BC>BAB?@8>:<><:<;?@??<??>BA???A?A@56;7857<?A<@@@A<@<??@@;
@T6XXI:00048:00685
GGGGAATCTAATACGACTCACTATAGGAGAG
+
;>>1=-1///,/444333397931110..//
@T6XXI:00048:00689
GGGAGAAGGGAGAAAGGAAGCTAGTCGACTCGACATCTGTGGCTTCACAGTACAGAGGACTGTCCAAAGCCGCAGAT
CTCG
+
CC?CCC@CC>@A@B=C?B5;;C:::BCDCCCC??@@B?CDA;=??BBGAAA@994..-
/;;;=@CAC;@@?AB@@A@<???
@T6XXI:00049:00650
GGGGAATTCTAATACGACTCACTATAGGAGAGGGAGAATAGCTACTCCCGATTAACGCAATTACACCCCTTCCTTTC
TCCCTTCTCCC
+
>A>5@,/,/6678>B@@;;<F@>?>00,00077.:=?;<@@?ABCBBA<???:?;???=?D>A???BD6A?B37718
=;A7>;><<<6
@T6XXI:00049:00671
GGGAGAAGGGAGAAAGGAAGGAGAAGTCGCCCCAAACGATTTGTTGACTATTCTCCCTCTCCCTATAGTGAGTCGTA
TTAGAATTCCCC
+
ECACC@@BC>BBBB=B?A?B@BCC@AAB<;;;5?@>BCC?>;>>>>9>:==;===<:::?;<;=====;=;=<<<68
??C@@==8;8;0
@T6XXI:00049:00674
```

```
GGGGAATTCTAATACGACTCACTATAGGGAGAGGGAGAATAGTTTTTTTTTCCCTTCACCAACCGCGAACTGCACTT
CCTTTCTCCCTTCTCCC
+
DHC:C:;;<CC@BBBBACCCCBB?=666.7=>?B=B@@=@@@@AAAAAA@"::-
427BB=B@C?@@CC?BBBBBA?<?488/8>>>.736161,
@T6XXI:00049:00675
GGGAGAAGGGAGAAAGGAAGGGGAGAAGGGAGAAAGGAAGACAGTCCATCTGTGGCTTCACAGTACAGAGGGACTGT
CCAAAGCCGCAGATCTCGAGTCGACTAG
+
BB>;98;=>:999@1958<??@7@AA>@A<:::C?C<?=?BAA@@@>@@@?@@A>@@?AAAAAACCCC@@@<?@@A@
D?BB>BC@CAAABB:::????@@ABCC<
@T6XXI:00049:00688
GGGGAATTCTAATACGACTCACTATAGGAGAGGGAGAATAGTCAACAAATCGTTTGGGGCGACTTCTCCTTCCTTTC
TCCCTTCTCCC
+
GCB9A=@=@AB@CBBBCBAA@:995/.,0<:=?=?CCAA@@@@A>@@@<BAAAC>@@C7??@@F?BBA<?;?<??<?
BCC=B;==@@<
@T6XXI:00049:00699
GGGAGAAGGGAGAAAGGAAGGTGAACAGGGTACCGTATACAGGCACGCTATTCTCCCTCTCCCTATAGTGAGTCGTA
TTAGAACTT
+
::8;@@<?A<>?>@098:=@>BAA@CCCB><;9<?987839=;::::;:BDACGAC<988@D<AA@==<==;:4003
42333244/
@T6XXI:00010:00700
CTAGTCGACTCGAGATCTGCGGCTTTGGACAGTCCCTCTGTACTGTGAAGCCACAGATGTCGAGTCGACTACTCCTT
+
9494444444444499BAA@C@8880858?CCCCB>BBCBCCBB@==A>AC@CC@@@BBBA::::;9>;;--,,*,*
@T6XXI:00010:00701
GGGAGAAGGGAGAAAGGAAGGCTAGTGGACTCGACATCTGTGGCTTCACAGTACAGAGGGACTGTCCA
+
CD?BBCBDD>@A@C>C@C8:9;B@@CB?BA@@<<<???BBBC?BB@CA?AB@C===44468A?A@@48
@T6XXI:00011:00701
GGGAGAAGGGAGAAAGGAAGCTAGTCACTCGACATCTGTGGCTTCACAGTACAGAGGGACTGTCCAAAGCCGCAGAT
CTCGAGTCGACTAG
+
BB>AAA=@@<@??A<@=?>AB:::??>:;>>00///./7;2:8?AAD@??A===<;<6<??@@A>@@<ACAA=====
@@AA===@@@<=<?
@T6XXI:00011:00713
GGGAGAAGGGAGAAAGGAAGTACACCGACATCTGTGGCTTCACAGTACAGAGGGACTGTCCAAAGCCGCAGAA
+
CCB@??@CC>?@@B>B@B@BBBCA@<9449999?BB?CBADDDCBABBBB>>>=>@@?C@@BB>CC@CCCCCC
@T6XXI:00013:00703
GGGGAATTCTAATACGACTCACTATAGGGAGAGGGAGAATAGCCTGATGCTTGGCCTTGGGTGCTTCTCCTTCCTTC
TCCCTTCTCCC
+
=@>4>696<A@<B@@@@@CCCDB@?777-
777==9?AA=??DA>BBC?<<=794824;=>46<??:=;<7:7:24/3300+0/044=1
@T6XXI:00013:00704
GGGAGAAGGGAGAAAG
+
94-49>59914/11,/
@T6XXI:00013:00709
GGGAGAAGGGAGAAAGGAAGTAGAGGGCACTGGATTGGACGGTCCAGCTATTCTCCCTCTCCCTATAGTGAGTCGTA
TTAGAATTCCCC
+
DCACBB?BB=BBBB?B@B@BCC??>@;@CA@?>;;>@>?==99>>>>>>>>>????;<<===;<:8=====AA@@<<
?>@@@?A=?::4
```

```
@T6XXI:00013:00724
GGGGAATTCTAATACGACTCACTATAGGGAGAGGGAGAATAGTCAACAAATCGTTTGGGGCGACTTCTCCTTCCTTT
CTCCCTTCTCCC
+
?=@4><@=ABCCCCBB<<<BBBB>B161-
7<<<?<?AA?A@@A@@>@AA<B@@?C>CCC8CBCBB?AA?9<:=>AA;@AAC<?:=<??;
@T6XXI:00014:00708
GGGGAGAAGGGAAGAAAGGAAGGAGAACGCGCACCCGAACCAACGGTGATCTATTCTCCCTCTCCCTATAGTGATGT
CGTACTTACGAATTCCCC
+
///*/,0*,,*,*//656589>;BBC7<;>84444-
44892/,/8,//::88>99;333,333992661...3,,,+,,5557:6/58@A@=;=5
@T6XXI:00014:00713
GGGAGAAGGGAGAAAGGAAGGAGAAGTCGCCCCAAACGATTTGTTGACTATTCTCCCTCTCCCTATAGTGAGTCGTA
TTAGAATTCCCC
+
BC@CB@@BBB==?B@C797;?BBC@CBBBBCC9CC>BBBBB>B@?BBCAAA><<==;<:?;===???=?::AA@A==
=>@AA?A>@@@5
@T6XXI:00015:00700
GGGAGAAGGGAGAAAGGAAGGGCCAGCCGGTGGGAGAAAACCGTGTGCTATTCTCCCTCTCCCTATAGTGAGTCGTA
TTAGAATTCCCC
+
?A:@9859919==C0;4:;>>:;?A@A@C@CCC;:::?=2=;@ABBBCCCD?BBCC>AA@@@9>AABBCCCCCBA@A
B@CDC>??@CC5
@T6XXI:00015:00706
TGGGGAATTCTAATACGACTCACTATAGGGAGAGGGAGAATAGTCAACAAATCGTTTGGGGCGACTTCTCCTTCCTT
TCTCCCTTCTCCC
+
DBBA7A@A?ABB@CCB>@<BBBCB>;66659??BE>A>>?AA@@A@><<63?????8?;?5????CBCCF?@=@>@C
>@@?C<A=>>>C=
@T6XXI:00015:00709
GGGAGAAGGGAGAAAGGAAGGAGAATGCACCGCAGGAAATTGCAGACTATTCTCCCTCTCCCTATAGTGAGTCGTAT
TAGAATTCCCC
+
CD@BBB@DC>ABAB>B@C@B@B??>=@BB?;>BB?>@?;B?@@@@?>>>?>@?;;:;;=;;;:;@<=A@A@@@@@@?
@BAA?A>AA@5
@T6XXI:00015:00714
GGGGAATTCTAATACGACTCACTATAGGGAGAGGAGAATAGTCAACAAAATCGTTTGGGGCGACTTCTCCTTCC
+
??=292424888=@@BB@@@B@?>;77=6::00,.220135344<====5888?@9=?@68861101..44420
@T6XXI:00015:00735
GGGAGAAGGGAGAAAGGAAGCTAGTCGACTCGACATCTGTGCTTCACAGTACAGAGGGACTGTCCAAAGCCGCAGAT
CTCG
+
CE@AAA@CB;?;996;@C>CAC?@@CBBBBBB?>777,,/+0027==BD@<;;;444-
4;;;;;38814;9?@A@@@@@@@
@T6XXI:00016:00701
GGGGAATTCTAATACGACTCACTATAGGGAGAGGGAGAATATCGTCCCTA
+
;BA5>-9594424484:7A>>9;;61162<==>@:@>>34/4.++00*+*
@T6XXI:00016:00707
GGGGAATTCTA
+
DCC:C@C@;44
@T6XXI:00016:00729
GGGGAATTCTAATACGACTCACTATAGGGAGAGGGAGAATAGCCAATCTCTCGTGGTCATTGCGCTTCTCTTCCTTT
CTCCCTTCCTCC
```

```
+
@?=6@=@=AGCBCBBCC@@@BA?97111.777>>=AAA>A???><><A?===?AA?@@@@>@@?B@>@@@?<>88;1
0,5515+,+,,+
@T6XXI:00016:00740
GGGGAATTCTAATACGACTCACTATAGGGAGAGGGAGAATAGTCCGCCAAGCCTTCTCCCCGGAACTCATCTTCCTT
TCTCCCTTCCTCCC
+
999/45958@B?@@@@@?@@AADC;<;D?CBBBB;?<?<?@@@D8<:2747><@@AAACE7@<?:==@@@?@=A>@@
;@?==:;343444-
@T6XXI:00017:00702
GGGGAATTCTAATACGACTCACTATAGGGAGAGGGAGAATAGTGACGTGTTGTGCGGCGCTCACTGTCTCCTTCCTT
TCTCCCTTCTCCC
+
949/9<A@CBB@BBBA<<<?BAB?9111.6<<AB?CAA<@:AA;;::;;=;::8==;=:<<==<:?:6<<8;;=:??
<?@CC=B>A@@@<
@T6XXI:00017:00714
GGGAGAAGGGAGAAAGGAAGGATAGTCACGGAAAGTTCAAGGGTAAACTATTCTCCCTCTCCCTATAGTGAGTCGTA
TTAGAATTCCCC
+
ED@AAAACCBBBBC>BAC?B?ABCAAAACC@CB=@A=@A@BB<BCC>C@@?>;;8;6;884A7<888878@AA@??A
A=>?B:A;AAA5
@T6XXI:00017:00715
GGGAGAAGGGAGAAAGGAAGGAGAAGTCGCCCCAAACGATTTGTTGACTATTCTCCCTCTCCCTATAGTGAGTCGTA
TTAGAATTCCCC
+
CC@CCCDCB>BCBC=@=>@A?CBB?BAABCCC9DD??@@BC;ABA@B@@@@>AAAA:?@AAB<@==?@==<<887<;
<?>==<=<AAA5
@T6XXI:00017:00732
GGGAGAAGGGAGAAAGGAAGGAGAAGTCGCCCCAAACGATTTGTTGACTATTCTCCCTCTCCCTATAGTGAGTCGTA
ATTCAGAATTCCCC
+
44-44424=-44;;049?4848<>4:;AACEC9DA=ACCB?;?:666/..33359<;=@AAA588;;3003111-
++*+*++010148884
@T6XXI:00017:00745
GGGAGAAGGGAGAAAGGAAGGAGAAGTCGCCCCAAACGATTTGTTGACTATTCTCCCTCTCCCTATAGTGAGTCGTA
TTAGAATTCCCC
+
CH?AADACA>B@@@:@>@<A?BCCBCBBCCBB8BD>A?=?C>CC@CCCAAA><@@@;@<<<A489@@AAACA@@@==
?>ABA?A>@@?4
@T6XXI:00018:00701
GGGAGAAGGGAGAAAGGAAGCTAGTCGACTCGACATCTGTGGCTTCACAGTACAGAGGGACTGTCCAAAGCCGCAGA
TCTCG
+
DDBBBB@DDACCBC>B?B=@@A???CCBA???:::?BAAAC=8:;:BBCBB@AA87431368>>@9=<7<A>A@AAA
@<<<<
@T6XXI:00018:00704
GGGGAATTCTAATACGACTCACTATAGGAGAGGGAGAATAGTCAACAAATCGTTTGGGGCGACTTCTCCTTCCTTTC
TCCCTTCTCCC
+
CBB4@5959>><CBBBCBBBB>=??//,0:0776:CA@AA@@@@@@@A<CBBBB>CCC7?@BBBAABC8:89?AA=?
@@A<@=@@@@:
@T6XXI:00018:00722
GGGGAATTCTAATACGACTCACTATAGGGAGAGGGAGAATAGTCAACAAATCGTTTGGGGCGACTTCTCCTTCCCTT
TCTCCC
+
?@@6?=@@B@@=@B@9;;;;:B@?????<?AAEE@?<<;744;33/364297666,111,11166696323/00+04
443553
```

```
@T6XXI:00018:00738
GGGAGAAGGGAGAAAGGAAGCTAGTCGACTCGACATCTGTGGCTTCACAGTACAGAG
+
490444249-4449587777<>:;;0......3669>788=>@B;;0,**((,*+--
@T6XXI:00018:00739
GGGAGAAGGGAGAAAGGAAGCTAGTCGACTCGACATCTGTGGCTTCACAGTACAGAGGGACTGTCCAAAGCCGCAGA
TCTCGAGTCGACTAG
+
CKDBBA@B>9@;;;7;>A<A>>0000////?=??A:;:??@38:?BBBCBB?=;:437-
8;;?@@>@@<@A=?===??@AAAA===<<>;<<
@T6XXI:00018:00743
GGGAGAAGGGAGAAAGGAAGGAGAACGCGCACCCGAACCAACGGTGATCTATTCTCCCTCTCCCTATAGTGAGTCGT
ATTAGAATTCCCC
+
DCACCB@DCC@@?B>C@B@C@BBB=AAA@?@AA=BECC::8<C?BBCDCBBBACBAA<;;;BB>BECA?????=ABC
CC@B@@>C@CDC9
@T6XXI:00018:00748
CTAGTCACTCGAGATCTGCGGCTTTGGACAGTCCCTCTGTACTGTGAAGCCACAGATGTCGAGTCGACTAGCTTCCT
TTCCTCCCTTCTCCC
+
A<999>4449===?@ABC@@=@CB<?=@@BBCCC>AAB?@?@AA???>@D?BBCCCDCCD??@?@A@;;:?//./:=
?,0,/8828-83884
@T6XXI:00019:00707
GGGAGAAGGGAGAAAGGAAGGAGAAGTCGCCCCAAACGATTTGTTGACTATTCTCCCTCTCCCTATAGTGAGTCGTA
TTAGAATTCCCC
+
AA<@@@=@B>@@@@=B?B?B?BBD@B??>>>>2>?8?9BCC;BB@CC?==A>@@A@;@@@?C>A@?@A<??8788<<
<>@<<;82744-
@T6XXI:00019:00733
GGGAGAAGGGAGAAAGGAAGGAGAAGTCGCCCCAAACGATTTGTTGACTATTCTCCCTCTCCCTATAGTGAGTCGTA
TTAGAATTC
+
44-
449<>9:==@@<@9?<>483711173333,33,1116?;?B>AA?=<@=888=7=?>??<?A@AAAAD<<<8873:8
4222,+
@T6XXI:00019:00737
GGGGAATTCTAATACGACTCACTATAGGGAGAGGGAGAATAGTCTTGCAATTTCCTGCGGTGCATTCTCCTTCCTTC
TCCTTCTC
+
=>>4><><@@@=@B@@AA@@?@A>>777.7===<9?@A>???AAA?@8:399-
8983992477442337l748485///,.5...
@T6XXI:00019:00739
GGGAGAAGGGAGAAAGGAAGCTAGTCGACTCGAGATCTGCGGCTTTGGACAGTCCCTCTGTACTGTGAAGCCACAGA
TGTCAGTCGACTAGCTTCCTTTCTCCCTTCTCCC
+
DC?BA@=>A198@@<@>B?ABBCBBBBAAABAAAAAABBAC@BAC>C;:<CCCAA<??=@55844::98<=;>>>?@
?<<=??@A@A@CA<<<<B<A@<@@@@;@@A@A@<
@T6XXI:00019:00740
GGGAGAAGGGAGAAAGGAAGCTAGTCGACTCGACATCTGTGGCTTCACAGTACAGAGGGACTGTCCAAAGCCGCAGA
TCTC
+
CC@AAA@DCABCBB>C??5;;A@@AAA@??CB?????=>:838:@CBCBBBA==;886466:=A<<==>??<?B=A<
=:??
@T6XXI:00019:00743
GGGGAATTCTAATACGACTCACTATAGGGAGAGGGAGAATAGTTTTTCCCTTCACTAACCGCGAACTGCACTTCCTT
TCTCCCTTCTCCC
+
```

```
DCA:D=<;<@@=AAC<;;BBBCCBB::6.7<777.7889;;44444,044==@@@@C@E???=A?AAA?AAC>C5::
5:==@<A>AAAA;
@T6XXI:00020:00716
GGGAGAAGGGAGAAAGGAAGAAATGGCAAGAATGAGTGAACGAACACACTATTCTCCCTCTCCCTATAGTGAGTCGT
AATTTAAAACTTCCCC
+
<<-9A@;?@<@@@BBB>A????:@C>BB@@A=@BBC@@@>;:::<7666616;=644,033384???@?8834./.-
-,-2224747667555
@T6XXI:00020:00718
GGGAGAAGGGAGAAAGGAAGGAGAATGCACCGCAGGAAATTGCAAGACTATTCTCCCTCTCCCTATAGTGAGTCGTA
TTAGAATTCCCC
+
DKDBBB@CC@CCCC>B?B@B@B??;;;??>;<<?>>>>;B@@===@BBCCB>BBAA:>@@@@<@?@?==<AA?>><;
<8<>>;?:?AA5
@T6XXI:00020:00721
GGGAGAAGGGAGAAAGGAAGTGCAGTTCGCGGTTAGTGAAGGGAAAAACTATTCTCCCTCTCCCTATAGTGAGTCGT
ATTAGAATTCCCC
+
BC>AA@@BCABBBB>C?A>@BABBBC@CBBBAB?@A>;?;?C>BCCC2CBCC=@@CE>AAABA=?A@@=<AAAAADB
??<?AA>A>AAA5
@T6XXI:00020:00726
GGGAGAAGGGAGAAAGGAAGCTAGTCACTCGAGATCTGCGGCTTTGGACAGTCCCTCTGTACTGTGAAGCCACAGAT
GTCGAGTCGACTAGCTTCCTTTCTCCCTTCTCCC
+
CNCCCC@BC<@@@@<>>A>AA@??A@:766::7>B?@BAA?BCC;?=?A???BB<@@=888<<<>@@;<<:=???CA
A???A888888==;9;3838B?;;;;58./.133
@T6XXI:00020:00740
GGGAGAAGGGAGAAAGGAAGGAGACAGTACCGCCCAAAATTGG
+
;>-44>5991988;-8231373378888/1,/..+/6609242
@T6XXI:00021:00716
AGTCACTCGAGATCTGCGGCTTTGGACAGTCCCTCTGTACTGTGAAGCCACAGATGTCGAGTCGACTAGCTTCCTTT
CTCCCTTCTCCC
+
<==CB?@?@@@CCCCCCB@BBB>B?BBBBAAA@@A>@BBCC@CBC?BB===@@==8<8=====8>=<=86?=A=?B>
BCCC=?>?AAA<
@T6XXI:00021:00723
TCTGCGGCTTTGGACAGTCCCTCTGTACTGTGAAGCCACAGATGTCGAGTCGACTAGCTTCCTTTCTCCCTTCTCCC
+
@;<;<<@BCE>B>BBBDBBB@BDCCCBBBBBEL:<=ADCBCA@@@@BBCBDDDDFC<;8>@=AA<???C<=;=;;;4
@T6XXI:00021:00727
AGATCTGCGGCTTTGGACAGTCCCTCTGTACTGTGAAGCCACAGATGTCGAGTCGACTAGCTTCCTTTCTCCCTTCT
CCC
+
AACCCBBBB@@CCAB>BEBCBBB>BCBBBBCCBAAC@DD>ABBCBBBBAABCDCD<<98444;=@BH=BBCC@C7<:
77.
@T6XXI:00021:00728
GGGGAATTCTAATACGACTCACTATAGGGAGAGGGAGAATAGTCAACAAATCGTTTGGGGCGACTTCTCCTTCCTTC
TCCCTTCTCC
+
CBB4>69244;8=>BBAAABCBBBAA@?9?7:;B<?<A=>;8///41224999998<<<7ACBCC?B>:7877,/,-
4433464./,
@T6XXI:00021:00743
GGGGAATTCTAATACGACTCACTATAGGGAGAGGGAGAATAGTACAAC
+
CDC:BAA?ACC@CCBABAAA@@>77111.7>>BC?A>877-//.00*+
@T6XXI:00022:00709
```

```
GGGGAATTCTAATACGACTCACTATAGGGAGAGGGAGAATAGCTCCCAATTGGATGACTCGCGCGTGCCCTTCCTTT
CTCCCTTCTCCC
+
=@>4>69698=<@BBBBBBBCBBA<777.7=>@A>AA?@A@=??<<6=4424/76=8;;66:8<=<ABC?A=@344,
49;;7=8;444,
@T6XXI:00022:00719
GGGAGAAGGGAGAAAGGAAGTGCAGTTCGCGGTTAGTGAAGGGAAAAACTATTCTCCCTCT
+
94-9?><>>:@>?B<B?B?BACBCBB>A?>><>=?>88::??9?BBB2BCAA>A@@@:>>=
@T6XXI:00022:00720
GGGGAATTCTAATACGACTCACTATAGGGAGAGGAGAAT
+
88008+96<@A@GA@@ABCDDCA??777.0:00,//.44
@T6XXI:00022:00729
CTAGTCACTCGAGATCTGCGGCTTTGGACAGTCCCTCTGTACTGTGAAGCCACAGATGTCGAGTCGACTAGCTTCCT
TTCTCCCTTCTCCC
+
1//,,,,//:::;BCCBA@?<888/:;>9=;676-
111611104400/0424937776:@@@??;;77778<=?@@@?;9300+0/00330
@T6XXI:00022:00731
GGGGAATTCTAATACGACTCACTATAGGGAGAGGGAGAATAGTCAACAAATCGTTTGGGGCGACTTCTCCTTCCTTT
CTCCCTTCTCCC
+
CEC:CFB@>>>@CCCBBCB?B@BB?66649??CC>B@A<@@C?=<9<?>:@=<<<.777.777:E@BA?>@?@=@CA
B===9==?ABA<
@T6XXI:00022:00735
GGGGAATTCTAATACGACTCACTATAGGGAGAGGGAGAATAGTCAACAAATCGTTCGGTGGCGACTTCTCCTTCCTT
TCTCCCTTCTCCC
+
999/49@>BCGDDCCBBBCCBBBCBBBC>B?:::57<;<???===>A>=60000,//6354..100/03=:=<=;:6
44303,02307=;
@T6XXI:00022:00741
GGGGAATTCTAATACGACTCACTATAGGGAGAGGGAGAATAGCCACCAACTATTGTTCGTCGCCGTTCTCTTCCTTT
CTCCCTTCTCCC
+
CDC7A>A@BBB@AAABAAAACA@>>777.777BCAD@@<??86768,/,-
16;;@@>AAA@@@>A@@888848=A@7==??:=<877<5
@T6XXI:00023:00702
GGGAGAAGGGAGAAAGGAAGCTAGTCGACTCGACATCTGTGGCTTCACAGTACAGAGGGACTGTCCAAAGCCGCAGA
TCTCGAGTCGACTAGCTTCCTTTCTCCCTTCTCCC
+
CD>AA>;>>199@A<BBBEDD@;:7,,,,,,,,433596138;?:?:::AB@>A?888-
4;;;<=9=H?AA>@@ABA@AA@@==8888=;;<@A>@?AA9=<?A1858===:
@T6XXI:00023:00705
GGGAGAAGGGAGAAAGGAAGGAGAAGTCGCCCCAAACGATTTGTTGACTATTCTCCCTCTCCCTATAGTGAGTCGTA
TTAGAATTCCCC
+
HH>B;;88=:>>>A>B?B@C@CCB@?>>>BBB6@A@@CAAA;???AACA??:==ACAA>=>A<AAA@@888??@@A@
@>AAA>@<@@?5
@T6XXI:00023:00708
GGGGAATTCTAATACGACTCACTATAGGGAGAGGGAGAATAGAGACGTATTCGTTTGCGCTCTGTTCACCTTCCTTT
CTCCCTTCTCC
+
ABA4@595988249@<;;CCCDCB@777.7==>@=AAH@A@AA99..///2728444<@@A@@AA@>@@@B7:344,
4444,/,---,
@T6XXI:00023:00714
```

GGGAGAAGGGAGAAAGGAAGAAGAGCCTACGCGCAATATAGCTGGGACTATTCTCCCTCTCCCTATAGTGAGTCGTA
TTAGAATTCC
+
:D331ABBB<;49?09479>9;;@@@49999444924449=>DEE?D=94489444-44444-
43977;66667@;==@8769;242
@T6XXI:00023:00730
GGGAGAAGGGAGAAAGGAAGGATGAGATTGGGGAAATAGAACTGTTGACTATTCTCCCTCTCCCTATAGTGAGTCGT
ATTAGAATTCCCC
+
CE@C@B@BC>BBBB>C@B@>=>??B@DC=?::6?B>CCB<;<<<?;=::>A=<====;=<?:;::<<=<;=AAA@@@
@A><@A?A?BBA5
@T6XXI:00023:00736
GGGGAATTCTAATACGACTCACTATAGGAGAGGGGAG
+
HFC:C>?;?<<,//6677;;>:7;./.,...111,10
@T6XXI:00024:00701
CTCGAGATCTGCGGCTTTGGACAGTCCCTCTGTACTGTGAAGCCACAGATGTCGAGTCGACTACGTCCTTT
+
9?B;;9CCC<=<C?BBC@B@BCCCCDF><<@999;@@@BDACC@BA@>>888988>>>791990..9,--*
@T6XXI:00024:00707
GGGGAATTCTAATACGACTCACTATAGGGAGAGGGAGAATAGTGACGTGTTGTGCGGCGCTCACTGTCTCCTTCCTT
TCTCCCTTCTCCC
+
@@?4><@=@CC@CA@BBBB;>?B?7166.7>?AC>BBA?B;;8??A<?8@?AA?:?=<===<88=A??8838<;;;;
66666,/,/;9;9
@T6XXI:00024:00717
GGGAGAAGGGAGAAAGGAAGGGGAGAAGGGAGAAAGGAAGGCTAAGTC
+
4<19@@;>>:999D9A=@?ABA7B@?<?B:@?AA:;::,0,.-.,.43
@T6XXI:00024:00729
GGGGAATTCTAATACGACTCACTATAGGGAGAGGGAGAATAGTCAACAAATCGTTTGGGGCGACTTCTCCTTCCTTT
CTCCCTTCTCCC
+
@@?4?692844244443377;1336111,111679=<=<<@@==<8>==1:9:<B>@@@6AA>>;9;0/..499;@6
;??A<A>@AAAA
@T6XXI:00024:00737
GGGGAATTCTAATACGACTCACTATAGGGAGGGAGGA
+
@=94><@@CBBABBBBAAABA?BCB?==6///,0*)*
@T6XXI:00024:00743
GGGGAATTCTAATACGACTCACTATAGGGAGAGGGAGAATAGACGCGACTTCTCCGATGCCACAACATTCTTCCTTT
CTCCCTTCTCCC
+
@@@6@8;;<DC@CCCCCCCB>=<97116.7==>@AAAA=@A888@@?AAB@DBB?CB@@B?BCB?BA>;>?<?<@C:
@<<<6;;=>@@<
@T6XXI:00025:00716
GCTAGTCACTCGAGATCTGCGGCTTGGACAGTCCCTCTGTACTGTGAAGCCACAGATGTCGAGTCGACTAGCTTCCT
TTCTCCCTTCCTCCC
+
/////44444449;ABCB><=:00,.,.444.11,111169?5;;8855=<>=;AB@@=????:<<A@>?@::8:@B
F>?>6828,/,/882
@T6XXI:00026:00724
GGGAGAAGGGAGAAAGGAAGGGAATAGCAGCAGTCGGCAACGTGAAACTATTCTCCCTCTCCCTATAGTGAGTCGTA
TTAGAATTCCCC
+
CC>BB@@CC>?A?B=@=>@CI>B?B?@BAAAB>>>??@@@AB?<<<;@BB777111.7?>;=;@@@AAAA<A=A===
=>@@@>@;>>>4

```
@T6XXI:00026:00728
GGGAGAAGGGAGAAAGGAAGCTAGTCACTCGACATCTGTGGCTTCACAGTACAGAGGGACTGTCCAAAGCCGCAGAT
CTCGAGTCGACTAG
+
BB>CCB?=:6>988-
9<><@DBAAB<????A777976@AB=@@@CBBA>CCA<633317;;?>>89944434;9;<@<<=989A@A@?@@A
@T6XXI:00026:00740
GGGAGAAGGGAGAAAGGAAGGAGAACAACACGATCGGTTAAACGTAGCTATTCTCCCTCTCCCTATAGTGAGTCGTA
TTAGAATTCCCC
+
CCADCD@BB>AA??=B?A@B@CBB@BB?>@BBBBAA@A@??;BB@@@BBBD@????;@@@@@;?<<?=<<<==<<<?
;=<==>A>A@@6
@T6XXI:00027:00726
GGGGAATTCTAATACGACTCACTATAGGGAGAGGGAGAATAGTCACATTCATGTTTGTGACCGTGTCTCCTTCCTTT
CTCCCTTCCTCC
+
ADD:C@G@CCC@CBAAABBCCCBB@7995:>?CC=A?AAA899?B>7:8:ABBCE>AA?AA????AA==;=><<BA<
AA;;28,.,//,
@T6XXI:00027:00743
GGGGAATTCTAATACGACTCACTATAGGAGAGGGAGAATAGTCAAACAAATCGTTTGGGGCGACTTC
+
444,9::7:>=11/66733370..4..,...11,15353411--/+--327>@@>8:6623.,,,*,
@T6XXI:00028:00703
GGGAGAAGGGAGAAAGGAAGTGCAGTTCGCGGTTAGTGAAG
+
490444244-4444-4979<??=;1<;11..,.446.3101
@T6XXI:00028:00705
GGGGAATTCTAATACGACTCACTATAGGAGAGGGAGAATAGCCCTGCTACCTAATGACATGAAACTCTCCCTTCCTT
TCTCCCTTCTCCC
+
CHH9BBC@@AA?BBCCBAAAC>=>?//,0;0777:<;?AA???<ABBCCC@C@@@??ABBA?C<@;;444,39;/43
136==:@>A@AA<
@T6XXI:00028:00706
GGGGAATTCTAATACGACTCACTATAGGGAGAGGAGAATAG
+
CAA8A>@?ABB?BB@@@BBB@CB;;661-6=<<:?//,,,,
@T6XXI:00028:00711
CTATCGACTCGAGATCTGCGGCTTTGGACAGTCCCTCTGTACTGTGAAGCCACAGATGTCGAGTCGACTAGCTTCCT
TTCTCCCT
+
CCA;/////666686B9978<887,377979667169?A????>;<<;>B>A@>>>>77777<<@>??A;666010.
30.-//,/
@T6XXI:00028:00731
GGGGAATTCTAATACGACTCACTATAGGGAGAGGGAGAATAGTCAACAAATCGTTTGGGGCGACTTCTCCTTCCTTT
CTCCCTTCTCCC
+
CDC9C?C@BCC@BB@@??BB?>@?6166.7==>A>A???AABBAA?@@@<B?=9989995ABBBC@AA@6868>AC<
AAAB<@=?@?C<
@T6XXI:00029:00703
GGGAGAAGGGAGAAAGGAAAGCTAGTCACTCGACATCTGTGGCTTCACAGTACAGAGGGACTGTCCAAAGCCGCAGA
TCTCGAGTCGACTAGCTTCCTTTCTCCCTTCTCCC
+
BB@B9959804444-
4233,379777761116110696779 2::<>>:::?B?AAAA@;@?<==A>@C=CC<@@>==A:99@@=;=@@989A
AA>A>AA;===D<A>AA@@9
@T6XXI:00029:00712
GGGAGAAGGAGAAAGGAAGGAGAAC
```

```
+
99-1/1,1,//68+/,377774731
@T6XXI:00029:00724
GGGGAATTCTAATACGACTCACTATAGGGAGAGGGAGAATAGATCACGTAGGCGTTCGTGTGTCGATTCTCCCTTCC
TTTCTCCCTTCTCCC
+
,--*/0?AB;;;?CCDCCBBB@BC?696.6>777.:=;:;<88336>00//,..7:8;?@A><<AA>88344-
48;244-4;;;:@488;::
@T6XXI:00029:00727
GGGAGAAGGGAGAAAGGAAAGCCTAGTCGACTCGACCATCTGTGGCTTCACAGTACAGAGGACTGTCCAAAAGCCGC
AGATCTCG
+
AC:A@@598049>>04233,331311661116111636666 8:589C@BBA>00//994857>;;;>8<<;.34433
00.----1
@T6XXI:00029:00731
GGGAGAAGGGAGAAAGGAAGAGAACGGCGACGAACAATAGTTGGTGGCTATTCTCCCTCTCCCTATAGTGAGTCGTA
TTAGAATTCCCC
+
CC?B?>=@>:>999096858?>?=AC@B@:99959?=@<<;>B>?@=BCCC@BBBB>A@BDC>@@@AAAAAAA@???
?>A@@=?/888-
@T6XXI:00029:00732
GGGAGAAGGGAGAAAGGAAGGAGAAAGCGACGTCGGGGAAACGTTTGCTATTCCTCCC
+
DE>AAB=@@<@AAB>@@C7;7;>>>268377961111,4:6::::-44///,/,///+
@T6XXI:00030:00701
GGGAGAAGGGAGAAAGGAAGGAGAAGTCGCCCCAAACGATTTGTTGACTATTCTCCCTCTCCCTATAGTGAGTCGTA
TTAGAATTCCCCC
+
CCACB@@BB>BBBB>B=?=@?ACD??A>BBCE8BB>BBBBC>CG@C??=@A?A???<?AA@C<@@>>A===<=@@9=
=>@@;9;/////+
@T6XXI:00030:00705
GGGAGAAGGGAGAAAGGAAGCTAGTCGACTCGACATCTGTGGCTTCACAGTACAGAGGGACTGTCCAAAAGCCGCAG
ATCTCG
+
BB:A@A69919911-1::,..4/11=;7011=00/5==7784395:@@BBB??AA>=>7777///,///*--
22455533400
@T6XXI:00030:00708
GGGAGAAGGGAGAAAGGAAGCTAGTCGACTCGACATCTGTGGCTTCACAGTACAGAGGACTGTCCAAAGCCGCAG
+
CE@@AA@CC@CA@?:@>@5;;?:;:CCCB>>;//0000:;94=?@DCCCBB<444..-/9;8;??AC;@@?A?;?
@T6XXI:00030:00717
GGGAGAAGGGAGAAAGGAAGGAGAAGTCGCCCCAAACGATTTGTTGACTATTCTCCCTCTCCCTATAGTGAGTCGTA
TTAGAATTCCCC
+
EE@CCC@CD>BBBB=?=>>A>ABC@CBBBCCC7::8:CCCD>CC?BBCC@@<??==;=<<@@;@@@@@@@@A@@@==
=>B?:7856761
@T6XXI:00030:00732
GGGGAATTCTAATACGACTCACTATAGGGAGAGGGAGAATAGCAAACGTTTCCCCGACGTCGCTTTCTCCTTCCTTT
CTCCCTTCTCCC
+
EHH:C=?=@BB@CCBA??AC?AB>>111-
6<<BC>CAA>@<=77718CCD>BBB9ACCCAAA?AA<AB>8;887884;=??;A<??@C?
@T6XXI:00030:00736
GGGGAATTCTAATACGACTCACTATAGGGAGAGGGAGAATAGTCAACAAATCGTTTGGGGCGACTTCTCCTTCCTTT
CTCCCTTCTCCC
+
```

```
AAA7A@B@CCC@CBB@BBCB@@>7=111.777>@=:99?CAA@AA>AAA<BCBAA<A??6AABBC?BB@:=;=>AC>
>??C=A<??AA<
@T6XXI:00030:00746
GGGAGAAGGGAGAAAGGAAGCTAGTCGACTCAAGATCTGCGGCTTTGGACAGTCCCTCTGTACTGTGAAGCCACAGA
TGTCGAGTCGACTAGCTTCCTTTCTCCCTTCTCCC
+
CC=AAC?AA:=>@@<@>A@CCBAAAA@:::AA?ACCBBB;;:666.7<@CCCCCD=?=??<<=A@@@@=@@99====
::?@A=<@?@@A@A@9999<==;@??>7<8;<<A;
@T6XXI:00031:00706
GGGGAATTCTAATACGACTCACTATAGGGAGAGGGAGAATAGATCACCGTTGTGCGGGTGCGCGTTCTCCTCCTTTC
TCCCTTCTCCCTATAGTGAGTCGTATTCAGAATTCCCC
+
CCH9B?AA@/1,///899AAB??87777.7<111,144/004000..4,,*,,,2236:5448322...3333==;5
48A:?<????478@@?@=<<<<88878<8A:9;9333.
@T6XXI:00031:00711
GGGAGAAGGGAGAAAGGAAGGGGAGAAGGGAGAAAGGAAGCTAGTCACTCGACAT
+
CB<;99;>>:?=>>9><?=???6?AA?BB9???A:>;@:>@00/55,,,/+++01
@T6XXI:00031:00715
GGGAGAAGGGAGAAAGGAAGGCACTGTGGCAGCAAGAATCGATGGGGCTATTCTCCCTCTCCCTATAGTGAGTCGTA
TTAGAATTCCCC
+
CC@B@@?BC@BBBB>BAB@B@AAABB@@=@CBBB?BEGBBBBACCC8B?>>>>??@;@>>:?7==A@ACA==<@@<@
A>>AA>@9<<<4
@T6XXI:00031:00734
GGGAGAGAATTTGGTAATGGGCTCTGCGGGATATTCGGGGTTGGGCCATTTCCCCTTATATCTCTCTCCCTATAGTG
AGTCGTATTAGAATTCCCC
+
AA<@B89;=@@<@?BBBCDG:<;BBDDNC>CC@@>9999/928;5=;=9;-
333,39977795>00/>;7;><=<A888<<>=?>;===8?=?BC9
@T6XXI:00031:00747
GGGAGAAGGAGAAAGGAAGGAGAATGCACCGCGAGGAAATTGCAAGACTATTCTCCCTACTCCCTTGATAGTAGTGT
C
+
<=,-,,*,*,,/8+/,37773739663310331099270 8+-
,,*,0,..?576@?9;@<??A9=9==87:.++0+++
@T6XXI:00032:00706
GGGAGAAGGGAGAAAGGAAGCTAGTCGACTCGACATCTGTGGCTTCACAGTACAGAGGGACTGTCCAAAGCCGCAGA
TCTCGAGTCGACTAG
+
CC@B;;2880999919;>;>A>==B::::>>???A??B=;88:6:C??@B@?;9444-
444447<@A<??<??@???AAB???AA===??A
@T6XXI:00032:00711
GGGGAATTCTAATACGACTCACTATAGGGAGAGGGAGAATAGACTGTTGTTAAAAAAACTTCAGTCTCGTTCTCTTC
CTTCTCCCTTCTCCC
+
4494<>BACCC@BBBA??@C:8;BB7765777@F>C@@>@@@AA=@?<>;3444444&8B>AABBBB=??>00,,00
+,++*00*./.5775
@T6XXI:00032:00719
CTAGTCACTCGAGATCTGCGGCTTTGGACAGTCCCTCTGTACTGTGAAGCCACAGATGTCGAGTCGACTAGCTTCCT
TTCTCCCTTCTCCC
+
D@9<<<::9CBBCBBAAABB?BBB<B@BB?;?@@>AA@@<<A?=<=A>@B@CCCBAB=?<=@BBBCBBBBBBBDB@C
D>?===;=;:===;
@T6XXI:00032:00720
GGGAGAAGGGAGAAAGGAAGCTAGTCACTCGACATCTGTGGCTTCACAGTACAGAGGGACTGTCCAAAGCCGCAGAT
CTCGAGTCGACTAGCTTCCTTTCTCCCTTCTCCC
```

```
+
@;69=@==@:A99909585;?>>>;00.....41./8@??@BBABCC?:;:?==<<;6;==??A?BC<A@>@A@@@A
9888@=;=@888<@>?9<9<B6<:==6<9<??C;
@T6XXI:00032:00725
GGGAGAAGGGAGAAAAGGAAGCTAGTCGACTCACATCTGTGGCTTCACAGTACAGAGGGACTGTCCAAAGCCGCAGA
TCTCGAGTCGACTAGCTTCCTTTCTCCCTTCTCCC
+
990499549584999,4231333..2,,,,,,,,..35879;66;9;??CDF@@?????<@@@@>?;=?7=>;@?>?@
?788?>=>?A@@@@AAA>?==>-4444-4388AC=
@T6XXI:00032:00749
GGGGAATTCTAATACGAC
+
AA@2?2424442444/..
@T6XXI:00034:00738
CGAGATCTGCGGCTTTGGACAGTCCCTCTGTACTGTGAAGCCACAGATGTCGAGTCGACTAGCTTCCTTTCTCCCTT
CTCCC
+
EBBBCDCBB@?9:775;<??BC??@=?CCCBBBEBBB@>@A>CAA?@??@AAACCCDBBCECCC@A=AB<@ACC=B<
;<DD>
@T6XXI:00034:00746
GGGAGAAGGGAGAAAGGAAGCTAGTCACTCGACATCTGTGGCTTCACAGTACAGAGGGACTGTCCAAAGCCGCAGAT
CTCGAGTCGACTAGCTTCCTTTCTCCCTTCTCCC
+
94-44;:;;58499042747?7=76=7611111069<>>>?AB@CCCBBBB??;444-
4;;=?@?AC<??<?@?@A?<<<888;;9::4730444253+0044+023888;
@T6XXI:00035:00710
GGGAGAAGGGAGAAAGGAAGCTAGTCACTCGAGATCTGCGGCTTTGGACAGTCCCTCTGTACTGTGAAGCCACAGAT
GTCGAGTCGACTAGCTTCCTTTCTCCCTTCTCCC
+
CB>AAA=@@:?999142843@>>?AA@??ACC@@BBBBBC@CCB>B?BCCCBBB>@???@???@@@@?A=<:;::;<
@@AA<<?<<;;<<<<=?B=@A<?>=A7<699@C?
@T6XXI:00035:00716
GGGAGAAGGGAGAAAGGAAGCAGTCGGACTCGACATCTGTGGCTTCACAGTACAGAGGGACTGTCCAAAGCCGCAGA
TCTCGAGTCGACT
+
DD>B?:248099990958 47?>66::36;633/1////8:=>ABCEDCBBBCCCBBBB88:BBAA=AA>ACBCCCB@
BA??ACCCBAAAC
@T6XXI:00035:00726
GGGGAATTCTAATACGACTCACTATAGGGAGAGGGAGAATAGATCACCGTTGGTTCGGGTGCGCGTTCTCCTTCCTT
TCTCCCTTCTCCC
+
EID:EC>=@A@?BCCACBBBB@CB=6664688?D>CCA>>9<<<=@@>>B?A?B?CBB@B?@>BBCCCBB?B>@;?=
6::9;6<=??@A<
@T6XXI:00035:00727
GGGGAATTCTAATACGACTCACTATAGGGAGAGGGAGAATAGTCAACAAATCGTTTGGGGCGACTTCTCCTTCCTTT
CTCCCTTCTCCC
+
AB>6B8;8=9B?B<=<CCCCCCBA?::7.7>>@@=B@?=@A@@?>9<;<6=?@AA=ABB6?;:;@@@?8?A?A?AA:
?@AA@A;=444-
@T6XXI:00035:00729
GGGGAATTCTAATACGACTCACTATAGGGAGAGGAGAATAGTAC
+
---*,2@@CCC@CBB?;;>B??@99?@A???@9>?..-/,+++*
@T6XXI:00036:00725
GGGGAATTCTAATACGACTCACTATAGGGAGAGGGAGAATAGTCAACAAATCGTTTGGGGCGACTTCTCCTTCCTTT
CTCCCTTCTCCC
+
```

```
CCE:C@B@DBB@B??>BBBBBAB96166.7==ACCCAA<@>AAAA;=?C=CBBBB>BBB9BCBBB?AA?>@>A>@A<
?@AA<A>????;
@T6XXI:00036:00738
GGGGAATTCTAATACGACTCACTATAGGAGAGGGAGAATA
+
CDC9A@A?999<449=;;@A;0045..,/33778<442-7
@T6XXI:00037:00702
GGGAGAAGGGAGAAAGGAAGAGATAAGGACGGGAGTTGACTGCAAAGCTATTCTCCCTCTCCCTATAGTGAGTCGTA
TTAGAATTCCCC
+
AA<?@@;=@<@>>B<@>@BCCDBB@@@@BBBC>C?=>?B@>@:::5:BBCD@BCEE?BBBCC?DBBBBBECAAAAA@
@<@@A>A<@@@5
@T6XXI:00037:00711
GGGGAATTCTAATACGACTCACTATAGGGAGAGGGAGAATAGATCACCGTTGGTTCGGGTGCGCGTTCTCCTTCCTT
TCTCCCTTCTCCC
+
BCF:D>@>@CCCBBBBACCCCCCBB961-
7=<<?<??=>@@999@BB=@@5:57;A@B>C@@A@CCE???@CAB=BC>CA@A;?;788;4
@T6XXI:00037:00712
GGGAGAAGGGAGAAAGGAAGCAGTCCATCTGTGGCTTCACAGTACAGAGGGACTGTCCAAAGCCGCAGATCTCGAGT
CGACTAG
+
EDABBB@DCABBBC>C?A>BCB?785:AAAAAC@CA>A9==??CCA@@@<;<=<=AA?=CA?::?:<?BA@@@===@
BBCCCBB
@T6XXI:00037:00721
GGGGAATTCTAATACGACTCACTATAGGGAGAGGGAGAATAGTTTTCCCTTCACTAACCGCGAACTGCACTTCCTTT
CTCCCTTCTCCC
+
@??4>5969AA>BBC@>@CC?ACA@:::8:??CC<A>C?CA<;5;0:;7>=@CCCC?C>@??@=;???<9766366.
7;;;:?;=?=@7
@T6XXI:00037:00724
GGGAGAAGGGAGAAAGGAAGAGGAAATTGACACAGGAGAATGGTTGTCTATTCTCCCTCTCCCTATAGTGAGTCGTA
TTAGAATTCCCC
+
DC>BB?@CCABBAB=?>A?AAG@CC>B?@BABCCB?BA?>??>>>>;<BBB@@>>?8<<???;?<<>9====<@<<<
?><AA;A>@A@6
@T6XXI:00037:00735
GGGAGAAGGGAGAAAGGAAGGAGAAGTCGCCCCAAACGATTTGTTGACTATTCTCCCTCTCCCTATAGTGAGTCGTA
TTAGAATTCCCC
+
EE>BBB@CC>>>?B>B<><@?BBC@BBABCBB7BB<@BCCC>BB@CAA?AA?=:;;;?AA@A=AA=AA==@AAAA<=
=>A??=?>@@A5
@T6XXI:00037:00737
GGGGAATTCTAATACGACTCACTATAGGGAGAGGGAGAATAGTTAACGCACCTTCCGTTACCTGTTCGCCTTCCTTT
CTCCCTTCTCCC
+
>>?4><>>@AC=AABC@@@CC@<76166.777==9???>AA@@>?<?@=;::<,0,/9907399<89=C7728=?>:
@?=>7>384@@:
@T6XXI:00037:00744
GGGAGAAGGGAGAAAGGAAGGAGAAGTCGCCCCAAACGATTTGTTGACTATTCTCCCTCTCCCTATAGTGAGTCGTA
TTAGAATTCCCC
+
AC@ECD@CB=BCCC=B>@=@=;;;>C?@BBCC9CC?BBAC@9?@26696>><?AAA;@BBCC9=999@@@@@?=<<<
<9886555555.
@T6XXI:00038:00715
GGGGAATTCTAATACGACTCACTATAGGGAGAAGGAGAAAGGAAGGAGAAGTCGAGC
+
```

```
CCD9CAC?AAB=@@@BABB@@?>>=777.771063133403232323238:861/----
@T6XXI:00038:00737
GGGAGAAGGGAGAAAGGAAGCTAGTCGACTCGAGATCTGCGGCTTTGGACAGTCCCTCTGTACTGTGAAGCCACAGA
TGTCGAGTCGACTAGCTTCCTTTCTCCCTTCTCCC
+
99144><>@0989919<?>;<;>;;000//:=>>CBBBBBC?BAB=B>B>>>>;;;<<<;;<?AA@??>@A<99===
=@@?@A===?@@@A=9=>@>AC;??AA<A689AC=
@T6XXI:00039:00702
GGGGAATTCTAATACGACTCACTATAGGGAGAGGGAGAATAGTCAACAAATCGTTTGGGGCGACTTCTCCTTCCTTT
CTCCCTTCTCCC
+
ABC:B@B@BBC@BBBBB??BB@BB?661-
166<?<?@@?A?@?AA?AAA<CBCAC=AAA7?@?<><9?<3745<AA<??@C<@=?@AC:
@T6XXI:00039:00704
GGGGAATTCTAATACGACTCACTATAGGAGAGGGAGAATAGTCAACAAATCGTTTGGGGCGTGTTCTCCTTCCTTTC
TCCCTTCTCCC
+
CDD:C@C?ABBBDAA@@BBBB:995/.,0><?C=B@C?C??@AA>@@@:ABAAA<A??6BBBBC?CBBA?>??CD:>
>DC=A588AA=
@T6XXI:00039:00715
GGGGAATTCTAATACGACTCACTATAGGGAGAGGGAGAATAGTCAACAAATCGTTTGGGGCGACTTCTCCTTCCTTT
CTCCCTTCTCCC
+
@@?4>695<AA?CDBABBBBCCB?=661.7??BE>BAA>A:??@A>A@A<C@@@C>DCG7??BBB>@@@3837=?@;
AAA@<@=@===9
@T6XXI:00039:00725
GGGAGAAGGGAGAAGGAGAAAGGAAGGAGAACAACACGATCGGTTAAACGTAGCTATTCTCCCTCTCCCTATAGTGA
GTCGTATTAGAATTCCCC
+
CG@CBB==956-,*-
+,,228888877?C?<>A@B@=BBBCC@CBCC?CCCBBC@AA@CDCCA@@@@@:?BB@?@BFAAABBAA@ACDAGAB
BB9
@T6XXI:00039:00726
GGGGAATTCTAATACGACTCACTATAGGGAGAGGGAGAATAGCCGACGTGTTCGTTTCGCTGTATTCTCCTTCCTTT
CTCCCTTCTCCC
+
CBB7A>B>BCC@C?B?>BBBB@?;;661.6==@B>A??>A<@<<@<<<A777/0/?9=79==<5=;;;<;=;=<@@@
@AAA<A>@???;
@T6XXI:00039:00730
GGGAGAAGGGAGAAAGGAAGAGAACAGCCCCTGTAAATGTGTGTGAACTATTCTCCCTCTCCCTATAGTGAGTCGTA
TTAGAATTCCCC
+
CCBBBA@BC>???B>B@B@BBBB?@BBAAA8ABBBB?BBBB?<<<?=BB?B>>>>>;?;??@;?<<A=<@<AAAA==
=>@AA>A>AA@5
@T6XXI:00039:00735
GGGAGAAGGGAGAAAGGAGTGCAGTTCGCGGTTAGTGAAGGGAAAACT
+
9>1944244-/,,2*,*,,00;@BB@;;:?<<7:;.0.445+...*.3
@T6XXI:00039:00740
CTAGTCGACTCGAGATCTGCGGCTTGGACAGTCCCTCTGTACTGTGAAGCCACAGATGTCGAGTCGACTAGCTTCCT
TTCTCCCTTCTCCC
+
:994:4444449B@B@B<110,00,.344677C@?ABCA@????<<<8<AACCAC<<<?BCCDBBBBCCCCBB?BCC
C>;:8B7><A??A?
@T6XXI:00039:00741
GGGAGAAGGGAGAAAGGAAGCTAGTCGACTCGACATCTGTGGCTTCACAGTACAGAGGACTGTCCAAAGCCGCAGAT
CTCGAGTCGACTAG
```

```
+
DD@CDB>??<?AAA;A8:8:C<???:;:???=:;:???DEC=<?>ABC???=544..-
/9;;;A?BB:BB>>>=AA==<<:<?=??????>
@T6XXI:00040:00703
GGGAGAAGGGAGAAAGGAAGCTAGTCGACTCGACATCTGTGGCTTCACAGTACAGAGGGACTGTCCAAAGCCGCAGA
TCTCGAGTCGACTAG
+
CE@CCB@DB;A>=85>;<;?BBABA9;;==??:::AAAFCA>@B@A<<:?B====44468?AAAA=AA:AA>A?<<<
<<==<<<<9<=????
@T6XXI:00040:00717
GGGGAATTCTAATACGACTCACTATAGGGAGAGGGAGAATAGCCACTAACTTA
+
CEB7B798<BBACCA??ABBB@B<?66659??CE>C@@?@4894----2--,+
@T6XXI:00040:00723
GGGGAATTCTAATACGACTCACTATAGGGAGAGGGAGAATAGTCAACAAATCGTTTGGGGCGACTTCTCCTTCCTTT
CTCCCTTCTCCC
+
BB@7B:;:<CCAB@@@CDCCB>@77116.7==BCA;99=??????@???;ABCBC?BCB8?AB?C@BBA>?<?>AA;
@=<<7<9<=AA;
@T6XXI:00040:00724
GGGGAATTCTAATACGACTCACTATAGGGAGAGGGAGAATAGTCAACAAATCGTTTGGGGCGACTTCTCCTTCCTTT
CTCCCTTCTCCC
+
CJD:D<;;<BB@CCCCBAA?@@>77111.7?==?<?@@?A=CAA=:;?A<B@???>?@?8?A<<C?BBA<?<?>AA<
A@@@;@>@@AC:
@T6XXI:00040:00733
GGGGAATTCTAATACGACTCACTATAGGGAGAGGGAGAATAGTTTTTTCCCTTCACTAACCGCGAACTGCACTTCCT
TTCTCCCTTCTCCC
+
CHI9C?A?BCB@B@@>?>>BBC?97111.7>>>@=ABB>B<?<=AAA-
@@<@<?AA?@@?;9?AC>A<@?>AB>C4::8:??C=A>@@@@;
@T6XXI:00040:00740
GGGAGAAGGGAGAAAGGAAGCTAGTCGACTCGAGATCTGCGGCTTTGGACAGTCCCTCTGTACTGTGAAGCCACAGA
TGTCGAGTCGACTCA
+
49144>599-449::?@B@BBB>:61116111119677<AA;=<?>@?B<=7744-
4443003548:6889988==<<878=://-++++++
@T6XXI:00040:00745
GGGAGAAGGGAGAAAGGAAGACTACGGCGCTGTCTGGGGAAATTACAGCTATTCTCCCTCTCCCTATAGTGAGTCGT
ATTAGAATTCCCC
+
44-44;:;;-449914=?<@@@@@BC@DCC?9994444,49-
424944444429<9A=BBBBB>BAACLAA??@@?865883445<===4
@T6XXI:00041:00712
GGGGAATTCTAATACGACTCACTATAGGGAGAGGGAGAATAGTCTTTGGCTACATCCCTTGGTTATTCCCTTCCTTT
CTCCCTTCTCCC
+
CKKBH;:;<CB?CA@@C@@@CCBD=666.7?=<=<?>><@?>>===7=>AAAAA;=<;=5869>@@=@@<@>A>=@:
=733-48:448-
@T6XXI:00041:00728
GGGGAATTCTAATA
+
444,4242444244
@T6XXI:00041:00730
GGGAGAAGGGAGAAAGGAAGGCTAGTCACTCGAGATCTGCGG
+
HK@AA@>@@:>9990423137;;>75</.....21311...,
```

```
@T6XXI:00041:00745
GGGAGAAGGGAGAAAGGAAGGAGAAAGCGACGTCGGGGAAACGTTTGCTATTCTCCCTCTCCCTATAGTGAGTCGTA
TTAGAATTCCCC
+
DDBBBB@BC>AAAD@C@B?B@CCCB=?BCCBB@????6ADCEDCCACCCBB@BDDF>BBBCCACBCBBBCCCCA@@=
A>AA@>@?AAB6
@T6XXI:00042:00707
GGGGAATTCTAATACGACTCACTATAGGAGAGGGAGAATAGCTACACACTATCTTCTCTCGCCTCCGCT
+
CCC9C@B?ABB?BBCCCCBB@:997//-0>>BE>CAB>A=<>@A@?<;;73338=:886//-,--000-
@T6XXI:00042:00711
GGGGAATTCTAATACGACTCACTATAGGGAGAGGGAGAATAGTTTTTCCCTTCACAAACCGCGAACTGCACTTCCTT
TCTCCCTTCTCCC
+
DCC7B8;:<49?@BBBCABBCBBA@???<?@BBB<A==:8835999.<;;?=<??CC<?>@BCC@CCA:<8>;>;>>
/81666;31111,
@T6XXI:00042:00713
GGGGAATTCTAATACGACTCACTATAGGGAGAGGGAGAATAGTCCCGACATCTTCAACGCGCTATTCTACTTCCTTT
CTCCCTTCTCCC
+
ELDAE;;;<;@<>9;;;???CAA?????9???BB>:8824433??<898@BCC@C@;>0//9/71>?913777133,
3311,101177;
@T6XXI:00042:00722
GGGGAATTCTAATACGACTCACTATAGGGAGAGGGAGAATAGCTACGTTTAACCGATCGTGTTGTTCTCCTTCCTTT
CTCCCTTCTCCC
+
A=92?598<CC@CBBBBCCC=<?==166.7>>@B>BAA>A<<<@@@@?@;=?;?A=<8:<==???;==A=@<>3771
8;<C<@=?AAA<
@T6XXI:00042:00725
GGGAGAAGGGAGAAAGGAAGGAGAAGTCGCCCCAAACGATTTGTTGACTATTCTCCCTCTCCCTATAGTGAGTCGTA
TTAGAATTCCCC
+
BC>?@>588198>@9A>@>A?@@@@D?@@CFF7@@<?::7?;AA?BAA@??<?@BC<@899AA?>?@<;;;488;88
7=<:65423891
@T6XXI:00042:00744
GGGGAATTCTAATACGACTCACTATAGGAGAGGGAGAATAGTCAACAAATCGTTTGGGGCGACTTCTCCTTCCTTTC
TCCCTTCTCCC
+
FDC:D>A>AGBAC<<<ADDCC><:<00,/::>=;A;<>=@AAA@<87417>=>B=BBB7AACBB?AB@5868@AB7=
=:87>69.495
@T6XXI:00043:00709
CTAGTCGACTCGAGATCTGCGGCTTTGGACAGTCCCTCTGTACTGTGAAGCCACAGATGTCGAGTCGACTAGCTTCC
TTTCTCCCTTCCTCCC
+
<@-
,,,,,//6;;99:AACBB=88708588AAACCC;<;CAAAD?==A=?BABB@AB;<;ABB??AB;87931136011,
1.--+-,-,---+
@T6XXI:00043:00711
GGGAGAAGGGAGAAAGGAAGTGCAGTTCGCGGTTAGTGAAGGGAAAAACTATTCTCCCTCTCCCTATAGTGAGTCGT
ATTAGAACTTCCCC
+
CB>:=<@CC?C<;;7;>A?BBBCGCC@B@?>4858>887:?@<ADEC2CAA@>BCCC>BBBBA=?A@??BAAAA<88
7724;@;??<=?=1
@T6XXI:00043:00721
GGGAGAAGGGAGAAAGGAAGCTAGTCGACTCGACATCTGTGGCTTCACAGTACAGAGGGACTGTCCAAAGCCGCAGA
TCTCGAGTCGACT
+
```

```
CCAABB?B?:?@@=:@=@:@@@???:::BCBA;:;AAACBA=@B@??@<<?=<<;<<<<<==AA=;==;<<;<<===
====<=<==?<?A
@T6XXI:00043:00738
GGGGAATTCTAATACGACTCACTATAGGAGAGGGAGAATAGTCAACAAATCGTTTGGGGCGACTTCTCCTTCCTTTC
TCCCTTCTCCC
+
@CH:C>?=@BB@CCBBBBBBB:997/.,0><?B>BAA>A<<=A@>??84??;;;-
6664:BBCD@BB@>A>A?AA<@AAA;A9<<AC>
@T6XXI:00043:00739
GGGGAATTCTAATACGACTCACTATAGGGAGAGGGAGAATAGTCTCCGCATGTTCGAAAGTTAGTTCTCCTTCCTTT
CTCCCTTCTCCC
+
@>>6@>A@BCCDCBCBAABA@AB7;111.7<<>?<@8838A@<==@?A@<ACB<;77:5;@=ACD?AAB;=:<477-
49994:7:;;<7
@T6XXI:00043:00741
GGGAGAAGGGAGAAAGGAAGCTAGTCACTCGACATCTGTGGCTTCACAGTACAGAGGGACTGTCCAAAGCCGCAGAT
CTCGAGTCGACTAG
+
FH@CCC@DB;@444-9<>>BCBBBB???:::::8@A???B=?@=?EEAAAC?44000-
4;;?A@>@A:@@?A?<??AAAAAA===<@?;<<
@T6XXI:00044:00717
GGGGAATTCTAATACGACTCACTATAGGGAGAGGGAGAATAGTCACTTATCATCAACCTGGTTCTACTTCCTTCC
+
AAA4@545<?@@CBEABCCCCCB97111.777AB>B@@?C=A898<;;<<56<;;8;8::56777,,,*,*,*,*
@T6XXI:00044:00740
GGGAGAAGGGAGAAAGGAAGCTAGTCGACTCGACATCTGTGGCTTCACAGTACAGAGGGACTGTCCAAAGCGCAGAT
CTCGA
+
CC@CBB@BC>BBBB?C@C8:8A7::???CBBCCBBC@@@@<;<@@AA?@?@=<;;68648<<<<=;==;=>><><<=
<=?A=
@T6XXI:00044:00743
GGGGAATTCTAATACGACTCACTATAGGGAGAGGGAGAATAGTCAACAAATCGTTTGGGGCGACTTCTCCTTCCTTT
CTCCCTTCTCCC
+
CDC7C8;:<AB>CBCBBBBBB@>97111.7==;=9;9;90..5777;;;;@>A?B>@@@7=@CCE?BA@3837:==:
@@@A;?=?@AC<
@T6XXI:00045:00710
GGGAGAAGGGAGAAAGGAAGCTAGTCGACTCGACATCTGTGGCTTCACAGTACAGAGGGACTGTCCAAAGCCGCAGA
TCTCGAGTCGACTAG
+
CE?AAA=@@<@>>>9A=?<?B?;;;/00<>>>1:::::??A:?A@CCCCBC@>9400014;;AA@=@A<AA=AA==<
<<@???AA===AA@@
@T6XXI:00045:00716
GGGAGAAGGGAGAAAGGAAGCTAGTCGACTCGACATCTGTGGCTTCACAGTACAGAGGGACTGTCCAAAGCCGCAGA
TCTCGAGTCGACTAG
+
AB>@@@>A>:>998095848?800<0..3737/66966@AA=@B?BCCBA?<<=<<<<;::<<==;<<;?C?===::
:9==>=A@???@@?A
@T6XXI:00045:00718
GGGAGAAGGGAGAAAGGAAGCTAGTCGACTCGACATCTGTGGCTTCACAGTACAGAGGACTGTCCAAAGCCGCAGAT
CTCGAGT
+
CC>D@@@@B:?;;96;?B7;;C;;:CBBAA??777:::@BA>@B@C???CBA555.../;;;BC=@@<@A=@?=<<<
===<8::
@T6XXI:00045:00722
GGGGAATTCTAATACGACTCACTATAGGGAGAGGGAGAATAGTCTTGCAATTTCCTGCGGTGCATTCTCCTTCCTTT
CTCCCTTCTCCC
```

```
+
CCC9B>A?@@AABBBAAAACC@A>=777.7>>>@>B@@>@AAA??=??BACEAD==<ACA@A;;;=A?><><?488/
716=7;696176
@T6XXI:00045:00724
GGGGAATTCTAATACGACTCACTATAGGGAGAGGGAGAATAGTACTCTGCTACTAGCCCAAGCCACTTTTCTT
+
@A;2924248888BC<<:BAA?871111,163552.--,-2224<855<<767?>97;85/0042----*--,
@T6XXI:00045:00745
CGACTCGAGATCTGCGGCTTTGGACAGTCCCTCTGTACTGTGAAGCCACAGATGTCCGAGTCGACTAGCTTCCTTTC
TCCCTTCTCCC
+
11/8877??9:4?9;:>ADC>A@@CCAAAA<@>;77;;;;779;;;:;>947:/..,,20,,-
73361731:53979111,1016676
@T6XXI:00046:00702
GGGAGAAGGGAGAAAGGAAGGAGAATGCTAACGCGCAGAGTTGGCGGACTATTCTCCCTCTCCCTATAGTGAGTCGT
ATTACGA
+
490444244-4488589;:;<<885117431373111199998987;:8868;;:<:=144;6:4;44488-----1-
--23100
@T6XXI:00046:00716
GGGAGAAGGGAGAAAGGAAGGGGAGAAGGGAGAAAGGAAGGGGAGAAGGGAGAAAGGAAGCTAGTCACTCGACATCT
GTGGCTTCACAGTACAGAGGGACTGTCCAAAGCCGCAGATCTCGAGTCGACTAG
+
BC9;<A>AB>;;;A<A=@@CCC9@==67760000,.33,/..*6<8:==:=;;9,4348=???;@@:6633486>?>
?==<==9;AD@===B;;444,49400684844453000B447>;884@>>877?
@T6XXI:00046:00733
GGGAGAAGGGAGAAAGGAAGCTAGTCGACTCGAGATCTGCGGCTTTGGACAGTCCCTCTGTACTGTGAAGCCACAGA
TGTCGAGTCGACTAGCTTCCTTTCTCCCTTCTCCC
+
CJADAACAB>@;88096858?877:777@@AAC@@@D??@C?CC?;?>@ACA?;<;<=<<????????>AA>AA@@A
A>=558A@@@@@??@?644200,48884979444,
@T6XXI:00046:00737
GGGGAATTCTAATACGACTCACTATAGGGAGAGGGAGAATAGTCAACAAATCGTTTGGGGCGACTTCTCCTTCCTTT
CTCCCTTCTCCC
+
DCJDE>@>ABB@DCCBBBBBBCCBC:::79AABC>B@A>A???A@>ACC<BBBAC>CCC8CCCCB?BB@>@>@>@A<
@@?@6:794441
@T6XXI:00047:00702
GGGAGAAGGGAGAAAGGAAGGAGAAGTCGCCCCAAACGATTTGTTGACTATTCTCCCTCTCCCTATAGTGAGTCGTA
TTAGAATTCCCC
+
DKBBBB?BB<BBBB=B@B?C@ECC?B:9<DCC9CC@CCCCC<??@CC@A<><<=<;:????A<AAA@@@AA@??===
=@AAA>A>@@@5
@T6XXI:00047:00718
GGGGAATTCTAATACGACTCACTATAGGGAGAGGGAGAATAGACGCTACTCGGTCCGCCCTTAACTATTCTTCCTTT
CTCCCTTCTCCC
+
@@>4><>=@AB@CBBBBBAAA=;96167.7>>BC?<:9<???899B@C;:;D?CB@@@C>B@B?AAAA?A?:=8<<7
<AAB<A=??@@;
@T6XXI:00047:00721
GGGAGAAGGGAGAAAGGAAGCTAGTCGACTCGACATCTGTGGCTTCACAGTACAGAGGACTGTCCAAAGCCGCAGAT
CTACGAGTCGACTAG
+
?C@GECBCC9;<BABB?B>AB;:::?88??A=???:;:A<=:<?>??@C@CA;;;//,/86..8:?=;==;==<;8/
////:883533..--
@T6XXI:00047:00728
GGGGAATTCTAATACGAC
```

```
+
:><0@,1,///,////..
@T6XXI:00047:00731
GGAGAAGGAGAAAGGAAGCTAGTCGACTCTGACATCG
+
92///,/,////+/,31399167<97730,,,,,2,,
@T6XXI:00047:00734
GGGGAATTCTAATACGACTCACTATAGGGAGAGGGAGAATAGATCACCGTTGGTTCGGGTGCGCGTTCTCCTTCCTT
TCTCCCTTCTCCC
+
CDC9CAB@BCC@CBB?BBBB>@?<961659@@@CBC==;=?>=?A??=@C@C=@>ABB=B@@@CCB@B??<?<?=?C
>A88858;>@AA<
@T6XXI:00048:00708
GGGGAATTCTAATACGACTCACTATAGGGAGAGGGAGAATAGTCAACAAATCGTTTGGGGCGACTTCTCCTTCCTTT
CTCCCTTCTCCC
+
AA@4@5959@@ABBBBBBBB?A@77111.777?BBC@D?A?BB??=?AA:A???B8???6?@CBC?BBA:===>AC>
A=AA>D9<<AA<
@T6XXI:00048:00733
GGGGAATTCTAATACGACTCACTATAGGGAGAGGGA
+
EDB6>29244424>A@??>>>7336111,1634424
@T6XXI:00048:00741
GGGAGAAGGGAGAAAGGAAGGAGAAGTCGCCCCAAACGATTTGTTGACTATTCTCCCTCTCCCTATAGTGAGTCGTA
TTAGAA
+
AA=BCC@CC=BBCC?B@C=@=@BA>ABBBBBB9BCACAAAC>BB@B@?=AA=@@@@<===AC<A==?C?@@AA@?>>
=;=664
@T6XXI:00048:00744
CTATCGACTCGAGATCTGCGGCTTTGGACAGTCC
+
AAEB@==:999=@===@@;98000,.,0......
@T6XXI:00048:00745
CTAGTCGACTCGAGATCTGCGGCTTTGGACAGTCCCTCTGTACTGTGAAGCCACAGATGTCGAGTCGACTAGCTTCC
TTTCTCCCTTCTCCC
+
>2,---
,,:>;BBBBABBBBA?ADC>C>AABC??@=?CCC<:?B@AAA=AC?A@?@CCCBBAAAABB?@@C;;:8:::C>CB@
@;@<?@@@;
@T6XXI:00049:00732
TCTGCGGCTTTGGACAGTCCCTCTGTACTGTGAAGCCACAGATGTCGAGTCGACTAGCTTCCTTTCTCCCTTCTCCC
+
BCCCCDDBBB>B?CBCBAAA>BBBBC?>ABBBC>AA@BBBA?@BBBBCBCCBA@@CCCC?B?BK>AB@C>A>A@@@<
@T6XXI:00049:00737
GGGAGAAGGGAGAAAGGAAGGTTAAACGGCTAGCGGGGAGCTCTAGCTATTCTCCCTCTCCCTATAGTGAGTCGTAT
TAGAATTCCCC
+
@A<@@@?@B=A@@C=B?A@CGG?BB>BC@CA??9999-
999AB@<<<EDDBAACC@BBBCD@BAAABCBBBC:::==>ABA?=;=AA6
@T6XXI:00049:00738
GGGGAATTCTAATACGACTCACTATAGGAGAGGAGAA
+
BKN:EAC@AAB@CBBBBA?>>>;;://,,7,0,.222
@T6XXI:00049:00742
GGGAGAAGGGAGAAAGGAAGACAGTCCATCTGTGGCTTCACAGTACAGAGGGACTGTCCAAAGCCGCAGATCTCGAG
TCGACTAG
+
```

```
CC?BBBBCCABB;;;::9-
.0073..,.7==@AA?CC?CCA@@@@@A=9999=?CC@@>??<?A?@@?A==<<<88<@BCCCCCC
@T6XXI:00021:00757
GGGAGAAGG
+
2=88+-*,*
@T6XXI:00021:00761
GGGGAATTCTAATACGACTCACTATAGGGAGAGGGAGAATAGTCAACAAATCGTTTGGGGCGACTTCTCCTTCCTTT
CTCCCTTCTCCC
+
=@>4?<><@@@ACCCCCBBB@>;77111.7>==?<?@@>B899AA????:A??<=6;??5?BAAA@BB?=?=?=@A<
?AAA<A>AAAA<
@T6XXI:00023:00759
GGGGAATTCTAATACGACTCACTATAGGAGAGGGAGAATAGTTGCCGTCATTGACGACTGGTCG
+
68808,/,/6656=?@AAA@@<<0//.,.4.11,10044<;<:::/--2//,.---203.7:;9
@T6XXI:00023:00762
GGGAGAAGGAGAAAGGAAGGAGACAGTGAGGCGCCGCACAATACGTCACTATTCTCCCTCTCCCTATAGTGAGTCGT
ATTAGAATTCCCC
+
9>:<11,1,,,,,*,*.,.3449=@<>;11,.44,3311101666==??BBC@CCBD?CC@??9?99;;;8?A>=?@
?;6??=:>4888.
@T6XXI:00023:00769
GGGGAATTCTAATACGACTCACTATAGGAGAGGGAGAATAGTCACATTCATGTTTGTGACCGTGTCT
+
8==+1,/,//67/448>>33167661101167><>4428830;?938:<77777,610042303--1
@T6XXI:00025:00764
GGGGAATTCTAATACGACTCACTATAGGGAGAGGGAGAATAGTCAACAAATCGTTTGGGGCGACTTCTCCTTCCTTT
CTCCCTTCTCCC
+
@@@6@@B?BBB@B>>>???B?AB<7111-
6==@B>B@C?@::;==<<<<;>@@??57775:@CCC?BB@;=;=?BCAB88818;=>AA<
@T6XXI:00025:00766
GGGAGAAGGGAGAAAGGAAGGAGAACAACGCGATCGGTTAAACGTAGCTATTCTCCCTCTCCCTATAGTGAGTCGTA
TTAGAATTCCCC
+
BDACCC@CB>9::B?B@B?@?ACC@BB?BBBBBBBB?B?BB@DC@@ABCBBBDCCC?CC;;99;CC@@>CCCCCBBC
C@CCD@E@A@@6
@T6XXI:00026:00758
GGACAGTCCCTCTGTACTGTGAAGCCACAGATGTCGAGTCGACTAGCTTCCTTTCTCCCTTCTCCC
+
@=@@==AAB?BCCC><=BBBAB@AA?ACBBBC@@ACCCCCBCCDBB?@;>59918>??08577775
@T6XXI:00026:00768
GGGGAATTCTAATACGACTCACTATAGGGAGAGGGAGAATAGTGACGTGTTGTGCGGCGCTCACTGTCTCCCTTCTT
TCCTCCTTCCTCC
+
99=6=@B@CBB@CECBB::87=>7//..+...5577///4666=774:=><@899=;<<@@??A<<<=8//./,//7
067//,-,479;7
@T6XXI:00026:00773
GGGGAATTCTAATACGACTCACTATAGGGAGAGGGAGAATAGTCAACAAATCGTTTGGGGCGACTTCTCCTTCCTTT
CTCCCTTCTCCC
+
CCE:C>A?ACECC???BBBBB@B96116.7<<<?<?AAAA@?@ACB@?>6<====.777.7>>>?=@?;8;:;8=>7
==CD@A=@@@@;
@T6XXI:00026:00774
CTAGTCACTCGAGATCTGCGGCTTTGGACAGTCCCTCTGTACTGTCGAAGCCACAGATGTCGAGTCGACTAGCTTCC
TTTCTCCCTTCTCCC
```

```
+
DD<9;@=99A@ABBCCBBAC@AAA<A@CBBB???;?CCCCBA93,,++36>?CBBBAABCCCBBABA@@@@AA?@C@
CD>?=A@;@8:8=C?
@T6XXI:00026:00779
GGGGAATTCTAATACGACTCACTATAGGGAGAGGGAGAATAGCCTCCCGCAGTGCACGGTTCTTGTATTCTTCCTTT
CTCCCTTCTCCC
+
===4><==@BB?BCCBBB??>BB??111.7:7BDAB@@=@@@?;??@;?>>???;;=;:;5:;;=888;==9=2431
3<8<;<887741
@T6XXI:00026:00780
GGGAGAAGGGAGAAAGGAAGCTAGTCACTCGACATCTGTGGCTTCACAGTACAGAGGACTGTCCAAAGCCGCAGATC
TCGAGTCGACTAG
+
44-448299-844;-
427;;;BCCCDA?77:9:3=@>A?B@BB?BB::;BA=>@7738AA@AA>@@<@A???ABA<<<=<8===<<@@@@
@T6XXI:00027:00764
CTATCACTCGAGATCTGCGGCTTTGGACAGTCCCTCTGTACTGTGAAGCCACAGATGTCGAGTCGACTAGCTTCCTT
TCTCCCTTCTCCC
+
CCCE<::@BECBBCBBB>>=@AA?B;>@CC==<;?BA>AC@@<??C>?B@CCCCCB?B@B?B:9:CAAFCBB=?>@D
ACBAA<A?AAAA<
@T6XXI:00027:00768
GGGAGAAGGGAGAAAGGAAGGAGAAGTCGCCCCAAACGATTTGTTGACTATTCTCCCTCTCCCTATAGTGAGTCG
+
<9-4AB@CB>B111,1-0,.6..086=CCCGG8BB<9966688888?@?>=9;444-03033+0366:8333...
@T6XXI:00028:00754
GGGGAATTCTAATACGACTCACTATAGGGAGAGGGAGAATAGTTCACGTTGCCGACTG
+
944,4:B?CDDACBB<;;;<??A?8?;=-76777-631041-/,-1113,00/03944
@T6XXI:00028:00755
GCTAGTCACTCGAGATCTGCGGCTTTGGACAGTCCCTCTGTACTGTGAAGCCACAGATGTCGAGTCGACTAGCTTCC
TTTCTCCCTTCTCCC
+
;44444444449===BBBBF?;888/87777=<??8;>>7;;:;936:23;?@??BB?:667661//...4333588
9?;==9=:4/0000+
@T6XXI:00028:00762
GGGAGAAGGGAGAAAGGAAGGAGAAGTCGCCCCAAACGATTTGTTGACTATTCTCCCTCTCCCTATAGTGAGTCGTA
TTAGAATTCCCC
+
CE@CCD?AA=>@>B>C?B?A@CCC>??BBBBB8AB=>BBC?;@?>?>>:=;:;;;;48:::=;==;;;:::=:;?=<
@?@AB=@3/..+
@T6XXI:00028:00776
GGGAGAAGGGAGAAAGGAAGGGGAGAAGGGAGAAAGGAAGCTAGTCGACTCGACAT
+
>=099><@@:>=?A196;=@BB9J@@=BE>AAA98?475:;7:::0...--000--
@T6XXI:00029:00751
GGGAGAAGGGAGAAAGGAAGGAGAAGTCGCCCCAAACGATTTGTTGACTATTCTCCCTCTCCCTATAGTGAGTCGTA
TTAGAATTCCCC
+
CCBB=?ACC>AAAC>B?C?B?BBC@CBB@?;;5?A<BCCCC<BB?B??<???CBBA:AA@<<;@@@@AAA??AAA:@
@>A@@;;3444,
@T6XXI:00029:00766
GGGAGAAGGGAGAAAGGAAGCTAGTCGACTCGAGATCTGCGGCTTTGGACAGTCCCTCTGTACTGTGAAGCCACAGA
TGGACTGTCCCTTCCTTTCTCCCTTCTCCC
+
FCBDAA>AB<>;;909<?7;<DDCBBBB?BC?:?CCCCBBB@BBB>B@CBAABAC<???AAA???AA@>AA>AA@@?
??@?@=@88858=?=AA7<<AB>C?=>=D<
```

```
@T6XXI:00029:00774
GGGGAATTCTAATACGACTCACTATAGGGAGAGGGAGAATAGCCCTTCTCCGGAGTAGAGTCGATTATTCTTCCTTT
CTCCCTTCTCCC
+
999/9<@?BBB@BBBBBBB?>@BDA77777=>C@>CAA:A;===:<=>CC@A9??CCAA@===@?:=?<<<:=>AD;
@@@B:>378==7
@T6XXI:00029:00778
GGGGAATTCTAATACGACTCACTATAGGGAGAGGAGAATAGTAGGTCCTA
+
@860<-95949<>@@CCAACCCA??777.7?775:9;:;///--,-3,-.
@T6XXI:00029:00780
GGGAGAAGGGAGAAAGGAAGCTAGTTC
+
@@:?9959@:>911,1:88--,,,.,.
@T6XXI:00029:00781
GGGGAATTCTAATACGACTCACTATAGGAGAGGGAGAAATAGACGAGCATCCTCCTGAGCTGTCTATCTCTTCCTTC
TCCTTCT
+
DBB:B=@=;99<A@AAF:;;B=<;;00,../@777000+0;::=@@888@@57313769<@@@??@AAA?>;@40,,
,,++*++
@T6XXI:00030:00753
TATCACTCGAGATCTGCGGCTTTGGACAGTCCCTCTGTACTGTGAAGCCACGAGATCGAGTCGACTAGCTTCCTTTC
TCCCTTCTCCC
+
@ECD::9BCBABCBBB@A?AAA?A@AA>;;>B>A@>@BB@BBB?30*(()((((,--
038?BCCCBA@:=:==.7777.47:99;4
@T6XXI:00030:00761
GGGAGAAGGGAGAAAGGAAGGAGAAGTCGCCCCAAACGATTTGTTGGCTATTCTCCCTCTCCCTATAGTGAGTCGTA
TTCAGAATTCCCCC
+
DE@ECD@CC@CDDC=B>A>@=ABB?BC;88>>2>?<?9<A@7>?4731404236?A<==<AD<A@999ABAA9889/
/,--000516666+
@T6XXI:00030:00768
GGGAGAAG
+
/1,/,/,/
@T6XXI:00030:00771
CTAGTCACTCGAGATCTGCGGCTTTGGACAGTCCCTCTGTACTGTGAAGCCACAGATGTCGAGTCGACTAGCTTCCT
TTCTCCCTTCTCCC
+
?99449449BBBBBCCBBBB?BCC?C>A@@?777.7>>ABCC@???D>ACABCABBB???@A@>;;;8871118886
>8A;<<;@<?=AA7
@T6XXI:00030:00778
CTAGTCGACTCGAGATCTGCGGCTTTGGACAGTCCCTCTGTACTGTGAAGCCACAGATGTCGAACTGCTTCCCTTTC
TCCCTTCTCCC
+
<==CECCCCDCCCCCBBBCCC@CCC>B=@??>???9?@@???A@?884:<A9;;AB@@>==?>>>;8660/0,00,0
<99,/,/---+
@T6XXI:00030:00779
GGGGAATTCTAATACGACTCACTATAGGGAGAGGGAGAATAGTCAACAAATCGTTTGGGGCGACTTCTCCTTCCTTT
CTCCCTTCTCCC
+
BCD:C@B?BBBACBBBCBBBB@;76111.7==>@>A??>B???A;:9A=;@>??A>CC@8????C@CCA9=;=<AA<
@AAA=B?B@AA<
@T6XXI:00030:00790
GCTAGTACGACTCGAGATCTGCGGCTTTGGACAGTCCCTCTGTACTGTGAAGCCACAGATGTCGAGCGACCTAGCTT
+
```

4/////////8;B@?ABA@@?@?<771,1017111877161555347454-.15447666666661....,.44..,
@T6XXI:00031:00755
GAGAAGGGAGAAAGGAAGAACATTGCCGGGGAGTCGAATTGTCGCTATTCTCCCTCTCCCTATAGTGAGTCGTATTA
GAATTCCCC
+
BDCB?BCBAAAC?B?A?BA>?::4:?<?CC9B@>?0,+-+--
,,.=@CABBBC@BBBCC?CBCCCB=B@CAABBB@A@?<65;;<1
@T6XXI:00031:00762
GGGAGAAGGGAGAAAGGAAGCTAGTCACTCGACATCTGTGGCTCCACAGTACAGAGGGACTGTCCAAAGCCGCAGAT
CTCGAGTCGACTAG
+
BB@CBB>>=5=444-
95848B>==@?;::BC::8CDCCBB?BD?=?A@@B@===88846:6;;;37717=:;@<<<<<?@???@@AAAAA@
@T6XXI:00031:00764
GGGGAATTCTAATACGACTCACTATAGGAGAGGGAGAATAGTCAACAAATCGTTTGGGGCGACTTCTCCTTCCTTTC
TCCCTTCTCCC
+
CCC:C@B@BB?@CB?ABBB>B:995/.,0>;>@@BAA>A99:?C<<@<;@A@??8<<<6<CAA?@??<47378??<@
AAA<@=@AAA;
@T6XXI:00031:00767
GGGGAATTCTAATACGACTCACTATAGGGAGAGGGAGAATAGATCACCGTTGGTTCGGGTGCGCGTTCTCCTTCCTT
TCTCCCTTCTCCC
+
CBB9C@B@DBB@BAABBBCB?@<79111.7>>?D>C@@>@<=9==??:?A:?:?:BAB>BCAABCCCAAA?B@B@EF
@@@@A<@>???CA
@T6XXI:00031:00771
CCTAGTCATCGAGATCTGCGGCTTTGGACAGTCCCCTCTGT
+
/,//888::49444999;9@<>>>0858>>87777,16355
@T6XXI:00031:00774
GGGGAATTCTAATACGACTCACTATAGGGAGAGAGATATAAGGGACGATGAATTGTGAGTGAAGTGTGGAAAACACC
CATTACCAAAGTTCTCTCCC
+
FCD9B?C>@A@689443737@<BBAAAD>DB?::8@@884<CD@A@998AAAA>AD@@@===<???<?<?DC6A=<<
64.,.2324...7733333,
@T6XXI:00031:00778
CTAGTCACTCGAGATCTGCGGCTTTGGACAGTCCCTCTGTACTGTGAAGCCACAGATGTCGAGTCGACTAGCTTCCT
TTCTCCCTTCTCC
+
CA944>444BABAABICCAA?ACBAB=?@CCCCC>BBB??@@=<@@@;=@?BCCCC;::BBBEBCC;;<BAA??B?B
C>B@@A<A>@@@?
@T6XXI:00031:00795
GGGAGAAGGGAGAAAGGAAGCTAGTCGACTCGACATCTGTGGCTTCACAGTACAGAGGGACTGTCCAAAGCCGCAGA
TCTCGAGTCGACTAG
+
@@<@@@?BD<@A@B9>=@=@@B>==0/../335?8869>=:8:=:????BCA@>?77717==??A>?@<@C>@@AAA
A@@@@???===@@??
@T6XXI:00032:00754
GGGGAATTCTAATACGACTCACTATAGGGAGAGGGAGAATAGCAGCCAACTCTCAAGTTGCCAAACTCTCTTCCTTT
CTCCCTTCTCCC
+
@??4><A>BCC@CBAA????>@B77111.7<<AB>C@@>@<AA=AA<<;?AAA@?=@DB@@=??;?A==??<?;@CA
=@@A<@><A@A;
@T6XXI:00032:00757
GGGGAATTCTAATACGACTCACTATAGGAGAGGGAGAATAGACAACGGCTTCCCA
+
AAA9AACABAA?CCCBB????:99?/.,0<<?A>BAC?D=<9==<8:,//,//+-

```
@T6XXI:00032:00776
GGGAGAAGGGAGAAAGGAAGCTAGTCGACTCGACATCTGTGGCTTCACAGTACAGAGGGACTGTCCAAAGCCGCAGA
TCTCG
+
CC@CCB===5=>=@<@@C7;<B:;:BBCBBBB::;;AACBA=?A?CC@??>=;8833317>>>@A>@@<@A>A@===
===><
@T6XXI:00032:00791
GGGAGAAGGGAGAAAGGAAGGAGATACAGACGCGATCGGACACGCAGCTATTCTCCCTCTCCCTATAGTGAGTCGTA
TTAGAATTCCC
+
@?<ACCBFH@AABBBB>B?B;<;D??@@@@BBB@BB;;9;B@@AAAACCBB>AAAA<A>A@C<AAA=<<;831.---
-335=;C@B?<
@T6XXI:00032:00794
GCTAGTACACTCGAGATCTGCGGCTTTGGACAGTCCCTCTGTACTGTGAAGCCACAGATGTCGAGTCGACTAGCTTC
CTTTCCTCCCTTCTCCC
+
4/////////498=;AAAB?>=4711,101666111,000068;6?85423B<?>B@B:;;BBAAAAA;<:?85564
5::,0,/7733-73664
@T6XXI:00032:00799
GGGAGAAGGGAGAAAGGAAGCTAGTCACTCGACATCTGTGGCTTCACAGTACAGAGGGACTGTCCAAAGCCGCAGAT
CTCGAGTCGACTAGCTTCC
+
BB>AA?<A@1989919485;@>>><000....310244..,.465699::>::;444,4;;:><<BA<?@<==@<=<
@<<;>=::94/-.--000*
@T6XXI:00033:00772
GGGGAATTCTAATACGACTCACTATAGGGAGAGGGAGAATAGTCAACAAATCGTTTGGGGCGACTTCTCCTTCCTTT
CTCCCTTCTCCC
+
;;;4<?CACBB?BA@@>?BBBBB>9116.677BC>A??>@@@@AA>??;:>>==>;<:<7?BBBC>BBD>A>@>@A<
AA@A<@>@@@B<
@T6XXI:00033:00780
GGGAGAAGGGAGAAAAGGAAGCTAGTCACTCGAGATCTAGTCCATCTGTGGCTTCACAGTACAGAGGGACTGTCCAA
AGCCGCAGATCTCGAGTCGACT
+
@@<@AA=>>:>9888/95847A><<@177617==?CA=?@8:544;;86<5;:8;<==?AAC>=844+0846::;A=
144888699;;0//;;;@@@@A
@T6XXI:00033:00782
GGGGAATTCTAATACGACTCACTATAGGGAGAGGGAGAATAGTCAACAAATCGTTTGGGGCGACTTCTCCTTCCTTT
CTCCCTTCTCCC
+
@@?8A@C@CBB@BBBBBBBBA@<97111.7>>@B>C@@=@<<=4858@@:@B===.777.7>>>@???==?;>9<<7
=@@@<@?AAAB:
@T6XXI:00033:00786
GGGAGAAGGGAGAAAGGAAGGAGATACCAACTGCGCGAAACGTTAGGCTATTCTCCCTCTCCCTATAGTGAGTCGTA
TTAGAATTCCCC
+
CCBCBB@CB?CBCB>C?B@C@?>ACBC?B?DBBB==766-
7??>CB?BBCCBDB??>?=@ED>C>>8;;====:::<<;<<=<A?AAA5
@T6XXI:00033:00793
GGGAGAAGGGAGAAAGGAAGCTAGTCG
+
A;:@;<89B:@>>>1958;9<1,,0**
@T6XXI:00033:00798
GGGAGAAGGGAGAAAGGAAGCTAGTCGACTCGACATCTGGTGGCTTCACAGTACAGAGGACTGTCCAAAGCCGCAGA
TCTCGAGTCGACTAG
+
```

```
@@699929<7;@@@9<9;>B?>AAB7::<0/00,,,0,,*,+*-
5599?8??=;;;==:=A?;;<8::44434::99;<>>@4444.--0+-
@T6XXI:00034:00752
GGGGAATTCTAATACGACTCACTATAGGGAGAGGGAGAATAGTCAACAAATCGTTTGGGGCGACTTCTCCTTCCTTT
CTCCCTTCTCCC
+
BBA7A=@?ACCCCCA?BCCBCC>=7111.7==BC@C@@@DA??;<<<==;@?===<>>>7BCCAB>AA?;=:=8<;7
=AAA<??CAAB7
@T6XXI:00034:00754
GGGAGAAGGGAGAAAGGAAGGACAGCTTGCCGTGTTTAAGTTGGGAGCTATTCTCCCTCTCCCTATAGTGAGTCGTA
TTAGAACTTCCCC
+
>?1949;>=5:/11,1,.34449079>>=?<??9==:=<=@>AA=B@@@CC?CDDGACBBBA<>===7734555;?<
@>A=<58:2444+
@T6XXI:00034:00773
GGGAGAAGGGAGAAAGGAAGGGAATAGCAGCAGTCGGCAACGTGAAACTATTCTCCCTCTCCCTATAGTGAGTCGTA
TTAGAATTCCCC
+
CCABBA@BB=@BBC=A>@@BB>B>BB@BBAAA@C@@@BA?@AB@?C<<<>?B@@@B><<???:?@@@@@ACAA??:?
?>AAA>A=???5
@T6XXI:00034:00774
GGGGAATCTAATACGACTCACTATAGGGAGGAGGAGATAGTTATCTACCC
+
;8858,1//65/49?;@BAA>>76111,1666601----++*++++.452
@T6XXI:00034:00776
GGGGAATTCTAATACGACTCACTATAGGGAGAGGGAGAATAGTTTTTCCCTTCACTAACCGCGAACTGCACTTCCTT
TCTCCCTTCTCC
+
CCC9B?B??>=@999>;77;887<6666211166263848734===.==:6501111010163:5<44399:5:>;>
88334+0/00-,
@T6XXI:00034:00780
GGGAGAAGGGAGAAAGGAAGCTAGTCGACTCGACCATCTGTGGCTTCACAGTACAGAGGACTGTCCAAAGCCGCAGA
TCTCGAGTCGACTAG
+
EA:A@>59809<=???13.0;@?=?7:00//:0,*,,,,,,3,./:>BBBBA=7////,/7666938817947////
/:8>>@@===@@BBA
@T6XXI:00035:00750
CTAGTCGACTCGAGATCTGCGGCTTTGGACAGTCCCTCTGTACTGTGAAGCCACAGATGTCGACGTA
+
=11/////A??AC<<<BA@@B@BCC@C?BB?????9?CCEBAA@?AAC:=@@DBCCCBB770**()(
@T6XXI:00035:00754
GGGAGAAGGGAGAAAGGAAGGAGAATACAGCGAAATGAACACGTCGCTATTCTCCCTCTCCCTATAGTGAGTCGTAT
TAGAATTCCCC
+
CC@CBB@BB>BBB?=B<;:@?CCC@CCCCCCDCC>BBB@CCBC>?<?BBD?BBBD>B@87717788BBAA???A<<<
89:@3445CC5
@T6XXI:00035:00757
GGGGAATTCTAATACGACTCACTATAGGAGAGGGAGAATAGTTTAACATTAACTC
+
ADC:C@C><9B@@?BCBCBDB?;>>00,/;077-74846=66913/0532122--
@T6XXI:00035:00762
GGGAGAAGGGAGAAAGGAAGTAATAGATCAATGCGGAGAAATGAGGACTATTCTCCCTCTCCCTATAGTGAGTCGTA
TTAGAATTCCCC
+
CC>CCC@BB=ABBC?CAAAABB@B@??ABD@CBBBAC@<;2<<7637=@@A?BDCC>@@>AA<ACCCCCBBBB@@?;
;7;??=?4888.
@T6XXI:00035:00766
```

```
GGGAGAAGGGAGAAAGGAAGGAGAAGTCGCCCCAAACGATTTGTTGACTATTCTCCCTCTCCCTATAGTGAGTCGTA
TTAGAATTCCCC
+
CDABBA@BC@BCCB?B@C@CGDAA?AA?7779-
:B>BBBBC>A>>A??<@@>@@AA;@>?:C=?@?@B===@@<<=7837=949/444-
@T6XXI:00035:00769
GGGAGAAGGGAGAAAGGAAGGAGAAGTCGCCCCAAAACGATTTGTTGACTATTCTCCCTCTCCCTATAGTGAGTCGT
ATTAGAATTCCCCC
+
CB<@?>59919>?A>ABCGJ?>>>31/99666*...*.4779;=>;<<?:;::>>>=988030+000864..55.//
755=944443000.
@T6XXI:00035:00770
GGGAGAAGGGAGAAAGGAAGGGGAGAAGGGAGAAAGGAAGCTAGTCGACTCGACATCTGTGGCTTCACAGTACAGAG
GGACTGTCCAAAGCCGCAGAT
+
CD?BB=;==:@A@C<A>?477:48??<?<6=771,7<?5:;ABBBBBCC=<;448;===??=@A<??@?C@<A<===
=;=?=AA@=@@<@A=A9=:<<
@T6XXI:00035:00777
GGGGAATTCTAATACGACTCACTATAGGGAGAGGGAGAATAGCCGTTTAATCTCCCACTGCGGCATATCCTTCCTTT
CTCCCTTCTCCC
+
;;94<>A>BBB@B@@@>>>?>@A9>11159?;CE>B@@?A8<<589;1859AAAC<A??:???A@A@@@>@>B>AC<
??@@;?=A<<=7
@T6XXI:00035:00783
CTATCGACTCGAGATCTGCGGCTTTGGACAGTCCCTCTGTACTGTGAAGCCACAGATGTCGAGTCGACTAGCTTCCT
TTCCTCCCTTCTCCC
+
99944444444/8611//.77777,313366111,17;<?A?<<?==4795897<<><61168:=?>B>>7//:555
5+.,-11049@@>=;
@T6XXI:00035:00788
GGGAGAAGGGAGAAAGGAAGGAGAACATGACCCGGCGCGATTGGTGTCTATTCTCCCTCTCCCTATAGTGAGTCGTA
TTAGAATTCCCC
+
DE?BB@<@@=BBCCAC@C?A?AAA?CCBB@@@=@ACBB<AA>CA?>8<<BB>??>C>A@@@@>==A@AAA=@<588?
@=??A:=56<=1
@T6XXI:00035:00792
GGGAGAAGGGAGAAAGGAAGCTAGTCGACTCGACATCTGTGGCTTCACAGTACAGAGGGACTGTCCAAAGCCGCAGA
TCTCGAGTCGACTAG
+
FC@CCCBBB>AACC<@?@?BC@9:9////779/:7<?<BCA69;>AAAABCAAAA8<87<??AAA?AA;??=AA<<<
====<<<<<<<@==A
@T6XXI:00035:00795
GGGAGAAGGGAGAAAGGAAGCTAGTCACTCGACATCTGTGGCTTCACAGTACAGAGGGACTGTCCAAAGCCGCAGAT
CTCGAGTCGACTAG
+
??6??>>@A<@@BB>B@D=@@C:?<?77617<;5=>877?A?@@?ABBAAA=;;444-
49:448;=@6<<:98=AC@@===@A?<=:98A@
@T6XXI:00035:00796
GGGGAATTCTAATACGACTCACTATAGGGAGAGGGAGAATAGATCACCGTTGGTTCGGGTGCGCGTTCTCCTTCCTT
TCTCCCTTCTCCC
+
CDD:C?B>BBC@CAAAADB??@>96116.7>>@C>F@?=?=ABA=:82:?375::@>><A;;;@AA?ACB@C@C?BB
@@@AA<A=?@?A7
@T6XXI:00035:00797
GGGAGAAGGGAGAAAGGAAGTGCAGTTCGCGGTTAGTGAAGGGAAAACTATTCTCCCTCTCCCTATAGTGAGTCGTA
TTAGAATTCCCC
+
```

```
DKACCB@BB>BBBB>BAB<><BBBBB@?@?@>@?AAACC=AA<AAA6ADBC>>BBB=?9?B>;=999;;===<<<<@
@>AAA>B>AAA6
@T6XXI:00035:00798
GGGGAATTCTAATACGACTCACTATAGGGAGAGGGAGAATAGTCAACAAAATCGTTTGGGGCGACTTCTCCTTCCTT
TCTCCCTTCTCCC
+
:?F6DBD@CBB@??@BCCC?CCB<6667.7>7776:;=;>;;9::74444+177799=9=5999672166/323566
4;@?A<@>@@AD<
@T6XXI:00036:00798
CTAGTCACTCGAGATCTGCGGCTTTGGACAGTCCCTCTGTACTGTGAAGCCACAGATGTCGAGTCGACTGCTTCCTT
TCTCCCTTCTCCC
+
B=111>8==CCDCBBBBBBB@BBB<B?AAAAAA@;@@A@@??<<?<=<=B<?ABDB>==CCAAABBBCCC::8;?AC
?AA>C?C===<?7
@T6XXI:00037:00756
GGGAGAAGGGAGAAAGGAAGTGCAGTTCACGGTTAGTGAAGGGAAAAACTATTCTCCCTCTCCCTATAGTGAGTCGT
ATTAGAATTCCCC
+
ABABBB@CC>C@>=:@=?<@CCCCCB?BBBB@B>AAAA@>;;,166:0;CCC?BBCC>C<?<=;=<<@AAAA====<
?@?@AA>A>@@@5
@T6XXI:00037:00764
GGGGAATTCTAATACGACTCACTATAGGGAGAGGGAGAATAGTCAACAAATCGTTTGGGGCGACTTCTCCTTCCTTT
CTCCCTTCTCCC
+
BB@7A798<BB@CBA??BBBB@;76611.7>>>ABBAA>A99@A=<<;<;>@>@@>@CC7=@CCC@AA?>@=@?AA=
>?@A=B>A???<
@T6XXI:00037:00774
CTAGTCGACTCGAGATCTGCGGCTTTGGACAGTCCCTCTGTACTGTGAAGCCACAGATGTCGAGTCGACTAGCTTCC
TTTCTCCCTTCTCC
+
A9944444449;=?CCCCDCC?BAC>B?A@>=7::5:BBCCCCC===@>@BABECCB@@??BB:;<A@A@@A:1073
77.774;7;88//,
@T6XXI:00037:00785
CTAGTCGACTCGAGATCTGCGGCTTTGGACAGTCCCTCTGTACTGTGAAGCCACAGATGTCGAGTCGACTGCTTCCT
TTCTCCCTTCTCCCCTTCCTTTCTCCCTTCTC
+
CA;;;;99CFCBBBCCBA>A>@BBB<B<>@CCAAA<???BBCCAA?AA>?A?BBC@@@CCCCCA?AAAB@?::7:<?
B@CB@A<;343444-48;344-49;@:?>??=
@T6XXI:00037:00798
GGGGAATTCTAATACGACTCACTATAGGGAGAGGGAGAATAGTCAACAAATCGTTTGGGGCGACTTCTCCTTCCTTT
CTCCCTTCTCCC
+
ECD:B?C@AAABKCCCBBBBB?<><777.7;;;79655568:?@A>@AA@CCAAA?ADC5@????:=>;3424:;94
8003+0/40031
@T6XXI:00038:00751
GGGGAATTCTAATACGACTCACTATAGGGAGAGGAGAATAGTACGAAC
+
;;;0;,/,/667/999BCDDCCC>9166.7777476646,++++++*+
@T6XXI:00038:00757
GGGAGAAGGGAGAAAGGAAGTAGTCGACTCGACATCTGTGGCTTCACAGTACAGAGGGACTGTCCAAAGCCGCAGAT
CTCGAGTCGACTAG
+
CB>A@>>BC?CCCC>C;:5:?0000..437:;<:::4A@@<<<8<BCBCC<;9?83308???BD?BG>DB?BBBBBB
B@@?AABAAACB@?
@T6XXI:00038:00758
CTAGTCACTCGAGATCTGCGGCTTTGGACAGTCCCTCTGTACTGTGAAGCCACAGATGTCGAGTCGACTAGCTTCCT
TTCTCCCTTCTCCC
```

```
+
://////49CCCCCCCBB@?>@AA?B=@?AA9::4:AABBAAC>>=;>?C@BBB;;<BBBCCCBBBCAAABBB?A?B
C>A??A<A>AAAA;
@T6XXI:00038:00788
GGGAATTCTAATACGACTCACTATAGGGAGAGGGAGAATAGTTCCGAGTTTTCCCGATTCGTTGTATTCTTCCTTTC
TCCCTTCTCCC
+
BC>B>A?BBC@CBB@@ACCB@B?=669.:@?CE@C@@<?;@C>@>???ADC5AA<99:>AAA=?@;<;;C@CBBC?D
BBC>B?ACDC9
@T6XXI:00038:00789
GGGGAATTCTAATACGACTCACTATAGGGAGAGGGAGAATAGACATTTGCCCTTCGCCATGTGTACTATCTTCCTTT
CTCCCTTCTCCC
+
BEB7A798;AA>BCBBAACBBCBB<<A?88??AB>BAA>AA889@@B?AAA9=;=AA>AA======@AA<=:8;@@;
@@>>1748=AC?
@T6XXI:00038:00797
AGTCACTCGAGATCTGCGGCTTTGGACAGTCCCTCTGTACTGTGAAGCCACAGATGTCGAGTCGACTAGCTTCCTTT
CTCCCTTCTCCCTTCCTTTCTCCCTTCTCCC
+
ACCBBBBCBBBCCBAC@?>@CCAC@CCCBCBB>@@@>>>@@CA@A;???CCC@<<@A===9==@B==?74:;?@CC>
BBCC>?9;444,49;3441409;497:444+
@T6XXI:00038:00798
CTAGTCACTCGAGATCTGCGGCTTTGGACAGTCCCTCTGTACTGTGAAGCCACAGATGTCGAGTCGACTAGCTTCCT
TTCTCCCTTCTCCC
+
@11////4=CCBCCFBBBB<>BCC>A>AABC???=@DGCCCC<599A>@C@BBCB??>AABBB@@@@BBCA??:?;?
A>A>??7>>;<=@<
@T6XXI:00039:00770
CTAGTCACTCGAGATCTGCGGCTTTGGACAGTCCCTCTGTACTGTGAAGCCACAGATGTCGAGTCGACTAGCTTCCT
TTCTCCCTTCTCCC
+
>://///49BBCBABA>><?>??9;?>?B@@@@@=@@@@A@@<:??A>AC@CCCCABABBBBB?@@DCCCAAA@C?D
B>@==C;?<??@A=
@T6XXI:00039:00788
GGGGAATTCTAATACGACTCACTATAGGGAGAGGGAGAATAGTCAACAAATCGTTTGGGGCGACTTCCTCCTTCCTT
TCTCCCTTCTCCC
+
BEC4B61,///,////333>B6776111,1><<=9=8:-/.5:7=<===588?>7,111,14333,.,.3,-
,.5444<88836::9;;6
@T6XXI:00039:00799
GGGAGAAGGGAGAAAGGAAGGAGAAGTCGCCCCAAACGATTTGTTGACTATTCTCCCTCTCCCTATAGTGTAGTCGT
ATTAGAATTCCCC
+
9@:?>>5990944;-4271399;B@??@@BAA7B>766111,117>ACBAA;==<A<@899A7<;?;/////4-.-
4665:8656566>4
@T6XXI:00040:00766
GGGGAATTCTAATACGACTCACTATAGGGAGAGGGAGAATAGTCAACAAATCGTTTGGGGCGACTTCTCCTTCCTTT
CTCCCTTCTCCC
+
EDC:B7;:<@A@CCCBB??B@@<76166.7==BCBD@@>A@@@A=<=A@;BBBBC8:8:6???@D>BBA>A>A>@A<
AAAA<A<??@@;
@T6XXI:00040:00771
GGGAGAAGGGAGAAAGGAAGCTAGTCGACTCGACATCTGTGGCTTCACAGTACAGAGGGACTGTCCAAAGCCGCAGA
TCTCGAGTCGACTAG
+
```

```
CC>B@@644-
9999096858>>::;/00<<>>0;:::9::@38;@CDCCCCA@?8733,4999=?>@A<@C@BBA@@@@@A@@A???
?@@??
@T6XXI:00040:00776
GGGGAATTCTAATACGACTCACTATAGGGAGAGGGAGAATAGTTTCGCGTCTTCAGCCTTTCGATTCTCCTTCCTTT
CTCCCTTCTCCC
+
999/99@>AAB@CBAABBBB>CB77111.777?B>B@A>A@@@A<??===AA>@===<>?:?@@A@@@@>@>@>@B;
@?@?7>;>??A=
@T6XXI:00040:00782
GGGGAATTCTAATACGACTCACTATAGGGAGAGGGAGAATAGCTACAACATGTTTGGGCGTGTCTCTACTTCTCCCT
+
@@@6@>B@DCB@@??BBBBB?BB99111-6==?B>B=95773+,,,0)*+,355+..+49977331111636116,3
@T6XXI:00040:00786
GGGAGAAGGGAGAAAGGAAGTGCAGTTCGCGGTTAGTGAAGGGAAAACTATTCTCCCTCTCCCTATAGTGAGTCGTA
TTAGAATTCCCC
+
9914999>A:@@AA<B>A=ABAAA@A=@ABB=@=@@AFC@CD>CCB6???@<AABA:?>777,04335;8<>@889@
A>A@@>=<AAA5
@T6XXI:00040:00788
GGGGAATTCTAATACGACTCACTATAGGGAGAGGGAGAATAGCACCAACCACTGTACAGAGGCCATTCTCCTTCCTT
TCTCCCTTCTCCC
+
A@@6@<==@@@=@BBBCCBB?CB>>111.7<<=A>A??>AA::8644,/,/68;@?ABBBB>B=??=B@@?A=A?BD
>A@@@<@=?;<<6
@T6XXI:00040:00798
ATACGACTCACTATAGGGAGAGGGAGAATAGTCAACAAATCGTTTGGGGCGACTTCTCCTTCCTTTCTCCCTTCTCC
C
+
@??CCCCCCCBA;;37708?ABC>AAA@BBBBAA?BBBBAAABB=?BB8B>>@D@@BB?B>?:::;:?=94>;????
;
@T6XXI:00041:00750
GGGGAATTCTAATACGACTCACTATAGGGAGAGGGAGAATAGTTTTCCCTCACTAACCGCGAACTGCACTTCCTTCT
CCCTTCTCC
+
999298?<>>>=@@A?BB;:;;;8:@??9>;>>?,0./.-
6>A==5==4536111017777?<=73463:::,/,.122220---,
@T6XXI:00041:00753
GGGAGAAGGGAGAAAGGAAGCTAGTCGACCTACATCTGTGGCTTCACAGTACAGAGGACTGTCCAAAGCCGCAGATC
TCGAGTCGAC
+
CD@AA?@CCCEAAB<@=@>@@<9:0/00<,,,,,,,/7775=??ABCBCCCBB@3327>>>A@@BB<@A;==???A@
A@AAAA@@??
@T6XXI:00041:00790
GGGAGAAGGGAGAAAGGAAGTAAATGCGAGTGTCAGTAAACGGGAGACTATTCTCCCTACTCCCTATAGTGAGTCGT
ATTAGAACTTCCCC
+
491444244-444:-4231377?;888>;;9;::?BBCC<AAC>@=<=CD>997;;,---1110144333--
.3...444684989/444+
@T6XXI:00041:00797
GGGGAATTCTAATACGACTCACTATAGGGAGAGGGAGAATAGTCATTGCCCCACATCAATTACCGTTCTCTTCCTTT
CTCCCTTCTCCC
+
CCC:C@C@BDDCCBBBCBCB;@B>;6665:??CE>B@@?A<<<=<A>?;<:4><8==?>A?8848??>?:><>3785
8AAD<@>@@AA;
@T6XXI:00042:00764
```

```
GGGGAATTCTAATACGACTCACTATAGGAGAGGGAGAATAGTCAACAAATCGTTTGGGGCGACTTCTCCTTCCTTTC
TCCCTTCTCCC
+
999/429;>AB?DBBBBCCAA>>@9//,0;;>A>CAC?@<???><<<<7?A===-766-
7??AB@BA@>A@H>??<?@@C<A9<<AC=
@T6XXI:00042:00770
GGGGAATTCTAATACGACTCACTATAGGGAGAGGGAGAATAGTCAACAAATCGTTTGGGGCGACTTCTCCTTCCTTT
CTCCCTTCTCCC
+
ABB7B;;9<BC@CBB@DBCCABAB?777.7>>>@?AC@>@@@==;8::<6?BCCC>CCC8CBBBB?CFC<?<?>BC<
@@@@;?;>@@@;
@T6XXI:00042:00776
GGGGGAATTCTAATACGACTCACTATAGGGAGAGGAGAATAGTACC
+
,,,,)/7999995488@AB@@@B???777.771;:647/304----
@T6XXI:00042:00779
GGGAGAAGGGAGAAAGGAAGGCGAACAGGGTACCGTATACAGGCACGCTATTCTCCCTCTCCCTATAGTGAGTCGTA
TTAGAATTCCCC
+
CD>CAA9<919@@@:B9;78@>>?<>777.16601797@B8838>>?@@@@?BAAA:??AAA<??899??@@@<===
@=@@A;<;;=A5
@T6XXI:00042:00780
CTAGTCACTCGAGATCTGCGGCTTTGGACAGTCCCTCTGTACTGTGAAGCCACAGATGTCGAGTCGACTAGCTTCCT
TTCCTCCCTTCCTCCC
+
>1111=:?AAACBCDCBCBB@A@@=A=AA?@@@C@BCBBABA@8<<?>?B?BGBBB@??@@77:@@>?AB://:755
5+.,+0005+/,/72+
@T6XXI:00043:00768
GGGAGAAGGGAGAAAGGAAGGAGAAC
+
94-444244-4444-/666=-0..,/
@T6XXI:00043:00790
GGGGAATTCTAATACGACTCACTATAGGGAGAGGGAGAATAGACAAGACAGTCAGTTGTCTTCTATTCTCTTCCTTT
CTCCCTTCTCCC
+
DCE:CAC@CCB@BBB?BBBBBB>97111.7==BE>B@@?A====>;@@AAAA@=?<:<88=>A=??ACCCB>A;==9
=DDC<A?===<7
@T6XXI:00044:00772
CTAGTCGACTCGAGGTCTGCGGCTTTGGACAGTCCCTCTGTACTGTGAAGCCACGATGTCGAGTCGACTAGCTTCCT
TTCTCCCTTCTCCC
+
>8,--
//,888;9=89DCCCHBCDC?B?BBBB?>>=@BCDB;;>;???>?B@BBBB<<;AAABCCC@@@CBBB?A?AAAAB@
D<@>@@@C=
@T6XXI:00044:00791
GGGGAATTCTAATACGACTCACTATAGGGAGAGGGAGAATAGTCAACAAATCGTTTGGGGCGACTTCTCCTTCCTTT
CTCCCTTCCTCCC
+
CBB6@=?<=89=@AAB;;;AC???>777-7>;;:6:--,-
8466@>?@@:A@@?@<@AB7AACDA>B:4378;8<C>=99;68,/,,,,+
@T6XXI:00045:00759
GGGAGAAGGGAGAAAGGAAGGAGAAGTCGCCCCAAACGATTTGTTGACTATTCTCCCTCTCCCTATAGTGAGTCGTA
TTAGAATTCCCC
+
94-
9?A?BC?AAAC?C>A?A?ABC?BACBCCC9CC?ABABB<??;?C?==C=@@AC:@@@@A=B@@@AAAA889A=<894
--,-,---+
```

```
@T6XXI:00045:00764
GGGAGAAGGGAGAAAGGAAGGAGAAGTCGCCCCAAACGATTTGTTGACTATTCTCCCTCTCCCTATAGTGAGTCGTA
TTCAGAATTCCCC
+
CE@BBB@CC@BBBC=@>B=@=@CC@CBBBBCC6@@<?<@AC>CC@BBCA??<?ABB<?<==C>@@@@@AA=@9=<::
:.0088464:980
@T6XXI:00045:00767
GGGAGAAGGGAGAAAGGAAGTGCAGTTCGCGGTTAGTGAAGGGAAAAACTATTCTCCCTCCCCTATAGTGAGTCGTA
TTAGAATTCCCC
+
DCACBA==99>>>><@>B?CBCCCCB@@@><?B=@@@BB:;;-
7777,8?>=:=777.:>>A9BAA???A=<<<<8:?>C@@<=3444+
@T6XXI:00045:00777
GGGGAATTCTAATACGACTCACTATAGGGAGAG
+
>>:5:;D@CBB>?B=>>>8711311111,1663
@T6XXI:00045:00789
CTATCGACTCGAGATCTGCGGCTTTGGACAGTCCCTCTGTACTGTGAAGCCACAGATGTCGAGTCGACTAGCTTCCT
TTCTCCCTTCTCCC
+
B;;6/////6;@?AACCCBA?ACC@C=@@CA:::7:@ABBBCD=A==;=B@CDBBEBCBAAABABBAA@?<=60737
><@=AA697:344-
@T6XXI:00046:00752
GGGAGAAGGGAGAAAGGAAGAATACGACACGGGAGCCAAACGTGCAACTATTCTCCCTCTCCCTATAGTGAGTCGTA
TTAGAATTCCCC
+
CB:;9;<>>:@=?D9;8;?@B@C@AACCBBBB>;99:;;:>?>@A@@CBA?=?>>>8><111,167@:89B???>=<
;<=48/02333.
@T6XXI:00046:00771
GGGGAATTCTAATACGACTCACTATAGGGAGAGGGAGAATAGAGACGTATTCGTTTGCGCTCTGTTCACCTTCCTTT
CTCCCTTCTCCC
+
DDD9B?B?A>><@CBBBACCAB@>=676.7>>>@<?<?<??????=;<<<9<AAA@@@@@@@A@@=@>=:>9=:=A<
AAAA<?>?@AC<
@T6XXI:00046:00774
GGGAGAAGGGAGAAAGGAAGGAGACAGTGAGCGCCGCACAACACGTCACTATTCTCCCTCTCCCTATAGTGAGTCGT
ATTAGAATTCCCC
+
CCBCBD@B@:499>9>;>>A5:77B999;49?A>=@<A??=@BBCCDEEBBDABBCC?CCCCC>CCCBBBCCCCCBB
BB?@CC?A?CBB7
@T6XXI:00046:00775
GGGAGAAGGGAGAAAGGAAGCAGTCCATCTGTGGCTTCACAGTACAGAGGGACTGTCCAAAGCCGCAGATCTCGAGT
CGACTAG
+
CF?BBDACC<<<;@9@68588877741???ABC@CA<>>??ADB?8:443-
4:938A>@@;>?:=;;878<==;;<<@@>??;:
@T6XXI:00046:00777
GGGGAATTCTAATACGACTCACTATAGGGAGAGGGAGAATAGTCAACAAATCGTTTGGGGCGACTTCTCCTTCCTTT
CTCCCTTCTCCC
+
CDC:@698;;9ACC@A@BBE::;BB77:5:??BCCA??>@===B@@CDC<BBAAA=ADD9CCCCC@BB@4868<AC=
BBAA:B8;;AD;
@T6XXI:00046:00794
CTATCGACTCGAGATCTGCGGCTTTGGACACCTCCTGACTG
+
@8661////89>?ABCDC;;6;>@8><>?70,.33...,00
@T6XXI:00046:00796
```

```
GGGAGAAGGGAGAAAGGAAGCTAGTCGACTCGACATCTGTGGCTTCACAGTACAGAGGGACTGTCCAAAGCCG
+
BCBABB?BB=BBBD>C@C>@?B>>>??@@?>>9????9=>=;:>=<@??;>==44000-4;;AAA>BB:BA?A
@T6XXI:00047:00758
GGGAGAAGGGAGAAAGGAAGGAGAAAAGTGATGCACAGAATACAAGACTATTCTCCCTCTCCCTATAGTGAGTCGTA
TTAGAATTCCCC
+
A?6@B?59919999095848=?8888.17?BC??BCBCB?BBBB?BBBBGICC@@@=@CCCC>CBBABBACBB????
?>@A=;68===1
@T6XXI:00047:00777
GGGAGAAGGGAGAAAGGAAGAAGAATAGCCCTGCGTGTGACAGAAAACTATTCTCCCTCTCCCTATAGTGAGTCGTA
TTAGAATTCCCC
+
DFDCCBABD?BBAB>B@B@BB@BB@AB@@@;ABB??>?AC@;6333-
8@A@?A<<<;<<<<=49<>@B<<<<<?A==<?@AA=B2444,
@T6XXI:00047:00785
GGGGAATTCTAATACGACTCACTATAGGAGAGGGAGAATAGTGACGTGTTGTGCGGCGCTCACTGTCTCCTTCCTTT
CTCCCTTCTCCC
+
CCEBC??=?CCDCCBBBBBB?:654./-
0>>BL>BBB@?88<===:===<888::<===8>?===887748=@?CC5<=AC<@<>@AC<
@T6XXI:00048:00752
GGGAGAAGGGAGAAAGGAAGGAGAAGTCGCCCCAAACGATTTGTTGACTATTCTCCCTCTCCCTATAGTGAGTCGTA
TTAGAATTCCCC
+
DD@BA@@CC>BBBB>B<<:;;<BB?BCCCCBB8BB>????@;><=BBA<<<;<<==;<<==A<<::::<==<:8===
A?A@?;;3443+
@T6XXI:00048:00769
GGGGAATTCTAATACGACTCACTATAGGGAGAGGAGAATAGTCAACAAATCGTTTGGGGCGACTTCTCCTTCTTTCT
CCC
+
@6606,1,///,/=889@:;;1376111,.3..,..+003...4586;.10/0:+/00+.6669</.-22,.--+-
2442
@T6XXI:00048:00771
GGGAGAAGGGAGAAAGGAAGCTAGTCACTCGACCATCTGTGGCTTCACAGTACAGAGGGACTGTCCAAAGCCGCAGA
TCTCGAGTCGACTAG
+
CA:A<<88>:?=?;8;-1,.::?97,*******)**,.7:@:=??ABBBBBDAB=773-
499;<?>@C=AC>@?????@CA===??AAA@?@
@T6XXI:00048:00773
GGGGAATTCTAATACGACTCACTATAGGAGAGGGAGAATAGCTACGTTAACCGATCGTGTTGTTCTCCTTCCTTTCT
CCCTTCTCCC
+
BBA6A9<:<@C>@@BBB@@@C;;;//0,0;:==6=44/788>BAAAC@A:=;=A@@AA=<9;;2843/02328<3<?
?@:?=???@<
@T6XXI:00048:00785
CTAGTCGACTCGAGATCTT
+
B0,,,-,,8::=ABB=6:,
@T6XXI:00049:00763
GGGAGAAGGGAGAAAGGAAGGGGAGAAGGGAGAAAAGGAAGCTAGTCGACTC
+
CCBCCC@B@=B@@@<@?B?BCC9BBB@C>967776.7=>;@>./.@0..,++
@T6XXI:00049:00766
GGGAGAAGGGAGAAAGGAAGCTAGTCACTCGACATCTGTGGCTTCACAGTACAGAGGGACTGTCCAAAGCCGCAGAT
CTCGAGTCGACTAG
+
```

```
CC>ABB@BB>BBBB:=;;5:;BBB???7777=?;AA<A@@;??=?BBCCCCA?8444-
44@@AA>@@<@A>@@AA==<<<=9=????@@@A
@T6XXI:00049:00778
GGGGAATTCTAATACGACTCACTATAGGGAGAGGGAGAATAGTTTGCCTGTCGGCCATCTAGCTTTTCTCCTTCCTT
TCTCCCTTCTCCC
+
@@?4@>A>BBBAB@@@@AABBABB>66657??CE>C??>@<666187=@CCBB?B?AA??:449991;===;8;388
-4=@A<A>@@@@;
@T6XXI:00035:00808
GGGAGAAGGGAGAAAGGAAGTTGAATAGCACGCCTGAGGATAGCAGTCTATTCTCCCTCTCCCTATAGTGAGTCGTA
TTAGAATTCCCC
+
CC?CCB@BB>@@AA=A@A@AA@BB?BBBBB@AA?CBBC@BCAAC8;;BC??=ADCC;BB;;=;A<>?@ACC???@@A
A?AAB<?=@CC6
@T6XXI:00037:00802
GGGAGAAGGGAGAAAGGAAGGTGAACAGAGCGCGAACGAATACGTCTCTATTCTCCCTCTCCCTATAGTGAGTCGTA
TTAGAATTCCCC
+
FEBCBC@BB>BBBC=A?B@B@BCC@CBBBBBCBAB?DCC@CCCCDCCC<<<BA99918>@CEADCCGCDDCBBC@@@
B@AAC=C?AA@6
@T6XXI:00037:00805
GGGGAATTCTAATACGACTCACTATAGGGAGAG
+
=BA4?<>:<99?ACBCAA@@><733311,11..
@T6XXI:00037:00807
GGGGAATTCTAATACGACTCACTATAGGGAGAGGGAGAATAGTCAACAAATCGTTTGGGGCGACTTCTCCTTCCTTT
CTCCCTTCCTCCC
+
9??4??A>BCD@CB@ABBBBBBBB>976-
6=7775:AA?BCB@??86683?;7=?6===.86=<<:?@A?A?B@BA:@@CD?C6968<A?
@T6XXI:00037:00808
GGGGAATCTAATACGACTCACTATAGGGAG
+
88808,1///76449=>744......4242
@T6XXI:00038:00815
GGGAGAAGGGAGAAAGGAAGGGGAGAAGGGAGAAAGGAAGGGGAGAAGGGAGAAAGGAAGCTAGTCGACTCGACATC
TGTGGCTTCACAGTACAGAGGGACTGTCCAAAGCCGCAGATCTCGAGTCGACTAG
+
FC@CBB@CC>?A?C?C@B@BBB:CBC>@@<A@>B=C@C=?B=7?AC@A@;??AA;?<<<55000:/./..:=<<588
8@AA>AA>A@@@@ABBBBB@C<@<@@@@>AA<?B@@==@AA<==A=<<CCA@@BB
@T6XXI:00039:00811
CTCGAGATCTGCGGCTTTGGACAGTCCCTCTGTACTGTGAAGCCACAGATGTCGAGTCGACTAGCTTCCTTTCTCCC
TTCTCCC
+
EACBCCB@@==9969@@:???@@@999199994444499=BA=>A=;8:833:/...../478==>@CC>?@?A5
824333,
@T6XXI:00039:00814
GGGAGAAGGGAGAAAGGAAGGGGAGAAGGGAGAAAGGAAAGCTAGTCGACT
+
DICBBB@BB?9<<H>B@@@CEH:CBB?BB:?<<>7>477,6=131917/2,
@T6XXI:00040:00812
GGGAGAAGGGAGAAAGGAAGCTAGTCGACTCGACATCTGTGGCTTCACAGTACAGAGGGACTGTCCAAAGCCGCAGA
TCTC
+
AA:>>C<>>:??>>9>;>69;@@@AAA>;;>=00/00/=>B=BB@DB><?CA?:6;<;5;?>788,//,//,-
78;?A?==
@T6XXI:00040:00816
```

```
GGGGAATTCTAATACGACTCATATAGGGAGAGGGAGAATAGTTTTTCCCTTCACTAACCGCGAACTGCACTTCCTTT
CTCCCTTCTCCC
+
BBB9?=@>ADCABBBBB;:01.64...+1766<;?CD?=<44444,3?<A?@BACC@C=BBCC?<<?<@A@?C=AA>
ACCC>B=AA?@<
@T6XXI:00041:00804
GGGAGAAGGGAGAAAGGAAGCTAGTCAGTCAGATCGTGACGACAGTCCATCTGTGCTTCACAGTACAGAGGACTGTC
CAAAGCCG
+
?@1944244-444;-
429<=;A?:::/........1116799;<<9=;>/..3./..,.991168:66?;8685334/--+--,-
@T6XXI:00041:00805
GGGGAATTCTAATACGACTCACTATAGGGAGAGGGAGAATAGTCAACAAATCGTTTGGGGCGACTTCTCCTTCCTTT
CTCCCTTCTCC
+
///*/,1,///77?@@@ABBBBB@;????9?=>>@9??@=????@@;?@A:@<717,7<<2>=>?>8=6?5:5:>?;:
=88848=ACD9
@T6XXI:00041:00807
GGGAGAAGGGAGAAAGGAAGTGCAGTTCGCGGTTAGTGAAGGGAAAACTATTCTCCCTCTCCCTATAGTGAGTCGTA
TTAGAACT
+
G?8<:BAHH;<;CC>A?B?<?<CCCG@@@CC=@=AB>>;999,111,37777;9791668;7666111043333000
32333/00
@T6XXI:00041:00826
GGGAGAAGGGAGAAAGGAAGAGAACAGTTGCGCCTAGTAGTTTAGTAACTATTCTCCCTCTCCCTATAGTGAGTCGT
ATTAGAATTCCCC
+
?D?AA@<@@<@@@C<A=A@C<;<?CCDC@BBBB@@@@B<;;B?DDBBEDD@@=?@@@:A@@@A<C?<<???ABB:91
74/033232546.
@T6XXI:00042:00807
GGGAGAAGGGAGAAAGGAAGAGAATATCGCGACCAGATAGGAAGTGACTATTCTCCCTCTCCCTATAGTGAGTCGTA
TTAGAATTCCCC
+
?B:>@A699198>@<@=A@CGBC@EBCBB??BA@AA@A@@=@?B?@<::BB?BBAB>:::BB9?=8<5;>:303535
::@<94;2999/
@T6XXI:00042:00813
GGGAGAAGGGAGAAAGGAAGGGGAGAAGGGAGAAAGGAAGCTA
+
BF=B<<;<A?@@@@<@=@@AEGACBA78808000,,*,*,2,,
@T6XXI:00042:00814
GGGAGAAGGGAGAAAGGAAGGAGAACGCGCACCCGAACCAACGGTGATCTATTCTCCCTCTCCCTATAGTGAGTCGT
ATTAGAATTCCCC
+
9969=BACD@CCCC@C?B@B<<<DABCBBCDCCACC@E@C@CC@BC@@<;9@<@9991994>?<@C;;::9661119
96799<=A@BBC:
@T6XXI:00042:00816
GGGAGAAGGGAGAAAAGGA
+
DDBBBB794-91111+--+,
@T6XXI:00042:00827
GGGAGAAGGGAGAAAGGAAGGAGAAGTCGCCCCAAACGATTTGTTGACTATTCTCCCTCTCCCTATAGTGAGTCGTA
TTAGAATTCCCC
+
CEECBA@BC>ABBC7;5;<?<?AA>AABCBBB5;;68?86617:>BBBC@?==@@@;@@@@@7<=?@@??=;88443
3455:;;;===4
@T6XXI:00043:00813
GGGAGAAGGGAGAAAGGAAGCTAGTCACTCGACATCTGTGGCTTCACAGTACAGAGGGACTGTCCAAAGGCCG
```

```
+
DE?CGDCEE>AA?=<@794;@888B:99:?@?BAB@@@<<:01,.55=?AA==?A@C:87/---,--+-0101
@T6XXI:00043:00814
GGGAGAAGGGAGAAAGGAAGCTAGTCGACTCGACATCTGTGGCTTCACAGTACAGAGGGACTGTCCAAAGTCGCAGA
TCTCGAGTCGACTAG
+
CC?AAC@CC>CDBB>C?A>AC?6==//0=>=>>BCBBBBBCABC@BBCBCB???>77717;?@??<?C?A???@@@A
A???C@@@@@@A@@@
@T6XXI:00043:00817
GGGGGAATTCTAATACGACTCACTATAGGGAA
+
4999+424:;AA??@B@B;;;88<<00.4+.,
@T6XXI:00043:00823
GGGAGAAGGGAGAAAGGAAGTGCAGTTCGCGGTTAGTGAAGGGAAAAACTATTCTCCCTCTCCCTATAGTGAGTCGT
ATTAGAATTCCCC
+
CC?BCCABB?B@==:@=@=@DDDCCB@BBBC?B?A?>;;:79/66670:BCC@ABCC>?:?CC>CCBB@?=>@AAA?
8868@B?D?ABA6
@T6XXI:00043:00826
ACTCGAGATCTGCGGCTTTGGACAGTCCCTCTGTACTGTGAAGCCACAGATGTCGAGTCGCTACCTTC
+
DDEC;<=HCABDEF?BCC:<8<CBCDCJBDE>=<<BB@AADEDDDDECBB@@BAC@>?=-,,**)*)*
@T6XXI:00044:00819
GGGGAATTCTAATACGACTCACTATAGGGAGAGGGAGAATAGTCAACAAATCGTTTGGGGCGACTTCTCCTTCCTTT
CTCCCTTCTCCC
+
9A?6A=A=ACC?BBB@@ACDCCCB????9?@@AB=AAB?BBA?B=;<@@<CCAAB>BBB9CDBBC?BAB7;78<??<
@A@A<@8;8881
@T6XXI:00044:00822
GGGGAATTCTAATACGACTCACTATAGGGAGAGGGAGAATAGATCACCGTTGGTTCGGGTGCGCGTTCTCCTTCCTT
TCTCCCTTCTCCC
+
B@@4>6969A=?CBBBBAACCGCCB:::59?@BC>BCC@A>=9=AA?;@B6:6:?BAC@C<<<BBC@CCD?@<?477
5:@@C>C@A??B=
@T6XXI:00044:00823
GGGAGAAGGGAGAAAGGAAGGGCCAGCCGGTGGGAGAAAACCGTGTGCTATTCTCCCTCTCCCTATAGTGAGTCGTA
TTAGAATTCCCC
+
GC?BCC@CCAB?BC>C?B?BC?B@BA>=>@BB?:9=CCC9C?CCBBBDDCC@B@@@<ABBCDAC@AABCDCCAAAB:
:7:BB>A=???6
@T6XXI:00044:00834
GCTAGTACGACTCGAGATCTGCGGCTTTGGACAGTCCCTCTGTACTGATGAAGCCACAGACTGACTGTCCTTCCTTT
CTCCCTTCTCCC
+
/,/771////8;>9999@A@CBC<0//+.655///:755/8869>9457932-
/,,00***,,,20445587;8662677:5:>?@=83
@T6XXI:00044:00835
GGGAGAAGGGAGAAAGGAAGAGAATGAGTGTGAGCGCCAGCTGGCAACTATTCTCCCTCTCCCTATAGTGAGTCGTA
TTAGAATTCCCC
+
DFEDCC@BB>AABC>B@B@BBBB@BBBB@@BBBBBBC@DCCAA?AC?BBBCACABB=ACB@@<?CB@AABBBDCCCB
A>BCCAC@BCE9
@T6XXI:00045:00827
CTAGTCACTCGAGATCTGCGGCTTTCGACAGTCCCTCTGTACTGTGAAGCCACAGATGTCGAGTCGACTAGCTTCCT
TTCTCCCTTCTCCC
+
```

@?9444444;4449=DBCGDBDCC>CECCCCCCE@B@@@BAABBCCC=?@=?BCDCBBBCA???BBBCCFC@@=??C
C>B???7=;==<A<
@T6XXI:00046:00800
CTATCGACTCGAGATCTGCGGCTTTGGACAGTCCCTCTGTACTGTGAAGCCACAGATGTCGACGTCGTACTACGTCC
TTTCCTTTCCCTTCCC
+
==AB;9:DCCBDCE<;9A;;8;@B<B@@??A877.7A@ABABECC??=;@=@@BA@@?=00-
,,,,,..44..11356;::200+0301011+
@T6XXI:00046:00820
GGGAGAAGGAGAA
+
8A8---*,***,,
@T6XXI:00046:00829
GGGAGAAGGGAGAAAGGAAGCTAGTACGACTCGACATCTGTGGCTTCACAGTACAGAGGACTGTCCAAAGCCGCAGA
CTCTCGAG
+
AAAACA@AB<@@@@<@@B@D;31>0..775566B@AAA<AA=:8?>>7:::615:9//-
/875:A<AA<??<=::8/./;;;797
@T6XXI:00046:00831
CTAGTCACTCGAGATCTGCGGCTTTGGACAGTCCCTCTGTACTGTGAAGCCACAGATGTCGAGTCGACTAGCCTTCC
TTTCTCCCTTCTCCC
+
==1////44C>??@BCCCBB?ABB<B8:;B????<?BBBBBCC???CAAD?BBDCC<<<CBCCB@@;;94..,.553
3;6:666,1016330
@T6XXI:00046:00836
CTAGTCGACTCGAGATCTGCGGCTTTGGACAGTCCCTCTGTACTGTGAAGCCACAGATGTCGAGTCGACTAGCTTCC
TTTCTCCCTTCTCCC
+
>8,---
,,6::?CCCCCCBA@?BDC>A>DAAB:::8:CCCAA?A?@@D@@C@CCCCBAABCCCC?AADDCC@@@9:7;B<@CB
C@B?CCCC>
@T6XXI:00047:00818
CTGCGGCTTTGGACAGTCCCTCTGTACTGTGAAGCCACAGATGTCGAGTCGACTAGCTTCCTTTCTCCCTTCTCCC
+
EEDDDACBC@B;;;AB;;<;;CBCAAAAAACC@BC@CBCCCCBBCCCABCDBBBB@@@?AABE@BBCC?D?AACC>
@T6XXI:00047:00820
GGGGAATTCTAATACGACTCACTATAGGGAGAGGGAGAATAGCCAAATTCCCTGTGCCCCGTTGTTCTTCTTCCTTT
CTCCCTTCTCCC
+
999/42924A@BCCBBBCCCAA@?>877.7>>@A=BCD@CFB?:==1727@:@@>0444-
4479;2::>>?=A<??7>?>?;?=??AA?
@T6XXI:00047:00821
GGGGAATTCTAATACGACTCACTATAGGGAGAGGGAGAATAGTCAACAAATCGTTTGGGGCGACTTCTCCTTCCTTT
CTCCCTTCTCCC
+
449/959?CCD@CBAA@AB?BCBB@76647??CE>B@A>?@@=??<?AA<CC@BB4916-
8>>?A?ACC>A?A??=:?<;<7<;>?A@<
@T6XXI:00047:00825
GGGAGAAGGGAGAAAGGAAGGAGACACGGTCACGAACATGAATGTGACTATTCTCCCTCTCCCTATAGTGAGTCGTA
TTAGAATTCCCC
+
AA>ACDACC@BB@B>B@B?A?A??CBBB@B@BBBB>BBBBB>A@@@BBC??<?8775:::CC>BBBCCCCCC;:;BA
A>AA=9=37:7.
@T6XXI:00048:00821
GGGAGAAGGGAGAAAGGAAGGGGAGAAGGGAGAAAGGAAGGGGAGAAGGGAGAAAGGAAGCTAGTCACTCGACATCT
GTGGCTTCACAGTACAGAGGGACTGTCCAAAGCCGCAGATCTCGAGTCGACTAG
+

```
FEBBAA@CC?@BBB>B@B@BBB:BBB?A@:;;::8?=@39::4:@B>A<8?743+434/4>:::CB:67???9;888
@AA>@A>?????A@CCCCAA;@A@@@@<@A=BD@@===@A@@@CAAA:;;DCCC
@T6XXI:00048:00844
GGGAGAAGGGAGAAAGGAAGCTAGTCACTCGACATCTGTGGCTTCACAGTACAGAGGGACTGTCCAAAGCCGCAGAT
CTCGAGTCGACTAG
+
ED@:<;@BC@BCCC>B@B@BBBA?;9:;:?=77:996A??<?A@CBBCCC8:97000,4;;9;<=?@;@A@A@@AA@
@@A@@@@A>><<<@
@T6XXI:00049:00830
GTCGACTCGAGATCTGCGGCTTTGGACAGTCCCTCTGTACTGTGAAGCCACAGATGTCGAGTCGACTAGCTTCCTTT
CTCCCTTCTCCC
+
<<ACADCD@<=AAACCCC@BBB@C?>B@@BBB>@ABBAACBCBBC=@@@CCCBCBBBAABCBB::::B::;AB68:/
8>==6=4:9?B>
@T6XXI:00049:00844
GACTCGAGATCTGCGGCTTTCGACAGTCCCTTTGTACTGTGAAGCCACAGATGTCGAGTCGACTAGCTTCCTTTCTC
CCTTCTCCC
+
==DBCABBBBCCCCC@CDNCCBBBCCCCC?BB>BBCCCDDDC>B?@BCCCC?A@ACB@@@B;;<A<<<?B?BC=><C
C?C=AFDCB
@T6XXI:00049:00846
GGGGAATTCTAATACGACTCACTATAGGGAGAGGGAGAATAGTTCTGACGGATTGCTCTGACTATCATC
+
FDD:C?F?@BB=@@@BBBBCBBBBA:66.777==9?A@=BB?=;=AAFBC>;:47?>=895.,,,,,,,
@T6XXI:00049:00847
GGGGAATTCTAATACGACTCACTATAGGGAGAGGGAGAATAGTTTTCCCTTCACTAACCGCGAACTGCACTTCCTTT
CTCCCTTCTCCC
+
DEB9C@D@BDDBCCBBBBBBBBB?<311.6>@CC>CCC@AB@<<<56<:;;=@CCC@C????CACCA==??;?;?=7
>777.7:?<AA@
@T6XXI:00052:00043
GGGGAATTCTAATACGACTCACTATAGGGAGAGGGAGAATAGACACGCAACATTCAG
+
CCC7B5969>>;@BB@AAD@>;>>0000,0:;>><ACC@<A??>55.**)***)***
@T6XXI:00054:00031
GGGAGAAGGGAGAAAGGAAGGTGCCGTCGCGGCGATCGAGATTGGGAGCTATTCTCCCTCTCCCTATAGTGAGTCGT
ATTAGAATTCCCC
+
//+//4244-444;-
4249;2339>99==9>;789///8//.,.404...4677611,11111,068=@@A@A@>888?<??@>=<=AA4
@T6XXI:00055:00036
GGGGAATTCTAATACGACTCACTATAGGGAGAGGGAGAATAGTTTTCCCTTCACTAACCGCGAACTGCACTTCCTTC
TCCCTTCTCCC
+
@B@4@5959999>BBBCCCCCBA??666.7><<=9?B@?;;4444-
33+023=6919931:9A==?<;>;9::,/,.5577A;?444+
@T6XXI:00059:00020
GGGAGAAGGGAGAAAGGAAGGACAGTCCATCTGTGGCTTCACAGTACAGAGGGACTGTCCAAAGCCGCAGATCTCGA
GTCGACTAG
+
AB>BCB@CG@CDBC?B?B@B;00;=01,.7:0769>??<?AAABACC@@<<<6<??@AC>AA<==;=@@@AAA888>
>=AAA@@@A
@T6XXI:00061:00013
GGGAGAAGGGAGAAAGGAAGCATAGCCAGGGCGTGAGTTTATCATCAGCTATTCTCCCTCTCCCTATAGTGAGTCGT
ATTAGAACTTCCCC
+
```

```
CD=;<A=@@:<9BB?B?D@CCCCBCC?ABC>BBBBA???6=>;>9@????@65748=;<;A@?:?;778>>==??88
8==>>?<??>ACC0
@T6XXI:00061:00018
GGGAGAAGGGAGAAAGGAAGG
+
AA8<9B@CC>A@==+*)*)*)
@T6XXI:00061:00025
GGGGAATTCTAATACGACTCACTATAGGGAGAGGGAGAATAGTCAACAAATCGTTTGGGGCGACTTCTCCTTCCTTT
CTCCCTTCCTCCC
+
DCC7B9;;<DD@CC@@@BBBAA@??777.7====9?AA?AAA@@@>@@@<BBA@?9???5=>>7:477=4424344-
4../,.,.,---+
@T6XXI:00061:00026
GGGAGAAGGGAGAAAGGAAGGAGAAGTCGCCCCAAACGATTGATTGACTATTCTCCCTCTCCCTATAGTGAGTCGTA
TTAGAATTCCCC
+
99-449294-4444-
42499;;@A><=>8011*..+.6..,...,.61667:63344//.45447=9555@@@A@AAA?@<<<=;:;81
@T6XXI:00063:00009
GGGAGAAGGGAGAAAGGAAGCTAGTCGACTCGAGATCTGCGGCTTTGGACAGTCCCTCTGTACTGTGAAGCCACAGA
TGTCGAGTCGACTAGCTTCCTTTCTCCCTCTC
+
EC>@A@>AA<AACEAD>@?ACB???:::???@B?@@CBBCC?BCC>C?AABBBCC>CA@???AA;??C@A@;==@??
@?AAAAA@A???A@84476,//,/+++*++++
@T6XXI:00063:00011
CTAGTCGACTCGAGATCTGCGGCTTTGGACAGTCCCTCTGTACTGTGAAGCCACAGATGTCGAGTCGACTAGCTTCC
TTTCTCCCTTCTCCC
+
C7,-,-
,,668?ABBCBBBCC?BBA>A=>@BC@?@=@CCCA>>C@BB@=?B@CDBCA@@@BBBBBDBCCBBBBA?A@CC>@??
B>B?AB?A=
@T6XXI:00063:00014
GGGAGAAGGGAGAAAGGAAGAACGACAGTAGCTG
+
ED>@AC@CD>BB>>:>:88--+,,,,02431113
@T6XXI:00063:00030
GGGGAATTCTAATACGACTCACTATAGGGAGAGGGAGAATAGTTTTCCCTTCACTAACCGCGAACTGCACTTCCTTT
CTCCCTTCTCCC
+
EED9B8;;;AA@BCBCBBBB>?BDD==<8??@@B>CBC@A?7734-
4;:?>ACCCB=?=?AAB@@A??ACB:=366.7=>?:A?A>==:
@T6XXI:00063:00031
GGGGAATCTAATACGACTCACTATAGGGAGAGGGAGAATAGTCAACAAATCGTTTGGGGCGACTTCTCCTTCCTTTC
TCCCTTCTCCC
+
DCC:E@EABACGBA@CBAACCECC:::5:@?BB>BFE????@AA>@@A<CEBBC>CCC9CAA??:<=>::7:244-
4===:?<@AAA<
@T6XXI:00064:00014
GGGGAATTCTAATACGACTCACTATAGGGAGAGGGAGAATAGTCGTTTGCTTTGCTGCCGCGCTATTGTCTTCCTTT
CTCCCTTCTCCC
+
CEECC?B@BCC@CCCCCBBBB@>9911159@BCC>CAB?@=::8;;;:;;;;4B@B<?<?<???@C@@7742833;:
3;<=7;7;;;@<
@T6XXI:00064:00027
GGGGAATTCTAATACGACTCACTATAGGGAGAGGGAGAATAGTCTTGCAATTTCCTGCGGTGCATTCTCCTTCCTTT
CTCCCTTCTCCC
+
```

```
BBA7A8;:<CD@CBBBBBCCCBCC@???<?ABCC>BBCAA=<<<?>?;A?CBAB?BBCC@CCABB@BAA<>;>4880
8?>@<@=====6
@T6XXI:00064:00048
GGGAGAAGGGAGAAAGGAAGCTAGTCGACTCGACATCTGTGGCTTCACAGTACAGAGGACTGTCCAAAGCCG
+
DDBEECACB>@@@C=B?B@CCCABCCCA<ACCCC?<>BCCC>?@?BBBBBAA::7..,/8889=?AD=BB<@
@T6XXI:00065:00014
CTTTGGACAGTCCCTCTGTACTGTGAAGCCACAGATGTCGAGTCGACTAGCTTCCTTTCTCCCTTCTCCC
+
FDD@C?BBBB???<@A@AA@@@A@@A@BB@CCCAAACC@@?CCCC@@@A;;=4848?9>>>>8>577AB:
@T6XXI:00065:00019
GGGGAATTCTAATACGACTCACTATAGGGAGAGGGAGAATAGAGACGCATTCGTTTTGCGCTCTGTTCACCTTCCTT
TCTCCCTTCTCCC
+
CCC9C?D?BBB@BBBBBBCCCCCBA777.777?B8;;B@@@BA<<<?<??@BAABB7???CDDAAA=?9968?A8>@
6;;;;4849043+
@T6XXI:00065:00026
GGGAGAGGAGAA
+
1/+/,,,*,*,*
@T6XXI:00065:00038
GGGAGAAGGGAGAAAGGAAGGGAGAGGA
+
EC@HDC@CB>;;9@:>:<:;;-0...,.
@T6XXI:00065:00043
CTAGTCGACTCGAGATCTGCGGCTTTGGGACAGTCCCTCTGTACTGTGAAGCCACAGATGTCGAGTCCGTCCTTCTC
CTTCCTTCCC
+
IBA@A<;<ADDBECCCCBBBB>???/77/88>>>?A<ACCBBA??>AB@>?B?BCCCCCCCCC@<:;,11.,.,..3
763636011,
@T6XXI:00066:00033
GGGGAATTCTAATACGACTCACTATAGGAGAGGGAGAATAGTCAACAAATCGTTTGGGGCGACTTCTCCTTCCTTT
+
CCD7@595944?BCCCCBBBB?@@DA?;>=666.7??<=>8744>@AD;ABBBC9???5?>>;;::<<,/,/,,,+
@T6XXI:00067:00027
GGGGAATTCTAATACGACTCACTATAGGGAGAGGGAGGAATAGTCACAAATCGTTTGGGGCGACTTCTCCTCCTTTC
TCCCTTC
+
///*/,/,///58>CCBBA@B?AA9777,1....+..,.,--00---
1106988:;=AA8?:866016600354423000+0/-
@T6XXI:00067:00039
GGGAGAAGGGAGAAAGGAAGCTAGTCGACTCGACATCTGTGGCTTCACAGTACAGAGGGACTGTCCAAAGCCGCAGA
TCTCGAGTCGACTAG
+
99:=AAABB8<;>>9?<A>@@?=>=110:9:0081116?=E?@C@C???CCE??=444-
4;;;=?;=@:8837;:;8;///7;:=@@>>===
@T6XXI:00067:00046
GGGAGAAGGGAGAAAGGAAGGGGAGAAGGAGAAAGGAAGCTAGTCGAC
+
@9599989D>CCBCAECC?BBB7@??4851611,1017761768667.
@T6XXI:00069:00035
GGGAGAAGGGAGAAAGGAAGCTAGTCGACTCGACATCCTGTGGCTTCACAGTACAGAGGACTGTCCAAAGCCGCAG
+
CDBEEC@CB>CBBC?B@B=;:;>>8::@@=>>11000,.334313111119<<?B@@@=@@@===@CD899>?<<<
@T6XXI:00070:00042
GGGAGAAGGGAGAAAGGAAGGAATAGCCAACCGCAAACAATGCTCGCGCTATTCTCCCTCTCCCTATAGTGAGTCGT
ATTAGAATTCCCC
```

```
+
CC?BCC@BB=A@BB>B=?=@@D?ABBC@C@C@CBBA>BB@BBBBBCCBABCC@BBBB>@@@CC>CCCCBBCBDBDCB
BB?BBCAF@BBB7
@T6XXI:00070:00044
GGGGAATTCTAATACGACTCACTATAGGGAGAGGGAGAATAGCCATACTTGCTCCCAGTTGGCGTGAAACCTTCCTT
TCTCCCTTCTCCC
+
ECC7B8;;<AABNCCB??@AA@>>>166.777=?9?B?==88834??@?<?CBAA:?@A>A889C>>@<?<=>;388
58;;A7=583==7
@T6XXI:00070:00048
GGGAGAAGGGAGAAAGGAAGGAGAAGTCGCCCCAAACGATTTGTTGACTATTCTCCCTCTC
+
AD?AB@=>>:>>@A<A>B?BABBB>?=>8888.8C<BABBCCCC>@@BBB@><==D=?<<<
@T6XXI:00071:00004
GGGGAATTCTAATACGACTCACTATAGGGAGAGGAGAATAG
+
888188@>BDB@BBAA?BBBB@>99111.7>>?>C00,,+,
@T6XXI:00071:00033
GGGGAATTCTAATACGACTCACTATAGGGAGAGGGAGAATAGTTCCAACCGTACTGAGCC
+
DCB7C8;;<CB?BG@?@@BCCCBCA99646??BC>BAA?>?;;2424348:;;411---,
@T6XXI:00071:00036
GGGAGAAGGGAGAAAGGAAGCTAGTCGACTCGACATCTGTGGCTTCACAGTACAGAGGACTGTCCAAAGCCGCAGAT
CTCGA
+
CEBCCC@EHAAAAC?CAC@BBB:;;??????B;;:::9???8AA=@@CCBCB==6./,/678;A?CC<88238;;==
848;;
@T6XXI:00071:00043
GGGGAATTCTAATACGACTCACTATAGGAGAAGGGAGAATAGTCAACAAATCGTTTGGGGCGACTTCTCCTTCCTTT
CTCCCTTCTCCC
+
HEH:G@B@BCC@@BBBBAAAD6446..,000,77-
6=9A=@@@@?:;;;4<==>>6===2=?@@A<??B6838<??;@AACAA<@?AA@
@T6XXI:00072:00005
GGGGAATTCTAATACGACTCACTATAGGGAGAGGGAGAATAGCAAACGTTTCCCCGACGTCGCTTTCTCCTTCCTTC
TCCCTTCTCCC
+
AA?4?;><@B@=AEECCCCCCCCBB???<?BBCB>BCC@AA66<A<ABBB<BCC9CCCAA?@:77.000,09:,/,-
2223778==C<
@T6XXI:00072:00008
GGGGAATTCTAATACGACTCACTATAGGGCTGAGCACGATCGATGTACAGCTACGTACGTCTGAGCATCGATC
+
?A>4>>B=AAB>BBBBDBBCDC>>-,,,*,,,,,,22///4;;1333:447339344000;393168//--1-
@T6XXI:00072:00030
GGGAGAAGGGAGAAAGGAAGGGGAGAGGGCTAGTCACTCGACATCTGTGGCTTCACAGTACAGAGGGACTGTCCAAA
GCCGCAGATCTCGAGTCGACTAG
+
9904@@<?>:>>@B>B?C@CCC:CBABB<CDAA;=4444499=>?BDBC>@@=@?CCCCDDA@<<;9;A@@@A<>>9
>>=@@@BB@?9;7;;;:?A====
@T6XXI:00073:00017
GGGGAATTCTAATACGACTCACTATAGGGAGAGGGAGAATAGTCAACAAATCGTTTGGGGCGACTTCTCCTTCCTTT
CTCCCTTCTCCC
+
CCC7B=@=@CCACBAAAA??>@>99111.7>>BC>BBB?@:A@@@?@@@<BCCCC>BBB7AAAAB=@??<<8<?AA<
A=<<6;383@@;
@T6XXI:00073:00023
```

```
GGGAGAAGGGAGAAAGGAAGGAGAAGACGATCCGACCATAAGGATGCCTATTCTCCCTCTCCCTATAGTGAGTCGTA
TTAGAATTCCCC
+
EI@CCC@CC>BCBB>B999<@A??<>79333.53//,///,/,//4969??@BBBE;;;;:807??????A??;==8
434:=8698CC6
@T6XXI:00073:00030
GGGGAATTCTAATACGACTCACTATAGGAGAGGGAGAATAGATCACCGTTGGTTCGGGTGCGCGTTCTCCTTCCTTT
CTCCCTTCTCCC
+
CCEBC?AAADB@CBBBBBBBB:996..,0;;?A>CBC@@=558A@?;?C;?>?@CCE=B<;;DCC?BBA<@<>:==6
=>==7?>@@@B?
@T6XXI:00074:00010
GCTATCACTCGAGATCTGCGGGCTTGACAGCCCCT
+
,/449994:9==@B>>>@988,77133371111,1
@T6XXI:00074:00016
GGGGAATTCTAATACGACTCACTATAGGGAGAGGGAGAATAGCCACGCACGTCACGACGCCAAATTCTCCTTCCTTT
CTCCCTTCTCCC
+
BAA7A>A>BBB@CBA??@<@BC?99166.777==9???@B@??=AACCA<:??AA@;:=<<??;?>@@B?>48/44,
4;;;6<8<<<?:
@T6XXI:00074:00031
GGGAGAAGGGAGAAAGGAAGTGCAGTTCGCGGTTAGTGAAGGGAAAAACTATTCTCCCTCTCCCTATAGTGAGTCGT
ATTAGAACTTCCCC
+
=?<?CCBJG@HBBA<A9;9<BBBCCBAB=<<@C@BBBA?<=<.7777,8?@@<?777.1616688::<A@@@=8888
3656695332333.
@T6XXI:00074:00035
GGGGAATTCTAATACGACTCACTATAGGGAGAGGGAGAATAGTCAACAAATCGTTTGGGGCGACTTCTCCTTCCTTT
CTCCCTTCTCCC
+
DDHBH@A>@DDDCCBBBB@A?8<9<666.7>>BCACBBA?AAAAA=AAC=AAAAE>B@@7A@A?@;>@@:;8;:==7
????;@9<77;6
@T6XXI:00074:00045
GGGGAATTCTAATACGACTCACTATAGGGAGAGGGAGAATAGTCAACAAGTCGTTTGGGGCGACT
+
BCA2@=@=@BB@CDCCC<;;A?C88776,/3//.+/.5548=774:9;9=44484+000+.44..
@T6XXI:00075:00025
GGGGAATTCTAATACGACTCACTATAGGGAGAGGGAGAATAGCTACGTTTAACCGATCGTGCTGTTCTCCTTCCTTT
CTCCCTTCTCCC
+
??B6@=AACDB??@AAAA??;@;99116.7==BC?EBC?AA??AACCCC>B?B@CBCCAA9;;BB7:;B?A=?<??<
?@CD=A688<;6
@T6XXI:00075:00035
GGGAGAAGGGAGAAAGGAAGAAGAGCAGCACGGTGAGACGTCCGTACTATTCTCCCTCTCCCTATAGTGAGTCGTAT
TAGAATTCCCC
+
DDBBBC=@@<@@@C@C@C@CB@BB?A?BBB@A?ABBBCBBAB????A@@@<:66;98=??@;@===AAAAA??>?<<
8;;;94/034-
@T6XXI:00075:00048
GGGAGAAGGGAGAAAGGAAGAGGACGAGAAGGTAGAAAAGAAACGAGCTATTCTCCCTCTCCCTATAGTGAGTCGTA
TTAGAATTCCCC
+
DDCAA@@CCACCBB>B@B@CBB@?AABBB@C@CCBBAA7BCC?BBCA@BBA>AABC?@??BB>B@@@CCC@@@@@A?
?<?AA<>5;;<4
@T6XXI:00076:00040
```

```
GGGAGAAGGGAGAAAGGAAGCTAGTCGACTCGACATCTGTGGCTTCACGTACAGAGGGACTGTCCAAAGCCGCAGAT
CTCGAGTCGACTAG
+
CDCABAA@=<@B@9:@7:6;B>9;>/10;;;;081661=?B2::=?CBBAACC>:4417??BCB@CC>BB>BCB@?A
AAA@BBB@@@@AA@
@T6XXI:00076:00042
GGGGAATTCTAATACGACTCACTATAGGGAGAGGGAGAATAGTCAACAAATCGTTTGGGGCGACTTCTCCTTCCTTT
CTCCCTTCCTCCC
+
BDC:C8:<<CBACCBBBBBAB@BCB???9?AACC>AAA@C???AA?@@A<CCCB@6<<<2<=@@A>A??68388;AA
B;==9=./.,551
@T6XXI:00077:00005
GGGGAATTCTAATACGACTCACTATAGGGAGAGGGAGAATAGTCAACAAATCGTTTGGGGCGACTTCTCCTTCCTTT
CTCCCTTCTCCC
+
AAA4@@@=@BB>BBBBBB;;;@@AA77:5:?BCD<AAC@>>>AAA;87717==?A>BBC7BA@@@477@4438:==:
?@@A<A>@@AB<
@T6XXI:00077:00016
GGGGAATTCTAATACGACTCACTATAGGGAGAGGGAGAATAGTACGACTCAACTCGTTTGGG
+
:BB6>C?>@DBBBBB@AABBA@AB96:?6<>7775:A?=;7444493003;7433000+044
@T6XXI:00077:00020
GGGGAATTCTAATACGACTCACTATAGGGAGAGGGAGAATAGTTTTTCCCTTCACTAACCGCGAACTGCACTTCCTT
TCTCCCTTCTCCC
+
CCC9>8;;;BB@CCB@ABBBBAB99111.7>>?A>ACC@A8=8==C1??;@><@@BC@B@A?ACDAAA@@B?:=369
5:@>B<B?:99DE
@T6XXI:00077:00046
GGGAGAAGAGAGAAAGGAAGGAGAAGTCGCCCCAAACGATTTGTTGACTATTCTCCCTCTCCCTATAGTGAGTCGTA
TTAGAACTTCCCC
+
AA?ADCCCCCBCCB?B@C?A>@CC@CCB:991,9@<@ACGCABB?B<<@CBBC@@@<;::BC<A@B@BBBCC@@?88
88<;;3773777-
@T6XXI:00078:00023
GGGAGAAGGGAGAAAGGAAGCTAGTCGACTCGACATCTGTGGCTTCACAGTACAGAGGGACTGTCCAAAGCCGCAGA
TCTCGAGTCG
+
DDABCB@CC>BBAC>C@C@BBBCCB:;1;>77/-
///,7375=>>@CCBBABAB?74417?????=??:>><???AAAAAAA=@==@
@T6XXI:00078:00046
CTAGTCACTCGAGATCTGCGGCTTTGGACAGTCCCCTCTGTACTGTGAAGCCACAGATGTCGAGTCGACTAGCTTCC
TTTCTCCCTTCTCCC
+
EC111=1::BBACBECBABC@CCC>C?B>??7777.7==@BBBAACB@==A=ACCCCBBCAABC?@A@@@C;:68?:
<?9?BBD=?<?AAD<
@T6XXI:00079:00012
CTAGTCGACTCGAGATCTGCGGCTTTGGACAGTCCCTCTGTACTGTGAAGCCACAGATGTCGAGTCGACTAGCTTCC
TTTCTCCCTTCTCCC
+
C8-
,,,,,7::?CCCDBA@@A>???<@?@@>@<::67B@ABCCCDBBB@AC@BCBCBCCDC@@?BBBABBA?=607316-
766?6;8:97=7
@T6XXI:00079:00022
AGGACCTTCGTATACGACTCACTATAGGAGAGGGAGAATAAGAGACGGATAATTTCGTTTT
+
9/-//,,*,,,,,//6399@???>?7747>===.766300/---1--,---,--+035;;4
@T6XXI:00079:00025
```

```
CTAGTCACTCGAGATCTGCGGCTTTGGACAGTCCCTCTGTACTGTGAAGCCACAGATGTCGAGTCGACTAGCTTCCT
TTCTCCCTTCTCCC
+
B;1//669=CCCECCBBABC@ABC>C@B@??766-
7A@@BCBB=??@>@CACCCAAABAAABBABBCCCCCDC?B?BB>???B;=:=AAB=
@T6XXI:00079:00033
GGGAGAAGGGAGAAAGGAAGCTAGTCACTCGACATCTGTGGCTTCACAGTACAGAGGGACTGTCCAAAGCCGCAGAT
CTCGAGTCGACTAG
+
CEABBCBCC>CBBB>@796:AA@ABBA799>776777?AC?BB?AAACCCCB@=77717>>@==;<C=8869AABCB
A===<<;<;<====
@T6XXI:00079:00044
GGGAGAAGGGAGAAAAGGAAGCTAGTCGACTCGACATCCTGTGGCTTCACAGTACAGAGGGACTCGTCCAAAGCCGC
AGATCTCGAGGG
+
??:>:77;;,1////*/78,47;3336674457.....,../6BAC@?:??BFCCA=<86166///..333+--
,004853522---20
@T6XXI:00080:00019
GGGGAATTCTAATACGACTCACTATAGGGAGAGGGAGAATAGCTAACCACACGGCTACTTCCCAAGTTCCTTCCTTT
CTCCCTTCTCCC
+
CDC4A54694424499ABCBCC???777.7=?BC>BBC@@@BACC@?<?@???88;8:?@CC?A@A@>A6849?AA<
====;=>@@AB?
@T6XXI:00080:00021
GGGGAATTCTAATACGACTCACTATAGGGAGAGGGAGAATAGCTCAACCGTCTCTGGCGCAACCATATTCTTCCTTT
CTCCCTTCTCCC
+
DHNBH<<<<AABCCCBC?>?BA@<<116.6>=BC>ABB?@@==@CCCE=??@A@@@>===AAA@AAAA>??;9/..,
/666,/,/1140
@T6XXI:00080:00031
GGGAGAAGGGAGAAAGGAAGCTAGTCGACTCGACATCTGTGGCTTCACAGTACAGAGGGACTGTCCAAAGCCGCAGA
TCTCGAGTCGATA
+
CCACCCACCBBAAA=A???BB99:A:;:AABB;;;;;:BBB5:;8:A@CCBBCCA@@?:?889=A@AA<@@>>==A?
??AC??>AA@;;=
@T6XXI:00080:00033
GGGGAATTCTAATACGACTCACTATAGGGAGAGGGAGAATAGTTTGCGCATTCGGTGCACTGTTATTCTCTTCCTTT
CTTCCTTTCTCCCTTCTCCC
+
CKK:C;:>;FCBCC??ABBBA>;99111.777>>:ABC@A@@>;:==<<@B?BB@CCBBBCBB@@@;@??B@C?BB>
C@;>26657?>@<A????C@
@T6XXI:00080:00037
GGGGAATTCTAATACGACTCACTATAGGAGAGGGAGAATAGTGACGCGTTGTGCGGCGCTCACTGTCTCCTTCCTTT
CTCCCTTCTCCC
+
DDB5<8=ABCC@CB@@ABBB>689;//,000>F@CEL@C???@A@??=<9=??@A>A@@@@@=>;43//,/,/,//,
/4670649344-
@T6XXI:00082:00029
GGGGAATTCTAATACGACTCACTATAGGGAGAGGGAGAATAGTCCGCCAATTCTGCGCGTTAGCATTCTCCTTCCTT
TCTCCCTTCTCCC
+
CDC:C?B@BBB?BDCCCCCB>=@?B7776::7CL>A@@>B?@@A:?<=B?C@BBCB?C:957=;<?=?>?:=5;47>
<@AAD>E@999C7
@T6XXI:00082:00031
GGGGAATTCTAATACGACTCACTATAGGGAGAGGGAGAATAGTTTTCGACCCCCATTACACTGCTTTTCTCTTCCTT
TCTCCCTTCTCCC
+
```

```
CDD9C:;;<CCACBBBB?>>AA@?;69:.7<<?A@ACC@A@<<6<4<?ABCCA1=B?BCB8@>4888-
777><=377.7>>>.747666-
@T6XXI:00082:00036
GGGGAATTCTAATACGACTCACTATAGGGAGAGGGAGAATAGCACTCACTTCTGAGTAATCTATTTCTTCTTCCTTT
CTCCCTTCTCCC
+
DEG:C?B?ABB?BBCBBABAA?<99166.7>>BD@CCCBA><<?=@A===>===@@BC<??@@AA<@??AA?C=??;
?@AD<?<?AAC=
@T6XXI:00082:00048
GGGGAATTCTAATACGACTCACTATAGGGAGAGGGAGAATAGCGCAATTTTGGACATGTGGCCGCTTCCCCTTCCTT
TCTCCCTTCTCCC
+
CCC9B=@=@AA>BCBBBBBAA<<?<667.7?>CE>BBB@@<@==<?:?@?7?@AAABBBB@@=@7824;9,4347::
4<99;6<5=9<C=
@T6XXI:00084:00025
CTAGTCGACTCGAGATCG
+
C994444/88///020*,
@T6XXI:00085:00006
CTATCACTCGAGATCTGCGGCTTTGGACAGTCCCTCTGTACTGTGAAGCCACAGATGTCGAGTCGTACCT
+
AABA@::;BA@;@@;;44993375777;B<>?B>BCCCCDAA@=8:<<E>BBCD@>>;CA>>:0*(()((
@T6XXI:00085:00014
GGGAGAAGGGAGAAAGGAAGGAGAAGACGCAGCCGTATATCGGGAAACTATTCTCCCTCTCCCATA
+
EEBCCB@CB>?===:==?@C@ECC?CBBBBAAB>@@???ABFE>CB>CCCC?<558044443,000
@T6XXI:00085:00019
GGGAGAAGGGAGAAAGGAAGCTAGTCGACTCGAGATCTGCGGCTTTGGACAGTCCCTCTGTACTGTGAAGCCACAGA
TGTCGAGTCGACTAGCTTCCTTTCTCCCTTCTCCC
+
CEABDC@CC>C;;809;>;98800;0..4473>>A???AA@>???=??BCBBB@B;BA=???@@@AAC@??<?A@@@
A???AAA@@A888===>:=9<C7<=@@7=588<C<
@T6XXI:00086:00004
CTAGTCGACTCGACATCTGCGGCTTTGGACAGTCCCCTGTACTGTGAAGC
+
C<1//1//4499998998899@<@BC<@=@@A897771///,,22...,..
@T6XXI:00086:00006
GGGGAATTCTAATACGACTCACTATAGGAGAGGGAGAATAGTTGAACCAATT
+
;99-96924989=?AAAAD@@;=::00./==BC>ABB>??88203/0/-,.,
@T6XXI:00086:00032
GGGGAATTCTAATACGACTCACTATAGGGAGAGGGAGAATAGCGCACAGGCTTGTCGCAGTTCCCTAATCTTCCTTT
CTCCCTTCTCCC
+
A??4@6969@@>BDCCCBCCCBA??777.777>@?CBD@AA?@@ABAAC5::>:8:?AA@A9<<7;;:=84343481
8@;@7=347::7
@T6XXI:00086:00040
GGGGAATTCTAATACGACTCACTATAGGGAGAGGGAGAATAGCCAATCCCTGCCCCGCGTAGCGCTGCCCTTCCTTC
TCCCTTCTCCC
+
DDC9C@B?ABC@CFCBBBABB?BC@@?E>??:995:?>;:;44/47444-49;AA6B@??A@??==9;>6978,/,-
2734@?>:885
@T6XXI:00086:00045
GGGAGAAGGGAGAAAGGAAGCTAGTCGACTCGA
+
EDACCC?BB=BB??=B8:6;B<79=/00@770*
@T6XXI:00087:00004
```

GGGGAATTCTAATACGACTCACTATAGGGAGAGGGAGAATAGTCAACAAATCGTTTGGGGCGTCTTCTCCTTCCTTT
CTCCCTTCCTCCC
+
DCD:C@A=@BC?BCDBBBDCCB@??777.;A@AB>FDD@==>@@@>??A=BCCCB.777.77@FB6;:?47/48::6
<54444/0/-222
@T6XXI:00087:00010
GGGGAATTCTAATACGACTCACTATAGGGAGAGGGAGAATAGCTAACTCCTCCCAAGCATCATCCTCCCTTCCTTCT
CCCTTCTCCC
+
CCC9C>A>ACCCC?=?BBBA@@?99111.7>>>B>BCCAA=88<@@@>77616,77868AA5:32433,4/4/3/00
00+0/0000+
@T6XXI:00087:00015
GGGGAATTCTAATACGACTCACTATAGGGAGAGGGAGAATAGTCAACAAATCGTTTGGGGCGACTTCTCCTTCCTTT
CTCCCTTCTCCC
+
CGE7B>B@BCC@CBAAABBBA>999166.7===?<?CC@AABBA=<<<<;A@?>?8???5???AB=??A>?<??CC@
===A;@:==DA=
@T6XXI:00087:00019
GGGAGAAGGGAGAAAGGAAGCTAGTCACTCGAGATCTGGCGGCTTTGGACAGTCCCTCTGTACTGTGAAGCCACAGA
TGTCGAGTCGACTAGCTTCCTTTCTCCCTTCCTCCC
+
IC<@<<:9@:@99919<@4;;@BBCCCCC;;:A@@???;>?>7;86;>??@BBCD>CA????@@999C@@@>===@A
AA===@A@AA=<<@888;;8;;1//9939,/,.775
@T6XXI:00087:00031
GGGGAATTCTAATACGACGTCACTATAGGGAGAGGGAGAATAGCAAACGTTTCCCCGACGTCGC
+
7770<,9244424/6///..86?;;7337,3>=>=,61101799?@;<<996666,11116661
@T6XXI:00088:00012
GGGGAATTCTAATACGACTCACTATAGGGAGAGGGAGAATAGCCACCAACTATTGTTCGTCGCCGTTCTCTTCCTTT
CTCCCTTCTCCC
+
@@?4><>=@BB@CCCAAABBCCB??666.777@C@CCC@@<==>AA<?<?@?@;==?BA889?<>B:==8837:;;:
=<994:8;<@C?
@T6XXI:00088:00023
CTAGTCGACTCGAGATCTGCGGCTTTGGACAGTCCCTCTGTACTGTGAAGCCACAGATGTCGAGTCGACTAGCTTCC
TTTCTCCCTTCCTCCC
+
E8-
,,,,,8::>DAA@CCBBB?ADD>C<AACE@??8???AAAA@@A::68@=@CDDGCCBBB??:;9987731136011,
133501,.,.--+
@T6XXI:00088:00034
GGGAGAAGGGAGAAAGGAAGCTAGTCGACTCGACATCTGTGGCTTCACAGTACAGAGGGACTGTCCAAAGCCGCAGA
TCTCGAGTCGACTAG
+
EHADDD@BA<@@@>9?598;?><>>0//:79:081161=8>8?@?BBBCCBCCC?733-
4::;<=>@C?AD=@@@A@?===<@@AA@@@===
@T6XXI:00088:00043
CTATCGACTCGAGATCTGCGGCTTTGGACAGTCCCTCTGTACTGTGAAGCCACAGATGTCGAGTCGACTAGCTTCCT
TTCTCCCTTCTCCC
+
BBB;99:=??;>BAAAAACC@CCC?C=?>>>777.77==ACCE@??ABAC@CCC@@@BBBABABBBAAABBB?;?:<
=6=;<>.4240399
@T6XXI:00088:00044
GGGAGAAGGGAGAAAGGAAGGAGAACGCGCACCCGAACCAACGGTGATCTATTCTCCCTCTCCCTATAGTGAGTCGT
ATTAGAATTCCCC
+

```
/1-//4249-
448;5=;=<=;;<A>AA@BBBCC?CH?B?B@?A=ACBBBA@@?A;;;8000999>>B@=9961111;6127?A@C@B
CD:
@T6XXI:00089:00021
GGGAGAAGGGAGAAAGGAAGCTAGGTCACTCGACATCCTGTGGCTTCACAGTACAGACGGGACTGT
+
HKCH<;8<C>@@@CAC?D?BC@@@=>>5...4.....,/757;7957?BAAA;=<//--2+---03
@T6XXI:00089:00022
GGGGAATTCTAATACGACTCACTATAGGGAGAGGGAGAATAGCCGATCCTTCGTCAACCTTGCGCTGTTCTTCCTTT
CTCCCTTCTCCC
+
DCB6@8;;<BB@CC@?@CCCCCA?>666.7==>@=BBB?A@@@>A===<>;>???A>A<B?AAB:;:??A?=@=??:
??@@:@=:::C?
@T6XXI:00089:00029
GGGGAATTCTAATACGACTCACTATAGGGAGAGGGAGAATAGTCTTCCAATTTTGGGCGGTACTGTCTCCTTCCTTT
CTCCCTTCTCCC
+
EBB/<7;@@AAADCCBBBB@@>A??66957@>CC>BBB???>>?@?A=B>BDD9CB<BB@CCBBBBBBC?A>A>AAA
ABBC>C<;;CC>
@T6XXI:00090:00031
CTAGTCGACTCGAGATCTGCGGCTTTGGACAGTCCCTCTGTACTGTGAAGCCACAGATGTCGAGTCGATAGCTTCCT
TTCTCCC
+
DBAAB;;<CFDCEDJCBBBBB@BBB>B?BABB:::5:@=A@B:;:::7:;@=@AA@@?@@?;;::;;@?:736360/
/+.222*
@T6XXI:00091:00009
GGGGAATTCTAATACGACTCACTATAGGGAGAGGGAGAATAGTTTGTACATCCCCCGTGCAACATGCTTCTTCCTTT
CTCCCTTCTCCC
+
C@@4>=@=@CC@CCA?BBBB?=??9116.777BC@BBB>@>>>=;AA?=;86611+7>@AA@BB?@@@>@D@A?AA<
@88859>AAAA<
@T6XXI:00091:00012
GGGAGAAGGGAGAAAGGAAGTGCAGTTCGCGGTTAGTGAAGGGAAAACTATTCTCCCTCTCCCTATAGTGAGTCGTA
TTAGAACTTCCCC
+
IEAAAC@CC@CCBB=B@C@CCBABCNB=<<C@D@CCB@@=?A<ABC9@@@C?B?AA=AA@AB7:786337;??B@<8
838;;8;:2444+
@T6XXI:00091:00015
GGGAGAAGGGAGAAAGGAAGGGACAGTCCATCTGTGGCTTCACAGTACAGAGGGACTGTCCAAAGCCGCAGATCTCG
AGTCGACTAG
+
BEABBBACC@BBBC8:;;@CCCCAAA::58>>>===:::5:CCBB?BC@B?<???AAAAA>AA<AA;@@?@@@??>8
87=@@@ABB;
@T6XXI:00091:00018
GGGAGAAGGGAGAAAGGAAGCTAGTCGACGTC
+
CECCAA899-9>==9>5959?883........
@T6XXI:00091:00048
GGGAGAAGGGAGAAAGGAAGCTAGTCGACTCGACATCTGTGGCTTCACAGTACAGAGGGACTGTCCAAAGCCGCAGA
+
@D?A@C>BB@BDEC@CDC=@@??>>?7:;A@AAB:::A@@>>;;8:CBABBBBB?444-/66/.-244224,-033-
@T6XXI:00092:00006
CTATCGACTCGAGATCTGCGGCTTTGGACAGTCCCTCTGTACTGTGAAGCCACAGATGTCGAGTCGACTAGCTTCCT
TTCTCCCTTCTCCC
+
CA@=944::94BCCGCCCCC@BCB>C@B@9>666-
7<=BBB?@?B@=<<A=@BA;;<AAAABC?AA;;<C@?=>B@BD=AAAD=>587==:
```

```
@T6XXI:00092:00017
GGGAGAAGGGAGAAAGGAAGCTAGTCACTCGACATCTGTGGCTCACAGTACAGAGGGACTGTCCAAAGCCGCAGATC
TCGAGTCGACTAGCTTCCTTT
+
AC@FFC@CC<>99909594883<777,,,,,00,,.11..3...7799@6168444,498;AB>AA<@@99<D?@88
86644.434-.-222,.-3;:
@T6XXI:00092:00031
GGGAGAAGGGAGAAAAGGAAGGTGAC
+
DE?B@@599091111*1,/,/,...4
@T6XXI:00092:00046
GGGAGAAGGGAGAAAGGAAGGGGAGAAGGGAGAAAGGAAGCTAGTCGACTCGACATCTGTGGCTTCACAGTACAGAG
GACTGGTCCAAAGCCGCAGATCTCGAGTTCGACTAG
+
AA?ACCBCC@A@@B@B@DBABC:>>>?CD<@???8?;<:???AFB:::@@A?;<<;;;;464/.,-
878;>44/7//,/7/.,-1246388556@;<<984;B8849??A@>:
@T6XXI:00093:00008
GGGAGAAGGGAGAAAGGAAGCTAGTCACTCGACATCTGTGGCTTCACAGTACAGAGGGACTGTCCAAAGCCGCAGAT
CTCGAGTCGACTAG
+
DDBCCCACD@CCBB>B896:BBBBCBBAAA@::9966B:?8=B?BBB@@@A?=;444-
4444>@>AB@AC?>@?AA@@@@<<<?=<<@C??
@T6XXI:00093:00009
GGGAGAAGGGAGAAAGGAAGGAGAAGTCGCCCCAAACGATTTGTTGACTATTCTCCCCTCCTCCTAAGTGAGTGT
+
ED?BBC@CI>BBBD?B?B@CCCBA?>8>>>>>2?A<BBCD@>CC=??CCB?<;4444-4;791,--,------3:
@T6XXI:00093:00037
CTAGTCGACTCGAGATCTGCGGCTTTGGACAGTCCCTCTGTACTGTGAAGCCACAGATGTAGCGGAAGCTTCCTTTC
CTCCCTTCTCCCCTTCCT
+
ECCCCCCCCCCBBBDBBBBBC@9998;@BCBCAAB=ACCC==?AABCB>@C@CCB@@@BDCCCC@CAC>>::9:9,0
,/::6:./.<<>5@;A?A
@T6XXI:00094:00007
GGGAGAAGGGAGAAAGGAAGCTAGTCGACTCGACATCTGTGGCTTCACAGTACAGAGGACTGTCCAAAGCCGCAGAT
CTCGAGTCGACTAG
+
DDADCC@AA:@=885>594;A>::=/00:;;;3;:@??CCD?;;<;CCCCBA?:344/4:=;>@<??;?A<@@@B??
<?;?==AA@@@@==
@T6XXI:00094:00008
GGGGAATTCTAATACGACTCACTATAGGGAGAGGGAGAATAGTCTTTGATCTCCGACTTTTACACTTTTCTTCCTTT
CTCCCTTCTCCC
+
BBB9A>B@CCB@CBB?BBCC@??>>777.777@C?DCC@B>@?@>CA?AABBBCCBBBCD9ABB????5;=5?<@A<
===A?A49.445
@T6XXI:00094:00014
GGGAGAAGGGAGAAAGGAAGTAGTACAAGGTGGGGAGGACACCTCGTCTATTCTCCCTCTCCCTATAGTGAGTCGTA
TTAGAACTTCCCC
+
DB<@<<89@:@9990;>A>BCCCEBBA>B;>>>?2>@;:;:B?B:;:?@BC@;:::5///882:::>;;<@<;99;7
7276B<@@>@BA4
@T6XXI:00094:00018
CTAGTCGACTCGAGATCTGCGGCTTTGGACAGTCCCTCTGTACTGTGAAGCCACAGATGTCGAGTCGACTAGCTTCC
TTTCTCCCTTCTCCC
+
B--,,-
,,/8:;BBCDB@@@D?BBC>C@BCBB:::7:CCCBBAA?BBA??C@BCCCABAABCCBABB@??A??=4:47?:=<<
=6=5:9@C=
```

```
@T6XXI:00094:00021
GGGAGAAGGGAGAAAGGAAGCTAGTCGACTCGACATCTGTGGCTTCACAGTACAGAGGGACTGTCCAAAGCCGCAGA
TCTCGAGTCGACTAG
+
CE@CCC@CC?BAA?=B==<@A@@BB:;;CCB@<;:::7?@@4:;?BBDCCCCC@;333-
4:;;@?@DD@AA>@CA@@@A@A@AA?===<=<?
@T6XXI:00094:00025
GGGGAATTCTAATACGACTCACTATAGGGAGAGGGAGAATAGATCACCATTGGTTCGGGTGCGCGTTCTCCTTCCTT
TCTCCCTTCTCCC
+
@AA6A@B?CBB@CC@>@BCB@?AAB9997:A?CE<BAB?@@@AAABB?BC?B@B@CBB>C@@?DDC>AAA4:3:=;;
9=88818;=?@C<
@T6XXI:00095:00004
GGGGAATTCTAATACGACTCACTATAGGGAGAGGGAGAATAGATCACCGTTGGTTCGGGTGCGCGCTCTCCTTCCTT
TCTCCCTTCTCCC
+
CCC9C>?>ADCACBA?BAAAAAAB:9:?9?@@AB>CCB?@====AAA>AC=?>@?CCC@CCCBB;;;CAA?A=?=?C
>BC?=:=;888A<
@T6XXI:00095:00006
GGGGAATTCTAATACGACTCACTATAGGGAGAGGGAGAATAGCGTGCCTGTATACGGTACCCTGTTCGCCTTCCTTT
CTCCCTTCTCCC
+
DCC9C>?=ADC@BBBBBBBA>><;69959@?AB>BBB??<?@A@AA?A>BBC@BB@BBCD?BBB?FAA<@?@;??6
????<????AA=
@T6XXI:00095:00011
GGGGAATTCTAATACGACTCACTATAGGGAGAGGGAGAAT
+
BAA7A?BBCCBABBBABBBBCBBA>///,/0/::6<>:96
@T6XXI:00095:00014
GGGAGAAGGGAGAAAGGAAGCTAGTCACTCGACATCTGTGGCTTCACAGTACAGAGGGACTGTCCAAAGCCGCAGAT
CTCGAGTCGACTAG
+
B@<A@@=@C>BBBB<@=@>BAABCCB@1//@00////57776348>@@CBCABB844-
4444;=;;=7:;:;787>895<6@@@????=<<
@T6XXI:00095:00016
GGGGAATTCTAATACGACTCACTATAGGGAGAGGAGAATAGTACGTCGTA
+
A>>5B===@BBADCC@BBBB>>ACC>??>>@>??A:331-//---1---3
@T6XXI:00095:00035
GGGAGAAGGGAGAAAGGAAGGGGAGAAGGGAGAAAGGAAAGCTAGTCGACTC
+
GGBCCB@CC>BB?@8969?BCD9BCB?@?:>777-1011,166979611111
@T6XXI:00095:00037
GGGGAATTCTAATACGACTCACTATAGGGAGAGGGAGAATAGTCACATTCATGTTTGTGACCGTGTCTCCTTCCTTT
CTCCCTTCTCCC
+
999-
48?;>B@ACCCCCBCB>=@GCBBB>BBCCC>CBC?@@?@@AA<B?BACCCC>BBB==:<C@=<<<9<<@?AHEC???
:>;==??:
@T6XXI:00095:00041
GGGAGAAGGGAGAAAGGAAGGGGAGAAGGGAGAAAGGAAGCTAGTCGACTCGACATCTGA
+
DD?BBA@BB>A@@B=?=@@BBC9CCC@CC>C??88?:?:?C:;:B:77700.--***.*+
@T6XXI:00096:00027
GGGAGAAGGGAGAAAGGAAGCTAGTCGACTCGACATCTGTGGCTTCACAGTACAGAGGACTGTCCAAAGCCGCAGA
+
BB>DCDEDD@AAACBB?B6;;@BCCEC?>>>=00000/33638<<?BCBCBDCB>8838==;=>=??<AA>AAAAA
```

```
@T6XXI:00096:00033
CTAGTCGACTCGAGATCTGCGGCTTTGGACAGTCCCTCTGTACTGTGAAGCCACAGATGTCGAGTCGACTAGCTTCC
TTCTCCCTTC
+
C--,,-
,,///;@==@A@@@B=?@C@B>BB<;<CC@DB???@67:??A<?CBBCBCBB:;;A@@@A@ABB?@:7374747777
./,0
@T6XXI:00096:00040
GGGAGAAGGGAGAAAGGAAGGAGAACGCGCACCCGAACCAACGGTGATCTATTCTCCCTCTCCCTATAGTGAGTCGT
ATTACGAATCCCCCC
+
CECBACACD>BCBD@C@B@B@CCC?CCCBBCCC?CDCD@C@CDADCCCB@@@>AAAA=;;<BDBCCCCEBBBBBB?<
<<:000?::<?666*
@T6XXI:00096:00043
CTAGGTCACTCGAGATCTGCGGCTTTGGACAGTCCCTCTGTACTGTGAAGCCACAGATGTCGAGTCGACTAGCTTCC
TTTCTCCCTTCTCCC
+
AAACACBDCDDFEDCBABABBABBB?C@BB<A69947==B::;C@??A>ACACCCBABBBCCCCABBCCBA;;9:>;
>>6???A<?9<6<?:
@T6XXI:00097:00004
GGGAGAAGGGAGAAAGGAAGAAAAGAGATGACGCACCGTTAGCATGATCTATTCTCCCTCTCCCTATAGTGAGTCGT
ATTAGAATTCCCC
+
9;79AAACC@BBBCAC@C@BBBB7AABAAAACBBCBACC?BBB@@@AA???@=@;;;7888ADAA?@@@ABAAA@A?
??>???AC@CCC8
@T6XXI:00097:00030
GGGGAATTCTAATACGACTCACTATAGGGAGAGGGAGAATAGTCAACAAATCGTTTGGGGCGACTTCTCCTTCCTTT
CTCCCTTCTCCC
+
==9-
969?AAB@BCBCBBBBBBAA?677.777775:@@@@===@@>AAA<BBBBC<AAA6?A@??:?<?3437;==:===@
<A==<<A7
@T6XXI:00097:00037
GGGAGAAGGGAGAAAGGAAGAGAAATCACGCGGGCCAACGTATGCGCTATTCTCCCTCTCCCTATAGTGAGTCGTAT
TAGAAT
+
A@6@<989@:?>@?9>=>;@?AAAABDBCCBBB>BAC@CAAAB@@@@@AB>AA@D?ACBBE=@@B?C:37/------
,044/-
@T6XXI:00097:00040
GGGGAATTCTAATACGACGTCACTATAGGGAGAGAGAGAATGCAACTTATCGATGTCGAA
+
>DD1@+,,00*,/6/4433777888333,331..3...,.4377771111111111166
@T6XXI:00097:00045
GGGGAATTCTAATACGACTCACTATAGGGAGAGGGAGAATAGTTTCCTACAACTCATTCGGGCGAA
+
CGC:C>A>ACBACBBABBBBB?997166.7==ABBCCD?@@AAA<@>?@>7,///--,-23+3445
@T6XXI:00098:00014
GGGAGAAGGGAGAAAGGAAGCTAGTCACTCGACCATCCTGTGGCTTCACAGTACAGAGGGACTGTCCAAAGCCGCAG
ATCT
+
DD>>?@59919>??9>8949?888@A;79::00,...,.55>><>=@AAA:::B@==CC?B@=;99244-
04203083736
@T6XXI:00098:00044
GGGGAATTCTAATACGACTCACTATAGGGAGAGGGAGAATAGTCAACAAATCGTTTGGGGCGACTTCTCCTTCC
+
999,42424>@@CCCCC;;0001>00.4+...17799><694000/000+11117999=5963333...,-,-,
@T6XXI:00099:00011
```

```
GGGGAATTCTAATACGACTCACTATAGGGAGAGGAGAATAGCCAGGAA
+
BBB6B@B@CDC@:;;AA>>@@?@@CBBB<@?7747..312--,-1012
@T6XXI:00099:00036
GGGAGAAGGGAGAAAGGAAGAATAATCAAACACGACACGGTGAAAGGCTATTCTCCCTCTCCCTATAGTGAGTCGT
+
BB@BBB@BC=BBCC>C?B?BC@CC>BBCC>BABCCECAB?ABBB6:6;@@?<@>>>8777;B=BA?@DAA@@@A@?
@T6XXI:00050:00081
GGGGAATTCTAATACGGACTCACTATAG
+
:;;2A6;7<@?;=611,//..9733..0
@T6XXI:00052:00065
GGGAGAAGGGAGAAAGGAAGCTAGTCACTCGAGATCTGCGGCTTTGGACAGTCCCTCTGTACTGTGAAGCCACAGAT
GTCGAGTCGACTAGCTTCCTTTCTCCCTTCTCC
+
CGACAA>AC>@@@=9>5;8;AA8;;?==68??8<<@@BGB??@@=?ACBABAAA<ABAAA@@>;83644;8;:<<==
;;<?>>>???@@@@@B==:<>:;///,/,///,
@T6XXI:00052:00088
CTAGTCGACTCGAGATCTGCGGCTTTGGACAGTCCCTCTGTACTGTGAAGCCACAGATGTCGAGTCGACCTAGCTTC
CTTTCTCCCTTCCT
+
F<94;9444:@@@@CCBBCBB?BBA=A6:;?@@@B>CDDCCBBBCBBA>@C@<<<BABB@@;:779@98437736>@
=9986111,10.,,
@T6XXI:00053:00088
GGGAGAAGGGAGAAAGGAAGAATATTGTGATGGTGAAAAACCGAGGACTATTCTCCCTCTCCCTATAGTGAGTCGTA
TTAGAATTCCCC
+
CD?>>A@CC>BBBB>B@C@BA?BAA?BBBBBCDA;@?@@29>B<==?BBDC=>>CC>?:?BC>CC>>>A=A??888<
;9<@@>@?ADA6
@T6XXI:00054:00083
GGGGAATTCTAATACGACTCACTATAGGGAGAGGGAGAAATA
+
DCC:CAE?B@A;>@>98;8777979=<>7=7166-1111,0-
@T6XXI:00055:00066
GGGAGAAGGGAGAAAGGAAGCTAGTCACTCGAGATCTGCGGCTTTGGACAGTCCCTCTGTACTGTGGAGCCACAGAT
GTCGAGTCGACTAGCTTCCTTTCTCCCTTCTCCC
+
9@<>@@<@@194>>9>>B@A@@BAA=7716;;==966?@@=@AB<B?BBCCAAB<AB@899AC@@@=?;;388?@@A
?A?B@====@??=87::9799-4444-424444,
@T6XXI:00055:00074
GGGGAATTCTAATACGACTCACTATAGGGAGAGGGAGAATAGTTTTCCCTTCACTACCGCGAACTGCACTTCCTTT
+
444,42424@B@BBBCCCB:87888776,11111,1677333443+040323;6111:=11623335;8::8;;;5
@T6XXI:00055:00088
GGGAGAAGGGAGAAAGGAAGAAGAATAACGAGTTAAAGGTAAACAGCTATTCTCCCTCTC
+
AA?ABBACC@BBBB=B@C@CC?@@@BB@BCBBB<>@:?>@9=9=0/1>>C?==<A@AA@@
@T6XXI:00055:00093
GGGGAATTCTAATACGACTCACTATAGGGAGAGGGAGAATAGTCAACAAATCGTTTGGGGCGACTTCTCCTTCCTTT
CTCCCTTCTCCC
+
CCB7B5959?>=AA@@CDBBAA>>>777.7===?<?BB@????AB>AAA<BB??;6<<<5?ABCC@CCC@A>@>AC7
==AC7<:<>?@5
@T6XXI:00056:00091
GGGGAATTCTAATACGACTCACTATAGGGAGAGGGAGAATAGTTACAACTAAG
+
GCB9B=B@CCC@CCBBBBBBBAA@><??=?BCCC>ABA?798:...30004-.
```

```
@T6XXI:00056:00097
GGGGAATTCTAATACGACTCACTATAGGGAGAGGGAGAATAGTCAACTGAAATCGTTTGGGGCGACTTCTCCTTC
+
@AA6@?B@CBB@CCBBABBBB@BB86679///@@9;>>;>>83--,----20344:=3:884<4333,,,,*,*,
@T6XXI:00057:00063
GGGGAATTCTAATACGACTCACTATAGGGAGAGGGAGAATAGTTTTCCCTTCACCACGACGACTAGCACTTCCTTTC
TCCCTTCTCCC
+
DCB7B79;<CB?GCBBBBCCCCCB@666.7>=@A>BCCAA<<;88-44-
48;00,0./.....333149999233,37>>6><?77:5
@T6XXI:00057:00074
GGGGAATTCTAATACGACTCACTATAGGGAGAGGGAGAATAGTCACGCATTTGCCCGTTTGACTCTCACCTTCCTTT
CTCCCTTCTCCC
+
AB?4>6;;<BBACBBBBBCCCBB??666.777>@@DCG@BA@@<=<<<@A79166.7==6:<<<<@@BD@A;>/30-
4::;9>8=;;;6
@T6XXI:00057:00080
GGGGAATTCTAATACGACTCACTATAGGGAGAGGGAGAATAGTTTTTCCCTTCACTAACCGCGAACTGCACTTCCTT
TCTCCCTTCTCCC
+
999/4:@>CBB@CBBABBBBB>ACC???;>?=>@;@>=:6<<;>;>0;;7<=?AB::;:@BABB@@@@ADCC:8466
.7A@A>C?;99D<
@T6XXI:00058:00085
GGGAGAAGGGAGAAAGGAAGCTAGTCGACTCGACATCTGTGGCTTCACAGTACAGAGGGACTGTCCAAAGCCGCAGA
TCTCGAG
+
DD@CDC?BB=BCBB>B@B8;:A@@@CCC@B@>AABABACCC>?@=?C?A@@CA@=44414<:<?<?==5===AA=;:
;;<<<<;
@T6XXI:00059:00061
GGGGAATTCTAATACGACTCACTATAGGAGAGGGAGAATAGTCAACAAATCGTTTGGGGCGACTTCTCCTTCCTTTC
TCCCTTCTCCC
+
IHF8B6;549;24449>83334.....,...16669?=>?CAA?<?AA<;;:7?-769-:;??C7;6?3634344-
4;;@<@;>844-
@T6XXI:00059:00066
CTAGTCACTCGAGATCTGCGGCTTTGGACAGTCCCTCTGTACTGTGAAGCCACAGATGTCGAGTCGACTAGCTTCCT
TTCTCCCTTCTCCC
+
:11////44A>AABFCA?@B?@?@<@8:<CCCCC>BBBBB@BGCCB@==@=ACBBBBBBCCCCBCCCAAA;:7<A?A
B8:79@<=9;055+
@T6XXI:00059:00089
GGGAGAAGGGAGAAAGGAAGGAGAAGTCGCCCTACGAAGTT
+
99-44>;>>8<;@@<B?B>B@BCBA>><000,0...55..,
@T6XXI:00059:00095
CTAGGTCACTCGAGATCTGCGGCTTTGGACAGTCCCTCTGTACTGTGAAGCCACAGATGTCGAGTCGACTAGCTTCC
TTT
+
CEDBFDBBCDCCCCCCBBB=;933708=@ADCCCC>CCAAAAAA???><<@=ACBBBCABECCBBAABAAAC@@>??
CF>
@T6XXI:00060:00060
GGGGAATTCTAATACGACTCACTATAGGAGAGGGAGAATAGTTCTCCAGATCTCTCACAGCATAGCCAACACTTCCT
TTCTCCCTTCTCCC
+
BCA7A8;;<CBCBBBCCBBBB:64;//,0::?C?CBB?@>@@>=<<:?BABBAA@??CC?@@ABBBIGICA;:47:=
=6?AAE@C;==BC<
@T6XXI:00060:00074
```

```
GGGAGAAGGGAGAAAGGAAGCTAGTCGACTCGAGATCTGCGGCTTTGGACAGTCCCTCTGTACTGTGAAGCCACAGA
TGTCGAGTCGACTAGCTTCCTT
+
ED?BBB>@B;;<CE@C@C@BBCB==000/.3...778:<??:<<;2<3;>>=777.7;>AAAABA?@B@??<??==?
?898<<@473500040-,-011
@T6XXI:00060:00075
GGGGAATTCTAATACGACTCACTATAGGGAGAGGGAGAATAGTCACTGGCTTGGTTGTCCGTTATTCTCCTTCCTTT
CTCCCTTCTCCC
+
BBA7A598;BB@CCCBCCBAAA@>>777.7>>BCBBBB?@<A;;====5<<==<=<@@?><=;?@;6=>3858>AA<
A@AA<A>AAAA<
@T6XXI:00060:00076
GGGGAATTCTAATACGACTCACTATAGGGAGAGGGAGAATAGATCACGACCCTTCTACCACGACTGAGCTTCCTTTC
TCCCTTCTCCC
+
??=6@?=?CBB@BBAAABBBBAB?B66646?9@C>CCC@B=<<<:??>777.730000,086/478666?9924809
8>><@>??>?:
@T6XXI:00060:00098
GGGAGAAGGGAGAAAGGAAGGAGAACGCGCACCCGAACCAACGGTGATCTATTCTCCCTCTCCTATAGTGAGTCGTA
TTACGAAATTCCCC
+
44-4449::-
99===??BADACCD=AABABABA<@?596:9?AD;;<9A;;@6999:63...,.6446<<>88:@7617611..+.,
...*
@T6XXI:00060:00099
GGGGAATTCTAATACGACTCACTATAGGGAGAGGGAGAATAGTCCAGCGCCCGTTGAAACGTAATTATTCTTCCTTT
CTCCCTTCTCCC
+
BBB9B?B@ABC@B@??BBBB?A@99111.777>@=AEDAD>>?=;=?==AC=AA>A@@7??BB@B@CC@@@>A>AA<
@@AA=A>B@@@<
@T6XXI:00061:00091
GGGAGAAGGGAGAAAGGAAGCTAGTCACTCGACATCTGTGGCTTCACAGTACAGAGGGACTGTCCAAAGCCGCAGAT
CTCGAGTCGACTAG
+
CC?CCC?AA=AB@@=A>A>BC?>AB<A>::7;::::7ABC=@@?BCCC@?@A?<44445<<A==:;<:<>>><;;;<
<<<888AAAA;A;=
@T6XXI:00062:00050
GGGGAATTCTAATACGACTCACTATAGGGAGAGGGAGAATAGTCAACAAATCGTTTGGGGCGACTTCTCCTTCCTTT
CTCCCTTCTCCC
+
@@@/959<@?A?BCCFB?@>B???7177.7==>@=????@???<:7:::4==777.7>>5@BBBB@CCC@A>?<?C:
?=AC?C;=>=@<
@T6XXI:00062:00070
CTAGTCGACCCGAGATCTGCGGCTTTGGACAGTCCCTCTGTACTGTGAAGCCACAGATGTCGAGTCGACTAGCTTCC
TTTCTCCCTTCTCCC
+
B1111=8=DCBCDCCBCCC@@>ACC@C?BB@@@CC@CBB?@CA@?AAA>AC@CCDCCBB<<<BCCCCCBBDDDB?B?
BG@@?CC>C?BCDD>
@T6XXI:00062:00071
GGGAGAAGGGAGAAAGGAAGGAATAGTCTGATGCTGCCGAGCGTGAGGCTATTCTCCCTCTCCCTATAGTGAGTCGT
ATTAGAATTCCCC
+
DCACCBAAA=ABCC>B@B@B@B@BBB@BBBCBABBBB?B::;E@?@C?A@AA>==9=;>>;;;;@C=>=<<???@?=
==?CCC>C>AAA6
@T6XXI:00062:00075
GGGGAATTCTAATACGACTCACTATAGGGAGAGGGAGAATAGTTTCACGTTGCCGACTGCTGCTATTCCCTTCCTTT
CTCCCTTCTCCC
```

```
+
BBB7A=?>ACC@CCB?BBBB@@<99111.7==AB>ECC@???==;<<<<=;<:<<<<<=<<<=?=>3771044/00,
0999499;444-
@T6XXI:00062:00088
GGGAGAAGGGAGAAAGGAAGGAGAACACGCCGCGGGATATTGGCGAACTATTCTCCCTCTCCCTATAGTGAGTCGTA
TTAGAATTCCCC
+
AD=BBB>B?>BBBC@B?B?B?BCC?BBCBC@CCCB<ABCC?B?BBCACECDEDGCC>BC@@@>@ECCCBCCCBAAAA
A>BAA?>>CDD8
@T6XXI:00062:00095
GGGGAATTCTAATACGACTCACTATAGGGAGAGGGAGAATAGTCAACAAATCGTTTGGGGCGACTTCTCCTTCCTTT
CTCCCTTCTCCC
+
DDD9C?B@BCB@BCA??BBBB?BCC=====CCA@;?C?>:::=BA?AAA>BABBC>CCC8BCBBB?AA?47335<=:
?AAA=A689<<7
@T6XXI:00063:00064
GGGAGAAGGGAGAAAGGAAGCTAGTCACTCGACATCTGTGGCTTCACAGTACAGAGGGACTGTCCAAAGCCGCAGAT
CTCGAGTCGACTAG
+
CHDAA@@BB:?@@=:@=@=@C>;@@6:7:?>7::?==BBD?BB?BC@?>>@A>:33317=>?A@?AB:?A?@><89=
<<<::::==<<8;;=
@T6XXI:00063:00087
GGGAGAAGGGAGAAAGGAAGTGCAGTTCGCGGTTAGTGAAGGGAAAAACTATTCTCCCTCTCCCTATAGTGAGTCGT
ATTAGAATTCCCC
+
CC>CCB@AA=AAAA;A@B>?>BBBCB@BBBB?B?AABCC@C>=C?CC2@@CC@CCCC>CCBBB>BAA??8==AA@@@
@@>@@C=@<@AC4
@T6XXI:00064:00076
GGGAGAAGGGAGAAAGGAAGGAGAAGTCGCCCCAAACGATTTGTTGACTATTCTCCCTCTGCCTCA
+
DD@CCC@BB>BBBB@B@B@B@BBB?A@BBBBB8CC>BBBBC?CC==@CCA?<::73247.,+*+0+
@T6XXI:00064:00082
CTATCGACTCGAGATCTGCGGCTTGGACAGTCCCTCTGTACTGTGAAGCCACAGATGTCGAGTCGTACTAGCTTCCT
T
+
?:44444449449BCBC?=>;10,0,.....11,1788<@@@C??=?CG<<;::;;776611.81100.44333636
6
@T6XXI:00064:00094
AGGAGAAGGGAGAAAGGAAGCTAGTCACTCGAGATCTGCGGCTTTGGACAGTCCCTCTGTACTGTGAAGCCACAGAT
GTCGAGTCGACTAGCTTCCTTTCTCCCTTCTCCC
+
CDABBC@DCC@?@B>B@C?BCDBCC@AAAECBCCCCAA@B@@?@<A@BCNCBBB?BCCC?;?;?CCC>AC?BBBBCC
C@@@CCBBBBCBAAB>>=;??<???C<A=@===6
@T6XXI:00064:00098
GGGAGAAGGGAGAAAGGAAGCTAGTCGACTCGAGATCTGCGGCTTTGGACAGTCCCTCTGTACTGTGAAGCCACAGA
TGTCGAGTCCGACTAGCTTCCTTTCTCCCTCTCCC
+
B?:?BB?BC<A>@@<@@C?BB@???:::?>@A@;;:AAABA@CBB?B=????<<?9<99;788<::<?788>@879?
?>=AA999AA@AA@>;442/,/8288:;,/.1110
@T6XXI:00065:00050
GGGAGAAGGGAGAAAGGAAGACTAGTCGACTC
+
>>:??>59914=>>9>;>4937773363..,,
@T6XXI:00065:00067
GGGGGAGAAGGGAGAAAGGAAGCTAGTCGACTCGAGATCTGCGGGCTTTGGACAGTCCCTCTGTACTGTGAAGCCAC
AGATGTCGAGTCGACTAGCTTCCTTTCTCCCTTCTCCC
+
```

```
CCCB1B@@=@>:>999095958<74440.......3668::@BC<?@E=CABC????B<@=?888==>>8768<8;<
@@@C99998?>A==<A;/.0;;;;?./8;94979344,
@T6XXI:00065:00087
GGGAGAAGGGAGAAAGGAAGGTGAACAGAGCGCAAACGAATACGTCTCTATTCTCCCTCTCCCTATAGTGAGTCGTA
TTAGAATTCCCC
+
CC?CCC@CA:8///+1,/68::999BBBBBBBBDD>BBCACECBBCAAFCFACCCD@CC???>@CCCAABC;;<BA@
?ADBC@CBCCC7
@T6XXI:00066:00088
GGGAGAAGGGAGAAAGGAAGGTCATGGCTATGCGGTCGGTCTGGGAGCTATTCTCCCTCTCCCTATAGTGAGTCGTA
TTAGAACTTCCCC
+
99149B5997;@@C@C>A?A?BAGCC=A@B888737>=:6?53305///66:>:;>6==///,/.---
.83;<><<;=;:;;844/000+
@T6XXI:00066:00090
GGGGAATTCTAATACGACTCACTATAGGAGAGGGAGAATAGTCAACAAATCGTTTGGGGCGACTCTCCTTCCTTTCT
CCCTTCTCCC
+
DB;2;29294;9449984733.444..,..366661100444;428553:??@@;<<<29:<00/.4,--
.9?A685:=;<55.393
@T6XXI:00066:00091
GGGAGAAGGGAGAAAGGAAGGAGAAGTCGCCCCAAACGATTTGTTGACTATTCTCCCTCTCCCTATAGTGA
+
CB=@?>;>@<@BCC@C@C@C=@7758793;BB8BB=@996>;6688666=?>AAAA7;<884,4;830---
@T6XXI:00066:00093
GGGAGAAGGGAGAAAGGAAGCTAGTCGACTCGACATCTGTGGCTTCACAGTACAGAGGACTGTCCAAAGCCGCAGAT
CTCGAGTCGACTAG
+
DC@:;;=@@<B@@C>A?A@BBBA@@6:;===?;<:CCCCDC@?@=????AC@::7//,/866:??CC;>?;=C???:
:==<@788;;<;;;
@T6XXI:00067:00089
GGGGAATTCTAATACGACTCACTATAGGGAGAGGGAGAATAGTGACGTGTTGTGCGGCGCTCACTGTCTCCTTCCTT
TCTCCCTTCTCCC
+
999/49B<99;@999BB<;0>>=@31/77>>>AD>CCB@@@?<;;;:89;383>>@>A?==AA===AB;;8:79244
-4:::-434444-
@T6XXI:00068:00066
GCTAGTCGACTCGAGATCCGCGGCTTTGGACAGTCCCTTTGTACTGTGAAGCCACAGATGGACTGTCCGACTAGCCT
TCCTTCTCCTCTCC
+
111//4:999@ACABBDDABBB>A@@:@=?999984-44-
484444948548889>>83356;BBBB??>>>000,/66343433111110
@T6XXI:00068:00067
GGGAGAAGGGAGAAAGGAAGGA
+
DEACBB==95=911,1:8+-+-
@T6XXI:00068:00079
CTAGTCACTCGAGATCTGCGGCTTTGGACAGTCCCTCTGTACTGTGAAGCCACAGATGTCGAGTCGACTAGCTTCCT
TTCTCCCTTCTCCC
+
B8///:/44@BBBCEB@?@C@BBB@A?ABBB@@C>AACC???CCDB@=@C@CCCCCBAB???BBBCCEEDC@@?@?B
B>BCCC>A=@@AB<
@T6XXI:00068:00087
GGGAGAAGGGAGAAAGGAAGCTATCGACTCAACTCTGTGGCTTCACAGTACAGAGGGACTGTCCAAAGCCGCAGATC
T
+
```

9914??<>?:@AAA<A>A>@@?????@A??????????@=@B????BBB@>:4444-
4;@@@A=??7=?377?89>?>
@T6XXI:00069:00066
GGGAGAAGGGAGAAAGGAAGCTAGTCACTCACTATA
+
B;:9942495949904242477776000..,,,,,0
@T6XXI:00069:00068
GGGAGAAGGGAGAAAGGAAGGAGAAGTCGCCCCAAACGATTTGTTGACTATTCTCCCTCTCCCTATAGTGAGTCGTA
TTAGAATTCCCC
+
EE?CCB@CDBDCBB>?@B=@>?CC@B;ACCCC8??=@CBCE>CC@FBBBA??AACC<<<<@C>=;?@@CC<<;<?A=
@>@AC?D>?>>4
@T6XXI:00069:00092
CTAGTCGACTCGAGATCTGCGGCTTTGGACAGTCCCTCTGTACTGTGAAGCCACAGATGTCGAGTCGACTAGCTTCC
TTTCTCCCTTCTCCC
+
C2,---
,,/1:ACCCCBBBBB@BBB<B>AA?B@??=?CCDCBBC?BBC?AC@C@AACCCCBABCABBDCCCABA@B>ABAAA@
B9<9?A=>6
@T6XXI:00071:00079
CTAGTCGACTCGAGATCTGCGGCTTTGGACAGTCCCTCTGTACTGTGAAGCCACAGATGGACTGCTTCCTTTCTCCC
TTCTCCC
+
DCBBBBBBBCDBABB?BBBBBA@AA=A?A@AC@??=?DCC@?@B?BB@>@CDCBBBC@@=ACCBCC;;8;C>AABC>
C@BBBD?
@T6XXI:00071:00090
GGGGAATTCTAATACGACTCACTATAGGAGAGGGAGAATAGCAAACGTTTCCCCGACGTCGTTTCTCCTTCCTTTCT
CCCT
+
499,48>549>59499:?BCC;::;00,0::=><BBC@A@>==@;@BBB<@AA6??????777.77746:=333+00
00+0
@T6XXI:00072:00066
GGGGAATTCTAATACGACTCACTATAGGGAGAGGGAGAATAGTCAAC
+
AB>0@-1,/667/44=997BB?;;;@>>:77777,111339994620
@T6XXI:00072:00069
GGGGAATTCTAATACGACTCACTATAGGGAGAGGGAGAATAGAGACGTATTCGTTTGCGCTCTGTTCACCTTCCTTT
CTCCCTTCTCCC
+
CCD:C@@>ABB@CCB?BBBB>@??;116.677=@<ABB?AA???@AAA?<;:<<A:AAAABB=<<>@?@=@>@>AA<
===A<A=><<<7
@T6XXI:00072:00072
GGGAGAAGGGAGAAAGGAAGTAGTGGGGTGGGAAGAACTGAGGGCAGCTATTCTCCCTCTCCCTATAGTGAGTCGTA
TTAGAATTCCCC
+
DC@CAA>BB>BBBCAC@C?BA?;?8885?@@;B;??>???:771;CAADDE@CCBB=BBBCC?BBBCCCCCCBAAAB
B@AAA>A<A@A5
@T6XXI:00072:00081
CGAGATCTGCGGCTCCATATCAC
+
CCBBBB?@BCD><00)****(,,
@T6XXI:00072:00084
GGGGAATTCTAATACGACTCACTATAGGGAGAGGGAGAATAGCAGTGCAATTACT
+
444,48656>@,111/;;641109=;:?6=7167-17=:97.-/--.-3363673
@T6XXI:00072:00086
GGGAGAAGGGAGAAAGGAAGCTAGTCGACTCGACATCTGTGGCTTCACAGTACAGAGGACTGTCCAAAGCCGCAG

```
+
GDACAA=@B?9;;@<@>B?BBB:1-,,****0*,,33776>=?@=????BBC>>;//./71478/33+00/0333
@T6XXI:00073:00086
CTAGTCACTCGAGATCTGCGGCTTTGGACAGTCCCTCTGTACTGTGAAGCCACAGATGTCGAGTCGACTAGCTTCCT
TTCTCCCTTCTCCC
+
DA;;;A==?CCCCBBAA@@BABBB<B@A@@BBCD>BB??@@@@>BB@>@B@CBCBAA@CCCCCBAA@CCFB??:=:=
=9?CCC>@<??AC@
@T6XXI:00073:00099
GGGGAATTCTAATACGACTCACTATAGGGAGAGGGAGAATAGCTTGTCTCCGTGTTGCGCCGATGTATTCTTCCTTT
CTCCCTT
+
CDCDD?AAADC@CBAABBBBBC>99111.7==@C>CCC@A:;<@>@A@ADCCB@@>8::B@AAA@@97469,,+,,+
,++,+/7
@T6XXI:00074:00079
GGGGAATTCTAATACGACTCACTATAGGGAGAGGGAGAATAGTCAACAAATCGTTTGGGGCGACTTCTCCTTCCTTT
CTCCCTTCTCCC
+
AAA6A@B@CCB@BB????<??@DBB66641??CC>CBB?@:@@BC>@@@<BB?BA9???5?@ABC@BBB=<:=<@A<
AAAA<A;==A@<
@T6XXI:00075:00087
AGTGACTCGAGATCTGCGGCTTTGGACAGTCCCTCTGTACTGTGAAGCCACAGATGTCGAGTCGACTAGCTTCCTTT
CTCCCTTCTCCC
+
CBBCCCCCBBBCCD?BCEB@@@?AACCCBCC?<>>=?@@?>=>@C@<<=B?@?EED?AA@@<<=???><???C?BB>
B???-6444494
@T6XXI:00076:00066
GGGAGAAGGGAGAAAGGAAGGAGAAGTCGCCCCAAACGATTTGTTGACTATTCTCCCTCTCCCTATAGTGAGTCGTA
TTAGAATTCCCC
+
CDBCCC?BB?BCCC?BABBB?BAA?BCCCCBB9CC?CCCCC<CC@C?8?CC>>?<<;?AA==<?<@?@@AAAA?@==
=>A@>9;/444-
@T6XXI:00076:00070
CTAGTCACTCGAGATCTGCGGCTTTGGACAGTCCCTCTGTACTGTGAAGCCACAGATGCT
+
449446/11;:;;;;CCBBB@BBB=B=@@AABBB=AB@=?=7::1634042994366.,,
@T6XXI:00076:00074
GGGGAATTCTAATACGACTCACTATAGGGAGAGGGAGAATAGTCATGAAATTTTCTCCCCGCGGCTGGTCTTCCTTT
CTCCCTTCTCCC
+
AEDBE@B@<<;69@BCCBCCCD@@?777,16666661123696<9==@B6<;;1<;;>>4<<<<@@>>;;;3837C=
@::9540.--33
@T6XXI:00076:00083
GGGAGAAGGGAGAAAGGAAGCTAGTCACTCGACATCTGTGGCTTCACAGTACAGAGGGACTGTCCAAAGCCGCAGAT
CTCGAGTCGACTAG
+
DD?ABB?BA:A@::5<:<,10>>@B::777>7:9B@>9@A6;;9;DCFCB@BB=<<<:<==AAA<===;?=?A@@@@
@@A@A@@A===?A@
@T6XXI:00076:00095
GGGGAATTCTAATACGACTCACTATAGGGAGAGGGAGAATAGTCAACAAATCGTTTGGGGCGACTTCTCCTTCCTTT
CTCCCTTCTCCC
+
:;9/9:BADCC@CCCCABAAA>B?;69959B@BC>CCC?@=AAAA?AA>;><<<=<BBC9C@@@C?BBB=<8;5<<7
=AAC<A;=?AA<
@T6XXI:00077:00055
GGGAGAAGGGAGAAAGGAAGTGCATGCTAGGAAATGAGAATAGGGGACTATTCTCCCTCTCCCTATAGTGAGTCGTA
TTAGAACTTCCCC
```

```
+
@@699>59919>?A>B>B?BBCCHHB@A>@59919>>>>;=9999,399@ACBBBB>A:::C<<<=AA@@@??>>78
3/-33-./75444
@T6XXI:00078:00089
GGGAGAAGGGAGAAAGGAAGGGGAGAAGGGAGAAAGGAAGGGGAGAAGGGAGAAAGGAAGCTAGTCGACTCGACATC
TGTGGCTTCACAGTACAGAGGACTGTCCAAAGCCGCAGA
+
:;7;B@@CD@BBBC>B@A?AAA9@@?@BD>B==<8?=?:???5?=B:?<;=@@=;@>@<??@AABCBBA?A??:87<
<?=:<?@>@AA@<=7777/.-/;//3A=@<;<C@B@@@A
@T6XXI:00078:00095
ACTCGAGATCTGCGGCTTTGGACAGTCCCTCTGTACTGTGAAGCCACAGATGTCGAGTCGACTACTCCTTTCCT
+
AEDCCCCE@@;<;A9;;B=CAB@9999919CC99999999954989;A@=@@;@A;;;@?>;;--,*),,*,*,
@T6XXI:00078:00098
GGGGAATTCTAATACGACTCACTATAGGGAGAGGGAGAATAGGCACCGTGTAGTTCATTGGTCGCCTCTCCTTCCTT
TCTCCCTTCTCCC
+
CCC8AAC@CBB@@>>BBBBAAA?;;16659??CD>CBB>A;;:;;=<??>>>;:<?AB>A<AC=9;=AA:56/0244
-4;=@:@;????;
@T6XXI:00079:00062
GGGAGAAGGGAGAAAGGAAGCTAGTCACTCGACATCTGTGGCTTCACAGTACAGAGGGACTGTCCAAAGCCGCAGAT
CTCGAGTCGACTAG
+
AA<AAC==>:>988095969@;8:B@???A@77:::9@@@;@@=AAA???BAB;;;;6;;=;;;244-
448444;@@?A?9:9A@@@:88;
@T6XXI:00079:00072
GGGGGGGAAATTTCTAAATACGACTCACTATAGGGAGAGGGGAGAATAGCCACCAAACTATTGTTCGTCGCCCGTTC
TCTTCCTTTCTCCCTTCTCCC
+
//////&//+//+4;;;?BB:::?<BA???:77=6777777-
76630;00/00/04444./,//,.788/44+0320304565:@<@@C?:99+.,6+
@T6XXI:00079:00074
GGGGAATTCTAATACGACTCACTATAGGGAGAGGGAGAATAGCCTGACAATGACAGCTCTCCGAGTATTCTTCCTTT
CTCCCTTCTCCCATCAC
+
DED:C?C>CADADICCCCC>><>??666.777?B>ABA@==<@>@@?CD@ECDBBAABABBE<<>DBA:<<=A<??:
?999195870>8>>>-*
@T6XXI:00079:00085
GGGAGAAGGGAGAAAGGAAGGAGAAGCGGCCACATGTCCAAATTGCGCTATTCTCCCTCTCCCTATAGTGAGTCGTA
TTAGAATTCCCCC
+
CD@CCC@BC>BBBB>C@C@B@BDD<>?B@B@ABBBB??>@@>CAAGAACICACDFC>99944-
3;;;?B===A===@@=98884/0000+
@T6XXI:00079:00089
GGGGAATTCTAATACGACTCACTATAGGGAGAGGGAGAATAGTCAACAAATCGTTTGGGGCGACTTCTCCTTCCTTT
CTCCCTTCTCCC
+
EEC:C99;;BB@BDCBA@@@BA?99111.7>>@C>CBB>B;;::::::=;??@@C>B@@9@CEAC?BBB??=?=?@;
??AA=B:<=??:
@T6XXI:00080:00055
GGGGAATTCTAATACGACTCACTATAGGGAGAGGAGAATATACGACCAAATCA
+
8::111<@@@A>AABCCBBACAA;30>;9000:-//7788500000/00+111
@T6XXI:00080:00069
GGGGAATTCTAATACGACTCACTATAGGGAGAGGGAGAATAGCCAACCTTACTCCCGCGAGCGACTGTTCTTCCTTT
CTCCCTTCT
+
```

```
999,4:B?CCC@CBBBBBBBD@?97166.7?777.7>>;=<<=<@<?;?<??:==4;6;<373568>><<=::5662
0.-133-.+
@T6XXI:00080:00084
GGGAGAAGGGAGAAAGGAAGCTAGTCGACTCGACATCTGTGGCTTCACAGTACAGAGGGACTGTCCAAAGCCGCAGA
TCTCGAGTCGACTAG
+
CC@CCC@DB=A>==:?>=<BCB;;;/00:::<0;9A<9AAA<?@?AACCBBCBA933317?A@@>>AA=@@?@@<::
<==<;:?===<<<=<
@T6XXI:00080:00092
GGGGAATTCTAATACGACTCACTATAGGGAGAGGGAGAATAGTCAACAAATCGTTTGGGGCGACTTC
+
944/9<?<AAC?BBABBBBB>=>@B@AA>===@@>BCC????AAA>AAA<A?===-777.7A??A=?
@T6XXI:00081:00051
GGGAGAAGGGAGAAAGGAAGTGCAGTTCGCGGTTAGTGAAGGGAAAACTATTCTCCCTCTCCCTATAGTGAGTCGTA
TTAGATTCCCC
+
DKBCB@;==:>9990;=>=@CBCBDHAB::<@C@BBBCC@BB9AAA6ABCC@C??:8?????<????AAAA@A888=
<9<B@@>@@@5
@T6XXI:00081:00052
GGGGAATTCTAATACGACTCACTATAGGGAGAGGGAGAATAGTTTTCCCTTCACTAACCGCGAACTGCACTTCCTTT
CTCCCTTCTCCC
+
BA?6@=A@ACC?BCCBBFEBCDC??677.777>>7>8:;9A@B;B-44-
429=B?;:;:0000,/69477136779177/777:///75
@T6XXI:00081:00061
GGGATTCTAATCACGACTCACTATAGGAGAGGAGATAGTAGACGCAATC
+
/6+//5///:944==@BCDBBB@@<8588//,........100001011
@T6XXI:00081:00081
GGGGAATTCTAATACGACTCACTATAGGGAGAGGGAGAATAGCTTGGCTATCTGACTAATGCGTATTCT
+
CCC9B>B@CBC@CCBBBBDBBBBBB66659??CC>BBB???@@E?C=??=:::0.,,,*/44.311016
@T6XXI:00081:00086
GGGGAATTCTAATACGACTCACTATAGGAGAGGGAGAATAGTCAACAAATCGTTTGGGGCGACTTCTCCTTCCTTTC
TCCCTTCTCCC
+
CCB9C9;<<CCDCCBBAA?AB7666..,0::BC@CBC@A?????=@CC:BBBB>;?@@6<<<?A@BBB@@>@>@A<A
@AA<@>=AAA;
@T6XXI:00081:00091
GGGAGAAGGGAGAAAGGAAGGGGAGAAGGGCTAGTCACTCGACATCTGTGGCTTCACAGTACAGAGGGACTGTCCAA
AGCCGCAGATCTCGAGTCGACTAGCTTCCTTTCTCCCTTCTCCC
+
@@?;<=AECABBBB?B=A>@DN:CBB?BB>CCBCCCA==?;4999;4@;;9;A?ABCBCBBBAAAAB=ABBBBB@CC
CBB?BBBBBC:;;BCBBDCCCAAABB@C@CD9<9CC?C@A???8
@T6XXI:00081:00096
GGGAGAAGGGAGAAAGGAAGCTAGTCGACTCGACATCTGTGGCTTCACAGTACAGAGGGACTGTCCAAAGCCGCAGA
TCTCGAGTCGACTAG
+
C@>CCB=?>:@>::5==?=@A>==>/0000/0:>6166??A@AC@ACCCCCBBA=73317?>>??>@@<@A=@???A
A???@@@@@@@@A?@
@T6XXI:00082:00056
GGGGAATTCTAATACGACTCACTATAGGGAGAGGGAGAATAGTCAACAAATCGTTTGGGGCGACTTCTCCTTCCTTT
CTCCCTTCTCCC
+
DIH9B@B@CCB@BABABBB>B@997116.777>>:A??@=AAA>>=@CC;BAABC>@AA7A:8;E@BBC<;78/44-
4;;?:@>@A@C?
@T6XXI:00082:00070
```

```
GGGGAATTCTAATACGACTCACTATAGGGAGAGGGAGAATAGTCCGCCAATTCTGCGCTTTAACATTCTCCTTCCTT
TCTCCCTTCTCCC
+
BBB7A@B?BBB@CCBAABCCA<@<?666.7>>>@>BCC@@=@==9<A<@>B?BBCBDCCC=@>???::88::9;248
14;;B:A>@@AB?
@T6XXI:00083:00050
GGGAGAAGGGAGAAAGGAAGAAGAATAGCCCTGCGTATGACAGGAAACTATTCTCCCTCTCCCTATAGTGAGTCGTA
TTAGAATTCCCC
+
ED?CBB@BA?>>AC>B@@?BC@@?=@>@??=ACCCCAABBCCCCBB>BBB@@BBBC>??@AC<????CCA@@@?==<
<:=>B9;/744-
@T6XXI:00083:00088
GGGGAATTCTAATACGACTCACTATAGGGAGAGGGAGAATAGTCAACAAATCGTTTG
+
@AA6@@C@CBB@BCB@BBBBA<;97166.777==9?AA?@@@BAA?AAA<CCCCC>C
@T6XXI:00083:00098
GGGGAATTCTAATACGACTCACTATAGGGAGAGGGAGAATAGCTAATTGTTCCATCCATCCGCCATGTTCTTCCTTT
CTCCCTTCTCCC
+
499/49@=ABB@ABB@@A???>BBB@@@====AC>CBC?????AC@C?BB?C??>@?A?=:7749@AA>BA>A;=99
9CCB==:;@><;
@T6XXI:00084:00065
GGGGAATTCTAATACGACTCACTATAGGGAGAGGGAGAATAGTTCAACGTTTATCCGCATGAACATCTCCTTCCTTT
CTCCCTTCTCCC
+
?DD8B;<8<AA>BCCCCBDAB??><777.777?A>?@@?@?>?=??=?@?D9=<<;656855555334=7823/30+
43333<<?;@D<
@T6XXI:00084:00096
GGGAGAAGGGAGAAAGGAAGCTAGTCGACTCGACATCTGTGGCTTCACAGTACAGAGGACTGTCCAAAGCCGCAGAT
C
+
ED?AAC@CCBBBBB=B@B6<<CBBBCCCAA@@99::::@BC@BB?AC@@@?A774.-
,/8;;;A>AA;@@>???AAA<
@T6XXI:00084:00097
GGGAGAAGGGAGAAAGGAAGCTAGTCACTCGACATCTGTGGCTTCACAGTACAGAGGGACTGTCCAAAGCCGCAGAT
CTCGAGTCGACTAG
+
DD@B@@>@A@BBBB>B?B?BCB>??7::;@@:;:;;:BCE:;<@CCDCC?@@>9000,388<AA?AA=@@>@=9=<<
<<<<AA@@@@@?8<
@T6XXI:00085:00050
GGGAATTCTAATACGACTCACTATAGGGAGAGGGACAATAGCGTCTCGATTCCCAAGCCGACCGTTTGTTCTTCCTT
TCTCCCTTCTCCCATCAC
+
94-424894824849:97::?@><<??;888A@;@>>::5555568==BB@B?.0,-
1,//.69:::BB@?>?ACJL>AA@?6:241040/----
@T6XXI:00085:00058
GGGGAATTCTAATACGACTCACTATAGGAGAGGGAGAATAGCTACACGCTATTTG
+
@AA4@>BACCB>?=BBBB>BB7664./,/9;?C<BAC@A;=717711100000+0
@T6XXI:00085:00060
GGGGAATTCTAATACGACTCACTATAGGAGAGGGAGAATAGCCACCCAGTTGTGACTCGCGCTATTGTCCTTCCTTT
CTCCCTTCTCCC
+
DDD9ACAAC@A?AA@CCBEBB=;::00,000==9?BB@CA<;7444-
44;<?@AA@=====@ABC@D00,/,/,..+.1110343000+
@T6XXI:00085:00064
```

```
GGGGAATTCTAATACGACTCACTATAGGGAGAGGGAGAATAGTTCTGGACTGTACTCTGGGGCTGTCTCCTTCCTTT
CTCCCTTCTCCC
+
9?>/96<@BBB@CC@ABAAAA?>?;697.7>777.77734400230027===;001<9==?5?@@AB<<;:@?4::6
:?=?>C=;::B<
@T6XXI:00085:00068
GGGAGAAGGGAGAAAGGAAGCAGTCCATCTGTGGCTTCACAGTACAGAGGGACTGTCCAAAGCCGCAGATCTCGAGT
CGACTAG
+
@C@AA=;=>:@AAB=D8;89>>>7846:::CCK@BB>BB=:==?CCC@@?;===??A?A=:@@>@???AA===???A
CCCBBB?
@T6XXI:00085:00072
GGGGAATTCTAATACGACTCACTATAGGGAGAGGGAGAATAGTCAACAAATCGTTTGGGGCGACTTCTCCTTCCTTT
CTCCCTTCTCCC
+
AAD7@<>=@A@=BBBBBBBBBBBB<79757BBCC>CBB>A;;=AA>???;?>=9>8:??5?:::C?BBB??<?>@A;
@@@C>A=?@BC;
@T6XXI:00085:00076
GGGGAATTCTAATACGACTCACTATAGGAGAGGGAGAATAGCCCATAACTCCATTGTCCGAACGTACTCTTCCTTTC
TCCCTTCTCCC
+
@AA6@=@ABBEECCBAABDBB:999//,0>:==9?@><>?<==9=AC>@>A=@C@@<::?<:A>>>?888<@;=?;?
@AC?B9<=ADA
@T6XXI:00086:00052
GGGAGAAGGGAGAAAGGAAGGAGAAGTCGCCCCAAACGATTTGTTGACTATTCTCCCTCTCCCTATAGTGAGTCGTA
TTAGAATTCCCC
+
CEADCC?BB>BBB=:@7:6;>A??<>>@8888.880896A<;AC?CBBBCA>???A=AA@<<;=;??@;???=====
=>A?=44/444-
@T6XXI:00086:00082
GGGGAATTCTAATACGACTCACTATAGGGAGAGGGAGAATAGCACCTAG
+
@@@,;7;>ABC@CCBCCCAA@BB@@???9?@@AB>ABA>76.---,---
@T6XXI:00087:00068
GGGGAATTCTAATACGACTCACTATAGGGAGAGGAGAA
+
//-.-8A>BCB@BABCCBDBB@@97166.7=7747/0,
@T6XXI:00087:00090
GGGGAATTCTAATACGACTCACTATA
+
499/42924:?99;9@>?;8-,-,,,
@T6XXI:00088:00056
GGGGAATTCTAATACGACTCACTATAGGGAGAGGGAGAATAGTCAACAAATCGTTTGGGGCGACTTCTCCTTCCTTT
CTCCCTTCTCCC
+
DEE:C>@C@BB?BCBBBA???CB99111.6==@BACCC?@?@@AA?===9?A?AC<AAA7ACCCC@CCC>?<?>AA<
@AAA<A899AD?
@T6XXI:00088:00059
CTATCACTCGAGATCTGCGGCTTTGGACAGTCCCTCTGTACTGTGAAGCCACAGATGTCGAGTCGACTAGCTTCCTT
TCTCCCTTCTCCC
+
DCCA@::9BABBBBBBBA>>BBB>B>BBB?:::5:CCCCBCC<?<A>AC?BBCCCCCBCCCBABB;:;?>=88?:?B
>AAAC>@8<<??:
@T6XXI:00088:00062
GGAATTCTAATACGACTCACTATAGGGAGAGGGAGAATAGTCAACAAATCGTTTGGGGCGACTTCTCCTTCCTTTCT
CCCTTCTCCC
+
```

```
C>A=ACCBB@CBBAAAAAB?<?7177.7=77;8:CC@CAAA==9=?;:@===C>CBC9CBBBC@CCD=<9<<??;?A
AA<A<?:;;9
@T6XXI:00088:00064
GGGAGAAGGGAGAAAGGAAGCTAGTCACTCGACATCTGTGGCTTCACAGTACAGAGGGACTGTCCAAAGCCGCAGAT
CTCGAGTCGACTAG
+
EC>AAA?CA>A@>>9>6969>>>>>=6111661116l679279<;BBBAAA?>4444-
44430323505556<@AAA?@@@A99:=<A?<<
@T6XXI:00088:00068
GGGAGAAGGGAGAAAGGAAGGAGACAGTGAGCGCCGCACAACACGTCACTATTCTCCCTCTCCCTATAGTGAGTCGT
ATTAGAATTCCCC
+
94-
49924404>>><A=>=@>AAACBCCCCC@@>>ABBAA@BBCBA@AADBA<A888/8????<@CCBDECCC@?>>:;4
:<?=@5999/
@T6XXI:00088:00083
GGGAGAAGGGAGAAAGGAAGCTAGTCGACTCGAGATCTGCGGCTTTGGACAGTCCCTCTGTACTGTGAAGCCACAGA
TGTCGAGTCGACTAGCTTCCTTTCTCCCTTCTCCC
+
DB=B@@=>B19;AA>A>B?BCB>@>/00;;==>>CCC?BBC@CCC>C@BBCCC@@>===???AAAAA@>?::?@<<<
@@?>????????A@@@@=?/3714;;;4924444,
@T6XXI:00088:00086
GGGAGAAAGGGAGAAAGGAAGCTAGTCACTCGACATCTGTGGCTTCACAGTACAGAGGGACTGTCCAAAGCCGCAGA
TCTCGAGTCGACTAG
+
CD?;44419>:>9999;>A>BC@@@AB?>00000////=>B@BB@BB@????@A=<<::8<858;333,7=9=AA==
=AAC?899999CC@A
@T6XXI:00088:00088
GGGAGAAGGGAGAAAGGAAGGAGAAGTCGCCCCAAACGATTTGTTGACTATTCTCCCTCTCCCTATAGTGAGTCGTA
TTAGAATTCCCCC
+
=B:ABC@DD?BDDC>@=@49688>9<7:8ACC9CFBBAAAC?AA@CB@BCA=@@AD?889==9://0:::=?<<=AA
B>A95-.-,555.
@T6XXI:00088:00090
GGGAGAAGGGAGAAAGGAAGGAGAAGTCGCCCCAAACGATTTGTTGACTATTCTCCCTCTCCCTATAGTGAGTCGTA
TTAGAATTCCCC
+
DB?BBA?BB?BCCB>B@BAB?@@@@B@BBBBB7?><AAAAB>BA@BBBBA??A===;88=AA<A@@@@@C=<==A9=
<=@<;883444-
@T6XXI:00089:00054
GGGAGAAGGGAGAAAGGAAGGGGAGAAGGGAGAAAGGAAGCTAGTCACTCGACAGTCTGATGG
+
CEABAA>=99>=@@9>=@5999/8??<?>:=777-73747@??:BA?>00,,+****.*++20
@T6XXI:00089:00058
GGGGAATTCTAATACGACTCACTATAGGGAGAGGGAGAATAGTCAACAAATCGTTTGGGGCGACTTCTCCTTCCTTT
CTCCCTTCTCCC
+
CCC9C>@>@CGBCB?=BBBB>@??;661-
6;66:5:BB@AAAA@@>@AA<CBCCC>CCC9BBC@C?<AA?@><;AA<AAAA<A;>>@CA
@T6XXI:00089:00059
GGGGAATTCTAATACGACTCACTATAGGAGAGGGAGAATAGCTCCCGTAGAA
+
@??4>696<BBAFCBBBBBBB:664..,0;;BB@BAA??9//0:<,0//..5
@T6XXI:00089:00079
GGGAGAAGGGAGAAAGGAAGCTAGTCACTCGACATCTGTGGCTTCACAGTACAGAGGGCTGTGAAAGCCGCAGATCT
CGAGTCGACTAGATCCAGCA
+
```

```
CC@CHC@B=69884-9;>;>?B@???>:..///.3334.107848ABBCBCB>8466,//.-
2779AA?<=>>988??<<==@>;4////+0*++0+
@T6XXI:00089:00090
GGGAGAAGGGAGAAAGGAAGGAGAAGTCGCCCCAAACGATTTGTTGACTATTCTCCCTCTCCCTATAGTGAGTCGTA
TTAGAATTCCCCC
+
CC>BBC@CCBCBBD=B=@=>=@BC@@@ACCCC9CE>BBCCC=C??C?=@B?>A@AC>AA=867;==??@@AAAAA@@
?=<4401,,,,,)
@T6XXI:00089:00091
GGGGAATTCTAATACGACTCACTATAGGGAGAGGGAGAATAGTCACTTCCTATCTGGTCGCGATATTCTCTTCCTTT
CTCCCTTCTCCC
+
ECE:C@A@@CEBCBAABAAAAAABB:<<;::8?A>BAA?@7:///::BB;;;@@@?<??AAA>A??=?AAA@C<??;
??>>:>:<;;;5
@T6XXI:00089:00097
CTAGTCACTCGAGATCTGCGGCTTTGGACAGTCCCTCTGTACTGTGAAGCC
+
B11/188=ACBBCCCBBBBC?BBCAC=A@AB:::7:BBCCBCC<AAA>?B?
@T6XXI:00090:00076
GGGGAATTCTAATACGACTCACTATAGGGAGAGGGAGAATAGTCAACAAATCGTTTGGGGCGACTTCTCCTTCCTTT
CTCCCTTCTCCC
+
99<4<@B@CBB>BBBBBBBBB@>93167.7=?CD>BBB>@99<==<986386666-
677.:::;L?DBC@A?A?@A<@<<<7<;=>??;
@T6XXI:00090:00099
GGGAATTCTAATACGACTCACTATAGGGAGAGGGAGAATAGACT
+
94-42/,/11,///44=?88:?::6AA666677661100-2---
@T6XXI:00091:00051
GGGAGAAGGGAGAAAGGAAGCTAGTCACTCGACCATCTGTGGCTTCACAGTACAGAGGGACTGTCCAAAGCCGCAGA
TCTCGAGTCGACTAG
+
DE@C?=68809999094954<>>7AA>::;;00,///.7>B=BC@BB@???@@@<443136489=33714@=@????
???@@??????@@=<
@T6XXI:00091:00064
GGGAGAAGGGAGAAAGGAAGATCAGGGAGCAGCTGGTGAAACAATAGCTATTCTCCCTCTCCCTATAGTGAGTCGTA
TTAGAATTCCCC
+
HI>AAA8991999909;>;>?>?888,6179?CCA:>777.7=:<C;9;BB@B???<?<<<C@A?>?CCAA=<;::4
4/4;;20/038.
@T6XXI:00091:00067
GGGAGAAGGGAGAAAGGAAGCTAGTCACTCGACATCTGTGGCCTCACAGTACAGAGGGACTGTCCAAAGCCGCAGTA
ACAT
+
DEBBA?584-999919495;C@?@@B???AAA???BBBAA?A@CCCB;?BCB74003-
49999979:4::7611.+*)***
@T6XXI:00091:00089
GGGGAATTCTAATACGACTCACTATAG
+
8@@0:,9244424888443331377766
@T6XXI:00092:00050
GGGAGAAGGGAGAAAGGAAGGAGAAGTCGCCCCAAACGATTTGTTGACTATTCTCCCTCCTCCCTATAGTGAGTCGT
ATTCAGAATTCCCC
+
DD?<<<=AC@DCCLBD=<;<?B?@=@BC@@??.8808?@<>>?@@AA<===<:66=444/4880;<;==>=<;<<;8
8:.//;;;;;@CH5
@T6XXI:00092:00073
```

```
GGGGAATTCTAATACGACTCACTATAGGGAGAGGGAGAAAGGAAGCTAGTCACTCGAGATCTGCGGCTTTGGACAGT
CCCTCTGTACTGTGAAGCCACAGATCTCGAGTCGACTAGCTTCCTTCTCCCTCTCC
+
BBB9E@DACCC@CF@?BABBD@B97166.7>===6767=7<5;9<?C?@A<,,,,,,++0155==;=??7>488@<@
@A<=?=?@@ADB@@@>@A>AAA@@?788=4488877>A633247646///,/.-/,
@T6XXI:00092:00079
AGTCACTCGAGATCTGCGCTTTGGACAGTCCCTCTGTACTGTGAAGCCACAGATGTCGAGTCGACTAGCTTCCTTTC
TCCCTTCTCCC
+
BBBCBBCBCCBBBCBB9:<CCDCCCCCCCBB>BB>>>>@@@?;B@??>?@?@AA@<===@B=?>>C=<<===>>E>B
BCC>A>=;;;6
@T6XXI:00092:00084
GGGAGAAGGGAGAAAGGAAGGAGAACAACACGATCGGTTAAACGTAGCTATTCTCCCTCTCCCTATAGTGAGTCGTA
TTAGAATTCCCC
+
DBBBBB@CD>B?>><@?A?B@BCB@BB?BBBDCEAB=@=??<@AAACCA@@@CCCC>BCBCC>CAA??>AAAA====
=:<AA?@=@AA6
@T6XXI:00092:00099
GCTAGTCGACTCGAGATCTGCGGCTTGGACAGTCCCTCTGTACTGTGAAGCCACAGATGTCGAGTCGACTAGCTTCC
TTTCCTCCCT
+
==6/////////4449;@=;11-
00,.,...648<;=ABCCCB@@=?>>=C:::CCDCBBBBCBBBB;88>7..,/9::,0,,,7+,
@T6XXI:00093:00051
GGGAGAAGGGAGAAAGGAAGGAGAACAACACGATCGGTTGAACGTAGCTATTCTCCCTCTCCCTATAGTGAGTCGTA
TTAGAATTCCCC
+
=B?AAA>ADA<<=C?B?B@C@;<<BCC?CBCDCBBB?B?BA>@@???@A@?;?87:6:@@CD>BA@???AAAA??>8
83488432333.
@T6XXI:00093:00075
GGGGAATTCTAATACGACTCACTATAGGAGAGGGAGAATAGTCAACAAATCGTTTGGGGCGACTTCTCCTTCCTTTC
TCCCTTCTCCC
+
CCD9CBEABCC@CBAABBB?B:999//,0;:=?=?GG@AAAA==;=AA:????;6?<?5?BBBC@CCC>=:<>@@<?
@AC<A;==??:
@T6XXI:00093:00088
GGGAGAAGGGAGAAAGGAAGTGCAGTTCGCGGTTAGTGAAGGGAAAAACTATTCTCCCTCTCCCTATAGTGAGTCGT
ATTAGA
+
DC@CBB@CB@>??B>B@B@BBB@BAB@>>>BAB@;;;?B@@@;???B2BCBB>?;??;@A@A@;@<<;@<=A>@AC@
==;=@@
@T6XXI:00093:00095
GGGGTCTAATACGACTCACTATAGGAGAGGGAGAATAGCAAACGTTTCCCCGACGTCGCTTTCTCCTTCCTTTCTTC
CTTTCTCCCTTCTC
+
999,4;@B=ACCCCBCCCCB9=0.,0<00/+677=@::::C=?CAD@CDC:BEDDAACBBBAC<<;<BCADD>AC@C
ACC?ECAB>AACBA
@T6XXI:00094:00065
GGGGAATTCTAATACGACTCACTATAGGAGAGGGAGAAT
+
BDD:C?AAABBDCCCCDDBC<01@>00,.47::7://,-
@T6XXI:00094:00089
GGGAGAAGGGAGAAAGGAAGCTAGTCGACTCGAGATCTGCGGCTTTGGACAGTCCCTCTGTACTGTGAAGCCACAGA
TGTCCGAGT
+
DB=B;;79A:A999142424?337B:::BCB:<;FDC?@@B@BBE=A>@@AAABC>CC888AAAA@@B@??88==BB
A<??<??@A
```

```
@T6XXI:00094:00090
GGGAGAAGGGAGAAAGGAAGGAGAACGCGCACCCGAACCAACGGTGATCTATTCTCCCTCTCCCTATAGTGAGTCGT
ATTAGAATTCCCC
+
CCBCCB?BB=?>=><@@C@C?B??@AAAAAACC@?A>A@C@CCBCECBAAAA@CCCCACBBAC=ACABBBCCBBAC@
@@@CBB>BABCC7
@T6XXI:00095:00051
GGGGAATTCTAATACGACTCACTATAGGGAGAGGGAGAATAGCAAACGTTTCCCCGACGTCGCTTTCTCCTTCCTTT
CTCCCTTCTCCC
+
999/9;@?CCB@A@??BBBB??>><111.7==@B>BBB@@5;77714>??9???7?CCDBBB@>725..,09;:8:6
::==7>9<<@C?
@T6XXI:00095:00059
GGGAGAAGGGAGAAAGGAAAGCTAGTCACTCGAGATCTGCGGCTTTGGACAGTCCCTCTGTACTGTGAAGCCACAGA
TGTCGAGTCGACTAGCTTCCTTTCTCCCTTCTCCC
+
EB?B<;79@:?99914244-
49337ABBBBC?@<;<BBCCC?BBB<@<<A?B?BC>@@<8?:A==@?@@==<:;:<6<<?===AAA@@@AC===>@>
@A689@@;@688@B:
@T6XXI:00095:00070
GGGGAATTCTAATACGACTCACTATAGGGAGAGGGAGAATAGTACACGTA
+
=@B6A=@@CBC?BCCCCCCCB@A?<677.7?==?<?B@=;B4430--+++
@T6XXI:00095:00088
GGGGAATTCTA
+
8886D;;;==8
@T6XXI:00096:00080
GGGAGAAGGGAGAAAGGAAGCTAGTCACTCGAGATCTGCGGCTTTGGACAGTCCCTCTGTACTGTGAAGCCACAGAT
GTCGAGTCGTCTAA
+
88561114;:C111,/,/,/66333=7111;=?9779@BA==??8=477:9:CC<>=6////.113=86656887=8
738446//---220
@T6XXI:00096:00081
GGGGAATTCTAATACGACTCACTATAGGAGAGGGAGAATAGACAGGCGCTATTCTTCCCAGGAGCTTATCTTCCTTT
CTCCCTTCTCCC
+
DCD:B?A@BCC@BBBBBAAAB:999//-
0>>BD>CKJE@<:;<==?=AA<<?85585<7=A>AA@A<==AB?C;=<6;<::4:348;<7
@T6XXI:00097:00056
GGGGAATTCTAATACGACTCACTATAGGAGAGGGAGAATAGCTCCGTACTCCGTGGACTGCACAATCCCTTCCTTTC
TCCCTTCTCCC
+
CDD:CACDCCBABBBCCCBBC:999//,0;;?@=AAA>@@88:A@B?A@DC?CAA>???@@@??<<<<7<=?<??7<
;==7=<=::;6
@T6XXI:00097:00065
GGGGAATTCTAATACGACTCACTATAGGGAGAGGGAGAATAGTAGCCGTTCC
+
CBE7B8;;<CCACAAAABBB>?;99111.7??BC=AAB@@326.--,--,-1
@T6XXI:00097:00066
GGGGAATTCTAATACGACTCACTATAGGAGAGGGAGAATAGTCAACAAATCGTTTGGGGCGACTTCTCCTTCCTTTC
TCCCTTCTCCC
+
CGC:C9;;<9;?BBBBBBBAC=;;?00,0;07767>>;=<<<??=@@@:@:;:A<@BC7CBBBC?ACE;989>AA<>
@@C<A899@@?
@T6XXI:00097:00087
```

```
GGGAGAAGGGAGAAAGGAAGCTAGTCACTCGACATCTGTGGCTTCACAGTACAGAGGGACTGTCCAAAGCCGCAGAT
CTCGAGTCGACTAG
+
DD>BCBA=99>988-9597<CCCCD?AA???::;???@?B;?@=BBBBCAAA@=733-
488;<A>AA<??<?A@@?<;<<<===AA==A=;
@T6XXI:00098:00063
GGGGAATTCTAATACGACTCACTATAGGGAGAGGGAGAATAGTTACTAACTACTAGGCGCAACTGTTCTCTTCCTTT
CTCCCTTCTCCCATCACCG
+
CGB9E@ACACGBCCCCBBBCCA>9>616.7>>>><???==@CC>@@==:<A@@AA@877//./:;;;;=?><?244-
4::94984444-49;4430
@T6XXI:00098:00068
CTATCACTCGAGATCTGCGGCTTTGGACAGTCCCTCTGTACTGTGAAGCCACAGATGTCGAGTCGACTAGCTTCCTT
TCTCCCTTCTCCC
+
DCCCAAA@AABDBBCBBBB@AAA>A@AA>A???8?AAAABBBAB?A<?BBCCBA>@@@@BCDCBBBBBB@?:;?=?C
@???C<=;==@@;
@T6XXI:00099:00065
CTAGTCACTCGAGATCTGCGGCTTGGACAGTCCCTCTGTACTGTGAAGCCACAGATGTCGAGTCGACTAGCTTCCTT
TCTCCCTTCTCCC
+
>8/////4:CBBDBCBB;886..,09;;;=0::5:BBCCBBBABB?>@C@CCICBA>@??AAA@@@@@@??85759C
<?=CC>A>@@@A<
@T6XXI:00099:00070
GGGGAATTCTAATACGACTCACTATAGGAGAGGGAGAATAGTCAACAAATCGTTTGGGGCGACTTCTCCTTCCTTTC
TCCCTTCTCCC
+
@?>4@?B@CDB@BBBBB???D:969/.,0>;>A?ADD@A88<A=;8864????B9???6?AAAC?BBB?@>@?AD=?
@@A=A9<<AC<
@T6XXI:00099:00096
GGGGAATTCTAATACGACTCACTATAGGGAGAGGGAGAATAGTTTTCCCTTCACTAACCGCGAACTGCACTTCCTTT
CTCCCTTCTCCC
+
;99/9<@@BCB@CBBBB@@ABAB<;696-
6=@BB>BCC@B94333+4:498=@CCD@B????C?<@AABBB>A;??<ABBB>B=A<<;6
@T6XXI:00050:00146
GGGGAATTCTAATACGACTCACTATAGGGAGAGGGAGAATAGACGTGGAACTGTTGCCAGTGTATTCTCCTTCCTTT
CTCCCTTCTCCC
+
;<9/9;@=ACF@CCCA@AAAAA>>?777-
7<<>B>B?B?<<<<<<<C=B?BA@@:??:?====??=?96995555=7:6=@4:740341
@T6XXI:00051:00127
GGGAGAAGGGAGAAAGGAAGCTAGTCACTCGACATCTGTGGCTTCACAGTACAGAGGGACTGTCCAAAGCCGCAGAT
CTCGAGTCGACTAG
+
DECDDB@@;59998096959A>>>@??77:>7:::::CBB?BB?BCC>AACCB=77714:;;?@:==7=A>@@@===
@@A===A@@@AA?@
@T6XXI:00051:00132
GGGAGAAGGGAGAAAGGAAGCTAGTCGACTCGACATCTGTGGCTTCCGTACAGAGGGACTGTCCAAAGCCGCAGATC
TCGAGTCGACTAG
+
DC>AA=;==9@@==9>594;A>::=/0///..071161;?@;?@@CAEBCCB?76656;88;;;==:@A>AA<<<<<
??A@@????@A@@
@T6XXI:00051:00134
GGGAGAAGGGAGAAAGGAAGCTAGTCGACTCGACATCTGTGGCTTCACAGTACAGAGGGACTGTCCAAAGCCGCAGA
TCTCGAGTC
+
```

```
DDCAA@@DGA@@@C@C@B@BBB0031/0=>>=>@?:::A@@6:;=?BC?@?BCCB@@@<?????@9;<7<>=@AAA@
@<<<?<<<@
@T6XXI:00052:00100
GGGGAATTCTAATACGACTCACTATAGGAGAGGGAGAATAGATCACCGTTGGTTCGGGTGCGCGTTCTCCTTCCTTT
CTCCCTTCTCCC
+
BAA7A@A?CBC@CBBABAAAB6446./,0;:==<?CC@AA888A@?:?B:?:?=BBC>C<;;CBA?ADCAC>A>CE>
CBBB>@>AA@@<
@T6XXI:00052:00114
GGGAGAAGGGAGAAAGGAAGCTAGTCGACTCGAGATCTGCGGCTTTGGACAGTCCCTCTGTACTGTGAAGCCACAGA
TGTCGAGTCGACTAGCTTCCTTTCTCCC
+
DB:E9968?0949904284473779711111l;7?;?>AAA?AAA=A@E<;:CCC>CAA999AA<6<>:785/..88
8//5===A=<<==6//-,-,-4434443
@T6XXI:00052:00124
GGGGAATTCTAATACGACTCACTATAGGGAGAGGGAGAATAGTCAACAAATCGTTTGGGGCGACTTCTCCTTCCTTT
CTCCCTTCTCCC
+
444,9<@@CBA?BBBCBABBB>;99111.7=<>A?AAABAAAAAA>???4?A6;;6;<<2<>@@?<<=<3838=@A?
B?@@7>;=>@@A
@T6XXI:00052:00145
GGGGAATTCTAATACGACTCACTATAGGAGAGGGAGAATAGCTGTACTTTCGGCCGACCGTTTCACCCCTTCCTTTC
TCCCTTCTCCC
+
CBC:C?@>?DC@CBA@BBAAA:966/.,0<<AD>DCCB@?:=@@BBBCCBBB?B@AAA<??A?@@@AA6@9<9<=49
:::,4/4000+
@T6XXI:00053:00111
GGGAGAAGGGAGAAAGGAAGCTAGTCGACTCGACATCTGTGGCTTCACAGTACAGAGGGACTGTCCAAAGCCGCAGA
TCTCGAGTCGACTAG
+
DDBCDD?A@:@@996>482;A>::</000/99081111>@@;AB?BCCBB?B?>;33317===AA>@?<@A>AA>99
=?@@AAA===@AA??
@T6XXI:00053:00121
GGGAGAAGGGAGAAAGGAAGGGACAGTCCATCTGTGGCTTCACAGTACAGAGGGACTGTCCAAAGCCGCAGATCTCG
AGTCGACTAG
+
DDACCB@BC?@@@B7:7:<@@<;88?787;@@@AAB?CB@BCC??=ACAAAAC>AB>>>A>AA;=<8<??@AA===@
??@BBBBCCB
@T6XXI:00054:00100
GGGGAATTCTAATACGACTCACTATAGGGAGAGGGAGAATAGTCAACAAATCGTTTGGGGCGACTTCTCCTTCCTTT
CTCCCTTCTCCC
+
CBA4B8;8<GCBCB@BBBBB@<;9>166.7==>BADDD@B=??@=;<==;>:889899A6ACCCC@AAA=?<??CC>
C===9==@AAA<
@T6XXI:00055:00115
GGGAGAAGGGAGAAAGGAAGTCACCGACATCTGTGGCTTCACAGTACAGAGGACTGTC
+
995999959@:@B>>8@:<-/.46633311....//7777?<><8304//..,----22
@T6XXI:00056:00104
GGGAGAAGGGAGAAAGGAAGCTAGTCACTCGACATCTGTGGCTTCACAGTACAGAGGGACTGTCCAAAGCCGCAGAT
CTCGAGTCGACTAGCTTCCTTTCTCCCTCTCCC
+
B@=@<<89B1999914<>=BCDBB?7771111116>A?9???@=@BBBCBC?<;774-
8@@@@@?@A<@@@????A>=?@887<?=785===A<577753666,/----+
@T6XXI:00056:00114
CTATCACTCGAGATCTGCGGCTTTGGACAGTCCCTCTGTACTGTGAAGCCACAGATGTCGAGTCGACTAGCTTCCTT
TCTCCCTTCTCCC
```

```
+
BCB=@::9=@BBCCCDBBC@CBB>B>>A@????;?CCCBAC@>B73249=>@@BBBBBBBBDCBBCCBDGCB>BACC
>BAAC>A>A@@@;
@T6XXI:00056:00123
GGGAGAAGGGAGAAAGGAAAGCTAGTCGACTCGACATCTGTGGCTTCACAGTACAGAGGACTGTCCAAAGCCGCAGA
TCTCGAGTCGACTAG
+
KH>@@@5990999914284-
49>><?6:7>===88166117@>@BB@?@AAAB@@>77349:::<;=?:???A??==<;<@;<;>=888==;
@T6XXI:00057:00101
GGGAGAAGGGAGAAAGGAAGTGCAGTTCGCGGTTAGTGAGGGGAAAAACTATTCTCCCTCTCCCTATAGTGAGTCGT
ATTAGAATTCCCC
+
CD@BBA@DB=C;8809695;BB@AAD@CBCB=@=;;;CCCJE7AAAA1@BBC@CCCE@B????9?;<=@ACAAAAA<
<<=?@C?C<???1
@T6XXI:00057:00138
GGGGAATTCTAATACGACTCACTATAGGAGAGGGAGAATAGTCTTGTATTTGTGCATCACTTTTCTCCTTC
+
BKKBC99:9449894=>:::C:88?7737@777,66;3898400/40300+04468=:::3370,++00++
@T6XXI:00058:00104
GGGGAATTCTAATACGACTCACTATAGGGAGAGGAGAATAGCTTAGTT
+
944,4:;:9<;24>====887;?777?>;///;,0.531130101111
@T6XXI:00058:00113
GGGAGAAGGGAGAAAGGAAGGAGAAGTCGCCCCAAACGATTTGTTGACTATTCTCCCTCTCCCTATAGTGAGTCGTA
TTAGAATTCCCC
+
DDADCB=?>:=B==:@?B=@=@AA>ABCBA@B7AA<BCCCC>C@?BB@BCA=@===9=@A@C=AAAAA=99AA???@
@>A>=;=>@@@5
@T6XXI:00058:00114
GGGAGAAGGGAGAAAGGAAGGAGAAGTCGCCCCAAACGATTTGTTGACTATTCTCCCTCTCCCTATAGTGAGTCGTA
TTAGAATTCCCC
+
DIBECC=@99===@<@>C@C@CCC@GA@;;772<>:@CCFD9??@CCCBB@?B@@<;A@AA@<@CCA@<==AA===@
A?A@877,///+
@T6XXI:00059:00132
GGGAGAAGGGAGAAAAGGAAGCTAGTCATCGACATCTGTGGCTTCACAGTACAGAGGGACTGTCCAAAGCCGCAGAT
CTCGAGTCGACTAGCTTCCTTTCTCCCTTCTCCC
+
DD?B<;79B:?1111+1,1,/..7740,,,,,,,22777?=>><@??AAA???@>==188?@?A<??<?A>AA@?=<
<<<888=@888<<<>><?<?>7??=>:@<>??A;
@T6XXI:00059:00140
GGGAGAAGGGAGAAAGGAAGCTAGTCACTCGACATCTGTGGCTTCACAGTACAGAGGGACTGTCCAAAGCCGCAGAT
CTCGAGTCGACTAG
+
DD@DCB>==6>844-
95949A???A?@A????AB?@???C>BE?BCCCABBA??33317>9>>@<??<?C>A@@@@?===A@@A@???A==
@T6XXI:00059:00141
GGGGAATTCTAATACGACTCACTATAGGGAGAGGGAGAATAGCGACTTACCTCCCTTACCGTTAGCTATCCTTCCTT
TCTCCCTTCTCCC
+
AAF6@<@@@@@DBBBCCBBBA?;97166.7=777.7>?;<=<;;;8824:3433+-0-.,-
222448==@@AB@>?C;@>=@98,/.886
@T6XXI:00060:00110
GGGAGAAGGGAGAAAGGAAGGGAATAGCAGCAGTCGGCAACGTGAAACTATTCTCCCTCTCCCTATAGTGAGTCGTA
TTAGAATTCCCC
+
```

```
BB?BCC@BB?BCCC>B?B@BC=A?ABABAAA@AA:;5:B??@@DDC@CCCB@BB>>;>ABCC>A===AAABA@>==8
837==:886:=1
@T6XXI:00060:00137
CTATCACTCGAGATCTGCGGCTTTGGACAGTCCCTCTGTACTGTGAAGCCACAGATGTCGAGTCGACTAGCTTCCTT
TCTCCCTTCTCCC
+
B;;9>::9AA@ABBDCBBB@CCG>C?CBBB???9?A???A9:::8@?AC@CCCBC;;;??@CCEA@BBB??8898;>
9;79?,/,/8686
@T6XXI:00060:00143
GGGGAATTCTAATACGACTCACTATAGGAGAGGGAGAATAGTCAACAAATCGTTTGGGGCGACTTCTCCTTCCTTTC
TCCCTTCTCCC
+
AA?6@5;:<BAABBBBCBDBC>==;/0,0;;@C:<:C?AAAA=;8444-
7A777.17728888A2:6?:65=>@@:@?>>7=347;;4
@T6XXI:00061:00102
CTAGTCGACTCGAGATCTGCGGCTTTGGACAGTCCCTCTGTACTGTGAAGCCACAGATGTCGGGTCGACTAGCTTCC
TTTCTCCCTTCTCCC
+
CA<;;A@CCCCCCCCCBBBBB?BBB<B>A@AA@??<?BCC?AA@@A@F@AC@B@@A?>?8833,1661616111772
16,033845485689
@T6XXI:00061:00113
GGGGAATTCTAATACGACTCACTATAGGGAGAGAGATATAAGGGTCTAC
+
---*,2B@BA@=@BBBBBCBBAACBB??;???>>?<<:>?;972//+((
@T6XXI:00061:00118
CTATCGACTCGAGATCTGCGGCTTTGGACAGTCCCTCTGTACTGTGAAGCCACAGATGTCGAGTCGACTAGCTTCCT
TTCTCCCTTCTCCC
+
C9994444:A@EB?BBBBBB@ABA=B@CC@@@CCCC;<<CCBBCBB@<?B@CCCBBBCCBBBCBBBBCCDBBB@B@C
C>CFDC=?<?AAC<
@T6XXI:00061:00123
AATCTAATACGACTCACTATAGGAGAGGGAGAATAGAGACGTATTATTCGTTCGCTCTGTTCACCTTCCTTTCTCCC
TTCTCCC
+
DABBBB@CBCBBBAA@:9990/,0><?B>BCC@C;4000+****)--,--
103568<<:><?8848?C=@@;?>>>:?><==C8
@T6XXI:00061:00135
GGGGAATTCTAATACGACTCACTATAGGGAGAGGGAGAATAGTGACGTGTTGTGCGGCGCTCACTGTCTCCTTCCTT
TCTCCCTTCTCCC
+
@B?4@=@ACDB?A@@@AA??BC<99166.777BC@BAB?@AAAAA@@@<<<><<;:8?BAA@??=<:><=8;<?788
43444-4:=??@;
@T6XXI:00061:00147
GGGAGAAGGGAGAAAGGAAGAGAACGGCGACGAACAATAGTTGGTGGCTATTCC
+
CB?BCA?AA:A;9909595<AC@@GC=@A@@CB?BB@BBB@<?<80,010.,,*
@T6XXI:00061:00148
GGGAGAAGGGAGAAAGGAAGTGCAGTTCGCGGTTAGTGAAGGGAAAAAACTATTCTCCCTCTCCCTATAGTGAGTCG
TATTAGAACTTCCCC
+
BDAAAA@CB<A@@@<@>@>BBCCCBB?BCCB@C@;;;DE@CE=BAAAB-
BBCEACBBB>BCCCC>C@@585ACAAA<8888;=?9==>AAA6
@T6XXI:00062:00147
GGGAGAAGGGAGAAAGGAAGGAGAAGTCGCACCAAACGATTTGTTGACTATTCTCCCTCTCCCTATAGTGAGTCGTT
ATAATGACCTT
+
```

```
DD?CBB@CB>?>==:@=@=@CE@@=@DCB>AA8::5:CCCC>B?>BBBBBCCCCCB=99;AB>BBBCCAABA@>710
...33.-.,,*
@T6XXI:00063:00117
GGGGAATTCTAATACGACTCACTATAGGGAGAGGGAGAATAGTTTTTCCCTTCACTAACCGCGAACTGCACTTCCTT
TCTCCCTTCTCCC
+
DCD:B?B>ABBAB@??AABBBCB>7111.6><<?<?CBB@?<;999.:=:?>@BACD@C@B>??=<?@ABBB?C@CC
>BBBB>B?CAAA<
@T6XXI:00063:00146
GGGGAATTCTAATACGACTCACTATAGGAGAGGAGAATAGCCACAATCGTGTTTGCGTTGGCATCTCCTTCCTTTCT
CCCCTTCTCC
+
HDH:K@@=;9;;=@ABB@@??:;<::>;::00,...,-
52334....66;48818F?A=?<?>>>>>=?<@>99271111,1311.4
@T6XXI:00064:00103
GGGGAATTCTAATACGACTCACTATAGGAGAGGGAGGAATAGAGACGTATTCGTTTGCGCTCTGTTCACCTT
+
CB;8A:;694/781/1//.37...4..,.2033+..,.3114:;:??AAA9<<<;4976/..1120333222
@T6XXI:00064:00108
GGGAGAAGGGAGAAAGGAAGTGCAGTTCGCGGTTGGTGAAGGGAAAACTATTCTCCCTCTCCCTATAGTGAGTCGTA
TTAGAATT
+
DNABBB?BB>BCBB>C@B?ABBBCCC@CCBA<?575:BC@CB>BDD6BACB@BBBB@A@@@@<?:::CBCBBFB???
?<?A@>@;
@T6XXI:00064:00128
GGGGAATTCTAATACGACTCACTATAGGGAGAGGGAGAATAGTCAACAAATCGTTTGGGGCGACTTCTCCTTCCTTT
CTCCCTTCTCCC
+
999/98?<>A@?BBBCCCCBCCB97166.7=7775:BB>@@@A=;=8771:@@AD@CFD7ABBAA>A@A5434244-
8=;>:>4:347,
@T6XXI:00064:00132
GGGAGAAGGGAGAAAGGAAGCTAGTCACTCGACATCTGTGGCTTCACAGTACAGAGGGACTGTCCAAAGCCGCAGAT
CTCGAGTCGACTAG
+
DEACCB=>=:@@=8:@=?<@C@@@B??:::====?<<==>;:;7;ACABBB?=9300-
4:::>A@AA<@@>A@AAAA9==A@@@@@@AAA@
@T6XXI:00065:00103
GGGAGAAGGGAGAAAGGAAGGAGAAGTCGCCCCAAACGATTTGTTGACTATTCTCCCTCTCCCTATAGTGAGTCGTA
TTAGAATTCCCCC
+
EDACCB@BC>ABEB>C@BDA?ACE??A@BCGG8CBAAA@998<A>BCDBB@?@@AA<A???@;@==<AA<<<>===<
<98<944/////+
@T6XXI:00065:00118
GGGAGAAGGGAGAAAGGAAGCTAGTCACTCGACATCTGTGGCTTCACAGTACAGAGGGACTGTCCAAAGCCGCAGAT
CT
+
AA<ACB@CC>C?=85>584;BA@@CC::::@77:::9AAC=?@@CBBCBCCB=4000-
4;;>AA?CC:@@>@?<?<<<@
@T6XXI:00065:00143
GGGGAATTCTAATACGACTCACTATAGGGAGAGGGAGAATAGTCAACAAATCGTTTGGGGCGACTTCTCCTTCCTTT
CTCCCTTCTCCC
+
=@A6@@EDCCC@C===BBBCA=;77166.777?@>BCC@AAAAA<;8853;=;;;-
777.7?@??=@BC?@=@<??:?@@@;@8;8881
@T6XXI:00065:00144
GGGGAATTCTAATACGACTCACTATAGGGAGAGGGAGAATAGTTACTAACTACTAGGCGCAACTGTTCTCTTCCTTT
CTCCCTTCTCCC
```

```
+
CCB9B>A?ABB@CCCBBBBBBA>97116.7==>B>BCB?@@@A?AA??<?AAAAAA>AA?<;?A@@<??8858;?@@
@@AD<?<??@C<
@T6XXI:00066:00126
GGGAATTCTAATACGACTCACTATAGGGAGAGGGAGAATAGTCAACAAATCGTTTGGGGCGACTTCTCCTTCCTTTC
TCCCTTCTCCC
+
=@<@;?>@AACCCCBBBBBB?<93177.7?>?B>BAA@ACAA<<8;;;4??>>@:@@@5AAAAC@CBB??<??AC:@
@AC=A:8?@C<
@T6XXI:00067:00106
GGGAGAAGGGAGAAAGGAAGGAGTAGCGCGTACGGAGTTTTCATTGGCTATTCTCCCTCTCCCTATAGTGAGTCGTA
TTAGAATTCCCC
+
DB=@@B@BB>BBBB>B@?@A@???AA@@AACBBD@BBBBA8CBB@B@DIA@=@@@>9>>>??:?CB??@BCBBCCBB
B?BCDAC@CCC9
@T6XXI:00067:00110
GGGGAATTCTAATACGACTCACTATAGGGAGAGGGAGAATAGTCAACAAATCGTTTGGGGCGACTTCTCCTTCCTTT
CTCCCTTCTCCC
+
BDB9B@C@CDC@CBA@BBBAB9<>?666.7==?B>CBB@AAAAAA?@@A;@CAA@;@@C9BCCCE@B@@>=AA?AA:
>@@A=A>@==<7
@T6XXI:00067:00120
GGGGAATTCTAATACGACTCACTATAGGAGAGGGAGAATAGTCAACAAATCGTTTGGGGCGACTTCTCCTTCCTT
+
:8808,/,49898=99898:75666./,...16666<<899<@@:<;:-677179=?@6;;;66211100/02-,
@T6XXI:00068:00106
GGGGAATTCTAATACGACTCACTATAGGAGAGGGAGAATAGTGACGTGTTGTGCGGCGCTCACTGTCTCCTTCCTTT
CTCCCTCTCCC
+
CED:C?B@BCBAB@>=BCCE@>:=9//,00067;BBB><BAAA@A@@A@<<@@@A>AA===AAAAAABA34/.,./,
////,/-1223
@T6XXI:00068:00111
GGGGAATTCTAATACGACTCACTATAGGAGAGGGAGAATAGTCAACAAATCGTTTGGGGCATCTCCTCCTTCC
+
999-9>CAGCB@CB@ABBB??7666..,0::?BBCBB?A===@A<??A<BBCCC>CCC9=<5...,..,-,+*
@T6XXI:00068:00125
GGGGAATTCTAATACGACTCACTATAGGGAGAGGGAGAATAGCGCAATTTGGACATGTGGCCGCTTCTCCTTCCTTT
CTCCCTTCTCC
+
999/98@ACCC@AAAAA?<<B@;99111.7??BC@CCC@B===A@B?CC>B?BCCBAAA>=;=<@>?><:=8;8;?;
888CAC?>>@A
@T6XXI:00068:00131
GGGAGAAGGGAGAAAGGAAGCTAGTCGACTCGAGATCTGCGGCTTTGGACAGTCCCTCTGTACTGTGAAGCCACAGA
TGTCGAGTCGACTAGCTTCCTTTCTCCCTTCTCCC
+
ED>CEB@BB>B<<;8;9;8<D@@@>0/0/..56.:688?@@>????9?>@@@B???9?A>??@A????A@@@>@C@==
=@?=<=AA??????989>A?CD7==>B14244::7
@T6XXI:00068:00135
GGGAGAAGGGAGAAAGGAAGCTAGTCGACTCGACATCTGTGGCTTCACAGTACAGAGGACTGTCCAAGCATCTCGAG
TCGACTAG
+
DD@DBB?AB=BAAB=B?B@CGB??>8;:CCCBBAABCCA>@@BA?>B@=<?ACA<9888????A>A@A???@@BAAA
???????A
@T6XXI:00068:00140
GGGAGAAGGGAGAAAGGAAGGTGAGTGCGCAGTGGGAGATTACGTGGCTATTCTCCCTCTCCCTATAGTGAGTCGTA
TTAGAATTCCCC
+
```

```
EEDBBA@DC?BBBD>D?B?B?AAA@>??CC@??@B@BBBB@BCAAA?BBBA>A;9;7;??BC?ECCCCABBBBBB==
?DGCB>C@CCB8
@T6XXI:00068:00144
GGGAGAAGGGAGAAAGGAAGCTAGTCACTCGACATCTGTGGCTTCACAGTACAGAGGGACTGTCCAAAGCCGCAGAT
CTCGAGTCGACTAG
+
DD?CBA;9=9>99909595;A?>BB::7;<=776778CCC?BB@CCA???A?9933317???@??AA=AA?@@<@<<
<=A@=AA??????<
@T6XXI:00069:00106
GGGAGAAGGGAGAAAGGAAGGAGAATGCACCGCAGGAAATTGCAAGACTATTCTCCCTCTCCCTATAGTGAGTCGTA
TTAGA
+
CC@CCE@BB>AB@B>C@B@A@>>>>BBB>B?BBB?>>>;D@B<<;@DGDBB>BC@A=@=???;?<?@@A???@>:;/
/./;8
@T6XXI:00069:00107
GGGAGAAGGGAGAAAGGAAGCTAGTCGACTCGACATCTGTGGCTTCACAGTACAGAGGGACTGTCCAAAGCCGCAGA
TCTCGA
+
CD@DCC>?>:@@=><@@C6;;A???????@A?::::::BAA<?@=?CC@@?A?>:333,399=??>AA<@@=@BACB
=<?9==
@T6XXI:00069:00110
GGGAGAAGGGAGAAAGGAAGAAAACAGTGCTGTGGGATAGCTTGAAGCTATTCTCCCTCTCCCTATAGTGAGTCGTA
TTAGAATTCCCC
+
9919AB@DD?CCAB>C?;79777-
8??A?>>>8880399>@B>BB@BBBBC?ABFKBBBBBB<A;;;@@@>>?>998?=?AC?B5999/
@T6XXI:00069:00141
GGGAGAAGGGAGAAAGGAAGGATATGCCGCAGTGGGAGATTAAACGGCTATTCTCCCTCTCCCTATAGTGAGTCGTA
TTAGAATTCCCC
+
AA<@AA>BC=@@>=:@>B>B?BCCC@@?BCBBCBB>ACBB87:5:K>CCCCBC;;;;<BCCDAAACBBBBBBCAAB:
857@B;:;@@?5
@T6XXI:00070:00101
GGGAGAAGGGAGAAAGGAAGCTAGTCGACTCGAGATCTGCGGCTTTGGACAGTCCCTCTGTACTGTGAAGCCACAGA
TGTCGGTCGACTAGCTTCCTTTCTCCCTTCTCCC
+
CC@BA@=AC>CBCG?C?B@BBBBBB:::A<AABCCCCBA@;:777.7>?7::A@A<??@AA@AAA87:>7839AA@A
@@@@@;==B<==A==9<A<@@:?@8828./..44
@T6XXI:00070:00113
GGGGAATTCTAATACGACTCACTATAGGGAGAGGGAGAATAGCTAGCATTATCCCGCTAGGTGCTTCTCCTTCCTTT
CTCCCTTCTCCC
+
EEG:D@C@AAAACA@ABBCBCC?9?661.7?>>@>ACBBC=@8?:;;@B=@;9988?@@@=@C??:?AA>A@C?BB>
AADB:948444-
@T6XXI:00070:00118
GGGGAATTCTAATACGACTCACTATAGGGAGAGGGAGAATAGTTTTTCCCTTCACTAACCGCGAACTGCACTTCCTT
TCTCC
+
DDB695959998444161......3111,1167799>983900000+0303/01111010169??<822...,.,..
+.555
@T6XXI:00071:00104
GGGGAATTCTAATACGACTCACTATAGGGAGAGGGAGAATAGACACTCCCTTACGAACCACGCGCGAATCTTCCTTT
CTCCCTTCTCCC
+
999/4:CACCB@BBBBBBBBBCB?;111.6==@B>BCCBA=9999<<AA<@>@@AA:=:===<::30/38848999:
===?:=8=8881
@T6XXI:00071:00107
```

```
GGAGAAGGGAGAAAGGAAGAAGAACGT
+
EABBB@BB>?BBC>B@B@CC@>><?DC
@T6XXI:00071:00125
GGGGAATTCTAATACGACTCACTATAGGGAGAGGGAGAATAGCCGCCGTTTCAATGACCGTACTATTCGCCTTCCTT
TCTCCCTTCTCCC
+
DDB8B8;:4885///886;>B9?>>776,16==B>A?B?A@@A?AA>@AC?@?::;0/,/<<;AA@>@AC>@=@?AA
????@:>:;444-
@T6XXI:00071:00142
GGGAGAAGGGAGAAAGGAAGTGCAGTTCGCGGTTAGTGAAGGGAAAACTATTCTCCCTCTCCCTATAGTGAGTCGTA
ATT
+
DB?=<=<<CACBBB>B>A>A:;=CCCACCCB@BBDCCAA=??;?EE9CBBBBCBBA=???BBBC@@@B<6////+++
*+0
@T6XXI:00071:00147
GGGAGAAGGGAGAAAGGAAGGAGAACGCGCAATCAGATACCTGTTGGCTATTCTCCCTCTCCCTATAGTGAGTCGTA
TTAGAATTCCCC
+
DB@CBB@BB?BBBB>B?B@B?A@ABBCCBAB?BC?AAAACCBCC7;<<BA@BA;;;8;AACE>CCCCCCCABABCBA
A?BBC?DCDLK9
@T6XXI:00072:00116
GGGAGAAGGGAGAAAGGAAGCTAGTCGACTCGAGATCTGCGGCTTTGGACAGTCCCTCTGTACTGTGAAGCCACAGA
TGTCGAGTCGACTAGCTTCCTTTCTCCC
+
::6944248-489904284488:970....4497777>>A@<???;?>CCC<;<C?CA@ABA@@@?=>444/0--
222...5;;9@@888;=::8:/44-0000+
@T6XXI:00072:00117
GGGAGAAGGGAGAAAGGAAGGAGAAGTCGCCCCAAACGATTTGTTGACTATTCTCCCTCTCCCTATAGTGAGTCGTA
TTAGAATTCCCC
+
HEACCB@BC??=?C@C?B@BA>@@BCBCBCCC9CB<BBBCB>BB>CBBBB@>A@A@;@???@;????????>>??=<
<>@@@>?5>=?4
@T6XXI:00072:00122
GGGAGAAGGGAGAAAGGAAGCTAGTCGACTCGAGATCTGCGGCTTTGGACAGTCCCTCTGTACTGTGAAGCCACAGA
TGTCGAGTCGACTAGCTTCCTTTCTCCCTTCTCCC
+
ND?B<;79@:>999147>;9;8>>?7::??BCGAAAD@?@?>8?:9?>ABC??@@;CA=<<<==<??B;==>AAA@?
??????AAAA<9=AC=9;=>@8:8773,37344=7
@T6XXI:00072:00128
GGGAGAAGGGAGAAAGGAAGGTTACGATGACGCTGACGGGTTATGGCTATTCTCCCTCTCCCTATAGTGAGTCGTAT
TAGAATTCCCC
+
CD><<>@B@:9;9919499<@C?CEECCABCCCCCCCAA?>=B11,1;11-
1A>>8@888>:877?=777771166?3611042444,
@T6XXI:00072:00133
GGGGAATTCTAATACGACTCACTATAGGGAGAGGGAGAATAGCAAACGTTTCCCCAACGTCGCTTTCTCCTTCCTTT
CTCCCTTCTCCC
+
999/9<A@CDB@AABCBCECC?<<<177.77777.7??>@???AA<ACCC>CCC8B?BBBBBBBC<A:937<:344-
4:::6<=?ACC;
@T6XXI:00072:00142
GGGAGAAGGGAGAAAGGAAGCTAGTCACTCGAGATCTGCGGCTTTGGACAGTCCCTCTGTACTGTGAAGCCACAGAT
GTCGAGTCGACTAGCTTCCTTTCTCCCTTCTCCC
+
CB<@A@=@@:>999042824@;;;AAAA<A??:;;BCCCB>@BC>C?BBCCCCC>CC@@AA@A<??B??C?CCA@@@
@???ACCA=8;9944438:=D;A@@A;?<?@@A;
```

```
@T6XXI:00072:00148
GGGAGAAGGGAGAAAGGAAGCTAGCGACCGACACCTGTGGCTTCACAGTACAGAGGGACTGTCCAAAGCCGCAGATC
TCGAGTCGACTAG
+
CECDCE@BB>B???=B>??BBA;;;5..,0>;0:;;BBA>AB@CCCBB?@?99611-
688<@A>CC>A@>@AA@@@<@?=<<<:;<===<
@T6XXI:00073:00108
GGGGAATTCTAATACGACTCACTATAGGGAGAGGGAGAATAGCCTTGGCGTTTGTGCCACGTTATTCTCGACTTCCT
TTCTCCCTTCTCCC
+
A::2?5<:<BBCGCB@BCCCCB>99111.777==9?@A@A899?E?B?BBBB<AA??<??;;8;;79:===>?=@?A
C<>=>A<@<=;994
@T6XXI:00073:00118
GGGGAATTCAATACGACTCACTATAGGGAGAGGGAGAATAGTCAACAAATCGTTTGGGGCGACTTCTCCTTCCTTTC
TCCC
+
CEHDC@C@@A>@CCBBBBBBAB<>6664699CC>CCCA???A==<54416?667,1194:@@BC@BBB==;=>AA=B
BAA=
@T6XXI:00074:00128
CTATCGACTCGAGATCTGCGGCTTTGGACAGTCCCTCTGTACTGTGAAGCCACAGATGTCGAGTGACGTA
+
C=<;AAA?AB?9<=BCCCCCABBA=??>A;@???8?BABCBBBCCB@=?C@BCA?BCCCB;;2,,,+)))
@T6XXI:00074:00129
GGGAGAAGGGAGAAAGGAAGCTAGTCGACTCGACCATCCTGTGGCTTCACAGTAC
+
CDABBCBEF@CCEE>B=@6433933176661133111101777067111761167
@T6XXI:00074:00136
GGGAGAAGGGAGAAAGGAAGGCGAACAGAGCGCAAACGAATACGTCTCTATTCTCCCTCTCCCTATAGTGAGTCGTA
TTAGAATTCCCC
+
BB?BDC@EEACBAA646:<B>ADC@AACC??>>BB>B@@>BCCBBBCDBBB>A???:;;;BB>CBCBBB???>AAAA
@>>783<?AAB9
@T6XXI:00074:00137
GGGGGAATTCTAATACGACTCACTATAGGGAAGAGGGAGAATAGTCAACAAATCGTTTGGGGCGACTTCTCC
+
9999-4898=AB@CCCDCB@@@@D?00..+.,....+..78:<<C@@><<<4?@@??6::7./:,,,*,77+
@T6XXI:00074:00143
GGGGAATTCTAATACGACTCACTATAGGGAGAGGGAGAATAGTCAACAAATCGTTTGGGGCGACTTCTCCTTCCTTT
CTCCCTTCTCCC
+
>>?4>=@ACCB@CBAAAABBBC@99111.777BC>CCC@@@@AAA????7??<;=-777-
7?AADACBC?=9<<@@<@ACC=A8;8881
@T6XXI:00074:00146
CTAGTCGACTCGAGATCTGCGGCTTTGGACAGTCCCTCTGTACTGTGAAGCCACAGATGGACTGCTTCCTTTCTCCC
TTCTCCC
+
DD=<;CDCCCCCCBCBAAABB>?AD>C;;;?????<?BBCBBBC?AAA<?BACCCCB@@=@B:;;C=@=@CA??@B=
B@:;:B6
@T6XXI:00075:00107
GGGAGAAGGGAGAAAAGGAAG
+
<<19@@599199111*1,/,,
@T6XXI:00075:00113
GGGAGAAGGGAGAAAGGAAGGAGAATAACGGACAACCAAGCCAGTGACTATTCTCCCTCTCCCTATAGTGAGTCGTA
TTAGAATTCCCC
+
```

```
=B<@AB<@@>BAAC=B>@>@=:;;>A?>BC?BBB=B6:5::>BCBCBC@@@>?@@C;94444+3><AACCCAA588?
A>AAA?A;@@A6
@T6XXI:00075:00124
GGGGAATTCTAATACGACTCACTATAGGGAGAGGGAGAATAGTCAACAAATCGTTTGGGGCGACTTCTCCTTCCTTT
CTCCCTTCTCCC
+
EIE:D@B?BAA?@A@BDDCCCCCCCBBB>:::CC=:;;@@@?A@A?@AB;BBBBC>CBB9CDDAA=<A?:=:=:>?;
?AAA<A899BA:
@T6XXI:00075:00125
GGGGAATTCTAATACGACTCACTATAGGGAGAGGGAGAATAGTCACG
+
CCC9CCC@?@@@CBBAAA???AB<<66649=?CC>CCC?@??<=6//
@T6XXI:00075:00144
GGGAGAAGGGAGAAAGGAAGAGTAACGCAACGAAGAGGAAAGTCTGGCTATTCTCCCTCTCCCTATAGTGAGTCGTA
TTAGAATTCCCC
+
DEABBC@CC?DCBC?C?B?BBBAAAACCC@CBB@DDE>?@>AB@B@@BBCCACBAA<BACCC>?@?=<<?=BC????
?<?AA?D>@@@5
@T6XXI:00076:00100
GGGAGAAGGGAGAAAGGAAGGGGAGAAGGGCTAGTCACTCGAGATCTGCGGCTTGACAGTCCCTCTGTACTGTCGAA
GCCACAGATGTCGAGTCGACTAGCCTCTT
+
@D9>>@599-49:;-46999::,337133,4944494449999==:>?9958843397768888:?@@@>::.---
24B@BBB;>000...718433333106110
@T6XXI:00076:00111
GGGAGAAGGGAGAAAGGAAGAAAAGGGAGCCGTGGTTAATTTGTCGACTATTCTCCCTCTCCCTATAGTGAGTCGTA
TTAGAATTCCCC
+
DD@BBB@CC>ABAB>B@A=?>BB7AA>ABB?BBC?A@A@CC>BCCCCBCCC=??ACA===AA;A@AA?@@AA???@?
?=@@@>@>@@@5
@T6XXI:00076:00139
GGGGAATTCTAATACGACTCACTATAGGGAGAGGGAGAATAGTCCCCACTTGATTGGTACCTACTTGTCCTTCCTTT
CTCCCTTCTCCC
+
999/9=A@DBB@BC???BC@AAB9>111.7=<BD>BAAABAA@@@@5@AA>AAA>?>?<44;010,/:::>6@@CC>
D===7<<==?@:
@T6XXI:00076:00143
GGGAGAAGGGAGAAAGGAAGCTAGTCGACTCGACATCTGTGGCTTCACAGTACAGAGGGACTGTCCAAAGCCGCAGA
TCTCGAGTCGACTTAG
+
CD@BB@=@=9>98809;>;>A?=@>/11???>A@@@@?CBB?@@@@CC@?>A@;8330,399>>@;==9=DAD?6<A
==A???AAAAA==:=?
@T6XXI:00077:00104
CTATCACTCGAGATCTGCGGCTTTGGACAGTCCCTCTGTACTGTGAAGCCACAGATGTCGAGTCGACTAGCTTCCTT
TCTCCCTTCTCCC
+
FAA?:::?BB?@?EDFAAC@CCC>C?BBBA:::57@@AABBBBBB@@>@CABBCCCCBABBB@:;;;;;<3968:::C
>B@AC@C>@@@A<
@T6XXI:00077:00114
GGGAGAAGGGAGAAAGGAAGCTAGTCACTCGACATCTGTGGCTTCACAGTACAGAGGGACTGTCCAAAGCCGCAGAC
TCTCGAGTCGACTAG
+
44-4::9AC<>>>>096969>?BB@>>0,,,,****,,,02499>::>AA?@>:444-
49:9;=>AC@AA>=<<=43440003333534463
@T6XXI:00077:00118
GGGGAATTCTAATACGACTCACTATAGGGAGAGGGAGAATAGTCAACAAATCGTTTGGGGCGACTTCTCCTTCCTTT
CTCCCTTCTCCC
```

```
+
CDB8A@B@BBB@BBB@BBAABAA?B79959@BCC>BBB?A8====>AAA<BBBBA<BAA7B;;:B@@??;;8<;>@<
A@@@:=;888=7
@T6XXI:00077:00140
GGAGAAGGGAGAAAGGAAGGACAGTCCATCTGTGGCTTCACAGTACAGAGGGACTGTCCAAAGC
+
C@BBB@BB?@?AB?B>A<==?@???C?B@@@BAA?BB?CCCCC9@A==9897<@@@AA>@@;87
@T6XXI:00077:00145
GGGAGAAGGGAGAAAGGAAGCTAGTCACTCGACATCTGTGGCTTCACAGTACAGAGGGACTGTCCAAAGCCGCAGAT
CTCGAGTCGACTAG
+
DDAB?=644-
999909495<CCBCC@A@??777:::CCC@@@?@CECB?C@B433314;9;9;37818@=AA@@A@@@AAAA@???
@@@@
@T6XXI:00078:00100
GGGGAATTCTAATACGACTCACTATAGGGAGAGGGAGAATAGCTACTGCATGTACATCAGTAACGTCTCCTTCCTTC
TCCCTCTCC
+
DED:DBB@BCG><;<GC@@@CA>>>777-
716=7;?<885:44:=?8?::6?A:;<BA??@?=@A;77A47409:,0000+.---,
@T6XXI:00078:00102
GGGAGAAGGGAGAAAGGAAGGGAAGGGAGAAAGGAAGCTAGTCGACTCGACATCTGTGGCTTCACAGTACAGAGGGA
CTGTCCAAAGCCGCAGATCTCGAGTCG
+
CDACCB@BB>@@EB?C@C@CC?E?BA<?>??;>>>>><?;;>120>::8;:@@BBCCBAB@?@@AAAA===A@@A<@
@@????AA;??;A??<=?<?AA777<9
@T6XXI:00078:00103
GGGGAATTCTAATACGACTCACTATAGGGAGAGGGAGAATAGC
+
CCE:D@@>ACCACC?BBBBAC<;7<1775:@ACC>B>>=93**
@T6XXI:00078:00111
CTAGTCACTCGAGATCTGCAGCTTTGGACAGTCCCTCTGTACTGTGAAGCCACAGATGTCGAGTCGACTAGCTTCCT
TTCTCCCTTCTCCC
+
B:1////44CCEDBCBBBCCCBBB?B@CCBBACD>CCBBBCBCCCCC?BB?B@@??==A<<<<<@@@@@CB@@>@?A
A=A@@A;A>;???7
@T6XXI:00078:00125
GGGGAATTCTAATACGACTCACTATAGGGAGAGGGAGAATAGCAAAACGTTTCCCCGACGTCGCTTTCTCCTTCCTT
TCTCCCTTC
+
CFC9CBC@BBB@BBB@BBBB@???;16959?@CC>BBB@A=?7433-7=AA<AAA7???C???:91-
6;;:<:<344-4:::4;;=
@T6XXI:00078:00128
GGGGAATTCTAATACGACTCACTATAGGGAGAGGGAGAATAGTTACGACGAATTCTTGGCGTACC
+
CDD:DAB@BDC?CBCCB@AA:9:?A777.77777,11100400/00003332553424/.....,
@T6XXI:00078:00140
GGGGAATTCTAATACGACTCACTATAGGGAGAGGGAGAATAGTTAATCTGACCCGACAGCCTACTTGTTCTTCCTTT
CTCCCTTCTCCC
+
CCC9B=?=ABC@CCCBB:::A???>777-
7>>>><AAA?AAAA>A><?@ABBC@BBBB@@<@AA@>BB>@@?A>BB:@A@A9@;??FB>
@T6XXI:00079:00112
GGGGAATTCTAATACGACTCACTATAGGGAGAGGGAGAATAGCTCCTAAACCATTAACACTGCACGCACCTTCCTTT
CTCCCTTCTCCC
+
```

```
CCC9C?A@BCBEBAB>>>@@@C>77111.7<<?CBBBB@AAAA===?CC>B@7;9;@BBB?@?A@BBBB?B5989>:
>99909;>AAB=
@T6XXI:00079:00143
GGGGAATTCTAATACGACTCACTATAGGGAGAGGGAGAATAGCCGTTTAATCTCCCACGCGGCATATCCTTCCTTTC
TCCCTTCTCCC
+
999,424249;2499=??B?B9?87777,67111,177;<<==<@@?;@=//-22+-..44469444134---.4+-
-553323000+
@T6XXI:00079:00146
GGGGAATTCTAATACGACTCACTATAGGGAGAGGGAGAATAGCTTGATGTCCTGAGGACCTTGCTTCTCCTTCCTTT
CTCCCTTCTCCC
+
@BA6@@B@CBB@BBBBAAABAA@?<66659?ACC>CBC?@=@<@==?BCBCCAA==:<@=@>AAA?????@=@9;;6
;===7><===AA
@T6XXI:00079:00147
GGGGAATTCTAATACGACTCACTATAGGGAGAGGGAGAATAGTCAACAAATCGTTTGGGAGCGACTTCTCCTTCCTT
TCTCCCTTCTCCC
+
A>?4?;@?ACAICCCAABCBB@;77667.7>===<???@@@@????@DC;>>;::,00,0//:;;A?B@C?B>A>A@
7==@@;?;=;<<6
@T6XXI:00079:00148
CGAGATCTGCGGCTTTGGACAGTCCCTCTGTACTGTGAAGCCACAGATGTCGTACTTCT
+
ABBBBDCCBBB<=77,748>?C8::7:CBCCB@BBCCC>=?>CA@858=9B=/,,22,,
@T6XXI:00080:00122
GGGAGAAAGGGAGAAAGGAAGGAGAAGTCGCCCCAAACGATTTGTTGACTATTCTCCCTCTCCCTATGAGT
+
;;:9949-44-4444-9@A@A9<;;78>@>???2??:?@ABC>DC@BCD@@=>@@@@:;84//,,5,,,++
@T6XXI:00080:00128
GGGAGAAGGGAGAAAGGAAGGAGAAGTCGCCCCAAACGATTTGTTGACTATTCTCCCTCTCCCTATAGTGAGTCGTA
TTAGAATTCCCC
+
BCACDEBCC@AA@B>C>@=@=@CC@CBBBBBB8BB?BCAAA=AB?CB?BCA>===<;??@BB<A<@?=@==@@===@
@>AA@<>3444-
@T6XXI:00080:00141
GGGAGAAGGGAGAAAGGAAGGATAGCATCGGAGGGGGCAGTCGAGAGCTATTCTCCCTCTCCCTATAGTGAGTCGTA
TTAGAACTTCCCC
+
44-
49><>@:@>>>9@;@=A:;;BBCBCBB?CCDDD2BBBAAAA::3:@BB?>>>@;331;;;:99BCCCCBBC>10/53
1135986992
@T6XXI:00080:00142
CTAGTCACTCGAGATCTGCGGCTTTGGACAGTCCCTCTGTACTGTGAAGCCACAGATGTCGAGTCGA
+
FC111:::?BAABBBBBC@@@@CC?C@CBBB9994:BABBABCACC@<?B@CCBBBABBCCBBCBB@
@T6XXI:00080:00143
GGGAGAAGGGAGAAAGGAAGAACATTGCCGGGGAGTCGAATTGTCGGCTATTCTCCCTCTCCCTATAGTGAGTCGTA
TTAGAATTCCCC
+
BB>BBB@BB>ABBB>B@B@CC@AA?@?A?BCC9B?B99?@;:66.0,0=AE>BEDC@BC;;;8;@@ABDCCCBA??:
:6;@=;:;<<A5
@T6XXI:00080:00147
GGGAGAAGGGAGAAAGGAAGCTAGTCACTCGAGATCTGCGGCTTTGGACAGTCCCTCTGTACTGTGAAGCCACAGAT
GTCGAGTCGACTAGCTTCCTTTCTCCCT
+
DB=C><8;A:A;;<9;=A>AC::;;@???AAA???>>>8;47@A:>>A?ABCCD=BA?>>788>88:=>=8=>;888
@@@@@@C@@@=;99474/78828///,/
```

```
@T6XXI:00081:00108
GGGAGAAGGGAGAAAGGAAGGAGAAGTCGCCCCAAACGATTTGTTGACTATTCTCCCTCTCCCTATAGTGAGTCGTA
TTAGAATTCCCC
+
GD?CBA@BA@@@@A;A@A@A>>>B?C>C@??@6@B>BKDCB>CC?BAABBD?@???<????A<A<@@AA<<<<??AA
AA@<>49/444-
@T6XXI:00081:00144
GGGGAATTCTAATACGACTCACTATAGGAGAGGGAGAATAGTGACGTGTTGTGCGGCGCTCACTGTCTCCTTCCTTC
TCCCTCTCCC
+
9994;@C@CBB@BB???BBABCBBB:94:77=>9@AA?@@CA@@@@CAA<<?@@A<??@@AAAA???AA>?<;5646
///,/.1220
@T6XXI:00081:00146
GGGAGAAGGGAGAAAGGAAGGAGAATCGAGGTGAGTTGTACTCATCACACTATTCTCCCTCTCCCTATAGTGAGTCG
TATTAGAATTCCCC
+
EJBAAA@BA>@A@C@C@C?B@CCB@BB?<<@A@DBB?@A@@DGGBBCDCBABB?BBCC@CA:::6:@@ACCCCC@@@
ACC?CBC?B=???7
@T6XXI:00082:00101
CTATCGACCCGAGATCTGCGGCTTTGGACAGTCCCTCTGTACTGTGAAGCCACAGATGTCGAGTCGACTAGCTTCCT
TTCTCCCTTCTCCC
+
DC;;94444-
4;;;BCCCBB@ACD>B>AACCAAA>A@@@A@?BACC@@AB@CCCEDBBBBBCCCCCCCCCDEC?@=@C>BBDB>C=?
@DF<
@T6XXI:00082:00102
GGGAGAAGGGAGAAAGGAAGCTAGTCACTCGACCATCTGTGGCTTCACAGTACAGAGGGACTGTCCAAAGCCGCAGA
TCTCGAGTCGACTAG
+
DD>BAB59919@@BBC?CBCBB@00/0///0//,.4868CC5:;?@??CBCBB@=77718B>@A@>@A<??<?@@@?
@888=<@@@@@@@@@
@T6XXI:00082:00109
GGGGAATTCTAATACGACTCACTATAGGGAGAGGGAGAATAGTCAACAAATCGTTTGGGGCGACTTCTCCTTCCTTT
CTCCCTTCTCC
+
444,49B;<<;=A@@@BBAAAB@@@BBA<A??>>6<773444;;;8<<@9B?===.777.7777A?B?@;85;:==7
?>?A:;34443
@T6XXI:00082:00112
GGGAGAAGGGAGAAAGGAAGGAGCACTAGACGGCACTTCTCGCCAATCTATTCTCCCTCTCCCTATAGTGAGTCGTA
TTAGAATTCCCC
+
GEBBBA@CC<>@CD?>;>?=1001>>CDABAA?@@BBABA8;;=@=@CBBB>B:77,444<<7=AAA@@A??????B
A<??A975699+
@T6XXI:00082:00114
GGGGAATTCTAATACGACTCACTATAGGGAGAGGGAGAATAGCTAATTATCTCCCATCGGCCGCGTGCCCTTC
+
CDC:C?A?BBB@CCBBBBB?@><99111.777BC@CBB>@??@??=?>@C@<<=<?@@A>A;==AB99:5644
@T6XXI:00082:00118
GGGGAATTCTAATACGACTCACTATAGGGAGAGGGAGAATAGTCAACAAATCGTTTGGGGCGACTTCTCCTTCCTTT
CTCCCTTCTCCC
+
AAA9A@B?CDC@CBA@BBBBB@BB>66646??>B>BBB?@=B=<<:<?C<CC@BB>BBC9BB??C@CCC==98:<=;
AABB=C?A8771
@T6XXI:00082:00127
GGGAGAAGGGAGAAAGGAAGGAGAAGTCGCCCCAAACGATTTGTTGACTATTCTCCCTCTCCCTATAGTGAGTCGTA
TTCAGAATTC
+
```

CD?BCC?BB=BBBB>C@C?B?CCC?BBBBCCC9??:;ABCC>??>ABBBC@?@@@@<@@@@C9=899AAC@@@>=86
7,..-+*+53
@T6XXI:00082:00142
GGGGAATTCTAATACGACTCACTATAGGGAGAGGGAGAATAGTCCTCCATTCTGTGGACCCACTATTCGCTTCCTTT
CTCCCTTCTCCC
+
CCD:C=@>?CC@CCABAB>>>CB?>66659::CD>CCC?@899A>AD>BC?B????=@EE>CCCAA?ACBC?C?BA;
?77718=@AAA<
@T6XXI:00083:00119
GGGGAATTCTAATACGACTCACTATAGGGAGAGGGAGAATAGCTATTCAGTCCTCGTCGCCTTGCTATTCTTCCTTT
C
+
AAA4><@=ABBACCBBBBCCD??>?666.7??BD>CCC@???8??B?BBAAA;??;;788>@B@?=@A@AA<?=@A:
=
@T6XXI:00083:00139
GGGAGAAGGGAGAAAGGAAGCTAGTCGACTCGACATCTGTGGCTTCACAGTACAGAGGGACTGTCCAAAGCCGCAGA
TCTCGGTCGACTAGCTTCCTTTCTCCCTTCTCCC
+
CE@BCB@CGCCBCB>B=@;?C<3:;::1>>>>11////==A>@BB@C@@@CCCB4000-
4;;@AA?A@<@AB@@<??=<@??<??@<<<@A@@>@>AA<??@@:>96///,
@T6XXI:00083:00147
GGGGAATTCTAATACGACTCACTATAGGGAGAGGGAGAATAGCCAACATCTCCCGATTGTCACTGCTCCCTTCCTTT
CTCCCCTTCCTCC
+
CDC9B8:;<GC=@AC;;<CCBBCCC::7779?BC<AAA>>4888<9<@@@@A@<??@>@5:7;?439;;2776011,
11111,17776<9
@T6XXI:00083:00148
GGGGAATTCTAATACGACTCACTATAGGAGAGGGAGAATAGTGCGCTCACAGTTGGCGTCCTTTTTTCCTTCCTTTC
TCCCTTCTCCC
+
BBA7A798<CC@CCCCB>@>C:99=//-0;=?B>BBC?@@@@===AAA@@AAA>@=<?AA>AAA@>-
>2434244+044;:@>???A<
@T6XXI:00084:00116
GGGAGAAGGGAGAAAGGAAGCAGTCCATCTGTGGCTTCACAGTACAGAGGGACTGTCCAAAGCCGCAGATCTCGAGT
CGACTAG
+
CD@@A?ADD>BBAB@B@B?BBBB795:@?@BBB@CD<<A<<<=@@@?<<<;<==<8<;==;88:=;;;9<<<<<@@@
CCCC@@@
@T6XXI:00084:00123
GGGAGAAGGGAGAAAGGAAGGAGAAGTCGCCCCAAACGATTTGTTGACTATTCTCCCTCTCCCTATAGTGAGTCGTA
TTAGAATTCCCC
+
DD?AA@@BC?ACAH?C@B@B@>>>@BACA@??7?A=CCCCC>BB>BBC@B@><?<=:;:?@A;=;::<===?<???@
@>AA@<?38770
@T6XXI:00084:00125
GGGAGAAGGGAGAAAGGAAGAGAAGGCCCACAAAACAAATATGCTAGCTATTCTCCCTCTCCCTATAGTGAGTCGTA
TTAGAACTTCCCC
+
ECACCD=>=:;;9@<AAA?AA@AAC4;;8;;???8:::57=<?A@@A???A=@=?=644444-
4998;@84/;8///.332=445;8;;-
@T6XXI:00084:00132
GCTTTGGACAGTCCCTCTGTACTGTGAAGCCACAGATGTCGAGTCGACTAGCTTCCTTTCTCCCTTCTCCC
+
CBAA=B>@BECCCB>CBB??ABAAA@B@BB@BBBBBB@??<AC@@@???@<<@@B@B?:>BCD=?=:?AA<
@T6XXI:00084:00134
GGGAGAAGGGAGAAAGGAAGTGCAGTTCGCGGTTAGTGAAGGGAAAACTATTCTCCCTCTCCCTATAGTGAGTCGTA
TTAGA

```
+
EC?AAA@BB?==BC@B@B>BACCCCC?A>@B@B?>>?BC@CB>CCC9EBECBA>BB<?????9??>??ACAAA@@?8
83499
@T6XXI:00084:00144
GGGGAATTCTAATACGACTCACTATAGGGAGAGGGAGAATAGTCAACAAATCGTTTGGGGCGACTTCTCCTTCCTTT
CTCCCTTCTCCC
+
EDE9C@B@BCB@C@==?????AB?<111.7<<ABBABB@C@@@=<:=@@;BBBBC=BCC8CBBBB?BCC??=??AA<
@ACA<A>@@@A;
@T6XXI:00084:00145
CTAGTCACTCGAGATCTGCGGCTTTGGACAGTCCCTTGTAC
+
@=1//7/44BBBBCDCCC::6:<>6?688?=000,0,....
@T6XXI:00085:00149
GGGGAATTCTAATACGACTCACTATAGGAGAGGGAGAATAGCCACGTTTAACCGATCGTGT
+
==96@<@?BAB?BCBBBBBB@@<><662:??BC>CCC@A===:;;?@A>@>??B?CBB?AA
@T6XXI:00086:00103
GGGGAATTCTAATACGACTCACTATAGGGAGAGGGAGAATAGCTGTCCTATTCTTCGATTGCCCGCGCGCTTCCTTT
CTCCCTTCTCC
+
BBB6:<>6<<A=BCCCCCAA?AAA:::A:>>777.:?@;?BB@BCBA>AAA>AB>AB<<:777.044:=??<?;?A7
===B:;3///,
@T6XXI:00086:00109
GGGGAATTCTAATACGACTCACTATAGGGAGAGG
+
BKD9B@C?<99?888>81011/74...4211111
@T6XXI:00086:00110
GGGGAATTCTAATACGACTCACTATAGGGAGAGGGAGAATAGTCAACAAATCGTTTGGGGCGACTTCTCCTTCCTTT
CTCCCTTCTCCC
+
CB;,95429>A@CBBBBBCB;;;AA77:6:??BC?CBB?A@@?>99343,7??FC>ECC9ABAAA=;::;<8<8;A;
A<<=>B:=<?>7
@T6XXI:00086:00113
GGGGAATTCTAATACGACTCACTATAGGGAGAGGGAGAATAGTCTGCAGATCTGACATGACTTTTTCTCCTTCCTTT
CTCCCTTCTCCC
+
@@@4A69599999;>DCCA@ACCBB::7.7??@B>B?>@;//////>ABAF@@@9;<@@98;A?A2BBA==;>377-
7>??:?:=444-
@T6XXI:00086:00121
GGGAGAAGGGAGAAAGGAAGCTAGTCACTCGACATCTGTGGCTTCACAGTACAGAGGGACTGTCCAAAGCCGCAGAT
CTCGAGTCGACTAGCTTCCTTTCTCCCTTCTCCT
+
KD<@<<59@:>499149?<BBA@@>:0//......166778:;78>A????@>:447179;<@@=@C=AA=A@A@?@
???===?@AA???AAA;=:=A<===A<A?AA@:4
@T6XXI:00086:00122
GGGGAATTCTAATACGACTCACTATAGGGAGAGGGAGAATATTCGTACTA
+
BBA7@598;;;?BB@AA@@@CCAAA??=6;<767.7==:981,-------
@T6XXI:00086:00127
GGGGAATTCTAATACGACTCACTATAGGGAGAGGGAGAATAGTCAGCAAATCGTTTGGGGCGACTTCTCCTTCCTTT
CTCCCTTCTCCC
+
?==6>6;8<DC@CCA???>>B@CBA77746;99>>BCC?A8:8AAA@@A<CBB??8?685?@ABC?BBB=?=@AAA<
?AAC;@<??AA<
@T6XXI:00086:00135
```

```
GGGAGAAGGGAGAAAGGAAGCTAGTCGACTCGACATCTGTGGCTTCACAGTACAGAGGGACTGCCAAAGCCGCAGAT
CTCGAGTCGACTAG
+
EKDDCB@B>:@@==:@>@?BBB>>>/11>>><3;:???ABB?AA>A???CACCA<<==;<<<8:866:;;8;?@;;;
=888@BB@ABBBBC
@T6XXI:00086:00141
GGGGAATTCTAATACGACTCACTATAGGGAGAGGGAGAATAGTCAACAAATCGTTTGGGGCGACTTCTCCTTCCTTT
CTCCCTTCTCCC
+
AAA6@=A@BAA@BBBBCCCC@??>9611.7>>?A>AAA@BAAA?<:<;;6?BBBB5:::4:?AAB;???;;8=;=?:
=<<=7=383?@;
@T6XXI:00087:00115
GGGGAATTCTAATACGACTCACTATAGGGAGAGGGAGAATAGTCAACAAATCGTTTGGGGCGACTTCTCCTTCCTTC
TCCTCTCC
+
CAA8AADDCCC@BBBBBBBBBABB@:::;<AACC>AAA>A===><?<==9?CCCE>BBB7BBBBC=??A;=8:8846
//,/-000
@T6XXI:00087:00116
GGGGAATTCTAATACGACTCACTATAGGGAGAGGGAGAATAGATCACGACCCTTCTACCACGATTGAGCTTCCTTTC
TCCCTTCTCCC
+
CBB9C@C@CCB@BBAAABBBBCBBB::866;19:8:CCCB?@@?@A<AAAA=A>A=992:;<>C??@?A@;@=>>:?
>>@1;9<CCLA
@T6XXI:00087:00134
AGTCACTCGAGATCTGCGGCTTTGGACAGTCCCTCTGTACTGTGAAGCCACAGATGTCGAGTCGCAC
+
CCCBBBBBBBBBBB??@B@AAB?A@BBBBBBB>BB><<>>@@??>=>?A>>????<:>B4376,+((
@T6XXI:00087:00148
CTAGTCACTCGAGATCTGCGGCTTTGGACAGTCCCTCTGTACTGTGAAGCCACAGATGTCGAGTCGACTAGCTTCCT
+
?11////44?==?CBCBBBC@CCB>B@B@?A67:-7>>AAACC?AAA=?B?@@@BA;;;AAABBCCCBAABCB@C>D
@T6XXI:00088:00100
GGGAGAAGGGAGAAAGGAAGCTAGTCGACTCGACATCTGTGGCTTCACAGTACAGAGGGACTGTCCAAAGCCGCAGA
TCTCGAGTCGACTAG
+
AA<@CC@BC>@@@B>@<?=:::AAA7::>>>>;<:???<<<;7847BBCBBCB?;444-
4::9;?<??;?A;>?=<<??@@A===A====A@
@T6XXI:00088:00103
GGGGAATTCTAATACGACTCACTATAGGGAGAGGAGAATAGT
+
BDB6>>B?BBB@CCBBB?@@C???@?==6==774700,/***
@T6XXI:00088:00118
GGGAGAAGGGAGAAAGGAAGCTAGTCGACTCGACATCTGTGGCTTCACAGTACAGAGGGACTGTCCAAAGCCGCAGA
TCTCGAGTCGACTAG
+
BB>BCB>@@:@=885>484;B?;;;/00::;?0;9:76;:C7;<?BCCAAAC@>944417??@A@==A<AA?@?;:;
>><<86<AAAA?<<<
@T6XXI:00088:00121
CTAGTCGACTCGAGATCTGCGGCTTTGGACAGTCCCTCTGTACTGTGAAGCCACAGATGTCGAGTCGACTAGCTTCC
TTTCTCCCTTCTCCC
+
>7-
,,,,,//8:==BCD;:<C?AAC>C@CCBCCBB=AABBCCBBBB@A=8;7;BBCCCCBBBBCB;;;@>?8713<:<=.
716=69/7668:
@T6XXI:00088:00125
GGGGAATTCTAATACGACTCACTATAGGAGAGGGAGAATAGACTGCTATCCTCAGGCGTGCTATTCAA
+
```

```
444,424249BECCBB9:;@878>96131><<<6<>>48888;:::;?7737400235334911,1>:
@T6XXI:00088:00126
GGGGAATTCTAATACGACTCACTATAGGGAGAGGGAGAATAGTCAACAAATCGTTTGGGGCGACTTCTCCTTCCTTT
CTCCC
+
AAA7B@C@CBC@B@=??BBBBAB<<66659AABC>BCC>A===:;::@A<CC@@?8:886?BBBC?BBB?@>A?AA<
AAA>;
@T6XXI:00088:00128
GGGAGAAGGGAGAAAGGAAGCTAGTCGACTCGACATCTGTGGCTTCACAGTACAGAGGACTGTCCAAAGCCGCAGAT
CTCGAGCT
+
BK@AAA>AA><=<C@CDB@BABCCCCCCA?@?887??<===3131166616<==8=A:??774:7::499799::;4
4,5,,+++
@T6XXI:00088:00139
GGGAGAAGGGAGAAAGGAAGCTAGTCACTCGACATCTGTGGCTTCACAGTACAGAGGGACTGTCCAAAGCCGCAGAT
CTCGAGTCGACTAGCTTCCTTTCTCCCTTCTCCC
+
BC<@A@=@@:@99909<@>CBA?<-
,,,,,,,,,35>>@@?CL@BBBBCCBA?;;;;6;9;;;;38814;;=???>=888<<;;;>7843;>;;?<?>7?@>
@;@600;;6
@T6XXI:00089:00100
GGGAGAAGGGAGAAAGGAAGGAGAATGCTAACGCGCAGAATTGGCAACTATTCTCCCTCTCCCTATAGTGAGTCGTA
TTAGAATTCCCC
+
CDDCCB@BB?BBBB>B>??B@B>>8<;BBDCCBBB?B??>??@@??>?BBB@BBBB=@@<<<;?BBBC==AA=:<9=
=>@@A=B?DCC4
@T6XXI:00089:00111
CTATCACTCGAGATCTGCGGCTT
+
BBB=9::;AABCCCBCCBA?ABC
@T6XXI:00089:00131
GACTACGGAGAAGATCTGCGGCTTTGGACAGTCCCTCTGTACTGTGAAGCCACAGATGTCGAGTCGACTAG
+
@1/////,,*-*,,,000@B>ADB?A>A<<;=>B;>B<73337379@@CDABCB>;:=;00.222///.54
@T6XXI:00089:00138
GGGGAATTCTAATACGACTCACTATAGGGAGAGGGAGAATAGTCTCCCACGGTAGGCATCGCTGCGGTCCTTCCTTT
CTCCCTTCTCCC
+
BA@7@8;8;CB@BBBABBBBA@A<;8?;6;;;<<9?@@>@@@@@A@@;B@><=7:5:?A???@@@=@CC@C=?=?B<
ABCB:=:====:
@T6XXI:00090:00120
GGGAGAAGGGAGAAAGGAAGGAGAAGTGTCAGTACATCGCGGTAACACTATTCTCCCTCTCCCTATAGTGAGTCGTA
TTATTC
+
EEABBC?B?=BBAE?B@C@B=>?B@B?><<ACCC@=?<<888=@>BCAAA?=?77418??AA<A@@@A???B@===@
@@??<?
@T6XXI:00090:00124
GGGAGAGAATTTGGTAATGGGGTATGAAGCTGTAGGGGAGGGAGAAAATGTGCCTTATATCTCTCTCCTATAGTGAG
TCGTATTAGAATTCCCC
+
EC@AA@AA@CB=BA?A<8887,8BBAC@CBBB@@?BB8CCC>?CCCC9BBB??;?AB@???>:8766,./--
04655556:00/000/02344.
@T6XXI:00090:00141
GGGGAATTCTAATACGACTCACTATAGGGAGAGGGAGAATAGCAGTTAACTCCCCTCCACGACGATCGCCTTCCTTT
CTCCCTTCTCCC
+
```

```
A?=6@>A?CBB@BBB@B?<<<@B?<66659??CD>BBB@A888===<=;8<;;6.5???=?@@==<<<=<;86;>>7
;;??497:444,
@T6XXI:00090:00143
GGGAGAAGGGAGAAAGGAAGCTAGTCACTCGACATCTGTGGCTTCACAGTACAGAGGGACTGTCCAAAGCCGCAGAT
CTCGAGTCGACTAG
+
DD@CBB>==6=>==5>5949B??ACA?====776791=?A5:;?BAAABBABB933317?><=<@BC?@@=?????A
@@@AAAAAA????<
@T6XXI:00091:00104
CTAGTCACTCGAGATCTGCGGCTTTGGACAGTCCCTCTGTACTGTGAAGCCACAGATGTCGAGTCGACTAGCTTCCT
TTCTCCCTTCTCCC
+
C8//111:=CBBAABCCCCC@BC?<B:>@BB@@@=@AA@A@??<AAC>@C9;<NNBCABBBCABBBBCCCCAA?A?A
C@CCBC>A=?@@B<
@T6XXI:00091:00113
CTAGTCGACTCGAGATCTGCGGCTTTGGACAGTCCCTCTGTACTGTGAAGCCACAGATGTCGAGTCGACTAGCTTCC
TTTCTCCCTTCCTCCC
+
999999444499=@@B@@@;86:@@<A?BBCAAAA<A??@CCCB???>;=C?BBCC??@@;;7>A@A988<781013
11,13356:,0,0893
@T6XXI:00091:00118
GGGAGAAGGGAGAAAGGAAGGAGAAGTCGCCCCAAACGATTTGTTGACTATTCTCCCTCTCCCTATAGTGAGTCGTA
TTAGAATTCCCC
+
CD?CBB@BBBBBBB=A@B@BAAAA?>>@8888.7>9>BBCC>BB?CBB@@=@@@??5;784:7=<<=@@@A???AA=
=:<00/0/000+
@T6XXI:00091:00123
GGGAGAAGGGAGAAAGGAAGCTAGTCACTCGACATCTGTGACTTCACAGTACAGAGGGACTGTCCAAAGCCGCAGAT
CTCGAGTCGACTA
+
;<8<AA@CD?B@=85>494;@@ABB=??::::????B=B:::9A@CC@@ABAA?888;:;===;;8;;58;7;>?<<=
A<<;<<;444800
@T6XXI:00091:00124
GGGAGAAGGGAGAAAGGAAGGACGGTGTGCTGGGAGAAATGCACAGAACTATTCTCCCTCTCCCTATAGTGAGTCGT
ATTAGAATTCCCC
+
DA?AA@>BB>BBCC?C@CDC>AAC@CCCBA@>@<@ACG@C@BFBCCC@CBBB?AA??6>@==?9?ABCDCCCCCBBA
AAABBE@@>BCC8
@T6XXI:00091:00136
GGGGAATTCTAATACGACTCACTATAGGGAGAGGGAGAATAGCGTTACGTTATGTTGTGCGCCTTTTTCTTCCTTTC
TCCCTTCTCCC
+
BDE7A<>=>>>;9;<<<=CCCB778777.777=?>C=?=9A@@@@>@@BA>A???>AA@?>;24444,4?;@=AH99
99A7=;<;<=:
@T6XXI:00091:00144
GGGAGAAGGGAGAAAGGAAGGAGAAGTCGCCCCAAACGATTTGTTGACTATTCTCCCTCTCCCTATAGTGAGTCGTA
TTAGAATTCCCC
+
CD>BCB@CB=BB??=B@B@B@BBB=>>@????8??<BBB@A=AA@@>>>@=;<<<<:<====;<<<<<<<==<<@??
?>???>@>AAA6
@T6XXI:00091:00148
GGGAGAAGGGAGAAAGGAAGGAGAATGCTAACGCGCAGAATTGGCGGACTATTCTCCCCCTCCCTATAGTGAGTCGT
ATTAGAATTCCCC
+
CFABBB?CH@BB?B>B@B@B@?775;:AAB@CCB??B@@>>>@@@@@B>@??>>::==;.33317;9=A===;;;@;
<@>A@@=?9;;;0
@T6XXI:00092:00108
```

```
GGGGAATTCTAATACGACTCACTATAGGGAGAGGGAGAATAGTCAACAAATCGTTTGGGGCGACTTCTCCTTCCTTT
CTCCCTTCTCCC
+
999/49@?ABB@CBBBBBCBA@A?<66659??CC>CBB>B;=?A=<4771:?==>9???5?BBBB>AAB==:=>AA=
A@AA<A>??AA<
@T6XXI:00092:00125
GGGGAATTCTAATACGACTCACTATAGGGAGAGGGAGAATAGTCGCACGACGTACTATTGCCACATTATCTTCCTTT
CTCCCTTCTCCC
+
AAA9ADC@CAA@?@?BBBB?A><99166.7?>>@=AAA?@588A@?BCCCA?BAAC::::C>??@B??=<=;=7;;7
<;?A:=:<;;;4
@T6XXI:00092:00142
GGGGAATTCTAATACGACTCACTATAGGAGAGGGAGAATAGTCAACAAATCGTTTGGGGCGACTTCTCC
+
A?:4?5469>@@@AABCCCCC>996..,0;;??<A??=<@@A??=?AA<BBBAB>BBB8B??>C@CBB5
@T6XXI:00092:00146
GGGGAATTCTAATACGACTCACTATAGGGAGAGGGAGAATAGTCAACAAATCGTTTGGGGCGACTTCTCCTTCCTTT
CTCCCTTCTCCC
+
999/4298@CCACB@@@AB?CAB93167.7>>AA>C??=====@@>???<BCCCC8?::6??AAC?CCDBA>@=@B=
A==<7<;=??C;
@T6XXI:00093:00101
GGGAGAAGGGAGAAAGGAAGGGGAGAAGGTAGTCGACTCGACATCTGTGGCTTCACAGTACAGAGGGACTTCCAAAG
CCGCAGA
+
CECAA@@BC@@@@E>@=@@BBC9B9>:9>>0009::::E@<<?88:CA=><>?>>><??==96330-
4:=;?@CD@BB?CBAAA
@T6XXI:00093:00114
GGGGAATTCTAATACGACTCACTATAGGGAGAGGGAGAATAGCGCGTTACCAGTGCCACTCCCCAAGTTCTTCCTTT
CTCCCTTCTCCC
+
CBB8@=@?BBB@BBBBABCCCCCB@666.7==BC>CDEA@???=<AA?8888@@@C=??BBCC8@?AA>??<?>AA<
@@AA<@=@@AB=
@T6XXI:00093:00121
GGGGAATTCTAATACGACTCACTATAGGGAGAGGGAGAATAGCCTTTATATCTCCCCTTGCGCGCTACACTTCCTTT
CTCCCTTCTCCC
+
CDC:HAABACCC<<<GAA@BAA>@877<6==<?@<BBB?@@>B377.7777>0000+02390/.:::///4555774
:@AA;@;<<;;5
@T6XXI:00093:00131
GGGAGAAGGGAGAAAGGAAGAACAGGCTCATTCGGAAGATGATGGGGCTATTCTCCCTCTCCCTATAGTGAGTCGTA
TTAGAATTCCCC
+
DB???=@AA====A?B@A@?B?BBB@@@BDC@BB?B?BBEBDBBBC:CCCC@CBBB?BBAAA>BBBBA@@CBBBAB?
A>@@B=?38880
@T6XXI:00094:00103
GGGGAATTCTAATACGACTCACTATAGGGAGAGGGAGAATAGAGACGTATTCGTTTGCGCTCTGTTCACCTTCCTTT
CTCCCTTCTCCC
+
BB@9B?B@BDC@CGBDBAAAA?9?8677.7>>>B=????@A@?@AAA?=8=?AA;4889;>?===<=999=?A>AA:
AA;=5;:<><<:
@T6XXI:00094:00105
GGGGAATTCTAATACGACTCACTATAGGG
+
688*/,/,///76444443.43.....30
@T6XXI:00094:00111
GGGAGAAGGGAGAAAGGAAGCATGAGTCG
```

```
+
;;649=?BC>CA>>11--8+******233
@T6XXI:00094:00113
GGGGAATTCTAATACGACTCACTATAGGGAGAGGGAGAATAGTCAACAAATCGTTTGGGGCGACTTCTCCTTCCTTT
CTCCCTTCTCCC
+
AAA6@@B@CCC@BBBBBBBCC?;77161.7>>>A>BDC@A:AA@@>AAA<B?>9;88685????C>?@A7859>AA<
@@@A<A<?@AA<
@T6XXI:00094:00117
GGGAGAAGGGAGAAAGGAAGCTAGTCACTCGAGATCTGCGGCTTTGGACAGTCCCTCTGTACTGTGAAGCCACAGAT
GTCGAGTCGACTAGCTTCCTTTCTCCCTTCTCCC
+
DDACCC>A@:>99918549@@@@??7777:@AAAC@CCCC@CCC?B?AAABBBA=BC:;;;///:8>4888<?AA@?
??AA?==?AA@;<<4744:>>:@>>>143488:4
@T6XXI:00094:00121
GGGGAATTCTAATACGACTCACTATAGGGAGAGGGAGAATAGTTACATCTTAGCCGGCGTCCCTCTATTCTTCCTTT
CTCCCTTCTCCC
+
@@?4><@@CBB@BB@@AA???AB?;111.9=?CE>CAA?@===;;;<=>A>===>A?AB@@@<??:6::5878/331
7@;A6;78444-
@T6XXI:00094:00140
GGGAGAAGGGAGAAAGGAAGCTAGTCGACTCGACATCTGTGGCTTCACAGTACAGAGGGACTGTCCAAAGCCGCAGA
TCTCG
+
CDCCB@644-999909<>;>?@@@BBBGCCCB@@?:::??B?BC@CBBB@@A;74000-
49;;;=9==7=C?AAA???@@@<
@T6XXI:00094:00144
GGGAGAGAATTTGGTAATGGGCCGAAGTATTACCGGGAACTGGGCCGCCGGATCCCTTATATCTCTCTCCCATAGTG
AGTCGTATTAGAATTCCCC
+
AHBCCAAB@BC>??=;9333,9?BFACDAA?AB@B?;?@BCCC>CABB@C?CCCA==;=>>:<@BA@AAC<AAACCC
CB;<<DCC@CCC:>4888.
@T6XXI:00094:00145
GGGAGAAGGGAGAAAGGAAGCTAGTCGACTCGACATCTGTGGCTTCACAGTACAGAGGGACTGTCCAAAGCCGCAGA
TCTCGAGTCGACTAG
+
BDAAEB>BB>BA@@:@=?<@B@;;=/0000/00811669BC?CD@CCBA<<ACA=9999=@@A@C?AA<@@>AA:<;
A=858@@@<==@===
@T6XXI:00095:00117
GGGGAATTCTAATACGACTCACTATAGGAGAGGGAGAATAGTTTTTCCCTTCACTAACCGAACTGCACTTCCTTTCT
CCCTTCTCCC
+
999/9597<A@@CD<<=DCCA>@>>0/-
0>>BE?BBDB?<<:::9.:;:=8<;:::,0,..,/>77111013;=.:69B:??878@:
@T6XXI:00095:00119
GGGAGAAGGGAGAAAGGAAGTGCAGTTCGCGGTTAGTGAAGGGAAAAACTATTCTCCCTCTCCCTATAGTGAGTCGT
ATTAGAACTTCCCC
+
DBABAA?@@=@@@BBB>=<=BBCCBB>BBBA>@?AAACB?>@;>?BB2CDBB?B???9??BB>;>;=<<<<=<@@<<
<;8:=:2443444-
@T6XXI:00095:00137
GGGAGAAGGGAGAAAGGAAGGAGAACGCGCACCCGAACCAACGGTGATCTATTCTCCCTCTCCCTATAGTGAGTCGT
ATTAGAATTCCCC
+
CC?CCDACC>?@CB>B?B?B@CB?>CCCCBBCCBBB?A=ABACBDCBCCCCC@C@??<@BBCC?CBAACAAAAA@A?
??;?AA?AABEC8
@T6XXI:00095:00141
```

```
GGGAGAAGGGAGAAAGGAAGCTAGTCACTCGAGATCTGCGGCTTTGGACAGTCCCTCTGTACTGTGAAGCCACAGAT
GTCGAGTCGACTAGCTTCCTTTCTCCCTTCTCCC
+
DE>AAA>AA<@BBB=BAC?A@CCCC:::>8??????@BDCBCCC?B?BBBCBCC>@C@888AA???B?@@<<<=@@@
===@AA==988??8788;8;A:??@A<A>???D;
@T6XXI:00096:00100
GGGAGAAGGGAGAAAGGAAGCTAGTCACTCGACATCTGTGGCTTCACAGTACAGAGGGACTGTCCAAAGCCGCAGAT
CT
+
EK@CBB>@@9@@@@<@=@=@A:78?79188=696777?BC@BG?BB??>>A@>8300-
4::=@@=@C?A@?????A<<?
@T6XXI:00096:00108
GGGAGAAGGGAGAAAGGAAGAATAGCTCACGGTGGGAGATGAAATGGCTATTCTCCCTCTCCCTATAGTGAGTCGTA
TTAGAATTCCCC
+
CC>B<98<C<ABBB=B?B?BB?BBBB@@CBB@BBB>BBBBBBB@BB@BBCB?BCCC@BBBCC>C:;;CCCCDC>:;;
;8::?;65:??0
@T6XXI:00096:00124
GGGGAATTCTAATACGAACTCACTATAGGGAGAGGGAGAATAGTCAACAAATCGTTTGGGGCGACTTCTCCTTCCTT
CTCCCTTCTCC
+
=@C9?=@A99;<81///,/4..1773111,11./:77=>///::@C>A@A7:::;C>CCC7A@?7741667934389
788=;=<4..3
@T6XXI:00096:00127
GGGGAATTCTAATACGACTCACTATAGGGAGAGGGAGAATAGTTGCGCTCTAAATTTCTCCGGACTATCCTTCCTTT
CTCCCTTCTCCC
+
CBB9C79;<CC@CCBBBBBBBB?@;666-6=76:7:AA@ACCC=@@8=9@@BA7??:778<?=898@=>8;7:/44-
4444-49;///,
@T6XXI:00096:00134
GGGAGAAGGGAGAAAGGAAGGAGAAGTCGCCCCAAACGATTTGTTGACTATTCTCCCTCTCCCTATAGTGAGTCGTA
TTCAGAATTCCC
+
DD@CCCBCC>CCCC?B?B796:@?;>?@8888.87/397BC:;;=??BBB@89:AC?=<<<C@AAA???@@@?@>9/
/,..1133,/.-
@T6XXI:00096:00137
GGGAGAAGGGAGAAAGGAAGGAGAACGCGCACCCGAACCAACGGTGATCTATTCTCCCTCTC
+
CC@CCC@CJ@AA@B>C>@=@CCCC:==BBCCBB?CE@C@CACCACDBBCC@A=?CCC>BAAB
@T6XXI:00096:00140
GGGGAATTCTAATACGACTCACTATAGGGAGAGGGAGAATAGCTGCCGATTGTCTCACGTGGCTCTTCTCTTCCTTT
CTCCCTTCTCCC
+
BD@7@6;:<BBAECCCCBB9::CAA6695:AACC>CAB@@=99=BA@BAA?A99:?A<?=C>@@AA?@????A>A=;
7775487.//:6
@T6XXI:00096:00144
GGGAGAAGGGGAGAAAGGAAGGACAGTCCATCTGTGGCTTCACAGTACAGAGGGACTGTCCAAAGCCGCAGATCCTC
GAGTCGACTAG
+
:909@>5999,4;;;:C?B=@<888?7857888@AD@CC?BBCC?ACA<<98=7<?>==?8;;6<=<@>888..,/:
87:;A@A>?;8
@T6XXI:00097:00110
GGGAGAAGGGAGAAAGGAAGCGTGTAGGTGGGTGGAATGATTGAAAACTATTCTCCCTCTCCCTATAGTGAGTCGTA
TTAGAATTCCCC
+
EE@BCB@CC?BCBB>B@CAA@ACBCDCB@@@<@C>A?A@;;;;BBB8AAAEBCCEC@BB@@?<>??B@@@CCC???:
:5:>?;<<==A5
```

```
@T6XXI:00097:00119
TGCGGCTTTGGACAGTCCCTCTGTACTGTGAAGCCACAGATGTCGAGTCGACCTAGCTTCCTTTCTCCCTTCTCCC
+
@CCCABBB>C?DFCBBCC@CC@@@BDCA@?@BCDACCCC@C@AAA@CAA?=?;?@<;>;<;>>8<=>>.747===6
@T6XXI:00097:00120
GGGGAATTCTAATACGACTCACTATAGGGAGAGGGAGAATAGCTCCCGATTGGATGACTCGCGCGTGCCTTCCTTCT
+
499299@<ABB?CCIBBBBA87:A@?=?6=>===677744==?BCC>:;;AC:::::@:==><<5110*+*+00*++
@T6XXI:00097:00124
GGGAGAAGGGAGAAAAGGAAGCTAGTCACTCGAGATCTGCGGCTTTGGACAGTCCCTCTGTACTGTGAAGCCACAGA
TGTCGAGTCGACTAGCTTCCTTTCTCCCTTCTCCC
+
94-
444249694999/954248777911111116669?>ABC@CDB=B?BAAA@@@;>B<588ABA?>A?AA<?===@@@?
@@????<<;??;;?;=:==7:8896;8;=AD>
@T6XXI:00097:00134
GGGGAATTCTAATACGACGTCACTATAGGGAGAGGGAGAATAGTCAACAAATCGTTTGGGGCGACTTCTCCT
+
444,424244488449993387788983З,3<:::,00.3.//.10/00307377>=@CD849...,,,,*,
@T6XXI:00097:00135
GGGGAATTCTAATACGACTCACTATAGGAGAGGGAGAATAGTCAACAAATCGTTTGGGGCGACTTCTCCTTCCTTTC
TCCCTTCTCCC
+
9>>4?69:<@A<A<<<BBBBB>A;;00,0<;>@<ABC=<<?><<:=?A=D><<C?ECC9CC??=:<00,/,/47616
/33,0/30353
@T6XXI:00097:00142
GGGAGAAGGGAGAAAGGAAGGGCAAAAACCTTGAGAATAGTTAGCAGCTATTCTCCCTCTCCCTATAGTGAGTCGTA
TTAGAATTCCCC
+
DCCCDC@BC?CAAA=A?AACC?BCCCC2C@C?AACD@CBAA>A@@ACAAADCEDCIBAA;;;8;@ABFC<<<AAD?@
@@BCD>B=AAA8
@T6XXI:00097:00148
GGGGAATTCTAATACGACTCACTATAGGAGAGGGAGAATAGTTTTTCCCTTCACTAACCGCGAACTGCACTTCCTTC
TCCCTT
+
DDB8B8959989ABBCCDACA>@<;00,.:066,16674403000+08+0/016667?=@8:?>;;86000,0,0,.
.;6274
@T6XXI:00098:00100
CTGCGGCTTTGGACAGTCCCTCTGTACTGTGAAGCCACAGATGTCGAGTCGACTAGCTTCCTTTCTCCCTTCTCCC
+
CCCCB@BBB>D>@@CBBBB>AECBAA?@@BCCACB@BBB@@?ABBBCCBBBBCCCBCA?BABC@::;C<@=?:771
@T6XXI:00098:00119
CTATCACTCGAGATCTGCGGCTTTGGACAGTCCCTCTGTACTGTGAAGCCACAGATGTCGAGTCGACTAGCTTCCTT
CTCCC
+
AAA?>=?AA=@?B@>;>11,-
8888888/1/7::7>ACCBBBA?<<<=C@CBACCBAA@@=?:<9?<830..,.,,*,,,,*
@T6XXI:00098:00128
CTAGTCACTCGAGATCTGCGGCTTTGGACAGTCCCTCTGTACTGTGAAGCCACAGATGTCGAGTCGACTAGCTTCCT
TTCTCCCTTCTCCC
+
C:///1/4=CBECBDBBBBB@BBB>B>BBBB?@C>?@@@@<<<?BBA>@C@BCCBBBBBAAAA@BBAAAAABB?B?A
@<????8;:<=>?7
@T6XXI:00098:00133
GGGAGAAGGGAGAAAGGAAGCTAGTCGACTCGACATCTGTGGCTTCACAGTACAGAGGGACTGTCCAAAGCCGC
+
DC?BBB@BC>BBB?:=;=<?@B:;;CCCB@CGDC::7:@>>;?@=@CDCCBC?=4000-4;;@AA>@@;@A@AB
```

```
@T6XXI:00098:00143
CTAGTCGACTTGAGATCTGCGGCTTTGGACAGTCCCTCTGTACTGTGAAGCCACAGATGTCGAGTCGACTAGCTTCC
TTTCTCCCTTCTCCC
+
C1111888?BAB:=<CCAA@CADCD8<;;<CABBD@C???BCCEC?@@?@B@CCCBBBCC@@@C<?=CCCCCCC?B?
BD?BCBE?D:;8AC:
@T6XXI:00099:00104
CTAGTCGACTCGAGATCTGCGGCTTTGGACAGTCCCTCTGTACTGTGAAGCCACA
+
EAAACC<>>FCCCBBB??>?B>@B@<B?A@@B:::5:?BBBBCC@B@@==FBDCC
@T6XXI:00099:00111
GGGAGAAGGGAGAAAAGGAAGCTAGTCGACTCGACATCTGTGGCTTCACAGTACAGAGGGACTGTCCAAAGCCGCAG
ACTCGAGTCGACTAGCTTCCTTCTCCCTTCTCCC
+
//+//6,//+/4444,4992433774......4.....1711067769::<@>996;;848447=?<?C=AA;888/
./8;;;<=<@844;4/1-1-1-1;;>,1,1444-
@T6XXI:00099:00118
GGGAGAAGGGAGAAAGGAAGGAGAGGTC
+
BEB>=<@CD@CCCC?>;<,-+-,,0*,,
@T6XXI:00099:00136
GGGGAATTCTAATACGACTCACTATAGGGAGAGGGAGAATAGCTACGCTCTATTCGACACATGATATTCTTCCTTTC
TCCCTTCTCCC
+
DDC:B=?=AAAACB@@CBBAB?><?667.7==?D@CBA?@=A???A??AB@@@>AA@A@AA@?>CA=<<694/33-
499:-434443+
@T6XXI:00099:00137
GGGGAATTCTAATACGACTCACTATAGGGAGAGGGAGAATAGGGCCGACGTCGCATGCGTCCCGG
+
999/42424<FBCCCCBBAAAACC@@@E=????C>BBB@=;;;-424444443.373../55335
@T6XXI:00099:00141
GGGAGAAGGGAGAAAGGAAGGGGAGAAGGGCTAGTCACTCGACATCTGTGGCTTCACAGTACAGAGGGACTGTCCAA
AGCCGCAGATCACGAGTCGACTAG
+
DD@AAB@BD=?AAC=B?B@CCB8DDB@??;BBBBCBCBA@>=449;;CCDBCB>BBBCAABEDAABB>BBBCFB@BB
=BB@BBBBBB?A>>>BBBB>ABCF
@T6XXI:00099:00144
GGGAGAAGGGAGAAAGGAAGGATAACCGGGAGCGTCAAAGTACTTGGCTATTCTCCCTCTCCCTATAGTGAGTCGTA
TTAGAACTTCCCC
+
DH?:<<@CC@AAAG@C?B>B@CCB>B?CCBCCB?@7996:A>?A8:::CCC?CCCC>@B???9;==?????@@@===
@>@BBBA@:<<;4
@T6XXI:00050:00168
GGGGAATTCTAATACGACTCACTATAGGGAGAGGGA
+
>>=6@=@=B@@;@AB<<:CBB9>;66662;300000
@T6XXI:00050:00176
GGGAGAAGGGAGAAAGGAAGGAGAAGTCGCCCCAAACGATTTGTTGACTATTCTCCCTCTCCCTATAGTGAGTCGTA
TTAGAATTCCCC
+
CC>DDCBCC=A;99-
9<><@>A?@=?@?8888.880896>@:@@>@???AA>AAA@;@@@@=;=:66====<<=@=9=>A=:8:/444-
@T6XXI:00050:00193
GGGGAATTCTAATACGACTCACTATAGGGAGAGGGAGAATAGTCAACAAATCGTTTGGGGCGACTTCTCCTTCCTTT
CTCCCTTCTCCC
+
```

```
CGC:C8;;<==@BCBBCCCBBAD?>777.7?7775:CA>A==>CC@?A?<B@?@E?DCC7@@@@>?>><././78;9
;9>C>C;>?@A;
@T6XXI:00051:00190
GGGAGAAGGGAGAAAGGAAGAACATGGCGCAGGAGGATCGGGATGCACCTATTCTCCCTCTCCCTATAGTGAGTCGT
ATTAGAATTCCCC
+
AA<@BB=@A>ABCC@C=?=@@<??@@>BB;;;?:;5;B@>@<B?8@CB@CBA>A@@@<@AABA<A;<:??@BC:::?
??:?:?>@<AAA6
@T6XXI:00051:00193
GGGAGAAGGGAGAAAGGAAGGGGAGAAGGGAGAAAGGAAGCTAGTCACTCGACATCTGAGC
+
EDBAAABCC>A@@C<@=A@CCE9BBB@CE>C>;70:,0,0>:96??9>?<600+++0++++
@T6XXI:00052:00166
GGGAGAAGGGAGAAAGGAAGGACACACGGCTGGGGAAAACGCACGCACTATTCTCCCTCTCCCTATAGTGAGTCGTA
TTAGAATTCCCC
+
CEDAA@>?B>BBCC>C@B>B?BBBBCBC@AAAB@4@A@6ACBBBCCBBA@@@BBBB>BBBCC>BBC?<<ABBB???B
B@A@@=A>BCA6
@T6XXI:00052:00197
GGGAGAAGGGAGAAAGGAAGGAGAAGTCGCCCCAAACGATTTGTTGACTATTCTCCCTCCT
+
CB@BB@@BB@BBBC?B?B?B?ACC@@@AAACB7BB<BCC>B:?9>?@@?:6240--+,55,
@T6XXI:00053:00164
GGGAGAAGGGAGAAAGGAAGCTAGTCACTCGACATCTGTGGCTTCACAGTACAGAGGACTGTCCAAAGCCGCAGATG
TCGAGTCGACTAGCTTCCTTTCTCCCTCTCC
+
BE?CBA=@@:>99909596;AABB>;00.......55;8<:8C@BBBCBBB>=://./8:85=<@A<A=988@AAA=
==@A@@888:44444/34:848883+0344/
@T6XXI:00054:00153
GGGGAATTCTAATACGACTCACTATAGGGAGAGGAGAATAGATCACCGTTGGTTCGGGTGCGCGTTTCTCCCTTCCT
TTCTCCCTTCTCCC
+
999/4:;:=BA@CABBBBCBC?A@@=?B9790/,.::687<?@?<?:660101;AACACA933311,1111,10101
1,16344=<555=:
@T6XXI:00054:00170
GGGAGAAGGGAGAAAGGAAGCTAGTCACTCGAGATCTGCGGCTTTGGACAGTCCCTCTGTACTGTGAAGCCACAGAT
GTCGAGTCGACTAGCTTCCTTTCTCCCTTCTCCC
+
BC?BCA>@@19999142823;<9;=B:889???7:7>AAB>:::11:?7::??C<B@AA998?<==B??CEAB=?<?
??888>==6?<<<?>@<?384-4444-4/300;4
@T6XXI:00054:00193
GGGGAATTCTAATACGACTCACTATAGGAGAGGGAGAATAGCTGTAATTTCCCCAGACAGCGCCGTAGTCTTCCTTT
CTCCCTTCTCCC
+
CDC9B?B@DCB@@@AAA?>>B:995//-
0>>BC@CC@=@9==BCCGADC;???7??@@<558@>@@@@@<;858=>:??==:=378@@:
@T6XXI:00055:00160
GGGAGAAGGGAGAAAAGGAAGCTAGTCACTCGACATCTATGGCTTCACAGTACAGAGGGAACTGTCCAAAGCCGCAG
ATCTCGAGTCGACTAGCTTCCTTTCTCCCTTCTCCC
+
@;599964;:@9999,4242373334:/.....444476110160116<><@@@:887,/,.1411,00+0446454
>>?<?===@@AB<<888<9<?AA;@AAC14/4330,
@T6XXI:00055:00162
CTAGTCACTCGAGATCTGCGGCTTTGGACAGTCCCTCTGTACTGTGAAGCCACAGATGTCGAGTCGACTAGCTTCCT
TTCTCCCTTCTCCC
+
```

A11//7/44BBB99<CCDA@=@DC?D<?ADCACB>BCDCB@@B=@=@>AC@CCCAAABBBBCCCCBBBBCCBD?B@C
C>CCCC<>99444,
@T6XXI:00055:00166
GGGGAATTCTAATACGACTCACTATAGGGAGAGGGAGA
+
:;;,429249=?>BA<;;7771997111,17111,110
@T6XXI:00055:00169
CTATCGACTCGAGATCTGCGGCTTTGGACAGTCCCTCTGTACTGTGAAGCCACAGATGTCGAGTCGACTAGCTTCCT
TTCTCCCTTCTCCC
+
A777/////8:BBBCBBBCB@BBB=B@AA?>AAA=AC@@@??>=A?A=?B?CCBBAAACBB@CCBBBCCBBBB@C@C
C>BCBC>@>@AAA<
@T6XXI:00055:00186
CTAGTCGGACTCGAGATCTGCGGCTTTGGACAGTCCCTCTGTACTGTGAAGCCACAGATGCGAGTCGACTAGCTTCC
TTTCTCCCTTCTCCCTTCCTTCTCCCTTCTCCC
+
C1111>=:>EDCCBCCBBBABC@CBC>B89:>>77:.7?AB?@@C@BBA;=AACBHCBCEEC::;A@CBBAB@@?@A
CC>B???6979444-39;7;89044-49:89;9
@T6XXI:00056:00185
GGGAGAAGGGAGAAAGGAAGCTAGTCACTCGACATCTGTGGCTACACGA
+
CC@CBB=?><@B=@=C=@=@DBBC@>AABB@;;::76?>>90-,,****
@T6XXI:00056:00195
GGGGAATTCTAATACGACTCACTATAGGGAGAGGGAGAATAGTCTTCAGCCCAACATTTGTCCGATTCTCTTCCTTT
CTCCCTTCTCCC
+
BAA7A@B?CCB@B@@???BB?@;97111.7==?B>B:837888?AA@BACC>C>CCCE=?;:8:BC@A??@>@>@D>
@@BD;A:==>=7
@T6XXI:00058:00183
GGGAGAAGGGAGAAAGGAAGCTAGTCACTCGACATCTGTGGCTTCACAGTACAGAGGGACTGTCCAAAGCCGCAGAT
CTCGAGTCGACTAGCTTCCTTTCTCCCTTCTCCC
+
EC>@>>59919999142843@A??A:00//....///?@@8:<?GDCA@?BAB?77717==>@@>AA;@@>@@@@@@
??????CCAA@????AAA>AA7;;>?9:0/0:;9
@T6XXI:00058:00199
GGGAGAAGGGAGAAAGGAAGAGGCGCACAGAAGGGTCGGGATTCGTGCTATTCTCCCTCTCCCTATAGTGAGTCGTA
TTAGAATTCCCC
+
AAAAECACC>CCBB>B?BABBB?@ACBBB@B?BC>CCBC?BBCBCBBBCCC@CCB@;BBCCC>CC@@@@AAAAA@AA
@>@AA>A;@@@6
@T6XXI:00059:00153
GGGAGAAGGGAGAAAGGAAGTATTTGGGAGCGTCGATTCATGTCGTGTCTATTCTCCCTCTCCCTATAGTGAGTCGT
ATTAGAATTCCCC
+
DE@CCB@BD>DBBB=?=?@A@AAC>BB>BCB?BC=?A@B@><<BBCCBBBBB@CBCB>>@@@@<>:::???@@AAA;
:;@CCC@EDCCC:
@T6XXI:00059:00159
GGGGAATTCTAATACGACTCACTATAGGGAGAGGGAGAATAGTTTTCCCTTCACGAACCGCGAACTGCACTTCCTTT
CTCCCTTCTCCC
+
ECC7@2459>>;AAABC@@@CCBAB9::.7<<?A>ABECDA@@@@5@A<A?ABBBC@C@BBCC?AAB@BAA@B6::7
:?@C>D@BAAAA
@T6XXI:00059:00177
GCTTTGGACAGTCCCTCTGTACTGTGAAGCCACAGATGTCGAGTCGACTAGCTTCCTTTCTCCCTTCTCCC
+
DDCKBBABDBBDCD@CCCB;;;BBBACCDC;<<DBB@?;;9AABBBBBBC??@=?>AB6;<BB>B:::BC<
@T6XXI:00059:00194

```
GGGAGAAGGGAGAAAGGAAGGAGAAGTCGCCCCAAACGATTTGTTGACTATTCTCCCTCTCCCTATAGTGAGTCGTA
TTCAGAATTCCCC
+
CB>?9929@:ABCH@CDD<<<;CC@CC@;888.8BADCB>B?BC?CB??<<9<>>AA@===C<@@?@=888==@;:8
;.//;;;?;@DC6
@T6XXI:00060:00172
GGGGAATTCTAATACGACTCACTATAGGGAGAGGGAGAATAGTCAACAAATCGTTTGGGGCGACTTCTCCTTCCTTT
CTCCCTTCTCCC
+
CDH:C?D@BAB=@ABBCBBBBBA?>777.7?7776:?@=A@@@AA>A@@;B:::B>BBC9CBFBC@CCB?>8:244-
8=;=:@=@@AC9
@T6XXI:00061:00165
GCTTTGGACAGTCCCTCTGTACTGTGAAGCCACAGATGTCGAGTCGACTAGCTTCCTTTCTCCCTTCTCCC
+
@A>?5=6;:AA@@@:@A;;:<CBBBBB@BB@BCI@@;::;AA@?@@BCCCB:<8;=>>:>8880;5;BCK>
@T6XXI:00061:00170
GGGAGAAGGGAGAAAGGAAGAGATAACGTACACATACACATGGGTAGCTATTCTCCCTCTCCCTATAGTGAGTCGTA
TTAGAATTCCCC
+
ED?BBC@BB??@?B=B?@??>880.,/.....369;9444444-
44433331376626616656BBCAAAA@ABB@@@=?>=9?5999/
@T6XXI:00061:00173
GGGGAATTCTAATACGACTCACTAGTAGGAGA
+
///*/,424@@>BBBCC@A88,,,,,,,*,0.
@T6XXI:00061:00181
GGGGAATTCTAATACGACTCACTATAGGGAGAGGGAGAATAGTCAACAAATCGTTTGGGGCGACTTCTCCTTCCTTT
CTCCCTTCTCCC
+
CBB7A=B>BCC@CCBBBBBAB<;9=166.7?BBC>BCC?A=??@@>@@A<CBBBC<BBB7::CCC?ABD@A?A<??;
?A@A<A9<<==7
@T6XXI:00061:00182
GGGGAATTCTAATACGACTCACTATAGGGAGAGGGAGAATAGTCAACAAATCGTTTGGGGCGACTTCTCCTTCCTTT
CTCCCTTCTCCC
+
CCC9C@C@CBB@BBBBBBBBBCB97111.7>>>@@BCA>A8>@@@?8871:=<<<6=>>5??CCC?BBC=<8;8;;7
<A@A;A8:9AA<
@T6XXI:00061:00188
GGGAGAAGGGAGAAAGGAA
+
AB<>9959@:>911-1,--
@T6XXI:00061:00196
GGGAGAAGGGAGAAAGGAAGCTAGTCGACTCGACATCTGTGGCTTCACAGTACAGAGGGACTGTCCAAAGCCGCAGA
TCTCGAGTCGACTAGGGGAGAAGGGAGAAAGGAAGGAGAAGTCGCCCCAAACGATTTGTTGGACTATTCTCCCTCTC
+
CC@CBA>@=9>?>>9>4958@>::>000<:::071116=?A=AA@ADFCCCBB?=5;;6;>@AAA>AA<AA>A=9=A
@======@@@@????@A6@@@>AB;??@C7633115,,,+,000@AA6AA<?31>@6<<,1,//;7>=B@=9-3733
@T6XXI:00062:00152
GGGAGAAGGGAGAAAGGAAGCTAGTCACTCGACATCTGTGGCTTCACAGTACAGAGGGACTGTCCAAAGCCGCAGAT
CTCGAGTCGACTAG
+
AA?ACC@CC:A;88-
9<>;>@;88>==77:77??AAACCC@CB?BCDAA?BBA>>><7>@???A>AC>??<?AA@A@???A???A???AAA
@T6XXI:00062:00161
GGGAGAAGGGAGAAAGGAAAGCTAGTCACTCGACATCTGTGGCTTCACAGTACAGAGGGACTGTCCAAAGCCGCAGA
TCTACGAGTC
+
```

```
EE>B<<89@:A99914244-
333336:...........67@<>??CA@AAA:::<@@A<??@@==;=C=?@:<<8963/++++**00
@T6XXI:00062:00165
GGGGAAGGGAGAAAGGAAGGAGAAAACGTGAGCCAAGATCTAAGCTCTATTCTCCCTCTCCCTATAGTGAGTCGTAT
TAGAATTCCCC
+
FCC8E=AA?AAAD>B>BDC?B@CCC9BBCCBBB?C@BBBBBB@CCCA@?A>B@@@:88443-
44;;;>????@@@A@@AAA<?<>?>4
@T6XXI:00062:00166
CTATCGACTCGAGATCTGCGGCTTTGGACAGTCCCTCTGTACTGTGAAGCCACAGATGTCGAGTCGACTAGCTTCCT
TTCCTCCCTTCCTCC
+
B;;6/////8=??AADBBA@=@BC?B?CCCBBCC>CCC?BBBBB@==>AC@BBCAA;<;A??=:;9<<777//5444
4+.,,2700*,*++*
@T6XXI:00062:00185
GGGGAATTCTAATACGACTCACTATAGGAGAGGGAGAATAGCCAGTACATTGGCAACTGAGCTGTTCCCTTCCTTTC
TCCCTTCTCCC
+
BGC6@=A?BBBACCCCC:;;C>>;=/0,0::>>:A?<9<998?ABAA@@@6869A>@BCCC;;:CBCD>C?@58818
?;?7=7:8441
@T6XXI:00063:00158
GGGAGAAGGGAGAAAGGAAGGAGAAGAGCTGCACACATTATGCAATGGCTATTCTCCCTCTCCCTATAGTGAGTCGT
ATTAGAATTCCCC
+
DE@FEC@CBBBBBB>B?@>BACAA>@@@@BBB>BB?>>>@@A??>?<=?AAA@CAAA=@?@ED>C?@@<A@@@@AA=
AA>AAA>B?BCC7
@T6XXI:00063:00165
GGGGGAATTCTAATACGACTCACTATAGGGAGAGGGAGAATAGTCAACAAATCGTTTGGGGCGACTTCTCCTTCCTT
TCTCCCTTCTCCC
+
9444+489:@HD@CCDCCEBBCB??=766.7??BC?DBA>A@@?@@>???;ACBBC<AAA7AAABC@BBC5424344
-333303/4000+
@T6XXI:00063:00167
GGGGAATTCTAATACGACTCACTATAGGAGAGGGAGAATAGCTTAGACCTTGGTGGCATTCTGTTCTCCTTCCTTTC
TCCCTTCTCCC
+
CB@7B8;8<CA@CCBABCCD8/0;://,0>>BC>CFB=B9<9>>>>?A?@=A=9738?@=@AAA>@AA;=;=>@B:A
77814344881
@T6XXI:00063:00197
GGGGAATTCTAATACGACTCACTATAGGGAGAGGGAGA
+
BBB9B@B?BCC@BAAAB????CB97111.7???C?DDA
@T6XXI:00064:00168
GGGAGAAGGGAGAAAGGAAGGAGCAATAACCGGTCAAAAAAGGCCAGGCTATTCTCCCCCTCCCTATAGTGAGTCGT
ATTAGAATTCCCC
+
CCA<<;ACE>BBBD@C@B=B@BBAB@BB>B=>B?899994*9<?=ACACCBB>?88888,7?@:?@@BBBCCCCCCB
B@?A@A<B=AAA7
@T6XXI:00064:00179
GGGGAATTCTAATACGACTCACTATAGGGAGAGGAGAATAGCAACG
+
BHH6>>C@@AADCBBBC>@>BACCBBBB<9?663<=974444+*+,
@T6XXI:00065:00150
GGGAGAAGGGAGAAAGGAAGCTAGTCGACTCGA
+
DB6?;964;-9//1,/,/,/444490..447/0
@T6XXI:00065:00151
```

```
GGGAGAAGGGAGAAAGGAAGCTAGTCACTCGACATCTGTGGCTTCACAGTACAGAGGGACTGTCCAAAGCCGCAGAT
CTCGAGTCGACTAG
+
EC?BBB>?=:=@==5>887;C@@@CAA@A@@;::AAABCG@CC@CBBBBBCCC<=@?<@A@@@A<??;?A>@@;?==
<@@=<<<@@AAA??
@T6XXI:00065:00167
GGGAGAAGGGAGAAAGGAAGCTAGTCACTCGACATCTGTGGCTTCACAGTACAGTCG
+
<:-4AA<>>:>988094958A??@CBA@???::7888BBB>BA@B=70000,0***+
@T6XXI:00065:00169
GGGGAATTCTAATACGACTCACTATAGGGAGAGGGAGAATAGTCTACCAAATTT
+
BBB9B>B@DCB@BBB?@@@B>@??966656=?BC>CBA@@<A<96003/7,10,
@T6XXI:00065:00174
GGGGAATTCTAATACGACTCACTATAGGGAGAGGGAGAATAGTTATACTGCACGTCCCAACAACCGCTATTCTTCCT
TTCTCCCTTCTCCC
+
DDC8@7959>@599AAA@ABB@C?:000+0.::;9>>@;:@:997///30003.--3+-,--
,02100112368:<BA:@@AA7=79444-
@T6XXI:00065:00184
GGGGAATTCTAATACGACTCACTATAGGAGAGGGAGAATAGTCAACAAATCGTTTGGGGCGACTTCTCCTTCCTTTC
TCCCTTCTCCC
+
CDD9C@GDCBC@CCCBA@@BA6463./,0<0775:?;56>>???>@AC;B???@:A?B7BBBBC@DCD;824/44-
8=;;:;/4--.,
@T6XXI:00066:00160
GGGGGAATTCTAATACGACTCACTATAGGGAGAGGGAGAATAGCACCAACTAATT
+
1///*/,499>@=@CABBDBCCBA?>776.777?BCCCA>@7/,,,+,5,++020
@T6XXI:00066:00165
AGTCACTCGAGATCTGCGGCTTTGGACAGTCCCTCTGTACTGTGAAGCCACAGATGTCGAGTCGACTAGCTTCCTTT
CTCCCTTCTCCC
+
BBCBADCCCBBACCCCCC@@>@<@>@CCCCCC>CBBA@@@CCCBB?BC@CC@BBCAAB@??@@?AAAA=<?;?=?A=
AABC9?44444-
@T6XXI:00066:00183
CTAGTCACTCGAGATCTGCGGCTTTGGACAGTCCCTCTGTACTGTGAAGCCACAGATGTCGAGTCGACTAGCTTCCT
TTCTCCCTTCTCCC
+
=;///6699BBBDCCBBBBB@BBB<B<?ABC===;?CC@B@BB>A??<?C@BBBCCBCBCCBCBBBCCCCBBB?B@C
C>CCCF?@====@;
@T6XXI:00067:00153
GGGAGAAGGGAGAAAGGAAGGAGAATGCGGCAAACTAAGTATGTCGCTATTCTCCCTCTCCCTATAGTGAGTCGTAT
TAGAATTCCCC
+
EHBAA@@CC>BBBB<?=?=@DCCC?BBBC@ACA<B>?=@CBBC>=??CCC??A?A>>>???<<??@AAAA@@???88
58?A>A;@AB6
@T6XXI:00067:00159
CTCGAGATCTGCGGCTTTGGACAGTCCCTCTGTACTGTAGCAAGCCACAGTGCGAGTCGACCTAGCTTCCTTTCTCC
CTTC
+
DCCCBCCBC;;<C@CCB>C@CCCBBCD?CBBBB;:8/---
,,*,,*....113;<96999753<@?A@ABED>ABCC?B?A
@T6XXI:00067:00161
GGGAGAAGGGAGAAAGGAAGAATAGTCCAGTGCAGGGAATCGCGAATCTATTCTCCCTCTCCCTATAGTGAGTCGTA
TTAGAATTCCCC
+
```

```
DDBBBB@BB>BBBB?B>B@@@>AABAA>ACCC????;>BBBB@8:47@@:::;CDDB@>??A<???@@@AAAA@?<<
<<>@A>A>@AB6
@T6XXI:00067:00172
GGGAGAAGGGAGAAAGGAAGCTAGTCACTCGACATCTGTGGCTTCACAGTACAGAGGGACTGTCCAAAGCCGCAGAT
CTCGAGTCGACTAG
+
KD?AAC@C>:A988-9;>;?AA@AAA>=;;:00////;>C;?@CCDA??@CCB?777-
4448@@?AB@AA<?<<<@??>?<<=?988??<<
@T6XXI:00067:00192
GGGGAATTCTAATACGACTCACTATAGGGAGAGGGAGAATAGCTTGTCATTTGATCAATGTCTACATCTCCTTCCTT
TCTCCCTTCTCCC
+
GCD:EAE?BBB@BBBBB@@AB@>97111.7===?<?CC?@@@@@?A@A:::8;CCEE?@====AAAA???<@;<;??
<?ABC<@<@8885
@T6XXI:00067:00194
GGGGAATTCTAATACGACTCACTATAGGGAGAGGAGAATAGTCGA
+
?BB4;79?@=8=@@@@@BBB>?<?;677.7>7736;344821--+
@T6XXI:00068:00150
GGGGAATTCTAATACGACTCACTATAGGGAGAGGGAGAATAGTTCCTCTGTGTCAAGAGCGAGTGTCTCCTTCCTTT
CTCCCTCTCC
+
499/499<@DC@BCDDBCCC;:8??777-
7>>BC>CCB=;;//..9;7;BB:77C>??<A?=<=7736444375.5428::,//.+*
@T6XXI:00068:00160
GGGGAATTCTAATACGACTCACTATAGGGAGAGGGAGAATAGTCACAATTCATTGGT
+
AA@7A<@=@CBBCCA@?>BBB@BB@66646??BC>CB?>>?9:/./9,747;;3;36
@T6XXI:00068:00163
CTATCACTCGAGATCTGCGTGGACAGTCCCTCTGTACTGTGAAGCCACAGATGTCGAGTCGACTAGCTTCCTTTCCT
CCTCCTCCTTCCTT
+
FEDAAAACBBABBCCBBBBBB>AACA@@@;B@B@<>ABBBBA>AC@CCCCC@@?BBBCBBBBBBBC??=>:><6:90
/,/95;:9;24243
@T6XXI:00068:00185
GGGAGAAGGGAGAAAGGAAGCTAGTCGACTTCGAAGATCTGCGGCTTTGGACAGTCCCTCTGTACTGTGAAGCCACA
GATGTCGAGTCGACTAGCTTCCTTTCTCCCT
+
C@>BAA=@C><==E?C@CACC;;;><77/.,...,.55../61018;;??AA???BD>A@@977<>;;;=8;;:?<<
<<<=>??<<<C999???>;77,//,/,,5+,
@T6XXI:00068:00186
GGGAGAAGGGAGAAAGGAAGAGAATAAACCAGGCGTAAACGTTAGATCTATTCTCCCTCTCCCTAATAGTGAGTCGT
ATTAGAATTCCCC
+
44-
49@<@@<999@<>;@>88:@?BBC>D@CC@BBA@?:99736111611099<77530+++*++*+039==<@@A???=
??@:=:=AA6
@T6XXI:00068:00197
GGGAGAAGGGAGAAAGGAAGAATAGCCTGAGGTTGGGTAGTGCGGAACTATTCTCCCTCTCCCTATAGTGAGTCGTA
TTAGAATTCCCC
+
BB>B@@;>A>999>9>;>;3713?AF=ACDHCCFCDAD==<AAA9;68>?>;=>>>6771;885AB=@@=BAAA::7
?584@<85555.
@T6XXI:00068:00198
GGGAGAAGGGAGAAAGGAAGCTAGTCACTCGAGATCTGCGGCTTTGGACAGTCCCTCTGTACTGTGAAGCCACAGAT
GTCGAGTCGACTAGCTTCCTTTCTCCCTTCTCCC
+
```

```
CD?AAA>AB1989914295:CCABBC::6::966>99AAA?ACCAC@ECCCBBB>BB@AAAAAA@@B??=;<<;<=>
=885@?=??@<;9444348;:49444,4/3000+
@T6XXI:00069:00161
GGGGAATTCTAATACGACTCACTATAGGAGAGGGAGAATAGATCACCGTTGGTTCGTGTGCGCGTTCTCCTTCCTTT
CTCCCTTCCTCCC
+
EFE9C;;9<BB@BCCCBCCBB6977//,000?B>CBA>AA==<;BB=AA?B?B?CBB;;;ABC??:?::59>B>BC<
>>;939,/,/993
@T6XXI:00069:00163
GGGGAATTCTAATACGACTCACTATAGGGAGAGGGAGAATAGGCGTTTCGTTTTTACGGCATCCTATTCTTCTTCCT
TTCTCCCTTCTCCC
+
CCC9C@B?BCCA@@>BCCC??@D?>111-
6<<BCABB@>@<@=?<<A<@A@@@@1???;??<<:<@@=<=:=@>@?AA;??@@;@=@===9
@T6XXI:00069:00170
GGGAGAAGGGAGAAAGGAAGCTAGTCGACTCGACATCTGTGGCTTCACAGTACAGAGGACTGTCCAAAGCCGCAGAT
CTCGAGTGGGAGAAG
+
CEACCB?BC>@@>>8;,1-
....37999@??<><;71166636;;=?BBBC??<?8838?@@@A?AA;@@>:7/..368844.///+---33
@T6XXI:00069:00195
CTATCGACTCGAGATCTGCGGCTTTGGACAGTCCCTCTGTACTGTGAAGCCACAGATGATCGAGT
+
EB=:111:::=?ABBCCCCC@CBB<B@BABBAAA>ACCCCCBBBC@A>?B?BC8490011..,,.
@T6XXI:00069:00198
GGGGAATTCTAATACGACTCACTATAGGGAGAGGGAGAATAGCCTGCAGGTCGAGTGTAGGACTGTTCCCTTCCTTT
CTCCCTTCTCCC
+
DJDDD>@>ACB@CCAAABBBB@<97111.7?ABD>DC@>@===<ABBCC?CCBB?<===A>A=>8848C7<:=?AA<
AAAB?A38844-
@T6XXI:00070:00155
GGGGAATTCTAATACGACTCACTATAGGGAGAGGGAGAATAGCGCAATTTGGACATGTGGCCGCTTCTCCTTCCTTT
CTCCCTTCTCCC
+
DDD9C=A?ACFBCCCCCCCC:::?>77:6:@@CC<@AC@C8==@=?:>>8=:=??BBBC@A>??>?<8858<=<?B:
>;<<7<<?AAH=
@T6XXI:00070:00156
GGGGAATTCTAATACGACTCACTATAGGGAGGGGAG
+
499/9<ABCBB@BABBCCCBAB@B717<6/000+,0
@T6XXI:00070:00177
GGGAGAAGGGAGAAAGGAAGGAGAAGTCGCCCCAAACGATTGTTGACTATTCTCCCTCCTCCTCATAGTGAGTCGTA
TTAGAATTCCCC
+
CEABBB@DD=BBBB>B?B?B@CBB>A?B7888.8A:><110110176100/00-40/.,./,-
2234559@@@AADAA;8665553271
@T6XXI:00070:00179
GGGAGAAGGGAGAAAGGAAGTGCAGTTCGCGGTAGTGAAGGGAAAAACTATTCTCCCTCCTCCCTAT
+
CD@AA@@DH@C;<<19<@8:;BBB@>;;;;<,1.4447977,1111+3777733330.,*,220,++
@T6XXI:00070:00180
GGGGAATTCTAATACGACTCACTATAGGGAGAGGGAGAATAGTCTTGTATTTTGTGCATCACTTTTCTCCTTCCT
+
CEC8B895;9;<ABBAA@CBB@?>=777.:AABD@?@=>@@?9667.-/-11.-006;;;61000+0//,/,/,/
@T6XXI:00070:00183
GGGAGAAGGGAGAAAGGAAGCTAGTCGACTCGACATCTGTGGCTTCACAGTACAGAGGGACTGTCCAAAGCCGCAGA
TCTCGAGTCGACTAG
```

```
+
@A8<<B?AA<BA>>9>8;7;B>;=>/00<00007??88@?@=?@=?AFCCCCC@>7331399>??<?A<A@>@A=@<
<=@<><;????@??>
@T6XXI:00070:00186
AGATCTGCGGCTTTGGACAGTCCCTCTGTACTGTGAAGCCACAGATGTCGAGTCGACTAG
+
C@@ACCBAA@ACCBCABBCDECC?ECABBCCC;<:B?AA?ABB??;<<CDCGCCBCCCC@
@T6XXI:00070:00196
GGGGAATTCTAATACGACTCACTATAGGAGAGGGAGAATAGTCAACAAATCGTTTGGGGCGACTTCCCTTCCT
+
BBB7B=@=@BB@DBBBCCBBB7664..,0;;>@>DCA>@CC???>@A@;A???A<AAA5??:::977+,+,+,
@T6XXI:00071:00159
GGGAGAAGGGAGAAAGGAAGGAGAAGTCGCCCCAAACGATTTGTTGACTATTCTCCCTCTCCCTATAGTGAGTCGTA
TTAGAATTCCCC
+
DDAAA@=@=9@@>>9A=@=E@CCC@A?>BBBB8AA<BDCCC<>A?BBCCB@?====;::?A=;=:::<ACAA99=??
?>@AB?A9<<<1
@T6XXI:00071:00167
GGGGAATTCTAATACGACTCACTATAGGGAGAGGGAGAATAGTCAACAAATCGTTTGGGGCGACTTCTCCTTCCTTT
CTCCC
+
BA@4@6;8<CC@DCA??>BBBCB?;66659?@DE>BB@=AAAA==:=@?:BB@@@9???6?CCCD@BAA?@>@>A=;
=@@?<
@T6XXI:00071:00191
GGGGAATTCTAATACGACTCACTATAGGGAGAGGGAGAATAGCCTCCGCATGTTCGAAAGTTAGTTCTCCTTCCTTT
CTCCCTTCTCCC
+
BA@4@<@=@BCBCBABBBBBBAB@A???97::BB>CB@>@888>BB?CCCCCC@ABBC?CB?CED@CAA>?;>3::6
:BAB=A?AAAA:
@T6XXI:00071:00192
GGGAGAAGGGAGAAAGGAAGCTAGTCACTCGACATCTGTGGCTTCACAGTACAGAGGGACTGTCCAAAGCCGCAGAT
CTCGAGTCGACT
+
CHCAAA@BA<A@==6;:<103>@BBBCCBA@:::;:7?==::;7;B@BBBBA@>773-
4;8;;@>@@<@@>@@@@AA@@@A778?;;;?
@T6XXI:00071:00197
GGGAGAAGGGAGAAAGGAAGGAGAACAACACGATCGGTTAAACGTAGCTATTCTCCCTCTCCCTATAGTGAGTCGTA
TTAGAATTCCCC
+
CDACCC?AA=@@@C>C?B?B@CBB=AAABBBCCCBC?A?BC>CBCBBBCCB?BBBB=BABCC?CA??@@A@@??=;;
<387@=:5;;;1
@T6XXI:00072:00153
GGGGAATTCTAATACGACTCACTATAGGGAGAGGGAGAATAGTTCTCGCCGCACAAAACGAACATT
+
A@@6@?B>AA@=@AABCCBBCCBA?===6777>@9@BBAAAAA><<<<<8;@877>>.:9978310
@T6XXI:00072:00154
GGGAGAAGGGAGAAAGGAAGCTAGTCGACTCGACATCTGTGGCTTCACAGTACAGAGGGACTGTCCAAAGCCGCAGA
TCTCCGAGTCCGACTAG
+
DCAAA?@BB<B@==<@@B?AB<;@@6;:B@@>;;:??<>?>::;5:>>?A;::@B;;<7<@?>??>@@<@A?@@?A?
???A<===<=<??AA<<
@T6XXI:00072:00159
GGGAGAAGGGAGAAAGGAAGCTAGTCTACTCGGACATCTGTGGCTTCACAGTACAGAGGGACTGTCCAAAGCCGCAG
ATTCTCGAGTCGACTAG
+
CD>A>AACD?BBBB=B>B=BCA>@@C@@?CBB<?87776?8C?CC@B@@?>>A@?=44417==@A@?AA<??>=979
/../;;;<==AC@@A@@
```

```
@T6XXI:00072:00180
CTATCACTCGAGATCTGCGGCTTTGGACAGTCCCTCTGTACTGTGAAGCCACAGATGTCGAGTCGACTAGCTTCCTT
TCTCCCTTCTCCC
+
DC@B<::9A@@BABBCB@@?@@@<@?AA=@:::57B?C??@EA?>@=?B?BCCCCBBBBCABBA@@BABA?88?;@A
:AA=@:;:44441
@T6XXI:00072:00186
GGGAGAAGGGAGAAAGGAAGCTAGTCGACTCGACATCTGTGGCTTCACAGTACAGAGGGACTGTCCAAAGCCGCAGA
TCTCGAGTCGA
+
CC?AAA@CCCC???<@@B@BBA:>>/11>>::0::?<;?==::;7:@CABBAA>><889=ABC@?=?A<AA>????A
A<@=589?@@?
@T6XXI:00073:00161
GGGGAATTCTAATACGACTCACTATAGGGAGAGGGAGAATAGCTTGTCATTTGATCAACGTCTACATCTCCTTCCTT
TCTCCCTTCTCCC
+
EFEDD@@@ADCBCB??BB@@<AB97111.7?>>@?AB@@B:??C@CB@@@@;@A@@?@?@@;;999DDCC@B@C@BB
>BBBB=B=;;;;6
@T6XXI:00073:00167
CTAGTCACTCGAGATCTGCGGCTTTGGACAGTCCCTCTGTACTGTGAAGCCACAGATGTCGAGTCGACCTAGCTTCC
TTTCTCCCTTCTCCC
+
BC111=:ABCBBCDBBBBCB@CCC>C?AABB???;?CCCB?CC?A@><@A?BAA;;;AAB????AA?B;>>@7637:
==9?>>A.4340884
@T6XXI:00074:00154
GGGAGAAGGGAGAAAGGAAGGACGGTGTGCTGGGAGAAATGCACAGAACTATTCTCCCTCTCCCTATAGTGAGTCGT
ATTAGAATTCCCC
+
DE@CCC@CB=@ABB>B=?=@@ECD@BBC@@><;2<67:5;BBCCBB@@ABBB@?@@C>CBBBB>BB@@@BCCCCBCC
??=?AA=A@CCC9
@T6XXI:00074:00179
GGGGAATTCTAATACGACTCACTATAGGGAGGGGGAGAATAGCAGCTGTTTTTACGCGCCTACTATTCCCTTCCTTT
CTCCCTTCTCCC
+
DCD9B=A=@@@=ACCBC:;:CBBAA:::67?=>A2:::>A>=ABBBBBBBCC2B@:88888>>??;53111+,+,50
,++333377==;
@T6XXI:00074:00183
GGGAGAAGGGAGAAAGGAAGGAGAAAGCGACGTCGGGGAAACGTTTGCTATTCTCCCTCTCCCTATAGTGAGTCGTA
TTAGAATTCCCC
+
CC@BCA@BB>BBBB>B@B?A@ABBC>>BAACCCBBBB7CCAFCCC@CECCDABCCC?CC;::9;BBBCCCCCB9;:?
;9<@CB=<AAB5
@T6XXI:00074:00190
GGGAGAAGGGAGAAAGGAAGCTAGTCACTCGACATCTGTGGCTTCACAGTACAGAGGGACTGTCCAAAGCCGCAGAT
CTCGAGTCGACTAG
+
DDBCA?>@A:?;;87;>?>ACBBBB????A@:;:::9@AC:@@BCCDBBBBCB=43317====?:==7=A<??@@@@
?AA====9==??<=
@T6XXI:00075:00156
CTATCGACTCGAGATCTGCGGCTTAGGACAGTCCCTCTGTACTGTGAAGCCACAGATGTCGAGTCGACTAGCTTCCT
TTCTCCCTTCTCCC
+
CCD>:;133>>>>>;CBBBC@CC@AA?@????@B>BCCCCC@CBC@<=8=;?AA@A@@@@AA;<B?@BBC??@6968
A<AAAA=A=?@@A>
@T6XXI:00075:00161
CTAGTCGACTCGAGATCTGCGGCTT
+
```

```
IB;<<<<>DHHKHCDCDAAAC@CCB
@T6XXI:00075:00164
GGGAGAAGGGAGAAAGGAAGGAGAAGTCGCCCCAAACGATTTGTTGACTATTCTCCCTCTCCCTATAGTGAGTCGTA
TTAGAATTCCCCAATCGAATTCCCGCGGCCGCCATGGCGGCCGGAGCATGCGACGTCGGGCCCTATTCTCCCTCTCC
TATAGTCGAGTCGTAATTAC
+
CC>BBA=@@<@@@A>C;<;;@CAB@A@<?;?@6@C>EEC@@;772:??@@??AAAA=<<<<<7<AAAAA????=A??
??@@A>;5;;;1;/00494455569::;=8<<;?@;;;;>=././777;;DAA???@@:??;?AA>@@@B:?7//,
/85,////:4396..+,5,,
@T6XXI:00075:00180
CTATCACTCGAGATCTGCGGCTTTGGACAGTCCCTCTGTACTGTGAAGCCACAGATGTCGAGTCGACTAGCTTCCTT
TCTCCCTTCTCCC
+
BA@==:::DCCBBCBCCBB@BBB?B@<76<777.7??CBBBBA??@>=A>AA@BCCCEABBCABBA@@?9A9<>=AC
>@?;;6;8<;=A;
@T6XXI:00075:00190
GGGAGAAGGGAGAAAGGAAGTGCAGTTCGCGGTTAGTGAAGGGAAAAACTATTCTCCCTCTCCCTATAGTGAGTCGT
ATTAGAACTTCCCC
+
EHABBB@BA<A;;96<@C@CABBBCB@BBBA>A>@ABCB>==-
6116,8BACGDBBB<BCCCC;@=A??;<A@??;;<<383B>@;3444,
@T6XXI:00076:00150
GGGAGAAGGGAGAAAGGAAGCTAGTCACT
+
1858/1,/6+//44-424247799;>97.
@T6XXI:00076:00151
GGGAGAAGGGAGAAAGGAAGGGGGAGAAGGGGAGAAAAGGAAGCTTGTCACTCGACATCTGTGGCGTCACAGTACAG
AGGGACTGG
+
:@6:9>===699@@9>5944999,33<1333,11111,13638??@E@@A76....--
11100*++++002488:443973/---,
@T6XXI:00076:00153
GGGGAATTCTAATACGACTCACTATAGGGAGAGGGAGAATAGATCACCGTTGGTTCGGGTGCGCGTTCTCCTTCCTT
TCTCCCTTCTCCC
+
994,9>C@CCE@BBBBCDCB>?>99111.777>><ABC?AA===@??:?@5:7:?CBD=B;;;CB@>C;:8:@B=@@
7>:44-4278@A<
@T6XXI:00076:00170
CTAGTCACTCGAGATCTGCGGCTTTGGACAGTCCCTCTGTACTGTGAAGCCACAGATGTCGAGTCGACTAGCTTCCT
TTCTCCCTTCCTCCC
+
F;9499449BBABCBCCCBB@BBBAB>?;97116-
7>>@ABCCBCAA<?B@CCCCBB@@?BBBCCBCBCC@?<>A@CC>A7=<68,/,/793
@T6XXI:00076:00182
GGGAGAAGGGAGAAAGGAAGGGGAGAAGGGAGAAAAGGAA
+
44-444:;;6999;6=<B?ABB8B<<:66661111,.44,
@T6XXI:00076:00184
GGGAGAAGGGAGAAAGGAAGGAGAAGTAGCGCAGGGGGGAATTGTCAGCTATTCTCCCTCTCCCTATAGTGAGTCGT
ATTAGAACTTCCCGGGAGAAGGGAGAAAGGAAGCTAGTCACTCGACATCTGTGGCTTCACAGTACAGAGGGACTGTC
CAAAGCCGCGATCTCGAGTCGACTAG
+
99-444249-444A:??B>A<@A@=;::79A>@@CCBB-B@C8;9999;9997;:4;:99444-
44333744......35694/11011,11,117011,11030323234944A8///666///498885..,.43368=
=B?@AB<==999><@B=>?@B===?988>>??9911;00
@T6XXI:00076:00186
```

```
GTCGACTCGAGATCTGCGGCTTTGGACAGTCCCTCTGTACTGTGAAGCCACAGATGTCGAGTCGACTAGCTTCCTTT
CTCCCTTCTCCC
+
?D>::BCHDBGBHCBCAG?BDD@C@AACBABD@CCCAAAB@@@DCADC@BBBBBBBCBBBBAA?:::@????B=AA<
ABBB=B?ACCC<
@T6XXI:00076:00188
GGGGAATTCTAATACGACTCACTATAGGGAGAGGGAGAATAGTCAACAAATCGTTTGGGGCGACTTCTCCTTCCTTT
CTCCCTTCTCCC
+
@>>4>;?@B?B?BBCCDCCBBBBBB7;:4:@@AC>CC@>?===3444<=;AAABB.77:,7=<@@=???<;8;8:94
9;;;:;/4000+
@T6XXI:00076:00192
GGGAGAAGGGAGAAAGGAAGGAGAACCGGCTTCTAACGTTAGTAAAGCTATTCTCCCTCTCCCTATAGTGAGTCGTA
TTAGAACTTCCCC
+
AC?BBB@CDAAAAB=@@C;;;;CC@CACBAA>A@@=ACDDGCCCDA@AAEC>AACC?AAAFG@@@@CCCCCCD:::?
?::@@277:=>>2
@T6XXI:00076:00195
GGGGAATTCTAATACGACTCACTATAGGGAGAGGGAGAATAGGCCGACGTC
+
@@>6@8;8<BB=@@CBB@@@C@BAAAA@<:::<=6=:646//,.7.++++-
@T6XXI:00077:00156
GGGGAATTCTAATACGACTCACTATAGGGAGAGGGAGAATAGTCAACAAATCGTTTGGGGCGACTTCTCCTTCCTTT
CTCCCTTCTCCC
+
BDC:D>?C?@@=ACBBBBB>>@B<?661-
6==?A>BCC@A8=?B=<<=<;ABAAA<A?A6AAAAC@BCC??<?>AC=@@@A;A>@@AB<
@T6XXI:00077:00163
GGGGAATTCTAATACGACTCACTATAGAGG
+
CCC9C>A?BCC@CDAA@>:222,-,,,.44
@T6XXI:00078:00158
GGGGAATTCTAATACGACTCACTATAGGGAGAGGGAGAATAGTCAACAAATCGTTTGGGGCGACTTCTCCTTCCTTT
CTCCCTTCTCCC
+
CFC9C?B@CCC@CC??ACBC>?>>>777.7>>?C?AAA@AAC@??=?B=;??@CC>BBB7ACCBC?BBB:;784994
;==?;@<?>>>7
@T6XXI:00078:00163
GGGAGAAGGGAGAAAGGAAGGGAATAGCAGCAGTCGGCAACGTGAAACTATTCTCCCTCTCC
+
??5999544-4444-4<==?C>B=><<=<887;766B===AA@@@@5;33177111,11..4
@T6XXI:00078:00165
CTAGTCGACTCGAGATCTGCGGCTTTGGACAGTCCCTCTGTACTGTGAAGCCACAGATGACTAGCTTCCTTTCTCCC
TTCTCC
+
DCBCCCCBCBCBBBBBBBBAA@BBC>B?@@>>:7:5:@@BCBBB8;:@>@B@EBCBAAC<:8B889596991;>>?8
>,001,
@T6XXI:00078:00166
GGGAGAAGGGAGAAAGGAAGTGCAGTTCGCGGTTAGTGAAGGGAAAAACTATTCTCCCTCTCCCTATAGTGAGTCGT
ATTAGAATTCCCC
+
CDACCB@BH?BBBB=AAB@B>;==BB@B=?@@B@ABAA@=?=6<=<?0?BBB@CBBB>ABABB<A??@@AACAA??8
8849ABBC:@@@5
@T6XXI:00078:00167
GGGGAATTCTAATACGACTCACTATAGGGAGAGGGAGAATAGATCACGACCCTTCTACCCACGAATTGAGCTTCCTT
TCTCCCTTCTCC
+
```

```
A?;6?;@=@@>?;;;;;;;:;;:??<777.7776=8?8555A878AB:::A@:?==<777.4400/79;::9;64>@A
9@9>>0949384
@T6XXI:00078:00170
GGGAGAAGGGAGAAAGGAAGCTAGTCACTCGACATCTGTGGCTTCACAGTACAGAGGGACTGTCCAAAGCCGCAGAT
CTCGAGTCGACTAG
+
DD?@496991999909596;A@AAA???::77799:7@AB>?@=@BA?ACC@?<6555;;===@=@@9<;>=889=>
@@A???A===@C@?
@T6XXI:00078:00192
GGGGAATTCTAATACGATTCACTATAGGGAGAGGGAGAATAGTTCTCCGATTTTCAACATGTTGCTGTTCTTCCTTT
CTCCCTTCTCCC
+
BBB6@<@=@AB@CCBBBB@AAA>;448919>>CNACBBBBBAB>>>9>9BBCC9CC@BBBCA?BBBCB@BB=B@BB:
>@BB>@>?B?@;
@T6XXI:00078:00199
GGGGAATTCTAATACGACTCACTATAGGGAGAGGGAGAATAGTCAACAAATCGTTTGGGGCGACTTCTCCTTCCTTT
CTCCCTTCTCCC
+
BAA9B@D@BBB@BAAABBBB?<?96166.7>==?<?CC?AA?AAA>???<AC@@@=AAA7ABBBC@BBB8868>AA<
@@AA<A889AA<
@T6XXI:00079:00168
GGGAGAAGGGAGAAAGGAAGCTAGTCACTCGACATCTGTGGCTTCACAGTACAGAGGGACTGTCCAAAGCCGCAGAT
CTCGAGTCGACTAG
+
BC@CAA?BB<A@=85>4848A>@AB7;;???:;;???CAA?AA>@BAABBBBA?<887<A??>>373,4;79;<;<>
??@?@@???@C@@@
@T6XXI:00079:00169
GGGAGAAGGGAGAAAGGAAGCTAGTCACTCGACATCTGTGCTTCACAGTACAGAGGGACTGTCCAAAGCCGCAGATC
TCGAGTCGACTAGCTTCCTTTCTCCCTTCTCCC
+
EEBB<<;<A:@@@@<@DCD@A@B>:0//...444569999??=@BBBCDCBA?88858;<<@B>AA;@A====@@??
BA::9@AA???AA@@>@=?=:====7=3,.561
@T6XXI:00079:00180
GGGGAATTCTAATACGACTCACTATAGGGAGAGGGAGAATAGTCTGACTGTTTCCAAAGCAGCTGTTCTCTTCCTTT
CTCCCTTCTCCC
+
CCC6@=@>BCC@CBBBBAAAA?@A7111.7>>BC=BAA?A<88;;=BAC?@@=@@CC>BABCCCA>=ACCC?B?@A:
??AA<A9<<<<7
@T6XXI:00079:00192
GGGGAATTCTAATACGACTCACTATAGGGAGAGGGAGAATAGTCAGCGCATATGCCCGTTTACAATTCTCCTTCCTT
TCTCCCTTCTCCC
+
CCC9C>B@BCC@BBBBC>>>B>;97166.7===><AB@>AA@@<==@==<?<66?C;AAC=A??=@?B<=:<;=;?@
;@@==9;.//::6
@T6XXI:00080:00151
GGGGAATTCTAATACGACTCACTATAGGGAGAGGGAGAATAGTCAACAAATCGTTTGGGGCGACTTCTCCTTCCTTT
CTCCCTTCTCCC
+
AAA9B@C@CBB@BB???BC?BCB99166.7>>BD>DBB@B<@@A=;<AA<CCA??9???5?BB::8:AB:<;=>AC<
@@AC=A>@??=5
@T6XXI:00080:00155
GGGGAATTCTAATACGACTCACTATAGGGGAGGGAGAATAGTCAACAAATCGTTTGGGGCGACTTCTCCTTCCTTTC
TCCCTTCTCCC
+
BA?7@548;AAACC@?@BBB@?CB?6111-116-
7???BB@@CC@BBC>B?==608380;=@@@?A@@>@?A8<876??<;??CCAA:
@T6XXI:00080:00157
```

```
GGGAGAAGGGAGAAAGGAAGCTAGTCACTCGACATCTGTGGCTTCACAGTACAGAGGGACTGTCCAAAGCCGCAGAT
CTCGAGTCGACTAG
+
DCBCAA=??>ACBB>C;96<C??@CCBAAAA::;?@?AAC=?@=@BAABBBBAA88868CAAAA?AB:??>@A=<<@
<<@AAA@@@@AAAA
@T6XXI:00080:00165
GGGGAATTCTAATACGACTCACTATAGGAGAGGGAGAATAGTCAACAAATCGTTTGGGGCGACTTCTCCTTCCTTTC
TCCCTTCTCCC
+
CCD:C@B@@A@>ABACBAACA;;<<00,/:<>A>BCA>@@?>>=<===7???BB:AAA5?@???;???:;8;<>>;?
AAA;@;=884-
@T6XXI:00080:00167
GGGAGAAGGGAGAAAGGAAGCTAGTCACTCGACATCTGTGGCTTCACAGTACAGAGGGACTGTCCAAAGCCGCAGAT
CTCGAGTCGACTAG
+
CKDCCB=>=:@@>96@7848B@?@F?:76?@::7??=ACCAAC@ECBA>?AA?<44417>>;<==@@;@A>@@AA@@
???A@@???AAA??
@T6XXI:00080:00169
GGGAGAAGGGAGAAAGGAAGCTAGTCACTCGACATCTGTGGCTTCACAGTACAGAGGGACTGTCCAAAGCCGCAGAT
CTCGAGTCGACTAG
+
CDCCBA894-
999909;>=@ABAAC::;:BA:::???CCB?BB@BA?A@@>>?====;<===AA;999=<>@?<?=<9==AAA===@
@@==
@T6XXI:00080:00181
GGGAGAAGGGAGAAAGGAAGTGCAGTTCGCGGTTAGTGAGGGGGA
+
BB?<<;>BC>CCCC>C=@=@BCCFCE@BC=;9;=:::0-,,,,),
@T6XXI:00080:00190
GGGAGAAGGGAGAAAAGGAAGTGCAGTTCGCGGG
+
FB>=99544-44444,49;9?A>=8/1,11-,,*
@T6XXI:00080:00196
GGGGAATTCTAATACGACTCACTATAGGAGAGGGAGAATAGTTCCCTACTACTTCTTTCCACTCTGCTCTTCCTTTC
+
CCB9C?A@ACB@CCBAA<@@B:964..,0;;>C@CB@>@A@?;<=;<=@A????==A<A=@?AAAA??@A?A9==9=
@T6XXI:00080:00197
GGGGAATTCTAATACGACTCACTATAGGGAGAGGGAGAATAGCCTGACTGTCACTAGTCTGCGCTTTCTCTTCCTTT
CTCCCTTCTCCC
+
@==6@@B@CBB@BBBBBBBBBBB>7111.6<<BC>CCA?A:?<:AA??=?@B@@@AAA==<@@AAC=A>??=>8;50
38884898444-
@T6XXI:00081:00156
GGGAGAAGGGAGAAAGGAAGGCAACAACGCAGAGAAATAGTACAAAACTATTCTCCCTCTCCCTATAGTGAGTCGTA
TTAGAATTCCCC
+
CCAA@>@BC@BB@@=C@B@A@BB@BB@ABBAAA@@A<@CB@D;;;;4<BBEECCFCACBBBC<@@BBBCCBBBCCBB
B???A?A?BAA6
@T6XXI:00081:00158
GGGAGAAGGGAGAAAGGAAGCTAGTCACTCGACATCTGTGGCTTCACAGTACAGAGGGACTGTCCAAAGCCGCAGAT
CTCGAGTCGACTAG
+
EL?BBA644-
999909695;C@@@A6:77:>7::B?ACAA:?@=@BBCCC@BA=3331799=<=;=A=AA?A@:??AAB=9=9::==
;988
@T6XXI:00081:00169
```

```
GGGAGAAGGGAGAAAGGAAGCTAGTCACTCGACATCTGTGGCTTCACAGTACAGAGGGACTGTCCAAAGCCGCAGAT
CTCGAGTCGATCA
+
CC@BB=644-
999909596;CBADC<>>00000////;>B<AA?BFGC??@974000,3;;;>@;==7==8=><<=?AAA8./..**
***
@T6XXI:00081:00173
CTAGTCACTCGAGATCTGCGGCTTTGGACAGTCCCTCTGTACTGTGAAGCCACAGATGTCGAGTCGACTAGCTTCCT
TTCTCCCTTCTCCC
+
11/////44@@>??BBA@;:6;?B?C@CCCC:::67>>>:::?@9;<8=CBCECCCBBBBBCCCBBCAABC;::;@C
C>@@?B>@598?A;
@T6XXI:00081:00185
GGGAGAAGGGAGAAAGGAAGGAGAAGTCGCCCCAAACGATTTGTTGACTATTCTCCCTCTCCCTATAGTGAGTCGTA
TTAGAATTCC
+
490449899-
444858787>;?;@=@:;;BBB5@@=?BAA@8::>==>?=A:==CCAA=<<@187=><65448/..8;;::445./-
@T6XXI:00081:00186
GGGGAATTCTAATACGACTCACTATAGGAGAGGGAGAATAGCCAACAGGTATCTGATTGCGCGTTCTCCTTCCTTTC
TCCC
+
DDD:CAC@BBB@CBB?CBBB<6465..,0;;?@>BCA>@=A=;<;?==?AA???@@@?<<<?:=?BAA:=;=<==;<
AAB9
@T6XXI:00081:00188
GGGAGAAGGGAGAAAGGAAGGAGAAACACGGGCCAAAGAGTTATTGGCTATTCTCCCTCTCCCTATAGTGAGTCGTA
TTAGAATTCCCC
+
CCACDC?BB?BBBB>C?B?B@BAAA>BCCA;6>48809@@B<@A7;19AAB@CCCC>CB@B?;??;;?@===??>>=
=;=::732;9<4
@T6XXI:00081:00196
GGGGAATTCTAATACGACTCACTATAGGGAGAGGGAGAATAGATCACGTAGCGTTCGTGTGTCGATTCTCCTTCCTT
TCTCCCTTCTCCC
+
CCD:C?B??BB@CA>>AB??>@BB;66659@ACD>BBA<@<;<<<AA@A??BBB>A@>><=<<A@@>@@@>@>@>AC
<>>@C?A689?@;
@T6XXI:00082:00152
GGGAGAAGGGAGAAAGGAAGAAAAGTCGATGCGGCGGAATACGTGACTATTCTCCCTCTCCCTATAGTGAGTCGTAT
TAGAATTCCCC
+
EECCCC@BB>CECC=?=@@CCCD7>@@CCCBA?@BB@B@<?BBBBBBBCC?@BCC>>>@??;?EBBCCCA??CCCCA
>ABA8@=@@@6
@T6XXI:00082:00157
GGGAGAAGGGAGAAAGGAAGGCAGAG
+
DD>CCB==95=98809<@<<,..,,*
@T6XXI:00082:00167
GGGAGAAGGGAGAAAGGAAGGTGGAAACGGTGTCGGG
+
=@>BAA=BC>@ABE>C@B@C@AA>CN=<72--,,,,,
@T6XXI:00082:00168
GGGAGAAGGGAGAAAGGAAGCTAGTCACTCGACATCTGTGGCTTCACAGTACAGAGGGACTGTCCAAAGCCCGCAGA
TCTCGAGTCGACTAG
+
CC@CB@===5>844-95958@>>??AC@AAA::;???@AB<?@?BDDC?CA@?65555;99988/44-444-
4;;=@@@AAA???@@@@@@@
@T6XXI:00082:00178
```

```
GGGAGAAGGGAGAAAGGAAGCTAGTCGACTCGACATCTGTGGCTTCACAGTACAGAGGACTGTCCAAAGCCGCAGAT
CTCGAGTCGACTAG
+
CD>@==;>>:>999095968>?<<<0//734:>??A9?@??;?B?BCDCCBA744..-
/8;77=>AA<??=?@@@A@@@AA@@@???A???
@T6XXI:00082:00185
CTAGTCACTCGAGATCTGCGGCTTTGGACAGTCCCTCTGTACTGTGAAGCCACAGATGTCGAGTCGACTAGCTTCCT
TTCTCCCTTCTCCC
+
DB1//11::BCBCBCBBBBAABCC>B?A@?=666-
7??AAAAC>A?@>@CACECBBB;;:??=7<<@BAB@?=>??BB>BBBD=?;=<;;6
@T6XXI:00083:00155
CTAGTCACTCGAGATCTGCGGCTTTGGACAGTCCCTCTGTACTGTGAAGCCACAGATGTCGAGTCGACTAGCTTCCT
TTCTCCCTTCTCCC
+
<11//7/44BCCCCCBBBAB@BBB>B>?BBBBCC>BBBBBB??@CCA=>C@CCCCCCBBAABBCCB>;:000/,/,/
/+/.11,0/04442
@T6XXI:00083:00158
CTAGTCACTCGAGATCTGCGGCTTTGGACAGTCCCTCTGTACTGTGAAGCCACAGATGTCGAGTCGACTAGCTTCCT
TTCTCCCTTCTCCC
+
88/////99==?ACBAAA;;7;AB=B=A@@@@EE@BABBCC?ABCCB89?DDCCCCDBBBAABBBBBCAAB;74747
7.7777-434444-
@T6XXI:00083:00160
GGGAGAAGGGAGAAAGGAAGCTAGTCGACTCGAGATCTGCGGCTTTGGACAGTCCCTCTGTACTGTGAAGCCACAGA
TGTCGAGTCGACTAGCTTCCTTTCTCCCTTCTCCC
+
AA:AAA>AB>@99909696;CB>>>/11>????@CD@@ABA=???9?6:;BECBC>AAA=888????A>AA=@@A==
=<8<=?===CCAAA:3337:=>;@ACC?C9788;6
@T6XXI:00083:00176
GGGAGAAGGGAGAAAGGAAGGTTATTCGGGAGTAAGGGCAGTTGTTGTCTATTCTCCCTCTCCCTATAGTGAGTCGT
ATTAGAATTCCCC
+
DGBAAB?BD>BB>A?A@A=B@B@>;:;;?:?@@A?CC>CBBC?A>>??ABCC?BCCCABBBCC>C@?>>>ADCBBB?
??=@??<?<@@@5
@T6XXI:00083:00191
GGGAGAAGGGAGAAAGGAAGCTAGTCACTCGAGATCTGCGGCTTTGGACAGTCCCTCTGTACTGTGAAGCCACAGAT
GTCGAGTCGACTAGCTTCCTTTCTCCCTTCTCCC
+
ND@EAA=@@:>99809695<CBAAA:::?ABBC@BBBBB@@@@@;@@AA@@@@@>@AAAA???AA@B??>:>>????
A@A@@???@AB@A?=<9<=?@;@@@@<A>@@BC<
@T6XXI:00083:00197
GGGAGAAGGGAGAAAGGAAGAAGAGCGCAAGTGCGGAGATCACATGTCTATTCTCCCTCTCCCTATAGTGAGTCGTA
TTAGAACTTCCCC
+
CC:@@@@BC?=<<B<@?B?@C@BC@@@?A=>>???5:699A??@AA==9=A?;;;43./.444577ABAA@A@@888
>;==@99:7:::1
@T6XXI:00083:00198
GGGGAATTCTAATACGACTCACTATAGGGAGAGGGAGAATAGCCGCGTCT
+
:AA4969<@@@ABAA<<;;;A@@>=/00,0/0;72431354338:44222
@T6XXI:00084:00151
GGGAGAAGGGAGAAAGGAAGCTAGTCCATCGACTCGC
+
HD=?9;59A<ABBE@D@B@BCB>B>?::::..,,,,*
@T6XXI:00084:00162
```

```
CTAGTCGACTCGAGATCTGCGGCTTTGGACAGTCCCTCTGTACTGTGAAGCCACAGATGGACTGAGCTTCCTTTCTC
CCTTCTCCC
+
CDBBBBBBCECCCCCBBBBBB@BBB>B?A?@@9994:A?BBCBA?CBA?@C@CCAACCC@BBBAAC;;8<?BBACCC
E@C8:<BB<
@T6XXI:00084:00164
GGGGAATTCTAATACGACTCACTATAGGAGAGGGAGAATAGATTCGCGATTCCCTGCACTGGACTATTCTTCCTTTC
TCCCTTCTCCC
+
CCC9C@C@BBB@BBBBDAA>>:997/.-
0::BC>CCA?@9<<<<;;@<@C@CC>@@CECCC?AACBB?BC?C@CB>????6;;=<<?6
@T6XXI:00084:00168
GGGAGAAGGGAGAAAGGAAGCTAGTCACTCGACATCTGTGGCTTCACAGTACAGAGGACTCGTCAAAGCCGCAGATC
TCGAGTCGACTAG
+
DKBC>>644-999909699<CBBCC8:;@@>7::??<BDE@B@=@C;;6::::;;;786--
...355777889=<@<==????=<<???>
@T6XXI:00084:00179
GGGAGAAGGGAGAAAGGAAGAAAGGAAGAAGAGGCCAACTGCAACAACAGCAGTGCTATTCTCCCTCTCCCTATAGT
GAGTCGTATTCAGAATTCCCC
+
CE>CCC@CB>BAAB=B@B@BB>;>>?>>?A??@?@?B?BCCBB?BC@CDBBB@@?;;;?:?A?A>BAA?C<@@AAA@
?@ABA@778,//8:78;@A@5
@T6XXI:00084:00199
GGGGAATTCTAATACGACTCACTATAGGAGAGGGAGAATAGCCAAAACATTATTCCCGTCGCTGTTCTCTTCCTTTC
TCCCTTCTCCC
+
=>>/969?A@@>@CBCCCBBB:993//-
0>=BC>CBA?A=;;:<==5==A<8??CC:??A??ACC@B:::>A??B=CAAD=A58788-
@T6XXI:00085:00153
GGGAGAAGGGAGAAAGGAAGTGCAGTTCGCGGTTAGTGAAGGGGAAAAACTATTCTCCCTCTCCCTATAGTGAGTCG
TATTAGAATTCCCC
+
EHACBB@BB?CCCC>C@B@BBBCBBB?BBBB@B>;77334777-
7666/7ABB?BBDD@DBBCC>B???@@@B@@@?@@@?BBB>B?ACC6
@T6XXI:00085:00155
CTATCACTCGAGATCTGCGGCTTTGGACAGTCCCTCTGTACTGTGAAGCCACAAGATGTCGAGTCGTCCTTAGCC
+
ECCCBCCCBBBCCBBBBBB?BBB<B@BBC@@@@;@AA@@@@@@@C@A><=8>984833977611144331313..3
@T6XXI:00085:00161
GGGAGAAGGGAGAAAGGAAGCTAGTCACTCGAC
+
HC?C@@59409>>@:B9<8;?>??>7776;<7/
@T6XXI:00085:00179
GGGAGAAGGGAGAAAGGAAGGGAATAGCAGCAGTCGGCAACGTGAAACTATTCTCCCTCTCCCTATAGTGGTCGTAT
TAGAATTCCCC
+
CC>BBA@BB>BBBB>B@B@BB<B@BB?BBBBBBC>>>AC@CCCCBB>ABCC@CCCC>BBBAB>?788@<<9=AAAAA
=?=<88/4440
@T6XXI:00085:00185
GGGAGAAGGGAGAAAGGAAGCTAGTCACTCGAGATCTGCGGCTTTGGACAGTCCCTCTGTACTGTGAAGCCACAGAT
GTCGAGTCGACTAGCTTCCTTTCTCCCTTCTCCC
+
BB?B<979@:A99919495888>>=7666;?ABABBBBAA@???=B@CA@?;>?;>>=:==::;;<A;==>@@@?B?
??AA???@A===A=<<<?<>?<?A=A;=689DC<
@T6XXI:00085:00197
```

```
GGGGAATTCTAATACGACTCACTATAGGGAGAGGGAGAATAGTCAACAAATCGTTTGGGGCGACTTCTCCTTCCTTT
CTCCCTTCTCCC
+
:;9/9<A?CDB@CAAAA???BBB<8616.7>@AC=BCA?@::===<9984??;;?8???5?AAAC@CCC?@>@>@A<
AAAA<@>@@AA<
@T6XXI:00085:00199
GGGGAATTCTAATACGACTCACTATAGGAGAGGGAGAATAGTCAACAAATCGTTTGGGGCGACTTCTCCTTCCTTTC
TCCCTTCTCCC
+
CCB9C9;;<BB?CDBABBBBC>=97/.,0@<?A>BB@?A???AA@87719?=;B8???5?::;B>A@C?>9=:=D=C
ACC6858.793
@T6XXI:00086:00158
GGGAGAAGGGAGAAAGGAAGGAGAACATCCCGACGAGAATCAGGTAGCTATTCTCCCTCTCCCTATAGTGAGTCGTA
TTAGAATTCCCC
+
CGACCB@CCB===A?A@A@A@???@BBBBB=?BABBBB@BBBB@CABBCCC@BCCB=CBCCC>CCCCCCCBAB?;9.
.,/7743/003+
@T6XXI:00086:00163
GGGGGAATTCTAATACGACGTCACTATAGGGAGAGGGA
+
4444+42424988=9449333333443333,331.57/
@T6XXI:00086:00164
GGGAGAAGGGAGAAAGGAAGCTAGTCGACTCGACATCTGTGGCTTCACAGTACAGAGGGACTGTCCAAAGCCGCAGA
TCTCGAGTCGACTAGCTTCCTTTCTCCCTTCTCCC
+
CB?A<;6<@19;@>9>4948?>::@,,,,,,,,4457@???@;;;:DEABB??;;<<<6<>=?>?<?@<AA@@@@A?
?=<<<<<=<<<=<=<@@<?<?B=@@@A<A>A@@@<
@T6XXI:00086:00170
GGGGAATTCTAATACGACTCACTATAGGAGAGGGAGAATAGTCAACAAATCGTTTGGGGCGACTTCTCCTTCCTTTC
TCCCTTCCTCCC
+
999/429244488>@@@B@?8000//.,...11,1845:A===A>@AA;BA@@B>CCC8AABA@:<==4434;>>:@
73222,/,/660
@T6XXI:00086:00175
GGGGAATTCTAATACGACTCACTATAGGGAGAGGGAGAATAGTCA
+
444,429=>AC>AACCBBBBBAB<?:77.6>>>B<??@?878@?=
@T6XXI:00086:00184
GGGGAATTCTAATACGACTCACTATAGGAGAGGGAGAATAGTCAACAAATCGTTTGGGGCGACTTCTCCTTCCTTTC
TCCCTTCTCCC
+
CHD9A@A?A>><@@BBCCCCC>997..,0<<?@=BDA>@@@??>=???<ABAAC;???6?:::B?BAA??>A>AC<@
@BA<A=@BAA<
@T6XXI:00087:00154
GGGAGAAGGGAGAAAGGAAGAGAAGCGCAATGACCACGAGAGATTGGCTATTCTCCCTCTCCCTATAGTGAGTCGTA
TTAGAATTCCCC
+
BB=@@B@BC=?BBB>B@B@BB@@?ABB>?=@@BC>>9???>>??>A>>>>@=@AAA>BBBCC>CBA?<=?A????AA
A?AAA=B?BCC8
@T6XXI:00087:00158
GGGGAATTCTAATACGACTCACTATAGGGAGAGGGAGAATAGATCACCGTTGGTTCGGGTGCGCGTTCTCCTTCCTT
TCTCCCTTCTCCC
+
EED9C@A>BBB?BDDCCCBBB@;77111.7==?@>BCC@A8==?A??:?B=?>@@CED?C@@@CCC>B??:?6;6:B
:B?=<7<@??@A<
@T6XXI:00087:00162
```

GGGAGAAGGGAGAAAGGAAGCTAGTCACTCGAGATCTGCGGCTTTGGACAGTCCCTCTGTACTGTGAAGCCACAGAT
GTCGAGTCGACTAGCTTCCTTTCTCCCTTCTCCC
+
CC>B@@=>@:@9998;>@>@@@ACDBBBCCAAA@BCCCBA@??@=A?BDCCBBB>BC<<<<9=<??A=??<>??A89
9A@@@@@@@@@@@AA=:;:==4933317;?@AC?
@T6XXI:00087:00168
GGGAGAAGGGAGAAAGGAAGCTAGTCACTCGAGATCTGCGGCTTTGGACAGTCCCTCTGTACTGTGAAGCCACAGAT
GTCGAGTCGACTAGCTTCCTTTCTCCCTTCTCCC
+
NNAAACACD?BBB@<@=@=?B??@B:;;BCCCCACB??@C@CBB>B?CBB@@@@;B@==889ABAAB===>AAAAA?
@=9=A@@@BAAAAA??=?;==;==<834/04:94
@T6XXI:00087:00170
GGGAGAAGGGAGAAAGGAAGGAGAAGTCGCCCCAAACGATTTGTTGACTATTCTCCCTCTCCCTATAGTGAGTCGTA
TTAGAATTCCCC
+
CC?BBB@DD>?A@B>B@B@B@BBC@?;?BBBB7?A=BCCCC>B?=?ACC98555553<>@@A<@???AAA<A===??
?>@@?=?<==?5
@T6XXI:00087:00184
GGGAGAAGGGAGAAAGGAAGCTAGTCGACTCGACATCTGTGGCTTCACAGTACAGAGGACTGTCCAAAGCCGCAGAT
CTCGAGTCACACACGACTC
+
49199>;>?-8499149>58:7;47./.455.01776111101911186667311--,-222-
3234+0323368868664--.-+*****00035
@T6XXI:00087:00191
GGGAGAAGGGAGAAAGGAAGCTAGTCACTCGACATCTGTGGCTTCACAGTACAGAGGGACTGTCCAAAGCCGCAGAT
CTACGAGT
+
DDBCCC@B>:A@=8:@>@<ABBBCC@BBBB????AAACCD?BB@CCB???>=9666<7=@@AAA9<<7<?:?@@78/
:/--00+-
@T6XXI:00087:00192
GGGGAATTCTAATACGACTCACTATAGGAGAGGGAGAATAGTCAACAAATCGTTTGGGGCGACTTCTCCTTCCTTTC
TCCCTTCTCCC
+
CCC9B?B?BBB?BBCCCBBB>57;7//,0::>A>ADA>B788AA?A@@<BB?<;-666-
7=??A=?@B<<9<=??;@A@A<A;==AA<
@T6XXI:00087:00193
GGGAGAAGGGAGAAAGGAAGGAGAAGTCGCCCCAAACGATTTGTTGACTATTCTCCCTCTCCCTATAGTGAGTCGTA
TTAGAATTTCCCCC
+
CGAEDC@CB?BBBB>BBB?A@BBB@??BBBBB7@@=BCBCC>??>?<AA?C?C::::?@A<=;66<AAAA@@???@@
?;=:6,--++055.
@T6XXI:00087:00197
GGGAGAAGGGAGAAAGGAAGCTAGTCGACTCGACATCTGTGGCTTCACAGTACAGAGGGACTGTCCAAAGCCGCAGA
TCTCGGTC
+
DC@ECC?BB=BBCCBC@C;<;>:><0//0./:0:16667113??;?@BAABBA>;77717=:;;@384-
8838AAAAAA@@@>::
@T6XXI:00088:00157
GGGAGAAGGGAGAAAGGAAGCTAGTCGACTCGACATCTGTGGCTTCACAGTACAGAGGGACTGTCCAAAGCCGCAGA
TCTCGAGTCGACT
+
CEABBB@CC@CCBA=B??=@D@?@C8::@B?=;;:::9@@C@AD@ABBCCCCCC@@@@<?AB@@@<?::?A>A@=A@
<=;<<<<;<<==<
@T6XXI:00088:00162
GGGAGAAGGGAGAAAGGAAGAGAATAAACCAGGCGTAAACGTTAGATCTATTCTCCCTCTCCCTATAGTGAGTCGTA
TTAGAATTCCCC
+

CCBBBB@DC????A=B@C@CCBB@B@@=C@BB@BA@@><?BB@CBBB@@?==AA>?:><==>7>>A@@@AAAAAA??
@?=><;93333-
@T6XXI:00088:00171
GGGGAATTCTAATACGACTCACTATAGGGAGAGGGAGAATAGACACCGATCGTGCCGGGTCATGTTCTCCTTCCTTT
CTCCCTTCTCCC
+
444,4:;=BEC@AA;;<>????>>?:::67?===9?7838AAAABA?BA<<8407476-7??BA:7397,-
,/,//,/444-479033+
@T6XXI:00088:00181
GGGGAATTCTAATACGACTCACTATAGGAGAGGGAGAATAGTCAACAAATCGTTTGGGGCGACTTCTCCTTCCTTTC
TCCCTTCTCCC
+
EDB7@5959499BCCCCBAAC7;65..,/;0774:BA;=???AA=AA@:BCCBB=?@A7@BBAA<>@B3434244-
4:;;7=:;4481
@T6XXI:00089:00159
GGGAGAAGGGAGAAAGGAAGGAGACACGGTCACAAACATGAATGTGAC
+
CD@CCB?BB>BBBB?D@C?B?ABACBCC>BCCAAA=ABBBB@CCCBBB
@T6XXI:00089:00165
GGGAGAAGGGAGAAAGGAAGGGGAGAAGGGAGAAAGGAGGTAAGTCG
+
@@<@CC@CD>CCCC@C@C@CCC8@@C=??<??>>:>370,.0,/0.1
@T6XXI:00089:00168
GGGAGAAGGGAGAAAGGAAGCTATCGACTCGACATCTGTGGTCACTAGTACAGAGGGACTGTCCAAA
+
CC>ABBADHABBAB?C?C@88;CCCBBBAA???????CCC?A@@800330444-//+----133454
@T6XXI:00089:00170
CTAGTCACTCGAGATCTGCGGCTTTGGACAGTCCCTCTGTACTGTGAAGCCACAGATGTCGAGTCGACTAGCTTCCT
TTCTCCCTTCTCCC
+
B://1=699BBCCBBBCCCB>BBB?C?A>;?9994:==@:::B??<A?@B?BCDCA>@@BBBAABBBCCDBB@:?:=
?9==?B<?;???@;
@T6XXI:00089:00183
GGGGAATTCTAATACGACTCACTATAGGGAGAGGGAGAATAGTCTCCCATTCGTCACAATTAACGTGCCCTTCCTTT
CTCCCTTCTCCC
+
BBB9B=?>ADCBCC???B>?BAB?9111.7<<BC>BB@?A::89A==;?B@@@@@?BA@C?C?@?@@AA<A=?>==;
<@@=;@=<@A@<
@T6XXI:00089:00195
GGGGAATTCTAATACGACTCACTATAGGGAGAGGGAGAATAGCTCTTCAATTACGCACTCCCGCATCTCACTTCCTT
TCTCCCTTCTCCC
+
AAA9B?BDCBB?AAAAABBBB@>96116.6??CE>CA@=@=@;?@@>B??B@BBBBBA>><<<<AABCB;;<=<;<H
?CCCC=B?BCCC@
@T6XXI:00089:00199
GGGGAATTCTAATACGACTCACTATAGGGAGAGGGAGAATAGTCAACAAATCGTTTGGGGCGACTTCTCCTTCCTTT
CTCCCTTCTCCC
+
;99,98?=ABBBCBBBCCBBB@BB?77646??AB>BB@?A;AAA;<<AA<@:88989996>BCBB@BCB?@?A?AC<
@==<7<9==AC?
@T6XXI:00090:00150
GGGAGAAGGGAGAAAGGAAGCTAGTCACTCGAGATCTGTGGCTTCACAGTACAGAGGGACTGTCCAAAGCCGCAGAT
CTCGAGTCGACT
+
<<69@B@CF=CB@>9@5959>>>>??88;8??:77?;;;>267=>>??767114333,000000233056/039555
8<<<??@==;<:
@T6XXI:00090:00163

```
GGGGAATTCTAATACGACTCACTATAGGGAGAGGGAGAATAGTTTTCCCTTCACTAACCGCGAACTGCACTTCCTTT
CTCCCTTCCTCC
+
999/9<A>BCC@CCBBBABBB@B?9661.6===?<AA>;>6;;<<0;C:?>?CBA=:>:000;-
/0AA????C<??9=>7767,.,//,
@T6XXI:00090:00169
GGGAGAAGGGAGAAAGGAAGGAGAACAACACGATCGGTTAAACGTAGCTATTCTCCCTCTCCCTATAGTGAGTCGTA
TTAGAATTCCCC
+
GC@HDC@CD?BBBB=B?A?A?ABB@CB?A>BBBCBB>?=??<?ABCA?BBB@BA??;?AAA@;?<>8A=AA?AA=<<
=;=@<=?<???5
@T6XXI:00090:00170
GGGGAATTCTAATACGACTCACTATAGGAGAGGGAGAATAGTCAACAAATCGTTTGGGGCGACTTCTCCTTCCTTTC
TCCCTTCCTCCC
+
A::29<A>@AA@CCCBBABBB6::?:747>777.7>;8=>A<<<<?>@<CAAAB5:::5:@@BB@BBB5837;??;A
@;939,/,/882
@T6XXI:00090:00179
GGGGAATTCTAATACGACTCACTATAGGGAGAGGGAGAATAGTCCTAT
+
<99/9596<AA>BA@@CBCBA;>>7777-7==BC>CB@>AA:0..**(
@T6XXI:00090:00183
GGGAGAAGGGAGAAAGGAAGCTAGTCACTCGACATCTGTGGCTTCACAGTACAGAGGGACTGTCCAAAGCCGCAGAT
CTCGAGTCGACTAG
+
GC?@?>5991999919499;BAAA@;777777616::?BE>@B@CB@@CCCBB>777-
4::=??>@@;??<?@???A788?;;;>788AB@
@T6XXI:00090:00188
GGGAGAAGGGAGAAAGGAAGCTAGGTCACTCGACATCTGTGGCTTCACAGTACAGAGGGACTGTCCAAAGCCGCAGA
TCTCGGTCGACTAG
+
@CACDC>@?<@B??=B?B?ABBAA?A?GBBBA:::::@CC@AC?AAA@=??BA?;667<?=>>@9==9=A?A@@==
=AA@@=??CABA?A
@T6XXI:00090:00192
CTATCACTCGAGATCTGCGGCTTTGGACAGTCCCTCTGTACTGTGAAGCCACAGATGTCGAGTCGACTAGCTTCCTT
TCTCCTTCCTTCTCCTTCTCC
+
D:::A:::CCBBBC??@BB@BBB>@>@CBBABB>ACCCAAACAA?@<8;7;BA@@ABA@A::;BBCCC@>:7;>377
.113/0/0/02400/0/0449
@T6XXI:00090:00195
GGGGAATTCTAATACGACTCACTATAGGGAGAGGGAGAATAGTCAACAAATCGTTTGGGGCGACTTCTCCTCCTTTC
TCCCTTCTCC
+
:::2@<@5<9;;?@<<<BBBCB@?>777.77777,610256==<<;<<<;???A@?A@A7:?9:73:69><><==;8
8650320335
@T6XXI:00091:00153
GGGAGAAGGGAGAAAGGAAGCTAGTCGACTCGACATCTGTGGCTTCACAGTACAGAGGACTGTCCAAAGCCGCAG
+
CCACBBBCC?:;9=6>4:5;;B;;<BBBCFCC;;;;::B@A<@@>ADBBBBB;:2--,/666;@?AA?AA=@@@A
@T6XXI:00091:00160
GGGAGAAGGGAGAAAGGAAGAGAATAGGCACGCATGTAAAATGGTAACTATTCTCCCTCTCCCTATAGTGAGTCGTA
TTAGAATTCCCC
+
CC>BBB@CD>AAAB>B?BBBBBB@C??>?>?BBDBAAABB8@A@@>>A???>>>>>;>>;==;<;;<<<==A<<<=<
<;<<=;A=A==4
@T6XXI:00091:00186
```

```
GGGAGAAGGGAGAAAGGAAGTGTATGAGGGGACGGGTGATGTGGGGGCTATTCTCCCTCTCCCTATAGTGAGTCGTA
TTAGAATTCCCC
+
CC>B=@;@@<@AAC>C?B?BB@@@AABBBA8BBBB>BCEEBACCDC3BCBB@@99919>>>A<>@@?@@ELCCCDCC
C?B@A5844<@6
@T6XXI:00092:00153
CTATCACTCGAGATCTGCGGCTTTGGACAGTCCCTCTGTACTGTGAAGCCACAGATGTCGAGTCGACTAGCTTCCTT
TCTCCCTTCTCCC
+
DAAAA:::CCCBBBBBBBB@BBB@B@BBB@@@@;@@@>>>@@AC@@>@C@BCBCCBBAABA??@BABBBBAA?A@CB
;@??=6979:=@:
@T6XXI:00092:00171
GGGAGAAGGGAGAAAGGAAGGAGAAGTCGCCCCAAACGATTTGTTGACTATTCTCCCTCTCCCTATAGTGAGTCGTA
TTAGAATTCCCC
+
DE@CCB@BC>BBBB>A>B?B@BBB@AAB????7?C?CCCAA;>>>B@?B?A>;;;;;<<<=>;=<;:====<<<??=
@>@@?;;/444-
@T6XXI:00092:00175
GGGAGAAGGGAGAAAGGAAGGACTCGACATCTGTGGCTTCACAGTACAGAGGGACTGTCCAAAGCCGCAGATCTCGA
GTCGACTAG
+
CC?@A??BC>A@@C@C@C@B@:::CAAAB::;CCC?B@<>ABB9AAB>;37714;;?>?;>?6<<>AAAAA???BB<
<:::<<@@<
@T6XXI:00092:00193
GGGGAATTCTAATACGACTCACTATAGGAGAGGAGAATAGT
+
111+/,95999698?>>?>@ABBB99101176316955580
@T6XXI:00093:00170
CTAGTCACTCGAGATCTGCGGCTTTGGACAGTCCCTCTGTACTGTGAAGCCACAGATGTCGAGTCGACTAGCTTCCT
TTCTCCCTTCTCCC
+
B:1////44@===?B>113?@ED?=???CBAABB>B>>>?@B@@@==;=D@CCGBAAAAA@BAABBBCCCAAA@C@C
B>CCCC>@8;;;94
@T6XXI:00093:00184
GGGAGAAGGGGA
+
4904442444,4
@T6XXI:00093:00196
GGGAGAAGGGAGAAAGGAAGGAGAAGTCGCCCCAAACGATTTGTTGACTATTCTCCCTCTCCCTATAGTGAGTCGTA
TTAGAATTCCCC
+
C@:@BB?BCA<<;B9>4;68;?BB?A@?8888.8808AACD>CC@BAAAAA>A===7;==>?7???@@@A@@???==
=>AA@>@8;<;1
@T6XXI:00094:00158
GGGGAATTCTAATACGACGTCACTATAGGGAGAGGGAGAATAGTCTTCGTATTTTGTGCATCACTTTTCTCCTTCCT
TTCTCCCTTCTCCC
+
ECC4@5969482446///0.0.6687333,33111,1163666110794473AA6=@?????::9CC7BBCAB:9/4
3+39994:7:8884
@T6XXI:00094:00165
CTAGTCACTCGAGATCTGCGGCTTTGGACAGTCCCTCTGTACTGTGAAGCCACAGATGTCGAGTCGACTTAGCTTCC
+
=:1//7/44??>>BBAAA@><88808;>?>;766-6;<B?@?@BB@@;>C9999>>8887==>>?<:00,.,,,22*
@T6XXI:00094:00166
GGGGAATTCTAATACGACTCACTATAGGGAGAGGGAGAATAGCTAATGTCTCTACCTCACGATGCTGCTCTTCCTTT
CTCCCTTCTCCC
+
```

EKK9A>A@CCC@CBB>><?BBCB>9111-
6==?B>@@=>AB@?AB@ABC=AA==<<=<=A>A===@<<<<<9;<>@:?>>?:?<?>@@7
@T6XXI:00094:00174
GGGGAATTCTAATACGACTCACTATAGGGAGAGGGAGAATAGTTTTCCCTTCACTAACCGCGAACTGCACTTCCTTT
CTCCCTTCTCCC
+
999/9;@=ACCAB@@@CCCBBCB<6111.6<7776:B@>A@???>1=?7<:=CCCCBB=?@BA>AAA@???:?:<?9
?@?B<?;:444-
@T6XXI:00094:00177
GGGGAATTCTAATACGACTCACTATAGGGAGAGGGAGAATAGTCAACAAATCGTTTGGGGCGACTTCTCCTTCCTTT
CTCCCTTCTCCC
+
===6@>A@CBB@CBBBB>>?@?AB@77757<<AC>CB?>A;ABAA;4771:???C<AAA7ABBCC?BBB77378886
;==?:?9;4481
@T6XXI:00094:00180
GGGAGAAGGGAGAAAGGAAGGAGAAGTCGCCCCAAACGATTTGTTGACTATTCTCCCTCTCCCTATAGTGAGTCGTA
TTAGAATTCCCC
+
CCACBA=>>:@B=>:A=B=@=@BB?BBCCCBB9BC>CCAAA>AB?C???AA?=<<<;<@A==;<8<=BA=@@@AA=9
=?A@=;;2///+
@T6XXI:00094:00198
GGGAGAAGGGAGAAAGGAAGGAGAACGCGCACCCACGAACCGATA
+
DDABBC@CC@ECCC=B>@@B?>@@@CCCCC>;;1/1--+-*,,,*
@T6XXI:00094:00199
GGGGAATTCTAATACGACTCACTATAGGGAGAGGGAGAATAGCCGACAATTCGACTCCCCGGCAATGTTCTTCCTTT
CTCCCTTCTCCC
+
AA@6A>A@BCCCDCCC@AABB<;77111.7>>BC=BBA;>88838C?@>B=??=;7777.7=A?699@>@A>A>@A<
AA@@;@<??@A;
@T6XXI:00095:00169
GGGGAATTCTAATACGACTCACTATAGGGAGAGGGAGAATAGCTTGCAGCTCGAGCCTGTGACTGTTCTCTTCCTTT
CTCCCTTCTCCC
+
@@>6A>B@CCB@??BBB?@?B@>;966959;??B>CBA>A<<<@?A???>A??B<?;?CCCCCE?=9<<8848/03,
4444-434444-
@T6XXI:00095:00170
CTAGTCACTCGAGATCTGCGGCTTTGGACAGTCCCTCTGTACTGTGAAGCCACAGATGTCGAGTCGACTAGCTTCCT
TTCTCCCTTCTCCC
+
DB1/1//44>==BCGBBAA@;?;;<@=@ABBBCB>BCCCC@>@=@=@>@B?BBBDBCCCCCCB:;<???B::7<<<A
C>BBCD=?<?@@@<
@T6XXI:00095:00184
CTATCACTCGAGATCTGCGGCTTTGGACAGTCCCTCTGTACTGTGAAGCCACAGATGTCGAGTCGACTAGCTTCCTT
TCTCCCTTCTCCC
+
FFHC<::CCCDCBCBBBAA?BDD>C?CBBB???9?AAACCCC9<=A?CB@BBBB:::??BB:;<BAB???7737477
.7?;;-43444:7
@T6XXI:00095:00192
GGGAGAAGGGAGAAAGGAAGAGAACAGCGACGAACAATAGTTGGTGGCTATTCTCCCTCTCCCTATAGTGAGTCGTA
TTAGAATTCCCCC
+
DE>ECC@CC>CB?=:@8:7;CCC@BBBCCCCDC@C?>BBBB<<=::7;BB?<?7111699>@;@?:;===A=<?8<6
=5<3323/0000+
@T6XXI:00095:00195
GGGAGAAGGGAGAAAGGAAGGAGAAGTCGCCCCAAACGATTTGTTGACTATTCTCCCTCTCCCTATAGTGAGTCGTA
TTAGAATTCCCCC

```
+
BB???>ACC?9<=C>C=A=@@CBB??A;@AAA7ACACBBBD@B@>BAABAA>@@?@:<<;<>:789AADCAA?@@A?
?<=:4,-,.///+
@T6XXI:00095:00196
CTAGTCACTCGAGATCTGCGGCTTTGGACAGTCCCTCTGTACTGTGAAGCCACAGATGTCGAGTCGACTAGCTTCCT
TTCTCCCTTCTCCC
+
DB9999444ABABCCBBACC@BBB>B?CAB@<:<;?B?BCBB??@@A>ACACCCCA@@@AABBBBBAAAA;:13<37
7.7<=>.434:::7
@T6XXI:00096:00173
GGGGAATTCTAATACGACTCACTATAGGGAGAGGGAGAATAGTCTTGCAATTTCCTGCGGTGCATTCTCCTTCCTTT
CTCCCTTCTCCC
+
AAA9B@CACCB@B@AABAAABA@<>6775:ABCC=CBA=A<<<<<<==C@BB>B@BBBA>ABBBB?BBD=<:;5775
699>8<86666-
@T6XXI:00096:00189
CTAGTCGACTCGAGATCTGCGGCTTTGGACAGTCCCTCTGTACTGTGAAGCCACAGATGTCGAGTCGACTAGCTTCC
TTTCTCCCTTCTCCC
+
C?99994444;;@@@ABBBBB@@@@<@?AABC???8?AAAACBAAAB@>@CCCCCCAA@@?BCD?AA@???::6>B?
AA<@??=6:34344-
@T6XXI:00096:00190
GGGAGAAGGGAGAAAGGAAGGAGAAGTCGCCCCAAACGATTTGTTGACTATTCTCCCTCTCCCTATAGTGAGTCGTA
TTAGAATTCCCC
+
BCBC@@@BB>BBBB>B@B@B@BBB>??BBBBB7AA<AC@AA;@@??<<AAA?A@<<;?:?=C;=:;;=<<<<<<@@@
@>A<<7:3444,
@T6XXI:00097:00177
GGGAGAAGGGAGAAAGGAAGCTAGTCACTCGACATCTGTGGCTTCACAGTACAGAGGGACTGTCCAAAGCCGCAGAT
CTCGAGTCGACT
+
@@<@A>>AA8<<BB>BBC@CCB@@?CCCABC;::AA???C@CC@C;:;BBBCCA@@@<@AA<?:<=;;==<:::;==
=<;<@@A<???A
@T6XXI:00098:00164
GGGAGAAGGGAGAAAGGAAGCTAGTCGACTCGACATCTGTGGCTTCACAGTACAGAGGGACTGTCCAAAGCCGCAGA
TCTCGAGTCGACTAG
+
GC@CB;244-
94=>9=>B>BCA00/////....11688????BB?CCBCCBBA=>77717<<@?@>@A;A@>A?>>AAAB@@@@A==
=@@>>
@T6XXI:00098:00168
GGGAGAAGGGAGAAAGGAAGGAGAATTTGGCGTCGTGACGTGCGTGGCTATTCTCCCTCTCCCTATAGTGAGTCGTA
TTAGAATTCCCC
+
BB>BBB@BB>BBBC?C@D@C=@AC?BB<?<@B@@@CCCCABCECCC@BB@=;=99=:;;;BB=B<><CBBB;;;BB?
?<???>BDGCD:
@T6XXI:00098:00173
GGGGAATTCTAATACGACTCACTATAGGGAGAGGGAGAATAGTCAACAAATCGTTTGGGGCGACTTCTCCTTCCTTT
CTCCCTTCTCCC
+
CCA9>697;AA@CDBBABBBB@B?9669-
6=;BC>BA??A===@=<<:839968888?A7BCCCC@CCC?@><<AA<<@AA<A>??@@<
@T6XXI:00098:00175
GGGAGAAGGGAGAAAGGAAGCTAGTCGACTCGACATCTGTGGCTTCACAGGGACTGTCCAAAGCCGCAGATCTCGGT
GACT
+
```

```
GEAAAA@CI@;<<E@C?B?BCC888@BB@CC@87:?@???C?BB?B?A?<<;?A???@?CC>B>>=CC@=;?<<<89
8=G>
@T6XXI:00098:00181
GGGAGAAGGGAGAAAGGAAGTGCAGTTCGTGGTTAGTGAAGGGAAAAACTATTCTCCCTCTCCCTATAGTGAGTCGT
ATTAGA
+
CCABBA@BB?CBBB=B?B?BB@AACC@CBCC@C::8B?><A?9????0?B@@<?777.77777-
7;;4588<:6443--,---
@T6XXI:00098:00187
GGGAGAAGGGAGAAAGGAAGGAGAAGTCGCCCCAAACGATTTGTTGACTATTCTCCCTCTCCCTATAGTGAGTCGTA
TTAGAATTCCCC
+
4979@@<>?:>9990;>C>@?C@@==>?8888.33,3769?=AC@@A@B=<;?:?A:887;8058344==<6=848@
?3446:42333.
@T6XXI:00098:00189
CTCGAGATCTGCGGCTTTGGACAGTCCCTCTGTACTGTGAAGCCACAGAT
+
999999444484429AA=C@ABCCDDD?A@A?99911///76656/444.
@T6XXI:00098:00191
CTAGTCGACTCGAGGATCTGCGGCTTTGGACAGTCCCTCTGACTGTGAAGCCACAGATGTCGAGTCGACTAGCTTCC
TTTCTCCCTTCTCCC
+
B7///1//;BA=?A=ABABBEB@ACC?C=@@CC???9?AAABBDCCCC@AC?CCCCCCDCBAACCBCBCCB=<<@C@
CC?DBCB=B>ABAB>
@T6XXI:00098:00193
GGGAGAAGGGAGAAAGGAAGGAGATACAGACACGCCAATAGAAGCGCGCTATTCTCCCTCTCCCTATAGTGAGTCTA
TA
+
CCCCBD?@=9=98809;>;>?C@A@CCC;;<E@??AA?AA??=@AACCCACB=A???9???@E@CCA@@8444/,,+
**
@T6XXI:00099:00171
GGGGAATTCTAATACGACTCACTATAGGGAGAGGGAGAATAGTCAACAAATCGTTTGGGGCGACTTCTCCTTCCTTT
CTCCCTTCTCCC
+
FFD:CDC@BBB@CBAAABBAA?CC@8::8:::BCCCCB>A@@@@@>@AA<BCBBB5:66-
67>774:@@<?<?>AA:@@??:?=@@@C<
@T6XXI:00050:00202
GGGGAATTCTAATACGACTCACTATAGGGAGAGGGAGAATAGTCAACAAATCGTTTGGGGCGACTTCTCCTTCCTTT
CTCCCTTCTCCC
+
CDB9G=A=ACGBCCBABBBBB>99=116.7>>>@?ABH@D=;A@@=@AA<CABBE>BBB7AAABB@AAA?@=@?AA>
?@@B:A=@@@@;
@T6XXI:00050:00228
GGGAGAAGGGAGAAAAGGAAGGAGAAGTCGCCCCAAACGATTTGTTTGACTATTCTCCCTCTCCCTATAGTGAGTCG
TATTAAGAATTCCCC
+
4:-9@B599118888*-
+,*,2200837@8888.87,37111,...+..4426;<=AA=@A@>?6:0/..134589:9994423=245/740
@T6XXI:00050:00230
GGGAGAAGGGAGAAAGGAAGCTAGTCGACTCGAGATCTGCGGCTTTGGACAGTCCCTCTGTACTGTGAAGCCACAGA
TGTCGAGTCGACTAGCTTCCTTTCTCCCTTCTCCC
+
BA>B111185<,11,/,/,.66667/11>>@E?>BBCCBBB?CBB5:5;;AACEC>B@@@@@;??@@C>@@=?@@<<
?A===9;====<>>99=>@>AA:??>>:?>@AAC<
@T6XXI:00050:00232
GGGAGAAGGGAGAAAGGAAGCAAAAGACCGGGGAAAAAATAGTGGAACTATTCTCCCTCTCCCTATAGTGAGTCGTA
TTAGAATTCCCC
```

```
+
CC@BAB>BB<ABBB?B@C@CAAAA7ABB?A??.77777)7??@D?B?BBB????:=;<<>AC;=?B?<<AAAAABAA
A68:@?B8<=<4
@T6XXI:00050:00233
GGGAGAAGGGAGAAAGGAAAGCCTAGTCACTCGACATCTGTGGCTTCACAGTACAGAGGGACTGTCCGAAAGCCGCA
+
?C6@>>59914;8858,//+..,.6611/55..//....479077@><<CB@B?<<AAC?=<9:44/000+044003
@T6XXI:00050:00244
GGGAGAAGGGAGAAAGGAAGCTAGTCGACTCGACATCTGTGGCTTCACAGTACAGAGGGACTGTCCAAAGCCGCAGA
TCTCGAGTCGACTAG
+
GHDAAAACB>CBCB>B>B>ACA>=?/00009:=???=:BAC?AA?ABBABACC>>77718@B?@@;=<7<@=@@@@@
AA@@<<<<;;<?>??
@T6XXI:00050:00246
GGGAGAAGGGAGAAAGGAAGAACAGTGGCAATTGGGATATGTCGAGACTATTCTCCCTCTCCCTATAGTGAGTCGTA
TTAGAATTCCCC
+
CDABBA@BB@?A@C?C@C?B@><<B??>?A>?=<?;?BBAAABBEBDCCCC@C@@@<@@@CC?CBBBAAABCCCCAA
A8:;E@DBCCD9
@T6XXI:00051:00200
GGGGAATTCTAATACGACTCACTATAGGGAGAGGGAGAATAGCGACTCGTTCAGTTGCCTCCCTTTGCTCTTCCTTT
CTCCCTTCTCCC
+
EHH:C::;<BB@C@@@B@@@BC?9=111.7>>?C>BCA?A<@C@<=;9==>AAA?=?;:?CC<??;BAA@@=@9<<7
<@@@;@>@???<
@T6XXI:00051:00222
GGGGAATTCTAATACGACTCACTATAGGGAGAGGGAGAATAGTTTTCCCTTACACTA
+
CEECC::;9CCACC???BBBBCB?961659??BE>AA>?A:6550.00+++++++04
@T6XXI:00051:00231
GGGGAATTCTAATACGACTCACTATAGGGAGAGGGAGAATAGATCACCGTTGGTTCGGGTGCGCGTTCTCCTTCCTT
TCTCCCTTCTCCC
+
CDC:CBC@CCC@@?ABBBBCCCBBB966.:@@BE>CBB=@?@@@ABB?AA;?;?=@BC@C@@@A>>47673101069
9??<?:?344884
@T6XXI:00051:00241
GGGGAATTCTAATACGACTCACTATAGGGAGCATCG
+
999,4295<BBCDBBCCECA@:??>700,00,,,,2
@T6XXI:00052:00215
GGGAGAAGGGAGAAAAGGAAGCTAGTCACTCGACATCTGTGGCTTCACAGTACAGAGGGACTGTCCAAAGCCGCAGA
TCTCGAGTCGACTAGCTTCCTTTCTCCCTTCTCCC
+
CB>?;;79@:@8999,42423733377761111111677>2::?AAABBBCCC@78818;>@A@?AA:@A=@@A@=
A9==@<;888888;<;D=@=@A<AAAA=A<??@@:
@T6XXI:00052:00236
GGGGAATTCTAATTACGACTCACTATAGGGAGAGGGAGAATAGCACTCCCATTGGCCGTGTCCCGGTTGACTTCCTT
TCTCCCTTCTCCC
+
/11+1,-
*,,,*,*///43;;;7991111,11BBC?::4>AAA=<<;=448;7:2424;4477.4/4237302623535=994=
89:<=6
@T6XXI:00054:00211
GGGAGAAGGGAGAAAGGAAGCTAGTCACTCGACATCTGTGGCTTCACAGTACAGAGGGACTGTCCAAAGCCGCAGAT
CTCGAGTCGACTAG
+
```

DD@DCB@B@;:844-95958@@8;:B???A@;:9???BCL@BB?BCCBAA?=74444-
4::;@BAAHEAA>A?@????==?===AA@??=<
@T6XXI:00054:00213
CTGCGGCTTTGGACAGTCCCTCTGTACTGTGAAGCCACAGATGTCGAGTCGACTAGCTTCCTTTCTCCCTTCTCCC
+
FDCBA?BCD@C?BDCD@>>=@BBB>>=ABCBB?BB?BA@@CC@@@DCCABBB@@??@:=A>@C6;6==7>5:69>:
@T6XXI:00054:00214
GGGGAATTCTAATACGACTCACTATAGGGAGAGGGAGAATAGTTCCTAC
+
BBB9A=?=ABB@CBBBCBBAA?997166.7>??CACBA>?7/,*+**.*
@T6XXI:00054:00222
GGGAGAAGGGAGAAAGGAAGCTAGTCACTCGAGTCTGCGGCTTTGGACAGTCCCTCTGTACTGTGAAGCCACAGATG
TCGAGTCGACTAGCTTCCTTTCTCCCTTCTCCC
+
CC@@AB=@>:>>?>9>9<9;CCFC???6668:<?AA=??::::5:>>==>===7==?==>ACA@@A@@@=@AA===@
?@@@===@?===888;=;==7====7=:==??:
@T6XXI:00054:00227
GGGAGAAGGGAGAAAGGAAGGAGAAGTCGCCCCAAAACGATTTGTTGACTATTCTCCCCTCTCCCTATAGTGAGTCG
TATTAGAATTC
+
44-4@A6991999B>C@B:;;;00,0;;00..*...*33111,110177;4:8:4433-
444494888037<>?:=<@<630**)*)(
@T6XXI:00054:00248
GGGGAATTCTAATACGACTCACTATAGGGAGAGGGAGAATAGCCTTACCTTTGCTAAATTCTCCGTTGCCTTCCTTT
CTCCCTTCTCCC
+
DEC9B>B?@@ADCCBBBBBBBA<97111.7>==?=?CBCB???=CAC>>>B=?:??A<A>?@@>@@<==;=@A<??;
?@@B<@=?@@C=
@T6XXI:00055:00200
GGGAGAAGGGAGAAAGGAAGGAGAAGTCGCCCCAAACGATTTGTTGACTATTCTCCCTCTCCCTATAGTGAGTCGTA
TTAGAATTCCCC
+
BDAEICB@>:@@896>=@=@=@BBBBCCCDCB8?A<AAABG@GC@BAAAAB>@@AA<???AA<A???@ABA<==<<<
<E@@@>B;@A@5
@T6XXI:00055:00202
GGGGAATTCTAATACGACTCACTATAGGGAGAGGGAGAATAGCAAAC
+
944,49?<ACB@FCBBCBBBC?>?A777-7=777-:979=A@???;?
@T6XXI:00055:00227
GGGGAATCTAATACGACTCACTATAGGAGAGGAGAATAGTCAACAAATCGTT
+
@<<0<,1///7849;@?;??6996110111101102360004/000+11111
@T6XXI:00055:00244
GGGAGAAGGGAGAAAGAAGCTAGTCACTC
+
44-444244-4888-4499333.,,,,,,
@T6XXI:00055:00248
GGGGAATTCTAATACGACTCACTATAGGGAGAGGGAGAATAGTGACTAGCTGGTCTACGTTGCTCTCTCCTTCCTTT
CTCCCTTCTCCC
+
@@@6A?C@CBB@CCB?BBCBBA<96116.7>>BD@BB@<@====9;;9====?@???@AA?@<@??=?A==:;233-
44::4:8<;>>7
@T6XXI:00056:00203
GGGAGAAGGGAGAAAGGAAGCTAGTCACTCGACATCTGTGGCTTCACAGTACAGAGGGACTGTCCAAAGCCGCAGAT
CTCGAGTCGACTAG
+

```
?@<@AA?BB<A@885>;=;?AA@>>?:776=776:<:??C?AB?BCCCCCBB?=000-
4;;?@@>A@;@A?@@AAC88=@878?<==??DB
@T6XXI:00056:00215
GGGAGAAGGGAGAAAGGAAGCTCAATCGTGGGTAGAAGGGTCGTGATCTATTCTCCCTCTCTCTATAGTGAGTCGTA
TTAGAATTCCCC
+
BDAECC?A?:>884-
9;>=@FCFD?CBBBCD>C:::@@A<A99:@@CHDA@>@BAC????===AAAA@@A@@<;<@;<8;=>;?>@AB6
@T6XXI:00056:00216
CTAGTCACTCGAGATCTGCGGCTTTGGACAGTCCCTCTGTACTGTGAAGCCACAGATGTCGAGTCGACTAGCTTCCT
TTCTCCCTTCTCCC
+
B=///6/44>=AABDCBB?;;;BB>B<?@CA??>;?CAAA@@@=?=A;=A@CCBBBCBBBBBCCBBBCCCCCC@C@C
C>???D>C?>??A=
@T6XXI:00056:00225
GGGGAATTCTAATACGACTCACTATAGGGAGAGGGAGAATAGCCACGTATCTTGCCCCTGGCTGTTCGCCTTCCTTT
CTCCCTTCTCCC
+
944,48>ACBB@CCBBBAAAA8<<;677.777>@=ADA@@@@@<A<A88:AA94400+4928>@@:==@:?:B5771
8=@@<@588??:
@T6XXI:00056:00229
GGGAGAAGGGAGAAAGGAAGGAGCATAACGACCAGATATACCTGCACGCTATTCTCCCTCTCCCTATAGTGAGTCGT
ATTAGAATTCCCC
+
BCBBBB@BC>DBBB?B@B?C?AACBBB@B???>BB?B??>B@BABABBCAAB@BABA=?>><A>A??>?CCAAA??>
???AAA>@9<;;0
@T6XXI:00056:00235
GGGAGAAGGGAGAAAGGAAGCTAGTCGTCTCGACATCTGTGGCTTCACAGTACAGAGGGACTGTCCAAAGCCGCAGA
TCTCGAGTCGACTAG
+
CD@CCC?A@=ABA>=B>B=AB@;AA8;;CCBB@??????BA?AB@CCCB??>@@=88898<<@@A>AA<??<?AA@A
>>AA==899D@?A@@
@T6XXI:00056:00244
GGGAGAAGGGAGAAAGGAAGAACCGGCCGCGGGAGTTAAAATAACGGCTATTCTCCCTCTCCCTATAGTGAGTCGTA
TTAGAATTCCGGGAACGTCT
+
94-9@A699-
4:>C9@=@?BB?C@C@B>AC@A;6;<3666.77B=A?BCCC@B4990449@@>B::797.444..../47>?:6630
0000*+++++
@T6XXI:00057:00210
CTATCACTCGAGATCTGCGGGCTTTGGACCAGTCCCTCTGTACTGTGAAGCCACAGATGTCGAGTCGACTAGCTTCC
TTTCTCCCTTCCTCCC
+
FAAA?AA@DBBDCBAABBBB<BBB>C@??<:?<<>>BCDAC???>@@B=?B?CCDCB?>??ECCB@@@CBBBBB@C7
;;7:>8928,/,/993
@T6XXI:00057:00212
GGGGTTCTAATACGACTCACTATAGGAGAGGGAGAATAGTCAACAAATCGTTTGGGGCGACTTCTCCTTCCTTTCTC
CCTTCTCCC
+
CCC9BACCE@DCCA@@BCCC>99..,0@:::9?@@?CA??BDAB@@;@@@@C<???5?@@AA@=8888?A;??:???
?:@>???A<
@T6XXI:00057:00221
GGGAGAAGGGAGAAAGGAAGGAGAAGTCGCCCCAAACGATTTGTTGACTATTCTCCCTCTCCCTATAGTGAGTCGTA
TTCAGAATTCCCA
+
CCABCBABB>9;9?9@<><>;>??=?A?>>>>.8808768@>BB?ACCCAB;??CC====AA<??????@==88340
8/07;74479555
```

```
@T6XXI:00058:00232
GGGAGAAGGGAGAAAGGAAGCTAGTCACTCGACATCTGTGGCTTCACAGTACAGAGGGACTGTCCAAAGCCGCAGAT
CTCGAGTCGACTAGCTTCCTTTCTCCCTTCTCCC
+
FKDCA@=AD@B@@>9>6958??>>::,,,,,,,,,2457>77;?BBBCBB?==3044-
4;;:<=>AA<@@;@@@?@><<<<<?>@>887;666,/,/7,/.4442383;;5
@T6XXI:00058:00237
GGGAGAAGGGAGAAAGGAAGCTCAATCGTGGGTAGAAGGGTCGTGGATCTATTCTCCCCCTCCCGTATAGTGATCGT
TCACTG
+
CDAAA><99911//+/,/,..797761116626:9:?@@:?887834;>778=?=====.333+003535<=3+++*
)**.**
@T6XXI:00058:00241
GGGAGAAGGGAGAAAGGAAGGAGAACGCGCACCCGAACCAACGGTGATCTATTCTCCCTCTCCCTATAGTGAGTCGT
ATTAGAATTCCCC
+
CDBA?>@CC@CCCC?B?B@B@?>;>BBBAACCH@BB>B=C=ACEDCCCCCCC@BBAA<ACCCC>CCB:::BA@???B
??;?@@=B?CCCB
@T6XXI:00059:00200
GGGAGAAGGGAGAAAGGAAGGAGAAGTCGCCCCAAACGATTTGTTGACTATTCTCCCTCTCCCTATAGTGAGTCGTA
T
+
DCACCC@CB<B@@@>F@C@C@DBB?BABBCCC9EC>CCBBC>CB?CBB@==>AAAC=@@???:?@???AAAA@A???
?
@T6XXI:00059:00222
GGGGAATTCTAATACGACTCACTATAGGGAGAGGGAGAATAGTCAACAAAATCGTTTGGGGGCGACTTCTCCTTCCT
TTCTCCCTTCTCCC
+
A??9A?C?BDDADBBBCCCBBCCBB:77.7===B5:73866343945355.87777,1111+11116276<@=;<<=
A9===A<@38.461
@T6XXI:00059:00227
GGGAGAAGGGAGAAAGGAAGGAGAAGTCGCCCCAAACGATTTGTTGACTATTCTCCCTCTCCCTATAGTGAGTCGTA
TTCAGA
+
AE@HED?CB@AA@B>C:<;;CCAC>ACBCCCC8BB?BCBBC>CC@CBBB=<9<===:888>A<?@???===;440.-
+*++++
@T6XXI:00059:00230
GGGAGAAGGGAGAAAGGAAGGAGAAGTCGCCCCAAACGATTTGTTGACTATTCTCCCTCTCCCTATAGTGAGTCGTA
TTCAGAATTCCCC
+
GG?BBC@DCADBBB>B?B?B?BBB@BB@@@AA7AA<????98AA?BCCC@@=@AAA<???@C<A=<<AA@A=<;:7/
/,-.2237,430+
@T6XXI:00059:00237
CTAGTCGACTCGAGATCTGCGGCTTTGGACAGTCCCTCTGTACTGTGAAGCCACAGATGGACTGTCCTTCCTTTCTC
CCTTCTCCC
+
FBAA@@ADCCCCCCBBBBCBB>@A@<A@BBAC:::7:><????A?<:=;?A?BB?B:97943333376010177117
886716/72
@T6XXI:00059:00244
GGGGAATTCTAATACGACTCACTATAGGGAGAGGGAGAATAGTCAACAAATCGTTTGGGGCGACTTCTCCTTCCTTT
CTCCCTTCTCCC
+
CCC9B?A?BCB@CCBBBBBBB>997111.7==>A@CCA@@8>AAC>???:A;;;=-666-
7?ABC@CCD??<?>AA<??AA=A689AA=
@T6XXI:00059:00245
CTAGTCAGATCTGCGGCTTTGGACAGTCCCTCTGTACTGTGAAGCCACAGATGTCGAGTCGACTAGCTTCCTTTCTC
CCTTCTCCC
```

```
+
DE:>=CCCBDDDCCD@CCC>C@CBBBBBB>@@??BBBBBBBB?AA@B?BB??:?:?@@@???@@@<7;;:@CD>BBC
B<?;????9
@T6XXI:00060:00205
CTATCACTCGAGATCTGCGGCTTTGGACAGTCCCTCTGTACTGTGAAGCCACAGATGCTCGAGTCGAC
+
999444444949BCAAA>>;88808587??@@B<@>>7777==0348:B@B@>?00.....644..,,
@T6XXI:00060:00206
GGGGAATTCTAATACGACTCACTATAGGGAGAGGGAGAATAGTCAACAAATCGTTTGGGGCGACTTCTCCTTCCTTT
CTCCCTTCTCCC
+
CCF9E@C@B@@@BBB>@<?B>@??;111.777BB?C@?;=???@A?@@@<BCCCC>BBB7BCB??>?CC?@=@=AA<
AAAA=A;>?AA<
@T6XXI:00060:00218
GGGAGAAGGGAGAAAGGAAGCTAGTCGACTCGACATCTGTGGGTTCACAGTACAGAGGACTGTCCAAAG
+
??>999??@044885878<>?888<76611113117;6669>8857396=<88;<<?8<<;/++33000
@T6XXI:00060:00229
GGGGAATTCTAATACGACTCACTATAGGGAGAGGAGAATAGTACGACCAAAT
+
>>86==@=@AB?DCCBBB????BBA??<89>112:=979/////-41000*.
@T6XXI:00060:00243
GGGAGAAGGGGAGAAAAGGAAAGGAGAAAGTCGCCCCCAAAACGATTTTGTTGACTATTCTCCCTCCTCC
+
44-4442444,49999,8444-34.00.+....4444/444044.111,11017610.448882//,/./
@T6XXI:00061:00214
GGGAGAAGGGAGAAAGGAAGGAGAAGTCGCCCCAAACGATTTGTTGACTATTCTCCCTCTCCCTATAGTGAGTCGTA
TTCAGAATTCCCC
+
BCACEC@CD?CBBB?B?B@C@BBB@BA?7788.8>699171,11016<6=?688AB=:98@C<@A===?@@?B@=::
:,//887:7=;=1
@T6XXI:00062:00215
GGGAGAAGGGAGAAAGGAAGGTCGTGTAT
+
KD?C;;69>:>?88888?88+-,,,,*(2
@T6XXI:00062:00243
GGGGAATTCTAATACGACTCACTATAGGAGAGGGAGAATAGTCAACAAATCGTTTGGGGCGACTTCTCCTTCCTTTC
TCCCTTCTCCC
+
CCC9B798;AB@CCCCBB@@A:695..,0@@?C>CC@>@<?<AA>A@@:@:77<9<>@5BBBBC@BBB>@<?>BA:A
BAA=A8<<CC<
@T6XXI:00063:00210
GGGAGAAGGGAGAAAGGAAGAGAACGGCGACGAACAACAGTTGGTGGCTATTCTCCCTCTCCCTATAGTGAGTCGTA
TTAGAATTCCCC
+
CCCAAACCC>AAAC@DAB?BCCI@BA?BCCCCB@CC@???B@@@775:?>>=@AAA<BAAAAAAAC@@@AA@@?===
A<?AC>@>@CC5
@T6XXI:00063:00217
GGGGAATTCTAATACGACTCACTATAGGAGAGGGAGAATAGTCAACAAATCGTTTGGGGCGACTTCTCCTTCCTTTC
TCCCTTCTCCC
+
CCCAC@A?ACB@C???BBBBB:997/.,0@07:8:CA>@@AA@@=@AC=CAABB>B=>7@BCBCCBBC7848<?C?A
AAA;@387??:
@T6XXI:00063:00218
GGGGAATTCTAATACGACTCACTATAGGGAGAGGGAGAATAGTCAACAAATCGTTTGGGGCGACTTCTCCTTCCTTT
CTCCCTTCTCCC
+
```

```
CEC:EAC@@A@@CCCBBBAC>87?=167.7>>BC?BB@?A:AAAC>@@@;BAAAC<BBB7BCCCB@DCC>=;=>AA<
A@@@;@>@@@A<
@T6XXI:00063:00231
GGGAGAAGGGAGAAAAGGAAGCT
+
:C6944249091111*/,,*,,,
@T6XXI:00063:00235
GGGAGAAGGGAGAAAGGAAGGAGAAGTCGCCCCAAACGATTTGTTGACTATTCTCCCTCTCCCTATAGTGAGTCGTA
TTAGAATTCCCC
+
BBBBDCACC@BAABBB@B?A@ABB@AAABBCC9CC>@@???9???BC??;;:8:<=;99=@A<@@A@@<AAA?@@88
9@????A9<;;0
@T6XXI:00064:00203
GGGAGAAGGGAGAAAGGAAGGAGAAGT
+
@A<ABA@CC>CBB@<B7;7/8888+((
@T6XXI:00064:00209
GGGAGAAGGGAGAAAGGAAGGGAATAGCAGCAGTCGGCAACGTGAAACTATTCTCCCTCTCCCTATAGTGAGTCGTA
TTAGAACTTCCCC
+
CB?BBB>@>:>9998;<A>BB>B?BB;<<BBACDCC?BC?BCCCCC8:;@?<?ABA9???@@<?@???8886////.
A?D??<?B>AAA6
@T6XXI:00064:00221
CTAGTCGACTCGAGATCTGCGGCTTTGGACAGTCCCTCTGTACTGTGAAGCCACAGATGTCGAGCTCCTT
+
CD;<=@@>?99998899448823<?9A?A???677-17=??CCCCC?=99@B>?=A;;73174./,*)*)
@T6XXI:00064:00236
GGGGAATTCTAATACGACTCACTATAGGGAGAGGGAGAATAGTCAACAAATCGTTTGGGGCGACTTCTCCTTCCTTT
CTCCCTTCTCCC
+
CAA6A@C@CCC@BBBBBABCBAAA??<<6;<<?A>??=>@A@@AA=@@D;?????9?BB9CCCCCACCC@A>A>AA:
AAAD<@489?@;
@T6XXI:00064:00239
GGGAGAAGGGAGAAAGGAAGGAGAAGTCGCCCCAAACGATTTGTTGACTATTCTCCCTCTCCCTATAGTGAGTCGTA
TTAGAATTCCCC
+
DCACCC?BB>9;;H?C@B@C@BBB@>>@BBEE8??<@BACB>BC@CAAAAA>AAAC;???>>5==>>????@===??
>?BBA>A=@@A5
@T6XXI:00065:00201
GGGGAATTCTAATACGACTCACTATAGGGAGAGGGAGAATAGCCTCCAATTCCCAGTTAACGTGTTCCCTTCCTTTC
TCCCTTCTCCC
+
@AA8A@ECBBBACCCCC@B@BCB;9111.677=@<AB@>@A??<AA?B?C@@?;B<=9<=@;;9;244-
48;8;=6<<@A;@>@@BA@
@T6XXI:00065:00208
GGGAGAAGGGAGAAAGGAAGACCATGATGGGGGTGAACAGCGAAGAACTATTCTCCCTCTCCCTATAGTGAGTCGTT
CAATTATGAACT
+
CC@BA@@BB>BBBB?C@CABAB@EC>=76611,7==>@AABAB?BC@BBAC=A@BC:=>88718@@AAA===@@8/-
/656,----,--
@T6XXI:00065:00219
CTAGTCACTCGAGGATCTGCGGCTTTGGACAGTCCCTCTGTACTGTGAAGCCACAGATGTCGAGTCGACTAGCTTCC
TTTCTCCCTTCTCCC
+
,////////6///,//8799;:73:6:=@@;::@@:?@??A:?<B@==8;A>@ABAB::;B@@@CCKAACDD??;?<
>?9??AB9<:?=AC@
@T6XXI:00065:00226
```

```
GGGGAATTCTAATACGACTCACTATAGGAGAGGGAGAATAGCTACGTTTAACCGATCGTGTTGTTCTCCTTCCTTTC
TCCCTTCTCCC
+
@@@6@>B?BBBACBAAABBBB@<<9664:@BBC>BBA>@<?<CAAACC>C?B??AA;??@C?A@99=B9;5<:==78
88A;@=@@@?:
@T6XXI:00065:00230
GGGAGAAGGGAGAAAGGAAGCGGAACAGCAAGCACCGAAATTGGAGCTATTCTCCCTCTCCCTATAGTGAGTCGTAT
TAGAACTTCCCC
+
DC@CCC@CB>BBBB?BAB?@<?@B@BB><??CCCC?CCD?CDC@BBBCCC@CCCC?CBBCC>C@@A@@ABACCB?==
=??B8;:@CED7
@T6XXI:00066:00231
GGGAGAAGGGAGAAAGGAAGAAAGGAAGGGAATAGCAGCAGTCGGCAACGTGAAACTATTCTCCCTCTCCCTATAGT
GAGTCGTATTAGAATTCCCC
+
CD@BBA?BB>BBBB>B@B>?>B;?=?>>>;>;????BCBBCC:::;B?ABCCCC>CDCB>@@@@<@CABB>BBB@@@
@@AEDCCDC?BB@>DBDCC:
@T6XXI:00066:00240
GGGAGAAGGGAGAAAGGAAGCTAGTCACTCGACATCTGTGGCTCACAGTACAGAGGGACTGTCCAAAGCCGCAGATC
TCGAGTCGACTAG
+
CE>AA?@HC?BBA=:@=@<@CA@@A?:7:@@:;:BBBBBC@BBDCCAAA@A?977717@>??A?@@:@A=A?889:;
=;;:::99==<<@
@T6XXI:00066:00247
GGGGAATTCTAATACGACTCACTATAGGAGAGGAGAAT
+
C??1<64?@99599488??@B@>?:71361..52,+*+
@T6XXI:00066:00248
GGGAGAAGGGAGAAAGGAAGGGGGAGAAGGGAGAAAAGGAAGCAG
+
44-499599-4449582423333+399;==.77767.7:666131
@T6XXI:00067:00200
GGGGAATTCTAATACGACTCACTATAGGAGAGGGAGAATAGAGACGTATTCGTTTGCGCTCTGTTCACCTTCCTTTC
TCCCTTCTCCC
+
EED9C@A@ACCBBBBCCCCBBAB>?994:BBCD>CBA=AA?<<<<==>><?>AC@===AC????>B8848=@?BC;@
CAA:?:=<>A<
@T6XXI:00067:00209
CTAGTCACTCGAGATCTGCGGCTTTGGACAGTCCCTCTGTACTGTGAAGCCACAGATGTCGAGTCGACTAGCTTCCT
TTCTCCCTTCTCCC
+
E:1/11144BBB@?ACBB?;:733,7<<>>>777.:??BCCAB?CAA>@CAA@@CCCABBCBBBBBA@@@BB?@CBC
CCCCBC>@7;844-
@T6XXI:00067:00218
GGGAGAAGGGAGAAAGGAAGCTAGTCACTCGAGATCTGCGGCTTTGGACAGTCCCTCTGTACTGTGAAGCCACAGAT
GTCGAGTCGACTAGCTTCCTTTCTCCCTTCTCCC
+
DDAKAA@AC<>99809595;CAAAC@AAAA::;@BCCCCB?BBB>B?CC@@@@@;>>====;:??@E=??=@AAAB<
<<=<<<<@<<=;??558358969777,/,////,
@T6XXI:00067:00221
GGGGAATTCTAATACGACTCACTATAGGGAGAGGGAGAATAGTCAACAAATCGTTTGGGGCGACTTCTCCTTCCTTT
CTCCCTTCTCCC
+
AAB9B?B@BBB@C@@@CABC>>AB?7675:AABE>CB?=@=B@<;8;;84<;766-
7>>2ABABB@BCC@A?A?AA;@@AA<@=@@AB<
@T6XXI:00067:00238
```

GGGGAATTCTAATACGACTCACTATAGGGAGAGGGAGAATAGTCAACAAATCGTTTGGGGCGACTTCTCCTTCCTTT
CTCCCTTCTCCC
+
BBB7A=>=@@@?B<<<CCCCCCB96111.777>>:BBA>@A@@@C=<;<7?:::C>CCC9CB>@>:;00,/,/5886
99<C=A9<<;;6
@T6XXI:00067:00245
GGGGAATTCTAATACGACTCACTATAGGAGAGGGAGAATAGCCTGCAGGTCGAGTGTAGGACTGTTCCCTTCCTTTC
TCCCTTCTCCC
+
CB@7B79;<AA?A@@AACCEEC@@?:747?777.7>;;>???;@@@:95:??A@@@@@@<?==::7:94:7<;=>:>
;;<6<:;4481
@T6XXI:00068:00225
GGGGAATTCTAATACGACTCACTATAGGAGAGGGAGAATAGCCAGAATCTGTTCTGAGTTCGTTCTCTCTTCCTTTC
TCCCTTCTCCC
+
CDC:C?B?ACD@CCBAB@B?B>==://-
0>>BK>CB@@A<9856<<8=AACA=??AAAC88=A>A<<<=A;A=AA;@:::9://0;=9
@T6XXI:00068:00226
GGGGAATTCTAATACGACTCACTATAGGAGAGGGAGAATAGTTTATCACTATCATTCCTCAAG
+
CFE:F@@>?CCBCCBBBBBB@6;;://,0<<?A>C@=;=@@==>A?@A>677611360.--,-
@T6XXI:00068:00241
GGGAGAAGGGAGAAAGGAAGCTAGTCACTCGACATCTGTGGCTTCACAGTACAGAGGGACTGTCCAAAGCCGCAGAT
CTCGAGTCGACTAG
+
AA:ACA?A>:>99909;>;>@;8.77,,,,,,0002....,..,3688:?CBB?<<<6884;;;8<;6;;8<=AA@@
@<<;?;;<>=>>84
@T6XXI:00069:00205
CTATCACTCGAGATCTGCGGCTTTGGACAGTCCCTCTGTACTGTGAAGCCACAGATGTCGAGTCGACTAGCTTCCTT
TCTCCCTTCTCCC
+
CDB?>===A@ACCCCDCBA>BDDAB<>@CCA?A>ACB@B?AB@@A@?@B?BBBCCCCCCCC@@FBBBBBCB@?B?AF
<;:8@;@;??@A;
@T6XXI:00069:00218
GGGAGAAGGGAGAAAGGAAGCTATCGACTCGACATCTGTGGCTTCACAGTACAGAGGGACTGTCCAAAGCCGCAGAT
CTCGAGTCGACTAG
+
DDACCE@DC>B@?>:?<?=@ACA@AB:::?@?=??????@;??=???AABCB?;433-
4;;>@@?AA;@@?A@<<<<=@<@@@@AAAAC==
@T6XXI:00069:00241
GGGAGAAGGGAGAAAGGAAGGTGAATAGTCAGAGCAATCCGTCGGAACTATTCTCCCCTCCCTATAGTGAGTCTATT
ATATCGGAGTCTAATACGACTCACTATAGGAGAGGAGGAATAGT
+
DDCCCB?B?=ABBB>B=@=@@B>A@@8::CCCCBC?>?@@@@====?B@BB?BB==90333-
8;;BA999=;;;0,+,+++...+...-546000000468:@:?9766,,+,*)*.*,+.
@T6XXI:00069:00244
GGGGAATTCTAATACGACTCACTATAGGGAGAGGGAGAATAGTCAACAAATCGTTTGGGGCGACTTCTCCTTCCTTT
CTCCCTTCTCCC
+
CCD7:248<A@BCDCBBACBC@?96166.7>7775:BB>A@>>>B<>?A>BCBBC>BBB7CCCBB?CCC==;=>@@<
@@AC<A;=<AA<
@T6XXI:00070:00212
GGGGAATTCTAATACGACTCACTATAGGAGAGGGAGAATAGCTCATGGCTCCAATGACCTTTGCTTCTTCTTCCTTT
C
+
BBB9B@B@CDB@CBBABCBAB:==;//-
0>>?B@D;969BBACCC:888?A>A>@@?A>BC>AA@>??=@A>C>?@<?

```
@T6XXI:00070:00217
GGGAGAAGGGAGAAAGGAAGCTAGTCGACTCGACATCTGTGGCTTCACAGTACAGAGGGACTGTCCAAAGCCGCAGA
TCTCGA
+
CDDCBC>@=:@B?A=BCI;;;C:;:BBBCBB@@:::::?@A;?@?ABBBBBA=94000-
4999@A?@A=AB>B@=?<=<;;98
@T6XXI:00070:00224
GGGAGAAGGGAGAAAGGAAGGCGAACAGGGTACCGTATACAGGCACGCTATTCTCCCTCTCCCTATAGTGAGTCGTA
TTAGAACTTCCC
+
?B:@99699-4888-
424777;>??BCCB<?::6;B?<?@::68==@@@@@=?>>=49:448+7>>;==???=<;:44349:899/44-
@T6XXI:00070:00238
GGGGAATTCTAATACGACTCACTATAGGGAGAGGGAGAATAGCCCTCATTTGCGCACATGAATTCTATTCTTCCTTT
CTCCCTTCTCCC
+
999/98>=AEB@CBBBBBBBBCB>>7665:BBBE>CA?<><<689?CCCC>??=A>8>;;=?=<:<<?=>6565684
888;689;4771
@T6XXI:00070:00242
GGGAGAAGGGAGAAAGGAAGGAGAAGAGCTGCACACATTATGCAATGGCTATTCTCCCTCTCCCTATAGTGAGTCGT
ATTAGAATTCCCC
+
BB@AAA@BB>BBBB>B@BAB>?BB?@??>@@BC?B@@C?>A@<=:7737>@ADCAAB;???CC>A==887<;;=???
?@=<<<9?;?@?5
@T6XXI:00070:00249
GGGAATTCTAATACGACTCACTATAGGGAGAGGAGAATAGAGCAACCGGTCGGTTCG
+
**),*/,/6676BCCCC@@@BA@???@:@@??=@BB;;7//.-17701011101017
@T6XXI:00071:00204
GGGGAATTCTAATACGACTCACTATAGGAGAGGGAGAATAGTCAACAAATCGTTTGGGCCGCTTCTCC
+
CEE:DDD@BBB>BAB@@@AACB>>>00,0>0777:B@@AA@=>=?<<;6<=<<<.77.0,000,.333
@T6XXI:00071:00210
GGGAGAAGGGAGAAAGGAAGGGGAGAAGGGAGAAAGGAAGCTAGTCACTCGAC
+
CDABAA>AA=A@?B>CBC@BCC9BBB>AD>C:99-74737A:77?=6..,,++
@T6XXI:00071:00211
GGGGAATTCTAATACGACTCACTATAGGGAGAGGGAGAATAGTTCTCCGATTTTCAACATGTTGCTGTTCTTCCTTT
CTCCCTTCTCCC
+
ABB9CAC@BBBDCFCBBBCCCB@?;677.7???A>A:838@=?>@@@>@BBCC8BB?B?@?D@CAAAA@CC@B@CC>
?@@E??<?@@@:
@T6XXI:00071:00227
GGGGAATTCTAATACGACTCACTATAGGAGAGGGAGAATAGCCAACGATTCCCTGATACGCTATTTTACTTCCTTTC
TCCCTTCTCCC
+
CDF:C>?>ACC@CBA??BBBB:997/.,0<<?B>CB@>AA@@<?<?CCC;??==@@DCC?==7677.7?;:@48718
===:@>>>>A4
@T6XXI:00071:00228
CTAGTCACTCGAGATCTGCGGCTTTGGACAGTCCCCTCTGTACTGTGAAGCCACAGATGTCGAGTCGACTAGCTTCC
TTTCTCCCTTCCTCCC
+
?11//,,///866;;C<;<C?ABC?D?B??>7777-
7?=??CCCBB=A=<@=AA>??@@@ALCCCCCBB<;:?877:??9?=:<68,/,/661
@T6XXI:00072:00202
GGGGAATTCTAATACGACTCACTATAGGGAGAGGGAGAATAGTCAACAAATCGTTTGGGGCGACTTCTCCTTCCTTT
CTCCCTTCTCCC
```

```
+
CDB9E>@=ACC?BBBBBBBBA?<96166.7>>@C@DB@>?;?@@A?=<<7??<<@59694:@ABD@AAA??<?>AA<
A???<?<??AA<
@T6XXI:00072:00207
GGGGAATTCTAATACGACTCACTATAGGGAGAGAGATATAAGGGCAAGAGCTTGGCAACCTGGGAAGACGTGTGACC
CATTACCAAATTCTCTCCC
+
CCC:B@C@BCC@CBCBB?@@@?<<?>?>;??@@@@<<<<=>==;<@>@@BB??C?@A>?<?;;7<>@@@CCA@A888
18@=@A>;>9;;;//0;;6
@T6XXI:00072:00215
CTATCACTCGAGATCTGCGGCTTTGGACAGTCCCTCTGTACTGTGAAGCCATAGATGTCGAGTCGACTAGCTTCCTT
+
DCAA9::ABCABBCBBBAA?ABB>B?BCBBBBC?CCC@B>A?@??A>?B?BCCCBBDBBBBBBCCBC@@;:7;?;?@
@T6XXI:00072:00243
GGGAGAAGGGAGAAAGGAAGCTAGTCACTCGAGATCTGCGGCTTTGGACAGTCCCTCTGTACTGTGAAGCCACAGAT
GTCGAGTCGACTAGCTTCCTTTCTCCCTTCTCCC
+
??6A@@=>A:@999184943@:87?CBBBBBA?=@BCBBC?:::7:@A@AA???9?@>???AAA??A?@@>A@@@<<
<=<@AA??447888=><?@CC=ACDD9=9<>==7
@T6XXI:00073:00201
GGGAGAAGGGAGAAAGGAAGAGAACAGCCCTGCGGTGGGGTCGTAGGCTATTCTCCCTCTCCCTATAGTGAGTCGTA
TTAGAATTCCCC
+
CCDCCCACB>AAA@;@6968A??=ACBAB?EB@@<999911==>00-
0=>A>BAAA<@CBCE@CAAABBCCCDBA???;==A=A>ACC4
@T6XXI:00073:00211
GGGAGAAGGGAGAAAGGAAGCTAGTCGACTCGACATCTGTGGCTTCACAGTACAGAGGGACTGTCCAAAGCCGCAGA
TCTCGA
+
CC?CCC?AA=@B?B=B@C=@@B>@<<??B??;:77::7BCC>?@@BBBB??@A;=44448A;@AA><@<==:=A=><
@AAA<<
@T6XXI:00073:00215
GGGGAATTCTAATACGACTCACTATAGGAGAGGGAGAATAGACACCAATCGTGCCGGGTCATGTTCTCCTTCCTTTC
TCCCTTCCTCCC
+
CCB7B>BABCCABBB?@ACCCBCCA9957=?@B>BCD@@?@@@A@=?=B:::@?8??9?AAAAD>==8;<;>;5:59
49706,/,///,
@T6XXI:00073:00217
GGGGAATTCTAATACGACTCACTATAGGAGAGGGAGAATAGCTAATGTCTCTACCTCACGATGCTGTTCCTTCCTTT
CTCCCTTCTCCC
+
A==6?596<;;<ABCAA@AAA@>>>::5:BBBD=AABEHA@?AA?BB@=:6,++*+++-
000333==;<;<;=56844444457//.54
@T6XXI:00074:00200
GGGGAATTCTAATACGACTCACTATAGGGAGAGGGAGAATAGTCAACAAATCGTTTGGGGGCGACTTCTCCTTCCCT
TTCTCCCTTCTCCC
+
444,4899499==@FB;;;@?=?><777,6;=??<9:753;8///,-22277111,1111+117>?=:97;:/44-
4;7;9830323---+
@T6XXI:00074:00204
GGGAGAAGGGAGAAAGGAAGGGGAGAAGGGAGAAAGGAAGCT
+
CDAD@@=?D>BCBB>C@C<?;72?;>>@B:=00/,,+,+,0*
@T6XXI:00074:00207
GGGGAATTCTAATACGACTCACTATAGGGAGGGGGAGAATAGTCAACAAATCGTTTGGGGCGACTTCTCCTTCCTTT
CTCCCTTCTCCC
+
```

```
:::-
9<A>BCC@DCBBBBBBAAABA:::57?@CC2CCC=@=?@AA>ABB>788??:>BB4==@?=:><<8;<?8;<6<???
;@>AA@A;
@T6XXI:00074:00212
GGGAGAAGGGAGAAAGGAAGGAAAACAGGCAAGCGTGATGTAAGTTGACTATTCTCCCTCTCCCTATAGTGAGTCGT
ATTAGAATTCCCC
+
CB@AAA?@@;@BBA;?<?@B@BBB7BBB@BB@BBBB@CCCCB@BC?BBCCCC@CAAA<ABBBB>BCC=<:<BCCBAA
CC@AAA=@=@AA6
@T6XXI:00074:00215
GGGAGAGAATTTGGTAATGGGGTGTTCACCAGCGGTGTTGAGGGAGGGCCGTCCTTA
+
CA?AB;;<<@;-924847888.33104777781669>;66167.1--+.42--,-3-
@T6XXI:00074:00227
GGGGAATTCTAATACGACTCACTATAGGAGAGGGAGAATAGCCCCGTGATTACGAATTTAGCTGTTCTCTTCCTTTC
TCCCTTCTCCC
+
C992>5;:=GC@CEAAAADCCBBBC::4:BABC>AA?<>=;<;;1;=?AB@C?==377.777>?@=@AAC@C@CC>C
BAB<@379@A<
@T6XXI:00074:00239
GGGGAATTCTAATACGACTCACTATAGGGAGAGGGAGAATAGTGACGTGTTGTGCGGCGCTCACTGTCTCCTTCCTT
TCTCCCTTCTCCC
+
CEC:C>?=@BB@BA@@CCCCCCA97166.7>>AB?ECA>@<<>===<<<=:<<A@@=<AAAB=<=@A@@A9<9<;??
;@AAC<A9<<BB>
@T6XXI:00075:00203
GGGAGAAGGGAGAAAGGAAGCTAGTCACTCGACATCTGTGGCTTCACAGTACAGAGGGACTGTCCAAAGCCGCAGAT
CTCGAGTCGACTAG
+
CC@BAA?@=:@@@=<@=?<@CBBBA?;::>;00.;65;>C=?@@CBAACCCBB:65676<@@@@=@C=?>;>A====
<<<558@A@????@
@T6XXI:00075:00242
GGGAGAAGGGAGAAAGGAAGGAGAAGTCGCCCCAAACGATTTGTTGACTATTCTCCCTCTCCCTATAGTGAGTCGTA
TTAGAATTCCCC
+
CEBCBB?BA=AA>@=B>B?B@BAA?BCCA@@@5;;:?A@AC>B@>BBBB??>A=AB:<??@A<A<??@@@<AA????
@>A@?;;3444+
@T6XXI:00076:00200
GGGGAATTCTAATACGACTCACTATAGGGAGAGGGAGAATAGTCTACGA
+
CCC9D@C@CCB@B???AB??A@;76116.7>>BD>DB>;>7++,,,++*
@T6XXI:00076:00211
GGGGAATCTAATACGACTCACTATAGGAGAGGGAGAATAGTGGCGTGTTGTGCGGCGCTCACTGTCTCCTTCCTTCT
CCTCTCC
+
2@@00)-
,,,00//44337767311101;;;<;9>::;7338/40000/0038;<>>AA@@B@?>=;;242/46,/,1+,+++*
@T6XXI:00076:00222
CTAGTCACTCGAGATCTGCGGCTTGGACAGTCCCTCTGTACTGTGAAGCCACAGATGTCGAA
+
BB:996///66////48843138584833AB?E>>:::C::::944409594;440,,2,,*
@T6XXI:00076:00223
GGGAGAAGGGAGAAAGGAAGCTAGTCACTCGACATCTGGTGCTTCACAGTACAGAGGGACTGTCCAAAGCCGCAGAT
CT
+
ABBBDC?A=:@>==5@>>=@AAAAABAAAA@@@>0000,....6688:>???B@?A@;@DAAAA48818@<@===@A
A@
```

```
@T6XXI:00076:00225
GGGGAATTCTAATACGACTCACTATAGGAGAGGGAGAATAGTCACATTCATGTTTGTGACCGTGTCTCCTTCCTTTC
TCCCTTCTCCC
+
?BB7A695948>A@AACABBC><>>00,0;077.7=::>@@@@@99:?CF@@@D>:87==;<<==878;=8556A<A
@@@;@;<5=A7
@T6XXI:00076:00235
GGGAGAAGGGAGAAAGGAAGCTAGTCACTCGAGATCTGCGGCTTTGGACAGTCCCTCTGTACTGTGAAGCCACAGAT
GTCAGAGCGACTCA
+
<<8;BA>AA:@988095958833<>B@@88:CA>A???BB?BCE>C@CCCBABC=AA?===A@@@@C@BA>A???AA
A@80----+****0
@T6XXI:00076:00240
GGGAGAAGGGAGAAAGGAAGCTAGTCGACTCGAGATCTGCGGCTTTGGACAGTCCCTCTGTACTGTGAAGCCACAGA
TGTCGAGTCGACTAGCTTCCTTCTCCCTTCTC
+
99149@;>9-8999142423977317776111311171l6=3677-
7;===@@@@5:8;4488776=@;==;<?AB9879884/.--..334//,/,/,-43455449=
@T6XXI:00076:00245
GGGAGAAGGGAGAAAGGAAGGGGAGAAGGGAGAAAAGGAAGCTAGTCGACTCGACATCTGTGGCTTCC
+
CDBBAA@BB>BBBB=?;82777-8AB@BB:@7766.737479...:/..43.10;9440003/0023/
@T6XXI:00077:00205
GGGAGAAGGGAGAAAGGAAGAGAACAGCGCTGTGGATAAATATGGAACTATTCTCCCTCTCCCTATAGTGAGTCGT
+
CC@CBB@BC@BBBB>B@B@BBBB?BBBBBBC@@?@ABBB<BBBB?A?@@BB>BCCC>BBBBB>BA@???@@@@@@A
@T6XXI:00077:00231
CTATCACTCGAGATCTGCGGCTTTGGAACAGTCCCTCTGTACTGTGAAGCCACAGATGTCGAGTCGACTAGCTTCCT
T
+
A9944449>==>?>@@B@?B@??8>.0,.4433506?::7BCCA?>?=7;9<97BCBB@B=?669988333.47542
2
@T6XXI:00077:00234
GGGAGAAGGGAGAAAGGAAGCTAGTCGACTCGACATCTGTGGCTTCACAGTACAGAGGACTGTCCAAAGCCGCAG
+
CCACCC@BB=?==>:@>A7;;C@A@CBCB?A>><?:::BCE?BB@BCC>>9@755.../;;;?@>@D@?A<===A
@T6XXI:00077:00249
GGGGAATTCTAATACGACTCACTATAGGGAGAGGGAGAATAGTCAACAAATCGTTTGGGGGCGACTTCTCCTTCCTT
TCTCCCTTCCTCCC
+
CCB4@6959989A@ABC;;<A@>?=761,6===?9??<=@@@@@?<;;<-
77777.7777,:A?>@877?832;3885854739./../892
@T6XXI:00078:00229
GGGAGAAGGGAGAAAGGAAGCTAGTCGACTCGACATCTGTGGCTTCACAGTACAGAGGGACTGTCCAAAGCCGCAGA
TCTC
+
DC@BBB@BC>DBBE=B?C8;;DBBBBBB@CA@88;BBBBBB?AA>ABB@A@A@==44448===<><;<;?A>AA===
=<@<
@T6XXI:00078:00232
GGGAGAAGGGAGAAAGGAAGCTAGTCACTCGACATCTGTGGCTTCACAGTACAGAGGACTGTCCAAAGCCGCAGATC
TCGAGTCGACTAGC
+
DD@AAA@BB=BBCC@B?B@BB>@@??????>7:::76=<<3:848B@A?@?@@B7737;9;;;38818@:>?AD@??
??AAAAAAA@::///
@T6XXI:00078:00237
GGGGAATTCTAATACGACTCACTATAGGGAGAGGGAGAATAGTCAACAAATCGTTTGGGGCGACTTCTCCTTCCTTT
CTCCCTTCTCCC
```

```
+
CBD:C>@>ACCCCB?=BBBBBCB<7111.7>7776:BA>AAA@@@?@AA<B????8?AA6??CCB=@@C@A>A<??;
?;;;69.//;;9
@T6XXI:00079:00224
GGGAGAAGGGAGAAAGGAAGGAGAAGTCGCCCCAAGCGATTTGTTGACTATTCTCCCTCTCCCTATAGTGAGTCGTA
TTAGAATCCCCAATCGAATTCCGCG
+
9<-
49B@CGACBBB<><>48588859:97?BB5;79?:BCDBCC@DCDCCCACCCC>BBDED>BBB@A???A::::??::
7;...550341-00/0232300
@T6XXI:00079:00241
CTATCACTCGAGATCTGCGGCTTTGGACAGTCCCTCTGTACTGAGCACGAA
+
AA@=9::ABBAABDB@@@D?BDEAC=@?>>77:.;BB?>=7,,,++++,,*
@T6XXI:00079:00249
GGGAGAAGGGAGAAAGGAAGTGCAGTTCGCGGTTAGTGAAGGGAAAAACTATTCTCCCTCTCCTATAGTGAGTCGTA
TTAGAACTTCCC
+
HE@DCC@CC;;<CB<A>>4888998=88887:;:?@CCC@BB.7677,8BBC@C>>=6;:00./997;=;:::4000
324002369<=;
@T6XXI:00080:00203
GGGAGAAGGGAGAAAGGAAGCTAGTCGACTCGACATCTGGTGGCCTTCACAGTACAGAGGACGTGTCCAAAGCCGCA
GATCTCGAGTCGACTAG
+
EB?BBB?BB=BBBC::::@CCCBBA@=7671....5...,.5,.,7111>666?716//,....9:@>AA<@@;@@@
A@???@@@;;///////
@T6XXI:00080:00215
GGGAGAAGGGAGAAAGGAAGTGCAGTTCGCGGTTAGTGAAGGGAAAAACTATTCTCCCTCTCCCTATAGTGAGTCGT
ATTAGAACTTCCC
+
@CACCB?BB=BBBD?C@C@C:9;CCC>@@@B?A?@@?BB777.7>>>1?C>>47777.7=771,1333788<<;;88
//,/./277,.-+
@T6XXI:00080:00228
CTAGTCACTCGAGATCTGCGGCTTTGGACAGTCCCTCTGTACTGTGAAGCCACAGATGTCGAGTCGACTAGCTTCCT
TTCTCCCTTCCTCCC
+
CC49;<:::CCBCCCBBBCB@>>><<>@A@?@CC>???CCB:???;=;?C@CCCBBBCB???C?@@??B;7:6=>9>
@=@6>:69,/,///,
@T6XXI:00080:00233
CTATCACTCGAGATCTGCGGCTTTGGACAGTCCCTCTGTACTGTGAAGCCACAGATGTCGAGTCGACTAGCTTCCTT
TCTCCCTTCCTCCC
+
B??=9::9BBBABCBBABB@CCC=B>BCCBBAC>C@??AC?@A??@>@B?BBBCCCCCCCCBBBBA?@@::7>@@BC
>C>;939,/,/882
@T6XXI:00081:00215
CTAGTCACTCGAGATCTGCGGCTTTGGACAGTCCCTCTGTACTGTGAAGCCACAGATGTCGAGTCGACTAGCTTCCT
TTCTCCCTTCTCCC
+
GA944944:BBBBBCBB?BB@CCC@B=@?A@???<?BBCBBBABCAA>?B@BBBCCBBBBBBBABB;;;@AB@B>@B
B<CCBB.424003-
@T6XXI:00081:00225
CTAGTCATCGAGATCTGCGGCTTTGGACAGTCCCTCTGTACTGTGAAGCCACAGATGTCGAGTCGACTT
+
A@994999::99=>BBBBCICDDBC;;;@@@@C@ACBBCCCCBBAA==A;==BBCBDA@;;331///,*
@T6XXI:00081:00227
GGGAGAAGGGAGAAAGGAAGGAGAACGCGCACCCGAACCAACGGTGATCTATTCTCCCTCTCCCTATAGTGAGTCGT
ATTAGAATTCCCC
```

+
A@@ABB@CCBCBBB>B@B@B@B??=@CDCBCCC@BC>B?BACC@BDCCCBBB@CCCC?AAACC@CBB@?@ABAAA??
?B@BCC?B@CDD:
@T6XXI:00081:00241
GGGAGAAGGGAGAAAGGAAGCTAGTCACTCGACATCTGTGGCTTCACAGTACAGAGGGACTGTCCAAAGCCGCAGAT
CTCGAGTCGACTAG
+
CCACCB?BB=B@==:@=?;?C@????:70=;00///.3575=?@B@BBCCCCB4033-
49;AAA=AB>??<???>@A???=<<@?<<===<
@T6XXI:00082:00201
GGGAGAAGGGAGAAAGGAAGGCGAA
+
CCACCB@BA;@@::;882**)*((*
@T6XXI:00082:00206
GAGAAGGGAGAAAGGAAGGAGAAGTGCTCACTACAGTAGTGATAGCTATTCTCCCTCTCCCTATAGTGAGTCGTATT
AGAATTCCCC
+
CCBC?BCBCBBB?B?B?A@ABB@?A?ACC@@BA@@@@??<9<AAA?>??>A???<?;?AA<<<@@@@A;AAA=559>
AAA>CCCDC:
@T6XXI:00082:00219
GGGGAATTCTAATACGACTCACTATAGGGAGAGGGAGAATAGTCAACAAATCGTTTGGGGCGACTTCTCCTTCCTTT
CTCCCTTCTCCC
+
BAA9A>B?BCC@CBBCC@@@CBB?@667.7===A>BCA?@AACBA>@@@;BCCBC>CCC6BBCBB?BAA:;9;;>@:
??@A:?387;:4
@T6XXI:00082:00226
GGGGAATTCTAATACGACTCACTATAGGGAGAGGAGAATAGCTAAGG
+
:>>9;;@@?BC@BCCBBACB@>ABAACD=??>=;=/++,1,+,,*.,
@T6XXI:00082:00227
CTAGTCACTCGAGATCTGCGGCTTTGGACAGTCCCTCTGTACTGTGAAGCCACAGATGTCGAGTCGACTAGCTTCCT
TTCTCCCTTCTCCC
+
::/1/;/44BBBCCCBB@@B=?B?;?;>?CC===;=AAC@???@@<=>=D?BBCCCBAAAAABCBBBCBBA::7:>A
A>AAAB>@=@@AA<
@T6XXI:00082:00231
GGGAGAAGGGAGAAAGGAAGGAGAAGTCGCCCCAAACGATTTGTTGACTATTCTCCCTCTCCTATAGTGAGTCGTAT
TTAGAATTCCCC
+
@@<@;969B1949B=?<C>B=;:;?AACCCBD9DCAC<:8?8>A>A?@934/44--+----,-
332365633000033+044/32344.
@T6XXI:00082:00232
GGGGAATTCTAATACGACTCACTATAGGGAGAGGGAGAATAGTCAACAAATCGTTTGGGGCGACTTCTCCTTCCTTT
CTCCCTTCTCCC
+
CCNDD@A>@CCDCBA@B?>>BAB?;111.6<<=?>ADA>@;?@?@=?AC<CBCBA<???5?AAAC@BCD<969?CC<
@@AA<A<??AA<
@T6XXI:00082:00239
GGGAGAAGGGAGAAAGGAAGGAGAAGTCGCCCCAAACGATTTGTTGACTATTCTCCCTCTCCCTATAGTGAGTCGTA
TTAGAATTCCCC
+
EDBB@@@BB>BBBB>B@B@B@BBB>??BBBBB6BB>BB@@@;??@CA=?==;<<<<;==@AA;=<AA???@@@AA??
?@A@A>@<???5
@T6XXI:00082:00245
GGGAGAAGGGAGAAAGGAAGCTAGTCACTCGACATCTGTGGCTTCACAGTACAGAGGACTGTCCAAAGCCGCAGATC
TCGAGTCGACTAGCTTCCTTTCTCCCTTCTCCC
+

```
44-4498995999919<?<@B;<8:7,,,,,,,,,25:8?;<@?BBCABAEAA>843//----,-32--
..88:<A?::////8768==9<=;444331466,/,-2223
@T6XXI:00083:00210
CTAGTCATCAGATCTGCGGCTTGGACAGTCCCTCTGTACTGTGAAGCCACAGATGGACTGTCGCAGATCTCGAGTCG
ATCCTAGCTTCCCTTTCTCCCTTTCCTCCC
+
AA949;:4:=944444/688868680..466+.61666678<88>5924444444424498849939 64.,,,*****
,,,*,--3;<8;:;=;:8<;+00+0/044,
@T6XXI:00083:00218
GGGGAATTCTAATACGACTCACTATAGGGAGAGGGAGAATAGTCAACAAATCGTTTGGGGCGACTTCTCCTTCCTTT
CTCCCTTCTCCC
+
CCB9C>@=ADC@CBBABBCBB@>96111.7==?B?EDA?A=@@@=;<<<;>@>?A>AEE7A@BBC?BBB?@>?:??:
:CCC5=:?@@C:
@T6XXI:00083:00219
GGGAGAAGGA
+
B?6;11-1-,
@T6XXI:00083:00235
CTAGTCACTCGAGATCTGCGGCTTTGGACAGTCCCTCTGTACTGTGAAGCCACAGATGTCGAGTCGACTAGCTTCCT
TTCTCCCTTCTCCC
+
AB<99A?::994494=@ABC@CCCAC=@@C:::B=AA???B???A===8<9;A>>?ABBAB???BA????@89;;:?
?<?BAC>@34344-
@T6XXI:00083:00236
GGGGAATTCTAATACGACTCACTATAGGGAGAGGGAGAATAGTCAACAAATCGTTTGGGGCGACTTCTCCTTCCTTT
CTCCCTTCTCCC
+
DHKDC>>:111,/199;?@;;7??>777,6777B>BBA?C==<<>5;;=9==<<<6;;62;<77736;;6428/001
3=;=;=384=C<
@T6XXI:00083:00239
GGGAGAAGGGAGAAAGGAAGA
+
EDCEBA69419?888880)**
@T6XXI:00083:00241
GGGAGAAGGGAGAAAGGAAGGGGAGAAGGGAGCTAGTCGACTCGACATCTGTGGCTTCACAGTACAGAGGGACTGTC
CAAAGCCGCAGA
+
@A<ACA?AA:@944-
9<>4888.8>>9:;90//@>88>>AABCB97;;::BCC@B@::=====@BBB??>=?BCCCB>>B;>>>????@
@T6XXI:00083:00248
GGGAGAAGGGAGAAAGGAAGTGCAGTTCGCGGTTAGTGAAGGGAAAAACTATTCTCCCTCTCCCTATAGTGAGTCGT
ATTAGAACTTCCCC
+
CCBBBB@BB>BBCC?C@C?AAABBCC@C@A@?A?A?@@B=@>.77770:CCC?AACC>CBBBB>B@@AAAA@AA?>>
<<8;@>8<;8;<<1
@T6XXI:00084:00208
GGGAGAAGGGAGAAAGGAAGGAGAAGTCGCCCCAAACGATTTGTTGACTATTCTCCCTCTCCCTATAGTGAGTCGTA
TTAGAATTCCCC
+
GGACBB@AA=@@@A=?>A@B@@??@@?BBCCC8BA=BBBCB>@@DA@@?@@?A===;=??<<;==<<<===<<<@;?
?>AAC>@<>>=1
@T6XXI:00084:00212
GGGAGAAGGGAGAAAGGAAGCTAGTCGACTCGACATCTGTGGCTTCACAGTACAGAGGGACTGTCCAAAGCCGCAGA
TCTCGA
+
```

```
CD@BA@@CC>B9998;@B=@@@BBA?B@CCC?:::::;BAB?BC>AC@8;;CC?=<6<7;<=?;55=?;?B>AA@@<
<==<<<
@T6XXI:00084:00223
GGGAGAAGGGAGAAAGGAAGGAGATGACGGTGCTGGGGAGACAACGACTATTCTCCCTCTCCCTATAGTGAGTCGTA
TTAGAATTCCCC
+
EDDBBB?BBABBBC@B?B@B@BBBBA@>>=@AACBCC9BBBCA@BBBA@AC>?BCC=??@@@;@@@A@?@AAA====
@=@A@?A=@AA6
@T6XXI:00084:00224
GGGGAATTCTAATACGACTCACTATAGGGAGAGGGAGAATAGTCAACAAATCGTTTGGGGCGACTTCTCCTTCCTTT
CTCCCTTCTCCC
+
@AA8A@BABBB@CBBBBBBBBA?96111.7>>CD>CBA?A=?@@A>@A=;A=<:>8::?7?DDBB?BCC@?<?>AA;
@@AA<A?A@@@<
@T6XXI:00084:00244
GGGAGAAGGGAGAAAGGAAGCTAGTCACTCGACATCTGTGGCTTCACAGTACAGAGGGACTGTCCAAAGCCGCAGAT
CTCGAGTCGACTAG
+
CC?B>>644-
998809695;F:8<A?;::????????BBC?BB@CC@<<<A@>433317<A@AA>@@;@A?AA9====???@@@@==
=A@@
@T6XXI:00085:00201
CTATCACTCGAGATCTGCGGCTTTGGACAGTCCCTCTGTACTGTGAAGCCACAGATGTCGAGTCGACTAGCTTCCTT
TCTCCCTTCTCCC
+
GBB@94:9==BBCCBCC:::;CC@C=@@BB???9?BABCBBCCA@@=@E@CBAACBBBCC::;<<AABBBBC::8;C
>BC@@:?588AA<
@T6XXI:00085:00213
GGGAGAAGGGAGAAAGGAAGTGCAGTTCGCGGGTTAAGTGAAAGGGGAA
+
44-44489959888582427;79=A=<>8/..+.,.,.....+.576A?
@T6XXI:00085:00214
GGGGAATTCTAATACGACTCACTATAGGGAGAGGGAGAATAGTCAACAAATCGTTTGGGGCGACTTCTCCTTCCTTT
CTCCCTTCTCCC
+
FCC:C>@>@EBBCCCAA@CABB@?=677.7>>>@=BC@>@@@?>=;<;;6????D=BAB7??@@EACCC;958?AA<
@@AC<?698==7
@T6XXI:00085:00215
GGGGAATTCTAATACGACTCACTATAGGGAGAGGGAGAATAGATCACCGTTGGTTCGGGTGCGCGTTCTCCTTCCTT
TCTCCCTTCTCCC
+
<:4,96969>><?@A@BADBBBCB?777.7===A>BC@>@@877=?=:==;?;??BCCBD<<<BAA?ABB@C?@>AE
@BBA@:=94444-
@T6XXI:00085:00219
CTAGTCATCGAGATCTGGCGGTCTTGGACAGTCCCTCTGTACTGTGAAGCCACAGATGTCGAGTC
+
:9B944444;9999111,/85//0,,*,//..06299>>:6668@6666968780..,,,,,,.54
@T6XXI:00085:00232
CTAGTCACTCGAGATCTGCGGCTTTGGACAGTCCCTCTGTACTGTGAAGCCACAGATGTCGAGTCGACTAGCTTCCT
TTCTCCCTCTTCCTT
+
=////6/44A==@@A@BBB@>ABB<B@B@A@::7-
:<<?A;;;CF??<@C@CCCCCBABEBB@?;;8>>>8713<;==6;767.33-30*++
@T6XXI:00085:00233
GGGAGAGAATTTGGTAATGGGTAGGT
+
D>9CCCCG@BB=?<@A<>;;,--(((
```

```
@T6XXI:00085:00241
GGGAGAGGAGAAAGAAGCATGAGTCAAAGGGGAATAGGTTGTGACGTA
+
?7+1///,,,//+//,.44786111198<??8@=999<:94..,,,,,
@T6XXI:00085:00244
GGGAGAAGGGAGAAAGGAAGCTAAGTCGAACTCGACATCTGTGGCTTCACAGTACAGAGGGACTGTCCAAAGCCGCA
GGTCTCGAGTTCAACTTAGCTTCCTTTCTCCCTTCTCCC
+
DC>@A@=>>:>99909<>>CCB@B@C111A@??@@?A;::CAA;?@=@@ABBBBC??77717>>@AA?AC=AA?<==
A?===@AAAA>==;<??A98<?B89919>BB19699991
@T6XXI:00085:00245
GGGAGAAGGGAGAAAGGAAGGAGAAGTCGCCCCAAACGATTTGTTGACTATTCTCCCTCTCCCTATAGTGAGTCGTA
TTAGAATTCCCC
+
FHACCB@CC@BB?B>B?B?>><@@=@CCCCDE8@@>@CCC@;??>??@B=B<?:==;;:?AB<AAA9==@@@???@@
@>AAA?A>AAA6
@T6XXI:00085:00249
GGGGAATTCTAATACGACTCACTATAGGAGAGGGAGAATAGTCACAACGTTTTTCCCCGTACGTACATTCTTCCTTT
CTCCCTTCTCCC
+
999/424:9@@?BCCCBAABB:99;//,0<;>@AAB@=@<??AA@@>@CCCBB2BCD8CCA<ABAA==:77378=>7
==@@;?;?@??;
@T6XXI:00086:00218
GGGGAATTCTAATACGACTCACTATAGGGAGAGGGAGAATAGCTTAACCCTTTCCCCGTGCTAACGTAGTCTTCCTT
TCTCCCTTCTCCC
+
CCD9C?B@BCC@BB@ABBAB?@CB>76:5:?BCD?CBA=A=::<?C@CE=>A<ACC9CB?@@@>A@CA@8835;85:
4?=AA<A?<<<<;
@T6XXI:00086:00221
GGGAGAAGGGAGAAAGGAAGCTAGTCGACTCGAGATCTGCGGCTTTGGACAGTCCCTCTGTACTGTGAAGCCACAGA
TGAGTCGACTAGCTTCCTTCTCCCTTCTCC
+
AA=?BB>A@9;9@B9;<>ACCC@@?=@@@@CCC???:::>@;>==-
736;>>???<<??@AAA@A=9999=;<A@@@A998@A@<<;:8//,/,/,-273+/,..-,
@T6XXI:00086:00233
ACCGAGATCTGCGGCTTTGGACAGTCCCTCTGTACTGTGAAGCCACAGATGTCGAGTCGACTAGCTTCCTTTCTCCC
TTCTCCC
+
AADADABCDDCBA?ACC@C=@@DDBAB>BCCCCCBB?@@C@CA>B@BAB???GEBBABBCBBA==<@CBEDAHCCBB
B:33>>>
@T6XXI:00086:00234
CTAGTCACTCGAGATCTGCGGCTTTGGACAGTCCCTCTGTACTGTGAAGCCACAGATGTCGAGTCGACTAGCTTCCT
TTCTCCCTTCTCCC
+
C8///1/44AAACCCBBABB?BBD>B>?AA@??C;@@@@<<=B?A?@=?B?CBCCC;;;CCDCB@@@BBBB@B=@@F
D<AADCBA;==@@:
@T6XXI:00086:00243
GGGAGAAGGGAGAAAGGAAGCTAGTCACTCGACATCTGTGGCTTCACAGTACAGAGGGACTGTCCAAAGCCGCAGAT
CTCGAGTCGACTAG
+
GEDAAAACC@CCCC>@?@@CCCCB?9?7:97776:<:?BB?CC?BCCBBBCB?633317=ABAC=AA<AA>AA<<<@
@@@?=<=???A@@@
@T6XXI:00087:00205
GGGGAATTCTAATACGACTCACTATAGGGAGAGGGAGAATAGTCAACAAATCGTTTGGGGCGACTTCTCCTTCCTTT
CTCCCTTCTCCC
+
```

```
CCE:C@C@DDC@CCBBA?BBBAB97111.7==?BACB@>@=BA==<9884:;;77,111-
9??BCACCC??<?>AA=A@@@<A;==@@;
@T6XXI:00087:00212
GGGGAATTCTAATACGACTCACTATAGGGAGAGGGAGAATAGCAACAGTATTTTTACACGTGCGTCTCCTTCCTTTC
TCCCTTCTCCC
+
999/499<BDB>BB<:<CBBB@@@BB@@=?:::EB??AB=8:8A;;44444444,00044::854::@7;73/44-
4889,/./--33
@T6XXI:00087:00225
GGGAGAAGGGAGAAAGGAAGGGGGAGAAGGGGAGAAAATACGTCTGAGCATCGATCATGACAGCTACGTACGTCTGA
GCATCCGATCGTG
+
11,149<>>-9448-42423333+3331111,1....*......-4444;:66/.822/..---1-
..44:9<9=854:;/.,-0500++
@T6XXI:00087:00233
GGGAGAAGGGAGAAAGGAAGGAGAAGTCGCCCCAAACGATTTGTTGACTATTCTCCCTCTCCCTATAGTGAGTCGTA
TTAGAATTCCCC
+
FI@D@?=?B=ABBC?B@C@C=@@@?B?@@DDD9BC>CECCD>BB@CCEC@?688?=:==;;;6<>>=@???A???@A
@>?@@?A=@@@5
@T6XXI:00088:00204
GGGAGAAGGGAGAAAGGAAGCTAGTCGACTCGACATCTGTGGCTTCACAGTACAGAGGGACTGTCCAAAGCCGCAGA
TCCTCGAGTCGACTAG
+
CD@CBB>AA=BCBA>C;96:C?0000..335:066666>>B::;:;BBCCCCB?6;555;;A?@@?AA<AA@???A@
AB=???898AA@@@AB
@T6XXI:00088:00208
GGGAGAAGGGAGAAAGGAAGCTAGTCACTCGACATCTGTGGCTTCACAGTACAGAGGGACTGTCCAAAGCCGCAGAT
CTCGAGTCGACTAG
+
CC>@=92440999909698;CAABDCA@???77:::9@AB?BBBC@??CCC??<333-
4;;>?@>@A<AA?????A=<><=??@@@@AA@@
@T6XXI:00088:00212
GGGGAATTCTAATACGACTCACTATAGGGAGAGGGAGAATAGCTCCCGACTCTCACCAGGCCAGGCGCCCTTCCTTT
CTCCCTTCTCCC
+
CCC9C?B?BCB?BBBBBB???@BB?77759B?BB>BCA><66=@@@;??@@B@@??=7458==;9?88718:;=AA8
99@A<A;==@@<
@T6XXI:00088:00217
GGGAGAAGGGAGAAAGGAAGTAGAATAGCACGTTGAAGATGTCGGGACTATTCTCCCTCTCCCTATAGTGAGTCGTA
TTAGAATTCCCC
+
CDACDB@DB=ABCC=B=@ABCBA>=A@??BBBB?BBACCCCCABB>BBABB@CCCC>@@@@@<@EBBBCDBBCBAAB
C?BAA<?4888.
@T6XXI:00088:00223
GGGGAATTCTAATACGACTCACTATAGGGAGAGGGAGAATAGTCAACAAATCGTTTGGGGCGACTTCTCCTTCCTTT
CTCCCTTCTCCC
+
CDC:D?@>ACC@CCBBBBBBB@B@966959?ACC>CA?=@A@@@@=@AA:@ACCC>B@>7>78:CAB?A58248:=7
>?@A7=;=@AA<
@T6XXI:00088:00224
GGGAGAAGGGAGAAAGGAAGAGAACAACGCGGGCGGATAGGCCTAGCTATTCTCCCTCTCCCTATAGTGAGTCGTAT
TAGAATTCCCC
+
HH@DAA@BCCBB=A;A?A@????@AA?AA@BB?BB@AAAB@E@C::<CCB?CBBB>BB@??>ACCCDA@@@B@??@@
=@?A=C@ECC4
@T6XXI:00088:00228
```

```
CTATCGACTCGAGATCATGCGGCT
+
444444444444//////2,,*.4
@T6XXI:00088:00229
GGGGAATTCTAATACGACTCACTATAGGGAGAGGGAGAATAGCTACGTTTAACCGATCGTGTTGTTCTCCTTCCTTT
CTCCCTTCTCCC
+
CBC9B?C?AABACCCCCBBBBCBBA???6;=7775:@==@@AACCBBBB<A>@=;??=><;;<<<:=??=>842331
39@A<A=@@BA?
@T6XXI:00089:00205
GGGGGAATTCCTAAATACGCTCACTATAGGGAGAGGGAGAATAGTCAAACAAAT
+
4444+4:;282444-444437731331111,1766611:7,/-+++00*+-303
@T6XXI:00089:00208
GGGGAATTCTAATACGACTCACTATAGGA
+
CFE:C;<8;99<?AB::;>88*****(((
@T6XXI:00089:00215
GGGGAATTCTAATACGACTCACTATAGGGAGAGGGAGAATAGCTATAC
+
BBA7A===@A@?CBBBBCECCBC>81665999CE>CC@>7+*****((
@T6XXI:00089:00223
GGGGAATTCTAATACGACTCACTATAGGGAGAGGGAGAATAGCTTGTGCGGGTGTGTAAGGCAATTCTCCTTCCTTT
CTTCCTTTCTCCCTTCTCCC
+
CCDAC?B@BDC@CBBBCCCBB@BB@77877B?BC>BBA>@<<<A@>A==;=;=;<<<A<<=??=?@AAA:=9<=@@;
A@>?<@C?A???;>887781
@T6XXI:00089:00230
GGGAGAAGGGAGAAAGGAAGGGAATAGCAGCAGTCGGCAACGGTGAAACTATTA
+
CH?CBB>BA=BACB=@@@@CC>C?C;:<F?;::;?87376..,....+,4..,.
@T6XXI:00089:00233
CTTCGACTCGAGATCTGCGGCTTTGGACAGTCCCTCTGTACTGTGAAGCCACAGATGTCGAGTCGACTAGCTTCCTT
TCTCCCTTCTCCC
+
CA>;=<AACAAACCCA>BB@AAA?A@AAA?<<B<BB@??;??@@?B>>????@@@@:<>==;;;=@@A<99?@C@@?
;?BCC>@=9=AA<
@T6XXI:00089:00234
CTAGTCGACTCGAGATCTGCGGCTTTGGACAGTCCCTCTGTACTGTGAAGCCACAGATGTCGTCGATGGAACCATTA
GGAGTAGCACCCACCATCGATGGGCCCTGCCTTCCTTTCTCCCTTCTCCC
+
DECCBBCBDCCBBBBBBBB@@=@CC?C@CC@@@CC>CCCCBA>C<@@C?AC?BBBB>70000.553366677.88@A
A>=??B?<;;A<<<8;<884984+44,444/05646::?A@@<A>???A<
@T6XXI:00089:00248
GGGGAATTCTAATACGACTCACTATAGGGAGAGGGAGAATAGTCAACAAATCGTTTGGGGCGACTTCTCCTTCCTTT
CTCCCTTCTCCC
+
BBB9B=B@BBBACCBBBBBBBCB?9111.7>>BD>BB@>@<<<?@>A@A<BBBBC>BBB7ABACCAEBB??=@?CC:
>@@C=C=??@?:
@T6XXI:00090:00211
GGGGAATTCTAATACGACTCACTATAGGGAGAGGGAGAATAGTCAACAAATCGTTTGGGGCGACTTCTCCTTCCTTT
CTCCCCTCTCCC
+
499499DACCC@BBBCCCBAA?<96166.7>>?A>B@>;=AAA??<?AB<CA@?;1616-
:@BBC@CCC@@C@=:828////+//---+
@T6XXI:00090:00212
```

```
GGGGAATTCTAATACGACTCACTATAGGAGAGGGAGAATAGTGACGTGTTGTGCGGCGCTCACTGTCTCCTTCTTCT
CCCTT
+
:::49?@?;;<@?@@BCD;;;@:::00,0<077.763234748../1111/-.287?AAAAAB@;<:::7:34424-
.-+-,
@T6XXI:00090:00213
GGGGAATTCTAATACGACTCACTATAGGGAGAGGGAGAATAGTCTCATGAAACAAACATGGCTATACCTCCTTCCTT
TCTCCCTTCTCCC
+
CCC9CBDABBB?BBCBBBBBBA?97111.7>>BC>AB@?A;@?:?@?BACC>C@@;=?BBD@CCCBB>;;48=?;??
<?@@C=A@BCCB>
@T6XXI:00090:00217
GGGGAATTCTAATACGACTCACTATAGGGAGAGGGAGAAAGGAAGGAGAAGTCGACCCCAAACGATTTGTTG
+
CCD:B@D@C??<?A@?@@@@B@A??777.7>??A?BA=C<@>@9<8:::798400000+0448:;::46232
@T6XXI:00090:00219
CTATCACTCGAGATCTGCGGCTTTGGACAGTCCCTCTGTACTGTGAAGCCACAGATGTCGAGTCGACTAGCTTCCTT
TCTCCCTTCTCCC
+
CDA=@::;AABCCDBBCAA?ABB>B?CA?@<<<8?AAAB@CB@@@?>?B?BBBBAAAAAAAAA@@?A8867106377
.7744-47948=:
@T6XXI:00090:00234
GGGGAATTCTAATACGACTCACTATAGGGAGAGGGAGAATAGACACTCCCTAGAAACATGCGCCATTTGCTTCCTTT
CTCCCTTCTCCC
+
GCF:B895949<>@A@@@CBB@B?>777.7>==?<?A?@BA@???C?@?188;<:4<@:::::79::4:43/4<?@<
@@@A7<7:444-
@T6XXI:00090:00235
GGGAGAAGGGAGAAAGGAAGCTAGTCGACTCG
+
BC?BBB@CCBDBBE?B@:66--**((****,,
@T6XXI:00091:00206
GGGAGAAGGGAGAAAGGAAGCTAGTCGACTCGACATCTGTGGCTTCACAGTACAGAGGGACTGTCCAAAGCCGCAGA
TCTCGACGACTAG
+
CCB@@>=>=:@CCC@C@C@BBA??=?@@CCB@96::::@DG?AC@CC?99?C?9933317BB@A??AA<AA@@????
?A@@@AA?>8===
@T6XXI:00091:00216
GGGGAATTCTAATACGACTCACTATAGGGAGAGGGAGAATAGCCTCCCACTTCTCAGCCGACCAGCTGATCTTCCTT
TCTCCCTTCCTCCC
+
999/49@=ABB@@??BB????@D<9661-
6==@B>BCA>A=A<:<<<;?C@?A@899A>@@A>@A<<<:6?C>=8865;42201,/,/771
@T6XXI:00091:00219
GGGGAATTCTAATACGACTCACTATAGGGAGAGGGAGAATAGTCTGTCGTTCGGTCGACGCTGCCTATCCTCCTTTC
TCCTTCCTCC
+
CBC9B=A>ACDBBBB?BBBBAA@>9166.677=A>@0/,////::88A@@>AA;===<;8;:74249//,/-
3372,,,++*+*+00
@T6XXI:00092:00210
GGGGAATCTAATACGACTCACTATAGGGAGAGGGAGAATAGTCAACAAATCG
+
8?818+1///,/48?@?A?B>976111,1>>@A>B>=;4845444000+111
@T6XXI:00092:00216
GGGAGAGG
+
?@1944/,
```

```
@T6XXI:00092:00221
GGGGAATTCTAATACGACTCACTATAGGAGAGGGAGAATAGTCGTATTTTTAGAAACGGCGCGTGCTTCCTTTCTCC
CTTCTCCC
+
944,42424BB>BBBBCCCBB>;>=/0-0@>BE>CCA;==@@???@?@@A1@@CA=AA>A;558A>:8:244-
4;;@6<7:444-
@T6XXI:00092:00228
GGGGAATTCTAATACGACTCACTATAGGGAGAGGGAGAATAGCCATCGTGTTCGCCCCGCGATGAGTCTCTTCCTTT
CTCCCTTCTCCC
+
CBB7B7;8;AB@CCCBBBBB@@A?>61659??BC>CB@>@==999A=>99=?@;<<<4<AAAAAA@@@@AA>A:=<7
<?>?;@<??AC7
@T6XXI:00092:00233
GGGGAATTCTAATACGACTCACTATAGGGAGAGGGAGAATAGACACCAGTCCCATCAGCCACAATTATTCTTCCTTT
CTCCCTTCTCCC
+
@==6@>B?BCC@BB???BBBB??;;111.677=A>BCA>@@@?@?@>AAAAB;<A@<<<;<9=<=<<<:4444>@C<
@@@A;>8;<<<7
@T6XXI:00092:00242
GGGGAATTCTAATACGACTCACTATAGGGAGAGGGAGAATAGAGACGTATTCGTTTGCGCTCTGTTCACCTTCCTTC
TCCCTTCTCCC
+
DDE9B=?=?BBACCBBBBBBBA>97111.7>>>@@BCB@A=;;<=<<<;::<===<A=<<@<==<><::7744,/,-
2232?:=;;54
@T6XXI:00092:00244
GGGAGAAGGGAGAAAGGAAGAGAATGTTATTTGCGTGGAAGCATAAACTATTCTCCCTCTCCCTATAGTGAGTCGTA
TTAGAATTCCCC
+
DDACCC@CC>@AAC>CAC@C@@@>AAA?BCCBCB@AA>A=>71111,69?6?C=<<7;784=:=8877775<<;68<
@=:865;5;=;1
@T6XXI:00092:00245
GGGGAATTCTAATACGACTCACTATAGGGAGAGGGAGAATAGTCTCCCTGCGTATGGAGCTGTCATCTCCTTCCTTT
CTCCCTTCTCCC
+
CKL:C@B>BBB@AACCCCBCCCB<;666.777>B<?@@@?@@BA???:>A:::@A@<???BDE<;<CCC?B?B?BD8
/01>,0,00/7,
@T6XXI:00093:00225
GGGGAATTCTAATACGACTCACTATAGGGAGAGGGAGAATAGACGTGGAACTGTTGCCAGTGTATTCTCCTTCCTTT
CTCCCTTCTCCC
+
:99/9<?<>AA?BBBCCBCBBBAD><>A<99<BB>BCA>@@<A<<<<;B@?AAB@875:A:9:AB?A??=@7:3471
4@AC<@>@@@<;
@T6XXI:00093:00226
GGAGAAGGGAGAAAGGAAGGAGAAGTCGCCCCAAACGATTTGTTGACCTATTCTCCCTCTCCCTATAGTGAGTCGTA
TTAGAATTCCCC
+
/,///,//+449>09247799;;;66??>>>2?A=A???811136175:;<BCCCA;A987;94444==6>@=999@
A>89=8<5;==1
@T6XXI:00093:00241
CTATGACTCGAGAATCTGCGGCTTTGGACAGTCCCTCTGTACTGTGAAAGCCC
+
:44///***0**,*,22:33579?7?4731111778877667773000+-,,*
@T6XXI:00093:00249
GGGAGAAGGGAGAAAGGAAGTGCAGTTCGCGGTTAGTGAAGGGAAAACTATTCTCCCTCCCTCCATATGAGTAG
+
:?6<;A@C@=A;;909<@>ACDBCA@=@AA?=B=>?888;=<-776,77;>@???@>@@E>B@5611-------
@T6XXI:00094:00224
```

```
GGGAGAAGGGAGAAAGGAAGGAGAATAGTAGGCCACCGAGCATAGTCTATTCTCCCTCTCCCTATAGTGAGTCGTAT
TACGAATTCCCC
+
BCADECBCC>9<;EAC=A?ACCCG@CCC@6649:>?=@AAAADBBB@@AC<<<CE@AAACC>CCBBBABC?@@D>>>
,000;9;,779,
@T6XXI:00094:00225
GGGAGAAGGGAGAAAGGAAGCTAGTCACTCGACATCTGTGCTTCACAGTACAGACGGAGTCGTCAAA
+
CCBA@?>?>:@B@@>B=@=@B>@BB???????BBAAAA777?4311666689833.011----1110
@T6XXI:00094:00237
GGGGAATTCTAATACGACTCACTATAGGGAGAGGGAGAATAGTGCCGCGCGATCCCCTGTTGACAACTA
+
444,4:;@CBC?BC@@@@A@@?CCAAAC:?<???6=@@;==@<4434430686:==465321---,.+*
@T6XXI:00094:00238
GGGGAATTCTAATACGACTCACTATAGGGAGAGGGAGAATAGTTTTCTCCTCGCCAAACTGGTCATGCCCTTCCTTC
TCCCTTCTCCC
+
CBB9B8;;<CDACCB@BBBB@>;97111.7>>AF>CCA?A=<>>>5559>@===<?>7>==<6<8<<=>;<>A242-
.--+.,/+503
@T6XXI:00094:00243
GGGAGAAGGGAGAAAGGAAGACTAGTCACTCGAGATCTGCGGCTTTGGACAGTCCCTCTGTACTGTGAAGCCACAGA
TGTCGAGTCGACTAGCTTCCTTTCTCCCTCTCC
+
;@:=@A?A>:>911-/6;6.00969/........-./766=:677-
7:=>BABCB=@>@888888@AA===>@A@A@@???A?@@==<@;<<<;886:828,,,+,,++*
@T6XXI:00094:00246
GGGAGAAGGGAGAAAGGAAGGGGAGAAGGGAGAAAGGAAGCTAGTCACTCGAC
+
EH@A@=78809?>>9><>5888.7@?<?C>:::C;A;?477771<61111100
@T6XXI:00095:00223
GGGGGAATTCTAATACGACTCA
+
9444+42/7811,/68@97;87
@T6XXI:00095:00229
AGTCACTCGAGATCTGCGGCTTTGGACAGTCCCTCTGTACTGTGAAGCCACAGATGTTCCGGAAG
+
;<<;=@@BCCBABBBCCCCA@?>@8:<BAA@C<?@AA???@A@:;9:C@D@?><>//,,*+*+*+
@T6XXI:00096:00202
GGGAGAAGGGAGAAAGGAAGTGCAGTTCGCGGTTAGTGAAGGGAAAACTATTCTCCCTCTCCCTATAGTGAGTCGTA
TTAGAATTCCCC
+
HCACCC?BA=BC:;;8:;;000>==@@CCCC@C?@@@<<:8?8?BB9CCCC@BBBB>CCCBC>B@AA<<?B=<<<>8
858AA=A>@??4
@T6XXI:00096:00220
GGGAGAAGGGAGAAAGGAAGCTAGTCGACTCGACATCTGTGGCTTCACAGTACAGAGGGACTGTCCAAAGCCGCAGA
TCTCGAG
+
990499<@@:>9;;89?@??@@<::ACBB@>79611166660171l669=BA@;;444-4;;;;?244-
48289@88999A@@@
@T6XXI:00096:00230
GGGAGAAGGGAGAAAGGAAGCTAGTCACTCGAGATCTGCGGCTTTGGACAGTCCCTCTGTACTGTGAAGCCACAGAT
GTCGAGTCGACTAGCTTCCTTTCTCCCTTCTCCC
+
CCB@@>>A>:A@@@<@?B@CCAABA@CCBCBA@=@CCCCC@@??;?;??A???B<@B@<===@@??B>8948@@@A@
@@<<====99===<<<=@>@@;@AAC>A=>?==;
@T6XXI:00096:00238
```

```
GGGAGAAGGGAGAAAGGAAGCTAGTCACTCGACATCTGTGGCTTCACAGTACAGAGGGACTGTCCAAAGCCGCAGAT
CTCGAGTCGACTAG
+
CD@=<;@CC;<<BB<@=@?BBBBBA??=7:77716611688?A?BCCBCBA>94000,38;;7868C:;=;=<<B?>
<<<>>??AAAAA??
@T6XXI:00097:00213
CTATCACTCGAGATCTGCGGCTTTGGACAGTCCCTCTGTACTGTGAAGCCACAGATGTCGAGTCGACTAGCTTCCTT
TCTCCCTTCTCCC
+
D::;@::9?BB?EBBBBAA>AAB<B8:;AAA@B=B@@@CBCCB@?A>>B>A@B?AABAAACBBABAA;::<636066
-7109-4/83349
@T6XXI:00097:00235
GGGAGAAGGGAGAAAGGAAGGGGAGAAGGGAGAAAGGAAGATGAACAGCACGGTACGGGAGATTAAGCTATTCTCCC
TCTCCCTATAGTGAGTCGTATTAGAACTTCCCC
+
<D@CDCCFC?:;<C@C@C@BBB7@?B?AA<AAAA<A>A=7:=CC@CBCBBDC?BBICCABA@>8<8<;BBCBCCCC@
CDCCCACBBBAA@AB@?;?1637660342333,
@T6XXI:00098:00211
GGGAGAAGGGAGAAAGGAAGCTAGTCACTCGACATCTGTGGCTTCACAGTACAGAGGGACTGTCCAAGCCGCAGATC
TCGAGATCGACGTAGCTTCCTTTCTCCCTTCCTCCC
+
CD@ECC?BC@CBCC@C=@=@CCACABBB>>;0/0000<772/0,07:=@AACCA@AA<A===??;=8:=34033003
36443..----++++++3555757744303232044+
@T6XXI:00098:00221
GGGGAATTCTAATACGACTCACTATAGGGAGAGGGAGAATAGCTACGTTTAACCGATCGTGTTGTTCTCCTTCCTTT
CTCCCTTCTCCC
+
::4/9?B@CBB@CBBB@@@BBACB>66616?8?C>BA?=A===@@@>??;>@A@=??=<<<=<==999:<?=?=@A<
@@?B<A;=?CCA
@T6XXI:00098:00248
GGGAGAAGGGAGAAAGGAAGGAGAAGTCGCCCCAAACGATTTGTTGACTATTCTCCCCTCTCCTA
+
CC?AAA@CC@BBBC>C@C@A?ABB??><77772>>:@DDBC>B@B>??@A;99:888..//...*
@T6XXI:00099:00200
GGGATCTATACGACTCACTATAGGAGAGGGAGAATAGTCAACAATCGTTTTGGGGCGACTTCCTCCTTCCCTTTC
+
//+///,,,,,,,,644173711317111,16448<;644/00/111666,111,116677971100/00+00+0
@T6XXI:00099:00221
GGGGAATTCTAATACGACTCACTATAGGGAGAGGGAGAATAGTCATGTGCCTCCCGACGTGACATTCGCCTTCCTTT
CTCCCTTCTCCC
+
FFF9C@A?BBB=AA@<;;BB:7887777.7==;76;;;;>??@@??898@:<<C<=>=AA@BCCC@@@@=?@C@CC>
DCCB<?;::9;:
@T6XXI:00099:00227
CTAGTCGACTCGGAGATCTGCGGCTTTGGACAGTCCCTCTGTACTGTGAAGCCACAGATGTCGAGTCGACTAGCTTC
CTTTCTCCCTTCTCCC
+
B6///1//:BBB@CCDDBCBBB@AAB>B?@>>>777.7?BBDCCB@CB@>@C@CCAA@BBCDCBCBCC;;;?:718;
=@B>A?AC7858@>>:
@T6XXI:00099:00241
CTAGTCACTCGAGATCTGCGGCTTTCGACAGTCCCTCTGTACTGTGAAGCCACAGATGTCGAGTCGACTAGCTTCCT
TTCTCCCTTCTCCC
+
>:11111::BCA;9=CDCCC@BBA=<A??BBABB=BAAADABACCBB8;;?CCCDCB@====??CCCBAAA@?4734
4-444;:>8<<??7
@T6XXI:00099:00243
```

```
GGGAGAAGGGAGAAAGGAAGGAGAAGTCGCCCCAAACGATTTGTTGACTATTCTCCCTCTCCCTATAGTGAGTCGTA
TTAGAATTCCCC
+
CBBCCC@BB=A>>><@@C@B@B?@89:?>>>?5?A<BBCCC:<;@C@=?@??CCCA;>??A=;==<????:@AAAA?
@=AAA>A>@A@5
@T6XXI:00050:00260
GGGAGAAGGGAGAAAGGAAGGAGAAGTCGCCCCAAACGATTTGTTGACTATTCTCCCTCTCCCTATAGTGAGTCGTA
TTAGAATTCCCC
+
CC@DCB>?>:@B?B?B@C@C@C@?><@CBBBB8AA=ACFCC>CC@CAAB@@;>;;;6888BAAB===@AAAA===99
9@A;978/444-
@T6XXI:00050:00270
GGGAGAAGGGAGAAAGGAAGCTAGTCGACTCGACATCTGTGGCTTCACAGTACAGAGGGACTGTCCAAAGCCGCAGA
TCTACGAGTCGACTAGCTTCCCTTTCTCCCCTTCTCCC
+
DDCDBB?BC<=@@@<>5948?>>>=7777//990....335,17:99=A??:74;444-
4;;9<=;=A=AA>@A?;=1,,,,++044347./.;>;;;9;;3;4444,49;=?@<
@T6XXI:00050:00287
GGGGAATTCTAATACGACTCACTATAGGGAGAGGGAGAATAGTCAACAAATCGTTTGGGGCGACTTCTCCTTCCTTT
CTCCCTTCTCCC
+
@@?6>6969>?<A;<<@CCDBBAA@767.777>>:@A@>CAAA@@=@??:@===>.777.7?AAA>@>@4838:@@:
???A@A;===C?
@T6XXI:00051:00277
GGGGAATTCTAATACGACTCACTATAGGGAGAGGGAGAATAGTCAACAAATCGTTTGGGGCGACTTCTCCTTCCTTT
CTCCCCTTCTCCC
+
CBB4><>698@=?=BBA@BCCC@@?:76.7>>>@?AA@@B==<;;:444-
777?@<@BB4;:::?9@@@??:=9899:3444-48;8==:
@T6XXI:00051:00279
GGGGAATTCTAATACGAC
+
999//572--,*,,,//4
@T6XXI:00051:00285
GGGAGAAGGGAGAAAGGAAGGAAAACAGTGGCACCTACAAGTGACCCACTATTCTCCCTCTCCCTATAGTGAGTCGT
ATAGAACTTCCCCATCACCGAACC
+
DIC@<;>B@<;99?9>;><?;>B?5?BBBBADCC@??@C>>8==AA=AAB::;:77@.443030303З??A@@>@::
//.3333A<=??4=;579.++00*
@T6XXI:00051:00292
GGGGAATTCTAATACGACTCACTATAGGGAGAGGGAGAATAGCTACGTTTAACCGATCGTGTTGTTCTCCTTCCTTT
CTCCCTTCTCCC
+
999/4298@CD@CCBB@@AAD@BBB???9??9=>>BB@>@<@ACCBBBE>C?B??BBAA@AA>@@:==?=?9;344+
339:;A9<<AC?
@T6XXI:00051:00299
GGGAGAAGGGAGAAAGGAAGTGCAGTTCGCGGTTAGTGAAGGGAAAACTATTCTCCCTCTCCCTATAGTGAGTCGTA
TAGAACTTCCCC
+
BBACDCABB:@BBBBB@A=BCB=BBC@CCCC=@>@CEDD@CB49164;AAB@CAAA9???@@:@>><==@AA?=77/
/-13,-0/433+
@T6XXI:00052:00251
GGGAGAAGGGAGAAAGGAAGCTAGTCACTCGACCATCTGTGGCTTCACAGTACAGAGGGACTGTCCAAAGCCGCAGA
TCTCGAGTCGACTAG
+
IN@CCC@BC?CCBB>B<=<@AAAACC@;9;>00,/555@>C=?@@CDEBBBA?=;444-
4;;??>?DD>AA>A@@@@===AAAAAC@@<<88
```

```
@T6XXI:00052:00255
GGGGAATTCTAATACGACTCACTATAGGGAGAGGGAGAATAGTTCCTCTGTGTCAAGAGCGAGTGTCTCCTTCCTTT
CTCCCTTCTCCC
+
@=@6>6;:<CB@CBBBBCCCABAA@:6:57????<A:869AAA?C@??<??BFCF?BBBBCAAA@A??B3434244-
4;AA?C<==<@7
@T6XXI:00052:00273
GGGAGAAGGGAGAAAGGAAGGGGAGAAGGGAGAAAGGAAGCTAGTCACTCGACAGCTG
+
CC@CBC@DD>BAAB=?<=7<<<2::;DBB9A:77.60737@716B<::00,+++----
@T6XXI:00052:00277
GATCTGCGGCTTTGGACAGTCCCTCTGTACTGTGAAGCCACAGATGTCGAGTCGACTAGCTTCCTTTCTCCCTTCTC
CC
+
:;BA@@CC@CCC>CA<;9<???=@BBBCBB@@=@@><?;?A?;;<<<<<@A@CCC;::B<<7;8;;7:9:9-4/4-
272
@T6XXI:00052:00278
GGGGAATTCTAATACGACTCACTATAGGGAGAGGGAGAATAGACCTTCGACGTTCAGAACCTATTCCTCCTTCCTTT
CTCCCTTCCTCCC
+
CCC:C@A=@BB=@@BB@@@BA?>?>777.7>?CC>CCC@@777;8;8<ACCCC@B@@@?@=@::6:?C@>A<?<>?9
4.-611,/,+000
@T6XXI:00052:00279
GGGGAATTCTAATACGACTCACTATAGGGAGAGGGAGAATAGTCCCCACTTGATTGGTACCTATTTGTCCTTCCTTT
CTCCCTTCCTCCC
+
CCC9D@B@AAA?CBBBC@@@@@?>>777.7?777.71323;00000+09@8<=?;;9;4848877.7?=3:8::??9
;76616,/,/733
@T6XXI:00052:00283
GGGAGAAGGGAGAAAGGAAGCTAGTCGACTCCGAGATCTGCGGCTTTGGACAGTCCCTCTGTACTGTGAAGCCACAG
ATGTCGAGTCGACTAGCTTCCTTTCTCCCTTCTCCC
+
AA:@<979@:?99909;?<B@?ABA9::?::6:??CAAACA?;:995:ABCBBBCC=?====<AA===A>AA>@???
@@@@@AAAAA===??<<99:9;;.//996:00.<=<
@T6XXI:00052:00284
GGGGAATTCTAATACGACTCACTATAGGGAGAGGAGAATAGA
+
88C9>BC@BBB@CCCCB@AB?>ACC@@>;>@A?>@.-,+,,*
@T6XXI:00052:00286
GGGGAATTCTAATACGACTCACTATAGGGAGAGGAGAATAGCAGGGACCT
+
>>=5>===@BB>BBCB;;<CCCCBBBBB<=C::=?;777.////-+-333
@T6XXI:00052:00296
GGGAGAAGGGAGAAAGGAAGCTAGTCGACACATCTGTGGCTTCACAGTACAGAGGGACTGTCCAAAGCCGCAGATCT
CGAGTCGACTA
+
CG@BAA@BB>BCBB>B@B?ABA@@@CCCCBBB:8;AAC<@@?BBCDCCDDBB99;9;@AABB?BB=BB?BCCCCAAA
CBCCCCB@:;:
@T6XXI:00052:00297
GGGAGAAGGGAGAAAGGAAGGGAAATGACATGTGTAGTCAGTGGGAGCTATTCTCCCTCTCCCTATAGTGAGTCGTA
TTAGAATTCCCC
+
CC>@@@?AA<AA@B>B?C?BC<AA<ABABBCCCCCBB@BBBB?@;@A@AAB????@;@@?AA<A???AA@@@A@@@@
@=@AC=@=@AA4
@T6XXI:00053:00250
GCTAGTACATCGAGATCTGCGGCTTGGACAGTCCCTCTGTACTGCTGAAGACCACAGATGGACTGTCCATCTCGAGT
CGACTAGCTTCCTTCTCCCTCTC
```

```
+
4///////,,,,,,,228=A@@@B=@<;;<<;9=9<:8;7886661776036384435562004400/000355666
7743400-/,/5656/..+.377
@T6XXI:00053:00258
GGGAGAAGGGAGAAAGGAAGGAGAAGTCGCCCCAAACGATTTGTTGACTATTCTCCCTCTCCCTATAGTGAGTCGTA
TTAGAATTCCCC
+
CCBCBB@BA=AAAB>B@B@A@CAA?>?BBCBB8BB?CB>>B<BA@@>9><=;<<>=;89<?C<A???@?@<@@@A??
?>@CC=@<@AA6
@T6XXI:00053:00260
GGGGAATTCTAATACGACTCACTATAGGGAGAGGGAGAATAGCCATTCCTTGTTTCAGTTCCCGCTGTTCTTCCTTT
CTCCCTTCTCCC
+
JDB9C798;@@>ACBCA@ACCBA>=777.7=?CJ>CCACA@8838=;=;?;>??;?@@@<??7<;==?588;=8<;6
<:::-7/74=B=
@T6XXI:00053:00264
GGGAGAAGGGAGAAAGGAAGCTAGTCACTCGACATCTGTGGCTTCACAGTACAGAGGGACTGTCCAAAGCCGCAGAT
CTCGAGTCGACTAG
+
CC@B@@644-999909<@>AA@@ABB@@=<?:::A>=BCC=?@CNCBBBBA@=9033-
4;>>???AA=AA>A@@A?????==<<<==@AAA
@T6XXI:00053:00269
GGGAGAAGGGAGAAAGGAAGCTAGTCGACTC
+
AA<@B?;=@<999@9>59588810,,,*0**
@T6XXI:00054:00252
GGGAGAAGGGAGAAAGGAAGGAGAACACACAGAGAGGACCCACTGAGCTATTCTCCCTCTCCCTATAGTGAGTCGTA
TTAGAATTCCCC
+
CD@BBB@BB?BBBC>AAB@B@BBB@BBBBBABBCCC@CCE;?B;<<BBBBB>;88?:;:;BC>CBBCDCCB@@BC@@
=>@BB?B@BBB:
@T6XXI:00054:00263
GGGAGAAGGGAGAAAGGAAGGAGAAGTCGCCCCAAACGATTTGTTGACTATTCTCCCTCTCCCTATAGTGAGTCGTA
TTAGAATTCCCC
+
AC>CDC@CABB@@@=B@B@?>>BB@@@A>;99,33,;?B?C@CC@B@@?CC@A<<;444444-
4>;<=AAAABAA===>A<;8;5;:<1
@T6XXI:00054:00264
GGGAGAAGGAGAAAGGAAGAACAGTACGTTTGGTGTTGTTGAGATGA
+
B;-/11,/,,,22*,*/666388>>;;1?>9>;0/.5..,11...--
@T6XXI:00054:00277
GGGGAATTCTAATACGACTCACTATAGGGAGAGGGAGAATAGTCAACAAATCGTTTGGGGCGACTTCTCCTCCTTTC
TCCC
+
DEC:EAD<;99EBCCCCBC?>?>>7777-7>777,66325>>68=9<>=4==777-
7;;2<???;;??=340/00+01110
@T6XXI:00054:00281
GGGAGAAGGGAGAAAGGAAGCTAGTCGACTCGAGCATCTGTGGCTTCACAGTACAGAGGGACTGTCCAAAGCCGCAG
ATCTCGAGTCGACTAG
+
DC???>@BC>BCCC=?==;?@8:;A:::BB@AC???;::===4773:>@@BB=;;:433-
4;;;;>;>@:@A>===@A;;<<888@AAAAAAA
@T6XXI:00054:00282
GGGCTACGACTCACTATAGGGAGAGGGAGAATAGTCAACAAATCGTTTGGGGCTG
+
ED@CCCCCCCCCBB@?@888,39377116=>ABB>@@;>>>044...+...*...
```

```
@T6XXI:00054:00283
GGGGAATTCTAATACGACTCACTATAGGGAGAGGGAGAATAGTTACAAACAA
+
AA@4@6;:<@@BD<<<CBBBBCBCB?::88;89A<AC9799//,////+--0
@T6XXI:00054:00290
CTATCACTCGAGATCTGCGGCTTTGGACAGTCCCTCTGTACTGTGAAGCCACAGATGTCGAGTCCGACCTACCGCCG
CT
+
6A@;444449449>A@A??=?>?:?58877776-
17//...//9<<<=BBCB889>B;99<98=;/..3...,..,..4
@T6XXI:00054:00292
CTATCGACTCGAGATCTGCGGCTTTGGACAGTCCCTCTGTACTGTGAAGCCACAGATGTCGAGTCGACTAGCTTCCT
TTCTCCCTTCCTCCC
+
A:8<2//11:@CCBBAA@?>?CCC>C=@@BAAAA<ACCCC>??<?;;87;7;AA?@@C@???A:;;BBA?A@?2:59
B<@59:66,/,/893
@T6XXI:00054:00294
GGGAGAAGGGAGAAAGGAAGGAGAAAT
+
8;8=1959@:981?,1,1,.,.99688
@T6XXI:00055:00263
GGGAGAAGGGAGAAAGGAAAG
+
KH@C@@644-9>;;,1-11+,
@T6XXI:00055:00278
GGGAGAAGGGAGAAAGGAAGGAGAAGTCGCCCCAAACGATTTGTTGACTATTCTCCCTCTCCCTATAGTGAGTCGTA
TTAGAATTCCCCC
+
FG@GCC@CC=;99>9>;><>;>>@:?;>8888.7<<@>>@@;=B=??::8=8;;994443=?;@@?@<<888==??@
@>9868823388.
@T6XXI:00055:00293
GGGGAATTCTAATACGACTCACTATAGGGAGAGGGAGAATATCTGTCGTTCGGTCGACGCTGC
+
<99299A>==<@C<<<CBBBBBBBCAAC>:::BB<@A=;=<733<8555544/3300444430
@T6XXI:00056:00256
GGGGAATTCTAATACGACTCACTATAGGGAGAGGGAAG
+
///*/,1,///76>@BCBB@@=>>7777,0/0:=-/-,
@T6XXI:00056:00262
GGGAGAAGGGAGAAAGGAAGCTAGTCGACTCGACATCTGTGGCTTCACAGTACAGAGGGACTGTCCAAAGCCGCAGA
TCTCGAGTCGACTAG
+
CC@=;<89>:?99908<?<@A@@AA770:9::00/5657;?377<?=>@@A?=:;444-
444444/4717>9?<==<@@>??@???===<=<
@T6XXI:00056:00281
GGGGAATTCTAATACGACTCACTATAGGGAGAGGGAGAATAGTCAACAAATCGTTTGGGGCGACTTCTCCTTCCTTT
CTCCCTTCTCCCATCACCGACCTGCCAC
+
999/92959489448A>??C;:8?777;5:BBCDBB@=9<@@???387;56;677,111,16;11061100/0/03+
4044+0/338:7:;;::7444/334200
@T6XXI:00056:00290
GGGGAATTCTAATAACGACT
+
;=;1;,1,/,,0,/,///44
@T6XXI:00057:00261
GGGAGAAGGGAGAAAGGAAGGAGACACGGTCACAAACATGAATGTGACTATTCTCCCCCCTCCCTATAGTGAGTCGT
ATTAGAACTTCCCC
```

```
+
CA=BEC@CC>A?;;6;::,0,0;;>CCD?B@A4;;7;A@@@@DCCC@??===>104444*4944389=<?@A>??@=
<;;=8:7442499.
@T6XXI:00057:00268
GGGGAATTCTAATACGACTCACTATAGGGAGAGGGAGAATAGCTCAACTTTTGTTGCATGTGATTTCTCCTTCCTTT
CTCCCTTCTCCC
+
CDC9C@C@ECB@BBB@@@BBBABB@:::8<??CC>CB@:?:==??>A??AB8@=<<98=A???=@;?A?8<968??:
??==6;8;<@A<
@T6XXI:00057:00287
GGGAGAAGGGAGAAAGGAAGCTAGTCGACTCGACATCTGTGGCTTCACAGTACAGAGGGACTGTCCAAAGCCGCAGA
TCTCGAGTCGACTAG
+
EIA<<9@CH>CACC?C=@=>C??@B::;BBAB;::::9@AC?ABCBBBBA?CECA===7<=?<==>AA=AA????A?
@?AA@@@A???AA@@
@T6XXI:00057:00293
GGGGAATTCTAATACGACTCACTATAGGGAGAGGGAGAATAGTCAACAAATCGTTTGGGGCGACTTCTCCTTCCTTT
CTCCCTTCTCCC
+
CLD9C@A@ACD@CA?AA>>>BAB>;661-
6=;;?=ABA>@@AA@@?@AA<BA@AB>BCC9CCCBB?<AA==;=<AA<A988178<<@A;
@T6XXI:00058:00256
GGGAGAAGGGAGAAAGGAAGCTAGTCACTCGACATCGTGTGCTTCACAGTACAGAGGGACTGTCCAAAGCCGCAGAT
CTCGAGTCGACTAG
+
DECAA@@BC?CCBC>B:96:B??@B?????A77///../....,.779=B???;;;;6;=B??@>@@<@A><=<?<<
<==A@@A===@===
@T6XXI:00058:00262
GGGAGAAGGGAGAAAGGAAGCTAGTCACTCGACATCTGTGGCTTCACAGTACAAGGAGGACTCGTC
+
CHBC=@798099990849437377;?65:7;//....0/?8?@@CBBCBB???4324;777/++++
@T6XXI:00058:00269
GGGAGAAGGGAGAAAGGAAGGAGAAAAGGCGTAAGCAAACATTACGGCTATTCTCCCTCTCCCTATAGTGAGTCGTA
TTAGAATTCCCC
+
BD?CBC=@=:@@??=B@C@C?BAABC8B>B?CB@BBCC@CB@@B;;89BBD8>;A?9=?>=85?>>BBB988JAA@A
A5:3688::==0
@T6XXI:00058:00279
GGGGAATTCTAATACGACTCACTATAGGGAGAGGGAGAATAGTTCTGTGCATTTCTCCCAGCACACCGTCCTTCCTT
CTCCCTTCTCCC
+
999/49BB>;;:;AAAC@@@B<@?=667.7><<<6=@=;?AAA?A@@AAA?BA9?===7>>@?@;;9;;:999;...
/;77-/.44A?:
@T6XXI:00059:00293
GGGGAATTCTAATACGACTCACTATAGGGAGAGGGAGAATAGCCGACATCCCGCGCCCGCAATTAC
+
DDE:CAC@DCC>AABBC@@@CC?<?7:7.7@@BC>BBA=@@77349:;877.1164524..,.,--
@T6XXI:00059:00297
GGGAGAAGGGAGAAAGAAGGA
+
9>9>99284-/,,,*,,*,*,
@T6XXI:00059:00298
GGGAGAGGAAG
+
11-/,,,**)*
@T6XXI:00060:00257
```

```
GGGAGAAGGGAGAAAGGAAGCTAGTCATCCTGTGGCTTCACAGTACAGAGGGACTGTCCAAAGCCGCAGATCTCGAG
TCGACTAG
+
AB><<;?BB<;99A<@=@@CCCCB7177737>==5:88<6?@@?>=;;///,////885475575AA=<<8888?A<
888>44/9
@T6XXI:00060:00266
GGGAGAAGGGAGAAAGGAAGGGGAGAAGGGAGAAAGGAAGCTAGTCA
+
1::77:,4919>A@1;9;>BB?7B;;7:A<::1?9?9:9:/.,,00*
@T6XXI:00060:00285
GGGGAATTCTAATACGACTCACTATAGGGAGAGGGAGAATAGACACCAATCGTGCCGGGTCATGTTCTCCTTCCTTT
CTCCCTTCTCCC
+
EDEDC>?=@BA>BECCBCBBB??;A116.6>=@B>BCC?A<>;?A@<?<A?==66077.7?>@?=9<AA2434344-
4;;A:@=@<<<6
@T6XXI:00060:00299
GGGGAATTCTATACGACTCACTATAGGGAGAGGGAGAATAGTCAACAAATCGTTTGGGGCGACTTCTCCTTCCTTTC
TCCCTTCTCCC
+
999,921,//1/8;@@@CCCBBB::;C6:;AAA=AB@>999AAA>@AA<CAAAD9?@A477?77377743249;;7<
6;:7627.496
@T6XXI:00061:00291
CTAGTCGACTCGAGATCTGCGGCTTTGGACAGTCCCTCTGTACTGTGAAGCCACAGATGTCGAGTCGACTAGCTTCC
TTTCTCCCTTCTCCC
+
B?499944::CBCCCBB?@?B?A@A<A>AACCBCB>BCCAAA@A@BB@>@C@BBB?@@@@??@@:;;??>>8713;:
;<67777.4/43ACA
@T6XXI:00062:00257
CTAGTCACTCGAGATCTGCGGCTTTGGACAGTCCCTCTGTACTGTGAAGCCACAGATGTCGAGTCGACTAGCTTCCT
TTCTCCCTTCTCCC
+
C:11111:?ECDCCC??@BB@BBB>B>AA@A99:8:CDECAAA?B@B?<CABBBAABABBAAABBCCCCEBBB@C;?
?9?@>B<=378==7
@T6XXI:00062:00260
GGGAGAAGGGAGAAAGGAAGGAGAAGTCGCCCCAAACGATTTGTTGACTATTCTCCCTCTCCCTATAGTGAGTCGTA
TTAGAATTCCCC
+
@A?ABB@CC?CBBB>B?A>?@BCB?BAAACCC8AA=ACCCC;BB@@<<A@5557775../7756;@@A@@AA??>@@
A?A?>:=8:<;1
@T6XXI:00062:00279
GGGAGAAGGGAGAAAGGAAGCTAGTCGACTCGACATCTGTGGCTTCACAGTACAGAGGGACTGTCCAAAGCCGCAGA
TCTCGAGTCGG
+
DCCCDC?CB>BCBC>@?@>A@CBA@8;1>>>?0/100/<>@5;:@CEAAABCB@A=<?<@???@@>?@:==:778<<
?=?>???AAA>
@T6XXI:00062:00290
CTCGAGATCTGCGGCTTTGGACAGTCCCTCTGTACTGTGAAGCCACAGATGTCGACTAGCTTCCTTTCTCCCTTCTC
CC
+
A><<AB;99A9;8=@@85@?@@@@AB@>@A999@@@A@@A@<959D99;BBBBBBDCAA@>;;377.777=9;/403
30
@T6XXI:00062:00291
GGGAGAAGGGAGAAAGGAAGCTAGTCGACTCGAGATCTGCGGCTTTGGACAGTCCCTCTGTACTGTGAAGCCACAGA
TGTCGAGTCGACTAGCTTCCTTCTCCTCTCCATCCGACC
+
KC?CBC@CC;9=BB>B?B?BDCCCA7:::::BDCDBCBBB=;667.7=????777.49:;=>@@A@@?=<<9;>>=8
88888=@<<=@@?=>>;6:9;.0/;9;66;6828,//+*
```

```
@T6XXI:00062:00298
GGGGAATTCTAATACGACTCACTATAGGAGAGGGAGAATAGTCAACAAATCGTTTGGGGCGACTTCTCCTTCCTTTC
TCCCTTCCTCCC
+
;E>5>,46944244=@@??BE79==//,.:7;;8?988:888;;;<=;477111,111,11111017700/0/00+0
12222,/,/640
@T6XXI:00063:00268
GGGGAATTCTAATACGACTCACTATAGGGAGAGGGAGAATAGTCAACAAATCGTTTGGGGCGACTTCTCCTTCCTTT
CTCCCTTCTCCC
+
ECE:C746989;=@@ACAAACCBA96::.7??BC@??=?@@@@?=;444-
:77??9=??5?@@BB>@?=::798=>;@@@C<A:==AD@
@T6XXI:00063:00272
GGAGAAGGGAGAAAGG
+
42499249-1111,1,
@T6XXI:00063:00280
GGGAGAAGGGAGAAAGGAAGCTAGTCGACTTGACATCTGTGGCTTCACAGTACAGAGGGACTGTCCAAAGCCGCAGA
TCTCGAGTCGACTAG
+
NH@AA@ACD@AA@B<?=@=?AB;;:AA>:;><00000//5609:>A<BDBB??<>444-
4::=?A>AA<AA@A??????@??<<<@?888<<
@T6XXI:00063:00287
GGGGAATTCTAATACGACTCACTATAGGGAGAGGGAGAATGAGTCAACAAATCGTTTGGGGCGACTTCTCCTTCCTT
TCTCCCTTCTCCC
+
CCC9B===@AA>@<<<CBBBCBBB?69:56A70000**)***..0<<<==;@AAAC>CCC9DCC??;6::342479:
6=?>C<A9<<AC=
@T6XXI:00063:00288
GGGGAATTCTAATACGACTCACTATAGGGAGAGGGAGAATAGCTTTTTGCTGCTCGACACCCTGATCCTCTTCCTTC
TCCCTTCTCCC
+
EHK9B?B?BBB?BCCAA@AAB@@@@@@@6::BCC=@@=@A??7:777,7>@??B@?:::A@7>AB::6;99,/,/,.
777,434444+
@T6XXI:00063:00290
GGGGAATTCTAATACGACTCACTATAGGGAGAGGGAGAATAGTCTGACCTGTG
+
CCC7B@@=ABB@CCCBAAABBBBB<:?A9?ABBB>BA?;>?;3041./,.3:/
@T6XXI:00063:00296
TAGTCGACTCGAGATCTGCGGCTTTGGACAGTCCCTCTGTACCTGTGAA
+
24;?=BB@@?99444879>@<=996924497777,11666110111133
@T6XXI:00064:00270
GGGGAATTCTAATACGACTCACTATAGGAGAGGGAGAATAGTCAACAAATCGTTTGGGGCGACTTCTCCTTCCTTTC
TCCCTTCTCCC
+
DDDDE@@=?CCCDCB>@@BBB:997//,0<;>@?ACB@A<@@@A?@@@<BA>>@=???7ABBAA<??@:;8;:==:?
B@A@A=@?AAA
@T6XXI:00064:00272
GGGAGAAGGGAGAAAGGAAGCTAGTCGACTCGACATCTGTGGCTTCACAGTACAGAGGACTGTCCAAAGCCGCAGAT
CTCGAAGTCGACTAG
+
CF@<<;@CC>AABB<@7;48>888@766==;7;:68:8B@A@CC?BAABCCA?>=7838===?@>@@<A@>@BAAA?
544..,//611.030
@T6XXI:00064:00274
GGGAGAAGGGAGAAAGGAAGTGCAGTTCGCGGTTAGTGAGGGGAAAAACTATTCTCCCTCTCCCTATAGTGAGTCGT
ATTAGAACTTCCCC
```

```
+
GK@C@?=@B>BDCC@B?B????@@BB@B???@B?<>@A@??@2?????2?CCB=@;::5:?=BC>@==@@AAAAA@;7
4434::8<??BHC5
@T6XXI:00065:00254
GGGGAATTCTAATACGACTCACTATAGGGAGAGGGAGAATAGATTGGCGAGAAATGCCGTCTAGTGCTCCTTCCTTT
CTCCCTTCTCCC
+
CFC8@=>5999;89ABBB@@CCBBAAAA:???CC>CB@=??=<=>A>AACB??:?7738?=?@?;<<8838;;9;>6
;<?=:?>>==C<
@T6XXI:00065:00257
GGGAATCTAATACGAC
+
99-42////,///4;9
@T6XXI:00065:00260
GGGGAATTCTAATACGACTCACTATAGGGAGAGGGAGAATAGTTTTCCCTTCACTAACCGCGAACTGCACTTCCTTT
CTCCCTTCTCCC
+
@@>6><@=@BB@CCCBA?BBBCBBB<<===CC@>;>>;;@<@<<=5<=;===?CBC@B?8:<CA===AB??:<477.
:77A<B????C<
@T6XXI:00065:00273
GGGGAATTCTAATACGACTCACTATAGGGAGAGGGAGAATAGTCAACAAATCGTTTGGGGCGACTTCTCCTTCCTTT
CTCCCTTCTCCC
+
CCC:B69:<AA>BDBCBBBBBA?97111.7===?<?CC>@>BAAA=@AA<B???B=BBB5ABCBB?AAA7837;??;
@@@AAB899BA<
@T6XXI:00065:00274
GGGAGAAGGGAGAAAGGAAGCTAGTCACTCGAGATCTGCGGCTTTGGACAGTCCCTCTGTACTGTGAAGCCCACAGA
TGTCGAGTCGACTAGCCTTCCTTTCCCCTTCTCCCCTTCCTTCTCCCTTCCTCC
+
44-949544-
4488589997;;;::B??:<=99586667;6668269=777777.770000003336:434+000400333394313
---2-0/3465699==;499554;;455523/00--+.,-,-22
@T6XXI:00065:00275
GGGAGAAGGGAGAAAGGAAGCTAGTCGACTCGACATCTGTGGCTTCACAGTACAGAGGGACTGTCCAAAGCCGCAGA
TCTCGAGTCGAC
+
CEDAAA@CC=BCAC<@=@@BBAAAA???????:::???B>>:;;7:BCBCBBA;;433-
4;;;==@CC=BA<?@AAAA???AAAA????
@T6XXI:00065:00276
CTATCGACTCGAGATCTGCGGCTTTGGACAGTCCCTCTGTACTGTGAAGCCACAGATGTCGAGTCGACTAGCTTCCT
TTCTCCCTTCTCCC
+
B?>949::?B=@BC<9;BBB?BCE>CBBBB@AAA=BCCCC?>>@A??<?B?DDCABBBBB>ABBBBCCECCBC?B;?
?9??A>:;;888@:
@T6XXI:00065:00283
GGGGAATTCTAATACGACTCACTATAGGGAGAGGAGAATATACGGAC
+
12112?AACDB>BBCBB>>>AAA<>66959?7::=0/,/50+--,-3
@T6XXI:00065:00289
GGGGAATTCTAATACGACTCACTATAGGGAGAGGGAGAATAGTCAACAAATCGTTTGGGGCGACTTCTCCTCCTTTC
TCC
+
CDD:F<<;<@A=ADCCDC@@@???:::;,0/:<<9>>?<@A==68<;==;CBAA?>ACD9AC>:7-//5-
..55435.30
@T6XXI:00066:00259
GGGAGAAGGGAGAAAGGAAGCTAGTCGACTCGACATCTGTGGCTTCACAGTACAGAGGGACTGTCCAAAGCCGCAGA
TCTCGAGGTCGACTAG
```

```
+
:?>=@A@CC@CDDC?B?B?BAAA@@?BBB@@??:::???<@=>A@B@@CBBCA>?AAC<@@A@>;344-
44244449;433=78/3;777446
@T6XXI:00066:00268
GGGGAATTCTAATACGACTCACTATAGGGAGAGGGAGAATAGTCAACAAATCGTTTGGGGCGACTTCTCCTTCCTTT
CTCCCTTCTCCC
+
CFNAC::;;CC@CCBBB@BBB@B97111.7?7776;?;<A<<=AA><=<7?@@@?9ABB7BBBBC=??D??<?;>>7
<=AA<A8:9AB<
@T6XXI:00066:00289
GGGGAATTCTAATACGACTCACTATAGGGAGAGGGAGAATAGATCACCGTTGGTTCGGGTGCGCGTTCTCCTTCCTT
TCTCCCTTCTCCC
+
BBC2A698<@@@CBBBBACABB><<777.7777A>BDA>AA@???B@;@@476:6;<C@DCBAA?<:6110101016
6;9>=7><847A5
@T6XXI:00067:00251
GGGGAATTCTAATACGACTCACTATAGGGAGAGGGAGAATAGTCTTGCAATTTCCTGCGGTGCATTCTCCTTCCTTT
CTCCCTTCTCCC
+
999/9<@>@@A@BBCCBCBAA>A@?69956=<??=@BA?B=A?@@>@:B=AB=B?CCA@=@CBAB?BA>5347477/
8>>?6<:7777.
@T6XXI:00067:00252
GGGAGAAGGGAGAAAGGAAGCTAGTCGACTCGAGATCTGCGGCTTTGGACAGTCCCTCTGTACTGTGAAGCCACAGA
TGTCGAGTCGACTAGCTTCCTTCTCCCTTCTCCC
+
?:6>9959@:A99;9:@@@@@;;:@7669667A@CCCCDBB?BBB>B@BBBBCCE;==?788@@@AAB>AA>@A@<;
;=448<878C>;;8///,/,/,.03403230344
@T6XXI:00067:00253
GGGGAATTCTAATACGACTCACT
+
666067:<>>?5444///.,22,
@T6XXI:00067:00266
ACGTGGCTATTCTCCCTCTCCCTAAGTA
+
BBE99594>A69999141/885//,//0
@T6XXI:00067:00272
GGGGAATTCTAATACGACTCACTATAGGGAGAGGGAGAATAGTCCAGCGACCCGTTTGAAACGTAATTATTCTTCCC
TTTCTCCCTTCTCCC
+
6<<0<-1,///,/488=7;;;6774...+..00000,-,-
1134235330044000+04407899=9==7363/00+044435535556<=;
@T6XXI:00067:00278
GGGAGAAGGGAGAAAGGAAGGAGAAGTCGCCCCAAACGATTTGTTGACTATTCTCCCTCTCCCTATAGTGAGTCGTA
TTCAGAATTCCCCATCACCGCACTGACCCATACGGTAACGAGGGTC
+
4A-
44@2499<AAC>C@CACBCAA>:::?@<<5B?27<766,16777?A84349996<<<?C>A998984===<<;::88
86:88:::8.61-..3399;?>AAB?@@A;:43333++,3333
@T6XXI:00067:00289
GGGGAATTCTAATACGACTCACTATAGGAGATGGAGAA
+
CEA1=+96998888=9;7;?@<>>=70,/>0..,...3
@T6XXI:00068:00256
GGGAGAAGGGAGAAAGGAAGGAGAACGCGCACCCGAACCAACGGTGATCTATTCTCCCTCTCCCTATAGTGAGTCGT
ATTAGAATTCCCC
+
```

```
<<6;ACACC@CCAA=B@B@B@@AAACCCBDGCC@CC?A?AADEACDHCA<<<?A>@@<@@@DJ=AABBCCCCAAA??
::5:=>:?2999/
@T6XXI:00068:00271
GGGGAATTCTAATACGACTCACTATAGGGAGAGGGAGAATAGCCAATCGTTCATCGTCCCCGGGCCACTCTTCCTTT
CTCCCTTCTCCC
+
DEC9B>A>ABA@DBBACBBBB@?97111.7>7776:A@<@@@@>?<?AAA>?AABA====1==7=<==8447:344,
344;;=<<<@?;
@T6XXI:00068:00272
GGGAATTCTAATACGATCACTATAGGGAGAGGGAGAATAGTCAACAAATCGTTTGGGGCGACTTCTCCTTCCTTTCT
CCCTTCTCCC
+
EC@E@?CABB?BB;;;:030779444-
4;44467>A@BCCCAA>BCD?CCBCC>CCC8BA@@C>A?B7:7:@CCE??@C?D<;/9;3
@T6XXI:00068:00276
GGGAGAAGGGAGAAAGGAAGGGAATAGCAGCAGTCGGCAACGTGAAACTATTCTCCCTCCTCCCTATAGTG
+
DC>BA??B?<A@@@<@?BBCC>C=@@?@@@@@CCBB???=@BACCC=DCBA:?==<6610111,0333355
@T6XXI:00068:00285
GGGAGAAGGGAGAAAGGAAGAGAACGGTAATTGATGTGAGGCAATGACTATTCTCCCTCTCCCTATAGTGAGTCGTA
TTAGAATTCCCC
+
BHACFC@CC?CBCC>@@A@CCBB@CCACCAC?AACCCCBCDDC@BBBBBA@<@9991888BC?CCCCCBBB???@@:
:8;BA>D?BBB7
@T6XXI:00069:00269
GGGGAATTCTAATACGACTCACTATAGGGAGAGGGAGAATAGTCAACAAATCGTTTGGGGCGACTTCTCCTTCCTTT
CTCCCTTCTCCC
+
?@@6@=@=ACGACCCBBBBBB@B<;66656B@AB>BB@>@===@@>@AA<BAA<?:=9B7AABBC?AAB5837:=9:
@@@@<A;==CD?
@T6XXI:00069:00272
GGGAGAAGGGAGAAAGGAAGGAGAAGTCCG
+
4;649=9=A:=9;B<A>@:;,---+,,,*,
@T6XXI:00069:00279
GGGAGAAGGGAGAAAGGAAGCTAGTCGACTCGACATCTGTGGCTTCACAGTACAGAGGACTGTCCAAAGCCGCAGA
+
GEABBC>@=:BBBB=BCE=@AC;<:FDCBBA@87;;::?BC>BBCBCC?@@A:77..,/::8;A@@A@AA>@@@AA
@T6XXI:00070:00262
GGGAGAAGGGAGAAAGGAAGAAGAGCAGCACGGTGAGACGTCCGTACTATTCTCCCTCTCCCTATAGTGAGTCGTAT
TAGAATTCCCC
+
DH?@A>@BC?BBAA?A@A=>B@B@>ABBCAAA@CCCCBB?@@@CEBB?7:7:@@B<<=@@@;A@??AA@A?????AA
>A@D>@;@BA6
@T6XXI:00070:00271
GGGAGAAGGGAGAAAGGAAGGAGAATAGCAGCAATAAAACCAGCTAGACTATTCTCCCTCTCCCTATAGTGAGTCGT
ATTAGAATTCCCC
+
BGCDCC@CC>BBBB<?=??A@CEC@C@@ACCBB?BBBB8B@CBBBBDCECCB@B9990999@A>BBBAACDBBBCBB
@@>@CC?DACBB4
@T6XXI:00070:00292
GGGGAATCTAATACGACTCACTATAGGGAGAGGGAGAATAGTACAAC
+
82208+1///789@2200.65667111,16777.7602383304-,-
@T6XXI:00071:00262
GGGAGAAGGGAGAAAGGAAGCTAGTCACTCGACATCT
+
```

```
?A:A@B>=96>48804242333396711........,
@T6XXI:00071:00284
GGGAGAAGGGAGAAAGGAAGCTAGTCACTCGACATCTGTGGCTTCACAGTACAGAGGGACTGTCCAAAGCCGCAGAT
CTCGAGTCGACTAG
+
CB>ABC@BC>BBBB<?=?@CCAAAB:10==>00/555=>B?CC?CBEBBBBC?8733-
4448?@@BC<AA>@???AAA@@===@===@@??
@T6XXI:00071:00288
GGGAGAAGGAGAAAGGAAGAATACGCGGGATCAACTCAAGGTGTGCTATTCTCCCTCTCCCTATAGTGAGTCGTATT
AGAACTTCCCC
+
8>88-1-
1,///7+/,423313;@@@@B0;94424444249;337;776>:8:A777@A@;===<<;=<<930000/0442332
344.
@T6XXI:00071:00298
GGGAGAAGGGAGAAAGGAAGTGCAGTTCGCGGTTAGTGAAGGGAAAACTATTCTCCCTCTCCCTATAGTGAGTCGTA
TTAGAACTTCCCC
+
AA>AAA>AA<A@@@<@>A?BBBCCCC@CC@@=@=@CCCCABB>BCC8BAAA?BBCE=???DDA?<<???AAA@@887
334048<8::<80
@T6XXI:00072:00252
GGGGAATTCTAATACGACTCACTATAGGGAGAGGGAGAATAGCTCCACAACAGCCCTCATTGTACTTTCCTTCCTTT
CTCCCTTCCTCCC
+
DCC7B696999<@?ABEABBA?<?;677.7?>@B>BCA>????AA=??E>==@@@:888=<>@?@?@6:79/434:7
9889-0/3/3;>;
@T6XXI:00072:00254
GGGGAATTCTAATACGACTCACTATAGGGAGAGGGAGAATAGTCAACAAATCGTTTGGGGCGACTTCTCCTTCCTTT
CTCCCTTCTCCC
+
GDE:CDC@CCC?BCCCCCBBBCB>869:.7>=??<?B?=@AAAAC>@BA<CAAAC>CCB9CCCBB>::7443479:6
<?>A=A6:.7;6
@T6XXI:00072:00276
GGGGAATTCTAATACGACTCACTATAGGGAGAGGGAGAATAGATCTAACGTTTACGCCTGGTTTATTCTCCTTCCTT
T
+
999,42929@@@CDCCCCCBB?>87777-7=777-
794=AB<<<<>?;@@?@9???A8;;@EL?@;9;/,+,10+++*
@T6XXI:00073:00256
GGGGAATTCTAATACGACTCACTATAGGAGAGGGAGAATAGACTCACCGT
+
HEE7B;;;<CCADCCBB9;;B:=<<00,0:077,6=926@A8884>>666
@T6XXI:00073:00273
GGGAGAAGGGAGAAAGGAAGCTAGTCACTCGACATCTGTGGCTTCACAGTACAGAGGGACTGTCCAAAGCCGCAGAT
CTCGAGTCGACTAGCTTCCTTTCTCCCTTCTCCC
+
DC@@<;9<@:@99919<@<;;;A?>=;6111111667>?>:??<??=AA????88818@=>??>AD?@@=CA@@;;
/00;;;@A@???@B<;8;;==7=??@7<9<;;@<
@T6XXI:00073:00278
GGGAGAAGGGAGAAAGGAAGCTAGTCGACTCGACATCTGTGGCTTCACAGTACAGAGGGACTGTCCAAAGCCGCAGA
TCTCGA
+
BBCBEC@CC>BBBBBB@B@@@AAA?:;;A=><00000/:9;3??=????BB?;98434-
4;;;>B=??;?C>998??<888AA
@T6XXI:00073:00281
GGGAGAAGGGAGAAAGGAAGGAGAAGTCGCCCCAAACGATTTGTTGACTATTCTCCCTCTCCCTATAGTGAGTCGTA
TTAGAATTCCCC
```

```
+
IIABBA?BHBAA@C>C@C@C@BBB?BBBCCBB8BC>CB7695;?:?BCC@?=<AAC<?<<=C=AA@A<==@AA@?=A
A>HA@;=8;:91
@T6XXI:00073:00287
GGGAGAAGGGAGAAAGGAAGCTAGTCGACTCGACATCTGTGGCTTCACAGTACAGAGGACGTGTCCAAAGCCGCAGA
TCTCGAGT
+
9:6:A@<>=199990949487@@>?@@?=77787=7616799<@<:BB??ABB@B@@=@888=;<8<@:@@?A@<<<
884;::30
@T6XXI:00073:00289
GGGAGAAGGGAGTAAAGGAAGAACGAGCGGTGGGTGGGTTGATAACAGAGCTATTCTCCCTCTCCCTATAGTGAGTC
GCTATTAGAATTCCCC
+
;B;:66,//-1////+,*,2,.,.....09:AB>B?@;964;//,.3003500.4444;68;444-
498;=>=<64553033/0382425=:0
@T6XXI:00074:00250
GGGGAATTCTAATACGACTCACTATAGGGAGAGGGAGAATAGTCAACAAATCGTTTGGGGCGACTTCTCCTTCCTTT
CTCCCTTCTCCC
+
499,92924989@BCCCBCCBB@?@77:6:??CD>B=:8;====A885@:@AAAA<ABB4:90//,/::,.,/461+
..8848273662
@T6XXI:00074:00278
GGGGAATTCTAATACGACTCACTATAGGGAGAGGGAGAATAGTCAACAAATCGTTTGGGGCGACTTCTCCTTCCTTT
CTCCCTTCTCCC
+
CCC9C@C@CCB@BCCAA?BBB?<<9166.7>>=?AACA>@ABA??<?ACAAAACC>FDD7??@??:=>>4428;>>;
@@?A<A69.9;6
@T6XXI:00074:00284
GGGAGAAGGGAGAAAGGAAGGAGACAGTGAGCGCCGCACAACACGTCACTATTCTCCCTCTCCCTATAGTGAGTCGT
ATTAGAATTCCCC
+
CDAFCC@CDACDCC@C@C@C@BBACBBBBBAABA?@BB::8:CC@??CNCAA?ADCC?CBBBC?BBBCCCCCCB@@?
??;?AD@DADDD9
@T6XXI:00074:00291
GGGGAATTCTAATACGACTCACTATAGGGAGAGGGAGAATAGCCGTTATTTTAACTCCCGCGGCCGGTTCTTCCTTT
CTCCCTTCTCCC
+
@??4@6969BB?CC@?@@@@B?>?>777.7?7777:BB@A@A@=9:73444-49;?AA:=:=.0./9;-
0/7:6=A;=99=>C==99=1
@T6XXI:00075:00257
GGGGAATTCTAATACGACTCACTATAGGGAGAGGGAGAATAGTCAACAAATCGTTTGGGGCGACTTCTCCTTCCTTT
CTCCCTTCTCCC
+
CEC:D>@>ADF@DCBABACC::;BB:7:5:@@AD?CDA<??@CAA?@AA@BBA??6<??5?@CCDABBB?=;=>AA<
@@?=7><>::=9
@T6XXI:00075:00269
GGGGAATTCTAATACGACTCACTATAGGGAGAGGGAGAATAGATCACCGTTGGTTCGGGTGCGCGTTCTCCTTCCTT
TCTCCCTTCTCCC
+
FB@8E;<;<GBBCBA@BBBDBAB?A791-
6;??B>BDC>AA<==@??:?A<?<?@BBB?BCCBBBDAAAA@D@C@CCCCCAA<@>???A:
@T6XXI:00075:00281
GGGGAATCTAATACGACTCACTATAGGGAGAGGGAGAATAGACCAAT
+
444,4244442448==;<;;@B;::BC:@@?==6=:641++++00*+
@T6XXI:00075:00287
```

GGGGAATTCTAATACGACTCACTATAGGGAGAGGGAGAATAGTCAACAAATCGTTTGGGGCGACTTCCTCCTTCCTT
TCTCCCTTCTCCC
+
CEB9B998;AA>ACCBBABBBAA?>777.77=?@=BCC>===?<97444-7==>=6<;;2?97::,0,00,-
24224-33442423044:
@T6XXI:00075:00289
GGGGAATTCTAATACGACTCACTATAGGGAGAGGGAGAATAGCTCACTCTTACCGGCATTATGCTCTTCTTCCTTTC
TCCCTTCTCCC
+
9=>4>692944248>>>?A@@?BBA;;:7:@ACE<??C@CA9:;C???4323;;@>??D>AA955<=;:=8>/7717
==A:B:=444,
@T6XXI:00075:00298
GGGGAATTCTAATACGACTCACTATAGGGAGAGGGAGAATAGCTGTAATTCCCCAGACAGCGCCGCAGTCT
+
8><0>,45448244BCC@@@BBABB77;.7=>?B>ECB@AAAAB@??3101662656444433/049773.
@T6XXI:00076:00283
GGGGAATCTAATACGACTCACTATAGGGAGAGGGAGAATGAGTTTCCCTCACTAACCGCGAACTGCACTTCCTTCTC
CTTC
+
88802*///6,/444437776776111,11111,1102355===;=<4554:43/0203334:8:<:8856//,.--
,+*+
@T6XXI:00076:00289
GGGAGAAGGGAGAAAGGAAGAGAATAACAATGCACCGAATGCGCAAACTATTCTCCCTCTCCCTATAGTGATCGTAT
TAGAACTTCCCC
+
CC>AAA=@@:@>>@<A>B=?@@?<?B8<<ECBBBCAFCCAACBDCDBBBBB<>387/376<<8?@ABCC@AA@=<<<
><=:;=70666,
@T6XXI:00076:00290
CTAGTCACTCGAGATCTGCGGCTTTGGACAGTCCCTCTGTACTGTGAAGCCACAGATGTCGAGTCGACTAGCTTCCT
TTCTCCCTTCCTCCC
+
DC<::BCFA@@@BABBDCA?=<8:6;?BBCCCCC>AAA??@CBCCBA;=A@BCCCCAAACCCCBCC@@???:62:;=
@9://:62,-,-110
@T6XXI:00077:00255
GGGAGAAGGGAGAAAGGAAGGAGGAAGTCGCCCCAAACGATTTGTTGACGTATTCTCCCTCTCCCTATAGTGA
+
:B>??B?AA7;9?@01,1,0,..,.4...88995;?;777<<66@;771111023334+00000+03000585
@T6XXI:00077:00268
GGGAGAAGGGAGAAAGGAAGCTAGTCGACTCGACATCTGTGGCTTCACAGTACAGAGGGACTGTCCAAAGCCGCAGA
TCTCGAGTCGACT
+
ENBBAA?BC>CBBB=B>B@CCA>>>/10><<;1:7776CCC>AA@CABABCBB@A@@@<?AA<A9>;;499?AA===
<9===<<<<<=?@
@T6XXI:00077:00273
GGGAGAAGGGAGAAAGGAAGCTAGTCACTCGACATCTGTGGCTTCACAGTACAGAGGGACTGTCCAAAGCCGCAGAT
CTCGAGTCGACTAG
+
BB?@>><>>:>>@@0969;>??BBABBBBBBBBBCC=?<B>?@>@CCBCAAAB><<<7<@@AAA>@@<AA@===@@A
A==<<<<878=<<<
@T6XXI:00077:00275
GGGAGAAGGGAGAAAGGAAGCTAGTCGACTCGACATCTGTGGCTTCACAGTACAGAGGGACTGTCCAAAGCCGCAGA
TCTCGAGTCGACTAG
+
9?9999599-448;-
4247333464//.334.01;;;6617713111779>B?8:444,4;=;==>AA<@A?AD@@???@84////--1116
@T6XXI:00077:00280

```
GGGAGAAGGGAGAAAGGAAGATGATCAAGGGGAAAGAGAGGCCAAGGCTATTCTCCCTCTCCCTATAGTGAGTCGTA
TTAGAATTCCCC
+
99194A?AB<A@@@<@>B@CBBADCCA=???6?C:??A::47=?4757===:?===74444=7=?@@=>>@;::;00
/975=88866=1
@T6XXI:00078:00271
GGGGAATTCTAATACGACTCACTATAGGGAGAGGGAGAATAGTCAACAAATCGTTTGGGGCGACTTCTCCTTCCTTT
CTCCCTTCCTCCC
+
CCD7@245999=ABABCCCCCCBB?7775:?BBC>@@=BA@@@@A?@AA<B???B>BCC9CB>@@;::<,/,/7879
:34359-/./;73
@T6XXI:00078:00272
GGGAGAAGGGAGAAAGGAAGCTAGTCGACTCGACATCTGTGGCTTCACAGTACAGAGGGACTGTCCAAAGCCGCAGA
TCTCGAGTCGACTAG
+
FC@AAA@BC>BBBC>BAB?@@@AAC:;:???=;;:???@AB5;;?BFBBBBCCA=<<<;<=>=<<;>=;<=;?<<?A
A?=====AAAA@@@@
@T6XXI:00078:00273
GGGGAATTCTAATACGACTCACTATAGGGAGAGGGAGAATAGTCACATTCATGTTTGTGACCGTGTCTCCTTCCTTT
CTCCCTTCTCCC
+
@==4>6969AAAABBB@@@CC@??<777.7>>>@=CCA@AA@@@===B@CCCBBB<????@>@@@A888;=:>;>?:
>?@A<@=??==7
@T6XXI:00078:00290
GGGAGAAGGGAGAAAGGAAGACAGTCCATCTGTGGCTTCACAGTACAGAGGGACTGTCCAAAGCCGCAGATCTCGAG
TCGACTAG
+
9@<@@@>@B><;;A<A=AB@@8887858>?;;:C@GG@CBAB=<?B????D?998@BA?A=;??>@AAAAA988??:
8BBC@@@>
@T6XXI:00079:00260
GGGAGAAGGGAGAAAGGAAGGAAGAGCAGCAGTCGGCAACGTGAAACTATTCTCCCTCTCCCTATAGTGAGTCGTAT
TAGAATTCCCC
+
AA<@CA@CC>@>;;8000)*)*)****0289BCAAABBAFCCCC@9??CC@BBBB>;:;CE?A<<<ACAAABAA===
9<<B;65>>=1
@T6XXI:00079:00261
GGGAGAAGGGAGAAAGGAAGGAGAAGTCGCCCCAAACGATTTGTTGACTATTCTCCCTCTCCCTATAGTGAGTCGTA
TTAGAATTCCCC
+
CDBDDC@BB>BBBB=B@B@B@BAA?@@BBBCC9BC?CC==?=@C?C@=@??;=;;;6;;<@A<AAAA???@@???@@
@?ACA>@>@AA6
@T6XXI:00079:00277
GGGGAATCTAATACGACGTCACTATAGGAGAGGGAGAATAGTCACAGTTCATGTTTGCGTACCGTGTCCTC
+
111*1,1//6,/4444333770686..,.64798:::21/:0.+++--,---28943333364444--,--
@T6XXI:00080:00276
GGGAGAAGGGAGAAAGGAAGGAGAAGTCGCCCCAAACGATTTGTTGACTATTCTCCCTCTCCCTATAGTGAGTCGTA
TTCAGAATTCCCC
+
CB<@<969B9<;DE=B@C?BBCBC@CCCCCBB9BC>BB?:8234,/..336:<=?;:8848C<@A@=<99=AAAA==
=899??:;3444-
@T6XXI:00080:00277
GGGGAATTCTAATACGACTCACTATAGGGAGAGGGAGAATAGCCAGTCGATATCCCTAAACTGGTGTGCTTCCTTCT
CCCTTCTCC
+
>>>1>,/,1//79?AABABAAA@>=777,77777-6=;:=@??;??A<<<==8894000,1;;:?AA:9;6:,/,--
//,48:34/
```

```
@T6XXI:00080:00278
GGGGAATTCTAATACGACTCACTATAGGGAGAGGGAGAATAGTACTCACGTGTGTGGATTCTCT
+
9;;,96924449:9;<@?<<@973116626=777-610/034468?=97766...5,--010++
@T6XXI:00080:00284
GGGGAATTCTAATACGACTCACTATAGGGAGAGGGAGAATAGTTCTGTGCATTTCTCCCAGCACACCGTCCTTCCTT
TCTCCCTTCTCCC
+
CCC9A=?=ACC@CCAAA???BC<97111.7>>>A>BCA@D????C??>>AACC>C???<?C@@?@A>@AA<;9;244
1794;;A489@C:
@T6XXI:00080:00289
GGGAGAAGGAGAAAGGAAGCGTAGTCACTGAGATCTGGCGGTTGGACAGTCCCTCTGACTGTGAACCACAGATGCGA
+
685;19595449969242393333331111..,,,,,,*,0046<;;>866:@:>??<<====4=92*+++++++---
@T6XXI:00081:00253
GGGGAATTCTAATACGACTCACTATAGGGAGAGGGAGAATAGCCGACAATTCGACTCCCCGGCAATGTTCTTCCTTT
CTCCCTTCTCCC
+
CCC:E;;;<CD@CCCBBABC>=?@=66657??AC>CB?=?888>AA@@=B?????????7?@CC????>AB>A>AB;
@AAC;@898@A<
@T6XXI:00081:00260
GGGAGAAGGGAGAAAGGAAGGAGAACGCGCACCGAACCAACGGTGATCTATTCTCCCTCTCCCTATAGTGAGTCGTA
TTAGAATTCCCC
+
DG>BBC@BB>CDDC?C@C@B@BBB?BBCCCCCC@?=@=?=ACACBCCB@?@@B9880888AA>BCBBBAA@A???CC
D@CCC@D@CCD:
@T6XXI:00081:00272
GGGAGAAGGGAGAAAGGAAGCTAGTCGACTCGACATCTGTGGCTTCACAGTACAGAGGGACTGTCCAAAGCCGCAGA
TC
+
HE@@@?@CC>BBBA=@?B=@@@BBBCCBBBC?717::7>?B7;:@DBBABBCCA;33314:;??@>@A;@@@C@@?<
==
@T6XXI:00081:00286
GGGAATCTAATACGACTCACTATAGGGAGAGGAGAATAGC
+
:K?@311:::8?@ABCBBAA@BA::7.07,,+,,+000--
@T6XXI:00082:00260
GGGGAATTCTAATACGACTCACTATAGGAAGAGGGAGAATAGTTTTTCCCTTCACTAACCGCGAACTGCACTTCCTT
CTCCCTTCTCC
+
BCA9B7;8;<;ABBACB9;;?=>89:97=;777=6<66:>@@@AAA1?@14/4;0/.,.449B@889=8::?:=3:5
11??.0,///,
@T6XXI:00082:00263
GGGAGAAGGGAGAAAGGAAGCTAGTCGACTCGACATCTGTGGCTTCACAGTACAGAGGGACTGTCCAAAGCCGCAGA
TCTCGAGTCGACTAG
+
AA>AA@>BEACDCC<@8;8:AAABC:;:700010....355-79@@<??BD?=::444-
4;;9<=>AA@AA?@A@A@@@==<@A>////-.1
@T6XXI:00082:00274
GGGGAAGGGAGAAAGGAAGCTAGTCGACTCGACATCTGTGGCTTCACAGTACAGAGGACTGTCCAAAGCCGCAGATC
TCGAGTCGACTAG
+
CCC9B>A@:>98809<>=BC@?==100::97<<<11/86658924==@AAB:::00,0::::@:??9??>BBBBBC@
<<8::CDDCC@@>
@T6XXI:00082:00278
CGAGATCTGCGGCTTTGGACAGTCCCTCTGTACTGTGAAGCCACAGATGTCGAGTCGACTAGCTTCCTTTCTCCCTT
CTCCC
```

```
+
?AA=<<ACCCG@CCG>C@>@BCCCC>ABAD@??<877?>8?6::?<B??@BBBCDCCCCBB44424244-4999-
42..462
@T6XXI:00082:00291
CTAGTCACTCGAGATCTGCGGCTTTGGACAGTCCCTCTGTACTGTGAAGCCACAGATGTCGAGTCGACTAGCTTCCT
TTCCT
+
499444499?==BAAACCA>>:88087;;BFDFC>CCBBBCCAA?;<9<A=@CC;;;BB@6:68@?;;;73//5342
7+,+,
@T6XXI:00083:00250
GGGAGAAGGAGAAGGAGAAGGAGAGGAGAGGAGAG
+
7A-19959549:28443317131..3....,..10
@T6XXI:00083:00253
GGGGAATTCTAATACGACTCACTATAGGGAGAGGAGAATAGTCAACAAATCGTTTGGGGCGACTTCTCCTTCCTTTC
TCCCTCTCCCTATAGTTATTAGAACTTC
+
???2?;@=@CB>BB@@@@ABCBA?<000,000/33.--0423//,-2229<9996666,11..74447-.-
.3333.+-10---.44338300/00/033234/0
@T6XXI:00083:00255
GGGAGAAGGGAGAAAGGAAGGAGAAGTCGCCCCAAACGATTTGTTGACTATTCTCCCTCTCCCTATAGTGAGTCGTA
TTAGAATTCCCC
+
CC@@@@@CC>BBBB=B@B@C@BBB@CCCCCCC7BB<>?:::5:B@CDBBAA?=AAC;=599DAA@@?===AA???<=
=CA@@::8==>1
@T6XXI:00083:00257
GGGGAATTCTAATACGACTCACTATAGGAGAAGGAGAATAGCTACGTTTAACCGATCGTGTTGTTCTCCTTCCTTTC
TCCCTTCTCCC
+
CEC:C>@>ADCCC@??BABCB7;;7//,0<<9=<?A?<?BBBBBB???=@AC@@AAAAA>=93727897;9;,//,/
--10345:@@:
@T6XXI:00083:00263
GGGGAATTCTAATACGACTCACTATAGGGAGAGGGAGAATAGTCCCCAAATAGCTACCGACGACACGCGTCCTTCCT
TTCCTCCCTTCCTCC
+
BDB:@798;=A=@@AAA88771331111,16111,110/08334==4=A;=?===8858<<8;@///3263255<28
403/./71778,//5
@T6XXI:00083:00267
GGGAGAAGGGAGAAAGGAA
+
991449;=9544//+/565
@T6XXI:00083:00275
CTAGTCACTCGAGATCTGCGGCTTTGGACAGTCCCTCTGTACTGTGAAGCCACAGATGTCGAGTCGACTAGCTTCCT
TTCTCCCTTCCTCCC
+
@:1/1:7:?C::;BCCCDBA?ABB?C?BBCCBBB>BABAB@CC=?=@=@B?BBCCCBBB???AABBB@@CB??=@@C
D=?>>>69./../993
@T6XXI:00084:00277
CTAGTCACTCGAGATCTGCGGCTTGGACAGTCCCTCTGTACTGTGAAGCCACAGATGTCGAGTCGACTAGCTTCCTT
TCTCCCTTCTCCC
+
499449999A==99<BB<<<;00,0,0.966?C>?@@CBA???A>=7;@8;;;BB?@?C>AA?AA;;:B;:78:>?B
5:66=68/30442
@T6XXI:00084:00280
CTAGTCACTCGAGATCTGCGGCTTGGACAGTCCCTCTGTACTGTGAAGCCACAGATGTCGAGTCGACTAGCTT
+
///////999494=@@@><>;10,0,049>>CA>BA;::CC????A8;A<<;@BB@B@B>B711/,,,,,**)
```

```
@T6XXI:00084:00281
GGGGAATTCTAATACGACTCACTATAGGGAGAGGGAGAATAGTACACAACTCG
+
C@;2@5<69>?<AA@@BBBBB@?<>777.7>7775:@>=786///++-,-111
@T6XXI:00085:00261
GGGAGAAGGGAGAAAGGAAGCTAGTCACTCGAGATCTGCGGCTTTGGACAGTCCCTCTGACTGTGAAGCCACAGATG
TCGACGTCCGCAACTCAGCCTTCCTTT
+
CC<><C@B@:@99908=@<8:7;;6699667:89>:88;93117,101116;?>;<>443309403/004666??60
0000000-2000*+++00325554840
@T6XXI:00086:00255
GGGAGAAGGGAGAAAGGAAGCTAGTCGACTCGACATCTGTGGCTTCACAGTACAGAGGGACTGTCCAAAGCCGCAGA
TCTCGAGTCGACTAG
+
BB>AB?==@9>>>>9A=@=@C?==>//,777,-0....7:A>>>@DCCAAACA99773-
4;;>@@>@@;?A@AAAA?===AA@@@A@@@@@A
@T6XXI:00086:00260
CTAGTCGACTCGAGATCTGCGGCTTTGGACAGTCCCTCTGTACTGTGAAGCCACAGATGGACTGCTTCCTTTCTCCC
TTCTCCC
+
CCCCCCCHAAACCECCCCC??<77708;>>CDCCC>CBBBBCCCAAB?>@DDDCCFB<;7;@?>>;3736=977:?.
:3:66;;
@T6XXI:00086:00279
GGGAGAAGGGAGAAAGGAAGGGGAGAAGGGAGAAAGGGGGAGAAGGGAGAAAGGAAGCTAGTCGACTCGACATCTGT
GGCTTCACAGTACAGAGGACTGTCCAAAGCCGCAG
+
BH>@@@;==<999B8;59;>>?5?BB>AAAA?@?8????2??D@CC????=;@;<>=BF=@@CCBBDCA<=;998;4
717316???BB><<<11,/6//4B>;;:;98444;
@T6XXI:00086:00281
GGGAGAAGGGAGAAAGGAAGCTAGTCGACTCGACATCTGTGGCTTCACAGTACAGAGGACTGTCCAAAGCCGCAGAT
CTCGAGTC
+
9C><9959919=>@096:??9;B@?98;166786666697736;:679:<=BC<A>A=AD?=39/44,00/000833
3:8466//
@T6XXI:00087:00263
GGGAGAAGGGGAGAAAGGAAGCTAGTCGACTCGACATCTGTGGCTTCACAGTACAGAGGGACTGTCCAAAGCCGCAG
ATCTCGA
+
FF?CCC@BBB:CBBC>B@B?ABBBBBCAAACB;:9:::7@AC8::ACCBBBBCC?>77718>;@@A9<=7=A>AAAA
===A==<
@T6XXI:00088:00254
GGGGAATTCTAATACGACTCACTATAGGAGAGGGAGAATAGCAAAACGTTTCCGC
+
EDD:C::<<CBBCBA?@BBBC648;//,0><@B>BC@>@@6635500,,,+,+,+
@T6XXI:00088:00259
GGGAGAAGGGAGAAAGGAAGGAGAACGCGCACCCGAACCAACGGTGATCTATTCTCCCTCTCCCTATAGTGAGTCGT
ATTAGAATTCCCC
+
A?6<;BAEEABBAA?B@B=?=>AA>BBCDCCCB=CC@C@B@CDA@@ADCAAA?BBBA?;<;@GAEDD?;;:BCAABB
??=@CB?D@CCC:
@T6XXI:00088:00262
GGGAGAAGGGAGAAAGGAAGCTAGTCACTCGACATCTGTGGCTTCACAGTACAGAGGGACTGTCCAAAGCCGCAGAT
CTCGAGTCGACTAGCTTCCTTTCTCCCTCTCCC
+
HDABBB>A@:>99904<>98;9@97?6111111166661112137;99:DGECCB88717>488?8;:6<=:==>>88
888?@@?@??989ADC>;;;>68879,/////,
@T6XXI:00088:00265
```

```
GGGGAATTCTAATACGACTCACTATAGGGAGAGGGAGAATAGTCAACAAATCGTTTGGGGCGACTTCTCCTTCCTTT
CTCCCTTCCTCCC
+
CEFAC;:<<CDBCCBABBBB@@<97111.7?==?<?B@>A@@A@@=@AC<CAAAB>BCC7BBBBB?BBL@?<?:==9
=A;96;,/,,011
@T6XXI:00089:00251
GGGGAATTCTAATACGACTCACTATAGGGAGAGGGAGAATAGTCAACAAATCGTTTGGGGCGACTTCTCCTTCCTTT
CTCCCTTCTCCC
+
EKK:E@ABACCBCCBBBBB?>AB?>666.6>>@B>BCB@AAA<@@>?@@;B???C>BBB7@?>>@;=>>./,/9776
;;@?;@=@@@A<
@T6XXI:00089:00262
GGGAGAAGGGAGAAAGGAAGCTAGTCGACTCGAGATCTGCGGCTTTGGACAGTCCCTCTGTACTGTG
+
94-44999;-8499142943377976666617176616 6;899>1606666899663533000 64/
@T6XXI:00089:00272
GGGAATTCTAATACGACTCACTATAGGAGAGGGAGAATAGTCAACAAATCGTTTGGGGCGACTTCTCCTTCCTTTCT
CCCTTCTCCC
+
AC@B79:<9=9A;<<C@@@C>@;;00,0<077.7>;<8:9A@@>@CC?DDBAB.77:4:ADKL=??B7858?AC<??
@CEB59/::6
@T6XXI:00089:00276
GCTAGTCACTCGAGATCTGCGGCTTGGACAGTCCCTCTGTACTGTGAAGCCACAGATGTCGAGTCGACTAGCTTCCT
TTCCTCC
+
/,,,,///449449@@@@A>@;88431337668;;?????BC??@@@:=A?A@BCC@@@AAA@:;;33397..,.,.
.+.,.33
@T6XXI:00090:00262
GGGAGAAGGGAGAAAGGAAGTGCAGTTCGCGGTTAGTGAAGGGAAAAACTATTCTCCCCTCCTCCATATGAGTA
+
?C@CDC@CB>ABBB>A>A?AAABCCCBBBCC8:7;B;;:>AB>CCCC2C@@?7:169;2;>:=60..11---++
@T6XXI:00090:00263
GGGGAATTCTAATACGACTCACTATAGGGAGAGGGAGAATAGATCACCGTTGGTTCGGGTGCGCGTTCTCCTTCCTT
TCTCCCTT
+
;@>0@,1,16679@CDFBBCCBB??777.777?@:@@;7:;4434??:?B=<;?@CCC@BBBCA?B@C<<;:5901>
2..-2000
@T6XXI:00090:00269
GGGAGAAGGGAGAAAGGAAGGAGAAGTCGCCCCAAACGATTTGTTGACTATTCTCCCTCTCCCTATAGTGAGTCGTA
TTCAGAATTCCCC
+
CNBAA?>BB>@@@D<=;<BD=@@B?BFCBGDE8A@=@A<>A;@@=>>@@><:;65303000333=>>747<<88788
:.//>>;;9???5
@T6XXI:00091:00258
GGGAGAAGGGAGAAAGGAAGCTAGTCGACTCGACATCTGTGGCTTCACAGTACAGAGGGACTGTCCAAAGCCGCAGA
TCTCGAGTCGACTAG
+
DC@BBB@CC>B??=:@=@?BBBCCC:;::;;>;;7??8A@C=@@@BBCBBBDCB?777-
4;;>@@?AA<AA>@@@A@@@??>@@@===??@@
@T6XXI:00091:00267
CTAGTCGACTCGAGATCTGCGGCTTTGGACAGTCCCTCTGTACTGTGAAGCCACAGATGTCGAGCGACTAGCTTCCT
TTCTCCCTTCCTCCC
+
@A<;;A@BBDCBBBBCDBBAA<77308=A@CDCDD@CDCCAAABCCCA>@D?DBBBBBBBDCC?AA<;;BA::5:>?
?.:.3761,-,-110
@T6XXI:00092:00259
```

```
GGGAGAAGGGAGAAAGGAAGGGAATTGTGCTGGAATCGTGCGTCGGAACTATTCTCCCTCTCCCTATAGTGAGTCGT
ATTAGAACTTCCCC
+
CC@CCC?BC?BBDC@N@CBAA<A=?<@BA9:>CH>@@B><;BBCC?B?CBCC?:69?8?9<>C===AA??:>7:7@8
55<755///7:544
@T6XXI:00092:00264
GGGAGAAGGGAGAAAGGAAGCTAGTCGACTCGACATCTGTGGCTTCACAGTACAGAGGGACTGTCCAAAGCCGCAGA
TCTCGAGTCGACTAG
+
AE===A@CD=BBBB=B>B=@@@>==0//:9900:8896??B;?@?BCDCCDCB>>88858AAA@@<?C<AA?<<<AC
@@?C==<887;===?
@T6XXI:00092:00272
GGGGAATCTAATACGACTCACTATAGGGAGAGGGAGAATAGTCAACAAATCGTTTGGGGCGACTTCTCCTTCCTTTC
TCCCTTCCTCCC
+
;??1<-
1/6878ABBBABDDCB>=676.7=777.:?;=AAAA==:@8=7?6117,111,66??>???@5738;=;195><;98
<9;;99
@T6XXI:00092:00299
GGGGAATTCTAATACGACTCACTATAGGGAGAGGGAGAATAGTCAACAAATCGTTTGGGGCGACTTCTCCTTCCTTT
CTCCCTTCTCCC
+
DCB:C9;;<BB@CBBBBBBBA><97166.7>>>><ABA?A@AAAA899C<BAAAE>DBC7;:BBC7;9@5828<??;
@7::97///759
@T6XXI:00093:00255
GGGGAATTCTAATACGACTCACTATAGGAGAGGGAGAATAGTTACAAC
+
CC@7?69;<GCBCCBBBB?B>:965//-0<??B>A@<:4;//,//+*+
@T6XXI:00094:00258
GGGGAATTCTAATACGACTCACTATAGGGAGAGGGAGAATAGTCAACAAATCGTTTGGGGCGACTTCTCCTTCCTTT
CTCCCTTCTCCC
+
CCB9B7;;<CC@DB@@BBC??@<97166.7?==?<?BA?B@?@AA?@AA<BAAAC>CCC9BABAC>=@@5838=AC?
>>@C=D587AC=
@T6XXI:00094:00262
GGGGAATTCTAATACGACTCACTATAGGGAGAGGGAGAATAGTCAACAAATCGTTTGGGGCGACTTCTCCTTCCTTT
CTCCCTTCTCCC
+
CB@4B7;;<CCCC@==A????C>97111.7?>>><AB@>@@AA==;?@@;E???C@CKK7B?CAA;==A6838>AA<
@?@C<A;==@C<
@T6XXI:00095:00251
GGGGAATTCTAATACGACTCACTATAGGGAGAGGGAGAATAGTGACGTGTTGTGCGGCGCTCACTGTCTCTTCCTTC
TCCTCCTCC
+
?C>6B:<6;=@9BBB<<;B;:1<<7167,6=777,6639>@998>A=488;844884;<;=:?>:4//,,+*,5533
99945547,
@T6XXI:00096:00263
GGGAGAAGGGAGAAAGGAAGGAGAAAGCGACGTCGGGGAAACGTTTTGCTATTCTCCCTCTCCCTATAGTGAGTCGT
ATTAGAACTTCCCC
+
FF@AA=;99:>>>@9>;>;?=@@AC>;<<CCCCC@BB9CB>B9999,9@C9929=9>199877:?A@??CB@@?:6:
6;28888770166,
@T6XXI:00096:00298
GGGAGAAGGGAGAAAGGAAGCTAGTCGACTCGACATCTGTGGCTTCACAGTACAGAGGGACTGTCCAAAGCCGCAGA
TCTCGAGTCGACTAG
+
```

EDACCC@DD>AA==::446::8;==/11>>>:3:::::@AC=A@@GBBBBB@?<@>>>;????A@>AA<@@>@A==@
=9=?A@@@@AA???A
@T6XXI:00097:00265
GGGGAATTCTAATACGACTCACTATAGGGAGAGGGAGAATAGTCAACAAATCGTTTGGGGCGACTTCTCCTTCCTTT
CTCCCTTCTCCC
+
CCB4@54699>9AAA@BBBBCCA?;677.7=777.;@>=@A@A@@>@CH9@@AAA:@A@6??AAC>BBB==:=@AA<
ABCC;?59.7;6
@T6XXI:00097:00297
CTGCGGCTTTGGACAGTCCCTCTGTACTGTGAAGCCACAGATGTCGAGTCGACTAGCTTCCTTTCTCCCTTCTCCC
+
EDFCD?;9;9<>ABGCCBB?BBBB?A@C@AAAACC@AACBBAB@@@CCBBBA@?B@?8:?=@B<???B<A;??@AA
@T6XXI:00097:00299
GGGGAATTCTAATACGACTCACTATAGGGAGAGGGAGAATAGACTATGCCTCGGTGGCCTA
+
ANCAC;<89>>;8@CBBBCCCCCA996-67<@A=BCA>AA999@;;//./8::8;5:90*
@T6XXI:00098:00298
GGGGAATTCTAATACGACTCACTATAGGGAGAGGGAGAATAGTTACACG
+
CCB8B64694498=@@@BB@B?BA>777.7=<@B=A?8765**)**(((
@T6XXI:00099:00250
GGGGAATTCTAATACGACTCACTATAGGGAGAGGGAGAATAGTTCCCACAGACATGCAACACTATTCTTCTTCCTTT
CTCCCTTCTCCC
+
C@?4>;@=BBB@CCCBBBCBCBCCBAA>;ABCCC>CBA<?@@@>@A9<<@B889@?AA=???<;=;4424;797;;6
<:@AAA><<=D?
@T6XXI:00099:00253
GGGGAATTCTAATACGACTCACTATAGGGAGAGGGAGAATAGTCAATAAATCGTTTGGGGCGA
+
CCB8@59599824448=;B>AA<?<761,9?@@C>BA?>==<@@=24364:3334+000+11.
@T6XXI:00050:00306
GGGGAATTCTAATACGACTCACTATAGGGAGAGGGAGAATAGTCAACAAATCGTTTGGGGCGACTTCTCCTCCTTTC
TCCCTTCTCCC
+
88802*/,16656=A<@<CECCB??777,7>===6==978:::44/034461111,1662663..,.55,-
.555575==;=44-172
@T6XXI:00050:00314
GGGAGAAGGGAGAAAGGAAGCTAGTCGACTCGACATCTGTGGCTTCACAGTACAGAGGACTGTCCAAAGCCGCAGAT
CTCGAGTCG
+
DDBBB=>?B@BB@A?A@B@BBCCCB:;;A@B@;::?===7706711AABCCB=;;/.-
/;;;;A>@@<@A<@A???A888?==;87
@T6XXI:00050:00342
GGGGAATCTAATACGACGTCACTATAGGGAGAGGGAGAATACGCTCGAC
+
ABG9>11///,/494433344486....+.64>@;<<9011111111--
@T6XXI:00051:00305
GGGGAATTCTAATACGACTCACTATAGGGAGAGGGAGAATAGAGACGTATTCGTTTGCGCTCTGTCACCTTCCTTTC
TCCCTTCTCC
+
C?>4@69599;9ABG<?<@@B@B@::;?9=?>@@<::8=>>@??=;;;4434440+0--+,+0+++444244-
30+033:-424000
@T6XXI:00051:00318
GGGGAATTCTAATACGACTCACTATAGGGAGAGGGAGAATAGAGCACCGTTGTTCGGTGCGC
+
//1+1,924448848;A@CC@@@BBBCD9=?777.7>;9;=884065,..,,,2,,*,4..1
@T6XXI:00051:00322

```
GGGAGAAGGGAGAAAGGAAGGAGAAGTCGCCCCAAACGATTTGTTGACTATTCTC
+
AF@CCCBCC<@>@?0969=B7;:C@BBADBCC7@?:>666621101610042111
@T6XXI:00051:00323
GGGGAATTCTAATACGACTCACTATAGGGAGAGGGAGAATAGTCAACAAATCGTTTGGGGCGACTTCTCCTTCCTTT
CTCCCTTCTCCC
+
EEC7A;;8;CC?BBBBBCCCCCBA?::7.7?=??=@AA>?=BAAA>BAA=C???C>CDD9B@???;=>?44347::4
<;;@6<687@C;
@T6XXI:00051:00325
GGGAGAAGGGAGAAAGGAAGGGGAGAAGGGAGAAAAGGAAGC
+
9404/::;>,1/6656565;;>7ABC@CB6=....*,*,*,,
@T6XXI:00052:00302
GGGAGAAGGGAGAAAGGAAGCTAGTCGACTCGACATCTGTGGCTTCACAGTACAGAGGGACTGTCCAAAGCCGCAGA
T
+
:::ADCBCE@@AAB<A>A988>>;79?=;9=;00....34661311111666633044,3333;<>AA<AA>AAAA:
:
@T6XXI:00052:00309
GGGAGAAGGGAGAAAGGAAGCTAGTCGACTCGACATCTGTGGCTTCACAGTACAGAGGGACTGTCCAAAGCCGCAGA
TCTCGAGTCGACTAG
+
94-
9=@BDL=D<CB<>=A>@>>?A>7::<??>?????89666171199?BBC@>;444,4:9;>??CA<AA>@@???878
88;9>??877??
@T6XXI:00052:00310
GGGGAATTCTAATACGACTCACTATAGGGAGAGG
+
8B808,45944888@@A??;71391111,1.33,
@T6XXI:00053:00307
GGGAGAAGGGAGAAAGGAAGCTAGTCGACTCGACATCTGTGGCTTCACAGTACAGAGGGACTGTCCAAAGCCGCAGA
TCTCGA
+
AB>BCC@CC?B;996;@B?BB@@ACCBCCCC@;777:7717067??<AACDA@=>77717=;==?<??7>A>===B@
@==<<<
@T6XXI:00054:00305
GGGAGAAGGGAGAAAGGAAGCTAGTCGACTCGAGATCTGCGGCTTTGGACAGTCCCTCTGTACTGTGAAGCCACAGA
TGTCGAGTCGACTAGCTTCCTTTCTCCCTTCTC
+
DCACCC@CC>BBBB;BAC@AAA@AA:::C???CCBBAAA??46:?9A?BBBCA?A<>778@@@@@@@AAA@=?@BB@
@@A===A???@?@?@==;=;;;:;444-4/4;;
@T6XXI:00054:00308
GGGGAATTCTAATACGACTCACTATAGGGAGAGGGAGAATAGCTCAGCTTTTGTTGCATGTGATTTCTCCTTCCTTT
CTCCCTTCTCCC
+
FDC9A89;;A@@DBBBBBCCCA@?=777.7>7776;A@@@??@CCA?4444-
7;=A@@A@@><<<4:::799;7::4::::4;8;;?A<
@T6XXI:00054:00311
GGGAGAAGGGAGAAAGGAAGCTAGTCGACTCGACATCTGTGGCTTCACAGTACAGAGGGACTGTCCAAAGCCGCAGA
TCTGAGTCGACTAG
+
CDAAA?@DC@CCBC<>=@=ABC@@@C?@?ECB;::????==;:;5:AABBBBB@?77717??@AB?AA:BB<@@@@?
?===DCEFDFCCBA
@T6XXI:00054:00322
GGGAGAAGGGAGAAAAGGCAAGCT
+
```

```
4904:>599-99999,42442433
@T6XXI:00054:00340
GGGAGAAGGGAGAAAGGAAGCTAGTCGACTCGACATCTGTGGCTTCACAGTACAGAGGACTGTCCAAAGCCGCAGAT
CTCGAGTCGACTACG
+
CC>AAEACC>@>>@0959;>;;<B?:<<666?977CA667?81756669;?=:884423;;345<A=;==<=487?7
873361------146
@T6XXI:00055:00301
GGGAGAAGGGAGAAAGGAAGCTAGTCACGCG
+
6<8<19599-48?B@C@>;;1..*((*****
@T6XXI:00055:00310
GGGGAATTCTAATACGACTCACTATAGGGAGAGGGAGAATAGCCGTTATTTAACTCCCGCGGCCGGTTCTTCCTTT
CTCCCTTCTCCC
+
@@>4>696<@>=BAA@ACCBBBCA?99959?ABC>AA==@ACC>7734:;:.=<?AAA:>==38389;7899978;6
0.0=9;9;7?A<
@T6XXI:00055:00316
GGGAGAAGGGAGAAAGGAAGAGAGCGCCAACGA
+
GC@AAAACC=BB>>?>;=1004../;;:7:;73
@T6XXI:00055:00321
GGGAGAAGGGAGAAAGGAAGCTAGTCGACTCGACATCTGTGGCTTCACAGTACAGAGGGACTGTCCAAAGCCGCAGA
TCTCGAGTCGACTAG
+
CC@ECC?BC<=@==5>;>;?A@>>>/11000>>?<:96=;;;:;6:BABBCC@??77717=>?@@>AA<@@>@@??7
8888>@<<<<<?;<<
@T6XXI:00055:00328
GGGGAATTCTAATACGACTCACTATAGGGAGAGGGAGAATAGTCAACAAATCGTTTGGGGCGACTTCTCCTTCCTTT
CTCCCTTC
+
9>?4>;><ABB?BBB@@@@@BA??????9?BABB>@==?@@ADBC<?@@<BBAAAAABB5<76110111645688:;
;423+-,-
@T6XXI:00056:00305
CTAGTCACTCGAGATCTGCGGCTTTGGACAGTCCCTCTGTACTGTGAAGCCACAGATGTCGAGTCGACTAGCTTCCT
TCTCCCTCTCCC
+
:444444449=949@>>?>>;888,3777;9?@C888>7::AAAA?A9<ABCBFC@;<;??BBBBBADBD>00,0,,
+,,,,++---10
@T6XXI:00056:00316
GGGGAATTCTAATACGACTCACTATAGGGAGAGGGAGAATAGTCAACAAATCGTTTGGGGCGACTTCTCCTTCCTTT
CTCCCTTCCTCCC
+
ACB7@8;8<AB?CBBBBBEABBB????=6=>=??<?B@=@@===C>=<=9????C=B@B5@7::?:=;=34/8;==;
@:9707-/./9;6
@T6XXI:00056:00340
GGGAGAAGGGAGAAAGGAAGGGAGAAGGGGAGAAGGGAGAAGG
+
>@<@@@?AB>999>095958807><7?CC-777011,11.,.,
@T6XXI:00056:00341
GGGGAATTCTAATACGACTCACTATAGGGAGAGGGAGAATAGATCACCGTTGGTTCGGGTGCGCGTTCTCCTTCCTT
TCTCCCTTCTCCC
+
CGC7B5969>>;@@@AA@CCCCCCC???9?@??A<A@A>AA===ABA=@A6747:99919AABBBC@?@@?B@B?BC
>CCCA1/,////,
@T6XXI:00057:00301
```

```
GGGGAATTCTAATACGACTCACTATAGGGAGAGGGAGAATAGTCAACAAATCGTTTGGGGCGACTTCTCCTTCCTTT
CTCCCTTCTCCC
+
EHH:C;;;<@@@CCBBCCCCBBB<7166.7>7776;B@>A@AA@?<?CC?DCAAA:@@@5AA@??=?@A44243441
8>AC<=484<;6
@T6XXI:00057:00313
GGGAGAAGGGAGAAAGGAAGTGCAGTTCGCGGTTAGTGAAGGGAAAACTATTCTCCCTCTCCCTATAGTGAGTCGTA
TTAGAACTTCCCC
+
BDDAA@@CCBBCBB=B@BA??BCCBB?BCCC?B>;9:00,00,...*.77;@ACCA88;;LJ>C@@@@@A@@@A@<<
=9<>@<??=@CC5
@T6XXI:00057:00326
CTAGTCGACTCGAGATCTGCGGCTTTGGACAGTCCCTCTGTACTGTGAAGCCACAGATGTCGAGTCGACTAGCTTCC
TT
+
DC<99999BCCCCBBACCC@><88808<?>87:?A9??BCDBBBAA==3495;9ACDDCBBBB=:1100.4.,,033
35
@T6XXI:00057:00338
GGGAGAAGGGAGAAAGGAAGCTAGTCGACTCGACATCTGTGGCTTCACAGTACAGAGGACTGTCCAAAGCCGCAGAT
CTCGAGTCGACTAG
+
CB>BDC@CCBDCCC>@=@@CCCCCC::::::>???BBBCCC@BB?BCCBBBB773--
,/8878??AA:BA8?@@A@@@?@A@@??>888<=
@T6XXI:00057:00348
GGGAGAGGATAAG
+
11,/4//,///76
@T6XXI:00058:00307
GGGGAATTCTAATACGACTCACTATAGGGAGAGGGAGAATAGCTCCCACTTATGAGTCGTCCTGACCGTCTTCCTTT
CTCCCTTCTCCC
+
EDC9B>A=@BB?BCBBB>@@CCEDBAAA:@@???<?B@9<<B????9???<?AAAA@DCCC:<;CB=B;8718;>>6
>777-7:611>;
@T6XXI:00058:00327
GGGAGAAGGGAGAAAGGAAGGAGAAGTCGCCCCAAACGATTTGTTGACTATTCTCCCTCTCCCTATAGTGAGTCCGT
ATTCAGAATTCCCC
+
9919=>599-
444C08242399<B@:8<@BBB8BB<>;711,1601198<@<@A@@;@887=5:99;;888:4434==@688AB8;3
444-
@T6XXI:00058:00332
GGGGAATTCTAATACGACTCACTATAGGGAGA
+
///*/7779995444?@8333133...5+,,0
@T6XXI:00058:00335
GGGAGAAGGGAGAAAGGAAGGAGAAGTCGCCCCAAACGATTTGTTGACTATTCTCCCTCTCCCTATAGTGAGTCCGT
ATTAGAATTCCCCC
+
BB?BDC@BA=BBBB=@=?=>:<@@>@???@@A7AB=B@::61969@ABA@@>@??@:<;<<A<@ACC==7;;//-
....4=<=;=55555.
@T6XXI:00059:00302
GGGGAATTCTAATACGACTCACTATAGGGAGAGGGAGAATAGTCAACAAATCGTTTGGGGCGACTTCTCCTTCCTTT
CTCCCTTCTCCC
+
CCC:C@D?BBB=A@BBC@@@CA>>=666.7?==?=?CB6:8@@A@?===9?CAAB>BA@5@@@??;==?4434344-
4999498:8894
@T6XXI:00059:00310
```

```
GGGGAATTCTAATACGACTCACTATAGGGAGAGGGAGAATAGCCTGCAGGTCGAGTGTAGGACTGTTCCCTTCCTTT
CTCCCTTCCTCCC
+
AA?6?598;BB@CCCBBBB<BABB=77759B@BC>BCA>A=@<<@B@BB@ACCCA@@@@@<?===C>AB8:89?BC;
?;>?6;./.;82
@T6XXI:00059:00317
GGGGAATTCTAATACGACTCA
+
:;;,4644//5/,,***,*2
@T6XXI:00059:00321
GGGGAATTCTAATACGACTCACTATAGGGAGAGGGAGAATAGCCGTGATATCTTCTCCGGCACGTTTCTCCTTCCTT
TCTCC
+
CBC7B=@=;<;BACABB@BAB@@????=6==777.7=;389@?;=>;?????AACAA>A7768=99:;771396,..
+..45
@T6XXI:00059:00322
GGGAGAAGGGAGAAAGGAAGGAGATAACGACATCGGGACAACAACAGTCTATTCTCCCTCTCCCTATAGTGAGTCGT
ATTAGAACTTCCCC
+
4<8;9B@CC>CABC=B?B@C>@@CBB=@@BB???AA<AAA>AA?AC@=:;//,-117-
..AAC;?AA?@@@@@<<;;66588=;:?<?@?4
@T6XXI:00059:00323
GGGGAATTCTAATACGACTCACTATAGGGAGAGGGAGAATAGTCAACAAATCGTTTGGGGCGACTTCTCCTTCCTTT
CTCCCTTCTCCC
+
DID:B8;;<@@=AACCC;<<@?@@?777-
7=777,14448??==<>@@?;AAABF?CCC7A@@77477744/4:;;7>88;:@;??CC?
@T6XXI:00059:00331
GGGGAATTCTAATACGACTCACTATAGGGAGAGGGAGAATAGTCAACAAATCGTTTGGGGCGACTTCTCCTTCCTTT
CTCCCTTCTCCC
+
BB?6:548<@@BBCC@@@BBCBBB?::7.7>>@A?B@ACAAA???>@@A<CBBBC?CCC9CC@AA;==?4434:==7
?;8;68-0.262
@T6XXI:00060:00300
CTAGTCACTCGAGATCTGCGGCTTTGGACAGTCCCTCTGTACTGTGAAGCCACAGATGTCGAGTCGACTAGCTTCCT
TTCTCCCTTCCTCCC
+
CA:9;BADBCCECABACCCC@@@@=@?ABDCCCC>C:::A9??:;8A=@C@CCCB>A?CBAAACBBABAC;::3747
7.7::>,/,/,///,
@T6XXI:00060:00302
GGGGAATTCTAATACGACTCACTATAGGGAGAGGGAGAATAGTCAACAAATCGTTTGGGGCGACTTCTCCTTCCTCT
TCTTCCTTTCTCCCTTCTCCC
+
EDC:C;;;<BB?CCCBBACCCCBBA???8???AA>ACA?@?@@AA;?=@<BBCCD>BCC7::?00,0/<,/,/7762
4;5282481889A6<9=@?@;
@T6XXI:00060:00318
GGGAGAAGGGAGAAAGGAAGCTAGTCGACTCGACATCTGTGGCTTCACAGTACAGAGGGACTCGTCCAAAGCCGCAG
ATCTCGAGTCGACTAG
+
::6@<95<B<@@BG>B>B>@@>?????><666;?<=>>716067111118<?<8;7//,/7/////,.724348:>=
?:84333333550--2
@T6XXI:00060:00327
GGGAGAAGGGAGAAAGGAAGGGGACGCCTCGGTGAGTGAGCTGGGAACTATTCTCCCTCTCCCTATAGTGAGTCGTA
TTAGAATTCCCC
+
A?:>>A<@@<@ABD<@;>5888.8B?A?DCC@BBBCCCB@@<<<79@CCAA>ABBB><<;BB<AA<:9BDAC@>?<3
311666:2444,
```

```
@T6XXI:00060:00339
GGGGAATTCTAATACGACTCACTATAGGGAGAGGGAGAATAGTTTTTCCCTTCACTAACCGCGAACTGCACTTCCTT
CTCCCTTCTCCC
+
BDC9B69;<BC@GCBBBBBCCCCBBAAB<?ABBB>BAA9;;44444,00+026>@CA?C?BB?<8;;;4000,0,/,
.3550630000+
@T6XXI:00061:00301
GGGAGAAGGGAGAAAGGAAGCTAGTCGACTCGAGATCTGCGGCTTTGGACAGTCCCTTCTGACTGTGAAGCCACAGA
TGTGAGTCGACTAGCTTCCTTCTCCCTCTCC
+
??:?@C@CF><<CC@C?C@BA9979666888<<??CAA@@?=777,1777886==-
600000356344?>?;==<=@@==9:?:;;@??<<7777,/,/115+,,//,
@T6XXI:00061:00333
GGGAGAAGGGAGAAAGGAAGGAGAATGCACTGCAGGAAATTGCAAGACTATTCTCCCTCTCCCTATAGTGAGTCGTA
TTAGAATTCCCC
+
CB@AAA@BB?BBBB?B>@@B=>@B?BBCDCCCBBB?BC<@=@DC@CCDCAA>A@@@<A??@@;???AA@@@<65343
3/3588;;:=C5
@T6XXI:00061:00345
GGGGAATTCTAATACGACTCACTATAGGGAGAGGGAGAATAGTCAACAAATCGTTTGGGCGACT
+
,--*-*1,///56444;:;9>6771111,1>77:5:;::?;;8002444261111,11,11555
@T6XXI:00062:00304
GGGAGAAGGGAGAAAGGAAGAATAGGGTGACTACAGTGGCCGTGTGGCTATTCTCCCTCTCCCTATAGTGAGTCGTA
ATTAGAACTTCCCC
+
DDABBB@BB>BBBC<@=@?AA?CBCD>B<:;AA:88@A?A=??@?@>A??<8;9951336:::<===?AAA<??;//
-/97790-0/000+
@T6XXI:00062:00308
GGGAGAAGGGAGAAAGGAAGTGCAGTTCGCGGTTAGTGAAGGGAAAAACTATTCTCCCTCTCCCTATAGTGAGTCGT
ATTAGAACTTCCCC
+
ADBCCC@CB;@444-
9<>>BBCDDCCAA<<<?BBCCCCD?AA:@@@@2BCCCABBBC>???AC9?<@<<<?@?===8889<@A@@;;;=;1
@T6XXI:00062:00321
GGGGAATTCTAATACGACTCACTATAGGGAGAGGGAGAATAGTTTTCCCTTCACTAACCGCGAACTGCACTTCCTTC
TCCCTTCTCCC
+
CCB9B9;9;CCBDCB??>BBBABB?77746B?AB>B@@=>=777707A<A??BBCD@@==B@@9<<A?>9:::,0,/
5:=9B?@@AD<
@T6XXI:00063:00308
GGGAGAAGGGAGAAAGGAAGCTGTCGACTCGACATCTGTGGCTTCACAGTACAGAGGGACTGTCCAAAGCCGCAGAT
CTCGAGTCGACTAG
+
CDAECB?BB899;;8;BB@BCE<:;CC:1/700.14444771464?<>?@?>>>7::5:@@>@@ALC@AA??74464
/---.-33304433
@T6XXI:00063:00321
GGGGAATCTAATACGACTCACCTATAGGAG
+
1;:0A111//7688=83...,*,,,,,*.3
@T6XXI:00063:00332
GGGAGAAGGGAGAAAGGAAGCTAGTCGACTCGAGATCTGCGGCTTTGGACAGTCCCTCTGTACTGTGAAGCCACAGA
TGTCGAGTCGACTAGCTTCTTCTCCTCTCC
+
//+//8566+/4991;@CADCCCA?:6166619116968690116898<<CCCCC<<<<888?>899A>AA=@:888
8//////.88AA;;4,,+,+00++*+2110
@T6XXI:00064:00307
```

```
GGGAGAAGGGAGAAAGGAAGTGCAGTTCGTGGTTAGTGAAGGGAAAAACTATTCTCCCTCTCCCTATAGTGAGTCGT
ATTAGAACTTCCCC
+
AC@GBB?CA<A@@=<@=>=???AB@@=@ABB=@=?A??@>AC>CCCC2BA??===>>.7766;9?>??=<@CA<86<
4427;B:<=9;;;0
@T6XXI:00064:00338
GGGAGAAGGGAGAAAGGAAGGAGAAGTCGCCCCAAACGATTTGTTGACTATTCTCCCTCTCCCTATAGTGAGTCCGT
ATTAGAATTCCCCCATCAAC
+
FE?CBD=@@:>@??<@=@@DAAAJBCAA@@@>7@>9>688626738:>8679;1110---34547:=AA9;9//-
.4887<==<=84443.10++*+
@T6XXI:00065:00310
GGGGAATTCTAATACGACTCACTATAGGGAGAGGGAGAATAG
+
CBB7B79:<AAACBBABACDBAA<=666.7=777.7<550**
@T6XXI:00065:00312
GGGAGAAGGGAGAAAGGAAGCTAGTCGACTCGACATCTGTGGCTTCACAGTACAGAGGACTGTCCAAAGCCGCAGAC
TCTCGAAGT
+
9@:>>><>>:>?@B<ACABCCA<;7111666171>5433..,.43366111663300/00486899=;:<;<<<:;:
8400*((((
@T6XXI:00065:00321
GGGGAATTCTAATACGACTCACTATAGGGAGAGGGAGAATAGTCAACAAATCGTTTGGGGCGACTT
+
BBB7B896<?@<@@@@BABBA@?>777=6===@@<?=<=@@>@88888847A9//-.==+0.,,,*
@T6XXI:00065:00333
GGGGAATTCTAATACGACTCACTATAGGGAGAGGGAGAATAGTCAACAAATCGTTTGGGGCGACTTCTCCTTCCTTT
CTCCCTTCCTCCC
+
BBA7A797<BB@DCA@@BBBD@B@>776-
1=???=@CC>@>>AAA?ABA<CBBCCACEE7BBBBC;;:C====>AA<A=@=9=././;;3
@T6XXI:00066:00332
GGGAGAAGGGAGAAAGGAAGGGAGAAGGAGCCAAAGGAAGTCA
+
CC@AA@BCC>?ABC>C>C8::/8>>473116011,10133...
@T6XXI:00066:00337
GGGGAATTCTAATACGACTCACTATAGGGAGAGGGAGA
+
8>=17+/,///,//6/1.444697....+..447.//=
@T6XXI:00066:00340
GGGAGAGAATTTGGTAATGGGGATAGACGAGTTGGGGAGGAGAATGGCACGGGTCCCTTATATCTCTCCTCCCTATA
GTGAGTCGTATA
+
CKAACCCC?BB=?<??;8888,73337>;?A@:===28706?B9>>8?=67:-
67>;?;=:>6<70/0,/73336=::83;51.----+
@T6XXI:00066:00342
GGGGAATTCTAATACGACTCACTATAGGGAGAGGGAGAATAGTTCCTGAATGGTGGAATCCCGGTGTTTTCTTCCTT
CTCCCTTCTCCC
+
?@B4A6959=>=CA@AG?@@CCC@????6<><<?9?==:=>=>;?:=<CABB?CC>A>::;7:@C??@@10/46,/,
---1034:===;
@T6XXI:00067:00323
GGGGAATTCTAATACGACTCACTATAGGGAGAGGGAGAATAGTCTCTCCGTTTGTTCTGTGCT
+
@HB8D6959449949;A:;;;???>777,777775:AA<@AB878;99:444-/87///..34
@T6XXI:00067:00343
```

GGGAGAAGGGAGAAAGGAAGAACAGTTTAGAGCGGCGGAGTTTATAACTATTCTCCCTCTCCCTATAGTGAGTCGTA
TTAGAACTTCCCCATCACCTCGACCATGCCCCAT
+
BC<@?@5999;AABBC@D@AD?AC???9?AA@AA@BB@BA@@:??66>?7727667174:8?:@@===A@<;998?;
<;==>;=?:@@?-40300/004332000000+03
@T6XXI:00068:00300
AGTCACTCGAGATCTGCGGCTTTGGACAGTCCCTCTGTACTGTGAAGCCACAGATGTCGAGTCGACTAGCTTCCTTT
CTCCC
+
ABACDBCB?@@ABBBBB@>@@><@=@AAAAAA<ABC??A@@@CCC@BB>CCCCCC==@==A@@@A?>9//0,0,00,
/177,
@T6XXI:00068:00309
GGGGAATTCTAATACGACTCACTATAGGGAGAGGGAGAATAGTTGTGACATGTAACGTCCTTCTATCTTCTTCCTTT
CTC
+
CCC8;2459989;=A@@;;;B@AA@???9=?<??9?B@>@@@C;?=<B??@<>B;@ABA9;78>>@9948848488,
0.,
@T6XXI:00068:00310
GGGAGAAGGGAGAAAGGA
+
8=8>1969901277+-**
@T6XXI:00068:00325
GGGAGAAGGGAGAAAGGAAGGGGAGAAGGGAGAAAGGAAGCTAAGTCACGTGC
+
AD?C@@598-94;>:>?@:@;;+.01,.575>?@9?=@;?//0:;00,,,**+
@T6XXI:00069:00305
GGGGAATTCTAATACGACTCACTATAGGGAGAGGGAGAATAGATCACCGTTGGTTCGGGTGCGCGTTCTCCTTCCTT
TCTCCCTTCTCCC
+
CGC9B7;:=BB?BGC:;;<;CA??>777.7>===9?A@8<===<@CB?AA<?:=5;;@<@ABABB?887@;>69266
40:87285/-..+
@T6XXI:00069:00320
GGGAGAAGGGAGAAAGGAAGGAGAAGTCGCCCCAAACGATTTGTTGACTATTCTCCCTCTCCCTATAGTGAGTCGTA
TTAGAATTCCCC
+
<<6<BBDCD@CBBB?CDB>@@BBB>?ACBBBC9BC?C???B>CD@CCB???478=B:=888>7==<<<@??=778<<
;<@8844/003-
@T6XXI:00069:00325
GGGAGAAGGGAGAAAGGAAGCTAGTCGACTCGACATCTGTGGCTTCACAGTACAGAGGGACTGTCCAAAGCCGCAGA
TCTCGAGTCGACTAG
+
BB>BBB@DB;@944-9;>;>?>===/11>>><1::::6?@A::;9;DBCCCCABB733-
4999=?<??<?C@H@@@=====AA@;;A@0/0;
@T6XXI:00070:00303
GGGGAATTCTAATACGACTCACTATAGGGAGAGGGAGAATAGTCAACAAATCGTTTGGGGCGACTTCTCCTTCCTTT
CTCCCTTCCTCCC
+
FCF:B8;;<BAACBBA?BCCBCB?9666.7?===9?C@<??AA<@;@??7A@@>>-777-7;:==-0.5,-
,.7776;35444-.-+550
@T6XXI:00070:00324
GGGAGAAGGGAGAAAGGAAGGAGAATCCACGGGCTCGAACCCTAGGACTATTCTCCCTCTCCCTATAGTGAGTCGTA
TAGAACTCCC
+
?D>@AA584-9A@BAC@CBC?BAA8;<@D>>>900.33337778493:::???>@A644--.+------
00355:688=8>874384
@T6XXI:00070:00333
GGGGAATTCTAATACGACTCACTATAGGGAGAGGGAGAATAGCGACCAATTGT

```
+
DED:D@B@BBB@CBBABBCBBACBB???;?BBCC>CAA<=<//.--37,60..
@T6XXI:00072:00329
GGGAGAAGGGAGAAAGGAAGTGCAGTTCGCGGTTAGTGAAGGGAAAAAACTATTCTCCCCTCC
+
CB@BBC?BB>CCCB>C@B@>@@ECCB?BCDC?B?ABBCEBEC=AAA??-@A@@=>0000+0:9
@T6XXI:00072:00330
GGGAGAAGGAGTGAAGAGGAAGGGAAGGAAGAAGGAGCGAGCGAA
+
CC@B9459594444244423133,37797>>>010....3....,
@T6XXI:00072:00331
CTAGTCGACTCGAGATCTGCGGCTTTGGACAGTCCCTCTGTACTGTGAAGCCACAGATGTCGAGTCGACTAGCTTCC
TTTCTCCCTTCTCCC
+
C:49;<99@DCDACCBBBBBC@A;A<A?BBCCCCC>BBCB??AA??=A>ACACCC@>@ACCBBBACCBBBBB??:?;
=??7777.47:;;C=
@T6XXI:00072:00344
GGGGAATCTAATACGACTCACTAGTAGGAGAGGG
+
8AA1A+1///5644;@?;6622,,,,,*,0.11,
@T6XXI:00073:00302
GGGGAATCTAATACGACTCACTATAGGAGAGGGAGAATAGATCACCTGTTCG
+
;>>1>1////7/44948378/.0...,.5/71,174=?A=933,0)**0)**
@T6XXI:00073:00324
GGGAGAAGGGAGAAAGGAAA
+
??6A9959>:999904244-
@T6XXI:00073:00329
CGAGATCTGCGGCTTTGGACAGTCCCTCTGTACTGTGAAGCCACAGATGTCGAGTCGACTAGCTTCCTTTCTCCCTT
CTC
+
BBA@@@@<=<CDDCC>C?BBCBCCD=@@?@@>:7:6658<=;BBB@<;<>CAACCCC=<<B=992959919>@B<A?
@BB
@T6XXI:00073:00344
GGGGAATTCTAATACGACTCACTATAGGGAGAGGGAGAATAGATCACCGTTGGTTCGGGTGCGCGTTCTCCTTCCTT
TCTCCCTTCTCCC
+
DBC7B8;;<BB@CCCCCBB@@CB>9616.7>7775:@@@AA@?@@CC?CC?B?B@BBD@CCBBBCC@AA>:=47477
.79<A6<9888?:
@T6XXI:00074:00302
GGGAGAAGGGAGAAAGGAAGGAGAAGTCGCCCCAAACGATTTGTTGACTATTCTCCCTCTCCCTATAGTGAGTCGTA
TTAGAATTCCCC
+
BH?CBB>@C<@A@B<@=@@C=@@B@??@ABBB7BB9?<711,1679>BC@B>@A@@;<99:A=AAB=988C?875??
?<<<<<=;:>>4
@T6XXI:00074:00319
GGGAGAAGGGAGAAAGGAAGGAGAAGTCGCCCCAAACGATTTGTTGACTATTCTCCCTCTCCCTATAGTGAGTCGTA
TTAGAATTCCCC
+
@C8;<@<>@<AABC?BAFBB@ACCGCCB@??>2>?8>7111,16369A><=;??AC<A989@<@AC=989B=889??
?>?@A>@8<<<4
@T6XXI:00074:00333
CTAGTCGACTCGGCGGCTTTGGACAGTCCCTCTGTACTGTGAAGCCACAGATGTCGAGTCGACTAGCTTCCTTTCTC
CCTTCTCCC
+
```

```
EC111>>>CDCEBC@<94:7;=?@??;;;89>>@AABBBBBB?BC@C?C?CC::;BBB@>>000>>>?>?=>@<??@
B>D<;;CD>
@T6XXI:00075:00333
GGGGAATTCTAATACGACTCACTATAGGGAGAGGGAGAATAGACGTTTCGTTTACGGCACCCTATTCTTCTCCTTTC
TCCC
+
8>>98*98;;;=@BBBBAAAB?@7;611,1>>AB=?<;;>;//////,./-2+..;:<>==;?536/0,/0-,--+-
-00+
@T6XXI:00075:00336
GGGGAATTCTAATACGACTCACTATAGGGAGAGGGAGAATAGCCGTGTCTCGATTCACACCTAGTTCTCCTTCCTTT
CTCCCTTCTCCC
+
@@>6><A>BBB@CBBBBBAAAAB>=666.7@@CC>BBB<?;<><<<<<<@@@A@>@@;@A=?@@@>@@@9;79344,
4;;;-4/499;7
@T6XXI:00075:00342
GGGGAATTCTAATACGACTCACTATAGGGAGAGGAGAATAGCCAGGTG
+
---*/5984499?BC<<<@@@@CB>===.77==:=4423--/,-2,--
@T6XXI:00076:00327
GGGAGAAGGGAGAAAGGAAGTGCAGTTCGCGGTTAGTGAAGGGAAAACTATTCTCCCTCTCCCTATAGTGAGTCGTA
TTAGAACTTCCCCC
+
BC@CBC;<8099997<@CADBBBCCC@C@@@@C?@@@AB?AA@AEE9BB@@=??>?9:::AC=A???A@@@@B;940
0/034244/0000+
@T6XXI:00076:00330
GGGAGAAGGGAGAAAGGAAGGGAGAGGAGAAAGAAGCAGT
+
//+/4;244-4444-42477727;<977111,11361116
@T6XXI:00076:00340
GGGGAATTCTAATACGACTCACTATAGGGAGAGGAGAATAGTAGA
+
A@B9C998;<;=@BCCCBBBB=@?A???9?@<;:?4424/,+***
@T6XXI:00077:00301
GGGGAATTCTAATACGACTCACTATAGGGAGAGGGAGAATAGCTCCCCATAGTCAATCGTGACTAGATATCTTCCTT
CTCC
+
GCD:D@@>ADC@CAAABABAB;737777.7?==?<?DB>@=;96777.777;@@@>@A???:99AAA?@;//,/,/,
+...
@T6XXI:00077:00320
GGGAGAAGGGAGAAAGGAAGGAGAAGTCGCCCCAAACGATTTGTTGACTATTCTCCCTCTCCCTATAGTGAGTCGTA
TTAGAATTCCCC
+
FC?CBB@BB@BBBB>B>A<@;;;;9:CCDFFF9CC@CBBCC>BB?B::9@@>@@?@;@===AAA@@@===AAA@A??
?>@AA?A;?>=1
@T6XXI:00077:00330
GGGGAATTCTAATACGACTCACTATAGGGAGAGGGAGAATAGTCCCTGTCTTCTCAACGTTTCCGTATGCTTCCTTT
CTCCCTTCCTCCC
+
CDB9B>B>BCEBCBBBBAAAA@><?66957??ABBC@@>AAAAAA<A====;=A@A?@CCC:?=@C<<<=<9<>@A<
A=;;69,/,/:;6
@T6XXI:00077:00338
GGGAGAAGGGAGAAAGGAAGCTAGTCGACTCGACATCTGTGCTTCACAGTACAGAGGGACTGTCCAAAGCCGCAGAT
CTCGAGTCGACTAG
+
DCACCB@BC@BBBB=?696;C@;=>/10>>>>1;;>>::00/4337=>??A?;;444-
4;;>@A>@D=BA?AAA@??:???@@@AAAA@@@
@T6XXI:00077:00343
```

```
GGGAGAAGGGAGAAAGGAA
+
?C>A<;,11,1///,,*,*
@T6XXI:00078:00322
GGGAGAAGGGAGAAAGGAAGTGCAGTTCGCGGTTAGTGAAGGGAAAAACTATTCTCCCTCTCCCTATAGTGAGTCGT
TAAT
+
EEBBBB@BB==@BB@C@C@?@@CCBA>A9995849AABB>??.7777,8?@@=@@BB?BBBDE?BB@=<@<6544--
,-,-
@T6XXI:00079:00326
GGGAGAAGGGAGAAAGGAAGGATAGCTGGCGTGGGGAAAGACGTCGTCTATTCTCCCCTCCTCCTATAGTGAC
+
DD?CCC=@=:>>>>9><A>A?ABC?@AA=@@9999,9;:>BC;99=744777;;>BB7<<>5330,,+++++-
@T6XXI:00080:00335
GGGGAATTCTAATACGACTCACTATAGGGAGAGGGAGAATAGCTTGCAGCTCGAGCCTGTGACTGTTCTCTTCCTTC
TCCCTCT
+
CBB4B8;:<@@@CBBBBBCCCCA?=767.7?>>A>BAA<??@?A>BBAAACBBB@B?BBC@BCA88244//78,/,/
///+--0
@T6XXI:00081:00301
GGGGAATTCTAATACGACTCACTATAGGGAGAGGGAGAATAGCCGATCCTTCGTCAACCTTACGCTGTTCTTCCTTT
CTCCCTTCTCC
+
CCB9B9;;;CB@CBABBBBCBCCC???B<?????9??;;=@?A5//68,/,.74355<9??AB;;;AA?@@;>477-
7.44,/,.--,
@T6XXI:00081:00319
GGGGAATTCTAATACGATCTCACTATAGGGAGGGAGGAATAAGCTTCTCCATGCGTAACGTCAATGTATCCTTCCTT
TCTCCCT
+
DCC7@6;:9A?<>A>>98833314?;;9?6000,,707*,,*,287=?@=?BBBAADCDCBCC@AAA???:=:=400
,0/,5+,
@T6XXI:00081:00333
GGGGAATTCTAATACGACTCACTATAGGGAGAGGGAGAATAGTCAACAAATCGTTTGGGGCGACTTCTCCTTCCTTT
CTCCCTTCCTCCC
+
BBB7A>A>BCB@CBBBBBBBB@BC88:A:??AAB=A?D<?>BDAA9<=B<BAAAB<A@@4;0::=,0.5,.-
/9:55=74426,/,/>92
@T6XXI:00082:00319
GGGAGAAGGGAGAAAGGAAGCTAGTCGACTCGACATCTGTGGCTTCACAGTACAGAGGACTCGTCAAAGCCGCAGAT
GTCGAGTCGACTACG
+
ED@BBB@CC=BA@B8;8;@CAABBB:;0<:::00.35///A;=>BCCBBBBBB@?8/-/7////--
133558::344=887@8866------
@T6XXI:00082:00321
GGGAGAAGGGAGAAAGGAAGCTAGTCGACTCGACATCTGTGGCTTCACAGTACAGAGGGACTGTCCAAAGCAGTCGA
CTAG
+
CD@BBB@DB=?@@=:@?A?BCBAACCBBBAA??@BBCBBBB@@@@?CCCBBC<;833317==?@@?AA<==><9;;0
00;;
@T6XXI:00082:00322
GGGGAATTCTAATACGACTCACTATAGGGAGAGGAGAATAGAAGGTAGC
+
?A=0?+9:<AAACBBBBBBBGCCCBBCE>AA764:;;9?//,6.)****
@T6XXI:00082:00327
GGGGAATTCTAATACGACTCACTATAGGGAGAGGGAGAATAGTCAACAAATCGTTTGGGGCGACTTCTCCTTCCTTT
CTCCCTTCTCCC
+
```

```
CB@4@5469>><ABCAA@CCCCCCC???9<>7775:;<<@AAAAA>AAD<BAA@@:@A@5A>7>7+,,2++*,5503
:7<=;=444<=;
@T6XXI:00083:00305
GGGGAATTCTAATACGACTCACTATAGGGAGAGGGAGAATAGCCGGCATCTATCCCGTGTCGGTTCGTACTTCC
+
CCB7@5469>><>@@BCEDCCCCCB???56777:5:A=;?@8848==@B??87:C>F@@=;8/4/403430/0/
@T6XXI:00083:00333
GGGGAATTCTAATACGACTCACTATAGGGAGAGGGAGAATAGTCAACAAATCGTTTGGGGCGACTTCTCCTTCCTTT
CTCCCTTCTCCC
+
CCB7B897;BB?BBBCBCCC@?@@?????9<??AA>AAA>AAAA@A>@@@<CCB@?.777.7?<<>.0/:,/,/4671
88=A<A495==;
@T6XXI:00084:00320
GGGGAATTCTAATACGACTCACTATAGGGAGAGGGAGAATAGCCAAACTTTTCCTGGTCCCCGGCTACTCTTCCTTC
TCCCTTCTCCC
+
DCC8@69:<489BAAABBCBBA@<=666.7>>>@=A@B9<<<<8:;:4444-4844/0000+023403.3137,/,-
-224577=:63
@T6XXI:00084:00334
GGGGAATTCTAATACGACTCACTATAGGGAGAGGGAGAATAGTCAACAAATCGTTTGGGGGCGACTTCTCCTTCCTT
TCTCCCTTCTCCC
+
:??6@=@@BCC@CBBBBBCCCDCB?996-
6=6697:CC>@>@@@A=ABB<CA@>>.7777+7>?>>471733/7;>;:?=:=7;283==:
@T6XXI:00084:00338
GGGGAATTCTAATACGACTCACTATAGGGAGAGGAGAATAGTTGCGCATTCGGTGCACTGTTATTCTCTTCCTTCTC
CCTTCTCC
+
499/96954989449;A???A>?>=777-0>00,.77,-
/=:/034368849237443=;<77993...,.,.,.133032300/
@T6XXI:00084:00339
GGGGAATTCTAATACGACTCACTATAGGGAGAGGGAGAATAGATACCGCACTGTTCC
+
;;:08,/,///56449@?BBBAB@:::@9==<==9?=@=CACA?:9566.----333
@T6XXI:00084:00345
GGGAGAAGGGAGAAAGGAAGGAGAAGTCGCCCCAAACGA
+
/::=?@>BD;<<GF@CAC@C???>57137444586244.
@T6XXI:00085:00310
CTAGTCGACTCGAGATCTGCGGCTTGGACAGTCCCTCTGTACTGTGAAGCCACAGATGTCGAGTCGACTAGC
+
444444449449;9ACCAB@@588584837<111,16116?<<A>9=4395848>@;;<91...1/,,,0..
@T6XXI:00085:00322
GGGGAATTCTAATACGACTCACTATAGGGAGAGGGAGAATAGT
+
7>@1<,969888449@BABBBCB@;;>>.00;>>60/43-0+,
@T6XXI:00086:00308
CTAGTCACTCGAGATCTGCGGCTTTGGACAGTCCCTCTGTACTGTGAAGCCACAGATGTCGAGTCGACTAGCTTCCT
TTCCTCCC
+
B9999<44:@999A<;;C@?=88808;>>?@AAA:@A?::;::7?==:>D=@@CCBACBBDCB?AA310;4,,*,57
7+,+,0:,
@T6XXI:00086:00322
GGGGAATTCTAATACGACTCACTATAGGGAGAGGGAGAATAGATCACCGTTGGTTCGGGTGCGCGTTCTCCTTCCTT
TCTCCCTTC
+
```

```
?A@4@69699>=@@A@@@CCDDCA?777.7=777.7::7;<898?A@:<<3101:984-
47777889=;;;;5626;1//530..*
@T6XXI:00086:00339
GGGGAATTCTAATACGACTCACTATAGGGAGAGGGAGAATAGTCAACAAATCGTTTGGGGCGACTTCTCCTCCTTTC
TCCC
+
?DA5>,/,1//76==?@@CBF?>87777,7>777-
7;;<?@@@<<;;<@;B@>>?.777.7=:;>:;>>./.333230220
@T6XXI:00086:00343
GGGAGAAGGGAGAAAGGAAGTCCTCGACATCTGTGGCTTCACAGTACAGAGGACTGTCCAAAGCCGCAGATCTCGAG
TCGACTAG
+
CDAAAACFCAAAAC@C@C>@??;>>=777777>>?;;<8;=@888:;////,/98:;;;AC<@@>B888A@@@@@A@
;<<;878@
@T6XXI:00087:00304
GATCTGCGGCTTTGGACAGTCCCTCTGTACTGTGAAGCCACAGATGTCGAGTCGACTAGCTTCCTTTCTCCCTTCTC
CC
+
BCCBBBA><9;;5:>ABCAAAE?AAABB@@??;==;<?=?@>=<<=@???@=@ABA@A?8434344-
4;;;4:344994
@T6XXI:00087:00305
GGGGAATTCTAATACGACTCACTATAGGGAGAGGGAGAATAGTTTTT
+
/11+1,92444999>;;;@@B??<88862;;777.7;8/4+55300)
@T6XXI:00087:00313
GGGAGAAGGGAGAAAGGAAGGAGAACAACACGATCGGTTAAACGTAGCTATTCTCCCTCTCCCTATAGTGAGTCGTA
TTAGAACTTCCCC
+
CFACBC?BD>CBCD>@=?=@6:;;?>>=@@>B@9995928;9::7<<<7:777766,11111,1746:<<8883333
5558=5:<8:64.
@T6XXI:00088:00316
GGGGAATCTAATACGACTCACTATAGGGAGAGGGAGAATAGATCACTGACCTCTACCACGATTCAGCTTCCTTCTCC
TCTCCC
+
:==1;,94442448493;776?;9661,6=777.7=97;<?><4=7/./.,..0000---
**)+++/424>@?CBB?;;433,
@T6XXI:00089:00300
GGGGAATTCTAATACGACTCACTATAGGGAGAGGGAGAATAGTCAACAAATCGTTTGGGGCGACTTCTCCTTCCTTT
CTCCCTTCCTCCC
+
AA@6@598;AA>BBBBCBCCCCCCC???8;;61649?=????@<<9=@@:@@@@>6===2=>>>@:=<<5838<?C9
=8=A=A698:CC:
@T6XXI:00089:00301
GGGGAATTCTAATACGACTCACTATAGGGAGAGGGAGAATAGCCGCAAACGCTCTCTCATACTCCTGTTCTTCCTTC
TCCCTTCTCCC
+
DCC9B8;9<CCBCCBBBBBBBAB>B966-
6=;;?8??<=@A??<?ACC>CCCB@BB??BCAABBAA>>;;:9:,0,0:9:,/,4034-
@T6XXI:00090:00305
GGGAGAAGGGAGAAAGGAAGGAGAAGTCGCCCCAAACGATTTGTGACTATT
+
BDADCC?CC=@;;;8<@FBC@CBB??:;:?<<6AB;?91..+...445144
@T6XXI:00090:00310
GGGAGAAGGGAGAAAGGAAGCTAGTCGACTCGACATCTGTGGCTTCACAGTACAGAGGGACTGTCCAAAGCCGCAGA
TCTCGA
+
```

```
?B=AAC@CDAAA@B=@=@>AAABCCCB@?>>;10/;7../?;:<?B=?@CBC?;;444-
4;;?@A>@@<@A;AABB@===@@@
@T6XXI:00090:00313
GGGGAATTCTAATACGACTCACTATAGGGAGAGGGAGAATAGTCTTCCCTTATTTCCAACTTCCCACGCACTTCCTT
TCTCCCTTCTCCC
+
CCB4@545<AA<ACBBBBCBA@CBA:::67><<?9?@>;>>;:44349484111,1363;669>8:::886666011
,1000,4793353
@T6XXI:00090:00316
GGGAGAAGGGAGAAAGGAAGTGCAGTTCGCGGTTAGTGAAGGGAAAAACTATTCTCCCTCTCCCTCATAGTGAGTCG
TATTAGAACT
+
ACCDCC@DE;;<CD@D@C?A@@@==?>@=>A9;9;BAAAACB6:9:7,8?8311666267111,110;698><6440
000/000/04
@T6XXI:00092:00309
GGGAGAAGGGAGAAAAGGAAGCTAGTCGACTCGAGATCTGCGGCTTTGGACAGTCCCTCTGACTGTGAAGCCACAGA
TGCGAGTCGACTCAGC
+
4<19@A<>?194999,49:23737;9888;9777977677<<;>>>1657:<9?C>8<73303549==555;<<=AA
AA887531+++++003
@T6XXI:00092:00314
GGGGAATTCTAATACGACTCACTATAGGGAGAGGGAGAATAGTCAACAAATCGTTTGGGGCGACTTCTCCTCCTTTC
TCC
+
499/42954989;9;;<;BCCCBBBAAA6777775:A=>???==988=;:B?6;6,111,11557-
//:...75444322
@T6XXI:00093:00306
GGGAGAAGGGAGAAAGGAAGCTAGTCGACTCGAGATCTGCGGCTTTGGACAGTCCCTCTGACTGTGAAGCCACAGAT
GTCGAGTCGACTAGCTTCCTTCCTCCCTC
+
BC>AADECC>C<<;79>@>A<7:A?:77?@@:;:BB?<?<?A??=;?>AAAAABB>?<<=888A???<?@BCA<<;>
44888=>@???==;44343424/333030
@T6XXI:00095:00303
CTCGAGATCTGCGGCTTTGGACAGTCCCTCTGTACTGTGAAGCCACAGATGAGTCGACTAGCTTCCTTTCTCCCTTC
TCCC
+
DDC<==BBCCCCCABBB?B@CCCB;;;099@AABFCCDCC@CC@CCCC:;;B@??C??@BAAAAK???<?=>@:@:;
<<;6
@T6XXI:00095:00329
GGGGAATTCTAATACGACTCACTATAGGGAGAGGGAGAATAGTTTAGTC
+
CCB7B598<AA?BAA@CCCBBBCB=:??9?<=??<:88988///,----
@T6XXI:00096:00300
GGGAGAAGGGAGAAAGGAAGCTAGTCACTCGACATCTGTGGCTTCACAGTACAGAGGGACTGTCCAAAGCCGCAGAT
CTCGAGTCGACTAGCTCCTTCTCC
+
49049@<@@1949918;>;87:@@?>==11111666678A@AB>B??CCBBCBAAAA7<;<<==;=@;@A>@AAAAA
=====<=>6;;993330/040002
@T6XXI:00096:00307
AGATCTGCGGCTTTGGACAGTCCCTCTGTACTGTGAAGCCACAGATGTCGAGTCGACTAGCTTCCTTTCTCCCTTCT
CCC
+
FDBBBCCCC?BBB?BCBBBDCCCABBBBCCBB@@?A?AA@BBCCBBCBCCCDBBBCCBA=4424244-
444408404666
@T6XXI:00096:00314
GGGGAATTCTAATACGACTCACTATAGGGAGAGGGAGAATAGCACATTCTGTTTCGTCGCGCCGTATTCTCCTTTCT
CCCTTCCTCCC
```

```
+
9>>4?696<AA?CBBBBCCCCBBA@???6<=;?A=AC@=??744;43/30104466665;773/7833...555577
882627///92
@T6XXI:00097:00308
GGGAGAAGGGAGAAAGGAAGGGGAGAAGGGCTAGTCGACTCGACATCTGTGGCTTCACAGTACAGAGGGACTGTCCA
AAGCCGCAGATCTCGAGTCGACTAG
+
CCACDC?BB?BCBC<?=>>>;B7BCCBBE>BABBB949@@A@@?><<;98;97313311699:;:99@>999BBB=@
B@99:9A=8=>773964./1:7::=
@T6XXI:00097:00310
CTAGTCGACTCGAGATCTGCGGCTTTGGACAGTCCCTCTGTACTGTGAAGCCACAGATGTCGAGTCGACTACCCTTC
TCCC
+
9<1111//8::9:9AA@ABA@=;8:0868:BCCCC?CCCCCCCCC@?==@B?CBBDCDCCC@@?:<:883333,847
7,,+
@T6XXI:00097:00312
CTAGTCACTCGAGATCTGCGGCTTTGGACAGTCCCTCTGTACTGTGAAGCCACAGATGTCGAGTCGACTAGCTTCCT
TTCCT
+
BB;;;A?A?CBBAA@@@@?><888/7;><@AAAB<A@@@@@???A=?<?B@CBBABCABABBB:<;>:969,,*,00
0*,+,
@T6XXI:00097:00313
GGGAGAAGGGAGAAAGGAAGGTAACCCACGAGAGAGATAGCGAATGGCTATTCTCCCTCTCCCTATAGTGAGTCGTA
TTAGAATTCCCC
+
CD@AB><==<@?@B8<:<@C>DA@CCACCCCBBBBB@@@ACCC>AAA@@AA@CCCD@<;;CCADDB@@?A?;79789
::<;64;2444,
@T6XXI:00097:00339
GGGGAATTCTAATACGACTCACTATAGGGAGAGGGAGAATAGTCTTCCCTTCACTAACCGCGAACTGCACTTCCTTC
TCCCTTCTCCC
+
9>>4>69599>9B:?<<;B@AAB@@???9???AA:?<<9<799//-
/;5544:7=@>@=788858@@@@@?:=38334;;4;24330+
@T6XXI:00098:00311
GGGAGAAGGGAGAAAGGAAGGAGAAGTCGCCCCAAACGATTTGTTGACTATTCTC
+
8>8<<>5990944;-42499133371133;;72;9277111,1736611-10+++
@T6XXI:00098:00334
GGGGAATTCTAATACGACTCACTATAGGGAGAGGGAGAATAGTCAACAAATCGTTTGGTGCGACTTCTCCTTCCTTT
CTCCCTTCTCCC
+
CCC8A7;:9449>@A<<<CCCDC@???=6<<777.:;@;=89878???=:?=777.7>???@B?@;:99,/,/4681
//44,49;6??:
@T6XXI:00098:00342
GGGGAATCTAATACGACTCACTATAGGGAGAGGGAGAATAGCCACGTAATCATCGCTCCCGGCGTATCCTCCTTTCT
CCCTT
+
88808*11//78449;83396776111,1;=>?<A;;585,,+++,,,*+0-100044436233:::<;=<;;924-
--+-,
@T6XXI:00099:00300
GGGAGAAGGGAGAAAGGAAGGAGAAGTCGCCCCAAACGATTTGTTGACTATTCTCCCTCTCCCTATAGTGAGTCGTA
TTCAGAATTCCCC
+
BA:ACA=@@<@@BC=B?B@C@CBC@AAACDCC8BA<?>777,6601666899;;::2//.86688<8=??@A==<?=
@3844/584666.
@T6XXI:00051:00387
```

```
GGGAATTCTAATACGACTCACTATAGGGAGAGGGAGAATAGTCAACAAATCGTTTGGGGCGACTTCTCCTTCCTTTC
TCCCTTCTCCC
+
//+1,1,/7778>AAAABCBCB?9111,6===@>BDC@ABC===:;=6:B:::B9???5;647;,/.5,.,.99759
9=A?A574=B>
@T6XXI:00052:00362
GGGGAATTCTAATACGACTCACTATAGGGAGAGGGAGAATAGTTCCGTCCTGTGTCAAGAGCGAGTGTCCTCCTTC
+
//1+/,/,///,/4499<<<?@871116263//.+.--0112224/000/06166>>>:887642++++*+000*0
@T6XXI:00052:00366
GGGGAATTCTAATACGACTCACTATAGGGAGAGGGAGAATAGCCTACGATAACGTGTCGTTGCTGTACTCCTTCCTT
CTCCTC
+
BB@4@548<9@?CAA@CAA@B687=777.7>>>@<A@@=@@<;8??:77>>9<=@@9:<B?BA?AACADC=:66+,+
,**)**
@T6XXI:00052:00368
GGGAGAAGGGAGAAAGGAAGCTAGTCGACTCGACATCTGTGGCTTCGA
+
8>:<<@59919>@@9<<<@CCBCBA:16;61176755....,.4,..1
@T6XXI:00052:00385
GGGAGAAGGGAGAAAGGAAGGAGATAGAAGCAGCAATGGTCAGACATCTATTCTCCCTCTCCCTATAGTGAGTCGTA
TTAGAATTCCCC
+
CD?AAA@BC@ECCCAC@C@B@B@@ABCC@CCCCCC@CB?CCCBBBCCCCCB>A:::7:CCA@;@AA@@@AABAB??A
?>AA@9<5<?@1
@T6XXI:00052:00393
GGGGAATTCTAATACGACTCACTATAGGGAGAGGAGAATAGTCAACAAATCGTTTGGGGCGACTTCTCCTTCCTTTC
TCCCTTCTCCC
+
BA@4@69599>9FAA@BCCCBCCGC???6<=663:=@>@AB???>B@@;A?===6===2==>>?:;;@./.0??>>;
;AA<A;==@@<
@T6XXI:00053:00361
GGGGAATTCTAATACGACTCACTATAGGGAGAGGGAGAATAGTCAACAAATCGTTTGGGGCGACTTCTCCTTCCTTT
CTCCCTTCCTCCC
+
BB@8;2459989=@BCB@@@;:8?>777.7>===9?=;;=;;;44/334976116,111,175455444,-
,/46939.//,/,/,/:94
@T6XXI:00054:00364
GGGAGAAGGGAGAAAGGAAGCTAGTCGACTCGACATCTGTGGCTTCACAGTACAGAGGGACTGTCCAAAGCCGCAGA
TCTCGA
+
?F@BBB>@@>@A@B=B@C@BBBAA?????<>::71?9167A88;@DBBBCCD@@>774-
4;;?A@>AC<??<??AAA===?>>
@T6XXI:00054:00366
GGGGAATTCTAATACGACTCACTATAGGGAGAGGGAGAATAGTCAACAAATCGTTTGGGGCGACTTCTCCTTCCTTT
CTCCCTTCTCCC
+
DCHAC::<<AABCBA?B>>??BB9=666.7?7775:?@?A@AB@B=@AA<BBBBC>BBB7@?BCE8:;B7859?CC<
=?@AA@6:8AA<
@T6XXI:00055:00362
GGGAGAAGGGAGAAAGGAAGGGGAGAAGGGAGAAAGGAAGCTAGTCGACTCGACATCTGTGGCTTCACAGTACAGAG
GGACTGTCCAAAGCCGCAGATCTCGAG
+
C@<@@A?BC@CCCC@B?B@BB?8???>>>;?@@C=B?A?A:8:BBAABAAAAAAA=8=@AC>@@=@AAA====:833
317=>?@?@CC<@@AA??888===?AA
@T6XXI:00055:00387
CTAGTCGACTCGAGATTTGCGGCTTTGGACAGTCCCTCTGTACTGTGAAGCCACAGATCTC
```

```
+
:B<9;CDCBEBCBB@@A>ABA@@AA@BACCCCCC@9>==000=00/AB<???AAAA>=>>=
@T6XXI:00055:00392
GGGGAATCTAATACGACTCACTATAGGGAGAGGGA
+
*0000),,//76444?877?677766623....+.
@T6XXI:00056:00362
GGGAGAAGGGAGAAAGGAAGTGCAGTTCGCGGTTAGTGAAGGGAAAAACTATTCTCCCTCTCCCTATAGTGAGTCGT
ATTAGAACTTCCCC
+
DCCCCC@CC>CCBC>B@B?BBBC@BCAC@@@?A?ABBCD@CC>B@??2ABBC?BBCE>AAACC>EB===@A@@<<<8
878;?B388>?A>6
@T6XXI:00056:00366
GGGAGAAGGGAGAAAGGAAGTGGAATTCTAGGGGTGTTAGTGGTGGCTATTCTCCCTCTCCCTATAGTGAGTCGTAT
TAGAACTTCCCC
+
FEACBA@BC>BBBB=?>B@C@@?A=?<?CB@AB6???<?:<<:00,.8;;>ABBC=>@BBB=CCBBBCCFFB<<<<5
55==:>B?BBA6
@T6XXI:00056:00368
GGGAGAAGGGAGAAAGGAAGCTAGTCGACTCGACATCTGTGGCTTCACAGTACAGAGGACTGTCCAAAGCCGCAGAT
CTCGAGTCGACTAG
+
/1+//4899-
449909249?@@==70.......1679688C@CD>>?@>AAB:;;//./;:57=;<<46688<>?@<<<<;;;4434
4033
@T6XXI:00057:00360
GGGGAATTCTAATACGACTCACTATAGGGAGAGGGAGAATAGTCAACAAATCGTTTGGGGCGACTTCTCCTTCCTTT
CTCCCTTCTCCC
+
?@=6@>A>BBBACCB?AABBB@BD@77757?B@C>B@A?@<<=@@<?@C<C?;??;@CC9CBCAA=<AB:;8;3741
7==?7?<?AAA=
@T6XXI:00057:00366
GGGAGAAGGGAGAAAGGAAGCTAGTCGACTCGACATCTGTGGCTTCACAGTACAGAGGACTCGTCAAAGCCGCAGAT
CTCGAGTCGACTAG
+
?B>BHFCFC>@ABB>B?B@CA@==;///75557A<A<7;:A=AB?BCBDCCD?999B8::888778=;==::BAAA@
==<<<<=874:888
@T6XXI:00057:00392
CTAGTCACTCGAGATCTGCGGCTTGGACAGTCCCTCTGTACTGTGAAGCCACAGATGTCGAGTCGACTAGCTTCCTT
TCTCCCTTCCTCCC
+
9;9449444?==8=BA<99>;88431339??A@?><:::???B???>?B?BBBBCAAAABBA:<<@?79;6677>BB
9@67:63,-,.673
@T6XXI:00058:00369
GGGAGAAGGGAGAAAGGAAGACGAT
+
CC>CCC@BB<A@@@9==>----,**
@T6XXI:00058:00387
GGGGAATTCTAATACGACTCACTATAGGGAGAGGGAGAATAGTCAACAAATCGTTTGGGGCGACTTCTCCTTCCTTT
CTCCCTTCTCCC
+
AA@4@6;8<@@@DBBBCBCCCA@>=777.7?>?B>BCC=?:@BAA>A@@;B???E>CCC9B??;747<<33/89==;
@@@A;@>@@BD@
@T6XXI:00059:00373
GGGGAATTCTAATACGACTCACTATAGGGAGAGAGATATAAGGGCGAGATATACGTACTGAGCCCGCCGCATGGGCC
CATTACCAAATTCTCTCCC
+
```

```
CC?6?298;BA?CCBBBBCCA>BBBBCC>==>@A99=?@@AAA<??@B@B?@@CCAAAAA;9;>:?A>BBCCCB>BB
<@@>@A>@@<>6::7:@@9
@T6XXI:00059:00381
GGGGAATTCTAATACGACTCACTATAGGGAGAGGGAGAATAGTTTCACGTTGCCGACTGCTGCTATTCCCTCCTTTC
TCCCTTCTCCC
+
C@?4?6;:=CAACCCCCAAAA?@<?999.7@<??=@@??BA77718==>@>@A@AA???AA@@@;;;;;.//9;957
5>>5<9<<AA<
@T6XXI:00059:00391
GGGAGAAGGGAGAAAGGAAGCTAGTCGACTCGAGATCTGCGGCTTTGGACAGTCCCTCTGTACTGTGAAGCCACAGA
TGTCGAGTCGACTAGCTTCCTTTCTCCCTTCTCCC
+
=?<ADCDCC<@99909<@<@A@BB?;::AAA:<;BBB?@AD@DCC>B@DCBBBBB<;;;888???@A@>@A>??@@@
@988@???@998??8888;344-4;;;-4/34943
@T6XXI:00060:00365
GGGAGAAGGGAGAAAGGAAGCTAGTCGACTCGACATCTGTGGCTTCACAGTACAGAGGGACTGTCCAAAGCCGCAGA
TCTCGAGTCGACTA
+
99049;2880499918297<?????:;:CCC;;:@:711455..-
.557?=???==;;7=888==<?@;?=5577368<655560004400
@T6XXI:00060:00374
GGGGAATTCTAATACGACTCACTATAGGGAGAGGGAGAATAGTCAACAAATCGTTTGGGGCGACTTCTCCTTCCTTT
CTCCCTTCCTCCC
+
CGD:C=A=@CC>BBCBBC@@@AAB@???<?AABC>B@@?AAA@@?=??D9?@ADE>BBB9BBAAA>AAA68347::6
8//93;-0./;;6
@T6XXI:00060:00382
GGGGAATTCTAATACGACTCACTATAGGGAGAGGGAGAATAGCACGGATCCTGCTCCCGC
+
=?>4>595444888488;:::?99=676,7<==>:?<;;>>=<<;?:?;;/0030--+--
@T6XXI:00060:00384
GGGAGAAGGGAGAAAGGAAGCTAGTCGACTCGACATCTGTGGCTTCACAGTACAGAGGGACTGTCCAAAGCCGCAGA
TCTCGAATCGACTAGCTTCCTTTCTCCCTCTCCC
+
CD>AAB7;919?@A@A>B@;;;?@?ABB???B:::AAA@BC>?@B@CCDDCA???<;;7<???A@?CA;@@?<=<9;
//0:BBBC<<<A@@@873/,//,///8,//6643
@T6XXI:00061:00368
GGGAGAAGGGAGAAAGGAAGGAGAAGTCGCCCCAAACGATTTGTTGACTATTACTCCTCTC
+
CFAAAB?BCACCCB>B@B@B@BBB>=>ACCCC8BB>BBBBD>C@=>AB=88-///67,++1
@T6XXI:00061:00372
GGGGAATTCTAATACGACTCACTATAGGGAGAGGGAGAATAGATCACCGTTGGTTCGGGTGCGCGTTCTCCTTCCTT
TCTCCCTTCTCCC
+
GGC:C=?=>AA@CCCC;8;CBBBC????9?ABBC>C??<??=8:<::5:C?B?B@CCC>CECCCCB>@@>3747477
.06692:6///;6
@T6XXI:00061:00390
GGGAGAAGGGAGAAAGGAAGGGAATAGCAGCAGTCGGCAACGTGGAACTATTCTCCCTCTCCCTATAGTGAGTCGTA
TTAGAATTCCCC
+
AC>BBB;999=99809<>5886;>ABABBBAAACBC@BB>BBBB?B@CCBA>???=.766668 6??>??@@@?==?<
<8<@A?=<=AA5
@T6XXI:00061:00393
GGGAGAAGGAGAAAGA
+
9>11//,/,,,,2*,/
@T6XXI:00062:00379
```

```
GGGAGAAGGGAGAAAGGAAGCTAGTCGACTCGACATCTGTGGCTTCACAGTACAGAGGACTGTCCAAAGC
+
99144B@A@:998809<@<@?@?B::7:7;;767?98666;6681166?AB?766//,.464-0/04+03
@T6XXI:00062:00380
GGGAGAAGGGAGAAAGGAAGGGGAGAAGGGAGA
+
4914;;244-444;7;9;2377,333111,111
@T6XXI:00062:00381
GGGAGAAGGGAGAAAGGAAGCTAGTCGACTCGAACATCTGTGGCTTCACAGTACAGAGGGACTGTCCAAAGCCGCAG
ATCTCGAGTCGACTAG
+
FCCCCC@BC>BCBB>A>@9;=9/0//,,27/..,.....799579@B:;:CGCAA@88858?@@@??AC=@@<@@AB
A@???@@@AAAAAAAA
@T6XXI:00062:00385
GGGGAATTCTAATACGACTCACTATAGGGAGAGGGAGAATAGTCAACAAATCGTTTGGGGCGACTTCTCCTCCTTTC
TCCCTTCTCCC
+
=>>/96929BB@BBBBCBCAAABCCBBB>BBBBB>BBC=@@AA??=?@A<CAA@@.777.77/:;3;5;-
..445577<=;B:?4996
@T6XXI:00063:00372
GCTAGTCACTCGAGATCTGCGGCTTTGGACAGTCCCTCTGTACTGTGAAGCCACAGATGTCGAGTCGACCTAGCTTC
+
444444;99944448444;877333,3777;>>?>;<500/...3000/0424443339966...//..,.631101
@T6XXI:00063:00386
GGGGAATTCTAATACGACTCACTATAGGGAGAGGGAGAATAGCCGCACCAGCGTTGACAATTTATTCTCCTTCCTTT
CTCCCTTCTCCC
+
?A?4@69589@9CCCA@@CAB@A87777.7?777.:=;8=@;;8<<?@?BBBBA==<?<9=A=BA9;<=3838@CC;
?@AA;?<@@@@<
@T6XXI:00064:00365
GGGGAATTCTAATACGACTCACTATAGGGAGAG
+
::807,924449;44;@;3391331111,11..
@T6XXI:00064:00366
GGGGAATTCTAATACGACTCACTATAGGGAGAGGGAGAATAGTCAACAAATCGTTTGGGCGCAGTACT
+
FHK9B?B@BBD@CCCCBBBBC@BCA>BC>BBBCC>CAA>@@@AAA<?@A<CCCCC9<<67,*******
@T6XXI:00064:00378
GGGGAATTCTAATACGACTCACTATAGGGAGAGGGAGAATAGTCAACAAATCGTTTGGGGCGACTTCTCCTTCCTTT
CTCCCTTCTCCC
+
ECC:B8;;<BB@CCBBBABBB@@BA???9?BAAB>A??>@<??AA=AAC?BA@@@;?CC9CCBBA:<==44248;=:
?A@C<@=@@@C?
@T6XXI:00064:00379
TCTAATACGACTCACTATAGGGAGAGGGAGAATAGTCAACAAATCGTTTGGGGCGACTTCTCCTTCCTTTCTCCCTT
CTCCC
+
@CFCBECDBBBBBBB>97377/7>==?<?DC?BA?@BC@CCC>A?@@@<AAA6AA@=<9<??;>9;/7718<=A?C?
@@@A@
@T6XXI:00065:00366
CTAGTCGACTCGAGATCTGCGGCTTTGGACAGTCCCTCTGTACTGTGAAGCCACAGATGTCGACTTCCTTTCT
+
<=<=<CCCEEDBBBBB?@@BA><??:?=@BCCCBC=BBC?@@AAAA?A;=A@CC@B@BBA>;;7600,,,+**
@T6XXI:00065:00369
GGGGAATTCTAATACGACTCACTATAGGGAGAGGGAGAATAGTCAACAAATCGTTTGGGGCGACTTCTCCTTCCTTT
CTCCCTTCTCCC
+
```

```
999/95928989CBBBC:<;CBBBCBBB<@@???=?AB????@@A;==D<C:::?.777-
7<=<;:<6<34/478;:?@@@;A89.9;6
@T6XXI:00066:00399
GGGAGAAGGGAGAAAGGAAGCTAGTCGACTCGAGATCTGCGGCTTTGGACAGTCCCTCTGTACTGTGAAGCCACAGA
TGTCGGGTCGACTAGCTTCCTTTCTCCCTTCTCCC
+
=@<>A@;@@<@A@N@C@C@;:;BCCCBBCCE@?@BCBAAAC@???<??AABCCCC>@?????AA===A>??????@A
AA???A<@@===???@A;=;=A6<;<;495388=4
@T6XXI:00067:00384
GGGGAATTCTAATACGACTCACTATAGGGAGAGGGAGAATAGGGCATGTCCCTTTCCCGTGTAACTCACCTTCCTTC
TCCCTTCTCCC
+
CCB:B;==@AA<ACB?@@CCECCC;:;B?BBBCC>@===@@?@:?===;;;-4418A>C;;8:.0/777+/,-,-,-
11245/44==5
@T6XXI:00067:00394
GGGGAATTCTAATACGACTCACTATAGGGAGAGGGAGAATAGCTAACGTTACCCAACTCGTT
+
CCB6@24698=<>>@<<;AB??@B;::C:;:JDK>?=;999///.,...,.22242000342
@T6XXI:00068:00364
GGGGAATTCTAATACGACTCACTATAGGGAGAGGGAGAATAGATCACCGTTGGTTCGGGTGCGCGTTCTCCTTCCTT
TCTCCCTTCTCCC
+
9>?4>69599>=BBBBBACCDBA>=666.7>7776:?=<@A===AEC>@@6747=999:;??B???:666313:=@B
=??;>9;7////,
@T6XXI:00068:00376
GGGGAATTCTAATACGACTCACTATAGGGAGAGGAGAATAGTA
+
;?:5:198<=@?CCCCBABBCA@>=777.7>77374424//--
@T6XXI:00068:00377
GGGAGAGGAGAAGAGAG
+
4;?<444://,*,,,,,
@T6XXI:00068:00378
CTATCGACTCGAGATCTGCGGCTTTGGACAGTCCCTCTGTACTGTGAAGCCACAGATGTCGAGTCGACTAGCTTCCT
TTCTCCCTTCCTCCC
+
BCB@99:ABBBCCDDBDBBB@CCC>C?BBCC???<?AAABBBB?<=A:=ADCCCCCC@@@CCB?@ACCB@@??:=:=
=9700>,/,/,//40
@T6XXI:00069:00358
CTAGTCGACTCGAGATCTGCGGCTTTGGACAGTCCCTCTGTACTGTGAAGCCACAGATGTCGAGTCGACTAGCTTCC
TTTCTCCCTTCCTCCC
+
CB<9<<9;?ABB9<<CBBAAA<;880;=@AEECCC?E;::AA???C<=:=DBCCCC@@@CCCCA:<<;;8AB?8:?=
@@<?;:<68,/,///,
@T6XXI:00070:00362
GGGGAATTCTAATACGACTCACTATAGGGAGAGGAGAATAGAACGACCAGTCC
+
@=94><==<=<BGAA@BCBCBCCCB::9.6=663:=;:86//,.---000000
@T6XXI:00070:00365
GGGAGAAGGAGAAAGGAAGAAAGGCAAGGTACCGAAGACTGAGT
+
6;,--1,1,//66+/,42333,39//,/5///,/63444.....
@T6XXI:00070:00383
CTATCGACTCGAGATCTGCGGCTTTGGACAGTCCCTCTGTACTGTGAAGCCACAGATGTCGAGTCGACTAGCTTCCT
TCTCCC
+
```

```
<@=8111:::=BA@@A;;;A>ACC>C@C@>@::7.:B??@CCCB@AA@=B>@CB@????????@ABA>87...,.,/
,.311+
@T6XXI:00070:00386
GGGAGAAGGGAGAAAGGAAGCTAGTCACTCGACATCTGTGGCTTCACAGTACAGAGGGACTGTCCAAAGCCGCAGAT
CTCGAGTCGACTAG
+
ACBCCC=@@<@AAC@C@C@;;;B;::>94390/////77>589=:?BB@??C??///,///.-.445577.43411-
---113334000--
@T6XXI:00071:00366
GGGAGAAGGGAGAAAGGAAGCTAGTCACG
+
44-44;244-4666+/,/,...15544..
@T6XXI:00071:00373
GGGAGAAGGGAGAAAGGAAGCTAGTCGACTCGACATCTGTGGCTTCACAGTACAGAGGACTGTCCAAAGCCGCAGAC
TCTCGAGTCGACTAG
+
:B:>>A698199A@>B?B>??::;@;::>@@?;::@@6:8>:<>@??>?::9778//,.8647==@@<@@>B876//
///483403355356
@T6XXI:00071:00376
GGGGAATTCTAATACGACTCACTATAGGGAGAGGAGAATAGTAC
+
:44,9<@>@@@?BBCCB>@?><@AAAAA<AB@@=A/,+,*****
@T6XXI:00071:00389
GGGGAATTCTAATACGACTCACTATAGGGAGCGGGAGAATAGTTTTCCCTTCACTAAACCGCGAACTGCACTTCCTT
CTCCCTTCTCCC
+
CJC:C;;:<@@;@@CBBBBBC???@777.7>>@B>CCC??><<<<4<@7?;=AA:::1:>A>?>?0/0;>7>:<,0,
....+100038+
@T6XXI:00071:00390
GGGAGAAGGGAGAAAGGAAGGAGAAGTCGCCCCAAACGATTTGTTGACTATTCTCCCTCTCCCTATAGTGAGTCGTA
TTCAGAATTCCCCC
+
:B?CBCACCABBBE?C@C@C@@@@@CBBCCBB8CB?B::68887;;:=868:3334,0000303003668@=@@<6<
523300/023333.
@T6XXI:00071:00394
GGGAGAAGGAGAAAGGAAGGAGACAGTGAGCGCCGCACACAGCACTATCTCCCTCTCCTATAGTGACGTCGTATAGA
TTCCCC
+
;K:7,1,1-//66+/,42313339444;483337333311111//,,,,,,2097@?>69933333..335.....-
10111,
@T6XXI:00072:00365
CTAGTCGACTCGAGATCTGCGGCTTTGGACAGTCCCTCTGTACTGTGAAGCCACAGATGTCGAGTCGACTAGCTTCC
TTTCTCCCTTCTCCC
+
>7-,--,,:C;CCDBCB<<;?=??B>B?@@<;966-
177777700..334C@CCCF@@@@?A???AB??@C8:699016,66;=6;8;<<A:
@T6XXI:00072:00371
GGGGAATTCTAATACGACTCACTATAGGGAGAGGGAGAATAGAGACGTATTCGTTTGCGCTCTGTTCACCTTCCTTT
CTCCCTTCTCCC
+
FDC9A9;;;@@?DBCCCAAAA?<9=166.7>>>@?AAA>AA===A@@>><:<55;6<>=?B@@@@>A??<?>A:>?;
998D?C>@@C@?
@T6XXI:00072:00372
GGGAGAAGGGAGAAAGGAAGCTAGTCGACTCGACATCTGTGGCTTCACAGTACAGAGGGACTGTCCAAAGC
+
CB:?9959>19444-42423333367177116635.//..55..-6777AB?::;444-/76/--272211
@T6XXI:00072:00374
```

```
GGGGAATTCTAATACGACTCACTATAGGGAGAGGGAGAATAGTTCCGTGCATTCTCCCAGCACACCGCCTTCCTTCT
CCCTTC
+
7;70<-
929449449;::?B96771111,167778834/043428233436424433,37777110110136010.113+-,-
@T6XXI:00072:00376
GGGAGAAGGGAGAAAGGAAGCTAGTCACTCGACATCTGTGGCTTCACAGTACAGAGGACGTGTCCAAA
+
::-44:<996>99909<><88883554.,,,0,,,,..,,0..,.779@B?74.--,++++++.4455
@T6XXI:00072:00379
GGGAGAAGGGAGAAAGGAAGCTAGTCGACTCGAGATCTGCGGCTTTGGACAGTCCCTCTGTACTGTGAAGCCACAGA
TGTCGAGTCGACTAGCT
+
AD@E=<<=CACCGC>B=A=?;87??:::AA?BBB:::AAA@?BBB>C@CECCBDCCA========??A>AA;==<<;
?><388/--16.03614
@T6XXI:00072:00391
GGGAGAAGGAGTAAGAAGAATGACATCGGACGCAACACGGAGACAGGCTATTCTCCCCTCTCCCTATAGTGAGTCGT
ATTAGAATT
+
;A::44585944424313777></....,..3..,331101.7...,.6653:1111,11667,0033355800053
002444532
@T6XXI:00072:00393
GGGGAATTCTAATACGACTC
+
8==0=+,*,,,*,///4333
@T6XXI:00072:00398
GGGAGAAGGGAGAAAGGAAGGAGAAGTCGCCCCAAACGATTTGTTGACTATTCTCCCTCTCCCTATAGTGAGTCGTA
TTAGAATTCCCC
+
CE@CCB@BB>BBBC>C?B@A@ABB@?>?????8??<D@>?A>AC?BBB@<<:=<AA<===@C?A=<=@AAABB>>>B
A<@AA;>28880
@T6XXI:00073:00350
GGGGAATTCTAATACGACTCACTATAGGGAGAGGGAGAATAGTCAACAAATCGTTTGGGGCGACTTCTCCTTCCTTT
CTCCCTTCTCCC
+
999/9595<A@DEDBBBCCCC@A>?611.7>>@A>BAA>@<AAAA?@AA<CABAB>BEE7BBBBB?@AC8989?CC?
@???<?<A?@C@
@T6XXI:00073:00361
GGGGAATTCTAATACGACTCACTATAGGGAGAGGGAGAATAGCCAAATTCTCTCCGCCGGCAAGTTGTTCTTCCTTC
TCCCTTCCTCCC
+
DDD:D>AA@BBACCCBBBBCC8?>?777.:@@CE@CAA<?===@AA=B?<<<8837@>A899>AA@?;.//58,/,+
00000+/,--22
@T6XXI:00073:00368
GGGAGAAGGGAGAAAGGAAGCTAGTCGACTCGACATCTGTGGCTTCACAGTACAGAGGGACTGTCCAAAGCCGCAGA
TCTCGAGTCGACTAG
+
CCACCB?BB>BCBB>C?A?ABB;;=/00;;;>0::::6@@@::;6:@B?@?B@;;444-
4:::;=:=A<AA>@@@@A@@@@A@@@??????@
@T6XXI:00073:00375
GGGAGAAGGGAGAAAGGAAGGAGACAAGCCCGCCAGTAAGAACGAAGCTATTCTCCCTCTCCCTATAGTGAGTCGTA
TTAGAATTCCCC
+
9A<@@BACDBBBBB=B>B@C=@@BCCACCC=BD@CA@@?BC@CEEB=<;<9?B111-
111=>;>>@@@@<<>><B@@><<>=>E@BBB8
@T6XXI:00073:00376
GGGGAATTCTAATACGACTCACTATAGGGAGAGGAGAATAGCAAC
```

```
+
CBA4@6969@@<BCCBBAAAB@BAB:::6:A>@>A8641,+++*+
@T6XXI:00073:00395
GGGAGAAGGGAGAAAGGAAGCTAGTCGACTCGACATCTGTGGCTTCACAGTACAGAGGACTGTCCAAAGCCGCAGAT
CTCGAGCGACTAG
+
CD@CCCACC@ABAB>B@B@BBBBCCCCCAAA?:::@B???B?AA?C@@@BBB797..,/8867=>A@<@A<@@@AA@
@??=<<==889?@
@T6XXI:00074:00360
GGGAGAAGGGAGAAAGGAAGCTAGTCACTCGAGATCTGCGGCTTTGGACAGTCCCTCTGTACTGTGAAGCCACA
+
NH<:=B?BE>CCCC>C?B>@???;::?<<9=98;89:??BACBB>B@DBBBBCD?CAA988@??>;65++)***
@T6XXI:00074:00374
GGGGAATTCTAATACGACTCACTATAGGGAGAGGGAGAATAGTCCAGAGCAAGTCCTCCCTAAGCTATTCCTTCCTT
TCTCCCTTCTCCC
+
BCB9B::<<CC@BBBCBBCAA?BB>166.7>>?A>AAA>??AAA?@??@AA?@@@>AAA<?==?<=??>A=?47477
-7149,3/3000+
@T6XXI:00074:00384
GGGGAATTCTAATACGACTCACTATAGGGAGAGGGAGAATAGCTAAACGCTGTTTGCAACCCTCTTACTCTTCCTTT
CTCCCTTCCTCCC
+
999/49?99;<?BBCCCAAACCCCB999.7<;;?9?=<>AA@?BBE@???<:6116;:;@A;CD@;:::://-
/7733436403/0/06>:
@T6XXI:00074:00386
GGGAGAAGGGAGAAAGGAAGCTAGTCGACTCGACATCTGTGGCTTCACAGTACAGAGGGACTGTCCAAAGCCGCAGA
TCTCGAGTCGACTAG
+
4<8<A@>BCABBCC<?=@@BBCCCB:;;=?==:76961?8?5:9?BAAABBBAAA=<=7<??@AA=AC=??=??;@=
AAAA===@@@@@>>>
@T6XXI:00075:00377
GGGGAATTCTAATACGACTCACTATAGGGAGAGGGAGAATAGTCTCATGGTCAAGATCCGGAGCTTCCCCTTCCTTT
CTCCCTTCCTCCC
+
BA@4@59694954>>AAABBAA@@@::::.7>@@A?A@@>AA@@AAAC@A>@CCACBA>?@>=;//./9;1;;;9;;6
?@9939,/,/.-+
@T6XXI:00075:00378
GGGGAATTCTAATACGACTCACTATAGGGAGAGGGAGAATAGTCTTGCAATTTCCTGCGGTGCATTCTCCTTCCTTT
CTCCCTTCTCCC
+
BCD:D:::9AA@CCCBB@ABA>A@AAAA9?@?AA>AAC>AA??8834;?39;9;@BDCC@BBBCD@CAA=?;>;>?:
?AAC>C@@@?C<
@T6XXI:00075:00382
GGGAGAAGGGAGAAAGGAAGGGGAGAAGGGAGAAAGGAAGCTAGTCGACTCGACAT
+
CC@CEB@DC==@@=:878477:4:?C@CC>B:77.7:=:=?311761161100400
@T6XXI:00076:00367
CTAGTCGACTCGAGATCTGCGGCTTTGGACAGTCCCTCTGTACTGTGAAGCCACAGATGTCGACTAGCTTCCTTTCT
CCCTTCTCCC
+
?A;<<BCEEDCBA@;<;A@@CAELC?E=@@BB@?A<???BBBBBAB?B>BC@CCBBBBA@??7::=;<>40,/3+/.
77,/,/-222
@T6XXI:00076:00368
GGGAGAAGGGAGAAAGGAAGGGCAAAAACCTTGAGAATAGCCACCAACTATTGTTCGTCGCCGTTCTCTTCCTTTCT
CCCTTCTCCC
+
```

```
CDAEHC@BB=BCCE=B>B@CC?CCCCC2C@CBCCCC@C<=<@BB?B?@@>>=@@>?@AA<;6;C=@@@C?B?AA:@@
A@:@?;<<@;
@T6XXI:00076:00373
GGGGAATTCTAATACGACTCACTATAGGGAGAGGGAGAATAGTCAACAAATCGTTTGGGGCGACTCTCCTT
+
:CB8B:<69=@=;;<C@@@CBABAAABC56:7::.:<<:=?@:55<===<?788<8:??5=;;00.00*++
@T6XXI:00076:00380
GGGAGAAGGGAGAAAGGAAGCTAGTCGACTCGACATCTGTGGCTTCACAGTACAGAGGGACTGTCCAAAGCCGCAGA
TCTCGAGTCGACTAG
+
C@<@CCBCC@CCBB=?=?@AB@@@B:;:ACB???@??:;:A:?@=?BBDCBA=::444-
4;;BAA>@@<@@B@@@????AA?AAA@@???>A
@T6XXI:00076:00384
GGGAGAAGGGAGAAAGGAAGGAGAAGTCGCCCCAAACGATTTGTTGACTATTCTCCCTCTCCCTATAGTGAGTCGTA
TTAGAATTCCCC
+
CC@DDB@DB?BCCC@B?C@C@BBB@?BBADEC9BC?BCAAA<AC7:;?<@@>@@AC<===?@;?@@@:99@@???AA
A>?@A>?8<<<4
@T6XXI:00076:00388
GGGAGAAGGGAGAAAGGAAGGGCACGGGCACATAACGTAGGAGAATACTATTCTCCCTCTCCCTATAGTGAGTCGTA
CTTAGAATTCCCC
+
49149@599094>>0949279:@BBCCAC:::67:;;66556@>:???????A@@@;888<<6:@@8875563444.
./77433;2488-
@T6XXI:00076:00396
GGGGAATTCTAATACGACTCACTATAGGGAGAGGGAGAATAGTCAACAAATCGTTTGGGGCGACTTCTCCTTCCTTT
CTCCCTTCTCCC
+
ADE7@@A@BABADCCCCABAA?ABAAAA<AABCC>BAA=A@@@AA????<ADDDE>BBB7A@???:;=<3837;=;:
?@>=7><878A:
@T6XXI:00077:00359
GGGAGAAGGGAGAAAGGAAGGAGAAGTCGCCCCAAACGATTTGTTGACTATTCTCCCTCTCCCTATAGTGAGTCGTA
TTAGAATTCCCC
+
CD@CFC@BB?BBBBAC@C@C@BCB@??BBBCC9CC?CCCBB>BC?CCC@@@=@AAA<???AA;<??===???>=:;/
/./;977,444-
@T6XXI:00077:00373
GGGGGAATTCTAATACGACTCACTATAGGGAGAGGGAGAATAGCCACCAACTATTGTTCGTCGCCGTTCTCTTCCT
+
CEB@-4249:9979=@BA@@CC:;<CBBB>BCCCD>C865;8987::7:388@@@AA:=9???;9;8-/++00...
@T6XXI:00078:00352
GGAGAAGGGA
+
1,1/1,//+,
@T6XXI:00078:00360
GCTAGTCACTCGAGATCTGCGGCTTGGACAGTCCCTCTGTACTGTGAAGCCACAGAGTGTCGAGTCGACTAGC
+
44444:::888////72,,,00,,*,*,..66:@=@ACCE<>8<@:658>>911>0...;:1616497733.,
@T6XXI:00078:00361
GGGAGAAGGGAGAAAGGAAGCTAGTCGACTCGACATCGTGTGGCTTCACAGTACAGAGGGACTGTCCAAAGCCGCAG
ATCTCGAGTCGACTA
+
CC>A@>589194>>9>498:;977.,,,,,,,,,000,//;??237:911666;844300+04446<:;<;;<:<<<9
96842-/----..03
@T6XXI:00078:00383
GGGGAATCTAATTGACGA
+
```

```
;>>5;--***)*)****,
@T6XXI:00079:00352
GGGAGAAGGGAGAAAGGAAGCATGTTGCACGGGCGATGTACAAACTATTCTCCCTCTCCCTATAGTGAGTCGTATTA
GAATTCCCC
+
DC@CBB@CC>BAAA=B=@=@CBGBB?ACBCBC>@CA@<>=99909>?@=AACB@@>99909>@CCBA@DDDD:;;@C
A><60117.
@T6XXI:00079:00353
GGGAGAAGGGAGAAAGGAAGGAGACAGTGAGCGCCGCACAACACGTCACTATTCTCCCTCTCCCTATAGTGAGTCGT
ATTAGAATTCCCC
+
4<6<CB@CF@CBBB>BBB?A@BBABB@AACBABB9<;;;:>BAA:::;999><>38808>>>?08999@ABBAAABA
??<?=>=B5888.
@T6XXI:00079:00356
GGGAGAAGGGAGAAAGGA
+
?:6:44244-44//+/,/
@T6XXI:00079:00361
GGGGAATTCTAATACGACTCACTATAGGGAGAGGGAGAATAGTCAACAAATCGTTTGGGGCGACTTCTCCTTCCTTT
CTCCCTTCTCCC
+
@DB8B:;58989;8;<A<;;B;88A777.7?77767==>AAAA@@=??@:A???@<@??2>>=7737<=44247:94
400494/3-222
@T6XXI:00079:00366
CTAGTCGACTCGAGATCTGCGGCTTTGGACAGTCCCTCTGTACTGTGAAGCCACAGAGGGACTG
+
BDCD<;@FGFCCDCDBABBBC@>>@>@@BBBC???8????BBCCBC@B<?CACCDD@@@:??@8
@T6XXI:00079:00370
GGGAGAAGGGAGAAAGGAAGGGGAGAAGGGAGAAAGGAAGCTAGTCGACTCGACATCTGTGGCTTCACAGGACGGAC
+
CCBB@@@AA=>BBC=ADB@BBB7>;;<<>>BCCE>C?@>><>AAAAA???BC889@@?@@A<??>?@9.-,,,,+,+
@T6XXI:00079:00372
GGGAGAAGGGAGAAAGGAAGCTAGTCGACTCGACATCTCGTGGCTTCACAGTACG
+
99-444:@C>@@CD;<;<=@@C???:6/75.....4444676237>>30-0+***
@T6XXI:00079:00373
GGGAGAAGGGAGAAAGGAAGCTAGTCGACTCGACATCTGTGGCTTCACAGTACAGAGGGACTGTCCAAAGCCGCAGA
TCTCGAGTCGACTAG
+
:C>CCCACCCBDA?=@=?=;;8@:;0//:777/0......338;<<ACDCC@<;;444-
4444:;37758A:=AA@@A===@B@??>>;;<<
@T6XXI:00079:00397
CTAGTCACTCGAGATCTGCGGCTTTGACAGTCCCTCTGTACTGTGAAGCCACAGATGTCGAGCGACTAGCTT
+
BB<449944499944444877<<7,33337111,11116688733=;<BABC@@B<:76>>9=>.008//,2
@T6XXI:00080:00359
GGGGAATTCAATACGACTCACTATAGGGGAAGAGGGGA
+
;BD4>61,18,////433...66...55*.,/37775:
@T6XXI:00080:00372
GGGAGAAGGGAGAAAGGAAGGAGAAGTCGCCCCAAACGATTTGTTGACTATTCTCCCTCTCCCTATAGTGAGTCGTA
TTCAGAATTCCCC
+
9959@A>AC=BCCD?B@D?B?BBC?BCDCDDC8AC>ABB?@;BB?BBA>86..+000+++44547<=<::@?==<::
8-..05000411.
@T6XXI:00080:00375
```

GCTATCACTCGAGATCTGCGGCTTGGACAGTCCCTCTGTACTGTGAAGCCACAGATGTCGAGTCGACTAGCTTCCTT
TCTCCCCTTCCTCCC
+
<:4;444444=>?CDCC=00,00,0344...662111669>=B@@@;=A@CBABDA@=:9?7699773971126017
711000+0/0/000+
@T6XXI:00080:00382
GGGGAATTCTAATACGACTCACTATAGGGAGAGGGAGAATAGTGATCCTATTTTCTCCTGTTCGCGTACCTTCCTTT
CTCCCTTCTCCC
+
BB?7A598;CB@CCCB@?@AC=@B???B>BBBCC>B??<?A=A===A>@BBCC9@AA>@??<?CAAA??>?>@<??6
99::4;384AB=
@T6XXI:00080:00388
GGGAGAAGGGAGAAAGGAAGCTAGTCGACTCGACATCTGTGGCTTCACAGTACAGAGGGACTGTCCAAAGCCGCAGA
TCTCGAGTCGACTAG
+
@C>ABB@CC@A@AC9;:<>ABABB@::;769>:77??9:6?>??=?AAACCB@;;444-
4;;?@A>@@?@A>AAA@@???@CAA@999?>=>
@T6XXI:00080:00391
GGGAGAAGGGAGAAAGGAAGCTAGTCGACTCGACATCTGTGGCTTCACAGTACAGAGGGACTGTCCAAAGCCCAGAT
CTCGAGTCGACTACGCTCC
+
=B>CAA>AB<@@AC<@=@>:;311:/.......4575>===?@B><65//..--
3000+044369<<=;89948899<<888;770787---+//,
@T6XXI:00081:00362
GGGGAATTCTAATACGACTCACTATAGGGAGAGGGAGAATAGTTCTGGACTGTACTCGTGGGGCTGTCT
+
:@?4@69294488=999878769366<989?===.74079;9?8303/1/.....57777440..0...
@T6XXI:00081:00370
GGGAGAAGGGAGAAAGGAAGTGCAGTTCGCGGTTAGTGAAGGGGAAAACTATTCTCCCTCTCCCTATAGTGAGTCGT
ATTAGAACTTCCCC
+
AC>CBC?BA=BECB>B@B@BBBBBBB@B??@8:::CBCD>=;;.716.8>@@?CAAA=AABDC?B@@BBBAAA?=87
8;7<@@5888<<:1
@T6XXI:00081:00379
GGGGAATTCTAATACGACTCACTATAGGGAGAGGGAGAATAGTCAACAAATCGTTTGGGGCGACTTCTCCTTCCTTT
CTCCCTTCCTCCC
+
DCE:E@C@BBBBCCDCCA@@AAB?88??9?@ACC<A>A?A====B=BBB:BBABB>CCC7@?BAA:??@3837<?@<
@@;92:././;;6
@T6XXI:00081:00380
GGGGAATTCTAATACGACTCACTATAGGGAGAGGGAGAATAGTGACGTGTTGTGCGGCGTCACTGTCTCC
+
999/96;:<BB@CCBABABCB@B@?99959??CE;?;B?A=9=A=<;<==<=<:894./.-..1661160
@T6XXI:00081:00398
GGGAGAAGGGAGAAAGGAAGC
+
CF@CBB=>=5=844-9594-*
@T6XXI:00082:00352
GGGAGAAGGGAGAAAGGAAGGTGAAAGTCAAACGGGCAAATGCGTGACTATTCTCCCTCTCCCTATAGTGAGTCGTA
TTAGAACTTCCCC
+
BC>=;;59@:@@AB<@=@?B>BBBB>BBBBA=@>>779909A?ABDCBBBB;<;BB6;<7>>8<B??ACCCAA@=<<
<8;=;7::3444,
@T6XXI:00082:00367
CTATCGACTCGAGATCTGCGGCTTTGGACAGTCCCTCTGTACTGTGAAGCCACAGATGTCGAGTCGACTAGCTTCCT
TTCTCCCTTCTCCC
+

```
CCB>99:?===BCCDBBBCB@B?B=B>@BCBBBB>B?AABCBB?==A:=A@CCBB@@@???@AABCBBBCA:73747
7.7<9;4<9=@@B<
@T6XXI:00082:00379
GGGGAATCTAATACGACTCACTATAGGGAGAGGGAGAATAGTTCTCGTCAGTTG
+
/11*1,94499;=@AB@@?@>>81661,1<776,13558>==:733340--10-
@T6XXI:00082:00388
GGGAGAAGGGAGAAAGGAAGGGCCAGCCGGTGGGAGAAAACCGTGTGCTATTCTCCCTCTCCCTATAGTGAGTCGTA
TTAGAACTTCCCC
+
DDA@AB@DC>CCBC=A>A@BBAB@BCC@B?BBC:::@DD9C?CCCCCCCCDBCEEB99999919>>>BA@?@?8877
;37199<>:9962
@T6XXI:00082:00390
GGGGAATTCTAATACGACTCACTATAGGGAGAGGGACGAATAG
+
/11*1,9599958;AAABGBBCC<<>CB:>>777,1.-101--
@T6XXI:00082:00393
GGGGAATTCTAATACGACTCACTATAGGGAGAGGGAGAATAGCCGTTTAATCTCCCACTGCGGCATACTCCTTCCTT
TCTCCCTTCTCCC
+
CCC8@=@>@@@?BBBB??@A@??AB???<?C@AA>B<;8;;4424::4974433949A?8909:88///22222011
055689;79034-
@T6XXI:00082:00396
GGGGAATTCTAATACGACTCACTATAGGGAGAGGGAGAATAGTTTTCCCTTCACTAACCGCGAACTGCACTTCCTTT
CTCCCTTCTCCC
+
>==4?6969@@>BBBB@@@AA@AA????;?CBBB<BBC=>>4344-
494:=@BCCC@C@@??@=<;;8:7=:=477.:?=B>C=<<>>7
@T6XXI:00083:00350
CTAGTCGACTCGAGATCTGCGGCTTTGGACAGTCCCTCTGTACTGTGAAGCCACAGATGTCGAGTCGACTAGCTTCC
TTTCTCCCTTCC
+
A8-
,,,,,77:;B<<<;;ABA>AAB>B;<<CCCCDAC@@?@>=>:63588@?A?=@??@??@@::<A?><331101066,
735501,-,
@T6XXI:00083:00351
AGTCGACTCGCGATCTGCGGCTTGGACAGTCCCTCTGTACTGTGAAGCCACAGATGTCGAGTCG
+
<77////118?@B>800?>910,06;49661A577//60+,+++4457:@9988865314.--+
@T6XXI:00083:00355
GGAGAAGGGAGAAAGAAGCT
+
45444244-////+//,.49
@T6XXI:00083:00364
GGGAGAAGGGAGAAAGGAAGGAGAAGTCGCCCCAAACGATTTGTTGACTATTCTCCCTCTCCCTATAGTGAGTCGTA
TTAGAATTCCCC
+
CD><99<>>:>=>@<>?B=@=@BA@;88AACC8CBACBBB?;AC@CBB:6;8743><<?:?CAC994/..444:==;
?;=644443494
@T6XXI:00083:00368
GGGAGAAGGGAGAAAGGAAGCTA
+
:C==AB>99694//+/686....
@T6XXI:00083:00379
CTAGTCGACTCGAGATCTGCGGCTTTGGACAGTCCCTCTGTACTGTGAAGCCACAGATGTCGAGTCGACTAGCTTCC
TTTCTCCCTTCTCCC
+
```

B:/1111/=DDBCCCBABC@A=@BD>C>@@A@???<?BB@@?CC>B?A>?B@BDBADBCCBFCC;<<@@@AAA?;@<
@@:??=B9:7:9994
@T6XXI:00083:00380
GGGGAATTCTAATACGACTCACTATAGGGAGAGGGAGAATAGTCAACAAATCGTTTGGGGCGACTTCTCCTTCCTTT
CTCCCTTCTCCC
+
CB?7A=@=@BB@CCCBBAABAAAAA???9?B?AA=ABB?@<<A==;=@A<CFBBB@BCC9BB@??:67:34248;;6
<?;?:?698BAE
@T6XXI:00083:00382
GGGAGAAGGGAGAAAGGAAGTGCAGTTCGCGGTTAGTGAAGGGAAAAACTATTCTCCCTCCTCCCTA
+
ELCCBA>B@<AABB?BCG@CCABBCC@BD@@>B?ABAAB<>>.7777,33331....+..,.550..
@T6XXI:00083:00386
GGGAGAAGGGAGAAAGGAAGCTACGTCA
+
4:-44:24909499047873....,,,0
@T6XXI:00083:00389
GGGGAATTCTAATACGACTCACTATAGGGAGAGGGAGAATAGCCTAACGTTTTGCACAGTTGGTGTCTCCTTCCTTT
CTCCCTTCTCCC
+
499/49A=ABB?BBBBBACABA@@?::7.7><??=?AA?A=@@:BB@CA@CC9@888<AA8;9;;888>:;:;244+
359A=A>@?@@<
@T6XXI:00083:00396
GGGAGAAGGGAGAAAGGAAGAACAGGGCACCGCAGGAAAACGTGAAGCTATTCTCCCTCTCCCTATAGTGAGTCGTA
TTAGAACTTCCCC
+
49599?;>A<A>>@<@;><>>7:777,79@<ACCC?CCB9BBBAA?A999959499-
44::::;;;;<:::?;998882338;@A?@@?6
@T6XXI:00084:00370
CTAGTCGACTCGAGATCTGCGGCTTTGGACAGTCCCTCTGTACTGTGAAGCCACAGACTGTCGACTCTTC
+
44449@@BCA@<<<;;@AB>@@;<;6;ACBCBA?@:@?=?:::::@;>78<91:;=0..0.77671110.
@T6XXI:00084:00373
AGTCGACTCGAGATCTGCGGCTTTGGACAGTCCCTCTGTACTGTGAAGCCACAGATGTCGGTCG
+
CCC:<;CEEBB?BCCBBBBAAAA>A@BB?BBCC>?::?CCCB=@@C?CC@BBBC:800+,++++
@T6XXI:00084:00376
CTAGTCGACTCGAGATCTGCGGCTTTGGACAGTCCCTCTGTACTGTGAAGCCACAGATGTCGAGTCGACTAGCTTCC
TTCTCC
+
@<1111//68/44999@BB@B?@A@<?<>??7177.7====??;0/.454;===8;>7?;89996==7833433,.,
.,.333
@T6XXI:00084:00377
GGGAGAAGGGAGAAAGGAAGGAGAAGTCGCCCCAAACGATTTGTTGACTATTCTCCCTCTCCCTATAGTGAGTCGTA
TTAGAATTCCCC
+
CB?BBB@BB>BBBBBB=@=>@BBB>??CCCCC9CC>CE??@=@B@CCCA<=>=<<<;????C<@===@@@AB@@@@@
@@AAA>A8;<;1
@T6XXI:00084:00388
GGGAGAAGGGAGAAAGGAAGGAGAAGTCGCCCCAAACGATTTGTTGACTATTCTCCCTCTCCCTATAGTGAGTCGTA
TTAGAATTCCCC
+
ECBCC@?BB>BBBB>B@B@B@BBB@?BBCCCC8BB=BBBBB<AA>B?=?A=<<8<<7<<<@D?A@@???AA@88:@A
A>AAA?@3888-
@T6XXI:00084:00389
GGGGAATTCTAATACGACTCACTATAGGGAGAGGGAGAATAGTCAACAAATCGTTTGGGGCGACTTCTCCTTCCTTT
CTCCCTTCTCCC

```
+
@@>4>=@?ACDCCCCBB@AACCCAB:6656=??A>B@A>@=?ABB>?AA;BCCCC<AAA6A@A@B=>==4428<@@<
A@@@:?587@A?
@T6XXI:00084:00396
GGGGAATTCTAATACGACTCACTATAGGGAGAGGGAGAATAGCAAACGTTCCCCCGACGTCGCTTTCTCCTTCCTTC
TCCCTTCTCCC
+
AAA7@=@=@AA@BCCCA?@BBCCCB???9???CC>BAA?AAA@@@;?@=:<???0?@BB:::777.0::9646,/,-
-3334355330
@T6XXI:00085:00355
GGGAGAAGGGAGAAAGGAAGGGGGAGAAGGGAG
+
/1-//9;>>5==>@094928333+33376626.
@T6XXI:00085:00389
GGGAGAAGGGAGAAAGGAAGCTAGTCGACTTGACATCTGTGGCTTCACAGTACAGAGGGACTGTCCAAAGCCGCAGA
TCTCGAGTCGACTAGCTCCTTCTCCCTTCTCCC
+
44-4>><>B199>?9><B>A<;;CCCBBBB>?888771111713179>>DCDDA@884-
4::=??>AC<@@=AAAA@@888;::6677/,*.***)+*+3335534333+
@T6XXI:00085:00392
GGAGAAGGGAGAAAGGAAGGAGAAGTCGCCCCAAACGATTTGTTG
+
:994964=:849904;=<?=8:7:667;;<;5;9,73111,1101
@T6XXI:00086:00357
GGGAGAAGGGAGAAAGGAAGGAGAAGTCGCCCCAAACGATTTGTTGACTATTCTCCCTCTCCCTATAGTGAGTCGTA
TTAGAATTCCCC
+
9C<>?A>?A>@@@C@C@B@C>;;;?@@BBCCC9CC?CCAAA;AC?CCC@AA>@AAA<A===@;@>??899B@?????
?B@@A?C=@E>4
@T6XXI:00086:00365
GGGGAATTCTAATACGACTCACTATAGGGAGAGGGAGAATAGTCAACAAATCGTTTGGGGCGACTTCTCCTTCCTTT
CTCCCTTCTCCC
+
C?:4?596<AA?CCCBBBBBCBA?>777.7>>AC?A@@?AAAAAA=?@B?D???E?CED7@?CAA<?@>44347::6
<<AB=A:==AA<
@T6XXI:00086:00374
GGGAGAAGGGAGAAAGGAAGTGCAGTTCGCGGTTAGTGAAGGGAAAACTATTCTCCCTCTCCCTATAGTGAGTCGTA
TTAGAATTCCCC
+
@B@BBB@BB=ABBBBA<?<<BBDC@?=@BB?@B@AAACB=77-666-
7?CB@DCCC>:::BC>BAAA@A@AA878><<9=A@=@38880
@T6XXI:00086:00395
GGGAGAAGGGAGAAAGGAAGGAGAAGTCGCCCCAAACGATTTGTTGACTATTCTCCCTCTCCCTATAGTGAGTCGTA
TTAGAATTCCCC
+
DD@CCC@BA;@@AA?B@A>@>@BB@@?CCCCB9BC>B???@;BC@CAA@CC;=88818==@DEA@@989AAA@@@@@
A>@>=8;3444-
@T6XXI:00087:00353
GGGGAATTCTAATACGACTCACTATAGGGAGAGGGAGAATAGTCTCCCCATTAATCGCGGCTTAATTGCCTTCCTTT
CTCCCTTCTCC
+
/11+/,1,/887:CC;;88871331111,1611116@@9<<>;40444+7?;;364700,0:673;33=360/33:6
/--2+-,+--,
@T6XXI:00087:00358
GGGAGAAGGGAGAAAGGAAGGAGAGCACC
+
BGCCCC@DC?ABBB?B?B@>:68-,,,*)
```

```
@T6XXI:00087:00359
GGGAGAAGGGAGAAAGGAAGGAGAAGTCGC
+
4914=><>>094?@9;8<>B;?>>;71333
@T6XXI:00087:00375
GGGGAATTCTAATACGACTCACTATAGGGAGAGGGAGAATAGTCCCTGTGTTCTCCCCACAGCGGTCAACTTCCTTT
CTCCCTTCTCCC
+
@@>4@<>=@B@?BBCCCCCCBCCBA???;?ABCC>CAA=AA@@@C<A???A>?>=HD6:99@@B?AAE;::;>4775
8;;A;@>A?=<7
@T6XXI:00087:00379
CTAGTCGACTCGAGATCTGCGGC
+
999444:A@CCD;4////,,,**
@T6XXI:00087:00397
GGGAGAAGGGAGAAAGGAAGCTAGTCACTCGACATCGTGTGGCTTCACAGTACAGAGGGACTGTC
+
11-///,//+//48587=<;<@A;;=111..3344....344.311616166644000+---1--
@T6XXI:00087:00399
GGGGAATTCTAATACGACTCACTATAGGGAGAGGAGAATAG
+
,,,*-8><@@@@CC::<BDDCCCC@@@@9:9/0,055555,
@T6XXI:00088:00350
GGGGAATTCTAATACGACTCACTAGGAGAGGGAGAATAGTCTCTGTCCCCTTGCTCTG
+
499/9596<@@ADBBBC:;:AAB;:67?>>?9?@>5:878;>==<..//+.,-1.--1
@T6XXI:00088:00374
GGGGAATTCTAATACGACTCACTATAGGGAGAGGGAGAATAGCCGTCAACAAATCTGGGGTCTATTCTGCTTCCTTT
CTCCCTTCTCCC
+
DED8B:;8;=>@CAAACCAAA@@?>777-
7>BDD@C@@=@@AA>@@@B=??@:@><77723844313;>;9995775777?;A?A><<;
@T6XXI:00088:00396
GGGAATCTATACGACTCACTAC
+
99142/////,,//...97331
@T6XXI:00089:00361
GGGGAATTCTAATACGACTCACTATAGGGAGAGGGAGAATAGCTACGTTAACCGATCGTGTTGTCTCCTTCC
+
;?>5>,469989ABBBDBDBB@??=777-7====9???=@=;::::00,.4234766<<::2334/82230+
@T6XXI:00089:00386
GGGGAATTCTAATACGACTCACTATAGGGAGAGGGAGAATAGTCAACAAATCGTTTGGGGCGACTTCTCCTTCCTTT
CTCCCTTCTCCC
+
CDC9B===>>><:;:@@CE<;:AB???A<@AAAA=AAA;==@AAA>A@@?@?@@L@DCC9CBAA?=>=>4424:;;:
<=<A<A6:.5;6
@T6XXI:00089:00399
GGGGAATTCTAATACGACTCACTATAGGGAGAGGGAGAATAGCCATTGTACCCCCAAATTCGGCGTCTACTTCCTTT
CTCCCTTCTCCC
+
DFC:C=@=9<;?@AABBCDCBAABCBBA<?B:::5:>>;??>>;8858A@@@@A1?>59=8978//.47.324;A94
;66;6;=>>>A<
@T6XXI:00090:00377
GGGGAATTCTAATACGACTCACTATAGGGAGAGGGAGAATAGTCAACAAATCGTTTGGGGCGACTTCTCCTTCCTTT
CTCCCTTCTCCC
+
```

/11+1,95999;@@B<<;>?>BCB=?=E>AABCB>B@@;?><<;=:===7>>7::59B>-
:7:89667700/37884;648562336<7
@T6XXI:00090:00378
GGGGAATTCTAATACGACTCACTATAGGGAGAGGGAGAATAGACTGCTATCCTCAGGCGTGCTATTCAACTTCCTTT
CTCCCTTCTCCC
+
CEC9@6;:<;;?CC@AAAAA>?@A@:::7:BCCC>CAA>@????@??8:;A>@@AA;==A887CB?AB@;<7;=??:
>>@>9?;;::C>
@T6XXI:00090:00387
CTATCGACTCGAGATCTGCGGTTGGACAGTCCCTCTGTCTGCGGCTTTGGACAGTCCCTCTGTACTGTGAAGCCACA
GATGTCGAGTCGACTAGCTTCCTTTCTCCCTTCCTCCC
+
BBBA;9:CCDDCBBABBBBB@C@C@@@@C???;?A?A???A@@;9;91;@BBBCCCC>BBBBCCBC<<;EACC>BBD
CBB;9=CBCGDC==<@=8999244-/644+.,.,.556
@T6XXI:00090:00392
GGGGAATTCTAATACGACTCACTATAGGGAGAGGGAGAATAGCCACCAACTATTGTTCGTCGCCGTTCTCTTCCTTT
CTCCCTTCTCCC
+
BBA9B?B@BBB?BBCCBABBAAABC7775:ABCC>CA?=@<AA9==>@?????;>?<???@@A>?>:==44348;;7
>@?@:?=??AC?
@T6XXI:00090:00393
GGGGAATTCTAATACGACTCACTATAGGGAGAGGGAGAATAGCTTATGTTTCTCGCGTGCCTATTCGTCTCCTTTCTC
CCTTCTCCC
+
:<?1?-1/:::;BCCC:;;??79711616B>>?=>88.//669336633+0305566644:.-,-22---
333457==::553;<4
@T6XXI:00090:00397
GGGAGAAGGGAGAAAGGAAGCGTAGTCGAC
+
9?:=@C@GF@CCCB71,/5.......1111
@T6XXI:00091:00374
GGGAGAAGGGAGAAAGGAAGTGCAGTTCGCGGTTAGTGAAGGGAAAAACTATTCTCCCTCTCCCTATAGTGAGTCGT
ATTAGAATTCCC
+
AD@CBB@BC@BDCC?D@CBCBBCCBB@B@@?=?<B;88?677.7===/?@@@?A===677116-
7777@CA=?:99A@A>@AC=?>@C<
@T6XXI:00091:00381
GGGAGAAGGGAGAAAGGAAGTGCAGTTCGCGGTTAGTGAAGGGAAAAAACTATTCTCTCTCTCCCTATAGTGAGTCG
TATTAGAACTTCCCC
+
CC<@@A>BB>BCELAC?B?BCCDCBC?B<<;?B>AAAAABBC@CCCCD-
DCAC=??@AB;::<@7=????A@@@@CB?;:30:>,00:;556
@T6XXI:00091:00388
GGGGAATTCTAATACGACTCACTATAGGGAGAGGGAGAATAGTCAACAAATCGTTTGGGGCGACTTCTCCTTCCTTT
CTCCCTTCTCCC
+
BAA7@=@=<==BDDN@@>AACCECABAB>BCCCB<B@C=@@AAA@=<;;6?@@AC=BAB7B???A;???<;8;7::6
<;;B:A>:89A<
@T6XXI:00091:00394
CTAGTCGACTCGAGATCTGCGGCTTTGGACAGTCCCTCTGTACTGTGAAGCCACAGATGTCGAGTCGACTAGCTTCC
TTTCTCCCTTCCTCCCATCACCGACTGACAGCTACGTACGCTGAGCATCGATCGAGTACACACTACGTCTGAGCATC
ACGATGTACAGCTACGACGTCGAGCATCGATCGAGT
+
4/////////69>9=AA;1/65.00+.,..6777::@BCEECC@??>=8;@>B??BBABABAA@@=>00.48556<A
>?8<64422,-,+000+.---2--
344:9=:644455586555556555:66466;703=6569<>==>><<8833343335.-
.33...5444743345333+++0+++333

```
@T6XXI:00092:00353
CTAGTCACTCGAGATCTGCGGCTTGGACAGTCCCTCTGTACTGTCGAAGCCACAGATGTCGAGTCGACCTAGCTT
+
44444449994449988610,.0,.,..5:7>>9886161633---12196949;?799?==71//..,.43333
@T6XXI:00092:00387
GGGGAATTCTAATACGACTCACTATAGGGAGAGGGAGAATAGTCAACAAATCGTTTGGGGCGACTTCTCCTTCCTTT
CTCCCTTCTCCC
+
@?>4?696<BB?BDBBBBBCCB@A?:::5:@BCD>BBA>@@@???@AAA:AABCD>BBB7AAB??:;@@3838;==:
==<A<A:==?C:
@T6XXI:00092:00390
GGGAGAAGGGAGAAAGGAAGGAGAAGTCGCCCCAAACGATTTGTTGACTATTCTCCCTCTCCCTATAGTGAGTCGTA
TTAGAATTCCCCCATCACCGAGACTCTGTACAGCACGACGCTGAGCTCATGATG
+
EDACCB@BB?BDBCBC@C@C@@@@=?????;>6@C>A9AAA8@@?A;:34;9;99948434>:<<==ABAAA?????
87://-.499;@.@;@@@@<970.+0333+..533363,+,00+++001366=;
@T6XXI:00092:00397
GGGAGAGGAGAAAGAGG
+
491444424//6+/,,2
@T6XXI:00093:00374
GGGGAATTCTAATACGACTCACTATAGGGAGAGGGAGAATAGGTGCAAAACCTCTCGACGATACCTGTTCTTCCTTT
CTCCCTTCTCCC
+
@@>4><@=@AA>BBBBBABABCBD=<<?88?BCC>BAA>A99:8==>>>8?>@?AA;:;BAAAA<???<8=;;/441
8;=B<A>A@@@:
@T6XXI:00093:00377
GGGGAATTCTAATACGACTCACTATAGGGAGAGGGAGAATAGTTTTCCCTTCACTGACCGCGAACTGCACTTCCTTT
CTCCCTTCTCCC
+
?=@6>=@=@BC@CCBBBBB@>9<>7777.7BBCD>DDB=@><<==5==9=<?ACBCCC????B????@::::=:==6
744;4:8<??B=
@T6XXI:00093:00378
GGGGAATTCTAATACGACTCACTATAGGGAGAGGGAGAATAGTCAACAAATCGTTTGGGGCGACTCTCCTTCCTTTC
TCCCTTCTCCC
+
CCB8@;A=@A@@BBBBBA@@BCA>@:::.7?ABB@BCH>@@C@@?;?>=7AB>>>.777-0::00.33,---
988499:;::24.-73
@T6XXI:00093:00379
AGGGAGAAGGGAGAAAGGAAGCAGTCCATCTGTGGCTTCACAGTACAGAGGGACTGTCCAAAGCCGCAGATCTCGAG
TCGACTAG
+
DBC?BBB@BB>BBBC>B=?<@AAA8;7;;;;CCC?BBACAA@BBCC?AAAA<AAA@??=<=;==<89=???@@@@AA
A@AA@@@A
@T6XXI:00094:00384
GGGAGAAGGGAGAAAGGAAGCTAGTCGACTCGACATCTGTGGCTTCACAGTACAGAGGGACTGTCCAAAGCCGCAGA
TCTCGAGTCGACTAG
+
CC?AAH@BB>B@@@<@8;8;CA<;;00/007,,0../,233*/0::>@BDJB?==744-
499:<<=?C@CD@@???=<<?????AAABCC@A
@T6XXI:00094:00399
GGGGAATTCTAATACGACTCACTATAGGGAGAGGGAGAATAGCAACA
+
==>4>6969A@=@A;:;A@@@@@LB@?C>BB@??9?;84;9///,//
@T6XXI:00095:00382
CTAGTCGACTCGAGATCTGCGGCTTTGGACAGTCCCTCTGTACTGTGAAGCCACAGATGTCGAGTCGACTAGCTTCC
TTCTCCCTTCTCCC
```

```
+
B;1/11///879994994849933357=9?<@=??8???:::BA::8A>@CDCCCB@@@AABB;:<CBAA@00/6:,
0,.44424-700;4
@T6XXI:00095:00387
GGGGAATTCTAATACGACTCACTATAGGGAGAGGGAGAATAGCCTAGTGGTGTACCGCCCAGCTGTCTCCTTCCTTT
CTCCCTT
+
999/9592;CB>BB@@@;;;BBA>=777-
7>>>@9?;=9;<@@5:;BBC@AB@@??????7>===?884;/4/0/00+0--3+-,
@T6XXI:00095:00390
GGGAGAAGGGAGAAAGGAAGGAGAAGTCGCCCCAAACGATTTGTTGACTATTCTCCCTCTCCCTATAGTGAGTCGTA
TTAGAATTCCCCC
+
CCCCCG@BB>??@B>B@C@C@CBB@??ACCCC7AD=AC???>@C@CCDA==;=?=?:778==:=@@?A@A@@===@@
A>A?=99/////+
@T6XXI:00096:00374
GGGGAATTCTAATACGACTCACTATAGGGAGAGGGAGAATGTGACGTGTGTGCGGCGCTCACTGTCTCCTTCCTTCC
TCCCTTCTCCC
+
DEC:D?>=<CC=A@BB;:<@B>??>777-7>>>@>@=@9444011-------
3323973355:88664/0/0/0/0/04444443222
@T6XXI:00096:00376
GGGAGAAGGGAGAAAGGAAGGAGAAGTCGCCCCAAACGATTTGTTGACTATTCTCCCTCTCCCTATAGTGAGTCGTA
TTAGAATTCCCC
+
BB@CCB@BB>BBBC@C>ADA?A?A?@B@BBBB8BC?CCB??;@C?BBB?AA>=A==;;;?==;<:::<<<==<<?::
?=@AA>A:<<<4
@T6XXI:00096:00377
CTGCGGCTTTGGACAGTCCCTCTGTACTGTGAAGCCACAGATGTCGAGTCGACTAGCTTCCTTTCTCCCTTCTCCC
+
CB=?DABBD?C@BDGECCC>B@?;;;A??<?C@KEBCFAEA@@CCCCCCCC@@@A;;7>@@CC>ABAB>B8:7?A:
@T6XXI:00096:00389
GGGAGAAGGGAGAAAGGAAGGGGAGAAGGGAGAAAGGGCTAGTCGACTCGACATCTG
+
CKCCCB?BB>BCBB>B=@=???2><<477.7???9??<?B>==100900316.....
@T6XXI:00097:00361
GGGGAATTCTAATACGACTCACTATAGGGAGAGGAGAATAGTACGA
+
---*-+1,//65649;;;AACBCBA::7.7=7737449;95,,,**
@T6XXI:00097:00378
GGGGAATTCTAATACGACTCACTATAGGGAGAGGGAGAATAGTCGACAAAATTAACCACGGCTCCCTTTCTTCCTTT
CTCCCTTCTCCC
+
999/959:<@@ABBBBC@@@AAABA???<?BCGC>B@A?A=A@@@???AA6C@C@C?BBB?A@>?-66-
7=::38858BBA7=;=;?A7
@T6XXI:00097:00381
GGGGAATTCTAATACGACTCACTATAGGGAGAGGGAGAAATAGTCAACAAATCGTTTGGGGCGACTTCTCCTTCCTT
TCTCCCTTCCT
+
:CC8B>B:<9=649BC<;;B?>>>1611,16161,1000+08884655562166665577-/,322*//74222,--
+033446;=52
@T6XXI:00097:00384
GGGAGAAGGGAGAAAGGAAGGAGAACAACACGATCGGTTAAACGTAGCTATTCTCCCTCTCCCTATAGTGAGTCGTA
TTAGAATTCCCC
+
<C>BCC@BB?BCCC@C?B@A@AAA@CC@CCCCDBBB;9<;C?B;<;@@@??=?@FC=BBCCC=?=<<??A@>==A??
?>?>B33/0881
```

```
@T6XXI:00098:00372
GGGAGAAGGA
+
11-///,/,,
@T6XXI:00098:00389
GGGAGAAGGGAGAAAGGAAGAATAGTGAATTGGAGATCAGTAGTGGACTATTCTCCCTCTCCCTATAGTGAGTCGTA
TTAGAATTCCCC
+
CCAIDD?BA?BBCE>C?C@CB=>>>>??>??=<CB?AACCBHDDCA@@ACA?@9991;>@@D@CBBBDCBBC?B???
?:?6?=A<???4
@T6XXI:00099:00355
GGGGAATTCTAATACGACTCACTATAGGGAGAGGGAGAATAGCCGTTTAATCTCCCACTGCGGCATATCCTTCCTTT
CTCCCTTCCTCCC
+
944,92959AA@BBBBBBBBCBB<B:::59B?AA=?AA?<570/433+4>;6:<<466=798<A7?;54-.-
/5433433106,/,,550
@T6XXI:00099:00356
GGGGAATTCTAATACGACTCACTATAGGGAGAGGGAGAATAGTTCAACCGTCTCTGGCGCAACCATACTTCTTCCTT
TCTCCCTTCTCCC
+
499/96959449A@AABBBCCA@@=777,77777.:77:=?@A695;?;<A8746;2330-
,/,+0+++0++*+663577:834/4000+
@T6XXI:00099:00363
GGGGAATTCTAATACGACTCACTATAGGGAGAGGGAGAATAGTTACCTCCTCGCCTACGAACGCTGTTCTTCCTTCT
CCCTTCTCCC
+
::808,1,///7:@BCCABAA@B?77::6:BABC>B@@>@@@C;=>EA@<??::999===;=?A<88;8746,/,/.
/.+-,0000+
@T6XXI:00099:00376
GGGGAATTCTAATACGACTCACTATAGGGAGAGGGAGAATAGATCACCGTTGGTTCGGGTGCGCGTTCTCCTTCCTT
TCTCCCTTCTCCC
+
CEF9CBCDCCC?BBCBBBBD?=@@@???<?BBBCB@==>AA=<=AED@@A=?<?=BBB@CCCDCCC@@B?;=:=:=>
:>777.7371692
@T6XXI:00099:00386
CTCGAGATCTGCGGCTTGGACAGTCCCTCTGTACTGTGAAGCCACAGATGT
+
/8899499944//7//,/7879?AB:6444446/////7,,,*////...4
@T6XXI:00099:00397
GGGAGAAGGGAGAAAGGAAGGAGAAGTCGCCCCAAACGATTTGTTGACTATTCTCCCCTCTCCCTATAGTGAGTCGT
ATTAGAATCCCCC
+
GC:B99594-
4;;A<@=?>><@@LBCBABCBB7BB=B@@@C;>A>CDFA>;9;4444+03338:;;==<?;;;00/;;302--,---
--*
@T6XXI:00050:00407
GGGAGAAGGGAGAAAGGAAGTGCAGTTCGCGGTTAGTGAAGGGAAAAACTATTCTCCCTCTCCCTATAGTGAGTCGT
TAATTAGAAAC
+
DD>CDD@CC>CCCC?B@B@BCCCBBB@CFCC?B>@@?886:?9?DEC1BBBD@CBAA>ABBBL>B?AA????@=;:;
34242.--10-
@T6XXI:00050:00414
GGGAGAAGGGAGAAAGGAAGGAGGAAGTCGCCCCAAACGATTTGTTGACTATTCTCCCTCTCCTATAGTGAGTCGTA
TTAGAATTCCCC
+
?C@CDC@B;-4911--+-+-*-,*,222299495;>676111,16011853355537321-
++++++133:>>==5432000/0/000+
```

```
@T6XXI:00050:00429
GGGGGAATTCTAATACGACTCACTATAGGGAGAGGGAGAATAGTTCCTACAAATCGATT
+
ACDH3C>?>@BB>CDCBB?>>BABB?77759@BCC>B@@?A:8866,//-110111110
@T6XXI:00051:00408
GGGGAATTCTAATACGACTCACTATAGGGAGAGGGAGAATAGTCAACAAACTCGTTT
+
999,49A>BCC@CCBBB@::8=?;;:?C>@??BEAC??<<;:;44/0--+------+
@T6XXI:00051:00409
CTAGTCACTCGAGATCTGCGGCTTTGACAGTCCCTCTGTACTGTGAAGCCACAGATGTCGAGTCGACTAGCTTCCTT
TCTCCCTTCTCCC
+
B=///:699ABBBBCCC;;9::::/03????@?=DCCCC>>>@<><><B@BBBBBBBB???@@BBBBCC@@@?ACCC
>BBBC>A>@@AA;
@T6XXI:00051:00413
GGGAGAAGGGAGAAAGGAAGCTAGTCACTCGAGATCTGCGCTTTGGACAGTCCCTCTGTACTGTGAAGCCACAGATG
C
+
AA<>>><@@<@CCE>A>AB<;0@0/.4...6660119=><=?C;@?B;8899>;633633348000/04/0000000
3
@T6XXI:00051:00423
GGGAGAAGGGAGAAAGGAAGACTACAGCGGCGGGGATTACGACCAAGCTATTCTCCCTCTCCCTATAGTGAGTCGTA
TTAGAACTTCCCC
+
/1,119244-4488-
42497<AA@C>>885866623427>>>@7737?>7101111,1114:46=???@===<:??<<8569/00/000+
@T6XXI:00051:00443
CTATCACTCGAGATCTGCGGCTTTGGACAGTCCCTCTGTACTGTGAAGCCACAGATGTCGAGTCGACTAGCTTCCTT
CTCCTCCT
+
CBA=;::;BBBBBCBA@AB@BBB<B>@A@C@@@=B@@A?>>@:AAC?:A<@ABB@@?BBCC>>ABBECBBBC@B:;9
:0/,/,+*
@T6XXI:00052:00411
GGGAGAAGGGAGAAAGGAAGGCAGA
+
FDA<<<ACH?BB>>;>;>:4,..**
@T6XXI:00052:00425
GGGAGAAGGGAGAAAGGAAGGAGGAAGTCGCCCAAAACGATTTGTTAACT
+
CC>BAA=@@:@A@@:<:;-0,00,.6546...+333,36111,.5,.,.1
@T6XXI:00053:00401
GGGGAATTCTAATACGACTCACTATAGGAGAGGGAGAATAGCCTAACGTTTTGCACAGTTGGTATCTCCTTCCTTTC
TCCCTTCTCCC
+
@@@6@=@=@BB@CCBBA??BB:997/.,0;<?B>@==?A?>?;??=@BBEE9@@@@@@@>@=@BBCCC>=9;244,4
9880948044-
@T6XXI:00053:00403
GGGAGAAGGGAGAAAGGAAGGAGAACGCGCACCCGAACCAACGGTGATCTATTCTCCCTCTCCCTATAGTGAGTCGT
ATTAGAATTCCCC
+
CC@CCB?BB>CCBB>B=@=@?BBC?CCBBBBBC@DIAC>D@ADFHDCDC;;<>ACCE>AAAAB>CCCA@@????B??
??:?@B?CBCCE:
@T6XXI:00053:00406
GGGAGAAGGGAGAAAGGAAGGGCACGCGCGAGTCATCCAATTGGGAGCTATTCTCCCTCTCCCTATAGTGAGTCGTA
TTAGAATTCCCC
+
```

```
BBABCBACBB==>B?B@A@??;;;>BB???BCBBBBC@C?B?CCACCBCCC@CCBB?CBBBB?CBBBCCCBBBBB>A
?<>@>:>;???4
@T6XXI:00053:00409
GGGAGAAGGGAGAAAGGAAGTAGAACAGGACGACCTATAAGGCTGAACTATTCTCCCTCTCCCTATAGTGAGTCGTA
TTAGAATTCCCC
+
HE?BBB>BC=BBBD@D@C@CCCCB>BDC@BBCBB?BAAA=?>@CEC@BBCC@CAAA>BC@@?;>>BB@AA===????
?<?AHBDACCC:
@T6XXI:00053:00417
GGGAGAAGGGAGAAAGGAAGGAGAACATGACCCAGCACGATTGGTGTCTATTCTCCCTCTCCCTATAGTGAGTCGTA
TTAGAATTCCCC
+
BCBCCE?BB=?BBC?C@C@B@AAB@CCAABBB>BBBBBB>?=??<A:8:BB@CBBA<>A>??<?AAA<<<=AA?@=<
<;>??:><?@@5
@T6XXI:00053:00426
GGGAGAAGGGAGAAAGGAAGCTAGTCGACTCGACATCTGTGGCTTCACAGTACAGAGGGACTGTCCAAAGCCGCAGA
TCTCGAGTCG
+
DG@BBB@BB>:<=C@CDC@CBC>>>111@>>>@?????@@@?AA?ABBBCBCAA=44417??@@=?AA:BB?AB???
<=?AA;??==
@T6XXI:00053:00432
GGGGAATTCTAATACGACTCACTATAGGGAGAGGGAGAATAGTGACGTGTTGTGCGGCGCTCACTGTCTCCTTCCTT
CTCCCTCTCC
+
AA>4>69544499=@ACBCDCECBB777.777==6??@<?AABABA@===?A@@@@;==C@@@===@@CC?C9;242
4///,///.,
@T6XXI:00053:00443
GGGAGAAGGGAGAAAGGAAGCTAGCTGACTCGACATCTGTGGCTTCACAGTACAGAGGGACTGTCCAAAGCCGCAGA
TCTCGAGTCGACTAG
+
DECAA@BCC>AAAA<@=@=@D;78,-
,,,,,..1179>999>::7;BCCCBB=;;400,4;;@A@?AA<AA>AA<:::===<;<<<@=<<<=
@T6XXI:00054:00408
CTAGTCACTCGAGATCTGCGGCTTTGGACAGTCCCTCTGTACTGTGAAGCCACAGATGTCGAGTCGACTAGCTTCCT
TTCTCCCTTCTCCC
+
DB1111/44:;9999HBBCB?BCE?C?AAAA???9?BACBBBC777?<?B@CCCBBBBB>@@@@@@BCCCCACAA@B
B=??BA9;9;<=A7
@T6XXI:00054:00420
GGGAGAAGGGAGAAAGGAAGCTAGTCACTCGACATCTGTGGCTTCACAGTACAGAGGGACTGTCCAAAGCCGCAGAT
CTCGAGTCGACTAG
+
?CC=><@CC<><@C<B>B>@?7777666111166611666676757:AAAA??;;44417==AAA>A@<??<@@?889
@@@A??????AAAA
@T6XXI:00054:00434
GGGGAATTCTAATACGACTCACTATAGGGAGAGGGAGAATAGCCACGTTCCTGCATCAGTGACCTATTCCTCCTTCT
CCTCTCC
+
8>=5>=@=@A@AACBBBBB@@?BBA???9?@>@@=B?>9;94434477360......551156530024/40/0226
9,/-//4
@T6XXI:00054:00446
CTAGTCGACTCGAGATCTGCGGCTTGGACAGTCCCTCTGTACTGTGAAGCCACAGATGTCGAGTCGACTAGCTTCCT
TTCTCCCTTCTCCC
+
44444444444:A@BBCC>11-
11,037464.11,166176::7787/7;?B==>;777888668=777;766;<@?A>?==?985=55;6
@T6XXI:00055:00409
```

```
GGGAGAAGGGAGAAAGGAAGCTAGTCACTCGACATCTGTGGCTTCACAGTACAGAGGGACTGTCCAAAGCCGCAGAT
CTCGAGTCGACTAGCTTCCTTTCTCCCTTCTCCC
+
CA=BBB@CC8;<;9194958B?>>=>711111116798:<??B?B;;;CBA@==;;;6;;=?@A?@DAAB>B@AA??
<;;;=8887>888577/3:>>;><<>14/4444-
@T6XXI:00055:00443
GGGAATTCTAATACGACTCACTATAGGGAGAGGGAGAATAGCTTTCCCTTCACTAACCGCGAACTGCACTTCCTTTC
TCCCTTCTCCC
+
CB@BEA@BBB@CBBABBB>>C>97111.7==?BAD@A>AAAAAA=@@;@=??CBB?C?A@A=<9==@=<;:>7;;7<
>>A<A>AA@@;
@T6XXI:00056:00401
GGGGAATTCTAATACGACTCACTATAGGGAGAGGGAGAATAGTCTTCCCATATTTTTGCACCGCTATTCGCTTCCTT
TCCTCCCTTCTCCC
+
999/42424@@@CD@@<;;BAABD;::C=??AKC>?<<86<<?@@?A@3=?77777,7?=?;???BB>A<?652272
,/,/992800/;72
@T6XXI:00056:00406
GGGAGAAGGGAGAAAGGAAGGAGAAGTCGCCCCAAACGATTTGTTGACTATTCTCCCTCTCCCTATAGTGAGTCGTA
TTAGAATTCCCC
+
AA?ACC@CC@BAAB>BAA?B>BBB@@@>@@EE8BB>BCCCB=>>>?<<8AA>A@AA<@===CAA??>99;:=<9<88
>>BAA@B>@??5
@T6XXI:00056:00407
CTATCGACTCGAGATCTGCGGCTTTGGACAGTCCCTCTGTACTGTGAAGCCACAGATGTCGAGTCGACTAGCTTCCT
TTCCTCCCTTCTC
+
B99444444;499BBD<<?B?BCCAC;;>@??BE=BAA???AAA?@?9=@>BAAAA;<:BBCCCCCCDBC>1/:>:?
>.0,,5515+//.
@T6XXI:00056:00425
CTAGTCGACTCGAGATCTGTCGGC
+
CA;;;A=@CFCBC<;9<8+*((*(
@T6XXI:00056:00438
GGGAGAAGGGAGAAAGGAAGGAGAAGTCGCCCCAAACGATTTGTTGACTATTCTCCCTCTCCCTATAGTGAGTCGTA
TTAGAATTCCCC
+
991444244-
48;A:B>B@B?@@@@EEAABDB6??676111,1676668552446;;=545=;==;@=<<??@@@@@B?AAA>A:=<
=4
@T6XXI:00056:00446
GGGGAATTCTAATACGACTCACTATAGGGAGAGGGAGAATAGTCAACAAATCGTTTGGGGCGACTTCTCCTTCCTTT
CTCCCTTCTCCC
+
CCC9B=?=ABD@CCBABBAA@>;97111.7<<==9?>??AAAAAA;<AA<C???C>CCB8;?BCC@DDF==9<:?@;
AAAA<@;<<@A;
@T6XXI:00057:00401
CTAGTCACTCGAGATCTGCGGCTTTGGACAGTCCTCTGTACTGTGAAGCCACAGATGTCGAGTCGACTAGCTTCCTT
TCTCCCTTCTCCC
+
@@1////44>==?BBCCC::8;BB<B?AACCCC>CBC?>??@@AA@=?B@BBBBC@@@CCCBBBBCCCBBBB?B?BB
>BAAB<?<?@@B=
@T6XXI:00057:00440
GGGGAATTCTAATACGACTCACTATAGGGAGAGGGAGAATAGATCACCGTTGGTTCGGGTGCGCGTTCTCCTTCCTT
TCTCCCTTCTCCC
+
```

```
A?;6>7<8<9;9A;;<@@AAA>??=777,7<777,13325:9::4766660101;;;A>9888@>>>887;<???BC
;?88::999300+
@T6XXI:00057:00441
GGGAGAAGGGAGAAAGGAAGCTAGTCGACTCACATCTGTGGCTTCACAGTACAGAGGACGTGTCCAAAGCCGCAGAT
CTCGAGTCGACTAGCTTCCTTTCTCCCTTCT
+
??:ABB@CB:>911-1:;,-,,,.2,,,....33..1667067:77;???<::7//,.4.--
.888<;;:;:;;51---.--486330553323/331.54444---
@T6XXI:00058:00400
GGGGAATTCTAATACGACTCACTATAGGGAGAGGGAGAATAGTCTCGCGATGGTCCGCTACTTGTTCTCCTTCCTTT
CTCCCTTCTCCC
+
444,42958999=BBAA@CCCCCC@:::57==?A<A??>@<@@A@@@@<<?<8:;<88<BBC@CC@BBB?B:;366.
7>>?6<:=::86
@T6XXI:00058:00417
GGGGAATTCTAATACGACTCACTATGGGAGAGGGAGAATAGTGGACACATCAAGGTCATCCACTTTCTCTTCCTTTC
TC
+
999267<:1//5///443333133111,37666261101111333-
++0,,,*,039==98944969A6666,..+..,
@T6XXI:00058:00426
GGGAGAAGGGAGAAAGGAAGCTAGTCGACTCGAGATCTGCGGCTTTGGACAGTCCCTCTGTACTGTGAAGCCACAGA
TGTCGAGTCGACTAGCTTCCTTTCTCCCTTCTCC
+
GJ?@AB?BB<BBBB=B@C?BB>9::000:::;>>BBC?A@B?BBC>C@C?@@BBB<@@A9:9@AAAAA=@@<A@?@@
@@@@ACA???==<@<<;;>8;<7;:;=1858==;
@T6XXI:00059:00400
GGGGAATTCTAATACGACTCACTATAGGAGAGGGAGAATAGTTAACGCACCCTCGTTACCTGT
+
:>?1;-1,///784488>77767761101<777,63444:00/0/443111,11117110111
@T6XXI:00059:00417
GGGGAATTCTAATACGACTCACTATAGGGAGAGGGAGAATAGTCAACAAATCGTTTGGGGCGACTTCTCCTTCCTTT
CTCCCTTCTCCC
+
A@?4>2<;=CC@DBBABBCC@=@B?667.7><<?<?AA?A@@@A@>???7AAAAC>BDD8BBBBB@AAA>@>@=AB>
B???<?68:AA<
@T6XXI:00059:00440
GGGAGAAGGGAGAAAGGAAGGAGAAGTCGCCCCAAACGATTTGTTGACTATTCTCCCTCTCCCTATAGTGAGTCGTA
TTCAGAATCCCC
+
=;699@=>@?@999094948>A?@<=CDB???.8803177>588<<9;3304444<5;;?=?4:744458<<;<<44
3*++36+++550
@T6XXI:00059:00441
GGGGAATTCTAATACGACTCACTATAGGGAGAGGGAGAATAGTCAACAAATCGTTTGGGGCGACTTCT
+
CDKAC>@>;<<@BECBBAAABAAC>777.77777.78326@;@???<<;6?@@AB>BAA500/,72**
@T6XXI:00059:00442
GGGAGAAGGGAGAAAGGAAGCTAGTCGACTCGAC
+
::+//8,49144CC<@<?<7780:9/.....20.
@T6XXI:00059:00443
GGAGAAGGAGAAGGAAGCTAGTCGACTCGACATCTGTGGCTTCACAGTGACAAGAAGGGACTGTCCAAAGCCGCAGA
TCTCGAGTCGA
+
1,///,,*,,/,/,/,.69;;7:76<6<::/477///75548881114..--,--,-
3323:8668??;?C?AA@<<=AD@@==9:9A
@T6XXI:00060:00412
```

```
GGGGAATTCTAATACGACTCACTATAGGGAGAGGGAGAATAGCACTTACATGTGAGGATTCGCTGTTCTCTTCCTTT
CTCCCTTCTCCC
+
BBB9BACACCC@CCBBBBBB?@;97111.7?>BC>C@@?@<<<<<=<=888==<@@?==<<<@@@A>@@@@>A>??;
?@@@;@?>>>>7
@T6XXI:00060:00415
GGGGAATTCTAATACGACTCACTATAGGAGAGGGAGAATAGTCAC
+
?B@9B=@=<;;>A;;<C;99A766//.,.4:=<9:40246//--1
@T6XXI:00060:00439
GGGGAATTCTAATACGACTCACTATAGGGAGAGGGAGAATAGTCAACAAATCGTTTGGGGCGACTTCTCCTTCCTTT
CTCCCTTCTCCC
+
CB?6?598<AA@BBBABCCBB@<97111.7>7776:@@>A@AAAA><<<5?@=@@<@AA7AAACF?BBC>=:=>AA<
@???;?<?A@@<
@T6XXI:00060:00440
GGGGAATTCTAATACGACTCACTATAGGAGAGGGAGAATAGTTTTTCCCTTCACTAACCGCGAACTGCACTCTTCTC
CTTCCTCC
+
BBA7A79;<CBADDBBB8::C;=>9/0,/:<?A>BAA>A888AAA1AA<?;=?CCC>B@EC?3/400-
.....,.,,*,*+*+00
@T6XXI:00061:00400
GGGAGAAGGGAGAAAGGAAGCTAGTCACTCGACATCTGTGGCTTCACAGTACAGAGGGACTGTCCAAAGCCGCAGAT
CTCGAGTCGACTAG
+
DDAAAC@BC>BBBB>B996:B@?????=7977:6<>>CDC@BB?BCC@@CCC==33317>@ABA?AA:AA<>><<==
<<<<====<<<<<<
@T6XXI:00061:00413
CTAGTCACTCGAGATCTGCGGCTTTGGACAGTCCCTCTGTACTGTGAAGCCACAGATGTCGAGTCGACTAGCTTCCT
TTCTCCCTTCTCCC
+
B<944=44499999;CCBDC?BBB>C?BCBC9994:@=BAB@@?AAA>ACABBBAA@@@BBCBBBBBBBC@??@CBC
C?@@?B<?;788=7
@T6XXI:00061:00430
GGGGAATTCTAATACGACTCACTATAGGGAGAGGGAGAATAGTCAACAAATCGTTTGGGGCGACTTCTCCTCCTTTC
TCCCTTCCTCCCA
+
499/92924489>99;A@A@AABCCCCC977666.7:;9@988<;;<@?;@<;;<-
777,7>>?B?AA@6402894:///,/,,*+00*+
@T6XXI:00061:00437
GGGGAATTCTAATACGACTCACTATAGGGAGAGGGAGAATAGTTCTGGACTGTACTCTGGGGCTGTCTCCTTCCTTT
CTCCCTTCTCC
+
4:4,9?B<@@A=@@A@@AB@@?BB?@@C>CBBB@;BBA?@?@C388=3877700009.777-
7<<<@AB?B@C@BB>BC@@6:9:0/,
@T6XXI:00061:00444
GGGAGAAGGGAGAAAGGAAGAATAGTGGGACGCGGTCAGATCGACGCCTATTCTCCCTCTCCCTATAGTGAGTCGTA
TTAGAATTCCCC
+
ICADDC>BA;;<BD?CBC@BB?A@AABC5::@:837=BBBABCDD@@=BCC@CBDC>D@@@@<??@@AA???@@@@<
=9<8A=<<<<A6
@T6XXI:00062:00420
GGGAGAAGGGAGAAAGGAAGGAGAAGTCGCCCCAAACGATTTGTTGACTATTCTAC
+
BC?CBCACB?BBBC>C?B@A?B@@=?DBBBDD9CC>BAAA<896776630443000
@T6XXI:00062:00425
```

```
GGGAGAAGGGAGAAAGGAAGCTAGTCACTCGACATCTGTGGCTTCACAGTACAGAGGGACTGTCCAAAGCCGCAGAT
CTCGAGTCGACTAG
+
ECBDBB=>=:=>>>9>>@=@BAAACC?????:79?<<ABA>AB@ABCCBBBC@B===9=<<<<<5887==?AAAA<<
=AA<<???@===AA
@T6XXI:00062:00426
GGGAGAAGGGAGAAAGGAAGCTAGTCGACTCGACATCTGTGGCTTCACAGTACAGAGGGACTGTCCAAAGCCGCAGA
TCTCGAGTCGACTAG
+
CCACCCBCC=?AAD>@=@?ACB=>;/00:;;=>ACAA?CCDCCDBCCBBBBC@>>77718??A@A>@@;??<?AAAA
A===877>;<488<;
@T6XXI:00062:00429
GGGAGAAGGGAGAAAGGAAGGATAGGGCACCAATGAACAAATGGCGGCTATTCTCCCTCTCCCTATAGTGAGTCGTA
TTAGAACTTCCC
+
BB<@>@59991/78+/,/,.,...11,3339<;9>B=AAC;<<A@?=?BB@28899-4:444-
49449@??=>;;;3333..,..,..+
@T6XXI:00062:00436
CTAGTCGACTCGAGATCTGCGGCTTTGGACAGTCCCTCTGTACTGTGAAGCCACAGATGTCGAGTCGACTAGCTTCC
TTTCTCCCTTCTCCC
+
4/////////74999@AAABA<88808588??AA@;BCABAD???F@>;8<8;8>?@;;:C?AABC@?;;7;9<>A?
BE>@??C<?4839:3
@T6XXI:00063:00414
GGGGGAATCTAATACGACTCACTATAGGGAGAGGGAGAATAGTTTTCCCTC
+
9444+42444824448877>>6331111,11177>>30/033030+00+00
@T6XXI:00063:00417
GGGGAATTCTAATACGACTCACTATAGGGAGAGGGAGAATAGTTCGCCAATATCCGGCGGCGTGTTCTCCTTCCTTC
TCCCTTCTCCC
+
444,42924449;88;:;:8??97660/+0/..503143.343=A<=9@@CCAB<@677:=774;556:44232324
35<699;444,
@T6XXI:00063:00436
GGGAGAAGGGAGAAAGGAAGCTAGTCACTCGACATCTGTGGCTTCACAGTACAGAGGACTGTCCAAAGCCGCAGAGA
TCGACTAG
+
@A?ACC@CC>@@>?=B@C@CCCBBABCCC@A88:AAAAAC@CC@CA==??A755..-
/8;;=@?AA>AA?AA?@?8>?????AB;
@T6XXI:00063:00447
GGGAGAAGGGAGAAAGGAAGCTAGTCACTGACATCTGTGGCTTCACAGTACAGAGGGACTGTCCAAAGCCGCAGATC
TCGAAGTCGACTAGCTTCCTTTCTCCCTTCTCC
+
44-
449:??09499147897>>>;4<0/.......1111013<>B;@>?<966377,3:8:;;38817>8<=@CA>8844
4/373;=?@@??>:=88848666,/,///,
@T6XXI:00064:00407
GGGGAATTCTAATACGACTCACTATAGGAGAGGGAGAATAGACTGTTATGCCGTCGCCTTCTATTTTCCTTCCTTTC
TCCCTTCTCCC
+
DDC:D>?=?BB?BBBBBBBBB:=;:/.,000775:AA?B>>>@AAA:<<BB>@@?8848?AAAAAA5@9<9<9<B?A
@?>6;0/0=;9
@T6XXI:00064:00409
GGGGAATTCTAATACGACTCACTATAGGGAGAGGGAGAATAGTCAACAAATCGTTTGGGGCGACTTCTCCTTCCTTT
CTCCCTTCTCCC
+
```

```
CCC9C9;9<AA>AABBBCBBBAA?=777.7>===6=9;;==???@>@DH<@@@AC=BBB7BAB??:=7740/48:=;
?AB=<?=??AC=
@T6XXI:00064:00411
GGGAGAAGGGAGAAAGGAAGCTAGTCGACTCGACATCTGTGGCTTCACAGTACAGAGGGACTGTCCAAAGCCG
+
CD@BBC@DD?ABBC>B@B@BBB===ACCCCCAAABBBBAAAACC?AACAAAC@>A77717>>AAA>A@<??<?
@T6XXI:00064:00431
GGGAGAAGGGAGAAAGGAAGCTAGTCGACTCGACATCTGTGGCTTCACAGTACAGAGGGACTGTCCAAAGCCGCAGA
TCTCGAGTCGACTAG
+
CCABB@===5=>>=9@=@=@A?9:</0//34:;?:<<<??C>?@@@CCCBAB@C=99999<<===;==;===<<<<=
<<<<::?@@AAA?>>
@T6XXI:00064:00447
GGGGAATTCTAATACGACTCACTATAGGGAGAGGGAGAATAGACCGAACTCCTTCTCCGCGACAATTTGCTTCCTTT
CTCCCTTCTCCC
+
FCF:CC?=@BB@CCBBBBBBBB<97111.7<<BD@C?@>@A??@>@@=???>@>@AA;9=====>A@<???<?9;<7
;>;?7<:=AAC=
@T6XXI:00064:00449
GGGAGAAGGGAGAAAGGAAGACTACGGCGCTGTCTGGGGAAATTACAGCTATTCTCCCTCTCCCTATAGTGAGTCGT
ATTAGAATTCCCC
+
94-49@=BC<=<CC?B?B@BBBBACB@DCC@AAB==9929:-
429:;;>;99<9>>?<@@8BC>:;7:ABBB@@A===?688A>=<<BB5
@T6XXI:00065:00414
GGGGAATTCTAATACGACTCACTATAGGGAGAGGGAGAATAGTCAACAAATCGTTTGGGGCGACTTCTCCTTCCTTT
CTCCCTTCTCCC
+
BCC:E;;<;AABCCBBB?B?ACB??9:7.7<77:6:?@?AABDAB>@@@<BBBCC>BCC9CBB??:?@@;>=??AA<
@@AA=A;>@AA<
@T6XXI:00066:00420
GGGAGAAGGGAGAAAGGAAGGAGAAGCGGCCACATGTCCAAATTGCGCCATTCTCCCTCTCCCTATAGTGAGTCGTA
TTAGAATTCCCC
+
BC?CBB>?B=ABCC>B@B@C@CCA?>BB@B>@@>BAAB@EC8707B9954;998>?98?>?B<@BCCBBBCDBAA??
@<?@A?A:??@5
@T6XXI:00066:00437
GGGGAATTCTAATACGACTCACTATAGGGAGAGGGAGAATAGTCAACAAATCGTTTGGGCGAC
+
DCHAC>@>@CC@CCBBB8::AB@??:66.7>777.7::79<9;8:7;8:7>7777.77..30,
@T6XXI:00067:00409
GGGGAGGA
+
---*,*)*
@T6XXI:00067:00443
GGGAGAAGGGAGAAAGGAAGAAGAATAGAGATGCGGCCGCGTGTCATCTATTCTCCCTCTCCCTATAGTGAGTCGTA
TTAGAATTCCCC
+
94-
4@@<>>:>>>>9>>@>BB=@@?@??@@>::7==>44433838@BB;;<@@BCC=;:;;7.7A@@?AAA??=9==D?=
;;5857881
@T6XXI:00068:00411
GGGGAATTCTAATACGACTCACTATAGGGAGAGGGAGAATAGTCTCCCTACTAC
+
???6A=A=@@@?A@@@@AAACAAA???@<???BC?BA@>@8883444+0.....
@T6XXI:00068:00442
GGGGAATTCTAATACGACTCACTATAGGGAGAGGAGAATAGTA
```

```
+
,22088DACCCACCCBBBCCCBBA@:::8:B??3:4/,////-
@T6XXI:00068:00445
GGGAGAAGGGAGAAAGGAAGCTAGTCACTCGACATCTGTGGCTTCACAGTACAGAGGGACTGTCCAAAGCCGCAGAT
CTCGAGTCGACTAG
+
CC>BBC?A=:@;84-
9594;B???CA???A@:::?=<CCC=??@CC?99>?@<><><;<665;65<?;?A>??@@@@?@@BAAA@@@AA?@
@T6XXI:00068:00447
GGGGAATTCTAATACGACTCACTATAGGGAGAGGGAGAATAGTCAACAAATCGTTTGGGGCGACTTCTCCTTCCTTT
CTCCCTTCTCCC
+
<<<4<@CCCCC@BBBCCCBB@?@BB:9:7:AABC>B@@?A?@@??<?AA<A;::B5:::4:B@BB?AA@68348996
<@@@<@9<;=>:
@T6XXI:00069:00400
GGGAGAAGGGAGAAAGGAAGTGCAGTTCGCGGTTAGTGAAGGGAAAACTATTCTCCCTCTCCCTATAGTGAGTCGTA
TTAGAATTCCCC
+
DDADCB?B?:?BB?=B?B@BCCCCBB@?@@B?B?AABCB@@A;@@?7BBBB>>???;AABBD>A??@@<<<AAAA=<
?<;AA?A@AAA4
@T6XXI:00069:00439
GGGGAATTCTAATACGACTCACTATAGGGAGAGGGAGAATAGTCAACAAATCGTTGGG
+
///*/,/,4=>;999998:A?=>>6617,7==<>>:45<898?==885720000,,22
@T6XXI:00069:00440
GGGGAATTCTAATACGACTCACTATAGGAGAGGGAGAATAGCCCCGGCCGATGTGACGCTTCCCTCCTCTTCCTTTC
TCCCTTCTCC
+
A@@6@>B@A@@=@@A@B@BBBAAA????AABCC>B@A?A83000+4:;40:::77:@?@B99<18>;??@=?;==6=
777.740/.,
@T6XXI:00070:00413
GGGGAATTCTAATACGACTCACTATAGGGAGAGGGAGAATAGTCTGTCGTTCGGTCGACGCTGCCTATCCTTCCTTT
CTCCTTCCTCC
+
CDD:C?B@BCBAB@@@@>BBB@>97116.7>>>>:>//,//-
.4557=;A@BC>@@@<<4735553;::2424244,444/0/0/032
@T6XXI:00071:00407
GGGAGAAGGGAGAAAGGAAGGAGAAGCTAGTTCGGCTTGAAACGTAGCTATTCTCCCTCTCCCTATAGTGAGATCAC
CC
+
11+//878:::994-
49999=<3311844//5667//1//851119988//,/888+1/88857;7765...,,,///+
@T6XXI:00071:00410
CTAGTCACTCGAGATCTGCGGCTTTGGACAGTCCCTCTGTACTGTGAAGCCACAGATGTCGAGTCGACTAGCTTCCT
TTCTCCCTTCTCCC
+
C@111::?@FDAAABCGCDC@CCC?CBBBB???B?AACCAA:;;88@37<?BABC;;;B???AAA@@@@A?710637
7-7677.49404=;
@T6XXI:00071:00416
GGGAGAAGGGAGAAAGGAAGCTAGTCACTCGGACATCTGTGGCTTCACAGTACAGAGGGACTGTCCAAAGCCGCAGA
TCTCGAGTCGACTAG
+
CD@CCC@BC?CCBB=??@@BCC@CC?BBBCB@;:;;<;AAL=ABBBBAABCCC??77717?>??@<??;>@?A???A
@@@@@AAA???@@@@
@T6XXI:00071:00426
CTATCACTCGAGATCTGCGGCTTTGGACAGTCCCTCTGTACTGTGAAGCCACAGATGTCGAGTCGACTAGCTTCCTT
TCTCCCTTCTCCC
```

+
AA?==::49=9=>@ABAB?;>>>8?688??BCC>DCA<;;B@>==>:4959>>880//.477/99B<3361101;>>
:><99-4/4++,+
@T6XXI:00071:00448
CTAGTCACTCGAGATCTGCGGCTTTGGACAGTCCCTCTGTACTGTGAAGCCACAGATGTCGAGTCGACTAGCTTCCT
TTCTCCCTTCTCCC
+
CC;<<@===AAAABBCBBA@>AAA>A?@AAAABB>BCCCCC@@@@@@>@C@CCCCCCBAAACBBBBBBDC;:7=B=A
AAAABC?@=???@;
@T6XXI:00072:00413
GGGAGAAGGGAGAAAGGAAGTGACGCTTGGTCGATTGGTGAAGGAGGACTATTCTCCCTCTCCCTATAGTGAGTCGT
ATTAGAATTCCCC
+
/1-1/9244-449;-
469<;;;;@@@@;9:@4444898=?=:;944244437731698<<ECA;<=A@@==:7779:85:8;<9998==5
@T6XXI:00072:00414
GGGAGAAGGGAGAAAGGAAGCTAGTCGACTCGAGATCTGCGGCTTTGGACAGTCCCTCTGTACTGTGAAGCCACAGA
TGTCGAGTCGACTAGCCCTTCTCCCCTTCCTTCTCCCTTCTCCC
+
CC>AAA@CC@BA@@<>494;B>;=>/11?@>>@BCCBCBBC@BBB>B>BB???@?;=<<<<<<<<<?<>?<=A==<<
<9==@@A@@@???@@AA<?98////+/46,/,----+-036:A7
@T6XXI:00072:00415
GGGGAATTCTAATACGACTCACTATAGGGAGAGGGAGAATAGTCAACAAATCGTTTGGGGCGACTTCTCCTCCTTTC
TCC
+
C;>5@-1,1667/:;AAA@B@??F?777.7>>??6:8<<?@@C@A>@@@;BA@??6??<2=><@>:000,/-013+-
-00
@T6XXI:00072:00416
GGGAGAAGGGAGAAAGGAAGTGCAGTTCGCGGTTAGTGAAGGGAAAAACTATTCTCCCTCTCCCTATAGTGAGTCGT
ATTAGAATTCCCC
+
CCBBA@@BB>@ABA?A@B<BBAEBDC@BA?><>=@BBAB?AC>CCCC2BBBB@@ACA;???CD?C@@<<<AAAB@A?
??>AAC>@<???4
@T6XXI:00072:00420
GGGGAATTCTAATACGACTCACTATAGGGAGAGGGAGAATAGTTCTAGTTCCTCGTCTAGTATATTCGCCTTCCTTT
CTCCCTTCTCCC
+
CEC9C@C@BBB@BA????BBB@B<9111.6>=>A@BAA>@AA=;A;;;<;=<<@@A@@A@@@<@@?=@<;A>A<??<
?AAA<A?A???<
@T6XXI:00072:00422
GGGAGAAGGGAGAAAGGAAGGAGAAGTCGCCCCAAACGATTTGTTGACTATTCTCCCTCTCCCTATAGTGAGTCGTA
TTAGAATTCCCC
+
;B099959>9>8>?<@=@@C@CCDBBABBBCC9DD@A@?88166AAAAAAA;==BC:>????:?@A?<==@<;;A88
7=<:8885388-
@T6XXI:00072:00424
GGGAGAAGGGAGAAAGGAAGAAGAATAGGACGGATGTGGCACGATCGCTATTCTCCCTCTCCTATAGTGAGTCGTAT
TAGAATTCCCC
+
:C121C698898@@<@A@>AA?BB?;::;===<77000,/6644304449;:;9;9486//,/666:==>>;886//
,/16,82448,
@T6XXI:00072:00425
GGGAGAAGGGAGAAAGGAAGCTAGTCGACTCGACATCTGTGGCTTCACGTACAGAGGGACTGTCCAAAGCCGCAGAT
CTCGAGTCGACTAGCTTCCTTTCTCCCTTCTCCC
+
AE<@AB@BD?D<<;89<>=;;;<<7,,,,,,,04456:9=?<7:67>>7:;>>>444,4?7779277-
7<=B@AABAAA@?B?A@@@@@@@AA<?<?@;@@@D<A>==;B;

```
@T6XXI:00072:00448
GGGAGAAGGGAGAAAGGAAGCTAGTCACTCGACATCTGTGGCTTCACAGTACAGAGGGACTGTCCAAAGCCGCAGAT
CTCGAGTCGACTAG
+
CC@EEDAB?:=944-
9494;A?>?AB:::AB:::;;:CCC=@@@BBBC@BBB@<==<;=<;:??<??;?A?@@@AA<<??===A??????A
@T6XXI:00073:00401
GGGGAATTCTA
+
CFC:CAC@A;9
@T6XXI:00073:00402
GGGAGAAGGGAGAAAGGAAGGGGAGAAGGGAGCTAGTCGACTCGACATCCTGTGGCTTCACAGTACAGAGGGACTGT
CCAAAGCCGCAGATCTCGAGTCGACTA
+
CD@BB@@CC@ABBB>A<>;;;;5??A<??;?>>==99///887;/0//0,16=>>CA:=<<<C=A@BCCDC<ABB8;
;ABC>AC>AAAA@CC?@??@@CCCCBA
@T6XXI:00073:00404
CTAGTCACTCGAGATCTGCGGCTTTGGACAGTCCCTCTGTACTGTGAAGCCACAGATGTCGAGTCGACTAGCTTCCT
TTCTCCCTTCTCCC
+
B8/////449@=>?DCDCBC@CCCAC@A@>>777.:BACCCCD<<;=38<?BCDNAAABCCEAABA@@AC<:9:@:?
?9?CCC>A>@<;;6
@T6XXI:00073:00405
GGGGAATTCTAATACGACTCACTATAGGGAGAGGGAGAATAGTTCTCCCATTGCTTGTCATCACCTGCACTTCCTTC
TCCTTCCTCC
+
:;;292;24>>549>;::CCBA?C7777,76776,144:;=<<<;;5639<>AAB??8;=774:8<=;=AA;=2424
/.,-,.,/.0
@T6XXI:00073:00412
GGGAGAAGGGAGAAAGGAAGAGAAAAGTGACACTCAAGGCGAAGGGACTATTCTCCCTCTCCCTATAGTGAGTCGTA
TTAGAATTCCCC
+
CHCCCB@BC?BBBB>B??<993333,7?ABBB?=772834;;:?@<@DDAA=@<==;?<@@A<???=??;;===A<<
?>=@A<@8<<<4
@T6XXI:00073:00443
GGGAGAAGGGAGAAAGGAAGGAGAAGATACTGACGTGTGTGT
+
44-4?@?BC<@@@=<@=@=@@;80;<0/0...;6////..44
@T6XXI:00073:00444
GGGAGAAGGGAGAAAGGAAGGAGAAGTCGCCCCAAACGATTTGTTGACTATTCTCCCTCTCCCTATAGTGAGTCGT
+
:;>AAA=@@:9;;;09=@48137776677;<<27763116786677 1:3;=;888@:=;;7716/-.3-+....44
@T6XXI:00074:00412
GGGAGAAGGGAGAAAGGAAGGAGAACAACACGACCGGTTAAACGTAGCTATTCTCCCTCTCCCTATAGTGAGTCGTA
TTAGAATTCCCC
+
EC8<9@<>>1944809482373339 4429;;;44542424A58=997784824433,7;<?@;CCB:;<CB?80000
02300/024484
@T6XXI:00074:00430
GGGGAATTCTAATACGACTCACTATAGGGAGAGGGAGAATAGCCTAGGCACACGATGGCGCCCTTCTCCACTTCCTT
CTCCCTTC
+
BDB4@51,1/7,/,0,/.9997331111,11176,100/0---2...,.3777;960/4444-
9@BBC@>9;9;,0,....+177
@T6XXI:00074:00440
GATCTGCGGCTTTGGACAGTCCCTCTGTACTGTGAAGCCACAGATGTCGAGTCGACTAGCTTCCTTTCTCCCTTCTC
CC
```

```
+
GFDC??AC>A@C?C@CBBC:::8:BBCCBBB>AA=;487:?@>??@@@A==:>>??>><>A><;<=<=<==;=;<??
A<
@T6XXI:00075:00436
GGGAGAAAGGAAGCTAGTCGACTTCGACATCTGTGGCTTCACAGTACAGAGGGACTGTCCAAAGCCGCAGATCTCGA
GTCGACTAGCTTCCTTCTCCCTCTCC
+
BB1949>19282499@=B<<<@@>B;:::::BAB>?<9<>=<<@A<;<<@@:@@A???@AC;@@>????AA@@A@=
==?>;8883,,*,+*,*333,,.996
@T6XXI:00075:00441
GGGGAATTCTAATACGACTCACATAGGGAGAGGGAGAATAGTTTTTCCCTTCACTAACCGCGAACTGCACTTCCTTT
CTCCCTTCTCCC
+
@?>4><@@BCC@CB@@BBBB967?777.7>>AA<A@@:==??AAA1AC:?;?CCCD>A=9==C>???A???;=37:5
:BCC>BC===A<
@T6XXI:00076:00405
GGGAGAAGGGAGAAAGGAAGGAGAAGTCGCCCCAAACGATTTGTTGACTATTCGTCCCTCTCCCTATAGTGAGGTCG
TATTCAG
+
6;8<19284-448;-969@B<??@@?;>8888.8803666>;@@?BC?448?8558:+00004+34===;74354--
+00*+-0
@T6XXI:00076:00414
GGGAGAAGGGAGAAAGGAAGACAAGATTGCCAGAGGGGAGTTCATGGCTATTCTCCCTCTCCCTATAGTGAGTCGTA
TTAGAATTCCCC
+
DCCEDC@CC>CBB@=@=>;?>>><;::6:7=A@9999/9>>;<;11,/./88=8A;733388/36161<99999677
:>>99;91778-
@T6XXI:00076:00429
GGGGAATTCTAATACGACTCACTATAGGGAGAGGGAGAATAGTCAACAAATCGTTTGGGGCGACTTCTCCTTCCTTT
CTCCCTTCTCCC
+
CCC9C@B?ABB@CAAAA?>>>C>97111.7==>@>B@@>@AAAAA??<<;?@@=>5777-
9B?BBBBBB==:=>AA<A@@?:@58/;;6
@T6XXI:00077:00415
GGGAGAAGGGAGAAAGGAAGCACAGGGCGAAGCGTGGGAAACTTGGCCTATTCTCCCTCTCCCTATAGTGAGTCGTA
TTAGAATTCCCC
+
CDCCCC@BB>??BB@B?B>AABCDCB>>>>>@@ABBB>BC>CA;=<=<===<<A==;::?AA;=:::88<====::?
@?=CC>@>A@@5
@T6XXI:00077:00426
GGGAGAAGGGAGAAAGGAAGGAGAAGTCGCCCCAAACGATTTGCTTGACTATCTCCCTCCTCCCTATAGTGAGTCGT
ATTCATGAATCCC
+
:?69=?@CD@CCFC>B;>7:7;?B>CAA@>>>.88/31...+...,.45-
034844433/004033030333=>668..,-.--421113
@T6XXI:00077:00428
GGGGAATTCTAATACGACTCACTATAGGGAGAGGGAGAATAGTGACGTGTTGTGCGGCGCTCACTGTCTCCTTCCTT
TCTCCCTTCTCC
+
444,4292989249>BBB<<;AA>>:::6:ABCC>C@@;<;9;///68/42400448:;;==737>><<?<=:;344
-4///,/4///,
@T6XXI:00077:00433
GGGAGAAGGGAGAAAGGAAGGGGAGAAGGGAGAAAGGAAGCTAGTCGAC
+
CC?BCB@BB?BBBB=?=?@BCC8BBC@B?;9:6;6=4737;//.:..,2
@T6XXI:00077:00436
```

```
CTAGTCACTCGAGATCTGCGGCTTTGGACAGTCCCTCTGTACTGTGAAGCCACAGGTGGACTATCCAAAGCCGAGAT
CTCGAGTCGACTAGCTTCCTTTCTCCCTTCTCCC
+
C9944944;=99;AAABBCD@DEC>C?BB??961,:??ABCCC????<?C@CCCC@CC@BCA?@C?BCABB?BCBBB
?@@CBAABCAABB@??:?;?F:@@AA9;8;=;;9
@T6XXI:00078:00419
GGGAGAAGGGAGAAAGGAAG
+
CC>DBB79809911,-+-+-
@T6XXI:00078:00432
GGGGAATTCTAATACGACTCACTATAGGGAGAGGGAGAATAGTCAACAAATCGTTTGGGGCGACTTCTCCTTCCTTT
CTCCCTTCTCCC
+
BBA7A=@>ACCCCCBB?BBBB>;77116.7==>B>CAA?@<?==<=???;ACBBB=BCC9CBBBCABBC@@>@<??;
??=A;@:==?@:
@T6XXI:00078:00437
GGGAGAAGGGAGAAAGGAAGCTAGTCACTCGACATCTGTGGCTTCACAGTACAGAGGGACTGTCCAAAGCCGCAGAT
CTCGAGTCGACTAG
+
CC>DCC?BA=ABBA=B7968?87:???<??=77:::6?AA:?@?ABCBBCEB>833317??AA@=@A<AA=?B888;
;;A8>>AAAA<<<=
@T6XXI:00078:00444
GGGAGAAGGGAGAAAGGAAGAGAACAACGCGGGCGGATAGGCCTAGCTATTCTCCCTCTCCCTATAGTGAGTCGTAT
TAGAATTCCCC
+
DECCCC?BB>BCBB>B=@=@BACBCC@@??BB?CC@BCBB>B?C<=>KDDABBCC?ABBBB=@BCCBAABBBECCBB
?BCC>B?AAA6
@T6XXI:00079:00400
GGGGAATTCTAATACGACTCACTATAGGGAGAGGGAGAATAGTCAACAAATCGTTTGGGGCGACTTCTCCTTCCTTT
CTCCCTTCTCCCATCACCGACTGCCCAGCTACTAGTC
+
999/48?;@CCBBBBB;;<AB@???776.7?>>@?A@@>?<?@BA>@AB=CAABC>BBB9BAAAA?BAA@C@C<??;
?AAC<A;=844-4;;<A>AA@3044-434;;;55111
@T6XXI:00079:00405
GGGAGAAGGGAGAAAGGAAGCTAGTCGACTCGACATCTGTGGCTTCACAGTACAGAGGACTGTCCAAAGCCGCAG
+
BC@CEC>@@>FCCB>B@B7;;C:;:CFDC>>;//0000>>?;@ABBC@@??A555..-/888;@>AA=AA@A>::
@T6XXI:00079:00407
GGGAGAAGGGAGAAAGGAAGGAGAAGTCGCCCCAAACGATTTGTTGACTATTCTCCCTCTCCCTATAGTGAGTCGTA
TTAGAATTCCCC
+
CC@AAA@CC>BCCB?CBB?A@BBB?ABAAAAA8BC>CBA;B=??A?996?9:999;466?==;<<<<===9?:???:
C?C??<>9<;<4
@T6XXI:00079:00416
GGGAGAAGGGAGAAAGGAAGGAGAAGTCGCCCCAAACGATTTGTTGACTATTCTCCCTCTCCCTATAGTGAGTCGTA
TTAGAATTCCCC
+
DCBCCB@BB?BBBB>C@A>A@CBB???BBBCB8AA=BCA@@;>>>C@@<;;8;===;88>;A;=<<::;@AA:??@@
@?AA@>?;=<>5
@T6XXI:00079:00432
GGGGAATTCTAATACGACTCACTATAGGGAGAGGGAGAATAGTCCAAAGAAACCCCGACTATTCACAAACTTCCTTT
CTCCCTTCTCCC
+
CCC:C@D@BBB@BBB?@@@BB@BB@77757A9@G>B?>>B====9=<6<::7===4@@@B??<?@<<<9<A;>9==7
<@AD<A898?A<
@T6XXI:00079:00444
```

```
GGGAGAAGGGAGAAAGGAAGGGAATAGCAGCAGTCGGCAACGTGAAACTATTCTCCTCTCCCTATAGTGAGTCGTAT
TAGAATTCCCC
+
AC?===@BC@BBBB?BDB@BC>B@BB?ABBA@ACBA?AC@GAA@@@;ABBC@A>?>>>AAA>?AAA=======<???
<?AB>A>BAB6
@T6XXI:00080:00402
CTATCACTCGAGATCTGCGGCTTTGGACAGTCCCTCTGTACTGTGAAGCCACAGATGTCGAGTCGACTAGCTTCCTT
TCTCCCTTCTCCC
+
CAB>;::9>@@?@ACCCDC@AAA>A@AA@@<:<;??BBCCCA<9999=B@BBBCABBBAACCCCCBBBDBBB?B?AB
>BBBB=A<??AA<
@T6XXI:00080:00403
GGGGAATTCTAATACGACTCACTATAGGGAGAGGGAGAATAGACTTTGGTTTGACCTTAAGTGACTATTCCCTTCCT
TTCTCCCTTCTCCTACTAGTGAGTCGTATTAGAATTCCCC
+
EED:C=@;>>?<A>ABB;;;BA@>>777.777@B>?==<:;:9443+0444468888565600339 83/331388;<
>;A=;;-4343:8:;8<?@@@@@@AA@9:89AB<@<?==1
@T6XXI:00080:00419
GGGGAATTCTAATACGACTCACTATAGGAGAGGGAGAATAGATCACCGTTGGTTCGGGTGCGCGTTCTCCTTCCTTT
CTCCCTTCTCCC
+
BCC8A?ACCBB?@BCCBBBBB7664./,0::=B@L@@<@A@@===<8?B5:5:ACAC?C:;:CCC?BBB@CCC@CC>
B@@C=A?=>>CB
@T6XXI:00080:00427
GGGGAATTCTAATACGACTCACTATAGGGAGAGGAGAATAGTACG
+
:;=5:,;:9@@B@<<<DDGCCCCA?<>966711074:7:8/////
@T6XXI:00080:00432
GGGAGAAGGGAGAAAGGAAGGAGAAGTCGCCCCAAACGATTTGTTGACTATTCTCCCTCTCCCTATAGTGAGTCGTA
TTAGAATTCCC
+
?>194@599-
84>C<BBB?C@CDC@CBABBBC9CC>B@::61669=BB@??<?AAA<?999A<?@@A===@@@A@A@@>@@@>A;??
7
@T6XXI:00080:00433
GGGGAATTCTAATACGACTCACTATAGGGAGAGGAGAATA
+
;::1><@=@CBBCABBBBAAABCBB8886;=663:4424/
@T6XXI:00080:00434
GGGAGAAGGGAGAAAGGAAGCTAGTCACTCGACATCTGTGGCTTCACAGTACAGAGGGACTGTCCAAAGCCGCAGAT
CTCGAGTCGACTAG
+
CC?AAA@CD>BCA>:>696:B@>@?9::???776:769CC=@@@BBBB??@A==96848::<AA?=A:AA?==89==
===:::==AA<<<=
@T6XXI:00080:00441
CTATCACTCGAGATCTGTGGCTTTGGACAGTCCCTCTGTACTGTGAAGCCACAGATGTCGAGTCGTACCTTA
+
DAAB<::@BBBBBCCCCCC@CCC?C@CBBBCCC>CCCBBB@>9==A>AC;;<C@@B;::A=<:8----*,*,
@T6XXI:00080:00445
GGGAGAAGGGAGAAAGGAAGCTAGTCGACTCGAGATCTGCGGCTTTGGACAGTCCCTCTGTACTGTGAAGCCACAGA
TGTCGATCGACTAGCTTCCTTTCTCCCTTCTCCC
+
CG?ABA>@C>A@@@<@>@<?@@??ADCABAABB::;CBBBC?BB?;===AAC@??;<<<<<<<===<<;==>A@@A@
@@889@AA@?@@==<<==<==448@C7<;=@?A;
@T6XXI:00081:00403
GGGGAATTCTAATACGACTCACTATAGGGAGAGGAGAATAGCAGGCCTTCATG
+
```

```
?BD9CAB@BDB@@@@BBCCCCBA?;677.7>775:==9@8998A94/-,---+
@T6XXI:00081:00404
GGGAGAAGGGAGAAAGGAAGCTAGTCACTCGACATCTGTGGCTTCACAGTACAGAGGGACTGTCCAAAGCCGCAGAT
CTCGAGTCGACTAG
+
DDBBAA69;7;A@@<B:;8;CA@?A7::??A:::::7ACC;?@?BBBBCCDCA?<557<>??CC>AA9===?@@@@?
@@@@<??A?@@AAA
@T6XXI:00081:00405
GGGAGAAGGGAGAAAGGAAGCATAGCCCGGTAGGGAATTCGTGTCGGCTATTCTCCCTCTCCCTATAGTGAGTCGTA
TTAGAATTCCCC
+
CCBB;;244194885=<A?B8;8BB@@;@@C<<?9??A8;9>9949649999:444-
499BC>BBAAA?>999=776>9658552444+
@T6XXI:00081:00417
GGGAGAAGGGAGAAAGGAAGCTATCGACTCGACATCTGTGGCTTCAACAGAGGGACTGTCCAAAGCCGCAGATCTCG
AGTCGACTAGGGGGAATTCTAATACGACTCACTATAGGAGAGGGAGAATAGTCAACAAATCGTTTGGGCGACT
+
CCACBB@BB<:44409;>=@CCB<;7/000::0./76>BC7<<BAB;==6677.79988@;<=;<<;@??==<@?><
<<??=:;;44444,49;7<777:/./;;;:B@;;<8724;44718=B=@@@@=<9<?=7;;444-44-49488
@T6XXI:00081:00423
GGGAGAGGAGA
+
11-////,,,/
@T6XXI:00081:00424
GGGAGAAGGGAGAAAGGAAGCTAGTCGACTCGACATCTGTGGCTTCACAGTACAGAGGGACTGTCCAAAGCCGCAGA
TCTCGAGTCGA
+
CH?@@>599194?@<A?B?BB@;;:/.....336616188>5;<@CDBBACC@;;444-
4;;>@@>AC<@@?@888@@@@A>==@AAA
@T6XXI:00081:00432
GGGGAATTCTAATACGACTCACTATAGGGAGAGGGAGAATAGATCACGTAGCGTTCGTGTGTCGATTCTCCTTCCTT
TCTCCCTTCTCCC
+
DKK:C?B?ABB?BBBBBB??BCB97111.777==9?@@>AA???BC@@=<??AA>AC@@A@@@BBA?D??9;8;7;;
7==<C<@=??@C;
@T6XXI:00081:00434
CTAGTCACTCGAGATCTGCGGCTTTGGACAGTCCCTCTGTACTGTGAAGCCACAGATGTCGAGTCGACTACTTCCTT
TCTCCCTTCTCCC
+
<:1//:/44?BA?CCDBBBC@CBB>B@AA@BBB@;AA>@@@@B@@@A<?B?CB@@@BECECBB;<<BBBCBB?B@EE
>CBBB>A??AAA:
@T6XXI:00081:00436
GGGAGAAGGGAGAAAGGAAGGAGAAAGCACCCAAGGCGTTAGATGGATCCGTCTTATTCCCTCCCCTCCCTCAT
+
BB@CCC@?=5=988-95968186;6+/00.636,.,,,.3.,,,2000>+/+6774658303400+00423333
@T6XXI:00081:00438
GGGGAATTCTAATACGACTCACTATAGGAGAGGGAGAATAGTCAACAAATCGTTTGGGGCGACTTCTCCTTCCTTTC
TCCCTTCTCCC
+
CCC:B>B>?BB@BA>@@BBBA:665..,0<;>@>AAA>AAAAAA<??A<CCCCC>CCC7?@CBC@CCC>=;=>AA<@
@@A<A;==BA=
@T6XXI:00081:00439
GGGGAATTCTAATACGACTCACTATAGGGAGAGGGAGAATAGCCTATTTCCAACCCACAGCCGCATGCTCTTCCTTT
CTCCCTTCTCCC
+
DCB9B>A>ADE@CCBBBBBBB@>96116.7?>@B>BBC?@<<=;?77646:;:?A7=748:7:BBDC?898?A3881
86?@<A>ABAA;
```

@T6XXI:00082:00412
GGGGAATTCTAATACGACTCACTATAGGGAGAGGGAGAATAGATCCATTTTTCTATTCGATTTCCTCACACTTCCTT
TCTCCCTTCTCC
+
??:2?<A=A@B>BCABBB;<;B??=777.:>BBC@A?A>B@A@@B=77666,6=?==?>444-
47:::;;::8:/44,434;,42403/
@T6XXI:00082:00433
GACTCGAGATCTGCGGCTTTGGACAGTCCCTCTGTACTGTGAAGCCACAGATGTCGAGTCGCTCCTTTCTCC
+
///4B9449444;9?>BCCBB@CCBBAAC<;;;?7:7=AA?A=@@=?F;;:C;::B@>;>0//-,++*++-,
@T6XXI:00082:00438
CTAGTCGACTCGAGATCTGCGGCTTTGGACAGTCCCTCTGTACTGTGAAGCCACAGATGTCGAGTCGACAGCTTCCT
TTCTCCCTTCTCCC
+
=994444/,2,,,,,,2/449239?:;899;99>B<AAABBBBBEB@?;?BACCCA@;;;A@@A:<<B?A@:94:=>
>9??==6849033,
@T6XXI:00082:00440
GGGAGAAGGGAGAAAGGAAGGAGAAAGTAGCGTCCATTAGGATCAGACTATTCTCCCTCTCCCTATAGTGAGTCGTA
TTAGAATTCCCC
+
DIADAA=@C=BBCC=B?B@C@CCCC>AAACBCCC@CDBCC@CBBB<<<DCB>B???9?BABC>B@@===ABCB@@@?
?=?AA=:8:CC6
@T6XXI:00082:00448
GGGAGAAGGGAGAAAGGAAGAGAATAGGCACGCATGTAAAATGGTAACTATTCTCCCTCTCCCTATAGTGAGTCGTA
TTAGAACTTCCC
+
6=6<<@5991944;-
429<7779@CCC@;;:<;@?@@@CC7@B=::56??677177,100084:8<<@@;887/./.4086=8:;3444
@T6XXI:00083:00401
GGGAGAAGGGAGAAAGGAAGTGCAGTTCGCGGTTAGTGAAGGGAAAAACTATTCTCCCTCTCCCTATAGTGAGTCGT
ATTAGAACTTCCCC
+
DCBDBC=>=5@@@@<@?B?BCBCCCC@CBBB?B?888AC?AB>BCCC2CBBB?????;B?B??;@<<<<<===<@<7
888;;?59:>ACC5
@T6XXI:00083:00406
GGGGAATTCTAATACGACTCACTATAGGAGAGGGAGAATAGCCGTTTAATCTCCCACTGCGGCATATCCTTCCTTTC
TCCCTTCTCCC
+
CEE9C?C@DBB@CBBBBBBBB6445..,0>>AK@D@@@A=@=;?AA7<9<?@@@;@A=858>A@@A?=8;8949;:=
=?>7?;=6882
@T6XXI:00083:00413
GGGAGAAGGGAGAAAGGAAGCTAGTCACTCACTCTGTGGCTTCACAGTACAGAGGGACTGTCCAAAGCCGCAGATCT
CGAGTCGACTAGCTTCCTTTCTCCCTTCTCCC
+
BC@><<89>9>=BB>C@CAC@???0000.....54403/0136667434888343+304356;==;><;977777;A
=<==?>>@===;::7:344-8???;@9776:2
@T6XXI:00083:00425
GGGAGAAGGGAGAAAGGAAGGATATGCCGCAGTGGGAGATTAAACGGCTATTCTCCCTCTCCCTATAGTGAGTCGTA
TTAGAATTCCCC
+
9<6;BA@CFABA@C>B?B?BACCBB8:9;CC<;:D@CCAA?BC>CB@CCCC=A@BB<9;;??<@DCBBACCCCAAA8
858AA<?:?CC5
@T6XXI:00083:00436
GGGAGAAGGGAGAAAGGAAGGAGAAGTCGCCCCAAACGATTTGTTGACTATTCTCCCTCTCCCTATAGTGAGTCGTA
TTAGAATTCCCC
+

```
CD@BBAAAA=BCBC>C??>?@BBB?@@BCABB9BE?BBBBBBBB@BBCBAA:=?==;<8==A<AA@@@@AA@=?A@?
><@=;8:2444-
@T6XXI:00083:00437
CTTTGGACAGTCCCTCTGTACTGTGAAGCCACAGATGTCGAGTCGACTAGCTTCCTTTCTCCCTTCTCCC
+
DDFBC=@ACBBBA;BBB@?A?BBBBB?@@@BDA@@@>AA?ABDCBBBCC@?BBB@AA@BBBB@A?@@@@=
@T6XXI:00084:00410
GGGAGAAGGGAGAAAGGAAGGAGAAGTCGCCCCAAACGATTTGTTGACTATTCGTCCCTCTCCCTATAGTGAGTCGT
ATTAGAATTCCCCC
+
/1-//4244-444;-
469>>=?>A@B:;:>99,77/36111,16368855553005535 6::=555<@::::866655555644421466.
@T6XXI:00084:00417
GGGAGAAGGGAGAAAGGAAGGAGAAGTCGCCCCAAACGATTTGTTGACTATTCTCCCTCTCCCTATAGTGAGTCGTA
TTAGAATTCCCC
+
CC>CEB@CB>??@B>C@C>@=@CC@C@?3333,8>8?BCDD>BB=BBCAAA>AAAB=@@@@A<A???AAAAAAA???
??@@?<=3488-
@T6XXI:00084:00418
GGGAGAAGGGAGAAAGGAAGCTAGTCGACTGACATCCTGTGGCTTCACAGTACAGAGGGACTGTCCAAAGCCGCAGA
+
9959@A59918A@@AC;;57>80.3/...20002,,*,,,22..-388769??973?=4=?=73356636433++++
@T6XXI:00084:00426
GGGGAATTCTAATACGACTCACTATAGGGAGAGGGAGAATAGCCACCTGTTTGTGGTTGAGCTATCTCCTTCCTTTC
TCCCTTCTCCC
+
449/9<@ACDB@B@@ABBBBBA>97111.7==?B?BAA?@998976,/---+-
44:C>???@BC???>;9:;:;;:::;;;=99=<<;
@T6XXI:00084:00433
GGGGAATTCTAATACGACTCACTATAGGGAGAGGGAGAATAGCCTAGTGATCTCCCACAACGTAACGTTCTTCCTTT
CTCCCTTCTCCC
+
:99/9<@=ABC@CBAABBBBBAB97111.7=<BC>CAA@?<??;@@BCB??=::777/../>>>=?>C@AA>A@CC>
BBBC9:8:444-
@T6XXI:00084:00439
GGGGAATTCTAATACGACTCACTATAGGGAGAGGGAGAATAGCCCGTGATTTTACCACATGGCCTATGCCCTTCCTT
TCTTCCTTTCTCCCTTCTCCC
+
CCDDD@B?BDBDCCCCCBBBB@<97111.777>?=C??=@???A=A@>@BCD9BB@@@BAA>A@?BBCDB6;6:<>?
=AA?B?CB>BCEE>B<?=BD<
@T6XXI:00085:00400
GGGGAATTCTAATACGACTCACTATAGGGAGGGGGAGAATAGTCTGCCAATTTTGGGCGGTACTGTCTCCTTCCTTT
CTCCCTTCTCCC
+
DCC9B9;;<BBACCBBBB???@?97111-
6??BE1@@B@DADB>>>@>B@AAA8>>@CA@>BCCC@?AC?B?B@GG>@@BB<B=@@BC>
@T6XXI:00085:00410
GGGGAATTCTAATACGACTCACTATAGGGAGAGGGAGAATAGCATTCGCTTAAATTCGTCCCAGCTACTCTTCCTTT
CTCCCTTCCTCCC
+
AEC6A>A@@@@ABBAAECBB@?887777-:BBCB>BAC=A==<?=;=;::244-
448344433308666999655533331227,/,-222
@T6XXI:00085:00438
GGGAGAAGGGAGAAAGGAAGGAGAAGTCGCCCCAAACGATTTGTTGACTATTCTCCCTCTCCCTATAGTGAGTCGTA
TTAGAATTCCCC
+
```

```
BA:@@@;>><@@@C=B?B@C?DCC@BBCBECE9BC>AB9:1,1=:??A???=??BC>A==<?7<<==ABA@??7888
83044/0/004-
@T6XXI:00086:00400
GGGAGAAGGGAGAAAGGAAGCTAGTCATCGACATCTGT
+
,,*,6859>-94==0424233?>317761111.33..,
@T6XXI:00086:00438
GGGGAATTCTAATACGACTCACTATAGGGAGAGGGAGAATAGCTCCCAACTGACTACAGTATAAATTCACTTCCTTT
CTCCCTTCTCCC
+
BCC9>698<@@ACCCBBABCB@@?7611.777776:>@>AAAAAAA<A<?;;BB???A@AA@@A<A>@@@?>A7::5
:@@B>B@CABA>
@T6XXI:00086:00440
GGGGAATTCTAATACGACTCACTATAGGAGAGGGAGAATAGTTTTCCCTTCACTAACCGCAAACTGCACTTCCTTTC
TCCCTTCTCCC
+
CCN:D>@>@CC@CBBBBBBBB>;;96107>>>A@BDC?A=<66648=;=?>?@BCA@>@???;<BB@BA?97137/8
??B=B?A???<
@T6XXI:00086:00447
GGGAGAAGGGAGAAAGGAAGCTAGTCGACTCGACATCTGTGGCTTCACAGTACAGAGGGACTGTCCAAAGCCGCAGA
TCTCGAGTCGACT
+
CD@BBC@BB>?ABB>B?B@CCA>>>0//;;;:>???BACBB?AC@ACCBBBB<8<73317B==?A>@@<@A>@AAAA
888?====;<<<=
@T6XXI:00087:00401
GGGAGAAGGGAGAAAGGAAGCTAGTCACTCGACATCTGTGGCTTCACAGTACAGAGGGACTGTCCAAAGCCGCAGAT
CTCGAGTCGACTAG
+
CD@CFC>??<@A@@<@=>>BBAB@?8;::?@:::>??BCC?AA@BCCC@@B@9933313=9?>@96<7<><?A<A==
<??::;<<@A<@@@
@T6XXI:00087:00406
GGGAGAAGGGAGAAAGGAAGGACAGGAGACTGCAGAATGTGCCGTAACTATTCTCCCTCTCCCTATAGTGAGTCGTA
TTAGAATTCCCC
+
CC>AB@@BB====A?A@A@?>???>>>>>>>???>?>AC@@C@CFCACCCF@BAAA<@BBBB?BBBAAABAAA@@?C
C@CCD@C?AAA6
@T6XXI:00087:00411
GGGGAATTCTAATACGACTCACTATAGGGAGAGGGAGAATAGTCAACAAATCGTTTGGGGCGACTTCTCCTTCCTTT
CTCCCTTCTCCC
+
CCC9C?C@CBB@B@A@BAAAB?<97111.7=7777;==?A==<@@>AAB>CBBAA<A@@7@BCCC?BBB>=9=<@@<
<AAA<@378;;6
@T6XXI:00087:00418
GGGGAATTCTAATACGACTCACTATAGGGAGAGGGAGAATAGCCCCCTATACTCTACTCCTCACCACTCTTCCTTTC
TCCCTTCT
+
999/429<>>@=@BBCCBA>>?CCCCCC==??BC<?<;844499990?@@BB@@@AAAA@@A;?9<<?:878,//,/
--303033
@T6XXI:00087:00428
GGGAGAAGGGAGAAAGGAAGGAGAAGTCGCCCAACGATTGTTCGAATC
+
BC>;:<@CE@=<;B8;8;>A<@@@;913779,3131166672116711
@T6XXI:00087:00444
GGGGAATTCTAATACGACTCACTATAGGGAGAGGGAGAATAGTCAACAAATCGTTTGGGGCGACTTCTCCTTCCTTT
CTCCCTTCTCCC
+
```

```
BAA7A<A>BBB@CBBBAAAAA@997111.777=?=@BB?BABBAC<9994?AABC>AAB9BBBB@?>BC>?<?>@@<
@@CCAC378AA<
@T6XXI:00087:00445
GGGAGAAGGGAGAAAGGAAGCTAGTCACTCGACATCTGTGGCTTCACAGTACAGAGGGACTGTCCAAAGCCGCAGAT
CTCGAGTCGACTAG
+
FC>BCC@BB<>@A@<@=@@CCAABAA>===>00/55///A@?A?ACEAAABB;;77718;;??@<?C<@@=?AACC=
9=?778=;<<<<<?
@T6XXI:00087:00446
GGGGAATTCTAATACGACTCACTATAGGAGAGGGAGAATAGTCAACAAATCGTTTGGGGCGACTTCTCCTTCCTTTC
TCCCTTCCTCCC
+
:;;2;2959449;=@BA;;;A:<=9//,..7=><:77>@@@CCB>???;A@@??6000+00,,,*,05,--
.444577;A6;2724;9,
@T6XXI:00088:00409
GGGGAATTCTAATACGACATCACTATAGGAGAGGGAGAAATAGTGACGTGTTGTGCGGCGCTCACTGTCTCCTTCCT
TTCCTCCTTCCTCCC
+
9>>4>61,/88,1,,,/.666.631166011111,100440000000466=:9=<AA>???AA@???A@@A>A;=;=
B?@>=;34//,/133
@T6XXI:00088:00410
GGGGAATTCTAATACGACTCACTATAGGAGAGGGAGAATAGTCAACAAATCGTTTGGGGCGACTTCTCCTTCCTTTC
TCCCTTCTCCC
+
CCB9C@CBCDC@BBBBBBBBB:997//-
0<<>A>B@D>A:??@A?AAA<C??<?8???6?@ABB?BCC@@>@?AA<A@@?<@>@@AA<
@T6XXI:00088:00413
GGGAGAAGGGAGAAAGGAAGCTAGTTGACTCGAGATCTGCGGCTTTGGACAGTCCCTCTGTACTGTGAAGCCACAGA
TGTCGAGTCGACTAGCTTCCTTTCTCCCTTCTCCC
+
:;094@<@@=BBBH?C@C@A@@CCB5:7=77677766667;=?BB>B???@CCBB>@=<=@>??><<<9=<>@@A::
/;;;0.,35+/-///442/47606888,/,/2222
@T6XXI:00088:00417
GGGAGAAGGGAGAAAGGAAGGAGAAGTCGCCCCAAACGATTTGTTGACTATTCTCCCTCTCCCTATAGTGAGTCGTA
TTAGAATTCCCCAATCGAAT
+
CD@CCA@CC>??@B>B?B?A?AAC@???BBCC8AA=@CEC@;=>>@@=9=A>AAAA<A??<=;<<@==?@AA99<@A
B>A@@>@>=;;4855.*+)*
@T6XXI:00088:00428
GGGGAATTCTAATACGACTCACTATAGGGAGAGGGAGAATAGTCAACAAATCGTTTGGGGCGACTTCTCCTTCCTTT
CTCCCTTCCTCCC
+
DID9C;<8<AA=@@BBB:;;B?>>>777.777?@8?4733?==?A>@?A<B;<;C>CCB8BBBCC>AAC@@9>344-
4400+0/02488:
@T6XXI:00088:00432
GGGAGAAGGGAGAAAGGAAGAGAATAGGCACGCATGTAAAATGGTAACTATTCTCCCTCTCCCTATAGTGAGTCGTA
TTAGAAT
+
CB?BCB@BB?BBAA=B>@>BBAA@BB@D??ACAAAAABBB8??>??>?===ACCCC>B<<<<;<@@@@?AAAA<@@=
=<?@A?=
@T6XXI:00088:00435
GGGAGAAGGGAGAAAGGAAGCTAGTCACTCTGCGGCTTTGGACAGTCCCTCTGTACTGTGAAGCCACAGATGTCGAG
TCGACTAGCTTCCTTTCTCCCTTCTCC
+
CECCCC?CA<>>>@9>;A@;;<C???=<:<>>==>8664855;B?;;;6<;<>@@888?8838<?AA@@<<<8<;BC
C@ACEC@??996,11+1.00,0,0354
@T6XXI:00088:00437
```

```
GGGGAATTCTAATACGACTCACTATAGGGAGAGGGAGAATAGTCAACAAATCGTTTGGGGCGACTTCTCCTTCCTTT
CTCCCTTCTCCC
+
CCC7A?B@BCD@B@@>BCCB?C@97111.7>==?<@AA?A:?????AAA<CAAAC>C@?6AACCC?CCC:848>@@;
@CCCAC689AA<
@T6XXI:00088:00438
GGGGAATTCTAATACGACTCACTATAGGGAGAGGGAGAATAGTACGACCAA
+
?BD9B=?=ADCDBB@?BABABCB>?661-6=666.7;;978.**+***)+0
@T6XXI:00088:00449
GGGGAATTCTAATACGACTCACTATAGGGAGAGGGAGAATAGTCCGAACTTCGCCCTCACATCTATTACTCTTCCTT
TCT
+
=994?5959449;8A@@@@@B@BB@??=6<>677.7;;;==;;9-//.0.44334-.0355378;;9//-
//,+00000+
@T6XXI:00089:00400
GGGGAATTCTAATACGACTCACTATAGGGAGAGGGAGAATAGTCAACAAATCGTTTGGGGCGACTTCTCCTTCCTTT
CTCCCTTCTCCC
+
CBB9B?BACCC@BBBBBBBBB@997111.7>>>ABC@C?@9>AAC<:<<;AA?==-666-
7==?B@BBL@@>@<?>7?@?@<@=??CA:
@T6XXI:00089:00413
GGGGAATTCTAATACGACTCACTATAGGGAGAGGGAGAATAGTCCTGTGCATTTCTCCCAGCACACCGTCCTTCCTT
TCTCCCTTCTCCC
+
C??6C>B>BCB@BCB?>@@B;9;@?77:6:??BD>C@@>@@@AB>===@??AB;=;;;7<=?@@AA>===;=:;388
1/9976;00/;@9
@T6XXI:00089:00417
GGGAGAAGGGAGAAAGGAAGCTAGTCACTCGACATCTGTGATTCACAGTACAGAGGGATGTCCAAAGCCGCAGATCT
CGAGTCGACTAG
+
CC>@A@@CCACBB@9>492;@@BCBB@@@E???AA>;=;0//73344-----138;4<;633244-
3424448=?@A@AAAA===AA89
@T6XXI:00089:00431
GGGGAATTCTAATACGACTCACTATAGGGAGAGGGAGAATAGTCAACAAATCGTTTGGGGCGACTTCTCCTTCCTTT
CTCCCTTCTCCC
+
CDD9C@C@DDB@BBB@BBBBBA>97111.7>>>A=AAA@A9>9BA?AA;:>=>@E=@@@7@CCBA?<BB:<98:??;
@AAA<A=@@CB;
@T6XXI:00089:00443
GGGGAATTCTAATACGACTCACTATAGGGAGAGAGATATAAGGGCAGAAAAGACGTT
+
?:?2@69249;94444437779>ABBCC<=0000/.-3268:9433....*....-3
@T6XXI:00090:00402
TTCTAATACGACTCACTATAGGGAGAGGGAGAATAGTCAACAAATCGTTTGGGGCGACTTCTCCTTCCTTTCTCCCT
TCTCCC
+
BEDDC?CAABBBBBBBB99<78807>>>><ACC?BB@ACC@B??;?====;=<<5=@@@A<;==;=?C=?@7<<??;@
>A@@A=
@T6XXI:00090:00408
GGGAGAAGGGAGAAAGGAAGTGCAGTTCGCGGTTAGTGAAGGGAAAAACTATTCTCCCTCTCCCTATAGTGAGTCGT
ATTAGAATTCCCC
+
CHBBA@@BC>ABAC>B?A?AAAABBB@AABC@A@??>BB@@@>CCBB2CBBB@C@@@=BBBCC>A>>AA==:?;@A@
@@>@@@?A=ABB6
@T6XXI:00090:00413
```

```
GGGAATTCTAATACGACTCACTATAGGAGAGGGAGAATAGCTACGTTTTAACCGATCGTGCTGTTCTCCTTCCTTTC
T
+
:9148?=ACCDCCCBB@B@BABB;665:??CE>B???B=846667711,100/0644777?:;;::::,/,-,.-+-
0
@T6XXI:00090:00424
CTAGTCGACTCGAGATCTGCGGCTTTGGACAGTCCCTCTGTACTGTGAAGCCACAGATGTCGAGTCGTAC
+
CC:<<<9<DIICBCCBBBC@?=@AB<C8<<BBAAA?ACCCC>??@@;<;<B@BBBBBB<??@@A70((((
@T6XXI:00090:00430
GGGGAATTCTAATACGACTCACTATAGGGAGAGGGAGAATAGTCAACAAATCGTTTGGGGCGACTTCTCCTTCCTTT
CTCCCTTCTCCC
+
@@@6@?C@CBB@BBBBBBB<<E?97111.7>>>A>BBB>@<<<@>=?AC:?>???;@@@7>>C?C?@CE>=;=<AA<
A@AA<A689AA:
@T6XXI:00090:00440
GGGGAATTCTAATACGACTCACTATAGGGAGAGGGAGAATAGTCAACAAATCGTTTGGGGCGACTTCTCCTTCCTTT
CTCCCTTCTCCC
+
BDC9B9;;<AA?DCBBC@@@CA>>=767-
777=<8?::<=ABAAA>A@?1::>=>6==?2=>>7747774424;>A<=94438./+356
@T6XXI:00090:00442
GGGAGAAGGGAGAAAGGAAGTGCAGTTCGCGGTTAGTGAAGGGAAAACTATTCTCCCTCTCCCTATAGTGAGTCGTA
TTAGAATTCCCC
+
CD?CCB@BB?@@>=<@?B?BCCCCCB@BBB@@C@A@ACC?AB>ACC8BBBB@CCCC;B@CBC>B<<<;=<<AAAA??
?<??@?A=@DC6
@T6XXI:00091:00407
GGGGAATTCTAATACGACTCACTATAGGGAGAGGGAGAATAGCCACAGCATCATCTCCTTCGCGCTTTTCTTCCTTT
CTCCCTTCTCCC
+
BAA9A>B@CDB@BBBBBBBBB@<96111.7>>AB?C@AAA>==;=@==<A???@??????<<<<====558<?9<=7
=BAA<A>A@@=<
@T6XXI:00091:00410
GGGAGAAGGGAGAAAGGAAGTGCAGTTCGCGGTTAGTGAAGGGAAAAACTATTCTCCCTCTCCCTATAGTGAGTCGT
ATTAGAATTCCCC
+
BD>A@9;>>:>@AA<A?B?AABAA@H@CB@@6:68>>>?==A.77:7,8???=?BBA<?????<???CBBBA??@@A
??<?AA;=5;<;1
@T6XXI:00091:00419
GGGGAATTCTAATACGACTCACTATAGGGAGAGGGAGAATAGCCCAACAATCTCCTCGATC
+
9BA/969249><?BDBCDCCC???7::>7>>711,100244000+-,-77////4434515
@T6XXI:00091:00422
GGGAGAAGGGAGAAAGGAAGTGCAGTTCGCGGTTAGTGAAGGGAAAAAACTATTCTCCCTCTCCCTATAGTGAGTCG
TATTAGAATTCCCC
+
CGDAAA@CC@CCCC?C@C@AAABACB@BBBC???AABBA<;<-
76666);CCCCDAAB>ABACC====@@@AAAA=:????AA=:?<?AA6
@T6XXI:00091:00425
GGGGAATTCTAATACGACTCACTATAGGGAGAGGGAGAATAGTCAACAAATCGTTTGGGGCGACTTCTCCTTCCTTT
CTCCCTTCTCCC
+
999-
9<A>BBB@CCA??BBBB@;97111.7>>AC>C@A?@88;;;:99=;AA@>>;>@@8@CCAA??CC?@=@=AA<=@@@
<@<??AA<
@T6XXI:00091:00431
```

GGGAGAAGGGAGAAAGGAAGCTAAGTCACTCGACATCTGTGGTCACAGTACAGAGGGACTGTCCAAAGCCGCAGATC
TCGAGTCGACAGCTCTTTCTCCTCTCCC
+
B;6@9968>9=49904244.:00,,0**,,,2236656767311446==@?>><<;5;;;56<;==;@A>?8//;//
/;;:=?==?=834..+000++++-1110
@T6XXI:00091:00443
GGGAGAAGGGAGAAAGGAAGGGCAAAAACCTTGAGAATAGTTAGCAGCTATTCTCCCTCTCCCTATAGTGAGTCGTA
TTAGAATTCCCC
+
BF@CBC???:=@@@:==@?BCACABBB2B@C@CBBB@BBBB@BBCCCCAAA@CBBB?AAAEEBDCBBBBBBABBFCD
N?BBB?EDDDC>
@T6XXI:00091:00444
GGGGAATTCTAATACGACTCACTATAGGGAGAGGGAGAATAGCCTAGCTTTGTACTACCGGCTGTTCGCCTTCCTTT
CTCCCTTCTCCC
+
DCC:D@D@BCB?ABBBBAA?A>>9;177.7==?A<A>?>@<==>???AB@8??@A=<<:=>AA@@>AAA>?<?>AA:
;777-4344<<6
@T6XXI:00091:00446
CTAGTCGACTCGAGATCTGCGGCTTTGGACAGTCCCTCTGTACTGTGAAGCCACAGATGGACTGCTTCCTTTCTCCC
TTCTCCC
+
CC@AABBDDCCAAACCBBBCB@8998;=@@CBBCC?BBBB>>>?@C@@>@C@CBBBC@@=ECCBCC7:7:B:>???9
?=??=A<
@T6XXI:00092:00413
GGGAGAAGGGAGAAAGGAAGGAGAAGTCGCCCCAAACGATTTGTTGACTATTCTCCCTCTCCCTATAGTGAGTCGTA
TTAGAATTCCCC
+
CE@B==@BC>>@>B>B?B?B?BCIB@BBBBCC9CC>CCBCC>C@?CC@A@<;<===;:999;;===899=@A?@@@@
@?A@@<>8;;;0
@T6XXI:00092:00430
GGGGAATTCTAATACGACTCACTATAGGAGAGGGAGAATAGTTCGCCAATATCCCGCGGCGTGTTCTCCTTCCTTTC
TCCCTTCTCCC
+
A@?4>6;:<BBACBBBCEC@@>=;:/0,0<:=?<?AB@AAAA=@@A<A:@=6664:=?>A@@=@?@??<??C>AA<@
<<;6999:<;6
@T6XXI:00092:00434
GGGGAATTCTAATACGACTCACTATAGGGAGAGGGAGAATAGTTACCTTCTCACGTATGTTGGCATTTTCTTCCTTT
CTCCCTTCTCCC
+
B??7?598;A@@CCBBBBBBBBB9>6665:@>BC>B@@>@<@??BA?A8<=AA@BBBBBB=?>@CEDC5AB@A;<<;
<@@A<A>A@@@<
@T6XXI:00092:00435
GGGGAATTCTAATACGACTCACTATAGGGAGAGGGAGAATAGTCAACAAATCGTTTGGGGCGACTTCTCCTTCCTTT
CTCCCTTCTCCC
+
CCB4@5969>>;@?CBBAAA@?>??777,7>>>><@?@=889AAA@@AA=CBBBA<ADD8CAABC@BBB??<?>@A;
@@AC<A>A???:
@T6XXI:00092:00439
GGGAGAAGGGAGAAAGGAAGCTAGTCACTCGACATCTGTGGCTTCACAGTACAGAGGGACTGTCCAAAGCCGCAGAT
CTCGAGTCGACTAG
+
BB<@?928809998096958@??????=@@@:;:???A@@;?@=?CCCCBA@=933313;@BAC=AA:AA?A?<<==
====<??AAAA@@A
@T6XXI:00092:00440
CTAGTCACTCGAGATCTGCGGCTTTGGACAGTCCCTCTGTACTGTGAAGCCACAGATGTCGAGTCGACTAGCTTCCT
TTCTCCCTTCTCCC
+

```
A@1/1=8==DCCCDCC?@AB?BCC@D::;A@:::5:??C@??CA??A=AB@ABBAA@@@AAABBBBA@@@B??=@BB
C>BAAA<=9=<;<7
@T6XXI:00092:00442
GGGGAATTCTAATACGACTCACTATAGGGAGAGGGAGAATAGTCAACAAATCGTTTGGGGCGACTTCTCCTTCCTTT
CTCCCTTCTCCC
+
CCC9B?C@CBBA@?@BBBCBB?<97111.7>>BC@DAA>A@A===>@@B?BBBBD<BBB7BBBBC?AAA7837:::7
=;996;8<<AA?
@T6XXI:00092:00443
GGGGAATTCTAATACGACTCACTATAGGAGAGGGAGAATAGTGACGTGTTGTGCGGCGCTCACTGTCTCCTTCCTTT
CTCCCTTCTCCC
+
?JA9@3;59992999;A?><BCBBB:937===?<?AAACAC999887;<<?AA@A@A888;<===??<<9<=A;==7
9444-434:9;9
@T6XXI:00093:00432
CTATCACTCGAGATCGTGCGGCTTTGGACAGTCCCTCTGTACTGTGAAGCCACAGATGTCGAGTCGACTAGCTCCTT
CT
+
/::49444944/6:211==98==@;@888@A@CE8:::A?:<<=669239>@>@>CCC:;:A@@?8004.....,.5
53
@T6XXI:00094:00409
GGGAGAAGGGAGAAAGGAAGCAGTCCATCTGTGGCCTTCCACAGGT
+
CC>@@@5991949914242333111011166663636 00/034425
@T6XXI:00094:00417
GGGAGAAGGGAGAAAGGAAGCTAGTCGACTCGACATCTGTGGCTTCACAGTACAGAGGGACTGTCCAAAGCCGCA
+
CC>AA>?AA<BBBB=B@C@CCA=?BCBBBBA???9777>>@?BB?BB??CBC@A?33317=;?@A>AA<??<?@?
@T6XXI:00094:00420
GGGGAATTCTAATACGACTCACTATAGGGAGAGGGAGAATAGTCAACAAATCGTTTGGGGCGACTTCTCCTTCCTTT
CTCCCTTCCTCCC
+
DDC9B>B>@AC?BBCC@?@C@:??;666.7@>@B>C@A>?:?AA=<8>A<CCCB@9???6?CCCC?AAA?@>?>AA?
@@@?:?8986655
@T6XXI:00094:00422
GGGAGAAGGGAGAAAGGAAGAATACGGTGGGGCTGCTTAGTTAACAGCTATTCTCCCTCTCCCTATAGTGAGTCGTA
TTAGAATTCCCC
+
BBCBCB@DC>BBBB>B@B@BB@BACB@>==>6@BCCB@CCB@B@BBB?CCCACBBB?BBBAA=CABB??@?@???<;
=?=<::>;>>?5
@T6XXI:00094:00429
GGGAGAAGGGAGAAAGGAAGCTAGTCACTGACATCTGTGGCTTCACAGTACAGAGGGACTGTCCAAAGCCGCAGATC
TCGAGTCGACTAG
+
:@5=>=<>@:?=>>9>594888==?77761111769999><@=@CA@A?:77?44814;;;8838B-
4/65//.77====:::<===<;6
@T6XXI:00094:00435
GGGAGAAGGGAGAAAGGAAGCTAGTCGACTCGACATCTGTGGCTTCACAGTACAGAGGGACTGTCCAAAGCCGCAGA
TCTCGAGTCGACT
+
CD>BAA?ACBABAC@B996;B87;A:::BBBA:;:::7???:?A?BBBCBBC??<<<<:<;:==?;??7>A>A@?@@
AA???:??@A===
@T6XXI:00094:00436
GGGAGAAGGGAGAAAGGAAGCAGAAAACAGTGCACAACAGCTGTGGACTATTCTCCCTCTCCCTATAGTGAGTCGTA
TTAGAATTCCCC
+
```

GCBCBE@BC>ABB@:=;@=?AB@AAA7BCCCBB@@@>??ABCCBB@CCCBC@CCCG?CC@??8?B@@@@CCCBBBBC
B?BAB;>3::7.
@T6XXI:00094:00437
CTAGTCACTCGAGATCTGCGGCTTTGGACAGTCCCTCTGTACTGTGAAGCCACAGATGTCGAGTCGACTAGCTTCCT
TTCTCCCTTCTCCC
+
BA999>===ABBBAACCCCB?CCC?B??B???@A<ABBBBBBB?<<@>@C@CCCABCBABCDDCCBBBCD@???B=B
B676=>.424;==:
@T6XXI:00094:00439
GGGAGAAGGGAGAAAGGAAGGAGAAGTCGTCCCAAACGATTTGTTGACTATTCTCCCTCTCCCTATAGTGAGTCGTA
TTAGAATTCCCC
+
FC@BAA@BB>BBBB>B999;@CCC>AACC@AA>AB>BDCC@>@@@C?@ACDACCCC>BBEBB=BCCBBBCCCC@???
?<?@@<?;@AA5
@T6XXI:00094:00444
GGGAGAAGGGAGAAAGGAAGGAGAACGCGCACCCGAACCTACGGTGATCTATTCTCCCTCTCCCTATAGTGAGTCGT
ATTAGAATTCCCC
+
CC>@A@?A@<BBBB?B@C@B@B<<;ABAA@:;;566,/,///87@ABAA@?@BB=@@<@CBCC>CCBCCCCBBBBBC
CC@CCC@CBCCC:
@T6XXI:00095:00402
GGGGAATTCTAATACGACTCACTATAGGGAGAGGGAGAATAGTCAACAAATCGTTTGGGGCGACTTCTCCTTCCTTT
CTCCCTTCCTCCC
+
999/98><@>=;@@@@@BAA@@@CC???6;=6665:?;=@@@A@=====7?AAAC>CBE7BCCDCABA@:;343340
532201,-,-222
@T6XXI:00095:00405
GGGAGAAGGGAGAAAGGAAGCACATCGCGCGGAGGCTAACGGGGAGACTATTCTCCCTCTCCCTATAGTGAGTCGTA
TTAGAATTCCCC
+
CC@CBA@AA=@@AB@B=@>AACCCCCBB???>AA=AB@?@@AA5???C@>?>@@AA:==733-
4:9;@AAAA=:??;;8;=><;AAA5
@T6XXI:00095:00409
GGGGAATTCTAATACGACTCACTATAGGGAGAGGGAGAATAGTCAACAAATCGTTTGGGGCGACTTCTCCTTCCTTT
CTCCCTTCTCCC
+
CEC:C>?=ACC@CCBBB@??B?996166.7??AB>BAA>@===AA<??@<CCBB@9??<2=>@AA?ACC?@=?=?A<
AAAA<A899@A<
@T6XXI:00095:00411
GGGAGAAGGGAGAAAGGAAGGAGAAGTCGCCCCAAACGATTTGTTGACTATTCTCCCTCTCCCTATAGTGAGTCGTA
TTAGAATTCCCC
+
CFAEEC>?>:@@===B?B?B@CBB>??BBBBB8AA=AAACC<??>?B>?@C?====;::><=;=:::;====88>?@
@>A@AAA>@@@5
@T6XXI:00095:00413
GGGGAATTCTAATACGACTCACTATAGGGAGAGGGAGAATAGACGTGTCATATCCCGATGCGTTGTCTCCTTCCTTT
CTCCCTTCTCC
+
BCC9B?B?ABB7;<CBBBBBCCBBBCCC>BBBCC>B@C>A@889?>>>?>@=<<<9=AABAAA>A99:@>@<A9==9
=A@A=A>?AA?
@T6XXI:00095:00425
GGGGAATTCTAATACGACTCACTATAGGAGAGGGAGAATAGATCACCGTTGGTTCGGGTGCGCGTTCTCCTTCCTTT
CTCCCTTCTCCC
+
BDC:D@B?BDE=@@BBBB>?@:997..-
0=>@C@C??=@=989=??:?C7:5:>@>@=B;;;CCC?BBA?A?A?AD>BA??;?????@;
@T6XXI:00096:00406

```
GGGAGAAGGGAGAAAGGAAGGGAAAACTGGGAGAAGGGAGAAAGGAAGTGCAGTTCGCGGTAGTGAAGGGAAAAACT
ATTCTCCCTCTCCCTATAGTGAGTCGTATTAGAATTCCCC
+
CC@CAA@BB>???B>B@B@BB<BBD8AA@B>?AC?@@;BBAA9B=A>?A=CCBB;@@BB:8:>>64244+0444,4:
:;<?CDC?BA???:===AAAAAAA???B@>AAC>@<@CC6
@T6XXI:00096:00414
CTAGTCACTCGAGATCTGCGGCTTTGGACAGTCCCTCTGTACTGTGAAGCCACAGATGTCGAGTCGACTAGCTTCCT
TTCTCCCTTCTCCC
+
>=1//11:=DDECCCBA@BB?BB?<B>A@;969:5:CCECCCC@??@><BABBBBBB?><8???BBAAAABCB?B?D
J@CAAB>?@===A7
@T6XXI:00096:00420
GGGGAATTCTAATACGACTCACTATAGGGAGAGGGAGAATAGTTCTCCCAGCTTGAGTTGTCAACGCGTCTTCCTTT
CTCCCTCTCCC
+
@@B/<8<?@>@=@@@B@BBC::8?<777.7>>>@>A@@>B@@@?888?;ABCC@?@@D@?<=@>?>>A@8848<;;9
;;;;,/--//,
@T6XXI:00096:00436
GGGAGAAGGGAGAAAGGAAGCTAGTCACTCGAGATCTGCGGCTTTGGACAGTCCCTCTGTACTGTGAAGCCACAGAT
GTCGAGTCGACTAGCTTCCTTTCTCCCTTCTCCC
+
@>:>@@=@B:>999095968A???B?????AA:8;@@BBB@CCC>A?@@CCC@C>B@@A@<@<<<;;;<<;<<=<<<
:<<==<==@<?==63878/44-4444-4:A<<<6
@T6XXI:00096:00437
GGGGAATTCTAATACGACTCACTATAGGGAGAGGGAGAATAGTCAACAAATCGTTTGGGGCGACTTCTCCTTCCTTT
CTCCCTTCCTCCC
+
?CA6B=A=@@B>BCCDBBBCBBA?=767.7>>AD?C???@@A@AD>B;=;EB777-666-
6=>@B=??A5738;;;6<93733,/,.--+
@T6XXI:00096:00440
GGGGAATTCTAATACGACTCACTATAGGAGAGGGAGAATAGTGACGTGTTGTGCGGCGCTCACTGTCTCCTTCCTTT
CTCCCTTCTCCC
+
CID:C>@>@BC@CAA@B@@@BCBB@772:A@@C>BAB?@<<><<=====><@@@A?==:??==98<:<<9<=@<@@;
@@AC<A=???A=
@T6XXI:00097:00410
GGGAGAAGGGAGAAAGGAAGCTAGTCGACTCGACATCTGTGGCTTCACAGTACAGAGGGACTGTCCAAAGCCGCAGA
+
DD@DCC@DC>B?>><@?@=@@B=?B@B>:777./000/77@>@A@BB@A@>@:8;34317?>?A@>@B;?>@AAA??
@T6XXI:00097:00420
CTAGTCACTCGAGATCTGCGGCTTTGGACAGTCCCTCTGTACTGTGAAGCCACAGATGTCGAGTCGACTAGCTTCCT
TTCTCCCTCTCCC
+
111,,,,//;;;;BBCBBBB??@?:?>887>777.77777<;;:00343;8;@@>8;;:<166:;<BBBB8:7;>83
30.../,/----+
@T6XXI:00097:00427
GGGAGAAGGGAGAAAGGAAAGCTAGTCGACTCACATCTGTGGCTTCACAGTACAGAGGGACTGTCCAAAGCCGCAGA
TGTCGAGTCGACTAGCTTCCTTTCTCCCTTCTCCC
+
CC>DAC@CB:?98809599-
3933397777131119<<9>9:@=@?ACBBBCB@?==:;778>>;>A:??>@@??@?<====DCC@?===A<;8;3
771/;9;6;000==9
@T6XXI:00097:00431
GGGAGAAGGGAGAAAGGAAGGGAGAAGGAGCAA
+
DKCCCC=>=:@@@A:@<>411,0==,0,.....
@T6XXI:00097:00434
```

```
GGGAGAGATGAGAGGAGGAGAGAAGAGAGCTAGATCGAGA
+
A=8*--,,,,//449431399810116661110033----
@T6XXI:00097:00438
GGGGAATTCTAATACGACTCACTATAGGGAGAGGGAGAATAGCTACGTTTAACCGATCGTGTTGTTCTCCTTCCTTT
CTCCCTTCTCCC
+
AA@7A=A=ACC?BB??ABBBA>;97111.7?>BC>B;6663+.//3336666555<=A<?8568@=;88385;=AB<
??AA<A<??AAB
@T6XXI:00097:00446
GGGGAATTCTAATACGACTCACTATAGGAGAGGGAGAATAGTCAACAAATCGTTTGGGGCGACTTCTCCTTCCTTCT
CCCT
+
999/959594824448=@@AC>;;//0,/00<68888::AA@@?=@@D?CB@??<?AA5>@A::5:>>:799,/,--
--+-
@T6XXI:00098:00408
GGGGAATTCTAATACGACTCACTATAGGGAGAGGGAGAATAGTCAACAAATCGTTTGGGGCGACTTCTCCTTCCTTT
CTCCCTTCTCCC
+
???5;7<CCDB@BB@@CBBB87:?;677.7??AD?D@@?A=A<@@>?@=;C>?>><BBB9CBAAC@??@>>:<9<<9
=AAD>A588AC<
@T6XXI:00098:00415
GGGAGAAGGGAGAAAGGAAGTGCAGTTCGCGGTTAGTGAAGGGAAAAACTATTCTCCCTCTCCCTATAGTGAGTCGT
ATTAGAATTCCCCCGGAGAAGGCAGAAAGGAAGGA
+
CDCBBA?BB>BCCC>D=@=@C>@@@@=@BBC@D@@@@CC@CB;ADDD2@<@A@CCCB=?AAC@;@<???:==AA?@@
@@<?@B9;/4444,4960155+,..6003333448
@T6XXI:00098:00418
GGGAGAAGGGAGAAAGGAAGGAGAAGTCGCCCCAAACGATTTGTTGACTATTCTCCCTCTCCCTATAGTGAGTCGTA
TTAGAATTCCCC
+
CD@CBB@BB>BBBC>B?B?A@BBB@@ACBBBB8BB=BBB@?;@?>@<<8<?>;;==:55<<=;=<:;;@<===<<:;
?>AAA>@=???5
@T6XXI:00098:00421
GGGGAATTCTAATACGACTCACTATAGGGAGAGGGAGAATAGT
+
;A?1>1111887<9<<BAADBDB??766.7====9?CC>@@@A
@T6XXI:00098:00423
GGGGAATTCTAATACGACTCACTATAGGGAGAGGGAGAATAGTCAACAAATCGTTTGGGGCGACTTCTCCTTCCTTT
CTCCCTTCTCCC
+
DEEAD74698899@CBCACCCBB?>777.7?>@A@BAA>AA@@CA>A@@;A???@<AAB7BB?AA=??C:<9=>AA<
A??A<B>@AAA:
@T6XXI:00098:00428
GGGAGAAGGGAGAAAGGAAGCTAGTCACTCGAGATCTGCGGCTTTGGACAGTCCCTCTGTACTGTGAAGCCACAGAT
GTCGAGTCGACTAGCTTCCTTTCTCCCTTCTCCC
+
CB>A<98<B<@AA@<@?B?BCB@@BACCCDDCD@CBCCCC?BCB>B?CCCB@@A>?;:;<=A=:??=<==<=<??A=
====<@@@989@@?>?<@:66:=8835;378BC?
@T6XXI:00098:00442
GGGAGAAGGAGAAGA
+
:@5>195951//,/,
@T6XXI:00099:00406
GGGAGAAGGGAGAAAGGAAGAGAACAGCGACGGGGATAACGTTTTAGCTATTCTCCCTCTCCCTATAGTGAGTCGTA
TTAGAATTCCCC
+
```

```
@@@ACCABA=@@@B@B>@>ABAA?CCAABBCCCC:B@>=@BBCC9@@@CCCABCCC>BCC@C;>>A???====:?:<
<=??A>A>@@@5
@T6XXI:00099:00408
GGGAGAAGGGAGAAAGGAAGGAGAAGTCGCCCCAAACGATTTGTTGACTATTCTCCCTCTCCCTATAGTGAGTCGTA
TTAGAATTCCCC
+
@@?ABA@DC>CBBB>B=?>@@B<>>@CCCDCC9CC>CBB>>;?>>?::<AA<====;::===:=9<<==<<<<<=<<
@>A??<>9;;<4
@T6XXI:00099:00409
CTAGTCACTCGAGATCTGCGGCTTTGGACAGTCCCTCTGTACTGTGAAGCCACAGATGTCGAGTCGACTAGCTTCCT
TTCTCCCTTCTCCC
+
C11111/4?CBBCCBAABBB@BCC>A>;<?=6694:BBBCBCC@@@A?AB?BBBBBBBBAA@?ABBCCCCBAB@C@C
C>CCCC>@?@@>A>
@T6XXI:00099:00413
GGGGAATTCTA
+
@@C9B?=@D99
@T6XXI:00099:00415
GGGAGAAGGGAGAAAGGAAGCTAGTCACTCGAGATCTGCGGCTTTGGACAGTCCCTCTGTACTGTGAAGCCACAGAT
GTCGAGTCGACTAGCTTCCTTTCTCCCTTCTCCC
+
DB>A@@=>@:>999095958??>@B???BBCEBABBCCBB?AAA=B?CCCCCBB>B??889@AAA@A?AA;=<<<<;
<@@CA;@@AA=??<<?><<==<====;<:<@==;
@T6XXI:00099:00420
GGGGAATTCTAATACGACTCACTATAGGGAGAGGGAGAATAGTCAACAAATCGTTTGGGGCGACTTCTCCTTCCTTT
CTCCCTTCTCCC
+
CCC9B7;8<AB>DCBBCCB@@@>97111.7>>BG=C?A?BBBBAA@@@=;BBAAA<AAB6?@@::47773424244-
4998484;334,
@T6XXI:00099:00436
GGGAGAAGGGAGAAAGGAAGCTAGTCATCGACATCTGTGGCTTCACATACAGAGGACTGCCAAAGCCGACAGATCGT
CACTCGACTAG
+
495449?AB=999918;>=CC@??:7761111166116888;<;>BB797;<7737<;..,.53..,.---
340000344@@:4478
@T6XXI:00050:00454
GGGGAATTCTAATACGACTCACTATAGGGAGAGGGAGAATAGTCAACAAATCGTTTGGGGCGACTTCTCC
+
///*/,424449499@@@?;;1331111,177=98A=:8;4449:9444-7>>;=6==?-775./,.,,2
@T6XXI:00050:00460
GGGGAATTCTAATACGACTCACTATAGGGAGAGGGAGAATAGTCAACTCCCATTTTAACGCACCATTGCCTTCCTTT
CTCCCTCTCCC
+
DFF9C8;8<89<@@@@@BBA@?>?=777.7?77:7:@@=@===<=8>300+0000+0449=;;7//,/8,/,/,,,+
,033-/.4886
@T6XXI:00050:00469
GGGGAATTCTAATACGACTCACTATAGGGAGAGGGAGAATAGATCACCGTTGGTTCGGGTGCGCGTTCTCCTTCCTT
TCTCCCTTCTCCC
+
999/99A?BBB@CCCCCBCBBA<97111.7>>>A@AAA@A;====?83;;365:@CCG?C???CCE@A@@=?@C?BB
>A?AA<A?;?:A=
@T6XXI:00050:00471
CTAGTCACTCGAGATCTGCGGCTTTGGACAGTCCCTCTGTACTGTGAAGCCACAGATGGACTGTCCCAAGCCGCAGA
TCTCGAGTCGACTAGCTTCCTTTCTCCCTTCTCCC
+
```

```
B<9<<A===?????ABBB@?<>><6>;>>???AA6:;:?==??748<9<A@BCBB;;;>@@@@??C<?=?CAKCCCC
BA@88888;F;;;B::6<<=AC;?8;?-737334:
@T6XXI:00050:00485
GGGGAATTCTAATACGACTCACTA
+
DDBAD595844244443700,,,,
@T6XXI:00050:00486
GGGGAATTCTAATACGACTCACTATAGGGAGAGGGAGAATAGAGACGTATTCGTTTGCGCTCTGTTCACCTTCCTTT
CTCCCTTCTCCC
+
BHB7B;;:9>><BBAAA:;:??>=?777.7???A>@9868A>>?888A<;8<???;@???A@@@A;=<>=@382494
98;>:@>@?@C=
@T6XXI:00050:00487
GGGAGAAGGGAGAAAGGAAGGAGAAGTCGCCCCAAACGATTTGTTGACTATTCTCCCTCTCCCTATAGTGAGTCGTA
TTAGAATTCCCC
+
EE>BBB@BB>BBBB=BAB?>=BB@>>>?ABDD9CB>BCCCC<BAAC??:<<;<<<<;@<<<=;;;??AAAA==@@@<
@=@AA?@3444,
@T6XXI:00050:00495
GGGAGAAGGGAGAAAGGAAGCTAGTCGACTCGACATCTGTGGCTTCACAGTACAGAGGGACTGTCCAAAGCCGCAGA
TCTCGAGTCGACTAG
+
CCBBB?@BB>?@AE>@=@?BB@>;;.00:000;=<??9;:=8<??ADCBBA@@>A33317;?AAA?AA<A@>???AA
>=99====A@@@@AB
@T6XXI:00050:00498
CTATCGACTCGAGATCTGCGGGCTTTGGACAGT
+
;B<7//////44444448...+.552434...4
@T6XXI:00051:00450
GGGGAAATTCTAATACGACTCAACCTATAGG
+
///*//+484442444443333110371111
@T6XXI:00051:00456
GGGAGAAGGGAGAAAGGAAGCTGAGTC
+
9;:@99284-4;:;;8;66,-,,,*22
@T6XXI:00051:00461
CTCGAGATCTGCGGCTTTGGACAGTCCCTCTGTACTGTGAAGCCACAGATGCGAGCGACTAGCTTCCTTTCTCCCTT
CTCCC
+
DDC<;<A@>>@999;AB>C?AADECDD><<B@@@AA@@BCAAB=AA@<;10//....;8;77799=<:=::99>0:7
;BBB>
@T6XXI:00051:00480
GGGGAATTCTAATACGACTCACTATAGGGAGAGGGAGAATAGATCACCGTCGTGTCGGGTGCGCGTTCTCCTTCCTT
TCTCCCTTCTCCC
+
==>4><A>BCCCCCCBBAAACCCBA:::8:BCCC=B?A?A;66:=:745./0009>;>9@????7828;;8<<@>AD
:;884-4;=>?C?
@T6XXI:00051:00485
GGGGAATTCTAATACGACTCACTATAGGGAGAGGGA
+
?CB6A=@58449>>BCBBBBB=311111,16111,0
@T6XXI:00051:00492
GGGAGAAGGGAGAAAGGAAGCTAGTCGACGTCGAACATCTGTGGCTTCACCAGTACAGAGGGACTGTCCAAAGCCGC
AGACTCTCGAGTCCGACTAG
+
```

```
CC>BA@?A>9>944-958488766611....3-.,......13/0188877431---1---
+068===<AA<??==;;;878=?A=;;::6:0//;;
@T6XXI:00051:00494
GGGAGAAGGGAGAAAGGAAGGAGAAGTCGCCCCAAACGATTTGTTGACTATTCTCCCTCTCCCTATAGTGAGTCGTA
TTAGAATTCCCC
+
BB:>99599-4444-
42313777676173333,39666111,1101<<:==<77:858/..442,+....559<99<A>=<@;<;AAA4
@T6XXI:00052:00454
GGGAGAAGGGAGAAAGGAAGGAGAAGTCGCCCCAAACGATTTGTTGACTATTCTCCCTCTCCCTATAGTGATCGTAT
TAGAATTCCCC
+
AE@CCB@CC>:<<B=B8<<<ACCC??788888.37277;??;BB@BBCAAA38333,0036=;=;:878;:;><73-
33--,-,---*
@T6XXI:00052:00455
GGGAGAAGGGAGAAAGGAAGCTAGTCGCTCGACATCTGTGGCTTCACAGTACAGAGGGACTGTCCAAAGCCGCAGAT
CTCGAGTCGACTAG
+
CJCBBB?BC>BBBB=B996:BC?@@?CBABA99:::;BBB7:;?BBBBCBCBA;3331788;@A>AA=@@>@???@@
<<=@@@ABA@@@<=
@T6XXI:00052:00470
GGGAGAAGGGAGAAAGGAAGCTAGTCGACTCGACATCTGTGGCTTCACAGTACAGAGGGACTGTCCAAAGCCGCAGA
TCTCGAGTCGACTACG
+
9:69@A=@B:>@AA@B>BBECB?@?0/,,,,,,..34...6367:898::@B8380//+.7//./775444/44::8
:A888AA7/./,+***
@T6XXI:00052:00471
GGGGAATTCTAATACGACTCACTATAGGGAGAGGGAGAAAT
+
8AA5:,9594424;;=?AA@A=<71111,1177767000+0
@T6XXI:00052:00490
GGGGAATTCTAATACGACTCACTATAGGGAGAGGGAGAATAGTGACCG
+
;>>5:198;449=?@@?@A@:7877777,76771,033254..1-.,-
@T6XXI:00052:00496
GGGGAATTCTAATACGACTCACTATAGGGAGAGGGAGAATAGTTTGCGCATTCGGTGGCACTGTT
+
CEF:C@C@BBBCCCA@@BBB?>?>>?@@?===@C>A@A?AC<<658;;441,/6,0.,/4449>:
@T6XXI:00053:00465
GGGGAATTCTAATACGACTCACTATAGGGAGAGGAGAATAGTAC
+
CEB8B5969449>@A@BABC@?@BCAA@:;:/0,0//,/664-/
@T6XXI:00053:00480
GGGGAATTCTAATACGACGTCACTATAGGGAGAGGGAGAA
+
C8707+/,//6,/48477>>??CBA>==<,0/...+...,
@T6XXI:00053:00482
GGGAGAAGGGAGAAAGGAAGGAAGGAGAAGTGTGCAGCCTCATTTAGCAGCTATTCTCCCTACTCCCTATAGTGAGT
CGTATAGAATCCCC
+
44-444244-///8+/,.,.6666,.110161111997361116,134888:84---22+-
..30033...413;>8313//+03*./774
@T6XXI:00053:00496
CTATCGACTCGAGATCTGCGGCTTTGGACAGTCCCTCTGTACTGTGAACGAGCCACA
+
CCC>;9:?AA=BAAACCC:::;BC>C>@?A????<?BBB??<@8001+,,++00++-
@T6XXI:00054:00465
```

```
GGGAGAAGGGAGAAAGGAAGGAGAAGTCGCCCCAAACGATTTGTTGACTATTCTCCCCTCCT
+
?BACCB@BB0999909>@=B@A;;516377775;9796111,16666730323304403323
@T6XXI:00054:00466
GGGGAATTCTAATACGACTCACTATAGGGAGAGGAGAATAGAC
+
111+/,424AA?CGCBBABBCCCC????6<;;;:?443/,,+*
@T6XXI:00054:00479
GGGGAATTCTAATACGACTCACTATAGGGAGAGGAGAATATAGGACA
+
---*-+9244424448=<>>?@AA;::B7::A>4744244300/-30
@T6XXI:00054:00481
GGGAGAAGGGAGAAAGGAAGCCTAGTCGACTCGACCATCTGGTGGCTTCACAGTACAGAGGACTGTCCAAAGCCGC
+
BD>BCC=@95=1--+-+-*,,*,..7678C:66;;:000./,.-,066611116444400/00000399<;;:2--
@T6XXI:00054:00482
GGGAAGAAGGGAGAAAGGAAGCGGGGCTAAGGTGTGGGTCGAAAGGTGAGTCCTA
+
67+/,//,/8+/444-423133333,1999:984448589333,31377111011
@T6XXI:00054:00495
GGGGAATCTAATACGACTCACTATAGGGAGAGGGAGAATAGCAAACGTTCCCCGACGT
+
888*1--,,,*,/68;DC:=>;;//.424:.61,10056;8:8906//,---*-..11
@T6XXI:00055:00460
GGGAGAAGGGAGAAAGGAAGGAGAAGTCGCCCCAAACGATTTGTTGGACTATTCTCCCTCTCCCTATAGTGAGTCGT
ATTAGAA
+
9904//,//+//68+/,/,39;>A@B@@@CBC.88036111,..,.,.3--
0/04441435;B7<6<<837<=44.6//,-300
@T6XXI:00055:00468
GGGGAATTCTAATACGACTCACTATAGGAGAGGGAGAATAGTTTTCGACCCCCATTACACTGTACGTCTGAGCATCG
ATCATGTACAGCTACGTACTCTGAGCATCGATCGATGTACACTACGACGTCTGAGCACGACGATGTACAGCTACGTA
CGCTGAGCATCATCGAGTACACACACGTCTACATCGACATTGTACAGCTACGTTACGCTGGAGGCACATCGGAGTTA
CACACACGTTCCTACAT
+
;AA5>,458449:>@ABBBBB>@=:/0,0:077.:;;48;4444+0444446.;;:??ABA@?>9888>>>998><<
<7716111711190000000666688600030004553333..54444...3333477755443333...554475
93478666,,+6...666:;;9<<???>??AAAAAA@::99::??999;;;:;;;;??@;;>>;;;6....33*,,,
333333333333333**
@T6XXI:00055:00469
GGGGAATTCTAATACGACTCACTATAGGGAGAGGGAGAATAGTCAACAAATCGTTTGGGGCGACTTCTCCTTCCTTT
CTCCCTTCTCCC
+
?=94>696<AA?CF@@BCCCCDC?8667.7>>@B>CAA>A<@@@@<??C;BBBCC>BBB9BAABC@AAC7858=@@<
A@@@;?<?CADA
@T6XXI:00055:00471
GGGAGAAGGGAGAAAGGAAGCTAGTCGACTCGACATCTGTGGCTTCACAGTACAGAGGGACTGTCCAAAGCCGCAGA
TCTCGAGTCGACTAG
+
CCBBAA@BB=?@@=:@=@>AB@===/00:;;;0:9A>>CCD@BC@ABBBBBC?99333-
4:;?@A>@@<AA@A?<????@@@@@AAAB@@@A
@T6XXI:00055:00475
GGGGAATTCTAATACGACTCACTATAGGGAGAGGGAGAATAGTCTTGCAATTTTCTGCGGTGCATTCTCCTTCCTTT
CTCCCTTCTCCC
+
CEE:B8;;<<<@CCCCC?@>B@B??9995:B?BC>B@@?@@@>@A?A@B?CCC:BBCAA?ACCCC@CBB?B@B<>>>
?BBC>C@?@@C>
```

```
@T6XXI:00055:00479
GGGGAATTCTAATACGACTCACTATAGGGAGAGGGAGAATAGAGACGTATTCGTTTGCGCTCTGTTCACCTTCCTTT
CTCCCTTCTCCC
+
CCC9C=?>ABC@CCBBB??AB?997111.7?==?>@CCAC=;<??=>;<<;=AAC=@@??B?===?A8888>@>@A<
A=<=7<8;<??;
@T6XXI:00055:00489
GGGGAATTCTAATACGACTCACTATAGGGAGAGGGAGAATAGTCAACAAATCGTTTGGGGCGACTTCTCCTTCCTTT
CTCCCTTCTCCC
+
@@>6@>B>BCC@CCB<BB?B?@B@<:8:88?ACC>AAA=A:=8CC??==;?<<<?;>CB8CBBBC??;;88784886
<??C?C=?@?A<
@T6XXI:00055:00499
GGGGAATTCAATACGACTCACTATAGGGAGAGGGAGGAATAGTC
+
8>=1=+-*,,00/33333766986669666177-010101----
@T6XXI:00056:00476
GGGAGAGGA
+
11-/,,,*,
@T6XXI:00057:00456
CTATCACCTGAGATCTGCGGCTTTGGACAGTCCCTCTGTACTGTGAAGCCACAGATGTCGA
+
99444//,/////8;44474337,377311997675.0.447;<=899:11/.66331117
@T6XXI:00057:00470
GGGAGAAGGGAGAAAGGAAGGAGAAGTCGCCCCAAACGATTTGTTGACTATTCTCCCTCTCCCTATAGTGAGTCGTA
TTAGAATTCCCC
+
CB@BBB@BB>AABB@B@B?B?BBB@>>?????8??;>>???9??>@===@@><<AC<?=?@C;=:>?@@@@@?>>AA
B>@?A=@<>>?5
@T6XXI:00057:00475
CTAGTCGACTCGAGATCTGCGGCTTGGACAGTCCCTCTGTACTGTGAAGCCAGCAGATGTCGAGTCGACTAGCTTCC
TTTCTCCCTTCTCCC
+
944444444499==AA>@;11-
00,.344466EA=CAA;;;AAAB@A<;??311>>>9BB@??88==997<;;6663668111716277944
@T6XXI:00057:00478
GGGAGAAGGGAGAAAGGAAGCTAATCGACTCGACATCTGTGGCTTCACAGTACAGAGGGACTGTCCAAAGCCGCAGA
TCTCGAGAG
+
99149@=@B><;<C>A=@@A;;:>>>7::;;>777<<<=;<8?A?@>?@887;55000+0330005=63552446--
-2468:88;
@T6XXI:00057:00498
GGGAGAAGGAGAAAGGAAGAGAAAGCGCAGACTAGTGGACAGTCAGGCTATTCTCCCTCTCCCTATAGTGAGTCGTA
TTAGAACTTCCCC
+
4901//,/,///7+/,39>>;>=96337336166633,...-433000,+-,-
11601133305556::A@@??88.30124-./75754
@T6XXI:00058:00476
CTATCGACTCGAGATCTGCGGCTTTGGACAGTCCCTCTGTACTGTGAAGCCACAGATGTCGAGTCGACTAGCTTCCT
TTCTCCC
+
:A@94449999=@A9918--+,2202*//24335-
669??AB::8;?;;A=@BB?@?@BBC::7@9883731636311,16662
@T6XXI:00058:00481
GGGAGAAGGGAGAAAA
+
```

```
BA:@>>599191***)
@T6XXI:00058:00492
GGGAGAAGGGAGAAAGGAAGGAGAAGTCGCCCCAAAC
+
9A?@?@>?C;<<CCADIN?B>AB=47173333,11,1
@T6XXI:00058:00495
GGGGAATCTAATACGACTCACTATAGGGAGAGGGAGAATAGGACCCTGTAATCCCGACCGGCTCTATC
+
18807*-,,,0088AB;00<......42679662600/000/000+033587774223,-,+,....-
@T6XXI:00059:00464
GGGAGAAGGGAGAAAGGAAGCTAGTCGACTCGACATCTGTGGCTTCACAGTACAGAGGGACTGTCCAAAGCCGCAGA
TCTCGAGTCGACTAG
+
DC>BBA=@@<@BAB=C;<;;CB==>/00:::;076966ACE>@@ACBCBCCC?99000-
4:=>AC?CC:?@=@A??@???@???@@?;<<<<
@T6XXI:00059:00469
GGGGAATTCTAATACGACTCACTATAGGGAGAGGGAGAATAGTCAACAAATCGTTTGGGGCGACTTCTCCTTCCTTT
CTCCCTTCTCCC
+
AB@6A=@=A@AAFBBBCCCBB@B?;111-
6;;<?<?AC@CAA@@@<??C<B?BBB=BBB8??A??@ABB@=;9<AA<AAAB;@<??AC?
@T6XXI:00059:00475
GGGAGAAGGGAGAAAGGAAGGAGAAGTCGCCCCAAACGATTGTTGACTATTCTCCCTCTCCCTATAGTGAGTCGTAT
TCAGAATTCCCCA
+
9>1949244-448;-
969<@5:7B=7133...*.6263..,..,.7103;443<=40335503343333468555334-..3342366648
@T6XXI:00059:00477
GGGAGAAGGGAGAAAGGAAGAGAATGCGGCCGGGTCAATAATTTTGGCTATTCTCCCTCTCCCTATAGTGAGTCGTA
TTAGAATTCCCC
+
DD@CCB@BBBBBBB>B?A>>BB?>?>??=>=>?<B;>?BB@BBB898<CCC@CCCD@CCCCC>CBBBBACCBBBBC@
@=?@@;>8<<;0
@T6XXI:00060:00456
GGGAGAAGGAGAAAGGAAGGAGATGCCAACGCAAACACGATTGTGGCTATTCTCCCTCTCCCTATAGTGAGTCGTAT
TAGAATTCCCC
+
8A87-
1,1,,,,,*,*/,.4..0BCC@C@@@@BB=B;;;BC@BCB;@A881333357<CCD>CCB>>=@@@>7777837??:
6:???5
@T6XXI:00060:00469
GGGGAATTCTAATACGACTCACTATAGGGAGAGGGAGAATAGTCAACAAATCGTTTGGGGCGACTTCTCCTTCCTTT
CTCCCTTCTCCC
+
CCC9B798<>>;A@@ABBDBDBBBAA?@:???AB>BAA@AAA@@@>@@@<BCCCF>BBB7::CAA>BCC?@;>3881
8>=A<A=@???;
@T6XXI:00060:00478
CTAGTCGACTCGAGATCTGCGGCTTTGGACAGTCCCTCTGTACTGTGAAGCCACAGATGTCGAGTCGACTAGCTTCC
TTTCTCCCTTCTCCC
+
ED9999449==?ABBCBBBBC>AAB<@=AB@??CCADBBBCBCD::9A69=DCDDL;;;;;B???DCCC<;:@96=:
==6=<;?9;/40340
@T6XXI:00060:00484
GGGAGAAGGGAGAAAGGAAGGAGAAGCACAG
+
CB@B>>@AC@BBA?:@<><;:;00,.....3
@T6XXI:00060:00493
```

```
GGGAGAAGGGAGAAAGGAAGGAGAAGCCGCCCCAAACGATTTGTTGACTATTCTCCCTCTCCCTATAGTGAGTCGTA
CC
+
BC>BBB>AA<@ABB=B?B?B@@@@ACC>@BCC8BB<A?=;6266899?A<;8;444-
4:99;9;440774=A<<=???>
@T6XXI:00060:00499
GGGAGAAGGGAGAAAGGAAGGACGCTGTCGTCGGTAGCTATTTGGGGACTATTCTCCCTCTCCCTATAGTGAGTCGT
ATTAGAATTCCCC
+
AB>CBB@BC==@=85>;?<@@CCBBDCDCC=<<@BB;=<AAA>BCB9CCDCB>A9991999;C:<>DAAACCBBBBA
BB:;:A;@5999/
@T6XXI:00061:00450
GGGGAATTCTAATACGACTCACTATAGGGAGAGGGAGAATAGCCGATAAAACTTCGCAGTCGTCCCATTCTTCCTTT
CTCCCTTCCTCCC
+
@@?4?5969AA>9;<<<ADDCCA?=777.7>>>?<A8837;<;9;;9:::1:;::488=784=AA<@@>88248994
;86616,/,//82
@T6XXI:00061:00454
GGGAGAAGGGAGAAAGGAAGGGGAGAAGGGAGAAAGGAAGGGGAGAAGGGAGAAAGGAAGGGGAGAAGGGAGAAAGG
AAGCTAGTCGACTCGAGATCTGCGGCTTGGACAGTCCCTCTGTACTGTCGAAGCCACAGAGTGATC
+
AE;=;CACCBBBBC8;;<@CCC8BBB@BB;?99>;?>B>BBB8>BA?;;:::;C<A>@AAA@5???<?C9==?D?A>
@=??488?334166.0000994455..,.,..442222-..332244...444;@B730,,,,,,,
@T6XXI:00061:00468
GGGAGAAGGGAGAAAGGAAGCATAAGCGAATGCCAGGGAGTTAGTGACTATTCTCCCTCTCCCTATAGTGAGTCGTA
TTACGAAGAT
+
//+//4244-444;-424777<@@<@B=<:611019;8<9=:65/...-.;:8444,---22+--
430034003:<=775735++++
@T6XXI:00061:00473
GGGGAATTCTAATACGACTCACTATAGGGAGAGGAGA
+
>AA1=+/,///78>@ACBBBBBAA?>==,6/*00***
@T6XXI:00061:00479
GGGAGAAGGGAGAAAGGAAGCTAGTCACTCGACATCTGTGGCTTCACAGTACAGAGGGACTCGTCAAAGC
+
FKDAA?=>=5@@>=<@=><?BA@@B<??AAB:;;BBACBC?AB@BB@?????<<<==4318,,++++*++
@T6XXI:00061:00481
GGGAGAAGGGAGAAAGGAAGCTAGTCACTCGACATCTGTGGCTTCACAGTACAGAGGACTGTCCAAAGCCGCAGATC
TCGAGTCGACTAG
+
@C?A@>=@@9AB@><A886;B?=:>:0/..-
.1179863:900,/..6@@<499//,.6666;244,482037=@@@@@?431-----10
@T6XXI:00061:00490
GGGAGAAGGGAGAAAGGAAGAGAACAGCCCCTGTAAATGTGTGTGAACTATTCTCCCTCTCCCTATAGTGAGTCGTA
TTAGAATTCCCC
+
CD@DEB@DD>BBBB>B=><@CA?:@@9988,9A99<:<AACCABBC>@@BCGEBBB>BBAAA<????@AAAA@@><
<9<@;:;2748-
@T6XXI:00062:00469
GGGAGAAGGGAGAAAGGAAGGGGAGAAGGGAGAA
+
99-449244-49;;;8888882*,,,*,20,.11
@T6XXI:00062:00473
GGGGAATTCTAATACGACTCACT
+
/8807;;:1;;24481/....,,
```

```
@T6XXI:00062:00475
GGGGAATTCTAATACGACTCACTAT
+
AA?699@994424444433....4/
@T6XXI:00062:00477
GGGGAATTCTAATACGACTCACTATAGGGAGAGGGAGAATAGTTCAACCGTCTCTGGCGCAACCATATTCTTCCTTT
CTCCCTTCC
+
D;@0>,/,//67649<@@@@C?>?A777-7>777-
64444;44/44249=;8344427;99<@;=<<<=??;=8::4:///,/,,+
@T6XXI:00062:00487
GGGGAATTCTAATACGACTCACTATAGGGAGAGGGAGAATAGCTACGTTTAACCGATC
+
9?92>592499244998>7<=?<=66??8?@>@?<:47<?;5;=17100+0/0/0442
@T6XXI:00062:00488
GGGAGAAGGGAGAAAGGAAGGAGAAGTCGCCCCAAACGATTTGTTGACTATTCTCCCCTCTCCCTAT
+
CH@D@?==@<@@@C>B=?<?;88637673333,1626688@;<:>;85533230000+0333;:334
@T6XXI:00062:00494
GGGAGAAGGGAGAAAGGAAGCTAGTCACTCGACATCTGTGGCTTCACAGTACAGAGGGACTGTCCAAAGCCGCAGAT
CTCGAGTCGACTAG
+
ACAA??=?>:@B@@=B=@=@C@CC@@CBBA?:88AA?ABB@BB@CDCC?=;<==988466<=A@>A@<AC?@@??@A
<<<;<<A@?<?A??
@T6XXI:00063:00452
GGGAGAAGGGAGAAAGGAAGCTCAATCGTGGGTAGAAGGGTCGTGATCTATTCTCCCTCTCCCTATAGTGAGTCGTA
TTAGAACTTCCCC
+
CDFDCDDCC>@BDC?C?B?BB@@@@CCCCBD@BB??:;;5;?BA@???@@C@AA@@:??BDD<A@@AA=@===A@AB
A=;;>0009;;81
@T6XXI:00063:00467
GGGAGAAGGGAGAAAGGAAGCTAGTCGACTCGACATCTGTGGCTTCACAGTACAGAGGGACTGTCCAAAGCCGCAGA
TCTCGAGTCGACTAG
+
FC@CCC?BB=?B=9:@642:A700=//0=;=>1::@??BBB=?@??CCCCCC@>833317=9>@A>@@;??<?A<;?
@@@@@AAAA99==@A
@T6XXI:00063:00471
GGGGAATTCTAATACGACTCACTATAGGAGAGGGAGAATAGCCGCGAAATTTGTGCCCTCTTACTATTCTTCC
+
ABC9CAC8;9=<949=@@@ABA@@B::5:?4:=,0/7,----,066?A>@C>88:>:67//,/.4043..,.,
@T6XXI:00063:00483
GGGAGAAGGGAGAAAGGAAGCTAGTCACTCGACCATCTGTGGCTTCACAGTACAGAGGGACTGTCCAAAGCCGCAGA
TCTCGAGTCGACTAG
+
CNADA?>@A<A@@>9@=>;?@???BB>=::80/,-
...;>A<AAABCBBACACCA===5;;;799>AC<A@@A@@@@?======AAAAAA@@
@T6XXI:00064:00454
GGGGAATTCTAATACGACTCACTATAGGGAGAGGGAGAATAGCGTAGCA
+
HCC9B8;;<CB@DA?>ABCCCACB>66656===A>A97475**(((((*
@T6XXI:00064:00488
GGGGAATTCTAATACGACTCACTATAGGGAGAGGGAGAATAGCCACCAACTATTGTTCGTCGCCGTTCTCTTCCTTT
CTCCCTTCTCCC
+
CEC:D=?BABBADCBB@@@B@@B;;6665:BBCC<?@A?A<??8>=:><???C>AA=AAA=<<;@?>????<?<?@;
???A<;9//0?9
@T6XXI:00064:00495
```

```
GGGAGAAGGGAGAAAGGAAGGGGAGAAGGGAGAAAGGAACGCT
+
9=:=::7::811185866,333,397=BD>C77702**)***.
@T6XXI:00065:00477
GGGAGAAGGGAGAAAGGAAGCTAGTCGACTCGACATCTGTGGCTTCACAGTACAGAGGACTGTCCAAAGCCGCAGAT
CTCGAG
+
EKABAC@CC>9<;D>B@C:;;;?>>>?????BBC?@@BA@@=:;8:CCC@?@;;;/.-
/;::;A>AC?BA>@?;;88484848
@T6XXI:00065:00481
GGGGAATCAATACGACTCACTATAGGGAGAGGGAGAATAGTCAACAAATCGTTTGGGGCGACTTCTC
+
ABB:B8<9B??@B973771131996986//,7+,++*++++--,-443;?8862666263477,/-1
@T6XXI:00065:00487
GGGAGAAGGGAGAAAGGAAGCTAGTCACTCGACATCTGTGGCTTCACAGTACAGAGG
+
496999244-4444-9<;=ABBB:::8;610100779787>;;26688663444000
@T6XXI:00065:00497
GGGAGAAGGGAGAAAGGAAGGGAACATGCCGCTGGAAGATAGCGAACTATTCTCCCTCTCCCTATAGTGAGTCGTAT
TAGAATTCCCC
+
DD@BBBABB?CBBB>B@B?BBABAAABBB@CCCC@C@D=AACDCCCCBBC@BCBC>CCCCC;==C?@@BCDBCCBBA
ACCC@C?@@@5
@T6XXI:00066:00453
GGGAGAAGGGAGAAAGGAAGCTAGTCACTCGACATCTGTGGCTTCACAGTACAGAGGGACTGTCCAAAGCCGCAGAC
TCTCGAGTCGACT
+
991449899588>>9>;><88;B<<?888898688889<99BB=@=BCCCCE@=<<<7<<AAAA<??<?A<=::://
//--000003---
@T6XXI:00066:00456
GGGAGAAGGGAGAAAGGAAGGAGAACGCGCACCCGAACCAACGGTGATCTATTCTCCCTCTCCCTATAGTGAGTCGT
ATTAGAATTCCCC
+
ACABBBACC@@B?B=A?BACBAAC@CCDCCCCCACE@C@B>AFA<;<CCAACACBBB><;<CD>CCBBAAACBABCB
CB@BBB>BACCC:
@T6XXI:00066:00467
TCTAATACGACTCACTATAGGGAGAGGGAGAATAGTCAACAAATCGTTTGGGGCGACTTCTCCTTCCTTTCTCCCTT
CTCCC
+
8?AAC>>>?@@@@???@;88>87777=6=??=<B?=>?=777.14444+000+033468888>B>@>=;56678,/,
/-335
@T6XXI:00066:00468
GGGGAATTCTAATACGACTCACTATAGGGAGAGGGAGAATAGCTAGCGTTTCCCCCGCAATCTATTTGTCCTTCCTT
TCTCCCTTCTCCC
+
DDC:C>?=>B@=@@@AABBCDCB@?966.9??CC@A??=@===@>B@@=@;@@@A1A@@>?5/09336//,-01,--
+-;;;-828;:;:
@T6XXI:00066:00469
GGGGAATTCTAATACGACTCACTATAGGGAGAGGGAGAATAGTTTTTCCCTTCACTAACCGCGAACTGCACTTCCTT
TCTCCCTTCTCCC
+
CDC:C?B@BBBACCB@B>?B>@BB?7777:CCDD>B>><?<;==CC1??;?>ACB?B@?;?>AC;=<<;=86:;366
.7>>A>C????A;
@T6XXI:00066:00492
GGGAGAAGGGAGAAAGGAAGCTAGTCACTCGACATCTGTGGCTTCACAGTACAGAGGGACTGTCCAAAGCCGCAGAT
CTCGAGTCGACTAG
+
```

```
GD>CCB@BB?BB@B<=>;;BB???BAA@AA=;88???CFB?BB@BCC>AA@A6633317?AA@@=@A=@@>@@<<<<
<=====:8::==<A
@T6XXI:00067:00468
GGGGAATTCTAATACGACTCACTATAGGGAGAGGGAGAATAGTCCTCGAGTGAGCGCCTCTGATTAGTTCTTCCTTT
CTCCCTTCTCCC
+
CA@4@6;:<<<AFCCBBACB@?BCCAAB>BCDCC>CAA?@@???>A@@BCDCB@@@D@BBBBCC@BBB@BB>A=@@<
@BBB8;;;CAB>
@T6XXI:00067:00471
GGGAGAAGGGAGAAAGGAAGCTAGTCGACTCGACATCTG
+
?A:ACC@GD>@ACC=B?B@CB<;43......344.....
@T6XXI:00067:00499
GGGGAATTCTAATACGACTCACTATAGGAGAGGGAGAATAGTCAACAAATCGTTTGGGGCGACTTCTCCTTCCTTTC
TCCCTTCTCCCGGGATTCTAACTACGACCTCACTATAGGGAGAGGAG
+
BCB4B:<7;=@@CBBBBCCB@@B=?6137=777.4;;=A@@@@@>@@C:@@BBB>BBC8CB???@CDD>@<=388-
4>==:?<;::8166,/.,.11,-03333353664000000+0004231
@T6XXI:00068:00454
GGGGAATTCTAATACGACTCACTATAGGGAGAGGGAGAATAGATCACCGTTGGTTCGGGTGCGCGTTCTCCTTCCTT
TCTCCCTTCTCCC
+
BBA4>59:<DC@CB>>>?BBBAB>9111.6=@CC>CAA>B=;===??=AC?B@B?CCC@CBAACB?>B??:?=A>FC
<AA??;?>898B<
@T6XXI:00068:00471
GGGGAATTCTAATACGACTCACTATAGGGAGAGGGAGAATAGCTCCGAAATCTCCGAATCAAGCAAGCCCTTCCTTT
CTCCCTTCTCCC
+
CCC9B=?>?CC@CBBABABBA><97111.7???D@DAA>A==<@@@AAA=?==><>=<A@<;<<;<===;::<3781
89;;4<4:444,
@T6XXI:00068:00491
GGGAGAAGGGAGAAAGGAAGCTAGTCACTCGACATCTGTGGCTTCACAGTACAGAGGGACTGTCCAAAGCCGCAGAT
CTCGAGTCGACTAGCT
+
4<19>@;>>1948;9><B<A><?><:3...-
.313.11110181179???@;;;444,4;;;@@>@A<AA<??@AA??>>8///////..113
@T6XXI:00069:00460
GCTATCACTCGAGATCTGCGGCTTGGACAGTCCCTCTGTACTGTGAAGCCACAGATGTCGAGTCGACTAGCTTCCTT
TCTCCCTTCTCCC
+
669:444444=>@BCFB<00,11,.,..477=>;<<?????8?5433-
/,/6444;?>=9@A?;;73376668889;;>?;=44/3003,
@T6XXI:00069:00462
GGGAGAAGGGAGAAAGGAAGCTAGTCGAACTCGACATCTGTGGCTTCACAGTACATAGGGACTGTCCAAAGCCGCAG
ATCTCGAGTCGACTAG
+
CC>@A@@CC@CBBC?B?BBBCCACAABC?ABBA::;;;;BBC?@@??AAAA==://.9:99:A@@?>AA<??<?@??
?@>???@@A???AAA?
@T6XXI:00069:00477
GGGAGAAGGAGAAAGGAAGAATAACGTAATGGCGACGGGTTGATAGCTATTCTCCCTCTCCCTATAGTGAGTCGTAT
TAGAATTCCCC
+
:@1111,1,////+/,4239179=?@@B?9;877=899-
42444;:987313777666116,9?:;<=@<=<>>=;;8<=>;8<@AA6
@T6XXI:00070:00474
GGGAGAAGGGAGAAAGGAAGTGCAGTTCGCGGTTAGTGAAGGGAAAAACTATTCTCCCTCTCCCTATAGTGAGTCGT
ATTAGAACTTCCCC
```

```
+
99149><??-
98>A<@>BBCBBCCCC@A>>>4858?8;:>AA<ACBB2C@@@>AAAA9=????9?@@@@@A@;::/.4462655<8
??=0
@T6XXI:00070:00489
GGGAGAAGGGAGAAAGGAAGGAGAAGTCGCCCCCAAACGATTTGTTGACTATTCTCCCTCTCCTAT
+
?B<>9959>9A@BD>C@CBCBBBB@<=>88888+372679>@;?@>::7<5525632222--,-22
@T6XXI:00070:00496
GGGGAATTCTAATACGACTCACTATAGGGAGAGGGAGAATAGTCAACAAATCGTTTGGGGCGACTTCTCCTTCCTTC
TCCCTTCTCC
+
A?92989<99988AAAAA@???B?????8?A99=95552335:;<<<<=;CA<8886662<?=@?=:..3333,-,-
---+-24332
@T6XXI:00070:00498
GGGAGAAGGGAGAAAGGAAGCTAGTCACTCGACATCCTGTGGCTTCACAGTACAGAGGGACTGTCCAAAGCCGCAGA
TCTCGAGTCGACTA
+
BD?BBA784099880;=?<@CAAAB=>>776,,,,,*,22:7AB?BD@??>====<==;;<<<?@>:;5<;8;><<=
@AAA???A@???AA
@T6XXI:00071:00462
GGGAGAAGGGAGAAAGGAAGCTAGTCACTCGACATCTGTGGCTTCACAGTACAGAGGGACTGTCCAAAGCCGCAGAT
CCGTCGAGC
+
GD?DCC?BB?BBAB>C@B@ACA???BBDBCB::::::AA@>:;7:@?@@A@>;;433-
4;::<?>ABEAB>@?;77/.,//---02
@T6XXI:00071:00471
GGGGAATTCTAATACGACTCACTATAGGAGAGGGAGAATAGTGACGTAGCTGGTCTACGCTGCCTCTCTCCTTCCTT
TCTCCCTTCTCC
+
:=<0<,1,/66,/4444377;3477//,.::?C>?==>??>@<;8774430423663736=44/00433323238=8
:A;;=6;7///,
@T6XXI:00071:00491
CTATCGACTCGAGATCTGCGGCTTTGGACAGTCCCTCTGTACTGTGAAGCCACAGATGTCGAGTCGACTAGCTTCCT
TTCTCCCTTCTCCC
+
@86611188;BHBBB???BB@B@?>B>ABBBBBB>BAA@@B>><===?AC@BBCCCAAA@BBBABBCCCBCAA>A?B
B>B@@C<@>???A<
@T6XXI:00071:00499
GGGGAATTCTAATACGACTCACTATAGGAGAGGGAGAATAGTCAACAAATCGTTTGGGGCGACTTCTCCCTTCCTTT
CTCCCTTCTCCC
+
DFC:C@CCCBC@CDC>>033>787:0.,.33662344/33655558684==776,66;29;96601000+0/0/00+
034405551122
@T6XXI:00072:00457
GGGAGAAGGGAGAAAGGAAGCTAGTCGACTCGACATCTGTGGCTTCACAGTACAGAGGGACTGTCCAAAGCCGCAGA
TCTCGAGTCGACTAG
+
CC?CBB8996;A@@9A8;48A>:::/00:;;;1:::;/===-
10866>CCCC@?;;;=7===??@AAA<@A@AA@===@<<=A888@=<<@A
@T6XXI:00072:00471
GGGAGAAGGGAGAAAGGAAGCTAGTCGACTCGACATCTGTGGCTTCACAGTACAGAGGGACTGTCCAAAGCCGCAGA
TCTCG
+
CDBA??@DB>BBBB=B@C@BB?=BAAABBBCC:9:::;??C7;;@DDBBBBC>;4000-
4:::>@>@A@@A>A@?@??<>>;
@T6XXI:00072:00472
```

```
GGGAGAAGGGAGAAAGGAAGTGCAGTTCGCGTATTAGGATGA
+
DC@CBB@BC>B@??=@@C@BBBBBC;:8//0004,331171.
@T6XXI:00072:00480
GGGGAATTCTAATACGACTCACTATAGGGAGAGGGAGAATAGTCAACAAATCGTTTGGGGCGACTTCTCCTTCCTTT
CTCCCTTCTCCC
+
499/99@@BBB@CBBAABCCCCCC?9::59??CC>@@A?@<?<<A>===9@BBBD=AAA7A@@@A;=:;3838<?A<
<<<B<A9<;BB?
@T6XXI:00072:00486
GGGGAATTCTAATACGACTCACTATAGGGAGAGGGAGAATAGCTCCCAAATCACGTTACGTGCGACCCACTTCCTTT
CTCCCTTCTCCC
+
DCA4@5459889??AAB@@@AA?>=777.7===C@@AA@C@@@BAA<??7>>???AAACCBBB@A@@:@99595991
9???0848888,
@T6XXI:00072:00488
GGGGAATTCTAATACGACTCACTATAGGGAGAGGGGAGAATAGTTTTCCCTTCACTAACCGCGAACTGCACTTCCTT
TCTCCCTTCTCCC
+
@>>/969>AAB?BBBBBAA@???=?=?=6=>::::16//,.72444.33439>?@@?@C?CB@@>@@??AAA>A>AA
>A@?C7=:<@@>;
@T6XXI:00073:00457
GGGAGAAGGGAGAAAGGAAGCTAGTCACTCGACATCTGTGGCTTCACAGTACAGAGGGACTGTCCAAAGCCGCAGAT
CTCGAGTCGACTAG
+
CC?@@@>AA<BBBB=B?B?BBBBCB:::?A@:::BBBBBC?@@@CCCBBBCCA:33317747AA>A><@A???:<<=
==?===A@A@@<==
@T6XXI:00073:00458
GGGAGAAGGGAGAAAGGAAGTGCAGTTCGCGGTTAGTGAAGGGAAAAACTATTCTCCCTCTCCCTATAGTGAGTCGT
ATTAGAATTCCCC
+
EC@BA@@BD>BAAA=B?B?@ACCDA?>@@@?=@=@AADC?BC>CCBB1BBBD8:;CE=@@@EDAC?@?BBA==<;;;
8838>A>=;@CC5
@T6XXI:00073:00464
GGGAGAAGGGAGAAAGGAAGCTAGTCGACTCGAGATCTGGCTTTGGACAGTCCCTCTGTACTGTGAGCCACAGATGT
CGAGTCGACTAGCTTCCTTTCTCCCTTCTCCC
+
AC:A<<;<A<AAA>9@598;A??>A:::??CCDCCB@@@?BBD>C@BBBCCCC>B9=====@AAA===?@@@A@@??
??>>>?<==?@ABB@A=@@;?<<<5>;788C?
@T6XXI:00073:00468
GGGGAATTCTAATACGACTCACTATAGGGAGAGGGAGAATAGTCAACAAATCGTTTGGGGCGACTTCTCCTTCCTTT
CTCCCTTCTCCC
+
BBC7B>B>@AA?CBCACBBB?@>97111.7>>CC>C@@<@9A;A=<?@@<BBABC>@A@9CCCCC?AAA57335=;:
??>@<A=??CC?
@T6XXI:00073:00498
GGGAGAAGGGAGAAAGGAAGGAGAAGTCGCCCCAAACGATTTGTTGACTATTCTCCCTCTCCCTATAGTGAGTCGTA
TTCAGAATTCCCC
+
CC?AAA@CC@CCCC?D@C?BABCC?CCDB@>>-88/7167>;???A77784/48996<;377+-/--
11135544115-..7978,444+
@T6XXI:00074:00451
GGGAGAAGGGAGAAAGGAAGCTAGTCACTCGACATCTGTGGCTTCACAGTACAGAGGGACTGTCCAAAGCCGCAGAT
CTCGAGTCGACTAG
+
CE?CCC@CC>CCBC=B>@>ABAAABBA????7:6?97?BB8:;@CBBBBBBB;9744-
4::<?@>AD<AA@9:9@@@@AAA@AAAC===??
```

```
@T6XXI:00074:00459
CTAGTCACTCGAGATCTGCGGGCTTTGGACAGTCCCTCTGGTACTGATGAAGCCACAGATGTCGAGTCGACTAGCTC
CT
+
///////999499;;@?9478,3<7,31336689>9998886766799:49;B8;99@;;B:77A>711--
,2,,,.,.
@T6XXI:00074:00460
GGGGAATTCTAATACGACTCACTATAGGGAGAGGAGAATAGAGCCACCGTTGTTCGGTGCGCGTTCTCCTTCCC
+
?A:29295;449A?ADCB@@@CCB?:77.7=774:;;9;44400/11011016711067766 33/044/02.22
@T6XXI:00074:00463
GGGAGAAGGGAGAAAGGAAGCTAGTCGACTCGACATCTGTGGCTTCACAGTACAGAGGGACTGTCCAAAGCCGCAGA
TC
+
CDAEAA7;909>@B>B?B???8::9769919;=71...3,,*,,*,35767:<::444-48;8;8===:7-,--
/./.3
@T6XXI:00074:00471
CTAGTCACTCGAGATCTGCGGCTTTGGACAGTCCCTCTGTACTGTGAAGCCACAGATGTCGAGTCGACTAGCTTCCT
TTCTCCCTTCTCCC
+
121/111:?BCBCDC@AACC>;;@6:78>???BD=BBC;:;@@?==A??BACCBB@AAB?@@CBCCCCDBCA?;?47
7.7>>?:=587;C;
@T6XXI:00074:00472
GGGGAATTCTAATACGACTCACTATAGGAGAGGGAGAATAGATCACCGTTGGTTCGGGTGCGCGTTCTCCTTCCTTT
CTCCCTTCTCCC
+
DJCAD798<89<>@A@BAAAC???@7747===B>A??>AA@===A?;>?3:6:@CBC?C><>GA@=?993177:<;6
=744-49:;;>7
@T6XXI:00075:00450
GCTAGTACGACTCGAGGTCTGCGGCTTTGGACAGTCCCTCTGTACTGTGAAGCCACAGATGACTAGCTTCCTTTCTC
CTCTCCC
+
4//771////7::9992499;888488,42439<;;?877CCAA?@@>>A9?B?A@CCCAACCA@?81,1,00,000
,.....+
@T6XXI:00075:00452
GGGGAATTCTAATACGACTCACTATAGGGAGAGGAGAATAGT
+
CCCCD=@=ABB@BC;;<BBCCCAABCCB6::00,.3333.33
@T6XXI:00075:00462
GGGAGAAGGGAGAAAGGAAGCTAGTCGACTCGACATCTGGTGGCTTCACAGTACAGAGGGACTGCCAAGCGAC
+
BGCDAA>@B99;88095958?8//....337./1611173167135688?AA?==AAAA<A;768,,*+++++
@T6XXI:00075:00465
GGGGAATTCTAATACGACTCACTATAGGGAGAGGGAGAATAGTCAACAAATCGTTTGGGGCGACTTCTCCTTCCTTT
CTCCCTTCTCCC
+
=@@6@@@ACCB@CCBAA<BBBA<97111.7>>>@@AAA>A=AA@><>AA;?<<<@>@@?8@AC?B=A??4738=@@<
???A<A9<<CC<
@T6XXI:00075:00468
GGGAGAAGGGAGAAAGGAAGCTAGTCACTCGACATCTGTGGCTTCACAGTACAGAGGGACTGTCCAAAGCCGCAGAT
CTCGAGTCGACTAG
+
FDBAA@===9@@>><A896;A???C?>;==@00////=>B=@@@CCCCBBBA>=33717?>@AA>@@<@A>AABB?>
8??A==A@@@===@
@T6XXI:00075:00470
GGGAGAAGGGAGAAAGGAAGCTAGTCGACTCGACATCTGTGGCTTCACAGTACAGAGGGACTGTCCAAAGCCGCAGA
TCTCGAGTCGACTAG
```

```
+
CC>BBB?BC>BB@>6>4948>>:9;0..3333586696?:;8?@@BAAABCBA@;<<<6;=>889@@DAAA>998BA
AA???@AA=<<>=88
@T6XXI:00076:00451
GGGGAATTCTAATACGACTCACTATAGGGAGAGGGACGAATAGTCAACAAATCGTTTGGGGCGAC
+
18808,/,4449>>99988886336;;<5:699>;9:7;333668<;88;111111,177-78/.
@T6XXI:00076:00452
CTATCGACTCGAGATCTGCGGCTTTGGACAGTCCCTCTGTACTGTGAAGCCACAGATGTCGAGTCGACTAGCTTCCT
TTCTCCCTTCTCCC
+
DBBAA@ACECBCBBB@@B;;8;AB=B@AADB:::8:CBCCCCC===A:<@ADBCCCCB>>>>//3>==ACBA<<A;?
?;?AAC>A;==AA<
@T6XXI:00076:00472
GGGAGAAGGGAGAAAGGAAGGGAGCGCCTAGGATAGTACTTATCGGGCTATTCTCCCTCTCCCTATAGTGAGTCGTA
TTAGAATTCCCC
+
11+//1,//+//44-
424233,3999=8:9A@<;<CCCCD@CC@??:?:;<@CBCC@<==CC?@B;<;;9AA?@AA@?<@795;@CCD:
@T6XXI:00076:00476
GGGAGAAGGGAGAAAGGAAGCTAGTCGACTCGACATCTGTGGCTTCACAGTACAGAGGGACTGTCCAAAGCCGCAGA
TCTCGAGTCGACTAG
+
>@:??A599199880;>A?BCA=>>/10;;;:071886@AB7;:@DDC@??CA@A77717?;@=@<??<?A>A@??A
@@@@???ABCA===@
@T6XXI:00077:00461
CTAGTCACTCGAGATCTGCGGCTTTGGACAGTCCCTCTGTACTG
+
4444444449==?@BBBA;:68>>8><><>;7::,0..,,,,4.
@T6XXI:00077:00498
GGGAGAAGGGAGAAAGGAAGGAGAAAGCGACGTCGGGGAAACGTTTGCTATTCTCCCTCTCCCTATAGTGAGTCGTA
TTAGAACTTCCCC
+
99-994244-444;-968@?;<@??8?@?>><=7777-
4;:4:;B:=?C@?@?CHB>CBA@@7>66668664:;/0.4444<;@A>AAA7
@T6XXI:00078:00454
GGGAGAAGGGAGAAAGGAAGCTAGTCGACTCGACATCTGTGGCTTCACAGTACAGAGGGACTGTCCAAAGCCGCAGA
TCTCGAGTCGACTAG
+
FCAB?A>?B>BBBB>B@B@BCB>=:/0000/;0::BA@BBD>:;9:BBBCCF@;;433-
4;;@@@>@A<AD@=====A@@=====>A@@@AA
@T6XXI:00078:00458
GGGGAATCAATACGACGTCACATAGGAGAGGAGAAT
+
/11*/,44424444433339653..,...10000/0
@T6XXI:00078:00466
GGGAGAAGGGGAGAAAGGAAGCTAGTCGACTCGACATCTGTGGCTTCACAGTACAGAGGGACTGTCCAAAGCCGCAG
ATCTCGAGTCGACTAG
+
EEA@=92989,9444042469CB>>?/00::::;??:87CCC=@@@BCCCBABA@<44417>A?@?=AA<AA>A@==
=??@A@<@AAA@@@@A
@T6XXI:00078:00467
GGAGAAGGGAGAAAG
+
42444244-////+/
@T6XXI:00078:00468
```

```
GGGGAATTCTAATACGACTCACTATAGGGAGAGGGAGAATAGTCAACAAATCGTTTGGGGCGACTTCTCCTTCCTTT
CTCCCTTCTCCC
+
CCD9B>A@ACCCCBAAAABBB@?99666.7=@BC>BBA?@::?AA>@@B:B??@A=@CB9BBBBC?BBB??<?>@?:
@=<<7=699BC<
@T6XXI:00078:00486
CTGCGGCTTTGGACAGTCCCTCTGTACTGTGAAGCCACAGATGTCGAGTCGACTAGCTTCCTTTCTCCCTTCTCCC
+
CCCCBBCBB=B?BBBCABA>BDCB@?>>=CCC@B??BBCBAABCCCCCBCCBBBECBB?BADD>?@?C<>;???@<
@T6XXI:00078:00491
GGGAGAAGGGAGAAAGGAAGCTAGTCACTCGACATCTGTGGCTTCACAGTACAGAGGGACTGTCCAAAGCCGCAGAT
CTCGAGTCGACTAG
+
BB>CDC?BB?EBA>=@675;C???C>A@AA=:;8?????C?:;::BBCBBBA>9333,088;;=8<<7=A>AA@@@A
@@@=<<?????AAA
@T6XXI:00079:00455
CTAGTCGACTCGAGATCTGCGGCTTTGGACAGTCCCTCTGTACTGTGAAGCCACAGATGTCGAGTCGACTAGCTTCC
TTTCTCCCTTCCTCCC
+
:9949444::;94444>898?=888/8;?>>B???<?@B::;ABBDAA<?B@CCDDBBCCCCBBABC@@?AA:137<
>>9?7::,/,/,...,
@T6XXI:00079:00466
GGGAGAAGGGAGAAAGGAAGCTAGTCACTCGACATCTGTGGCTTCACAGTACAGAGGGACTGTCCAAAGCCGCAGAT
CTCGAGTCGACTAG
+
CEACCBBCB>BBCB?A=@=>@A?BB<?????::::::BAA?AB@CC@B?@CA=:99948==?><?A;49>>9<<<==
;?=?????@AAAAA
@T6XXI:00079:00486
GGGGAATTCTAATACGACTCACTATAGGGAGAGGGAGAATAGTCAACAAATCGTTTGGGGCGACTTCTCCTTCCTTC
TCCCTTCTCCC
+
===6?@C@BCC=A@C@@@@@B???=777,7;<B@>B<@<<?A??=<<;;7???@C>CCC9CABAA=?;8,/,/,/,-
43345465553
@T6XXI:00079:00489
CTAGTCACTCGAGATCTGCGGCTTTGGACAGTCCCTCTGTACTGTGAAGCCACAGATGTCGAGTCGACTAGCTTCCT
TTCTCCCTTCTCCC
+
<:1////49BABBBCB@@AB@B??=B@BCCCBBB;@AC@A@<<<??C=?B@CCCBABAAACC@@ACCCCCD??:=47
75:>>C:@<<<<<6
@T6XXI:00079:00493
GGGAGAAGGGAGAAAGGAAGCTAGTCGACTCGAGATCTGCGGCTTTGGACAGTCCCTCTGTACTGTGAAGCCACAGA
TGTCGAGTCGACTAGCTTCCTTTCTCCCTTCTCCCCTTCCTTTCTCCCTTCTC
+
DC<AAB>AA<A@>>9>5848?=>AF:;:DDCBBCCBB<8:C@ABABBCCBB?@AA<AA@@@@@@@@??<??=?;;<<
;<;==<<<<<?=??=<777467.//;=9;68/444,434/44-4///,/56--
@T6XXI:00080:00454
GGGGAATTCTAATACGACTCACTATAGGGAGAGGAGAATAGTTACGTACAC
+
,**)*)1,///7:BDBBACBBBB?>777.7???=@4424///,---11123
@T6XXI:00080:00458
GGGGAATTCTAATACGACTCACTATAGGGAGAGGGAGAATAGCTATCGCACCTGA
+
BA@4@<>=@AB@CCCCC@@@@>;?7116.7>@CC>C==:<93479:0,+..)*++
@T6XXI:00080:00459
GGGGAATTCTAATACGACTCACTATAGGGAGAGGGAGAATAGATCTTCTCGTCCGAGCTTGCTATTCACCTTCCTTT
CTCCCTTCTCCC
+
```

```
DDLBF>;><AAINBBBBBBBDAA@?=766.7=>BC@C??=?A?::8655;;@AB@CCBBB@AAACC@CCC?B=A=AB>
BBBE>C=A?<<6
@T6XXI:00080:00469
CTAAGTCACTCGAGATCTGCGGCTTTGGACAGTCCCTCTGTACTGTGAAGCCACAGATGTCGAGTCGACTAGCTTCC
TTTCTCCCTCTCCC
+
,,,*,,,;;;A;999;@@@>?<888/8487??BBC;?@@AAAAAA@?@=?B?BB@@;;;:????@CCCC;;:A97?3
00.000>,//1222
@T6XXI:00080:00475
GGGGAATTCTAATACGACTCACTATAGGGAGAGGGAGAATAGTCAACAAATCGTTTGGGGCGACTTCTCCTTCCTTT
CTCCCTTCTCCC
+
CCC9B>A>ACCDCDCCCBBB@?>>@777.7?>>A=BAABBA???@>@AA<BBAAA=ACC9C??@C?CBC>>;=3886
8BAC;@>@@AA<
@T6XXI:00080:00481
GGGGAATTCTAATACGACTCACTATAGGGAGAGGGAGAATAGCTACAACGCA
+
BCC9A=@=BA@AEAA@CCCCCBB???8?;?BBCC>C??:=<//,,,,*+***
@T6XXI:00080:00498
GGGAGAAGGGAGAAAGGAAGCTAGTCACTCGACATCTGTGGCTTCACAGTACAGAGGGACTGTCCAAAGCCGCAGAT
CTCGAGTCGACTAG
+
:C@CCC>A=:@?88:@8:6;C???B9:7??;787::6?AC5;:?AGABACA=43444-
499889@AA<@A>A@AA@A?????@899BAAA@
@T6XXI:00081:00458
GGGGAATTCTAATACGACTCACTATAGGGAGAGGGAGAATAGATCACCGCTGGCTCGGGTGCGCGTTCTCCTTCCTT
TCTCCCTTCTCCC
+
CCC9B7;;<@ADDBB?CBBBBBB>816657>@CC>C?@>@=99=@CC@AAAB=@@BBC>C;99?>=;?8857<;8=?
688@D;@=??@A<
@T6XXI:00081:00467
CTATCGACTCGAGATCTGCGGCTTTGGACAGTCCCTCTGTACTGTGAAGCCACAGATGTCGAGTCGACTAGCTTCCT
TTCTCCCTTCTCCC
+
C::811/6689?ADBACC;;8;CC>C?BACBAAA>ACC@CB@@?@@A>@B@BBBBBAACBBBB?@@???@;:688:?
B>BCCC?A;=<@B<
@T6XXI:00081:00473
GGGAGAAGGGAGAAAGGAAGAGAAAGCCGACTTTGCCACAAATGAAGCTATTCTCCCTCTCCCTATAGTGAGTCGTA
TTA
+
==:>@A>@?199>@1969;>@ABCACD@?>888/77:>>?A8ABB@DDFDC?BBEC>;;9;72<:766?>=>BBAAA
@>@
@T6XXI:00081:00478
GGGAGAAGGGAGAAAGGAAGGAGAAGTCGCCCCAAACGATTTGTTGACTATTCTCCCTCTCCCTATAGTGAGTCGTA
TTAGAATTCCCC
+
DD@?A?@CB@?BBB@C@C>A?AAB?ABBBCCC9CB<BB8??9?@>BAA?CB>A@A@;><<<@;?@@A???A@@@??@
A?@@A>@9<;<4
@T6XXI:00081:00479
GGGAGAAGGGAGAAAGGAAGGAGAATGCCCCGCAGGAAATTGCAAGACTATTCTCCCTCTCCCTATAGTGAGTCGTA
TTAGAATTCCCC
+
CA>BBB@BA=@@AA?B@B@B@B@>:>????6@ABC@CC>B?BBC@CAAA??<?AAA<????@;@@@@AAAAA@@<88
=>AA@>@<>=>4
@T6XXI:00081:00480
GGGGAATTCTAATACGACTCACTATAGGGAGAGGGAGAATAGTCAACAAATCGTTTGGGGCGACTTCTCCTTCCTTT
CTCCCTTCTCCC
```

```
+
EIB7>245989<@BBBCCCCC@@??:77.7>>@C>DAA>??AAAC<>>C?CB@??<?BB7BALDC?A@=34347:;:
??@A;@<@<<<7
@T6XXI:00081:00496
GGGGAATTCTAATACGACTCACTATAGGAGAGGGAGAATGAGTCAACAAATCGTTTGGGGCGACTTCTC
+
AKK:C=><>992449>????=010:00,.:07716:89784;80023:44=7677-6>=2>:0//,/--
@T6XXI:00082:00459
GGGGAATTCTAATACGACTCACTATAGGGAGAGGGAGAATAGTTACACCTCTGTTGATTGCTATATCTCCTTCCTTC
TCCCTTCTCCC
+
CDH:DACCBBBCDDCBBAA8::>>?777.7??@B?BCC?@8@A=AA@A;==???>A@@?@@@@AA@@@;;;9;./,-
-/.,479444-
@T6XXI:00082:00469
GGGGAATTCTAATACGACTCACTATAGGGAGAGGGAGAATAGTCATTGCCTCACATCAATTACCGTTCTCTTCCTTT
CTCCCTTCTCCC
+
CCB7A>B>@BB@BCCBBABAB@?97161.7==@C>B?A?A=?AAAA=??=AAAA@@@@>@?;:9:94.///,/4663
546=17/4444-
@T6XXI:00082:00474
GGGGAATTCTAATACGACTCACTATAGGGAGAGGGAGAATAGTCAACAAATCGTTTGGGGCGACTTCTCCTTCCTTT
CTCCCTTCTCCC
+
CDKBE;:9;AA8;<CCBCCCA@>?8166.6==@B>CAA>@<@A@@>@CC>AAA??7=??5?@AA?;???<<:=>AA=
>>?A<A68:@A<
@T6XXI:00082:00486
GGGGAATTCTAATACGACTCACTATAGGGAGAGGGAGAATAGCACATCCTTCTCCACAGCACACTTCATCTTCCTTT
CTCCCTTCTCCC
+
=@>4@>B?BBB@BA??>BBB@CB;966659BBCC??BB>B;====>==;=;<AB>==8:;<=<<<>@<A?>:<8;;6
<<BA;@>@???;
@T6XXI:00082:00491
CTATCGACTCGAGATCTGCGGCTTTGGACAGTCCCTCTGTACTGTGAAGCCACAGATGTCGAGTCGACTAGCTTCCT
TTCTCCCTTCTCCC
+
A@@=99:=AA@DCCCBCBDD?AAB>B?A??=777.7777?B>>?@>@>?B@BCCCBBCB@B@CABA@@?AA?=:=47
7.7:::4974444-
@T6XXI:00082:00494
GGGAGAAGGGAGAAAGGAAGGGAATAGACGGGAGTCGGGTCTGGTTACTATTCTCCCTCTCCCTATAGTGAGTCGTA
TTAGAATTCCCCC
+
AE?CCD@BB=BCBB?A=?@BB<B@BA=<@BB6::?BBC?CBBC@CAA>>?A?AA@A;<;<;;6;??@???@AAAA==
=>A<;8;/////+
@T6XXI:00082:00495
GGGAGAAGGGAGAGGGAGAATAGTTACCATTTTACATGCGTGCCTATTCTCTTCCTTTCTCCCTTCTCCC
+
BCABCB@DD>BBBBB<BBB@>;??@CC?:773.7>???CCCCB@B997;???>B?BB>BCBC>B;:?AA8
@T6XXI:00082:00496
GGGGAATTCTAATACGACTCACTATAGGGAGAGGGAGAATAGTCCGCCAATTCTGCGCGTTAGCATTCTCCTTCCTT
TCTCCCTTCTCCC
+
CBG9B=@=@AA>BBB@@@DBCAA??777.7====6477;??@@A;==AC=?=??==7:7=:<;;68:?@><=07466
,68;;:?344//,
@T6XXI:00082:00499
GGGGAATTCTAATACGACTCACTATAGGGAGAGGGAGAATAGCCGTCTC
+
<>;2;6<294;2448988387678<99@;@?@BE>B@C><//,1*****
```

```
@T6XXI:00083:00455
GGGGAATTCTAATACGACTCACTATAGGAGAGGGAGATAGCCACATGGCAGCTGTCCCGCGGTTCTTCTTCCTTTCT
CCCCTTCTCCC
+
EDH9C@BAABD@CCBBBBBBB:997/.,0::==<?>A????<<=B7748@@>?@@@@:???;>:==;;<9=?A;:;4
444-47:;@A<
@T6XXI:00083:00475
CTATCGACTCCAGATCTGCGGCTTTGGACAGTCCCTCTGTACTGTGAAGCCACAGATGTCGAGTCGACTAGCTTCCT
TTCTCCCTTCTCCC
+
EEBCBBCCCC@CBBBBCCCC@CCB>B@BBBCB@@;@AC@@@???B?A;=A@BBBBCBBBCCCCBBBDDCDDBB?CAB
C>BAAA9=8;<=?:
@T6XXI:00083:00477
GGGAGAAGGGAGAAAGGAAGCTAGTCACTCGACATCTGTGGCTTCACAGTACAGAGGGACTGTCCAAAGCCGCAGAT
CTCGAGTCGACTAG
+
JDBBBB?AC>=@@C8::<@BBB@@?AA@@CB:;:::7ABB=:<9:BCAAAB=::333-
4;;<=<?DD?DD?A?:?@ABBA???@@@@@@@A
@T6XXI:00083:00489
GGGGAATTCTAATACGACTCACTATAGGGAGAGGGAGAATAGATCACCGTTGGTTCGGGTGCGCGTTCTCCCTTCCT
TTCTCCCTTCTCC
+
AA?7A=A>BBC@CCBB??@BA>ABBAAA>ABBBE>C??=??89:@B?:;;,/,/,11=8;;B;?6636111,17706
7.43380;956<=
@T6XXI:00083:00490
GGGGAATTCTAATACGACTCACTATAGGGAGAGGAGAATAGAG
+
;A;9@798<@A>BCBBABBBBBCCCBBB>BB?@=@//,,,,,+
@T6XXI:00083:00494
GGGAGAAGGGAGAAAGGAAGAAAAACACACAAACAAAGAAGGGACAGACTATTCTCCCCCTCCCTATAGTGAGTCGT
ATTCAGAATTCCCC
+
AC>CAB=@A<@@@CAC@C@BBCCB2AA?@@@?;@@@;<>>AC;<?=@=A@CC?AAABBA2@@@:@???AA@CAAA;6
//,//99489<?<4
@T6XXI:00083:00497
GGGGAATTCTAATACGACTCACTATAGGGAGAGGGAGAATAGCCGCCATTTACCCCCCGGACACGTGTTCTTCCTTT
CTCCCTTCTCCC
+
BCC:B::;9CCBCB<<BBBB>@;77111.7>?CD>AAA?A::;;AA=BBB>BBB?>@-
>>==?>??99974438=AB9<<CC?C9<<@@;
@T6XXI:00084:00458
GGGAGAAGGGAGAAAGGAAGGAGAA
+
CC@CCC@?:6>844-951-0,0..,
@T6XXI:00084:00474
GGGAGAAGGGAGAAAGGAAGTGCAGTTCGCGGTTCGTGAAGGGAAAAAACTATTCTCCCTCTCCCTATAGTGAGTCG
TATTAGAACTTCCCC
+
991444244-
44485878777;@@88;<11.,.622,..,..+.....(/:99299@A;B@@>A<=????:?;97000042455587
:4440
@T6XXI:00084:00476
GGGAGAAGGGAG
+
/756,,+,2*,,
@T6XXI:00084:00491
```

```
GGGGAATTCTAATACGACTCACTATAGGGAGAGGGAGAATAGTCAACAAATCGTTTGGGGCGACTTCTCCTTCCTTT
CTCCCTTCTCCC
+
AA@4@<@=@@@ACCCABBBB?@<7=111.7?==?=BHDB@99ABB<?@A<BBCCC>BBB7BB@AC?CCA:<9<8;;6
;==>:?>@AAA<
@T6XXI:00084:00494
GGGAGAAGGGAGAAAGGAAGCTATCGACTCGACATCTGTGGCTTCACAGTACAGAGGGACTGTCCAAAGCCGCAGAT
CTCGAGTCGACTAG
+
CCBBAA@BB>BB?B?A=>@BB????@89:B?:7;:::::D8<;@CCBBBBAAA=44417@@ACA?@@<AA>@@::::
8==A@AA@@@@@=<
@T6XXI:00084:00497
GGGAGAAGGGAGAAAGGAAGGAGAAGTCGCCCCAAACGATTTGTTGACTATTCTCCCTCTCCCTATAGTGAGTCGTA
TTAGAATTCCCC
+
CC?DAA>AC@CCCC>C@B@B@BB@>A@BAAAA7A?<?B???=@C@CC@<??=@@?B<A@@@A<A@@@???@A@@===
A>@@@<?8:::1
@T6XXI:00085:00461
GGGGAATTCTAATACGACTCACTATAGGGAGAGGAGAATAG
+
A889?1:<;CBACBA@BBBB>AB?>661-6===;@//,/,,
@T6XXI:00085:00469
GGGGAATTCTAATACGACTCACTATAGGGAGAGGGAGAATAGTCAACAAATCGTTTGGGGCGACTTCTCCTTCCTTT
CTCCCTTCTCCC
+
CBA9>7;;<CCACCBAA>>>BC?97111.7>>?C@B@@>@<@AAA?===9@CBBC>CBB7AACBB?AAA:;8<<??<
@AAA<B9<<DC;
@T6XXI:00085:00471
GGGAGAAGGGAGAAAGGAAGGGGAAGGGAGAAAGGAAGCTAGTCGACTCGACATCTG
+
GNDCCC@CB?@AFE?B?B@CCC9C?BB=B<968?592:@?<<=11-8--..3,..3.
@T6XXI:00085:00478
GGGAGAAGGGAGAAAGGAAGCTAGTCACTCGACACCTGTGGCTTCACAGTACAGAGGGACTGTCCAAAGCCGCAGAT
CTCGAGTCGACTAG
+
CC@CCB@BC>?A?C=@=@ACC@=?CC@?AC@??C=<B???<AB@CEBABBC;4433317?>@A@=AB:AC?A<68<A
@@A888@<<<@@??
@T6XXI:00085:00491
GGGAGAAGGGAGAAAGGAAGCTAGTCACTCGACATCTGTGGCTTCACAGTACAGACGGGACTGTCCAAAGCCGCAGA
TCTCGAGTCGACTACGCTTCCTTTCTCCCTCTCCC
+
AC8;;@;@@:<9AC>B@B@@@?A@>;:00/-/--34466601637A??@B;;99////,.--
03555;:68:44363988884;;;9573...577555533332+--1110
@T6XXI:00085:00494
GGGAGAGGAGAAA
+
,-+-,,,*,,44-
@T6XXI:00085:00495
GGGAGAAGGGAGAAAGGAAGCTAGTCGACT
+
:C894@6991941@8<-1,--,**2,,,33
@T6XXI:00085:00499
GGGGAATTCTAATACGACTCACTATAGGGAGAGGGAGAATAGCCGTTATTTTAACTCCCGCGGCCGGTTCTTCCTTT
CTCCCTTCTCCC
+
CDC9C=?>ADCCCB?>>>B??BB7;111.7<<ABA@A@>@?<<9<?=?ABA5A>@???7==>3738>A8==<???D;
<<<?6;8<===7
```

@T6XXI:00086:00451
GGGAGAGG
+
<:+1,,,*
@T6XXI:00086:00459
GGGGAATTCTAATACGACTCACTATAGGGAGAGGGAGAATAGACACCAATCGCGCCAGGTCATGTTCTCCTTCCTTT
CTCCCTTCTCCC
+
AA?7A=@=@CCBCBBBB?@@B?>?>777.7??ABCB==>@@AAA@@>@<??@CBB??A>AAAAAA>@@@9<7;244-
744;:>=;<<?7
@T6XXI:00086:00460
GGGAGAAGGGAGAAAGGAAGGAGAATCAGCC
+
491444244-4444-47823133313,,,22
@T6XXI:00086:00463
GGGAGAAGGGAGAAAGGAAGTGCAGTTCGCGGTTAGTGAAGGGAAAACTATTCTCCCTCTCCCTCATAGTCGAGTCG
TATTAGAATTCCCC
+
DFDCBB?BB?BCBB?B@A<;ABCC;;99>@>4848337976888996AACB=@???6=>>@A>;73966/----
111355:<=A?;<CCD5
@T6XXI:00086:00467
GGGAGAAGGGAGAAAGGAAGCTAGTCACTCGACATCTGTGGCTTCACAGTACAGAGGACTGTCCAAAGCCGCCAGAT
CTCG
+
4A6>9959@:@>?>1949>;;;??:0....3...5569>?588@@BB??:6/.//.,-222365340112--,-
334844.
@T6XXI:00086:00493
GGGAGAAGGGAGAAAGGAAGACTAGTACGACCCGACATCTGTGGCTTCACAGTACAGAGGACTGTCCAAAGCCGCAG
ATCTCGAGTCGACTAG
+
/1-
/;><>A1941>9;:>;00.7571116111,11111999?@?@A>C????:59>977377396=<@@;??<??@@A@?
>>==88873AA>8
@T6XXI:00087:00456
GGGAGAAGGGAGAAAGGAAGTGCAGTTCGCGGTTAGTGAAGGGAAAAACTATTCTCCCTCTCCCTATAGTGAGTCGT
ATTAGAATTCCCC
+
HH>BB@@BC>@AAB?B?A?@B>BBCC?AAAB?A?AABCC@@@8::88/?BBB@?@@@;BBBCC>?:??AAAAAA?@?
??>AAA=?8<<;1
@T6XXI:00087:00470
CTAGTCGACTCGGAGGATCTGCGGCTTTGGACAGTCCCTCTGTACTGTGAAGCCACAGATGTCGAGTCGACTAGCTT
CCTTTCTCCCTTCTCCC
+
C8/11111@CCD?AAABAAABBB@@AA>B@BBB?@@@;A@@@>>?@@CAB>?B?BCCCCBBBBBCCBBBCBAAC;:7
:?ADCBBCC=>378<<6
@T6XXI:00087:00476
GGGGAATTCTAATACGACTCACTATAGGGAGAGGAGA
+
;>=5<,469>>;ABBBCCCCBCB?6166./<00,/00
@T6XXI:00087:00490
GGGAGAAGGGAGAAAGGAAGCTAGTCGACTCGACATCTGTGGCTTCACAGTACAGAGGGACTGTCCAAAGCCGCAGA
TCTCGAGTCGACTAG
+
;C>CFCACB>BBBB?B>><>B9/0>0//977/07<:66@@>88;8;ECCCCB;98444-
4;;;>A9<<7=@>ACCAA@9==@@@@???>>?>
@T6XXI:00087:00493
GGGAGAAGGGAGAAAGGAAGGAGGAAGTCGCCCCAAACGATTTGTTGACTATCT

```
+
=A6<;@;>?<@>>B<@=><?48311711799DD8CC977171,17366<633.4
@T6XXI:00087:00494
GGGGAATTCTAATACGACTCACTATAGGGAGAGGGAGAATAGTCAACAAATCGTTTGGGGCGACTTCTCCTTCCTTT
CTCCCTTCCTCCC
+
??>4?<@>ABB@CBCCCAABCCB>@777.7???L@DAB>====@@>AAC?@@?AB<ABF6@@B:;57<92424344-
444704,/,.122
@T6XXI:00088:00461
GGGAGAAGGGAGAAAGGAAGGAGAAAGCGACGTCGGGGAAACGTTTGCTATTCTCCCTCTCCCTATAGTGAGTCGTA
TTAGAATTCCCC
+
HECCCC@BB>BBBBBB@B>??ADDC?=@@CB@@????5AC?BDBD?BDCIH@CCCCBCCCBC>BBCBBBBCCCAAA7
734==:::?CC5
@T6XXI:00088:00470
CTATCACTCGAGATCTGCGGCTTTGGACAGTCCCTCTGTACTGTGAAGCCACAGATGTCGAGTCGACTAGCTTCCTT
TCCTCCCTTCTCCC
+
44=4444499==?A?AA:84;>>8@?BBCBCCB>AAAC@@CA<@=:88;8;;CCDCAACCDBABA;88763565555
+/,/7737-70330
@T6XXI:00088:00474
GGGAGAAGGGAGAAAGGAAGCTAGTCACTCGACATCTGTGGCTTCACAGTACAGAGGACTGTCCAAAGCCGCAGATC
TCGAGTCGACTA
+
685:19699-848;-
429977@?;??761111..43...77388:88??AA@==8858@@??>8<<6;;:@@@?;0//8616/.-----
@T6XXI:00088:00489
CTAGTCACTCGAGATCTGCGGCTTTGGACAGTCCCTCGTACTGTGAAGCCACAGATGTCGAGTCGACTAGCTTCCTT
TCTCCCTTCTCCC
+
@8///889?ADDBBBBBBBBBA@@=A??=>B???=?BBBB@@@@@@@<<;???@?AAA888AA@@===@??>>=;??
9<<AC9=:=@@C<
@T6XXI:00088:00491
GGGAGAAGGGAGAAAGGAAGA
+
DC>A9859>:>111-/,0,.4
@T6XXI:00088:00492
GGGAGAAGGGAGAAAGGAAGCTACGTCGACG
+
@C:<99>A@9>99919<?=@::0....50,,
@T6XXI:00088:00497
GGGAGAAGGGAGAAAGGAAGAAAGGAAGAGGGCCCCGGCTGCAATAAGTCAGAGACTATTCTCCCTCTCCCTATAGT
GAGTCGTATTAGAATTCCCC
+
=?69@A69<8;A@C?B?B@CCK@C?B>??=?.77:.7:?;?<ECCFAAAACAB@AAAAA598>727<>>?:??B??@
A@98=>?>77400/488;;7
@T6XXI:00089:00461
CTAGTCACTCGAGATCTGCGGCTTTGGACAGTCCCTCTGTACTGTGAAGCCACAGATGTCGAGTCGACTAGCTTCCT
TTCTCCCTTCTCCC
+
AB1//6699==949;@@AA?=?AA=B@DCCEBBB<A:::AAACBA==9<B=@@BB;;;@?CCBCAA@BAA??83<:=
=6=777.47:344-
@T6XXI:00089:00472
GGGAGAAGGGAGAAAGGAAGGATACTTGTACTGCGCAGGGTTGGGAACTATTCTCCCTCTCCCTATAGTGAGTCGTA
TTAGAATTCCCC
+
```

```
DCBBBA=>><@A@C8;;<@C@BBBBB@9;;<BCCBBCCC=A?@E>D?ABBC@B@@A;A@??A;A=B@@@A@???@?@
@@CAA:89=DD6
@T6XXI:00089:00484
GGGAGAAGGAGAAAGGAAGAACAGAAAAGGGATAACCCGCGGAAAGCTATTCTCCCTCTCCCTATAGTGAGTCGTAT
TAGAATTCCCC
+
280--1,/,//44-
4231399771677,1=6777016,1335<@;@@888=;<<=;=?@@C=8;:<;<;<8?89>33/4882;=@CC6
@T6XXI:00090:00463
GGGAGAAGGGAGAAAGGAAGGAGAAGTCGCCCCAAACGATTTGTTGACTATTCTCCCTCTCCCTATAGTGAGTCGTA
TTAGAATTCCCC
+
HCAB=A@BC>BBCC>C@C@C@BAA?AAABCCC9CDAC????8?B>BBA?CC@A==;4::433-
4448@??@@<<=888;==:4428891
@T6XXI:00090:00473
GGGAGAAGGGAGAAAGGAAGCTAGTCGACTCGACATCTGTGGCTTCACAGTACAGAGGGACTGTCCAAAGCCGCAGA
TCTCGAGTCGACTAG
+
@D>BA@=@B<@@AB7;9;>@@>===/00;0/0071;?7:8B?CC?BAA@@@C;;9333-
4;;?@A?DA=@@=@@@@AB===?<?===;784=
@T6XXI:00090:00485
GGGAGAAGGGAGAAAGGAAGCTAGTCACTCGACATCTGTGGCTTCACAGTACAGAGGGACTGTCCAAAGCCGCAGAT
CTCGAGTCGACTAG
+
BCBAA@@DB?DDBA=B675:A@@BCB>;;>:00/;;;>@C?BB@BAAA@@@<<<43317:?<A@?AA<AA>===?AA
@A@@@???@???AA
@T6XXI:00091:00469
GGGAGAAGGGAGAAAGGAAGTGCAGTTCGCGGTTAGTGAAGGGAAAAACTATTCTCCCTCTCCCTATAGTGAGTCGT
ATTAGAACTTCCCC
+
GDBCCB>==<@ACC@C@C@BBBCBCC@B@@@<?<???BB?BCBCCCC2C>>B@BCCC>AAABB>BAA9::?>>@@89
8@<@AD8::>@@?5
@T6XXI:00091:00487
GAGGAGAAGGGAGAAAGGAAGCTAGTCGACTTGAGATCTGCGGCTTTGGACACGTCCCTCTGTAC
+
449244999:699<@<A@C<@<;;@@@=@==999444487777111,1366613333,16611.3
@T6XXI:00091:00494
GGGAGAAGGAGAAAGGAAGGTGAACAGAGCGCAAACGAATACGTCTCTATTCTCCCTCCCTC
+
8>88-1,1,,,,,*,*.,.34..,4998=>@@ACCBEC<<;ABA989494:;99964//+//
@T6XXI:00092:00450
GGGAGAAGGGAGAAAGGAAGGTTAGTTGGGGGCAGACTTAGGTATCGGCTATTCTCCCTCTCCCTATAGTGAGTCGT
ATTAGAACTTCCCC
+
EF<A@A>@@<AABC@C@C@E?B?BCC@CCCC2CBB=???BB?B???C=@B@@<??@C<??A??<?CC@@@@@AA@@@
@@:==A<==9<<<1
@T6XXI:00092:00462
CTAGTCACTCGAGATCTGCGGCTTTGGACAGTCCCTCTGTACTGTGAAGCCACAGATGTCGAGTCGACTAGCTTCCT
TTCTCCCTTCTCCC
+
@@1//7699BABCBCCCBBC@CAB>A=>@BBBCC>BBCCC@@@A@@@;=ABAACCBBBCBBBA?@@A;889662:>A
C@CCFD?@587=>:
@T6XXI:00092:00467
GTCGACTCGAGATCTGCGGCTTTGGACAGTCCCTCTGTACTGTGAAGCCACAGATGTCGAGTCGACTAGCTTCCTTT
CTCCCTTCTCCC
+
```

```
BC=::CBCBBECECCCCCDCCC>C@CCDBCDB?BCC?@@CBAAAC?BB>AABBBCBBB@?ACAA88:C????A>AA:
<=>>.:6;CBC@
@T6XXI:00092:00471
GGGAGAAGGGAGAAAGGAAGAATACGGTGGGGCTGCTTAGTTAACAGTATTCTCCCTCTCCCTATAGTGATCGT
+
:B199>699-4888-424733179;@=9888/9999>9==??B<99<999@B<;<:;>===6=66111..2---
@T6XXI:00092:00481
GGGGAATTCTAATACGACTCACTATAGGGAGAGGGAGAATAGTTCTGGACTGTACTCTGGGGCTGTCTCCTTCCTTT
CTCCCTTCTCCC
+
CCB7A;;;<CC?BBCCCCCC==@CA???;?CCCC>A??=A:???AAA;>>?@6/11D@EEC8B@CCCCB@B2629?8
;?BB>B?A??C:
@T6XXI:00092:00489
GGGGAATTCTA
+
CDE7;749949
@T6XXI:00092:00495
GGGGAATTCTAATACGACTCACTATAGGGAGAGGGAGAATAGTCAACAAATCGTTTGGGGCGACTTCTCCTTCCTTT
CTCCCTTCTCCC
+
BCC:C?B@BCC?BBCBBBBB@AD@>776416??A=AAA>AA@@@?=?@A>BAAAB;CCC9CBBBB=??B=@=@>@A<
??BB=A;==AA<
@T6XXI:00092:00498
GGGAGAAGGGAGAAAGGAAGAATAATTACGTTTCAACGGGCGCTGGACTATTCTCCCTCTCCCTATAGTGAGTCGTA
TTAGAATTCCCC
+
@A@ACCABA?@??B>B@B@BB?A??@@BBBCC?A?>?CC>BBBAB@B@@AA>A@=>;A9==B<A???@@AAA=A=68
=>B<<8;8<:;1
@T6XXI:00093:00459
GGGGAGAAGGGAGAAAGGAAGGGGAGAAGGGAGAAAGGAAGC
+
BCD9ECC@A=69444-9<@<ABB7A??576-7000,0,/,.3
@T6XXI:00093:00460
GGGAGAAGGGAGAAAGGAAGCTAGTCGACTCGACATCTGGTGGCTTCACAGTACAGAGGGACTGTCCAAAGCCGCAG
GATCTCGAGTCG
+
49-4;B<>@<@BBC@C?B?BA769???=>@@770000..,.5-/0:@BCJCDC;88344-
7===AC9<;4::34444/4;;443..112
@T6XXI:00093:00488
CTAGTCACTCGAGATCTGCGGTTCTGGACAGTCCCTCTGTACTGTGAAGCCACAGATGTCGAGTCGACTAGCTTCCT
TTCTCCCTTCTCCC
+
=<1/111:@HCBBABB;;4634,./9699::A@?;@CCBB@??A@@?>8;8<CBCBCBCCB?<=A@<:?A::8>@?B
C???@A<@;=;<;6
@T6XXI:00093:00490
GGGGAATTCTAATACGACTCACTATAGGGAGAGGGAGAATAGTCAACGAATCGTTTGGGGCGACTTCTCCTTCCTTT
CTCCCTTCTCCC
+
DCB9A99<<BBBCBBBCAABBBBBB???;?ABCC>CAA=A<<<==<A??;??:==7>=>4AABBB>BAB>@>@?AC:
BBAA<A99=??;
@T6XXI:00094:00450
GGGAGAAGGGAGAAAGGAAGGAGAAGTC
+
CCACBCAEG@CB;;5;::,0,0..65..
@T6XXI:00094:00464
GGGGAATTCTAATACGACTCACTATAGGGAGAGGGAGAATAGTCACCAAACCCCCCATGGTGCCTATTGCCTTCCTT
TCTCCCTTCTCCC
```

+
CCB8A54694499=@@CA@@B>?>=777,7>===9?>?=@88877A;@@:?CDDD-
D@?;=8:177?>@@>C?C?A@7:444-47:8;;6
@T6XXI:00094:00481
CTAGTCGACTCGAGATCTGCGGCTTGGACAGTCCCTCTGTACTGTGAAGCCACAGATGGACTGCTTCCTTTCTCCCT
CTCCC
+
444499@<:;BA@?94949337771313766111,.......13335238;998;;8?>B8;<??@B=?@9//,7+,
,++00
@T6XXI:00094:00492
GGGGAATTCTAATACGACTCACTATAGGGAGAGGGAGAATAGTCAACAAATCGTTTGGGGCGACTTCTCCTTCCTTT
CTCCCTTCTCCC
+
CCE9C=?>?CCBCB>>BBBB@A>76116.7==?B@AA@?A:?AAB????<ACBBB>BBB7BCCBB?:87:=;=<??;
@AAC<A=@@@A<
@T6XXI:00094:00496
GGGAGAAGGGAGAAAGGAAGGAGAAGTCGCCCCAAACGATTTGTTGACTATTCTCCCTCTCCCTATAGTGAGTCGTA
TTAGAATTCCCC
+
CC?CBB@BBBBBBB@B@C@A@ACA??BBBCEC8BB>BBAAA@AB@FA??AA?@@@B=??@AA<A===?@AAA@@@@@
@?A@?<=8;;;4
@T6XXI:00095:00456
GGGAGAAGGGAGAAAGGAAGGGGAGAAGGGAGAAAGGAAGGGGAGAAGGGAGAAAGGAAGCTAGTCACTCGACATCT
GTGGCTTCACAGTACAGAGGGACTGTCCAAAGCCGCAGATCTCGAGTCGACTAG
+
BB<AAB>BCCAB@B?B?B@BBB9CBB@AA=888?9?=@>AAA6BB@;<<48<553;;=;??A<?BA;6:?<==??A?
AAC?858:AAAA@AB==74718B===?38718>><=?=AA<==???@CCB@@?A
@T6XXI:00095:00458
CTAGTCACTCGGAGATCTGCGGCTTGGACAGTCCCTCTGTACTGTCGAAGCCACAGATGTCGAGTCGACTAGCTTCC
T
+
@99449,,,,,*,00,/49:9937131336666;;776<9=7//---2,-
4884437;;?A997711.......,101
@T6XXI:00095:00487
CTATCGACTCGAGATCTGCGGCTTTGGACAGTCCCCTCTGTACTGTGAAGCCACA
+
@;94444;999=848>998847==9A8;66=6611,166>==988./-,-75/.,
@T6XXI:00095:00494
GGGGAATTCTAATACGACTCACTATAGGGAGAGGGAGAATAGTCACACATTTACCCAAATTTCTATTCTCTTCCTTT
CTCCCTTCTCCC
+
BCC9A=>=;<;>ACBDAACGBA@==661.7==AC@CDA@D9>?::?@?@AA:??A<AA<AA99==<8;;44247:94
9;;;-434;==7
@T6XXI:00095:00499
GGGAGAAGGGAGAAAGGAAGCTAGTCACTCGACATCTGTGGCTTCACAGTACAGAGGGACTGTCCAAAGCCGCAGAT
CTCGAGTCGACTACGCTTCCTTTCTCCCTTCTCCC
+
995==@>@@9A999095858@@>=:<00..-.--
35999?59:7::::AA=;;;444,4444=?;=C;==9=@AACA===@?==<3461---27-.-
.448>?@<??B@@?1
@T6XXI:00096:00454
GGGGAATTCTAATACGACTCACTATAGGAGAGGGAGAATAGTCAACAAATCGTTTGGGGCGACTTCTCCTTCCTTTC
TCCCTTCTCCC
+
BCCAC=?=?CC@BBBBBABAA>AA?9937?<<<<?>>>AAA?@@;??D=B???C>DDC7@@C;;8;CC=@;>;?=7@
@AH=D?@@?C;
@T6XXI:00096:00458

```
GGGAGAAGGGAGAAAGGAAGGGACTGCGGGTAGGGAAGAACGTTGTGCTATTCTCCCTCTCCCTATAGTGAGTCGTA
TTAGAATTCCCC
+
DCACCB@DD=BBDE=B>B@CC>CBCBBBCBCCAA>A@B@?BBB@ACCCCCE?BADN?BBBCD@CCC@@ACBBBAAAA
A?BAH>=;@AA5
@T6XXI:00096:00468
GGGGAATTCTAATACAGACGCTATAGGGAGAGGGAGAATAGTGACAATCTTG
+
8?=1=+/,///7881100...894444-44433,3777711161777///,/
@T6XXI:00096:00480
GGGGAATTCTAATACGACTCACTATAGGGAGAGGGAGAATAGTCAACAAATCGTTTGGGGCGACTTCTCCTTCCTTT
CTCCCTTCTCCC
+
A;?9>1:<<BBIDBBBBCCB@C@=<777.7>777.48;<@?@?A;?<;8461111,177,11444,0.7,/,/,//,
/.4;:8243553
@T6XXI:00096:00483
GGGAGAGGAGAAAG
+
11,1,,02,*,,*,
@T6XXI:00096:00493
GGGAGAAGGGAGAAAGGAAGCTAGTCACTCGACATCCTGTGCTTCACAGTACAGAGGGACTGTCCAAAGCCGCAGAT
CTCGAGTCGACTAGCTTCCTTTCTCCCTTCTCCC
+
CD@CCCBCC@@A@B=B=@=@CA@>:77,,,0,****),,,...67678@@A==;444,44:==><?C8:9?AAAAA@
???A@@@@@@899@@@>@?AB:?@@C9=:<ACD=
@T6XXI:00096:00497
GGGGAATTCTAATACGACTCACTATAGGGAGAGGGAGAATAGTTTTTCCCTCCACTAACCGCGAACTGCACTTCCTT
TCTCCCTTCTCCC
+
=>>4?<>?CBB@CCBB>>>B?A?77111.7>>@D>CAA@@?====;.=>7==?CF@@=@???=A><<<??<<;>477
.7:;;:?=???@7
@T6XXI:00097:00450
CTAGTCACTCGAGATCTGCGGCTTTGGACAGTCCCTCTGTACTGTGAAGCCACAGATGTCGAGTCGACTAGCTTCCT
TTCTCCCTTCTCCC
+
CB:99<44:AA=BBBBCBB?;7776:>ABBBBBB>BCCB?@@A=8<<48<@CDCAAACCAAACCCCBCABAAA>@>A
@:AA@A<@598?A<
@T6XXI:00097:00453
GGGAGAAGGGAGAAAGGAAGCTAGTCACTCGACATCTGTGGCTTCACAGTACAGAGGGACTGTCCAAAGCCGCAGAT
CTCGAGTCGACTAG
+
<9-9AA>BCBCBBC<@=@>@@@BBA@7422,,,,///367:=?=??@@AACB;;444-
4::<<=>@C@AB>AA=<<<===<<<=>>=;<<<
@T6XXI:00097:00486
GGGAGAAGGGAGAAAGGAAGC
+
CB@B<;79@:?111,1,0,..
@T6XXI:00097:00487
GGGGAATTCTAATACGACTCACTATAGGAGAGGGAGAATAGCTTGATGTCCTGAGGACCTTGCTTCTCCTTCCTTTC
TCCCTTCTCCC
+
@>?4>6;:<CCCCC@@@BBB@@<?;6107>77758B@>C?AAC@BA=?AA>@@BA??><@?C@@>@==899;244-
4:;;:@?===C<
@T6XXI:00098:00451
GGGAGAAGGATAAGAGAG
+
--+,/1,/,//;883777
```

```
@T6XXI:00098:00454
GGGAGAAGGGAGAAAGGAAGCTAGTCACTCGACATCTGTGGCATCACAGTACAGAGGGACTGTCCAAAGCCGCAGAT
CTCGAGTCGACTAG
+
CB>BBB@CG?A@@C7:8;?A@AC@?????@B??@B?>:770,,,+++039569=@AAA??A?BA;>>7;;74444=;
<=AAAAA=<<<<<;
@T6XXI:00098:00460
GGGAGAAGGGAGAAAGGAAGCTAGTCGACTCGAGATCTGCGGCTTTGGACAGTCCCTCTGTACTGTGAAGCCACAGA
TGTCGAGTCGACTAGCTTCCTTCTCCCTCTCC
+
99099@=A@19999184827730.70.....37369>6::C=??B<@@@?=???=69;;48844;;4438=;>@@@@
A<<<?==;<<==;;4442/,/44...+----,
@T6XXI:00098:00482
GGGGAATTCTAATACGACTCACTATAGGAGAGGG
+
444,4242499299B<?>;76131166011111,
@T6XXI:00098:00486
GGGAGAAGGGAGAAAGGAAGGGGAGAAGGGAGAAAGG
+
DD@AABADD@@A@C=?<;:<<>2887377.7000,/,
@T6XXI:00098:00488
GGGAGAAGGGAGAAAGGAAGGATCGTTGAATACGGATGTTGCGATTGAAATAGATATGGATGATCCTATCGACTCGA
CATCTGTGGCTTCACAGTACAGAGGACTGTCCAAAGCCGCAG
+
ECAAAAACC>BBBC>C@C@C@CCA@A@BB?BCA??A@<><===;@>AAA<@AAAA9==>AA@@@A;:;?@A@AAA@7
388;9:89;;;9<@@@AB@;;9./,/88844/3717@=AAAA
@T6XXI:00099:00452
GGGGAATTCTAATACGACTCACTATAGGGAGAGGGAGAATAGTCAACAAATCGTTTGGGGCGACTTCTCCTTCCTTT
CTCCCTTCTCCC
+
CCC:C;;<<CC@CBBBCAA@?8<?=666.7??>B>FAA@@9??@B>@@@;CBBBC>BBB7BBBBC?AAA=<9<<?@<
@@AC?C;==@?<
@T6XXI:00099:00469
GGGAGAAGGGAGAAAGGAAGCTAGTCGACTCGACATCTGTGGCTTCACAGTACAGAGGGACTGTCCAAAGCCGCAGA
TCTCGAGTCGACTAG
+
KC>AAEBDB>?@@@6>=?=?A?9=;000<<>:0:9??8BCC>@@@CBBB???=;;;;;7<@?@AA=AA<AA:<====
99=@@@AA????=88
@T6XXI:00099:00472
CTAGTCACTCGAGATCTGCGGCTTTGGACAGTCCCTCTGTACTGTGAAGCCACAGATGTCGAGTCGACTAGCTTCCT
TTCCTCCCT
+
444444444;4444:=98499338,313379=?A=9775/000034>23;>CAABB@@?AAAABBCBAA@@//,0,0
0,0,,7715
@T6XXI:00099:00475
GGGAGAAGGGAGAAAGGAAGGACAAATAGATTGCGGGGGAAACGCTAGCTATTCTCCCTCTCCCTATAGTGAGTCGT
ATTAGAATTCCCC
+
HDACCB>@@==@@C>A=@CE?AACC>BBCBA?BBBBB?1BB>:::BCCCCBB?BCCE<A>>?C=????AAAA@@@AA
AA>AA@>@>@A@5
@T6XXI:00099:00483
GGGAGAAGGGAGAAAGGAAGAATAATCAAACACGACACGGTGAAAGGCTATTCTCCCTCTCCCTATAGTGAGTCGTA
TTAGAATTCCCC
+
DC>BCC@BB>?@@B>B@B@BB>A@DBCAA>BC@A>==AB>A:77/7:=>BC=@ACD>BBBCC>C@@==<C@@AA@@8
869?C>A=@DA6
@T6XXI:00099:00484
```

```
GGGAGAAGGGAGAAAGGAAGGAGAAGTCGCCCCAAACGATTTGTTGACTATTCTCCCTCTCCCTATAGTGAGTCGTA
TTAGAATTCCCC
+
CD?BBA?AB?BBBB>B?A?C?AB@>>BBB?BB8??<??>BC@BC?B?@=?@=??=?:?===D?B@@@@@@<@AAA@@
@>@A@>@9<<<4
@T6XXI:00099:00487
CGAGATCTGCGGCTTTGGACAGTCCCTCTGTACTGTGAAGCC
+
:@A<<;93;4;87797<597;999<8<76<3.....-4211,
@T6XXI:00099:00488
GGGGAATTCTAATACGACTCACTATAGGAGAGGGAGAATAGTCAACAAATCGTTTGGGGCGACTTCTCCTTCCTTTC
TCCCTTCTCCC
+
DLCAD::;<@@ADBB@?B??>AD@?7607><=?<?@@>@@@BBA?@@A<B?==A=@CC9?:::B==B@3837:==7=
==C?B<??AB@
@T6XXI:00050:00502
CTATCGACTCGAGATCTGCGGCTTTGGACAGTCCCTCTGTACTGTGAAGCCACAGATGTCGAGTCGACTAGCTTCCT
TTCTCCCTTCTCCC
+
<1=7////68;BBBBBCCCB?BB@;>=>BBBCCC>B>B@??9=789A69<@CCCBAAACCCCCABACCCB???@D@C
B<B@AA<@9;;==6
@T6XXI:00050:00506
GGGGAATTCTAATACGACTCACTATAGGAGAGGGAGAATAGTTTTCCCTCACTAACCGCGAACTGCACTTCCTTCTC
CCTTCTCC
+
444,42924=@<CBBACCCCC>;::// ,.:.76,044/0...--*--+--
44668::::;=<=<<>@A=;=4747343+34000/
@T6XXI:00051:00519
GGGGAATTCTAATACGACTCACTATAGGGGAGAGGAGATACAT
+
DDG:C?@?ACCDCC@@AA@@AABCA:???5<99:;8//.,,,,
@T6XXI:00051:00522
GGGAGAAGGGAGAAAGGAAGGATACGCGGAGGCTGGCAGGGGAAAAAAACTATTCTCCCTCTCCCTAATAGTGAGTC
GTATTAGAATTCCCC
+
94-
499:;>699AA?A=?=?<>>A>;9B@BB@B>:244444,444444&479999111,114000**)****+24558:<
=;<<9/0/034-
@T6XXI:00051:00531
GGGAGAAGGGAGAAAGGAAGGAGAAGTCGCCCCAAACGATTTGTTGACTATTCTCCCTCTCCCTATAGTGAGTCGTA
TTAGAATTCCC
+
A?=AAB?BG?BCGBAA>B=?=@@A=AAAAACA7AD=A<BB>576A<A???A>@?AC>=888@178@=AA@@@999A@
;.--0*+**..
@T6XXI:00052:00505
GGGGAATTCTAATACGACTCACTATAGGGAGAGGGAGAATAGCTATCAACCGTCGCCATTACGTTATTCTTCCTTTC
TTCCTTTCTCCCTTCTCCC
+
<;9/9<@=ACB@CBA?BBBB?@@77111.:>>CE<A???A<<<@?<8?5607?:4438?;?@@@>@A>==;=>@C;@
@>@>@A7>=>>784:4471
@T6XXI:00052:00507
GGGAGAAGGGAGAAAGGAAGGAGAAGTCGCCCCAAACGATTTGTTGACTATTCTCCCTCTCCCTATAGTGAGTCGTA
TTAGAATTCC
+
491449599-
448908239?<>@C@?@@BDCC8BA<?;<888::>@>CABC@999A:::743+34037;;;<743833/000/0211
@T6XXI:00052:00519
```

```
GGGAGAAGGGAGAAAGGAAGGAGAACAACACGATCGGTTAAACGTAGCTATTCTCCCTCTCCCTATAGTGAGTCGTA
TTAGAATTCCCC
+
CCBBAA@BB>BBBC>B@B@B@BA?>BB@BBBCCC??=@=@C?CCBCCCCA??>ACA===?BB>@AA@@@B??@@@??
?=@@@?A=@@@5
@T6XXI:00052:00521
CTAGTCGACTCGAGATCTGCGGCTTTGGACAGTCCCTCTGTACTGTGAAGCCACAGATGTCGAGTCGACTAGCTTCC
TTTCTCCCTTCTCCC
+
CA99994449;;@;<9C=<<CBCCC>B?AA>?77:5:??AABEA898A>=A>ADDDA@CCCCA@ACBBBBBCBB=@:
??<?A=@9;84444-
@T6XXI:00052:00527
GGGGAATTCTAATACGACTCACTATAGGGAGAGGGAGAATAGTACGACTAACTCGTTCGGGCCATACTTCTCCTTCC
CTTTCT
+
444,48>?GCCACCC;:<FDCCDCFBBC>BBCDC>A==>AAA;:::@//.7:54599044-4/00-2201--,-,-
3011333
@T6XXI:00052:00529
GGGGAATTCTAATACGACTCACTATAGGGAGAGAGATATAAGGGCGTCAAATCTTTCTCTCCACAACCAACCTTCCC
ATTACCAATTCTAC
+
999-
42928AA>BDBBBCCCBBBBB???;>@@AA===<@@98875??;<<7?>;;6;<;;=;?@A>A>@=@6000?9;;53
3+,++*++++
@T6XXI:00052:00534
GGGGAATTCTAATACGACTCACTATAGGGAGAGGGAGAATAGACATGTGATCTCCGCACTTGCGCTCTTCTTCCTTT
CTCCCTTCTCCC
+
CCC9B=>=@BB@CCB???BB?C@77111.7>?CC>BAA?C=::888====<<<<:;=AAC<==::?A@>@@=@=?@;
@@@@<@?@@@A<
@T6XXI:00052:00542
GGGGAATCTAATACGACTCACTATAGGGAGAGGGAGAATAGTCTTGCAATTTCCTGCGGTGCATTCTCCTTCCTTCT
CCCTTCTCCC
+
111*/,1/6676?@<;;?@:7:@<?<?9????C>@?@>998?A;37872;A?B9:8@@?BB@?@?AAA>97701011
11,1611030
@T6XXI:00052:00546
GGGGAATTCTAATACGACTCACTATAGGAGAGGGAGAATAGCCACCAACTATTGTTCGTCGCCGTTCTCTTCCTTTC
TCCCTTCTCCC
+
444,42424448887?<A@BD/0/.//,.33;>;8>@648?A<??<<97344/00/334435233555:<:?<?C<@
?AA?D@@B@A:
@T6XXI:00053:00503
GGGAGAAGGGAGAAAGGAAGCTAGTCACTCGACATCTGTGGCTTCACAGTACAGAGGGACTGTCCAAAGCCGCAGAT
CTCGAGTCGACTAG
+
AA<@BC>BB=@@?@<@>B??@?A???;//.///-
.....;9778:CCCA?=???77714;;;8834;74437;;=?AAAAA@;8/..///3
@T6XXI:00053:00504
GGGAGAAGGGAGAAAGGAAGCTAGTCACTCGACATCTGTGGCTTCACAGTACAGAGGGACTGTCCAAAGCCGCAGAT
CTCGAGTCGACTAG
+
9914489@C?BCCC=B@A@@@:::=7661101606688:=>AB?AABBCBCABB88814;;?@@>?D:<<9<<<?@A
@???@@=>=AAC??
@T6XXI:00053:00513
GGGAGAAGGGAGAAAGGAAGCATAGTCGACAGTCAAGACGCTTGGACATCCTCCTCTCTGAGCTAGATGC
+
```

```
;<8;@@BBHBD@>>5;;<,0..52../...466342118///,/,/8444:4424:87/,,,**,,,,06
@T6XXI:00053:00514
GGGGAATTCTAATACGACTCACTATAGGGAGAGGGAGAATAGTCACACATTTACCCAAATTTCTATTCTCTTCCTTT
CTCCCTTCTCCC
+
?A@6>=@>BCC?BBB?@BBB@@@>9167.7>>@B>BAC@A<<<==<>=???;?==;==9984686?<?@?@>@>AB;
@@AA<@:==AC<
@T6XXI:00053:00515
GGGGAATTCTAATACGACTCACTATAGGGAGAGGGAGAATAGTTTCCCTCCACTAACCGCGAACTGCACTTCCTTCT
CCCTTCTCCC
+
CEB7B79:<@A?BFCCCCBBCC@=7777.7>>@B>@BA=;4000+4;,442:B?CAC@CC?<>=>A<:7447-/-
0;;;6@;>;;;7
@T6XXI:00053:00541
GGGGAATTCTAATACGACTCACTATAGGGAGAGGGAGAATAGTCTGTCGTTCGGTCGACGCTGCCTATCCTTCCTTT
CTCCCTTCTCC
+
===6>>A>BBC@C@?>BBBB9>A??6665966BC==9;;;.//:666>8>;==3448444<<<<8;;993434344-
4//.+.,---,
@T6XXI:00054:00504
GGGGAATTCTAATACGACTCACTATAGGGAGAGGGAGAATAGCCGTCAACAAATCTGGGGTCTATTCTGCTTCCTTT
CCTACG
+
/11+/7@<ABB@GBBBCCCCCBBB???B<@ABBCB@AB>:99@;>=:>;116,39777;5649=;<>B;??<?:4=6
7+****
@T6XXI:00054:00505
GGGGAATTCTAATACGACTCACTATAGGGAGAGGGAGAATAGTCAACAAATCGTTTGGGGCGACTTCTCCTTCCTTT
CTCCCTTCTCCC
+
944,9595<BCAECCBBBCCA@<77111.7>==?>@CA@D;A?@?=A;;:>;;??;CCC7AABBB?BCC8968?BA=
????;@9==A@;
@T6XXI:00054:00513
GGGGAATTCTAATACGACTCACTATAGGGAGAGGGAGAATAGCTACGTTTAACCGACCGTGTTGTTCTCCTTCCTTT
CTCCCTTCTCCC
+
AAA8A@CACCC@CCCBAACB=:?==666.7>@AC?C@@>@<<<???@==;<;<>???><<@<;?=:<;;8;9<=@A;
>??C=A>A;;=;
@T6XXI:00054:00514
GGGGAATTCTAATACGACTCACTATAGGGAGAGGGAGAATAGTCAACAAATCGTTTGGGGCGACTTCTCCTTCCTTT
CTCCCTTCTCCC
+
?>@4>6959BB@AA@BCCCCBAA9;66659??BC>A??<?==<<<>=??<BBACC8?:?6??BCD?A@?>@=@=BA=
B@@@<@?A@@A<
@T6XXI:00054:00521
GGGGAATTCTAATACGACTCACTATAGGGAGAGGGAGAATAGTTCGCCAATATCCCGCGGCGTGTTCTCCTTCCTTT
CTCCCTTCTCCC
+
ABC:C?B?BCCDBBAABBBB?AB?@77788CCAC;A>A?=;;<>=<<;A?BC?==;@BB?B884?;=??=@>@>@@;
@@AC<A>>===9
@T6XXI:00054:00527
GGGAGAAGGGAGAAAGGAAGGTAACGGCAGCGACGTAGGGAGATCAGCTATTCTCCCTCTCCCTATAGTGAGTCGTA
TTAGAATTCCCC
+
CE@CCC@BB=?ABBBA@B@B@BB@CB@CCCA@@BA@@@B=@ABFBBAAABA>ABB@<>@8::6:@@>>;9998?8;;
C=??@=@5888.
@T6XXI:00054:00533
```

```
GGGAGAAGGGAGAAAGGAAGAGAACAGTCACAGGCTCGAGCTGCAAGCTATTCTCCCTCTCCCTATAGTGAGTCGTA
TTAGAATTCCCC
+
/1-//4284-448:-
423777???CCCBCCCBB?B??@??ABAAC@FLCBB?BBBD>CC@@@;?AACCA?@B@???8838==:=8<<<4
@T6XXI:00054:00536
CTAGTCGACTCGAGATCTGCGGCTTTGGACAGTCCCTCTGTACTGTGAAGCCACAGATGTCGAGTCGAGTAGCTTCC
TTTCTCCCTTCTCCC
+
BCDCC:><FCCCCCCBAB@CB@BBBAB@BB@BBC@>@@@?AAAA???A<?B@BCBBBBBBCCCB?@@CCCCBBB@C@
DC>BC@C<@>???A<
@T6XXI:00055:00500
GGGGAATTCTAATACGACTCACTATAGGGAGAGGG
+
28207+959;;?CBCGEC;;:7316611,.....+
@T6XXI:00055:00517
GGGGAATTCTAATACGACTCACTATAGGGAGAGGGAGAATAGTCAACAAATCGTTTGGGGCGACTTCTCCTTCCTTT
CTCCCTTCCTCCC
+
111*1,1,///,/433<BAAAA?77777-
716=796<3037//.4546;:777BC;@EE7;;>>>::77,/,/,//,/01101,-,---+
@T6XXI:00055:00519
GGGGAATTCTAATACGACTCACTATAGGGAGAGGGAGAATAGTCAACAAATCGTTTGGGGCGACTTCTCCTTCCTTT
CTCCCTTCTCCC
+
999/4:B@CCCACBBBCCCE;:8>>777.7?777-4;=<@AAA@@>@AA<CCCB?.777-
7??@B?B@A;?<?<?A<AAADAB9;;<:4
@T6XXI:00055:00521
CTAGTCGACTCGAGATCTGCGGCTTTGACAGTC
+
;2,,,-,,//8;B??ABB;8887770---****
@T6XXI:00055:00540
GGGGAATTCTAATACGACTCACTATAGGAGAGGGAGAATAGCCGCACCAGCGTTGACAATTTATTCTCCTTCCTTTC
TCCCTTCTCCC
+
DCC9B>B?BCCBCCCBBBBA?:977//-
0==AB>B@@=@;A=<=<><<<@@A=<A<<<=A=;A=;:?>8<9<=@A<@@@C<A>A==84
@T6XXI:00055:00547
GGGGAATTCTAATACGACTCACTATAGGAGAGGGAGAATAGTCTAGCTCCCAGTCAAACGATGTTCGTCTTCCTTTC
TCCCTTCTCCC
+
?<<0>-
1,1//768>?A;77B@7166101:7?B>744:6;9;8738=68::???<<<7=?>>BD9<5500/04662///:287
8///,
@T6XXI:00055:00549
GCTAGTCTACTCGAGATCTGCGGCTTTGGACAGTCCCTCTGTACTGTGAAGCCACAGATGTCGAGTCGTA
+
4/,,,,,,,//6/4448844477333,31331716666777@BAAB:99;>B?@>;BB:::??;;;<000
@T6XXI:00056:00502
GGGGAATTCTAATACGACTCACTATAGGAGAGGGAGAATAGTTTGCGCATTCGGTGCACTGTTATTCTCTTCCTTTC
TCCCTTCTCCC
+
444-92959@@@BBBBCBBBB>=;;//-
0>>@B>AAA?===A@;????@BACC@CC@BB>??=>?>?<AA?BACC>=?<<;?><AA=:
@T6XXI:00056:00534
GGGATTCTAATACGACTCACTATAGGGAGAGGGAGAATAGCTAGCGTTATTCCGCTAGGTGCTTCTCCTTCCTTTCT
CCCTTCTCCC
```

```
+
9919><AACCCBBABBBA@CB==616.7==BC@AA@>?<?A?>AA=B>==:<:;<<@@?CB?<<==<=?8<37717A
AA<A??>>A<
@T6XXI:00056:00540
GGGAGAAGGGAGAAAGGAAGCTAGTCGACTCGACATCTGTGGCTTCACAGTACAGAGGACTGTCCAAAGCCGCAGAT
CTCGA
+
@@<@@@@CC@CBBB?B@D?CAA=??BBCBBB?99::::@AA==@=?BB@=?=994.../9;;=A=AA<AA=@@=<<<
488AA
@T6XXI:00057:00505
GGGAGAAGGGAGAAAGGAAGGAGAAGTCGCCCCAAACGATTTGTTGACTATTCTCCCTCTCCCTATAGTGAGTCGTA
TTAGAATTCCCC
+
@@<>985;B>AABC<@=@>A=ACCCCCCBBAA5?<6=>>??8AB?BB@A@@=?AAA<??@AA<A@@@@AAA=;;;0/
/682924/003,
@T6XXI:00057:00510
GGGAGAAGGGAGAAAGGAAGCTAGTCGACTATCTGCGGCTTTGGACAGTCCATCTGTGGCTTCACAGTACAGAGGGA
CTGTCCAAAGCCGCAGATCTCGAGTCGACTA
+
AA?ACC@CC?ABBB>C@B@BB>>B@CCCCB??==711,111,1><<?A<958=?;AACCCC>BBBBB>CA@<===9=
@@AAA===;==;?;:?AAAAA<==@@@?@==
@T6XXI:00057:00513
GGGGAATTCTAATACGACTCACTATAGGGAGAGGGAGAATAGCCCTGTTATTTTGCGGCGCTGTCTTTGTCTTCCTT
TCTCCCTCTCC
+
944,4242499<9101.=>07637716619?;?D>AAAC<;;;:4==<363880888533366646640003/0/00
+----+----,
@T6XXI:00057:00528
GGGAGAAGGGAGAAAGGAAGGAGAAGTCGCCCCAAACGATTTGTTGACTATTCTCCCTCTCCCTATAGTGAGTCGTA
TTAGAATTCCCC
+
DD?BBB@BB>BBBB=BBC@A@BB?>A?A@@@@7?C>CBBBC>B?>@??;@@>AAAA;@???A<AA@@@ABA@><=?C
C?B@@@B<>==4
@T6XXI:00058:00518
GGGGAATTCTAATACGACTCACTATAGGGAGAGGGAGAATAGTCAACAAATCGTTTGGGGCGACTTCTCCTTCCTTT
CTCCCTTCCTCCC
+
944,9:A@DBCDFCCBBBBB??ABC???<?AAAE>@@@>@@@ADA>@@@?ABABD>DDD7??C??:??A343479:4
:88;7:/43444,
@T6XXI:00058:00520
GGGAGAAGGGAGAAAGGAAGGAGAAGTCGCCCCAAACGATTTGTTGACTATTCTCCCTCTCCCTATAGTGAGTCGTA
TTAGAATTCCCCC
+
BC?BCD@CC?BCCC>B?A=@=;;9;?=??>??2??<?CCA@>??5::<<@@?BCCC489ACC9==ABA@AA@9:9@A
A>A@@<?=ADCC1
@T6XXI:00058:00522
AGATCTGCGGCTTTGGACAGTCCCTCTGTACTGTGAAGCCACAGATGTCGAGTCGACTTAGCTTCCTTTCTCCCTTC
TCCC
+
A999@;<4D<?@A=@>BBBCBBD@DBDBBBC@<;;@=9>7::?86685;;9?8868//,/8:::B?BB=?977,313
1662
@T6XXI:00058:00524
GGGGAATTCTAATACG
+
;:808,,*,,*)*,,,
@T6XXI:00058:00532
```

```
GGGAGAAGGGAGAAAGGAAGGAGAAGTCGCCCCAAACGATTTGTTGACTATTCTCCCTCTCCCTATAGTGAGTCGTA
TTAGAATTCCCC
+
EECCCB?A?=BB??=B=@>@@CB@?C?@??BB7BB>CEBBB>BC?CB<@@<8:<==;9==?A<AA@?@@@@A@@@>>
??CAA@@<?>>5
@T6XXI:00058:00544
GGGAGAAGGGAGAAAGGAAGGAGAACAACACGATCGGTTAAACGTAGCTATTCTCCCTCTCCCTATAGTGAGTCGTA
TTAGAATTCCCC
+
CC@B@@@AA?>>>B<B@B@B@BB?@BB>??ACDBBB?B@AC>BB>ACCC?>=?ACC>BAAAB>A=::?8<@AA@@?@
@>A@?=?>BAA6
@T6XXI:00058:00547
GGGAGAAGGGAGAAAGGAAGGTGAACAGAGCGCAAACGAATACGTCTCTATTCTCCCTCTCCCTATAGTGAGTCGTA
TTAGAATTCCCC
+
CB>BBB>BC>BBBB>B@B595;@><>>999477=C?BBBBBBBBBCCCCC@CBBA===98809???@@??????A;
;6:=>:>5999/
@T6XXI:00059:00510
GGGGAATTCTAATACGACTCACTATAGGGAGAGGGAGAATAGTCAACAAATCGTTTGGGGCGACTTCTCCTTCCTTT
CTCCCTTCTCCC
+
BA?4@5;8<BCACCB????>>AB>9111.6=77:8:AA@A??AAA?;@<:????A;>>?7ABCCCA?@=;=;9<CC>
>??C=A<??AA<
@T6XXI:00059:00512
GGGGAATTCTAATACGACTCACTATAGGAGAGGGAGAATAGTCAACAAATCGTTTGGGGCGACTTCTCCTTCCTTTC
TCCCTTCTCCC
+
DCC9CAC@BCC@BAAA?<<<>:977//-
0>;>A?AAA>B===AA<<@A<CC>=?8<996<BBBE@BB@>A>@>@@<@A@@<@>@@AB>
@T6XXI:00059:00516
GGGGAATTCTAATACGACTCACTATAGGGAGAGGGAGAATAGTCAACAAATCGTTTGGGGCGACTTCTCCTTCCTTT
CCTCCCTTCTCCC
+
999/42924>><A;;<D@@@CDCA>777.77===6??@8<89884:48417>>>A<ABB7BDBCC@A>:5756788,
/,,5500*-+000
@T6XXI:00059:00543
GGGGAATTCTAATACGACTCACTATAGGGAGAGGGAGAATAGTCAACAAATCGTTTGGGGCGACTTCTCCTTCCTTT
CTCCCTTCTCCC
+
=@=4><@=@BBBCB@<BBBB?BA9966659??BC>@CC=@<???@>@AB?CBAA?8?=?5A@ABC?A?A>?>??AA<
???@<A?@@@A;
@T6XXI:00059:00545
GGGAGAAGGGAGAAAGGAAGGAGACAGTGAGCGCCGCACAACACGTCACTATTCTCCCTCTCCCTATAGTGAGTCGT
ATTAGAATTCCCC
+
CDBDDC?AA=BBBC?C@C@C?BA@DBAA@@BBAC?ACC@@>??????BBCCC@B::95;CCCC>CBCBBBDAACCBB
BB@BBB@EABBB8
@T6XXI:00059:00549
GGGGAATTCTAATACGACTCACTATAGGGAGAGGGAGAATAGTATACGCTTTTCTAACGCTACTATCTTCTTCCTTT
CTCCCTTCTCCC
+
CB?7A59:;@A=@NCCCBBB@BBAA???9???BC>@@@=@=AA8=<;94444+44=<@???A>=888<9<??A;>@7
884;497:444-
@T6XXI:00060:00503
ACTCGAGATCTGCGGCTTTGGACAGTCCCTCTGTACTGTGAAGCCACAGATGTCGAGTCGACTAGCTTCCTTTCTCC
CTTCTCCC
+
```

CCBCB???979;9:9444-4244AA?==:=999@<<<<<BB@BD@BBBCDCAB;:887668>:79;><>598-
48>@9>4:339:
@T6XXI:00060:00512
GGGGGAATTCAATACGACTCACTATAGGGAGAGGGAGAATAGT
+
::8:-96;8>;886......57761117,11111,000/0556
@T6XXI:00060:00525
GGGAGAAGGGAGAAAGGAAGTGCAACTTGTCGCGCAAAATAGGGAGACTATTCTCCCTCTCCCTATAGTGAGTCGTA
TTAGAATTCCCC
+
/1,/6@?BC<=<BC>B>C?F;;<B@BB@BBGCC>>===5100=9>7>:BBB?>7742...1101338?????A<<<<
<9;?A=?;@AA4
@T6XXI:00061:00501
GCTAGTCACTCGAGATCTGCGGCTTTGGACAGTCCCTCTGTACTGTCGAAGCCACAGATGTCGAGTCGACTAGCTTC
CTTTCTCCCTTCCTCCC
+
444444444494=AB=A=@00,0/.+.,./:@BAA;<?:::=:88---
2,.9@BCBCBBBBABBC@A@?881711016;?5:/4333-.--272
@T6XXI:00061:00517
GGGAGAAGGGAGAAAGGAAGGGGCAGGTCCGTGATCAGGGAGCTATTCTCCCTCTCCCTATAGTGAGTCGTTATTCA
+
DDAA@?@A@=@?=@<??A?BB@7@875:>;>@A??A@=>:???@AA?===A<AA@A@<@??=<<@:667/,+,0*+-
@T6XXI:00061:00520
GGGAGAAGGGAGAAAGGAAGGGAC
+
9919>@>AA<A@88000=)**)**
@T6XXI:00061:00524
GGGGAATTCTAATACGACTCACTATAGGAGAGGGAGAATAGTCAACAAATCGTTTGGGGCGACTTCTCCTTCCTTTC
TCCCTTCTCCC
+
949/4292488249ACBADBF>>::00,//0774:66<=ABA>><?AC<A??=?.777-7>=BC8:7<30/4344-
49996<:>?@@;
@T6XXI:00061:00529
GGGAGAGGGA
+
/1+/////+,
@T6XXI:00061:00530
GGGAGAAGGGAGAAAGGAAGACGATAG
+
9;7<A@?AB<@@==9;:@;10,,,2,,
@T6XXI:00061:00531
GGGAGAAGGGAGAAAGGAAGCTAGTCGACTCGACATCTGTGGCTTCACAGTACAGAGGACTGTCCAAAGCCGCAGAT
CTCGAGTCGACCTA
+
BB>AA@=AE?BBCCAC?B?AA;7;7777719=67699917420626?<B@A====8838D@@@A?@@:@?<???;:/
///,)******)*0
@T6XXI:00061:00536
TTTGGACAGTCCCTCTGTACATGTCGCAAGCT
+
;9-9:994999909>BDB>61//./.,,0*,,
@T6XXI:00061:00542
GGGAGAAGGGAGAAAGGAAGTGCAGTTCGCGGTTAGTGAAGGGAAAAACTATTCTCCCTCTCCCTGATAGTGAGTCG
TA
+
@C>BBB5;;7;;9C=C:;::A?@B;924:3313137767011,1666/73368766?97//:77-
++0+**++++2200
@T6XXI:00062:00517

```
GGGAGAAGGGAGAAAGGAAGGGGAGAAGGGAGAAAGGAAGCTAGTCGACTCGACATCTGAT
+
<;8;HB?CD<@@@@<A?C@CCC9@@??BB>A?@>.747:=?4766166630003-++++++
@T6XXI:00062:00523
GGGGAATTCTAATACGACTCACTATAGGG
+
999-48984;9=@>;<;8.63../,000)
@T6XXI:00062:00531
GTCGACTCGAGATCTGCGGCTTTGGACAGTCCCTCTGTACTGTGAAGCCACAGATGTCGAGTCGACTAGCTTCCTTT
CTCCCTTCTCCC
+
49:99>@CBBDCBA=<>CBDCC?C@CCCC;::89>?<:;BCCCADBCC@CA????????AB@@@BBBE@@@@C>AA9
?=>>5:;:DJE>
@T6XXI:00062:00533
GGGGAATTCTAATACGACTCACTATAGGGAGAGGGAGAATAGTCAACAAATCGTTTGGGGCGACTTCTCCTTCCTTT
CTCCCTTTCCTCCC
+
999/42429DB?A>320==>ABA??7::67??BB?C=;8;:4403/003066111,166266=AB=A=9;=;=;==9
;;;;-44+4/-23+
@T6XXI:00063:00513
GGGGGAATT
+
////*/,/,
@T6XXI:00063:00517
GGGAGAAGGGAGAAAGGAAGAACGAGGCAATGGGGGACGGAGCATATGGCTATTCTCCCTCTCCCTAA
+
/1-//87881/4@?0953977331103343799>27774...3...-,16669888=55.4332--,
@T6XXI:00063:00525
GGGGAATTCTAATACGACTCACTATAGGGAGAGGGAGAATAGCTTTTGCGTTAAGGCATTCTCCGTGGACTT
+
///*/,/,/,,*,/..447B<>AA888C>@A@?>200323348;?@788==>A>A>A44230440035331,
@T6XXI:00063:00540
CTCGAGATCTGCGGCTTTGGACAGTCCCTCTGTACTGTGAAGCCACAGATGTCGAGTCGACTAGCTTCCTTTCTCCC
TTCTCCC
+
:;;@@BCCCBBBB9;;@<@@CBBEDDDBCCCA@ABBBAAB@@@=@C@@@ACAB@@@BCBBA?>>8969599199990
713333,
@T6XXI:00063:00545
GGGGAATTCTAATACGACTCACTATAGGGAGAGGGAGAATAGTCACGCAGTCTACGCTCCGTCTATTCCCTTCCTTT
CTCCCTTCTCC
+
BA@4@5459449;@D@A@CCBCC@@776.7>>>A??BC=@?@???AA889AA???<=<=;=888==8;;-434344-
4444-434443
@T6XXI:00063:00548
GGGAGAAGGGAGAAAGGAAGGAGAAGTCGCCCCAAACGATTTGTTGACTATTCTCCCTCTCCCTATAGTGAGTCGTA
TTAGAATTCCCC
+
HHBB@?=>B>BBBH?B@B@C@BB?>>;<<?BB8>>;?C?CC>BB?BBB@@A><<<<;@AAA=;=:::?<<=A::?@A
@>A@@=?;===4
@T6XXI:00064:00515
GGGAGAAGGGAGAAAGGAAGACGAAAAGACAGGCGCAGATTGCATGGCTATTCTCCCTCTCCCTATAGTGAGTCGTA
TTAGAATTCCCC
+
DC?@A?@BC>BCBB>B@B@B???>>>8>>???>>>?B?@@>?>>AC=?ACA?A<<=;<=<<<<<<<@@=A@A@A@A@
@=?AB>C>@@@5
@T6XXI:00064:00549
GGGGAATTCTAATACGACTCACTATAGGGAGAGGGAGAATAGTCTGACTGTTTCCAACAGCTGT
```

```
+
444,424244888:49:A??8>A@>@CA8:;>@?,0--,-224<==@6>63303,.,...4453
@T6XXI:00065:00500
GGGGAATTCTAATACGACTCACTATAGGGAGAGGGG
+
/88*/19>@@>?@=11.78-0.4...772691111,
@T6XXI:00065:00529
GGGAGAGAATTTGGTAATGGGGAATAAAGGAAGGTTGGAAAGGAATTCGGGACCTT
+
4914449=@JC?C?AB<>>>>.86777,10101013336:56:<-1-//6+//,/,
@T6XXI:00066:00529
GGGGAATTCTAATACGACTCACTATAGGGAGAGGGAGAATAGTAGACCTAAATCGTTTGGGCCGTCTTCCTCC
+
7;<1<-95844244897;AC@ABA?<<<167>@@9?<@=?:4400002334433553034+.,....55354-
@T6XXI:00066:00547
GGGGAATCTAATACGACTCACTATAGGGAGAGGGAGAATAGTCAACAAATCGTTTGGGGCGACTTCTCCTTCCTTTC
TCCCTTCTCCC
+
/11+1,1/665649;?@AABCCCCAAA7;7>>>.74978;8:332366088777,111,1645559:2,-,-
7776;:AA<@>@?==7
@T6XXI:00067:00502
GGGGAATTCTAATACGACTCACTATAGGGAGAGGGAGAATAGTTTTCCCTTCACTAACCGCGAACTGCACTTCCTTC
TCCCTTCTCCC
+
?A;2B8<6488949<BAABBA@>?>777.7>===9?:84944444-04+0/3?;75:=-
0/.4444<<>;:::,0,/6862:9;4471
@T6XXI:00067:00507
CTAGTCGACTCGAGATCTGCGGCTTTGGACAGTCCCTCTGTACTGTGAAGCCACAGATGTCGAGTCGCTAGCTTCCT
TTCTCCCTTC
+
EEJJC9<<FDCCACCBBBB@>:333/7=>A?AABB>BBBCB?@=??>@;=A@CCBBAAA??????AA@BB@@?=B47
:.7==?:A>>
@T6XXI:00067:00510
GGGAGAAGGGAGAAAGGAAGCTAGTCACTCGACATCTGTGGCTTCACAGTACAGAGGGACTGTCCAAAGCCGCAGAT
CTCGAGTCGACTAG
+
CC>BB@699-
9>==9@=?<@B???A700:;800./.../@;@@DABBB>?@=<<86646::<=<>==<<@>@A<><==<<?;@AAA?
8=;=
@T6XXI:00067:00512
GGGAGAAGGGAGAAAGGAAGCTAGTCACTCGACATCTGTGGCTTCACAGTACAGAGGACTGTCCAAAGCCGCAGATC
TCGAGTCGACTAG
+
BC?C@@;>><@CCB=C8;8;A@>@@?????<@C?BB@A??;?A=????@>>4444424;@885;?A===;=AA@AA@
@@==<?@@@????
@T6XXI:00067:00523
GGGGAATTCTAATGACGACTCACTATAGGGAGAGGGAGAATAGCCAGCTTCTCCTTC
+
444,424244424344488664./.....+31111,111;966:2.-110---33,-
@T6XXI:00067:00526
GGGGAATTCTAATACGACTCACTATAGGAGAGGGAGAATAGTTTCCTTCACTAACCGCGAACTGCACTTCCTTT
+
28818+1,///,/488;;<>BB>>>77477>?A<F@?7:6///,-,-,-.111363170444>776==36011,
@T6XXI:00067:00529
GGGGAATTCTAATACGACTCACTATAGGGAGAGGGAGAATAGGCGA
+
?;;1<-1,///788737:7;86977116,1<766,0002331041-
```

```
@T6XXI:00067:00536
GGGGAATTCTAATACGACTCACTATAGGGAGAGGGAGAATAGTCAACAAATCGTTTGGGGCGACTTCTCCTTCCTTT
CTCCCTTCTCCC
+
@@>4?6;:=CG@DBBBBABBBCB==777.7==?A>FABBA@@@AA>@@@;BCCCC>CCC7BBBCC?AA82744;AA<
A@AA<@;==AB>
@T6XXI:00067:00538
GGGAGAAGGGAGAAAGGAAGCTAGTCGACTCGACATCTGTGGCTTCACAGTACAGAGGGACTGTCCAAAGCCGCAGA
TCTCGAGTCGCTAG
+
CHABBA@CB>BBBB?C?B>@B@:::/11?>?@1;;@A?BBC;<??BC@>>>;;=<<<=:;===<<;:<;:;<?=<<?
=;;;=9====;;8;
@T6XXI:00068:00522
GGGGAATTCTAATACGACTCACTATAGGGAGAGGGAGAATAGTCAACAAATCGTTTGGGGCGACTTCTCCTTCCTTT
CTCCCTTCTCCC
+
@@>6>=@=ABB@BBAACBCCCDB>>111.7<<AB>AAA?BA????=?AC>CBBBB>BCA8<<A@C@B@@9;7;8;;7
=AAC=A>@@@@<
@T6XXI:00068:00524
GGGAGAAGGGAGAAAGGAAGGAGAAGTCGCCCCAAACGATTTGTTGACTATTCTCCCTCTCCCTATAGTGAGTCGTA
TTAGAATTCCCC
+
DDACCB@BB>@A@B>CBC?A?ABE@@=?@BBB9BC>BCC@C<B?B?<:>AA<:?AA<@@A@A<A@@<@<@AA?????
@?A@=;;2444-
@T6XXI:00068:00530
GACTATCACTCGAGATCTGCGGCTTGGACAGGTCCCTCTGTACTGATGAATGGCCACAGTA
+
1///8/44444448==???8857733136110166,119;664903344/0026,///...
@T6XXI:00069:00517
GGGAGAAGGGAGAAAGGAAGGAGAAGTCGCCCCAAACGATTTGTTGACTATTCTCCCTCTCCCTATAGTGAGTCGTA
TTAGAATTCCCC
+
ABAAA@=@C=BBBC?DBC?B?AAA8?=?8877.77.1;;?C;BBBBB??@A>A===9=AAAB=A@@AAA@899AAAA
A><<542,.//+
@T6XXI:00069:00523
GGGAGAAGGGAGAAAGGAAGCTAGTACAC
+
CC8=:CABF9>99908<><<7111111,,
@T6XXI:00069:00537
GACTCACTATGAGGGAGAGGAG
+
CBCCCDC?=449990433.,..
@T6XXI:00069:00541
GGGGAATTCTAATACGACTCACTATAGGGAGAGGGAGAATAGTCAACAAATCGTTTGGGGCGACTTCTCCTTCCTTT
CTCCCTTCTCCC
+
>>@4=295989>;;<CGDCBCCCB?@?C>ABA@@548?<????CD<?@C;BAAAC>BBB8CCC:936::/424/44-
498848289;;4
@T6XXI:00070:00502
GGGAGAAGGGAGAAAGGAAGGAGAAGTCGCCCCAAACGATTTGTTGACTATTCTCCCTCTCCCTATAGTGAGTCGTA
TTAGAATTCCCC
+
CD>BBA?BB<@@CC@B@A@E@CAAAAAA@???.77.6167;;?D?::4<:=:?:??:>A;<<7887;@:6///6146
97555558433-
@T6XXI:00070:00523
GGGGAATTCTAATACGACTCACTATAGGAGAGGGAGAATAGTCACATTCATGCTTGTGACCGTGTCTCCTTCCTTTC
TCCCTTCTCCC
```

```
+
ACIDC<:<<AAADDCBB?BAC7.////,0=>AE>BAD>A488AA??A?CCC?=?@C@@@CBBA@AC21,0:;,00,0
.4427-/6796
@T6XXI:00071:00511
GGGAGAAGGGAGAAAGGAAGGAGAAGTCGCCCCCAAACGATTTGTTGACTATTCTCCCTCTCCCTATAGTGAGTCGT
ATTAGAATTCCCCC
+
=@5>@@5991999919<?<><887077<78333+11,1116?;???B=68<@;=<CA944033+0400050006666
8<@=<<;<8:==?1
@T6XXI:00071:00517
GGGAGAAGGGAGAAAGGAAGGGGAGAAGGGAGAAAGGAAGCTAGTCGACTCGACATCTGTGGCTTCACAGTACAGAG
GGACTGTCCAAAGCCGCAGA
+
995=AADEE@<<<E>B>A<???5?BB?BC>?996,7=?:=<<:::CCDB;@>..////4;=;BA>?@@====>@@<<
<7;=;;:;8<<6;A=@@@AA
@T6XXI:00071:00523
AGTCACTCGAGATCTGCGGCTTTGGACAGTCCCTCTGTACTGTGAAGCCACAGATGTCGAGTCGACTAGCTTCCTTT
CTCCCTTCTCCCTTCCTTCTCCCTTCTCCC
+
AAACCBDBABBLBCAABB@CBC?B@BAAA>>><<??BBBC@C@@?>??;>>?==;:<<<<=??@AAAA:<A@@;<<8
?=9=17/4999-4343434:;;4:7:444,
@T6XXI:00071:00535
GGGAGAAGGGAGAAAGGAA
+
AA<@@A?@A>@@@B>B@C?
@T6XXI:00071:00539
GGGAGAAGGGAGAAAGGAAGGAGACAGTGAGCGCCGCACAACACGTCACTATTCTCCCTCTCCCTATAGTGAGTCGT
AATAGATTCCCC
+
CCDCEB@BB>AAAB@B@B@?=>C@BBAA?BBBB?>?>9>>@@?@<<?CBCCC@CAA?<AACCC>BCC@@@BBCBC=,
,+,,,,222280
@T6XXI:00072:00503
GGGAGAAGGGAGAAAGGAAGCTAGTCGACTCGACATCTGTGGCTTCACAGTACAGAGGGACTGTCCAAAGCCGCAGA
TCTCGAGTCGACTAGC
+
;B:=?A6991999@9@5860900../..1.54........3,01011666640000884677 4--,-
101455<==545=<=<999;6633/-
@T6XXI:00072:00506
GGGGAATTCTAATACGACTCACTATAGGGAGAGGGAGAATAGTCAACAAATCGTTTGGGGCGACTTCTCCTTCCTTT
CTCCCTTCTCCC
+
499,499<=>>?=@@@BBBCCBBB???@9??ABB>@DD?A===@@>@AA;AAA@@8??<5?>::74/--,-,-,-4-
-36424/00344
@T6XXI:00072:00518
GGGAGAAGGGAGAAAGGAAGCTAGTCGACTCGAGATCTGCGGCTTTGGACAGTCCCTCTGTACTGTGAAGCCACAGA
TGTCGAGTCGACTAGCTTCCTTTCTCCCTTCTCCC
+
DFACBBBCC=BBCC?C?A>:::=<>0////7:>1:7?<???????<?@BBC@@AA<AAA@@><<<778;=@<?????
?====>CBA??=<<8879;244-4:::-4/4444,
@T6XXI:00073:00500
GGGGAATTCTAATACGACTCACTATAGGGAGAGGGAGAATAAGTCATTAACTCCCAAATGAATTCCACCGTCTTCCT
TTCTCCCTTCTCCC
+
6=;1<,959492844477;;:8>776661/::=?11.43--
,000009=544;;17818888=:<<<=:?@8434344,4:;94?;?<@@:
@T6XXI:00073:00504
```

```
GGGAGAAGGGAGAAAGGAAGGAGAACGCGCACCCGAACCAACGGTGATCTATTCTCCCTCTCCCTATAGTGAGTCGT
ATTAGAATTCCCC
+
CJDCCE?BB=BCBB>C?B?A@CAA?CCBCCCCB>AA>B9:9<GACCECCBBB>BBB?=BCCCB>BCCBA;BCC?AAC
CC?@@@=B@CCC:
@T6XXI:00073:00508
GGGAGAAGGGAGAAAGGAAGGAGAAGTCGCCCCAAACGATTTGTTGACTATTCTCCCTCTCCCTATAGTGAGTCGTA
TTAAGAATTCCCC
+
//+///,//+/79;:?=>??@>@?>><>;@??.8866;;?A;@B?BBB?@C>===D@BA@;A6;;/+++++244111
1,-,-22357554
@T6XXI:00073:00510
GGGAGAAGGGAGAAAGGAAGGAGAAAGCGACGTCGGGGAAACGTTTGCTATTCTCCCTCTCCCTATAGTGAGTCGTA
TTAGAATTCCCC
+
BC@CAA=?><@CBB>C@C@B=BB@@88;CCCCBBB?@8CE@CGBA>BBCCB@BBBB>BBCAA=A@@@?>@@CC???@
@>AAA?A;???5
@T6XXI:00073:00515
GGGAGAAGGGAGAAAGGAAGCTAGTCACTCGACATCTGTGGCTTCACAGTACAGAGGGACTGTCCAAAGCCGCAGAT
CTCGAGTCGACTAGCTCTTCTCCTC
+
CC:BC@5990994=98<A::;:??<:000.-/--
./69998??:=@@:::?<<<?AA;??@@=<:=C7<=<>847///..4446;<;:4250,++*+++302
@T6XXI:00073:00531
CTAGTCACTCGAGATCTGCGGCTTTGGACAGTCCCTCTGTACTGTGAAGCCACAGATGTCGAGTCGACTAGCTTCCT
TTCTCCCTTCTCCC
+
>8/////49CCDCCBAA@>B><?>;B?B?>>?@@;@>@@@@<;<@@A=:B@BBAACBAAABB@@A>CCCCBBB?B?B
C>CAA@;?<??@A=
@T6XXI:00073:00533
GGGGAATTCTAATACGACTCACTATAGGGAGAGGGAGAATAGTCAACAAATCGTTTGGGGCGACTTCTCCTTCCTTT
CTCCCTTCTCCC
+
BB?7@598;@@@CB@@@CBBBBBCCCCC>BBB>>;<@@>@AAA@@=@AA<CCCCC>??C8CCCBA=A???C=A=AB<
A@@?:=::4441
@T6XXI:00073:00534
CTAGTCGACTCGAGATCTGCGGCTTTGGACAGTCCCTCTGTACTGTGAAGCCACAGATGTCGAGTCGACTAGCTTCC
TTTCTCCCTTCT
+
EB99<<9:ACBBBBAA@@D@@=@CD9<<<BAECDC?CCC@@@C@<<=@68;=?@BAACCCCCBB?@A>??>111010
16267884423-
@T6XXI:00073:00535
GGGAGAAGGGAGAAAGGAAGTGCAGTTCGCGGTTAGTGAAGGGGAAAACTATTCTCCCTCTCCCTATAGTGAGTCGT
ATTAGAACTTCCCC
+
49-
4=>;>>9>=@B<A>@AFBBBCC>;<:00,1,0;65?>BCC8B>?8BBAB?BCCBAAAAAA>?AA@@@ACA@???==9
=CC9<<9<<;1
@T6XXI:00073:00544
GGGGAATTCTAATACGACTCACTATAGGGAGAGGGAGAATAGTCAACAAATCGTTTG
+
/11+/5>=@BB@C@@@EECB@@B@<<<966;666.:;;9?@987653110....4+.
@T6XXI:00073:00547
GGGGAATTCTAATACGACTCACTATAGGGAGAGGGAGAATAGTGACGTGTTGTGCGGCGCTCACTGTCTCCTTCCTT
CTCCCTTCTCCC
+
```

```
A??7@=@=@BBABBABCCCBBBB==669.:>?CC>@@@?A===A:::@@<<;A===;:;<==<<:@<@@9B99:..-
/8885999ACC<
@T6XXI:00074:00511
GGGAGAAGGGAGAAAGGAAGGAGAAGTCGCCCCAAACGATTTGTTGACTATTCTCCCTCTCCCTATAGTGAGTCGTA
TTAGAATTCCCC
+
CH@CCBBDD>BBBB>B?C?B?@BC?AA@<<<<2>>:?BDCC>C@?BAA?AA>A???<===AC<A??????==A????
?=@@ACC:;;;.
@T6XXI:00074:00543
GGGGAATTCTAATACGACTCACTATAGGGAGAGGGAGAATAGCTACGTTTAACCGATCGTGTTGTTCTCCTTCCTTT
CTCCCTTCTCCC
+
?@@6@>A>BCC@CCB?@@@B@CB;969959?ACE>A>>=?;;;>>??<<;<;<<;::99<==<<<999==?=@=@@<
???A<A>@AAA<
@T6XXI:00074:00545
CTAGTCACTCGAGATCTGCGGCTTTGGACAGTCCCTCTGTACTGTGAAGCCACAGATGTCGAGTCGACTAGCTTCCT
TTCTCCCTTCTCCC
+
>>11111:?BBDCCCBBB:88:CE>C?BBCCCCC>BCCBC@===A@@:=AACCCCCCCCCCBBBBBBCACA:95:=@
A<@==;4:7:89=:
@T6XXI:00075:00503
GGGGAATTCTAATACGACTCACTATAGGGAGAGGGAGAATAGTCAACAAATCGTTTGGGGCGACTTCTCCTTCCTTT
CTCCCTTCTCCC
+
CDHAC?A?ABB@CCBBBBBB?@B>?966.7>777.4:;8<>AAAC@===7?ABBB9???5??BCC=?<<9<;@;?D?
@@@C?C898@@:
@T6XXI:00075:00507
GGGGAATTCTAATACGACTCACTATAGGGAGAGGGAGAATAGTCAACAAATCGTTTGGGGCGACTTCTCCTTCCTTT
CTCCCTTCTCCC
+
499,49A>BDCFCBBBBBCB:<?A@::?;?AABD>@??=@@@@AA>???<ABCCC;<<?7?BCCC@?<<3737>AA<
AAAA<A>????<
@T6XXI:00075:00515
GGGGAATTCTAATACGACTCACTATAGGGAGAGGGAGAATAGATCACCGTTGGTTCGGGTGCGCGTTCTCCTTCCTT
TCTCCCTTCTCCC
+
444,42488BHB<<;CC@@A@?@BC??@=?CCBA<?4424;::;<??;?B5:5:?B@B?B:;;??9;?>97701011
,04443858 8??:
@T6XXI:00075:00523
GGGAGAAGGGAGAAAGGAAGGAGAAGTCGCCCCAAACGATTTGTTGACTATTCTCCCTCTCCCTATAGTGAGTCGTA
TTAGAATTCCCCC
+
CB@AAA@BB@CCCC>C@C@C?ABC@B??8888.7><B@BBC>CCAC:6<AA@@@AA<?==<C:899A@?AC@999@A
A<<6823/.///+
@T6XXI:00075:00528
GGGAGAAGGGAGAAAGGAAGCTAGTCGACTCGACATCTGTGGCTTCACAGTACAGAGGGACTGTCCAAAGCCGCAGA
TCTCGA
+
CBAAB@===<@@@@<@@C>AB@8::8;:BAA?99:::7>B@9=@@CCC@?;<<8;03317=>@AA=BA:?A?A@<<=
=;;:@<
@T6XXI:00075:00541
GGGGAATTCTAATACGACTCACTATAGGGAGAGGGAGAATAGTTACGGCACATTCTGCAGTCTCCTGTTCTTCCTTT
CTCCCTTCTCCC
+
CCB9D:;;;BB@BBBABADB@CA>>6665:AACC<?>>=@<<A>A??<?@@@@?BCCACCC@>B>?BEACC=?<<?:
??AC;?;=844-
@T6XXI:00075:00542
```

```
GGGGAATTCTAATACGACTCACTATAGGGAGAGGGAGAATAGCTCACCTGTGACCAGTGCGAGACTGCTCTTCCTTC
TCCCTTCTCC
+
999/49@?BBB@CCBBAACDCCBBBAAA89??AA<A@@>@=AAB?=A>@@@AAA>@@@@ABBA@@?@@?<<999977
///,/,///,
@T6XXI:00075:00543
GGGGAATTCTAATACGACTCACTATAGGGAGAGGGAGAATAGTCAACAAATCGTCGGTGGCGACTTCTCCTTCCTTT
CTCCCTTCTCCC
+
CCB9C>?=@EECCCBBBBBB;@B>>66659ABCD?BBA=?:;;A;:=57455..00:<<6>6:<<<===>A;>;=A:
;:;:4:8;=?D?
@T6XXI:00076:00503
GGGGAATTCTAATACGACTCACTATAGGGAGAGGGAGAATAGTCAACAAATCGTTTGGGGCGACTTCTCCTTCCTTT
CTCCCTTCTCCC
+
CCB9C=@=@CC@CCBBBBBB@A<77111.7>>>A>BA@@@<<;?@?@@?;>>966,111-
7>???>?CA<?=?>@?<?AAA<??AAA;4
@T6XXI:00076:00504
CTAGTCACTCGAGACTGCGGCTTTGGACAGTCCCTCTGTACTGATGAAGCCAGCAGATGTCGAGTCGCACGTCTCCT
+
>>9444444=:;8=4449998885884449799577:.55./,,,,,228,999===>987>77194//6/....,.
@T6XXI:00076:00511
GGGAGAAGGGAGAAAGGAAGCGCAATAAGGCAGTGAATAGGGTTAACCTATTCTCCCTCTCCCTATAGTGAGTCGTA
TTGAGAATTCCCC
+
=C@CFCACB>BB??<>;?>B::;F;9949;=9;<AB?DCCC@D?C=@8<<A>@@A?088777,16699BBB8888/9
9,//.22226>>.
@T6XXI:00076:00528
GGGAGAAGGGAGAAAGGAAGCTAGTCGACTCGACATCTGTGGCTTCACAGTACAGAGGGACTGTCCAA
+
?;:=985991999919;>=;;:B=7:6??ACCCDB;:9A@;3473777:?7448849=78888//./7
@T6XXI:00076:00539
GGGAGAAGGGAGAAAGGAAGGAGAAGGTCGCCCCAAACGATTTGTTGACTATTCTCCCTCTCCCTATAGTGAGTCGT
ATTCAGAATTCCCC
+
@@<@AA?AB<@@AC>C@E@>:;::,0,.4444458886>DDCABB@AACAA@>@A==;;:?@@<ACA???=:///.5
00+--//,-03480
@T6XXI:00077:00500
CTATCGACTCGAGATCTGCGGCTTTGGACAGTCCCTCTGTACTGTGAAGCCACAGATGTCGAGTCGACTAGCTTCCT
TTCTCCCTTCTCCC
+
4678/////79==ABD<;8?=876,377667111,176669=94000/048877994...43..9?77331110136
98<9==;=<<7442
@T6XXI:00077:00507
GGGGAATTCTAATACGACTCACTATAGGGAGAGGGAGAATAGATCACCGTTGGTTCGGGTGCGCGTTCTCCTTCCTT
TCTCCCTTCTCCC
+
CCDDF?B?BBB@CCCCCBBB?@<==611.7777B?????AA@@@ABA>AC@B?B@BBB=C@@@CDD@B;;::?B?CC
A@@A@:><788?:
@T6XXI:00077:00515
GGGAGAAGGGAGAAAGGAAGCTAGTCGACTCGACATCTGTGGCTTCACAGTACAGAGGGACTGTCCAAAGCCGCAGA
TCTCGAGTCGACTACG
+
CH?A@@=@>9@ABB>CDC@CAAAA@7::700<00......200121777683339444+399444/494;?;<C@@@
@<<<=;::44440300
@T6XXI:00077:00517
```

```
GGGGAATTCTAATACGACTCACTATAGGGAGAGGGAGAATAGTCAACAAATCGTTTGGGGCGACTTCTCCTTCCTTT
CTCCCTTCTCCC
+
AAA9A>A@BCCACCCB???B@ABA<:??8???BC>AAA>AAAA888<CCACCBBB>CCC7:????<A@@=?=@=@@<
@@@@;@@@@@A<
@T6XXI:00077:00528
GGGAGAAGGGAGAAAGGAAGCTAGTCACTCGACATCTGTGGCTTCACAGTACAGAGGGACTGTCCAAAGCCGCAGAC
TCTCGAGTCGACT
+
490949594-
44>C8;8:@BCBCCCCCC??<??6::<B?A>AD?AAA?@@A?AA@@@7==@@@A>@?:<<8<<;B4888844;;473
8::
@T6XXI:00077:00542
GAGTCCTACTGT
+
/1,**)****(,
@T6XXI:00078:00500
GGGGAATTCTA
+
ACB9D?B@BBC
@T6XXI:00078:00504
GGGGAATTCTAATACGACTCACTATAGGGAGAGGGAGAATAGTCACATTCATGTTTGTGACCGTGTCTCCTTCCTTT
CTCCCTTCTCCC
+
999/99A?BBB@BB??AABB@?A=7111.7==@D@??@?A<??=;@@BAC>>9>?;@=<@=<=<?;<==;=>D?AA?
A===9=>=AA=;
@T6XXI:00078:00517
GGGGAATTCTAATACGACTCACTATAGGGAGAGGGAGAATAGCCGCAACTGACGTCATCCCTGGTTACTCTTCCCTT
TTC
+
CCB6@8@;>@@<>@CCCBACA<@==777.7>>BC?CAA>A9==;@@B:;<BCDA9=3034-
0323984830/030122.6
@T6XXI:00078:00547
GGGGAATTCTAATACGACTCACTATAGGAGAGGGAGAATAGTTTTCCTTCAC
+
499/42959>>;>A@@BBB@?;;;;00,0<:==<?=@>;9////+-,-,-51
@T6XXI:00079:00501
GGGGAATTCTAATACGACTCACTATAGGGAGAGGGAGAATAGTTTTCCCTTCACTAACCGCGAACTGCACTTCCTTC
TCCCTTCTCCC
+
999/4295;HCDCBBBCCCCCBA==777.7>77758@@>@?@BAD6AA;@>@BBBC?B@BJBA?999@?>?,0,0,/
4673@9===A;
@T6XXI:00079:00509
GGGAGAAGGGAGAAAGGAAGGAGAAGTTGCCCCAAACGATTTGTTGACTATTCTCCCTCTCCCTATAGTGAGTCGTA
TTAGAATTCCCC
+
BC>BBBACC>CCBC>B?A?>::97://,....*..+..7@A>BB?BBCACC=>@AA;===@A<A???@A@@A@@@<=
=>A@?;;2444-
@T6XXI:00079:00517
GGGGAATTCTAATACGACTCACTATAGGGAGAGGGAGAATAGTGACGTGTTGTGCGGCGCTCACTGTCTTCTTCCTT
TCTCCCTTCTCCC
+
CCB7@<>=@@@@CCBBBBBB@@>77111.7>>>C@@??@A=AA:::@@@@?=;:?<;<=<<===8865;8;<:=8;;
6784>:@=@AAA<
@T6XXI:00079:00531
GGGAGAAGGGAGAAAGGAAGAGAATAACCGGAAACTGAAAGTGTTGACTATTCTCCCTCTCCCTATAGTGAGTCGTA
TTAGAATTCCCC
```

```
+
CCBDCD@BC?A@@B?B?B@CC@=;?B:?;@>AA<AB?@@<ABAB@CAAADC@ECBD?<:<DD>CBBCCA@>>>;00.
99;:@><;<AA5
@T6XXI:00080:00503
GGGAGAAGGGAGAAAGGAAGACAGCTAGCGTGTAAGAGTTGAGAGAACTATTCTCCCTCTCCCTATAGTGAGTCGTA
TTAGAATTCCCC
+
DD@HBB?BCACCCC>B?A?BAABCCCCDDCBCBCBCCBBADBBBCC@CCBB??==C>AA@@@<@CBBBCCBBBCBB?
@=?BB?C@CCC9
@T6XXI:00080:00510
GGGAGAAGGGAGAAAGGAAGGATGCGCGACAGTCGAAAATTGGAGAACTATTCTCCCTCTCCCTATAGTGAGTCGTA
TTAGAATTCCCC
+
CD@CBA?AB>?B88888888+///8:@CCBABCCCCCB8A@A?BBC?B>>;=>>>>;;;;?@;=:???AAAAA???A
A=;?A=@=@AA6
@T6XXI:00080:00511
GGGAGAAGGGAGAAAGGAAGCTAGTCACTCGACATCTGTGGCTTCACAGTACAGAGGGACTGTCCAAAGCCGCAGAT
CTCGAGTCGACTAG
+
49049>;>><?@AB<A=@@DABBBCA?=?8471069116;213<988????;;;444-
4;=>?A=AC:@@?A??@A??<<884=;=884;6
@T6XXI:00080:00514
GGGGAATTCTAATACGACTCACTATAGGGAGAGGAGAATAGCAAAGTACGAAC
+
944/98>@CBB@BBBBCBB@B@CA@?>96661108::<@===@C:41/.-*)*
@T6XXI:00080:00518
GGGAGAAGGGAGAAAGGAAGGAGAACGCGCAATCAGATACCTGTTGGCTATTCTCCCTCTCCCTATAGTGAGTCGTA
TTAGAATTCCCC
+
<;8<@@=ACCDCCC>C@C@B>AA?>BCCCCDBCECGBBCD@CCC7;8;AAA?AAAG@BBAAC>CBAABC:::@@??C
J@A?A@EBDCC:
@T6XXI:00080:00522
GGGAGAAGGGAGAAAGGAAGAAGAATAACGAGTTAAAGGTAAACAGCTATTCTCCCTCTCCCTATAGTGAGTCGTAT
TAGAATTCCCC
+
AN@CBC=@=:@@@B=B@C@CC@CC?CA=@ABBA;=?=@?BBC>B:::@@@>@AAC<=9=?A<A???@@A=9====<A
?A<<8;8<<;4
@T6XXI:00080:00529
GGGGAATTCTAATACGACTCACTATAGGGAGAGGGAGAATAGCTAACTATTCTTCTGGTAAGTTATTCGCCTTCCTT
TCTCCCTTCTCCC
+
999/4:B=BBBBBBBCCBBB=:?==667.7?>>><A?=;>?<=AB?BBCC?@98<<C?@?=@A>AB>@??>?@D9==
7<@@@;A>@AAA<
@T6XXI:00081:00501
GGGGAATTCTAATACGACTCACTATAGGGAGAGGAGAAT
+
/11+/7;9:;964>888@@B::;A:::C>::77+,*.)*
@T6XXI:00081:00525
GGGAGAAGGGAGAAAGGAAGGAGACACGGTCACAAACATGAATGTGACTATTCTCCCTCTCCCTATAGTGAGTCGTA
TTAGAATTCCCC
+
EC@AA@ACC@BBBB>B<A5:8;BBDCCC@C>>>BB>BBBBB@AA>BBCCCC????>7>==?@<??A@AACA???A88
9@CAA>A<???5
@T6XXI:00081:00535
CTATCGACTCGAGATCTGCGGCTTTGGACAGTCCCTCTGTACTGTGAAGCCACAGATGGACTCGAC
+
BBCB99:??B=BCBB@@@CC@CCC<B=?===777.7===??;==<;525@==796;-,*,,,,,,,
```

```
@T6XXI:00082:00513
GGGAGAAGGGAGAAAGGAAGCTAGTCACTCGACATCTGTGGCTTCACAGTACAGAGGGACTGTCCAAAGCCGCAGAT
CTCGAGTCGACTAG
+
CC>CAA>AA=BBAB>C?A?AB><<C?:::@;777:76@BC=?@@BBBACCCA<<8887=====A<==:@@>@AA???
AAA===@AAA@@<=
@T6XXI:00082:00522
CTAGTCGACTCCAGATCTGCGGCTTTGGACAGTCCCTCTGTACTGTGAAGCCACAGATGTCGAGTCGACTAGCCTTC
CTTTCTCCCTTCCTCCC
+
DC><;CDCDCDBCCCCCCBBB@@@E?C@CCAB:::-
7>>:::CC:9:H>@C=@@A;:;???@AAA@@:<::/0,/93::>:B>9333,/,.122
@T6XXI:00082:00536
GGGGAATTCTAATACGACTCACTATAGGAGAGGGAGAATAGTCAACAAATCGTTTGGGGCGACTTCTCCTTCCTTTC
TCCCTTCTCCC
+
CGB9B8;8<CB@CCCBBBBB<7977//,0?:=?=?DC>A888AA:;;=;?@@AA=ACC9@CCAC?>??<?<?>@@<A
@AA<@=??AB:
@T6XXI:00083:00506
CTATCGACTCGAGATCTGCGGCTTTGGACAGTCCCTCTGTACTGTGAAGCCACAGATGTCGAGTCGACTAGCTTCCT
TTCTCCCTTCTCCC
+
B::6/////8;>ABCC?@@C@CDC>B>?@CC@@>;BAAAA@;<<@@A><B@AABCCBABBBBBCCCCCCCCC@C@C
C>B@AA<A>@???:
@T6XXI:00083:00508
GGGGAATTCTAATACGCTCACTATAGGG
+
944,42/56;@-/,0,....3711111,
@T6XXI:00083:00509
TCTGCGGCTTTGGACAGTCCCTCTGTACTGTGAAGCCACAGATGTCGAGTCGACTAGCTTCCTTTCTCCCTTCTCCC
+
@B;9;95;@A<B@@A@BCDC@CCCA>ACC??=@;:<8;BBBADCC:::??A@DDDDEB@=>377.71=>.434444-
@T6XXI:00083:00515
GGGGAATTCTAATACGACTCACTATAGGAGAGGGAGAATAGTCAACAAATCGTTTGGGGCGACTTCTCCTTCCTTTC
TCCCTTCTCCC
+
DCB7B;<;<CC@CDCBCBCCB>=@=//-
0?;>A??AA>???AAB=?AA=CABAC8???6?BCBB?B@@>@<@?AC:???A:D>@@@@<
@T6XXI:00083:00518
CTATCGACCGAGATCTGCGGCTTTGGACAGTCCCTCTGTACTGTGAAGCCACAGATGTCGAGTCGACTAGCTTCCTT
TCTCCCTTCTCCC
+
BBAAABCBBBBB>>ABBCB@BBD<A?AAABA??;@@@>??A@>ABA>@C@CCCCBBBCCBCBBCCBBBCDBB>C=??
<?CCC>B?BCCC>
@T6XXI:00083:00532
GGGGAATTCTAATACGACTCACTATAGGAGAGGGAGAATAGTCAACAAATCGTTTGGGGCGACTTCTCCTTCCTTTC
TCCCTTCCTCCC
+
?CC9CCCB<1171//1..37639:900,/::<<9?;?;@@@?>;9444-
7=;AD>C@A8;:69?4:7888<=;<<:632201,-,-110
@T6XXI:00083:00535
GGGAGAAGGAGAAAGGAAGGAGAAGTCGCCCCAAACGATTGTTGACTATTCTCCCTCTC
+
49-///,/,///8+/,313999>866>80..*.5244..,..447463...233+..-2
@T6XXI:00083:00549
GGGGAATTCTAATACGACTCACTATAGGGAGAGGGAGAATAGTTCCACCTTTGGAACCGCTCCCTCGTTCTTCCTTT
CTCCCTTCTCCC
```

```
+
999/9<@>@AAACCBBAAAB@@<77166.7==?BB????AAA@=?>AA>A;:=;<;<;@AAA@<AAAA;=<;=;==7
;<<A@A?@BBA=
@T6XXI:00084:00502
GGGGAATTCTAATACGACTCACTATAGGGAGAGGGAGAATAGTCAACAAATCGTTTGGGGCGACTTCTCCTTCCTTT
CTCCCTTCTCCC
+
999/42928>>=BBBBB@@@B@?>>777.7===?<<@@>@@@@AA:;894779?C>CCC7??BBB?B@@:<9<>@@<
@=>>9:-/.340
@T6XXI:00084:00503
GGGAGAAGGGAGAAAGGAAGGAGAAGTCGCCCCAAACGATTTGTTGACTATTCTCCCTCTCCCTATAGTGAGTCGTA
TTAGAATTCCCC
+
BC@CBB@BC>B@@B?B@>=>@BB?<>?CAAAA8AC>BCBBB>BB>AAA?AA??><=;=9==@<AA@@AAAAA8??AA
A???@>A9<<<0
@T6XXI:00084:00506
GGGAGAAGGGAGAAAGGAAGCTAGTCGACTCGACATCTGTGGCTTCACAGTACAGAGGACTGTCCAAAGCCGCAGAT
CTCGAGT
+
@=<AA@@BD>BBBA9@7:7;;BBCCCBBBBBA::;?????@<?B?BBAACCA999//,/666:@>@A<@@?@AA@@?
CA?@:::
@T6XXI:00084:00509
CTATCACTCGAGATCTGCGGCTTTGGACAGTCCCTCTGTACTGTGAAGCCACAGATGTCGAGTCGACTGCTTCCTTT
CTCCCTCTCCC
+
B@@:11118:::>=A;;;A?@?B7;9:?A?AAC<A?;;:@>>>@<<9<B@AAA?87;777?A?A@@@C888@E>772
.---+--0000
@T6XXI:00084:00523
GGGAGAAGGGAGAAAGGAAGCTAGTCACTCGACATCTGTGGCTTCACAGTACAGAGGGACTGTCCAAAGCCGCAGAT
CTCGAGTCGACTAGTACTA
+
CE>AA@>BB>BCBH?B?B?BC??@A=@?BC@::9@@?BCE>@@@AABC@@@<==;:::::8>?A<??<?A?@@@AAA
<?<:????@:86/////++
@T6XXI:00084:00524
GGGGAATTCTAATACGACTCACTATAGGGAGAGGGAGAATAGTCAACAAATCGTT
+
49929@B8<=A?B=AA;;;8778<?6798???A>8384533333320-33....5
@T6XXI:00084:00526
GGGAGAAGGGAGAAAGGAAGCTAGTCGACTCCATCTGTGGCTTCACAGTACAGAGGGACTGTCCAAAGCCGCAGATC
TCGAGTCGACTAG
+
DD>CCB===:@@>9:B8:8;CCBB>:555..,0007ADDACB@BCCCCCDCBBAAA?BCBBCB@BB>BB@BAAB?==
AABCBCBBBBCCC
@T6XXI:00084:00530
GGGGAATTCTAATACGACTCACTATAGGAGAGGGAGAATAGCCTACTATCCCTTAGAAACCCTTTGTTCTTCCTTTC
TCCCTTCTCCC
+
FBB9B79;<CEBCB>BBBBB?:977//-
0>>CE>AAA=@<<<<>>?A=<<=;<>;@@@;?@:?=:?@=@@>A>A<<@@@A<A<@@@@;
@T6XXI:00084:00531
GGGAGAAGGGAGAAAGGAAGCTAGTCACTCGAGATCTGCGGCTTTGGACAGTCCCTCTGTACTGTGAAGCCACAGAT
GTCGAGTCGACTAGCTTCCTTTCTCCCTTCTCCC
+
CK@BBC@CD>BBCB=A?B=@@A???A???A>A>?CCBBBC@B@B>B?CCCBCCC>???AA=9=<<@<<==;<<@@@@
@@AA@@@??;;;==::8:/44-4444-48;:=?:
@T6XXI:00084:00538
```

```
GGGGAATTCTAATACGACTCACTATAGGGAGAGGGAGAATAGCCTGTACGTTTGTACACGTTATTCGCACTTCCTTT
CCTCCCTTCTCCC
+
@@>4><@=@CC@CC@?@CCB???@=777.7>>AC>@@@=A=AA:>?<<9AA@<@@@AA==9;=A>AA8/0=;>;==.
/00;;6B898==9
@T6XXI:00084:00547
GGGGAATTCTAATACGACTCACTATAGGAGAGGGAAGAATAGTCAACAAATCGTTTGGGGCGACTTCC
+
944,/56,1//7668=881:0,,22,,*,0255+.,-2211343424AC<???@>6===.705..,,*
@T6XXI:00085:00500
GGGGAATTCTAATACGACTCACTATAGGGAGAGGGAGAATAGTCAACAAATCGTTTGGGGCGACTTCTCCTTCCTTT
CTCCCTTCTCCC
+
;99/9<A?BCC@CCB@A>>?@CCCA???9???CC>C@@=@AAAAA?@@@;CC?;716664:BCCC@BB@<?<??AB=
ABAA=A?A@@@;
@T6XXI:00085:00510
GGGGAATTCTAATACGACTCACTATAGGGAGAGGGAGAATAGTCAACAAATCGTTTGGGGCGACTTCTCCTTCCTTT
CTCCCTTCTCCC
+
CEE:C>@=@A@?CBCCBBBBCB?==666.7>>AC>BAA>AAAA==;<@@;BCCCC>@?@7<ABAA>ACA>@>@>AA<
??CC<A=@<<;5
@T6XXI:00085:00522
GGGAGAAGGGAGAAAGGAAGTGCAGTTCGCGGTTAGTGAAGGGAAAAACTATTCTCCCTCTCCCTATAGTGAGTCGT
ATTAGAATTCCCC
+
BBAECC?BA=BC@B=B?B?CCABBBB@BBCA?B?@@@CC?@@>BBBB2BCB?A??@@;@@@@@;===:::;;;=AA<
==98BB?A>BBB6
@T6XXI:00086:00503
GGGGAATTCTAATACGACTCACTATAGGGAGAGGGAGAATAGTCTACGTTGCGTGCAATCTTAGTCTTTCCTTCCTT
TCTCCCTTCTCC
+
>>>2>5959?>;@BAACCA@@CCCBBBB>???@@9=;<8;9;B=887><56=<<===@A@@>AA@87714:;8:/44
-4689,/,/144
@T6XXI:00086:00520
GGGAGAAGGGAGAAAGGAAGCTAGTCACTCGACATCTGTGGCTTCACAGTACAGAGGGACTGTCCAAAGCCGCAGAT
CTCGAGTCGACTAG
+
CC>A=98>>9>>>?9A9;7<A??@A:;:AB>;99@@@BCC?BB?ACBBCB@=99770,4;=?@??AC<AA>????=;
;;<888=95;<;5;
@T6XXI:00086:00524
GGAGAAGGAGA
+
?;1//,,**((
@T6XXI:00086:00530
GGGGAATTCTAATACGACTCACTATAGGGAGAGGGAGAATAGTCAACAAATCGTTTGGGGCGACTTCTCCTTCCTTT
CTCCCTTCTCCC
+
@@@6@?A?@A@@@@@@@BBCCA;=;666.7===?<<AA>===AA@?===9@CCCC=AAA6??ABB=?=@<>;?;>?:
?BAA<A=??@@;
@T6XXI:00086:00532
CTAGTCACTCGAGATCTGCGGCTTTGGACAGTCCCTCTGTACTGTGAAGCCACAGATGTCGAGTCGACTAGCTTCCT
TTCTCCCTTCTCCC
+
>:11111:?BBBBBBBCFAA>A@>;C@AB@??BC>AA>@AA999AAA??B>BCCCCBBBBCBC<AB:8;AABB?B?B
D>A>>A=A>?@@A<
@T6XXI:00086:00539
```

```
CTCGAGATCTGCGGCTTTGGACAGTCCCTCTGTACTGTGAAGCCACAGATGTCGAGTCGACTAGCTTCCTTTCTCCC
TTCTCCC
+
BCCBBBBBAB?AB@BBA?B@BEAACEECDCDAA?AHBDBA?AB@BB@>BBBB@AABB?ACCBBCAB==;=89=8840
7488886
@T6XXI:00086:00545
GGGGAATTCTAATACGACTCACTATAGGAGAGGGAGAATAGTCAACAAATCGTTTGGGGCGACTTCTCCTTCCTTTC
TCCCTTCTCCC
+
BAA7A:;;<BB@CA@@CAAAB>=77/.,0>07:8:BBBDAAAAA<@@A<BBBBB>BCC9BBBCC@CCA;=:<;??<?
@AC;@=?@AA;
@T6XXI:00087:00505
CTAGTCACTCGAGATCTGCGGCTTTGGACAGTCCCTCTGTACTGTGAAGCCACAGATGTCGAGTCGACTAGCTTCCT
TTCTCCCTTCTCCC
+
>//////44@=@ABBCBC@@=@DE>D<?A@@@BC>CCC@<<?==AAA;<B?BAACCCBBBCCB?@@BBCCBAA?A@C
C>B@@A;<96<==7
@T6XXI:00087:00520
GGGAGAAGGGAGAAAGGAAGCTAGTCGACTCGACATCTGTGGCTTCACAGTACAGAGGGACTGTCCAAAGCCGCAGA
TCTCGA
+
CC>=9429919BBCBC?B9;;A@@@:;;B@@>:::7=<>@=;?A?BCC<??ACA=77717@@A@?<@CAAA>A@@@A
A888>=
@T6XXI:00087:00527
GGGAGAAGGGAGAAAGGAAGGAGAAGTCGCCCCAAACGATTTGTTGACTATTCTCCCTCTCCCTATAGTGAGTCGTA
TTAGAATTCCCC
+
CC?BDCCCC>BBBC=A=?=@?CBB?AA93333,7>:@CCA@;CC@C?6<AA?<?<=;=:::=;<<?<?==AA<??@?
@>A@@;;3444,
@T6XXI:00087:00541
CTATCACTCGAGATCTGCGGCTTGACAGTCCCTCTGTACTGTGAAGCCACAGATGTCGAGTCGACTAG
+
44;44444449889982--+-,*,,,243355:889>==7748:88C@:99AA6679666133.....
@T6XXI:00087:00542
GGGGAATTCTAATACGACTCACTATAGGGAGAGGGAGAATAGTTACTACCGTT
+
@@?4>695<@@AABAA@CCB??>=>777.7>@CE>C?@=@=9;78//++*+*)
@T6XXI:00087:00545
GGGAGAAGGGAGAAAGGAAGGGAATAGTACTTAAGGCAGATCAAAGTCTATTCTCCCTCTCCCTATAGTGAGTCGTA
TTAGAATTCCCC
+
ECCCCB@BB@BBBB=C?A?AA>A@A@@?BBB@B@B>?CB?BBAB@BBAABCABCCC@CCCBC>BCCBBBCCCC@??A
A>?@@>A?AA@5
@T6XXI:00087:00546
GGGAGAAGGGAGAAAGGAAGCTAGTCACTCGACATCTGTGGCTTCACAGTACAGAGGGACTGTCCAAAGCCGCAGAT
CTCGAGTCGACTAG
+
>>=ABC=@>:@@885@8:6;CBBBC?>>;;800-
...35;:?>=@@?????<;?84814;;=??<?C?@@<@A@@D=888@=;;<5;8330
@T6XXI:00088:00503
GGGGAATTCTAATACGACTCACTATAGGAGAGGGAGAATAGTTTTCCCTTCACTAACCGCGAACTGCACTTCCTTTC
TCCCTTCTCCC
+
DCC9B>@=@BBDCBBBBBBB<6877//-0>;>A=AACBA>7744-
4;9=79:=0/,/7>>???BAAABBA:?5::5:@@DAA?===A=
@T6XXI:00088:00514
```

```
CTAGTCACTCGAGATCTGCGGCTTTGGACAGTCCCTCTGTACTGTGAAGCCACAGATGTCGAGTCGACTAGCTTCCT
TTCTCCCTTCTCCC
+
;://1=8==CCDCCCBBBBB?B@?;B>A@A@BBB>B???@?==<@@A;??@BBBBBB??<<<@@BBBBBC????C?B
C@CA@@;@>AAAA>
@T6XXI:00088:00517
GGGAGAAGGGAGAAAGGAAGGCGAAATGTCACGTCGGGAGGTACATGACTATTCTCCCTCTCCCTATAGTGAGTCGT
ATTAGAACTTCCCC
+
9=:=CCADCABAAA8;8;<><?877.111376766<@;A@@@BACC<8:?::2/7230-/-
110144=??<;68///.54-.4:::;<@>0
@T6XXI:00088:00533
GGGGAATTCTAATACGACTCACTATAGGGAGAGGGAGAATAGATCACCGTTGGTTCGGGTGCGCGTTCTCCTTCCTT
TCTCCCTTCTCCC
+
CCC:C<;<<ECACC@@@CCCCCB=;666.7777A=AAA>A@@@@@??:?A6:7;@CCD@B;;;A??;?CB<@<A;??
<=@@A=A9<<>?7
@T6XXI:00088:00534
GGGGAATTCTAATACGACTCACTATAGGAGAGGGAGAATAGTTACTAACTACTAGGCGCAACTGTTCTCTTCCTTTC
TCCCTCTCCCAT
+
CCC:C>@>B@A=@A?@ACCCDCBB@:737>==<9;8>8<@AA>@A@@;==AA@@A>??@@>??@@?@987568<<7;
;;;.//466,30
@T6XXI:00088:00542
GGGAGAAGGGAGAAAGGAAGGAGAAGTCGCCCCAAACGATTTGTTGACTATTCTCCCTCTCCCTATAGTGAGTCGTA
TTAGAATTCCCC
+
DD>BCB@BB>ABAB@B@B??@BB?>?>>BBBB8@@=@A>>A>AB?CC<@@<8;;==;<<<@A9==A@@@@AA:66:=
=<?@A?A<???5
@T6XXI:00088:00544
GGGAGAAGGGAGAAAGGAGCTAGTCGACGTCGAACATCTGTGGCTTGACAGTACAGAGGACTCGTCAAA
+
>>5998244-4448582337777/.......-43....164456=5667711000000/-------.45
@T6XXI:00088:00545
CTAGTCACTCGAGATCTGCGGCTTTGGACAGTCCCTCTGTACTGTGAAGCCACAGATGTCGAGTCGACTAGCTTCCT
TTCTCCCTTCTCCC
+
=8//1;/44AABBBBCBBCC@?<<;>?A@@C??C=;?>???<<<<<<::?ACCCAAAAABCBCCCCCCCCAAB@C?B
B>B@AA:@<:??@:
@T6XXI:00088:00546
GGGAGAAGGGAGAAAGGAAGGAC
+
DDBCCD?A?=@@88800,)))((
@T6XXI:00089:00516
CTAGTCACTCGAGATCTGCGGCTTTGGACAGTCCCTCTGTACTGTGAAGCCACAGATGTCGAGTCGACTAGCTTCCT
TTCTCCCTTCTCCC
+
;:///1/49AAABBCBAAAA?A@?;?@BAC@?AA=A>?@888A===A=?B@BDAAAA@@BBBCCCCBBBCCCC>@@@
ECB@@A=A=@@AA<
@T6XXI:00089:00528
GGGAGAAGGGAGAAAGGAAGGACAAATAGATTGCGGGGGAAACGCTAGCTATTCTCCCTCTCCCTATAGTGAGTCGT
ATTAGAATTCCCC
+
:B<@@>;>=:>?>A>B?BAC@CBBB>BA?AA>AADCCC1BB<CCCCBBB?@@@BBBB99944418B@AA@@@@A===
=<9<==:=<ACC4
@T6XXI:00089:00534
GGGAGAAGGGAGAAAGGAAGAGTGGGACGACTGCGAAGTTTTATCGGCTATTCTCCCTCTCCCTATAGTGAGTCGTA
```

```
+
BB>BCCABB>BBBB=B@B@?>>@@@>?AAA@@@A=?;>>;91;<//-/=@@?B@AA<@@@@A<A@???C@@@??@@A
@T6XXI:00089:00537
GGGAGAAGGGAGAAAGGAAGCTAGTCACTCGACATCTGTGGCTTCACAGTACAGAGGGACTGTCCAAAGCCGCAGAT
CTCGAGTCGACTACG
+
BA>BCC?CC<<;<E<A=@B@@?FCDB?==?:B?@B@AAAC???8:;B@BB@===8771899;==:=?7<<CA@@@@A
DAA@?888:788///
@T6XXI:00090:00500
GGGAGAAGGGAGAAAGGAAGATCGGTTTGCGGAGAGTAAGCTTAGGGCTATTCTCCCTCTCCCTATAGTGAGTCGTA
TTAG
+
49-949244-4;;;-423133881011,1999?@??:747=?;;A@7=::64.---+--++0*--.-
43335504438466
@T6XXI:00090:00504
ACAGTCCCTCTGTACGTGTGAAGCCACAGATGTCGAGTCG
+
/////@B=?B>=66/////.37744/..44...9;/////
@T6XXI:00090:00505
GGGAGAAGGAGAAAGGAAGTAGTCGACTCGACATCTGTGGCTTCACAGTACAGAGGGACTGTCCAAAGCCGCAGATC
TCGAGTCGACTAG
+
6650--*,*,,//11:?=?700:1---
,,,/,,20////,//,4499999997337,3337673:@<@B;?AB@??B?<;9661..3...
@T6XXI:00090:00524
GGGAGAAGGGAGAAAGGAAGTGCAGTTCGCGGTTAGTGAAGGGAAAAACTATTCTCCCTCTCCCTATAGTGAGTCGT
ATTAGAATTCCCC
+
CD?CCHACC>BBCB@B@B@BABBCBB@CCB??C@BBCCC=??;?CCC2CBBA>ACCD>BBBBC>@@<<=@AAAA>>;
<<8;;=;;<ABC5
@T6XXI:00090:00530
GGGGAATTCTAATACGACTCACTATAGGAGAGGGAGAATAGTGACGTGTTGTGCGGCGCTCACTGTCTCCTTCCTTC
TCCCTTCTCCC
+
DEC9B=@=@AB>CBCCDCC@?>@@=00.0>;?C@???=@@@@@BAA=<<;<>AAC=;?@@A@@@A??<95677,.,/
6761776;==7
@T6XXI:00090:00535
GGGGAATCTAATACGACTCACTATAGGGAGAGGAGAATAG
+
>8202*1///5644433771137788826;610100/-22
@T6XXI:00090:00549
GGGGAATTCTAATACGACTCACTATAGGAGAGGGAGAATAGCTACCGTCCGTTCCCTGCTATGCTGTTCTTCCTTCT
CCCTTCTCCC
+
AA?6A===@CCACC@@>????:977//-
0>>BE>C@A?@<<=@@@?@<><<<=??:>>==>@<==AB=AA<=5427444-4799;;7
@T6XXI:00091:00506
GGGGAATTCTAATACGACTCACTATAGGGAGAGGGAGAATAGTCAACAAATCGTTTGGGGCGACTTCTCCTTCCTTT
CTCCCTTCTCCC
+
CDCDD::<;BB@DCCBCBCCBBB@?9:7.7>77:5:@@?C===AA?A@@<CDBBC>BBB7BBBBC???=:=;=?AA:
@AAA>C;=<@>7
@T6XXI:00091:00520
GGGGAATTCTAATACGACTCACTATAGGAGAGGGAGAATAGCCAACAGGTATCTGATTGCGCGTTCTCCTTCCTTTC
TCCCTTCCTCCC
+
```

```
>A>1>,/,1//,/4447>790/./...,11177996>;><<<=@??@A@??<<<<@@>A==9/0;;;;.0./7:;6;
6;;69-/.0;95
@T6XXI:00091:00525
GGGAGAAGGGAGAAAGGAAGCTAGTCGACTCACATCTGCGGCTTTGGACAGTCCCTCTGTACTGTGAAGCCACAGAT
GTCGAGTCGACTAGCTTCCTTTCTCCCTTCTCCC
+
9><@@>?A@1949904231331.../....3386998???377@9?;???A::;.4;;888>>>>@?37789;;98:
844484005<6??D@;9;/44-4444-4/--222
@T6XXI:00091:00534
GGGAGAAGGGAGAAAGGAAGTGCAGTTCGCGGTTAGTGAAGGGAAAACTATTCTCCCTCTCCCTATAGTGAGTCGTA
TTAGAATTCCCC
+
<<:<BB@CG@BBAB>B@B@BBBC@BA@A?>>@B?AACBC@CC?BBC8C?AB?CBCC>??@BC>A?????@@@@@@A?
?>?@B?A9==<4
@T6XXI:00092:00501
CTAGTCACTCGAGATCTGCGGCTTTGGACAGTCCCTCTGTACTGTGAAGCCACAGATGTCGAGTCGACTAGCTTCCT
TTCTCCCTTCTCCC
+
>:///1/49BEBDCCAAABB@BAC=A<<??????;?CCCBA=A<??A<?B?BBBBBC>A?CCBBBABBCCBBBAA?A
C>DAAA<@>?@AA?
@T6XXI:00092:00504
GGGAGAAGGGAGAAAGGAAGCTAGTCACTCGACATCTGTGGCTTCACAGTACAGAGGGACTG
+
99-4B@=@A>ACCD?C@C@@@:::AAA>?CABAACC:9:><7747??6;A@74.4972,,**
@T6XXI:00092:00508
CTAGTCACTCGAGATCTGCGGCTTTGGACAGTCCCTCTGTACTGTGAAGCCACAGATGTCGAGTCGACTAGCTTCCT
TTCTCCCTTCTCCC
+
>:/1111:9CCBBBDB@@@C?BCC>C<@????CC>CAAABB==5:9C?AC?BCDC@@@???CBBBCBBBB::7;?=?
E>B@@?7>9<79;3
@T6XXI:00092:00509
GCTATCACTCGAGATCTGCGGCTTTGGCTCTGTACTGTGAAGCCACAGATGTCGAGTCGACTAGCTTCCTTTCTCCC
TTCTCCC
+
///8:9=====9<;BBAB??;877.8<?A>?????B;::A@CC@BBBBCC==@CB@???BBCA=@A?>=<>97;661
65:@AA=
@T6XXI:00092:00517
GGGGAATTCTAATACGACTCACTATAGGGAGAGGGAGAATAGTCAACAAATCGTTTGGGGCGACTTCTCCTTCCTTT
CTCCCTTCTCCC
+
ACE:DACAAAA@CC@A@@@@A?>==777.7???@@B@@>@?@AAA?A@B:BBBBB?CCC9BBBBB?BBA;=;=?AA<
@@AA<A378<;6
@T6XXI:00092:00522
GGGAGAAGGGAGAAAGGAAGCTAGTCGACTCGACATCTGTGGCTTCACAGTACAGAGGGACTGTCCAAAGCCGCAGA
TCTCGAG
+
AB>BCB@BC<@;44-
95858>@<????AABBB@@@CCCCBA<?A?CB@??@===<8664688<@A>::99:7//0//4;><<<?
@T6XXI:00093:00517
GGGAGAAGGGAGAAAGGAAGGAGAAAGCGCCA
+
991444244-4444-4979977377,13.6,.
@T6XXI:00093:00522
GGGGAATTCTAATACGACTCACTATAGGGAGAGGGAGAATAGTTCCTCTGTGTCAAGAGCGAGTGTCTCCTTCCTTT
CTCCCTTCTCCC
+
```

```
>>>4>59:<@ABDBBBBBCBBCC@>=<:8:AAAC>CAB>@<@@>@><<BBBAC??59888>?@@A@@??;=9;3885
8AAC;@>;;<<6
@T6XXI:00093:00525
GGGGAATTCTAATACGACTCACTATAGGGAGAGGGAGAATAGCCACCTGTTTTGTGGTTGAGCTATCCCCTTCCTTT
CTCCCTTCTCCC
+
ABB:C>A>?BBABBB?BBBB><;67116.777BE>@@@@A9??<==:::<><6<>?8<9<<==B@BBBC>C::/44-
4:;A<A;==A@;
@T6XXI:00093:00527
GGGAGAAGGGAGAAAGGAAGCTAGTCGACTCGACATCTGTGGCTTCACAGTACAGAGGGACTGTCCAAAGCCGCAGA
TCTCGAGTCGACTAG
+
DF>@@@78809999094858>=:/00../..4/96666@@>:;?;????BC=::;444-
4;;;AA>AC:??=@AAAAA???A==<>>;7888
@T6XXI:00093:00528
GGGAGAAGGGAGAAAGGAAGCTAGTCGACTCGAGATCTGCGGCTTTGGACAGTCCCTCTGTACTGTGAAGCCACAGA
TGTCGAGTCGACTAGCTTCCTTTCTCCCTTCTCCC
+
CC;=<CBDE@BBCC>C@CGAA?;::0/0/..377@A==ABB@EDD;?=@CECBAA<AB====<<???@>??<?????
?AAAB@?<4888=@@==;8,//,////,/,//..+
@T6XXI:00093:00530
AGTCACTCGAGATCTGCGGCTTTGGACAGTCCCTCTGTACTGTGAAGCCACAGATGTCGAGTCGACTAGCTTCCTTT
CTCCCTTCTCCC
+
:><CBBBBCCCCBBBBB@>:999;?@?>@;;:6:BB??@CC@??C=AA>AAC@AA@@:::====<<:=44:=?=?A=
AB@@<=344:;7
@T6XXI:00094:00505
GGGGAATTCTAATACGACTCACTATAGGGAGAGGGAGAATAGTCAACAAATCGTTTCGGGGCGACTTCTCCTTCCTT
TCTCCCTTCTCCC
+
/11*/,/,///7/499<>A>>???0000+/7/7116:=:=@A@@@@A>?5>>>>>1//::6::<==866623238;D
=AACC9=9=8886
@T6XXI:00094:00507
GGGAGAAGGGAGAAAGGAAGCTAGTCGACTCGACATCTGTGGCTTCACAGTACAGAGGGACTGTCCAAAGCCGCAGA
TCTCGAGTCGACTAG
+
CKBABAADCABCCC@C@B@BBA665/00;;:?;???=<CCA>ACA;:;C::8===9;;6;=>?@A?AA>BB<????A
C:@@A@===@@@A??
@T6XXI:00094:00508
GGGAGAAGGGAGAAAGGAAGCTAGTCACTCGAGATCTGCGGCTTGGACAGTCCCTC
+
4;:999289094;;-42377319=:<0/./4315>/768>;773101166111,00
@T6XXI:00094:00513
GGGAGAAGGGAGAAAGGAAGTAGTCGACTCGACATCTGTGGCTTCACAGTACAGAGGGACTGTCCAAAGCCGCAGAT
CTCGGGTCGACTAG
+
BKBAAA>@C:89;;9;BBBCC;<>CBBCCCC<<=AAA@@B?BB@ECCECCA=9733308??ABB@CCACC@CA=AB?
;=9==:?588AAAA
@T6XXI:00094:00522
GGGGAATTCTAATACGACTCACTATAGGGAGAGGGAGAATAGTCAACAAATCGTTTGGGGCGACTTCTCCTTCCTTT
CTCCCTTCTCCC
+
??@0?,1,188,19CCABABCBCC99:<.77777.44437?@@==;=CC?@?@CC>CCD9BBCCC<<8<=?<=<=@1
849<;@8:8AA?
@T6XXI:00094:00539
GGGGAATTCTAATACGACTCACTATAGGAGAGGGAGAATAGCACCAATATTGACAATGCCTGCTTACTCCTTCCTTT
CTCCCTCTCC
```

```
+
B>;4=5959>>?CBBBCBCCB@@996637>>>>9?<@>@@AC??<?>@;@>778?;?AA<?@AA?@A@@8:7:344-
////,////,
@T6XXI:00094:00545
GGGGAATTCTAATACGACTCACTATAGGGAGAGGGAGAATGAGTCAACAAATCGTTTGGGGCGACTTCTCCTTCCTT
TCTCCCTTCTCCC
+
;D>5:,45999289A@BCABBBBF???>:>>777.7;;7;<<<<69<===;A888@99A>7?85>>-/-
3,.,/46616/43,49;7771
@T6XXI:00094:00546
GGGAGAAGGGAGAAAGGAAGCTAGTCACTCGACATCTGTGGCTTCACAGTACAGAGGGACTGTCCAAAGCCGCAGAT
CTCGAGTCGACTAG
+
CF@BBA>?=:BB@@:@7:5:B???A8??@A;=<;:::CLL=??@CC@@<?=AA=8999=AAA@A?;;4::<=::6==
==<::?AAAAAA==
@T6XXI:00094:00549
GGGAGAAGGGAGAAAGGAAGATGAGGGAGGAGCATGAATGTCGATGACTATTCTCCCTCTCCCTATAGTGAGTCGTA
TTAGAATTCCCC
+
=@:999<>>0949909<>>BC@@????9?@=@@AABBB>BBDCCCCCCBCAB>A???9??=@A=AAACCCBBBAA@@?
?;=:;8>:===2
@T6XXI:00095:00509
GGGAGAAGGGAGAAAGGAAGAACAGTCGGGGAATGTGGATCAGTAGACTATTCTCCCTCTCCCTATAGTGAGTCGTA
TAGAACTTCCC
+
94-
49>;>A<AAAB8;9;=A@<?<::BCDL>CAA:;;;?@=?:C==@CCED@AB@C>BBBBB>BCCBBBB;:0;:<00,,
,*,.,--+
@T6XXI:00095:00512
GGGAGAAGGGAGAAAGGAAGGAGAACAACACGATCGGTCAAACGTAGCTATTCTCCCTCTCCCTATAGTGAGTCGTA
TTAGAATTCCCC
+
49-
94928;:=@AA>ADNBC@CAA=@B>@@@B@@;9697?9599<@BBB>B@::;D9;;661,000000035=;>;8858
?=@C;??@5
@T6XXI:00095:00514
GGGGAATTCTAATACGACTCACTATAGGGAGAGGGAGAATAGTTTTTCCCTTCACTAACCGCAATAGCACTTCCTTT
CTCCCTTCTCCC
+
:;;2@89:<??59;@A@B@@?>?=00..+/....+.14258=AABA1AA<A>A<;:C@C5::9/00--
..344577./4<944500034
@T6XXI:00095:00517
CTAGTCGACTCGAGATCTGCGGCTTT
+
94944447711//7/////.46...+
@T6XXI:00095:00529
GGGAGAAGGGAGAAAGGAAGGAGAAATGACACCATTCGTGATTAGGGTCTATTCTCCCTCTCCTATAG
+
4;694924@<@@@C@C=A8;9;<BB<;87>B5,/.3...455,77>;=@A?@>@??>6000-,-00++
@T6XXI:00095:00530
GGGAGAAGGGAGAAAGGAAGCTAGTCACTCGACATCTGTGGCTTCACAGTACAGAGGACTGTCCAAAGCCGCAGATC
TCGAGTCGACTAG
+
@A=ABB@CC>BB?B>B@B>@AACC<?;:967??:>@@CBCA@@=?ACAABB997..-
/;;;?A>AA=AA<??@A@889AAAAA@@???@@
@T6XXI:00095:00538
GGGGAATTCTAATACGACTCACTATAGGGAGAGGAGAATAGTACGGCCATT
```

```
+
?LA6A>D=@@@=@A;;;AABBBBBCA???6;<<<:=::7:444443242000
@T6XXI:00095:00540
GGGGAATTCTAATACGACTCACTATAGGAGAGGAGAAT
+
AC@4B54584424449<@BBAB@?9110.7,,*,++00
@T6XXI:00096:00510
GCTTTGGACAGTCCCTCTGTACTGTGAAGCCACAGATGTCGAGTCGACTAGCTTCCTTTCTCCCTTCTCCC
+
;CCCAD?AABBAAB<BCBBBCCCCCBB?AACC@@?BCCCCBBB<<AC???A?@=@@CD>CBBB<?;?@@?:
@T6XXI:00096:00517
GGGAGAAGGGAGAAAGGAAGCTAGTCGACTCGAGATCTGCGGCTTTGGACAGTCCCTCTGTACTGTGAAGCCACAGA
TGTCGAGTCGACTAGCTTCCTTTCTCCCTTCCTCCC
+
DC@@;959?:>ABC@C@CBBG??<<<777??B@CAA?A=7747==6=;?@BB>8826/----
116699<=A=>8889;;:37488<<@@A=:::>A>AA699=;7;3838@A:
@T6XXI:00096:00520
GGGAGAAGGGAGAAAGGAAGGGGAGAAGGAAGCTAGTCGACTCAACATCTGTGAAGCCACAGATGTCGAGTCGACTA
GCTTCCTTTCTCCCTTCTCCC
+
CCB@??@BB@BCCC?C@C@BAA8ABC@C@B?>>>>>?1/069078;CCBBBBBC?BB@CCCAB?C@<<<BAA@?=?A
ABB?B???:=766-744444+
@T6XXI:00096:00530
GGGGAATTCTAATACGACTCACTATAGGGAGAGGGAGAATAGCCGCAATTGACGTCATCCCTGGTTACTCTTCCTTT
CTCCCTTCTCCC
+
944/9<>=@CC@BBBBBB?>>@B;>111.7<<AB>AAA>A588<>;>5:7:@@@??@D@A=AA=@?AAA<;79243,
09994979444-
@T6XXI:00096:00536
GGGGAATTCTAATACGACTCACTATAGGAGAGGGAGAATAGTCAACAAATCGTTTGGGGCGACTTCTCCTTCCTTTC
TCCCTTCTCCC
+
ACB9B797;A?>AAAABBBAA>=><00,000>@??AB@AA===@=@@@;CDCCC>CCC7;;;;;?B@A6958=@A<@
@@C=C=@@AA7
@T6XXI:00096:00547
GGGGAATTCTAATACGA
+
88717+-*,,0*,///3
@T6XXI:00096:00548
GGGGAATTCTAATACGACTCACTATAGGAGAGGGAGAATAGTCAACAAATCGTTTGGGGCGACTTCTCCTTCCTTTC
TCCCTTCTCCC
+
999/9<A?BCC@C?>>?????:955//,0=>@B=?CC?A<<<?;:;AC=C???B=>>>8@AC::56<<5;8;8;;7=
@BC<@CAAAA;
@T6XXI:00097:00501
GGGAGAAGGGAGAAAGGAAGGTGAACAGAGCGCGAACGAATACGTCTCTATTCTCCCTCTCCCTATAGTGAGTCGTA
TTAGAATTCCCC
+
CDBBBB@BB?CCBB@C@B@B@BBB@CBBBBBBB?A>@CB@CBCCBBBBBBB?BBBD>BB???>?BBBA>ACB?CBC?
B??CC?C@CBB9
@T6XXI:00097:00510
GGGAGAAGGGAGAAAGGAAGCTAGTCGACTCGACATCTGTGGCTTCACAGTACAGAGGGACTGTCCAAAGCCGCAGA
TCTCGAG
+
CD@A@??B?=BBBB=B@B>AA><ABABAACCC??ACCAAAA<?B@??@??@<>>=999964484468C=@A<@@@@A
A???@C=
@T6XXI:00097:00513
```

GGGAGAAGGGAGAAAGGAAGGGAATAGCAGCAGTCGGCAACGTGAGACTATTCTCCCTCTCCCTATAGTGAGTCGTA
TTAGAATTCCCC
+
CCAAA@@BB=BBBC=B>A@BB?B?ABBCBAABBAAC>C?>>>@>@A>AAC?@>>;=;=<44448@AAA==A=<9==?
?>@AA>A>AAA5
@T6XXI:00097:00515
GGGAGAAGGGAGAAAGGAAGCTAGTCGACTCGACATCTGTGGCTTCACAGTACAGAGGGACTGTCCAAAGCCGCAGA
TCTCGAGTCGACT
+
CC@ABA=@@<@@@A<B?B?BB9>>5/11>>>?1:::::@@@9<?=?BCCCCAAA=<<<:<<<<==<<<:;<>A===<
<8<=<<==<A???
@T6XXI:00097:00516
CTAGTCACTCGAGATCTGCGGCTTTGGACAGTCCCTCTGTACTGTGAAGCCACAGATGTCGAGTCGACTAGCTTCCT
TTCTCCCTTCTCCC
+
=8/111/449@BBBBBAA<>>???;A@A:8?????9?CBBBB@@=AAA=AC@BCCCA@??AAAA?@@CDCB:;75757
C>C@@@?A:==AA;
@T6XXI:00097:00517
CTATCGACTCGAGATCTGCGGCTTTGGACAGTCCCTCTGTACTGTGAAGCCACAGATGTCGAGTCGACTAGCTTCCT
TTCTCCCTTCTCCC
+
B?;A1////8868;;A@BBB?AAA<A=@>>=777.7==??A888;==:?D?CDB@@@CBABCCCCC::;:7713601
1,139;9;;:988,
@T6XXI:00097:00518
GGGGAATTCTAATACGACTCACTATAGGGAGAGGGAGGAATAGATCACCGTTGGTTCGGGTGCGCGTTCTCCTTCCT
TC
+
@@;6@6;8;889=?B<;;B@A?>71166,6:/00,0-,-
337:?;??>@???A?>=;990888876771163739,0,/
@T6XXI:00097:00522
GGGGAATTCAATACGACTCACTATAGGAGAGGAGAATA
+
,--*,*924424999;;?<969711101677;;9923-
@T6XXI:00098:00519
GGGGAATTCTAATACGACTCACTATAGGAGAGGGAGAATAGCTCAGCCTTTTGTTGCATGTGATTTCTCCTTCCTTT
CTCCCTTCTCCC
+
BCC:D@B>BCB@BBB?BCC?>:9:7//.1>>CE>B@A=A=;:>>888:;==4:;:;?ACABBBAA<@??=@>B?A@:
@BAC<A>@???:
@T6XXI:00098:00530
GGGAGAAGGGAGAAAGGAAGGAGAAGTCGCCCCAAACGATTTGTTGACTATTCTCCCTCTCCCTATAGTGAGTCGTA
TTAGAATTCCCC
+
=B?CDCDBB>BCBB:;2318588:=ABCBBBB8BC>A@ADD@AA>ABAABA=@@AA<@@???:@@???>>//0;66.
/..33336:881
@T6XXI:00098:00549
GGGAGAAGGGAGAAAGGAAGCTAGCATCGACATCTGTGGCTTCACAGTACAGAGGGACTGTCCAAAGCCGCAGATCT
CGAGTCGACTAG
+
CCACCDACC>?@?B>BA?=>B????AAC@88:@@@DCC@BC@CCCC@?C><<7664788<<=9=CA??<?@@@AAA@
>@@<A9==??<;
@T6XXI:00099:00516
GGGAGAAGGGAGAAAGGAAGCTAGTCACTCGACATCTGTGGCTTCACAGTACAGAGGGACTGTCCAAAGCCGCAGAT
CTCGAGTCGACTAG
+
BB>@A@?BB<@@A@<A>@=@C:::B@;7:?<::7CC::;C?BB@CBBAC?A==933317>>@AA?AA<A@?@A====
=<<==AA===A@AA

```
@T6XXI:00099:00521
GGGAGAAGGGAGAAAGGAAGAGAAAGCCGCACGACAGATAAA
+
DCABAB784-99990958487BC@;AA>8331111111111,
@T6XXI:00099:00522
GGGGAATTCTAATACGACTCACTATAGGGAGAGGGAGAATAGCCCAAGCATCAGTGCACCGTTGTTCTCCTTCCTTT
CTCCCTTCTCCC
+
BA@4@595<@@?BCCCCBCCBC?7=616.7?777-7::45;=??;?AA@?BA7::?AA@;??@CB@A<<899;344-
44;;-4343441
@T6XXI:00099:00523
GGGAGAAGGGAGAAAGGAAGCTAGTCGATCGACATCTGTGGCTTCACAGTACAGAGGGACTGTCCAAAGCCGCAGAT
CTCGAGTCGACTAG
+
EN?BBA@BA>BBBCAC@C@BB@<==CDBBBB???BBBBB@>=?>@ECA@?=:6;3331799=??<?A<??<?A@AAA
=@@???A@@@@@@A
@T6XXI:00099:00524
GGGAGAAGGGAGAAAGGAAGGACGACAGT
+
CKBHCB@B@:9>::5;::9:,0......1
@T6XXI:00099:00531
GGGGAATTCTAATACGACTCACTATAGGAGAGGGAGAATAGCCGTCTCGTTTGGCGCTCCCTTCTTGTTCTTCCTTT
CTCCCTTCTCCC
+
>>;0;,/,/000,/...47?<?BB>::5::7@B>898585<<>:?747<<=:@>@@@BBC>CABB?BB???:=444-
4;;?6;8;;;<7
@T6XXI:00099:00535
GGGAGAAGGGAGAAAGGAAGGATAGGCAGCGTCGACCGAACGACAGACTATTCTCCCTCTCCCTATAGTGAGTCGTA
TTAGAATTCCCC
+
<96;BA@CD@DBBC@C@B>B@BB@@>AABCCBBBCB@BB@ECCBB@@@B@?<>777.7BBCD>B@@@AAACA>AA:7
8374:88/444-
@T6XXI:00099:00547
CGGCTTTGGACAGTCCCTCTGTACTGTGAAGCCACAGATGTCGAGTCGACTAAGCTTCCTTTCTCCCTTCTCCC
+
CB=?AD?C@CCCC@??=@DDGCBB?BBCB@BB@CBAA>????>?@A<>??6647=?=@?AD<?@BA<A=@@BE@
@T6XXI:00050:00555
GGGGAATTCTAATACGACTCACTATAGGGAGAGGAGAATAGT
+
C886;;A@AEA@CCBBBAAA@=@=CACC>@B:7:7+++,***
@T6XXI:00050:00558
GGGAGAAGGGAGAAAGGAAGGGAATAGTCGCGCTTGTTATCAACGTGCTATTCTCCCTCTCCCTATAGTGAGTCGTA
TTAGAATTCCCC
+
EDBCEB?BB;@9996;=?5::8;>ABBBBBBABB@CC@CCCC@CCCCCCCB?A;;;7;@@AB@CCBAACCCBBCCBB
B@C??=B?AKKD
@T6XXI:00050:00570
GGGAGAAGGGAGAAAGGAAGGGAATAGCAGCAGTCGGCAACGTGAAACTATTCTCCCTCTCCCTATAGTGAGTCGTA
TTAGAATTCCCC
+
?A<@=A>BC?GCBC>C?D?@B:>4;0000...55335/659?CCCD>AA?=:=777.7966;;884488689=?@>=
<5;>;94/000+
@T6XXI:00050:00591
GGGAGAAGGGAGAAAGGAAGCTAGTCGACTCGACATCTGTGGCTTCACAGTACAGAGGACTGTCCAAAGCCGCAGAT
CTCGAGTCGACTAG
+
```

```
9<89BA?CC>AACH>@?@@CC????::::669::9AA88::568899??@CA9;;//.0;;;@@?AA=@@=@@C@@?
??@AAAA:88@>88
@T6XXI:00051:00558
GGGGAATTCTAATACGACTCACTATAGGGAGAGGGAGAATAGTCCCTGTGTTCTCCCCACAGCGGTTAACTTCCTTC
TCCCTTCTCCC
+
@@@6A@C@CBB?BBBAAABBA??;;677.7?==?<@@A>@@A???;>???=;=;;;;1;BAAAB>@>@?@@=@5858
6=;:@>?;??;
@T6XXI:00051:00561
GGGAGAAGGGAGAAAGGAAGCTAGTCACTCGACATCTGTGGCTTCACAGTACAGAGGGACTGTCCAAAGCCGCAGAT
CTCGAGTCGACTAG
+
CC@CCC?A?:=?==5>584;????DCCCDB=:87:67AAD@AA>ACCCBB@?=933717=>>???AA=BA@@@@@A?
?@@@@===ABAA@A
@T6XXI:00051:00572
GGGAGAAGGGAGAAAGGAAGGAGAAGTCGCCCCAAACGATTTGTTGACTATTCTCCCTCTCCCTATAGTGAGTCGTA
TTAGAATTCCCC
+
CD@BB?@BB=ABAA?B@B>??AB@>>>???BB6@A<@CACC>CCBB?<=BB>AAAA<====A<AA@@???AAAAA88
9?A@A@A>@A@5
@T6XXI:00051:00582
GGGGAATTCTAATACGACTCACTATAGGGAGAGGGAGAATAGTTTTCCCTTCACTAACCGCCGAACTGCACTTCCTT
TCTCCCTTCTCCC
+
?==6=>BAABB=:;AD;<;BBBCBA:::7:CCCD@B@A<=:4444-
4:7::?AAA:9:900,/9784:9??C>7017-709949930384
@T6XXI:00051:00590
GGGGAATTCTAATACGACTCACTATAGGGAGAGGGAGAATAGATCACGACCTTCTACCACGA
+
999/92489>@?A@@AB>8;67311111,17669;:66;<AA==<<;7,,+,**2,+*-123
@T6XXI:00052:00550
GGGGAATTCTAATACGACTCACTATAGGGAGAGGGAGAATAGCTACTACG
+
CCB7B89;<CC@CCAAABBA@BA?B6695:=<CC>@@A>>8/./00,+++
@T6XXI:00052:00567
GGGGAATTCTAATACGACTCACTATAGGGAGAGGGAGAATAGCCTGATGACTTGGC
+
?A?+1,1,/8876=;;;;:;B@<7?9:7-6;>@A;?8A=9@?>;>97-----0000
@T6XXI:00052:00569
GGGGAATTCTAATACGACTCACTATAGGGAGAGGGAGAATAGCTTACTT
+
CCC9C999;BB@CCCCCAAA==@??99:7:AACCCC==:<55,,+,,,*
@T6XXI:00052:00587
GGGGAATTCTAATACGACTCACTATAGGGAGAGGGAGAATAGCAAACGTTTCTCCGACGTCGCTTTCTCCTTCCTTT
CTCCCTTCTCCC
+
CEB9B9;<<CC@CCA@@@@@@CB76116.7>>@C@CAA?DB@@@@;?A@C>6<=?@@@@AD??AA:?7734:;79;5
88BB=A<===B<
@T6XXI:00053:00551
GGGAGAAGGGAGAAAGGAAGAAAAAGATAGGGCCAAGTATCAATTGGGAACTATTCTCCCTCTCCCTATAGTGAGTC
GTATTAGAATTCCCC
+
CE?BA=<>>9>88809;:9::<<@+0/..0000*,*.....77>;=@8?=?:;;A?<<A<884??7<===AA@A@@@
??<<7388/323;@6
@T6XXI:00053:00552
GGGAGAAGGAGAAAGGAAG
+
```

```
8:50-1-1-////+/,313
@T6XXI:00053:00567
GGGGAATTCTAATACGACTCACTATAGGGAGAGGGAGAATAGTCAACAAATCGTTTGGGGCGACTTCTCCTTCCTTT
CTCCCTTCTCCC
+
999/95929ABICCBBB???A@<=;6775:?BCC>@@A<?:AAAA<??A=CCBCB>BBB9BBBBB?A=?9=;=>ADA
@@AA;?;==@C?
@T6XXI:00053:00575
GGGAGAAGGGAGAAAGGAAGCTAGTCACTCGACATCTGTGGCTTCACAGTACAGAGGGACTGTCCAAAGCCGCAGAT
CTCGAGTCGACTAG
+
@@<AAC@BB<@99909;?58;?@A@B>=<=8>::@@<AAA>@@?@??@CEA@@@77417@?@AA?AA=AA>988?@?
=;;<;<<==><<<@
@T6XXI:00053:00585
GGGAGAAGGGAGAAAGGAAGGAGAAGTCGCCCCAAACGATTTGCTGACTATTCTCCCTCTCCCTATAGTGAGTCGTA
TTCAGAATTCCCC
+
?C>A@C@CC>BBBC?C?B?B?C??:=783333,177;?@BC>=??CBAAA@;;:88,//-1101-
3366299//.382,3300/0/000+
@T6XXI:00053:00595
GGGGAATTCTAATACGACTCACTATAGGGAGAGGGAGAATAGTTAGCGTTT
+
/11+1,4244488=?:::AAA?A<9?A?4:7===9<<>9;9//,/,+0000
@T6XXI:00054:00558
GGGGAGAAGGGAGAAAGG
+
///*/6988;-4444-42
@T6XXI:00054:00564
GGGGAATTCTAATACGACTCACTATAGGGAGAGGGAGAATAGTTCCCCACGATTAGGCCGGTCGTTTTCTTCCTTTC
TCCCTTCTCCC
+
CEDBD>@AACCACCCB@@BBAA>9?666.7>>AB>@@A?AB@@=?AA6?8<<C>AA=B@B?B???CD8AA<=37717
=<C<@=>ACC>
@T6XXI:00054:00581
GGGGAATTCTAATACGACTCACTATAGGGAGAGGGAGAATAGTCAACAAATCGTTTGGGGCGACTTCTCCTTCCTTT
CTCCCTTCTCCC
+
BEE9B9;8<@@=@@@AA@@@BBC<;;@D<@@???9?==<@@AAAA>BAA<CCCBB<AAA7ACCCC???;3858<;;9
4333,0/03333
@T6XXI:00054:00583
GGGAGAAGGGAGAAAGGAAGCTAGTCGACTCGACAC
+
>BACHCABB<@>@@0968<>?=0...,,,,,,,,...
@T6XXI:00054:00589
GGGAGAAGGGAGAAAGGAAGCTATCGACTCGACATCTGTGGCTTCACAGTACAGAGGACTGTCCAAAGCCGCAGATC
TCGAGTCGACTAG
+
@@<AAA?CH@DCCC;;9;=?>??<=>77616;;6666177717516;=?::4;;8838@;745>AA;=:;6;88844
3400000000004
@T6XXI:00054:00593
CTAGTCACTCGAGATCTGCGGCTTTGGACAGTCCCTCTGTACTGTGAAGCCACAGATGTCGAGTCCGACTAGCTTC
+
7:///6699=444=>>>>884877,31316866826668??>?@@@@?AC>A@CCC@;<;?<<01,000.41..33
@T6XXI:00055:00566
GGGAGAAGGGAGAAAGGAAGGAGAAGTCGCCCCAAACGATTTGTTGACTATTCTCCCTCTCCCTGATAGTGAGTCGT
ATTAGAATTCCCCCATCACGT
+
```

```
?B:@9969>:>>>>9@=>=C?;;7@B?@@BBB8CD>C::7>9??@AAC?=<4466717,,+00+++3...8=B@A@=
=<;>665421146./.--200
@T6XXI:00055:00584
GGGAGAAGGGAGAAAGGAAGCTAGTCACTCGACATCTGTGGCTTCACAGTACAGAGGGACTGTCCAAAGCCGCAGAT
CTCGAGTCGACTAG
+
DCB???@BB>BCCC=B?A@AA><??AA@AB?::8BBBDDC?AA@CCCA==C==844417?AAAA=AA<AA=@?:;==
<<<=<<<=B<=<<=
@T6XXI:00055:00585
CTAGTCGACTCGAGATCTGCGGCTTTGGACAGTCCCTCTGTACTGTGAAGCCACAGATGTCGAGTCGACTAGCTTCC
TTTCTCCCTTCTCCC
+
B94999449=A=AAAACCCCC@CCB?B?AA@@@@?;@@A@==?A@AAA>AC?BBBCCCCBAAACCCBAABC@@@@C<
??9?<==:=9<<<@:
@T6XXI:00056:00562
GGGGAATTCTAATACGACTCACTATAGGGAGAGGGAGAATAGATCACCGTTGGTCCGGGTGCGCG
+
BBA4@5959>>244????@???>:::@@9?@???8;66:=6480317476013142//+....33
@T6XXI:00056:00564
GAGGAGAAGGAGAAGGAAGCTAGCGACTCGACGATCTGTGGCTCACAGTACGAGGACTGTCCAAAG
+
611-111,/,//.,31377444:4448///6//...47;>>76696000366..,....11011,1
@T6XXI:00056:00576
GGGAGAAGGGAGAAAGGAAGATGACGCGATCGATACATGGGGAGTAGCTATTCTCCCTCTCCCTATAGTGAGTCGTA
TTAGAATTCCCC
+
AFABBBACC@CBBB@BAB@BCBB@AACDCCBBCCCBBCCED9C;<<CBABC?BBAB<::::B<AB@?@CC???C@B=
@==5;8@=@BB8
@T6XXI:00056:00590
GGGAGAAGGGAGAAAGGAAGCTAGTCGACTCGAGATCTGCGGCTTTGGACAGTCCCTCTGTACTGTGAAGCCACAGA
TGTCGAGTCGACTAGCTTCCTTTCTCCCTCTCCC
+
CF?AAC>>>9>99909<><>?=:;;0//=>>11/CC98??@<@AA<B@BBB>=?C<??@888<<<<<=:==>AA@@@
@888@88348=8;<88486,/.+-111+--1222
@T6XXI:00057:00555
GGGGAATTCTAATACGACTCACTATAGGGAGAGGGAGAATAGCGCAATTTGGACATGTGGCCGCTTCTCCTTCCTTT
CTTCCTTTCTCCCTTCTCCC
+
CCC:C?B?BBBABCAAAAAAAAAABB???;?BBCC>>@A?AA>??AB;??8??BCCBB@@>@>@??<887899;344-
44347:;7=@AH=A?A@A@;
@T6XXI:00057:00566
GGGAGAAGGGAGAAAGGAAGCACAGGGCGAAGCGTGGGAAACTTGGCCTATTCTCCCCCTCCCTATAGTGAGTCGTA
TTAGAATTCCCC
+
CC@AAA@AA===?B>B@B@BBBB???;??;>?@AA@A=@@;?A<?9=9=?A>ABBAAA1@A=;=9==@AA=@@A=@<
?>??=;=;===1
@T6XXI:00057:00573
GGGAGAAGGGAGAAAGGAAGGAGAACGCGACTAGTACCGGCACATGGCTATTCTCCCTCTCCCATAGTGAGTCGTAT
TAGAATTCCCC
+
?B<9985991488;:@<;;?;:;:;?@BCCA@@@@:878;=<::677403373..5+....3033000300440000
2344/42444,
@T6XXI:00057:00574
CTAGTCACTCGAGATCT
+
444449/////8///>/
@T6XXI:00057:00578
```

```
GGGAGAAGGGAGAAAGGAAGTCGACCGACATCTGTGGCTTCACAGTACAGAGGGACTGTCCAAAGCCGCAGAT
+
BC@BEB?BB?BDBB>C@A@@A?A??;<//000//?>:A@?@@@@AAA===:::4:======??<@A>A@=<==
@T6XXI:00057:00583
GGGAGAAGGGAGAAAGGAAGCTAGTCGACTCGACATCTGTGGCTTCACAGTACAGAGGACTGTCCAAAGCCGCAGAT
CTCGAGGCGACTAG
+
CN@<<<=@@<@AAC<A=@@CBBBCCB:::AA@:::@@>>?;9<A@CBBABB@A==4424::448;==7>?<<==@?=
?;<888<?A<<<@?
@T6XXI:00057:00584
GGGGAATTCTAATACGACTCACTATAGGGAGAGGGAGAATAGTTATTCGCTTGGTCACCGCTTCTATTCCTTCCTTT
CTCCCTTCTCCC
+
DCC9C@C@CBBACCB=B@BBCCB=?6115:@@CD>CBB;=:=A=AA?@<==;=>?@@A>??A@CBC@?@888:@CC?
CB@@<@>AAA<;
@T6XXI:00057:00585
GGAGAAGGAGAAGAAGGAGAGGAGAAGAAGGAGAGGAGAAGAAGTA
+
/,///,/,//8243131331679@@6:1:=:=74.3685+,+0,++
@T6XXI:00057:00589
GGGGAATTCTAATACGACTCACTATAGGGAGAGGGAGAATAGTCCCGACATCTTCAACGTGCTATTCTACTTCCTTT
CTCCCTTCTCCC
+
@=94?<@=@BB@CCCBBBCCCCC==611.6==>A>A@D>@<A@@@;@@@AC?@@@C@C@@@<;7:59??>@=?=@>:
>>==6?:?BCC>
@T6XXI:00058:00568
GGGAGAAGGGAGAAAGGAAGAGAACAGCGACGGGAATAATGTTTTAGCTATTCTCCCTCTCCCTATAGTGAGTCGTA
TTAGAATTCCCC
+
99-
49@@CD?A@@C@C@C@CBBB><<<CCBGCC>C>@>=>8888,99>?AC@CBCC@=<9=?:?B;?B::;A@>==74/4
8;/324==0
@T6XXI:00058:00585
GGGAGAAGGGAGAAAGGAAGCTAGTCGACTCGACATCTGTGGCTTCACAGTACAGAGGGACTGTCCAAAGCCGCAGA
TCTCGAGTCGACTAG
+
CCACDC@BA=BB@==B=@=@DA>@@1//===>1::BBCAA@<=?=@BBCCA??A><;<7<??@A@?CC<?@>@A>>?
?<;;<<<>@@<==@@
@T6XXI:00059:00550
GGGGAATTCTAATACGACTCACTATAGGGAGAGGGAGAATAGCCTGCAACCATATTGCCGGTCACGTCTCCTTCCTT
TCTCCCTTCTCCC
+
88207+1,///,/9@<;;BBCBB@B::;6:@@BC>BAA?AA>?<AC??<?=>>99/0424997;:<<?:?;777043
+044;;=<=85;5
@T6XXI:00059:00557
GGGGAATTCTAATACGACTCACTATAGGGAGAGGGAGAATAGTCAACAAATCGTTTGGGGCGACTTCTCCTTCCTTT
CTCCCTTCTCCC
+
?B@4A5969@@<>@@;;;BBAA?CA???9?@@BB>??A>AAAA@@=??=:>>777.777,67::@:0-
.,/,/,//,/7>A;=;@<@@;
@T6XXI:00059:00560
GGGAGAAGGGAGAAAGGAAGTGCAGTTCGCGGTTAGTGAAGGGAAAAAACTATTCTCCCTCTCCCTATAGTGAGTCG
TATTAGAATTCCCC
+
991944244-
44;;5=;<<@@CCCCC?AA8868<>>311011,11111(399>@::77.////72377=<<?@@=;;<<<:=CC@A=
AAA4
```

@T6XXI:00059:00577
GGGGAATTCTAATACGACTCACTATAGGGAGAGGGAGAATAGTCAACAAATCGTTTGGGGCGACTTCTCCTTCCTTT
CCTCCCTTCTCCC
+
ECD:C@B>BCC:<<;<;ACCBBB@777=.77@BC>@@@>@@A??A?A>=9878@@;A@C9DEC??;://4646,//,
/,/23+--458;;
@T6XXI:00059:00587
GGGAGAAGGGAGAAAGGAAGAAGATGCCTCGGGGAGTTCTCGGTAGACTATTCTCCCTCTCCCTATAGTGAGTCGTA
TTAGAATTCCCC
+
CC?CCC@BB????B=A?A@CA@@?ACCBBBCCC9CCC?BAAC@CBBAAACC@BBBC=999AC<???A?@@AA===@?
=<@?>;?;>@?5
@T6XXI:00059:00596
GGGAGAAGGGAGAAAGGAAGTGCAGTTCGCGGTTAGTGAAGGGAAAAACTATTCTCCCTCTCCCTATAGTGAGTCGT
ATTAGAACTTCCCC
+
CC?BBB@CC?BCBB>B@B@BBBBBBB@CCCB?A?@@@CA>@B<ACCC1B?B@>@BBB>BBCCC>A@A@@@AAAAAA?
8838?A7992444-
@T6XXI:00060:00576
CTAGTCACTCGAGATCTGCGGCTTTGGACAGTCCCTCTGTACTGTGAAGCCACAGATGTCGAGTCGACTAGCTTCCT
TTCTCCCTTCTCCC
+
>:11121:=?CBBABCCBBB<<?C>C=@?AA:::5:CA???@@889@>AC@CCCC:<:BBB@A?@@AA@AA@=:>47
7.79:;-4340384
@T6XXI:00060:00582
GGGAGAAGGGAGAAAGGAAGGAGAAGTCGCCCCAAACGATTTGTTGACTATTCTCCCCTCCTCCTAGAT
+
DC@BBBCDC?AAAG@@=@@B>AAA=A::;CCC9CC>:;;BA8AA=??==;<8;;;??1==;910-++**
@T6XXI:00061:00555
GGGGAATTCTAATACGACTCACTATAGGGAGAGGGAGAATAGTCAACAAATCGTTTGGGGCGACTTCTCCTTCCTTT
CTCCCTTCTCCC
+
DHH:C>?=@BB@CDCBBBBBBAA>A9995:?@BC>@AC=??@AAA>@AA@BBBBK<???5::BBB;?;<34342441
8==C<A=A<<<7
@T6XXI:00061:00559
GCTAGTCATCGAG
+
44444444/////
@T6XXI:00061:00569
GGGGAATTCTAATACGACTCACTATAGGGAGAGGGAGAATAGTTTCTCGCTAGGCGCCCTGTGACTGTTCTTCCTTT
CTCCCTTCTCCC
+
CDH9C@E@BBBEBBBBBBBB@@;7?666.7>=>@:=??=?@AA@<AAA=<<AC>A???<?@@A@@A@A?@?:<3441
4;;@7=:=AAA<
@T6XXI:00061:00573
CTAGTCGACTCGAGATCGCGGCTTTGGACAGTCCCTCTGTACTGTGAAGCCACAGATGTCGAGTCGACTAGCTTCCT
TTCTCCCTTCTCCC
+
B9999944?CBCCAA@BCCB>;BB>C?BBCCCBB<@@BBBBAACBBB?BB?BCB@:;;A@@@@:<<AA@B::68?:=
=9?<9;7=;888@:
@T6XXI:00061:00578
GGGAGAAGGGAGAAAGGAAGGAGAAGTCGCCCCAAACGATTTGTTGACTATTCTCCCTCTCCCTATAGTGAGTCGTA
TTAGAATTCCCC
+
CHDCCB@BB>BBBB>A@B@B?BA@>B@CCCBC8BC>CCAAB@BC@CBCA??<?AAA<????C<A@>?AAAAA??@??
?>A?=;;2444-
@T6XXI:00061:00580

```
GGGGAATTCTAATACGACTCACTATAGGGAGAGGGAGAATAGCCATCGTGTTCGCCCCGCGATGAGTCTCTTCCTTT
CTCCCTTCTCCC
+
BAA9B=?=ACCABBBBBBBB@CB99611-
6>=?A>BAA?A<?<:?AA=::@@C????5<@A@@@AAA==>@>B38858<AB;?<?BAC;
@T6XXI:00061:00589
GGGAGAAGGGAGAAAGGAAGGAGAAGTCGCCCCAAACGATTTGTTGACTATTCTCCCTCTCCCTATAGTGAGTCGTA
TTAGAATTCCCC
+
AD@ECC@CB<;99A9>;>4;13;63679?BBB8@C>C@B??8<A8??8000530094445<=;<:::0..749<458
<;<6654299:-
@T6XXI:00061:00590
GGGGAATTCTAATACGACTCACTATAGGGAGAGGGAGAATAGCTGCCACGTTTGGACAATTGCAGTTCTCCTCCTTT
CTCCCTTCTCCC
+
AAA4@695944299CCBBBB9:8==777.7=777.798785<499;079@>>9@<?@B?B@CCF;3-
085,//75503111,19655=1
@T6XXI:00062:00552
GGCAGAAGGGAGAAAGGAAGGAGAAGTCGCCCCAAACGAT
+
9EA;66,//+///85658-7133?96176333555244..
@T6XXI:00062:00554
GGGAGAAGGGAGAAAGGAAGGATATGCCGCAGTGGGAGATTAAACGGCTATTCTCCCTCTCCCTATAGTGAGTCGTA
TTAGAATTCCCC
+
==:@CC@CC@BAAB>B@CBC@BBBBBBABCBBABC>CCBB@BB>AC?BCCDEBBBGB=<;CC?CAAACCCCBCDA==
=BAAB<?;?AB5
@T6XXI:00062:00582
GGGGAATTCTAATACGACTCACTATAGGGAGAGGGAGAATAGTCAACAAATCGTTTGGGGCGACTTCTCCTTCCTTT
CTCCCTTCTCCC
+
AA?7@8;:<CC@CBAABABA@BD?:699-
6=;??9<@@=A@@??@@AAA:CCCCC=AA?5??AAB>@=A38387994844>:=;??@?;
@T6XXI:00062:00595
TATCGATCGAGATCTGCGGCTTTGGACAGTCCCTCTGTACTGTGAAGCCACAGATCGTAGCGACTAGCTTCCTTTCT
CCCTTCTCCC
+
CCCD@@@BCCCBBA>BBBE@??<@=@@CBCCC@BBBBBBBC????:?@=BBC;5.*((((((336:=BC@C?AA=A=
=B7=;=AAC<
@T6XXI:00063:00565
GGGAGAAGGGAGAAAGGAAGGAGAAGTCGCCCCAAACGATTTGTTGACTACTCTCCCTCTCCCTATAGTGAGTCGTA
TTAGAATTCCCC
+
CC>BCD@BB=BBBB;?=>>?>?B@@@><@@A@7@B:>>771,177?>??A????@B9;9430+0333037;;<??>>
?;9==<C8=<<4
@T6XXI:00063:00585
GGGGAATTCTAATACGACTCACTATAGGGAGAGGGAGAATAGTACACGTGTCTGATCGTGCTACTTCCCTTCCTTCT
CCCTTCTCCC
+
@A@4@5959>>59;@CCCEDC??<>777.7==?A>>??=888<<;=8=8=@@<<<=?@@@:7733/0844::23204
44+0/0004,
@T6XXI:00063:00590
GGGAGAAGGGAGAAAGGAAGGGGAGAAGGGAGAAAGGAAGCAGTCACTCGTACACG
+
FC>BBD@BB=?@@A<@=A@CDC8BBB>CC>C=??8?4737=;6766;11++0++++
@T6XXI:00063:00592
GGGGAATTCTAATACGACTCACTATAGGGAGAGAGATATAAGGGGAGTGCTAATTGGGCATATCTTGGTCCCATTA
```

```
+
CCB7A798<BA?CCCBBBBCB@?9966649==?A888:;;3444-49:;686<=<??188=;;;8/0/04446431
@T6XXI:00063:00596
GGGAGAAGGAGAAAGA
+
8>,1//,/,,,//+/.
@T6XXI:00064:00555
GGGAGAAGGGAGAAAGGAAGAGAAAGCCGACTTTGCCACCAATGAAGCTATTCTCCCCTCCTCCCTATAGTGAGTCG
TATTAAG
+
4;8<BC@CH@A9991958;>BABB8019:00.303344738011731////,/////*/42333,39766:?8547.
,+0*+*+
@T6XXI:00064:00559
GGGGAATTCTAATACGACTCACTATAGGGAGAGGGAGAATAGCTATCGTCACTTGACATGGTGCGTTCTCCTTCCTT
TCTCCCTTCTCCC
+
@A@6@@A?BDB?BCBAABBB@@B@><::8:ABBC>C?@>@=@=B?BA@A<<=B>A@;?@=>AA=<<99=@<@698:D
?AB@C<?;???>;
@T6XXI:00064:00566
GGGGAATTCTAATACGACTCACTATAGGGAGAGGGAGAATAGTCAACAAATCGTTTGGGGCGACTTCTCCTTCCTTT
CTCCCTTCTCCC
+
CFC9BC?>ACCCCCB>?B?B?AD>;666-
6=;?A>?AA>AA??@A=AAA=BAAAC>CCC9CCCBB?A??;?<?>AA<=<=A<A<>?CC<
@T6XXI:00064:00573
GGGGAATTCTAATACGACTCACTATAAGGGAGAGGGAGAATAGGTCGTAGTCCGCCCCTTCTCCTCCATGTTCTT
+
499292924882444A?790?7;7./,.5344797,1--,---,----1138:8:?>1=33//4--,---32-32
@T6XXI:00064:00585
GGGAGAAGGGAGAAAGGAAGCTAGTCGACTCGACATCTGTGGCTTCACAGTACAGAGGACTGTCCAAAGCCGCAGAT
CGTACGTCAAGCTCGAACGTACGCATC
+
KC>BBBADB?BBBB?B?CAAAA>;;/0000=;>?BBB@CDB@=A=@CCCBBA999..-
/998;A>AD?CA>@=66////.-11113534440.,------1334
@T6XXI:00064:00590
GGGAGAAGGGAGAAAGGAAGTGCAGTTCGCGGTTAGTGAAGGGAAAACTATTCTCCCTCTCCCTATAGTGAGTCGTA
TTAGAACTTCCCC
+
@D?BBC?AA<@AAA<A?B?BBCBCCC@CCBB=@=@BBBBACC?CCC9CBBA=@777.77776,3>;=?A?>@A>;<8
838==8;;3444-
@T6XXI:00064:00592
GGGGAATTCTAATACGACTCACTATAGGGAGAGGGAGAATAGTTTCCTTCACTAACCGCGAACTGCACTTCCTTCTC
CCTTCTCC
+
CHE:C899<@A=B@@@@@BBBAD?>777.7?777.4;;99;444-
0/0/0=;1101:673323=:399?3:0::;:::7:8:442
@T6XXI:00065:00571
CTAGTCGACTCGAGATCTGCGGCTTTGGACAGTCCCTCTGTACTGTGAAGCCACAGATGTCGAGTCGACTAGCTTCC
TTTCTCCCTTCCTC
+
;:44444499;9=498@>>885888/77<7>C>??9999:96@>=34458ADDCCCCCBA??BB79;:737611269
999=64403/0/++
@T6XXI:00065:00574
CTAGTCGACTCGGAGATTTGCGGCTTTGGACAGTCCCTCTGTACTGTGAAGCCACAGATGTCGAGTCGACTAGCCTT
CCTTCTCC
+
```

:9444989?@?@9>A999-4?@<894-
42377;;?;,16661166?88B>BB?BCLC@@===AA<==?=;;;4437474747//,
@T6XXI:00065:00585
AGGAAGCTAGTCGACTCGACATCTGTGGCTTCACAGTACAGAGGACTGTCCAAAGCCGCAGATCTCGAGT
+
00*1,//8:9AAAA@CDB@@@???BCC@CL>;;9988BB780/,/88///,//+.64///88;?==<B88
@T6XXI:00066:00554
GGGAGAAGGGAGAAAGGAAGGAGAAGTCGCCCCAAACGATTTGTTGACTATTCTCCCTCTCCCTATAGTGAGTCGTA
TTAGAATTCCCC
+
CC@CCB@BB>BB??=B=@>@@CB@?CBB>;;;-
611:B@A@>A@@C@====<=8863;5;=?;@AAA@@?:::<<9=A?A?>;;3444+
@T6XXI:00066:00566
GGGAGAAGGGAGAAAGGAAGCTAGTCGACTCGAGATCTGCGGCTTTGGACAGTCCCTCTGTACTGTGAAGCCACAGA
TGTCGAGTCCACTAGCTCCTTCTCCCTCTCC
+
49-
44;9@A8<9CC@CBBDB;::86119;<<66577//..54111,1769<@??@477;;4833368@>@@>AA@AA@A@
998<>>587034+++*+*++++*+1358
@T6XXI:00066:00574
GGGGAATTCTAATACGACTCACTATAGGGAGAGGGAGAATAGGGCCGACGTCGCATGCGTCCCGGCCGCCACTGGC
+
BBB9CAC@BCC?BBBCCBBAAAB=??@C<ABBBB>@@A=9030,4/4::;<=?==B@996<=355638883100*+
@T6XXI:00066:00584
GGGAGAAGGGAGAAAGGAAGGAGAAGTCGCCCCAAACGATTTGTTGACTATTCTCCCTCTCCCTATAGTGAGTCGTA
TTCAGAATTCCCC
+
7@8?<@699099@@?C@C?B=@?A@C?@?A99,77-
11116899;886:8899><C<@988=7:6880/.94.73443-..88567===4
@T6XXI:00066:00588
GGGGAATTCTAATACGACTCACTATAGGGAGAGGGAGAATAGTCAACAATCGTTCG
+
:>=5=199<BB@CCBBBBBB@AB;;661-6=766.8;=;>?@=44200/1111011
@T6XXI:00067:00557
CTAGTCACTCGAGATCTGCGGCTTTGGACAGTCCCTCTGTACTGTGAAGCCACAGATGTCGAGTCGACTAGCTTCCT
TTCTCCCTTCTCCC
+
EC<;;CD?@CAABBBBBBBB?CCC>C=@@CBBCC>CBB=@?@C???A?AC?AA>>CBBBBBBCBCC;;;A::6;??B
CA@==@:@387;;4
@T6XXI:00067:00573
GGGAGAAGGGAGAAAGGAAGGACGGTGTGCTGGGAGAAATGCACAGAACTATTCTCCCTCTCCCTATAGTGAGTCGT
ATTAGAATTC
+
CDACCC=@?>ACAC<@=@>A>C@A>ABBAABBC>BBCC=AABBCDCCAAAAB<?;@B>BBBDD?CDCBB@@AAA???
::07<3/427
@T6XXI:00067:00574
GGGGAATTCTAATACGACTCACTATAGGGAGAGGGAGAATAGTCCGTGCGTTTATCG
+
FDC:B89<<BBDCBA@?ACCCCCA@:77.7==AB>B@@=?=>;;848443.3411--
@T6XXI:00068:00557
GGGGAATTCTAATACGACTCACTATAGGGAGAGGGAGAATAGTCAACAAATCGTTTGGGGCGACTTCTCCTTCCTTT
CTCCCTTCTCCC
+
DCI:C899;;;?FCCBAACCCABAC???6;<;<?9?@@>A>>>CC@@A@<BBA@B9?<?.77;;>.009,/,/7771
768;668?;AA<
@T6XXI:00068:00573

```
GGGAGAAGGGAGAAAGGAAGCTAGTCGACTCGACATCTGTGGCTTCACAGTACAGAGGACTGTCCAAAGCCGCAGAT
TCGAGTCGACTAG
+
CC@CAB@BB??BBB?BBA?ABAABB:;:@A@7::?:::BBA?@D@BBBBBB<455.../;;9;A9<<7<@=@AA@@@
>@A===???AA@@
@T6XXI:00068:00577
GGGGAATTCTAATACGACTCACTATAGGGAGAGGGAGAATAGTTTCCCTGATCTCCTAACTGGCTATGTTTCTTC
+
BA?6A=>=@BBBDCB?>?CB?AB@@66757??EE>A>>=A=@?@;@=<?@BBCCC@A?:<00,0/.3377,1101
@T6XXI:00068:00578
GGGGAATTCTAATACGACTCACTATAGGGAGAGGGAGAATAGCCGACGTCGTTCGTTTCGCTGTATTCTCCTTCCTT
CTCCCTTCTCCC
+
>@@4>69:<BBADCCBBBCB@=;7=666.7==?BAAAA=@@A;9666,.**..**.3312363436;==:7877,/,
-1334827333+
@T6XXI:00068:00583
GGGGAATTCTAATACGACTCACTATAGGGAGAGGGAGAATAGCTATCAACCGTCGCCATTACGTTATTCTTCCTTTC
TCCCT
+
9A@4@59599959?@AA@@@BBCBFBF@<@????9?@@>@@@@@??;B574:?9899==388@@>99/00/023440
00303
@T6XXI:00069:00557
GGGGAATTCTAATACGACTCACTATAGGGAGAGGGAGAAATAGCTA
+
999/959594488:>>>@A@@@A777<<.71;66,0000+0---3.
@T6XXI:00069:00564
GGGGAATTCTAATACGACTCACTATAGGGAGAGGGAGAATAGTCAACAAATCGTTTGGGGCGACTTCTCCTTCCTTT
CTCCCTTCTCCC
+
@@@6A@C@CBB@BBBBBBBB?@A?=67759@@BC>B@A>@;@@AA=@@B:BCCCC>AAA7BBBBB??A?3737:==7
??=@;@<??HC?
@T6XXI:00070:00553
GGGAGAAGGGAGAAAGGAAGCTAGTCGACTCGACATCTGTGGCTTCACAGTACAGAGGGACTGTCCAAAGCCGCAGA
TCTCGAGTCGACTAG
+
CC>AA@@CC>BABD;<<;@CCC>>>/00:000078999A?<:8:5:@B?@@@?;;444-
4;;@AA?@@<AD?=>?AA===@@>=>??>>><<
@T6XXI:00070:00556
GGGGAATTCTAATACGACTCACTATAGGGAGAGGGAGAATAGTCAACAAATCGTTTGGGGCGACTTCTCCTTCCTTT
CTCCCTTCTCCC
+
@==4>596;@@>BCBBCBBB@CB;7111.6<77:88CCAAAAA@@>@AA<BBBAB.777-
77?A@:?;?3738>AA<@;@>9>00.8;6
@T6XXI:00070:00558
CTGCGGCTTTGGACAGTCCCTCTGTACTGTGAAGCCACAGATGTCGAGTCGACTAGCTTCCTTTCTCCCTTCTCCC
+
?A>>>;999-4888BBBBB<BCCCBB@??A@@>?A>@?@@?;;;?9@A:<<BBA<@:62:=@B=@@?DAC;==@C<
@T6XXI:00070:00570
GGGAGAAGGGAGAAAGGAAGGTGAACAGAGCGCAAACGAATACGTCTCTATTCTCCCTCTCCCTATAGTGAGGCGTA
TTAGAATTCCCC
+
BC<AAG?BB?BAAC>@?@CE@CBC@CBAABAAACC?CCB;<<CCCBBCBBB@BDDC>BBBCD>CBBBBCBBB?BCD?
?=@AA?CADCC9
@T6XXI:00070:00574
GGGGAATTCTAATACGACTCACTATAGGGAGAGGGAGAATAGTCTTGTGCCCTTGCCCCCGTTGCTGTTCTTCCTTC
TCCTTCTCCC
+
```

```
944,9;><?AA?ABB;:<DCC:;7?777.7>==?>?AD@C=??==9;;844-
4844444,497447=B9;678,/,//.,-,-1330
@T6XXI:00070:00582
GGGAGAAGGGAGAAAGGAAGAAAAGTGGGCAAACAGGTACAATGGAACTATTCTCCCTCTCCCTATAGTGAGTCGTA
TTAGAATTCCCC
+
CC@CCB>??=>@?B=B>A?A@AA8BBBD>CBB=ABD@CC?B=AC>B@BCBB>AAAB>B@B@D@DDCA==?AA=====
=AA@@;@;AAA6
@T6XXI:00071:00550
GGGAGAAGGGAGAAAGGAAGGAGAAGTCGCCCCAAACGATTTGTTGACTATTCTCCCTCTCCCTATAGTGAGTCGTA
TTAGATCCC
+
6:,119598-
///8+/,034699::@8<;CDD8CA:76711,16366<:@B@@AAC?A<<<C<A@=<<8888447;8594-..335
@T6XXI:00071:00565
CTATCGACTCGAGATCTGCGGCTTTGGACAGTCCCTCTGTACTGTGAAGCCACAGATGTCGAGTCGACTAGCTTCCT
TTCTCCCTTCTCCC
+
D;;>111:::=?AAFCCCCC?BB@;B<??CC====?GD?A??A=AAA::A>ABDBBBBCBBABABABBBCACC@CBE
C=@=@@;@>@@BA<
@T6XXI:00071:00568
GGGGAATTCTAATACGACTCACTATAGGGAGAGGGAGAATAGTCAACAAATCGTTTGGGGCGACTTCTCCTTCCTTT
CTCCCTTCTCCC
+
6660<-
959=>599@BBBAACB<7=776,6>7776;?;=A8985=>AAC=BCBCB:@@@5A@?AB;??=3838>AD:?>6=65
5;4??7
@T6XXI:00071:00571
GGGGAATTCTAATACGACTCACTATAGGGAGAGGGAGAATAGCCACCAACTATTGTTCGTCGCCGTTCTCTTCCTTT
CTCCCTTCTCCC
+
BEC:C@A>ABB@CCCCCBCCBAB@B7::5:::BCCAAA>@@<;238;>=<=>A;==>AA980>>E6..+++*+3333
445848/74:@;
@T6XXI:00071:00599
GGGGAATTCTAATACGACTCACTATAGGGAGAGGGAGAATAGACATATAACCGATCGTGTGTCTGATCTCCTTCCTT
TCTCCCTTCACC
+
499/92928?@<@A@@@@CCCDCCA::956?8>C>??A>A<<<;=;<78348;;899?==;88403043558>@>@A
6:///,/4/,,1
@T6XXI:00072:00570
GGGGAATTCTAATACGACTCACTATAGGGAGAGGGAGAATAGTCTATTAGTCAACCACGTAGACATCTCCTTCCTTT
CTCCCTTCTCCC
+
6=:0;-
;:<<<IDCAACABAC?@9?677.7>>>@:>>?<?AA?888859@ADC>@=@??@AAA<<==;=3730:<:,/-
12223..3;3
@T6XXI:00073:00550
GGGAGAAGGGAGAAAGGAAGCTAGTCGACTGACATCTGTGGCTTCACAGTACAGAGGGACTGTCCAAAGCCGCAGAT
CTCGAGTCGACTAG
+
DB?BBB@BC>BBBB>B?A@BCCBBB:::AA@::::;:@?@=AA?ACLCBBCB99333-
4::;?@@AA:BB=B???BAAA@???A@@@@AA@
@T6XXI:00073:00554
GGGGAATTCTAATACGACGTCACTATAGGGAGAGGGAGAATAGTACGACAAATCGTTTGGGCGTACTTC
+
=A@4@6924442446/....99;:BB;;;27>===.:6>;=7:.-----13377311,1663...55,.
@T6XXI:00073:00575
```

```
GGGAGAAGGGAGAAAGGAAGCTAGTCGACTCGACATCTGTGCACAGTACAGAGGGACTGTCCAAAGCCGCAGATCTC
GAGTCCGACTAG
+
?B>BBA@CC=BB@@:@>@:@@?;;;/00;:::>?@AABCCAA@@@@@@A8833317:::;=8:;6;;5<B==<:;:9
978//,/888:=
@T6XXI:00074:00566
GGGGAATTCTAATACGACTCACTATAGGGAGAGGGAGAATACGTAGTCCGAACCCATGT
+
499,454888=9;B:<877<;<7611176>=/0/,+,,+++,,0,.--,049:;-3337
@T6XXI:00074:00578
GGGGAATTCTAATACGACTCACTATAGGGAGAGGGAGAATAGTGACGTGATTGTGCGGCGCTCACTGTCTCCTTCCT
TCTCCTCTCC
+
;>@0?-1,/66,/4@@@@BBCBA?;777-7=777.4;;:????53:86/--
211110/00009:=;<5;8;7<8<8;;=::78///,
@T6XXI:00075:00571
GGGAGAAGGGAGAAAGGAAGTGCAGTTCGCGGTTAGTGAAGGGAAAAACTATTCTCCCTCTCCCTATAGTGAGTCGT
ATTAGAACTTCCCC
+
AA@ABC@CC=BBBCBCAD@BBBCCCC@B@??<?=?A@?@@FC>CCCC2CBBB>ABCD>BBBCC?AA?>>AACDAC@?
==9<AB8::3444-
@T6XXI:00075:00575
GGGGAATTCTAATACGACTCACTATAGGGAGAGGGAGAATAGC
+
8?808+469>?<@A@@@@CBBBCCCCCC<??8888;7675.))
@T6XXI:00075:00582
GGGAGAAGGGAGAAAGGAAGCTAGTCGACTCGACATCTGTGGCTTCACAGTACAGAGGGACTGTCCAAAGCCGCAGA
TCTCGAGTCGACTAG
+
A@<@AA?AA<@?>>9>;><;;7@A@:::CAAA??????AA@=?B?BCCAA@CB;;444-
4;;>>;;;@6/0./8:>@@??B998<;;788==
@T6XXI:00076:00551
GGGAATTCTAATACGACTCACTATAGGGAGAGGGAGAATAGTCTCCCATTGTTACGTTCCATCACGTTCTTCCTTTC
TCCCTTCTCCC
+
CH>B>?=@BB@DB??BBCB>A<77111.7?>>A=ABB?@:?@<AAA<>A?BC?BB@B?B?BBCCCBB?BB=>477.7
=AB<B?;;8@:
@T6XXI:00076:00552
GGGGAATTCTAATACGACTCACTATAGGGAGAGGGAGAATAGTCAACAAATCGTTTGGGGCGACTTCTCCTTCCTTT
CTCCCTTCTCCC
+
A??4@698<BBDNBBBBBCB?AA9?666-
1;;??==DC>A;AA@@>A@@;BCCCC>CCC7BBBBC?B@A;=;=?@@<@AAA<A:=<AH?
@T6XXI:00076:00563
GGGGAATTCTAATACGACTCACTATAGGGAGAGGGAGAATAGCTGCTAACTATTCTCAAGGTTTTCGCCCTTCCTTT
CTCCCTTCTCCC
+
??@8@?A?@A@?BBBBDBBB;@C??66646;;?A>B@@?CA??>>><?>@@?@>?===>@>@AA5@AA@<A?A3771
4;;;6=8:8@C=
@T6XXI:00076:00570
GGGAGAAGGGAGAAAGGAAGGAGAAGCGAGACGAA
+
49049>ABB;<9<C?B?B<A:<9701........3
@T6XXI:00076:00577
GGGAGAAGGGAGAAAGGAAGCTAGTCGACTCGACA
+
497<@>=@@19999187>;>;68661....,,,*0
```

```
@T6XXI:00077:00567
GGGAGAAGGGAGAAAGGAAGGAAGCTAGTCGACTCGACATCTGTGGCTTCACAGTACAGAGGGACTGTCCAAAGCCG
CAGATCTCGAGTCGACTAG
+
991999599-844;-
823;>;<<899999>>>><94998=?::7>=??=?AAB==AAAA999098833313928@@BB>>>>0//:011===
@==<
@T6XXI:00077:00571
GGGGAATTCTAATACGACTCACTATAGGGAGAGGGAGAATAGTAGGACCGTGTTGTCGCCGCTGCCTCACTGTCTCC
CTTCCTT
+
111+/,95;@@@CC@>@BBBB@CB???@7;>777.4999;889<@==>688=941-
+++****++*+2227=8:044-443232
@T6XXI:00077:00578
GGGAGAAGGGAGAAAGGAAGCTAGTCGACTCGACATCTGTGGCTTCACAGTACAGAGGGACTGTCCAAAGCCGCAGA
TCTCGAGTCGACTAG
+
CC>BA>;==199>@9;;;@B?=;;00..5../:B??@=A?;8:?:=69:?B@@AA88858??@B@>@@;@A>AA@@@
?:?C??><<<888;>
@T6XXI:00078:00579
GGGGAATTCTAATACGACTCACTATAGGGAGAGGGAGAATAGCTGGTGTCTCGAAGGCCTCCTCCTCTTCTTCCTTT
CTCCCTTCTCCC
+
4;;2?=@=>AA=AADGBBBBCBB:::AA<ACCCC>B@?=@===@C?C<<<<<<<<<?>@;==>A@<888<==:?5==7
=8::7;8;89:4
@T6XXI:00079:00557
GGGAGAAGGGAGAAAGGAAGGAGAACGCGCACCCGAACCAACGGTGATCTATTCCTCCCTCTCCCTATAGCTGA
+
//+/<>699-4444-477>???>=@CCLBB>B>11/56,/11:@A99/76//,/,.6628>?@>76.,,,,,,,
@T6XXI:00079:00577
GGGAGAAGGGAGAAAGGAAGCTAGTCGACTCGACATCTGTGGCTTCACAGTACAGAGGACTGTCCAAAGCCGCAGAT
CTCGAGTCGAC
+
44-999598-
//66+/,.4467:69:616861611;;6679936277<?AC@==>8838?@:=>9<=7=@?@@@AA???A@@?@=<<
<
@T6XXI:00079:00578
GGGGAATTCTAATACGACTCACTATAGGGAGAGGGAGAATAGTTTCTCGCTAGGCGCCCTGTGACTGTTCTTCCTTT
CTCCCTTCTCCC
+
CDE:CAAAABB@B@B@?BBB?@<77111.7>>@B>BAC?A====4::===;;;:;;;=;=88:?@BBA=AA>A<?=<
<@<=78:<8771
@T6XXI:00080:00579
GGGGAATTCTAATACGACTCACTATAGGGAGAGGGAGAATGACGCAGTGCATTGTTACCGCCCGTTTTCTCCTTCCT
TTCTCCCTTCTCCC
+
BAA7A=@=@CC@CBA?BBBB?AB@><::88===?8;6646/,,,,***+++33142096883,39??4;7?;?:?;?
@<@FAA<?9<<=@<
@T6XXI:00080:00580
GGGAGAAGGGAGAAAGGAAGGAGACACGGTCACAAACATGAATGTGACTATTCTCCCTCTCCCTATAGTGAGTCGTA
TTAGAACTTCCCC
+
AB<999;>>9>>>@:@>@=?=@CDCCBC@CCBBCC@CAB@B?A???A???C>?<?@<@CADC>@@@@?@@@@@A@@@
@B;;;00/9?;;4
@T6XXI:00080:00589
GGGAGAAGGGAGAAAGGAAGGAGAAGTCGCCCCAAACGATTTGTTGACTATTCTCCCTCTCCCTATAGTGAGTCGTA
TTAGAATTCCCC
```

```
+
DKCCCB?BB>BBCB=?=>=@=@CC@CBBBCCC9CC>BC???;?C@DCB@AA?@@@@<@???C<AA@@===AA?????
>?DCC?A>A@@5
@T6XXI:00080:00599
GGGGAATTCTAATACGACTCACTATAGGGAGAGGGAGAATAGTCAACAAATCGTTTGGGCGACCTTC
+
499/95954889499=;>7;96>>>777,7>161,134246;300/0444=;6;6,11,17..,.33
@T6XXI:00081:00552
GGGGAATTCTAATACGACTCAGCTACGTACGTCTGAGCA
+
999,42499@@@BBBCCCAA??9;88>>>61113.//30
@T6XXI:00081:00556
GGGGAATTCTAATACGACTCACTATAGGGAGAGGGAGAATAGTCAACAAATCGTTTGGGGCGACTTCTCCTTCCTTT
CTCCCTTCTCCCA
+
DCC:C697;>>;>BA<;<CCECCA::77.7>>?A>AAA@CB@@@@>@?@<C@??B9???6??BCJ@@@@:<8<=??:
@A@A<A9<=??7=
@T6XXI:00081:00565
GGGAGAAGGGAGAAAGGAAGCTAGTCGACTCGACATCTGTGGCTTCACAGTACAGAGGGACTGTCCAAAGCCGCAGA
TCTCGAGTCGACTAG
+
FC>BBC@BB==;84-
9;>;?B@=>B111>>>>@BAAA:::@=@B@BBCAAAAA??77717>>?@@>AB>A@=@AA@<???@<;<<<<@<<<?
@T6XXI:00081:00582
GGGAGAAGGGAGAAAGGAAGCTAGTCGACTCGACATCTGTGGCTTCACAGTACAGAGGGACTGTCCAAAGCCGCAGA
TCTCGAGTCGACTAG
+
FCCAA@@CC>BCBB===;:??<:=;0/0:7;9076667AAC;@B@CBCBBB@@@?<<<7<@@???>AC<@@>@@AAA
A=AA858@@@???>>
@T6XXI:00081:00587
GGGAGAAGGGAGAAAGGAAGCTAGTCGACTCGACATCTGTGGCTTCACAGTACAGAGGACTGTCCAAAGCCGCAGAT
CTCGA
+
9919?>>@A<@@@A=A>B?BCB?@?BBBBCB???????>;92775:@BBCBA@AA8889@BAAA>@A<@@BAA@A@@
===AA
@T6XXI:00082:00553
GGGAGAAGGGAGAAAGGAAGGAGAAGTCGCCCCAAACGATTTGTTGACTATTCTCCCTCTCCCTATAGTGAGTCGTA
TTAGAATTCCCC
+
BC?BBB@EEAAAAD@C@C?B?BCD@??@CCCC7@?:?@ABB=BBCDCC@@@?AAAA;@899@;@CA@@@@?A888??
?>AAA=?;ACC5
@T6XXI:00082:00560
GGGGAATTCTAATACGACTCACTATAGGGAGGGAGGAAGAA
+
CCC9BAA@BAB>BBBBA>>>@@D@@@?;8166-66343--,
@T6XXI:00082:00570
GGGAGAAGGGAGAAAGGAAGGAGAACAACACGATCGGTTAAAACGTAGCTATTCTCCCTCTCCCTATAGTGAGTCGT
ATTAGAACTTCCCC
+
CC@CEC@CB>B???<@@B?B@BBB@AA=@CF@>@994423995BAAABB@>@?A???6=>???<<???A@AAAD<;<
88/439248/000+
@T6XXI:00082:00578
GGGAGAGAATTTGGTAATGGGTAAGTAGGTAGATCGTGTTGGTTTATTGGCAGCCCTTATATCTCTCTCCCTATAGT
GAGTCGTATTAGAATTCCCC
+
EC>BB;=82;;99@CC@AAC>CDACCCC@BBBBFCEFAA@C>AA<CB?AAAA@@A>AACCCCCA@8888808>>?BC
BABBBBABA@CAA>C<???7
```

```
@T6XXI:00082:00583
GGGGAATTCTAATACGACTCACTATAGGGAGAGGGAGAATAGTCTTCCCTTCACTAACCGCGAACTGCACTTCCTTC
TCCCTTCTCCC
+
CDB9D?B?ACBABAA?BBBB@AB@<:??9;=??A>AAA>AA??==;=C<@;==@<<:=>???A<??A@97658,/./
8;?9A>A8881
@T6XXI:00082:00584
AGTCGACTCGAGATCTGCGGCTTTGGACAGTCCCTCTGGACTGTGAAGCCACAGATGCGAGTCGAGCTTCAG
+
;>B<;;===484?4379@DBCDC@B?A@?@;;7.7@6:2:?>>?:;47>:778<<?==<<B;;6/---,-++
@T6XXI:00082:00592
GGGGAATTCTAATACGACTCACTATAGGAGAGGA
+
>DA17+459449?@A@@@CCC>;::00,.//730
@T6XXI:00083:00550
GGGGAATTCTAATACGACTCACTATAGGGAGAGGGAGAATAGTCCGTAAGTCTCCCGCGGCCTAATTCCCTTCCTTT
CTCCCTTCTCCC
+
CBC7D>B>BCC@CCBBA??B@@@;?19956=?@B>B??<?=AAA>AA<<AA9==B<AAA<?>AA>B:??9::;8;<7
<AAA<@=@=AD;
@T6XXI:00083:00555
CGACTCGAGATCTGCGGCTTTGGACAGTCCCTCTGTACTGTGAAGCCACAGATGTCGAGTCGACTAGCTTCCTTTCT
CCCTTCTCCC
+
EDCA@ACCBAADCCEC@CCC?C?CBBCCCC>CCBA@@AB@@@C@CC@CCCCCCBBA>AACC<87:B@<>;>487/8>
>>.76:>>@<
@T6XXI:00083:00560
GGGGGAATTCTAATACGACTCACTATAGGAGAGGGAGAATAGTCAACAAATCGTTTGGGGCGACTTCTCCTTCCTTT
CTCCCTTCTCCC
+
////*/,424449:=ABA@@@B000;00,.7:??-
93323;>@<=998:4?777<6;<<2111660114/0/02440333;468:3000
@T6XXI:00083:00569
GGGGAATTCTAATACGACTCACTATAGGGAGAGGGAGAATAGTCAACAAATCGTTTGGGGCCGACTTCTACTTCCTT
TCTCCCTTCTCCC
+
,22*,*/,///7:9;;B;;8@=>=>777-
7>>AB>A8858A??>=:=AA=C@?;<,000+0,0:::67=?A97=31:-44776;///442
@T6XXI:00083:00573
GGGGGAATTCTAATACGACTCACTATAGGGAGAGGGAGAATAGTCCTAGCTACTCG
+
9994+42424449=ABBCBBBAABB@:99.6<<<?9<AA?@@;:86,,+.***+++
@T6XXI:00083:00599
GGGAGAAGGGAGAAAGGAAGCTAGTCGACTCGAACACTGTGGCTTCACAGTACAGAGGGACTCGCA
+
49-449:;@69488:877?8;:=;<1...43..,....3335<9>><:<<B;99;4//+.60,+++
@T6XXI:00085:00559
GGGAGAAGGGAGAAAGGAAGGAGATAATTGGGCAATTGGGAGTCGTAACTATTCTCCCTCTCCCTATAGTGAGTCGT
ATTAGAACTTCCC
+
@C?CCC@CC?AADGBCACCCBBBACD=?>?B<BBBB?BD@C<==@@@=@?@@=@@CE>AAABECC???<<<@A?68>
33/+00*++*++*
@T6XXI:00085:00567
GGGAGAAGGGAGAAAGGAAGGAGAAAGCGACGTCGGGGAAACGTTTGCTATTCTCCCTCTCCCTATAGTGAGTCGTA
TTAGAATTCCCC
+
```

```
DC@CCDBBB?BBCCAC@C@D@CBAB;?@BCCBA????5ACBCCCD@BBCCCBAAEC?C@@BC@C???BBBCBB@A>8
838>>;8;?AA6
@T6XXI:00085:00570
GGGAGAAGGGAGAAAGGAAGTGCAGTTCGCGGTTAGTGAAGGGAAAAACTATTCTCCCTCTCCCTATAGTGAGTCGT
ATTA
+
CB>BBC@CD>BBBB=B@B?ACBDBBC?B@@@=@>@CCCC>BB?B>?@2BABA@BBCD=@@BCC>A:888A;313-
++++*+
@T6XXI:00085:00571
GGGAGAAGGGAGAAAGGAAGCTAGTCACTCGACATCTGTGGCTTCACAGTACAGAGGACTGTCCAAAGCCGCAGATC
TCGAGTCGACTAG
+
CDDCCBACB?DBBC<@=@?BC?@@CC>;5760/-
...35;:::?B:;>CBB;;:/../;;;?@>AB@==;=AAAAA:????@>7889<:;
@T6XXI:00086:00555
GGGAGAAGGGAGAAAGGAAGTGCAGTCCACAGGGGCAGATCGGTGAACTATTCTCCCTCTCCCTATAGTGAGTCGTA
TTAGAACTTCCCC
+
BHAACA@CC@CCCC?B?B@CCCCCBDB>?>7888.8?>@AACBCDD?DCBB?AAAC>BBA@@;@BBBA@@@@@??@8
959@A=@@=@@@5
@T6XXI:00086:00560
GGGAGAAGGGAGAAAGGAAGGAGAAGTCGCCCCAAACGATTTGTTGACTATTCTCCCTCTCCCTATAGTGAGTCGTA
TTAGAATTCCCC
+
AEACCC?B?=B@=?=B@C@C@BB??@?BCBAA5??9?BCDC>CC?CA??@@>A@@@;<===A<AAA@@@@@@AAA==
=>A??<85;;<4
@T6XXI:00086:00568
GGGAGAAGGGAGAAAGGAAGGAATAGCACGGAAGCACAGGGCACAGCTATTCTCCCTCTCCCTATAGTGAGTCGTAT
TAGAATTCCCC
+
AA>BCB@BB>ABBB>A?ABC?C>AA?@@CC@C@CBCCBEE?BBBBBBAAB?BDCE>BBAAA<??A?@@???>>??>>
<?@@>@>AAA6
@T6XXI:00086:00576
GGGAGAAGGGAGAAAGGAAGCTAGTCGACTCGACATCTGTGGCTTCACAGTACAGAGGGACTGTCCAAAGCCGCAGA
TCTCGAGTCGACTACG
+
491949;>>91/1@-110999;CB?7776716?<69?7:7:<@B?BBCABC@;;;444-
4;;;?@<?>7;;9?;<<@888>><<<<99;:447
@T6XXI:00087:00552
GCTAGTCGACTCGAGATCTGCGGCTTGGACAGTCCCTCTGTACTGTGAAGCCACAGATGTCGAGTCGACCTTAGCC
+
44/6/////8::BDCBBBCB>>;8853733666;?;887;1610033348>=?=;??:::?:71694..,.,...,
@T6XXI:00087:00561
GGGGAATTCTAATACGACTCACTATAGGGAGAGGGAGAATAGCCAAGCTCCCGCGGATTGTTTCGTCATCTTCCTTT
CTCCCTTCTCCC
+
BFC:C;<;<=@<A@@ABBGBC?@????<6<=>@C<ABB?A???;><@@?=9:?07/442000+1177663328>AA<
@===788<2782
@T6XXI:00087:00568
CTAGTCGACTCGAGATCTGCGGCTTTGGACAGTCCCTCTGTACTGTGAAGCCACAGATGTCGAGTCGACTAGCTTCC
TTTCCTCCCTTCCTCCCCTTCCTTCTCCCTTC
+
49944444;@B?@AGCCCCB?=77708;>?CCBBD>ABAAAAAA@@@@<?B@CCGCC:00000.469798B?6675,
..+.,.2201,0/3000+04423568663643
@T6XXI:00087:00571
GGGAGAAGGGAGAAAGGAAGGAGAATAAGCTGACACAATGAGACAACTATTCTCCCTCTCCCTATAGTGAGTCGTAT
TAGAATTCCCC
```

```
+
8>;@>A5980998>092313733136;368;?AA@??;?>>::11,/666:?@@A64:<??2>@?:::@=7711100
/0468<<===4
@T6XXI:00087:00590
GGGGAATTCTAATACGACTCACTATAGGGAGAGGGAGAATAGTCAACAAATCGTTTGGGGCGACTTCTCCTTCCTTT
CTCCCTTCTCCC
+
BBA9B=>=ACC>BBC@@@CCCCCCC??:8;=6695:?A>AA@@@A=AAA<B@@@C?CEE7?@BBB?AAA:<9<>@@<
@@@B=CC@???<
@T6XXI:00088:00552
GGGGAATTCTAATACGACTCACTATAGGGAGAGGGAGAATAGCTGGCACACACACGTCAGTATCTTCTCCTTCCTTC
TCCCTTCTCCC
+
EJC:C;:<<DDDDBBBBBAA?@?7=991.7?77758@@C@??@BC@FCCCA?@@@@A@@?===8838<@997;,/,/
///,/,/8884
@T6XXI:00088:00567
GGGAGAAGGGAGAAAGGAAGTTTGTGACGGGAGACTTATTAGCGAGACTATTCTCCCTCTCCCTATAGTGAGTCGTA
TTAGAATTCCCC
+
:B9:<@5981949>09271333,3766337,37=6.,./,.5@=BBCCBBB>A888088877.7??<????<;?AA?
<9=?94:2999-
@T6XXI:00088:00573
GGGGAATCTAATACGACTCACTATAGGAGAGGGAGAATAGCTATCAACCGTCGCCAT
+
88807+/1/6789;<@????=>>>7747>777.7887840011106363863332..
@T6XXI:00088:00598
GGGGAATTCTAATACGACTCACTATAGGGAGAGGGAGAATAGCCGCCAGTAGTATTGGCCCACTATCTCCTTCCTTT
CTCCCTTCTCCC
+
999,959:;BB@BBAAEBBBA>>7=166.77777.4==:@AAA>@A>CBB?B@CL?A=??9>>A>>??=<>47477.
7499-4/4033+
@T6XXI:00089:00554
GGGAGAAGGGAGAAAGGAAGCTAGTCGACTCGACATCTGTGGCTTCACAGTACAGAGGGACTGTCCAAAGCCGCAGA
TCTCGAGTCGACTAG
+
DD@BA>;==5=@>>6@7:5;B=:==/00;;;;;??::7AAD5:<AACCBBAAAA934317==AAA=?A=AA?==8==
==<<66=CAA===@@
@T6XXI:00089:00560
GGGAGAAGGGAGAAAGGAAGGAGACACGGTCACAAACATGAATGCGACTATTCTCCCTCTCCCTATAGTGAGTCGTA
TTAGAATTCCCC
+
CHACBA>@=<=A@C<@<@<?<@@?BCBB@CCBBBC>B@A?@=?ABCBAAACAA@?C>BDAAA@A@AAAAACA@????
?=?@A><;?BB6
@T6XXI:00089:00565
GGGGAATTCTAATACGACTCACTATAGGGAGAGGGAGAATAGTTGTGACATGTAACGTCCTTCTATCTTCTTCCTTT
CCTCCCTTCTC
+
494,4295;@@=@BAABBBAAB@@;166.7=<@B>AAC=??A=;AA?>B?BCBB>BBBB@B>CBAB9858?<;,11,
0,00/,/::36
@T6XXI:00089:00575
GGGGAATTCTAATACGACTCACTATAGGGAGAGGGAGAATAGCCGCACCAGCGTTGACAATTTATTCTCCTCCTTTC
TCCC
+
8>>17+/,16678=??B@@@B@>>?777.7==?@>@>;995,,+,+.16666632303323;4:<5449/00/00+0
4444
@T6XXI:00090:00560
GGGGAATTCTAATACGACTCACTATAGGGAGAGGGAGAATAGTCAACAAATCGTTTGGGCGACTTCCT
```

```
+
CIDAD7989489@BCCCABAAA@?==?=.77777-48446<<?:85644411111,11,177110100
@T6XXI:00090:00567
GGGAGAAGGGAGAAAGGAAGTGCAGTTCGCGGTTAGTGAAGGGAAAAACTATTCTCCCTCTCCCTATAGTGAGTCGT
ATTAGAACTTC
+
?B<@AA@CC>BACD?B?B?BBBAAADACCCC9;7;A????DC>CGCD2DC@?=?@B?.76111,366:<<:663300
00/088448/0
@T6XXI:00090:00595
GGGGAATTCTAATACGACTCACTATAGGGAGAGGGAGAATAGTTTTTTCCCTTCACTAGCCGCGAACTGCACTTCCT
TTCCTCCCTTCTCC
+
@@>4?6959=>9@BBBB@@@BAB=>777.7>>>?AAAA<;;444444)4:4855<<@>BB@BFBC6::7780-
,/464+/,/11+-,-00/
@T6XXI:00091:00561
GGGGAATCTAATACGACTCACTATAGGAGAGG
+
88808*3111::BAAA:;1:/.....333111
@T6XXI:00091:00576
GGGAGAAGGGAGAAAGGAAGGAGAAGTCGCCCCAAACGATTTGTTGACTATTCTCCCTCTCCCTATAGTGAGTCGTA
TTAGAATTCCCCATCACCGACTTCGC
+
@B:>>?;>?1949;-8277<7376>??@BBCC9CD@@@761,1769::<??698@A=B989@:=<@=84/62/.-
211388=;=<<==5=999=>=30.)***
@T6XXI:00091:00595
GGGAGAAGGGAGAAAGGAAGCTAGTCGACTCGACATCTGTGGCTTCACAGTACAGAGGACTGTCCAAAGCCGCAGAT
CTCGAGTCGACTACG
+
9?6@@@6991944;58<>>>>9ABA911611111779688<<=C?@@C::;???@8858@=99<8=<5;6555533.
/-2-+-112---311
@T6XXI:00092:00559
CTAGTCACTCGAGATCTGCGGCTTTGGACAGTCCCTCTGTACTGTGAAGCCACAGATGTCGAGTCGACCTAGCTTCC
TTTCTCCCTTCCTCCC
+
A@9;<CCDBBBBBABBBCBB?@??;@?BBCBBCC>BBBBAA@@AAAA>@C@CBAAADBAAACDABCBB>@CA::;:B
CE?B;>=6;././882
@T6XXI:00093:00560
CTGCGGCTTTGGACAGTCCCTGTACTGTGAAGCCACAGATGTCGAGTCGACTAGCTTCCTTTCTCCCTTCTCCC
+
BDCBB?999099>9@A???:?;;;ABC::;@AA<AB@:::?89:6@:;<;76;0709:,00,,,27+,*,,--+
@T6XXI:00093:00570
GGGGAATTCTAATACGACTCACTATAGGAGAGGGAGAATAGCCAAATCCCTTCTTCCAGGTGGCTCTCCTTCCTTCT
CCCTTCTCCC
+
BCCAD::><A@ACA@@@BBBB>:9://,0>:=?=@AA>@>AA?AA:??A1737=8;><<9=@:>??@?;=:;34249
996<>@AAC<
@T6XXI:00094:00554
AGTCACTCGAGATCTGCGGCTTTGGACAGTCCCTCTGTACTGTGAAGCCACAGATGTCGAGTCGACTAGCTTCCTTT
CTCCCTTCTCCC
+
CCCCBBBAACCCC@@?995999-
4997??78807>=79:BCAAAC@BB?BCB@@@???ABBAA><6;;847::,00,0:66,/,.---+
@T6XXI:00094:00558
GGGGAATTCTAATACGACTCACTATAGGGAGAGGGAGAATAGTCAACAAATCGTTTGGGGCGACTTCTCCTTCCTTT
CTCCCTTCTCCC
+
```

```
BKK:C;:;<BA@CCCBBA@@AAB79661-
6?=?B>@AA>@?A@@@=?@B=CB@@B>CC@7AABBD@A??9;8;:==7>>?C;@>@@@@<
@T6XXI:00094:00559
GGGAGAAGGGAGAAAGGAAGCTAGTCGACTCGACATCTGTGGCTTCACAGTACAGAGGGACTGTCCAAAGCCGCAGA
TCTCGAGTCGACTAG
+
CC@BBB@CC>@@AB8;9;@@@@???/10:;>01::BC6:74;6?;BAACCCA@AA88818==>???AA<AA9<<<;;
<>===AAAAA@AAA@
@T6XXI:00095:00557
GGGAGAAGGGAGAAAGGAAGGAGAATACGCGCACAAACGCTGGAGGCTATTCTCCCTCTCCCTATAG
+
:A11/8,//+/88?0923139<B@@CCA@@?:;9==:=?999<8877>8813317,11111,1:643
@T6XXI:00095:00566
GGGAGAAGGGAGAAAGGAAGGACAAATAGATTGCGGGGGAAGCGCTAGCTATTCTCCCTCTCCCTATAGTGAGTCGT
ATAGAATTCCCC
+
<<6<CC@DD?CBBB<=<;>@?@@BC@AAA:737<;<<</;:9ABBBCBBBCC===@D689@A<4@<9999==@?8/0
0/.29242333.
@T6XXI:00096:00562
GACTCGAAGTGATCTGGCGGCTTTGGACGAGTCCCTCTGTACTGTGAAGCCACAGATGTCGAGTCGCACCTAGCTTC
CTTTCTCCTCTCCCCTTCCTTCTCCTCT
+
//060,,*,,,,<66869::;FC::69@999BBA:>;333337731423313311161116661000000/0336577
74424.-,---333.3/0/0/000/000
@T6XXI:00096:00597
GGGGAATTCTAATACGACTCACTATAGGAGAGGA
+
A8202*/,//6,/48887;;>4644..,,7,0,.
@T6XXI:00097:00552
GGGGAATTCTAATACGACTCACTATAGGGAGAGGAGAATAGTACG
+
8820:1959>?==@BBCBBBBBB@B???9?A<<:?//,///////
@T6XXI:00097:00577
GGGAGAAGGGAGAAAGGAAGGGGAGAAGGGAGAAAGGAAGCTAGTCGACTCGGACATCTGTGGCTTCACAGTACAGA
GGGACTGTCCAAAGCCGCAGATCTCGAG
+
?G<@@A;@@<@A@C<A=@?BBB8BCC@CD>C??88?<@=A@?BABAA@@=8424;>;;==8838<<@@A@AA?6380
33-4;4444244+799=A====<<;<>;
@T6XXI:00097:00582
GGGGAATTCTAATACGACTCACTATAGGGAGAGGGAGAATAGTGGATCACTTGTAGGTGCCACCGTTTTCCTTCCTT
CTTCCTTTCTCCCTTTCTCCC
+
@BB7A7;:<AA>BCCBCBBB>>;79166.7>>?B>B@A>@<??A:<<@?;6466//,///,+000111.30101,/.
/7977;;-439:-44-49;@:
@T6XXI:00097:00583
GAATTCTAATACGACTCACTATAGGGAGAGGGAGAATAGTCAACAAATCGTTTGGGGCGACTTCTCCTTCCTTTCTC
CCTTCTCCC
+
;B797;BBACDCBBABA@@?77161.7?=<?==@A@@@@A@A>@@@;B????6???5?ABCC=?<@598:@CC7==C
C<A;>>@C<
@T6XXI:00098:00553
GGGAGAAGGGAGAAAGGAAGTGCAGTTCGCGTAGTTAGGAAAGAGG
+
EDCCCC@BB>BBBC=B??@BBBCBBB@BA;78888411011,1..,
@T6XXI:00098:00571
GGGGAATTCTAATACGACTCACTATAGGAGAGGGAGAATAGTCAACAAATCGTTTGGGGCGACTTCTCCTTCCTTTC
TCCCTTCTCCC
```

```
+
BCB7;69698=299@@@?@@;00::00,.99?A7:5<>AA@@@@>A@A:::;?=6=?A5??BCC>A?;7;3838A=@
@>@<AEA???<
@T6XXI:00098:00580
GGGAGAAGGGAGAAAGGAAGATTAGCAATGTGGAGGAGAAGGTGCCGACTATTCTCCCTCTCCCTATAGTGAGTCGT
ATTAGAATTCCCC
+
CC?CCC?BB:9<CC=B?A@;;<?BDDD@CCBA=A@@@@@=A?BBFBBBBBBB>AAAA<ACCCC>@@@A@CC?;::?;
==:=<<98<@AA6
@T6XXI:00098:00585
CTAGTCACTCGAGATCTGCGGCTTGGACAGTCCTCTGTACTGTGAAGCCACAGATGTCGAGTCGACTAGCTTC
+
,//////4444==?BGC8885-,+-**000*,*.5669<<<?>:87/1-1.469B@@=7:114333331..,,
@T6XXI:00099:00552
GGGGAATTCTAATACGACTCACTATAGGA
+
///*/,424442448640..446....,,
@T6XXI:00050:00624
GGGGAATTCTAATACGACTCACTATAGGGAGAGGGAGAATAGATACAA
+
CC@2;2459449A@@@AABBB??=>7775:@@BD>AAB=?8//--1,(
@T6XXI:00050:00633
GGGAGAAGGGAGAAAGGAAGCTAGTCGACTCGAGATCTGCGGCTTTGGACAGTCCCTCTGTACTGTAAAGCCACAGA
TGTCGAGTCGACTAGCTTCCTTTCTCCCTTCTCCC
+
@C>CCC?CC@C@@@<><>;8877?????BBBBBB@@@CBAB@::;8:?BDCBBAC9==@@?=@??>?A7=<<?>284
1.--14833;;434;4424344-44;;-424889:
@T6XXI:00050:00647
GGGAGAAGGGAGAAAGGAAGGAGAAGTCGCCCCAAACGATTTGTTGACTATTCTCCCTCTCCCTATAGTGAGTCGTA
TTAGAATTCCCC
+
9@<@BBACN>ABEC?CAA@B?BCA@?;>8888.8>8<6611,1=5::CAAA=@?@B=A889C@A???===ACA>===
==<;::8:@@@5
@T6XXI:00051:00601
GGGGAATTCTAATACGACTCACTATAGGGAGAGGGAGAATAGCTCGCCACATCTTGCGTGCCTAATTTCTCCTTCCT
TTCTCCCTTCTCCC
+
CCB9B798;BB?BBBAABBB@@B>A?::88AABC>@BB>A<=<:8?9317777434??>886787>>6?BB@C@C@B
B>BC@@99:=633,
@T6XXI:00051:00613
GGGGAATTCTAATACGACTCACTATAGGGAGAGGGAGAATAGTCAACAAATCGT
+
4;;2=59248824437<833@:77><9>;?;;;C>A>:5///---3-00+1111
@T6XXI:00051:00614
GGGGAATTCTAATACGACTCACTATAGGGAGAGGGAGAATAGTTTCCCTTCACTAACGCGAACTGCACTTC
+
:B@8B;<8<@@;@@BBBBBBB?@????>7>><=?9:87998=?4+44-323=::;;///4=:68653.,*,
@T6XXI:00052:00632
GGGAGAAGGGAGAAAGGAAGCTAGTCGACTCGACATCTGTGGCTTCACAGTACAGAGGGACTGTCCAAAGCCGCAGA
TCTCAGTCGACTAG
+
CB???=@BBB==?B?BAB@C?=<>=???AAAAAAA???A@==8:6:BBBCCAAAA@<<488<;???AC:=>?AAA@:
???@A@@@@@=<<A
@T6XXI:00054:00602
CTAGTCGACTCGAGATCTGCGGCTTTGGACAGTCCCTCTGTACTGTGAAGCCACAGATGTCGAGTCGACTAGCTTCC
TT
+
```

```
9944444/666/////688777778,31336666726811161003044558;88373769166614973397..,.3
02
@T6XXI:00054:00604
GGGGAATTCTAATACGACTCACTATAGGGAGAGGGAGAATAGCTACGTTTAACCGATCGTGTTGTCTCCTTCCTT
+
?CB4@592844889><;;ACABB?::9?8??BCC:98B=?===>76630+0/048<<99;663...35653:,/,
@T6XXI:00054:00613
CGAGATCTGCGGCTTTGGACAGTCCCTCTGTACTGTGAAGCCACAGATGTGCGACGTG
+
BBBCCCBBBCGABAA>A@CBBAABC>B@?@CDCCAA?A?@@<A>6446//.--++-1-
@T6XXI:00054:00634
GGGGAATTCTAATACGACTCACTATAGGGAGAGGGAGAATAGCTATCGTCACTTGACATGGTGCTTCTCCTTCCTTC
TCCCTCTCCC
+
=>>4?595;BA?CDCCCBCCDCDCB???<??BBCCAAA<??><???;;=>??C>@@@@@@<??==55==34/4/4/-
../+.-2455
@T6XXI:00054:00639
GGGGAATTCTAATACGACGTCACTATAGGGAAGAGGGAGAATAGTTCGACACACATTTACAGGGCTGTTCTCTTCCTT
TCTCCCTTCTCCC
+
88807+-
,,,*,/6/3377;>A;<4..0,0,/4:4+./6883799...1689566<48?:::9:@>@6;;??8?9@?<BAAA<?
;=???7
@T6XXI:00054:00640
GGGGAATTCTAATACGACTCACTATAGGGAGAGGGAGAATAGTCAACAAATCGTTTGGGGCGACTTCTCCTTCCTTT
CTCCCTTCTCCC
+
CEB7B79:<AA@CA@@CBBAC??;?777.7>=<?<?@AAA@@@@@>@AA:A?8;;6<==5?@=>>:;:7,/,/4661
6/44-8;??@A<
@T6XXI:00054:00642
GGGGAATTCTAATACGACTCACTATAGGGAGAGGGAGAATAGTCAACAAATCGTTTGGGGCGACTTCTCCTTCCTTT
CTCCCTTCTCCC
+
>@>1<,1,/667:>AACBBAAA@<=777.7>>@B>C@A??<????>@@A<CCCBCCCCC9CBBBC=??@38378<<9
=AAD?A8:8A@;
@T6XXI:00055:00616
ACTCGAGATCTGCGGCTTTGGACAGTCCCTCTGTACTGTGAAGCCACAGATGTCGAGTCGACTAGCTCCTTCTCCTC
TCCCTCCTTCTCCTCTCC
+
9??=444433947;949969649AB<<=19?C9<<BCABADFHH@CGBB<<;;;CF@?@BB@??7899285444233
777,11013333,....3
@T6XXI:00056:00600
GGGGAATTCTAATACGACTCACTATAGGGAGAGGGAGAATAGCCAATCTCTCGTGGTCATTGCGCTTCTCTTCCTTT
CTCCCTCCTCC
+
CCB4@545999<CBBBB@ACEBBAA???9?@?AB>?AA>@@@@>@;>>>888>>@:=???<;;;87/7>;97:343+
/*..)**)**)
@T6XXI:00056:00617
AGTCGACTCGAGATCTGCGGCTTTGGACAGTCCCTCTGTACTGTGAAGCCACAGATGTCGAGCTCTTACTCCTCTCC
CATC
+
?@BCDCCBBBBBCCBCCCC@;;;8;>????>==.77771198::?C@CC?CCB===8739;93000/0004446---
+---
@T6XXI:00057:00601
GGGGAATCAATACGACTCA
+
:>>9>1<982443.4..,2
```

```
@T6XXI:00057:00647
GGGGAATTCTAATACGACTCACTATAGGAGAGGA
+
82208+1,/66,/9=>>877<00....,.33631
@T6XXI:00058:00600
GGGGAATTCTAATACGACTCACTATAGGGAGAGGGAGAATAGTCAACAAATCGTTTGGGGCGACTTCTCCTTCCTTT
CTCCCTTCTCCC
+
@>>4?6969?><A@@@CCBCACB?@9995:BBCC>ABB>???@@@>@@A=CAAAC>CCC7??>>>9;88,/,/7886
;:==6;384?A<
@T6XXI:00058:00601
GGGGAATTCTAATACGACTCACTATAGGGAGAGGGAGAATAGTCCAGTGTTTAATTTCTTGGCTTTCTCTTCCTTCT
CCCTTCTCCC
+
BCB7@54:<A?<BC@@@BBDDCCBBAAA<AA>BC>CAA>@AA@@===:444-
49;;9:;343444,044424/3/0000+0233==;
@T6XXI:00058:00616
GGGGAATTCTAATACGACTCACTATAGGGAGAGGGAGAATAGCCACCAACTATTGTTCGTCGCCGTTCTCTCCTTTC
TCCCTTCTCCC
+
B?:292959>@<AB;;<ECCC<<:????9???AA=?AB>A@@A=@ABA=;844744/3=6<8B<::6::/..45544
9=@:;<@5::9
@T6XXI:00058:00618
GGGGAATTCTAATACGACTCACTATAGGGAGAGGGAGAATAGTTTTCCCTTCACTAACCGCGAACTGCACTTCCTTT
CTCCCTTCTCCC
+
D>?5>,1,///76=7;:;::@:97?@CE>BBBBC?@@@==@;:98.34,4/0=6168:::<8:;844<;9:63,..+
.--303533394
@T6XXI:00058:00623
GGGAGAAGGGAGAAAGGAAGGGGAGAAGGGAGAAAGGAAGCTAA
+
?C7;9?<>A8;;@@<@=@?BCC9CBA>CE>C//,++)+)****)
@T6XXI:00058:00647
GATCTGCGGCTTTGGACAGTCCCTCTGTACTGTGAAGCCACAGATGTCGAGTCGACTAGCTTCCTTTCTCCCTTCTC
CC
+
AACCCBBA?ABC>C@BBCBABC>CAABBBB@@?@@?BC@C?===@@@@A@@ABCCAA?@8838=AA<899C7>;>==
=7
@T6XXI:00059:00637
GGGAGAAGGGAGAAAGGAAGGTGAACAGACGTGCGATGGTAGATGCTATTCTCCCTCTCCCATAGTGAGTC
+
/8+1/9244-448:6=<>@B799<;>>94444...///,/,2.....442499;-99//85...44471.,
@T6XXI:00060:00602
GGGGAATTCTAATACGACTCACTATAGGGAGAGGGAGAATAGTCAACAAATCGTTTGGGGCGACTTCTCCTTCCTTT
CTCCCTTCCTCCC
+
A@@4@5959449:B;;;ACCCCCC@@@B;??BBB>@@B=@@AAAA<?@C=B????8?@A6BBBBB?@>=34249;;:
?:8927,/,/642
@T6XXI:00060:00640
GGGGAATTCTAATACGACTCACTATAGGGAGAGGAGAATAGAAGGAC
+
AC807+969=>;?BA;;<BABBBBCCCB9??773::;:;84/46,./
@T6XXI:00061:00621
GGGAGAAGGGAGAAAGGAAGCTAGTCGACTCGAGATCTGCGGCTTTGGACAGTCCCTCTGTACTGTGAAGCCACAGA
TGTCGAGTCGACTACGCTTCCTTCTCCCT
+
```

```
:;;?BB@BC;;<CC<A>@=@???CA676888666;B666977171,1;6??;<881778///7788@A<???A@AAA
A8887><;8=;;8////3/0/0/0000+0
@T6XXI:00061:00635
GGGAGAAGGGAGAAAGGAAGAATAGCGGTTGATGGGCGTGCAGATAACTATTCTCCCTCTCCCTATAGTGAGTCGTA
TTAGAATTCCCC
+
@A<?@A?BB?BBBC>C@C?BB?BB@B@@C?BBBBB<@@A?AABAAA@CCDCBBAACABBBBB=ABABBBDDFADB;;
;?@884;2449/
@T6XXI:00061:00645
CTAGTCACTCGAGATCTGCGGCTTGGACAGTCCCTCTGTACTGTGAAGCCACAGATGTCGAGTCGACTAGCTTCCTT
TCCT
+
6999444999444444442,,*,,*,*,,233377?777<>=99===249999;C@@?@B???9:::73966656;77
,100
@T6XXI:00062:00608
GGGAGAAGGAGTGA
+
AC@7//,1,,****
@T6XXI:00063:00607
GGGAGAAGGGAGAAAGGAAGCCGACCGAA
+
8;;>>DBCG@CBA@9;-1,0.,..22,.,
@T6XXI:00063:00612
CTAGTCGACTCGAGATCTGCGGCTTTGGACAGTCCCTCTGTACTGTGAAGCCACAGATGTCGAGTCGACTAGCTTCC
TTTCTCCCTTCCTCCC
+
:4949444::49;@@@B;;8?<8880;=?@EBBBB>BBDB????===A>AC@CBAC@@@AA???:<<BAAA?:937<
>>:?;6737,/,.//,
@T6XXI:00063:00633
GGGAGAAGGGAGAAAGGAAGCTGTAACTGTGCGGGAATAGAAAATTGGTCTATTCTCCCTCTCCCTATAGTGAGTCG
TATTAGAATTCCCC
+
6:6:;>69919>?@1948<>>?788>A@<>;6==6=:==7777.177268>?::?@@913355536<=?@@???A;5
;<<8<AA@C>@@A5
@T6XXI:00063:00638
GGGAGAAGGGAGAAAGGAAGGCGACGGGAGTGCACAAGG
+
8>8:<?599-4448-423137776316,661113..,.,
@T6XXI:00063:00639
GGGAGAAGGGAGAAAGGAAGGAGAAGTCGCCCCAAACGATTTGTTGACTATTCTCCCTCTCCCTATAGTGAGTCGTA
TTAGAATTCCCCCAATG
+
?C@CAB?@@9>>@A=B?B@C@CCC@=@@BBCC9CE?D??<?;?A5::<33846.//,----
23345@===AAAAA???>@;843/3344,4481
@T6XXI:00064:00609
GGGAGAAGGGAGAAAGGAAGGAGAATGCACCGCAGGAAATTGCAAGACTATTCTCCCTCTCCCTATAGTGAGTCGTA
TTAGAATTCCC
+
CK@CBB@BC=BBCB>B@B@BAABB=@@CCC@CCBA>>>;<=?CD@BBBBBA>A>>=6;;<;;7<???AA@@@?>>><
<8<@B>@@DC<
@T6XXI:00065:00635
GGGAGAAGGGAGAAAGGAAGAAAATGTGGGTGGTGCAAAATGAGGAGCTATTCTCCCTCTCCCTATAGTGAGTCGTA
TTAGAACTTCCCC
+
?CCDCCCHC>ABBB?B>B=7777-
8>>?;8:?@777BBB8BBBB>A?899>;>7;>897@>>18887?@@<<:66330/0442683677,
@T6XXI:00067:00625
```

```
GGGAGAAGGGAGAAAGGAAGCTAGTCGACTCTACATCTGTGGCTCACAGTACAGAGGGACTGTCCAAAGCCGCAGAT
CTCGAGTCGACTAGCTTCCTTTCTCCCTTCTCCC
+
49694469;7@>@@<?4858>==71111711O1666111312-./5586<:===784-
4::88:?AC?AA>==<<<<<;>887;;<6<<=??=9;244,43;;494988:5
@T6XXI:00067:00631
GGGGAATTCTAATACGACTCACTATAGGGAGAGGGAGAATAGTCAACAAATCGTTTGGGGCGACTTCTCCTTCCTTT
CTCCCTTCTCCC
+
CDC9A698;AA?CBBBCBBBCCCCBBBC=<A>>>;<AA>@@@AAA?AAA:BBBBB>BBB6ABBBC?A??9<8<:==:
998D<A;==AC@
@T6XXI:00068:00601
GGGGAGAAGGGAGAAAGGAAG
+
///*//:244-4478670720
@T6XXI:00068:00608
GGGAGAAGGGAGAAAGGAAGCTAGTCGACTCGACATCTGGTGGCTTCACAGTACAGAGGACGTGTCCAAAGCCGCA
+
6:+1/9299-44=B?C@C@;:;CAAA>@@75..,,,,,,*,0*-/:@878CC7//;,,+,,++++-0144468:43
@T6XXI:00068:00619
GGGGAATTCTAATACGACTCACTATAGGGAGAGGGAGAATAGCCTAGTCCCCTAAGCCGTCTGCACTTCCTTCTCCT
CTCC
+
999292924449;=997:7:???<9???8>==?A=A@A?B???;>==7444-
0.4447//.311100/0/4/000/00032
@T6XXI:00068:00621
GGGAGAAGGGAGAAAGGAAGGAGAAGTCGCCCCAAACGATTTGTTGACTATTCTCCCTCTCCCTATAGTGAGTCGTA
TTAGAATTCCCC
+
9B>@?@<>A<;<;B<@=@@C?BEC@:788888,89,11111,1601174558478;9:/./:9779?844@9779./
.444588;<<>5
@T6XXI:00068:00625
GGGAGAAGGGAGAAAGGAAGGAGAAGTCGCCCCAAACGATTTGTTGACTATTCTCCCTCTCTCTAGAGTGAGTCGTA
AT
+
@C@CCB@CB>??BB>B@B?B?ABA==?CCCCC9CB:==771,:A>A??;7838;7736/./+00+--
2113400000/3
@T6XXI:00068:00628
GGGGAATTCTAATACGACTCACTATAGGGAGAGGGAGAATAGTCAACAAATCGTTTGGGGCGACTTCTCCTTCCTTT
CTCCCTTCTCCC
+
DCC9A8;8;BA>BBACACBCA@B@B@BC>B@A@?;:>><???AAA>@@@;BBBBC>BCC9C???C>?>?9<8;;==7
>===:@>@@AC<
@T6XXI:00068:00636
GGGGAATTCTAATACGACTCACTATAGGGAGAGGGAGAATAGTCAACAAATCGTTTGGGGCGACTTCCCCTTCCTTC
TCCCTTCTCCC
+
AEB7A8;:;>@>CBBBCABB@>AAA???:;<???<<@C>@@@AAA=???7@@@@A9?=?5?87732777*,+,+,++
+++*-,00442
@T6XXI:00069:00603
GGGAGAAGGGAGAAAGGAAGGAGAAGTCGCCCCAAACGATTTGTTGACTATTCTCCCTCTCCCTATAGTGAGTCGTA
TTAGAATTCCCC
+
CC@CCA@AA?>>>B?B@B?B@BBB>><BAA@A7AA<ACABB?BC@CB?@AA?====;<:?<=;<:?:<@AAA???@=
<9=@@>A>AAA6
@T6XXI:00069:00610
GGGAGAAGGGAGAAAGGAAG
```

```
+
;B1999599-/,00*,*,*,
@T6XXI:00070:00627
GGGAGAAGGGAGAAAGGAAGGAGAATGCACCGCAGGAAATTGCAAGACTATTCTCCCTCTCCCTATAGTGAGTCGTA
TTAGAATTCCCC
+
:CACGCACB=@BBB>A?A@A?@AA=?@CCB?BCCC@FC::;:BA>AAA@::47::;-
44::=7>;;<?@@@@A@AA==9;=B5455=B6
@T6XXI:00070:00634
GGGGAATTCTAATACGACTCACTATAGGGAGAGGGAGAATAGTCTGTCGTTCGGTCGACGCTGCCTATCCTC
+
CHDAC;<;<;;?CC@@@BBBCCCBBAAA<AABBC>A9997////4--03337@8;;=;98400323343/00
@T6XXI:00071:00602
GGGAGAAGGGAGAAAGGAAGCTAGTCGACTCGACATCTGTGGCTTCACAGTACAGAGGGACTGTCCAAAGCCGCAGA
TCTCGA
+
=@<@BB>AA:?>>>9>=?>ABBAAAB:::AA?::777661323;:?AACBC@C@@774-
4:::<<=?@;@?=?@>>88888==
@T6XXI:00071:00618
GGGAGAAGGGAGAAAGGAAGCGTAGTCGACTCGACATCGTGTGGCTTCAACAGTACAGACGGACTGTCCAAAGCCGC
AGATCTCGAGTCGACTAG
+
4<89@B<>>19988-
8239937698869996677??::0/...2,+,0..,.3366644804200044 6:774755.0/48<===9>=A@@?=
:;:
@T6XXI:00072:00609
GGGAGAGGAA
+
16+/**0)*)
@T6XXI:00072:00613
GGGAGAAGGGAGAAAGGAAGCTAGTCGACTCGACATCTGTGGC
+
4?:==B@BB8<<AA<A=@=@A9>9811..,,0,,,,,,,01,0
@T6XXI:00073:00622
GGGGAATCTAATACGACTCACTATAGGGAGAGGGAGAATAGTTTCCCTTCACTAACCGCGAACTGCACTTCCTTTCT
CCCTTCTCC
+
;;;5:,//6656=A@CBCCCCCBBAAA=A??AB=?==9::444-
00+0/0776688<8?;A=@?=@A>>:=377,1444224.--,
@T6XXI:00074:00600
GGGAGAAGGGAGAAAGGAAGAGAAAACCGGGGCCGAAAAATGTCGCGCTATTCTCCCTCTCCCTATAGTGAGTCGTA
TTAGAACTTCCC
+
9A=BCHBCB=ABBB>B@B>B<BBBC8C@CDC:C@BDEEE0<;??@>779?A=<<<E?AACCC;<>AADDCDDDCB::
:54;;788488/
@T6XXI:00074:00633
GGGGAATTCTAATACGACTCACTATAGGGAGAGGGAGAATAGATCACCGTTGGTTCGGGTGCGCGTTCTCCTTCCTT
TCTCCCTTCTCCC
+
BBA7A899<BBAGCC?>?AA@@A=C:6646=;??=?CC>@:<<@=B@>BC>@>?@CDC=B@@BBBB????>B;?;?B
<=>?=7=?89:A<
@T6XXI:00075:00604
GGGAATTCTAATACGACTCACTATAGGGAGAGGGAGAATAGTCAACAAATCGTTTGGGGCGACTT
+
/1-/,924449:=ABBAAAA:<:AAA@9>>????9?@@?@@@=88;<<=;C9666,111,11...,
@T6XXI:00075:00605
GGGGAATTCTAATACGACTCACTATAGGGAGAGGGAGAATAGTTAGGTC
```

```
+
/11+/,924449;@@;;;AACCBA@BCL>@?999.4;;98511++,*++
@T6XXI:00075:00616
GGGGAATTCTAATACGACTCACTATAGGGAGAGGGAGAATAGCTGACACCCTTTCGTTAGCCGTTCCTCCTTCCTTT
CTCCCTTCTCCC
+
FKC9B;;;<BBACCCBA?@@CCCABABB>CBBCC>C@@>AA@@BBBB@@D<@@<@;98<8838D;=;?@8:79244+
4>@A=A;>?@C<
@T6XXI:00075:00628
CTAGTCGACTCGAGATCTGCGGCTTTGGACAGTCCCTCTGTACTGTGAAGCCACAGATGTCGAGTCGACTAGCTTCC
TTTCTCCCTTCTCCC
+
911/////8::AEC9<<;9?A=888,3999;8:892611161?;734==?BBB==>100;;;??BBCCBA?<71013
11,6109,4/4---+
@T6XXI:00075:00634
GGGAGAAGGGAGAAAGGAAGGAGATAGAGCCGGTCGGGATTACAGGTCTATTCTCCCTCTCCCTATAGTGAGTCGTA
TTAGAATTCCCC
+
DCBCCC?BB>BBBB?C@A@B@@>?AC?=?B6;78?AC;??=????6:9AA@9<;?B5786?>5<=@@===AA?=>@8
838AB=A:@AA6
@T6XXI:00075:00644
CTAGTCACTCGAGATCTGCGGCTTGGACAGTCCCTCTGTACTGTGAAGCCACAGATGTCGAGTCGACTAGCTTCCTT
TC
+
44444449444999@A>@885885813766111,11116??9<88=48@=;;8BC>??:8??:;933376110.444
+/
@T6XXI:00076:00611
GGGAGAAGGGAGAAAGGAAGTGCAGTTCGCGGTTAGTGAAGGGGAAAACTATTCTCCCTCTCCCTATAGTGAGTCGT
ATTAGAACTTCCCC
+
;C>CDCACCABBCC>B?B@C@@ABBC@C;89<><?B;663777.7==2>?@@?;::C@ABECC<>888;===<6;=8
889<@;7992444+
@T6XXI:00076:00616
CTAGTCACTCGAGATCTGCGGCTTTGGACAGTCCCTCTGTACTGTGAAGCCACAGATGTCGAGTCGACTAGCTTCCT
TTCTCCCTTCTCCC
+
CC;<<CD?@CBA?A@@@@>?<8776;7;:DBBCE>B@????<==??C>@C@CCBA:<;77>>??AB??A=>00,0,0
0,0/4=4;/80334
@T6XXI:00076:00643
GGGAATTCTAATACGACTCACTATAGGGAGAGGGACGAATAGTGACGTGTTGTGCGGCGCTCACTG
+
/1-/,/,///,/8988377317766>?;===A@;<98:566=888<@@AA=:4.//,.136445;/
@T6XXI:00077:00603
GGGAGAAGGGAGAAAGGAAGCTAGTCGACTCGAGATCTGCGGCTTTGGACAGTCCCTCTGTACTGTGAAGCCACAGA
TGTCGAGTCCGACTACGCTTCCTTCTCCCTCTCCC
+
?A=ABB@BA<AA@@<AAB@<>;EBBCCCEBB<::@@???=?=777-
7:;;=;=@@6<<?<;888/..99999??@@AA??>=?44423641.--/4/4/3/0334+00000+
@T6XXI:00078:00603
CTAGTCGACTCGAGATCTGCGGCTTTGGACAGTCCCTCTGTTGTGAAGCCACAGATGTCGAGTCGACTAGCTTCCTT
TCTCCCTTCTCCC
+
C>;9<<9:CCECCBBB@@@>><88808<?@FAAAC<===776:88CEAA=@@A@@@===@@A?@?778=;;=>>:67
56888,/,/6660
@T6XXI:00079:00619
GGGGAATTCTAATACGACTCACTATAGGGAGAGGAGAATAGTTACG
+
```

```
>>60;-9:;AABCA@@AACCCBB?B966-1=663444344//778-
@T6XXI:00080:00607
GGGAGAAGGGAGAAAGGAAGCTAGTCGACTCGACATCTGTGGCTTCACAGTACAGAGGACTGTCCAAAGCCGCAGAT
CTCGAGTCGACTAG
+
?A:@AAACC<@@A@<>;>>:;:@@>66666668?6768963969<?A@BCC@;;;//,/76/09@AD?@@=@A@A@@
8::AAA@???????
@T6XXI:00080:00619
GGGGAATTCTAATACGAACTCACTATAGGGAGAGGAGAATAGTA
+
>>802+/,///7////.,.4459A@A@@B::;A?<>//,,,+++
@T6XXI:00080:00639
GGGAGAAGG
+
991///,/,
@T6XXI:00081:00606
GGGGAATTCTAATACGACTCACTATAGGGAGAGGGAGAATAGTCAACAAATCGTTTGGGGCGACTTCTCCTCCTTTC
T
+
4992?5924449::A<;;@@CCCB<<<@>BCBBC?B@?<>@885A>AAC?CBBBB>BBC7B@>00,0.4,.-,--+-
-
@T6XXI:00081:00608
GGGAGAAGGGAGAAAGGAAGAGAACGGCGACGAACAATAGTTGGTGGCTATTCTCCCTCTCCCTATAGTGAGTCGTA
TTAGAATTCCCC
+
CCBBBB@BB>CCBB>B?A@BCBB@CC?BBCCCB@CC?BBBE@C=@@>ABBB@AACD<??DEE=>@??CBAA??@AA?
?=??C<?>?AA5
@T6XXI:00082:00602
GGGAGAAGGGAGAAAGGAAGCTAGTCGACTCGACATCTGTGGCTTCACAGTACAGAGGGACTGTCCAAAGCCGCAGA
TCTCGAGTCGACTAG
+
?B<@AC@BB>BBBB=B?A?AB?@@A;67<<????=???:6444:2:??AAA@;;;444-
4;;;@A>AH@A@>@>;;<=;<<<==?@@@=<<@
@T6XXI:00082:00610
TCGACTCGACATCTGTGGCTTCACAGTACAGAGGGACTGTCCAAAGCCGCAGATCTCGAGTCGACTAGCTTCCTTTC
TCCTCTCC
+
244:49449949B<91<;111188BAA@BA@8;;0;AB?@?@CC@CC@???@@A???@BBBBAAAAA@=:9:,00,0
0/,-----
@T6XXI:00083:00607
GGGGAATTCTAATACGACTCACTATAGGGAGAGGGAGAATAGCTACACGCT
+
?@;2@5959488=@CBCCCCCCCBBBBB=AAA?A9;::844//...----1
@T6XXI:00083:00618
GGGGAATTCTAATACGACTCACTATAGGGAGAGGGAGAATAGTCAACAAATCGTTTGGGGCGACTTCTCCTCCTTTC
TCCCTTCTCCC
+
944,4242448249@A@<;<B@A@????8?>@BB9?<?8::;44437>;9>>777,16626=:7:90//,/+*++*+
+-222248:<;
@T6XXI:00083:00645
GGGAGAAGGGAGAAAGGAAGCTAGTCGACTCGACATCTGTGGCTTCACAGTACAGAGGACTGTCCAAAGCCGCAGAC
TCGAGTCGACTA
+
9@?999599-
///8+/,.34499?:976111111666111440126899<@=<66;5489989979;6;?<A@@AAA@;;//.//.5
55
@T6XXI:00084:00601
```

```
GGGGAATCTAATACGACTCACTATAGGGA
+
944,9294442448:93..9/......+.
@T6XXI:00084:00604
GGGGAATTCTAATACGACTCACTATAGGGAGAGGGAGAATAGTCAACAAATCGTTTGGGGCGACTTCTCCTCCTTCT
CCCTTCTCCC
+
499,42959;;=BBCBDCCACAA???@@<??@CD>A@A9<<<<=C?@@@:@@===6===2=><00,0,,+,+*+*+0
12+-,034:;
@T6XXI:00084:00623
GGGGAATTCTAATACGACTCACTATAGGGAGAGGGAGAATAGCCCACGCGTAGC
+
;:80?-1,1//7688=;>?>@77711668<?=>=6?74234033,08;300---
@T6XXI:00085:00606
GGGAGAAGGGAGAAAGGAAGCTAGTCATCTGTGGCTTCACAGTACAGAGGGACTGTCCAAAGCCGCAGATCTCG
+
?C@AA@@CC>FABD?D@C@AAA@@<<???<BCC@@B>AA@@@AA@??<867<AAA@?@AA=??=@AA@@@===@
@T6XXI:00085:00619
GGGAGAAGGGAGAAAGGAAGGAGAAGTCGCCCCAAACGATTTGTTGACTATTCTCCCTCTCCCTATAGTGACGTCGT
ATTAGAACCCC
+
:B@>>C5;;99@@B<A?@@C?BBB>B@@@CBB7::57<=66266011334443000+0000423354......4554
46<;3329::2
@T6XXI:00085:00632
GGGGAATTCTAATACGACTCACTATAGGGAGAGGGAGAATAGTCAATCGCGATATTGCTTGGCCCACTTCTCCTTTC
TCCCTTCTCCC
+
9A@4@6959449A?@AB:;;CCB==777.77>BC8<9A<??@@@<;<4546640737;;;?>@A<=>B9//.48807
9;;-4/4000+
@T6XXI:00085:00646
CTAGTCGACTCGAGATCTGCGGCTTGGACAGTCCCTCTGTACTGTGAAGCCACAGATGTCGAGTCGACTAGCTTCCT
TTCTCCCTTCTCCC
+
99444494B?=?BBBAA@<10,00,.,..5//7>996616:???6:A<=A=AA@?@@ABECBC@AA@B>??:65:>B
CA@>7;67,/+++*
@T6XXI:00086:00615
GGGAGAAGGGAGAAAGGAAGACGAGTCCAT
+
/2,/<A<>>1944856546.....334340
@T6XXI:00086:00642
GGGAGAAGGGAGAAAGGAAGCTAGTCGACTCGACATCTGTGGCTTCACAGTACAGAGGGACTGTCCAAAGCCGCAGA
CTCTCGAGTCGACTACG
+
9@<@AB@BC<<<BC>B?B???>==;1116661116661114/013889777;:6=77717>=778>@@<@@>>////
--1110.-222------
@T6XXI:00087:00600
GGGGAATTCTAATACGACTCACTATAGGGAGGG
+
BCB7B7<594424499:3;7=;711111,1.53
@T6XXI:00087:00604
GGGAGAAGGGAGAAAGGAAGTGCAGTTCGCGGTTAGTGAAGGGAAAAACTATTCTCCCTCTCCCTATAGTGAGTCGT
ATTAGAACTTCCC
+
=A>BBC@CD>CCBB=B@B@ABBCCCB?BCBA@C@CCCBB?BC?BBBB2BCCB?AAAC9?????<=ACDA???A@?@<
8838@;79:344-
@T6XXI:00087:00609
```

GGGGAATCTAATACGACTCACTATAGGGAGAGGGAGAATAGTCAACAAATCGTTTGGGGCGACTTCTCCTTCCTTTC
TCCCTTCCTCCC
+
;EK5B111//78>@BEAAABA??????7>?=??9<;?9<<CC??<??=9>>777-
711,7>=760733/0/3244,4003034:84575
@T6XXI:00087:00620
GGGAGAAGGGAGAAAGGAAGGAGAATACAGCGAAATGAACACGTCGGCTATTCTCCCTCTCCCTATAGTGAGTCGTA
TAGAATTCCCC
+
>B<>>>;@A@BGFD@DAC@C@BBB?BCAAACCCE:8:@9961111100111;966>130344+0333000433311-
--345<;<==5
@T6XXI:00087:00622
GGGAGAAGGGAGAAAGGAAGTCGATGTGCGTGACATGGGAGATAACAGCTATTCTCCCTCCTCCCTATAGTGACGTC
GTATTAGAACTCCCC
+
9=:9@F>@@>ABAC?C@C@;;;CAA;:8CDA=A;000;64.//6777::649943332-.--
4324778889655;00000/0044033660
@T6XXI:00087:00623
GGGAGAAGGAGAAA
+
11-1//,/,/,//+
@T6XXI:00087:00624
GGGAGAAGGGAGAAAGGAAGCTAGTCGACTCGAGATCTGCGGCTTTGGACAGTCCCTCTGTACTGTGAAGCCACAGA
TGTCGAGTCGACTAGCTTCCTTCTCCTC
+
995498>CC09488989;:>>??@@:6166711178616673166,176699?664=8=777<=@@BA>==:==<>@
@899>?==?=<;;;8//,.,-21---.2
@T6XXI:00087:00647
GGGGAATTCTAATACGACTCACTATAGGGAGAGGGAGAATAGCTACGTTTAACCGATCGTGTTGTTCTCCTTCCTTC
TCCCTTCTCCC
+
AB@7@5469>>5;;BBAAAAB?@?BABB>BCCCC>CAB<A???A?A?AA??>A>A@AAAA65747/4.32778,/,.
3545=74==?:
@T6XXI:00088:00600
CTAGTCGACTCGAGATCTGCGGCTTTGGACAGTCCCTCTGTACT
+
:11/////888499=>>>?885888,377;>@><<86;;//.0+
@T6XXI:00088:00611
GGGAGAAGGGAGAAAGGAAGCTAGTCGACTCGACATCTGTGGCTTCACAGTACAGAGGGACTGTCCAAAGCCGCAGA
TCTCGAGTCGACTAG
+
49149>;>>7;4@B<BBC@@@BBBA:66611166678666885:;<@B??A@B;;444-
4;;;>@=@@;@A>@AAAA??@@=<;;4374733
@T6XXI:00088:00626
GGGGAATTCTAATACGACTCACTATAGGGAGAGGGAGAATAGTCGACAAATCGTTTGGGCGACTTCTCCTCCTTTCT
C
+
8860:,1,/77768>8;;@@@BCBBBBE=AA@@?;<==;=;:4303000+11111,162666110111000/00+00
0
@T6XXI:00088:00642
GGGAGAAGGGAGAAAGGAAGGGCACGTTAATTGTGACGAATGGGAGACTATTCTCCCTCTC
+
BC<@?>59919@@BBC@B?CC>B;;80,.,.,...445<;==A;<55<;,,*+***)*(((
@T6XXI:00088:00649
GGGAGAAGGGAGAAAGGAAGGAGAACGCGCACCCGAACCAACGGTGATCTATTCTCCCCTCCCTACTA
+
CE>AA@>@A>@BCC>CAC@CABBC?BBCCC@@@<>@<A?C@DH@BBCC@@@><@9888.800+00-,,

```
@T6XXI:00089:00618
GGGGAATTCTAATACGACTCACTATAGGGAGAGGGAGAATAGCTCGACGACTTTTTGCCTAGCTATTCCCTTCCTTT
CTCCCTTCTCCC
+
CCB9B797;@@>BBBBBCC>>>>7/00;9;>?CD=A?A;==AABBCB@@AAAAAA1AA898AA?>?8;;6;;;8==1
78=@;@>===C=
@T6XXI:00089:00636
GGGAGAAGGGAGAAAGGAAGAGAATAACAGTGCACCGAATGCGCAAACTATTCTCCCTCTCCCTATAGTGAGTCGTA
TTAGAACTCCCC
+
@D@BAA8991999919<@<?@?B=??;?>>7;9;;;ABC?ADEHCEAA@;;688376616<?;?@CBB@A=<:;;66
72,20*+++++*
@T6XXI:00089:00643
GGGGAATTCTAATACGACTCACTATAGGGAGAGGGAGAATAGTCAACAAATCGTTTGGGGCGACTTCTCCTTCCTT
+
??:2?59284488=CA@>??@:;;@@AB<>@???<?AC>A@@@@@?@@@<CBAAB<AAA6?C>/0.0:6,/,/,//
@T6XXI:00090:00638
GGGAGAAGGGAGAAAGGAAGCAGAATAGACCCCAGATTTGTTGACGGCTATTCTCCCTCTCCCTATAGTGAGTCGTA
TTAGAACTTCCCC
+
?C<@@A>A@>@AAB69;;@CCCCC?B?77777.799<=8?63339;8<BCCAGGCD@@@DCC>BBBBCBBBC==<<<
<8;8;2773777,
@T6XXI:00091:00610
GGGGAATTCTAATACGACTCACTATAGGGAGAGGGAGAATAGCAGCGAGCACTGTTTGCGGTTGGCTATTCCTTCCT
TCTCCCTCTCC
+
8:85:1;8;AA>AAABA@@@CCCBBBBE;?????9?=;9666///-188;=554=C<888;<5<9<;?B:<3/,-
,.,++++*++--,
@T6XXI:00091:00611
GGAGAAGGGAGAAAGGAAGCTAG
+
96444244-4448-423133711
@T6XXI:00091:00615
GGGAGAAGGGAGAAAGGAAGAATACGTCGGTTGTCGGGAGCGAAAAACTAT
+
6<,1/>599-4444-4231397;<CB>>;24233377698833333+33..
@T6XXI:00091:00632
GGGGAATTCTAATACGACTCACTATAGGGAGAGGGAGAATAGCTACGTTTAACCGATCGTGTTGTTCTCCTTCCTTT
CTCCCTTCTCCC
+
@A@7@8;:<AA5;<@@@CCCCBBA????88?BBB>B@@<??A;AAB?;;7<<?<988BB848744/47>:;9;/441
834A7?<???B<
@T6XXI:00092:00628
GAATCTAATACGACTCACTATAGGGAGAGGGAGAATAGTTTTCCCTTCACTAACCGCGAACTGCACTTCCTTCTCCC
TTCTCCC
+
//,1/6:76?B@@@DBBBCEC???9?<===9;;:8;;4444-
44,02379><>B@::9A=@?A=>>?,0,0,/1642:8:444+
@T6XXI:00092:00632
GGGGAATTCTAATACGACTCACTATAGGGAGAGGGAGAATAGTCAACAAATCGTTTGGGGCGACTTCTCCTTCCTTT
CTCCCTTCTCCC
+
CCE:B899<BB?CFCBBBCCBCB?@::6-
9<?AB>@@A?AAA=AA>@A@;BBA@@9???5A@BCE;:8A9;7<8;;7=ABA<A;==B@;
@T6XXI:00092:00647
GGGAGAAGGGAGAAAGGAAGGAGAATAAATTGTCAACGCTGGTGCGGCTATTCTCCCTCTCCCTATAGTGAGTCGTA
TTAGAATTCCCC
```

```
+
CD@CBB?BD>BCBC=@>>@D@BBB=@=@;BABABBABCCEC=@@CC>@@BB?B@@@<:::BD@CC<<=AAA@@@@@@
A@AAA<B>AAA7
@T6XXI:00093:00601
GGGGAATCTAATACGACTCACTATAGGGAGAGGAGAATAGTAAGG
+
88808,1///789:;AAB@B=<?8;;62<=>>=:4/,///--,+0
@T6XXI:00093:00633
GGGGAATTCTAATACGACTCACTATAGGGAGAGGGAGAATAGTTTTCCCTTCACTAACCGCGAACTGCACTTCCTTC
TCCCTTCTCCC
+
CDC7B8;;<@@@DCBBCAAAEA@;=777.7>===9;>>9;;4444-
4;7;;?ADED@C@AB?@>???@>>799,/,06792=8;444-
@T6XXI:00094:00621
GGGGAATCTAATACGACTCACTATAGGGAGAGGGAGAATAGTCAACAAATCGTTTGGGGCGACTTCTCCTTCCTTTC
TCCCTTCCTCCC
+
8C902+/1//789<<BBBBBBC@@??=6==<<=.74;;888<888;<=;@@>77+...*.....,.10/02544914
45444/0/0340
@T6XXI:00094:00626
GGGAGAAGGGAGAAAGGAAGCTAGTCGACTCGACATCTGTGGCTTCACAGTACAGAGGACTGTCCAAAGCCGCAGAT
CTCGAGTCGACTACG
+
AB>BB@?BB>@@CD>B?C@CCB>>=000:000=@?@A<???=<?=??CBBCA888//-
/::::@=A?:@@=@@AAA???===:444////..
@T6XXI:00095:00605
GGGAGAAGGGAGAAAGGAAGCTAGTCGACCCGACATCTGTGGCTTCACAGTACAGAGGGACTGTCCAAAGCCGCAGA
TCTACGAGTCCGACTAGCTTCCTTTCTCCCTCTCC
+
:B:?>@599-
94;;0:69;<7??:8?==<=6<>777>==996/0,.3569?C@;;844,4;;899@AH@@@=@?>=@///.-
3..,,+++++044:::489,4/8939;//.
@T6XXI:00095:00608
GGGGAATCAATACGACTCAC
+
BGA5@19448844433.,2,
@T6XXI:00095:00646
GGGAGAAGGGAGAAAGGAAGCTAGTCGACTCGACATCTGTGGCTTCACAGTACAGAGGACTGTCCAAAGCCGCAGAT
C
+
CC@BBCBBB=A;996;>A?CCC???AAAA@@?;;:??<<6823736;<?AB@@>>8738;;;<=>AB??>;=A@@A@
A
@T6XXI:00096:00600
GGGAGAAGGGAGAAAGGAAGCTAGTCGACTCGACATCTGTGGCTTCACAGTACAGAGGACTGTCCAAAGCCGCAGAA
CTCGTCGC
+
>B<>9859@198?B<A=@@CB@>>>0./7/.//169?6:8=<=C>BCB@@??988//./;@;;??AA:BA>;;8/,+
,,,,+---
@T6XXI:00096:00623
GGGGAATTCTAATACGACTCACTATAGGGAGAGGGAGAATAGTCTTGTATTTTGTGCATCACTTTTCTCCTTCCTTC
TCCCTTCCT
+
?@@4@6924::9A<<<CBBBBBBA?7:7.7>777.7::;;988//,---
300+04476:8;?8<@4<;9793434330--+.,/,/
@T6XXI:00098:00646
GGGGAATTCTAATACGACTCACTATAGGGAGAGGGAGAATAGCTCCAAACTGTTTGGCACGAACACGCCCTCCTTTC
TCCCTTCTCCC
```

```
+
CCA9A99<<BBCDB??BBBB?AB9?616.7>>ACBCAA;=;?;::57>:488@?:=>@=?=;;;;;::9-
./0640786=;@8=8??:
@T6XXI:00098:00647
GGGGAATTCTAATACGACTCACTATAGGGAGAGGGAGAATAGTCAACAAATCGTTTGGGGCGACTTCTCCTTCCTTT
CTCCCTTCTCCC
+
?B@4@695949<AAABABAAAA@?????6=>??A=A@A>A@@??A@AAA>CCBBB9???-
67<00,00/,/,/78726-00,0/3000+
@T6XXI:00099:00649
GGGGAATTCTAATACGACTCACTATAGGGAGAGGGAGAATAGTCAACAAATCGTTTGGGGCGACTTCTCCTCCTTTC
TCCCTTCTCCC
+
CEB8B79:<?@>B?<;CCCCCCB6=777.7>==?<?CA>A@@@??;?>@:@777>.777,71:776>77///77799
9@B?A69.886
@T6XXI:00050:00657
GGGAGAAGGGAGAAAGGAAGTGCAGTTCGCGGTTAGTGAAGGGAAAACTATTCTCCCTCTCCCTATAGTGAGTCGTA
TT
+
;C==:A89919999187<9@;BBC<31/;;;,0,....7;>78:??2>3318666=56764:6;/..433365322-
-,
@T6XXI:00050:00661
GGGAGAAGGGAGAAAGGAAGGAGAAGTCGCCCCAAACGATTTGTTGACTATTCTCCCTCTCCCTATAGTGAGTCGTA
TTAGAATTCCCC
+
CC@CCC@BB?BBBB>B?BAAACAB??<>@GEG8@@<?@?AA;AC?BBB@AA>A@@@<@???A<@===???CC@@@==
<;?@>;>8>=>4
@T6XXI:00050:00664
GGGAGAAGGGAGAAAGGAAGGAGAAGTCGCCCCAAACGATTTGTTGACTATTCTCCCTCTCCCTATAGTGAGTCGTA
TTAGAATTCCCC
+
BCABBBACB@=??C=BAA@B@CCC?@BBBCCC7AA<@@@AA=BC@CCCA??<?@?@;@===CAB?=?@@@?@===AA
@>@@@>@>AAA6
@T6XXI:00050:00677
GGGGAATTCTAATACGACTCACTATAGGGAGAGGGAGAATAGTCAACAAATCGTTTGGGGCGACT
+
9@;2@592444249=>>@BABCCCBABE>@@DBB5:8;7;;58662336466661,111,163/.
@T6XXI:00052:00670
CTAGTCGACTCGAGATCTGCGGCTTTGGACAGTCCCTCTGTACTGTGAAGCCACAGATGTCGAGTCGACTAGCTTCC
TTTCCTC
+
;4444994ABBB@@A@;;9@?<888/8;>?C???C56669==8<=??@@BCBCCA;;<BCAAAA?A@BA?A;//.0:
:=,0,,5
@T6XXI:00052:00683
GGGGAATTCTAATACGACTCACTATAGGGAGAGGGAGAATAGTCAACAAATCGTTTGGGACGACTTCTCCTTCCTTT
CTCCCTTCTCCC
+
944,969598=244=@ACCJDCBBA???6;=<<<9?===@AAA@@=@AA=CCBCC>CFBAAB>/1.0@<,/,/,//+
.1344<8=9=@;
@T6XXI:00053:00664
GGGAGAAGGGAGAAAGGAAGGAGAAGTCGCCCCAAACGATTTGTTGACTATTCTCCCTCT
+
1;;=:@69919>>@<B@C@D?AAB=:633777,33,11111,1777783332111101--
@T6XXI:00053:00680
GGGAGAAGGGAGAAAG
+
A;-4//,//+//,,*,
```

```
@T6XXI:00054:00658
GGGGAATTCTAATACGACTCACTATAGGGAGAGGGAGAATAGCCGTATTTTAACTCCCGCGGCCGGTTCTTCCTTTC
TC
+
9992@59244488=BCC@@@CAB?????6>@=??9?<<=@89;7.-----*-0-
03;4::<;79?999,.+,+,,*,++
@T6XXI:00054:00669
GGGAGAAGGGAGAAAGGAAGGAGAAGTCGCCCCAAACGATTTGTTGACTATTCTCCCTCTCCCTATAGTGAGTCGTA
TTAGAATTCCCC
+
AF@CCD?BA=BBBC>B?A@C?@@C@::;@???6@B=A@??88?;8?AA>@A>==<A<<755=;=<:A@A;?;//0=9
99A=<<=;???4
@T6XXI:00054:00678
GGGGAATTCTAATACGACTCACTATAGGGAGAGGGAGAATAGTACGACTAAATC
+
?CB9B=@=>8>;?@AAB@@@BBBBBBBB:>>777.78;9@?41-///--++*+-
@T6XXI:00054:00679
GGGGAATCTAATACGAC
+
8C808*////,/4//..
@T6XXI:00054:00688
CTAGTCGACTCGAGATCTGCGGCTTTGGACAGTCCCTCTGTACTGTGAAGCCACAGATGTCGAGTCGACTAGCTTCC
TTTCTCCCTTCCTCCC
+
::444444::99999@A@@@@<888/8;<<=?AAB8??:::?AA?==A<?CABDBCBBBCBBBB:=<BA>?@:75:>
BB<@69722,-,--.,
@T6XXI:00055:00652
GGGAGAAGGGAGAAAGGAAG
+
49699=>@C;11=7+-+-*,
@T6XXI:00055:00682
GCTAGTCGACTCGAGATCTGCGGCTTTGGACAGTCCCTCTGTACTGTGAAGCCACAGATGTCGAGTCGACTAGCTTC
CTTTCCT
+
44//////////44948866/.,...+.,..6661726667:@@?@=8988C@CCFCB>?;61611443339;/.-
.555+.,.
@T6XXI:00056:00656
GGGAGAAGGGAGAAAGGAAGCTAGTCGACTCGACATCTGTGGCTTCACAGTACAGAGGGACTGTCCAAAGCCGCAGA
TCTCGAGTCGACTAG
+
@@<@@@>@?:>99909>?>AB????:66???:::?ABBBB@>ACBBCCBBBA@;;444-
4;;AAB?AA?AA=??@@AA<==?==<<<<888=
@T6XXI:00056:00662
GGGGAATCTAATACGACTCACTATAGGGAGAGGGAGAATAGCGACA
+
,--*-+1///56:998;:8==<666<999ABFB<B;9,..--++++
@T6XXI:00056:00676
CTAGTCGACTCGAGATCTGCGGCTTGGACAGTCCCTCTGTACTGTGAAGCCACAGATGTCGAGCGACTAGCCCTTCT
CCCCTTCCTTCCTC
+
DC=<?BCDHEDBCBBB<=2>=;00,.666777>A=ABBA@:;:;77?:?A?AA;;;??==;7:;:::9CACA;B?B;
;::16,/,/,/,**
@T6XXI:00056:00679
GGGGAATCTAATACGACTCACTATAGGGAGAGGGAGAAT
+
8881?+1///,/444977:BBA>::672<;161,12222
@T6XXI:00057:00685
```

```
GGGGAATTCTAATACGACTCACTATAGGGAGAGGGAGAATAGCTACGTTTAACCGATCGTGTGTC
+
999-42929>>;A@@BBBCCCCB?>>>@;?ACCE>B@@>@=@@A??:::78=A>=<9;997/-++
@T6XXI:00058:00662
GGGGAATTCTAATACGACTCACTATAGGGAGAGGGAGAATAGCCGCACCAGCGTTGACAATTTATTCTCCTCCTTTC
TCCCTTCCTCCC
+
6<<1<-9598>;@CCCCBBBD@@?@?==6==777.7;;2377723<B==?96132331324;4574443---
3444757444--,/460
@T6XXI:00058:00678
GGGAGAAGGGAGAAAGGAAGGGAGAAGGAGCCCTA
+
IC>BBC@CC?@A@C@B?B@CC>CC>>?,/***)**
@T6XXI:00058:00681
GGGAGAAGGGAGAAAGGAAGAAAAAGTTGGGATGAGCAAGAGTAAGCTATTCTCCCTCTCCCTATAGTGAGTCGTAT
TAGAACTTCCCC
+
BE>BBA=@@<@@@@<@=@=@CCCC2CFCBB?BBCCAAA@C@??A?C@CCB@CCCC8;;@@@<ACCDCDDBC???884
79==36:8<<81
@T6XXI:00058:00683
GGGGAATTCTAATACGACTCACTATAGGGAGAGGGAGAATAGTCTCCTTTGAACCAGCGACACCATCGTCCTTCCTT
TCTCCCTTCCTCCC
+
9==4>6959=>;=?@;;;B?A@AA:;:C<BB???9?::89:9:444233+189?<<>BB776766161673356564
43..222,.-.544
@T6XXI:00058:00684
GGGGAATTCTAATACGACTCACTATAGGGAGAGGGAGAATAGCTGTACGATGGATACTGCAGCCTTCTCTTCCTTTC
TCCCTTCTCCC
+
?>>4?6959@@<ABBBBBBCCCC??::959@CCC>C@@>@AA=E:<<B@BA?<@@?AAA@;<;<>;>877:=:=>50
/:=,/,/--10
@T6XXI:00060:00678
GGGGAATCTAATACGACTCACTATAGGGAGAGGGAGAATAGCCACCAA
+
8861;-1///7849;A??>B737;776,6<=>>9?96778,,+,+*+*
@T6XXI:00060:00683
GGGGAATTCTAATACGACTCACTATAGGGAGAGGGAGAATAGTCTTGTATTTTGTGCATCACTTTTCTCCTTCCTTT
CTCCCTTCTCC
+
?A?4A8<;=DA?F<;:;;BBA@AA=777.77>BA>B@@=?>=><;2844444+4;;???>>7677-
4448:/4/43+04444201--,
@T6XXI:00061:00688
GGGAGAAGGAGAAAGAGATAAAACAGGAAGCAAGCAGGGAAGAGCTA
+
8>-119292////+/.....6604442499799444 33,3777672,
@T6XXI:00062:00656
GGGGAATTCTAATACGACTCACTATAGGGAGAGGGAGAATAGTCAACAAATCGTTTGGGGCGACTTCTCCTTCCTTT
CTCCCTTCTCCC
+
BB@4?5459=>=A@@ACAAACBA=>:77.7?77:7:@B=@@AA@A?@AA<C??=>.777.7>?AA?@@=34347994
99994:8;9;;6
@T6XXI:00062:00657
GGGAGAAGGAGAAAGGAAGGAGAGACACGTTGGGAGGAAACGATTGCTATTCTCCCTCTCCCTATAGTGAGTCGTAT
TAGAACTTCCCC
+
8=67-1-1-,,,,*,*.,.4499?>C@?@A?AB6>?59909>9924:94424494-
44433,37668?886711100/03349;6999,
```

```
@T6XXI:00062:00677
GGGAGAGGAGAAGAGAGACGCGCACCG
+
99-4///,///78.33334//////,4
@T6XXI:00063:00655
GGGGAATTCTAATACGACGTCACTATAGGAGAGGGAGAATAGTCAACAAATCGTTGG
+
6=:0;-924449886100..442264..,...61,1677?CB?5343--+.4..,..
@T6XXI:00064:00654
CTAGTCGACTCGAGATCTGCGGCTTTGGACAGTCCCTCTGTACTGTGAAGCCACAGATGTCGAGTCGACTAGCTTCC
TTTCTCCCTTCTCCC
+
:,,---
,,7::=?<<<AA@@@=@AB?C=@@CCCCB>BBC8::BCCAAA??B>BBBBABBBBAAABBBBCBBC::7:=?@<::8
A5;278::4
@T6XXI:00065:00657
GGGAGAAGGGAGAAAGGAAGCTAGTCGACTCGAGATCTGCGGCTTTGAGTCCCTCTGTACTGTGAAGCCACAGATGT
CGAGTCGACTAGCCTTC
+
99-
49@<@@:>99908<<1733667676996676687167>?>CC>>>///;+..4338CCBBB@6998;:;;;999499
98==444;41,/,,
@T6XXI:00065:00660
CTAGTCGACTCGAGATCTGCGGCTTTGGACAGTCCCTCTGTACTGTGAAGCCACAGATGTCGAGTCGACTAGCTTCC
TTTCTCCCTTCCTCCC
+
99444444;;;4949=>49884888/8<?@?@?BE>BBBBBAAAB@@@>AD@AAAECEEADECD@AA<;8?@965:>
??<?;6611,.,---+
@T6XXI:00065:00680
GGGAGAAGGGAGAAAGGAAGCTAGTCGACTCGAGATCTGCG
+
?CABB@>A@:>99909;?;?A@?;>96/...3...533311
@T6XXI:00065:00681
GGGAGAAGGGAGAAAGGAAGGAGAACGCGCACCGAACCAACGGTGATCTATTCTCCCT
+
/:577@99A194>>0923;<=BBD?BB=98442442424:4424/7/////76,..+.
@T6XXI:00065:00697
GGGGAATTCTAATACGACTCACTATAGGGAGAGGGAGAATAGTTCACACACATTTGACCAA
+
AEA4@>A>A@@>BBBBAAA>@?BBABBC>AA?@B>B@C>AA==:=@@@;@=;77,//*)*0
@T6XXI:00066:00659
GGGAGAAGGGAGAAAGGAAGGAGAAGTCGCCCCAAACGATTTGTTGACTATTCTCCCTCTCCCTATAGTCGAGTCGT
ATTCAGAATCCCC
+
?C<@@?59919=>@095;<?<?BBA???;;;;4;C:@??:88898<<?<@@?997968/..55535=0/..8:;;A/
//..-994/444+
@T6XXI:00067:00652
GGGAGAAGGGAGAAAGGAAGCTAGTCGACTCGACATCTGTGGCTTCACAGTACAGAGGGACTGTCCAAAGCCGCAGA
TCTCGAGTCGACTAG
+
@B<@@B;>@8;9AB:<><AECCB@@B76666//,223,//33347=7777B@@AA===7<@@@A@>@A=CB>?===A
@@?@884>7049344
@T6XXI:00067:00653
GGGAGAAGGGAGAAAGGAAGTGCAGTTCGCGGTTAGTGAAGGGAAAAACTATTCTCCCTCTCCCTATAGTGAGTCGT
ATTCATTCCCC
+
```

```
49149@=BB<@;;;09=?<@;<<CCC@B@??4848??@?@CC>CCCC2CDEC@:::B9?=8998::<==???=AA=/
.-,-.22234-
@T6XXI:00067:00697
GGGAGAAGGGAGAAAGGAAGCTAGTCACTCGACATCTGTGGCTTCACAGTACAGAGGGACTGTCCAAAGCCGCAGAT
CTCGAGTCGACTAG
+
@@:>@@;>>:>@>=<@>A?CBCCBBBC:66<770161;678:;8:CCECDC@@@844-
49::>?;>?:??>===AB=<=@?A?CAA???@B
@T6XXI:00068:00650
GGGGAATTCTAATACGACTCACTATAGGGAGAGGGAGAATAGTGCAAACCATTGACCCGCAACTATTTTCCTTCCTT
TCTCCCTTCTCCC
+
;><08,/,16676@B@@@CECCBAAAAC>AAC@@;<<@<====@ACB=A;@@>A@@=7==;8<A@BBC7;67=>477
,48444=8;6??:
@T6XXI:00068:00653
GGGAGAAGGAGAAA
+
9901/1,/,,,,,*
@T6XXI:00068:00676
CTAGTCGACTCGAGATCTGCGGCTTTGGACAGTCCCTCTGTACTGTGAAGCCACAGATGTCGAGTCGACTAGCTTCC
TTTCTCCCTTCTCCC
+
4/1/////877499@A@@@984888/84837=6688898??A?@@AAA>AC?BBCCCBBBCCCB?@A<;8?A:78:@
CC?<;8@7:493884
@T6XXI:00068:00678
GGGGAATTCTAATACGACTCACTATAGGGAGAGGGAGAATAGTTTTTCCCTTCACTAACCGCGAACTGCACTTCCTT
CTCCCTTCTCCC
+
999/48><>@@=BBBBBCCCCCBBAAAB>??BDE>?<<=@AAAA?A1AB;@>@CBAA?B@@@=C>@@?A>?:9:,0,
/1472=266484
@T6XXI:00068:00698
GGGAGAAGGGAGAAAGGAAGCTAGTCGACTCGACATCTGTGGCTTCACAGTACAGAGGGACTGTCCAAAGCCGCAGA
TCCCGAGTCGACTAG
+
AA<@AA?BB>DBBB?B@B@AA????7;:@@@=::::::BDD>@A?BCCBBBCAA6333,4;;==?9<=7=@?@AAA=
==@<@@A@?===@B@
@T6XXI:00069:00663
GGGGAATTCTAATACGACTCACTATAGGGAGAGGGAGAATAGTCTTCCAATTTTGGGCGGTACTGTCTCCTTCCTTT
CTCCCTTCTCCC
+
:>>1<,1,16676?BBBCCCBBBBAAAA<AABCD>C@A>A@??8838;@=@AA5??<?C@CCDDC@??B7:7:?AA<
A>;;9;.00;>9
@T6XXI:00069:00682
GGGGAATCTAATACGACTCACTATAGGGAGAGGAGA
+
/220@,1///7/444377796331166,11670114
@T6XXI:00069:00683
GGGAGAAGGGAGAAAGGAAGCTAGTCGACTCGACATCTGTGGCTTCACAGTACAGAGGACTGTCCAAAGCCGCAGAT
CTCGA
+
99149><@>098>A:@>B>BBB::7;661861116999<?=;=??A?A@@@@;;;/0.0;;;?A?AA<AA?899@A8
88>8=
@T6XXI:00069:00699
GGGAGAAGGGAGAAAGGAAGCTAGTCGACTCGACATCTGTGGCTTCACAGTACAGAGGGACTGTCCAAAGCCGCAGA
TCTCGAGTCGACTAG
+
```

```
FCABBC=995>98809<?<ACB>>>/11>>>>1;:??9::=5<?=ABBCEC@@>?877-
4;;;<=>@@<@C?@@@AA????>>>=<;944//
@T6XXI:00070:00667
CTATCGACTCGAGATCTGCGGCTTTGGACAGTCCCTCTGTACTGTGAAGCCACAGATGTCGAGTCGACTAGCCTCCC
TTCT
+
@A999?9;;BBA?AAA;;8>?88;5:>>A@AA?A<@???@AA@???ABCC=@AA;;;ABCAB??AB000;/0,000,
0903
@T6XXI:00070:00677
GGGAGAAGGGAGAAAGGAAGGAGAAGTCGCCCCAAACGATTTGTTGACTATTCTCCCTCTCCCTAT
+
EDACB?>BC>BBBB?B?A?C@BBB@@BBCCCC9CC@DBAAA=BE@CAA?@@=@@@C<A898@:>=>
@T6XXI:00070:00693
GGGAGAAGGGAGAAAGGAAGCTAGTCGACTCGACATCTGTGGCTTCACAGTACAGAGGGACTGTCCAAAGCCGGCAG
ATCTCGAGTCGACTAG
+
:C@BCC@DHBFCCC@C=@=@A@B@A:::?B::9?8666663/012176877388:444,4;;//.7992//,-,-
233544437;;6686854
@T6XXI:00070:00697
TGCGGCTTTGGACAGTCCCTCTGTACTGTGAAGCCACAGATGTCGAGTCGACTAGCTTCCTTTCTCCCTTCTCCC
+
EDCB?CCD>C@BBCCCBD>@@@;:;AA<;;D@CCABCCBBCBAA?ABBCACBBB@:840,00,0:99,/,..--+
@T6XXI:00071:00659
GGGAGAAGGGAGAAAGGAAGCTAGTCGACTGACACTGTGGCTCACAGTACAGAGGACTGTCCAAAGCCGCAGATCTC
GAGTCGAC
+
6;:88=;@@9<9CCCDCD?@@9663443...44..1166313366667:=@<<=;<?=434/44,799=??>?:865
372+++++
@T6XXI:00071:00662
GGGGAATTCTAATACGACTCACTATAGGGAGAGGGAGAATAGTTTATCATCTCCCCATCAGTCTCCTGCTTCC
+
DCB7@5928=89=B<<<@A@CCCAAABB<AAAAA=ABA588<::4730=?>7777-7>==44400201.101,
@T6XXI:00071:00687
GGGGAATCTAATACGACTCACTATAGGAGAGGGAGAATAGCTTGTCATTTGATCAATGTCTACATCTCCTTCCTTTC
TCCCTTCTCCC
+
>::1;,1/665/449AB??A;>::00,/:077-
7304444770166;??9?BA@>38877=;5<<8374:43/00+044449285482
@T6XXI:00071:00688
GGGGAGAAGGGAGAAAGGAAGACGTGGCATGTTAGCAAAATGGGAGAACTATTCTCCCTCTCCCTATAGTGAGTCGT
ATTAGAACTTCCCC
+
;FC:BCCAA@<@BBB=B@C>A@>9::;>>?874:???CE7CBBA@@@@@B?A=88:666=///+.----
.3:=B;86//,/697//,---*
@T6XXI:00071:00694
GGGGAATTCTAATACGACTCACTATAGGGAGAGGGAGAATAGTCAACAAATCGTTTGGGGCGACTTCTCCTTCCTTT
CTCCCTTCCTCCC
+
@BA7@696999<@B@@@CCCBCBBBBBC>BBBBA8?8738@@?AA>8881:AAAA<@@@5????B>??=<?<?>AA;
@;;93;-/./992
@T6XXI:00072:00675
GGGAGAAGGGAGAAAGGAAGCTAGTCGACTCGACATCTGTGGCTTCACAGTACAGAGGGACTGT
+
?C>@?>59819@@B<@=@>A@AABBCBAAAB:::@@???<;>8:8:CDCBA?@@?7771799;;
@T6XXI:00072:00683
GGGAGAAGGGAGAAAGGAAGTGCAGGGAGTTACAGGTTCGTTATCGTCTATTCTCCCTCTCCCTATAGTGAGTCGTA
ATTAGAACTTCC
```

+
?A=BCCBCC=ABBB>B@C@?@@AA>>044423;>;>A@??@?BBA???B=>4>37717<<>A=A=@@???=;667//
,/2000*..444
@T6XXI:00072:00699
GGGGAATTCTAATACGACTCACTATAGGGAGAGGGAGAATAGCCACCTGTTTTGTGGTTGAGCTATCTCCTTCCTTT
CTCCCTTCTCCC
+
>>>4>6959>><=>@A???BB;;8====9?@AFE>C@A<??@A<<<8;;;;;1;;@?@<@@<8::A>=A5869>BB;
=8BB9=/0/7?9
@T6XXI:00073:00662
GGGGAATTCTAATACGACTCACTATAGGGAGAGGGAGAATAGTGCCGCCAATTCTGCGCGTTAGCATTCTCCTTCCT
TTCTCCCTTCTCCC
+
>994>6959?><>B@@@BCCBBAAA@@@<BBBBC>B@@<>>>888>@@>B@C@AB@CBBCC@CB?@@@CCC?@;>48
807>>>:><7777.
@T6XXI:00073:00671
GGGAGAAGGGAGAAAGGAAGTAGGCGACCGGACATCTGTGGCTTCACAGTACAGAGGACTGTCCAAAGCCGCAGATC
TCGAGTCGACTAG
+
>C<99959909444-
4777377?>BABB@C?A>BAA9949899>?AB@?@C>;:00,09936=@CC?CC@BA??C::77;::00///..6
@T6XXI:00074:00651
CTAGTCGACTCGAGATCTGCCGTTCTGGACAGTCCCTCTGTACTGTGAAGCCACAGATGTCGAGTCGCTACGCTCT
+
D=<<B@@@GAA9<<@>@<;;:::,..63440087@;@CBBBA?::?A@?=A?B<;:DCABACB?=8,,++******
@T6XXI:00074:00658
TTTAATACGACTCACTATAGGGAGAGGGAGAATAGTCAACAAATCGTTTGGGGCGACTTCTCCTTCCTTTCTCCCTT
CTCCC
+
99,48=@BCCCAAAA>>>888/8?==?<?@B@BBBBAA=@BA:>>977,8>>2>?@@A=??C@D?C<??<??;B:?;
884:4
@T6XXI:00074:00661
GGGAGAAGGGAGAAAGGAAGCTAGCGACTGACATCTGGTGGCTTCACAGTACAGAGGGACTGTCCAAAGCCGCAGAC
TCTCGAGTCGA
+
49044424:-
99@C>B?B@BBBB::67666;444...,.45..,3;7667>===444+3::334>@@:@@<78483339530.-2--
-
@T6XXI:00074:00672
GGGGAATTCTAATACGACTCACTATAGGGAGAGGGAGAATAGTCACATTCATGTTTGTGACCGTGTCTCCTTCCTTT
CTCCCTTCTCCC
+
@=92>6959489A@AACAAAAA@>@777.7><??<?@@>@?@@@@@@BACBB:::5:;8AC>@@AA999?A9;388-
49;@7=8=7081
@T6XXI:00074:00689
GGGAGAAGGGAGAAAGGAAGCTAGTCGACTCGACATCTGTGGCTTCACAGTACAGAGGGACTGTCCAAAGCCGCAGA
TCTCCGAGTCGACT
+
CC@CCC@CCBCBBE?C@B@CCCB:;;::?@B?::666177453656BAB?@=A==<<<6;<<<??>AA;AA<===47
88;;9=??;:8:0/
@T6XXI:00074:00695
CTCGAGATCTGCGGCTTTGGACAGTCCCTCTGTACTGTGAAGCCACAGATGTCGAGTCGACTAGCTTCCTTTCTCCC
TTCTCCCCTTCCTTTCTCCCTTCTCCC
+
BBA@@@BB@AACC@BBB>BBCCCCCCC?AACCCCBBBCCC@CC@CBCBCBBBB@@>@?CBBBCBBB@BCCD?DBBB6
7478888.737477.77<=6=47166,
@T6XXI:00075:00661

```
GGGGAATTCTAATACGACTCACTATAGGGAGAGGGAGAATAGCCGTTTAATCTCCCACTGCGGCATATCCTTCCTTT
CTCCCTTCCTCCC
+
999/9595999<BCCCC@?>?@C???A?9???@@<@8838?==<??@7=;=;<<?:?@@===>?;:846,-
,/9934733224,/,/993
@T6XXI:00075:00662
GGGGAATTCTAATACGACTCACTATAGGGAGAGGGAGAATAGCCGCACCAGCGCTGACAATTTATTCTCCTTCCTTC
T
+
?BA4B545989;=C@A@DCCCBCBC???<?CBBC>A??<>87738;@C?CCCCDBD@@@?BB<?>;>80,-
20)*)**
@T6XXI:00075:00666
GGGGAATTCTAATACGACTCACTATAGGGAGAGGGAGAATAGCTCCTAACTATCCCGCCACGCTATTCGCACTTCCT
TTCTCCCTTCTCCC
+
?A@4@5924889?>AAA@@@B>?87777,7<777-
744:6>==::77?>:6:8<846=:<=999;;../4313,0/03+044446:6444+
@T6XXI:00075:00695
GGGAGAAGGGAGAAAGGAAGGGGAGAAGGGAGAAAGGAAGCTAGGTCACTCGACATCTGTGGCTTCA
+
BC>DED@B=:=9996;=?<?@@7@BCACCB@??@7>:=4763687999661400+++++++*00/03
@T6XXI:00076:00669
GGGGAATTCTAATACGACTCACTATAGGGAGAGGGAGAATAGTCAACAAATCGTTTGGGGCGACTTCTCCTTCCTTT
CTCCCTTCTCCC
+
BC@4@2459889?>@ADAAACCCBA:77.7=<@@>BAA????@AA>@AA<E@??C9???5?;??93714/0/4<<=;
=<<<;<444==;
@T6XXI:00076:00694
TCACTATAGGGAGAGGGAGAATAGTCAACAAATCGTTTGGGGCGACTTCTCCTTCCTTTCTCCCTTCTCCC
+
6:AADB@AA@>ACCBC>>@@@C@AA??;?@@:ABBBB>BBB8BBB@A=??>587:<@A>;::C<>:=677.
@T6XXI:00076:00698
GGAGAAGGGAGAAAGGAAGGGAGGAGAAGAA
+
1,/66,//+4444-423133,3713110113
@T6XXI:00077:00660
GGGAGAAGGGAGAAAGGAAGGGGAGAAGGGAGAAAGGAAGAATACGCCGGTACCACTTATTTTGGAGCTATTCTCCC
TCTCCCTATAGTGAGTCGTATTAGAACTT
+
=A=BBC@CC?@A@C=@<>488:4;BCECC>CBBB>C@C>??:;;9@>:@>::7=B?;37777.:>BCHDDDBBBAB<
<;;AA<B@@@ACC996111110034464,
@T6XXI:00079:00689
GGGAGAAGGGAGAAAGGAAGAAGAGCGCACAGCGGGAGGACCATTGGCTATTCTCCCTCTCCCTATAGTGAGTCGTA
TTAGAATTCCCC
+
BD@CDD?BB?ABBB?A=?=BB@BA?AAAABC@??;;;?A?A=@@>A>CCBB?<<;>18788B7>888??@@BA@@A;
;8;::7828891
@T6XXI:00080:00665
GGGAGAAGGGAGAAAGGAAGCTAGTCGACTCGAGATCTGCGGCTTGGACAGTCCCTCTGTACTGTGAAGCCACAGAT
GTCGAGTCGACTAGCTTCC
+
99-
499<=@<>=>@<>;>=;;7977166999999886117990/,.444=??;;499:444;;<>@A@AA>@@=<<<873
<>;88./-11-3021,
@T6XXI:00080:00694
GGGGAATTCTAATACGACTCACTATAGGGAGAGGGAGAATAGTCAACAAATCGTTTGGGGCGACTTCTCCTTCCTTT
CTCCCTTCTCCC
```

```
+
944,424:9>>=@AA@@ACABBB???AA=ABBCC=B??;;<?A@@=?>>:AA@BB>BBB7BBAAA?::88<9<;>@;
9:9C<@????>:
@T6XXI:00081:00651
GGGGAATTCTAATACGACTCACTATAGGGAGAGGGAGAATAGTTTGGACGTCTCCCAGTCCTCGTTACGCTTCCTTT
CTCCCTTCTCCC
+
CEE8B8;8<=@<A@@<;<CCCCB??@@@9??@BC>A??>AAAAA<A<??AD===A<AA@@=@AAA?AC88344/44,
4444-4/03854
@T6XXI:00081:00657
GGGGAATTC
+
///*,*,*/
@T6XXI:00081:00680
GGGGAATTCTAATACGACTCACTATAGGGAGAGGGAGAATAGTACGCC
+
=;;,4642488248>?<33787:7:@?@>C===@;<8995500000-,
@T6XXI:00082:00680
CTAGTCGACTCGAGATCTGCGGCTTTGGACAGTCCCTCTGTACTGTGAACCACCAGAGTGT
+
@;1//111ADCCCCBAADCBA??CC>C?BBBBBBB>BAAABB>A?=<B:;23,*+******
@T6XXI:00082:00684
GGGGAATCTAATACGACTCACTATAGGGAGAGGGAGAATAGTCAACAAATCGTTGGGG
+
:880:-1///,/9@A:;;?@>?=886666666;;94644000002422+....,.55*
@T6XXI:00082:00685
GGGAGAAGGGAGAAAGGAAGCTAGGTCGACTTGACATCTATGGCTTCACAGTACAGAGGGACTGTCCAAAGCCGCAG
ATCTCGAGTCGACTAG
+
:<9<CC@CC>CBBB>B>@:7771657>??7:5:??<<?:61676:5:CAAA@A@@?888-
4;;:==;==6<<<>>>@????>>>????????A
@T6XXI:00082:00697
GGGAGAAGGGAGAAAGGAAGGAGAAGTCGCCCCAAACGATTTGTTGACTATTCTCCCCTCTCCCAGAC
+
4=:=BD@BC@CCCCADCB?B@DCD;::888<<2>726999>;<?8?::30;493333+33004+0030
@T6XXI:00083:00651
GGGGAATTCTAATACGACTCACTATAGGGAGAGGGAGAATAGTCTATCAATATTGGCCGGTAGCTTTCTCTTCCTTC
TCCCTTCTCCC
+
:AA5=,959489A>@A@@@?AA?76676,7><<?8?:9<?@???<888<8?3824/959<?=03098///344,/-
13342420000+
@T6XXI:00083:00659
GGGGAATTCTAATACGACTCACTATAGGGAGAGGGAGAATAGTCAACAAATCGTTTGGGGCGACTTCTCCTTCCTTT
CTCCCTTCTCCC
+
?@>2=8>=AAB>BBBBBBADCCCAA???9?@?BB>BAA@A???@A?@@A9@?AAB9???5=>>::67742838:;;7
877;7=8947A:
@T6XXI:00083:00697
GGGAGAAGGGAGAAAGGAAGGGGAGAAGGGAGCTAGTCG
+
995=>?<@A<@BCC=B>@=>>>2>9:,0/+/,,,,00,,
@T6XXI:00084:00657
GGGGAATTCTAATACGACTCACTATAGGGAGAGGGAGAATAGTCAACAAATCGTTTGGGGCGACTTCTCCTTCC
+
499/424244424984333<<;;<:<:C=@@BBEBC@@>A@A>>>@AAA<CBBBB>BED8A;,,7+,,+*+*+0
@T6XXI:00084:00662
```

```
CTATCGACTCGAGATCTGCGGCTTTGGACAGTCCCTCTGTACTGTGAAGCCACAGATGTCGAGTCGACTAGCTTCCT
TTCTCCCTTCTCCC
+
B??>99:?BBADBCB?BC::8;CC>C?AABCCCCBDC?????<<??A@@C@CCCB@A:::BBBACC@@@ECBB?B?A
A=A==;7=7844;:
@T6XXI:00084:00677
GGGGAATTCTAATACGACTCACTATAGGGAGAGGGAGAATAGTTTTTC
+
9A;2;5-*,,,*,,,/73337677667>;7>6661133234/4544.2
@T6XXI:00084:00696
GGGGAATTCTAATACGACTCACTATAGGGAGAGGGAGAATAGCTTTCCCTTCACTAACCGCGAACCGCACTTCCTTT
CTCCCTTCTCCC
+
999/99A@B;;8<CBBBCDDAABBBBBE@CC@@?9?==:>?=;??.77.7;>BAA?@C>A@@@>@?>>@8868677.
7<=?<@7:6?9:
@T6XXI:00085:00651
GGGAGAAGGGAGAAAGGAAGGAGAAGTCGCCCCAAACGATTTGTTGACTATTCTCCCTCTCCCTATAGTGAGTCGTA
TTAGAATTCCCC
+
ACAECC@CB>?ABC>C@C@C@@@@???@@GDD9CCBD@@=A=AC?CBCA??=??>C;@9:9C;@@@@@???A???==
=>AAA>@>@AA6
@T6XXI:00085:00669
CTAGTCGACTCGAGATCTGCGGCTTTGGACAGTCCCTCTGTACTGTGAAGCCACAGATGTCGAGTCGACTAGCTTCC
TTT
+
94444444;9994489>><10,001,0,0.567777999:9@?=>63555=>ABBCBBBBBCCBBBB310;6,,*,5
77+
@T6XXI:00087:00666
CTAGTCGACTCGAGATCTGCGGCTTGGACAGTCCCTCTGTACTGTGAAGCCACAGATGTCGAGTCGACTAGCTTCCT
TTC
+
96//////666444==@>;;:911,03747::>?9>666168?8;:;AC@AACDBBBBB@@@@BB97397..,.30
0*,
@T6XXI:00087:00694
GGGAGAAGGGAGAAAGGAAGA
+
AC?BAB=>95>9--+-+-+**
@T6XXI:00088:00660
GGGGAATCTAATACGACTCACTATAGGGAGAGGGAGAATAGCCAACGACTGCATTTCCTCAGGCCTATCCCTTC
+
8A>1=+1///76:;::7;45...///4245/77-630/00-2010-...-134575;<6333/4/10000+024
@T6XXI:00088:00663
GGGGAATTCTAATACGACTCACTATAGGGAGAGGGAGAATAGTTTTTCCCTTCACTAACCGCGAACTGCACTTCCTT
TCTCCCTTCTCCC
+
;;4/95;:<CC@CCB?BBBBB@B9::AA<?BCCC>CAA<@<>>>>>1>A;>>@BBBC@@>:??C>=@@???7:;366
.7;;;;:@;><;;6
@T6XXI:00088:00695
GGGAGAAGGGAGAAAGGAAGCCGATGGCGCGGTAGATGAGTGAGAGACTATTCTCCCTCTCCCTATAGTGAGTCGTA
TTACGAATTCCCCC
+
44-
4;;:AC>B@??<@?B@AA<@A::7<CCK@BBCBBBBBB<AABCCBABC@@<=B7=;::94<<;?AAA@<<<<=@=;:
631,-,----*
@T6XXI:00089:00656
GCTAGTCGCCTCGAGATCTGCGGCTTGGACAGTCCCTCTGTACTGTGAAGCCACAGATGTCGAGTCGACTAGCTTCC
T
```

```
+
//***,,,,*,,//98=>@<:;:00,.,.443.11,1669ABBBBB@@;<@=@?@AB?????@7:;:00.33,,*,0
0
@T6XXI:00089:00695
GGGGAATTCTAATACGACTCACTATAGGGAGAGGGAGATAGTCCCTGTGTTCTCCCCACAGCGGTTAACCTTCCTTC
TCCCTTCTCCC
+
9@@4>=AABBC@BBBCC@@@B?@????B<BABBB>C???????@:?@@<=9=AABB6990;830155+,+,50*+*+
+-3477998C;
@T6XXI:00090:00651
GGGGAATTCTAATACGACTCACTATAGGGAGAGGGAGAATAGTCAACAAATCGTTTGGGGCGACTTCTCCTTCCTTT
CTCCCT
+
CDC9@24598=9>@A<;<BBB@@@<=<=6===??<?@A=@@D===;=?A<B::6=-777,7;::53731,-,-
2224533332
@T6XXI:00090:00664
GGGGAATTCTAATACGACTCACTATAGGGAGAGGAGAATAGTAGACA
+
C8808+45<;;?DBBBBBBCCBB@B???<?B@@>C8834////-++.
@T6XXI:00090:00685
GGGGAATTCTAATACGACTCACTATAGGGAGAGGGAGAATAGTCAACAAATCGTTTGGGGCGACTTCTCCTTCCTTT
CTCCCTTCTCCC
+
999-424:;>@=@BCBACCCCAABA???9?A?AA<A@@;=<AAAA>???;AB;=>.777-
7>>?@???;8<9<?@A<@@@B;A====C?
@T6XXI:00090:00691
GGGAGAAGGGAGAAAGGAAGAAAGTATATCGTCAGTGGGCAGATCGGGAGACTATTCTCCCTCTCCCTATAGTGAGT
CGTATTAAGAATTCCCC
+
99149>5991999908<?=88808>??ABB;;;CCCCC>CCCCCCCC@AAACCDC:?:77-
789:=6<ACDDDCJCC@::1/,/,.468,///*
@T6XXI:00091:00693
GGGGAATTCTAATACGACTCACTATAGGGAGAGGGAGAATAGTCAACAAAAAAATCG
+
:>@1@-1,///,/4;890009/..1111,11111,1845445<8656322222&33.
@T6XXI:00092:00696
GGGGAATTCTAATACGACCTCACTATAGGGAGAGGGAGAATAGTCCTTCCCCTGCTCGACATGA
+
CCC4@6;:<=>9?C<<;@=??BA711111,167=72633/0-2--,-3333-..1117777;61
@T6XXI:00093:00665
GGGGAATCTAATACGACTCACTATAGGGAGAGGGAGAATAGTCG
+
F>?5>,////,/4443:;;9?;?9?862=;000,0--01-++--
@T6XXI:00093:00680
GGGAGAAGGGAGAAAGGAAGTGCAGTTCGCGGTTAGTGGAGGGAAAAACTATTCTC
+
4@?>AB?BCACBBC?B=>=>00/8//,49331313777577>9=997/3.,,*,,,
@T6XXI:00094:00691
GGGGAATCTAATACGACTCACTATAGGGAGAGGGAGAATAGACACCAATCGTGCCGGGTCAAT
+
>>>5>,,,,,*,/8:<39;@:88??A@<@C??@<?@B388?6449/4211169775564..,*
@T6XXI:00095:00651
GGGGAATCTAATACGACTCACTATAGGAGAGGG
+
8AA17+1/77784;;;?;794644..,.43668
@T6XXI:00095:00691
```

```
GGGGAATTCTAATACGACTCACTATAGGGAGAGGGAGAATAGCTGACAATTCGGCGACTGATCTTCTCCCTTCCTTT
CTCCCTCTCC
+
999-48>?BBBACCCCCCCB@AB?B::?8:B>CC>CAB>@<?<ABCC@AA@CB@B>>>BBBB??=??CC:@<:344-
/111+,+++*
@T6XXI:00096:00664
GGGGAATTCTAATACGACTCACTATAGGGAGAGGGAGAATAGTTACACACACATTTACAGGGCTGTTCTCTCCTTTC
TC
+
9==4>6958449@?BBBAAAB@?>>777.7>777.7::97888,-.-24444004-
4>===9<>;;,.....44424.0
@T6XXI:00096:00679
GGGAGAAGGGAGAAAGGAAGCTAGTCACTCGACATCTGTGGCTTCACAGTACAGAGGGACTGTCCAAAGCCGCAGAT
CTCGAGTCGACTAG
+
BA?@A>58809111-
/,0,.6;>?@?@==>>11.//.:367977:AACCCCA??=<<5;865;;8;;6<?<??AA=====@@>==AA???@
@T6XXI:00098:00681
GGGAGAAGGGAGAAAGGAAGCTAGTCGACTCGACATCTGTGGCTTCACAGTACAGAGGACTGTCCAAAGCCGCAGAT
CTCGATCGACTAG
+
CEDCBB?AA=BB>>=B>><ABA@CC@@@@@@C=<???????;?A@BCCBCCA994..-
/8:8;@>@A<??>?@=??<788@@@A@@@@AC
@T6XXI:00098:00682
GGGAGAAGGGAGAAAGGAAGGAGAAGTCGCCCCAAACGATTTGTTGACTATTCTCCCTCTCCCTATAGTGAGTCGTA
TTAGAATTCCCC
+
=C@DDC@CB>BBBB=B@A?A@DAA>>>@CCCC7BB@BDAAAAAC@ECB@==;=@?A=889?@:=899AAAAA===@@
@>A@@>?5;;<1
@T6XXI:00099:00657
GGGGAATTCTAATACGACTCACTATAGGGAGAGGGAGAATAGTGCGTACCTTAGACCAACACTTTTCTCCTCCTTTC
TCCCTTCTCCC
+
BCB:C;;;<AA?BBBCB@AACCC??9:7.7><<?9?@A=@@??@@BA??<?>?964,/,/2.///+/44259<==4<
<;;4:3403;6
@T6XXI:00099:00683
GGGGAATTCTAATACGACTCACTATAGGGAGAGGGAGAATAGTGACGTGTTGTGCGGCGCTCACTGTCTCCTTCCTT
TCTCCCTTCTCCC
+
@>@4?6969>?;@A@@AABCCBB?C:::57==?B>BAA>AA:@@A<999=>???AA<??@?@@??777:92424799
4444:-424;=>;
@T6XXI:00099:00685
GGGGAATTCTAATACGACTCACTATAGGGAGAGGGAGAATAGACCAACCTGTTTTTGTCGGTCACTCTCCTTCCTTT
CTCCCTTCTCCC
+
999/49>:8;;>CAABB@@@@>AACBCCBBBBCC>C@@>AAAAA6848<>=?@?=.;;4837;>488@@;;8;344-
4;:;4;8<5;A=
@T6XXI:00099:00687
GGGGAATTCTAATACGACTCACTATAGGGAGAGGGAGAATAGTGACGTGTGTGCGGCGCTCACTGCTCCTTC
+
ABA4B695<9;=@BA@<;;?B@@:79@A<?@CDC>C@@>??===@@::5//---5388=:8:@==<620*+**
@T6XXI:00099:00697
GGGGAATTCTAATACGACTCACTATAGGGAGAGGGAGAATAGTCAACAAATCGTTTGGGGCGACTTATCCTTCCTTT
CTCCCTTCTCCC
+
999/489:9>@=A999?AA???BA???C>BBBBB<B??<?@A<;;;8841:>??>9?==2=====47443434344,
4444565<5=>:
```

```
@T6XXI:00050:00702
GGGGAATTCTAATACGACTCACTATAGGGAGAGGGAGAATAGTTTTTCCCTTCACTAACCGCGAACTGCACTTCCTT
TCTCCCTTCTCCC
+
999/9695<CCCCCBBAAAAA@@99166.6<?AB>BAA?AA@AAAA1@@;@<?BBBC@C?BB?@?<AA=@97:=377
.744B<A>??AA<
@T6XXI:00050:00717
CTAGTCGACTCGAGATCTGCGGCTTTGGACAGTCCCTCTGTACTGTGAAGCCACAGATGTCGAGTCGACTAGCTTCC
TTTCTCCCTTCTCCC
+
B:1111///1:=?@CDBBB>D=88808;>>?BABA=BFCBB@@?@===>@C@CCBABCCCBBBB?AA<;;BB::::@
BB<::4;-4248;=:
@T6XXI:00050:00721
GGGGAATTCTAATACGACTCACTATAGGGAGAGGGAGAATAGTCTTGTATTTTGTGCATCACTTTTCCCCTTCCTTT
CTCCCTTCTCCC
+
BA?7A89;<CC@BBAAABBBB@B?869959?ACC?CBB=>::=@<<<<@=>>5A??ACCBB:;::.4;;1;8<;>@:
@B@@;@899AC@
@T6XXI:00050:00727
CTAGTCGACTCGAGATCTGCGGCTTTGGACAGTCCCTCTGTACTGTGAAGCCACAGATGTCGAGTCGACTAGCTTCC
TTTCTCCCTCTCCC
+
E@999944:?A=ABBCCCBBB?BDC?C88;BBBBB>BBBBAAACCA@@<@C@BBBBBAABCCCBABBDDCCC??;?:
::690//,//--.,
@T6XXI:00051:00705
GGGAGAAGGGAGAAAGGAAGCTAGTCGACTCGACATCTGTGGCTTCACAGTACAGAGGGACTGTCCAAAGCCGCAGA
CTCTCGAGTCGACTAG
+
ED@BBB@CC=ABB@<>=?<?B?;>C1//?///>??@B?@@A?@C@CCBBBC@=99333-
8@?@AA=@@;@@@A;::/00>@@AAAA<;<;773
@T6XXI:00051:00721
GGGAGAAGGGAGAAAGGAAGAATAATTACGTTTCAACGGGCGCTGGACTATTCTCCCTCTCCCTATAGTGAGTCGTA
TTAGAATTCCCC
+
DC@AA@?BB>BBBC?B?A@BB=@?;?=?BDCECCA?AAA=ABCCC:<9?@>=>=>?:??@BA?AAAA===@?<====
=>A@;5434661
@T6XXI:00051:00723
CTAGTCGACTCGAGATCTGCGGCTTTGGACAGTCCCTCTGTACTGTGAAGCCACAGATGTCGAGTCGACTAGCTTCC
TTTCTCCCTTCTCCC
+
A99448444:A9@BBA@A@>?=888/8588=@:::7:CCDBBBCB==B?@D@CFCBAAABAAA??@B;;;CAAA?C@
CC>CB@D?A9<<<:4
@T6XXI:00051:00726
GGGGAATTCTAATACGACTCACTATAGGGAGAGGGAGAATAGATCACCGTTGGTTCGGGTGCGCGTTCTCCTTCCTT
TCTCCCTTCTCCC
+
999/42424;;9?@@A@?@AB:;8?777-7?777-:;8<@@===>>><773747:444-
477766106777736011,-333443----+
@T6XXI:00051:00733
GGGAGAAGGGAGAAAGGAAGGACGCTGTCGTCAGTAGCTATTGGGACTATTCTCCCTCTCCTATAGTG
+
495988,/1+//885654,313788499==>=@=?>A999B@CC>BBB4424444-4;4429:4////
@T6XXI:00051:00747
GGGAGAAGGGAGAAAGGAAGAATAATTACGTTTCAACGGGCGCTGGACTATTCTCCCTCTCCCTATAGTGAGTCGTA
TTCAGAATTC
+
```

```
AB?A><:<=111=>?>?>;@@=;B<?88;7<72310111,11117260558:==AC;>5044-
343>@C8:9@=;777-..000.)+
@T6XXI:00052:00705
GGGAGAAGGGAGAAAGGAAGGAGAAGTCGCCCCAAACGATTTGTTGACTATTCTCCCTCTCCCTATAGTGAGTCGTA
TTCAGAATTCCCC
+
?C@A?=59919?@@1948<?;?@B=??CCDGG9CC>B@;61117278?<><<A=?@:=844;4:666/----100-
44-./;;9?;A@@5
@T6XXI:00052:00731
GGGAGAAGGGAGAAAGGAAGCTAGTCGACTCGACATCTGTGGCTTCACAGTACAGAGGGACTGTCCAAAGCCGCAGA
TCTCGAGTCGACTAG
+
ADBCCC?B?<>@@@<@=?;@C::;A::;A???;;;?????@;>AABBBBCBA?A9333,498;<?>@@;@C>A<==@
A@@@@@@A==<??>>
@T6XXI:00052:00741
GGGGAATTCTAATACGACTCACTATAGGGAAGA
+
:BA4B59544424889:@799633....+.,,2
@T6XXI:00052:00742
GGGGAATTCTAATACGACTCACTATAGGGAGAGGGAGAATAGCTGTCACGTTTGGACAATTGCAGTTCTCCTTCCTT
TCTCCCTTCTCCC
+
A?@4>545<AA@B@@@BBDCBCC?B696.7=7775:>>=@@??A??????996;:6981377898:6:AA>B?@<@B
7;;BE>>?>;8:2
@T6XXI:00053:00730
GGGAGAAGGGAGAAAGGAAGCTAGTCGACTCGACATCTGTGGCTTCACAGTACAGAGGACTGTCCAAAGCCGCAGAT
CTCGAGTCGACTAG
+
44-
4=C@DL>AABB<A>@<@@;::>:6//.3///.33../95568869:ACA8;;//,/68::?>AA<9969AAAA@@AA
???@@@???AA
@T6XXI:00053:00739
CTAGTCGACTCGAGATCTGCGGCTTTGGACAGTCCCTCTGTACTGTGAAGCCACAGATGTCGAGTCGACTAGCTTCC
TTTCTCCCTTCTCCC
+
C@;999449999>@@ADBBCC@CCB?CBBCBCCCC>BBBBBCCCCAA@=?B?BBCCBBBBBCCB?@A;;8?<7107;
>>9??>B:;/4000,
@T6XXI:00053:00741
GGGGAATTCTAATACGACTCACTATAGGGAGAGGGAGAATAGTCAACAAATCGTTTGGGGCGACTTCTCCTTCCTTT
CTCCCTTCTCCC
+
CCB7@===<99=AA@A@BBBBBB@@:::.7=767.::::=??@@A>AAAACBCBB<BBB7A@?>>:9;9,/,/9896
;;@>:?9<6<A=
@T6XXI:00054:00741
GGGAGAAGGGAGAAAGGAAGGAGAAGTCGCCCCAAACGATTTGTTGACTATTCTCCCTCTCCCTATAGTGAGTCGTA
TTAGAATTCCCC
+
49094>5990988B<@=@?B:;;CGD???CCC9CCAAAA?C>>??CCB@@@>@@AA@?>==C9<=@A@??A=<<<88
8>AAA<A8==<4
@T6XXI:00055:00705
GGGGAATTCTAATACGACTCACTATAGGGAGAGGGAGAATAGTTTTCGACCCCCATTACACTGCTTTTCTCTTCCTT
TCTCCCTTCTCCC
+
999/9<>=@AA?BBBBCCCBCCCAA<:888;;??<AA@<;94334-
4;44444,4BBCCBBCCB@AA7773777066.7>>>6?;?F?@;
@T6XXI:00055:00723
```

```
CTGAGATCTGCGGCTTTGGACAGTCCCTCTGTACTGTGAAGCCACAGATGTCGAGTCGACTAGCTTCCTTTCTCCCT
TCTCCC
+
CA@AACBBBBDDBBBB=B@CCDBBCE?BBBBBCBCCBBB@C@@@@BBBB>@@BBBBBBBBCC>>A==<>C=BBBC<@
=@BBC<
@T6XXI:00055:00731
GGGAGAAGGGAGAAAGGAAGGACAGTCCATCTGTGGCTTCACAGTACAGAGGGACTGTCCAAAGCCGCAGATCTCGA
GTCGACTAGCTTCCTTTCTCCCTTCTCCC
+
C>:DBB>BD@CCCEAC;:5:?@>@@??<???????@??=ACCACA@@@@@CB;@A@@>=;>?7@A?@@BA???@@=@
@@CCBCCCB@@>@?BCAAB?C<A;==@D;
@T6XXI:00055:00735
GGGAGAAGGGAGAAAGGAAGG
+
00001989A:A911,-8888+
@T6XXI:00055:00738
GGGAGAAGGGAGAAAGGAAGGAAGAAGTCGCCCCAAACGATTTGTTGACGTATTCTCCCTCT
+
/;;;;B?BJDCD>@>B:;;?,0,0.3...73995;<:8>8::8:7668740113-.+000++
@T6XXI:00055:00740
GGGGAATTCTAATACGACTCACTATAGGGAGAGGGAGAATAGTCAACAAATCGTTTGGGGCGACTTCTCCTTCCTTT
CTCCCTTCTCCC
+
DKKAC998<AA<ABBBAACCCCB@?661.7==@A>BAA>A=>>AA?AAA:AAAAD>BCB7BBBB@<>7424349;;:
;<<<6<;>?AA<
@T6XXI:00056:00701
AGTCGACTCGAGATCTGCGGCTTTGGACAGTCCCTTCTGACTGTGAAGCCACAGATGTCGAGTCGACTAGCTTCCTT
TCTCCCTCTCCC
+
>>>111;;;;;>CDBBBCB?:999;BBB??>;;8:,....47@777>A?<ABB>@?@<==@@AAA?884:4747477
.70//,/.---+
@T6XXI:00056:00704
GGGAGAAGGGAGAAAGGAAGCTAGTCGACTCGACATCTGTGGCTTCACAGTACAGAGGACTGTCCAAAGCCGCAGAT
CTCGAGTCGACTAG
+
DCACCD@CC>BBBB=B675:B><?@9::???>:7::::::A9=@@CCE???A@@@8838AB;<=>AA<@C@@@@A@@
@@@???@@@@@@@@
@T6XXI:00056:00706
TGACGCGCGGCTCAATGATGATTGAAGGACGAATTGCGCGCTTTGTATATATCTCGCTATACCGAATGCATCAGATT
GCGCTGCATGGTTACTTTAAAACCGGATTAATGATGCTGGTGGGGAGTATTAACCGCGTTATCCGTCCGCGT
+
@CCBCBCCC@BCCC@BBBCCCB=??>?=?CBB=?=@CC@AACC>AAAAAB<A888AAAA@@@>@A>==8==@@AA@>
@AAA=99==A>@<??@@;@AA5@=A>AD?C>A<<<>888=@AAA6>=?0000;>9;?<<9<?@>AAC?A=<<
@T6XXI:00056:00719
GGGGAATTCTAATACGACTCACTATAGGGAGAGGGAGAATAGCCCTTTCCGTACGTGTCGCAGTTTTCCTTCCTTCT
CCTCTCC
+
BCB7@2;59888=@BBB;;;C:77;761,67777,6::889336.,,+,+,,0+++146=???DD5=;?;;9958/
/,-1--,
@T6XXI:00056:00720
GGGGAATTCTAATACGACTCACTATAGGGAGAGGAGAATAGCC
+
AGB8@545944248110>=;@B?:11>>.77<<37--44.**)
@T6XXI:00056:00728
GGGAGAAGGGAGAAAGGAAGCTAGTCGACTCGAGTCTGT
+
KIDBBB?BD<@ABB?BEJIC@@>?>0..33../4457/.
```

```
@T6XXI:00056:00733
TGGACAGTCCCTCTGTACTGTGAAGCCCAGATGCTCGAGTCGACTAGCTTCCTTTCTCCCTTCTCCC
+
?B?BDCCBBB>BCDCBBBBBBBC@C?B;?????????<AACCAAAD@AA@CCBE?CCJG?B=??@@<
@T6XXI:00056:00748
GGGGAATTCTAATACGACTCACTATAGGGAGAGGGAGAATAGAGCTTAGATCTTGGCTCACGTTTTCTCCTTCCTTT
CTCCCTTCCTCCC
+
BCG9B>B@CBB@BCBA@C><??><=777.7=7775:?A>BA?@@AA=A::;BB?C?BAAA?7444-4;;3434244-
40/728,/,/.-+
@T6XXI:00057:00708
GGGAGAAGGGAGAAAGGAAGTGCAGTTCGCGGTTAGTGAAGGGAAAACTATTCTCCCTCTCCCTATAGTGAGTCGTA
TTAGAACTTCCCC
+
FH@?A@=@@<@BAA<@>@BCCCCCEE?BFCD?B?BBCB@???>ACC8CBCC?BCCC>????B>@???@@@AAA==>8
838BB7::2444,
@T6XXI:00057:00743
GGGAGAAGGGAGAAAGGAAGCTAGTCACTCGACATCTGTGGCTTCACAGTACAGAGGACTGTCCAAAGCCGCAGATC
TCGAGTCGAC
+
<<8<;B?BG>A@==:@>@@CCCBCCABA?BA::4>98?<B?BBBBCCDBBA::;//./;;;;@=@B@AD>@@@@@??
?A@@@A==<@
@T6XXI:00057:00745
GGAATTCTAATACGACTCACTATAGGGAGAGGGAGAATAGTCAACAAATCGTTTGGGGCGACTTCTCCTCCTTTCTC
CCTTCTCCC
+
B@B@D@BBB@CC?BBDB@<=@CB???9?B??A<A@@>AA@BAAA???<ACCEC>CCC9BB>>>:=>;./.59;2;9<
<7<:==CC<
@T6XXI:00057:00747
GGGAGAAGGGAGAAAGGAAGGAGAAGTCGCCCCAAACGATTTGTTGACTATTCTCCCTCTCCCTATAGTGAGTCGTA
TTAGAATTCCCC
+
=B?B@@=@@<@ACC>C@C@C=@@B?BBACCBB2DBCBCAB@>BB?BBBAAB>AAAA<===@C<AAB===AAA=====
=B@@A>A>A@@5
@T6XXI:00058:00711
GGGGAATTCTAATACGACTCACTATAGGGAGAGGGAGAATAGTCTACGCAATTCG
+
@@@6@=@=ACCCCB@@B?AAB@>96111.7>>@B>A??;;;4././---1,.,.0
@T6XXI:00058:00736
GGGAGAAGGGAGAAAGGAAGGAGAAGTCGCCCCAAACGATTTGTTGACTATTCTCCCCTCCTCCTATAGTGAGTACT
TAGATTCCATAATCCCGTCCA
+
EEBBA@@CB@@A@A=B@B@?>AAD?@BBBCCC8AB<AB???<?A>BB@@@A>@@@CC5??:3/,-.----+-----
23334=:<8:64/003+---3-
@T6XXI:00058:00746
GGGAGAAGGGAGAAAGGAAGGAGAACAACACGATCGGTTAAACGTAGCTAGTTCTCCCTCTCCCTATAGTTGAGTCG
TATTAAGAACT
+
//+1/4244-444;-
423:;179C=B@?@@>>>8442423927;7.......,...53/78>@;668833/344000--2,-,-2311
@T6XXI:00059:00714
CTAGTCGACTCGAGATCTGCGGCTTGGACAGTCCCTCTGTACTGTGAAGCCACAGATGGACTGTCCTTCCTTTCTCC
CTTCTCCC
+
D>?<BCBBBCC==<BCBB><;;00,1:000=;??9????BB??????>@C@C@@???>488>776360101626777
,1011-22
@T6XXI:00059:00721
```

```
GGGAGAAGGGAGAAAGGAAGGATGAGATGCATCGATAAACGGTGGGACTATTCTCCCTCTCCCTATAGTGAGTCGTA
TTAGAATTCCCC
+
AC<AAA?@A<>>?D1958<?4:9;99A???A@BBBBCGAAA>;9918AABC@CCCB<7::::.7?@@AA>>@;;8=7
424:;4:;==92
@T6XXI:00060:00701
GGGAGAAGGGAGAAAGGAAGCTAGTCGACTCGACATCTGTGGCTTCACAGTACAGAGGGACTGTCCAAAGCCGCAGA
TCTCGAGTCGACTAGCTTCCTTTCTCCCTTCTCCC
+
BA<@<959A:@999084847A?<<</000....66;@>>><9<?=?BBBBC@@99444-
4;;A@@>@CE??>?A@?@?@AA???A@@@@@@@@@8;8;=677;;6;000;;6
@T6XXI:00060:00703
GGGAGAAGGGAGAAAGGAAGGAGAAGTCGCCCCAAACGATTTGTTGACTATTCTCCCTCTCCCTATAGTGAGTCGTA
TTAGAATTCCCC
+
CE?ABB?BC>AACC<?=@>><>??<?@A@@@>5??:?AB@A;CC@BA@=8434;;;-
7777;:?<<<>>;<;@999AA@=5420/3370
@T6XXI:00060:00720
GGGAGAAGGGAGAAAGGAAGGAGAAGTCGCCCCAAACGATTTGTTGACTATTCTCCCTCTCCCTATAGTGAGTCGTA
TTAGAATTCCCC
+
BC>A@@;>>:>>>@0;9;>B?BCC@BAABCCC9CC?AA?::;<B?CBCADA=>@@B=@??@AA@AAA?==BAAA@<<
<@AAA=@=ABA6
@T6XXI:00060:00723
CTAGTCACTCGAGATCTGCGGCTTGGACAGTCCCTCTGTACTGTGAAGCCGCAGATGTCGAGTCGACTAGCTTCCTT
TCT
+
444444444444498=8<::900,0,0.4:.11,16668?96<<<::8>111;:99CBBBB??A@111;8//,.446
083
@T6XXI:00060:00743
GGGGAATTCTAATACGACTCACTATAGGGAGAGGGAGAATAGACTACCGT
+
>99299A>BBB@DCBBB@@@CBB?@777.77>>@<@=;<75,.,+++*+*
@T6XXI:00060:00747
GGGGAATTCTAATACGACTCACTATAGGGAGAGGGAGAATAGTCAACAAATCGTTTGGGGCGACTTCTCCTTCCTTT
CTCCCTTCTCCC
+
@=94><>=ABB@CCCBABCCCBCB?:::.6=;??=@AD?A>>>AA?BAA=?<==C>B@B9CCCBB=;=9/42448;:
??AC=A9<<AA=
@T6XXI:00061:00746
GGGGAATTCTAATACGACTCACTATAGGGAGAGGGAGAATAGTGACGTGTCGTGCGGCGCTCACTGATCTCCTTCCT
TCTC
+
;>;2@2;:9AA<@@@<;;@A>7771111,1>777,13344@>8.///.//--0044244444241...33004--,-
2211
@T6XXI:00062:00705
GGGAGAAGGGAGAAAGGAAGGGGTGAAGGGAGAAAGGAAGCTAGTCGACTCGACATCTGTGGCTTCACAGTACAGAG
GACTGGTCCAAAGCCGCAGATCTCGAGTCGACTAG
+
CC?CCC@BB?BA;;;;;::9=>-
00994424.11,10101?868?7116333;9;:9873827727>=@?@888;::7::::78;/43+00/06>?????
===>;;;;;;<8
@T6XXI:00062:00707
GGGAGAAGGGAGAAAGGAAGGAGAAAACGCTAGAGCCAAGATCTAAGCTCTACT
+
BC>A9969>:949?:@<@<><>8888,994444444249;94333113/7////
@T6XXI:00062:00737
```

```
GGGGAATTCTAATACGACTCACTATAGGGAGAGGAGAATAGTTA
+
;;:111<@CBB@BB??>BBBB@BBAA@?>?AA?>@30/3/,,+,
@T6XXI:00062:00742
GGGAGAAGGGAGAAAGGAAGGAGAAGTCGCCCCAAACGATTTGTTGACTATTCTCCCTCTCCCTATAGTGAGTCGTA
TTAGAATTCCCC
+
CD@CBB@CC@ABEB=B@B@A?ABB@@@AAABB8BB>A@@BC>BB?BA@=AB>@@AA=@898A?A@@@AAA@A=??==
=AA@@>@=?@?5
@T6XXI:00063:00715
GGGAGAAGGGAGAAAGGAAGAGGAAGACGCCAAAGATTGTGACGTTGCTCTT
+
CD>AAHACCBBB>>6<::,0.4344...37777:111>>:8:>94/-.***)
@T6XXI:00063:00744
GGGGAATTCTAATACGACGTCACTATAGGGAGAGGGAGAATAGCATAG
+
9;;2:29244;9888=8333337>>;<@B<<;:<<,00.4444.0000
@T6XXI:00063:00745
GCTATCGACCGAGATCTGCGCTGAGCATG
+
//44444:?;9==>999;;4434...,,,
@T6XXI:00063:00749
TCGAGATCTGCGGCTTTGGACAGTCCTACTGTACTGTGAAGCCACAGATGTCGAGTCGACTACGTCCTTTCCTTCCT
CCTTCCTTCC
+
AC@@<<;>@@;<=<NG?C?>87777+,,,22165<==;@>@?9<=@AAAA@BBCBAB>>77,,,,,+//,4248;8:
97924/344/
@T6XXI:00064:00743
GGGGAATTCTAATACGACTCACTATAGGGAGAGGGAGAATAGACACGACATGAATCGACGCTCCCAAATACTTCCTT
TCTCCCTTCTCCC
+
BBB9A>@>BCBABAAABAAA???;C661-
6<;<?<?@@?A@@@AA======<<;<<<A=@@@@@;@A=A>>>?C?AA<??>A5878444-
@T6XXI:00064:00744
GGGGAATTCTAATACGACTCACTATAGGGAGAGGGAGAATAGTCAACAAATCGTTTGGGGCGACTCTCCTTCCTTTC
+
999,4242444;==BGA;<;@><61166288?BC8:4@=????<?::?>7>?777-7>?2<:7,,,,+*,+++/301
@T6XXI:00064:00746
GCTAGTCGACTCGAGATCTGCGGCTTTGGACAGTCCCTCTGTACTGTGAAGCCACAGATGTCGAGTCGTACCT
+
44///6//////499==AB@>>;733,31339;999899A????A=88527<8;<@?@B;;:7?=71,,**)*
@T6XXI:00065:00706
GCTAGTCACTCGAGATCTGCGGCTTTGGACAGTCCCTCTGTACTGTGAAGCCACAGATGTCGAGTCGACTAGCTTCC
TTTCTCCCTTCTCCC
+
44444499::949;;>>>@986888,3777;@>@@99BA:::?;;4644;@=@@BBCBCABCBB;<<@B>??:127;
>>9??<?:8273330
@T6XXI:00065:00717
GGGGAATTCTAATACGACTCACTATAGGGAGAGGGAGAATAGATACCTAATCCC
+
9??-969289=@CBBBAABBBCCC????9=?@?A=A@?=@?3////,.-33333
@T6XXI:00065:00733
GGGAGAAGGGAGAAAGGAAGGGGAGAAGGGAGAAAGGAAGCTAGTCGACTCGACAT
+
CC@CCC@AA=@@@B>B?A?ABB8BBC@CC<A::7.7374:@9=?B:6/2++++0+-
@T6XXI:00065:00742
```

```
GGGAGAAGGGAGAAAGGAAGCTATCGACTCGACATCTGTGGCTTCACAGTACAGAGGGACTGTCCAAAGCCGCAGAT
CTCGAGTCGACTAG
+
CC>BBB@CC>CBBB=B?B?BDCBAB@;711;;68<<<>AB>?@?@DFCCBB@>?77717>>?@@>@@;@A>AAA@A=
==?@AAA:99A@AB
@T6XXI:00065:00745
GGGAGAAGGGAGAAAGGAAGGTGAACAGAGCGCAGACGAATACGTCTCTATTCTCCCTCTCCCTATAGTGAGTCGTA
TTAGAATTCCCC
+
CD?CCC=>><@AAC=B=@=@6;;@=BCCBBCCBBBBCCC?B@AAA@@@AAA@A@@C=CAABGACCCBBBA:77>??=
=:?777?5999/
@T6XXI:00065:00747
GGGAGAAGGGAGAAAGAAGGGAGA
+
9@:999599-//11+/.,,,*,,,
@T6XXI:00066:00733
GGGAGAAGGGAGAAAGGAAGGAGAAGTCGCCCCAAACGATTTGTTGACTATTCTCCCTCTCCCTATAGTGAGTCGTA
TTAGAATTCCCC
+
9@<AHEBCC@CCCDBB8;7;=@BF@CABBBCC9CC>B?BEE<>93//.-2443044-0003333/---.-
2323856:<884440000.
@T6XXI:00066:00739
GGGGAATTCTAATACGACTCACTATAGGGAGAGGGAGAATAGTCAACAAATCGTTTGGGGCGACTTCTCCTTCCTTT
CTCCCTTCTCCC
+
@AA6@8<8<AB>B@@@@@AAA@@>777=9?@ACCCA??>A>@@@B?AAA<CCCCB?BBF5?>>9957>;2434244-
4;;;4824444,
@T6XXI:00066:00745
GGGAGAAGGGAGAAAGGAAGCTAGTCGACTCGACATCTGTGGCTTCACAGTACAGAGGACGTGTCCAAAGCCGCAGA
TCTCGAGTCGACTAG
+
995449;@@:@AAA<B@C=@ADBA7,,,,,,,,,*00...-3-.,/8888B>766,,++,++++-,-
10144655566434<:65<6;30-3
@T6XXI:00067:00705
GGGAGAAGGGAGAAAGGAAGGAGATATTAGCATGCACCATGGAATGCCTATTCCTCCCTCCTCCTATAG
+
AB>BBB>BB;<9AD196;;?;?ACBAA?@@<><<999>?BB?<9<3311170101552..,.-,-4644
@T6XXI:00067:00714
GGGGAATTCTAATACGACTCACTATAGGGAGAGGGAGAATAGCTAACGATTCTCTAGCTCCCAGTTAAACTTCCTTT
CTCCC
+
ACC:D<;9;AA>BBCDDCCCA?@?@7776:=?BA<B<;8<??>BB@???=9<==;<<<?==7888466,/-22,--
+-134+
@T6XXI:00067:00740
GGGAGAAGGGAGAAAGGAAGCTAGTCACTCGACATCTGTGGCTTCACAGTACAGAGGGACTGTCCAAAGCCGCAGAT
CTCGAGTCGACTAGCTTCCTTTCTCCCTTCTCCC
+
CC?BAA=@@:@99909<?<8;8>>>7771111101677110784 7????@A=;>884-
4:986>;=>:;<56<<<@@AA===<;;=8833933/0/30+04440;588994
@T6XXI:00067:00744
GGGGAATTCTAATACGACTCACTATAGGGAGAGGAGAATATACGGA
+
8886>>C?@AA@CCCCCBCCCBCAA???9??==;>;;7:80+++**
@T6XXI:00068:00703
GGGAGAAGGGAGAAAGGAAGCTAGTCGACTCGACATCTGTGGCTTCACAGTACAGAGGGACTGTCCAAAGCCGCAGA
TCTCGAGTCGACT
+
```

```
44-
9?@@BB1999909;>@ABABBA:::???:::?CB???823706?9?AB@@;;48814;;@@?>@C???<@==99///
/+***+++++
@T6XXI:00068:00717
GGGAGAAGGGAGAAAGGAAGTGCAGTTCGCGGTTAGTGAAGGGAAAAACTATTCTCCCTCTCCCTATAGTGAGTCGT
ATTAGAATTCCCC
+
?B<AA@?CCABBBG>@?@@BBBB=11,11;;378>AAAB?BB>BBBB1BBCB?BBCC>ABDEEBAA???@@AHD?A?
?@?@HC?@9>AD6
@T6XXI:00068:00746
CTCGAGATCTGCGGCTTTGGACAGTCCCTCTGTACTGTGAAGCCACAGATGTCGAGTCGACTAGCTTCCTTTCTCCC
TTCTCCC
+
ACBBFCCCCBBBB?BCB@B=@=AADDD?BBBBCBBD?AAC>AAACCC@BBCCBABAB@C@@@I@@A@C?BB?B@BB7
:6:>??9
@T6XXI:00068:00748
TCTGCGGCTTTGGACAGTCCCTCTGTACTGTGAAGCCACAGATGTCGAGTCGACTAGCTTCCTTTCTCCCTTCTCCC
+
@:;94@;444-49:?BCBBB<@@@@???A:::?=?CCCCCC@????@@@ABBBBBCCB>>@477.7<=?.4/40030
@T6XXI:00069:00702
GGGGAATTCTAATACGACTCACTATAGGGAGAGGGAGAATAGCACGCAGCTCGAGCCTGTGGCTGTTCTCTTCCTTT
CCTC
+
:@=0<,924889;9;;;8>7;1337111,166<<6<88248833386;??95865?;7700/000440---,-,-
101035
@T6XXI:00069:00709
GGGAGAAGGGAGAAAGGAAGGAGAACATGTTGTGGGACAAAGCCAAACTATTCTCCCTCCTCCCTATAGTGAGTCGT
ATTAGAACTTCC
+
?CCDCDBDD<<<BH<@>@<?;888;BBABD@;:<@6;7AB>@@B?D=A==<>=<=@801,000,/738:?:8>:400
-1,--3,-.511
@T6XXI:00069:00737
GGGGAATCAATA
+
:8806-,,2*,,
@T6XXI:00069:00738
GGGAGAAGGGAGAAAGGAAGCTAGTCGACTCGACATCTGTGGCTTCACAGTACAGAGGGACTGTCCAAAGCCGCAGA
TCTCGAGT
+
CH@BBA>@=9@@@=:@>A<?@@???BBCBBCA:::::;AAA;=@@CCCCCCA=94000-
4;;AA@?AC>>><@A=<<=<<<858A
@T6XXI:00069:00742
CTAGTCGACTCGAGATCTGCGGCTTTGGACAGTCCCTCTGTACTGTGAAGCCACAGATGTCGAGTCGACTAGCTTCC
TTTCTCCCTCTCCC
+
@11////49??9=>BCBA@A?=88808;>>;?9?9:?==7776./-
.345C?BBB@??B???BA?@AA@>;@7101300,0../,/----+
@T6XXI:00070:00702
GGGAGAAGGG
+
//+//8500*
@T6XXI:00070:00712
GGGAGAAGGGAGAAAGGAAGGAGAAGTCGCCCCAAACGATTTGTTGACTATTCTCCCTCTCCCTATAGTGAGTCGTA
TTCAGAATTCCCC
+
DD?@ACABC=BBBA=@@C=@>@CC@A?@CCCC8AA>BCBBB>BC@BBCA9=;===C<AA@@@:????@@@@?><;<=
=<=68=/3/4::.
```

```
@T6XXI:00070:00729
GGGGAATTCTAATACGACTCACTATAGGGAGAGGGAGAATAGTTTTCCTTCACT
+
ABA9A>B?BCC@CAAA????B@BB>666-1;;??;?AA>@56555*,++*+,.1
@T6XXI:00070:00738
GGGAGAAGGGAGAAAGGAAGTGCAGTTCGCGGTTAGTGAAGGGAAAAACTATTCTCCCTCTCCCTATAGT
+
99-44C59914899194843334444429???@B@BBB:::;E>E?>@2B<7706161-1666>5448885
@T6XXI:00070:00739
GGGAGAAGGGAGAAAGGAAGTGCAGTTCGCGGTTAGTGAAGGGAAAACTATTCTCCCTCTCCCTATAGTGAGTCGTA
TTAGAAC
+
CD@=9429919999149>>BCCECCC=@<:;?B9;8BBC@DD>CBB7A<<;@@LEE=11/5;66?9<<@@977421-
-,+00*+
@T6XXI:00070:00746
GGGGAATTCTAATACGACTCACTATAGGGAGAGGGAGAATAGTCCGCCAATTCTGCGCGTTAGCATTCTCCTTCCTT
TCTCCCTTCTCCC
+
CCC:CAC@CCC@BABBB?A?@CBB?666.7<<>B?CA@?A>@AC?==;??CA@C???B??=@C?AA@CCC>B>B@CC
>BB??;?>@@@C<
@T6XXI:00070:00749
GGGAGAAGGGAGAAAGGAAGGAGAAGTCGCCCCAAACGATTTGTTGACTATTCTCCCTCTCCCTATAGTGAGTCGTA
TTAGAATTCCCC
+
CC@CCB@BB>??AC>B@B@B@A@B?@BBCCCC9CC?CCACC>BB=AAB?AA>A???:???@A<A9==A????=<<@@
@>@@@@A=AAA5
@T6XXI:00071:00708
GGGGAATTCTAATACGACTCACTATAGGAGAGGGAGAATAGTTAACGCACCCCCCGTTACCTGTTCACCTTCCTTTC
TCCCTTCTCCC
+
4BB6?=><999689>A@@AB@79::00,/::=B>A>?;778><@<=44111777)7?;110111018838>>88:88
344+0230344
@T6XXI:00071:00711
GGGGAATTCTAATACGACTCACTATAGGGAGAGGAGAATAGTTACGACAAC
+
9@B9>C?=ABB@C@@AB?@@CCA@@:::.7?==;@4434444288000.-.
@T6XXI:00071:00731
GGGGAATTCTAATACGACTCACTATAGGGAGAGGGAGAATAGAGACGTATTCGTTTGCGCTCTGTTCACCTTCCTTT
CTCCCTTCTCCC
+
BCC7B:<<=DDECCCCCCAB@?AAAB???=?CCCC<A?A>@:98<??@????AAAA<AA????AAA>A:;9;>;9::/
//;9,434444+
@T6XXI:00071:00733
GGGAGAAGGGAGAAAGGAAGGAGAAGTCGCCCCAAACGATTTGTTGACTATTCTCCCTCTCCCTATAGTGAGTCGTA
TTAGAATTCCCC
+
DDCBBB=@@<@@@B<@=@?B?ACCBBBBCCCC8BB<ABAAA;AA?BDCAAB>???B@A:::B@@@@A???AA===@A
@>@??;=34480
@T6XXI:00071:00734
GGGGAATTCTAATACGACTCACTATAGGGAGAGGGAGAATAGCCGACAATTCGACTCCCCGGCAATGTTCTTCCTTT
CTCCCTTCTCCC
+
CCB7A===@AAABBBBCCCCCA@>;666.777?BACBA>B888;????>C@@@@BCCCC8C@BB===@>?>;?:=<9
=@@@;A;==AA<
@T6XXI:00071:00736
GGGAGAAGGGAGAAAGGAAGGAGAAAAGTGATGCACAAAATACAAGACTATTCTCCCTCTCCCTATAGTGAGTCGTA
TTAGAACTTCCCC
```

```
+
CCAKCC;99-
98880;885;>?>???2=777<947116626716010111145558A443>94+33558858<>96///,-772-
7/399.
@T6XXI:00072:00702
GGGGAATTCTAATACGACTCACTATAGGGAGAGGGAGAATAGTTATCTGCACGCCCAACAACCGCTATTCTTCCTTT
CTCCCTTCTCCC
+
CB@7A54:<@A?BCCCCBBBCB@==666.7>7777;AA>@@AC>@@@?BBCBBBCCC@;?>@@@?@@@@A@<@6:76
:>>>:><>777.
@T6XXI:00072:00734
GGGAGAAGGGAGAAAGGAAGAAAATGAGGAGAACGAAAAAGGCACGCGCTATTCTCCCTCTCCCTATAGTGAGTCGT
ATTAGAATTCCCC
+
CHCCCD@CB>?ACG?C@C@AA@A7;::F@DCC@BBBBBB1B>CCCC=<<BBBACGDD>BBACC>BBCCCFCBBAAA;
;<8<<@>@?AAA5
@T6XXI:00072:00735
GGGAGAAGGGAGAAAGGAAGCTAGTCGACTCGACATCTGTGGCTTCACAGTACAGAGGGACTGTCCAAAGCCGCAGA
TCTCGAGTCGACTAGCTTCCTTTCTCCCTTCTCCC
+
EB<D<<89@:@111-
/,.,.4...50//97/./96?99??<39:?ACCBBBAA>>77713;;>?@>@A<==;=AA???@@=899ABA:99AA
@?=?8;;6<?=A<A;==AA;
@T6XXI:00072:00736
GGGAGAAGGGAGAAAGGAAGATGAAGATAGCAAGGGGAAGCTACTATCTATTCTCCCTCTCCCTATAGTGAGTCGTA
ATTACG
+
99-444249-
49BE@E@C?@@@@B=BGBAABC@DCC9BBDCAAAA@===99598880:<;>?;:<>><8:?4133..,+000+
@T6XXI:00072:00739
GGGAGAAGGGAGAAAGGAAGTAAACGTATGCGGGACAAGGGTGGTATCTATTCTCCCTCTCCCTATAGTGAGTCGTA
TTAGAACTTCCCC
+
<<8;AA@BC:<<AA>B@B@@?@C>CB@>9948809>C>BC?BCCCDCCH;96888808@@BC>CCBBDCDA?@@@??
B@??>1/09;;94
@T6XXI:00072:00747
GGGGAATTCTAATACGACTCACTATAGGGAGAGGGAGAATAGTCAACAAATCGTTTGGGGCGACTTCTCCTTCCTTT
CTCCCTTCTCCC
+
944,429<@AA?BBBBCCCCDBB@?:9:5:>@AB=AAA@C@@@?>;<<<7?CB??6:::.7????=??<8<9<<??<
?AAC<A<9//82
@T6XXI:00073:00701
GGGGAATTCTAATACGACTCACTATAGGGAGAGGGAGAATAGTCTCGTCTGAGCACGATCATGTACAGCTACGTACG
TCTGAGCAT
+
@@=6@<@>BBB@CBAAABBB?@A@>:8?88ABBC>CCC?@::?;?>444400000333356344<>A==@>666444
77755333,
@T6XXI:00073:00703
GGGAGAAGGGAGAAAGGAAGAATAGAGTGGGCGTGTCGGGTTGGTAACTATTCTCCCTCTCCCTATAGTGAGTCGTA
TTAGAACTTCCCC
+
CA@==??BB?BBBC>A>A@BB@BC:::AAA9??ABBAAA<A>A??B?CEGD>ABBE>BB@@@<@@?@CCCCAAA::8
<<?@C9??3777.
@T6XXI:00073:00712
GCTATCACTCGAGATCTGCGGCTTTGGACAGTCCCCTACTGGT
+
449999=?AA=ABAC@=>884311,37769==:??811.,,*,
```

```
@T6XXI:00073:00718
GGGAGAAGGGAGAAAGGAAGTGAAAATGTAGAACAGGCCAGGTGCGGTGACCTATTCTCCCTCTCCCTATAGTGAGT
CGTATTAGAACTTCCCC
+
CB=AA>24819?>@0;9;=@>@ABB9CABB??<?==:?6::@@@@C@ABAA?===<====6;;;<<6<A????@@@@
;;;8837==9;;3444-
@T6XXI:00073:00731
GGGGAATTCTAATACGACTCACTATAGGGAGAGGGAGAATAGTCTTTCATCCACGAGTTTCAGCATATTCTTCCTTT
CTCCCTTCTCCC
+
CA>9==?=ACC@CBAABBBB@?<77166.7>>@B>BAA@A=??<<<;<@@@?BBCCCCC>CC@??>??@@@?<;<A<
AAAC;:;?>??:
@T6XXI:00073:00741
GGGGAATTCTAATACGACTCACTATA
+
@DD/9642444844443370..64..
@T6XXI:00074:00702
GGGAGAAGGGAGAAAGGAAGCTAGTCGACTCGACATCTGTGGCTTTACAGTACAGAGGGACTGTCCAAAGCCGCAGA
TCTCGAGTCGACTAG
+
BC;<=A?CB<B@@@<@>@>ACB>>>/01:777,0..//453239:8:CCFCCBAA88818B???=?CB@BC?AAC@A
AA?=<?AA?<=????
@T6XXI:00074:00713
GGGAGAAGGGAGAAAGGAAGACGTAGT
+
4969;A@@@19999-4231333313..
@T6XXI:00074:00718
GGGAGAAGGGAGAAAGGAAGAGAATAACAGTGCACCGAATGCGCAAACTATTCTCCCTCTCCCTATAGTGAGTCTAT
TAGAT
+
BC>@@@;>><@>>@<A=@?@@@@DDC<;>FGB?@@@CB=@@B;<<C=BBCC@CCCE<AA???9??@AB87;17=6.,
*,*((
@T6XXI:00074:00737
GGGAGAAGGGAGAAAGGAAGCTAGTCACTCGACATCTGTGGCTTCACAGTACAGAGGGACTGTCCAAAGCCGCAGAT
CTCGAGTCGACTAG
+
CDBBB?@BCCBBBC?C@A@BC?==ACBBBEC::9;;:BDB@BA=@CCAA?B844000-
4999<>>BC<@@?@@@@@@A@@===A@@AAA@@
@T6XXI:00075:00704
GGGGAATTCTAATACGACTCACTATAGGGAGAGGGAGAATAGCCACCAACTATTGTTCGTCGCCGTTCTCTTCCTTT
CTCCCTTCTCCC
+
CCB9A<==@BB?DCCBB??AB:;7;677.777AC@BAA?A==?<AC<=;????>@@=AA@@@A>9744//-
0144454:?A7=;=<;<7
@T6XXI:00075:00723
GGGAGAAGGGAGAAAGAAGCTAGTCACGTGACATCTGTGGCTTCACAGTACAGAGGACTGTCCAAAGCCGCAGATCT
CG
+
6=6<<@699-//68+/.3467943:3......33.1684556-////699422-.-
.5547<<==4865556865544-
@T6XXI:00076:00744
GGGAGAAGGGAGAAAGGAAGGAGAAGTCGCCCCAAACGATTTGTTGACTATTCTCCCTCTCCCTATAGTGAGTCGTA
TTAGAATTCCCC
+
CD@BB>@BB>BCCC>B?A?A@BAA>A??BBBB6>><<@@?>;B>@CA<?A@=?<<64<?@?CA@?==A====89=58
9?B@@=>8<;;1
@T6XXI:00076:00745
```

```
GGGAGAAGGGAGAAAGGAAGCTAGTCACTCGACCATCCTGTGGCTTCACAGTACAGAGGGACTGTCCAAAGCCGCAG
ATCCTCGGAG
+
4959::;@B<@@BH<BAA9;887111.4.....,-..,.447787763../74244000+000056;==;=;8640-
+**)***)*+
@T6XXI:00077:00713
CTAGTCACTCGAGATCTGCGGCTTTGGACAGTCCCTCTGTACTGTGAAGCCACAGATGTCGAGTCGACTAGCTTCCT
TTCTCCCTTCTCCC
+
BB9<<A===B??99<CBB??<@??:?=@@BBCCC>BBBBBCC@@@AA>?B?BCCCBB?BBCBCBBCABBBB@?=@@C
C>B?>A1434:::4
@T6XXI:00077:00732
CTAGTCGACTCGAGATCTGCGGCTTGGACAGTCCCTCTGTACTGTGAAGCCACAGATGTCG
+
B@-,,,,,///;;=>@BCA@C4885813776111,676??AAAC?;85/1-/686.37611
@T6XXI:00077:00748
GGGGAATTCTAATACGACTCACTATAGGGAGAGGGAGAATAGCCTGTCGTTCGGTCGACGCTGCCTATCCTTCCTTT
CTCCCTTCTCCC
+
JHH9A>A@BCC@CCBB>BBBB>;9=667.:@@CC>CBC>@898;@@BBB@>@A@BA@@@>>@7744777:=::344-
4444-474444,
@T6XXI:00078:00702
GGGAGAAGGGAGAAAGGAAGGAGAAGTCGCCCCAAACGATTTGTTGACTATTCTCCCTCTCCCTATAGTGAGTCGTA
TTAGAATTCCCCC
+
4=:49<@CC>B@@>9?6848=???;<=>8888.33,111666644...--
0/056;:8885432/..359>@@=899=<6643300000.
@T6XXI:00078:00705
GGGAGAAGGGAGAAAGGAAGCTAGTCGACTCGACATCTGTGGCTTCACAGTACAGAGGGAAAGCCGCAGATCTCGAG
C
+
>9544;<AB?<<=DAD?C?AA<:00.33../>?<?A@;;8A<?A>ABB???>;;;444-4=-
489<<>?CBAAD@B==
@T6XXI:00078:00707
GGGGAATTCTAATACGACTCACTATAGGGAGAGGGAGAATAGTCAACAAATCGTTTGGGGCGACTTCTCCTTCCTTT
CTCCCTTCATCC
+
B:@4=59248888=@@@AAB@???@>@@7::A@B>C@A;==AA=A>A<<<?????8;795??AAA576830/4344-
3111014/--11
@T6XXI:00078:00709
GGGGAATTCTAATACGACTCACTATAGGGAGAGGGAGAATAGCCGATAAAACTTCGCAGTCGTCCCATTCTTCCTTT
CTCCCTCTCCC
+
FNH9B;;8;AA>BDCBACCCBAACCCCC>B<>AE>A?::?=AA<??@@CC4?@?B?===A@999A<74374347761
6///,/.-..+
@T6XXI:00078:00712
GGGAGAAGGGAGAAAGGAAGGGGAGAAGGGAGAAAGGAAGCTAGTCGACTCGACATCTGTGGCTTCACAGTACAGAG
GACTGTCCAAAGCG
+
AA=AA@<>>:999@09684;BB9CCC?BB<??8>:A>B?BB?B??:::A:=;;;/0//4495==;?@@@888@<<;7
788,,,,5550+++
@T6XXI:00078:00730
GGGGAATTCTAATACGACTCACTATAGGGAGAGGGAGAATAGTCAACAAATCGTTTGGGGCGTCTCCTCCT
+
>BB4@6924;;9?BBCCBBBA?@?AABD>BBAAA=AAA:==?>=B387<5:7:AA7;66,71....,.-,-
@T6XXI:00078:00737
```

```
CTAGTCACTCGAGATCTGCGGCTTTGGACAGTCCCTCTGTACTGTGAAGCCACAGATGTCGAGTCGACTAGCTTCCT
TTCCT
+
A99444449;@==9;;;98<<88757<@ACB@@A;?@@FAAA@988A>AGFCCBCCCBCBCBBBCC;;;;6..33,.
.+.,+
@T6XXI:00078:00739
GGGGAATTCTAATACGACTCACTATAGGGAGAGGGAGAATAGTCAACAAATCGTTTGGGGCGACTTCTCCTTCCTTT
CTCCCTTCTCCC
+
AAA7A>B@CCB@CBBBBBBBB@BDA<:<;<<<CE>C??=?AAA:=;<<<;?B@;>1616,7>=?A??@=>A>@>AA=
???@<@>@@@A=
@T6XXI:00078:00742
CGAGATCTGCGGCTTTGGACAGTCCCTCTGTACTGTGAAGCCACAGATGTCGAGTCGACTAGCTTCCTTTCTCCCTT
CTCCC
+
EBBBBBABBDBA>>><><@@CCAAA=ADCCBCC@B>B?=?A?A=99=??@@AABBBCAA@AA<<;=BKEACCCB=B?
AACCA
@T6XXI:00078:00743
GGGAGAAGGGAGAAAGGAAGCGTAGTCGACTCGACATCTGTGGCTTCACAGTACAGAGGGACTGACCAAAGCCGCAG
ATCTCGAGTCGACTAG
+
44-
4:>;>>194?@<@>A>A:9:@A?=:99???:::AA:::C:?A?B:;:A?<<<@;;;489:8<;64>64<>=EGBCDB
@@@AABBCCCA?0
@T6XXI:00079:00702
CTAGTCACTCGAGATCTGCGGCTTTGGACAGTCCCTCTGTACTGTGAAGCCACAGATGTCGAGTCGACTAGCTTCCT
TTCTCCCTTCTCCC
+
C=49;@99=BECCBCBB@>?>?BB<B;;;>>>?>;?<>?@@>?===A=?B@BBC@@@@C>@BCCBBAAABDDC?B?B
D>B??A=A;>>@C<
@T6XXI:00079:00705
GGGAGAAGGGAGAAAGGAAGGACACAGTACGAAGGGCGGATAGAAAGCTATTCTCCCTCTCCCTATAGTGAGTCGTA
TTAGAATTCCCC
+
BDCCDDACB>?@BC<>=@?C?CCC@????B??=@C?CC@BBBBCC>AACCC@CCCC@<<<@A<?@@@DDCCCFAA??
?=;=;:4/378-
@T6XXI:00079:00706
GGGAGAAGGGAGAAAGGAAGGAGAAGTCGCCCCAAACGATTTGTTGACTATTCTCCCTCTCCCTATAGTGAGTCGTA
TTAGAATTCCCC
+
CC@BCCACH>BBBB?C?B?B@CCC@CAB<;77,77/:BCCC>@C?BBBA>A=@@??;???AA<@??C:;<<==<???
@?AAA;A28880
@T6XXI:00079:00733
GGGGAATTCTAATACGACTCACTATAGGGAGAGGGAGAATAGTCAACAAATCGTTTGGGGCGACTTCTCCTTCCTTT
CTCCCTTCTCCC
+
EBC7B595<@@?ACBBBBBBCCEBBAAA<888>A>B@A>@@@@AA=@@A<CBBBBACFF7A@?77477;34/48;;7
>?:;;@>@@AA=
@T6XXI:00079:00737
CGAGATCTGCGGCTTTGGACAGTCCCTCTGTACTGTGAAGCCACAGATGTCGAGTCGACTACTCTTCCTT
+
CCBBDBB@?@C@BBB<B<>?ABAAC>CCCCA<ABCCC@=@@=CA==<<98@BABBBCB>>111-,*,*,*
@T6XXI:00079:00743
GGGAGAAGGGAGAAAGGAAGCCGG
+
9?69;@599199991/,0,..,,*
@T6XXI:00080:00704
```

```
GGGAGAAGGGAGAAAGGAAGGAGAAGCGGCCACATGTCCAAATTGCGCTATTCTCCCTCTCCTATAGTGAGTCGTAT
TAGAACTTCCCC
+
99:=AB@CG>BBBB@CCJ<<9;BB;70.3434...566389288:ABBDCB?A111,/,,,*,,,0339?AB7.///
,-132460111,
@T6XXI:00080:00711
CTAGTCGACTCGAGATCTGCGGCTTTGGACAGTCCCTCTGTACTGTGAAGCCACAGATGTCGAGTCGACTAGCTTCC
TTTCTCCCTTCTCCC
+
B--,,-
,,6::=CA@ACCCCC@A@A<A8:<CCCCB>AAAAB??????A?@C@BBCBABABBB???BB@@@BB??=?>AB>BB@
@;@9==@C=
@T6XXI:00080:00748
CTAGTCGACTCGAGATCTGCGGCTTTGGACAGTCTCTCTGTACTGTGAAGCCACAGATGTCGAGTCGACTAGCTTCC
TTTCTCCCTTCTCCC
+
B999@9449==>BCCBB@@@@?A@C<@=@?====>>>:77;>888>@A<@@>=<<888====888@@==;;;;<8;;
?B<=<<C==7/..40
@T6XXI:00081:00709
GGGAGAAGGGAGAAAGGAAGGAGAAGTCGCCCCAAACGATTTGTTGACTATTCTCCCTCTCCCTATAGTGAGTCGTA
TTAGAATTCCCCCAATGAATTCCC
+
CH@ACA>BB>@@@C>B@B?B?ACN@CA@ABEE8@@<?B@@A=BB?CCCAAA>>>?CAA<<=CA?===@@@AA???@A
A>@AB9;/4444,476,,*+*+0*
@T6XXI:00081:00719
GGGAGAAGGGAGAAAGGAAGGAGAAGATACTGACGTGTGTGTGCCAGCTATTCTCCCTCTCCCTATAGTGAGTCGTA
TAGATTCCCC
+
:@:>99699-4448-
42313999<;76:::779>999783711104:AA>A?BCCC>BAAAD=@@C;::::@<0000,+++,23636
@T6XXI:00081:00720
GGGGAATTCTAATACGACTCACTATAGGGAGAGGGAGAATAGCCGTCAACAAATCTGGGGTCTATTCTGCTTCCTTT
CTCCCTTCTCCC
+
DHIAC<:<<BB@CBBBCCCC?@B>B666.7<<@C>B@C?@@@@;?@AC@CCC>CCCCCC9CCCCC@CBCCC@C=@>>
@CBE>C:?:77.
@T6XXI:00081:00722
GGGGAATTCTAATACGACTCACTATAGGGAGAGGA
+
;D>6>-9544499A=B?B;;8>776111,1663,.
@T6XXI:00081:00734
GGGAGAAGGGAGAAAGGAAGCTAGTCGACTCGACATCTGTGGCTTCACAGTACAGAGGGACTGTCCAAAGCCGCAGA
TCTCGAGTCGACTAGCTTCCTTTCTCCCTTCTCCC
+
99049969B:@999094843?80/7,,,,,,0,....1663276:????DBA@??844-
4448@??AH?@@<??A@A@???@@=<<@@889@>;9>38858B;B7===?@B<
@T6XXI:00081:00745
ACTCGAGATCTGCGGCTTTGGACAGTCCCTCTGTACTGTGAAGCCACAAGATGTCGAGTCGACTAGCTTCCTTTCTC
CCTTCTCCC
+
CCCCCCBBBBBBBB@BBB=A@BB9<<CCCDECFCCCBCBB>B;65:11-
1:1...7797:9@@@CBBC@C@CB99<B@<A>@@@E?
@T6XXI:00082:00713
GGGGAATTCTAATACGACTCACTATAGGGAGAGGGAGAATAGATTGGCGAGAAATGCCGTCTAGTGCTCCTTCCTTT
CTCCCTTCTCCC
+
```

;;;,424=>@ABBBCCCBCCC?<<<777.7>@AB>B??<?A9::?D?==?444-48655./--------,-
330133..3;485:8<<3
@T6XXI:00082:00747
GGGAGAAGGAGAAAGGAAGGAGAAGTCGCCCCAAACGAGTTTGTTGACTACTT
+
996///,/,,,02*,*0666499911377775;?>C?:?CA;:::811000-,
@T6XXI:00083:00702
CTAGTCGACTCGAGGATCTGCGGCTTGACATGGT
+
>//11111AD@>?A=@@@>>>>401,00.,,,*,
@T6XXI:00083:00711
GGGAGAAGGGAGAAAGGAAGATGAGGTTTGCGCTCTATATCGGGAGTCTATTCTCCCTCTCCCTATAGTGAGTCGTA
TTAGAATTCCCCC
+
@=-949598-48??<@?B?AAAA7747>:>@@?<<:<;887=A;<843>89466661./--
333./5<<:<==558@@<??@::566<=1
@T6XXI:00083:00716
AGTCGACTCGAGATCTGCGGCTTTGGACAGTCCCTCTGTACTGTGAAGCCACAGATGTCGAGCGACTCA
+
9=D<94===?>@A?73799<=BB?B@CBBB@@@:?A??AAA@?A??<?C=??C@=8:///--2--++++
@T6XXI:00083:00730
GGGAGAAGGGAGAAAGGAAGGAGAAAGACACAGCATGGAACTGCTTGGCTATTCTCCCTCTCCCTATAGTGAGTCGT
ATTACGAATTCCCC
+
44-
49:<BE?BCCH?B;<9;@B??@<?788:::?;BD<<:<BCAC?A6;<@@=@<<;80009:6:;??CBBBB:77=773
744654,---*
@T6XXI:00083:00734
GGGAGAAGGGAGAAAGGAAGGAGAAGTCGCCCCAAACGATTTGTTGACTATTCTCCCTCTCCCTATAGTGAGTCGTA
TTAGAATTCCCCC
+
CEAEDC@CFACBB=:@7848=88;=?AACECC9BCACB??8866367>4472098=;@@@CD<A<==A@@@@A??=<
677/.,-00000.
@T6XXI:00083:00735
GGGAGAAGGGAGAAAGGAAGGGGATAGCAGCAGTCGGCAACGTGAAACTATTCTCCCTCTCCCTATAGTGAGTCGTA
TTAGAACTTCCCC
+
CD>GBA@CC?ABBC>B?B@BBB9CBB@@@CBBBBCB@DC@CCBCBC>BBBC@CBCC;:::==:;848AAAAA@<<=@
@>@>@>@;2444-
@T6XXI:00083:00736
GGGGAATTCTAATACGACTCACTATAGGGAGAGGGAGAATAGTCTTGCAATTTCCTGCGGTGCATTCTCCTTCCTTT
CTCCCTTCTCCC
+
BBB9B>@?BCB@CBBAABBBC@>;9616-
6=?AB>BAA?A<@@@@>AAC=BB>B@CCCC@CCABB@BBB=?;>;>?:?@BC9?:=777.
@T6XXI:00083:00740
GGGAGAAGGGAGAAAGGAAGGAGAAGTCGCCCCAAACGATTTGTTGACTATTCTCCCTCTCCCTATAGTGAGTCGTA
ATTAGAATTCCCC
+
CC@AACCCC99;@@9><?<?6:77;?AAACCC9BC>BAA>=899578>=AA>@??=7::434,49;;>@@@;667//
,/,--6<=<=;;4
@T6XXI:00083:00742
GGGAGAAGGGAGAAAGGAAGTGCAGTTCGCGGTTAGTGAAGGG
+
?C8=:A@CB=B;;<9:>A?AAACB;21110<,0,....6367,
@T6XXI:00083:00747

```
CTAGTCGACTCGAGATCTGCGGCTTTGGACAGTCCCTCTGTACTGTGAAGCCAAGATGTCGAGTCGACTAGCTTCCT
TTC
+
D@99<<9:?AAADCCCBBBBB@CBC?C>@@AA???;?CBCBBA=///85/9592443339737796111116601O1
626
@T6XXI:00083:00749
GGGGAATTCTAATACGACTCACTATAGGGAGAGGGAGAATAGACACCAGTCCCATCAGCCACAATTATTCTTCCTTT
CTCCCTTCTCCC
+
ECB6@8<7<@A>BBC<<838<=816111,16679;><<;<@@<==B344444-
3449@@<AA<<?<?>;==3837?4=?<=;?5=3731
@T6XXI:00084:00711
GGGAGAAGGGAGAAAGGAAGGAGAACA
+
9A:;9<58@:949904231313666//
@T6XXI:00084:00734
GGGAGAAGGGAGAAAGGAAGGAAACACGCGCGACGAATAGGTGGAAACTATTCTCCCTCTCCCTATAGTGAGTCGTA
TTAGAATTCCCC
+
DCACCD@DC>BBBB>@?@@B@BB<>>@DCCC;<=BB>BBC@CC=@@<@?<>;>:::5:@??A<A@@<<<ABBBA@@?
?<?AA>@=@CB5
@T6XXI:00084:00737
GGGGAATTCTAATACGACTCACTATACGTCTGAGCATCGATCGATGTACAGCTACGTACCGTCT
+
/11+1,959@?<@BB<;;CAAAE@??@:::AA@C???<<68<99?>A?<3333/--1./,--++
@T6XXI:00084:00741
GGGGAATTCTAATACGACTCACTATAGGGAGAGGGAGAATAGGCCGACG
+
=;;,924899979;AB@?@@@?@@:::A<A???C8;7=;;4/,/76---
@T6XXI:00084:00744
GGGAGAAGGGAGAAAGGAAGGAAACTGCGCGCGCAAGTCAGCTGGGAGCTATTCTCCCTCTCCCTATAGTGAGTCGT
ATTAGAATTCCCC
+
;<69@BAFC@BB?B>B@B>A=@@<???BCBCC;::=??>>=899919>>AAB>AA@@:999>?:@AABCBA???@7:
::=@7>7:::BB6
@T6XXI:00085:00702
GGGAGAAGGGAGAAAGGAAGCTAGTCGACTCGACATCTGTGGCTTCACAGTACAGAGGGACTGTCCAAAGCCGCAGA
TCTCGAGTCGAC
+
94-49>;>A<AABBAC@C@BB?<<776//000,,,,/,22304756;AB?@<::;444-
4;>@AA?BB=@@;?<;;8783366866300
@T6XXI:00085:00711
GGGAGAAGGGAGAAAGGAAGTGCAGTTCGCGGTTAGTGAAGGGAAAAACTATTCTCCCTACTCCCTATAGTGAGTCG
TATTCAGAATTCCCC
+
99-944244-448:-
4231377==?>@;;7@5310;00.,..+.555/:;;9961172666977178:8<:6663330388:<<=<<<=BA5
@T6XXI:00085:00728
GGGAGAAGGGAGAAAGGAAGAGCACAGAGATGACTGGGAGACGACAGCTATTCTCCCTCCTCCCTATAGTGAGTCGT
ATTAGAATTCCCC
+
DC@CCBABCABBBB>C?B@BBBBBABCCC?A@AC;;;;;BBBCBBBCCCCC>A>::6/.,/8724>ACBB?A=A@@@
??<A@;:?4777.
@T6XXI:00085:00729
GGGAGAAGGGAGAAAGGAAGCTAGTCGACTCGACATCTGTGGCTTCACAGTACAGAGGACTGTCCAAAGCCGCAGAT
CTCGAGTCGACTAG
+
```

990<99@@C9>999084848>8..5......./66;>966320106>>ABB=?>B??<?@?===37858A>A@@@A@
9:9@;;;/../-+-
@T6XXI:00085:00730
GGGAGAAGGGAGAAAGGAAGGGGAGAAGGGAGAAAGGAAGGGGAGAAGGGAGAAAGGAAGCTAGTCGACTCGACATC
TGTCGGCTTCACAG
+
4:-44><@@<;;<B?CADABBB2888577-
7711,136367758<6:?C9988C@C>B67761133016?003<837=B403204/00033
@T6XXI:00085:00734
GGGAGAAGGGAGAAAGGAAGA
+
490449599-44<A,1,/+-,
@T6XXI:00085:00736
GGGAGAAGGGAGAAAGGAAGCTAGTCGACTTGACATCCATC
+
BE>BBB@CC@AAAC>@?@BBBB?B<A==6747831160...
@T6XXI:00085:00748
GGGGAATTCTAATACGACTCACTATAGGAGAGGGAGAATAGTCAACAAAATCGTTT
+
4;;,4249:99549997>888/..3..,.337>;>8<53;8///,//77+67111,
@T6XXI:00086:00703
GGGAGAAGGGAGAAAGGAAGCTAGTCACTCGACATCTGTGGCTTCACAGTACAGAGGGACTGTCCAAAGCCGCAGAT
CTCGAGTCGACTAG
+
BB>BCC@BB>CCCH?C@@=@C@:?B:<:CCC:;8:91?773:@=@CCC???B;8334-
499:<<>@@<AA>@@@@??===?@@A@@@AA==
@T6XXI:00086:00712
CATTCTAATACGACTCACTATAGGAGAGGGAGAATAGTCAACAAATCGTTTGGGGCGACTTCTCCTTCCTTTCTCCC
TTCTCCC
+
CBB@@BB@BA??BBBB?:997/.,0>07::;CC>A=AA@@:;==;A>>>@;@@?8@@A@@?>BB:<8<8>?<?@AC<
A@@@@@<
@T6XXI:00086:00746
GGGGAATTCTAAACTACG
+
>=92><888---*,,**,
@T6XXI:00087:00717
GGGGAATTCTAATACGACTCACTATAGGGAGAGGGAGAATAGATCACCGTTGGTTCGGGTGCGCGTTCTCCTTCCTT
TCTCCCTTCTCCC
+
CCCAF::99BA>BBBBA?@@CB>;<777.777775:AA?DA???AC?;??=?=?@CCC>CAAACCC@CFCAA?A?AB
:?88718=<==A?
@T6XXI:00087:00734
GGGGAATTCTAATACGACTCACTATAGGGAGAGGGAGAATAGCCGATTCCTGGCTGACCCGCAATTTCTCCTTCCTT
TCTCCCTTCTCCC
+
BCC:B89;;BBCCBAA???BB@>;?1165:??CC>C@A?<=<A?@@@>A>@C?B@BAAA<@??;=?:;;;8::;247
17>9;;@=???D=
@T6XXI:00087:00738
GGGAGAAGGGAGAAAGGAAGGGGAGAAGGTAGAAAGGAAGCTAGT
+
A?<@A>29;19>>>9@<>5888.8>?=?;=888808110111111
@T6XXI:00087:00740
GGGAGAAGGGAGAAAGGAAGCTAGTCGACTCGAGATCTGCGGCTTTGGACAGTCCCTCTGTACTGTGAAGCCACAGA
TGTCGAGTCGACTAGCTTCCTTTCTCCCTTCTCCC
+

```
99194958;-
949918<>=@@????::7@@@:;;CCFBBBF????9??BBBBCAA=>@@A@A9==????@@>@@@AAA===@@?=>>
;;;=84488,//,/888,/,/2799
@T6XXI:00087:00742
GGGGAATTCTACGACTCACTATAGGGAGAGGGAGAATAGTCAACAAATCGTTTGGGGCGACTTCTCCTTCCTTTCTC
CCTTCTCCC
+
CCB7A=@=ACCCCBA?>?A>>9666-
6=7?B>BCC?B88=CC@C@A<AAAAC<@@?5@====;=?@;?<<8??;???C;A=<>>CA
@T6XXI:00088:00703
GGGGAATTCTAATACGACTCACTATAGGGAGAGGGAGAATAGCTCCCTGTTGTGTGGCTGCAACATACCCTTCCTTT
CTCCCTTCTCCC
+
@@>6?>A>ABACCCC?BBBBBAB??6665:AACD?BBC?@<<<?@B>B@?=@===A>CCCCC?BBABBC8:37:<<6
?@AA>B?AA?@:
@T6XXI:00088:00711
GGGAGAAGGGAGAAAGGAAGGAGGAAGCGACGTCGGGGAAACGTTTGCTATTCTCCCTCTCCCTATAGTGAGTCGTA
TTAGAATTCCCC
+
BB=AAA@DC@BBBD?CBC@B@BC@C@>BBCBEBBBCC9CC?CCCC@CCCCC@ECCC>BBABB?BBAABBBCCF?@@=
=;=CD?C=@@@5
@T6XXI:00088:00717
GGGAGAAGGGAGAAAGGAAGCTAGCCGACTCGACATCTGTGGCTTCACAGTACAGAGGGACTGTCCAAAGCCGCAGA
TCTCGAGTCGACTAG
+
CDADCD@AA=?B??=B@B>BAAAAA>ACBBBB;::AAABCE?AB@C:;;CC??<?77718=?@AA>@@;@A@@?@AA
A888AAA@@A????@
@T6XXI:00088:00730
CTAGTCGACTCGAGATCTGCGGCTTTGGACAGTCCCTCTGTACTGTGAAGCCACAGATGTCGAGTCGACTAGCTTCC
TTTCTCCCTTCTCCC
+
B@1///1/:@A=?AAA@ABBCBBCD?D>@B@BACCBBBAABC@A?AAB@AC@CCECBAAACBCB:<<;889361887
;;;?:8;48/30384
@T6XXI:00088:00739
GGGAGAAGGGAGAAAGGAAGAAAGGAAGGAGAACACGCCGCGGGATATTGGCGAACTATTCTCCCTCTCCCTATAGT
GAGTCGTATTAGAAATTCCCC
+
DGAGCB@CC>BB??=B@B@8888;@C@C>A@@>?CC?<8;;886;;;<<A8B;968@@;:=734,4=;?B????AAA
A@???<83343000+044:=4
@T6XXI:00088:00740
GGGGAATTCTAATACGACTCACTATAGGGAGAGGGAGAATAGCTACGTTTAACCGATCGTGTTGTTCTCCTTCCTTT
CTCCCTTCTCCC
+
BB@,959599A?BBBCCCCC@@CC:9:?9???BD>C@C@@?98;:BBCC==99:;?>;;<7837?:@88385<:8;9
;:;;9;.//::6
@T6XXI:00088:00749
GGGGAATTCTAATACGACTCACTATAGGGAGAGGGAGAAATAGTCAACAAATCGTTTGGGGCGACTTCTCCTTCCTT
TCTCCCTTCCTCCC
+
999,454249@@BCCCCDGBBB;::@@@9ABB@>9>444+011---
0111+1177788882333..,.42724449=;=77431,-,...,
@T6XXI:00089:00702
GGGGAATTCTAATACGACTCACTATAGGGAGAGGGAGAATAGCTCTGACGGACTGCTCTGACTATTCACCTTCCTTT
CTCCCTTCTCCC
+
CBB7A=@=@BB@CCBBBBBBA?<97111.7<<BC>C@@>@@AACBB@BBB>ACB@@@BA?@@@:85;==:><?588/
8366,1011662
```

```
@T6XXI:00089:00709
CTAGTCGACTCGAGATCTGCGGCTTTGGACAGTCCCTCTGTACTGTGAAGCCACAGATGTCGAGTCGACTAGCTTCC
TTTCTCCCTTCCTCCC
+
B9949894ABAACECBGGCCB?AA><A?BBBCCCB>BBC@@???<??A??B@BCCBBB@??A???BB???@;:686:
<?9?47716,/,///,
@T6XXI:00089:00717
GGGAGAAGGGAGAAAGGAAGGAGAAGTCGCCCCAAACGATTTGTTGACTATTCTCCCTCTCCCTATAGTGAGTCGTA
TTCAGAATTCCCC
+
ECDBBB@CC@BBBB>C@C@B@BBB>>;>>BBB7@A=@A?BA=AC@CBB@AA>AAAA<@?@ACA?<<<AAAC@???//
/.0/8858,444-
@T6XXI:00089:00722
GGGGAATTCTAATACGACTCACTATAGGGAGAGGGAGAATAGTCAACAAATCGTTTGGGGCGACTTCTCCTTCC
+
CCC9?=@>99;>@@@GBAABB?<?=776.7?777-733/4AAC=;:ABA<CDCCC>CCB.71<710752,.,-,
@T6XXI:00089:00726
GGGAGAAGGGAGAAAGGAAGGAGAAGTCAGCCCAACGATT
+
CB>BBCABB=BBBC>A?@@B@B>917633339,10.3..,
@T6XXI:00089:00730
GGGGAATTCTAATACGACTCACTATAGGGAGAGGGAGAATAGATCACCGTTGGTTCGGGTGCGCGTCCTCCTTCCTT
TCTCCCTTCTCCC
+
@@>4@<@=ACC@CCBBBBBBB@<97111.7<<=?>AAB@AA??<=??;@B?B?A>@BA?BB;>=ACC>BB>C@C?BC
>@??>7>;788A<
@T6XXI:00090:00736
GGGAGAAGGGAGAAAGGAAGGGAATAGCAGCAGTCGGCAACGTGAAACTATTCTCCCTCTCCTAT
+
CI@AAC>A@<@@CC<@<?47808<@9374....557::@@CCBCBD<@<::==777+,,,+*+++
@T6XXI:00090:00743
GGGGAATTCTAATACGACTCACTATAGGGAGAGGAGAATAGCCAACCGAC
+
6;<1<-9594;<?BG<<<DBB6337111,11179<:<;4303*+*+*,,,
@T6XXI:00091:00711
GGGAGAAGGGAGAAAGGAAGGAGAATTTGGCGTCGTGACGTGCGTGGCTATTCTCCCTCTCCCTATAGTGAGTCGTA
TTAGAATTCCCC
+
@C;<;C?A@<@>>B<@=??A?GCB@CD>C>BBBBB??@CBBBEDDCEBGDE@CCCC?<<=DD@BDC@;::AA?@CC@
@>??>:>;??>4
@T6XXI:00091:00738
GGGGAATTCTAATACGACTCACTATAGGGAGAGGGAGAATAGACACCAATCGCGCCGGGGTCACTCTGAGCATCGAT
CGATGTACA
+
;AA1>,1,16679BCCCCCC?>@?=666.777BD>CBB@C998;;;3/,0:/..442244::::<<=;@>;161000
0----2216
@T6XXI:00091:00741
GGGAGAAGGGAGAAAGGAAAGGAGAAGTCGCCCCAAACGATTTGTTGACTATTCTCCCTCTCCCTATAGTGAGTCGT
ATTAGAATTCCCC
+
CCABBB@CD?BBCB>@510+0;;00,.46....*..+1111666=:?;:4AA>888@:887==79;778<7879/./
745=84433111.
@T6XXI:00092:00700
GGGAGAAGGGAGAAAGGAAGCTAGTCGACTCGAGATCTGCGGCTTTGGACAGTCCCTCTGTACTGTGAAGCCACAGA
TGTCGAGTCGACTAGCCTTCCTTTCTCCCTTCTCCC
+
```

```
==094><@@194>>9=4848:3737/......56@@@@EBB>:::7:@CBCBCAA<A@AA?????889>@A>A@@<=
=AA???A<<<844404//44014+.---+-011330
@T6XXI:00092:00723
GGGAGAAGGGAGAAAGGAAGCTAGTCACTCGACATCTGTGGCTTCACAGTAGGGACTGTCCAAAGCCGCAGATCTCG
AGTCGACTAG
+
AB?AAB@CB>A@>=9><@=ACCCBAA@711111099999?::;7;BA@@@?;;6<ABCBA@CCCDC@AAABDBBBDB
?=>CB==>CB
@T6XXI:00092:00743
GGGAGAAGGGAGAAAGGAAGGAGAAAGCGACGTCGGGGAAACGTTTGCTATTCTCCCTCTCCCTATAGTGAGTCGTA
TTAGAACCTTCCCCC
+
66+/49:;;-
849>9>;>;<97339:679;9976166,4;6?ACB@CCB@>331:::110<<:;7669888=<<?A57---
3,.,.,....*
@T6XXI:00093:00742
GGGGAATTCTAATACGACTCACTATAGGGAGAGGAGATAGAAG
+
>>80>;A?BCI@CBBBAABBB@CCA@@@<<<5567++,***)+
@T6XXI:00093:00743
GGGGAATTCTAATACGACTCACTATAGGGAGAGGGAGAATAGTCTAACGTTTTGCACAGTTGGTATCTCCTTCCTTT
CTCCCTTCTCCC
+
CCEBF>A?ACGBBBBCCCCBBB@?7166.7==>@=AAA>AAAA8==>A=<@@5===@@@@>A>??@A??:;8:244+
0000+4;==AA<
@T6XXI:00093:00745
GGGGAATTCTAATACGACTCACTATAGGGAGAGGGAGAATAGCTATCGTCACTTGACATGGTGCTTCTCCTTCCTTT
CTCCCTTCTCCC
+
BBA7A89;=CC@BCBBABCCCCCA?9::.7>>@A=B@@>AAA@BBBBA===?A>@AAA@A<??AB<88<899;/40+
3444+4989<?:
@T6XXI:00093:00746
GGGAGAAGGGAGAAAGGAAGTAAGCCGGGATGCAGGATAGTGGTGAGCTATTCTCCCTCTCCCTATAGTGAGTCGTA
TTAGAATTCCCC
+
CC>BBC@CC?BAAA=B?A@BBB@CC@CC>CCB@@@@@BBBCC@A=9===BAB>9990999BBAAAACACCEBED@AA
@@BBD?D?DELC
@T6XXI:00094:00720
GGGGAATCTAATACGACTCACTATAGGGAGAGGAGA
+
:=>0>-1///76449;@>BCB@@?777.0000,/,,
@T6XXI:00094:00723
GGGGAATTCTAATACGACTCACTATAGGGAGAGGGAGAATAGTTTTCCCTTCACTAACCGCGAACTGCACTTCCTTT
CTCCCTTCTCCC
+
>=92>695<AA@BBBBABCBBBBB?88866;;??<A@@??=8778-
4;6<:=CCCE?B??@@A>?@@?BA=;=377.7;;B<A>@=<=7
@T6XXI:00094:00739
GGGGAATTCTAATACGACTCACTATAGGGAGAGGGAGAATAGACTACCGTGCT
+
@>>4><@>BCC@CCBBAAAB??>BB916.7<<<?<AAA>@988//.-,++0+-
@T6XXI:00094:00747
GCTAGTCGACTCGAGATCTGCGGCTTTGGACAGTCCCTCTGTACTGTGAAGCCACAGATGTCGAGTCGACTAGCTTC
CTTTCTCCCTCTCCC
+
////:C994@?@9<<9==@@@B@@?@<@=@@CCCDEB@@@@BCBCE===;<C@CDA>>883116768=377<76177
0./+..55+---110
```

```
@T6XXI:00095:00709
GGGGAATTCTAATAAGACTCACTATAGGGAGAGGGAGAATAGTGACGTGTTGTGCGGCGCTCACTGTCTCCTTCCTT
TCTCCCTTCTCCC
+
CCC7D:<<=CECDB@A>>B>?ABBB66656==?B>BBD@A;???AA;AAA?===AA>A???AAAA@@?@@8979/33
-4;;=1838==;7
@T6XXI:00095:00716
GGGAGAAGGGAGAAAGGAAGGG
+
9@:999599-/68;,1,.,00.
@T6XXI:00096:00716
TCTGCGGCTTTGGACAGTCCCTCTGTACTGTGAAGCCACAGATGTCGAGTCGACTAGCTTCCTTTCTCCCTTCCTCC
C
+
:??4498884-
4244?BABD>DBBCAB@@=86621?7;8??@:;;CAAA@AAB@?;977>@@?C=B:7:67,/,/882
@T6XXI:00096:00724
GGGGAATTCTAATACGACTCACTATAGGGAGAGGGAGAATAGCTTATCCCGTACATT
+
88707+-*,,,*,/6/33><;9@<///7-//===91/4?A?98;9:,,,*+++++0*
@T6XXI:00096:00739
GGGGAATTCTAATACGACTCACTATAGGGAGAGAG
+
BCC7B7;699>594>983;>;8;7;111,7=::0/
@T6XXI:00097:00701
GGGAGAGAATTTGGTAATGGGAATCACAAGAGGGAACGCAGAACCAAATTCGCCCTTATATCTCTCTCCCTATAGTG
AGTCGTATTAGAAT
+
EC?AAAA@@CDCB?BB@DDC>C?BBABB?ACCD>C@?????B=@;AA:><;444-
49==??@?>8:9AA?CBBCAC??<9.....,.-2,.
@T6XXI:00098:00735
GGGGAATTCTAATACGACTCACTATAGGGAGAGGGAGAATAGTCAACAAATCGTTTGGGGCGACTACTCCTC
+
BBA7A=@=@CC?BBBBBCCCCDCB?996-6<<@B>BAC>A@???@?BC;:>99<C>CCC8>777,,,**)**
@T6XXI:00098:00736
CTAGTCGACTCGAGATCTGCGGCTTGACAG
+
44444444444444>:>96/0,..,..6..
@T6XXI:00099:00709
GGGGAATTCTAATACGACTCACTATAGGGAGAGGG
+
CEC:C>@=@AA?@@@@AAABCB?=7///,00,,,*
@T6XXI:00099:00719
GGGGAATTCTAATACGACTCACTATAGGGAGAGGGAGAATAGTCAACAAATCGTTTGGGGCGACTTCTCCTTCCTTT
CTCCCTTCTCCC
+
DDC:C9:<;CDBCBA?>>??>@A9>661-
6==AB>BAA?A===@@?@AA<CAAAC>@CC6AABBB?BAA;=:=>AA<@@@B=A9;<AC>
@T6XXI:00099:00730
GCTATCACTCGAGATCTGCGGCTTGGACAGTCCCTCTGTACTGTGAAGCCACAGATGTCGAGTCGACCTAGCTTC
+
/19;999=444=@=8?@?00,00,.,...449@;???::93<<6==<8?=?<@@A><=?@?96;;.0,04.,,*.
@T6XXI:00099:00735
GGGAGAAGGGAGAAAGGAAGCTAGTCGACTCGGCATCTGTGGCTTCACAGTACAGAGGGACTGTCCAAAGCCGCAGA
TCTCGAGTCGACTAG
+
```

```
CC>CCBABB;BCCH::;:@CCB?@B8;;CCCC=@@ABBBBB@A@>@CCBBBAA?A77714;;;7869D?BA=@@@AA
@@@@@?@@???@???
@T6XXI:00099:00736
TGATCGAGATCTGCGGCTTTGGACAGTCCCTCTGTACTGTGAAGCCACAGATGTCGAGTCGACTAGCTTCCTTTCTC
CCTTCTCCC
+
@GCCB@AACBEBBCC@CCC>C@ECBBBBC>BBABBBCC@@?C@CC>ABCCCA:::B???BAA@@@:78;>488088>
>6=:====6
@T6XXI:00050:00763
GGGAGAAGGGAGAAAGGAAGCTAGTCGACTCGACATCTGTGGCTTCACAGTACAGAGGGACTGTCCAAAGCCGCAGA
TCTCGAGTCGAC
+
CDADAB?A=6:994-;884:C87:?:::CCCB;;;AA@>7438:47BBBBB@>;;300-
499:>@>AA<AA>@AAAA?<@@858@??>@
@T6XXI:00050:00777
GGGAGAAGGGAGAAAGGAAGCTAGCGACTCGACATCTGTGGCTTCACAGTACAGAGGACTGTCCAAGC
+
ED@AAABCC:>99919<?<@<;:CB::;CBFAA@@::99?9AC@BAABBB@B>>5558@7000+*)+*
@T6XXI:00050:00783
GGGAGAAGGGAGAAAGGAAGACGTGAGTCGA
+
BB>BCC?BB=BB>@;>;=>---,,,,43331
@T6XXI:00050:00786
CTAGTCGACTCGAGATCTGCGGCTTTGGACAGTCCCTCTGTACTGTGAAGCCACAGATGTCGAGTCGACTAGCTTCC
TTTCTCCCTTCTCCC
+
D44949444:@=CBBBBDBCC?CBB>B=??CBBAB>CCA@>>>@@<<=;<B@BABBBCBB@@@BCBABCCCBBB?B@
CC>BB@C;@=<@AC;
@T6XXI:00050:00791
TCGAGATCTGCGGCTTTGGACAGTCCCTTGT
+
@DBBBBCCCCCB@BCD@C?BA=://0,0,..
@T6XXI:00051:00754
GGGGAATTCTAATACGACTCACTATAGGGAGAGGGAGAATAGCGCAATTTGGACATGTGGCCGCTTCTCCTTCCTTT
CTCCCTTCTCCC
+
BBB9B?A?ABB@CCCCCABBC@BAA99659A?CE>A>==AAAAA@B>AC>C????CBB@=B=BAC>998;=:=;=@:
??@@<A>AA@B;
@T6XXI:00051:00768
GGGGAATTCTAATACGACTCACTATAGGAGAGGGAGAATAGTTTTTCCCTTCACTAACCGCGAACTGCACTTCCTTT
CTCCCTTCTCCC
+
CCC:DBB@A@@8;;;<@;:;BA999162688;>;>=<85300000+04+0/39888>?@??:83444/:00,.,..+
.13303/0000+
@T6XXI:00051:00770
GGGAGAAGGGAGAAAGGAAGCTAGTCACTCGAACATCTGTGGCTTCACAGTACAGAGGGACTGTCCAAAGCCGCAGA
TCTCGAGTCGACTAG
+
CCACAA99919>??9>5868?877>7//....,.-5556>A77;?CA@AABB=;;488-
4;;<<=?@D<@?=??888?@@@AAAA@@@=<<<
@T6XXI:00051:00778
GGGAGAAGGGAGAAAGGAAGCTAGTCACTCGACATCTGTGGCTTCACAGTACAGAGGACTGTCCAAAGCCGCAGATC
TCGAGTCGACTAG
+
DCADCC>BC><;;B<A=@?AACBBCB@@>?>10/;5/0/B5:9@BBBCCCA::;//,/677/838C<A@<A@@@@AA
@@===@@@?@??=
@T6XXI:00051:00784
```

```
GGGAGAAGGGAGAAAGGAAGGAGAAGTCGCCCCAAACGATTTGTTGACTATTCTCCCTCTCCCTATAGTGAGTCGTA
TTAGAATTCCCC
+
9@<?>@;>>1948@9>;>7>1333999;@AA@8AD>@A?<:8:?<AACAAA=?@AD?@@@@D9<=@@=<=@@888??
?>>AA;A=AAA6
@T6XXI:00051:00787
GGGGAATTCTAATACGACTCACTATAGGGAGAGGGAGAATAGCTACCAAATTTCTCTCCTCACGTGACCTTCCTTTC
TCCCTTCTCCC
+
BCC:B@CACCCACCBBBCC???@>>7665:?<CE@A>?>@A99;BBABC>AC9=>;;;9///87=8@=:::9/7717
39A<A?@@AA<
@T6XXI:00051:00793
GGGGAATTCTAATACGACTCACTATAGGGAGAGGGAGAATAGTTTTTCCCTTCACTAACCGCGAACTGCACTTCCTT
TCTCCCTTCTCCC
+
CDC9B>B@CBBAB???ABCB??BCC===;=><CA>B>@??>>>AAA1AA:;:;AAB@>A>A>?@?@@A8::A>A=BB
>B@@A<A?????:
@T6XXI:00051:00794
GGGAGAAGGGAGAAAGGAAGGTGAACAGGTAACGGAGGGTGCGTTAACTATTCTCCCTCTCCCTATAGTGAGTCGTA
TTAGAATTCCCC
+
CHACCEABC>?@CCBB@?>B@BBB@BBC@BB@CB@BBB>?@@BB@C=ABCC@CCCC>CCCCC>C@CC???CDA?@?@
@@AAA==:@@A5
@T6XXI:00052:00757
GGGAGAAGGGAGAAAGGAAGTAGAATAGCACGTTGAAGATGTCGGGACTATTCTCCCTCTCCCTATAGTGAGTCGTA
TTAGAATTCCCC
+
99194>599-
84@@8;;<@BCDCA?ABBBCCAABDDBBB@>999909?B?@>A@CC?CCA@@<@CCCDAAABB@?A;;58>?4;9;D
D8
@T6XXI:00052:00759
GGGGAATTCTAATACGACTCACTATAGGGAGAGGGAGAATAGTCACATTCATGTTTGTGACCGTGTCTCCTTCCTTT
CTCCCTCTCCC
+
999,49::=BC;:;9:7;;;76>>7777,1134533..,-
33135556799677?886855/338888<5323/00+044439554=:
@T6XXI:00052:00771
GGGAGAAGGGAGAAAGGAAGCTAGTCGACTCGACATCTGTGGCTTCACAGTACAGAGGGACTGTCCAAAGCCGCAGA
TCTCGAGTCGACTAG
+
@A<>@?5880999909685;@800@/00>><<0;:::6CCB>@A?CC@?>?==;933317<?AAA=?A:??=AA<<<
==;;;;<<<@@=A8>
@T6XXI:00052:00775
CTAGTCGACTCGAGATCTGCGGCTTTGGACAGTCCCTCTGTACTGTGAAGCCACAGATGTCGAGTCGACTAGCTTCC
TTTCTCCCTTCTCCC
+
EB999<9:?ABCCBBBCCDBB?BBB>B?AACCCCB>BBCCCCCAA?AA>AC@E@A@BDBACCCCBCCCCDAAAA>AA
BE?AA@A7=3438:4
@T6XXI:00052:00796
GGGGAATTCTAATACGACTCACTATAGGGAGAGGGAGAATAGGGCTTGCATCCCCGAGTCGTGTACCTATTCTTCCT
TTCTCCCTTCTCCC
+
@@>6@<@?CBB@CBBBA@@B@CB>866659??CC>CBB>B;;;:<?>@<9====6?@BABBCCCCC@@:98:=;>:?
?6<<<<6<;>7481
@T6XXI:00053:00774
GGGAGAAGGGAGAAAGGAAGGAGAAGTCGCCCCAAACGATTTGTTGACTATTCTCCCTCTCCCTATAGTGAGTCGTA
TTAGAATTCCCCC
```

```
+
DFACBC@CB>BCBB>B@B=@=<AA?BCCCCCC7BB>BBBBC?@@>@BDCAB>BAAA=A???>7==>?@@@A@A@@@;
;8530/021146.
@T6XXI:00053:00777
GGGGAATTCTAATACGACTCACTATAGGGAGAGGGAGAATAGCTTCGTTCTTACTGGCGGGCTTATCTCCTTCCTTT
CTCCCTTCTCCC
+
CCC9C@A>B@@=AB@@@CCCBBB@A766.:==BB>C@@>@A??A?B::;;A?CCCC@B@@<@C;?:88837=A=@@;
?<@@;@9;<=>7
@T6XXI:00053:00787
GGGGAATTCTAATACGACTCACTATAGGGAGAGGGAGAATAGTTGACAATC
+
DCE9C>A=ABB>AB<;;ABCAAA@F:::8:@ACC=A?A?=;43/000-,.3
@T6XXI:00053:00796
GGGGAATTCTAATACGACTCACTATAGGGAGAGGGAGAATAGTCAACAAATCGTTTGGGGCGACTTCTCCTTCCTTT
CTCCCTTCTCCC
+
ECF:C?AAACBABB@@?CCBB@BBB?====CC?>=>AA<>9:<==>???<BCCCB>BBB8B?AAC?::83737>??<
@@@B<B>@ABA=
@T6XXI:00054:00755
CTAGTCGACCTCGAGATCTGCGGCTTTGGACAGTCCCTCTGTACTGTGAAGCCACAGATGTCGAGTCGATTAGCTTC
CTTTCCTCCCTTCTCCC
+
CBA@@<@--
*,,,2228BDDBC@BBB?BABDCCCCC>CC@@@@?AA;964/1,/889;?BBBACDA>>00,0.,,33255+.,.33
34/54664
@T6XXI:00054:00756
GGGAGAAGGGAGAAAGGAAGCTAGTCACTCGACATCTGTGGCTTCAAGTACAGAGGGACTGTCCAAAGCCGCAGATC
TCGAGTCGACTAGCTTCCTTTCTCCCTTCTCCC
+
A@<ACCACC?A;99095858@A@@==/,,,,,,+0255.1013=AA?BCB@>;:84-
4?B?@<>CC;BB?CCBB@AAAA9;;CBC884;6478656=;??@A7=:=7341
@T6XXI:00054:00757
GGGAGAAGGAGAAAGGAAGGACG
+
491//8,/,////+/,3131333
@T6XXI:00054:00782
GGGAGAAGGGAGAAAGGAAGCTAGTCGACTCGAGCATCTGTGCGTTTGACAGT
+
AA>ACB@CC=B@@@:@@A=@@@>;=0/0::900.6177>755<;664644+00
@T6XXI:00054:00783
GGGAGAAGGGAGAAAGGAAGGGAGAGTCG
+
BC>CCC@CA:=@==5>;><;=8;80****
@T6XXI:00054:00786
GGGAGAAGGGAGAAAGGAAGCTAGTCACTCGACATCTGTGGCTTCACAGTACAGAGGGACTGTCCAAAGCCGCAGAT
CTCGAGTCGACTAG
+
DC@CFDACC>B@@@<@>A?;;<BBAA>=;;000-
57///A>?@@CCCB@BCAA????;?@@@@A>@@;<<9===AA@AAA?????@><<<?
@T6XXI:00054:00792
GGGGAATTCTAATACGACTCACTATAGGGAGAGGGAGAATAGTTAGCGTTTTCTGACCCCGACATTTCTCCTTCCTT
TCTCCCTTCTCCCCTTCCTTTCTCCCTTCTCCC
+
CCC:CC?>ACC@CBAAABCC=??==666.7>@CE>C@@=@===<>9==AAA5@@ABAA@5@@A===9=@@>@>A9<<
7<?=@:@9;4444-4:;8=?6<;@?:@=888A7
@T6XXI:00054:00794
```

GGGAGAAGGGAGAAAGGAAGGAGAAGTCGCCCCAAACGATTTGTTGACTATTCTCCCTCTCCCTATAGTGAGTCGTA
TTAGAATTCCCC
+
490444244-444;-
427:777;;?::;BBCG9CC611111,110116664443885./.44574<<444@@???@@@>@@A>@;>=>1
@T6XXI:00055:00778
GGGGAATTCTAATACGACTCACTATAGGGAGAGGGAGAATAGTTCCTCACCTGCACTCTCCCCAGCGTCGCTTCCTT
TCTCCCTTCTCCC
+
DDE:C?B?BCC?BCAA@BBBBCCBB:965:@?BC>CAA>@@@@?A>AA@?;?AA??888AED9BBFAAA;8:B?477
-7103,44/--33
@T6XXI:00055:00779
GGGAGAAGGGAGAAAGGAAGCTAGTCACTCGACATCTGTGGCTTCACAGTACAGAGGGACTGTCCAAAGCCGCAGAT
CTCGAGTCGACTAG
+
CDACBB>?==@AAA:@=@=@CCCCC=@?AB?::9???BCL>?@?BCBB>BCAA=44317?AA@=?AA:===@@<===
:::;;<>>@?=???
@T6XXI:00055:00782
GGGGAATTCTAATACGACTCACTATAGGGCAGAGGGAG
+
9@@/9242444249;=<;9:B9><8897,1..,,,*,*
@T6XXI:00055:00799
GGGAGAAGGGAGAAAGGAAGGATAGCGTGGTGGTGAGGACACTGGTGCTATTCTCCCTCTCCCTATAGTGAGTCGTA
TTAGAATTCCCC
+
AA?AAC@DG@CDBB?B@B?A>ACDCBBBB@CB@A??A?CCB???>@AA@@@=AAA==AA@@@<@AAA@@@AAA?A?A
A8:=::@?AAA6
@T6XXI:00056:00766
GGGGAATTCTAATACGACTCACTATAGGGAGAGGGAGAATAGTCAACAAATCGTTTGGGGCGACTTCTCCTTCCTTT
CTCCCTTCTCCC
+
B@A-95924>B>BCCA@@BAA@@>>7775;A@CC>?==?@888?;7444-
77??EACDC8C?@@E@BA@<@;=;==6<94A1838@@@<
@T6XXI:00056:00767
GGGGAATTCTAATACGACTCACTATAGGGAGAGGGAGAATAGTTTTTCCCTTCACTAACCGCGAACTGCACTTCCTT
TCTCCCTTCTCCC
+
CCB9B=>=ACCCCCB?????BAB?8166.7>=?A>A@>9;433344,044;<@BCCC??????=<==<<@@@:8:8A
8?<?C;@>@@@@;
@T6XXI:00056:00774
GGGGAATTCTAATACGACTCACTATAGGGAGAGGAGGAA
+
?;;292424:;94999A@9<?:97/445+,,,,*,0022
@T6XXI:00056:00775
GGGAGAAGGGAGAAAGGAAGTGCAGTTCGCGGTTAGTGAAGGGAAAAACTATTCTCCCTCTCCCTATAGTGAGTCGT
ATTAGAATTCCCC
+
DA=ABB@BC>BBBB=BAB@B<<>BBBEAB@A?B?BCDCC@BB>BCCC2CBBB@BAAA;AB>>>=;99<<<<>A?==;
8838@B>@=@CD6
@T6XXI:00057:00769
GGGGAATTCTAATACGACTCACTATAGGGAGAGGGAGAATAGCCGTTTAATCTCCCACTGCGGCATATCCTTCCTTT
CTCCCTTCTCCC
+
BA@4@69:<@@?BCCBCBCCCCB??999.7==?B>CAA>A@@?=@CC=B?B898<7<AA@A@<??=;;;-
/,/58626133+4/439;6
@T6XXI:00057:00774

```
GGGAGAAGGGAGAAAGGAAGTGCAGCTCGCGGTTAGTGAAGGGAAAAACTATTCTCCCTCTCCCTATAGTGAGTCGT
ATTAGAATTCCCC
+
DC>A>>@AA=AAAB=B@B@BB>BABB@BBBB>@=?BB?A?BC>CCCC2CCCC@BBBB>@C@@@>B?BBB@AAAAA@@
==;=AB?C=AAA5
@T6XXI:00057:00779
GGGGAATTCTAATACGACTCACTATAGGGAGAGGGAGAATAGAGACGTATTCGTTTGCGCTCTGTTCACCTTCTTTC
TC
+
444,42424895444477;;:;977111,17666,103240.//-.2239;454<;;AAA=<<A;;;8646,/-
101-.
@T6XXI:00057:00795
GGGAGAAGGGAGAAAGGAAGGGCAAAAACCTTGAGAATAGTTAGCAGCTATTCTCCCTCTCCCTATAGTGAGTCGTA
TTAGAATTCCCC
+
BB>B>>;>?<@@@C=B?A?BCABCACA2AACCCEBB@CCCC@CCECDEBBB?BCCDAAAAA@<@DCCCBCC@@ACCC
E@CCD@CACCC:
@T6XXI:00058:00783
GGGAGAAGGGAGAAAGGAAGGGACAGTCCATCTGTGGCTTCACAGTACAGAGGGACTGTCCAAAGCCGCAGATCTCG
AGTCGACTAG
+
@A:>>>;>@:>>>@8;9;>BB=BAAB?@;?@B:::B?BB@CCCA@@===???CA?<??<=;==;@<;<<=<<@????
??@BBBGCBB
@T6XXI:00058:00791
GGGGAATTCTAATACGACTCACTATAGGAGAGGGAGAATAGTCGAACAAATCGTTTGGGG
+
444,42424899:>CB@@BAC>=:3//,.35778=885588940323-349///:,...*
@T6XXI:00059:00754
GGGGAATTCTAATACGACTCACTATAGGGAGAGGGAGAATAGATTGGCGAGAATGCC
+
CCC:B697<>?<@A<;;AB<<8BDAGCC>BA???9?=<:=???;;3655,,,+++..
@T6XXI:00059:00762
GGGAGAAGGGAGAAAGGAAGGGAAAGCCGCGGTCTTAGTTCTGCAAGCTATTCTCCCTCTCCCTATAGTGAGTCGTA
TTAGAATTCCCC
+
IJ?AA@;>=:9<;B?B@C@DD?BBABB@??@@CBA>AAD@BBBBB?DBBCB@BAAC=@?CCC>CC>AACCCA@????
?=?AB>:8?CC>
@T6XXI:00059:00785
GGGAGAAGGGAGAAAGGAAGGGCATGATCGGCGATGCGGAGAAGGTTGGCTATTCTCCCTCTCCCTATAGTGAGTCG
TATTAGAATTCCCC
+
BB@BCD@BB?CCBB>B<;5775:A@BC=@?>ACCB?BB>B?@@C=>2737@CC@CB??7;;;=>:?A@@??CAA?>?
AAA=?@@>EDCCC9
@T6XXI:00059:00789
GGGGAATTCTAATACGACTCACTATAGGGAGAGGGAGAATAGTCAACAAATCGTTTGGGGCGACTTCTCCTTCCTTT
CTCCCTTCTCCC
+
111+/,1,///768=AB@<>>?@@AA@?;=?777.7::79430034433-
/.445+...*177162174/344<=;;;:882424---+
@T6XXI:00060:00756
GGGAGAAGGGAGAAAGGAAGACTTATAGTGCAATAGAATAGTTGGAAACTATTCTCCCTCTCCCTATAGTGAGTCGT
ATTAGAATTCCCC
+
CB>ABC@CB=ABAB>B@B??@@C@CCBCBB?@=@CBC@C<<;BC@CC@CCA@>@CCB><B9::6:?B????BBABCC
AA?@=<@D:<<</
@T6XXI:00060:00758
```

```
GGGAGAAGGGAGAAAGGAAGGAGAAGTCGCCCCAAACGATTTGTTGACTATTCTCCCTCTCCCTATAGTGAGTCGTA
TTAGAATTCCCC
+
CD@AA?>BC>@AAC?C@A@C@BCC@C?<CDCC7@@<?AAA@;BB@ABA@AABAA@A<@>?=C=AAA?????@???@@
@>@@A>A<???4
@T6XXI:00060:00763
GGGAGAAGGGAGAAAGGAAGGAGAAGTCGCCCCAAACGATTTGTTGACTATTCTCCCTCTCCCTATAGTGAGTCGTA
TTAGAATTCCCC
+
CD?BBBACC@ABBC?B?A?A@CAA@CCCA?;>6;;:?AABC>CC?BBB@@@>@AAA=A@>?A<AA<A???@AAAA>=
=?ABB@B;=<<4
@T6XXI:00060:00782
GGGGAATTCTAATACGACTCACTATAGGGAGAGGGAGAATAGTTTCTCCGTTATCGTAGATCGCTGTTCTCTTCCTT
TCTCCCTTCTCCC
+
HHC8@;><99<=@CDCCCBB@>976167.7>?BE>BAA>A;??B===<?AA@A@@@@@@@@???@BB@E?@>;>;==
7=;<<6;8::;;6
@T6XXI:00060:00790
GGGGAATTCTAATACGACTCACTATAGGGAGAGGGAGAATAGTTTCTCGTTAGGCGCCCTGTGACTGTTCTTCCTTT
CTCCCTTCCTCCC
+
AAA7A>@?AAB?BAAABBCCCBCAB???9???AA<B==;=AACC<@888>;===?88=9=@@@@@@>=;:;7:8;=7
?==;6;./,/782
@T6XXI:00061:00750
GGGGAATTCTAATACGACTCACTATAGGGAGAGGAGAATAGTACGAACA
+
?BA18*4699><>;<<@@BBCBA>=777.7==?=B4834,,,,+++000
@T6XXI:00061:00751
GGGAGAAGGGAGAAAGGAAGGAAAATAGGCCGAGGTAACGTGCTAAACTATTCTCCCTCTCCCTATAGTGAGTCGTA
TTAGAATTCCCC
+
?B?AACAFC>BBBD?B@B@C@CDD8BBB?B@CCC@EC@CBBBBCCB>BBAC=@@@@:=<<<@;@@@CA??A@=>=@@
@>A?B=B?AAA6
@T6XXI:00061:00756
GGGAGAAGGAGAAAGAGGAGAATACAGCGAAACGGAACACGTCGCTAGTTCTCCCTCTCCTATAGTGAGTCGTATAG
AC
+
;A0/11,/,,**0)*,,*,,22.7:;494;;600,.33..66111111101111,1300/00000344946-
/,,++++
@T6XXI:00061:00780
GGGGAATTCTAATACGACTCACTATAGGGAGAGGGAGAATAGTCAACAAATCGTTTGGGGCGACTTCTCCTTCCTTT
CTCCCTTCTCCC
+
?>>4?<>=@BB@DBAABBBBAAA?966656==?B>BAA>@:;=CC=???7?????;?CC7AACCC>ABC<?<>?@C<
AAAA;A>AA@A<
@T6XXI:00061:00788
GGGGAATTCTAATACGACTCACTATAGGGAGAGGGAGAATAGCTTGATTGAAGCTAGTCCCCTGTTTACTCTTCCTT
TCTCCCTTCTCCC
+
AA?6A=>=@CB@BBBBACCCBBB?=666.7=<BCBF==:=@@B?@??C@=B>??@C??>CC5@@@@;????@?@?AA
<AAAA<@9<<<<7
@T6XXI:00062:00762
GGGGAATTCTAATACGACTCACTATAGGGAGAGGGAGAATAGCAGCTGTTTTAACGCGCCTACTATTCCCTTCCTTT
CTCCCTTCTCCC
+
ABA7A===@CC@DAAAABBB?AB>>111-
6<;CF>C@B>B;;<<<@===AA5@<??@@@>@<===<8;C1738=??<A@AA7<9<<@@;
```

```
@T6XXI:00062:00781
GGGAGAAGGGAGAAAGGAAGCGCAGTTCGCGGTTAGTGAAGGGAAAAACTATTCTCCCTCTCCCTATAGTGAGTCAT
ATTAG
+
DCCCBB58419>?A1;:<@CJCBBBB?CCBC?B?BC>>?>A9,0000,7?>;:>884-4;44417=;=?@7868/--
--,01
@T6XXI:00063:00775
GGGGAATTCTAATACGACTCACTATAGGGAGAGGGAGAATAGCCTCAGGTCCCGTCTTTGTCGCTATGTCTTCCTTT
CTCCCTTCTCCC
+
CCC:C@C@BBB@BAABBBCB?ACAA?8?9:::BE>C@@?@@@@>BBB@=@CC<>==ACAC:<<AB??@>762::?A>
A=@>>>.0.775
@T6XXI:00063:00785
GGGGAATTCTAATACGACTCACTATAGGGAGAGGGAGAATAGAGACGTATTCGTTCGCGCTCTGTTCACCTTCTTCT
CCCTTCTCC
+
8B808*1,/67,/44883;;;9977111,1>777.78845@??<=<;?7646//,.1613333;9-..746,/-,-
133032300/
@T6XXI:00063:00787
CTAGTCGACTCGAGATCTGCGGCTTTGGACAGTCCCTCTGTACTGTGAAGCCACAGATGTCGAGTCGACTAGCTTCC
TTTCTCCCTTCTCCC
+
?61/////6674444;;899?<8886<=@@CCKCC@@@<<<DCBCBBA>AB@><<AC;<;DDDE?AD>>==9//-
0:<>9>;<B-4/3000,
@T6XXI:00063:00790
GGGAGAAGGGAGAAAGGAAGCTAGTCGACTCGACATCTGTGGCTTCACAGTACAGAGGGACTGTCCAAAGCCGCAGA
TCTCGAGTCGACTAG
+
4919;@=@@199;919>B=@@B???:9:@:::::??B:;7<8<@;?669?A=>@@88818AA??>?AA<AA>@@@@@
@@@@?=<<?====;<
@T6XXI:00064:00772
GGGGAATTCTAATACGACTCACTATAGGGAGAGGAGAATAGTCACAAATCGTTTGGGGCGACTTCTCCTTCCTTTCT
C
+
===4><><>@@=@CCBAAAA??@=7111.7>775;?A>@A;;//.882>:::C>C@C76:@::376420/4244+30
0
@T6XXI:00064:00778
GGGGAATTCTAATACGACTCACTATAGGGAGAGGGAGAATAGCCTTGGCGTTTGTGCCACGTTATTCTCGACTTCCT
TTCTCCCTTCTCCC
+
CCB7A89;<BBIDBBBB@@@?@>77111.7>>>B>CAD>?487?C@C=?AAA:?@BA?888B>@A>AA==<?;2424
4-4@?A<A?A<=<7
@T6XXI:00064:00782
GGGGAATTCTAATACGACTCACTATAGGGAGAGGGAGAATAGCTGTAATGTTATTCACGCGGCCATCTTCTTCCTTT
CTCCCTTCTCCC
+
CJC:E::;<BB@CCBA@@CB?@C=7111.7>7777:A@?@===@??B@===?@@=@?=<;<8<:;A@@=@B=@=@@<
?@@>:>=8777.
@T6XXI:00064:00788
GGGAGAAGGGAGAAAGGAAGGAGAAACACGCGCCAAAGAGTTATTGGCTATTCTCCCTCTCCCTATAGTGAGTCGTA
TTAGAATTCCCC
+
8=6:18599-4444-
4231399337,4@?>9;;59D:C8;4247131/6/.,....+.3111,17668<=:44003032300/02344.
@T6XXI:00065:00756
GGGGAATTCTAATACGACTCACTATAGGGAGAGGGAGAATAGTCAACAAATCGTTTGGGGCGACTTCTCCTTCCTTT
CTCCCTTCTCCC
```

```
+
BEC:B7<6;889?>@@BC@@@A@>7777.77=BC>?99<;86<;;5<;?:BBB??.777-7;=6606@4/0/0/34-
438949796460
@T6XXI:00065:00757
GGGGAATTCTAATACGACTCACTATAGGGAGAGGGAGAATAGACGTGGAACTGTTGCCAGTGTATTCTCCTTCCTTT
CTCCCTTCTCCC
+
CDD:D@B?BCC@BBBCCBBBB@BCCAAA<9:9??<?<<<@@?=<<==8?:?AAB@AA?ABBB@??@B==:9<=>AA<
BAAA7=8;;;=7
@T6XXI:00065:00784
GGGGAATTCTAATACGACTCACTATAGGGAGAGGGAGAATAGTCAACAAATCGTTTGGGGCGACTTCTCCTTCCTTT
CTCCCTTCCTCCC
+
DCB7A=>=@BC@C@A@AACCCCB?=111.7?===<?@@>@@A<=9::6;4?:7672;<?5???A@=AA:2738>@@<
@:9726,/,//72
@T6XXI:00065:00798
GGGAGAAGGGAGAAAGGAAGCTAGTCACTCGACATCTGTGGCTTCACAGTACAGAGGGACTGTCCAAAGCCGCAGAT
CTCGAGTCGACTAGCTTCCTTTCTCCCTTCTCCC
+
CC<A<<:<B>BBBBAB=@=@CB?:77,,,,,,2027?@@@;?B?BBBCAA@;;;444-
4==????CC:>?:AAAA=>>A@@AAC@@@@?=>@@=?=>=7=444-47::9;4
@T6XXI:00066:00756
GGGAGAAGGGAGAAAGGAAGAATAGCTGACTGGGATGTCGTGAAGGGTCTATTCTCCCTCTCCCTATAGTGAGTCTA
TTAGAACTTCCCC
+
::54/1:;;,1/,200002..,.3..4888///+//////44969B<B888B@@@@C;?<<<8834---2-
1111004/003200/3990
@T6XXI:00066:00757
GGGGAATTCTAATACGACTCACTATAGGAGAGGGAGAATAGCCTCGCCCTTTTCTACGCTATCGTAATCTTCCTTTC
TCCCTTCTCCC
+
BCC:C::<<CGACBAA?????:997/.,0<<>B>BCD>@<@A?C>>>>6111..//7;B@@?<<<>>@??:655;6;
=;=6878444-
@T6XXI:00066:00763
GGGGAATCTAATACGACTCACTATACG
+
444,4244442444473336.......
@T6XXI:00066:00767
GGGGAATTCTAATACGACTCACTATAGGGAGAGGAGAATAGTACGACAAATCGTTGG
+
6860<=A?BBB@BBBBBBBBA@;97111./://,0;;7;:=44884000*,,,*)**
@T6XXI:00066:00768
GGGGAATTCTAATACGACTCACTATAGGGAGAGGGAGAATAGTCAACAAATCGTTTGGGGCGACTTCTCCTTCCTTT
CTCCCTTCTCCC
+
BBB9B@C@CBBACBB?BBBB??<77111.7>>>@?AAA>@?AAAA88=A=CCBBB<ACC9AC@<?>:@B/424/33,
3==?:?9;<<=9
@T6XXI:00066:00776
GGGAGAAGGGAGAAAGGAAGGCTAGTCACTCGACATCTGTGGCTTCACAGTACAGAGGGACTGTCCAAAGCCGCAGA
TCTCGAGTCGACTACG
+
@C<@@?;>>194@A9>;>484887==>6711111077167:::>@CBBBBB@;;;444,4;;=@??CC>BA<:@@@@
@@@@?@=>==:::444
@T6XXI:00067:00750
GGGGAATTCTAATACGACTCACTATAGGGAGAGGGAGAATAGTCAACAAATCGTTTGGGGCGACTTCTCCTTCCTTT
CTCCCTTCCTCCC
+
```

```
CCC9B=>=@AAACCCCC>BBB?BABB??9>?C@C>===>@@A@@?@AAA;BCAAC:@AA5??@AA?BA><?;=;=C?
CAAC;@5838AA:
@T6XXI:00067:00798
GGGAGAAGGGAGAAAGGAAGCTAGTCGACTCGAGATCTGCGGCTTTGGACAGTCCCTCTGTACTGTGAAGCCACAGA
TGTCGAGTCGACTAGCTTCCTTTCTCCCTTCTCCC
+
CC>CCC@BB:?984-
96858B?::>/10???@?@CCGBB@??<<==?@DC@?@<=;888;??A==@?<;@A>AA=AA???<;?<CA@@?==7
3434344-4;;>:>8;;;<6
@T6XXI:00068:00752
GGGAGAAGGGAGAAAGGAAGTGCAGTTCGCGGTTAGTGAGGGGAAAAACTATTCTCCCTCTCCCTATAGTGAGTCGT
ATTAGAATTCCCC
+
CC>BB?@BC>BBAA=B=>=?CCCCCB@BB@A?B@@@@CB@@B7BCCC2B??>>>?>?;@@CCC>@=@=@<=AA==<:
@@>A=;8:3444+
@T6XXI:00068:00753
GGGAGAAGGGAGAAAGGAAGAGAACAGCGACGGGGATAATGTTTTAGCTATTCTCCCTCTCCCTATAGTGAGTCGTA
TTAGAATTCCCC
+
KHCCAA===<=>>@<@=@@CCCC@CBBBCBBA?A8ACC@AAABB8CDCCCB@DBBB>CABBB?CAAABBCCCCBC@B
B?@AA?D?BAA4
@T6XXI:00068:00764
GGGGAATTCTAATACGACTCACTATAGGGAGAGGGAGAATAGATGTCTGACCATTGCTGCTTCTATCTCCTTCCTTT
CTCCCTTCTCCC
+
,--*-+1,///56=@@CAAACA;77777-
7==@A=AAC>@A878:;44330/03200366856<?=<<@=@9;388148;@7=;;444-
@T6XXI:00069:00755
GGGGAATTCTAATACGACTCACTATAGGGAGAGGGAGAATAGTCAACAAATCGTTTGGGGCGACTTCTCCTTCCTTT
CTCCCTTCTCCC
+
@AA8AAD?BA?>BBBBACCC???@=666.777>@<A@A>@@@@AA?AAA<CCCCC;???5?@?==377:8;;>?BH@
A@??7=387AA<
@T6XXI:00069:00757
GGGGAATTCTAATACGACTCACTATAGGGAGAGGGAGAATAGTCAACAAATCGTTTGGGGCGACTTCTCCTTCCTTT
CTCCCTTCTCCC
+
BBB9B=@=@BB?BCCCBBBCBB@B;111.7>>AB>BAA?A=AAAA?77717==?B>BCC7A???>:<7837<@@CD?
B@AA<A899?H<
@T6XXI:00069:00758
GGGGAATTCTAATACGACTCACTATAGGGAGAGGGAGAATAGTTGTCAAATCCCGCGCATAACTATTCCCTTCCTTT
CTCCCTTCTCCC
+
CCC6==A>AAC=@BDBA@@@?788>777.7==<B>@==>A@?@>@:9;C>AAA>ABB@AAA587><::@1728;=>;
???C7<9=?@@;
@T6XXI:00069:00781
CTAGTCACTCGAGATCTGCGGCTTTGGACAGTCCCTCTGTACTGTGAAGCCACAGATGTCGAGTCCGTACCTTAGCC
TTCCTTTTCCTCCCCTTCCTCCC
+
7<1////4499==BBD<;8@>888/81737??@A;888CCBB@@??@88;9;@ABC><=>79/,-
*,,,,*,*,..,.7;9>@?6978555.58882220
@T6XXI:00070:00763
GGGGAATTCTAATACGACTCACTATAGGGAGAGGGAGAATAGTCAACAAATCGTTTGGGGCGACTTCCTCCTTCCTT
TC
+
CCE:DCE@AAAACECBBBB><?BC?ACC>AAABC>B??<?>@A==;===;BBBBD?CBC6?7977,/,.3,--
/7544<
```

```
@T6XXI:00070:00764
CTAGTCGACTCGAGATCTGCGGCTTTGGACAGTCCCTCTGTACTGTGAAGCCACAGATGTCGAGTCGACTAGCTTCC
TTTCTCCCTTCTCCC
+
444494444499BBBBCBBBD>;880858;?AABCCCABAAA???@@@<@C@@@@@AAC@<<:<8==<>AAAEC@C=
??6=;894:94444-
@T6XXI:00070:00771
GGGGAATTCTAATACGACTCACTATAGGGAGAGGGAGAATAGTACGAA
+
?BB9A>G@AAA?BCCBACB;;;@J;;;D??>777,600/0------+*
@T6XXI:00070:00781
CTATCACTCGAGATCTGCGGCTTTGGACAGTCCCTCTGTACTGTGAAGCCACAGATGTCGAGTCGACTAGCTTCCTT
TCTCCCTTCTCCC
+
CCC99::;@=??CGCBBA?<877.8<??FCDCB>BBBAA><BB???>@C>BAC@@;::CAAABCC@??>=6107;>?
<:78A6;8;5499
@T6XXI:00071:00761
GGGGAATTCTAATACGACTCACTATAGGGAGAGGGAGAATAGCCAC
+
@BB6@=><>@@;>@C@?@BBBAAFB:::57>=AB>BAA;==AB><<
@T6XXI:00071:00779
GGGAGAAGGGAGAAAGGAAGCTAGTCATCTGTGGCTTCACAGTACAGAGGGACTGTCCAAAGCCGCAGATCTCGAGT
CGACTAG
+
4954//,/65///6+/,.6373166661106164423 4...311330000+04446?:83+--,.--
2224:6558866<6373
@T6XXI:00071:00780
GGGGAATTCTAATACGACTCACTATAGGGAGAGGGAGAATAGCTACGTTTAACCGATCGTCGTTGTTCTCCTTC
+
;;;292924442448;>ACCA@??=776,61777974488AA<??><<=6:28236426/--22--,-004;44
@T6XXI:00071:00783
GGGAGAAGGGAGAAAGGAAGCTAGTCGACTCGACATCTGTGGCTTCACAGTACAGAGGGACTGTCCAAAGCCGCAGA
TCTCGAGTCGACTAG
+
CC>BBB@BB1;;>>9>>A=A@BAA?:69;<<<::699;@@=;=C?ABF@;;8@;;88818;;==@<??:?B>AAA@A
???@@<;;844;844
@T6XXI:00072:00776
GGGAGAAGGGAGAAAGGAAGGAGAAGTCGCCCCAAACGATTTGTTGACTATTCTCCCTCTCCCTATAGTGAGTCGTA
TTAGAATTCCCC
+
::5499;>@199AB<@=@>A?AAC@CAABCCC6@@<?:8:@;?A?BBBA@?:;;;;6;<AAB====?==<@@@@@@@
A@A@@<<5:;;1
@T6XXI:00073:00764
GGGGAATTCTAATACGACTCACTATAGGAGAGGGAGAATAGTCAACAAATCGTTTGGGGCGACTTCTCCTTCCTTTC
TCCCTTCTCCC
+
CDD:C@B?ABBEBBBBB@AAB:997/.,0::>@AAAA>@8??AA<<<<;?>@@A;>>?7AAABB???<9=;=?AB=A
@@@<@@AAAA=
@T6XXI:00073:00784
GGGAGAAGGGAGAAAGGAAGCTCAATCGTGGGTAGAAGGGTCGTGATCTATTCTCCCTCTCCCTATAGTGAGTCGTA
TTAGAACTTCCCC
+
EDABBC?B?>>@AC>@=@@CDCB>>>>?@@@?@@@<<>=;=<<@@@<A@@A>???@<@@@@A=AAB?@@A@===A==
=<;;>.00;;991
@T6XXI:00073:00787
GGGGAATCTAATACGACTCACTATAGGGAGAGGGAGAATAGCTGGCCTTTTACCGGTTATTACTCC
+
```

```
88808*1///7/4443<;@A<@@@777-7>>>><@>=:;<88:B?C>C@C8?;5626742,-..+0
@T6XXI:00074:00754
GGGGAATTCTAATACGACTCACTATAGGAGAGGGAGAATAGCCTTCATCGGTGTTGAGCATCACTATTCTTCCTTTC
TCCCTTCTCCC
+
CAA8A@B@CCC?BBBCC@@@C76:7//-
0>>BC>CAD??8772:<>A@@@>@AA>AA@@??===A@A@AACA=??<?AA@7>;788@:
@T6XXI:00074:00768
GGGGAATTCTAATACGACTCACTATAGGAGAGGGAGAATAGTTCCCTTCTAACCCGTACGCAACAATGCTTCCTTTC
+
BBA9B?C?A@@>ABBB@;8870.....,...779930/0:0328@:>?@@C@BB>BB6:177,//,.-110124646
@T6XXI:00074:00777
GGGAGAAGGGAGAAAGGAAGGAGAAAGCGACGTCGGGGAAACGTTTGCTATTCTCCCTCTCCCTATAGTGAGTCGTA
TTAGAATTCCCC
+
CC@BA=@BC@???B>B@B?B@BBBB@=?@CC@??BBB8BB?BDCC@BDDBB?BCCC?CBBBD>BA??@@CA@BBBB?
?<?AA?C>AAA5
@T6XXI:00075:00763
GGGAGAAGGGAGAAAGGAAGCTAGTCGACTGACATCTGTGGCTTCACAGTACAGAGGACTGTCCAAAGCCGCAGATC
TCGAGTCGACTAG
+
BB><=<?BB<;;9A9>;?=@ABAAA?????=887777<??=BC?CCCBBBC==;//,///68=>AA<@A>AAAAA>>
>AAAA??ACA@@=
@T6XXI:00075:00766
CTAGTCGACTCGAGATCTGCGGCTTTGGACAGTCCCTCTGTACTGTGAAGCCACAGATGGACTGCTTCCTTTCTCCC
TTCTCCC
+
CDCCCCCCBDCBCBBBAAADC@A@A=A@BCAA@@A>?@<=???CC??@=>B>BBBCCCC@CCCAAC5:5:@<???C>
C@??=C?
@T6XXI:00075:00778
GGGGAATTCTAATACGACTCACTATAGGGAGAGGGAGAATAGTACGGCTCGAAAGTGCAGTCAGTTCTCCTTCCTTC
TCCCTTCTCCC
+
CCC:C@B@BBB@AAAA??????DB@?<<;<<==C>CA?=A==A=<<<@AA77418BB@AA@<>;::8775879,/-
.544599@<@A<
@T6XXI:00075:00780
GACTATCACTCGAGATCTGCGGCTTGGACAGTCC
+
***/8/44444498=>A?=<<:0/,.,.553033
@T6XXI:00075:00798
GGGAGAAGGGAGAAAGGAAGCTAGTCGACTCGACATCTGTGGCTTCACAGTACAGAGGGACTGTCCAAAGCCGCAGA
TCTCG
+
BB?BCB@BB<@ABB=B?B?CBB?AA@CC@AA?:::???BDA>?B?CC>>>?=844000-
4;;;<=?AA=BB@@@<=<<<<<<
@T6XXI:00076:00751
GGGGAATTCTAATACGACTCACTATAGGGAGAGGGAGAATAGCTAGCATTATCCCGCTAGGTGCTTCTCCTTCCTTT
CTCCCTTCTCCC
+
BCE:C?B?BCC?BBCAB@<<A@<96116.7>>BD>C@B?A;::>>==@B@B9898:CBCB@CB?B=?BC=>>>>==<
=AAC;A;<==87
@T6XXI:00076:00769
GGGAGAAGGGAGAAAGGAAGCTAGTCACTCGACCATCTGTGGCTTCACAGTACAGAGGGACTGTCCAAAGCCGCAGA
TCTCGAGTCGACT
+
CDBA@@?A=<@@@=9@8:5;CBBCCB?>:=;00,-./.>:C8:;DBBBAACC@?>344-
4:;>>A=??<?D@AA@@A@???@@@A@@@AA
```

```
@T6XXI:00076:00780
GGGAGAAGGGAGAAAGGAAGCTAGTCACTCGACATCTGTGGCTCACATACA
+
44-44;99=899>B9<;<@DCCC>>>0...3...58>7777/0//.768..
@T6XXI:00076:00782
GGGGAATTCTAATACGACTCACTATAGGAGAGGGAGAATAGTCGTATACTACCCCTCACGCATTATTCTCTTCCTTT
CTCCCTTCTCCC
+
/11*/,924@@?9;;BACCCBAAA?7747>77767;;;4//6.--
111330000+0;;>=771710/300023/00+0000+3230384
@T6XXI:00076:00785
CTATCGACTCGAGATCTGCGGCTTTGGACAGTCCCTCTGTACTGTGAAGCCACAGATGTCGAGTCGACTAGCTTCCT
TTCTCCCTTCTCCC
+
DA?=99:?ABBDCCCBBBBB@?BB<B<>>==>@?;??????@<;;;=<=B?BBAAAA@?>>>A?AABBBDCDC?A?B
C>CAA@<A=A<<<7
@T6XXI:00076:00786
GGGGAATTCTAATACGACTCACTATAGGGAGAGAGATATAAGGGTCCTATGTCTTTCTTCCCATCATCTGGTCGTCC
CATTACCAAATTCTCTCCC
+
CCB7@5959>?<@BBCCAAACBBAA:::7:BBBB===@@??AA>AA????@<<?C>BB@CC>@@?;?;8838BAAAD
=??;=?444-7=>888BC;
@T6XXI:00077:00759
GGGAGAAGGGAGAAAGGAAGGAGAAGTCGCCCCAAACGATTTGTTGACTATTCTCCCTCTCCCTATAGTGAGTCGTA
TTAGAATTCCCC
+
CDACCB@CB?BBBC>B@B>@?@BB@@<?@BBB7BB<?B?BB>CC>BAB@AC>@???<?@A@C<@@@@@<@@@AAA@=
<8;74/05::=1
@T6XXI:00077:00772
GGGAGAAGGGAGAAAGGAAGGAGAAATCACATGCAACAAAAGCTGAGCTATTCTCCCTCTCCCTATAGTGAGTCGTA
TTAGAACTTCCCC
+
;;6;AA@CC>AABB@C@C?A?BBCDA?@?BAAA?A=A@AA6:;:BAA:?;9/44==:>??==;@?877==7346559
<545=<=@>@CC5
@T6XXI:00077:00773
GGGGAATTCTAATACGACTCACTATAGGGAGAGGGAGAATAGTCAACAAATCGTTTGGGGCGACTTCTCCTTCCTTT
CTCCCTTCTCCC
+
999/49@@GCC@CBB?BCCB??CAC?:?9???AC>B@@>@AAAAA>@@@:?=666-
777.7??@A?AA?<A<?>AA<@@AA<A=??@CA
@T6XXI:00077:00778
CTAGTCGACTCGAGATCTGCGGCTTTGGACAGTCCCTCTGTACTGTGAAGCCACAGATGGACTGCTTCCTTTCTCCC
TTCTCCC
+
EAABBBCCFBBABBAB>>?BB@BBB>C?CB?????9?BBCCCDD@???>ACBBCCCC;;9;C@?AB6:5;B???@A=
AB<;8C?
@T6XXI:00078:00757
GGGGAATTCTAATACCACTCACTATAGGAGAGGGAGAATAGCCTGACCTTTCCAGTGCGCGCTGTTTTCTTCCTTTC
TCCCTTCTCCC
+
CCCDE@CACBB@BBB=BBBD<=896..,1::=?<?CCACBB@;BA>9366-
637?BCA<<<@@AAAA4=<<?38818@@A;@>???CA
@T6XXI:00078:00765
GGGGAATTCTAATACGACTCACTATAGGGAGAGGGAGAATAGTTACATCTTAGCCGGCGTTCCTCTATTCTTCCTTT
CTCCCTTCTCCC
+
```

```
@A@4@=@=ACC?BBBBBBBBBA@<;611.6=;@C>B@@?A;A=;=<=<<?<?@C>A>@AA>@>@???@>@@>A>AA<
A???;>:<;;<7
@T6XXI:00078:00770
GGGGAATTCTAATACGACTCACTATAGGGAGAGGGAGAATAGCACTTACATGTGAAGATTCGCTGTTCTCTTCCTTT
CTCCCTTCTCCC
+
CHH:C;;;<<<@CDCCCBBEAB?>>777.7==BD>BAA>A????@A>BA>::?8869BC@A<<=??@<<<@5;8;@4
84AA464//..,
@T6XXI:00079:00759
GGGGAATTCTAATACGACTCACTATAGGGAGAGGGAGAATAGTCAACAAATCGTTTGGGGCGACTTCTCCTTCCTTT
CTCCCTTCTCCC
+
CHK9C@C@BCC@CCCBAAA??AB>8666.7<=>@<:88=@@@AA@>==<7?@??A<AAB7BCCCB@BBA;=9=?CC<
@@@A;@=?AAC:
@T6XXI:00079:00785
GGGAGAAGGGAGAAAGGAAGGAGAAGTCGCCCCAAACGATTTGTTGACTATTCTCCCTCTCCCTATAGTGAGTCGTA
TTAGAATTCCCC
+
CC?CCC@CD>BBBC>C@A?A?ABB@@@AAAAA8AE>C@ABC>B?>BAA?@@>@===;<<?88:?;??@@@AA?<?==
=?A@?=;3444,
@T6XXI:00080:00751
GGGAGAAGGGAGAAAGGAAGCTAGTCGACTCGAGATCTGCGGCTTTGGACAGTTCCTCTGTACTGTGAAGCCACAGA
TGTCGAGTCGACTAGCTTCCTTTCTCCCTTCTCCC
+
?=9=9959@:@111,1-
0,.007167:7@=?:::AACBB@?;761.7;?>:00,/5::;;>CC@==?=;<<>@<=<<<@A@@?AA@@@889A@?
:?37768>;A7=888<A6
@T6XXI:00080:00755
GGGGAATTCTAATACGACTCACTATAGGGAGAGGGAGAATAGCTCACGATTTTTCTGAGCCTCCCTGGGCTTCCTTT
CTCCCTTCTCCC
+
DCD:C@C@BBB@BBBBBBBB@?@B=:??9?B??A=?==>A==;>BA=:00000+388=??<CCB>;;;68><><>A6
::AC>D:==AC<
@T6XXI:00080:00756
GGGGAATTCTAATACGACTCACTATAGGGAGAGGGAGAATAGTCAACAAATCGTTTGGGGCGATTTCTCCTTCCTTT
CTCCCTTCTCCC
+
CEE:CC?=@BB@CCCCBBBB;@B?>666-
1;;;?9?@A>AAAA@@=<<<6?@@@@<A@@6B@?@L@AAA;=;=?CD<@@AA;@9<=@A;
@T6XXI:00080:00758
GGGGAATTCTAATACGACTCACTATAGGGAGAGGGAGAATAGTCAACAAATCGTTTGGGGCGACTTCTCCTTCCTTT
CTCCCTTCTCCC
+
BCC9B@CBBBB@CCC?BBBB??>9?1695:??CC=A?@>B???@A?AAA<CCBBB>BBB7BB?>A>AB@>@=@>@@<
@AAA<A889@A=
@T6XXI:00080:00764
GGGGAATTCTAATACGACTCACTATAGGGAGAGGGAGAATAGTTTTTCCCTTCACTAACCGCGAACTGCACTTCCTT
TCTCCCTTCTCCC
+
BCC:B@C@ABB@BCCBBCBAA>@?A9965:???D?B@@<4444444,44-
44?CAAABE@??=@;==ACCCB>B@CC>:98C<A>8:8A:
@T6XXI:00080:00766
GGGGAATTCTAATACGACTCACTATAGGGAGAGGAGAATAGTCAACAAATCGTTTGGGGCGACTTCTCCTTCCTTTC
TCCCTTCTCCCGGGAATTCTAATACGACTCACTATAGGAGAGGGAGAATAGTTTTCCCTCACTAACCGCGAACTGCA
CTTCCTTCTC
+
```

```
CCC9B=@=@@A?BDDCCBBB?>977111.7?776:@@9;3////,///,7@@AB>BDG9BB?>>?==8-
/,.,//,//44-4:;;;;-44-4799;:;8<==?888==::::8838A>=>7>?=34/////+//,/-
113344463345684553.--4640,,
@T6XXI:00080:00768
GGGGAATTCTAATACGACTCACTATGGAGAGGGAGAATAGTCGACTACTAGTTTGTCTTCAGATTCTCCTTCCTTTC
TCCCTTCTCCC
+
CDG:C>ABACCCCA???>B>@797//.3>>BD>BBB?BCCC998@@A==A@==;<;<<<=<<<C@BBB?C?B?AA>B
CCC>A>>???8
@T6XXI:00080:00778
GGGGAATTCTAATACGACTCACTATAGGGAGAGGGAGAATAGCTTTACTAACGTTAGAAGCCGGTTCTCCTTCCTTT
CTCCCTTCTCCC
+
CCC9C>@=@AA=AACCBBBBCCCB=666.77===6=4420;::??9??@@>=>>;899<@@=@=?8<;>;=8=7;;7
888B:@:4448-
@T6XXI:00080:00779
GGGGAATTCTAATACGACTCACTATAGGGAGAGGGAGAATAGTCAACAAATCGTTTGGGGCCGACTT
+
AAA9B@C@CCC8998877337977666688666B8?;<337898;244<;??A::7555*.,.0,,*
@T6XXI:00080:00783
GGGGAATTCTAATACGACTCACTATAGGGAGAGGGAGAATAGCTACGTTTAACCGATCGTGTTGTTCTCCTTCCTTT
CTCCCTTCTCCC
+
CCE:C@C@BBB@CBBB>?@BCA@@A996.7?>@ABBAA>A@@?BBCAAA>?<?>AA@?@A==?A=:;?<>@?A;:;<
===A?A=???B>
@T6XXI:00080:00797
GGGGAATTCTAATACGACTCACTATAGGGAGAGGGAGAATAGTCAACAAATCGTTTGGGGCGACTTCTCCTTCCTTT
CTCCCTTCTCCC
+
CCB6@:;;<CCACCB??<<<?AB?9111.7>==?<A@@>@?@AAC???@;BC@@@>@CC9CB@@??@CA9=;=<AA<
@AAA=A?@@AA<
@T6XXI:00081:00755
GGGGAATTCTAATACGACTCACTATAGGGAGAGGGAGAATAGATCACCGTTGGTTCGGGTGCGCGTTCTCCTTCCTT
TCTCCCTTCTCCC
+
499/9;??BBC@CCB@<<@B?B>9961659??BE>A???A<<<==???BC@B?B?CCC?CBBBCCC?B@?=@@C@ED
>@===7<?@AAA<
@T6XXI:00081:00758
GGGGAATTCTAATACGACTCACTATAGGGAGAGAGATATAAGGATTTCGGGTCCCTCCCTTATTGGTAACCTGGCCC
ATTACCAAATTCTCTCCC
+
@CB6@=@<?>@799B:9:CCDDCBB;:7.7>>>70044338;:;B=4<64+344-
4;;6;9<=8:;<=<=988<=;44824699<;<999<89=9
@T6XXI:00081:00762
GGGGAATTCTAATACGACTCACTATAGGGAGAGGGAGAATAGCCATTGCATAATGTGTGCAGCTCTTCTCCTTCCTT
TCTCCCTTCTCCC
+
DCB9B@B@BBB@BBBAA?A?BCB<;111.6<<>@<A@A>B@@@>@@<=;?>@@A@?:;?AA@<;<<:<?=;A?=<;;
3:<==4656>>>:
@T6XXI:00082:00751
GGGAGAAGGGAGAAAGGAAGCTAGTCACTCGACATCTGTGGCTTCACAGTACAGAGGGACTGTCCAAAGCCGCAGAT
CTCGAGTCGACTAG
+
CCABBA>?A<@@@=9@;?=@CCCEE811>>>01.>>6>?C:;;?BBCABBC<88333-
4:;?@@@CC;@@>@???AA===@@@AAAC?@@@
@T6XXI:00082:00799
```

```
CAGTCGACTCGAGATCTGCGGCTTTGGACAGTCCCTCTGTACTGTGAAGCCACAGATGTCGAGTCGACTAGCTTCCT
TTCTCCCTTCTCCC
+
DBBBBBBBAA@@ACCCCBBB@BBB<?=@@BBBBC>BB;>>>???@=@>>?>?@::;7884;:3033336<AAB@CAB
B;>@AA:@><AA=;
@T6XXI:00084:00751
GGGGAATTCTAATACGACTCACTATAGGGAGAGGGAGAATAGTCAACAAATCGTTTGGGGCGACTTCTCCTTCCTTT
CTCCCTTCTCCC
+
BHH:C@B@BBB@CABA>>>??@CB@77757??CC>A?>?C:@@A<>8<<5???AB>BCC9CBBBB?AA>8<9;8==:
????:@>AA@A<
@T6XXI:00084:00756
GGGAGAAGGGAGAAAGGAAGAATGGGACGACTGCGAAGTTTTATCGGCTATTCTCCCTCTCCCTATAGTGAGTCGTA
TTAGAATTCCCC
+
EC@DEC@CB>BCBB>B?>>BC@BBB>BCCCCBBAAB>>>>B1=;00-
0>@B@CCCC?CCBCC>C?@@BCAAAA@@?7737=@9656<<4
@T6XXI:00084:00766
GGGGAATTCTAATACGACTCACTATAGGGAGAGGGAGAATAGTCAACAAATCGTTTGGGGCGACTTCTCCTTCCTTT
CTCCCTTCTCCC
+
CCC:C?C@BBB@DBBBBBCB@>;67111.7?>>B>BAA>AAAA<65<<;5??<<=.777.7==?A?BA?@C>@>??<
?AAC<A<?8662
@T6XXI:00084:00770
GGGAGAAGGGAGAAAGGAAGGGGAGAAGGGCTAGTCACTCGACATCTGTGGCTTCACAGTACAGAGGGACTGTCCAA
AGCCGCAGATCTCGAGTCGACTAG
+
EC@AA@@AA5499>09489>9<5777:??9@DCDGCC1//7/////44977;?6::BBBBCBAA<;<7;BCCCCABB
ABC=@ADDD@;;:;;86::@=B??
@T6XXI:00084:00772
GGGAGAAGGGAGAAAGGAAGCTAGTCGACTCGACATCTGTGGCTTCACAGTACAGAGGGACTGTCCAAAGCCGCAGA
TCTCGAGTCGACTAG
+
CHCBBBCCC<B<;8-
9584:@>>@@9;:700<00/0/.;654=?@CC@@@C@==;;;;4944888;=B?<<9<@?=@A????AAA@@@AA?=
@T6XXI:00084:00776
GGGAGAAGGGAGAAAGGAAGGTACGCGCGGGGCCAATCAATCATCGGCTATTCTCCCTCTCCCTATAGTGAGTCGTA
TTAGAATTCCCC
+
BHBCCC@CC?CBBB>B@A=?=001166:>=?6A@C@BCC@CCCCBB?BDCCACCCC>A@CCC>B@@@CDCCC@8?66
7449::92;;;1
@T6XXI:00084:00779
GGGAGAAGGGAGAAAGGAAGCTA
+
BA-49>59918111,/,0,.444
@T6XXI:00084:00789
GGGAGAAGGGAGAAAGGAAGAACAGGTCGGGAGCGCAAAGGCGAAAGCTATTCTCCCTCTCCCTATAGTGAGTCGTA
TTAGAATTCCCC
+
CD>AAG?BBABBBC>B?A@CC@BCC?AA=@9=0//7775666ACA><ADDC?AACCAHCCBDA@@@CCAAAA???:8
8=@AA@=<==C5
@T6XXI:00085:00757
GGGAGAAGGGAGAAAGGAAGGAGAACGCGCACCCGAACCAACGGTGATCTATTCTCCCTCTCCCTATAGTGAGTCGT
ATTACGAATTCC
+
BA?;=@79909899047>77:;;?<@C@>>99909?<>AB@AA:99999488<@99<6387>>8<;6668:?:9=:7
777...3...00
```

```
@T6XXI:00085:00764
GGGGAATTCTAATACGACTCACTATAGGGAGAGGGAGAATAG
+
=996=9@@B;;9;AAA@AAC@=>?=777-7>::969--,+00
@T6XXI:00085:00769
CTAGTCACTCGAGATCTGCGGCTTTGGACAGTCCCTCTGTACTGTGAAGCCACAGATGTCGAGTCGACTAGCTTCCT
TTCTCCCTTCTCCC
+
>7/1/1/44A@=A@@CCCBB?BIC=B@BB???@@<@:::B??;888<<?AAGCAACA@@B::9?AA7<>>::99=89
98104;4974444-
@T6XXI:00085:00780
GGGGAATTCTAATACGACTCACTATAGGGAGAGGGAGAATAGATCACCGTTGGTCGGTGGT
+
CCG9B@B@BBB@BBBBBBBBB@BB<<??9?CBBB<AAA?C;==<?7737747:<11,14,.
@T6XXI:00085:00787
GGGAGAAGGGAGAAAGGAAGTGCAGTTCGCGGTTAGTGAAGGGAAAAAAAAAACTATTCTCCCTCTCCCTATAGTGA
GTCGTATTAGAATTCCCC
+
GD@CCC@CB?BBB?=B=<<@ACCCCC@BBBB@B@??@B?:==-
676666777"8FFB>BBBB@BCCCC>@?@?AA==A???????AA?9;2444-
@T6XXI:00085:00789
GGGAGAAGGGAGAAAGGAAGCTAGTCGACTCGACATCTGTGGCTTCACAGTACAGAGGGACTGTCCAAAGCCGCAGA
TCTCGA
+
CDABBB?BB=BBBC>CAC=@@<:?????ABB>::::::B@?;?B?BCCC@C=:44000-
4;;=?A>AB=@@=@A==<;;=;??
@T6XXI:00086:00777
GGGAGAAGGGAGAAAGGAAGCTAGTCACTCGACATCTGTGGCTTCACAGTACAGAGGGACTGTCCAAAGCCGCAGAT
CTCGAGTCGACTAGCTACTTC
+
DE?BB=<@@9>@@@<@=?=@CB?@@;=77676609>?99A=:;7:@@?AAA=:;47418==AAA>@@<@A?BB====
99=???<==@@@B80..,55,
@T6XXI:00086:00783
GGGAGAAGGGAGAAAGGAAGTGGGAGCTGGAACATTGTGCGCTATTCTCCCTCTCCCTATAGTGAGTCGTATTAGAA
CTTCCCC
+
A@<ABB@CD?D<;809687;DCG@@?BDEBCCBCC@CBBACCBBB@BFBC?CCCCB>B@@@BCCECC;;;??:<@=3
88;>>>5
@T6XXI:00086:00785
GGGGAATTCTAATACGACTCACTATAGGGAGAGGGAGAATAGAGACGTATTCGTTTGCGCTCTGTTCATCTTCCTTT
CTCCCTTCTCC
+
944,424:49;89B@BCD@@@??>>777.777?C@;99<>?>?<<<@<<<=??@@<@@ADAA@@A99=<<A:=:=D<
?;@A9946//,
@T6XXI:00087:00771
GGGGAATTCTAATACGACTCACTATAGGGAGAGGGAGAATAGCCCACATTCCCATTTCGTTGCTACGTTTTCCTTCC
TTTCTCCCTTCTCCC
+
994,49@>ABCB>==ECA@A:78==777-
6166626444400000+0333/03+0777>9999BBBCCBCC9C?A8;79>8;6777;-//777
@T6XXI:00087:00773
GGGGAATTCTAATACGACTCACTATAGGGAGAGGGAGAATAGTCAACAAATCGTTTGGGGCGACTTCTCCTTCCTTT
CTCCCTTCTCCC
+
EFC:CACABDC@CCCBBBA>=<ABB???6777=?<?AA>AA888838=>:=====.777.7?>7767=;3838;>?<
?A@CAA===@A;
@T6XXI:00087:00782
```

```
GCTAGTCACTCGAGATCTGCGGCTTTGGACAGTCCCTCTGTACTGTGAAGCCACAGATGTCGAGTCGACTAGCTTCC
TTTCTCCCTTCTCCC
+
449444:4449449@=ACCA@>87708487?BCBC<ABCGCB?<<56656B>BBCBECBBBCCC@AE@@?B???;?;
?C>BB?C:=28344-
@T6XXI:00087:00787
GGGGAATTCTAATACGACTCACTATAGGAGAGGGAGAATAGTCCTTTGGTGTTTGCCCGACTGTTCTCCTTCCTTTC
TCCCTTCTCCC
+
@@A7A@CBCBB?CBDCCCBB>:=??//-0>>BE@DAA<??@==:=A<A>@AAA<7871888?@A>@A@>A=B374-
499948298;@7
@T6XXI:00087:00795
GGGAGAAGGGAGAAAGGAAGCTAGTCGACTCGAGATCTGCGGCTTTGGACAGTCCCTCTGTACTGTGAAGCCACAGA
TGTCGAGTCGACTAGCTTCCTTTCTCCCTTCTCCC
+
;9199959?9?@@@<A=?=@B@:=@/00;;;;>>BBBAAAB=7::5:<@BBB?<;6;=;;;<@A<==B>@@>@????
>?<==@@@@A@@??<<;8;;>@:@@>@:@589@@<
@T6XXI:00087:00798
GCTAGTCACTCGAGATCTGCGGCTTTGGACAGTCCCTCTGTACTGTGAAGCCACAGATGTCGAGTCGACTAGCTTCC
+
4444449///6/////744499378,3133>@@@@;>>??@D@==<:6249>@BAABC@?====711...6.,,*.3
@T6XXI:00088:00764
GGGAGAAGGGAGAAAGGAAGCTAGTCGACTCGACATCTGTGGCTTCACAGTACAGAGGGACTGTCCAAAGCGCAGAT
CTCGAGTCGACTA
+
DCAAA@BBC@BB@@:@=><>A8/0=000==>>1;;CCBBA>;<?<?BBBCCAAAA8655;;<=>??AA<98:AAA@?
@AA@@@@888>>?
@T6XXI:00088:00765
CTAGTCACTCGAGATCTGCGGCTTTGGACAGTCCCTCTGTACTGTGAAGCCACAGATGTCGAGTCGACTAGCTTCCT
TTCTCCCTTCTCCC
+
>>111=8==CBCA?@BBBB?>?B?<B>AAAA@@@=@CA??>@=<@@@<@B@BBBBBA@B@CBBBBBBCCCCDC?F>A
A<?<<A<A<??AA<
@T6XXI:00088:00766
GGGAGAAGGGAGAAAGGAAGGAGAAGTCGCCCCAAACGATTTGTTGACTATTCTCCCTCTCCCTATAGTGAGTCGTA
TTAGAATTCCCC
+
CFCCCB@BB?BBBC>B@B=@@?CC@BBACCBB8>>;>???C<BB?B::9AA>AAAA;@@@AA<A:?@AAB;??:???
??HB@<>3444-
@T6XXI:00088:00776
GGGAGAAGGGAGAAAGGAAGGAGAAGTCGCCCCAAACGATTTGTTGACTATTCTCCCTCTCCCTATAGTGAGTCGTA
TTAGAATTCCCC
+
DD?@A@@BC@BBAB>C@C?A@ABC@???????8??;>??CB9??@BAA>AA?AAAA:??AAA<@<>A;AAAA888==
=CAA>;;2444-
@T6XXI:00088:00783
GGGGAATTCTAATACGACTCACTATAGGGAGG
+
C@;2>59699>549@BDDC301>;00/4+,*0
@T6XXI:00088:00792
GCTAGTCGACTCGAGATCTGCGGCTTTGGACAGTCCCTCTGTACTGTGAAGCCACAGATGACTC
+
/1866?4999==ACCCBDBA>?;888,7133<?@A@;?BCBBBCBA66>8;@=@A@A<-)((((
@T6XXI:00088:00794
GGGGAATTCTAATACGACTCACTATAGGGAGAGGGAGAATAGTCCGCGATTTGTGCATGCGGCCGGCTCCTTCCTTT
CTCCCTTCTCCC
+
```

```
DHE:C<:;;CC@CBBAACCB@?A;@666.7==>>:@=?>@@@@A>==:;;C>CCAAABCA@<@:?:?A>9;9;3881
8==A6:7:444-
@T6XXI:00088:00799
GGGAGAAGGGAGAAAGGAAGACAGT
+
DD>BBB>AB:@99809;:90,))((
@T6XXI:00089:00750
GGGAGAAGGGAGAAAGGAAGGAGAAGTCGCCCCAAACGATTTGTTGACTATTCTCCCTCTCCCTATAGTGAGTCGTA
TTAGAATTCCCC
+
CCAC@B@BB>?@@B>C?A?B?BCC@ACB>@><6@C?CCBBC>C@?B??=AA=@?AC>?=?;=;<<???@@@@===??
?@C<;43/444-
@T6XXI:00089:00756
GGGAGAAGGGAGAAAGGAAGGAAAAAGCGACGTCGGGGAAACGTTTGCTATTCTCCCTCCTCCATA
+
DEACBA@CB>?B?B=C@B@B<??;;0?BBCCCBBABB9CF@CCBC@CCBCC@C;;:0099*)))))
@T6XXI:00089:00766
CTATCACTCGAGATCTGCGGCTTTGGACAGTCCCTCTGTACTGTGAAGCCACAGATGTCGAGTCG
+
CBB@<;:9@;;A??B@>??5877-313116111,1111110311--,-87877333611166786
@T6XXI:00089:00772
GGGGAATTCTAATACGACTCACTATAGGAGAGGGAGAATAGCTTACGT
+
CCD:C@C@BBB@BBBBBBBBB:665..,0@<AC>D@A>;0+,,+,,,,
@T6XXI:00090:00765
GGGAGAAGGGAGAAAGGAAGGAGACGTGAGGGTGCAGCCAAAAGTGTTGAC
+
44-4999=A=?ABC;<;:@C>BEB@A=@=9=:=333331111,1111;@@6
@T6XXI:00090:00774
GGGAGAAGGGAGAAAAGGAAGC
+
99149>=CC094111*/54,..
@T6XXI:00090:00777
GGGGAATTCTAATACGACTCACTATAGGAGAGGGAGAATAGAACCTCTGGATGCGGGGACTAGACTCCTTCCTTTCT
CCCTTCTCCC
+
BCC:C>@>@CCACB@@BBBB?7997/.,0::>>7:9@>AB@@3738;=@?DCGCCCC7BBBAABCCC875:;??AAB
@D@D699A?:
@T6XXI:00090:00790
GGGGAATTCTAATACGACTCACTATAGGAGAGGAGAATAGTACA
+
88808+954449;9:;AA?@??>B?76067774744//60,++*
@T6XXI:00091:00767
GGGGAATTCTAATACGACTCACATAGGGAGAGGAGAATAGTACGACCAATCTTC
+
>886>?B@CCC@CDCBA@A@@;@>777.7=::8877>=88884404/0/1117.
@T6XXI:00092:00751
GGGAGAAGGGAGAAAGGAAGGAGAAGTCGCCCCAAACGATTTGTTGACTATTCTCCCTCTCCCTATAGTGAGTCGTA
TTAGAATTCCCCC
+
CD@BBA@BC@BBBB>B@CBA?AAA@ACCCCCC9EC>BBBBB>BB@CBB>??=?;==;=8:;=;>=?@?@?A@>888<
<=<=944/--//+
@T6XXI:00092:00778
GGGAGAAGGGAGAAAGGAAGGAGAAAGTCGCCCCAAACGA
+
1>;?@?@BB:@===:=<=9917878,164499:6840400
@T6XXI:00092:00792
```

```
GGGGAATTCTAATACGACTCACTATAGGGAGAGGGAGAATAGTCAACAAATCGTTTGGGGCGACTTCTCCTTCCTTT
CTCCCTTCTCCC
+
944,48@=ADNCBBBBCDCB?@@A>777.77777.7=>?A???@H>@;;6=000>9>>?5>?>760768/3247::4
;<>?;@;==@C?
@T6XXI:00093:00751
GGGAGAAGGGAGAAAGGAAGCTAGTCGACTCGACATCTGTGGCTTCACAGTACAGAGGGACTGTCCAAAGCCGCAGA
TCTCGAGTCGACTAGCTTCCTTTCTCCCTTCTCCC
+
A?:@?B@BB?B;99096868@>::7********22236:@=55;@CDBBCC888;;::4<;;6;;;@@<??:@@@@@
?=788487:3335743549/7844?CD=B=@@AD@
@T6XXI:00093:00752
GGGGAATTCTAATACGACTCACTATAGGAGAGGGAGAATAGCGATCGTGCCACATCCGTCCTATTCTTCTTCCTTTC
TCCCTTCTCCC
+
BBB8B?@?BBB@AAABAAAAA:997./-
0>=BL>BBB@B====;;:::B=<<9=@@?C=99=BB@CC@CC@C?CB>BBDD>C??BBB>
@T6XXI:00093:00773
GGGAGAAGGGAGAAAGGAAGCTAGTCGACTCGACATCTGTGGCTTCACAGTACAGAGGACTGTCCAAAGCCGTCCTC
GAGT
+
ED@AAAACD@@@@@<>;?=?@?A@A:::@AC:::BB@@774/4737BC:;:A@@@??=????AA>9:6664///,-
11344
@T6XXI:00093:00780
CTAGTCACTCGAGATCTGGCGGCTTTGGACAGTCCCTCTGTACTGTGAAGCCACAGATGTCGAGTCGACTAGCTTCC
TTTCTCCCTTCTCCC
+
>>111>8==FBCAAABBB?AB@BBB;B>?B@@@@B>B@>@AAA<<@@@>AC?BBDBB@>>?AACCBB::<CCBC@C>
@?<?AAA<A>@@AA<
@T6XXI:00093:00797
GGGGAATTCTAATACGACTCACTATAGGGAGAGGGAGAATAGTCAACAAATCGTTTGGGGCGACTTCTCCTTCCTTT
CTCCCTTCTCCC
+
CCC9C@C?CDC@B???????>AB77111.7>>BC>B@@?A;@@@A>A==;A@@>?;@BB7BCC@?AAA?;=;9<AA<
A@AA<A;==A@<
@T6XXI:00095:00751
GGGAGAAGGGAGAAAGGAAGCTAGTCACTCGACATCTGTGGCTTCACAGTACAGAGGGACTGTCCAAAGCCGCAGAT
CTCGAGTCGACTAGCTTCCTTTCTCCCTTCTCCC
+
DB>CAA>A@:@999044843;7642,,,,,,,,+27>99996:5:BA@BFC<<<88818@@ABA?AA<AA<888<<8
87;>>>@@???@@@@A>@=@?:==;=6<9=ACH@
@T6XXI:00095:00756
GGGAGAAGGGAGAAAGGAAGCTAGTCGACTCGACATCTGTGGCTTCACAGTACAGAGGGACTGTCCAAAGCCGCAGA
TCTC
+
CCAAAA@CC>@@@@9><A8;;DCCBBBBAC@B88;AAABB@>@C@CBBAC?==<<44417AA?==;=A<AB@<<<A@
@@A@
@T6XXI:00095:00783
GGGGAATTCTAATACGACTCACTATAGGGAGAGGGAGAATAGTGACGTGTTGTGCGGCGCTCACTGTCTCCTTCCTT
TCTCCCTTCTCCC
+
AA@2@=A=<9;ACCCCD@@;::;:A@@A9???B?<@8834444;;:===?<?A@@C>@@=<878=888@@;???AAB
<???C:?833111
@T6XXI:00096:00762
GGGAGAAGGGAGAAAGGAAGGAGAACGCGCACCCGAACCAACGGTGATCTATTCTCCCTCTCCCTATAGTGAGTCGT
ATTAGAATTCCCC
+
```

```
CDACCB@CC?BBBB?C@C@C@BBB>AAAAAAAA=AB@C@DDDG@CCCDA@AB?BBBB=BBBDC=BAABAAACA?BBB
BC@AAA>D@CDC:
@T6XXI:00096:00763
GGGAGAAGGGAGAAAAGGA
+
E@:B99584-44///*/,.
@T6XXI:00096:00772
GGGGAATTCTAATACGACTCACTATAGGGAGAGGGAGAATAGTTCTCGTCAGTTGTTGCTAACTATTCCCTTCCTTT
CTCCCTTCTCCC
+
AAA6A=@AFBB@B@AABAAA>@>77111.:A>CC?A????<<<;@@@@A=9=?>@A@AACC?BBCE?BB>B?C?CC>
BACC>B9<<AA<
@T6XXI:00096:00778
AGGAGAAGGGAGAAAGGAAGCTAGTCACTCGAGATCTGCGGCTTGGACAGTCCCTCTGTACTGATGAAGCCACAGAT
GTCGAGTCGACTAGCCTTCCTTT
+
>>24/6,//+////+.99::BDCBBAAAAABBB@=@@666510,/,.6488AB?CAC>>;//00.4444B=??BCB@
B@A@@@?AA11/6.,*,22222+
@T6XXI:00097:00755
GGGAGAAGGGAGAAAGGAAGCTAGTCGACTCGACATCTGTGGCTTCACAGTACAGAGGACTGTCCAAAGCCGCAGAT
CTCGAG
+
CC>BCCAHNAB;99-9<?688>:9:BBCCBCCCCB@??CB@>?A?ACCCBBA998/.-
/6998=;=A<AA>AA<=98888@@@
@T6XXI:00097:00757
GGGAGAAGGGAGAAAGGAAGCTAGTCGACTCGACATCTGTGGCTTCACAGTACAGAGGACTGTCCAAAGCCGCCAGA
TCTCGAGT
+
991444:;;-
9999047>;>?????BCC@:9777111179:::A@C;;;AA=8:;//.0;=;;A>;?9;99//./;;A@?>?@@A
@T6XXI:00097:00758
GGGAGAAGGGAGAAAGGAAGAATATTGCGCGTTTAACGGGGTGATGGCTATTCTCCCTCTCCCTATAGTGAGTCGTA
TTCAGAATTCCCC
+
BC>B;98<B?B;<<<<@B@BB?BDC@CCCCB@@?@=@?CC9CBB::7:?A?;>???:??@AA<A99:A@A?@888>=
>8<878445355.
@T6XXI:00097:00770
GGGGAATTCTAATACGACTCACTATAGGAGAGGGAGAATAGTCAACAAATCGTTTGGGGCGACTTCTCCTTCCTTTC
TCCCTTCTCCC
+
>A;,42424442449=<BCBC>?>:00,000?C>C@A<9======@?B??777=6=>>5A?;;68?8658:@>AC7<
6<;63443222
@T6XXI:00097:00771
GGGGAATTCTAATACGACTCACTATAGGGAGAGGGAGAATAGTCAACAAATCGTTTGGGGCGACTTATCCTTCCTTT
CTCCCTTCTCCC
+
@=@6??A>AAAACCCCC;<;B?>=>777,7<=>?9?4443;?;442344-
7>7775:>>6@::9B4938565;8;=;=44555...47,
@T6XXI:00097:00774
GGGGAATTCTAATACGACTCACTATAGGGAGAGGGAGAATAGTCACATTCATGTTTGTGACCGTGTCTCCTTCCTTT
CTCCCTTCTCCC
+
CCB9B@A?BDB@AB@@@BBCCCCCA99956<;?B>BAA>A8??BB??A?BB@BBC>@A???<?@=??8968<A>AC;
@@AA<A>AAAA<
@T6XXI:00097:00775
GGGGAATCTAATACGACTCACTATAGGGAGAGGGAGAATAGTCAACAAATCGTTTGGGGCGACTTCTCCTTCCTTTC
TCCCTTCTCCC
```

```
+
8>>0=+1///,/44437<A@@B??667-
777=?9?8?;@=99<=<@A@<CBABB<???5?BB?A=??;3727<>>:???;:<<???@:
@T6XXI:00097:00781
GGGAGAAGGGAGAAAGGAAGTGTTTGTCGGAGCAGCCGGTTTAGAGGCTATTCTCCCTCTCCCTATAGTGAGTCGTA
TTAGAACTTCCCC
+
BFACCC?BB=BA@B9<5;>@:<9B>C:>:@CCCCBB@B@CA<A@9948@>@?CCEC>BB?LE<?::98600333000
4/0068668:<<.
@T6XXI:00097:00797
GGGAGAAGGGAGAAAAGGAAGCTACGTCGACTCGACC
+
;;69A@599199999/958137313311116611110
@T6XXI:00098:00750
GGGGAATTCTAATACGACTCACTATAGGGAGAGGGAGAATAGCTACGTTTAACCGATCGTGTTGTTCTCCTTCCTTT
CTCCCTTCTCCC
+
CCC:C@C@CCB@BCCCA::;91377111,1777BC@===@===BCBAAA=?;?=@===A@@??A=>@<<>@>@<??<
?AA=;:<>9==;
@T6XXI:00098:00756
CTAGTCGACTCGAGATCTGCGGCTTTGGACAGTCCCCTCTGTACTGTGAAGCCACAGATGTCGAGTCGACTAGCCTT
CCTTTCTCCCTTCTCCC
+
:--
,,,,,8>:=?@AAAABBB@;<;68=887=7777,1666;?>>?;7857@ADA;;;?877??<=@>00.4..,.3344
::6=8=;=;=:<84
@T6XXI:00098:00764
GGGAGAAGGGAGAAAGGAAGCTAGTCACTCGACATCTGTGGCTTCACAGTACAGAGGGACTGTCCAAAAGCCGCAGA
TCTCGAGTCGACTAG
+
CH@B;969>:>99909484788=8????<AA::8::6@AC:@@@CCCBBBC@@<<==;==:9;9/333-
4434:;;<<<==AA@@@=<<??;
@T6XXI:00098:00774
GGGAGAAGGGAGAAAGGAAGCTAGTCACTCGAGATCTGCGGCTTTGGACAGTCCCTCTGTACTGTGAAGCCACAGAT
GTCGAGTCGACTAGCTTCC
+
=959=@>@@198>?9>;?<8;;>@@AAA?AAA?ACB8;;E?AAC>A=ABCCBCC>99:@@?@@???@:=<>:88??8
7439;8;4374440442/,
@T6XXI:00098:00782
GGGAGAAGGGAGAAAGGAAGCTAGTCGACTCGACATCTGTGGCTTCACAGTACAGAGGGACTGTCCAAAGCCGCAGA
TCTCGAGTCGAC
+
CG@CCD@BB?BBBB@B>A?CC><<<7::BBB@::;???A@=;<?=?CCCCCCA?9000-
4;;@AB?AA:BA>??;<<<<??AA@@@@@A
@T6XXI:00098:00790
GGGGAATTCTAATACGACTCACTATAGGGAGAGGGAGAATAGTTTTCCCTTCACTAACCGCGAACTGCACTTCCTTT
CTCCCTTCTCCC
+
CCB7B>B?BCC@CBB?>>BB>@>77111.7@@CC@A??>@?====4>?:?>ABBAC@C>888A?A@@@CA?:=3776
7;=A7=;=A@A<
@T6XXI:00098:00795
GGGGAATTCTAATACGACTCACTATAGGGAGAGGGAGAATAGAGACGTATTCGTTTGCGCTCTGTTCACCTTCCTTT
CTCCCTTCTCCC
+
?@@6@AB@CCB@BCBBB>B>BA>97111.7>>BC>C@A>@<558@@A<==>@AAC<AAAA=<<<=>A4448=@=@A<
A???<??AAAA<
@T6XXI:00099:00752
```

```
GGAGAAGGGAGAAAGGAAGGAGAAGTCGCCCCAAACGATTTGTTGACTATTCTCCCTCTCCCTATAGTGAGTCGTAT
TAGAATTCCCC
+
<6<BB@CC@B>==5>;<;??BBB?BB@@@@<2>>:?ABCC>CA>AAA?AA>B=<<599:;<7=AAAA???A===A@A
>A?=9;2444-
@T6XXI:00099:00755
GGGAGAAGGGAGAAAGGAAGAACAGCAACATGTTGAGAATCGGAGAACTATTCTCCCTCTCCCTATAGTGAGTCGTA
TTAGAATTCCCC
+
CC>ABA@BA=@@@B=B@B@BC@BA=@@@CBB@C=AAC@>BAB@A::>??@?>????;>:<==;=9=@AAAAA;:;::
:<8?B@B38880
@T6XXI:00099:00770
GGGGAATTCTAATACGACTCACTATAGGGAGAGGGAGAATAGTCACATTTATG
+
DDC:C;99;BB@CCBBB???@A@97111.7==>AA@?>>A????99:3//,*0,
@T6XXI:00099:00772
GGGGAATTCTAATACGACTCACTATAGGGAGAGGGAGAATAGTTCGCCAATATCCCGCGGCGTGTTCTCCTTCCTTT
CTCCCTTCTCCC
+
AAA7A>A?BBC@CBBBA??B?>;?=666.7>?CE>B@@>A<<<?A==9?>?B???=@BCACB@@A<??A>?<A?AC<
B@@@:?8;<==7
@T6XXI:00099:00773
GGGGAATCTAATACGACTCACTATAGGGAGAGGGAGAAATAGTCAACAAATCGTTTGGGCGAC
+
444,4244>@<>@@;88??77811111,111.4242222-/---103350899>>;@C1..0*
@T6XXI:00099:00774
GGGGAATTCTAATACGACTCACTATAGGGAGAGGGAGAATAGTTCTCTTTTTCTGCTGCACCGTTTACGCCTTCCTT
TCTCCCTTCTCCC
+
BBA7A798<CC@CCB@@@?BBCB>;111.6=<<?<?CC>A???<;944444,4405=@@A<;===;==<<;:89/33
-44:;7><???A?
@T6XXI:00099:00791
GGGAGAAGGGAGAAAGGAAGTGCAGTTCGCGGTTAGTGAAGGGAAAAACTATTCCTCCCTCTCCCTATAGTGAGTCG
TATT
+
A9649;??;:>89;849B@;<;BA>?=99379?57777>47:49?>?2?3873/,/::6///777577<996:8431
-++*
@T6XXI:00050:00804
GGGAGAAGGGAGAAAGGAAGCTAGTCGACTCGAGATCTGCGGCTTTGGACAGTCCCTCTGTACTGTGAAGCCACAGA
TGTCGAGTCGACTAGCTTCCTTTCTCCCTTCTCCC
+
ICB<=<>@@:?99918<><A@>:=<000:9:000:??8?@C?BAB<?<?@@@????9::8>??@@???A??A>@@A??
?@878A=88487;;8448;244-44994;<==>@:
@T6XXI:00050:00810
GGGAGAAGGGAGAAAGGAAGCTAGTCGATCGAGATCTGTGGCTTCCATCTGTGGCTTCACAGTACAGAGGGACTGTC
CAAAGCCGCAGATCTCGAGTCGACTAG
+
BBE@@B?BB>BCCC>B?@ABBB>>>@@@C?>?>???>?@@>=?=>==<@A@>???@?A@<=====<;88878@====
:;;:;=;==57777//0===>;BAAA>
@T6XXI:00050:00817
CTAGTCGACTCGAGATCTGCGGCTTTGGACAGTCCCTCTGTACTGTGAAGCCACAGATGTCGAGTCGACTAGCTTCC
TTTCTCCCTTCTCCC
+
>2,---
,,//8;BAABCCCBC@CCC>C@DB??877.7A?BCDEB=@@A??B@CCCDBBC@@@CBCCCCCBAABB?B@FDBBAA
A=A>BCCC>
@T6XXI:00050:00829
```

```
GGGAGAAGGGAGAAAGGAAGGAGAAGTCGCCCCAAACGATTTGTTGACTATTCTCCCTCTCCCTATAGTGAGTCGTA
TTCAGAATTCCCC
+
991944244-
499;588=<@?BBC@B:;;???.;;9;<BAC88:?AA?@?D??AAC=;////,//68/.8A????::;.//::7:7?
?=1
@T6XXI:00050:00831
GGGGAATTCTAATACGACTCACTATAGGGAGAGGGAGAATAGTCAACAAATCGTTTGGGGCGACTTCTCCTTCCTTT
CTCCCTTCTCCC
+
BEIAC::;;CC@BBBBBAAAAA999311.7>>?C>CCC????<@C>???;AC@@A>?>?7AABBE?CBA?B?A>AA:
>>@C<A9<=AA;
@T6XXI:00050:00837
GGGGAATTCTAATACGACTCACTATAGGGAGAGGAGAATAGTCTACAA
+
DDC9C797;<<7<BBBBBCBBBCB>767./000,.55,./?6/..---
@T6XXI:00051:00806
GGGGAATTCTAATACGACTCACTATAGGGAGAGGGAGAATAGACATGTGATCTCCGCACTTGCGCTCTTCTTCCTTT
CTCCCTTCTCCC
+
BBA7A:;;<CD@BCBCBBAE:8;AA:::5:@BBC>BBB?B??5;784;=889??EAAAA@?D@@@???>@?=@=@@<
@@AC:>;<;:;7
@T6XXI:00051:00811
GAATTCTAATACGACTCACTATAGGGAGAGGGAGAATAGTCAACAAATCGTTTGGGGCGACTTCTCCTTCCTTTCTC
CCTTCTCCC
+
GB?A@CBCACB??BBBBBA>;;76959@@CC>BBB>>>>::;=AAA<BA@>?8:8:6;<<?B?BCC>@>?@AA<AAA
A<A>A@A@;
@T6XXI:00051:00831
GGGAGAAGGGAGAAAGGAAGGAGAAGTCGCCCCAAACGATTTGTTGACTATTCTCCCTCTCCCTATAGTGAGTCGTA
TTAGAATTCCCC
+
GDBB@@=@A:?AAA<A?ABKAG@@@@BCBBBB8BB=BCAA<8649;00.4674000+03389;<;8<<=<@A@=<<C
C>@A74434461
@T6XXI:00051:00832
GGGAGAAGGGAGAAAGGAAGCTAGTCGACTCGAGATCTGTGGCTTCACAGTTCAGAGGACGTAGTCCAAAGCCGCAG
ATCTCGAG
+
CD?CCC>?><@AAA<@=@:<;CCCBBBCC@@>9::7769=>888@C@=?774777040/-
++++++*.4+..7999<>>;8??AA
@T6XXI:00051:00833
GGGAGAAGGGAGAAAGGAAGT
+
FDBEDC?AA:=9//+1,1,-,
@T6XXI:00051:00837
GGGGAATTCTAATACGACTCACTATAGGGAGAGGGAGAATAGATCACGTAGCGTTCGTGTGTCGATTCTCCTTCCTT
CT
+
99929295<A>?BBBDBCCCCCCC@@@D=AACDC<B??;?@:9:ACEFBBBAAB????B@==9=;89933/022,-
,--
@T6XXI:00051:00844
GGGGAATCTAATACGACGTCACTATAGGGAGA
+
8:8+/,9444244444443;97444387,...
@T6XXI:00051:00845
GGGGAATTCTAATACGACTCACTATAGGGAGAGGGAGAATAGTCTCTTCTCGTTCGGCATCACGGTGTTCCTTC
+
```

```
=EE:D?A?BAA?@@A;::>>>>733311,16111,16601./././.5=9::@>B@CC?AA::66441--,-3322
@T6XXI:00051:00849
CTAGTCGACTCGAGATCTGCGGCTTTGGACAGTCCCTCTGTACTGTGAAGCCACAGATGGACTGTCCTTCCTTTCTC
CCTTCTCCC
+
ECECBBBBBCC@BBBBBBBBB@9776:=>?BCCBB>BC@??;??>BBC??C?CCCCCCC=@@CC;87;<?=@CACAA
BBA@BCBBA
@T6XXI:00052:00800
GGGGAATTCTAATACGACTCACTATAGGGAGAGGGAGAATAGCCGTCAAT
+
999-4:A?BAB?BCBBA@@BBB@@;766.:::CDABBB=@7/.,+++((*
@T6XXI:00052:00801
GGGGAATTCTAATACGACTCACTATAGGGAGAGGGAGAATAGCTACGCTTAACCGATCGTGTTGTTCTCCTTCCTTT
CTCCCTTCTCCC
+
CCC7DCB?BCC;<<AAACCCCCCC@@@C?AABCCACBCCBBA=@@CBBB=@=@CE?@AC??<:;;8<??<?=@<??:
====7>=@@@@<
@T6XXI:00052:00805
CGAGATCTGCGGCTTTGGACAGTCCCTCTGTACTGTGAAGCCACAGATGTCGAGTCGACTAGCTTCCTTTCTCCCTT
CTCCC
+
=?=9998AA>A=@BB>B@CCBBBCC>BBCCCB@?:::@>B?<AC@??BBAECBBCCCCECBA996969919@@@195
9988/
@T6XXI:00052:00817
GGGAGAAGGGAGAAAGGAAGGATCGAGAAATATGGGCCGGTGTTGGACTATTCTCCCTCTCCCTATAGTGAGTCGTA
TTAGAACTTCCCC
+
DHBA4429919@AA>B@CAC?A>CBB@@@;?BBBC>C?B?=?>;?<?BBBC?BCBB>;;9@B<?@@@===??=?883
0/4055342333.
@T6XXI:00052:00821
GGGGAATTCTAATACGACTCACTATAGGAGAGGGAGAATAGACAACAATTCTACCGTCCGCTATGTACCTTCCTTTC
TCCTTCTCCC
+
BB?4@5959445449;8=>>>8466..,.4579787;>B@7336400216668::89>=:86==88348824244-
33313116662
@T6XXI:00052:00831
CTAGTCACTCGAGATCTGCGGCTTTGGACAGTCCCTCTGTACTGTGAAGCCACAGATGTCGAGTCGACTAGCTTCCT
TTCTCCCTTCTCCCCTTCCTTTCTCCCTTCTCCC
+
C=111>;DDCBBBB@AAABA@???;B@BBAAA@@>@ACA>==?@CCC>:B@BBBBBBAAAA?=<BBCB@@@A?;A?A
C>DBBD>B=?7444-48:8:<4<==>7=8;444-
@T6XXI:00052:00844
GGGAGAAGGGAGAAAGGAAGCTAGTCGACTCGAGATCTGCGGCTTTGGACAGTCCCTCTGTACTGTGAAGCCACAGA
TGTCGAGTCGACTAGCTTCCTTTCTCCCTTCTCCC
+
DF?@?><>>:>?>?<@?A?BCB@@@:;:::AACDCC@BBCE?BBB>B@CCCCCCC>CCAAA@@@@A@B?@A<?>AAA
AAA548@?>??===<77/49;;:888A:@=@@A@;
@T6XXI:00052:00848
GGGGAATTCTAATACGACTCACTATAGGAGAGGGAGAATAGACACCAATCGTGCCGGGTCATGTTCTCCTTCCTTTC
TCCCTTCTCCC
+
AAA6@:;;<BC@EAAABBB?@>=;;0/,0;;?A>BED?@@?<?@@;><A???=B5995:CCCCB>BAC@D;=;=@7>
@@@;@<=;;;4
@T6XXI:00053:00800
GGGAGAAGGGAGAAAGGAAGCTAGTCACTCGACATCTGTGGCTTCACAGTACAGAGGGACTGTCCAAAGCCGCAGAT
CTCGAGTCGACTAGCTTCCTTTCTCCCTTCTCCC
+
```

```
CC?CCC@BA<@@AA>A:;:<B@>>?700/...3345599>2:;;?>=;:;@>??477.44449;/8817;28=8888
8304>73335000564265664688;10/3403+
@T6XXI:00053:00831
GGGGAATTCTAATACGACTCACTATAGGGAGAGGGAGAATAGTCCTTCATACTTTGTAAGCGCTATCTTCTTCCTTT
CTTCCTTTCTCCCTTCTCCC
+
?@@4>596999:>AAAB<<<BB?DA877-
7>>>?>ABA>:774434/0?><777.7<==B?A@ACC@@>@A@@?BC>BD@A>AA;@@CC<B588;:4
@T6XXI:00053:00836
CTAGTCGACTCGAGATCTGCGGCTTTGGACCCCTCTGTACTGTGAAGCCACAGATGTCGAGTCGACTAGCTTCCTTT
CTCCCTTCTCCC
+
B@-
,,,,,686;BCCBBBB::8;BC>C@CCCC9BCCBBBB@BBBC@CC?B@?@GDCB?BBCBABC>>A::<?B>AA<@?>
>8<:<==?9
@T6XXI:00053:00848
GGGAGAAGGGAGAAAGGAAGGGCGTGTTCGTGC
+
4B:449249-44991/7;,1/+.0.99-/0973
@T6XXI:00054:00814
GGGGAATTCTAATACGACTCACTATAGGGAGAGGGAGAATAGTCAACAAATCGTTTGGGGCGACTTCTCCTTCCTTT
CTCCCTTCTCCC
+
@??6?>A@<949=;9;=A;;>>@B>>BB577777.7>@=AAB@<<9<:<;CBC>B?BCC9C@?:737>>:=:=444-
49944644000+
@T6XXI:00054:00824
CTAGTCGACTCGAGATCTGCGGCTTTGGACAGTCCCTCTGTACTGTGAAGCCACAGATGTCGAGTCGACTAGCCTTC
CTTTCTCCCTTCTCCC
+
@--
,,,,,/689499=>>@995988,7579BCBBB>BDCCCBBBBCB?>8;8<@AABBCBB>@@BBB;=1...,.65,./
-/1>>2636...+
@T6XXI:00054:00828
GGGAGAAGGGAGAAAGGAAGGAGAAGTCGCCCCAAACGATTTGTTGACTATTCTCC
+
:D@AAE@B@:8999149999;=331364337;579773316611016616/:4444
@T6XXI:00054:00834
GGGAGAAGGGAGAAAGGAAGCTAGTCGACTCGACATCTGTGGCTTCACAGTACAGAGGGACTGTCCAAAGCCGCAGA
TCTCGAGTCGACTAG
+
DDBDDC>@=9>988095959?800?00,7777-
0.33535?>AAABCCC@@??;930317>>@AA>A@<@@>@@????@@@A@@AAACC???
@T6XXI:00055:00804
GGGAGAAGGGAGAAAGGAAGTGCAGGTGGAGAGCAGTAATTTAACTGCTATTCTCCCTCTCCCTATAGTGAGTCGTA
TTAGAATTCCCC
+
EFBD@@@BB>>AAC@C@B@BBACCB@CCABBBBBBABA>AA>BACCBBBCC@CAAA>99;AA>BBBCCCCCCCBCAA
AGCBD?B@EGEB
@T6XXI:00055:00833
GGGAGAAGGGAGAAAGGAAGAGAACAGCTGCTTTGGAAACAGTCAGACTATTCTCCCTCTCCCTATAGTGAGTCGTA
TTAGAATTCCCC
+
DEBCBC@CD@BBBC>CBC@CBBA>BCCCCCCBB>B@CCAEBDECBBBBBBB?BDCG>@??@@9???BBBCCAA@???
?;>>??@<@@@4
@T6XXI:00055:00839
GGGAGAAGGGAGAAAGGAAGCTAGTCGACTCGACATCTGTGGCTTCACAGTACAGAGGGACTGTCCAAAGCCGCAGA
TCTCGAGTCGACTAG
```

```
+
DD@BCD@CC?BCCC>B@B?CBB999/00::;@@B?:;:BBD@BB?BC??>>?@C<988468:<<@==;:;A>99==<
<889==<<@@AAAA;
@T6XXI:00056:00811
AGTCACTCGAGATCTGCGGCTTTGGACAGTCCCTCTGTACTGTGAAGCCACAGATGTCGAGTCGACTAGCTTCCTTT
CTCCCTTCTCCC
+
DDBA@?CBBBFA>>;>995844-
49;=>?CDC>BCCCCBBBCB?B@CC@BBCCBAB<??<@??@BA??76:7:>BC=BABB=B?@;;<6
@T6XXI:00056:00814
GGGGAATCTAATACGACTCACTATAGGGAGAGGGAGAATAGTTTTTCCCTTCACTAACCGCGAACTGCACTTCCTTT
CTCCCTTCTCCC
+
9<<4<BBBBC@CCCBBB@@@DEB@>??86=???9?A>;=>74334,49-
4/07999?B@AAAC@CC?8::>:=477.77>@<B?A<<:6
@T6XXI:00056:00835
GGGGAATTCTAATACGACTCACTATAGGGAGAGGGAGAATAGTCAACAAATCGTTTGGGGCGACTTCTCCTTCCTTT
CTCCCTTCTCCC
+
BDE:D>@@@CC@GABBBBBAAA<99311.7==?B>AAA@BBC@AA;<;<;AD>AA<A??6ABBBB=A??=?=<=AA<
@AAA;@<??AA<
@T6XXI:00056:00836
GGGGAATTCTAATACGACTCACTATAGGGAGAGGGAG
+
944,49?994424449/6.009000:>9642,,,+,,
@T6XXI:00057:00805
GGGAGAAGGGAGAAAGGAAGGAGAAGTCGCCCCAAAGAGTTAT
+
CCECC=<=A7;;;B<@=A@C=A>BAB?;8777277233310,,
@T6XXI:00057:00808
GGGAGAAGGGAGAAAGGAAGCTAGTCGACTCGACATCTGTGGCTTCACAGTACAGAGGGACTGTCCAAAGCCGCAGA
TCTCGAGTCGACTAG
+
CB?<<;@CC>BAA?:>=@=@B@AAD::7>==7;<?BA6?<<;:<@A??@BA@??;444-
4;;AAA?CA<@@688@@AAA@@@?>>;<<:<<;
@T6XXI:00057:00814
GGGAGAAGGGAGAAAGGAAGCGTCAATCGTGGGCAGAAGGGTCGTGATCTATTCTCCCTCTCCCGTATAGTGAGTCG
TATTAGAATTCCC
+
9=5@AA>AA<@>=95;,1-10..7734@@?976788747?:>>??::7>;<<<==4;686///,----
38<6<;;730665444/0/00+
@T6XXI:00057:00820
GGGAGAAGGGAGAAAGGAAGGACAGTTTTTGGGAGAAGGGAGAAAGGAAGGAGACAGTACCGCCCAAAATTGGAAGA
CTATTCTCCCTCTCCCTATAGTGAGTCGTATTAGAATTCCCC
+
DCBAA?BDD>BBBB<@;?<?=ABCDDCBB2BC<BBC?AA;BBBC>C@A9=9=??ACCB@B@@AA<BCC9C?CBC?C?
BABB=?77717>;<<9=DAAA@@@@@<<<@A699C?A:???5
@T6XXI:00057:00822
GGAGAAGGGAGAAAGGAAGCTAA
+
929496;C>B>B>,1,/,,,,,.,
@T6XXI:00057:00827
GGAGAAGGGAGAAAGGAAGGAGAATCGACACACGAACGCCTACGTGATCTATTCTCCCTCTCCCTATAGTGAGTCGT
ATTAGAATTCCCC
+
C@CCB@CD>AA@@=B?B@CCCBB?CBAA@?BBBCG@GBB?CCDCGBBCCEBB?BBID@A@ACC>CCBBBCBB?BCBB
;?>>>>>B?BBB8
```

```
@T6XXI:00057:00832
GGGGAATTCTAATACGACTCACTATAGGGAGAGGGAGAATAGCTACGTTTAACCGATCG
+
444,4:;:;BG@<99=;8773333331798<=CA;@BA:?774<666676601011110
@T6XXI:00058:00815
GGGGAATTCTAATACGACTCACTATAGGGAGAGGAGAATAGCTTT
+
/11+1,96<A@@DCCCC?>>CCCCBAB=94:./,0<>:<70-000
@T6XXI:00058:00829
GGGAGAAGGGAGAAAGGAAGCTAGTCGACTCGACATCTGTGGCTTCACAGTACAGAGGGACTGTCCAAAGCCGCAGA
TCTCGAGTCGACTAG
+
CCBCCB899-9844-
;>@=BBAAAA677>777????>ABCB??@@CBBBB>@AA=9884666?A@>@C<AA=@@=@?;;??@@@AAABBBBA
@T6XXI:00058:00830
GGGAGAAGGGAGAAAGGAAGGAGAACA
+
EDABCB?BC?CB;;;878,/11==2-/
@T6XXI:00058:00835
TCGAGATCTGCGGCTTTGGACAGTCCCTCTGTACTGTGAAGCCACAGATGTCGAGTCGACTAGCTTCCTTTCTCCCT
TCTCCC
+
BGDDA@@@=@=B=@@C?D?BBCCBCC>CBBBB@B?????:?;8<<;><74344449;;;;4444424244-4444-
499433,
@T6XXI:00058:00841
GGGAGAAGGGAGAAAGGAAGAGCAAAAGGGAGGCAATGAACGAGTCGCTATTCTCCCTCTCCCTATAGTGAGTCGTA
TTAGAATTCCCC
+
FEBBBA@CC>BCCC>A?@ABBABAAA7AB?CCACB@BBB?BB@@BBBBBBB@BBBB<BBBBB;?=AA??@ABBB??@
A>@@@>B<???5
@T6XXI:00058:00845
CTATCGACTCGAGATCTGCGGCTTTGGACAGTCCCTCTGTACTGTGAAGCCACAGATGTCGAGTCGACTAGCTTCCT
TTCTCCCTTCTCCC
+
C8661/118:@CBBBBBC@>=333/8;>???87757A?CBBAA?C?>=8@@ABBABB>>?ABA?BBCBAB<;:=A?C
E@CBBB>C@@@=@;
@T6XXI:00058:00847
GGGAGAAGGGAGAAAGGAAGGAGAAGTCGCCCCAAACGATTTGTTGACTATTCTCCCTCTCCCTATAGTGAGTCGTA
TTAGAATTCCCC
+
BB@CCB@BB=BBBB>B@B?B?BCA?ABCBCBB8BB=BBB>B<AA@BBB>>>>99==;<<<?<;?9==AAAAAA?@??
?>AAA?A<???4
@T6XXI:00059:00817
GGGAGAAGGGAGAAAGGAAGCTACGTCGACTCGAGATCTGCGGCTTTGGACAGTCCCTCTGTACTGTGAAGC
+
?B6<4=89964488042424.....69856996:66688<@C@CFD>B511777;6234000004000/,-1
@T6XXI:00059:00839
GGGAGAAGGGAGAAAGGAAGCTAGTCGACTCGACATCTGTGGCTTCACAGTACAGAGGGACTGTCCAAAGCCGCAGA
TCTCGAG
+
BBAA@@;@@<@>AA<@>@7<;@BBBCBCCBBBAAB??:::B>AB@CCCC>BBBB????<?@@@A??C=5::><====
AAAAAA@
@T6XXI:00059:00843
GGGGAATTCTAATACGACTCACTATAGGGAGAGGGAGAATAGTCAACAAATCGTTTGGGGCGACTTCTCCTTCCTTT
CTCCCTTCTCCC
+
```

```
DFD:C@CDECC>AACA@@;;;;88@877-:6=BE>CCC?AAA<<<>@AA<BA@@@.766-
8=::<,0//,/:;::=9;@@A<A?@888-
@T6XXI:00059:00844
CTATCCGACTCGAGATCTGCGGCTTTTGGGACAGTCCCCTCTGTACTGTGAAGCCACAGATGTCGAGCGACTGC
+
6--,,*,,,8844944>999427333,33,33777666211166999977?==BABB@9900..442///4426
@T6XXI:00060:00829
GGGAGAAGGGAGAAAGGAAGAGAGCGC
+
DFDCCA@BA?@@::;;;:,,-,,,*,,
@T6XXI:00060:00835
GGGAGAAGGGAGAAAGGAAGCTAGTCACTCGACATCTGTGGCTTCACAGTACAGAGGGACTGTCCAAAGCCGCAGAT
CTCGAGTCGACTAGCTTCCTTTCTCCCTTCTCCC
+
==:=BBAA@>@99908<><AC;:397111111131119>8<@@987?<96666011,00034737?9==8778===
888?<<@<;<<==<<=<=<AA<???<48553342
@T6XXI:00060:00837
GGGGAATTCTAATACGACTCACTATAGGGAGAGGGAGAATAGCCAATGGTCCTCCACTGTGCGCTCTTCTTCCTTTC
TCCCTTCTCCC
+
CBB7B7;;<CC=BBBCCCCCCC<77766.777BK@DCC?BCCA8859>9887//,-
3434<?A@@@A>AA=;244+48;90948;=>:
@T6XXI:00060:00840
GGGAGAAGGGAGAAAGGAAGCTAGTCGACTCGACATCTGTGGCTTCACAGTACAGAGGGACTGTCCAAAGCCCGCAG
ATCTCGAGTCGACTAG
+
CDEDDC?AA=BCCC>C:98;B@>=>;/00>>>>@>CC:::B=?@@CCBBBBCCC><<>;=<;:=>8884444-
4===@@@???@@@A???@@C
@T6XXI:00060:00842
GGGAGAAAGGAAGGGGAGAAGGGAGAAAGGAAGGGGAGAAGGGAGAAAGGAAGCTAGTCACTCGACATCTGTGGCTT
CACAGTACAGAGGGACTGTCCAAAGCCGCAGATCTCGAGTCGACT
+
FHBC@@@<@=C58;;49>@>BC>@@@E>C@C=@@@6?CD@@@;@@AA;@>>=<ABBCABCB@@@<<:9AACEC@BB?
?A??@@BB==9998;??@DB@CE>CC@BBCCBC?BBC?@@CDCDC
@T6XXI:00061:00809
CTAGCGACTCGAGATCTGCGGCTTTGGACAGTCCCCTCTGTACTGTGAAGCCACAGATGTCGAGTCGACTGCTTCCT
TTCTCCC
+
GCEC;;<?999@>A@@;<<CACDC@C@B@?>8777,6661117.0.0.44A=@==>>01000;00999973310101
1,16662
@T6XXI:00061:00813
GGGGAATTCTAATACGACTCACTATAGGGAGAGGGAGAATAGTCAACAAATCGTTTGGGGCGACTTCTCCTTCCTTT
CTCCCTTCTCCC
+
?=A6@>B@CCC@BB???BBBBBB?;76656=?BB>BBB>B@?;@A?AAA<CCCBA;@>@7A??BC@@CB?B=?5771
8AAA=A>@@@@;
@T6XXI:00061:00825
GGGAGAAGGGAGAAAGGAAGGGGAGAAGGGAGAAAGGAAGCTAGTCACTCGACAGTCGTG
+
DCBCDH@AA:====5><>4999/9??=?>:>00/,0,0,.5345887=70,,,+++00-0
@T6XXI:00061:00826
GGGAGAAGGGAGAAAGGAAGGGCGACG
+
BB?ADB>AA:?;88-95959@1....,
@T6XXI:00061:00833
CTAGTCACTCGAGATCTGCGGCTTTGGACAGTCCCTCTGTACTGTGAAGCCACAGATGTCGAGTCGACTAGCTTCCT
TTCTCCCTTCTCC
```

```
+
>8/111/4:BBBCCDCCCAB=?B?<B>AB@AAAA<ACBCBBBB>AAC@@C@BBCBBAAAABBBBCBBBAA@?8=@>@
A<@@@@9?:??B>
@T6XXI:00062:00808
GGGAGAAGGGAGAAAGGAAGCTAGTCGACGTCGGACAT
+
BD?CCA?AC04<>>:?69598887311111...,.3..
@T6XXI:00062:00821
GGGGAATTCTAATACGACTCACTATAGGGAGAGGGAGAATAGTCAACAAATCGTTTGGGGCGACTTCTCCTTCCTTT
CTCCCTTCTCCC
+
B??4@5969?>;A@@ABBBBBB??7366.777775:BB@CCC???<?@?:AAAAB=BBB7BB>>>::<<,0,/7996
;7;;497:344,
@T6XXI:00062:00827
GGGGAATTCTAATACGACTCACTATAGGGAGAGGGAGAATAGTCAACAAATCGTTTGGGGCGACTTCTCCTTCCTTT
CTCCCTTCTCCC
+
BBA6A=A>BBCBBB@AA@>>?CB99311.7>>BC>CCC>?>>===<::::?BACC>BBB9CBAAC?CCC>@>=>BC:
ABBA=A<???B<
@T6XXI:00063:00828
GGGGAATTCTAATACGACTCACTATAGGAGAGGAGAATAGTCACATTCATGTTTGTGACCGTGTCTCCTTCCTTTCG
CCTTCT
+
CFE:C@C@CAA=@BBBBBBAA8883363/...,.5577:B?80003136667:5:B@A@?@??@98/9=;;7551,+
+*+02-
@T6XXI:00063:00831
GGGAGAAGGGAGAAAGGAAGCTAGTCGACTCACATCGTGTGGCTTCACAGTACAGAGGGACTGTCCAAAGCCGCAGA
TCTCGAGTCGACTAGCTTCCTTTCTCCCTTCTCCC
+
AC>AB@<@B1949958889=;3334..........3....3,..3887?>@>86<7::54;;===;=D<AA<858<;
@>;98////-22--388;<;<940003032348:6
@T6XXI:00064:00825
GGGAGAAGGGAGAAAGGAAGAAAGGAAGGAGAACGCGCACCCGAACCAACGGTGATCTATTCTCCCTCTCCCTATAG
TGAGTCGTATTAGAATTCCCC
+
CEBCCC@BB@BABA=A>?>BB>:B898<BCCD>BBB>?B@A;BCDA?B@BCBJCECC;;;@CCCC@CCAAA>ACB??
=?C???CBBB@B@A?CACCC9
@T6XXI:00064:00832
GGGGAGAAGGGAGAAAGGAAGACTGCTTCCAATGGAGATTAGTTAACTATCTCCCTCTCCCTATAGTGAGTCGTATT
AGAACTTCCCCT
+
499,44424@:=AFC@E@CACC88011,/,.,..,.77777660.,.6...446+..97969:::<89::4446688
<<:842,---*+
@T6XXI:00064:00836
GGGAGAAGGGAGAAAGGAAGTGCAGTTCGCGGTTAGTGAAGGGAAAACTATTCTCCCTCTCCCTATAGTGAGTCGTA
ATCGAT
+
9<14@B6991949?:A>B?A??==9964;;;2969?;7B:?;:8665;;>?:7;86,1166689;88?7339000--
,-----
@T6XXI:00064:00846
GGGGAATTCTAATACGACTCACTATAGGGAGAGGGAGAATAGTCAACAAATCGTTTGGGGCGACTTCTCCTTCCTTT
CTCCCTTCTCCC
+
EBC9C>B>BCCDCCBBBBBBBBBB>97641=??B>BCC@@@@?<68<??:BB@@>5::94:BCBB>BCD@C?B?@@<
AAAA<?<?AA@;
@T6XXI:00065:00800
```

```
AGGAGAAGGGAGAAAGGAAGCTAGTCACTCGAGATCTGCGGCTTTGGACAGTCCCTCTGTACTGTGAAGCCACAGAT
GTCGAGTCGACTAGCTTCCTTTCTCCCTTCTCCC
+
BB>AAA>AA<ABBB=B@C@CCBBABA<99====ABECECDBBBB>B?BBBBBBC?CBAABBCCCCCC?BCBDCCCCF
AABBBC<>=CAA@BBA<?@@EACBBA9=47677.
@T6XXI:00065:00801
GGGAGAAGGGAGAAAGGAAGGAGAAGTCGCCCCAAACGATTTGTTGACTATTCTCCCTCTCCCTATAGTGAGTCGTA
TTAGAATTCCCC
+
9?199?599-
449A<B?B@D?BBC@BABECCC9CC>B@?86,1188:>ACC@@@@@<???AA;??AA==<@@CAA@@@=?AA?A388
80
@T6XXI:00065:00804
GGGGAATTCTAATACGACTCACTATAGGGAGAGGGAGAATAGTCAACAAATCGTTTGGGGCGACTTCTCCTTCCTTT
CTCCCTTCTCCC
+
CDC7B69;<AC@C?@@CBCBBCB93377.7??BE=AACACCCAAA<@@A=BBBCC>CCC9CBBCB=??B5:57:=;:
???@:?8;<<=7
@T6XXI:00065:00816
GGGGAATTCTAATACGACTCACTATAGGGAGAGGGAGAATAGTTTCCTACAACTCGATC
+
FDE:D?D>@@@?A;<<BCDDCCB??866.7>>??<A::5;::?@;@>@?=.)******(
@T6XXI:00065:00820
GGGAGTAGGA
+
--+,***0)*
@T6XXI:00065:00821
CTTTGGACAGTCCCTCTGTACTGTGAAGCCACAGATGTCGAGTCGACTAGCTTCCTTTCTCCCTTCTCCC
+
;<<7<?B@@A<<<19@@;:;BBB>?B@<<;<BBBBCA@ACCCBA?@@CC@@BBC?@@<>>>>/847777.
@T6XXI:00065:00849
GGGGAATTCTAATACGACTCACTATAGGGAGGGAGGAAG
+
CCC9C6<8<4499;9;BCCDBBCD;;:?5:.5373,,**
@T6XXI:00066:00821
GCTCGAAGATCTGCGGCTTTGGACAGTCCCTCTGACTGTGAAGCCACAGATGTCGAGTCGACTAGCTTCCTTTCTCC
CTCTCCC
+
4/////,//444?AC@DBB?@64444444-
4448=?BBBCB:99;<;?@AC:79CC???AA@@??=>;>58808888087777-
@T6XXI:00066:00827
GGGAGAAGGGAGAAAAGGAAGGGGAGAAGGGCTAGTCACTCGACCATGCCTTGTGGCTTCACAGTACAGGAGGGACT
GACTCCAAAGCCGCAGATCACGGAGTCGTACCTTAGCCTTTCCTTTCCTCCCTTCCTCCC
+
CA6@9964904////*/,/,/880644,..+4;;;4:///////,////,42488974446..,,,0,/,/.63688
>9997@??<?A>>>9...,,,,*,2./44431377331172601l,10111,1777633+
@T6XXI:00066:00828
GGGAGAAGGAGAAAGGAAGGAGATACGCAGTTCATAAAACATAACGCTATTCTCCCTCTCCCTATAGTGAGTCGTAT
TAGAACTTCCCC
+
6:508<-1-,,,,*,*/,/689:ABBBB>>?5779969600..33../5<=>CEE==943101.--
14448:447999000200/000+
@T6XXI:00066:00835
GGAGAAGGGAGAAAGGAAGCGTAGTCGACTCGAGATCTGCGGCTTTGGA
+
?599@6991999967,/,/...,,........4466113746;7,/,,
@T6XXI:00066:00848
```

```
GGGAGAAGGGAGAAAGGAAGTAGTCGACTCGACATCTGTGGCTTCACAGTACAGAGGGACTGTCCAAAGCCGCAGAT
CTCGAGTCGACTAG
+
CDBCBA?AC@BBBC>C?B>B=@@@:<;AAA?<<<<<<A@B@==>@LLECCCA>>84418?C>AC?CG@DC@CA?BBB
?@@@@CCBBBBBBB
@T6XXI:00067:00801
GGGAGAAGGGAGAAAGGAAGGAGTATAGCCGCACCCCGAATAACGCAACTATTCTCCCTCTCCCTATAGTGAGTCGT
ATTAGAATTCCCC
+
FICCCB?DB>B?BB=B>A@B@BBCBBBBBACC????9@D@CCBBCBC?BCBB@9;;C?CDCCC>CCBAAACCCCCBC
CB@CCC=?;?CD8
@T6XXI:00067:00804
GGGGAATTCTAATACGACTCACTATAGGGAGAGGGAGAATAGCGCACCATCAATTTTGTCGTGCCGTTCTCCTTCCT
TTCTCCCTTCTCCC
+
EHE:C=<;<CC@CBB?>@BCCGCAA311.777AG@CCB?B??:??CC>@=773777.7<<;BBCEECA;>;?B>B??
?<?AAA<A698=?:
@T6XXI:00067:00820
GGGAGAAGGGAGAAAG
+
BEB999699-/667+,
@T6XXI:00067:00826
CTAGTCGACTCGAGATCTGCGGCTTTGGACAGTCCCTCTGTACT
+
9999994944994499CCBCC=@>?/743;;;B@?;611/...,
@T6XXI:00067:00834
GGGGAATTCTAATACGACTCACTATAGGAGAGGGAGAATAGTCTCCCCATTAATCGCGGCTTAATTGCCTTCCTTTC
TCCCTTCTCCC
+
CCC8B595944<@BBBCCAAA>79;00,0;07:8:CD@CBBA@@@@?5BB@B@B>A><=AC?A?B@CB?B@C?CC>B
A?A8:9;444-
@T6XXI:00067:00841
GGGGAATTCTAATACGACTCACTATAGGGAGAGGGAGAATAGTCAACAAATCGTTTGGGGCGACTTCTCCTTCCTTT
CTCCCTTCTCCC
+
CCF:E?A?ABC@CBAABBBBBBBB;766-
1==?A>BCC@@B?A@?<<<<5?A?@?<AAA7CCBBD?CCC>@=??@@<@AAA<A=@@AC<
@T6XXI:00068:00804
CTAGTCGACTCGAGATCTGCGGCTTTGGACAGTCCCTCTGTACTGTGAAGCCACAGATGTCGAGTCGACTAGCTTCC
TTTCTCCCTTCTCCC
+
:--
,,,,,/1:?FBBCBBBAA??<@<@?BCBBCCB>BB>>>>>9>BCC?@C?BBBDCCBBBCCG?@@CCCCCA@@@@BBC
CCCB<@4:7==6
@T6XXI:00068:00807
GGGGAATTCTAATACGACTCACTATAGGGAGAGGAGAATAGTACG
+
@99-92929A>?A?@FCCCCBCCBCA@?999610:7:47771,,+
@T6XXI:00068:00825
GGGAGAAGGGAGAAAGGAAGCTAGTCGACTCGACATCTGTGGCTTCACAGTACAGAGGGACTGTCCAAAGCCGCAGA
TCTCGAGTCGACTAG
+
?A=;?@=>>:>999195948<.....,,200...4...5888:>A><=@ADB>>;444,4;::8868C=??;?@;;;
<484=:800063308
@T6XXI:00068:00826
GGGAGAAGGGAGAAAGGAAGCTAGTCGACTCGACATCTGTGGCTTCACAGTACAGAGGGACTGTCCAAAGCCGCAGA
TCTCGAGTCGACTAG
```

```
+
94-4>>?BC>CBBB=B@B<4788887;76161731688<>A;=@@CCCCAAA??=444-
44493<5;9977233>==??=73733335344:
@T6XXI:00069:00813
GGGAGAAGGGAGAAAGGAAGGAGAAGTCGCCCCAAACGATTTGTTGACTATTCTCCCTCTCCCTATAGTGAGTCGTA
TTAGAATTCCCCC
+
BB@CBC@BC@ABAA=B@B@C@CBB?BCBCBCC9CC?C@@<?;@BADCBBBC@@??@;88:BC@C???A>>=844444
8734423/---.+
@T6XXI:00069:00817
GGGGAATTCTAATACGACTCACTATAGGGAGAGAGATATAAGGGGCCCATTCT
+
?A?4><><@AB@B@@@ABBACAB??877.7??ACBDB@;:2444-43+..,..
@T6XXI:00069:00828
GGGAGAAGGGAGAAAGGAAGGAGAACGCGCACCCGAA
+
44-44489>:944=-424249933144444///+//,
@T6XXI:00069:00831
CTAGTCACTCGAGATCTGCGGCTTTGGACAGTCCCTCTGTACTGTGAAGCCACAGATGTCGAGTCGACTTAGC
+
4444444///////67/4449779,357>>;>?>11667671....4,-42449=979933761,77-,*,,,
@T6XXI:00069:00833
TCTGCGGCTTTGGACAGTCCCTCTGTACTGTGAAGCCACAGATGTCGAGTCGACTAGCTTCCTTTCTCCCTTCTCCC
+
@CAABBACCC>??BBBBAA@<ADBBA@@??BB@>?A?BBBBBBABBBCCACCBBBCCCC?B?BD>AAAD>B?BAAA=
@T6XXI:00069:00834
GGGGAATTCTAATACGACTCACTATAGGGAGAGGGAGAATAGTCCGCCAATTCTGCGCGTTAGCATTCTCCTTCCTT
CTCC
+
ACF:C8<24=9549949=AABBB@7777.7=777.79;:>00.4-
..7C@C@D::;??=>:;1677<7660/,/,/,...3
@T6XXI:00069:00836
GGGAGAAGGGAGAAAGGAAGGAGAACTGACTGCGCTTCGAGCCGTGCTATTCCTCCCCCTCCTATAGTGAGTCGTAT
TACGAATTCCCCC
+
AA<@BB@CC?==<D=B?B?B?DCC@BCCD<;8@<11-
14477<??@::.0,0,0?=57/885486348@877<768;9///8,-,0000+
@T6XXI:00069:00846
GGGGAATTCTAATACGACTCACTATAGGGAGAGGGAGAATAGCTCATTGCAATAGCAGTGCGTTGTTCCTTCCTTCT
CCCTTCTCCC
+
999/4:CACBC9<;;;99;;A;;8A?=?<????E>BBB@C@B.0000,..5446379637>??<?@9B4301,-,-
12226;565=1
@T6XXI:00070:00813
GGGGAATTCTAATACGACTCACTATAGGGAGAGGAGAATAGTTAGGCGTACTC
+
DII9>C@>?CC@CA@ACCBAACCC<>??6;=665:@><>77/,/20----122
@T6XXI:00070:00814
GGGAGAAGGA
+
49-4442/,/
@T6XXI:00070:00816
GGGAGAAGGGAGAAAGGAAGGAGAACGCGCAATCAGATACCTGTGGCTATTCTCCCTCTCCCTATAGTGAGTCGTAA
TTAGAATTCCCC
+
:B:>99599-4999-
4249:7=<>?DCCCCEACB>?94//,////,///4249::6:9988,7>7;8999<344..,.,.577F;==92
```

```
@T6XXI:00070:00844
GGGGAATTCTAATACGACTCACTATAGGGAGAGGGAGAATAGTCTCCACCTTAACGCCTGACACTTGGCCTTCCTTT
CTCCCTTCTCCC
+
CCD9DCBBCCC?BBCBCBBBBBBCCCCC><A<@C>BAA:;<C@????DE?A?BA????BBABCCC@B?B@B@C?@>:
;444-49448A;
@T6XXI:00070:00847
GGGGAATTCTAATACGACTCACTATAGGGAGAGGGAGAATAGTCAACAAATCGT
+
444,489<@>>599>>993373333;<A><<?CC>DCC571700020--+....
@T6XXI:00071:00824
GGGGAATTCTAATACGACTCACTATAGGAGAGGGAGAATAGTCCGCCAATTCTGCGCGTTAGCATTCTCCTTCCTTT
CTCCCTTCTCCC
+
FED9C>@>ACFBBBBBB?;;B:9990/,0<:=?=@CC@CB??>:<68@=@@CCBC???A>AABBB?BBE@D>@>@C>
BBBB<A>A???:
@T6XXI:00071:00829
GCGACTCGAGATCTGCGGCTTTGGACAGTCCCTCTGTACTGTGAAGCCACAGATGTCGAGTCGACTAGCTTCCTTTC
TCCCTTCTCCC
+
444499;99:?===AADADCC@C?CBCCDBB>:<;@??@C;;<DBCC=>??8711111?@B?=<?BD::<BB=>>8>
877.737777;
@T6XXI:00071:00830
GGGAGAAGGGAGAAAGGAAGCTAGTCGACTCGACATCTGTGGTCTCGAC
+
=BAAAA>@A:?9990959;>?@@@?BA???B@A??:7777<0.....--
@T6XXI:00071:00836
GGGAGAAGGGAGAAAGGAAGGAGAAGTCGCCCCAAACGATTTGTTGACTATTCTCCCTCTCCCTATAGTGAGTCGTA
TTAGAATTCCCC
+
DF@GCD@CC>BB@@=@=?=@?BBC@CBBBBBB8BD>BB?BB<CC?BAABBC?A@AA;@???CA??=@@@@A<<<C??
?=@@A>A?@>=1
@T6XXI:00071:00838
GGGGAATTCTAATACGACTCACTATAGGGAGAGGGAGAATAGCGATCGTGCCACATCGTCCTATTCTTCTTCCTTTC
TCCCTTCTCCC
+
:;;4<CCBCBB?A@?@AA@B>A;99311.7>>BE@DCB?@@@<===<<@CC>===@AAA@?@CC@BC@CC?A?BC>C
BAB>A@BBBC=
@T6XXI:00071:00843
GGGAGAAGGGAGAAAGGAAGGAGAAGTCGCCCCAAACGATTTGTTGACTATTCTCCCTCTCCCTATAGTGAGTCGTA
TTAGAATTCCCC
+
CDBCCCACD@BBCC>A?B=@?@CC@AABAA@@7AA<AA;:::;C@DCABBC???@C>A===A<>=??<<<@@???>=
=BAAA>@9;<<1
@T6XXI:00072:00800
CTAGTCACTCGAGATCTGCGGCTTTGGACAGTCCCTCTGTACTGTGAAGCCACAGATGTCGAGTCGACTAGCTTCCT
TTCTCCCTTCTCCC
+
<11////44C@BBBDBBABB>ABB=B?A@?A:9:8:CCEB@AC?BCC@AC@CCCBCCCCCBCB?@@<<;B??8;?:?
C8:8@C<A>@?AC>
@T6XXI:00072:00818
CTATCACTCGAGATCTGCGGCTTTGGACAGTCCCTCTGTACTGTGAAGCCACAGATGTCGAGTCGACTAGCTTCCTT
TCTCCCTTCTCCC
+
CBC?;:::BBBCBJABBBB?BCD>C>AACCCCB>CBB?????@CCC;=A?BBBCDDCCABACB;<<BBB@B?AB;AA
>??@B6;5;?==7
@T6XXI:00072:00824
```

```
GAGAAGGGAGAAAGGAAGCTAGTCGACTCGAGATCTGCGGCTTTGGACAGTCCCTCTGTACTGTGAAGCCACAGATG
TCGAGTCGACTAGCTTCCTTTCTCCCTTCTCCC
+
IDAA@@B:@999095959@>;;>0/0;;;=>>BBBBBBC????;??BABA@BC>BB?A@@A=;;?@><=;=<;@@==
=;::<A=A>588A?>9>333-48994;;>@BD<
@T6XXI:00072:00835
GGGGAATTCTAATACGACTCACTATAGGGAGAGGGAGAATAGTCAACAAATCGTTTGGGGCGACTTCTCCTTCCTTT
CTCCCTTCTCCC
+
DKK:C?@>?CC@BCBCBBBBBC@99311.7==>@=ADBCB@B@@A?AA=;B>==A;@A?8B>>?@@BBB=?:6::?<
:ACB>C<?<<<6
@T6XXI:00073:00821
TGCGGCTTTGGAACAGTCCCTCTGTACTGTGAAGCCACAGATGTCGAGTCGAACTAGCTTCCTTTCTCCCTCTCCC
+
><9B?994-4242498=@C@C@@?;87?;<:C@CCB<;0:>>?00/0,22./,....18;:9:@-0/57+..5552
@T6XXI:00073:00826
GGGAGAAGGGAGAAAGGAAGCTAGTCACTCGACATCTGTGGCTTCACAGTACAGAGGGACTGTCCAAAGCCGCAGAT
CTCGAGTCGACTAGCTTCCTTTCTCCCTTCTCCC
+
CB:=@C@CCAA<;809598;@?>=8,,,,,,,,,,/77;;96;7;@@@BBCCDCABB<<;<877=?@;@B<<<<<@@
==<<<<==>>444444/4:>>;@@@@:=/4444,
@T6XXI:00073:00828
GGGGAATTCTAATACGACTCACTATAGGGAGAGGGAGAATAGTCTCACCGGTGACGGTATACGCTTTCTCCTTCCTT
CTCC
+
DCA6>6969849@B@A;88>888>:000,0./:969;696;66=<776=;53678841716199>A;;7440*+*+*
+***
@T6XXI:00073:00833
GGGGAATTCTAATACGACTCACTATAGGGAGAGGGAGAATAGTTCACACACATTTACAGGGGCTGTTCTCTTCCTTT
CTCCCTTCTCCC
+
BAA4@598<A@@CBBBAACBBB?97377.7>>>@>BAC@BBB;:;;668:?AA=;=C????7ACBB?BCCC@B@CC>
BBCC>C=A???9
@T6XXI:00073:00840
GGGGAATTCTAATACGACTCACTATAGGGAGAGGGAGAATAGC
+
:;;2;29244::4444;93333377316,17666261101774
@T6XXI:00073:00842
GGGAGAAGGGAGAAAGGAAGCTAGCGACTCGACATCTGTGGCTTCACAGTACAGAGGGACTGTCCAAAGCCGC
+
>C?CCE@DC>C>68+1,1;100999???///>><01/:3431313768:<?>6;677-49940055536=5:8
@T6XXI:00073:00847
GGGGAATTCTAATACGACTCACTATAGGGAGAGGGAGAATAGATCGTCTATTCTCTTAACCACGCGTCAGCTTCCTT
TCTCCCTTCTCCC
+
:99/9<?=@BC@BBCBBBBBBBB>>311.6>>>@=ABA?BCA<?AAA=?BB?C===>=;<;==A@@@BB????A<>=
49=??;?9==AA<
@T6XXI:00074:00804
GGGAGAAGGGAGAAAGGAAGGAGACAGTGAGCGCCGCACAACACGTCACTATTCTCCCTCTCCCTATAGTGAGTCGT
ATTAGAATTCCCC
+
AC:499699-89:;-
8247878>@BBCCBBBBCC?A>?00,0:::=>BAB@@=ACCA9@>88808@@BCDBBBC;;;@>=>::5;>AFF9
@T6XXI:00074:00821
GGGAGAAGGGAGAAAGGAAGCTAGTCACTCGACATCTGTGGCTTCACAGTACAGAGGACTCGTCCAAAGCCGCAGAT
CTCGAGTCGACTACG
+
```

```
AA<CA>>AB<A@AA89::@BBCB==>//.....79<<??B=AA?@??CBBA===40,/8////.,-
3224434766;==?<76666,,++++
@T6XXI:00074:00837
TCTGCGGCTTGGACAGTCCCTCTGTACTGTGAAGCCACAGATCGCGAGTC
+
BDA88=8,,*,*,,2,,1:5;7:77797661.,/1,///006.....334
@T6XXI:00075:00800
GGGAGAAGGGAGAAAGGAAGGAGAAGTCGCCCCAAACGATTTGTTGACTATTCTCCCTCTCCCTATAGTGAGTCGTA
TTAGAATTCCCC
+
CB?CAA=AC@AA@C@D@B?C@BBA@A?A3333,88/8?ABDABA?B??@AA<=;;;7<<<<C<@@@@===@@@898@
>;==;99/444-
@T6XXI:00075:00804
CTAGTCACTCGAGATCTGCGGCTTTGGACAGTCCCTCTGTACTGTGAAGCCACAGATGTCGAGTCGACTAGCTTCCT
TTCTCCCTTCTCCC
+
B=1/111:=CCBBCCBBBBB?BBB<B<?@>B:9:7:BBCCABB?BBC?@B@CCBBBCACCBA??@@@BBCBBB?BBD
C>BCBC>C?AA?A<
@T6XXI:00075:00806
CTATCGACTTCGAGATCTGCGGCTTTGGACAGGTCCCTCTGTACTGTGAAGCCACAGATGTCGAGTCGACTAGCTTC
CTTTCTCCCTTCTCCC
+
=???4444424499CBAAC<<8;??8>688>><?@@<?????BBCBCBBA=ABCC@AA@?A?>869;8877733136
011,986=9=37133,
@T6XXI:00075:00814
GGGAGAAGGGAGAAAGGAAGCTAGTCGACTCGACATCTGTGGCTTCACAGTACAGAGGGACTGTCCAAAGCCGCAGA
TCTCGAGTCGA
+
EE?CCB=@>:@@>@<@>B@BCCCDB:;:CCCC;;:;;:@@@;::5:AAB:;;CC>??=6:99:44349488284337
34:;4//14..
@T6XXI:00075:00839
GGGAGAAGGGAGAAAGGAAGGAGACAGTGAGCGCCGCACAACACGTCACTATTCTCCCTCTCCCTATAGTGAGTCGT
ATTAGAATTCCCC
+
DDBCBB@BB?@@AC>B>@@C?:;;A@ABCCCBB?=BBBB?>?<<<>>CCCCC@BB@@=BBCCD>CECBBBBCCBA?>
87477>=@5999/
@T6XXI:00075:00843
GGGAGAAGGGAGAAAGGAAGCTAGTCGACTCGAGATCTGCGGCTTTGGACAGTCCCTCTGTACTGAAGCCACAGATG
TCGAGTCGACTAGCTTCCTTTCTCCCTTCTCCC
+
DDBBBCCCC@DCB?=B896;C>:::/0////=>>ACABABC>B?B>C?BB@BB?B<BA?===??A@>AA>@@??><=
<BCCCDBAACCCBAA=@>BB@BDFD?A@ACCDA
@T6XXI:00076:00805
GGGGAATTCTAATACGACTCACTATAGGAGAGGGAGAATAGTCAACAAATCGTTTGGGGCGACTTCTCCTTCCTTTC
TCCCTTCTCCC
+
DFFBE<:;<CB?CBBBBBB??99990/,0::=?;?CCACBC@??=?@A<BCCCC>CCC9CBBCC@BAA=?==>AC=@
@@A>C=?@@@;
@T6XXI:00076:00806
GGGGAATTCTAATACGACTCACTATAGGGAGAGGAGAATAGCTAAGTCCCTGACCTAT
+
=8:1?696<A@?BCA@@CCBB?B?BB@A977//-0>>:D?@<??47111,17..,...
@T6XXI:00076:00829
GGGGAATTCTAATACGACTCACTATAGGGAGAGGGAGAATAGATCACCGGTTGGTTCGGGTGCGCGTTCTCCTTCCT
TTCTCCCTTCTCCC
+
```

```
/11+/,/7811-///849:B>>?1--
,,*,,,..+.7777B:83842*,*,*,*,222202/..4444996772126>166@@8636000+
@T6XXI:00076:00838
GGGAGAAGGGAGAAAGGAAGCTAGTCGACTCGACATCTGTGGCTTCACAGTACAGAGGGACTGTCCAAAGCCGCAGA
TCTCGAGTCGACTAG
+
DKBCCC@CBBDCBB<><?<?A>9;;//0<=;=>AA???@::5;A>ABBCCBBBC=;;;177=>>><??<??BCAA@A
A??????@=<=@AAA
@T6XXI:00076:00845
GGGAGAAGGGAGAAAGGAAGGAGAATGCACCGCAGGAAATTGCAAGACTATTCTCCCTCTCCCTATAGTGAGTCGTA
TTAGAATTCCCC
+
DDBCCB894-
99980;?B@C?BBC@CC<;9;;;;;;BB<A?ABA?CBABBCA;;:C>C??>>@00/9>>C==B@?<//,/./,-
0122.
@T6XXI:00076:00848
CGAGATCTGCGGCTTTGGACAGTCCCTCTGTACTGTGAAGCCACAGATGTCGAGTCGACTAGCTTCCTTTCTCCCTT
CTCCC
+
AAAAACBDCCC=A@C<@?ADCAAA@<@?973?>8677??B@>BBB???@@FFCCCCA<99=44489899-4444-
424444-
@T6XXI:00077:00831
GGGGAATTCTAATACGACTCACTATAGGGAGAGGGAGAATAGAGACGTATTCGTTTGCGCTCTGTTCACCTTCCTTT
CTCCCTTCTCCC
+
CCC9C@C@BCB@A@@@??<BBB?99311.7>>BC>BAB?CC@=@=A@?=?>AAAA;@<@=@@<<<;<=<;<858<>7
>?AA<A<????;
@T6XXI:00077:00842
GGGGAATTCTAATACGACTCACTATAGGGAGAGGGAGAATAGTTGTAACCATTTCCCTGCCAACGTATCTCTTCCTT
TCTCCCTTCTCCC
+
CEA9B@C@BBB@A??AB@???C?99311.7>>?B?ABA?BACA?===<?<>BC>BB<@??;?<?BB@????;:>=@@
<BAAB<A;=888-
@T6XXI:00077:00845
GGGAGAAGGGAGAAAGGAAGGGATTCTTCCGGTCCCAGGAGAGAGTACTATTCTCCCTCTCCCTATAGTGAGTCGTA
TTAGAATTCCCCGGAGAAGGCAGAAAGGAAG
+
?A<@A@C@C?C@@><@=?<@C<@@?BB@C@C@BBC>CC?BBCCCAAEC=<<ACBCE@<<;CCBCCBBBBCCBFBBBB
C?@@B:8/000+3/-2746-..222243103
@T6XXI:00078:00809
GGGAGAAGGGAGAAAGGAAGGAGACACGGTCACAAACATGAATGTGACTATTCTCCCTCTCCCTATAGTGAGTCGTA
TTAGAACTTCCCC
+
IHBBBBDDC>AABC<@?A7;8<@@CCCC@DBCBBB>BBB?@?@ACCCAABC?BBBC>BAAAA<A@?@@AAB@AAA?=
=87;8,//48674
@T6XXI:00078:00811
GGGAGAAGGGAGAAAGGAAGACTCCGACATCTGTGGCTTCACAGTACAGAGGGACTGTCCAAACTCTCGAGTCGACT
ACG
+
DDBBAA@AA@@@@A=A@B@BB>@@@C>??BBDAAA@CC??<=AAA988<<<=:==:988,//,/-.---
066====:433
@T6XXI:00078:00817
GGGAGAAGGGAGAAAGGAAGGAGAAGTCGCCCCAAACGATTTGTTGACTATTCTCCCTCTCCCTATAGTGAGTCGTA
TTAGAATTCCCC
+
CDBCDC@EE>BCCB>A<><>???A?BCBBBBB8AA>BDCC@9>AACAA???A=<<<;99=@B>A<?<8<===<8<8:
?>A??;=34440
```

```
@T6XXI:00078:00820
GGGAGAAGGGAGAAAGGAAGGAGAAAGCGACGTCGGGGAAACGTTTGCTATTCTCCCTCTCCCTATAGTGAGTCGTA
TTAGAATTCCCC
+
CCBCCB?@@=BCCD?BAB?A>ABBB<>?BAAABA@>A.;;:<BBDBDBBEC@BCCN@CDA@@<@A@@@CCBCBBAA?
?:??@=?<AAA5
@T6XXI:00078:00839
CTAGTCACTCGAGATCTGCGGCTTTGGACAGTCCCTCTGTACTGTGAAGCCACAGATGTCGAGTCGACTAGCTTCCT
TTCTCCCTTCTCCC
+
A?994=444CBBBBCBBB@?=@CE>C?AAAA:::5:CCAAACC?BBB??B@CDCCC@@@CCCDCCCBBBCC??;=:=
>5:9BB<@8:78>:
@T6XXI:00079:00800
GGGGAATTCTAATACGACTCACTATAGGAGAGGGAGAATAGTCAACAAATCGTTTGGGCGACTTCTCCTCCTTTCTC
CCTTCTCCC
+
?B?4@5924448888=>@;;;4666.0,/..11,1160169533/004277666,11,315..,.44,/.4432446
634/03553
@T6XXI:00079:00808
GGGGAATTCTAATACGACTCACTATAGGGAGAGGGAGAATAGTCAACAAATCGTTTGGGGCGACTTCTCCTTCCTTT
CTCCCTT
+
=FB7@5959>9<@AAAA>8888773366274/00,0/:9//74;<<=CC6;<BBB9===2870:7373:,/,/4660
--03084
@T6XXI:00079:00813
GGGAGAAGGGAGAAAGGAAGACCGGGGACAGGTATAAGAAGAAGAGACTATTCTCCCTCTCCCTATAGTGAGTCGTA
TTAGAACTTCCC
+
DC@@@@;@@<@ABD@C@C@CCC@BBB8BBBB>B><<>;?>>>>;;==BB??>AAA@;BBAAA<?@@@@@AAAB<==@
@>@AA344344-
@T6XXI:00079:00818
GGGAGAAGGGAGAAAGGAAGCTAGGTCACTCGACATCTGTGGCTTCACAGTACAGACGGGACTGTCCAAAGCCGCAG
ATCTCGAGTCGACTAG
+
9B:999294-///8+/,/699ABB@B>1111111171111101913169>@7775-.--+---
6==<==;==;998;<<<<<;65553776=6
@T6XXI:00079:00820
GGGGAATTCTAATACGACTCACTATAGGGAGAGGGAGAATAGTCACTGGCTTGGTTGTCCGTTATTCTCCTTCCTTT
CTCCCTTCTCCC
+
FCI:C;<;<@=9;;ADA@@@BBB?@???9::;AB>BCC@CEC@@????=@A>A>A;=8837;9;;793;555:;@C;
:::=;=;=4454
@T6XXI:00079:00822
CTAGTCATCGAGATCTGCGGCTTTGGACAGTCCCTCTGTACTGTGAAGCCACAGATGCTCGAGTC
+
944//////77/99AA@>@5100+/,/......+.576166666673146499;66///6///,/
@T6XXI:00079:00832
GGGGAATTCTAATACGACTCACTATAGGAGAGGGAGAATAGCTACGTTAACCGATCGTTGTTGTCTCCTTCCTTCTC
CCTTCTCCC
+
944-
959244:;>>A?@@??;10;;00,.7077.196>BC;8:>=174101011,,++*++*+++597B8;8;8;88;3=8
;///,
@T6XXI:00079:00844
GGGAGAAGGGAGAAAGGAAGCTAGTCGACTCACATCTGTGGCTTCACAGTACAGAGGGACTGTCCAAAGCCGCAGAT
CTCGAGTCGACTAGCTTCCTTTCTCCCTTCCTCCC
+
```

DC?F@<99A:>111,/,/,/....6/......7711166736989BDDAAADDCCCC>====@?>AA<@@5530000
0036465535530004/0/3:48894132323;=;
@T6XXI:00079:00845
GGGAGAAGGGAGAAAGGAAGCTAGTCGACTCACATCTGTGGCTTCACAGTACAGAGGGACTGTCCAAAGCCGCAGAT
CTCGAGTCGACTAGCTTCCTTTCTCCCTTCTCCC
+
<<89DB6991999918594:B??70+******00*,17110131378>>99966616640000323445555543311
---1++-22.--1213/0/030356=;=553330
@T6XXI:00080:00819
GGGAGAAGGGAGAAAGGAAGGGCATGCTGCAACTGCGCACGGGGAGACTATTCTCCCCTCCTATAGTGAGTCGTATT
AGAATTCCCC
+
DICCCB@CC>BCCD?B@B@BB@B?????ABB@BB?B@B@?AAA9AABFCCC@CB=8;011,1===BCDCECBCAACA
BCC@C;<;;4
@T6XXI:00080:00820
GGGAGAAGGGAGAAAGGAAGTGCAGTTCGCGGTTAGTGAAGGGAAAACTATTCTCCCTCTCCCTATA
+
9?694@=A@8<;AB>BDC@CBDFE<<<<BAA?B@A@@CCBDC>BBA5A;;;=>=<<64//..+.44.
@T6XXI:00080:00829
GGGAGAAGGGAGAAAGGAAGCTAGTCACTCGAGATCTGCGGCTTTGGACAGTCCCTCTGTACTGTGAAGCCACAGAT
GTCGAGTCGACTAGCTTCCTTTCCTCCTTCCTCC
+
CC<<:9=>=5>499149><BD<;;BA::6871163111377;BB<A;866677=6<<6000003336769433;46:
:444.....-388447::7:;7;74437<;7887
@T6XXI:00080:00830
GGGGAATTCTAATACGACTCACTATAGGGAGAGGGAGAATAGCGCAATTTGGACATGTGGCCGCTTCTCCTTCCTTT
CTCCCTTCTCCC
+
EEB9B>@?ADDABB??BBBBBBB9>311.7>>BC@CBB?B??=9??>??7?=>>;?>>>>?;C=B?777898;8=?:
??AC@A<??AC=
@T6XXI:00080:00832
GGGAGAAGGGAGAAAGGAAGTGCAGTTCGCGGTTAGTGAAGGGAAAACTATTCTCCCTCTCCCTATAG
+
::644424@6>>@B=B@B>B@@AAA;84498249=798@=99,111,3333>:9772.....+.4466
@T6XXI:00080:00847
GTCGACTCGAGATCTGCGGCTTTGGACAGTCCCTCTGTACTGTGAAGCCACAGATGTCGAGTCGACTAGCTTCCTTT
CTCCCTTCTCCC
+
DC=::CCDB@ACB@@@@A@BDEAF=@?ACCCC?BCBAAAAAA@AB?BC@CCCCA@8::DCAABA:::B??B@B?BB>
BCCC>C>AACEA
@T6XXI:00081:00808
GGGGAATTCTAATACGACTCACTATAGGGAGAAGGAGAATAGTCAACAAATCGTTTGGGGCGACTTCTCCTTCCTTT
CTCCCTTCTCCC
+
BB@7@548<BB@BCBBBBAAB<7793//,000,/,.447:EDA@?<?AA=BA;;>;>?>8B===@@CBC=?;68?@<
@BAC<A=A@@@<
@T6XXI:00081:00811
GGGAGAAGGGAGAAAGGAAGCTAGTCACTCGACATCGTGTGGCTTCACAGTACAGAGGGACTGTCCAAAGCCGCAGA
TCTCGAGTCGACTAG
+
@@0999544-
///8+/11:88@DCA>771.....4...33356D@A??AAA@===444+0006>B;A?55;334033--
.3334:<<444=3
@T6XXI:00081:00813
GGGGAATTCTAATACGACTCACTATAGGGAGAGGGAGAATAGTCAACAAATCGTTTGGGGCGACTTCTCCTTCCTTT
CTCCCTTCTCCC
+

```
CCD:DAC@BCC@CBBBBBB>?AB>;76959@@BC>CBB>@CC@@?=BBA:CBBBC<A<>7@BBBBCBBC;;;:@CC>
==?A=A=@@AA<
@T6XXI:00081:00815
GGGAGAAGGGAGAAAGGAAGGAGAACAAGTGGCCGCATAGCGCAGGACTATTCTCCCTCTCCCTATAGTGAGTCGTA
TTAGAATTCCCC
+
FE@@CA?BC>AAAE@C@B@C@CBB=@A@AAA@B@BBBCBBBCCCC@CCCCC@BCBC@CBBCB>CCCCAAABAAACBB
B?ACB@EACDD:
@T6XXI:00081:00820
GGGAGAAGGGAGAAAGGAAGACAT
+
AA<>@B599199991421,//...
@T6XXI:00081:00825
GGGAGAAGGAGAAA
+
8>,///,/,,*,,*
@T6XXI:00081:00830
GGGGAATTCTAATACGACTCACTATAGGGAGAGGGAGAATAGTCAACAAATCGTTTGGGGCGACTTCTCCTTCCTTT
CTCCCTTCCTCCC
+
BEC9CFHACCC>AACCCCBBB@?<>877.777??=ACC@CCB===>A@AABBBBB>BBB6AAA::67?A5:77;??<
@75443,.,+000
@T6XXI:00081:00832
GGGGAATTCTAATACGACTCACTATAGGGAGAGGGAGAATAGCACAACAGCATGTTCTACTCCCATTTACTTCCTTT
CTCCCTTCTCCC
+
944,49E>AA@@=<>CCBBBCCA??766.7??BC>BBB@BCCAA??=?A???CCC:=<;=;;;5;;?6887:42=>1
7034+3/3000+
@T6XXI:00081:00835
GGGGAATTCTAATACGACTCACTATAGGGAGAGGGAGAATAGTTACCATTTTACATGCGTGCCTATTCTCTTCCTTT
CTCCCTTCTCCC
+
CDB9B=?>ACB?CAAACCCCBCCBB7995:?=BE@;;:@CCC@>@@:788808AAAAADA<757//,/9//,/5985
;===:?<?>?A<
@T6XXI:00081:00844
GGGAGAAGGGAGAAAGGAAGAGAAGCA
+
CDBC<<<<C@CCCC@CBC;1280)(((
@T6XXI:00081:00847
GCTCGAGATCTGCGGCTTTGGACAGTCCCTCTGTACTGTCGAAGCCACAGATGTCGAGTCGACTAGCCTTCCTTTCT
CCCTTCTCCC
+
4444////////;>2444-
49::99===699:83441//..65//,/=;;;>@<?AACBB??>=?7739;>588/8733,3116;68
@T6XXI:00081:00848
GGGAGAAGGGAGAAAGGAAGTGCAGTTCGCGGTTAGTGAAGGGAAAAAC
+
44-444244-4///+/,/,/6499445991/686/....,..+....*3
@T6XXI:00081:00849
GGGAGAAGGGAGAAAGGAAGCTAGTCGACTCGACATCTGTGGCTTCACAGTACAGAGGACTGTCCAAAGCCGCAGAT
C
+
FIBAA@?BB>BCCD>C@C;;;B@@@BCAA@::;;::7:?@@:?@=@CCBBBB::1-
.,/:99;A=?A?AA>AA@@@@A
@T6XXI:00082:00809
TAATACGACTCACTATAGGGAGAGGGAGAATAGTGTCTCCACAGCGCTACCTGCCACCTCTTCCTTTCTCCCTTCTC
CC
```

```
+
?GBCBBBBBBBBB>;9888,3><>@<ABC@CBBB><=9758?>@AAABBB@BBC@AA>AAE@FCAG;>=BB?@5999
90
@T6XXI:00082:00810
GGGGAATTCTAATACGACTCACTATAGGGAGAGGGAGAATAGTCAACAAATCGTTTGGGGCGACTTCTCCTTCCTTT
CTCCCTTCTCCC
+
CFD:DAA>@CCCBB>>@@AABB<99377.7==>?>ACC@CBB?A;:;AA:?B>??;@@@8CBBBB?AAA:?:?:;;6
<?>?;@:==@A<
@T6XXI:00082:00811
GGGAGAAGGGAGAAAGGAAGGAGAACGCGCACCCGAACCAACGGTGATCTATCTCCCTCTCCTATAGTGAGATCGTA
TT
+
:E@CBB@BB=@ABBA@5;58249?<9444499959@<B?A@BB@<<91<<1111:856333137731/55/..30,+
-,
@T6XXI:00082:00815
GGGGAATTCTAATACGACTCACTATAGGGAGAGGGAGAATAGTCAACAAATCGTTTGGGGCGACTTCTCCTTCCTTT
CTCCCTTCTCCC
+
CCA6?6;9;CK@BBBBBBBBBBBBB76641;;@B>CBC?CCB>?A<@AC;B;;:C>CDB8BCCCC?BAC=@>=>@B<
A@@@;@>AA@C<
@T6XXI:00082:00823
GGGAGAAGGGAGAAAGGAAGGAGAAGTCGCCCCAAACGATTTGTTGACTATTCTCCCTCTCCCTATAGTGA
+
44-49:9=C>CCBC>C=@=@<>>>481477772772;;>@A>BC?BB@@>A>??=;9440./+-,++++*0
@T6XXI:00082:00830
GGGGAATTCTAATACGACTCACTATAGGGAGAGGGAGAATAGATCTAACGTTTGCGCCTGGTTTATTCTCTTCCTTT
CTCCCTTCTCC
+
:GB7D?CDDCCAGCCG<:<B;88>8877,6667;;677566<7735;<>>=<-
1166666011,16369=844/00+0009,0/0000
@T6XXI:00082:00831
GGGGAATTCTAATACGACTCACTATAGGAGAGGGAGAATAGTCAACAAATCGTTTGGGGCGACTTCTCCTTCCTTTC
TCCCTTCTCCC
+
BEDBC?A?BEC?BBB@BBB>?:9990.,0<<?@>BBB?BBBAA@?@@@<CCCCC>CCC7@?A??:?@A5:78>@@<A
A??:>8<;<<7
@T6XXI:00082:00832
GGGAGAAGGGAGAAAGGAAGCTAGTCACTCGACATCTGTGGCTTCACAGTACAGAGGGACTGTCCAAAGCCGCAGAT
CTCGAGTCGACTAG
+
491444244-499:-
42499>@AA?>7711166688<:<==?@=@@ACBBCDCC88818=:==>;=?:==;><<<@@@888;:::;:<9;8
@T6XXI:00082:00835
GGGAGAAGGGAGAAAGGAAGGATAGTGCGTGTCGGTGGGTGGGCTATTCTCCCTCTCCCTATAGTGAGTCGTATTAG
AATTCCCC
+
DFDA@@@CC>BBBB>B@B@B@BBBB????>>BC@@B<<4>>>7BACC@CBBB=ACAAA<AAAABBCCCCBBB@@<??
A?A<ABA5
@T6XXI:00082:00839
GGGAGAAGGGAGAAAGGAAGCTAGTCACTCGACATCTGTGGCTTCACAGTACAGAGGGACTGTCCAAAGCCGCAGAT
CTCGAGTCGAC
+
BCAD@@59;8;9AA<B@C;<<B@@?@A<66;671;8:CCD=?@@CCCBBCCB=7011.4;;?@@@BC=AD9==AC??
???@A@A@@??
@T6XXI:00083:00802
```

```
GGGGAATTCTAATACGACTCACTATAGGGAGAGGGAGAATAGTTTTTCCCTTCACTAACCGCGAACTGCACTTCCTT
TCTCCCTTCTCCC
+
FIC9E@A=@AA>BBBCCCBB:::A?87:.7<<=B8?::;:A>@AA>.AA<A>9;;BB@BA:;:>:=<>?AAA=?377
-66>=9?4::771
@T6XXI:00083:00819
GGGGAATTCTAATACGACTCACTATAGGAGAGGGAGAATAGTCAACAAATCGTTTGGGGCGACTTCTCCTTCCTTTC
TCCCTTCCTCC
+
DCC:C8=6;==29;B@@@BBB000>00,..47>>@@@=??A=3323694A;::C>A?A4:116;016@7888@==45
37444-.,-22
@T6XXI:00083:00822
GGGAGAAGGGAGAAAGGAAGTGCAGTTCGCGGTTAGTGAAGGGAAAAACTATTCTCCCTCTCCCTATAGTGAGTCGT
ATTAGAATTCCCC
+
DDBBBB@CC>C;;;69>?>BGCBCDCABBBB=@=@B@@@@CC<BBBA2B?AA>@@??6<=777-
:??=@?>?@@????=;=88335=>@5
@T6XXI:00083:00827
GGGGAATTCTAATACGACTCACTATAGGGAGAGGGAGAATAGTCAACAAATCGTTTGGGGCGACTTCTCCTTCCTTT
CTCCCTTCTCCC
+
A??6A?B@CBD@CAAABA>B?CC?>311.777>?<CAAAACCAAA>@@?:BA@>=9???5?BCBB?AAA>AAA@DD>
A===7<;==>>7
@T6XXI:00083:00831
GGGGAATTCTAATACGACTCACTATAGGGAGAGGGAGAATAGTCCTAGCCTTTTCCGCTGACCTGTCTCCTTCCTTT
CTCCCTTCTCCC
+
BCC9C@C@BBB@BBBBBBBBBBB>;799.6=@BC>BCB>>>>=A>@@AA?AAA6@?CCCC@?=????AC@BA@>@@<
?:99,/,./882
@T6XXI:00083:00834
GGGGAATTCTAATACGACTCACTATAGGGAGAGGGAGAATAGCTTCCGCTTAATTACCCTCCAAATATCCTTCCTTC
TCCCTTCTCCC
+
CDIAF>@?ACC@BBABBCAAA?@??766.777>@>BCB?BB?;>>>>BBB>B>>>CCC>@@?AA;@67777,/,/,-
---+-0336@<
@T6XXI:00083:00835
GGGAGAAGGGAGAAAGGAAGTCGACGACATCTGTGGCTTCACAGTACAGAGGGACTGTCCAAAGCCGCAGATCTCGA
GTCGACTAG
+
DFF@D@@BB>ABAB<@>B8<<BBBBB:::::CCC?BB@BAAA=<<=6633718A@A@A?CA7=>@@@A=<<@@@CA
=::===A;?
@T6XXI:00083:00843
GGGGAATTCTAATACGACTCACTATAGGAGAGGGAGAATAGTCAACAAATCGTTTGGGGCGACTTCTCCTTCCTTTC
TCCCTTCTCCC
+
EEB9B?C@BBCCB???BBBBB:966..,/:579>??A>BACA<@;?<:;@A>>@>CCC9CCBBB=??@=>=?;<<6;
@?@;@>@@CA;
@T6XXI:00084:00805
GGGAGAAGGGAGAAAGGAAGGAGAAGTCGCCCCAAACGATTTGTTGACTATTCTCCCTCTCCCTATAGTGAGTCGTA
TTAGAATTCCCC
+
FD?BBA@BC@BBBB>B?A>A@BBB@>>@????8?B>BBB@B>B@?BCCCC?;9===;<<?@@<<=<<<==<<:::<A
<;@AA>A=???5
@T6XXI:00084:00808
GGAGAAGGAGAA
+
95///,/,,***
```

```
@T6XXI:00084:00823
GGGAGAAGGGAGAAAGGAAGGAGA
+
??:@DA@BB:@999195961:;0-
@T6XXI:00084:00825
GGGGAATTCTAATACGACTCACTATAGGGAGAGGGAGAATAGTCAACAAAATCGTTTGGGGCGACTTCTCCTTCCTT
TCTCCCTT
+
///*/,4:;ABCDBCCA@@DC???8877-
7?7775:9>:<=C;5874444+87:CA;CBC8A?=77371636332330533330*
@T6XXI:00084:00829
GGGAGAAGGGAGAAAGGAAGGAGAACAACACGATCGGTTAAATGTAGCTATTCTCCCTCTCCCTATAGTGAGTCGTA
TTAGAATTCCCC
+
?C?CBA?AC<@BBB<@=@5989>?<9<;;=AAAB@656,1/+/.688@AA@@B=?B=9999967737>??>@?6611
1000099;<<8.
@T6XXI:00084:00835
GGGAGAAGGGAGAAAGGAAGTGCAGTTCGCGGTTTGTGAAGGGAAAACTATTCTCCCTCTCCCTATAGTGAGTCGTA
TTAGAATTCCCCC
+
BD?AAAADHABBBB?B?B>?BBCBBD@866:,11,..66,33,111,44449B==A>@@@CNACBBAAACCCCAAAA
B<>;:73/0344,
@T6XXI:00085:00802
GGGAGAAGGGAGAAAGGAAGTGCAGTTCGCGGTTAGTGAAGGGAAAAACTATTCTCCCTCTCCCTATAGTGAGTCGT
ATTAGAACTTCCCC
+
BEBCCCACBAAA=ABB@B=@ABCCED@B?==698;AACC@CD?BBBB2CCCC@CCCC;AAAA?9=??;<<@AAA@==
==9<AA5888;;;4
@T6XXI:00085:00817
GGGGAATTCTAATACGACTCACTATAGGAGAGGGAGAATAGTTTCACGTTGCCGACTGCTGCTATTCCCTTCCTTCT
CCCTTCCTCC
+
DEB7A7;:<@@@BBBCCBCBC>;;;0.,//.178:8>>??:44-4:98555585;;=;;;;==;7469,/,/,/,-
+++*-,-,--0
@T6XXI:00086:00821
GGGGAATTCTAATACGACTCACTATAGGGAGAGGGAGAATAGATCAGAACCTTTCTGCATCCCACTATTCTTCCTTT
CTCCCTTCTCCC
+
EFF:D@@@ADHACAAAABB?BC@<;311.7>>?A>ACD@@?>:?@CBB?B@CC>ABABCCBC>BBBBB=?<;>377.
7744-4344494
@T6XXI:00086:00825
GGGGAATTCTAATACGACTCACTATAGGGAGAGGGAGAATAGTCAACAAATCGTTTGGGGAGCGACT
+
DDF9CBE?CBCDGBBCCBBAB>;<?366.7=7776:99BCCB?642000+.63330344*..3,,..
@T6XXI:00086:00832
GGGGAATTCTAATACGACTCACTATAGGGAGAGGGAGAATAGATCACGTAGGCGTTTGTGTGTCGATTCTCCTTCCT
TTCTCCCTTCTCCC
+
CFICI@@>@CB@CBCCBBBBBBB?9311.7=77719>?@CAA?AAC=;://,0:::9>ABBCAA???>@@@;=8:24
4-4:;;-858<AAA
@T6XXI:00086:00837
GGGAGAAGGAGAAAGGAAGGAGAAGCGCCATGTCAAGTGACGATAG
+
490///,/,,,,,*,*/,/6869:A?@@:??76.353.......5=
@T6XXI:00087:00803
GGGAGAAGGGAGAAAGGAAGCTAGTCACTCGACATCTGTGGCTTCACAGTACAGAGGGACTGTCCAAAGCCGCAGAT
CTCGAGTCGACTAG
```

```
+
FFBCCC@EB=?B=96>7:6;BAAAC?B:::?;;:??=CCC?BB?BBBBCCA@;;43317==?A@?AA:??=@@;:<<
<<<=:<?A?@@@A@
@T6XXI:00087:00808
GGGGAATTCTAATACGACTCACTATAGGAGAGGGAGAATAGTTCATCTGTCCCTAGCTTGAACGTGCTCTTCCTTTC
TCCCTTCTCCC
+
EJIFC?@=?DD@AAAAAA???:9990/-0>>BC>CBB>>998;=A<?:?66;7<A@@A=@@>A???<A?=9;244-
4:;;:;348;;6
@T6XXI:00087:00812
GGGAGAAGGGAGAAAGGAAAGCTAGTCACTCGAGATCTGCGGCTTTGGACAGTCCCTCTGTACTGTGAAGCCACAGA
TGTCGAGTCGACTAGCTTCCTTTCTCCCTTCTCCCTT
+
=A6:9>;=>:>48904244-43333766611166679:?<?=888/7;>=??@@>5::?<;<;A;;/0,00,.444-
-004000304440--/.664673.349-4/4-22220
@T6XXI:00087:00831
GGGGAATTCTAATACGACTCACTATAGGGAGAGGGAGAATAGTTTTCCCTTCACTAACCGCGAACTGCACTTCCTTT
CTCCCTTCTCCC
+
DCB7@798<==@;<<BDCCCCC?97377-
7<61116??@CAA@CC5@A<@>?BEDC?@=AABC@CB>767=:=477.777=9A?B@AC;
@T6XXI:00087:00832
GGGAGAAGGGAGAAAGGAAGCTAGTCGACTCGACATCTGTGGCTTCACAGTACAGAGGGACTGTCCAAAGCCGCAGA
TCTCGAGTCGACTAG
+
CDBCCC@CC>AA@B9;:<=@B>?@B:::AAA?;;::::@AB=?@@DCCAAACCB<37714;;?8858D:=:9:0//;
;?<==@AA???A@AA
@T6XXI:00087:00833
GACTCACTATAGGAGAGGGAGAATAGTCAACAAATCGTTTGGGCGACTTCTCCTTCCTTTCTCCCTTCTCCC
+
<::::;;;?11/,/66668<999<796663111,11111,11,0031101110177011,1644+7275344
@T6XXI:00087:00834
GGGAGAAGGGAGAAAGGAAGGAGACAGTGAGCGCCGCACAACACGTCACTATTCTCCCTCTCCCTATAGTGAGTCGT
AT
+
DHB@>>59919@@@:?<>695;??ACAAADCCBB?BCCCCBBB;:;ABBBCCAFCCC=B:;;A<ABA@@;8..0,,,
,*
@T6XXI:00087:00838
GGGAGAAGGGAGAAAGGAAGTGCAGTTCGCGGTTAGTGAAGGGAAAAACTATTCTCCCTCTCCCTATAGTGAGTCGT
ATTAGAATTCCCC
+
DDBCCCADC?BA?><@?A?BADCCCB@B??@@CACCCCC@CD=@@@@2AABC@CCCC>AABCCAAAA@@AA@@AAA@
??<?AA>A>AA@5
@T6XXI:00087:00841
GGGAGAAGGGAGAAAGGAAGCTAGTCGACTCGAGATCTGTGGCTTCACAGTACAGAGGGACTGTCCAAAGCCGCAGA
TCTCGAG
+
CD?B@9;>@:?99809;>5;;BBBBCBBAAAA;::?<:?@@;=@@C@@?BBC?=9333-
4;;=AB>BA?AA<@@=A:??@@@@A
@T6XXI:00088:00808
GGGGAATTCTAATACGACTCACTATAGGGAGAGGGAGAATAGATCACCGTTGGTTCGGGTGCGCGTTCTCCTTCCTT
TCTCCCTTCTCCC
+
B??6B@DACBC@AA?ABBBB?A;99366.7>7776:@@>BCB@??AA:?A5:5:=AAB>B<<<CBC?DAB?A?B?BB
>BCBC>C?;9:A<
@T6XXI:00088:00811
CTATCACTCGAGTTGCGCTTGGAC
```

```
+
CCFBBADB=998/,/11/,*,*,,
@T6XXI:00088:00813
GGGGAATTCTAATACGACTCACTATAGGGAGAGGAGAATATAGACCTCA
+
CHEAC8;8<@=?CBBBBAAA?A>??7665:B>>=B;:7:C;;111,--,
@T6XXI:00088:00821
GGGAGAGTGAGAAGGGGAATGGGCG
+
//+//////4442444,/,//8+//
@T6XXI:00088:00825
CTATCACTCGAGATCTGCGGCTTTGGACAGTCCCTCTGTACTGTGAAGCCACAGATGTCGAGTCGACTAGCTTCCTT
TCCTCCCTTCTCCC
+
D994444499449>CBBGDIBBB<@=<>>?777.7===??===610/0::?=@A@BBBB<;:?AACBBBA>>;>:<<
,0,0;>6>,7/--+
@T6XXI:00088:00841
GGGGAATTCTAATACGACTCACTATAGGGAGAGGGAGAATAGTGACGTGTTGTGCGGCGCTCACTGTCTCCTTCCTT
TCTCCCTTCTCCC
+
CCA7A8;:<CC@BBBBBB<?BB?93366.7>>>?<ACC@CBC@;:::=AA?;<<AC?A???A???A===?>??C=@@
7==DD?A8;<AD=
@T6XXI:00089:00811
GGGGAATTCTAATACGACTCACTATAGGGAGAGGGAGAATAGCCGTGTTTCGATTCACACCTAGTTCTCCTTCCTTT
CTCCCTTCTC
+
999/489:ABB>A@?=9988?888C877.777>@:???:??@>;==>=>:=:??>???;?:>944483777771//+
....+.,...
@T6XXI:00089:00821
CTAGTCGACTCGAGATCTGCGGCTTTGGACAGTCCCTCTGTACTGTGAAGCCACAGATGTCGAGTCGACTAGCTTCC
TTTCTCCCTTCTCCC
+
B7----,,/8:;CCCA@A@@C@CCC>C=@???766-
7?>@CDCCBAAB>8<<>CCCDCABCBAB@AA;=;:00.83337./69>882678;;
@T6XXI:00089:00831
GGGAGAAGGGAGAAAGGAAGCTAGTCACTCGACATCTGTGGCTTCACAGTACAGAGGGACTGTCCAAAGCCGCAGAT
CTCGAGTCGACTAG
+
CC?BBD@BC?DB?=:=696;CBBBC??:66<7768:89@C>?@BCCCBBBCA>>000,4;;=@A>@@<@A?A@AAA@
====??DA===AA@
@T6XXI:00089:00837
GGGAGAAGGGAGAAAGGAAGGAGAACAACACGATCGGTTAAACGTAGCTATTCTCCCTCTCCCTATAGTGAGTCGTA
TTAGAATTCCCC
+
8<,1/9594-444:-
42424789;;AB>BA@>@8446499A:?><;77977:;<??266111,1768:=;;68338;;8;8>8827780
@T6XXI:00090:00807
GGGAGAAGGGAGAAAGGAAGGAGAAGTCGCCCCAAACGATTTGTTGACTATTCTCCCTCTCCCTATAGTGAGTCGTA
TTAGAATTCCCCC
+
CA=<99<>@<@AAC<A>A8;8;@CCBACABBB9BC>BBAA@8A?;??CCCC>@ACC<889@@;@=>=@@;;>//...
.4:9444/0000+
@T6XXI:00090:00834
GGGGAATTCTAATACGACTCACTATAGGGAGAGGGAGAATAGTTTGCGCATTCGGTGCACTGTTATTCTCTTCCTTT
CTCCCTTCTCCC
+
```

```
ADD9<;>B<<<?BA;<<A@@@B@@8877.77777,66;;??@>?;<<==?DCCDAAAABCBDCBCC?;::68969?8
6617263:004:
@T6XXI:00090:00842
GGGAGAAGGGAGAAAGGAAGAGAATGAGCGATGGATGGAATAGACAGCTATTCTCCCTCTCCCTATAGTGAGTCGTA
TTAGAATTCCCC
+
BD?@C@@BC>BBBD>B?B?BBAA?ACAAAC@B@@B@?=>@C>BABCCCAAA@A<@@;@AAAC;?<==ACA@@A@@?@
?<??>:=7<;;0
@T6XXI:00090:00847
GGGGAATTCTAATACGACTCACTATAGGGAGAGGGAGAATAGTTTTTTCCCTTCACTAACCGCGAACTGCACTTCCT
TTCTCCCTTCTCCC
+
CCC9C?C@CCB@CCBBAA?>BBB?;76659??BD>BBC??@733334*494:=ACCBB>@?B@??>>@=8=<?>A3:
:7:AAC@C@CC@A<
@T6XXI:00091:00803
CTATCGACTCGAGATCTGCGGCTTTGGACAGTCCCTCTGTACTGTGAAGCCACAGATGTCGAGTCGACTAGCTTCCT
TTCTCCCTTCTCCC
+
A=86/////89=>BCCCBBB@CBBAB<?>B?<<?=?CCCA?AA?CCC@@C?CHECB@@@BCCCBCCCCCDBBB@@>?
C?BBBC=A=@AEEA
@T6XXI:00091:00808
GGGGAATTCTA
+
999,49@<@99
@T6XXI:00091:00814
GGGGAATTCTAATACGACTCACTATAGGGAGAGGGAGAATAGCCATGTCGCGTCTGGCCCCACGTTCTCCCTTCCTT
TCT
+
999/49@99;98=BADFBCCCCCA@377.7777;2<11016942744446<<<554<===06:AA>;///,/46466
+.+
@T6XXI:00091:00821
GGGAGAAGGGAGAAAGGAAGTGCAGTTCGCGGTTAGTGAAGGGAAAACTATTCTCCCTCTCCCTA
+
44-4/6778+//8;,1115>=?<<<@88844242477<>999,166-333313311,11117764
@T6XXI:00091:00828
CTAGTCGACTCGAGATCTGCGGCTTTGGACAGTCCCTCTGTACTGTGAAGCCACAGATGTCGAGTCGACTAGCTTCC
TTTCTCCCTTCTCCC
+
A11//1////84;9@@CBBBB>BBB>B@B@>?87757@?BCBBCCCCC?@C@CCDCBBBBCCCCCCCAA@C???;?;
?D@BDCC>C5::??9
@T6XXI:00091:00848
GGGGAATTCTAATACGACTCACTATAGGGAGGAGGGAGAATAGTCATCGC
+
DCB6@=A@ABECCCC@@ABCBBBD?:7:.7>=777.71101110000003
@T6XXI:00092:00800
CTATCGACTCGAGATCTGCGGCTTTGGACAGTCCCTCTGTACTGTGAAGCCACAGATGTCGGAT
+
EAAA;9;DDB=@AABCBDBC@BBA=@>@ACCBBB>BCCCCBBB@BBB?@CACCC@><0,(((((
@T6XXI:00092:00811
GGGGAATTCTAATACGACTCACTATAGGAGAGGGAGAATAGTGACGTGTTGTGCGGCGCTCACTGTCTCCTTCCTTC
TCCCTTCTC
+
DDD9C@D@CCFBBBBECCCBBB???::5:FFDD>CBB>B??==<=99=??@?@8738<<<A@@??<788;=;:5676
///,/,-11
@T6XXI:00092:00818
GGGGAATTCTAATACGACTCACTATAGGGAGAGGGAGAATAGATCACCGTTGGTTCGGGTGCGCGTTCTCCTTCCTT
TCTCCCTTCTCCC
```

```
+
CCC7B=@>ACFABBAA??>BBB<99311.7==AB?CBB?C@@<<<>::?A:?:?;@@B?BCCCCCC@B@@>??BAFG
=BABA>B@B@@A=
@T6XXI:00092:00821
GGGGAATTCTAATACGACTCACTATAGGGAGAGGGAGAATAGCTTGTGCGGGTGTGTAAGGCAATTCTCCTTCCTTT
CTCCCTTCTCCC
+
?=92><@=@CC@DBBBBBBBBBBBB76657::CD>CAA=A@@=?>>>>>>>>;>999>B@C>@@>C@B<>3838?AC<
@@@@<@;><<=9
@T6XXI:00092:00823
GGGGAATTCTAATACGACTCACTATAGGAGAGGAGAATAGTAGACCAATCTT
+
ECB0;,45999<A@A<<<A@B>=;;10,0::<:8=?>A??8;000/-,,,,*
@T6XXI:00092:00829
GGGGAATTCTAATACGACTCACTATAGGGAGAGGGAGAATAGTCAACAAATCGTTTGGGGCGACTTCTCCTTCCTTT
CTCCCTTCTCCC
+
DDC9C@C@EBB@BB??AAAAA?997361.7???F>BBD@A?B??@>AAA<CBBBC>CCC8CAAACABBB=@>=>AA<
A@@@<@;==AA=
@T6XXI:00092:00837
GGGAGAAGGGAGAAAGGAAGCTAGTCGACTCGACATCTGTGGCTTCACAGTACAGAGGGACTGTCCAAAGCCGCAGA
TCTCGAGTCGAT
+
CCBCBC@CB?B?>=:@>?=BC@8>>/11>??>>@?:::@@A?AA>ABBBBBCB>:333,3;9=???AA<AA>@@=::
?<<A?<?AA>48
@T6XXI:00092:00841
GGGGAATTCTAATACGACTCACTATAGGGAGAGGGAGAATAGTCAACAAATCGTTTGGGGCGACTTCTCCTTCCTTT
CTCCCTTCTCCC
+
FII:FAC@BBA@BEBDB?@?AA@?B:96-
6=;;<9:::?CFC@@@>AAA;BB???9???5A???C?AAB?B?@>AA<@AAA<A:==BA=
@T6XXI:00092:00845
GGGAGAAGGGAGAAAGGAAGCTAGTCACTCGACATCTGTGGCTTCACAGTACAGAGGGACTGTCCAAAGCCGCAGAT
CTCGAGTCGACTAGCTTCCTTTCTCCCTTCTCCC
+
DEBBAA@@AAA999195954AA?9;<,,,,,,,,,,,/57;8:;<?::;CC@?>>477.4;;:<=>@@;@B>C9<<=9
43000049:37.7./.2424858:;>9A;?@AC<
@T6XXI:00093:00802
GGGAGAAGGGAGAAAGGAAGGATAAGGCAACGGGCGAGAAAGTTGGTCTATTCTCCCTCTCCCTATAGTGAGTCGTA
TTAGAATTCCCC
+
CDBCBB@BB>AAAB>A@A@A@BCB=@>@C@CCB@CCE@?@>?C?B??BBCC@BBBB>AACBB>B@>?@@@@BBCBBB
@=?>>;>8?@@4
@T6XXI:00093:00820
GGGGGAATTCTAATACGACTCACTATAGGA
+
4999+4:;::;959<;11/00..44.0,*,
@T6XXI:00093:00823
GGGGAATTCTAATACGACTCACTATAGGGAGAGGGAGAATAGCCGTTATTTTAACTCCCGCGGCCGGTTCTTCCTTT
CTCCCTTCTCCC
+
CCA6D:96<@=;@AB<<=BBB????877.777?B>BAB?ABB@>AC=@@A@5?:<;<@;999>A@A?A>;?9978=9
/0/>9A;;;AA<
@T6XXI:00093:00829
GGGGAATTCTAATACGACTCACTATAGGGAGAGGGAGAATAGTTTTTCCCTTCACTAACCGCGAACTGCACTTCCTT
TCTCCCTTCTCCC
+
```

```
::4,9<==@BB?CBBBBBBBBB<99311.7>>BD>BBB>>>>;;=A1@@<@=?>?B?B?>::?@@CC=:>?@@A?@>
;@CBC;???@@=;
@T6XXI:00093:00838
GGGGAATTCTAATACGACTCACTATAGGGAGCAGGGAGAATAGCTCGGGAAAACCTTCCCCACCGGGCATTGTCTTC
+
4;;,/,/5///7///:::30:<110/,2*,,//.553665/7186111,111,1010166244/11,04420004/0
@T6XXI:00093:00849
GGGAGAAGGGAGAAAGGAAGGACGGTGTGCTGGGAGAAAAAAATGCACAGAACTATTCTCCCCTCTCCCTATAGTGA
GTCGTATTAGAACTTCCCC
+
BD?A:<<;B<AACA<A9;9;BB@A?AAC??@AA9?8888888&9B@@AC<969@::9:4333,3336688888@??@
>>CA<<A8///,./45555
@T6XXI:00094:00806
GGGAGAAGGGAGAAAGGAAGGAGAACGCGCACCCGAACCAACGGTGATCTATTCTCCCTCTCCCTATAGTGAGTCGT
ATTAGAATTCCCC
+
EFBDCC@DB>BAAA=B@C@C@CAB?BBCCBBCC>BB?BAC@CBAAA@CB@AAAC@@A<@BBBC?CCBBBBBABBBAB
BC@CCC@CACCC:
@T6XXI:00094:00811
GGGGAATTCTAATACGACTCACTATAGGAGAGGGAGAATAGTCAACAAATCGTTTGGGGCGACTTCTCCTTCCTTTC
TCCCTTCTCCC
+
EBC9C>B@CCC@CBB@B?>?B9666./,0<;>@>BCC>B@CA?<;?A<;BCBBB<BB?2;:/00,0::,/.9?DC>A
?==;==AAAA<
@T6XXI:00094:00817
GGGGAATTCTAATACGACTCACTATAGGAGAGGAGAATAGAAGGTCGTACCTTCCCCATCGCCGTACTCCCTCCTTC
TCCTCCTC
+
AC818+96<@=?BCCCCBBBB:9990.,0<:=:?7::>00/,-
33...33535399000000/0000000+0444/000/00/01
@T6XXI:00094:00844
GGGGAATTCTAATACGACTCACTATAGGGAGAGGGAGAATAGATCACCGTTGGTTCGGGTGCGCGTTCTCCTTCCTT
TCTCCCTTCTCCC
+
BB?2A8;;<CC@CBABBBBBBC>;@:66.7?>@A?BDC@CB848?;:5:B474:<@@A>BDBBBCC@DCD@C@D@CC
>BBAC>C?A<;;6
@T6XXI:00095:00807
GGGAGAAGGGAGAAAGGAAGCTAGTCGACTCGAGATCTGCGGCTTTGGACAGTCCCTCTGACTGTGAAGCCACAGAT
GTCGAGTCGACTAGCTTCCTTTCTCCCTTCTCCC
+
FF>AAA=>B:>999185959>783361111117111119;A;?BB87769666662113><:::=8<377<>=?>??
?784<>7730300000/0/00+0004+0243644
@T6XXI:00095:00808
GGGGAATTCTAATACGACTCACTATAGGAGAGGAGAATAGCTAACCAATTTCCATCGGCACCGTATCTTCCTTTCTC
CCTTCTCCC
+
CAB4>6969>>;@@@@B@@@A000>00,,0,/,.33556<=BB=97?377,17776:5:?9710,,++00*++*+-
00+0/04443
@T6XXI:00095:00822
GGGAGAAGGGAGAAAGGAAGAGAATGCCACTGTAATTTTAAGCCGTGACTATTCTCCCTCTCCCTATAGTGAGTCGT
ATTAGAATTCCCC
+
DEBCBC@CC>BDDD?B?B@BBBB?B9:9;CBBBB5777.7>@@?CCBBBCAA=???=7>;444,8==@AAAAAA<<<
8838@@==;AAA5
@T6XXI:00096:00813
TTCTAATACGACTCACTATAGGAGAGGGAGAATAGCCAACAGGTATCTGATTGCGCGTTCCCCTTCCTTTCTCCCTT
CTCCC
```

```
+
D>CCC?AABBCCCBBB?7900,0=100,7??=ABBB?B@BBC?AABBACBB?AAAA@>8;;<0;=?:>?:?@?@<@>
AAAA;
@T6XXI:00096:00817
GGGAGAAGGGAGAAAGGAAGCATAGTCACTACATCTGTGGCTTCACAGTACAGAGGGACTGTCCAAAGCCGCAGATC
TCGAGTCGACTAGCTTCCTTTCTCCCTTCTCCC
+
CEBCCC@CB:>98809;;:;1..462.....3355<88896737??7::;:::88818>>?@@>AA;@@@A?><<<8
3399777<===;<74/448803333,3/438;4
@T6XXI:00096:00822
GGGAGAAGGGAGAAAGGAAGGAGAAGTCGCCCCAAACGATTTGTTGACTATTCTCCCTCTCCCTATAGTGAGTCGTA
TTAGAATTCCCC
+
/1-1:>5990944404889924339314377 7,33,33319899=8867112..-
333...331346555<3447;;;==;<;<:???4
@T6XXI:00096:00828
GGGGAATTCTAATACGACTCACTATAGGGAGAGGGAGAATAGTTTCCTTCGACTAACCGCGAACTGCACTTCCTTCT
CCTTCTC
+
///+/,-*,00+-
/6/44<<>;<<;???597>>@9??><=<744,023/003311363111101653777010101110101 44
@T6XXI:00096:00845
GGAGAAGGGAGAAAGGAAGCTAGTCACTCGACATCTGTGGCTTCACAGTACAGAGGACTGTCCAAAGCCGCAGATCT
CGAGCTCACGATAC
+
1-
/8878:598480959698379766111117731111 7067579?@CA?;>>/0./;;87=?AA<AA<?>==;666++
++++02--.139
@T6XXI:00096:00847
GGGAGAAGGGAGAAAGGAAGGAGAACTGCAATTGTCCAAACGTGGCAGCTATTCTCCCTCTCCCTATAGTGAGTCGT
ATTAGAATTCCCC
+
CH?BECCCE>BBCC>B@B@BABAA?BBCBC???B@>=AC?BDCEACBCCCDFACA??>ACECB?ABBBAACCCGCCC
BA>ABA>A=???8
@T6XXI:00097:00803
GGGGAATTCTAATACGACTCACTATAGGGAGAGGGAGAATAGGCCGACGTCGACATGCGTCCCGGCCGTCCCAC
+
AAA6A@C>ABB=@@A<;<BGCBDCA@?B<???BB>B;76://,.2-----3366::@<<==B/.,/4///-+..
@T6XXI:00097:00814
CTATCGACTCGAGATCTGCGGCTTTGGACAGTCCCTCTGTACTGTGAAGCCACAGATGTCGAGTCGACTAGCTTCCT
TTCTCCCTTCTCCC
+
B:66///667:BBBA@@BBB>ACC>E?CBCC@??=?AABB??B?BCC??BACBBBB@@@CBB@A;;;:;?8;6>A>A
B=AABB>B:;:@@7
@T6XXI:00097:00817
GGGAGAAGGGAGAAAGGAAGTGAACGTTACCGTGGGAGAATTGGTAACTATTCTCCCTCTCCCTATAGTGAGTCGTA
TTAGAATTCCCC
+
HHCCCD@BB89<@@<A?B?BBAA?CA?=AJ@CCCCAC<<;>ADADCACCBB?BFNHBBB<;;0998;AABABA?7::
;5:@C>?>@@@5
@T6XXI:00097:00829
GGGAGAAGGGAGAAAGGAAGGAGAAGTCGCCCCAAACGATTTGTTGACTATTCTCCCTCTCCCTATAGTGAGTCGTA
TTAGAATTCCCC
+
:;6999299-499;-
989@C@FB@CC?@?>>>.88083311,16366><AB??ABA<A=88817887@@?>?A9988@<58999;<<=5
@T6XXI:00097:00830
```

```
GGGGAATTCTAATACGACTCAGCTAATAGGAGAGGGAGAATAGTCAACCAAATCGTTTGGGGCGACTTCTCCTTCCT
TTCTCCCTTCTCCC
+
CFB08,1,/,,2,///66777774423331331 1799;@>@@;:<8,/,-
329<@=?8>>?7ACDDE@B@?474747?:>:::6:.0.5:9
@T6XXI:00098:00803
GGGGAATTCTAATACGACTCACTATAGGAGAGGGAGAATAGCCAACAGGTATCTGATTGCGCGTTCTCCTTCCTTTC
TCCCTTCTCCC
+
CFC:C?A?ACCACBBBBBBBB:996..-
0>>BCAE??;@CCA>@?A<<?<@<<=<<><==99=A?AAA9<9<8=@:???=:=8<<?A<
@T6XXI:00098:00804
GGGGAATTCTAATACGACTCACTATAGGAGAGGGAGAATAGTTCTGTGCATTTCTCCCAGCACACGGTCCTTCCTTT
CTCCCTTCTCCC
+
BBG7A>B>BBB@GBCBBBCB>??C>7927>>>@?ACC@C?B??<<9A;==??;@@<=;<<<<<=<><====9;2771
8@AH@A<===C=
@T6XXI:00098:00807
GGGAGAAGGGAGAAAGGAAGCTAGTCGACTCGAGATCTGCGGCTTTGGACAGTTCCTCTGTACTGTGAAGCCACAGA
TGTCGAGTCGACTAGCTTCCTTTCTCCCTTCCTCCC
+
CB?B>>699-
89>>:@=@=@DCBCB:67;611;89;<<?@A>???9?>A;::C=@=@7444433944723323 89663446;<;@@@
@@?A8889<;;A1//..,.,/,-22+
@T6XXI:00098:00810
GGGGAATTCTAATACGACTCACTATAGGGAGAGGGAGAATAGTCAACAAATCGTTTGGGGCGACTTCTCCTTCCTTT
CTCCCTTCTCCC
+
ADHAD=@>ADDBBA@ABBBBBB977366.7==?A>BBB?CBB>BA:;;;;BB??@?@AA8B@>>C?BBC>A>A@BA:
AAAC:?<?@@C;
@T6XXI:00098:00812
CTAGTCGACTCGAGATCTGCGGCTTTGGACAGTCCCTCTGTACTGTGAAGCCACAGATGTCGACTAGCTTCCTTTCT
CCCTTCTCCC
+
8=111>;?BBCCE??ABBCCC@CCB=B>CCBB;:61:@@ABA?BBCCC;9<ACC@FABBBBBBAAAA@A<>;==6==
?A<B6:;??9
@T6XXI:00098:00817
GGGGAATTCTAATACGACTCACTATAGGAGAGGGAGAATAGTCAACAAATCGTTTGGGGCGACTTCTCCTTCCTTTC
TCCCTTCTCCC
+
A9?4>6969@>>@ACBFCECC>;6700,/9:@C>CCCABBBAAA>AAA;AA???;@DD>G::;A;>=?37447996<
ABA<A:==CC=
@T6XXI:00098:00819
GGGGAATTCTAATACGACTCACTATAGGGAGTGGGAGAATAGTCAACAAATCGTTTGGGGCGACTTCTCCTTCCTTT
CTCCCTTCTCCC
+
CCB9B@C@CDC@AABBBCCC@@??<777.7>>@BC@@@@CCCC@@<??C;AA@AB@CCB6@@@@B:=>>,0,09::9
96;?9?./.355
@T6XXI:00098:00820
GGGAGAAGGGAGAAAGGAAGGAGACAGCCCCAGAGTACAGTCCAGAACTATTCTCCCTCTCCCTATAGTGAGTCGTA
TTAGAATTCCCC
+
AC7<<@@AB=AAGG@CAD@C:<<BBCCDCC9A<<<BBBBCCB9<:A?@B@@=@BCB>BC???9??;<@AACAA7888
85;?@><<=AA5
@T6XXI:00098:00821
GGGAGAAGGGAGAAAGGAAGGAGAAGTCGCCCCAAACGATTTGTTGACTATTCTCCCTCTCCCTATAGTGAGTCGTA
TTCAGAATTCCCCC
```

+
LHECCC>A>:99991959=B@BAA@CBBBCCC9CC>CCCC@=B@>AAA<AD==<9940335535==CA@7:8./-
+++*-.-.,-03333.
@T6XXI:00098:00834
GGGGAATTCTAATACGACTCACTATAGGAGAGGGAGAATAGCTACCTGATGTGTGGCTCGCTCCTAGTCTTCCTTTC
TCCCTTCTCCC
+
??@4?;><>AA?DCCCB?ABB:999..,0;;?@<ABB?BBC@CCC>??BABA???=?ADCCBB?ACABCB?B?BD>B
BBC9?;?AA@:
@T6XXI:00098:00843
GGGAGAAGGGAGAAAAGGAAAGCTAGTCACTCGACATCTG
+
DBB@9429904999=,8544-43777;?77****(((((
@T6XXI:00098:00845
GGGGAATTCTAATACGACTCACTATAGGGAGAGGGAGAATAGTCAACAAATCGTTTGGGGCGACTTCTCCTTCCTTT
CTCCCTTCTCCC
+
CFC9@?A?BCDDCA@?BBBBBB>99311.7>>?B>BBB>>>>9AA>??;:AB??98???5@??@C?BBB@F@@>@@<
@AAA<A;?>AC:
@T6XXI:00099:00804
GGGAGAAGGGAGAAAGGAAGGAGACAGCCCCAGAGTACAGTCCAGAACTATTCTCCCTCTCCCTATAGTGAGTCGTA
TTAGAATTCCCC
+
DD?CCC@CC>CCAA=B?B@CDA@@B@9899/9999>A<;;CCBBBBABBAA?>877.7=;;686889484<;:9;;;
;:=;:78/444-
@T6XXI:00099:00810
GGGGAATCTAATACGACTCAGCTATAGGAGAGGGAGAATAGTCAACAAATCGTTGGG
+
499,499449<=88==9333334433377316=,61101116632030+39310.30
@T6XXI:00099:00815
CTAGTCACTCGAGATCTGCGGCTTTGGACAGTCCCTCTGTACTGTGAAGCCACAGATGTCGAGTCGACTAGCTTCCT
TTCTCCCTTCTCCC
+
CB;;;B9=?AAAAACCCCCC@BBB>B>AABB:::5:CCABACC=?@B??BBCCCCCCBB@ABCABB??@CCCCBG@B
B>???A:?:??AB>
@T6XXI:00099:00833
GGGGAATTCTAATACGACTCACTATAGGGAGAGGGAGAATAGAGCTTAGATCTTGGCTCACGTTTTCTCCTTCCTTT
CTCCCTTCTCCC
+
CCC9C=@=@CC>BABBBBBB>?<A9366.7>>AB>;;<@BBB@???>CBBBCC@@@CBAABBBBC7AAA=>;>3881
8;;;9;79444-
@T6XXI:00099:00836
GGGAGAAGGGAGAAAGGAAGGAGAAGTCGCCCCAAACGATTTGTTGACTATTCTCCCTCTCCCTATAGTGAGTCGTA
TTAGAATTCCCC
+
CCBCCCAAB=@A?C?B?B?B@CCCABBCCCCC9BD@CBB@@;AA@CC?BBB>:===;;<@AA<@@@@@@@AAA@@??
??A@@C@>AAA6
@T6XXI:00053:00853
GGGAGAAGGGAGAAAGGAAGCTAGTCGACTCGACATCTGTGGCTTCACAGTACAGAGGGACTGTCCAAAGCCCGCAG
ATCTCGAGTCGAC
+
CC?B99;@@:>AAA<B8:7<B>;=>/11@D@??BCDBC@@@@<@>AC===CA@?;8885>?A;;<37717@@<@@A@
??@@A==<<<?AA
@T6XXI:00054:00857
GGGGAATTCTAATACGACTCACTATAGGGAGAGGGAGAATAGCTGACACATTGAGCGCACCTGTCCTCCCTTCCTTT
CTCCCTTCTCCC
+

```
CDFFDEE@BBC@CBBBA@@BBB>99311.7??CD>BBB>>>>8;???????>;;:ABBBD@A?@B?@EC>C?B?B><
AA?A<A:5;>>7
@T6XXI:00054:00859
GGGGAATTCTAATACGACTCACTATAGGAGAGGGAGAATAGACCTCGCAAAGGCAATGTCGTCCCACTTCCTTTCTC
CCTCTC
+
?BA4@69599;2499998@AA;>?000,.3379=@A>>?:0//,./--..+.3..,-00003=<;<=C<9976+/-
.-+----
@T6XXI:00055:00851
GGGGAATCGTAATACGACTCACTATAGGGAGAGGGAGGAATAGTGACGTGTTGTGCGGCGCTCACTGTCTCCTTCCT
TTCTCCCTTCTCCC
+
77708+,,,,,2,/48843399>>776<6??99?;==;=@C>@=8//----
,03688:;488<<>=>?@=98;97,..+../.+.,-3455
@T6XXI:00055:00859
GGGAGAAGGGAGAAAGGAAGGAGAAGTCGCCCCAAACGATTTGTTGACTATTCTCCCTCTCCCTATAGTGAGTC
+
CB>B@@=@B:ABBA>C=@=A?A@C@BA>8;;8.8B>BC?CC=@@>BBBBCC@@@@A144444-444:?@//.9/
@T6XXI:00055:00861
GGGAGAAGGGAGAAAGGAAGGAGAAGTCGCCCCAAACGATTTGTTGACTATTCTCCCTCTCCCTATAGTGAGTCGTA
TTAGAATTCCCC
+
CC@CBABDGABBBA=@?C=?@ACC@CCCBBBB8BB<BCCCB>@?@???BBC@=;==;====A?A@@@@@AAA=AA?@
@>AAA=@38880
@T6XXI:00055:00862
GGGGAATTCTAATACGACTCACTATAGGGAGAGGGAGAATAGCTAACACTCAAATTCCCAGCATTATTC
+
66606,/-/::;?CCCCAB:;;88C877,7777><:::@A@@;/0,..../7535,..+1017701101
@T6XXI:00056:00856
GGGGAATTCTAATACGACTCACTATAGGAGAGGGAGAATAGCTACGTTTAACCGATCGTGTTGTTCTCCTTCCTTTC
TCCCT
+
DDD:CAC@CCCCCBBBCCCCBBBCC8:8:CCBC>CBB>@@?3::@?<686:=8:@@@=744443/3;87979,//+-
-+000
@T6XXI:00056:00859
GGGGAATTCTAATACGACTCACTATAGGGAGAGGGAGAATAGTTTCTCCGTTATCGTAGATCGCTGTTCTCTTCCTT
TCTCCCTTCTCCC
+
CDF9C?B@BCCACA@ABBBBBBB99311.:@@CE>CCC>>??<=4999:;;:;;:::;888;:::>?>CAAA@C@BB
>@CCC:?>=?<:4
@T6XXI:00057:00855
GGGAGAAGGGAGAAAGGAAGG
+
FEBBBB@BA:>8,,**)*)*)
@T6XXI:00058:00857
GGGGAATTCTAATACGACTCACTATAGGGAGAGGGAGAATAGCCAAACTTTTCCTGGTCCCCGGCTACTCTTCCTTT
CTCCCTTCTCCC
+
@==6@@@ABBB@CCCCCBBBBBBB<76659??CD>CBB?CB?<;?@;=??@5@>AD>AAAA6A><:<<<>=;;8;;4
87AC:@<?<<;7
@T6XXI:00058:00860
GGGGAATTCTAATACGACTCACTATAGGGAGAGGGAGAATAGATCACCGTTGGTTCGGGTGCGCGTTCTCCTTCCTT
TCTCCCTTCTCCC
+
CDC9C8;;<CC@BBBBA@>BBC?99311.7>>>@>BCC@B??===BB?BD=@=@ACCB?C;<;CBB?BCB@C?B?BC
>CBBB>C@B<=83
@T6XXI:00058:00864
```

```
GGGGAATTCTAATACGACTCACTATAGGGAGAGGGAGAATAGATACAAGACC
+
EEC9A?B@CCC@BA@ABBBBBB>99361.7???BCEEB9=5//--13,-032
@T6XXI:00059:00854
GGGGAATTCTAATACGACTCACTATAGGGAGAGGGAGAATAGGGCCGACGCGA
+
EKH:@;=699=<BBBBBBBBCC:;;;:>7>=777.17?;661664862221-.
@T6XXI:00059:00855
GGGGAATTCTAATACGACTCACTATAGGGAGAGGGAGAATAGTCAACAAATCGTTTGGGGCGACTTCTCCTTCCTTT
CTCCCTTCTCCC
+
CFD:EEC@BBB@BBBBBBBBBCBB>76659??CF>CBB>>;;<==;<<=:@?666,667-
8ABBC@CBB?B?B?@A<>AAA;@;==CB<
@T6XXI:00059:00856
GGGGAATTCTAATACGACTCACTATAGGGAGAGGGAGAATAGTCAACAAATCGTTTGGGGCGACTTCTCCTTCCTTT
CTCCCTTCTCCC
+
=A?4?<@=@CC@CBBB?CCBBDB>;761-
6??CF>CBB;C@@><:<???:ABBBC>AAA7ABC??@?BB?C@A=@@<@AAB;@>@@@@<
@T6XXI:00059:00858
GGGGAATTCTAATGACGACTCACTTATA
+
444,49924448444;994/////,/00
@T6XXI:00059:00865
CTATCGACTCGAGATCTGCGGCTTTGGACAGTCCCTCTGTACTGTGAAGCCACAGATGTCGAGTCGACTAGCTTCCT
TTCTCCCTTC
+
A;;///////449=9?99;;8;BCAC=@@<;;CC;?@CAAACBCB??3/1-
//888<AA?;@B:;;/8;4...,.,..+.6776636
@T6XXI:00059:00866
GGGAGAAGGGAGAAAGGAAGCTAGTCGACTCGACATCTGTGGCTTCACAGTACAGAGGGACTGTCCAAAGCCGCAGA
TCTCGAGTCGACTAGCTTCCTTTCTCCCTTCTCCC
+
IEBC=<99A:>999185948>>>>>77711113768<?CA@ABB@AAABCCB??9333-
4444=?=?A@AA=@ABDC?====???AAA@???@@>?>AA;?>==:;244;;7
@T6XXI:00060:00863
GGGAGAAGGGAGAAAGGAAGGGGAGAAGGGACGCAAAGGAAGTCTAGTCACTCGA
+
:D<@AC@BC@<<<B<@=@@AAC8@:9,01,./....+.66,31161111111133
@T6XXI:00060:00864
CTAGTCGACTCGAGATCTGCGGCTTTGGACAGTCCCTCTGTACTGTGAAGCCACAGATGTCGAGTCGACTAGCTTCC
TTTCTCCCTT
+
B0,,,,,,//:?AABFC;:<CECCD>G@BBCBBBB@CFFBBBBCBBBD@AC?BBBDC@@?BCCCCAAFAA@BBBBDA
CB=??EE@C?
@T6XXI:00061:00851
GGGGAATTCTAATACGACTCACTATAGGGAGAGGGAGAATAGTTCAACGTTTATCCGCATGAACATCTCCTTCCTTT
CTCCCTTCTCCC
+
DDEEC@C@BBC@BAAAAA?B>AA<<76656==AB<BCD@@??<;<=>???A;??C?BA???@=@A==AB>@<>300-
4;;?;@>BAAA<
@T6XXI:00061:00870
GCTAGTCACTCGAGATCTGCGGCTTGGACAGTCCCTCTGTACTGTGAAGCCACAGATGTCGAGTCGTA
+
4/////6/44B==<CEFC>>8:00,0,.4488@@;<=?BBA:::611704249449?97376..,,,*
@T6XXI:00061:00872
GGGGAATTCTAATACGACTCACTATAGGGAGAGGGAGAATAG
```

```
+
A770<,1,1/87:;BBCC@@@@>>>877.00935+//755.6
@T6XXI:00062:00853
GGGAGAAGGGAGAAAGGAAGGAGACAGTGAGCGCCGCACAACACGTCACTATTCTCCCTCTCCCTATAGTGAGTCGT
ATTAGAATTCCCC
+
BB@CBB?BB>CBBB>B?A?B@B@@ECBB?BBBBB@?>BBB@>>==?@CBABB@BBA@<=;>?A>ACHBB@AACBABA
BA>A@?=>=@@@6
@T6XXI:00062:00871
CTCGAGATCTGCGGCTTTGGACAGTCCCTCTGTACTGTGAAGCCACAGATGTCGAGTCGACTAG
+
DCBBBBBBB@@@A@BBB<B?BBCDDBB>BCCBB@@@AAAC>==@ABBBB?>>AABBBB<BBBBB
@T6XXI:00062:00875
GGGGAATTCTAATACGACTCACTATAGGGAGAGGGAGAATAGCCCGACATTAGGCCGCATCAATACATTCTTCCTTT
CTCCCTTCTCCC
+
499/9<A@BCCAC@@?BBBB?BBBB97646?@CC>CBCB@@><;4655<A>@C>A?ACCCB@?@CBBB?BB?B?@A<
AAAA:A:;A;;:
@T6XXI:00062:00876
GGGGAATTCTAATACGACTCACTATAGGAGAGGGAGAATAGTCAACAAGTCGTTTGGGGCGACTTCTCCTTCCTTTC
TCCCTTCTCCC
+
CDF:C?A?ABD@BBBBBBBBB:664./-
0>>BE>BBA>??@=900+,+++00000000.8?BAD@CCDAB??>@C=@???7=388@A<
@T6XXI:00063:00852
GGGGAATCTAATACGACTCACTATAGGAGAGGGAGAATAGATCACGACCCTTCTACCACGATTGATCTCTTCCTT
+
EB>1:-1///78449497<73333367665330300,...-0331167726361668<<B>=931---01,-,-,
@T6XXI:00063:00858
GGGAGAAGGGAGAAAGGAAGCTAGTCACTCGACATCTGTGGCTTCACAAGTACAGAGGACTGTCCAAAGCCGCCAGA
TCTCGAGTC
+
CCB@?=5991999A:A<C<B??;8,02,,,,,22226116313135..,....110100444073784448//,---
--2330300
@T6XXI:00063:00861
GGGGAATTCTAATACGACATCACTA
+
:EB8>6;2444844/////.4....
@T6XXI:00064:00851
GGGAGAAGGGAGAAAGGAAGAGAACGGCGACGAACAATAGTTGGTGGCTATTCTCCCTCTCCCTATAGTGAGTCGTA
TTAGAATTCCCC
+
A??AAA@CC@BBBC>CDC@BAAA@CC@BB@BCB@ABACB?@=@@>;=@@BB<?@@@<?BBCD>AAABAAAA<<=@@@
A>AAA>A<???5
@T6XXI:00064:00866
GGGAGAAGGGAGAAAGGAAGTGCAGTTCGCGGTTAGTGAAGGGAAAACTATTCTCCCTCTCCCTATAGTGAGTCGTA
TTAGAATTCCCC
+
EB@CBBAAA=ABBB?C@B@BBBBCCD?B::;@C@CBBCC@CB?@CC8CBAB?ABBB>BBBCC>BBBB?<<@AA?@?@
@<??A>B?AAA6
@T6XXI:00064:00870
CTATCGACTCGAGATCTGCGGCTTTGGACAGTCCCTCTGTACTGTGAAGCCACAGATGTCGAGTCGACTAGCTTCCT
TTCTCCCTTCTCCC
+
B::6/1//78;B:9<CDCCC?BBB<B@BBBAA??>?@AC@===C???=<AACCABBBBBBBBA?@A@BABDBB@C@C
B>BBBC>C?BBCC=
@T6XXI:00064:00875
```

```
CTAGTCACTCGAGATCTGCGGCTTTGGACAGTCCCTCTGTACTGTGAAGCCACAGATGTCGAGTCGACTAGCTTCCT
TTCTCCCTTCTCCC
+
>:/111/4:CCCBABBAA??<<?B<B?BBBBB??;??>;?>?????@>=B@BBBBBAB?>>C@>ABBBCCCCC=B>B
D=AABC;BA;A9;;
@T6XXI:00064:00878
GGGAGAAGGGAGAAAGGAAGGAGAAGTCGCCCAACAAGTAT
+
/1,/44244-444=-4692499>><>;>883,313.43.,*
@T6XXI:00065:00867
GGGAGAAGGGAGAAAGGAAGGAGAAGTCGCCCCAAACGATTTGTTGACTATTCTCCCTCTCCCTATAGTGAGTCGTA
TTAGAATTCCCC
+
<<:9B@<@@8<;>>:?595;8;77>?:<:CCC9CDACB:71,163116>9A>?==>-
44300+354431++++++0322688585=>>0
@T6XXI:00065:00868
GGGAGAAGGGAGAAAGGAAGGAGAAGTCGCCCCAAACGATTTGTTGACTATTCTCCCTCTCCCTATAGTGAGTCGTA
TTAGAATTCCCC
+
CC?@?=<=@<BBCC@C@C@B@B7717=>8888,3967777:,:689?>>BD<<;=;9477=>7=AC??>?@ABAAA@
@?@@@>@>AAA5
@T6XXI:00065:00877
GGGAGAAGGGAGAAAGGA
+
CCB99969919=000000
@T6XXI:00066:00855
GGGGAATTCTAATACGACTCACTATAGGAGAGGGAGAATAGTCAACAAATCGTTTGGGGCGACTTCTCCTTCCTTTC
TCCCTTCTCCC
+
949/99A>A;<6;BBABBBBB=;;;0/,///=;>A@@@C@CB@A>===9@DCCC>CCC9CCCBB?BAA:?;;7894;
=;=7>9<=?B<
@T6XXI:00066:00856
GGGAGAAGGGAGAAAGGAAGCTAGTCACTCGACATCTGTGGCTTCACAGTACAGAGGACGTCCAAAGCCGCAGATCT
CGAGTCGACTAGCTTCCTTTCTCCCTTCTCCC
+
E@:@99649699>>:=5944>8834/..........111188<:>@A??@A@<<//,///-
.7@?04499944999488999AACBCCB@@;?5990999@0959@=;?
@T6XXI:00066:00860
GGGAGAAGGGAGAAAGGAAGGAGAAAGCGACGTCGGGGAAACGTTTTGCTATTCTCCCTCTCCCTATAGTGAGTCGT
ATTAGAATTCCCC
+
CDEC@@@AA?AABB>C@C=A?@CCB<@@@?;;;@CCC8CC>AAADF9BBCIC;<=DGA;<<@@<?BB@@ACCBCC@?
?B=?<@>=;<CC5
@T6XXI:00066:00862
GGGAGAAGGGAGAAAGGAAGGAGAAGTCGCCCCAAACGATTTGTTGACTATTCTCCCTCTCCCTATAGTGAGTCGTA
TTAGAATTCCCC
+
CEBCBB@BDBCCBB?B@C?B?:;;=@CBBBFF7@@=@CBCEAAA=??????@???@:44444,4:900088;;<=A@
@;997777:;;4
@T6XXI:00067:00865
GGGGAATTCTAATACGACTCACTATAGGGAGAGGGAGAATAGTCAACAAATCGTTTGGGGCGACTTCTCCTTCCTTT
CTCCCTTCTCCC
+
EEB9C>@@ACCABA@ABBBBBB?99311.7?7771988@CCC@@?=BBA:BA?AD>BB?7ACCBB?ABB4:599==:
@AAA<A9<<??7
@T6XXI:00067:00879
GGGAGAAGGGAGAAAGGAAGGAGAAGTCGCCCCAAACGATTTGTTGGACTATT
```

```
+
FB>?@@=@A><<<C@B?C@EBBBB@;<<BCDC7@@<@;797,..,.,.54.//
@T6XXI:00068:00852
GGGGAATTCTAATACGACTCACTATAGGGAGAGGGAGAATAGTCAACAAATCGTTTGGGGCGACGTTCTCCTTCCTT
TCTTCCTTTCTCCCTTCTCCC
+
DBG4D=@=@BCACB@>BBBBAA<99311.7>==?>@DE@CCC@A@>==<7@BABC>CCC9CAB;;<@AAA::8:?@@
;AA>@>@@<@@ACAC>????;
@T6XXI:00068:00853
GGGGAATTCTAATACGACTCACTATAGGGAGAGGAGAATAGTACGACAAA
+
CEK6>>B@B=@=A@@@CBAACBC?<;??6?==?;A??;?00//.---...
@T6XXI:00068:00854
GGAGAAGGGAGAAAGGAAGCTAGTCGACTCTGA
+
95444244-/788+/,/564933311..33...
@T6XXI:00068:00861
GGGAGAAGGGAGAAAGGAAGCTAGTCACTCGAGATCTGCGGCTTTGGACAGTCCCTCTGTACTGTGAAGCCACAGAT
GTCGAGTCGACTAGCTCCTTTCTCCCTTCTCCC
+
CBBC<<;<BAA<<919596;???AABBCCBBBBFC@@@CC@CCC<A>::16666266635378@CCC@BB?@<;5;;
;<<<<<@@@@=89:/0/,/426///+-2844=:
@T6XXI:00068:00864
GGGGGAATTCTAATACGCTCACTATTAGG
+
4;;;/98959942444447......,...
@T6XXI:00068:00865
GGGAGAAGGGAGAAAGGAAGCTAGTCGACTCGACATCTGTGGCTTCACAGTACAGAGGGACTGTCCAAAGCCGCAGA
TCTCGAGTCGACTAG
+
BC>ADB@CBBBCBB<B8949?888@;::A:::@??:::=67387:;7688<<=><===;<=B?>>;=<5>;:<AA@@
@=<<><;;8//./..
@T6XXI:00068:00871
GGGGAATTCTAATACGACTCACTATAGGGAGAGGGAGAATAGCCATCACCCCGTTAAACGCGCAATATTCTTCCTTT
CTCCCTTCTCCC
+
;<;4<>B?CCCAC@A@BBBBAABB>76656??BC>CCC>?>>9;><9888807B?AA:BCCCCC@CCCCBB?B@CC=
BBBC>C?BBBB<
@T6XXI:00068:00879
GGGGAATTCTAATACGACTCACTATAGGGAGAGGGAGAATAGTTTTCCCTTCACTAACCGCGAACTGCACTTCCTTT
CTCCCTTCTCCC
+
BEF:C::;:CC@CABBBBCCCC;77366.7>>?A>DCC?B??==C5@A<@>?@<@@=?>A?BC@AA?@@@B>?;<==
?BA?;>;<?>=7
@T6XXI:00068:00890
GGGAGAAGGGAGAAAGGAAGGA
+
DD>CBA=>>:><8887+*)(((
@T6XXI:00069:00857
GGGAGAAGGGAGAAAGGAAGGTGAACAGAGCGCAAACGAATACGTCTCTATTCTCCCTCTCCCTATAGTGAGTCGTA
TTAGAATTCCC
+
FICDCC@CC@A@==:@=?<@@CCC@ECCBBBB???<@CCECCDECBABCDE>A@@@<@BABC@CCCCBBB@@?@BB@
@=?BB@DCDE@
@T6XXI:00069:00858
GGGGAATTCTAATACGACTCACTATAGGAGAGGGAGAATAGCTCCCTTGTCGATAACCGCGGACTCCCCTTCCTTTC
TCCCTTCTCC
```

```
+
994,4:;@BCC@CCCC<<<AA;=?>10,.336799666:@;8:CC5:5:99776B2424B=9440333+0/0/04+0
000+043032
@T6XXI:00069:00869
GGGAGAAGGGAGAAAGGAAGGAGAACCATTGCCAAGCCTGATGGCAACTATTCTCCCTCTCCCTATAGTGAGTCGTA
TTAGAATTCCCC
+
FH@BBA@CD>ABBE>B?B@C?BBC@C?AA>??54599<?@A<<9<D@CCBB?BBBA<???@@9@@?:;;BB?;;6=8
838??=A>ACH5
@T6XXI:00069:00871
GGGGAATTCTAATACGACTCACTATAGGGAGAGGGAGAATAGTCAACAAATCGTTTGGGGCGACTTCTCCTTC
+
A@@,424244424;<<<CDJBCBBBAAA=AABCD<??E?CC:84?:68=;887@@?B?>24....,.3333,.
@T6XXI:00069:00876
GGGAGAAGGGAGAAAGGAAGGAGAAAGCGACGTCGGGAAAACGTTTGCTATTCTCCCTCTCCCTATAGTGAGTCGTA
TTAGAATTCCCC
+
FDBCCB@CCBBBBB?B@B?B?BAAA>ABBBBCCCCB>CCB8BCBB?BBCAD@@CCC@CCBBB>BC@B@CCDDCAAAC
C@CAA>A>AAA5
@T6XXI:00069:00879
GGGAGAAGGGAGAAAGGAAGAGAACAGCTGCTTTGGAAACAGTCAGACTATTCTCCCTCTCCCTATAGTGAGTCGTA
TTAGAATTCCCC
+
FFBCBB@BD>9;;CBD@B?BABC@@@BBDCBBB>B@CCACCCC?@@CCBBB><<?B>CC???;A>ABBACC@<????
?=?@A>A?@AA5
@T6XXI:00069:00887
GGGGAATTCTAATACGACTCACTATAGGAGAGGGAGAATAGCTATTTCTTGTCCGTCCGTCTCATTCT
+
IKB6@8969@>>BCDCCEGB@@:>>0/-/55?B;@<:;?::8@BB?=AA><::,0//,.,,.---,--
@T6XXI:00069:00891
GTCGACTCGAGATCTGCGGCTTTGGACAGTCCCTCTGTACTGTGAAGCCACAGATGTCGAGTCGACTAGCTTCCTTT
CTCCCTTCTCCC
+
AA>:9>>>@CCBHEBBBB?CAA<@ACCBBBCC?CCC<@<?BBB>B@CC@C>BBCCBC@@?BAAABBCCA?A?A@CC>
CCC@;A?@BB@=
@T6XXI:00069:00892
GGGAGAAGGGAGAAAGGAAGCTAGTCACTCGACATCTGTGGCTTCACAGTACAGAGGGACTGTCCAAAGCCGCAGAT
CTCGAGTCGACTAG
+
CCBCCB===5@@@=:@>@>BC@@?B6777777?=896?AA<AB@@A?BCBBBA?77764::>@?=?C<??9<<>>==
<<=@@@@DA@@<<<
@T6XXI:00070:00852
GGGGAATTCTAATACGACTCACTATAGGGAGAGGGAGAATAGATCACGACCCTTCTACCACGATTGAGCTTCCTTTC
TCCCTTCTCCC
+
CFC:D@C@DDDBBBABBBBBAA;99311.7==BEACBC?CA@<<AC@CBB@=>;;?6707=:/0,/:2@BE@=BC>B
BCC@C;<:CC>
@T6XXI:00070:00853
GGGAGAAGGGAGAAAGGAAGGAGACAGCCCCAGAGTACAGTCCAGAACTATTCTCCCTCTCCCTATAGTGAGTCGTA
TTAGAATTCCCC
+
CB@ACBACCABBBB?E@B@BAA>>>>>ABA8A@@?AAA???B@B===>BBB@@A>@=@@?<?9?A=??@@?@@@@@A
@>@BA=@<?AA1
@T6XXI:00070:00857
GGGAGAAGGGAGAAAGGAAGCTAGTCGACTCGACATCTGTGGCTTCACAGTACAGAGGGACTGTCCAAAGCCGCAGA
TCTCGAGTCGACTAG
+
```

GB>AB=6;;09===9@594;B801A////...4799>?AAC@?@>@BCCCCC??;73317?=@AA?AA<??=AAA?@
@<@@???A???AAAA
@T6XXI:00070:00870
GGGGAATTCTAATACGACTCACTATAGGGAGAGGGAGAATAGCAGCTGTTTTTACGCGTCTACTATTCCCTTCCTTT
CTCCCTTCTCCC
+
999/9<A?CCC?@@ABBBB>BBB9>31157>>BC>BAA>C@@=<<<<<>><A1AAAA<<<=<@???>@A<A>@?@@;
@@??:?;>>>>7
@T6XXI:00070:00874
GGGAGAAGGGAGAAAGGAAGTGCAGTTCGCGGTTAGTGAAGGGAAAAACTATTCTCCCTCTCCCTATAGTGAGTCGT
ATTAGAATTCCCC
+
FFB@@@@E@:=;;96;?A@CBCCCCB@BBBB?B@@@A@@?AB>A@CC1BBBC@CCBB>CCCCB>BCC??=@AAAAAA
??<?A=>B>@@@5
@T6XXI:00070:00876
GGGGAATTCTAATACGACTCACTATAGGGAGAGGGAGAATAGT
+
?992=29544988:=@;;;@@@@@BBDEA:</>?,0,,+,,,*
@T6XXI:00070:00878
GGGGAATTCTAATACGACTCACTATAGGGAGAGAGATATAAGGGCAGATTGTCTCCTCCAC
+
AA?4?6969>969;AABDCDCCCCAAAC>A?:7:?@>=33/44-4?;;?=?:7775,,+,,
@T6XXI:00070:00882
GGGGAATTCTAATACGACTCACTATAGGAGAGGGAGAATAGCCGCCATTTGTTCATTGGTGCCCTATCCTTCCTTTC
TCCCTTCTCCC
+
AAA6A>B?BBC@BBBBBBBAA:9990/-
0??CE>BBB@@E8428=8>>@<>><>;:47=>777.377?:?;?:??9?A@C>C===??7
@T6XXI:00070:00885
CGAGATCTGCGGCTTTGGACAGTCCCTCTGTACTGTGAAGCCAGCAGATG
+
CBBBDCBBB??=>BB>B?BBDC@==;@CCCC?B?@@;>90,+,,,,311+
@T6XXI:00071:00854
GGGGAATTCTAATACGACTCACTATAGGAGAGGGAGAATAGTCAACAAATCGTTTGGGGCGACTTCTCCTTCCTTTC
TCCCTTCTCCC
+
BBB7B=?>ACJCCA>ABBBBB:9990/,0>;>A@ADD@@??<==>?@@<BB???88886@BBBCACBB?B?>?CCA=
??;;?>AAAA;
@T6XXI:00071:00862
GGGAGAAGGGAGAAAGGAAGCTAGTCGACTCGACATCTGTGGCTTCACAGTACAGAGGGACTGTCCAAAGCCGCAGA
TCTCGAGTCGACTAG
+
CC?BB=64409>>>:@;>;@?>::>0../../0311116998<>?BCCBCBB?=8333-
49;;=@<??<?A@A@@@AA<@>@@@C======@
@T6XXI:00072:00851
GGGAGAAGGGAGAAAGGAAGCTAGTCGACTCGAG
+
/1,//9244-444=-469999?8887..444.,,
@T6XXI:00072:00852
CTATCGACTCGAGATCTGCGGCTTTGGGTCCCTCTGTACTGTGAAGCCACAGATGTCGAGTCGACTAGCTTCCTTTC
TCCCTTCTCCC
+
B::6/////8;@BBAAACCC@CCC<@@<@AA>CAA?CB@@@CCB=>>;???@A@?<>>>@@==??@B@?A???>@;>
BBB;>=9???;
@T6XXI:00072:00853
GGGGAATTCTAATACGACTCACTATAGGGAGAGGGAGAATAGCTTAACTCTTTATGTTTCTGCTCTCCTTCCTTCTC
CAT

```
+
CCC9C?B=@A@=@C@==><>>@AABBBD>@<::?8911717.-
.44,.,,,,*,,44576=?@A@910023/0/000242
@T6XXI:00072:00857
CTAGTCACTCGAGATCTGCGCTTTGGACAGTCCCTCTGTACTGTGAAGCCACAGATGTCGAGTCGACTAGCTTCCTT
TCTCCCTTCCTCCC
+
B;1////44?;;>B991<11...+.,..4;6553...33..19;:?>=FABCCCA@@CC;;;?ACBBBCBBB;?=>@
?>777+,+,+***)
@T6XXI:00072:00863
CTAGTCGACTCGAGATCTGCGGCTTTGGACAGTCCCTCTGTACTGTGAAGCCACAGATGTCGATCGACT
+
B@:<9;9;BCCBBBCBBBBAA?BBB<><@AA@???;?AACBBBB9???58;?BB@@A>=)))(((((*1
@T6XXI:00072:00865
GGGAGAAGGAGAAAGGAAGGAGAACGCGCACCCGAACCAACGGTGATCTATTCTCCCTCTCCCTACTAGTTGACGTC
GTACTTAGAATTCCCC
+
11,-,,*,*,,/8+/,47899;><@>?>=444-
44242424::;:9444//5///6+..?99,733136333.....33..199332:=@;@,
@T6XXI:00072:00871
GGGGAATTCTAATACGACTCACTATAGGAGAGGGAGAATAGTCAACAAATCGTTTGGGGCGACTTCTCCTTCCTTTC
TCCCTTCTCCC
+
AAA8A@B@CBCABBCBBBCBB9666./,0:077-
6??;?CC@@@>@@@<BCBBC>BBB6BBC::567>:=;<=@@;@>>>:?=?@AA<
@T6XXI:00072:00872
GGGAGAAGGGAGAAAGGAAGCTAGTCGACTCGACATCTGTGGCTTCACAGTACAGAGGGACTGTCCAAAGCCG
+
CD?AAA@CC=B@??<@@C=@@CBBBBBBCC??@AC@@@CCB?A?=?>>>>>@A?=33317>>???>AA<??<?
@T6XXI:00072:00876
GGGAGAAGGGAGAAAGGAAGGAGAAGTCGCCCCAAACGATTTGTTGACTATTCTCCCTCTCCCTATAGTGAGTCGTA
TTAGAATTCCCC
+
DE@@@?@CH@BBBC@C@C@B@BAA?A@BBBDB7BC>CECAA;???CA>ABC@<<<=;99=A@<A=;??AAAA==;:?
A>@@@<@;===1
@T6XXI:00072:00881
CTAGTCACTCGAGATCTGCGGCTTTGGACAGTCCCTCTGTACTGTGAAGCCACAGATGTCGAGTCGACTAGCTTCCT
TTCTCCCTTCTCCC
+
B=49<A9::CBBBBCBBBBB@?BB<B>?AAAA@?;@@@@@@>>@@@@>:A@BBCBCAACDBBCCCCCCCBBBE@B?B
C>BBCC>C@CBAA<
@T6XXI:00072:00882
GGGAGAAGGGAGAAAGGAAGCTAGTCGACTCGACATCTGTGGCTTCACAGTACAGAGGGACTGTCCAAAGCCGCAGA
TCTCGAGTCGACTAG
+
CEBCCD?B=:=;84-
9=@=@C@;;;.00:99::?8:<:ABB?AB?BBCC???A?;3331739;=?>@@;@C=??@@@A@@@?:?AAA@@@@@
@T6XXI:00072:00883
GGGGAATTCTAATACGACTCACTATAGGGAGAGGGAGAATAGCTAAAGCCTCGGCAACTGCTAACTCCCCTTCCTTT
CTCCCTTCTCCC
+
BBG7A>B?CCC@CCCBA@BBBCB><766.7?BCC>BBC?A::4337,..,.445<B?@@<@;=9:;;A@19,/,--
+-123262336;9
@T6XXI:00072:00896
GGGGAATTCTAATACGACTCACTATAGGGAGAGGGAGAATAGCAACTTACCCTC
+
DDC7B<?5994244?;@@@BBBBA;;;C>BBCCC>???:??747440/000+00
```

```
@T6XXI:00072:00899
GGGGAATTCTAATACGACTCACTATAGGAGAGGGAGAATAGTCAACAAATCGTTTGGGGCGACTTCTCCTTCCTTTC
TCCCTTCTCCC
+
CCC:C?B?CBB>B@@@BBBAB=;;;0/,0::>@=ACE@C?@?AA?@??:BCAEA;@@@7BABBE@BCB?A>>9<<7=
AAA<A?@@AA9
@T6XXI:00073:00855
GGGGAATTCTAATACGACTCACTATAGGGAGAGGGAGAATAGATCACCGTTGGTTCGGGTGCGCGTTCTCCTTCCTT
TCTCCCTTCTCCC
+
DDF9C?B?BCDDCA@?BBBBBB<97311.7>>>C?ECC>B??<<=CC?CC?B?A?BEC>B9;:CBB>A::569=9=C
>B???<?@B@@A;
@T6XXI:00073:00856
GGGGAATTCTAATACGACTCACTATAGGGAGAGGGAGAATAGTCAACAAATCGTTTGGGGCGACTTCTCCTTCCTTT
CTCCCTTCCTCCC
+
B9929<><>AA9;;B<;;BBA>>>8877.7<677.6???AA@>8958;C6?@??88?BB6CCEBB?ACC@D@B9<<6
;79937,/,/882
@T6XXI:00073:00861
GGGAGAAGGGAGAAAGGAAGCTAGTCGACTCGACATCTGTGGCTTCACAGTACAGAGGACTGTCCAAAGCCGCAGAT
CTCGAGTCGAG
+
IHBC:;7;A<@ABC>A?ABCCBCCB:;;????@??@>::9?:?B@C@@@BBB==;//,/8//.;:==:?C<BA>>@A
AA???@?<;;=
@T6XXI:00073:00865
GGGAGAAGGGAGAAAGGAAGGAGAACGCGCACCCGAACCAACGGTGATCTATTCTCCCTCTCCCTATAGTGAGTCGT
ATTAGAATTCCCC
+
991999299599=?<@=@DC?A@@ACCAA@@@=59;2424244<?DBCA<<<@CBBB=ABA??:??A;:7?711111
666866392444,
@T6XXI:00073:00868
GGGAGAAGGGAGAAAGGAAGCTATCGACTCGACATCTGTGGCTTCACAGTACAGAGGGACTGTCCAAAGCCGCAGAT
CTCGAGTCGACTAG
+
CD?CC=@CC?A?>><=<?>BCCB8300*****0000799>>?@=@CCC@BA@?<5::86>>>>@48868A?AAA>8>
??>=;;=;==:::;
@T6XXI:00073:00870
GGGAGAAGGGAGAAAGGAAGGAGAAAGCGACGTCGGGGAAACGTTTGCTATCTCCCCCTCCCTATAGTGAGTCGATT
CAGAATTCCCC
+
CC?B;;8;A<>AAA>B?B@C@C@>?:>?;88>>8833,44-4444-
4/////66666////+44333166686688,//8888:B?92
@T6XXI:00073:00875
GGGAGAGGAGAAAGGAA
+
44-44442///6+/,/,
@T6XXI:00073:00886
GGGGAATTCTAATACGACTCACTATAGGGAGAGGGAGAATAGTACACCA
+
BA?6A>B@CBB@C@?@AA???BBB@76659??AB>C@?872,,,++0*+
@T6XXI:00073:00893
GGGAATTCTAATACGACTCACTATAGGGAGAGGGAGAATAGCCTTGATGTGGTCGGCCCCGGTTTTCTCTTCCTTTC
TCCCTTCTCCC
+
CCBB@C@CCC@BBBB??@CBAABA766.6<<=@<A@@?B:::?C@CCFC?=@=AA?CDC6D;<<<4887B:;388-
4;??;@>?@@@4
@T6XXI:00074:00852
```

GGGGAATTCTAATACGACTCACTATAGGGAGAGGGAGAATAGTTACCGCGTATCTGAACGGGCTGTTCCCTTCCTTT
CTCCCTTCTCCC
+
DII:D?B@BCC@B@@ABBBBBA??7377-
7>=<68886:?@?>;?>:<<<889887?:@??<?AAB?BE>C;>377.74::4;8;??A<
@T6XXI:00074:00856
GGGGAATTCTAATACGACTCACTATAGGAGAGGGAGAATAGCTCTTA
+
DCC9@<A6949999=A>A@;;8>>88107:7BD>??>>00,,,,,**
@T6XXI:00074:00863
GGGAGAAGGGAGAAAGGAAGGAGAAGTCGCCCCAAACGATTTGTTGACTATTCTCCCTCTCCCTATAGTGAGTCGTA
TTAGAATTCCCCCAA
+
DD???=BBC>BBDB=>897:@CCC@CCBBBBB6<<<@CCCC>CA?A@@BCA>====;;;??>7>?===CB@@@@A??
?;@<<79/////+/7
@T6XXI:00074:00864
GGGGAATTCTAATACGACTCACTATAGGGAGAGGAGAATAGAACGACG
+
,--*,2@@C@@@A@?@AA????B?BB?@>A@>98?:740<0.,-----
@T6XXI:00074:00876
GGGAGAAGGGAGAAAGGAAGCTAGTCACTCGAGATCTGCGGCTTTGGACAGTCCCTCTGTCTGTGAAGCCACAGATG
TCGAGTCGACTAGCTTCCTTTCTCCCTTCTCCC
+
94-
4999996>999149><@B???@==77:??@@AA??ACBBCD?C>@@BBBBB>BB???===ACC===;=<???<==@@
@@@@@@A@@<6?:;8;<7=CBB<A8;8841
@T6XXI:00074:00880
GGAGAAGGGAGAAAGGAAGAGAACAGCTGCTTTGGAAACAGTCAGACTATTCCTCCCTACTCCCTATAGTTGACGTC
GTATTAAG
+
,*,,/,//+449=-429;89;B5<<B@A@A==:A@BC@CCCCBB9949931313772377@;@8A998/0,0.--
22++..4444
@T6XXI:00074:00883
GGGAGAAGGGAGAAAGGAAGGAGAAGTCGCCCCAAACGATTTGTTGACTATTCTCCCTCTCCCTATAGTGAGTCGTA
TTAGAATTCCCC
+
DD?CCC?BC>BCBA>B=@>A@CCB>>=ABBB?8B>;>B?>>;>>>>>>>>>?><<<<;====<;<====:999:999;
;:AAA>A>@==5
@T6XXI:00074:00897
GGGGAATTCTAATACGACTCACTATAGGGAGAGGGAGAATAGCCGTTCGCCCTTTGACTGCAGATTATTCTTCCTTT
CTCCCTTCTCCC
+
@?9,42489>:<=A@@<<<CBBBDCEBB>BCDDC>BBB=99:89<999000+00+16698@===A?AA>@;-
/,/60/--103477775
@T6XXI:00075:00865
GGGAGAAGGGAGAAAGGAAGCTAGTCGACTCGACATCTGTGGCTTCACAGTACAGAGGGACTGTCCAAAGCCGCAGA
TCTCGAGTCGCTAG
+
CC>CA>644-
99990;?B?BCCCCC8;:AAA>:;;;::@AB?CB@CCC???CA?<<:;;======;;:;==>=??@@AAAA@;@=::
:;;=
@T6XXI:00075:00873
GGGGAATTCTAATACGACTCACTATAGGAGAGGGAGAATAGTTAACAAATCGTTTGGGGCGACTTCTCCTTCCTTTC
TCCCTTCTCCC
+
CCC9B>B?BCC@CBBBBBB??:9990/.1>>BE>CBB??@@<;=><@A<AAAAA<AAA6AAAA@>AAA>@<?9<<7<
AAA<?=?@AC=

```
@T6XXI:00075:00894
GGGGAATTCTAATACGACTCACTATAGGGAGAGGGATGAAT
+
CFD9FFD@CCC?<>CDCCAA?BB>;<?;679///,0.,,*,
@T6XXI:00076:00850
GGGAGAAGGGAGAAAGGAAGCTAGCGACTCGACATCTGTGGCTTCACAGTACAGAGGGACTGTCCAAAGCCGCAGAT
C
+
CC@@@@@B@:=844-
9<?>@CCCDCAC>>>977;8777:?3???BCBBCBA?==166.4;999=8;;7<A>@@?BBBA
@T6XXI:00076:00855
GGGGAATTCTAATACGACTCACTATAGGGAGAGGGAGAATAGCCTTCGAAACTC
+
444,424:DABBCCBBBBABA?;;;<9@,61666,166777743741111,144
@T6XXI:00076:00860
GGGGAATTCTAATACGACTCACTATAGGGAGAGGGAGAATAGTCACATTCATGTTTGTGACCGTGTCTCCTTCCTTT
CTCCCTC
+
499/49@<><<8;@>?A@?>>?>87811,166679<<<?AAA=<:?:C9;:BBCC?BA@:837::3400/0/0/0;4
5375---
@T6XXI:00076:00861
GGGAGAAGGGAGAAAGGAAGCTAGTCGACTCGAGATCTGCGGCTTTGGACAGTCCCTCTGTACTGTGAAGCCACAGA
TGTCGAGTCGACTAGCTTCCTTTCTCCCTTCTCCC
+
CNBB@@=>>:>>>?<A@B<;;88887111666;:>?ABCCC@CCC=@AA@JEEDB>?@==<<<<<<<A?>><><<;;
;<>=??888<<=<@=;8,/,//,/-22+-,-1330
@T6XXI:00076:00880
GGGAGAAGGGAGAAAGGAAGACAGTCCATCTGTGGCTTCACAGTACAGAGGGACTGTCCAAAGCCGCAGATCTCGAG
+
CCAAA@@EB>@@=99=;?<@CCCCAA?DCECBBB@@@??AABCACB><47718=@<==>AA<@@?AAAA<????AAA
@T6XXI:00076:00885
GGGAGAAGGGAGAAAGGAAGCTAGTCACTCGACATCTGTGGCTTCACAGTACAGAGGGACTGTCCAAAGCCGCAGAT
CTCGAGTCGACTAG
+
CC??:9244-
999919596;BA?BA9;:=?=779:96???;?@?ACCCCBCA?936657AAABB>AA=??<?A=><>=<:======A
A???
@T6XXI:00076:00887
GGGAGAAGGGAGAAAGGAAGTCATAGACTAAGGGCGAAAGGTGTTGTCTATTCTCCCTCTCCCTATAGTGAGTCGTA
TTAGAACTTCCCC
+
/1-//4244-449;-
469<=;::;A?><<=477.1133,677=?7:6@DAA>A@C@999661,069;;<884::44382304/03/044+
@T6XXI:00076:00888
GGGGAATTCTAATACGACTCACTATAGGGGAGAGGGAGAATAGGTCAACAAATC
+
944,42424442444444622,-,,,,2204./17-71;0111004=2677/00
@T6XXI:00077:00856
GGGGAATTCTAATACGACTCACTATAGGGAGGAAGGGAG
+
9AA6ABA:<11-//89==CCB@A@:9:56/.,.,..+.,
@T6XXI:00077:00870
GGGAGAAGGGAGAAAGGAAGGTTATGGTCACGCGGGAAAATGGAGGTCTATTCTCCCTCTCCCTATAGTGAGTCGTA
TTAGAATTCCCTC
+
CEBBAA>AC>BACC<@=@=B>B>@@D@FD@@@AAA<BDD:CC@;;8;?>?B9;;?B<::;BC>BCAAA??@A@8884
4249;8;<?;440
```

```
@T6XXI:00077:00883
GGGGAATTCTAATACGACTCACTATAGGGAGAGGGAGAATAGCTCGAAAATCTCCGTCGTCTGCTGCTCCTTCCTTT
CTCCCTTCTCCC
+
DCCECBC@BBC@CB???B?B??BBBA@@;??>AC>BBA@CBC=?>6111,7==?>CAA@?:?A>>?>@D@C@C>AA5
::@B<>;?:::.
@T6XXI:00078:00850
GGGGAATTCTAATACGACTCACTATAGGGAGAGGGAGAATAGCTACCGACGCTCCGAACACTTTATTATCTTCCTTT
CTCCCTTCTCCC
+
AAA6C?B@CCCACBBAAB<<BB>99311.7>>AC>BCC>??@<AAB@BBBCA??=??>?A===;<<;:;<=<=;<<<
:@>@:=788771
@T6XXI:00078:00854
GCGATGGGGAATTCTAATACGACTCGACTATAGGAGAGGAGAATAGTTTTTCCCTTCACTAACCGCGAACTCGCACT
TCCTTTCTCCC
+
44494444,4242444244:;996///////9?9::44249988444 9?9/9A6969@@99;924444244444///
78,//+//685
@T6XXI:00078:00857
GGGGAATTCTAATACGACTCACTATAGGGAGAGGAGAATAG
+
;:;+11<ACCC@BAAABBB??BBDBB?<89?6;:?=<:<,)
@T6XXI:00078:00866
GGGAGAAGGGAGAAAGGAAGGAGAAGGTCGCCCCAAACGATTTGTTGACTATTCTCCCTCTCCTATAGTGAGTCGTA
TTAGAATTCCCC
+
DEBCCB@CB>=944-
95949=@881816>8833,37:;CCCA9?@@@A@@@?74000,4;;==@@@@B==<:;:A99<>A883424480
@T6XXI:00078:00871
GGGAGAAGGGAGAAAGGAAGCTAGTCGACTCGACATCCTGTGGCTTCACAGTACAGA
+
94-47;:<85111;6:;>;B@><997///332,,,..,.44446;7;611637/.--
@T6XXI:00078:00873
CTATCACTCGAGATCTGCGGCTTTGGACAGTCCCTCTGTACTGTGAAGCCACAGATGTCGAGTCGACTAGCTTCCTT
TCTCCCTTCTCCCA
+
D@A@;:::?=ADBCBBB@@?@BB<?;>?DB@@?;AA@@@?@>@BB<8=?@BBBBBBBBBBAC=A@BBACCBC@B@BE
>CCBB<@:??==7=
@T6XXI:00078:00883
CTAGTCACTCGAGATCTGCGGCTTTGGACAGTCCCTCTGTACTGTGAAGCCACAGATGTCGAGTCGACTAGCTTCCT
TTCTCCCTTCTCCC
+
B<1////44??=BBCCCCBB?ABB?C@?@BBAAA=ABBC?=BC?BBC@@C?BDCCCBAACDCA?A@;;8>78136:;
?;?A?A<>379;;4
@T6XXI:00078:00884
GGGAGAAGGGAGAAAGGAAGGGGAGAAGGGAGAAAGGAAGCTAGTCACTCGACAGTCTGATGGCTTCCACAGTACC
+
CC@AAA@DC>@A?B=@=?;=??6>?A<??6>871,747477636>>6997661000000000423,-,.2235747
@T6XXI:00078:00898
GGGGAATTCTAATACGACTCACTATAGGAGAGGGAGAATAGTCAACAAATCGTTTGGGGCGACTTCTCCTTCCTTTC
TCCCTTCTCCC
+
CC@4@5959998499==@ACBBABA:737=777-
7;;3<88;>@>@AA<@??<<6:::1000==>@>@=A;?:;?:?<@@:@9<<@@;
@T6XXI:00078:00899
GGGGAATTCTAATACGACTCACTATAGGGAGAGGGAGAATAGCTCCCAACTAC
+
```

```
444,49@@<<=ACCCCDCC@@@EGCAAC::;?CC=AAC>?773111,.5.../
@T6XXI:00079:00876
GGGAGAAGGGAGAAAGGAAGGAGAAGTCGCCCCAAACGATTTGTTGACTATTCTCCCTCTCCCTATAGTGAGTCGTA
TTAGAATTCCCC
+
FC@BBB@BB>BBBB?B@B>A>?AB>@?>887748?:B@AAB=AA@CCCBCE>?<<<5;==?@;@AA???AA=<=???
?>@CB?A=@@@6
@T6XXI:00079:00877
GGGGAATTCTAATACGACTCACTATAGGGAGAAGGAGG
+
:>A1?,1,///,/444447779777367-1110101.,
@T6XXI:00079:00883
GGGGAATTCTAATACGACTCACTATAGGGAGAGGGAGAATAGTCAACAAATCGTTTGGGGCGACTTCTCCTTCCTTT
CTCCCTTCTCCC
+
A?A8A=A@ACCABAA@@?BBBB<99311.7>>BC?DCC@@??<==;4881;B===-766-
8>@BC?BAC@C@CAAA<A@@@<@>@@AA<
@T6XXI:00079:00888
GGGGAATTCTAATACGACTCACTATAGGGAGAGGGAGAATAGCGCTACAATTTGTAATCCGTTTATTCTCTTCCTTT
CTCCCTTCTCCC
+
CDD:C@B@BCCACBBB??>??DB?9311.777?B>CBC?B??===<CB@@CA?@<<;<=>>@B>BC?AA??<<<<;;
?:@@;@=@<<<7
@T6XXI:00080:00874
GGGGAATTCTAATACGACTCACTATAGGGAGAGGGAGAATAGTCAACAAATCGTTTGGGGCGACTTCTCCTTCCTTT
CTCCCTTCTCCC
+
BBB7A>B>BBC@CDBBBCBABCDBC;:886:9@B?CCE@BBB@@A>AAC<BBBBA<ACC7B??@FABAA?A<A;=?:
@<<<7=889CC<
@T6XXI:00080:00876
GGGGAATCTAATACGACTCACTATAGGGAGAG
+
///*/,//7756844443333733311,1111
@T6XXI:00080:00877
GGGAGAAGGGAGAAAGGAAGGAGAACGCGCACCCGAACCAACGGTGATCTATTCTCCCTCTCCCTATAGTGAGTCGT
ATTAGAATTCCCC
+
CDEBBB?CH?DBCD>C@C?@ABBB>@BBBBBAA>BCAB>A>AD@FCBBA@?@@CDBB<@@@CC>BB?AAABBAAACC
BB>ABB@C@CCC:
@T6XXI:00080:00881
CTAGTCACTCGAGATCTGCGGCTTTGGACAGTCCCTCTGTACTGTGAAGCCACAGATGTCGAGTCGACTAGCTTCCT
TTCTCCCTTCTCCC
+
//////44??AABCCCC<<;;CC?C?BBCCCBC>BCBD@??BBABB@8<8;ABAAABCECCC@AADBBACDC@D?B
B8;:BB>B8::@@:
@T6XXI:00080:00883
GGGGAATTCTAATACGACTCACTATAGGGAGAGGGAGA
+
///*/,4:;>>;498>>=??AA@?776;66666621..
@T6XXI:00080:00887
GGGGAATTCTAATACGACTCACTATAGGGAGAGGGAGAATAGTCTTGCAATTTCCTGCGGTGCATTCTCCTTCCTTT
CTCCCTTCTCCC
+
BDE9B@B@CCD@CBBBCBBBBB?><777.7>==?=?CC?CBB@@<;<<A@DD?B?BBBC@BBBBB>?@C?B?B@CC@
CBBB>B?BBBB>
@T6XXI:00081:00858
```

GGGAGAAGGGAGAAAGGAAGGAGAAGTCGCCCCAAACGATTTGTTGACTATTCTCCCTCTCCCTATAGTGAGTCGTA
TTAGAATTCCCC
+
CC@CCB@CC>BBCC>B>?=@@CA?;;>ABBBB7BB>AAAAA;A@>???@@@@=8899=A@AB=A99>::;99=<<::
?>A@@>?38770
@T6XXI:00081:00864
CTATCACTCGAGATCTGCGGGCTTTGGACAGTCCCTCTGTACTGTGAAGCCACAGATGTCGAGTCGACTAGCTTCCT
TTCTCCCTTCCTCCC
+
4::994:?99==>>?@@99908>>8>6;:@BBBB>??::7?<>:::?:?CAABCBBA;;;;::7;<B??7931013;
;9?;::,/,/,.440
@T6XXI:00081:00872
GGGAGAAGGGAGAAAGGAAGGAGAAGTCGCCCCAAACGATTTGTTGACTATTCTCCCTCTCCCTATAGTGAGTCGTA
TTAGAATTCCCC
+
BD?BEC@CC@B???<@?A?B@BBB@?>@>BDC8AA>ACBCA;??>?@@@?>=;===;::??C;===99:;;=<<;<@
@>AA@>A>@AA5
@T6XXI:00081:00873
GGGAATTCTAATACGACTCACTTATAGGGAGACGGGAGAATAGTCAACAAAATCGTTTGGGGCGACTTCTCCTTCCT
TTCTCCCTTCCTCCCT
+
991424:49<=@@@BA@88888133311,111139:;79066866676777,49779277;-
94<<D7:9?263::??8AAC?=3,/,0.-+-
@T6XXI:00081:00874
GCTAGTCACTCGAGATCTGCGGGCTTTGGACAGTCCCTCTGTACTGTGAAGCCACAGATGTCGAGTCGACCTAGCTT
CCTTTCTCCCTTCTCCC
+
/////444449994449><111+.0.+.,..6....+17B@AAB?:8:::5>>BBBBCCCCC;;:71111,0;../7
?@@A>=66724,/.//,
@T6XXI:00081:00880
GGGAGAAGGGAGAAAGGAAGTACGGGAGCTGGAGTTATGTTTGCGTCTATTCTCCCTCTCCCTATAGTGAGTCGTAT
TAGAATTCCCC
+
IIBCCC@CB?BCBB>B@B@BC@AAB>BCCCDANBB?AAABC@CCCCDECD@CCBB>CBBBB>B@@@BBB<<<ACCAA
?A@?<??BDD9
@T6XXI:00081:00890
GGGGAATTCTAATACGACTCACTATAGGGAGAGGGAGAATAGTCAATCTGTCAGCCCCTTTAGACTCTTCTTCCTTT
CTCCCTTCTCC
+
999299@6<<C69;AAADFCCC<<;@?B9??A@>:77;5:>BA8969<<;8:?;;;;-
99048>>=3713311011,66.53?,0..,
@T6XXI:00081:00895
GGGGAATTCGTAATAACG
+
A:;1<-1,/,,,*,/,//
@T6XXI:00081:00896
GGGGAATTCTAATACGACTCACTATAGGAGAGGGAGAATAGTCAACAAATCGTTTGGGGCGACTTCTCCTTCCTTTC
TCCCTTCCTCCC
+
DDD9BAB?AAA59111117:=<0001/,...11,167:?:>:>=56684<7667,111,3111101163686566l3
.3333,-,.640
@T6XXI:00082:00860
GGGGAATTCTAATACGACTCACTATAGGGAGAGGGAGAATAGTCAACAAATCGTTT
+
494,4248494?CCBBBA?:;877977307./..-/5577<7338/004077111,
@T6XXI:00082:00866

```
GGGGAATTCTAATACGACTCACTATAGGGAGAGGGAGAATAGAGACGTATTCGTTTGCGCTCTGTTCACCTTCCTTT
CTCCCTTCTCCC
+
:;;4=CB@CBB@BBBBBBBBBBB99311.7>>BC=AAC@CAA?A<@??<?<?CBC>>>@A??<==;=7437:=:>@<
@AAC>C=??;<;
@T6XXI:00082:00871
GGGGAATTCTAATACGACTCACTATAGGGAGAGGGAGAATAGTCAACAAATCGTTTGGGGGCGACTTCTCCTTCCTT
TCTCCCTTCTCCC
+
AAA6=>B@BBB@CBBBBB@@>>>>8877-7667>9>BBCBBC?==:784-
8>===.7777+77=7799660164/04-4444-0248==7
@T6XXI:00082:00874
GGGAGAAGGGAGAAAGGAAGGAGATGTTTTGATCAAGGGAATACAAAGCTATTCTCCCTCTCCCTATAGTGAGTCGT
ATTAGAACTTCCCC
+
AE?BBB69919>>>:>==<=@BAAA????6??CCE@DD>B7;;>>?69849=6::@>:44444-
44333..44...//98==C>@@<9962
@T6XXI:00082:00888
GGGAGAAGGGAGAAAGGAAGCTAGTCACTACATCTGTGGCTTCACAGTACAGAGGGACTGTCCAAAGCCGCAGATCT
CGAGTCGACTAGCTTCCTTTCTCCCTTCTCCC
+
EE?@@@=@@:@@@@<A?B?BEC@;;00...33.1178838986::ABBCA?;888148;@@A===9=B?A@@<<;87
77734:44548;<@;<;;?;??AA<A6987@:
@T6XXI:00082:00889
GGGGAATTCTAATACGACTCACTATAGGGAGAGGGAGAATAGTTTTTCCCTTCACTAACCGCGAACTGCACTTCCTT
TCTCCCTTCTCCC
+
9994<>CACCB>BBBBBBAABB99<777.77711,17>>@>7=4441AA:A;?>==B@C@7::B6:;B8:7>:=477
.7>>A>B>A?<;6
@T6XXI:00082:00892
GGGGAATTCTAATACGACTCACTATAGGAGAGGGAGAATAGCTAACGTTCCCCGACGTCCGTTTCG
+
DFI:DCB?BBC@BCBDBCBA900;:00,0==BE>AAA?A997:736..,.55*..//577:??;7.
@T6XXI:00082:00894
GGGGAATTCTAATACGACTCACTATAGGAGAGGGAGAATAGTCAACAAATCGTTTGGGGCGACTTCTCCTTCCTTTC
TCCCTTCTCCC
+
ABB8C>B@BBB>?BBBBBB>>:9990/,0::?B>BCE@C>?<<=:<AA<B976786685@BBCD@CCC8:8:?@A=A
@@@;?=@@BA:
@T6XXI:00083:00850
GGGAGAAGGGAGAAAGGAAGGAAAGATAGCGACCGGACAAGTACGAACTATTCTCCCTCTCCCTATAGTGAGTCGTA
TTAGAACTTCCCC
+
BA>AA@?BC<@ACB?CAC@B@CC@BAB@@@@@A<?;?BC@CCCCCC@CCB@>@888/8?>@A=BLECCCC@@@BB::
:70..-//:>776
@T6XXI:00083:00861
GGGAGAAGGGAGAAAGGAAGGGGAGAAGGGAGAAAGGAAGGGGAGAAGGGCTAGCGACATCTGTGGCTTCACAGTAC
AGAGGGACTGTCCAAAGCCGCAGATCTCGAGTCGACTAG
+
BDB@@?@CB=?@@B=B>B@CCC:A@@?BA=;;:B<A@C?BBB7BBB===;?@8>?@<;;@@@BCC>AABDCBBBBCD
CDBBB>@@CBBB?BB?BC?BAAABABBCBBABBCBAAAC
@T6XXI:00083:00864
GGGAGAAGGGAGAAAGGAAGCTAGTCGACTCGACATCTGTGGCTTCACAGTACAGAGGGACTGTCCAAAGCCGCAGA
TCTCGAGTCGACTAG
+
EDB???@BC?BBCC>C?B?BB;=>>/00><00081167BCC?BB@CC@@?@?;74003,4;;=?@>@@<@B>@@<==
AA@@===AAA@@AAA
```

```
@T6XXI:00083:00875
GGGGAATTCTAATACGACTCACTATAGGGAGAGGGAGAATAGCCACCAACTATTGTTCGTCGCCGTTCTCTTCCTTT
CTCCCTTCTCCC
+
BBB9C@C?CCC@CCBBBBBCB?;;@767.77:BC@GCC@F??<8=@8;8;<?==@A?AAC=:<;?=>@@A@>@?AA<
AABB<A88:@A<
@T6XXI:00083:00876
CTAGTCGACTCGAGATCTGCGGCTTTGGACAGTCCCTCTGTACTGTGAAGCCACAGATGGACCTTCCTTTCTCCCTT
CTCCC
+
DCAA@BCCBDCCBBBBABBCC@BBB<B>ABCC@?=;?BBBBBCC7:::78BBBBBDDBG@BEAC@B=@@<@A?B/84
77776
@T6XXI:00083:00892
GGGAGAAGGGAGAAAGGAAGGAGAAGTCGCCCCAAACGATTTGTTGACTATTCTCCCTCTCCCTATAGTGAGTCGTA
TTAGAATTCCCC
+
AA<999<>>19999149><??;;;>8798888.78,3976>:??;????CC578;94443::7887??@@A@?=<=?
=;65323/344-
@T6XXI:00084:00858
GGGAGAAGGGAGAAAGGAA
+
AA:?;<-11+////+/,/,
@T6XXI:00084:00887
GGGGAATTCTAATACGACTCACTATAGGAGAGGGAGAATAGTCAACAAATCGTTTGGGGCGACTCTCCTTCCTTCTC
CCT
+
999/99@699=8888884337.0.00.,...17166<<9;96662004077667,111,366661770104/42000
0+0
@T6XXI:00084:00895
GGGGAATTCTAATACGACTCACTATAGGGAGAGGGAGAATAGTCACTGGATTCTCCCGCAGTACGTTTATCTTCCTT
TCTCCCTTCTCCC
+
9==6@>A>BCC@CBABBAB?BDCB;761-
6=77:7:CC@BBB>=8300/00988;>7>??@BA???A<@?7748=@AAAAAA:?8;68:6
@T6XXI:00085:00854
GGGAGAAGGGAGAAAGGAAGAATAGTGGGATGCAGAAAGGTTCTGATCTATTCTCCCTCTCCCTATAGTGAGTCGTC
+
DD=<=B@AA9>499149;9@B>>>>A?66611679;>;A>A>?7760.7/-,.133444:6636:6644-.---1--
@T6XXI:00085:00859
CTAGTCACTCGAGATCTGCGGCTTTGGACAGTCCCTCTGTACTCGTGAAGCCACAGATGTCGAGTCGACTAGCT
+
EC<<9@44444444444449999;<<:<:;;;;;998??=::::/...--,0989;:947;;3331144/844.,,
@T6XXI:00085:00875
GGGGAATTCTAATACGACTCACTATAGGAGAGGGAGAATAGTTCCTCCCACCTTACCGTAAATTTTCCCTTCCTTTC
TCCCTTCTCCC
+
ICC:@6;:<99@CCC=<<CCCAAA>:937=??A<B::8;DB@=@=@@>7ACACACCBDA@@6===.77.:@C@CC>A
A@@7=8:3384
@T6XXI:00085:00878
GGGGAATTCTAATACGACTCACTATAGGGAGAGGGAGAATAGTCAACAAATCGTTTGGGGCGACTTCTCCTTCCTTT
CTCCCTTCTCCC
+
BBB7D>A@BBCBCCCCCBBBCCCA>>?E><==BB>BBC@CCB?;;8::;7@@??DBCCC9EECBB?ACB?A>B?ACA
@@?@:>377;;7
@T6XXI:00085:00886
GGGGAATTCTAATACGACTCACTATAGGGAGAGGGAGAATAGTCAACAAATCGTTTGGGGCGACTTCTCCTTCCTTT
CTCCCTTCTCCC
```

```
+
CCB9B=?=@BB?BBBBB@@@:88@8877,76161,166;@;:8;;7;;=:BBAAC>BCB8BAAAC?BBD@B>?>@A;
@???:?68:AHA
@T6XXI:00085:00889
GGGAGAAGGGAGAAAGGAAGGAGCACTAGACGGCATTTCTCGCCAATCTATTCTCCCTCTCCCTATAGTGAGTCGTA
TTAGAATTCCCC
+
CFECBB@CA:;444-
1:<:<:101@=BBA@AC@CCCDBCCB;97;@AABA?;==<=9?ACCCAA@???@@BBB?????<?=<9<;?=@5
@T6XXI:00085:00899
GGGAGAAGGGAGAAAGGAAGGAGAACGCGCACCCGAACCAACGGTGATCTATTCTCCCTCTCCCTATAGTGAGTCGT
ATTAGAATTCCCC
+
FFBCBB@CC>CBBB>B>@=A@BBB@BBBAAAAA>AA@B?BEAA@EDDAA@?>?@@@@<@BBCC?CCCBBBBCBBBCB
BB>BCC@C@BCC:
@T6XXI:00086:00853
GGGGAATTCTAATACGACTCACTATAGGGAGAGAGATATAAGGGCCCAATTTCATCCGTCTCCCGCCTTCACCCTGC
CCTTTC
+
DCB8;296944244:499BABBB??877.7>777:68744/00+0307779531110111111,110101167,100
0+0424
@T6XXI:00086:00854
CTATCACTCGAGATCTGCGGCTTTGGACAGTCCCTCTGTACTGTGAAGCCACAGATGTCGAGTCGACTAGCTTCCTT
TCTCCCTTCTCCC
+
EAA=;:::BB@BACCCCA@=@CE>C>?BCC:::7:CCCCCBBABA?<=A?ACCCCBBBCABBAA@@AAAABB@CCED
=AABC>B=????:
@T6XXI:00086:00859
GGGAGAAGGGAGAAAGGAAGGAGAAGTCGCCCCAAACGATTTGTTGACTATTCTCCCTCTCCCTATAGTGAGTCGTA
TTAGAATTCCCC
+
CBABCCBCH@DBDB=?897<@CCC@CCBCCCC7BB?BCABB=@?@CFBAAC?<<==;<<?@=;>A@@;@?===;>8?
=<A==9<;===4
@T6XXI:00086:00861
GGGGAATTCTAATACGACTCACTATAGGGAGAGGAGAATAGAGTCACCGTTTGGTTCGGTCGCGCGTTCTCCTTCCT
TCTCCCTTCACC
+
==A6A=@@ABB@BAAACCCAAAAAAACC>BB??:?774000/---
767311,1010331...5...7756:8222,.-.244474444/
@T6XXI:00086:00866
CTATCACTCGAGATCTGCGGCTTTGGACAGTCCCTCTGTACTGTGAAGCCACAGATGTCGAGTCGACTAGCTTCCTT
TCTCCCTTCTCCC
+
CAAA<::ACDCBBCBBBBA>A@@<@>>@@@99:4:?ABCBBB<??A=?C@BBBBBBAA???C?BB@@@@BB>>A?AC
>CCCD>B=??@CA
@T6XXI:00086:00871
GGGGAATTCTAATACGACTCACTATAGGGAGAGGGAGAATAGCCACAATCGTGTTTGCGTTGGCATCTCCTTCCTTT
CTCCCTTCTCCC
+
BAB7A8;;<DC@B@BBBB?@?B<99311.7>>>@=BBB@BBCA>C=99=@<?@A@;@BAB?BCCDCCCB@C@D8;;8
;CBC>C=?:::5
@T6XXI:00086:00876
GGGAGAAGGGAGAAAGGAAGGAGAACAACGCGATCGGTTAAACGTAGCTATTCTCCCTCTCCCTATAGTGAGTCGTA
TTAGAATTCCCC
+
CD@CBB@BB>@@AC>B@B@B@BCB@BC@BBBBBBAC>B>BB=BDCCCCBAB@CCDD?CCAAA>ABCBBACCCCCCBB
B@CBB?BACCC:
```

```
@T6XXI:00087:00877
GGGGAATTCTAATACGACTCACTATAGGAGAGGGAGAATAGTCAACAAATCGTTTGGGGCGACTTCTCCTTCCTTTC
TCCCTTCTCCC
+
499/9595<;9@CAAABBCDC><=>0/,0>:=?=?CC@C?@==<<???<BBBBC>CDC6BBBBB@D;<;;@C?@@<@
A@@<A895AB>
@T6XXI:00087:00881
GGGGAATTCTAATACGACTCACTATAGGGAGAGGGAGAATAGTTACCAATGTTTGCTCCCTGTAACGGCCTTCCTTT
CTCCCTTCTCCC
+
CCD:C?@@AAA@CCC@?ABBBB<99311.7>?BC>CDB>>;;<;==>=;<;;?<?@@AA<AA@@>@A?B>@?@?AA<
A@@@;@>@@@@;
@T6XXI:00087:00884
GGGGAATTCTAATACGACTCACTATAGGGAGAGGGAGAATAGCTGCGTGTCCGATCGCGTCTGTATCTCCTTCCTTC
TCCCTTCTCCC
+
AAA6=>BCCCC@CCC@@@BBBBB>;7995:BBBC>A??;?A?<?CCCA?@@?B@@@@@AA===@?===?7676,/,/
6660778:=?6
@T6XXI:00087:00890
GGGAGAAGGGAGAAAGGAAGGTTACGTCGGCTGGGGGAAGGATAGATCTATTCTCCCTCTCCCTATAGTGAGTCGTA
TTAGAATTCCCC
+
CC?CHB@BC@DCCC=B?B@CAB?AEGC@=;=98999,9=BBECCCEDCBBB?BBBB>BBBBB<BBABBBCCBB??;:
:@CAA;70044-
@T6XXI:00088:00865
GGGGAATCTAATACGACTCACTATAGGAGAGGGAGAATAGTCAACAAAATCGTTTGGGGCGCACTCTCCTTCCTTTC
TCCCTCTCCC
+
888*/,1/66,/44844360--8,-
,*,0066261131160335555;4033166,111,,,,,,,,22226::7755543,/----+
@T6XXI:00088:00868
GGGAGAAGGGAGAAAGGAAGAACATTGCCGGGGAGTCGAATTGTCGGCTATTCTCCCTCTCCCTATAGTGAGTCGTA
TTAGAATTCCCC
+
CIBBBC@BC>BBBE=B?B@CC@CBB@>?=@CB9BBAA@A>>;=;00.1>BCDECCKDDBA@@<@C@@@CCABB:::?
?<?AG@C@AAA5
@T6XXI:00088:00879
GGGAGAAGGGAGAAAGGAAGGACAACGAACAGCGGGGAATTAAAGAGCTATTCTCCCTCTCCCTATAGTGAGTCGTA
TTAGAATTCCCC
+
DB=AAA@BB>>>>B=A??@BAC??=?BB@CC?9991.7:<:??9?CCCDFD@CBBB>BBBBB=B@@@AAA@@@????
?@AAA>=;=@@5
@T6XXI:00088:00881
GGGGAATTCTAATACGACTCACTATAGGGAGAGGGAGAATAGCGCAATTTGGACATATGGCCGCTTCCTTTCTCCCT
TCTCCC
+
CCC9C@C@CBB@B@?AAA??BBB><76659AAEE>B@@;???==AA?AB>B@BECA<<<?@@@<<9<=??:@AAA<A
=?@AA:
@T6XXI:00088:00892
GGGAGAAGGGAGAAAGGAAGCTAGTCGACTCGACATCTGTGGCTTCACAGTACAGAGGGACTGTCCAAAGCCGCAGA
TCTCGAGTCGACTAG
+
DEFBBB===:@@==9@896;C>9;>/00<:;:0;::77DCDAAA@CB???@AC@:333+08;>AA>A@<@A>A@@@=
<<==99=AA@@?@@@
@T6XXI:00089:00860
GGGAGAAGGGAGAAAGGAAGGAGAAGTCGCCCCAAAACGATTTGTTGACTATTCTCCCCT
+
```

```
;;-4:>>=====>@195444247713183333,333,399772110116611244;;;03
@T6XXI:00089:00864
GGGAGAAGGGAGAAAGGAAGGGGAGAAGGGCTAGTCACTCGACATCTGTGGCTTACACAGTACAGAGGGACTGATCC
AAACGCCGCAAGACTC
+
99044959A098>>:>;82444,433133,4;4444449999:949499;273533773113333335O355.//77
4566597711011001
@T6XXI:00089:00876
GGGGAATTCTAATACGACTCACTATAGGGAGAGGGAGAATAGACACCAATCGTGCCGGGTCATGTTCTCCTTCCTTT
CTCCCTTCTCCC
+
EEICEAD@ABBAB@@@CCCCBCB99311.7==>@>BDF@C@B=??@;=;?CB@@A8::4:BBCFC@C@A>A>@=?=:
=<;;7<=?@AA<
@T6XXI:00089:00880
GGGAGAAGGGAGAAAGGAAGCTAGTCGACTCGACATCTGTGGCTTCACAGTACAGAGGGACTGTCCAAAGCCGCAGA
TCTCGAAG
+
CA:=996991<C@@>@12,/.;;;>@@AECCC@@@@?CA@@;7848???BBC@?<:::4444400/04233200035
3:46//,-
@T6XXI:00089:00896
GGGGAATTCTAATACGACTCACTATAGGGAGAGGGAGAATAGCCGATCGCTCCCTATCCTCACATGTTTCTTCCTTT
CTCCCTTCTCCC
+
AAA6@ACDCCC@CBBB?ABBBB<99361.7>>CJ>CBC@BA=999;?@A@AAA<A@@<:;<==AEABAAA@=@>AA<
??CC>A>@A?B<
@T6XXI:00090:00862
GGGAGAAGGGAGAAAGGAAGCTAGTCACTCGACATCTGTGGCTTCACAGTACAGAGGACTGTCCAAAGCCGCAGATC
TCGAGTCGACTAG
+
AA>ABA>BB=@>@@08595988>@AB:78ABCCA@?BAAA?AC@CDCCCCC===//-
0;>=>A@HH698:@@@@A@=88///:;8;;@AA
@T6XXI:00090:00863
GGGGAATTCTAATACGACTCACTATAGGGAGAGGAGATATAGAACG
+
FDF6;;A@BCC@@?@@BBB;;<CCCBBB>@?;=;>00.:2-00/0-
@T6XXI:00090:00864
GGGAGAAGGGAGAAAGGAAGCTAGTCACTCGACATCTGTGGCTTCACAGTACAGAGGGACTGTCCAAAGCCGCAGAT
CTCGAGTCGACTA
+
CD>;<;@AA:A98809>A<BCCBBB@B@AA@:::???BCB?BD@CCCCCCCC>B???:??@AAA?A@<AA?@;;=<A
@??<<;>>==><<
@T6XXI:00090:00867
GGGAGAAGGGAGAAAGGAAGGGGAGAAGGGAGAAAGGAAGCTA
+
94-944294-444C082:=>@D9:;;<>?67100,.,.,.,,,
@T6XXI:00090:00870
GGGGAATTCTAATACGACTCACTATAGGAGAGGGAGAATAGTCAACAAATCGTTTGGGGCGACTTCTCCTTCCTTTC
TCCCTTCTCCC
+
BBD:D>A>@CC?BCBCBBBBB:8880/,0::>@>BDC@CBBA@<;<>>;B>==A>@B@9CBBBB??@@5:7:>@@?@
?@B<A=??AD@
@T6XXI:00090:00877
GGGAGAAGGGAGAAAGGAAGGAGAAGTCGCCCCAAACGATTTGTTGACTATTCTCCCTCTCCCTATAGTGAGTCGTA
TTAGAATTCCCC
+
EEBCCB@CB>BBDB>B>@?@ACBB>B?BBBBB8BBABBBAA;?<?CCCCBB??AAA<;8===;A=??@@@<<<<A<@
A>A@?;=8;;;0
```

```
@T6XXI:00090:00879
GGGAGAAGGGAGAAAGGAAGTATACGTCGTGGGAGAAAAAACGCTCGACTATTCTCCCTCCTCCCTATAGTGTAGTC
GTATT
+
4:6:;<@FG>CBCD?B?B@B9;;ABAAABA@>:@999999*999?@@BBBB????@?811,/.535753:,,++++0
0213/
@T6XXI:00090:00884
GGGAGAAGGGAGAAAGGAAGAG
+
44-4:;9::6:911,1=8+-,,
@T6XXI:00090:00886
GGGAGAAGGGAGAAAGGAAGCTAGTCACTCGACATCTGTGGCTTCACAGTACAGAGGGACTGTCCAAAGCCGCAGAC
TCTACGAGTCCGACTAG
+
491444244-4449-42498>>@?>=77111666769<<886D;8166666===433+088@?@>;?6//-
///666444331.***)****04
@T6XXI:00090:00888
GGGAGAAGGGAGAAAGGAAGAGCAGCTGGCCGTTAGCGATATTTTAGCTATTCTCCCTCTCCCTATAGTGAGTCGTA
TTAGAATTCCCC
+
DDEBB>@CC>?AAC?E@B@CCCCBBBCC=?=AB?BCGCCC<<;;5?ABBACBC??A<A?@@@<A?==@A@A?:?A>>
>?ACA<;/478-
@T6XXI:00090:00891
GGGGAATTCTAATACGACTCACTATAGGGAGAGGGAGAATAGCCGTTTAATCTCCCACTGCGGCATATCCTTCCTTT
CTCCCTTCTCCC
+
AA?6C@@=@CB=>@@@BBACCCB?B;76.7777CBDCB@C??98??C:@>AB<<53;;3435;?=>>==8;8<;=?;
@ADC?A>A///-
@T6XXI:00090:00892
GGGGAATTCTAATACGACTCACTATAGGGAGAGGGAGAATAGTCAACAAATCGTTTGGGGCGACTTCTCCTTCCTTT
CTCCCTTCTCCC
+
EBB9C=@>ACCABB?@@@>??CB?9311.7?>@B>BCC>@??<AA>@@?:><966,111-
:?ABC?BBB>@=?@A=;=AAA=B>@???;
@T6XXI:00090:00899
GGGAGAAGGGAGAAAGGAAGCTAGTCACTCGACATCTGTGGCTTCACAGTACAGAGGGACTGTCCAAAGCCGCAGAT
CTCGAGTCGACTAGCTTCCTTTCTCCCTTCTCCC
+
FD=B<<:9@:>111-
/,/,/44776::0......43879988?>???ABBBBAB;??99444::7::4::8;===899AA@@A??@>>==;:
:8;8:;7;;;;7=3//681
@T6XXI:00091:00851
GGGGAATTCTAATACGACTCACTATAGGGAGAGGGAGAATAGCTTGCAGCTCGAGCCTGTGACTGTTCTCTTCCTTT
CTCCCTTCTCCC
+
CCC:B=@?@BB@BB:9<<CCCCB?9311.7>>?A>BCC>B>?<?=AB??C?B?B???B<??CAC??><==?>A;<<:
:=9=5<488:=7
@T6XXI:00091:00855
GGGAGAAGGGAGAAAGGAAGGAGAACGCGCACCCGAACCAACGGTGATCTATTCTCCCTCTCCCTATAGTGAGTCGT
ATTAGAATTCCCC
+
DEBCD@=>@=;<<@:>;>;>;?@B@DCCCBCCDBBC?CACBCDBDDCCB<<=BCA@B>@CCGD?CEDAAA@@DCCDB
BB@CCD?CBCDC:
@T6XXI:00091:00867
GGGGAATTCTAATACGACTCACTATAGGGAGAGGGAGAATAGTCAACAAATCGTTTGGGGCGACTTCTCCTTCCTTT
CTCCCTTCTCCC
+
```

```
EED9C>A?BBBBCA@>AA???CB99311.7>>AB@BBC@C?C=@<;@@=;AC@@@;????7ACCA@?@@@?@?@@==:
:AAC;A=?@AA;
@T6XXI:00091:00871
GGGAGAAGGGAGAAAGGAAGCTAGTCACTCGACATCTGTGGCTTCACAGTACAGAGGGACTGTCCAAAGCCGCAGAT
CTCGAGTCGACTAGCTTCCTTTCTCCCTTCCTCCC
+
BA6@9<:9A?@999142824??8700,,,,,,,,,/.57@788@CDDCCBBCBC777-
4::;==<?A<?C@?==<<<==>89:AAAAACA@@@>@>@B=88:959-../893
@T6XXI:00091:00873
GGGAGAAGGGAGAAAGGAAGGAGAAGTCGCCCCAAACGATTTGTTGACTATTCTCCCTCTCCCTATAGTGAGCCGTA
TTAGAATTCCCCC
+
DB@CCF>>=5=844-
;=@=@IEBB?A;87788.87/399>C>BC?CCCBB@@=<<;4:====;<6==AA>>7837<=;>A5523/-///+
@T6XXI:00091:00889
GGGAGAAGGGAGAAAGGAAGGAGAAATCACGTGCAACAAAAGCTGAGCTATTCTCCCTCTCCCTATAGTGAGTCGTA
TTAGAATTCCCC
+
CDBCBC@CC>ABBCBC@C@B==7776;?B?CDCBB?BBBB8BCBABBA:::8::BB::;734+3;8=?@@???;787
<8;6A<><<==.
@T6XXI:00091:00894
GGGGAATTCTAATACGACTCACTATAGGAGAGGGAGAATAGTCAACAAATCGTTTGGGGCGACTTCTCCTTCCTTTC
TCCCTTCTCCC
+
GEA9B@C@CBBACAAABBBB<:9990/,0<:=?=@CC@CB?<@<;=AA<BA@>?88886@BBBC@CCC?B@A>?;:<
??=;@>@@AA:
@T6XXI:00092:00853
ACAGTCCCTCTGTACTGTGAAGCCACAGATGTCGAGTCGACTAGCCTTCCTTTCTCCCTTCTCCC
+
DCDCBBBBCCCCCCCDEAA@<@?:AAAC@@@ABBBCBBA??>111,/,/,/6+,//858111885
@T6XXI:00092:00859
GGGAGAAGGGAGAAAGGAAAGCTAGTCACTCGACATCTGTGGCTTCACAGTACAGAGGGACTGTCCAAAGCCGCAGA
TCTCGAGTCGACTAG
+
IHAB>>5991844804244-47><3379/./:0/....446063;;9?AA;;7>?477-
44449=<=;:==<=:878<888?B@A@@@BAA@
@T6XXI:00092:00874
GGGGAATTCTAATACGACGTCACTATAGGAGAGGGAGAATAGTCAACAAATCGTTTGGGCGACTCTCC
+
88808,1,///,/4;44443:3.///..,...776111011111100044?799<6996........,
@T6XXI:00092:00882
GGGAGAAGGGAGAAAGGAAGCTAGTCACTCGACATCTGTGGCTTCACAGTACAGAGGGACTGTCCAAAGCCGCAGAT
CTCGAGTCGACTAG
+
CCBCCB==95=98809595;ABBBC7;:?==776661?AC8;<ACBCB@?AB?>33317=9=@@>AA<AA>A?:?=<
<<=:<<=<88<AAA
@T6XXI:00092:00883
CTCGAGATCTGCGGCTTTGGACAGTCCCTCTGTACTGTGAAGCCACAGATGTCGAGTCGACTAGCTTCCTTTCTCCC
TTCTCCC
+
DCCDAAACCGAA@=@BB<@=@A@@@AB?GBAAB<<=BCCEBCCCCCF@;;;?;;;B??@;94478959<>>9>>885
=28333,
@T6XXI:00092:00897
GGGAGAAGGGAGAAAGGAAGGAGAACGCGCACCCGAACCAACGGTGATCTATTCTCCCTCTCCCTATAGTGAGATCG
TATTACGAATTCCCC
+
```

```
CCBCAA644-
999909;>;>;?>?>BCCBC@@@<@@=@BBAAA>B=BBD<<<ACCAA988877,3?776554....02030..--
01,000+
@T6XXI:00093:00874
GGGGAATTCTAATACGACTCACTATAGGGAGAGGGAGAATAGTCAACAAATCGTTTGGGGCGACTTCTCCTTCCTTT
CTCCCTTCTCCC
+
CCE:C@C@BBB@@@?AAA???BB99311.7==>B@CDC?C@@<AA>@@?:;<6662;;;5?BCBB>?@C>A==>AA=
A@@@;@:==CC;
@T6XXI:00093:00882
GGGAGAAGGGAGAAAGGAAGCTAGTCGACTCGACATCTGTGGCTTCACAGTACAGAGGGACTGTCCAAAGCCGCAGA
TCTCGAGTCGACTAG
+
EDBCBB==95=///+1-1-
1000.;0..977:0:6886>=?5:;?BEBBACCB?6300,388;AA>A@<@@>@@AC<<8==AAAA@@AAA@?
@T6XXI:00093:00885
GGGGAATTCTAATACGACTCACTATAGGGAGAGGGAGAATAGACGTTTCGTTTACGGCATCCTATTCTTCTTCCTTT
CTCCCTTCTCCC
+
=AA6CCB?CBC@C@AAAA??BB>97311.7??BL>CA@>>@@<><<:4?<;<7=@@>@A??<?@@9<=>@@>??AA:
BACC:?<@@@B<
@T6XXI:00093:00889
GGGGAATTCTAATACGACTCACTATAGGGAGAGGGAGAATAGTCAACAAATCGTTTGGGGCGACTTCTCCTTCCTTT
CTCCCTTCTCCC
+
BBB7ACB?BDC@BBBB@A?BBC?97311.7>>?A>BCC@B>;<@=<9=84;@???8???6ABBBD@BBC::8:@AA<
@@@A<A=???A=
@T6XXI:00093:00890
GGGGAATTCTAATACGACTCACTATAGGGAGAGGGAGAATAGCCCTGTATTATGTAGTCCGCAGCTATCCTTCCTTT
CTCCCTTCTCCC
+
DIK9CA@?@99548494=?@????887=27771>178@;77100+166668=111463;8;;;;<<>@A9<8;:==1
88:;9;898;;-
@T6XXI:00093:00896
GGGGAATTCTAATACGACTCACTATAGGGAGAGGGAGAATAGTCAACAAATCGTTTGGGGCGACTTCTCCTTCCTTT
CTCCCTTCTCCC
+
BEHBC?B@BCCBCBBBBBBBBB>99311.7==>@>ACCAC>>===<9994>B;?B8:885@???CADCB?B?A?BA7
<<CD=A88:AA<
@T6XXI:00094:00856
GGGAATTCTAATACGACTCACTATAGGAGAGGGAGAATAGTGACGTGTTGCGCGGCGCTCACTGTCTCCTTCCTTTC
TCCCTTCTCCC
+
DI?A>AABCCDDCCCCCCBA944=0/-
1>=BC>AAA@ACCA===???A>@@AAA>A?888==:8843?8<9<>@@;@???;@>@ACC;
@T6XXI:00094:00865
GGGAGAAGGGAGAAAAGGAAGGAGAAGTCGCCCCCAAAACGATTTGTTGACTATTCTCCCTCTCCCT
+
A?:?C@@@@199999,89:9=?B>?<;@@88888+733,39311,17779>>:885000,00000+-
@T6XXI:00094:00878
GGGAGAAGGGAGAAAGGAAGCTAGTCGACTCGACATCTGTGGCTTCACAGTACAGAGGGACTGTCCAAAGCCGCAGA
TCTCGAGTCGACTAG
+
CEBCCC@BB>;444-
9;>;@A@;;;/00//770868:<CCC?BB?BCC>><ABC=:::7<<=?@@>AA<@@=@@@AA<==<<:??@?@@A==
@T6XXI:00095:00850
CTCGAGATCTGCGGCTTTGGACAGTCCCTCTGACTGTGAAGCCACAGATGTCGAGCGACTAGCTTCC
```

+
DA<<<<<7=?>??9499-89>@ADAAC<AA99499998924424448879;=7744.//4...8,/,
@T6XXI:00095:00856
GGGAGAAGGGAGAAAGGAAGGAGAAAAGTGATGCACAAAGCACAAGACGTAT
+
::5>44244-444;58887878<<@>2<?:9677<<BC:8/700)*******
@T6XXI:00095:00858
GGGGAATTCTAATACGACTCACTATAGGGAGAGGGAGAATAGTCTGCAGATCCGACATGACTTTTTCTCCTTCCTTT
CTCCCTTCTCCC
+
:44/9<A>BCC@BA@ABBBBBC<99311.7>>BD>DCCA???<:;;=?????>::@???>==AAA1BAA>?;>3781
8B;B:?<?@@@<
@T6XXI:00095:00863
GGGAGAAGGGAGAAAGGAAGGAGAAGTGCCCAAGC
+
9A:>>><>@<<<;C<A=@?>;=;7?00.00*,*,.
@T6XXI:00095:00873
GGGAGAAGGGAGAAAGGAAGCGTAGTCGACTCGACATCTGGTGGGTTCACAGTACAGAGGGACTGTCCAAAGCCGCA
GATCTCGAGTCGACTAG
+
4;7;CB@BC8;949-429<=;;?;7773116113444/0.,...+.4220----
1113::48;:83=8:;:;;55:<<=?=A@@@A<==?<::4
@T6XXI:00095:00875
GGGAGAAGGGAGAAAGGAAGGGCACGCGCGAGTCATCCAATTGGGAGCTATTCTCCCTCTCCCTATAGTGAGTCGTA
TTAGAATTCCCC
+
DEBCDC@CC>AAAC>B?A?BC?AAABBA@BBCCCCCC@BBB@BB@BBBCFC@A;9969C@BB>BECCCCCCCCBAAA
A?B=:8:/444-
@T6XXI:00095:00876
GGGGAATTCTAATACGACTCACTATAAGGGA
+
?9929;@@<=9998@=843....4..,.524
@T6XXI:00095:00878
GGGGAATTCTAATACGACTCACTATAGGGAGAGGGAGAATAGCACGTTAATAACAAGCGCGACTATTCCCTTCCTTT
CTCCCTTCTCCC
+
?@?2929249;?BCCCCCBBBB@?<777.7?777,166>??==AADG@C?AB=::58>>=?@A?>@:<;665=:><;
>?>?:=7;;:;4
@T6XXI:00095:00887
GGGAGAAGGGAGAAAGGAAGTGACAGTTCGCGGTTCAGTGAACGG
+
CIBB@?294-9911-/,/,//../444244889;64/,,,,*,,*
@T6XXI:00095:00891
GGGGAATTCTAATACGACTCACTATAGGAGGAGGGAA
+
999/9>@@AAABCBBBCBCAB=;;;00,,70055+.,
@T6XXI:00095:00895
GGGAGAAGGGAGAAAGGAAGGAGAAGTCGCCCCAAACGATTTGTTGACTATTCTCCCTCTCCCTATAGTGAGTCGTA
TTAGAATTCCCC
+
HD?CCB@CC>CCBD>B@B?B@BBB?B>@BDCC8>?<?A???>@C@CB>?C?>====;=<<<<;<==<<;;;<=?A=;
?<??=8;3447,
@T6XXI:00096:00850
GGGAGAAGGGAGAAAGGAAGGAGAAACACGGGCCAAAGAGTTATTGGCTATTCTCCCTCTCCCTATAGTGAGTCGTA
TTAGAATTCCCC
+

```
AA>AENBCC@BBBB>B>A>A?BBBC?DCCBB>B=>><@C>=;;;,0,1>>FBCBBA>ABBCC>B@C@????@=??==
<:=?B=;/884-
@T6XXI:00096:00868
GGGGAATTCTAATACGACTCACTATAGGGAGAGGAGAAT
+
,,,*/7;:ACC@CCCC<<;CCB??8877.0@00,.222,
@T6XXI:00096:00875
GGGGAATTCTAATACGACTCACTATAAGGGAGA
+
<;;+1,/76<<;9;;<;996......,..+...
@T6XXI:00096:00879
GGGAGAAGGGAGAAAGGAAGGAGAAGTCGCCCCAAACGATTTGTTGACTATTCTCCCTCTCCCTATAGTGAGTCGTA
TTAGAATTCCCC
+
==<=FDCCC>CBDC>B=@=@@BBB@BCBBBBB8AA=BCCCC>BC@CCCB?BA@@@B;==<AB?B=<<@@@<A=AA?@
A?A=<8<899;0
@T6XXI:00096:00888
GGGGAATTCTAATACGACTCACTATAGGAGAGGGAGAATAGTTTAGCACGTTACCTCGGCCTATTTTCCTTCCTTTC
TCCCTTCTCCC
+
CFD:CBD@CCD@BBBBBBBBB:9990/-
0>>BC>CCC>?=96;:==CCBBB>?D@BAA@@?BBEEE9C>A>BCEE=@@AB<@:????<
@T6XXI:00097:00851
GGGAGAAGGGAGAAAGGAAGCTAGTCACTCGAGATCTGTGGCTTCACAGTACAGAGGGACTGTCCAAAGCCGCAGAT
CTCGAGTCGACTAG
+
CEBCCB=>=:@@>>6@786;A@@@B8??@;;;::<==@CC8:<@CBBABBBBB@=@B=:64488/34,4;:?@@@@@
@@@><??@@@@B<>
@T6XXI:00097:00853
GGGGAATTCTAATACGACTCACTATAGGGAGAGGGAGAATAGATCACCGTTGGTTCGGGTGCGCGTTCTCCTTCCTT
TCTCCCTTCTCCC
+
BDE:C>@=?CFBB?>>BBBB?CB99311.777==9?BC@CBCA==A><AA;?;?@DCC?C<<;ECC@@??>A>A?AK
>C@@@=@@B@@@<
@T6XXI:00097:00854
GGGAGAAGGGAGAAAGGAAGGAGAAGTCGCCCCAAACGATTTGTTGACTATTCTCCCCTCTCCCTATAGTGAGTCGT
ATTCAGAATTCCCC
+
CC?CEA?AHCC@AFAC<<:9=B8848=>8888.33,3931767=:=7:666640000+0338846==??@?@@@888
;;388<;9;;:::1
@T6XXI:00097:00857
GGGGAATTCTAATACGACTCACTATAGGGAGAGGGAGAATAGTCAACAAATCGTTTGGGGCGACTTCTCCTTCCTTT
CTCCCTTCTCCC
+
AAA6=?BACBD@BCBCDCCCCBA??776.7==?><??B?BBCA<<8;==6@@@>?5:::4;@@DE@;:;?C=>5881
8>@B<A898<A?
@T6XXI:00097:00859
GGGGAATTCTAATACGACTCACTATAGGGAGAGGGAGAATAGTCAACAAATCGTTTGGGGCGACTTCTCCTTCCTTT
CTCCCTTCTCCC
+
IHK9C@A=@B@>BA@A@BBB@@@AB???9:::@@=:::>@BA@@@588A<GABCK<@>>.87717267A?@?C??=:
659:49/40;;4
@T6XXI:00097:00860
GGGGAATTCTAATACGACTCACTATAGGGAGAGGGAGAATAGTTTTTCCCTTCACTAACCGCGAACTGCACTTCCTT
TCTCCCTTCTCCC
+
```

CDC9C=A?ACC@BBBBBBBBBBBBB97757??DE>BBB?BCB@@??1>=:?=@CCCC?C??<?B@BA@58:C>B5::
::BCE>B?BB@@<
@T6XXI:00097:00870
GGGGAATTCTAATACGACTCACTATAGGGAGAGGGAGAATAGTCAACAAATCGTTTGGGGCGACTTCTCCTTCCTTT
CTCCCTTCTCCC
+
CCD:C@B@BCB@B???BBBB?BBBB76646?@CC>BBBA???<@<;=A=;A@?;=-
111,8=AAB?BBB?B?@>AA<<?@?;@>A@A?;
@T6XXI:00097:00894
GGGAGAAGGGAGAAAGGAAGGAGAAGTCGCCCCAAACGATTTGTTGACTATTCTCCCTCTCCCTATAGTGAGTCGTA
TTAGAATTCCCC
+
DIBC@@=@@:>88809<@6;9<FC>BAC??<<2>>:?CDCC>BB?BBBBCC?@@@C<???AA<A>??CC??@=<?==
=?A>><>;==@5
@T6XXI:00098:00860
CTATCGACTCGAGATCTGCGGCTTTGGACAGTCCCTCTGTACTGTGAAGCCACAGATGTCGAGTCGACTAGCTTCCT
TTCTCCCTTCTCCC
+
@7771////8;BBBDBBBBB@BAA>A>;97>76:4:BBEBAAC>BBA><B@@AAABB?@@CA??@@@BAC@@??C?A
ABACCC>B?BBEEB
@T6XXI:00098:00866
CTAGTCGACTCGAGATCTGCGGCTTTGGACAGTCCCTCTGTACTGTGAAGCCACAGATGTCGAGTCGACTAGCTCCC
TTCTCCCTTCCTTCTCCCTTCTCCC
+
B994444499;49;99B@BBC@CCC>C@CB@B@??9?AABBBBC??@C@@CADECBB@@@AAA@:;;??><7<311-
7371;;663<;>:=034-4/3000+
@T6XXI:00098:00867
GGGAGAAGGGAGAAAGGAAGCTAGCCGACTCGACATCTGTGGCTTCACAGTACAGAGGACTGTCCAAAGCCGCAGAT
CTCGAGTCGACTAG
+
;A:@>>59919449-
4249;937771311116333;9999=<AA>@A@B???<<;00,/7;8:@>AB?AA>??@AAABAA???788<==88
@T6XXI:00098:00876
GGGGAATTCTAATACGACTCACTATAGGGAGAGGAGAATAGTAGGACGT
+
---*1:@ACDC@CBBA?BBBBBB?>311.6<<?=B::5::74;8,++++
@T6XXI:00098:00883
ACTCGAGATCTGCGGCTTTGGACAGTCCCTCTGTACTGTGAAGCCACAGATGTCGAGTCGACTAGCTTCCTTTCTCC
CTTCTCCC
+
DCCCBCBEC@A@?D<99909<@>>>99914;ADCC<:;B<<8=C>@@AB@AAAC<<;@ACBBAB?;1-1:><-
21:;;;8;8BC?
@T6XXI:00098:00895
GGGAGAAGGGAGAAAGGAAGGGAATAGCAGCAGTCGGCAACGTGAAACTATTCTCCCTCTCCCTATAGTGAGTCGTA
TTAGAATTCCCC
+
AA?ECCBHD>@?@C=@>=;??<@?BCBBBCBABBBB?BB@EGDAB@<AB@@=????9:::BB>CAB@@?BBAA???@
A>A@@;;/444-
@T6XXI:00099:00852
GGGAGAAGGGAGAAAGGAAGGAGAAGTCGCCCCAAACGATTTGTTGACTATTCTCCCTCTCCCT
+
99:=AACCC>@111,1,/,/6648:9198833,38/3331799?DC887@:/..433-----+-
@T6XXI:00099:00872
GGGGAATTCTAATACGACTCACTATAGGGAGAGGGAGAATAGTCAACAAATCGTTTGGGGCGACTTCTCCTTCCTTT
CTCCCTTCCTCCC
+

```
AA9299?>A?B>ABBB@@AAA@@83861,6/...+...77::5648400+331162666,3161106998<>65661
3.2222,.,.672
@T6XXI:00099:00879
GGGGAATTCTAATACGACTCACTATAGGGAGAGGGAGAATAGTCGTCCCTTATGCTGCTCCTATTTCATCTTCCTTT
CTCCCTTCTCCC
+
CCD:CAC@BCC@CA@AABBBBBB<;361.9??BC>BBBAC?CA9;443,42>?BCDB?BC@B@@@>@BBCC@C=AA5
::BC<B;?:::5
@T6XXI:00099:00885
GGGGAATTCTAATACGACTCACTATAGGGAGAGGGAGAATAGTCAACAAATCGTTTGGGGCGACTTCTCCTTCCTTT
CTCCCTTCTCCC
+
BBDAD=@>?CCACA@?BBBBBCB99311.777=?=?CC@BAA?AA>???;B@<=A=@?B7B???B=?@C7:7:?@@<
@AAA=A9<<AA<
@T6XXI:00099:00887
GGGGAATTCTAATACGACTCACTATAGGGAGAGGGAGAATAGTTTTTCCCTTCACTAACCGCGAACTGCACTTCCTT
TCTCCCTTCTCCC
+
=AA6C?C?CBC@CC?@@CCCGBA@@777.777AA>AA@<>774433+0305<=CCCC?B?AAAB?AA?@:::?A=@B
>AAAC>C@A??C<
@T6XXI:00099:00891
GGGAGAAGGGAGAAAGGAAGCTAGTCGACTCGACATCTGTGGCTTCACAGTACAGAGGGACTGTCCAAAGCGCAGAT
CTCGAGTCGACTAG
+
DB?CCC>>=:@@99:@=?<@D:00=/00:==;>@????BBC?AA@CC@??@A@?<44413>>?@A>AA<889AAAA?
??@A==<<<<<===
@T6XXI:00099:00897
GGGAGAAGGGAGAAAGGAAGCTAGTCGACTCGACATCTGTGGCTTCACAGTACAGAGGGACTGTCCAAAGCCGCAGA
TCTCGAGTCGACTAG
+
IIEAAA=@@:@@@@<@:;6<C?;;>/00<>;;08::96??B7;<?ACCCBBCAA;86846:<AAA>AA<??<?A<>=
<<@@AA@A@A@@AA=
@T6XXI:00074:00901
GGGAGAAGGGAGAAAGGAAGGAGAATCGAGGTGAGTTGTACTCATCACACTATTCTCCCTCCTCCCTATAGTGAGTC
GTATTAGAATTCCC
+
CDBBCC=@@<BB??:A5949;>>><;::6=>?;B@A:;9AAAADG<9999=44248885/.,...+.//8777??:6
/552+,,,*,2222
@T6XXI:00076:00907
GGGAGAAGGGAGAAAGGAAGGGGAGAAGGGAGAAAGGAAGGGGAGAAGGGCTAGTCGACTCGACATCTGTGGCTTCA
CAGTACAGAGGGACTGTCCAAAGCCGCAGATCTCG
+
BB@CCB@CB=>>>?=@??<=>>8@@A?BB?AA<>9?;=3666-
7<=:?A:??B@AAAA;5<;968888>@A>@C?CA=<<<=:84000,3999>@=@@<@A>A@??A<@@@A
@T6XXI:00077:00908
GGGGAATTCTAATACGACTCACTATAGGGAGAGGGAGAATAGTCAACAAATCGTTTGGGGCGACTTCTCCTTCCTTT
CTCCCTTCTCCC
+
EDE:CEDBCBB?CB@@@AABCB??3377-7==>?>BBB@CCB=@<:???:BA??=4969-
8<<??>::;?C@B=AA<AAAA<A<?@AA=
@T6XXI:00078:00900
GGGGAATTCTAATACGACTCACTATAGGGAGAGGGAGAATAGACACCAGTCCCATCAACCACAATTATTCTTCCTTT
CTCCCTTCTCCC
+
;<9/9<B?CCC@BBBBBBBBBBB9>311.7>>BC>BBB@E=@=???>AAAAA<@7788<<@@A?A>AAA??<?>AC<
?@@AAC<?8871
@T6XXI:00078:00911
```

```
GGGAGAAGGGAGAAAGGAAGCTAGTCACTCGACATCTGTGGCTTCACAGTACAGAGGGACTGTCCAAAGCCGCAGAT
CTCGAGTCGACTAG
+
99-999>ABAA<<<99;>;;:;;;B??=:777:6>66><=:=>587>@;::??=477.4:;444344-
79:AA@@@88/8///5000+/.2
@T6XXI:00079:00903
CTATCACTCGAGATCTGCGGCTTTGGACAGTCCCTCTGTACTGTGAAGCCACAGATGTCGAGTCGACTAGCTTCCTT
TCTCCCTTCTCCC
+
CAAA::::FHDCABBBBBB@A??<A>>>?B<<==?CCC@9=>@@@<:?B@BABBBBAABA?@ACBBBBCCCC?B?AA
<ABCC>B?BB@A<
@T6XXI:00079:00907
GGGGAATTCTAATACGACTCACTATAGGAGAGGGAGAATAGACATGTGATCTCCGCACTTGCGCTCTTCTTCCTTTC
TCCCTTCTCCC
+
DCB9B@A@ACDDCCCCCCBBCBAA?77377777.766;??@?===AAAA@?A?>@?898;==:;934333/4:;;98
78=:8351220
@T6XXI:00079:00910
GGGAGAAGGGAGAAAGGAAGGATAAGCACGCTGAGGGAATGGGTGTACTATTCTCCCTCTCCCTATAGTGAGTCGTA
TTAGAATTCCCC
+
CCBCCCACCBBCBB>B?B?BBBAC?ABBCCBB@===:@=@CE@CCCBCBCECDAAB=BCABB>B@ABCBBCC@?BBB
A>B?<8<8<;<4
@T6XXI:00080:00902
GGGAGAAGGGAGAAAGGAAGCTGGAGGATAGCCGGGATGTAAACGTACTATTCTCCCTCTCCCTATAGTGAGTCGTA
TTAGAATTCCCC
+
BB?BCC@BB?BB@@=@@A@A?>>?BB@A???A>AC>B;>C?@;>@AAABBC@C:887:B?@A<@@A@@<C?@??@==
=>AAA>A>@@@6
@T6XXI:00082:00901
GGGAGAAGGGAGAAAGGAAGCTAGTCGACTCGACATCTGTGGCTTCACAGTACAGAGGGACTG
+
FFCCCB@BA=B@>><@?B?BCC:;;CCFBBBA@@?AA???A>?@=@CCBAAACCA<887=AAA
@T6XXI:00082:00912
GGGGAATTCTAATACGACTCACTATAGGGAGAGGGAGAATAGCTGTAAGCTAACACTTGCTCTATTCACCTTCCTTT
CTCCCTCTCCC
+
DEF:E@C>AACBCCCAABCAA;7<<367-
77776,167=ABB@BABCAC?=?5888838@A===B?@9;34347::7:788./.3455
@T6XXI:00083:00901
GGGAGAAGGGAGAAAGGAAGAACTGACAGCTGTAGTTGAGTAAGGGAGCTATTCTCCCTCTCCCTATAGTGAGTCGT
ATTAGAATTCCCC
+
BB?@@@@CDB@A?A=A@C@CC@@@@CCABBAAB?>>8<<??@@@A=@??BBC@E???>?@@@A<@<@???A@@?@@?
???AAA>A=@@@5
@T6XXI:00083:00908
TGCGGCTTTGGACAGTCCCTCTGTACTGTGAAGCCACAGATGTCGAGTCGACTAGCTTCCTTTCTCCCTTCTCCCCT
TCCTTTCTCCCTTCTCCC
+
@CDDBCAA>B?@ACBBBB?BBAA@AACEAAA>AA@CBA>???A>=B?B@>??@C::;GG@CB?BBBE>B:;7777.7
<?=;>-44994:7:9:94
@T6XXI:00084:00916
CTATCACTCGAGATCTGCGGCTTTGGACAGTCCCTCTGTACTGTGAAGCCACAGATGTCGAGTCGACTAGCTTCCTT
TCTCCCTTCTCCC
+
CBCCA:::CBBA@ABBBCC@AAB>AAA>BB7788:CCC@=AB?CCA=?A<@BBCB>>@CCCBB?@ACCCBBB?B@BC
>BBBC>C@CB@@;
```

```
@T6XXI:00084:00917
GGGGAATTCTAATACGACTCACTATAGGGAGAGGAGAATATAGACCGAAA
+
FCC6;;?=@CC@BBBBBCCBB@9>9366.7>>>;@::47:3-..5.-+00
@T6XXI:00085:00900
GGGGAATTCTAATACGACTCACTATAGGGAGAGGGAGAATAGTCGACAACTCCCCAAACGACGCATGCACTTCCTTT
CTCCCTTCTCCC
+
AAA4><==@AA?CBBBBBAAAB>99311.7==?@>BBC@C??<<<<=A?AAAAA6==56777<;=@@998?9;3881
8>;>:@9<<<<7
@T6XXI:00085:00906
GGGAGAAGGGAGAAAGGAAGCTAGTCACTCGACATCTGTGGCTTCACAGTACAGAGGGACTGTCCAAAGCCGCAGAC
TCTCGAGTCGACTAG
+
C@?@<<;<C@CCBDAE=A<>>87331111111..43///:::::CA@????CA?8<<634340024=;==<<<<477
390000000000666
@T6XXI:00085:00908
GGGAGAAGGGAGAAAGGAAGGAGAAGTCGCCCCAAACGATTTGTTGACTATTCTCCCTCTCCCTATAGTGAGTCGTA
TTAGAATTCCCC
+
FF?CBB>AA<BAAC>B>B?B:<<B?BA@B?EL6=?9300/777A>@@BBB@;;::82//--.+-
799?<;87443348:66424/000+
@T6XXI:00085:00915
GGGGAATTCTAATACGACTCACTATAGGGAGAGGGAGAATAGTCAACAAATCGTTTGGGGCGACTTCTCCTTCCTTT
CTCCCTTCTCCC
+
DEB9C?A?BDCBBBBAAA@@BCB99311.7==?B>BBB@C>?===;<<<;BB@@@;>>@7BCCCC?@BB>A=>>A=<
>>?=;A=??@A;
@T6XXI:00085:00919
GGGAGAAGGGAGAAAGGAAGGAGAATGCACCGCAGGAAATTGCAAGACTATTCTCCCTCTCCCTATAGTGAGTCGTA
TTAGAATTCCCC
+
FFBBBB@CC>BAA@=A>@>@?BA@@B@CCE?BBAC?AB<@?BCC@CBBBBB>AABC<B?@@@<@@AAAAA@@@<<<<
<;>@@>?<ACD4
@T6XXI:00086:00901
GGGAGAAGGGAGAAAGGAAGGGGGAGAAGGGATAAGGGA
+
=A@@DEIHG?=;=@6469<>?;;2833133,31101626
@T6XXI:00086:00909
GGGAGAAGGGAGAAAGGAAGGGAATAGTTAGCAACAACTGACGAGAACTATTCTCCCTCTCCCTATAGTGAGTCGTA
TTAGAATTCCCC
+
DEBA?>@BC?BBBB>B@B@BB>B@BBB@@BD??=@C@CCBBBABC?A??BB@B@@B;??9977:@@AA<=<<<:??A
A=?@A>A=@@@5
@T6XXI:00087:00925
GGGGAATTCTAATACGACTCACTATAGGGAGAGGAGAATAGTA
+
>>>6>AC@CCCACABABBBBBBBCCA>?>??<??@//./*)))
@T6XXI:00088:00900
GGGAGAAGGGAGAAAGGAAGCTAGTCGACTCGACATCTGTGGCTTCAACAGAGACA
+
DHFGCC?A@:==995@786;C@;;=.000:;;086666BCC;?@@<:30>0,**((
@T6XXI:00088:00910
CTATCGACTCGAGAGCTGCGGCTTTGGACAGTCCCTCTGTACTGTGAAGCCACAGATCTCGACGT
+
D?B?B944994449499:9959@@:@=@==DACC>??@?;;;@BCBB?BDAAABCCDAA>>133>
@T6XXI:00088:00911
```

```
GGGGAATCTAATACGACTCACTATAGGAGAGGAGAATAGTCGAACAAATCGTTT
+
DJE6C+318:819<CBBBCF=1000/,.3.1017778:84400/434+333.6+
@T6XXI:00088:00919
GGGAATTCTAATACGACTCACTATAG
+
44-424249;:;=?@@@?>?>?8800
@T6XXI:00088:00922
GGGGAATTCTAATACGACTCACTATAGGGAGAGGGAGAATAGTACTCTTTAC
+
?99292929>9;99;@@BCCCDDC@@??8=?>>>:=6<711100043./,..
@T6XXI:00089:00902
GGGGAATTCTAATACGACTCACTATAGGAGAGGGAGAATAGCTACGTTTAACCGATCGTGTTGTTCTCCTTCCTTTC
TCCCTTCTCCC
+
DJE:D>?=@BC?CCC@?@CCC>>;=00,/99668?166:::8BCAAAC8;58>A?A?A@@@;==;?AD@C>@=:864
666,/,-1110
@T6XXI:00089:00908
GGGAGAAGGGAGAAAGGAAGATCATGACGCGTAGGGAGATTTACGGTCTATTCTCCCTCTCCCTATAGTGAGTCGTA
TTAGAATTCCCC
+
ED>BABBCH@CA@@>ABC?BBBBBCBBBBBBCCCC>?ABB@;CCC@CB>>?@=<<@;?A;<<9=@@@@=:??A?@??
?=@@A>A>@AA6
@T6XXI:00089:00910
GGGGAATTCTAATACGACTCACTATAGGGAGAGAGATATAAGGGGAGAGACTTTGTATTGTGCATGCCGCATGGACC
CATTACCAAATTCTCTCCC
+
EEE:C@C@BBD?BBBBBBBCBBBBBA@A>@@>?@A=@?A@=???5=<9BCAAA<588A@ECBBB@<?=@BCCC@A??
7=?@CFCDC<A>ACCC@@;
@T6XXI:00089:00917
GGGGAATTCTAATACGACTCACTAT
+
DD000)/,///,/44444333.44.
@T6XXI:00089:00926
GGGGAATTCTAATACGACTCACTATAGGGAGAGGGAGAATAGTTGACGACTTC
+
=A?4@6959>9;>@A@ABA@@???>877-7>>>C>@@@@DB>;7,+,+**+*+
@T6XXI:00090:00915
GGGGAATTCTAATACGACTCACTATAGGAGAGGAGAATAGTCAACAAATCGTTTGGGGCGACTTCTCCTTCCTTTCT
CCCTTCTC
+
AA@4?<@>ABB?<<<A@CCCD@?>000,,,,.,...,.7988==<<;6>9;??=?CC9@??FC@???;=::344-
4330,499,,
@T6XXI:00090:00918
GGGGAATTCTAATACGACTCACTATAGGGAGAGGGAGAATAGCTGTTTGCAATCCCACAGTTCTCCTACTCTTCCTT
TCTCCCTTCTCCC
+
?@@4?696<AC@BA??BBCB@BB>9311.7==?A>BCBCBC@:A9@C=C::5:?=6?ABBB?BAA;<@@AAA>@>@A
9==DC7;378441
@T6XXI:00090:00932
GGGAGAAGGGAGAAAGGAAGGAGCATAACGACCAGATATACCTGCACGCTATTCTCCCTCTCCCTATAGTGAGTCGT
ATTAGAATTCCCC
+
DE?A?A@BDBCBB?:@797;ABAECEC@CBAA?BBABCBBB@BBCBDCCBBB@BBEB=ABBB?;??B===??@@==:
:;;AC@>;3443+
@T6XXI:00091:00912
```

```
GGGGAATTCTAATACGACTCACTATAGGGAGAGGGAGAATAGTCAACAAATCGTTTGGGGCGACTTCTCCTTCCTTT
CTCCCTTCTCCC
+
DCC:C?A?BEC@BBBB?A>>>CB99311.7>==?=@EC@B?A===<<??<ACBCE>@@@7CCA@???BB>B>?>AA=
??@=;A>@@AA;
@T6XXI:00091:00919
GGGGAATTCTAATACGACTCACTATAGGGAGAGGGAGAATAGTCAACAAATCGTTTGGGGCGACTTCTCCTTCCTTT
CTCCCTTCTCCC
+
DBB6B=?>BBC@BB??BBBB<BB?<766.6?>@B>BBCA>>>:?A=@@@<CCBBC>BBB9CC@B?>ABF>@@?@@C<
AABB<A689AC<
@T6XXI:00091:00924
GGGAGAAGGAAGTGAAGA
+
DH?800+-+*)(((((*
@T6XXI:00092:00910
GGGAGAAGGGAGAAAGGAAGTGCAGTTCGCGGTTCGTGAAGGGAAAAACTATTCTCCCTCTCCCTATAGTGAGTCGT
ATTAGAATTCCCC
+
DDFC?>694-
9>>>:@>B?BCCCBCBA;;9868,100996;@;<A9<2BBCBACDDC<AABCC>?>BCB?@BCCCCA??>@AA>B>A
@@5
@T6XXI:00092:00914
GGGGAATTCTAATACGACTCACTATAGGGAGAGGGAGAATAGCACAACTAATTGT
+
DBA4@5969>9;>>A;;<CBBBB??866.7==>>9>??<=??83309330000++
@T6XXI:00092:00916
GGGAGAAGGGAGAAAAGGAAGCTAGTCACTCGACATCTGTGGCTTCACAGTACAGAGGGACTGTCCAAAGCCGCAGA
TCTCGAGTCGACTAGCTTCC
+
99049969C9?9999,4242477944,,,******,,,3577AB@AA??==777=:996;???AA>@@;@C=??@88
770043333336==66621,
@T6XXI:00092:00917
GGGGAATTCTAATACGACTCACTATAGGAGAGGGAGAATAGCTAAGCCATTTGGGACTGCTGTTTATCCTTCCTTTC
TCCCTTCTCCC
+
78:5?;:>B9969===@CC;;100;10,...6788=A@>9A?::2177699044-
487<BCBCC@<;;?B@E@FC9?617-737643+
@T6XXI:00092:00929
GGGAGAAGGGAGAAAGGAAGCTAGTCACTCGACATCTGTGGCTTCACAGTACAGAGGACTGTCCAAAGCCGCAGATC
TCGAGTCGACTAG
+
4974?BADE>>@BC@C@C@DA899A7........44556=>675766:?A=:::/0./;997A>AC888:@===?AA
AA@@??888@@@@
@T6XXI:00093:00907
GGGGAATTCTAATACGACTCACTATAGGGAGAGGGAGAATAGCCAAGTTCACTCCCTGCGGCGACATCTTCTTCCTT
TCTCCCTTCTCCC
+
BBB9DBD@BBB@CABA?B>?BCB?>766.7??CC>CCC@A@B?;<;@C<<<@@==;A@@@>@@<<ACCC?BC@C=AA
<ABBC>@>@A@A;
@T6XXI:00093:00913
GGGGAATTCTAATACGACTCACTATAGGGAGAGGGAGAATAGACAACCAAATC
+
AA?4>5<8<CC@BABABCCBBC>77366.7>?AB<@@B?A=7034/0/03077
@T6XXI:00093:00928
GGGAGAAGGGAGAAAGGAAGTGCAGTTCGCGGTTAGTGAAGGGAAAAACTATTCTCCCTCTCCCTATAGTGAGTCGT
ATTAGAATTCCCC
```

+
CD?BCB>@@>ACCE@C@C@BAACCCB?CBBB@C@CCCAB?A?:88??2@CCC@C@??8?A@AB>@@@AAAAAAA@@?
<<9;>>;>8<<;1
@T6XXI:00094:00921
GGGGAATTCTAATACGACTCACTATAGGGAGAGGGAGAATAGCACCGTTGGCCATAATGGTACTATTCCCTTCCTTT
CTCCCTTCTCCC
+
FEI:D@B@BBC@C@BBBBBB@A<99311.7==BE@????C@B?A<>BC@A@C?ABA@<<=?CCC?>>?@>?77366,
43442=387AA<
@T6XXI:00094:00938
GGGGAATTCTAATACGACTCACTATAGGGAGAGGGAGAATAGCCGTTTAATCTCCCACTGCGGCATATCCTTCCTTT
CTCCCTTCTCCC
+
BBB9BBCABBB@CBBBBBBBBBB99311.7>>?BBCCC?B6738444-
429;4=?;?AAAA@>@@A??<;@=?>@@<A@AA;?9<<?@;
@T6XXI:00095:00900
GGGAGAAGGGAGAAAGGAAGCTAGTCACTCGAGATCTGCGGCTTTGGACAGTCCCTCTGTACTGTGAAGCCACAGAT
GTCGAGTCGACTAGCTTCCTTTCTCCCTT
+
AA>?:989@:>999142824?783@:96<@BBBCCCAABD@BBB>B@CCBBBAA9?AA<==ACAAAD>@@>=@AAAA
@@::?==@@;<==<9?==<=CAA===7<?
@T6XXI:00095:00907
GGGGAATTCTAATACGACTCACTATAGGGAGAGGGAGAATAGATCACCGTTGGTTCGGGTGCGCGTTCTCCTTCCTT
TCTCCCTTCTCCC
+
DEB9A>A?ABBCCBB@AA??BC>97316.7>>?@=AAA?CCB=A?BB?BC7::;@DCC?C;<;CAA=AAA?A@C@CC
>BBCC>C@A>>A<
@T6XXI:00095:00916
GGGAGAAGGGAGAAAGGAAGGTGAACAGAGCGTAAACGAATACGTCTCTATTCTCCCTCTCCCTATAGTGAGTCGTA
TTAGAATTCCCC
+
CDEDCCACD>B??=:@7:8<@CCC@CCB@?BBBBA<BBB@@AACCBCBADD@BBBB>CB@@@<@CBCBBCACBAAB?
C@?BB@CDCCC7
@T6XXI:00095:00919
GGGAGAAGGGAGAAAGGAAGCTAGTCGACTCGACATCTGTGGCTTCACAGTACAGAGGGACTGTCCAAAGCCGCAGA
TCTCGAGTCGACTAG
+
FD?CAA>AB>AAA@:?595988;000...44..3111179@888@BB:;;?@??;444,430066:==555<=<>??
A888C888???>@@@
@T6XXI:00095:00933
GGGGAATTCTAATACGACTCACTATAGGGAGAGGGAGAATAGTTCCACGATTCCCTGTGTTGGCCTACTCTTCCTTT
CTCCCTTCTCCC
+
ECB7B=?>@CF@BBB>@>??BCB?;311.7==BC@CCC>B>>:<=<=<<<<<@A<@AAAA?A=;:<===<;8=8;84
;==?;@>AAA;4
@T6XXI:00096:00904
GGGAGAAGGGAGAAAGGAAGCTAGTCGACTCGACATCTGTGGCTTCACAGTACAGAGGGACTGTCCAAAGCCGCAGA
TCTCGAGTCGACTAG
+
CC?@AA@AA:@98809596988;4;0..3334;>8966???:?BA@?:::B??=;88418=;<<@@>>9>;;000;;
889A@=<=@@><=<@
@T6XXI:00096:00905
GGGGAATTCTAATACGACTCACTATAGGAGAGGGAGAATATCAACAAATCGTTTGGGGCGACTTC
+
///*/5><499:994==@>><793331011111,1777684844000+36666,111,1111101
@T6XXI:00096:00931
GGGGAATTCTAATACGACTCACTATAGGGAGAGGAGAATAGTTTACC

```
+
IFE6>===@@A=ACCCBBB>BBCCB76646?=?=B==97/,+.....
@T6XXI:00096:00939
GGGAGAAGGGAGAAAGGAAGAGCGCATGACGACAATTTTGTGGGAGCTATTCTCCCTCTCCCTATAGTGAGTCGTAT
TAGAATTCCCC
+
JIBCCB@CC@@@@@<@=@=ACCCCABB?B@BCCC@CCB8B:::://1@ECACCCC?AAADD>CBBB@@@A@@B@999
CA@@<BABBB8
@T6XXI:00096:00940
GGGGAATTCTAATACGACTCACTATAGGGAGAGGGAGAATAGATCACCGTTGGTTCGGGTGCGCGTTCTCCTTCCTT
TCTCCCTTCTCCC
+
CDC9CBC@CBB@CBBBAA??BB<99311.7==>@=ADC@C??<<=BA=??778:?CBCAC;<;CCC@C??=?>A>AC
=BBAA=A@CACA>
@T6XXI:00097:00904
GGGGAATTCTAATACGACTCACTATAGGGAGAGGGAGAATAGTCAACAAATCGTTTGGGGCGACTTCTCCTTCCTTT
CTCCCTTCTCCC
+
=A?4><@=ACDAC??=?@>>?CB99311.7==>A?BCC@B??A<<<<A@;CAA<>;==>7BCCCC?BBCAC?A>AA<
A@??:?699BD?
@T6XXI:00097:00908
GGGGAATTCTAATACGACTCACTATAGGGAGAGGGAGAATAGTCAACAAATCGTTTGGGGCGACTTCTCCTTCCTTT
CTCCCTTCTCCC
+
ECB9B=A@ABB@ACBCCCCBCDBBA????9:::CC>BAB@CCC@A?<@==7>877>6===2?>??B=??C=?>=BBD<
@@AA7<9<6895
@T6XXI:00097:00918
GGGAGAAGGGAGAAAGGAAGGGGAGAAGGGGAGAAAAGGAAGCTAGTCACTCGACAGTCTGATGGCTTCAC
+
DDBCCB@A=6=949-9=@5999/9>>5888-87677-70106???:??116711100000000444/333
@T6XXI:00097:00925
GGGGAATTCTAATACGACTCAC
+
EEB:C7<:94499448843...
@T6XXI:00097:00926
GGGAGAAGGGAGAAAGGAAGGAGAAGTCGCCCCAAACGATTGTTGACTATTCTCCCTCTCCCTATAGTGAGTCGTAT
TAGAATTCCCC
+
990444244-449;-429999=;;58<>8100*063660/-/.557?:6734300+00000+0222-
..4336<886830-,-,-22.
@T6XXI:00098:00900
GGGAGAAGGGAGAAAGGAAGGTTATGTGGCCAAAAGGGAATGGAGAACTATTCTCCCTCTCCCTATAGTGAGTCGTA
TTAGAATTCCCC
+
DDBA??@CC?ACBD>E?A>@@B@CCCB=973666-
7=9>=AA>ABB>CC?@@@CAA<=9=AB;===@@@AAA<=AAAA?=@@>@9<<<4
@T6XXI:00098:00920
CTATCACTCGAGATCTGCGGCTTTGGACAGTCCCTCTGTACTGTGAAGCCACAGATGTCGAGTCGACTAGCTTCCTT
TCTCCCTTCTCCC
+
CBBA;:::BAABBDBBBBA>ABB>B=>@BB???=?CCCA<ABACB@>@C@CCBCBBBBBBCCCCBBBEBBBC?A?AA
<AAAC>E@CCAA<
@T6XXI:00098:00922
GGGGAATTCTAATACGACTCACTATAGGAGAGGGAGAATAGTTTCACGTTGCCGACCGCCGCTATTCCCTTCCTTTC
TCCCTTCTCCC
+
```

```
CCC:C=@>?CC@B?@?BBBBBA;;<7107==>@?ADC?CC@==;<<?AA898>@@?>??<?@ABB?AA<A>A<<<<<
@AA<=<??A=;
@T6XXI:00098:00927
GGAGAAGGGAGAAAGGAAGGAGAAGTCGCCCCAAACGATTTGTTGACTATTCTCCCTCTCCCTATAGTGAGTCGTAT
TAGAATTCCCCCAATCGAATTCCCGCGGCCGCA
+
AAACD>BB=@===:@?@?BACCB@CCB;;;;5?@<ACECC<AA@AAABBC@B@<<;<>@@=;<=;::=<<<===99=
=@=:8:/4444,494//:./,/7+-+00.)+,+
@T6XXI:00098:00936
GGGAGAAGGGAGAAAGGAAGGAGAAAACGTGAGCCAAGATCTAAGCTCTATTCTCCCTCTCCCTATAGTGAGTCGTA
TTAGAATTCCCC
+
BC?ABDBCB<BA@@<A?B>B@@8:::.9A>>@@@?CABDCDBB?BBBACA?9@99909<9BCBCCBBB;;<AB;;<<
;;;;D@B@CCD8
@T6XXI:00099:00909
GGGAGAAGGGAGAAAGGAAGGGAGA
+
IFACAA@CC>BB>>6888+**)***
@T6XXI:00099:00916
GACTATCACTCGAGATCTGCGGCTTTGGACAGGTCCCTCTGTACTGTGAAGCCAC
+
1///6/4444499===>@?994...+.,....,.330..111116999;:5=64/
@T6XXI:00099:00917
GGGGAATTCTAATACGACTCACTATAGGGAAGAGGGAGAATAGATACCTTCTCTAGTCTCTTGCGCTCT
+
CDD:D8;;<9984;99=AA@@???00/0+0,..55767<89?:88;>8;384<<8:=?;11+))((((*
@T6XXI:00099:00930
GGGAGAAGGGAGAAAGGAAGCTAGTCACTCGACATCTGTGGCTTCACAGTACAGAGGGACTGTCCAAAGCCGCAGAT
CTCGAGTCGACTAG
+
AA?ABB;==099?>:>599;BBAAB;666616667988<98?B9;:A???@>>>477.8??@AA>AA<@A?@@AAAA
@<<;888888=@@@
@T6XXI:00099:00939
GGGGAATTCTAATACGACTCACTATAGGGAGAGGGAGAATAGATCACCATTGGTTCGGGTGCGCGTTCTCCTTCCTT
TCTCCCTTCTCCC
+
949/49@=BECBCAAACCCBBB<<8877-
7>>?A>B??=?AC@AACC@CD@B?A>AAA<B<<<CCE?AA?>ADD=AB>?@AAAB@><<<6
@T6XXI:00100:00024
GGGAGAAGGGAGAAAGGAAGATGAACTAGCGGGGAGGATGCGCTCGTACTATTCTCCCTCTCCCTATAGTGAGTCGT
ATTAGAATTCCCC
+
DB>ABB@BB>BDDC>@>A@ECDBC@DECCCCAB8AA=@BDCCCDECCBA>>9>@99919>9998;BBBBBCCDBCBB
BC@CCC?C=@@@6
@T6XXI:00100:00039
CTAGTCGACTCGAGATCTGCGGCTTTGGACAGTCCCTCTGTACTGTGAAGCCACAGATGTCGAGTCGACTTCCTTTC
TCCCTTCTCCC
+
=;1111//@CCCBCCBABABA@???;B?CB??:::5:BBCBBBC@BBC@AD?AACCB@@@AABB:;443313133,3
667;C;?=<A;
@T6XXI:00100:00044
GGGAGAAGGGAGAAAGGAAGCTAGTCACTCGACATCTGTGGCTTCACAGTACAGAGGGACTGTCCAAAGCCGCAGAT
CTCGAGTCGACTAGCTTCCTTTCTCCCTTCTCCCATCACCGACTGCCATC
+
B;=?;<;9C=@99914242473<<;7,,,,,,,,,2256880:;>ABBDBBBB@A77717==;<=>A@<@A>A???A@
;88///34457?<65948/43,3>@AAA;>>AC?C988=9444//6,-//
@T6XXI:00100:00049
```

```
GGGAGAAGGGAGAAAGGAAGCTAGTCACTCGACATCTGTGGCTTCACAGTACAGAGGGACTGTCCAAAGCCGCAGAT
CTCGAGTCGACTAG
+
DDACEC=>=5=>==5>5949A?>?@??=<>>779977CCC?CB@CB@?@AACC999<4:688??>@@<AA>A@=AA=
;<<>>=<8<<><<9
@T6XXI:00101:00011
CTAGTCACTCGAGATCTGCGGCTTTGGACAGTCCCTCTGTACTGTGAAGCCACAGATGTCGAGTCGACTAGCCCTTC
TCCCCTTCCTTTCTCCCTTCTCC
+
C>111=8==CBCAAABBCCC@@@><@???>919947BBCAAAC?AA@>=@=@DCBCBBBBBBCCBBBBBBCBB>C@B
::;7.7:<333+0444-434343
@T6XXI:00101:00023
GGGAGAAGGGAGAAAGGAAGAAGAACAACGGGGCACAGGGAATTTGGCTATTCTCCCTCTCCCTATAGTGAGTCGTA
TTAAGAA
+
9914>?69914@ABAC@C@CB@BC@CD=AABC8BCEBBD>B8;909<999A>>>?B088788,1666;?67:770--
-,-,-22
@T6XXI:00101:00026
GGGAGAAGGGAGAAAGGAAGCTAGTCACTCGACATCTGTGGCTTCACAGTACAGAGGGACTGTCCAAAGCCGCAGAT
CTCGAGTCGACTAG
+
BD?AAA?BB=BCCC@C>@=@?;88@6..,0,,,,,,,000036-0/>;BAC??<<<;6<==?;B344-
4974484844344//67//.9/7
@T6XXI:00101:00040
GGGGAATTCTAATACGACTCACTATAGGAGAGGGAGAATAGCTCATATGTGTGCCCCCATGACTATCCTTCCTTTCT
CCCTTCTCCC
+
B??4>>A>BCE@CCBAAABCB748;//,0<<>C>BBB?@;?:A@@@@?@:6400000+49::@888?;=9;7;;58/
9;28,////,
@T6XXI:00102:00004
GGGGAATTCTAATACGACTCACTATAGGGAGAGGGAGAATAGTCCGCCAATTCTGCGCGTTAGCATTCTCCTTCCTT
CTCCCTTCTCCC
+
ECC7A=@=@>@>@A@ABACD<:;??77:5;AACC>BBBAA@==<?==;A>@>A@ACBBBB=AA@?@=@@A@>>>111
/>=@9B:;/44,
@T6XXI:00102:00019
GGGAGAAGGGAGAAAGGAAGCTAG
+
GKDBBC994-9988095959@???
@T6XXI:00102:00025
GGGGAATTCTAATACGACTCACTATAGGGAGAGGGAGAATAGCCAAGCGGTTCCATGCTGTGTCTTTCTCCTTCCTT
TCTCCCTTCTCCC
+
@@B6@=@ACBB?BCCBBB@@A????666.777=?;?CC@A?==;=?AAA>A>@=AABCA@A?@@AA7;<>3828;>>
<?=?>9=./++,+
@T6XXI:00102:00028
GGGGAATTCTAATACGACTCACTATAGGGAGAGGGAGAATAGTCAACAAATCGTTTGGGGCGACTTCTCCTTCCTTT
CCTCCCT
+
AAA6@BCAAAA>@BCCCCCCAABDCAA@.77777-7113333303336=;CBBBD;???2<77::5:@>;:5;977-
/-,0500
@T6XXI:00102:00032
AGTCACTCGAGATCTGCGGCTTTGGACAGTCCCTCTGTACTGTGAAGCCACAGATGTCGAGTCGACTAGCTTCCTTT
CTCCCTTCTCCC
+
BBBCBBBBBBABBCCBAA>ABB?C@CCCBBBB>BC@@==?BCCCC@BB>B@@?@??<??;<<<A@A@=<<<==@CC>
BCCC>B===AA<
```

```
@T6XXI:00102:00035
CTAGTCACTCGAGAT
+
:>1//2,///87778
@T6XXI:00102:00043
GGGAGAAGGGAGAAAGGAAGGAGAAGTCGCCCCAAACGATTTGTTGACTATTCTCCCTCTCCCTATAGTGAGTCGTA
TTCAGAATTCCCC
+
99-
494>AB>BABB=B@B@B>A;;6;?B@CCC8CB>AAB@@=>C>A:;8B@>@A@A<AADBB98.//887==:<<<<=<=
=;:5423340
@T6XXI:00102:00044
GGGAGAAGGG
+
88+///,44-
@T6XXI:00103:00004
GGGAGAAGGGAGAAAGGAAGCTAGTCACTCGACATCTGTGGCTTCACAGTACAGAGGGACTGTCCAAAGCCGCAGAT
CTCGAGTCGACTAG
+
CD?BBA?A@:>99909<>;ABBABB?>:00000./..47>8AA@CBCCC????;88;689;==>;>=7:=;>878@>
??A=<<A899@AAA
@T6XXI:00103:00014
CTAGTCGACTCGAGATCTGCGGCTTTGGACAGTCCCTCTGTACTGTGAAGCCACAGATGACT
+
DA:<<<9<CEECCCGBBBC??=@BB>C?B@??:::5:BBCFCBBACAA>=@=AB@<8*(&&&
@T6XXI:00103:00018
GGGAGAAGGGAGAAAGGAAGAAAACGTGCCGGCAAAATAGTGAGGAGCTATTCTCCCTCTCCCTATAGTGAGTCGTA
TTAGAATTCCCCC
+
<<69A@=@@<>>>@>B?@=@???5?@@AA>@<88884;ACCDCCAAAAAAA?A???<?===A=A=<<@CC?>>?@AA
A>A?=8:24444,
@T6XXI:00103:00020
GGGAGAAGGGAGAAAGGAAGGAGACAGTGAGCGCCGCACAACACGTCACTATTCTCCCTCTCCCTATAGTGAGTCGT
ATTAGAATTCCCC
+
DDACDC@CB>BCBB>B@C@C@@@>CCBBBBCCBB@??<<;>???@??BBBCC@B@@@=@BBED=AAABBCCBBBBBC
CC@@CC>D4999/
@T6XXI:00103:00045
GGGGAATTCTAATACGACTCACTATAGGGAGAGGGAGAATAGTCAACAAATCGTTTGGGGCGACTTCTCCTTCCTTT
CTCCCTTCTCCC
+
CDB7B=@>ACD@CCB@BBBB>@>99111.7=<AB>BBB@AAAA@?:;;;5?<=??9???6???@C?BBC@@>@>@@;
@@@=;A><@@@;
@T6XXI:00104:00005
GGGGAATTCTAATACTACTCTAGAGGAGGAGGAAGAAT
+
DCH9@>?=@B;?=>//,2,..8557><5534,.,..,-
@T6XXI:00104:00007
GGGAGAAGGGAGAAAGGAAGGGGAGAAGGGCTAGTCGACTCGACATCTGTGGCTTCACAGTACAGAGGGACTGTCCA
AAGCCGCAGATCCTCGA
+
GEBAAA@CC>?A?C?B?B===?6>??4:;9<CFDCCBDBBA@998;<<ABD?BA@CCBBBBCCBB;;;:<AAABB@B
D?BC@CCICB@@@@AAA
@T6XXI:00104:00025
GGGAGAAGGGAGAAAGGAAGGAGAAGTCGCCCCAAACGATTTGTTGACTATTCTCCCTCTCCCTATAGTGTAGCGTT
ATTCAGAATT
+
```

```
EDCCCC@CC=;;;A9>=@?B?BDC@CBBAAAA7AC>EBA><;<??BCCCA>:<333,8;:??;@=<8;;///000.)
++*++57445
@T6XXI:00104:00043
GGGAGAAGGGAGAAAGGAAGTGCAGTTCGCGGTTAGTGAAGGGAAAAACTATTCTCCCTCTCCCTATAGTGAGTCGT
ATTAGAATTCCCC
+
CCCCCBAA@:@@==:@@B?CCBBBBBABBBB@BB888CC??B9????0?ACC?BBCC>AAACC>C<<<AAACCA>>>
?@?AAA?B3844-
@T6XXI:00104:00044
GGGAGAAGGGAGAAAGGAAGCTAGTCGACTCGACATCTGTGGCTTCACAGTACAGAGGGACTGTCCAAAGCCGCAGA
TCTCGAGTCGACAG
+
DEDA@?@CD>DCBB=B=>;>B;;@A7::A>=:00000/@>>;BB@CA>>@@@AA=<<<:;:68688::4>A>@??A=
=;::<<<<<<<<==
@T6XXI:00104:00049
GGGAGAAGGGAGAAAGGAAGCTAGTCACTCGACATCTGTGGCTTCACAGTACAGAGGGACTGTCCAAAGCCGCAGAT
CTCGAGTCGACTAG
+
@?:>?=59919111,1-1-
/..<737/737;00.///7:@8::@CECBCCCCC666;5;<>>AA>@@;=<9=??@AAAAA@=A=9==@AA=
@T6XXI:00105:00017
GGGGAATTCTAATACGACTCACTATAGGGAGAGGGAGAATAGACTCACTTCTCACCGCTCCTGTATCGCCTTCCTTT
CTCCCTTCTCCC
+
99929>=?BBCACCCCBBAB@?>?>777.7>777-
6==;6??A878A>>:????A>@@CC<?=<<9;442737<@=;>ACC=A<===B4
@T6XXI:00105:00031
CTAGTCACTCGAGATCGCGGCTTTGGACAGTCCCTCTGTACTGTGAAGCCACAGATGTCGAGTCGACTAGCTTCCTT
TCTCCCTCTC
+
;11////4444494=AACB9;8?8@<@@<;;9C?CCDAA<;8>73747858;:=;?A@;7;4/1/...4.1189645
33...+----
@T6XXI:00106:00011
CTAGTCACTCGAGATCTGCGGCTTTGCGACCAGTCCC
+
>://///49ABB?@BDCBBC<777/8833313....+
@T6XXI:00106:00014
GGGAGAAGGGAGAAAGGAAGCTAGTCACTCGACATCTGTGGCTTCACAGTACAGAGGGACTGTCCAAAGCCGCAGAT
CTCGAGTCGACTAG
+
BB>AA@@DD@BDBC?C@C@CAA;<;?????:::8@CBCBC?BD?BBCCCBB?=9333-
4:;;?@>BA?@A>@@@A@A@@@@<??788====
@T6XXI:00106:00027
GGGGAATTCTAATACGACTCACTATAGGGAGAGGAGAATAGTCAACAAATCGTTTGGGGCGACTTCTCCTTCCTTTC
TCCCTTCTCCC
+
EDCDC=?>@A@@CCBABBAABBB?A696./:/0,/?>7=;ACAC>9=<7???9988;?5?AABC?CCC=<9=>CC;?
?@A<A>@@@@;
@T6XXI:00106:00045
GGGAGAAGGGAGAAAGGAAGGGGAAAGGGAGAAAGGAAAGCTAGTCACTC
+
@?6>9954=54444-49;<?BB6<;<7979311,1011,1119:88873.
@T6XXI:00106:00047
GGGGAATTCTAATACGACTCACTATAG
+
BDE7B?B?B;;7;;9??8873...2,,
@T6XXI:00107:00005
```

```
GGGGAATTCTAATACGACTCACTATAGGGAGAGGGAGAATAGCACTAAACTCCCTAGCGCTTCATTTCACTTCCTTT
CTCCCTTCTCCC
+
444-
9<@ABDB@BBBB@A???@CBB66959??CD>BAAA?A@;8;37718@@@;@????CA@AAAA:B==??C>AA<===@
<@>A@@@;
@T6XXI:00107:00012
GGGGAATTCTAATACGACTCACTATAGGGAGCAGGGAGAATAGTCAACAAATCGTTTGGGGCGACTTCTCCTTCCTT
TCTC
+
///*/,/,///76==>@=@ACAAAA>@<60/..552557440----,---
+.1111,67729611101677974234O300
@T6XXI:00107:00013
GGGGAATTCTAATACGACTCACTATAGGGAGAGGGAGAATAGTCAACAAATCGTTTGGGGCGACTTCTCCTTCCTTT
CTCCCTTCTCCC
+
;;;4<?BAC@?=@CCCCCBCAA@?>677.7==AC?CDCBBAA>?>>AAB=C????9???5ACCCC@BBB8968?AA<
@@@@<B;==AD<
@T6XXI:00107:00014
CTAGTCGACTCGAGATCTGCGGCTTTGGACAGTCCCTCTGTACTGTGAAGCCACAGATGGACTGTCCTTCCTTTCTC
CCTTCTCCC
+
FBDBBCCCCCDCCBBBBBCBB@99979=;?AB;::8:DDEB=BB>BBC@=A>ACCBCBC?BBBC@@=@=A=AB>BBB
C>C????D>
@T6XXI:00107:00017
GGAGAAGGGAGAAAGGAAGGAGAAGTCGCCCCAAACGATTTGTTGACTATTCTCCCTCTCCCTATAGTGAGTCGTAT
TAGAATTCCCC
+
DABBA=@A<AAAA<@=A9;8;BB?@?@8888.7727;=A>;??<?B???B?A;<<7;;<>>:?899AAA@@@AA===
>=<884/444-
@T6XXI:00107:00028
GGAGAAGGAGA
+
72-,,*,*,,4
@T6XXI:00107:00035
GGGAGAAGGGAGAAAGGAAGCTAGTCGACTCGACATCTGTGGCTTCACAGTACAGAGGGACTGTCCAAAGCCGCAGA
TCTCGAG
+
DD?BBB@BB>CCBC>B@B7<;CCBCBBB@???AAA@@==?C?BB@CCCC@?@>;833317?9==?37718;7:;:4/
/.-.-++
@T6XXI:00107:00039
GGGAGAAGGGAGAAAGGAAGCTAGTCACTCGACATCTGTGGCTTCACAGTACAGAGGGACTGTCCAAAGCCGCAGAT
CTCGAGTCGACT
+
CC@AA@==95=844-
9695;BBCDC?@@?E>01////;>E>AA@CCC>>>@AC<988466888<:884??<?@@@@748;888?>==??
@T6XXI:00107:00047
GGGAGAAGGGAGAAAGGAAGAGTAGCCGGGGACCAGGAAAAGTTTGGCTATTCTCCCTCTCCCTATAGTGAGTCGTA
TTAGAACTTCCCC
+
99-
9@@?CG@CBBB?B@B?BACCABA@CCC:AA=>=<;@@7BBB?B::;CCAB;<;7<AACC@CBBBBBBBB@>A8;;@C
CD@AA@@CA7
@T6XXI:00108:00005
GGGGAATTCTAATACGACTCACTATAGGGAGAGGGAGAATAGTTCAACGTTTATCCGCATGAACATCTCCTTCCTTT
CTCCCTTCTCCC
+
```

```
<99/98@=ABB@CBBBCBBBBCC?@666.6<67757BA?B@@A?AA<@@@A<A@@>@<<<??<?A@?@A<>;>;>>:
?@@@<A>@@AC<
@T6XXI:00108:00041
GGGAGAAGGGAGAAAGGAAGAACAGGTATCGTCGAGAGGTTTTGCACTATTCTCCCTCTCCCTATAGTGAGTCGTAT
TAGAACTTCCCC
+
BB:999599-448;-
429;89;BAD?ACBB@@;944;;2494,/7///77,/1:::999=A<<88:BB966?717;;00035:<@@@@7
@T6XXI:00108:00044
GGGGAATTCTAATACGACTCACTATAGGGAGAGGGAGAATAGTCTCCTTTCCTCTCATGACCACGTGCCCTTCCTTT
CTCCCTTCCTCCC
+
ABA7A>B@BEBBBCBBBB??BABB>66646??CC>BBB@@;?:=@?9=<7<<=??A<==<C;::@>111,4/49?>;
@42406,/,///,
@T6XXI:00109:00016
GGGAGAAGGGAGAAAGGAAGTGCAGTTCGCGGTTAGTGAAGGGAAAAACTATTCTCCCTCTCCCTATAGTGAGTCGT
ATTAG
+
44-449<@@-944;582424334>AB=@?BD?A?B@@>?<=>:@@??/?83848777-66111,14448??8744--
++*++
@T6XXI:00109:00025
GGGGAATTCTAATACGACTCACTATAGGGAGAGGGAGAATAGTCAACAAAATCGTTTGGGGCGACTTCCTCCTTCCT
TTCT
+
CCDAC@C@CBB><<<A@?@88783771>9:8<976711230344;84444+1>776-1778>5333,.,.54224-
..-4=
@T6XXI:00109:00043
GGGAGAAGGGAGAAAGGAAGGAGAAGTCGCCCCAAACGATTTGTTGACTATTCTCCCTCTCCCTATAGTGAGTCGTA
TTAGAATTCCCCC
+
CCCCCB?BB=BBBB@B?A?B@BBB??AA7788.8>8?ABBC>C@>@@CBC@>>===;@@@@><A?@@A==<@?;<<<
=9=@>99/////+
@T6XXI:00109:00047
GGGAGAAGGGAGAAAGGAAGAACATGCGAAAGAGTTAACAACTAGC
+
1/+//856656411-1:;:11,,700,...+..337@>99611106
@T6XXI:00110:00010
GGGAGAAGGGAGAAAGGAAGGGGAGAAGGGAGAAAGGAAGCTAGTCGACTCGACATCTGTGGCTTCACAGTACAGAG
GGACTGTCCAAAGCCGCAGATCTCGAGTCGCTAG
+
490999294-
///8+/,/568:6::>BCC<:::?6=;>4::9@?ABB???<=<<<@=<8745668<<<@>===84444-
4::==><@A;@@>@@@@???77788///88;;
@T6XXI:00110:00015
GGGAGAAGGGAGAAAGGAAGCTAGTCACTCGAGATCT
+
DDAC<::;A>C999085944@>>7>0//7//..,444
@T6XXI:00110:00026
GGGAGAAGGGAGAAAGGAAGGAGAACACGCCGCGGGATATTGGCGAACTATTCCTCCCTCTCCCTATAGTGAGTCTA
TTAGTAAA
+
//+///,4954444-4242493..6/////,/433,44//,/,///56879292444-
477;@<>??A??::7<;4..,.*0033
@T6XXI:00110:00030
GGGGAATTCTAATACGACTCACTATAGGGAGAGGGAGAATAGTCAACAAATCGTTTGGGGCGACTTCTCCTTCCTTT
CTCCCTTCTCCC
+
```

```
CCD:C@D@EBB@BBBBBBB<?AD?B666-
6==@ABCCC@A@@?>>?A==;BB?AA<ABC9CBCBC?BBE@A?@>@B:?887,4247=@:
@T6XXI:00110:00032
GGGGAATTCTAATACGACTCACTATAGGGAGAGGGAGAATAGTTTTCCCTTCACTAACCGCGAACTGCACTTCCTTT
CTCCCTTCTCCC
+
DDD9C@C@CCB@BA@@BBBBA@@93167.777>A>FCC@A?@@CC07718;=BCBC@C?A>A@?==?<?>B?B;??9
????9A@@==A?
@T6XXI:00111:00021
CTATCACTCGAGATCTGCGGCTTTGGACAGTCCCTCTGTACTGTGAAGCCACAGATGTCGAGTCGACTTAGCCTTTC
CCTTTCCTCCCTTCCTCC
+
DB?@9////86//1>>=?A?88808479311688888?:::6A944204244433778689914433313310l6,1
6-77-10177,0/-,--,
@T6XXI:00111:00031
GGGAGAAGGGAGAAAGGAAGTGAACGTTACCGTGGGAGAATTGGTAACTATTCTCCCTCTCCCTATAGTGAGTCGTA
TTAGAATTCCCC
+
@=:=EB@DC?CBBB>B>BDBB@@?BCC@@@>BBDD?CEC?C?C@CDAC@AAAECCC>CCCEC>BAABCCCBBBCCBC
B>@?A=9/888-
@T6XXI:00111:00034
CTATCACTCGAGATCTGCGGCTTTGGACAGTCCCTCTGTACTGTGAAGCCACAGATGTCGAGTCGACTAGCTTCCTT
TCTCCCTTTCTCCC
+
CBCBBAA?A@=BBAAACBB?AAA<>4888777@;@AAACA???A==;<@>AAAAAB@????779988733113688A
;:877,00+0044+
@T6XXI:00111:00046
GGGGAATTCTAATACGACTCACTATAGGGAGAGGGAGAATAGCCGTAATGTTTGCTTACGCCTTTTCTCCTTCCTTT
CTCCCTTCTCCC
+
DB@6>698<CC>BCBBA@@@B@B??777-777>@=ACC@A???=@=<:=89;6<<<?AAA@=@CC6@@?;;79244-
4;;;4<587AC9
@T6XXI:00112:00019
GGGGAATTCTAATACGACTCACTATAGGGAGAGGGAGAATAGTCAACAAATCGTTTGGGGCGACTTCTCCTTCCTTT
CTCCCTTCTCCC
+
=994><?=@BBACBBBBCCBBCB?9111.7>>?A>ACC@A=:<=<;8<=7?BABB>BBB7AAA??=?CC==;=>@C=
AAAA<A<??BC<
@T6XXI:00112:00040
TGCGGCTTTGGACAGTCCCTCTGTACTGTGAAGCCACAGATGTCGAGTCGACTAGCTTCCTTTCTCCCTTCTCCCGG
AGAAGGAC
+
@CCC@;;969;AACCCCC>AAABBBCCCBBB@CC?BBCBBB???B@A@:7//0=;?>=??AB?BBCDCDA>>>>6<:
7,,*,2**
@T6XXI:00113:00010
GGGAGAAGGGAGAAAGGAAGGCGAACAAGTGCGAAAGAGAATAGTGCTATTCTCCCTCTCCCTATAGTGAGTCGTAT
TAGAATTCCCC
+
DECDFD@CC@CCBC>C@C@C@A:::<D@CDDC;;;6;???>BBBCA?AAC>89994:A>AA<@@===@@A???=<;=
8;<:73233;1
@T6XXI:00113:00015
GGGAGAAGGGAGAAAGGAAGCTAGTCGACTCGACATCTGTGGCTTCACAGTACAGAGGGACTGTCCAAAGCCGCAGA
TCTCGAGTCGACTAG
+
DD>CCC@BB=?>==:@>?=AC<9==/00:0/0;@<AA@CEC?BB@C@@?@?AA?<44414===@A=??<?A>AA==;
==:;::;=;?<<<==
@T6XXI:00113:00019
```

```
GGGAGAAGGGAGAAAGGAAGCTAGTCACTCGACATCTGTGGCTTCACAGTACAGAGGGACTGTCCAAAGCCGCAGAT
CTCAAGTCGACTAG
+
CE?BBC@CCABBAA=B<@?BBBBCC?@>>>?01////=>C>BC@BC@A@BCB>633313;9>@A>A@<AA=A?:?B<
<<4447?@@???@@
@T6XXI:00113:00028
GGGAGAAGGGAGAAAGGAAGCTAGTCACTCGACATCTGTGGCTTCACAGTACAGAGGGACTGTCCAAAGCCGCAGAT
CTC
+
CG=B=929919999095958A??@A?/.,77,,,,,,035-
79>A???CCBBA?<;;6;:::;;;==:?@>@AAA??===
@T6XXI:00113:00042
GGGAGAAGGGAGAAAGGAAGGCGAAGCGGGATCTTGTGGTAGGATAGCTATTCTCCCTCTCCTATAGTGAGTCGTAT
TAGAATTCCCC
+
DB@AAA@AA?>AAB>B@B@B@BAA?ABBC=?>BC?CBB?AAB@A??>??DC@CCCE?BBCC@CCCC@@ABBBBBCDF
BDBCABAIII:
@T6XXI:00113:00048
GGGGAATTCTAATACGACTCACTATAGGGAGAGGGAGAATAGTCCGCCAATTCTGCGCGTTAGCATTCTCCTTCCTT
TCTCCCTTCTCCC
+
BA>6@<A@A=?=AABBCBB?BA>99111.7><=?<?CC@@@@@@?@A;@>B????@?@??>?@A@A@BB;8?;?=?B
;C???=@?@ABB9
@T6XXI:00114:00011
GGGAGAAGGGAGAAAGGAAGCTAGTCACTCGACATCTGTGGCTTCACAGTACAGAGGGACTGTCCAAAGCCGCAGAT
CTCGAGTCGACTAG
+
EC?A9924909999142824;888A??=???7:9?::BCC?BB@CBC<<ABAA9033-
49;;>@>AA<@A>A@@@@@<<<CAA??B@@@@@
@T6XXI:00114:00012
GGGGAATTCTAATACGACTCACTATAGGGAGAGGGAGAATAGTCTTGTATTTTGTGCATCACTTTTCTCCTTCCTTT
CTCCCTTCTCCC
+
CDD9B?BACDD?BB@AABBBB@<99166.7==BC>CBBD@AA===?;9@<==4;;;?@@BC:::94:>?>@@A?DA:
??AA<A659AC<
@T6XXI:00114:00018
GGGAGAAGGGAGAAAGGAAGCTAGTCGACTCGAGATCTGCGGCTTTGGACAGTCCCTCTGTACTGTGAAGCCACAGA
TGTCGAGTCGACTAGCTTCCTTTCTCCCTTCTCCCCTTCCTTTCCTCCTT
+
DD>CDC@BC<@;;8-
9=>;@C>9:=/00==>>?>B???BAA=ABB>B@CCAAACC>C>9==?AAABAC=<=>AAA???@;6<889=B@@AA4
47?A?CC7==@A;?794444-/,/,//,/,//,.0
@T6XXI:00114:00023
GGGGAGAGGAATTTGGTAATGGGGATGGGCCAACAGCTAGGAAAGAGATAGTACACCCTTATATCTCTCTCCCTATA
GT
+
///*/44424248589>?<8888.8><726566600/5946;?07311611176111,04110344430000+0711
66
@T6XXI:00114:00035
GGGGAATTCTAATACGACTCACTATAGGGAGAGGGAGAATAGTCCCGATATACGTCGTGGGCTCTTCGCCTTCCTTT
CTCCCTTCTCCC
+
BBB9CAB@BCB@CCBAA?>>BA>97111.7==>B=BDD@A8==;;:==:;<;;<=<<<<<;====<=BB?B9;8<G>
BFDD>B?BBBB<
@T6XXI:00114:00037
GGGGAATTCTAATACGACTCACTATAGGGAGAGGAGAATAGTTTT
+
```

```
AGE9B?A>@9954>>998>?87;BB?=C87.,00,,,*++00330
@T6XXI:00114:00042
GGGAGAAGGGAGAAAGGAAGGAGAAGTCGCCCCAAACGATTTGTTGACTATTCTCCCTCTCCCTATAGTGAGTCGTA
TTAGAATTCCCC
+
DC@DDB?BB=BB?==B=@=@@CCC@CCB@??@8@B>BB???;?@?@>>ABB774444..0;=9;;>>@@@C@@@@??
?>A<<8559:<1
@T6XXI:00114:00043
GGGAGAAGGGAGAAAGGAAGCAGTCCATCTGTGGCTTCACAGTACAGAGGGACTGTCCAAAGCCGCAGATCTCGAGT
CGACTAG
+
EC?AAA@CC>CBAA=B?A?@969..-
0000LCB@BB@BB??<=C@>?44417A=?@C>@@<??>AAAA@A@@?===?CBCCCBB
@T6XXI:00115:00007
GGGAGAAGGGAGAAAGGAAGCTAGTCGACTCGACATCTGTGGCTTCACAGTACAGAGGACTGTCCAAAGCCGCAGAT
CTCGAGTCGACTAGCTCCTTTCTCCCTTCTCCC
+
AA?>=959C09111,/,/,/644460./9737;:1166=;?:?@?BBCBCCB==9//,/6667=>AA=@@999@@@@
CBB===@@@@;88:00.99706---+-03000+
@T6XXI:00116:00016
GGGAGAAGGGAGAAAGGAAAGGGGGAGAAGGGCTAGTCACTCGACATCTGTGGCTTCACAGTACAGAGGGACTGTCC
AAAGCCGCAGATCTCGAGTCGACTAGCCCTTCTCCCCTTCCTTCTCCT
+
LD>CA>;>>1949904244-4444+33377@:@BCCB999994//////68=<>>>A:666;999:999-
8>88=B@CC>CDAFC<=<BBBC@@@BHDECBB@@@9><?8888.0,0,0,***)*
@T6XXI:00116:00024
GGGGAATTCTAATACGACTCACTATAGGAGAGGGAGAATAGAGACGTATTCGTTTGCGCTCTGTTCACCTTCCTTTC
TCCCTTCTCCC
+
@AA8A@C@CCCBCCBBBBCCC>==;/.,0::?B>BCCAB=89=@@@@???@ABC;@;<?>A?@@>AAA>=<?<?=<<
::=;<;<A@A;
@T6XXI:00116:00033
GGGAGAAGGGAGAAAGGAAGGAGAAGTCGCCCCAAACGATTTGTTGACTATTCTCCCTCTCCCTATAGTGAGTCGTA
TTAGAATTCCCC
+
DCABBB?BA:????=B?B?B?BCC@CCB>;;;5??<@CCCC;A?@BA>9C=<=;<<;<=>==;<<======<;;?@A
A>A@A<>3847+
@T6XXI:00116:00038
GGGGAATTCTAATACGACTCACTATAGGGAGAGGGAGAATAGTCAACAAATCGTTTGGGGCGACTTCTCCTTCCTTT
CTCCC
+
@=?4@>A>BCC@BB@BB@@<BAD?@666-
1=;;?<ABBAA<@AAA>@@=;@?>?C>@BC9C@@@CACCC>=;=>AA<A==<4
@T6XXI:00116:00047
CTATCACTCGAGATCTGCGGCTTTGGACAGTCCCTCTGTACTGTGAAGCCACAGATGTCGAGTC
+
C::9>::;CDHBBBCCBB@?@DB<>=@@>=7::5:?@BBCCC8;9A?@C@CBAABBAA??@CCC
@T6XXI:00117:00010
GGGAGAAGGGAGAAAGGAAGCTAGTCGACTCGACATCTGTGGCTTCACAGTACAGAGGGACTGTCCAAAGCCGCAGA
TCTCGAGTCAACTAGCTTCCTTTCTCCCTTCTCCC
+
FE?AAC?A@:>99909?BACACDDD:;;A>>:11000.3577AB>>>BBBCCAA?@AC<???A?=8;94::7888??
>>?@<;<@>=;>@<<<@>@=@A:;444-434444-
@T6XXI:00117:00013
GCTATCGACTCGAGATCTGCGGCTTTGGACAGTCCCTCTGTACTGTGAAGCCACAGATGTCGAGTCCGACCTAGCTT
CCCTTTCTCCCTTCCTCCC
+
```

```
4444944444@@AAA@>>?995733,3137@BAC@;97/..3...61-,-
/,//664;ABB<55./,/64348311011,11,1111,04446640
@T6XXI:00117:00019
GGGAGAAGGGAGAAAGGAAGCTAGTCACTCGAGATCTGCGGCTTTGGACAGTCCCTCTGTACTGTGAAGCCACAGAT
GTCGAGTCGACTAGCTTCCTTTCTCCCTTCTCCC
+
ED>BA@=@@:>99919<?<@@:;:B;77@?::8BAACCLEE@B@=??BBBAAAC>BCAB???@888B??@>@AA===
AA889=AAAA?;;4//,/7876;89=6:-838:4
@T6XXI:00117:00023
GGGAGAAGGGAGAAAGGAAGCTAGTCGACTCGACATCTGTGGCTTCACAGTACAGAGGGACTGTCCAAAGCCGCAGA
TCTCGA
+
BDB??=@BC>@@@C>B@C9<;B@@@@@BCBB?@::::AAC8;<BBCBAACCCB533317>AACA<??:?C???:<<
<;;<==
@T6XXI:00118:00013
GGGAGAAGGGAGAAAGGAAGCTAGTCGACTCGACATCTGTGGCTTCACAGTACAGAGGGACTGTCCAAAGCCGCAGA
TCTCGAGTCGACTAG
+
DD??A?@CC=BBBG@C?B?BCA>>>1//>>>>B@?::9??A7<<GBBBBBBB@>;447-
8??AA@?AA>BA=A?:;<AAAA???@888@@@>
@T6XXI:00118:00033
GGGAGAAGGGAGAAAGGAAGTGCAGTTCGCGGTTAGTGAAGGGAAAAACTATTCTCCCTCTCCCTATAGTGAGTCGT
ATTAGAATTCCCC
+
HKDBBA@BC>CBCC@C?C@BBCCCCC@@@@C@C?<<;?>;<?.7777,8@??=@CCC>@@@CD>C?@@@????A<;;
>>;>8:;=8<<?4
@T6XXI:00118:00035
CTATCGACTCGAGATCTGCGGCTTTGGACAGTCCCTCTGTACTGTGAAGCCACAGATGTCGAGTCGACTAGCTTCCT
TTCTCCCTTCTCCC
+
B11////68:>BCCA@ACCC@CBC>C>A@?9111,7>>@CAAA>AAA?AC@CCCCCBBBBCEEB@@@AAB;:95:7:
CACBAC>A>A@@@<
@T6XXI:00118:00042
GGGAGAAGGGAGAAAGGAAGGAGAAGTCGCCCCAAACGATTTGTTGACTATTCTCCCTCTCCCTATGTGAGTCGTAT
TAGAATTCCCC
+
CHA:<:@CCBAABD@C@C@B@BBB@B?BBBBB8>?=@CCCE;@?@C?=@BA>AAAB<@@@@A=<<<:??<AA<??A@
>AAA99/333+
@T6XXI:00118:00047
GGGAGAAGGGAGAAAGGAAGCTAGTCGACTCGACATCTGTGGCTTCACAGTACAGAGGGACTGTCCAAAGCCGCAGA
TGTCGAGTCGACTAGCTTCCTTTCTCCTTCTCCCATCCACCGACTCGCCATAGAGTACAGGCTTACGGAACGTCTGA
AGC
+
DC>A9959A:@111-/,/,/4...6,,,,,,,,64567?>>29;57<<=@B@>>;444-
44:::=<?@;@A>??@?78888;;<9;>=99;;4/,/,//,///,.,/.4626;978;2//6//..49?A@?@////
746878//,.433355@6565
@T6XXI:00119:00017
GGGAGAAGGGAGAAAGGAAGCTAGTCGACTCGACATCTGTGGCTTCACAGTACAGAGGGACTGTCCAAAGCCGCAGA
TCTCGAGTCGACTAG
+
<<14CA59919999094959880.60....44076777?AA>AB?ACC@A?AA@>777,7;;;??@AA<@@699@@>
>;<<<<<=>>><<<?
@T6XXI:00119:00038
GGGGAATTCTAATACGACTCACTATAGGAGAGGGAGAATAGACCTGAATTGGACACAGACTATTCCTCCTTCCTTTC
TCCCTTCTCCC
+
```

```
/11+1=CCECB@???BBBBBB7664..,0>>AD>CCC@D:::<;<@?>>;>>>B>CBBBBB==>=9=9<A>A>@@;@
@@B7;4;430,
@T6XXI:00119:00041
GGGAGAAGGGAGAAAGGAAGGAGAAGTCGCCCCAAACGATTTGTTGACTATTCTCCCTCTCCCTATAGTGAGTCGTA
TTAGAATTCCCCAATGAA
+
<<6<BB@BC><<<B<A>B?B?BAA?BFCCCCD9CE>ECAAC<BA?::;?BB384?>7:=<;;6<=<<<A@><<<?<<
<<>>844449909,++.)
@T6XXI:00119:00044
GGGAGAAGGGAGAAAGGAAGTGCAGTTCGCGGTTAGTGAAGGGAAAAACTATTCTCCCTCTCCCTATAGTGAGTCGT
ATTAGAACTTCCCC
+
DD?CCB?>><@@@@<@@C@CCCBBBBEAABC@A@@@??B>>?>ACCC2BBCC@>;776000::6:;;?@@AAA@?;;
<<8;>A;???@@>4
@T6XXI:00119:00045
GGGAGAAGGGAGAAAGGAAGGAGAAGTCGCCCCAAACGATTTGTTGACTATTCTCCCTCTCCCTATAGTGAGTCGTA
TTAGAATTCCCC
+
CDBCCE?BB=BB??=A=?>@@BBB@BAB;;775?@<ADCC>;>B@>99?B;:;;==;88>==;<<?<<<<<===B;;
?>@AA>A<>66.
@T6XXI:00119:00047
GGGAGAAGGGAGAAAGGAAGCTAGTCACTCGACATCTGTGGCCTCACAGTACAGAGGGACTGTCCAAAGCCGCAGAT
CTCGAGTCGACTAG
+
CD?A==;=@<@B@B=C7;8;@>>>>7777===<?88????<?=@@CCABACC?;;;;68;9====@A<@@>A<<<78
7;;<=>@A@@@AA@
@T6XXI:00120:00014
GGGAGAAGGGAGAAAGGAAGGAGAAGTCGCCCCAAACGATTTGTTGACTATTCTCCCTC
+
44-449>CD?BBBC@D>B9;4839136?;??B573,33116117>?87674-..43332
@T6XXI:00120:00016
GGGGAATTCTAATACGACTCACTATAGGAGAGGGAGAATAGTTTTTCCCTTCACTAACCGCGAACTGCACTTCCTTT
C
+
ACC6ACD?BBC@BBBBBBCBCCBB:::57@@BC>CCC@A?@ACAB,43,434==>><@>::;B??@A?AAC@B?CB=
?
@T6XXI:00120:00021
GGGGAATTCTAATACGACTCACTATAGGGAGAGGAGAATAGTTT
+
944,4242499;=@@BBBBCC;:<@???677610111001111+
@T6XXI:00120:00022
GGGAGAAGGGAGAAAGGAAGTGCAGTTCGCGGTTAGTGAAGGGAAAACTATTCTCCCTCTCCCTATAGTGAGTCGTA
TTAGAATTCCCC
+
CC@CCC?BA=ABBB>C@B@BBBBBBBE???BAD@>@@CC>998???5@CCC@BCBC>AAABB>B@<;?8====<<<?
?><A@;?=?=?4
@T6XXI:00120:00035
GGGGAATTCTAATACGACTCACTATAGGAGAGGGAGAATAGCCGTTATTTTAACTCCCGCGGCCGGTTCTTCCTTTC
TCCCTTCTCCC
+
DCC9E@A>@CC?BBBBBBBBA:999//-
0==CC>BBCAA<999=C>AAAA5@>@A@<;=@<;<>A9=;=@=?>AA<@BBA<A=ADBC:
@T6XXI:00120:00048
GGGAGAAGGGAGAAAGGAAGCTAGTCGACTCGACATCTGTGGCTTCACAGTACAGAGGGACTGTCCAAAGCCGCAGA
TCTCGA
+
```

```
CC=A@928809>>>9>>B=@@?AAB:::@@??:::???B@A<?@?BCCCCC@@;;300,3;9>@A>AA<AA=@@AAA
@?@@@@
@T6XXI:00121:00021
GGGAGAAGGGAGAAAGGAAGCTAGTCACTCGACATCTGTGGCTTCACAGTACAGAGGGACTGTCCAAAGCCGCAGAT
CTACGAGTCGACTAG
+
B:=AA@<@B=;9;B9;8<@CCBBBD???BBB:::::7>>>5:;=?@@BBBCBB9300,399BAA>@A;AB>A@?=85
,,,,00+33886463
@T6XXI:00121:00024
CTAGTCACTCGAGATCTGCGGCTTTGGACAGTCCCTCTGTACTGGAAGCCACAGATGTCGAGTCGACTAGCTTCCTT
TCTCCCTTCTCCC
+
>:///1/44BBBBABAAAAA<<BB>B?AABBBBB>BBCBBBBCB@C@CC?@???AAAA<<=:<;;;;===<A?B?BC
>C?661024:::6
@T6XXI:00121:00029
ATCTGCGGCTTTGGACAGTCCCTCTGTACTGTGAAGCCACAGATGTCGAGTCGACTAGCCTTCCTTCCTCC
+
CCCBBHD@BBB?B?AABA??@:?AABCCCC@CCA;<?>BCACC@;@@==<=>??BB>;764,++,55+,,+
@T6XXI:00121:00035
GGGGAATTCTAATACGACTCACTATAGGAGAGGGAGAATAGCAAACGTTTCCCCGACGTGGCTTTCTCCTTCCTTTC
TCCCTTCTCCC
+
?AA9A?C@CCB@CBBBAA?B>64;6//,0<:==<?AB@A=999B@ACBB.777.7?@@@@@::7.77737??444-
4:994;9<<?A<
@T6XXI:00121:00046
GGGAGAAGGGAGAAAGGAAGGAGAAGTCGCCCCAAACGATTTGTTGACTATTCTCCCTCTCCCTATAGTGAGTCGTA
TTAGAATTCCCC
+
CC@BBB@BB>AAAC@C?A?B?ACC@BCCCBBB7BD>BB<;B>?@B?<<?C=<=;<=;<<?<<<?@A@@???CA??==
<?A<:79/444-
@T6XXI:00122:00007
GGGAGAAGGGAGAAAGGAAGGAGAAGTCGCCCCAAACGATTTGTTGACTATTCTCCCTCTCCCTATAGTGAGTCGTA
TTAGAATTCCCCC
+
DD>CCH=@@9>944-;<A>BAECC@@=A7777-
87/3997A=BBACCCCBA>AA@@<????CA?===???????????>A<;792////+
@T6XXI:00122:00010
GGGAGAAGGGAGAAAGGAAGCTAGTCACTCGACATCTGTGGCTTCACAGTACAGAGGGACTGTCCAAAGCCGCAGAT
CTCGAGTCGACTAG
+
CC?B@;244-
9@>>9A:;:<CCBBC7;:?=?779966?BC?AD?BCA??>?@@=<==;<>====<<?;?A>?@?A@A@@??;:=<<<
<<:<
@T6XXI:00122:00025
GGGGAATTCTAATACGACTCACTATAGGGAGAGGGAGAATAGTCAACAAAAATCGTTTGGGGCGACTTCTCCTTCCT
TTCTCCCTTCTCCC
+
BAA7A>A>BCC@CCBAAA??>C>99111.7>>>@=BDJBA:==AA?ABBAA1CAA<989996AACCDCBBC??<?>@
@<?@@C=A<>>@B=
@T6XXI:00122:00028
GGGAGAAGGGAGAAAGGAAGTGCGGTTACGCGGTTAGTGAAGGGAAAACTATTCTCCCTCTC
+
99-44>5990949?<@@C@<:;;;:6111;:;;:8><733167,111,337713111,1111
@T6XXI:00122:00042
GGGAGAAGGGAGAAAGGAAGAAGAACGTGCCTAGGAGAGCTGGGATGGCCATTCTCCCTCTCCCTATAGTGAGTCGT
ATTAGAATTCC
+
```

```
>D@CEC@CB9=;4809695;C?BB@CCGA@<@995999899@@6@992424?9B@BB?>@999-
44>>88;>AAA;::A7;177:9?>
@T6XXI:00123:00009
GGGAGAAGGGAGAAAGGAAGGACAAATAGATTGCGGGGGAAACGCTAGCTATTCTCCCTCTCCCTATAGTGAGTCGT
ATTAGAATTCCCC
+
CD@BCB@BB>BBCC=B?@?B@B>??=@C?9?:;76666+8F>CCBAAABBB?>????;?====;===<<<==AAAAA
@@>@AA?A>@@@5
@T6XXI:00123:00014
GGGGAATTCTAATACGACTCACTATAGGGAGAGGGAGAATAGTTTTCCCTTCACTTAACCGCGAACTGCACTTCCTT
TCTCCCTTCTCCC
+
CDD:D@C?BBC@BA@@BB@@@BB???==6====?:?7767<;=CD6AA:=79=7747>B@CCBC?@@A@BBB?C?BB
=??BA<@>:88@:
@T6XXI:00123:00027
GGGGAATTCTAATACGACTCACTATAGGGAGAGGGAGAATAGACACCAATCGTGCCGGGTCATGTTCTCCTTCCTTT
CTCCCTTCTCCC
+
999/9<?<@@@>BCBBBCBBBAC>>6665:AACC>CBB@>8899AA?A?@<?>>98??8?AABJC?AAH@A:=:=@?
ADDD<A>===A;
@T6XXI:00123:00038
CTAGTCGACTCGAGATCTGCGGCTTTGGACAGTCCCTCTGTACTGTGAAGCCACAGATGTCGAGTCGACTAGGGCAT
CCAGCGTGGCGCTTCCTTTCTCCCTTCTCCC
+
B;;9A<49;4:@A@@@GBB@A?AAA=@799>>>BA;ABC????B??AB53998448333333179@?=>>88807>9
959@@@9;67;76;;;;?8:76B;A=;:8C?
@T6XXI:00124:00006
TCTGCGGCTTTGGACAGTCCCTCTGTACTGTGAAGCCACAGATGTCGAGTCGACTAGCTTCCTTTCT
+
5444498@8=6=6999994=:>:979311....,.6,/4988;>?>DB@=B??43373101011,1.
@T6XXI:00124:00023
GGGAGAAGGGAGAAAGGAAGGAGAAAAAGTCATGTCAGATCTGCAGACTATTCTCCCTCTCCCTATAGTGAGTCGTA
TTAGAATTCCCC
+
DCBBBB@BB?BBCB>B>A>BAB?<>>>2@CBBBBBBA>>@CCCCB@>A?@@>@?::8?@@AA<AAA@99A?@@????
?<>AA>?<?CC6
@T6XXI:00124:00024
GATCTGCGGCTTTGGACAGTCCCTCTGTACTGTGAAGCCACAGATGTCGAGTCGACTAGCTTCCTTTCTCCCTTCTC
CC
+
EEDCBABB?DCC@C@C>BACCC>???BB?@A@B>>;<?=?CCAA@??@??B@BBBBBBB@@=?=?=9=88818;==?
A;
@T6XXI:00124:00029
GGGGAATTCTAATACGACTCACTATAGGAGAGGGAGAATAGCAAACGTTTCCCCGACGTCGCTTCTCCTTCCTTTCT
CCC
+
444,489?BCC@DCA@@<;1:78-
,,,*,2279;?A@><D999@:;===.777,17711111101110101646345222
@T6XXI:00125:00010
GGGAGAAGGGAGAAAGGAAGCTAGTCGACTCGAGATCTGCGGCTTTGGACAGTCCCTCTGTACTGTGAAGCCACAGA
TGTCGAGTCGACTAGCTTCCTTTCTCCCTTCTCCC
+
DD>C<<;9B:@111-
/,1,/0.;6=000;=::@>::98?BB;??B<A>ACCCBCC>CB??@???@@@B?AA?AAAA@??@@@@@A??ABBAA
@?6847;9;444-47433=;
@T6XXI:00125:00017
GGGAGAAGGGAGAAAGGAAGCGTAGTCGACTCGACATCTGGTGGCTTCACAGTACAGAGGACTGTCCAAAGCC
```

```
+
44-4998995444;585:56.....4688:9863...3..,.44448886:6776=0335540003/33,082
@T6XXI:00125:00023
GGGAGAAGGGAGAAAGGAAGACAGTCCATCTGTGGCTTCACAGTACAGAGGGACTGTCCAAAGCCGCAGATCTCGAG
TCGACTAG
+
HH?BAC@CD>BBBB@B@C?BB:;;:;7;AAAAAC?CC@CBBBCACC@@@AB@A@<<882440343344377>;;340
084;<BA?
@T6XXI:00125:00031
GGGAGAAGGGAGAAAGGAAGCTAGTCACTCGACATCTGTGGCTTCACAGTACAGAGGGACTGTCCAAAGCCGCAGAT
CTCGAGTCGACTAGCTTCCTTTCTCCCTTCTCCC
+
DEACCC?BB:;844-9595;BBBBB3000/..33377?;;:?@=?@?:::@>=:444-
4447<?>@A<@C>@@????999DA===AA99:A??;?:<<7<?==:@689>C?
@T6XXI:00125:00040
GGGAGAAGGGAGAAAGGAAGGGGAGAAGGGAGAAAGGAAGCTAGTCACTCGAGATCTGCGGCTTTGGACAGTCCCTC
TGATACTGTCGAAGCCACAGATGTCGAGTCGACTAGCTTCCTTTCTCCCTT
+
DDCAAA@CC@<=<D=B>B?BCC9A@A@CB8<000,0,0,.:345BAAAAA??999AC???>AAA7<9888=;;;49;
;44434333400/00/-221...1::9;::=@@>@=4424244-4///+.3
@T6XXI:00126:00005
GGGAGAAGGGAGAAAGGAAGGAGAAGTCGCCCCAAACGATTTGTTGACGTAT
+
DDBECC?BA:>9990959642377779>7777,33,37777883,.....00
@T6XXI:00126:00014
GGGAGAAGGGAGAAAGGAAGAATG
+
DD?BAA@AA=@@:;;880)**)**
@T6XXI:00126:00018
GGGAGAAGGGAGAAAGGAAGGAGAAGTCGCCCCAAACGATTTGTTGACTATTCTCCCTCTCCCTATAGTGAGTCGTA
TTAGAATTCCCC
+
CC?CCE@CD>B@?><@=@=ACDCC@CBA>;;;5AA<ACC@?;>>;>>>>>;:====:<<<<=;==<<=<==A=AA88
9>AA@>@8<;;0
@T6XXI:00126:00019
GGGGAATTCTAATACGACTCACTATAGGGAGAGGGAGAATAGATCACCGTTGTTCGGGTGCGCGTTCTCCTTCCTTT
CTCCCTTCTCCC
+
999/48=8=BA@CBBBCEBB;:397611,116668:?>944-/--
/,*,,+,,*,8856979;9776/.44,.,.524.--+04335<1
@T6XXI:00126:00021
TCTGCGGCTTTGGACAGTCCCTCTGTACTGTGAAGCCACAGATGTCGAGTCGACTAGCTTCCTTTCTCCCTTCTCCC
CTTCCTTTCTCCCTTCTCCC
+
BDCABB?BBB?BDCCCBBBB=>BBBBBB?@CCB?BC@CCBBB?AB>@@?@BBBEDCBBB?CACD=ABBC>B>@?<<;
1;:>;?@489AC?D899@?9
@T6XXI:00126:00034
GGGAGAAGGGAGAAAGGAAGAAAAGGTGTGTATTGTGGAGCTAGGGGCTATTCTCCCTCTCCCTATAGTGAGTCGTA
TTAGAATTCCCC
+
CG@CBB@BC>BBBB>BAB??>>>7>@BBC9A@C@@BA>DBBBBBBB9BABA>?BCC>C=@AA;@?A=A=AA?@@@?@
A;=AC?D38770
@T6XXI:00126:00037
GGGGAATTCTAATACGACTCACTATAGGGAGAGGGAGAATAGTCAACAAATCGTTTGGGGCGACTTCTCCTTCCTTT
CTCCCTTCTCCC
+
```

```
A=94>6;8<CCACCBBBBBB?@>9?111.777BD@CBA?@??A==<<<<;A@==989995999:B?AAB@@>@=AA:
@@@A>C<??@@;
@T6XXI:00126:00045
GGGAGAAGG
+
11-,,,*,*
@T6XXI:00127:00010
GGGAGAAGGGAGAAAGGAAGCTAGTCACTCGACATCTGTGGCTTCACAGTACAGAGGGACTGTCCAAAGCCGCAGAT
CTCGAGTCGACTAG
+
AB>BB@?AA<A@==:@=@>BCCCCC?AABA>:::::6A>@<>A@CCCCAABC>934417??BA?=?A:AA?AA;;;;
;;:=<<<;@@A=9=
@T6XXI:00127:00021
GGGAGAAGGGAGAAAGGAAGGAGACAGTACCGCCCAAAATTGGAAGACTATTCTCCCTCTCCCTATAGTGAGTCGTA
ATTAGAA
+
DD>BCC>?=6;<@C>CAC@C=@;:<>9;;B>AAB>CCC9B@A=@=@BCCBB>BBAA?A@@?B>???::::7198./-
,-,-222
@T6XXI:00127:00030
CTAGTCACTCGAGATCTGCGGCTTTGGACAGTCCCTCTGTACTGTGAAGCCACAGATGTCGAGTCGACTAGCTTCCT
TTCCTCCCTTCTCCC
+
C=1/111:?FCCCBCBAABE@BBB>B@BA??677-
7????@ABB@A><?B>BBFBB>>0/0>=;@BB??;;6675555,/,/00,/7:;;=;
@T6XXI:00127:00032
GGGGAATTCTAATACGACTCACTATAGGGAGAGGGAGAATAGATCACCGTTGGTTCGGGTGCGCGTTCTCCTTCCTT
TCTCCCTTCTCCC
+
?=96@>A>BBB@C???BA???AB?<111.7>>@CBCBC@A=999=AB=AC?A>A?B@B=B;;:CCD@CABABAB?BC
<A?<=9=>AAAA<
@T6XXI:00127:00045
GGGAGAAGGGAGAAAGGAAGCTAGTCACTCGACATCTGTGGCTTCACAGTACAGAGGGACTGTCCAAAGCCGCAGAT
CTCGAGTCGACTAG
+
CCCCDB@BB=@@A?5?784;A@@ABAAAFA@::::::CCC?AB@BCCBBBBB?=44448@BCCC<>?;@A?@@<===
<<<===;;:==<<<
@T6XXI:00128:00005
GGGGAATTCTAATACGACTCAACTATAGGGAG
+
080*,*/78881988>>1..0,.6600320..
@T6XXI:00128:00027
GGGAGAAGGGAGAAAGGAAGGGGAGAAGGGAGAAAGGAAAGCTAGTCACTCGAC
+
CJBCBA@BC>??BB>C?BDBCC8<@;:>?8=777.1017,1779777611...-
@T6XXI:00128:00029
GGGAGAAGGGAGAAAGGAAGCTAGTCGACTCGACATCTGTGGCTTCACAGTACAGAGGGACTGTCCAAAGCCGCAGA
TCTC
+
KH@CCC==95>98809B@9<;CCDBBBB@ACC88:BB???B?BB?AA????<==:444-
4:444;28417<268@?=?@@<
@T6XXI:00128:00033
GGGAGAAGGGAGAAAGGAAGCTAGTCGACTCGACATCTGTGGCTTCACAGTACAGAGGACTGTCCAAAGCCGCAGAT
CTCGAGT
+
GD@BBA59909>??:>;>24388>@@=??:0011000.547,79???AB??=<0///,/.2726555355558==A@
888887A
@T6XXI:00128:00038
```

```
GAGGTAATTCTAATAGACTCACGATAGGAG
+
@11,//,/7//:;==>><7--,,,,//,..
@T6XXI:00128:00039
GGGAGAAGGGAGAAAGGAAGCTAGTCACTCGACATCTGTGGCTTCACAGTACAGAGGGACTGTCCAAAGCCGCAGAT
CTCGAGTCGACTAG
+
EECAAA==95>988095949A???A?6669???<::A@@A?AB?BCCCCCBA??333-
499=AA@AB:BB=A?;?<@AAA???@AAAABA@
@T6XXI:00128:00042
GGGAGAAGGGAGAAAGGAAGCTAGTCACTCGACATCTGTGGCTTCACAGTACAGAGGGACTGTCCAAAGCCGCAGAT
CTCGAGTCGACTAGCTTCCTTTCTCCCTTCTCCC
+
A>:>9969>9>899144829<;BBB@:7116616<<<>?A6:;=?@@ACAAAA??@@;@AA???@AC<AA>@878==
888;<=<?6;<<====3837=:=>@@:@>::9D?
@T6XXI:00128:00049
GGGAGAAGGGAGAAAGGAAGGAGAAGTCGCCCCAAACGATTTGTTGACTATTCTCCCTCTCCCTATAGTGAGTCGTA
TTCAGAATTCCCC
+
CDACAA@BC?BBBB>B?B?B?A?A?B?A7777-
8>8@ABBB>A@=A:7:?8273==;384?:3<>?A??@@<;:44404333449/444+
@T6XXI:00129:00004
GGGAGAAGGGAGAAAGGAAGGAGAAGTCGCCCCAAACGATTTGTTGACTATTCTCCCTCTCCCTATAGTGAGTCGTA
TTAGAATTCCCC
+
CD@CCC>BB=?>==:@8:8<@CBB?CCB?@AB8BC?CCCCC>CB?BBCBB@>?@<=;===?A<???@AAAAB==A==
<;?=;8:2444-
@T6XXI:00129:00005
GGGGAATTCTAATACGACTCACTATAGGAGAGGGAGAATAGTCAACAAATCGTTTGGGGCGACTTCTCCTTCCTTTC
TCCCTTCTCCC
+
944,4999=BBACCCCECC@@;;;:/0,000???B:0,-8-.--,.4;479661,1775BCCCC>A<<8424/44-
300424882233
@T6XXI:00129:00012
GGGAGAAGGGAGAAAGGAAGCTAGTCGACTCGACATCTGTGGCTTCACAGTACAGAGGGACTGTCCAAAGCCGCAGA
TCTCGAGTCCGACTAG
+
CC@BBB@BA:@888:@>@?BBB@A@:;;C???;;:?@@BBC@BB?BCBB@>@;74003,7>;>@A<??<?C>@@?88
8898AA@@@>AAA@??
@T6XXI:00129:00017
GGGAGAAGGGAGAAAGGAAGGAGAAGTCGCCCCAAACGATTTATTGACTATTCTCCCTCTCCCTATAGTGAGTCGTA
TTAGAATTCCCC
+
DDACBB@BC=ABCC@C@C@C@BBA@B@CCCCB7@@<@CCCC:;;BDCCBBB?;@A=;=@@@=<A599AA@@AAAA??
?>@DA@A3444,
@T6XXI:00129:00035
GGGGAATTCTAATACGACTCACTATAGGGAGAGGGA
+
?@C:CACCCCCDCBAAA8888;73..34+..42333
@T6XXI:00129:00039
GGGAGAAGGGAGAAAGGAAGGAGAAGTCGCCCCAAACGATTTGTTGACTATTCTCCCTCTCCCTATAGTGAGTCGTA
TTAGAATTCCCC
+
DD?BBB@CD?BBCB=B?B@B@BA??B?A3333,8><ACDCC<BB?CC@BBA>A@@<;?@@AA;===<<<A=A<<?:;
@?A@@=?3444+
@T6XXI:00129:00049
```

```
GGGAGAAGGGAGAAAGGAAGCACACCAGTTTTAGGGATATCGACTGGCTATTCTCCCTCTCCCTATAGTGAGTCGTA
TTAGAATTCCCC
+
BB@A>A@AA===?B?A@A@A?<@@?A@@@AA8@A@;?B?@@CAB@@=ABDD@CDCC<;;98:08ABBBBBBBBADBB
D@BCB?C@CGC:
@T6XXI:00130:00025
GGGAGAGGAGAAGAGAGCAAAAGGAGCGAACTGAACGAGTCGCTA
+
94-9///,///,//4334==:2994443424449244:../////
@T6XXI:00130:00029
GGGGAATTCTAATACGACTCACTATAGGGAGAGGGAGAATAGTCAACAAATCGTTTGGGGCGACTTCTCCTTCCTTT
CTCCCTTCTCCC
+
???2>9A?BBC@CBBBB@>>??CCC88877<<AC>BAA@A===>88>C=;CA<??6;;;2<???C@?@@?@>A>AA<
@@?@7<9=@AA<
@T6XXI:00130:00030
GGGAGAAGGGAGAAAGGAAGTGCAGTTCGCGGTTAGTGAAGGGAAAAACTATTCTCCCTCTCCCTATAGTGAGTCGT
ATTAGAATTCCCC
+
996<CA@CC@AAAB=B@C@C@@ABCC@CBBB@C@AAA;;;??9?ABC2BBCC@CBBB>B@BBB>B@A@@?<;<>???
==;=@A?A>@@?5
@T6XXI:00130:00038
GGGGAATTCTAATACGACTCACTATAGGAGAGGGAGAATAGCCACCAACTATTGTTCGTCGCCGCTCTCTTCCTTTC
TCCCTTCTCCC
+
B@=6=<BABCCACDCCA@@BB:696./-
0>>BC>CCC@A88:<>=;=;?@??<?A>??@:<<8;>@?===?B<@@;=<=A<@=A8771
@T6XXI:00130:00039
GGGGAATTCTAATACGACTCACTATAGGGAGAGGGAGAATAGCTACTAAACGCCTCGCTCCACATTGATCTTCCTTT
CTCCCTTCCTC
+
999299A@ACCB@AFEA@BCB>>>8777.7BBCDAC;;88==:>@@???9?@>=@?998A?@=>=>@@???<@<??<
?CCD;@9<8<<
@T6XXI:00130:00043
GGGAGAAGGGAGAAAGGAAGGATATGCCGCAGTGGGAGATTAAACGGCTATTCTCCCTCTCCCTATAGTGAGTCGTA
TTAGAACTTCCCC
+
CD>CCC?BB>BBCC>C@B@C>AA@A::9;???BBB>CBAC@CC>B?>BCDE@CCCC>@@@??:?@>9AA???B><<6
3/04426;0166,
@T6XXI:00130:00049
GGGAGAAGGGAGAAAGGAAGGAGACAGTGAGCGCCGCACAACACGTCACTATTCTCCCTCTCCCTATAGTGAGTCGT
ATTAGAATTCCCC
+
FDABBB@BB?>>>A?A@B@B?>@@CCCCCBAA??;<<??>>>>>;??@@BCB@B9995:A@AA>BCCCCC@@@CBBB
AA?AA@;@499;4
@T6XXI:00131:00010
GGGGAATTCTAATACGACTCACTATAGGGAGAGGGAGAATAGGTGCAAAACCTCTCGACGATACCTGTTCTTCCTTT
CTCCGTTC
+
994,42499AA?BAA@BBABCBBB>;:7-
7<777.71774=>344:CCC9C@E;::A????:6689969<<998542+++*++0*
@T6XXI:00131:00018
GGGGAATTCTAATACGACTCACTATAGGGAGAGGAGATAGTAGACTA
+
BBB9;;A@AAC>BAA@CCAAACA:;;@@9=?:<::,,+++++--124
@T6XXI:00132:00009
```

```
GGGAGAAGGGAGAAAGGAAGCTAGTCACTCGACATCTGTGGCTTCACAGTACAGAGGGACTGTCCAAAGCCGCAGAT
CTCGAGTCGACTAGCTTCCTTTCTCCCTTCTCCC
+
B;:C9<;;A@@???095424<77.27,,,,,,,,03869936>;;::BBBB@@=??C<<=<<;;8<;6<A;@?AAA@
?>??@<<<@@===AAA=?<=>-44::-424=>?<
@T6XXI:00132:00020
GGGGAATTCTAATACGACTCACTATAGGGAGAGGAGAATAG
+
944-969:<CB?BBAABBBBBAB>>111-1<6737773//,
@T6XXI:00132:00021
GGGGAATTCTAATACGACTCACTATAGGGAGAGGGAGAATAGTTCTGACGGATTGCTCTGACTATTCACCTTCCTTT
CTCCCTTCTCCC
+
BBA7A>B?BCC@CB==BBBBBBB99111-
6<<AC>BBB>@9AA>A@@ABB?AC>CBA=??BB?::8:A>:<36366.7666,700000+
@T6XXI:00132:00045
TGGAGAAGGGAGAAAGGAAGCTAGTCACTCGACATCTGTGGCTTCACAGTACAGAGGGACTGTCCAAAGCCGCAGAT
CTCGAGTCGACTAGCTTCCTTTCTCCCTTCTCCC
+
@@9B9959@:@/11,/,/,/..997=861111169<A@@C;=@<??=??AB@AB;;;184>=@@>AC<AA@?@@@@>
><<<<<@@888<>>=?>@>@A<AAAC;@588==:
@T6XXI:00132:00046
GGGAGAAGGGAGAAAGGAAGCTAGTCACTCGACATCTGTGGCTTCACAGTACAGAGGGACTGTCCAAAGCCGCAGAT
CTCGAGTCGACTAG
+
DBBCC@644-
999909596;C@?@C?;::B@:::::9@BD?CCBCCCAAACC?644417==@@@?AA<@A>AA=<<<<<<<<?A@??
?@A@
@T6XXI:00133:00020
GGGAGAAGGGAGAAAGGAAGCTCAATCGTGGGTAGAAGGGTCGTGATCTATTCTCCCTCTCCCTATAGTGAGTCGTA
TTAGAATTCCCC
+
DDAFCC@CC>?@?C@B@B?B?B?B>@>;;AC?@8:7;<A<AAAAA===?@@;:;;=;==?<<;@=B@<<<@@@AA??
?>A@A>@>@AA5
@T6XXI:00133:00025
GGGGAATTCTAATACGACTCACTATAGGAGAGGGAGAATAGTTGTACCGCTTCCGCTATATGCTTTTCTTCCTTTCT
CCCTTCTCCC
+
CCC7B7;8<BB@DCBB@???B:999//-
0==AB>BCC@A<<<:;;<<;===>A><==@@99:ABAD5@@8:9<<7<>>?7>:<;;;5
@T6XXI:00133:00026
GGGGAATTCTAATACGACTCACTATAGGTCTGAGCACGATCG
+
///*/,/,/777:BCCAA@BB@C?877311133116111144
@T6XXI:00133:00047
GGGAGAAGGGAGAAAGGAAGCTAGTCTCGACATCTGTGGCTTCACAGTACAGAGGGACTGTCCAAAGCCGCAGATCT
CGAGTCGACTAG
+
CH>CB=;==5>>>><@ACDCBBCBC>?A?@;;;@@AGC@CBBCBBACCCCBA99409>>>>>49919@<A@@@AAAB
B@AFCCCCABCC
@T6XXI:00133:00048
GGGAGAAGGGAGAAAGGAAGAAGAGGCAGGGGGCATGTAGTGGCAGTCTATTCTCCCTCTCCCTATAGTGAGTCGTA
TTAGAATTCCCC
+
F@199>=@@<949@1959<>><A@><@88888+7<>BBCCCC@BCC@??;;3434403;<==:<;<?=/////.1-
00244=>=;9;=6
@T6XXI:00134:00031
```

```
GGGGAATTCTAATACGACTCACTATAGGGAGAGGGAGAATAGTCAACAAATCGTTTGGGGCGACTTCTCCTTCCTTT
CTCCCTTCTCCC
+
<<<4<@CBCAB@BBBB@?ACCCB>>111.7<<>A>AAACAAA@@@?@AA=CB@@B>B>@7BCCBB????:;8;=AA<
@@@A=AAAA??<
@T6XXI:00134:00045
GGGAGAAGGGAGAAAGGAAGTTGTGTGCCATGTCGTTCCCAGGGAGACTATTCTCCCTCTCCCTATAGTGAGTCGTA
TTAGAATTCCC
+
DB?A?@ACD=BBAA=B@C@CC?AAABAA<?AAB=@=>AA?AAA<@@@@@AA?AAAA<<=@@?<?AA@88====8==@
@>ADB=@<?C:
@T6XXI:00134:00048
GGGGAATTCTAATACGACTCACTATAGGAGAGGGAGAATAGCCTGGCCTTTTTTGACCGGTTATTACTCCTTCCTTT
CTCCCTTCTCCC
+
CCC9C?A?BCC@CCBBBBBBB:994./-0>>BL>FCE@B9=9;@@@?>ABBBB-
BBCAC?B?CDDAABC@C?B@@C=888=;=<;@<=;
@T6XXI:00135:00008
GGGAGAAGGGAGAAAGGAAGGATAAACAGCAGTCCCAAATGGCTTGGCTATTCTCCCTCTCCCTATAGTGAGTCGTT
ATTAGAACT
+
EC>AAA@CCAC;;;7<>@<@;?8;7,4A>>>@99909>9>>98;<@@CBBB?BAAC<@A???<?BB??;86,,,++*
-100032..
@T6XXI:00135:00014
GGGAGAAGGGAGAAAGGAAGGAGAAGTCGCCCCAAACGATTTATTGACTATTCTCCCTCTCCCTATAGTGAGTCGTA
TTAGAT
+
@B?BBB@BBBCCCB>B@@@BABBB@B7;3333,8?:@BBCE>AA=ACCCC?>@<<<;<<@@A=????@@AA@@?A>?
?><<<@
@T6XXI:00135:00015
GGGGAATTCTAATACGACTCACTATAGGGAGAGGGAGAATAGTCAACAAATCGTTTGGGGCGACTTCTCCTTCCTTT
CTCGCTTCTCCC
+
9<9/<?C@CBBABAA@@;;:GDCCC???87:6?B>AAA?@===@@>@@C;CCBBB<>==6>BBBC@CCC??=@>@A<
@@@<<?@CCDC>
@T6XXI:00135:00025
GGGAGAAGGGAGAAAGGAAGCTAGTCACTCGAGATCTGCGGCTTTGGACAGTCCCTCTGTACTGCGAAGCCACAGAT
GTCGAGTCGACTAGCTTCCTTTCTCCCTTCTCCC
+
DC>CBA=>@0899904242433337>61189?8:@CCCCC@AAA<A?BA>?@@@>@B@@@@??===?:<<=@@CAA@
===AA?@?<<<<<<<<;<;<=;@@?@1738=AC?
@T6XXI:00135:00028
CTAGTCACTCGAGATCTGCGGCTTTGGACAGTCCCTCTGTACTGTGAAGCCACAGATGTCGAGTCGACTAGCTTCCT
TTCTCCCTTCTCCC
+
C8//1:14:CBBEBDBBBCB@BBB?B??;9>9994:BCCAAAC<AAA<?B@CCCBB??B>AADBBBBBBDBAB=@=?
C@FBDC>A:=<@C?
@T6XXI:00135:00029
GGGAGAAGGGAGAAAGGAAGGGAATAGCAGCAGTCGGCAACGTGAAACTATTCTCCCTCTCCTATAGTGAGTCGTAT
TAGAATTCCCC
+
CD@@@>ACC?BAAB898:@CC>B?BCAB?@AACCBB>;>>>???>?;BB@A??@@>9BA??=?@A@@A==AA<@=??
<?>A9;/444-
@T6XXI:00136:00016
GGGGAATTCTAATACGACTCACTATAGGGAGAGGGAGAATAGTCAACAACTCGTTTGGGGCGACTTCTCCTTCCTTT
CTCCCTTCTCCC
+
```

```
999/424:9998:AACDCBBA>;97177.777:C?CCC===A@;77//,/677795777.<;<8?:??B>>=@<??7
>?@A<@;>;<<6
@T6XXI:00136:00039
GGGGAATTCTAATACGACTCACTATAGGGAGAGGGAGAATAGTCAACAAATCGTTTGGGGCGACTTCTCCTTCCTTT
CTCCCTTCTCCC
+
CCD:C@C@BBB@CAAABBBBB@B?;66659??CE>BAB>@<<<@@>@A=;AA@??8<<<6ABB@@@@BB>?<?>AC<
A@@A<@<??AA<
@T6XXI:00136:00041
GGGGAATTCTAATACGACTCACTATAGGGAGAGGGAGAATAGCCTGAGGTTGTTCTTCATTGGCTTTCTCTTCCTTT
CTCCCTTCTCCC
+
499/4:BAHBB@BCBBB@<<>@BBB6664699CE>CBB@AA@@:???@DC@>>=?=<><<;:<??A;@<@B>B><<;
<?AB<<<?@CC<
@T6XXI:00137:00009
GGGAGAAGGGAGAAAGGAAGGAGAAGTCGCCCCAAACGATTTGTTGACTATTCTCCCTCTCCCTATAGTGAGTCGTA
TTAGAATTCCCC
+
CDBCEC@CC>B@=@<@>A?B@BCC?BCCDEDD7;;8<B9@C;@?>>>>CCA>:99@;==>>@:??@@B@<<;=8478
025555556660
@T6XXI:00137:00013
GGGGAATTCTAATACGACTCACTATAGGGAGAGGGAGAATAGTCAACAAATCGTTTGGGGCGACTTCTCCTTCCTTT
CTCCCTTCTCCC
+
===4><?<@AA=@ACBBCBB?@CBB666-
1;;?B>BBB@@@@A@?87871:BBBB<???6?BCCC=AACBA>A>@@<<AAA=A>@AA@;
@T6XXI:00137:00016
GGGGAATTCTAATACGACTCACTATAGGGAGAGGGAGAATAGTCACCAAACCTCCCATGGTGCTATTACCTTCCTTT
CTCCCTTCTCCC
+
999/49?:ACC@CCCBCBBBAA@<<66959??BB>CCC?@<?AACC=AA;@>@AA<==<=???BCC@<;8;=@<@B>
CCCC>B@CBBB>
@T6XXI:00137:00018
CTCACTATAGGGAGAAGGA
+
9==CC>;=4//+/60*,*,
@T6XXI:00137:00024
GGGAGAGGGAGAAAGGAAGCTAGTCACTCGAGATCTGCGCTTTGGACAGTC
+
>959994;:>999142948B???@???<<B???A?=?.....+.,.5441.
@T6XXI:00138:00010
GGGAGAAGGGAGAAAAGGA
+
CD?>>>599194111*/,/
@T6XXI:00138:00019
CTAGTCACTCGAGATCTGCGGCTTTGGACAGTCCCTCTGTACTGTGAAGCCACAGATGTCGAGTCGACTAGCTTCCT
TTCTCCCTTCTCCC
+
C8/111/49BBBDBCBCCBB>ACC<A=@??>:::7:BBCCBCC@BB@;@C@CCC::;C@@@ECCCAA@AAAAA?B@C
B<@?==698;;=A=
@T6XXI:00138:00033
GGGAGAAGGGAGAAAGGAAGAGAACAGCCCCTGTAAATGTGTGTGAACTATTCCGTC
+
<<6<AA>AC:>99909595;AA>9>>9988,49;BD>@BBCCDCCB9;/--+*)***
@T6XXI:00138:00035
GGGAGAAGGGAGAAAGGAAGCTAGTCACTCGAGATCTGCGGCTTTGGACAGTCCCTCTGTACTGTGAAGCCACAGAT
GTCGAGTCGACTAGCCCTTCTCCCTTCTCCCCTTCCTTTCTCCCTTCTCCC
```

```
+
A?>@;;7<B>BBBA<A698;@>>>>///>>?>>>@BBBBB?BBB>B@DCABAAA<AB@@@@ACAA?B@?@?ABAA@@
@@AA@@@A899===<C;B>=:::4:7:::;;1:79/44+036606,/---+
@T6XXI:00139:00010
GGGAGAAGGGAGAAAGGAAGCTAGTCGACTCGACATCTGTGGCTTCACAGTACAGAGGGACTGTCCAAAGCCGCAGA
TCTCGAG
+
DD?ABA@CC>:<;B<B@C;<;CACBCBB@@B>AABBBBCBB?AC@C?;;??@@=6333138=AAA?AA:>><>C===
<::;===
@T6XXI:00139:00020
GGGAGAAGGGAGAAAGGAAGGAGAAGTCGCCCCAAACGATTTGTTGACTATTCTC
+
ED?B?A:9919999142949<<<B>?<=;>>>6>B<><<?;16.333//.00+--
@T6XXI:00139:00047
GAGATCTGCGGCTTTGGACAGTCCCTCTGTACTGTGAAGCCACAGATGTCGAGTCGACTAGCTTCCTTTCTCCCTTC
TCCC
+
DCBBBBBBAA>BDD@B>ABBCA@>=@IBBBCCCAAAC?B@@>@@>BBC@CBBCCCCCCCEBCC@CADC@DECE@@A@
AJCA
@T6XXI:00139:00048
GGGAGAAGGGAGAAAGGAAGTGCAGTTCGCGGTTAGTGAAGGGAAAAACTATTCTCCCTCTCCCTATAGTGAGTCGT
ATTAAGAACTTCCC
+
?B>BA@=@B:A@@@<>;@:=001=8=;CCCC=@=?@>8847=6====/>B??=@BAB>B???C<:87=>;;<<;::6
/.,/,/6,//4830
@T6XXI:00140:00004
GGGAGAAGGGAGAAAGGAAGCTAGTCGACTCGACATCTGTGGCTTCACAGTACAGAGGGACTGTCCAAAGCCGCAGA
TCTCGAGTCGACTAG
+
DDABB@694-9844-
9594;C?;<=.0000::0;6777BCC?AB@BC>999@C@=55554==A??=?@<@C>@?;<<B=@??@@A@@??A=<
@T6XXI:00140:00008
GGGGAATTCTAATACGACTCACTATAGGGAGAGGGAGAATAGTCAACAAATCGTTTGGGGCGACTTCTCCTTCCTTT
CTCCCTTCTCCC
+
999/9<@@BBC@A?A?BBBBB@B?9111.6==BC>CBB>B;====;88638==7=-166-
7@@BC@BCB??>??A=;??@=;A<??@@;
@T6XXI:00140:00014
GGGGAATTCTAATACGACTCACTATAGGGAGAGGGAGAATAGTCAACAAATCGTTTGGGGCGACTTCTCCTTCCTTT
CCTCCCTTCTCCC
+
999/4295<@A<ABBAAABBCB;;8777.:ABBD@CDC@==988;98881:BABC>BCC9DAAAC;:00,/,/,//,
/,/3301355563
@T6XXI:00140:00022
GGGGAATTCTAATACGACTCACTATAGGGAGAGGGAGAATAGTCAACAAATCGTTTGGGGCGACTTCTCCTTCCTTT
CTCCCTTCTCCC
+
DCC:C?A>?@@?BCCCC?>>BCB><661-
6=;CC>CCC@?8?A==<<?<:<?<<<;>BB9CBBBC@CBB?@>@>@=;=?AC=A9==AA<
@T6XXI:00140:00023
GGGAGAAGGGAGAAAGGAAGCTAGTCACTCGACATCTGTGGCTTCACAGTACAGAGGGACTGTCCAAAGCCGCAGAT
CTCGAGTCGACTAG
+
EC>CCB59909>@@9B8;8:<<>;B:/..5.......499<>@=@?A@@BCCBB7771899?@A?@C>AA>@AA988
AA?=>?887<=<<B
@T6XXI:00141:00009
CTATCACTCGAGATCTGCGGCTTTGGACAGTCCCTCTGTACTGACTGAAGCCAC
```

```
+
CDB?9::?BBBCBGBB@9:5:??:@@BBBB:::57>><<777771000/-2*++
@T6XXI:00141:00024
GGGGAATTCTAATACGACTCACTATAGGGAGAGGGAGAATAGTTTTTCCCTTCACTAACCGCGAACTGCACTTCCTT
TCTCCCTTCTCCC
+
?==6@@A?CDB@CA@@BBBBBC>99111.7==>@>BCC@?944333,4;:@49;CBC@C@BBBB@<==<><@@C?BC
>BAAA>A@CAAA=
@T6XXI:00141:00046
GGGGAATTCTAATACGACTCACTATAGGGAGAGGGAGAATAGTCATCGAGTATTCCTCACTGCTATTTCCTTCCTTT
CTCCCTTCTCC
+
449/48>?<<<ACBBCCCBABC?;@616-
6::?B>C::58;::;<889AA==<?A>AAA<??@@<@@;@>@=?>AA<==74330.,,,
@T6XXI:00142:00027
GGGGAATTCTAATACGACTCACTATAGGAGAGGGAGAATAGTCAACAAATCGTTGGGGCGACT
+
999/9:A><99@?@>BBBA@>78887101111>;:8<;303333;=<=;>6110111,11...
@T6XXI:00142:00035
GGGAATTCTAATACGACTCACTATAGGGAGAGGGAGAATAGTCAACAAATCGTTTGGGGCGACTTCTCCTTCCTTTC
TCCCTTCTCCC
+
CE@E@@>@CCACCBBBBBBBCB?<111.6<<CC>CBB>B;;===<5441:?===8?;?5?@BCC@CCC;988?AC>B
@@@<@>AAAA<
@T6XXI:00142:00047
GGGGAATTCTAATACGACTCACTATAGGGAGAGGGAGAATAGCCCTGAATTCATCGGCGGCAACTTCTCCTTCCTTT
CTCCCTTCTCCC
+
DDA4@<@=ACDACCBB@ABAC?B@B9::5:??CE>CCC?@===C=CCC?C@;:;BC@CA?AA9==?A=98;9;244-
/88628,/--/,
@T6XXI:00143:00004
GGGAGAAGGGAGAAAGGAAGGAGAAGTCGCCCCAAACGATTTGTTGACTATTCTCCCTCTCCCTATAGTGAGTCGTA
TTAGAATTCCCCCAA
+
DDAA@?@CB>BBBC?C@B?B@CA@?ABA3377-
8>:@CCCC?A?>BABBCA><<><;<8=<?:????@@A=AAAA888<?8843/-.//+/5
@T6XXI:00143:00006
GGGGAATTCTAATACGACTCACTATAGGGAGAGGGAGAATAGCTACTACC
+
A@B6A=@=@DCACCAAABBBBABDA<=<;:=>?@>C>7250+,,,,,,,*
@T6XXI:00143:00022
GGGAGAAGGGAGAAAGGAAGCTAGTCGACTCGACATCTGTGGCTTCACAGTACAGAGGGACTGTCCAAAGCCGCAGA
TCTCGAGTCGACTAG
+
AA<ACA?CDACBBB>B@B@BBB:::/0;0./>A769676;:7847?@BCCB@>;443-
4;;;4838<6<B>A998?==888==<>@@???A
@T6XXI:00143:00027
GGGAGAAGGGAGAAAGGAAGCTAGTCACTCGACATCTGTGGCTTCACAGTACAGAGGGACTGTCCAAAGCCGCAGAT
CTCGAGTCGACT
+
CCABBB@CC>B@@85>594;??BGDCCCECC:;;A?9B:;5:C?@?@CC@?<@:444-
4::;@@?CC:883444;873A?6;8;<4=;;
@T6XXI:00143:00038
GGGAGAAGGGAGAAAGGAAGTACGCAGAGATGCCGGCTGCCAGGGAGCTATTCTCCCTCTCCCTATAGTGAGTCGTA
TTCAGAATTCCCC
+
```

```
DHAAA@BCC@A@BC>C@C?B@BCCDCCBB>A@CBC@@AAB@A@A<@A@AAB>A;;;89AABCADEDBCBB@?>>;//
/,//88787;;;.
@T6XXI:00143:00040
GGGGAATTCTAATACGACTCACTATAGGGAGAGGGAGAATAGTCAACAAATCGTTTGGGGCGACTTCTCCTTCCTTT
CTCCCTTCTCCC
+
CAA6@@A>BCC@CB@@AA???AB>>111.7??AC>BCC@@;;===?===7?B?7=49664:@BBC?CED?=;=>AC>
C???<?=?AAA;
@T6XXI:00143:00042
GGGAGAAGGGAGAAAGGAAGGAGAAAGCGACGTCGGGAAGAAGTTTGCTATTCTCCCTCTCCCTATAGTGAGTCGTA
TTAGAACTTCCCC
+
ED><<@5991<9ABACBEBB?@@@@<A@;:881-,,*,*,,*,0000==9?59984-
44433,37766??<?@?9:7@<:;<5660111,
@T6XXI:00143:00048
GGGAGAAGGGAGAAAGGAAGCTAGTCACTCGACATCTGTGGCTTCACAGTACAGAGGGACTGTCCAAAGCCGCAGAT
CTCGGCA
+
CD>B996;@:>AA><A898;A::;B??8<9@::7661?@@7;<ACCCCCC;::A55;5;;;??@=@@;<;8878848
88//,+.
@T6XXI:00144:00013
GGGAGAAGGGAGAAAGGAAGGGGAGAAGGGAGAAAGGAAGCTAGTCACTCGACATCTGTGG
+
CGBBBB@CC>CBDB?C?A;>>>4>>>=??:8877.136:?B?CCC?869900.--22100/
@T6XXI:00144:00016
GGGGAATTCTAATACGACTCACTATAGGAGAGGGAGAATAGATTGGCGAGAAATGCCGTCTAGTGCTCCTTCCTTTC
TCCCTTCTCCC
+
677*/,4849499@@AAB@B@@??>8747<:777777.0/52444/001111,1634:<:494-----,-,-
,3=144==::::644+
@T6XXI:00144:00031
GGGAGAAGGGAGAAAGGAAGAGAATGCTAGAGGGGCGGACCATCAGGCTATTCTCCCTCTCCCTATAGTGAGTCGTA
TTAGAATTCCCC
+
CC?CCC@CC>BBCB>B@B@BB>@<@B@@AECGDC9BB=@@ACGCAA@CBBC@BCCC>B>BCC=BABB@>AA?@@88:
@?@@A>=<=AA5
@T6XXI:00144:00035
GGGAGAAGGGAGAAAGGAAGTGCAGTTCGCGGTTAGTGAAGGGAAAAACTATTCTCCTCTCCTCCATAGTG
+
CCBCCC?DC>BBBB>B@B?A?>ACDCBCAAA=@=@BBCC@CC>B?AA2B>>?@?@?>?<==:70,/.---1
@T6XXI:00144:00046
CTATCACTCGAGATCTGCGGCTTTGGACAGTCCCTCTGTACTGTGAAGCCACAGATGTCGAGTCGACTAGCTTCCTT
TCTCCCTTCTCCC
+
CBCA<::ACCCBBCBBBBC@@?@:?=>>??:::7:BCCCBBCBCAA??B>BBBBCBCBBBB??@@>?B?::6<<<<A
>B?CC>C:?9771
@T6XXI:00145:00004
CTAGTCACTCGGATCAT
+
@::99@87;//,///,,
@T6XXI:00145:00012
GGGGAATTCTAATACGACTCACTATAGGGAGAGGGAGAATAGAGACGTATTCGTTTGCGCTCTGTTCACCTTCCTTT
CTCCCTTCTCCC
+
AAA8AACDCBC@CABBBBA@?B>99311.7??@C:::C@CCA??@AA@@?@AAAA<AA@@BCB:899//./6:9>?9
==CH9===?AD;
@T6XXI:00145:00027
```

GGGAGAAGGGAGAAAGGAAGCTAGTCGACTCGACATCTGTGGCTTCACAGTACAGAGGGACTGTCCAAAGCCTGCAG
ATCTCGAGTCCGACTAG
+
BE@CDC@CC>CCBB89;;?BCB:;;8<:BCCBBBCAAB>A?=::8;ABBBBCCBA73417??779-
..,/9,//;;;@@A@@A@??@;<<<===
@T6XXI:00145:00039
GGGAGAAGGGAGAAAGGAAGACTGGGCTGATGTAAATA
+
AD=>?>599-49>>0;8:=?>?888,4>11..,220,.
@T6XXI:00146:00019
GGGGAATTCTAATACGACTCACTATAGGGAGAGGGAGAATAGTCAACAAATCGTTTGGGGCGACTTCTCCTTCCTTT
CTCCCTTCTCCC
+
?==6@>B?BCC@A>BBBBBBBBB?<76659@@BC>BBC>>>>:?A=AA=;BB???;?@?8B???@@@BC=@>=<AA=
=@AA<A88:?A?
@T6XXI:00146:00023
GGGAGAAGGGAGAAAGGAAGTGCAAAACAGCTAAATCGTTGGGAGGACTATTCTCCCTCTCCCTATAGTGAGTCGTA
TTAGAATTCCCC
+
DB?CCB?BB<AB@A=BDC@CB?99991;BB@@AC?>>>>>>>61112><>CBCCCD@BB???9?AA?@?CCA@?=>8
838=?;==@AA5
@T6XXI:00146:00031
GGGAGAAGGGAGAAAGGAAG
+
CB>A@?59909911--08+-
@T6XXI:00146:00034
GGGAGAAGGGAGAAAGGAAGCTAGTCGACTCCCATCTGTGGCTTCACAGTACAGAGGGACTGTCCAAAGCCGCAGAT
CTCGAGTCGACTAG
+
FDBAA@@BB>CBCCAGDC@BBB>>>>@?AAAE@CAAAAAC@CB=BBBBBCCBA?99969>@?BB?BB>BC@B??BCB
BB?=BBAABBBBAA
@T6XXI:00146:00045
GGGGAATTCTAATACGACTCACTATAGGGAGAGGGAGAATAGCCGTCAACAAATCTGGGGTCTATTCTGCTTCCTTT
CTCCCTTCTCCC
+
?AA6A<@=ACC@BBABABBBBBB?>311.7==ACBCBB?B@@=<==:@>BC@;AAABBB8BBBBB@CBBBC@C=@@<
@CCCAC@B9::5
@T6XXI:00147:00005
GGGGAATTCTAATACGACTCACTATAGGGAGAGGGAGAATAGCTACGTTTAACCGATCGTGTTGTTCTCCTTCCTTT
CTCCCTTCTCCC
+
:99/9<@>BBC@CBBBB@??BBB9;766.6@@BC>CCC>??????@@@C>C@B@A?ACB???>?@;A??<?=@>@C;
>=<A:A:=>AC;
@T6XXI:00147:00036
GGGGAATTCTAATACGACTCACTATAGGGAGAGGGAGAATAGATCTCTCCTCAGCAATAACGCGCAATTCTTCCTTT
CTCCCTTCTCC
+
999/95959@@?CBCCCB<<AAB?7366.777?B>B@@?@CA?????AA?A@@@??:::57???8;7<?@@=A@B?:
?000,1?:00,
@T6XXI:00148:00006
GGGAGAAGGGAGAAAGGAAGCTAGTCACTCGACATCTGTGGCTTCACAGTACAGAGGGACTGTCCAAAGCCGCAGAT
CTCGAGTCGACTAGCTTCCTTTCTCCCTTCTCCC
+
9959=9<9969499149:9=??880<0/,,,,2,,,.4333:>><>B????@>>7:;.4>;888<>>:8844;?>A>
<<==<:9:=777;73044288,73484<544:42
@T6XXI:00148:00014

```
GGGAGAAGGGAGAAAGGAAGGAGAAGATGCTGACGTGTGTGTGCCAGCTATTCTCCCTCTCCCTATAGTGAGTCGTA
TTAGAATTCCCC
+
9A<@B@A@A<@@@A>B@A?C=<;:>BBB???>A@?<;;BCCDCC>BBDCCC?BBCE>??;::6:@BCCCACGC@>==
;:=;;374666-
@T6XXI:00148:00018
GGGAGAAAGGAAGCTAGTCACTCGACATCTGAGT
+
DCB@99909;><>A@@@@>8=88333...3....
@T6XXI:00148:00020
GGGAGAAGGGAGAAAGGAAGGAGAAGTCGCCCCAAACGACTTGTTGACTATTCTCCCTCTCCCTATAGTGAGTCGTA
TTCAGAATTCCCC
+
DDBCDCACC?B999-9<@>@?A@@?B?@8000+1;8;;0..,.5559B;;1;>;;;6//-
6424.34==BAHC=<788,//674609:4.
@T6XXI:00148:00028
GGGAGAAGGGAGAAAGGAAGGAGAGTGCGGCGTGGGCAATTAGACGACTATTCTCCCTCTCCCTATAGTGAGTCTAT
TAGAATTCCCC
+
4A=::<@CB:@999185959>@??BDBBC?BBCCE>BC8;6:@??@DBBAB==776-
0444636333=55366300323048854440
@T6XXI:00148:00035
CTAGTCACTCGAGATCTGCGGCTTTGGACAGTCCCTCTGTACTGTGAAGCCACAGATGTCGAGTCGACTAGCTTCCT
TTCTCCCTTCCTCCC
+
11/////44?=?>BBBBB99588808;>>?@@BC>BCC?@@BBCCCE@?ECBADCCDCCCAGFA@@;<8@;><=A@F
E>C>?>6<,0,0661
@T6XXI:00148:00046
GGGGAATTCTAATACGACTCACTATAGGGAGAGGGAGAATAGTTCCCTGACAACAATTACCGCAAGCCTCTTCCTTT
CTCCC
+
DBB7B9;<<EH@BBBBBBBB@@;77311.7??BBAAAA@BAAA>>>7?@ABA@BB>B>BC?BAA>AC@CCC;:8:C?
??BC?
@T6XXI:00149:00006
GGGGAATTCTAATACGACTCACTATAGGGAGAGGGAGAATAGTCTTGTATTTTGTGCATCACTTTTCTCCTTCCTTT
CTCCCTTCTCCC
+
AA?6A@B?BBB@BAAAAA>>BB>97311.7??BC>CBB>>>?<<<;;?::=C6AAA>>@BB????5?@?=@?C:==9
=@@D=A>@@@@<
@T6XXI:00149:00012
GGGGAATTCTAATACGACTCACTATAGGGAGAGGGAGAATAGTCAACAAATCGTTTGGGGCGACTTCTCCTTCCTTT
CTCCCTTCTCCC
+
CCB7B=@=@BB?BBBBBCCCBC99311.7=?BG>BBC@CCA@AA>===9@CCCC>BBB7BAAAC>AAB?B?@=??7
====6<9<@?A<
@T6XXI:00149:00016
GGGGAATTCTAATACGACTCACTATAGGGAAGGCAATCGGGGAAT
+
999/48><@9954999A@BBBB@@>>:<64,.,.534....*.53
@T6XXI:00149:00036
GGGAGAAGGGAGAAAGGAAGCTAGTCACTCGACATCTGTGGCTTCACAGTACAGAGGGACTGTCCAAAGCCGCAGAT
CTCGAGTCGACTAG
+
FFBCBB?BB=BBB@>B896:CB@AB8:7:77=<6777?CC?BBCBBB@@@?><95888<?<>AA;==9=@>@@@@;;
<AA;;?A@@@@AA@
@T6XXI:00149:00046
```

```
GGGGAATTCTAATACGACTCACTATAGGGAGAGGGAGAATAGTCACATTCATGTTTGTGACCGTGTCTCCTTCCTTT
CTCCCTTCTCCC
+
AAA6><@=@BB@AAABBCBCCCC?9311.7>>>@<ACC@BBBAACA?A?????@B=C@==CBA???A??:=:;243,
4:9;-434344-
@T6XXI:00149:00048
GGGAGAAGGGAGAAAGGAAGGAGAAGTCGCCCCAAACGATTTGTTGACTATTCTCCCTCTCCCTATAGTGAGTCGTA
TTAGAATTCCCCC
+
DE@BBD@CC>DDDD?B@B?B?BCD@B?BBBBB8BB<BBBA@;?B?BBBBC>>888;;=====;;<;=<<<;==@@??
?>@@@>@8?===1
@T6XXI:00100:00052
GGGAGAAGGGAGAAAGGAAGGAGAAGTCGCCCCAAACGATTTGTTGACTATTCTCCCTCTCCCTATAGTGAGTCGTA
TTAGAATTCCCC
+
DHAA@?@DB?CCBB>C?BBB@BDD@B@B@?;;5?@<@CLCC<B?>@>>ACA>><<=;@AAAA<=9==A@@@A@@@@A
@>A<978/444-
@T6XXI:00100:00064
CTATCACTCGAGATCTGCGGCTTTGGACAGTCCCTCTGTACTGTGAAGCCACAGATGTCGAGTCGACTAGCTTCCTT
TCTCCCTTCTCCC
+
BBB=9::9B>??BCCCBBC@CBB>B>B@B=:??;?CCCBAAC@CAA<=?=@CCECCCAAABBABBAAAAAA>?B@BD
?BBCD>@:==@@;
@T6XXI:00100:00075
GGGGAATTCTAATACGACTCACTATAGGGAGAGGGAGAATAGTCAACAAATCGTTTGGGGCGACTTCTCCTTCCTTT
CTCCCTTCTCCC
+
949/9;>>@@@=@CBBB@@@ABBAB???9???@C=???;878888=@A?<BBBBB>BAA7BBA@?::::58?A@HC<
983448/4-//,
@T6XXI:00100:00087
CTATCGACTCGAGATCTGCGGCTTTGGACAGTCCCTCTGTACTGTGAAGCCACAGATGTCGAGTCGACTAGCCTTCC
TTTCCTCCCTTCTCCC
+
DECB:::CCCCBGDBC99;@>@??:A<>>??777.7?@AACCCCB@87/1,1:<008BBBCCBAB@00100,+,+,+
,7+,+0<:2248533+
@T6XXI:00100:00091
GGGAGAAGGGAGAAAGGAAGGAGAAGTCGCCCCAAACGATTTGTTGACTATTCTCCCTCTCCCTATAGTGAGTCGTA
TTAGAATTCCCC
+
DD?BBC@CD>BBDB=B@C?B?AAA???BBBBB6BB<BBBA@>@@>???>?=;====;==<==;========@?:?<A
A>@@A?A>@AA5
@T6XXI:00101:00054
GGGAGAAGGGAGAAAGGAAGCTAGTCACTCGACATCTGTGGCTTCACAGTACAGAGGGACTGTCCAAAGCCGCAGAT
CTCGAGTCGACTAG
+
BDADDD?BB=@AA@<A=@=@B;;:B??????@9<?A>?>@@BC>BCABBCCCA?<<<7<?A@@@:=<7<?=@AAAAA
AAA;<<>=>><<<<
@T6XXI:00101:00081
GGGAGAAGGGAGAAAGGAAGGGGAGAAGGGCTAGTCGACTCGACATCTGTCGGCTTTGGAAGTCCCTCTGTACTGTG
AAGCCAAGATGGACTGTCTTCCTTTCTCCCTTCATACC
+
CD@BCB@BB>BCCC<>=?@BBB:BAA<@@<@BCCECC>>CBA111827-
//,8888888888>CC?BBBA???AABBB?BC@C@CBAB??BCC@@B@C?A@7==CD7:5///--,
@T6XXI:00102:00052
GGGGAATTCTAATACGACTCACTATAGGGAGAGGAGAATAGTTTACCGTTCACTCAACCG
+
BBB6D@A@@BC@CCBBBBBBB?997111.7===:?7738788@174<032431163,.,.
```

```
@T6XXI:00102:00083
ACTCGAGATCTGCGGCTTTGGACAGTCCCTCTGTACTGTGAAGCCACAGATGTCGAGTCGACTAGCTTCCTTTCTCC
CTTCTCCC
+
ABBCCCDB??BBBB>A?B>B@ABBBBBB>CCBBBB@@BBBCEAA@BCBBBBBBBA@CAABBBBA@@@<A5;;7<CBC
@C;;;AA<
@T6XXI:00102:00084
GGGAGAAGGGAGAAAGGAAGCTAGTCACTCGACATCTGTGGCTTCACAGTACAGAGGGACTGTCCAAAGCCGCAGAT
CTCGAGTCGACTAG
+
AA<@?==@@<@A??<B>A>BCBBAA?==1161619@7::C;;>?CAFAAACCB;33317999=?8<;6;><@@@@??
?@A@?@AA===<<@
@T6XXI:00103:00050
GGGAGAAGGGAGAAAGGAAGCTAGTCGACTCGACATCTGTGGCTTCACAGTACAGAGGGACTGTCCAAAGCCGCAGA
TCTCGAG
+
CKCCAA>@=6@@=?=B@C@CBA9::/11?>>>3;:::7?=?:;<7:BBBBBBBB;<<<998==?;<:?;?A?A<<A=
<;?:===
@T6XXI:00103:00057
GGGGAATTCTAATACGACTCACTATAGGGAGAGGGAGAATAGCGACCTGATTTTCAGTGCCGTGCTTCTCTTCCTTT
CTCCCTTCTCCC
+
CCC9C=?=ABBCCB???@??B@CB@77757BBCC>AAB?A<::?AA>A<@@@B7B@?><?>===<<<=<:><?:>?;
===A?A?===@<
@T6XXI:00103:00067
GGGAGAAGGGAGAAAGGAAGGTGAACAGAGCGCAAACGAATACGTCTCTATTCTCCTCTCCCTATAGTGAGTCTAT
+
ED=A<;79@:>@B@9@8;8<?CDC?CCB?ABBBBB>BBCABBBDCBBCCB;:;11,1<>>><A@?00000/..333
@T6XXI:00103:00087
GGGGAATTCTAATACGACTCACTATAGGGAGAGGGAGAATAGTCCTTTCCCTTCCTTATTGCGCATTCTCTTCCT
+
?A:4@54699958=>>ABBABACBB:9959@?CC>CBC@<<<77/39488-4349;:9B<?98///76717,/,.
@T6XXI:00103:00090
GGGAGAAGGGAGAAAGGAAGCAAATGGCGCATGTTTCCAGGGAGTGTCTATTCTCCCTCTCCCTATAGTGAGTCGTA
TTAGAATTCCC
+
BC?CCC@CC>A@==:==@=@@@CACC@B@??AABC;?>??@;<??A@:8??;==<<6=>9;;6;=>>@?A<<84444
424:;20/-..
@T6XXI:00104:00050
GGGAGAAGGGAGAAAGGAAGCAGTCCATCTGTGGCTTCACAGTACAGAGGGACTGTCCAAAGCCGCAGATCTCGAGT
CGACTAGCT
+
CK?B;96;A:AB@@<B:;8<ABB?<<?:::CCCABB?BA??<=@CCC;:<<<=A???>AA?==:=@@@AA======@
CC>>=>000
@T6XXI:00104:00061
GGGGAATTCTAATACGACTCACTATAGGGAGAGGGAGAATAGCCAAGATCTCCCTGGCCGCATCATACACTTCCTTT
CTCCCTCTCCC
+
CCC:C=@=@BA@BCBBBBBBBAB>;116.6><<?<?AA>=787:=:=?@???C<AA?@????AAA<@AAAA;=:<A:
8888,//6660
@T6XXI:00104:00080
GGGGAATTCTAATACGACTCACTATAGGGAGAGGGAGAATAGTCAACAAATCGTTTGGGGCGACTTCTCCTTCCTTT
CTCCCTTCTCCC
+
BBD9B@CACBB@CA>>BACBB@BBA:::8899?B>?@@@A=AAA@>???;A?997-666-
7>@?A?BBB?@>A?AC>C<<;4:74444-
@T6XXI:00104:00088
```

```
GGGAGAAGGGAGAAAGGAAGAGAACGGCGACGAACAATAGTTGGTGGCTATTCTCCCTCTCCCTATAGTGAGTCGTA
TTAGAATTCCCC
+
BC=B<;9<C;<<CK@C?A>@AAA?BB?B;;<@@=@C=@@CF;>;8848?>?<B@??;?AAAC>BA??@@@@@?=@A8
8349:558:;=4
@T6XXI:00104:00092
GGGGAATTCTAATACGACTCACTATAGGGAGAGGGAGAATAGTCAACAAATCGTTTGGGGCGTCTC
+
CBB2?@@=@@@DCDBBBBAAAAA>?79657??@B>BBB@@<@@==:=@@:A@:7757>>2/,****
@T6XXI:00104:00094
GGGGAATTCTAATACGACTCACTATAGGGAGAGGGAGAATAGTCAACAAATCGTTTGGGGCGACTTCTCCTTCCTTT
CTCCCTTCTCCC
+
DCC:C@B?BBB>BBCDDCCC@????777.7???C?CKE@A?>>>>?AAA>CC?>?.777.7>>?@>AAA>><@9<<7
;>>>6;79:994
@T6XXI:00105:00095
AGTCACTCGAGATCTGCGGCTTTGGACAGTCCCTCTGTACTGTGAAGCCACAGATGTCGAGTCGACTAGCTTCCTTT
CTCCCTTCTCC
+
AAACDCCACCCCBAAAB@<9998;@CCCBCCC>BBC@@@>>ACCC@CC@BBBBBDD@CBA@@@ABBA?8::>A>AA=
A@>>5:64;=9
@T6XXI:00106:00054
GGGAGAAGGGAGAAAGGAAGCTAGTCGACTCGACATCTGTGGCTTCACAGTACAGAGGGACTGTCCAAAGCCGCAGA
TCTCGAGTCGACTAG
+
DC?B>>;995>988096959B800;/00//7:;?????ABC;:;@CCCB>BCCC=44448<9@@A>??<?C?@??@@
=<??::>ACAA:::<
@T6XXI:00106:00088
GGGAGAAGGGAGAAAGGAAGGAGACACGGTCACAAACATGAATGTGACTATTCTCCCCCTCCCTATAGTGAGTCGTA
TTAGAATTCCCC
+
CCACCB@BB>BBBC?B@B::;8CCDCBB@BBBBDD>BBCCA?CCA@?BABB=@?@C==199968@@AA<<@A:???A
B>?@@?@3773-
@T6XXI:00106:00098
GGGGAATTCTAATACGACTCACTATAGGGAGAGGGAGAATAGATCACGCCTTCTACCACGACTAGCTTCCTTTCT
+
9@?6=>??@@;:4894487...4.0...+...6989<<=9=89//,7..,.,.1160110.----1986568+//
@T6XXI:00107:00057
GGGAGAAGGGAGAAAGGAAGCTAGTCGACATCTGTGGCTTCACAGTACAGAGGGACTGTCCAAAGCCGCAGATCTCG
AGTCGACTAG
+
DCABAA@BB?CCCB?B?B@BACABA@=77:;;<BCD?BB@CCCCCC@?@?>@@<@CCCBB=@?<@B@CBBACCBBBC
BBBBB?@CA@
@T6XXI:00107:00075
GGGGAATTCTAATACGACTCACTATAGGGAGAGGGAGAATAGATCACCGTTGGATTCGGGTGCGCGTTCTCCTTCCT
TTCTCCCTTCTCCC
+
CCC9B79;<BBACCCBB?>>A@?99111.7>>BC>BBC?@=988=::7:?;?4773777.6<8;??@?=<<9<<=;=
?:;48818==?@A<
@T6XXI:00107:00077
GGGGAATTCTAATACGACTCACTATAGGGAGAGGGAGAATAGTTCTCG
+
CBD9B=@=@CC@CCBBBBBBB@BCC@@>99999<;ABB?>436+++**
@T6XXI:00107:00096
CTAGTCGACTCGAGATCTGCGGCTTTGGACAGTCCCTCTGTACTGTGAAGCCACAGATGGACTGTCCCTTCCTTTCT
CCCTTCTCCC
+
```

```
FCCBBBBBBBCCDEBBBBBBB>;9333/8<>ABBBBD>AABBC@?><BBC@BC=@@CCCAC@CBCCAAA<A?A?AD>@:
::5:?::1L1
@T6XXI:00107:00098
GGGGGAATTCTAATACGACTCACTATAGGGAGAGGGAGAATAGTCAACAAATCGTT
+
/111*/59:=BA?BDCCCBAAA@C@@@DC?;=666.7==:840000/004066111
@T6XXI:00108:00054
GGGGAATTCTAATACGACTCACTATAGGGAGAGGGAGAATAGTTTCACGTTGCCGACTGCTGCTATTCCCTTCCTTT
CTCCCTTCTCCC
+
BCC:D@D@ECC@CCBBBB?>>@B99111.7>>>@BBBB@A<@==;>====;@=<AA@?@<==8>>C>AA;@=@?CC6
;<AD<A689@C<
@T6XXI:00108:00059
GGGGAATTCTAATACGACTCACTAGGGAGAGGGAGAATAGTCACGATCATCTCCAATCTCCAGCACGCTTCTTCCTT
TCTCCCTTCTCCC
+
BBE7A=?=@BB?BBCCBBBBC>A>>>=>B>@E=??B?CCAABBABBEB:::BB@C@==<@=;;;<=;<;?BB>F=?>
8>777.747@BE@
@T6XXI:00108:00060
GGGAGAAGGGAGAAAGGAAGCTAGTCGACTCGACATCTGTGGCTTCACAGTACAGAGGGACTGTCCAAA
+
GDCCCB?@=6@9998<@C@CCD>@@@B@ACC>?ABB@><A?A?B@???999@CA=55544<9===3333
@T6XXI:00109:00082
GGGGAATTCTAATACGACTCACTATAGGGAGAGGGAGAATAGCCACCAACTATTGTTCGTCGCCGTTCTCTTCCTTT
CTCCC
+
AAA9B@B@BAA@CCBBBBBBBBB99111.777@DACCC@@???<AA>@>@A<=;;;:;@A:?;;AA>=@:?;<5=>;
>>?A:
@T6XXI:00110:00051
GGGGAATTCTAATACGACTCACTATAGGGAGAAAGGAAGTGCAGTTCGCGGTTAGTGAAGGGAAAAGCTATTCTCCC
TCTCCTATAGTGAGTCGTATTAGAATTCCCC
+
BBD1>:969995898>A@?@CB:;<::D<;><C>C=@=?@:771<:;;<;3324304456936=:-
33<<321+++*++++*+0026?888=887<;8;3843/444-
@T6XXI:00110:00061
GGGGAATTCTAATACGACTCACTATAGGAGAGGGAGAATAGTTCTGTGCATTTCTCCCAGCACACCGTCCTTCCTTT
CTCCCTTCTCCC
+
DCD9C9;9;AA@BBBBBCEAA:944..,0::>A>CCD?@@AA;=::?@A=AC>DA??<?A=@=AB>A5548:;3894
8BBA;?>?@@A;
@T6XXI:00110:00082
GGGGAATTCTAATACGACTCACTATAGGGAGAGGGAGAATAGTTCTGTGCATTTCTCCCAGCACACCGTCCTTCCTT
TCTCCCTTCTCCC
+
?==6@=@@CBB@CBBBAA?BBCBBB66646?ACC>CBBA@<<=;;;;@@A<@@;@<<<;<====<=<=999=>B>A@
<@A=A;=:<@<=;
@T6XXI:00111:00065
GGGAGAAGGGAGAAAGGAAGCTAGTCACTCGACATCTGTGGCTTCACAGTACAGAGGGACTGTCCAAAGCCGCAGAT
CTCGAGTCGACTAG
+
CDAA==<>>:>999094969B;88B1777<=779?::?CC>AA?ACBBBB@B74000-
4::=@??AA<AA<??>>>=999A><=9==@??>
@T6XXI:00111:00067
GGGGAATTCTAATACGACTCACTATAGGGAGAGGGAGAATAGCCTGACCTTTCCAGTGCGCGCTGTTTTCTTCCTTT
CTCCCTTCTCCC
+
```

```
999/99@>BDC@BBBB@@@BB@>97166.7>>BC<BCCAA=??;AB==366.747;@@B===??AABB5==<?<?A@
===A;@==?<A=
@T6XXI:00111:00089
GGGGAATTCTAATACGACTCACTATAGGGAGAGGGAGAATAGCTATCACACGGCATGCACTCCCATTATCTTCCTTT
CTCCCTCTCC
+
999/9<B=AAAADCBBBCCAA@>;A9:7.7?===6=7743;;=ACABACBBB@BBBA@===?A<AA<@AAA:=:;>9
6///,////,
@T6XXI:00112:00077
GGGAGAAGGGAGAAAGGAAGCTAGTCACTCGACATCTGTGGCTTCACAGTACAGAGGGACTGTCCAAAGCCGCAGAT
CTCGAGTCGACTAGCTTCCTTTCTCCCTCTCC
+
C@=BBB>A@:;;;@:A6;9<??>>>;7776111169>9;;26:5:A@BBBBA@><<<6;?@===?AA<AA>AA@@@@
AA@@???????==<??<;;;936///,/...,
@T6XXI:00112:00088
GGGGAATTCTAATACGACTCACTATAGGGAGAGGGAGAATAGTCAACAAATCGTTTGGGGCGACTTCTCCTTCCTTT
CTCCCTTCCTCCC
+
?99,959=@BCBBBBCCCB?BA???777-7>>>C>AAA@A@A@??<?AB?CCA?;-666-
:@@BE=AAA@C?B?AA<?;:916,/,///,
@T6XXI:00113:00055
GGGGAATTCTAATACGACTCACTATAGGGAGAGGGAGAATAGTCAACAAATCGTTTGGGGCGACTTCTCCTTCCTTT
CTCCCTTCTCCC
+
ABB9CADBCCB@A@?BBB??A?<99166.777>><ACB@@B@@@:8;AB=CC?;;-
777.7@AAB@C?@>==?>AA=>?@A=B<??AA:
@T6XXI:00113:00069
GGGGAATTCTAATACGACTCACTATAGGGAGAGGGAGAATAGCCGCAATTGACGTCATCCCTGGTTACTCTTCCTTT
CTCCCTTCTCCC
+
=@C9A>C?<<<ADDGCCCBBBAA>>777.777?C@@>>?;0./987>5846;===??:7:67=;?8;<><;5;:9;4
6///,/,/.--+
@T6XXI:00113:00096
CTAGTCGACTCGAGATCTGCGGCTTTGGACAGTCCCTCTGTACTGTGAAGCCACAGATGTCGAGTCGACTAGCTTCC
TTTCCT
+
>7-,,-
,,8:>BBCBBBBBBA=>BB>B>@@:;;DD>CC?@@???B???;<A>AB@A@BBBCCB??;;9>A?80.,.,..+.,.
@T6XXI:00114:00061
GGGAGAAGGGAGAAAGGAAGGAGAAGTCGCCCCAAACGATTTGTTGACTATTCTCCCTCTCCCTATAGTGAGTCGTA
TTAGAATTCCCC
+
CD@GCC@B>:====:@>@=B>ABB?A?A<<<<2?@<ADDCC>@@>AAAACC>A=999=AAAC<<8>?A===99=A==
=><<::655;;1
@T6XXI:00114:00083
GGGAGAAGGAGAAAGGAAGGGGAGAAGGGAGAAAGGAAGCTAGTCGACTCGTACGGATCTGCTG
+
AA11//,/,,002*,*/56880...47761///,.,.,.....7:@887CAA743:54662+++
@T6XXI:00114:00087
GGGAGAAGGGAGAAAGGAAGCTAGTCATACATCTGTGGCTTCACAGTACAGAGGACTGTCCAAAGCCGCAGATCTCG
C
+
9?1949594-
444969999=93730/,,,,,02...4201017767;@AAB8858???CB>AA<@A<<;98/,,++++
@T6XXI:00114:00089
GGGAGAAGGGAGAAAGGAAGGAGAAGTCGCCCCAAACGATTTGTTGACTATTCTCCCTCTCCCTATAGTGAGTC
+
```

```
CDADCC@CCBBBBB>C@C@C@BBB@BABAA@@6<@<@CCCC;@@=>77:CB?AAAA<??@==;<:;<====A@@
@T6XXI:00114:00090
GGGAGAAGG
+
CD@A?=@BB
@T6XXI:00114:00099
GGGAGAAGGGAGAAAGGAAGCTAGTCACTCGACATCTGTGGCTTCACAGTACAGAGGGACTGTCCAAAGCCGCAGA
+
EE@BAA=@A:@AAA>BACBCDA?BCBBA>>://000///75?@?AABBBAA>?;444-4;;444/30+00/04443
@T6XXI:00115:00054
GGGAGAAGGGAGAAAGGAAGGAGAAGTCGCCCCAAACGATTTGTTGACTATTCTCCCTCTCCCTATAGTGAGTCGTA
TTAGA
+
DC?BBA@CC>D@??<@?B>B@BCB?BB=<7<<2>>9?@@?A<??@BAAAB=<;===;66<A=;=::?=AB<<<<?<=
A>A@A
@T6XXI:00115:00057
GGGAGAAGGGAGAAAGGAAGGAGAAGTCGCCCCAAACGATTTGTTGACTATTCTCCCTCTCCCTATAGTGAGTCGTA
TTAGAATTCCCC
+
BB???=@AB===BC>B>B?B?ADB@BCB<<<<6<B>CCCBB;;;>>>>>>>=;=<<<;<<?==;==9?=A==A<<?9=
<?A<;88/4440
@T6XXI:00115:00073
GGGGAATTCTAATACGACTCACTATAGGAGAGGGAGAATAGTACAACGGACCGCCGATTGCATCCTCTTCTTCCTTT
CTCCCTTCTCCC
+
DBB8BACACBB?BCCBBBCBB7;6;//,0>>?B>B??>=ABAA8869?<@A=???A??<CC??::?==<<:<:;<=;
A@@@;@;788@:
@T6XXI:00115:00074
GGGGAATTCTAATACGACTCACTATAGGGAGAGGGAGAATAGTCGTAAACTGCAGCTATCGACACTCCCTTCCTTCT
CCCTTCTCC
+
944,4295<CA>CFA@AEBBCABB87:7.777AB?AAB@A888888=@:;<;878<;;;;548-----+-,-
2244<<=485<8<:
@T6XXI:00115:00079
GGGGAATTCTAATACGACTCACTATAGGGAGAGGGAGAATAGTCAACAAATCGTTTGGGGCGACTTCTCCTTCCTTT
CTCCCTTCTCCC
+
EDB9B9;;<ABBBBBCCCCBBABBB66616?9??<BAA??=AAAA=?@A;B?;;;-777-
7@@@A?B??<<<@=@@;?AAA=A=?@@@;
@T6XXI:00115:00095
CGAGATCTGCGGCTTTGGACAGTCCCTCTGTACTGTGAGCCACAGATGTCGAGTCGACTAGCTTCCTTCTCCTT
+
AAA@ACAAA>>;988085377;>BE>AB;;<C??777>40/1799588:?A=A>==@?998442/5656//,/,
@T6XXI:00116:00077
GGGGAATTCTAATACGACTCACTATAGGGAGAGGGAGAATAGTCAACAAATCGTTTGGGGCGACTTCTCCTTCCTTT
CTCCCTTCTCCC
+
DED:CBC@BBB@DBBBBBBAABBA@666.77777.7>>@><8844249;6?@???-
777.7>>?B@AAA??:?;??;>>;B183/+01+
@T6XXI:00116:00079
GGGAGAAGGGAGAAAGGAAGTGCAGTTCGCGGTTAGTGAAGGGAAAGCTATTCTCCCTCTCCCTATAGTGAGTCGTA
TTAGAACTTCCCC
+
DDDCCC@BB>BBBC?B@?=?BBCBAB?BBBB>A?AA@@B8:::;E>BA@BC@CBBB>B@CCB?C=?=A@@A??8444
4343;9;?3444-
@T6XXI:00116:00090
```

```
GGGGAATTCTAATACGACTCACTATAGGGAGAGGGAGAATAGCAATCGCTCTGGTATTCTCTCCTTTACTTCCTTTC
TCCCTTCTCCC
+
DDDCD?@@ACC@CCB@ABCBB@BBB66646?8AB>BBB@@AA@=;?<@<@C8448??;??AABBCC7<=A>@=@@;?
>884<9<<<A;
@T6XXI:00116:00093
GGGAGAAGGGAGAAAGGAAGTGCAGTTCGCGGTTAGTGAAGGGAAAAACTATCTCCCTCTCCCTA
+
44-999594-4449-4249=<BHFCC=@>996718>337>=@>@CCE2E?31.477,//111,10
@T6XXI:00116:00098
GGGAGAAGGGAGAAAGGAAGCTAGTCACTCGACCATCTGTGGCTTCACAGTACAGGAGGACTCGCAAAG
+
99-9>><@A:A@@@<A@C@C@;:8?7....3..,....7:@=:@@DCCBBBBB>;7443//-3--++*+
@T6XXI:00117:00051
GGGGAATTCTAATACGACTCACTATAGGGAGAGGGAGAATAGCCACCAACTATTGATTCGTCGCCGTTCTCTTCCTT
TCTCCCTTCTCCC
+
999/9;?ABBB@CBCBB@@@BAB9<111.7>>>@>BAA??<??:?@9;497++++++000349<>AA>@@@@6889C
?@@@A<A<??AA<
@T6XXI:00117:00060
GGGGAATTCTAATACGACTCACTATAGGGAGAGGGAGAATAGCCACCAACTATTGTTCGTCGCCGTTCTCTTCCTTT
CTCCCTTCTCCC
+
999/4:BACCB@@>>?BBB?BCB99111.7<<>>:@AA=>=88444/438;=??@A>@@@<??8?A8;;==8;9<A?
@@AA<A>AAA=;
@T6XXI:00117:00066
GGGGAATTCTAATACGACTCACTATAGGGAGAGGGAGAATAGTTTTTCCCTTCACTAACCGCGAACTGCACTTCCTT
TCTCCCTTCTCCC
+
BCB9C@D@CBBEBBBBBBBBBBAB?>66646??CC>BBC@A=A<:8=0;;7<=@BBBB@C@AC??=<?A<??B>@:??
<?BBC>C@B@@@;
@T6XXI:00117:00070
GGGGAATTCTAATACGACTCACTATAGGAGAGGGAGAATAGTCAACAAATCGTTTGGGGCGACTTCTCCTTCCTTTC
TCCC
+
;<9/9=A?CCC@CCBBBBBBA7;;6./,0@:=?<?CC@AAAA@?<<;;4<><<<6<<<2<@?AB?BBB?@>A>AC<@
@@A;
@T6XXI:00117:00072
GGGGAATTCTAATACGACTCACTATAGGAGAGGGAGAATAGTCACCTTAAA
+
BAA9B@C@<<<;9;AA;;9BAA?AA773777==9?A?>>85++++*+0...
@T6XXI:00117:00093
GGGGAATTCTAATACGACTCACTATAGGGAGAGGGAGAATAGTCAACAAATCGTTTGGGGCGACTTCTCCTTCCTTT
CTCCCTTCTCCC
+
DDD9CCC?CCB@CBAABBBBBAB?<661-
6<;CC>CCC>A===<<;<<=;@@>>>8<<<7ACCCC@ACC>?<?:@@<@AAB<@>@@?@<
@T6XXI:00118:00050
GGGGAATTCTAATACGACTCACTATAGGGAGAGGGAGAATAGTGTTCATCCCCCCGGAACCTAGATCTCCTTCCTTT
CTCCCTTCTCCC
+
444-99BACAB?BCBBBBABAAA<>66659??BB>CBB??<<<;??BCCCBB@A-
?>?777::B@@===24347994;?@@<A698AA<
@T6XXI:00118:00055
GGGGAATTCTAATACGACTCACTATAGGGAGAGGGAGAATAGTCCATTCCTGTTCGTCGCTCCGCTATTCTTCCTTT
CTCCCTTCTCCC
+
```

```
ABA7A>B@CCBEBB@?BBBAA@B?@616.9::CC@CBB?@<@A=<888;<=:;:?===<??A>@A@AA>@@>@<?>7
8<<<5<:9;;;7
@T6XXI:00118:00077
GGGGAATTCTAATACGACTCACTATAGGGAGAGGGAGAATAGTCAACAAATCGTTTGGGGCGACTTCTCCTTCCTTT
CTCCCTTCTCCC
+
BAC7A>B>BCBDCA@?BBBBB@BBB88888??ACCBBB@A=<=AA?@?@;BBA<<;<?C7AACBB?BBC@AA@>@@<
@A@@;@9<<?@;
@T6XXI:00118:00098
GGGGAATTCTAATACGACTCACTATAGGAGAGGGAGAATAGTCAACAAATCGTTTGGGGCGACTTCTCCTTCCTTTC
TCCCTTCTCCC
+
CCC:C@CCCCB@ABAABBB?>:994..,0::AC>CCC?A<<=;:8777,7=<;<-
777.7>@BC=??C@A>A?AA=???A<A?AAA;:
@T6XXI:00118:00099
GGGGAATTCTAATACGACTCACTATAGGGAGAGGGAGAATAGCTTGATGTCCTGAGGACCTTGCCTCTCCTTCCTTT
CTCCCTTCTCCC
+
BBB7A<???448;BCBCCBBBCBB>88886=??A<BCB?@===?B>????A?==<<:;=;=::;;<<<=;854300,
7=>>;A>===A<
@T6XXI:00119:00053
GGGAGAAGGGAGAAAGGAAGGAAAATAGTTGCGGGTCAATGGTTTGCACTATTCTCCCTCTCCCTATAGTGAGTCGT
ATTAGAATTCCCC
+
9919>><@C>AADE@C@CCD=@@@5?@@B=A??<6?:;9<D@CD=BDBBDDD@CBBB<@@@BB=BCBBBBCBBBBBB
AA>A@>;:86:?5
@T6XXI:00119:00054
GGGGAATTCTAATACGACTCACTATAGGGAGAGGGAGAATAGTCAACAAATCGTTTGGGGCGACTTCTCCTTCCTTT
CTCCCTTCTCCC
+
BBB9B?CACBCACBA??BBBBA>93166.7==>A?DBC>@?BAAA>===9@CCCC;???5???@C@DCF>=;==@A<
AA@C;?9<<==6
@T6XXI:00119:00087
GGGAGAAGGGAGAAAGGAAGGAAAATAGTTGCGGGTCAATGGTTTGCACTATTCTCCCTCTCCCTATAGTGAGTCGT
ATTAGAATTCCCC
+
DDACCC@BB=???B>B@B@B=<<<6?BAA;??<<;?BB@CC@BB>BCBBAAD@CBBC=BBCCD@CAAABBBCBBB@?
?B?A==8<8;<;1
@T6XXI:00119:00094
GGGGAATTCTAATACGACTCACTATAGGAGAGGGAGAATAGTCAACAAATCGTTTGGGGCGACTTCTCCTTCCTTTC
TCCCTTCTCCC
+
DDDDC?B@BBB@CBBBCCBB@:=96/.-
0>;?C@DBB>@AAA==;=@A:@@===.777.7>==<=>7750289?@<AABA;@=@@AC;
@T6XXI:00120:00077
GGGGAATTCTAATACGACTCACTATAGGGAGAGGGAGAATAGTCAACAAATCGTTTGGGGCGACTTCTCCTTCCTTT
CTCCC
+
DGG9B?B@CCC@BBAAAA>>BA?93166.7>>AC@BBC@A<??AA>???7>?777.777.7??AC?AAB=<9=>AA;
?ABC?
@T6XXI:00121:00051
GGGAGAAGGGAGAAAGGAAGGAGAAGTCGCCCCAAACGATTTGTTGACTATTCTCCCTCTCCCTATAGTGAGTCGTA
TTAGAATTCCCC
+
AC<ABA?CC<A@@C?C?BAA=??@<>>@??>>.8808@9AA>BB?BBAAAA>@@?@<@9::C?D=<=@@?@=<<??8
868@?8828480
@T6XXI:00121:00061
```

```
GGGAGAAGGGAGAAAGGAAGTGTACAGTTAACAGCCGGGGGGAATTGGCTATTCTCCCTCTCCCTATAGTGAGTCGT
ATTAGAATTCCCC
+
EEABBB@BB:@@>=:@>A@CBBBCCDCC@E?BC;;544444)9<@ACADEBB?B???8?AACD@CBB@@@AAA@@@?
==>=<=;927880
@T6XXI:00121:00086
GGGGAATTCTAATACGACTCACTATAGGGAGAGGGAGAATAGTCAACAAATCGTTTGGGGCGACTTCTCCTTCCTTT
CTCCCTTCTCCC
+
BBB9B@C@CBCABB>@@BBBBAB99111.7<<?ACBBBAA=A??::;AC:9999>;>>>8@@@===:BB=>;>8;;6
<==?;@>AA@@;
@T6XXI:00121:00099
GGGAGAAGGGAGAAAGGAAGCTAGTCACTCGAGATCTGCGGCTTTGGACAGTCCCTCTGTACTGTGAAGCCACAG
+
DD<<;<?AA:A@>>9>694;A?>>A?;:66B?=?A9978>;78:6:@CCCCCCD>?@=A@???=;<?;<><>?@?
@T6XXI:00122:00053
GGGGAATTCTAATACGACTCACTATAGGAGAGGGAGAATAGTCAACAAATCGTTTGGGGCGACTTCTCCTTCCTTTC
TCCCTTCTCCC
+
499/9<@AAAB?BCFBA@@BB:996..,0::>AACCC?AAA@=<:773,7>>==-
777.:@ABC@CBC@=:=?CC=A==<7<;=>@A<
@T6XXI:00122:00057
GGGAGAAGGGAGAAAGGAAGGAGAAGTCGCCCCAAACGATTTGTTGACTATTCTCCCTCTCCCTATAGTGAGTCGTA
TTAGAATTCCCC
+
DD?BBC@CC?BBBB>B@A=?=@BC@CCCBBAA8AC>CCCBD>CC@BCBCB?=:;;=;A@@AA:@?????BAAA@@==
=>AA>94/444-
@T6XXI:00122:00067
GGGAGAAGGGAGAAAGGAAGGAGAAGTCGCCCCAAACGATTTGTTGACTATTCTCCCTCTCCCTATAGTGAGTCGTA
TTCAGAATTCCCC
+
EDABBA@CC??A@C=@=@<@>AAB@CBCAAAA7AC>CCEDC>CD?BAAABA9<:<<6880A;3885A@@@A@<<;//
....4:484<44.
@T6XXI:00122:00072
GGGAGAAGGGAGAAAGGAAGGATGTCGTAGGGTGAGAGAGTTGCGATCTATTCTCCCTCTCCCTATAGTGAGTCGTA
TTAGAATTCCCC
+
@C>AA@@BB>BBBC>B?B@C@CABDBB@@AC>A@CCC@A@A<>><;9>@AB?A@@>:>@99909@?>@AAA=@BBBB
B?A<<>A7;;<4
@T6XXI:00123:00054
GGGGAATTCTAATACGACTCACTATAGGGAGAGGGAGAATAGTCTTCCAATTTTGGGCGGTACTGTCTCCTTCCTTT
CTCCCTTCTCCC
+
999-
49?@CCB@??@CCCCBB@?;;6??9??ACE>@@@@AAA<??>?>C@CDD9BB>CC?BBDDBBA@@?B?B@CD<??CE
>B5::??6
@T6XXI:00123:00057
GGGGAATTCTAATACGACTCACTATAGGGAGAGGGAGAATAGCTAGCTCATACTCCCAGGCTAACGCGACTTCCTTT
CTCCCTTCTCCC
+
CIK9C>A=@BCCGKCCB@AAB@;99111.:B?BC>C@?=<=<6?BBBBBCCCE=?B=?C@BB@?=@@@BB@<?<?@:
?;;;498448=7
@T6XXI:00123:00059
CTAGTCACTCGAGATCTGCGGCTTTGGACAGTCCCTCTGTACTGTGAAGCCACAGATGTCGAGTCGACTAGCTTCTA
TTCTCCCTCTCCCTATAGTGAGTCGTATTAGAATTCCCC
+
```

```
C8//1=/4:CBABBCAAA?=<<<@<@<@???:::5:@@BBCCC???<;<C@CCCBCBBBABBCCCBBCCCCCC?ACB
B>A@AB=>>777-4??=??CBB@???74/434/7:<??7
@T6XXI:00123:00070
GGGAGAAGGGAGAAAGGAAGCTAGTCACTCGACATCTGTGGCTTCACAG
+
AC<>@@59909999095959A@>>>>76111111688>@??BCBCBC??
@T6XXI:00123:00085
GGGGAATTCTAATACGACTCACTATAGGGAGAGGGGGAATAA
+
AAA7A>A?CCC@CCBBC>@@BABB?:8?6779::::/:,0,*
@T6XXI:00123:00088
GGGAGAAGGGAGAAAGGAAGAATACGGTGATGGGAAGCTGAAAAGACGGCTATTCTCCCTCTCCCTATAGT
+
KIAAAC@CC@BBAA?A@B@?><ABCCACBBCBB=B@A><7777-8@??=@BCC?BBBBBBDDCC>B@@@B@
@T6XXI:00123:00090
CTAGTCACTCGAGATCTGCGGCTTTGGACAGTCCCTCTGTACTGTGAAGCCACAGATGTCGAGTCGACTAGCTTCCT
TTCTCCCTTCTCCC
+
C;999@44:CDCCBCA??AA@??B<B>AA@@9994:??BBBBC=??B>@C?BBCCCCCBCBBACBBAAAA??<>B?B
LE@@@E>@<@???:
@T6XXI:00123:00091
GGGAGAAGGGAGAAAGGAAGCTAGTCACTCGACATCTGTGGCTTCACAGTACAGAGGGACTGTCCAAAGCCGCAGAT
CTCGAGTCGACTAG
+
CDACDC?BB>@>>=:?>==BCBBBC<????=77:::9BCC?AA@CCCC@?CB?933317>>@AA=BC=AA<??<===
=<<99=AC@@@AA?
@T6XXI:00124:00077
GGGAGAAGGGAGAAAGGAAGCTAGTCACTCGACATCTGTGGCTTCACAGTACAGAGGGACTGTCCAAAGCCGCAGAT
CTCGAGTCGACTAG
+
EDBBAA@CE>BB==:@=@<@CB<@A7:7=?>:;:???BDG?AB?ABB@@?AC?=4441399 8??>@A<@A>???@@@
???A===8==A@AA
@T6XXI:00124:00090
GGGAGAAGGGAGAAAGGAAGGAGAAGTCGCCCCAAACGATTTGTTGACTATTCTCCCTCTCCCTATAGTGAGTCGTA
TTAGAATTCCC
+
CCACCC@BB>BB?A=B>@@B@CCB?CCBBBCC9CC>CCB@@>@A@C?><?=>AAAA>>>>A=;=;;=<==488????
@=@AA?A?AA9
@T6XXI:00124:00095
GGGGAATTCTAATACGACTCACTATAGGGAGAGGGAGAATAGTCAACAAATCGTTTGGGGCGACTTCCCTTCCTTCC
TTCCTC
+
==?4?<@=ACC@CBAAACCBBBB><666-6;;?B>BBD@A=?@@@>@@?497166-777-
7@>?>:<<,0,0,/,,5555+/-
@T6XXI:00124:00099
GGGGAATTCTAATACGACTCACTATAGGGAGAGGGAGAATAGCACGGCTCGAAAGTGCAGTCAGTTCTCCTTCCTTT
CTCCCTTCTCCC
+
AAA9B>A?BBB@AAAAA?<<??BCB>>>;888AE>BAB?A@@@<<@@A??77757?CAA?;;:==<===;<=@;@?<
?A==;=>=A><;
@T6XXI:00125:00051
GGGAGAAGGGAGAAAGGAAGCTAGTCACTCGACATCTGTGGCTTCACAGTACAGAGGGACTGTCCAAAGCCCGCAGA
TCTCGAGTCGACTAG
+
BB>@@>58809111,1-1-1;:>?CA???A@???BBBBBB@BB?AA@@@@@@A<=<<:<:66662384334-
4>>?AAA???<<=<??AA==
@T6XXI:00125:00073
```

```
GGGAGAAGGGAGAAAGGAAGTTGAAGAACAAGCTGCAGAGAAGAAGACTATTCTCCCTCTCCCTATAGTGAGTCGTA
TTAGAATTCCCC
+
CD?CCB@BCBA?=BBB@A@BB@>>=@B@BB@BCCB?B????>@@>?<<<C@@B@A@;@????:?@@@AAAAAAAA==
=>@A@>@9===4
@T6XXI:00125:00087
GGAGAAGGGAGA
+
1,///,//+//1
@T6XXI:00125:00089
GGGAGAAGGGAGAAAGGAAGCTAGTCACTCGACATCTGTGGCTTCACAGTACAGAGGGACTGTCCAAAGCCGCAGAT
CTCGAGTCGACTAGCTTCCTTTCTCTCTTCTCCC
+
DB@B9959>:>99919>><AF;88876111111111179<5:;?DBBBCBBBA=;;;6;;>@@@>AB>??<?@888@
AAA???;;;=000;;9,/,44-4<<=@@?A=>>9
@T6XXI:00125:00097
GGGGAATTCTAATACGACTCACTATAGGAGAGGGAGAATAGCCAATCGATTTCTCCTGCCAGGCTGCCCTTCCTTTC
TCCCTTCTCCC
+
GDE9C8;;<@@@EGCCC9;:;00?<00./=>@B?C@?=<???>A>???88814;;4888;=?;?@@@@;@?A<>>5;
;=>:?>AAAA@
@T6XXI:00126:00066
GGGAGAAGGGAGAAAGGAAGGAGAAGTCGCCCCAAACGATTTGTTGACTATTCTCCCTCTCCCTATAGTGAGTCGTA
TTAGACC
+
BB@@@B?BB>BBBB?B@C@B@BB>@B@B7733,8>:?CCCC>CC@CCCCB=<=@<<:<??AA<A@???BA====@=A
A?AAAC@
@T6XXI:00126:00077
GGGAGAAGGGAGAAAGGAAGGGAATAGTCGCGCTTGTTGTCAACGTGCTATTCTCCCTCTCCCTATAGTGAGTCGTA
TTAGAATTCCCC
+
DB=ACC@CCADCBC>B@B=@@>@@CCBAFCCCBB@CC@AA@A@CBBBCBCEDFBBB<;;<AB>BDDBBBD@@?@@@@
@BBDECDCDDD:
@T6XXI:00126:00082
GGGGAATTCTAATACGACTCACTATAGGGAGAGGGAGAATAGCCGTTATTTTAACTCCCGCGGCCGGTTCTTCCTTT
CTCCCTTCTCCC
+
BAD7A8;9;CCDCCBBBA@@BC?99111.7==>@>@@@ABA?@;=?;A>==5::?@AB;@@?;<:>=@>AA>A>AA<
===@;@>A@@@<
@T6XXI:00126:00085
GGGAGAAGGGAGAAAGGAAGCTAGTCACTCGACATCTGTGGCTTCACAGTACAGAGGGACTGTCCAAAGCCGCAGAT
CTCGAGTCGACTAG
+
D?6@@@;>>:>@>=9>594988?>>711474793...3577AB@C??????==9333-
44;:<?BAD@==:==<<@A>>?????@@AAAA@
@T6XXI:00126:00090
CTATCACTCGAGATCTGCGGCTTTGGACAGTCCCTCTGTACTGTGAAGCCACAGATGTCGAGTCGACTAGCTTCCTT
TCTCCCTTCTCCCCT
+
8994>949444@94488877777,313336111,17116???BC==98@?BBBCCB@??C@??BD?;:39660626>
;;;@@10/0----*-
@T6XXI:00126:00092
GGGAGAAG
+
??5;=<=A
@T6XXI:00126:00097
GGGAGAAGGGAGAAAGGAAGGAGAAGTCGCCCCAAACG
```

```
+
CD?AAC@CC?CCCB=B?BAB@BCB@B?@@BCC6@@:?A
@T6XXI:00127:00073
GGGGAATTCTAATACGACTCACTATAGGAGAGGGAGAATAGTCAACAAATCGTTTGGGGCGACTTCTCCTTCCTTTC
TCCCTTCTCCC
+
@??4?<?<@@@?BCCCCCB@B:66;..,0<;>?>BCC@A:?=AA?@AA<B????:???5??@@@>A??=<=@>A@<@
AAA<A;==@A?
@T6XXI:00127:00082
GGGGAATTCTAATACGACTCACTATAGGGAGAGGGAGAATAGCCGACAACCACCAC
+
ABA6A8;8<BB@CCCCCCCBC?>>>777.777==9?AA@A@@@::6..,/,/.300
@T6XXI:00127:00083
GGGAGAAGGGAGAAAGGAAGGAGATATAGACACGCCAATAGAAACGCGCTATTCTCCCTCTCCCTATAGTGAGTCGT
ATTAGAATTCCCC
+
GG@EB@@AA=@@@A?B@B?B@AAAA???BBBCCBBDB@C?BAA=ACCBBBCC@CBAA>@?@CC>B>?BBCG8::???
??;???<AACBC9
@T6XXI:00127:00087
GGGGAATTCTAATACGACTCACTATAGGGAGAGGGAGAATAGCTCCTTTCTCCTCTGACGGCGGTTGCCCTTCCTTT
CTCCCTTCTCCC
+
ECD:C;:<;CB@CCB@@@BBB@A@B66659??CC>CBB>?;==?>>@@?=??>??A@@@@><<9<=@@@;><@3881
8??>;@>@@@C=
@T6XXI:00127:00097
GGGGAATTCTAATACGACTCACTATAGGGAGAGGGAGAATAGTGACGTGTTGTGCGGCGATCACTGTCTCCTTCCTT
TCTCCCTTCTCCC
+
BCC:C@D@B??=@CBAB@@@C@A?<667.777>@=BCBBCAA?;?=<<<<;<<<@<;<<<;<<<<====<;<86555
45=?A:?<????;
@T6XXI:00128:00051
GGGAGAAGGGAGAAAGGAAAGCTAGTCACTCGACATCTGTGGCTTCACAGTACAGAGGGACTGTCCACGCAGATCTC
GAGTCGACTAGCTTCCTTTCTCCCTTCTCCC
+
EC>B;969@:?99904244-49996600/..7/./7;;;@A<?@=?@@@BBA==:444-
499;;9<A==<AAA@<<<;>@>9999@@@ADBE@DD;<<A?097;@?B<
@T6XXI:00128:00067
GGGGAATTCTAATACGACTCACTATAGGAGAGGGAGAATAGCAAACGTTTCCCCGACGTCGCTTTCTCCTTCCTTTC
TCCCTTC
+
@BB4><@=A@AAC=<<CCBBA>>99/.,00077-
77798>>>@?;?CCE>BBB9BCCC:::??>6<=:9:9:,//,,+0003*+
@T6XXI:00128:00081
GGGGAATTCTAATACGACTCACTATAGGGAGAGGGAGAATAGCCATCCCCTCTTACATCATTCGCTTTTACTTCCTT
TCTCCCTTCTCCC
+
999/49AACBBADBBABBCAAAA??111.7==>A=AAA>@@<<8<><<=1=;;8?@==<<<9<;?AA@5;;;8;8;@
:<;<@7<8;??=:
@T6XXI:00128:00087
GGGGAATTCTAATACGACTCACTATAGGGAGAGGGAGAATAGTCAACAAATCGTTTGGGGCGACTTCTCCTTCCTTT
CTCCCTTCTCCC
+
CCB9C>A?BCC@CBBABCCBCEA?9116.7==BE>CBC@A8??@A>@@@<BCAA988885?BBBCAC@?==>C?BA:
@@@A<?<?ABB;
@T6XXI:00129:00063
GGGAGAAGGGAGAAAGGAAGCATAGCCAGGGCGTGAGTTTATCATCAGCTATTCTCCCTCTCCCTATAGTGAGTCGT
ATTAGATCCC
```

```
+
CCACCC?BC>BBBB>B@@@BBBA>>>@@@@;BCB>??>>;??@@C@>>BCCA><@@@;@====;@<@@@@@AAA@?:
??>AAA@@A;
@T6XXI:00129:00066
CTAGTCACTCGAGATCTGCGGCTTTGGACAGTCCCTCTGTACTGTGAAGCCACAGATGTCGAGTCGACTAGCTTCCT
TTCTCCCTTCTCCC
+
C8/////4:CCCCCCBB@AA=??B<B<@AAA::=;?@@A><<@>AA@><D?BBBBBBAA@AAC@BBBABB@@??C@B
C>BBBB>A>A@@@<
@T6XXI:00129:00071
TCGAGATCTGCGGCTTTGGACAGTCCCTCTGTACTGTGAAGCCACAGATGTCGAGTCGACTAGCTTCCTTTCTCCCT
TCTCCC
+
DCCDCCBBBBBB@B??;?=BBBBB@@>AA@AAB?@@@AC???:@AA@<<==BBBCBBBCABBAAAAC@CC>BBEHAC
?BAAA<
@T6XXI:00129:00079
GGGAGAAGGGAGAAAGGAAGCTAGTCACTCGACATCTGTGGCTTCACAGTACAGAGGGACTGTCCAAAGCCGCAGAT
CTCGAGTCGACTAG
+
DCACAA6998<BCB=B=@=@C>>AB<@???@:::???B??=?A@ECCCCBA??933313<999>37717>;?@@AAA
???<<@<=@<?A=A
@T6XXI:00129:00083
GGGAGAAGGGAGAAAGGAAGCTAGTCACTCGAGATCTGCGGCTTTGGACAGTCCCTCTGTACTGTGAACCACAGATG
TCGAGTCGACTAGCTTCCTTCTCCCTTCTCCC
+
CB<@<959@:@999042824A<>;B:::BAAACCCCA::;>???<??@A@@?==677430030003;3733400030
038555?;;::::/1-1-1,/6665656444-
@T6XXI:00129:00099
GGGGAATTCTAATACGACTCACTATAGGGAGAGGGAGAATAGTCAACAAATCGTTTGGGGCGACTTCTCCTTCCTTT
CTCCCTTCCTCCC
+
DF@6@<>>A@@>BC<;<CCDEAB??766.7>>>@=ADC@B@A===?A@@<CBBBC<BBB7AACBC@CCB@A>@>AA<
A;9728,/,/761
@T6XXI:00130:00052
GGGAGAAGGGAGAAAGGAAGGTAAAACGTGAGGGGAGCTAGGTGTCAACTATTCTCCCTCTCCCTATAGTGAGTCGT
ATTAGAATTCCCC
+
CCA@?=@CH@9<;B<@=@?B?BCBB7CCBBBBBB9A@ABBB@CBBBB@@BBB@B??A=88;CE>CBB???ACA???=
==>AAB?C>@@@5
@T6XXI:00130:00056
GGGAGAAGGGAGAAAGGAAGTGCAGTTCGCGGTTAGTGAAGGGAAAAACTATTCTCCCTCTCCCTATAGTGAGTCGT
ATTAGAATTCCCC
+
9904499>:-
849918?B@CBBBCCB@?BBA@C@BCCCC;882?;??2ADDE?BBBB>BBCKE>;98=AACBAA???8888AA>B38
880
@T6XXI:00130:00075
GGGAGAAGGGAGAAAGGAAGCTAGTCACTCGACATCTGTGGCTTCACAGTACAGAGGGACTGTCCAAAGCCGCAGAT
CTCGAGTCGACTAGCTTCCTTTCTCCCTTCTCCC
+
ECCAA@@CB=B;;;09=@6;;??>B777767761116>8:8?B?;:;CCCCCB@88818;;<A@>AA<@@=@??=<<
?=><<<>>;<=???AA>?;>>589DC=C69:AA<
@T6XXI:00130:00084
GGGAGAAGGGAGAAAGGAAGGAGAACGCGCAATCAGATACCTGTTGGCTATTCTCCCTCTCCCTATAGTGAGTCG
+
CD@BBA@BC>BBBB=B?BDB@BBB;BCCBAA?BCDDGBCDACDE>ACACCC@C@@@<@>@CC?CCCCCA@@@BBB
@T6XXI:00130:00090
```

```
CTAGTCACTCGAGATCTGCGGCTTTGGACAGTCCCTCTGTACTGTGAAGCCACAGATGTCGAGTCGACTAGCTTCCT
TTCTCCCTTCTCCC
+
@99444444499499@AABC@CCD=A?AB>?777.7>?@ABBBBBB<58<@CCCCBA;;:7;>>9988773112627
@;===>68/4000+
@T6XXI:00131:00051
GGGAGAAGGGAGAAAGGAAGGAGACAGTGAGCGCCGCACAACACGTCACTATTCTCCCTCTCCCTATAGTGAGTCGT
ATTAGAATTCCCC
+
CEACCB@DD>BBAB>C?B>B?:;;ACCCDCCCBB>>>BB?@CCC???CCCCC@B;;:6;>@AB>BBBBCBBBCECAA
AC?A@@<@5999/
@T6XXI:00131:00061
GGGGAATTCTAATACGACTCACTATAGGGAGAGGGAGAATAGCCGTGAATTAGTTCACGATCTGCTTCTCTTCCTTT
CTCCCTTCTCCC
+
@==6><@>B@@=ACDBABBBB@<99111.7??CE>BBC@A<?::<AAC><=<@C?CBBBCAAABCC@E<;;?CAFC?
@AAA=A=@@AA;
@T6XXI:00131:00063
CTAGTCACTCGAGATCTGCGGCTTTGGACAGTCCCTCTGTACTGTGAAGCCACAGATGTCGAGTCGACTAGCTTCCT
TTCTCCCTTCTCCC
+
EA;<<A:49@=999?B@AADBDBB<A=??AA=??9;;:ABBBCBA@@:8969>A@BCCDCC@@;=<B>>9;110166
99@B@A>=585===
@T6XXI:00131:00073
GGGGAATTCTAATACGACTCACTATAGGAGAGGGAGAATAGTCAACAAATCGTTTGGGGCGACTTCTCCTTCCTTTC
TCCCTTCTCCC
+
;<<49?B@CDB@CBA@BBBB?7996..,0::>ABBGD@A>>??@<9=<7?=677-777-
7>@AC?AAB??<?>AC===?A>D9<8881
@T6XXI:00131:00077
GGGAGAAGGGAGAAAGGAAGAATAGCTCACGGTGGGAGATGAAATGGCTATTCTCCCTCTCCCTATAGTGAGTCGTA
TTAGAATTCCCC
+
AA<ADDDDD>CAA?=B>@>BC@CCBBCCCCB?;;;89>@@>@@<AA;ACCC@BBBCACC;::6:@@ACCBBBCCC@8
858@>@=<<<A6
@T6XXI:00131:00080
GGGGAATTCTAATACGACTCACTATAGGGAGAGGGAGAATAGTTTTTCCCTTCACTAACCGCGAACTGCACTTCCTT
TCTCCCTTCTCCC
+
?==6@>A>BECACAA@@@???AB?;661.6==?A>BCC@AA@A<<>0;;6<<?BCCC?C?:7:?;;>A<??A>A=AB
<?@?E?CAB@@@;
@T6XXI:00131:00087
GGGAGAAGGGAGAAAGGAAGCTAGTCGACTCGACATCTGTGGCTTCACAGTACAGAGGACTGTCCAAAGCCGCAGAT
CTCGAGTCGACTAG
+
EEBCBE=>=:@@@==B>@>@B800:0//;7;:0;7877CEK?CB?BB>>@@=:;7..-
/;;;?C>@@;@A>AA@A?;:===AB???@@@A@
@T6XXI:00131:00090
GGGAGAAGGGAGAAAGGAAGGAGAAGTCGCCCCAAACGATTTGTTGACTATTCTCCCTCTCCCTATAGTGAGTCGTA
TTCAGAATTCCCC
+
CD>BBB@CC??>?C@C??>B@CAA88;CCCED7AB@ABA@B?BB?BCBBC@;==?A:<<=?A:@??<<;===>?>=A
@378<=9<>CBA5
@T6XXI:00131:00093
GGGGAATTCTAATACGACTCACTATAGGGAGAGGGAGAATAGTCAACAAATCGTTTGGGGCGACTTCTCCTTCCTTT
CTCCCTTCTCCC
+
```

```
CCC9C@C@CBA?BCCCCCCBBCB;;661-
6=<<?<?CCAAAA?@@>A@@;A@@@>6<??5??B@@>???>?>A?A@:>84418387AB=
@T6XXI:00131:00095
TAGTCGACTCGAGATCTGCGGCTTTGGACAGTCCCTCTGTACTGTGAAGCCACAGATGTCGAGTCGACTAGCTTCCT
TTCTCCCTTCTCCC
+
ADGCBBBBBBBCBBBCBBBB@BBC?B@BCBBBBB>A@@AA?ACBBBB>AA<ABBBBB@?B??A@<==?A@@AC?@@A
C====?;?><@@A;
@T6XXI:00132:00052
GGGAGAAGGGAGAAAGGAAGGAGAAGTCGCCCCAAACGATTTGTTGACTATTCTCCCTCTCCCTATAGTGAGTCGTA
TTAGAATTCCCC
+
CC@BBAACC>?AAC>C?B@B@CBB?@?@@@>@5??<@KJEE?BB?CAAAA>;====:<==AA<A???AA@@@@@@@?
>;:6424/034-
@T6XXI:00132:00064
CTAGTCACTCGAGATCTGCGGCTTTGGACAGTCCCTCTGTACTGTGAAGCCACAGATGTCGAGTCGACTAGCTTCCT
TTCTCCCTTCTCCC
+
B8/11=8=?DCBCBCBBBBB@BBB>B?@A@B:::5:BBCBBBCABBA>@C@BBABCC?A<BAAABB@@@DBBA?B?B
C<AACC@B9==@A<
@T6XXI:00132:00065
CTATCACTCGAGATCTGCGGCTTTGGACAGTCCCTCTGTACTGTGAAGCCACAGATGTCGAGTCGACTAGCCTTCCT
TTCTCCCTTCTCCC
+
C==?@::;BBBBBCBBFA@>@CC?C?ABCCBBB>BBCBABBBBB?A>AC=@@CCCCCC??@BBAA1104..,.555;
>:@BAA;:566684
@T6XXI:00132:00067
GGGAGAAGGGAGAAAGGAAGCTAGTCACTCGACCATC
+
CC>AA@@BHC;<;C?C=@=@@@?@BB7007,,*)*((
@T6XXI:00132:00075
GGGAGAAGGGAGAAAGGAAGTGCAGTTCGCGGTTAGTGAAGGGAAAAACTATTCTCCCTCTCCCTATAGTGAGTCGT
ATTAGAATTCCCC
+
49149A@CH@AABCCCAD@B88:CCD=@@?@7858>773116,6>>?2@AA<:78;;261111,146>@??>=<466
3327<A<=>AAC5
@T6XXI:00132:00076
GGGAGAAGGGAGAAAGGAAGGAGAAGTCGCCCCAAACGATTTGTTGACTATTCTCCCTCTCCCTATAGTGAGTCGTA
TTCAGAATTCCCC
+
BDBBBB@BB?AA?B:=<@7;8;@@@NABACCC9CB?BAABFBBB?B;;<B?=?ACC=@?@@A<@89:A@@@??<;88
7,////,/,444+
@T6XXI:00132:00088
CTAGTCACTCGAGATCTGCGGCTTTGGGACAGTCCCTCTGTACTGTGAAGCCACAGATGTCGAGTCGACTAGCTTCC
TTTCTCCCTTCTCCC
+
:7///6/44@=A@@AGCCBC?@@;6?<6>?8;:AA=ACCBBAAA>AAD?AC?BCCAA8:;B@BCC<<<B@@@??<??
@B<AABB>@<??@A;
@T6XXI:00132:00093
GGGGAATTCTAATACGACTCACTATAGGGAGAGGGAGAATAGTCCTTATTTTGTCTCCCCGTCACTGTGCTTCCTTT
CTCCCTTCTCCC
+
CDD:C?A?BCCBCCBBBBBBB@<99111.7==AC@BCB@A=AA?=:8?>A;4=?:@@<<5<=;;:====;;<=;<<;
<<<<;<<==<<;
@T6XXI:00133:00055
GGGGAATTCTAATACGACTCACTATAGGGAGAGGGAGAATAGCCGGTCACCTGTCCCCTGCC
+
```

```
66:+2-9AB>?>@ABCB@?BBACC><??66;666.7?A<=???;=><4110111111,1131
@T6XXI:00133:00078
GGGAGAAGGGAGAAAGGAAGCTAGTCACTCGAGATCTGCGGCTTTGGACAGTCCCTCTGTACTGTGAAGCCACAGAT
GTCGAGTCGACTAGCTTCCTTTCTCCCTTCTCCC
+
DB<EA@=>A:>99809;><AC??@B6:7@@:::BCCBCBB?ACC>C=?;;>?@?;@@=<<<?@@<<?<<@;@@A@@?
??AAAA@A@<??5;7957<>>:>988-43444:4
@T6XXI:00133:00084
GGGAGAAGGGAGAAAGGAAGCTAGTCACTCGAGATCTGCGGCTTTGGACAGTCCCTCTGTACTGTGAAGCCACAGAT
GTCGAG
+
DD?CA@=@B:@@@>9?5;8;?>>?A::96?A@ACCCBBBB?9776:>BAAACCC>CC<???AAA@?B?AA8:=;===
=<<<<;
@T6XXI:00134:00051
GGGGAATTCTAATACGACTCACTATAGGGAGAGGGAGAATAGTCAACAAATCGTTTGGGGCGACTTCTCCTTCCTTT
CTCCCTTCTCCC
+
@AA6A@A@CBE?BCBBBBBBBAB<;11159@@CC>BBB>A::<A@=78719A;??<A??6B???C?A@A<<9<?AA<
AAAA<A:<<AA<
@T6XXI:00134:00063
GGGGAATTCTAATACGACTCACTATAGGGAGAGGGAGAATAGTCAACAAATCGTTTGGGGCGACTTCTCCTTCCTTT
CTCCCTTCTCCC
+
DCC9C9;;<CB@CCBB?A?BB@>97111.777?@>BCF@@@@@@@>@<<:>BACA>@@@7BAABC@BCD@@=?>AC<
AA@?7<588@A:
@T6XXI:00134:00067
GGGGAATTCTAATACGACTCACTATAGGGAGAGGGAGAATAGTCAACAAATCATTTGGGGCGACTTCTCCTTCCTTT
CTCCCTTCTCCC
+
CCC9C@C@BCBEBBAAABBBB@;99111.777BC@GCC@B=AA=<<=984?BAAA9?885<@???=???:?=<>AA<
A???;??AA@@<
@T6XXI:00134:00070
GGGGAATTCTAATACGACTCACTATAGGGAGAGGGAGAATAGCACCATTTAATCCCGTACCTGTTTTGTTCTTCCTT
TCTCCCTTCTCCC
+
:44,9>@>BCC@CCBBB?BBBCB>;16656??CE>BAA@A=A@<<;>=C9=9=AA<A==??CCCCC8BB@BB>A@AB
=A@@@;@>@@AA=
@T6XXI:00134:00078
GGGAGAAGGGAGAAAGGAAGCTAGTCGACTCGACATCTGTGGCTTCACAGTACAGAGGACTGTCCAAAGCCGCAGAT
CTCGAGTCGACTAG
+
?A:BBB@CC<@@>>9>6959B7<?@71161117<6>;///7-
/09>>>CCCB>>9..,/8868=>@@:<<:=@A@C@AA?===C=<=A@==
@T6XXI:00134:00080
GGGAGAAGGGAGAAAGGAAGGATATGCCGCAGTGGGAGATTAAACGGCTATTCTCCCTCTCCCTATAGTGAGTCGTA
TTAGAATTCCCC
+
;;8<AA?BGABB?BBB@C@B@B<<;7;8;BBBBBB>CCCC@??;@C@CCDC?C;;969>>?@@A@B@CCCCCCA???
<8<BA8<8<?@1
@T6XXI:00134:00089
GGGAGAAGGGAGAAAGGAAGCTAGTCACTCGACATCTGTGGCTTCACAGTACAGAGGGACTGTCCAAAGCCGCAGAT
CTCGAGTCGACTAG
+
DC@DBB@CB?BCBB>C>?=@BAAAB?BAAAA::;ABABBC?BB@CA><?@C?>9333,399388;>@988338>???
BAA===A===??A@
@T6XXI:00134:00099
```

```
CGAGATCTGCGGCTTTGGACAGTCCCTCTGTACTGTGAAGCCACAGATGTCGAGTCGACTAGCTTCCTTTCTCCCTT
CTCCC
+
CCBBBBAAABB@BBB>B@BBBBB??;?@@BBB??>=AB=>>?>>?B@@<@AAACCCCBBBCCAA@A@CG?CBAB<@<
??AB<
@T6XXI:00135:00062
GGGAGAAGGGAGAAAGGAAGGAGAAGTCGCCCCAAACGATTTGTTGACTATTCTCCCTCCTCCCTATAGTGAGTCG
+
EKAAA@@CC@@@@@9><>>B?BCC?BBBBCCC9B@9>>871,177B;;7C>;;;::2//,///,/689>@@???==
@T6XXI:00135:00067
GGGGAATTCTAATACGACTCACTATAGGGAGAGGGAGAATAGTCAACAAAATCGTTTGGGGCGACTTCTCCTTCCTT
TCTCCCTTCTCCC
+
CCE9A@AACCC@CCDCC;;8ABABB???6=>77:5:CC>@@@;::74444+17==>;ABB9BCDBB?BCB68348::
7==??;@>@CAB;
@T6XXI:00135:00074
GGGGAATTCTAATACGACTCACTATAGGGAGAGGGAGAATAGTCAACAAATCGTTTGGGGCGACTTCTCCTTCCTTT
CTCCCTTCTCCC
+
@>?4>696<@@@CCCBBBG@@?@?;///,000::695356>778;8;<;6?B???.777.7=<<@=????=8;344-
49;;7;7:444-
@T6XXI:00135:00088
GGGGAATTCTAATACGACTCACTATAGGGAGAGGGAGAATAGATCACCGTTGGTTCGGGTGCGCGTTCTCCTTCCTT
TCTCCCTTCTCCC
+
DCC9A?A@CDB@BBB@BBBB>@?>>61659AACE?BBB@A=<<==AA>AB>A>A@BBB=B<;;ACC@DBB@B@C?B@
<??;;7<=@ABA=
@T6XXI:00137:00064
CTATCGACTCGAGATCTGCGGCTTTGGACAGTCCCTCTGTACTGTGAAGCCACAGATGTCGAGTCGACTAGCTTCCT
TTCTCCCTTCTCCC
+
D;8811188:@BCBBCBBBA??>><A>@<??77:5:A@BCBCCACA<<?CBBCBBAB?@@CCCCBBBCCDDCC?C@B
C>@?@C@C:;;AA<
@T6XXI:00137:00085
GGGGAATTCTAATACGACTCACTATAGGGAGAAGGG
+
999/49ADCBB@BBBABBB@B@A;;6..,//,+,70
@T6XXI:00139:00052
CGCGGCTTTGGACAGTCCCTCTGTACTGTGAAGCCACAGATGTCGAGTCGACTAGCTTCCTTTCTCCCTTCTCCC
+
FDCC@BBB>DACDBB<<<8;AACCCCDBAAA?@@=@AA@AAA@AAAA;<=AAAABBB=@=@D?B@?C<A8::AC<
@T6XXI:00139:00055
GGGGAATTCTAATACGACTCACTATAGGGAGAGGGAGAATAGCAACATCAC
+
BBA7B=@>ACC@CBBAAABCC><33766.7??BC>BBC>93/./,.---.3
@T6XXI:00139:00066
GGGGAATTCTAATACGACTCACTATAGGGAGAGGGAGAATAGTCAACAAATCGTTTGGGGCGACTTCTCCTTCCTTT
CTCCCTTCTCCC
+
999/<?B@DCB@BBBBBBBAB>?99166.6==BC>AAA@B<@A@?=7771:?=?A=ACC9BBCCC@@??5737:<<7
?@@C>D;==B@<
@T6XXI:00139:00077
GGGAGAAGGGAGAAAGGAAGGAGAAGTCGCCCAAACGATTTGTTGACTATTCTC
+
DH@CCD@A@:@99914242499;;:76>783,37279?@A87686611.2,---
@T6XXI:00139:00087
```

```
GGGAGAAGGGAGAAAGGAAGGAGAATTTGGCGTCGTGACGTGCGTGGCTATTCTCCCTCTCCCTATAGTGAGTCGTA
TTAGAATTCCCC
+
AABACCABA:>>AABB@B?B@BCC@CC>B@CBBAABBBBBB>@@AB@BBAB?AA@@=;;<CC@CCCCCBCBBB?;@7
95:??=B<ACC6
@T6XXI:00140:00058
GGGGAATTCTAATACGACTCACTATAGGGAGAGGGGA
+
CCD:C?B@BBB@CBBBBABBBCB>?167./:@000)*
@T6XXI:00140:00059
GGAGAAGGGAGAAAGGAAGCTAGTCACTCGACATCTGTGGCTTCACAGTACAGAGGGACTGTCCAAAGCCGCAGATC
TCGAGTCGAATCGA
+
B=B<<89B??99909599<BB@@@7776111119??@BA;;<?BBCCBBBBB@@AA<??AAAA>@@<AA>==<??=<
=@A;555,,+,,++
@T6XXI:00140:00068
GGGAGAAGGGAGAAAGGAAGAGTAGCCGGGGACCAGGAAAAGTTTGGCTATTCTCCCTCTCCCTATAGTGAGTCGTA
TTAGAACTTCCCC
+
CA>ADCBCB?BBBB@B@B@@@@DBBB?BNEABC?AA=@CE9ECC?C>BBCFACDCCACCDDC?CBBECBB@@?AB?<
=@@7=7492444,
@T6XXI:00141:00067
CTAGTCACTCGAGATCTGCGGCTTTGGACAGTCCCTCTGTACTGTGAAGCCACAGATGTCGAGTCGACTAGCTTCCT
TTCTCCCTTCTCCC
+
>1/////4:B==@ABCBDBC@CCC>B@B>;@9994:??AABBBBAAA?@D@CCCCBBABDCCBBCC@@@A::6::;?
C<A<BC<=8<;:=7
@T6XXI:00141:00089
GGGGAATTCTAATACGACTCACTATAGGGAGAGGGAGAATAGTCACATTCATGTTTGTGACCGTGTCTCCTTCCTTT
CTCCCTTCTCCC
+
ABB9B@CACBB@ABBBBBBBBBCCAA<<<<788CD>A@AAA???;??A@>?===@@;>=B@@>=@>>3442058=AA=
ABCC9=;9@@?4
@T6XXI:00141:00091
GGGAGAAGGGAGAAAGGAAGGAGATGTTCATGCGGATAAACGTTGAACTATTCTCCCTCTCCCTATAGTGAGTCGTA
TTAGA
+
CC?BCB@BC>BBBA;A@BAB@98:C@@=BDCCA?=@@@B>A@@=@ECEAAA>BBCE>?==??;?AAAAAAA?@??>7
434::
@T6XXI:00141:00099
GGGAGAAGGGAGAAAAGGAAGCTAGTCACTCGACATCTGTGGCTTCACAGTACAGAGGGACTGTCCAAAG
+
DEADCB@>;:;8444/959247337B?>37>=00///.47@588@CBAAACCBA:<<<7;;;;4424:-4
@T6XXI:00142:00072
GGGGAATTCTAATACGACTCACTATAGGGAGAGGGAGAATAGTCAACAAATCGTTTGGGGCGACTTCTCCTTCCTTT
CTCCCTTCTCCC
+
CBC9B?B?BCBABA@@BBBBB@;99111.7==>@BADDB@;@@A@?==<5?=;;;-
677.:ABBB@BBB@@>A<??<?AAA<A>A@@@<
@T6XXI:00142:00078
GGGAGAAGGGAGAAAGGAAGCTAGTCGACTCGACATCTGTGGCTTCACAGTACAGAGGGACTGTCCAAAGCCGCAGA
TCTCGAGTCGACTAG
+
BB?ABB@BB=B?==:=642;:8=>;//00/7:=>?:77@@B<?@?@DCCCCBBA?73317>>@@A>@@<@D?@???A
==@@888AAA@@???
@T6XXI:00142:00083
```

```
GGGGAATTCTAATACGACTCACTATAGGGAGAGGGAGAATAGTGACGTGTTGTGCGGCGCTCACTGTCTCCTTCCTT
TCTCCCTTCTCCC
+
DED9B=?==@@@BBBCBBACCA?99111.777?BACBC@@@@@???@@AA?AA?A;::====<=<??@@@>@>A>AA
7==AB<A?@AAA=
@T6XXI:00143:00057
GGGGAATTCTAATACGACTCACTATAGGGAGAGGGAGAATAGACACTCCCTAGAAACATGCGCCATTTGCTTCCTTT
CTCCCTTCTCCC
+
949/9>@@BAA@CDBBBBCBBCBB;111-
6<?CC@CCC@@@@<<<?AAA<AAA==;A==A<?@<=;=:>443479:6<=>A:@====@<
@T6XXI:00143:00062
GGGAGAAGGGAGAAAGGAAGCTAGTCACTCGAGATCTGCGGCTTTGGACAGTCCCTCTGTACTGTGAAGCCACAGAT
GTCAGCTACG
+
44-
44954=9=999148878???@A????@A>@A????AD=?AA:88?ABBECC<BA<;;;;9:<;?<;=;><=99;;96
//---++
@T6XXI:00143:00066
GGGGAATTCTAATACGACTCACTATAGGGAGAGGGAGAATAGCCTCCCGCTTATCGACAATAACATACTCTTCCTTC
TCCCTTCTCCC
+
999/9<@>AAB?BCBBBBBBA?<99111.777>F?C??@;///5;;>:>?@;BCCBCBC@CC@C@@::;;879,/,-
113454999@:
@T6XXI:00143:00073
CTATCACTCGAGATCTGCGGCTTTGGACAGTCCCTCTGTACTGTGAAGCCACAGATGTCGAGTCGACTAGCTTCCT
+
BA@=9::@DBABBGABBAA?AAB>B?BBAA:::7:CCBBBBC<?=A?AC@BBCBBBCCCCAAA<;064.,,,*,*,
@T6XXI:00143:00081
GGGAGAAGGGAGAAAGGAAGGAGACAGCGCCGAAGTCCAGGGTTGAGCTATTCTCCCTCTCCCTATAGTGAGTCGTA
TTAGAATTCCCC
+
DECBBBBDC?BBBB>F@C>A?>@@ACECBBDBC?ABB?BDE?C?BBCCCCB@B::97;CCCD>AAABCCA@;<<@==
A><?A;=8<<<0
@T6XXI:00143:00099
GGGGAATTCTA
+
EED:CAEAC<<
@T6XXI:00144:00051
GGGAGAAGGGAGAAAGGAAGGGAATAGCAGCAGTCGGCAACGTGAAACTATTCTCCCTCTCCCTATAGTGAGTCGTA
TTAGAACTTCCCC
+
CC?AAC@CC@BBAC998:?@@=B=@@DCAACBB881666644....+.47?788A@868661,14000544453333
32348444;===5
@T6XXI:00144:00057
GGGAGAAGGGAGAAAGGAAGTAGTCGACTCGACATCTGTGGCTTCACAGTACAGAGGGACTGTCCAAAGCCGCAGAT
CTCGAGTCGACTAG
+
=D??@@=ABABCCE@C?B?;;:@@;;;@AA?@@@;;::=;?@@=ABBABCAA@@99908>>733133,799;977:?
8??::9>>@<A???
@T6XXI:00144:00083
GGGAGAAGGGAGAAAGGAAGCTAGTCGACTCGACATCTGTGGCTTCACAGTACAGAGGGACTGTCCAAAGCCG
+
DD?D;;244-9@>>:>?>313?>@@BBB>67788111177>288BDBCCCCCBB533317>@AAA>BA:??<@
@T6XXI:00144:00085
GGGAGAAGGGAGAAAGGAAGCTAGTCACTACATCTGTGGCTTCACAGTACAGAGGGACTGTCCAAAGCCGCAGATGT
CGAGTCGACTAGCTTCCTTCTCCCTCTCC
```

```
+
IC>BCA>@>089>>:>>BACCBBA@?776111199>9<<>B=@@????@::;444-
49;>@@?AC<@@?A@@@CB===A??=;;9444:443/,/,////,.-222
@T6XXI:00145:00064
GGGGAATTCTAATACGACTCACTATAGGGAGAGGGAGAATAGTCAACAAATCGTTTGGGGCGACTTCTCC
+
DEC9C=A=@BC@DBBBB?BBAA;97366.7>==?<???@DCCA@>83331:><<@9???6@BBCC@????
@T6XXI:00145:00073
GGGGAATTCTAATACGACTCACTATAGGGAGAGGGAGAATAGTCAACAAATCGTTTGGGGCGACTTCTCCTTCCTTT
CTCCC
+
EBB9C=?>ACD@BAAABAA?>A<99311.7>>>@?BCC@C@CB@@>@AA<BBAAA<A??5BBAA@>A??>@BCAAB:
BBAA>
@T6XXI:00145:00096
GGGGAATTCTAATACGACTCACTATAGGGAGAGGGAGAATAGATCACGACCCTTCTACCACGATTGAGCTTCCTTTC
TCCCTTCT
+
A?=6@>A?BDE@CBAABBBBAAA?;76659>@CC>BAC>?@<88<A@CB??;<;>?::47?A::7:@?AA;>699:<
A@B<@=>>
@T6XXI:00146:00052
GGGGAATTCTAATACGACTCACTATAGGGAGAGGGAGAATAGCTAGCATTATTCCGCTAGGTGCTTCTCCTTCCTTC
TCCCTTCTCC
+
999/48><>A:<@;;<CCKBBB@>?877.7>77:7:CCACBBBBBA::;>@@<@@CBCBB>@?::39,,*,*,*,*.
...+100008
@T6XXI:00146:00053
GGGAGAAGGGAGAAAGGAAGCTAGTCACTCGACATCTGTGGCTTCACAGTACAGAGGGACTGTCCAAAGCCGCAGAT
CTCGAGT
+
:65611-
/756/4804999=B<<;?7.........55/117184:?ABBBBB>>477.4::8??<?D<AA9==@<<;788;<;=
@T6XXI:00146:00054
GGGAGAAGGGAGAAAGGAAGGAGGACAGTGAGCGCTGCACAACACGTCACTATTCTCCCTCTCCCTATAGTGAGTCG
TATTAGAATTCCCC
+
CD@CDD?=964989-
15:56,/.,.66:;CBC<<<DCBBCEC@?A@@ABDCCD?BCCC?BB;;;8:BBBCBBBBCBAB@?=?774969994
@T6XXI:00146:00074
GGGAGAAGGGAGAAAGGAAGTCCTCGACATCTGTGGCTTCACAGTACAGAGGGACTGTCCAAAGCCGCAGATCTCGA
GTCGACTAAG
+
EE?CB==AA<ACBC>BCD;<<A>AB?:::::7@AA6::??==@@@@AA?<==7<???;;8<=7==;@@@888@>==@
<<8699//--
@T6XXI:00146:00091
CTATCACTCGAGATCTGCGGCTTTGGACAGTCCCTCTGTACTGTGAAGCCACAGATGTCGAGTCGACTACGCTCCTT
CT
+
BAB?;:::AB=BBBBBBBB?BCDAC?ABA?7::5:>>@@A???AA=<@C=@?@@@????B::;C=888--
,,2200003
@T6XXI:00146:00094
GGGAGAAGGGAGAAAGGAAGCTAGTCACTCGACATCTGTGGTTCACAGTACAGAGGGACTGTCCAAAGCCGCAGATC
TCGAGTCGACTAGCTTCCTTTCTCCCTTCTCCC
+
AD?BCB;9909999095958>>>>>>77777?::9@?B??7:6:@B@@@CC=<=?>7====;<9;>:;<8=@AAA==
=????=8;;///;:;7;.8818@@@;@=;;;=7
@T6XXI:00147:00066
```

```
CTAGTCACTCGAGATCTGCGGCTTTGGACAGTCCCTCTGTACTGTGAAGCCACAGATGTCGAGTCGACTACTTCCTT
TCTCCCTTCTCCC
+
>>11188?ADCDAAACCCCC@CCB>B?ABBB@??8?BBCCCCC=??C?@C@CCCBBCBDAABB@AADDDA@@@C@BB
>CCCB=?=?BDK?
@T6XXI:00147:00082
GGGGAATTCTAATACGACTCACTATAGGGAGAGGGAGAATAGTCAACAAATCGTTTGGGGCGACTTCTCCTTCCTTT
CTCCCTTCTCCC
+
EEB9C@CBCBB?BBAA@B<<BBB?;711.6>>@C>BCC@@@?<@@=<<<7@B@@@9???5?::;C@CCC=@>=>AA;
?AA=;B:==?A<
@T6XXI:00148:00051
GGGGAATTCTAATACGAACTCACTATAGGGAGAGGGAGAATAGTCAACAAATCGTTTGGGGCGACTTCTCCTTCCTT
TCTCCCTTCTCCC
+
CDE9D?A>@A@@@<<11116669;73377-71111,16;8<AB;9;4044-
3377788885;88?@>@AA?@?@>?=4;96553/4-110
@T6XXI:00148:00055
GGGGAATTCTAATACGACTCACTATAGGGAGAGGGAGAATAGTTTTTCCCTTCACTAACCGCGAACTGCACTTCCTT
CTCCCTTCTCCC
+
499/429244488444=@?BCBBB;???5::ABB<BAB>>CB@@@>-
77,498>888=9=AA@B?BCA@>>>,0,0,//00,7;=7431
@T6XXI:00148:00072
GGGGAATTCTAATACGACTCACTATAGGGAGAGGGAGAATAGTCAACAAATCGTTTGGGGCGACTTCTCCTTCCTTT
CTCCCTTCTCCC
+
:;;4<?C@CCC@CABB??BBBBB>>761-
6>=?A>ACC??9AABB>@@@<BB9?=8;;;2?@?AC@BCC?B?B@AA=AA@@;?:==CC<
@T6XXI:00148:00074
GGGAGAAGGGAGAAAGGAAGGAGAAGTCGCCCCAAACGATTTATTGACTATTCTCCCTCTCCCTATAGTGAGTCGTA
TTAGAATTCCCC
+
CDBCBA@BC>CBBB=?<>=?>@BB@CCBBBAA8AC>C>>:>///::::BB@@@B=>;?<?@A<A???AAAA@@@A@@
@?@@?9;2444,
@T6XXI:00148:00085
GGGAGAAGGGAGAAAGGAAGCTAGTCGACTCGACATCTGTGGCTTCACAGTACAGAGGGACTGTCCAAAGCCGCAGA
TCTCGAGT
+
DDA@@@?BC:>>>>0959<@?>@A><==777=??=7:6A7B8:;8;@@AACBA?;444-
4::9:<<?A;@B<??@@@?<==777;
@T6XXI:00148:00092
CTAGTCGACTCGAGATCTGCGGCTTTGGACAGTCCCTCTGTACTGTGAAGCCACAGATGTGAGTCC
+
D?:9<<44=CCBCBEBBBDBB@@@@=@?A>>?88;67??ABA?AA@??;?A@BCCDC>77,*((((
@T6XXI:00149:00068
GGGAGAAGGGAGAAAGGAAGGAGAAGTCGCCCCAAACGATTTGTTGACTATTCTCCCTCTCCCTATAGTGAGTCGTA
TTAGAATTCCCC
+
BC?BDCADH@@@=ABC@B?A?BAA?A@BBBBB8BB<BBB?C999;;;?>>>;99==;<;;;=;<<==:;<<>=<???
?>A@A>@@AAC6
@T6XXI:00149:00081
GGGAGAAGGGAGAAAGGAAGCTAGTCACTCGACATCTGTGGCTTCACAGTACAGAGGGACTGTCCAAAGCCGCAGAT
CTCGAGTCGACTAG
+
EFBCCD@BA:A@==9>594;BB>?@@???A@:;:@??AAA??@=@CBBBBCB=7116.4;;;==>AA<@@=??@@@B
@???AAAA@@@@@A
```

```
@T6XXI:00149:00087
GGGGAATTCTAATAGACTCACTATAGGAGAGGGAGAATAGTGACGTGTTGTGCGGCGCTCGACTGTCTCCTTCCTTT
CTCCCTTCTCCC
+
A?B4>;@@B<>?=)((((((,8893959A?DD<@AB;>B>9=BCAA@@>=AAAA>@366..++3335<<;<<=>AA;
?===6;8;=>@<
@T6XXI:00149:00095
GGGGAATTCTAATACGACTCACTATAGGGAGAGGGAGAATAGTCAACAAATCGTTTGGGGCGACTTCTCCTTCCTTT
CTCCCTTCTCCC
+
=AA6C?A@CCC@CBCBABBBBB<99311.7==?@>BCC>C>?@==<9=87@B@@@9???5?BCCC@CCC>B@A@AA<
@AAA=A;??AA<
@T6XXI:00149:00096
GGGAGAAGGGAGAAAGGAAGTGCAGTTCGCGGTTAGTGAAGGGAAAAAACTATTCTCCCTCTCCCTATAGTGAGTCG
TATTAGAATTCCCC
+
DKBCCC@BA=ABAB?BAC@BBBBBBB?BBBB@B@AABBB@BB<88:::+@CCC@BB?@>CAAAA<AAA?@A?@@@>=
>8858@@>A>@CD4
@T6XXI:00149:00098
GGGAGAAGGGAGAAAG
+
CF?B=9299091--+,
@T6XXI:00100:00103
CTAGTCACTCGAGATCTGCGGCTTTGGACAGTCCCTACTGTAC
+
:6///66==CCCCCCBBBBB?ABD?B?A>?;000,0..00,,.
@T6XXI:00100:00105
GGGAGAAGGGAGAAAGGAAGTGATGGGTAAAGGCGATGGGTGAAAGGCTATTCTCCTAGCTTTCCTCA
+
DECDBB@BD@BCBB>B@B@@??ABBCACDB?B@B>>ACD>CBBB>B?BCB@<>995894444-49433
@T6XXI:00100:00115
GGGAGAAGGGAGAAAGGAAGCTAGTCGACTCGAGATCTGCGGCTTTGGACAGTCCCTCTGTACTGTGAAGCCACAGA
TGTCGAGTCGACTAGCTC
+
?@:@9959A:@99918<@>BAB>?=/0//..497?C?=?@B@CCEBC:;:CBA>B<?:88<:;;;44744;;?<<==
<@<<65;55331------
@T6XXI:00100:00116
GGGAGAAGGGAGAAAGGAAGCTAGTCGACTCGAGATCTGTGGCTTCACAGTACAGAGGGACTGTCCAAAGCCGCAGA
TCTCGAGTCGTA
+
CE?CBC@DC>CCBB?BAB>?>B:::/11@>>>>>????BBC>BA@BB??????@=<<<;=<===<:<=;==;888<@
=;?:<<<=;<<=
@T6XXI:00100:00120
GGGGAATTCTAATACGACTCACTATAGGGAGAGGGAGAATAGCTAAGACATTGCCGAAACTTAGCGCCCCTTCCTCT
TCCTTCTCCTCCTCC
+
:99/9;@?AICACB>??BBBBAB>?111-
6==@BB@A@AA?@?BB@C?=@C@BB@B>>9787:B;9;<;1<8:8=83/7343/55+,,+,5+
@T6XXI:00100:00121
GGGGAATTCTAATACGACTCACTATAGGGAGAGGGAGAATAGATTTCCCTTCGACTGCACAACCATATTCTTCCTTT
CTCCCTTCTCCC
+
@==6@?B@CBB@BBAA@BBB?AB<<111.7>>CE>BBB@A===<<:;:4:=<=@@AA????<?>AAAC=AA;=:<<:
<===;:567301
@T6XXI:00100:00144
GGGGAATTCTAATACGACTCACTATAGGGAGAGGAGAATAGCA
+
```

```
BID:C@C@CCB@CBABBBB@@?BBB???9?A;::;,,+*)))(
@T6XXI:00100:00145
CTAGGTCACTCGAGATCTGCGGCTTTGGACAGTCCCTCTGTACTGTGAAGCCACAGATGTCGAGTCGACTAGCTTCC
TTTCTCCCTTCTCCC
+
CDCC@BABBBBBCHDCCCBBB?AAB>B@BBBA<AA<ABBABBBC?=@=>AD@CBBBB>?B?CBB?ABCA@@@BA>B?
BC>BBBE=?;?>>=7
@T6XXI:00100:00148
GGGAGAAGGGAGAAAGGAAGAGAATAACAGTGCACCGAATGCGCAAACTATTCTCCCTCTCCCTATAGTGAGTCGTA
TTAGAATTCCCC
+
ED>CBC@BC>ABAC>B?A>BBCB?>B@BBB?AACC?CC@CBCCBBC@CC??;?@AA<BCCCC>B>BBCCCB@@@BBB
B?BBB<?;<@@5
@T6XXI:00101:00105
GGGAGAAGGGAGAAAGGAAGCAAGGTGTGATGCGAGACGGAGACAAACTATTCTCCCTCTCCCTATAGTGAGTCGTA
TTAGAATTCCCC
+
EDACBA@BA=>>>A?A@B@BBB@B@??B@BC@BCCAADBADCCBCB>BBCC?B@@?6>>8::7:CBCCCAAABB@@@
B;?>?:>4888.
@T6XXI:00101:00122
GGGAGAAGGGAGAAAGGAAGGAAATAGCAAGGATGTGAATCGCAGGGCTATTCTCCCTCTCCCTATAGTGAGTCGTA
TT
+
DDBECC@BA<BBBB>B@CBC@AD>;9<<A>A=@?:::?<??@:::?6?@A@=ACCC@BCCDE>?BAAA:00:00..,
,*
@T6XXI:00101:00127
GGGGAATTCTAATACGACTCACTATAGGAGAGGGAGAATAGTCTTCCCATATTTCGCACCGCTATTCGCTTCCTTCT
CCCTTCTCCC
+
CEE:C;;;<BB?BDCCBBBBB7666..,0>>BJ>BBB>B:?@AA>@<;@BCCC;>A@C@BABBCC>7770,0,0,/.
//,/,/.44,
@T6XXI:00101:00134
GGGGAATTCTAATACGACTCACTATAGGAGAGGGAGAATAGTCTTGTATTTTGTGCATCACTTTTCTCCTTCCTTTC
TCCCTTCTCCC
+
A@?4>6954449;=@AABAAC?>>>00,0::?><@::47>>?@A>@;877704;@@AAA?@=@@5:849;/4244-
4444-48:444,
@T6XXI:00101:00137
CTAGTCGACTCGAGATCTGCGGCTTTGGACAGTCCCTCTGTACTGTGAAGCCACAGATGTCGAGTCGACTAGCTTCC
TTTCTCCCTTCTCCCCTTCCTTCTCCC
+
BA;9<999ABBCDCCCCBBBC@BBA=A>@@ECCCC>CB@>@@@@?BCA>@C?BBCCCECBBBBCBBBC@@@???3:5
:A<@BBC>?;?7444-4242424444+
@T6XXI:00101:00146
GGGGAATTCTAATACGACTCACTATAGGGAGAGGGAGAATAGTTTTTCCCTTCACTAACCGCGAACTGCACTTCCTT
CTCCCTTCTCC
+
999/48=8>@A599@@@@@;;:??A777.7===?<??CC??>ABAB1@@;@@ACCCC572:??==>>@8:7?37373
6777,/:/-.,
@T6XXI:00102:00103
GGGAGAAGGGAGAAAGGAAGCTAGTCACTCGACATCTGTGGCTTCACATAGGACTGTCCAAAGCCGCAGATCTCGAG
TCGACTAG
+
DECCBB894-
998809<>;@CBABB:?=::7::7>@@CLE@@@=@DCAC<A>BCBBBC@BB@BB@BAAABABBCBBBCCFCCCCC
@T6XXI:00102:00111
ACTCGAGATCTGCGGCTTTGGACAGTCCCTCTGTACTGTGAAGCCACAG
```

```
+
A994444:<>99399444-49:444444-4444999999::2442/220
@T6XXI:00102:00119
GGGGAATTCTAATACGACTCACTATAGGGAGAGGGAGAATAGTTTTTCCCTTCACTAACCGCGAACTGCACTTCCTT
TCTCCCTTCTCCC
+
A?=6@?B@BBBCCCA??BB???CDB78757??AA;@CCAAA?AABB1??;?;9=@CC@C>B@C@@<<;<>??@@=BA
=BCBA;?=9===;
@T6XXI:00102:00121
TCTGCGGCTTTGGACAGTCCCTCTGTACTGTGAAGCCACAGATGTCGAGTCGACTAGCTTCCTTTCTCCCTTCCTCC
C
+
?B<99>9884-9<=ABDBCB?BAAAC@@@BBEDBCC?BBC@@;;;@>B>:;;998933136016-
7./:6<,0,///,
@T6XXI:00102:00127
CTGCGGCTTTGGACAGTCCCTCTGTACTGTGAAGCCACAGATGTCGAGTCGACTAGCTTCCTTTCTCCCTTCTCCC
+
AAABB?CCC?B?CCCCBBB>BAAABBB;;;?@>@B>??BBBB<<BBB??BBBBBCCEC?A>BD>CCCG>B?@C@@;
@T6XXI:00102:00136
GGGGAATTCTAATACGACTCACTATAGGGAGAGGGAGAATAGCAACAGTATTTTTTACACGTG
+
BCC:C@B@BAA@AABBB?>?B@BBA??<<9>8:<;=C@?A<??<89:444::::9*9:=>><7
@T6XXI:00102:00139
GGGAGAAGGGAGAAAGGAAGCTAGTCACTCGACATCTGTGGCTTCACAGTACAGAGGGACTGTCCAAAGCCGCAGAT
CTCGAGTCGACTAG
+
DB?BCC>@@=@@===B?BACCDCCC6:76?>7:7===BAA?AB@BBABAA?;<;43418BBAA@@BD?@@>@@=<<C
AAA858A=<<?<<<
@T6XXI:00102:00148
GGGAGAAGGGAGAAAGGAAGAACGCGGTGACATAGGTCAGGGATTAGCTATTCTCCCTCT
+
DDACCC@BBABBBC>C?B@BB@BBAA?A@CCBBBCBCDBBA=AC?BBC@@@=AABB@DCC
@T6XXI:00103:00105
GGGAGAGAATTTGGTAATGGGGAAGGAAGTCGTGCAGGGAGATTGATACGGGCCCTTATATCTCTCTCCCTATAGTG
AGTCGTATTAGAATTCCCC
+
BB?BBBBB>AB<@=?A>?@@@7@CC@C=@?CCCCB???;;???>@CC@???<AC=A?A@=<=A@@@@A@<??AAAAA
@@?;:44838?A?=<AAA1
@T6XXI:00103:00108
GGGAGAAGGGAGAAAGGAAGGAGACCGCGCACCCACGAACACGGTGATCTATTCT
+
BD?BAB=@@<@BBD?C@>;=67,,-*,288A>A19999248;;<?@>?ABA?<?7
@T6XXI:00103:00114
GGGGAATTCTAATACGACTCACTATAGGGAGAGGGAGAATAGCTCAACCCTTTTCCCTCGGTGCGTACCCTTCCTTT
CTCCCTTCTCCC
+
CED:C::;;BB@C@@?BBBCBCEB?6695:@@AB>BBC@A===;11077.7BC9CD>==?<@8;;BBBA<A>A?BC@
CCCC>C=<?=<4
@T6XXI:00103:00132
CTAGTCGACTCGAGATCTGCGGCTTTGGACAGTCCCTCTGTACTGTGAAGCCACAGATGTCGAGTCGCTACTTCCTT
TCTCCCTTCTCCC
+
CCCCB:<=CCDCDCCDDDABBBCCC>B@AAAB???<???CCCBB?BCC@AC?CBBBBCCAAAAC:<<ABABC=@=@C
?CBBDAF=?@BB>
@T6XXI:00103:00148
CTATCGACTCGAGATCCGGCTTTGGACAGTCCCTCTGTACTGTGAAGCCACAGATGTCGGGTCGACTA
+
```

```
CBA@@BBFCCBAAABB@B@BBB=A?ABBCCCC>?AAC@BBCCCA@>>>>@A@?A<44111.73000--
@T6XXI:00104:00111
GGGAGAAGGGAGAAAGGAAGCTAGTCACTGACATCTGTGGCTTCACAGTACAGAGGGACTGTCCAAAGCCGCAGATC
TCGAGTCGACTAGCTTCCTTTCTCCCTTCTCCC
+
DC?BBC594-4444-
424249887=0/.......07;;?B=?AACCBBBCDBABBB<?<<<@@>BC>AA>@@A=;;8444<;9;A@888<@>
:><=;97///,/,//893
@T6XXI:00104:00119
GGGGAATTCTAATACGACTCACTATAGGGAGAGGGAGAATAGTCAACAAATCGTTTGGGGCGACTTCTCCTTCCTTT
CTCCCTTCTCCC
+
BBB9B@CCBBB@BBAABBBBB?997116.777AC>AAA@???AA=;<@=;@?86;6;;;2?@BBC@BBB??;?>@@<
@@AC;?>@@@@:
@T6XXI:00104:00120
GGGAGAAGGGAGAAAGGAAGCTAGTCACTCGACATCTGTGGCTTCACAGTACAGAGGGACTGTCCAAAGCCGCAGAT
CTCGAGTCGACTAG
+
DC?ABB?BA=BB==:@;=;?@@AAB?:7:7777666168>2:;B@CBECABBB833417?8<==>@A<=<8;=7448
<847<80333343=
@T6XXI:00104:00145
GGGGAATTCTAATACGACTCACTATAGGGAGAGGGAGAATAGCCGTAATGTTTGCTTACGCCTTTTTTCTCCTTCCT
TTCTCCCTTCTCCC
+
;<</9?B?AABDCCCBBBBCCBB@@9::5:?ABC>AAA@A?7328>><?CCD<A9856///,/;9;;+///4649/4
3+0000+0448@A<
@T6XXI:00105:00105
GGGGAATTCTAATACGACTCACTATAGGGAGAGGGAGAATAGCTCTTTCTTCTCATTGCACCTGCCTATTCTTCCTT
TCTCCCTTCTCCC
+
CDE:C>@>@DB@CCAAA?BBB@A@@69956?=?B>BBB@C<>9;;;>;>>>;888<<=A988<DCBCAA??<>B@CC
59;::/7:<777.
@T6XXI:00105:00118
GGGAGAAGGGAGAAAGGAAGAGAAAGCTGTGACAGGTGATGGAGAAGCTATTCTCCTCTCCCTATAGTGAGTCGTAC
TTAGAATTCCCC
+
9904=9244-
444:692469;CCN>BBBBA=<44;9499==699@AB>>;8;A11,.4333,37666:111111111636117;244
4,
@T6XXI:00105:00119
GGGGAATTCTAATACGACTCACTATAGGGAGAGGGAGAATAGTCAACAAATCGTTTGGGGCGACTTCTCCTTCCTTT
CTCCCTTCTCC
+
@AA6@?B@CCC?BBCBBBAAB@ABB@BB>=B=CC>CCC>??==<A<<<;6==<==.777,16779;<:?83232394
44494988+,+
@T6XXI:00105:00125
GGGAGAAGGGAGAAAGGAAGCTAGCGACTCGACATCTGTGGCTTCACAGTACAGAGGGACTGTCCAAAGCCG
+
CA<AAB?CC@CBBB=@=@@BBCBCB;;?@AC???><?@?A??@=@BCBBC@BCA<8<7<>BA==;=A<>?=?
@T6XXI:00105:00126
GGGAGAAGGGAGAAAGGAAGCTAGTCACTCGACATCTGTGGCTTCACAGTACAGAGGGACTGTCCAAAGCCGCAGAT
CTCGAGTCGACTAG
+
DDC?==644-
999909599<LCCCC=>>;>=00/>6>?BB?BC<>?@@@@@@=633317=>@@@>AA:AB??=6==AC=?@AA????
A===
@T6XXI:00105:00131
```

GGGAGAAGGGAGAAAGGAAGCTAGTCACTCGACATCTGTGGCTTCACAGTACAGAGGGACTGTCCAAA
+
EG@CCC@CBB???B=BAB@A@@B?>7;:AAAAAAABACBCBCC@CC:????@=;444-444/99;561
@T6XXI:00105:00136
GGGAGAAGGGAGAAAGGAAGCTAGTCGACTCGAGATCTGCGGCTTTGGACAGTCCCTCTGTACTGTGAAGCCACAGA
TGTCGAGTCGACTAGCTTCCTTTCTCCCTTCTCCC
+
;909?>=@C=AACC?C@B>BB?;;00//.33385>?<7;;B?ABB?BADCBB?::8??9>>778<==>=<<389A?>
>??999<A@=8449444/48:;7:88827,/-110
@T6XXI:00106:00103
GGGGAATTCTAATACGACTCACTATAGGGAGAGGGAGAATAGACACCAACTCGT
+
@@@6@=@@CCB@BBBBBBBBA@ABB88:;8:8EE>EAA==68//.-2507/--/
@T6XXI:00106:00111
GGGAGAAGGGAGAAAGGAAGCTAGTCGACTCGACATCTGTGGCTTCACAGTACAGAGGGACTGTCCAAAGCCGCAGA
TCTCAGTCGACTAGCTTCCTTCTCCCT
+
CC?CBB?A@:@99908=@>B@@@BC:;;ADEC<<:??:77A:::51===??>>>9444,4:;:==:??<@<<8??@@
=<<888@<<<A8////,/,/,-22345
@T6XXI:00106:00117
GGGGAATTCTAATACGACTCACTATAGGGAGAGGGAGAATAGTTGACCTCTGTCATCTATGTCCCTTTTTCTTCCTT
TCTCCCTTCTCCC
+
CEE:CAA?AAA@A@??@ABBB@BBB66616??CE>CBB?@@@@<=<<;<<<@?=;;;;;BAAAA<A===.9999<==
;<=<<5846;;;5
@T6XXI:00106:00125
GGGGAATTCTAATACGACTCACTATAGGGAGAGGGAGAATAGTCAACAAATCGTTTGGGGCGACTTCTCCTTCCTTT
CTCCCTTCTCCC
+
DCGBE@B>ABBABBB@@@BBBAA@@66646???B>BCC@A<@<==<=@@;BA???8??86?CCDC=AAB@@<?5530
3330+3994471
@T6XXI:00106:00128
CTAGTCACTCGAGATCTGCGGCTTTGGACAGTCCCTCTGTACTGTGAAGCCACAGATGTCGAGTCGACTAGCTTCCT
TTCTCCCTTCTCCC
+
E8/111/4?BBBDBDBBBBBABBA?A?@A@A:::5:ACBBBCC::;@=@CBCBBBBBBCBBBBBCBBBDCBBB?A?B
ECCDCC>@689@A<
@T6XXI:00107:00111
GGGAGAAGGGAGAAAGGAAGGAGACAGTGAGCGCCGCACAACACGTCACTATTCTCCCTCTCCCTATAGTGAGTCGT
ATTAGAATTCCCC
+
CDDCEC@CB>BBBC>B?A>?>9::BBBBCCCABB@B?>>;>>>>>>>ABBBB@BCBB?BBCCB<BBBBBBAABBCCB
BA?B@=;>4999/
@T6XXI:00107:00115
GGGGAATTCTAATACGACTCACTATAGGGAGAGGGAGAATAGTCAACAAATCGTTTGGGGCGACTTCTCCTTCCTTT
CTCCCTTCTCCC
+
BBB9B@CABBB@BBAA?BBBB?BBA::888889B>ABA@A=<<AA????<BBAAA5966-
:???C@BCC>=;=?CC>;;AA:C<??@@;
@T6XXI:00107:00117
CTATCACTCGAGATCTGCGGCTTTGGACAGTCCCTCTGTACTGTGAAGCCACAGATGTCGAGTCGACTAGCTTCCTT
TCTCCCTTCTCCC
+
CBB;9::=A@B?BBA@?@CACCC?C@@@CABBC>C?;;>????B@@;?B@CBC@@@CCBCCCAB?@@;<;A@=@>BC
?ABBJ>A=@@AA<
@T6XXI:00107:00141

```
GGGAGAAGGGAGAAAGGAAGTGCAGTTCGCGGTTAGTGAAGGGAAAAACTATTCTCCCTCTCCCTATAGTGAGTCGT
ATTAGAATTCCCC
+
BB?BBB?BB>BBBB=B@B?BBBBBBB@BBBB@B@A??@A@AC;@BBB2BCCC>BCCC:@?;??;96699;<>?===<
==9<@A>A>@CC6
@T6XXI:00108:00104
GGGAGAAGGGAGAAAGGAAGAGACAGCCTG
+
EHACBB@BD>DBCB??<=<9:0--2,*)*(
@T6XXI:00108:00105
GGGGGAATTCTAATACGACTCACTATAGGGAGAGGGAGAATAGAGACGTATTCGTTTGCGCTCTGTTCACCTTCCTT
TCTCCCTTCTCCC
+
4444+424:;9999@AABCBBBACBB<:?9???BB?BBB@@===?AAAA??8;===;<<::A?<@A<==<::2337B
7>?@A<A>AA@@9
@T6XXI:00108:00123
CTAGTCACTCGAGATCTGCGGCTTTGGACAGTCCCTCTGTACTGTGAAGCCACAGATGTCGAGTCGACTAGCTTCCT
TTCTCCCTTCTCCC
+
DA;<<@;:;@==CB<<=CBB?BBB?B@CBBB???8??AAAACCAAA?<?GCCCBAA;<;A@AA?@A??837965:@E
E>CCEK<<8<9::4
@T6XXI:00109:00114
CTAGTCACTCGAGATCTGCGGCTTTGGACAGTCCTCTCGTATACTGAGAGCCACAGATGTCGAGTCGACTAGCTTCC
TTTCTCCCTTCTCCC
+
86///6/44A>BBBBBB@?@=@??<A9;<DCCB>A7,,,,,,,.04321..-
.445=>@AAA=777<744>888>?>AC;@==<6:996661
@T6XXI:00109:00117
GGGGAATTCTAATACGACTCACTATAGGGAGAGGGAGAATAGTCAACAAATCGTTTGGGGCGACTTCTCCTTCCTTT
CTCCCTTCTCCC
+
AAA6@?B@CBA?ECBBBBBBBABBB77777@?CC>???@A<@@A@>=<;5;;;;;-
777.7?BCC=@@C@@?@>AA<A@A@<@<???AB
@T6XXI:00109:00121
GGGGAATTCTAATACGACTCACTATAGGGAGAGGGAGAATAGTCTCCCTATTTTGCGCGACAAGTTGCACTTCCTTT
CTCCCTTCTCCC
+
999,48@@CBB@CC@@BCCBB@BBB76757B?@C>CCC==598AACC;?@@@?5?C??@DDC?AA>@@AAC?AAA@7
<;>>4323443-
@T6XXI:00109:00126
GGGAGAAGGGAGAAAGGAAGGAGAAGTCGCCCCAAACGATTTGTTGACTATTCTCCCTCTCCCTATAGTGAGTCGTA
TTAGAATTCCCC
+
ED>BCB@CB?BAA?=B?B?B@DBB?@>AABDC9CC>BBB??;@@@@==?CA>A???<?A@AA<<<<==<<<::?A<@
@=AAA?@3887,
@T6XXI:00109:00128
GGGAGAAGGGAGAAAGGAAGCTAGTTCGATC
+
ED@AA@=@99>>>@<@@C?BBAB;000**((
@T6XXI:00109:00142
GGGGTTCTAATACGACTCACTATAGGGAGAGGGAGAATAGCTCGTGCTTTTTCCATCCCTGGTTTTATTCTTCCTTT
CTCCCTTCTCCC
+
CDC:CCCDCACBBBBBBBBB>99333/8?>>@AAAA=><??AAAABBBCCC3B?BDDD?CBBBAB9CDACC@C=@@>
@CCC>C>@@CC=
@T6XXI:00109:00145
```

GGGAGAAGGGAGAAAGGAAGGAGAATGCACCGCAGGAAATTGCAAGACTATTCTCCCTCTCCCTATAGTGAGTCGTA
TTAGACT
+
7<8<19599098885824242377 9BBBAA@CB?C577,37311761116666176,10000+000008833437;6
877//-0
@T6XXI:00110:00132
GGGAGAAGGGAGAAAGGAAGCTAGTCACTCGACATCTGTGGCTTCACAGTACAGAGGGACTGTCCAAAGCCGCAGAT
CTCGAGTCGACTAG
+
CD@CCB@BB?BBBB>B@B@BBBABC8;:@@>7:::96BCE?BB@CA;;>>?AC=86646::<==;==;??<??=B=<
::<==<=@?@@A==
@T6XXI:00110:00137
GGGAGAAGGGAGAAAGGAAGGAGAAGTCGCCCCAAACGATTTGTTGACTATTCTCCCTCTCCCTATAGTGAGTCGTA
TTAGAATTCCCC
+
CD@BBB@BB>@@BBBB?@?A@BBB@B@ABBCC9CC>CCCBB>AA=A::;C@=@@@@<@@@:;:;;;;===;::?@AA
A?AA@<;/444-
@T6XXI:00110:00145
GGGGAATTCTAATACGACTCACTATAGGGAGAGGGAGAATAGTTTTCCCTTCACCAACCGCGAACTGCACTTCCTTT
CTCCCTTCTCCC
+
::;4;@CADBB@CBBBBBBBB@B??16659??CE>BBB???@AAA5<=;=>A@@?A?A>BACC=>@BBB@B@B;?;:
??AB<@=B9995
@T6XXI:00111:00103
GGGGAATTCTAATACGACTCACTATAGGGAGAGGGAGAATAGCCAATAGTCATGAGACCGTCGGCTGCTCCTTCCTT
TCTCCTTCTCCC
+
CDD:C?C@BEC@C??=AA???@CB>66656??BC>@@@?@???<?>?@@@@AB??=:@?>@<8;?B???C?B>?:88
,.//,-,----+
@T6XXI:00111:00134
GGGGAATTCTAATACGACTCACTATAGGGAGAGGGAGAATAGTTTTTCCCTTCACTAACCGCGAACTGCACTTCCTT
TCTCCCTTCTCCC
+
999/9<A>BBC@CB??BBBB>CB<<66659@@DD>@AA>B;A;;;;.;=;=;:>>>?A>>9>?@?<<;<@@@;?277
5:A?C>B=<??B=
@T6XXI:00111:00143
GGGGAATTCTAATACGACTCACTATAGGGAGAGGGAGAATAGTTCACGTCTAAACATCCCATCACTGTTCTTCCTTT
CTCCCTTCTCCC
+
::419<@=ACGDCCBBBB>>>AC?91695:@@AD>BAA??=@=;<<@@@;;@@>@?@?@;@B?==?AA>AA<=;<<;
????7?;?@@<;
@T6XXI:00111:00147
GGGAGAAGGGAGAAAGGAAGCTAGTCGACTCGAGATCTGGCGGCTTTGGACAGTCCCTCTGTACTGTGCAAGCCACA
GGATGCGAGTCGACTAGCCTTCCTTC
+
C@?BCC@CGAC@@@8;99>FCA=>>0//.455..1111103;89BC>C??@?@<6:7633.----111147//,---
-,--046<:<<<87783.,+00+,++
@T6XXI:00112:00114
GGGAGAAGGGAGAAAGGAAGGAGACACGGTCACAAACATGAATGTGACTATTCTCCCTCCTCCTATAGTGAGTCGTA
TTAGAACTTCCCC
+
FE?CCC?BC@BBAC>B@B@B=?@CCCCCACAB;8809@@AB>@ABB@@91/94.,,+++*++*+049<<<<<894>?
A4311,--22220
@T6XXI:00112:00140
GGGGAATTCTAATACGACTCACTATAGGGAGAGGGAGAATAGTCAACAAATCGTTTGGGGCGACTTCTCCTTCCTTT
CTCCCTTCTCCC
+

```
;;<4;:B@BBB>BBBBBCCCCCB>>111.777BC@AAA@A@@@AA>AA@;BBBFEBCBB7ABB::49??:9897;;6
<@@A?A>A6650
@T6XXI:00112:00144
CTAGTCACTCGAGATCTGCGGCTTTGGACAGTCCCTCTGTACTGTGAAGCCACAGATGTCGAGTCGACTAGCTTCCT
TTCTCCCTTCTCCC
+
EC111=8==BBCDBCDCBA@=@CC>C<>@CC:::7:CCCC@CC@CC@>?B?BBCCCCCCBBB??@@A@@@BBB?B?B
C=AABEAC;==<@:
@T6XXI:00112:00147
CGGCTTTGGACAGTCCCTCTGTACTGTGAAGCCACAGATGTCGAGTCGACTAGCTTCCTTTCTCCCTTCTCCC
+
CEEEFDBC@BEB?@FD@CCCCCCCCCBAB@CC?AADBBBBBAAABBAB??AE?=@@B>AA<ACBC>C>?@CD>
@T6XXI:00113:00103
GGGGAATTCTAATACGACTCACTATAGGGAGAGGGAGAATAGTCAACAAATCGTTTGGGGCGACTTCTCCTTCCTTT
CTCCCTTCTCCC
+
C@B6><@=ABB@CDBBCBBABA???666.7====<?BBB?=>ACB;<??;AABBB>BCC9BBBBC;??A58378;;7
=?@A<B;;444-
@T6XXI:00113:00109
GGGAGAAGGGAGAAAGGAAGGTGGGTCGAGCTTTGGCCTATGCCGCAACTATTCTCCCTCTCCCTATAGTGAGTCGT
ATTAGAATTCCCC
+
CFCCCC?CB>BBBB?A@A@A<<<?<BBBBBBBC>C?B?ABBCB<BBC@CCB>;@?;;:?AABC>CCBBB;;BBCCCB
BB?BDC@A>@@?4
@T6XXI:00113:00112
GGGGAATTCTAATACGACTCACTATAGGGAGAGGGAGAATAGTGACGTGTCGTGCGGCGCTCACTTCTCCTTCCTTT
CTCCCTTCTCCC
+
BAC9B?B@C??=ACBBBCBBBAB?<161-
6<=?A>BBC@AA=?;A=>=<9==AAA=>A888@@<<9=@@=@=@;>=7=>==7?><==A<
@T6XXI:00113:00113
GGGGAATTCTAATACGACTCACTATAGGGAGAGGGAGAATAGTCTCATGAAACAAAAACATGGCTATACCTCCTTCC
TTTCTCCCTTCTCCC
+
ABB9BAC@CCB@CCBBBBBBBA<99111.7>>BK@CCC@B;<;<<;?BCCD>BBBCC2CBA>=AABBBC@@@=?>B=
?>8>==<6<:<777.
@T6XXI:00113:00122
GGGGAATTCTAATACGACTCACTATAGGGAGAGGGAGAATAGCCAACACATTCTTCTCCTTTTCGCGCACTTCCTTT
CTCCCTTCTCCC
+
:49/9>A?CCCACBBBBBBBBA@<<1665:??CE<A?A@A=??8?>=====>:656558<??4?@@@A@@A>A>AA<
????;?>@@@A=
@T6XXI:00113:00126
GGGGAATTCTAATACGACTCACTATAGGGAGAGGGAGAATAGTCAACAAATCGTTTGGGGCGACTTCTCCTTCCTTT
CTCCCTTCTCCC
+
DDC9C>?=ACC@CB@?BBBB?AA<<66659??CC>BAB@A<<=??<<<<5??;<<-777-
:@ABC=??A=<9=>AA<@AAA<@>@@AA<
@T6XXI:00113:00132
TTCTAATACGACTCACTATAGGGAGAGGGAGAATAGCTTCGACCCGGTTATCGCAAACTGTTCTTCCTTTCTCCCTT
CTCCC
+
C?AADBDCDBCBCCCB<??88808====6=>=:????AACBCCE@CBCCDAAACCE>@ABB=@@>B>BC@A@BD@D=
@@CC?
@T6XXI:00113:00135
GGGAGAAGGGAGAAAGGAAAGCTAGTCACTCGAGATCTGCGCTTTGGAC
+
```

```
;;-944244-////+/,//+/6333;?:8;;>611111133133,1011
@T6XXI:00113:00149
GGGAGAAGGGAGAAAGGAAGGGACGAGG
+
A@<@@@ABC>BA>?>???000)*****)
@T6XXI:00114:00102
GGGAGAAGGGAGAAAGGAAGGAGAAGTCGCCCCAAACGATTTGTTGACTATTCTCCCTCTCCCTATAGTGAGTCGTA
TTAGAATTCCCC
+
CD@BA;244-
94=>9>?A<A=???<>>@8888.880:BBCC>?B>@AAACA>A<<=;???A=;<@?@@AA=??===@?@A<;88/44
4-
@T6XXI:00114:00110
GGGAGAAGGGAGAAAGGAAGGGAATAGCAGCAGTCGGCAACGTGAAGCTATTCTCCCTCTCCCTATAGTGAGTCA
+
DCBAA?BDDB?@@B<?;?<@@>@@CCBB>>BBBB?>>AA?C>>>>///::CB>??C>AAB??67***((**+++-
@T6XXI:00114:00119
AGTCACTCGAGATCTGCGGCTTTGGACAGTCCCTCTGTACTGTGAAGCCACAGATGTCGAGTCGACTAGCTTCCTTT
CTCCCTTCTCCC
+
<<>C??ABBBBBCCBBBB@CCC@CDCCCCBBB>BBBB??@BBBBC@BB?BCCCB>B?CA@@A@@BBB@9:::;9::6
:000,0,/---+
@T6XXI:00114:00124
GGGGAATTCTAATACGACTCACTATAGGGAGAGGGAGAATAGCCACGAGTTTGCTTGCGTGGTTTACTCCTTCCTTT
CTCCCTTCCTCCC
+
EDD9CAEC<<=BGCDBCDC?><@???@@=ACBCD>:::@AA@A;77:A=??<:::@A@@<<9<?:;<=@>A>A>::9
::8727,/,///+
@T6XXI:00114:00131
GGGGAATTCTAATACGACTCACTATAGGGAGAGGGAGAATAGTCAACAAATCGTTTGGGGCGACTTCTCCTTCCTTT
CTCCCTTCTCCC
+
<<<4;@CACAA@CCCA@@CBC@B@A9965:A?AE>BAB@AA==<;=;<<6??===6===.77A>><?7:678;>BB7
=<?=:;;:;<>7
@T6XXI:00114:00143
GGGAGAAGGGAGAAAGGAAGCTAGTCGACTCGACATCTGTGGCTTCACAGTACAGAGGGACTGTCCAAAGCCGCAGA
TCTCG
+
CB?BBA@DD@BBBC>C?B=@@CBBB?CC@@@@99:????@C>:;::??BBBBA??33717=;B@@=@@588?888@@
@@???
@T6XXI:00114:00148
GGGGAATTCTAATACGACTCACTATAGGGAGAGAGATATAAGGGTGCCCTTCGTACGTCTTCTCCACCACG
+
CDD:CAC@CCCABBBBBBBBBBC?;;66649??CCCB@<AA?==;=??:7<8;AAAAAA@@:<:6,--+-,,
@T6XXI:00115:00101
GGGAGAAGGGAGAAAGGAAGGGACGCGACGAGCTGGGATAAACTGCTGCTATTCTCCCTCTCCCTATAGTGAGTCGT
ATTAGAATTCCCC
+
AA?@CB?BC>BBBB>B@C@BB?B<><B?@@?@@@BA<BBBC>BCCBBBC@??>BBCC>CCA=<:?@BA@<?=<<>><
<=>??=;A<???4
@T6XXI:00115:00114
GGGGAATTCTAATACGACTCACTATAGGGAGAGGGAAGAATAGTCAACAAATCGTTTGGGCG
+
CCE:D@B=A@@<9;;BCBBGBB@@@??A9;0/.3+.,..,--33365644461116,11,11
@T6XXI:00115:00115
GGGGAATTCTAATACGACTCACTATAGGGAGAGGAGAAT
+
```

```
/11*99@<9895948>=9788=>33166235,,*,,,*+
@T6XXI:00115:00123
GGGGAATTCTAATACGACTCACTATAGGGAGAGGGAGAATAGCTGTAATTTCCCCAG
+
BBB9?<==;<<@CCCCC?>>A?>>?777.7===B>??????>@???@;>>:@@@5A?
@T6XXI:00115:00124
GGGGAATTCTAATACGACTCACTATAGGGAGAGGGAGAATAGCAAACGTTCCCGACGTCGCTTCTCCTTCCTTCTCC
TTCTCCC
+
@AA6@<A>BBB>BDBBBCEBBBBAA99:5:@@ACCBBA>=47.//,/00,..+.5.1166./,.:=67673058//,
/246<@6
@T6XXI:00115:00125
GGGGAATTCTAATACGACTCACTATAGGGAGAGGGAGAATAGTGCGCTCACAGTTGGCGTCCTTTTTTCCTTCCTTT
CTCCCTTCTCCC
+
C@@6@@BBCBB@B@??BBBB>>ABB66646??BC>CBB@A==<588?@@A@AAA?=588==<<=<;:-
:;=:=:<=7>??@<?>A@A=;
@T6XXI:00115:00129
GGGAGAAGGGAGAAAGGAAGCTAGTCACTCGACATCTGTGGCTTCACAGTACAGAGGGACTGTCCAAAGCCGCAGAT
CTCGAGTCGACTAG
+
BDAHBB=>=5===85@784;C@?@@??????:;:AAACBB?BB@BA>>9=>AA=89948<=<=@;==;=C=@@<<<<
:::<<<<<AA==9<
@T6XXI:00115:00134
GGGAGAAGGGAGAAAGGAAGCTAGTCACTCGAGATCTGCGGCTTTGGACAGTCCCTCTGTACTGTGAAGCCACAGAT
GTCGAGTCGACTAGCTTCC
+
AA>ABB@CC>BAAA=DCC@BBA?>;9;:???BBBBBBBCC@?AA<@>AAAA@>B>AA?@A888A??A?@@:>>7788
====744>684833+,**)
@T6XXI:00115:00136
GGGGAATTCTAATACGACTCACTATAGGGAGAGGGAGAATAGTCAACAAATCGTTTGGGGCGACTTCTCCTTCCTTT
CTCCCTTCTCCC
+
944,48=?BCC@BBBBBCCBBAA<;166-
6;;;<9?AA@AAA@@@>@@A<A@@@C>CCC8CCCBB>AAB>?=A;??;>=;@6;588@@;
@T6XXI:00115:00145
GGGGAATTCTAATACGACTCACTATAGGGAGAGGGAGAATAGTCAACAAATCGTTTGGGGCGACTTCTCCTTCCTTT
CTCCCTTCTCCC
+
@@@6B=A=@BB@CCBBBCB@@?CB@????9<????=@BB?A=AA@@=@@D?CC@??9?@@5AABBD>@@F==9=>@@<
AAAA;?98444-
@T6XXI:00116:00117
GGGGAATTCTAATACGACTCACTATAGGGAGAGGGAGAATAGTCAACAAATCGTTTGGGGCGACTTCTCCTTCCTTT
CTCCCTTCCTCCC
+
AAA4@6;8<BB?CCCCCCCCCB@>>677.777>B<A?>@=ABBAA<?:;:>>@CA=ACC7?;00.,0.331244444
:8:::6442044+
@T6XXI:00116:00123
GGGAGAAGGGAGAAAGGAAGGGGAGAAGGGAGAAAGGAAGCTAGTCGAC
+
CC@B?=699-9?>@9>;@=>BB9@@@@CB:>776-73747</..3/...
@T6XXI:00116:00131
GGGAGAAGGGAGAAAGGAAGCTAGTCACTCGACATCTGTGGCTTCACAGTACAGAGGGACTGTCCAAAGCCGCAGAT
CTCGAGTCGACTAG
+
DDCCBB>==:@@==:@=?=@BBBBCAAA@@?779?8?ABD@CC@BA?@@@@=74000,4;;=@@?AA<AA<??=<<<
>>>558AAAAAA??
```

```
@T6XXI:00117:00111
GGGGGGAAATTCTAAATACGACTCACTATAGGGAGAGGGAGAATAGTCAAACAAATCGTTTGGGGGCGACTTCTCCT
TCCTTTCTCCCTTCTCCCGGATCACT
+
/8888(//+/,////+/;;;=?@@@BBCCAAE@BA>;:,/00,.611030+044476111,1666/99999896633
/02042468<<A<@::9,,+,*****
@T6XXI:00117:00113
GGGAGAAGGGAGAAAGGAAGAATACAACGAATCGGGAAAACTCGGAACTATTCTCCCTCTCCCTATAGTGAGTCGTA
TTAGAATT
+
CDBCCG@CB>BCBBBB<B?BB>B@BB>B?B?BBBA?AAA8BCCD>A=@CCA>B@@@<@CCCC>CAAA@@@AAAAA88
8?<6454/
@T6XXI:00117:00115
GGGGAGAAGGGAGAAAGGAAGGAGAATACAGCGAAAATGGACACGTCGGCTATTCTCCCTCTCCCTATAGTGAGTCG
TATTA
+
444,449:;;594998;?CBC@CAA?AB@>883777,1103699C@>>>900.,....+.55:=;=AA@77030000
--23-
@T6XXI:00118:00117
GGGGAATTCTAATACGACTCACTATAGGGAGAGGGAGAATAGTTCTATTATCCCAAAACGACCGCAGCCCTTCCTTT
CTCCCTCTCCC
+
944/42924AC@CCCC<;8>>7837611,17666889:77@@B<@?884768A6;;;0;::;9444444-
47979:78///,--2230
@T6XXI:00118:00118
GGGAGAAGGGAGAAAGGAAGCTAGTCACTCGACCATCTGTGGCTTCACAGTACAGAGGACTGTCCAAAGCCGCAGAT
CTCGAGTCGACTAG
+
A?:@@98>>:>A>>9?59598883>=43333..,......7267>BABBBBC@@2--
,/688:?BBB?AB>@@?@AA===88=@=<<AA89
@T6XXI:00118:00136
GGGAGAAGGGAGAAAGGAAGCTAGTCGACTCGACATCTGTGGCTTCACAGTACAGAGGGACTGTCGAAAGCCGCAGA
TC
+
DCBECB?CB=BB?=:@>@<?@A:;;CCCCCCA<7::::AAC?BB?BCBA@@<964000,4:;=?@??C=AA;>>@??
=8
@T6XXI:00118:00137
GGGGAATTCTAATACGACTCACTATAGGGAGAGGGAGAATGAGTCAA
+
:CD:C:=?<BB@B@@AB@:79?>>>@@?>BCD>@10.33---***+*
@T6XXI:00119:00102
GGGAGAAGGGAGAAAGGAAGGAGAAGTCGCCCCAAACGATTTGTTGACTATTCTCCCTCTCCCTATAGTGAGTCGTA
CTTCAT
+
CKBCBA@BC?BCBB>B@B?B?ACBAABBCCDC8BC=ACBBB>BCACBCCCA<??AC<===AD<???@@@AA@<.///
-+000.
@T6XXI:00119:00108
GGGAGAAGGGAGAAAGGAAGCTAGTCACTCGACATCTGTGGCTTCACAGTACAGAGGACTGTCCAAAGCCGCAGATC
TCGAGTCGACTAG
+
::7<B@<AB8;;A?<@9;:<AAABCA:::BB:::::7BBB@:;9;BBBCBB::7.-
,/688?A>AA:AA<@@@@????@@9:9AAAAA??
@T6XXI:00119:00112
GGGGAATTCTAATACGACTCACTATAGGAGAGGGAGAATAGTCAACAAATCGTTTTGGGCGACTTCTCCTTCCTTTC
TCCCTTCTCCC
+
```

```
BDC:C;:<<BBCEBB@CCCBB:996..,000>@>BBB?@AAAA=;9965?=??:?7?D>DCCBB?BAA:=;>:=?:?
@?@;@=??A@;
@T6XXI:00119:00116
GGGAGAAGGGAGAAAGGAAGTCGAGAATAACGTGGCACAAACGCCAAGCTATTCTCCCTCTCCCTATAGTGAGTCGT
ATTAGAACTTCCCC
+
CHDCBB?BC@BBBC>C@C@CCCCAAA?CC?BA@?=??=?@9000,0,/.88>177?;67>777-
7=<?BC@@@C=<<;=/4055665688-
@T6XXI:00119:00136
CTAGTCGACTCGAGATCTGCGGCTTTGGACAGTCCCTCTGTACTGTGAAGCCACAGATGTCGAGTACTTCCTTTCTC
CCTTCTCCC
+
IEAAACCCCCCCCBBBCBBBC@CCCBC::<CCCCCABA???:;:ABBB=?B><<CCB@@@B@@ABA;;59<>>9>88
80858???:
@T6XXI:00120:00104
GGGAGAAGGGAGAAAGGAAGGAGAATGTACATGAATGGAGGAGCGATCTATTCTCCCTCCTCCCTATAGTGAGTCGC
TATTAATGAACTTCCC
+
GB?D<;9<B<AAA@<A>A<@;>8848B9;;CC::8;C@DC@CBAA?>=:88,,+++*++*+--+-
2249:<;<;;686::54/000/002330
@T6XXI:00120:00116
GGGAGAAGGGAGAAAGGAAGCTAGTCGACTCGACATCTGTGGCTTCACAGTACAGAGGGACTGTCCAAAGCCGCAGA
TCTGTCGACTAGCTTCCTTTCTCCCTTCTCCC
+
KH>CA@=@@<@@AC@C@C;=<BCBBBCC@BBB>@?CCA6:7<7857==>>@@>>:444-
4;;9;<>@@<@A=????????899AA;999495969909999090924444-
@T6XXI:00120:00118
GGGGAATTCTAATACGACTCACTATAGGGAGAGGGAGAATAGTCAACAAATCGTTTGGGGCGACTTCTCCTTCCTTT
CTCCCTTCTCCC
+
9;;49?B@BAA@CCCBB?;;BABB;69:.7>=?A>ABB@A<@?@@=@AA<CBBBC>@@@8CABAA:6<?5838<@@<
@@BD?B;==@@:
@T6XXI:00120:00120
GGGGAATTCTAATACGACTCACTATAGGGAGAGGGAGAATAGTCAACAAATCGTTTGGGGCGACTTCTCCTTCCTTT
CTCCCTTCTCCC
+
EEE:C@@=ACC@C@@?BCBBBCC?;66659BBDD>?AA@A?@@AA=?@@<BCCDD@BBC9CC?@@?BBB?@>A>AA;
@;<;582899;6
@T6XXI:00120:00131
GGGAGAAGGGAGAAAGGAAGGAGAAGTCGCCCCAAACGATTTGTTGACTATTCTCCCTCTCCCTATAGTGAGTCGTA
TTAGAATTCCCC
+
CGACAA644-
999908<@<A@BBG?BCCCCCC8BA=BBBCC>@C=>8:;A==?;;<7<<<AA<AA@@@@@@A@@@858?@?=;;799
91
@T6XXI:00120:00143
GGGAGAAGGGAGAAAGGAAGGAGAAGTCGCCCCAAACGATTTGTTGACTATTCTCCCTCTCCCTATAGTGAGTCGTA
TTCAGAATTCCCC
+
DA>BBB?BC@CBBB>C@C@C@BCB@8;<@@ID8BB?CC;::4:?;@ADBB?9<<>?:<==AA<A@888@<<<??=8;
;000;;77,444-
@T6XXI:00120:00148
CTAGTCACTCGAGATCTGCGGCTTTGGACAGTCCCTCTGTACTGTGAAGCCACAGATGT
+
DB1////44499=@=AA@>>;888/8;<><??8>88????7777113/-/,///.4437
@T6XXI:00121:00117
```

```
GGGGAATTCTAATACGACTCACTATAGGGAGAGGGAGAATAGTCAACAAATCGTTTGGGGCGACTTCTCCTTCCTTT
CTCCCTTCTCCC
+
CCC:C@DAEBB@BBBBB?@>BCB?9166.7>>?A:A??<<@@====@@C=CBBBC<AAB7BBBCD@BBB>@<@=@C;
:?@C<A88:A@:
@T6XXI:00121:00119
GGGAGAAGGGAGAAAGGAAGTGCAGTTCGCGGTTAGTGAA
+
9914449:;59999042444334;B>=1111-0,.22,.,
@T6XXI:00121:00124
GGGGAATTCTAATACGACTCACTATAGGGAGAGGGAGAATAGCTTTCCTCCCGGTGACGTTAAATTATTCTTCCTTT
CTCCCTTCTCCC
+
999/49A>ABB?BCCBBBBB@?ABB77777??CC>AAA>?AA@BB>B@CCC>C=@@CCBC?@?<@>AA>??;88<<7
?@@@<@????C<
@T6XXI:00121:00130
GGGGAATTCTAATACGACTCACTATAGGGAGAGGGAGAATAGCGCAAAACTAGCCCGCGCCACACTCCTCTTCCTTT
CTCCCTTCTCCC
+
AAA8A@C@BAB@BA=??BBBB@BBB661-166?@>BCB@@???@ACCC8AAAC??;??CC>BBBCAA>A?>;;344-
8;;@AA=@??>7
@T6XXI:00121:00132
GGGGAATTCTAATACGACTCACTATAGGGAGAGGGAGAATAGTCAACAAATCGTTTGGGGCGACTTCTCCTTCCTTT
CTCCCTTCTCCC
+
BBC9B>B?BCD@C@?>BBBBBBB?;16659=?BC>BBB@A=@@AA>@@@<BB?B=4996-
7=?BB@CCD??<?@BC<@?@@<A;:???4
@T6XXI:00121:00133
GGGAGAAGGGAGAAAGGAAGGAGAACGCGCACCCGAACCAACGGTGATCTATTCTCCCTCTCCCTATAGTGAGTCGT
ATTAGAATTCCCC
+
BD?CCBACBBCBCC?B?B@C@CCC@CBC9;;CC>BB@BBBABDACCAAACCCECCCC>@@@BC?BBCCAAABACC@@
@C?B@A<@=@BB9
@T6XXI:00121:00145
GGGAGAAGGGAGAAAGGAAGCTAGTCACTCGAGATCTGCGGCTTTGGACAGTCCCTCTGTACTGTGAAGCCACAGAT
GTCGAGTCGACTA
+
EC@EBB?BC<@@??0959<?>>>@;::?;6<???ABCBBC@::98:>?ABAAADBCJC989???==@CAA>@@=;;;
;//-77--0----
@T6XXI:00121:00146
GGGAGAAGGGAGAAAGGAAGAAAGGAAGGAGAATGCACTGTTTCGTATCCAGGGTCTATTCTCCCTCTCCCTATAGT
GAGTTCGTATTACGAATTTCCCCC
+
DD@C?@=>>:>@@B?CACACCC?C@C@C@CC@>?<AAA;:;D@AAABB@>::958==>A>@@DC7==777-
7>;>;7//./,/888=888?;933+----*
@T6XXI:00121:00149
GGGGAATTCTAATACGACTCACTATAGGGAGAGGGAGAATAGTTTTCTCCTAGTTTGCTGCGCTTTGCCTTCCTTTC
TCCCTTCTCCC
+
;;<4;?CCCCC@CC@@BCCBB@;77116.7==BC>DAADB;;===4::<;9B???;?BBBCCBCC>BCDC?@>@?;>
???;:56<884
@T6XXI:00122:00108
GGGAGAAGGGAGAAAGGAAGCTAGTCACTCGACATCTGTGGCTTCACAGTACAGAGGGACTGTCCAAAGCCGCAGAT
CTCGAGTCGACTAG
+
DEADCB>==5999909;>;AFBBBCA@>>>=00/;>;=>C?AA@CCC?>>@@?=44447?AAA@=@A=AB=??<==<
<=9=??????@??A
```

```
@T6XXI:00122:00112
GGGAGAAGGGAGAAAGGAAGGAGAAGTCACCCCAAACGATTTGTTGACTATTCTCCCTCTCCCTATAGTGAGTCGTA
TTAGAATTCCCCC
+
DDABBBBCC;;=BC@C@C@CDC>>;=:/..44*..+.446>;=B=??A<@B>@AAD<9:9AA<AAA@A@?988<<<@
A><<<;<;@@@?1
@T6XXI:00122:00128
GGGAGAAGGGAGAAAGGAAGAATACAACGAATCGGGAAAACTCGGAACTATTCTCCCTCTCCCTATAGTGAGTCGTA
TTAGAATTCCCC
+
EE@GCC?CCABBBB>B@B@BB@BABB@CAA>BB==:BBB9BCCC>B>ACCC@BBBB<B>AAC>B??>;;=;A<@@89
=>A?>;=8<;;0
@T6XXI:00122:00134
GGGAGAAGGGAGAAAGGAAGTGCAGTTCGCGGTAGTGAAGGGAAAACTATTCTCCCTCTCCCTATAGTGAGTCGTAT
TAGAATTCCCC
+
DD@CCA@BB>==?C@C>?>ABBCBB;;;;;:100888<@AA;@CC7BBCC@DBBB>@@@CC>B===@AAAAA@==AA
>@@?<;3848-
@T6XXI:00122:00140
GGGAGAAGGGAGAAAGGAAGCTAGTCACTCGACATCTGTGGCTTCATACAGAGGACGTGTCCAAAGCCGCAGATCTC
GAGTCGACTAG
+
BB>BBA?AB<@@>>9>=@=@AACCBAA=??7771166?:==AA>@==77776636111666399:9;:>::;?@CBC
BCBBABBBAA@
@T6XXI:00122:00142
GGGAGAAGGGAGAAAGGAAGCTAGTCACTCGACATCTGTGGCTTCACAGTACAGAGGGACTGTCCAAAGCCGCAGAT
CTCGAGTCGACTAG
+
=@<ABB?BE@CBBC8::<>BAC@@??::6:?797788<AK?BB?BBC@??BBB????<?@@AAC9884<?;?AAA@=
==@A@@A??==<?=
@T6XXI:00122:00146
GGGGAATTCTAATACGACTCACTATAGGGAGAGGGAGAATAGATGCGATCTCCCAGCCTCAACTTAAGCTTCCTTTC
CTCCCTTCTCC
+
DED:D?A?ABB>BBBBBBB?>=BA?666.7>>@@>BCCAB@7:..0:;;<7775384<CCC?CC8033=:;=,00,0
,.352701110
@T6XXI:00122:00147
GGGGAATTTC
+
///*/,//+/
@T6XXI:00123:00109
GGGGAATTCTAATACGACTCACTATAGGGAGAGGGAGAATAGTGACGTGTTGTGCGGCGCTCACTGTCTCCTTCCTT
TCTCCCTTCTCCC
+
CDD9DBD@EBB@BBAABBBB?A;?>3695:??CC>AAA@C<??@???A==999:::;:::=;==>98<>5<8<:<<
5;;;@:?;?<<<6
@T6XXI:00123:00126
GGGGAATTCTAATACGACTCACTATAGGGAGAGGGAGAATAGTCAACAAATCGTTTGGGGCGACTTCTCCTTCCTTT
CTCCCTTCTCCC
+
CCC9C>A>ADCACBA@BCCBAA@9<116.7>=?A>ACC@@<@@@?:???:ACCCB>BCC7A?BBC@CBB?@=@>AB;
>?ACAC589BA<
@T6XXI:00123:00133
GGGAGAAGGGAGAAAGGAAGCTAGTCACTCGACATCTGTGGCTTCACAGTACAGAGGGACTGTCCAAAGCCG
+
==:?>=;>>:>?>>9>698;;;BCCBAAAA>:::::6@BB?AC@BCCA@?A97403317?BAAA=@@;@A>@
@T6XXI:00123:00140
```

```
GGGGAATTCTAATACGACTCACTATAGGGAGAGGGAGAATAGTTCTGTGCATTTTCTCCCAGCACACCGTCCTTCCT
TTCTCCCTTCTCCC
+
999/;:BBFBB@BBBBBBBBBB@99111.777@B>BCC@C9?A;<<<====@@@7@C==;==<<<=<;=998<?A8<
<7<=>@;@;=>AC?
@T6XXI:00123:00142
GGGGAATTCTAATACGACTCACTATAGGGAGAGGGAGAATAGAGACGTATTCGTTTGCGCTCTGTTCACCTTCCTTT
CTCCCTTCTCCC
+
AAB9B@B@CBBEBB???BBBBBB99111.7<<@C@BBA@@<?A===<999;<<<=;@==?@?===<=@=;:568;;6
8;9=6878444-
@T6XXI:00123:00144
GGGAGAAGGGA
+
6681,-+-,*,
@T6XXI:00123:00145
GGGGAATTCTAATACGACTCACTATAGGGAGAGGGAGAATAGTCAACAAATCGTTTGGGGCGACTTCTCCTTCCTTT
CTCCCTT
+
@::2=;ACCCBACHCAAB@@@?CCCAAA:????C>CCB@@@AA@?9<<;6?>>?>.777.7;===;>>>:97:8:;7
:344--,
@T6XXI:00123:00146
GGGGAATTCTAATACGACTCACTATAGGGAGAGGGAGAATAGTCAACAAATCGTTTGGGGCGACTTCTCCTTCCTTT
CTCCCTTCTCCC
+
?==6@@B@CCB@BA??BBBBB@BBB67756?8CC>CAB??=AABB;<?<:=7666-666-
7>@@C@CAA??>A>AA=@@AA<A;9>@A;
@T6XXI:00124:00105
GGGAGAAGGGAGAAAGGAAGGAGAACGCGCACCCGAGCCAACGGTGATCTATTCTCCCTCTCCCTATAGTGAGTCGT
ATTAGAATTCCCC
+
@B?BCC?BA=B?===B=@=@@CBB?BCBBBBBCBCBBA@CACD@CCCCCCBB=ABDC?B@@BB=AAAAAABBC@CCA
AA?C?<9??BBB7
@T6XXI:00124:00109
GGGGAATTCTAATACGACTCACTATAGGGAGAGGGAGAATAGTCCAACATTCCTTGGTTCCCGGCTATTGCTTCCTT
TCTCCCTTCTCCC
+
;99,9595<BBADCBBCBB?BAB?;666.6=;=>9???<=@@@C?A?BBC>B?C@C?B>AC>C@B?@B>B:737;>>
<???A<@<@8871
@T6XXI:00124:00114
GGGAGAAGGGAGAAAGGAAGGAGAAGTCGCCCCAAACGATTTGTTGACTATTCTCCCTCTCCCTATAGTGAGTCGTA
TTAGAATTCCCCC
+
DCAEEC@CB>BBBB=B@B?BACBA?CCB?>>;5<<6?A@A@<AA>BBBBCA>BA@A<888??<?@A@AA@<<=ACA@
@=?;979/////+
@T6XXI:00124:00126
GGGGAATTCTAATACGACTCACTATAGGGAGAGGGAGAATAGTCAACAAATCGTTTGGGGCGACTTCTCTACCTTCC
T
+
AD@4A=@=ACCECCBACBBBA@BB@166.7>>@C>DAA??@A@@A>@@?;B<<<?;AAA7ABBBB?=77,,,++)*)
*
@T6XXI:00124:00130
GGGAGAAGGGAGAAAGGAAGGGAATAGCAGCAGTCGGCAACGTGAAACTATTCTCCCTCTCCCTATAGTGAGTCGTA
TTAGAATTCCCC
+
GC@CAB?BC>@BBB>A>A>BB>A@BCBBBBAAABBB?BC@AAA@??9@BBC@BBBE>AA???9?>?>??@@@A988?
?=?@@<95<;;1
```

```
@T6XXI:00124:00144
CTAGTCACTCGAGATCTGCGGCTTTGGACAGTCCCTCTGTACTGTGAAGCCACAGATGTCGAGTCGACTAGCTTCCT
TTCTC
+
B=1/1=8=@DCCDCBBBCBB?BCCBC@CCBBBCC>CBB>?@AAC??;8495999>><8866777;;79733110101
1,11;
@T6XXI:00124:00147
GGGGAATTCTAATACGACTCACTATAGGGAGAGGGAGAATAGCACAAACTCG
+
999/9<ABCBA@AAA@@BBBBBB>;111.677@BACBC@@A../.79,..-+
@T6XXI:00125:00107
CTAGTCACTCGAGATCTGCGGCTTTGGACAGTCCCTCTGTACTGTGAAGCCACAGATGTCGAGTCGACTAGCTTCCT
TTCTCCCTTCTCCC
+
E8111:89=CCCCCCBBBCB@BBB=B?CAAC@@@;B@AAA@@B>AA?9=@@CDCBBCBCCCCCAACBBDDDB@C@F
F>@@@C>A=@A@@:
@T6XXI:00125:00109
GGGAGAAGGGAGAAAGGAAGTGCAGTTCGCGGTTAGTGAAGGGAAAACTATTCTCCCTCTCCCTATAGTGAGTCGTA
T
+
EB@AAA@CC>CCBD=@=@@CDCB;<<ACBBB@C@AAACC@CC=AAA7BCCE@CBBB>BBBBC>CAAA?<@@@?8784
4
@T6XXI:00125:00119
GGGAGAAGGGAGAAAGGAAGGAGACAGCCCCAGAGTACAGTCCAGAACTATTCTCCCTCTCCCTATAGTGAGTCGTA
TTAGA
+
CCCCCC@CC@CCBB=A>AABAB>>@AAAAB8BBBCCCBCC@>=@BB?CCCC@BAAB<A?AAA=B?@@??@?888<<<
;<===
@T6XXI:00125:00134
CTAGTCGACTCGAGATCTGCGGCTTTGGACAGTCCCTCTGTACTGTGAAGCCACAGATCTG
+
<=<ADBBBCBC@AAB;<;BFF=<@@:@<<><88662;<<<>><77116336,//,-,,,,0
@T6XXI:00125:00135
GGGGAATTCTAATACGACTCACTATAGGGAGGGGGAGAATAGACCTGTAATCCCGACCGGCCCTATCTCCTTCCTTC
TCCTT
+
BHKBC;97<9959AABBCDCCCDCCA<9868888/?CA?BA<@B>?BBA=AAC>BCB?B??@:><;88841+,+,++
++*+*
@T6XXI:00125:00136
GGGAGAAGGGAGAAAGGAAGGAGAAGTCGCCCCAAACGATTTGTTGACTATTCTCCCTCTCCCTATAGTGAGTCGTA
TTAGAATTCCCCC
+
BB>BCC?BB?BCBB>D>?=A@CAA?C@@;;;;5??<@CDCC>B@?BCC??:;<<<=;;;?==;==<<<====;<?<A
@=@<:792,,,,)
@T6XXI:00126:00108
GGGAGAAGGGAGAAAGGAAGAGAATAGGCACGCATGTAAAATGGTAACTATTCTCCTCTCCCTATAGTG
+
ED<@A@>AA>AAAD@C@B@BBCB@B??==@CDA@>;?;<<2><;?B?@C@=:?:731764413;;85.*
@T6XXI:00126:00123
GGGAGAAGGGAGAAAGGAAGCTAGTCGACTCGACATCTGTGGCTTCACAGTACAGAGGGACTGTCCAAAGCCGCAGA
TCTCGAGTCGAC
+
CC?CCB?BB=B?==:B=@<@C@8>>/11>?B=3<:???A@@=?@?BBBBCCC?=4000-
4;;:=?<?>7>@;>><<;?=>?==<@@@@@
@T6XXI:00126:00134
TCGAGATCTGCGGCTTTGGACAGTCCCTCTGTACTGTGAAGCCACAGATGTCGAGTCGACCTAGCTTCCTTTCTCCT
CTCC
```

```
+
@CBBABBBBBCB@BBB<B=@@CCDCC>BBBBBB??;;;>>;;:>??<::<<CBBCCCC<;7;@99<??;>@;@>>11
000,
@T6XXI:00126:00135
GGGAGAAGGGAGAAAGGAAGCTAGTCGACTCGAGATCTGCGGCTTTGGACAGTCCCTCTGTACTGTGAAGCCACAGA
TGTCGAGTCGACTAGCTTCCTTTCTCCCTTCTCCC
+
?D=B@A=@B:@99909;>;>?888>76767=?@?@CAB@@@ABAEACAB@?@BAE=AB@AA===??AC@8868@;;=
=87798:488>???===?@9;<7;??@<A>988?:
@T6XXI:00126:00139
GGGGAATTCTAATACGACTCACTATAGGGAGAGGGAGAATAGTCAACAAATCGTTTGGGGCGACTTCTCCTTCCTTT
CTCCCTTCTCCC
+
999/9296<BB=ABCCCCCB>CB99111.7?>@B>BBB@A=====;4781:===B9???5?@AAB>AAA<<9=>AA<
AAAA<A=????:
@T6XXI:00126:00142
GGGAGAAGGGAGAAAGGAAGCTAGTCGACATCTGTGGCTTCACAGTACAGAGGGACTGTCCCAAAGCCGCAGATCTC
GAGTCGACTAG
+
BB@BBC@BB=BB?B=B?B@BBB?AAA?76:;;<ACBADC@CCECDBCB;944409???88:6;A<AD@CAAACDBBB
BBBBCCBCBCC
@T6XXI:00127:00100
GGGGAATTCTAATACGACTCACTATAGGAGAGGGAGAATAGTGGACTGCTCTCACCACATACCTTATTCTTCCTTTC
TCCCTTCTCCC
+
CCB9A=@C@BBCDB??BBBBB:999//-
0==AC=BBC@A<<<=<<<=?<????AA=?:9>>;:;;@@?@@>@>AA<@?=63448444-
@T6XXI:00127:00110
GTCGACTCGAGATCTGCGGCTTTGGACAGTCCCTCTGTACTGTGAAGCCACAGATGTCGAGTCGACTAGCTTCCTTT
CTCCCTTCTCCC
+
CC:::BBBCABBBCBBDC@CCC?C=??BB88800******024>B@AB@ABBBBBCBBB???CB?AACB?A?B@CB=
AA@?86:;777.
@T6XXI:00127:00117
GGGAGAAGGGAGAAAGGAAGTGCAGTTCGCGGTTAGTGAAGGGAAAAACTATTCTCCCTCTCCCTATAGTGAGTCGT
ATTAGAACTTCCCC
+
DC>AAA=>>1999909;>598ABCCDACBBB:;9;@@BC?BC>CCCC2@@@B?DABDACBBBC>BA@@@@>884404
4348345555;>>+
@T6XXI:00127:00144
GGGAGAAGGGAGAAAGGAAGCTAGTCGACTCGAGATCTGCGGCTTTGGACAGTCCCTCTGTACTGTGAAGCCACAGA
TGCGAGTCGACTAGCTTCCTTTCTCCCTTCTCC
+
BA>A@@=>@:?99909595;CB>?D/11??????BBC;:;C?CDC<A>BBBBBBD>BC@@@=====<?@<<9=@@@@
@A???A@@@AAAA8778:24818ACC@A>0//4
@T6XXI:00128:00126
GGGGAATTCTAATACGACTCACTATAGGGAGAGGGAGAATAGTCAACAAATCGTTTGGGGCGACTTCTCCTTCCTTT
CTCCCTTCTCCC
+
:;9/9<A>BBB@CBAA@A?BBAB?<111.6<<<?=?DE@A=AAAA;;@=:>9666,111-
:BCBC?BBC?@>@@AA:?@AA=B<??@@<
@T6XXI:00128:00134
GGGAGAAGGGAGAAAGGAAG
+
>>:A@>599144//+/,/,/
@T6XXI:00128:00146
GGGAGAAGGGAGAAAGGAAGGAGAAAGCGGCAGACGAAACATT
```

```
+
DE?BBA===<@AAA<@<>4958??>8>9813333...:800.,
@T6XXI:00129:00134
GGGAGAAGGGAGAAAGGAAGTGCAACTTGTCGCGCAAAATAGGGAGACTATTCTCCCTCTCCCTATAGTGAGTCGTA
TTAGAATTCCCC
+
CC>C@@=>>:@A>>:B=@=@BB?@>AEADBBCDBAAA?6?@CCBCCECCCC?BDCC>:::??8?>=>@@@@@A===?
?<==B79/4440
@T6XXI:00129:00140
GGGAGAAGGGAGAAAGGAAGCTAGTCACTCGACATCTGTGGCTTCACAGTACAGAGGGACTGTCCAAAGCCGCAGAT
CTCGAGTCGACTAG
+
@D>BCC@E>:?988-
9<@>ACCCBBAAAAA@:;::::CCD@?@@DCCAAAA?;977717>A>??=@@<@A>@@AAA@????@???@?A>?=
@T6XXI:00129:00145
GGGGAATCTAATTACGA
+
88217+-,,,*,*///4
@T6XXI:00129:00149
GGGAGAAGGGAGAAAGGAAGGAGAAGTCGCCCCAAACGATTTGTTGACTATTCTCCCTCTCCCTATAGTGAGTCGTA
TTAGAATTCCCC
+
EE@ABA@BC>AAAB>B?A?BABA@>ACBAAAA8CBBCEC@?;??B??;;;;:====:<=@<@<AA@<====<:::<=
=<A?>;;3444,
@T6XXI:00130:00120
GGGGAATTCTAATACGACTCACTATAGGGAGAGGGAGAATAGTCAACAAATCGTTTGGGGCGACTTCTCCTTCCTTT
CTCCCTTCTCCC
+
@@?4><A>BBB@BBAABBBA@>@99166.7=7777;BB@A===@A<<==9?CCCC=AAA7ACCCCACCBA?=?>A=;
=A==;=99=@@<
@T6XXI:00130:00131
GGGAGAAGGGAGAAAGGAAGAACAGGAGACTGCAGAATGTGCCGTAACTATTCTCCCTCTCCCTATAGTGAGTCGTA
TTAGAA
+
EEACCC@CC@BBBB?B?A@CB@???=?B?>@A@A==>@@@@E@CCC@BBCEBC??@=@CBBB>BCCCC@@@@@CBCA
A@C??@
@T6XXI:00130:00133
GGGGAATTCTAATACGACTCACTATAGGAGAGGGAGAATAGTCAACAAATCGTTTGGGGCGACTTCTCCTTCCTTTC
TCCC
+
;99/9;@=@BBCBBBBCBBB>:966..,0<:==9?AA?A===AA?@?@<BC?9989??5ABBCE@AAB??<?>@@<@
@@A<
@T6XXI:00130:00142
GGGGAATTCTAATACGACTCACTATAGGGAGAGGGAGAATAGTCAACAAATCGTTTGGGGCGACTTCTCCTC
+
DDC9B=@CACC@CCB?BBBB?@><<111-6<<@B>BBBAA=?@A@=<<<7?BBBC>CCC9;:0**))))))(
@T6XXI:00131:00108
GGGAGAAGGGAGAAAGGAAGCTAGTCGACTCGACATCTGTGGCTTCACAGTACAGAGGGACTGTCCAAAGCCGCAGA
TCTCGAGTCGACTAG
+
CECCCB894-
99880;=>=@CA;;;/00:;=;0;::;8CCC@AB?ACA@@@C@;83331399<=?=@@<@B>A===@@@A====<9=
=@AAA
@T6XXI:00131:00110
GGGAGAAGGGAGAAAGGAAGCTAGTCGACTCGACATCTGTGCTCACAGTGACAGACAGGGCAGTCT
+
GH?B94299199991854243744.......5..311611111766696668;96004000+0-1---
```

```
@T6XXI:00131:00118
GGGAGAAGGGAGAAAGGAAGCTAGTCACTCGAGATCTGCGGCTTTGGACAGTCCCTCTGTACTGTGAAGCCACAGAT
GTCGAGTCGACTAGCTTCCTTTCTCCCTTCTCCC
+
ID<A@A=>@:A999048878CBACC?:::>777:99;AAA@CBC>C@CBBBB>>6=://,,++++4774;;<<<;;@
888?=;<<988;@880,/588./.77,/,--00+
@T6XXI:00131:00119
GGGAGAAGGGAGAAAGGAAGGAGA
+
;;:444249-4444-4242/5...
@T6XXI:00131:00120
GGGAGAAGGGAGAAAGGAAGA
+
;4-9BB@CBA@1**))))(((
@T6XXI:00131:00127
CTATCACTCGAGATCTGCGGCTTTGGACAGTCCCTCTGTACTGTGAAGCCACAGATGTCGAGTCGACTAGCTTCCTT
TCTCCCTTCTCCC
+
C???9::====ABBCCCBB?A@A<@@BBBBBBB>BBBAA@@@@B@A=AC@CBBBAAAB???BBBBAABAAAB=?=@C
ADCCC>A=?@@?<
@T6XXI:00131:00140
GGGAGAAGGGAGAAAGGAAGC
+
DE>B@>;>>:>9990951,/,
@T6XXI:00132:00112
GGGAGAAGGGAGAAAGGAAGCTAGTCACTCGAGATCTGCGGCTTTGGACAGTCCCTCTGTACTG
+
DD=@A@=>@:>999149><?@@@@C?A@??????C?=@AD@KCB?E@CB00000,/.-112300
@T6XXI:00132:00131
GGGGAATTCTAATACGACTCACTATAGGGAGAGGGAGAATAGCCACACGTTATTCCCATTCTTGGCTGTCCTTCCTT
TCTCCCTTCTCCC
+
C@B6@@BACBB@A@??BBBA@@>99111.7==BC>AAABD=A?<=:::<=<;;5<<7<=4>>4858AA?@>@?@56<
7<@@A<@=@??;:
@T6XXI:00132:00133
GGGAGAAGGGAGAAAGGAAGCTAGTCACTGACATCTGTGGCTTCACAGTACAGAGGACTGTCCAAAGCCGCAGATCT
CGAGTCGACTAGCTTCCTTTCTCCCTTCTCCC
+
CD>@@C>BB;<<CC?CAC?A@A>:8,*******,254033./,.4;?AA><?B??:;;;444377.499;=888A98
8@@<888=;44774/4344,7;9;6<8;<>@:
@T6XXI:00133:00105
GGGGAATTCTAATACGACTCACTATAGGGAGAGGGAGAATAGTCAACAAATCGTTTGGGGCGACTTCTCCTTCCTTT
CTCCCTTCTCCC
+
@A?6@@A@CBBABBB=?=?BBCB?9111.7>>BC>BAA@A=AA@?=<<63?B=7749694:@@?A?BAA;=<?8<;6
<66616,/-./,
@T6XXI:00133:00108
GGGAGAAGGGAGAAAGGAAGGAGAACGCGCACCCGAACCAACGGTGATCTATTCTCCCTCTCCCTATAGTGAGTCGT
ATTAGAATTCCCC
+
DB@CCC>?=:@;446<?B?B@CBB?BBAA@@BCAAB?B@C@CE@CCCCC;;<@CBBB=@@@CE>CCBBAAABBBBCB
BB?BAB?DACCF9
@T6XXI:00133:00119
GGGAGAAGGGAGAAAGGAAGGAGAAGTCGCCCCAAACGATTTGTTGACTATTCTCCCTCTCCCTATAGTGAGTCGTA
TTAGAATTCCCC
+
```

```
DDB@AA@CC@CCCC@C@C?B?ABB@B?BBBBB8AA@ACCCC<>A@CBBABA>B???;?@@@B=AA=??<>>>;:?:<
<8<;:792444-
@T6XXI:00133:00121
GGGAGAAGGGAGAAAGGAAGCTAGTCGACTCGACATCTGTGGCTTCACAGTACAGAGGGACTGTCCAAAGCCGCAGA
TCTCG
+
DEACCB=@@9>@@@<@@C;<;CCBBBCC@==977::??BAC>;<8:CCBBBCA=4000-
4;;=?A>@@<@@?@@@AAA<?@?
@T6XXI:00133:00128
GGGAGAAGGGAGAAAGGAAGCTAGTCGACT
+
@:-4448=?5@=A@:?8968?80.00,,,,
@T6XXI:00133:00136
GGGGAATTCTAATACGACTCACTATAGGGAGAGGGAGAATAGCCATTGTCCCTACGAACTAATCCTGTTCTTCCTTT
CTCCCTTCTCCC
+
CBB9B?BACBB@BBBBBBBBAA@99111.7>>AC>BCC?@<A=;<<<<===;8<<>>>?=A<<@>@AA=>>?A=;;:
;AA=:<;<?A<;
@T6XXI:00133:00146
GGGGAATTCTAATACGACTCACTATAGGAGAGGGAGAATAGTCAACAAATCGTTTGGGGCGACTTCTCCTTCCTTTC
TCCCTTCTCCC
+
BBA7A@B?CDH@BBBBBBBBB:999/.,0::==<?CC@A@@AAA?AAA<CC@BB>@@@8@@???@A@C?@>@<AA<?
??>3655;>?:
@T6XXI:00133:00147
CTAGTCACTCGAGATCTGCGGCTTTGGACAGTCCCTCTGTACTGTGAAGCCACAGATGTCGAGTCGACTAGCTTCCT
TTCTCCCTTCTCCC
+
C;;;;@===CBB@?AC;;<C@BBB?B?AABBAAA=BCCCCBC>>BBA?AC=@@BCCBBBBCCC??@;;;B@???C@B
J>BBBCCA=??AA<
@T6XXI:00134:00120
GGGAGAAGGGAGAAAGGAAGAAAACAAGGGACAGCGCTAACACAGGACTATTCTCCCTCTCCCTATAGTGAGTCGTA
TTAGAATTCCCC
+
DDBBB@@BC>DBBB<?=@@CCCB9BC@CD<B>BBBB??B>???>>;?BBBB=;?>>;;;;>?;>::=====@=<@??
?<?@A@B>BAA6
@T6XXI:00134:00127
GGGGAATTCTAATACGACTCACTATAGGGAGAGGGAGAATAGCCGCGTGTCCGATCGCGTCTGTATCTCCTTCCTTT
CTCCCTTCTCCC
+
?>?4><@@BCBDCCBBBA@@BBB99111.7=<ADAECCA@=<==??@????>@@?=?<????????A@?;=8:243,
3844:=<788=:
@T6XXI:00134:00147
GGGAGAAGGGAGAAAGGAAGTAGTACAAGGTGGGGAGGACACCTCGTCTATTCTCCCTCTCCCTATAGTGAGTCGTA
TTAGAATTCCCC
+
DEBCA?>@C=@BBB?B@CCCCCBBCCC?B>????5@A?CBBB?A:::B???@@@??:==;;<7<AAAA@AA??<<<?
?<=>B8;5<>=1
@T6XXI:00135:00109
GGGGAATTCTAATACGACTCACTATAGGGAGAGGGAGAATAGTCAACAAATCGTTTGGGGCGACTTCTCCTTCCTTT
CTCCCTTCCTCCC
+
BB@2@=@==9@<9;9A??ACC?B?>777-77771,166;9@<59::444-
7777A<?9=6<77:B?AAB>=;>@AA<A;;816,/,/772
@T6XXI:00135:00149
GGGGAGAAGGGAGAAAGGAAGGAGGTGGCAGGGCACGAATAGAAAGGTCTATTCTC
+
```

```
999,444244-///8+/,/,/6..-799;333,397331111166666100321--
@T6XXI:00136:00109
GGGAGAAGGGAGAAAGGAAGCTAGTCGACTCGACATCTGTGGCTTCACAGTACAGAGGACTGTCCAAAGCCGCAGAT
C
+
CD@@@@=>>:>99;0<BB8<<B@@@CCCCCB?@7::7:@=?;@B?BCCCBBA774..-
/;;;>A=AC=?@>B@A???@
@T6XXI:00136:00118
GGGAGAAGGGAGAAAGGAAGGAGAAGTCGCCCCAAACGATTTGTTGACTATTCTCCCTCTCCCTATAGTGAGTCGTA
TTAGAATTCC
+
44-
4;;:AH?<<<C=@;>49588848793377,33,3;;?C>BB@;<8@?<?@===7444:87487;;>>=988373-
33--,-,++
@T6XXI:00136:00127
GGGGAATTCTAATACGACTCACTATAGGGAGAGGGAGAATAGCGTGCCTGTATACGGTACCCTGTTCGCCTTCCTTT
CTCCCTTCTCCC
+
GA?9A===@BA?BBBBBBBBC?A9?166.7==>A?CDC@B@@@=>=99=CCC?<BB@<7775:9?@B?><A5:7:C>
CCCC>C>??@<;
@T6XXI:00136:00141
GGGGAATTCTAATACGACTCATATAGGGAGAGGGAGAATAGTCAATAAATCGTTTGGGGCGACTTCTCCTTCCTTTC
TCCCTTCTCCC
+
AA?292929==@BACCC330..63...+1677?;@@A=@78/88,//8288/44+033.666B?::8<888=?AA<A
A@@:;8:444-
@T6XXI:00137:00101
GGGAATTCTAATACGACTCACTATAGGGAGAGGAGAAT
+
991424::48599>>@BBCBB@?>=<<-71..5542*+
@T6XXI:00137:00107
GGGGAATTCTAATACGACTCACTATAGGGAGAGGGAGAATAGTCCCGAATTTATCCGCGCGTCTGTCTCCTTCCTTT
CTCCCTTCTCCC
+
999/4:A?ABABCCBBBBB@A@<97166.7==BC?BBBBAAA87417=9=?<@@@?@@@@@@88?888<=@;@<??<
?DHC<@=AAAA;
@T6XXI:00137:00117
GGGGAATTCTAATACGACTCACTATAGGGAGAGGGAGAATAGCGCGTTCCTATTGGCGTGCCTATATCTCCTTCCTT
TCTCCCTTCTCCC
+
DDD:E?A@@A@@CBBBDBA?><?99116.7====<?AB@A@@@@@@@<?<<??<?==BAAA9<<@AAA@@>A>@<>=
7;9:9-4/44.-+
@T6XXI:00137:00122
GGGAGAAGGGAGAAAGGAAGCTAGTCGACTCGACATCTGTGGCTTCACAGTACAGAGGGACTGTCCAAAGCCGCAGA
TCTCGATCGACTAGCTTCCTTTCTCCCTTCTCCC
+
HD@ECCACC=A<;4-
9598<AAABB@B??BCA::::::>>>;:;9;DCCDCCCC???@:@A@>>>9<<7<@>@@AA===?>=@>888<<<<=
@>A>@?:444:-4244494
@T6XXI:00137:00127
GGGAGAAGGGAGAAAGGAAGGGAATAGCAGGCGTCGGAACGACGAACTATTCTCCCTCTCCCTATAGTGAGTCGTAT
TAGAATTCCCC
+
DD?ABB@DB>@?:75;,1,11-
1,.,22,,*....44:2444944249;;:=A@?:=ABBB>BAAAAA>A<<99B8834;=;688=A0
@T6XXI:00137:00129
```

```
TCGAGATCTGCGGCTTTGGACAGTCCCTCTGTACTGTGAAGCCACAGATGTCGAGTCGACTAGCTTCCTTTCTCCCT
TCT
+
?DA@ADCCBABB@BBB<B=BBBBB??;?@@DB?@BCC??>=@=BCCB?===CCCDCCCAAACC<=:<>A@:AAAA<A
8:;
@T6XXI:00137:00134
TCGAGATCTGCGGCTTTGGACAGTCCCTCTGTACTGTGAAGCACAGATGTCGAGTCGACTACTCCTTCCT
+
ADCAABBBCCBB?@??:@=??CCAAA?ABBCBBBC:70>://1??BA@@@CF?DD32/==1//,*/,/,/
@T6XXI:00138:00115
GGGAGAAGGGAGAAAGGAAGCTAGTCACTCGACATCTGTGGCTTCACAGTACAGAGGGACTGTCCAAAGCCGCAGAT
CTCGAGTCGACTAGCTTCCTTTCTCCCTTCTCCC
+
BB1999699-4111-/,/,/6477?77771666111666067=@?????B@@=?AD@A=<<;;244-
4;7;<=;78/..94.04444;???B?A>AA<??@DAA9<<DDA
@T6XXI:00138:00121
GGGGAATTCTAATACGACTCACTATAGGAGAGGGAGAATAGCTCTTCTCATACTCACCACCAGCAACCTTCCTTTCT
CCCTTCTCCC
+
DDB9CADACCC@CCBBBAAAB:999//-
0;;BCCEBB@@AAABAA?C@@?CCBABAA>?@>@???>@=?<=8;<7;=@@6;89:::4
@T6XXI:00138:00148
GGGAGAAGGGAGAAAGGAAGTGCAGTTCGCGGTTAGTGAAGGGAAAACTATTCTCCCTCTCCCTATAGTGAGTCGTA
TTAGAATTCCCC
+
CC@CCB?BB>BBBB?B@C@B??@@BB@B??AAB@?>>BB@@@;@BB9BBBB>?>>>;>>>@C=C=<<<<>AAA@@@@
@=@@@>A>AB@5
@T6XXI:00139:00100
GGGAGAAGGGAGAAAGGAAGGGGAGAAGGGAGAAAGGAAGCTAGTCACTCGACATCTGTGGCTTCACAGTACAGAGG
GACTGTCCAAAGCCGCAGATCTTCGAGTCGACTAG
+
CD=@>>5991999919;>;>>>488848807777.60747=@?8?BA???=744773949844233330?>;:::4//
,//...577755555=A@A??>9<<788>873;;5
@T6XXI:00139:00110
GGGGAATTCTAATACGACTCACTATAGGGAGAGGGAGAATAGCGCACCATCAATTTTGTCGTGCCGTTCTCCTTCCT
TTCTCCCTTCTCCC
+
DDD9GCB@DCC@BCCBBB>BBA?97111.777?B>CCC@A??@AC?A?BBAC@CCD9CCCCCCC?BC@::;=<?A>A
@;@AAA<A>@???<
@T6XXI:00139:00121
GGGGAATTCTAATACGACTCACTATAGGGAGAGGGAGAATAGTTTTTTCCCTTCACTAACCGCGAACTGCACTTCCT
TTCTCCCTTCTCCC
+
BBB:B@B@BCB@CBBBBBB<B@?99111.7==?C@AAA@AA<;;888)3318<@BCCC@C@?=@B??@A8::A>B;?
?9?A@@<A@CAAAA
@T6XXI:00140:00102
GGGGAATTCTAATACGACTCACTATAGGGAGAGGGAGAATAGTTTTTCCCTTCACTAACCGCGAACTGCACTTCCTT
TCTCCCTTCTCCC
+
DDGBC?C?BCC@CCBBBA??B@<97116.7>>?D@BBB@@844433,4;:=?ACBABB@>@@B@?====@@@>?>AA
>???@;>?@AAA<
@T6XXI:00140:00117
GGGAGAAGGGAGAAAGGAAGTGCAGTTCGCGGTTAGTGAGGGGAAAACTATTCTCCCTCTCCCTATAGTGAGTCGTA
TTAGAATTCCCC
+
DHABBA@CC>CCBB=B8:6<BCCECC@BBBB>A@@@@B@66;-
777.;ABB?BCCC>BBBCC;B<?@AAAAAAA????;=<A?95;;<1
```

```
@T6XXI:00140:00125
GGGAGAAGGGAGAAAGGAAGGAGAAAAGTGATGCACAAAATACAAGACTATTCTCCCTCTCCCTATAGTGAGTCGTA
TTAGAATTCCCC
+
CECAAA@BA<B;996<?@>@=>8788.8>BCCC@BB@@@8<<<?BB??BB>>>;;=;=933718?@BA=@A=<<;;;
;;>>@?A8???5
@T6XXI:00140:00127
GGGAGAAGGGAGAAAGGAAGTGCAGTTCGCGGGTTAGTGAAGGGAAAAACTAATTCTCCCTC
+
/1-//4244-448:-42499779998844444-3173333111,1111+...,.995::7.1
@T6XXI:00140:00129
GGGAGAAGGGAGAAAGGAAGCTAGTCACTCGAGATCTGCGGCTTTGGACAGTCCCTCTGTACTGTGAAGCCACAGAT
GTCGAGTCGACTAGCTTCCTTTCTCCCTTCTCCC
+
DGBBA@=@B:@@@@<@>A>BCA@AB:::?BCBCBBBBDBC@@AA@B@ABCCB@B>@B???488@@@C?@@>A<A;<A
@@???@@AA@@@<??B??>AA;?;8848/34;;6
@T6XXI:00140:00135
GGGGAATTCTAATACGACTCACTATAGGGAGAGGGAGAATAGTCAACAAATCGTTTGGGGCGACTTCTCCTTCCTTT
CTCCC
+
BBB8A>A@CCB@AABBBCCAA@B>>66659??CE@CBB?@??@AA>????7==666-
777.7=??A;??@;97<8;;7=@@A<
@T6XXI:00140:00145
GGGGAATTCTAATACGACTCACTATAGGGAGAGGGAGAATAGTCAACAAATCGTTTGGGGCGACTTCTCCTTCCTTT
CTCCCTTCCTCCC
+
D??6B?CADDB@BBBBBBB??@BBB:::8:<<AC>AA?>A:????=AA;:>;??@;@AB7BCB@B@CCC>?<?>AA<
@;:827,.,/661
@T6XXI:00141:00117
GGGGAATTCTAATACGACTCACTATAGGAGAGGGAGAATAGCTCCCGGGTATGACAACAGATCGCGCATCTTCCTTT
CTCCCTTCTCCC
+
B?:6A=@=ABC>BBCCCCCBB>>96./,000==6=@>:;<<<?CFCFD<??AACEA>A@===@@@A==?BC@B=<<7
;88811+,+,,+
@T6XXI:00141:00131
CTAGTCACTCGAGATCTGCGGCTTTGGACAGTCCCTCTGTACTGTGAAGCCACAGATGTCGAGTCGACTAGCTTCCT
TTCTCCCTTCTCCC
+
=11////44A?>@BBCCBCC?BCC>C?BBAB9994:@?BCBBBACB@>@CACCCCCCCBBCCCCBB@@AC@@=?B@B
C<????6:7:8==7
@T6XXI:00141:00138
GGGAGAAGGGAGAAAGGAAGCTAGTCGACTCGACATCTGTGGCTTCACAGTACAGAGGGACT
+
CB@AAB@BB=BBBC=B>B>BACBBBABBAAA?@:::??@AA@CC@CAA???CA=9033-4;:
@T6XXI:00141:00145
GGGGAATTCTAATACGACTCACTATAGGGAGAGGGAGAATAGATCACCGTTGGCTCGGGTGCGCGTTCTCCTTCCTT
TCTCCCTTCTCCC
+
CCC:C;:><CECCBA?BBBBA@>;@666.7<<BC>CBC@A@===ABA>AA;?=ABG@@>>333>EEAC??=@@B?BB
<A???9<9<A@A<
@T6XXI:00142:00114
GGGAGAAGGGAGAAAGGAAGGGGAGAAGGGAGAAAAGGAAGCTAGTCACT
+
GC@CBBACC>?BCC=B?B?BDD9@@C@BC:77777.74701713196755
@T6XXI:00142:00117
GGGAGAAGGGAGAAAGGAAGCTAGTCACTCGACATCTGTGGCTTCACAGTACAGAGGGACTGTCCAAAGCCGCAGAT
CTCGAGTCGACTAG
```

```
+
CD?CBA>==:@@885>884;A:8;BA???A@??????BBB?BA@CC@<<A@AA=98655<8<==::=7;;8;<<@AA
?@@==<<@?>??@@
@T6XXI:00142:00125
GGGAGAAGGGAGAAAGGAAGGAGAAGTCGCCCCAAACGATTTGTTGACTATT
+
AA<>=<BEGBAA@C?C@D@CBACC=>>@8833,33,39311,1101661102
@T6XXI:00142:00129
GGGGAATTCTAATACGACTCACTATAGGGAGAGGGAGAATAGCCGCTATTTTAACTCCCGCGGCCGGTTCTTCCCTT
TTCTCCC
+
CCC7A8;8;AB?BBCBBBCBB@;99111.777>ABCCC@D898>??;????5?=AABC>AAA>A??>=:;;244-
444-4449<
@T6XXI:00142:00134
GGGGAATTCTAATACGACTCACTATAGGGAGAGGAGAATAGAAGGA
+
>>80=198;CA<AA9844333179>9>?8AC?=:?//,,,,,*+*+
@T6XXI:00142:00140
GGGGAATTCTAATACGACTCACTATAGGGAGAGGGAGAATAGCCACAATTCTCACCACGCTTCTATGCGCTTCCTTT
CTCCCTTCTCCC
+
494,49@ACBBABDBBB?@@B688?777.7<=?B>BAB?@@<<9<?@=@?A===@;>>7//97///::99;3833;:
?@@A=B588<>:
@T6XXI:00142:00146
GGGGAATTCTAATACGACTCACTATAGGGAGAGGGAGAATAGTCAACAAATCGTTTGGGGCGACTTCTCCTTCCTTT
CTCCCTTCTCCC
+
BBA9A=@=@CB@CCBBBBBCCB?<<776.7==>A=BBC?@@@@AA=AAB<CAAA@;?@?8>BCBB?CBB;<98:?B?
AAAA=A378;<6
@T6XXI:00142:00148
CTAGTCACTCGAGATCTGCGGCTTTGGACAGTCCCTCTGTACTGTGAAGCCACAGATGTCGAGTCGACTAGCTTCCT
TTCTCCCTTCTCCC
+
IB;99D:::CDDHCCBAAABAACA=AAACBCBBC>C@@@@@A??AAA=?B?BBCCCC@@@BBB?@@B@??::7>>>B
C<BABC@A>A8881
@T6XXI:00143:00101
CTATCACTCGAGATCTGCGGCTTTGGACAGTCCCTCTGTACTGTGAAGCCACAGATGTCGAGTCGACTAGCTTCCTT
TCTCCCTTCTCCC
+
DDCC;::ACCDCCCBBBBB?<;;:?;<>><777.7@?CBAAC@C@@=@C@CCCBBCAABBBBBCBBBBC@@?@C@CE
?@@@C?A>@@AA<
@T6XXI:00143:00102
GGGAGAAGGGAGAAAGGAAGCTAGTCACTCGAGATCTGCGGCTTTGGACAGTCCCTCTGTACTGTGAAGCCACAGAT
GTCGAGTCGACTAGCTTCCTTTCTCCCTTCTCCC
+
B;:@99:9@:@111-
/,1,/647;AAAAAB@?????@BBB@CCC>C@CC?@?BA9??788<=AAA@B?@A<<;;===<?@A@@@???@??@8
:588;;7;84418384=B<
@T6XXI:00143:00104
GGGAGAAGGGAGAAAGGAAGTGCAGTTCGCGTAGTTGAGGAATAGGAAAACTTATTCCTCCC
+
BB>AA@@CC?CCCC@C@C@BBBBB?B?BA?=70--+,,,22*//8B>7>66101600/0442
@T6XXI:00143:00107
GGGGAATTCTAATACGACTCACTATAGGGAGAGGGAGAATAGCCTAGTGGTGTACCGCCCCAGCTGTCTCTTCCTTT
CTCCCTTCTCCC
+
```

```
999-48>=DBB?BCCBBAABB?;77111.777?BAABA=><473:BBCD?DBA8440000-
4;;?@<=<@@4768B9;874,47448@<
@T6XXI:00143:00109
GGGGAATTCTAATACGACTCACTATAGGGAGAGGGAGAATGGCCGTTCATTTTCTCCCGCGGCTGTGAGCTTCCTTT
CTCCCTTCTCCC
+
=@@6@@C@CCB@CBBBBBBB>CB9?111.7>>BC>CCE@?;<A>@@?CA===5@A==:<;?><AABBCCCC>A=AE>
CBBB>B=?@CB>
@T6XXI:00143:00124
GGGAGAAGGGAGAAAGGGAAGC
+
AC?BBA<>>19167+//+,*,,
@T6XXI:00143:00128
CTATCACTCGAGATCTGCGGCTTTGGACAGTCCCTCTGTACTGTGAAGCCACAGATGTCGAGTCGACTAGCTTCCTT
TCTCCCTTCTCCC
+
IBBAA::;AAB@@ABBBCC@CCD?C:;;A@???<?CAAAAAB::9?ACC?B@@999@??CAAAAA?@@A3665621>
,169988:=2272
@T6XXI:00143:00129
GGGAGAAGGGAGAAAAGGAAGCTAGTCACTCGACATCTGTGGCTTTGGACAGTCCCTCTGTACTGTGAAGCCACAGA
TGTCGAGTCGACTAGCTTCCTTTCTCCCTTCTCCC
+
@B:@<<89@:@9999-
8482497<7<::0//....345889;:?@<@?BBCAAAE8;:A?<???B@@C>@?ADAA889AAAA@@AAA==<@@;
<:<444-4444-479304+
@T6XXI:00143:00144
GGGGAATTCTAATACGACTCACTATAGGGAGAGGGAGAATAGCTAGTGTTCA
+
999/9=A@CDB@BA@@??BB?@CDB<<:88?A;A>C@>6700+,,,,**)*,
@T6XXI:00143:00148
GGGAGAAGGGAGAAAGGAAGCTAGTCACTCGACATCTGTGCTTCAC
+
EC=B><8<B:@999095959@@@FKA;:::::96;;77/,**)***
@T6XXI:00144:00124
CTAGTCGACTCGAGATCTGCGGCTTTGGACAGTCCCTCTGTACTGTGAAGCCACAGATGTCGAGTCGACTAGCTTCC
TTTCTCCCTTCTCCC
+
B--
,,,,,/6:ADCBBCABBB?@@@>@@B?<>888/7BBBCB?AAD??=@C@CCEBBBBBCDCBABB;;;?<?7:?:?B<
ABCC<A;?:??9
@T6XXI:00144:00125
TCGAATCTGCGGCTTTGGACAGTCCCTCTGTACTGTGAAGCCACAGATGTCGAGCTTCCTTTCTCCCTTCTCCC
+
CCCD@CBBDCC@CCH>B=?ACCCBB>BCBB@CBBB@AA?BB?BBA???:9;<AB@@AB@DC>@@@C>B@C???<
@T6XXI:00144:00147
GGGGAATTCTAATACGACTCACTATAGGGAGAGGGAGAATAGCAACTAC
+
999/9?BACDDBBABBBBCABABB;3995:@?CC>CBB?C?7+*)****
@T6XXI:00145:00118
GGGAGAAGGGAGAAAAGGAAGCTAGTCACTCGAGATCTGCGGCTTTGGACAGTCCCTCTGTACTGTGAAGCCACAGA
TGTCGAGTCGACTAGCTTCCTTTCTCCCTTCTCCC
+
::5>9969=9>8999,82824<373<A99<>AA<BBB?@ACABDGAE?AA;;;CC?@@:888;<=>?C@BC@AAA??
@=<889@??@?===@@B?@>@@<??AA<A899<D?
@T6XXI:00145:00120
GGGGAATTCTAATACGACTCACTATAGGGAGAGGGAGAATAGACACACGTTATGTCTGCTCCTGCTGCCCCTTCCTT
TCTCCCTTCTCCC
```

```
+
AAB9B?C@CCC@BBBAAA??<B<99311.7==?B>BBB@C<A:?AAA@CC?BBB???AAA@>@@@???DD5=9<9<<
9=@DD>C<???A<
@T6XXI:00145:00128
GGGAGAAGGGAGAAAGGAAGTGCAGTTCGCGGTTAGTGAAGGGAAAAACTATTCTCCCTCTCCCTATAGTGAGTCGT
ATTAGAATTCCCC
+
FECCCD?A@:BB===B?B?BCBBAAA@A@?B@B@@>>BB@CC<<<AA2ACCC@?CCB;?9ACC>AAA@@ACBA@@A?
??>A@@=>8;<<1
@T6XXI:00145:00130
GGGGAATTCTAATACGACTCACTATAGGGAGAGGGAGAATAGATCACGACCCTTCTACCACGATTGAGCTTCCTTTC
TCCCTTCTCCC
+
AAA6C@B?BCC???BBB@@@BB<99311.7>>AD>DBB>??@<<<C@BBBC>A;7=//,0::;A?C@AD@=@69919
@@A:?<>@BC<
@T6XXI:00145:00148
GGGAGAAGGGAGAAAGGAAGGAGAAGTCGCCCCAAACGATTTGTTGACTATTCTCCCTCTCCCTATAGTGAGTCGTA
TTAGAATTCCCC
+
DKDBBB?CB=AAAG@CDC>@=@BB?BBCECCC8BB>BCCCE@CC?ABBBBB?@AAA<@A@?@<@@@@A???A>>>?<
<8<==56599:1
@T6XXI:00146:00116
CTAGCGACTCGAGATTTGC
+
A@AAA@@=888---,,*,*
@T6XXI:00146:00128
CTAGTCACTCGAGATCTGCGGCTTGGGACAGTCCCCTCTGTACTGTGAAGCCACAGATGTCGAGTCGACTAGCCT
+
111////44:449BB<==AFA??588087>73111,16666611<61100:<=>>>=B@?>?>7799686,,,*,
@T6XXI:00146:00132
CTAGTCACTCGAGATCTGCGGCTTTGGACAGTCCCTCTGTACTGTGAAGCCACAGATGTCGAGTCGACTAGCTTCCT
TTCTCCCTTCTCCC
+
:1//1=1::DDDCCBBBBAB@BBBAB@BBBB>==;?AAA@>@@AB@@?@C@CGCBCBBBBBBCBBBCCCCCBA=?=?
B>BDCFCF@@@CE>
@T6XXI:00146:00134
GGGGAATTCTAATACGACTCACTATAGGGAGAGGAGAATAGAACGGTACCAA
+
BDC9C=@>ACC@CAAAAA??BBBB<;?;6;=7:5;A?<<:,,+++*+++000
@T6XXI:00146:00147
GGGAGAAGGGAGAAAGGAAGCTAGTCACTCGAGATCTGCGGCTTTGGACAGTCCCTCTGTACTGTGAAGCCACAGAT
GTCGAGTCGACTAGCTTCCTTTCTCCCTTCTCCC
+
BB:@99599199991959 49???@;;:<??@???CC??;;:;CE?C?BBBBCBB>BCB???AAAAAA???>;<=>><
AAAA@A??<;<=;;;?>;7;;188CC;@=?8841
@T6XXI:00147:00101
GGGAGAAGGGAGAAAGGAAGGGGAGAAGGGAGAAAAGGCTCTGACATCATCGATGTACAGCTACGTACGTCTGAGCA
TCGATCGATGTAC
+
BB=A=<;<B>A@@@:><@5999/9??588088777.7467776976617668:?638300000033;<669=?A<<A
==A@:;33++*.(
@T6XXI:00147:00111
GGGGAATTCTAATACGACTCACTATAGGGAGAGGGAGAATAGTCTTGTAATTTCCTGCGGTGCATTCTCCTTCCTTT
CTCCCTTCTCCC
+
DEJECBC?BCBABBBBB>><BB?97376.7==?A>BAA>BBBBAA>@@@=@A<=<<?BB?CCCBB@BCC@C@B?BC>
CCCCCC?<<<<6
```

```
@T6XXI:00147:00124
GGGAGAAGGGAGAAAAGAAGTGCAGTTCGCGGTTAGTGAAGGGAAAAACTATTCTCCCTCTCCCTATAGTGAGTCGT
ATTAGAATTCC
+
DBE@@B@CD=:111/+1/6899C???@CCCC@C?BBBBC@CD>?????2BCBB>??@@>:::AC>?@@AAA@???>=;
442489845=:
@T6XXI:00147:00125
GGGAGAAGGGAGAAAGGAAGGAGAAGTC
+
CDFCB>6996<A@B>C@C?B?BCD@B<?
@T6XXI:00147:00126
GGGGAATTCTAATACGACTCACTATAGGGAGAGGGAGAATAGAGACGTATTCGTTTGCGCTCTGTTCACCTTCCTTC
TCCCTTCTCC
+
AAA8A?B@DBB@CBBBCBAAA@@A@77:6:::CD@GCC?CA@<99=AA@@=@AAA<A@@?@AA7858;;7946,/,-
+,,+,+,++*
@T6XXI:00147:00131
GGGGAATTCTAATACGACTCACTATAGGGAGAGGAGAATATTTTACCGGTCA
+
9AA8C?C@CCD@CBBCBBCCBCCB@776-6><<:=??;?=775011,-,--,
@T6XXI:00147:00148
GGGGAATTCTAATACGACTCACTATAGGGAGAGGGAGAATAGATCACCGTTGGTTCGGGTGCGCGTTCTCCTTCCTT
TCTCCCTTCTCCC
+
BGA4D=@=@CC@BABBBBBBBCB<<761.6=>?A=ABB?BC=9=ACC?BB=?=??CCC@B;;;CBB?BCC@C@C@EC
CCBBC>B@B??A>
@T6XXI:00148:00101
CTAGTCACTCGAGATCTGCGGCTTTGGACAGTCCCTCTGTACTGTGAAGCCACAGATGTCGAGTCGACTAGCTTCCT
TTCTCCCTTCTCCC
+
>://111:?DCCCBBBBBBB@BBB>B>@>><79:5:?BCBBBCACAA>=A>ADDCCBBBCCA@?A@BBBC@@?>A=A
A?ABBD=@@@DAA<
@T6XXI:00148:00111
GGGAGAAGGGAGAAAGGAAGGAGAACGCGCACCCGAACCAACGGTGATCTATTCTCCCTCTCCCTATAGTGAGTCGT
ATTACGAATTCCCC
+
FIBCCBACCCCBBB?B@C@B@BAA@BBCDCCCC>CA>A=@=@B@CBBBC<<<@C@@A>@@@GNBCBABAACABAA?>
00,0007132757-
@T6XXI:00148:00124
GGGGAATTCTA
+
IIE9B>@<>99
@T6XXI:00148:00131
CTATCGACTCGAGATCTGCGGCTTTGGACAGTCCCTCTGTACTGTGAAGCCACAGATGTTCGGACTACT
+
DCDBABADFCDCAAACBBCB@BBB<B@BB;?:9:4:CCCBBCC@BBB?@D@CCGDC@00,0-+,,,**,
@T6XXI:00148:00133
GGGAGAAGGGAGAAAGGAAGCTAGTCACTCGACATCTGTGGCTTCACAGTACAGAGGGACTGTCCAAAGCCGCAGAT
CTCGAGTCGACTAG
+
EEECCCA@=:=@@96@7:6;CB@BB:??>A>7::@??DLE?AA?CC@?@@BA>958886?@@AD<??<?A>A@?@A@
@@@A===888?@??
@T6XXI:00148:00139
GGGAGAAGGGAGAAAGGAAGCTAGTCACTCGACATCTGTGGCTTCACAGTACAGAGGGACTGTCCAAAGCCGCAGAT
CTCGAGTCGACT
+
```

EDCCCB?A@=@B==:@896:CAACC@D?@?>00/;33<>A=BB@ECC>>?@C@<8::8;>?@AA>@@<@A>A@@@@@
=?????AA@@?A
@T6XXI:00148:00140
GGGAGAAGGGAGAAAGGAAGTGCAGTTCGCGGTTAGTGAAGGGAAAAAACTATTCTCCCTCTCCCTATAGTGAGTCG
TAT
+
GC@@9969@:@>>>:><@>A@;><CCCCAA@>A>;;;BB>@?/88888)9>??<?888067117;666660004000
003
@T6XXI:00148:00148
GGGAGAAGGGAGAAAGGAAGTGCAGTTCGCGGTTAGTGAAGGGAAAAACTATTCTCCCTCTCCCTATAGTGAGTCGT
ATTAGAACTTCCCC
+
CC@CCB>=96@988096959>?BBCC@BB;;8;>A@?B@>AC:?@@?0ABAAABBBG>AA???<?ABCCA@@>>>88
7;7:8;2447:::1
@T6XXI:00149:00109
GGGAGAAGGGAGAAAGGAAGCTAGTCACTCGACATCTGTGGCTTCACAGTACAGAGGGACGTCAAAGCCGCAGATCT
CGAGTCGACTAG
+
CC?BAA=@@:@999096959BAABCAA@B::;?=><?AAB?CC@CCCB??A@;;611-
3344=AB<AB?A<<>AABBCDC=>>AAABBC
@T6XXI:00149:00115
GGGAGAAGGGAGAAAGGAAGGAGACGTGACCGGCAATATGGTTGCAGCTATTCTCCCTCTCCCTATAGTGAGTCGTA
TTAGAATTCCCC
+
AAAADA>AC@ACAH>B@C?B?BCDDCCBBB?DABC?BBC>;>;>>003??>=D>AA<AA@@A<A???BAA@@@A@@?
?<??==458891
@T6XXI:00149:00118
GGGAGAAGGGAGAAAGGAAGAAGA
+
CHBCAA==@9@@==6887+--+**
@T6XXI:00149:00123
GGGGAATTCTAATACGACTCACTATAGGAGAGGGAGAATAGATCACCGTTGGTTCGGGTGCGCGTTCTCCTTCCTTT
CTCCCTTCTCCC
+
CCE:CAD@EBC@BABBBBBBB:9990/-
0<<>C>BBC>???<<<BA?CC;;;:@CEH@C@@@CCCCD?@=??B@BC@CDCC>B>?<<@;
@T6XXI:00149:00124
GGGGAATTCTAATACGACTCACTATAGGGAGAGGAGAATAGC
+
DDD6>;@>@CC@CBBBBBBBBBBBB<:866=66377740,,+
@T6XXI:00149:00135
TCGACTCGAGATCTGCGGCTTTGGACAGTCCCTCTGTACTGTGAAGCCACAGATGTCGAGTCGACTAGCTTCCTTTC
TCCCTTCTCCC
+
>AB;><CCBECCCCABB@BBB>B@CDCDDBBABBCBB??BBA?A???@A<B<?B?@A@??BBBBBBC==@@B@BC=A
BCC>>?AAA<<
@T6XXI:00149:00143
GGGGAATTCTAATACGACTCACTATAGGGAGAGGGAGAATAGTTTTTCCCTTCACTAACCGCGAACTGCACTTCCTT
TCTCCCTTCTCCC
+
CDD:GCCABCC@CB???BBAAAAAA?>A<AAACC>AAA@CBBBBBB2?@<A??AACC@C?B@A>?ACA=A?B=A:??
9???D>C@??<<6
@T6XXI:00100:00154
CTATCGACTCGAGATCTGCGGCTTTGGACAGTCCCTCTGTACTGTGAAGCCACAGATGTCGAGTCGACTAGCTTCCT
TTCTCCCTTCTCCC
+

```
CA;@99:::;@CBBBBAABC@BC?;?@BBBA??@;AA@@>>>??BCA=AC@CCCBBCBBBBBCCCC@@BC@???C>A
A=A@BC<=;=ACC<
@T6XXI:00100:00156
GGGAGAAGGGAGAAAGGAAGCTAGTCGATCGACATCTGTGGCTTCACAGTACAGAGGGACTGTCCAAAGCCGCAGAT
CTCGAGTCGACTAGCTTCCCTTTCTCCCTTCTCCC
+
CD@CAA?BD>BBBA<A=A=@@@@CCCB???:;76?;?;9=:8;:;69:?A???<=@@<@=<<<<8;;497,///.77
8300000033300004/00+0446:;<;<;=???7
@T6XXI:00100:00157
GGGAGAAGGGAGAAAGGAAGAATAGGGACGGTGCCAAATATTGTGTAGCTATTCTCCCTCTCCCTATAGTGAGTCGT
ATTAGAATTCCCC
+
CEBCCC@CC@@??C>B?B>BA?ABBC>ABD@CBB@CC?BCD@CBCABABC??=@CCCBCCCCB>A??@CCCCBCCC?
==:=@@>A>@A@5
@T6XXI:00100:00160
GGGGAATTCTAATACGACTCACTATAGGGAGAGGGAGAATAGTCAACAAATCGTTTGGGGCGACTTCTCCTTCCTTT
CTCCCTTCTCCC
+
=@?6@>A>BCBABBBBBBBB?AB97111.7>>>B@DD@BB9?AAC<<??<ACCBA;@@@9BBBBB?@CC?@>=<=<;
>AA=;A><??;:
@T6XXI:00100:00161
GGGGAATTCTAATACGACTCACTATAGGGAGAGGGAGAATAGATCACCGTTGGTTCGGGTGCGCGTTCTCCTTCCTT
TCTCCCTTCTCCC
+
:44/9<@AABB?BCCBBB@AAAB;91695:??CD>@?=?AA==<CCC@CC?B>A?BCE=BBB:;;CABBB?C@C@CB
@DCAA=B<??AA;
@T6XXI:00100:00164
GGGAGAAGGGAGAAAGGAAGGAGAATGCGGTACCGGTTACGTCTTGGCTATTCTCCCTCTCCCTATAGTGAGTCGTA
TTAGAATTCCCC
+
BB>@@@>BB>BBBB>C@B@B@AAA=@@CCBCCC?A@@@???AAA>A@CCC=889@?:4787858CAAAA@=@>4444
424;;9;;>>>5
@T6XXI:00100:00171
CTAGTCGACTCGAGATCTGCGGCTTTGGACAGTCCCTCTGTACTGTGAAGCCACAGATGTCGAGTCGACTAGCTTCC
T
+
=:1//1//:BCBCCGBBBBBB@BBB<BA>;9;6994:?BC?@@C@BB??AC@BCBBB;88.00/:;>;B<;<<::8=
=
@T6XXI:00100:00173
GGGGAATTCTAATACGACTCACTATAGGGAGAGGGAGAATAGTCAACAAATCGTTTGGGGCGACTTCTCCTTCCTTT
CTCCCTTCTCCC
+
AA?9B?B@BDB@B??BBB??>@BB?66641;;AB>BCA?@99<A=<9984??;67,111-
6>@AC@CCC??=??BC<@@@?:?<??@A<
@T6XXI:00100:00186
GGGGAATTCTAATACGACTCACTATAGGGAGAGGGAGAATAGTCAACAAATCGTTTGGGGCGACTTCTCCTTCCTTT
CTCCCTTCTCCC
+
BBC9C@D@BBBAB@@>BBBBB@>97111.7<<>@>@@=BDAAA@A>??636;;;;-
777.7>>>??B:;:8>B>A@<A@@@:?=@8886
@T6XXI:00100:00191
GGGGAATTCTAATACGACTCACTATAGGAGAGGAGAATAGTCAACAAATC
+
A??699@<9448888==??@D?><7666888888968643300/02224.
@T6XXI:00100:00196
GGGGAATTCTAATACGACTCACTATAGGGAGAGGAGAATATTAGGCGTCCT
+
```

```
CA>6>?C?BCD@CCBCCBB>@?AC???C>BBBB?CA>::66,--,----,-
@T6XXI:00101:00171
CTAGTCGACTCGAGATCTGCGGCTTTGGACAGTCC
+
C0,,,-,,6::@CAABCBA@B@AA><A>><<>777
@T6XXI:00101:00182
GGGGAATTCTAATACGACTCACTATAGGGAGAGGGAGAATAGTGACGTGTTGTGCGGCGCTCACTGTCTCCTTCCTT
TCTCCCTTCTCCC
+
???,929=@ACCCDBBBCBB:68>7777,777>?9AA?>A@@@===AAAA>AAA@@>@A@<<<<<<===@;>;><??
7<<??:=;9<;=6
@T6XXI:00101:00194
GGGGGAATTCTAATACGACTCACTATAGGGAGAGGGAGAATAGATCACCGTTGGTTCGGGTGCGCGTTTCTCCCTTC
CTTTCTCCCTTCTCCC
+
4444+42424999;CBBA@@BBBCC:::A<???BB>??<;>??@@@BC@CC?B@C@CBB>@:88?777.7777.747
;=C>BBA=;>9;444-
@T6XXI:00101:00195
GGGGAATTCTAATACGACTCACTATAGGGAGAGGGAGAATAGTCAACAAATCGTTTGGGGCGACTTCTCCTTCCTTT
CTCCCTTCTCCC
+
?@@6A@B?CCC@BBA??>BBBA?97111.7==>A>BB@@B8=<@@=@A=;B??><8<BB7BBBCC@BCE==;=>AA<
@@@C<A;==@@<
@T6XXI:00101:00197
GGGGAATTCTAATACGACTCACTATAGGAGAGGGAGAATAGTCAAACAAAATCGTTTTGGGGGCGACTTCTCCC
+
499/49@<;<<@DCBBCCCCA>;;9//,0;:==6<;823403000136;=5??CBBB9BKEJ0:0000,,,,,*
@T6XXI:00102:00152
TCGAGATCTGCGGCTTTGGACAGTCCCTCTGTACTGTGAAGCCACAGATGGACTGCTCTTCT
+
?AA@AACCCCNC?@@@<??ABABAAC>??:;;B@BB?::78>;B???@44/-///6///:86
@T6XXI:00102:00157
CTATCGACTCGAGATCTGCGGCTTTGGACAGTCCCTCTGTACTGTGAAGCCACAGATGTCGAGTCGACTAGCTTCCT
TTCTCCCTTCTCCC
+
D?99/////88::;;CBBBD@>@@=@@@@@A:::5:??AABBBAAAC:=ADCCCCCAABACBBBBCC???;:689:;
B9?<@B>@>?<?A:
@T6XXI:00102:00159
GGGAGAAGGGAGAAAGGAAGCTAGTCGACTCGACATCTGTGGCTTCACAGTACAGAGGGACTGTCCAAAGCCGCAGA
TCTCG
+
HE>CB@79409@>A<B@B8;;;;;???@=;;001//..47?:=?CBB@@@CBA=<333-
4;;;=@9<<7;B;?===?AAAAA
@T6XXI:00102:00160
CTAGTCACTCGAGATCTGCGGCTTTGGACAGTCCCTCTGTACTGTGAAGCCACAGATGTCGAGTCGACTACTTCCTT
TCTCCCTTCT
+
E?:<<@===BCBBBBBBBBA?AAAAA?@>>=777.7==??CCD??<>9<@?BCAABAAA@??=:;9<<?798DA@@>
;CCCC7:5:4
@T6XXI:00102:00167
GGGAGAAGGGAGAAAGGAAGGAGACAGTGAGCGCCGCACAACACGTCACTATTCTCCCTCTCCCTATAGTGAGTCGT
ATTAGAATTCCCC
+
CC@CCC@DD>DBBB?BAB@B@?@@CCCBCCBBBB=>B>C>>???:??CCBCB@CBBB>BECCC>BBBAAABBCBAB?
CC@CBA=>;===4
@T6XXI:00103:00164
```

```
GGGAGAAGGGAGAAAGGAAGGAGACACGGTCACAAACATGAATGTGACTATTCTCCCTCTCCCTATAGTGAGTCGTA
TTAGAACTTCCCC
+
CCABBB@CD?BBBB@C=?=@=@CCCCBA>BBCBBB=BBCBA?@@@BBBBA?8@:3317>>?DAC@@@@@@AAA@@==
=<;;;.0/7:944
@T6XXI:00103:00165
GGGGAATTCTAATACGACTCACTATAGGGAGAGGGAGAATAGTTAGCGTTTTCTGACCCCGACATTTCTCCTTCCTT
TCTCCCTTCTCCC
+
DFD:C@C@CCC@C@@?BBAA@BA77777.7=7776:?=;????;=9;4433,4;>>>@?1;878?@:??D@C=A>AC
7888<4:9===?:
@T6XXI:00103:00174
GGGAGAAGGGAGAAAGGAAGCTAGTCACTCGACATCTGTGGCTTCACAGTACAGAGGGACTGTCCAAAGCCGCAGAT
CTCGAGTCGA
+
CC@CCB>=:6>944-96958B@@ABA?=???:::C?CBBC?BC@C?=?AACB?9333-
4;;;>@=@@<@A?A?::=<<><=<?@@@@
@T6XXI:00103:00177
GGGAGAAGGGAGAAAGGAAGCTAGTCACTCGACATCTGTGGCTTCACAGTACAGAGGGACTGTCCAAAGCCGCAGAT
CT
+
DCA@@?>=>9>98809694;B??@A<@@???:;::::DCC?BB@CC?>9=>C@;44447>>9<A8664;?<@?:???
==
@T6XXI:00103:00186
GGGGAATTCTAATACGACTCACTATAGGGAGAGGGAGAATAGTCAACAAATCGTTTGGGGCGACTTCTCCTTCCTTT
CTCCCTTCTCCC
+
AAA7A@B@BBB@CBBBBBBB<AB?>661-
6=<<?=@ED>A??=AA?===9?C???8???6?CCCC@BBCC@>@>AA?AA@@;@688AD<
@T6XXI:00103:00191
GGGGAATTCTAATACGACTCACTATAGGAGAGGGAGAATAGTCAACAAATCGTTTGGGGCGACTTCTCCTTCCTTTC
TCCCCTC
+
999/4:;:@BB?BBBBCBB@A;664..,0<<?@@BA??A=AA@A>???::97>>.777.:B@CB@CAA>:9;344-
30000+0-
@T6XXI:00104:00173
GGGGAATTCTAATACGACTCACTATAGGAGAGGGAGAATAGATCACCGTTGGTTCGGGTGCGCGTTCTCCTTCCTTT
CTCCCTTCTCCC
+
CBB7C=@=@@@?BCCCBBBBB7663./,/:;>?>ACC>A<<<<<?<8?D7:8;ACCC>C;;;ABA>A;:7:>A?BC>
C:8818;=>;>6
@T6XXI:00105:00154
GGGAGAAGGGAGAAAGGAAGAACAGGGCACCGCAGGAAAACGTGAAGCTATTCTCCCTCTCCCTATAGTGAGTCGTA
TTAGAACTTCCCC
+
490444244-4488-
42499<>BBCD?BCC>ACBB=@@@6@>@@B@CCAAAAGCED@BBBA@9??@??@B?BA:::??8?8?8AB>ABB7
@T6XXI:00105:00162
GGGAGAAGGGAGAAAGGAAGCTAGTCACTCGACATCTGTGGCTTCACAGTACAGAGGGACTGTCCAAAGCCGCAGAT
CTCGAGTCGA
+
CC@BBC@BB=A>=85>484;@@>@B8?=::77::?<<CCCB?@=@E@@?>@@?933417@AAAA<??<?A>A=9=A=
==;<<<=<<<
@T6XXI:00105:00184
GGGGAATTCTAATACGACTCACTATAGGGAGAGGGAGAATAGCTCCCAAATCACGTTACGTGCGACCCACTTCCTTT
CTCCCTTCTCCC
+
```

```
999/49A@GBB?CB??BBBBA?<97111.7>>?C@@@=>?===CCD>BB>B@BBBBAACCCBBBDDD>BBC?B?BCC
D>??>>.00::8
@T6XXI:00105:00185
GGGGAATTCTAATACGACTCACTATAGGGAGAGGAGAATA
+
888188A>BCE@CBAAA???B@CB>:8888?9A>B//-/+
@T6XXI:00105:00190
GGGAGAAGGGAGAAAGGAAGGAGAAGCACCAAC
+
CC>AAA?BC?AABB?B@C@>;=87+,,,,*,2*
@T6XXI:00105:00192
GGGGAATTCTAATACGACTCACTATAGGAGAGGGAGAATAGTTTTCCCTTCACTAACCGCGAACTGCACTTCCTTTC
TCCCTTCTCCC
+
BCD:D@A?ACC@CCBAAAAAA@<<9660777777:A?BD@@@CC4@@;@>??@BB?B=?:??;=>@=@@C@C@CC>A
ABC>C>???B<
@T6XXI:00106:00152
GGGAGAAGGGAGAAAGGAAGGAGACAGTACCGCCCAAAATTGGAAGACTATTCTCCCTCTCCCTATAGTGAGTCGTA
TTAGAACTTCCCC
+
AE?AAC@CC=?A@C>C@C@C@CCBAAABBCBBBB>BBA:C@E?B?BBBCCC?BBCC>CBBCC>BBBBBCBCBA<<<<
<;:;=3::?=AA6
@T6XXI:00106:00165
GGGAGAAGGGAGAAAGGAAGGATG
+
DCBCDC@BB?BB??8000)*)***
@T6XXI:00106:00169
CTATCGACTCGATGCGGCTTTGGACAGTCCCTCTGTACTGTGAAGCCACAGATGTCGAGTCGACTAGCTTCCTTTCT
CCCTTCTCCC
+
CD;;11188:=@AADDACCC@C@DCCCCCB=A?@:;<BCCDCC@CC@CCC?>ACA@??BBCCBAAC<=@@C@CC>CB
BB?B>?@BC?
@T6XXI:00106:00174
GGGGAATTCTAATACGACTCACTATAGGAGAGGGAGAATAGTCAACAAATCGTTTGGGGCGACTTCTCCTTCCTTTC
TCCCTTCTCCC
+
BBB5<8<BBCC@CBCCICCC:/0;<00,0<>?F<???:==@@?A;884-
7?777.7@@5BB;::7::?=99;38818?AC=A8:+,,+
@T6XXI:00106:00176
GGGAGAAGGGAGAAAGGAAGCTAGTCACTCGACATCTGTGGCTTCACAG
+
CC>?995991999919<@>BCBBBC7:::;777:9769BC=@@@CC@BB
@T6XXI:00106:00177
GGGGAATTCTAATACGACTCACTATAGGAGAGGGAGAATAGTTGCGACATGTAACGTCCTTCTATCTTCTTCCTTTC
TCCCTTCTCCC
+
DFD:C@B>ABB?BCCCCC@>>C737:737?==?>?BA?A998>AAACCDC@::49>>@>@AC;=<BB@BC@C@EG@B
;;7.7:7777.
@T6XXI:00106:00178
GGGAGAAGGGAGAAAGGAAGCTAGTCGACTCGACATCTGTGGCTTCACAGTACAGAGGACTGTCCAAAGCCGCAGAT
CTCGAGTCGACTAG
+
C@:==><>>:>99909;>;>A800:.......89>>?CCAA???=?:::AAB>;://-
/99;;?=?A<AA;==@@AA@@A?===???AAAA
@T6XXI:00106:00194
GGGAGAAGGGAGAAAGGAAGTGCAGTTCGCGGTTAGTGAAGGGAAAAACTATTCTCCCTCTCCCTATAGT
+
```

```
BB?BCB@CC>CBBB=B?B?ABBBACC@@>@B@C@999>?;;@.7777,8?CC>B:::5:??DJ@C???AA
@T6XXI:00107:00153
GGGGAATTCTAATACGACTCACTATAGGGAGAGGGAGAATAGTCAACAAATCGTTTGGGGCGACTTCTCCTTCCTTT
CTCCCTTCTCCC
+
DCC:C9;;;CB@DBA?@@CB?ABB@661-
6;;?A>ACE@@???AA>AAA<BBBBC>B@@7>BCCC::;C??<?>AA<?ABC<A:====9
@T6XXI:00107:00156
GGGAGAAGGGAGAAAGGAAGCTAGTCACTCGACATCTGTGGCTTCACAGTACAGAGGGACTGTCCAAAGCCGCAGAT
CTCGAGTCGACTAG
+
DE@CCC@A=:=944-
;897;C@?@EBAA???;;:::6@AB?BB@CBA>??C?>9000,3;8=?@>AA<?@=@@<@====<:;;@AAA@@@@
@T6XXI:00107:00166
GGGAGAAGGGAGAAAGGAAGCTAGTCACTCGACATCTGTGGCTTCACAGTACAGAGGGACTGTCCAAAGCCGCAGAT
CTCGAGTCGACTAGCTTC
+
A?>B;<<;@:A999142943B>>;7*******00,//9;;2:<=@@C???BA@<;<;4;<<;9;8:;7;;:>888A@
>8?4.-33-.--1000/0
@T6XXI:00107:00168
GAGATCTGCGGCTTTGGACAGTCCCTCTGTACTGTGAAGCCACAGAAGT
+
/18==@<<9=>99999??A@B;;;6:@@BCAA???960135,****)**
@T6XXI:00107:00172
GGGAGAAGGGAGAAAGGAAGCTAGTCGACTCGACATCTGTGGCTTCACAGTACAGAGGGACTGTCCAAAGCCGCAGA
TCTC
+
BB@CBB699-9=8=5@>A:<<CCCD???CBBB::<CCCA@@=?B@B???BBBCA8777149;;99244-
4424;<<>@@>=
@T6XXI:00107:00175
CTAGTCACTCGAGATCTGCGGCTTTGGACAGTCCCTCTGTACTGTGAAGCCACAGATGCTCGAGTCGACTTAGCTTC
CTTTCTCCCTTCCTCCC
+
:1//18===BCDCAACCCCB>BBB=C?BCAA???;?CBBCC@@@B@A>@CACC;78?88813991..4..,.3935>
;<@8843222,-,...+
@T6XXI:00107:00178
GGGGAATTCTAATACGACTCACTATAGGGAGAGGGAGAATAGCAAACGTTTTCCCCGACGTCGCTTTCTCCTTCCTT
TCTCCCTTCTCCC
+
?@@6@@B@CCB@BBBBBBBBBBB>;111-
6==@BBBA?@A?===C?@B@CC8CCC8BBCCCC;::C9?::5:<:7;<7=AAA<A=??@A<
@T6XXI:00107:00180
GGGAGAAGGGAGAAAAGGAAGCTAGTCGACTCGACATCTGTGGCTTCACAGTACAGAGGACTGTCCAAAGCCGCAGA
TCTCGAGTCGACTAG
+
BB<@@B>>>:>9111*1,/,..4244...3...46666666??AC@CCCCBB?;7;//.0===AD>BA<AA?@@@@A
A@@@A@@@?======
@T6XXI:00107:00181
GGGGAATTCTAATACGACTCACTATAGGGAGAGGGAGAATAGCCAACGGCTCGCTCATGGCTGGTTCTCCTTCCTTT
CTCCCTTCTCCC
+
CED:C9;;<@?<A@A@@@@@CCCBB::7.777>>:@@=:;;8838<>A>AAB?=>==8723;;58986A;=8=>@A:
??>>4:24444+
@T6XXI:00107:00184
GGGGAATTCTAATACGACTCACTATAGGAGAGGGAGAATAGTCCGCCAATTCTGCGCGTTAGCATTCTCCTTCCTTT
CTCCCTTCTCCC
+
```

```
DDD:F@A=ACC@CBBBCCCCC>==://,0;;?B@BCC>AAA=<588<@?B@BB;;<BCFACBBBC=??C@C@C7::7
:@>B<B878>>4
@T6XXI:00107:00193
GGGAGAAGGGAGAAAGGAAGAGCAAGACTAAAGGGCTAATGGAAGAAACTATTCTCCCTCTTCCCTCATAGTGAGTC
GTATTAGAACTTCCCC
+
;B:>99598-44;;-
969=338@>;>@@:99,37/7./566,/,/6632111::82202//,/85/..9977;116866188656/110111
,
@T6XXI:00107:00197
GGGAGAAGGGAGAAAGGAAGCTAGTCACTC
+
HL@CBB=@=9>>==9@8;7;CBAAB:?:::
@T6XXI:00108:00161
GGGAGAAGGATGAAGAGATGGAACAGAGCGAAA
+
;:::1/-1-1/66,//4443131484443388+
@T6XXI:00108:00164
CTATCGACTCGAGATCTGCGGCTTTGGACAGTCCCTCTGTACTGTGAAGCCACAGATGTCGAGTCGACTAGCCTTCC
TTTCTC
+
B<<6////669?BBB>@@<;9;BB=CB@@BBAAA<ABBABBAAAA==<<A=ABBBDECAA@???AB::76..,.333
3:///7
@T6XXI:00108:00171
CTATCGACTCGAGATCTGCGGCTTTGGACAGTCCCTCTGTACTGTGAAGCCACAGATGTCGGTCGACTAGCTTCCTT
TCTCCCTTCTCCC
+
CBBB<::EDDCCBB???ABC@AAA>A=<@@A???9???AC?@@?@=A?AD@C@A@CAA@::5:@@7::C=?:>C578
68A@D;@>???A:
@T6XXI:00108:00172
GGGAGAAGGGAGAAAGGAAGCTAGTCGACTCGAGATCTGCGGCTTTGGACAGTCCCTCTGTACTGTGAAGCCACAGA
TGTCGAGTCGACTAGCTTCCTTTCTCCCTTCTCCC
+
>@6>11-/656/44-
42423;9>7;6:777>>@:::99@??:???9??BCCCCCC>A?<9==@AAABA>>>;@@AAA?::@<<=A???AAAA
CC;=;=A<@@A@<@<??@@1
@T6XXI:00108:00174
GGGAGAAG
+
ND<??>59
@T6XXI:00108:00175
GGGAGAAGGGAGAAAGGAAGGAGAAGTCGCCCCAAAACGATTTGTTGACTATTCTCCCTCTCCCTA
+
BB<<<<?@A:A>>>9>;>;?<?@B;==>8888.833,37677;9A::?:8<67////,..--3--.
@T6XXI:00109:00164
GGGGAATTCTAATACGACTCACTATAGGGAGAGGGAGAATAGTTCCCGCATTCTCCCCGGAGGGTTGTACTTCCTTT
CTCCCTTCTCCC
+
BBB6A=A?CCCACCBBB?A@BBBBB61659:9CC>B@??AAAA?==:::BB@CBBCC9C????9?>=?@?@>@?AA<
A@AA<?>?AAC<
@T6XXI:00109:00183
GGGGAATTCTAATACGACTCACTATAGGG
+
AAA7A=A@ACCACBBBBBBBBA@@A6764
@T6XXI:00109:00193
GGGGAATTCTAATACGACTCACTATAGGGAGAGGAGAAT
+
```

>88688?=@BB?<<<CBAABDCA@8977.7@>>>C0/,,
@T6XXI:00110:00150
GGGGAATTCTAATACGACTCACTATAGGGAGAGGGAGAATAGACTCCACCAGCATGAACTGCGAATCCCTTCCTTTC
TCCCTTCCTCC
+
EDC:C?A?ACB@C@?BBBBBB@;77166.7>>>A?ACCAAA;;;4427=:=@CAABC?CCC@=@<777.744344,4
---+-,-,-10
@T6XXI:00110:00164
GGGGAATTCTAATACGACTCACTATAGGGAGAGGGAGAATAGTCTTGTATTTTGTGCATCACTTTTCTCCTTCCTTT
CTCCCTTCTCCC
+
=@@8AACDCBB@BB@>@BBBBABB866656??CC>CCA?A<@@<<;<<?===4999>@CCC????5?<<9=@A>AA:
??@CAC>A???;
@T6XXI:00110:00171
GGGAGAAGGGAGAAAGGAAGCTAGTCACTCGACATCTGTGGCTTCACAGTACAGAGGGACTGTCCAAAGCCGCAGAT
CTCGAGTCGACTAG
+
CK@DCC>>=59844-
9;>=@B@@@CCF@A@@:;:AAACCD?BB@C@@>>>@CA=98646:699=37718B=@A???BAA@8:?BAA<<=<<
@T6XXI:00110:00178
GGGAGAAGGGAGAAAGGAAGCTAGTCACTCGACATCTGTGGCTTCACAGTACAGAGGGACTGTCCAAAGCCGCAGAT
CTCGAGTCGACTAG
+
DG>BB>=@=<@@88:@=?<?C;;??8?=?@A7::???@@@>?@=@BCCCB@A@<8664686==A<??<?A>AA=A:=
===;::<======<
@T6XXI:00110:00187
GGGGAATTCTAATACGACTCACTATAGGAGAGGGAGAATAGTCAACAAATCGTTTGGGGCGACTTCTCCTTCCTTTC
TCCCTTCTCCC
+
CCB6:2<7;==9?>@ACBCCCCB@>:737=7776:?<?CAD@@A>@@@<BBBBC?CBB9C;;;E?AAB?=9;34413
88CAA>@?@C9
@T6XXI:00110:00193
GGGGAATTCTAATACGACTCACTATAGGAGAGGGAGAATAGACCGTGATTTTTCAGTCCCGGGCTCTCCTTCCTTCT
CCCTTCTCCC
+
:>?1@-
1,/677849:9;787198111011776,17448996<843313333+111111,11,167;;;7860100000+044
3564
@T6XXI:00110:00199
GGGAGAAGGGAGAAAGGAAGCTAG
+
>?:>>>;>>:>111-/,/,.6..,
@T6XXI:00111:00173
GGGGAATTCTAATACGACTCACTATAGGAGAGGGAGAATAGTTTTCCCTTCACTAACCGCGAACTGCACTTCCTTCT
CCCTTCTCC
+
999/4:A@CBB=@@B@@?BBB<<<;00,/:077,16344;;<<;05;4;8<@::::?;?AA@>A@@=;::,0,0,.4
742966..,
@T6XXI:00111:00175
GGGGAATTCTAATACGACTCACTATAGGGAGAGGGAGAATAGCTACGTTTAACCGATCGTGTTGTTCTCCTTCCTTT
CTCCCTTCTCCC
+
CCC9C?B?ABB@BB@BB;;>BC?97111.7=<>A>BDA>A=@:>?????;?>?><??<<<<>>;?9<<=;=;>:=>7
888C:?;???=7
@T6XXI:00111:00179
GGGGAATTCTAATACGACTCACTATAGGAGAGGGAGAATAGTCAACAAATCGTTTGGGGCGACTTCTCCTTCCTTTC
TCCCTTCTCCC

```
+
HCC:EBC@BBB@BBBBB?@@B:997//-
0>@>B>BBA>A=AB@@=@AA<BAAAC>CCC9BBBBB?BCC>?<?>AB=AAAA<A;==@@<
@T6XXI:00111:00183
GGGGAATTCTAATACGACTCACTATAGGAGAGGGAGAATAGTTGGAGGCATTGCTACCCCGACTCTTCTCTTCCTTT
CTCCCTTCTCCC
+
DEF9A?BABBB@BBBBBBBCC>==?//-
1>>BL>CCD>=9=@>@>??>@BG<=<B?>>@6>>????;A:77;?377,68;>:@>=8881
@T6XXI:00111:00187
GGGAGAAGGGAGAAAGGAAGAAACGATAACGGTGGGACCAGTGTTAGCTATTCTCCCTCTCCCTATAGTGAGTCGTA
TTAGAATTCCCC
+
CC@BBC@BB>BBBB=B?BCCCB>BCCC@>BB?BBBABB>??@A@>@::;CCBFCCC@CCCCC>CCDCBB<@?CC:::
:5:7?<4/0340
@T6XXI:00111:00191
GGGGAATTCTAATACGACTCACTATAGGAGAGGGAGAATAGCCTAACCCACGGCCTTATCGACTTATTCTTCCTTTC
TCCCTTCTCCC
+
GDD9C?B>BBB@CCCBBB@?>:995..,00077.7=:48844:>>377,7710056274=====>@?C8848=A=;=
?<<44444444
@T6XXI:00111:00193
GGGAGAAGGGAGAAAGGAAGCTAGTCACTCACATCTGTGGCTTCACAGTACAGAGGGACTGTCCAAAGCCGCAGATC
T
+
HC@CCB?BC@9<<B<A<A?A;;;BBCBAC@888@>=A=?8??=?AACCCCCCCCBB9;88444244+0020003330
-
@T6XXI:00112:00161
GGGAGAAGGGAGAAAGGAAGCTAGTCACTCGACATCTGTGGCTTCACAGTACAGAGGGACTGTCCAAAGCCGCAGAT
CTCGAGTCGACTAGCTTCCTTTCTCCCTTCTCCC
+
EHAAAD?BA<>988095988BC@8:777.........4444675:BDBCCB:74444-
4::4488;<6;>9=;<8838873338849;880002456=465663523.444
@T6XXI:00112:00173
GGGAGAAGGGAGAAAGGAAGGAGAAGTAGCCCCAAACGATTTGTTGACTATTCTCCCTCTCCCTATAGTGAGT
+
EC?BBB@BB>ABEB>B@B@::99997/009996::::>CC?CCAAAAB@=;=@CCA@??AA<A???AAA???
@T6XXI:00112:00174
GGGGAATTCTAATACGACTCACTATAGGGAGAGGGAGAATAGCTGGTTTTGTTTCCAGTGTCTCCATCGTCTTCCTT
TCTCCCTTCTCCC
+
CCB9C?A>ACCABBCCCCB>B@?96166.7>>>A>DB@>A===CCACCC8BB@>@@AAAAA@@@=@AAB==<:>378
18AAA?A<<==C>
@T6XXI:00112:00181
GGGAGAAGGGAGAAAGGAAGGAGAACGTACGTGCAAATATCGCATGGCTATTCTCCCTCTCCCTATGTGAGTCGTAT
TAGAATTCCCC
+
BE@ECCAAA=BBAB@B@@@B@B995;C@@ACBAABC?BCCBB@@@@=@@AA@CCCCACDCCC>CCBBBC@@?C<<<C
@CCC@CACCC9
@T6XXI:00113:00164
GGGAGAAGGGAGAAAGGAAGCTAGTCGACTCGACATCTGTGGCTTCACAGTACAGAGGACGTGTCCAAGCCGCAAGA
T
+
:B>BAB=>>:?99904986;0.....11111191..54...,.4,/6;:????@@<<9<<<<;=;7<8:641,,+**
*
@T6XXI:00113:00167
```

GGGGAATTCTAATACGACTCACTATAGGAGAGGGAGAATAGTCAACAAATCGTTTGGGGCGACTTCTCCTTCCTTTC
TCCCTTCTCCC
+
BDECE@A?A???BBBBCCCCB:694..,0>>BJ>CBD@@;<<@<>@AA<CC???9?<<5???AC@???=<9<>@@<@
CCC<@=?@AA<
@T6XXI:00113:00168
CTAGTCACTCGAGATCTGCGGCTTTGGACAGTCCCTCTGTTGTGAAGCCACAGATGTCGAGTCGACTAGCTTC
+
C:49;944:CCBCCC?BABB?BAB=?7:;@??BB>CBBCC?AABB?@@>A@@AAAAA???@@@A@???==C?C
@T6XXI:00113:00174
GGGGAATTCTAATACGACTCACTATAGGGAGAGGAGAATAGTAGT
+
7>>6??C@BBC@B@@@A??>B@B?9111./:/0,0:77716/./-
@T6XXI:00113:00180
GGGGAATTCTAATACGACTCACTATAGGGAGAGGGAGAATAGTCAACAAATCGTTTGGGGCGACTTCTCCTTCCTTT
CTCCCTTCTCCC
+
@??4>596<@@>B<<<CCCCBCCBB611.777<=9?A??CBBBAB>==94?A?AB<BBB7AAAAB?BBB>?<?>AA<
@@CA=D8<<CC<
@T6XXI:00113:00182
GGGGAATTCTAATACGACTCACTATAGGGAGAGGGAGAATAGCTTCTAGCATTGTCAGAGCTGTCCTCCTTCCTTTC
TCCCTTCTCCC
+
BAA7A=A>ACE@CA?BBBBAC>337677.7?AAB?BBA=@A?>A?A??A>CCAC<<;BBCBCB:947?370136<8<
=AA:;89444-
@T6XXI:00113:00189
GGGGAATTCTAATACGACTCACTATAGGGAGAGGGAGAATAGTCACTTCCTATCTGGTCGCGATATTCTCTTCCTTT
CTCCCTTCTCCC
+
>@@6@>A@CDB@BCBBBBBCA@CCC<:<;<<<BC>B?=9<::///=>9=;??C@@?>AA=<?>>AC?A??@=A=AC<
@@AA<A8<<<<6
@T6XXI:00113:00196
GGGGAATTCTAATACGACTCACTATAGGAGAGGGAGAATAGTTGGAGCATTGCGTACCCGACTTTTCTCTTCCCTTT
CTC
+
DDC9A>AABBBABBBB;:;;@>??=7747A==?<?AA<==:7,/,/++,,*///.344-
34499:69./.,..+.30.11
@T6XXI:00113:00198
GGGGAATTCTAATACGACTCACTATAGGGAGAGGGAGAATAGTTCACACACATTTACAGGGGCTGTTCTCTTCCTTT
CTCCCTTCTCCC
+
GED9C>?C@BB@CCB>?@CDA?<97166.7==@A?BCB@A:??=?@@@AA@??@<@CB@BB7BBBC@CCCA>@;<?;
?BBC>C?BB@;5
@T6XXI:00114:00159
GGGGAATTCTAATACGACTCACTATAGGAGAGGGAGAATAGATCAAGTCTAAAGTACGCC
+
<99/96954989444944990.0:...,.3.11,17798<=6./0,......+1019<:=
@T6XXI:00114:00163
CTGCGGCTTTGGACAGTCCCTCTGTACTGTGAAGCCACAGATGTCGAGTCGACTAGCTTCCTTTCTCCCTTCTCCC
+
ICDFA?BCD@C?BBCCCCC>CCBBAAAC?@BF@BC?BBBBBBBABBBAABB@@@B@@@8;6:E@BBBD=A5::==:
@T6XXI:00114:00178
GGGAGAAGGGAGAAAGGAAGGGGAGAAGGGAGAAAGGAAAGCTAGTCGACTCGACATCT
+
DD>DCB?BB=BBCB>CAB@ACB9BBB?>966611,7377,6:555=///...1031111
@T6XXI:00114:00181

GGGGAATTCTAATACGACTCACTATAGGGAGAGGGAGAATAGATCACCGTTGGTTCGGGTGCGCGTTCTCCTTCCTT
TCTCCCTTTCTCCC
+
ABA7A=@=@CC@CBBBBBAAB?>;9196.6<;??;?C@?AA===@BB=AA=?=??CCC@C@@@ECCAAAB@C@B@A?
:>744-44-4::A?
@T6XXI:00114:00182
GGAATTCTAATACGACTCACTATAGGAGAGGGAGAATAGTCAACAAATCGTTTGGGGCGACTTCTCCTTCCTTTCTC
CCTTCTCCC
+
ABCBC@CB@=@@A<<<CCB;:::00,/<0775:@?=@=>>AA=@?C<CC;::5:@>5AAA@F476733/4:;>:873
848588;=;
@T6XXI:00114:00183
GGGAGAAGGGAGAAAGGAAGGAGGACAGACGCAAATAATTGCCTGCTATTCTCCCTCTCCCTATAGTGAGTCGTATT
AGAATTCCCC
+
DDABBA@CC>BBCC?C@D?@>CC?BCC;<<CCCCABB?BADB?CDAAAD?AABB<AB??@<?@BB??????????A?
CA@>BA@=B7
@T6XXI:00114:00188
GGGGAATTCTAATACGACTCACTATAGGAGAGGGAGAATAGTCAACAAATCGTTTGGGGCGACTTCTCCTTCCTTTC
TCCCTTCCTCCC
+
CCE9C@ECCCCDAEACCCCCBA@@>76377777.:A??A@::/-//73<???@;@CC7<?CCB5::?:87:344-
444;78/4/033+
@T6XXI:00114:00193
GGGAGAAGGGAGAAAGGAAGGAGAATCGACACACGAACGCCTACGTGATCTATTCTCCCTCTCCCTATAGTGAGTCG
TATTAGAATTCCCC
+
CCAAA@@BC=@@@@<@>B>@@C@@=@CBBCCBBB;:2:::5<BDEAAADFCBB=@@>>99999908?@BB@@@BBAA
ABB?BB@;?4777-
@T6XXI:00115:00155
GGGGAATTCTAATACGACTCACTATAGGGAGAGGGAGAATAGCCCTGACCTGATTTTCGCTCTCTTGACCTTCCTTT
CTCCCTTCTCCC
+
AA@4@<@=ACC@CCBB@AAAB@;@@9995:@==@<A::34;4471:?BC@C???CC9C::8AA7858BB>B@C?BB<
@???<@@BCCC>
@T6XXI:00115:00157
CTGCGGCTTTGGACAGTCCCTCTGTACTGTGAAGCCACAGATGTCGAGTCGACTAGCTTCCTTTCTCCCTTCTCCC
+
ECBA@=@AC>C;<<DGCCC>CEBB?>=??CBB>BC@BBBBBBBBAADCABEBCBBA@@AC@CC>CCCC;?<?A?A<
@T6XXI:00115:00159
GGGAGAAGGGAGAAAGGAAGCTAGTCGATTCGACATCTGTGGCTTCACAGTACAGAGGGACTGTCCAAAGCCGCAGA
TCTCGAGTCGACTACG
+
491499599-4444-42499993......333443886669>><@B@<?@?A>?;444-
4;?@@B?AC=@@=@A@BB@?<<888==;888///
@T6XXI:00115:00162
GGGGAATTCTAATACGACTCACTATAGGGAGAGGGAGATAGTTGCGTGTCCGATCGCGTCTGTGTCTCCTTCCTTTC
TCCCTTCTCCC
+
@?=6@>B?BBBACCC??<BBBCBB966659?77767/,++333<<::@@A;<<<<<AA<<?<@@A=??<?>B38968
?@C<A=899D9
@T6XXI:00115:00164
GGGGAATTCTAATACGACTCACTATAGGGAGAGGAGAATAGT
+
:AC6>?DDCCC@CCCCCABAA<A?=666.7=774;:741,**
@T6XXI:00115:00169

GGGGAATTCTAATACGACTCACTATAGGGAGAGGGAGAATAGCTCCCAGACCGACCGCATAGCCATGACCTTCCTTT
CTCCCTTCTCCC
+
CEC9EBC@BBCAB@@@CBBBC?>>>777.7==@C>BBA>@>88:@@9@BBC@ECC=@@BBB??<@B@@@7;48=@@<
ABBB<B>:::A<
@T6XXI:00115:00179
GGGGAATTCTAATACGACTCACTATAGGGAGAGGGAGAATAGCTGCCACGTTTGGACAATTGCAGTTCTCCTTCCTT
TCTCCCTTCTCCC
+
?@@2@;?=@ABABCCBCC@@@A887777-
7=77619B@9==A=?@E@CBBBC@C@BCC@C>A@BA>,00>,0,0,00,0666,601100+
@T6XXI:00115:00181
GGGAGAAGGGAGAAAGGAAGTGGATAAAAAAGCCAAAGAGATTCGGAGCTATTCTCCCTCTCCCTATAGTGAGTCGT
ATTAGAACTTCCCC
+
DHACCB=@A<<;<E@C@C?ACB?BBBCBBB-@>:??9?@BCD@CF@?;<=BB@CCCC>BB@@@<???B@CBA//..-
--3339477==??7
@T6XXI:00115:00183
GGGGAATTCTAATACGACTCACTATAGGGAGAGGGAGAATAGATCACCGTTGGTTCGGGTGCGCGTTCTCCTTCCTT
TCTCCCTTCTCCC
+
BAA7@@A>@B@ABCA@ACBAA@>>=966.7===@<??@?AA@=<=CC>::57:=;9990988;CCB>BBA>B=A>BD
>BA>C<A>999@:
@T6XXI:00115:00187
GGGAGAAGGGAGAAAGGAAGGAGAACGCGCACCCGAACCAACGGTGATCTATTCTCCCTCTCCCTATAGTGAGTCGT
ATTAGAATTCCCC
+
CDCDCC@DC>BBBB?BBI@B@@BB@BAABCCCC@CC@C?B@CCADCBABBCCAFBBB=::;CG?EABBBCCBBBBD;
;;@EAA?BCCCC:
@T6XXI:00115:00189
GGGGAATTCTAATACGACTCACTATAGGGAGAGGGAGAATAGTCAACAAATCGTTTGGGGCGACTTCTCCTTCCTTT
CTCCCTTCTCCC
+
CCE:CCD@BCB@C@@@B>;;BCB?9111.6<<?A>AFA@@;@A<;;<@?:?@;;;-
777.7===B@EAA??@A>AA<A@A@<@=?<==7
@T6XXI:00116:00173
GGGAGAAGGGAGAAAGGAAGCTAGTCACTCGACATCTGTGGCTTCACAGTACAGAGGACTGTCCAAAGCCGCAGATC
TCGAGTCGACTAG
+
GD>BBA=@>:>99809695;CC@@AA>000:00////::C>@@@C@??CD?::4..,/799;>?AC<??:?A===??
>><<<??AB@@@A
@T6XXI:00116:00181
GGGAGAGAATTTGGTAATGGGAGTCGGGAGGGGTCAGGAGAGAGGTGAAGGACCTTATATCTCTCTCCCTATAGTGA
GTCGTATTAGAATTCCCC
+
CC>B@499699-42477;BB>>>=BF@:8888.8?BCB><111/56022460/-
/777@;;ACAA;::68BB@@@CAA;88B773:?A>C@EHD:
@T6XXI:00116:00186
GCGGATCTAATACGACTCACTATAGGAGAGGAGAATAGTCCTTTATTCTTTCTCTCCATCGTGGTCTTCCTTTCTCC
CTTCTCCC
+
CAB694444244444999:9998@?=8000,..5551111011,0443455///775357.,,*,-
,27:7D8>@==:92449=:
@T6XXI:00116:00192
GGGGAATTCTAATACGACTCACTATAGGGAGAGGGAGAATAGAACACTGCTCCCCTAAATATGTCTATTCTTCCTTT
CTCCCTTCTCC
+

```
CCD:C?B?BCCACBAACCCB>???=666.7?@?D@;;8>B@@?>AA?====<AA6@A@;??@C====<<;>3734;4
:444-4/.122
@T6XXI:00117:00155
GGGGAATTCTAATACGACTCACTATAGGGAGAGGGAGAATAGTCAACAAATCGTTTGGGGCGACTTCTCCTTCCTTT
CTCCCTTCTCCC
+
AA@7A=@=ACC@CB@@@BBBB@B?;661.6@=@B>BCC@A===@?=<<;5?@@@@:AAA7BCCCC?BBB?@>@>AC:
ABBA<A;??AA<
@T6XXI:00117:00162
CTAGTCACTCGAGATCTGCGGCTTTGGACAGTCCCTCTGTACTGTGAAGCCACAGATGTCGAGTCGACTAGCTTCCT
TTCTCCCTTCTCCC
+
D:/111/44ABCBBBBBBB?>???;B@B>;9=??;>>>@@@@@@B??=@B@BCDDCC@@?BAAACCCCCCC??=?>A
B>BBBC>A====?;
@T6XXI:00117:00180
GGGAGAAGGGAGAAAGGAAGGAATAGTGAGGGGCAAAAAGTGGGTAGCTATTCTCCCTCTCCCTATAGTGAGTCGTA
TTAGAACTTCCCC
+
CD=??D@DC>BBBC;;;<@CBC@BBBBBFCCC9ABBBB2@??B<;;:?@@A?AADC>BB@@>9;:1000?;///...
.,.-0/1736665
@T6XXI:00118:00152
GGGGAATTCTAATACGACTCACTATAGGGAGAGGGAGAATAGTCAACAAATCGTTTGGGGCGACTTCTCCTTCCTTT
CTCCCTTCCTCCC
+
CBA6@>A>BBB@CCBBBDDBA@?<;16959:7AB>:<<?@@@AAA?@@C7?@CCB5:::5:BBBC=@?C?A:=;?D@
@;;;68,/,/881
@T6XXI:00118:00163
GGGAGAAGGGAGAAAGGAAGGAGAACATGACCCGGCGCGATTGGTGTCTATTCTCCCTCTCCCTATAGTGAGTCGTA
TTAGAATTCCCC
+
CDABA@=>=:@@=?=B@C@B@BBB?BCCBBBB?B@BBBAAA@BA@@>?BCC@C@@@;>>ACC>B??@@AC=AA;?==
@>@@A>A>@@@5
@T6XXI:00118:00164
GGGAGAAGGGAGAAAGGAAGCAGTCCATCTGTGGCTTCACAGTACAGAGGGACTGTCCAAAGCCGCAGATCTCGAC
+
CC@CDB@DC?BBBH?B?B@B<771317@A??@LABB?B@@@??>>>>;;;:<<<<@@>AA<A=;<;<<==843**)
@T6XXI:00118:00170
GGGGAATTCTAATACGACTCACTATAGGGAGAGGGAGAATAGCTCCAACCTCACGATCTATCGCGAACTCTTCCTTT
CTCCCTTCTCCC
+
CCD:C@B?ABB@BC@@@BB?BAD?9661-
6=;?A>BCA>A=????>?>@@CA@A<=?@:///;;0;;<CCC?B>A@9??@?:=:;????9
@T6XXI:00118:00172
GGGGAATTCTAATACGACTCACTATAGGGAGAGGGAGAATAGTCAACAAATCGTTTGGGGCGACTTCTCCTTCCTTT
CTCCCTTCTCCC
+
499/9?B@CCCAC???BBEDAB@9=111.7>===<?CB>AAAABB=BAA>CBAAA=AEE9CAA@@>?CE?A>@>@A<
@@AA<@;==@@<
@T6XXI:00118:00180
GGGGAATTCTAATACGACTCACTATAGGGAGAGGGAGAATAGTCTCCCAATCGTTCGCATCAGCCGCGCTTCCTTTC
TCCCTTCTCCC
+
CCC7B@A?@AA@CCBCCBBBA@B?=6665:??BD>BB@=@B?>>7771:=A???@CAAACC?7737;?;;9<=@>:@
=;;7;7::::4
@T6XXI:00118:00185
GGGGAATTCTAATACGACTCACTATAGGGAGAGGGAGAATAGTTTTTCCCTTCACTAACCGCGAACTGCACTTCCTT
TCTCCCTTCTCCC
```

```
+
ABB9BABADDB>BBBBCEC@A??>=777.:>@@D:<;A>@===AAA1@@;@69<CCC@E@AA:8344;;@@A>B>??
:?777.7;99;A;
@T6XXI:00118:00187
GGGAGAAGGGAGAAAGGAAGCTAGTCGACTCGAGATCTGCGGCTTTGGACAGTCCCTCTGTACTGTGAAGCCACAGA
TGTCGAGTCGACTAGCTTCCTTTCTCCCTTCTCCC
+
DB>B<989@:>999042423334430//7;;;>>CBBBBCC@??@;@?AAA@BDF===A==>BB@@@@?AC=???>?
?C=;:;<::?<?@AA=A==;==5<AAA5;78444-
@T6XXI:00118:00190
GGGAGAAGGGAGAAAGGAAGCTAGTCGACTCGACATCTGTGGCTTCACAGTACAGAGGGACTGTCCAAAGCCGCAGA
TCT
+
DC@A?>===<@@@@>B=@=@C<..00//7334.66676BCC;:;@DCCBBCCCA====99=AA??37717?=A@@@A
@@@
@T6XXI:00118:00196
GGGAGAAGGGAGAAAGGAAGCTAGTCGACTCGACATCTGTGGCTTCACAGTACAGAGGACTGTCCAAAGCCGGAGAT
CTCGAGT
+
FF>AAB@BB?ABAA=BDC;;;D@@@CCCA>>@AABCBAABC@AA@ACA@>>@744..-
/8:8;@>@@:?@<?<?@A@===A???
@T6XXI:00119:00155
GGGGGAATTCTAATACGACTCACTATAGGGAGAGGGAGAATAGCCAACAGGTATCTGATTGCGCGTTCTCCTTCCTT
TCTCCCTTCTCCC
+
AA@@/@>A>BCC@CA??ABBBB@<99111.7>>>BBDDC@A:<<9==@AB=@@AA>?>@?AAAAAA?AA@:=9<;<@
<@@BC7;37/882
@T6XXI:00119:00158
GGGAGAAGGGAGAAAGGAAGTGCAGTTCGCGGTTAGTGAAGGGAAAAACTATTCTCCCTCTCCCTATAGTGAGTCAT
ATTAGAATTCCCC
+
DDABAA@CB>BAAA=B?B?BCCCCDBBB@@?@C@AAABA;;6,6777,8??A>B???9?@>@@<A??AAA@C==98:
?;9444/023494
@T6XXI:00119:00164
CTAGTCGACTCGAGATCTGCGGCTTTGGACAGTCCCTCTGTACTGTGAAGCCACAGATGTCGAGTCGACTAGCTTCC
TTTCCTCCCTTCTC
+
:444444944999@@@AAA;;:;BCAC@B???777.7>>7::CCFFB@<?B@BA;98883111668877333..,.4
45+.,.5522-303
@T6XXI:00120:00155
GGGGAATTCTAATACGACTCACTATAGGGAGAGGGAGAATAGTTTTTTCCCTTCACTAACCGCGAACTGCACTTCCT
TTCTCCCTTCTCCC
+
DDD:C@A@ACB@CCBAAAAAB@<9<666.7>>BC>AAA@A=>>><==-
=@;@=?ACCC@@>>BBC?=??<>?<9926957>=B9?:?;;=7
@T6XXI:00120:00157
GGGAGAAGGGAGAAAGGAAGCTAGTCACTCGACATCCTGTGGCTTCACAGTACAGAGGGACTGTCCAAAGCCGCAGA
TCTCGAGTCGACTAGCTTCCTTTCTCCCTTCTCCC
+
CDBC@@>AB>C;9909595;A@@>=*))((((((((*0057?A?BAABCBAAB@88814;;=<<9=C<AA>AAAA9
99@??>?>:776///77565775949>5<5;88;7
@T6XXI:00120:00171
GGGAGAAGGGAGAAAGGAAGGGGAGAAGGGAGAAAGGAAGCTAGTCGACTCGACAT
+
CE?BBB@CB>AA@C=B>B>>AA8BCC@CC>C@>999410:>00/>0..43/10311
@T6XXI:00120:00178
GGGGAATTCTAATACGACTCACTATAGGAGAGGAGGAATAG
```

```
+
8881<:><?8854889@ABAB>>>=7747671011004333
@T6XXI:00120:00183
GGGAGAAGGGAGAAAGGAAGCTATCGACTCGACATCTGTGGCTTCACAGTACAGAGGACTGTCCAAAGCCGCAGATC
TCGAGTCGACTAG
+
A>:?9959?:9;9@9B9;9;B???AB?==>777166771786?<?@@??@C>?9//-
/8888<9<?:?@>@@@@??==;:944430846:
@T6XXI:00120:00186
GGAATTCTAATACGACTCACTATAGGGAGAGGGAGAATAGTCAACAAATCGTTTGGGGCGACTTCTCCTTCCTTTCT
CCCTTCTCCC
+
D@C@C??AB@DCCBBBCCGCB<<777.7>>??9?:837@@<==@@?=:@:::?<?CE9C@@?A<>>?44349;;7==
=;7;8444:7
@T6XXI:00120:00188
GGGGAATTCTAATACGACTCACTATAGGGAGAGGGAGAATAGCAGTTCAAATAAATTGAGCCGGCGTTCTCTTCCTT
TCTCCCTTCTCCC
+
BBB9B@C@CBD@CCBBCBBBA@A<98??9??ACC>CB@>A>A?<?:;?==;?<:3<=778:?A<?@@=?@?=8;9;?
:=?@@:>248;;6
@T6XXI:00120:00189
GGGAGAAGGGAGAAAGGAAGGAGAAAAGTGATGCACAAAATACAAGACTATTCTCCCTCTCCCTATAGTGAGTCGTA
TTAGAATTCCCC
+
EIACCC@B>:;444-
9<@=?>?88;8.7????A;?7777.7?BC?BBDFBB>?>=?:9:9BC<<?:<???A?=;8449244;94/000,
@T6XXI:00120:00191
CTATCGACTCGAGATCTGCGGCTTTGGACAGTCCCTCTGTACTGTGAAGCCACAGATGTCGAGTCGACTAGCTTCCT
TTCTCCCTTC
+
B:8<1///1:@BCBCBCBBA>ABB=B@B@B?9994:A@B???C@B@A=@C@CCCC@AABCBBB?@@@;;8>>70737
@:@ABD<<8<
@T6XXI:00120:00198
CTAGTCACTCGAGATCTGCGGCTTTGGACAGTCCCTCTGTACTGTGAAGCCACAGATGTCGAGTCGACTAGCTTCCT
TTCTCCCTTCTCCC
+
CA49<<444BAAABCCABBC@BBB>BBAABB??@=?CCCBBBB@B@@=?C@BBBAABABCCCCBBBBAAA@@B?C@C
C>AABD>@>@AAA;
@T6XXI:00121:00152
GGGGAATTCTAATACGACTCACTATAGGGAGAGGGAGAATAGTCAACAAATCGTTTGGGGCGACTTCTCCTTCCTTT
CTCCCTTCTCCC
+
DCB9EAC@CBB@CCB?AABBBAA<<111-
6;;@B>CBB@A=AAA==@>=;A8::B9???7@BBFE@;;;?@>A?AA=@@@@<A>???:4
@T6XXI:00121:00164
GGGAGAAGGGAGAAAGGAAGCTAGTCGACTCGACATCTGTGGCTTCACAGTACAGAGGGACTGTCCAAAGCCGCAGA
TCTCGAGTCGAC
+
CC>BA=;=99>98809=>=@C@==>/00>0000:6688@BC?BDBBAAA@@A@9833317;;=AA?CA:AA>@@558
>>??===A???A
@T6XXI:00122:00163
GGGGAATTCTAATACGACTCACTATAGGGAGAGGGAGAAATAGTCAACAAATCGTTTGGGGCGACTTCTCCTTCCTT
TCTCCCTTCTCCC
+
DGB8B7;:4687///8667>>8<<AAAE;@@BB?67600+033034:::=;@<=<?999=588:A?=?;>232838A
?AAAA<=:=744-
@T6XXI:00122:00166
```

```
GGGAGAAGGGAGAAAGGAAGCTAGTCGACTCGACATCTGTGGCTTCACAGTACAGAGGGACT
+
BB>BBAAAB=BCBB>B=?<@A;4=;/00;:::17:::6>>A=:<:;BDCCCBBA@?<<;:=;
@T6XXI:00122:00167
GGGGAATTCTAATACGACTCACTATAGGGAGAGGGAGAATAGCTTCCTTCTCTTCACATGCAACGTGCACTTCCTTT
CTCCCTTCTCCC
+
999/49A@CCDCDBBBBABAA@AB?66959??CE>CA@>AA==@>?@C?@?@@>@AA???BB??AA@?BBB?@>==;
=@@@;@?@@>A=
@T6XXI:00122:00173
GGGAGAAGGGAGAAAGGAAGCTAGTCGACTCGACATCTGG
+
BB@AAHACC?CBCC@BBG=?B??>=77771113......,
@T6XXI:00122:00174
GGGAGAAGGGAGAAAGGAAGTGCAGTTCGCGGTTAGTGAAGGGAAAAACTATTCTCCCTCTCCCTATAGTGAGTCGT
ATTAGAATTCCCC
+
DE@BBA@BB>BCBBBB=A?<BBBBBB@BBAB<;;<?BBB@CD>CCCC2CCCC@CBBB>BBBDC>C<==@@@@@AA@?
??>@AA=?=@??4
@T6XXI:00122:00178
GGGAGAAGGGAGAAAGGAAGCTAGTCGACTCGACATCTGTGGCTTCACAGTACAGAGGGACTGTCCAAAGC
+
BB>999<>>:999@<A>B?BDCBBBCCB????;:7==<<;?::87:????CB@;;444-4;;?@@>AB;@@
@T6XXI:00122:00180
GGGAGAAGGGAGAAAGGAAGGAGAAGTCGCCCCAAACGATTTGTTGACTATTCTCCCTCTCCCTATAGTGAGTCGTA
TTAGAATTCCCC
+
BC?AAA@CC@CDCC?CDCAB?BBB@?=>8888,397>B@EC;BB?BCBA??;999H@BA@AA?@?<<<=;<@A9:9C
C>=<77776661
@T6XXI:00122:00182
TCTGCGGCTTTGGACAGTCCCTCTGTACTGTGAAGCCACAGATGTCGAGTCGACTAGCTTCCTTTCTCCCTTCTCCC
+
BFA@AC@CDE@C@CCCC?>>=@BBCB@>>?CCC?AB?CCCCBBBBBBAA@BBBCCCCCC=?=?A<@@@@<@:<=;=7
@T6XXI:00122:00192
GGGGAATTCTAATACGACTCACTATAGGGAGAGGGAGAATAGATCAACTGTTGGGTT
+
999/49A?CDC@CB@BBBBB?@AB@:::88?79A<?@?;;;<88-,*,,,0002*,*
@T6XXI:00123:00154
GGGAGAAGGGAGAAAGGAAGGGAATAGTTATGTGCGGGATTTGACAACTATTCTCCCTCTCCCTATAGTGAGTCGTA
TTAGAATTCCCC
+
CD@BBB@BDADEBA=B@B@BB@B@BBC@>AAABB@@B?@@@<ABCC@BCCC@B:::6;@ADCCCCCBBCBBCC@::?
?5::B@<;AAA5
@T6XXI:00123:00163
CTATCGACTCGAGATCTGCGGCTTTGGACAGTCCCTCTGTACTGTGAAGCCACAGATGTCGAGTCGACTAGCTTCCT
TTCTCCCTTCTCCCCTTCCTTCTCCCTTC
+
CA@=99:?ABAECGCCCBBB?AAC>B>A@>7::?9?CCCBBBC@@??<=@=@CC;:>BCBBC??;;;??????7;;:?
?=@DDD@C>@99991677,.-/8882:46
@T6XXI:00123:00165
GGGGAATTCTAATACGACTCACTATAGGAGAGGGAGAATAGTCAACAAATCGTTTGGGGCGACTTCTCCTTCCTTTC
TCCCCTTCTCCC
+
BCC:E@D@CDC@AABBBAABB>>@:00,/:/77-
:?::;?BA7857=;:>????9?@?5AA:::=?<C?@>@>>=9;3444-434-244
@T6XXI:00123:00167
GGGGAATTCTAATA
```

```
+
944,4298=CCCCA
@T6XXI:00123:00173
GGGGAATTCTAATACGACTCACTATAGGGAGAGGGAGAATAGTCAACCAACCTCCATGGTGCTATTGCCTTCCTTTC
TCCCTTCTCCC
+
GCD:D@A>ACCCC???BCCB??><;111-6<;;<9?@=<=997.+*+00*+*-
222223355=ACABD@C@C@BB>BCAB=A=?ABB>
@T6XXI:00123:00176
GGGGAATTCTAATACGACTCACTATAGGGAGAGGGAGAATAGTCAACAAATCGTTTGGGGCGACTTCTCCTTCCTTT
CTCCCTTCCT
+
>>?/95489995:ABABBB@@???>777,7=777,174:=@??AA>??=::::??<?BC8?::9?:;?@=@>A?AB<
9/.735+*)*
@T6XXI:00123:00179
GGGGAATTCTA
+
FEI9B>=2448
@T6XXI:00123:00197
CTATCACTCGAGATCTGCGGCTTTGGACAGTCCCTCTGTACTGTCGAAGCCACAGATGTCGAGTCGACTAGCTTCC
+
G@>@A::;BBBCCDCCCAA>A@A<@?AAACBCE>:;;BA?>@:/.-4,/9?A;>;@:3316111443337111013
@T6XXI:00124:00150
CTAGTCACTCGAGATCTGCGGCTTTGACAGTCCCTCTGTACTGTGAAGCCACAGATGTCGAGTCGACTAGCTTCCTT
TCTCCCTTCTCCCC
+
C>111>8==A@@ACCCBBCCAA@A<A@@??:::5:CCCCCCC?AA@>@BCCCCBBBAABCCAABB@@@BBBB>A?AC
>CBAB:;240344,
@T6XXI:00124:00165
GGGGAATTCTAATACGACTCACTATAGGGAGAGGGAGAATAGCTATACGTTCTCCGGCGAGTCACTTCCCCTTCCTT
TCTCCCTTCTCCC
+
ECB7C8;8;A@@CGDCCAAAB;9>;677.7A?BE@AA@=@===@@A?<<>8;;?;=;???@@@@??<>@@4?<?>AA
<AAAA99;=@AA=
@T6XXI:00124:00178
GGGAGAAGGGAGAAAGGAAGCTAGTCACTCGACCATCTGTGGCTTCACAGTACAGAGGGACTGTCCAAAGCCGCAGA
TCTCGAGTCGACTAG
+
BC>BBBACH>@@@@9?595887777661.....,.....775=?@CCDBBBCBB?<<<7<???@A>@@;@C>AA==<
?888?>><<<==>88
@T6XXI:00124:00182
GGGAGAAGGGAGAAAGGAAGCTAGTCGACTCGACATCTGTGGCTTCACAGTACAGAGGACTGTCCAAAGCCGCAGAT
CGACTAG
+
CDCCCB?AA=@BBB>CAC@CCC?@=@BBCBBA::;???B@?<?@=?BCCCCCCB=5527A?;<=>@A<AA>@@@?>?
CBC===?
@T6XXI:00124:00190
GGGAGAAGGGAGAAAGGAAGGAGAAGTCGCCCCAAACGATTTGTTGACTATTCTCCCTCTCCCTATAGTGAGTCGTA
TTAGAATTCCCC
+
CC?>>>@BC>BBBB>B?B?A@B?A><9777::4:D>CCC??;???B??B@=<==<<:<==?A<A????@BBA@???A
@>@=<8;5;;<4
@T6XXI:00124:00197
GGGGAATTCTAATACGACTCACTATAGGAGAGGGAGAATAGTCAACAAATCGTTTGG
+
KDF9@?B>BBDB<<<CCB@@@?@@?9737=777.7>:378335<9;:;-7777<,/.
@T6XXI:00124:00198
```

GGGGAATTCTAATACGACTCACTATAGGGAGAGGGAGAATAGTTATAACCTCCCTAAAGCGTGCATTGCCTTCCTTT
CTCCCTTCTCCC
+
CBB9C=?=@CC@BBBBCCCBB@>97116.7==?C@BB@>@??@;@AB?=<88468BCCAAAA?<?=;=99=>A>AA:
>@AB<<88=@C>
@T6XXI:00125:00161
GGGGAATTCTAATACGACTCACTTAATTAAG
+
CCD4@51,1//,///997.00/.,.300063
@T6XXI:00125:00164
GGGAGAAGGGAGAAAGGAAGGATATGCCGCAGTGGGAGATTAAACGGCTATTCTCCCTCT
+
CC?CCC?CC?BBBB>B@B?A@BCB>=@?BCCCCCC>BB@B@CC>AA?CCCC@CCCC=AA:
@T6XXI:00125:00171
GGGGAATTCTAATACGACTCACTATAGGGAGAGGGG
+
BBB4A8;8;==@CCBBBCDC@??;:7<;6:7,,,,*
@T6XXI:00125:00180
GGGAGAAGGGAGAAAGGAAGAGAACAGTCACAGGCTCGAGCTGCAAGCTATTCTCCCTCTCCCTATAGTGAGTCGTA
TTAGAATTCCCC
+
BC@CCC@BB=BB===B=@=@CCC@CCCB?@@@@=@CCCCBBBBCC@ABABA=????8?8?@A<@CECB@@?BA==>7
737==;@>@@@5
@T6XXI:00126:00158
CTATCGACTCGAGATCTGCGGCTTTGGACAGTCCCTCTGTACTGTGAAGCCACAGATGTCGAGTCGACTAGCTTCCT
TTCTCCCTTCTCCC
+
B866/////:@BA@ABBBCBABBB=B=>@CCAAB>AB@B@@@BCCA?=<@=@CCBBCBAAACAABBAAAA@??:?;@
A=AAAE?@=?@@C=
@T6XXI:00126:00168
GGGAGAAGGGAGAAAGGAAGCTAGTCGACTCGACATCTGTGGCTTCACAGTACAGAGGACTGTCCAAAGCCGCAG
+
99149>;>>9989C9>4;23>?01000>=:=>>:77@>ACC@??<?????AA@;<<<9<@@@A@?AA=BB?AAA?
@T6XXI:00126:00170
GGGGAATTCTAATACGACTCACTATAGGGAGAGGGAGAATAGTTTTTCCCTTCACTAACCGCGAACTGCACTTCCTT
TCTCCCTTCTCCC
+
CCG9E>@AACCCCCBAAAB@A>B<9166.67:CC>CBA<@?BBA;;.:=;=:;>?BCAA>78;C>A@?@@AC??:?8
8???A>B>@@@@:
@T6XXI:00126:00193
GGGGAATTCTAATACGACTCACTATAGGGAGAGGGAGAATAGTGCGTGCGTTTT
+
CCC:B@B@BCB?ABBBBBBBBCB;????6<>===9??=:;66///./-00000+
@T6XXI:00126:00197
GGGAGAAGGGAGAAAGGAAGCTAGTCACTCGACATCTGTGGCTTCACAGTACAGAGGACTGTCCAAAGCCGCAGATC
TCGAGTCGAC
+
CCBCCCA@=:=>=>9>;@=?BC@A@CCAABB;:::::?@@;?@?ABBBBBC;;4.../;;;;A>@@;@@699@A@@A
@@@A@@====
@T6XXI:00127:00155
GGGGAATTCTAATACGACTCACTATAGGGAGAGGGA
+
AA?6@?B?CCC@CCBBBBBB??><<6195/9777+,
@T6XXI:00127:00162
GGGAGAAGGGAGAAAGGAAGGTGAACAGAGCGCAAACGAATACGTCTCTATTCTCCCTCTCCCTATAGTGAGTCGTA
TTAGAATTCCCC
+

CC>CCB@BB>BBBB=B?A@B@BBB@B??BBBB???>@DD@BBCDCBBBBCCACCBB>BBCBB?BBBAA@ACCCBBBB
B@CAA>B@CCC:
@T6XXI:00127:00180
GGGAGAAGGGAGAAAGGAAGCACAGGGCGAAGCGTGGGAAACTTGGCCTATTCTCCCTCTCCCTATAGTGAGTCGTA
TTAGAACTTCCCC
+
HK?AA?@DCBBBBB>B?B69;A@ABB<A6:5::::BDCDB>CA>B<>;>AC?AAAA<=<<@@7?>?;>000==;600
.9;8:77;/444+
@T6XXI:00127:00186
GAGAGAAGGGAAGAAAGGAAGCTAGTCACTCGACATCCTTGTGGCTTCACAGTACAGGAGGGACTCGACTCCAAAGC
CGCAGATCTCGAGTCGACCTTAGCCTTTCCTTTCCTCCCTTCCTCCC
+
944944244-42444-
313134;449=9;;;4//8//,/,..99>?>?ABA988944248;6;83779999ABB@BB@B@>;77311111644
44249443133,3111,177;A;:::./,23
@T6XXI:00127:00191
GGGAGAAGGGAGAAAGGAAGGGGAGAAGGGAGAAAGGAAGCTAGTCACTCGACAGTCTG
+
AB>BCB?BB?ABAB?B@C?BB>6@??;662<777-7;>=FBCDAB?;;;/.--------
@T6XXI:00127:00198
GGGAGAAGGGAGAAAGGAAGCTAGTCACTCGACATCTGTGGCTTCACAGTACAGAGGGACTGCAAAGCCAGACGCAT
CTCGAGTCGACTAG
+
FF@B?A@BC>BCCB=@<?;@A@ACBA?????:::???BCD@BB?BCCCBBBCB=77714;;9<<=4:9230333000
330049;94::=:9
@T6XXI:00127:00199
GGGAGAAGGGAGAAAGGAAGCTAGTCGACTCGACATCTGTGGCTTCACAGTACAGAGGACTGTCCAAAGCCGCAGAT
CTCG
+
CCACCB>==6:849-9@B8;<B@@?BCCCAA?:9:???ABC>?B=?C@@@A;6034424;::99244-
482;@A@@@AAA=
@T6XXI:00128:00150
GGGAGAAGGGAGAAAGAAAGCTAGTCACTCGACATCTGTGGCTTCACAGTACAGAGGGACTGTCCAAAGCCGCAGAT
CTCGAGTCGAC
+
4?:??B@CE@<;9@1944-479333:116667688:611B5:;<>?>AAA::6;444-44;;473/93665/--
2444555568589;
@T6XXI:00128:00152
GGGGAATTCTAATACGACTCACTATAGGGAGAGGGAGAATAGCCGTTTAATCTCCCACTGCGGCATATCCTTCCTTT
CTCCCTTCCTCCC
+
CCG9D@C@CCB?CCB@ABBBB@?99166.777BC?CBB@A898?AAA:A?A@AA@;?>>;;<;===@<<:=>@>@A=
A?:826,-,+150
@T6XXI:00128:00154
GGGGAATTCTAATACGACTCACTATAGGGAGAGGGAGAATAGCCAACGACTCTCCCGCCGTACAGTTCTCTTCCT
+
?>>4>;@ABCB@CCCCBBBAAAA>;666-6=<??;?BB@A@@@;<9<B???A?>@<?;8<;8:8<D?@==9;856
@T6XXI:00128:00155
GGGGAATTCTAATACGACTCACTATAGGGAGAGGAGAATGTTT
+
BCD9B7;;<CBBCCB?BBBBBA<99111.7?>><@:0,/6551
@T6XXI:00128:00158
GGGGAATTCTAATACGACTCACTATAGGGAGAGGGAGAATAGCACATTTCTGCTTCGTCGCGCCGTATTCTTCCTTT
CTCCCTTCTCCC
+
<<</9>CACBB@CCBBBBBCCBBA@:::5:CCCC?BBC@@<=9=???A9<;A??;877>@B@A>A=9<=@@8<9=@:
?@@A<@>@@@@:

```
@T6XXI:00128:00159
GGGAGAAGGGAGAAAGGAAGCTAGTCGACTCGACATCTGTGGCTTCACAGTACAGAGGGACTGTCCAAAGCCGCAGA
TCTCGAGTCGACTAG
+
CD@DGCFCC@CABB=B@C@CC?:@8,,,,****,,,,.33351716;777??@@?=??;@@A??A9=<7<=<;;788
=;:::://-----335
@T6XXI:00128:00168
GGGAGAAGGGAGAAAGGAAGTGCAGTTCGCGGTTAGTGAAGGGAAAAACTATTCTCCCTCTCCCTATAGTGAGTCGT
ATTAGAATTCCCC
+
FFACCC?BA?BBBC@C@C?BCCABBB@ABBA@A?ABCBC@@@;@???2BB>B@BBB>;C@B@A==9=AAAAAAC@@<
==:<@A=@>AAA5
@T6XXI:00128:00170
GGGGAATTCTAATACGACTCACTATAGGAGAGGGAGAATAGTGGACGCCTTCTCCACCGGTCACGTAATCTTCCTTT
CTCCCTCTCCC
+
CCH:C;;;<BB?BBBBBBCB>6;6:..,000?CACC@=B??:=<==998<?A??<?C>A>@@??===>@@A>A?=:9
9994-/.0000
@T6XXI:00128:00183
GGGGAATTCTAATACGACTCACTATAGGGAGAGGGAGAATAGTCAACAAATCGTTTGGGGCGACTTCTCCTTCCTTT
CTCCCTTCTCCC
+
999/4999@CC@CCCCCCCAA<<?;667.7==BC@CB@>AAA@=?>?==7>:::A<@BD7A?@@C?CBBA?;=;>A7
<9;;6;./.8;6
@T6XXI:00128:00191
GGGAGAAGGGAGAAAGGAAGGGGAGAAGGGCTAGTCGACCATCTGTGGCTTCACAGTACAGAGGGACTGTCCAAAGC
CGCAGATCTCGAGTCGACTAGCTTCCTTTCTCCCTTCTCCC
+
DD?CEC@CC>BBBA<A?B?BCD5;;A77:59AD99999964:?>ABCACCCGBBBBCCBAA@@@:?BB??@>BA<?;
:8?;;88944=A>@B<<>>877@?@?@C;@=:8852343=4
@T6XXI:00128:00194
GGGAGAAGGGAGAAAGGAAGGACAATGGCCCGACGGAGACTAATCGGCTATTCTCCCTCTCCCTATAGTGAGTCGTA
TTAAGAACTTCCCC
+
@@<ACB?CCCCBBB>B?A>>>AAB>BB?AA@ABBB@A>?@AC@@??>>BBB=>@CC>?<???9?@???AAC888:68
8,/,.32237===5
@T6XXI:00129:00152
GGGGAATTCTAATACGACTCACTATAGGGAGAGGGAGAATAGATCACGTAGCGTTCGTGTGTCGATTCTCCTTCCTT
TCTCCCTTCTCCC
+
BCB8A>ABCBB@ABBBBBBBBCB>>66659@?AB>BCC?@;?::ACBB@A>A??=?CA<;8???AC?C@@;=9<=@@
<:=?=9=/.0;;6
@T6XXI:00129:00178
GGGAGAAGGGAGAAAGGAAGAGGTTGTGGAAAGTAGATAGCGAGAAGCTATTCTCCCTCTCCCTATAGTGAGTCGTA
TTAGAATTCCCC
+
CB>CEC@BA=ABBB:><>@BBB?B@B@C@@@??AAC@CCBCBBBC@B???@ACBBB<:::AA<BCAAAAA===BA==
=>D=:8>5999/
@T6XXI:00129:00184
GGGAGAAGGGAGAAAGGAAGCTAGTCACTCGACATCTGTGGCTTCACAGTACAGAGGGACTGTCCAAAGCCGCAGAT
CTCGAGTCGACTAGCTTCCTTTCTCCCTTCTCCC
+
FCAEFCACC>BBCB=B@C@AA>;></00......44577901;7;@@AEBBBA@78818@;<=@>AA<AA>@@@AAA
899AA@@A@<<<<;;@8<9<@;@???;?:=<@@;
@T6XXI:00129:00193
GGGAGAAGGGAGAAAGGAAGGAGAAGTCGCCCCAAACTATTTGTTGACTATTCTCCCTCTCCCTATAGTGAGTCGTA
TTAGAATTCCCC
```

```
+
BBACCC@BB?BBBB?B@B@B@BAA@@@988885;=800/::::B?C<<AAA?=<<<;;<?@B>B;???@@==;?=@@
@?@AD@B3487+
@T6XXI:00130:00155
GGGAGAAGGGAGAAAGGAAGGAGAAGTCGCCCCAAACGATTTGTTGACTATTCTCCCTCTCCCTATAGTGAGTCGTA
TTAGAAT
+
DCBAAA@CE>BCBB>C?B>B@BCB@BABAAAA8AB>CCBBB>BB?BBBCB@>@AA=;=8=AA<A<<<@@AA@@???A
A>AAA=@
@T6XXI:00130:00156
GGGGAATTCTAATACGACTCACTATAGGGAGAGGGAGAATAGTCACATTCATGTTTGTGACCGTGTCTCCTTCCTTT
CTCCCTTCTCCC
+
CEICC@B@BCC@BCBBABBBBBB<<661.7?>>@=???<<@;<=A@@C@???BBB<ACB==:=?==?9968@A>A@<
@===14/3:996
@T6XXI:00130:00164
GGGAGAAGGGAGAAAGGAAGGAGAAACACGGGCCAAAGAGTTATTGGCTATTCTCCCTCTCCCTATAGTGAGTCGTA
TTAGAACTTCCCC
+
CC@CCB@BB>@?AC>B@B@B@BBBB>BBA??:=<==<@BBA>A@5:7<ACC@CCCC?CC@@@>@AA>>>==@A@A??
?>??B7=<4777.
@T6XXI:00130:00167
GGGGAATTCTAATACGACTCACTATAGGGAGAGGGAGAATAGTCTTCCCATATTTTGCACCGCTATTCGCTTCCTTT
CTCCCTTCTCCC
+
==>4@<@=ACCCCCB?BBBBBB<97111.7>>AC>BB@?A==<=A>?<;?BB@@@8?@@?A?>ABC?CCCC=?=?B?
;::949844/;6
@T6XXI:00130:00173
GGGAGAAGGGAGAAAGGAAGCTAGTCGACTCGACATCTGTGGCTTCACAGTACAGAGGACTGTCCAAAGCCGCAGA
+
@C@CDC>?=5>99;8;@B=@@A;;:CBBBBCBA??:::???::;6:@AAAAB<99/.,/88689;;;6:8-..:::
@T6XXI:00130:00183
CTAGTCACTCGAGA
+
;11//8/::CEBBC
@T6XXI:00130:00184
GGGAGAAGGGAGAGGGAGAATAGGTTACAACTCCCCCTTGTCAACCTTCTCTTCCTTTCTCCCTTCTCCC
+
CE>B@@=@B=BCCCC<AAB>??>>@@BAA<@>66880?AAABD@@>@ACC@@;=<<=<<?AA<A>@@@A<
@T6XXI:00130:00188
GGGAGAAGGGAGAAAGGAAGGAGAACGCGCACCCAAACCAACGGTGATCTATTCTCCCTCTCCCTATAGTGAGTCGT
ATTAGAACTTCCCC
+
FEADCC@AA=AAAA?A@A<<;<BB@CAA>@BCC?BC@B?B>AB@BBBABABA?BBBB>BCAAA;@@?@<=AA@@=<<
==76//,//79744
@T6XXI:00130:00194
GGGGAATTCTAATACGACTCACTATAGGAGAGGGAGAATAGCTGTGCACTCACATCCGGCTTTGTCTCCTTCCTTTC
TCCCTTCTCCC
+
CDF:D?B?BCB@CBBBBCCC@>;;://,0::?B>AA?=??@8::C??:<:AAAA@<=?>@@A<@889=9<=A;==7:
444-434/660
@T6XXI:00130:00195
GGGAATTCTAATACGACTCACTATAGGAGAGGGAGAATAGTCCAGTGTTTAATTTCTTGGCTTTTCTCTTCCTTTCT
CCCTTCTCCC
+
CC@C@C@CCB@CBB??>>??:997..,0::BK>CB@=@=AA=;<???@C<B?@@;@A=@=BBAA6@@??<??AA:??
AA;A9==AA<
```

```
@T6XXI:00130:00196
GGGAGAAGGGAGAAAGGAAGGAGAACATGTTGTGGGACAAAGCCAAACTATTCTCCCTCTCCCTATAGTGAGTCGTA
TTAGAATTCCCC
+
DE?BBBBDCAABAB?B@C@C@BBBACBCDC@BBA@<>???:@C?CB=BCCBABBBBCBBAA@<:::?BB@BB?=67/
/,///,3<===0
@T6XXI:00130:00199
GGGAGAAGGGAGAAAGGAAGTGCAGTTCGCGGTTAGTGAAGGGAAAAACTATTCTCCCTCTCCCTATAGTGAGTCGT
ATTGAA
+
CD@CBBACB@???B=B@BADAACABB@CBCB@C?BABAC?>@<<AAA2ADAA?AAAA<ACBFC>B??A@@@AA@@@@
8948@@
@T6XXI:00131:00166
GGGAGAAGGGAGAAAGGAAGGCGAATAGACTTTTATCAGAAGGGAAAACTATTCTCCCTCTCCCTATAGTGAGTCGT
ATTAGAATTCCCC
+
AB>DCD@CB>BBBB>B@B@B>AABABBBBCCCC9@B@DBC<>>.777.6BBD?A<<64;<<=@<;<<?===??CA@<
;:7300/0/004-
@T6XXI:00131:00172
CTAGTCGACCGGCTTTGGACAGTCCCTCTGTACTGTGAAGCCACAGATGTCGAGTCGACTAGCTTCCTTTCTCCCTT
CTCCC
+
EBBBBECFC@C@CDD@C@DCDCBA@<@BBCAA@ABCCC?CC>??>>99>?CCBBBBBBBBCAAA@C@CC@CBDDAC=
@@CC@
@T6XXI:00131:00174
GGGAGAAGGGAGAAAGGAAGGAGAAGTCGCCCCAAACGATTTGTTGACTATTCTCCCTCTCCCTATAGTGAGTCGTA
TTAGAATTCCCC
+
EC@CCC?BB=BA?C<?=@@C@CCC@CBBAA@@7@B>BCC@C>BB?CAAA@A>B???;???AA=@???AAA=<<@@==
=>A?=8:/444-
@T6XXI:00131:00176
GGGAGAAGGGAGAAAGGAAGCTAGTCACTCGACATCTGTGGCTTCACAGTACAGAGGGACTGTCCAAAGCCGCAGAT
CTCGAGTCGACTAG
+
::-9?>599148>?9A8;:;CA@AC??=717711677==A@@B?BCCABAB=;8333-
4;;=?>?AD@@B>==<<<<????A@A@@@@888
@T6XXI:00131:00191
GGGGAATTCCTAATACGACTC
+
7000027,1,//,/684433.
@T6XXI:00131:00194
GGGAGAAGGGAGAAAGGAAGGAGAAGTCGCCCCAAACGATTTGTTGACTATTCTCCCTCTCCCTATAGTGAGTCGTA
TTAGAATTCCCC
+
EC@CB@@BB>BBBB>BAB?A?AAA?@ACAAAA8CE>CBBBC>C@>???B>A:8::=4:???C<A???@@@<AA9==@
@=?@>89/444-
@T6XXI:00132:00152
GGGGAATTCTAATACGACTCACTATAGGGAGAGGAGAATAGAAGCACCTGTTGT
+
===5@:A>BCD@CCBCABBBBAACBBBC><?:?:?<<:8444/.-..,...331
@T6XXI:00132:00158
CTATCACTCGAGATCTGCGGCTTTGGACAGTCCCTCTGTACTGTGAAGCCACAGATGTCGAGTCGACTAGCTTCCTT
TCTCCCTCTCCC
+
==B=9::9@==??CCDLDD@A@@<@7;;BBBCC<CCCCCB??@B=B<8<:<@@@B;;:??BACAA???;;<9>A@:>
9:379,/-1110
@T6XXI:00132:00164
```

```
GGGGAATTCTAATACGACTCACTATAGGGAGAGGGAGAATAGTCAACAAATCGTTTGGGGCGACTTCTCCTTCCTTT
CTCCCTTCTCCC
+
BBB9B@C@CBB@BB@@AAAB?<<A????9???BB>BCA>@AAAAB?@@A;>;8662;662;????>BBBDA?A>AA<
AA@@<@>@@@@:
@T6XXI:00132:00167
GGGGAATTCTAATACGACTCACTATAGGGAGAGGGAGAATAGATAGCGTCCTCCCAAAACCAGTCTGTTCTTCCTTT
CTCCCTTCTCCC
+
CCC9C>A>ADDBCCBBBBBBBA@97111.7>>BC>CCA>A><<;<<<ABC?@@?7>==4><??@@@AB?@@9<8<?;
?@@C<A;==AD<
@T6XXI:00132:00177
GGGAGAAGGGAGAAAGGAAGCTAGTCACTCGACATCTGTGGCTTCACAGTACAGAGGGACTGTCCAAAGCCGCAGAT
CTCGAGTCGACTAG
+
CC>ABC@B>:?944-
95948A???@?A=???:::AB???D?AB@BBBC??@B@=4431366<?@<??<?C>A=<<ABBAA>??AAA<<<<<
@T6XXI:00132:00194
GGGAGAAGGGAGAAAGGAAGTGCAGTTCGCGGTAGTGAGGGGAAAAACTATTCTCCCTCTCCCTATAGTGAGTCGTA
TTAGAACTTCCCC
+
:C:?@A:9809999049>7>?;;;@>B<@=?.1.6443111,1===/888847777-
671178634;==?@9:9CH@?=8..,-+*+000
@T6XXI:00132:00197
CTAGTCACTCGAGATCTGCGGCTTTGGACAGTCCCTCTGTACTGTGAAGCCACAGATGTCGAGTCGACTAGCTTCCT
TTCTCCCTTCTCCCCTTCCTT
+
>:111=8=?ABBBCDCCCBB?BCC<B>BBA?==?=?CCA@C@C@BA@<?BCCCCABCBC???BB<<;CCCAA<:?:?
>7>;??6::;////+/,/,/,
@T6XXI:00133:00158
GAGATCTGCGGCTTTGGACAGTCCCTCTGTACTGTGAAGCCACAGATGTCGAGTCGACTAGCTTCCTTTCTCCCTTC
TCC
+
BAAA@BBBBB?ACC@B?BBBBCCCBCCCCCCCC@??C@BD?BBCBBCCBCCCCBBA@>>=44424244-
466669,0..,
@T6XXI:00133:00161
GGGGAATTCTAATACGACTCACTATAGGGAGAGGGAGAATAGTCAACAAATCGTTTGGGGCGACTTCTCCTTCCTTT
CTCCCTTCTCCC
+
A>92=6;6;CC@CCCBCCCCA?@B?===97::B?>A:967@<>8:8434-
7>>>?.777.6;>@C?BBC?@=@@AC:>>>A:@=?ACC<
@T6XXI:00133:00180
GGGGAATTCTAATACGACTCACTATAGGGAGAGGAGAATAGAG
+
DCE:C@B@BCC@CBBBB@??A@>?9699.6=;;;?0/,,,***
@T6XXI:00133:00199
GGGGAATTCTAATACGACTCACTATAGGGGAGAGGGAGAATAGTTTTTCCCTTCACTAACCGCGAACTGCACTTCCT
TTCTCCCTTCTCCC
+
;99/9;>=BCB@C@@?ABBDBCB761111.777?BABA@>@?:9888,44-
4;=BCBD@B?A;;9;<=<<?>97=36:.7@@CAC@@@@A<
@T6XXI:00134:00162
GGGAGAAGGGAGAAAGGAAGAAAGGAAGAGATCAGTTACAGCGGCACGCGCGGGACTATTCTCCCTCTCCCTATAGT
GAGTCGTATTAGAATTCCCC
+
FF>AB@@BCA>@>B=B@B@AAA<B@C@????B?BBB?BAAACB?BCCCCCBBC<@@CBB=???<6==@@A>BCCCAA
ABCCBBB??<?@@>B?AA@6
```

```
@T6XXI:00134:00171
GGGGAATTCTAATACGACTCACTATAGGGAGAGGGAGAATAGTCCATGACTCTACACACCCCATCTTTCTTCCTTTC
+
BCC:C@C@BBB@BBBBB>??@@<97111.7>>AB>BC@B@9==A>@???A@??BCAAAAAA6;;444,4;9;:??;?
@T6XXI:00134:00187
GGGAGAAGGGAGAAAGGAAGGAAAACAGCTATTTAGTCAGGAGTAAGCTATTCTCCCTCTCCCTATAGTGAGTCGTA
TTAGAATTCCCC
+
CDCCEC@DD>CCBB?AAB@D>AAA8CB@@A?@@=ACCCCC@CCCCA?AACC@CDBC@BD??=<@@@ABCCCCEA???
A=@<;855::?5
@T6XXI:00134:00190
GGGGAATTCTAATACGACTCACTATAGGGAGAGGGAGA
+
AAA7A;==@BA?BCAAABBBB?BB>??@86;166-70/
@T6XXI:00135:00155
GGGAGAAGGGAGAAAGGAAGCTAGTCACTCGACATCTGTGGCTTCACAGTACAGAGGGACTGTCCAAAGCCGCAGAT
CTCGAGTCGACTAG
+
DD?BB@644-
998809695;F??B@????@@:::BBB@BB=@@?BBBCBABAA<;<<99=<@@@>??;?@:>?====?@@889AA@A
@@??
@T6XXI:00135:00156
CTATCACTCGAGATCTGCGGCTTTGGACAGTCCCTCTGTACTGTGAAGCCACAGATGTCGAGTCGACTAGCTTCCTT
TCTCCCTTCTCCC
+
DAA?A::;AAABBGCCCBA>A@B<B=@?BB:::8:CAAACCCBB@@?AC?BBCCCAA>@A@<?>?A@@@AA<>@?BC
@CBCD?A<??@@;
@T6XXI:00135:00159
GGGAGAAGGGAGAAAGGAAACTAGTCGACTAGATCTGCGGCTTTGGACAGTCCCTCTGTACTGTGAAGCCACAGATG
TCGAGTCGACTAGCTC
+
KHCH<<>>C@CECC=A7:908>??BCCCCBCCCCDCCCC@CHH>C@B@A@CCC?CCCCCCCBB@@A@AB?CBCCCBA
A>;:000/00661,,,
@T6XXI:00135:00162
GGGGAATTCTAATACGACTCACTATAGGGAGAGGGAGAATAGTCAACAAATCGTTTGGGGCGACTTCTCCTTCCTTT
CTCCCTTCTCCC
+
=@@6@>A@CDC@CCA?A?????BB??@A>>>@@A=@B<;<<@AA;:3331:???B<???7?BBBB@CBC?@>A>@@<
@ABC<A85444-
@T6XXI:00135:00172
GGGGAATTCTAATACGACTCACTATAGGGAGAGGGAGAATAGTCAAGCCTTCCGCGCCTAATTACTGTTCTTCCTTT
CTCCCTTCTCC
+
CKK:D?B?ABB?BB@@@BCC@??>=766.7==BB>AA?<>==;44344302877??:76;?<@>AA?@@>A@7:344-
4444-434:::
@T6XXI:00135:00173
GGGAGAAGGGAGAAAGGAAGGAGAAGTCGCCCCAAACGATTTGTTGACTATTCTCCCTCTCCCTATAGTGAGTCGTA
TTAGAATTCCCC
+
DF>BBCACDABB?=:@=?=@@CCB@BBABBCC9CC>CBBBB=???BBBC??9=<??;@@@@A<AAAA@???@???==
?AAAA>A:>==1
@T6XXI:00135:00182
GGGAGAAGGGAGAAAGGAAGCTAGTCACTCGAGATCTGCGGCTTTGGACAGTCCCTCTGTACTGTGAAGCCACAGAT
GTCGAGTCGACTAGCTTCCTTTCTCCCTTCTCCC
+
CF?D@@=@A<@BCC>D>@=@;;8?;:0/..43.-
/69>??::77.7=?777==?6???877=884=A<;:7676///88928?;??@AAA===;;=@A-4///,/,/---+
```

```
@T6XXI:00135:00188
GGGGAATTCTAATACGACTCACTATAGGAGAGGAGA
+
1/1+4:ABB;;7<BBABBBBB:88?7740<00,./-
@T6XXI:00135:00191
GGGAGAAGGGAGAAAGGAAGGAGAAGTCGCCCCAAACGATTTGTTGACTATTCGTCCCTCTCCCTATAGTGAGTCGT
ATTAGAATTCCCCC
+
44-444244-4488-
424=>=AAB?A:;8>>>,77/376698<<>AAB?88;0//7759:7;::::<A@AAAC9888<@<<>=<=<::;;+
@T6XXI:00136:00162
GGGGAATTCTAATACGACTCACTATAGGAGAGGGAGAATAGTGCAAACCATTGACCCGCAACTATTTTCCTTCCTTT
CTCCCTTCTCCC
+
CDCBD>@@@DC@CCBBBAA@A:;;?./-
0>>@D>CCA?D==AAA??;?>CC=?BAC><??==BBDDC9D=?:?:?<9?A@@<@>A@AA<
@T6XXI:00136:00172
GGGGAATTCTAATACGACTCACTATAGGGAGAGGGAGAATAGTCAACAAATCGTTTGGGGCGACTTCTCC
+
999/959:<BB@FDDCCB??@???=7::.77==?<?CA>@@???A?ADA?AAACF>CBB7BEEBB??@>3
@T6XXI:00136:00175
GGGGAATTCTAATACGACTCACTATAGGGAGAGGGAGAATAGTTTTCCCTTCACTAACCGCGAACTGCACTTCCTTT
CTCCCTTCTCCC
+
;<9/9<A>BCCDCCCACBCBBAB@9611.6>>>?=AA?<?AAACA6AA<A?>AAAC?B?<AAB?@@@=>?A?B=??8
AA??8A;===D?
@T6XXI:00136:00197
GGGGAATTCTAATACGACTCACTATAGGAGAGGGAGAATAGTTTTCCCTTCACTAACCGCGAACTGCACTTCCTTTC
TCCCTTCTCCC
+
:49/9<B?CCC@BBCCCB???:997//-
000BK@DA@=BAAB==5999=>=@@?@>@>>>AA>==<=@@@;<;??;????8?>A???;
@T6XXI:00137:00154
CTATCACTCGAGATCTGCGGCTTTGGACAGTCCCTCTGTACTGTGAAGCCACAGATGGACCAGATCTCGAGTCGACT
AGCTTCCTTTCTCCCTTCTCCC
+
E:444444449499BCCCC@CCC=@=?A<B???8???AACBCBB??>@C@CDFDBBC@CC@C@@@BCCC:;;@@@BD
D:<<?BBEDADCCC>A@@BDC@
@T6XXI:00137:00172
GGGAGAAGGGAGAAAGGAAGGATAGCGTTGCGGGGAAAAGTGTTGAGCTATTCTCCCTCTCCCTATAGTGAGTCGTA
TTAGAACTTCCCC
+
=?:>>>;@C?BAAN?B?B@C@CCB?AAB>?7778.9BB8CCCCBCBBBDCC?BBCD@CCCCB=B@@@AAAAAAABBA
A??@@=BB?BBB6
@T6XXI:00137:00180
GGGGAATTCTAATACGACTCACTATAGGGAGAGGGAGAATAGCAAACGTTTTCCCCGACGTCCG
+
999,98?>:88:11188.39979<?A;5+.....+.322286664+01666,111,16111101
@T6XXI:00138:00153
GGGGAATTCTAATACGACTCACTATAGGGAGAGGGAGAATAGTCAACAAATCGTTTGGGGCGACTTCTCCTTCCTTT
CTCCCTTCTCCC
+
BAA7A>B?BCDDBBABBAA?B@<99166.7==?B?CFBA@@@@AA;=AA=@@B@@88885?ACBCCCBB??=@?CA:
>@AA>C??@@@;
@T6XXI:00138:00188
CTAGTCGACTCGAGATCTGCGGCTTTGGACAGTCCCTCTGTACTGTGAAGCCACAGATGTCGAGTCCGTCCTT
+
```

```
CB:<<;;;@CCCCEBBBBBCB@BBB<B8::?>777-7?>>7::::@@@>ACACCCBBBBDCC?:01,-**)*)
@T6XXI:00139:00178
TCGAGATCTGCGGCTTTGGACAGTCCCTCTGTACTGTGAAGCCACAGATGTCGAGTCGACTAGCTTCCTTTCTCCCT
TCTCCC
+
@CAAABBBAAAB@BBB>A?BACCB@>;B@@BB>>???;<99;<?>?6::;;A@>CCCBBABFAAA@B@CCACDCC>C
=@@BC>
@T6XXI:00139:00180
GGGAGAAGGGAGAAAGGAAGGGGAGAAGGGAGAAAGGACTCGACATCTGTGGCTTCACAGTACAGAGGACTGTCCAA
AGCC
+
:;8<<<BDJC=?<C>B@B@BCB8CCC?<96;111,7:=>76:;::?=<?;:5:CABBBCCC<997//,/:::@@?HH
@==;
@T6XXI:00139:00184
GGGAGAAGGGAGAAAGGAAGCTAGTCGACTCGACATCTGTGGCTTCACAGTACAGAGGGACTGTCCAAAGCCGCAGA
TCT
+
CC@C@@699-9>>@<B@C=@@B:;:??@BAA@;9;;:7==?:?@?ABFCCBC994000-
4::=AA?AB:?@@A@<====:
@T6XXI:00139:00192
GGGAGAAGGGAGAAAGGAAGCTAGTCGACTCGAGATCTGCGGCTTTGGACAGTCCCTCTGTACTGTGAAGCCACAGA
TGTCCGGA
+
44-
44424959111,/,/,.443311111111388866;AA8:;D>C?A????==9;833683030004532688::843
2,.,-
@T6XXI:00140:00153
GGGAGAAGGGAGAAAGGAAGGAGAATGCACCGCAGGAAATTGCAAGACTATTCTCCCTCTCCCTATAGTGAGTCGTA
TTAGAATTCCCC
+
EEAFCC@BB>ABAB?BBC@B@B???BBCAB?ACBC@??;B<><?>@@??@@@AA@>910444-
4<;<CAAAA@???@A<A?A??5<<=4
@T6XXI:00140:00164
GGGGAATTCTAATACGACTCACTATAGGGAGAGGGAGAATAGCCGTCAACAAAATCTGGGGTCTATTCTGCTTCCTT
TCTCCCTTCTCCC
+
CCC9B?B?BBC@CCBB?>>BBABB=67646??BC>AA??A=@>;<=<A869664;BCCCC9CCCCC@BBBBB@B@CC
>CCAA<A?BBBC?
@T6XXI:00140:00168
GGGAGAAGGGAGAAAGGAAGCTAGTCACTCGACATCTGTGGCTTCACAGTACAGAGGGACTGTCCAAAGCCGCAGAT
CTCGAGTCGACTAG
+
CF?CCC@B@:@@==5>594;??>?BA??:::;::???ABB?BB?BCBCBBB>9933317@AAA@?AA<=<9<=6<=<
;<<???AAA@A@@@
@T6XXI:00140:00170
CTAGTCACTCGAGATCTGCGGCTTTGGACAGTCCCTCTGTACTGTGAAGCCACAGATGTCGAGTCGACTAGCTTCCT
TTCTCCCTTCTCCC
+
D:111=8==CCCCACBBB@@>@BB<B@BB@AACC>B@@@@B@?ACA==?B@BBBBBA?ABGDCBBBBBBC@@@@B?B
D?CBBC>A?AAAA<
@T6XXI:00140:00183
GGGAGAAGGGAGAAAGGAAGCTAGTCACTCGACATCTGTGGCTTCACAGTGAGGACGTGTCCAAAGCCGCAGATCTC
GAGTCGACTAG
+
DDBDBBA@@<@@=85@897;B???A900:9:00.///56?:@@@BAB>;000:4+,.....,4;:9;::EFCBBAA@
@><<CGCC=<=
@T6XXI:00140:00188
```

```
GGGAGAAGGGAGAAAGGAAGCTAGTCGACTCGACATCTGTGGCTTCACAGTACAGAGGGACTGTCCAAAGCCGCAGA
TCTCGAGTCGACTAG
+
A?>1121185:/44049958;>>>>777=767><;;997::901,.7/76A??====>:>?=@>><??:=A388<88
7<?>48/.-055,..
@T6XXI:00140:00192
GGGAGAAGGGAGAAAGGGCTAGTC
+
BB?@@@59909999144-466,,,
@T6XXI:00140:00196
GGGAGAAGGGAGAAAGGAAGCTAGTCACTCGACATCTGTGGCTTCACAGTACAGAGGGACTGTCCAAAGCCGCAGAT
CTCGAGTCGA
+
ACABEB?BB89999095968?B?A>?:711111169988<A?@=@BBBBBA;;9333-
4;;>@@>AC<@@>@@<<?A@@@?8<=888
@T6XXI:00141:00153
GGGAGAAGGGAGAAAGGAAGGAGAAGTCGCCCCAAACGATTTGTTGACTATTCTCCCTCTCCCTATAGTGAGTCGTA
TTAGAATTCCCC
+
CA<@BC@CC>BCBB=B=@=A@CAB?B?;7788.8808<?CC=?C=AABBCA=AAAC;A===@<A@@@CB@@@@AAAA
@>AAA=@9<:9.
@T6XXI:00141:00159
GGGGAATTCTAATACGACTCACTATAGGGAGAGGGAGAATAGTCAACAAATCGTTTGGGGCGACTTCTCCTTCCTTT
CTCCCTTCTCCC
+
EFC:D@CABAA=@BABCC@@@CCB>//0,000;;9;=>;?<<<?@=>@>7>?==?6=??.::@??:?AB?@=@=??:
????:?=?8:;6
@T6XXI:00141:00168
GGGGAATTCTAATACGACTCACTATAGGGAGAGGGAGAATAGTCAACAAATCGTTTGGGGCGACTTCTCCTTCCTTT
CTCCCTTCTCCC
+
?@@6@@B@CCC@CBBABBBBBBBCA96959??BC>BCA?A;;?A<:8864??;;=-677-
7>@AC?@@@?@=@=AC<A???<?>@@AA<
@T6XXI:00141:00174
GGGGAATTCTAATACGACTCACTATAGGGAGAGGGAGAATAGTTTGCGCATTCGGTGCACTGTTATTCTCTTCCTTT
CTCCCTTCTCCC
+
==@6@>BBCCC@B@@B?>BB?AB??66656==BC>BB@>@=BAC=????@B@BBABAABBBBB?CA?ABBB?B@CD>
A?AC>C@9>>A:
@T6XXI:00141:00181
GGGGAATTCTAATACGACTCACTATAGGGAGAGGAGAATAGCCATTTAGCGTCTGGCCCCA
+
CDC8B:;699>9@@A@BBBBB@?>>777,/<00,.:724:;:8;;9499440300/000+0
@T6XXI:00141:00189
GGGAGAAGGGAGAAAGGAAGAATATTGCGCGTTTAACGGGGTGATGGCTATTCTCCCTCTCCCTATAGTGAGTCGTA
TTCAGAATTCCCC
+
??:?@@69911<?>-1,16..,..37677;;>A:><6611,167663898?=@@AC;<<784-
444335554555333568=B>B?AAA4
@T6XXI:00141:00195
GGGAGAAGGGAGAAAGGAAGAGTGACAGCCGTCCGGGCAATAGGGAGCTATTCTCCCTCTCCCTATAGTGAGTCGTA
TTAGAA
+
FF>BAA@BBBB@BB=B?B?BBBBB?BBAA?A?>?BE>?=;@CDC>C@@;:;?BBBB<>>77:67@?=?@<??=8<6<
;89;;7
@T6XXI:00142:00150
```

```
GGGGAATTCTAATACGACTCACTATAGGGAGAGGGAGAATAGTCAACAAATCGTTTGGGGCGACTTCTCCTTCCTTT
CTCCCTTCTCCC
+
CCC9CBC@BBB@BBBA??>??@CB?66646?8BE>CCB@A<@@@@><8638;6;;-666-
7>@ABEECB?@=@@AC=??@A;?:6<<>7
@T6XXI:00142:00171
CTAGTCACTCGAGATCTGCGGCTTTGGACAGTCCCTCTGTACTGTGAAGCCACAGATGTCGAGTCGACTAGCTTCCT
TTCTCCCTTCTCCC
+
CC/1111::CCCDBCBBBB<>?BB;B?AA@AABB<BCBBBCBB@B@A>@C@CCCBCCBCBCC@ABBBBBB<>8?C?B
B>B@ADAA698>?:
@T6XXI:00142:00188
GGGGAATTCTAATACGACTCACTATAGGGAGAGGGAGAATAGTCTTGTATTTT
+
CCCAC;:<;CCBDBAAABCBB@;96116.7>>BD>BF@@AA???@:;<@@A=4
@T6XXI:00142:00190
CTAGTCACTCGAGATCTGCGGCTTTGGACAGTCCCTCTGTACTGTGAAGCCACAGATGTCGAGTCGACTAGCTTCCT
TTCTCCCTTCTCCC
+
B:/111/44BB@BBBBCBBB@AAA>A@A??=6775:AABCBBB>A?A;>B@CCCBAABECCCBBBBAA@@BA?:?=?
B?>=??9<9<@HC<
@T6XXI:00142:00196
GGGAGAAGGGAGAAAGGAAGGTGAACAGGTAACGGAGGGTGCGTTAACTATTCTCCCTCCCTCATAGTGT
+
CC?@AACCC@BBBC>CAB@B@BBC>BBBBAA>BC?BBC>CCCCC?C8;<BB@BBAB>???81-,,*****
@T6XXI:00143:00181
GGGGAATTCTAATACGACTCACTATAGGGAGAGGGAGAATAGTCAACAAATCGTTTGGGGCGACTTCTCCTTCCTTT
CTCCCTT
+
BBA9A=@>AAACCCCAA<BBBA<99111.6==@ABBB@@A=@@@=<<??;ACCCE>B@@7=?A??=?CC@A?A?AA<
@@@?:=5
@T6XXI:00143:00184
GGGAGAAGGGAGAAAGGAAGGTTATGGTGCCGATGCATAGTGGGAGACTATTCTCCCTCTCCCTATAGTGAGTCGTA
TTAGAATTCCCC
+
CB>===@CCAAA@@<@>B>CBB@CAA?ACC@CC@<??CBCAAA=AAACCA?=B444,4;;BA;AA@@?@888@<<<=
?ABAA?A38880
@T6XXI:00143:00196
GGGGAATTCTAATACGACTCACTATAGGGAGAGGGAGAATGGTCAACAAATCGTTTGGGGCGACTTCTCCTTCCTTT
CTCCCTTCTCCC
+
CCC9C>A>ACCCCB@AA>>>BCB>9666.6===?>AA??@<=?BC?@??;=====;<<<5<<<<<>=@A;?;:5667
<?8@;@=@???;
@T6XXI:00144:00159
CTATCGACTCGAGATCTGCGGCTTTGGACAGTCCCTCTGTACTGTGAAGCCACAGATGTCGAGTCGACTAGCTTCCT
TTCTCCCTTCTCCC
+
@866////18ACB?BCCCBB?BCB>B>AAAB@??;?CCCCCB@@CBB??C@BBBBBABACCCCCCB@@@A>>60736
=9>;??9?=A>;=9
@T6XXI:00144:00175
GGGAGAAGGGAGAAAGGAAGCTAGTCACTCGACATCTGTGGCTTCACAGTACAGAGGGACTGTCCAAAGCCGCAGAT
CTCGAGTCGACTAG
+
IDBBBA@BB>BCDC>C?AABBB>@@???<??::6777BBE=?@@CCBBBCC@@?@@C;=8:433/484865;<<??@
@?????A===AA??
@T6XXI:00144:00183
```

```
CTAGTCACTCGAGATCTGCGGCTTTGGACAGTCCCTCTGTACTGTGAAGCCACAGATGTCGAGTCGACTAGCTTCCT
TTCTCCCTTCTCCC
+
>>111=1::DDCCBBBCCCB@CCB>A>@?>>8::57@@ABCBCACBB>@C@CBBBBB@ACBAA?@@@:;;:;7=?=A
E>AAAB9?6::;?:
@T6XXI:00144:00186
GGGAGAAGGGAGAAAGGAAGGAGACACGGTCACAAACATGAATGTGACTATTCTCCCTCTCCCTATAGTGAGTCGTA
TTAGAACTTCCCC
+
BA>BFECDC@DBBB:=<@6;8<AACNCCDCCC;;;9<CFCC@B@@@ABBBEAC@B@==@@AA<??@???ACAAA@@9
93/;9,./7664.
@T6XXI:00144:00198
GGGGAATTCTAATACGACTCACTATAGGAGAGGGAGAATAGCACAACGTTCTTCCTTTCTCCCTTCTCCC
+
BEC9C@C?CBC<<<BBB@??80.44..,.5368;<=<>ABC===?<?@A9<<;65684688;46273;=1
@T6XXI:00145:00157
GGGAGAAGGGAGAAAGGAAGCTAGTCACTCGACATCTGTGGCTTCACAGTACAGAGGGACT
+
IECAA@@CHCHBBB=B7:6;@@@@AAB=::;:::AAAAAB?BB@CAAABBB?=7666-4=;
@T6XXI:00145:00167
CGAGTCTGCGGCTTTGGACAGTCCCTCTGTACTGTGAAGCCACAGATGTCGAGTCGACTAGCTTCCTTTCTCCCTTC
TCCCCTTCCTTTCTCCCTTCTCCC
+
CCBBBBBD@F?@BB@B@BBBBBBB<BBBBBABBCCCC?BC@CC@AAACCBBBBBCCGDDDBCA?CCED@CCCC@B<?
8888.8;>477-77>>:>47777.
@T6XXI:00145:00176
GGGAGAAGGGAGAAAGGAAGGAGAAGTCGCCCCAAACGATTTGTTGACTATTCTCCCTCTCCCTATAGTGAGTCGTA
TTAGAATT
+
CC@CBB@CCCCBBB=A@B@B@B@A?BCCFDCC9CB>BBBBC:A9>>9>????<<=>;99=<<;;<?@@?A====:8=
=?A??<>8
@T6XXI:00146:00151
GGGAGAAGGGAGAAAGGAAGCTAGTCGACTCGACATCTGTGGCTTCACAGTAGAGAGGGACTGTCCAAAGCCGCAGA
TCTCGAGTCGACTAG
+
FFFCBA?A?:?984-
9;>;@B801<//0//370:7<=<CCC@CC@CC@@BA8::@@@?:=;4300/00+049<==<@@@@@?<?@@???A@@
@T6XXI:00146:00159
GGGGAATTCTAATACGACTCACTATAGGGAGAGGGAGAATAGTCAACAAATCGTTTGGGGCGACTTCTCCTTCCTTT
CTCCCTTCTCCC
+
=??4??A?BCC@CBBBBBBBBBB<<366.6>>@B>BCC>?;;<AC??@>:<B?CC>@@@8ABBCA>=BB8::8<AC=
>?@A<A=@@@B<
@T6XXI:00146:00177
GGGGAATTCTAATACGACTCACTATAGGGAGAGGGAGAATAGTCAACAAATCGTTTGGGGCGACTTCTCCTTCCTTT
CTCCCTTCTCCC
+
ABB9B?CACBC@CBB:8-
*****02,,/*17;;?<????BBB@?>;<<;6?B???9?BB7BBCCE@CBB?B=<9=C=A@@@<A899AA;
@T6XXI:00146:00189
GGGAGAAGGGAGAAAGGAAGTGCAGTTCGCGGTTAGTGAAGGGAAAAACTATTCTCCCCCTCCTCATAGTGAGTCGT
ATTAGAATTACCCC
+
DD@CCC>??>ACBB>B?BDBCBBAAA@A==?@B@??>@<:772<>>@1ACB?=?87777,7=:=<??643366589:
::56332320000+
@T6XXI:00146:00194
```

```
GGGGAATTCTAATACGACTCACTATAGGGAGAGGGAGAATAGTTTTTCCCTTCACTAACCGCGAACTGCACTTCCTT
TCTCCCTTCTCCC
+
999/4295<CC@CB@ABBBBAA;97367.7==AC@CCCAB?BACCA077148;BCCCAB>BBBC>::8487=37371
,16==9>;7744-
@T6XXI:00146:00196
GGGGAATTCTAATACGACTCACTATAGGAGAGGAGAA
+
9;;2;2964449=>>CDCECC@;;00/,,,,.,.553
@T6XXI:00147:00153
GGGGAATTCTAATACGACTCACTATAGGGAGAGGGA
+
4;;298989>9889;ABB@AA@??7811,..,,2*,
@T6XXI:00147:00157
GGGAGAAGGGAGAAAGGAAGCTAGTCACTCGAGATCTGCGGTTTTGGACAGTCCCTCTGTACTGTGAAGCCACAGAT
GTCGAGTCGACTAGCTTCCTTTCTCCCTTCTCCC
+
B@9><989A:A99919;><ACD<<<C@??B???BB@>>>>4888.7@BBBBBBB>BC<:????@<<??BB?@@@A@@
@@@AA@?@??===:4737:996;=??6;8;;996
@T6XXI:00147:00160
GGGAGAAGGGAGAAAGGAAGGGTTATAGAATGCCGAGGGAAAGTTGGCTATTCTCCCTCTCCCTATAGTGAGTCGTA
TTAGAATTCCCC
+
DC@AAA@CB?BBBC>B?B?AB@B@BBBABB?AA?AB:>::=674,/,1>BC?CBCD@DBCCC>CBBBBBBBCB????
?<?CA?A;ACC4
@T6XXI:00147:00166
GGGGAATTCTAATACGACTCACTATAGGGAGAGGGAGAATAGAGACGTATTCGTTTGCGCTCTGTTCACCTTCCTTT
CTCCCTTCTCCC
+
=AA6A?@?BBB@CCCCCB@@@B?99311.777==9?@@<@??=??>A===>?AAA=@@@A?==?A?A;?>@?@>AA<
898C;@<@8885
@T6XXI:00147:00170
GGGAGAAGGGAGAAAGGAAGGAGAAGTCGCCCCAAACGATTTGTTGACTATTCTCCCTCTCCCTATAGTGAGTCGTA
TTTCAGAATCCCC
+
DEBCCCACCBCEBB>C=@=A@CCC@CCBBAAB8ABABAABC=@?@CCCC@B@=AAC==>?BB;@??@AAA@AB??88
/;-//46,--340
@T6XXI:00147:00174
GGGAGAAGGGAGAAAGGAAGCTAGTCACTCGACACCTGTGGCTTCACAGTACAGAGGGACTGTCCAAAGCCGCAGAT
CTCGAGTCGACTAG
+
DKCCCB?CC>DECB?C=?<?@<@>@9::??BBCB@?A8878:;7<FBBBCBA?????9;===<=:=A<@@>A@788;
;>?888A??==888
@T6XXI:00147:00185
GGGGAATTCTAATACGACTCACTATAGGGAGAGGGAGATAGCCTGTTTACCTTTAACTCGTTATTCTTCTTCCTTTC
TCCCTTCTCCC
+
CCB1>,/,//6,/499AAAAA@??>877,66;668<;?@B?<9?@>>:77011,1016663566566;8>9>244,4
00446485553
@T6XXI:00147:00186
GGGAGAAGGGAGAAAGGAAGGAGAAGTCGCCCCAAGAAC
+
ECBBEC=@?>ACCC@B<=>A@CAA>::;8282*,*,,*,
@T6XXI:00147:00196
GGGAGAAGGGAGAAAGGAAGCTAGTCGACTCGACATCTGTGGCTTCACAGTACAGAGGGACTGTCCAAAGCCGCAGA
TCTCGA
+
```

DD?CCC@EA:>9996;?A<BBC@@@???A??<::;;::>@A;AC>ABBB@B?977000,7>;>@@>@@;@A>@@<AA
==???A
@T6XXI:00148:00166
GGGGAATTCTAATACGACTCACTATAGGAGAGGGAGAATAG
+
??@4?<@@BBC@DBBBBBCBC==;000,.3:@7;@882/:7
@T6XXI:00148:00169
GGGAGAAGGGAGAAAGGAAGCTAGTCGACTCGACATCTGTGGCTTCACAGTACAGAGGGACTGTCCAAAGCCGCAGA
TCTCGA
+
CEEBEB@CC>BBBC>B@C=@@B>AA:::@@?=?:;:::ABC>BC@CC@@@@?>;733317=9A=@<??<?A?AA<<=
=588AA
@T6XXI:00148:00181
CTAGTCGACTCGAGATCTGCGGCTTTGGACAGTCCCTCTGTACTGTGAAGC
+
BBBBBBBBBBDCCBBBCDBBBB?BAC>C8;8?@8880:BBDGCCC?AAA??B
@T6XXI:00148:00184
GGGAGAAGGGAGAAAGGAAGCTAGTCGACTCGACATCTG
+
G?:@@B>A>9>984-95959@>::</00;;<<0;::77B
@T6XXI:00148:00192
GGGGAATTCTAATACGACTCACTATAGGGAGAGGGAGAATAGTCAACAAATCGTTTGGGGCGACTTCTCCTTCCTTT
CTCCCTTCTCCC
+
CCC:C?A?ABC@CEAABBBBBBA?9366.777>?>:::?BBBAAA>@@A;BAAAA8ABB7@?AAA7::B?BA@>AC<
@@BD=A899@@;
@T6XXI:00148:00193
GGGAGAAGGGAGAAAGGAAGAGAATATCGCGACCAGATAGGAAGTGACTATTCTCCCTCTCCCTATAGTGAGTCGTA
TTACGAATTCCCCC
+
CC?B@?=?B>CCCC>C@B@BBAA?AAA>>>BCE@CB?@@@@D@BBBBACCC@CBBB=BBCCC>CB@@???A??A=;;
;;0//9,/,7444.
@T6XXI:00148:00199
GGGAGAAGGGAGAAAGGAAGCTAGTCACTCGAGATCTGCGGCTTTGGACAGTCCCTCTGTACTGTGAAGCCACAGAT
GTCGAGTCGACTAGCTTCCTTTCTCCCTTCTCCC
+
CDBBBB>AA:>>>>9>=@=@BAABC::::AA@??@BAACC@CCB>B@@C8::CC>BG?==?ACAAAC?BB><<=@A@
@A@?@======@AAAB?A;<<;>=::13/34::4
@T6XXI:00149:00164
GGGAGAAGGGAGAAAGGAAGCTAGTCACTCGACATCTGTGGCTTCACAGTACAGAGGGACTGTCCAAAGCCGCAGAT
CTCGAGTCGACTAG
+
DKB<<;?BB<BBB@<A:;6;C@@@D<>;;;:00000/>>D@AB?BC?@??A@?=<8885558@@>AA<AB>??:?A<
=??A<<<888@=<=
@T6XXI:00149:00184
GGGGAATTCTAATACGACTCACTATAGGGAGGG
+
AAA9B?CBCBB@BBBBBBBBBBBA977>./,,+
@T6XXI:00149:00186
GGGGAATTCTAATACGACTCACTATAGGGAGAGGGAGAATAGTCAACAAATCGTTTGGGGCGACTTCTCCTTCCTTT
CTCCCTTCTCCC
+
GBB9B>BAEDB@BBCBBBABAA>?;97:5:BBCC>???@B>>;BA=??A<CC@@>;>??7C@@@E@AAB@C?@<AA<
A???7>378?@:
@T6XXI:00149:00189
CTATCGACTCGAGATCTGCGGCTTTGGACAGTCCCTCTGTACTGTGAAGCCACAGATGTCGAGTCGACTAGCTTCCT
TTC

```
+
99944444:??=>AA;999>488?:799<7776767>A??8???677:6=<B=9980....4338==9997998530
2*,
@T6XXI:00149:00190
GGGAGAAGGGAGAAAGGAAGGAGAAGTCGCCCCAAACGATTTGTTGACTATTCTCCCTCTCCCTATAGTGAGTCGTA
TTAGAATTCCCC
+
CF@CCC@CC@CCDB>C?A?A@CAA@A;:4333,8?<BCDC?;@@>@::<CB?@???<?@@BC<@:?:<<<<AAA@?@
@>A@@<;/444-
@T6XXI:00149:00195
GGGATCATACGACTCACTATAGGGAGAGGGAGAATAGTCAACTCCCTTTCAAATCGATCGTATTCTTCCTTTCTCCC
TTCTCCC
+
B@9>1/////8;ABBBB@?9366.7<@AC>CCC=@=AA@@>@???;?A<?@@:???@@>>@>?;=>;=;>@:;444,
4348991
@T6XXI:00149:00198
GGGAGAAGGGAGAAAGGAAGCTAGTCACTCGACATCTGTGGCTTCACAGTACAGAGGGACTGTCCAAAGCCGCAGAT
CTCGAGTCGA
+
CBB@@@=>>:>99919;>=@@CC?=:/0/.....4499::8:C>@@?:::AA@@A@@<>874<<;?=;==<9==77/
8/////6844
@T6XXI:00100:00226
GGGAGAAGGGAGAAAGGAAGCTAGTCGACTCGACATCTGTGGCTTCACAGTACAGAGGGACTGTCCAAAGCCGCAGA
TCTCGAGTCGACTAG
+
CC@CCB894-8988-
9695;B>::;/00;:9:;?????CBC@BB@BB@@>>B?9933317=9;<=>@@;?C>@@AAA<:???@@A@AA???A
@T6XXI:00100:00244
GGGAGAAGGGAGAAAAGGAAGCTAGTCACTCGAGATCTGCGGCTTTGGACAGTCCCTCTGTACTGTGAAGCCACAGA
TGTCGAGTCGACTAGCTTCCTTTCTCCCTCTCC
+
DC?B=<89@:A9999,49:97A7378:669<ABB@B???BB?AAE>A?ACBBBCC>AA===<<<?@@B>=<=@?===
@AAAA===@988??=;;76,//,/,,,+,+++*
@T6XXI:00101:00207
GGGAGAAGGGAGAAAGGAAGCTAGTCACTCGACATCTGTGGCTTCACAGTACAGAGGGACTGTCCAAAGCCGCAGAT
CTCGAGTCGACTAG
+
EDAB9924809999142947C@?@D?A@AA?:::::BBB?BB@CC??>;?@C=889988<AAB<??:@C>???@;<
<<==<<<<<<@A<<
@T6XXI:00101:00208
GGGGAATTCTAATACGACTCACTATAGGAGAGGGAGAATAGCCATGAGCTACTCCATACTCCATCCCACTTCCTTTC
TCCCTCTCCC
+
>99299A@BBBAEDCBCCCC?:<>>7737==>@=CB@>@@==;=@@AA@???>>;==>???>@@??:?8838<;968
677,///,+*
@T6XXI:00101:00227
GGGAGAAGGGAGAAAAGGAAGCTAGTCACTCGAGATCTGCGGCTTTGGACAGTCCCTCTGTACTGTGAAGCCACAGA
TGTCGAGTCGACTAGCTTCCTTTCTCCCTTCTCCC
+
BB>;<<>A@:>9999,4999;CD@@?CCBB????=????A@<>@@9?;?@?:::C>BB@@@@@A998B@7748<<<@
A@@A@<<<:8//-7/448<:>@;@===-4/4444-
@T6XXI:00101:00233
CTATCACTCGAGATCTGCGGCTTTGGACAGTCCCTCTGTACTGTGAAGCCACAGATGTCGAGTCGACTTAGCTTCCT
TTCTCCCTTCTCCC
+
CA@?BBCBEBBBBBBCCBB?BBC>C?BBCCCCC>C@>>>@AC@@@A>@C?BCCCB@@@BBBB?;;:10,0;<47>;B
E>BB?A<@;???=:
```

```
@T6XXI:00101:00236
GGGGAATTCTAATACGACTCACTATAGGGAGAGGGAGAATAG
+
CCD:D@E?BBB@BBBCCAAAD>9<<777.7>777.7;84=??
@T6XXI:00101:00247
GGGAGAAGGGAGAAAGGAAGTGCAGTTCGCGGTTAGTGAAGGGAAAAACTATTCTCCCTCTCCCTATAGTGAGTCGT
ATTAGAATTCCCC
+
CCCBBB@CC<@AB@=B?B?ACCBAAA@B?@@@B@BCCCC?=@-
6111+8?@??CFCC>???BC>?===AA@AA@888==:=>?<;:@@@5
@T6XXI:00102:00214
CTAGTCGACTCGAGATCTGCGGCTTTGGACAGTCCCTCTGTACTGTGAAGCCACAGATGTCACTAGCTTCCTTTCTC
CCTTCTCCC
+
99944999==?CBBBB@@@;<:;ADAD@@?;36862;?@766:77/.2,-
/,/688;>7337611...,.555+//54244..11,
@T6XXI:00102:00227
GGGGAATTCTAATACGACTCACTATAGGGAGAGGGAGAATAGTCAACAAATCGTTTGGGGCGACTTCTCCTTCCTTT
CTCCCTTCTCCC
+
499/48>;@CB?BBCBBBCCBA?97111.7==?A>BCA?@@AAA9278:4==??C>CDC9C=@?C?BBB?@=??AC?
A@@@<A;><<<6
@T6XXI:00102:00230
GGGGAATTCTAATACGACTCACTATAGGGAGAGGGAGAATAGTCCCGACATCTTCAACGTGCTATTCTACTTCCTTT
CTCCCTTCTCCC
+
@::2@;>@@===@BBABBABB@>;966659==AC>AA?>A====CA@A==@BB@CC?BABBBBCC?BBCCDBECBC>
A@@@5637<<<6
@T6XXI:00102:00233
GGGGAATTCTAATACGACTCACTATAGGGAGAGGGAGAATAGCTTTACACTCATTAG
+
CCB4<8<@ABB@CBBBB@@@BACB<66959??AC>B?=8:78/00,.....--1010
@T6XXI:00102:00235
GGGGAATTCTAATACGACTCACTATAGGGAGAGGGAGAATAGTTTTTCCCTTCACTGACCGCGAACTGCACTTCCTT
TCTCCCTTCTCCC
+
444,48989=@=@@CBB@LCB?>>=777.7=777-
76>89==A===.:610/0....,00,,.20116588=9=9=@>==@E>C?=?CCA
@T6XXI:00103:00208
GGGGAATTCTAATACGACTCACTATAGGGAGAGGGAGAATAGCCCTATGACCTTCGCCGCGTTCCTATTCTTCCTTT
CTCCCTTCCTCCC
+
CCC:EAB@CCC@;9;>>88;;7??>777-
7>>@C?AA@9<;<<;:?76./.,.,.42036:=555533204/3/34,3649:=3838AA:
@T6XXI:00103:00209
GGGAGAAGGGAGAAAGGAAGTGCAGTTCGCGGTTAGTGAAGGGAAAAACTATTCTCCCTCTCCCTATAGTGAGTCGT
ATTAGAACTTCCCC
+
CHAEEC=@95=9887;>A@CCCCCCB@???C@C@CAABC?>B-
6:::0;CCCBCBBB>;:;BE??=<@@@@A@@>;744349;8<>CDCC4
@T6XXI:00103:00215
GGGGAATTCTAATACGACTCACTATAGGGAGAGGGAGAATAGTCAACAAATCGTTTGGGGCGACTTCTCCTTCCTTT
CTCCCTTCTCCC
+
:;;4<?B@CCB@A??BBBB?BAB?9161.7?>@B>??=>@@A?AA?===7???==46694:BCCC@CCB?@?@=@A<
@@??:@<??AA=
@T6XXI:00103:00225
```

```
GGGGAATTCTAATACGACTCACTATAGGAGAGGGAGAATAGTGACGTGTTGTGCGGCGCTCACTGTCTCCTTCCTTT
CTCCCTTCTC
+
BBA4@<@=@A@?CBBCECCCB;;;://,000?C>CBB=A??@<::@AAC>;????<?@AA===@@@????C>A>@?:
788?7<:=DC
@T6XXI:00103:00230
GGGGAATTCTAATACGACTCACTATAGGGAGAGGGAGAATAGTCAACAAATCGTTTGGGGCGACTTCTCCTTCCTTT
CTCCCTTCTCCC
+
===6@>A@BCC@CCBBA?BCC@??7111.7?>>A=ACA?C?@@@@>???<BBBBC;???7?ABCB?BDD??<?>A=;
@@@A>C>@9881
@T6XXI:00103:00234
GGGGAATTCTAATACGACTCACTATAGGAGAGGGAGAATAGTTTTCCCTTCACTAACCGCGAACTGCACTTCCTTTC
TCCCTTCTCCC
+
>>A6A>A@CBB@BAAABBB?B:9:7//-
0==@B>BDA>@???DD5@@<@;=@BBE???@CB<:88==???>B:??6====9?>??AC=
@T6XXI:00103:00242
GGGAGAAGGGAGAAAGGAAGGAGAAGTCGCCCCAAACGATTTGTTGACTATTCTCCCTCTCCCTATAGTGAGTCGTA
TTAGAATTCCCC
+
BC@DCC>=95>98809599;@CCC@JC?3333,88/897=?<A?:????<A?@@@C<===AA<@@@@@A?;<=AA??
?<?=824/444-
@T6XXI:00104:00204
GGGAGAAGGGAGAAAGGAAGCTAGTCACTCGACATCTGTGGCTTCACAGTACAGAGGGACT
+
DD@:<9>=@:A><<6<-1,1>@>@C<:::?=7:::::BBBABB?BD@BBBCB74000-4::
@T6XXI:00104:00226
CTATCACTCGAGATCTGCGGCTTTGGACAGTCCCTCTGTACTGTGAAGCCACAGATGTCGAGTCGAC
+
C?@=9::;BEBBBCBCBAA?BBE@C@BB??:77.7==?@BBDB@@@>@DCBBDDCBBBA70//-,**
@T6XXI:00104:00228
GGGAGAAGGGAGAAAGGAAGGAGAAGTCGCCCCAAACGATTTGTTGACTATTCTCCTCTCCCTATAGTGAGTCATTA
TGATACG
+
CC>A@@=@@:@>>>9>595;>BAB?BBBBCCC9DD?BABCB=CC?AADDCC3844348030+040305330004/5/
//,,2,,
@T6XXI:00104:00231
GGGAGAAGGGAGAAAGGAAGAGAACGGCGACGAACAATAGTTGGTGGCTATTCTCCCTCTCCCTATAGTGAGTCGTA
TTAGAACTTCCCC
+
CE?BCC@DD>BDAB>B>B?C@@@GBB>BBBBBB@CC@BBBB@C@@@=@BAA?C???9?C?<<<A<?@A@AAAAAAA8
848?=7::3444-
@T6XXI:00105:00203
GGGGAATTCTAATACGACTCACTATAGGGAGAGGGAGAATAGATCACCGTCGGTTCGGGTGCGCGTTCTCCTTCCTT
TCTCCCTTCTCCC
+
BAA6BAC@DCC@CCCBBABABABB@77756?9?@>BA???:;===BA?BBA?;?=?;;8<778;<<<AAB?@=?=@H
@C===9=>@@AA<
@T6XXI:00105:00210
GGGAGAAGGGAGAAAGGAAGAGAACAGTTGCCCTAGTAGTTAGTAACTATTCTCCCTCTCCCTATAGTGAGTCGTAT
TAGAATTCCCC
+
CC>BCC@BD>BBBB>B@B@BBBB@BBBB@CCC>CBCBBB?@@AAB?BBCC@CBBB;BBCCC>BB??<<>CBBAAAA?
=@AA>@?@DC6
@T6XXI:00105:00213
```

```
GGGGAATTCTAATACGACTCACTATAGGAGAGGGAGAATAGTCAACAAATCGTTTGGGGCGACTTCTCCTTCCTTTC
TCCCTTCTCC
+
A??6?;@=ABBIJBBBCBCBC>;:9//,00077-:A@=@@@@AA>AAA<BCBBC<BBB477A774:7748::8::-
40384322--,
@T6XXI:00105:00222
GGGAGAAGGGAGAAAGGAAGCTAGTCACTCGAGATCTGCGGCTTTGGACAGTCCCTCTGTACTGTGAAGCCACAGAT
GTCGAGTCGACTAGCTTCCTTTCTCCCTTCTCCC
+
885711,/656,//+/,/,.647;@@@?ACA?:8:@AABB?BCC>B?BCCCBBCBBC@@===A@@@C@AA9<=@CCA
???B=<<A988?B===>B>AA<@==:-4344;;6
@T6XXI:00105:00234
GGGGAATTCTAATACGACTCACTATAGGGAGAGGGAGAATAGTCAACAAATCGTTTGGGGCGACTTCTCCTTCCTTT
CTCCCTTCTCCC
+
DBB6B8;8;@@=@A@@CCBBB@<97111.77777-
7<856??@AB@?AA<AABCD>CBC8CCCBB?BB@:978/33,3;;>;@9<<??:
@T6XXI:00105:00241
GGGGAATTCTAATACGACTCACTATAGGGAGAGGAGAAT
+
/11+17BADBB@@BBBB???BBCBC::7.0:00,.7003
@T6XXI:00105:00242
GGGGAATTCTAATACGACTCACTATAGGGAGAGGGAGAATAGCCCTTGATCCTTTAACACGTTATTGCTCTTCCTTT
CTCCCTTCTCCC
+
@>>4><A>BCCAC?AABBCBB?991177.777BE>CBA=@?@@=;?>>@==???:B?A@@@A>??<===AA>A:<<6
;===:?=?@BA:
@T6XXI:00105:00245
GGGGAATTCTAATACGACTCACTATAGGGAGAGGGAGAATAGTTTTTCCCTTCACTAACCGCGAACTGCACTTCCTT
TCTCCCTTCTCCC
+
@@@4?;><>>@=@BBBBBCCCCA9=666.7=777-
79456=;;:::.:=7=<?BAAA?B?ABAA>ABA8::>;>477.7>>B<B788??:
@T6XXI:00106:00212
GGGAGAAGGGAGAAAGGAAGGAGAAGTCGCCCCAAACGATTTGTTGACTATTCTCCCTCTCCCTATAGTGAGTCGTA
TTAGAATTCCCC
+
CCABBB@BB?C?==:@8;8;BDDC@CCBAABB7BB>BDCDC>AB?BBCB@=;====;::?<=;=86<<@@@@A@A58
8>B?=8:2444,
@T6XXI:00106:00213
GGGAGAAGGGAGAAAGGAAGCTAGTCGACTCGACATCTGTGGCTTCACAGTACAGAGGACTGTCCAAAGCCGCAGAT
CTCGAGTCGACTAG
+
CCACB@844-
998809;?<>A888?7:777>>;7:771=7707847=@@@BA?<<4434888BA;;=744/78;;A888A888@>88
8>87
@T6XXI:00106:00218
GGGGAATTCTAATACGACTCACTATAGGGAGAGGGAGAATAGTCAACAAATCGTTTGGGGCGACTTCTCCTTCCTTT
CTCCCTTCTCCC
+
449/49BBDBB?CBBBBB??BAB?;111.777>A=ACA>A@@@AA>AAA<CB?=?-666-
7>BCCBBBB==:<>AC;@@AA<A698AC@
@T6XXI:00106:00239
GGGAGAAGGGAGAAAGGAAGTGCAGTTCGCGGTTAGTGAAGGGAAAACTATTCTCCCTCTCCCTATAGTGAGTCGTA
TTAGAACTTCCCC
+
```

```
EC?AA@>BB=BDHD?C?@=@AABBBC@BBBC@C@BBBBB>AA=ACC9BBBB@BB?A>?=?AA9?<<<==??==8487
;8;=B588=@@@4
@T6XXI:00106:00243
GGGAGAAGGGAGAAAGGAAGTGCAGTTCGCGGTTAGTGAAGGGAAAAACTATTCTCCCTCTCCCTATAGTGAGTCGT
ATTAGAACTTCCC
+
CC?CBA;>><;;;C=B>B?@@@CCCBCAAAA?B?<<<DCA??.7777,8>>>=ACCC>@@@BC>C?@>?@844;404
0628342384884
@T6XXI:00106:00247
GGGGAATTCTAATACGACTCACTATAGGGAGAGGAGAATAGTAGACTACTTCCAT
+
ABA9B@C@DBBBC@@>CCCCCBBAB??;67://,/97798?8834---22,-,03
@T6XXI:00107:00207
GGGGAATTCTAATACGACTCACTATAGGGAGAGGGAGAATAGCTTGATGTCCTGAGGACCTTGCTTCTCCTTCCTTT
CTCCCTTCTCCC
+
CDD:DEC@:<<ADBBCD@@@A?<<>777.777>A<AA@<==>8:5:??@AA>A@@A>@@=@?ADC=?78387:9;?1
87<A7<;=@@C9
@T6XXI:00107:00224
GGGGAATTCTAATACGACTCACTAGGGAGAGGGAGAATAGTCAACAAATCGTTTGGGGCGACTTCTCCTTCCTTTCT
CCCTTCTCCC
+
@@A6@@A?BBB@BBBBBBBBBAB>?><8999<;=??@C@<<@@?@AA;<::;<;?AB5@???A<?==;<=@=?@;?=
=?7?<?@AA<
@T6XXI:00107:00225
GACAGTCCCTCTGTACTGTGAAGCCACAGAGGGACTGTCCAAAGCCGCAGATCTCCGAGTCGACTAG
+
ABCCCCCC>CCCCCAABCCCB?BC@CCCCCBB;?@AADC@CC>@@=@BBBCCBCBAB<<<DBCC@??
@T6XXI:00107:00238
GGGAGAAGGGAGAAAGGAAGGAGACACGGTCACAAACATGAATGTGACTATTCTCCCTCTCCCTATAGTGAGTCGTA
TTAGAACTTCCCC
+
BB>@@;28819>>>9>596848;CCDCB@CCCBAE=BBB@@=@AAACBDC@><?;;6;=?@B<BAA@@<A=?=????
@;889,//78934
@T6XXI:00107:00240
AGTCGACTCGAGATCTGCGGCTTTGGACAGTCCCTCTGTACTGTGAAGCCACAGATGGACTGCTTCCTTTCTCCCTT
CTCCC
+
CCCCBBBBBBB??@CBBBB@BBB>B>@@BBBBBABBBBBBBC@@BC@A?>BDCBB?A<??C@@@=@>BB;@AAA<AA
@BCD?
@T6XXI:00108:00201
GGGGAATTCTAATACGACTCACTATAGGGAGAGGGAGAATAGCCAGTTCCTGCAAGCCAATCGGTTATCCTTCCTTT
CTCCCTTCTCCC
+
:99/9<@?BCC@CB@>>???>?BB@77746??BC>BCA??::<;<<<<=<=??@>AD;=9<@@>B;=<>:;8:8;;6
;@@A;@=@@AA<
@T6XXI:00108:00203
GGGAGAAGGGAGAAAGGAAGAT
+
DE@CA@=@A9>9--+-+-+--,
@T6XXI:00108:00207
GGGAGAAGGGAGAAAGGAAGCTAGTCACTCGACATCTGTGGCTTCACAGTACAGAGGGACTGTCCAAAGCCGCAGAT
CTCGAGTCGACTAG
+
EH@B@A79809>>>9?695;C?>?B9:77?=77:???@AA?BB@CBCC??@@9833317=9=?@>@@<@A>@A@A=<
===AA@@@@AB@@@
@T6XXI:00108:00211
```

```
GGGGAATTCTAATACGACTCACTATAGGAGAGGGAGAATAGATCACGACCCTTCTACCACGATTGAGCTTCCTTTCT
CCCTTCTCCC
+
BBC:DCCACCB@BBBB?>ABB:997/.,0?077.7=:48<<<A@BCCDCC>B=::0/,/8;:?@@C@CC@CACC>BB
DDCC=A@CC>
@T6XXI:00108:00212
GGGGGAATTCTAATACGACTCACTATAGGGAGAGGGAGAATAGTTTTTCCCTTCACTAACCGCGAACTGCACTTCCT
TTCTCCCTTCTCCC
+
DCDC3B===@@@@BCBCBBBCCC??=777.7>>>@>ABB?B;44444,042=<?BBBD?B>@@CD>@@A8::7=?:<
?8?ABC>B;>AAA:
@T6XXI:00108:00215
GGGGAATTCTAATACGACTCACTATAGGGAGAGGGAGAATAGTCAACAAATCGTTTGGGGCGACTTCTCCTTCCTTT
CTCCCTTCTCCC
+
CCEAC@C@?AA@D@@@CA@A?CB9>111.777>@@DB@>AA@@A@><??<ACCCC?BBB8CCCCB@=??:<96;@@<
@@@CAC=?@@A<
@T6XXI:00108:00222
CTAGTCACTCGAGATCTGCGGCTTTGGACAGTCCCTCTGTACTGTGAAGCCACAGATGTCGAGTCGACTAGCTTCCT
TTCTCCCTTCTCCC
+
B8///6/44AA@CBDBCBBB@BBB<B<<@CCBBA<BC@?;;>>?@AA>?A?DCCCCBBBCCCBB@ABBBFC???@@C
E>BDBE;<8<<;94
@T6XXI:00108:00223
GGGAGAAGGGAGAAAGGAAGGGCACGTGCAAGTCGGGAGAGATTAGCTATTCTCCCTCTCCCTATAGTGAGTCGTAT
TAGAATTCCCC
+
CC@BBB@DD?ABBB=?>A>@@>BBBC?@:;;<CGED@ICCBBC@CCBBBC@CCCBA;;<CH?CBABCCCCCBCBBBC
@ABA@C=BA@8
@T6XXI:00108:00224
GGGGAATTCTAATACGACTCACTATAGGGAGAGGGAGAATAGACTCCTTTTATAACACGACGTACGCTCCTTCCTTT
CTCCCTTCTCCC
+
BCB9C?B@BCB@BBBBBBBBBCB<9661-
6=<;?=@C@?B=@<<@<;<==5@DD?BBCBBBC@@=AA@@?CBD=??7=<?@;@<??@@<
@T6XXI:00108:00228
GGGAGAAGGGAGAAAGGAAGCTAGTCACTCGACCATCTGTGGCTTCACAGTACAGAGGGACTGTCCAAAGCCGCAGA
TCTCGAGTCGACTAG
+
CB=BBCBDC@DCB>:=6927@<8877000;0//77::::CC=?@=@BBCCBBA@9337-49;99;344-
4427=?>A???AA==<??><<88
@T6XXI:00108:00230
GGGGAATTCTAATACGACTCACTATAGGAGAGGGAGAATAGTCAACAAATCGTTTGGGGCGACTTCTCCTTCCTTTC
TCCCTTCTCCC
+
CBB9E?C@DBDBC>?>BBBBB:665..,0::BE@CB@=A=AA<;8774,7?>>>.777.7==?@>@CE@?<?>CB=@
@AA<A>@@@@<
@T6XXI:00108:00236
GGGGAATTCTAATACGACTCACTATAGGGAGAGGGAGAATAGTCAACAAATCGTTTGGGGCGACTTCTCCTTCCTTT
CTCCCTTCCTCCC
+
;99/9<A?CDB@BBBBB@@@B@<97111.777?C?DCA>A@@@AA>===9@BBBC>AAA7ACCCC@CCB??:=:<64
>;;737,/,/882
@T6XXI:00108:00239
GGGAGAAGGGAGAAAGGAAGCTAGTCACTCGACATCTGTGGCTTCACAGTACAGAGGACTGTCCAAAGCCGCAGATC
T
+
```

```
DC>B@>58809111,/,/,.6.33;@?<;;<776?88@AA@CC@CCCCCC@CB@4424>@????BA>CC>@<==AA@
@
@T6XXI:00108:00242
GGGAGAAGGGAGAAAGGAAGGAGAAGTCGCCCCAAACGATTTGTTGACTATTCTCCCTCTCCCTATAGTGAGTCGTA
TTAGAATTC
+
CC8;;A=>?<<99B<@=@@C?AAA@BAA@?B?.880397;?;<:;??<<<=<>;@@9@0/.99554;655:3534..
.442444/-
@T6XXI:00109:00208
GGGAGAAGGGAGAAAGGAAAGCTAGTCGACTCGACATCTGTGGCTTCACAGTACAGAGGGACTGTCCAAAGCCGCAG
ATCTCGAGTCGACTAG
+
44-49;2491499914244-33333766/...3.,,,,,,,,4,..9;;;777>94:443+343033234442-----
--0056456<;;5033
@T6XXI:00109:00219
GGGGAATTCTAATACGACTCACTATAGGGAGAGGGAGAATAGGGCCGACGTCGCATGCGTCCCGGCCGCCATGGCCG
GCCGCGGGAATTCGATTGGGAATCTATA
+
BBB9CCDBCBC@@A?BBBB>??BB?AA@>777??:@;756889,/,/6/..:A;:>600000+024266866900/0
/0/034436:;;?977,./,-,----00
@T6XXI:00109:00244
CTATCGACTCGAGATCTGCGGCTTTGGACAGTCCCTCTGTACTGTGAAGCCACAGATGTCGAGTCGACTAGCTTCCT
TTCTCCCTTCTCCC
+
B=:6///668=?@AACCCCC@CEB@B=>@BA???=?CCCBBBC@B@A>@BACBBBCAAACDCB@AABCDDCBB?B?A
A?A@>B68349:;7
@T6XXI:00110:00240
GGGAGAAGGGAGAAAGGAAGTGCAGTTCGCGGTTAGTGAAGGGAAAAACTATTCTCCCTCTCCCTATAGTGAGTCGT
ATTAGAATTCCCC
+
A@<ABB@CCABBBC@C@C@CBBBCCC@BBBC=@=@@@CC@CC>CCCC2AACC@DBBB>@CBCC>A??===A@@@;<<
8858ACEB=AAA5
@T6XXI:00111:00202
GGGAGAAGGGAGAAAGGAAGCTAGTCACTCGACATCTGTGGCTTCACAGTACAGAGGGACTGTCCAAAGCCGCAGAT
CTCGAGTCGACTAG
+
CDACCB=@95=>>>9>898;C?>?@6777;=7768<:ABLA?@@CC@;;;AA>6333,089>AA>@@<@A?A>88;;
===<<<<=??>@AA
@T6XXI:00111:00204
GGGAGAAGGGAGAAAGGAAGGGGAGAAGGGAGAAAGGAAGCTAGTCACTCGAACAGTCTGAG
+
BCBCDB?BCABBAB>C@C@EBB8888?>?9?0//,0,0,0:/./CA;=?<64/0---33---
@T6XXI:00111:00208
GGGGAATTCTAATACGACTCACTATAGGGAGAGGGAGAATAGATCACCGTTGGTTCGGGTGCGCGTTCTCCTTCCTT
TCTCCCTTCTCC
+
BCA7B>@=@998<CCCCCBBB@<96166.7>>>@=BB@>@@@@??A?:;;495:ACCE>B;<<DCF?AAB<?<?ACC
>BA??:?>===D
@T6XXI:00111:00210
TACGACTCACTATAGGGAGAGGGAGAATAGCTCATCTTTCCCCACTAGACTCATTTCTTCCTTTCTCCCTTCTCCC
+
ACBCGCCEABA@A>@@<ABBCC?CCC@D@AA@B@A99909@A4>>>B@?@ACCCC>BB=?<?>8>>>>7><>777.
@T6XXI:00111:00223
GGGGAATTCTAATACGACTCACTATAGGGAGAGGGAGAATAGTTTTTTCCCTTCACTAACCGCGAACTGCACTTCCT
TTCTCCCTTCTCCC
+
```

```
9>?4@>@@B@=<@:<;BBBAAAA@77:=6==>>@<A?==@<<<@@@@-
>A13/0616688567CB899A@;77:=477.7@CB>CD888<6
@T6XXI:00112:00206
GGGGAATTCTAATACGACTCACTATAGGAGAGGGAGAATAGTCAACAAATCGTTTGGGGCGACTTC
+
CCB9A>A>@@@@DCBABBBBB7665..,0<<AC>DCB?@:=AA;2444-7>777.777.611160/
@T6XXI:00112:00207
GGGGAATTCTAATACGACTCACTATAGGGAGAGGGAGAATAGCCAACTTCTTGGCCGCGCTCGACTGTTCTTCCTTT
CTCCCTTCTCC
+
DCC6A;=;<AC@CCCAAABBA???=777.7=>?@<BB@>@AAA?A>AA=@@>A<?=?A@@@@A@@???=>>;=8;;6
;666,/,///,
@T6XXI:00112:00210
GGGAGAAGGGAGAAAGGAAGGAGAAGTCGCCCCAAACGATTTGTTGACTACTTCTCCCTCTCCCTATAGTGAGTCT
+
CC>A?@59909<<<8;-1-.6;<=.16;8899,73,397>@<AF>?<<0/..311245:<<@@@===AAAB??=;4
@T6XXI:00112:00215
GGGAGAAGGGAGAAAGGAAGCTAGTCACTCGACATCTGTGGCTTCACAGTACAGAGGGACTGTCCAAAGCCGCAGAT
CTCGAGTCGACTAG
+
DDCCDB?@=6@@885>594:BB>???::9?@::7<>ABBB@AA?BBA@@?@C@:655137=?AA?AB=AC?@???;=
==A?:?=8==AA==
@T6XXI:00112:00217
GGGAGAAGGGAGAAAGGAAGGAGAACAACACGATCGGTTAAACGTAGCTATTCTCCCTCTCCCTATAGTGAGTCGCT
TATT
+
BC@CCB@BCBBAA@:8;8;?=?CECCC@BBACCCAA>B?BC>C;;;BAA@B7;;A@:AA@AA=AA??ACC;55,,**
)*.)
@T6XXI:00112:00230
GGGAGAAGGGAGAAAGGAAGCTAGTCGACTCGACATCTGTGGTCACAGTACAGAGGGACTGTCCAAAGCCGCAGATC
TCGAGTCGACTAG
+
CD>@@A;>>:>99919292333620,///,22856/77B@@=A:5<<8?=@@@??@:?==884798433/007366?
=?874<6:8:565
@T6XXI:00112:00237
GGGAGAAGGGAGAAAGGAAGGACAGGACCGCGGCAAGTGTTAGGTAACTATTCTCCCTCTCCCTATAGTGAGTCGTA
TTAGAA
+
CEACCC@DC@BBBB?B?B@C@CCA>=:858??=AD@CDCC@CCACCBBBBB?B@@@=;:;AC>@?CCCC@BBCA<88
8:>>A5
@T6XXI:00113:00206
GGGGAATTCTAATACGACTCACTATAGGGAGAGGGAGAATAGCTCTTCCCCAAACTGCCCTGTTCTTGTCTTCCTTT
CTCCCTTCTCCC
+
@AA8AAC@CDB?ABBBCBBAC=9?;677.7==BD@D?=;=<?@<AA>AAA7BE@CCACC>>743497:88799:?@9
==CC<@<@@AB;
@T6XXI:00113:00210
GGGAGAAGGGAGAAAGGAAGCTAGTCACTCGACATCTGTGCATCAC
+
EEABBA8998;CCCAC;98;C@;AA:???BB??@AA9?0,,,,++.
@T6XXI:00113:00231
GGGGAATTCTAATACGACTCACTATAGGGAGAGGGAGAATAGTTTTCCCTTCACTAACCGCGAACTGCACTTCCTTT
CTCCCTTCTCCC
+
499/9?BACCC@BBBBB?B>BA<97111.7>>BC>::8?ABAACC5==7<9<@CCC@C@??AA????@CBB?B@BA9
????9?@?@@@<
@T6XXI:00113:00232
```

```
GGGGAATTCTAATACGACTCACTATAGGAGAGGGAGAATAGCTACGTTTAACCGATCGTGTTGTTCTCCTTCCTTTC
TCCCTTCTCCC
+
BA@2@5<7;=B?BBBCB@@@@;:;<0/,0;:=<9?B@?A@==?A???>9>3730?@=???A>??4788565655:44
488+444033+
@T6XXI:00113:00247
GGGAGAAGGGAGAAAGGAAGCTAGTCGACTCGACATCTGTGGCTTCACAGTACAGAGGGACTGTCCAAAGCCGCAGA
TCTCGAGTCGACTAGATCCT
+
FDBCBA?@A<A@=85>8948B>9:;/00<<<00:6666@CC=?@@CCCC@@@944033-
4;;;=?<@>:>?<??ABAAA==<<;;78884;95,,**
@T6XXI:00114:00201
GGGAGAAGGGAGAAAGGAAGGAGAACGCGCAATCAGATACCTGTTGGCTATTCTCCCTCTCCCTATAGTGAGTCGTA
TTAGAATTCCCC
+
CC@DCB@BB>CBBB>B?B?BADAB?BBBBBC@CCCABEAA<?==5989@@B@C@A@<@BBCC?BBCBBBCCBBBB??
??@@@?EDDBB8
@T6XXI:00114:00211
GGGAGAAGGGAGAAAGGAAGCTAGTCGACTCGACATCTGTGGCTTCACAGTACAGAGGACTGTCCAAAGCCGCAGAT
CTCGAGT
+
CCADEC?=969844-9<>5;;D><<CCC@<??:9::::BBJ?BC?BBBB@@A754..-
/;::;C>@@:@B@@??C?;:::<=A>
@T6XXI:00114:00227
GGGGAATTCTAATACGACTCACTATTCTCTCTCT
+
CCC8A=><@9959?>>A@BB>@@87+,,,,**,+
@T6XXI:00114:00237
GGGAGAAGGGAGAAAGGAAGCTAGTCGAACTCGACATCTGTGGCTTCACAGTACAGAGGGACTGTCCAAAGCCGCAG
ATCTCGAGTCGACTAG
+
BB@B>>@BBA??@C?B?B@BAB@@?CCB?CCCC:8:@???@A=:;9:AABCBBA;9300-
4;;AAA?AA<@@>@=9=AAAAA???A:?;===?
@T6XXI:00114:00239
GGGGAATTCTAATACGACTCACTATAGGGAGAGGGAGAATAGTCAACAAATCGTTTGGGGCGACTTCTCCTTCCTTT
CTCCCTTCTCCC
+
CCB9C@C@BCB@BBBBBB>>?AB?;661-
6;;?B>BCA@A9?AAA<<<=:=>6662?;;5?BBBC@BBA==9=<CC>?@@@<@<?@AA<
@T6XXI:00114:00240
CTATCACTCGAGATCTGCGGCTTTGGACAGTCCCTCTGTACTGTGAAGCCACAGATGTCGAGTCGACTAGCTTCCTT
TCTCCCTTCTCCC
+
CDCD:::ABCBAAABBBBB@>>>=>?<@BA:::5:??A>?BCAAAA>AC@BBBCACCBBBBCBBBBCCC;:7<A>?B
<B?==9=>@@@B=
@T6XXI:00114:00247
CTATCACTCGAGATCTGCGGCTTTGGACAGTCCCTCTGTACTGTGAAGCCACAGATGTCGAGTCGACTAGCTTCCTT
TCTCCCTTCTCCC
+
C9944::===@BBCCBBBN?BBC>C@BB??:::5:@@BBBBC@@@@>@CACCBCAACBBBBCCBBBBBD;<8?B@CC
>CCAC7<9<;;;7
@T6XXI:00115:00235
CTATCACTCGAGATCTGCGGCTTTGGACAGTCCCTCTGTACTGTGAAGCCACAGATGTCGAGTC
+
;AA:94449;999BBBBBC?ABB:@=?><6711,1116;711603325=?947=>00,22,../
@T6XXI:00115:00239
```

```
GGGGAATTCTAATACGACTCACTATAGGGAGAGGGAGAATAGTCAAACAAATCGTTTGGGGCGACTTCTCCTTCCTT
TCTCCCTTCTCCC
+
CHH:F@A=ACCCCCACC8;:A@88;=>>9???AB<B?<9:;44444,4830;=677-677-
7=??C=@BB?>9B344-4;9;7?;=8BC9
@T6XXI:00116:00212
GGGAGAAGGGAGAAAGGAAGGCTAGTCGACTCGACATCTGTGGCTTCACAGTACAGAGGACTGTCCAAAGCCGCAGA
TCTCGGTCGACTAG
+
CC>BBA=@95>>=>9>594;>@>@AA:::A@@B;:::::?@F?AC?ACEC?><522--
,/677;A?AA;??:@@AA?887/.,///62-00
@T6XXI:00116:00230
GGGAGAAGGGAGAAAGGAAGCTAGTCACTCGACATCTGTGGCTTCACAGTACAGGAGGACTCGTCAAAGCCGCAGAT
CTCGAGTCGACTAG
+
DDADCC==95>988-95958??>?BB>:==>00////=>B;@@BBB@?60000/,..,--
0000000+0656<>=?@AAA=9=@???AABA
@T6XXI:00116:00235
GGGGAATTCTAATACGACTCACTATAGGGAGGGGGAGAATAGTTTGTACATCGCCCGTGCAACATGCTTCTTCCTTT
CTCCCTTCTCCC
+
BBB9BBC@CCB@BCBB@>???AB>;66616>>CC2?@F@C898>7=:===888>@:@A9;;@CCAB@@:==>C>CB9
==@?:?<<=<@;
@T6XXI:00116:00238
GGGGAATTCTAATACGACTCACTATAGGGAGAGGGAGAATAGTTACCATTTTACATGCGTGCCTATTCTCTTCCTTT
CTCCCTTCTCCC
+
CCH:CAC@BBB?BBA?ABBBA@><9116.777BC?AB?>???@>@A>AAAA6=A;<?=@@>=:=7758;;;3858@:
>BCBA@9;<;;6
@T6XXI:00117:00200
GGGGAATTCTAATACGACTCACTATAGGGAGAGGGAGAATAGTACACGTC
+
999/9;@=@CDBCCBBBBBBCDD?C661.6<<?A>BC@@@:,,*******
@T6XXI:00117:00204
GGGGAATTCTAATACGACTCACTATAGGGAGAGGGAGAATAGTCAACAAATCGTTTGGGGCGACTTCTCCTTCCTTT
CTCCCTTCTCCC
+
:::1<@B@DBB?BAABB???B@BB9111-166=@=BBA?@@@AA=<9=63?=<<<-
777.7>@AB@CBB?@>A?AB:BBBA:B<>?AA;
@T6XXI:00117:00206
GGGGAATTCTAATACGACTCACTATAGGGAGAGGGAGAATAGTCAACAAATCGTTTGGGGCGACTTCTCCTTCCTTT
CTCCCTTCTCCC
+
DCDAC@B@BBB@BAAABBBBB@CB966646??CC>BB@=@=BAAA>???;ABB@A8???6?AACC@CCC@?>?<?C=
A???<?>A???;
@T6XXI:00117:00224
CTAGTCACTCGAGATCTGCGGCTTTGGACAGTCCCTCTGTACTGTGAAGCCACAGATGTCGAGTCGACTAGCTTCCT
TTCTCCCTTCCTCC
+
CB99;>:44A=@BBBCCBBB@CCC>B?BBCCBCB>BCBBBBBC:>:@>ACACCDDB@@@?BBBBCCBABC;:7=><<
C>B:;:68,/,//5
@T6XXI:00117:00230
GGGAGAAGGGAGAAAGGAAGGAGAAGTCGCCCCAAACGATTTGTTGACTATTCTCCCTCTCCCTATAGTGAGTCGTA
TTCAGAATTCCCC
+
??6;;B@CC>BCCB@C@C>A@BCCBDDDBB@B6?>8><111,11766997838??A@@@@DD;<=<=>>?;::8///
+*+--.,-,---*
```

```
@T6XXI:00117:00239
GGGAGAAGGGAGAAAGGAAGTGCAGTTCGCGGTTAGTGAAGGGAAAACTATTCTCCCTCTCCCTATAGTGAGTCGTA
TTAGAATTCCCC
+
BEAB@@594-
9999149B>BBBCCCB@BCBB?B@CBBBE???=@DC9CBBB@CCCC>:::?=6<:83003;;;9=<;;8:;;3747A
C6
@T6XXI:00117:00240
GGGAGAAGGGAGAAAGGAAGAGAACAGTGGCACAGGGGATTTGCGAACTATTCTCCCTCTCCCTATAGTGAGTCGTA
TTAGAACTTCCCC
+
CC?CCB@DD@CBCB>C?A>ABBC@D@@@A?BCCCCCC:BBB=@BAA?ECBB?B@@@<?CAAB=BCDC@B@@??BBAA
A>=>=1//9=;94
@T6XXI:00117:00246
GGGAGAAGGGAGAAAGGAAGCTAGTCACTCGACATCTGTGGCTTCACAGTACAGAGGGACTGTCCAAAGCCGCAGAT
CTCGAGTCGACTAG
+
CHABBB@CC>B@>=:@4828AA@@@?@@;:;???AABCBB?@@=@B>>@A???=;;;6;;>?@@38818;==<<<?=
;<=??@@A@@@@AA
@T6XXI:00117:00247
GGGGAATTCTAATACGACTCACTATAGGGAGAGGGAGAATAGTCAACAAATCGTTTGGGGCGACTTCTCCTTCCTTT
CTCCCTTCTCCC
+
:99/9<A>BCB@C@@@BBBBB@<97111.777>AAABA>A???AA>???;A??7726;;5?@BBCADCB??<??CC;
>?AA=A<?@AA;
@T6XXI:00118:00203
GGGGAATTCTAATACGACTCACTATAGGGAGAGGGAGAATAGTTTTTCCCCTTCACTAACCGCGAACTGCACTTCCT
TTCTCCCTTCTCCC
+
DDD9C?BACCC@CCBBBCBBAA>??777-
7==?B@;85<===<;;;+334+0236;99065:AB?>@@?ACDC>@:?<6==>>:>=;<<<6
@T6XXI:00118:00205
GGGAGAAGGGAGAAAGGAAGCTAGTCACTCGAGATCTGCGGCTTTGGACAGTCCCTCTGTACTGTGAAGCCACAGAT
GTCGAGTCGACTAGCTTCCTTT
+
CB>B9959>:?999142423;@@?CB?=<AAA:8:9>BABABCE>B?ACCCCCC>AA=<<<?AA??A@??<?A===A
AAAA===AAA??@878349;;1
@T6XXI:00118:00207
GGGGAATTCTAATACGACTCACTATAGGAGAGGGAGGAAT
+
CEF:C@B@BBA@AAABBBC??6:8===:70.79600,/76
@T6XXI:00118:00212
CTAGTCGACTCGAGATCTGCGGCTTTGGACAGTCCCTCTGTACTGTGAAGCCACAGATGTCGAGTCGACTAGCTTCC
TTTCTCCCTTCTCCC
+
C61111//4::9>ABCBCBBD@CDE?B?BA?8777.7?@ABBBC@BBA>@CBCFCBBCCCCCCBBB@;;873777=;
?@:@AAB??9877;4
@T6XXI:00118:00214
GGGATTCTAATACGACTCACTATAGGGAGAGGGAGAATAGCTCCCAAATCACGTTACGTGCGACCCACTTCCTTTCT
CCCTTCTC
+
CC?A======@CCCCAABA<;96167.7>@CE>BBA=A<==??>;>A;@=@@BB?@BBBBB>AAD@CCB@B@BB=BB
BC<=<;??
@T6XXI:00118:00215
ACTCGAGATCTGCGGCTTTGGACAGTCCCTCTGTACTGCGAAGCCACAGATGTCGAGTCGACTAGCTTCCTTTCTCC
CTTCTCCC
+
```

```
CEEGAAAECCCC>??BBA?A@ABBBBCC>AADBBAAABBGHACC@CBBCBBA@ADCCCC@@?@ACBB@B@CH?CCID
:=478880
@T6XXI:00118:00220
GGGGAGAAGGGAGAAAGGAAGGATAGACAGGGGCCAG
+
@@@6@CB@CCACCCB>C@>;=;0-,,,,,,,,*,*,.
@T6XXI:00118:00223
GGGAGAAGGGAGAAAGGAAGGAGAAGTCGCCCAAACGATTTGTTGACTATTCTCCCTCTCCCTATAGTGAGT
+
99-4;;298-41//+1,1,.34..,.18773,39673111,16778?844<=8??753333+0300317//.
@T6XXI:00118:00232
GGGGAATTCTAATACGACTCACTATAGGAGAGGGAGAATAGCTCAGGATTATCATCCGCGCTGTCCTCCTTCCTTTC
TCCCTTCTCCC
+
CCC:C?A?BCC@BB@@BBBBB:964..,0::?C>BA@<@=@<>?@A@=@=?@@A::8;AACCCCC@CC@B?B>BB>>
BB@9999744-
@T6XXI:00118:00241
GGGGAATTCTAATACGACTCACTATAGGGAGAGGGAGAATAGATCACCGTTGGTTCGGGTGCGCGTTCTCCTTCCTT
TCTCCCTTCTCCC
+
;<9/;?BACCBAC===A????AB97111.777?B@?@=>@@@@@ABA>AC=?;?=@>@<B;;;CBC?B?@=@@C@CC
9??<;6;?<==C?
@T6XXI:00118:00249
GGGGAATTCTAATACGACTCACTATAGGGAGAGGGAGAATAGTCAACAAATCGTTTGGGGCGACTTCTCCTTCCTTT
CTCCCTTCTCCC
+
@=@6@=@?ACCABA@@@BBBBA<966195:ABCE>CA?<?=AAAB>8881:?<<=6<<<2?BBBCACBB@=;=>AA<
AAAA;@>@@AB<
@T6XXI:00119:00212
GGGAGAAGGGAGAAAGGAAGGAGAAGTCGCCCCAAACGATTTGTTGACTATTCTCCCTCTCCCTATAGTGAGTCGTA
TTAGAATTCCCC
+
IKACCC?BB>CDBB@BAC998:;:7:B?8888.8>8?BBCC>CD@A@??AA>A@@>;?<?A@<A===@BCCA===@A
A>@@A?A<;::4
@T6XXI:00119:00225
GGGAGAAGGGAGAAAGGAAGTGCAGTTCGCGGTTAGTGAAGGGAAAAACTATTCTCCCTCTCCCTATAGTGAGTCGT
ATTAGAATTCCCC
+
DD@CBE@BB=?B??=B@BBCCBCCCC@BBBB@C@BAACC@AB-
6777,8?AB?@CCC>A<ABC>B?@?@=<<<=@@@??=??A>A?ADA5
@T6XXI:00119:00244
GGGGAATTCTAATACGACTCACTATAGGGAGAGGAGAATAGTT
+
/11+1:AADBB@AB==?BBBA?AAB<<A89?663:?979/,,+
@T6XXI:00120:00201
CTATCGACTCGAGATCTGCGGCTTTGGACAGTCCCTCTGTACTGTGAAGCCACAGATGTCGAGTCGACTAGCTTCCT
TTCTCCCTTCTCCC
+
D=9=;4:::@@BBBDBBBBA@AAA>A@BB@B???;?ABC@B>@>B?<=@C@CCCBBBBBBCB@AABABCBBAC@C@C
C<AAAC==>488A<
@T6XXI:00120:00216
GGGAGAAGGGAGAAAGGAAGGAGAAGTCGCCCCAAACGATTTGTTGACTATTCTCCCTCTCCCTATAGTGAGTCGTA
TTAGAATTCCCC
+
BDADCC?A=:@@==<@@B@C@CBB???BBBBB8AA=ACC??99>>????88:;;=;;::;<=;><;<<<A<@<;A:;
;;@A@=?8;;;.
@T6XXI:00120:00218
```

```
GGGAGAAGGGAGAAAGGAAGCTATCGACTCGACATCTGTGGCTTCACAGTACAGAGGGACTGTCCAAAGCCGCAGAT
CTCGAGTCGAC
+
CC>A?9288099990;>A>ACC@?:?79:?@???:;;A@@<?@=@CCCCB@?=4300-
4999>@=@@<AB>B?===A@@@AAAC@???
@T6XXI:00120:00232
CTATCACTCGAGATCTGCGGCTTTGGACAGTCCCTCTGTACTGTGAAGCCACAGATGTCGAGTCGACTAGCTTCCTT
TCTCCCTTCTCCC
+
CBC??:::CCCCBBABBBB@>>><@>A?==9::5:BBBBAACC@@@>@BBCCCABBBCABBBBBBBBBBAAB8;::C
>AA??:><@@@@;
@T6XXI:00120:00246
CTAGTCACTCGAGATCTGCGGCTTTGGACAGTCCCTCTGTACTGTGAAGCCACAGATGTCGAGTCGACTAGCTTCCT
TTCTCCCTTCTCCC
+
:6/111/44BBBCBCBBABC@ABA=A>>@@????9?BBCBBBC>B@A><B?BDDCBBBBCCCAABCCCBBCCC?A?A
K@CDDC>A>@@AA<
@T6XXI:00121:00201
CTATCACTCGAGAT
+
CCBA@::;CBBBCC
@T6XXI:00121:00212
GGGGAATTCTAATACGACTCACTATAGGAGAGGGAGAATAGTCAACAAATCGTTT
+
999/48@ACCB?BBCBBBCB>>AB@8759??9A=BA?>A:?=BC?A@A<CCBBB>
@T6XXI:00121:00220
GGGAGAAGGGAGAAAGGAAGGAGAAGT
+
@B?@=92991999184823@BCC?>>
@T6XXI:00121:00223
CTAGTCACTCGAGATCTGCGGCTTTGGACAGTCCCTCTGTACTGTGAAGCCACAGATGTCGAGTCGACTAGCTTCCT
TTCTCCCTTCTCCC
+
:8/11;/44BB@BBDCCBCC=>>?;B@>@ABABB>BBB@BB@C?CAA;>B@BBBABCBCBBABACCCCBGCCC?B@B
D>BBDDCA>???A<
@T6XXI:00121:00226
CTATCACTCGAGATCTGCGGCTTTGGACAGTCCCTCTGTACTGTGAAGCCACAGATGTCGAGTCGACTAGCTTCCTT
TCTCCCTTCTCCC
+
DA@=9::;BBBCBCBCBBB?BBC>C?ABBBAAA=ABBBCCCC:99A>@CABBBAAAAABABAABBCBBB::8;=;?B
<??<;7<?A@@C@
@T6XXI:00121:00242
GGGAGAAGGGAGAAAGGAAGCTAGTCACTCGACATCTGTGGCTTCACAGTACAGAGGGACTGTCCAAAGCCGCAGAT
CTCGAGTCGACTAGCTTCCTTTCTCCCTTCTCCC
+
CC?B@@=@@:@99909;>;>@>?>:00/....334477662:;=@CBBBBCA@=777149;;<<:=?:@D;=>A???
AA@A????@>===;<<<??AC;@<<<6;388@C@
@T6XXI:00121:00248
GGGAGAAGGGAGAAAGGAAGGAGAAGTCGCCCCAAACGATTTGTTGACTATTCTCCCTCTCCCTATAGTGAGTCGTA
TTAGAATTCCCCC
+
EDACCD@DC>BBAA=B@B@A?ABB@??AACAA8AB>BBBBC>C@@@???@=<><=<;?<@==;=588@@@?@???<<
<<?=:48/////+
@T6XXI:00122:00203
CTAGTCACTCGAGATCTGCGGCTTTGGACAGTCCCTCTGTACTGTGAAGCCACAGATGTCGAGTCGACTAGCTTCCT
TTCTCCCTTCTCCC
+
```

```
C99949444@=99:CDGCBE?BBA>BB@ACCCCB:ABC@==??CC@@?8<8;@@>@@@@AAAA?A@B;<:;888:::
C>B?BC==;=8881
@T6XXI:00122:00222
CTATCGACTCGAGATCTGGCGGCTTTGGACAGTCCCTCTGTACTGTGAAGCCACAGATGTCGAGTCGACTAGCCTTC
CTTTCTCCCTTCTCCC
+
A999=====?AAA@@@>;-
209;==,0,0......+11119???BBA<<?B8949:;37>>78:::;0;;;00,0,.,..+.11779544394
@T6XXI:00122:00223
GGGAGAAGGGAGAAAAGGAAGGAGAACGCGCACCCGAACCAACGGTGATCTATTCTCCCTCTCCCTATAGTGAGTCG
TATTAGAATTCCC
+
::6499584-
44888,4249?@CCC@CCBCBCBC>AB<?5<69E@999>9998249><633333,317611681..5...,.57692
44-
@T6XXI:00122:00224
GGGAGAAGGGAGAAAGGAAGAATAATTGTGGCTGATGGGACTGGTGTCTATTCTCCCTCTCCCTATAGTGAGTCGTA
TTAGAATTCCCC
+
CEABBA@CC>AA>B=B>A?AB?AA>>=@BB?CCBB7777:ABBBCCBBBAC@CCCC?@==>>7>??@@AAA@@@>><
<:<?B=4/034-
@T6XXI:00122:00227
GGGAGAAGGGAGAAAGGAAGGAGAAGTCGCCCCAAACGATTTGTTGACTATTCTCCCTCTCCCTATAGTGAGTCGTA
TTAGAATTCCCC
+
DC>AA@>BB=AAAD@D@C@B?BBA@CCABAAA8AC>CBAAA=ACBCAAAAC?C?==;@===CAAAA>>??;BCAA?=
=9=?=:=38880
@T6XXI:00122:00229
CTATCACTCGAGATCTGCGGCTTTGGACAGTCCCTCTGTACTGTGAAGCCACAGATGTCGAGTCGACTAGCTTCCTT
TCTCCCTTCTCCC
+
CA?=9::9BBBBBCBBBAA@ABB=B?ACC@@B@=BB?@AA@@@@@?>?B?BBCCCBBBCCCCC@A@BBC@FA8:;:E
@CCAB<A=??ACA
@T6XXI:00122:00234
CTAGTCGGACTCGAGATCTGCGGCTTTGGACAGTCCCTCTGTACTGGAAGCCACAGATGTCGAGTCGACTAGCTTCC
TTTCTCCCTTCTCCC
+
B>=49;@2<;??ACCCDFCC@=;88808;?B;<<FCADCBCBB<::5:>BB?AA@@@?@@===@@?==<;;:./,09
::6:888174:88:6
@T6XXI:00122:00238
GGGGAATTCTAATACGACTCACTATAGGGAGAGGGAGAATAGTCTCGCGATGGTCCGCTACTTGTTCTCCTTCCTTT
CTCCCTTCTCCC
+
999/9298<DC@CCBBB?@@CCB??777.7==>@>BEC>A?>>@AAAA=???;;=;=>DDCC?BC@ABB@C>@>@C;
?@CD>B67744-
@T6XXI:00122:00240
GGGAGAAGGGAGAAAGGAAGAGTAGCCGGGGACCAGGAAAAGTTTGGCTATTCTCCCTCTCCCTATAGTGAGTCGTA
TTAGAATTCCCC
+
;;59495990948;-
469<;;@BCCCBDDD9A@=>@;>>?5BBC@D@ECCIBCCHFCBCBCC@CBBBBBCCB@:::160666::;<AA5
@T6XXI:00122:00248
GGGAGAAGGGAGAAAGGAAGCTAGTCAACTCGACATCTGTGGCTTCACAGTACAGAGGGACTGTCCAAAGCCGCAGA
TCTCGAGTCGACTAG
+
FF>@@@AA@=BB>A>C?B>?DA>@BBB?BCEB=?@@??BBD?AA@BB????A?;933317>>@A??AA:BB?B@?==
:<@<@;@AAA<;;;<
```

```
@T6XXI:00123:00217
GGGAGAAGGGAGAAAGGAAGGGGAAAGGGAGAAAGGAAGCTAGTCGACTCGACAT
+
CCAAA?>??=BBAB<@>@>@>>2>?8>>:?B??<@5737?61/1//..4.311..
@T6XXI:00123:00223
GGGAGAAGGGAGAAAGGAAGAGAGCCTTTGCAATCGCACGTGGGAGACTATTCTCCCTCTCCCTATAGTGAGTCGTA
TTAGAATTCCCCC
+
DDABBB@BA=@98809<>>BBBCCC@BB>BCCAB@BADBCBEB>ABBCBBBCECCC>BF;;;8:B@>@A<<9FA@@B
BACBA=A59999,
@T6XXI:00123:00229
GGGGAATTCTAATACGACTCACTATAGGGAGAGGGAGAATAGCTCACGATTTTTCTG
+
AAA7A598;CB?BCBBB?>?C@<96166.7=<>@<AB?=@@A?AA@>766667,7;;
@T6XXI:00123:00233
CTAGTCACTCGAGATCTGCGGCTTTGGACAGTCCCTCTGTACTGTGAAGCCACAGATGTCGAGTCGACTAGCTTCCT
TTCTCCCTTCTCCC
+
://1/1/4:BBBCCBAA@@?=;;:5:;<776111,1;;?@BBBAA?@;>A@CBA@;<;A@ACC?AABBBBBA?:>47
7.777=.434444-
@T6XXI:00123:00244
GGGGAATTCTAATACGACTCACTATAGGAGAGGGAGAATAGTCAACAAAATCGTTTGGGACGA
+
CCC:E@C@BAA=@CBB?<>>>13311106?>>@;7742304438333490961117,11,116.
@T6XXI:00124:00215
CTAGTCGACTCGAGATCTGCGGCTTTGGACAGTCCCTCTGTACTGTGAAGCCACAGATGTCGAGTCGACTAGCTTCC
TTCTCCTTCTCC
+
C0,---
,,/1:@CCCBBA@??@??B;B<?@AC@AA=@@@AA@@??BBA?;B@CCCA@AACB?700110;00.17010631663
02--/,
@T6XXI:00124:00222
GGGGAATTCTAATACGACTCACTATAGGGAGAGGGAGAATAGATCACCGTTGGTTCGGGTGCGCGTTCTCCTTCCTT
TCTCCCTTCTCCC
+
C@@6@?B@A@A?BBC;<<@@B??>=777,7=7774:BB<@@@@@@BE@CC?C?B@FDC<@;;;@>><?>>;;4101>
16688359=AAB?
@T6XXI:00124:00226
GGGGAATTCTAATACGACTCACTATAGGGAGAGGGAGAATAGCGCCGAAATTTGCTCCCCTGCGCTCATCTTCCTTT
CTCCCTTCTCCC
+
444,9<@@CCBACB@@BB?BB@>9;666.7?>?B?BCB>@<?8>A>AFB>BB=BBCCCC9BB??<?;<<==:;384-
4;::7:89444-
@T6XXI:00124:00228
CTATCGACTCGAGATCTGCGGCTTTGGACAGTCCCTCTGTACTGTGAAGCCACAGATGTCGAGTCGACTAGCTTCCT
TTCTCCCTTCTCCC
+
?=<7/////89ABACBCC;::;CD?C=@ABAAA@<:;::;??@B?<<;=A=<<<BC@BBBBBA:<<<;;6433,.,.
57566::44:6563
@T6XXI:00124:00239
GGGAGAAGGGAGAAAGGAAGGGACGCGACGAGCTGGGATAAACTGCTGCTATTCTCCCTCTCCCTATAGTGAGTCGT
ATTAGAATTCCCC
+
FFCCBB?BC>BBBB>B@B@BB<A@@@B@@@A@?:??<?BBCACCCCBBCBBB@CCCC>C>?==;:;?A@@@@@@@A=
==>AAA?CACCC:
@T6XXI:00124:00244
```

ACTCGAGATCTGCGGCTTTGGACAGTCCCTCTGTACTGTGAAGCCACAGATGTCGAGTGACTAGCTTCCTTTCTCCC
TTCTCCC
+
CCBDBCCC?;=??B@BAA;B@BBAA???>ADCDCCCCBBBB@BB@BBBBBBA?ABBBB?@??B??B?BACC>BBCB>
@67;??:
@T6XXI:00124:00247
GGGAGAAGGGAGAAAGGAAGTGCAGTTCGCGGTTAGTGAAGGGAAAACTATTCTCCCTCTCCCTATAGTGAGTCGTA
TTAGAATTCCCC
+
AA<@AA=BB=BBBC?CAC?BBCCBBB@A@AA@B@888BB;;88???5@CCC@CCCC>AAACC>B=?AAB=AAC<<<9
958AC=A=A@>4
@T6XXI:00125:00203
GGGAGAAGGGAGAAAGGAAGTGCAGTTCGCGGTTAGTGAAGGGAAAAACTATTCTCCCTCTCCCTATAGTGAGTCGT
TATTAGAACTTCCCC
+
ENADBC@CCAAAAC>B?B?BEBBBBCBCC<;9;?AAACCABB<BDDC1AABC@CCCC>B=@CD>C@@<==A?;88//
,//,/4=:<A<???5
@T6XXI:00125:00207
CTATCGACTCGAGATCTGCGGCTTTGGACAGTCCCTCTGTACTGTGAAGCCACAGATGTCGAGTCGACTAGCTTCCT
TTCTCCCTTCTCCC
+
GA?;444//66///888ACC@CCC?C?@@@CCBC>CCBBBA@><A@?<?C=AACCCBBBCCCCBBBAA@?:9166:?
?;??AB974/./60
@T6XXI:00125:00211
GGGAGAAGGGAGAAAGGAAGGAGAAGTCGCCCCAAACGATTTGTTGACTATTCTCCCTCTCCCTATAGTGAGTCGTA
TTAGAATTCCCCC
+
CC?@@@@BB@???B>B?@?@@BBB=;;>BBBB9BB<CCCCC@C@?@BCC@@>@ABB:???@C=A<<<@@AA@@@@?@
@?@A@=;/////+
@T6XXI:00125:00213
GGGAGAAGGGAGAAAGGAAGCTAGTCACTCGACATCTGTGGCTTCACAGTACAGAGGGACTGTCCAAAGCCGCAGAT
CTCGAGTCGACTAG
+
CC@B>>644-
9>==9>598;A:8;@?::6?A:::A<??CC=@@@CC???@AC@=98848:8=>>37718@=AAA@@@BB@<<<<<<<
<::<
@T6XXI:00125:00220
GGGAGAAGGGAGAAAGGAAGGGGAGAAGGGAGAAAGGAAGCTATCGACTCGACATCTGTGGCTTCACAGTACAGAGG
GACTGTCCAAAGCCGCAGATCTCGAGTCGACTAGCTTCC
+
EEBGBB@CB>BCCC>B?B@BBB8B>><??7;766-
:>A>AACBAB96:;:>>=?@@ACAA<???A@@=:?AA==AAA<AA@@??<>@:=>;>>??@@@@A@@@@;=6.,.5,
,*++
@T6XXI:00125:00221
GGGAGAAGGGAGAAAGGAAGAAAGGAAGGAGAACGCGCACCCGAACCAACGGTGATCTATTCTCCCTCTCCCTATAG
TGAGTC
+
94-4498995949@9?495888031311;;;@@=?6=>776,44892424;:4499439=<>@B?738877-
7=/.....-+4
@T6XXI:00125:00227
CTAGTCACTCGAGATCTGCGGCTTTGGACAGTCCCTCTGTACTGTGAAGCCACAGATGTCGAGTCGACTAGCTTCCT
TTCTCCCTTCTCCC
+
B=499>444AAB?ACB::<C@CCC>C@CB@?:::5:??BCBBCAC@@>@CACDCCC@@@CCCB:;<@@@C@?<;?=?
FBCCBC@B?@@@@;
@T6XXI:00125:00228
CTAGTCGACTCGAGATCTGCGGCTTTGGACAGTCCCTGTG

```
+
BA;<<ACDEFDECBBBBBBBB?BBD>C?AA?;0,,+,,,(
@T6XXI:00125:00229
GGGAGAAGGGAGAAAGGAAGAAAACGCGCAAACAAAGAAGGGACGGACTATTCTCCCTCTCCCTATAGTGAGTCGTA
TTAG
+
DCACCC@CC?CCBCAC?B>ADEE8@@@CCCB>:::4:?:AB>B@C>AABAA=AB@?:6<;8848<;;<A@A@@@@@A
A>AA
@T6XXI:00125:00233
GGGGAATTCTAATACGACTCACTATAGGGAGAGGGAGAATAGCTTAGATCTTTCCACCGCCCGGCTTTTCTTCCTTT
CTCCCTTCTCCC
+
999/9<AACDB?BB?ABBB?>?;97111.7>>BD>??<?B<<<C@CBABB@@:@>@@?A@A<A?=???8A=;=;=;:
=;;;389;4441
@T6XXI:00125:00237
GGGAGAAGGGAGAAAGGAAGAGTCCATCTGTGGCTTCACAGTACAGAGGGACTGTCCAAAGCCGCAGATCTCGAGTC
GACTAG
+
CC@:<:@CC@BBBD@D?B>><;396;@@@CCC@BC@CAA???BBBD@??>?BCDCA>@@;?>@B@@@C@>A?AC@AC
CCCCAC
@T6XXI:00126:00203
GGGGAATTCTAATACGACTCACTATAGGGAGAGGGAGAATAGCTATACGCACCG
+
7=<1<,92899?>ACCA@@@CA836666,6>777,67=:8?;///....---,1
@T6XXI:00126:00227
GGGAGAAGGGAGAAAGGAAGGATAAATTCATGGGGGGCGTGCCATGACTATTCTCCCTCTCCCTATAGTGAGTCGTA
TTAGAATTCCCC
+
ADBCCB@CC>BCB?=B=@=?@B:;;8<>??777777)7<<BC@CBBBBBCB>A:7717;;;@<@>===AAAB@@@??
@;::6568===1
@T6XXI:00126:00228
GGGGAATTCTAATACGACTCACTATAGGGAGAGGGAGAATAGCAAACGTTTCCCGACGCCGCTTTCTCCTTCCTTTC
TCCCTTCTCCC
+
=@@6@>AACCB?@BBBB>><B@CB>:<<;<B@E@9=AA>A94.....::=,//,..36636117,16=4424244,4
89808/009>6
@T6XXI:00126:00231
GGGGAATTCTAATACGACTCACTATAGGGAGAGGGAGAATAGATGACACGCGGCCGCATCTCTATT
+
444,424:;AC>AAD@A@CBCAB?=676.7>>>A=@@A>A@@===B@?@A;;6:@BBAA?B:9668
@T6XXI:00126:00234
GGGAGAAGGGAGAAAGGAAGCTAGTCGACTCGACATCTGTGGCTTCACAGTACAGAGGACTGTCCAAAGCCGCAGAT
CTCGAGTCGAACT
+
BDBCCD?B@<>>=>9>;?<?BB??>67776617>9<9<?<=;784:@???CA<88//,/8641656648:;:<<<;=
888<664--+*+0
@T6XXI:00126:00235
GGGGGAATTCTAATACGACTCACTATAGGGAGAGGGAGAATAGTCAACAAATCGTTTGGGGCGACTTCTCCTTCCTT
TCTCCTTCTCCC
+
DBBD2A>ABCCCACCBCA@@;;:;:?<?B9==?==6=7438@@@@@?BBA:CCBCC>BAA5??;>@:;>>,/,,+,,
+,+.,-013483
@T6XXI:00126:00237
GGGAGAAGGGAGAAAGGAAGCTAGTCACTCGACATCTGTGGCTTCACAGTACAGAGGGACTGTCCAAAGCCGCAGAT
CTCGAGTCGACTAG
+
```

```
CC?B@@=@@<@CCC?C;:6;B:8;???====776976BCC:;;@ECCBBCCCC====5;;=<:<8864<B>AA@@@@
@@@:::A===:::=
@T6XXI:00126:00239
GGGAGAAGGGGAGAAAGGAAGCTAGTCGAC
+
6;86,-+,,,*,,/858,1137>?8000,,
@T6XXI:00126:00243
GGAGAGGAGAAG
+
/,////,/,/,/
@T6XXI:00127:00204
GGGGAATTCTAATACGACTCACTATAGGAGAGGGAGAATAGTTTTTCCCTTCACTAACCGCGAACTGCACTTCCTTT
CTCCCTTCTCCC
+
CCD9C@CABBB@BBBBABBBB7444./,0@<?A>BCC>C=@<<=A1AA<A=?BCCC@C?BBA?<==A;>?C>@5765
:??C>C?A???;
@T6XXI:00127:00217
CCTAGTCACTCGAGATCTGCGGCTTTGGACAGTCCCTCTGTACTGTGAAGCCACAGATGTCGAGTCGACTAGCTTCC
TTTCTCCCTTCTCCC
+
FA?4<<999;CBCECCBBB@@>@BB<B;@AA@@@@;@@@@B@@@?B@A=<B@BCBBB?@@BABBBBECCBDCBB?B@
CC>BBCC>@:<<AC<
@T6XXI:00127:00218
GGGAGAAGGGAGAAAGGAAGGGGAGAAGGGCTCGCCGACATCTGTGGCTTCACAGTACAGAGGACTGTCCAAAGCCG
CAGATCTCGAGTCGACTAG
+
@@<ADC@CC@C>>=:;,1,0;;+0.033309949989@A===B@?>;99699>A@AA<<;10,100..6033,3911
1161110337611111161
@T6XXI:00127:00221
GGGGAATTCTAATACGACTCACTATAGGGAGAGGGAGAATAGTCAACAAATCGTTTGGGGCGACTTCTCC
+
999/99A>BCC@CBAABBCBBAB?966656;;?@<BB?<?A@@<<9<>=7AA@AA<BBB9BCBBB>@AB?
@T6XXI:00127:00226
GGGGAATTCTAATACGACTCACTATAGGGAGAGGGAGAATAGCTACGTTTAACCGATCGTGTTGTTCTCCTTCCTTT
CTCCCTTCTCCC
+
@CA6@@A@BCCBBBBBBBBB>=;9=116.777>><BB?=@@AABCCBBA=B====C@B=;;=;<<;<<=<A>A<?>7
<<<A<A>BAAA<
@T6XXI:00127:00232
GGGGAATTCTAATACGACTCACTATAGGGAGAGGGAGAATAGCGACTCGTTCAGTTGCCTCCCTTTTGCTCTTCCTT
TCTCCCTTCTC
+
999/98>@CBB@AA????B<A@B?966659??CC>BB@?A=@@<??A@A=?AAA=;?<>BBB.::94:@BB@>A9<<
7<>@?<@;>?@
@T6XXI:00127:00238
CTATCACTCGAGATCTGCGGCTTTGGACAGTCCCTCTGTACTGTGAAGCCACAGATGTCGAGTCGACTAGCTTCCTT
TCTCCCTTCTCCC
+
BGB=9::9A>ABBCBBB:88:BC>B?BCBBBCC>BBBBBB@D@AA@>@B?BCCCC;;;BBBA:;<@?@A??:?B>AB
=@?@A<@<988@A
@T6XXI:00127:00248
GGGAGAAGGGAGAAAGGAAGCTAGTCACTCGACATCTGTGGCTTCACAGTACAGAGGGACTGTCCAAAGCCGCAGAT
CTCGAGTCGACTAG
+
;<19?>?AA:A999095958?>>?A??=A??;;:@@BB@@=:;8;BCCCABABA300,4;;;<=?AA<AA>A@@@@?
??@AA@@@@@@@A@
@T6XXI:00128:00207
```

```
GGGAGAAGGGAGAAAGGAAGCTAGTCACTCGAGATCTGCGGCTTTGGACAGTCCCTCTGTACTGTGAAGCCACAGAT
GTCGAGTCGACTAGCTTCCTTTCTCCCTTCTCCC
+
FC@C<98;@:@998095968>888A???CCCDC@CDDBB@@C@@>@?@GAA???>?A==<<<888;>;99:;;:8::
<<==9:;====<9==?>A>A@<@@@C8888AAC>
@T6XXI:00128:00212
CTATCGACTCAAGATCTGCGGCTTTGGACAGTCCCTCTGTACTGTGAAGCCACAGATGTCGAGTCGACTAGCTTCCT
TTCTCCCTTCTCCCTTCCTTTCCTCCCT
+
EA??:9:AB;99;C@@AAAC@CCC>C@CD;;;CC>CBBBBA>9?BBA=?B?@?ABBBBBBBBBBBBBBBGCCB>?=?
K=?@CC@B?B==>9=99,//,/,///,/
@T6XXI:00128:00215
GGGAGAAGGGAGAAAGGAAGGGGAGAAGGGAGAAAGAAAGGGGGAGAAGGGAGAAAGGAAGGGGAGAAGGGAGAAAG
GAAGGGGAGAAGGGAGAAAAGG
+
9=:=AA?BB<B@@@<@AC@CDC7B??;<627777.177,7777,77176A?=<=C8985<AAA6998=?@1888A<@
@A8;;;-4484661/////+//
@T6XXI:00128:00216
GGGGAATTCTAATACGACTCACTATAGGGAGAGGGAGAATAGTTCCCTTCTAACCCGTACGCAACAATGCTTCCTTT
CTCCCTTCTCCC
+
=@@6@=@@DDB@C@@@@BBBB@AA<8::;<BBEC>C?>=>;:::?;:;:;A?>??;?@?@???>AA@@=:?;;/371
7BBA<A=??@@<
@T6XXI:00128:00218
GGGGAGAAGGGAGAAAGGAAGCTAGTCACTCGAGATCTGCGGCTTTGGACAGTCCCTCTGTACTGTGAAGCCACAGA
TGTCGAGTCGACTAGCTTCCTTCTCCCTTC
+
444,499899-
84991;>F?B@@@BBC?@?BBA:8:A??@BAAAD>C>@@BCCCC>AA?A@>?<888B?@@>@@@@@AAAAA?>>>88
8;::6647,/,/666,/,/
@T6XXI:00128:00226
GGGAGAAGGGAGAAAGGAAGGAGAAGTCGCCCCAAACGATTTGTTGACTATTCTCCCTCTCCCTATAGTGAGTCGTA
TTAGAATTCCC
+
BC?BBB@BB>BBBB>C@B>?>?BB@>>?BBBB8AA=ACCCC>@@>@????<==A==;8:?;=;<;<;;<===:8>?A
A?DC?;;,//,
@T6XXI:00128:00236
GGGAGAAGGGAGAAAGGAAGGAGAAAGTTCTGCGCGAAACTGAGACACTATTCTCCCTCTCCCTATAGTGAGTCGTA
TTAGAATTCCCC
+
@A>ABC@CC@DCBB>B@CAB?ABBB>AB?BCDECCCCB>BBBCCC@BBA@B?BCCC>C?A==;<<9==AAAA@@A=<
<<???<?;AAA5
@T6XXI:00129:00203
GGGGAATTCTAATACGACTCACTATAGGGAGAGGGAGGAATAGCTCAC
+
?CC9C?CC<=<DB;;<@?>>B?887777-7===<67600/030-.33.
@T6XXI:00129:00229
CTAGTCGACTCGAGATCTGCGGCTTTGGACAGTCCCTCTGTACTGTGAAGCCACAGATGTCGAGTCGCTAGCTTCCT
TTCTCCCTTCTCCC
+
FCCCC:<<BCBCCDCCBB?>=7><>/:7;BBBBBC@BCECCBB>@CBA=?B?AACDCC@A@<=9?;;:@77777711
1,6<<=6=:=<==6
@T6XXI:00129:00245
GGGAGAAGGGAGAAAGGAAGCTAGTCGACTCGACATCTGTGGCTTCACAGTACAGAGGGACTGTCCAAAGCCGCAGA
TCTC
+
```

```
CC@CCC@BB?AB??:@?C8;;CBBBBCC@CAA@=???@CDD@AA?ACA@?;?@===<<;<<==><;=<;==>A@=AA
==??
@T6XXI:00129:00246
GGGAGAAGGGAGAAAGGAAGCTAGTCGACTCGACATCTGTGGCTTCACAGTACAGAGGACTGTCCAAAGCCGCAGAT
CTCGA
+
FDABAA=@=9>98;09?@8;;?@@@:;;BAA?;::00/://,.69;ABCCAA?<;<<8<<<>>@;==78838@@@?=
<<@<<
@T6XXI:00130:00212
GGGGAATTCTAATACGACTCACTATAGGGAGAGGGAGAATAGACACCAATCGTGCCGGGTCATGTTCTCCTTCCTTT
CTCCCTTCTCCC
+
ADD9D@CBCCC@CCCBBBCBB@;97111.777>@>BCA>@@?????<?>B@@CEE>A?;<BAAB??AAB>@<@<>?:
?<<=7=>AA@?9
@T6XXI:00130:00238
GGGGAATTCTAATACGACTCACTATAGGGAGAGGGAGAATAGCAGCCTGAGT
+
KKC9C@D@ABB?;;;CB;;;B@>>=777.7=776,1745;>689/,+*****
@T6XXI:00130:00242
GGGGAATTCTAATACGACTCACTATAGGGAGAGGGAGAATAGTGACGTGTTGTGCGGCGCTCACTGTCTCCTTCCTT
TCTCCCTTCTCCC
+
@>>6@@B@CDB@BBA@@BB??AB?>111.777?B?BC@@BAA@::;=C==;===<<;<=<<@@<=A=@??<?>A>A@
;@@@A=A?<?@@<
@T6XXI:00131:00202
GGGGAATTCTAATACGACTCACTATAGGGAGAGGGAGAATAGTCATGTGTTCAATCATCTCGCTTTCTCTTCCTTCT
CCCTTCTCC
+
?=94@<@@ACC@CC@@@<<<BAB??76659??BC>CDC=@;;<<;?@?;;9;;8;?@>=99:443,3;799;,/,/-
-.,4798:;
@T6XXI:00131:00208
GGGGAATTCTAATACGACTCACTATAGGGAGAGGGAGAATAGTCTGTCGTTCGGTCAACGCTGCTATCCTTCCTTCT
CCTTCCTCC
+
AGC:C@B@@@@=@BAABABCB@>?7777.7???B<?:658///...3<<@C983884/4866//--
1323242376,,+,+,++00
@T6XXI:00131:00209
GGGGAATTCTAATACGACTCACTATAGGGAGAGGGAGAATAGTTCGCCAATATCCCGCGGCGTGTTCTCCTTCCTTT
CTCCCTTCTCCC
+
AAA7A=A>AJE@C@@@BBBB><;76666.7>>>@?AA?AAA@?<?@A?C?B@???;?<A@C??=A>AA@>A>A>AA;
@@AC<A>@??:4
@T6XXI:00131:00218
GGGGAATTCTAATACGACTCACTATAGGGAGAGGGAGAATAGTCAACAAATCGTTTGGGGCGACTTCTCCTTCCTTT
CTCCCTTCTCCC
+
CDCDC?B@BBB@CCBB@>>BBCB99111.6<<AC>CBB>@<?<==;9==9?BBBE>@@B9?BCCC@AAA=:::<AC>
???A=A=<8771
@T6XXI:00131:00220
GGGGAATTCTAATACGACTCACTATAGGGAGAGGGAGAATAGTCAACAAATCGTTTGGGGCGACTTCTCCTTCCTTT
CTCCCTTCTCCC
+
CFF:CBC@BCC@C@@A@CCCAAAA?7695:@@CF>BB@=A=@@@=5377,7>>>>6=<<5?BBBC??@@5824<;;6
<;==7<8;5=?;
@T6XXI:00131:00224
GGGGAATTCTAATACGACTCACTATAGGGAGAGGGAGAATAGCTATTTCTCCAGCACACCAATCCTCTCCTTCCTTT
CTCCCTTCTCCC
```

```
+
CCD:D@C?BCB=A@BBBBBB?=@@A???;?BBBC>BA?<??<<?BBB>BBB?BBCA=5837=;98<@@?<??B>A@6
58BA18687;;4
@T6XXI:00131:00229
GGGGAATTCTAATACGACTCACTATAGGGAGAGGGAGAATAGCCAGACCATTTGTGTTTGAGGCATCTCCTTCCTTT
CTCCCTTCTCCCTTCCTTTCCTCCTTCC
+
=@>4>695<>A9BBBBBBD@@?B??777.7==>>=AB@>A@@@=??<<588A<AACAA8:8A>@;<;=<3438;=?<
@@CC<B>@86606,/,..,/,/601,-,
@T6XXI:00132:00208
GGGGATTCTAATACGACTCACTATAGGGAGAGGGAGAATAGTTAGCGTTTCCCCCGCAATCTATTTGTCCTTCCTTT
CTCCCTTCTCCC
+
CCC9CC@CCC@CB@BBB?BBB@97111.7<<@B>@?>@AA??;==A>>=5;<;;0<@@>AB=8=A<A7848<?;?C;
?@@@;==?7331
@T6XXI:00132:00220
GGGAGAAGGGAGAAAGGAAGCTAGTCATACATCTGTGGCTCACAGTACAGAGGGACTGTCCAAAGCCGCAGATCTCG
AGTCGA
+
94-
499:;;:;89904999?EC:7/:.,,,,,,.3000./.559897?==;;;48;:;5<:==5>>3338853333840-
164
@T6XXI:00132:00222
GGGGAATTCTAATACGACTCACTATAGGGAGAGAGATATAAGGGCCATTTCTCTGCTCCCATTGCTCGCACGATTCC
CATTACCAAATTCTCTCCC
+
CDD:E@C@BCB@BBCBBBBAB@CBBCCG>::8BBBAA??:;==<@>BCC>CCBAAB;::6//,/699:@995=><@B
?CCAA@=?K<@>>1//:;6
@T6XXI:00132:00225
GGGAGAAGGGAGAAAGGAAGAATATTCGGAGAGCTGTCATTGTCAGGCTATTCTCCCTCTCCCTATAGTGAGTCGTA
TTAGAATTCCCC
+
BBCDHD@DC?BBBB>B@?@BB>?>B@B>=>>>>>?@?@@=<@@BCC@CECC@CBBB@BBAAB>AAABCCCCC@@@:<
?>A?<=?:>??5
@T6XXI:00132:00226
CTAGTCACTCGAGATCTGCGCTTGGACAGTCCCTCTGTACTGTGAAGCCACAGATGT
+
:1///8/4:CBCBCEB=7-,--+,*,,,3.11,1;9=BECCCB<:86?=??AABBBB
@T6XXI:00132:00246
GGGAGAAGGGAGAAAGGAAGGTTAGCGTCGTGCTAGATACTGGGAGACTATTCTCCCTCTCCCTATAGTGAGTCGTA
T
+
FD????@BC?DAA@=@@C?A?A@BABAB>>?B???>;;;>>;=:;888;==<=?<<;?=A==;A===8<<AAAAA??
?
@T6XXI:00133:00208
GGGGAATTCTAATACGACTCACTATAGGGAGAGGAGATAGTAGGACAATCTTG
+
CCD9C8;8<;9=@@ABBDNCCAAA@:66.7777:=743:::44/000/11101
@T6XXI:00133:00210
GGGGAATTCTAATACGACTCACTATAGGGAGAGGGAGAATAGTCAACAAATCGTTTGGGGCGACTTCTCCTTCCTTT
CTCCCTTCTCCC
+
:::4<?CAC@@@AAAAA>>>?AB?;6665:BBCC>BCC>@<===@>@@A<BCBBC8?:?7?ACCC@ABB@@>@?@C>
???A;A:==BB<
@T6XXI:00133:00219
GGGAGAAGGGAGAAAGGAAGGAGAAAGCGACGTCGGGGAAACGTTTGCTATTCTCCCTCTCCCTATAGTGAGTCGTA
TTAGAATTCCCC
```

```
+
FC@AA@@BB>???BBB@B@>=BB?B;;?<BBBBA@@@7CC?C::::<EECH@CCCD?B@BA@<@AABBBBBABA::;
A@@CA>=88<<4
@T6XXI:00133:00229
GGGGAATTCTAATACGACTCACTATAGGGAGAGGGAGAATAGCTACGTTTAACCGATCGTGTTGTTCTCCTTCCTTT
CTCCCTTCTCCC
+
=@@8@BECCAB@B@A@BBB???BB@<::888?@C>CB@?A=???@@???;?>@>=9<8====>A<448?;=;@;>?:
>@==:=998;84
@T6XXI:00133:00230
GGGAGAAGGGAGAAAGGAAGATGTACGTCAGGGAGATGTGGTGAGTTGCTATTCTCCCTCTCCCTATAGTGAGTCGT
ATTAGAATTCCCC
+
49199969?:@9;;9;>BDBBC@@AEAAEGCCAABBBBBA?BAAAA488>9<:<DDC@CCBBB<?@A98;@;;;GF;
;<?B9=5<8;A@7
@T6XXI:00133:00239
GGGGAATTCTAATACGACTCACTATAGGGAGAGGGAGAATAGTTCTGTGCATTTCTCCCAGCACACCGTCCTTCCTT
TCTCCCTTCTCCC
+
BCC:C?B?BCC@CCBBBBBBB@B97111.777>@>BBA@A?@?:;;;?===@?;@<<<;<====8998<<;<?C>@:
5:===;=;:?;=;
@T6XXI:00134:00204
CTATCGACTCGAGATCTGCGGCTTTGGACAGTCCCTCTGTACTGTGAAAGCCACA
+
CBB9//////7////444431333,377331111,1111116111000+08,/1/
@T6XXI:00134:00223
GGGAGAAGGGAGAAAGGAAGGGAATAGCAGCAGTCGGCAACGTGAGACTATTCTCCCTCTCCCTATAGT
+
//+//9:@C>B;;::911,.62288-,1....../799>267;3111111601;7766----+--22-0
@T6XXI:00134:00225
CTTTGGACAGTCACATGAGGA
+
:::6;?@BA?---,,,,,,*,
@T6XXI:00134:00248
GGGAGAAGGGAGAAAGGAAGGAGATGTTCATGCGGATAGACGTTGAACTATTCTCCCTCTCCCTATAGTGAGTCGTA
TTAGAATTCCCC
+
CD?CBB@BC>DBBB>BDB?A@BA?A>?@??B?>>>?B>==BBB?BA??@@CABB@C>B?9::5:CA@@@AA<<<@88
8>ACC>C@CCD9
@T6XXI:00135:00203
GGGAGAAGGGAGAAAGGAAGGAGAAGTCGCCCCAAACGATTTGTTGACTATTCTCCCTCTCCCTATAGTGAGTCGTA
TTAGAATTCCCCC
+
BB?CBC@BB?BBBB=B@CAE@BBB@C?@@CBB4;:68?>9A>AC@C@??A@?A???;>????;<<<@=<<AA9::AA
=9:44-.,.468.
@T6XXI:00135:00214
GGGAGAAGGGAGAAAGGAAGCTAGTCACTCGACATCTGTGGCTTCACAGTACAGAGGGACTGTCCAAAGCCGCAGAT
CTCGAGTCGACTAG
+
DG>A9928909;;:6;-1-
080,,,***(*,,,,,,/577:?BCBB?>>>>@@=88876A?77737718=:@=????@@@???@@??@@=<
@T6XXI:00135:00223
GGGGAATTCTAATACGACTCACTATAGGGAGAGGGAGAATAGCCAATTACTCCCTACAGCAGCGACATTCTTCCTTT
CTCCCTTCTCCC
+
444-9<A@CCC@BBB@@????@B?;661-
6;;?A>BC@>A=A@:;;<<=;===;;>;=<<@88=@@<@<@A?B><A<:?==;B<;==<7
```

```
@T6XXI:00135:00238
GGGGAATTCTAATACGACTCACTATAGGGAGAGGGAGAATAGTCAACAAATCGTTTGGGGCGACTTCTCCTTCCTTT
CTCCCTTCTCCC
+
ABB9B@C@CBB@BBAAABBBBCB?7111.6<<BCAAA??AAA<AA;<<63;=;;=-
7774:ACCC?BBB?@=@>BA:AACC;?;??AA=
@T6XXI:00135:00240
CTATCGACTCGAGATCTGCGGCTTTGGACAGTCCCTCTGTACTGTGAAGCCACAGATGTCGAGTCGACTAGCTTCCT
TTCTCCCTTCTCCC
+
B:4499444;=A<<<CBBBC@CCC>C=@?>=7794:@@CCBBC=B@@>@B?BCBABB>@BCC@A@BBBBCB@?>@=?
C>@@@D@A<??>>7
@T6XXI:00135:00241
GGGAGAAGGGAGAAAGGAAGGTAATGGCTGTTGCGGGGAGTGAGTGGCTATTCTCCCTCCTCCCTATAGTGAGTCGT
ATTAGAACTTCCCC
+
/1+/:;244-4?@A9>9;839?A6977849;<?<?BC9>?>=;;7726779=9>>7,110111,000005533660-
.4-.-3222,000+
@T6XXI:00136:00205
GGGAGAAGGGAGAAAGGAAGGAGAAGTCGCCCCAAACGATTTGTTGACTATTCTCCCTCTCCCTATAGTGAGTCGTA
TTAGAATTCCCC
+
BB?BBB@BBB?==A;B@A@?>?BB@@?A@ABB7BB>BBBBB><<;?CCC@C?C<<<;88>CC;;888;<<<=548==
<<@=:88/444-
@T6XXI:00136:00219
GGGAGAAGGGAGAAAGGAAGCTAGTCACTCGAGATCTGCGGCTTTGGACAGTCCCTCTGTACTGTGAAGCCACAGAT
GTCGAGTCGACTAGCTTCCTTTCTCCCTTCTCCC
+
CH@C<;7<@:>99909;>=?@??@??@?CCB@@=CCBBCB@BCB>B?C?>;@@A>888==ACCA??A??>:=?<<<<
?@@@?@DDC@@???9989?CA<A<<;6;8887B<
@T6XXI:00136:00226
GGGAGAAGGGAGAAAGGAAGGAGAAGTCGCCCCAAACGATTTGTTGACTATTCTCCCTCTCCCTATAGTGAGTCGTA
TTAGAATTCCCC
+
CC?BBB@BB?BAAA=B@C@B?AAA?BAB?>;;5<<<@CCC@;??>???;9;:;===;:::<=;===:::;;==:;:=
A<??B=B3743+
@T6XXI:00136:00231
GGGGAATTCTAATACGACTCACTATAGGGAGAGGGAGAATAGTTTTTCCCTTCACTAACCGCGAACTGCACTTCCTT
TCTCCCTTCTCCC
+
499/9<AACBB<BABBA??BBCB>966656??CC>A@??@@?>644.994938:BBB?B???@@>@?A?BBB>A:AA
<AAAC>C?A@AA<
@T6XXI:00136:00235
GGGAGAAGGGAGAAAGGAAGCTAGTCACTCGACATCTGTGGCTTCACAGTACAGAGGGACTGTCCAAAGCCGCAGAT
CTCGAGTCGACTAG
+
GG?AA>2880999909596 8B@@ABC@>???00//./;=@8?@=@ABBCBB?B<73317=??@>?ACA@@>??<??8
8888<<@@?????=
@T6XXI:00136:00236
GGGAGAAGGGAGAAAGGAAGAGAATAACAGTGCACCGAATGCGCAAACTATTCTCCCTCTCCCTATAGTGAGTCGTA
TTAGAATTCCCC
+
GKBBBB@CC@?BBB>C?B?BCAA?B@>BAAB?AAC@A@>ACABECC@ECBB>@:::5;BABC>BCC@@@<@B?BBBA
B@ACC@=<AAA5
@T6XXI:00136:00240
GGGAGAAGGGAGAAAGGAAGGAGAAGTCGCCCCAAACGATTTGTTGACTATTCTCCCTCTCCCTATAGTGAGTCGTA
TTAGAATTCCCCC
```

```
+
BGCCCB@BC>BBBB?B?B@C@AAA>?@?>>>>2>>:?BDCC>CA@A==@AA>A@@A????@@;?==<@@@<==@??>
>;=665658878.
@T6XXI:00136:00246
GGGAGAAGGGAGAAAGGAAGCTAGTCACTCGAGATCTGCGGCTTTGGACAGTCCCTCTGTACTGTGAAGCCACAGAT
GTCGAGTCGACTAGCTTCCTTTCTCCCTTCTCCC
+
DD@CAA=@@:C98809596;BBABC?@BCCCBB@BBBABA@?@@;@@A@@?@@@;@@=<<<8==@?B?AC=???<<A
=@==9==A@=?A=99=>@>AA7:<<;6898444-
@T6XXI:00137:00208
CTAGTCACTCGAGATCTGCGGCTTTGGACAGTCCCTCTGTACTGTGAACACCAGAGTGTC
+
C:11/<1::CECGCBABCBA@CCC?C@CABA???<?BBBBBAAA=;9-//,55.*+++++
@T6XXI:00137:00210
GGGGAATTCTAATACGACTCACTATAGGGAGAGGGAGAATAGTCAACAAATCGTTTGGGGCGACTTCTCCTTCCTTT
CTCCCTTCTCCC
+
@@@6>=@=@BB?BBBBCCCBB@?;911657==BC>BB@?@:??AA>@@@;BCCCA;??@7AAA@A>@CC?A?A?A=;
=AAA;@<>???:
@T6XXI:00137:00215
GGGGAATTCTAATACGACTCACTATAGGGAGAGGGAGAATAGTCAACAAATCGTTTGGGGCGACTTCTCCTTCCTTT
CTCCCTTCTCCC
+
CED:C@B@BCB>??BBB>>>?@D?;111.6<<BC>BB?=@@AABA>AA@:?=666-
7=?5ACCCC?BBC?@>@;AA<AAAA;?689AA<
@T6XXI:00137:00232
GGGAGAAGGGAGAAAGGAAGAGCGGGCTGTGATGGGAAATAGGTTAGCTATTCTCCCTCTCCCTATAGTGAGTCGTA
TTAGAATTCCCC
+
DD?BBA>A=<AAA@<B?B@CCCCCC>CCBAABBCHACB<@AC@B@BBCCCC?ABCA=ABBFF>BBBBBC@@??===<
<9<AA@A;ABB6
@T6XXI:00137:00242
CTAGTCACTCGAGATCTGCGGCTTTGGACAGTCCCTCTGTACTGTGAAGCCACAGATGTCGAGTCGACTAGCTTCCT
TC
+
CB:<<<:::BBB<;;CBBBB@CCC>C=>@AAABB>BAABBBBCBCAA>?B?BCCCCAAABBBCC@@=::80**)*)*
)*
@T6XXI:00137:00247
GGGAGAAGGGAGAAAGGAAGGACACGCTGTTGGAGAATAACGGAGAGCTATTCTCCCTCTCCCTATAGTGAGTCGTA
TTAGAATTCCCC
+
CEADDC@BC@BAAA=B>B?B@BCCBBBBBB@?=?>B@BB?A?>??@@??????>BB>@BAAA<?<@@@@@A@@@?<?
@<??A=@38880
@T6XXI:00138:00206
GGGAGAAGGGAGAAAGGAAGCTAGTCACTCGACATCTGTGGCTTCACAGTACAGAGGGACTGTCCAAAGCCGCAGAT
CTCGAGTCGACTAG
+
CCBBB>699-
9>>>9@=@=?A@@A?6:;7@@;;:AAACCD@@A@CCC@@?@C@=44414<<;??<==<AA>@@<8==???AAA???B
B???
@T6XXI:00138:00209
GGGAGAAGGGAGAAAGGAAGCTGAGTCACTCGACATCTGTGGCTTCACAGTACAGAGGACTCGTCCAAAGCCGCAGA
TCTCGAGTCGACTAG
+
=A<@B@?AB:<;>A:=<?<9737163333,,,,,,,/,,,2*..35//7@C>455.+*+0+++3757>6::7=<@@@
A@@<<88///8;::@
@T6XXI:00138:00211
```

```
GGGAGAAGGAGTGAAGAG
+
;::?//-1-1////,//4
@T6XXI:00138:00220
GGGGAATTCTAATACGACTCACTATAGGGAGAGGGAGAATAGTCAACAGGTGTCTGATTGCGCGTTCTCCTTCCTTT
CTCCCTTCTCCC
+
BBA7A>B>BBD?BBBBCB?BBAB<;66656?;?B>@B=?A???@@>@@B>=<<===9999===AA>A@@>@>@?AB<
??@C<A;>;;;6
@T6XXI:00138:00234
GGGGAATTCTAATACGACTCACTATAGGGAGAGGGAGAATAGTCAACAAGTCGTTTGGGGCGACTTCTCCTTCCTTT
CTCCCTTCTCCC
+
999/9<AACBB@CCA@ABBB?@;97116.7>>CC>CCA>@<@@==<@@>@;;;==7===1@?A??=?CB@A>@=@C<
A@@A<A;==@@:
@T6XXI:00139:00204
GGGAGAAGGGAGAAAGGAAGGTGAACAGAGCGCAAACGAATACGTCTCTATTCTCCCTCTCCCTATAGTGAGTCGTA
TTAGAATTCCCC
+
CD@CCB@BB?BBBB?BAC@B@B>B=BB@ABBBAAA=ABB@ECCCBBCCBCC@DAA@:===>?<?BCCAABCCCCCBB
B@CBB?C@BCC8
@T6XXI:00139:00206
GGGAGAAGGGAGAAAGGAAGCTAGTCGACTCGACATCTGTGGCTTCACAGTACAGAGGGACTGTCCAAAGCCGCAGA
TCTCGAGTCGACTAGCTTCCTTTCTCCCTTCTCCC
+
DG@B@=;>>:>999042943<<949/000/...111679>?=ADBBAAAA@====8<<7<@?>>?:==9=C>A@@@A
A=??==<<;@888=<<@BA>AA<A???<?>@@@?:
@T6XXI:00139:00210
GGGAGAAGGGAGAAAGGAAGGAGACAGTACCGCCCAAAATTGGAAGACTATTCTCCCTCTCCCTATAGTGAGTCGTA
TTAGAACTTCCC
+
DDCECC@CD@CCCC=B6:8:<>??A@@@@@54:;:@BB4<<<>A?BCBBBCACBBB<??777.7?=::;BB?;4./-
-,-11316000+
@T6XXI:00139:00211
GGGAGAAGGGAGAAAGGAAGCTAGTCGACTCGAGAATCTGCGGCTTTGGACAGTCCCTCTGT
+
EF>BAA>AC>BBB@196947<7?76711.......,...36::<@>;?461111..+.2---
@T6XXI:00139:00220
GGGAGAAGGGAGAAAGGGAAGAAAGGAAGGAGAACGCGCACCCGAACCAACGGTGATCTATTCTCCCCCTCCTACTA
GTGAGTCGTATTACGAAATTCCA
+
CK>BBB@DG@B;9908A01,000,0,0,.557::?CCCCAAB?CD@AAACCCACC<<<;;;<?>>???/:;91000/
...175443--,-----+02429
@T6XXI:00139:00223
GGGAGAAGGGAGAAAGGAAGGGGAGAAGGGCTAGTCGACTCGACATCTGTGGCTTCACAGTACAGAGGGACTGTCCA
AAGCCGCAGATCTCG
+
BB?BBBBCC@ABBC?@<=2333,8>>;??<@CCECBBBDCCB;9<<BCHDD@BB>BB????BFBB???<@CCCCDDC
CABC@CDCCCC8;;C
@T6XXI:00139:00224
GGGGAATTCTAATACGACTCACTATAGGGAGAGGGAGAATAGATTACCGCTCATGTGCCCTGTACTATTCTTCCTTT
CTCCCTTCTC
+
<99/9;@@ACC@CB@@BBBBBAB9>111.7>>BC<AAA?A====;<;<:???@;;;476.7>>@C?;;8;<>B;865
<;;;6;9;47
@T6XXI:00139:00234
```

```
GGGGAATTCTAATACGACTCACTATAGGGAGAGGGAGAATAGTCAACAAATCGTTTGGGGCGACTTCTCCTTCCTTT
CTCCCTTCCTCCC
+
CCD:CEA@BBB@BBBB?>>BBBB>;666.7@=AB>;<:>@AA?<<8<==7@@@AC>BBC9BBBBD?AABC@>?=?A:
<86606,.,/671
@T6XXI:00139:00235
GGAGAAAGGAAGCTAGTCGACTCGACATCTGTGGCTTCACAGTACAGAGGGACTGGTCCAAAGCCGCAGATCTCGAG
TCGACTAGCTCC
+
B>AA@>9>695<><>=>1//44.31>>776>>6610/03300984478442./..,-
222447777<AAAA@??@;888>;//-0+,*)
@T6XXI:00139:00236
GGGGAATTCTAATACGACTCACTATAGGGAGAGGGAGAATAGTCAACAAATCGTTTGGGGCGACTTCTCCTTCCTTT
CTCCCTTCTCCC
+
999/99A@BBC@CBBBCBBBBCCB?661.6;6777:CAEAA??@@=ABA=B???E>BBB7AAC:;:;CC?@>@>@@:
?===697:444-
@T6XXI:00139:00248
CTATCGACTCGAGATCTGCGGCTTTGGACAGTCCCTCTGTACTGTGAAGCCCAGAGTGTCGATCGACTAGCTTCCTT
TCTCCCTTCTCCC
+
CA@@AAACCCCCCBAABBCB?BBC<B@BB>A:::5:CCCAAAC?@44//11-
1///..4;;BBBAAA9??:;8;ABB@>77707+***00
@T6XXI:00139:00249
GGGAGAAGGGAGAAAGGAAGCTAGTCACTCGACATCTGTGGCTTCACAGTACAGAGGGACTGTCCAAAGCCGCAGAT
CTCGAGTCGACTAG
+
CF@CCC@B?:=>==:@786;@?AAA9???@A;::B@@FCC8:<@CCCAAACCC=99946::;???AA<??:==AA:=
===;;<====<<@A
@T6XXI:00140:00211
GGGAGAAGGGAGAAGGAAGGAGAACATGTTGTGGGACAAAGCCAAACTATTCTCCCTCTCCCTATAGTGAGTCGTAT
TAGAATTCCCC
+
CHADCB@?;:;442949484833149=BB@B@BB;A??B>BB?AA>BBBBABCBB?:8;CC?BBBCC@@BCC@<<@@
>AAA>@<??>4
@T6XXI:00140:00217
GGGAGAAGGGAGAAAGGAAGCTAGTCACTCGACATCTGTGGCTTCACAGTACAGAGGACTGTCCAAAGCCGCAGATC
TCGTC
+
AB>BCC@FCABBBH@C?B?BD@@@AA0//7;00/57/0/B=?@?BBBBBAA?BA7738A;:;;344-
442434304355<..
@T6XXI:00140:00224
GGGAGAAGGGAGAAAGGAAGCTAGTCGACTCGAGATCTGCGGCTTTGGACAGTCCCTCTGTACTGTGAAGCCACAGA
TGTCGAGTCGACTAGCT
+
99149;:?>:@@>@9@=@=??>:900.....343611177=:===8?=@AABBEC?C@A??999@<<<9<<<??=@@
>877;;988340004-.
@T6XXI:00140:00227
GGGGAATTCTAATACGACTCACTATAGGGAGAGGGAGAATAGTCAACAAATCGTTTGGGGCGACTTCTCCTTCCTTT
CTCCCTTCTCCC
+
AA@4A=@=ACC@CCBBC>>>B@BB@8::8777AC>CCB=AAA===9=@?:ABBBB>BCC9BBCCC?CBB782448;6
<A@A<A=?@AA=
@T6XXI:00140:00236
GGGGAATTCTAATACGACTCACTATAGGGAGAGGAGAAT
+
,,-*,0>?CCB@CCBBBBBBB@BB@<<<8::55:;,++,
```

```
@T6XXI:00140:00238
GGGGAATTCTAATACGACTCACTATAGGGAGAGGGAGAATAGTCAACAAATCGTTTGGGGCGACTTTTCCTTCCTTT
CTCCCTTCTCCC
+
=@@6A?B@DCC@BAABBBB<;=D?>6795:??BC?BB@>A<B?==;=@A1::::?8ABC9DAABDDD7?=?=?444-
449?<A>@@@A?
@T6XXI:00140:00247
GGGGAATTCTAATACGACTCACTATAGGAGAGGGAGAATAGCCGATTCCTGGCCGACCCGCAATTTCTCCTTCCTTT
CTCCCTTCTCCC
+
999/9<A@CBB>B@?BCBBBB@@;;6637>@@BBBCA>@:<<<774224-
/,0:<0//.8<<>AA<@A@<>887;;6;?CC@C9<=?A@
@T6XXI:00141:00200
GGGGAATTCTAATACGACTCACTATAGGGAGAGGGAGAATAGTCTACCCATCTC
+
499,429244424444443?7;8;74...+//:=?=/19?@A;83-322+-...3
@T6XXI:00141:00209
CTAGTCACTCGAGATCTGCGGCTTTGGACAGTCCCTCTGTACTGTGAAGCCACAGATGTCGAGTCGACTAGC
+
6:1////49??BBCDCBBBA?ABC?B?ABCC???9?AAABBBBB=98;=C=@@<>;CCBAAAA?@A>??;;5
@T6XXI:00141:00217
GGGAGAGAATTTGGTAATGGGGTGAA
+
CG@GBBBB@BB=BA;:9.,,,*--22
@T6XXI:00141:00218
GGGAGAAGGGAGAAAGGAAGGAGAAGTCGCCCCAAACGATTTGTTGACTATTCTCCCTCTCCCTATAGTGAGTCGTA
TTAGAATTCCCC
+
BBABCB?BB?BCBB>B>A>??A?@?ABBAAAA8CC>CC@CC>=?=?BBB@A>@@@A<??@AC<A@????AAA@?@=<
<<@=:8:/444-
@T6XXI:00141:00220
GGGAGAAGGGAGAAAGGAAGACTACGGCGCTGTCT
+
DC>BBA@BC?AABB?B@C@BAAABBB@B@@ABCB9
@T6XXI:00141:00225
CGACTCGAGATCTGCGGCTTTGGACAGTCCCTCTGTACTGTGAAGCCACAGATGTCGAGTCGACTAGCTTCCTTTCT
CCCTTCTCCCCTTCCTTTCTC
+
==?CCCGHCCCCCC;;8;@@:ACCBBDCCCACCCA@@CCACBB@BB?BBBCCCBBA@:;:???=>>77968<?C<;;
;C<>:>6777.747477-0/+
@T6XXI:00141:00229
GGGGAATTCTAATACGACTCACTATAGGGAGAGGGAGAATAGCTTAGCACGTTACCTCGGCCTATTTTCCTTCCTTT
CTCCCTTCCTCC
+
CCD9DCCABB@@CCCC@>@BBA<>=766.7==?B>@@=>A?<=??BBABD@@=@B>>@D486;AABC9CAC@C@CB0
8000,0,0,0;9
@T6XXI:00141:00239
GGGAGAAGGGAGAAAGGAAGCTAGTCACTCGACATCTGTGGCTTCACAGTACAGAGGGACTGTCCAAAGCCGCAGAT
CTCGAGTCGACTAG
+
CC?B@;244-
9A??9A9968BABBB<A@??=771166??@;?AAACEEBABB;8003,4;;=@?@AA=AC;>>?@A=<@<==<<<??
?@@?
@T6XXI:00141:00241
GGGAGAAGGGAGAAAGGAAGGAGACACGGTCACAAACATGAATGTGACTATTCTCCCTCTCCCTATAGTGAGTCGTA
TTAGAATTCCCC
+
```

```
CHACDE@CB?A?>>:><>6:9;BBGCBB@CCCAAA=ABCBD?BBBBBBBCC?B?=99=AABC:?>?@ACA@@@@A@?
?<=;9784;::1
@T6XXI:00141:00243
GGGGAATTCTAATACGACTCACTATAGGGAGAGGGAGAATAGTGACGTGTTGTGCGGCGCTCACTGTCTCCTTCCTT
TCTTCCTTTCTCCCTTCTCCC
+
ACD:C?B?BBB@BB@@@BBBBCB>;111.7>=BC>CCA>A===8::;;;<;<====;===<<<===<<<<<><><>>
;==;<;<<:<@@@<<<AAAA<
@T6XXI:00141:00244
CTAGTCACTCGACATCTGCGGCTTTGGACAGTCCCTCTGTACTGTGAAGCCACAGATGTCGAGTCGACTAGCTTCCT
TTCTCCCTTCTCCC
+
C:///<6994449=>AGDDDECCC?C@BBBCBBB>BBCCA=?=<??A;=A@CCCCBB@@@CCCCBB@@@A@AA=@=@
C>CCED>@9<<<<7
@T6XXI:00141:00248
GGGGAATTCTAATACGCTCACTATAAGGGAGAGGGAGAATACGCAAAACGTTCTTCCCCATTTGCCTACT
+
A::,1,1,/,,0,,,/.3336771601>16763520,+*++,,,+3307:::6<<86602442..,.4..
@T6XXI:00142:00201
GGGAGAAGGGAGAAAGGAAGAGAACGAAACGGCCGGCGAATTGGGAACTATTCTCCCTCTCCCTATAGTGAGTCGTA
TTAGAATTCCCC
+
CC@@@@@BC>?BBB>B@B@A?AB@B;;;-
9?<@>BDBBCAC@CB?A?BBAA?BBBG>CAAA@>CA@;<<=?<==?9:5<<<><=88<;1
@T6XXI:00142:00202
GGGAGAAGGGAGAAAGGAAGCTAGTCGACTCGACATCTGTGGCTTCACAGTACAGAGGGACTGTCCAAAGCCCGCAG
ATCTCGAGTCGATAGGGGAGAAGGGAGAAAGAAAG
+
CC>AAB?BA<AB@==B=@=@C>99>/11>>>>@?BBB?@@D?BB@BBBBBBB?=;;<=<>98<==8<<7<AA====?
?@A@B8==A@>;;<==:.;8;8::76///,/.404
@T6XXI:00142:00206
GGGAGAAGGGAGAAAGGAAGCACAGGGCGAAGCGTGGGAAACTTGGCCTATTCTCCCTCTCCCTATAGTGAGTCGTA
TAGAATTCCCC
+
?B>EAA=AC>AAAC@CDC@A@@B@AA<????BBC@>>>>=3?;./..5;7A<??AA:;>444-
4444<<==<;<:;///:95574440
@T6XXI:00142:00211
GGGGAATTCTAATACGACTCACTATAGGGAGAGGGAGAATAGTCAACAAATCGTTTGGGGCGACTTCTCCTTCCTTT
CTCCCTTCTCCC
+
AAA7E=@?ACBACBB@@BBBBABB?6664:AACE>B@?=@<<<@A>@@@;BACAC;<:<6?@CCCCBCC??<?>AA=
ABAA=A<??AA<
@T6XXI:00142:00214
GGGGAATTCTAATACGACTCACTATAGGGAGAGGGAGAATAGTGTACAAC
+
CCC9C@C@CDB@BBBBBAA>@?CB?99:5:?<@B>BB;795,,++00..+
@T6XXI:00142:00220
GGGAGAAGGGAGAAAGGAAGCTAGTCACTCGAGATCTGCGGCTTTGGACAGTCCCTCTGTACTGTGAAGCCACAGAT
GTCGAGTCGACTAGCTTCCTTTCTCCCTTCTCCC
+
CC>B@@=@A<AB==:@786:A?<?@?:::AACCACC?@@C????>?@CCAA===;=@===<<====@><=<=<=9==
AA@@?AA=@==@A===>@>@B;????7>:==CD?
@T6XXI:00142:00224
GGGAGAAGGGAGAAAGGAAGCTAGTCGACTCGAGATCTGCGGCTTTGGACAGTCCCTCTGTACTGTGAAGCCACAGA
TGTCGAGTCGACTAGCTTCCTTTCTCCCTTCTCCC
+
```

```
CA<@>>;>>:>999094957BC>=>/00;;?>B@CCC???@@@@?;?>@??????;@<?@@A@?A<7758?<?>??@
@@BAA<@<<A??>??6;8;;==9=377179;=?A<
@T6XXI:00142:00233
GGGAGAAGGGAGAAAGGAAGCTG
+
CC?E<;;<BA@111,1-1,,,,,
@T6XXI:00142:00249
CTAGTCACTCGAGATCTGCGGCTTTGGACAGTCCCTCTGTACTGTGAAGCCACAGATGTCGAGTCGACTAGCTTCCT
TTCTCCCTTCTCCC
+
CA:<;A:::ECCCBBBBBBB>BBB>B>AACAAA@;@AAA@???@CAA=?B@BAB@ABBBCCCEA@@@AACC;;<;?B
C>???C9979444-
@T6XXI:00143:00202
GGGAGAAGGGAGAAAGGAAGCTAGTCACTCGACATCTGTGGCTTCACAGTACAGAGGGACTGTCCAAAGCCGCAGAT
CTCGAGTCGACTAG
+
CE@DCC@DD>DBB?=A=?=>CBABB8;:???:::@????C?AB@CC???@@@A<<:;:::::<<;=<<@C?A?:?<=
======AA?><<==
@T6XXI:00143:00203
GGGAGAAGGGAGAAAGGAAGCTAGTCACTCGACATCTGTGGCTTCACAGTACAGAGGGACTGTCCAAAGCCGCAGAT
CTCGAGTCGACTAG
+
AA<>@@58809>>>9A>B@CBBBCB7:;AAABAC??????<?B@CCBA>?A@>:43317>=@AA?AA9==<@?@@??
?AA@@@?@@A@===
@T6XXI:00143:00210
GGGGAATTCTAATACGACTCACTATAGGGAGAGGGAGAATAGTTCTGGACTGTACTCTGGGGCTGTCTCCTTCCTTT
CTCCCTTCTCCC
+
?>>2=8@<?@A>;;;@@@@:;7???777.777>?;BB@>A===>A@@=CB?>66::>==AB7CDDEDDC??;=377.
7>>?>C?;;0?,
@T6XXI:00143:00219
GGGAGAAGGGAGAAAGGAAGGAGAAAGCGAACGT
+
>@<@AADCNBCBBB>B@C@C@C@@?8:<0*)*((
@T6XXI:00143:00226
GGGAGAAGGGAGAAAGGAAGAAGAAAGCCGTTGACCTGATCAAAGGGCTATTCTCCCTCTCCCTATAGTGAGTCGTA
TTAGAATTCCCC
+
BC@CCB@CC?CCCB>B@B@BB@BBB<?B=??>???=>>>BB@?5777<BBCACCCC@A?FBA>AAABBCGDBBBB@B
B?BAC?C:@@@5
@T6XXI:00143:00232
GGGGAATTCTAATACGACTCACTATAGGGAGAGGGAGAATAGCCTAACGTTCCCTCGCAGTATGGTATTCTTCCTTT
CTCCCTTCTCCC
+
944,4:BACBA@A@@B??B??@B<>111.6<<=B>?@<;>>884:B>A>>4::6:@?@BA@@@A>@@@>AA=@>@A<
???A78676686
@T6XXI:00143:00246
GGGGAATTCTAATACGACTCACTATAGGGAGAGGGAGAATAGTCAACAAATCGTTTGGGGCGACTTCTCCTTCCTTT
CTCCCTTCTCCC
+
DDD:C=?=ABB@CCB?A????CB97111.777?B@CCA?AA@A@:8<<<5?B;;;8???5?BBCC?BBB?@=@?A==
?@@A<A>@???;
@T6XXI:00144:00201
CTAGTCGACTCGAGATCTGCGGCTTTGGACAGTCCCTCTGTACTGTGAAGCCACAGATGACTAGCCTCTTC
+
DECC@?AHHCDAAACCCBBBC@BBC>B?BECB@@@<?@???AA???FC?@CBCCCBBBBA>?A0-+***)*
@T6XXI:00144:00204
```

```
GGGGAATTCTAATACGACTCACTATAGGGAGAGGGAGAATAGCCGTTTAATCTCCCACTGCGGCATATCCTTCCTTT
CTCCCTTCC
+
CDC9C@D@CBC@B;;;110=>=???8::67?>@B>ABB@DCC@>;;86898../?9899=<<<<<<@@A;99>384-
422228,/,
@T6XXI:00144:00224
GGGGAATTCTAATACGACTCACTATAGGAGAGGGAGAATAGCACGGATCCTGCTCTCGCCGTGTTTCTCTTCCTTTC
TCCCTTCTCCC
+
BDD:D8;;<>@?BEDDBDB@;88?>8747>@AC>BBE@CB??@AC>?BC@C;;;????ABAAAAH<????=??AA=>
>?A<?/3444-
@T6XXI:00144:00228
GGGGAATTCTAATACGACTCACTATAGGGAGAGGGAGAATAGTTCTGACGGATTGCTCTGACTATCACCTTCCTTTC
TCCCTTCTCCC
+
EEE:CAD@CCCBCBCCBBA@@@@@@AAAAAB?CC8::?>@::89<<=ABB?B?=?@:::=<<:00///5777066,6
111,101000+
@T6XXI:00144:00236
GGGAGAAGGGAGAAAGGAAGTGTGGTGTACCTGTAGTGATGCGGGAGCTATTCTCCCTCTCCCTATAGTGAGTCGTA
TTAGAATTCCCC
+
EEADDC@CC>ABAD>B@B@BBBBB@B@===@CBCCC@==?AC@C>A??@CE@C@@@:878<<7=AACA@AAAA@@??
?>@@@>C@CCC9
@T6XXI:00144:00246
GGGAGAAGGGAGAAAGGAAGCTAGTCGACTCGACATCTGTGGCTTCACAGTACAGAGGACTGTCCAAAGCCGCAG
+
IDBC<<59@198=@=A>B?@????877771667<3111111779>;99?7777=743/000000/00+044044;
@T6XXI:00145:00200
GGGAGAAGGGAGAAAGGAAGGAGAAGTCGCCCCAAACGATTTGTTGACTATTCTCCCTCTCCCTATAGTGAGTCGTA
TTAGAATTCCCC
+
FDA@AAAGD>@@@@<@@C=@>@BA?BBCCCBB8BB>AAA??;CC@B:8;BB>A<<<7888AA<AA===A===@??>@
?;86444/444-
@T6XXI:00145:00207
GGGAGAAGGGAGAAAGGAAGGGTCACATTGCGGGAGTACAGGAGGTAGCTATTCTCCCTCTCCCTATAGTGAGTCGT
ATTAGAATTCCCC
+
DDDCCD>??>ADCC?B@B?BB=BBBBCC@CB@>;BBCBBBBABB?BA;;A@98=99909?>BCACCCCCCCCBBBCB
BD?BBEIKBECC:
@T6XXI:00145:00231
GGGAGAAGGGAGAAAGGAAGCTATCGACTCGACATCTGTGGCTTCACAGTACAGAGGGACTGTCCAAAGCCATCTGT
GGCTTCAC
+
CBBCDC>?=5=444-
9597<BBAABB7::?@AAB@BBBBB?BB?AC@@@@@>@@@@@>999A@@=?=:<<8;;<?=?@899>A??
@T6XXI:00146:00213
GGGAGAAGGGAGAAAGGAAGCTAGTCACTCGACATCTGTGGCTTCACAGTACAGAGGGACTGTCCAAAGCCGCAGAT
CTCGAGTCGACTAG
+
DEBDCB=>=:@AAA>B=@=@BA;AB<??@BA:::;::CCC@AB@CC@??@?AAC@@@><<<<==<=>;==><<<<=<
<<;9<<99888:==
@T6XXI:00146:00231
GGGAGAAGGGAGAAAGGAAGCTAGTCACTCGACATCTGTGGCTTCACAGTACAGAGGGACTGTCCAAAGCCGCAGAT
CTCGAGTCGACTAG
+
EE@@@?===:>988095949BB@BD@@>>>@000>>;=@C@?@@CCCBBBCCCC===;=@???@>A@<@A>AA====
=:::==AA???AAA
```

```
@T6XXI:00146:00232
ACTCGAGATCTGTGCGTTTGGACAGTCCCTCTGTACTGTGAAGCCACAGATGTCGAGTCGACTAGCTTCCTTTCTCC
CTTCTCCC
+
DGBAA<<47994444444-
42449999<1;BBGCCCAAACC@CC@DCB>97;011.333.33376>?<?899199880848317;
@T6XXI:00146:00236
CTATCACTCGAGATCTGCGGCTTTGGACAGTCCCTCTGTACTGTGAAGCCACAGATGTCGAGTCGACTAGCTTCCTT
TCTCCCTTCTCCC
+
:A99444499=@@AA;99@=888/377997111,1;@>@A????@@=<@=@C;;;C;<;A???A@?>;<;?899>AB
<@???6=?@744-
@T6XXI:00146:00241
CTATCGACTCGAGATCTGCGGCTTTGGACAGTCCCTCTGTACTGTGAAGCCACAGATGTCGAGTCGACTAGCTTCCT
TTCTCCCTTCTCCC
+
@::6///11:ACAAACCBCC@CAA>A?@@>A:::5:BBCBCCB=@@C@AC;<<CBB;;;CCCABBB???;786999B
C>A?BC>C@@@=A;
@T6XXI:00147:00206
GGGGAATTCTAATACGACTCACTATAGGGAGAGGGAGAATAGCTCTACTGTTTTCCCAGGCGTATCGTTCTTCCTTT
CTCCCTTCTCCC
+
A==6@BCBCBC@CBBAABBDBB;@@761.9?=?D@AAA@BCB;B@AC>>A@@@5@>:;;3111.../55::6===A>
?ACD@@;=884-
@T6XXI:00147:00210
GGGGAATTCTAATACGACTCACTATAGGAGAGAGATATAAGGGGTGCATGTATCTCTCTTCCTCCAGCCACG
+
CCC:C@C@BCB@B;111>>@C?==>1.,0;;>@CCCB@@>@@<5==ABCBB@@A>=;;;,0,00:0074776
@T6XXI:00147:00222
GGGGAATTCTAATACGACTCACTATAGGGAGAGGGAGAATAGTCACATTCATGTTTGTGACCGTGTCTCCTTCCTTT
CTCCCTTCTCCC
+
DFF9C@CABBB@CBBBBBBA>;<?<877.7>>?@<??C>B??=AAAAC@BBB@@?;?CB>;;88///1641430;<4
84AC;@>???@:
@T6XXI:00147:00242
GGGGGAATTCAATACGACTCACTATAGGGAGAGGGAGAATAGTCACATTCATGT
+
4444+48989B@?==:9110;4;03777-7;>>>.777477799443.,..433
@T6XXI:00147:00247
GGGAGAAGGGAGAAAGGAAGGGGAGAAGGGAGAAAGGAACTAGTCGACTCGACATCTGTGGCTTCACAGTACAGAGA
CTGCCAAAGCCGCAG
+
FF?@==5991999?:>595999/4<7777279776757+,,,2256=?@?=7784678,,+,,++229;;//,,,0+
+-335574445<<<<
@T6XXI:00148:00201
GGGAGAAGGGAGAAAGGAAGCTAGTCACTCGAC
+
<B>ACC@CCAA?>B<>=@>A;;00-,***,0,*
@T6XXI:00148:00209
GGGGAATTCTAATACGACTCACTATAGGGAGAGGGAGAATAGCTACGTTTAACCGATCGTGTTGTTCTCCTTCCTTT
CTCCCTTCTCCC
+
BBB9BACCCCB@BAAABBBBBA;99311.7==CC>BBB@C@>:=?@@A@;?>B?BABABA@A>@8:67733482<?;
?@<>;??A;;=;
@T6XXI:00148:00213
GGGGAATTCTAATACGACTCACTATAGGGAGAGGAGAATAGT
+
```

```
444-9<A@BCC@CBBBBBBBBBBBBC@>9=>6106//,0,,,
@T6XXI:00148:00223
GGGGAATTCTAATACGACTCACTATAGGGAGAGGGAGAATAGCGCAATTTGGACATGTGGCCGCTTCTCCTTCCTTT
CTCCCTTCTCCC
+
AA?6A=A>BCD@BBBBBABB>BBBB><<;<<<@C>BBB?B@C==>B@BB=A?BCBBBBBAC?G=A<773289;7;<7
<@AC?C?B@@@;
@T6XXI:00148:00225
GGGGAATTCTAATACGACTCACTATAGGGAGAGGGAGAATAGTGACGTGTTGTGCGGCGCTCACTGTCTCCTTCCTT
CTCCCTT
+
A?A8A?C@CCC@BBBBBB>>>B?99316.7>>>A>BCF@B>>;;<>><888488?@>@CAA@????=:99243/,/,
/888,/,
@T6XXI:00148:00230
GGGAGAAGGGAGAAAGGAAGAACATTTGCATCATGAAAGGGATTTGGCTATTCTCCCTCTCCCTATAGTGAGTCGTA
TTAGAATTCCCC
+
DDBCBB7;969B?A>C>A?AC@C?B?;BBAA??<@BC>CC>B@@=B@ABCC>B;;;;:<@A>;=???AAAAAA??98
7<:5323/003,
@T6XXI:00148:00233
GGGAGAAGGGAGAAAGGAAGGAGAGACACGTTGGGAGGAAACGATTGCTATTCTCCCTCTCCCTATAGTGAGTCGTA
TTAGAATTCCCC
+
GGBBGBABA@=?BC=A?A@B@BBB>>?CCAA?AA>BB?BC@CDCC@CCCDC@BBCC>BBBCC?BABBCCCCCC?@?A
B?BAA>A;AAA6
@T6XXI:00149:00205
CTATCACTCGAGATCTGCGGCTTTGGACAGTCCCTCTGTACTGTGAAGCCACAGATGTCGAGTCGACTAGCTTCCTT
TCTCCCTTCTCCC
+
C@BB<:::BBABBCBBB??=@CC?B>?AA@???=?CCC?:?A=ACC@AB?@BBBB@@>?@C@@BBCCAABAB>@<??
9:77?6?::?B>;7
@T6XXI:00149:00206
GGGGAATTCTAATACGACTCACTATAGGGAGAGGGAGAATAGACACCAATCGTGCCGGGTCATGTTCTCCTTCCTTT
CTCCCTTCTCCC
+
AAA6A@C@CCC@CCBBBBBCCCCCB>=<777BB>BCC@CBB?????A>C>@>?A5675:CCCCC?CAA<=:=;==6
;<AA<A><<<?1
@T6XXI:00149:00209
GGGGAATTCTAATACGACTCACTATAGGGAGAGGGAGAATAGTCAACAAATCGTTTGGGGCGACTTCTCCTTCCTTT
CTCCCTCTCC
+
EBC9C@C@CBA?BDCBBA?BBBB?;76659==BC>BBB?B@CBCC@@@@;A?777,116-
8>?BC@BBB@CAA>==9:;;;..0776
@T6XXI:00149:00210
GGGGAATTCTAATACGACTCACTATAGGGAGAGGGAGAATAGTCAACAAATCGTTTGGGGCGACTTCCTCCTTCCTT
TCT
+
CCD9C=@<<;9<AHCCBBCCCB@@>877.777=?:B::7:BB@A@>@@B@BBBA@:>>@5@:@<>,0,0<9:98544
28-
@T6XXI:00100:00258
GGGAGAAGGGAGAAAGGAAGCTAGTCGACTCGACATCTGTGGCTTCACAGTACAGAGGACTGTCCAAAGCCGCAG
+
CC::=BABB9<;;909;>;?@?BBB;10>>;010.;45><@@<@=?BB;;;CBB@8834:9335234+3354002
@T6XXI:00100:00265
GGGGAATTCTAATACGACTCACTATAGGGAGAGGGAATAGCCACGGCTCGCTCATGCTGGTTCTCCTCCTTTCTCCC
TT
+
```

```
8B>18+4699><@@@@CBBBCCBA@:97.7>777.0:0--
.,../549779;;88633911016601697761000+-,
@T6XXI:00101:00261
GGGGAATTCTAATACGACTCACTATAGGGAGAGGGAGAATAGTCAACAAATCGTTTGGGGCGACTTCTCCTTCCTTT
CTCCCTTCCTCC
+
?C?6B;@=<;;?B;;;A;7:76<<1667,..///,/.-347====569=;96616266621..44,..3,-,-,-
24;===;;/-,--,
@T6XXI:00103:00257
GGGAGAAGGGAGAAAGGAAGAGAACGGCGACGAACAATAGTTGGTGGCTATTCTCCCTCTCCCTATAGTGAGTCGTA
TTAGAAACTT
+
?C>CCB>@@=;99D1959<<<777>@;=>@F<<><G@EC@L??=00-
0;;460.11,166668<:::<:93333333343---+--,
@T6XXI:00105:00253
GGGGAATTCTAATACGACTCACTATAGGGAGAGGAGATATAGGACC
+
88708+;:=CCBCE@@@@BBBCBBBCGL>BA=>:>/.-33-02-22
@T6XXI:00106:00260
GGGAGAAGGGAGAAAGGAAGGAGCATAACGACCAGATATACCTGCACGCTATTCTCCCTCTCCCTATAGTGAGTCGT
ATTCAGAATTCCCC
+
CE>BBB@CC>BBBC>@?@@C@CCCBBBDCCBA?BCAA@A?A:<9ABA<==BB<>9998;A?BGED@@@@@DA@AA>/
//.//88,/,444,
@T6XXI:00107:00272
CTAGTCACTCGAGATCTGCGGCTTGGACAGTCCCT
+
7:1//::A:::BCBBBB<<;900,.,..43.11,1
@T6XXI:00108:00275
GGGGAATTCTAATACGACTCACTATAGGAGAGGGAGAATAGTCAACAAATCGTTTGGGGCGACTTCTCCTTCCTTTC
TCCCTTCCTCCC
+
CB@4@546<AA?CCBBCCCBB>>=;./,0<<?@=BB@>@@CAAA=@@A<B@@?D<@@B6???A?377730/49>A;?
7:733,/-/;73
@T6XXI:00109:00273
CTAGTCACTCGAGATCTGCGGCTTTGGACAGTCCCTCTGTACTGTGAAGCCACAGATGT
+
A:<;;B==ACABAB<;<A>@<888/8487<7:6;;?:::@A:::142,-/,/8644777
@T6XXI:00110:00254
GGGGAATTCTAATACGACTCACTATAGGGAGAGGGAGAATAGTACGACAAATCG
+
///+1:><@BB?BCBBBAA@?@AC@:86666177.7>;:==5,,,,,++00311
@T6XXI:00110:00258
GGGGAATTCTAATACGACTCACTATAGGGAGAGGGAGAATAGTCAACAAATCGTTTGGGGCGACTTCTCCTTCCTTT
CTCCCTTCCTCCC
+
BBB7C=@=@BB@BBCCCBB@BCB>;111.677>>:@B@>@AAAA@>@A@;BBBBC>CCC9C???@;==?;;9=CAC?
@;::68-/./;;6
@T6XXI:00110:00277
CTAGTCGACTCGAGATCTGCGGCTTGGACAGTCCCTCTGTACTGTGAAGCCCACAGGAGTACTCGATCGTT
+
BB@>@<9;AACCECCC@C>>>;00,09;;;>>BB>BCBAABA???=:7499094882333........33,
@T6XXI:00111:00251
GGGGAATTCTAATACGACTCACTATAGGGAGAGGGAGAATAGTTTTTCCCTTCACTGACCGCGAACTGCACTTCCTT
TCTC
+
```

```
8A717+/,/667649;@@CCCBCB@?==.77@AB>CCA=;;44444,4948/01;888C569:><;>3368666011
,11.
@T6XXI:00111:00272
GGGAGAAGGGAGAAAGGAAGGGGAGAAGGGAGAAAGGAAGCTAGTCGACTCGACATCTGTGGCTTCACAGTACAGAG
GGACTGTCCAAAGCCGCAGATCTCG
+
BC>AACACB<;;;;09>@>BBA7B@@=?B9=7:9-
7;><@???ABBECBBC=338888=AA>@?>A@A>?@AB93444-4;;<<=;=C;@@;===?=@>888
@T6XXI:00111:00278
GGGAGAAGGGAGAAAGGAAGAGAACAGGCACGCATGTAAAATGGTAACTATTCTCCCTCTCCCTATACGTG
+
CD<@>>6990944C08242333<;CCE@CBDCAAA=;9BA6;@6948844427799676197,116111..
@T6XXI:00111:00279
GGGGAATTCTAATACGACTCACTATAGGGAGAGGAAGAATAGTCCCTGTGTTCTCCCCACAGCGGTTAACCTTCCTT
C
+
FID:B79:<489A;<<C@@@@??>>777-,7,,+,*,00006330+0946<;<57774./+3630003*+*-
220343
@T6XXI:00111:00295
GGGGAATTCTAATACGACTCACTATAGGGAGAGGGAGAATAGTTTTCCCTTCACTAACCGCGAACTGCACTTCCTTT
CTCCCTTCTCCC
+
BA?7A=?>ADD@CB@AB@AAB@>97111.7>>>ABAFA=;84444-
4;9?>?CBCD@@=:8<?@;;<<??><>477.7<=?7A>888A;
@T6XXI:00111:00298
GGGGAATTCTAATACGACTCACTATAGGGAGAGGGAGAATAGTTTCCCTA
+
FDC:C@A>@@@=ABBBBCCCB@BCA9::5:??AB<A:834:899-44-/8
@T6XXI:00112:00251
GGGGAATTCTAATACGACTCACTATAGGGAGAGGGAGAATAGTTTGTCGTTTTCCCCGCAGCTCTGTTCTTCCTTTC
TCCCTTCTCCC
+
CBC7B798<A@BGCCCBBBBBA>97111.7>>BC>AA??A<<<=<===@;;;-
444,4=;<<=@?=?9;<:;24818;@A;@>@???7
@T6XXI:00112:00264
GGGGAATCTAATACGACTCACTATAGGGAGAGGGAGAATAGTCAACAAATCGTTTGGGCGACTTCT
+
8AA0A*//665699;DBBBCA??=777-7=>>@9AB?<???834244=;B=945+..+..0,,*,3
@T6XXI:00112:00276
GGGGAATTCTAATACGACTCACTATAGGGAGAGGGAGAATAGTCAACAAATCGTTCGGGGCCGACTTC
+
BBB6B6969>><ABA@@@@@@@B?7777.7=777.;?=?@A@B=A>???:???573333*.,.,00*+
@T6XXI:00112:00277
GGGGAATCTAATACGACTCACTAT
+
;@A6=,1///,/4>>>>787..6.
@T6XXI:00113:00261
GGGGAATTCTAATACGACTCACTATAGGGAGAGGGAGAATAGTTTTCCCTTCACTAACCGCGAACTGCACTTCCTTT
CTCCCTTCTCCC
+
BAA7A;==@B@@CCCCCCCBAB@?=677.777?B?BB@?AA@@??5>>:?<?ACCC?C>AACC>@@A=@?A>B;??=
?CBC<B?=@?A<
@T6XXI:00113:00297
GGGAGAAGGGAGAAAGGAAGGAAAATAGCGGAACTTGGAGTTCAGATCTATTCTCCCTCTCCCTATAGTGAGTCGTA
TTAGAACTTCCCC
+
```

```
BC@C@@=@>:>>>@<A=@<?5888.8?=@@=@?AC@C?CBC@CCCCDBCCFDBBBC<;::AA:??????BB@=837;
;8;8A>AC>AA@5
@T6XXI:00114:00255
GGGAGAAGGGAGAAAGGAAGGGACAGTCCATCTGTGGCTTCACAGTACAGAGGGACTGTCCAAAGCCGCAGATCTCG
AGTCGACTAG
+
DDACCC@BB?CCCB=@>?<;@<=;;=00,0010@BC@BA?ABCBAABB??>>?7?@@@AA>ADE>=:=AAAAA===?
??>BBA@AAC
@T6XXI:00114:00294
GGGGAATTCTAATACGACTCACTATAGGAGAGGGAGAATAGCTACGTTTAACCGATCGTGTTGTTCTCCTTCCTTTC
TCCCTTCTCCC
+
CEC9B8;69>?;@BBBBBBCB7663./,0<07::;CA?A@@?BABA@A<?;?=>??=AA@?===8;;;7:9;344+4
39:7>;899C<
@T6XXI:00115:00254
GGGAGAAGGGAGAAAGGAAGCTAGTCGACTCGACATCTGTGGCTTCACAGTACAGAGGGACTGTCCAAAGCCGCAGA
TCTCGAGTCGACTAG
+
CCABAA=>=5@@@@<B?@>BB?;;;/00:::<0:6776>@B5:;9:CBCBBCCAA87717?=?AA>@@<@A>A???A
A???>>==<;78443
@T6XXI:00115:00274
GGGGAATTCTAATACGACTCACTATAGGAGAGGGAGAATAGATCACCGTTGGTTCGGGTGCGCGTTCTCCTTCCTTT
CTCCCTTCTCCC
+
CDC9B7;;<@@AC<<<CBBBC>;6://,00077-
7?=9@@888???5;:??=?@CCC?CCCGC??;?7746:<:??9;;8:6:9/./90
@T6XXI:00115:00275
GGGAGAAGGGAGAAAGGAAGGAGAAGTCGAAGGCACATCGATGCCAGCTATTCTCCCTCTCCCTATAGTGAGTCGTA
TTAGAACTTCCCCATCACCTGACCT
+
CF@BBB>@A<;;;C>B?C@C@CCC@@<>9:58=:9<AAA><9998;BBCCB;?994-
444;@>CBCA@@A>7733111888@@BB>;?@7@7333,....,.
@T6XXI:00115:00294
CTATCACTCGAGATCTGCGGCTTTGGACAGTCCCCTCTGTACTGTGAAGCCACAGATGTCGAGTCGACTAGCCTTCC
+
AAAB<::BDBBCC?;<CCB?:::68=>>777===2=7777:@@?;;?8;@=@A@BBCCACCCC?@@110,,,*,*,*
@T6XXI:00116:00273
GGGAGAAGGGAGAAAGGAAGGGGAGAAGGGAGAAAGGAAGCTAGTCGACTC
+
CJCCCCCDC>BBCN;<;<@DCC9BBB8:7.7777.70747=611711134.
@T6XXI:00116:00279
GCTAGTACGACTCGAGATCTGCGGCTTGGACAGTCCCTCTGTACTGTGAAGCCCACAGATCGACTAGCCTTCCTTTC
TCC
+
/,/66/////6;BBBBCBBA888+,,*,*,,2,,.776116600030004044-
339931616666110/66,..+....
@T6XXI:00117:00270
GGGAGAAGGGAGAAAGGAAGAGAACAGCGACGGGAATAATGTTTTAGCTATTCTCCCTCTCCCTATAGTGAGTCGTA
TTAGAATTCCCC
+
DD>A@@;=>:999@8;9<BDCCC?CAABCCCCE@C@CB?CCCBC9CCCCEF>@<<<89@ABC>CBB@@ACAAA:::A
B?B@@@=56<<1
@T6XXI:00117:00298
GGGAGAAGGGAGAAAGGAAGTGCAGTTCGCGGTTAGTGAAGGGAAAAAACA
+
@D@<><@BB>BBCC@C@C@CCCDCCB?BBBA?B@A>>88400,.....(.,
@T6XXI:00118:00258
```

```
GGGGAATTCTAATACGACTCACTATAGGGAGAGGGAGAATAGCACGTTGATAACAAGCGCGACTATTCCCTTCCTTT
CTCCCTTCTCCC
+
CCB9C8;:<AA>BCCBBBBDBBB<899959??CC>AA?=@???A=AA?BBCC?>@??????<<=?@=@@7=8:2441
79:;4;388;;:
@T6XXI:00118:00265
GGGAGAAGGGAGAAAGGAAGGAGAACATGACCCGGCGCGATTGGTGTCTATTCTCCCTCTCCCTATAGTGAGTCGTA
TTAGAACTTCCCC
+
EDACBB@BC@BBBC?C@D@B?BCC@CBBBCBC@C?BBCCCC=?=?CBCBIB57::7.7??AB>C@??????>>==<<
<9<@D=A@<???5
@T6XXI:00118:00269
GGGGAATTCTAATACGACTCACTATAGGGAAGG
+
BCH:G;;<<AB=;<<@A??@BB@B:000,,+,2
@T6XXI:00118:00275
GACTCGAGATCTGCGGCTTTGGACAGTCCCTCTGTACTGTGAAAGCCCA
+
=@BCB;<:B?ABCDGAHCBAD>><<CCBC?CC<9<?8::93//+/68+,
@T6XXI:00118:00281
GGGGAATTCTAATACGACTCACTATAGGGAGAGGGAGAATAGTCAACAAATCGTTTGGGGCGACTTCCTCCTTACCT
TTCTCCCTTCCTCCC
+
6<<0;,954449?BBBCCDCDBBB>777.7=777.7?;=?===<<:::=;>8667,111,1.452*,*,2*+*,,22
3675775544-..43
@T6XXI:00119:00256
CTAGTCGACTGAGATCTGCGGCTTTGGACAGTCCCTCTGTACTGTGAAGCCACAGATGTGGATGCTTCCTTTCTCCC
TTCTCCC
+
EEBCBBCBBBBBCCCBCCCC?ABI?C?BACB:775:A?BA@AA=??A>?B?BCCCCCBBB@B>?9969588/8>>?0
8476=@9
@T6XXI:00119:00270
GGGGAATTCTAATACGACTCACTATAGGGAGAGGGAGAATAGTCAACAAATCGTTTGGGGCGACTTCTCCTTCCTTT
CTCCCTTCCTCCC
+
CBD7B5;:<AB@CCCCBAACCA@>=677.7=<@B?CCA>@<==CC;==A<C???C8?==5?:;:@>B??:;8;7;B6
;43622,/.,551
@T6XXI:00119:00273
GGGGAATTCTAATACGACTCACTATAGGGAGAGGGAGAATAGTTGTCAGCTGCGCTCACACTCATTCTCTTCCTTCT
CCTCTCCC
+
:??6?>B?<<;>A;;<A??8;7<>;676,1117799>:/3300/0001666166997696111OO/0000/023255
=999>>>4
@T6XXI:00119:00280
GGGAGAAGGGAGAAAGGAAGCTAGTCGACTCGACATCTGTGGCTTCACAGTACAGAGGGACTGTCCAAAGCCGCAGA
TCTCGA
+
@@<ABA@CC>AABB=B@C@CCC:<;CCAA???;::??=B??;?A>@AA@@A@?;;444-
4;;;?@=@B;@@<@A???A?A?@@
@T6XXI:00120:00256
GGGGAATTCTAATACGACTCACTATAGGGAGAGGGAGAATAGTCAACAAATCGTTTGGGGCGACTTCTCCTTCCTTT
CTCCCTTCCTCCC
+
CCG:F?ABADFACCCCCCBBBBA?=77:67>>AB>B?=:=@@@@@@=<<7?BABC>CCC9AAAA?;6:7;;8:/44-
8:8827,/,/673
@T6XXI:00120:00258
```

```
GGGGAATTCTAATACGACTCACTATAGGGAGAGGGAGAATAGTTTCTCGTTAGGCGCCCTGTGACTGTTCTTCCTTT
CTCCCTTCCTCCC
+
CCB6B@A@ACCCCBBBBCCBB@BB@77749??BC>BB@<??@@B<@?>>A?AA><8884<??A@AA@A899?A>AC7
<5=?9?00..65+
@T6XXI:00120:00266
GGGGAATTCTAATACGACTCACTATAGGGAGAGGGAGAATAGTCAACAAATCGTTTCGGGGCGACTTCTCCTTCCTT
TCTCCCTTCCTCCC
+
8==1=+1,/66,/9=@CCCCCCBBB???9??@BCCCB@>@@@AAA;8//-/337?-
/.55*./63;/004/0/356;;<74701,.,-773
@T6XXI:00120:00273
GGGGAATTCTAATACGACTCACTATAGGGAGAGGGAGAATAGTTTTTCCCTTCACTAACCGCCGAACTGCACTTCCT
TTCTCCCTTCTCCC
+
DCC:C799;=<@CDDBBBCC@?@BC???<:::ACACB@?B@AABCD0<<7;<?AAA?>@:0/,08,/.>>AAA>>01
7-717>6?:<88=;
@T6XXI:00120:00281
GGGGAATTCTAATACGACTCACTATAGGGAGAGGGAGAATAGATCACCGTTGGTTCGGGTGCGCGTTCTCCTTCCTT
TCTCCCTTCTCCC
+
CLD:C>@=ABB@CB@@?BCBB@BB?696-
6==?B>BCA>A=<???CC:??8:5:>BBB?BBBBCBCACBB@B?B?BJCCB@A;@?888>7
@T6XXI:00120:00282
GGGGAATTCTAATACGACTCACTATAGGGAGAGGAGAATAGATCACCT
+
BDC7B798<=99@CBBBADCCCCAA??=.777737@;8=?8800,*)*
@T6XXI:00120:00293
GCTAGTCGACTCGAGATCTGCGGCTTGGACAGTCCCTCTGTACTGTGAAGCCACAGATGTCGAGTCGACCTAG
+
44//////////494444499943313133333119;?<??>::776942392944;<7787761194,,*,0,
@T6XXI:00121:00261
GGGAGAAGGGAGAAAGGAAGCTAGTCGACTCGACATCTGTGGCTTCACAGTACAGAGGGACTGTCCAAAGCCGCAGA
TCTC
+
CK@ECBADDA:;;C?C=A=@BBBBBBCCB>>>>>>//0@::97816=?@?@?B;;478-4:;444344-
4928377;<966
@T6XXI:00121:00262
GAGATCTGCGGCTTTGGACAGTCCCTCTGTACTGTGAAGCCACAGATGTCGAGTCGACTCGAGATCTGCGCTTG
+
AAAACAAACC<<<C?D@CA>>888088899>>>>=610117?@?@?D@?@AAAA@@@@@999>83777..,0*,
@T6XXI:00121:00268
GGGAGAAGGGAGAAAGGAAGCTAGTCACTCGAGATCTGCGGGCTTTGGACAGTCCCTCTGTACTGTGAAGCCACAGA
TGTCGAGTCGACTAGCTTCCTTCTCC
+
BH@@@@7;;9<DCDDCAA=@A@@AA?<967??<C???A877.777.7:=?@AABC5:::834;;38<B===;=<@@;
;<<<;;A@<<A@;97/.,+*+*++00
@T6XXI:00121:00269
GGGGAATTCTAATACGACTCACTATAGGGAGAGGGAGAATAGTCACAATTCATCGTTTGTGACCGTGTCTCCTTCTC
CCTATAGTGAGTCGTATTAGAACTTCCCC
+
@==2@:B699;58?ABCCBACBA?=777.7=777.76;78989933.,,000,,,00*,,03337<<65<>3869AB
C@@<<=AAA?A?;;;48/4442449=A;0
@T6XXI:00122:00254
CTAGTCGACTCGAGATCTGCGGCTTTGGACAGTCCCTCTGTACTGTGAAGCCACAGATGGACTGTCTTCCTTTCTCC
CTTCTCCC
+
```

```
A?=@<<9BCEDGEDCD@A@BB=?AA<@?AA?A77:5:BBCCCBB@CB@=8;;<CBBC<<<<CB?88737411,166;
667=;;?6
@T6XXI:00122:00258
GGGAGAAGGGAGAAAGGAAGGGGAGAAGGGAGAAAGGAAGCTAGTCGACTCGACATCTGTGGCTTCACAGTACAGAG
GGACTGTCCAAAGCCGCAGATCTCGAGTCGCTAG
+
CEACBB@CC@BBBB=B@CBCCD>@@??>>9AA?A8A;?;?>?>>>/11>??B=9=<<?@@A??=<<<<=====<<<<
=;==<;??:8857738AAC@><=====??===@?
@T6XXI:00122:00273
GGGGAATTCTAATACGACTCACTATAGGGAGAGGGAGAATAGTCATGTGTCAATCATCTCGCTTCTCTTCCTTCTCC
CTCTCC
+
A??6A<@<@>A54;AAA?AAA:;8=777-76=;;2?888:<::5///:/779;AA?==<<=<A?884=58264/--
2221--,
@T6XXI:00122:00279
GGGGAATTCTAATACGACTCACTATAGGGAGAGGAGAATAGTACGA
+
C871<-95;=@?CCCBCBBBCCBA?996.7>>>=B;756/++++++
@T6XXI:00123:00262
GGGGAATTCTAATACGACTCACTATAGGGAGAGGGAGAATAGTACGACAA
+
7>>5>>B@CBB@CBBBA??BAAAB<<?866=6695:B;9;0,,,,++000
@T6XXI:00123:00268
GGGGAATTCTAATACGACTCACTATAGGGAGAGGGAGAATACGTGACGTGTTGTGCGGCGCTCACTGTCTCCCTTCC
TTTC
+
499/4292444944:<;;BC@?@B???A<AAACCAD@>780//5755>@@@;830332300004383000 4<78750
0010
@T6XXI:00123:00273
GGGGAATTCTAATACGACTCACTATAGGGAGAGGGAGAATAGTTTTTCCCTTCACTAACCGCGACTGCACTTCCTTT
CTCCCTTCTCCC
+
@>>4>698<GFADCCBB?@@A?>?>777.;BABD=;<9>AABCDCC0771028@BBB@>=>//,,00/::=770174
67>?.7340344
@T6XXI:00124:00267
GGGGAATTCTAATACGACTCACTATAGGGAGAGG
+
/1/*/,1,///,/49;433776336666,11111
@T6XXI:00125:00252
GGGAGAAGGGAGAAAGGAAGTGCAGTTCGCGGTTAGTGAAGGGAAAAACTATTCTCCCTCTCCCTATAGTGAGTCGT
ATTAGAATTCCCC
+
CC>CFC@A=6>;;;6<?A@C@@ABBB?B@@?>A>@@@EC@BB>CCCC2CCAA;8???:::;CLAD???@???<9:::
44/43823/003,
@T6XXI:00125:00253
GGGAGAAGGGAGAAAGGAAGCTAGTCACTCGACATCTGGTGC
+
ED?AABBCE>AABB<>=@=???BDCBBCBBD0,*****)***
@T6XXI:00125:00254
GGGGAATTCTAATACGACTCACTATAGGGAGAGGGAGAATAGTCAACAAATCGTTTGGGGCGACTTCTCCTTCCTTT
CTCCCCTTCTCCC
+
CCC:C=@>ACCBCAA@@>BBBABB;66649BACC>AA?<??@??A?AAA<C???C>@@?8@CA?A=9@A5888?A;9
;4444-43434=4
@T6XXI:00125:00263
GGGGAATTCTAATACGACTCACTATAGGGAGAGGGAGAATAGTCGTGTCACAGGCACCTCCCAGTCGTCTCCTTCTC
CCTTCTCCC
```

```
+
444,42499>?;>?ABA>??;7886111,767A>89<;84477>===?<::8=::;;8/11,.......1197216;
716235544
@T6XXI:00125:00268
GGGAGAAGGGAGAAAGGAAGGAGAATACAGCGAAATGAACACGTCGGCTATTCTCCCCCTCCTC
+
IH@BCA@CD@CCCC<@=@>?;?@A?ABB@A????:??BD>;733..,.4<>:=61111+110-2
@T6XXI:00125:00270
GGGGAATTCTAATACGACTCACTATAGGGAGAGGGAGAATAGCCATTCCTTGTTTCAGTTCCCGCTGTTCTTCCTTT
CTCCCTTCTCCC
+
CCC9B>B@BCC@BB>>@BBBA@@99116.6?=BC>BB@>AA??<??=@>@>AAA:????=?;;===99;<<99/781
4;;?:@;><;;6
@T6XXI:00125:00276
GGGGAATTCTAATACGACTCACTATAGGGAGAGGGAGAATAGTTACTAACTACTAGGCGCAACTGTTCTCTTCCTTT
CTCCCTTCTCCC
+
@@@4@;><>>@=@CA@@AACCBAA=676.7==@CCBB@>AA??>?A@@>===AA@@899@@<??AA<?=<<:=;>>:
=8::4:7<;<A<
@T6XXI:00125:00292
CTAGTCACTCGAGATCTGCGGCTTTGGACAGTCCCTCTGTACTGTGAAGCCACAGATGTCGAGTCGACTAGCTTCCT
TTCTCCCTTCCTCCC
+
BA;<<A9==??=@@AA@@@A@ECCAC=@?8777:5:BBCB@@@BB??<@B?BBBAA;;;B??=:;9888;;867706
75:54:64,/,.//,
@T6XXI:00125:00294
GGGGAATTCTAATACGACTCACTATAGGGAGAGGGAGAATAGACACCAATCGCGCCGGGTGATGTTCTCCT
+
CCC:A6<59448888;;7@;>000....+..1662610/0803000/331?;7=;2:956444330---,-
@T6XXI:00125:00297
CTATCACTCGAGATCTGCGGCTTTGGACAGTCCCCTCTGTACTGTGAAGCCACAGATGTCGAGTCGACTAGCCTTCC
TTCC
+
?B?99444=9A>>=@AA?@=@@CCCECC???BEE9CAABBFCCB@@@38;>;;8@?@@@@@@:79>>8733.,.554
4,.,
@T6XXI:00126:00250
GGGAGAAGGGAGAAAGGAAGGAGAAGTCGCCCCAAACGATTTGTTGACTATTCTCCCTCTCCCTATAGTGAGTCGTA
TTAGAATTCCCCC
+
HIBCCB@BCA?@=BBB@B@C@D@@=?BA??@@7@C>ECBBB>CC@CBBBAC:677718>>@A<A@@@@<AA@?>>?@
@>A@@=?88666.
@T6XXI:00126:00254
CTAGTCGACTCGAGATCTGCGGCTTTGGACAGTCCCTCTGTACTGTGAAGCCACACGATGT
+
A@99;<99ABCDBBCCCCBBB@CCDAC@B??>777.777?AB?@<>0/,-2206///6447
@T6XXI:00126:00276
GGGGAATTCTAATACGACTCACTATAGGGAGAGGGAGAATAGTACTCCGC
+
ADC8A=<==BCAEBBABCCCCCCC@87756A?BB>B?;8;7../.--,--
@T6XXI:00126:00279
GGGGAATTCTAATACGACTCACTATAGGGAGAGGGAGAATAGTCAACAAATCGTTTGGGGCGACTTCTCCTTCCTTT
CTCCCTTCTCCC
+
AA@7@=@=@BBADBBAAABCB@A97111.7>>?BAB@=?AA@@=;9444-
7>>>A9???5=????;=??6859?CC>A98857<=?@B;
@T6XXI:00126:00290
ACTCGAGATCTGCGGCTTTGGACAGTCCCTCTG
```

```
+
CBBDABBCA>@@AB>BBB=BA@@@B111,1>--
@T6XXI:00126:00292
GGGAGAAGGGAGAAAGGAAGGAGAAGTCGCCCCAAACGATTTGTTGACTATTCTCCCTCTCCCTATAGTGAGTCGTA
TTCAGAATTCCCC
+
CB>CCC@CC<A;;8096958<>8841=?8888.37,37@BD>E>=>11/842:444,000>A<A999AB;9;/0/78
8-77?@794988.
@T6XXI:00126:00295
GGGAGAAGGGAGAAAGGAAGCTAGTCACTCGACCATCTGTGGCTTCACAGTACAGAGGGACTGTCCAAAGCCGCAGA
TCTCGCAGCTCACGTAGC
+
HKC<<;>@>:?>>>9>5958?>>???>;5=:00,....;:@888@C<;:CCECBB333-
49::;><??:?@><<<?<<9:4444/-------21-
@T6XXI:00127:00252
GGGAGAAGGGAGAAAGGAAGAGAAAAGCAGTGTAATGGGGTCGAAAACTATTCTCCCTCTCCCTATAGTGAGTCGTA
TTAGAATTCCCCGGAG
+
AA?ACCACB@CBBB=B@B@>;@AAA7ACD@B@DD?AAAA7>AA@??8?CB@<?@AD====??:?@@AAAA@@AA@?=
<986444/000+-0--
@T6XXI:00127:00292
CTATCACTCGAGATCTGCGGCTTTGGACAGTCCCCTCTGTACTGTGAAGCCACAGATGTCGAGTCGACTAGCCTTCC
TTTCTCCCTTCTCCC
+
CCCB@AACCCBCCCBBBBB@CCC>B?@>>>7777.7>>BBCCC?==A=?B?BCBBBGBCCDD::;A000...,.,.,
.77:98;13230444
@T6XXI:00127:00298
GGGAGAAGGGAGAAAGGAAGCTAGTCGACTCGACATCCTGTGGCTTCACAGTACAGAGGGACTGTCCAAAGCCGCAG
A
+
CC@?><@CB>;;22-1:<-0000::BBA@>=:00000,.5557<>=????@A@?::444-
4:::4434:4:;:8//;;
@T6XXI:00128:00256
GGGGAATTCTAATACGACTCACTATAGGGAGAGGGAGAATAGTTTTTCCCTTCACTAACCGCGAACTGCACTTCCTT
CTCCCTTCTCCC
+
:@:,95924988=BCBDBCCCCCA?777.777>>9??A?A????>=1=B7<:=BBAC@D?A@A?>AA@=??@=@;??
?>>>,0,/...+
@T6XXI:00128:00270
GGGAGAAGGGAGAAAGGAAGCTAGTCACTCGACATCTGTGGCTTCACAGTACAGACGGGC
+
9=59@A<>>:>9991949588887>=3.........5470131366668>7700333+0
@T6XXI:00128:00271
CTCGAGATCTGCGGCTTTGGACAGTCCCTCTGTACTGTGAAGCCACAGATGTCGAGTCGACTAGCTTCCTTTCTCCC
TTCTCCC
+
EDCDCCCB@@@@AACCE@GCCAAAAA@>AA@ABDCCBCBC?;;9<DBABE@@ABCAACB;;;?899<>599199>@>
C?BBBL@
@T6XXI:00128:00275
GGAGAAGGAGG
+
1-/,,*,*,/,
@T6XXI:00128:00285
GGGGAATTCTAATACGACTCACTATAGGGAGAGGGAGAATAGTCTTGTATTTTGTGCATCACTTTTCTCCTTCCTTC
TCCCTTC
+
```

??>9>1969>><>@A@@@CCCCECA:::7:BCCC>BBA?A<A@;;4988883.38;??AB=6167-79779,/,/,-
2222778
@T6XXI:00128:00297
GGGGAATTCTAATACGACTCACTATAGGGAGAGGGAGAATAGTCAACAAATCGTTTGGGGCGACTTCTCCTTCCTTT
CTCCCTTCCTCCC
+
FCF:C@C@DCD@BBCCCBCC>=@B????<???CC>??=>A???@@<??C?AAABD@CCC7AA???7;9=:8/837?:
?437,.,/,--10
@T6XXI:00128:00299
CTATCGACTCGAGATCTGCGGCTTTGGACAGTCCCCTCTGTACTGTGAAGCCACAGATGTCGAGTCGACTAGCCTTC
+
<9@;4449444@=>>><;@?58;;6:@??><7777.7>>?>:::;;3440;79=?ACAAA=9?;184...6..,.,.
@T6XXI:00129:00250
GGGAGAAGGGAGAAAGGAAGCTATCGACTCGACATCTGTGGCTTCACAGTACAGAGGGACTGTCCAAAGCCGCGATG
C
+
CC?AA@@AA<BA@B>B@C@CCCCB??7::?=???AAA??A6:;=@CCBECBA@?444-4:;;;;344-
4;28343331
@T6XXI:00129:00253
GGGAGAAGGGAGAAAGGAAGGAGGACAGACGGCAAGTAATTGCATGCTATTCTCCCTCTCCCTATAGTGAGTCGTAT
TAGAATTCCCC
+
CCAAAABCC@CCCC>C?B?B@CD@BBCA@AC@DD@BBB@C@BCBBBCCCA<@988/888?A<ABCCCCCBCCBBCBB
@A??>C@CCC8
@T6XXI:00129:00258
GGGGAATTCTAATACGACTCACTATAGGGAGAGGGAGAATAGCCTGCAGGTCGAGTGTAGGACTGTTCCCTCCTTTC
TCCCTTCCTCCC
+
8>>5@1;:=BB?BGDCBADCBACC???A=???CC>CC@;=988>B???A<@A??<@845=;7::::9:9,/-,--+-
244+0/0/04:7
@T6XXI:00129:00260
GGGAGAAGGGAGAAAGGAAGGGGAGAAGGGAGAAAGGAAGAGTCCAC
+
DC@BAA=>=:@@@B=B?B>AAA7AAC>@?7>777.70631688;;50
@T6XXI:00129:00276
CTATCGACTCGAGATCTGCGGCTTTGGACAGTCCCTCTGTACTGTGAAGCCACAGATGTCGAGTCGACTAGC
+
4999444444499449;?==<;?<?:713331111,144...33.103/04<==?@>;7<1111144,,,,,,
@T6XXI:00129:00296
GGGAGAAGGGAGAAAGGAAGCTAGTCGACTCGACATCTGTGGCTTCACAGTACAGAGGGACTGTCCAAAGCCGCAGA
TCTCGAGTCGACTAG
+
AD?CAB;<909999096968?800=0/.5544066666?BB2:95:BACCCCB>;444-
4:;448;>>7?D>A??AA@===???AAC????@
@T6XXI:00129:00299
GGGAGAAGGGAGAAAGGAAGGAGAAGTCGCCCCAAACGATTTGTTGACTATTCTCCCTCTCCCTATAGTGAGTCGTA
TTCAGAATTCCCCC
+
CDCCDC@CC=BBBC>A>B:;9;BB>AF?8877-8?8?;9@A<AA?B?????:;;88444444-
4;:===AA999<;;;.///,+,11110.
@T6XXI:00130:00250
CTAGTCACTCGAGATCTGCGGCTTTGGACAGTCCCTCTGTACTGTGAAGCCACAGATGTCGAGTCGACTAGCTTCCT
TTCTCCCTCTCC
+
C:11121:ADCCBBCBBBBB@BBC?B?A?;A9665:BBCCCCC@@=><=D?CCFCBBBBDCCCBBBAAA??773740
0,0;;>,/.223
@T6XXI:00130:00253

```
GGGGAATTCTAATACGACTCACTATAGGGAGAGGGAGAATAGTCAACAAATCGTTTGGGGCGACTTCTCCTTCCTTT
CTC
+
:A;296<249824444337?7331111,116>?;B@A??:8./.33845B@??>;>>>6>>:7>:0/0,/,/,//,
/35
@T6XXI:00130:00258
GGGGAATTCTAATACGACTCACTATAGGGAGAGGGAGAATAGTCAACAAATCGTTTGGGGCGACTTCTCCTTCCTTT
CTCCCTTCTCCC
+
CC?6?=@=@BB@CCCCBB>>B@BB?8888191?A>AA??A?A???;?@B<A???@;@EC8CAABB>>??5838<@@<
A??@7><<<<@:
@T6XXI:00130:00274
GGGGAATTCTAATACGACTCACTATAGGGAGAGGGAGAATAGACTGTTGCTAAACTTCAGTCCCGTTCTCTTCCTTT
CTCCCTTCCTCCC
+
CDC:C@A@ACC@CCBB@?>BBBB?966659??CC>CB@=@A888?AA<>><<<7<@8;9=78858B=A8942333>7
=44727,/,/::6
@T6XXI:00130:00279
GGGAGAGAATTTGGTAATGGGCACTGGTTACCAACGTCAAGGGGCGCAGGTTCCCTTATATCTCTCTCCCTATAGTG
AGTCGTATTAGAATTCCCC
+
CJ@DCCCC>AA?A?BC@BAB=AAAAD@DBAA@B@BBBAB?BCC:CCCDD>A>@C=B?BAA@@;;;@@CD?CCCED<<
<A>>>>00,0::,0,///+
@T6XXI:00130:00294
GGGGAATTCTAATACGACTCACTATAGGGAGAGGGAGAATAGTCACTGGATTCTCCCGCAGTGCGTTTATCTTCCTT
TCTCCCTCTCCC
+
AA@7@8;;<CCCCCBABBBB>=>CA99959??BC>B@=;=@@A@@AAA>A<?@<684=@@AAAA@@?5;;;<8::>>
77669./.7575
@T6XXI:00131:00252
CTAGTCGACTCGAGATCTGCGGCTTTGGACAGTCCCTCTGTACTGTGAAGCCACAGATGTCGAGTCGACTAGCTTCC
TTTCTCCCTTCTCCC
+
BB=@@;9<CEDDCCCBBB?BCAEBB>A??A@AAAA>ACECBB?@@CB@>=A=ACCCC@B@CBB@A>=000>@:::>:
=?9?<=?6;383399
@T6XXI:00131:00253
GGGAGAAGGGAGAAAGGAAGGGGAGAAGGGAGAAAGGAAGCTAGTCGACTCGACA
+
CC>BCB@CC>BCBB?B?B?AAA8BBB@AA;BA<98A;?8?>;;;>00,0**,+0*
@T6XXI:00131:00254
GGGGAATTCTAATACGACTCACTATAGGGAGAGGGAGAATAGTCAACAAATCGTTTGGGGCGACTTCTCCTTCCTTT
CTCCCTTCTCCC
+
BCHAD::<;CCACB???B?>B@BB?66656=?AB>BDC>B???AA:??A<C@@CC>CCB9BBDAA>ABB=>;=8;;7
<@>?:@<??CA<
@T6XXI:00131:00262
GGGGAATTCTAATACGACTCACTATAGGGAGAGGGAGAATAGTCAACCGAAATCGTTTGGGGGCGAACTTCCTTCC
+
?BC9B>A;>44248888777767316;;8<?=??;<<5;>=440423234443448::6;==.77/64555,.,.,
@T6XXI:00131:00270
CTAGTCACTCGAGATCTGCGGCTTTGGACAGTCCCTCTGTACTGTGAAGCCACAGATGTCGAGTCGACTAGCTTCCT
TTCTCCCTTCTCCC
+
BA<;;A?A::;AA?BCC<;;?BBEACBB@>>777.7@?CECCB@B=<><@=@AAC@@@BBCCCCBBBB??8710737
7.7=>?:?<=6<C:
@T6XXI:00131:00278
```

```
GGGAGAAGGGAGAAAGGAAGCTAGTCGACCGAGATCTGCGGCTTTGGACAGTCCCTCTGTACTGTGAAGCCACAGAT
GTCGAGTCGACTAGCTTCCTTTCTCCCTCTCCC
+
HC@CA@=BB:>>>>9>;><?;;;@;:<00.0;:>>BB@@@@BBCAC?BAB@:::5:BB@@@?A???C=AA@@A@===
???@@BAA?>>>=847=8,//,/899,//4672
@T6XXI:00131:00279
GGGAGAAGGGAGAAAGGAAGTGCAGTTCGCGGTTAGTGAAGGGAAAAACTATTCTCCCTCTCCCTATAGTGAGTCGT
ATTAGAACTTCCCC
+
GCACCCA@=:@@@@<@>B?BBBCDHEBA??@@C@CBBCC>=7-
1667,8??@?B::;.6=<>@<A@@@AACAB@@?<<<8<>A8<<<>@>4
@T6XXI:00131:00289
GGGGAATTCTAATACGACTCACTATAGGGAGAGGGAGAATAGTCCAACACTTGCTGGTCGTCCGCTATCCTTCCTTT
CTCCCTTCTCCC
+
999-
9<?=BCCACCBBAABBB>;9;11159::CE>A@=?C????<?<?AAA>???A>=>>8858?;;::34/473308349
:?4948==
@T6XXI:00131:00293
GGGAGAAGGGAGAAAGGAAGGGGAGAAGGGAGAAAGGAAGCTAGTCACTCGACATCTGCTC
+
BB>AA@@DC@CBBB>C@C@BBB8BAA??=97777-73747<?8:AA>A??;94..,+++++
@T6XXI:00132:00264
GGGAGAAGGGAGAAAGGAAGCTAGTCACTCGACCATCTGTGGCTTCACAGTACAGAGGGACTGTCCAAAGCCGCAGA
TCTCGAGTCG
+
BC@ECC@CB?DAA?=B=@=@CCCBBB=:1111601611>>>3787;?@AFCCCCB?<<;:;<:??:==97738??@8
98:88,,,,+
@T6XXI:00133:00259
GGGGAATTCTAATACGACTCACTATAGGGAGAGGGAGAATAGTCAACAAATCGTTTGGGGCGACTTCTCCTTCCTTT
CTCCCTTCTCCC
+
ACB7B7;;<CCACCBBBBAA@ABBA???9???@@<B;86:AAA@B=@@A;BBBCC>CCC9CCC??:;>=34247994
;>;=:?384@@;
@T6XXI:00133:00262
GCTAGTCGACTCGAGATCTGCGGCTTTGGACAGTCCCTCTGTACTGTGAAGCCACAGATGTCGAGTCGACTAGCTTC
CTTTCTCCCTCTCCC
+
//0076///77>A<;=BDCAB@>;7:68<>@BC???<?@@@A?@@DCCD>@C?CCCDBBBCCCBBAA@AA?><7637
400,0.45+-----+
@T6XXI:00133:00266
CAGTCCCTCTGTACTGTGAAGCCACAGATGTCGAGTCGACTAGCTTCCTTTCTCCCTTCTCCC
+
D;;444-4;?B@??CCCAB=@?;@BBBBBBBCCAA@CB;;<B<<;>A=@C<BA@@<@8<:AB:
@T6XXI:00133:00281
GGGGAATTCTAATACGACTCACTATAGGGAGAGGGAGAATAGTCAACAAATCGTTTGGGGCGACTTCTCCTTCCTTT
CTCCCTTCTCCC
+
DDC:C>A>AFC@CCBBBBBB?@>?9669.6=>?A>BB@>AAAABA?@@<:?@BEE>BBB7AA???:?>=:98;;>@;
AAAA<@=A@AC?
@T6XXI:00133:00283
GGGGAATTCTAATACGACTCACTATAGGGAGAGGGAGAATAGAGACAACC
+
?==6@?B@CCCABB@@ABBBB?>;;66649??BB<BBA?C=43/4/,+*)
@T6XXI:00133:00285
GGGAGAAGGGAGAAAGGAAGCTAGTCGACTCGACATCTGTGGCTTCACGTACAGAGGGACTGTCCAAAGCCGCAGAT
CTCGAGTCGACTAG
```

+
DGDBBA@CB>?@@C=B>B@BB@00////9==<0:7696A@A>AA?ABBA@?B==633-
4>77:B@CC>CC@BB@AAC<==<?;87373034
@T6XXI:00133:00288
GGGGAATTCTAATACGACTCACTATAGGGAGGGAGGAAG
+
CDB9B?A?BCBDCCBBCAAAAAAC==?C>C>?6/,+*)(
@T6XXI:00133:00294
GGGAGAAGGGAGAAAGGAAGCTAGTCGACTCGACATCTGTGGCTTCACAGTACAGAGGGACTGTCCAAAGCCGCA
+
CDAEDB@B>:>==><@@B?@@@CDBCCBB;>>97///0><<-:;?BAAACCCCB=44417;9>=?<>>7==;>@@
@T6XXI:00134:00254
GGGAGAAGGGAGAAAGGAAGACTAGGAGCGTGCCACATATCAGGTAGCTATTCTCCCTCTCCCTATAGTGAGTCGTA
TTAGATTCCCC
+
CDBBB@@CCCBCCC?CAC@C?@ACB?@@ACAA>>AC===@@@A?@===@@A>@A==;88<?C=A>>>AAA@?@@???
A>AA@>9;;;.
@T6XXI:00134:00269
GGGGAATTCTAATACGACTCACTATAGGGAGAGGGAGAATAGTCACATTCATGTTTGTGACCGTGTCTCCTTCCTTC
TCCCTCT
+
499/95929>A;@ACCCCCCCBA??777.7====9?B@=@@B884::<:7753330...13457<<::<34242420
389-400
@T6XXI:00134:00291
GGGAATTCTAATACGACTCACTATAGGGAGAGGGAGAATAGCTTTCCCCTTCACTAACCGCGAACTGCACTTCCTTT
CTCCCTTCTCCCATC
+
//+/,/,49;8=?DDBCA@@??87777-
7=6;<671044800?7,611,177=@AA>A>@?@@>@@??<<;8;78849444-474444-99?
@T6XXI:00134:00297
GGGAGAAGGGAGAAAGGAAGCTAGTCACTCGACCATCTGTGGCTTCACAGTACAGAGGGACTGTCCAAAGCCGCAGA
TCTCGAGTCGACTAGACGCTCTGCATC
+
CKDCCB=@@9@@==9@8;7;BAAAB:10:<>00,....77>0:7=@ADCEBDBB=;;;6;=>==><>?;?A>AAAA@
????777@?<<=@===///66/---++
@T6XXI:00135:00251
CTAGTCGACTCGAGATCTGCGGCTTTGGACAGTCCCTCTGTACTGTGAAGCCACAGATGATGAGTCGACTAGCTTCC
TTTCTCCCTTCTCCC
+
CB<==<@=GIDCDCCBAAABB@BBB>B@B@?>777-
7===A???AAA=<=A>@@;;;DFCCECB@@@;;;>>><4847>.71672;071662
@T6XXI:00135:00255
GGGGAATTCTAATACGACTCACTATAGGGAGAGGGAGAATAGCCCCCACTATTCGAACTACAGCATCTCCTTCCTTT
CTCCCTTCTCCC
+
CCC:C6;9;BB@CBCBBABBA@B@?9995:??CC>BB@?;944330+4;;;=<?@@=@?@@@<<<;;;=::8;344+
44944A384>?7
@T6XXI:00135:00261
GGGAGAAGGGAGAAAGGAAGTGCAGTTCGCGGTTAGTGAAGGGGAAACTATTCTCCCTCTCCCTATAGTGAGTCGTA
TTAGAATTCCCC
+
DCBBBA@BB>===A?A@A<<<BBCBE?@==;5:6;@@CD@CBB8BB?CCBB>@@AC=BBCCC>B???@@@@?@@@?@
@>@@A=?=@@@5
@T6XXI:00135:00267
GGGGAATTCTAATACGACTCACTATAGGGAGAGGGAGAATAGTGACGTGTTGTGCGGCGCTCACTGTCTCCTTCCTT
TCTCCCTTCTCCC
+

```
CDH:C;;;<AAABA@ABAACCAA<<776.777?A>BA?=?@@AA@@@@AC?@???@>BA???<<;<;;;>8:7:7<<
6;>;A6;28734-
@T6XXI:00135:00273
GGAGAAGGGAGAAAGGAAGGAGAAGTCGCCCCAAACGATTTGTTGACTATTCTCCCTCTCCCTATAGTGAGTCGTAT
TAGAATTCCCC
+
A?ACCACC@AABC>B=?=@??BC@CC?77975:B>CCCAA;@@?BCCB<A>A@?;4;;;==7=???@AACC<<<?@@
>AAA<;2444,
@T6XXI:00135:00274
GGGAGAAGGGAGAAAGGAAGCTAGTCGACTCGACATCTGTGGCTTCACAGTACAGAGGGACTGTCCAAAGCCGCAGA
+
CCABBC@BA:>@=>:@@B7;;B@@@CCFC>>;0/000/=9;5?@>ACCBBBGB44000-4;;:>?>AC?A@>???@@
@T6XXI:00135:00280
GGGGAATTCTAATACGACTCACTATAGGAGAGGGAGAATAGATATCTCATCTACCCTATCCCGCTATCCTTCCTTTC
TCCCTTCTCCC
+
A@?6@8;:<DC@CCBCBA@AB;7<;6737777:7;CA>@@@@@??A@AAAB??BC>BA??>7=;444424/4/44+7
6776;///:96
@T6XXI:00135:00281
GGGAGAAGGGAGAAAGGAAGCTAGTCGACTCGAGATCTGCGGCTTTGGACAGTTCCTCTGTACTGTGAAGCCACAGA
TGTCGAGTCGACTAGCTTCCTTTCTCCCTTCTCCC
+
CA@@?@=@B<DB?A:?-1-
100::?:::ABBBDBDD??@CC>:::67<>>====@=?<<??????AAA>??>?@A?@@@@?@A989?=<:<B//..
66666666..+0>:?9
@T6XXI:00136:00255
GGGAGAAGGGAGAAAGGAAGGATATGCCGCAGTGGGAGATTGAACGGCTATTCTCCCTCTCCCTATAGTGAGTCGTA
TTAGAATTCCCC
+
<=6<BA@CC>CCBCBB=?=@@ECAAAB@BCCBBBC>BBCB@CC@CC=?ECB?@98;09A@CC@CCAABCBBBCBBBB
A<??@:4/004-
@T6XXI:00136:00271
GGGGAATTCTAATACGACTCACTATAGGGAGAGGGAGAATAGTTTTTCCCTTCACTAACCGCGAACTGCACTTCCTT
CTCCCTTCTCCC
+
CCEDC@D@BCB@CBAAA??BAA@?86695:>@AC@CCA?B<@@AAA1??;?>ADCCCAB>?99=?=@??>>:::,/-
0>?@9A=<<?>:
@T6XXI:00136:00288
GGGAGAAGGGAGAAAGGAAGGAGAAGTCGCCCCCAAAACGAT
+
=?:=9A>A@:@9990951,0;=00-/7;;=966*0..*33..
@T6XXI:00137:00252
CGGCTTTGGACAGTCCCTCTGTACTGTGAAGCCACAGATGGACTGCTTCCTTTCTCCCTTCTCCC
+
EC@CCEEC@CBCC;;;79>>@AAAAAAAB@BB@CCBBBBB?BC???B:=:?@547@@:@@989C<
@T6XXI:00137:00256
GGGGAATTCTAATACGACTCACTATAGGGAGAGGGAGAATAGTCAACAAATCGTTTGGGGCGACTTCTCCTTCCTTT
CTCCCTTCTCCC
+
ACC7C@C<>@CBCCDC?>@A@?>C?7::5:??CE>BA@?AAA??=:9984?==>F6==?2=====:=7744/49;;7
??@@;@9=9@C:
@T6XXI:00137:00262
GGGGAATTCTAATACGACTCACTATAGGGAGAGGGAGAATAGTCACGC
+
ACB6B=@<?>?<99@A@??@A@AB???B<??>@>6>0/,/666:8//-
@T6XXI:00137:00270
```

```
GGGGAATTCTAATACGACTCACTATAGGAGAGGGAGAATAGTTACATCTTAGCCGGCGTTCCCTCTATTCTTCCTTT
CTCC
+
CGB8B59589824489>>;7;/6;://,.::>><??;=>888<==:=755669,/,.37,//,4:;44344/43//,
/555
@T6XXI:00137:00271
GGGGAATTCTAATACGACTCACTATAGGGAGAGGGAGAATAGTCAACAAATCGTTTGGGGCGACTTCTCCTTCCTTT
CTCCCTTCTCCC
+
AAA6@=A@CBB@BB@?ACAAAB@A?9995:AACD?CBA@A=A===>@AD=CCBBA>B?@8?A>>C@<AA;;885884
8::96;;>@AA<
@T6XXI:00138:00250
GGGGAATTCTAATACGACTCACTATAGGGAGAGGGAGAATAGTCAACAAATCGTTTGGGGCGACTTCTCCTTCCTTT
CTCCCTTCTCCC
+
CDD:D@@>?CC@CCBBBBBB>@AB?66757A?CE>A@>?BA?>?A=@@A;B@@BB>BBB7BBBBB?@BB??=@9<<7
<>==7?===AB=
@T6XXI:00138:00251
GGGGAATTCTAATACGACTCACTATAGGGAGAGGGAGAATAGTAGCAATAAAATGATTGGGCGATTCTCCTTCCTTT
CTCCCTTCTCCC
+
CHH:C?A>@BBACDBBBCCBB@BB;111.677@B>??=>A@BAAA?A?CCCE9CCBB?BE>BBBB@A>?6868@AC;
@<;;6:387=@:
@T6XXI:00138:00263
GGGAGAAGGGAGAAAGGAAGCTAGTCACTCGACATCTGTGGCTTCACAGTACAGAGGGACTGTCCAAAGCCGCAGAT
CTCGAGTCGACTAG
+
CB>B@@=@A:@AAA>B?B@@@?@@@:/077@,/////3676BB@CEBBCBA==8333-
4;;AAA>??;@A>???B===A@@@@@@@@@@@@A
@T6XXI:00138:00275
GGGAGAAGGGAGAAAGGAAGAATAGTACAGGGAACATGAGCGGTAATCTATTCTCCCTCTCCCTATAGTGAGTCGTA
TTAGAATTCCCCC
+
DDCBCC@CBADBBB?B@B>A>@CB??AABB?<B>><@@CABA@BC?BBB@@8;;;;6<==?AAA@@@@@AA=@A==<
=9;4423/0034,
@T6XXI:00138:00276
GGGAGAAGGGAGAAAGGAAGAAAGGAAGGAGAAAGCGACGTCGGGGAAACGTTTGCTATTCTCCCCTCTCCCTATAG
TGAGTCGTATTTAGAATTCCCC
+
9<6;@@DDLADCCCAB@A>???<@?B?B@C???9?=<9=@::9AA6<>=@BA?:??7=>;>4444,4443?<B@?83
000.,,,,,...+..1692444,
@T6XXI:00138:00286
GGGAGAAGGGAGAAAGGAAGATGAAGATAGCAAGGGGAAGCTACTATCTATTCTCCCTCTCCCTATAGTGAGTCGTA
TTAGAATTCCCC
+
B;:>=<8<B?@AAC@CBCACCBBB@C@CBBBC@CCC:C@BBBBCCCCCBBB>A888/;;;BC?CCAAABGDDAD;;:
A?B<@>C?BBA2
@T6XXI:00138:00287
CTAGTCGACTCGAGATCTGCGGCTTCGGACAGTCCCTCTGTACTGTGAGCCACAGATGTCGAGTCGACTAGCTTCCT
TTCTCCCTTCTCCC
+
<8-
,,,,,6119CCCCCB@@@=@@@DEDA@>@99909@@@CBDDBABA:;8:BBBCBB@A@<?=@@BBB::7=3744;4:
433,02856:9
@T6XXI:00138:00288
CTATCGACTCGAGATCTGCGGCTTTGGACAGTCCCTCTGTACTGTGAAGCCACAGATGTCGAAGTCGACTA
+
```

```
:@AA994F@9499@;994477383,373336111,1668::>>?633/0488:8773.44..,../43337
@T6XXI:00138:00289
GGGGAATTCTAATACGACTCACTATAGGGAGAGGGAGAATAGCCAATAAGTCTCGCAGTCTTGGCTC
+
CCD9C@C@CDCBDCCBBACD>=@AA666.7?==?<?A=;><<<9=?AA>AA@>==A@??:6,+*+**
@T6XXI:00139:00280
GGGGAATTCTAATACGACTCACTATAGGGAGAGGGAGAATAGTCACTCCCACTAGCTTGTCGTACTCGCCTTCCTTT
CTCCCTTCTCCC
+
CFE:C@A>ACCCCBAAABBBB@;97167.7==?B@:;9=@@@@@@AA@@;@=C>?:?>@B@BB???CA?;=8;3441
8==@<A>989H?
@T6XXI:00139:00282
GGGGAATTCTAATACGACTCACTATAGGGAGAGGGAGAATAGTTCCGCTATTTTCCACACCAAGTTAACCTTCCTTT
CTCCCTTC
+
CEC:D@B>@@@=@ABA;:;@@=>>=777.7====9?@=<=@==3834;87766-
177>::7:?BB>>>B34/3343+0004+0/0
@T6XXI:00139:00283
GGGGAATTCTAATACGACTCACTATAGGGAGGGGGAGAATAGCTCGTAATAGAATCACGACTGACTGTCCTTCCTTT
CTCCCTTCTCCC
+
BAA7A=A=@BBDCCBBBBBB>@AB?76746;ABC2CCC?B??CABBBB<@@>>=>>>1//;7;?B:??C4747444-
44;=:=588??:
@T6XXI:00139:00284
ACTCGAGATCTGCGGCTTTGGACAGTCCCTCTGTACTGTGAAGCCACAGATGTCGAGTCGACTAGCTTCCTTTCTCC
CTTCTCCC
+
CBBBBCCCBAAABB@BBB>B@BCBBAAA=ABACCAAADCCFAAA@CCCBCBABBBBAAAA;;9?;996<=@@>BBBD
>C@ABBC>
@T6XXI:00139:00287
CTCGAGATCTGCGGCTTTGGACAGTCCCTCTGTACTGTGAAGCCACAGATGTCGAGTCGACTAGCTTCCTTTCTCCC
TTCCTCCC
+
EAAABBCC<<<BB>BCC@C?BA>@99909@@99<AACABC?A@=BBA@BA@@@BAC<<<BBAB;;<:<59F:;211-
1-1,0;;3
@T6XXI:00139:00290
GGGGAATTCTAATACGACTCACTATAGGGAGAGGGAGAATAGCTAGCGTTTCCCCCCGCAATCTATTTGTCCCTTCC
TTTCTCCCTTCTCCC
+
BCE9B@BACBBDDFCCBCACC?<96177.777BC@@?=?AC??AB::7>@-
44444(0334=<989A9;78313848?C69444-3/0349;
@T6XXI:00139:00294
GGGAGAAGGGAGAAAGGAAGCTAGTCACTCGACATCTGTGGCTTCACAGTACAGAGGGACTGTCCAAAGCCGCAGAT
CTCGAGTCGACTACG
+
94-
444?BC<A;;;09@@8;;B@<::66533///../;7;5AA:<;@@BBBB?>77317=A??@>@@;@A9<<<<;7803
488553174/--
@T6XXI:00139:00295
GGGAGAAGGGAGAAAGGAAGGAGAAGTCGCCCCCAAAACGA
+
CEBCCE@BA:A@@@<@>B>A@CCC@B?@@AA>>,888.10*
@T6XXI:00139:00296
GGGGAATTCTAATACGACTCACTATAGGGAGAGGGAGAATAGCCGTTTAATCTCCCACTGCGGCATATCCTTCCTTT
CTCCCTTCCTCCC
+
```

```
???6>;@>@@@@@@ABAA@CCCCAA691-
6<;?ABBFA>A===8<@?:><<<5;@:=??@??==867//,.,/4695:54301,/,/882
@T6XXI:00140:00253
GGGGAATTCTAATACGACTCACTATAGGGAGAGGGAGAATAGCTCAGTAACTCGCTCAGCTTCAATCTCCTTCCTTT
CTCCCTTCTCCC
+
A@?6@7<=<CCCCCCDBAAAB=9?=677.7>===9?B?;????BCBBBB>AA==:???A??>?A>A???==9;/33-
4::;-434444,
@T6XXI:00140:00260
GCTTTGGACAGTCCCTCTGTACTGTGAAGCCACAGATGGACTGTCCAAGACGGGAGCTTCCTTTCTCCCTTCTCCC
+
::<CCCCEDB=9996;AABCBBBCCCC?BB@A@@CCBA>A:;;?A?@>ACBBC>CCEC?B?BB8??@@<A:::CE>
@T6XXI:00140:00264
GGGAGAAGGAAGAAAGGAAGCTAGTCGACTCGACATCTGTGGCTTCACAGTACAGAGGGACTGTCCAAAGCCGCAGA
TCTCGAGTCGACTAG
+
CC?AAB@C@B@>=85=646;CA=>>/11;7>7/001//;6:.:8@CBDBCAB@;9300-
4;;489@DC@@@=@AAAAA@=<<<?A???@@?A
@T6XXI:00140:00267
GGGAGAAGGGAGAAAGGAAGCTAGTCGACTCGACATCTGTGGCTTCACAGTACAGAGGGACTGTCCAAAGCCGCAGA
TCTCGAGTCGACTAG
+
CDACCB?AA=BB@@<B7:6;B?<=>//0::<<0:6776@AC5;;?AABBBBBBB9737-
4==?A@?AA<==:<@??@@AAAA@@A????AA?
@T6XXI:00140:00273
GGGAGAAGGGAGAAAGGAAGCTAGTCGACTCGAGATCTGCGGCTTTGGACAGTCCCTCTGTACTGTGAAGCCACAGA
TGTCGAGTCGACTAGCTTCCTTTCTCCCTTCTCCC
+
DD@A<<;<B:AA@@9>5953>><7;::67?==@B>AABB@>=::7-
:=>@@B???9:;:8888@@==?><<9<@A???????AA@?=;:4-.,+*+0003377757444593
@T6XXI:00140:00279
GGGAGAAGGGAGAAAGGAAAGCTAGTCGACTCGAGATCTGCGGCTTTGGACAGTCCCTCTGTACTGTGAAGCCACAG
ATGTCGGTCGACTAGCTTCCTTTCTCCCTTCTCCC
+
CFBDCC@FC@C984-9599-
7><>7?:::BBBADBCCBB@<<:777.7>?ABCAAA??>>??==<===A>AA>AA@@A@@@@@=@CC///9//-,-
224554@>14344030
@T6XXI:00140:00286
GGGAGAAGGGAGAAAGGAAGAACGACAGTATTCGGGATTGGTAACGTCTATTCTCCCTCTCCCTATAGTGAGTCGTA
TTAGAATTCCCC
+
DE@CBB6440899?<A>BADD@CCEA@BB<?;???9@@?B@CC@CCAAAAB?A98809>99918?>>AA@@@@;;;?
@<?CC;<;=CC9
@T6XXI:00140:00296
GGGAGAAGGGAGAAAGGAAGCACATCGCGCGGAGGCTAACGGGGAGACTATTCTCCCTCTCCCTATAGTGAGTCGTA
TTAGAATTCCCC
+
CC?CCE@BB>BBBBBA@A=>BBCA@=??A?@@??<??A?ADCD6=>@DBB@>@AAA:<;;;;6<@@ACB@@@=<<?<
<9<@CB<:@CC5
@T6XXI:00141:00250
GGGAGAAGGGAGAAAGGAAGGTGAACAGAGCGCAAACGAATACGTCTCTATTCTCCCTCTCCCTATAGTGAGTCGTA
TTAGAATTCCCC
+
CDCCCB@BD>BCBB>A?A@B?AAC@CBBBBBBBBB>BCBAABFDAABCCCCACBBB=@@@CC>CCCCCDBC??@CAA
A@C@@?C?BCC9
@T6XXI:00141:00257
```

```
GGGAGAAGGGAGAAAGGAAGCTAGTCACTCGACATCTGTGGCTTCACAGTACAGAGGGGTCCAAAGCCGCAGATCTC
GAGTCGACTAG
+
CC?BB@@CD@CCDE>B=??@BBAABB@?BBD:;:;;:BBB=:;7;@AAABBB?;4300-44344-
4;::55655344?C=AA===??=
@T6XXI:00141:00285
GGGGAATTCTAATACGACTCACTATAGGGAGGGGGAGAATAGCTTTTCGCTTCCCTGACTATTGTTTTCTTCCTTTC
TCCCTTCTCCC
+
BCC9B=?>@CB@CCBBBBBBDCBBBBBB>ACCBB1BBB=@<>999949D>><@@<@CCCCCB>CCCC6@@@B7::7;
?@B6;7:ACB=
@T6XXI:00141:00293
GGGAGAAGGGAGAAAGGAAGCTAGTCGACTCGACATCTGTGGCTTCACAGTACAGAGGGACTGTCCAAAGCCGCAGA
TCTCGAGCTCGCT
+
EDBBBB?A@:@@@=:@>B?BCBAAA7;:@BA?;:::::>@@:?@?A??@CCBB?A@=A;@A=944/00+44344444
498684-...-00
@T6XXI:00141:00299
GGGAGAAGGGAGAAAGGAAGCTAGTCGACTCGAGATCTGCGGCTTTGGACAGTCCCCTCTGTACTGTGAAGCCCACA
GATGTCGAGTCGACTAGCCTT
+
DC>E;;99>:>999147=;>?>:93/..445596<:>>@@@=:::69>????????2744400003400/000+034
35555;=<94/----3-0/0/
@T6XXI:00142:00253
CTAGGTCACTCGAGATCTGCGGCTTTGGACAGTCCCTCTGTACTGTGAAGCCACAGATGTCGAGTCGACTAGCTTCC
TTTCTCCCTTCTCCC
+
CCBC@C@CDDECCCCCBBABA@AAA>ABBB?><:<8?BB@BC@C>B@A>ACACCCCBCCCCCCB;<<???B;:6993
;?9<?>@<=8<6??:
@T6XXI:00142:00261
GGGAGAAGGGAGAAAGGAAGCTAGTCGACTCGAGATCTGCGGCTTTGGACAGTCCCTCTGTACTGTGAAGCCACAGA
TGTCGAGCGACTAGCTTCCTTTCTCCCTTCTCC
+
DIACCC?B@<@;8809594;;8ABB:::???AAAAAB@??=;777.7>???@DCC>88888@?@@AAC6::????AA
@???@?<<8889<<@A:=:<?:9///,/,/-11
@T6XXI:00142:00269
GGGGAATTCTAATACGACTCACTATAGGGAGAGGGAGAATAGTCAACAATTTATCTTCGTCGGCTTTCTCTTCCTTT
CTCCCTTCTCCC
+
CCB9B=@>@BBACCBB@??AB@B?869659??CD>BB@@@<?@AA>A@>AA?@>>B?CBB@@>6663487;9;/881
8;;A;?<?A@C<
@T6XXI:00142:00273
GGGAGAAGGGAGAAAGGAAGCTAGTCACTCGACATCTGTGGCTTCACAGTACAGAGGGACTGTCCAAAGCCGCAGAT
CTCGAGTCGACTAG
+
CDBCBA@BC>BBB?=B=>=@CBBBB?AACBB::::;:BBC8;<?ABBBBBCCB9300-
4;::=?>AA<AC@?>?A@@888@@@@A@@A@?=
@T6XXI:00142:00279
GGGAGAAGGGAGAAAGGAAGGAGAAGTCGCCCCAAACGATTTGTTGACTATTCTCCCTCTCCCTATAGTGAGTCGTA
TTAGAATTCCCC
+
CC@CCB@DB?AB??=B>B?A@C@@=?=?7777-
8>:@BCC@;@@@A??A@@=????:>;;;>:==>>@>??A???@@@@@@@?=2444-
@T6XXI:00142:00281
CTAGTCACTCGAGATCTGCGGCTTTGGACAGTCCCTCTGTACTGTGAAGCCACAGATGTCGAGTCGACTAGCCTTCC
TTT
+
```

```
B<1////4:BB>BAB@@@@@=@CD>C?BB>>777.7===@;;:@?;==8<:<AA?@@BCAABBADB=::4.,*,*,*
,2*
@T6XXI:00142:00285
GGAGAAGGGAGAAAGGAAGGGGAGAAGGGAGAAAGGAAGGGGAGAAGGGAGAAAGGAAGGGGAGAAGGGAGCAAACG
GAATCGGG
+
CBCBB>A?<@B@@<B=@=@BB7@@A?A>:>777.7:>>CCD9BBBA@A<===A;A9<9;==1>@??A?7:9444-
4;2420-110
@T6XXI:00142:00287
GGGGAATTCTAATACGACTCACTATAGGGAGAGGGAGAATAGTTTTCCCTTCACTAACCGCGAACTGCACTTCCTTT
CTCCCTTCTC
+
999-
49@>=<<@CDDCBCACCACBBBCC=A@A@@.77=378?A94+00+024=7;;2626:99=??99?B@;;066,1333
+/,---
@T6XXI:00143:00277
CTATCGACTCGAGATCTGCGGCTTTGGACAGTCCCTCTGTACTGTGAAGCCACAGATGTCGAGTCGACTAGCTTCCT
TTCTCCCCTTCTCCC
+
BB?;944:===BCCGBCCCB?AAC>B=@?>?777.7===@ACC@@=A>?B>BDCDBAAAAA:;;AA88:A;888886
76=1167,38:4444
@T6XXI:00143:00284
GGGAGAAGGGAGAAAGGAAGGAGAAGTCGCCCCAAACGATTTGTTGACTATTCTCCCTCTCCCTATAGTGAGTCGTA
TTAGAATTCCCCGGGAGAAGGGAGAAAGGAAGATCCA
+
EC?B=92991<@@GBDIC>A>BCC@@<@7777-
8>8?9@BB>BB@C::;<<=<<<@;<<<@A7<999AABC@@@@@@A>?>=:656:?4@C<@;??AA=A@6666555,,
*)),
@T6XXI:00143:00290
GGGGAATTCTAATACGACTCACTATAGGGAGAGGGAGAATAGTCAACAAATCGTTTGGGGCGACTTCTCCTCCT
+
@EC7B:<:<=@;@@A<<;???>B>=?<98???BB>CCA;=<AAAA699A<BA@F?.777,164..,.53,--,-
@T6XXI:00144:00252
GGGGAATTCTAATACGACTCACTATAGGGAGAGGGAGAATAGCTACGTTTAACCGATCGTGTTGTTCTCCTTCCTTT
CTCCCTTCTCCC
+
FDKBF?A@ACC?DCCBBA@?BC@<;796.7<<<?9????BB?=?@@?=<9?>><ABBCB@@A>AC;===;=9;3881
4:;B:?<?@CC?
@T6XXI:00144:00255
GGGGAATTCTAATACGACTCACTATAGGGAGAGGGAGAATAGTCAACAAATCGTTTGGGGGCGACTTCTCCCTTCCT
TTCTCCCTTCCTCCC
+
A=A8AADAAAA@CECCCCCC@@@AA><?9:::BC>?@?A????@@:==C=C;::D>CCCC0::77>;7111,133/0
0+0---+-,-,-232
@T6XXI:00144:00267
GGGAGAAGGGAGAAAGGAAGCTAGTCACTCGACATCTGTGGCTTCACAGTACAGAGGGACTGTCCAAAGCCGCCAGA
TCTCGAGTCGACTAGCTTCCTTTC
+
CA=B<<89A:>99919595988>9;0........7889<<:8?>:87:@@>@@A???9<884::348-
4;9:<<<??:443684661..+0++---,-4-.
@T6XXI:00144:00285
GGGGAATTCTAATACGACTCACTATAGGGAGAGGGAGAATAGCGTGATCTTTGTTACCGGGCACCTGTCCTTCCCTT
TCTCCCTTCCTCCC
+
EB@4@5459@;8@@@B@@@@CA@@A8776:77??9::7:=6135449?877.7738><?D>??=;<?7777011,04
246;;0323/0042
@T6XXI:00144:00296
```

```
GGGGAATTCTAATACGACTCACTATAGGGAGAGGGAGAATAGTCAACAAATCGTTTGGGGGCGACTTCTC
+
999-424844429;DAAACCA>>?887<676167266826696885666 3;6;77,1111+366110167
@T6XXI:00145:00256
GGGAGAAGGGAGAAAGGAAGCTAGTCGACTCGACATCTGTGGCTTCACAGTACAGAGGACTGTCCAAAGCCGCAGAT
CTCGA
+
44-44424969=@@<@?@>@@<0,7,,,,,22,,,33....44437611:=><<://,-----
3444400/0300044400-
@T6XXI:00145:00260
GGGAGAAGGGAGAAAGGAAGAAAATGGCTCAAATGATACCAGGGTAGCTATTCTCCCTCTCCCTATAGTGAGTCGTA
TTAGAATTCCCC
+
FBBCCB?BB?BBBB?B@A?BBBA7AB@CBBBC@CCACC;;;;;;9110?>>=?>AA<AAAAB>BBBBCCBCB@>><<
<:===8;5;>>1
@T6XXI:00145:00272
GGGGAATTCTAATACGACTCACTATAGGGAGAGGGAGAATAGTCACATTCATGTTTGTGACCGTGTCTCCTTCCTTT
CTCCCTTCTCCC
+
FFB7C8;8<@>@DCCBCBBBAA;99366.777BE>CBC@C:;:@AA@B?BBB@CA>??BB?=?@?>?==9<?A9<=7
;;;;1858AAC@
@T6XXI:00145:00282
CTAGTCACTCGAGATCGCGCGTCTTGGACAGTCCCTCTGTACTGTGAAGCCACAGATGTCGAGTCGACTAGCTTCCT
TTCCTCCCTTCCTCCC
+
A>99444:ACCC>>>2,-
,,0,,.,.68=>??;9:;881111111168:?@CC@B@CCCBB>B::3111616699748132.1320*+*++00
@T6XXI:00145:00285
GGGAGAAGGGAGAAAGGAAGCTAGTCGACTCGACATCTGTGGCTTCACAGTACAGAGGGACTGTCCAAAGCCGCAGA
TCTCGAGTCGACTAG
+
DEB@@@=@>:>>>>:@5949>>>??7::?@@?<<;::9A@A:?@?@??BCCCCBB77717=88:=244-
4;8<AAA@A999=@AAAA@AB??
@T6XXI:00145:00296
GGGGAATTCTAATACGACTCACTATAGGAGAGGGAGAATAGCTTGTAACTCCCCAATCACAGCCTATCCTT
+
CFC:C=?=<99<9<9BBBBBI@7770/,0<077-7=@9BCC@B>???=?>>>=5<>00//.-4/7@<:,.,
@T6XXI:00145:00299
GGGGAATTCTAATACGACTCACTATAGGGAGAGGGAGAATAGTCAACAAATCGTTTGGGGCGACTTCTCCTTCCTTT
CTCCCTTCCTCCC
+
?B?2=;?=@BCBCCCCCCCBCBA@@:96.777<?<?CB@C??<8838==:AAAAC>CKC7<;CBAD?::37<;344-
404444/3/44B4
@T6XXI:00146:00253
GGGAGAAGGGAGAAAGGAAGGAGAAGTCGCCCCAAACGATTTGTTGACTATTCTCCCTCCTCCCTCATAGTGAGTCG
TATTAGAATTCCCC
+
AB=CBC?BA:=944-
9;@=@@DCC??=>8788.797993..+..,.44.67>473@77476665/..88449==?AAAAA?>8624/000+
@T6XXI:00146:00267
GGGAGAAGGGAGAAAGGAAGAATAACGATCTGCGGGGAAAAAAGAAACTATTCTCCCTCTCCCTATAGTGAGTCGTA
TTAGAACTTCCCC
+
CKBCCB@CC>BBBB?C@C@CB>B?>BBCBBEBCCCG8BBBBB-
CBB>CCCC@CBBB=@@@BEACBABBBCCCC??7888;?B9?A?ACC9
@T6XXI:00146:00274
```

```
CTAGTCACTCGAGATCTGCGGCTTTGGACAGTCCCTCTGTACTGTGAAGCCACAGATGTCGAGTCGACTAGCTTCCT
TCTCCCTT
+
>:111=:?ADDDBCCBBBBB@BBB>B@A;9?:::47??AAABB@CBD=<@@CCCCCCBBBCBB:;;B@?>:10,0,0
,/000,/,
@T6XXI:00146:00277
GGGAGAAGGGAGAAAGGAAGGGCAGCGGGGAGAAAGAGATGGCAGTCTATTCTCCCTCTCCCTATAGTGTAGTCGT
ATTAGAACTTCCCC
+
;<6<AB=@A=AGGG>B<@59908>?8888-
8>>>:@?????D>AA>86::?<>>@A7=877B1775170+++0+++3365664400/000+
@T6XXI:00146:00279
CTAGTCACTCGAGATCTGCGGCTTTGGACAGTCCCTCTGTACTGTGAAGCCACAGATGTCGAGTCGACTACGCCCTT
TCC
+
>>111=:=?@BCCCA@@@@@>ACC>C@A@??;;76;@@AACBBBCCB>8<9;@AAA@@BCCC??A;;;6,,,02*,,
*,+
@T6XXI:00146:00286
CTAGTCGACTCGAGATCTGCGGCTTGGACAGTCCCTCTCGTACTGTGAAGCCACAGATGGACTGCTTCCCTT
+
/4449<99=CCC<<<@981//,/.,/544,,,66+.,///7777:<??549;>4448331444799133,.6
@T6XXI:00147:00250
GGGGAATTCTAATACGACTCACTATAGGGAGAGGGAGAATAGCACGGCTCGAAAGTGCAGTCAGTTCTCCTTCCTTT
CTCCCTTCTCCC
+
111*/,/788;;??@CBBCCCCA?>;??99::CC>CBB?BCB@?=A@>BB????9<AA@@@@@@8858=?:;8;3441
744;3<8<?@?:
@T6XXI:00147:00254
GGGAGAAGGGAGAAAGGAAGGAGAAGTCGCCCCAAACGATTTGTTGACTATTCTCCCTCTCCCTATAGTGAGTCGTA
CTTCATGAA
+
AA?ADEIDB<AABC>B=@=@?BCCGCC?=;;;5?A>CCDCC>CC@C?:?DC>=744,44444-
4??AAAAAAA=66///,.10...
@T6XXI:00147:00257
GGGGAATTCTAATACGACTCACTATAGGGAGGAG
+
A@?,4592444248>AACCD@?>>:10/,..,.,
@T6XXI:00147:00259
GGGGAATTCTAATACGACTCACTATAGGGAGAGGGAGAATAGTCAACAAATCGTTTGGGGCGACTTCTCTACCTTTC
CT
+
FKK:C?B@BCD@CBBBABBB?A>?>76659@@CC>CBB>B>>9AB???;;ACCCC>BBB7BAAA>:://000,/.+.
33
@T6XXI:00147:00261
GGGAGAAGGGAGAAAGGAAGCTAGTCGACTCGACATCTGTGGCTTCACAGTACAGAGGGACTGTCCAAAGCCGCAGA
TCTCGAGTCGACTAG
+
CCBBBC@CC>BB>;:@=?<@CA==>/10>>><0;;???CCC>?@@CBB??BBCC@8887<>>???<?@;@@>???AA
@???@AAA???A@@?
@T6XXI:00147:00265
GGGAGAAGGGAGAAAGGAAGCTAGTCACTCGACCATCCTGTGGCTTCACAGTACAGAGGGACTGTCCAAAGCCGCAG
ATCTCGAGTCGACTA
+
CC?B=<<;A:@<<<89595;;;8;;=77777,,*,,,*,,245;?597::><??AAACD@BA@<8858B?>@>?>=;
44434300433330-
@T6XXI:00147:00274
```

GGGAGAAGGGAGAAAGGAAGCTAGTCGACTCGACATCTGTGGCTTCACAGTACAGAGGGACTGTCCAAAGCCGCAGA
TCTCGAGTCGACTAG
+
DEBCCC@CC>C@@=<@=@=@CB>??0//9../0731117663:848@ABBBBA=?444-
444;;@7<<6;=:889HAA@@@A==<?>>==<<
@T6XXI:00147:00277
GGGGAATTCTAATACGACTCACTATAGGGAGAGGGAGAATAGATCTGAACTCCAAGTTCCGCTATTTTTTCCTTCCT
TTCTCCCTTCTCCC
+
DBA6?798;AA@CCCCCAB?>CD?B7775:??CE>BBC=??A><<<BFIBAAFBBBB?C@BBABB@@@@+<;><>48
8,7611,13617:5
@T6XXI:00147:00278
GGGAGAAGGGAGAAAGGAAGCTAGTCACTCGACATCTGTGGCTTCACAGTACAGAGGACTGTCCAAAGCCGCAGATC
TCGAGTCGACTAAG
+
CB=?@@=>>:>=;<6;,1-
/769337....4../4445.10131379977:<<<00,/8666<9<B<?A44487=9;8;044330040324
@T6XXI:00147:00280
GGGAGAAGGGAGAAAGGAAGGTGAACAGAGCGCAAACGAATACGTCTCTATTCTCCCTCTCCCTATAGTGAGTCGTA
TTAGAATTCCCC
+
DB>BCB@CB>CCCC=A<><><@@C@CBACCEB@??<@BB@CBCCEDDCCCC?AABC>BBBED?CCB@@@;;;@BC;;
<@C@B?C@CCC:
@T6XXI:00147:00285
GGGGAATTCTAATACGACTCACTATAGGGAGAGGGAGAATAGTCAACAAATCGTTTGGGGCGACTTCTCCTTCCTTT
CTCCCTTCTCCC
+
AAA6ACA?BBBACCCCCBBAB?;99311.777>@=BCE@DCC@@@?=<<7@AAAC?CCC7AABBB?AAA>A;=8;96
;=;;:?484CD;
@T6XXI:00148:00250
GGGAGAAGGGAGAAAGGAAGCTAGTCGACTCGACATCTGTGGCATCACAGTACAGAGGGACTGTCCAAAGCCGCAGA
TCTCG
+
CDECCB@BB>BBBB>B@B9;:B:;:BBCDCC@>>@B>BC??>@CCCA;8:8==99300-
4;;?@=?CC>@@>@@AAA=<?:@
@T6XXI:00148:00254
GGGAGAAGGGAGAAAGGAAGTGCAGTTCGCGGTTAGTGAAGGGAAAAACTATTCTCCCTCCTCCCTATAGTGAGTCG
TATTAGAACTTCCCC
+
CEBCEFCA@:?9991424788;=@=CD<<<;=A=;<<BB>BB07777+3<88400..+..,.55+//69==A@AB;3
440/06<<=9<===5
@T6XXI:00149:00253
GGGAGAAGGGAGAAAGGAAGCTAGTCACTCGACATCTGTGGCTTCACAGTACAGAGGGACTGTCCAAAGCCGCAGAT
CTCGAGTCGACTAGCTTCCTTTCTCCCTTCCTCCC
+
AA<@CB@CC?CCCC@C@C@<;<B?<0//......43671106817766=8?<<=777.8==?@A>AA<BH>@@AAAA
888<<878><;9>655532461..3704,.,-133
@T6XXI:00149:00261
GGGAGAAGGGAGAAAGGAAGGAGAACGCGCACCCGAACCAACGGTGATCTATTCTCCCTCTCCCTATAGTGAGTCGT
ATTAGAATTCCCC
+
FIBBBB@CC@BCBC?C@CAC@CCC?BBBCDECC>CCAC@C@CCECCCCA<<<@B@@@>BACGD@DCCABAAAAAAAB
BB?B@C<B?BBB7
@T6XXI:00149:00263
GAATTCTAATACGACTCACTATAGGGAGAGGGAGAATAGTATTCCACTACACAACGGCCGGCTGTTCTTCCTTTCTC
CCTTCTCCC
+

```
//,/,/9988@@@A@C@@@@?A89617??CC>C;:::AA>?@=?=???:::BC?BA@C@C@@@?A@BC@C?BB?CB;
;9;./.770
@T6XXI:00149:00267
GGGGAATTCTAATACGACTCACTATAGGGAGAGGGAGAATAGCCACCAACTATTGTTCGTCGCCGTTCTCTTCCTTT
CTCCCCTTCTCCC
+
DID9B?B?BCDAEDCC99:@@788>877.777??<@A@<@==:7::3434====@AB@@A@8848A?<<=?<?>>;9
;4444-/7///>9
@T6XXI:00149:00268
GGGGAATTCTAATACGACTCACTATAGGGAGAGGGAGAATAGCCGCACCAGCGTTGACAATTTATTCTCCTTCCTTT
CTCCCTTCTCCC
+
EEF:C@A>@CC@BBBAAA???CB?9311.7><<?<?CC@CCC=<==?=;<@@BB?C::<ACD>DA=<==3838?@C<
A???<?>??BA=
@T6XXI:00149:00273
CTATCGACTCGAGATCTGCGGCTTTGGACAGTCCCTCTGTACTGTGAAGCCACAGATGTCGAGTCGACTAGCTTCCT
TTCTCCCTTCTCCC
+
DA??;9;?CCBDCDCBCCCC@BCC>C@AB??:::5:CBCBBBB=@@DA@C?BDCCBBBBAAABBBBBBBC;;65:5:
B9?:BB>B;???=:
@T6XXI:00149:00277
GGGGAATTCTAATACGACTCACTATAGGAGAGGGAGAATAGATCACCGTTGGTTCGGGTGCGCGTTCTCCTTC
+
CBD7B=@>@CCCBBABBAA?B:666..,0;;>BACBB?BBB??A::4:B5:8<@CCB>;988870,000,0,0
@T6XXI:00149:00285
GGGGAATTCTAATACGACTCACTATAGGGAGAGGGAGAATAGTCGGCGCCCAAACTTCGTACTCATTCTCTTCCTTT
CTCCCTTCTCCC
+
FDF9A=A=@BC@CBABBBCBBBCDA><?;???CC>BBB?C:?<4737>>>;BJBBB?BB@@BBCBB?B;:7=?377.
7<==6=:??>A:
@T6XXI:00149:00292
GGGGAATTCTAATACGACTCACTATAGGGAGAGGGAGAATAGTCAACAAATCGTTTGGGGCGACTTCTCCTTCCTTT
CTCCCTTCTCCC
+
ABB7A>A>BBCACCBBCBCA>;<><777.777?@>BCC@BBC???=???:BBAAA<AAB7BABAA;?>>4744244-
4;;>:?484@A:
@T6XXI:00149:00295
GGGGAATTCTAATACGACTCACTATAGGGAGAGGGAGAATAGTGACGTGTTGTGCGGCGCTCACTGTCTCCTTCCTT
CTCCCTTCTCCC
+
CIE:C;;;<B?@CDDA@ABC;:8@?877-
777?B=@@C@A?@=@@@?@@A9888=>;?@@@@=<;=;44/4741+,+,055+,+,.//,
@T6XXI:00149:00296
GGGAGAAGGGAGAAAGGAAGGAGAACATGGTGCCACAGATTGGTGGTCTATTCTCCCTCTCCCTATAGTGAGTCGTA
TTAGAACTTCCCCATCACCGC
+
BB?BA@=@C@<=<ECE?B?B?DBD>AACC@CBB>BBB@@@@DAAB=:;:@B;@>BC<7766988774BAAA@?????
@=@AA>??>AAA5?8/,5+*(
@T6XXI:00149:00299
GGGAGAAGGGAGAAAGGAAGCTAGTCGACTCGACCATCCTGTGGCTTCACAGTACAGGAGTCTCGC
+
BB?<:=@CE@CCBG8;9;9;;7???1.,2*****)***)**,55135796??@::075,*******
@T6XXI:00100:00310
GGGAGAAGGGAGAAAGGAAGCTAGTCGACTCGACATCTGTGGCTTCACAGTACAGAGGGACTGTCCAAAGCCGCAGA
TCTCGAGTCG
+
```

```
990999<>?9@@@B<B?A=@CBBBB???=??=<::@?:?<>=8?;?:::::>?::444-
4:;;:B9==7<?>888@@8435333300
@T6XXI:00100:00335
GGGGAATTCTAATACGACTCACTATAGGGAGAGGGAGAATAGCCACCAACTATTGTTCGTCGCCGTTCTCTTCCTTT
CTCCCTTCTCCC
+
CDECC::<<AABDDB@@??BBCB97111.7<<@B>BAA?B:?@<=<9=9=@AA>AB>AA=9=?>@B=@@88375=>;
A@@C?C>???@<
@T6XXI:00101:00307
GGGGAATTCTAATACGACTCACTATAGGGAGAGGGAGAATAGTTACACTAAAC
+
BBA9BAC@CDB@@@@CCCCCB@BB76:>.77777-6744944384900003+-
@T6XXI:00101:00309
GCTAGTACATCGAGATCTGCGGCTTGGACAGTCCCTCTGTACTGTGAAGCCA
+
/,2,,////4;44444;4844243142379;;>?;6616111177773-1,/
@T6XXI:00102:00300
GGGGAATTCTAATACGACTCACTATAGGGAGAGGGAGAATAGTCAACAAATCGTTTGGGGCGACTTCTCCTTCCTTT
CTCCCTTCTCCC
+
ABA7A===@CC@CBBBBBBBBA<97111.7=7775:A@>AA@@@@>???:BBBBC>CCC8CCBBB@CCC>?<?:@@<
@AAA@A899AA<
@T6XXI:00102:00302
GGGAGAAGGGAGAAAGGAAGGATATGCCGCAGTGGGAGATTAAACGGCTATTCCTCCCTCTCCCTATAGTGAGTCGT
ATTAA
+
CC@CB@@BC>B?=85>4948483771313333311,391311666;?9@@@6;64;;544333,331116661111.
--,-,
@T6XXI:00102:00304
GGGAGAAGGGAGAAAGGAAGCTACTCGACATCTGTGGCTTCACAGTACAGAGGGACTGTCCAAAGCCGCAGATCTCG
AGTCGACTAG
+
HK?<=<>AA<<;9@9>=@=@ACCCCCA88;CBCFECBDC@CAAACFCB??8990999;BB@CD?BB@BCCCCBBBCB
<;;A@@A?::
@T6XXI:00102:00305
CTAGTCACTCGAGATCTGCGGCTTTGGACAGTCCCTCTGTACTGTGAAGCCACAGATGTCGAGTCGACTAGCTTCCT
TTCTCCCTTCTCCC
+
BB;<<@=A>?@>@A@CBBA@>A@A<A=@ABABCC@BBAAACCCCB@?=<@=AACBBBCCDDAAABB<;8@<662:=B
?9??>>69793886
@T6XXI:00103:00302
GGGAGAAGGGAGAAAGGAAGGAGAAGTCGCCCCAAACGATTTGTTGACTATTCTCCCTCTCCCTATAGTGAGTCGTA
TTAGAATTCCCC
+
CD@CB=@BBBAAAB=A?@@B@BBB@??BBBBD8BB<BCCCC:?AACC@B@A>;<<=;;:?>=;===::;=>A<=?==
=>BDD>@<>>?4
@T6XXI:00103:00342
GGGAGAAGGGAGAAAGGAAGGAGAAGTCGCCCCAAACGATTTGTTGACTATTCTCCCTCTCCCT
+
BHABCB=@@<;<<CAC@DAB?AA@<??;;;CD9DC@@??C?;EA@==<55323003030030+0
@T6XXI:00103:00345
GGGGAATTCTAATACGACTCACTATAGGGAGAGGGAGAATAGTTACACCTCTGTTGATTGCTATATCTCCTTCCTTT
CTCCCTTCTCCC
+
;99/9<?=@CC@CBAABBBBBCB>9111.6==@B>BA@>@<@@>A===>ACA@@>@@@>???@C@@???<?BC<??<
?AAA<A<???A<
@T6XXI:00103:00346
```

```
GGGGAATTCTAATACGACTCACTATAGGGAGGGAGGA
+
CHH:C;;7<8>;>@@BBABCCAABA?;6,11623200
@T6XXI:00104:00300
GGGGAATTCTAATACGACTCACTATAGGGAGAGGGAGAATAGTCAACAAATCGTTTGGGGCGACTTCTCCTTCCTTT
CTCCCTTCTCCC
+
;B>9;1:<<CC@CCBBBBBBB?<97111.7>??@>BCA?AACC@@688@;BEBBB>BCD7?>A:8366;3727=AA<
:=?C>C9;;AB=
@T6XXI:00104:00303
GGGGAATTCTAATACGACTCACTATAGGGAGAGGGAGAATAGATCACGACCCTTCTACCCACGATTGAGCTTCCTTT
CTCCCTTCTCCC
+
BAA9B=?>ACC@BAA@A@CBB?<97111.7>>BD>BBB>B=:;;<@@B???;A@>B961.4;;;>9<<;@@=A=@B?
CCCC?F?A@EEA
@T6XXI:00104:00308
GGGGAATCTAATACGACTCACTATAGGGAGAGGGAGAATAGTTTTCCTCCGACTAACC
+
:::292444888448;83>>1331111,11676,6A@>@ABCCC4;8;:3370/.44,
@T6XXI:00104:00312
GGGGAATTCTAATACGACTCACTATAGGGAGAGGGAGAATAGTTTTTCCCTTCACTAACCGCGAACTGCACTTCCTT
CTCCCTTCTCCC
+
@@>4>6;8<BB@C@??BBBBA@B;9616-6=<<?9?B@9;944444,44-
4/0?;::>A<???=;?@@@>>:,0,0,/77:6>;;443+
@T6XXI:00104:00336
GGGGAATTCTAATACGACTCACTATAGGGAGAGGGAGAATAGTCAACAAATCGTTTGGGGCGACTTCTCCTTCCTTT
CTCCCTTCTCCC
+
BEC:B6;:9>@;@AAACB@@@B??=166.7=77757<><??@@;555553?ABB@;B;;-
::??A:??@77388986;;9?:@3848>4
@T6XXI:00105:00301
GGGGAATTCTAATACGACTCACTATAGGGAGAGGGAGAATAGATCACCGTTGGCTCGGGTGCGCGTTCTCCTTCCTT
TCTCCCTTCTCCC
+
@@>4@>A>BDC@BC@@ABBAA>;97111.7>>?B?CA?<?<437A??:?B?@<A@CCC>C@@@@??<@:;7:=@?DE
>BBDF==;=AAAA
@T6XXI:00105:00331
GGGAGAAGGGAGAAAGGAAGGAGAAGTCGCCCCAAACGATTTGTTGACTATTCTCCCTCTCCCTATAGTGAGTCGTA
TTAGAATTCCCC
+
CD>A@@<=@<@@@@>A@D@B@CCBAB?@BCCC9BB>BCCC@>CBABBB@==:=??BA???@A<??AA899@?A????
?>A@>:;/444-
@T6XXI:00105:00335
GGGGAATTCTAATACGACTCACTATAGGGAGAGGGAGAATAGTCAACAAATCGTTTGGGGCGTACTTCCTCC
+
DHN:E@A>ABBBCCAAABDBBAB97111.7><<?<????B?@@AA>===9?AAAC>CC>57.....,.,,,2
@T6XXI:00105:00338
GGGAGAAGGGAGAAAGGAAGCTCAATCGTGGGTAGAAGGGTCGTGATCTATTCTCCCCTCTCCCTATAGTGAGTCGT
ATTAAGAACTTCCCC
+
CCCDCC@DC>BBBB=B?B@CCBAB>BB@CCC>B??<;??:@AAAA@@@??=9;4444-
4;996;<<====@?;88//-2,/,.44347:=90
@T6XXI:00105:00345
GGGAGAAGGGAGAAAGGAAGGAGAAGTCGCCCCAAACGATTTGTTGACTATTCTCCCTCTCCCTATAGTGAGTCGTA
TTAGAATTCCCC
+
```

```
CD@CBB@CA=@@@B>B@B?A?@BB@=?@@CDD9BC>B?9::5:A@CCB=??@AAAA;<<<<A<@AA@===?@?=>?9
985==<=;@@@5
@T6XXI:00105:00349
GGGGAATTCTAATACGACTCACTATAGGGAGAGGGAGAATAGTTTCGTGCATTTCTCCCAGCACACCGTCCTTCCTT
TCTCCCTTCTCCC
+
CDC:C::<<CC@CBAAB???BA>97111.7>>BC>A@@?A:?6A::=AA=?BB=ABCC>BBBBCBA>B::5:=?47:
1:=;>:=348<<6
@T6XXI:00106:00305
GGGGGAGAAGGGAGAAAGGAAGGAGAAGTCGCCCCAAACGATTTGTTGACTATTCTCCCCTCCTCCTATAGTGAGTA
CTT
+
CCCC3CDC@CB>CCCC?B@B@B<>AC@@@ABAAA8AC>BCB?C>@?A?;;;:::::===54420;8:43000-
00****)
@T6XXI:00106:00306
GGGGAATTCTAATACGACTCACTATAGGGAGAGGGAGAATAGTCAACAAATCGTTTGGGGCGACTTCTCCTTCCTTT
CTCCCTTCTCCC
+
CCB9B8;;<CCACBBBBBCBBA?97111.7>>>A>BGBB@>>AAA??@@;B?B?E>BBB7BBBBC?BBB8968>AA<
>@AC<A898@@;
@T6XXI:00106:00341
GGGGAATTCTAATACGACTCACTATAGGGAGAGGGAGAATAGTCCTACATT
+
DDC9C=@>ADD@CA??A????AB97111.7>>BE>CAA>=0.**)***,33
@T6XXI:00107:00306
GGGAGAAGGGAGAAAGGAAGCTAGTCACTCGACATCTGTGGCTTCACAGTACAGAGGACTGTCCAAAGCCGCAGATC
TCGAGTCGACTAG
+
9914498=9-44>>1949;;::AB<?96661619;<788??BCBBBBB:;:<>://./;:88@344-
4;9=====A@AABA88884:::9
@T6XXI:00107:00315
GGGGAATTCTAATACGACTCACTATAGGGAGAGGGAGAATAGCTACAACAATC
+
DDC:B6;9<@@@D<<<CBBBCBB?=666.777>CACCB>@76/0//.442253
@T6XXI:00107:00326
GGGGAATTCTAATACGACTCACTATAGGAGAGGGAGAATAGTCAACAAATCGTTTGGGGCGACTTCTCCTTCCTTTC
TCCCTTCTCCC
+
GEC9B=?>BBB@CCCBBABCA7477/0,0>:=?=?AB?@@@AAA=@@A<BBBCC?CC@7BABBB=??C==:=?AA?A
AAA<A9<<>=7
@T6XXI:00107:00342
GGGGAATTCTAATACGACTCACTATAGGGAGAGGGAGAATAGTCAACAAATCGTTTGGGGCGACTTCTCCTTCCTTT
CTCCCTTCTCCC
+
BDC:C=?=?AAADCCBBB<BBA>97111.7==>BABCC?A?@@@@>??<;?===@;?@?8CBB???@BB=?<:<AC:
A?AA;@?@@@A<
@T6XXI:00108:00301
GGGGAATTCTAATACGACTCACTATAGGGAGAGGGAGAATAGACAACAATTCTACCGTCCGCTATGTACCTTCTTCC
TTTCTCCCTTCTCCC
+
CDG:C?A?@AACDBA?B??AD@<97111.7=<AB>BCC@C<::?<;@=<A;=?B?>?788:BBBCC@@@DC@CC?A?
BC@CCCC>B?BCCC>
@T6XXI:00108:00312
GGGGAATCTAATACGACGTCACTATAGGGAGAGGAGAATAGTAGCCACTTATCA
+
BA>17+-,,,*,,,,/...667=?;;;;:@>>?;777:<711110/...63...
@T6XXI:00108:00325
```

```
GGGAGAAGGGAGAAAGGAAGCTAGTCGACTCGACATCTGTGGCTTCACAGTACAGAGGGACTGTCCAAAGCCGCAGA
TCTCGAGTCGACTAG
+
CD>BBA8<4-
9>>>9>598;A><=@/0/99900:8666B@@>?C@ABBBBDA=;;44418=;>AA>A@<AA@A888=878@888888
898??
@T6XXI:00108:00340
GGGAGAAGGGAGAAAGGAAGCAGTCGACTCGACATCTGTGGCTTCACAGTACAGAGGGACTGTCCAAAGCCGCAGAT
CTCGAGTCGACTAG
+
BC?AAA@BA=>>AB>B?A?A@ABC:;;CCCB<;ACAACFEDBB@BDCCDDB?==;;;:??@???>@CBCE@CBBBAA
?C==B>>:????@@
@T6XXI:00109:00306
GGGAGAAGGGAGAAAGGAAGTGCAGTTCGCGGTTAGTGAAGGGAAAAACTATTCTCCCTCTCCCTATAGTGAGTCGT
ATTAGAATTCCCC
+
CC?AAC@CD>BBBC>A?@@?A?BBBB@BCBB?A?ABBCC@DC>BBB>2BCBB@BCCB<?????9?@@@A@@AAA???
??<?BA>A;@@@5
@T6XXI:00109:00331
GGGGAATTCTAATACGACTCACTATAGGGAGAGGGAGAATAGTCTCCCAGTATCTAGCACGACGCTAACCTTCCTTC
TCCTTCTCC
+
6==5=?B@CDB@C@@@BABBB?AA@AA@;8616657897////31145677?::8::56>AAB>BC?;?;<:<,,+,
+++-,--11
@T6XXI:00109:00349
GGGGAATTCTAATACGACTCACTATAGGGAGAGGGAGAATAGCCGCGACATTTTCCCAGCTGGCCAGCTCTTCC
+
BBB:GAB?BBB>BBBBBABBA?>?<777.7>7775:6646///4674::8BEG:CC<??9992/67-,,,,*,*
@T6XXI:00110:00304
GGGAGAAGGGAGAAAGGAAGCTAGTCGACTCGACATCTGTGGCTTCACAGTACAGAGGACTGTCCAAAGCCGCAGAT
CTCGAGTCATCAGA
+
BD>AA@>@>:>>>>9A>B=@@A>>=0//::9;3:6==6:9C5::@BCBC@A?;89//,/8888?>AA<@@<9=>@A;
;88884;954630.
@T6XXI:00110:00317
CTAGTCGACTCGAGATCTGCGGCTTTGGACAGTCCCTCTGTACTGTGAAGCCACAGATGTCGAGTCGACTAGCTTCC
TTTCTCCCTTCTCCC
+
C2,---
,,6>:BCCCBBBBBB@BBB=B?AABA@@@=@AC@>>A>CCCA9<A@BBBBBBBBACCCBBBB@@BDDC@C?BC>BBB
C>@:==A@;
@T6XXI:00110:00322
GGGGAATTCTAATACGACTCACTATAGGGAGAGGGAGAATAGTCAACAAATCGTTTGGGGCGACTTCTCCTTCCTTT
CTCCCTTCTCCC
+
ABA7B=?=ACBABBB?><<?B@CB>777469>@C>BAA?A888;=<<<<;BBBBC>@BB9BCCCC?ACC==;9<>A<
<AAA<A;==@@;
@T6XXI:00110:00331
GGGGAATTCTAATACGACTCACTATAGGAGAGGGAGAATAGTCAACAAATCGTTTGGGGCGACTTCTCCTTCCTTTC
TCCCTTCTCCC
+
?>?4?;??ABB@DCCBCBCBB7663..,0;;AB>CAA?A=AAA=;===;?>>@C=@@@7>CAAB@BCC?@=@>AA<@
@AA<A>??@@;
@T6XXI:00110:00340
GGGGAATTCTAATACGACTCACTATAGGGAGAGGGAGAATAGCCACCAGACAACTCCCTCAGGTGTGTACTTCCTTT
CTCCCTTCTCC
+
```

```
CDC9C>@@ADC@CBAABCBAA@B8<;CE>@??CC>A?@>A???<AA>=@<@A?<@<=;AAA=<A@@@???@?B>AA7
??CC<@>@@@>
@T6XXI:00111:00303
GGGGAATTCTAATACGACTCACTATAGGGAGAGGGAGAATAGTCACCAAACCTCCCATGGTGCTATTGCCTTCCTTT
CTCCCTTCTCCC
+
499/9<>=BCCBCBAABBBBC>@>=666.7>>BC>BBA@A???ABC<??<<;<<<;A??<??@BBCABDBB@CACC>
CCCC>C??AAA<
@T6XXI:00111:00306
GGGAGAAGGGAGAAAGGAAGCTAGTCGACTGACATCTGTGGCTTCACAGTACAGAGGACTGTCCAAAGCCGCAGATC
TCGAGT
+
AB>BBB@CC=BBBC>C@C;<<CBBBC@??B?::;;:;AAL??@@CBBA@AB??7..,/887;@>@D<@@>@@@AA@?
??AAAA
@T6XXI:00111:00309
GGGGAATTCTAATACGACTCACTATAGGGAGAGGGAGAATAGCCACCAACTATTGTTCGTCGCCGTTCTCTTCCTTT
CTCCCTTCTCCC
+
?BA4@546<AA>ABCBBBECCCCA@777.7?;?C>BBA?@?8868A>A?A@??9<;<@A??AA>A?3788838>AA<
A@AA<A=@@AA<
@T6XXI:00111:00316
GGGGAATTCTAATACGACTCACTATAGGGAGAGGGAGAATAGTCAACAAATCGTTTGGGGCGACTTCTCCTTCCTTT
CTCCCTTCTCCC
+
944,48?<ABB@CBBABCBD>=@CCAA><<A<AC>CC=<A<?@??=???<BFDDE>BBB9CAAAA@DDC==;=?AA;
??AD<A888@@;
@T6XXI:00111:00339
GGGAGAAGGGAGAAAGGAAGGAGAAGTCGCCCCAAACGATTTGTTGACTATTCTCCCTCTCCCTATAGTGAGTCGTA
TTAGAATTCCCC
+
DB>AAA@DE@CCCC>C@C@B@BBB???BBBDD7??<BC@BB?C@@@778AB>BAAA<@@@?A<A===AAAA@@AAA@
@>@@>:;3444-
@T6XXI:00112:00300
GGGAGAAGGGAGAAAGGAAGGAGAAGTCGCCCCAAACGATTTGTTGACTATTCTCCCTCTCCCTATAGTGAGTCGTA
TTAGAATTCCCCC
+
BB>ABB=AA>ACGD>A>?@B@B@A?@C?@@CC7AA>CDCFE=BB@BAAA@C?@==<59;999499:<<<@@@B==<@
>;34488<7777.
@T6XXI:00112:00308
GGGAGAAGGGAGAAAGGAAGTGCAGTTCGCGGTTAGTGAAGGGAAAAACTATTCTCCCTCTCCCTATAGTGAGTCGT
ATTAGAATTCCCC
+
:B>@@>?AA<@?>?9@;>=@999CCC:;;@A=B=@BCBB?BC9??880?ABB=??@@97::??8???<==?@@=437
3323AA<=;=AA6
@T6XXI:00112:00310
GGGAATTCTAATACGACTCACTATAGGGAGAGGGAGAATAGCCAACAAATCGTT
+
,,*,0>;=AB@BBBBBBCCBBBCAAAB966667.7;978///,.4+++*++--3
@T6XXI:00112:00322
GGGGAATTCTAATACGACTCACTATAGGGAGAGGGAGAATAGTTTTTCCCTTCACTAACCGCGAACTGCACTTCCTT
TCTCCCTTCTCCC
+
BA@4@59694498=@BBABBB??>7777,7???C>C@@>@<@@ABB1BA=A>AB:::>@<??BA6::CACBB>B=??
<?CCC>D?>;;;6
@T6XXI:00112:00328
GGGAGAAGGGAGAAAGGAAGGGGACGTCGGCTTCACTGAAGAGCGAACTATTCTCCCTCTCCCTATAGTGAGTCGTA
TTAGAATTCCCC
```

```
+
HCCCCC@CB?@ABB>B=@=@CC9C@ABAC??@>@CCCCC@BABBBB?AABA@==>?:???CD@@?>>?@BCA<<<A?
<<:A>9;/444-
@T6XXI:00112:00334
GGGGAATTCTAATACGACTCACTATAGGAGAGGGAGAATAGTCAACAAATCGTTTGGGGCGACTTCTCCTTCCTTTC
TCCCTTCTCCC
+
CCD:C::;:CC@C@@?BBBB?:997/.,0<;>@>AAD?CA@@@@=@BB<CBA?=6?;;5??AAFABBB7858;@C@@
@@D=D9;<DD<
@T6XXI:00112:00339
CTATCGACTCGAGATCTGCGGCTTTGGACAGTCCCTCTGTACTGTGAAGCCACAGATGTCGAGTCGACTAGCTTCCT
TTCTCCCTTCTCCC
+
DA?=99:==?=A<<?DCCCB@CCD>B?CB??99:7:CCCB?BBAA==9<@=@CCCEACCBBBCBBBAAABBBB?C?B
DACCAA<A;==?C:
@T6XXI:00112:00345
GGGAGAAGGGAGAAAGGAAGCTATCGACTCGACATCTGTGGCTTCACAGTACAGAGGACTGTCCAAAGCCGCAGA
+
:;79A@<@@1999909<><?@@B=AB??557:/..0.5577CB<??BBBA9:8=8838=B<;<=??:?@>???;>
@T6XXI:00112:00347
GGGAGAAGGGAGAAAGGAAGTGCAGTTCGCGGTTAGTGAAGGGAAAACTATTCTCCCTCTCCCTATAGTGAGTCGTA
TTAGAACTTCCCC
+
HD@BAA@BB>BCCB>C@C?AABBBBB@BBAA@C@CCCCC@CA>ACC9CBBB?BBBB>BCCFC>B@@?<<=AAA?788
:7:;;7:<;===4
@T6XXI:00113:00307
GGGGAATTCTAATACGACTCACTATAAGGGAGAGGGAGAATAGTCAACAAATCGAT
+
499/9296<99@A@=BC;;0:::0..,..+.57@@?@@<;>73444/044+111..
@T6XXI:00113:00314
CTAGTCACTCGAGATCTGCGGCTTTGGACAGTCCCTCTGTACTGTGAAGCCACAGATGTCGAGTCGACTAGCTTCCT
TTCTCCCTTCTCCC
+
BA999944:@=@??@BAB@@=@@A<A?AABBBBB>BCB@?????BAA?AC@CCBBBBBBCCBB@BBAAABCAA?AEC
D>BBBD5837<<?7
@T6XXI:00113:00322
GGGAGAAGGGAGAAAGGAAGAAGATGTCGCCGCAGGGAGTGAACTTGGCTATTCTCCCTCTCCCTATAGTGAGTCGT
ATTAGAATTCCCC
+
CC@@@@@BB>BBBB>B@A@??>??>??B>>>B@CDB<778CC?CC@B?BCBA>B???=;:;CI>DB@@?CCBBAABB
BCBBAA?BABAB9
@T6XXI:00113:00329
GGGAGAAGGGAGAAAGGAAGTCGCAGTTCGCGGTTAGCTGAAG
+
BA=@@>;=>19911,1-1-0.....477661101011103101
@T6XXI:00113:00337
GGGGAATTCTAATAACGACTCACTATAGGGAGGAGGGAGAATAGTTACGCCAATGATCCCCGTCGG
+
DCC8B@C@AA?@BB@BAB?B?BBBB>>;>6,,+,,,*,++0225556885<@@///74440.,,,2
@T6XXI:00113:00341
GGGAGAAGGGAGAAAGGAA
+
CA199454;6>///+/,/,
@T6XXI:00113:00345
GGGGAATTCTAATACGACTCACTATAGGGAGAGGGAGAATAGTCAACAAATCGTTTGGGGCGACTTCTCCTTCCTTT
CTCCCTTCTCCC
+
```

```
DHC8@6;8<<<DGBBAABBBB@>96111.7====9?>@>AA@@@@>@AA<CCCBC9???7?BBCC@ABA;988?AC=
@AAA<@889@C=
@T6XXI:00114:00311
GGGGAATTCTAATACGACTCACATAGGAGAGGGAGAATAGCACG
+
>;808,/,///7/4484833976611016111,0449;44//-.
@T6XXI:00114:00333
CTAGTCACTCGAGATCTGCGGCTTTGGACAGTCCCTCTGTACTGTGAAGCCACAGATGTCGAGTCGACTAGCTTCCT
TTCTCCCTTCTCCC
+
B99499444@==999C<<=C@CDC>C?AA@@???<?ABCCCCCC===9<@?BCCCCCCAABCCB@@@;;:7:7?@??
B9?667.44433==
@T6XXI:00114:00335
GGGAGAAGGGAGAAAGGAAGCTAGTCGACTCGACATCTGTGGCTTCACAGTACAGAGGACTGTCCAAAGCCGCAGAT
CTCGAGTCGACTAG
+
CE@AA@?AA<@@@B>A?B?@@?;97/00//39;?8666@??:?@=?ACCCCB999/../;;;=@=AC<@@=?@=AA:
<??878@@>??A=<
@T6XXI:00114:00336
GGGAGAAGGGAGAAAGGAAGCTAGTCACTCGAGATCTGCGGCTTTGGACAGTCCCTCTGTACTGTGAAGCCACAGAT
GTCGAGTCGACTAGCTTCCTTTCTCCCTTCTCCC
+
CD<E=<;<@:>99909;>;>>888@:66<?AAAABBBAAE?BBB>B@BB??@@A;>::::=A=:@@=<999=AA??@
@A858@@@<889A<<=>A?@A9998827,//776
@T6XXI:00114:00347
GGGAGAAGGGAGAAAGGAAGCTAGTCGACTCGACATCTGTGGCTTCCACAGAG
+
FDDBBB@DD?BBCB>B=@;;96,,,,,,2354/67:??CB>9:8+,**((((*
@T6XXI:00115:00301
GGGAGAAGGGAGAAAGGAAGGCTAGTCGACTCAACATCTGTGGCTTCACAGTACAGAGGGACTGTCCAAAGCCGCAG
ATCTCGAGTCCGACTAG
+
CC@AA@@BB?BCCB==<=676:>7;=/00>>;;.;:???ABC;<<BCBBACCCC>9000-
4::=@@?AA=AA>===@BAA?AAAA8868?>=??
@T6XXI:00115:00302
GGGAGAAGGGAGAAAGGAAGCTAGTCGACTCGACATCTGTGGCTTCACAGTACAGAGGGACTGTCCAAAGCCGCAGT
CTCGAGTCGACTAG
+
CC@BBB@DB?@==>6:8:>CCBAAA:::@A@:<:@CBAAAB?CC@CBBBBC@96433317=AABA?AA:?@>A@=??
<<77777///;??=
@T6XXI:00115:00305
GGGGAATTCTAATACGACTCACTATAGGGAGAGGGAGAATAGTCAACAAATCGTTTGGGGCGACTTCTCCTTCCTTT
CTCCCTTCCTCC
+
999-
49@>CCC@CCBCCCBBA>;@?677.7><<<9?B@>AAAAAA@AAA;B@@?C@CCC9BBC@?=?@>5858=@C<@@AA
<?987//,
@T6XXI:00115:00323
GGGGAATTCTAATACGACTCACTATAGGGAGAGGGAGAAATAGTCAACAAAATCGTTTGGGGCGACTTCTCCTTCCT
TTCTCCCTTCATCCC
+
CBB:CACCAA@>>9998;7A@AAD???B.77761,1000+042222,-
/..*.;667,111,1777786663323259;5554+0/0--330
@T6XXI:00115:00334
GGGGAATTCTAATACGACTCACTATAGGGAGAGGGAGAATAGTCAACAAATCGTTTGGGGCGACTTCTCCTTCCTTT
CTCCCTTCTCCC
+
```

```
@@A6@@B@CBBACCB@BBBBBAB>>66659==ABB@==?A@@>>>?AAA<ABBCC>CCC7???BB>AAA>><??AA<
???B<A889@A:
@T6XXI:00115:00335
GGGGAATTCTAATACGACTCACTATAGGAGAGGGAGAATAGTCAACAAATCGTTTG
+
CCD:C?B?BBB@CCBBBBBBB:665..,0::==9?@A?A???AA:=?A=CCAAA<B
@T6XXI:00115:00348
GGGAGAAGGGAGAAAGGAAGCTAGTCGACTCGACATCTGTGGCTTCACAGTACAGAGGGACTGTCCAAAGCCGCAGA
TCTCGAGTCGACTAG
+
CCBCBB894-
999909695;CA@AC:::@@@>77:::6?>C8;:@DCCAAAAA=;777149:;;;34814=;?===ACAB===?@@@
@???@
@T6XXI:00116:00300
GGGGAATTCTAATACGACTCACTATAGGGAGAGGGAGAATAGTCAACAAATCCTTTGGGGCGACTTCTCCTTCCTTT
CTCCC
+
CDK9A?A@CCC@CBAAAABCBA;97111.777?BEBA?>A@@@A@=866100,.5257:6@AA<?998:85;>;?@<
@A@C<
@T6XXI:00116:00324
GGGGAATTCTAATACGACTCACTATAGGGAGAGGGAGAATAGTCAC
+
9@@8A?B?BAA?BCDBC@@@BCB<;661-6=667.7;;8=:::4--
@T6XXI:00116:00326
GGGAGAAGGGAGAAAGGAAGAGAACAGACGATTATTCATTAATAACAGCTATTCTCCCTCTCCCTATAGTGAGTCGT
ATTAGAATTCCCC
+
CCBCBB@BB>BBDB?B?B?ACBB?CCCCCCBB@?@BAAA>A?BC@GB@@@DE?>???<?BBCEA@===AAAA@?@@?
@@?AAA?@9<<<4
@T6XXI:00116:00332
GGGGAATTCTAATACGACTCACTATAGGGAGAGGGAGAATAGTCAACAAATCGTTTGGGGCGACTTCTCCTTCCTTT
CTCCCTTCTCCC
+
CCA8CAB@==>@BBB@@@BBA?<96111.7<<>@=A@C>B???AA?AAA<C???C>@CB8BBCCC?A??=<<>8<=7
=?@A<@:==CC=
@T6XXI:00117:00306
GGGGAATTCTAATACGACTCACTATAGGGAGAGGGAGAATAGCCTGCAACCATATTGCCGGTCACGTCTCCTTCCTT
TCTCCCTTCTCCC
+
CBB8@=@@A?B@BCCCA?@ABBBCC9::59??BB>CCA>@<@?8=B??=?ACBBB??@=@=@CCB@AA:75:@C@CJ
>@=@?:?>???A?
@T6XXI:00117:00307
GGGGAATTCTAATACGACTCACTATAGGGAGAGGGAGAATAGTCAACAAATCGTTTGGGGCGACTTCTCCTTCCTTT
CTCCCTTCTCCC
+
@@?6?<@=@A@@CCAAABB@@@B?;66659@@CC>CA?<?=B@?>:=?A=C@><<<AAA7BCCCC@?AA@C=?;?B;
@@AA<A688??:
@T6XXI:00117:00309
GGGGAATTCTAATACGACTCACTATAGGGAGAGGAGAAT
+
8:806,42844248==@A???@??;9996,,,,*,2001
@T6XXI:00117:00321
GGGAGAAGGGAGAAAGGAAGCTAGTCACTCGACATCTGTGGCTTCACAGTACAGAGGGACTGTCCAAAGCCGCAGAT
CTCGAGTCGACTAG
+
```

```
@@@CCB:99-
;@==5@497;BAABC=A>>>>001///>>E?BB@BB@@??@@<=99948;:488>@A<@@>AAA@@??;@558AAAC
@@??
@T6XXI:00117:00329
GGGAGAAGGGAGAAAGGAAGAGAATAACAGTGCACCGAATGCGCAAACTATTCTCCCTCTCCCTATAGTGAGTCGTA
TTAGAACTTCCCC
+
CE@CBE@BB?BBBB=B=>;?A::8<C?BB@@@CCC@BA>BCCCBBB@CCB@<>888039668;<@@BBBADCCBB::
89777.//55574
@T6XXI:00117:00335
GGGAATTCTAATACGACTCACTATAGGGAGAGGGAGAATAGCCTAATCTCCCAGCAAACCGTACGTGCCCTTCCTTT
CTCCCTTCTCCC
+
CCBC@B@BBB@DCCC?>@CD@BBA99646??BB>B@@>A?==9?@=>::8<7<@@@C=A>A@A??>=?@6;782441
8<@@<A<??AD<
@T6XXI:00117:00347
GGGGAATTCTAATACGACTCACTATAGGGAGAGGGAGAATAGTCAACAAATCGTTTGGGGCGACTTCTCCTTCCTTT
CTCCCTTCTCCC
+
///*/5659@@6;;99@@;;;??>87:;-
77>>?6::?=;BAA4844?>:BCBDC>CDE9C???A<771363478844000+0231232
@T6XXI:00118:00310
GGGAGAAGGGAGAAAGGAAGTGCAGTTCGCGGTTAGTGAAGGGAAAAACTATTCTCCCTCTCCCTATAGTGAGTCGT
ATTAGAACTTCCCC
+
9<8<@@?BG>AABB?BDD@CBBBCCC@CBBA8;8;B;;;?AA<ABBB2DCBA@CCCD=@@BBB<BA999@@===>88
7>;?AA>@@>A@?1
@T6XXI:00118:00314
CTAGTCGACTCGAGATCTGCGGCTTTGGACAGTCCCTCTGTACTGTGAAGCCACAGATGGACTGCCTTCCTTCTCC
+
/////667:?:?9<<>@AAA@<88808;>?@NLED=@??;::?BBCCA>>B>;999?8844<77;7339,,+*000
@T6XXI:00118:00317
GCTAGTCACTCGAGATCTGCGGCTTTGGACAGTCCCTCTGTACTGTGAGCCACAGATGTCGAGTCGACTAGCTTCCT
TTCTCCTTTCTCCCCTTCC
+
;494444999?@B@AAA<;;B>???:<:;;:88BC>CBAAB@@@;0...:8=?CCCCCBAA????@8887316663;
<-717333+3-224.646,
@T6XXI:00118:00324
GGGGAATTCTAATACGACTCACTATAGGGAGAGGGAGAATAGTCTCCTTGTCTTTGACGGTGCAATTTTCTTCCTTT
CTCCCTTCTCCC
+
CCF:CAE?B@@=AABBBCCCCBBCA???9??ACCBB??>ACC889?>@=BBBBLACA@@CBBBC@BAB8<;9<ACB>
C;::.7:===?9
@T6XXI:00118:00333
GGGGAATTCTAATACGACTCACTATAGGGAGAGGGAGAATAGTTCTGGACTGTACTCTGGGGCTGTCTCCCTCCTTC
CTTTCTCCCTTCTCCC
+
CCC9B?=>ABB>BCCCCCBBB@BC88:A<????B>B@C@AAAA>898>B@@B::::@@@BB9CBB?B;::5:A;>>B
3::5::>?>C<00;=9
@T6XXI:00119:00303
CTATCGACTCGAGATCTGCGGCTTTGGACAGTCCCTCTGTACTGTGAAGCCACAGATGTCGAGTCGACTTAC
+
4::9/////8877,,//1646;66+347776111-16;>=77:767958>7;;::???>?;17160,,,*,,
@T6XXI:00119:00317
GGGAGAAAGGGAGAAAGGAAGAGAACAGTTGCGCCTAGTAGTTAGTAACTATTCTCCCTCTCCCTATAGTGTAGTCG
+
;;:4444-4:-44;;-42477>>B@C==<?;94A=899>=3859=??=@@?C>:668266667,30111----22--
```

```
@T6XXI:00119:00321
GGGGAATTCTAATACGACTCACTATAGGGAGGGAGGAAGAACT
+
ABB7@698<99=A@@ADCCCCBA?=777.077177+*)**)*,
@T6XXI:00119:00331
GGGAGAAGGGAGAAAGGAAGGGGAGAAGGGAGAAAGGAAGTCGACCGACATCTGTGCTTGCACAC
+
=?<@CHDCC>CCCC>A=@@CDD9C@@=@C>B?>?,0,0,.44467:A@BCFCB0/.--,--1130
@T6XXI:00119:00334
GGGGAATTCTAATACGACTCACTATAGGGAGAGGGAGAATAGCTATTGTTTCACCAGCTGCTCCCTGATCTTCCTTT
CTCCCTTCTCCC
+
BBC8@=A?BBCABBBBCBBBCGC??:67.7@?BE>CAA9<<@@BBC@CAA;@8738@>>@@???588CBA?<?488/
8@??.747777,
@T6XXI:00119:00342
GGGAGAAGGGAGAAAGGAAGGAGAAGTCGCCCCAAACGATTTGTTGACTATTCTCCCTCTCCCTATAGTGAGTCGTA
TTAGAATTCCCC
+
@A>ABB?BC>DCCB>B@D@B@BBB@;977788.88087?ACABB?B@@=@A<?88718==@A<AAA???<@?<===A
B=??A<A28880
@T6XXI:00120:00302
GGGGAATTCTAATACGACTCACTATAGGGAGAGGGAGAATAGTCAACAAATCGTTTGGGGCGACTTCTCCTTC
+
AA@4@54:<ADACEDBBAAAA>@??777-7?==?<?B?=@=AA??<?CC=C???C>BBB7A?;>>:9::,/,/
@T6XXI:00120:00303
GGGAGAAGGGAGAAAGGAAGCTAGTCACTCGAGATCTGCGGCTTTGGACAGTCCCTCTGTACTGTGAAGCCACAGAT
GCGAGTCGACTAGCTTCCTTTCTCCCTTCTCCC
+
CIACA@=@>:>>>><A;>>>;8<??:99;BA?A?AA?A?><777.7=AAABAA@<::7===;<<@@C>@@;==@AAA
@887;<<;<<>@?@@>@@AA=BAAA<A:99=96
@T6XXI:00120:00312
GGGAGAAGGGAGAAAGGAAGGAGAAGTC
+
AD=AAA59819>>@9@=><?58;?=@@B
@T6XXI:00120:00317
GGGAGAAGGGAGAAAGGAAGCATCGACTCGACATCTGTGGCTTCACAGTACAGAGGACTGTCCAAAGCCGCAGATCT
CGAGT
+
FD@CA@=@>1999919<?4373......../////66654469>AACBCBBB;857===77377.7?>AAAB::89
::>:7
@T6XXI:00120:00336
GGGGAATTCTAATACGACTCACTATAGGGGAGAGGGAGAATAGATCACCGTTGGTTCGGGTGCGCGTTCTCCCTTCC
TTTCTCCCTTCTCCC
+
944,48=?CFH?AAACCBBC;:8?>7777-
7@?CE?@?@?B778777=4773737<>?><@?>>?<;01111,13;36?9?88=;=;<===;
@T6XXI:00120:00343
CTAGTCACTCGAGATCTGCGGCTTTGGACAGTCCCTCTGTACTGTGAAGCCACAGATGTCGAGTCGACTAGCTTCCT
TTCTCCCTTCTCCC
+
D:49;<:::CCBBABBBBBB@BBB<B@CC<?:::4:BBBBBCC<==A>@C@CBCCABABBBBB@BBBBCCAAA?B?B
CABBBC>@>???D>
@T6XXI:00120:00346
GGGAGAAGGGAGAAAGGAAGCTAGTCGACTCGACATCGTGGTGCTTCACAGTACAGAGGGACTGTCCAAAGCCCGCA
GATCTCGAGTCGACTAG
+
```

```
997;@>?AD>AA@A<>=A>@A>9:;///9;;<>>7772,,+,,0002///6=>;;:444,4;;;8834;9444+34;
BBA:89?>>?<<<@888
@T6XXI:00121:00305
GGGGAATTCTAATACGACTCACTATAGGGAGGGAGGAAGAATACGCG
+
A@>4@=@=@CB?BBBBBBBB?<<;667>6677.7731010/001...
@T6XXI:00121:00315
CTAGTCACTCGAGATCTGCGGCTTTGGACAGTCCCTCTGTACTGTGAAGCCACAGATGTCGAGTCGACTAGCTTCCT
TTCTCCCTTCTCCC
+
D>111=899BBBBBBCCABD?ACC?C?BBB?:::7:CCCDBBB:?=B<<B?BBBBBBBC??B?>ABB@@@A774737
7.777>697;==?:
@T6XXI:00121:00318
GGGGAATTCTAATACGACTCACTATAGGGAGAGGGAGAATAGTCAACAAATCGTTTGGGGCGACTTCTCCTTCCTTT
CTCCCTTCCTCCC
+
444,48?<<;;?BBFCCAA>@;<<7777-
7>777.710===>@;;:<<=;A@@CC>CCD7BBBCC?BBA78589<C<A;?939,/,/872
@T6XXI:00121:00321
GGGAGAAGGGAGAAAGGAAGCTAGTCGACTCGACATCTGTGGCTTCACAGTACAGAGGGACTGTCCAAAGCCGCAGA
TCTCGAGTCGACT
+
CC?BCC?BB:=@==:@==<?B@==>/11??>@1:::::BBB?CB@CCC@BB@>><<<=;<==<8<23317;8<AA@?
????@<?@@@BBA
@T6XXI:00121:00329
GGGAGAAGGGAGAAAGGAAGCTAGTCACTCGAGATCTGCGGCTTTGGACAGTCCCTCTGTACTGTGAAGCCACAGAT
GTCGAGTCGACTAGCTTCCTTTCTCCCTTCTCCC
+
BA>BAA?ALBCCCC>C?B?ABCCB?:961688<8>@?BBA>???;??BCCBBBB9?<??@AAC??>@=A=<=<<;<=
<<===???A@?@C=@@>@>?A<@;;;6;378@C?
@T6XXI:00121:00330
CTAGTCACTCGAGATCTGCGGCTTT
+
B811/789=@@CCCCCCCBB@BBB@
@T6XXI:00121:00334
GGGAGAAGGGAGAAAGGAAGCTAGTCACTCGACATCTGTGGCTTCACAGTACAGAGGACTGTCCAAAGCCGCAGATC
TCGAGTCGACTAGCTTCCTTTCTCCCTTCCTCCC
+
DE@CBA?AB>@111,1,1-
.=4==@?@::;:7@=<<;71107711=;??A70/8//,/61138:<=;;=<==A?===@@@AA???><<;;=:8=8;
<7<@CC>C8;9=CC;
@T6XXI:00121:00335
GGGAGAAGGGAGAAAGGAAGGAGA
+
CB?CFC??=6>844-9;88888))
@T6XXI:00121:00341
GGGAGAAGGGAGAAAGGAAGGAGAAGTCGCCCCAAACGATTTGTTGACTATTCTCCCTCTCCCTATAGTGAGTCGTA
TTAGAATTCCCC
+
CC>CBA===5>;998<@C@CGC??=<=>8888.8<678<@D>B@?B??=AA?A@@?:>>>?@<AA@@A@@<=<<?=<
?;?;978/444-
@T6XXI:00122:00303
GGGAGAAGGGAGAAAGGAAGCTAGTCACTCGAGATCTGCGGCTTTGGACAGTCCCTCTGTACTGTGAAGCCACAGAT
GTCGAGTCGACTAGCTTCCTTTCTCCCTTCTCCC
+
DK@CBB>@>:=>>>9>;@=?>>?87711676674?@=@AF@AAA=A?AFCCCCC>BB??@878B@@B?@C?AAA=<<
<<>=??@@@8888>=878,//,//7717994;=4
```

```
@T6XXI:00122:00318
GGGAGAAGGGAGAAAGGAAGGAGAAGTCGCCCCAAACGATTTGTTGACTATTCTCCCACTCCCTATAGTGAGTCGTA
TTAGAATTCCCC
+
CC@BAA@BC>CBBB?B@B>?>ABB?BCCCC??8??;??>>B>?@?CBBB@@>AAAA<C@@AC@DAA===BBBBBBAA
A?ABB:>4777-
@T6XXI:00122:00323
GGGAGAGGAGAAAGAAGGAGAACAACACGATCGGTTAAACGTAGCTAGTTCTCCCTCTCCCTCATAGTGAG
+
77+1///,,,/6+//,.3...,4988;666///,/5688:A>666....44...+.1111,11033==548
@T6XXI:00123:00304
GGGAGAAGGGAGAAAGGAAGGTGAACAGAGCGCAAACGAATACGTCTCTATTCTCCCTCTCCCTATAGTGAGTCGTA
TTAGAATTCCCC
+
CC>BA@=@C@BBBC?C@C@B>?>B@BBAAAAA?AA>BCC>@@EAA@BB@@B<>>>>:9999919BBBBBCC@?@C@A
@BB@@>C>BBB7
@T6XXI:00123:00305
GGGAATTCGTAATGACGACTCACTATAGGGAGAGGGAAGAATAGCAGAGCTAAATAGTCCCCCTCGCTG
+
/1-,06,///87///944;9987883337,33111,1777788881139/--20--036666/6660-.
@T6XXI:00123:00324
GGGAGAAGGGAGAAAGGAAGCTAGTCACTCGAGATCTGCGGCTTTGGACAGTCCCTCTGTACTGTGAAGCCACAGAT
GTCGAGTCGACTAGCTTCCTTTC
+
CD@CCC=@@<@BAB?D=@=??>==@:::88ABB?A?CCCB>BAC>C@CAA@????9?===>??@@@AD>A@>??@;;;
=888====>888=888>A?BH88
@T6XXI:00123:00325
GGGAGAAGGGAGAAAGGAAGTGCAGTTCGCGGTTAGTGAAGGGAAAACTATTCTCCCTCTCCCTATAGTGAGTCGTA
TTAGAACTTCCCC
+
CHACDC@CC>DBBC@F@C=@>CCCCC@B@A@?C@BCCB@<?98??A7ACCC?ABCCBBBBCC>B??@A@@@AA?<<<
<8;?A8<<<>>=4
@T6XXI:00123:00326
GGAATTCTAATACGACTCACTATAGGGAGAGGAGAATAGTTACGATACTTTC
+
-+,0656BB@FCCDCCBBBAA?>777.7=774:9978///,-.-2-0000+0
@T6XXI:00123:00328
GGGAGAAGGGAGAAAGGAAGAACCGGCCGCGGGAGTTAAAATAACGCTATTCTCCCTCTCCCTATAGTGAGTCGTAT
TAGAATTCCCC
+
CC@DBBAAB=EBCB?C@C@?A?A@B@B???B@>@CC?CCC8AA>:::CDD?CBCC@CCCCC?CBABBBBBBBA??@@
=AAC@A>AA@5
@T6XXI:00124:00304
CTAGTCACTCGAGATCTGCGGCTTTGGACAGTCCCTCTGTACTGTGAAGCCACAGATGTCGAGTCGACTAGCTTCCT
TTCTCCCTTCTCCC
+
CC11131::CDDCCCBBBBA?ACC>D>AABA???9?BBCGB>B>B@C>@C@CCB<B9;;::CCCDCBBBA@?<;?<?
C@??@C<?<?A?A=
@T6XXI:00124:00314
GGGGAATTCTAATACGACTCACTATAGGGAGCGG
+
FEE9D?CC?AC@DCCCA?:::78?>000+...,*
@T6XXI:00124:00323
GGGAGAAGGGAGAAAGGAAGCTAGTCACTCGACCATCTGTGGCTTCACAGTACAGAGGGACTGTCCAAAGCCGC
+
EH>>;<89>:>111,/,/,.6.116==7111110111168;267?????AAAA?@@@@7====>D@AA???<?@
@T6XXI:00125:00300
```

```
GGGAGAAGGGAGAAAGGAAGGGGAGAGGGAC
+
DI@A@?@CC?BBBC@?<;,000+0--,20**
@T6XXI:00125:00316
CTAGTCACTCGAGATCTGCGGCTTTGGACAGTCCCCTCTGTACTGTGAAGCCACAGATGTCGAGTCGACTAGCTTCC
TTTCTCCCTTCTCCC
+
CC:9<<::=@BBCDBCCFCC@CCC>CAB?>>7777,7>>>A???::7=;>B@CCCC;<;;;@@A?@@CA@>?><;><
AB=??BB:;24344-
@T6XXI:00125:00318
GGGGAATTCTAATACGACTCACTATAGGGAGAGGGAGAATAGTTTTTCCCTTCACTAACCGCGAACTGCACTTCCTT
TCTCCCTTCTCCC
+
DCD9B?B@CCCABB??ABBBAAA>966659??CC>BA>>BABA;==.<=;=99=ACC@BA???=;<<<==:877316
.7>>><A????A<
@T6XXI:00125:00333
GGGGAATTCTAATACGACTCACTATAGGGAGAGGGAGAATAGTCAACAAATCGTTTGGGGCGACTTCTCCTTCCTTT
CTCCCTTCCTCCC
+
999/9@DDCCC@CBBBBBCC@??D@7:7.7><<=6=;:;>?@@@A?AAA<BBBAB>BBB6BBCBB>AAA<<:=>AA<
A76616,/,,110
@T6XXI:00125:00340
GGGGAATTCTAATACGACTCACTATAGGGAGAGGGAGAATAGTCAACAAATCGTTTGGGGCGACTTCTCCTTCCTTT
CTCCCTTCTCCC
+
CDC9C>@>ABBACC???BBBB@A@?6775:B?CC>A???@==A@=;@A@;BB>?A8???6?ABBC@BBB?@>A?CA:
>@@C;@=?@AB=
@T6XXI:00126:00309
GGGGAATTCTAATACGACTCACTATAGGGAGAGGAGAATAGTTAG
+
088088CAA@@@CCCB@?ACCAB?=611.7<77477/,/1,,+++
@T6XXI:00126:00318
GGGGAATTCTAATACGACTCACTATAGGGAGAGGGAGAATAGTCAACAAATCGTTTGGGGCGACTTCTCCTTCCTTT
CTCCCTTCTCCC
+
999-
4<A>BCCCCBBABBBBC@BCA?AC>?B?:?8??=;@AA@@?=AAA:BCCCC;???6?BBAA>???=>;=8;;7=?>C
>C898?C=
@T6XXI:00126:00328
GGGGAATTCTAATACGACTCACTATAGGGAGAGGGAGAATAGTTTTCCCTCCACTAACCGCGAACTGCACTTCCTTT
CTCCTTCTCCC
+
CHD:DCCABBB@CBCCCCCCAB@B969959?@CC>B???AAA<==5@@;??<AADC@C@AAA@?????C?=;>300,
0/.,.,--144
@T6XXI:00126:00345
GGGAGAAGGGAGAAAGGAAGCTAGTCGACTCGAGATCTGCGGCTTTGGACAGTCCCTCTGTACTGTGAAGCCACAGA
TGTCGAGTCGACTAGCTTCCTTTCTCCCTTCTCCC
+
CB>@A@=AC?CCCC?C@C@BA;;;;/00;;==>@FCCBBBC?CCC>C?BCCBBBB=A==;;<=<=??>=??;??A<=
====;:8//.764;:;;9;8:;-4444-4798;CB
@T6XXI:00127:00306
GGGAGAAGGGAGAAAGGAAGGCTGTCATAGCGAGGTAGAGGGTTTAGCTATTCTCCCTCTCCCTATAGTGAGTCGTA
TAGAA
+
CCAAAC@CC@C;;90969681739;BEAAA>BCC@CC???C>AA<B?B@CC;??BB>@CAAC<@@@@@@;876/+++
+--++
@T6XXI:00127:00308
```

```
GGGGAATTCTAATACGACTCACTATAGGGAGAGGGAGAATAGCAAACGTTTCCCCGACGC
+
DCC9><A><4894:9;:?@;;8;;76<88:::CCCCCA>A@@>=;74778887726/...
@T6XXI:00127:00318
GGGAGAAGGGAGAAAGGAAGTGCGGTTCGCGGTTAGTGAAGGGAAAAACTATTCTCCCTCTCCCTATAGTGAGTCGT
ATTAGAACTTCCCC
+
CF@AAA@BC@CBBB=B=@=@CCDC@C@@>==6:6;@@CD@CC>BBCC2CCBB?B@??8:::@@<A888???@AAA@@
9838?=7::3444-
@T6XXI:00127:00320
GGGAGAAGGGAGAAAGGAAGTGCAGTTCGCGGTTAGTGAAGGGAAAAACTATTCTCCCTCTCCCTATAGTGAGTCGT
ATTAGAACTTCCCCATCACCGACTGCC
+
BB?BBC@BB>A@@@<@@C@CAABCEC@BBBA@C@CBCCC@@?899991@AAA?ACCC>B@AAC>B@@???A@@=<;;
;;8;=B:==8;99.9444=<4444000
@T6XXI:00127:00338
GGGGAATTCTAATACGACTCACTATAGGGAGAGGGAGAATAGTCAACAAATCGTTTGGGGCGACTTCTCCTTCCTTT
CTCCCTTCTCCC
+
CCD:C@D@?A@?BCBBBAAAB?<??6965:??CE?C@@>@?A@??<?@?:AA?;=6?<;2<???A?BA@=>8;8<>;
?AAC;?689@@:
@T6XXI:00127:00339
CTAGTCGACGAGATCTGCGGCTTTGGACAGTCCCTCTGTACTGTGAAGCCACAGATGTCGAGTCGACTAGCTTCCTT
CTC
+
GB<<;@@<999994<<<ACABA@<A=>???88807=>>>A;::::?>@C@CBAAA<<<@B@>?999>84//.34,.,
...
@T6XXI:00127:00343
GGGGAATTCTAATACGACTCACTATAGGGAGAGGGAGAATAGCTCGGAAACCTCCCACCGGGCATTGTCTTCCTTTC
TCCCTCTCCC
+
499-49@>>>>599999379<7931111,11116261101---
..4,..+.3349155<@?;==A@844:24343+0044030000+
@T6XXI:00127:00349
GGGGAATTCTAATACGACTCACTATAGGGAGAGGGAGAATAGTCACATTCATG
+
CCB9B>B>BCCACCCBB???BA<96111.7==?B@B=?;=@@@BA@<B>?@A@
@T6XXI:00128:00300
GGGGAATTCTAATACGACTCACTATAGGGAGAGGGAGAATAGTCAACAAATCGTTTGGGGCGACTTCTCCTTCCTTT
CTCCCTTCTCCC
+
CB?7B8;:<BC@BCEDBB@@@CBC?777.7==BCACA?<?AAAAA>===9@CCFC>CCC9CCAAA@:::<<9<=@?7
=9:<44/7--.,
@T6XXI:00128:00303
CTAGTCGACTCGAGATCTGCGGCTTTGGACAGTCCCTCTGTACTGTGAAGCCACAGATGTCGAGTCGACTAGCTTCC
TTTCTCCCTTCTCCC
+
E0,---
,,/78?BAAACBBBB@BBB<B?A>><69:5:CCCBAAC@??<;<B?AABBAB@CBCA?@AACCCD@@@?C?BC>BAA
C9<8<<<>:
@T6XXI:00128:00308
GGGAGAAGGGAGAAAAGGAAGCTAGTCACTCGACATCTGTGCTTCACAGT
+
?:694954>:99999,4249773331111677788776774,,*,,,,.1
@T6XXI:00128:00310
GGGGAATTCTAATACGACTCACTATAGGGAGAGGGAGAATAGCACGTTGATAACAAGCGCGACT
+
```

```
CDD:CAE@BBB@BBBBAAAAC@A?@>>>>?@@CA;@B@<99=AAA9747777011011164220
@T6XXI:00128:00316
GGGAGAAGGGAGAAAGGAAGGAGACAGTTCCCGTTCTGAAATCGAGACTATTCTCCCTCTCCCTATGAGTG
+
785///,/85:8;858,/-.6000/:::7;;611,/622=821111::?BC>A>>>:44///+.....552
@T6XXI:00128:00319
GGGGAATTCTAATACGACTCACTATAGGGAGAGGGAGAATAGTCAACAAATCGTTTGGGGCGACTTCTCCTTCCTTT
CTCCCTTCTCCC
+
@=@6@@B?BBB@BBBBCCCB@?;97116.7>>>A>BBA>@?@AAA<???<BAAAB88??5????B>@::588;;?@<
@CCD<A9==HC<
@T6XXI:00128:00330
CTAGTCGACTGGAGATCTGCGGCTT
+
=:1//:00111;;;;;BBBBC@???
@T6XXI:00128:00332
GGGAGAAGGGAGAAAGGAAGGAGAAGTCGCCCCAAACGATTTGTTGACTATTCTCCCTCTCCCTATAGTGAGTCGTA
TTAGAATTCCCCC
+
DFACCC@CC@BBBCAC@CAC?BBD?AAECECC9CC?CB?@@=?C@D;;9AA>A@?@:=A88818;<<ACAAA@?778
:750323566=?1
@T6XXI:00128:00345
GGGAGAAGGGAGAAAGGAAGGAGAAGTCGCCCCAAACGATTTGTTGACTATTCTCCCTCTCCCTATAGTGAGTCGTA
TTAGAATTCCCC
+
CC>BCC@DD>@BBC>C=@=@?>;=;=00099:6:::::@BB@AA=ACC??;<==<;46;;;<5>?AAA@@<AA@@@@
@>AA@=?3848-
@T6XXI:00129:00301
GGGGAATTCTAATACGACTCACTATAGGGAGAGGGAGAATAGCGCCCAGTTGAATCCGTCTAGC
+
BBB9A@A?BBB@DDCCCBBBB@@>>777.7===>7>74344406==;>>E@D?10090/0...+
@T6XXI:00129:00305
GGGAGAAGGGAGAAAGGAAGGAGAAGTCGCCCCAAACGATTTGTTGACTACTTCTCCCTCTCCCTATAGTGAGTCTA
T
+
<<6<CA@CD;<:BB?C=@=@>AAC=@@BBBBB7AA=BDCC@>BC@C>00///7459=9A<6;>,4//./--
1113166
@T6XXI:00129:00313
GGGGAATTCTAATACGACTCACTATAGGGAGAGGGAGAATAGCTGTTCCTTCTGCCGCATGCCCATTGTCTTCCTTT
CTCCCTTCTCCC
+
AA@7@=@=BCC@CCCB?>@CC;??7677.7>>@C@AA??A?==@CC@D@C>A>>A?BAA<;33044244435>:<?;
?@@D<B<??@@<
@T6XXI:00129:00319
GGGAGAAGGGAGAAAGGAAGGAGATGCCAACGCAAACATGATTGTGGCTATTCTCCCTCTCCCTATAGTGAGTCGTA
TTAGAATTCCCC
+
CC@CCCACB>BBCCAD?@=@@CCCCCC?B?ABAAA<A@@@7747770188@<BCCC>CCA?A<?:::CBACC=888>
>8899,-,-11.
@T6XXI:00129:00322
CTAGTTCACTCGAGATCTGCGGCTTTGGACAGTCCCTCTGTACTGTGAAGCCACAGATGTCGAGTCGACTAGCTTCC
TTTCTCCCTTCTCCC
+
CCCCB@?ADFDEAABBABBCC?@@@>@@??77777.7>>@B@@@899?=?BACCCB@@@??A@::<B@@@@@?67=3
77.7;=B@D>===A:
@T6XXI:00129:00325
```

```
GGGAGAAGGGAGAAAGGAAGCTAGTCACTCGACATCTGTGGCTTCACAGTACAGAGGGACTGTCCAAAGCCGCAGAT
CTCGAGTCGACTAGCTTCCTTTCT
+
99594954;:@999184823><>><=:/......55...101378;;@@>B@;;444-
4448AC@DD;@@;998<<;888;;;9=884:=;//,/,//,--
@T6XXI:00129:00326
GGGAGAAGGGAGAAAGGAAGCTAGTCACTCGAGATCTGCGGCTTTGGACAGTCCCTCTGTACTGTGAAGCCACAGAT
GTCGAGTCGACTAGCTTCCTTTCTCCCTTCTCCC
+
BC>B<;9<B:@999195958887:CFB?AA???<?@@@BA>AAA=A@BCCCCCC>A?????AAA???>??<?@@@@@
AA===?===??@@A<@<?9<<7=AAB<A>==<A<
@T6XXI:00129:00333
CTATCGACTCGAGATCTGCGGCTTTGGACAGTCCCTCTGTACTGTGAAGCCACAGATGTCGAGTCGACTAGCTTCCT
TTCTCCCTTCTCCC
+
?;:999:?ABBCCCCCBBBC?CCC?A?A@@@???;?AAAA???688:8;B=A@BABBBBAAAB?@@@CCBBAB@D@C
D>:::@:;7999;7
@T6XXI:00129:00335
GGGAGAAGGGAGAAAGGAAGAATTTAAGTGCTGTCAGGAGTTTAGACTATTCTCCCTCTCCCTATAGTGAGTCGTAT
TAGAATTCCCC
+
CC@EBE@CC>BBBB?B@B@BBABC>C?@CCCCCAAAB@?C??<BB>>>AC@@<<<;===DD=A@>>>@@AA@AA@??
<???>;3888-
@T6XXI:00130:00304
CTAGTCGACTCGAGATCTGCGGCTTTGGACAGTCCCTCTGTACTGTGAAGCCACAGATGGACT
+
DA:<<@AACFECCAA@BCCCC@CEB>B>AA@B::88:DAAB;?@@CB?;@EACC?;;-*))(&
@T6XXI:00130:00308
GGGGAATTCTAATACGACTCACTATAGGGAGAGGGAGAATAGCTCATCCCCATTCATAACACCTGTCATCTTCCTTT
CTCCCTTCTCCC
+
BBB9BAC@BCC@BBBBA????@C?;611-
6<?CE>CBB>B;9=AA?:5550??>BBCA>CCC@CCABBBCC@C=@?6<<<<6><>777.
@T6XXI:00130:00309
GGGGAATTCTAATACGACTCACTATAGGGAGAGGGAGAATAGTCAACAAATCGTTTGGGGCGACTTCTCCTTCCTTT
CTCCCTTCTCCC
+
DCC:C>@C@BB?BDCCBCCCA>B976965:BBCC<AA@>A>AAAA>???;ACCCCACCC7BBCCCCBBA;;8;:=;9
>84814247>@:
@T6XXI:00130:00329
GGGAGAAGGGAGAAAGGAAGCTAGTCGACTCGACATCTGTGGCTTCACAGTACAGAGGGACTGT
+
16+///,//+/4990478777796>@@::7<A:::@@;77><784761777?8:4499-+++0+
@T6XXI:00130:00334
GGGGAATTCTAATACGACTCACTATAGGGAGAGGGAGAATAGCTACGTTTAACCGATCGTGTTGTTCTCCTTCCTTT
CTCCCTTCTCCC
+
CKK:C@A>ABC@BBCCCBBB@>;96166.7<<>@<A??>B@@@BBCCCB>A>?=@???AAAB?AA?@@?<>:>;=?;
===C@;9;6661
@T6XXI:00130:00344
GGGGAATTCTAATACGACTCACTATAGGGAGAGGGAGAATAGTTCAC
+
@==6@>A>BBB@BAA@BBBBBBB97111.7>>@D@C?;680**)*((
@T6XXI:00131:00305
GGGGAATTCTAATACGACTCACTATAGGGAGAGGGAGAATAGCCTATTCCTATTCTGCTCCCTGCCACGCTTCCTTT
CTCCCTTCTCCC
+
```

```
CED:D>@>@CB?<<<BCCCBCCB9>111.777>@=BCA>@AAA?BBB?C@CBB??===766.77;8>AB@?=B=AB>
BCBB>B@@@?CA
@T6XXI:00131:00306
GGGGAATTCTAATACGACTCACTATAGGGAGAGGGAGAATAGATCACAACAAAGACCTATCTCCAGCCGCTTCCTTT
CTCCCTTCTCCC
+
@@@6@<@=@BB@CBBABBCBB@BB>::866=;??;?B?>AA@@@@C??>B??;<<<?@AA@CA?AA??A<<=A>>><
>B?>;B??@=C<
@T6XXI:00131:00307
GGGAGAAGGGAGAAAGGAAGCTAGTCACTCGACATCTGTGGCTTCACAGTACAGAGGGACTGTCCAAAGCCGCAGAT
CTCGAGTCGACTAGCTTCCTTCTCCCTTCTCC
+
CG@CA@=@A<@BBB>B?B?BCAA@=9:0/...333387688?@=???BCCCB?>77717;;;==>@C<AA<??@AAC
===@@=;<;;84/7//,/,/,/,+,++*+.-,
@T6XXI:00131:00326
GGGGAATTCTAATACGACTCACTATAGGGAGAGGGAGAATAGCTACACGACTCGCAAAATGGTTATCTCCTTCCTTT
CTCCCTTCTCCC
+
CCC9B>A?BCC@CCBBBAA?BA@9;111.7<<?CA;99@C@@@BBBC@=?<<=@<<@@5@?=@=????@=?;>8;;6
<@@@<A;>=?A=
@T6XXI:00131:00336
GGGAGAAGGGAGAAAGGAAGAATAACGCGGAGTGGACGAAAAGAAGAACTATTCCTCCCTCCTCCCTATAGTGA
+
DCAAA??CCBABBD@C@C@BB@BA<8811,///65694444,4424:9444=;;=BBB;313777,11166663
@T6XXI:00131:00338
GGGAGAAGGGAGAAAGGAAGCTAGTCACTCGAGATCTGCGGCTTTGGACAGTCCCTCTGTACTGTGAAGCCACAGAT
GTCGAGTCGACTAGCTTCCTTTCTCCCTTCTCCC
+
@?199954=:44990424938::6<?<:==@@?<?ABBAA>AAA<B?BBBBBBC>:8887743>>==<=<9=AA@AA
A?@?@;;;/./.34>@=@;==188==7>:<<>@6
@T6XXI:00131:00340
GGGAGAAGGGAGAAAGGAAGGGGAGAAGGGAGAAAGGAAGCTAGTCGACTCCGACA
+
DDCCCC@CB=?BCC>B:9:;CD9AAAACC>BBB?9?592:@/00>0.,2,,*----
@T6XXI:00131:00341
GGGAGAAGGGAGAAAGGAAGCTAGCGACTCGACATCTGTGGCTTCACAGTACAGAGGACTGTCCAAAGCCGCAGATC
+
CD@CBB>@=6@@==<@@C?AA>:01/::77@>><787AAB?@@?AECA@?@774..-/999;?=??<??>@@==<<<
@T6XXI:00131:00342
GGGAGAAGGGAGAAAGGAAGGAGACACGGTCACAAACATGAATGTGACTATTCTCCCTCTCCCTATAGTGAGTCGTA
TTAGAATTCCCC
+
CCAECB@BB>BBBB>><=676:BCCCCC@CBCDBB>ABA@@?ABBABBBAB>B??<;@@@==;?:??<<ABAA?@??
?>@CC9;8<;;0
@T6XXI:00131:00346
GGGAGAAGGGAGAAAGGAAGCTAGTCGACTCGACATCTGTGGCTTCACAGTACAGAGGACTGTCCAAAGCCGCAGAT
CTCGAGTCGACTAG
+
4<8;<CABC<;9>A198;>??99000..334./1667176>77847@@@BB@88://./9998<8<>:=?>?=?><<
::::4304433358
@T6XXI:00132:00301
GGGGAATTCTAATACGACTCACTATAGGGAGAGGGAGAATAGATCACCGTTGGTTCGGGTGCGCGTTCTCCTTCCTT
TCTCCCTTCTCCC
+
@?=6@8;;<DC@BBABBBBBB@B>8696.7>>?A>AB?>AA@<@=>@@@C==;:BBBC<A?@@BAB>AAA?A>@>AA
<>?;;6<==??@:
@T6XXI:00132:00304
```

```
GCTATCACTCGAGATCTGCGGCTTTGGACAGTCCCTCTGTACTGTGAAGCCACAGATGTCGAGTCGACCTTCCTTTC
TCCCTCTCCC
+
,/;C===:////1//88;47537>6@?BBBAABB.7/7,,,,,,,.-
423=<==??;77866711443313771..+.335+///.52
@T6XXI:00132:00318
GGGAGAAGGGAGAAAGGAAGGAGAAGCGGCGGTGGGAGATTGTGCAGCTATTCTCCCTCTCCCTATAGTGAGTCGTA
TTAGAATTCCCCCATCAC
+
BKADDC?B@:@===:B?C?B?BBD@CCB?B<>??B<CBCC?A@>?D>944989444-
47;??<<?:::BBA;;:A?;;5=5344/0033+4443:
@T6XXI:00133:00300
GGGGAATTCTAATACGACTCACTATAGGGAGAGGGAGAATAGATCACCGT
+
@>@6@@B?BBB=@@BC@@@CDCCBB:::5:AABD>CCA>???A??B>:;=
@T6XXI:00133:00301
GGGAGAAGGGAGAAAGGAAGGAGAAGTGACGTACGGGGGTTTAGCCACTATTCTCCCTCTCCCTATAGTGAGTCGTA
TTAGAATTCCCC
+
CHCBBC@DIABBAB>C@B@B>>>?@@?>AC???A>>>?1?C@@?????@A@?<<;=7;:::<6;<<@@A@AA???@@
?>AAA?A37770
@T6XXI:00133:00304
GGGGAATTCTAATACGACTCACTATAGGGAGAGGGAGAATAGTCAACAAATCGTTTGGGGCGACTTCTCCTTCCTTT
CTCCCTTCTCCC
+
999/98?@CDCACBBBAAACA97?=776.7???B>BB@>A@@77484441:>>F?.777.7===?476?:82979:6
<===7?;>?@@:
@T6XXI:00133:00307
GGGAGAAGGGAGAAAGGAAGGGACTGAGGAGAGATAATGGGAAATTGCTATTCTCCCTCTCCCTATAGTGAGTCGTA
TTAGAACTTCCCC
+
CC>DCB>BB=BCBD>B@B@B?;>>>?>>>>>?>=?>?CCC>BC>B>ABAAA?BBCC?ABBCF>@@???@@@A???@@
?<??@;==>ADD5
@T6XXI:00133:00336
GGGAGAAGGGAGAAAGGAAGCTAGTCACTCGACATCTGTGGCTTCACAGTACAGAGGGACTGTCCAAAGCCGCAGAT
CTCGAGTCGACTAGCTTCCTTTCTCCCTTCTCCC
+
BC<@@>=?@:A999149>9>?;:6>>761111118889;?5:;?AAAACCB@?=77718>A==<?AA=@@=@@@ACC
??????@@@?=<<<@?=ABCC<@AAA<?<???@:
@T6XXI:00133:00341
GGGGAATTCTAATACGACTCACTATAGGAGAGGGAGAATAGCTGACACTCTTTCGTTAGCCGTTCCTCCTTCCTTTC
TCCCTTCTCCC
+
@@@6>696<@@=@DBBBBAAB76;:/.,0::==9?==;?AAACBBCAA==;:;??=;???;?C:<9=A<?>?>@D<A
@@A<@349??7
@T6XXI:00133:00342
CTAGTCGACTCGAGATCTGCGGCTTTGGACAGTCCCTCTGTACTGTGAAGCCACAGATGGACTGTCCTTCCTTTCTC
CCTTCTCCC
+
CDCC?>@CCCCBBBBBBCBB;=77308??ABCBBB>BBBAAAAABB@@=?B?ABCDDDC@BA@B<8573747=6=77
7.7=?@:;6
@T6XXI:00133:00344
GGGAGAAGGGAGAAAAGGAAG
+
>86>11-/85//444,42423
@T6XXI:00134:00309
```

```
GGGAGAAGGGAGAAAGGAAGCTAGTCGACTCGAGATCTGCGGCTTTGGACAGTCCCTCTGTACTGTGAAGCCACAGA
TGTCGAGTCGACTAGCTTCCTTTCTCCCTTCTCCC
+
CFADBB>BNACBBB=@8;7:B?:::0//3447>>??@BBBC?BAC>C?BBBCCCC=???=<<<==??@>AA>A@@88
99C@>?@?A?@AA??=:/4244-43995<;====;
@T6XXI:00134:00312
GGGGAATTCTAATACGACTCACTATAGGGAGAGGGAGAATAGCTTGGCTCTGTGCCATCCGCGTCCTCCCTTCCTTT
CTCCCTTCTCCC
+
DCCBC?@>@CC@DBBABBAAA>;97111.7?>>@>BB@>A===?>?=>?@=?:::<===:<<<<<;<CC;A><2441
8??A=C>???@:
@T6XXI:00134:00334
GGGGGAATTCTAAATACGACTCACTATAGGGGAGA
+
AA;;/:2959444-4;444;<010.6,,,00*...
@T6XXI:00134:00335
GGGGAATTCTAATACGACTCACTATAGGGAGAGGGAGAATAGCTTTTTCTTATTCTTCTCGGCAATGTTCCTTCCTT
TCTCCCTTCTCCC
+
94:4<<AACBB@B@CCBBCB@@AB?6695:??DE>?<<@AA@ABBBA1@@=:738@>@AAA>@@>@AAB@>A>A>@@
<@A@A7<9=?=@<
@T6XXI:00134:00336
GGGGAATTCTAATACGACTCACTATAGGGAGAGGGAGAATAGTCAACAAATCGTTTGGGGCGACTTCTCCTTCCTTT
CTCCCTTCTCCC
+
DDC:CCC@BCB?BBCAA????AC@966646??CC>B@A?A:=?A=<<@<;@>=999=>@7>CC=?>?BB;=;=<AA<
@??>7?<===CA
@T6XXI:00134:00343
GGGAGAAGGGAGAAAGGAAGCTAGTCACTCGACATCTGTGGCTTCACAGTACAGAGGGACTGTCCAAAGCCGCAGAT
CTCGAGTCGACTAG
+
CDADDD?AA=??::6>4428????CC@>>>>111111??C;:;@CCCBBCCCA<<<<;::==865665<>=@AAAAA
===<<<<===AAAA
@T6XXI:00134:00346
GGGGAGAAGGGAGAAAGGAAGGAGAACAACACGATCGGTAAAC
+
///*///788+/444-42423993314424444466 8,//6+.
@T6XXI:00134:00348
GGGAGAAGGGAGAAAGGAAGCTAGTCACTCGACATCTG
+
BB><=<@CC@BBBC<@=@<??@BA@@@=7777711166
@T6XXI:00135:00316
GGGAGAAGGGAGAAAGGAAGCTAGTCACTCGACATCTGTGGCTTCACAGTACAGAGGGACTGTCCAAAGCCGCAGAT
CTCGAGTCGACTAG
+
CI?AA?=@=<@@==<@=@<@CBAAA7::7:=7::::7BBB?AC@BB@?@@A@?:55535<>@A@>AB<??<?@@<8=
AA@AAAAA====A@
@T6XXI:00135:00320
GGGAGAAGGGAGAAAGGAAGCTAGTCGACTCGACATCTGTGGCTTCACAGTACAGAGGGACTGTCCAAAGCCGCAGA
TCTCGGAGTCGACTAG
+
CC@CBB@BC>BCBB?B>ABCBB>>>@C@@CA?88::::BCC?BB?BCC@??@<<<44317<<=>A=??:??<?@@A@
@AAAA888AA???A@@
@T6XXI:00135:00324
GGGAGAAGGGAGAAAGGAAGGAGAAGTCGCCCCAAACGATTTGTTGACTATTCTCCCTCTCCCTATAGTGAGTCGTA
TTAGAATTCCCC
+
```

```
CC@CCB@BB>BAA?=B>B>B@CBC?AAB<;;;5?@<ABBB@;C?;?@?<<<;==<<;<<@=C;>9==A@=<=;@@AA
A<?<:79/444-
@T6XXI:00135:00328
GGGAGAAGGGAGAAAGGAAGCTAGTCGACTCGACATCTGTGGCTTCACAGTACAGAGGGACTGTCCAAAGCCGCAGA
TCTCGAGTCGACTAG
+
DDAAA@BDC>DCCC?BBB?ABA>;?/00::;;07:?????A=:;9:CBBCBB?;;333-
4::9<=@AA<@@>?==AAAA@A@@@899=ABA@
@T6XXI:00135:00332
GGGGAATTCTAATACGACTCACTATAGGGAGAGGGAGAATAGATGTCTGACCATTGCTGCTTCTATCTCCCTTCCTT
TCTCCCTTCTCCCAGGCTTCCTTCTCCCTTCTC
+
CHE:C@B?BCC@A@@BBCCBB>997116.7??AC>C@ACC=888@@88;?A>AA>@@@@?=8;9444444+044248
466<=<@?A?>>:9998;79,/,.-110646.,
@T6XXI:00135:00333
GGGGAATTCTAATACGACTCACTATAGGGAGAGGGAGAATAGAGCTCCCGTAGTGCTAGCACGTCACATCTTCCTTT
CTCCCTTCTCCC
+
;<<4;>BACCC@CCA????B@?CB@77756AABC>B@A?A=868<@@A<AC??:<=?@C@@ACB?ABC@?@@D@BB=
BBCE>B>??AA:
@T6XXI:00135:00348
GGGGAATTCTAATACGACTCACTATAGGGAGAGGGAGAATAGTAAACGTTTCCCCGACGTCGCTTTCTCCTTCCTTT
CTCCCTTCTCCC
+
CCC9C?B@BCCDCC@?BBCBBAB?961659??CC>B@@?@=A@@@<?@@;177708BB?A?CCBB9?::5:;@<AB>
?@CC?D=@@@@;
@T6XXI:00136:00322
GGGGAATTCTAATACGACTCACTATAGGGAGAGGGAGAATAGTCAACAAATCGTTTGGGGCGACTTCTCCTTCCTTT
CTCCCTTCTCCC
+
CCB:C>@>ACBBCC@@ABBBA?<96111.7>>>@?A@@@A<??AA>AA<;@@@@A=@@@7@CC?B>9??4837?AC;
???B=A>?@@A<
@T6XXI:00136:00339
GGGGAATTCTAATACGACTCACTATAGGGAGAGGGAGAATAGTCAACAAATCGTTTGGGGCGACTTCTCCTTCCTTT
CTCCCTTCTCCC
+
CCB7D=A=ABB@CCBCCBBBBCB@<88866;?AB>BAA?AA@@@A>A@@:A<9<<<ABC9BCCBA@ABB==:=<AA<
A@@@;@?AAAC;
@T6XXI:00137:00316
TCTAGTCGACTCGAGATCTGCGGCTTTGGACAGTCCCTCTGTACTGTGAAGCCACAGATGTCCGAGTCGATACTTCC
TTTCTCCCTTCTCCC
+
AACCCB@CAIFDCCBBBABBBC@BBB>B=???B:::8:CBBBBBBBBBA<?BCFCBCAA;<:?@AA?AA@BAAB:;;
;D>>=B>.0,08882
@T6XXI:00137:00319
CTAGTCACTCGAGATCTGCGGCTTT
+
CC:;;@===CBBCBBBCCBB?@@@<
@T6XXI:00137:00325
GGGAGAAGGGAGAAAGGAAGGAGAAGTCGCCCCAAACGATTTGTTGACTATTCTCCCTCTCCCTATAGTGAGTCGTA
TTCAGAATTCCCC
+
DCACDC@DD>@A?B>C>C=@@@BEBCBB:833-
8808B?DC>CC@C??=AC<?<;;6888@C?A>>>AABAA???:::/00=;97.778-
@T6XXI:00137:00327
GGGAGAAGGGAGAAAGGAAGGACAGTCCATCTGTGGCTTCACAGTACAGAGGGACTGTCCAAAGCCGCAGATCTCGA
GT
```

```
+
EGCBBB@BB=;<;F?CCE;:;;A??6:6;8;7?BB>:::;CBCABB:=;33017>;;;9/03+3;9BC@@?@<=A=<
=@
@T6XXI:00137:00334
GGGGAATTCTAATACGACTCACTATAGGGAGAGGGAGAATAGTCCAGTGTTTAATTTCTTGGCTTTTCTCTTCCTTT
CTCCCTTCTCCC
+
>C>6@>BACCC@CCCBCBBBBA<97111.777BB@C>=:<<>>?>@BA<==99>>?9;=97,////+/9/4288==7
?@@C?B:==@@<
@T6XXI:00137:00340
GGGGAATTCTAATACGACTCACTATAGGAGAGGGAGAATAGTCTGTCCCTCACTTCTCCGACATTGTTCTTCCTTTC
CTCCCTTCTCCC
+
CDG:C;;;<BA>BBBBB@@@B=<<:00.0>>BC@A??>A===A@@>??7;;@<<8<<<9=AA@A?==;==9;476,/
,,11115,//75
@T6XXI:00137:00341
GGGAGAAGGGAGAAAGGAAGACAGTCCATCTGTGGCTTCACAGTACAGAGGGACTGTCCAAAGCCGCAGATCTCGAG
TCGACTAG
+
CEAA@??BB=AA@B?C?B>ACBBB885;AA<??@=??;?BCA??C==?:;;:==::?@>AA<??>???@AAA@<<<<
CCCCBBBB
@T6XXI:00138:00310
GGGGAATTCTAATACGACTCACTATAGGGAGAGGGAGAATAGCTTCAAAATTTGCATCTCCCCTGGGTCGCCCTTC
+
999-98><>AAC<<<CC@?;;788777;6=>777-76444000101666,11,000176668448:266///+.,-
@T6XXI:00138:00324
GGGAGAAGGGAGAAAGGAAGCTAGTCGACTCGAGATCTGCGGCTTTGGACAGTCCCTCTGTACTGTGAAGCCACAGA
TGTCGAGTCGACTAGCTTCCTTTCTCCCTTCTCCC
+
GC?AAB>BC=BBCG@C@C@BBCA@?:::@@@AB?B?ACCDE????;??BCCDBBB>?<<888?>>@??<??>AAB=A
@@?=???@AA@A@=?6656;>?;@AAA;?:<<??:
@T6XXI:00138:00329
GGGGAATTCTAATACGACTCACTATAGGGAGAGGGAGAATAGTCAACAAATCGTTTGGGGCGACTTCTCCTTCCTTT
T
+
CCD:CBB@BCD>BB@@<;;ABB???776.77777-74456;;;;;8;>?7ACCBB5:::4:>@?A::1;:858,..-
*
@T6XXI:00138:00333
GGGAGAAGGGAGAAAGGAAGCTAGTCCATCTGTGGCTTCACAGTACAGAGGGACTGTCCAAAGCCGCAGATCTCGAG
T
+
DD>ACB@DC>???B?B@B@BA>996637:::ABB???>AAA@@@AA@@===9=BAAAA>AA9==>AAAAA@???AA=
A
@T6XXI:00138:00334
GGGGAATTCTAATACGACTCACTATAGGGAGAGGGAGAATAGTCAACAAATCGTTTGGGGCGACTTCTCCTTCCTTT
CTCCC
+
CCE9A>A@CCB@CCBBBBBB?=@A????;?CCED>A??=A<??AA>@@@:?AAA@;<<<7?BBBB?BBB>=;=>AA<
?BAA<
@T6XXI:00138:00336
GGGGAATTCTAATACGACTCACTATAGGGAGAGGGAGAATAGCCGACGTGTTC
+
@BB6@=A?CCB@CBBBBBBAAAA@?9995:BACC>B@@>A<=85<???@@A>A
@T6XXI:00138:00337
GGAGAAGGGAGAAAGGAAGCTAGTCGACTCGAGATCTGCGGCTTTGGACAGTCCCCTCTGTACTGTGA
+
1,///,/8588BB8969<<A@=><0...43334111111699?@=>>91116766,00033111--+0
```

```
@T6XXI:00138:00344
GGGAGAAGGGAGAAAGGAAGGAGAAGTCGCCCCAAACGATTTGTTGACTATTCTCCCTCTCCCTATAGTGAGTCGTA
TTAGAATTCCCCC
+
BHBAAA?BC>@AAI@I?B?A@CBB@?793333,8?9A?BCC>CC@B::9=;9;444-
498:A:><==?<<<AAA@@@A?=:<94/.///+
@T6XXI:00139:00318
GGGAGAAGGGAGAAAGGAAGGGGAGAAGGGAGCAGTCACTCGAGATCTGCGGCTTTGGACAGTCCCTCTCGTACTGT
GAAGCCACAGATGTCGAGCGAC
+
:65611-/856/44-424237;2777117,7=>11663.../..-
5885B;2488+39;440300+033307388;69;;:7743.//-.000030006
@T6XXI:00139:00329
CTAGTCACTCGAGATCTGCGGCTTTGGACAGTCCCTCTGTACTGTGAAGCCACAGATGTCGAGTCGACTAGCTTCCT
TTCTCCCTTCTCCC
+
>:111=8?AFCHBBBC;;<B@BBC>B?A?><:::5:BCCCBBB<??C;=A@BGCBBBBCBBB<<@AACBC;::>C@D
B<BBCB>A=@@AA;
@T6XXI:00139:00332
GGGAGAAGGGAGAAAGGAAGCTAGTCGACTCGACATCTGTGGCTTCACAGTACAGAGGGACTG
+
BB>@@?58809>@@:?5958>>==??;:::@00.2,,,,..,.4,.18:?>B?AAAAB@A@??
@T6XXI:00139:00334
GGGGAATTCTAATACGACTCACTATAGGGAGAGGGAGAATAGTCAACAAATCGTTTGGGGCGACTTCTCCTTCCTTT
CTCCCTTCTCCC
+
?@@6@?B@BBB@CBBBBBBBB@B<9666-
6;;<?9???=@???>=<>>?;ACCCC>CCC8BBCCC????5868?CC<@ADD9=698@@?
@T6XXI:00139:00340
GGGAGAAGGGAGAAAGGAAGGGGAGAGGCTAGTCACTCGACATCTGTGGCTTCACAGTACAGAGGGACTGTCAAAAG
CCGCAGATCTCGAGTCGACTAG
+
CC?AAA@AA=>??C=?=?>??@8BCCC?BBCCCCDA@A=999;<<BBC@CC@CCC>??BCBB99978?;<10000*.
;:;BBBBCABAAAADFCCBBBC
@T6XXI:00139:00341
GGGAGAAGGGAGAAAGGAAGGAGACAGTGAGCGCCGCACAACACGTCACTATTCTCCCTCTCCCTATAGTGAGTCGT
ATTAGAATTCCCCC
+
CD@CCC@CC>C@??<@@B>B?:;;<1111//..667<?<;7:BB@A?AABA@<?8880887>>:?AA@AEA@>C@-
,/3*,,54854844/
@T6XXI:00140:00308
GGGAGAAGGGAGAAAGGAAGCTAGTCACTCGACATCTGTGGCTTCACAGTACAGAGGGACTGTCCAAAGCCGCAGAT
CTCGAGTCGACTAGCTTCCTTTCTCCCTTCTCCCT
+
CE?B;;79@19999094958;8??F6:711677116677>8::9;@@AAACBAA77717=?@AA?AA<@@<??@@AA
599AAA@AA@?@@@@><?>AA<A@@@:?8;;;;6;
@T6XXI:00140:00334
GGGGAATTCTAATACGACTCACTATAGGGAGAGGGAGAATAGTTTTTCCCTTCACTAACCGCGAACTGCACTTCCTT
TCTCCCTTCTCCC
+
BAA7A@B@CCCACB@@ABBBAAAB::??9?BBCC>C@@>@=<655;0564?>?AABC@?><<?A<;<A=?@A@@;<<
8?A??:?:;;;;7
@T6XXI:00140:00347
CTAGTCACTCGAGATCTGCGGCTTTGGACAGTCCCTCTGTACTGTGAAGCCACAGATGTCGAGTCGACTAGCTTCCT
TTCTCCCTTCTCCC
+
```

```
CA94;@===BBB@@ABBBBB@ABC=AAA@??9:94:@@BBCCC===A?@C@C@@@AAAAAABBA;<;@@@@@?<A>A
C>BBBC>@;==@D=
@T6XXI:00141:00300
GGGAGAAGGGAGAAAGGAAGGAGAAGTCGCCCCAAACGATTTGTTGACTATTCTCCCTCTCCCTATAGTGAGTCGTA
TTAGAATTCCCC
+
AC?AAA@BC?ABBC@C@C@C?<@<@CCC@@@@7@C>CCB??;?B@CCCB?<>====;?<?AA;>9==A@@??:BA??
?>A@@;>2448-
@T6XXI:00141:00305
GGGAGAAGGGAGAAAGGAAGGAC
+
CCBAA@>==9>81/+1:;::,0,
@T6XXI:00141:00306
GGGGAATTCTAATACGACTCACTATAGGGAGAGGGAGAATAGTCCGCCAATTCTGCGCGTTAGCATTCTCCTTCCTT
TCTCCCTTCTCCC
+
CGD9C;:<<BA@BB@AABBBCCB?A611.777BCEDCA:==@AA>AA>C@C?????????;?C?@ADCBB=?<?>AA
=BBAA8?;<>>A<
@T6XXI:00141:00308
GGGAGAAGGAGTGAAGAGATGGCAGTCGCGTTGAGTGG
+
EB:8,,+-+,,,,,*,/4433144443333373333.4,
@T6XXI:00141:00311
GGAGAAGGGAGAAAGGAAGGGAATAGTTATGCGCGGGATTTGACGACTATTCTCCCTCTCCCTATAGTGAGTCGTAT
TAGAATTCCCC
+
B?BCD@BB?BBBB>A>A?AB>B?BABB?@@@CCBAB?:7767==????BC@CCCC>><<<<7<?AAAB@@A?>>888
9=@;99/344-
@T6XXI:00141:00323
GGGGAATTCTAATACGACTCACTATAGGGAGAGGGAGAATAGATCACCGTTGGTTCAGGTGCGCGTTCTCCTTCCTT
TCTCCCTTCTCCC
+
999/98><ACC@CCCBBBBBBCB9>1115:@>CE>A@@>A<=<<=??=?B?B>B>BBB@BCCBCBB@BAA=A?B>@B
9@88718????A=
@T6XXI:00141:00325
GGGGAATTCTAATACGACTCACTATAGGAGAGGGAGAATAGCCAGCCCTTCGATAACCCCTGATTCTCCTTCCTTTC
TCCCTTCCTCCC
+
?==4>598<BB@CBBACBBBB:665..,0;;?C>CAA>A===<=A==;<<===AA>AAA5@A@A>???::9;/44-
////,/,/,/9;6
@T6XXI:00141:00328
GGGAGAAGGGAGAAAGGAAGAGCAAAAGGGAGGCAACTGAACGAGTCGCTATTCTCCCTCTCCCTATAGTGAGTCGT
ATTAGAATTCCCC
+
BC>>@><>?:?@?@8;:;>??8;;@>6@B<@A@DCABBCC@CCBCCC@BBCC@C::;68>;999AAABACHCA???@
AA>A?=84/344-
@T6XXI:00141:00330
GGGAGAAGGGAGAAAGGAAGAGATAGACAGCTCAGGAGGATGTTTGTCTATTCTCCCTCTCCCTATAGTGAGTCGTA
TTAGAACTTCCCC
+
CC?ABB@CD>BCCC>C@B@BB@?@BBBBCCC@AA><9=;=AB@A<?>887;9;3;949;034+3:;9<<@>>=;;<<
<;:87,//,///+
@T6XXI:00142:00301
GGGAGAAGGGAGAAAGGAAGCTAGTCGACTCGACATCTGTGGCTTCACAGTACAGAGGGACTGTCCAAAGCCGCAGA
TCTCGAGTCGACTAG
+
```

```
AC>BBB@CC>BB??=B?A?AB<00/.00<==<3::???BBC@CC@CC??>9>@==<<=999=A<><==;<<????@=
<<@<?<?AAA===AA
@T6XXI:00142:00303
GGGGAATTCTAATACGACTCACTATAGGGAGAGGGAGAATAGTCAACAAATCGTTTGGGGCGACTTCTCCTTCCTTT
CTCCCTTCTCCC
+
999/9596<CC@CBBBBBB?B@BB>:::;<BBDC>BB@>????=;:788-
7?===.777.7??BA<???;;8<?@C@AA??;?:<=AD;
@T6XXI:00142:00306
GGGAGAAGGGAGAAAGGAAGCTAGTCGACTCGACATCTGTGGCTTCACAGTACAGAGGGACTGTCCAAAGCCGCAGA
TCTCGA
+
CB@CB@694-
99980;>CABCB>@?=@?BCB?:::::;BBB<?@?AABBCCCA<83331799>?@=?A<AB??>?@==9==AA
@T6XXI:00142:00308
GGGAGAAGGGAGAAAGGAAGCTAGTCACTCGACATCTGTGGCTTCACAGTACAGAGGGACTGTCCAAAGCCGCAGAT
CTCGAGTCGACTAGCTTCCTTTCTCCCTTCTCCC
+
A:6@9989A:@99909494888>>@70//..//.33577@;?@@BBBBCCC?=4033-
4::;;<9<?:?@>@B@@==888????????A@@@A?A>AA;?>??7>;>??@;
@T6XXI:00142:00311
CTATCGACTCGAGATCTGCGGCTTTGGACAGTCCCTCTGTACTGTGAAGCCACAGATGTCGAGTCGACTAGCTTCCT
TCTCCCTTCTCCC
+
A?>94444=?=BDDCBBBBC@BCC?CA<;>=777-
7<==@BBCBB@C>=A=@CCCCCAB?A<799=>>880:347,0,.44:,/,4444-
@T6XXI:00142:00320
GGGAGAAGGGAGAAAGGAAGCTAGTCGACTCGACATCTGTGGCTTCACAGTACAGAGGACTGTCCAAAGCCGCAGAT
CTCG
+
GJ@ECC?BB:?>=8:@784:B>;==/10=>?>>AA;>:BBC=@@@BB@???@333--,/888;A>@@:=>/0090-
++***
@T6XXI:00142:00324
GGGAGAAGGGAGAAAGGAAGCCGGGAGGTCCGTCGCTAAGTTCGGGACTATTC
+
CC9;=@>BB?BBBC=@<@6::<>?:870131377366?>@A>?=:8990*(((
@T6XXI:00142:00328
GGGGAATTCTAATACGACTCACTATAGGGAGAGGGAGAATAGCCTCCATTCTTCCGCTACTGACCAAGTCCTTCCTT
TCTCCCTTCTCCC
+
CCF:D@C@BBB?BBCCCBBAA=;?;677.777@B>BAB@BA??;@B@?<;<=>A>@@A@@?@@A@A=?@A>A>@>?A
14844-48;;=@:
@T6XXI:00142:00339
CTAGTCGACTCGAGATCTGCGGCTTTGGACAGTCCCTCTGTACTGTGAAGCCACAGATGTCGAGTCGACTAGCTTCC
TTTCTCCCTTCTCCC
+
B9999944=AACDDBBBEBBCACBA>C=>@AA:::8:CCCCBBB?CAC?=?>BCCBBBBBCCCCAB@??@@<:7BC?
BB<???@:=387?@:
@T6XXI:00142:00342
GGGAGAAGGGAGAAAGGAAGCTAGTCGACTCGAGATCTGCGGCTTTGGACAGTCCCTCT
+
AA<AA@=@B:>99919497>>>;==011>>@>@?CC?::7?=777,6366;>777./65
@T6XXI:00142:00348
CTATCACTCGAGATCTGCGGCTTTGGACACTCCCTCTGTACTGTGAAGCCACAGATGTCGAGTCGACTAGCCTTCCT
TTCTCCCTTCTCCC
+
```

CDCC=::@CCBCBC?@@CD@9998=;==000>=6>BBBC999@@@@?@B?BCBB??AABAB==?A;///./,/795:
;6;@@A;A>=99B<
@T6XXI:00143:00301
GGGGAATTCTAATACGACTCACTATAGGGAGAGGGAGAATAGCCTTGCACTATCCCGCCGTAAACTTTTCTTCCTTT
CTCCCTTCTCCC
+
BBB:B@C@BBB@BBABBAABAAAB@::<;<@@A@>CC=;<:::;@?@@@@?;@@A?8888==C:;;==4<A;<8899
=ABC;?<@@C=;
@T6XXI:00143:00306
GGGGAATTCTAATACGACTCACTATAGGGAGAGGGAGAATAGTTTTTCCCTTCACTAACCGCGAACTGCACTTCCTT
CTCCCTCTC
+
DCC:C>@AACCACCBABABBCBA>=777.777>?:::79;;44444,44-
344??==@D?BBB@<??>=@==:=4737/,,*,+++
@T6XXI:00143:00313
AGGGAGAAGGGAGAAAGGAAGCTAGTCGACTCGACATCTGTGGCTTCACAGTACAGAGGGACTGTCCAAAGCCGCAG
ATCTCGAGT
+
ACC@BCC?BB?BBAC?B=?:<<CCCCCDCCBB://111/68=?BB@@@@CBBCCBD99919@A@?D?BC>BDBDDBB
BB<;78833
@T6XXI:00143:00323
GGGAGAAGGGAGAAAGGAAGGTGAATAGTCAGAGCAATCCGTCAGAACTATTCTCCCCCTCCTC
+
CC>BAA=>=:@BAA:B=@=@?BAB?ABB?>>???====?B@@CCAA>BCCC>A:;:88.,,***
@T6XXI:00143:00325
GGGGAATTCTAATACGACTCACTATAGGGAGAGGGAGAATAGTCAACAAATCGTTTGGGGCGACTTCTCCTTCCTTT
CTCCCTTCTCCC
+
EED:D@B@BBB@CCBBA@@BBCB<;111-
6<<=?>A??@C?A@AA<??A<CCFFC=AAA7ABCAB><AA5738>@?;@???7>;==>?:
@T6XXI:00143:00339
GGGAGAAGGGAGAAAGGAAGCTAGTCGACTCACATCT
+
CCBDCC;;4-9>>><A@B>AA::;@::7<0/...,,,
@T6XXI:00143:00341
GGGGAATTCTAATACGACTCACTATAGGGAGAGGGAGAATAGTCAACAAATCGTTTGGGGCGACTTCTCCTTCCTTT
CTCCCTTCTCCC
+
EDD9CBD@BBBBDCBA?ABDC@B;>66156==@B>C@@>?;?AAA>???<BAABC8???6??@@B<???5837=AA<
@AAA;@:==@@;
@T6XXI:00144:00306
GGGGAATTCTAATACGACTCACTATAGGGAGAGGGAGAATAGTCAACAAATCGTTTGGGGCGACTTCTCCTTCCTTT
CTCCCTTCTCCC
+
BCB9C>B?BCFACCCAAA???BBBB76646?BCC>CCB>B??=<=<=??<BCCCC>@@@9BCCCA>>BB>A??>AA<
A@@@<A889AA<
@T6XXI:00144:00314
GGGAGAAGGGAGAAAGGAAGAGAACAACAGAGGCAACACAAGACCTGCTATTCTCCCTCTCCCTATAGTGAGTCGTA
TTACGAATTCCCC
+
CDBB@==@A>AAAB<@7:8;FAB?ED@CBBAA@BB?BBBC?@@B@CF===DBDCCC@<<<CC@CDBB@@@BBBB?>>
>=100?9;9===2
@T6XXI:00144:00315
GGGGAATTCTAATACGACTCACTATAGGGAGAGGGAGAATAGTCAACAAATCGTTTGGGGCGACTTCTCCTTCCTTT
CTCCCTTCTCC
+

```
NKK:C@B@BCCACCC@@@CCCBBBA?=<.77>>A:@AC>BBA??@8<<<7?BAAA6===.87=7737;;:664/...
.267,/,////
@T6XXI:00144:00316
GGGGAATTCTAATACGACTCACTATAGGGAGAGGGAGAATAGTCAACAAATCGTTTGGGGCGACTTCTCCTTCCTTT
CTCCCTTCTCCC
+
=??4C?A>BEC@CBBBBBBB>B>?>7665:BBCE<BBB?A=?=@?>@AA<BBBBC>CCB8CCCBB>AAB=@>=?CC9
==@@:>378=>7
@T6XXI:00144:00328
GGGGAATTCTAATACGACTCACTATAGGGAGAGGGAGAATAGTCAACAAAATCGTTTGGGGGCGACTTCTCCTTCCT
TTCTCCCTTCTCCC
+
999/4:;?CCC?BBBB;<<BCEBBBCBC>B@CCB;;7747==;4424444+3@>=;,6166+3;;9A@BBA;?:;;:
;:=<;<6;9<<@B4
@T6XXI:00144:00332
GGGGAATTCTAATACGACTCACTATAGGGAGAGGGAGAATAGCTTCCGTTACTCCCGCGGCGGCATGTGCTTCCTTT
CTCCCTTCTCCC
+
DJD9C897;AA>BCBBCCBB>CB99311.7>>?BCBDC@B>=>9:?:??=@@AAB>BC@>@=<=<=@?===2837A:
;;<?7><AACD@
@T6XXI:00145:00308
GGGAGAAGGGAGAAAGGAAGAATAATGCTGGGAATTTGAAGTGTTAGCTATTCTCCCCTCCTCATAGTGAGTCGTAT
TATGAATACCC
+
CGBBDC@CB>@BAA=A@A@AA@???BBBBCC@C8::6;A?BBBB@CBBCCC@@:444,9@<911//......11111
7>::<57066,
@T6XXI:00145:00309
GGGAGAAGGGAGAAAGGAAGGAGA
+
DDBB@@@AA?BB;A;;7860)*(,
@T6XXI:00145:00312
GGGAGAAGGGAGAAAGGAAGCTAGTCGACTCGAGATCTGCGGCTTTGGACAGTCCCTCTGTACTGTGAAGCCACAG
+
IFBCBB?BC@BCFF@C@B@BA:00@00/>>>>@BCCC@A@D@?@@<?>ABCBCBE=@@?A@<==??=BA;<9<?==
@T6XXI:00145:00316
GGGGAATTCTAATACGACTCACTATAGGGAGAGGGAGAATAGTGGCTAAACCCCCGTACGTCACTTCTCCTTCCTTT
CTCCCTTCTCCC
+
999/9>B@CDC@CB??=A???BCB>76646?BCC?BBB@C@<;?>@?AB=AEEC2AAABBBBCGC@:;8;=9;/771
7==C?C?;<<C<
@T6XXI:00145:00324
GGGGAATTCTAATACGACTCACTATAGGGAGAGGGAGAATAGTCAACAAATCGTTTGGGGCGACTTCTCCTTCCTTT
CTCCCTTCTCCC
+
DII:C?B?BCDCCCCBBBBBBB<99316.7>77:7;AA@BAB@AA<??A<BCCAC9???5><<;?:<=<:?;;;>>:
@A@@;@989:A<
@T6XXI:00145:00325
GGGGAATTCTAATACGACGAGT
+
CBE9BBD59442448//1/.44
@T6XXI:00145:00335
GGGAGAAGGGAGAAAGGAAGGAGACAGTGAGCGCCGCACAACACGTCACTATTCTCCCTCTCCCTATAGTGAGTCGT
ATTAGAATTCCCCC
+
8BCFCC?BB>BCCB?B:97<@BAACCCBBAA@BB?BCCCD=@@C@??CAAAA>AABA:@@9;;9;@BBA<<<A@@@B
<<:<BA=A69999,
@T6XXI:00145:00337
```

```
GGGGAATTCTAATACGACTCACTATAGGGAGAGGGAGAATAGCCATGTGCCGGTACCTGTCGCGTTCTCCTTCCTTT
CTCCCTTCTCCC
+
FED9C?B?BCD@CBBAB@>>BC?99311.7==BD>AAAAB@?=9=A=A@A>A>@@;;@@AA@@@A>A?>/4347::4
9::94;8;?@D?
@T6XXI:00145:00338
GGGGAATTCTAATACGACTCACTATAGGGAGAGGGAGAATAGACAACC
+
CFC:C798<@=?BBBBBCB@@@@ABABE@BBA?>;<A?>74,++*)*)
@T6XXI:00146:00301
CTAGTCGACTCGAGATCTGCGGCTTTGGACAGTCCCTCTGTACTGTGAAGCCACAGATGTCGAGTCGACTAGCTTCC
TTTCTCCCTTCTCCC
+
B7-,--
,,6>CCECCCBBBAC@CCC?A?CB@?:::5:BBCCBBC>AA@?AC>BBCDCCBBAABBABBBBBBAAA:?:?@:???
B<A;??AB<
@T6XXI:00146:00304
GGGAGAAGGGAGAAAGGAAGCTAGTCGACTCGACATCTGTGGCTTCACAGTACAGAGGGACTGTCCAAAGCCGCAGA
TCTCGAGTCGACTAG
+
DEBCB@899-
9>>><A>@=;;:==>/11>;;>0;;::6BBC:?@?ABABCCB??9333,4;;<??>@C=@@<?????A@@@AA?@@A
A@===
@T6XXI:00146:00308
AGTCACTCGAGATCTGCGGCTTTGGACAGTCCCTCTGTACTGTGAAGCCACAGATGTCGAGTCGACTAGCTTCCTTT
CTCCCTTCTCCC
+
CECA?@CBBCCCCBABDC@??@>@@CBBBCCB>BB?AAC@CBBBB@BB@BB>B>;BBBB><@>A@9;;91747;>?:
AACLAFAAB@@:
@T6XXI:00146:00313
GAATACGACTCACTATGAGGGAGAGGGAGAATAGTCACAAATCGTTTGGGGCGACTTCTCCTTCCTTTCTCCCTTC
+
/,2,,,-1:886:8;1..0000,..566>D@CC13321962498<><:@>8:<661:777:==?477-7000+020
@T6XXI:00146:00314
GGGAGAAGGGAGAAAGGAAGTGCAGTTCGCGGTTAGTGAAGGGAAAAACTATTCTCCCTCTCCCTATAGTGAGTCGT
ATTAGAACTTCCCC
+
GICFCC@CB>DAAA?BBC?BBCECCC@CCCC@C?BBCCC@CEAB>>9/7<88488>>:AB@@A>AA@>???A@@?<8
847:::8;;98991
@T6XXI:00146:00328
GGGAGAAGGGAGAAAGGAAGCAAATGGCGCATGTTTCTAGGGAGTGTCTATTCTCCCTCTCCCTATAGTGACGTCAT
A
+
AC=?A@=>>:>9990;<@?B;;<:<C@CCCCCBBB>BBBCC>CCBAAACCC@FBBB=:88@A;@A@;880,++++++
0
@T6XXI:00146:00338
GGGAGAAGGGAGAAAGGAAGGAGAAGTCGCCCCAAACGATTTGTTGACTATTCTCCCTCTCCCTATAGTGAGTCGTA
TTAGAATTCCCC
+
CCBCCC@BB>BDDC>B@A@B@BAA?A@BCAAA7AD>BBB@C>CA@CB@BCC?<<<=;86<CC=A@@@<@@===A==;
@?A<<8<8:9;0
@T6XXI:00146:00340
GGGAGAAGGGAGAAAGGA
+
CFABCB894-98//+-+-
@T6XXI:00146:00345
```

GGGAGAAGGGAGAAAGGAAGCTAGTCGACTCGACATCTGTGGCTTCACAGTACAGAGGGACTGTCCAAAGCCGCAGA
TCTCGAGTCGACTAGCTTCCTTTCTCCCTTCTCCC
+
FFEB==;>>:>99919<@>BA?=@?0000/...33111799<>B@CCCCBBB?=7000-
4999=><@@9==?DDBAA@==<@==;<@A78884;8;34418@@A<@<@?ACE
@T6XXI:00146:00348
GGGAGAAGGGAGAAAGGAAGCTAGTCACTCGACATCTGCGGCTTTGGACAGTCCCTCTGTACTGTGAAGCCACAGAT
GTCGAGTCGACTAGCTCCTTCTCCC
+
EKFECC@BB1948804242433;;?<777//.538//7??=7;<9:?AACCCED=;::884406../7;?:99@???
@@@AA@@@@@<;99,,,+,++***)
@T6XXI:00147:00328
GGGGAATTCTAATACGACTCACTATAGGGAGAGGGAGAATAGTCAACAAATCGTTTGGGGCGACTTCTCCTTCCTTT
CTCCCTTCTCCC
+
CCC9C798;@@=@A@ACBBBCCA??761.7=7775:AC@DCCA@@=@AB;<<<CC>CBB9CBBBB==>>37449==;
@AAC<A>A?@A@
@T6XXI:00147:00331
GGAATTCTAATACGACTCACTATAGGGAGAGGGAGAATAGACTCCAAGTCTTTTGCTCCCACCT
+
E?A>ABAAB@BBBCCCC@@@ACA???9=<===6=7740<0////,8<=>7611,16.00,/:9.
@T6XXI:00147:00333
GGGGAATTCTAATACGACTCACTATAGGGAGAGGGAGAATAGCGCATACGTTGGCCCGCGTGATTTCTCTTCCTTTC
TCCCTTCTCCC
+
FHH:C?B?ACA<>>:899>>88:>A@A@:>>/00,0//,.3528=DCCCAA=A>AA<ACBB@BBB>B@@?@C@CB<A
A@C?A>887?5
@T6XXI:00147:00349
GGGAGAAGGGAGAAAGGAAGCTAGTCACTCGACATCTGTGGCTTCACAGTACAGAGGGACTGTCCAAAGCCGCAGAT
GTCGAGTCGACTAGCTTCCTTTCTCCCTTCTCCC
+
EIFDBB@BB:?;;809695;BABBB7;;//..4.../9;?>?A@BBBBCBCCCB???9;888@A@CD?BB=@B@@@@
===A===AAA?778//./;;;9;;?;9;9@>>?:
@T6XXI:00148:00304
GGGAGAAGGGAGAAAGGAAGAACAGAAGGACACGTTACTGCGGGAGACTATTCTCCCTCTCCCTATAGTGAGTCGTA
TTAGAATTCCCC
+
CDBCCC@BE>BBBB>B@A=AA@BB>?=???ABC?C>BB??@;;:<==A@A@=<A<=5;;;>>:???@@CDAAA===@
@>@AC>A>@@@5
@T6XXI:00148:00307
GGGGAATTCTAATACGACCTCACTATAGGGAGAGGGAGAATAGCCATTGCATAATGTGTGCAGCTCTTCTCCTTCCT
TCTCCCTTCTCC
+
CDD9DEIABAB@B:77//-000.79B;>A<AA?A=<???=:::<9=A>AABAA=@???A??AAAA@@=?8:-
.,/,/,,,,,,+/,/.-,
@T6XXI:00148:00329
GGGAGAAGGGAGAAAGGAAGTGCAGTTCGCGGTTAGTGAAGGGAAAACTATTCTCCCTCTCCCTATAGTGAGTCGTA
TTAGAACTTCCCC
+
CB?AAA@BC>DBBB?D@C@BBBCCCC?ABB@>B>BAACC@CE?CCC9CBBB?BDCC>?@@BB>BBBBCAAAAAA<<<
;8;;?=@@?CCC5
@T6XXI:00148:00342
GGGGAATTCTAATACGACTCACTATAGGAGAGGGAGAATAGTCAACAAATCGTTTGGGGCGACTTCTCCTTCCTTTC
TCCCTCTCCC
+
9;;2:24294424::8=??@B<777710167778:8899AA;<:9444-876<<.777-
3=5335//.477473550111+-----+

```
@T6XXI:00149:00303
GGGGAATTCTAATACGACTCACTATAGGGAGAGGGAGAATAGCCTGACCTTTCCAGTGCGCGCTGTTTTCTTCCTTT
CTCCCTTCTCCC
+
A?:4@5;8<CD@BBBBB@@<?C>99311.7>>?CBBCD@CBA?<A?<?=??595:ACCC???A<<;<<5;:<?;<65
;9994:;<<<C;
@T6XXI:00149:00324
GGGGAATTCTAATACGACTCACTATAGGGAGAGGGAGAATAGTTTTCCCTTCACTAACCGCGAACTGCACTTCCTTT
CTCCCTTCTCCC
+
EBB7B7;9<CBACBBBBBBBBEB99311.7???F>CDCABBB;==6@@<@==B>>B>>;>>>>><<?=B:6:;366.
7??A>B?BA>?:
@T6XXI:00149:00325
GGGAGAAGGGAGAAAGGAAGGAGAAGTCGCCCCAAACGATTTGTTGACTATTCTCCCTCTCCCTATAGTGAGTCGTA
TTAGAATTCCCCC
+
DB?CCB@BB>BBBB?@<?@A@A??@B@>7777-
8?<ABCB?9ACBCABBCC>??@D<???@C9==AA??==<<@<89:>@;A:4/4444,
@T6XXI:00149:00330
GGGGAATTCTAATACGACCTCACTATAGGGAGAGGGAGAATAGTCAACAAATCGTTTGGGGCGACTTCTCCTTCCTT
TCTCCCTTC
+
CGC9C@C@BB?@911111,....3773694:BCCC>C??;?>@84833770...330333079999=?:8888523;
43/.606,.
@T6XXI:00149:00337
GGGGAATTCTAATACGACTCACTATAGGGAGAGGGAGAATAGTCAACAAATCGTTTGGGGCGACTTCTCCTTCCTTT
CTCCCTTCTCCC
+
AA?6A>A?BBC@CBBBB@B?BCB99311.7>>AB>CCB?B??<==<<@@<CB;A=-966-
:@@BC@BBB=?==>AA<?ACC=B;???C<
@T6XXI:00149:00340
GGGAATTCTAATACGACTCACTATAGGGAGAGGGAGAATAGTCAACAAATCGTTTGGGGCGACTTCTCCTTCCTTTC
TCCCTTCTCCC
+
CC?D@B@BBB@CBBBAB@B?A?BB76656=>?A=ACC@?;;<>A?8871:@?AC>CCC9CCCCB=<?B?A@??CC<=
==A<A>@CCC<
@T6XXI:00149:00342
GGGAGAAGGGAGAAAGGAAGCTAGTCGACTCGACATCTGTGGCTTCACGAGTACAGAGGGACGTGTCCAA
+
FEBCAA=@A<AABB=B?B?BCB=;;/0//....331977685779971666766610 17,0000004/00
@T6XXI:00100:00353
GGGAGAAGGGAGAAAGGAAGGAGAAAGCGACGTCGGGGAAACGTTTGCTATTCTCCCTCTCCCTATAGTGAGTCGTA
TTAGAACTTCCCC
+
DD>BBC@CC>AAFC>A?@@C?BBCD>BC@@???7777.9B>CDBCBEHFCB>AACB<@A;;;/8B>???AA???9A<
<;=AC=CC:???2
@T6XXI:00100:00370
GGGGAGAAGGGAGAAAGGAAGAGAA
+
444,44;9::6:911,-88+,,,,*
@T6XXI:00100:00378
GGGGAATTCTAATACGACTCACTATAGGGAGAGGGAGAATAGTCAACAAATCGTTTGGGGCGACTTCTCCTTCCTTT
CTCCCTTCTCCC
+
===2>6959>>;;;<CDCCCCC77;677.777??>B==;=ACAAA<??@;BBABA>BEE7BBBBB=?@D?@>@<?>7
==;;6<8;>@A;
@T6XXI:00100:00380
```

```
GGGGAATTCTAATACGACTCACTATAGGGAGAGGGAGAATAGTTACCAATTCCCCCACGGTAACGTTCACTTCCTTT
CTCCCTTCTCCC
+
ACF9CBC@CAA>ACCFCCBBB=<;?6776:??DE?B?A>@@//,/4,-,-,----
*.299=>3::A<@@AAAB@AA;@@BA<A<??CC<
@T6XXI:00100:00386
GGGGAATTCTAATACGACTCACTATAGGGAGAGGGAGAATAGCCGACGTGTTCATTT
+
999/42959949?<121.627344....+...17886<<<::=<4454344234030
@T6XXI:00101:00352
GGGGAATTCTAATACGACTCACTATAGGG
+
9@?4?69544424444437;;6;;1116,
@T6XXI:00101:00353
GGGGAATTCTAATACGACTCACTATAGGAGAGGAGAAT
+
---*,*924989;9<<BCC@@>@>=//,0:0737//,-
@T6XXI:00101:00363
GGGAGAAGGGAGAAAGGAAGGAGAAGTCGCCCAAACGATTTGTTGACGTATTACTCCCTCTCCCTATAGTGAGTCGT
ATTAGAA
+
:=:;A@<AB>@@AB<@@A?>;:888,,,,,2*,,*,,.3:6=>:====834/00000+---24-
.+0+++.334===<@>@@@9
@T6XXI:00101:00366
GGGGAATTCTAATACGACTCACTATAGGGAGAGGGAGAATAGTCAACAAATCGTTTGGGGCGACTTCTCCTTCCTTT
CTCCCTTCCTCCC
+
DHD:E;;;<@@=@<<<DCCCCCBA>777,7>777.:=>?BABB=A;=9=;AAABC<AAA7??AAB@???8838=@@;
@@;939,/,/422
@T6XXI:00101:00382
GGGGAATTCTAATACGACTCACTATAGGGAGAGGGAGAATAGCACGGATCCTGCTCCCGCCGTGTTTCTCTTCCTTT
CTCCCTTCTCCC
+
;99/9>A>BCB@BBBA?BBBD>;76116.7??BL>A==>B;;===C=<?;::::9::4;<8<AAAB7??AA>@>@A<
===A<A?@@@D<
@T6XXI:00101:00385
GGGAGAAGGGAGAAAGGAAGTGCAGTTCGCGGTTAGTGAAGGGAAAACTATTCTCCCTCTCCCTATAGTGAGTCGTA
TTAGAATTCCCC
+
CC@@BB@BB=?BBB=B@@@BBBBBBB@B@@@?C@AABCC@CA;??B7BCB?>>>>?9=ABCC>C@@AA<@@AA@@??
?>ACC>A<>>>1
@T6XXI:00101:00396
GGGAGAAGGGAGAAAGGAAGGAGAAGTCGCCCCAAACGATTTGTTGACTATTCTCCCTCTCCCTATAGTGAGTCGTA
TTAGAATTCCCC
+
CC@CCC?BB>BBBB>B=@=>@BBB>99<?BBB6BB<BAA@@8<<ACC?A==;=<<;:>8>??;?CA?@?AAA@@@@@
@>@AA=@38880
@T6XXI:00102:00371
GGGAGAAGGGAGAAAGGAAGCTAGTCGACTCGAGATCGTGTGGCTTCACAGTTACAGAGGGACTGTCCAAAGCCGCA
GACTCTCGAG
+
490444244-448;5824237>716111166111161116677031111111101489<==:<<?===>==6;:7.--
23356888<<
@T6XXI:00102:00377
GGGAGAAGGGAGAAAGGAAGGGGAGAAGGGAGAAAGGAAGGGGAGAAGGGAGAAGAAGCTAGTCGACTCGACATCGT
GGCTTCACAGTACAGAGGGACTGTCCAAAGCCGCAGATCTCGAGTCGACTAGCTTCCTTTCTCCCTTCTCCC
+
```

```
BBCBBBACA@BBBB=@=?@BBB8AAA??B>A?AC>C=?;???2;<;;==7:;;;883/89:7>../::?A???==<>
?;<=:=@@CC<<?CA@@C=?<???@?AC<@@;==<=?@@??@@@8<;?B@@::7??=?C8:4;:4=;837@<
@T6XXI:00102:00378
GGGAGAAGGGAGAAAGGAAGGGGAGAAGGAAGAAAGGAAGGGGAGAAGGGAGAAAGGAAGGGGAGAAGGGAGAAAGG
AAGACTAGTCGACTCGACATCTGTGGCTTCACAGTACAGAGGGACTGTCCAAAGCCGCAGATCTCGAGTCGACTACG
CC
+
CCC<;9>BB>BBBC@C@B6:77,8??463737=?9?;?;?AA5ABB??=4>?@@:;4:2444-
34;9>>5848>9;8;34337777../////,,,,....171127<>>AAFCCBCCC>:::@9;6?@<?@@CBCCCC;
;;>777A9822,,,,*
@T6XXI:00102:00383
GGGAGAAGGGAGAAAGGAAGTGCAGTTCGCGGTTAGTGAAGGGAAAACTATTCTCCCTCTCCCTATAGTGAGTCGTA
TTAGAACTTCCCC
+
CB>CCCACC=?BBC>C@C?AABCCCC@B@@@<?<?ABBC@BABBEE9BBBB>?@A@9=????9?@@??@ABDA9998
=?AAB<??@AAC5
@T6XXI:00102:00392
GGGGAATTCTAATACGACGTCACTATAGGAGAGGGAGAATAGTACA
+
8BA00)1,//6,/4444......88...,37111,16633;4..-2
@T6XXI:00103:00365
GGGAGAAGGGAGAAAGGAAGGAGAAGTCGCCCCAAACGATTTGTTGACTATTCTCCCTCTCCCTATAGTGAGTCGTA
TTAGAATTCCCC
+
@@?ABB@CEA@@?B=@=B>A?ACCABB?BCBB8BC>BC@@><BC@C:8:@A>ABBB=@?@<=;<=<AA???@@@A@@
@<??D<;2444-
@T6XXI:00103:00366
GGGGAATTCTAATACGACTCACTATAGGAGAGGGA
+
A@A8@BC?<;;=@949AA;@:63711101:/,,*,
@T6XXI:00103:00391
GGGAGAAGGGAGAAAGGAAGCTAGTCACTCGAGATCTGCGGCTTTGGACAGTCCCTCTGTACTGTGAAGCCACAGAT
GTCGAGTCGACTAGCTTCCTTTCTCCCTTCTCCC
+
CC>@@><@@:>99909;>;?BBBCCBCBBA???@CCCCCC?B@B>B?BB?CBB@>@A=><89=>==@?A@?@?@@AA
???A=AA@??=9;43476,//,////,/,/...+
@T6XXI:00103:00395
GGGAGAAGGGAGAAAGGAAGGGCCAGCCGGTGGGAGAAAACCGTGTGCTATTCTCCCTCTCCCTATAGTGAGTCGTA
TTAGAATTCCCC
+
CCACCC@CB>BBBB>B@B@BB<?@BBB@B>=@C;?:<<<6<?BCB@@@ABB?AAABADC:9979=@BBBCCDDBBB?
?=?CC@C@CCC8
@T6XXI:00104:00365
CTAGTCACTCGAGATCTGCGGCTTTGGACAGTCCCTCTGTACTGTGAAGCCACAGATGTCGAGTCGACTAGCTTCCT
TTCTCCCTTCTCCC
+
:8/////44BBBCCDBBBBB>BDB<B;?A@@===;??<>?@BB@@@@?AC@CCBBAAABCCCCABBBAAA<;8?C?B
C>DBCC>@>@AAA<
@T6XXI:00104:00369
GGGAGAAGGGAGAAAGGAAAGCTAGTCACTCGACATCCTGTG
+
BA:9995891999914288-333117:/.337,,,,,*,005
@T6XXI:00104:00389
GGGGAATTCTAATACGACTCACTATAGGAGAGGGAGAATAGATCACCCGTTGGGT
+
:99/959598>9;;<EDBCBB=70000,0:077,600/3=;;;;777-11017,.
@T6XXI:00104:00399
```

```
CTAGTCGACTCGAGATCTGCGGCTTTGGACAGTCCCTCTGTACTGTGAAGCCCAGATGTCGAGTGACTAGCTTCCTT
TCTCCCTTCTCCC
+
B;;;9CBCDECCCBBB??>BB=???;B<?ABBAAB>BCDBBB>>?C@A<?BCABCDCCCBCCCCB@BBBB?B=?:??
8;=@@9?;?A@A=
@T6XXI:00105:00380
GGGAGAAGGGAGAAAGGAAGTGCAGTTCGCGGTTAGTGAAGGGAAAAACTATTCTCCCTCTCCCTATAGTGAGTCGT
ATTAGAATTCCCC
+
CC?BBB@BD>CBBB?B@B@AAABCCBBBBAA@C?BAACC@@?>?BBC2CC??><?@A;BBBBB>BA==;;;9==CA=
==;=AA?A?AAA6
@T6XXI:00105:00381
CTAGTCACTCGAGATCTGCGGCTTTGGACAGTCCCTCTGTACTGTGAAGCCACAGATGTCGAGTCGACTAGCTTCCT
TTCTCCCTTCTCCC
+
>=1//7/44BBBCCFCBCBA?AAA?A>ABCBBCC>CCCCCBBAA??A>AC=AADCC;;;CCCCBCC@@@@A??=??A
D=??BE>@387884
@T6XXI:00105:00382
GGGAGAAGGGAGAAAGGAAGCTAGTCGACTCGAGATCTGCGGCTTTGGACAGTCCCTCTGTACTGTGAAGCCACAGA
TGGATGTCCTTCCTTCTCCCTTCTCCC
+
CHABBB@CC?B;;;8;A@?@B@ABBCEDCCA@B@BBBBBBC>AAC>C?B?@@CCC>@@@AA@@@AAA=8;?:@<@?>
>A?AA99:9701,.,-22223456<A7
@T6XXI:00105:00384
GGGAGAAGGGAGAAAGGAAGCTAGTCGACTCGAGCATCT
+
BD@CA@=@@>@ABE?@?AB;:;BBA::1;<<:0/.....
@T6XXI:00105:00392
GGGGAATTCTAATACGACTCACTATAGGGAGAGGGAGAATAGTTTTCCTTCACTAACGGCGAACTGCACTTCCTTCT
CCCTTCTCC
+
444/4898999599@>@@B@@?BD>777.7>777.7999?0//77+/,-,-
..63533,..2233;:77<9:,/,/99:6>4444/
@T6XXI:00105:00397
GGGGAATTCTAATACGACTCACTATAGGAGAGGGAGAATAGTACGGACGTCTCACCGTGCTGCTCTTCTTCCTTTCT
CCCTTCTCCC
+
BBA7A8;8<BB@BCCBBABBB7667//,0><?A>BAA@A9???@B<;==>A???A@@@@A==@A8837>2424;7;9
<<4:::4471
@T6XXI:00106:00363
CTATCGACTCGAGGATCTGCGGCTTTGGACAGTCCCTCTGTACTGTGAAGCCACAGATGTCGAGTCGACTAGCTTCC
TTTCTCCCTTCTCCC
+
B:::1118:;BBC@CCBBBBB@A@@=A?;;7969947=ACBBBC===C<?B?BBBBDAAA@@AA@ABACCCBAB>B?
BC>BB?A<A?8=8;;
@T6XXI:00106:00370
GGGAGAAGGGAGAAAGGAAGCTAGTCGACTCGACATCTGTGGCTTCACAGTACAGAGGGACTGTCCAAAGCCGCAGA
+
CDACCB=>=5=844-;?A8;;C;<<CCCCCA>:6::::AAD@BB@CB>>>>?===88999<====:::;?B?@@@?<
@T6XXI:00106:00383
GGGGAATTCTAATACGACTCACTATAGGGAGAGGGAGAATAGTCTGTCGTTCGGTCGACGCTGCCTATCCTTCCTTT
CTCCCTTCTCCC
+
AA?8A>B@BCC@CBB?@@CCCCCC:::B=ABCBB<@664/.//7887@@@>@@>@@@>;9::44/09;;3824244-
4444-424444,
@T6XXI:00106:00387
```

```
GGGGAATTCTAATACGACTCACTATAGGGAGAGGGAGAATAGTTTTTCCCTTCACTAACCGCGAACTGCACTTCCTT
TCTCCCTTCTCCC
+
9AA8A@C@BBB@BAA?@ABBBAB<96665:CCCC;B?A?A;;;;;A1AB<?<?AAAA?C@BBB@@@??ACCC=A>AA
<ADCE>A>6777-
@T6XXI:00107:00360
GGGGAATTCTAATACGACTCACTATAGGAGAGGGAGAATAGACTAGTAATCCTCCACTAAACTGTTCCCTTCCTTTC
TCCCTTCTCCC
+
ADD7B9;;=BBAGB>ABBBB@6997//,0;;@C@DAA?AA@@@<<@@@>AA>??<??BAC>BA>A@EE?A<?<?>:>
<<:4:896686
@T6XXI:00107:00361
GGGGAATTCTAATACGACTCACTATAGGGAGAGGGAGAATAGTCAACAAATCGTTTGGGGCGACTTCTCCTTCCTTT
CTCCCTTCTCCC
+
=@@6@?BADBB@AA@@@>>>A@AB769959AACD?BBB=?<8<A=>==<7?@@@A<BBB7BAABF@BBC?@=??AA<
?BAA=A<??@@<
@T6XXI:00107:00391
GGGGAATTCTAATACGACTCACTATAGGGAGAGGGAGAATAGACTGTTGCTAAACTTCAGTCTCGTTCTCTTTCCAT
TTCTCCCTTCCTCCC
+
?AA,46;6448?>@BBB@B>><<711178??@AA5:77=<<8?AB<<744444-
482434=@=?AA?8003:5:...55433002,/,.722
@T6XXI:00107:00393
CTATCACTCGAGATCTGCGGCTTTGGACAGTCCCTCTGTACTGTGAAGCCACAGATGTCGAGTCGACTAGCTTCCTT
TCCTC
+
AA@994449;:>;98>????@BD@D:;;A@@@C=CBBB><;@@B@A>@DCCCCAA@@?A???@@B>8831..44,..
+.,,0
@T6XXI:00107:00396
GGGGAATTCTAATACGACTCACTATAGGGAGAGGGAGAATAGCTTTTTCCTGCCTGGACTGCGTATTCTCTTCCTTT
CTCCCTTCTCCC
+
CAA6A=@=@CC@DBAABCCBBAB>>666-
9;;BC>B@A?A888>?@@2>>ABA:;;8<>>??@C<=;?=<?=@<??;?AAA?@=@@BC;
@T6XXI:00108:00354
GGGAGAAGGGAGAAAGGAAGCTAGTCGACTCGAGATCTGCGGCTTTGGACGTCCACTCGTCTCTGTGACACAGATGT
CGAGTCGACTAGCTTCCTTTCTCCCTTCTCCC
+
=?:>9959@:>;;91;=A=@CBAAA:::CBB:;<ECCCBBC>>7767277,**)*****-
22034;<=89=A===??998@>>87444449<@69<;=CBB>@;>9990
@T6XXI:00108:00358
GGGGAATTCTAATACGACTCACTATAGGGAGAGGGAGAATAGTTTTCCCTTCACTAACCGCGAACTGCACTTCCTTT
CTCCCTTCTCCC
+
999/92958=@?A@BACCCA@?BB::::57>BBC>C@A8;84444-44-
4/3ABCC@D@@@?@;=>?8::?<?477.7>>B@CA?<<<7
@T6XXI:00108:00379
GGGGAATTCTAATACGACTCACTATAGGGAGAGGGAGAATAGTCAACAAATCGTTTGGGGCGACTTCTCCTTCCTTT
CTCCCTTCTCCC
+
CBB9B>B@EBCABB@>@BBBB@B<9111.6<<=@>BAA>A?@@AA<@@@<BAAA@;?@@7BBBBB?:??;<:=>AA<
@@@A<A>@@@@<
@T6XXI:00108:00385
GGGAGAAGGGAGAAAGGAAGGAGAAGTCGCCCAAACGATTGTTGGACTATTCTCCCTCTCCCTATAGTGACGTGTAT
TAGAATTCCCC
+
```

```
991444244-448:-
469@B><AB5713333,39697110..,.,.422423484440000+00433555688<=<:<<54445222.
@T6XXI:00108:00393
GGGGAATTCTAATACGACTCACTATAGGGAGAGGGAGAATAGATCACGACCCTTCTACCCACGACTGAGCTTCCTTT
CTCCCTTCTCCC
+
@>?4=;>=@BB?DCCCCC????A<;66656;;AB>CAA>A=?::=??@@?B>A;7;611.4:;=@@@AC@C@C?AA<
@@>><?<?ABC?
@T6XXI:00108:00397
GGGAGAAGGGAGAAAGGAAGCTAGTCGACTCGACATCTGTGGCTTCACAGTACAGAG
+
CD@BBB>@=:@@=?:B?C8;;CCCCCCCCCC88:CCCCBCC???=??CBB??=@@AA
@T6XXI:00109:00350
GGGGAATTCTAATACGACTCACTATAGGGAAGAGGACGAAT
+
111+1,9548=299;<<9BBCCBBCBBC50,000,..-33-
@T6XXI:00109:00358
GGGAGAAGGGAAGAAAGGAAGCTAGTCGACTCGAGATCTGCGGCTTTGGACAGTCCCTCTGTACTGTGAAGCCACAG
ATGTCGAGTCGACTAGCTTCCTTTC
+
991466566+/,///+/5657;?88??????<====766=<<:776,1366/9::96//----
13000437=<?BDAA@=844443<6;>@5///75+,,+,
@T6XXI:00109:00363
GGGGAATTCTAATACGACTCACTATAGGGAGAGGGAGAATAGTCAACAAATCGTTTGGGGCGACTTCTCCTTCCTTT
CTCCCTTCTCCC
+
DDC9B@C@DBB?ABBBBAAAB@<97111.777?A?C@@>AAAA??<?AA<CBBAA8???5?AABE@BBB=<8;;>?<
?BBC>A;==AA=
@T6XXI:00109:00364
GGGAGAAGGGAGAAAGGAAGGAGAAGTCCCCCCAAACGATTTGTTGACTATTCTCCCTCTCCCTATAGTGAGTCGTA
TTAGAATTCCCC
+
DIDCCE@DC>@@AC<@=@>A9;;@@CCCCDCC-
CD?CCC?@;@@>@@>?BB?@@AC<@898?7>BAA@??@A?==@@@?AAA?A<?>>5
@T6XXI:00109:00366
GGGGAATTCTAATACGACTCACTATAGGGAGAGGGAGAATAGTCAACAAATCGTTTGGGGCGACTTCTCCTTCCTTT
CTCCCTTCTCCC
+
499-49@AGBC@BBBBB@@@B@<97166.7>>BE@?<<>AAA=<;;784-
7777>6=<<2?@@AB?BBB?@>A>AA=AAAA<A;==@D<
@T6XXI:00109:00374
GGGAGAGGGAGAAAGGAAGGAGAAGTCGCCCCAAACGATTTGTTGACTATTCTCCCTCTCCCTATAGTGAGTCGTAT
TAGAATTCCCC
+
CFCCBBCC>B@@@=A@C@?>???@?@BAAAA8AC>@AB??;??>?>>?CC><9<=;=<<<=;=<<<==<;;<AAAA@
>B<<792444-
@T6XXI:00109:00380
GGGGAATTCTAATACGACTCACTATA
+
:A@4@69648;28////.9225....
@T6XXI:00109:00390
GGGGAATTCTAATACGACTCACTATAGGGAGAGGGAGAATAGTCAACAAATCGTTTGGGGCGACTTCTCCTTCCTTT
CTCCCTTCTCCC
+
CAB9A===@BB@BBBBBBBBB@B97111.7<7777:AA>AA@@@@?@<<;@><99888?6?BBBC?A?A7837=AB<
?@@C<A>A???;
@T6XXI:00109:00398
```

```
CTAGTCACTCGAGATCTGCGGCTTTGGACAGTCCCTCTGTACTGCGAAGCCACAGATGTCGAGTCGACTAGCTTCCT
TTCTCCCTTCTCCC
+
99444444494449@CBBCB>88808<>>@BBBC>BCDD?>>?1/.777B@BBBBBCCBBAAACDCA@@@??<>>=?
B.70384328000,
@T6XXI:00110:00351
GGGGAATTCTAATACGACTCACTATAGGGAGAGGGAGAATAGAGACGTATTCGTTTGCGCTCTGTTCACCTTCCTTT
CTCCCTTCTCCC
+
CGECEBA@ACCACBAABBCBB@<<;667.7?>>@>A@@>A>99=A???@@?@?8;:8889>;AAB>A=<9<<?<@A?
AAA@;>:==<>:
@T6XXI:00110:00358
GGGAGAAGGGAGAAAAGGAAGCTGAGT
+
:<14A@599199999/96940,,,,,,
@T6XXI:00110:00359
GGGGAATTCTAATACGACTCACTATAGGAGAGGGAGAATAGCGCACCATCAATTTTGTCGTGCCGTTCTCCTTCCTT
TCTCCCTTCTCCC
+
@@>4><@=BBB@DB@?BBBBA:99:/.,0==AC?CAB=????AABA?ABA?=AAA7ACCCBBC@CCB@B=;=@C>AB
?@@@C<@=>??C>
@T6XXI:00110:00361
GGGAGAAGGGAGAAAGGAAGGAGAAGTCGCCCCAAACGATTTGTTGACTATTCTCCCTCTCCCTATAGTGAGTCGTA
TTAGAATTCCCCC
+
BB>BBC@DD>BCCC?B@B?>>>BB@A@ABB@<7AA<ABCCC>CC?CAA?AA?=AA=:@AA@@;@???@AAAAA====
==??922,+,,,)
@T6XXI:00110:00368
GGGAGAAGGGAGAAAGGAAGGAGAACCGCAATTGTCCAAACGTGGCAGCTATTCTCCCTCTCCCTATAGTGAGTCGT
ATTAGAATTCCCC
+
CB@AAA?BB>BEA?=@=>=>@BAABB?BCB@C@B;<<@@<@CED@BCCCBBB@BBBB@BBBBA=A@BBB@AABB???
??=?CC>A<???5
@T6XXI:00110:00370
GGGAGAAGGGAGAAAGGAAGTGCAGTTCGCGGTTAGTGAAGGGAAAAACTATTCTCCCTCTCCCTATAGTGAGTCGT
ATTAGAACTTCCCC
+
AB>AA@@CC@CCB?=B;99:IACDCC@BBBB@C@@@@CC@CC<AAAA2AACB@BBBCACCAAA<A?@@AAAAAA??8
99>@BABAA;>>=0
@T6XXI:00110:00380
GGGAGAAGGGAGAAAGGAAGCTAGTCACTCGAGATCTGCGGCTTTGGACAGTCCCTCTGTACTGTGAAGCCACAGAT
GTCGAGTCGACTAGCTTCCTTTCTCCCTTCTCCC
+
:B>@><8<B:?;;9194958888A@A9988::97A>8?@A?A@B=B@BCBBBCB9?>;44444358>;?A>A????@
@@BA@AA@@<<<;;;;8<9<A<@@@A;@688AA7
@T6XXI:00110:00382
GGGGAATTCTAATACGACTCACTATAGGGAGAGGAGAATAG
+
8880=:A@CCB@BCCBA???>?BC?==<8:?757;+*)*))
@T6XXI:00110:00387
GGGGAATTCTAATACGACTCACTATAGGGAGAGGGAGAATAGTCAACAAATCGTTTGGGGCGACTTC
+
?DD:C?B=@=>;>>?AA;99B?997161,177=;2;4425556688;=C1:6666266621....,.
@T6XXI:00110:00389
CTAGTCACTCGAGATCTGCGGCTTGGACAGTCCCTCCTGTACTGTGAAGCCACAGATGGACTGTCCAAAGCCGCAGA
TCTCGAGTCGACTAGCTTCCTTTCTCCCTTCTCCC
+
```

```
;/1////4444499;@==**)**)*)*000444266011116693335:3969?733BB7793976011,110118:<
A==<??<<@BACBCDCC?B?BB?AACEAH<==<A:
@T6XXI:00110:00391
GGGGAATTCTAATACGACTCACTATAGGGAGAGGGAGAATAGTTCGCTATCTTCCAGCGGCATGTTCCCCTTCCTTT
CTCCCTTCTCCC
+
BAA7A8;;<CCCDB>>ABBBBA>97111.7==>A<A@@>@A@A;==@@BBBC@C?CBBB?BCADC>@@@0837<@@<
@?AC>B388<<7
@T6XXI:00110:00392
GGGAGAAGGGAGAAAGGAAGGAGAAGTCGCCCCAAACGATTTGTTGACTATTCTCCCTCTCCCTATAGTGAGTCGTA
TTAGAATTCCCC
+
DDBCCC@BC>@A??=B?B@B@CCC@AAACCBB4:7/8?=9?=BB?BCC???>@AAC???=<<7<?@@@@@DA???@@
A?AAA=?8<<;1
@T6XXI:00111:00350
GGGAGAAGGGAGAAAGGAAGCTAGTCACTCGACATCTGTGGCTTCACAGTACAGAGGGACTGTCCAAAGCCGCAGAT
CTCGAGTCGACTAGCTTCCTTTCTCCCTTCTCCC
+
C@@B<<;<@:>999194958@;77>=77111111;?@@@@?CB>AAB??ACB@A??>7<<>?@A>@@;@A=??@@AA
?@@???@@@@A@@?@@:=;=D=AAAD<@8784:4
@T6XXI:00111:00360
GGGGAATTCTAATACGACTCACTATAGGGAGAGGGAGAATAGATCACCGTTGGTTCGGGTGCGCGTTCTCCTTCCTT
TCTCCCTTCTCCC
+
DKK:C@C@AAC@DCCBAABCC?@@BBBB>BB>;:9=//,/://4@::5:?<?5;;<????333???;@?A>B=@A?C
<A===7<@@;;;6
@T6XXI:00111:00362
CTAGTCGACTCGAGATCTGCGGCTTTGGACAGTCCCTCTGTACTGACGAAGCCACAGATGTCGAGTCGTACCTTA
+
>2,---,,68:BCBBBBABBA?ABB>B>AAD??AA<A?7:711111111100924446.......11444331313
@T6XXI:00111:00364
GGGGAATTCTAATACGACTCACTATAGGAGAGGGAGAATAGATCACCGTTGGTTCGGGTGCGCGTTCTCCTTCCTTT
CTCCCTTCTCCC
+
999/49A?AAB?CBBBCCAAA:=6:/.,0;:==<?AA>@@@??=:607?3747;>>@=@;;;AAA@CAA??5:47>7
>:<;7<>===C=
@T6XXI:00111:00369
GGGAGAAGGGAGAAAGGAAGCTAGTCGACTCGACATCTGTGGCTTCACAGTACAGAGGGACTGTCCAAAGCCGCAGA
TCTCGAGTCAACTAG
+
CBAA?=@CB=A994-
9;><@BA=>>/10>000:=<716@@B?CG@CC???@?844000,4:::?@:==7=A=@@@@@@A@@A@@==<;<???
@T6XXI:00111:00384
GGGAGAAGGGAGAAAGGAAGAATAAGATCGGGACGGCCAGGCGGCATCTATTCTCCCTCTCCCTATAGTGAGTCGTA
TTAGAATTCCCC
+
EF@AAA5888;?@B<@>ACAF?DBCCCBBBB>BBB@C@BB>BB@CEDC::8;===@69;88817@>@A@@@@A????
?<?CC?C=@@@5
@T6XXI:00111:00387
CTAGTCACTCGAGATCTGCGGCTTTGGACAGTCCCTCTGTACTGTGAAGCCACAGATGTCGAGTCGACTAGCTTCCT
TTCTCCCTTCTCCC
+
B?999=444>===?BB@@@C?BCC?C?BBCBBBC>C@???B?@?@@C>@C?BCDCCBBBCCCD@AA<;;@<<??B?B
E8<:CB583835A=
@T6XXI:00111:00397
GGGAGAAGGGAGAAAGGAAGGAGAAGCGACGTCGGGGAAACGTTTGCTATTCTCCCTCTCCCTATAGT
+
```

```
9904499:;6949914999?<83311399?<>=@@@7CA?C:44-4944994444-48433,3.....
@T6XXI:00111:00398
GGGGAATTCTAATACGACTCACTATAGGGAGAGGGAGAATAGTCAACAAATCGTTTGGGGCGACTTCTCCTTCCTTT
CTCCCTTCTCCC
+
CDC:C?CABCD?BCCCC?BBB@@<6166.6==?A>BAA>@@CAAA>=<84<?777.777-
7CCCCAA@@=<5=;=>7?@>=:A8:8DD<
@T6XXI:00112:00352
GGGGAATTCTAATACGACTCACTATAGGGAGAGGGAGAATAGATCACCGTTGGTCCGGGTGCGCGTTCTCCTTCCTT
TCTCCCTTCTCCC
+
?=94><>=@BB@CBBBBCCBBCA<7166.7>>@C>C@A@A=888=CC@CC?C?BC@FC>C:::CBA>B??=?@C?CK
>:;89497444;7
@T6XXI:00112:00354
GGGAGAAGGGAGAAAGGAAGCTAGTCGACTCGACCATCTGTGGCCTTCACAGTACAGGACGGATCCAAAGCCGCAGA
CTCTCGAGTCGACTAG
+
;B<@@@?A@<?A>>0969<88377676117716701111711010371 67;;77++*.***)*++*4;7;@@4////
///88:BDD@AA@B=9
@T6XXI:00112:00356
CTAGTCGACTCGAGATCTGCGGCTTTGGACAGTCCCTCCTGTAA
+
BA?=9944?AACCCCCBA;;;58AC<@>A>91177,1.,....,
@T6XXI:00112:00371
GGGGAATTCTAATACGACTCACTATAGGGAGAGGGAGAATAGTCTTCGCTAATCACCATGCCCG
+
?=92>;?<>995994>@A>??>887776,711676600/01----,-----,0004569=>@27
@T6XXI:00112:00386
GGGGAATTCTAATACGACTCACTATAGGGAGAGGGAGAATAGTTTTTCCCCTTCACTAACCGCGAACTGCACTTCCT
TTCTCCCTTCTCCC
+
999/48?;>9;8<CCCCCBB77871111,177;@=@<@===<>>>;.999-
434?>7747<===<9<AC@AA?5:57<6=>??<A?@@AA<
@T6XXI:00112:00394
GGGGAATTCTAATACGACTCACTATAGGGAGAGGGAGAATAGTCAACAAATCGTTTGGGGCGACTTCTCCTTCCTTT
CTCCCTTCTCCC
+
CCC9C>@>ACC@C@@@BCCCBBCB?77659??AA>AAA>@=@?A?>AAC<AAABC>BCC9BCCCC>?@C??=?>@D<
AAAA<@<??@@;
@T6XXI:00112:00396
GGGGAATTCTAATACGACTCACTATAGGGAGAGGGAGAATAGTCAACAAATCGTTTGGGGCGACTTCTCCTTCCTTT
CTCCCTTCTCCC
+
?A@4@6959>@;AA@ABBCCCCB>=777.7>777.7;;:?@@@>>34/820..45+.//*1771621660024:;;:
;444-4/43=>7
@T6XXI:00113:00351
GGGAGAAGGGAGAAAGGAAGTGCAGTTCGCGGTTAGTGAAGGGAAAAACTATTCTCCCTCTCCCTATAGTGAGTCGT
ATTAGAATTCCCC
+
CDBCEB@BA:=@@@<@>C?BBBBCCB@BBB?>A<@@AA><=;6<==?0BFCC@CCCC>B@CCC>B=<@@AAAAA===
??<?CC?A=AAA1
@T6XXI:00113:00353
GGGGAATTCTAATACGACTCACTATAGGGAGAGGGAGAATAGTCATGTGCCTCCCGACGTGACATTCGCCTTCCTTT
CTCCCTTCTCCC
+
CCC9CAD@BCB@AAAAA>@@@A?97111.7==BD>B@@CB=@<<<==A@?=?AB=A???AACCCC@CA@>@@C@CC>
BBBC>C@AA@A<
```

```
@T6XXI:00113:00363
GCTAGTCACTCGAGATCTGCGGCTTTGGACAGTCCCTCTGTACTGTGAAGCCACAGATGTCGAGTCGACTAGCTTCC
TTTCTCCCTTCTCCC
+
/,,,,///44999<@AAAB>>;888,7133>>@AA===BAA?;::>5556?>??CBC><<BCCBAA@@???A??:?:
==6777>68/40445
@T6XXI:00113:00366
CTAGTCACTCGAGATCTGCGGCTTTGGACAGTCCCTCTGTACTGTGAAGCCACAGATGTCGAGTCGACTAGCTTCCT
TTCTCCCTTCTCCC
+
D:11/1/49BBCCACBBBBB@BBB?B?A@;;66:5:BBCCBB>;8>A>?@?BCBBB@@?@@B@C@@@CCC@??@C@E
C>CBDB<?<<<<@;
@T6XXI:00113:00368
GGGGAATTCTAATACGACTCACTATAGGAGAGGGAGAATAGTTTTCCCTTCACTAACCGCGAACTGCACTTCCTTTC
TCCTCTCC
+
@@?4@<@?ABB?BCCCCCB@@;;;://,/9:=BCB@??AAAA=A5@?<?9=?BCD@C@B?A?;>>=;??A=A?AB9?
0/,0.443
@T6XXI:00113:00382
GGGAGAAGGGAGAAAGGAAGTGCAGTCG
+
CHACCC@CC>CB:::<,1,00.///444
@T6XXI:00113:00387
GGGGAATTCTAATACGACTCACTATAGGGAGAGGGAGAATAGTACACA
+
AAA9B>B>BCC@CBBBCBB>BA?97111.7==BC>B@;;90+,,++0.
@T6XXI:00113:00389
GGGAGAAGGGAGAAAGGAAGCTAGTCACTCGACATCTGTGGCTTCACAGTACAGAGGGACTGTCCAAAGCCGCAGAT
CTCGAGTCGACTAG
+
DDA@?>===9@@==<@=@<@BA@A?9?????;;:BCBBBC?AB@BBBBBB@?9;33717A?AAA=@@<@A>AA=<<=
===???A@@@@BAA
@T6XXI:00113:00390
GGGAGAAGGGAGAAAGGAAGGTGAACGCGCGGTTGGCGATATGGGAGCTATTCTCCCTCTCCCTATAGTGAGTCGTA
TTAGAATTCCCC
+
BBBBCBCCB@>??B>B@B@B@BBB@BBBBBB@B@B>A?BBCC?@;B?;ABBBBAAA>?@BBE>BBA?AA====<===
?>@@@=@8;<;1
@T6XXI:00113:00394
GGGGAATTCTAATACGACTCACTATAGGGAGAGGGAGAATAGTCAACAAATCGTTTGGGGCGACTTCTCCTTCCTTT
CTCCCTTCTCCC
+
999/9:@@CBC@C@AACCCABB>96166.7==>B?BDD@D>>?<<>?==;B???A<A?A7BBBBC@@@@??<?<@@<
@@AC<A:==??:
@T6XXI:00113:00397
GGGAGAAGGGAGAAAGGAAGCTAGTCACTCGAGATCTGCGGCTTTGGACAGTCCCTCTGTACTGTGAAGCCACAGAT
GTCGAGTCGACTAGCTTCCTTTCTCCCTTCTCCC
+
DCCCCC@BB@C;94-
9<@>ABBCC:::?88??:78?@CCC>BBB=B>?@BB?<?8?<<??A?@===@?AA>@?@@@===A@@AAA=99=A??
?>A<?<;?@@@;@<??A@<
@T6XXI:00114:00355
GGGGAATTCTAATACGACTCACTATAGGGAGAGGGAGAATAGTCAACAAATCGTTTGGGGCGACTTCTCCTTCCTTT
CTCCCTTCTCCC
+
@?@6@=@>ACCACCA@ABBBB@BC@><<;<?:@A=?<A?AAA===>?@@;A?;;;,666-
7<??B@BBB==;=?A=AC@@@;@>A@@@;
```

```
@T6XXI:00114:00358
GGGGAATTCTAATACGACTCACTATAGGGAGAGGGAGAATAGCTATCGTCACTTGACATGGTGCTTCTCCTTCCTTT
CTCCCTTCTCCC
+
BE@7A=@=@BB@BCCCBABAA<;<=666.7>>AC>@==>A=@@BBA@B@@@@A>A888AA>A;:;49//,.060144
74==1834;;=7
@T6XXI:00114:00376
GGGAGAAGGGAGAAAGGAAGGAGAACATGACCCGGTACGATTGGTGTCTATTCTCCCTCTCCCTATAGTGAGTCGTA
TTAGAATTCCCCC
+
CDCCBA694-
;AAA=B?B@B@CCB@BBBBBBB>B=CBBBBB@B@8::@BBB?BBBB>BCAAC;;;?<<<==AA;=:??=?@>94/34
44,
@T6XXI:00114:00377
GGGAGAAGGGAGAAAGGAAGGAGAAGG
+
GHDCCB@CCBBB;;5::6,0,0;;67.
@T6XXI:00114:00382
GGGAGAAGGGAGAAAGGAAGGAGAATGCACCGCAGGAAATTGCAGGACTATTCTCCCTCTCCCTATAGTGAGTCGTA
TTAGAATTCCCC
+
BB>CCC@BB>BBBB>BAB?A@B@A?BCCCA?ABBB@>?;?>B????>@@@@>AAAA;<<AAA;<<<<<<<<@AAAAA
@>@??<?>A@@5
@T6XXI:00114:00386
GGGGAATTCTAATACGACTCACTATAGGGAGAGGGAGAATAGTCAACAAATCGTTTGGGGCGACTTCTCCTTCCTTT
CTCCCTTCTCCC
+
<><5<BKCCAC@CCCB@>ABBCB@969959CBCC>?=<>@=AA@@=<<<7?BBCE>CCC9CBAAA?AAA?@>A?AA;
@@AA:@599@@<
@T6XXI:00114:00389
GGGGAATTCTAATACGACTCACTATAGGGAGAGGGAGAATAGTTTAGCACGTTACCTCGGCCTATTTTCCTTCCTTT
CTCCCTTCTCCC
+
AFIAF::;;DC@DBAABBAAA@<97111.7==>><AAA>BA@@@<@=ACCCC@CC=??C@E?CBBBB7B@C@C@FC<
?000,/,//661
@T6XXI:00114:00399
GGGGAATTCTAATACGACTCACTATAGGGAGAGGGAGAATAGTCAACAAATCGTTTGGGGCGACTTCTCCTTCCTTT
CTCCCTTCTCCC
+
CDD:D?B?ABBACCBBBBBBB@CB<:<:8:AACC>CAA=A=AA;87444,7?=<=.777.7==?D?AAB?A>A=??<
?@AA<A?A@A@<
@T6XXI:00115:00352
GGGAGAAGGGAGAAAGGAAGCTAGTCACTCGACATCTGTGGCTTCACAGTACAGAGGGACTGGTCCAAAGCCGCAGA
TCTCGAGTCGACTAG
+
BB@CCC?B@:=;44-
;886;CC@BC<:;:??;::BBBDBD@AB>=?@@@?@B<:888:899=<;C64417?<@A<=<A@A@=::?@A@@@>?
@T6XXI:00115:00359
GGGGAATTCTAATACGACTCACTATAGGGAGAGGGAGAATAGTCAACAAATCGTTTGGGGCGACTTCTCCTTCCTTT
CTCCCTTCTCCC
+
999/9<@@BBB@CBBBBBBB@A@97111.777?B=A??>AA@@@C>==63;=666-
777.7@@BC@BBB<;8;8;;7=AAABA<?=<<7
@T6XXI:00115:00384
GGGGAATTCTAATACGACTCACTATAGGGAGAGAGATATAAGGGAGTTCTCTCTC
+
>?;6>>D@CCC@BBBBCAAA?8>>=777.7?>@A?A@<::5885//-494530.,
```

```
@T6XXI:00115:00387
GGGAGAAGGGAGAAAGGAAGAATACAAAACGTGTCGAGATAAGAACGGCTATTCTCCCTCTCCCTATAGTGAGTCGT
ATTAGAATTCCCC
+
AA>BAA=@B>BCCC>C?B?BC=9?9844,9@??ACCCCCCC@::8<B>BDCDBBBBB@BECAC>A@BBBBCCCA@@=
==;=<B;4/304-
@T6XXI:00115:00394
GGGGAATTCTAATACGACTCACTATAGGGAGAGGGAGAATAGAGTACAGAACCTTTCGTGCATTCCCACTACTTCTT
CCTTTCCTCCCTTCCTC
+
7>>6>?A?BCCCDC???>>?B@BB:5576.//00,067796,,++++-03375775....--
1034+03309987864::=;<;33000*+*++
@T6XXI:00115:00396
GGGAGAAGGGAGAAAGGAAGAATAGAGAAAGTCTGGGAAAATAGCTGACTTCTCTTCCGTCAAGGCTAAACTCCTTC
CTTTCTCCCTTCTCCC
+
9909?><AC>BCCCAC@CABC@C<??:::5:BBBDC?EDD9D==@CCDDC@<<<C>B>AAAC@C@CCFI@AAB>A?A
=@@<@ACCACBA@@EB
@T6XXI:00116:00350
ACTCGAGATCTGCGGCTTTGGACAGTCCCTCTGTACTGTGAAGCCACAGATGTCGAGTCGACTAAGTCTTTC
+
<=<BCA@A@@ACED@BBDBFABBBBBBEAFCCCCBAAAAHB@CD>?@CBCB<<=@@@CB@></0,..,,,*.
@T6XXI:00116:00351
GGGAGAAGGGAGAAAGGAAGGCAATGTGGGCGGAAGGAGCGCTAGGCTATTCTCCCTCTCCCTATAGTGAGTCGTAT
TAGAATTCCCC
+
EDDECB894-
99887<ACAC@CC?B>>?C>BB>BA?>B>@B>===;<A@B?<??B>@AACC<@<<<AAAAAA@@@7757=@:?9<<<
4
@T6XXI:00116:00352
GGGAGAAGGGAGAAAGGAAGCTAGTCACTCGACAATC
+
BC?<=<@CC@BBCA=F:;8;B@>>=7////,,,,*,,
@T6XXI:00116:00359
GGGAGAAGGGAGAAAGGAAGAACACACGGCCGTCGGAGAGCGCAGAGCTATTCTCCCTCTCCCTATAGTGAGTCGTA
TTAGAATTCCCC
+
CCACCB@CB>BBBC?B>AADD@@BBBBC@C@CCDC;<=HBCCCBBBB?<AB@B>>><@B@BCBCCCCBBBCBBC@@?
B?BCB@B@CCC:
@T6XXI:00116:00365
GGGGAATTCTAATACGACTCACTATAGGGAGAGGGAGAATAGTCTTCCCTTCACTAACCGCGAACTGCACTTCCTTT
CTCCCTTCTCCC
+
CCB7A899<BCBDCB?BB??B@B?966659??BE<B???A=====<=<;<<=@=<<;<::<<=<:<=99=?>A:?@7
==?B<A<??AC<
@T6XXI:00116:00366
GGGAGAAGGGAGAAAGGAAGGAGAAGTCGCCCCAAACGATTTGTTGACTATTCGTCCCTCTCCCTATAGTGAGTCGT
ATTAGAATTCCCC
+
490444249-
448858789;<>>B>==>8888.77/36667286=:;:;894/..444477<=;::=?878989355565333/0/0
00+
@T6XXI:00116:00377
GGGGAATTCTAATACGACTCACTATAGGGAGAGGGAGAATAGTCAACAAATCGTTTGGGGCGACTTCTCCTTCCTTT
CTCCCTTCTCCC
+
```

```
9?>2=<AACCD@BBBBCAAA>:?<;677.7==AB?B??>B????@>@@@:?=776-
777.:@ABCAAAD@A?@?AD?D==<7<8;;@@;
@T6XXI:00116:00379
GGGAGAAGGGAGAAAGGAAGGAGAAGTCGCCCCAAACGATTTGTTGACTATTCTCCCTCTCCCTATAGTGAGTCGTA
TTAGAATTCCCC
+
CC@@@@@BB=@@@ABB@B>A?AAA?BBCBB>>6BB>BABBC@CC?C??=??<?AAA====?@:@AA@@@@@==<???
?<<AA:42444-
@T6XXI:00116:00380
CTATCACTCGAGATCTGCGGCTTTGGACAGTCCCTCTGTACTGTGAAGCCACAGATGTCGAGTCGACTAGCTTCCTT
TCTCCCTTCTCCC
+
AABA;::@CCHCCCBBBAA>ABB>B?AA@@>AB=ABCA>=AC=AA<;;A?BCCBBAAACCCC@ABBBCBBBB?B>BB
>BCAA@A=??AA<
@T6XXI:00116:00392
GGGGAATTCTAATACGACTCACTATAGGAGAGGGAGAATAGTTTCCGCCGATCGAAGCTAATTTTTCCCTTCCTCTT
CCTTTCTCCCTTCTCCC
+
CEC:C8;8<?@=AAABCCCBC>>=7//,0>;>@<A?@;==;;;6;;=@>=@@DCD@;;:C@DDDD2AA<A>@=@@=;
=8<:4<==?:?=@???:
@T6XXI:00116:00394
GGGGAATTCTAATACGACTCACTATAGGGAGAGGGAGAATAGACTAC
+
CCC9C?B?BCCACCCBB?BBAAAB>669-6<<=>9@88245,,+*(,
@T6XXI:00116:00396
GGGAGAAGGGAGAAAGGAAAGCTAGTCACTCGACATCTGTGGCTTCACAGTACAGAGGGACTGTCCAAAGCCGCAGA
TCTCGAGTCGACTAGCTTCCTTTCTCCCTTCTCCC
+
CC>A@>5991999904244-
3779940/......../699>>AC?ABC?@@A@>:77717;AAA@=AC:AA=A@===@@@@???AA=====@?@8<9
;=7:86658.//5:5
@T6XXI:00117:00354
GGGAGAAGGGAGAAAGGAAGGAGAAGTCGCCCCAAACGATTTGTTGACTATTCTCCCTCTCCCTATAGTGAGTCGTA
TTAGAATTCCCC
+
CC>CCC>?>:@@===B?B?C@CBB@B@A;7<<-
786;ABDE;??ABBB===?A@@@<@@@@A;@@@@A<<<<<===AA>A@?:;3444+
@T6XXI:00117:00374
GGGGAGAAGGAGAAAGGAAGGAGAAGTCGCCCCAA
+
/11*//9242444;-42477133791133333,33
@T6XXI:00117:00376
GGGAGAAGGGAGAAAGGAAGCTAGTCACTCGAGATCTGCGGCTTTGGACAGTCCCTCTGTACTGTGAAGCCACAGAT
GTCGAGTCGACTAGCTTCCTTTCTCCCTTCTCCC
+
CC>B<<89@:?111,/,/,..7668;::CCAAA?AB@?@A>BBB>B@CCCBBBE>C==<@?<899AA?A@>AA@??8
88?@>===74479;9;9;38818;;;6<9<99;4
@T6XXI:00117:00380
GGGGAATTCTAATACGACTCACTATAGGGAGAGGGAGAATAGTCAACAAATCGTTTGGGGCGACTTCTCCTTCCTTT
CTCCCTTCTCCC
+
DECCC?B?BBB@B@?@CCDC?<?<;677.7>>AC=@=BBB???@@>@AA<CAA@A9???5?ABCC@CCC@=;<<>;9
;444-4/43>A:
@T6XXI:00117:00382
GGGGAATTCTAATACGACTCACTATAGGGAGAGGGAGAATAGTTTTTCCCTTCACCAACCGCGAACTGCACTTCCTT
TCTCCCTTCTCC
+
```

```
999/98@BCBB?CBBB@?@@@?BBB???9?AACC>B@@????@@@?1@A<A>?B@?@>?>AABB?BAAAC@@>@@CC
>CCBE>?>BCCC
@T6XXI:00118:00357
GGGGAATTCTAATACGACTCACTATAGGGAGAGGGAGAATAGTCAACAAATCGTTTGGGGCGACTTCTCCTTCCTTT
CTCCCTTCTCCC
+
;<<4<?C@DBB@BBBB@?@BC?A?=611.7<<<?<?AAEB@@@@@=?AC=CBB@@>CCC9BBCBB@@CC5737:==;
?@@C=A?@@@A6
@T6XXI:00118:00358
GGGAGAAGGGAGAAAGGAAGCTATCGACTCGACATCTGTGGCTTCACAGTACAGAGGGACTGTCCAAAGCCGCAGAT
CTCGAGTC
+
CB?BBB?BC>BCBB?B?B?ABBBC@@;::@>:7:::7>>B;?@=?CCA@?B?8833414?;A>@8;;5;;8<@A?@?
A@@@<<=<
@T6XXI:00118:00359
GGGAGAAGGGAGAAAGGAAGCTAGTCGACTCGACATCTG
+
AA>ADCACC>@@@>9>=>=>??=;;1/0=@B>1;:A??@
@T6XXI:00118:00364
GGGGAATTCTAATACGACTCACTATAGGGAGAGGGAGAATAGTCAACAAATCGTTTGGGGCGACTTCTCCTTCCTTT
CTCCCTTCTCCC
+
CCC9C9;<<CDBCA>>A?BBB@<97116.7???@>C@@>A;@@BA>@@@<BAAAD?CCC9C::;D?BBB?A>A>@@<
@AAA;@=@@AB=
@T6XXI:00118:00367
CTAGTCGACTCGAGATCTGCGGCTTTGGACAGTCCCTCTGTACTGTGAAGCCACAGATGGACTGCTTCCTTTCTCCC
TTCTCCC
+
=A?ACCCFDDC<;<BBBBEDD@BA@6:7;ACCBCD>:;<DKA?@A@=A;AC@@?AA<77497>3768?:?B<???C>
C>;;8C7
@T6XXI:00118:00369
GGGAGAAGGGAGAAAGGAAGCTAGTCGACTCGACATCTGTGGCTTCACAGTACAGAGGGACTGTCCAAAGCCGCAG
+
CCB@@?@CC@@@@@<@>@8;;C:;;CCCE>>>//00/0<:=5AB?BABACBA999344-4;;=?A>AC<AA<>??A
@T6XXI:00118:00386
GGGGAATTCTAATACGACTCACTATAGGGAGAGGGAGAATAGTTGTAACCATTTCCCTGCCAACGTATCTCTTCCTT
TCTCCCTTCTCCC
+
999/4:CABBB@CCC????B?@BB;66659?;BD>?<<>@@AA?A??<?>@BB>BB>BAA>A@BBA???=<<?A>@A
<@???:?<=???:
@T6XXI:00119:00363
GGGAGAAGGGAGAAAGGAAGCTAGTCACTCGAGATCTGCGGCTTTGGACAGTCCCTCTGTACTGTGAAGCCACAGAT
GTCGAGTCGACTAGCTTCCTTTCTCCCTTCCTCCC
+
CDDCCD@CB?>999142823??ABBCBCCBB@?<?B:;;C@CCD?B=@@CCCFC>A??AA????@@D>@@<??@@@?
===@@@A??=;788<<;;<?@80/9917,/,.110
@T6XXI:00119:00367
GGGAGAAGGGAGAAAGGAAGCTAGTCGACGCGACATCTGTGGCTTCACAGTACAGAGGGACTGTCCAAAGCCGCAGA
TCTCGAGTCGACTAG
+
BC>AAA@CCCBBDC=B>B?CB>9;::;;ABC@;::?????A=?@=?BBBCBCCA>=<::8:;<@<;==>AA?????@
???@AA@@@@@AA??
@T6XXI:00119:00369
GGGGAATTCTAATACGACCTCACTATAGGGAGAGGGAGAATAGATCACCGTTGGTTCGGGT
+
999/<<A@CDB@B?;;43488?=>D@:::67>=<?9?===@AAAAAAA;@B5:7:>ABD@B
@T6XXI:00119:00376
```

```
CTATCGACTCGAGATTCGTTGGAC
+
CCCCCC:>=ECC>>?;,-,*,***
@T6XXI:00119:00388
GGGGAATTCTAATACGACTCACTATAGGGAGAGGGAGAATAGCAGACCG
+
:99/9:B?BCCBCCBBBBB>BBB97111.:>>BD<@<@>;99/////,,
@T6XXI:00120:00363
GGGGAATTCTAATACGACTCACTATAGGGAGAGGAGAATATACGTCGCAGTCTGACCTCACTTTCTCCATCTTC
+
ECFBD@B?BBCABBACBBBBAAAB>8?;6;<774:==8;9//./------0000049977333,3110111.,.
@T6XXI:00120:00364
GGGAGAAAGGGAGGAAAAGGGAAAGCTA
+
675///8+491485888,44-44-3333
@T6XXI:00120:00380
GGGGAATTCTAATACGACTCACTATAGGGAGAGGGAGAATAGTTTACCG
+
8870=+9698>594@9<:@>A?@<;767-771==6=;8427141+--,-
@T6XXI:00120:00391
CTCGAGATCTGCGGCTTTGGACAGTCCCTCTGTACTGTGAAGCCACAGATGTCGAGTCGACTAGCCGCAGATCTCGA
GTCGACTAGCTTCCTTTCTCCCTTCTCCC
+
DC<==CCCCDBBC@BBB>B@DCBC@A?<@BBBCCCCCCCB?BB@CCCCCCC8:;BBBBC::;?;:6;B@@@CCCBBC
DC@A@BAA;;;@B?BB7::@A<@6::??9
@T6XXI:00120:00393
GGGAGAAGGGAGAAAGGAAGTGCAGTTCGCGGTTAGTGAAGGGAAAAAACTATTCTCCCTCTCCCTATAGTGAGTCG
TATTAGAACTTCCCC
+
CC?BBB@CC=B>==:@>B>ACCCCBA@???B@D>??@BB=>=-
67777*8?;>./1>@9011??9?==@????===488;387A?A@<@BC6
@T6XXI:00120:00395
GGGGAATTCTAATACGACTCACTATAGGGAGAGGGAGAATAGTCACTCCCACTAGCTTGTCATACTCGCCTTCCTTT
CTCCCTTCTCCC
+
::9/9<@@BBBACBCCCCBB>=?B?8:?;???CE>C??<?=A?;:<@A=<===?<?B?BCAA?BBBCBB>C?C?@@<
@AAA<@?AAA=;
@T6XXI:00120:00396
GGGGAATTCTAATACGACTCACTATAGGAGAGGGAGAATAGCCACCAACTATTGTTCGTCGCCGTTCTCTTCCTTTC
TCCCTTCTCCC
+
@@C8A@C@CDC@BBBABBCBB7664./,/;;>B>C@@>A;??>@@<?<????>@A>@AAA?<;@=99=AC=?>?C<?
??=5878::96
@T6XXI:00121:00350
GGGGAATTCTAATACGACTCACTATAGGGAGAGGGAGAATAGCCCTTCTCAAGTATTCGCTAATCATGCCCTTCCTT
TCTCCCTTCTCCC
+
CCD:C?B@BCCDCBBBB@AABA;97166.7=<BC>CAA=?<A==:?=>>BE?CC?<9;>7::>@?@@DB?0:5:37?
:>@@@6=:=744-
@T6XXI:00121:00355
GGGGAATTCTAATACGACTCACTATAGGGAGGGGGAGAATAGTCAACAAATCGTTTGGTGCGACTTCTCCTTCCTTT
CTCCCTTCTCCC
+
CGE:D=?=?BB@CCBABBBBBACD=<??9:;BCC2BBB?CAA<<<<<@@>=8===;;<?A;;<==:=DC@A@A:<<6
;===:@>AACC:
@T6XXI:00121:00375
```

GGGGAATTCTAATACGACTCACTATAGGGAGAGGGAGAATAGTCAACAAATCGTTTGGGGCGACTTCTCCTTCCTTT
CTCCCTTCTCCC
+
;;<4<?B@CCB@BABCCBB@A<9A6166.777>A>A??>AA@@AB><<<7?AACCCCCC9CBAAC@???7868?ACA
ABBA<A<?@AA=
@T6XXI:00121:00397
GGGGAATTCTAATACGACTCACTATAGGGAGAGGGAGAATAGCTTCATATCTACCTCCACCTGTTCGCCCTTCCTTT
CTCCCTTCTCCC
+
BAB9B@D@BBB@AAAABBBB@@A>;6675:BBDC>BAA=?:;;@@==96894.112B;=?B@@@@BBB7-
95747?.7:@@9@;<=@@<
@T6XXI:00122:00350
CTATCGACTCGAGATCTGCGTCTTTGGACAGTCCCTCTGTACTGTGAAGCCACAGATGCCGAGTCGACTAGCTTCCT
TTCTCCCTTCTCCC
+
B?=999::DCCCCC<9<CB@?:;:8;@B???88806?>>>:::???C??C?BBB@@@AA?BB?@@A@BBA::::97;
?7<=AD=B;:444-
@T6XXI:00122:00355
GGGAGAAGGGAGAAAGGAAGGAGAAAGCGACGTCGGAGAAACGTTTGCTATTCTCCCTCTCCCTATAGTGAGTCGTA
TTAGAATTCCCC
+
DIACBA@BH@BBAA=B>B>B@BBBC>?@CABCCC?@DCBC@DAAA=BCCBBC@@BC>BBBBB=B@@@CCCCCCAB<A
?>A@@<>28880
@T6XXI:00122:00375
GGGAGAAGGGAGAAAGGAAGGAGAAGTCGCCCCAAACGATTTGTTGACTATTCTCCCTCTCCCTATAGTGAGTCGTA
TTAGAATTCCCC
+
CDACCD=@95=84809<A>ABCCC@CCD@@@@7@B<AA???9?C?BBBAAA<>77718>>>?;===AAAA???????
A?AAA?@3444,
@T6XXI:00122:00384
GGGAGAAGGGAGAAAGGAAGGAACAGGGAGGTCTGGAACCCAGAAGACTATTCTCCCTCTCCCTATAGTGAGTCGTA
TTAGAATTCCCC
+
CC?BBA=@@<@@CC?B?B@C?B?BECC>CA?@<?;:=59919>>=@BABCC?BCDE>CCBDB=ACBAAACCBC;;:@
?=?A@?=:AAA6
@T6XXI:00122:00396
GGGGAATTCTAATACGACTCACTATAGGAGAGGGAGAATAGCCGTTTAATCTCCCACTGCGGCATATCCTTCCTTTC
TCCCTTCTCCC
+
DDG:CECCBBB@CBBABBBBB:995//-
0>>BC>BAA>A8<52888,424?=?@<@B@???>A@@@??<?=@?AA<A@AA<@<?8881
@T6XXI:00123:00363
GGGGAATTCTAATACGACTCACTATAGGGAGAGGGAGAATAGTCAACAAATCGTTTGGGGCGACTTCTCCTTCCTTT
CTCCCTTCTCCC
+
999/49AAHBB@BAA??>>>B@CBA77757::@C>A??=@AA@<63774-
7?777.777.7>@BLB????@CA?AA=A?@?<@=??AA<
@T6XXI:00123:00366
GGGGAATTCTAATACGACTCACTATAGGAGAGGGAGAATAGC
+
BBB7@?@=<<;=?B@BABCCCCBB@::5:7:??,0//,-2++
@T6XXI:00123:00378
GGGGAATTCTAATACGACTCACTATAGGGAGAGGGAGAATAGTCAACAAATCGTTTGGGGCGACTTCTCCTTCCTTT
CTCCCTTCTCCC
+
999/98?<@BB>BCBBCBABBBBB@:::5:::CD>C@@>BAAAA@>=<;6?@@@A>ADF9DFBBB?AAA@@>@?CC:
>>?C<A;==AA<

```
@T6XXI:00124:00365
GGGGAATTCTAATACGACTCACTATAGGGAGAGGGAGAATAGTTGCTCG
+
///*17B@@@@>;;9;;B;>;BBA7777.77677-794446--,++0++
@T6XXI:00124:00375
GGGAGAAGGGAGAAAGGAAGAGAAGCGCAATGACCACGAGAGATTGGCTATTCTCCCTCTCCCTATAGTGAGTCGTA
TTAGAATTCCCC
+
CC@CEC@ECBCBBB?B@C@CBBC@CBBBB@B@@@>?@@AA?CB@?B===BA=@???;@CBC@;@<<<=<<><;?@A@
@>@AA>B?BDD8
@T6XXI:00124:00376
GGGAGAAGGGAGAAAGGAAGCTAGTCACTCGACATCTGTGGCTTCACAGTACAGAGGGACTGTCCAAAGCCGCAGAT
CTCGAGTCGACTAG
+
BC?CBB=>=5=844-
9594;BBABB>A@AAA:::BBBBBC=?@@C@A<<<A?8:33313;9?>@<??<?A>@@==<<<<<<<<==<<<<<<
@T6XXI:00124:00379
GGGAGAAGGGAGAAAGGAAGGAGAACAACACGATCGGTTAAACGTAAGCTATTCTCCCTCTCCCTATAGTGAGTCGT
ATTAGAATTCCCC
+
CC@CCC@BB>BBBB?A@A@?>;;>@BB@BAABBBBB@C@;;820**)***0002/./-
666BEAC@@AA@@?@@BA???;==A=?8<;<4
@T6XXI:00124:00385
CTATCGACTCGAGATCTGCGGCTTTGGACAGTCCCTCTGTACTGTGAAGCCACAGATGTCGAGTCGACTAGCTTCCT
TTCTCCCTTCTCCC
+
EB=:1//6679@AABCCCCC>>;;<>=??@BAAA>AAACCAC@AAAA>@C@CCCCCCBBBCCCBBB@@?B??;:=:=
?9?B@A:>8=444-
@T6XXI:00124:00386
GGGGAATTCTAATACGACTCACTATAGGGAGAGGGAGAATAGTTTTTCCCTTCACTAACCGCGAACTGCACTTCCTT
TCTC
+
AAA7A>B@CDB@BBBAA?ABBABD@@@A>@BBCD>B?@?B943344,44-
49:@ACB?B@ABAA>A???ABB?C?CC>B??
@T6XXI:00124:00393
GGGAGAAGGGAGAAAGGAAGCTAGTCACTCGACATCTGTGGCTTCACAGTACAGAGGGACTCGTT
+
BB><<<>@@:@99904244377779@???@@AA;::A@AA??@=?AAABC??;@884-/6////,
@T6XXI:00125:00354
CTAGTCGACTCGAGATCTGCGGCTTTGGACAGTCCCTCTGTACTGTGAAGCCACAGATGGACTGTCCCTTCCTTTCT
CCCTTCTCCC
+
CCBCCBBBCCCBBB???ABBB@???>@@BB@@@BC>BCCBBB@@@CAA?@C@CFBAA@A>AB@@88807<>=??:?:
:75;@@A?D<
@T6XXI:00125:00364
GGGGAATTCTAATACGACTCACTATAGGGAGAGGGAGAATAGCAAACGTTTCCCCGACGTCGCTTTCTCCTTCCTTT
CTCCCTTCTCCC
+
ABB9B>B@BCC@B>>AABB?>@>97111.7>>>><A@@>@A@@@@;@CBB=BBB8B???>>>?@A>@<<8;484884
898;7=>@@AA?
@T6XXI:00125:00386
GGGGAATTCTAATACGACTCACTATAGGGAGAGGGAGAATAGCCCTGAATTCATCAGTGGCAACTTCTCCTTCCTTT
CTCCCTTCTCCC
+
@@@8AAC@DCC@CBB??B?B?@B97111.7==?C>CAAC@489@;BCC@B?BAABCAAA?@@>@C>@@@=@>A?@@<
@AAA<A>AAA@<
@T6XXI:00125:00394
```

GGGAGAAGGGAGAAAGGAAGGAGAGACACGTTGGGAGGAAACGATTGCTATTCTCCCTCTCCCTATAGTGAGTCGTA
TTAGAACTTCCCC
+
CDABCB@BC>BCBB>B>?=>@BB?;;BBCA?=??9AC@CC?CBBB@CCECD?ABDD@A@ABC?B@A@BAECCBAABA
??A@@;@@@DCC7
@T6XXI:00125:00396
GGGGAATTCTAATACGACTCACTATAGGGAGAGGGAGAATAGCTGACTTCTCCCCAACTGCTGTGTGCACCTTCCTT
TCTCCCTTCTCCC
+
99919:B@AAA@BCCCC>??B@B<;111.6<<@A>CAA>@<<=@@?A@=95<650;<>????AAAB??==;=?B=@A
688@@;@<??AA=
@T6XXI:00126:00354
GGGAGAAGGGAGAAAGGAAGGAGAACACGCCGCGGGATATTGGCGAACTATTCTCCCTCTCCCTATAGTGAGTCGTA
TTAGAATTCCCC
+
CC>CDC?BA<BBBB?D@D@C@@@@GCHBBB>A@@@:AACCBGADDDBBCHCDAA@C@CCBCC>BCBBADCBBA????
?;?@@?<<ACC7
@T6XXI:00126:00355
GGGAGAAGGGAGAAAGGAAGCTAGTCGACTCGACATCTGTGGCTTCACAGTACAGAGGACTGTCCAAAGCCGCAGA
+
CB@B>>644-999909<?<>@B@:771111111111111>@>AA?BAAABBB799..-/;99;A:==7=@;@A@??
@T6XXI:00126:00388
GGGAGAAGGGAGAAAGGAAGCTAGTCACTCGACATCTGTGGCTTCACAGTACAGAGGGACTGTCCAAAGCCGCAGAT
CTCGAGTCGACTAGCTTCCTTTCTCCCTTCTCCC
+
NIBDBB?BB=?988095953?<777=/......../57>067;??@ABCA@;;444-
44;;:>;==7<;8;>=>>A888<<<:;::000::=;;;@AAA@??:;79444-
@T6XXI:00126:00390
GGGAGAAGGGAGAAAGGAAGCTAGTCACTCGACATCTGTGGCTTCACAGTACAGAGGGACTGTCCAAAGCCGCAGAT
CTCGAGTCGACTAG
+
@A>ACC@CC@B?>><@=B=ACC@@?A:::?@:::AAACDC?BB?BBBBBBB?9977717?>?AA;==7<A?<==@@>
<;;873688:==<<
@T6XXI:00127:00357
GCTAGTCACTCGAGATCTGCGGCTTGGACAGTCCCTCTGTACTGTGAAGCCACAGATGTCGAGTCG
+
444444444444;9>?AC><;900,.,...57??;>@B?@@CBB?===:B?A@BCBBBBCCLCBCB
@T6XXI:00127:00362
GGGAGAAGGGAGAAAGGAAGAGTAGGCAAATGGGGAAGAACGTTTTGCTACTCCTCCCTCTCCT
+
CC>DDC@BB<>@@@<@>B=@@CBAABACF@CBCC:C>BA@DCCCC9BBBAB91,---+--,,*,
@T6XXI:00127:00363
GGGAGAAGGGAGAAAGGAAAGGAGAAGTCGCCCCCAAAACGATTTGTTGACTATCTCCC
+
CC?@<;94=:>89904244-3999;561933333+333,31111,1101664-----22
@T6XXI:00127:00386
GGGAGAAGGGAGAAAGGAAGA
+
DD@BCC@CB<A@820708+**
@T6XXI:00127:00389
GGGAGAAGGAGTGAA
+
>?:B1/-1,1,,00)
@T6XXI:00128:00358
CATCGACTCGAGATCTGCGGCTTTGGACAGTCCCTCTGTACTGTGAAGCCACAGATGTCGAGTCGACTAGCTTCCTT
TCTCCCTTCTCCC
+

```
DB=9<<99>BBDB@B<BBBA@AB=B?AABBB@B=@C=??BCCCBB?>??@@@A999<@AAAA<<=<<<=86?>BB@?
;=><=;=;:???:
@T6XXI:00128:00361
GGGAGAAGGGAGAAAGGAAG
+
CA>AAA=>>:>A8880))))
@T6XXI:00128:00370
GGGGAATTCTAATACGACTCACTATAGGGAGAGGGAGAATAGTCCCAAACTATTCTGTACCTGGCATGACCTTCCTT
TCTCCCTTCTCCC
+
=994>698<CCADCBBBBBBBAB?966659??CC>B>?=@<====14417AAA>AA??AC@BA??BBCBB?BABAAE
@CCBC@A?===@:
@T6XXI:00128:00386
GGGGGAATTCTAATACGACTCACTATAGGGAGAGGGAGAATAGATCACCGTTGGTTCGGGTGCGCGTTCTCCTTCCT
TTCTCCCTTCTCCC
+
9999+49:89;979@;<<B@CCB??=777.7????:>4423<;;@@:;;:C@C@C@C>=9;001@>>:???@C?C?B
C>CBCA183888;:
@T6XXI:00128:00394
GGGGAATTCTAATACGACTCACTATAGGGAGAGGGAGAATAGCAAACGTTTCCCCGACGTCGCTTTCTCCTTCCTTT
CTCCCTTCTCCC
+
CCB9B?A@DDB@BBBBBBB@@A?97111.7>>BC@B??=@=AA<<;<??@;@CC8B@?@==<@AC?@@@;68555;5
<???;A>===A=
@T6XXI:00128:00396
GGGAGAAGGGAGAAAGGAAGGATACCAGACGGCGGAATAGCGCGTAGCTATTCTCCCTCTCCCTATAGTGAGTCGTA
TTAGAATTCCCC
+
HH@CEC@CE?BBBH>A=A?B?BBCC<>=DCC=AABC@CBCCGCAA@CC@@@=@AAABACCDE=@;::?=@;88:??;
>>A<89B5999,
@T6XXI:00129:00350
GGGAGAAGGGAGAAAGGAAGAGAACAGTCACAGGCTCGAGCTGCAAGCTATTCTCCCTCTCCCTATAGTGAGTCGTA
TTAGAATTCCCC
+
FC?BB@@BA=@@BC@B>@>?AAA@BCBCBBA?>=@BABBBABCCC?AACCC?B?<:8???@A<ACC?@@BBB?;;<<
<9=AB?A>@@@5
@T6XXI:00129:00356
GGGGAATTCTAATACGACTCACTATAGGGAGAGGGAGAATAGTTAGTCATTTGACGGCCGCCTATTCTTCTTCCTTT
CTCCCTTCTCCC
+
999/4:B?BBBACCCBB<BBBBB>9666-
1;?BC>CAA=@9==<=:::B@C>AAA@<<=?::@A>=AB?BC>?;>>;>;;;;=;====4
@T6XXI:00129:00368
GGGGAATTCTAATACGACTCACTATAGGGAGAGGGAGAATAGTCAACAAATCGTTTGGGGCGACTTCTCCTTCCTTT
CTCCCTTCTCCC
+
??A9B>BADBB@BBBBBABBA<7?6677.7??CK>D@@>B==;6557771:????.777.7>ACC@CBB==;==@?<
?@AA<A374:94
@T6XXI:00129:00374
GGGAGAAGGGAGAAAGGAAGCTAGTCACTCGACATCTGTGGCTTCACAGTACAGAGGGACTGTCCAAAGCCGCAGAT
CTCGAGTCGACTAG
+
CC?AAA=>>:>888096958B>777700;;;00////=@C@BC@CC>;;;A@9933317;9>@@=??;??=?AAAA=
==?A;;:??AAA;=
@T6XXI:00129:00378
GGGAGAGAATTTGGTAATGGGGCACGCAATCGGGCTCAGGGAGAAAGGCAACACCCTTATATCTCTCTCCCTAT
+
```

```
CCBCBAAB@BC?B@BC@BBBB8CCBBAA?BBBB>ABBBBB>BBBB>B@CC@BBCC>D?A858:??@?@AC>@??
@T6XXI:00129:00382
GGGGAATTCTAATACGACTCACTATAGGGAGAGGGAGAATAGTTTTCCCTTCACTAACCGCGAACTGCACTTCCTTT
CTCCCTTCTCCC
+
AAA9AAEAC@B@BBBBA????AB<;111.:A?AD>B@@=@=AACC5<<7;8;?BBB@C@@?@A>@A??BCC@C8::5
:???<B??@@B<
@T6XXI:00129:00385
GGGAGAAGGGAGAAAGGAAGCTAGTCACTCGAGATCTGCGGC
+
B@>@;;;<B>@;;9095968@?88::::?BCC:9:BAAAC?B
@T6XXI:00129:00386
GGGGAATTCTA
+
CBB4A61,111
@T6XXI:00129:00395
GGGGAATTCTAATACGACTCACTATAGGAGAGGGAGAATAGTGACGTGTTGTGCGGCGCTCACTGTCTCCTTCCTTT
CTCCCTTC
+
DCC:C?A@ACC@CCCBB?B>BCDD@888:A?BC>A==>AA@A@@@@AAA>?===@>@@?@D@@@888>>;>9<:<?:
;<<<6<=8
@T6XXI:00130:00357
GGGGAATTCTAATACGACTCACTATAGGAGAGGGAGAATAGCATCACGT
+
499/9;>@BCCACCCBBBBBB:664..,0<<?@>B>=983+.+++00--
@T6XXI:00130:00378
GGGGAATTCTAATACGACTCACTATAGGGAGAGGGAGAATAGCCGATACTTCTCCTATATCGCGTTGCCCTTCCTTT
CTCCCTTCTCCC
+
999-
4:B>BBB@BBBBBBBABB@?=666.7>>>@>::9>AA>?;==@@@C>A?::?AA@@?@@AA>@??1738<==9>888
149;=>@7
@T6XXI:00130:00381
GGGAGAAGGGAGAAAGGAAGGAGAACGCGCACCCGAACCAACGGTGATCTATTCTCCCTCTCCCTATAGTGAGTCGT
ATTAGAATTCCCC
+
CCACCB?BC?CBBB>B@B@B@BBB@BBBBBB@B>BCBC@DCDDCCDCCC@AB@CBBB<@@??@<@CCBBBCCBB???
CC?B9;;=?CCC9
@T6XXI:00130:00383
GGGAGAAGGGAGAAAGGAAGGAGAAGTCGCCCCAAACGATTTGTTGACTATTCTCCCTCTCCCTATAGTGAGTCGTA
TTAGAATTCCCC
+
BC@BAA=>=:@BAB>C?B@B?AAA?BABA???7?A@BCCCB<>>AAAA?A;:;;==;<<<<=;=<<?<<=<===@??
??A>=8;3444,
@T6XXI:00130:00394
GGGGAATTCTAATACGACTCACTATAGGAGAGGGAGAATAGTTTTTTCCCTTCACTAACCGCGAACTGCACTTCCTT
TCTCCCTTCTCCC
+
CDD:C>A>@AADCCCCCB?>?:995/.,0::=?<?A@9;;444444)04248=@BCF@C>AAAA>B=A8<AC@C=<<
8????8?=;;<?:
@T6XXI:00131:00364
GGGGAATTCTAATACGACTCACTATAGGGAGAGGGAGAATAGTCAACAAATCGTTTGGGGCAACTTCTCCTTCCTTT
CTCCCTTC
+
@==6>6;8<CC@CC@?@BAA@>@>=6665:??CC>C@@?@=A???>@@@<BBB@@;AAA7BCADE@BAA;>3737<6
8?@@;@??
@T6XXI:00131:00379
```

GGGGAATTCTAATACGACTCACTATAGGGAGAGGGAGAATAGTTTTCCCTTCACTAACCGCGAACTGCACTTCCTTT
CTCCCTTCTCCC
+
BBC9CAC@BBB@BBCCCCBAA@>97111.7>>?A>B@@>@@AACC5??;?>@CCCC@@><<?A>>>=<<=?@B:??;
?ACC@C@<<<A=
@T6XXI:00131:00382
GGGGAATTCTAATACGACTCACTATAGGGAGAGGGAGAATAGCTACGTTTAACCGATCGTGTTGTTCTCCTTCCTTT
CTCCCTTCTCCC
+
DDG:C?A?BCC@CCBBBBBBA>;97166.7AABE>B@@=@;;<@>?8888?===@A@@A=;=;==99=A>A><;<=:
====:;:<<@B<
@T6XXI:00131:00385
GGGAGAAGGGAGAAAGGAAGCTCTCGACATCTGTGGCTTCACAGTACAGAGGGACTGTCGAAAGCCGCAGATCTCGA
GTCGACTAG
+
CCB@@@@CC?DBBC?C@C@BBBBCCA87:@@ABDDBCC@CBBBCBCDDB???<?A@<?B7997:A>CCBBBCCCC::
;BBBBCCDA
@T6XXI:00131:00389
GGGAGAAGGGAGAAAGGAAGGGGAAAGCGACGTCGGGAAAACGTTTGCTATTCTCCCTCTCCCTATAGTGAGTCGTA
TTAGAACTTCCC
+
CB<@@A>AA<AA@@<A:;8;CC7@A<;;<@CBAAA?:@@@4??>><@>@AA:<9BB9888;;67??@::::>==787
>:=64/11011,
@T6XXI:00131:00394
GGGAATTCTAATACGACTCACTATAGGGAGAGGGAGAATAGTTCGCCAATATCCCGCGGCGTGTTCTCCTTCCTTTC
TC
+
A@6@=@=BCCBCCBABB>?<A@97111.777BE@B>@@@@??<>??9?;?@@A@<CBB>ABB<<5;5;555642201
+*
@T6XXI:00131:00397
GGGAGAAGGGAGAAAGGAAGGAGAACGCGCACCCGAACCAACGGTGATCTATTCTCCCTCTCCCTATAGTGAGTCGT
ATTAGAATTCCCC
+
CCACCC?AA=ABBB>B@BAB@BA??BCCCBBCC>CCAE@DBCBACCCCB>?ADCCCC<B@>>?=?EEBB?B?ABCCA
AAACCC@C@CCC:
@T6XXI:00131:00398
GGGAGAAGGGAGAAAGGAAGCTAGTCACTCGACATCTGTGGCTTCACAGTACAGAGGGACTGTCCAAAGCCGCAGAT
CTCGAGTCGACTAG
+
CC>ABA694-9>==9>595;???@D???A@@7;:???@BC?BC@AAACBBCB>>333-
4;::;>38818><@A@?AAAAA::;==A<<?@@
@T6XXI:00132:00355
GGGAGAAGGGAGAAAGGAAGCTAGTCACTCGACATCTGTGGCTTCACAGTACAGAGGGACTGTCCAAAGCCGCAGAT
CTCGAGTCGACTAG
+
CKCCAA==95=@==<@=@=@BB?BC?AAACA:::;;:BBE?@@@CC?>9>A?:4000,3;9>@A>AA<AA>@@<<<<
===;<???@@@@B@
@T6XXI:00132:00360
GGGGAATTCTAATACGACTCACTATAGGGAGAGGGAGAATAGCTGCTAAAAAAATGAGGCTGCACACTTCTCCTTCC
TTTCTCCCTTCTCCC
+
444,429589888=@ABBC@A?BB@777.77===6?;=;<=9@B??7777777&7>6;9@@@BBBBBF@CAA?@=?4
77.77==6=;;444-
@T6XXI:00132:00362
GGGAGAAGGGAGAAAGGAAGGAGAACATGTTGTGGGACAAAGCCAAACTATTCTCCCTCTCCCTATAGTGAGTCGTA
TTAGAACTTCCCC
+

```
BE?BCC?BA:>98809;><??B@@=ACB>A>AB>><@@@@6>><@@<ABBDAC@@@>@AACG>BBBBBCBBBCA???
A>?@A8;:????5
@T6XXI:00132:00364
GGGAGAAGGGAGAAAGGAAGCTAGTCACTCGACATCTGTGGCTTCACAGTACAGAGGGACTGTCCAAAGCCCAGATC
TCGAGTCGACTAGCTTCCTTTCTCCCTTCTCCC
+
C:6?94298046::66,1,0>?C?>.0////.55?@A=??@CC?AACAAABB@@?==:;;::;;34818=@<AAA@?
??AA@@?@?A?@@7;5;=@?<ACBCAC=@@@@9
@T6XXI:00132:00377
GGGGAATTCTAATACGACTCACTATAGGGAGAGGGAGAATAGCGTGCCTGTATACGGTACCCTGTTCGCCTTCCTTT
CTCCCTTCTCCC
+
999,48>?BCC@CCCBBBBB@??;=8??;?AAEE>C@@>@<<<@@AA>AA>:<AB?;;7::5;==>@B?>B?C@@@;
?===9=>AAAA;
@T6XXI:00133:00353
GGGAGAAGGGAGAAA
+
995999289044485
@T6XXI:00133:00372
GGGAGAAGGGAGAAAGGAAGCAGTCCATCTGTGGCTTCACAGTACAGAGGGACTGTCCAAAGCCGCAAA
+
CC>A?:2480999909699;CCBB@@888;CCC@CC@@>>>==AC==<<;::<:??@>@@;@@>@@@C>
@T6XXI:00133:00373
GGGAGAAGGGAGAAAGGAAGGAGAAGTCGCCCCAAACGATTTGTTGACTATTCTCCCTCTCCCTATAGTGAGTCGTA
TTAGAATTCCCC
+
CC@BBB@BA:=@==<@@C@C?CCC@ACB>>>>6<C?CCCCC>AA>@BB?A;:;9<<;<<@<=;=<<<=<<AA@@@=<
<9<=;9;/444-
@T6XXI:00133:00380
GGGAGAAGGGAGAAAGGAAGCTAGTCACTCGAGATCTGCGGCTTTGGACAGTCCCTCTGTACTGTGAAGCCACAGAT
GTCGAGTCGACTAGCTTCCTTTCTCCCTTCTCCC
+
CC>BBB?>@:=99909<@<BBBADCA??????A@B@@@;:47??.75:@@B???<?>=??@@?A@<;8<<8;@><==
A@@A@??<?<?A@??<;=<=;:;:==;=89===;
@T6XXI:00133:00383
GGGGAATTCTAATACGACTCACTATAGGGAGAGGGAGA
+
;@B9>1969=>594>;;:99B9776111,1?777.74/
@T6XXI:00133:00389
GGGGAATTCTAATACGACTCACTATAGGGAGAGGGAGAATAGTCAACAAATCGTTTGGGGCGACTTCTCCTTCCTTT
CTCCCTTCTCCC
+
CCC8@=A?AAB@<<<D<<<DEA>>7777.77===9?@A>===C@@>?AB=CCCCC;???5????A;?@@5858=AC<
>>@C@A=?:881
@T6XXI:00133:00393
CTATCGACTCGAGATCTGCGGCTTTGGACAGTCCCTCTGTACTGTGAAGCCACAGATGTCCGAGTCGACTAGCTTCC
TTTCTCCCTTCTCCC
+
C=88111>;;BBCCCBAA@@=@BC>C::;?A7::7;BBCCBBC@@@A>=A=ABBBCBBBB@CBC@BB::<BAAA@C@
CLAB@@B=?<<==A<
@T6XXI:00134:00365
GGGGAATTCTAATACGACTCACTATAGGGAGAGGGAGAATAGTCAACAAATCGTTTGGGGCTACTTCTCCTTCCTTT
CTCCCTTCCTCCC
+
==@6@>B@CBB@BBBBAABCBA@>=66967??BC>B@@?A=::?@>??>5?=677.777.7??@@>AAA<?==>AA;
?;>;69,/,///,
@T6XXI:00135:00355
```

```
GGGAGAAGGGAGAAAGGAAGCTACGGCGCTGTCTGGGGAAATTACAGCTATTCTCCCTCTCCCTATAGTGAGTCGTA
TTAGAATTCCCC
+
CE@BBB@CD>AB>>?>;>100>@=@ACDDCCCCDDDD:DEAC?BBBBABBD?BCCCAGCCCC@E@A@CCCC???AAA
B?@>;:;/444-
@T6XXI:00135:00356
AATACGACTCACTATAGGAGAGGGAGAATAGTCAACAAATCGTTTGGGGCGACTTCTCCTTCCTTTCTCCCTTCTCC
C
+
@>ACBBGCBBBECB?::48>===9?@@=A@@@AA@CCG>BBBCC<@@@6@>:@?300=:;:B;AB>=777.4/0000
+
@T6XXI:00135:00357
GGGAGAAGGGAGAAAGGAAGGGGAGAAGGGGACTCGACATCTGTGGCTTCACAGTACAGAGGGACTGTCCAAAGCCG
CAGATCTCGAGTCGACTAG
+
HNBBBC@BB><<<C>A?ABCCF8<>@AJLE9BB;;;888?@<<=B=@A@CCAACC@>>>89918CABCD@DE?CC>A
@ADDCABA9;;AAAABAAA
@T6XXI:00135:00364
GGGGAATTCTAATACGACTCACTATAGGGAGAGGGAGAATAGTCAACAAATCGTTTGGGGCGACTTCTCCTTCCTTT
CTCCCTTCTCCC
+
:99/9<A@BCCCCB??BBBBB@B?>66656<;?C>CAA?A;;888;=AA<CB?66,666-
:BBBC?BCDA?<??AA=A@@@;@=@@CC9
@T6XXI:00135:00370
GGGGAATTCTAATACGACTCACTATAGGGAGAGGGAGAATAGTCAACAAATCGTTTGGGGCGACTTCTCCTTCCTTT
CTCCCTTCTCCC
+
CDH:C?A?ADC@CCCBBBB>?><96166.7==>A=B@@>@AAA@@>@@A<CC;;;-
777.7==?C@CCCA@=A>AA<??@A=A=@@@@<
@T6XXI:00135:00371
GGGAGAAGGGAGAAAGGAAGCAGTCCATCTGTGGCTTCACAGTACAGAGGGACTGTCACGC
+
??:<99;@@>@A@C>A>A@EEBB>???AA<=@B?@C?CA=AA=?<9:444,4;75,.++03
@T6XXI:00135:00374
CTCGAGATCTGCGGCTTTGGACAGTCCCTCTGTGAGCACAGATGTCGAGTCGACTAGCTTCCTTTCTCCCTTCTCCC
+
C::<CCCCBBBBD@BBB?B@CCCDAEA?A@A;410,****,,,,9@AADCCBCDBC@AA?ACCC?CCBB=@=?@BB<
@T6XXI:00135:00378
GGGAGAAGGGAGAAAGGAAGCTAGTCACTCGACATCTGTGGCTTCACAGTACAGAGGGACTGTCCAAAGCCGCAGAT
CTCGAGTCGACTAG
+
BEACCC994-9988096958B@>???000::00////;>@;?ACBBCCCCC@>==<<;;:8444/44-
4424:?>?;<;:==<<;;=>=<<
@T6XXI:00135:00399
GGGGAATTCTAATACGACTCACTATAGGGAGAGGGAGAATAGCTACGTTTAACCGATCGTGTTGTTCTCCTTCCTTT
CTCCCTTCTCCC
+
BAA9B=A>ADC@B@=AA?B?BC@96116.7==@B>BAA?A8>8??@@@A;@>?>@@=;;;;=;<<888<>A>>;<<<
<?<A<A?AAAA<
@T6XXI:00136:00352
GGGGAATTCTAATACGACTCACTATAGGGAGAGGGAGAATAGTCAACAAATCGTTTGGGGCGACTTCTCCTTCCTTT
CTCCCTTCTCCC
+
;99/48?=ACCBCDCBCCCBB@;<6177.7?BCC>B@@>@=====<=@?:?????<?@@6CCCCCACBB?@:=;=AA
AAAA=B:===A<
@T6XXI:00136:00353
```

```
GGGGAATTCTAATACGACTCACTATAGGGAGAGGGAGAATAGAGACGTATTCGTTTGCGCTCTGTTCACCTTCCTTT
CTCCCTTCTCCC
+
CCB9B?B??AF@CCCBBBBBA?<97111.7==>@>BAA?A>AA<=<@=9=>A@A@;@AAA??<@A>@?==?>@>?A=
@@>A=A=@?<=;
@T6XXI:00136:00361
GGGAGAAGGGAGAAAGGAAGGAGAATACAAGC
+
CC>BCD>?>:====:@=;:;;;00,....,,*
@T6XXI:00136:00379
GGGAGAAGGGAGAAAGGAAGGGAATAGCAGCAGTCGGCAACGTGAAAACTATTCTCCCTCTCCCTATAGTGAGTCGT
ATTAGAATTCC
+
@@>AACABB:999919<>;??6<;;;::A@<@0/.5,..,.40,...*..6555666,1666;933388:;:9;;/+
++*++---244
@T6XXI:00136:00387
GGGAGAAGGGAGAAAAGGAAGGCATGAGCCTACGACTTGACATTCG
+
CC?;;;244-84/11*/,/,.,.4...11033339@?@@C?57*,*
@T6XXI:00137:00355
GGGAGAAGGGAGAAAGGAAGTCGAGCATCTGTGGCTTCACAGTACAGAGGGACTGTCCAAAGCCGCAGATCTCGAGT
CGACTAG
+
ED@@@@@BB@BBBC>C@C@BBBABA@A:::BBB?AB@CCCB<@CAA<=??<?ABAA;:;==<<:;<<===<<@?@@A
CBBBBBB
@T6XXI:00137:00360
GGGAGAAGGGAGAAAGGAAGCTAGTCGACTCGACATCTGTGGCTTCACAGTACAGAGGGACTGTCCAAAGCCGCAGA
+
DI@BBA;=95>>@@<@@C=B@CAAA?CC@::75/000/;7>:B@CCB?>>>@===44444=;?@A<@?:?A?@@<;=
@T6XXI:00137:00375
GGGAGAAGGGAGAAAGGAAGCTAGTCACTCGACATCTGTGGCTTCACAGTACAGAGGGACTGTCCAAAGCCGCAGAT
CTCGAGTCGACTAG
+
CC>AAA:94-
998809695;CAAAC=>;??=001>>>?@C=?@@CC?999@C==98848:88==;=<;==>A=<999AAA<AAA<<<
<>>>
@T6XXI:00137:00376
CTATCACTCGAGATCTGCGGCTTTGGACAGTCCCTCTGTACTGTGAAGCCACAGATGTCGAGTCGACTAGCTTCCTT
TCTCCCTTCTCCC
+
44@444:9A@DBBGCCC:84888/8>AAACABC>??@CBBBAA@@@;=B@CBBCA<;;BAAADBABCCC@??@C@CD
@CCCA<@=@<=<7
@T6XXI:00137:00381
GGGGAATTCTAATACGACTCACTATAGGGAGAGGGAGAATAGACAAGACAGTCAGTTGTCTTCTATTCCCTTCCTTT
CTCCCTTCTCCC
+
CCD9@>AACBB?BAAAA??<BAB<9169.7>>CC>CAA?A<<<<<<@<?@@AAC@=<@<<?>AABC@CC>D?B?BC<
@A==7=278==7
@T6XXI:00137:00386
GGGAGAAGGGAGAAAGGAAGCTAGTCACTCGACATCTGTGGCTTCACAGTACAGAGGGACTGTCCAAAGCCGCAGAT
CTCGAGTCGACTAG
+
DE?CCB>>=5=884-
9;>;?CB???9000>;00////;>C=@@@BB@?>?C?44000,4;;;:>;=>7>@>ABAA???@@@??<?@@==<<
@T6XXI:00137:00388
GGGGAATTCTAATACGACTCACTATAGGGAGAGGGAGAATAGTCACGCATTTGCCGT
+
```

```
AAD7A@B@BBBACCCAA>A>?@BC?661.67777.7;;9@=<<=;9:9777.70,.,
@T6XXI:00138:00355
TCCCGAGATCTGCGGCTTTGGACAGTCCCTCTGTACTGTGAAGCCACAGATGTCGAGTCGACTAGCTTCCTTTCTCC
CTTCTCCC
+
7990999<BCBCBC=@@CAC:;;C@??B>BCBBBACCCCBA<??=CB@@AA==AB@@@BCBAAA@@??B?CC@CCDG
BD=<<AB?
@T6XXI:00138:00356
GGGAGAAGGGAGAAAGGAAGGAGAACGCGCACCCGAACCAACGGTGATCTATTCTCCCTCTCCCTATAGTGAGTCGT
ATTAGAATTCCCC
+
CD@DDD?BB?BBBB>C?B?B@AAA@BBBBBBBB>CFAD@CBCE@CCCCBAAB?A?;<<@ABCC>B???C>ABBBCCB
CC?BAA>BCCCC9
@T6XXI:00138:00369
GGGGAATTCTAATACGACTCACTATAGGGAGAGGGAGAATAGATCACCGTTGGTTCGGGTGCGCGTTCTCCTTCCTT
TCTCCCTTCTCCC
+
CDD:C@CBBBB?BCCBBAAAB>>B76:?9???BC>CAA>@<???<A<<A?;?:?;@AC>C:;;CBA>BBCAB?C@EG
=A???:?>@A==<
@T6XXI:00138:00376
GGGAGAAGGGAGAAAGGAAGGAGAAGTCGCCCCAAACGATTTGTTGACTATTCTCCCTCTCCCTATAGTGAGTCGTA
TTAGAATTCCCC
+
CC?CCC@DC>AAAB?B>@>@@CBBA@BA>;;;5@@<ABCCC>C@??::9?C=@@AA<???AC>A?@?AA??<589@@
A@@@?:;/444-
@T6XXI:00138:00377
TAGTCGACTCGAGATCTGCGGCTTTGGACAGTCCCTCTGTACTGTGAAGCCACAGATGTCGAGTCGACTAGCTTCCT
TTCTCCCTTCTCCC
+
AEDBBCCA@ACCBBBCBAB?@BBB?B?B>>>BBB<<<<?;??>>>>>>??>?>>><<<<=9====:=;<=<<?>?>?
?;>>??;=<=<:::
@T6XXI:00138:00381
GGGGAATTCTAATACGACTCACTATAGGGAGAGGGAGAATAGTCAACAAATCGTTGGGCGGACTCTCTCTTTCTCCC
T
+
ACC9CAC@DCC@CCCAA?B??@A<7111.7=<BC>C@A>=4+,++*+-
10371.,.:,0.633331103333588;13
@T6XXI:00138:00387
GGGGAATTCTAATACGACTCACTATAGGGAGAGGGAGAATAGTCAACAAATCGTTTGGGGCGACTTCTCCTTCCTTT
CTCCCTTCTCCC
+
999,99A?BBB?DAA@@CCCBA@@?6995:CCCE>A??<?@@@@@>@AA<BCDBF>BDC8CBBBC?BCC8848>@><
@===1834===:
@T6XXI:00139:00353
GGGAGAAGGGAGAAAGGAAGCTAGTCACTCGACATCTGTGGCTTCACAGTACAGAGGGACTGTCCAAAGCCGCAGAT
CTCGAGTCGACTAG
+
AB><<<AB@:>99908;=;>ABBA@::7<;=7711111698:>@BCCBAAAB?65;<7=;;9;;38818=9?898A?
??@989@@@@@<<;
@T6XXI:00139:00373
GGGGAATTCTAATACGACTCACTATAGGGAGAGAGATATAAGGGACCACTCTCTTCCAACTCCATAGTCGACCACCC
CATTACCAAATTCTCTCCC
+
@@@8AAC@BCC@BBAABAABBABB@A@A====AA=<<88<<==:===A==<;::<<<=;=?<:?===A@BBC?<;;;
.88489/00,434:9444-
@T6XXI:00139:00374
```

```
GGGAGAAGGGAGAAAGGAAGGGAATAGCGGCAGTCGGCAACGTGAAACTATTCTCCCTCTCCCTATAGTGAGTCGTA
T
+
CD@CBA@CBB??AB>B@B@BC?B?AA@AA?ABBGBA?AC@CCCBBB>BBB@>AA@A>??BCC>A???@<=B@<@<>>
<
@T6XXI:00139:00383
GGGAGAAGGGAGAAAGGAAGGCAACAACGCCAGCATCGATCGATGTACAGCTACGTACGTCTGAGCATCGATCGATG
TACAGC
+
GCACBB@BCABEBE?B@B@B@CCB746:11,///669444444444444;>>A@A>@777769?7>@<9115=:344
344-++
@T6XXI:00139:00384
GGGAATTCTAATACGACTCACTATAGGAGAGGGAGAATAGTTTGTGCATTTCTCCCAGCACACCGTCCTTCCTTTCT
CCCTTCTCCC
+
FH@C:;9;@@=ABBBC?BBB?7711637>>>@=CAA?A@@AA<A@<<BBC@@A@C@BAABB??;@773:;?;?C>@@
@@:?:=<@C<
@T6XXI:00139:00389
GGGGAATTCTAATACGACTCACTATAGGGAGAGGGAGAATAGTCAACAAATCGTTTGGGGCGACTTCTCCTTCCTTT
CTCCCTTCTCCC
+
CBC:CBCACBA@CBBBBB<<B@CD@7774699BC>C@@=A:::==<<?@;@@977,111,6AACC@BBB?@=@=@C>
@@@A;A>?@AA;
@T6XXI:00139:00399
GGGGAATTCTAATACGACTCACTATAGGGAGAGGGAGAATAGTTTGTATTTTGTGCATCACTTTTCTCCTTCCTTTC
TCCCTTCTCCC
+
CCC9C8;;;BB@CCBBCCCCBBCB?:<:88??>?>@@@>B=@@A7==AAAA6:?;AAAAA??;:4>=?=@>@>>A<A
AAA9=;=AAA<
@T6XXI:00140:00351
GGGAGAAGGGAGAAAGGAAGGGGAGAAGGGAGAAAGGAAGCTAGTCGACTCGACATCTGTGGCTTCACAGTACAGAG
GACTGTCCAAAGCCGCAGATCTCGAATCGACTAG
+
CDDBBB@BA:A999-9>@4777-8??577.7777-
70747B<??B;;:CC?<=??@@@AAA>??=?A=<=??=6?7738;;;<A=@@@AA@AAAA??:<<==9==B???A?
@T6XXI:00140:00362
GGGAGAAGGGAGAAAGGAAGCTAGTCACTCGACATCTGTGGCTTCACAGTACAGAGGGACTGTCCAAAGCCGCAGAT
CTCGAGTCGACTAG
+
FKDDCB==95=844-
9595;F??@B>A@???::::::BCC@?@@BB@B?ACA<:::846<<>>?8<<7;>=A====ACCB?@@@@?:8==<
@T6XXI:00140:00368
ACTCGAGATCTGCGGCTTTGGACAGTCCCTCTGTACTGTGAAGCCACAGATGTCGAGTCGCGACTAGCTTCCTTTCT
CCCTTCTCCCGGGAGAAGGGAGAAAGGAAGGAGAAGTCGCCCAAACGATTTGTTGA
+
BCBCBBCC@ABCCCBBBB>B@BBBBBBB>DB@@@A@@BBBCDAA@BBCBBBAACCDCC8;;AAABB?<@>A>AB=AA
@A7=;<777,61,111011,166>7=;>34389;79;;444-44,43311,11011
@T6XXI:00140:00369
CTATCGACTCGAGATCTGCGGCTTTGGACAGTCCCTCTGTACTGTGAAGCCACAGATGACTC
+
DCBB:9:BCCCBCCBB??@C@AAC>A=<??=:::5:BBBBBBB<??A>@B>@@A??0-**((
@T6XXI:00140:00372
GGGGAATTCTAATACGACTCACTATAGGGAGAGGGAGAATAGATCACCGTTGGTTCGGGTGCGCGTTCTCCTTCCTT
TCTCCCTTCTCCC
+
499/99@AEDB@C@BABBBB??AB;6195:???D>C@@>A=?8:<??:;@3:5:=@AB>B;;;CCC@CBB?B@BABK
@CC@@;@>AA@<;
```

```
@T6XXI:00140:00376
GGGAGAAGGGAGAAAGGAAGCTAGTCACTCGACATCTGTGGCTTCACAGTACAGAGGGACTGTCCAAAGCCCGACA
+
GDAB995991499904242 3?ABBB:::???:::???BDE::;@CCCCCCCC?::?>7<<>;;;344-0442422-
@T6XXI:00140:00383
GGAGAGGAG
+
*)***0006
@T6XXI:00140:00389
GGGGAATTCTAATACGACTCACTATAGGGAGAGGGAGAATAGTCCCCTGGCTTTTGCCCATTGCTGTTCTTCCTTTC
TCCCTTCCTCCC
+
CEEACAD@A@@@C@@@BBBAB?<<6177.7>>?A>B@@>==9;;;91//...444:>7:777995>A?=C;=9;;9;
444,434/4;;4
@T6XXI:00140:00392
GGGGAATTCTAATACGACTCACTATAGGGAGAGGGAGAATAGTCAACAAATCGTTTGGGGCGACTTCTCCTTCCTTT
CTCCCTTCTCCC
+
@@@6@>B@CDB@B??AA?<<<AB>;666-
6??CC>B?C?A;99<=;<<=;?986;6;<<5?BCCC@CBC?A>A>A=;=@AA<?<?ADC;
@T6XXI:00141:00366
GGGGAATTCTAATACGACTCACTATAGGGAGAGGGAGAATAGTCAACAAATCGTTTGGGGCGACTTCTCCTTCCTTT
CTCCCTTCTCCC
+
==>4?<>=@CC@CCB??@DCB?>?;677.777AC>CAA?A==<<;8;==7AA?>?.:77.777:?=?BB>?=@>@?:
@???:?>;<<<7
@T6XXI:00141:00390
GGGGAATTCTAATACGACTCACTATAA
+
ABA7A>A=B@@=A;;;@8800/44,,*
@T6XXI:00141:00391
GGGAGAAGGGAGAAAGGAAGGATAGTCACGCAAAGTTCAAGGGCAAACTATTCTCCCTCTCCCTATAGTGAGTCGTA
TTAGAATTCCCC
+
99-
9?><>>:?>>><@=@=@@CCDAA>????AA?BB@BC@B?;9996;CAA@C@@@<@AADC>CBB?>>????9=<<@;<
<=<@9<;;0
@T6XXI:00142:00379
GGGGAATTCTAATACGACTCACTATAGGGAGAGGGAGAATAGATCACGACCCTTCTACCCACGATTGAGCTTCCTTT
CTCCCTTCTCCC
+
999,48>8;<CBBBCBBBCBBBCBA:::5:??CD>B>A????A>@B?==<<6<:=?777.4::4438<ABCAC@EC?
CBBB=B@B?@@<
@T6XXI:00142:00394
GGGGAATTCTAATACGACTCACTATAGGGAGAGGGAGAATAGCTTTGATAATTCCCGCCAATCTATTCACCTTCCTT
TCTCCCTTCTCCC
+
A?A6@=@@CCD@BBCBBB<>>@CB@67646??CD>B>??A;::>>;>;;;>>>>>;99<=<<:;<><>BB@B=;;?@
59:AA<B@CBBC>
@T6XXI:00143:00355
GGGAGAAGGGAGAAAGGAAGAGCATAGCGGGTTGAGGAATCGTTTGGCTATTCTCCCTCTCCCTATAGTGAGTCGTA
TTAGAATTCCCC
+
AA>DCDACC>BBAH>C>B>@??>@@@<<;EBG@BBB@B?@>;880849<BE@ECCD@CBBBB>B:<;BBCCCC898?
?<?@>:;38880
@T6XXI:00143:00357
```

```
CTAGTCACTCGAGATCTGCGGCTTTGGACAGTCCCTCTGTACTGTGAAGCCACAGATGTCGAGTCGACTAGCTTCCT
TTCTCCCTTCTCCC
+
D:/111/44AA@BBCCBBA?<<<<<@@BAAA:::8:CCCCCCA@@@@>@CDCDCAAACCCCBBBCCBBBCDAA@B?F
D?AABC>A>===@;
@T6XXI:00143:00368
GGGAGAAGGGAGAAAGGAAGCTAGTCGACTCGACATCTGTGGCTTCACAGTACAGAGGGACTGTCCAAAGCCGCAGA
TCTCGA
+
BB>@@@=@B>BCCC>C@C8;;B?????????=::;;;:BBC?AC@CCB@???:44000-
4;;;?@<??<?A>AA???<=AA@@
@T6XXI:00143:00372
GGGAGAAGGGAGAAAGGAAGCTAGTCGACTCGACATCTGTGGCTTCACAGTACAGAGGGACTGTCCAAAGCCGCAGA
TCTCGAGTCGACTAG
+
JDA@??;==9@>>@<A>B>ACA;;;//0:;<<1:9==8??C?AA?ABBB@BA;<:44447AAAAA<??;?A>A@<<=
=<;@<@A@???@AAA
@T6XXI:00143:00377
GGGAGAAGGGAGAAAGGAAGCTAGTCGACTCGAGATCTGCGGCTTTGGACAGTCCCTCTGTACTGTGAAGCCACAGA
TGTCGAGTCGACTAGCTTCCTTTCTCCCTTCTCCC
+
CB<@=<;<BA@;;9095967??<=<0/0;;>>?@FBAAABC?AAC>C?A?AAACCAA@@AAAA99=@B?AA>==<<<
<>==>>788=>=>===A>@<><46///,/,//999
@T6XXI:00143:00395
GGGAGAAGGGAGAAAGGAAGGAGAAGTCGCCCCCAAACGATTTTGTTGACTATTCTCCCTCTCCCTATAGTGAGTCG
TTATT
+
99-999:9@:A111-
1,1,.,...956<78888+33,361111,16369?89=>AAAA898?==4;::9:44366/++*,+*
@T6XXI:00143:00398
GGGAGAAGGGAGAAAGGAAGCTAGTCACTCGAGATCTGCGGCTTTGGACAGTCCCTCTGTACTGTGAAGCCACAGAT
GTCGAGTCGACTAGCTTCCTTTCTCCCTTCTCCC
+
CE@C;<<=B:?99919;>?AB@??>>666855313//.44566757;9>BCCCC?A?AAA????@<;9;<8<<@=<=
??@@@?><<<877A?><?>AA<??>>7><=?=A1
@T6XXI:00144:00371
GGGGAATTCTAATACGACTCACTATAGGGAGAGGGAGAATAGTCAACAAATCGTTTGGGGCGACTTCTCCTTCCTTT
CTCCCTTCTCCC
+
444/99A>ACD@CBBBBBB?BBD??76659??CD>BBB>>>>;==<<?@;AB99;88886@BCCC@AAA>A??>AA<
A>>>7><=?@A<
@T6XXI:00144:00376
GGGAGAAGGGAGAAAGGAAGCTAGTCACTCGACATCTGTGGCTTCACAGTACAGAGGGACTGTCCAAAGCCGCAGAT
CTCGAGTCGACTAGCTTCCTTTCTCCCTTCTCCC
+
::6:4954904499042424B>>>B6777761613117110 67:???CCCCB@?9::.7>><==>AA<AA>B@@@@?
;;888;=????AA@@@<?;>?:>?>A<A689<A9
@T6XXI:00144:00387
GGGAGAAGGGAGAAAGGAAGCTAGTCGACTCGACATCTGTGGCTTCACAGTACAGAGGGACTGTCCAAAGCCGCAGA
TCTCGAGTCGACTAG
+
CCFC@;244-
9999195958>>::=/00:0/00;:?<<@BC8><BCCABACC?=933317>>@AC;==9=@@???@@A@@@CA@???
?A?@@
@T6XXI:00144:00393
GGGAGAAGGGAGAAAGGAAGCTAGTCACTCGACATCTGTGGCTTCACAGTACAGAGGGACTGTCCAAAGCCGCAGAT
CTCGAGTCGACTAG
```

```
+
CDACCB===5=9995>5;7<CAABC8;:??=7:::87CEE=?@@CB>=?@@AA?>>@;9=?@AA><@<@@??888??
@@@@@@AC<<<CAA
@T6XXI:00144:00395
CATCGACTCGAGATCTGCGGCTTTGGACAGTCCCTCTGTACTGTGAAGCCACAGATGGTCTACTTCCTTTCTCCCTT
CTCCC
+
AAEBCCBAFACCCCDCAAA@BCCAC?BBCDB@@<AB<??BBBCCBA?BC@BB????=99>>:9=;=;=A<A???<??
@@?A=
@T6XXI:00145:00355
TCTGCGGCTTTGGACAGTCCCTCTGTACTGTGAAGCCACAGATGTCGAGTCGACTAGCTTCCTTTCTCCCTTCTCCC
+
?BBBBC@CCC?D;<;?@99;8;BBCCCBBBBCC?BB@CCCCCBAAAABCCDBBBBB@@@7:5;B<BBBA=BABBBCB
@T6XXI:00145:00364
GGGAGAAGGGAGAAAGGAAGGAGAAGTCGCCCCAAACGATTTGTTGACTATTCTCCCTCTCCCTATAGTGAGTCGTA
TTAGAATTCCCC
+
BBBCAA@BE>AABD>B@A?@@BBB@B@@@ABB7AA<@BAAA?AB=B::;::?@@CC;@@@@=;>9==AAAA@@@A89
9@B==:8/444-
@T6XXI:00145:00377
GGGGAATTCTAATACGACTCACTATAGGGAGAGGGAGAATAGTCAACAAATCGTTTGGGGCGACTTCTCCTTCCTTT
CTCCCTTCTCCC
+
AAA6@CB?CCC@CBBB??BBBBB<<366-
6==@B>AAA@CCCA@@?AA>;B?9?=6?;;2<???B@BBB@C@A=AA<A@@@;?<@?@@;
@T6XXI:00145:00394
GGGGAATTCTAATACGACTCACTATAGGAGAGGGAGAATAGTCAACAAATCGTTTGGGGCGACTTCTCCTTCCTTTC
TCCCTTCTCCC
+
BBB6A=@=@CC?BACBBBBBB:996./,0::=?;?CC@CCB@@@>???:A@===-666-
8>>>@?AAA>B@@>AC>???A=A>A???:
@T6XXI:00145:00396
GGGAGAAGGGAGAAAGGAAGCTAGTCACTCGAGATCTGCGGCTTTGGACAGTCCCTCTGTACTGTGAAGCCACAGAT
GTCGAGTCGACTAGCTTCCTTTCTCCCTTCTCCC
+
A?:>9979B:@999195959?AABC@C@@@CABACADCBA?ABB>B@@@AAACC>AAAAAAAB@@@C@BB>@=A><<
<<<<=====:;<==<><88<<5<===:9/3444-
@T6XXI:00145:00398
CTAGTCACTCGAGATCTGCGGCTTTGGACAGTCCCTCTGTACTGTGAAGCCACAGATGTCGAGTCGACTAGCTTCCT
TTCTCCCTTCTCCC
+
C8/11</4:BBBBBCBB?BB@???;B?BB>@@CC>B?CABA<ABCCC==A@BCCCCB?AABCBBBBBCCDA@?@C@C
D<BBCE>A@@@@A:
@T6XXI:00146:00361
CGAGATCTGCGGCTTTGGACAGTCCCTCTGTACTGTGAAGCCACAGATGTCGAGTCGACTAGCTTCCTTTCTCCCTT
CTCCC
+
CBBBCCBCBCB?BBC?B@CDBB@<><?CCCCBBCCCCC>B@>C@<<ABBAAAABBCBDBCBAAA?BDDDBDAAA<>5
89880
@T6XXI:00146:00364
GGGAGAAGGGAGAAAGGAAGCTAGTCGACTCGACATCTGTGGCTTCACAGTACAGAGGGACTGTCCAAAGCCGCAGA
TCTCGAGTCGACT
+
DIBCBB>BA<@B@><A7:6<CA>>>/11B>><0;:::6@@C=ACBBBBC@@AB?<44417==<==>AA<AA=@@==<
=<<=@A=<@A===
@T6XXI:00146:00365
```

```
GGGAGAAGGGAGAAAGGAAGCTAGTCGACTCGACATCTGTGGCTTCACAGTACAGAGGGACTGTCCAAAGCCGCAGA
TCTCGAGTCGACTAG
+
AA>ABB@CC>BBCC@B>@<@B7.000///,,770.//.5:?-
;;?BCABBBA?==33314;;;>@:==9=A>A@@@AA===???@@@@@@A@
@T6XXI:00146:00378
CTATCGACTCGAGATCTGCGGCTTTGGACAGTCCCTCTGTACTGTGAAGCCACAGATGTCGAGTCGACTAGCTTCCT
TTCTCCCTTCTCCC
+
/,,,**,,,,/::;;AA=?<=@??8:6777779986577,,,,,..60048889=<>BCCBBAB;99>8333736:6
=-6111,1016394
@T6XXI:00146:00393
GGGGAATTCTAATACGACTCACTATAGGGAGAGGGAGAATAGTTTTTCCCTTCACTAACCGCGAACTGCACTTCCTT
TCTCCCTTCTCCC
+
BFD:C?B@BCB?BBAA@BAA?888?877,7=17:8:?B@AAA>==;1<<3<8;@@BA=>=70//,.311<?:;<;=E
>AABB.747743+
@T6XXI:00146:00394
GGGAGAAGGGAGAAAGGAAGAAGAATAGAAGAACACATGTCGGTTGGCTATTCTCCCTCTCCCTATAGTGAAGTCGT
ATTAGAACTTCCCC
+
@B7<<9<@A8<9CD@C?B=;;98@=>>8741<:;>::09>;::=-/-.5<>5::??.74000+033422-.-
.333=9848=@9=<5333.
@T6XXI:00146:00398
GGGAGAAGGGAGAAAGGAAGCTAGTCACTCGACATCGTGGCTTCACAGTACAGAGGGACTGTCCAAAGCCGCAGATC
TCGAGTCGACTAG
+
DC?CBB89919999195958A??AB:;;BCDDCBDCC@@=@B@BBBCBB@@@@8865;;;9:?38818B9<<=<<<<
==@A@@@@@??@=
@T6XXI:00147:00370
GGGAGAAGGGAGAAAGGAAGCTAGTCACTCGACATCTGTGGCTTCACAGTACAGAGGGACTGTCCAAAGGCC
+
BB:@=929909@@BAH;<;;EB?88,,,,0,,,006.57:./0::9=????BB@?@?=00./.-,//,4/02
@T6XXI:00147:00372
GGGGAATTCTAATACGACTCACTATAGGGAGAGGGAGAATAGTCAACAAATCGTTTGGGGCGACTTCTCCTTCCTTT
CTCCCTTCTCCC
+
=AA6A=@@CDB?B@AA?B>?BBB>;7695:@BCC>CBB;?AA=<687871:B=>>.777-
8>@BD?CCC?A??>BA=>>@C<A=@@@C:
@T6XXI:00147:00381
CTAGTCACTCGAGATCTGCGGCTTTGGACAGTCCCTCTGTACTGTGAAGCCACAGATGTCGAGTCGACTAGCTTCCT
TTCTCCCTTCTCCC
+
DA:<=<;:ACHCCCD?AABB@BBB<B>BBCBBB@;@@>@>@@@=??C?AC@CCCCB@AAACBBBBBCABB@@@7:5:
B>??@C>B>BB@A<
@T6XXI:00147:00397
GGGAGAAGGGAGAAAGGAAGCTAGTCGACTCGACATCTGTGGCTTCACAGTACAGAGGGACTGTCCAAAGCCGCAGA
TCTC
+
DFCBBB@BB:@A@@:@@B;<;CAACCCB@BA@????BACBB?BB@CCA@@@@??<6664686==?;><5>@:==AAA
@@?@
@T6XXI:00147:00399
GGGAGAAGGGAGAAAGGAAGCTAGTCGACTCGACATCTGTGGCTTCACAGTACAGAGGGACTGTCCAAAGCCGCAGA
TCTCG
+
CCBCA><=96>>>>9>>@8<<CECCBBCACC89:::??CDD>A@@CC?9==C?;4330,4;;;?A9<<7<@>AA@@@
<<@?=
```

```
@T6XXI:00148:00350
GGGAGAAGGGAGAAAGGAAGTGCAGTTCGCGGTTAGTGAAGGGAAAAACTATTCTCCCTCTCCCTATAGTGAGTCGT
ATAGAA
+
EDBDCI@BB?ACCB=B?B@C@?@A;<8<HCGBFAA?>88173,1111+33331397769968?88?@=;;////-
0+---112
@T6XXI:00148:00352
GAGACTCGAGATCTGCGGCTTTGGACAGTCCCTCTGTACTGTGAAGCCACAGATGTCGAGTCGACTAGCTTCCTTTC
TCCCTTCTC
+
91///,****,,228CDACCC@D@BBBBBBB=BC;;;BBCCCCCBCC?ABBDA>A>???ECCC?<?C?=@@C@CC=@
@BC>C@8::
@T6XXI:00148:00369
GGGAGAAGGGAGAAAGGAAGCTAGTCGACTCGAGATCTGCGGCTTTGGACAGTCCCTTTGTACTGTGAGCCACAGAT
GTCGAGTCGACTAGCTTCCTTTCTCCCTTCTCCC
+
GEBA9969>:>111-
1,/,/..........334999:<?CD@BCC6;37777>>>.77,6=@>>=:11180778/11:::::;BCBACE<<<<
@<@>A@:>???:@<?@@@:
@T6XXI:00148:00371
GGGGAATTCTAATACGACTCACTATAGGGAGAGGGAGAATAGTCACATTCATGTTTGTGGCACGTGTCTCCTTCCTT
TCTCCCTTCTCCC
+
HCC:ECC@BBCBDBBBABBBBBB973115:?ACE>BBB>?@B?AAA=B@B?@B>?57//,0.-
697:;;@6:69:@@188>>:>588@@;
@T6XXI:00148:00379
GGGAGAAGGGAGAAAGGAAGTGCAGTTCGCGGTTAGTGAAGGGAAAAACTATTCTCCCTCTCCCTATAGTGAGTCGT
ATTAGAATTCCCC
+
DE?A:@59919A@B>GAC@CCBCEBB?ABAB@B?BAC@@=?B8<<??0>BBC9<:BC>?@@CCBBDC@?=AAC==99
8@>AA@@=<ACC>
@T6XXI:00148:00389
GGGAGAAGGGAGAAAGGAAGGGGAGAAGGGAGAAAGG
+
DFBCCC@BA=@?BB>B?A>AAA7;;;>AC/8.,,+,*
@T6XXI:00148:00391
GGGAGAAGGGAGAAAAGGAAGCTAGTCACTCGACATCTGTGGCTTCACAGTACAGAGGGACTGTCCAAAGCCGCAGA
TCTCGAGTCGACTAGCTTCCTTTTCTCCCTTCTCCC
+
;A=A<<:9B:A9999,424247733.0.........3668=27:@<;:CEBBBBC???;<AAA@@>@@;<<9<?<<=
@@@@???A@;;=0//7677,///+43044<8<65;7
@T6XXI:00148:00397
GGGGAATTCTAATACGACTCACTAGTAGGAGGAGGAGAATAGCTTTTCGCTTCCCTGACTATTGTTTTCTTCCTTTC
TCCCTTCTCCC
+
AA?4?<@=ABC@DCBBAAA:::::030>:00,7;,/,,*,,,+,220666@;BB>?CAAA<=:;;;<1<;=@>AC<@
<666;8;@AA<
@T6XXI:00148:00398
GGGAGAAGGGAGAAAGGAAGAGAAG
+
CBABDC@CC>A@>@;>B;1/-00)(
@T6XXI:00149:00365
GGGGAATTCTAATACGACTCACTATAGGAGAGGAGAATA
+
//1+1:><ABB@CCCABBCBA:6;6..,0;0731/.,.0
@T6XXI:00149:00366
```

```
GGGAGAAGGGAGAAAGGAAGGAGAAGTCGCCCCAAACGATTTGTTGACTATTCTCCCTCTCCCTATAGTGAGTCGTA
TTAGAATTCCCC
+
BB?CDC?BB?BCDD>B?B>A@BCB@BAA>;;;5;@<@ECC@9>B?CBBBCCD=<<=;??@AC>;;A888;;=<<<;<
?>@?>:;3444,
@T6XXI:00149:00375
AGTCACTCGAGATCTGCGGCTTTGGACAGTCCCTCTGTACTGTGAAGCCACAGATGTCGAGTCGACTAGCTTCCTTT
CTCCCTTCTCCC
+
EGBBAABBBABBDDCCCC@BBB>BEBAA@BBB>BB@@@@>@>@@B@@@>???@CAA==?=AAAAB@A@:>A>A?BC>
BBBB>B>?@DB<
@T6XXI:00149:00382
CTATCACTCGAGATCTGCGGCTTTGGACAGTCCCTCTGTACTGTGAAGCCACAGATGTCGAGTCGACTAGCTTCCTT
TCTCCCTTCTCCC
+
CCA=;:::BCCCBBBAAABAAAA@A?>>;@9::5:BCCBAAB>BBC?@C@BBBBBBBCCCBCABBBBAACCBAC?BB
>BCCC>B49:==7
@T6XXI:00149:00390
GGGGAATTCTAATACGACTCACTATAGGGAGAGGGAGAATAGCGCAATTTGGACATGTGGCCGCTTCTCCTTCCTTT
CTCCCTTCTCCC
+
EBB9CCB?BCC@CCBBBBBBAAA??31659?=BC>CCC>C>>:?AB@CB<@?ADB??CC@D@D?=:<3738;=;=@:
?@@A<A?@A@A:
@T6XXI:00100:00400
GGGGAATTCTAATACGACTCACTATAGGGAGAGGGAGAATAGTCAACAAATCGTTTGGGGCGACTTCTCCTTCCTTT
CTCCCTTCTCCC
+
CCC9B=@=@CBACCB?????BE?97111.7>==?>@CC>@8AAAA<<@@<AAAAA=ACB9CBBBB>ABB47375;85
5::=4878444-
@T6XXI:00100:00401
GGGGAATTCTAATACGACTCACTATAGGGAGAGGGAGAATAGTCCGAGCATTTCCCCGCCCATGGCTATCTTCCTTC
TCCCTTCTCCC
+
DCC9A?B?BBB@B@@@BBBBA>;96116.7>>AB?CBA?A<<<=<:<<<@BB>CCC9BB@<@??=??<<00,0,/,.
555+/.74=A<
@T6XXI:00100:00404
GGGAGAAGGGAGAAAGGAAGGAGAAGTCGCCCCAAACGATTTGTTGACTATTCTCCCTCTCCCTATAGTGAGTCGTA
TTAGAATTCCCC
+
AA:?DA5;;9;ABB>B?@=?=8889=;<?>>>2>@:87677,1>7;=@;443444;944-..+---22358:30-
4//,-12010355.
@T6XXI:00100:00438
GGGAGAAGGGAGAAAGGAAGGAGAACGCGCACCCGAACCAACGGTGATCTATTCTCCCTCTCCCTATAGTGAGTCGT
ATTAGAATTCCCC
+
CE@CCB@DB??AFG@C?B?B@CCC@BBBBBEBB=@BAC@C?BDBCDBBC;;<ACCCB=BBBBB=CCCCBBBCBBBDC
BA?ABC@BBBCB8
@T6XXI:00101:00404
GGATTCTATACGACTCACTATAGGGAGAGGAGAAT
+
42442499;BCBCBBBB@BB>661-/9//,07741
@T6XXI:00101:00407
GGGGAATTCTAATACGACTCACTATAGGGAGAGGGAGAATAGTCAACAAATCGTTTGGGGCGACTTCTCCTTCCTTT
CTCCCTTCTCCC
+
CB?6>698<AABCFCBBBBBB@A?7116.7>>>@@BAA>@:?@==;<@@<C?@@C>DDC7BBCBB>@AB6858=AA<
??@A@A>=>=AA
```

```
@T6XXI:00101:00417
GGGGAATTCGTAATACGACTCACTATAGGGAGAGGGAGGAATAGCTCCC
+
///*,*,*,,,,*,/444337<>BB???@<<:7::60/,.2222-.0<,
@T6XXI:00101:00426
GGGAGAAGGGAGAAAGGAAGCTAGTCGACTCGACATCTGTGGCTTCACAGTACAGAGGGACTGTCCAAAGCCGCAGA
TCTCAAGTCGACTA
+
DD@CCB@CC>ABAB>B?A@DBB666/11??K?>@@AAA?A?=AA?ABBBBBC=>;;:::8=888?<??:?A9==?AA
?@@8889AAA@??B
@T6XXI:00101:00435
CTCGAGATCTGCGGCTTTGGACAGTCCCTCTGTACTGTGAAGCCACAGATGTCGAGTCGACTAGCTTCCTTTCTCCC
TTCTCCC
+
CDCDCBCAB;;<B?CDC@C?BBBAACCBCBBAAABBBABD:;;@CCCCCBAEBCCBAACBBBBABC@B@CC?CBBB?
B@BBBB=
@T6XXI:00102:00401
GGGAGAAGGGAGAAAGGAAGGGGAGAAGGGAGAAAGGAAGCTAGTCGA
+
CC>BCC@CC=9;;@<@=@>BBC7A??676-7777.60736.-.....,
@T6XXI:00102:00407
GGGAGAAGGGAGAAAGGAAGGAGAAGTCGCCCCAAACGATTTGTTGACTATTCTCCCTCTCCCTATAGTGAGTCGTA
TTAGAAT
+
7<6;19599-
844;587=<;7;@>=@?@@BEC9CB=@8;8@;=<8:8@C@@=@AAC<A998?:?<=???AD@=<<@@C?AAA>A
@T6XXI:00102:00408
GGGGAATTCTAATACGACTCACTATAGGGAGAGGGAGAATAGATCACCGTTGGTTCGGGTGCGCGTTCTCCTTCCTT
TCTCCCTTCTCCC
+
<<<4<?A?BCB?BB@?@BCCAB@A?611.7=<>@<A@@<@?=<<@AA=AE;;;;@CCC>CBBBBBB>ABB@D?B?BC
>B?=<7<????C>
@T6XXI:00102:00409
CTAGTCGACTCGAGATCTGCGGCTTTGGACAGTCCCTCTGTACTGTGAAGCCACAGATGTCGAGTCGACTAGCTTCC
TTTCTCCCTC
+
@99449444444;444>999@=?CC?CC??86111,16679@97<<--,-
/,/;437<;;;99<7::;<AAA<9770..+/000+++
@T6XXI:00102:00412
GGGAGAAGGGAGAAAGGAAGGAGAAGTCGCCCCAAACGATTTGTTGACTATTCTCCCTCTCCCTATAGTGAGTCGTA
TTAGAATTCCCC
+
;B9=9959?:>>@B8;8;=?<@BCCAAA@@@@7@D>CCBBB;CC@CCC@@@?===D<A==>A<@?@@==<AA>>?99
9>AAA>B;AAA5
@T6XXI:00102:00414
GGTTATCAAATCGTAGTCGTTTCAGGTCGTGAAAGTGGAACTAAAGAAGACCCAACGAAATATTATCGTATGACCCG
TAAATGGGTTGAGGACATTGCTGGCGTTCCATTAGTTATGCAATGTCAGCGCGAACAAGGCGATACCCGTAA
+
CBC@CBBBC>BB>>@BBCBBCBCBB?B@<<7::5;@B>B?BCBB=BB>???C<@:????9=AA=::8>>????AC?C
@@@<@@A<?>?@A>@AAA>???@698?=?=@@;==A>AAAAB;==A??@==<>>==@<?=@@???ABAA@@A
@T6XXI:00102:00426
GGGAGAAAGGAAGTAGTCGACTCC
+
CHACECD@B==;@>1121..2-*)
@T6XXI:00102:00429
GGGGAATTCTAATACGACTCACTATAGGGAGAGGGAGAATAGTCTTGCAATTTCCTGCGGTGCATTCTCCTTCCTTT
CTCCCTTCTCCC
```

```
+
CBD:C@B@BDD@CA?=AB???AB96111.7>>>A>BBB?@=@A4738;?49919<@@9;8<BBCDACCC=>;>4880
7===6=:777>6
@T6XXI:00103:00402
GGGAGAAGGGAGAAAGGAAGCTAGTCGACTCGACATCTGTGGCTTCACAGTACAGAGGGACTGTCCAAAGCCGCAGA
TCTCGAGTCGACTAG
+
DCAAA====<@A>>9?8;6;C?@>>000/.47/67<<:?@C?AA@CCCB>BCC94300-
4;;?BC=??;@C=@>AAAA<9966?ACC=<=?@
@T6XXI:00103:00427
GGGGAATTCTAATACGACTCACTATAGGGAGAGGGAGAATAGCCCACACTTTTGCGACTCCGGCCGTTCACTTCCTT
TCTCCCTTCTCCC
+
8880<-959449?@@@B@@;<:???777-7==>A<BAC===844-
4::9;AA4<;;7///,/999:777=;844/00+034448280001
@T6XXI:00103:00432
GGGAGAAGGGAGAAAGGAAGGCTAGTCGCTCGACATCTGGTGGCTTCACAGTACAGAGGACTGTCCAAAGCCGCAGA
TCTCGAGT
+
::-
49?69909@DD>@6948<<76;;?76161111111626?<<@@@CAAACA??;<=;;<<@@@>AA:==233034...
53336
@T6XXI:00103:00434
GGGAGAAGGGAGAAAGGAAGTGCAGTTCGCGGTTAGTGAAGGGAAAAAACTATTCTCCCTCTCCCTATAGTGAGTCG
TATTAGAATTCCCC
+
CDAAAGACC>BBCC?C@C@CAABABB@CCCB@C@AACBB?AA9???AA-
ACEC@CCCC>AAACC>CAA???A@@@@@@@A?AAB>B?AAA6
@T6XXI:00103:00444
GGGGAATTCTAATACGACTCACTATAGGGAGAGGAGAATAGCTAC
+
>A@6A=@=@@@ACCCCB?>>BABBA997.7<<>;@//,//,+,,,
@T6XXI:00103:00447
GGGAGAAGGGAGAAAGGAAGCTAGTCGACTCGACATCTGTGGCTTCACAGTACAGAGGGACTGTCCAAAGCCGCAGA
TCTCGAGTCGACTAG
+
EBCBB@;=95>>>>9>595;B?000/00::79071677ABC?BB@CBBAAAC===86648:?@AC?<@<@A>??AA<
<<<<<<<====@@??
@T6XXI:00104:00405
GGGGAATTCTAATACGACTCACTATAGGGAGAGGGAGAATAG
+
4>>6@8=899@<B??=@100736.1166,1.///+.--33--
@T6XXI:00104:00409
GGGGAATTCTAATACGACTCACTATAGGGAGAGGAGAATAGCAACGAAGCGCTTGTTG
+
DDB9A6;7;AC?BBCBBCCBBCB@?666.6===;@==8:4//.,+,,*,+++004574
@T6XXI:00104:00410
GGGAGAAGGGAGAAAGGAAGCTAGTCACTCGACATCTGTGGCTTCACAGTACAGAGGGACTGTCCAAAGCCGCAGAT
CTCGAGTCGACTAG
+
CCADCB@BD>BB?=6@6428?B>??A????????:96>>@::;9;BBCBACC??7441799;;<9<=7=>;>@@888
=@@@ABA@@@AAAA
@T6XXI:00104:00411
GGGAGAAGGGAGAAAGGAAGGGGAGAAGGGAGAAAGGAAGCTAGTCACTCGACAGTCTG
+
BC@CCB@CC>?A@C?C@C@BBB9BAB?<C=?:96-7;><@B@@AB>7./0,+0***+++
@T6XXI:00104:00418
```

```
GACTCGAGATCTGCGGCTTTGGACAGTCCCTCTGTACTGTGAAGCCACAGATGTCGAGTCGACTAGCTTCCTTTCTC
CCTTCTCCC
+
AAABB@@<;<<;<BB@??@:>699==@>A>BBBACB@AACCC=A@A;;;BBBBB@@@AAAA;::>7795858>:>>>
?/:2:<?C>
@T6XXI:00104:00419
GGGAGAAGGGAGAAAGGAAGGAGAAGTCGCCCCAAACGATTTGTTGACCTATTCTCCCTCTCCCTATAGTGAGTCGT
ATTAGAATTCCCCATCACCGA
+
4:6999289199AB<CABC?>@==;0/..0..*0689>CCB>@45://,-
144599=;968<<698:8/..445755==<==531,---*---.344=
@T6XXI:00104:00448
GGGGAATTCTAATACGACTCACTATAGGGAGAGGGAGAATAGTCAACAAATCGTTTGGGGCGACTTCTCCTTCCTTT
CTCCCTTCTCCCT
+
DDE:DABCAAA@C<<<B@@@@????=>@:@@?AB>BA@=@?=<;=;<;;6?B??@=@EE9CBBAA>@@@:<9<<@@;
854<49/4-//,/
@T6XXI:00105:00403
GGGAGAGAATTTGGTAATGGGCAACCTGTCCCCCCATGGCTAAATTATTCTCCCCTTATATCTCTCTCCCTATAGTG
AGTCGTATTAGAATTCCCC
+
CB<AACFC@BA?B@BBACAA=BDBB@CCCDDCBA-
CAAAACCCAC@CB=?8899/9<>@BCCA??C888/8@@>@ABBDG<<<BC?AA@;>5999/
@T6XXI:00105:00409
GGGAGAAGGAGAAAGGAAGGAGATGCCAACGCGAAAACACGATTGTGGCTAGTT
+
66,1//,/,//4:-424237779;1743997799B?8?;:;3311110./0..,
@T6XXI:00105:00425
GGGAGAAGGGAGAAAGGAAGCTAGTCGACTCGACATCTGTGGCTTCACAGTACAGAGGGACTGTCCAAAGCCGCAGA
TCTCGAGTCGACTAG
+
AC>E@B59919@@?:@5948?3339111111111111161101311179;9?884344,4;;3588<94:?5<<=<<
<888?<<;837;:43
@T6XXI:00105:00426
GGGAGAAGGGAGAAAGGAAGCTAGTCACTCGACATCTGTGGCTTCACAGTACAGAGGGACTGTCCAAAGCCGACGA
+
HNCCBA>@?9>98809<@=@@CBBBBB:77??A?@>@CCD=?@@CB????C@9933317??AA?9556..))))((
@T6XXI:00105:00431
GGGAGAAGGGAGAAAGGAAGAAGATGTCGCCGCAGGGAGTGAACTTGGCTATTCTCCCTCTCCCTATAGTGAGTCGT
ATTAGAATTCCCC
+
CF>AAC?A;-
891<8;::500,..644=BA>B:;0=9>>//.4//,424444244;;588433,39779?=<<>B:66<:7110424
44,
@T6XXI:00105:00433
GGGGAATTCTAATACGACTCACTATAGGAGAGGGAGAATAGACACCAATCGCGCCGGGTCATGTTCTCCTTCCTTTC
TCCCTTCTCCC
+
A@>6>598<BA?CA@?BABBB>=95..,0><BC>A??>A9==@@@<?>B@@?CC=??=@AAABA@ABA>@<?<?C?@
@@B=B?????:
@T6XXI:00105:00443
CTATCACTCGAGATCTGCGGCTTTGGACAGTCCCTCTGTACTGTGAAGCCACAGATGTCGAGTCGACTAGCTTCCTT
TCCTCCCTTCCTCCC
+
B99994444449=@FDA@@@???:?<?@??CDD>BBBBCECB???@>AC@BCCCC;<;BB???AA88771//64445
+.,.3322,4/4880
@T6XXI:00105:00445
```

GGGGAATTCTAATACGACTCACTATAGGAGAGGGAGAATAGTCAACAAATCGTTTGGGGCGACTTCTCCTTCCTTTC
TCCCTTCTCCC
+
CCA4@54544824499@?AAA=<::00,/<<?A=:88<;<9;83/004261117,111,16544,..3,-,-
33739.37178;<AC?
@T6XXI:00106:00401
AGGGAATTCTAATACGACTCACTATAGGAGAGGGAGAATAGTGACGTGTTGTGCGGCGCTCACTGTCTCCTTCCTTT
CTCCCTTCTCCC
+
ABB>A=@=AAA?CDCCCCCCBB>@<00,0?:::9???=@CCCCB@998A=A889@>@?@@<;;=8887?=A:=:=D?
C@@A?A;=9;92
@T6XXI:00106:00410
GGGAGAAGGGAGAAAAGGAAGG
+
4904@@599194111*/,/,.,
@T6XXI:00106:00412
GGGAGAAGGGAAGAAAGGAAGCTAGTCACTCGACCATCTGTGGCTTCACAGTACAGAGGGACTGTCCAAAGCCGCAG
ATCTCGAGTCGACTAG
+
::-
4//566+/,///+/::;>?FAB?700//0//,....46;5:;@CCCBCCCC@;8887<?====8;;5<@>ACAAAA?
??<<<<:??AA<A
@T6XXI:00106:00423
GGGAGAAGGGAGAAAGGAAGGATAGCGAGTC
+
CCABBA@BB>CBBB?A>@>><;A@@;1.44.
@T6XXI:00106:00428
GGGAGAAGGGAGAAAGGAAGGGCACGCGCGAGTCATCCAATTGGGAGCTATTCTCCCTCTCCCTATAGTGTAGTCCG
TATTAGAACTTCCCC
+
991944244-///8+/,/,..+.47@@@::99994442499244-44444;99444-
4;877:<<?3.,,,,2,,*,.-04===5:677::6
@T6XXI:00106:00436
GGGGAATTCTAATACGACGTCACTATAGGGAGAGGGAGAATAGCTACGTTTAACCGATTCGTGTTGTTCT
+
@BC4@596944244<11100=>>>CCCCC9<:777.7766699BB=@>?>97567,,,*+0**+0++*--
@T6XXI:00106:00438
GGGAGAAGGGAGAAAGGAAGTGCAGTTCGCGGTTAGTGAAGGGAAAACTATTCTCCCTCTCCCTATAGTGAGTCGTA
TTAGAATTCCCC
+
DD@ABB@BB=BCCC>C?A?@CBBBBB@ABBB@C@A@ACC@??>??>8BBBB@@>>@;BCCCB>B@@??<><@>>;;<
;8<@A?A?ADA6
@T6XXI:00107:00406
GGGAGAAGGGAGAAAGGAAGTAGGGTGCATGCCAGGGGCGTTAGTGCTATTCTCCCTCTCCCTATAGTGAGTCGTAT
TAGAATTCCCC
+
DEAAAA@CD@AACC?F@B?>@>>>9//1>>==>DCBB7BAA?A??=ABCD?AAAA<A@???:?@==?BBAA@@===?
<?AA??8?DC6
@T6XXI:00107:00413
CTCGAGATCTGCGGCTTTGGACAGTCCCTCTGTACTGTGAAGCCACAGATGTCGAGTCGACTAGCTTCCTTTCTCCC
TTCTCCC
+
ABA==<CCB@AAA@CCC@C@CCBBBFD@CCC@@@BB@ABB=AA?BBCCCCCCBBCBAAA>?@B;:;@@59<89?AA=
B?A@@A<
@T6XXI:00107:00414
GGGGAATTCTAATACGACTCACTATAGGAGAGGGAGAATAGCCAACAGGTATCTGATTGCGCGTTCTCCTTCCTTTC
TCCCTTCTCCC

```
+
ECC9B8;;<CCBCCBCC?@@B;777//,0>:=?<?@A@@;@A>=;@@@>@@@@@@@@A;?=@@@@>@@A38347886;
<@A:@=@???<
@T6XXI:00107:00417
GGGAGAAGGGAGAAAGGAAGCTAGTCACTCGACATCTGTGGCTTCACAGTACAGAGGGACTGTCCAAAGCCGCAGAT
CTCGAGTCGACTAG
+
CB>@@@@CC@BCBB>C@CBBB?>@A:;7:@>7::?88?AA?AB?@????@@=88333-
4;;;=@<?@:?@>AAAAAA??888<;@=>?@99
@T6XXI:00107:00428
GGGGAATTCTAATACGACTCACTATAGGGAGAGGGAGAATAGACTCGACT
+
DED:B::;;CCACCA@ABBBA?<96166.7===?<?A@?;5*****(**,
@T6XXI:00107:00433
GGGAGAAGGGAGAAAGGAAGGGAATAGTCGCGCTTGTTATCAACGTGCTATTCTCCCTCTCCCTATAGTGAGTCGTA
TTAGAATTCCCC
+
GC>AA@=@?:>>>@<>;@>BB=B@BBCCBBBC@C@CD?BB=;8;B998B>?<>9991999>><ACBBBBBDBA@@?@
??AAB@CFCBB:
@T6XXI:00107:00442
GGGAGAAGGGAGAAAGGAAGGAGAAGTCGCCCCAAACGATTTGTTGACTATTCTCCCTCTCCCTATAGTGAGTCGTA
TTAGAATTCCCC
+
CKAECC>BB>;<<C@C@C@C@CBB=?=>7777-
8808=?CA>AC@><A?BC?A???<?@@CC>A8==@A@A@@??===?A@?<A/444-
@T6XXI:00107:00448
GGGAGAAGGGAGAAAGGAAGCTAGTCGACTCGACATCTGTGGCTTCACACAGAGACGTGTCCAAAGCCGCAGATCTC
GAGT
+
??:@A><AA<@@@@:@@C>AAB<??ABBB?A??::;:7@@B=@?=?7*)((((((,,,...,//+/67::?======
>ABB
@T6XXI:00108:00400
GGGAGAAGGGAGAAAGGAAGGAGAAAGCGACGTCGGGGAAACGTTTGCTATTCTCCCTCTCCCTATAGTGAGTCGTA
TTAGAATTCCCC
+
FC>BBB@BB>ABBB>BAB@B@BCCC>@BBBCBBACCC8CC?CCCD@BDCCC@CCCCBCBCAB=ACBBBBBDBBB???
?9<<C>@=?CC6
@T6XXI:00108:00409
GGGGAATTCTAATACGACTCACTATAGGGAGG
+
9=92?6969@@599@;;7;4;/0.....+..3
@T6XXI:00108:00423
GGGAGAAGGGAGAAAGGAAGCTAGTCACTCGACATCTGTGGCTTCACAGTACAGAGGGACTGTCCAAAGCCGCAGAT
CTCGAGTCGACTAG
+
?B>BAB=@99>>==9@>A>AACBAAA;:770/.....56?<?@@C@@@BFFC???AA<????7734849;8<@A@@@
C==<>;;488====
@T6XXI:00108:00432
GGGAGAAGGGAGAAAGGAAGGAGTTCCGGTGTTGGAGAAAGCATAACGCTATTCTCCCTCTCCCTATAGTGAGTCGT
ATTAGAATTCCCC
+
BB>CCC?CB=BCBB=?=?@C?@AB?C@A@BBB@C@C@@@>@CCDC??BBCCC@C:::8;AACC@DDDABBCCB@@@=
==>@AA?A27780
@T6XXI:00108:00438
GGGGAATTCTAATACGACTCACTATAGGGAGAGGGAGAATAGTCAACAAATCGTTTGGGGCGACTTCTCCTTCCTTT
CCTCCCTTCCTCCC
+
```

```
BDA7A=A=@BBACBA@BBB>?C?97111.7>==?=@AD@D;AABB=@AA=CAAAB>BCC69:16601/57322,--
+-,--333,0/0330
@T6XXI:00108:00447
GGGAGAAGGGAGAAAGGAAGTGCAGTTCGCGGTTCATGAAGGGAAAAACTATTCTCCCTCTCCCTATAG
+
4469AA@CC<B<<<;<BBBB;;;@=:5868634,.////,4=:=ACC.;;9989444-44///+/...4
@T6XXI:00109:00405
GGGGAATTCTAATACGACTCACTATAGGAGAGGGAGAATAGACACCAAATATTCCCCAGTTCGCCCAGCTTCCTTTC
TCCCTTCTCCC
+
CCC8@>A?BCC@CC?AACCBBBAA@::37?==?>?AC>A?@AAAA>A@:=>>4777.7>;;ABDD>B@AA?C69968
B;;1858<=@<
@T6XXI:00109:00414
GGGAGAAGGGAGAAAGGAAGAGGACATTCGTGTTGGGGAGCTATAGGCTATTCTCCCTCTCCCTATAGTGAGTCGTA
TTAGAATTCCCC
+
CC@BBB@BB>AAAB>B?B=?@B@CCBCACCA@@ACDD9BBB<<;00,0=>@?BBCC>EBCCC>BABABBA??@====
?<?>@<;/444-
@T6XXI:00109:00415
GGGAGAAGGGAGAAAGGAAGGCTGCTGTGGCTTCATGTGTAGGGAGACTATTCTCCCTCTCCCTATAGTGAGTCGTA
TTAGAATTCCCC
+
CGAAA?@CBBBBBB=B?B@C@BBBBBAA@@@A?A@@@@=B@CC>ABABBBB?BFED>AAACC>A@??@AA@@??==;
;9;>@=BCCEC7
@T6XXI:00109:00426
GGGAGAAGGGAGAAAGGAAGGAGAAGTCGCCCCAAACGATTTGTTGACTATTCTCCCTCTCCCTATAGTGAGTCGTA
CTTAGAATCCCC
+
BD>CAABAC?BBBB?B?B?B@BBB@??BBBBB8BBAB@@@B>B?>@@@<<<8<<==5:8<<=;=<88===AA=799.
//,/664----*
@T6XXI:00109:00428
GGGGAATTCTAATACGACTCACTATAGGGAGAGGGAGAATAGTCAACAAATCGTTTGGGGCGACTTCTCCTTCCTTT
CTCCCTTCTCCC
+
@@@6@>B?BCC@CCB??>BBBAB96111.7AACC>B@@>A===;;:?<=;C==<>8<<>7BCCCC?AAA?@><<CC<
@@AA<A>@@AB<
@T6XXI:00109:00436
GGGAGAAGGGAGAAAGGAAGCTAGTCGACTCGAGATCTGCGGCTTTGGACAGTCCCTCTGTACTGTGAAGCCACAGA
TGTCGAGTCGACTAGCTTCCTTTCTCCCTTCTCCC
+
BD?BAA=@@<@BCC@D@B@BCB>;>1/.35////BB=::;?<?AC>C@CBBCCCE>889??AAA@@?@>@A>@@===
@@@@AAA@?@@@@?889>@>AC9=<@@18588=B;
@T6XXI:00109:00438
GGGGAATTCTAATACGACTCACTATAGGGAGAGGGAGAATAGCCAACTCACCACATCTCCCTGACGTACATCTTCCT
TTCTCCCTTCTCCC
+
CCB9B=A>ACB@B@@@BBBBBC<97111.7>>>@>B@@>A<@A999=AA??;@888?@@C=AA???@@@AA??;=9;
94;99;6;8;;;;6
@T6XXI:00109:00442
GGGAGAAGGGAGAAAGGAAGGAAGGGAGAAAGGAAGTAGTCGACTCGACATCTGTGGCTTCACAGTACAGAGGGACT
GTCCAAAGCCGCAGATCTCGAGTCGACTAG
+
CC?DB@<??<>@@B>B?B?A>A<??:?A@?<B:?:;0/0:0..5;55/08899<=C???<?@@===EBAB???<<@8
88?<?A<@CA;99?@@@AA===?AA@@CC;
@T6XXI:00109:00447
GGGAGAAGGGAGAAAGGAAGGGAAGAGCCGGGAACAAAACGTCGATGGCTATTCTCCCTCTCCCTATAGTGAGTCGT
ATTAGAATTCCCC
```

```
+
CCACDB@BB?999@0969688/;>?A@B?BA:?<8777.7@;=:77<<<<>A>???@;<=9?A<ACAA??8436688
<=;===<B9===4
@T6XXI:00110:00407
GGGAGAAGGGAGAAAGGAAGG
+
//+/8;89=6//=A,1,/,-,
@T6XXI:00110:00423
GGGAGAAGGA
+
11-,**)*)(
@T6XXI:00110:00431
GGGAGAAG
+
CC?CCC@C
@T6XXI:00110:00437
GGGAGAAGGGAGAAAGGAAGGAGACACGGTCACAAACATGAATGTGACTATTCTCCCTCTCCCTATAGTGAGTCGTA
TTAGAATTCCCC
+
CC@BB@@BBB?AAB>B=B698:AECCBB?BBA@BB>CBB@@=@BAAAADCC@C===7<AAAA<AAAAAAA>@@A@?<
?>A??<?>AAA6
@T6XXI:00111:00414
GGGGAATTCTAATACGACTCACTATAGGGAGAGGGAGAATAGTCACTGGCTTGGTTGTCTGTTATTCTCCTTCCTTT
CTCCCTTCTCCC
+
CCB9A=?=@BB@CBB@BAAAA?<97116.7==?C>CAA>D;AA<<??=;;=?A=?=?;;<>?<=@=;<<8<;;9>@:
==<C<A388<:4
@T6XXI:00111:00423
GGGAGAAGGGAGAAAGGAAGGAGAAGTCGCCCCAAACGATTTGTTGACTATTCTCCCTCTCCCTATAGTGAGTCGTA
TTAGAATTCCCC
+
AA?AAB@BC?B@??<@>@>B@BCB@C?>BBDC8AA=ACCBC<ABACA==@A?====;::??=;=:>>;AA==:;???
?>A??<@?CCA6
@T6XXI:00111:00426
GGGAGAAGGGAGAAAGGAAGGAGAAGTCGCCCCAAACGATTTGTTGACTATTCTCCCTCTCCCTATAGTGAGTCGTA
TTAGAATTCCCC
+
CCACCB@BBBAAAB>B@C?@><BB@??BBBBB8BB<B??CC>CC?CA>?@@>@@AA<?>??C>AA?AAA@@@AAA==
=?CA@>@9<<<4
@T6XXI:00111:00438
GGGGAATTCTAATACGAACTCACTAT
+
499/4:;:==82411,,*,,,,...1
@T6XXI:00111:00440
GGGGAATTCTAATACGACTCACTATAGGGAGAGGGAGAATAGTTACGGCACATTCTGCAGTCTCCTGTTCTTCCTTT
CTCCCTTCTCC
+
CCB9A=?CABBCE@@>BBBBBC<97111.7>>@B>BAA=@;<=<:5587:::@@??=CC@BBCCA<88@?A;?577.
70//,/,/-.,
@T6XXI:00111:00442
GGGGAATTCTAATACGACTCACTATAGGGAGAGGAGAATATAGGACCTAATC
+
880088@=@CC@CBBBBAA@BABB@77:8:CBB@?8837B;3.--30-.,--
@T6XXI:00112:00416
GGGAGAAGGGAGAAAGGAAGCTAGTCACTCGACATCTGTGGCTTCACAGTACAGAGGGACTGTCCAAAGCCGCAGAT
CTCGAGTCGACTAG
+
```

NF@AA>@BCCBBBB?C?A?AB>>@C=A?CED;;;;<;ABE@AA?BDC??>@?:433317>?AAA<??<?A<??AAA@
?@A@A@AA=9=@@@
@T6XXI:00112:00424
GGGGAATTCTAATACGACTCACTATAGGGAGAGGGAGAATAGTTCCAGACATCG
+
999,4;@DCCB@CBBB???@A@>97111.6<777.7;;99;44/4/00330000
@T6XXI:00112:00435
GGGAGAAGGGAGAAAGGAAGCTAGTCGACTCGACATCTGTGGCTTCACAGTACAGAGGGACTGTCCAAAGCCGCAGA
TCTCGAGTCGACTAG
+
DBCBCC@CC?BBAA=B@BACBB////00//...119?@@BC@AB?BBCBCBCAAA===7<>C??@>@@<@A?@@@AA
AA?????????@AAA
@T6XXI:00112:00439
GGGAGAAGGGAGAAAGGAAGGCGATAGCACCATGGGAGGTTTGGTGACTATTCTCCCTCTCCC
+
DC>@AB=>>19911-1:;,0,.9957B@AA;>166,763668<>@???;8131111,16111,
@T6XXI:00112:00441
GGGAGAAGGGAGAAAGGAAGCTAGTCGACTCGACATCTGTGGCTTCACAGTACAGAGGGACTGTCCAA
+
LCAAAC>@@<@A>><@=@<>??:::B???CDBBBBCCCCAC@BA?AA???@?A?@<<<6;?>9:<388
@T6XXI:00112:00446
GGGGAATTCTAATACGACTCACTATAGGAGAGGGAGAATAGTGACGTGTTGTGCGGCGCTCACTGTCTCCTTCCTTT
CTTCCTTTCTCCCTTCTCCC
+
BAA9A>A>BCC@CCBBB@A@B:885//,0;;?C?C@@>@??@=@A??<A>@A<A=888:;<><<<;<?A>=:=333,
3;9;244-4444-47:=?D<
@T6XXI:00112:00448
GGGAGAAGGGAGAAAGGAAGCTAGTCACTCGACATCTGTGGCTTCACAGTACAGAGGGACTGTCCAAAGCCGCAGAT
CTCGAGTCGACTAG
+
DD>BBB@DC>BB==:=;8;?@AABBAA@??=7:9?::CBB@BB@CCA???@=4;33017;=?@A?BC>BA=?@<@<A
=@????@A@===@@
@T6XXI:00113:00419
GGGAGAAGGGAGAAAGGAAGTGCAGTTCGCGGTTAGTGAAGGGAAAAACTATTCTCCCTCTCCCTATAGTGAGTCGT
ATTAGAATTCCCC
+
CB?BBB@BB?BBBB?CAB@A?ABBBB@B??@@C?BBCBB?BC>CCBC2CCCC@CC??;@?@@@;>::==;=AAA?@@
@@>@@@>H>A@A5
@T6XXI:00113:00421
GGGAGAAGGGAGAAAGGAAGGAGACAGTGAGCGCCGCACAACACGTCACTATTCTCCCTCTCCCTATAGTGAGTCGT
ATTAGAATTCCCC
+
FGAEEC@CB>BBBB>B@B@B?@AABCDCBCBBBB>????>;;??>>>@@ACB@CA??<@CCDB<>ABBBABCBDBBB
BC?AA?:?4733,
@T6XXI:00113:00424
GGGGAATTCTAATACGACTCACTATAGGAGAGGGAGAATAGTCAACAAATCGTTTGGGGCGACTTCTCCTTCCTTTC
TCCCTTCTCCC
+
CCD:C@B@BCC@CCBBA<BBB:997/.,0>07:8:BAB@?@@@@;9==;CB>B@>BCA7AABBC?CBB>=;=>AA<A
???<?;==AC:
@T6XXI:00113:00436
GGGAGAAGGGAGAAAGGAAGGAGAAAGCGACGTCGGGGAAACGTTTGCTATTCTCCCTCTCCCTATAGTGAGTCGTA
TTAGAACTTCCCC
+
9919448995888:-469<><?783,1779<<;<<995::69444-4::4424444-
44;99:<79668889966565686994110111,
@T6XXI:00114:00404

```
AATACGACTCACTATAGGGAGAGGGAGAATAGTCAACAAATCGTTTGGGGCGACTTCTCCTTCCTTTCTCCCTTCTC
CC
+
B@BBBB@@@@@CCB?9976:?>?B>BBA@BBBCC=>=BC@CCBB@<???5?ACCC>@@B?B@B@BB>BB?@;C???@
CA
@T6XXI:00114:00410
GGGAGAAGGGAGAAAGGAAGGCAGGGCCATCTGTGGCTTCACAGTACAGAGGGACTGTCCAAAGCCGCAGATCTCGA
GTCGAC
+
CB>BCB@BB==??B>B@B>;74...+0,/9<<BBB@CC>>>=888@@@?8667<?@@AA?CC<=99==A@A@<<=@@
?@BCCB
@T6XXI:00114:00413
GGGAGAAGGGAGAAAGGAAGAAGAATAGAAGAACACATGTCGGTTGGCTATTCTCCCTCTCCCTATAGTGAGTCGTA
TTAGAATTCCCC
+
490449584-444:-
424239777<:??CADD@?@BBB?88636010111636100+00000+04446=4333++++002262344==5
@T6XXI:00114:00423
GGGAGAAGGGAGAAAGGAAGCTAGTCGACTCGAGATCTGCGGCTTTGGACAGTCCCTCTGTACTGTGAAGCCACAGA
TGTCGAGTCGACTAGCTTCCTTTCTCCCTTCTCCC
+
FE>BAA=AC>CBBB=B=@=@B>7;;/00<;;;>>CBBBB??:????9A?CBBBCCC>A??<@@@AA???>@A>@???A
@===@@AAAA99=A???>:9;:99666,/,/8;;6
@T6XXI:00114:00427
GGGGAATTCTAATACGACTCACTATAGGGAGAGGGAGAATAGTTACATCTCCCATCACGGCGAACTACTCTTCCTTT
CTCCCTTCTCCC
+
DCD:CACACBC@CBCBABCAC><9761659??CC>B@?=?===?A???:??=;<<<=<<:::=<==::;<A?A>AC<
AA@@:>9==AA<
@T6XXI:00114:00437
GGGAGAAGGGAGAAAGGAAGTAATTGCCATGGAGAAGGGAACTTGGCTATTCTCCCTCTCCCTATAGTGAGTCGTAT
TAGAACTTCCCC
+
DC?AAA@AA=???B=B@BA@@@=B?@C?;;;7378477.7:66698337>2:6AB>:;8<<<>AAADBA@<;:4448
/4099945553.
@T6XXI:00114:00443
CTATCGACTCGAGATCTGCGGCTTTGGACAGTCCCTCTGTACTGTGAAGCCACAGATGTCGAGTCGACTAGCTTCCT
TTCTCCCTTCTCC
+
A:8811111:@CCCBBBB?><@CC>B<@BCAAAB>ACCBBAB@@@@@=@C@CBBBBBBABBBB;<<CCDBBBB:;7;
C<AA?@<@<??A>
@T6XXI:00114:00448
GGGAGAAGGGAGAAAGGAAGCTAGTCACTCGACATCTGTGGCTTCACAGTACAGAGGGACTGTCCAAAGCCGCAGAT
CTCGAGTCGACTAG
+
9@<<<9@BB9<;BB<@=@ABCCA@@A7111111177667<<<B?ABCBAA?=;;444-
4:;;;?<?@7>?<??@AAA@@@><=8444::::
@T6XXI:00115:00411
GGGGAATTCTAATACGACTCACTATAGGGAGAGGGAGAATAGTCAACAAATCGTTTGGGGCGACTTCTCCTTCCTTT
CTCCCTTCTCCC
+
CCC:D?B?BCB@BBAABCBB87:?;677.7==@B?B@@?@<?A==<9==7?A??C;@@B7BCCCC?BCD>=;=>AA<
??@A=A=@@@A<
@T6XXI:00115:00418
GGGGAATTCTAATACGACTCACTATAGGGAGAGGGAGAATAGTCTCTTATTCTCTCTACAACTCCCATCATCGTCTA
CTTCCTTTCTCCCTTCTCCC
+
```

```
BBB9B>A?BBB@BA??A@BBBAB97111.7<<BC@CBB?A;::<<9=:??999;B?CC?A?=<<>;<><==9=<?;;
8979/34-49::4:8;3894
@T6XXI:00115:00426
GGGAGAAGGGAGAAAGGAAGCTAGTCGACTCGACATCTGTGGCTTCACAGTACAGAGGGACTGTCCAAAGCCGCAGA
TCTCGAGTCGACTAG
+
CC>@9@299099997;AC@BBB>>;.00:0000:6787ABCAAA?BBCC@?A=9<33313=6@AA>AA<AA>@?:?@
@:?????AA@@@@@@
@T6XXI:00115:00430
GGGGAATTCTAATACGACTCACTATAGGGAGAGGGAGAATAGCAAACGTTTCCCCGACGTCGCTTTCTCCTTCCTTT
CTCCCTTCTCCC
+
FDDAC8;7;@@>AACDCBCCCCB?=666.7=>>A@BCC>A???A<:@AA?>ACC9BBBC::;AAB>A::34:;344-
4:;:7=9=;<=9
@T6XXI:00115:00435
GGGGAATTCTAATACGACTCACTATAGGGAGAGGGAGAATAGCCTC
+
CCB9B=AC@CCACCBBBBB?>ABB<77759?ACC>C96650+*)*)
@T6XXI:00115:00436
CTATCGACTCGAGATCTGCGGCTTTGGACAGTCCCTCTGTACTGTGAAGCCACAGATGTCGAGTCGACTAGCTTCCT
TTCTCCCTTCTCCC
+
B77<1///68:BBBCBBBBA?ABB?B=?@AA???;?BBCCCBB@?===8<8<CB@ABDCCCCB:<<@@@CBB??C?C
D>@?<B6<8<89;-
@T6XXI:00115:00440
GGGAGAAGGGAGAAAGGAAGCTAGTCACTCGACATCTGTGGCTTCACAGTACAGAGGGACTGTCCAAAGCCGCAGAT
CTCGAGTCGACTAG
+
DC@;<;?BB>BBCC@C@CABAA>A=??=<>>776999@AB?BB@BBECCC@?;>77418==?@@>@D<A@:==AAAA
@@@???=787;;;=
@T6XXI:00115:00442
GGGGAATTCTAATACGACTCACTATAGGGAGAGGGAGAATAGTCAACAAATCGTTTGGGGCGACTTCTCCTTCCTTT
CTCCCTTCTCCC
+
BCB7B>B?BBB@C???BBBBB@>96116.7>==?<@CC>@?AAAA=A==;A=<<>9>;B7BAAAD@CCC==:=?CC:
BBBB:A;8>AA<
@T6XXI:00116:00415
CTAGTCACTCGAGATCTGCGGCTTTGGACAGTCCCTCGTACTGTGAAGCCACAGATGTCGAGTCGACTAGCTTCCTT
TCTCCCTTCTCCC
+
B/1//1/44AA=>?CDBBBC@CCC>C@BC?@@CD?BAAABCC@?=A@@@@B<===AA@A@;=@==86<>>;?>=>AC
:>>CC<@:=<<=7
@T6XXI:00116:00441
GGGAGAAGGGAGAAAGGAAGGAGAAGTCGCCCCAAACGATTTGTTGACTATTCTCCCTCTCCCTATAGTGAGTCGTA
TTAGAATTCCCC
+
CCAECC?AA<;;;C>CAC@C@CCC@A=>@@AA5@@<@CCEC=BB?BBC@@?=@@CC<A<<=B>@A@@@@@@A??@@=
=9<AB:=8;<:1
@T6XXI:00116:00444
CGACTCGAGATCTGCGGCTTTGGACAGTCCCTCTGTACTGTGAAGCCACAGATGTCGAGTCGACTAGCTTCCTTTCT
CCCTTCTCCC
+
AAA@AC@AACCFDCCC@CCC>CACECBBBC?BCB@@@CCCCCC?BB@BBAA?@>BBBB?C99?@AC88;@C@CG>BB
@@9;:<=<<6
@T6XXI:00117:00412
GGGAGAAGGGAGAAAGGAAGCTAGTCACTCGACATCTGTGGCTTCACAGTACAGAGGACTGTCCAAAGCCGCAGATC
TCGAGTCGACTAGCTTCCTTTCTCCCTTCTC
```

```
+
>:6?>?6990999914787;@;71=776111111688696068;=7;:::B;88//,/:48:?>AA<@@;<;=;66/
...++111--.--2444/3310004442331
@T6XXI:00117:00415
GGGAGAAGGGAGAAAGGAAGCTAGTCACTCGACATCTGTGGCTTCACACAGAGGACGTGTCCAAAGCCGACAGACTC
GCGAGTCGACTAGCTTCCCTTTCTCCCCTTCTTCCC
+
DC?CA@=>@:?99914242344...,0,,........667067>@>?B:777774611111049-
4484////,,,,,,66/44449=999:2//+/857////*/,/.4442
@T6XXI:00117:00427
GGGAGAAGGGAGAAAGGAAGCTAGTCGACTCGACATCTGTGGCTTCACAGTACAGAGGACTGTCCAAAGCCGCAGAT
CTCGAGTCGACTAG
+
CA?BCB@CC>ABAB=B?B@CC900=0..34../66687:7?@BC@BBBBC>?:A<8847>>???=?D@AA>@@@===
@@@?@@@@@@??>?>
@T6XXI:00117:00444
GGGAGAAGGGAGAAAGGAAGGAGAAGTCGCCCCAAACGATTTGTTGACTATTCTCCCTCTCCCTATAGTGAGTCGTA
TTAGAATTCCCC
+
DH@BAA@BD?BCBB?B@B@B@DAA>>?BBB??8??;B@??>9?@@@>><<<:<;==::>>8=;<<?=====><<??A
A>A@@>@?AAA6
@T6XXI:00118:00424
GGGGAATTCTAATACGACTCACTATAGGGAGAGGGAGAATAGACTGGTCGTCAAGGACAGTGTGTTCTTCTTCCTTT
CTCCCTTCTCCC
+
>>?2=;A@CCC@CBBB@@ABB@>;966659?ACC>CAA?A:;;=<<;;<@@AA=A=A@??>AA==<AB>BB>B>BB:
>?AA;A>@==<7
@T6XXI:00118:00431
GGGGAATTCTAATACGACTCACTATAGGGAGAGGGAGAATAGCTACCTAGTCTGTTAGCAGTGGCGTTCTCTTCCTT
TCTCCCTTCTCCC
+
BBB9C?B@CCCBDBBABCCCBA;97111.7>>CE>C>?<@=::<@@B@<??>>?<;=A==<;;;<<<;@@<=>A>==
;;?<=:;:;@??<
@T6XXI:00118:00442
CTAGTCACTCGAGATCTGCGGCTTTGGACAGTCCCTCTGTACTGTGAAGCCACAGATGTCGAGTCGACTAGCTTCCT
TTCTCCCTTCTCCC
+
:8///6/44BBBDBBBBCBA?CAA<A=>@CBBBB>BCCCB>>>?=>>;9A>@CCAABCBBACC?@AABBB@?:7:5:
B>???A=>;@?>@7
@T6XXI:00118:00443
GGGGAATTCTAATACGACTCACTATAGGGAGAGGAGAATAGCAGTGCAGGT
+
/11+17BACCC@BB@?@CCCCCCCAAA?954..,066467::44-///.,-
@T6XXI:00119:00405
GGGGAATTCTAATACGACTCACTATAGGGAGAGGGAGAATAGTCAACAAATCGTTTGGGGCGACTTCTCCTTCCTTT
CTCCCT
+
>>9292424;?29:@@><BCBBBCCCCD@BB???9?<@=@==@@@>@@@<BBAA@.777-
76611376;:449/44,4000+0
@T6XXI:00119:00415
GGGGAATTCTAATACGACTCACTATAGGGAGAGGGAGAATAGTTATGCCACCAGCCCAGTCTATTTCTCCTTCCTTT
CTCCCTTCTCCC
+
=@?6@>A?BCC@CBBBCBCCC@>96116.7>>AB>BBAAA=999=<@<>@@?@===;<<=<=@A@7<<?=@>@>AC:
>=?CAC9<<AA<
@T6XXI:00119:00417
```

GGGAGAAGGGAGAAAGGAAGGAGAAGTCGCCCCAAACGATTTGTTGACTATTCTCCCTCTCCTATCAGTAGAGTCGT
AT
+
BCBCDC@BA=ABAB=@?B=>=@CC@BCCCCBB8>B<B@@@@;B@?C?==B=;<<74+..//,/33--
300033334030
@T6XXI:00119:00426
GGGAGAAGGGAGAAAGGAAGACAGTCCATCTGTGGCTTCACAGTACAGAGGGACTGTCCAAAGCCGCAGATCTCGAG
TCGACTA
+
44-444244-
444;697:@CBB??::48>>00./,.:5/:79=;=;?88764=788<<9<=:>><<<;<<:;444900449<>>
@T6XXI:00119:00429
GGGAGAAGGGAGAAAGGAAGTGCAGTTCGCGGTTAGTGAAGGGAAAAACTATTCTCCCTCTCCCTATAGTGAGTCGT
ATTAGAATTCCCC
+
DFDCCB@BBBAAAA?A@A@>>>A>AC@BA?>@B@BBE@@@=B=B@?B1BBBC?ABCC>BBBCC>B<@@@==AAAAA=
==>@AA=??ADC6
@T6XXI:00119:00437
GGGGAATTCTAATACGACTCACTATAGGGAGAGGGAGAATAGCCATACTTGCTCCCAGTTGGCGTGAAACCTTCCTT
TCTCCCTTCTCCC
+
CCE:CAC@BCC@B@@?BBCABAB<;111-
677>A>BAA>@888:==<==?@AA@A=A@@?<888<@AC:B@A?B344-49::4<=@@@C<
@T6XXI:00119:00447
GGGGAATTCTAATACGACTCACTATAGGGAGAGGGAGAATAGCTAGCGTTTCCCCCGCAATGTATTTGTCCTTCCTT
TCTCCCTTCTCCC
+
CCC9B=@=;=<@CGCCCBABBCDCB:::5:A?BE>A??;==A@BCAA74459CCC1@==;=<7=;;2;-/,-
0103?6:===6<8;=>@:
@T6XXI:00120:00404
CTAGTCACTCGAGATCTGCGGCTTTGGACAGTCCCTCTGTACTGTGAAGCCACAGATGTCGAGTCGACTAGCTTCCT
TTCTCCCTTCTCCC
+
B@9999444;BBDBCBBBBB?CBC>B?CB@?@CC>CABBBB???=<@>@C?DCDCBDBEBBCCBBBBCCBAAB@D@C
C=??@?6:744494
@T6XXI:00120:00406
CTAGTCGACTCGAGATCTGCGGCTTTGGACAGTCCCTCTGTACTGTGAAGCCACAGATGTCGAGCGTTCCTT
+
CA<999499<;BB@ABC<<<CACEC@CCBBB;::B>:::::>==7103204248=@AABA<666,,,*,*,,
@T6XXI:00120:00444
GCTAGTCACTCGAGATCTGCGGCTTTGGACAGTCCCTCTGTACTGTGAAGCCACAGATGTCGAGTCGACTAGCTTCC
TTTCTCCCTTCTCCC
+
//444444449;CCCECCB>>;888,31337679=8<<AACBAAA:6556====9:::;;AACB;<<;;8??:67:>
AB=@:?B932;3334
@T6XXI:00121:00402
GGGAGAAGGGAGAAAGGAAGCTAGTCACTCGACATCTGTGGCTTCACAGTACAGAGGGACTGTCCAAAGCCGCAGAT
CTCGAGTCGACTAG
+
CCABBAACB?ABBC<@=@?BC@??????@AA:::???AAA?AB?ABAAACC?;;33317;9;=?:==9=CEDA@??@
<<<88<?744:944
@T6XXI:00121:00435
GGGGAATTCTAATACGACTCACTATAGGGAGAGGGAGAATAGTCAACAAATCGTTTGGGGCGACTTCTCCTTCCTTT
CTCCCTTCTCCC
+
CCB9C=?>@DB@BBBBA????AB?966659??CE>A>>>@9@??:::==;?:::>;>?>8>BB>C?CCC77348??<
?AAB=A<?@AA<

```
@T6XXI:00121:00444
GGGAGAAGGGAGAAAGGAAGCTAGTCACTCGACATCTGGTGGCTTCACAGTACAGAGGGACTGTCCAAAGCCGCAGA
TCTCGAGTCGACTAG
+
=A=ACA@CC>AABC<@=@?BB@><554......45516267017577?<<<<69;444-
4;;?AA>AC?@?<?@@CBAA;:/,,,,+0388<
@T6XXI:00121:00445
GGGAGAAGGGAGAAAGGAAGCTAGTCACTCGACATCTGTGGCCTTCACAGTACAGAGGACTGTCCAAA
+
11+///,//+/7:;58242.;00,2,,...3../66179;285:=?=?:;:=?;;483/66/-.,+0*
@T6XXI:00121:00448
CTATCGACTCGAGATCTGCGGCTTTGGACAGTCCCTCTGTACTGTGAAGCCACAGATGTCGAGTCGACTAGCTTCCT
TTCTCCCTTCTC
+
B=66///688=?@BCDCCCC@CAB>A>ABCCBBB>BCA@@?@?@@AA>@B>@@CCCBBBBBCCABB@@@FBCA>A>A
C=AA@A<A<???
@T6XXI:00121:00449
GGGGAATTCTAATACGACTCACTATAAGGAGAGGGAGAATAGTCAACAAATCGTTTGGGGCGACTTCTCCTCCTTTC
+
:::29=@=@==;8=>A;:;@?>>C67010137440.--43347==<<A@<:99?966;;-76555,./5-.-44447
@T6XXI:00122:00410
CTAGTCACTCGAGATCTGCGGCTTTGGACAGTCCCTCTGTACTGTGAAGCCACAGATGTCGAGTCGACTAGCTTCCT
TTCTCCCTTCCTCCC
+
C:/111/44AAACBCBBBBB?AAB>B>@BBA===;?DAB@@@AA@@@>@D>BFCDBBABCCCBBBB@@@CBA?;>47
7.7777.4342///,
@T6XXI:00122:00413
GGGAGAAGGGAGAAAGGAAAGGCGTCGTTCGGGGGAGTAGTATTGGTAACTATTCTCCCTCTCCCTATAGTGAGTCG
TATTAGAATTCCCC
+
94-444244-449904244-
31399976:77777,7@BBBAAA@D>=?=@AAC@C???6<<777.4;==>?@@@@A==;<9<<@?=;AAA6
@T6XXI:00122:00419
CTATCGATCGAGATCTGCGGCTTTGGACAGTCCCTCTGTACTGTGAAGCCACAGATGTCGAGTCGACTAGCTTCCTT
TCTCCCTTCTCCC
+
CCCBBBDBBCBCCBB@@@C@CCC>C?BBCDCCC=@@CCCCCCABBA>AC?EBCCBBBAAACCCCCCDCDAB@@C@DD
@@??B<A?ACCC>
@T6XXI:00122:00426
GGGGAATTCTAATACGACTCACTATAGGAGAGGGAGAATAGCTTGCAGCTCGAGCCTGTGACTGTTCTCTTCCTTCT
CCCTCTCCC
+
@?:58,/,/66789;;<88?878>;77377711,14426644@3777777;6147:===;>>?::7;844:8,/,//
//,/./--+
@T6XXI:00122:00428
GGGAGAAGGGAGAAAGGAAGCACAGGGCGAAGCGTGGGAAACTTGGCCTATTCTCCCTCTCCCTATAGTGAGTCGTA
TTAGAATTCCCC
+
ECB@@@?@A=AAAC>B>B?ABBBBCC?C@@>>>?99:5;B<BA>A>A>A==:=AAC<849@>7=@???@AA@@@AA@
@>?@A?B?AAA6
@T6XXI:00122:00433
GGGAGAAGGGAGAAAGGAAGGAGAAGTCGCCCCAAACGATTTGTTGACTATTCTCCCTCTCCCTATAGTGAGTCGTA
TTAGAATCCC
+
;<9=DHACB=CCBC<><=;><>?@=?CCBBBB8BC>BBBBB>BF@BBBAAA>A@@@;?A@AC:BAA>?@AA?===@A
A?AA@>?>>7
@T6XXI:00122:00434
```

```
CTAGTCGACTCGAGATCTGCGGCTTTGGACAGTCCCTCTGTACTGTGAAGCCACAGATGTCGAGTCGACTAGCTTCC
TTTCTCCCTTCTCCCCTTCCTTTCTCCCTTCTCCC
+
B<1111///789ABADDCBBC?AAB=?>???>>>>;??@@@@A??A>>;8;8;AA@@@CCCC:::@A@@BB;::7:8
:C>CCBB>@>BA???4=9;244,4:::4:79444-
@T6XXI:00122:00437
GACTATACACTCGAGATCTGCGGCTTGGACAGGT
+
///1///44444:4377796..,..,.,.3..,.
@T6XXI:00123:00401
GGGATCTAATACGACTCACTATAGGGAGAGGGAGAAATAGCTATGAATCTTCATGAGCCCATCTCCTC
+
;B5449//,/////.33317<666;8;:;;@11+30003../777?@===A?5504444,398//,/-
@T6XXI:00123:00403
GGGGAATTCTA
+
BDF:C?B?@66
@T6XXI:00123:00416
GGGAGAAGGGAGAAAGGAAGCTAGTCGACTCGACATCTGTGGCTTCACAGTACAGAGGGACTGTCCAAAGCCGCAGA
TCTCGA
+
CCABAA>A@=BAAB@C@N>B@BBBBBBBE???:7::::BCC?@B=?BAAB?>>?93331799;88/44,4=;?A@AA
A@@@@>
@T6XXI:00123:00418
GGGGAATTCTAATACGACTCACTATAGGGAGAGGGAGAATAGTCAACAAATCGTTTGGGGCGACTTCTCCTTCCTTT
CTCCCTTCTCCC
+
CCC9C?A?BDD@BA>@@?>>BCB97111.7??BC>A???A?@@AB>@@@<BCBBC>@@B9BCCCC?CCC==;=>AA<
??@A=A>??>A<
@T6XXI:00123:00432
GGGAGAAGGGAGAAAGGAAGGAGACACGGTCACAAACATGAATGTGACTATTCTCCCTCTCCCTATAGTGAGTCGTA
TTAGAACTTCCCC
+
BB>ABB>BD@BAAB>B?B=?=?CBCBBB@BBCCBB=@A@AB?BBBCCC@B?:;43013?>CC?BB@<<;<???=<=@
@;8;9,//78870
@T6XXI:00124:00400
GGGGAATTCTAATACGACTCACTATAGGGAGAGGGAGAATAGTTTTCTGTCATACGCAGGGCTATTCTTCTTCCTTT
CTCCCTTCTCCC
+
?@@6@=@>ACC@CB@@@????@B<;111.6>=CF>CAA?A6;<==5<??@?AADAAA@@<;;;?=<==99;:::==;
=99999;:?AB<
@T6XXI:00124:00420
GGGGAATTCTAATACGACTCACTATAGGGAGAGGGAGAATAGATTATTCCTAACCACAGCCCGCTATTCTTCCTTCT
CCCTTCTCCC
+
AAA6@<A@BCC@CCCAABBBB@<97111.7??AB>BAA>@<????AA>A<=C>824444/71655,,+,+*+0000-
--+0233380
@T6XXI:00124:00425
GGGGAATTCTAATACGACTCACTATAGGGAGAGGGAGAATAGTGACGTGTTGTGCGGCGCTCACTGTCTCCTTCCTT
TCTCCCTTCTCCC
+
499/42929AB@CCBAABBBA>@?=111.7==?BBD??<?AA@<:?@A==<<@<?:8;:=======<9=9;<<=<==
;8>?C;=>?@@A;
@T6XXI:00124:00429
CTAGTCGATCGAGATCTGCGGCT
+
B99449444;44444444442/.
```

```
@T6XXI:00124:00430
GGGAGAAGGGAGAAAGGAAGGAGAAGTCGCCCCAAACGATTTGTTGACTATTCTCCCTCTCCCTATAGTGAGTCGTA
TTAGAATTCCCC
+
CC?CDB@BB>BBBB>B=@=>@BCC@BB>BBBB6>><ACBBC>BA?@@@<<>=<===;88<@A=A999;;A999:::;
?>A@A=?8;;;0
@T6XXI:00125:00408
GGGGAATTCTAATACGACTCACTAAGTAGGAGAGGA
+
:AA6BAB?C?A?BBBCCC>;;:00,0/..,171.,/
@T6XXI:00125:00412
GGGAGAAGGGAGAAAGGAAGGAGAAGTCGCCCCAAACGATTTGTTGACTATTCTCCCTCTCCCTATAGTGAGTTCGT
ATTCAGAATTCCCC
+
CC?AA@>BB>ECCI@C@C@B@A@@;;788888,7>;B?>A@;CC@C@?=@@8<<==7<<<=?:>?@A?=:;//,/60
14,..7777,444+
@T6XXI:00125:00416
GGGGAATTCTAATACGACTCACTATAGGGAGAGGGAGAATAGCTATCGTTATTCTCATGCCGCCGTGGCCTTCCCTT
TTCTCCCCTTCTCCC
+
EDCCC>A@@CC@CCBABBCCC??>=677.7>>@C@A??>B===CBBBB@>@@;::;<@@=;=;::30/424344-
4;;0;7444-47:;=?:
@T6XXI:00125:00417
GGGAGAAGGGAGAAAGGAAGCTAGTCGACGACATCTGTGGCTTCACAGTACAGAGGGACTGTCCAAAGCCGCAGATC
TCGAGTCGACTAG
+
CC@CBB@BC>BBBB=B=?=>CA?@?@GCCC==?CC@@BC?BB?BDCCAA@A?933417>>;<=>@@<@A>A@@?AAA
A=<:;;<==<<<=
@T6XXI:00125:00425
GGGAGAAGGGAGAAAGGAAGGAGAATTGTTTACGCCATGATCGCTAACTATTCTCCCTCTCCCTATAGTGAGTCGTA
ATTAGAACTT
+
DC?CBC>?B?9;;=9><@>A?BBCBC?AAA8@@??<??:::AAAAB@BCBCABCAC;>><<<6;@<9=CAB=;96//
,-32242+.,
@T6XXI:00125:00428
GGGGAATTCTAATACGACTCACTATAGGGAGAGGGAGAATAGCTGCAAGTTCCCAGACTTCTAGTTATTCCTTCCTT
TCTCCCTTCTCCC
+
DCC7B79;<@@BCECABBBB@>;97111.777=?<?BB>@>@@???>;>>>?@;@;<BB@BBBBB?@@=@@B=?;??
6;===6<:<777.
@T6XXI:00125:00430
GGGAGAAGGGAGAAAGGAAGGGAAAGTCGATTTGGGATCGGAGTCGACTATTCTCCCTCTCCCTATAGTGAGTCGTA
TTAGAATTCCCC
+
AKDDBB?BB>?A?B>B@?>>B<BB<BBCDCCC?CC>CCCC@DBBBAAADCCABBBB=BBBDC@CCBCCCCBACBBCB
B?BAB?A@DCC6
@T6XXI:00126:00410
GGGAGAAGGGAGAAAAGGAAGCTAGTCGACTCGACATCTGTGGCTTCACAGTACAGA
+
@AA<;<@A@:98999,49;9;A?A7177777:7B?8:<:9==:7846/0,,,,0--0
@T6XXI:00126:00412
GGGGAATTCTAATACGACTCACTATAGGGAGAGGGAGAATAGCTGTCAACCCGTCGCCATTACGTTATTCTTCCTTT
CTCCCTTCTCCC
+
BBB9BAC@CBB@BCCCCCCBB?<96166.7??AB>B@@>@<<<?BBBC>B>>AAC8888?;?AAA?@C?AA=@;@A<
@<<CAC?@???7
@T6XXI:00126:00421
```

```
GGGAGAAGGGAGAAAGGAAGGACAATAGGCATCAACCACAAGACGAAGCTATTCTCCCTCTCCCTATAGTGAGTCGT
ATTAGAATTCCCC
+
CDACBB?BB=BBBB?B?B?A@BDB@B@@>@?>99295:AA@CCBB?>>;6ABCDBBB@AAACD@BBBCEBBBBBCBB
BC>BBB>B@BBA8
@T6XXI:00126:00428
GGGAGAAGGGAGAAAGGAAGGGAATAGCAGCAGTCGGCAACGTGAAACTATTCTCCCTCTCCCTATAGTGAGTCGTA
TTAGAATTCCCC
+
HEABAA>==9=>>>0959;>>:@@DCBCBBBBBBCC@BA>ABB?AA<ABDC@CC?C>BBAAA:887888>>??888>
>=?@B>A>@AA6
@T6XXI:00126:00440
GGGAGAAGGGAGAAAGGAAGCACATGCCGCCGCGGGGGTAACGGAAGCTATTCTCCCTCTCCCTATAGTGAGTCGTA
TTAGAATTCCCC
+
FD@BBB@CC>ABBD>G?B?@CCBCC??>?C?BBCCCC2@A?BBCD=@A@AAACAAB>=<<CCBCCDCAABBBBDBCC
D@CBA>BABDC:
@T6XXI:00126:00442
GGGGAATTCTAATACGACTCACTATAGGGAGAGGGAAGAATAGTTTTTCCCTTCACTAACCGCGAACCGCACTTCCT
TTCTCCCTTCTCC
+
9AC6@>A>@BAAC@>@@?;;;BBA???B800/77+/,-
101246>?>.8;369=?BB::=:0/0/7949/..4,.,//,0789,/,/--,
@T6XXI:00126:00447
ATACGACTCACTATAGGAGAGGGAGAATAGACAGGCGCTATTCTTCCCAGGAGTTATCTTCCTTTCTCCCTTCTCCC
+
BCA@;;<@?BB>@A;;58?7774:A?;AB<;<<<>ABBAAAACBACLCAA?ACC@C@@B@@477.7>>>:A?BABC?
@T6XXI:00127:00420
GGGGAATTCTAATACGACTCACTATAGGGAGAGGGAGAATAGTCTCCCATTCGTCACAATTAACGTGCCCTTCCTTT
CTCCCTTCTCCC
+
DCE:C9;;<CC@CCAABBBBB@<9=611.7==?B>B??<?@@@AAAA8:;@BA?A???:?>A?BBB===1837=AB?
AABC=A69.9;6
@T6XXI:00127:00426
GGGGAATTCTAATACGACTCACTATAGGGAGAGGGAGAAAGGAAGCTAGTCACTCGAGATCTGCGGCTTTGGACAGT
CCCTCTGTACTGTGAAGCCACAGATCTCGAGTCGACTAGCTTCCTTCTCCCTCTCC
+
BA?6A695;>>=AA@@CC;;<C>>>777.7>????B844-
4497;=???CA@<<00/..34;@A@:==C;@>=;;>74417>>>AAA??==<>==;=@B>=88878:=899?AAA99
578,,+,000+,---0
@T6XXI:00127:00440
GGGAGAAGGGAGAAAGGAAGCTAGTCGACTCGACATCTGTGGCTTCACAGTACGAG
+
4919??<>>1999919489<<<?>//......33335611>01376....200+--
@T6XXI:00128:00417
GGGAGAAGGGAGAAAGGAAGCTAGTCACTCGACATCTGTGGCTTCACAGTACAGAGGGACTGTCCAAAGCCGCAGAT
CTCGAGTCGACTAG
+
FC@CEB?B=6=944-
9494;@?=?CA????@776677AAC?BD@BBBB@@@@<=86648?@@AC>@@;AA>@===?BAAA888==<@@@AA
@T6XXI:00128:00424
GGGAGAAGGGAGAAAGGAAGTGCAGTTCGCGGTTAGTGAAGGGAAAACTATTCTCCCTCTCCCTATAGTGAGTCGTA
TTAGAATTCCCC
+
DD@@A@@DC>CBCB>B?BB@AAECCB@B::;@C@AAABC@CC>BBB9CCCC@AADD>ABBBC>CAA@;:=AAA@???
?>AAA@A:=<<4
@T6XXI:00129:00414
```

```
GGGGAATTCTAATACGACTCACTATAGGAGAGGGAGAATAGTCAACAAATCGTTTGGGGCGACTTCTCCTTCCTTTC
TCCCTTCTCCC
+
ABB9BCD@CCE@BBBBBCB<<1@@:00,0>0776;=;?@@@@@@>@?A<C???B9???6?A@BB@;<>BA?@?@@:?
?==:=383=;4
@T6XXI:00129:00417
GGGAGAAGGGAGAAAGGAAGGAGAAGTCGCCCCAAACGATTTGTTGACTATTCTCCCTCTCCCTATAGTGAGTCGTA
TTAGAATTCCCCC
+
<<8<CD@DC@GBA?=?<><@?BBC@BCC@@<>7@B>CCCCC<BB?CA@=@@>@AAA<88:AC>A@@@A77/0/;;;;
@><6424/3444,
@T6XXI:00129:00419
GGGAGAAGGAGAAAGGAAGAATATGCAGGAGACGAACAACGCCGAACTATTCTCCCTCTCCCTATAGTGAGTCGTAT
TAGAACTTCCCC
+
08+-/42424448-
4242391333=?80,0.....555356621686::4445==;<:=AA<AA998=:;:<555754211,++*+00,
@T6XXI:00129:00427
GGGAATTCTAATACGACTCACTATAGGAGAGGGAGAATAGCGCGTTTCTATTGGCGTGTCTATATCTCCTTCCTTTC
TCCCTTCTCCC
+
DB>>6459>?;ACBBBBCCB76;7//,0<<?C>B@C?AA??@A@@A;@<??@A?>@@588;<;;<<<?:>8;8;<7=
>@@;@=@???;
@T6XXI:00129:00431
CTAGTCACTCGAGATCTGCGGCTTTGGACAGTCCCTCTGTACTGTGAAGCCACAGATGTCGAGTCGACTAGCTTCCT
TTCTCCCTTCTCCC
+
B=/11=6==CBCCBCBCBCC@CCC>B?BBCCCCC>CB@=?=@?B@@@>@CDCCCCCCCABBCCABBCCCC<;:=A>A
B=B@@@<<9<>@@<
@T6XXI:00129:00436
GGGAGAAGGGAGAAAGGAAGCTAGTCACTCGACATCTGTGGCTTCACAGTACAGAGACTGTCCAAAGCCGCAGATCT
CGAGTCGACTAG
+
DDACAB@DC<>@B@<@=@>@@@BBBB@?<@C::8B==BB?<:;116=>???@==<;334300/0303458;<<<@@@
@<;<<::::00-
@T6XXI:00130:00411
GGGGAATTCTAATACGACTCACTATAGGGAGAGGGAGAATAGCAAACGTTTCTCCGACGTCGCTTTCTCCTTCCTTT
CTCCCTTCTCCC
+
999/9<A@CBCACB???BBBBBB?966659@@CC>C@A>@;;:??:?AAB>@@=@AAA???AAAA??:<;=<?>BC=
A>@@<A>??@;4
@T6XXI:00130:00420
GGGAGAAGGGAGAAAGGAAGCTAGTCGACTCGACATCTGTGGCTTCACAGTACAGAGGGACTGTCCAAAGCCGCAGA
TCTCGA
+
DD@@A?@BB>C@?@=@@C=@@A@@?:::BA@>::::::@BC>?@=?BBBCCC?=644317??>??>AA<@A>@@@??
@A==??
@T6XXI:00130:00443
GGGAGAAGGGAGAAAGGAAGCTAGTCGACTCGACATCCTGTGGCTTCACAGTACAGAGGGACTGTCCAAAGCCGCAG
ATCTCGAGTCGACTAG
+
@=<@@@?BB<@>>>0969<>@800>1//=77,,00..,.557:@B?AACB;;:@;;48818>>@AA=@@;@A>A@<@
@@?A@=7778489:;;
@T6XXI:00131:00404
GGGAGAAGGGAGAAAGGAAGGGGAGAAGGGAGAAAGGAAGGGGAGAAGGGAGAAAGGAAGGAGAATATTGTGTCCTC
GCCCCTGGGACTATTCTCCCTCTCCCTATAGTGAGTCGTAT
+
```

```
BDBA@?@BB=B@??<@<@<???5??@@CC>???>;>@C>???7??=<==4688=;@>=>?=???=<<A====8;89@
>>??289909D@BCAAAAEBCC<<<;:CBBBBBAACCCC;;
@T6XXI:00131:00409
CTATCACTCGAGATCTGGCGGCTTTGGACAGTCCCTCTGTACTGTGAAGCCACAGATGTCGAGTCGACTAGCTTCCT
TTCTCCCTTCTCCC
+
CAA=@::4=9:999CBBBCC@CCC>C?@@==7::5:CCDFCC@=<::7498;AABCD;;:@??<9988831113636
7,11644<:877A:
@T6XXI:00131:00410
GGGGAATTCTAATACGACTCACTATAGGGAGAGGGAGAATAGTTAGCGATCATGGCGTAAACAATTCTCCTTCCTTT
CTCCCTTCTCCC
+
CCC9B@B@CBC?BCCCCCCB878?>7776:BBCJ>C@@>@<==<A???BBCBBB?BBBBC>C>@>@B?A@A<?3771
4AAA;B<==>A;
@T6XXI:00131:00419
GGGGAATTCTAATACGACTCACTATAGGGAGAGGGAGAATAGTCAACAAATCGTTTGGGGCGACTTCTCCTTCCTTT
CTCCCTTCTCCC
+
HGD:E@C@CCC@CCCBBBBBBAB?;66659BBCD>A??=?:;;AA?<@@<?@AAA;@@@7?AA@??ACD87375<>;
?ACD=A388AC<
@T6XXI:00131:00440
GGGGAATTCTAATACGACTCACTATAGGGAGAGGGAGAATAGTCAACAAATCGTTTGGGGCGACTTCTCCTTCCTTT
CTCCCTTCTCCC
+
C@?4>698<BBAEA@@BBBBBA>97111.7====<?@A@A@@@@@>@@@;B???B>BCC9BACBB@CCC;988?AC<
>@AA=A9<<AA<
@T6XXI:00131:00442
CTAGTCACTCGAGATCTGCGGCTTGGACAGTCCCTCTGTACTGTGAAGCCACAGATGGACTGTCCAAAGCCGAGATC
TCGAGTCGACTAGCTTCCTTTCTCCCTTCTCC
+
9A9444/////7::;BC<::;8748<>?>>???9?BBBCCB::8<;89A=@@A?B<;7<@@?@?@CCBEC@BCCCF=
=<>;;498737331106>>7;6..4,-,+++*
@T6XXI:00132:00411
GGGAGAAGGGAGAAAAGGAAGAGT
+
44-44424956////*/,/,...3
@T6XXI:00132:00418
GGGAGAAGGGAGAAAGGAAGACTACGGCGCTGTCTGGGGAAATTACAGCTATTCTCCCTCTCCCTATAGTGAGTCGT
ATTAGAATTCCCC
+
BC@B@@@BCCAABB?B@CACCBBBCB@CCBCCCDBAAB:CC?B?B@@AAADC?BBBA<@B@AA>ABBCAABCC?=<<
@A9=<@9;2444-
@T6XXI:00132:00421
CTATCACTCGAGATCTGCGGCTTTGACGTCCA
+
CCCB;::@CCC:9=C?;:;:010,0..,,,*.
@T6XXI:00132:00429
GGGGAATTCTAATACGACTCACTATAGGGAGAGGGAGAATAGTTTTTCCCTTCACTAACCGCGAACTGCACTTCCT
TTCTCCCTTCTCCC
+
@@?8AACCEBC@BBBBBACCCB<??677.7<?AB>B@@;=<CCCAAA-
AA<A>@B@???B>???=:<@A@@@@?C@CC5::??9?>;<<A1
@T6XXI:00133:00400
GGGGAATTCTAATACGACTCACTATAGGGAGAGGGAGAATAGTCAACAAATCGTTTGGGGCGACTTCTCCTTCCTTT
CTCCCTTCTCCC
+
```

```
@@@6@@A?BBB@BCBBBCCB?:<?=666.7>>AC@C@@<@===@A>???;AB@@B<???6?BBBC@AAA??<?>AC?
@@@A;@588>?:
@T6XXI:00133:00405
GGGGAATTCTAATACGACTCACTATAGGGGAGAGGGAGAATAGTTCACACACATTTACAGGGGCTGTTCTCTTCCTT
TCTCCCTTCCTCCC
+
DBC2@5959442448;:;:BCC??>7777-
7616626332333979=>=>?A??;+011111,1;77779310101621..525,.,.-2+
@T6XXI:00133:00411
ACTCGAGATCTGCGGCTTTGGACAGTCCCTCTGTACTGTGAAGCCACAGATGGTACGTGC
+
CBCDDDBBBAA@?A=AAA?A@A@@>>ACADCAAACBDCCBC@BB@B;800**)******4
@T6XXI:00133:00423
GGGAGAAGGGAGAAAGGAAGGGGAGAAGGGAGAAAGGAAGCTAGTCGATCGACATACT
+
BCBABB@BD>CBBB?C@C@CBB8?>>?999=766,7<>4::?A>7777...-,+++++
@T6XXI:00133:00433
GGGAGAAGGGAGAAAGGAAGTGCAGTTCGCGGTTAGTGAAGGGAAAAACTATTCTCCCTCTCCCTATAGTGAGTCGT
ATTAGAATTCCCC
+
CC@CBB@AA=BBBB=@=@@BB?AABB@B@AA@B@A??BB@?7-
6667,8??B@DCCD@C@@@@;@<<<;;?A@@<==@@>@CC>A>AAA5
@T6XXI:00133:00435
GGGGAATTCTAATACGACTCACTATAGGGAGAGGGAGAATAGAGACGTATTCGTTTGCGCTCTGTTCACCTTCCTTT
CTCCCTTCTCCC
+
AC@4>;>=ABB?BFEACCCCCBBA=677.7<<=@=@?B>BA?AAA=:;<=;<<6=;@?8=B;;;;<=<;4424;>A=
A@@@<A????<;
@T6XXI:00134:00408
GGGAGAAGGGAGAAAGGAAGGAGAAGTCGCCCCAAACGATTTGTTGACTATTCTCCCTCTCCCTATAGTGAGTCGTA
TTAGAATTCCCC
+
DDBBBA@CC=A?=89>8948BA?@==?A7788.8?9?>@BB=??:=::8<?>AAAC<@??>A>BAAA>>?@AA@@?@
A>A?=;:2444-
@T6XXI:00134:00415
GGGAGAAGGGAGAAAGGAAGGAGAAGTCGCCCCAAACGATTTGTTGACTATTCTCCCTCTCCCTATAGTGAGTCGTA
TTAGAATTCCCCC
+
EF@CCC@CC=??==:@>@=A?ADC?BABBBBB7A@<@??>>;>?>>>B????C<==9=@@AC=A@???<<<A<;?88
8?H8834/////+
@T6XXI:00134:00419
GGGGAATTCTAATACGACTCACTATAGGGAGAGGGAGAATAGTCAACGAAATCGTTTGGGGCGACTTCTCCTTCCTT
TCTCCCTTCTCCC
+
==@6@@CCCBB@AAAA=;BBBCBB<<??9<BBCC>C==9=<@?<:99444-
44;;;498;0?CBBBACCC@=;=<@C>?===7<>A<==9
@T6XXI:00134:00425
GGGAGAAGGGAGAAAGGAAGGAGAAGTCGCCCCAAACGATTTGTTGACTATTCTCCCTCTCCCTATAGTGAGTCGTA
TTAGAATTCCCC
+
BBA@@@@BBB==>B@B?A>B@BBB@@@B<<;;5?A<BBBBD>CB>BBC=:;;<===;===AA<?;?AAA==<<?@?@
@?A84243///+
@T6XXI:00134:00430
GGGGAATTCTAATACGACTCACTATAGGGAGAGGAGAATAGT
+
111*/5>ACDB@CCCBBBBBBAB<;666-6=;;:?//,/,,,
@T6XXI:00134:00432
```

```
GGGAGAAGGGAGAAAGGAAGCTAGTCACTCGACATCTGTGGCTTCACAGTACAGAGGGACTGTCCAAAGCCGCAGAT
CTCGAGTCGACTAG
+
CC>BCB>=A9@@885>886:B@?@CB@??C@::::::CBFABB@CBC???>@=<<::999====;;::?=?=:66<<
==<<:??<?;;===
@T6XXI:00135:00439
CTAGTCACTCGAGATCCTGCGGCTTTGGACAGTCCCTCTGTACTGTGAAGCCACAGATGTCGAGTCGACTAGCTTCC
TTTCTCCCTTCTCCC
+
;=1//6699?:444997;BBBBCAAAA=@@CCAAA<ABBCCCCB@@AA;=A?BBBAA@@@CCCC;<<BA@@B::7:?
BBBBBCC>@583>>6
@T6XXI:00136:00405
GGGAGAAGGGAGAAAGGAA
+
GE@B4429909911,/,//
@T6XXI:00136:00408
GGGAGAAGGGAGAAAGGAAGCTAGTCGACTCGACATCTGTGGCTTCACAGTACAGAGGGACTGTCCAAAGCCGCAGA
TCTCGAGTCGACTAG
+
FH>AAD;980999809594777<76777==>>777<;67:@=A@?ACBBBBB?=?77717<<<>?=?@;>><?===A
A???@@@???@@@AA
@T6XXI:00136:00433
GGGGAATTCTAATACGACTCACTATAGGGAGAGGGAGAATAGCGTGCCTGTATACGGTACCCTGTTCGCCTTCCTTT
CTCCCTTCTCCC
+
BDD:D=?=@AA?BECCCCCBBBB@B966.7?>?ABAAA>A=<<?????@@?=@@BB>B;9:9;B@>ABA>?@B<?@=
AAAA<A>A@??:
@T6XXI:00137:00403
GGGAGTAGG
+
88+-,,,*)
@T6XXI:00137:00418
GGGAGAAGGGAGAAAGGAAGGAGAACGCGCACCCGAACCAACGGTGATCTATTCTCCCTCTCCCTATAGTGAGTCGT
ATTAGAATTCCCC
+
CGDCCE?B=:=9996;?B?B@CCC@CBBBBBBB?BC@C?D@CD@CAABBCBB?B9775:AAAG>BBBBCCBBAAACC
CC>B?A>B@BCC8
@T6XXI:00137:00419
CTAGTCGACTCGAGATCTGCGGCTTTGGACAGTCCCTCTGT
+
,/////44444999;99A@@@=8880313331111,16111
@T6XXI:00137:00436
GGGGAATTCTAATACGACTCACTATAGGGAGAGGGAGAATAGTTTTTCCCTTCACTAACCGCGAACTGCACTTCCTT
TCTCCCTTCTCCC
+
AAC9BACACBA?CBBB??@CB9>>;677.777>>:@@><;;44444,4;9?9=@BFD@C?AAA@<>>?>?<<:=477
.7??B>D>999@;
@T6XXI:00137:00444
GGGAGAAGGGAGAAAGGAAGGAAAATAGAAGGCGACGGCATAACAGTCTATTCTCCCTCTCCCTATAGTGAGTCGTA
TTAGAACTTCCC
+
;;6;99@AA>;99@194958=??B8@A<<;?;?><::900.667669:;:::???C===AB@;@@??=;::...233
1-..4311,000
@T6XXI:00137:00447
CTAGTCACTCGAGATCTGCGGCTTTGGACAGTCCCTCTGTACTGTGAAGCCACAGATGTCGAGTCGACTAGCTTCCT
TTCTCCCTTCCTCCC
+
```

```
99944944444==BBCCC>9973708<??@CAAA<:::AABC?@=AA>?C@A@@BC;<<A@C@;=>@@@CCA?>??B
B7;/;<68,/,///,
@T6XXI:00138:00402
GGGAGAAGGGAGAAAGGAAGAGAACGGCGACGAACAATAGTTGGTGGCTATTCTCCCTCTCCCTATAGTGAGTCGTA
TTAGAATTCCCC
+
DDAFCC@CC=BBBB>B@B@BBBB@BB?A@A@CC@BB?BBBC>C?:::;CCG@@@@B>B>B?C>?6<<B@AA@A@@@@
@>@AB?A38880
@T6XXI:00138:00408
GGGAATTCTAATACGACTCACTATAGGGAGAGGGAGAATAGCTACGTTTAACCGATCGTGTTGTTCTCCTTCCTTTC
TCCCTTCTCCC
+
CC?B@B@CDC@CAAAAABBB@<96166.7<<CF>CBA>A;=<99=AAA;>=?>?=?=====<==<<<<>?=@38818
>=C?A>A=967
@T6XXI:00138:00416
GGGGAATTCTAATACGACTCACTATAGGGAGAGGGAGAATAGTCAACAAATCGTTTGGGGCGACTTCTCCTTCCTTT
CTCCCTTCTCCC
+
@?=2>=@=@CCACB@@BBBBB?<9;677.7??CC>BBB=@:8<<=?<@@;?>8868;;;2?AAAC>=??7858?AB<
@@@A<@>AAAC:
@T6XXI:00138:00417
GGGAGAAGGGAGAAAGGAAGGTG
+
BDACCA?BD>B9/,*-+-+-+,,
@T6XXI:00138:00418
GGGAGAAGGGAGAAAGGAAGGAGAAGTCGCCCCAAACGATTTG
+
CB>CCC@CC>CCB@:B8:6;B@@A@@=?8888.77,31111,1
@T6XXI:00138:00427
GGGGAATTCTAATACGACTCACTATAGGGAGAGGGAGAATAGTCAACGAATCGTTTGGGGCGACTTCTCCTTCCTTT
CTCCCTTCTCCC
+
AED:C>A>ABBACBB?BBBB?>;97116.7==BC>CBA@A:??;;;<<=;<;<==;<<=5BC@@@?AAA=?<?>AB=
===?;?@AA@@;
@T6XXI:00138:00429
GGGGAATTCTAATACGACTCACTATAGGGAGAGGGAGAATAGTCAACAAATCGTTTGGGGCGACTTCTCCTTCCTTT
CTCCCTTCTCCC
+
ABA7A=@>ADDBBBBBBBBBB@;96116.7>@BC>B@@?A<<<<><996387;;;-667-
7?BAB@BBB==;=>AA<===C<A:<<BC<
@T6XXI:00138:00430
CTAGTCACTCGAGATCTCGGCGTTCTGGACAGTCCCTCTGTACTGTGAAGCCACAGATGTCGAGTCGACTAGCCTTC
CTTTCTCCCTTCTCCC
+
BB999@==9B=>99;@;;;;100,...44666366,1>?==:::@??=;=@>ABBBBC@@@BA=;10,,,0,,*+02
2243355303233330
@T6XXI:00138:00434
GGGAGAAGGGAGAAAGGAAGCTAGTCGACTCGACATCTGTGGCTTCACAGTACAAAGGGACTGTCCAAAGCCGCAGA
TACTCGAG
+
:>=D3<??@:9999149:2377>>7717711119?<==CACB8=6000/..1000+00+0004485=>:==99996/
///78/44
@T6XXI:00138:00443
GGGAGAAGGGAGAAAGGAAGCTAGTCACTCGACATCTGTGGCTTCACAGTACAGAGGGACTGTCCAAAGCCGCAGAT
CTCGAGTCGACTAG
+
```

```
AA<ABBACD@CCBB?C?BAA?>?A??;::??:::BBAAABABB@C<<<FCA>:;444-
4;;;=?=@@:@A>???AAC@@?????888?=:=
@T6XXI:00139:00405
GGGAGAAGGGAGAAAGGAAGAGAAGAAACAAGCTGCACAAAGCGAGTCTATTCTCCCTCTCCCTATAGTGAGTCGTA
TTAGAATTCCAC
+
CCDCCB@CC?AAAB=AAB@BBBB===@;AB@BCCC@?BCC>B>BBCAB?BC@A===9=@@AA<AA???AAA@@?@@@
@>?@@<?<??AA
@T6XXI:00139:00408
CTGCGGCTTTGGACAGTCCCTCTGTACTGTGAAGCCACAGATGTCGAGTCGACTAGCCTTCTCCCCTTCC
+
CBDAA@ADD@C@BBAA;;;6;??BBBD?=?B@<795999>@?A???@;:<BCC?B7<41011111,.444
@T6XXI:00139:00409
GGGAGAAGGGAGAAAGGAAGCTAGTCGACTCGACATCTGTGGCTTCACAGTACAGAGGGACTGTCCAAAGCCGCAGA
TCTCGAGTCGACTAGCTTCCTTTCTCCCTTCTCCC
+
ED?DGC@BC=A;;9095958@><;=0//;=99;79:66>=?=:;9:B@@BBCAAAA@@9=<<<??<?A<??=?@@@@
AA???A9:9AAA@899=;=@AC:;9;>9>//00;6
@T6XXI:00139:00433
GGGAGAAGGGAGAAAGGAAGGAGAATCGGCCGCAAAGGGGAAGTTGGCTATTCTCCCTCTCCCTATAGTGAGTCGTA
TTAGAACTTCCCC
+
CD>DDBBBB>@;;;8;>A?B@BCC@BA@5:6:?BC?BCB4;477;>:ADCCFJCBC@AAABC>?;::BBBAA>8./-
-,-234:@@AAA7
@T6XXI:00139:00434
CTAGTCACTCGAGATCTGCGGCTTTGGACAGTCCCTCTGTACTGTGAAGCCACAGATGTCGAGTCGACTAGCTTCCT
TTCTCCCTTCTCCC
+
C>11188==AAABBB@@@@?>AAA=B@C@@@CCC>BAABCCCB;:9A>AC@CCECBBBBCCCCD@A@AAB;:6<?;>
A.71;<.428<=A=
@T6XXI:00139:00446
CTAGTCGACTCGAGATCTGCGGCTTTGGACAGTCCCTCTGTACTGTGAAGCCACAGATGTCGAGTCGATAGCTTCCT
TTCCTCCCTTCCTCCC
+
::C@=999:A9=??AGAB@994888/84839@691111119?:AAA>>8;A=AA@B?8;;777<:;;<999..,.44
4,/,/::62,0/000+
@T6XXI:00140:00404
GGGGAATTCTAATACGACTCACTATAGGGAGAGGGAGAATAGTCAACAAATCGTTTGGGGCGACTTCTCCTTCCTTT
CTCCCTTCTCCC
+
:;;4<@A?BBB?BB@?AABB@?>?<777.7====9???>@@A>>>=??A:BB?CE====7=@@@E@BBA>?;>=??;
@A@A<A888AA@
@T6XXI:00140:00405
GGGAGAAGGGAGAAAGGAAGGGGAGAAGGGAGAAAGGAAGCTAGTCGACTCGACATCTGTGGCTTCACAGTACAGAG
GGACTGTCCAAAGCCGCAGATCTCGAGT
+
EEAECC?AA?@A?B?C@C@BCC9@>>@A@;=?=@9B@C7;;@BBBCCBC??856;788>@C????@B;;;;==6633
0-4?>@@A>@@;@@>A@==;;:<<<===
@T6XXI:00140:00411
GGGGAATTCTAATACGACTCACTATAGGGAGAGGGAGAATAGTCAACAAATCGTTTGGGGCGACTTCTCCTTCCTTT
CTCCCTTC
+
499,42424AA=@B@@@@BCBAAAA???9?C?AB>?==>@??@==;=AA<CCBBA<A@@5@???B?AAB:;7:344-
4400+0/-
@T6XXI:00140:00415
GGGGAATTCTAATACGACTCACTATAGGGAGAGGGAGAATAGCCAATACCTCTTATGAACCAACCTCCCCTTCCTTT
CTCCCTTCTCCC
```

```
+
6;<1<-95949544999:BB@?@;9:?A<AACBD>@==9<@>@<@9<;=::882847734;A>A=BBCC9C:<377-
40942823044,
@T6XXI:00140:00425
GGGGAATTCTAATACGACTCACTATAGGGAGAGGGAGAATAGTTTTTCCCTTCACTAACCGCGAACTGCACTTCCTT
TCTCCCTTCTCCC
+
@@C6@@A@BCC@ABBB@@@CGEDB????9??ACC>BAA?A=7337708D@C>@BBBC@B?AAAA?AAAA<<A?C?BB
=AAB@>B>788@5
@T6XXI:00141:00404
GGGAGAAGGGAGAAAGGAAGGAGAAGTCGCCCCAAACGGTTTGTTGACTATTCTCCCTCTCCCTATAGTGAGTCGTA
TTAGAATTCCCCC
+
BC@C>>644-
9>>><B?CACBCCC?A?93333,880777;?>>B?B???AA?>@>=7:;44818>>@=??=?=;;;4838;;9;244
44+
@T6XXI:00141:00412
GGGAGAAGGGAGAAAGGAAGCTAGTCACTCGACATCTGTGGCTTCACAGTACAGAGGGACTGTCCAAAGCCGCAGAT
CTCGAGTCGACTAG
+
A@<?@@7;;0;A>>9A8;7;BA@AC=>>>>=//1111?BD<;<@CDCB=BBB>83331789;;<9;>7=><@???A@
???C??????@A@@
@T6XXI:00141:00414
GGGAGAAGGGAGAAAGGAAGTGATAGGCACGCCAGATA
+
7@9<:9584-448;6:<:99333319961133133133
@T6XXI:00141:00418
CTATCACTCGAGGATCTGCGGCTTTGGACAGTCCCTCTGTACTGTGAAGCCACAGATGTCGAGTCGACTAGCTTCCT
TTCTCCCTTCTCCC
+
CAAA;::?BA>A>@GCCBCB?@AA=A@CCAA?@@;?DCA>A?99=@A>@C@CCCBB88:BBBBBBDCCBB;;8>A>A
D>BB@AAA=@@??:
@T6XXI:00141:00420
GGGAGAAGGGAGAAAGGAAGCTAGTCACTCGACATCTGTGGCTTCACAGTACAGAGGGACTGTCCAAAGCCGCAGAT
CTCGAGTCGACTAG
+
CE?AAA?BBABDCDAB?C?@@@CCBB@??A@:::???AAA<?@@BBAGBBB?;9333-
4>:==@=@@<@?=@B889@=<<AA=<===44,*
@T6XXI:00141:00425
GGGGAATTCTAATACGACTCACTATAGGGAGAGGGAGAATAGCTACGTTTAACCGATCGTGTTGTTCTTCTTCCTTT
CTCCCTTCTCCC
+
BBB9B@C@CCC@BBCCCCCBB@<96116.7>@CC>B@@=@=<<==?@B@>B?A?AAA999=57432--,/-
3303444=>B==>=;<<;
@T6XXI:00141:00429
GGGGAATTCTAATACGACTCACTATAGGGAGAGGGAGAATAGCTTTTTGCGTCGCTGTTAGCCGCTCGCCTTCTTCT
CCCTTCTCCC
+
;CB/95<=@BB@CC;9<C@@@BBAB:::.7?77:6:=;==???C@@@/==7748=;;5986<877/86848,/-22-
---3:46?B9
@T6XXI:00141:00438
GGGAGAAGGGAGAAAGGAAGAGAAGCGCAATGACCACGAGAGATTGGCTATTCTCCCTCTCCTATAGTGAGTCGTAT
TACGAATTCCCC
+
;@>BBB>AB>999919<?<;;:<>6873713633116669>><;>@53369216;;66?774064.-
.4336==::<<554=52,---*
@T6XXI:00141:00443
```

```
GGGAGAAGGGAGAAAGGAAGCTAGTCGACTCGACATCTGTGGCTTCACAGTACAGAGGGACTGTCCAAAGCCGCAGA
TCTCGAGTCGACTAG
+
9409>?=@B<@@@@9>=@=>??0100..333368:@@@BBC@CC?CCCA@@?;9;47718BBAA@=BC<@A=@@@@A
<<<@988<A@=>=;7
@T6XXI:00142:00414
GGGAGAAGGGAGAAAGGAAGTGCAGTTCGCGGTTAGTGAAGGGAAAAACTATTCTCCCTCTCCCTATAGTGAGTCGT
ATTAGAATTCCCC
+
ED@CCC?BB=BBBC@B?@?@BBCDBB?B@A@@C@AAAA@=;=6;<==/?BBF@C?@@=@BACD>?<<@AAAA???A?
??>;AC?A?@AA5
@T6XXI:00142:00435
GGGAGAAGGGAGAAAGGAAGCTAGTCACTCGACATCTGTGGCTTCACAGTACAGAGGGACTGTCCAAAGCCGCAGAT
CTCGAGTCGACTAG
+
BDBCCC=>=5=984-
9594;BBABB<??@A>7::988ABB@BBBCAAAAACBA=77717>?@@?=@CA@@=@?<?A@@@@@AA?@@AA@??
@T6XXI:00142:00436
GGGAGAAGGGAGAAAGGAAGGAGAAGCACCCAGCTTAGA
+
CDCBBA==95>98809<A>A@C==:5...6+..,.,///
@T6XXI:00143:00402
GGGAGAAGGGAGAAAGGAAGGAGATATAGACACGCCAATAGAAACGCGCTATTCTCCCTCTCCCTATAGTGAGTCGT
ATTAGAATTCCCC
+
CC@CCC=>=:@=:::B8<;;CECBB??@@@B@BBBBB@CCBABABB??>>AA@BBBB<BBB??;@?@@@@?@B@AA=
=??AAA>C@CCC:
@T6XXI:00143:00405
GGGAGAAGGGAGAAAGGAAGCTAGTCACTCGAGATCTGCGGCTTTGGACAGTCCCTCTGTACTGTGAAGCCACAGAT
GTCGAGTCGACTGCTTCCTTTCCTCCCTTCTCCC
+
>95999584-4444-
478237?BBCB>BBCCBB@;;:A@B>????9?=>>>?????9?==>@=<<<=<@>@A>CAAAB::9==A<==A@?====
2424;-434::4::;8;<6
@T6XXI:00143:00421
GGGAGAAGGGAGAAAGGAAGCTAGTCACTCGAGATCTGCGGCTTTGGACAGTCCCTCTGTACTGTGAAGCCACAGAT
GTCGAGTCGACTAGCTTCCTTTCTCCCTTCTCCC
+
CDACCBAB@<@@@=<@=@;?BA;;;??:7:CBCACCCECA>ABB>C@DCCC???;?@?@:??@@@AA>@@>???@@?
?=<<@A???B@@@44424/4;-48??;@:<=@@7
@T6XXI:00143:00422
GGGGAATTCTAATACGACTCACTATAGGGAGAGGGAGAATAGAGACGTATTCGTTTGCGCTCTGTTCACCTTCCTTT
CTCCCTTCTCCC
+
CCB9B=@=@CC@CCBBBBBBBA<97111.7>>?B>C@B@B=:<==<999:<?AAA<@A@@B==AA>A==;=<?<?A=
AA?C<A=;7771
@T6XXI:00143:00429
GGGGAATTCTAATACGACTCACTATAGGGAGAGGGAGAATAGTCAACAAATCGTTTGGGGCGACTTCTCCTTCCTTT
CTCCCTTCCTCCC
+
DCC9C@C@DDB@BBBBBBBBBBB97111.6<<?A>BAA?C=AA<=<<??;AB??=49694:@?BDABBC;988?CC=
>;?;69,/,/882
@T6XXI:00143:00442
GGGAGAAGGGAGAAAGGAAGTGCAGTTCGCGGTTAGTGAAGGGAAAAACTATTCTCCCTCTCCCTATAGTGAGTCGT
ATTAGAATTCCCC
+
```

```
?C>@@@>@@>;;<C=B>CBBBACDCC@BB@?<?=?@@@@CCB?ACDC2CBBB9::==6=?>??8A@???AA?>=::;
44/4::26555;4
@T6XXI:00144:00401
GGGAGAAGGGAGAAAGGAAGATCGCGGGGTCAAGCTCAAAGGTATGGCTATTCTCCCTCTCCCTATAGTGAGTCGTA
TTAGAACTTCCCC
+
IC?B:;79A:@111-1-1-/<9748888.3663166?=B<A>=<<A58::@9;444-
3;;;B7><=888@=8848>9:8;<=;>A>@AA5
@T6XXI:00144:00403
GGGAGAAGGGAGAAAGGAAGCTAGTCGACTCGACATCTGTGGCTTCACAGTACAGAGGACTGTCCAAAGCCGCAGAT
CTCGAGTCGACTAG
+
44-49989A19999149:9:;74440...,,,,44.....3,131317167;:::/-,-311-
0/00+0424387<>??@@@@==;@::// 
@T6XXI:00144:00420
GGGAGAAGGGAGAAAGGAAGGGGAGAAGGGAGAAAAGGAAGCCTAGTCGACTCGACCATCTGTGGCTTCACAGTACA
GAGGGACCTGTCCCAAAAGCCGCGAG
+
DB:@44299149>>:8;>;=>>/98?<??:@10//+0:>,..,.643389=<:<0/,-12236;4--
,++000+++34344133/044440333+--01---1
@T6XXI:00144:00425
GGGAGAAGGGAGAAAGGAAGGAGAACGCCAGCTACCCTGAACCGACGGTTGACTCCTACT
+
CFBCDC?CB>BBBB=B@BAB@BCAACCC>?996///+///56682/.,.,.....33111
@T6XXI:00144:00439
GGGGAATTCTAATACGACTCACTATAGGGAGAGGGAGAATAGTTTTTCCCTTCACTAACCGCGAACTGCACTTCCTT
TCTCCCTTCTCCC
+
AAB9ACB?CBD@BBBBBBBBAA><93165:AACC>CBB?@??8888,44-
48;@BB@=A?:::C>??<7::?;?<@@<AABE@C@AA@B=
@T6XXI:00144:00444
GGGAGAAGGAGAAAGGAAGGAGAAGTCGCCCCAAACGATTTGTTGACTATTCTCCCCTCCTACTCA
+
491///,/,//66+/,47899;A;76;@@@@5<9673311,..,...336>===;=574,-.----
@T6XXI:00145:00404
GGGAGAAGGGAGAAAGGAAGCTAGCCGACTCGACATCTGTGGCTTCACAGTACAGAGGGACTGTCCAAAGCCGCAGA
TCTCGAGTCGACTAG
+
DDDAA@?BB>BB@B<@8;49?>8;;7;:@???;<;?@?AAD@ED?BBCCBBCBCC???9<@@@@@?AA<@A?A=<<?
7788===????@@==
@T6XXI:00145:00422
GGGGAATTCTAATACGACTCACTATAGGGAGAGGGAGAATAGCACGGCTCGAAAGCGCAATCAGTTCTCCTTCCTTT
CTCCCTTCTCCC
+
CEE:D?B?CDCBCBB?AA>BBBBAA>:<;<A>CD>CBBAC??<==?@C@@77714;;443736444833388;8<<;
A@CC=A;>?@C<
@T6XXI:00145:00424
GGGAGAAGGGAGAAAGGAAGCTAGTCACTCGACATCTGTGGCTTCACAGTACAGAGGGACTGTCCAAAGCCGCAGAT
CTCGAGTCGACTAG
+
CD@@A?@CB>>444-
969;988?>>?;:9;;00/0/.45=8??@A;:;CCBBB@9::57;?>?@>@@<@A>@??A===::==<>9==888?
@T6XXI:00145:00431
GGGAGAAGGGAGAAAGGAAGGAGAAGTCGCCCCAAACGATTTGTTGACTATTCTCCCTCTCCCTATAGTGAGTCGTA
TTCAGAATTCCCCC
+
```

```
CGBCCBACCA>>?D>B=@=AACCC@B>A?BBB6@@<ACCCA>@A?BB@CCC=8<><443444-
4448>??=??=;<;=3349;9;2////*
@T6XXI:00145:00438
GGGAAATTCTAATACGACTCACTATAGGAGAGGGAGAATAGTTCCCAACCTCATTACTCCCTGTGTATTCTTCCTTT
CTCCCTTCTCCC
+
??199-9?ABB@BBAA?BBBB:9990/-
0<<@C>CCC>?@CA=<;485<<?@@A>A@@@@<<AAA====AA>A?@@;???@:9484771
@T6XXI:00145:00441
CTATCACTCGAGATCTGCGGCTTTGGACAGTCCCTCTGTACTGTGAAGCCACAGATGTCGAGTCGACTAGCTTCCTT
TCTCCCTTCTCCC
+
B===9::=?=A@?@CCBBC@CBB?B>AA<A9994:BACCCBB>BC@>?B?BBCCCB@@CCBBBCBBABB;:7>B?BC
?CCDF@C=??A@:
@T6XXI:00146:00404
GGGAGAAGGGAGAAAGGAAGAG
+
BBBCCD@?>:@@;;5<,-+***
@T6XXI:00146:00419
GGGGAATTCTAATACGACTCACTATAGGGAGAGGGAGAATAGTCGCGCAGTAACTCCCTTTGCCATCATCTTCCTTT
CTCCCTTCTCCC
+
?@@4C@@@CBCACCBBBBBBBCB?7311.777=?<?CC?C??=@AAA@ABB@>A@?@7<<7>?<BDDFCBC@C@CC>
A@AA<?=8;:@<
@T6XXI:00146:00423
GGGAGAAGGGAGAAAGGAAGGAGAAGTCGCCCAACGACTTGGT
+
CKFCCB844-9988095959?@??4314333,31333177774
@T6XXI:00146:00433
GGGGAATTCTAATACGACTCACTATAGGGAGAGGGAGAATAGCTGTTAACTAAGCAGCCCCACCGTATTCTTCCTTT
CTCCCTTCTCCC
+
IED:C@B@CCCA==<BBBB@@?888877,61167;667=A:7477736366999;??<??5?A@>>=?00/74447/
/.<=4>:?<@?;
@T6XXI:00146:00439
GGGGAATTCTAATACGACTCACTATAGGGAGAGGGAGAATAGCTGTAATGTTATTCACGCGGCCATCTTCTTCCTTT
CTCCCTTCTCCC
+
ABB7@=@=@BB@CBB@BBBB?A;99311.7===A=BDC@C??<??@@>B??==@>A@??<=;==BCEDACC?B@CC?
CCCC?B@ABBC>
@T6XXI:00146:00443
GGGGAATTCTAATACGACTCACTATAGGGAGAGGGAGAATAGTTTTTCCCTTCACTAACCGCGAACTGCACTTCCTT
TCTCCCTTCTCCC
+
CCD:E@B?CDC@BBBBB@@@BB?99311.7?>>@<ABB?CCCAAAA1@@;?;>BCAA>A?>BAC@BB@??BA@A?@@
;=?BC>C>?B?CA
@T6XXI:00146:00447
GGGAGAAGGGAGAAAGGAAGGACAGACGGCTGGGGAAAACGCACGCACTATTCTCCCTCTCCCTATAGTGAGTCGTA
TTAGAATTCCCC
+
CD@BBC@CCBBBBC>B?A?BABBBAABB@BABAA8BCC9CBBBCA?>CCAA=AAAA<A?>?@;@AA@@<=@@@@@@?
?<?AA>@>@CC5
@T6XXI:00147:00412
GGGGAATTCTAATACGACTCACTATAGGAGAGGGAGAATAGATCACCGTGTTGCGGGTGCGCGCTCTCCTTCCTTTC
TCCCTTCTCCC
+
```

```
BBD:CBDDEDDCBBBBCCCBA:9990.,0<:=?>@DD@CB=;<=944,,,0*,,00033660444443/35=<??9;
888-4799<<7
@T6XXI:00147:00414
CGGCTTTGGACAGTCCCTCTGTACTGTGAAGCCACAGATGTCGAGTCCGACTAGCTTCCTTTCTCCCTTCTCCC
+
CE@BBD?CBCCCCCCB>BBCB>>@BBBB?=BB@BBBA??AA@>><763:?>>BAAB@C@BD88<CC?C?ABCD>
@T6XXI:00147:00424
GGGAGAAGGGAGAAAGGAAAGGG
+
AB7;<9<>A044990/,//+///
@T6XXI:00147:00431
GGGGAATTCTAATACGACTCACTATAGGGAGAGGGAGAATAGTCAACAAATCGTTTGGGGCGACTTCCTCCTTCCTT
TCTCCCTTCTCCC
+
G??4@695999;>@AAAC@;;;8>>876,1<<=@>?@@@BCB?=;2444-8?<<<6<;6,7.::=-
0,0:,0,/7;=9AAAA<@?@AAA<
@T6XXI:00147:00445
GGGGAATTCTAATACGACTCACTATAGGGAGAGGGAGAATAGTCAACAAATCGTTTGGGGCGACTTCTCCTTCCTTT
CTCCCTTCTCCC
+
DDD:C@C@AAB@BBA??BBBBB@<9311.7><<?<?BA@C?@=AA>AAA<C==?C=@BB7BCCCC@CCC>?=<<@@<
@A@C<A9==AA<
@T6XXI:00147:00446
CCTTAGTCACTCGAGATCTGCGGCTTTGGACAGTCCCTCTGTACTGTGAAGCCACAGATGTCGAGTCGACTAGCTTC
CTTTCTCCCTTCTCCC
+
D?D?ABB;=>CCAAACDCBBBB?BB?;?=>>BBA?>;>???@???>>BC>?B@CCCBCCCBAAB@@BBBAAC@@??A
?AD@BBBB<@5::==:
@T6XXI:00147:00448
GGGGAATTCTAATACGACTCACTATAGGGAGAGGGAGAATAGTCCTACACACAACAGCGGGCTTGTTCTCTTCCTTT
CTCCCTTCTCCC
+
EAA9B@C@CBB@B?BBBBBBBAA;?7665:A?CE>CBB>B@A==<::?<<>995;4803418=8<<>=<;>;=;@@<
BAAA=A<8::=:
@T6XXI:00148:00417
GGGGAATTCTAATACGACTCACTATAGGGAGAGGGAGAATAGTCCCGATATATGTCGTGGGCTCTTCGCCTTCCTTT
CTCCCTTCTCCC
+
CCC9C@D@BBCACBBAABCBAAA<<311.677@B>BCC@B>>??C<<<:;==<<8>@:9:7:ABBACBB?B8:8:C@
CDCC>C=@=AC<
@T6XXI:00148:00418
GGGGAATTCTAATACGACTCACTATAGGGAGAGGGAGAATAGCAAACGTTTCCCCGACGTCGCTTTCTCCTTCCTTT
CTCCCTTCTCCC
+
A?@4>>A?CCC@AA@@@@BBBBBB@96646??AB>BCC?B??;<=;=>?>;ACC9?@@C??????6???:??B>@A;
@?ACAC;>>AB<
@T6XXI:00148:00425
CTAGTCGACTCGAGATCTGCGGCTTTGGACAGTCCCTCTGTACTGTGAAGCCACAGATGTCGAGTCGACTAGCTTCC
TTTCTCCCTTCTCCC
+
@--,,-
,,68:?CCCCB??@C@CCB=?<???>888/7>@CCBA@>BBB>?B@ABBBAB?@@CA@@BBB@@>?874747?6?BB
D@C=A@AC@
@T6XXI:00148:00426
GGGAGAAGGGAGAAAGGAAGCTAGTCACTCGACCATCTGTGGCTTCACAGTACAGAGGGACTGTCCAAAGCCGCAGA
TCTCGAGTCGACTAG
+
```

```
CDAF;;79>:>>>>:>698<CBBGCB>>::<00,....5:C888@CCCBBJCB>7777.44;;>@<??<?A?@@@==
=?988??A899A@AA
@T6XXI:00148:00427
GGGAGAAGGGAGAAAGGAAGTGCAGTTCGCGGTAGTGAAGGGAAAAACTATTCTCCCCTCCTCATATGT
+
EDB@@??BC>CEEC>C@B@@@>AABB@BABB>A@@@CB@CB<?CCC2AABB@C>>>>5799,,,,,,++
@T6XXI:00148:00428
TAATACGACTCACTATAGGGAGAGGGAGAATAGCCTCTCGCGCTTAGCTCCCACGTTATACTTCCTTTCTCCCTTCT
C
+
@AACCCCBCCCCCA??83708?>>?<?KC=BBBB?C;<<ACAACCCCGDDG@DAAA?@>94424244-
4999,31331
@T6XXI:00148:00433
GGGAGAAGGGAGAAAGGAAGCTAGTCGACTCGACATCTGTGGCTTCACAGTACAGAGGGACTGTCCAAAGCCGCAGA
TCTCG
+
CF@AAA@BB>BBBB>B@B9<:B:;;CCBAAA@88;@@@CLDABB?BBA@>=@@;>44317>9>@A>AA<AA?@A=;;
;===<
@T6XXI:00148:00439
GGGAGAAGGGAGAAAGGAAGAACAAAACTGGAGCGGGGAGTATAAGACTATTCTCCCTCTCCCTATAGTGAGTCGTA
TTAG
+
:D1119599144/856,/,//,....*.44:=;9:CB:BB@A@<<;7@@94247<7279317,7667>>:<:30094
4/03
@T6XXI:00148:00445
GGGAGAAGGGAGAAAGGAAGCTAGTCGACTCGAGATCTGCGGCTTTGGACAGTCCCTCTGTACTGTGAAGCCACAGA
TGTCGAGTCGACTAGCTTCCTTTCTCCCTTCTCCC
+
DIBEBB@CDAB;;;7<@@=>?>0110...577::A@@CBBB@BBB>B@BCBBBBC>CB<??<<<?==@@AD?AA@@@
@AAAA@9=<@<<;@88:???A@9@844-4347<D=
@T6XXI:00148:00446
GGGAGAAGGGAGAAAGGAAGAAAACAGCGCGCACTGGAAAGGTCAGGCTATTCTCCCTCTCCCTATAGTGAGTCGTA
TTAGAATTCCCC
+
CB?BFCABB>AA@A<A=E@CCCC:CBBBBBBBB?BBCBB@A@BBBBBCBBB;;<@@<@BABB=@@@CEECBCC????
?:?9;94/003,
@T6XXI:00149:00417
GGGAGAAGGGAGAAAGGAAGAAT
+
FDBBCB>==<@@8707+-+--*,
@T6XXI:00149:00420
GGGGAATTCTAATACGACTCACTATAGGGAGAGGGAGAATAGCCTTCCCTGCATGTATCGCGACGTTCTCTTCCTTT
CTCCCTTCTCCC
+
DDD:D@B@BFGBBBBBBB@@@C<999311.7>>?C>???@CAC<<A?B@;BBB===@:9;:?@AAAE@A>;:>B:??9
?@>>;@>===A<
@T6XXI:00149:00432
GGGAGAAGGGAGAAAGGAAGCTAGTCGACTCGACATCTGTGGCTTCACAGTACAGAGGGACTGTCCAAAGCCGCAGA
TCTCGAGTCGACTAG
+
CDBBB;2996<CBBAE;<;<C>;;;.0///::087611=?A4:;@DCCCC???;9337,4::4447<<6<B?A@:99
??<<=?><<=@@@@D
@T6XXI:00149:00435
CTAGTCACTCGAGATCTGCGGCTTTGGACAGTCCCTCTGTACTGTGAAGCCACAGATGTCGAGTCGACTAGCTTCCT
TTCTCCCTTCTCCC
+
```

```
CB:9<>4::?AA>@@BBBCC@CCB>B@CCCCBBB>BB@BA?>>?AAC??B@BBCCCA?@CAA>79:;;<C@@??B?B
C@GBCC?C8::>@7
@T6XXI:00100:00454
GGGGAATTCTAATACGACTCACTATAGGGAGAGGGAGAATAGACGGCAGTTCGCCCACCTCGTCATGTTCTTCCTTT
CTCCCTTCTCCC
+
DCB7B54:<@@?CDBBBBCBB>AAA@AA=@?>@@=@AA<=<858C:=?BC@BA@B>BC@CCB;:<@@@=@C?B@BC?
CCDC<?=;::C>
@T6XXI:00100:00471
GGGGAATCTAATACGACTCACTATAGGGAGAGGAGAATAGTGACGTGTTGAGCGGCGCTC
+
999,95944/56///8:9:9/.1//..+,,,.,.45344==54;8.**)**++0/031-.
@T6XXI:00101:00463
GGGGAATTCTAATACGACTCACTATAGGGAGAGGGAGAAATAGCCTAACGT
+
1?>+1-1,///,/449>:88>78897==9==777.7444+0222,..3...
@T6XXI:00101:00471
GGGAGAAGGGAGAAAGGAAGTCGACTCGACATCTGTGGCTTCACAGTACAGAGGGACTGTCCAAAGCCGCAGATCTC
GAGTCGACTAG
+
AEABBB@CCACCCC?C@C>:88873333768999=?C?AA@B:::AAABB@99;1;ABCCCCDCBBB@BBCACBAAA
B;;;?@@?AA<
@T6XXI:00101:00475
GGGGAATTCTAATACGACTCACTATAGGGAGAGGGAGAATAGTCTTCGACCCCCATTACACTGCTTTTCTCTTCCTT
CTCCCTTCTCCC
+
FCC:C@A>AHBCDCBBBCCBB@?BA???9;==??9?:;998::<<7;8@@ABB2AB>AAAAB@@AAA8;>0>:?,0.
/7;:7C@;::B>
@T6XXI:00101:00487
GGGGAATTCTAATACGACTCACTATAGGGAGAGGGAGAATAGCCGTAATGTTTGCTTACGCCTTTTCTCCTTCCTTC
TCCCTTCTCCC
+
CA;2;545;===CCABBCCCBBBAB:995766?B>A@A?A@@@<=<<;>>?@;@AA?@?@@27770/;;9978,/,/
6666;9@@@B<
@T6XXI:00101:00494
GGGGAATTCTAATACGACTCACTATAGGGAGAGGGAGAAT
+
,8808+1,///769;<CC@@@??=?777.77000,/77,-
@T6XXI:00101:00499
GGGGAATTCTAATACGACTCACTATAGGGAGAGGGAGAATAGTTTGGCTTTGTCCCACAACATGTCTCTCCTTCTCC
TTCCTCCACT
+
;B>9>:@=;;<ABCBBBBBBCCB@?777.7=>@A=@@A>@???>:;2443+1116,676010000434042870000/
0/02382533
@T6XXI:00102:00455
GCTAGTCGACTCGAGATCTGCGGCTTGGACAGTCCCTCTGTACTGTGAAGCCACAGATGTCGAGTTCGACTAGCTTC
CTTTCTCCCTTCCTCCC
+
444449494:A@<<=<@@@?<<:00,.,..9::A??@CC???AA@85894<9<;AAB;<0>::00,11.03;;8888
;;;57/4533,-,-772
@T6XXI:00102:00468
GGGGAATTCTAATACGACTCACTATAGGAGAGGGAGAATAGCCTGATACTAACCAGCTTGACTATCTCCTTCCTTTC
TCCCTTCTCCC
+
DCC7B=@=@@A?CBCCCCCBB7=;://,0;:=?<?ACBA@AA>BBBBCB@@?B>A??BBCBBABB@@?=@<>:?B:?
@@B:@<777<6
@T6XXI:00102:00490
```

```
GGGGAATTCTAATACGACTCACTATAGGGAGAGGAGAATAGTACGT
+
>?=1=+959889ACCCCCC@@?BB?:::6:@A?>@7734.////,+
@T6XXI:00103:00451
GGGGAATTCTAATACGACTCACTATAGGAGAGGGAGAATAGCCGTTCTATGTCACATATCGCTTTCTCCTTCCTTTC
TCCCTTCTCCC
+
CCC:C=?>ABB@CBB@CCCBB:664..,0>:==<?>A>A???>@@=@@BBDCCB@B@BA?=;<<6;=@8;7:344-
4;;;4979444-
@T6XXI:00103:00463
GGGGAATTCTAATACGACTCACTATAGGGAGAGGGAGAATAGCTATCCAATCTCCT
+
;99/9<@=ACDCDBB=BBBBB@<96166.7>>?B@B??<?ABAA;:7,,*,2**)+
@T6XXI:00103:00464
GGGGAATTCTAATACGACTCACTATAGGGAGAGGGAGAATAGCCACCAACTATTGTTCGTCGCCGTTCTCTTCCTTT
CTCCCTTCCTCCC
+
999/48?=@CC@CBBBDAA@@?>>>777.7>>>@=A@@>@===>@@=@>@@@@>@@AA@@@@@=??:::44347::6
<9;;6;,/,///,
@T6XXI:00103:00465
GGGAGAAGGGAGAAAGGAAGGAGAAGTCGCCCCAAACGATTTGTTGACTATTACGTCCCTCTCCCTATAGTGAGTCG
TATTAGAATTCCCC
+
//+//4244-448;58;=<;=AAA?ABABDGG8AA<@7769899=BAC@;=.//..930---
00+004433446;;6595<==;44/000+
@T6XXI:00103:00469
GGGGAATTCTAATACGACTCACTATAGGGAGAGGGAGAATAGTCAACAAATCGTTTGGGGCGACTTCTCCTTCCTTT
CTCCCTTCTCCC
+
66606,/1<BBBCDCCCCCCB?@A<166.7>>@A>BCC@AAAA@@>?==7@;;;C?CEE7;;>>>9:>?,0,/,//-
0:C=;=48.555
@T6XXI:00103:00472
GGGAGAAGGGAGAAAGGAAGGAGATATAGACACGCCAATAGAAACGCGCTATTCTCCCTCTCCCTATAGTGAGTCGT
ATTAGAATTC
+
;B1999599-
88>@9@=@=?>AAA?:777>766=>;=@B?>@C>;;68?1449;37:57@BBB?BAAA@??BB@BCAAA?=9<=B=@
@T6XXI:00103:00473
CTAGTCACTCGAGATCTGCGGCTTTGGACAGTCCCTCTGTACTGTGAACCACAGATGTCGAGTCGACTAGCTTCCTT
TCTCCCTTCTCCC
+
C8111=8=ACBBCDCBBABC@BDC>B@BA>?<==;?EA@B;;>;99;:=888==<>;;;<;<AAA??:=@@<;=;==
;<::8469<<<B?
@T6XXI:00103:00481
GGGGAATCTAATACGACTCACTATAGGAGAGGGAGAATAGTCAGCGCATAGCCCGTTTACAATTCTC
+
/7707,1///,/47;;AB@?;:?:00,.4.11,044/3499===??<@<>8::13391..2010---
@T6XXI:00103:00488
GGGGAATTCTAATACGACTCACTATAGGGAGAGGGAGAATAGCTTAGACCTTGGTGGCATTCTGTTCTCCTTCCTTT
CTCCCTTCTCCC
+
BBB7A=@=@@@@CAA@CCBAAAC?=667.7>>BC?C@B>AAA?B?BAA?>A>@?8868@@8;<>>8;;D:=;=>AA<
A@@@<@===DAA
@T6XXI:00103:00492
GGGAGAAGGGAGAAAGGAAGCTAGTCGACTCGAGATCTGCGGCTTTGGACAGTCCCTCTGTACTGTGAAGCCACAGA
TGTCGAGTCGACTAGCTTCCTTTC
+
```

```
AE<><98<C@ACCC>BAB?GC@?@?::7=<=::7@@66:9=:6?:8:>99=@://,/87/-
.4488?A;==>AA??>@889==ACB????;///,,+,,+,
@T6XXI:00104:00452
GGGAGAAGGGAGAAAGGAAGGAGAAGTCGCCCCAAACGATTTGTTGACTATTCTCCCTCTCCCTATAGTGAGTCGTA
TTAGAATTCCCC
+
ED@DCB?BC@BBAA=B?B=>@>??;99??BB?5@A=A@<AA>AC?CB@<<<8:<==;9<<?C>CAA??@@AA???@A
A>A??;<5;;<1
@T6XXI:00104:00454
TGCACCGAGTCTGCGGCTTTGGACAGTCCCTCTGTACTGTGAAGCCACAGATGTCGAGTCGACTAGCTTCCTTTCTC
CCTTCTCCC
+
DDCCBAAAAECDDBB?BCE>B>BBBB@@@@@DCCCC@@?ABC@BB?BAAA?@?@DCBCB@@AAAB:;9<?488/8<>
>/737676-
@T6XXI:00104:00465
GGGAGAAGGGAGAAAGGAAGAGAATAACAGTGCACCGAATGCGCAAACTATTCTCCCTCTCCCTATAGTGAGTCGTA
TTAGAA
+
ECADCC@BC>BBBB?C?B?BCCB@B@>ABBDCBCB<AA@BCCCCCC>CCCB??;::8;BBDD>BBABBB@@???BAA
B@BBFC
@T6XXI:00104:00474
GGGAGAAGGGAGAAAGGAAGGGGAGAAGGGAGAAAGGAAGCTAGTCGACTC
+
CH>CCC?BA=ABBC>B@B>@>>7ABB?@?:>777.101016111111144.
@T6XXI:00104:00476
GGGAGAAGGGAGAAAGGAAGGGGAGAAGGGAGAAAGGAAGCTAGTCGACTCGACATCTGTGGCTTCACAGTACAGAG
GGACTGTCCAAAGCCGCAGATCTCG
+
ECAAAA@DD>@AAE>B?@@?B?8BBB?=9619688?BC=?@BBABCCCDDA=558=<<AAA>@@>AA@<<===<<44
417>8?@A?AA<@@>@@<====:;;
@T6XXI:00104:00480
GGGAGAAGGGAGAAAGGAAGGAGAAGTCGCCCCAAACGATT
+
44-4998=A:;:CC<A=>48583>7<;73...*.503...,
@T6XXI:00104:00481
GGGAGAAGGGAGAAAGGAAGCTAGTCGACTCGACATCTGTGGCTTCACAGTACAGAGGGACTGTCCAAAGCCGCAGA
TCTCGAGTCGACTAG
+
DC@AAA@BD>BBCB=A6:5;B>:>?/00<0/00719<9@?>38:5:A???B@=;;444-
4::@@A>@@;@B>AAAAA===?A==?B====<<
@T6XXI:00104:00493
GGGAGAAGGGAGAAAGGAAGGAGAAGTCGCCCCAAACGATTTGTTGACTATTCTCCCTCTCCCTATAGTGAGTCGTA
TTAGAATTCCCC
+
BB=AAC@CC<@A@@<A@C@C@CCC@??B????6@B=BAAA?>AA@CCC@<<9<>>@<@????<?@@@???A@@@A99
9>??A>A>>?;4
@T6XXI:00105:00452
GGGGAATTCTAATACGACGTCACTATAGGAAGAGGGAGAATAGTCAACAAATCGTTTGGGGCGACTTCTCCTCCTTT
CTCCCTT
+
8=70=+1,/6678449>:77>@;;?;00,0,0//.+.3638<<6304000+37333,111,376455574,..3330
--0424/
@T6XXI:00105:00455
GGGGAATTCTAATACGACTCACTATAGGGAGAGGGAGAATAGTCAACAAATCGTTTGGGGCGACTTCTCCTTCCTTT
CTCCCTTCTCCC
+
```

```
EDE8A=?<>9959BA@ABB@@???=777.7=AADACCA:=<BAAA=?@@<BBAAC>CCB6ABCCC>??<34247996
=AAA<B9<=CD@
@T6XXI:00105:00465
GGGAGAAGGGAGAAAGGAAGCAGTCCATCTGTGGCTTCACAGTACAGAGGGACTGTCCAAAGCCGCAGATCTCGAGT
CGACTAG
+
=D?BBCACC>BCCC>C?B>AA@;8;:88888:A>BB@A??@8776::444+38;A7969=;=A>@:0/;;/..::?:
CC;;;;;
@T6XXI:00105:00475
GGGGAATTCTAATACGACTCACTATAGGGAGAGGGAGAATAGTCAACAAATCGTTTGGGGCGACTTCTCCTTCCTTT
CTCCCTTCTCCC
+
8A=08+1,///,/44447;<A=>>?66616;:AB<@<<9;=;;<?<=443///772566597666011100/0/044
4033+32:3;;4
@T6XXI:00105:00480
GGGAGAAGGGAGAAAGGAAGGAGAAGTCGCTCCAAACGATTTGTTGACTATTCTCCCTCTCCCTATAGTGAGTCGTA
TTAGAATTCCCC
+
DH@BA@@BCABBAB>C@B@B@BB????@@B>>>>>;>????;@@>@<AACC@BBCCB@@@BB>B@@@BBCCCDBBB:
:78?A<<<<=C5
@T6XXI:00105:00488
CTATCGACTCGAGATCTGCGGCTTTGGACAGTCCCTCTGTACTGTGAAGCCACAGATGTCGAGTCGACTTAGCCTT
+
9994444==ACBBCB9;4771311,31316>?@@=A@779A8776:648@897;BAAACCBC?711,-,*,,*)*0
@T6XXI:00105:00496
GGGAGAAGGGAGAAAGGAAGGAGAAGTCGCCCCAAACGATTTGTTGACTATTCTCCCTCTCCCTATAGTGAGTCGTA
TTAGAATTCCCC
+
BKBBA@=@C=@AAG?B?A@D@CCB?@A?ACDD9CC>BABBB=@A@GCCAAA>????:????A<@@A@===@?>=@<=
=;=BB:93484-
@T6XXI:00106:00456
GGGAGAAGGGAGAAAGGAAGGCGAATACGTT
+
4:699>59919@@A<=:;-0,...64.....
@T6XXI:00106:00457
GGGGAATTCTAATACGACTCACTATAGGGAGAGGAG
+
BDB8D8929442448@A;::B@>>;776,//00,.-
@T6XXI:00106:00460
GGGAATTCTAATACGACTCACTATAGGGAGAGGGAGAATAGATCACCGTTGGTTCGGGTGCGCGCTCTCCTTCCTTT
CTCCCTTCTCCC
+
@95?5954999499997;@@?>B?777,7;;=7886656@@???BC>AB7:7:<A@C?CCCBCBBBBBB@C?B?DB<
AA>=7:94444-
@T6XXI:00106:00462
GGGGAATTCTAATACGACTCACTATAGGGAGAGGAGAATAGTTACGTCCCGTCACTATCCTTCCAGCAGCGTCTACT
TT
+
88808+;8<CB@CCBBBB?>BA?96116.7>774:44248888:;:030+000330000232320000000000000
0+
@T6XXI:00106:00473
GGGGAATTCTAATACGACTCACTATAGGGAGAGGGAGAATAGTACGGACGTCTCACCGTGCTGCTCTTCTTCCTTTC
TCCCTTCTCCC
+
CCC:CACBBCC@BBAAB<<<A@>?:8??9???AE@@=<>AA@=9=A;==CC@@@@A<<@===><=BA898=?37817
;;@6<8<;;<6
@T6XXI:00106:00480
```

GGGGAATCTAATACGACTCACTATAGGGAG
+
,27*-+1///,/97>>;7316911677,.,
@T6XXI:00106:00488
GGGGAATTCTAATACGACTCACTATAGGAGAGGGAGAATGGCCAACAGGTATCTGATTGCGCGCTCTC
+
;>>08,/,/7778=@@@@CAA;::<00,/;077.4775//,0,/,--3223680034/0356533422
@T6XXI:00106:00492
GGGAGAAGGGAGAAAGGAAGGAGAAGTCGCCCCAAACGATTTGTTGACTATTCTCCCTCTCCCTATAGTGAGTCGTA
TTCAGAATTCCCC
+
996>??599-
948?9@<@>B?BCD@BBBAAAA8AC>@B:::5:C>CB@@AA????A<@888>:4877=9283.......55==:;27
77+
@T6XXI:00106:00493
GGGGAATTCTAATACGACTCACTATAGGGAGAGGGAGAATAGTTCCCCAGTATAAAACCGGCTATCCGCCCTTCCTT
TCTCCCTTCCT
+
999/92959;;=BBBBA@@@?=?=A777,7>7777:@CAAAA@388708==?=777099;,/,2,,*.5503499;;
6;74101,-,+
@T6XXI:00107:00460
GGGAGAAGGGAGAAAGGAAGCTAGTCACTCGACATCTGGGCTTCACAGTACAGAGGGACTGTCCAAAGCCGCAGATC
TCGAGTCGACT
+
CB@@@@@AA==?BC?B?A>@A@=?CCCCCCBBBBDECCC>CC@CCDBBCD@??==<7<A=??@>AA<@B>AAAAAA?
?????AAA@??
@T6XXI:00107:00466
GGGAGAAGGAGAAAGGAAGCTAGTCGACGTCGAAGATCAGGCGGCTTTGGGACAGT
+
/6502;,1,////+/5973331116711111110111116110111,17611...
@T6XXI:00107:00468
GGGGAATTCTAATACGACTCACTATAGGAGAGGGAGAATAGCGCAATTTGGACATGTGGCCGCTTCTCCTTCCTTCT
CCCTTCTCCC
+
CDF:B=?=@A@?BB@@@CCCC>>=:/.,0;;>>:A>>>???AA@C8::7:=@BCBBB@>A>CAA?=<57746,/,.9
;99A@A9885
@T6XXI:00107:00492
GTCGACTCGAGATCTGCGGCTTTGGACAGTCCCTCTGTACTGTGAAGCCACAGATGTCGAGTCGACTAGCTTCCTTT
CTCCCTCTCCC
+
99:?ACCEBCHCCC9<:B?ABC>B?B@@@>@@0;;?@AACDBBBC@CC@BBCCCC;>;CAA>BBA@@B::8;:,00,
0;;;,0.4442
@T6XXI:00108:00459
GGGAGAAGGGAGAAAGGAAGCTAGTCACTCGACATCTGTGGCTTCACAGTACAGAGGGACTGTCCAAAGCCGCAGAT
CTCGAGTCGACTAG
+
CCADBB=??>ACCC>B>?=@C>??CCC?>>;00000.9573?@>ABCBBBCC@?84417<<=A@?A?<AB?A?===A
CAA@?@A???AA?@
@T6XXI:00108:00466
GGGGAATTCTAATACGACTCACTATAGGGAGAGGGAGAATAGTCAACAAATCGTTTGGGGCGACTTCTCCTTCCTTT
CTCCCTTCTCCC
+
CDB8B8;:;449;9;<@?A@A>>>6716,7<>>@>BAA@@@@@;<:<<=;??;;626<;,11611016600/04444
444C;<5=8:94
@T6XXI:00108:00482
GGGAGAAGGGAGAAAGGAAGCTAGTCACTCGAGATCTGCGGCTTTGGACAGTCCCTCTGTACTGTGAAGCCACAGAT
GTCGAGTCGACTAGCTTCCTTTCTCCCTTCTCCC

```
+
DC;=;B?BA9;8>>9ACA?BCBBBBBAA@@8:8;?BBBBB@CCB>B?AAACCCC>AAA@===C?????AA;9;;===
<<;??@@@@A@?==33/4/44-4:::-424434+
@T6XXI:00108:00484
GGGAGAAGGGAGAAAGGAAGGAGAAAACGTGAGCCAAGATCTACGCTCTATTCTCCCTCTCCCTATAGTGAGTCGTA
TTAGAACTTCCCC
+
ED?@AA>@>9>>>><@=@>B=@@ACD9FCBCBBC9;9;BBA?@ACEEDBEDAB;;;7<ABCD?CCCCABAB;;;A@@
?=>=7.//=:776
@T6XXI:00108:00487
CTAGTCACTCGAGATCTGCGGCTTTGGACAGTCCCTCTGTACTGTGAAGCCACAGATGTCGAGTCGACTAGCTCCTT
T
+
449944444=@A99<BB@??5877-84819ABCC>AC@A@C@@9;//--
756;AACAABCA@>>98....../5550
@T6XXI:00108:00492
GGGGAATTCTAATACGACTCACTATAGGGAGAGGGAGAATAGCAAACGTTTCCCCGACGTCGCTTTCTCCTTCCTTC
TCCCTTCTCCC
+
BEC8A79:;AA>BBBACBCCCBB=96:7.7?77:78?B@H?>?@C<ABBB<<<<4<CBBAAA?@@6:8=8:9;2423
8886<:=8@@;
@T6XXI:00109:00450
GGGAGAAGGGAGAAAGGAAGGAGAATACAGCGAATGAAAC
+
CC@C@@?BC@?@@C@C@C@C@@@@4713333331111626
@T6XXI:00109:00455
GGGAGAAGGGAGAAAGGAAGCTAGTCGACTCGACCATC
+
BD>A@928819?>@<@=@=8;;A:16;5333...,,02
@T6XXI:00109:00465
GGGGAATTCTAATACGACTCACTATAGGGAGAGGGAGAATAGTTCCTAGCGACCTCCCGTA
+
DDHAC89:9988499>AB<<;:??>777,7;777.7;;9;;;:73233333332000+000
@T6XXI:00109:00483
CTAGTCGACTCGAGATCTGCGGCTTTGGACAGTCCCTCTGTACTGTGAAGCCACAGATGTCGAGTCGACTAGCTTCC
TTTCTCCCTTCTCCC
+
@9944444:?AABAAACCC::8;BC>C=@@CBCCC>AAA?CA@??AAA=?B@CCCCB@B@AABBCCC@@@FB??>?@
CC=A>=>:=8;;;@A
@T6XXI:00109:00491
GGGAGAAGGGAGAAAGGAAGGACATGAGTAG
+
9@:>=@69909>?>8@:;:<,.....4...,
@T6XXI:00110:00455
GGGAGAAGGGAGAAAGGAAGGAGAAGTCGCCCCAAACGATTTGTTGACTATTCTCCCTCTCCCTATAGTGAGTCGTA
TTAGAATTCCCC
+
EC@CBB@BB>AAAC>B?A?B@BBB@??BB???8?B>CA8::7:D@GCCC<@=:??=;C6;<B=A????@@AA@@?<<
<?DCH>A58870
@T6XXI:00110:00470
CTATCGACTCGAGATCTGCGGCTTTGGACAGTCCCTCTGTACTGTGAAGCCACAGATGTCGAGTCGACTAGCTTCCT
TTCC
+
BAA944:::@@CBBBCCD;:6:>>8?9;;CCDCC?C?:::AABB@@A=@CACBAA@;;;?ACBBBB;8733..,.55
5+/,
@T6XXI:00110:00475
```

```
CTGCGGCTTTGGACAGTCCCTCTGTACTGTGAAGCCACAGATGTCGAGTCGACTAGCTTCCTTTCTCCCTTCTCCCC
TTCCTTTCTCCCTTCTCCC
+
?A:49<998-4888BBAAB>ACCCBAAAA:??9AA=A@BBBBCCCCBA@AADCFBB;:5:<AC>BBBE>B:=6344-
4:;344-4444-47;3555
@T6XXI:00110:00482
GGGGAATTCTAATACGACTCACTATAGGGAGAGGGAGAATAGTCAACAAATCGTTTGGGGCGACTTCTCCTTCCTTT
CTCCCTTCCTCCC
+
ACB8@5954449;9;<BBBBKBA==777,7>77717;;;=??@<<9<@?:?A???6=??5??B??:?==8<:=>@C=
=98616,/,///+
@T6XXI:00110:00491
GGGAGAAGGGAGAAAGGAAGGGGAGAAGGGAGCTAGTCGACTCGAGATCTGCGCTTGGACAGTCCCTCTGTACTGAT
AAGCCACAGA
+
CF?AAA??@198>?9>8;7:CC5??@077,711779111.4...43..144/0-1,-,---
06:=;;<@<;;843000/06366610
@T6XXI:00110:00492
GGGAGAAGGGAGAAAGGAAGCTAGTCGACTCGACATCTGTGGCTTCACAGTACAGAGGGACTGTCCAAAGCCGCAGA
TCCTCGAGTCGACTAG
+
9@0;;>=BB>@@AB=B?A@BBABAA961111111179167:236=::8<=94444333+0448@@>@@;@@<?=:66
//,/646870000000
@T6XXI:00111:00451
GGGAGAAGGGAGAAAGGAAGGAGAAGTCGCCCCAAACGATTTGTTGACTATTCTCCCTCTCCCTATAGTGAGTCGTA
TTCAGAATTCCCC
+
9:0944244-4444-42477777;=679>??>7A?:76111,173689668:665;03333303311..-
328=8472,-.66359AC=4
@T6XXI:00111:00454
CTAGTCGACTCGAGATCTGCGGCTTTGGACAGTCCCTCTGTACTGTGAAGCCACAGATGTCGAGTCGACTAGCTTCC
TTTCTCCCTTCTCCC
+
C6/111//7::;BAAA@BBCC@CCC>C>@@ECCCC>C@@@@@>>?B@A=?B@BBBAA??@BAAAABBCCCCCAA?A>
@B<???@9=;>@@@;
@T6XXI:00111:00457
GGGGAATTCTAATACGACTCACTATAGGGAGAGGGAGAATAGTTCACGTGACG
+
?@;2B5959>>>BAAAE@@@C?>>>777.7==>AAAAH@??630++,,,+,+6
@T6XXI:00111:00462
CTAGTCGACTCGAGATCTGCGGCTTTGGACAGTCCCTCTGTACTGTGAAGCCACAGATGTCGAGTCGACTAGCTTCC
TTTCTC
+
A?9999449?CACCBCBCBAA>:7:/8<??CDDCCCEAB@??<AAB@@>@C@CBB>>@@@?BCBBBBBCBBC::47:
<<6777
@T6XXI:00111:00463
GGGAGAAGGGAGAAAGGAAGAGAACAGTCACAGGCTCGAGCTGCAAGCTATTCTCCCTCTCCCTATAGTGAGTCGTA
TTAGAATTCCCC
+
CE?CCB?BB>@AAB<@=@@DCCB?CBBBDBBECACDBBBAAA;;<=>??::8:BCE?CC@==7<@@@????CA:;;4
4/4332556==5
@T6XXI:00111:00464
GGGAGAAGGGAGAAAGGAAGCTAGTCGACTCGAGATCTGCGGCTTTGGACAGTCCCTCTGTACTGTGAAGCCACAGA
TGCCGAGTCGACTAGCTTCCTTTCTCCCTTCTCCC
+
DG>A@@59919?@B>B@CBBB?>>=0/0>>>>>@CBBBCBCA:::7:>@A@A???9<??<<=AA???C=@?????AA
AA=;<=???===AA=<<<;247144;;-838;@?<
```

@T6XXI:00111:00466
CTATCGACTCGAGATCTGCGGCTTTGGACAGTCCCTCTGTACTGTGAAGCCACAGATGTCGAGTCGACTAGCTTCCT
TCTCCCTCTCC
+
GCB=99:?ABBBBCDBA@@@>?>?:@?@@?B;;:7:CCCLCDBA??A=?B?BBCCAAAAA<:??@@??@>8745:-
,+,000*+--23
@T6XXI:00111:00475
GGGAGAAGGGAGAAAGGAAGAGAACAAGCCCGCTGTTGTGTGTAGGACTATTCTCCCTCTCCCTATAGTGAGTCGTA
TTAGAACTTCCCC
+
DHAAAA@CC>BBBD@D@C@CCBA?CG@BBB?BCCCD?BBBBCC@@=@BBBB?ABAC>CCB@@;@AAAAAA???B@??
@?;;9,//4764.
@T6XXI:00111:00481
GGGAGAAGGGAGAAAGGAAGCTAGTCACTCGACCATCTGTGGCTTCACAGTACAGAGGGACTGTCCAAAGCCGCAGA
TCTCGAGTCGACTAG
+
CCACCC?BA7;;?>9?584;B:::GC>;7780/,-
333;6A;B?ACCCBBB@@?@43717>>?AA?AC:>>=AAC<???AA@@?A@A@@==<
@T6XXI:00111:00495
GGGAGAAGGGAGAAAGGAAGTGATAAGCCCGGGAGCAATGAGGGATGATCTACTTCTCCCTCTCCCTATAGTGAGTC
GTATTAGAATTC
+
A@=AEB@CC;;;A@<>>;9000..44...+17268<;799444-
499449;973:7766266111,1666;=:=6/.....5..-,0/-
@T6XXI:00111:00496
GGGAGAAGGGAGAAAGGAAGGAGAAGTCGCCCCAAACGATTTGTTGACTATTCTCCCTCTCCCTATAGTGAGTCGTA
TTAGAATTCCCC
+
??:BA@=@C9<<BCBD@C@C?BBC@@?@@CCC9CD??????9?B@BAAAAA?@@@A<???@C<AA@A@@@@@99:AD
A>AAA=A?AAA6
@T6XXI:00112:00451
GGGGAATTCTAATACGACTCACTATAGGGAGAGGGAGAATAGTCAACAAATCGTTTGGGGCGACTTCTCCTTCCTTT
CTCCCTTCTCCC
+
CC@9B69;<AAAC<;;CBABCCB9=111.7>>@C>BAC?A9=9??=@@A:ABBCC>CCC8CBCBB>>AB5737:??;
@@AA<@>???A<
@T6XXI:00112:00484
GGGGAATTCTAATACGACTCACTATAGGAGAGGGAGAATAGTCCCTCAATAGCTAGTTCCACGTACCTCTTCTCCTC
TCC
+
HDK:C>A=@@A@CCCCCABA?7654..,0<<AC>@AABB=633317;?;>@;7833<<>;0,,,,2*3379;88866
110
@T6XXI:00112:00489
GGGAGAGGAGAAG
+
990944/,/,*)*
@T6XXI:00113:00452
GGGAGAAGGGAGAAAGGAAGACATGTACAGTGGAGGAATAGTTAGCGACTATTCTCCCTCTCCCTATAGTGAGTCGT
ATTAGAATTCCCC
+
?B>AA@<>>1999>9><A?@@@BC?=?CB><?90/-
/78BCC?BBCB@@@:;87;AA;:::??9=@=73468:8:;<<@=@:??C@CCC9
@T6XXI:00113:00453
GGGGAATTCTAATACGACTCACTATAGGGAGAGGGAGAATAGCTACGTTTAACCGATCGTGTTGTTCTCCTTCCTTC
TCCCTTCTCCC
+

```
DGB8B7;;=>@@CCCBBBBBCBA?>777.77===9?@@>@@AADCCBBB>BBC???????@@<;<580;9977,.--
--30334>==7
@T6XXI:00113:00455
CTAGTCACTCGAGATCTGCGGCTTTGGACAGTCCCTCTGTACTGTAGCCACAAGATGTCGAGTCGTACCTTAGCCTT
CCTTTCCTCCCTTCCTCC
+
A?1//78==BBB@ABBBBBC=@@B>B?AABB???<?@@A:::>:///-
+,/.,.6446<6116444442488889913579699977,1010..3
@T6XXI:00113:00457
GGGGAATTCTAATACGACTCACTATAGGGAGAGGGAGAATAGTTTGCGCATTCGGTGCACTGTTATTCTCTTCCTTT
CTCCCTTCTCCC
+
@=94>59:<BC>BCBBCBBBBCB97166.7???B>D@A>AA@@@<@A@@BC@CC@CBBCBCCC@CC@@@?B<A<AB>
BAAD?H8::@@:
@T6XXI:00113:00458
GGGAGAAGGGAGAAAGGAAGTGCAGTTCGCGGTTAGTGAAGGGAAAGACTATTCTCCCTCTCCCTATAGTGAGTCGT
ATTAGAATTCCCC
+
DDABBA@BC>BBCB?C@C@B??BBBBBCCBB?B@CC>>>;;:,/.+...45;@CAA@;=9?AA<AAA@@@AAAC===
@@>AAA>A:???5
@T6XXI:00113:00472
GGGAGAAGGGAGAAAGGAAGCTAGTCGACTCGACATCTGTGGCTTCACAGTACAGAGGACTGTCCAAAGCCGCAGAT
CGGGGAATTCTAATACGACTCACTATGAGGAGACGGG
+
DIAAAA@CC>BBBC?C@C@>?@???CB@??AA?:::@@>>@=:;9:CCDCCCA==5548AA????A@<AA>===?AA
@@AA5?=@=?@@<<==AAAAAA?7+****)***3994
@T6XXI:00113:00480
CTAGTCGACTCGAGATCTGCGGCTTTGGACAGTCCCTCTGTACTGTGAAGCCACAGATGTCGAGTCGACTAGCTTCC
TTTCCTCCCTTCTCCC
+
67-,,,,,//89>>B@>>?884888/84839BBCC>CAAAB@@=AAAA=8<;<CBBB@A?AAA@BCB@?>?6//-
.:::,0,/66,/,3---+
@T6XXI:00113:00485
GGGAATTCTAATACGACTCACTATAGGGAGAGGGAG
+
9?:>6958889@;;;?@A@??B?>000,00.//,/2
@T6XXI:00113:00491
GGGAGAAGGGAGAAAGGAAGCTAGTCACTACATCTGTGGCTTCACAGTACAGAGGGACTGTCCAAAGCCGCAGATCT
CGAGTCGACTACGCTTCCTTTCTCC
+
@B694?599-
16:>,110999C??::0.....44666896<89??888>;;;88814;;=;<9<@;>@>@<<<<7833740330333
3553422,--+---2
@T6XXI:00114:00461
GGGAGAAGGGAGAAAGGAAGGAGAACAACACGATCGGTTAAACGTAGCTATTCTCCCTCTCCCTATAGTGAGTCGTA
TTAGAACTTCCCC
+
BKCAAA?B?>;;;C<@>AAC@BBB@BB?BCCCCCCC>AA@CAB@@@BAB;;;;?BB6:;?AA8A?@@A?>8<73037
7278;:==8;;:1
@T6XXI:00114:00465
GGGGAATTCTAATACGACTCACTATAGGGAGAGGGAGAATAGAGACGTATTCGTTTGCGCTCTGTTCACCTTCCTTT
CTCCCTTCTCCC
+
DEE:C;;9;AA@BCBBBBCBB@BB@<<<8:AABB>BAA>A?=9=?AA@@@=AAA=;88<?BCC<<9<BC?A8;377-
4>>?:?;=<<<6
@T6XXI:00114:00472
```

```
GGGAGAAGGGAGAAAGGAAGCTAGTCGACTCGACATCTGTGCTTCACAGTACAGAGGACTGTCCAAAGCCGCAGATC
TCGAGTCGACTAG
+
?C>CBB?AB<;;;B<@=@?BB@>=?0//.,,7770******002468:7::=@>=?<>>>878>@@;AA>??AAB??
????@A???A=<<
@T6XXI:00114:00478
GGGGAATTCTAATACGACTCACTATAGGGAGAGGGAGAATAGTCACATTCATGTTTGTGACCGTGTCTCCTTCCTTC
+
?BA7@<=<99969>89;@@BBAACB??<6<=7775:BA?@@@AA??AA>===677-7103575433-.+*+00*+*-
@T6XXI:00114:00483
GGGAGAAGGGAGAAAGGAAGCCGCAGATC
+
775//9598-4888587793.,.,,,,,,
@T6XXI:00114:00486
GGGAGAAGGGAGAAAGGAAGCTAGTCCGACATCTGTGGCTTCACAGTACAGAGGGACTGTCCAAAGCCGCAGATCTC
GAGTCGACTAG
+
BB?BBD?BB=?@@C=CBB@CC?>CCC@77::999??><@A>BDDFCCCCBA:::7:ABBCB?BC?CC@BBBBCBCBB
@BBCAA:;;DD
@T6XXI:00115:00457
GGGAGAAGGGAGAAAGGAAGGAGAAGTCGCCCCAAACGATTTGTTGACTATTCTCCCTCTCCCTATAGTGAGTCGTA
TTAGAATTCCCC
+
CC@CA?=@C>BDCCAC@C@CABBB@BBCBCCC9CC?C??@@=BC@CCC@@@>@@@@;<<<<?:=?@===@@@===@A
B?D?>::/444-
@T6XXI:00116:00458
GGGAGAAGGGAGAAAGGAAGCTAGTCACTCGACATCTGTGGCTTCACAGTACAGAGGGACTGTCCAAAGCCGCAGAT
CTCGAGTCGACTAG
+
CC>BCC@CC<?ACB>B?B?ABB8;:CCCCCC8::???AAE?BB@BC@<<<A?8;33317=A@A?>AA:AA???===<
<<<=>><588<<<?
@T6XXI:00116:00460
GGGGAATTCTA
+
?::292959=>
@T6XXI:00116:00470
CTAGTCACTCGAGATCTGCGGCTTTGGACAGTCCCTCTGTACTGTGAAGCCACAGATGTCGAGTCGACTAGCTTCCT
TTCTCCCTTCCTCCC
+
?D9449444444498@@@985888086888@@AB<BBB@A@CDCAAA>@C;=<@CCCCDDBBB@AA@?>A79656;>
C;A>@;;8232.133
@T6XXI:00116:00497
GGGAGAAGGGAGAAAGGAAGCTAGTCGACTCGACATCTGTGGCTTCACAGTACAGAGGACTGTCCAAAGCCGCAGAT
CTCGAGTCGACTAG
+
CLDAAA@CC=@AAC>B?B@CCBBBB::;?777:71;86:698<?=?ADCCCA555..-
/=;;@C=@@<@A>A@?@CAAAA@@@?<<<>===
@T6XXI:00117:00451
GGGGAATTCAATACGACTCACATAGGAGAGGGAGAATAGCTACCA
+
***)*),*,,288=@BABAA:44..,.33344000/----...47
@T6XXI:00117:00452
GGGGAATTCTAATACGACTCACTATAGGGAGAGGGAGAATAGCTACGTTTAACCGATCGTGTTGTTCTCCTTCCTTC
TCCCTTCTCCC
+
;EC9EBAAACEBC@??BBAAACB>7616.6====6?==<??AAB=>777-700403038883/0025389744-/.-
2224443;;;6
```

```
@T6XXI:00117:00454
GGGAGAAGGGAGAAAGGAAGGCGAATTAACATAGTAGCGATCTGGGAGCTATTCTCCCTCTCCCTATAGTGAGTCGT
ATTAGAATTCCCC
+
GDABBB@BC?ABBD>B?B@CBBBB?A?B@CCBBBAAAB;:;AAAD?CABBEE@CCCC=??BDD?AA?@@AAAAA@@A
@@>@@@>@?@A@5
@T6XXI:00117:00457
GGGGAATTCTAATACGACTCACTATAGGGAGAGGGAGAATAGTCAACAAATCGTTTGGGGCGACTTCTCCTTCCTTT
CTCCCTTCTCCC
+
;;:5;,4699><A@AABBCCCA??>777.77776,1348;A=?;?>@@@<B;;:C>CDB5@ABBB?BBD;969?AA=
B@@@<@;==AA<
@T6XXI:00117:00485
GGGGAATTCTAATACGACTCACTATAGGGAGAGGGAGAATAGTTAGCTAACACGACGATACTCATTCTCCTTCCTTC
TCCCTTCTCCC
+
6<70<,1,/8::>CCCAABBCABABAA?6;;667.4;;8<<=<9<<=9??BBDCCAABAABBA??<?;;7:7:3430
89:4:798;=:
@T6XXI:00117:00497
GCTTTGGACAGTCCCTCTGTACTGTGAAGCCACAGATGTCGAGTCGACTAGCTTCCTTTCTCCCTTCTCCC
+
=499-4244@B99<1;AB;::CCCCCC?BB>AAAABC<:8CCC@B?::9?::;?A>AC@AABB9?;???B9
@T6XXI:00118:00483
GGGAGAAGGGAGAAAGGAAGTAGGCGCTCATCTGTGGCTTCACAGTACAGAGGACTGTCCAAGCCGCAGATCTCGAG
TCGACTAG
+
;B:9995990949>0948173:@?CCCECCBBBBAA?AC>?<8888;;88//,/05100+/,--,++/-
34;999<4387>>?@A
@T6XXI:00118:00490
GGGGAATTCTAATACGACTCACTATAGGGAGAGGGAGAATAGCTCCCTCATCTACAAGTTGTCTCACCTTCCTTCTC
CCTTCTCCC
+
1@>0>-9244488;?;;<@@BBCBBAAAA???DC>AAA<>=;;777.1479744,-
*,7+,,6772774,.,.,..553;544550
@T6XXI:00118:00499
GGGAGAAGGA
+
49-4442/5/
@T6XXI:00119:00468
GGGGAATTCTAATACGACTCACTATAGGGAGAGGGAGAATAGTCAACAAATCGTTTGGGGCGACTTCTCCTTCCTTT
CTCCCTTCTCCC
+
GDB8@64:9449@@AACDCCCA><;677.7=777-:>>>AAAA@@<>AA=C?<;;.777.7>>>@<@@@44/4344-
8;;;:@685AC<
@T6XXI:00119:00487
GGGAGAAGGGAGAAAGGAAGGGAGAGGAGAAAGGAAGC
+
?A:@@@=AA>@?@C<@=@9::80...52,,,*.,.55-
@T6XXI:00120:00451
GGGAGAAGGGAGAAAGGAAGCTAGTCGACTCGACATCTGTGGCTTCACAGTACAGAGGGACTGTCCAAAGCCGCAGA
TCTCGAGTCGACTACGCTTCCTTTCTCCCTCTCCC
+
?B<@@@<>>:>99909;>588871:0../........11198<>@CCBCC@;99@@@B<A===AC>A@<A@>@@@@@
@>>=<;;8843000004422,--+-112+--2224
@T6XXI:00120:00457
GGGGAATTCTAATACGACTCACTATAGGGAGAGGGAGAATATCTGTCGTTCGGTCGACGCTGCCTATCCTTCCTTTC
TCCC
```

```
+
999/48?=AEECCA?BBBBBB@AAB?AC;<?AA?8?==:778>?@<>>A>BA>A888;884<@:=:=>3424//.++
***)
@T6XXI:00120:00467
GGGGAATTCTAATACGACTCACTATAGGGAGAGGGAGAATAGTTACTCC
+
FDC:C79:<8=9A<<<CCCC@????777.7>>>A=@>;955,,+++++*
@T6XXI:00120:00468
GGAGAAGGGAGAAAGGAAGCTAGTCACTCGACATCTGTGGCTTCACAGTACAGAGGGACTGTCCAAAGCCGCAGATC
TCGAGTCGACTAG
+
;EBA@=@C<AABC<@=A>ACCAAA@?711;;;677666998?=89???@AA;=444-
4;9:<@>@@<AAB@@@AA???AAA@@@@<;888
@T6XXI:00120:00476
GGGAGAAGGGAGAAAGGAAGCTAGTCGACTCGACATCTGTGGCTTCACAGTACAGAGGGACTGTCCAAAGCCGCAGA
TCTCGAGTCGATAG
+
AA<@@@<@B:<;CC<@>AABCBBBG:::>>>7::8>>7:8A?AB?ABDABBBB;;333-
4;?@@B>BA:@@?<==@A???A@;<<;94400
@T6XXI:00120:00481
GGGGAATTCTAATACGACTCACTATAGGGAGAGGGAGAATAGTTTTCCCTTCACTAACCGCGAACTGCACTTCCTTT
CTCCCTTCTCCC
+
@=94>695<AA@CB@@CCCB@A>76111.7>>>@=B?=9;94444-
494<>AGCCC@C?::8B<??A@BAB;=377.7;;?:@<=;;<6
@T6XXI:00121:00451
GGGAGAAGGAGAAAGAAGGGGACCGAGGTATAGAATAACTGAGCAGCTATTCTCCCTCTCCCTATAGTGAGTCGTAT
TAGAATTCCCC
+
990///,/,,,00*,/,...*.999:C==ABBBB>::A>66683222,,,*/+030033553?4<777<4665;6?
<@;=;444561
@T6XXI:00121:00475
GGGAGAAGGGAGAAAGGAAGCTAGTCGACTCGACATCTGTGGCTTCACAGTACAGAGGACTGTCCAAAGCCGCAGAT
CTCGAGTCGAC
+
6<8<;>699-4444-
424774..,,2,,,,,...//6;69<?789@?@?CBB:88>>?<?@@?=>8;<6<<8<A===CC883666/---
@T6XXI:00122:00450
GGGAGAAGGGAGAAAGGAAGCTAGTCGACTCGACATCTGTGGCTTCACAGTACAGAGGGACTGTCCAAAGCCGCAGA
TCTCGAGTCGACTAG
+
CC>;;;=@A<AABB<A>A<::801?0/0;;;>1::BBAAAD?BB@BCCBBAA>::47717>>>??>AA<AA>A?=<?
@AA@AA@A@@@@@@A
@T6XXI:00122:00452
GGGAGAAGGGAGAAAGGAAGGAGAACGCGCACCCGGACCAACGGTGATCTATTCTCCCTCTCCCTATAGTGAGTCGT
ATTAGAATTCCCC
+
AB<@@B?BC?FBAC?C@B@?>BB??BB?BACCC@CACD>A?BC?AACC@@@@=B@??:?B@AAABCCCCCBBBCCAA
::8:BC@CACCC:
@T6XXI:00122:00458
GGGGAATCTAATACGACTCACTATAGGGAGAGGGAGAATAGCCAGCAGTCATTTTGCAGCTATGTCGCCTTCCTTTC
TCCCTTCTCCC
+
0=708+1///,/4488737;9<>:8:888@@==9;//,,,*.)**+-
2240000+069?BB@@;>711363601117::?;>75000+
@T6XXI:00122:00461
GGGGAATTCTAATACGACTCACTATAGGGAGAGGAGAATAGTACGAC
```

```
+
@660>5959>>=A<<<BBBBC?>>>777.7===:?;;89/////,,+
@T6XXI:00122:00466
GGGGAATCTAATACGACGTGCACTATAGGAGAGGGAGAATAG
+
=@>2>;1///,////4393343733377011111,111014-
@T6XXI:00122:00474
CTAGTCACTCGAGATCTGCGGCTTTGGACAGTCCCTCTGTACTGTGAAGCCACAGATGTCGAGTCGACTAGCTTCCT
TTCTCCCTTCCTCCC
+
999444444944448;=>>?=88808;>?CCBBB>BCFC???CCAAA;?A8;<ACBBA@@BBCCBCCCCDF@?=?;A
A>A?<?66,/,/661
@T6XXI:00123:00464
GGGGAATTCTAATACGACTCACTATAGGGAGAGGGAGAATAGTCAACAAATCGTTTGGGGCGACTTCTCCTTCCTTT
CTCCCTTCTCCC
+
CC@2;24588899=A@@@CCCCCBA:::.7>>@A>B@A>AAAA@@>@@A7??AAA9??;2<>>>@<>>A5838;>>1
88@@;>=B8885
@T6XXI:00123:00472
GGGGAATTCTAATACGACTCACTATAGGGAGAGGGAGAATAGTTCGCGTC
+
BCC8B746<@><ACCCCCCCBBBB@766.7>=AB>CA@<;;44/440---
@T6XXI:00123:00482
GGGGAATCTAATACGACTCACTATAGGGAGAGGGAGAATAGTCCTCGGTTTT
+
28818+1///,/49;;:BB???<;711,7?777.:::9;8:/-,---,----
@T6XXI:00124:00452
GGGAGAAGGGAGAAAGGAAGGAGAAGTCGCCCCAAACGATTTGCTGACTATTCTCCCTCTCCCTATAGTGAGTCGTA
TTAGAATTCCCC
+
CG?BBB?B?=BBBB>B@B?A@CBB@??BBBBB8??<B@A777////772::9977;6//.3333:<?@@A989???@
@@A:968>ACD6
@T6XXI:00124:00463
GGGAGAAGGGAGAAAGGAAGCTAGTCGACTCGACATCTGTGGCTTCACAGTACAGAGGGACTGTCCAAAGCCGCAGA
TCTCGA
+
AA<AA@@CC<BA@@<@@C@CEA>AAAA???@=::::;:AAD@<<<<FCCCBC?<830314;;@?@>@A<AA>@@@CA
@;@AAA
@T6XXI:00124:00473
GGGAGAAGGGAGAAAGGAAGGAGAGAGCAACGTAGACCAGCTAGTCACTATTCTCCCTCTCCCTATAGTGAGTCGTA
TTACGAAGATTCCCCC
+
/A9<<>69919?ABBB?B@C?CCBCCCBB>?>;:6?9?AB???CC===??C=988A=388==79:9=;;;;=888@A
@;=<77,/..,----*
@T6XXI:00124:00498
GGGAGAAGGGAGAAAGGAAGGGGAGAAGGGAGAAAGGAAGCTA
+
:B:9996990949>094:>CCC9CDCACB>B/,,+*)*)****
@T6XXI:00125:00454
GGGGAATTCTAATACGACTCACTATAGGGAGAGGGAGAATAGTCAACAAATCGTTTGGGTGCGACTTCTCCTTCCTT
TCTCCCTTCTCCC
+
CFD:C<;9<BB?BC==::;B@A@C;;:C>D???C>B@A;<<=8848;@?;BCBBA9??9:67?<<=,//6,/,/782
454:9142848CA
@T6XXI:00125:00462
CTATCACTCGAGATCTGCGGCTTTGGACAGTCCCTCTGTACTGTGAAGCCACAGATGTCGAGTCGACTAGCTTCCTT
TCTCCC
```

```
+
4E<99?:99=484>989>>;888,37<77;??<6<@777A744;<<5=A@BA@??;:18888:9706643//-
/76667317,
@T6XXI:00125:00464
GGGAGAAGGGAGAAAGGAAGTGCAGTTCGCGGTTAGTGAAGGGAAAACTATTCTCCCTCTCCCTATAGTGAGTCGTA
TTAGAACTT
+
?BA<;<BFEABCCC?CBDABA@@A<<8:???4;5::33?<??<?B>8BBBC?>=;?88666=76734@???888;34
45594230*
@T6XXI:00125:00469
GGGGAATTCTAATACGACTCACTATAGGGAGAGGGAGAATAGTTTTTTCCCTTCACTAACCGCGAACTGCACTTCCT
TTCTCCCTTCTCCC
+
CBC:C>?=?AA?BDFCB@BBBABB<<??;:ACDC>CAA?@@@@A>AA-
@A<A>@BAAC@C><<?A>==A@BBA?B5::8:CCC>C????@;
@T6XXI:00125:00478
GGGGAATTCTAATACGACTCACTATAGGGAGAGGGAGAATAGTCAACAAATCGTTTGGGGCGACTTCTCCTTCCTTT
CTCCCTTCTCCC
+
AEF:C;:;<BC@CCBBBBBB?@;97111.7>>>A@BAA@A==?@@?AAA=CBBBC>CCC7BBCAA=?BB4737:==7
?A?@<@=@?AD?
@T6XXI:00125:00479
CTATCACTCGAGATCTGCGGCTTTGGACAGTCCCTTGACTGT
+
44;444449449?>A@@885888/848:A>;:5+.,..442.
@T6XXI:00126:00455
GGGGAATTCTAATACGACTCACTATAGGGAGAGGGAGAATAGTTTTTCCCTTCACTAACCGCGAACTGCACTTCCTT
TCTCCCTTCTCCC
+
DCB9A=@=@BB@BCCBCCBBA@BA?99757ACCC>C@@=?:>?@AA1AC<@>@BBBF@C?>AAA>AA@=?@CAC=??
<?@@@:@<;4881
@T6XXI:00126:00460
GGGAGAAGGGAGAAAGGAAAGCTAGTCGCA
+
CC-44;2991489969244-3333971114
@T6XXI:00126:00466
GGGAGAAGGGAGAAAGGAAGTTCTAGCTTACGCGTCAGTTTGGGAGACTATTCTCCCTCTCCCTATAGTGAGTCGTA
TTAGAATTCCCC
+
CCCDCC@DD>@AGB>B@B@BC@?@ABBC@CCCCBBB@@@@>@C=@@?B@CC?CCCC>B<AAA<<@@@@@ABAB???=
<9<@@><<BBB5
@T6XXI:00126:00477
GGGGAATTCTAATACGACGTCACTATAGGGAGAGGGAGAATAGCACTGGTCCCTCCCAGC
+
CDC8B51-1//,//6/433377<>88337,7>>7;?@@@?B://94/.,---+--44466
@T6XXI:00126:00479
GGGGAATTCTAATACGACTCACTATAGGGAGAGGGAGAATAGTCAACTCAATCGTTGGACGAT
+
@>?4>6959:>@BAA@CCCAAAA?=666.7>>CC>B@A>=5/,++*+++101--0/3/07111
@T6XXI:00127:00460
GGGAGAGGGAAG
+
//+////852*/
@T6XXI:00127:00463
GGGAGAAGGGAGAAAGGAAGTAGAATAGCACGTTGAAGATGTCGGGACTATTCTCCCTCTCCCTATAGTGAGTCGTA
TTAGAATTCCCC
+
```

```
CC?BBC@BCBBBBB>B@B@A@ABA@A???BCCHBAA@ACCBBDCC@CCCBB@BCCC@??@CC?CCCCCCCCCCBBB@
@=@CCAC=@@@8
@T6XXI:00127:00467
GGGAGAAGGGAGAAAGGAAGGAGAAGTCGCCCCAAACGATTTGTTGACTATTCTCCCTCTCCCTATAGTGAGTCGTA
TTCAGAATTCCCC
+
44-
449249544;B>C@B@CABAB@@AAACCC7@@:><=66266278@<AA=<<<C=B@@@A=@@???AAAA?>;876,/
/;;9;6>=>0
@T6XXI:00127:00489
GGGGAATTCTAATACGACTCACTATAGGGAGAGGGAGAATAGTCAACAAATCGTTTGGGGCGACTTCTCCTTCCTTT
CTCCCTTCTCCC
+
HDC9B798;@@?ACBBCCBCBCB==666.7?=<?==AA>@<<=AA>@@A<CAAAC>CCC7AAA??:?::3858>@B<
@@@A>D@@@??:
@T6XXI:00128:00455
GGGAGAAGGGAGAAAGGAAGAGAAATGGAGTGCCGGCGAGGAGATGCCTATTCTCCCTCTCCCTATAGTGAGTCGTA
TTAGAATTCCCC
+
CKCCCC?AA=BBBC@C@C@CCBBA=BB=AACBB?A@BBBB@BCCB?A?A??:=:7758;<<A;@===AAAAA@????
@=?@@<?;?AA6
@T6XXI:00128:00461
CTAGTCGACTCGAGATCTGCGGCTTTGGTCAGTCCCTCTGTACTGTGAAGCCACACATGTCGAGTCGACTAGCTTCC
TTTCTCCCTTCTCCC
+
B9949944ABBABAACCCCCC@CCC>A@BBB??BB?B?AACBBBBA??<?@=@CCCCCCCB??=ACCCCCCCAA>A@
CE>BB?>;A>@@AA:
@T6XXI:00128:00467
GGGGAATTCTAATACGACTCACTATAGGGAGAGGGAGAATAGTCAACAAATCGTTTGGGGCGACTTCTCCTTCCTTT
CTCCCTTCTCCC
+
@A@6@=@>ABB@CCCCCBCBB@CBA69959?CCC>CAA?B988>;=AAA=AA?BB=CCC8BAAAB@?CC8837:==;
?BAC<B<>>AC=
@T6XXI:00128:00468
GGGAGAAGGGAGAAAGGAAGTGCAGTTTGCGGTTAGTGAAGGGAAAAACTATTCTCCCTCTCCCTATAGTGAGTCGT
ATTAGAACTTCCC
+
=C:999699198>?9><@=;:;NECDE?B;;587>?7;;;?;8?AAA2ACCJABBBB<??ABB>BABA@A@A888?8
89>AAC<??@C@9
@T6XXI:00128:00473
GGGGAATTCTAATACGACTCACTATAGGGAGAGGGAGAATAGATCACCGTTGGTCGTCGGTGCGCTGTTCCT
+
DFF9B8;;;@A@CBB=BBBBBBB>>111-6===?<A@@=@?<;;=:737=474799988477?=///.,.,.
@T6XXI:00128:00482
GGGGAATTCTAATACGACTCACTATAGGGAGAGGGAGAATAGTCAACAAATCGTTTGGGGCGACTTCTCCTTCCTTT
CTCCCTTCTCC
+
9;;2;2959449:=:;:BBBDCB>>766.77==?<=DD?A@@@?<;<ACADBBBB>BBC9BCBBB><98/3/34884
99;;-42/-/,
@T6XXI:00129:00452
GGGAGAAGGAGTAAGA
+
@B@=9461-,***)((
@T6XXI:00129:00457
GGGGAATTCTAATACGACTCACTATAGGGAGAGGGAGAATAGCATATAACTCGGCGCCG
+
==>4?>@?@@ABCCCBBBBBB@BB?:::;<BBBE>C??<>==340030000++***.)+
```

```
@T6XXI:00129:00462
GGGGAATTCTAATACGACTCACTATAGGGAGAGGGAGAATAGTCTGACTGTTTCCAAAGCAGCTGTTCTCTTCCTTT
CTCCCTTCTCCC
+
CDD:CBA>ACC@CCBBBABBB@BB966659?BCC=BBA=A;;9::????CCC>C?B?;?BBB>>>?B?@??<<9??;
@AAD<A>@@AA<
@T6XXI:00129:00489
GGGAGAAGGGAGAAAGGAAGCTAGTCGACTCGACATCTGTGGCTTCACAGTACAGAGGGACTGTCCAAAGCCGC
+
CB@@@@@BB@?@AB=B?A=@@D???CCC@?@@@?::7:@@;38;=@BCCCBBBA;3331399?@@>AC<@@>??
@T6XXI:00130:00477
GGGAGAAGGGAGAAAGGAAGAGAACGGGGCCCGCCGATGCATAACAGGCTATTCTCCCTCCTCCCTATAGTGAGTCG
TATTAGA
+
99-444244-48885==@<;73314444,4:-44249944488249=<?@BB@11/8+..,.44+./599544..--
--02444
@T6XXI:00130:00480
CTAGTCGACTCGAGGATCTGCGGCTTTGGACAGTCCCTCTGTACTGCTGAAGCCACAG
+
4444444444499924499439933 3,3133?CAA@=:<1>:90/...4:86855334
@T6XXI:00130:00488
GGGAGAAGGGAGAAAGGAAGGATAAGTCGCCCCAAACGATTTGTTGACTATTCTCCCTCTCCCTATAGGTGAGTCG
+
?@:99;>>A094990948=@=@CD?BADA???.77.11111,16:<>843=;888?:<<5990,,+++*+344889
@T6XXI:00131:00463
GGGGAATTCTAATACGACTCACTATAGGGAGAGGAGAATAGCAGGA
+
?:=6==A@@A@DCCB?BBCD>=@CC?<?8?B??:?0000,,,,,**
@T6XXI:00131:00464
GGGAGAAGGAGAAAGGAAGGAGAAGTCGCCCCAAACGATTTGTTGACTATTCTCCCTCTCCCTATAGTGAGTCGTAT
TAGAATTCCCC
+
8?8---
*,***,,*,*/,.,.69666?8888,396971111177;>?99A<===C<A989D>><<7846?ABA=6<=>=<>54
/444-
@T6XXI:00131:00467
GGAATTCTAATACGACTCACTATAGGGAGAGGAGAATAGTTACGGCGCTACTCT
+
@=A:98<@@=ACCBB?A@A?ABAAAA9?=66379979=<;8<;9:/------22
@T6XXI:00131:00487
GGGGAATTCTAATACGACTCACTATAGGAGAGGGAGAATAGCAAGT
+
CEE:D@@@@CCDCCBBBBBB?:977//-0>>?B@===;80.**)+*
@T6XXI:00132:00451
TCACTATAGGGAGAGGGAGAATAGTCAACAAATCGTTTGGGGCGACTTCTCCTTCCTTTCTCCCTTCTCCC
+
AHCBDB>489-9?>=?>@BB@CBBBCC@BBB=BBABB=BCC9CCCBB?BBC=@=@?BB>BBAB<A::;EE:
@T6XXI:00132:00453
CTATCGACTCGAGATCTGCGGCTTTGGACAGTCCCTCTGTACTGTGAAGCCACAGATGTCGAGTCGACTAGCTTCCT
TTCCTCCCTTCCTCCC
+
AA@=99:A@@=?AA@@@>984;>?:@@BBCBCCD@AA:::??B@?>A9<@>BBAABBCAAABB?@AAA@A>65645:
:,0,,,515+/,//.+
@T6XXI:00132:00461
GCTAGTCACTCGAGATCTGCGGCTTTGGACAGTCCCTCTGTACTGTGAAGCCACAGATGTCGAGTCGACTAGCTTCC
TTTCTCCCTTCTCCCCTTCCT
+
```

```
44444999999999==ABA@@>888/87<79?:<:88?:::?>>>834204248=;>CBBBCBB:;;?>??>:92:=
?B<@?<@999;.///+/,/,/
@T6XXI:00132:00464
CTAGTCGACTCGAGATCTGCGGCTTTGGACAGTCCCTCTGTACTGTGAAGCCACAGATGTCGAGTCGACTAGCTTCC
TTTCTCCCTTCTCCC
+
>2,---
,,/:::BAAACCCCC@?AA<A@BB<????;?CCBAA@@=?<C=@C@BBBBBCAA8:;CBBBC@@@;;7<<=AD>BBC
C>@=@@DD?
@T6XXI:00132:00474
GGGGAATTCTAATACGACTCACTATAGGGAGAGGGAGAATAGCCCAACATTCGCTCCTCAGGTTGCTATCTTCCTT
+
499/4:;<@AB@BCCDDBCCBAAB@ADB.77700,066,-----+-44353144//,/723,-,373.34979,/,
@T6XXI:00132:00478
GGGAGAAGGAGAAGA
+
2=8-11,/,///,//
@T6XXI:00132:00479
CTAGTCGACTCGAGATCTGCGGCTTTGGACAGTCCCTCTGTACTGTGAAGCCACAGATGTCGAGTCGACTAGCTTCC
TTTCTCCCTTCTCCC
+
=;1/////8889>;<<A@@@CADCC>C=@@CCAAA=ABAAABBBCCAA<?B@BBBCCCCBBBCCBCABCEND??<?=
@A>ABAE>@>?@AA<
@T6XXI:00133:00454
GGGGAATTCTAATACGACTCACTATAGGGAGAGGGAGAATAGTCAACAAATCGTTTGGGCGACTTCTCCTCCTTTCTC
CCTTCCTCC
+
;@>0@11186:;BBECBA@@CCBA:::.7>777.7::8<=7;49./.307<=<=,11,1777?=:86000/04-
411101,+*++*
@T6XXI:00133:00465
GGGAGAAGGGAGAAAGGAAGAATAACAGGCAATGGGGGATCATGTGGCTATTCTCCCTCTCCCTATAGTGAGTCGTA
TTAGAATTCCCC
+
AB=BBC@CCAA@@C?B@B@CC@BB?CCC@AA>@?;;;/?ABBCBBBACBCC@ABAC@A@ABG?CDCDCCAC@CBC@;
;9:??=;<><A6
@T6XXI:00133:00468
GGGGAATTCTAATACGACTCACTATAGGGAGAGGGAGAATAGAGACGTATTCGTTTGCGCTCTGTTCGCCTTCCTTT
CTCCCTTCTCCC
+
CEC8A>A@BBB@BBBBCCCBBBBBA661-
6>77:5:@A?A===@>;;94434444,7>>@B@@@A?===>@:;3441489;9;94433+
@T6XXI:00133:00469
GGGAGAAGGGAGAAAGGAAGCTAGTCGACTCGACATCTGTGGCTTCACAGTACAGAGGGACTGTCCAAAGCCGCAGA
TCTCGAGTCGACTAG
+
BCBAAA?BBACBBB>A??>>A@8;;/00:::<071666??C?BC?ACCC??A994000-
4;;>@@>@C<AA?B@=@??===ABAA@@@@===
@T6XXI:00133:00476
CTAGTCACTCGAGATCTGCGGCTTTGGACAGTCCCTCTGTACTGTGAAGCCACAGATGTCGAGTCGACTAGCTTCCT
TTCTCCCTTCTCCC
+
B8/111/44?BBBBCCCBBB?BCE>C>BBDCBCC>B?@?CCCBB@AA>?B?BDGCBBBBBC@@@ABBCCBAAA=@=@
A<???B9;8;===7
@T6XXI:00133:00482
GGGGAATTCTAATACGACTCACTATAGGGAGAGGGAGAATAGTCAACAAATCGTTTGGGGACGACTTCTCCTCCTT
+
>BB4A59594488;8?;AA??::789=?=8;;CE:;9AB@==377273;5>9977,111,166;>>7>63*++*+*
```

```
@T6XXI:00133:00497
GATCTAATACGACTCACTATAGGGAGAGGGAGAATAGCCGACAATTCGACTCCCCGGCAATGTTCTTCCTTTCTCCC
TTCTCCC
+
/11/6678=BAABCABAA>>777-
7=77758;99@<8858>>>>CA@??BBBCC7@>??>=68:4424;::4;8663324003+
@T6XXI:00134:00450
GGGAGAAGGGAGAAAGGAAGCTAGTCGACTGACATCTGTGGCTTCACAGTACAGAGGGACTGTCCAAAGCCGCAGAT
CTCGAGTCGACTAG
+
9<89A@ABH=AA@C>C@C@CCCCB:/000......4616>;9;>>AABAA@??;444-
4;;??A>@A;?A=A?@ABA@AA@?<;;=774:;
@T6XXI:00134:00453
GGGAGAAGGGAGAAAGGAAGCTAGTCGACTCGAGATCTGCGGCTTTGGACAGTCCCTCTGTACTGTGAAGCCACAGA
TGTCGAGTCGACTAGCCTTCCTTTC
+
9A<>>B599999>@:A>B@CDB?>>/00:/./>;BB>7:7?:777,67==>@:::.4@>799AAAABA=@@;>>A@A
@999???@@844;8//,/,/,//+-
@T6XXI:00134:00454
GGGAGAAGGGAGAAAGGAAGCTAGTCGACTCGACATCTGGTGGCTTCACAGTACAGAGGACTGTCCAAAGCCGCAGA
TCTCGAGTCGAC
+
8=6;19599-4444-
4999;999<996661811.333..,.554285///55?;;;47333599=<<=;<<:<:?@A998@>76//-++
@T6XXI:00134:00462
GGGGAATTCTAATACGACTCACTATAGGGAGAGGGAGAATAGTCAACAAATCGTTTGGGGCGACTTCTCCTTCCTTT
CTCCCTTCTCCC
+
CCB9B;;<<BBACBBBBBCBBABB?66649??BB>C??<????AA=AAA:???CC>@@@7@C@@B>AAC==:=?@@<
@@@A<A<?@AA<
@T6XXI:00134:00468
TTGATACGACTCACTATAGGAGAGGAGAAGTAGTCAACAAAGTCGTTTGGGGACGACTTCTCCCT
+
B?:9948444444443337763..,,,,*,,4-0431333,3768<@=C@A77761660....+.
@T6XXI:00134:00475
CTCGAGATCTGCGGCTTTGGCAGTCCTCCTGTACTGTGAAGCCACAGATGTCCGAGTCGACTAGCTTCCTTTCTCCC
TTCTTCCC
+
C<<<;;A@844892444-
49944442442444444444=92448944488434244477 9;;444442489A<;888+/,//,/85
@T6XXI:00134:00494
GGGAGAAGGGAGAAAGGAAGCAGTCCATCTGTGGCTTCACAGTACAGAGGGACTGTCCAAAGCCGCAGATCTCGAGT
CGACTAG
+
??:999;@B>BCBC?C@C@BAAA882:@@:::@C@@?@AA@A@C@???AA<@@==::244+4?;=????????@??=
ABBCBCD
@T6XXI:00135:00463
GGGGGAATTCTAATACGACTCACTATAAGGGAGAGG
+
,,,,)/,/,///7::=BB@?@::110/,.5244,..
@T6XXI:00135:00464
GGGGAATTCTAATACGACTCACTATAGGGAGAGGGAGAATAGCAACTCCATCTTCTCTACGAACCTGCCCTTCCTTT
CTCCCTTCTCCC
+
@AA6@@B?CCC@CB@@A???>>@B;6675:AACC>C??=@<<<<;<==:;:==<;::99===;:86;;84328/34-
48;@:>8;;;<6
@T6XXI:00135:00481
```

```
GTCTAATACGACTCACTATAGGGAGAGGGAGAATAGTCAACAAATCGTTTGGGG
+
BCCCCC@ABBCCBABA?93177.7777=9ABC@DA@@BB>;5535576663333
@T6XXI:00135:00489
GGGAGAAGGGAGAAAGGAAGGAGACAGTGAGCGCCGCACAACACGTCACTATTCTCCCTCTCCCTATAGTGACGTCG
TATAGAATTCCCC
+
44-
44;9996>99909585848379>@;;;@A@><?5...3......499:9?BBB?277311,1:;;7787330..3..
.44,.-0995
@T6XXI:00136:00469
GGGAGAAGGGAGAAAGGAAGCTAGTCGACTCGACATCTGTGGCTTCACAGTACAGAGGACTGTCCAAAGCCGCAGAT
CTCGGTAGCTCACG
+
DC>A@@>@@:@@@@<@@C@CCDAAA::7===777777179?8?@@AECCCCC;99..,/9878=>AA<@D>@?@A@9
87//,/+++++---
@T6XXI:00136:00498
GGGGAATTCTAATACGACTCACTATAGGGAGAGGGAGAATAGTCAACAAATCGTTTGGGTCGACTTCTCCTTCCTTT
CTCCCTTCCTCCC
+
EFE:E<;<<AAADDB>?BBB?>A<?66959>>@B>AAA?A?@>CC>@@A9@@CCC>BC>CBAAAC?BBA6837:=>:
?;;93;,/,/893
@T6XXI:00137:00453
GGGAGAAGGGAGAAAGGAAGGCATGATGACG
+
@@<@GC@CD@BBCB>A>>40,00..,**,,,
@T6XXI:00137:00460
GGGGAATTCTAATACGACTCACTATAGGGAGAGGGAGAATAGTCTTCCCCTGCTCGACATGATCATTCGCTTCCTTT
CTCCCTTCTCCC
+
BCB7B;;<<CC@CAAABBBBCCCCB99959?@CE>B?@>A@@==@>?=C5@???CCAAACCBAA??:?AA@>A9<<7
;>;>4878444-
@T6XXI:00137:00466
GGGAGAAGGGAGAAAGGAAGCTAGTCGATCGACATCTGTGGCTTCACAGTACAGAGGGACTGTCCAAAGCCGCAGAT
CTCGAGTCGACTAGCTTCCTTCTCCCTCTCCC
+
CD>AA@?AA<@AAB>C?B?B@@???BB?:::;77?????A=??=??@@BCDC@A87718@?@@?=@A=A@>@AAAAA
=A@?<<;;;=444:443/,/,//77,/.//.+
@T6XXI:00137:00486
GGGGAATTCTAATACGACTCACTATAAGGGA
+
///*/,42444888=@@AA00000,,*,0*,
@T6XXI:00137:00488
GGGGAATTCTAATACGACTCACTATAGGGAGAGGGAGAATAGTGACGTGTTGTGCCGGCGCTCACTGTCTCCCTTCC
TTTCTCCCTTCTCCC
+
/11+17A@CGC@CCBBABCC??@@@??;6;=767.7;;:?=://./.44406634/-42--.4+++4--
.452;99/44-3444+3275662
@T6XXI:00137:00492
CGAGATCTGCGGCTTTGGACAGTCCCTCTGTACTGTGAAGCCACAGATGTCGAGTCGACTAGCTTCCTTTCTCCCTT
CCTCCC
+
DCCCCCBABBC@CCI>A>ABDCCCB<BBCCCBBBAA@C?AA=B<=;;;<<CBBCBCB@?@CBA@=@=@A<?>>>-1-
0,1;;8
@T6XXI:00138:00460
GGGGAATTCTAATACGACTCACTATAGGGAGAGGGAGAATAGTTCACACACATTTACAGGGGCTGT
+
```

```
999/92969>><9;;A@?BBF;88>777-76=>@<?==>A@A@;==@=::9444,0>/...*.844
@T6XXI:00138:00497
GGGAGAAGGGAGAAAGG
+
BA:@@>59911/**)*)
@T6XXI:00138:00499
GGGGAATTCTAATACGACTCACTATAGGGAGAGGGAGAATAGTCAACAAATCGTTTGGGGCGACTTCTCCTTCCTTT
CTCCCTTCTCCC
+
AA@6?5;8<@@>BCBAABCB?AB;;6665:@@CC?CBB>?99999:;AC<CAB>A>BCC7??CAA?ABA8;8;8;;7
=A@DAC;=>AA<
@T6XXI:00139:00455
GGGGAATTCTAATACGACTCACTATAGGAGAGGGAGAATAGTCAACAAATCGTT
+
C>>5>,1,/777:BBBBBB;<0@;;00,/<:==6;7>:?@C=;998//,0000,
@T6XXI:00139:00482
GGGAGAAAGGAAGCTAGTCGACTCGACATCTGTG
+
CE?BB@@:A8;7<CBABB;8877811611101.5
@T6XXI:00139:00488
GGGAGAAGGGAGAAAGGAAGCTAGTCGACTCGACATCTGTGGCTTCACAGTACAGAGGGACTGTCCAAAGCCGCA
+
CB@BBB@CD>BCCB?B@B?CAA=<<AC::;C@>>:::7=>;38:?BECBCCA===44413668??>@C<AA=??A
@T6XXI:00139:00489
GGGGAATTCTAATACGACTCACTATAGGGAGAGGGAGAATAGATCACCGTTGGTTCGGGTGCGCGTTCTCCTTCCTT
TCTCCCTTCTCCC
+
BA?6A798<BBADCBBBBBB?@AC?:8888???C>AAA?@<AA@<AA:??5:5:=BBG>CBBBBBC@CCB?A?A>AB
>@@@@<@<888>7
@T6XXI:00139:00495
GGGAGAAGGGAGAAAGGAAGGGAGAAAGGAAGCTAGTCGACTCGAACATCTGGTGGCTTCACAGTACAGAGGTGTCC
AAAGCCGCAGATCTCGAGTC
+
490448244-488;-42:10?8:100,/,.,..449DC?.000/.,.442.0,..5---
48535?A797BB?ABA@:>>9889//.34334666633
@T6XXI:00139:00496
GGGAGAAGGGAGAAAGGAAGCTAGTCACTCGAGCATCTGTGC
+
CDDCCDBGG>@@@=5>;>;?BAAACC77375,+++222,.04
@T6XXI:00140:00455
GGGGAATTCTAATACGACTCACTATAGGGAGAGGGAGAATAGCTACATGTCCGCTGGCATCGCTATCTCCTTCCTTT
CTCCCTTCTCCC
+
GC@6>698<@A=CCBAA@CCD@@@CBAA<:88BC>C==9<?>=AABB?<==<??AA>@??????@@AAA>@<?8;84
6///,/7///:6
@T6XXI:00140:00459
GGGGAATTCTAATACGACTCACTATAGGGAGAGGGAGAATAGATCACCGTTGGTTCGGGTGCGCGTTCTCCTTCCTT
TCTCCCTTCTCCC
+
ACB8B6959=>;8CCBDBBBBCCCBAAA<???BC>B@@?AA===AAA?AB?B@C>BBB=CCCCBAA?A@@;>;=377
.7;>@7=8:4441
@T6XXI:00140:00462
GCTAGTCGACTCGAGATCTGCGGCTTTGGACAGTCCCTCTGTACTGTGAAGCCACAGATGTCGCGACTAGCTTCCTT
CTCCCTTCTCCC
+
44999??==?AA@@@ABCDB@@;888,3137>B@CC;><;:77?88:=A?@C@BBCC?:;;?>;;00/.3.101060
366;:?:=;;;4
```

```
@T6XXI:00140:00464
GCTAGTACGACTCGAG
+
446/////////8444
@T6XXI:00140:00483
GGGAGAAGGGAGAAAGGAAGTGCAGTTCGCGGTTAGTGAAGGGAAAAAACTATTCTCCCTCTCCCTATAGTGAGTCG
TATTAGAATTCCCC
+
=C>AAB?BB>;<;E@C@C@C;:<B;;8:AAB>B?B;96B:=<6<;<<<+>???8;;ED>BB@??9<888;888???@
@@@<??C?;7:::.
@T6XXI:00140:00493
GGGAGAAGGGAGAAAGGAAGGGGAGAAGGGAGAAAGGAAGCTAGTCGACTCGACATCTGATGGC
+
CC>CBC@CB=?@?C>B@B?AAA7B;>:?;6<666-73747;//.>0..41.0073303430340
@T6XXI:00140:00494
GGGGAATTCTAATACGACTCACTATAGGGAGAGGGAGAATAGTCAACAAATCGTTTGGGGCGACTTCTCCTTCCTTT
CTCCCTTCTCCC
+
CEB7B548;>>=BA@@C@@@CBA=?997.7@<??==@B?BA@@@A>@@A<CBBBC>BCB8CC>>>;;77-
/./9==9;;CC?A588BC<
@T6XXI:00142:00455
GGGGAATTCTAATACGACTCACTATAGGGAGAGGGAGAATAGTCAACAAATCGTTTGGGGCGACTTCTCCTTCCTTT
CTCCCTTCTCCC
+
CCB9BAB@BBBCCCB??BBBBA>96166.7>>@B>BAB@BA;;><;<@A<CCAAA=ACC7AABBC=??C=<9=>@@<
@AA@<@<?@AA<
@T6XXI:00142:00456
GGGAGAGGA
+
11,////,,
@T6XXI:00142:00457
GGGGAATTCTAATACGACTCACTATAGGGAGGGGGAGAATAGGTCTCAACTGA
+
///*/,4244484444493336;?=>;6,11111+1163600/0666701001
@T6XXI:00142:00461
GGGAGAAGGGAGAAAGGAAGTGCAGTTCGCGGTTAGTGAGGGGAAAAACTATTCTCCCTCTCCCTATAGTGAGTCGT
ATTAGAATTCCCC
+
DC@CBB@BB>@BBB=B<??BBBCBBB@BBBB@B@?>><<??B8>>>>/B>>?>?A@A;?B@CCBC@AAAA=AAAA?<
<<9<<@>A5<<=4
@T6XXI:00142:00465
GGGAGAAGGGAGAAAGGAAGGAGAAAGCGACGTCGGGGGAAACGTTTGCTATTCTCCCTCTCCCTATAGTGAGTCGT
ATTAGAATTCCCC
+
AC<9995991999>9><@>@>AABB>BBBCCCCBCBBB1BD@CCCC@CEDBDCCCDF:<<BAA<AFAAACBBAA???
<<8;>>;:;<<@5
@T6XXI:00142:00490
GGGAGAAGGGAGAAAGGAAGTCAAAAGAGACAAGACAACGATGGGAGCTATTCTCCCTCTCCCTATAGTGAGTCGTA
TTAGAACTTCCCC
+
<C@DDC@CB?BBBB?B@C?A@@@GG9???CDC@B@@=>@B@AAC;A@AE<<:<>AB><<<BB<@@@A?@AB::7;?<
6474;46<3766,
@T6XXI:00143:00488
GGGAGAAGGGAGAAAGGAAGCTAGTCGTCGACATCTGTGGCTTCACAGTACAGAGGGACTGTCCAAAGCCGCAGATC
TCGAGTCGACTAG
+
```

```
GC@AA@?BA=DEBB>C?BBBBA@@AA?A=?;;<;;<@AE@@?=AABAACDCB=9;919AABBA@BB@BC@CBBBCC@
A@=@@CEBABABB
@T6XXI:00143:00493
GGGGAATTCTAATACGACTCACTATAGGGAGAGGAGAATAGTACGACA
+
8C81?.;;<BB@DCCCBABB<>ACC??>89988:<8888////---2.
@T6XXI:00143:00494
GGGGAATTCTAATACGACTCACTATAGGGAGAGGGAGAATAGACCACGACCCTTCTACCCACGATTGAGCTTCCTTT
CTCCCTTCTCCC
+
@=94?<><99;>ACBBAACC=<@BA???;?ABDC>B?C?C=899>=AAA=A<@999444-
4;;>A>@@@@??A>AC:??AA:><<<<@;
@T6XXI:00144:00453
CTAGTCGACTCGAGATCTGCGGCTTTGGACAGTCCCTCTGTACTGTGAAGCCACAGATGTCGAGTCGACTAGCTTCC
TTTCTCCCTTCTCCC
+
>7----
,,/68;BBDCCBA@A@AEG>C>AACCCBC>BBBAAA???ABC>?B@CGCDBBABCCCCBBB@;;;@@??B@CD=??@
B9?7::BB>
@T6XXI:00144:00484
GGGAGAAGGGAGAAAGGAAGCTAGTCGACTCGAGATCTGCGGCTTTGGACAGTCCCTCTGTACTGTGAAGCCACAGA
TGTCGAGTCGACTAGCTTCCTTTCTCCCTTCTCCC
+
BB>B;;8<C@B@?><@?@?;9:>?A;;:AAADCBBBCCC@>=?BB<B?BBBBCCC=@@BAAAAC@??BAC?A===;<
?=A??????AA???@<8:;58;6<@?A<A<??@@;
@T6XXI:00145:00450
GGGAGAAGGGAGAAAGGAAGCTAGTCGACTCGACATCTGTGGCTTCACAGTACAGAGGGACTGTCCAAAGCCGCAGA
TCTCGAGGTCGACTAG
+
EDFAAA@CB@AABB?B?B>A@@>@@CBBBCCB8:;;;:BCDAAB@CBBBBCBBBB;??<?AA<<9:;;<@<;::?A?
??@AA@A>A@@@???A
@T6XXI:00145:00451
GGGGAATTCTAATACGACTCACTATAGGGAGAGGGAGAATAGTTTTCCCTTCACTAACCGCGAACTGCACTTCCTTT
CTCCCTTCTCCC
+
CII:CCC@BBB@CCBBB?>>BB>97777.7>>AB>BAA?AB?;;;0;84=8<@?@B@C@::;A?AA??BA@=?477.
777?:@5::@C;
@T6XXI:00145:00452
CTCGAGATCTGCGGCTTTGGACAGTCCCTCTGTACTGTGAAGCCACAGATGTCGAGTCGACTAGCTTCCTTTCTCCC
TTCTCCCCTTCCTTTCTCCCTTCTCCC
+
CCCAAABBBBBBB@BBB<B@@@@@@CC@CCBB@@@CBBBC@CC@CCCBBBBBBDCBBBCBB@@99;?A?AD?B@@@<
@<>>>>>.848477.80;<6@,0///,
@T6XXI:00145:00458
CTAGTCACTCGAGATCTGCGGCTTTGGACAGTCCCTCTGTACTGTGAAGCCACAGATGTCGAGTCGACTAGCTTCCT
TTCTCCCTTCCTCCC
+
:8/////4:CCBCABAA>AA=???;?=>BBCBB@=B?A?CCBBACCB>:A@BBBBB@@@BBBBCCCBAA@@@?=A=A
B<?;:>6;,0,0660
@T6XXI:00145:00463
GGGGAATTCTAATACGACTCACCTATAGGGAGAGGGAGAATAGTCAACAAATCGTTTGGGGCGACTTCTCCTTCCTT
TCTCCCATTCCTCCC
+
DC@4B6954//7//////....,......+16666266669974042006З;8<<@=@@@7A<<=C=??;263/6:8
68333+.-0147>?;
@T6XXI:00145:00489
```

```
CTAGTCGACTCGAGATCTGCGGCTTTGGACAGTCCCTCTGTACTGTGAAGCCACAGATGTCGAGTCGACTAGCTTCC
TTTCTCCCTTCTCCC
+
C44999444:ABDCCCB@@AB@BBB<B?AA@AACC>C>??????>BBC?BC?BBBBBAABCCCCBCCCCCCCBB?B@
CD>BCBCAC?AABB=
@T6XXI:00145:00491
GGGGAATTCTAATACGACTCACTATAGGGAGAGGGAGAATAGCTCTCATTTCCCCGACGTCAGCCGTGTCTTCCTTT
CTCCCTTCCTCCC
+
CB@4@546<A=?BBCBCBBB>@A@B9998:BBBC>CBC?BBCCC?:779909>>2=9433779B@BGKBA?=<<<@;
B>;917,/,/660
@T6XXI:00145:00492
CTAGTCGACTCGAGATCTGCGGCTTTGGACAGTCCCTCTGTACTGTGAAGCCACAGATGGACTGCTTCCTTTCTCCC
TTCTCCC
+
CBCBCCCBBEDCCCBCABBA;99776;>ABCCCCB>BBCC@DBBBBBA=AB@A@@CC@@=AC@>:<,0,0;60//;9
A@@?@D>
@T6XXI:00146:00455
GGGGAATTCTAATACGACTCACTATAGGGAGGGAG
+
CFC:C>@>?BB@CABABBCBAABCAA?=6/,,+*,
@T6XXI:00146:00457
GGGAGAAGGGAGAAAGGAAAGGACAGTA
+
DC:@@@5990889914244-4743.../
@T6XXI:00146:00483
GGGAGAAGGGAGAAAGGAAGGAGAAGTCGCCCCAAACGATTTGTTGACTATTCTCCCTCTCCCTATAGTGAGTCGTA
TTAGAATTCCCC
+
CIFCCB?BBAACBB=A@A@B>AAB@BBBBCBB8BB?CBDAC>AA@BBBCBB?BAA=;9==@A<@AAA???;??AA??
?>AA@=?=?@?5
@T6XXI:00146:00489
GGGAGAAGGGAGAAAGGAAGCTAGTCGACTCGACATCTGTGGCTTCACAGTACAGAGGGACTGTCCAAAG
+
EDEBBA@CCAEBBB>BCB@BAA???BCACCC?ABBBBBCCC>AA?B@@??>@AA@<<<;8;<<<A><<:@
@T6XXI:00146:00491
GGGAGAAGGGAGAAAGGAAGGAGAACAACACGATCGGTTAAGCGTAGCTATTCTCCCTCTCCCTATAGTGAGTCGTA
TTAGAATTCCCC
+
DFBDCC@CC@BCBB>B@A@B@BAA?BCACCCCCCBB?B@C@@@ACCCBBCB@A;;;7;@@CDCFCAAACCBCBCCCC
CDECC@CBDDH9
@T6XXI:00146:00496
GCTAGTCGACTCGAGATCTGCGGCTTGGACAGTCCCTCTGTACTGTGAAGCCACAGATGACTCG
+
49999>4:49=?9<9A@A@<<<:10,/,/.9::AC?@@BABBCCCDCC?AB?A@?=<1-,*(((
@T6XXI:00146:00498
GGGGAATTCTAATACGACTCACTATAGGGAGAGGGAGAATAGCGTGCCTGTATACGGTACCCTGTTCGCCTTCCTTT
CTCCCTTCTCCC
+
CCD:E=:<<CC@CCCBBBBBBBBBB99757??AB>BCC@C@A@;??B?CAA???CC@B@><<@??=@BB>A>B59:7
:AAD@C?A@@?;
@T6XXI:00147:00481
GGGAGAAGGGAGAAAGGAAGTGCAGCTTCGCGGGTAGTGAAGGGAAAACTATTCCTCCCTCTCCTATAGTGAGTCGT
ATTAGAACTTC
+
49199924959=BC>C@C??:8>>1//,/6///+/99988233,333,44882/,/440....,.459>A@B;:---
--,--0/0664
```

```
@T6XXI:00147:00488
GGGGAATTCTAATACGACTCACTATAGGGAGAGGGAGAATAGTCAACAAATCGTTTGGGGCGACTTCTCCTTCCTTT
CTCCCTTCTCCC
+
AED9@6:8<94=ABDCCBBBB>;7>33308?===9???=?BBCAA=ABB=A@@?C>CCC9@@?:;67@@;?;?<??<
=ABA@A?B@@@:
@T6XXI:00148:00450
GGGAGAAGGGAGAAAGGAAGCTAGTCACTCGACATCCTGTGGCTTCACAGTACAGAGGGACTGTCCAAAGCCGCAGA
TCTCGAGTCGACTAG
+
<D>:@B:<;9<BBB>B>B>A?????@@=<=777131016667135 7?CBCDAA?????<<??@AA>AA<??>@@88/
0077=C=<<BCA??@
@T6XXI:00148:00452
GGGGAATTCTAATACGACTCACTATAGGGAGAGGGAGAATAGTCAACAAATCGTTTGGGGCGACTTCTCCTTCCTTT
CTCCCTTCTCCC
+
A@?4><@=@AADCCB@@@??BBBB<76959?ACC>CBB>B>A@@C@?@?:BCCBC?BCC8CAAAC?AAA=?;=3785
8@>CAC387781
@T6XXI:00148:00457
CTAGTCACTCGAGATCTGCGGCTTTGGACAGTCCCTCTGTACTGTGAAGCCACAGATGTCGAGTCGACTAGCTTCCT
TTCTCCCTTCTCCC
+
D?:<;A===DDDBBBBCCCC@CBB@B?AABCCCC;@@@>>>>?@CCC==B@BBBBBBACCCECBCCCCBBABAAA@C
DB@@BC=A?AA@A<
@T6XXI:00148:00458
GGGAGAAGGGAGAAAGGAAGCTAGTCGACTCGACATCTGTGGCTTCACAGTACAGAGGGACTGTCCAAAGCCGCAGA
TCTCCGAGTCGACTAG
+
DCB@?=5991949A?B@B@@@>010/...,,2,.....3453677317??@AAC???C;888@==;@A;@A>987?;
:433/033930030--
@T6XXI:00148:00463
GGGGAATTCTAATACGACTCACTATAGGGAGAGGGAGAATAGCTCTCTTCCTGGTATGACGCTACTATCCTTCCTTT
CTCCCTTCCTCCC
+
FFJ:C?B?BBC@CECBABABBCBBB@??<?BCCC>BBB>AAB=??BCC@C?@@@CAAA==@BBBDFCB?865<;>@7
=9;739,/,/642
@T6XXI:00148:00489
GGGAGGAGGGAGAAAGGAAGCTAGTCACTCGAGATCTGCGGCTTTGGACAGTCCCTCTGTACTGTGAAGCCA
+
4:69?5949-4444-49;98988>:1/...335...71381333,376766//.+.:0/-222,--.,.57?
@T6XXI:00148:00492
GGGGAATTCTAATACGACTCACTATAGGGAGAGGGAGAATAGCCACCAACTATTGTTCGTCGCCGTTCTCTTCCTTT
CTCCCTTCTCCC
+
IED9D>A=@AD=ABCDBBABB@>>B?;;:777@@<@??:?AA@>@C6869@@@?@@>AAAA8868C9;;==3424;4
:588:;8:38:7
@T6XXI:00148:00495
GGGAGAAGGGAGAAAGGAAGTAGTCGACATCTGTGGCTCACAGTACAGAGGG
+
9:-44?;>>:?99919<@4443334477<<@BB7>=0,,7,,...49858=4
@T6XXI:00149:00463
GGGGAATTCTAATACGACTCACTATAGGGAGAGGGAGAAA
+
/77*1,92444889999@8889774477,/....+..11,
@T6XXI:00149:00466
GGGAGAAGGGAGAAAGGAAGCTAGTCGACTCGACATCTGTGGCTTCACAGTACAGAGGGACTGTCCAAAC
+
```

```
CC?BB:899:>?@@<@=AABFD@@@C@??CC;<<CCB?@=C;?@?B:::A??=AA48818A?@@B@CD?<
@T6XXI:00149:00469
GGGGAATTCTAATACGACTCACTATAGGGAGAGGGAGAATAGTCAACAAATCGTTTGGGGCGACTTCTCCTCCTTTC
TCCCTTCCTCCCT
+
CCB7B546<>9<A@@@BBBAA@>>>877.7?==?<?CC;??A83055887><700,...*..222*//7-
/.77555464+0/-0147,.
@T6XXI:00149:00496
GGAGAAGGGAGAAAGGAAGCTAGTCGACTCGACATCTGTGGCTTCACAGTACAGAGGACTGTCCAAAGCCGCAGATC
TCGAGT
+
9<999699-
999C:?5;2:9A>??B;:;777>><;;667=:??=?BBBCCC@??::58@A@@@@BA<AA@<<=A@????878?
@T6XXI:00100:00529
GGGAGAAGGGAGAAAGGAAGCTAGTCGACTCGACATCTGTGGCTTCACAGTACAGAGGGACTGTCCAAAGCC
+
CCABBBACD@CBBB?B@C@C@??CCB@@?FF:;;DEC::9?8473:@@AABA@;;444-4;@DCA=BB:AA>
@T6XXI:00101:00508
GGGAGAAGGGAGAAAGGAAGGAGAACAGAATGCCACCAAGGTCTAAGCTATTCTCCCTCTCCCTATAGTGAGTCGTA
TTAGAATTCCCC
+
FD@BAB@AA=AAAB=B?A?A@BCD@CB@@=A?A>BCACAC?BBBB@BBCA??8??98??@BC>???@A???ABA>?<
<9<<@?<:=@@5
@T6XXI:00101:00529
GGGAGAAGGGAGAAAGGAAGCGGGACGTGGGTTAGGGTTAAGCTATTCTCCCTCTCCCTATAGTGAGTCGTATTAGA
ATTCCCC
+
BB>>>>;>>9>>@B<B>A>@???:???AAD@C?A@@6;37<>??<<<?AAD;@<==D@===AAA@@@??>?=>387=
;>=<==5
@T6XXI:00102:00542
GGGAGAAGGGAGAAAGGAAGCTAGTCGACTCGAC
+
=C?BBC>BB>BBBC>C>?9;1.....1111116.
@T6XXI:00102:00545
GGGGAATTCTAATACGACTCACTATAGGGAGAGGGAGAATAGTCAACAAATCGTTTGGGGCGACTTCTCCTTCCTTT
CTCCCTTCTCCC
+
AA@4@<>=;<<ADCCCB???@?AABAAA:?@?AA=A@@>??AAAA>@@@:@@:;:8;LJ9CC@>>:00/,/,/4661
6794,7/766=:
@T6XXI:00103:00509
GGGAGAAGGGAGAAAGGAAGCGTAGTCGACTCGA
+
8:5//959909>=?=B?9>B831111133333..
@T6XXI:00103:00513
GGGAGAAGGGAGAAAGGAAGCTAGTCGACTCGACATCTGTGGCTTCACAGTACAGAGGGACTGTCCAAAGCCGCAGA
TCTCGAGTCGACTAG
+
9B?;<<@BC=BBBC=B>A?@@?>>>1/1>>>=1:::88AAA<?A@ABDBCB@>?;777-
4>;;==>AA<AA?@@<=9<<==@@@A???>?<<
@T6XXI:00103:00515
GGGGAATCTAATACGACTCACTATAGGGAGAGGGAGAATAGCTAGCATTATTCCGCTAGGTGCTTCTCCTTCCTTCT
CCCTCTCC
+
1880>121:::9A@@@@@@@@??=;7771:B@CC>@AA>A??9::B??A>:;57=B;<0=:;;0/,.33,.,0/0/00
00+00032
@T6XXI:00103:00541
```

```
CTAGCGACTCGAGATCTGCGGCTTTGGACAGTCCCTCTGTACTGTGAAGCCACAGATGTCGAGTCGACTAGCTTCCT
TTCTCCCTTCTCCC
+
=AACDCGCCCCBBBAABBCC@A?A9@6::BABAB=A???@A88899C>AC?BBCCCCABABBB:<;@@@D??:=A;@
@<?<;A:=8:444-
@T6XXI:00104:00510
GGGGGAATTCTAATACGACTCACTATAGGGAGAGGGAGAATAGCAAACGTTTCCCCGACGTCGCTTCTCCTTCCT
+
EBDC2A69:;49<ACBBAAAA????@???<??DDK>?@@<??@?DC:;777.444,4==>776/0,,050.)*)*
@T6XXI:00104:00518
GGGGAATTCTAATACGACTCACTATAGGGAGAGGGAGAATAGTCAACAAATCGTTTGGGGCGACTTCTCCTTCCTTT
CTCCCTTCCTCCC
+
BCB7A8;8<BA=BBB>@@A@???>77:@:@@@AB>@@@????CCC:==D@CCBCB=BCC7AACAA<:883838<>>:
@;7722,/,.772
@T6XXI:00105:00515
GGGAGAAGGGAGAAAGGAAGCTAGTCGACTCGAGCATCTGTGGCTTCACAGTACAGAGGGACTGTCCAAAGCCGCAG
ATCTCGAGTCGACTAG
+
9?6=@A=@CA<==C@C?B?A??:::117<66688679?7938858;<??@AA?=;;444-
4;;=AA?AA<AA>@??@@<<==?@?A???;964
@T6XXI:00106:00508
GGGAGAAGGATGAAGAGAT
+
=<8<11,1-1/665//437
@T6XXI:00106:00509
GGGAGAAGGGAGAAAGGAAGCTAGTCGACTCGACATCTGTGGCTTCACAGTACAGAGGGACTGTCCAAAGCCGCAGA
TCTCGAGTC
+
?C>DA>5941999@197:;ABB?@@C@???@:7:668666653627;???CA=;;444-
4;;;8858@:>AB@@@@A888@@?=@@
@T6XXI:00106:00510
GGGGAATCTAATACGACGTCACTATAGGGAGA
+
9AA4@69944888733833.../....60...
@T6XXI:00106:00512
GCTATACACTCGAGATCTGCGGCTTGGACAGTCCCTCTGTACTGTGAAGCCACAGACTGTCGAGTCGACTAGCTTCC
TTTCT
+
/,/8////68899;A@A@;00,//,.,.....1660000034589<=<=D@BABA7668?8?A99;:7716611013
66,00
@T6XXI:00107:00523
GGGGAATCTAATACGACTCACTATAGGAGAGGGAGAATAGTTTTCCTTCACTAACCGCGAACTGCACTTCCTTCTCC
CTTCTCCC
+
8@@17+////789:<C;;;E000<00,.7:=<;=@@>A9=8>;+/.-0136868981:7=5;<98>;:63,/-
.23338553330
@T6XXI:00108:00509
GGGAGAAGGGAGAAAGGAAGGAGAACGGCACGACAAAATTGATGGTGCGCTATTCTCCCTCTCCCTATAGTGAGTCG
TATTAGAATTCCCC
+
BDCCCC@CB>BBBB=B?A?B?@@?@CCDCCBBBCCCC:E@AAAC@CDCCCB>@<@@?C88:CCEAA?@A@@@@A@<<
>7758@A?A<A@@5
@T6XXI:00108:00541
GGGAGAAGGGAGAAAGGAAGCTAGTCGACTCGACATCTGTGGCTTCACAGTACAGAGGGACTGTCCAAAGCCGCAGA
TCTCGAGTCGACTACG
+
```

```
9A<>?>;>>:>>>@9;:<@CCBCCC:::7617:77A8691420:29@BAAA@?;;433-
4;;?@A>@@<@B@ABAAA===AA<<8884;;448
@T6XXI:00109:00512
GGGGAATTCTAATACGACTCACTATAGGGAGAGGGAGAATAGACCCTAATCAGAATGGTGTCATTTCTCCTTCCTTC
TCCCTTCTCCC
+
;JA5>11,/6679>AACAAAB???@:77.7===?<?AA=@??88818@:=;;640-.,-2233330137,/--
0101114094304>5
@T6XXI:00109:00517
GGGAGAAGGGAGAAAGGAAGTTGTGTGCCATGTCGTTCCCAGGGAGACTATTCTCCCTCTCCCTATAGTGAGTCGTA
TTAGAACTTCCC
+
?B>BAA>@@<@>>@<@=@@CG?BBBBBC>BCA@;9;38818@C@@@DAA==9;888-
44444,4A9;<=<;;=7787737;B:;<8;;9
@T6XXI:00109:00539
GGGAGAAGGGAGAAAGGAAGCTAGTCGACTCGACATCTGTGGCTTCAAC
+
@E@ABDCCC>B;:96;>>>BCABBB9::???=:777:1768/3176601
@T6XXI:00110:00513
GGGGAATCTAATACGACTCACTATAGGGAGAGGGAGAATAGGCCAAG
+
:AA1<,1///76489783311366111,6=?BC8;/96887,/,-2-
@T6XXI:00110:00541
GGGAGAAGGGAGAAAGGAAGGAGAATTTGGCGTCCGGCAAGGACTGTCTATTCTCCCTCTCCCTATAGTGAGTCGTA
TTAGAACTTC
+
BD@BCB?BB>>@BB8;::?B>?@?;??8?=BBBBAB@CC@D@C????@@9959???088877,7<>??@CC?=6333
55686975,.
@T6XXI:00111:00519
GGGAGAAGGGAGAAAGGAAGGAGAACGGCGTGCTCCAAAAGGCGATGCTATTCTCCCTCTCCCTATAGTGAGTCGTA
TTAGAACTTCCCC
+
8>-119599-
88=A0;687>48;7>CB?BB;98313666266166679>;@=?9=7,11111,6<?@@AA@::7//.4044>88>49
::1
@T6XXI:00111:00527
GGGGAATTCTAATACGACTCACTATAGGGAGAGGGAGAATAGACCATGAACCTTCTCCGCACCTTTACGCTTCCTTC
TCCCTTCTCCC
+
CGB8@5459889;?@@@@@@???F>777.7>77768?;>>@=<<9;;;;3838?@BA?<<<>;==:=<=<==;3424
998498<AAA<
@T6XXI:00113:00500
GGGGAATCTAATACGACTCACTATAGGGAGAGGGAGAATAGTCACATTCATGTTGTGACCGTGTCTCCTTCCTTTCT
CCCTT
+
,220=+1///76444783311711111,16166,144/38;=:65<63077,,+,++00347::4848444/00+04
2322,
@T6XXI:00113:00526
GGGGAATCAATACGACGTCACTATAGGGAGAGGGAGAATAGCTATCGTCA
+
=::2@:<489:4443333796733111,166;<8??82334061110000
@T6XXI:00113:00548
GGGGAATTCTAATACGACTCACTATAGGGAGAGGGAGAATAGTCAACAAATCGTTTGGGGCGACTTCTCCTTCCTTT
CTCCCTTCTCCC
+
?E@4@6959889CBCBCCCCCCA6=777.7>>>><?<<>@@@AAA>AA@;B@@>>.777.7>>?B=>=;./,/7886
;;@@;@<?=A@;
```

```
@T6XXI:00114:00508
GGGGAATTCTAATACGACTCACTATAGGGAGAGGGAGAATAGATCCT
+
/11+1,9248988=AAACCCEECC::;=6==>>>6;;:9;;4/--,-
@T6XXI:00114:00525
GGGAGAAGGGAGAAAGGAAGGAGAAGTCGCCCCAAACGATTTGTTGACTATTCTCCCTCTCCCTATAGTGAGTCGTA
TTAGAATTCCCC
+
=@?AAA>AA<@AA@9>;>;>=88:@??@@CCD9CC>@@:61,176?AC==A>@A@@<@859D@?@@@999AA???AA
A>@@@<?:?@@1
@T6XXI:00115:00501
GCTGTCGACTCGAGATCTGCGGCTTGGACAGTCCCTCTGTACTGTGAAGCCACAGATGTCGAGTCGACTAGCTTCC
+
//6/4494::?<<=CBBC@;:910,.,..34.11,111668;;??@@CAC=@ABCCB<<:?@>7;9...44..,.3
@T6XXI:00115:00502
GGGGAATTCTAATACGACTCACTATAGGGAGAGGGAGAATAGTCACAAATCGTTTGGGCGACTTCTCCTCCTTTCTC
CCTTCTCCC
+
499/92924449A9<<EBCCCBA==666.7>7776:==>>?;//.---+1111688>;@<65333.-,--
01334.4310495:;4
@T6XXI:00115:00535
GGGAGAGGAGAAGA
+
8@-144/,/,,*,,
@T6XXI:00116:00525
GGGAGAAGGGAGAAAGGAAGCTAGTCACCGCGATCGTCT
+
?CAABB@CC>BAB@<@=@@C@???9900,0.-,,0,...
@T6XXI:00117:00501
CTAGTCGACTCGAGATCTGCGGCTTTGGACAGTCCCTCTGTACTGTGAAGCCACAGATGTCGAGTCGACTAGCTTC
+
9944444499444444448371338,31336866888;88?::8?@?@;=A:<;?BB??@BBB@:32/-,00**),
@T6XXI:00118:00511
GGGGAATTCTAATACGACTCACTATAGGGAGAGGGAGAATAGTCAACAAATCGTTTGGGGCGACTTCTCCTTCCTTT
CTCCCTTCTCCC
+
CC@8;24598=9@;<<<CCCCCCB@796.7>?AC>BAA?A?@@@@>AAA;A?=<>.777.7?>:;::<;,/./9993
:79949283@A<
@T6XXI:00118:00512
GGGAGAAGGGAGAAAGGAAGCTAGTCGACTCGACATCTGTGGCTTCACAGTACAGAGGGACTGTCCAAAGCCGCAGA
TCCTCGAGTCGACCTAG
+
?C>AA@?AA<;;;B<A>A@CB@==>0./733;>?9??????><??@BBBBB@==?747-
499:<=9<C=??<?@>>@8838;4//611-.,-11
@T6XXI:00118:00519
GGGGAATCTAATACGACTCACTATAGGGAGAGGGAGAATAGTCAACAAATCGTTTGGGGCGACTTCTCCTTCCTTTC
TCCCTTCTCCC
+
8880@.2111:8=?@B??;:7><;766,7====67448;<@A<<9==C>C;;;B>BBC9CF>>>;:76,/,/7889=
:@A7=:=788-
@T6XXI:00118:00541
GGGGAATTCTAATACGACTCACTATAGGGAGAGGGAGAATAGACACTCCCTTACGAACCACGCGCGAATCTTCCTTT
CTCCCTTCTCC
+
999/96959@>=BBBBCDBBBCCCAAAA<AABCC>BAA?A<@89=B@@@9=;=AAA>A?@???==<@;=::7:344-
4///,/,/-.,
@T6XXI:00119:00518
```

```
GGGAGAAGGGAGAAAGGAAGGAGAAGTCGCCCCAAACGATTTGTTGACTATTCTCCCTCTCCCTATAGTGAGTCGTA
TTAGAATTCCCC
+
>B>AA@>@A<BBBCAC@D@C=@@CCBAABCCC9CCACBBB?;BC?ABEAAA=@@@C<A===A<AAA@?@?A@@@@@@
@=@@@?D>AAA6
@T6XXI:00119:00521
GGGAGAAGGGAGAAAGGAAGGGGAGAAGGGAGAAAGGAA
+
78+11959@1948=09471788,336368887472222*
@T6XXI:00119:00537
GGGAGAAGGGAGAAAGGAAGCTAGTCGACTCGACATCGTGTGGCTTCACAGTACAGAGGACTGTCCAAAGCCGCAGA
TCTCGAGTCGACTACG
+
7<8119598-444:-
423139111171111....57////.447?>>:888?8;;:8837B;;<@;>>588=@?@AA998;;6///--1--
00
@T6XXI:00120:00518
GGGGAATTCTAATACGACTCACTATAGGGAGAGGGAGAATAGTCAACAAATCGTTTGGGGCGACTTCTCCTTCCTTT
CTCCCTTCTCCC
+
CC@:@69:;>?<BBCCCDDBB<;6=666.7==@B>@AA?@:>>DA>@B@:BBBBC>CCC7??B@@=>:924347996
<A?C@C9<<@C=
@T6XXI:00120:00522
GGGAGAAGGGAGAAAGGAAGCTAGTCGACTCGACATCTGTGGCTTCACAGTACAGAGGGACTGTCCAAAGCCGAGAC
TCGAGTCACT
+
6=9219<AB:<:CC<@=@=@@AA?>>?;9::??<=A:7776<6>>>>>@CCAA;;444-
4::;=?=@@;@@;443440000333335
@T6XXI:00121:00522
GGTCTAATACGACTCACTATAGGAGAGGGAGAATAGCTCACGATTTTCTGAGCCTCCCTGGCTTCCTTT
+
,+,,//,/444;449B?>>400,./...+.770133699:4449==2=BB>;;=911,11,11,0,,,*
@T6XXI:00122:00521
GGGAGAAGGGAGAAAGGAAGGAATATCCTCGGGAGACACAGCGCTGGACTATTCTCCCTCTCCCTATAGTGAGTCGT
ATTAGAATTCCCC
+
@EA@>=58419>@A<ACA>B>A?AABA>BCB@<@@???AA?EAADFDAACCB@CBBC?<<<CC>CCCBAABCBBBBC
::;;B@=A6999/
@T6XXI:00122:00535
GGGGAATTCTAATACGACTCACTATAGGGAGAGGGAGAATAGCCGCCATTTGTTCATTGGTGCCCTATCCTTCCTTC
TCCCTTCTCCC
+
@>>4?695;=@@CBBB>@@@B@?>?777.7====9?<<<?@;;8;<8@@B=BDCCDC=?;<<<;7?CBB:<:<4744
444+04499=4
@T6XXI:00123:00514
GGGAGAAG
+
665//1,,
@T6XXI:00123:00517
GGGGAATCTAATACGACTCACTATAGGGAGAGGGAGAATAGTCAACAAATCGTTTGGGGCGACTTCTCCTTCCTTTC
TCCCTTCTCCC
+
88A68*111178?CBBCBBCCCBB:::5:?ACD@==?>@???==;<<=;C7:9B6666,115333711,-
,/464497?A<=584==A
@T6XXI:00123:00533
GGGGAATTCTAATACGACTCACTATAGGGAGAGGGAGAATAGCCACACTATTTGTTCCACTGCCGTAACCCTTCCTT
CTCCCTTCTCCC
```

```
+
BCB4@59598>?CCBBBBBA?>ACBCCE>BBAAA<?@A>AC@@56;:9:::949=:==@@BBC@CAC6;7.7;=34/
4:::7:798<65
@T6XXI:00123:00535
GGGGAATTCTAATACGACTCACTATAGGGAGAGGGAGAATAGATAAACTATTTCCACGGCGAGTGTTGCTTCCTTCT
CCCTTCTCCC
+
;A?1>,/,1//78@@@CACBCBD>>777.7?===9;::8;::9444-4//.-+-,---
,3::6<;?478;7934339994:9;444-
@T6XXI:00124:00535
GGGGAATCTAATACGACTCACTATAGGAGAGGAG
+
,--*,*1///78?;<;;::=:7....,,2,/,.1
@T6XXI:00124:00539
GCTAGTCGACTCGAGATCTGCGGCTTGGACAGTCCCTCTGTACTGTGAAGCCACAGATGTCGAGTCGACTAGCTTCC
+
,,///4444444>;<;@@@<;:901,.,..3..16263...531165;:=E?AA@DBBBBA??>7;900.66..,.,
@T6XXI:00124:00545
GGGAGAAGGGAGAAAGGAAGAACATGTCACAGCTGGGAAAAAGCAGGCTATTCTCCCTCTCCCTATAGTGAGTCGTA
TTAGAATTCCCC
+
?C>>>>59919?@A9A=?>BB?@??>:;761178??;?AAC2C@<A?ABCCB@@@A<@9::B@BAAA99:AA???@A
ABBAC:><@CC6
@T6XXI:00125:00515
GGGGAATTCTAATACGACTCACTATAGGGAGAGGGAGAATAGTCAACAAATCGTTTGGGGCGACTTCTCCTTCCTTT
CTCCCTTCTCCC
+
CCC9@69;<;;@CA@@CCCCCBA77166.7?77768=?;??@@@@>AAA?C@??A6==?2==<>?::68,/,/,//,
/8:=7?485AB<
@T6XXI:00125:00540
GGGAGAAGGGAGAAAGGAAGAATACGTGGGGACAGGAAAGTTCAACGCTATTCTCCCTCTCCCTATAGTGAGTCGTA
TTAGAATTCCCC
+
CH?DCC@CI;;?CCAC@B@CC?BCEAAADC9BBBC@CD?BB?BCACBBBCB?ABAE::9@@C7====AA@ACCA@@:
958@A9=:=CC6
@T6XXI:00126:00505
GGGAGAAGGGAGAAAGGAAGGAGAAGTCGCCCAAACGATTGTTGACTATTCTCCCTCTCCCTATAGTGAGTCGTATT
AGAATTCCCC
+
B::994599-////+/,.,.34435118>:9,00+.3..,.4449BA>;2////+..---+-
124377A?>=?@<<96??:?<???4
@T6XXI:00126:00509
GGGAGAAGGGAGAAAGGAAGGAGAAGTCGCCCCAAACGATTTGTTGACTATTCTCCCTCTCCCTATAGTGAGTCGTA
TTAGAATTCCCC
+
BC<=@@=>@:999@1;68=@6;:69??ABBBB8CG>@A:9616;;?CC@@A>?@=>:?999D?A???===AA?@=AA
A?=??<A2444-
@T6XXI:00126:00516
GGGGAATCTAATACGACTCACTATAGGGAGAGGAGAATAGCAGTGCAACTCATGCCTTGCCGATTCTCCTTCTCCT
+
ABB8C:<;448847:?87371316111,1?7744::24444030400/03330010606:>89>;<.-,-,+++*+
@T6XXI:00126:00547
GGGAGAAGGGAGAAAGGAAGCTAGTCGACTCGACATCTGGTGGCTTCACAGTACAGAGGGACTGTCCAAAGCCGCAG
ATCTCGAGTCGACTAG
+
9;79>@<@A<A@>@7:7:?BCBBCBAAAA@A?:00;;/0,.1225:?878CC@>>@74414;;>?@<??:??====A
AB;884::44303088
```

```
@T6XXI:00127:00511
GGGGAATTCTAATACGACTCACTATAGGGAGAGGGAGAATAGTCAACAAATCGTTTGGGGCGACTTCTCCTTCCTTT
CTCCCTTCTCCC
+
FHC:C89:;AA?DGBABABA@<;67116.7>>@B>@AB=@<;;@@=@@D<CAAA?9?=?5??BBD?A=?3838;==:
@@?@<A588>>7
@T6XXI:00127:00515
GAGTCGGACCGAGATCTGCGGCGACAGTCCTCGATGTACTGT
+
4<11/85//,///./439AA>BBA@660/,........6676
@T6XXI:00127:00517
GGGAGAAGGGAGAAAGGAAGGGGAGAAGGGAGAA
+
7?8<?>59909448-82377772797011,1000
@T6XXI:00127:00540
GGGGAATTCTAATACGACTCACTATAGGGAGAGGGAGAATAGACACTATTTCTTGACCTCCCGCGCCTCTTCCTTCC
TCCCT
+
?CC4>5924999=?CBB;;<C:87A7775:@@CC@CA@9<8;//-83483000/00731776161555223301,-
,--103
@T6XXI:00128:00516
GGGAGAAGGGAGAAAGGAAGCTAGTCGACTCGAGATCTGCGGCTTTGGACAGTCCCTCTGTACTGTGAAGCCACAGA
TGTCGAGTCGACTAGCTTCCTTCCTCCCT
+
49144?;>>-
84?@<A?B?@>:C?867166611161111176111,177>988::5;;;/..48::?A;=====@AA@9:8?>>884
;9;;34-,-,-,-,---+-
@T6XXI:00128:00534
GGGAGAAGGGAGAAAGGAAGGAAACTGGGAAAACGCAGGGCGAGGCTATTCTCCCTCTCCCTATAGTGAGTCGTATT
AGAATTCC
+
>C>BBB=@>9>>>?9>;?=@?BCAAA??-777.373116666..,.3..,...5363033+0779=899<<8860-
,---,-,--
@T6XXI:00129:00527
GGGAGAAGGGAGAAAGGAAGAAGAGTTGATTACAGCGGGTTAAGAAC
+
4A599929919844-477:;;136::57965:<;9884+320242,.
@T6XXI:00129:00528
GGGAGAAGGGAGAAAGGAAGCTAGTCGACTCGACATCTGTGGCTTCACAGTACAGAGGGACTGTCCAAAGCCGCAGA
TCTCGAGTCGACTAG
+
CE?CBC=??<@CBB=B=@=@B?;;>/11>>>>1::??::9=5>A@ABCCCCAAA?77317<<>?A@AA;@@>A????
@===AAAA@@@@@@A
@T6XXI:00129:00539
GGGGAATTCTAATACGACTCACTATAGGGAGAGGGAGAATAGTTTCCCTATCCGTTCCCCTAGCGTCCTCTTCCTTC
TCCCTTCTCCC
+
499/92924889>CBBBBCBACBBBBBB9??AAB>A@@8:9444-
4;:;858;==<=AA5??===BAC??A>@8<8;;884::444=7
@T6XXI:00129:00542
GAATTCTAATACGACTCACTATAGGGAGAGGGAGAATAGCCACATCGTCTGCTCCCTAGTTCTCCT
+
,0*1,///79BB?><9:?6711111,1=>>@<B@?34444/4330088444<700,...-,---01
@T6XXI:00130:00502
GGGAGAAGGGAGAAAGGAAGGAGAATACAGCGAAATGAACACGTCGCTATTCTCCCTCTCCCTATAGTGAGTCGTAT
TAGAATTCCCC
+
```

```
4914AA=BC<@A@A<A>@>B=<<;@CBC><;@?@:AAA?>>>=7/.-
4??:?BCC<<<88818A@AAA???A@@@==:<CC>?8>CC6
@T6XXI:00130:00509
GGGAGAAGGGAGAAAGGAAGACAGTCCATCTGTGGCTTCACAGTACAGAGGGACTGTCCAAGCCGCAGATCTCGAGT
CGACTAG
+
7?8<19598-448;-
467;;7<?>:8576:...34..,.110003689;@CA993222,.,.44698AA>???884;A@@A@?;
@T6XXI:00130:00510
GGGGAATTCTAATACGACTCACTATAGGGAGAGGGAGAATAGGCCGAACCTCCTCCATCGTGCCATGCGCTTCCTTT
CTCCCTTCTCCC
+
CB:4?545;==9CCCBBBAAB?>==777.7>>>B@A@@>AAA:=9==>?=@@:=<=?====5?;;;////.,-
373453;4084989:4
@T6XXI:00130:00539
GGGAGAAGGGAGTG
+
9>:?99611+,,,*
@T6XXI:00131:00510
GGGAGAAGGAGTAAG
+
BB:@91-1-,***)*
@T6XXI:00131:00526
GGGGAATTCTAATACGACTCACTATAGGGAGAGGGAGAATAGTCGACACCAGCCTCCCGCCGACCTTTCTTCCTTTC
TCCCTTCTCCC
+
DC@6;69:<;;?CCBBCBDAC?@?A???<?BBCC>C@@?@;??@A@@@A>@@@;==A<AA>A>>344-
44/8>AD:@<::-438;CC@
@T6XXI:00131:00527
GCTAGTCGACTCGAGATCTGCGGCTTTGGACAGTCCCTCTGTACTGTGAAGCCACAGATGTCGAGTCGACTAGCTCC
TT
+
4444444444444998;:48984333,31331667;6611161103:==;=B?A@BBDCC@??BAAB@00.0....,
,,
@T6XXI:00131:00535
GGGAGAAGGGAGAAAGGAAGGTAATGCGGGTTGGGTATTAGATGTGAGCTATTCTCCCTCTCCCTATAGTGAGTCGT
ATTAGAATTCCCC
+
AC>BBE?BB=BBBB>B@C@C?BC?ABECE>B>A@<@::7:CBB;;<C?=?::8:ABB<;;;984:;;<AA@@@AA9;
//./;;9:<<=A6
@T6XXI:00131:00537
GGGAGAAGGGAGAAAGGAAGAAGGAATAAGGATGCCGTAAACGAAAGCGTCTATTCTCCCTCTCCCTATAGTCGAGT
CGTATTAGAACTTCCCC
+
BJCCBB=>>9>944-
477779:7767173::679=?=99=::565106111433233385;<===;<>>=A977><443433/000/00/04
4+
@T6XXI:00131:00538
ACTCGAGATCTGCGGCTTTCGACAGTCCCTCTGTACTGTGAAGCCACAGATGTCGAGTCGACTCATGCTTC
+
=A?=A@?@?;::7@=9990444;;;9??6:9=@@@@GB@ADCECECCBCAB<;;AA@@:7-2,,,,,.31.
@T6XXI:00131:00547
GGGAGAAGGGAGAAAGGAAGCTAGTCGACTCGACATCTGTGGCTTCACAGTACAGAGGGACTGTCCAAAGCCGCAGA
TCTCGAGTCGACTAG
+
9?9@@>;>@0;;@@8<::,00.4471/.......3.../6685:;=??ACCAAB@===7<@@@??>AA<??<??<<>
888??A<<<@884/6
```

```
@T6XXI:00132:00509
GGGGAATTCTAATACGACTCACTATAGGGAGAGGGAGAATAGTCAACAAATCGTTTGGGGCGACTTCTCCTTCCTTT
CTCCCTTCTCCC
+
:==1<,1,///78@BDBBCAC???<A??<AABCB>DAA>@AA@@@>@@A<CB:::5:BB8BCCAA7:7;24/4344-
499;4;8;1234
@T6XXI:00132:00522
GGGGAATCTAATACGACTCACTATAGGGAGAGGGAGAATAGCAAACGTTCCCCGACGTC
+
1::08,1///5/43>783711311111,1@???<<AA499A?==38610000+044...
@T6XXI:00132:00526
GGGAGAAGGGAGAAAGGAAGCATGTTGCACGGGCGATGTACAAACTATTCTCCCTCTCCCTATAGTGAGTCGTATTA
GAATTCCCC
+
@CBCBB==89=99919;><@CEBBA@CBB@?C>ABBA=@?@CC>BBB@<=ABB>AAAAA<ACCBBDDFCCCBA??;?
AA?@@>AA6
@T6XXI:00132:00533
GGGAGAAGGGAGAAAGGAAGTGCAGTTCGCGGTTAGTGAAGGGAAAAACTATTCTCCCTCTCCCTATAGTGAGTCGT
ATTAGAATTCCCC
+
DD@ECB?AA=AAAA;B@B@BBBCCBB?BBBB=@=@CBABCBC>C@@C2BBBB@CBBB>BADCC@???@@@AAAA@@@
@@=@@A>A;@@@5
@T6XXI:00132:00537
GGGAGAAGGGAGAAAGGAAGCTAGTCGACTCGACATCTGTGGCTTCACAGTACAGAGGGACTGTCCAAAGCCGCAGA
TCTCGAGTCGACTAGCTTCCTTCTCCCTTCTCCC
+
44-49>;>?9=49904231336666711;667;;6899?;;:7:7:???BB<;:;444-
44449;;=?:@@>@A@@??>><<<<@@@===;;:858,/,/77705004640
@T6XXI:00132:00538
GGGAGAAGGGAGAAAGGAAGCTAGTCGACCGACATCTGTGGCTTCACAGTACAGAGGGACTGTCCAAAGCCGCAGAT
CTCGAGTCGACTAG
+
7>:<<>;>>9>@>@<B>B@CCCBBBB::;>?:77>=7716713166669>?>::444+399489@CC?BB=<==BB?
===@@?=;<;::85
@T6XXI:00132:00544
GGGGAATCTAATACGACTCACTATAGGGAGAGGAG
+
;>B5>,////5/48??>;<?6766111,.7..,-2
@T6XXI:00133:00503
GGGAGAAGGGAGAAAGGAAGCTAGTCGACTCGACATCTGTGGCTTCACAGTACAGAGGGACTGTCCAAAGCCGCAGA
TCTCGAGTCGACTAG
+
BB>CCDCCB??BAB@C=@=@A?ABB:::????:::???DCA>?A?A???AA?ACC???<???AA@>@CAAA=@@@A9
=9BAAA@A===AAAA
@T6XXI:00133:00511
GGGGAATTCTAATACGACTCACTATAGGGAGAGGAGAA
+
C8708+9598><ABBBCAAAABCBCBBA.0000,/,,+
@T6XXI:00133:00547
GGGGAATTCTAATACGACTCACTATAGGGAGAGGGAGAATAGTCAACAAATCGTTTGGGGCGACTTCTCCTTCCTTT
CTCCCTTCTCCC
+
CB@7A5469>>>BBCCC@A@CAB?A???9??ACD>@?C?>>>;AA=>@@<CCBBB<BEE9CCBAA=?=?3724:::7
>@@A;@>@@AB=
@T6XXI:00134:00529
GGGAGAAGGGAGAAAGGAAGCTAGTCTCGACATCTGTGGCTTCACAGTACAGAGGGACTGTCCAAAGCCGCAGATCT
CGAGTCGACTAG
```

```
+
ADBCCH?BC=@ABB=B@C?BBC@>>0//3//449444;29759=@BCCFCAA<9919>AADEBCCACC@CCDDCCBB
BABA;;;;:?;?
@T6XXI:00135:00503
GGGGAATTCTAATACGACTCACTATAGGGAGAGGGAGAATAGCAAACGTTTCCCCGACGTCGCTTTCTCCTTCCTTC
TCCCTTCTCCC
+
CC@6=2469>>?CBBBC@AA@????:::8:BBEC>???=A==<><49611.4>;1?BCBBAA<?;69754678,/,/
;;79@9???A<
@T6XXI:00135:00509
GGGGAATTCTAATACGACTCACTATAGGGAGAGGGAGAATAGTACACAA
+
@@?4>696;@A?CCBBBBB?@@BBAAAA<<BBBC>AAB90.*****.00
@T6XXI:00135:00512
AGTCGACTCGAGATCTGCGGCTTTGGACAGTCCCTCTGTACTGTGAAGCCACAGATGTCGAGTCGACTAGCTTCCTT
TCTCCCTTCTCCCT
+
<??211;;;>;>CCA@@@A?;;<;<@BCBBBCC?CCC????B???B@AA>BCCAA@===@==<=A@@>@;;?:?;>>
:=999-434///,+
@T6XXI:00135:00513
GGGGAATTCTAATACGACTCACTATAGGGAGAGGGAGAATAGTCAACAAATCGTTTGGGGCGACTTCTCCTTCCTTT
CTCCCTTCTCCC
+
CEC8B5459449;@BCBCCBCBBAA???<?BBBE?A??<?A@AAA;==A<CBBA@:>>@27:/774/./,/,/4640
546=:=9?484-
@T6XXI:00135:00527
GGGGAATTCTAATACGACTCACTATAGGGAGAGGGAGAATAGCGTCTCGATTCCCAGCGACCGTTTGTTCTTCCTTT
CTCCCTTCTCCC
+
7??1<-
9244488878::>><9;9716616?BCCA===>==<==<>?>447344+0497;@>@AD<>B;:8=A>@A9@99949
7:9<;7
@T6XXI:00135:00541
GGGGAATTCTAATACGACTCACTATAGGGAGAGGGAGAATAGTCTAACAAATCGTTTG
+
88808+1///7643:77;;@?><<:::8<?BAE@===>A=;=000/03306//.5+.
@T6XXI:00135:00545
GGGGAATTCTAATACGACTCACCTATAGGGAGAGGGAGAATAGTTTTTCCCTCACTAACCGCGAACTGCACTTCCTT
CTCCCTTCTCCC
+
:>@1<,4594498=>A@>::00,.3445:6<<<=99:>>;>;48877+--+--
4.1199567688665=>7736060144428230042
@T6XXI:00136:00517
GGGAGAAGG
+
9@:111,-*
@T6XXI:00136:00524
GGGGAATTCTAATACGACTCACTATAGGGAGAGGGAGAATAGTCAACAAATCGTTTGGGGCGACTTCTCCTTCCTTT
CTCCCTTCTCCC
+
CCCAB::;<@@@DBCBC?@?>>;7A9995:A?CC>ABB=?>?BBA=?@A<CCCBB<B??6A???C?B@@<?<?=@@<
@@@A=A687AC@
@T6XXI:00136:00525
GGGAGAAGGGAGAAAGGAAGGAGAAGTCGCCCCAAACGATTGTTGAC
+
99-999599-4444-4231313366117;999,33,1611015,...
@T6XXI:00136:00530
```

```
AGTCGACTCGAGATCTGCGGCTTTGGACAGTCCCTCTGTACTGTGAAGCCACAGATGTCGAGTCGACTAGCTTCCTT
TCTCCCTCTCCC
+
6771/1:999;?A@>>;@@<999-
4777<<@;@.7666116166<C?CC?AACA@@=<<??888@;<9=847::,00,0/77,/-1110
@T6XXI:00136:00533
GGGGAATTCTAATACGACTCACTATAGGGAGAGGGAGAATAGTCTCGACTGTACATACGTGGGCCTGC
+
A@@4@598<AB@CCCBBCCCC?AAA?:?=?CCCB=BAB9:7../8/,0//.4411111116623/016
@T6XXI:00137:00514
GGGAGAAGGGAGAAAGGAAGAGAACAGCGACGGGAATAATGTTTTAGCTATTCTCCCTCTCCCTATAGTGAGTCGTA
TTAGAACTTCCCC
+
@B:999699-4444-
42377?B@?CC@>99933,4787133333,4;;;4:6444@899BBB>C@@@B96111116868;<<<<?;?AA6
@T6XXI:00137:00515
GGGGAATCTAATACGACTCACTATAGGGAGAGGGAGAATAGTCAACAAATCGTTTGGGGCGACTTCTCCTCCTTTCT
CCCTTCTCCC
+
8@868*1111:843::;7391317111,1<>@C@@AC@AAAA@;9444-7=777,111,17:/0-027,.-
,//,//44-323000+
@T6XXI:00137:00524
CTAGTCGACTCGAGATCTGCGGCTTTGGACAGTCCCTCTGTACTGTGAAGCCACAGATGTCGAGTCGACTAGCTTCC
TTCTCCCTCTCCC
+
/44444449999494444437733,3133666662633.....-
34=25:=AFDCBCAD?????@@;;8A;/.,/,,+,,+0*+----+
@T6XXI:00137:00535
GGGAGAAGGGAGAAAGGAAGGATAGGCAGCGTCGACCGAACGACAGACTATTCTCCCTCTCCCTATAGTGAGTCGTA
TTAGAATTCCCC
+
CCCCECCCB=>ABC@C?B?B=@@CD@@@BBCCBCCC@CC@CAAACBBCCCA<????<?CCCC<B?@@C@AD@??B@=
=?AAD<::?AA6
@T6XXI:00137:00540
CGACTCGAGATCTGCGGCTTTGGACAGTCCCTCTGTACTGTGAAGCCACAGATGTCGAGTCGACTAGCTTCCTTCTC
CCTTCTCCC
+
ABCCCCCDCABAAABA?99::<>ABDECCB>@@@@@@BACCAC@CC?BBCCCC?@?CBCCC@BA@@:/0,-+-+-
,,,*,*,...+
@T6XXI:00137:00546
GGGGAATCTAATACGACGTC
+
2=@1=+1///,/46/....0
@T6XXI:00137:00549
GGGAGAAGGGAGAAAGGAAGCTAGTCGACTCGACACTGTGGCTCACAGTACAGAGGGAC
+
/;;;;A>ACACBCC@B>@B;::::61??96677761110341169996748533346+00
@T6XXI:00138:00508
GGGAGAAGGGAGAAAGGAAGCTAGTCGACTCGACATCTGTGGCTTCACAGTACAGAGGACTGC
+
9B<==B?CC>A>@B<A=?<>?6796672003,,**00**,-2-.,1999>A<322-/,-4003
@T6XXI:00138:00513
GGGGAATTCTAATACGACTCACTATAGGGAGAGGGAGAATAGTCAACAAATCGTTTGGGGCGACTTCTCCTTCCTTT
CTCCCTTCTCCC
+
4>>4>5959;<>@@ADCACABBB?@:::57?@@B>BAA>AA?@AA><;;4===??<?DE9DCEAA>==:3838=AB@
BAAA9=;=?A@;
```

```
@T6XXI:00138:00522
GGGGAATTCTAATACGACTCACTATAGGGAGAGGGAGAATAGAGACGTACTCGTTTGCGCTCTGTTCGCCTTCCTTC
TCCCTTCTCCC
+
;@;2A5928449>@;>;@@@?:87=777-7>>>A=?AA>@@A@AAA===??><<=7<@@@@@AAA>@;?;878,/,-
--3+724:::;
@T6XXI:00138:00525
GGGGAATTCTAATACGACTCACTATAGGAGAGGGAGAATAGTCAACAATC
+
///*/,/,///79=AAA>88700/000,.4/67,603:5<;;4/,/,+,,
@T6XXI:00138:00528
GGGAGAAGGGAGAAAGGAAGGAGAAGTCGCCCCAAACGATTTGTTGACTATTCTCCCTCTCCCTATAGTGAGTCGTA
TTAGAATTCCCC
+
@C><9959919?@@9A=B>A?ABA?:;<CCCC9BC>BCAA<8A<8??>?==;>=;>:?889CAAAA????A@@A@??
?>A@@>?8>>>1
@T6XXI:00139:00501
GGGGAATTCTAATACGACTCACTATAGGGAGAGGGAGAATAGTCAACAAATCGTTTGGGGCGACTTCTCCTTCCTTT
CTCCCTTCTCCC
+
CB@7:245;AA@CCBBB@@@>?ABA9::57>??B>@AA?A??AAA??@>7>>=??9?CC8CCBAA>:887979/34-
4;;;7=38/893
@T6XXI:00139:00503
GGGGAATTCTAATACGACTCATTATAGGGAGAGGGAGAATAGTGACGTGTTGTGCGGCGCTCACTGTCTCCTTCCTT
CTCCCTCTCCC
+
<CB6A8<8;@@=@@BBA???7747>==?9?BBAA<AAA<??===A@>=<<9<>?AA?CA@A??>==99::243/,/,
/666,/-7454
@T6XXI:00139:00512
GGGAGAAGGGAGAAAGGAAGGGCTTAGAGCGATCGTGGGGGGAAAACTATTCTCCCTCTCCCTATAGTGAGTCGTAT
TAGAATTCCCC
+
AA<@@?;>>199@A9>=B?DG?DGBC@@?::<9AA?@@@@?,???4>BCDBDECD@CBCCDBDCBBBBCCC@@@BBB
?BAA>A>AAA6
@T6XXI:00139:00514
GGGGAATTCTAATACGACTCACTATAGGGAGAGGGAGAATAGTCAACAAATCGTTTGGGGCGACTTCTCCTTCCTTT
CTCCCTTCTCCC
+
CCB9@698;@@>CDCBCBBB??ABA???9?ABBD>CAA=A===AA=@@A<AAACE<AAA7?@@AA;??=/4247996
<<<A7?<??@@;
@T6XXI:00139:00515
GGGGAATCTAATACGACTCACTATAGGGAGAGGGAGAATAGTTAGCGCACTTCGGTG
+
7>@1<,94448887777;79676711116?@CD=<<>?>??=00/:44304754233
@T6XXI:00139:00535
GGGGAATTCTAATACGACTCACTATAGGGAG
+
@8800)/,///,/43:;:;786763...+,,
@T6XXI:00139:00536
GGGGAATTCTAATACGACTCACTATAGGGAGAGGAGAATAGTAGACAATCTT
+
@=94?6924999:@B@@@BBBBBBB???6;=66379:3444433400/1111
@T6XXI:00140:00501
GGGAGAAGGGAGAAAGGAAGGAGGAAGTCGCCCCAAACGATTTGTTGACTGATCT
+
4;:=?>@BB1;;44-/54393.0,.3...9:::6BC>@C:875776661-----+
@T6XXI:00140:00506
```

```
GGGAGAAGGGAGAAAGGAAGGAGAAGTCGCCCCAAACGAGTTTGTTGAC
+
/1,//4244-444;-467<<<>>C@?:;9;9926626111662670..-
@T6XXI:00140:00535
GGGAGAAGGGAGAAAGGAAGCTAGTCGACTCGAGATCTGCGGCTTTGGACAGTCCCTCTGTACTGTGAAGCCACAGA
TGTCGAGTCGACTAGCCTTCCTTCTCCTC
+
49144;284-
448904<;@CBBBB?711111166679666>266>88389?AA@@:?@@999BD>>?@9<<58/8887..-2---
33004:00/02323/000563
@T6XXI:00141:00500
GGGGAATCTAATACGACTCACTATAGGGAGAGGGAGAATAGATCACGACCCTTCTACCCACGATTGAGCTCCTTCTC
CCTTCTCCC
+
,8802*1///7/449333767798:?A>CBACD@C@@>@AA@>=@CCCCD9=:=6644,4;B@A?8491/,*,00,,
,*,*.4463
@T6XXI:00141:00510
GGGAGAAGGGAGAAAGGAAGCTAGTCGACTCGACATCTGGTGGCTTCACAGTACAGAGGGACTGTCCAAAGCCGCAG
ATCTCGAGTCGACTAG
+
?B><=<?AGBGCCC?BBB?BB@>;:000:99:0/.34..,.3327:@==@CB?B;;88818@;@?@:==9=A>A@??
@889?CAAB???;:::
@T6XXI:00141:00518
GGGAGAAGGGAGAAAGGA
+
785668,8819A888880
@T6XXI:00141:00529
GGGGAATCTAATACGACTCACTATAGGGAGAGGGAGAATAGTCAACAACTC
+
1::5?11///7683333776?B=>??>26<777-4::8:?640-,.-,--1
@T6XXI:00141:00549
CTGCGGCTTTGGACAGTCCCTCTGTACTGTGAAGCCACAGATGTCGAGTCGACTAGCTTCCTTTCTCCCTTCCTCCC
+
===BB@BBB=B=@@BBBBC8;;;;==??:7:D@BDADDGB@@;<:AABBCCCLCD<:::=:==67/0@61+,+***)
@T6XXI:00142:00525
GGGGAATCTAATACGACTCACTATAGGGAGAGGGAGAATAGTACAC
+
>@A5>11///7/44437;@>><88177-7??CE;B@<?8821--+*
@T6XXI:00142:00531
GGGGAATTCTAATACGACTCACTATAGGGAGAGGAGAATAGATCACCGTTGGTTCGGGTGCGCGTTCTCCTTCCTTT
CTCCCTTCTCCC
+
;>>1>,/,1//7;CFCCABECAA@B??=6:<00,.2234799869>===:<:=;ABB>BCC@???:89?:<:=477.
/668267/---+
@T6XXI:00142:00549
GGGAGAAGGGAGAAAGGAAGGAGAAGTCGCCCCAAACGATTTGTTGACTATTCTCCCCTCTCCCTATAGTGAGTCGT
ATTAGAATCCCC
+
/;;>=C?AA<@@@B=B@C@CG<<;?BCCCCBB7@?:896998<9=>@=559430000+000-
231...557AA@@??@;9422,--11.
@T6XXI:00143:00515
CTAGTCGACTCGAGATCTGCGGCTTTGGACAGTCCCTCTGTACTGTGAAGCCACAGATGTCGAGTCGACTAGCTTCC
TTTCTCCCTTCTCCC
+
>?9999444:?@@@BAA@@;;7;??:A9;;@A???<?AAACCBA@A@A>=A>ADBBBC;<<CCCBBBCA@DA;:5::
=?9?<<A7>;<<<=7
@T6XXI:00143:00516
```

```
GGGGAATTCTAATACGACTCACTATAGGGAGAGGGAGAATAGTTTTCTGTCATACGCAGGGCTATTCTTCTTCCTTT
CTCCCTTCTCCC
+
BB@4@:;8<888=?BDACFCAACA:;:B7:;EFF?CAA=@;4034-
4449=?@===@@@C>@??A>AA>8838;=;7887?:?8<6<?:
@T6XXI:00143:00523
CTAGTCGACTCGAGATCTGCGGCTTTGGACAGTCCCTCTGTACTGTGAAGCCACAGATGTCGAGTCGACTAGCTTCC
TTCTC
+
::444444:44498=88=>>?<888,7<>;?A?>C;>??????=>8?A>BDABBBB@ABCCCCCDCCDA@A=0.,0,
,+,00
@T6XXI:00143:00546
GGGAGAAGGGAGAAAGGAAGGAGAAGTCGCCCCAAACGATTTGTTGGACTATTCTCCCTCTCCCTATAGTGAGTCGT
ATTCAGAATTCCCC
+
=95=9>;>>:>8>@9@=?@C=@@B???@BBBB9CD<@?:11,.5,.,.5226;:<8>:@885=;==<=<88886532
33-..44459@??4
@T6XXI:00143:00548
GGGAGAAGGGAGAAAGGAAGCTAGTCGACTCGACATCTGTGGCTTCACAGTACAGAGGGACTGTCCAAAGCCGCAGA
TCTCGAGTCGACTAG
+
:C@CEC?CC@BCCE@C?B@A@??:00/00..://...111043658@BBCCABA?88818>@@@@@AC=AA=???@?
??????544==545=
@T6XXI:00144:00504
GGGGAATTCTAATACGACTCACTATAGGGAGAGGGAGAATAGTCAACAAATCGTTTGGGGCGACTTCTCCTTCCTTT
CTCCCTTCTCCC
+
DDC9D7:8<@@@CAAACAAABB>9@77308>>@B?CCC?AABCAA?AAA<CCCCC>BDC9CBBAA;?@@5:6:=@A>
@CAH=A====A<
@T6XXI:00146:00502
GGGAGAAGGGAGAAAGGAAGCTAGTCGACTCGAGATCTGCGGCTTTGGACAGTCCCTCTGTACTGTGAAGCCACAGA
TGTCGAGTCGACTAGCTTCCTTTCTCCCTCTCCC
+
44-
4AA@AB<@<<<8;>@>BC9::C@@?BBBAA@BB?:::?=7;;5:=?@BB???6===488<???@C=?????@A??=<
899@@<=A@@?8473/,//+-111+-----+
@T6XXI:00146:00523
GGGAGAAGGGAGAAAGGAAGCTAGTCGACTCGACATCTGTGGCTTCACAGTACAGAGGGACTGTCCAAAGCCGCAGA
TCCTAG
+
?A>BDCBCCAB@@@<@>A@BBBBAAAAA@@AB?B?::699988;:<:??CEBB??777.4;;=??>DC;@@=@@@A@
@@:<<?
@T6XXI:00146:00544
CTAGTCGACTCGAGATCTGCGGCTTGGACAGTCCCTCTGTACTGTGAAGCCACAGATGTCGAGTCGACTAGCTTCCT
TTCTCCCTCTCCC
+
444444444444499=9@<<<:10,/544699;;26611161....3559<====999B;>@A=@@@@><<731730
0,0344+....30
@T6XXI:00147:00517
GGGAGAAGGGAGAAAGGAAGGAGAAGTCGCCCCAAACGATTTGTTGACTATTCTCCCTCTCCCTATAGTGAGTCGTA
TTAGAATTCCCC
+
:BBA?>69909=>>1959<@>@AA@;898733,79699311,16011;99?=?=@@;@:9:DEC???988@B===@@
@==?C<A/744-
@T6XXI:00147:00549
GGGAGAAGGGAGAAAGGAAGGAGAATGCACCGCAGGAAATTGCAAGACTATTCTC
+
```

```
AB?CBB=>=9>999195949<@@A<;7=3759337:?A<>;440*,,,,,.,...
@T6XXI:00148:00506
GGGGAATTCTAATACGACTCACTATAGGGAGAGGGAGAATAGCTGTCTCTGAGTCGGGCCAATTATTCTCTTCCTTC
TCCCTTCCTCCC
+
:@@4F595944<ABA@@A@@@DCBBBCC>CBBBB>AAA>A@B>=61111163400313:458;86568669;/4//
---+-,.,-.-+
@T6XXI:00148:00536
GGGAGAAGGAGAAAGGAAGACGAATAGGCACGCGAGAAACATAAGCTATTCTCCCTCTCCCTATAGTGAGTCGTATT
AGAATTCCCC
+
;B::11-1-
,,,,*,*/,/6...9;;?:;@;777>333,3111777;4813377,11111,6>;>?@:8:633333/38:8=<===
5
@T6XXI:00148:00539
GGGGAATCTAATACGACTCACTATAGGGAGAGGGAGAATAGTTCGCCAATTCTGCGCGTAGCATTCTC
+
>8808,1///,/9@CC;<;A??;97<?;?<<<>89AA7:8@@@=AC?>:@-00.227///75.31376
@T6XXI:00148:00540
GGGAGAAGGGAGAAAGGAAGTGCAGTTCGCGGTTGGTGAAGGGAAAACTATTCTCCCTCTCCCTATAGTGAGTCGTA
TTAGAATTCCCC
+
ACBHDCDCCBCBBB=B@CA@A@GCCB?BC@>=@@C@@@@?BBABEE8AACD@@A@C=B@@@CAC@@@@??A??=@?8
834;B3858CC>
@T6XXI:00148:00541
CTAGTCGACTCGAGATCTGCGGCTTTGGACAGTCCCTCTGTACTGTGAAGCCACAGATGTCGAGTCGACTAGCTTCC
TTTCTCCCTTC
+
>@944999=BBCDCCBAA@@><@??:@=?>ABAAD6::::77?8?8:=>AC?BC@@<<<;;CBAABBA@??883163
0/+/..5,/,.
@T6XXI:00148:00549
GGGGAATTCTAATACGACTCACTATAGGGAGAGGGAGAATAGCAACTTTCCGCAAC
+
A??4?<@=ACC?BBCDB@A@A@?@AAAC=@?CCC>BBB=@A::0233303200101
@T6XXI:00149:00501
GGGGAATTCTAATACGACTCACTATAGGGAGAGGGAGAATAGCCCGAGACCATCCGGCATCCCTTCTGCTTCCTTTC
TCCCTTCTCCCATCACCGACTGCCCCAGA
+
DCB8A546999?GGCCD?@@CBBAB;;:8;BBCC>CBB@BBCBE:99???>@BBBEAB>;;<,0,.77/-
,0/00+044405274==;@@??>;8864....*--+
@T6XXI:00149:00534
GAATCTAATACGACGTCACTATAGGGAGAGGGAGAATAGTTCTCCCGAGTTTGAGTTGTCAACGCCGTCTTCCTTTC
TCCCTTCTCCC
+
,,*//4424444444449734444433,.6669.//453.1101111,187?A?>>=;6....,..6011163325638
74557///754
@T6XXI:00149:00536
GGGAGAAGGGAGAAAGGAAGGAGAAGTCGCCCCAAACGATTTGTTGACTATTCTC
+
:A=B?B?AC?BBAA=B?><@69:A<;;>=;9;57727776626677666..,.--
@T6XXI:00149:00546
GGGAGAAGGGAGAAAGGAAGGAGAACGCGCACCCGAACCAACGGTGATCTATTCTCCCTCTCCCTATAGTGAGTCGA
TTAGAATTCCCC
+
4959=?=@C@BBBC=B?B@B@BAAABA@@====:==<=>?69:9::44444424///+///..+..33667688>77
8;755,/,/660
@T6XXI:00100:00555
```

```
GGGAGAAGGGAGAAAGGAAGGGGAGAAGGGAGAAAGGAAGGGGAGAAGGGAGAAAGGAAGCTAGTCGACTCGACATC
TGTGGCTTCACAGTACAGAGGGACTGTCCAAAGCCGCAGATCTCGAGTCGACTAGCTTCC
+
DC@CCB@CCB?BBC?B?A?ACC8B@A>AA?CA@@;>>@?@@B7@>@>@@;@@??:@<=:<>::8:4000/---2--
4;?;;8342443046;?;>88713669<<;=<:<=;683856/--64003790..4..,.6
@T6XXI:00100:00560
GGGGAATTCTAATACGACTCACTATAGGGAGAGGGAGAATAGTCAACAAATCGTTTGGGGCGACTTCTCCTTCCTTT
CTCCCTTCTCCC
+
A?A7@<>=@><:=100>>>@CCCA?:67.6>?AC>A@@=@?AAAA=@AA=C???C>CCB5@BCCC8:8@3838=@@:
A=<<7<;=?@@@
@T6XXI:00100:00566
CTATCGACTCGAGATCTGCGGCTTTGGACAGTCCCTCTGTACTGTGAAGCCACAGATGTCGAGTCGACTAGCCTTCC
TTTCCTCCCTTCCTCCC
+
?887////889999;@A?>><877.;>AACBBBD>BAAACB@@=AAC>=B>@@@CCCCAB@A?BBB000:/,*,275
77+,+,5500*-,-233
@T6XXI:00100:00568
CTAGTCGACTCGAGATCTGCGGCTTGGACAGTCCCTCTGTACTGTGAAGCCACAGATGGACTGTCCACTTCCTTTCT
CCCTTCTC
+
CDBCCBBEDBCBCCBB?C>=;;..,1,0<:>>CC>CBBBBBB@@A@?=<@=@@AAAAB<@@AAA@@::9<@,11,1/
11+.,...
@T6XXI:00100:00573
GGGAGAAGGA
+
4911,,*,*,
@T6XXI:00101:00565
GGGGAATTCTAATACGACTCACTATAGGGAGGAGG
+
CCC9B>;:7//,//69<BD@?=>=....+,,*,00
@T6XXI:00101:00569
GGGAGAAGGGAGAAAGGAAGCTAGTCGACTCGACATCTGTGGCTTCACAGTACAGAGGACTGTCCAAAGCCGCAGAT
CTCGAGTCGACTACG
+
99149>;@C<AACC>B@C?BBC>??0//7.../66;?7:7;=;?=????DBA;;;//.0=;;;C>A@<@@>@@AA@=
<998>>?66574433
@T6XXI:00101:00571
GGGGAATCTAATACGACTCACTATAGGGAGAGGAGGA
+
BE>98*////,/8?;;<B??6;61116,1.//,--,-
@T6XXI:00101:00577
GGGAGAAGGGAGAAAGGAAGGAGAAGATACTGACGTGTGTGTGCCAGCTATTCTCCCTCTCCCTATAGTGAGTCGTA
TTAGAATTCCCC
+
66+119598-
///6+/,.,.3445599@==77989487;;<777>?;?@<<<<@BC>@@:::.7??8::?@9788663235326997
92
@T6XXI:00101:00586
GGGAGAAGGGAGAAAGGAAGGAGAACGCGCACCCGAACCAACGGTGATCTATTCTCCCTCTCCCTATAGTGAGTCGT
ATTAGAATTCCCC
+
49-499244-
////+/,.,.,.433>C@D@99919A@BCD?BC?BBBFDBBB@CCDG@;;;@?:=@@A???BB=?A@::574;4>29
99/
@T6XXI:00102:00553
GGGGAATTCTAATACGACTCACTATAGGAGAGGAGAATAGCCACTAGTCTCCCT
```

```
+
CBA00)/,/66,/9A@@@BBD=:::00,,7,0,.2201103/044000000403
@T6XXI:00102:00566
GCTAGTACGACTCGAGATCTGCGGCTTGGACAGTCCCTCTGTACTGTGAAGCCACAGATGGACTCGCTTCCT
+
96666///666@@@@@B@>>;00,0.,.,..4558?;555666455===;?B?AA??B//,.....44,101
@T6XXI:00103:00566
GGGGAATTCTAATACGACTCACTATAGGGAGAGGGAGAATAGTCAACAAATCGTTTGGGGCGACTTCTCCTTCCTTT
CTCCCTTCCTCCC
+
CDC9B=?=ACBBCCBAA>@>A@<77166.7><<?<?AA>@AA@@?<<<;6????B>BBC7??B??;???3838<@@?
A@;>6;./.;;6
@T6XXI:00103:00595
GGGAGAAGGGAGAAAGGAAGAGGACGTGGGAGTAGAGGAACATTGAGCTATTCTCCCTCTCCCTATAGTGAGTCGTA
TTAGAATTCCCC
+
@A<@>@;>>9>>?>098;C;;<BCCCCBB<=9;;BBC?B?BAB?BCCBB::6:=??6==8;;5<@@AAAA???=884
=;==?:;;<AA6
@T6XXI:00103:00596
GGGGAATTCTAATACGACTCACTATAGGGAGAGGGAGAATAGCACACGGTTTTCTCCCACCGGCGGGCCCTTCCTTT
CTCCCTTCTCCC
+
AA@4@54:<@@@DCBBC?@@B?><<777.7>77:7:@@>A@@??@@?;3444-
443;46;/4/334+484;=A>AA887;;-4/4000+
@T6XXI:00104:00556
GGGGAATTCTAATACGACTCACTATAGGGAGAGGGAGAATAGTCAACAAATCGTTTGGGGCGACTTCTCCTTCCTTT
CTCCCTTCTCCC
+
@@>4>5969AA>BCCCCCCABAA==766.7><<?<=AA<?=A??A<>>;:CA??B>CDD7;;B??:<=>3869?CD?
??@A<A:<=AC=
@T6XXI:00104:00571
GCTAGTCACTCGAGATCTGCGGCTTGGACAGTCCCTCTGTACTGTGAAGCCACAGATGTCGAGTCGACTAGCTTCCT
TTC
+
4444444444999>@=@A@??5884313367;@A=AA=?:><8734420;844>D@@???BBB:;<77376..,.44
4+.
@T6XXI:00104:00587
GGGAGAAG
+
:A87,,+-
@T6XXI:00105:00556
GGGGAATTCTAATACGACTCACTATAGGGAGAGGGAGAATAGTCAACAAATCGTTTGGGGCGACTTCTCCTTCCTTT
CTCCCTTCTCCC
+
>BB4=595999=>@@<;<ABCAB?=777.7=777-
734:=AA?8>>BDC=C@@?B<A@@5@???=:=>:2438;@@<@AAC?B698AA;
@T6XXI:00105:00571
GGGAGAAGGAGAAAGAAGGAGATAGCTCAACCACAAAACAGGTGGCTATTCTCCCTCTCCCTATAGTGT
+
9@5?99595/,,,*,,*,002,/7<977601011111,..5,--,.7/424488587333,361..4,,
@T6XXI:00106:00550
GGGAGAAGGGAGAAAGGAAGCTAGTCGACTCGACATCTGTGGCTTCACAGTACAGAGGACTGTCCAAAGCCGCAGAT
CTCGAGTCGACTAG
+
AA@ABA@CC<@@@>9>=?>BC???C::;?===?=???;???:=A?ABCBBBA995..-
.577;A>@@;@A?@@@@@>=@@@???===????
@T6XXI:00106:00552
```

```
GGGAGAAGGGAGAAAGGAAGCAGAATAGACCCGAGATTGTTGACGGCTATTCTCCCTCTCCCTATAGTGAGTCGTAT
TAGAACTTCCCC
+
44-444244-////+/,.,.6666:8?;66=-./.-
.,.45337757<9>>@99>674344+0446:@@?:::585<;=>@<<?5;99.
@T6XXI:00106:00560
GGGAGAAGGGAGAAAGGAAGGAGAAGTCGCCCCAAACGATTTGTTGACTATTCTCCCTCTCCCTATAGTGAGTCGTA
TTAGAATTCCCC
+
CK@AAC=@=8;9@B<@=@@B??@?=:78????6?C>CBBBB=BBACCAAA@:=<=?:?788@;@AAA===@@<;<<<
<;?@?=?<???5
@T6XXI:00106:00563
TCGAGATCTGCGGCTTTGGACAGTCCCTCTGTACTGTGAAGCCACAGATGTCGAGTCGACTAGCTTCCTTTCTCCCT
TCTCCC
+
@CBB<<;AAAA@?ADG@C?AABBB??>CBBBBBBC@@=@>BA=B???AAAACCBCCCCDCCC<<<59<>?:?>?>19
600<<8
@T6XXI:00106:00564
GGGGAATTCTAATACGACTCACTATAGGGAGAGGGAGAATAGCTGTCTCTGAGTCGGGCCAATTATTCTCTTCCTTT
CTCCCTTCTCC
+
9??4>5969@A?BBBCCCCCBBBBBBBCCBBB@@>@<<;===<@>?A????>@A;<=7<;==@=??<?<;98;244,
48830;5;?A<
@T6XXI:00106:00571
GGGGAATTCTAATACGACTCACTATAGGGAGAGGGAGAATAGTCCTCCTTTCCCGATGCACCGACGTACGCTTCCTT
TCTCCCTTCCTCCC
+
AB@4A8;8<BB?CE@?BCCCBA@;=767.7><<<9?@@>ABB@A=?:37718A6<<?@@;757,..5110,,*-
2224755444-/-.193
@T6XXI:00106:00573
GGGGAATTCTAATACGACTCACTATAGGGAGAGGGAGAATAGCGATGTGCCGGTACCTGTCGCGTTCTCCTTCCTTT
CTCCCTTCTCCC
+
CDN:C::;<CC@C@@@BAAAB??;;667.7>???<C@@>@AA???==<@@<><?<<9<A@A@@@A>A??;;24244-
4<DC<@=8888-
@T6XXI:00106:00595
GGGAGAAGGGAGAAAGGAAGGAGAAGTCGCCCCAAACGATTTGTTGACTATTCTCCCTCTCCCTATAGTGAGTCGTA
TTAGAATTCCCC
+
BKCECC@BB<>@@C<@=?>A=;;:>??@ACBB4;;6:@A=>;BC?@@@AAA=??AD=@=?=A<??A?=<<@<<=A@A
@>@@@=@>ABB5
@T6XXI:00106:00599
GGGGAATTCTAATACGACTCACTATAGGGAGAGGGAGAATAGTCAACAAATCGTTTGGGGCGACTTCTCCTTCCTTT
CTCCCTTCTCCC
+
:>=5;,45<>><B@@<;<FCCBB@?7:7.7>7775:<<?@@@954:<:8:B7617,166,6;447-//4-.-
/99;9;:?A<A;=9AA<
@T6XXI:00107:00592
GGGAGAAGGGAGAAAGGAAGCTAGTCACTCGAGATCTGCGGCTTTGGACAGTCCCTCTGTACTGTGAAGCCACAGAT
GTCGAGTCGACTAGCTTCCTTCTCCCT
+
A@<@@A?BB<@<;;7;>B>AAABBA@??88363;896@@A5::B.76:??>@AB688>888?????B>@A=@@????
788?<<<AA?===?:855+,+,***)*
@T6XXI:00108:00552
GGGAGAAGGGAGAAAGAAGTGCACGTTGCATGTGGAGAGAAGGAAGCTATTCTCCCTCCTCCCTATAGTGAGT
+
9>5999599-////+/.,...44///,////9=B@EA===6424884344247797331366661111..4--
```

```
@T6XXI:00108:00561
GGGGAATTCTAATACGACTCACTATAGGAGAGGGAGAATAGCAAACGTTCCCGACATCGATCTGTACGCTACGTACG
TCTGAGCATCGATCGATGT
+
>==4>6959>><AB@@@:;;B>?<>00,0<0775:<B<@???BB9;773//,-:/...---
.3.43=:8?87355334=8?<>>68..33622:.+
@T6XXI:00108:00594
GGGAGAAGGGAGAAAGGAAGCTAGTCGACTCGACATCTGTGGCTTCACAGTACAGAGGGACTGTCGAAAGCCGCAGA
TCTCGAGTCGACT
+
CC@DCB@BB@DBBB=?=?@BB?:::/11>>>=>??;::@A@8<??A???BB?=>>77717;;>789?A<AC>@@@@A
@A???===@@@@@
@T6XXI:00108:00597
GGGAGAAGGGAGAAAGGAAGTGCAGTTCGCGGTTAGTGAAGGGAAAACTATTCTCCCTCTCCCTATAGTGAGTCGTA
TTAGAACTTCCCC
+
CDBBC@;==<=>@@0958;B@@BCCB@BBAA=@=@CBBA@CC>CCC8B@@?=@??=.77777,3>>@@@AA@?=;<4
424;;7993444,
@T6XXI:00110:00559
GGGAGAAGGGAGAAAGGAAGGAGAAGTCGCCCCAAACGATTTGTTGACTATTCTCCCTCTCCCTATAGTGAGTCGTA
TTAGAATTCCCC
+
AA<ABB@CC>CDCC>C@C@B@BC?A?><BCCC7BA>BBBCB>CB?BBBA@@=?@@CA?<<<C=AB@@?@@AA<<<?<
<9=AA;A9<<<4
@T6XXI:00110:00562
GGGGAATTCTAATACGACTCACTATAGGGAGAGGGAGAATAGTCACCAAACCTCCCATGGTGCTATTGCCTTCCTTT
CTCCCTTCTCCC
+
BBB7A7;8<CCBCBA@BBBB?AB>966659=@BD>@@A>A=@@?=B377149;;9:=;<9<>@BBA=@B6:7:@CDA
DCCC>@9<<>?;
@T6XXI:00110:00586
GGGGAATTCTAATACGACTCACTATAGGGAGAGGGAGAATAGTTTTCCTTCACTAACCGCGAACTGCACTTCTTCTC
CCTTCTCC
+
8>=17+1,/667843:;:>>@9<;6116,7777758>@?BA@@?=1=8;79?<773647700/06837::///33-
30+328;;9
@T6XXI:00111:00559
GGGGAATTCTAATACGACTCACTATAGGGAGAGGGAGAATAGTCAACAAATCGTTTGGGGCGACTTCTCCTTCCTTT
CTCCCTTCTCCC
+
GCB7A698;@@CDCCBB?>?@BB=BAAA<?@ABB>@@@>@=A?@@=@@A<BA???9?>@5@@BBB7:8;3859AAA?
>>@C<@9<<AD?
@T6XXI:00111:00560
GGGGAATTCTAATACGACTCACTATAGGGAGAGGGAGAATAGATCACCGTTGGTTCGGGTGCGCGTTCTCCTTCCTT
TCTCCCTTCTCCC
+
CCB9B=>=@A@@CBBBB@BBCCB?=777.7?>>@?AAA?A@===?AA>BC=?=??BBC>CBBBCCD@D@?=?=@=@E
@?==C>C@<=9=9
@T6XXI:00111:00594
GGGAGAAGGGAGAAAGGAAGGGGAGAAGGGAGAAAGGAAGCTAGTCGGACTCGACATCTGTGGCTTCACAGTACAGA
GGACTGTCCAAAGCCGCAG
+
4919996990999A1958;?>>2>?>:>>6777>:>:?:?@;???::5:=49:8878408>>:?@<===A@@@@787
;8:;;4447:9444249;;
@T6XXI:00112:00591
GGGGAATTCTAATACGACTCACTATAGGGAGAGGGAGAATAGCCTAACGAACCCAACGATCACAGCTATCTTCCTTT
CTCCCTTCTCCC
```

+
CCB7@5459>><A@@@CCCCCCCBB???9?==??<A?@>@A@@<AB@@?<?CD=A=@BBB@<==?@A@=5323/00+
0333+0/089<;
@T6XXI:00113:00560
GGGAGAAGGGAGAAAGGAAGCTAGTCGACTCGACATCTGTGGCTTCACAGTACAGAGGACTGTCCAAAGCCGCGATC
CGAAC
+
44-449599-
4:>@0948433>>>@???@AA::;A@?AB?=;=C@BBECBB@===8838@@@AC>@@;??<====8738@9<
@T6XXI:00113:00594
GGGAGAAGGGAGAAAGGAAGGAGAACGCGCACCGAACCAACGGTGATCTATTCTCCCTCTCCCTATAGTGAGTCGTA
CTTAGAATTCCCC
+
9>:999599-4449-42313773124@@@11/,/6,/,/59:>=9=@9999@BBBB;37333,11.....33-
11111103444:2444,
@T6XXI:00114:00550
GGGGAATTCTAATACGACTCACTATGGAGAGGGAGAATAGTCACAATTCGTTGGGCGACTCTCTCTT
+
=@>4>696;@A?B@@@CCBCC=@=00,09/775:AA>?:39///+*,*/..344087733......5
@T6XXI:00115:00568
GGGGAATTCTAATACGACTCACTATAGGGAGAGGGAGAATAGTTTTCCCTTCACTAACCGCGAACTGCACTTCCTTT
CTCCCTTCTCCC
+
AA@4@6969AA>BDCBBBBB?@CEB=??;?BBCC>B@A?940000+44,49@CDCG?B?A<?=><<<:?<9773165
7:;A7?<?@?=:
@T6XXI:00115:00570
GGGGAATTCTAATACGACTCACTATAGGGAGAGGGAGAATAGTCAACAAATCGTTTGGGGCGACTTCTCCTTCCTTT
CTCCCTTCCTCCC
+
A??7?;@=@CC@CB@@BBBB?AB>9666-
6===?<A>@>A@@@@A@AAA:AABBC>CCC7?@CCC>AA@9<8<<@@<?;;739,/,/882
@T6XXI:00115:00576
GGGGAATTCTAATACGACTCACTATAGGGAGAGGGAGAATAGCTTTCCTTCGCGTGGCAACAGTTATTCCTTCCTTT
CTCCCTTCTCCC
+
BB@4@2459889B@AACBBCBA>==777.7?==?=@AA?A>>767.74744;477=;=?589A@>@A??;=:;344,
4833055:;=C<
@T6XXI:00115:00586
GGGGAATTCTAATACGACTCACTATAGGGAGAGGGAGAATAGTCTCCCACGGTAGGCATCGCTGCGGTCCTTCCCTT
T
+
/11*/,9244499?@ABAA;::B???BC>CCCCF@C==;<888877;:71.,,,,*,,,,01599<89;660./,//
+
@T6XXI:00115:00590
GGGAGAAGGGAGAAAGGAAGGCGAAGAGCCCACGACGTATATCGGGACTATTCTCCCTCTCCCTATAGTGAGTCGTA
TATGAACTC
+
9@:999584-49::-4231313<>>A@::@:??><00.//111996454--,-133344:::489;<?A=A543---
-+++34../
@T6XXI:00116:00556
GGGAGAAGGGAGAAAGGAAGAGTAGTCATTACGTTATGAGGGTTGGACTATTCTCCCTCTCCCTATAGTGAGTCGTA
TTAGAATTCCCC
+
AE@AAB@CC=;<<B>B?B@CA@?CFCCBB?AA;86;??@AD?CBCBCB<<;@B@@A<999@@;@???ABDAA@AA@@
@=@@C?C@CCD:
@T6XXI:00116:00567

```
GGGAGAAGGGAGAAAGGAAGGAGAACGCGCACCCGAACCAACGGTGATCTATTCTCCCTCTCCCTATAGTGAGTCGT
ATTAGAATTCCCC
+
CC>BCA@AA=AAAA;A=?>?>?>;;BBBBBECC>CCAC@CADC@CCEB@@@@=@A@A=@?@BB>ACCCBBBCA?A@A
AC@ACC?C@@@@4
@T6XXI:00117:00554
GGGAGAAGGGAGAAAGGAAGGAGAAGTCGCCCCAAACGATTTGTTGACTATTCTCCCTCTCCCTATAGTGAGT
+
?B<?@CBKDABBBB?B@B>>>@977:AAACCC9CC>AAALE>??;??A=>@>AAAC<>>773+4;;;;;71**
@T6XXI:00117:00555
GGGAGAAGGGAGAAAGGAAGCAAGGAATTGTGTTGTGAGGATAGAGACTATTCTCCCTCTCCCTATAGTGAGTCGTA
TTAGAATTCCCC
+
DC@CBA@AB=BBBB@B@B?AAA>>=A?C@CB@B??<<<=;=999;;:====886884??@ACAA?>>AAAAA@@@??
?>@??>BCBEH:
@T6XXI:00117:00562
CTAGTCGACTCGAGATCTGCGGCTTTGGACAGTCCCTCTGTACTGTGAAGCCACAGATGTCGAGTCGACTAGCTTCC
TTTCTCCCTTCCTCCC
+
@:1111//99;;9<;BBBA@@=@BB>B?BBCCCCC<BBB??@@=AA@@<?BABBABAB@????B?@@@??@::7;?@
CE>B>;928,/,///,
@T6XXI:00117:00567
GGGAGAAGGGAGAAAGGAAGGAGAAGTCGCCCCAAACGATTTGTTGACTATTCTCCCTCCTCCTA
+
BC?CA@=@@<@@CB>B=@=@>@AA??@@CBBB8AB<?98888899<><<:;8..-10--,--,--
@T6XXI:00117:00568
GGGAGAAGGGAGAAAGGAAGCTAGTCACTCGACATCTGTGGCTTCACAGTACAGAGGGACTGTCCAAAGCCGCAGAT
CTCGAGTCGACTAG
+
BB>BBB@CC?DBCC<@=@?BCCBBB?;::B=;;8::7AAC=@A@BCCB??@<<=33317>>?@@=?C>CC;==AA==
<?<<>>A=<<?><<
@T6XXI:00117:00571
GGGGAATTCTAATACGACTCACTATAGGGAGAGGGAGAATAGCCATCACCCCGTTAAACGCGCAATATTCTTCCTTT
CTCCCTTCTCCC
+
A@@/95959@C>CDAA@@@AABBAAAAA<ACCCC>A@A>@@==::994444-
4434;7BBBBBBADBC?@?;?>BB>CCDC<A?;;9H?
@T6XXI:00117:00574
GGGGAATTCTAATACGACTCACTATAGGGAGAGGGAGAATAGTTTGCGCATTCGGTGCACTGTTATTCTCTTCCTTT
CTCCCTTCTCCC
+
999/48>=ACCCCB@@>>BB??BABAAA>ABCCC>CAA>A?::;:::9<<B@BA?AABBCCCC@A?=ABBB@C?BB>
@AAA:;:<BBC;
@T6XXI:00117:00576
GGGGAATTCTAATACGACTCACTATAGGGAGAGGGAGAATAGTTTTTCCCTTCACTAACCGCGAACTGCACTTCCTT
TCTCCCTTCTCCC
+
@@>4><@=@BC@BBA?B?>>??BA@@@?;AAACC=A<<:?8;;;;;09@7?<?CCBB@B?AB?C>@@??BAB:;367
.7;;;:=:<<<@:
@T6XXI:00117:00589
GGGAGAAGGGAGAAAGGAAGGAGAACAACACGATCGGTTAAACGTAGCTAGTTCTCCCTCTCCCTATAGTGAGTCGT
AATTAGAAC
+
;@:A@>594194?D01,044,...5//,1=44484424777233.,00,***00000),/3553.4333366444--
-,-012443
@T6XXI:00117:00591
```

```
GGGAGAAGGGAGAAAGGAAGGAGAAGTCGCCCCAAACGATTTGTTGACTATTCTCCCTCTCCCTATAGTGAGTCGTA
TTCAGAATTCCCC
+
//+//9249-888:-423771336999?A?BB7??9?;<A<8AB7::?6598;;<;:>887>:;47784/67//-
003+..99995;;;.
@T6XXI:00118:00567
GGGGAATTCTAATACGACTCACTATAGGGAGAGGGAGAATAGTCAACAAATCGTTTGGGGCGACTTCTCCTTCCTTT
CTCCCTTCTCCC
+
CCB7A89;<BB@CBBABBBB?@B??77646??AA>AAA>@8AAAA;<@<<BBB>@>?@?7@BDBB?BBA6888<AA<
?@@A=A;=>AA<
@T6XXI:00118:00578
GGGAGAAGGGAGAAAGGAAGCTAGCGACTCGACATCTGTGGCTTCACAGTACAGAGGGACTGTCCAAAGCCGCAGAT
CTCGAGTCGACTAG
+
DC@AA@@BB>BBBB>A?A@BA><>>@@CCAA88:CCBBBC@BB?B@C@??@===99999==A==<>><??>@A99=@
AA@@@A@??<=<<>
@T6XXI:00118:00595
GGGAGAAGGGAGAAAGGAAGCTAGTCACTCGACATCTGTGGCTTCACAGTACAGAGGACTGTCCAAAGCCGCAGATC
TCGAGTCGACTAG
+
BC?BCC@CB=B@==:@>>>BB???@??7:64765>>6?8?:??>@BB@@@C@@A==;=CCAAB?@@<AA@===@CC9
99<==?;6<<<==
@T6XXI:00119:00550
CTAGTCGACTCGAGATCTGCGGCTTTGGACAGTCCCTCTGTACTGTGAAGCCACAGATGGACTGCTTCCTTTCTCCC
TTCTCCC
+
CCCDDDBCDBBBCCBBAB@??<778/8;>>=@ABB>BBCCBBA?<==@=@BABACDB@@=?@>>=>:=477.7<<?.
7370344
@T6XXI:00119:00561
GGGAGAAGGGAGAAAGGAAGATGACGCAGACG
+
BC>CCC??=6>:996<@C@CCC;77**(((((
@T6XXI:00119:00565
GGGAGAAGGGAGAAAGGAAGGAGAATTTGGCGTCGTGACGTGCGTGGCTATTCTCCCTCTCCCTATAGTGAGTCGTA
TTAGAATTCCCC
+
44-949284-
488A0;6:=@@CCC@C@>B>ABCCC::<BBA@BCGG@A@AC=A;=>:131>>=>>:;8BBBB<<;CBB?ACC@CAEI
DC
@T6XXI:00119:00581
GGGAGAAGGGAGAAAGGAAGAGAAAGCGCAGACTAGTGACAGTCAGGCTATTCTCCCTCTCCCTATAGTGAGTCGTA
TTAGAATTCCCC
+
AE@CCC@BC@BBBB>B@B@B>>>>;>BBBB?@@BBCBBCCCAAA==>==8@>;<:303303436;<6@AA???===8
858??>@:8<>4
@T6XXI:00120:00550
GGGAGAAGGGAGAAAGGAAGCTAGTCGACCCGACATCTGTGGCTTCACAGTACAGAGGGACTGTCCAAAGCCGCAGA
TCTCGAGTCGACTAG
+
NC?@A@>?B=@@@C<A?@@CCC@@@;<<CE>BAAAFD;;;C@?@>@BBBCABABA77717??AA??AA:AA:??@AA
A9==A@@AABAA@@A
@T6XXI:00120:00568
GACTCGAATCTGCGGCTTTGGACAGTCCCTCTGTACTGTGAAGCCACAGATGTCGAGTCGACTAGCTTCCTTTCTCC
CTTCTCCC
+
```

```
CCCDBBB@DBBCBB?BEEAC>AACCCC?9BBCB><=B@@@@>AA?CCA????@CBBBCCBABBCBAA=@=@@<@BBB
<@8:<CC>
@T6XXI:00120:00586
GGGAGAAGGGAGAAAGGAAGTGCAGTTCGCGGTTAGTGAAGGGAAAACTATTCTCCCTCTCCCTATAGTGAGTCGTA
TTAGAACTTCCCC
+
AC@CCB@CB>B@@@<@@C@BBABDCC@B@@@<?<@BBBC@CC>BBB9BBCC?BBDC<?????9<??AAAA@A@?><;
;8<;;8;=8;;;1
@T6XXI:00120:00596
CTAGTCACTCGAGATCTGCGGCTTGGACAG
+
99444444444444886<00,,,+,0-,,.
@T6XXI:00121:00551
GGGGAATTCTAATACGACTCACTATAGGGAGAGGGAGAATAGCTCTCCGGTCTGCCGTTTAGCTCTCGTCTTCCTTT
CTCCCTTCTCCC
+
?>@4?6;;<BB@CCBBBBCAB@BAA???;?BBCC>A@@>?<@=A=?=;>;=887>:>;;6;/./;//4@8738;@>;
A;;;57.//886
@T6XXI:00121:00575
CTAGTCACTCGAGATCTGCGGCTTTGGACAGTCCCTCTGTACTGTGAAGCCACAGATGTCGAGTCGACTAGCTTCCT
TTCTCCCTTCCT
+
::944999:B>>?GEAA@BA=711.8>?>ABBCB=BC@B@CA@;???<>B>BAABB;:;B???:<;@@;>?888:@E
E>C978,/,/,*
@T6XXI:00121:00585
CTATCACTCGAGATCTGCGGCTTTGGACAGTCCCTCTGTACTGTGAAGCCACAGATGTCGAGTCGACTAGCTTCCTT
TCTCCCTTCTCCC
+
BBBB@?@C??@CCCCBBAA>ABB<B@CABACCB>BB??????<==@>@C@CCBBCCCCAAABABCBAAA?:62:=@A
<A=>>6<7;===:
@T6XXI:00121:00596
GGGGAATTCTAATACGACTCACTATAGGGAGAGGGAGAATAGTCAACAAATCGTTTGGGGCGACTTCTCCTTCCTTT
CTCCCTTCTCCC
+
AAA6A?A?BBB@CBBBBBDB?AB?9666-
6==?A=AAA>AAAABB>@@@:BBCCC>BBB9C???C@CCA8888>AA=>>?C<A<=?AC>
@T6XXI:00122:00562
GGGAGAAGGGAGAAAGGAAGGAGAAGTCGCCCCAAACGATTTGTTGACTATTCTCCCTCTCCCTATAGTGAGTCGTA
TTAGAATTCCCC
+
EE?BBB?BB?BBBE?C@C?@>:::=>>?8888,1769@@BA?CC=@@AB@@>@A@@<A===C<@A@@???A@@@@88
8=@?><?>@@@5
@T6XXI:00122:00583
GGGAGAAGGGAGAAAGGAAGAATGCGTTAACGGTGCCAAACAGGGAACTATTCTCCCTCTCCCTATAGTGAGTCGTA
TTAGAATTCC
+
FC@CCB@BCBBBBC>B>A7;;@B@BBF@CBGD@CCC@GC?C;;;:;>?B@A<A@>>:77111,066:;=A@?=;9:3
/,--+*+000
@T6XXI:00122:00584
GGGAGAAGGGAGAAAGGAAGCTAGTCGACTCGACATCTGTGCTTCACAGTACAGAGGGACTGTCCAAAGCCGCAGAT
CTCGAGTCGACTAG
+
?B>DCCCCC>C@@@=A@CBCCD??@1//;559;<??;6?63368988:?A<=9?78814;;=@@>@A<@@>AAAA==
=CA=<<==8;7333
@T6XXI:00122:00588
GGGAGAAGGGAGAAAGGAAGCTAGTCACTCGACATCTGTGGCTTCACAGTACAGAGGGACTGTCCAAAGCCGCAGAT
CTCGAGTCGACTAG
```

```
+
9914>>;@@<@@>><B>@=AAA<AA9@@@@>;87AAAAAB=@@@CCCCBB@@=:33317>>@AA=?A:AB=A@<<<?
=@@@@@???===>?
@T6XXI:00123:00550
GGGGAATTCTAATACGACTCACTATAGGGAGAGGGAGAATAGTCAACAAATCGTTTGGGGCGACTTCTCCTTCCTTT
CTCCCTTCCTCCC
+
499/95924889=@@@DC@@@AB;<777.7A>@A?AAB>???B@@=?AD?E@@@C>BCC7;:AA@:==934248;=7
?@;939,/,///,
@T6XXI:00123:00553
GGGGAATTCTAATACGACTCACTATAGGGAGAGGGAGAATAGTCAACAAATCGTTTGGGGCGACTTCTCCTCCTTTC
TCCCTTCTCCC
+
BB?2:24599>9@@@@CBBDCCA=?996.7=76:88AA>AAA@A@=?>=7@?==>.777.7<?@>11/9-
/.5599;;?A<A394??:
@T6XXI:00123:00568
GGGAGAAGGGAGAAAGGAAGGGAGAAGGTAG
+
CC>BB?@BB>BBBB>B?A@88888233,,,,
@T6XXI:00123:00572
GGGGAATTCTAATACGACTCACTATAGGGAGAGGGAGAATAGCCCTTCACTCCGGACTGTATAGCGCAACTTCCTTT
CTCCCTTCTCCC
+
CBB7A=@=@CC@CCBA>>BB?AA??77616A??C>BAA?A<::::<<A>??A9=:=@@@A@@AAA?=@=B?=?:??9
?@BB>A<@?<<6
@T6XXI:00123:00582
GGGAGAAGGGAGAAAGGAAGCGCAGGTTAGCGAAGCCGAGGAGATAGCTATTCTCCCTCTCCCTATAGTGAGTCGTA
TTAGAATTCCCC
+
BC@CCB@BB>CBBB?B?A?@@?>>>>>;???@B@BB@BAA?ABBCCBBBCDBGGDC>AA@??<?BBCBCCC@@CCC@
=;=AA7>6888.
@T6XXI:00123:00587
GGGGAATTCTAATACGACTCACTATAGGAGAGGGAGAATAGTCTTGTATTTGTGCATCACTTTTCTCCTTCCTT
+
9=929292488?B@ADD@@@B;::;00,/::<;8;><:6;;8//,/-.00+0447177<<333.3432222,-,
@T6XXI:00123:00591
CTATCGACTCGAGATCTGCGGCTTTGGACAGTCCCTCTGTACTGTGAAGCCACAGATGTCGAGTCGACTAGCTTCCT
TTCTCCCTTCTCCC
+
B>=:111:::=B<<<AA@8;4;=?9??AACBBCB>CAA>>><==@@@<?B@CBB@@@BBBBBC@A@BBABB@@=A@D
E>A<<;-43449:7
@T6XXI:00123:00593
GGGAGAAGGGAGAAAGGAAGGAGAAGTCGCCCCAAACGATTTGTTGACTATTCTCCCTCTCCCTATAGTGAGTCGTA
TTAGAATTCCCCC
+
8?;>BB@CD@BBBB<?<?>B=:;:?BBCGBCC9CC?CC??:8898?@=AAA=@=<;6:444;7;;=:===AAAA@==
=><::24/////+
@T6XXI:00124:00558
GGGGAATTCTAATACGACTCACTATAGGGAGAGGGAGAATAGTCAACAAATCGTTTGGGGCGACTTCTCCTTCCTTT
CTCCCTTCTCCC
+
999/42959@@>BBCDCECE::;CBBBB>BCCCC>A@=;<<@?@@>@@@;BCCCC>CCC7BBBBB=?=@3838=?@:
?<<<6;5;5>?:
@T6XXI:00124:00570
GGGAGAAGGGAGAAAGGAAGCTAGTCGACTCGACATCTGTGGCTTCACAGTACAGAGGGACTGTCCAAAGCCGCAGA
TCTCGAGTCGACTAGCTTCCTTTCTCCCTCTCCC
+
```

```
@@:>>@<@A19999042847:144:0.......6699<99=55?:?@@AAA@?@@???:?@@A@A?AB:@@?BAA@@
@==>@@@@==844499979/44+3000+003353
@T6XXI:00124:00574
GGGAGAAAGGAGAAAGGAAGAC
+
9>:?111-1-////+/699333
@T6XXI:00125:00562
GGGGAATTCTAATACGACTCACTATAGGGAGAGGGAGAATAGTCAACAAATCGTTTGGGGCGACTTCTCCTTCCTTT
CTCCCTTCTCCC
+
CBB7A798;AA?CBBBAACCC@CBBBCC>>BAB@>?@@>B<A<@@=@C=;?B>BB>CCC6BBCBB?<A>8;8;8;86
787@<C:<<AD=
@T6XXI:00125:00567
GGGAGAAGGGAGAAAGGAAGGAGAAGTCGCCCCAAACGATTTGTTGACTATTCTCCCTCTCCCTATAGTGAGTCGTA
TTAGAATTCCCC
+
CC@CBA?BB>CCCB@C@C@B?BB@>@<>?BDD7??;BB@BCACB?>B?C??<>@CC>@?@@@<@AAA@@@AA===AA
A>@@D>@<>>>4
@T6XXI:00126:00554
GGGGAATTCTAATACGACTCACTATAGGGAGAGGGAGAATAGCTAGTCACTCGACATCTGTGGCTCACAGTACAGAG
GGACTGTCCAAAGCCGCAGATCTCGAGTCGACTAG
+
AAA7A89;<CCABBBCCBBBA??::;BB>BCAAAA@@A<=<<=:::B@???A?8880005695//----
350:8>>>?<?AAA@@>@C<@@>@<<;=;;;<887;??BAA?@
@T6XXI:00126:00560
GGAGAAGGGAGAAAAGGAAGCTAGTCGACTCGACATCTGTGGCTTCACAGTACAGAGGGACTGTCCAAAGCCGCAGA
TCTCGAGTCGACTAGCTTCCTTTCTCCCTCTCCC
+
A:A>>;>>9?8999,4999;CC;;;0000/......4...1,-
.59778CGAAAA@@?:?<<<=<>@A5889<=:<<@877>>@?<;;=994448;7:<7<@@A6;:444-
@T6XXI:00126:00572
GGGGAATTCTAATACGACTCACTATAGGGAGAGGGAGAATAGTCTTGTATTTTGTGCATCACTTTCTCCTTCCTTCT
CCCTTCTCCC
+
CCB:D<<;<CC?BBA99;BCBAA?@:::5:@CCD>@@@>@?:67424;4444+44477776603,0423322-.-
.2434?::444-
@T6XXI:00126:00573
GGGGAATTCTAATACGACTCACTATAGGGAGAGGGAGAATAGTCAACAAATCGTTTGGGCGACTTCTCCTCCTTTCT
CCCTTCCTCCC
+
9>>4>695988999<BABBD@?@AB777.7?77;7:@@<=888;;79894=616=-
71,1757:::75...43737/33,4242/640
@T6XXI:00127:00556
GGGAGAAGGGAGAAAGGAAGGAGAAGTCGCCCCAAACGATTTGTTGACTATCTCCCTCTCCCTATAGTGAGTCGTAT
TAGAATTCCCC
+
9>:>>>;>@<@@ACBB?B?B9;;:?BABBBBB8BC?C@???>>@>;?@7///75557//5=;=4::===@A?@?@>=
:=AA?@<@CC6
@T6XXI:00127:00569
GGGAGAAGGGAGAAAGGAAGGAGAACGCGCAATCAGATACCTGTTGGCTATTCTCCCTCTCCCTATAGTGAGTCGTA
TTAGAATTCCCC
+
DE@BA@<==<9>@@=B?A@DBBBAACBBCCC@CCAAABCC@>;<,1-
1<<<;>===:7;;BD@BED@ABCBBBFFB:;;;:A=A7;99/
@T6XXI:00127:00573
GGGAGAAGGGAGAAAGGAAGAGCAATTGGGTTCTGGAGATTTTGGGACTATTCTCCCTCTCCCTATAGTGAGTCGTA
TTAGAATTCCCCC
```

```
+
99199959=5=9?@<A=?=;;<BBAC@BB?B?DCDCCB;999/9@:AABDC?AA@C<A@@?<89@AB?<=??<<=??
>;64423/0344,
@T6XXI:00127:00582
CTAGTCGACTCGAGATCTGCGGCTTTGGACAGTCCCTCTGTACTGTGAAGCCACAGATGGACTGTCTTCCTTTCTCC
CTTCTCCC
+
CCCCCCBCCDDCCBBDBBB;;7<776>=@ABCBBD@DBAAABCA<AAA?=A=AEICC@B>@ABB???;@<>>:>===
9?A?<=D8
@T6XXI:00127:00583
GGGGAATCTAATACGACTCACTATAGGGAGAGGGAGAATGAGCCGGCACCAGCGTT
+
9>>2?69444244788:;79?B8//77.//;75+.+-,--1--,-,-002346865
@T6XXI:00127:00587
GGGGAATCTAATA
+
444,/,///,22,
@T6XXI:00128:00557
GGGGAATCTAATACGACTCACTATAGGGAGAGGAGATATAGG
+
>AA1@+1///7/99>;<?967<:667?8:?7717,,+++04/
@T6XXI:00128:00562
GCTATCGACTCGAGATCTGCCTTTGGACAGTCCCTCTGTACTGTGAAGCCACAGATGTCGAGTCGACTAGCTTCCTT
TCTCCCTTCCTCCC
+
444;=9>?CBCBBECBBA@?488,4249;?BBB>BDBCBBBBCBBA??@;=<BBBAAACCBB;==<<;B99989@BB
8;/4464,/,../+
@T6XXI:00128:00582
CAGTCCCTCTGTACTGTGAAGCCACAGAGGGACTGTCCAAAGCCGCAGATCTCGAGTCGACTAG
+
DCBBBB?CCBA>@@CBBBCAAA?BA>>777.7AAABCAEDBCCBCCEBBBABBDCDBAAACCBC
@T6XXI:00128:00584
GGGGAATTCTAATACGACTCACTATAGGGAGAGGGAGAATAGCTACGTTTTAACCGATCGTGTTGTTCTCCTTCCTT
CTCCCTTCTCCC
+
444-9595;BBACBA?BBBB@CB?>88888?ABB>@??<?@A896;644404?C@@?@<<=A@<7858/;7979-
/,.2633:55???7
@T6XXI:00129:00552
GGGAGAAGGGAGAAAGGAAGGAGAAGTCGCCCCAAACGATTTGTTGACTATTCTCCCTCTCCCTATAGTGAGTCGTA
TTAGAATTCCCC
+
CC@CCB@CC=?B?BBB@?>?>???>?;>>BBB8B?<BBCCC>CCA?<<>AA>A@@A<???AA<AAA@AAAAA===@@
@=@A@=@8<<<4
@T6XXI:00129:00553
GGGGAATTCTAATACGACGTCACTATAGGGAGAGGGAGAATAGTCAACAAATCGTTTGGGGCGACTTCTCCTTCCTT
TCTCCCTTCTCCC
+
>=>4>695444244>88:77BB;;99333,316=?<A>>?BAB@AA<<AA<B??<>-
166,7>??B;??B>A:=;=>599@D?A;>888-
@T6XXI:00129:00556
GGGGAATTCTAATACGACTCACTATAGGGAGAGGGAGAATAGTTGCGACATGTAACGTCCTTCTATCTTCTTCCTTT
CTCCCTTCTCCC
+
@=>4>;>=@A@@BC@@@BBCCBBBAAA?<??ABC?CAB=?:AA>A==@CBA@@@=@@BB?B?;;<CCC?BA<>4880
7=??<@=:;7=6
@T6XXI:00129:00559
GGGGAATTCTAATACGACTCACTATAGGGAGAGGGAGAATAGAACTATTCCCTTCTCATCCAGTGT
```

```
+
//1+1,95989<???BC;<<CBBBBAAB>A???B578824;4438;;::==:=57666//,---+2
@T6XXI:00129:00567
CTAGTCGACTCGAGATCTGCGGCTTTGGACAGTCCCTCTGTACTGTGAAGCCACAGATGTCGAGTCGACTAGCTTCC
TTTCTCCCTTCTCCC
+
?8-
,,,,,/88;>BBB@@@AA>???<@>ACCCCDD>B??????><<=@?AC@BCDDDBAABBBB?@@@@@AAAA?C@CC=
@=?@:@898@C7
@T6XXI:00129:00589
GGGAGAAGGGAGAAAGGAAGGAGAAGTCGCCCCAAACGATTTGTTGACTATTCTCCCTCTCCCTATAGTGAGTCGTA
TTAGAATTCCCC
+
49044>;>>0949@0948<>;:77AA?@@BBA7@@<>6889699:<<9=@@=??AA<A999A<?>AA?==A@<==@@
??AAA=B=A@A6
@T6XXI:00130:00572
GGGAGAAGGGAGAAAGGAAGGCATGAGTA
+
99149>599049990420,.6......45
@T6XXI:00130:00576
GGGGAATTCTAATACGACTCACTATAGGGAGAGGGAGAATAGCCGTTTAATCTCCCACTGCGGCATATCCTTCCTTT
CTCCCTTCTCCC
+
CCB8@698;@@@FGBBBBBA?AB;B66659???B>???<<<<@9=?CAA?A?=<<6;=>488<A5=:77////;=;5
::AA<@;=9;>9
@T6XXI:00130:00595
GGGAGAAGGGAGAAAGGAAGGGGAGAAG
+
:K;==>;>=111;;,1,0,...*..3,1
@T6XXI:00131:00553
GGGAGAAGGGAGAAAGGAAGCTAGTCGACTCGAGATCTGTGGCTTCACAGTACAGAGGACTGTCCAAAGCCGCAGAT
CTCGAGT
+
CC?CA><==5=>>=9><>=?@?BBABAAA@@??:::??@>@8<?=?CCBBB@575..-
/?;;;>>BA>BB==?<=<=588@?@?
@T6XXI:00131:00555
GGGGAATTCTAATACGACTCACTATAGGGAGAGGGAGAATAGTCAACAAATCGTTTGGGGCGACTTCTCCTTCCTTT
CTCCCTTCTCCC
+
@=92?<=<@@@?BCCBC>>>@@B?@<:<;?ACCA>=?A>A;;9>>?ABA:B@@@B>BBB8BCBBB598=3838;??<
?CCD=B;>>AA>
@T6XXI:00131:00560
GGGGAATTCTAATACGACTCACTATAGGGAGAGGGAGAATAGTTTTTCCCTTCACTAACCGCGAACTGCACTTCCTT
TCTCCCTTCTCCC
+
===4?<>=BBBCEBA?BBBBAAA9=77659CBCC>C??;@<A>><A1AA<A>@BBCC?@>778A>@AA@B?;;=377
.7::;-434444+
@T6XXI:00131:00593
GGGAGAAGGGAGAAAGGAAGGAGAATCGAGGTGAGTTGTACTCATCACACTATTCTCCCTCTCCCTATAGTGAGTCG
TATTAGAATTCCCC
+
49-
9>A6998999@<@=@?B=@@C@@@???>::;AA>?@@@CCBCEHFDCCCC8<;AB>;;;AB::<<BBABBABB?>8?
8?382;03330
@T6XXI:00131:00594
GGGGAATTCTAATACGACTCACTATAGGGAGAGGGAGAATAGCAACGTTTGCTCCACGATC
+
```

DCB6=7959@@=CB==;<>8:7767666,16777-600/0400323111,0000/0--457
@T6XXI:00131:00597
GGGGAATTCTAATACGACTCACTATAGGGAGAGGGAGAATAGATCACGACCCTTCTACCCACGACTGAGCTTCCTTC
TCCCTC
+
444-95959@@<BCCBBBCBCCCCBBBB>BAAA@;=<=<@?<<<>@@B???<==??744-44388<684-
/72*,*,,,,*,,
@T6XXI:00132:00552
CTAGTCACTCGAGATCTGCGGCTTTGACAGTCCCTCCTGTACTGATGAGACCACAGATGTCGAGTCGACTAGCTTCC
TTTCTCCCTTCTCCC
+
A=111>:??A@?B@?@998858=<-83111...+..,.4..-
00000000424=877;::9AACABC@@@B;710737<6<=?A9<9<@?A<
@T6XXI:00132:00557
GGGGAATTCTAATACGACTCACTATAGGGAGAGGGAGAATAGTCAACAAATCGTTTGGGGCGACTTCTCCTTCCTTT
CTCCCTTCTCCC
+
944,48>?BBB@CBBBBBBB?@B@@::<8<>>CC>CCC8<<?A??<8AA:BBBBB;BCC9A>=:?:;<;/4238;;7
=CCH<@=??A@<
@T6XXI:00132:00585
GGGGAATTCTAATACGACTCACTATAGGGAGAGGGAGAATAGCCAACAGGTATCTAATTGCGCGTTCTCCTTCCTTT
CTCCCTTCTCCC
+
BBA7A8;;;BB@CCBBBBBB?BB77111.7=<>@<A@?<?AAA>??AAA=A<@@??=@>@<?;?A;=@C8834:>><
?AAD<A9;;;<6
@T6XXI:00132:00593
GGGGAATTCTAATACGACTCACTATAGGGAGAGGAGAATAGC
+
88808+96<AC?CCCBBAA@??B?CCCC>=>@?>>-++,)))
@T6XXI:00132:00594
CTAGTCACTCGAGATCTGCGGCTTTGGACAGTCCCTCTGTACTGTGAAGCCACAGATGTCGAGTCGACTAGCTTCCT
TTCTCCCTTCTCCC
+
>>1/16699====BB@AB?><???9@@CCBCCCC>CBBBAA?AAAAA>@B@BCCCCBBACCCC@@@;;;CB??=@?B
JBB@@C<@=@??B<
@T6XXI:00132:00597
GGGGAATTCTAATACGACTCACTATAGGGAGAGGGAGAATAGTCAACAAATCGTTTGGGGCGACTTCTCCTTCCTTT
CTCCCTTCTCCC
+
ACB7B7;8;AA>ABBBADCC=?@CBBAB;:?ABB>B@C@???@@@=@@C?D;;:B577:4:=A@B7;:>5838>@@<
@@AH<A695AA?
@T6XXI:00133:00551
GGGAGAAGGGAGAAAGGAAGGAGAACGCGCACCCGAACCAACGGTGATCTATTCTCCCTCTCCCTATAGTGAGTCGT
ATTAGAATTCCCC
+
CKABBA@BB>BBBA;A@?=B>BB@?BBBBBBBC?BB?G@EADD@DCEB@@@@=@>@@<AA@AB>CBBCBABCA@CBB
B?>?AA>CAGCC:
@T6XXI:00133:00560
GGGGAATTCTAATACGACTCACTATAGGGAGAGGGAGAATAGTCAACAAATCGTTTGGGGCGACTTCTCCTTCCTTT
CTCCCTTCTCCC
+
@=94>696<BA@DDBBACCCA@CBBBA@;>?<<=;=AA;@=BABA?>@@<BBBBE>???7<AAAA>??<8<9=>AA=
@@@@;@<??CA>
@T6XXI:00133:00575
GGGGAATTCTAATACGACTCACTATAGGGAGAGGGAGAATAGTCAACAAATCGTTTGGGGCGACTTCTCCTTCCTTT
CTCCCTTCTCCC
+

```
>99,95959>>>BBBBCCCCBBBAB???;?BBCC>@@B>@=A@@@>@@C?CBBCC<AAA5?=?@@;=9:3838>BB@
@@@CAA<>>@CA
@T6XXI:00133:00584
GGAGAAGGG
+
1-///,//+
@T6XXI:00133:00587
CTAGTCGACTCGAGATCTGCGGCTTTGGACAGTCCCTCTGTACTGTGAAGCCACAGATGTCGAGTCGACTAGCTTCC
TTTCTCCCTTCTCCC
+
F:4999449?AACCAFCCCCB?BCB?B>ABC>??C>@?@@@A=99A@A>?B?BCBAADCCBBBBCCCBCBCA??=?;
@C>@==C<?<??@A<
@T6XXI:00133:00592
GGGAGAAGGGAGAAAGGAAGTGCAGTTCGCGGTTAGTGAAGGGAAAAACTATTCTCCCTCTCCCTATAGTGAGTCGT
ATTAGAACTTCCCC
+
=BACBB?BBABBBH@C@B@?BACC9;:;9:8=@=@CDEC@BC>CA@A2ABB@;;777-
7===?9<@@@A@A@@@>=;8834;;8;;3444-
@T6XXI:00133:00593
GGGAGAAGGGAGAAAGGAAGCTATCGACTCGAGATCTGCGGCTTTGGACAGTCCCTCTGTACTGTGAAGCCACAGAT
GTCGAGTCGATAGCTTCCTTTCTCCCTCTCCC
+
9A8<;A<@@1;8>B<A@DBAB?C?>>00//54427/6666:;6686366??A@A<=<<88887@898>AA:==A<<<
887;??897===74348/44+00440331110
@T6XXI:00134:00563
GGGGAATTCTAATACGACTCACTATAGGGAGAGGGAGAATAGCCACCAACTATTGTTCGTCGCCGTTCTCTTCCTTT
CTCCCTTCTCCC
+
999/95959>@=BAA@@@@?A9<16111,6=7776:AA>A@@;9:;7:7.00;99?;<<:==<??@8<8835;>@A7
<59;-43409;9
@T6XXI:00134:00564
GGGGAATTCTAATACGACTCACTATAGGGAGAGGGAGAATAGCCACAACATGTTTGCATGGCCGTTCACCTTCCTTT
CTCCCTTCTCCC
+
449/9695;AA@CBAAADAA?@<77111.7>>BC>AA@=A=?9999::<<<===;:>BCC=A<AC@@@=;=?A=@@;
@AAC<?<===A<
@T6XXI:00134:00582
GGGGAATCTAATACGACTCACTATAGGGAGAGGGAGAATAGTCAACAAATCGTTTGGGCGACTTCTCCTTCCTTTCT
+
:>?1=-944424/4.0099:;>;7111,6=>>?788@>@@;>33,--203111626 7661./7,.-3,-,-,/731.
@T6XXI:00134:00590
GGGAGAAGGGAGAAAGGAAGCTAGTCGACTCGAGATCTGCGGCTTTGGACAGTCCCTCTGTACTGTGAAGCCACAGA
TGTCGAGTCGACTAGCCTTCCTTCTCCTCTCC
+
44-444244-4444-
497:;@>>>00/0935///8>66:6?76;6,101113=?C==;<88444;4889<<=@@@?::0//.;888A?;:7/
/,/,/440133555:@=
@T6XXI:00135:00561
GGGAGAAGGGAGAAAGGAAGCTAGTCGACTCGACATCTGTGGCTTCACAGTACAGAGGGACTGTCCAAAGCCGCAGA
TCTCGAGTCGACTAG
+
BC@CCB@CB@CBBB=CAA@BB?6>6/11>==>1;:???@@?38:????CCCAAA?74417>?<==?AB<@@=@@@==
=::;<<==<??<@??
@T6XXI:00135:00565
GGGGAATTCTAATACGACTCACTATAGGGAGAGGGAGGAATAGTCAACAAATCGTTTGGGGCGACTTCTCCTTCCTT
TCTCCCTTCTCCC
+
```

?>?1<,/,/,,2,/...633187676662633442--,-22247<<<8444//.3424555995667423,-,-,-
222-00+3562455
@T6XXI:00135:00575
GGGAGAAGGGAGAAAGGAAGAATAGTCCAGTGCAGGGAATCGCGAATCTATTCTCCCTCTCCCTATAGTGAGTCGTA
TTAGAATTCCCC
+
EC?BB?;==<=?>@<@=??AA>ACCBB?BCCCAAAC>C@CCCCBB?BCA::;9@@C<@@@@@;@@A???@@@@998A
@=@@@>@<AAA6
@T6XXI:00135:00589
GGGAGAAGGGAGAAAGGAAGTATACATGGGAACCGCCTCGTGTTAGGCTATTCTCCCTCTCCCTATAGTGAGTCGTA
TTATGAACTTCCCC
+
9914@@<@D<<;<C>C>BAB<<;B@@@BB6?6606;0119888>@BDC@@B:;:BC869444+3;88;<;;56336/
/,---10344455.
@T6XXI:00136:00553
GGGAGAAGGGAGAAAGGAAGCTCAATCGTGGGTAGAAGGGTCGTGATCTATTCTCCCTCTCCCTATAGTGAGTCGTA
TTAGAATTCCCC
+
DFACCC@DC>AAA?=B?B?BCBBB?BB@BBB>CC@<;?A<===<<<<==<@=<=<<7=???B=A???@@@@@@AAA?
?<?AC;=:?CC5
@T6XXI:00136:00557
GGGGAATTCTAATACGACTCACTATAGGGAGAGGGAGAATAGTGCATTAACCTG
+
449-42928BB=:9<CDBB>=/00..330776<;8?5?>?86400/,*,*,*,,
@T6XXI:00136:00560
GGGAGAAGGGAGAAAGGAAGCTAGTCGACTCGACATCTGTGGCTTCACAGTACAGAGGGACTGTCCAAAGCCGCAGA
TCTCGAGTCGACTAGCTTCCTTTCTCCCTTCTCCT
+
@?:?>>;>@9>99914231371..50.//.../6116199=8=@?BECBBA@?=A77717888;<9=A<AA?BBA?=
=:??===??C@@@AA@@<@8;;6;:;;6;8;==8=
@T6XXI:00136:00576
GGGGAATTCTAATACGACTCACTATAGGGAGAGGGAGAATAGATTACGACCCTTCTACCCACGATTGAGCTTCCTTT
CTCCCTTCTCCC
+
:>?1<,958889;@@AA@DCFAA@?777.7=<>@:?AA>A@A@@<=:9444-
4:;;48818>89=;999=923/031066=;=:;5884
@T6XXI:00136:00585
GGGGAATTCTAATACGACTCACTATAGGGAGAGGGAGAATAGTCAACAAATCGTTTGGGGCGACTTCTCCTTCCTTT
CTCCCTTCTCCC
+
CCH:C@??@BB>CBBBCBCBCAB?@:<<;=>>AA>B??=@AA@==9=AC>C??AC>CCC7BB?==?BB@;=:=?AA<
@BAC=B689AA<
@T6XXI:00136:00589
GGGAGAAGGGAGAAAGGAAGCTAGTCACTCGACATCTGTGGCTTCACAGTACAGAGGGACTGTCCAAAGCCGCAGAT
CTCGAAGTCGACTACGCTTCCTT
+
<<;;BB@CC<AAAA>@@CA<<:@=::/...-.313/61663<?<??@BBB?::;444-
4;;478>@@:AA<AAA==84444424880---+++00*+*+5
@T6XXI:00136:00592
GGGAGAAGGGAGAAAGGAAGGAGAAGTCGCCCCCAAACGATTTGTTGACTATTCTCCCTCTCCCTATAGTGAGTCGT
ATTAGAATTCCCC
+
4919>?<?>9>>@@9>=?>A@BBB@BBA@@>>>,77.7666688;89<@6<=;;;8=:?888C<??@A?>?A?8737
30566424/0000
@T6XXI:00137:00591
CTGCGGCTTTGGACAGTCCCTCTGTACTGTGAAGCCACAGATGTCGAGTCGACTAGCTTCCTTTCTCCCTTCTCCC
+

```
CCCBB@99:;<?BBCBCCB=BCBB@@???CC?>CB@BBBCADBCCCCCBBBBCCCC;;;;@CC>BBAC>>9;4881
@T6XXI:00138:00552
GGGGAATTCTAATACGACTCACTATAGGGAGAGGGAGAATAGTCAACAAATCGTTTGGGGCGACTTCTCCTTCCTTT
CTCCCTTCTCCC
+
=?>4?<>>CCCACCAAAAAA@AB@@==<88B<>?;:AA?C;==;<:<@A<CCBBB<>AA7>AAAB=?==8;7;:==:
???@<@=??AB@
@T6XXI:00138:00556
GGGGAATTCTAATACGACTCACTATAGGGAGAGGGAGAATAGCTCCCGCAGTTTGACCATGTTCATCTTCTTCCTTT
CTCCCTTCTCCC
+
999/4:A@BBB@BC88:CCCCACACA><<>>>BC>A===?<AA<<><A@BCCE>A??<?AAA>AA@?@>@?8<9<>7
=@@@<A?AAA@;
@T6XXI:00138:00561
GGGAGAAGGGAGAAAGGAAGCTAGTCGATTCGAGATCTGCGGCTTTGGACAGTCCCTCTGTACTGTGAAGCCACAGA
TGTCGAGTCGACTAGCTTCCTTTCTCCCTCTCCC
+
DKBCBB@CD>BBCC>C=@=@BAA@>A@::2:@@ACB???CC@???9?>AACCB??6;888:;=>88:C=??>A@@=<
<<<<?@???@@?==;;;8477706666,//6893
@T6XXI:00138:00563
GGGAGAAGGGAGAAAGGAAGCTAGTCACTCGACATCTGTGGCTTCACAGTACAGAGGGACTGTCCAAAGCCGCAGAT
CTCGAGTCGACTAG
+
;;;;;B?BB?BBAB@B=>=@BBABA?AABB@::8?@???C;;>?CDFCBABA@<44418???CC=A?<AA???<<<=
=??@@@@AA@@@@@
@T6XXI:00138:00571
GGGAGAAGGGAGAAAGGAAGGAGAACAACACGATCGGTTAAACGTAGCTATTCTCCCTCTCCCTATAGTGAGTCGTA
TTAGAATTCCCC
+
BCACCB>?=:=@@=:B>A@C@CCB;@@=?@AAAAAB=A=@BA@@@BBA;;;=?BCD=AAAAAA?AA@@@A@@@@@@@
A>@@@;65533.
@T6XXI:00138:00586
GGGAGAAGGGAGAAAGGAAGGAGACAGTGAGCGCCGCACCACACATCACTATTCTCCCTCTCCCTATAGTGAGTCGT
ATTAGAATTCCCC
+
BB?BCBABA?@?BB>BAB@B@BB@BB@AAA?BBB@@>AB?CCC@888CBC@@?@AAA<@A<<=;=@=?=AAAA@AA@
@@>@AA>A>@@@5
@T6XXI:00138:00592
GGGGAATTCTAATACGACTCACTATAGGGAGAGGGAGAATAGTCACATTCATGTTTGTGACCGTGTCTCCTTCCTTT
CTCCCTTCTCCC
+
949/9;>>ABB@CCBA@BCB?@BCD==<;:==@@;<AC?A;==A@==@@CCCBBD>B@A@@=??>?;8833888AAA
BAAC7=;=A@@<
@T6XXI:00139:00561
GGGAATTCTAATACGACTCACTATAGGGAGAGGGAGAATAGACCCGACCCGAACTGCTAC
+
<<04:@89448887;;@@;:;;::>@@<BBBBB=??A>A???CC8:0?=6;;./--0.++
@T6XXI:00139:00565
GGGAGAAGGGAGAAAGGAAGCTAGTCGACTCGACATCTGTGGCTTCACAGTACAGAGGGATGTCCAAAGCCGCAGAT
CTCGAGTCGACTAG
+
CC@CCA@BB=BBC?;A875:@6/0=/00>>>>>?????CCA59:=?CBBBBA>94000,4;:9?9==9=;;<<@===
@@@???@@AAA@??
@T6XXI:00140:00558
GGGGAATTCTAATACGACTCACTATAGGGAGAGGGAGAATAGTTCTGACGGATTGCTCTGACTATTCACCTTCCTTT
CTCCCTTCTCCC
+
```

```
999/99@<ABB>BBBABBBB@@GCC?==;=>>EC>A>><>AAA=99=?BB???@>>B????B@BB?A==9?=B;??-
779:-43408:7
@T6XXI:00140:00561
GGGGAATTCTAATACGACTCACTATAGGGAGAGGGAGAATAGTTTTTCCCTTCAC
+
999/4:CAA@@>BCBBBCCABBC??:::7:CCCC>CA@9<;89888.3408230,
@T6XXI:00140:00563
GGGAGAAGGGAGAAAGGAAGCTAGTCGACTCGACATCTGTGCTTCACAGTACAGAGGACTGTCCAAAGCCGCAG
+
9914:;8=@089>>0958;??AA???CCBBBBBBC001>10/@=@@??CCA?@@==;=A@A@@?AA<AA>888<
@T6XXI:00141:00550
GGGAGAAGGGAGAAAGGAAGCTAGTCGACTCGACATCTGGTGGCTTCACAGTACAGAGGGACTGTCCAAAGCCGCAG
ATCGCG
+
CH>BBC@CB=BBCC=B>A?AA@?==716;;6;6/.44..,.22-.-6161>?:=>==?@:?@@877:<63644440-
++++++
@T6XXI:00141:00559
GGGGAATTCTAATACGACTCACTATAGGGAGAGGGAGAATAGATCACCGTTGGTTCGGGTGCGCGCTCTCCTTCCTT
TCTCCCTTCTCCC
+
999/49@>BCCBCBCCCCCC??BCCCAA>@@BCC;=:;;?=?<?ACC?BB@C@C@CCC?BBBB>CCCE;;::>A:??
97866287///:6
@T6XXI:00141:00562
GGGGAATTCTAATACGACTCACTATAGGGAGAGGGAGAATAGTCAACAAATCGTTTGGGGCGACTTCTCCTTCCTTT
CTCCCTTCTCCC
+
999/48989@A@B==@7830./.....5+//6:?8>:=;9353732369;BCA77+...*/.433,.132323/00+
04494955300,
@T6XXI:00141:00563
GGGGAATTCTAATACGACTCACTATAGGGAGAGGGAGAATAGCAACAAGA
+
444,42488??>@@AB?;;;FFAAABBC@BBBCE@@@@>>;8//,//,-*
@T6XXI:00141:00567
GGGAGAAGGGAGAAAGGAAGGTGACAGAGCGGCAA
+
/8571959>9>9990947131333349666..,.8,
@T6XXI:00141:00568
GGGGAATTCTAATACGACTCACTATAGGGAGAGGGAGAATAGTCAACAAATCGTTTGGGGCGACTTCTCCTTCCTTT
CTCCCTTCTCCC
+
499/42924889;4;;;;A@??CBA:::5:@>AA>@==>AAC@??<==A:ABB?@-
611,6=::;::37,.,/7896;;@@;@;?:??7
@T6XXI:00141:00572
GGGGAATTCTAATACGACTCACTATAGGGAGAGGGAGAATAGTTTTTCCCTTCACTAACCGCGAACTGCACTTCCTT
TCTCCCTTCTCCC
+
CBA4B59:<@>=BAAAABCBBBBBB???;?ABCA;??C<9430003,4:7;8:??110161103/0485::=<>377
-7=;=:?=;<<@7
@T6XXI:00141:00573
GGGGAATTCTAATACGACTCACTATAGGGAGAGGGAGAATAGCAAACGTTTTTCCCCGACGTCGCTTTCTCCTTCCT
TCTCCCTTCTCCC
+
ABA1>,/,///7/97>@AB@@?B??777.77===9<>>;>@>9::4471111+044.4;;711106?6=;;/4/3/3
/033338<==A=;
@T6XXI:00141:00590
CTAGTCACTCGAGATCTGCGGCTTTGGACAGTCCCTCTGTACTGTGAAGCCACAGATGTCGAGTCGACTAGCTTCCT
TTCTCCCTTCTCCC
```

```
+
311//1/44;A;A;=9110067330899556111,16177?>>=?<<8<B>BBBCCCBABCC:;<A;88>7710136
@;<<AB<@=@AAA<
@T6XXI:00142:00553
CTAGTCGACCCCGAGATCTGCGGCTTTGGACAGTCCCTCTGTACC
+
66//////444,499999844424776923337766266022,,*
@T6XXI:00142:00557
GGGAGAAGGGAGAAAGGAAGGAGACAGCCCCAGAGTACAGTCCAGAACTATTCTCCCTCTCCCTATAGTGAGTCGTA
TTAGAATTCCCC
+
CDACCB@BD>@@@B>B@B=>=>>>AAAAAA:BBB>@@CCB@>>=>>>AAA@???BA;BAC==<??@@@AACAA@A<<
<>?A>>B<???5
@T6XXI:00142:00558
GGGAGAAGGGAGAAAGGAAGGGGAGAAGGGAGAAAGGAAGCTAGTCGACTC
+
BE?CCC@CB>?@@B@F?B?BCC7:::>A?:BA??9@6737=477<71..-.
@T6XXI:00142:00569
GGGAGAAGGGAGAAAGGAAGTGCAGTTCGCGGTTAGTGAAGGGAAAAACTATTCTCCCTCTCCCTATAGTGAGTCGT
ATTAGAACTTCCCC
+
9@<@>=;>>:999@9A;?<?889@@@=@AAB=@@@CEBB?BB>BCCB1CBBB<?AKE=?@ACC>A@@@@@A?7/.-
/--0119233/000+
@T6XXI:00142:00576
GGGGAATTCTAATACGACTCACTATAGGGAGAGGGAGAATAGTGTACCGTGCTTCGC
+
CCB4@54699><ABBCCCCCCB@==661.7>>?B>AAA?@997++++*++++20111
@T6XXI:00142:00590
AATTCTAATACGACTCACTATAGGGAGAGGGAGAATAGCTTATGTTTCTCGCGTGCCTATTCGTCTTCCTTTCTCCC
TTCTCCC
+
B@CAAA@@CCCCCBBB><@BCB@?;@@A@A;@<<;???@B@@?@==<===??==;;8;==;=>;9:2428=:?HHD?
@>?>>=:
@T6XXI:00142:00597
GGGAGAAGGGAGAAAGGAAGCTAGTCGACTCGAGATCTGCGGCTTTGGACAGTCCCTCTGTACTGTGAAGCCACAGA
TGTCGAGTCGACTAGCTTCCTTTCTCCCTTCTCCC
+
9914:@@CC<@A@@<@@A?@@?BBC;<;BCB;;;ACBAAAB@BAB>B>BABBCAA=A??===@A???A<>??BB???
@898??A@@@????<<<=??AD9=<?@17588@D?
@T6XXI:00143:00567
CTAGTCGACTCGAGACTGCGGCTTTGGACAGTCCCTCTGTACTGTGAAGCCACAGATGTCGAGTCGACTAGCTTCCT
TTCTCCCTTCTCCC
+
A@999994:??=?BBCDEBB@BGE>F@BBB@@@C@BBBBBBBBCCCC??A?ACDCBBBCBBBCAAA@?>>;965:>B
C>??=C<A9<9<=<
@T6XXI:00143:00591
GGGAGAAGGGAGAAAGGAAGCTAGTCACTCGACATCTGTGGCTTCACAGTACAGAGGGACTGTCCAAAGCCGCAGAT
CTCGAGTCGACTAGCTTCCTTTCTCCCTTCTCCC
+
9;7<DA?A@:@98809=?<;<:@>>7:7118673888>9=9994:@@@BB@@@@A@@6;444447::49;=>>=A;7
/./6668830306633777:BB,++533*+++50
@T6XXI:00143:00592
GGGAGAAGGGAGAAAGGAAGCTAGTCACTCGACATCTGTGGCTTCACAGTACAGAGGGACTGTCCAAAGCCGCAGAT
CTCGAGTCGACTAG
+
<<19B@59919??><B=@=@C@@BB>BBCCA::8::9@BB>BBBCCBBBCCAA<8;;5;<<>??>BC:BB?C?@=9=
888?@@?===@@@A
```

```
@T6XXI:00144:00555
GCGGCTTTGGACAGTCCCTCTGTACTGTGAAGCCACAGATGTCGAGTCGACTAGCTTCCTTTCTCCCTTCTCCC
+
11:::@;;>1;9A@AAJBBCA@@ACCCBBB@CC@CBB???BA?:?B:::???C;;<@D@CE:;;AA:@;;;DK@
@T6XXI:00144:00569
CGACTCACTATAGGGAGAGGGAGAATAGTCAACAAATCGTTTGGGGCGACTTCTCCTTCCTTTCTCCCTTCTCCC
+
ECCCBBBCCBB99974C8::;<CC?CCCCCC@CFH>CC@?>1999/9AAAB=AAB??=?48808???>C???@D>
@T6XXI:00144:00591
GGGGAATTCTAATACGACTCACTATAGGGAGAGGGAGAATAGCAGACCTAAACTTTGTTT
+
CCC9BB@?AGC@C@@A@@@B?CAB>7798:BBCE>CAB>>;864438466;::351000+
@T6XXI:00144:00595
GGGAGAAGGGAGAAAGGAAGGAGAAGTCGCCCCAAACGATTTGTTGACTATTCTCCCTCTCCCTATAGTGAGTCGTA
TTAGAATTCCCC
+
CDBCCC@CB>EBDC?B=?=@@?<>>@@BCCCD9CC>BCBCB>CC>DBB@@?@CAAA<===@@:><<<<<<???<==?
=<>;843/444-
@T6XXI:00145:00551
GGGAGAAGGGAGAAAGGAAGAGAACAGTCACAGGCTCGAGCTGCAAGCTATTCTCCCTCTCCCTATAGTGAGTCGTA
TTAGAATTCCCC
+
:;:;99:<@<@DEE>A@C@CB??@BABBDCBA@=@BA??@BBAAC@CCC@@>@BB?:==777.78?7?A@???=<<7
4244437>ABB7
@T6XXI:00145:00558
GGGGAATTCTAATACGACTCACTATAGGGAGAGGGAGAATAGTCAACAAATCGTTTGGGGCGACTTCTCCTTCCTTT
CTCCCTTCCTCCC
+
EDC7B?@=@A@>BDGCCBB@BAABA<??:????B>BCF@CBBB@@>AAA=BEEAA>BCC9CBBBC?CEC?A:?:?@:
=;;939,/,/882
@T6XXI:00145:00561
GGGGAATTCTAATACGACTCACTATAGGGAGAGGGAGAATAGATCACCGTTGGTTCGGGTGCGCGTTCTCCTTCCTT
TCTCCCTTCTCCC
+
>DC:E?BABBB>BBCCBBBCAABCBBCC?CBBBB<BBB?BBB=@@CC@CC?C?CEDCC<@<<;@@C>DA@>@=A=AB
9=777.7:734:4
@T6XXI:00146:00553
GGGGAATTCTAATACGACTCACTATAGGGAGAGGGAGAATAGTTTTCCCTTCACTAACCGCGAACTGCACTTCCTTT
CTCCCTTCTCCC
+
BAA4>6969B@=A@BB@@ACCCCA@@@C>CCCCC>BA?>AC?<==.44-49;@@DC@C@;:;C@:;;<@==:=477-
76<?9@5::BB<
@T6XXI:00146:00561
GGGGAATTCTAATACGACTCACTATAGGGAGAGGGAGAATAGTTTGCGCATTCGGTGCACAGTTGTTCTCTTCCTTT
CTCCCTTCTCCC
+
AAA6A>B>AABCDCCBBA@@AABBB:::8:CCCC>ABB=>??AD>@A@ABB@BB@BBBBBBBB@BB@>A9?;=:<<6
=>>@9?<<??A;
@T6XXI:00146:00569
GGGGAATTCTAATACGACTCACTATAGGGAGAGGGAGAATAGTTTGGACGTCTCCCAGTCCTCGTTACGCTTCCTTT
CTCCCTTCTCCC
+
EED:C?AABBB@CBBACBCBBBB@>;;@:@ABCC>CCC>??>CD>A9<<@@@@@C<A??:<???A?AA@AA>A3871
7?>@<A?@@@C;
@T6XXI:00147:00553
GGGGAATTCTAATACGACTCACTATAGGGAGAGGGAGAATAGCAAGACGTCTTCCCCAT
+
```

```
444,/787111;;;;?@BCBB88837772<66?@:?AA?A??>:,/.7,++330000++
@T6XXI:00147:00557
GGGAGAAGGGAGAAAGGAAGCTAGTCGACTCGACATCTGTGGCTTCACAGTACAGAGGGACTGCCAAAGCCG
+
DIBCAB@DD>CCCB>B997;CBBBCB?;::0000....446067:;88:?<?<=B>77:5.--2,--+-222
@T6XXI:00147:00570
CTAGTCGACTCGAGATCTGCGGCTTTGGACAGTCCCTCTTACTGTGAAGCCACAGATGTCGAGTCGACTAGCTTCCT
TTCTCCCTTCTCCC
+
B<1111//66::BBBBCBBDC@CCD@D?@@?@?>>:???=ACBCCBB?BB@@C>BBA?====@A@A=<>AA495::<
<6=:77.8388=@:
@T6XXI:00147:00572
GGGAGAAGGGAGAAAGGAAGGGTATGCGATCTCATAGGGGTCTGGAGCTATTCTCCCTCTCCCTATAGTGAGTCGTA
TTAGAATTCCCC
+
CDBDDB@BA?AAAA=A?A=BB@B>@@CCCCCCAAABBBB9BBBB?BECBBC@;;:E>:9:??8?AABCBB@@@BBB@
A?@??<A=@@@8
@T6XXI:00147:00593
GGGGAATTCTAATACGACTCACTATAGGGAGAGGGAGAATAGCCACGAGTTTGCTTGCGTGGTTTACTCCTTCCTTT
C
+
DCC4F6959=9;8>999>>>?;;;8:AD>CB@BE@@??8:7<>>99?9111,1689>==@@>@=;<684--,-
0110-
@T6XXI:00147:00595
GGGAGAAGGGAGAAAGGAAGACAATCGCCGCGCGAGAGAAGGGTTGACTATTCTCCCTCTCCCTATAGTGAGTCGTA
TTAGAATTCCCCC
+
CIDCCB@EC>BBBB?B@B@BBCA?BA@A?BBBCDCC?A>?BB<B@B;;;CB=>BBB>===DC@A>??@@@AAAB?@@
A>@=;99/3444+
@T6XXI:00148:00552
GGGAGAAGGGAGAAAGGAAGGAGAAGTCGCCCCAAACGATTTGTTGACTATTCTCCCTCTCCCTATAGTGAGTCGTA
TTAGAATTCCCCC
+
AA<CBBBDD>AABCAH>B>B@CDC?BBBBACC9CC>BBBAA<@B>????710638840000403344=<A@@@@9:9
@A=<<94/.///+
@T6XXI:00148:00555
GGGAGAAGGGAGAAAGGAAGGAGAAGTCGCCCCAAACGATTTGTTGACCTATTCTCCCCTCTCCCTATAGTGAGTCG
TATTCAGAATTCCCC
+
:?>:BC?CCACDDD>B?B?B@CCC@BB@@@@@6@C>CDFCC<70,...,.57663033+0403305565@>><<;=:
44438888@B>AAA6
@T6XXI:00148:00574
GGGAGAAGGGAGAAAGGAAGCTAGTCGACTCGACATCTGTGGCTTCACAGTACAGAGGGACTGTCCAAAGCCGCAGA
TCTCGAGTCGACTAGCTTCCTTTCTCCCTTCTCCC
+
II?AA@?A>:@999196969A801=--
,,,,004456<966;784:<<??CCBAA???9;;;;?@<@@<@@>@@@@>>78844;;443<<<?AA>A>@@;@A@@
<@<??@C=
@T6XXI:00148:00591
GGGGAATTCTAATACGACTCACTATAGGGAGAGGGAGAATAGTTAACGCACCCTCCGTTACCTGTTCACCTTCCTTT
CTCCCTTCTCCC
+
AB?4?6;8<=<DCCCCCCCCDAA@<77767?<??=@BC?CBBB>=<=@9:;A<??=?BCCC@CEB<?;;6:=A<AA<
ABAB=A?AABC>
@T6XXI:00148:00593
GGGAGAAGGGAGAAAGGAAGCTAGTCGACTCGACATCTGTGGCTTCACAGTACAGAGGGACTGTCCAAAGCCGCAGA
TCTCGAGTCGAC
```

```
+
BDBDCB>??<@CBB>C>@=AC@BBB:<;AACB;;;;::>@@:???BAAABCB@@A:77.499889>AA@AB>AA@A9
9999AA????@@
@T6XXI:00148:00597
GGGGAATTCTAATACGACTCACTATAGGGAGAGGGAGAATAGTCAACAAATCGTTTGGGGCGACTTCTCCTTCCTTT
CTCCCTTCTCCC
+
BEA9B@C@BBB@CCBBB?@?BBBAC>>><>??CC>ABB>B@@AAA=@@A<CCECC<???6@@??C??AA=?=@CED>
??@B;@=@@AB=
@T6XXI:00149:00561
GGGGAATTCTAATACGACTCACTATAGGGAGAGGGAGAATAGTGACGTGTTGTGCGGCACTCACTGCTCC
+
/::0:5>8><964>>?>>>8888737@>79;6::57=::////.22238=3745313.....224430-0
@T6XXI:00149:00563
GGGGAATTCTAATACGACTCACTATAGGGAGAGGGAGAATAGCACTCCTTGCACTGTAGCTGCCGCAGCCTTCCTTT
CTCCCTTCTCCC
+
CCC:C?A?@AAACCDCCBBBCBBB;;??:@ABCE>CBA=B@AA<;??:<>BB@=<<@@<@BCC@BABBB?A@A8<=9
999DEB>@@AA<
@T6XXI:00149:00570
GGGAGAAGGGAGAAAGGAAGGAGAAGTCGCCCCAAACGATTTGTTGACTATTCTCCCTCTCCCTATAGTCGAGTCGT
ATTCAGAATTCCCC
+
491944244-
44;;5?>@?B@CDC?B@@AA??.88/77766,11888?A@>:>6662...55477==8865;:9;;;==/378843/
433+
@T6XXI:00149:00571
GGGGAATTCTAATACGACTCACTATAGGGAGAGGGAGAATAGCCTGTGCGGGTGTGTAAGGC
+
DDC:F@C?AAA;99CBBBB@?>>??888/8?>@A>C>=4;=//-0><<;==9://,+,*,+.
@T6XXI:00149:00572
GGGGAATTCTAATACGACTCACTATAGGGAGAGGGAGAATAGCTCCCAAATCACGTTACGTGCGACCCACTTCCTTT
CTCCCTTCTCCC
+
B?B4?8?=ABC@CCCBBCBBAAAA@@@CABBB@C>CA>>?A@A@?@>@A;BBBCBC@BCBCCBBBCC@CCC@C@CB>
CCCC:?;=9880
@T6XXI:00149:00577
GGGGAATTCTAATACGACTCACTATAGGGAGAGGGAGAATAGTCAACAAATCGTTTGGGGCGACTTCTCCTTCCTTT
CTCCCTTCTCCC
+
499/98>?BBB@BCCECABBBCAA?@?B>ACC@?;?B?>@A@A<<>??:;ACCBC>BBB9BBBBB?BBC=?=??BB>
AA@@<A:==@A<
@T6XXI:00149:00581
GGGAGAAGGGAGAAAGGAAGGAGAAGTCGCCCCAAACGATTTGTTGACTATTCTCCCTCTCCCTATAGTGAGTCGTA
TTAGAATTCCCC
+
EDBCBBABC?BBBB>>?A=@=@DC@CBBBBBB7BCBCCCCC>CC@C;;<CC@C@@@<???@A;<???@CAA@@AA@A
A>@=<8;24480
@T6XXI:00149:00596
GGGAGAAGGGAGAAAGGAAGGAGAACGCGCACCCGAACCAACGGTGATCTATTCTCCCTCTCCCTATAGTGAGTCGT
ATTAGAATTCCCC
+
DEBDEC@CC@BBBB?B@B@B@BBC@BBBBBCEC@DCAC?CACD@CCCC;<<C@BACA>;;<@C?CGCBBBBBBA@@@
BB@AAA:@;>>?4
@T6XXI:00108:00613
GGGAGAAGGAGAA
+
```

```
7=0---*,*,,/7
@T6XXI:00109:00606
GGGAGAAGGGAGAAAGGAAGGCGAACAGCCGGTAGACAAAGCGTAG
+
68+//><?@894:;9>=?;@?998>A@>@>;26661.4403----2
@T6XXI:00115:00648
GGGAGAAGGGAGAAAGGAAGCTAGCGACTCGACATCTGTGGCTTCACAGTACAGAGGGACTGTCCAAAGCCGCAGAT
CTCGA
+
44-4;;244-
49991;?B?@;000..5...67611111171356>>>??@A==<;<4:44888?BD<A?>>?99,,,++02
@T6XXI:00117:00643
GGGAGAAGGGAGAAAGGAAGCTAGTCGACTCGAGACA
+
/;;::@>BB>BBCD@CBCE@<<;;/.47/./544468
@T6XXI:00117:00647
GGGGAATTCTAATACGACTCACTATAGGGAGAGGGAGAATAGTCAACAAATCGTTTGGGGCGACTTCTCCTCCT
+
4992929244488849783361316666>;==B@@;773233566<<::611116616??68:6110110/0-22
@T6XXI:00118:00609
GGGAGAAGGGAGAAGGAGAAGGGAGAAAGGAAGCTAGTCGACTCCGAC
+
/;;;;C@HC:?89595733139;?A?B>C@C@@7144437773311..
@T6XXI:00118:00614
GGGAGAAGGGAGAAAGGAAGAGAATAACAGTGCACCGAATGCGCAAACTATTCTCCCTCTCCCTATAGTGAGTCGTA
TACGAACTTCCC
+
6:5<:>;@A>A@B@9><>;><>=0668;9776677144244=@=:::1///,...403./6>167=<86<??543,,
,,,,,*,.,.30
@T6XXI:00122:00645
GGGGAATTCTAATACGACTCACTATAGGGAGAGGGAGAATAGTCAACAAATCGTTGG
+
9::2@69284488849:;:7D?<988?A>BAABB>A??698854=;<54+../.,.4
@T6XXI:00124:00603
GGGGAATCTAATACGACTCACTATAGGGAGAGGGAGAATAGTCCACACA
+
1:80<,1///,/9?@B;;;A::666<9;<CCCA=BA9,-++++*+0022
@T6XXI:00126:00643
GGGGAATTCTAATACGACTCACTATAGGGAGAGGGAGAATAGTGACGTGTGTGCGGCGCTCACTGCTCCTTCCTTCT
+
8>=1=,92844888>;;;BAC?@:88@@;BCBBB<A?<33373344---.---4456>734>=:8--+*+*+*-323
@T6XXI:00129:00611
GGGGAATTCTAATACGACTCACTATAGGGAGAGGGAGAATAGCATGCATAATGT
+
08818+1,///7647;?>;796766111,1>77758;;3444430000163674
@T6XXI:00134:00609
GGGAGAAGGGAGAAAGGAAGAAGAACGGCTCGATGGTCAGTCGAGAACTATTCTCCCTCTCCCTATAGTGAGTCGTA
CTTAGAATTCCC
+
68+//C5990949909;>7831387==<=994444248777;:444;=8==@>888+...440446161977...4.
..,.6669244-
@T6XXI:00136:00609
GGGGAATTCTAATACGACTCACTATAGGGAGAGGGAGAATAGCTACGCTAACCGATCGTGTGTT
+
8>=17+1,///568443377717688?>8??????9?>>388::=7660016677674;;643..
@T6XXI:00138:00601
GGGGAATTCTAATACGACTCACTATAGGGAGAGGGAGAATAGTCACTGG
```

```
+
9@@4@692444999?A@;;;B?@:88@A=BBCCC>?<;3884/.2--+*
@T6XXI:00140:00620
GGGAGAAGGGAGAAAGGAAGGGGAGAAGGGAGAAAGGAAGGGGAGAAGGAGAAAGGAAGCAGTCGACTCGACATCTG
TGGCTCACA
+
//+/6><>@099@@9>=@;?>>.88=:=A:>777.747<>>>5@:858??=??<?=?>;7300033344:::00000
344551220
@T6XXI:00142:00620
GGGGAATTCTAATACGACTCACTATAGGGAGA
+
6?<1<-924442447;;?<?B:311166233.
@T6XXI:00142:00627
GGGAGAAGG
+
9@:<11--+
@T6XXI:00148:00628
GGGAGAAGGGAGAAAGGAAGAAAGGGAGGAGATAGAGCCGGTCGGG
+
:C?DCC@CC>B@@@9><>;88=:;><A<@99///663367-//552
@T6XXI:00101:00659
GGGAGAGGAG
+
44-4///,//
@T6XXI:00102:00661
CTAGTCGACTCGAGATCTGCGGCTTGGACAGTCCCTCTGTACTGTGAAGCCACAGATGTCGAGTCC
+
69994449:99444>>944:97771.3443....+.........--42398444933386111142
@T6XXI:00102:00663
GGGAGAAGGGAGAAAGGAAGAAGCGCCAAACGTGTTAGAGACAAGGGCTATTCTCCCTCTCCCTATAGTGAGTCGTA
TTAGAACTTCCCC
+
8?-11>699-449:-4231399;=A>=AA9>>99824877799167,394424444-
43333,36167@@;;961100/06959988862
@T6XXI:00102:00692
GGGGAGAAGGGAGAAAGGAAGAACAGGTCACG
+
///*//;:;B::<DGAD>A=8868310..4**
@T6XXI:00103:00695
GGGAGAGAATTTGGTAATGGGCAGTGGTCGGAGAGGGAAAGGAAGAAGGGCCCTTATATCTCTCTCCCTATAGTGAG
TCGTATTAGAACTT
+
4?:AAAAA=@>9>;974333,49:::244424:8>><=;6977783133,33,42///....3336911166334>8
6611110-42,-.-
@T6XXI:00104:00692
GGGGGAATTCTAATACGACTCACTATAGGGAGAGGGAGAATAGTTTTCCCTCACTAACCGCGAACG
+
////*/,42444244483830<0......+.57=9894334/.---*-1+--7799>@>7/+00++
@T6XXI:00109:00653
CTAGTCGACTCGAGATCTGCGGCTTTGGACAGTCCCTCTGTACTGTGAAGCCACAGATGTCAGCTTCCTTTCTCCCT
CTCCC
+
4;;@@BGECABBA@@>999986888,35:9C???A8<A::6671656658B<<<CCAA@???:9951-
11,1/11,1/8668
@T6XXI:00112:00655
GGGAGAAGGGAGAAAGGAAGCTAGTCGACTCG
+
```

```
/:8;;B?CC<@;;;09>@588366..22,,,,
@T6XXI:00113:00653
GGGGAATCTAATACGACTCACTATAGGGAGAGGGAGAATAGTCAACAAATCGTTTGGGGCGACTCTCCTCCT
+
8>>17+1///,/44:78:7>97666?>;>>???9?;;9==??<=:;;=466111,6>7299811110/0-20
@T6XXI:00113:00697
GGGAGAAGGGAGAAAGGAAGGAGACGCCGATTTCGTATAAGCTCGGACTATTCTCCCTCTCCCTATAGTGAGTCGTA
TTAGAATTCCCC
+
9B>BEC@CB>BBBB=?>>@B@BBBBBB<?88918111..,./34A@A999:94999144444-
44449=>=B>:99992399142444,
@T6XXI:00114:00652
GGGAGAAGGGAGAAAGGAAGCTAGTCGACTCGACATCTGTGGCTTCACAGTACAGAGGGACTGTCCAAAGCCGCAGA
TCTCGAGTCGAC
+
4;8;AC@ED@BBAB<?=@?B@@?AAABB@?@?776;1116425::::<<>>8649./.+./..947B45955458A>
7333666442--
@T6XXI:00114:00653
GCTAGTCGACTCGAGATCTGCGGCTTGGACAGTCCC
+
449:99444::?=4994448431331377731117,
@T6XXI:00115:00654
GGGGAATTCTAATACGACTCACTATAGGGAGAGGGAGAATAGTCAACAAATCGTTTGGGGCGACTTCTCCTCCTTTC
TCCCTTCCTCCC
+
///*/,1,///,/4898870=0....57666CCE>B@?388<<6::;==4<<AAB9AAA5??9;53353-
./5775..5=::2554=;;
@T6XXI:00116:00653
GGGGAATTCTAATACGACTCACTATAGGGAGAGGGAGAATAGAGACGATCGTTGC
+
///*/,424442448=<87.=/....330379>>71403008<;=62+++++*+5
@T6XXI:00117:00651
GGGGAGAAGGGAGAAAGGAAGCGTAGTC
+
///*//4:;A:?@@@<=;@+--,****,
@T6XXI:00117:00659
GGGGAATTCTAATACGACTCACTATAGGGAGAGGGAGAATAGTCAACAAATCGTTTGGGGCGACTTCTCCTCCTTTC
TCCCTTCCTCCC
+
444,4242444249483877;6711111,16;=?<:843448733:::;477111,166,11=710115234<==;;
=:;362423>A5
@T6XXI:00118:00661
GCTAGTCGACTCGAGATCTGCGGGCTTGGACAGTCCCTCTGTACTGTGAAGCCA
+
//444444444999444444433,331313331116,1....3...4432-/,/
@T6XXI:00118:00669
GGGGAATCTAATACGACTCACTATAGGGAGAGGGAGAATAGAGTCACCGTTGGTTCGGGTGCGCGT
+
,--*-+1///768443877>;73116626===?9?=:24///---03,..,.,.4...+166688:
@T6XXI:00119:00655
GGGGAATTCTAATACGACTCACTATAGGGAGAGGGAGAATAGTCGACATTCATG
+
,,,*,*/,///568???8:8>7311177116;>>.74:34...--.32664///
@T6XXI:00119:00656
GGGGAATTCTAATACGACTCACTATAGGGAGAGGGAGAATAGTCAACAAATCGTTTGGGGCGACTTCTCCTCCTTTC
TCCCCTTCTCCC
+
```

```
22202+1,///,/9ABC:;;@:;<77>?>BCCD@9>98-
///..586==388611,1772=?<0/,/.2,.+3000++---*-2249=;
@T6XXI:00119:00668
GGGAGAAGGGAGAAAGGAAGGAGAAGTCGCCCCAAACGATTTGTTGACTATTCTC
+
7?8<<@599194?@09487<938:97188833,39771611,1101163324300
@T6XXI:00120:00669
GGGAGAAGGAGAAAG
+
9;6;11,1,,***)*
@T6XXI:00123:00654
GGGAGAAGGA
+
491///,/,,
@T6XXI:00123:00698
GGGAGAAGGGAGAAAGGAAGGAGAAGTCGCCCCAAACGATTTGTT
+
44-4;;:CE?BB@A<?=>;==010=0/0996654424.111,110
@T6XXI:00125:00659
ACTCGAGATCTGCGGCTTTGGACAGTCCCTCTGACTGTGAAGCCACAGAGCGAGTCGACTAGCTCCTTCTC
+
//449491..//486444-49;;6////+////,,////777:;=>?@@;==;3333944449-,*,*,,,
@T6XXI:00127:00660
GGGGAATTCTAATACGACTCACTATAGGGAGAGGGAGAATAGTCAACAAATCGTTTGGGGCGACTTCTCCTCC
+
,--*,*1,///,/48=7888?7331166,116;661442338<6:;<<<399111,6;726616101104420
@T6XXI:00128:00668
GGGGAATTCTAATACGACTCACTATAGGGAGAGGGAGAATAGATCACCGTTGGTTCGGGTGCGCGTTCTCCTTCCTT
TCTCCCTTCTCCC
+
499/4292444244443<?>A:;:::BB>B@@@B>CAA;=====<AA>::5::=:@BB<@@CCBCD?:::==3<:<=
7/-.4,.,/-+50
@T6XXI:00128:00674
GGGGAATTCTAATACGACTCACTATAGGGAGAGGGAGAATAGTTTGCCTGTCGGCCATCTAG
+
08808+1,///7688?@???B?@:88BC>BDCCC=;999<<@B@7>>301661017116642
@T6XXI:00128:00699
GGGAGAAGGGAGAAAGGAAGGCGATCAACGAGTACG
+
9:6?99244-4448-423137333666311111...
@T6XXI:00130:00657
GGGAGAAGGGAGAAAGGAAGCTAGTCGACTCGACATCGTGTGGCTTCACAGTACAGAGGGACTGTCCAAAGCCGCAG
ATCTCGAGTCCGACC
+
44-
49;:@A8;9@B1;8;=?;;98888866611.34.....12247>>:<=?;64?73313;;=6;:??:@;:=@@@@43
043----01--,
@T6XXI:00132:00679
GGGAGAAGGGAGAAAGGAAGCTAGTCGACTCGACATCTGTGGCTTCACAGTACAGAGGGACTGTCCAAAGCCGCAGA
TCTCGAGTC
+
//+/8@;@@<@>>@<@=?>A@@?:77>>711111611110247998::<>6688343+344447=AA<AA<884;6
6/+++++00
@T6XXI:00135:00670
GGGAGAAGGGAGAAAGGAAGGAGATATAGACACGCCAATAGAAACGCGCTATTCTCCCTCTCCCTATAGTGACGTCG
TATTAGAATTCCCC
+
```

```
49049?@CC>BBAA696:68<?@<::779?99<>==;<<>ABC>@?<;778824339,33316268888>?745::=
8:888555=<9992
@T6XXI:00136:00669
GGGAGAAGGGAGAAAGGAAGGTGAACAGAGCGCAAACGAATACGTCTCTATTCTCCCTCTCCCTATAGTGAGTCGTA
TTACGAATTCCCC
+
44-
4>>;?>:>?@B<B>@>C9;;A?CA=<<;;;AA:999544::;8499:?>=:=A6999@A;AB888AA>>>>99533.
./0,/,111,
@T6XXI:00136:00673
GGGGAATTCTAATACGACTCACTATAGGGAGAGGGAGAATAGCCAATGTCT
+
6660;,1,///7644;@;::A?@869??8=@=??<?;8588//,+0000+.
@T6XXI:00137:00669
GGGGAATTCTAATACGACTCACTATAGGGAGAGGGAGAATAGTTTTC
+
6<<1<-9244499:>A@?@???>777@>8;:000,0.-,-1----*0
@T6XXI:00140:00669
GGGAGAAGGGAGAAAGGAAGAATATTGCGCG
+
4@<@AA>BG>BB>>:=:9,00,..3336964
@T6XXI:00141:00696
GGGGAATTCTAATACGACTCACTATAGGGAGAGGGAGAATAGCTACGTTAACCGATCGTGT
+
999/4292499;8@ABG@@@CAC@??BD>CBBBB>B?A9==<;===774424467.-99//
@T6XXI:00144:00692
GGGGGAATTCAATACGACTCACA
+
:>>>+92/,//,////4430A?.
@T6XXI:00145:00665
GGGAGAAGGGAGAAAGGAAGGAGAAGTCGCCCCAAACGATTTGTTGACTA
+
?D@CBB?BC?BBBB?C@A?A?7:;>86377775;9699111,11016866
@T6XXI:00146:00651
GGGAGAAGGGAGAAAGGAAGCTAGTCGACCGGACATCTGTGGCTTCACAGTACAGAGGGACTGTCCAAAGCCGCAGA
TCTCGAGTCGACTA
+
//+/69899-
48@@<A=A?B:8:::7333,.,..46.116689B@BA>>?>@CAC9=<;88<>@A<??;?A>???AA<7448662--
---+
@T6XXI:00146:00669
CTAGTCGACTCGAGATCTGCGGCTTTGGACAGTCCCTCTGTACTGTGAAGCCACAGATGTCGAGTCGACTAGCTCCT
TTCTCC
+
4999944>@@@@@?>>999995773,377;;977?8999911769676:<CAAAAC@@@A@@@;:<@?>=>>3//77
50/,.4
@T6XXI:00147:00666
GGGAGAAGGGAGAAAGGAAGGAGAACAGAATGCCACCAAGGCTAAGCATTCTC
+
44-49ABHG>AACC>C@C@B@BBB@>===-//.367792424437;4222,,,
@T6XXI:00148:00650
GGGGAATCAATACGACTCACATAGGGAGAGGGAGA
+
,--*,*//6,/4999;3044...330379::7743
@T6XXI:00148:00663
GGGAGAAGGGAGAAAGGAAGGGGGAGAAGGGAGAAAGGAAGCTAGT
+
```

```
44-4;B?DCADBBB<?=??ACEI2C@@=@EACB>;6:,0,00....
@T6XXI:00148:00680
GGGGAATTCTAATACGACTCACTATAGGGAGAGGGAGAATAGCGCACCATCAATTTGTCGTGCCGTTCTCCTTCCTT
C
+
499/95924449844:@>?>?<77366616?>AB?A>=:;9/---
1101111011,113778?>::5332*,*+*+*+
@T6XXI:00149:00662
GGGAGAAGGGAGAAAGGAAGGAGAAGTCGCCCCAAACGATTTGTTGACTATTCTCCCTCTCCCTATAGTGAGTCGTA
TTAGAATTCCCC
+
//+//95991989@9A896;<8;:>;69B<>>-73,3766626701668634.--101----+-
002...494<=@@=<>;600+,,,*
@T6XXI:00149:00664
GGGGAATTCTAATACGACTCACTATAGGGAGAGGGAGAATAGCCGGAACATCTCCGC
+
///*/,1,///,/4;AC900;0....33066<>?8???=<<?@8;4879,-,,,*,0
@T6XXI:00100:00707
GGGAGAAGGGAGAAAGGAAGAATATCGCCGCGTGGAAGAGATGTTGCTATTCTCCCTCTCCCTATAGTGAGTCGTAT
TAGAATTCCCC
+
CC@DAA?AC@CCCC>C@B@BA?@CCCBA>A>?C?;A;???CE@@8::@??=ABCC=ABBA@<@@@@@C@@@AAA@??
<?@@=@>ACA5
@T6XXI:00100:00709
GGGAGAAGGGAGAAAGGAAGCTAGTCGACTCGACATCTGTGGCTTCACAGTACAGAGGACTGTCCAAAGCCGCAGAT
CTCGAGTCGACTAGCTTCCTTTCTCCCTTCTCCC
+
44-49><AB<@@AA:@;>=?A>:77,,,,,,,,.4471114/0101968:A:429//,/611-
0/0446<;;<==780/0/,,96/..-11268856403304244400031
@T6XXI:00100:00712
GGGGAATTCTAATACGACTCACTATAGGGAGAGGGAGAATAGCCACGCAGTATCTCCCTGGTTCGTCCTCTTCCTTT
CTCCCTTCTCCC
+
@@@6@=@=ABB@CCBA>BBB?@?9>111-
6<7775:@@>A@@@>A?AA@BA=<@@B=;=@>@9<=@A>AAA>A=??7=:::4;89;;@:
@T6XXI:00100:00725
GGGAGAAGGGAGAAAGGAAGGAGAAGCGCCCCAAACGATTTGTTGACTATTCTCCCTCTCCCTATAGTGAGTCGTAT
TAGAATTCCCC
+
@@<@AA?AC>AA@C=B?B@C@>>>=/30994599897?B=9AB?AACACC;==@C=B???A9==AAAAA@AA@@@@?
?AAC>A=???5
@T6XXI:00101:00739
GGGGAATTCTAATACGACTCACTATAGGGAGAGGGAGAATAGCTACGTTCC
+
CCB7A>A>BBB?CAA@BBCC@AE@:69956<@CCAB@=;;9-/000..,-3
@T6XXI:00101:00749
GGGAGAAGGGAGAAAGGAAGGAGACAGTGAGCGCCGCACAACACGTCACTATTCTCCCTCTCCCTATAGTGAGTCGT
ATTAGAATTCCCC
+
CDCCCD?BB=ABBC>B@B@B@>>>BBBBAA?<<?@BB>?>>??????CBBBB>BB??<BBCCB<BBBB>>BBCCBB?
???ADE@D=@@@7
@T6XXI:00102:00703
GGGGAATTCTAATACGACTCACTATAGGGAGAGGGAGAATAGCTCCCGAGAACGGAACACCGCCGCCTGCTTCCTTT
CTCCCTTCTCCC
+
BBB9B=A>BBC@CCBBAAEBA>AB?99:5:ABCD?C==;=AA?AAC>CBBC?AC@C@CCC@BB?CB?CCCB484880
8=?B>C@;;:B=
```

```
@T6XXI:00102:00712
GGGAGAAGGGAGAAAGGAAGGAGAACGCGCACCCGAACCAACGGTGATCTATTCTCCCTCTCCCTATAGTGAGTCGT
ATTAGAATTCCCC
+
CCACCB@CD?BCBB>C@C=>=>?B?BBBCCCCD?CCBC@A?AEBCBBBB<<;?BAAA<;;;BA<@BBAAACCC@BB?
??=<AA>C?CDD:
@T6XXI:00102:00724
GGGGAATTCTAATACGACTCACTATAGGGAGAGGGAGAATAGC
+
@@@6@=@=@CB<@@@AAAA@A@?=<777.77;<<6<3420/-/
@T6XXI:00103:00707
GGGAGAAGGGAGAAAGGAAGCTAGTCACTCGACATCTGTGGCTTCACAGTACAGAGGGACTGTCCAAAGCCGCAGAT
CTCGAGTCGACTAG
+
@=9>?>;>B9;;AA<A>@?BBCBBCC?711611069678@?CC?BBDBBBB?;;78814;;@@?=?B<@A>::8@@A
C@@@C@@=<888<<
@T6XXI:00103:00711
CTAGTCGACTCGAGATCTGCGGCTTTGGACAGTCCCTCTGTACTGTGAAGCCACAGATGTCGAGTCGACTAGCTTCC
TTTCTCCCTTCTCCC
+
E99999444:A=A<<=C@@AB@CDC?C?ACBBAAB>BCBBB>>?@=<=;<A>ABCBCBABBBBBBBB@@@CB??AC@
DD<AA?A<A>AAA@<
@T6XXI:00104:00718
GGGGAATTCTAATACGACTCACTATAGGGAGAGGGAGAATAGCCACCAACTATTGTTCGTCGCCGTTCTCTTCCTTT
CTCCCTTCTCCC
+
=>>4>696<AA?BFCCCCCC??A?B777.7==BC@CAA>A===>@@;?9<<?><@@<A??@@C@?>;;;//,/4660
7459;<8::??;
@T6XXI:00104:00720
CTAGTCGACTCGAGATCTGCGGCTTTGGACAGTCCCTCTGTACTGTGAAGCCACAGATGTCGAGTCGTAC
+
B@944444::?=A==<AAB;;8<BC>C<<<CBAAA=ABBCCCBB9944249584744......1.//...
@T6XXI:00104:00721
GGGGAATTCTAATACGACTCACTATAGGGAGAGGGAGAATAGCTTACT
+
CEC:B=>=>BB>BCCCBCBBBACB>66659??CC>C??<?93.0)*+*
@T6XXI:00104:00736
GGGAGAAGGGAGAAAGGAAGGGGAGAAGGGAGAAAAGGAAGCTAG
+
BA>BBB@CC>BBBB>C@C@BBB8BBB?966;7776-731015-..
@T6XXI:00105:00713
GGGAGAAGGGAGAAAGGAAGCTAGTCGACTCGAGATCTGCGGCTTTGGACAGTCCCTCTGTACTGTGAAGCCACAGA
TGTCGAGTCGACTAGCTTCCTTTCTCCCTTCTCCC
+
CCCBBBACC=BBBB=B@C@BBB>;;/10>>>>@@CFBBCCE?B??=B@CCCB@<<;===<??@B???@>@@>@@@@@
@@A@@@???A???@?<:99/33-4;;;-434444,
@T6XXI:00105:00722
GGGAGAAGGGAGAAAGGAAGCTAGTCGACTCGAACATCTGGTGGCTTCACAGTACAGAGGACT
+
//+//87BC>BCBB898:7;;A>>7,/,,,,,,*.55311013447361111744683-,---
@T6XXI:00105:00735
GGGAGAAGGGAGAAAGGAAGCTAGTCGACTCGACATCTGTGGCTTCACAGTACAGAGGGACTGTCCAAAGCCGCAGA
TCTCGAGTCGACTAG
+
CC?CCB@DD?BBBB>B=><>@?;=?/00:::;071166??A:@C@BBCB??><9;33314;9>@@>@A;@@?A@<<<
<=?<<<?A===AA@@
@T6XXI:00106:00708
```

GGGAGAAGGGAGAAAGGAAGGCGAGAG
+
GE<A@=;>?:>911,1,0,0,....53
@T6XXI:00106:00717
GGGGAATTCTAATACGACTCACTATAGGGAGAGGGAGAATAGTCACATTCATGTTTGTGACCGTGTCTCCTTCCTTT
CTCCCTTCCTCC
+
999/959594954:;<CCCDCA@>7777.777?CB@==>AA@@@@AAC@BBBCCB>AA>??;=?;4833239=<==;
===935+,+,,*
@T6XXI:00106:00718
GGGAGAAGGGAGAAAGGAAGGGAATAGAGGACAACTGACTGTCTTGTCTATTCTCCCTCTCCCTATAGTGAGTCGTA
TTAGAATTCCCC
+
@CACDJ?B=<=@AA>B?BBDG?C?AAACD=???<????BB@AAA>?=>;0/,/4622.-
004+04446>==8878440270;99/344-
@T6XXI:00106:00730
GGGAGAAGGGAGAAAGGAAGGAGAAGTCGCCCCAAACGATTTGTTGACTATTCTCCCTCTCCCTATAGTGAGTCGTA
TTCAGAATTCCCC
+
9A<@@@<>?19948-
/,.66,.00633499945;<666111,16779=<<<;=<<=;=555=;;;==86566==4748.//;;;=;A@@4
@T6XXI:00106:00742
GGGGAATTCTAATACGACTCACTATAGGGAGAGGGAGAATAGTCAACAAATCGTTTGGGGCGACTTCTCCTTCCTTC
T
+
@B@6A8<8948?9::BBBCBBDA??777.7?777-749258AAAAB9985:B@AB?BDD9CAA::57;8,/,/,/,-
0
@T6XXI:00107:00708
GGGAGAAGGGAGAAAGGAAGTGCAACTCGTCGCGCAAAATAGGGAGACTATTCTCCCTCTCCCTATAGTGAGTCGTA
TAGAATTCCCC
+
@@<ACB@CC>BB??=@>A@CCC??=ADDCCCCDDBBBB8@?>C>;::6:7169>;;217100+084;<=8779814/
/.457=;=990
@T6XXI:00107:00714
GGGGAATTCTAATACGACTCACTATAGGGAGAGGGAGAATAGTATCTCCCTTGATCGGACGCGATCGTGCTTCCTTT
CTCCCTTCTCCC
+
CCB4;9<=@@@?DCBBBBAAA>A@99:?9?@ACCBB@@>A=A@8<9AAA<?<?BAAA>@@???AAAAA<==@A69:8
9BAC;@=?<<<6
@T6XXI:00107:00725
CTAGTCGACTCGAGATCTGCGGCTTTGGACAGTCCCTCTGTACTGTGAAGCCACAGATGTCGAGTCGACTAGCTTCC
TTTCTCCCTTCTCCC
+
BA1//////89>>BCACCE@=@GE>C8:;CBAAB=ACFCCCCCB@@@>@C@CCCCCCCBBBCC@@ABBBBBBB?B@
EC=@@AC7<9<7692
@T6XXI:00107:00728
CTAGTCGACTCGAGATCTGCGGCTTTGGACAGTCCCTCTGTACTGTGAAGCCACAGATGTCGAGTCGACTAGCTTCC
TTTCTCCCTTCTCCC
+
B?99994444;AABBDCCCBB@BBB=B=@@???B>;????>@@AA???>@D@CCBBBCCBCCCC?@@@@@DEBC?F@
CC>BCAA<@689?@<
@T6XXI:00107:00730
GGGAGAAGGGAGAAAGGAAGCTAGTCGACTCGGACATCTGTGGCTTCACAGGGGAGAAGGAGAAAGGA
+
CD>BAB788199@A>BBC?BBCBBCC???CCC>AAACCCCA?<?C=??BB>==1666,/,///-+-34
@T6XXI:00107:00737

```
CTAGTCGACTCGAGATCTGCGGCTTTGGACAGTCCCTCTGTACTGTGAAGCCACAGATGTCGAGTCGACTAGCTTCC
TTTCTCCCTTCCTCCC
+
A<1//1///66:=B?AAAACC@CCC?C;;;@?==<9??=??@:;:88=;>B@BB;;;;@>;::??@B@AAB;:8?A>
@A=B:8828,/,/661
@T6XXI:00107:00740
CTATCACTCGGACGTG
+
C<<9=682,,*,,,,,
@T6XXI:00107:00744
GGGAGAAGGGAGAAAGGAAGCTAGTCACTCGACATCTGTGGCTTCACAGTACAGAGGGACTGTCCAAAGCCGCAGAT
CTCGAGTCGACTAG
+
DC?CDB?BB==@::5=4:5:ACBBC@@???B??<:::B@B;??=?AABBBCBAA74317=9=@@<??:?@=@@AAA@
8>>@@@BA?????>
@T6XXI:00107:00746
GGGAGAAGGGAGAAAGGAAGGGGAGAAGGAGAAAGGAAGCTAGTCGACTCGACA
+
EKAAA@@DC@BEBE?CAB@BBB8BBB>966111,7474:?/0/>0..0++--11
@T6XXI:00107:00749
GGGAGAAGGGAGAAAGGAAGACGAATAGGCACGCGAGAAACATAAGCTATTCTCCCTCTCCCTATAGTGAGTCGTAT
TAGAATTCCCC
+
CC>AA@>BC>BBBC=BBCADDCAA=ACC@CBBCBBBCBC>CB??>@BAAC89:A@<??BCECB???@@@??@988<A
9<=A@=:=@@5
@T6XXI:00108:00728
GGGAGAAGGGAGAAAGGAAGGAGAAGTCGCCCCAAACGATTTGTTGACTATTCTCCCTCTCCCTATAGTGAGTCGTA
TTAGAATTCCCC
+
CC@AAA?BCCABBCAC@C?ABAEC@CBCCEBB8AA=BCCCC>CC?C;:9AA>AAAA<A@@@A=@@@@@<<<?@A@@@
@?A@@;>3444-
@T6XXI:00108:00736
CTATCGACTCGAGATCTGCGGCTTTGGACAGTCCCTCTGTACTGTGAAGCCACAGATGTCGAGTCGACTAGCCTTCC
TTTCTCCCTTCTCCC
+
C11/1/////9=?ABCCCCC>@??:?<>>>>77:6:BABCBB@;;;;38;?ADB@?;000000099074...,.335
576>655338556<4
@T6XXI:00108:00749
GGGGAATTCTAATACGACTCACTATAGGGAGAGGGAGAATAGTCAACAAATCGTTTGGGGCGACTTCTCCTTCCTTT
CTCCCTTCTCCC
+
999/9;>>BAB@CBBBB>BBBA?;9666-
6>>?A>A???@@@A@@>@AA<CBBBC>CCC7BAA??:?@=:=;>=??;@A@@<A>AAAA<
@T6XXI:00110:00719
GGGGAATTCTAATACGACTCACTATAGGGAGAGGGAGAATAGCCAATCTCTCGTGGTCATTGCGCTTCTCTTCCTTT
CTCCCTTCTCCC
+
AA?6A=>=ACC@CB@@BBBBBEB97111.7=<CE>A@@>@<??<=>A<@@??AAC<==?A>AA@<=;=C?>;?9<;7
<@@@7<9<@@>:
@T6XXI:00110:00734
CTAGTCACTCGAGATCTGCGGCTTTGGACAGTCCCTCTGTACTGTGAAGCCACAGATGTCGAGTCGACTAGCTTCCT
TTCCTCCCTTCTCCC
+
A?944=444;44999BBCBC@CCC?C=@@AABBB<BBB:;:A?@@=<9=B=@@CCCBAAEBBB@AA@@?B?>67;:>
>,0,/99,/,4300+
@T6XXI:00110:00739
```

```
GGGAGAAGGGAGAAAGGAAGCAGTCCATCTGTGGCTTCACAGTACAGAGGGACTGTCCAAAGCCGCAGATCTCGAGA
CGACTAG
+
CC@CCB@BB??@?C>B>A>A@CC788:???@?@@CA=??AA@@C@<=43317>>?AA?CA>A=;;<=@AA?<<<@89
<CCCBAA
@T6XXI:00111:00732
GGGAGAAGGGAGAAAGGAAGTCCAGCCATAGGGAGTCAGTGAATGGCTATTCTCCCTCTCCCTATAGTGAGTCGTAT
TAGAATTCCCC
+
9919>>;>>:999@<A=@BCDC@C?@<@BBCC<??CCBBB@@@@C?CC@A=?ACB9;;;;;6;=?@@@AB=====<A
>@@@=?8>??5
@T6XXI:00111:00744
GGGAGAAGGGAGAAAGGAAGGTGAACAGAGCGCAAACGAATACGTCTCTATTCTCCCTCTCCCTATAGTGAGTCGTA
TTACGAA
+
@E?BA@59919=>BAD@C@B?A@?=@@<;;BBBBB=?AA>?=BB?89;;99<@99918>>BC?CC>@?@;618;643
35...,*
@T6XXI:00112:00707
GGGGAATTCTAATACGACTCACTATAGGGAGAGGGAGAATAGCCGTTTAATCTCCCACTGCGGCATATCCTCTTCTC
CTTCTCC
+
CCC:D?B>8492444433<>???>?777.7>>?A<A==;<<<@>A@A@A;>=@@@;@=<<;;8;;;688,/+*)***
)*)+344
@T6XXI:00112:00713
GGGGAATTCTAATACGACTCACTATAGGGAGAGGGAGAATAGTTCTGACGGATTGCTCTGACTATTCACCTTCCTTT
CTCCCTTCTCCC
+
DDC:C?A>@ED@CCBB>?ABB@A>?6665:BBEE>B??;?<;<<;@?AAC>BA@BBBCCCCC??;???:==>477.
71;<.4243335
@T6XXI:00112:00722
GGGGAATTCTAATACGACTCACTATAGGGAGAGGGAGAATAGCCACATTCTTGTCTCCCTGGTAATAGTCTTCCTTT
CTCCCTTCTCCC
+
;99-
9>@?CBB?BBBBBBBB=@A;966659??CE>A???A<=<;<<==<==;<@@@@A<@@=@C>AAA???<>374,4;;=
:@===AC=
@T6XXI:00112:00726
GGGGAATTCTAATACGACTCACTATAGGGGAGG
+
BBB7A=@=@BA>BBBBB@@@A@@777611,/22
@T6XXI:00112:00736
CTATCGACTCGAGATCTGCGGCTTTGGACAGTCCCTCTGTACTGTGAAGCCACAGATGTCGAGTCGACTAGCCTTCC
TTTCTCCCTCTCCC
+
;11/////88:BA@ACCC@@=???8>7;;@A@CF@;;;A:::A=<<C<?BABB@@?@???@@??A@9;44..,.5::
7737454+--1110
@T6XXI:00113:00728
GGGAGAAGGGAGAAAGGAAGGATAAATTCATGGGGGGGCGTGCCATGACTATTCTCCCTCTCCCTATAGTGAGTCGT
ATTAGAATTCCCC
+
CEBAA@CBC:>99904274313333,1:??7777777&7?B??=??C@?A?<:8444,8??@D>@<<<AAAA?=?@=
==<?A?=;3444-
@T6XXI:00114:00704
GGGAGAAGGGAGAAAGGAAGGAGAAGTCGCCCCAAACGATTTGTTGACTATTCTCCCT
+
77+//7,//+/4::6=<?:@@@BB???;;;775;<<>@@AA>@?2661004/--+00*
@T6XXI:00114:00728
```

```
GGGGAATTCTAATACGACTCACTATAGGGAGAGGGAGAATAGTTTTCCCTTCACTAACCGCGAACTGCACTTCCTTC
TCCCTTCTCCC
+
CCB7A:;8<BBACCCCBBB?>CB77111.777==9?@=;;;4444-
040;9=ACBC@C?BBAB>A@A@>>:::,/,08886?>AA@C?
@T6XXI:00114:00739
GGGAGAAGGGAGAAAAGAAGCTAGTCGACTCGACATCTGTGGCTTCACAGTACAGAGGACTGTCCAAAGCCGCAGAT
CTCG
+
AB>BCB?@@094448,4377;@<<?C???????:9:=00.33/0,1166?A?988//,///9;;;;;6;9../;;;@
999@
@T6XXI:00115:00711
GGGGGAATTCTAATACGACTCACTATAGGAGAGGG
+
CKKB2@6959449@99;A888=0./...,.3.111
@T6XXI:00115:00716
GGGAGAAGGGAGAAAGGAAGCTAGTCACTCGGACATCTGTGGCTTCACAGTACAGAGGGACTGTCCAAAGCCGCAGA
TCTCGAGTCGACTAG
+
CDABBBBDB>BCBB=B@B@AAAA==?AAB??;;:7@A???C?AB@CCCAAAEC:943317==>???AA<AA?@@@A@
@???@@@@AA@@@@A
@T6XXI:00115:00720
GGGGAATTCTAATACGACTCACTATAGGGAGAGGGAGAATATCTGTCGTTCGGTCGACGCTGCCTATCCTTCCTTCT
CCCTCTCC
+
>>>4?69:<CCBCBBBBBBBCB887777.7==?B>B@A>A?<;73033348<26;;5>;;;44243/-,-,-,-
,++++*--134
@T6XXI:00115:00723
GGGAGAAGGGAGAAAGGAAGGAGACAGCCCCAGAGTACAGTCCAGAACTATTCTCCCTCTCCCTATAGTGAGTCGTA
TTAGAATTCCCC
+
CC@BBB@BA;BBBB=A>?@B@B>>BBBBBA8??BBCB>;;B?>?BBABBBC?@<<<8?A@ACBA???@@@AAAAA89
9??A@>=<==@5
@T6XXI:00115:00731
GGGGAATTCTA
+
999/48>8=@?
@T6XXI:00115:00732
GGGAGAAGGGAGAAAGGAAGGTGAACAGAGCGCAAACGAATACGCCTCTATTCTCCCTCTCCCTATAGTGAGTC
+
44-44;244-488;-969;;=><<:@@=4447344-4;;@CCC@@24444424439,33677,....33333..
@T6XXI:00116:00704
GGGGAATTCTAATACGACTCACTATAGGGAGAGGAGAAT
+
@<<*1-959889@?BBB:;;B?>=?777,6=7707*)((
@T6XXI:00116:00705
GGGGAATTCTAATACGACTCACTATAGGGAGAGGGAGAATAGTCAACAAATCGTTTGGGGCGACTTCTCCTTCCTTT
CTCCCTTCTCCC
+
CCB7A=@=ACC@CB??BBBBBCB?8166.7>=?A>BAA?@9;;AC<:??:ACCCC>AAA7A???A=?@@>@>@>AA<
@@AA<A;=?AAB
@T6XXI:00116:00747
GGGAGAAGGGAGAAAGGAAGGAGAAGTCGCCCCAAACGATTTGTTGACTATTCTCCCTCTCCCTATAGTGAGTCGTA
TTAGAATTCCCC
+
AB:@@A;>>199@A<A>@>B??@@??@@CGCC7<;/7<<66267688<8==?;;;E@A000@9;;;?877888;;<?
?=;<:;;:@@@5
```

```
@T6XXI:00117:00723
GGGAGAAGGGAGAAAGGAAGCTAGTCGACTCGACATCTGTGGCTTCACAGTACAGAGGGACTGTCCAAAGCCGCAGA
TCTCGAGTCGACTAG
+
CC>BBB@A@:B@=8:>474;@>==7,,,,,,,,,,,/6998:?A?BBBBBC@>93334-
8@@AAA=BB:@@>A?<<<;:>??>?@A;<=?99
@T6XXI:00117:00725
GGGAGAAGGGAGAAAGGAAGCGAAAGAGGGATAAGTAGAGTTGTAGACTATTCTCCCTCCCCTATAGTGAGTCGTAT
TAGAACTTCCCC
+
AA>?A>>AC@FAAH=@=@?BCCCC>CC@@>???=?AA??@A>@??@AA=A;<<@@=:877307?AA@?@===<;;@A
;@@C;=;/444-
@T6XXI:00117:00727
GGGGAATTCTAATACGACTCACTATAGGGAGAGGAGAATAGTAACG
+
---*-+9>A8==@@BCCEBB@@CC88:B9?=;<:=443/6,**)**
@T6XXI:00117:00731
GGGGAATTCTAATACGACGTCACTATAGGGAGAGGGAGAAATAGCAAACGTTTCCCCGACGTCGCTTTCTCCCTTCC
TTCTCCCTTCTCCC
+
?AA08,/,///,/4843333976887338,8>==<6<666,11116:::@AC=A==5476@::647=-
0.//+.01,-,-111034:68:4
@T6XXI:00117:00736
GGGAGAAGGGAGAAAGGAAGCCTAGTCATCGACATCTGTGGCTTCACAGTACAGAGGGACTGTCCAAAGCCGCAGAT
CTCGAGTCGACTAG
+
CD@CB?@CC@>@>B=B@>@BB@B7::CAA@>79::9:?AC?AACCCCB>>?@=====;====9=23317<:><==AA
@@A===@@@@@@@@
@T6XXI:00117:00737
GGGGAATTCTAATACGACTCACTATAGGGAGAGGGAGAATAGTCTACAAC
+
CCC9B=9;9488889@>>B;;7?>===>6==:::6://,-705....-,-
@T6XXI:00117:00743
GGGAGAAGGGAGAAAGGAAGCTAGTCGACTCGACATCTGTGGCTTCACAGTACAGAGGGACTGTCCAAAGCCGCAGA
TCTCGAGTCGACTAG
+
DCB@@@@BA9=99909<?<>?>::>0000//9;??<<>@@@=8:7:CC?@@;99:444-
4;;=@A>CA<@@>@AAAB===A=<<@=<<@@@@
@T6XXI:00117:00744
GGGGAATTCTAATACGACTCACTATAGGGAGAGGGAGAATAGTCAACAAATCGTTTGGAGGCGACTTCTCCTTCCTT
TCTCCCTTCTCC
+
CCC8?=@=@BB@BBB=9:;AB@D?>777-
7===?<@BC?B=<=@A>@A@;>>>@>9?,00,/7979/400/0/37::4;<<A<:58./.
@T6XXI:00118:00711
GGGAGAAGGGAGAAAGGAAGGAGAAGTCGCCCCAAACGATTTGTTGACTATTCTCCCTCTCCCTATAGTGAGTCGTA
TTAGAATTCCCC
+
BC:;<A>AA>BCCD@C@C@C?>@@@B:;<CCC9CC>CCCBC>CC?CAA?AA=AAAC9=898B;??@@AAABB???A@
@=@@?8;2444-
@T6XXI:00118:00714
GGGGAATTCTAATACGACTCACTATAGGGAGAGGGAGAATAGCTAACATCCCCCCGCAGCTCGAGTTCTCCTTCCTT
TCTCCCTTCTCCC
+
CCB9A=@=AGC@DCAA?CCB@@B?966646??CC>C?A=?;><;?@B@855555*;??AAAAAAAA>A><37244==
:?@@D<@=??AA=
@T6XXI:00118:00727
```

```
GGGGAATTCTAATACGACTCACTATAGGGAGAGGGAGAATAGTTTTCCCTTCACTAACCGCGAACTGCACTTCCTTT
CTCCCTTCTCCC
+
CCFBNAA=@BB@BDCBCACBB@CC88??9<<?BBCCAA>AAA@@@5??:?;==CBB?BACBB@?===?A?=;=377.
7;;;1838<<<7
@T6XXI:00118:00737
GGGGAATTCTAATACGACTCACTATAGGGAGAGGAGGAATAT
+
---*-0><>@@<@A;<;BCCCCCC???>:=;00,0/,-0100
@T6XXI:00119:00708
GGGGAATTCTAATACGACTCACTATAGGAGAGGGAGAATAGTCAA
+
6<<1<-1,16656>@@BBCCC>=;<00,00077,144444----,
@T6XXI:00119:00722
GGGGAATTCTAATACGACTCACTATAGGGAGAGGGAGAATAGTTCTGTGCATTTCTCCCAGCACACCGTCCTTCCTT
TCTCCCTTCTCCC
+
999/9<@>ABB?BCCCCBBBB@B98116.7>=?B>BAA@A=<<;:::<<====49>AA:=A==89=>@==:<=@8<=
9=AAC<?=?AAC=
@T6XXI:00119:00725
GGGGAATTCTAATACGACTCACTATAGGGAGAGGGAGAATAGTCAACAAATCGTTTGGGGCGACTTCTCCTTCCTTT
CTCCCTTCTCCC
+
CDC:C=>=?@@=AB@@@BCCCCECB:9959??BB?C@@<?=>@AD>===9?CBBC>CCC7BBBBB>?A>8=;=>BC>
@@@C=A9<<?C?
@T6XXI:00119:00728
GGGAGAAGGGAGAAAGGAAGAGAATAACAGTGCACCGAATGCGCAAACTATTCTCCCTCTCCCTATAGTGAGTCGTA
TTAGAACTTCCCC
+
CD@BBE?CB>BCBB?B@A@CABBBC@=>@@DB@AC?;;9<BBBCCC@BBCD@CAAA<@???B>BCCCCB@CAAAA:;
:><<8.//./;94
@T6XXI:00119:00737
GGGAGAAGGGAGAAAGGAAGA
+
CD@BBB?B?=?B88800,(()
@T6XXI:00119:00740
GGGGAATTCTAATACGACTCACTATAGGGAGAGGGAGAATAGTCACATTCATGTTTGTGACCGTGTCTCCTTCCTTT
CTCCCTTCTCCC
+
999/9<@>9;;=BBBBBBBCCAA?=777.7>>>@=AAA?B???AA=<B?B@BBBC>?@=??>AAAA????B>A>@@<
@AAA9=:=ACD?
@T6XXI:00119:00742
GGGGAATTCTAATACGACTCACTATAGGGAGAGGGAGAATAGCCTATGCCCTTCGCCCCACGGTTGTATTCTTCCTT
TCTCCCTTCTCCC
+
999/9<@>BCC@CBBBBB>>?@CB?77659BBCC>C@@?@=;<;>5:../,/,-13440388===;;/.,.-,-
4413446=;=487;@:
@T6XXI:00119:00745
GGGAGAAGGGAGAAAGGAAGGAGAAGTCGCCCCAAACGATTTGTTGACTATTCTCCCTCTCCCTATAGTGAGTCGTA
TTAGAATTCCCCC
+
CC><<;@CC?CBCE?B?B?B?ABC@CBACBDD8CCAACBBB;CC@CBC@AAAC???;?888A;>>?A===A@@@@@@
@:=<>84/////+
@T6XXI:00119:00747
GGGGAATTCTAATACGACTCACTATAGGGAGAGGGAGAATAGTCAACAAATCGTTTGGGGCGACTTCTCCTTCCTTT
CTCCCTTCTCCC
+
```

```
CB?2?54:<AB?CCBBAABCBBBBB996.6=;??=@DD>?8AAAA>???7??ABB@BDE9BBCCC=??@3838=@@<
?AAA=A9<<>C=
@T6XXI:00120:00700
GGGGAATTCTAATACGACTCACTATAGGGAGAGGGAGAATAGTCAACAAATCGTTTGGGGCGACTTCTCCTTCCTTT
CTCCCTTCTCCC
+
CCC9B899;CC@CDCBBBBEA?@>=777.7?===7:7??D???<?=?<:499666,167-
7=??A=??A4838?A@<A?>>7=247=>7
@T6XXI:00120:00704
GGGAGAAGGGAGAAAGGAAGGAGAAGTCGCCCCAAACGATTTGTTGACTATTCTCCCTCTCCTATAGTGAGTCGTAT
TAGAATTCCCCATCGAGATCTGCGGCTTTGGACAGTCCCTCTGTACGTGTGAAGCCACAGATGTCGAGTCGAC
+
44-49;:>B>A@AA8;8:@C=@@D@B?>;888.8?:88<><8<@>878=6<9=?;9388//,-
11104383385030564444/000+0000355AA9:9=<;==:>>77766166643......--
26<<@>@?900000003340468
@T6XXI:00120:00706
GGGGAATTCTAATACGACTCACTATAGGGAGAGGGAGAATAGAGACGTATTCGTCTGCGCTCTGTTCACCTTCCTTT
CTCCCTTCTCCC
+
A@?6A?A@CBB@BBBBAAB?@@?7869957BBCC>CAA>A<<=??@@===>AAAAA@@?AAAA@@>@@@>A?A>A@;
@?>?:?<??BC<
@T6XXI:00120:00716
GGGAGAAGGGAGAAAGGAAGGAGAACGCGCACCCGAACCAACGGTGATCTATTCTCCCTCTCCCTATAGTGAGTCGT
ATTAGAATTCCCC
+
HK@AA@@CC@CCCC@C?B@B@AAB@BBBCCCCC@CC@C?BBCC@CCEBB;;<?B@AA<ABBBB>BBBCCCBBBACBB
AA?@??<B>BBB9
@T6XXI:00120:00719
GGGAGAAGGGAGAAAGGAAGAACAAACGGTCGCTGGGAATCGAGACGCTATTCTCCCTCTCCCTATAGTGAGTCGTA
TTAGAACTTCCCC
+
CCBCCEDDC<A999149<<>>=::3/:B=??EKCDC@C=>>CCDFBBBCCCAC;;9-
9>>>>8;;@BBCCCAB?::968?58:>;>>BA6
@T6XXI:00120:00720
CTAGTCGACGCGAGATCTGCGGCTTTGGACAGTCCCTCTGTACTGTGAACACAGTGTA
+
DCAA@CCCCBAAABBBB??AC@CCC=C>BCBB???;???@===AA;56++*****++-
@T6XXI:00120:00726
GGGAGAAGGGAGAAAGGAAGCTAGTCGACTCGACATCTGTGGCTTCACAGTACAGAGGGACTGTCCAAAGCCGCAGA
TCTCGAGTCGACTAG
+
;<8<B@>@@<@>==:@>A@@@?;==/00:9791:6??7;:@<>A?ABBBCBAA?A77717==??>?CA:@@=AA@@?
A<@==9=A???AA??
@T6XXI:00120:00728
GGGGAATTCTAATACGACTCACTATAGGGAGAGGGAGAATAGACGTTACCAACTCCCGAATGCTGTCGTTCTTCCTT
CCTCCTTCCTCC
+
999/9;>;>>><A@AB@ABB::;@@==?<?AACD>A?A?A<??>=>2888344003134866????77/::7,-
,.,-,--,-012--,
@T6XXI:00120:00729
GGGAGAAGGGAGAAAGGAAGGGGAGAAGGGAGAAAGGAAGGGGAGAAGGGAGAAAGGAAGCTAGTCGACTCGACATC
TGTGGCTTCACAGTACAGAGGACTGTCCAAAGCCGCAG
+
44-444244-449>0/,06:::00.06552..11,1770169-7<9343,4::;97064/-...-22;;;;9//---
0389444/488;@@=@<<<;387300456636644421
@T6XXI:00120:00735
```

```
GGGGAATTCTAATACGACTCACTATAGGGAGAGGGAGAATAGTCAACAAATCGTTTGGGGCGACTTCTCCTTCCTTT
CTCCCTTCTCCC
+
C@;2@7<:<ABFLBBBBBBBBBCB?77777CBBC>BAA>?:?ACA?ABB:BCCCC>@B>7BAAAB@B@=3858=@D<
A@BA<A>???A<
@T6XXI:00121:00704
GGGGAATTCTAATACGACTCACTATAGGGAGAGGGAGAATAGTTCGCCAATATCCCGCGGCGTGTTCTCCTTCCTTT
CTCCCTTCTCCC
+
CA?4@5469>>;@@BBAACCCAA?>777.7==?@?@==@A@@@?A>@3:7:B?BC=?AA@CC@@@@?@>?A@C;==7
<===:@<?@BA:
@T6XXI:00121:00728
GGGAGAAGGGAGAAAGGAAGGAGAAGTCGCCCCAAACGATTTGTTGACTATTCTCCCTCTCCCTATAGTGAGTCGTA
TTAGAATTCCCC
+
CD>BBB=BA>@AAE?C@B@B?BAA?ACCECCC9BCBC>:??8?B>BBCAAA?@@<=;88>AA=@@@@A@A@AA?@?A
A>@@A??38880
@T6XXI:00121:00733
GGGGAATTCTAATACGACTCACTATAGGGAGAGGGAGAATAGCCATGAGCTACTCCATACTCCATCCCACTTCCTTT
CTCCCTTCTCCC
+
CCC:C>@>ACCACCBABBBB?@<77111.7>>@B>BAA>A===:<?@A@@@ACAC=?@A==;=@88818;;8:344-
/666,/,///65
@T6XXI:00121:00739
GGGGAATTCTAATACGACTCACTATAGGGAGAGGGAGAATAGCTATCTTTTCACCACTTTACGCCTATTCTTCCTTT
CTCCCTTCTCCC
+
CDD:E?A?AAAACCBBBCCDB>B?99:?9?@ABC@B@@>@@@C?=@BAAA5@AA?A@@@<@CBC@CC@=@?<>377-
4;;;:?>@A@A<
@T6XXI:00121:00741
GGGAGAAGGGAGAAAGGAAGGAGAACGCGCACCCGAACCAACGGTGATCTATTCTCCCTCTCCCTATAGTGAGTCGT
ATTAGAATTCCCC
+
CD@BB@?BBB???B>B@A?A?ABB@BBBCCCBB?@@=A@CACE@CDCCC<<<@CAAB?BBBCC>CCCBBBCBB@BBB
BD?@?;8@5999/
@T6XXI:00122:00705
GGGAGAAGGGAGAAAGGAAGGAGAAGTCGCCCCAAACGATTTGTTGACTATTCTCCCCTCTC
+
99599>5991948@9<:;6:,00.6444....*37,111699=>>??970/,/.---*--++
@T6XXI:00122:00719
GGGAGAAGGGAGAAAGGAAGCTAGTCACGACAC
+
AA;<:B@CB:@99904431379722,,,,,0,+
@T6XXI:00122:00724
CTAGTCGACTCGAC
+
B:1111/,::1///
@T6XXI:00122:00729
GGGGGAATTCTAATACGACTCACTATAGGGAGAGGGAGAATAGTTTTTCCCTTCACTAACCGCGAACCGCACTTCCT
TTCTCCCTTCTCCC
+
9999+499:?;<:<BBBBBBBDCCBA9665699CC>B?@>AA@@@@@1@C@@>ACCBC@C@:8<A>A=??BBC?B;?
?9;=;?:?8;:;;6
@T6XXI:00122:00733
GGGGAATTCTAATACGACTCACTATAGGGAGAGGGAGAATAGTCAACAAATCGTTTGGGGCGACTTCTCCTTCCTTT
CTCCCTTCTCCC
+
```

```
@>?4><><@;;8<CBBCAAABA@<?666.7=<AA>C??<?AB@@@>???:A::;C>BEC8CBBAA;;<87:784::7
=;<;7=>===D=
@T6XXI:00122:00745
GCTAGTCATGAGATCTGCGGCTTGACAGTCCTCTGTACTGTCGAAGCCACAGATGTCGAGTCGT
+
94444444449;49:;;:=1/.,/,,,2..,.361117111111004244444//////..///
@T6XXI:00123:00703
GGGGAATCTAATACGACTCACTATAGGGAGA
+
82208*111778:@;;87>=631....+..3
@T6XXI:00123:00711
GGGGAATTCTAATACGACTCACTATAGGAGAGGGAGAATAGTTTTTCCCTTCACTAACCGCGAACTGCACTTCCTTT
CTCCCTTCTCCC
+
DEB8B79:9>?<?A@@B@@@B;;::00,0>>BE>C@A?@930000,399?9>@CBB?B?@B@@>A=AA::;@C>AA>
B??A=A>@???;
@T6XXI:00123:00712
GGGAGAAGGGAGAAAGGAAGGAGGAAGTCGGCCCCAAACGATTTGTTGACTATTCTCCCTCTCCCTATAGTGACGTC
GTATTAGAATTCCCCC
+
8>;@@B>>>:89//+,2200*,*)*000*,*,220::::77>B9??@;;7==:2..1101--
/8589=@;;6.,******+03468<<<<<A1
@T6XXI:00123:00730
GGGGAATTCTAATACGACTCACTATAGGGAGAGGGAGAATAGACCAGCACTCCATGTTGTCATGTTCTCCTTCCTTT
CTCCCTTCTCCC
+
CCA4B596<AA>BDCBAAA@A9>?<777.777>>:@@@>@@?@A@AA????@>===A>@88:@@?;><<8;8;8;<7
<A@@<@8;444-
@T6XXI:00124:00710
CTAGTCGACTCGAGATCTGCGGCTTTGGACAGTCCCTCTGTACTGTGAAGCCACAGATGTCGAGTCGACTAGCTTCC
TTTCTCCCTTCTCCC
+
B11111//6689=@@ACBCCC@CCB=C?BBC???A<AAAACCCC@>>=8<@=@ACCCCDBCDCB89<;BABA??=?=
@A:::8;4:74344-
@T6XXI:00124:00716
GGGGAATTCTAATACGACTCACTATAGGG
+
@@@4><@=@CCAECCBCCCB?AA?@777.
@T6XXI:00124:00725
GGGGAATTCTAATACGACTCACTATAGGGAGAGGGAGAATAGTTTCCTACCACT
+
;;9/9<@=ACC@CC@?>>BBBAB<9111.6==CF>B@A>@<++++,5,**)**+
@T6XXI:00124:00730
CTCGAGATCTGCGGCTTTGGACAGTCCCTCTGTACTGTGAAGCCACAGATGTCGAGTCGACTAGCTTCCTTTCTCCC
TTCTCC
+
//4444:444448889::?89:DB@AB044444444444>:4489>=7>>A;;:?876688<;.0178788+/444-
31....
@T6XXI:00124:00733
GGGGAATTCTAATACGACTCACTATAGGGAGAGGGAGAATAGTCAATCGCTCAGCCCTTTA
+
BDE:B<;:1//,/6687<AA?788;677,7?777-74423;8477566---2--00+1723
@T6XXI:00124:00739
GGGAGAAGGGAGAAAGGAAGATTATGGGCGGTAGAAGGAGTGAATAGCTATTCTCCCTCTCCCTATAGTGAGTCGTA
TTAGAACTTCCCC
+
```

```
BC<@@>588199@B<B?B@BBB?B???<@CC@@@B?B?AAC??:?:70B@A=@@CC8?A???9?@>?@???BA<<=<
<8<@@;@?<???5
@T6XXI:00124:00745
GGGAGAAGGGAGAAAGGAAGCTAGTCGACTCGACATCTGTGGCTTCACAGTACAGAGGGACTGTCCAAAGCCGCAGA
TCTCGAGTCGACTAG
+
CC>@@=;999>99909585;AA<>=/00::;:>??BBBBB@9<??BBCCCCAAA<33317A??AC;==9<A<@@A@@
===A@===?????AA
@T6XXI:00125:00718
GGGGAATTCTAATACGACTCACTATAGGAGAGGGAGAATAGTCAACAAATCGTTTGGGGCGACTTCTCCTTCCTTTC
TCCCTTCTCCC
+
ABCBC=@=AAABDDCCCAA?B7663./,0::=>9A??<??@?<<9<??:BCA@@9???5?BCCF@@@=<?<?=@A<=
==C=B=??@C?
@T6XXI:00126:00705
GGGGAATTCTAATACGACTCACTATAGGGAGAGGGAGAATAGTCTGTCCCTTCTTTGTTTGCGCGTTTTCTTCCTTT
CTCCCTTCTCCC
+
A@@6@8;;<CC@CCBBBBBBBBB?9111.7>=@B>BAC?C;8;@AA???<?>ABC=@@@<===?AACD5==9<;>?<
?@@C>A=@?@A>
@T6XXI:00126:00709
GGGAGAAGGGAGAAAGGAAGCTAGTCGACTCGACATCTGTGGCTTCACAGTACAGAGGACTGTCCAAAGCCGCAGAT
CTCGAGTCGACTAG
+
CC?BBA=@@9;<ADAC@C@CCCCAA:::@BBC;<;CCDBCA;=??B@?@CBA>@A4427====@<??<?C>@@@@@<
<@=<<<<9<<<<??
@T6XXI:00126:00729
GGGGAATTCTAATACGACTCACTATAGGGAGAGGGAGAATAGTCAACAAATCGTTTGGGGCGACTTCTCCTTCCTTT
CTCCCTTCTCCC
+
DDEBC99;<@@?C@AACBBBBBBBB11659??CC>B@@=?<<<AB?@@A<CA@@B;?@?6ABBBD?CCA<?<?>BC:
??AA<A9<<AC<
@T6XXI:00126:00736
GGGAGAAGGGAGAAAGGAAGGCAACGATGATTTTGGCGAATAGTAAACTATTCTCCCTCTCCCTATAGTGAGTCGTA
TTAGAACTTCCCC
+
CCCBBBACC>BCBB>B@B>?>?C@DCB;;9999/9?@CCCCAAACDACCCCBCCBC>CBBBC>BBABCBCB;::???
?::>>,//,///*
@T6XXI:00127:00703
GGGGAATTCTAATACGACTCACTATAGGGAGAGGGAGAATAGAGACGTATTCGTTTGCGCTCTGTTCACCTTCCTTT
CTCCCTTCTCCC
+
CBC9B>B?BBBACB@@>?B?>@CCA999.7==?A=AAB>A?@?@@@888@>@CBB=@@@???AA@>A==9=<?<?CE
A@@@;@<999C=
@T6XXI:00127:00710
GGGGAATTCTAATACGACTCACTATAGGGAGAGGAGAATAGTACGA
+
88808+9598=9>A@BAABBBAAA?777.777747988:60,++**
@T6XXI:00127:00720
GGGAGAAGGGAGAAAGGAAGCTAGTCGACTCGACATCTGTGGCTTCACAGTACAGAGGGACTGTCCAAAGCCGCAGA
TCTAGTCGACTAG
+
CKBBAA@DD>@AAB>B@B@B@@==?BBCCCCBBBC?<?@CA>===?CCA@?<=<666635<@AAA>AA;@@>A@@@<
445.//;:;;@AA
@T6XXI:00127:00725
GGGGAATTCTAATACGACGTCACTATAGGAGA
+
```

```
444,/,/,///,/60660009948;333131.
@T6XXI:00127:00727
GGGGAATTCTAATACGACTCACTATAGGGAGAGGGAGAATAGCTCCGCTTCTTACAAACTGGACCTGCTCTTCCTTT
CTCCCTTCTCCC
+
CKK:E?A?BCC@CCCBCBBBCCCBA9995:??CC>CAA>@???BA@CCA>AA;<<==;==<>@@>@@ABBA<@=@A<
@@@;+334444,
@T6XXI:00127:00728
CTAGTCACTCGAGATCTGCGGCTTTGGACAGTCCCTCTGTACTGTGAAGCCACAGATGTCGAGTCGACTAGCTTCCT
TTCTCCCTTCTCCC
+
E=49<@449BBAAACBCBCB@AAB@B=>@CCCCC>AA<????;:?@B=?B@BB?????????@?ABBBDEAAB?CCC
C>A??CAC;>>@A<
@T6XXI:00127:00729
CTAGTCACTCGAGATCTGCGGCTTTGGACAGTCCCTCTGTACTGTGAAGCCACAGATGTCGAGTCGACTAGCTTCCT
TTCTCCCTTCCTCCC
+
=<944449?B==BBD@@BA@>AAA<A9<<CBCCC>BBBCBBB@??==:=DABDDBBBCCCAAA?@@<;8<888<<>A
C>C79767,/,///,
@T6XXI:00127:00738
GGGAGAAGGGAGAAAGGAAGACTAATGGGAGGCCGAACTACAGATGCTATTCTCCCTCTCCCTATAGTGAGTCGTAT
TAGAATTCCCC
+
CC?CCB@CC=?@@@<@?B?CAAAC@CCE>;;;:@BC@BCA@@888<<?BB@B<<919>99909@?>?@@@??BCDBB
>FCD@C=@@@6
@T6XXI:00128:00722
GGGGAATTCTAATACGACTCACTATAGGGAGAGGGAGAATAGTCAACAAATCGTTTGGGGCGACTTCTCCTTCCTTC
TCCCTTCCTC
+
CCC9BAB>BBB@FCA?@CCB>?ABA66659??BE>?;;?A;AAAA<8?A:BBBBB?BCC8@>>AB?ACA888;/3/4
444-434344
@T6XXI:00128:00724
GGGGAATTCTAATACGACTCACTATAGGGAGAGGGAGAATAGTTTTTCCCTTCACTAACCGCGAACTGCACTTCCTT
TCTCCCTTCTCCC
+
BED:DAB?@995948>?AAAA@><<777,777@B>C@@>@???BBA1@@5838ABCC@C@C?<<?AB@?BBB>A>AC
>===C<A>?@?@:
@T6XXI:00129:00705
GGGGAATTCTAATACGACTCACTATAGGGAGAGGGAGAATAGATCACCGTTGGTTCGGGTGCGCGTTCTCCTTCCTT
TCTCCCTTCTCCC
+
BCB4@54598888499;:?<B=<><767-
7=777.:>>>AACD?9;09;;,0,0:;;;9;88:?<?<>CADC@C>ABBA@@D@DA:88@:
@T6XXI:00129:00707
TGACGACTCACTATGAGGAGAGGGAGAATAGCTCGTTATTCTTCTCCTTTTGGTCTGTCCTTCCTTCTCCCTTCTCC
C
+
,///<99894:66///1,/8...+37<<@984;;;BACC@@@=<@97??<2<>7802,,+,22*,*,,..+.36449
4
@T6XXI:00129:00721
GGGAGAAGGGAGAAAGGAAGGAGAAGTCGCCCCAAACGATTTGTTGACTATTCTCCCTCTCCCTATAGTGAGTCGTA
TTCAGAATTCCC
+
=C?@@@;>@8;9:A,1,0,0,..66..0>>BB8BG@CCA?>;>?:::<88;:;:==:;4444+0333466337=837
4455.300****
@T6XXI:00129:00725
```

```
GGGGAATTCTAATACGACTCACTATAGGGAGAGGGAGAATAGTCAACAAATCGTTTGGGGCGACTTCTCCTTCCTTT
CTCCCTTCTCCC
+
@?>4><><@@@=@BBBCBBABCCBA996.7<7776:=?>A@A@??;?AA<CCCCB>CCC9CCBBC?BCA>A>@>@@<
@A@A<A9==AA<
@T6XXI:00129:00730
GGGAGAAGGGAGAAAGGAAGCTAGTCACTCGACATCTGTGGCTTCACAGTACAGAGGGACTGTCCAAAGCCGCAGAT
CTCGAGTCGACTAGCTTCCTTTCTCCCTTCTCCC
+
CC<ACC@CB<@;;809685;A?==?7771111639@@?AA8:<@AAACBBB@>934417;=?DD>@@<@@;==AAAA
9==@@?>?@==<A@>=;>;>>:>>>B6;344::4
@T6XXI:00129:00740
GGGAGAAGGGAGAAAGGAAGACGACATGCACATCGGGGGAAGGTTTGGCTATTCTCCCTCTCCCTATAGTGAGTCGT
ATTAGAACTTCCCC
+
ED?ADA@BC@BBBH@C@C>@@BCBBBCCBBBBBCBCCC2C@BCBB>B@CCCC?BBBA<>>???:?AAACEDCCCCB8
88584A>CCACCC5
@T6XXI:00130:00708
GGGAGAAGGGAGAAAGGAAGTGCAGTTCGCGGTTAGTGAAGGGAAAAAACTATTCTCCCTCTCCCTATAGTGAGTCG
TAT
+
;B:>>@5991948;9?<@:;;@AACC>BB@@=??AC<<;@CC>BBBBB-
CCCC8::AA<ABBDFCBB===@????<=<??
@T6XXI:00130:00731
GGGGAATTCTAATACGACTCACTATAGGAGAGGAGAATAGTCAACAAATCGTTTGGGGCGACTTCTCCTTCC
+
994,429244424:?BB@@??010/0/,,,,.,.24457<<==>AAA<BCAAA=AEC8<>7,,+,70++*++
@T6XXI:00130:00732
GGGAGAAGGGAGAAAGGAAGGAGAAGTCGCCCCAAACGATTTGTTGACTATTCTCCCTCTCCCTATAGTGAGTCGTA
TTAGAATTCCCC
+
BDBCBB@BA;@@AABB@B@B@B@@??@B;;<@4:86:BBCC>CC@BAA?@D>@@AA<===@C?C=<=@AA@A@A@@@
@?A?;9;9:8:1
@T6XXI:00131:00702
GGGGAATTCTAATACGACTCACTATAGGGAGAGGGAGAATAGTCAACAAATCGTTTGGGGCGACTTCTCCTTCCTTT
CTCCCTTCTCCC
+
8C817+/,/6778=A@@@@@@?>>>777-7?777.7::7;=<7345659481117,111,164353331,-,-
017+--00+0233353
@T6XXI:00131:00710
GGGGAATTCTAATACGACTCACTATAGGGAGAGGGAGAATAGCGTCAACAATCTGGGTCTACTCTGCTTCCTTCTCC
CTTCTCCC
+
/11+1-
9598>;>@A@@@CCDBA??777.7=777.:A;<><887037;?<:;@@@><;;998@@:449131313111,10111
1,
@T6XXI:00131:00720
GGGGAATTCTAATACGACTCACTATAGGGAGAGGGAGAATAGTCAACAAATCGTTTGGGGCGACTTCTCCTTCCTTT
CTCCCTTCTCCC
+
999/98?>ABB@BBBB?????@B?9661-
6=;?A>AAA?A;:=AA<;<;:<ACCE>BBB7BAAAB?AA@<?<?>@A<@BAB<A;??AC<
@T6XXI:00131:00731
CTAGCGACTCGAGATCTGCGGCTTTGGACAGTCCCTCTGTACTGTGAAGCCACAGATGTCGAGTCGACTAGCTTCCT
TTCTCCCTTCTCCC
+
```

ABABBBABAECAAACCBBBB?BCC>B?BBAAABB<AAAABAA<<=8858C@CCCCCCBCBBBDBBB@@?A??8:?=@
B6;4==7:384@A<
@T6XXI:00131:00733
GGGAGAAGGGAGAAAGGAAGGGGAGAAGGGAGAAAGGAAGCTAGTCGACTCGACATCTGA
+
EC>BCB<@=<@B=?=B=@=@BA5??B??=9=776,7473:>6:;>0//4//004---1--
@T6XXI:00131:00735
GGGAGAAGGGAGAAAGGAA
+
EDCCCC==95>911,-+-+
@T6XXI:00131:00745
GGGGAATTCTAATACGACTCACTATAGGGAGAGGAGAATAGTCACAAATCGTT
+
CCC9B>=?B;;7;@>@@@@@A@@??::7.7>773:;@;;;87.--++*....4
@T6XXI:00132:00701
GGGAGAAGGGAGAAAGGAAGGAGAAGTCGCCCCAAACGATTTGTTGACTATTCTCCCTCTCCCTATAGTGAGTCGTA
TTAGAATTCCCC
+
HC@CCC@BB=?BBB>B@B?A?ACB@@?AABBB8BB>@@@@C>BB@CBBAA<<8300+49:;A<A@@?@AAB?==<AA
A?AAA?C=?@@5
@T6XXI:00132:00702
GGGAGAAGGGAGAAAGGAAGGAGAAGTCGCCCCAAACGATTTGTTGGACTATTCTCCCTCCTCCCTATAGTGAGTCC
GTATTAGAATTCCCC
+
6=6<<@;>>19888565466-000976999::6CC>::1176=>;?;?B?>:9;.//,-.,---+--
58<<<=4..,----0113232333.
@T6XXI:00132:00735
GGGGAATTCTAATACGACTCACTATAGGGAGAGGGAGAATAGTCAACAAATCGTTTGGGGCGACTTCTCCTTCCTTT
CTCCCTTCTCCC
+
:;;,95424449:?AC@@@BA???7777-
7==@E<B@@>A8885843884<><;?88?@2??=770710949<>@@;?@>>9;/4034-
@T6XXI:00133:00726
GGGAGAAGGGAGAAAGGAAGCTAGTCACTCGACATCTGTGGCTTCACAGTACAGAGGGACTGTCCAAAGCCGCAGAT
CTCGAGTCGACTAG
+
DDDCCB@CC?BBBB>B=>=>BAAAC?@?AAA:;8?@?BBC@@@=?CCCCBC@:633317>>???>@@;@A>??:===
<<<=<<<8::<<;?
@T6XXI:00133:00729
CTAGTCACTCGAGATCTGCGGCTTTGGACAGTCCCTCTGTACTGTGAAGCCACAGATGTCGAGTCGACTAGCTTCCT
TTCTCCCTTCTCCC
+
@:4449444====ABDCC@@=?>@<@8;;CCBCB>CCCAABB@?@@@>@DCCCCBBBAAAAB@;;;887<777@@@A
C>?8=A<A8<6<<7
@T6XXI:00134:00724
GGGAGAAGGGAGAAAGGAAGGAGAAGTCGCCCCAAACGATTTGTTGACTATTCTCCCTCTCCCTATAGTGAGTCGTA
TTAGAATTCCCC
+
AC>DDC@CC>@@?B=B>A@C?AAC?BBC@@@@8@CACC???=?A@EJCA??>?@@@;A??=>18888==?>AA???A
@=@..,-,../+
@T6XXI:00134:00727
GGGGAATTCTAATACGACTCACTATAGGGAGAGGGAGAATAGTCTAGCTGGCTTTATTGCTGCTATTCTCCTTCCTT
TCTCCCTTCTCCC
+
CDE:D<:<;CB=ADBBBBCACCCBB9775:??CC>CA@>????@C@==<?;;<=7<C<=?>?C@@@?@A@<=86,//
,,,,5111.++30
@T6XXI:00134:00744

```
GGGAGAAGGGAGAAAGA
+
9>5949594-///6+/.
@T6XXI:00135:00704
GGGAGAAGGGAGAAAAGGAAGACGAT
+
BB7<;@=>@094---*-87*-,,,,,
@T6XXI:00135:00705
GGGAGAAGGGAGAAAGGAAGCTAGTCGACTCGACATCTGTGGCTTCACAGTACAGAGGGACTGTCCAAAGCCGCAGA
TCTCGAGTCGACTAG
+
BB>BCB?BB=BB=::@675;B?:>>/00<::00:???9AA@=@B?BACCBBA@>>77717@>@CA=@@<@A?@@===
=999==<@=<<@@@?
@T6XXI:00135:00708
CTAGTCACTCGAGATCTGCGGCTTTGGACAGTCCCTCTGTACTGTGAAGCCACAGATGTCGAGTCGACTAGCTTCCT
TTCTCCCTCTCC
+
;<1////44;9949A@>>?>;888/848366;888??:::B?<;?@@<@C>AABCCBAABBBB?@@B??8:::7:@@
>9;668,/+++*
@T6XXI:00135:00727
GGAGAAGGGAGAAAGGAAGATAGTTGCGGGTCAATGGTTTCGCACTATTCTCCCCCTCCTATAGTGAGTCGTATTAG
AATTCCCCCATCACCGACTCTCGAGTACAGCACTA
+
9249989=:=8>@<@=><><7:1666676,1687889?@67377689=;8:>>==/00,...37734<=545AA:68
<8653322.6--.1012334442244...330000
@T6XXI:00136:00732
GGGGAATTCTAATACGACTCACTATAGGGAGAGGGAGAATAGACACTAAC
+
999/9<@?CBB@BBBBB<<<?@B@=<??==CCA@>C@=;=4-,,,+++*+
@T6XXI:00136:00742
GGGAGAGAATTTGGTAATGGGCCAAAACACGA
+
CA?AAAAB@BB=B?BC>>>>//,///*/////
@T6XXI:00136:00745
GGGAGAAGGGAGAAAGGAAGAGAACAGCGACGGGAAATAATGTTTTTAGCTATTCCCTCCCTCCTCCTATAGTG
+
DK?BBBACB:@A@@<A=?<@C@?=AC@=>>988,9909C<>37888,9;@>94244-444-442/65......6
@T6XXI:00137:00707
GGGGAATTCTAATACGACTCACTATAGGGAGAGGGAGAATAGCTGCCCGTTGTCTCTATTCCCATCCCTCCTTCTCC
TTCTCCC
+
@>>4?=@?BBB@B???<<<>?@B?=6665:==BC>B@@>@AACCBBB;CC=@@@A?>>=;=;4:444,.+*+++++*
+0249=;
@T6XXI:00137:00731
GGGGAATTCTAATACGACTCACTATAGGGAGAGGGAGAATAGTACGAACAAATCGTT
+
/11+/5>8@BCIEBB?B?AA@BB>9666-6=6665:@B9;76////.0-00+11117
@T6XXI:00137:00739
GGGAGAAGGGAGAAAGGAAGACAGAATAAATGTTAGATTGACGGGGACTATTCTCCCTCTCCCTATAGTGAGTCGTA
TTAGAATTCCCC
+
CCBBBB@DD@CBBC?B?A??BBBBB@?=<;=@?>A@=B?BAB@@@6AAC??>;<==;668===?8=?====<=<<8<
<;>??<@>@@B5
@T6XXI:00137:00744
GGGAGAAGGGAGAAAGGAAGCTAGTCACTCGAGATCTGCGGCTTTGGACAGTCCCTCTGTACTGTGAAGCCACAGAT
GTCGAGTCGACTAGCTTCCTTTCTCCCTTCTCCC
+
```

```
JCABBBCFGBBBBB>B9;7;@CABCCA888?A?ABCBCCB?:::7:=@BD;;;C<;<@?<;<A>???>@@<??@===
@@????989@?@@A??<??AB7:444-48:8463
@T6XXI:00138:00707
GGGGAATTCTAATACGACTCACTATAGGGAGAGGGAGAATAGTCAACAAATCGTTTAGGGCGACTTCTCCTTCCTTT
CTCCCTTCTCCC
+
AAA7@:;;<BB@DA@@AACCCCCB?996.7==?B>BCC>@<@@AA?@@@<BBBBC>BBC>CBBB@<;;;3737:==:
???C<A>@@@A<
@T6XXI:00138:00711
GGGAGAAGGGAGAAAGGAAGCTAGTCGACTCGAGATCTGCGGCTTTGGACAGTCCCTCTGTACTGTGAAGCCACAGA
TGTCGAGTCGACTAGCTTCCTTTCTCCCTTCTCCC
+
>>:>9959@:@99918=?=@A?::;/0/;===>>ABCBBBE@BCB>B@BEBBA?A<=C<588?????A?AA<@@AA?
??===AAAA>@@BB778;=;;=7=@@@18349:;:
@T6XXI:00138:00738
GGGAGAAGGGAGAAAGGAAGGACGCTGTCGTCGGTAGCTATTTGGGGACTATTCTCCCTCTCCCTATAGTGAGTCGT
ATTAGAATTCCCC
+
BB=AHC?CB?BBA?=B=@=@?BBBBABABABAA=A@BBB99919>>4AEGBE?CACL@BCCDD@DDDBBBA@A@<<;
01,100,1;==>1
@T6XXI:00138:00745
GGGGAATTCTAATACGACTCACTATAGGGAGAGGGAGAATAGCTTAGCCTTCCGTCACCGCGGACCTCCCTCCTTTC
TCC
+
444,4242499<=BBB::;CB@@CAAAAAACCCC<BAA<?<<<A=????@>@;7788853@;:48344;,30/00+-
--,
@T6XXI:00138:00748
GGGGAATTCTAATACGACTCACTATAGGGAGAGGGAAGAATAGCCACAATCGTTGTTCGTGCAGT
+
944,48>=ABA?BBCBBBBB@@@9?666./:9:<,0,//4468:585<;:68>;;31001,,++.
@T6XXI:00139:00716
GGGGAATTCTAATACGACTCACTATAGGGAGAGGGAGAATAGTTACCAATGTTTGCTCCCTGTAACGGCCTTCCTTT
CTCCCTTCTCCC
+
AA@4@5959@?<ABBBBBCCBGC?;111.7<<BC>C@B>A;;;5::<A>??@B;??@CC;@@AA>@>;=3428?BB>
??AA<A<??CC<
@T6XXI:00139:00725
GGGAGAAGGGAGAAAAGGAAGCTAGTCGACTCGACATCTGTGGCTTCACAGTACAGAGGGACTGTCCAAAGCCGCAG
ATCTCGAGTCGACTAGCTTCCTTTCTCCCTTCTCCC
+
>95999544-
44889,4231333..4/.....4.118887BB98:7:B@BCCA???@??:?=>>@@=@A<B@?D@@?@A???888??
>@ABA@@@>A?AC699@@:@=?@CC;
@T6XXI:00139:00726
GGGAGAAGGGAGAAAGGAAGATAAGAACAGCTCTGGGCAGGGAATGAGACTATTCTCCCTACATCCCTGATAGTGTA
GTCGTTCATTAGAACCTTCCCC
+
991444244-444;-
423777;;>>>=A@?>;6111,111626311003369:.//320,,,,,.5+//77=AA@@?61112,---,-
22,0/0/04;0
@T6XXI:00139:00744
GGGGAATTCTAATACGACTCACTATAGGGAGAGGGAGAATAGCTAACATTTCGCGTCTCCCGATCCTC
+
999-98><@@ACDCCBBBCBBAB<;111-6;;;?<A@?=@A@@BB>@?<<7<====;6111,,++0**
@T6XXI:00140:00711
GGGAGAAGGGAGAAAGGAAGGAGAATAGCAGCAATAAAACTAACTAGACTATTCTCCCTCTCCCTATAGTGAGTCGT
ATTAGAATTCCCC
```

```
+
CC@CCB?AA=BBAB>B?C?A?ABB@C@===CBCABCBB:BBB>@B?@@@CCC@@@@C>CC<<>;?ADCBBB@@@CAA
?>?AAA?B?BCC:
@T6XXI:00140:00718
CTAGTCGACTCGAGATCTGCGGCTTTGGACAGTCCCTCTGTACTGTGAAGCCACAGATGTCGAGTCGACTAGCTTCC
TTTCTCCCTTCTCCC
+
=@1/////9=A49<CBBBBB@=@@@<@@DCB@@BB>AAA<??ABA?>@>=@>ACCCCBBCCCCB?@@;;;7;9=>@?
BC<??<;6;384:==
@T6XXI:00140:00731
GGGAGAAGGGAGAAAGGAAGAGGTGCGCGACTGATCCCCTGAGTCAGGCTATTCTCCCTCTCCCTATAGTGAGTCGT
ATTAGAATTCCCC
+
CCACBA@BB?BBBB?A@?>???@??BAA?>=ACCA<><7A@@BBCC@@B@A<>@???;?=?;?<===AAAA@??@A=
==;===>A9<<<0
@T6XXI:00140:00743
GGGAGAAGGGAGAAAGGAAGCTAGTCGACTCGACATCTGTGGCTTCACAGTACAGAGGGACTGTCCAAAGCCGCAGA
TCT
+
CCBDCB@DD>@A?A>B?B?BC@7;;/00>>><>??;;:CA?;8:7:ABBCBAB;8333-
4;;;?@?@C<AA?A@@@@>8=
@T6XXI:00141:00707
GGGGAATCTAATACGACGTCACTATAGGGAGAGGGAGAATAGTCTAATCAAGTCCTT
+
07718+-,00*,///3333779>><;<@<@?BCD<???566<=0002000/000/0/
@T6XXI:00141:00713
GGGGAATTCTAATACGACTCACTATAGGGAGAGGGAGAATAG
+
DHD:C<1-1<=,///177??CAA?@777.777>?.700/002
@T6XXI:00141:00724
GGGAGAAGGGAGAAAGGAAGCTATCGACCGACATCTGTGGCTTCACAGTACAGAGGGACTGTCCAAAGCCGCAGATC
TCGAGTCGACTAG
+
IE@AACACC<@@@@:B>A?ACB?<<;00,/002,..7743?A?C@??CCC??;333-
4;;?AA>AB<??<?===A@@@AA@@@A@@??88
@T6XXI:00141:00732
GGGGAATTCTAATACGACTCAC
+
B@;,9695944244486-,,,*
@T6XXI:00141:00745
GGGAGAAGGGAGAAAGGAAGCTAGTCGACTCGACATCTGTGGCTTCACAGTACAGAGGGACTGTCCAAAGCCGCAGA
TCTCGAGTCGACTAG
+
DEAECB?B@=>@885>4848@>:;;/00>===1::BBBCCA:=@@@CCCBB@@?933317;<=?A<??:>?=???@B
A@@>AAAA???AA@B
@T6XXI:00142:00700
GGGAGAAGGGAGAAAGGAAGCTAGTCGACTCGAGATCTGCGGCTTTGGACAGTCCCTCTGTACTGTGAAGCCACAGA
TGTCGAGTCGACTAGCTTCCTTTCTCCCTCTCCC
+
EC>A<;79@:>8>>9=<?<?CA@>>777777>AAAAC8???:?@@;B>AABBB@@<@AA===@?>>??>@A?AA@?8
88<==ADHC<9=AA=<=>;9;;67///,/,5500
@T6XXI:00142:00709
GGGAGAAGGGAGAAAGGAAGCTAGTCGACTCGACATCTGTGGCTTCACAGTACAGAGGGACTGTCCAAAGCCGCAGA
TCTCGAGTCGACTAG
+
9914=@>AB<@A@><?=@<?B>;::/000/.//61667A@=:AD@C:::ACA?99344-
4;;<<=?BA?AC>@<<=AAA@@@?>=9:444:9
```

```
@T6XXI:00142:00720
GGGGAATTCTAATACGACTCACTATAGGGAGAGGGAGAATAGTCAACAAATCGTTTGGGGCGACTTCTCCTTCCTTT
CTCCCTTCTCCC
+
CDC:C@C@CCC@CABBBBBB>@?>96675:BBCD>@>>>A<@?@@>@AA<A@=A?8???5?AAAC@A@>9<9<=AA@
@@@A<@=@@AA;
@T6XXI:00142:00721
CTATCGACTCGAGATCTGCGGCTTTGGACAGTCCCTCTGTACTGTGAAGCCACAGATGTCGAGTCGACTAGCTTCCT
TTCTCCCTTCTCCC
+
B::=1118;;BBCBC@@@9:8;CC>C@CCBBABC>BBCCBB@=<@A@>AB@ABBBB@>BBBBCBCCBCBCC?@?@?B
C>B@@A;?;>8871
@T6XXI:00142:00725
GGGGAATTCTAATACGACTCACTATAGGGAGAGGGAGAATAGAGACGTATTCGTTTGCGCTCTGTTCACCTTCCTTT
CTCCCTTCTCCCATCACCCGACTG
+
BCC9C=@=@<;6;BA@@@BBCBA?A776.7<<@B?A?@>@>878;;;899>ABAD<A?????@AA>A@@=@?A>AH<
???@7>?=::;,//8///+-3330
@T6XXI:00142:00735
GGGGAATTCTAATACGACTCACTATAGGGAGAGGGAGAATAGTCAACAAATCGTTTGGGGCGACTTCTCCTTCCTTT
CTCCCTTCTCCC
+
CCB9B=?=ACB@CAAA>>>?B@BD<:::88=??A>B@@?A=@AA@>???;ABBBC=AAA7ACCCG@BB@<?<?>AC<
@@AA<A:=<AC<
@T6XXI:00142:00736
GGGAGAAGGGAGAAAGGAAGCTAGTCACTCGACATCTGTGGCTTCACAGTACAGAGGGACTGTCCAAAGCCGCAAGA
TCTCGAGTCGACTAGCTTCCTTTCTCCCTTCTCC
+
BA>BA@;@@:>99909585;BAB@?<77111160117716078177:@CDB?>><;;6;;;<@A>AA<AA;===:=?
?=??888;B@899@AAA>@@AC;@AAA7<9<>>;
@T6XXI:00142:00738
TCGAGATCTGCGGCTTTGGACAGTCCCCTCTGTACTGTGAAGCCACAGATGTGAC
+
@D<:?DCDCCCC????:?>??>77777,17=ACBBCCBB@>@A>BA93..*)))(
@T6XXI:00142:00740
GGGGAATTCTAATACGACTCACTATAGGGAGAGGGAGAATAGCTCCCAGACCTTTCACAGCGGCATTGCCTTCCTTT
CTCCCTTCTCCC
+
?>>4>;?>@@@=ACCCCCBB?@C@>69959??BC>B??<@??>BBC>B@AA;==<A@@@??@?A==;=A>AC??CC?
??@A;@8;;;;6
@T6XXI:00142:00741
GGGAGAAGGGAGAAAAGGAAAGCTAGTCACTCGAGATCTGCGGCTTTGGACAGTCCCTCTGTACTGTGAAGCCACAG
ATGTCGAGTCGACTAGCTTCCTTTCTCCCTTCTCCC
+
@@<;9;?>;:8//11*/,..+.63119>>=;716006677>@@<AB?A?AABBDLE>@===@@@@???>?8838@@8
89?BA@@@A@@??>==<;9;=??7<=@>:><888@A
@T6XXI:00142:00743
GGGGAATTCTAATACGACTCACTATAGGGAGAGGGAGAATAGCCAAAATTATTGACCCGGCCGCATTCTCTTCCTTT
CTCCCTTCTCCC
+
999/424:;AB?BBBBBBBBABBCCA<A<799==9<4434;44349919=>>9>:881:=?>@?@@?===A>@>@?:
?<;;6<8:9;;-
@T6XXI:00142:00747
GGGAGAAGGGAGAAAGGAAGCTAGTCACTCGACATCTGTGGCTTCACAGTACAGAGGGACTGTCCAAAGCCGCAGAT
CTCGAGTCGACTAG
+
```

```
DCACDB?BB=BB=>:A675;C@??CC>;>>?00.666>>C?AG?ACC@??A==64331389>??=@@;>A?CAA???
===A@@@?@@@AAA
@T6XXI:00143:00711
GGGAGAGAATTTGGTAATGGGGTGGCAATGTGTGAGGTCCAACGTATTTCTCTCCCTTATATCTCTCTCCCTATAGT
GAGTCGTATTAGAATTCCCC
+
BC@ECBBB@BB<?=AC@CCCC9BC@CB@BAAA><<99A?=A?BBBCCC>@<5;==698<?888@@889AA<=A@BBB
BCCCA<8889<:;:;3444-
@T6XXI:00143:00718
GGGAGAAGGGAGAAAGGAAGGGCCAGCCGGTGGGAGAAAACCGTGTGCTATTCTCCCTCTCCCTATAGTGAGTCGTA
TTAGAACTTCCCC
+
BB>BCB?AA=?A@C>B>B=@@<@@CBB>>>@CD>BBB?@7B@BBAABCBAB>BCDI@BBBBB>BBBBCCCCCBBA::
::?AB5:;?BFA7
@T6XXI:00143:00721
GGGGAATTCTAATACGACTCACTATAGGGAGAGGGAGAATAGTCATTGCCTCACGATCAATTACCGTTCTCTTCCTT
TCTCCCTCTCC
+
9??6=@CCBBBABBCA@@CBBBBCC??@=@ADED>B@A>A:8888><<95888///333335777559////44444
32000+03222
@T6XXI:00143:00726
GGGGAATTCTAATACGACTCACTATAGGGAGAGGGAGAATAGTCAACAAATCGTTTGGGGCGACCTCTCCTTCCTTT
CTCCCTTCTCCC
+
999/9;>=ACC@CBBBABBD@:??=666.7>>@A>B@@?ABAA@<:=<53??;;=-
777.7?775:AA?>A?A>@@;???@<A;==AA<
@T6XXI:00143:00729
GGGAGAAGGGAGAAAGGAAGGAGAAGTCGCCCCAAACGATTTGTTGACTATTCTCCCTCTCCCTATAGTGAGTCGTA
TTAGAATTCCCCC
+
CD@CCC@CC>ABEC>>>@574:>><>??>>??.880677?B>BC@CBC@AA:<=@@:?=<<A;???@@@AAA?===<
<<;<<;<<77:?.
@T6XXI:00143:00734
GGGAGAAGGGAGAAAGGAAGCTAGTCGACCGACATCTGTGCT
+
CC<A<;9<C>?@@@<@>A>BD;::B??:7+****((((*.6
@T6XXI:00144:00706
GGGAGAAGGGAGAAAGGAAGCTAGTCGACTCGAGATCTGCGGCTTTCGACAGTCCCTCTGTACTGTGAAGCCACAGA
TGGACTGTCCCTTCCTTCTCCCTTCTCCC
+
@C199@699094>?:@>B?BBDCDDDCCAAA?@?AB???AA????<?CCBBBBAB<?A>899???AAA?AA=@A@@@
8838;;:444-4/4/323::=::8>8>=7
@T6XXI:00144:00714
GGGGAATTCTAATACGACTCACTATAGGGAGAGGGAGAATAGCAAACGTTTCCCCGACGTCGCTTTCTCCTTCCTTT
CTCCCTTCTCCC
+
BBB9A>A>BBB@CCBBBAABBBBB;66646?ACD>CBC@A<99==9=CCC>BBB8BBCBB>>::88897:;;;334-
4::;7?>@AAA<
@T6XXI:00144:00715
CTATCACTCGAGATCTGCGGCTTTGGACAGTCCCTCTGTTAGTGAGC
+
CCCBA::;BABAADBBCA@=@DC>B>ABBBAAA<?::/,+,,,,,++
@T6XXI:00144:00716
GGGAGAAGGGAGAAAGGAAGGAGAAGTCGCCCCAAACGATTTGTTGACTATTCTCCCTCTCCCTATAGTGAGTCGTA
TTAGAATTCCCC
+
```

```
CE?AAA@CD@BBBB>C@C>B>AAA?A@BBCBB8BC>CCCCC>BB@CCBBB@>A@@?:<<<@A<A???===@@@?@??
?=<<<:;27880
@T6XXI:00144:00719
GGGGAATTCTAATACGACTCACTATAGGGAGAGGGAGAATAGTTATATGCCGGTCTGCAGTCTATTCTCCTTCCTTT
CTCCCTTCTCCC
+
???4>8>@ABB@CCCCC?@>BCBB=69959??AD>???@AAAC<588???:?<====@??@????<?==3838:==7
>>???7>:=<<>:
@T6XXI:00144:00749
GGGAGAAGGGAGAAAGGAAGGAGAATGCTAACGCGCAGAATTGGCGGACTATTCTCCCTCTCCCTATAGTGAGTCGT
ATTAGAATTCCCC
+
CKDBBB=>=:@@@@<B=>;>;>=>.0/>?@;>CBB?<;>;>;?@@@=@@CCC>@==>;A=A>><<<?@@AABA???=
==<??A?A5<<<4
@T6XXI:00145:00714
GGGGAATTCTAATACGACTCACTATAGGGAGAGGGAGAATAGCAAACGTTTCCCCGACGTCGCTTTCTCCTTCCTTT
CTCCCTCTCCC
+
?AA7A>@<@;;8:CAB@E@A@AAB::::7:BCCC>@??>@???@D<=??C>CDD9DBA@@:777>9=?C?B@B>A@;
;::;,/////,
@T6XXI:00145:00716
GGGGAATTCTAATACGACTCACTATAGGAGAGGGAGAATAGCCTTTGTCTCCCGGACGCTAATTTTCCTTCCTTCTC
CCTTCTCCC
+
DCC:C@CBCB?>@=;:;ABB@;:;<00,.37;6888774:7437=7=:77>=4703;0003/---*-3333-.-
.4445944:=B<
@T6XXI:00145:00721
GGGGAATTCTAATACGACTCACTATAGGGAGAGGGAGAATAGTCAACAAATCGTTTGGGGCGACTTCTCCTTCCTTT
CTCCCTTCTCCC
+
ECB9E@A>@CCABBBBA>@@BCB99116.7==>A=AAA@AA@@@?=AA=;A@???;?@@8@>>>>=9??2:57;AA<
@@AB<A<?:8:6
@T6XXI:00145:00729
GGGAGAAGGGAGAAAGGAAGAGAACAGCGACGGGAATAATGTTTTAGCTATTCTCCCTCTCCCTATAGTGAGTCGTA
TTAGAATTCCCC
+
CCDCCB@CC>BAEB>B@B@BBAA?BCCBBABBC@C=@@@BBBCC9BBBCCCFCDCC?BBBCD@BBBBBCBCC??@::
:;?843028891
@T6XXI:00145:00749
GGGAGAAGGGAGAAAGGAAGTGCAGTTCGCGGTTAGTGAAGGGAAAACTATTCTCCCTCTCCCTATAGTGAGTCGTA
TTAGAATTCCCC
+
DDCCEC@CC@DCBB?B?B?ABBCCBB@CBBB?B?BCEDC?77,111,8??B@CCCC>BBBBC>?==@??A=AAAA@=
=;=@A?A<???4
@T6XXI:00146:00700
GGGAGAAGGGAGAAAGGAAGGAGAAGTCGCCCCAAACGATTTGTTGACTATTCTCCCTCTCCCTATAGTGAGTCGTA
TTAGAATTCCCC
+
CH>BAA5941999@:@<A=A=@BCACAAABCC9DD?@@@A?9AB>@?CCA?=?A@@<@899D<????AA@@?888<;
;=:::56255;.
@T6XXI:00146:00720
GGGGAATTCTAATACGACTCACTATAGGGAGAGGGAGAATAGATCACGACCCTTCTACCACGATTGAGCTTCCTTTC
TCCCTTCTCCC
+
BB@7A>B>BCC>@?BBBCCA977>>777.777?A>??@@AA===@BDBAAC>B>=;//,0>8;A???@<95969911
1<<8;-1/00,
@T6XXI:00146:00724
```

```
TCGAGATCTGCGGCTTTGGACAGTCCCTCTGTACTGTGAAGCCACAGATGTCGAGTCGACTAGCTTCCTTTCTCCCT
TCTCCCCTTCCTTTCTCCCTTCTCCCTTCCTTCTCCCTTC
+
BCAAABBBBBBC@CCC?C?BBBCBBA<ACCBB>;;>>>?;@<:>?@A===?AAA?AAECBAAA<<89@@@<@>@>09
598888.8=?577.7777.73735563,/,/,----+-,4
@T6XXI:00146:00736
GGGAGAAGGGAAGAAAGGAAGCTAGTCGACTCGACATCTGTGGCTTCACAGTACAGAGGGACTGTCCAAAGCCGCAG
ATCTCGAGTCGACTAG
+
CD>B@;244195999042429<>:==/00<<<;>??AAACCC>?@@C@@@??@A?<44444====A=555559.//9
=@@AB=??B=?:>?==
@T6XXI:00146:00737
GGGAGAAGGGAGAAAGGAAGCTAGTCGACTCGAGATCTGCGGCTTTGGACAGTCCCTCTGTACTGTGAAGCCACAGA
TGTCGAGTCGACTAGCTTCCTTTCTCCCTTCTCCC
+
@=5@BB?BB<A;99096968B?><>/00;7;;>>AA=<???<>>?.:5:@B@BBD<?<<<?@==4//6,//4998::
?=9<@@@@@@?>=@?8838;;;98///,/./.775
@T6XXI:00146:00748
CTAGTCACTCGAGATCTGCGGCTTTGGACAGTCCCTCTGTACTGTGAAGCCACAGATGTCGAGTCGACTAGCTTCCT
TTCTCCCTTCTCC
+
C:49;@:4:CBBBBCBCBCA@BBBAB<@BCCAAB>BGCAC??@@@CA>?B?DEDCBBBBCCDCBBBCCCB@@@?A?A
D@@??C>A<??@?
@T6XXI:00147:00700
TTCGACTCGAGATCTGCGGCTTTGGACAGTCCCTCTGTACTGTGAAGCCACAGATGTCGAGTCGACTAGCTTCCTTT
CTCCCTTCTCCC
+
CBCBBCCB??BABEBBDC@CCCCC@CCBBBBB>BBA??BBCAAAB?BB@BBBBB?>>>>@B@A@@>>@?AC@CBAA<
?@<@<><<BCC?
@T6XXI:00147:00709
GGGGAATTCTAATACGACTCACTATAGGGAGAGGAGAATAGTACGACAAATCGTTTGGGCGACTTCTCTCCTTCTCC
TTCTCC
+
AC<0;,/,//6,/448;;?>BBBA?7:7.0;00,0;;98/44000001304....+.30.111101111323/0002
323532
@T6XXI:00147:00711
GGGGAATTCTAATACGACTCACTATAGGGAGAGGGAGAATAGTTTCTT
+
@??4>69:<GC@BAA@ABBBBEB>7111.7>>BC@AABD;0***)**)
@T6XXI:00147:00720
GGGAGAGG
+
--+,***)
@T6XXI:00147:00721
GGGGAATTCTAATACGACTCACTATAGGAGAGGGAGAATAGTCAACAAATCGTTTGGGGCGACTTCTCCTTCCTTTC
TCCCTTCTCCC
+
BDB8BAB?CBB@BBAAA?<<?:666..,0;;>C>CCC>@8>@=<<9==9@CCBB5:66-
9???C?CCC@C@A>AA9==BA:=378==7
@T6XXI:00147:00723
AATTCTAATACGACTCACTATAGGGAGAGGGAGAATAGATCACCGTTGGTTCGGGTGCGCGTTCTCCTTCCTTTCTC
CCTTCTCCC
+
D@C@BBB@CBBBCBBBBCBB>761-
6;7775:AA@DBBAAABB>BB=?>?@GDD?C@@@CCC?AAC@N@D:;;6:???8?ACBBB>
@T6XXI:00147:00726
```

```
GGGAGAAGGGAGAAAGGAAGCTAGTCGACTCGAGATCTGCGGCTTTGGACAGTCCCTCTGTACTGTGAAGCCACAGA
TGTCGAGTCGACTAGCTTCCTTTCTCCCTTCCTCCC
+
A:5>@@=@B@BDBB=B=@=@@?>??:::???7;:???>AB@>;::5;>ACCCCCC;?<AAAA==788@388;<AAA:
::A???@@AC888??????BDC800;928,/,/772
@T6XXI:00147:00739
GGGGAATTCTAATACGACTCACTATAGGGAGAGGGAGAATAGTCAACAAATCGTTTGGGGCGACTTCTCCTTCCTTT
CTCCCTTCTCCC
+
DDC:C@C@CCCBCCCBCCBBBBBB>77757??CC>AAA?A<@@AA???:39;;66-666-
7>@AC@BCB?A>>=@@<@BAA=B689@@<
@T6XXI:00147:00749
GGGAGAAGGGAGAAAGGAAGTGCAGTCGCGGTTAGTGAAGGGAAAACTATTCTCCCTCTCCCTATAGTGAGTCGTAT
TAGAACTTCCCC
+
CE@CBC@CC=BBC@6859;>881<11/666599>><;;<998A@@9CCCC@DCCB<@@???9?>=@@AAA?><7736
568:8665533.
@T6XXI:00148:00702
GGGGAATCTAATACGACTCACTATAGGAGAGGGAGAATAGTCAACAAATCGTTTGGGGCGACTTCTCCTTCCTTTCT
CCCTTCCTCC
+
>::5>121//7894999333.46...,.4.11,1177335830/34829666=9=>@7::7569:35,.,.97737+
--+-,-,-13
@T6XXI:00148:00711
GGGGAATTCTAATACGACTCACTATAGGGAGAGGGAGAATAGTCAACAAATCGTTTGGGGCGACTTCTCCTTCCTTT
CTCCCTTCTCCC
+
CED:C?B@BBC@CBB@@>BBBCBB966659?ACC>B@BAA==:===>@C:@?@@B>BBB8@BBBB?A@B49435<=;
?@AC>B>A???<
@T6XXI:00148:00728
GGGAGAAGGGAGAAAGGAAGCTAGTCACTCGACATCTGTGGCTTCACAGTACAGAGGGACTGTCCAAAGCCGCAGAT
CTCGAGTCGACTAG
+
BC=@>=59919@??9A=@=@CBABB:??::;:;7::7??@;?@=BBBBCBBBB@<<<6;===;=38859C@H???CA
===A???@@@???@
@T6XXI:00148:00732
GGGGAATTCTAATACGACTCACTATAGGGAGAGGGAGAATAGTCAACAAATCGTTTGGGGCGACTTCTCCTTCCTTT
CTCCCTTCTCCC
+
DDD9A?A@CCC@CA@@BBBB<BB?>666-
6===@<AAA@?ACCAA>??<;>@A@C=>??8B:::A=??A5:77?@@<@@AA<A<?@BA<
@T6XXI:00148:00734
GGGGAATTCTAATACGACTCACTATAGGGAGAGGGAGGAAATAG
+
BD@4@6954//7///88/37;7::ABC?=??967,110176442
@T6XXI:00149:00702
GGGAGAAGGGAGAAAGGAAGGAGAAGTCGCCCCAAACGATTTGTTGACTATTCTCCCTTCTCCCTATAGTGAGTCGT
ATTAGAATTCCCC
+
9=:@BAABB<@ABB=B@C@C?A>@=;:<;CCC8CB<BBBCB<AB?AAABB@;>:99-
43444A:@?@<437==888??@><;=8?8>>=1
@T6XXI:00149:00709
GGGGAATTCTAATACGACTCACTATAGGGAGAGGGAGAATAGCTCTCAAGTCCT
+
CGC9C?C@CDE?BABBBBBBBB?97111.7>>>@>BBA<;977:7.5:,..5,.
@T6XXI:00149:00716
```

```
GGGGAATTCTAATACGACTCACTATAGGGAGAGGGAGAATAGCGACTCGTTCAGTTGCCTCCCTTTTGCTCTTCCTT
CTCCCTTCTCCC
+
BB?4@548<@@?CCBBBAAAAAA?966656=?AC>CCC?@9<<==<<::;?CA??;;87877.777-
7?>:::8,/,-1781:7:444,
@T6XXI:00149:00739
GGGAGAAGGGAGAAAGGAAGGAGACAGTGAGCGCCGCACAACACGTCACTATTCTCCCTCTCCCTATAGTGAGTCGT
ATTAGAATTCCCC
+
BD>ECC@CDABCBB>B@B?B?ABAJCAABCBBAA?BBAC@>@@@?@@BBBCC@@@>><@AAND>CBCBBBDBAC@@@
@@=<==:>5999/
@T6XXI:00149:00740
GGGAGAAGGGAGAAAGGAAGAGATAGTGAAACACGCAAAGTGCGTAGCTATTCTCCCTCTCCCTATAGTGAGTCGTA
TTACGCAAT
+
EN=<<C>AA@@@@C?D?B@CCDBBB@@@DC@DCCEECC?<:;@CAABAAAB?;;:@:?>777-
7<<<6@>733872410----+50
@T6XXI:00149:00744
GGGGAATTCTAATACGACTCACTATAGGGAGAGGGAGAATAGCTAATGTCTCTACCTCACGATGCTGTTCTTCCCTT
TCTCCCTTCTCCC
+
D@@4><?<>?@>AABB@@@@B@AAB@A?9::9AA>BCB@@?>>::57=>;8;;999;;;;@;;<=?69983/33+/7
,--33,.,-1110
@T6XXI:00100:00759
GGGAGAAGGGAGAAAGGAAGCTAGTCGACTCGACATCTGTGGCTTCACAGTACAGAGGGACTGTCCAAAGCCGCAAG
ATCTCGAGTCGACTAG
+
BB>ABB9<<8<BA@?B8858>8....,,,,,,.......0/0636677C@<<<@;::4:444443/90600+++*+
*+--.--.9898<==<
@T6XXI:00100:00761
GGGAGAAGGGAGAAAGGAAGCTAGTCACTCGACATCTGTGGCTTCACAGTACAGAGGGACTGTCCAAAGCCGCAGAT
CTCGAGTCGACTAG
+
DD@CBB>BB<BBB@=B=@=@C>??B<>;;>>00.>66;>C8;;?BCCCBCCAA:8<<7<??;@@<??7??=@@@AAA
@@@@<<?===@BBB
@T6XXI:00100:00773
GGGGAATTCTAATACGACTCACTATAGGGAGAGGGAGAATAGTCAACAAATCGTTTGGGGCGACTTCTCCTTCCTTT
CTCCCTTCTCCC
+
?A@4@6959>?<ABB@ABBCCBA?=661.7>77:7:AA?A@@@BB>AAB<C:::>.777.:>>@C;;:=>@=@>@@:
>844-3/409;4
@T6XXI:00100:00777
GGGAGAAGGGAGAAAGGAAGCTAGTCGACTCGACATCTGTGGCTTCACAGTACAGAGGACTGTCCAAAGCCGCAGAT
CTCGAGTCGACTAG
+
490449999-
4499042313??>??9/.333//..44./8=<>@@==<??B=>=?<=;=AAAAA=??:??9<<=@;<884:76//--
4166
@T6XXI:00100:00791
GGGAGAAGGGAGAAAGGAAGGAGAAGTCGCCCCAAACGATTTGTTGACTATTCTCCCCTCTCCCTATAGTGAGTCGT
ATTTCAAGAATTCCCCCATCTA
+
?C?DDEACC?BBBB@CDC@CAAAA?A:;8???-
77/177>?=CA;<;B;:::00000+00044:474466244...4223000/0355;::95.4+++0
@T6XXI:00100:00793
GGGGAATTCTAATACGACTCACTATAGGGAGAGGGAGAATAGTCAACAAATCGTTTGGGGCGACTTCTCCTTCCTTT
CTCCCTTCCTCCC
```

```
+
999-
42424AA>BBBBACBCCCCBA::7.7=777.788/03368=<===;><?@>66685???9A=@A?8;8;;=?7=9;9
39,/,///,
@T6XXI:00101:00753
GGGAGAAGGGAGAAAGGAAGCTAGTCACTCGACATCGTGTGGCTTCACAGTACAGAGGGACTGTCCAAAGCCGCAGA
CTCTCGAGTCGACTAG
+
991499244-4489-4237338=6;;6611166476168888:B<99?::::=>>@@D<;;;//.5472653-
003033533376675//.:;
@T6XXI:00101:00757
GGGAGAAGGGAGAAAGGAAGGAGAAGTCGCCCCAAACGATTTGTTGACTATTCTCCCTCTCCCTATAGTGAGTCGTA
TTAGAATTCCCC
+
DC@BBB>BBB>=<LBDBD<<:<CC?CCBAAAA7AD>B@???;@G@DBB?>>;;;9@6;;<<>:@==<<@@788==<<
<><;:8858991
@T6XXI:00101:00762
GGGGAATTCTAATACGACTCACTATAGGGAGAGGGAGAATAGATGACACGCGGCCGCATCTCTATTCTTCTTCCTTT
CTCCCTTCTCCC
+
CCC:GA@>ACC@CB??ABBBB@B?966659@BCD>BAA=A=:;<<A@@@?>?@B@?>?@@@=<<A@CC?AA=@>@A<
A<=84=;:??C=
@T6XXI:00101:00767
GGGGAATTCTAATACGACTCACTATAGGAGAGGGAGAATAGTCACGCAAATTC
+
CEB7A8<:<BDADCBBBBBB?:=;=//,0;;?A>BAA?AA9;//.----+,22
@T6XXI:00101:00768
GTCGACTCGAGATCTGCGGCTTTGGACAGTCCCTCTGTACTGTGAAGCCACAGATGTCGAGTCGACTAGCTTCCTTT
CTCCCTTCTCCC
+
11111:>?>AACDCCCCB@@@A>ABCCCCBBB=ABEACCC@@@BC@BB?BC???B:::@;:7;8779;/10;>9=>9
>?BB8?;?<AA<
@T6XXI:00101:00770
GGGGAATTCTAATACGACTCACTATAGGGAGAGGGAGAATAGTCTTAACTCCCCC
+
999/489:9;<;<DCCCCAAAA@>777=677;@A<B?@>??;;8855.,,0000)
@T6XXI:00101:00788
GGGGAATTCTAATACGACTCACTATAGGGAGAGGGAGAATAGAGACGTATTCGTTCTCGCTCTGTTCACCTTCCTTC
TCCCTT
+
,,-*,0?<>@@<ABCBBBCCBACB???A:6;6695:?;9;;8784;;:4/,///,/--
4443:86556678,/,/,.///,/5
@T6XXI:00102:00785
GGGGAATTCTAATACGACTCACTATAGGAGAGGGAGAATAGTCCTGGACTGTACTCTGGGGCTGTCTCCTTCCTTTC
TCCCTTCTCCC
+
AA@4@;@=ABCACBBBCAAAA>;;:00,0<:==<?@@>AAAAA>AA6::CB@??>?7777.7=?88:@;>;>377,6
7??;B?:3344
@T6XXI:00102:00796
CTAGTCGACTCGAGAATCTGCGGCTTTGGACAGTCCCTCTGTACTGTCGAAGCCACAGATGTCGAGTCGACCTAGCT
TC
+
=4444//////888119@@@@?>@<:@4831111666776611/.----,-
49==477>;;88611//..,.442203
@T6XXI:00102:00797
GGGAGAAGGGAGAAAGGAAGCTAGTCACTCGACATCTGTGGCTTCACAGTACAGAGGGACTGTCCAAAGCCGCAGAT
CTCGAGTCGACTAGCTTCCTTTCTCCCTTCTCCC
```

```
+
CB>AA@=>>:>?>>9?<><?@?<:::00...3315<<@@@>AC?BBBBBBCAAA==<7<??@AB?AA<AA=?@=<<=
<???<?AA=<=?<;;@<?<?D?A@@@<@<??@A<
@T6XXI:00103:00778
GGGGAATTCTAATACGACTCACTATAGGGAGAGGGAGAATAGTCACAACTG
+
949/429244829@?@?BBB???B:7:@<;;;CE?CAA>>;6/-+++*-22
@T6XXI:00103:00790
GGGAGAAGGGAGAAAGGAAGAAGAATAGGACGGATGTGGCACGATCGCTATTCTCCCTCTCCCTATAGTGAGTCGTA
TTAGAATTCCCC
+
GI>=<FDCC@CDBH=A>?@CC@??@?@C?A>>;??BCA>AAAAAAAAA@@?;>;;;6778@A<AACCA???999???
?>@><;@69;;/
@T6XXI:00104:00779
GGGAGAAGGGAGAAAGGAAGGAGAAGTCGCCCCAAACGATTTGTTGACTATTCTCCCTCTCCCTATAGTGAGTCGTA
TTAGAATTCCCCC
+
44-449244-444;:@>A@CBDBB??@B@<??-
73,17111,1769>A>>>;??>?9;4444+04000555AA@@A@@>?:684/////+
@T6XXI:00104:00782
GGGGAATTCTAATACGACTCACTATAGGGAGAGGGAGAATAGTCAACAAATCGTTTGGGGCGACTTCTCCTTCCTTT
CTCCCTTCTCCC
+
BBB9B>BACBB@BBBBBBBB@@@99661-
6=<??=@AC>A=A@@@=<>C>CCBBB=AAA7ACCCC?@CA;=;=<AC=???C;@>@AA@;
@T6XXI:00105:00772
GGGAATTCTAATACGACTCACTATAGGGAGAGGGAGAATAGTCAACAAATCGTTGGGCGACTTCTCCTTCCTTTCTC
CCTTCTCCC
+
99-
99@>BBB@BBBB??@AA>99;676.7==?CACAA>AA=;:;24//,.4..,..+..11;<9<=8999>AA<@AAA@A
699AD<
@T6XXI:00105:00781
CTAGTCACTCGAGATCTGCGGCTTTGGACAGTCCCTCTGTACTGTGAAGCCACAGATGTCGAGTCGACTAGCTTCCT
TTCTCCCTTCTCCC
+
B:/11=144BBBCCCCBBAC@CCC>C=@@BB:77.7===???;?=;>;8<:<BDCDCCCCAAAB@@?AAA::8@C>B
B>@;;<4:799;;7
@T6XXI:00105:00789
GGGGAATTCTAATACGACTCACTATAGGGAGAGGAGAATATAGACAATCGTTGGGC
+
>BB9J>@>ACB>BBC@@@BBCCCEC???9??66378838A;03300/1111367,1
@T6XXI:00105:00793
GGGGAATTCTAATACGACTCACTATAGGGAGAGGGAGAATAGCGATCGTGCCACATCCGTCCTATTCTTCTTCCTTT
CTCCCTTCTCCC
+
999/9<A@CBB@BAAABAAB@=?==677.7===?=@AB@DC@@<???<AAA=?AAAA???;;?BBCBB?BC@C@BB>
BCCC>C?ABBC>
@T6XXI:00105:00794
GGGGAATTCTAATACGACTCACTATAGGGAGAGGGAGAATAGATCACCGTTGGTTCGGGTGCGCGTTCTCCTTCCTT
TCTCCCTTCTCCC
+
BAA7B>B?BDD@C???@BBBBA@97111.7??BC>B@A>@<<====<:??;?:?:@@@=B;;;DFD@BBB?CAC@CD
>A===7<?<==A:
@T6XXI:00105:00797
GGGAGAAGGGAGAAAGGAAGGATAGCAGAATGCTGTGACATTGAACGGGCTATTCTCCCTCTCCCTATAGTGAGTCG
TATTAGAATTCCCC
```

```
+
99-
9996<B<BBBC>B>A?B@CCCBBBBB@CCBBBBABBBC?@=>@@@;@AA@>AAAA<A<@AA<A@ABAAAA@AAA=<<
<?;979/444-
@T6XXI:00105:00799
CTAGTCGACTCGAGATCTGCGGCTTTGGACAGTCCCTCTGTACTGTGAAGCCACAGATGTCGAGCGACTAGCTTCCT
TTCTCCCTTCTCCC
+
BA<<=HCDDCCCCBBBBBBBB@BBB>B>@A@BBCC>AA>?B?@?=??=;<B@BBCCABABB??@BBBBBCCAA?A@C
C>A??D<A<??@C<
@T6XXI:00106:00753
GGGAGAAGGGAGAAAGGAAGGCGAACAGGGCACCGTATACAGGCACGCTATTCTCCCTCTCCCTATAGTGAGTCGTA
TTAGAATTCCCC
+
HDDCCD@BB=?B??:@>@8:8;CC@CBBB>BCA;@@C=::/.,0@>@?@@@=??==;=<<<=;===<?A?@@AA===
A>A@@<>8>>>1
@T6XXI:00106:00756
GGGGAATTCTAATACGACTCACTATAGGGAGAGGGAGAATAGTCAACAAATCGTTTGGGGCGACTTCTCCTTCCTTT
CTCCCTTCTCCC
+
999/42959>>;A<<<CCCCBCC>9116.7<<;<9?@@?A===AA>@@@;B???B<@@A7??FFB>@@>38389>@:
@@?@;@9<=@B?
@T6XXI:00106:00761
GGGAGAAGGGAGAAAGGAAGCTAGTCGACTCGAGATCTGCGGCTTTGGACAGTCCCTCTGTACTGTGAAGCCATAGA
TGTCGAGTCGGCTAGCTTCCTTTCTCCCTTCTCCC
+
CDBCCB@BC>BBBB=B@B>@>:6:@:;;CCDAAACCCB?AA@<::8:<<AC@CAA<AA=======::::;;;<>><?
===@@AA@@>?BBCA@@<?<?A=B@@C;@>@@@@:
@T6XXI:00106:00771
GGGGAATTCTAATACGACTCACTATAGGGAGAGGGAGAATAGCTATGATCTCGTCGATTGTCGTATTCTCTTCCTTT
CTCCCTTCTCCC
+
DDB9A=>=@CC@CBB@BBBB?@A>966659@ACC>B?B=B;;;>@@<@<>>==;:6;=<=<89=@A<??A@>@>@A<
AAAA<@>@A@A;
@T6XXI:00106:00777
GGGGAATTCTAATACGACTCACTATAGGGAGAGGGAGAATAGTCAACAAATCGTTTGGGGCGACTTCTCCTTCCTTT
CTCCCTTCTCCC
+
111*1;A>BBB?BBCCC>>>B@<9?167.7<<@B>BAA?AAAA<;9;;84<>777.777.7>@AB@DBA=@=??AC<
@@AA;?378>>7
@T6XXI:00106:00782
GGGGAATTCTAATACGACTCACTATAGGGAGAGGGAGAATAGTCAACAAATCGTTTGGGGCGACTTCTCCTTCCTTT
CTCCCTTCTCCC
+
;;9/<@A@CBB@BABB@<<??AB@>766-1=166.7;>;>;88--,-11+11116266626>=9:4:?A9;=;344-
499;9;24444-
@T6XXI:00107:00752
GGGAGAAGGGAGAAAGGAAGGTGATGAGTTGGGTGAAGAGGAGCAAACTATTCTCCCTCTCCCTATAGTAGTCGTAT
T
+
CIDAAA@CB>BBBC>B@B@C@BBBFBBAA>AC>BBB?BBA@CBBBB=BAAA@BBCB>CCCAA;=??>4448730330
*
@T6XXI:00107:00771
GGGAGAAGGGAGAAAGGAAGCGACTCGACATCTGTGGCTTCACAGTACAGAGGGACTGTCCAAAGCCGCAGATCTCG
AGTCGACTAG
+
```

```
CC@@A@BCC@?=<CAC?B?BA@;:8;6166944;99244249:;>CA>>?99909>>>?C?CB>AA?ACCAAAAACA
=;;=///55@
@T6XXI:00107:00794
GGGAGAAGGGAGAAAGGAAGGAGAAGTCGCCCCAAACGATTTGTTGACTATTCTCCCTCTCCCTATAGTGAGTCGTA
TTAGAATTCCCC
+
CD>ABCACC??BAB>B@C@B@B@><:??@BBB8BE@CCB?B>@@??>>;=A?><>=;=88?C=??>@AAAAA=====
=>A@?;=88781
@T6XXI:00108:00753
GGGAGAAGGGAGAAAGGAAGCTATCGACTCGACATCTGTGGCTTCACAGTACAGAGGGACTGTCCAAAGCCGCAGAT
CTCGAGTCGACTAG
+
CD@CCD@CD>BCAC@C@A@BCGBA?A?:7:????BC:::A@CBBBLCAAAA;::444-
4>;==@:==7=A>A@@AAA@@@AA=<884:8//
@T6XXI:00108:00760
GGGAGAAGGGAGAAAGGAAGGAGAAGTCGCCCCAAACGATTTGTTGACTATTCTCCCTCTCCCTATAGTGAGTCGTA
TTAGAATTCCCC
+
CC@DDC?BD>A@@E?B@B?B?BCC@>;<<BBD7AA<BCCCC>CC?C??=AA>@?BA?A????;??@@AAB@@@@@@A
A>A?=9;/444-
@T6XXI:00108:00771
CTATCGACTCGAGATCTGCGGCTTTGGACAGTCCCTCTGTACTGTGAAGCCACAGATGTCGAGTCGACTAGCT
+
D><7/////89>>?BB@B;;8;CC?D?BABC??@=?CCCBAB@<AAA>AC@CBBBA::;CDCFCCCCCBBBAB
@T6XXI:00108:00780
GGGAGAAGGGAGAAAGGAAGTGGGTCGCACGTAACGTGATTTGGGAGCTATTCTCCCTCTCCCTATAGTGAGTCGTA
TTAGAATTCCCC
+
CC>BBB>BC@BBAA=B>A?BCCC>BCBA@@B<<;@GECCDD;<<;<BBBBB>@@@@<@??AA<@???CCCCBC?778
88:;A8689A@4
@T6XXI:00108:00791
GGGAGAAGGGAGAAAGGAAGCTAGTCGACTCGACATCTGTGGCTTCACAGTACAGAGGGACTGTCCAAAGCCGCAGA
TCTCGAGT
+
CC@=<;ACCCCBBB>B@C8;;CAAABABAAAA?7::??>>=;8:5:BBBBBA<>>73317@@ACA=??;?C?AA<<<
;<???>AA
@T6XXI:00108:00793
GGGGAATTCTAATACGACTCACTATAGGGAGAGGGAGAATAGATCACCGTTGGTTCGGGTGCGTGTTCTCCTTCCTT
TCTCCCTTCTCCC
+
999/929<A@B@CCCCCAAAC@B?96:7.7?>AB<B@@?@9<<<=B>8AB5:7:@CCC=BBCCCBB@BCC@B=@<??
<?CDC<A>?=<<7
@T6XXI:00108:00798
CTAGTCACTCGAGATC
+
<11//,///////,2/
@T6XXI:00108:00799
GGGAGAAGGGAGAAAGGAAGGAGAAGTCGCCCCAAACGATTTGTTGACTATTCTCCCTCTCCCTATAGTGAGTCGTA
TTAGAATTCCCC
+
>>:>>@<@@:99;A<@=@?C?DCD@CAAACCD9CB<@@::619?>@ACA@@<>>ACAC<<<C;@@@@AAA@@===@A
A>BAB=B?AAA5
@T6XXI:00109:00768
GGGGAATTCTAATACGACTCACTATAGGGAGAGGGAGAATAGCGTGCAGGTATATCTGGTCGTTATGCTCCTTCCTT
CTCCCTTCTCCC
+
```

```
999295959>>?BBBBCB@@@FCCCBBC>@=611-
64:7;@A???A?A?=<<<@889A@@AAC;===@@@;=;:243888?;@?B==?9
@T6XXI:00109:00775
CTAGTCACTCGAGATCTGCGGCTTTGGACAGTCCCTCTAGTACTGTGAAGCCACAGATGTCGAGTCGACTAGCTTCC
TTTCTCCCTTCTCCC
+
>8/111144BB?BBCCBBAA>@BB>B<?A@@??9642,///7305A@@;<B?BCCCCCCB>>@@?A@BBBBBBB>B>
BB>CAAA;A?9==A<
@T6XXI:00109:00791
GGGGAATTCTAATACGACTCACTATAGGAGAGGGAGAATAGATCACCGTTGGTTCGGGTGCGCGTTCTCCTTCCTTT
CTCCCTTCTCCC
+
A@A8A?BACDBACCBBBCCCB>=;:/.,0:0775:>??@@===@?;9:>,0.1:>?I>C@@@CCC@A::79:?=BB>
A@@@;@<<<<@:
@T6XXI:00110:00750
GGGAGAAGGGAGAAAGGAAGCTAGTCGACTGACATCTGTGGCTTCACAGTACAGAGGGACTGTCCAAAGCCGCAGAT
CTCGAGTCGACTAG
+
CC?AAA@CC@FA@C>@=@@CBBACC=??ACA88;CC@@?C@CC>CCCCBB@@=933317@>@@?=?A<AA?A<<=@@
C<=<<<>;<<<<<;
@T6XXI:00110:00759
GGGAGAAGGGAGAAAGGAAGTCCCTCCCATCTGGTGGCTTCACAGTACAGAGGGACTGTCCAAAGCCGCAGATCTCG
AGTCGACTAG
+
2888FC=@=:9999189>93337,333,117110110..,----
247:77==A:@AAA:968;38:374666:/..44<=@@>@888
@T6XXI:00110:00771
GGGAGAAGGGAGAAAGGAAGCTAGTCGACTCGACATCTGTGGCTTCACAGTACAGAGGGACTGTCCAAAGCCGCAGA
TCTCGAGTCGACTAG
+
BA?AAAACEABBBB>B?A>AD@>;7/0,,,7,,0../.56=458@D@@?BCA@@<44448@AABC;==9=B<??@@@
AAAA9==A=<<@@@@
@T6XXI:00110:00776
GGGGAATTCTAATACGACTCACTATAGGGAGAGGGAGAATAGATCACCGTTGGTTCGGGTGCGCGTTCTCCTTCCTT
TCTCCCTTCTCCC
+
999/9?@AAAA@BBBBAACCCAA?B661.7>==ABC@@?C=@@<<@A=AA=@>?@CCC>C@@@CAA>ACA?A8;:;E
AA@@B<@=;;;;4
@T6XXI:00110:00784
GGGGAATTCTAATACGACTCACTATAGGGAGAGGGAGAATAGATAGCGTCCTCCCAAAACCAGTCTGTTCTTCCTTT
CTCCCTTCTCCC
+
9>?4>@@>BBB@BCBBCCCGBACAABAB9??@@B>;99>@@A???@@@@@?@AA<AAA4<9<;;;4747944=:>>:
<=<C<A7:.//,
@T6XXI:00110:00789
GGGGAATTCTAATACGACTCACTATAGGGAGAGGGAGAATAGTTTTCCCTTCACTAACCGCGAACTGCACTTCCTTT
CCCTCCTTCCTCC
+
@@@6A@C@DBB@CBB?>>??B@B>96667:CCEC?B@@?@<<<<@5A<;A;=?CCC?B?B?@=>AAA?BBC@?;;;-
67.48,/,/,--0
@T6XXI:00110:00798
GGGGAATTCTAATACGACTCACTATAGGGAGAGGGAGAATAGTCACAACGCGTGATACCCAATTTTGCCTTCCTTTC
TCCCTTCTCCC
+
999,92959=@=@ACAB@CCBBDCA:::.777@A>BAA>@@@AAA<;8;???@??>444,044460::5449/30+3
4999;:;444-
@T6XXI:00111:00776
```

```
GGGGAATTCTAATACGACTCACTATAGGGAGAGGGAGAATAGTCGTGCAATTTTCGACGCGTCTCTAGTTCTTCCTT
TCCTCCTTCCTCCA
+
CBB9B799;CC@CCBBBBB?BACB669?6;<<??;@@A>A==8A@>;442999/9;;CLCCCCC@@@;@<>>;>477
.0,,,+,*,*,,*,
@T6XXI:00111:00786
GGGAGAAGGGAGAAAGGAAGGGAGAAGGGAGAAAGGAAGCTAGTCGACTCGACATCTGTGGCTTCACAGTACAGAGG
GACTGTCCAAAGCCGCAGA
+
CC@CCB@CC?@AAB>C@B@BC?BB?>966;611,7;?4;;DAAABBB@===4498===@@=@@?AA99====88433
14===??<@@9>>AA@@@A
@T6XXI:00111:00794
GGGGAATTCTAATACGACTCACTATAGGGAGAGGGAGAAATAGTCAACAAATCGTTTGGGGCGACTTCTTCCTTCCT
TTCTCCCTTCTCCC
+
>BB4=2424::2448933733177116;8?6?7=,1333+044359989;477111,111,1666631600442355
424000+0/00444
@T6XXI:00112:00756
CTAGTCACTCGAGATCTGCGGCTTTGGACAGTCCCTCTGTACTGTGAAGCCACAGATGTCGAGTCGACTAGCTTCCT
TTCTCCCTTCTCCC
+
B=1//1/44B>>@@CCCCBC@BB?=?;>ACA>>?>>?@C@>>=====;<B@BBABABBBBBCCCBBBCCBBBB@C?B
B>A??A<A>@@AA<
@T6XXI:00112:00775
GAGAGAAGGAGAAGGAAGCTAGTCGACTCGACATCTGTGGCTCACAGTACAGAGGACTGTCCAAGCC
+
4<949;24244343137794949999994444444//...,..161110449>;948>0,,,*,*,7,
@T6XXI:00112:00788
GGGAGAAGGGAGAAAGGAAGAGGAGTCTAGGCTAGGAGAATTCGTGCCTATTCTCCCTCTCCCTATAGTGAGTCGTA
TTAGAATTCCCC
+
CC@AAC@CD>BDCB?B??@BBB@B@AA??@@BCDCABBA?A?ACCCC?BCCAB@@@>@CCCC>C?BA@@A====A==
=>@@?:>38880
@T6XXI:00112:00793
GGGAGAAGGGAGAAAGGAAGCTAGTCCATCTGTGGCTTCACAGTACAGAGGGACTGTCCAAAGCCGCAGATCTCGAG
TCGACTAG
+
CC?ECBCCC>9<;C>CAC@BBB?B994:AA?FCB@AA?A@???@A?88377-
7==@AA>@@<AA?@@@AA?===AAAAAAA@@@A
@T6XXI:00112:00798
GGGAGAAGGGAGAAAGGAAGCTAGTCACTCGACCATCTGTGGCTTCACAGTACAGAGGACTGTCCAAAGCCGCAGAT
CTCGAGTCGACTAG
+
AH>A@C89809999042713331117?=711111001116698<@=?CCCCCA???<;9<;9;::/44,0323034;<
@AA@@@AA=9=@@@
@T6XXI:00113:00753
GGGGAATTCTAATACGACTCACTATAGGGAGAGGGAGAATAGCCAACATCTCCCGATTGTCACTGTTCCCTTCCTTT
CTCCCTTCTCCC
+
A==6@6;;<CC@CCBAACCB@BB>966659??CE>C@@=@<<<;<;<===@@@<@A@;@B>>>A??>BB>@;<111.
7>>>:A?BAAA<
@T6XXI:00113:00761
GGGGAATTCTAATACGACTCACTATAGGGAGAGGGAGAATAGTCAACAAATCGTTTGGGGCGACTTCTCCTTCCTTT
CTCCCTTCTCCC
+
CDJAC?A>9<<@CCCCCBBA?>>=?666.7===A=AAA>A???@@:==A@CFDCC9??<-
77:=?:;=;./.0;;;9;?BA<A>@@@@7
```

```
@T6XXI:00113:00762
GGGAGAAGGGAGAAAGGAAGCTAGTCGACTCGACATCTGTGGCTTCACAG
+
BB<@@@<@@:989@:>4713>;?<?CB@A??777;66111325636;9::
@T6XXI:00113:00764
GGGGAACTTCTAATACGACTCACTATAGGGAGAGGGGAATAGTCAACAAT
+
/8807,//,/66:99=:11//6449><<C<:::>>>.73114011000/0
@T6XXI:00113:00767
GGGGAATTCTAATACGACTCACTATAGGGAGAGGGAGAATAGTCAACAAATCGTTTGGGGCGACTTCTCCTTCCTTT
CTCCCTTCTCCC
+
@==6@=@=@CDBCCB??>>>;AB>;111.7>=BC>B???A<<<AA>??::<96662;;;5??AAE?BB@>A>@>AA=
???A:A@@@@<;
@T6XXI:00113:00787
GGGGAATTCTAATACGACTCACTATAGGGAGAGGGAGAATAGCTGTGTTCTTTCCCACCTAGCCCTGTTCTTCCTTT
CTCCCTTCTCCC
+
999/99@ACBD@BCCBAACB@@B@<:?:98?ACC>CAA?A=?:=>>>?>AAA<@@<@A>@@<<=7<?A889?C9<=9
<<@A:=388<<6
@T6XXI:00113:00793
GGGGAATTCTAATACGACTCACTATAGGGAGAGGGAGAATTAGTTTCACGTTGCCGACTGCTGCTATTCCCTTCCTT
CTCCCTTCT
+
EDC:C=@=@=9994997:7988>76666266:>>9<:;-/-.30033..-
1011240003330685699=;:9<2420000*+*++
@T6XXI:00113:00795
GGGGAATTCTAATACGACTCACTATAGGGAGAGGGAGAATAGTCAACAAATCGTTTGGGGCGACTTCTCCTTCCTTT
CTCCCTTCTCCC
+
999/99A@BBB@CAA@BBBB@A>76111.7==?C@B??>A???@A>A@@<CBBBB<BBB7BCCCDACB@=@=??AA:
ABBA<A><@@A<
@T6XXI:00113:00799
GGGGAATTCTAATACGACTCACTATAGGGAGAGGGAGAATAGCCACCAACTATTGTTCGTCGCCGTTCTCTTCCTTT
CTCCCTTCTCCC
+
999/99@?CBB@BBBBABBB@A>77111.777>BABA@>A???>CC9=9===<>@C?AA????<?C:<=??>@>@A;
@<==7<=@@@@;
@T6XXI:00114:00752
GGGAGAAGGGAGAAAGGAAGCTAGTCGACTCGACTGCGGCTTTGGACAGTCCCTCTGTACTGTGAAGCCACAGATGG
ACTGCTTCCTTCTCCCTTCTCCC
+
49144>;>>094>>9@?B?BDCCCBBBCCBA;;;CBBA?:;<:<?@@?@;<;8<BBBBBCCCCCE@BC@CCAB@??=
?>><;;9;,1,/:::6>;?@BC>
@T6XXI:00114:00756
GGGAGAAGGGAGAAAGGAAGCTAGTCACTACATCTGTGGCT
+
9404/1,1/+//6856999;;@><77,,,,0,,.6333/01
@T6XXI:00114:00761
CTATCGACTCGAGATCTGCGGCTTTGGACAGTCCCTCTGTACTGTGAAGCCACAGATGTCGAGTCGACTAGCTTCCT
TTCTCCCTTCTCCC
+
;<C:4/,--
,,2228@@B::8;BB=B=@@CCCCC>@@@BBBBAAAAA>@B?BB@;@@??AAC@?;<;;<CCCC>@=@D??=>A<@<
?===:
@T6XXI:00114:00772
```

```
GGGGAATTCTAATACGACTCACTATAGGGAGAGGGAGAATAGTTCGCGCTCCTCTTACCTCAACATATTCTTCCTTT
CTCCCTTCTCCC
+
@>>4>=@=ACI@CBB@AAAA?@<77111.777@KAC@>>@AAA::;<<<==;@AA><@DCBB?@@BBC@BA>A>@?;
@@@@<A>===B<
@T6XXI:00114:00783
GGGGAATTCTAATACGACTCACTATAGGAGAGGGAGAATAGCCACCAACTATTGTTCGTCGCCGTTCTCTTCCTTTC
TCCCTTCTCCC
+
BBB9B@D@CBBABABBBCCBB:99:..,0::>B>CBA?@??A=??<?<????>@@?AA???@=@A>AA==;=>AA=A
DCD9=;==<@<
@T6XXI:00114:00798
GGGGAATTCTAATACGACTCACTATAGGGAGAGGGAGAATAGCTATTACTCAA
+
DB>9A=@=@BCBCCC?????>@C?9661-6=;;<9?=;=95//00.44----,
@T6XXI:00115:00760
GGGAGAAGGGAGAAAGGAAGCTAGTCGACTCGACATCTGTGGCTCACACATGATCGATGT
+
DC>B9959914444-42377779668:?:7::7//....,+*-..336611611043036
@T6XXI:00115:00766
GGGAGAAGGGAGAAAGGAAGCTAGTCGACTCGACATCTGTGGCTTCACAGTACAGAGGGACTGTCCAAAGCCGCAGA
T
+
CC>@@=;9=9>>>>9>?B7;<B???CCBBBB?99::::BBA>@B@BCA?>>==:;33317>>@@??AA:>>?AA;::
:
@T6XXI:00115:00770
GGGAGAAGGGAGAAAGGAAGCTAGTCACTCGACATCTGTGGCTTCACAGTACAGAGGGACTGTCCAAAGCCGCAGAT
CTCGAGTCGACTAG
+
?@-99>:;?9<9=EBC@C@<<<?B>@76699664==99<A:?>=@????@@>88444-
474;<<>AA;???BA??@A788888;???<==@
@T6XXI:00115:00786
GGGAGAAGGGAGAAAGGAAGGAGAAGTCGCCCCAAACGATTTGTTGACTATTCTCCCTCTCCCTATAGTGAGTCGTA
TTA
+
9?6>>><>@:AABC@CCC@C@CBB@?<>@BIC8AA>BCCCC=AA@AAA@AB>?@==;@?@@CA@@@@A@@@???A==
=>@
@T6XXI:00115:00789
GGGGAATTCTAATACGACTCACTATAGGGAGAGGGAGAATAGCTCAGCTTTTGTTGCATGTGATTTCTCCTTCCTTT
CTCCCCTTTCCTCCCC
+
444,42428@@?DBBBBCC;::B@@=??8?@=<<6<//,-777=BA??@@@4@;9:://-1--
00+03;<=<<;==;95333+00+040000+
@T6XXI:00115:00797
GGGAGAAGGGAGAAAGGAAGCTAGTCGACTCGACATCTGTGGCTTCACAGTACAGAGGGACGTCCAAAGCCGCAGAT
CTCGAGTGGACTAG
+
CC?ECB@BHBCBCB?B?B@:8;CCBBCCCCCCCAAAAAAA@>?A?ABCBBBA@>;733-
499::9ACBCCBADDCCBA;;9>??@<>@>>?
@T6XXI:00116:00764
GGGGAATTCTAATACGACTCACTATAGGGAGAGGGAGAATAGCTGGCACACACACGTCAGTATCTTCTCCTTCCTTC
TCCCTTCTCCC
+
999/95969>><ABBDBBBBBCDB?7:7.7>>>@9?;;8;;;=@A>BAAA???AAAA@AAAAA8738?=9;;;3424
444-479:9<7
@T6XXI:00116:00765
```

```
GGGAGAAGGGAGAAAGGAAGCTAGTCGACTCGACATCTGTGGCTTCACAGTACAGAGGACTGTCCAAAGCCGCAGAT
CTCGA
+
BB@BCD@DD>CBBB=?@B5;;CAAABAA@BB?69:::7A@?<@B@BBB@?@=545.../9;:;?>BA=@@>@@=A==
===A=
@T6XXI:00116:00766
GGGGAATTCTAATACGACTCACTATAGGAGAGGGAGAATAGATCGCGCTGTTCGGGTTGCGGCGTCTCCTGCTTTCT
CCCTTCCTTCCACCTC
+
AA@6A=@=@BB@CCA?@ECCC>=;7/.,0::>A=CAD>@@@=804.//.--,--
4574746/0740002335;=144==;=;<<=54..,+,+
@T6XXI:00116:00767
CTAGTCACTCGAGATCTGCGGCTTTGGACAGTCCCTCTGTACTGTGAAGCCACAGATGTCGAGTCGACTAGCTTCCT
TTCTCCCTTCTCCC
+
=<1////49B@@BBB@?>?@=@CC>C?DBBCAAA=BCEA<<?@<?<=;=C@CCCBBB@BBCCCBBB@@@CCCC:;>;
E>A<=<7<=>??A<
@T6XXI:00116:00769
GGGAGAAGGGAGAAAGGAAGGAGAAGTCGCCCCAAACGATTTGTTGACTATTCTCCCCTCCTCCT
+
6=9<<><>?1944C<?<@@C>@?@@AACBCCC9DC876889,66<788===999<;806620-20
@T6XXI:00116:00791
GGGAGAAGGGAGAAAGGAAGGAGAACGCGCACCCGAACCAACGGTGATCTATTCTCCCTCTCCCTATAGTGAGTCGT
ATTAGAATTCCCC
+
CH>BBBBCC>@@@>9>;>;><@AC@CEHBABCEACCAC@CACECCCCAA@@@?BCCC>BBBCAAAC???AAA?<?A?
???A@@>BACCC7
@T6XXI:00116:00793
CTATCGACTCGAGATCTGCGGCTTTGGACAGTCCCTCTGTACTGTGAAGCCACAGATGTCGAGTCGACTAGCTTCCT
TTCTCCCTTCTCCC
+
D777/////8:BBBCCEB>><@CC>C88;BBCCC>CBBB?===9==@?@B?@@BBB>9ACCCAABBCCCCBBB?B@C
C>B?AA;A;8>AA<
@T6XXI:00116:00797
GGGGAATTCTAATACGACTCACTATAGGGAGAGGGAGAATAGTTTTTCCCTTCACTAACCGCGAACTGCACTTCCTT
TCTCCCTTCTCCC
+
999/49@>CCC@CC?A?BEBB@>;7116.7>>>@=A??>@@>><<=1==:?69;CCC?C=B=CA>AAA?A??6;367
57>?A7?<===@;
@T6XXI:00117:00761
CTAGTCACTCGAGATCTGCGGCTTTGGACAGTCCCTCTGTACTGTGAAGCCACAGATGTCGAGTCGACTAGCTTCCT
TTCTCCCTTCTCCC
+
C@9<<@===BAAAAADCBBC@BBB<B>AA@?<:?;?BBBCBC=>=AA<?B@CCCCCCAA=??@@BBBBCCBBB@C@C
C=@?=C;@>===@;
@T6XXI:00117:00765
GGGAGAAGGGAGAAAGGAAGCTAGTCGACTCGAGATCTGCGGCTTTGGACAGTCCCTCTGTACTGTGAAGCCACAGA
TGTCGAGTCGACTAGCTTCCTTTCTCCCTTCTCCC
+
99149><>@0999909;>;?B@=;@0////7///CCABCAA==??9?;?@@@A?>7===>??AAAA@A<??=?@AAA
@@@@AA<??===>A@??>?8<<178::7:378?@;
@T6XXI:00117:00769
GGGAGAAGGGAGAAAGGAAGGAGAACATGACCCGGCACGATTGGTGTCTATTCTCCCTCTCCCTATAGTGAGTCGTA
TTAGAACTTCCCC
+
CD?=992481999@19685848>>;@BBBBEN@A>@CEBBB=@=@BBBBCC>??CC>AB?@@<?@A?===@AA===@
@383;88978??0
```

```
@T6XXI:00117:00787
GGGGAATTCTAATACGACTCACTATAGGGAGAGGGAGAATAGTCAACAAATCGTTTGGGGCGACTTCTCCTTCCTTT
CTCCCTTCTCCC
+
?A@6A@C@BBB?BBBCCCBAAACB:9:A<???CC>CAA>A???BB=?@A<BBBBC>@C?8>B>?C@DBA;=9<=@A;
?AAC<A689AA<
@T6XXI:00117:00793
GGGGAATTCTAATACGACTCACTATAGGGAGAGGGAGAATAGTCAACAATCGTTGGGCGACTCTCCTCCTTTC
+
DCC7A>A?ABA7;<BC@@B@@?BBA??@=?BBCC=B?A8;;840423358666?@@;773....-,--,00+0
@T6XXI:00118:00775
GGGGAATTCTAATACGACTCACTATAGGGAGAGGGAGAATAGATCACCGTTGGTTCGGGTGCGCGTTCTCCTTCCTT
TCTCCCTTCTCCC
+
999/9:B@CBB@BB?BBBBAA=997166.7??AC>CA@=A===<;>?>?E?B?B?CBB@B9;;??=:A??=??C@CC
>@@@@<A?===@<
@T6XXI:00118:00786
GGGGAATTCTAATACGACTCACTATAGGGAGAGGGAGAATAGTCAACAAATCGTTTGGGGCGACTTCTCCTTCCTTT
CTCCCTTCTCCC
+
>992=8?=@BB@CC@>@CEC=<?=66776:@@DE>@==?A=@@@@>????7?A??<9?@@5@@C??=?A@3837:==7
?@@@<A688@@;
@T6XXI:00118:00787
GGGAGAAGGGAGAAAGGAAGAATATTGCGCGTTTAACGGGGGCGATGGCTATTCGTCCCTCTCCCTATATGTGAGTC
GTATTAGAATTC
+
491449244-44:;6=<;?AA?ACC=AA@?>=>.7311111+1111>;=6.-3-----+-
115=444A<8==98=<<34332211,-,*
@T6XXI:00118:00789
GGGAGAAGGGAGAAAGGAAGTGCAGTTCGCGGTTAGTGAAGGGGAAACTATTCTCCCTCTCCCTATAGTGAGTCGTA
TTAGAATTCCCC
+
BCBBBB@BB>BBBB>BAB?@<?@BBB@B?>;576::8D?3:::4;B@DCBBACBBB>BCCCC;=988<===<<???@
@?<A=<A=???5
@T6XXI:00118:00791
CTATCGACTCGAGATCTGCGGCTTTGGACAGTCCCTCTGTACTGTGAAGCCACAGATGTCGAGTCGACTAGCTTCCT
TTCTCCCTTCTCCC
+
C>::1111/8;?@AACBBBC@CNN>C>@?>>:::7:ABBCBB@<@AA>@D@CCCEBCAAACBCABBDDD@@?A=?;?
C=?==A<B====D@
@T6XXI:00118:00794
GGGGAATTCTAATACGACTCACTATAGGAGAGGGAGAATAGTCACGC
+
999-92928>A?BCCBCCCB?:975/.,0::==9?>8466,,+.***
@T6XXI:00119:00781
GGGAGAAGGGAGAAAGGAAGGAGAAGTCGCCCCAAACGATTTGTTGACTATTCTCCCTCTCCCTATAGTGAGTCGTA
TTAGAATTCCCC
+
CC@CCC@CC@@@?B<@=@@C@CCC@CBBBBCC8BB=BBBBC=AA?CBBA@@>@AAA<A@AAA<A@@@?@@=AA<@@@
@<???992844-
@T6XXI:00119:00785
GGGAGAAGGGAGAAAGGAAGCTAGTCACTCGACATCTGTGGCTTCACAGTACAGAGGGACTGTCCAAAGCCGCAGAT
CTCGAGTCGACTAGCTTCCTTTCTCCCTTCTCC
+
9909>>;@B>BCCC>C?A=AA>?==67776666369??<<?AB=?@CCCB?;8934418;;@??<?A<>?>????AA
@@@@@@CC???>>>=?=@>AA<AAAD9=:=@A?
@T6XXI:00119:00795
```

GGGGAATTCTAATACGACTCACTATAGGGAGAGGGAGAATAGTCCTCGGTTTTTCACCATCACAATATTCTTCCTTT
CTCCCTTCTCCC
+
CHN:D@@>?CCBCCBBB>>>?@?77111.7?>BC>B@D@A88<=?A@@<@AAA1A??<?AAAAA>A@@=@A>A<??<
?AAB@A?@??C<
@T6XXI:00119:00798
GGGGAATTCTAATACGACTCACTATAGGGAGAGGGAGAATAGCTTAGACCTTGGTGGCATTCTGTTCTCCTTCCTTT
CTCCCTTCTCCC
+
FCC9C@C@CBB@CCBBBB??A@A97166.7==BF>B@@?A:::@>@@?@?@>A?AA?@@@?<<7734:=7<8<;>>:
<<<@:=;=;;<6
@T6XXI:00120:00763
GGGAGAAGGGAGAAAGGAAGCTAGTCACTCGAGATCTGCGGCTTTGGACAGTCCCTCTGTACTGTGAAGCCACAGAT
GTCGAGTCGACTAGCTTCCTTCT
+
<4-
9@@=AKAB@@@<A>A?CEBBCCCC@??C?88:??AACBDDC>C?BBBBBCC>A===AA@@@@@A>@@>===?DAA@@
A???878==;=//,/,/,++
@T6XXI:00120:00784
GGGAGAAGGGAGAAAGGAAGGAGAAGTCGCCCCAAACGATTTGTTGACTATTCTCCCTCTCCTAT
+
AFADEE@CC=BBBHCD@A<?<;73=9<@@BBC8CC996166266368?::??<;760//+,++++
@T6XXI:00120:00797
GGGAGAAGGGAGAAAGGAAGTGCAGTTCGCGGTTAGTGAAGGGAAAAACTATTCTCCCTCTCCCTATAGTGAGTCGT
ATTAGAATTCCCC
+
CCCCCB@DB>BBBB?B@B@@ABEBCC@CA@@?B>AAAA@<>>.7777,8ABC@CBBB>BCCCA;<8<<<<AAAAAA@
==;=AA>A=@DC6
@T6XXI:00121:00759
GGGGAATTCTAATACGACTCACTATAGGGAGAGGGAGAATAGTCTTGCAATTTCCTGCGGTGCATTCTCCTTCCTTT
CTCCCTTCTCCC
+
CBB6C@A@ADE@CCBCCBBAA@B@B999.7==>@<A??>@@???C>@@A>BC@C@DBBBABCADBEDBA??=?488/
7777.7376=;6
@T6XXI:00121:00770
GGGGAATTCTAATACGACTCACTATAGGGAGAGGGAGAATAGTCAACAAAGTCGTTTGGGGCGACTTCTCCTTCCTT
TCTCCCTTCTCC
+
DCD:C>@>ACCCCCBB?<<@BAB>9661-
6=777.700/00045858==455;844+000+116676?:=/4/7::;7;8881499//.
@T6XXI:00121:00773
GGAGAAGGGAGAAAGGAAA
+
42449244-4499-4244-
@T6XXI:00121:00790
GGGAGAAGGGAGAAAGGAAGGAGAAGTCGCCCCAAACGATTTGTTGACTATTCTCCCTCTCCCTATAGTGAGTCGTA
TTAGAATTCCCC
+
BB@CBB6996;A?A=C?D@E@CAA?BACB>BB7BB<?AAAA=AC@CB;>==;=<<<;==<<<;<<<<=<<<===???
?>B?=;;3444-
@T6XXI:00121:00794
GGGGAATTCTAATACGACTCACTATAGGGAGAGGGAGAATAGTCAACAAATCGTTTGGGGCGACTTCTCCTTCCTTT
CTCCCTTCTCCC
+
CCC9C@C?BCC@CB??>???>@B?>66659??CD>A===A===@@>?>?;ABBBC;<<=7?BBCC??A=<?:8:?>:
?AAA<@=@ABB<
@T6XXI:00122:00765

```
GGGGAATTCTAATACGACTCACTATAGGGAGAGGAGAATAGTACGACAATCCGT
+
>;A6CDCCDAA?B@@@ABBDDCAA?@@D>?<:7379::;44444400.0355./
@T6XXI:00122:00772
GGGAGAAGGGAGAAAGGAAGGAGAAATGCCCATCTAACGGTTAGTAGCTATTCTCCCTCTCCCTATAGTGAGTCGTA
TATGAACTTC
+
CB>B@@9<<:;CCC>C?A=@>B??@<?BBB<B;::A<<A>@>?9=737776066?>666116,0889?@@????:6/
//..3322,-
@T6XXI:00122:00784
GGGAGAAGGGAGAAAGGAAGGAGAACGGGCGGGTCACAAAAACATGAGCCTATTCCTCCCTCTCCCTATAGTGAGTC
GTATTAAGAACTTC
+
;;-49424;6==BC==:;,0,0,,*-
,,*,/85//444444+4:4437A:;::2424;;>E???B?B@7777761;?<66101000/1101
@T6XXI:00123:00760
GGGAGAAGGGAGAAAGGAAGTGCAGTTCGCGGTTAGTGAAGGGAAAACTATTCTCCCTCTCCCTATAGTGAGTCGTA
TTAGAATTCCCC
+
BC@CCCBCB>A???<@@C?>;BBBBB@C@?>@C@BBBCE@CA>AA@7CCB@>?@@@>BBBCC>?:?====@@?@@@@
@>?@@>C;==>0
@T6XXI:00123:00778
GGGAGAAGGGAGAAAGGAAGCTAGTCACTCGAGATCTGCGGCTTTGGACAGTCCCTCTGTACTGTGAAGCCACAGAT
GTCGAGTCGACTAGCTTCCTTTCTCCCTTCTCCC
+
B>:@@B>@B>@<;;7;>A?BC@@@CBB;::CA@@@:77774777,66;BBABACA>888<<<>>@>?;@>;>??<<=
=<@@@<====AAA@87378884;:::7::;444-
@T6XXI:00123:00788
GGGGAATTCTAATACGACTCACTATAGGGAGAGGGAGAATAGCGCACCATCAATTTTGTCGTGCCGTTCTCCTTCCT
TTCTCCCTTCTCCC
+
@@@6@>A>BBB@CBBBBBBBBA>96111.7>>CC@B@@>A==<<<B@@@>?7261649@AAC@?=?C=??<8;8:8;
<7=ABA?A>A???;
@T6XXI:00123:00794
GGGGAATTCTAATACGACTCACTATAGGAGAGGGAGAATAGTCAACAAATCGTTTGGGGCGACTTCTCCTTCCTTTC
TCCCTTCTCCC
+
BCC:C?A?ACC@CBB?BBCBB:995..,0::=?=@AC@C;?A?=5333,:<<<@.77:4:BBCC@AA?>A>A=AA<A
???<??A???<
@T6XXI:00123:00798
GGGGAATTCTAATACGACTCACTATAGGGAGAGGGAGAATAGTCAACAAATCGTTTGGGGCGACTTCTCCTTCCTTT
CTCCCTTCTCCC
+
?@=4?<@=ACC@CCB?<<??BCB>9111.6<<@C>B@@?A???AA?@@@;BBA@@;????7>CB@B?AA<58432335
6??C>D?A???<
@T6XXI:00124:00763
CGAGATCTGCGGCTTTGGACAGTCCCTCTGTACTGTGAAGCCACAGATGTCGAGTCGACTAGCTTCCTTTCTCCCTT
CTCCC
+
DCBBCCCCCCC@AAA>AAA???:8:8?AABCCBBBBBA8<<;@==<?@==AAACCCCCCCC@<<8<?ACBCBCC@E?
A@@@<
@T6XXI:00124:00767
GGGGAATTCTAATACGACTCACTATAGGGAGAGGGAGAATAGATCCGGGCTTACTTCCGGCGAATTCTCCTTCCTTT
CTCCCTTCTCCC
+
@>>6@9A?BCCACBB?BCBB@A@971165:BBDE<@?@>B<<;==33317><@AA>A;=:=??<??A@@<>:<8;55
<??@;@;?;<<7
```

```
@T6XXI:00124:00774
GGGAGAAGGGAGAAAGGAAGCTAGTCACTCGACATCTGTGGCTTCACAGTACAGAGGGACTGTCCAAAGCCGCAGAT
CTCGAGTCGACTAG
+
CCBCBB@DC>B?>=<@?;>AB@??B?000::00-
../7>B?CD@@@CCB@@==;866466;89;37718=>AA@@@AAAA==@@@AAAA@A
@T6XXI:00124:00782
GGGAGAAGGGAGAAAGGAAGATAAGCACAAGGGAGTCAAGACTAGAGCTATTCTCCCTCTCC
+
9:-949244-4444-47797;;==@@@??;=B:@:::C>BB?<A@CC<<<?58/77270++*
@T6XXI:00124:00789
GGGGAATTCTAATACGACTCACTATAGGGAGAGGGAGAATAGTTTTTCCCTTCACTAACCGCGAACTGCACTTCCTT
TCTCCCTTCTCCC
+
BBB7A=@=@CD@CCBBB??AB?<97111.7>>?B?A??>B<@?AAA1AB<A=?ABCC??>=>B=;=@<<>@@@C?@?
99730+398;;>7
@T6XXI:00125:00762
GGGAGAAGGGAGAAAGGAAGTGCAGTTCGCGGTTAGTGAAGGGAAAAACTATTCTCCCTCTCCCTATAGTGAGTCGT
ATTAGAATTCCCC
+
AA>DDC?BD=??=@:B=@=@ABCBCC@CCCC@C?@@@CA===-
7777,8ABC@CCBB>AACCC>@@@AA??;BAA@???=@@A>A9<<<0
@T6XXI:00125:00765
GGGGAATTCTAATACGACTCACTATAGGGAGAGGGAGAATAGTCAACAAATCGTTTGGGGCGACTTCTCCTTCCTTT
CTCCCTTCTCCC
+
BAB9B@D@CBB@BBB?BBBB>@;7711657@@DD>B@@?A====68<=?:BBBBC>CCC9CC@@C?@B@;@>=<AA<
@@??4=8;;<;6
@T6XXI:00125:00766
GGGGAATTCTAATACGACTCACTATAGGAGAGGGAGAATAGTCAACAAATCGTTTGGGGCGACTTCTCCTTCCTTTC
TCCCTTCTCCC
+
CBC9A<@>@BB@@AABBADBA>995..,0<<>B?BAA?B<@A@@:<<=;@>??A;<<A6?@BAA>ACA>A>A>@?7>
<<63><?AACA
@T6XXI:00125:00772
GGGGAATTCTAATACGACTCACTATAGGGAGAGGAGAATAGTAG
+
111+1,9ACBBA??@BBCCE@?@CCAAA<AA;79=,++,,,**+
@T6XXI:00125:00791
GGGGAATTCTAATACGACTCACTATAGGGAGAGGGAGAATAGATCACCGTTGGTTCGGGTGCGCGTTCTCCTTCCTT
TCTCCCTTCTCCC
+
?@@6@>A>BCC@CCBA?@@<?AD>9111.7<<CE>CAA=?8@A====;?C=A=A>B@@=B;;;BBB@AAB@C?B?DD
AAA@C<A>888A<
@T6XXI:00126:00762
GGGAGAAGGGAGAAAGGAAGCTAG
+
CB>A;;79@:>911-/,0,.0.55
@T6XXI:00126:00770
GGGGAATTCTAATACGACTCACTATAGGGAGAGGGAGAATAGAGACGTATTCGTTTGCGCTCTGTTCACCTTCCTTT
CTCCCTTCTCCC
+
ECC7B>B>BBC?BBCCBA@@?@>79166.7<<CE>B@@>C???<A@AA@@><=@A<?;<<===@?=@==;>?C>A@;
?<;;5<<<==B>
@T6XXI:00126:00771
GGGGAATTCTAATACGACTCACTATAGGAGAGGGAGAATAGCCACCAACTATTGTTCGTCGCCGTTCTCTTCCTTTC
TCCCTTCTCCC
```

```
+
BCD:C@B?BCC?BBCCC@@@A?>>=7737:::E@DBACA899>AA;=9=ABA<AA8?<<=;;:?C<AA<A<?<?CAB
AAA<A=?@@A=
@T6XXI:00126:00772
GGGAGAAGGGAGAAAGGAAGGAGAAGTTGCCGCCGATGAATTCAGGCTATTCTCCCTCTCCCTATAGTGAGTCGTAT
TAGAATTCCCC
+
CCDCCCACB>AAAB=B@B?A?ABB@@?>@@@@@>9@ABB?B@CCC@BBBB?CBCCADCBBB=B@@@CCCCCCBBBBB
@B@A?A9<=<4
@T6XXI:00126:00788
GGGAGAAGGGAGAAAGGAAGAGCACGTTGCAGGAGTAAATCGATAGACTATTCTCCCTCTCCCTATAGTGAGTCGTA
TTAGAATTCCCC
+
DCB@??@BC?BBBB=B?ABDA@ABBBB@B>DABBBBBB>AABBBB;>;>@AA@7777:CCCA:=BCBCBBAAB@@@C
C@CCC@C=@@@6
@T6XXI:00127:00760
CTAGTCGACTCGAGATCTGCGGCTTTGGACAGTCCCTCTGCACTGATGAGCCACAGATGTCGAGTCGACTAGCTTCC
TTTCTCCCTTCTCCC
+
@//1/1////:=BBCDBBBBC@DCC=A>;<<>7775:>>@?==94444434779;@?AAAC@??BAAACCG<<<@B@
CFACCDL>B?BBCE>
@T6XXI:00127:00762
GGGGAATTCTAATACGACTCACTATAGGGAGAGGGAGAATAGCCACCACAACTCCGACACTGCTATTGCCTTCCTTT
CTCCCTTCTCCC
+
999/9;@=ABCACCBBB?>>BCB97111.7>>@C>B@@@C=668=C<=<<::?A>AA;;?@@@???<?@8<9<<?A9
==A=;><9==A=
@T6XXI:00127:00763
GGGGAATTCTAATACGACTCACTATAGGGAGAGGGAGAATAGTGGCTAAACCCCCGTACGTCACTTCTCCTTCCTTT
CTCCCTTCTCCC
+
@??8A@CBCCC@AA>>B???;AB77111.7>>BE>A==?A==;?>?<66,1111+7@ABCCBCCC@@??<?=?=@@<
AABB=B<??@A<
@T6XXI:00127:00774
GGGGAATTCTAATACGACTCACTATAGGGAGAGGGAGAATAGTCAACAAATCGTTTGGGGCGACTTCTCCTTCCTTT
CTCCCTTCTCCC
+
DDD9C@C@BBB?BBBBBBCBDAB<8696.6===?<?<<?A===AA@BAA<DCCBB>BCC8B?@@C=?@?=?@A>@@;
A@AB7<9<===:
@T6XXI:00127:00778
GGGAGAAGGGAGAAAGGAAGAGAACGGCGACGAACAATAGTTGGTGGCTATTCTCCCTCTCCCTATAGTGAGTCGTA
TAGAATTCCCC
+
EDADDC?AA=ABBB>C@A?BCBB@@@>?C?B@A=@B@CCBB?ABAA?ACCB?B::78;CCED>??====A=A???@A
@@@@:;37770
@T6XXI:00127:00780
GGGAGAAGGGAGAAAGGAAGCTAGTCGACTCGACATCTGTGGCTTCACAGTACAGAGGGACTGTCCAAAGCCGCAGA
+
CC>BBB>@@=B@=>:@?B5;;C:;;CCCCCCC?9:;:7>>>;<?=?CCB@?><44000,4;;>AA=AA:@A>BBAAA
@T6XXI:00127:00788
GGGGAATTCTAATACGACTCACTATAGGGAGAGGGAGAATAGTCAACAAATCGTTTGGGGCGACTTCTCCTTCCTTT
CTCCCTTCTCCC
+
444,4899>AB@CBCEBBDDBA>?=677.7??CE=BAA=?;<<;<8<@>:?A777.777.7??@C@CB@@B>A>AB<
?==<7<;==>>7
@T6XXI:00128:00755
```

```
GGGGAATTCTAATACGACTCACTATAGGGAGAGGGAGAATAGCCACGCGATAAACTCCCTGTAGCGCATACTTCCTT
TCTCCCTTCTCCC
+
999/98@=ADC@CBBBAABBBAD??766.7=777.7998=<<=>AAD?<?CCC>B?AB?@????>>AA99:D;=;=A
=;;:B996///85
@T6XXI:00128:00756
GGGAGAAGGGAGAAAGGAAGGGGTGAACCGCGCGTTAATTTAGGGAGCTATTCTCCCTCTCCCTATAGTGAGTCGTA
TTAGAACTTCCCC
+
990499294-
84;?09531333,37;<AACCCC<<8;>@=:ACB:@?@>?>111<<810.77:;B>BBEB@=<=7400/03=<@@@C
CC7
@T6XXI:00128:00759
GGGAGAAGGGAGAAAGGAAGGAGATAGAGCCGGTCGGGATTACAGGTCTATTCTCCCTCTCCCTATAGTGAGTCGTA
TTAGAATTCCCC
+
BB>CCCBCB>CBBC=@=>@B@CBAB@??A@?B?BCA<<BB?BC?=>@CCBB?@A>A<@====;::=A====>>9=88
8>@@A=@/888-
@T6XXI:00128:00770
GGGAGAAGGGAGAAAGGAAGCTAGTCACTCGACATCTGTGGCTTCACAGTACAGAGGACGTGTCC
+
<4-4;?59804=>@:>4843<>=AC@AA????8589966607711111?:=375,,+++0+++-,
@T6XXI:00128:00779
GGGGAATTCTAATACGACTCACTATAGGGAGAGGGAGAATAGTGTGATGAGTACAACTCACCTCGATTCTCCTTCCT
TTCTCCCTTCTCCC
+
BBB4@5959?>;>ABBACCCCCB>=777.7???C>?==>ABAA???C?B??????>ABBA@>?8::B@GGC?B?B>A
A>ACDC>@<778=7
@T6XXI:00128:00787
GGGGAATTCTAATACGACTCACTATAGGGAGAGGGAGAATAGATCACCGTTGGTCGGGTGCGCGTCTCCTTCCTTTC
TCCCTTCTCCC
+
999,42429DC>CBDBCCCCBBBAB:9956;;?B>A??>A@@@@@BB?>727+,-*0)*--
0:/.6<<;>===99688;84894444-
@T6XXI:00128:00788
GGGAGAAGGGAGAAAGGAAGTGCAGTTCGCGGTTAGTGAAGGGAAAAACTATTCTCCCCCTCCTCCATATGT
+
CC==:A@BB<@@@@<@ACCC@@AADBABCBB?C@<<<<C@BC9????/??874777777,09333,------
@T6XXI:00128:00792
GGGAGAAGGGAGAAAGGAAGAAGATGTCGCCGCAGGGAGTGAACTTGGCTATTCTCCCTCTCCCTATAGTGAGTCGT
ATTAGAATTCCCC
+
CD@AA=@BC>9;;C@C?A>AA@CB>?@BAA>>>BBB<7::AA>AC?A?ABA@@CCCC>CBBAB<BBCC??A???ABB
BC?BBB>CABBB:
@T6XXI:00128:00796
GGGGAATTCTAATACGACTCACTATAGGGAGAGGGAGAATAGCCAATCTCTCGTGGTCATTGCGCTTCTCTTCCTTT
CTCCCTTCTCCC
+
@=@8A=ABDBB@CBBBBB<B@AB77111.7<<AB>C@@=@4778;:;;<=A@AAA????@>AAA????AAA>@>AA;
??AA7<:=?@@<
@T6XXI:00129:00757
GGGAGAAGGGAGAAAGGAAGGAGAAGTCGCCCCAAACTATTTGTTGACTATTCTCCCTCTCCCTATAGTGAGTCGTA
TTAGAATTCCCC
+
DC@BBAACB@???B=B@B@B?AAB@@@999996:::///::::?@C??;<<<<>>>;<:?==;99=???AAA???@@
@?@@@@B3477+
@T6XXI:00129:00772
```

```
GGGAGAAGGGAGAAAGGAAGTGCAGTTCGCGGTTAGTGAAGGGAAAACTATTCTCCCTCTCCCTGGGAGAAGGGAGA
AAGGAAGGAGACAGTGAGCGCCGCACAACACGTCACTATTCTCCCCTCTCCCTATAGTAGATC
+
DK@CAA>=>9>98809<>=@ACCCDC@CCBB>A=?@???;@B9???5>?>@>???C=AABB?:003+403/03,48:
=;?@@>>98.,,,,,//8:;;56=AAA>===A???@@=A<;0000+0889<;====<43+++*
@T6XXI:00129:00778
GGGGAATTCTAATACGACTCACTATAGGAGAGGGAGAATAGTTCGCGCTCCTCTTACCTCAACATATTCTTCCTTTC
TCCCTTCTCCC
+
A@>4?<@=@A@CCBA?BBBBA:997//-
0==AB>B@@=?===:8:<=<<<;?A=<<@?CBB@<?CBB?B?9;/33,389948;6<<?;
@T6XXI:00129:00792
GGGGAATTCTAATACGACTCACTATAGGGAGAGGGAGAATAGTCAACAAATCGTTTGGGGCGACTTCTCCTTCCTTT
CTCCCTTCTCCC
+
444/9<AACBB@CBB@BBBBB@>97111.7>>@B>B@@@A@>>AA?<?=;=99998???5?AABBCBC@>@=?<AB=
A@@@;@>AAAA;
@T6XXI:00129:00794
GGGGAATTCTAATACGACTCACTATAGGGAGAGGGAGAATAGTCTCCTATTCCCTGACGTTAATTTGCTCTTCCTTT
CTCCCTTCTCC
+
CCIBD=?=ACE?BBABBBB>>=>??@@?>=>@BE>?==>A<;<<8838??;@E@DCCBBC@F?BB>BB@@@>A8;;4
:9:94;388?>
@T6XXI:00130:00757
CGAGATCTGCGGCTTTGGACAGTCCCTCTGTACTGTGAAGCCACAGATGTCGAGTCGACTAGCTTCCTTTCTCCCTT
CTCCC
+
CA@ACCCBBBB@BBB>B>AA@C@BB>BCCIA>BBCCB@:==>B@@@@@@@BBBDCCBAAAD<<;<A=>@<@@AA>A6
88880
@T6XXI:00130:00779
GGGAGAAGGGAGAAAGGAAGGAGAACGCGCACCCGAACCAACGGTGATCTATTCTCCCTCTCCCTATAGTGAGTCGT
ATTAGAATTCCCC
+
EE@CCBBCB=ABBB=B?B?C?BBC@BBA@?BBC>CC?C>AAECCDBBBBBCC@B:795;AACD>BBBBBAABB@CB?
?@@CAA?EADCC9
@T6XXI:00130:00783
GGGAGAAGGGAGAAAGGAAGTGCAGTTCGCGGTTAGTGAAGGGAAAAACTATTCTCCCTCTCCCTATAGTGAGTCGT
ATTAGAATTCCCC
+
DFCBA@@BB>AAAB>B@B?BBBBBBB@BBC:9=990005555+....*3BCC@CCCD>A>ABC>@?@@@@AA@A???
@@=@@@=>;>>>4
@T6XXI:00130:00786
GGGAGAAGGGAGAAAGGAAGGAGAAGTCGCCCCAAACGATTTGTTGACTATTCTCCCTCTCCCTATAGTGAGTCGTA
TTAGAATTCCCC
+
CD?BBC@CB>BBBB?C?C?A?ACC?AA@>>>>.880768:?=CC@B??<AA?@>?C<A==<?;?<==C???@A????
?>@?=99/444-
@T6XXI:00130:00798
CGGCTTTGGACAGTCCCTCTGTACTGTGAAGCCACAGATGTCGAGTCGACTAGCTTCCTTTCTCCCTTCTCCC
+
BB@BFF>C@BBBBBBB?BBBBAAAAAAAB@BB@BB???@@@A<ACC=@B@BC?=@@C?BC>BBCC>CACCCC>
@T6XXI:00130:00799
GGGGAATTCTAATACGACTCACTATAGGGAGAGGGAGAATAGCCCCAAGCATCAGTGCACCCGTTGTT
+
4??6A@C@CBB@BCBDBCCCCBA8:777.777>>:7347440000+0/044==700000--+.43369
@T6XXI:00131:00755
GGGAGAAGGGAGAAAGGAAGCTAGTCGACTCGACATCTGTGGCTTCACAGTACAGAGGACTGTCCAAA
```

```
+
990444249-4444-49913331167888773/////...1001014/./73022.-,-32113/000
@T6XXI:00131:00780
GGGAGAAGGGAGAAAAG
+
9?5>?@59914/---*-
@T6XXI:00131:00781
GGGGAATTCTAATACGACTCACTATAGGGAGAGGGAGAATAGACGTACCGTTTTCGGCACCGACTTCTCCTTCCTTT
CTCCCTTCTCCC
+
CCC9B=@=ACC@BCBB@<<B?@<77111.7?>>@=A@@>A@@@?AAAA=@@AA6@@>@@@<@A@@=44424782344
66@@:?>@DCC<
@T6XXI:00131:00783
GGGGAATTCTAATACGACTCACTATAGGGAGAGGGAGAATAGTCAACAAATCGTTTGGGGCGACTTCTCCTTCCTTT
CTCCCTTCTCCC
+
BAA7A>B>A@A@CBB?BBBB?AB;>661-
6;;?A>BAA>@@B@@@=AAA<CBBB@;>@@7AAAAA7::=;?;@=@@<@@AA<A>@@AB=
@T6XXI:00131:00784
GGGAGAGAATTTGGTAATGGGAAACGCGTTCGTGGCAGGGGGAGAGCAGGCACCCTTATATCTCTCTCCCTATAGTG
AGTCGTATTAGAATTCCCC
+
HK?AA@A@=@C@CACB?BBEABBABBBBF@BBCD?BBCBBB2AAACCB?<?8880<ABBDCB:6767==6487===<
=788A888>?<<<BABCC9
@T6XXI:00132:00761
GGGGAATTCTAATACGACTCACTATAGGGAGAGGGAGAATAGTGCTCATCCCTTACAAAGTCCCCGTATTCTTCCTT
TCTCCCTTCTCCC
+
BBBAB@B?ABB@CBBBBBAAA@7<;666.7????<?;<378A@AC@A?966.7;>=??<?DEDC9BB??=8=885<<
7<@@@<A>AA@@;
@T6XXI:00132:00770
GGGAGAAGGGAGAAAGGAAGGAGAAGTCGCCCCAAACGATTTGTTGACTATTCTCCCTCTCCCTATAGTGAGTCGTA
TTAGAATTCCCC
+
BEABBA=@><ACCCAC?B?B@BAA@ACBAAAA7AB<BBB?C>A>=ABB?AA?<9899=???C<A@@@===@@@@A89
9>A@;-.,////
@T6XXI:00132:00772
GGGAGAAAGGGGAGAAAGGAAGACTAGCGAATGGGGGAAAATCAATTGGCTATTCTCCCT
+
4:64444-444,44/756,.,...556667011111+333,333131104688,,,,,*,
@T6XXI:00132:00789
GGGAGAAGGGAGAAAGGAAGAGAATACCGCGATGGGAGAAACCACGCTATTCTCCCTCTCCCTATAGTGAGTCGTAT
TAGAATTCCCC
+
BBBCBB@CA:=;44-
9;><?ABB?BBCBCCBBBBC>CBBC>C?C;;9BBC@@A@C<?8??@;@AAAAAA<@99=?AA;==A?B:???5
@T6XXI:00132:00796
GGGGAATTCTAATACGACTCACTATAGGGAGAGGGAGAATAGCAATCTCTTTTGCGTTCGCCGTGTCTCCTTCCTTT
CTCCCTTCTCCC
+
CDC9C@C@BCB@BBBBB>>?@@>76116.7=?CE>B;A?C===99=AAAAAA6@@??=?4427@??@@A>A>A>AA<
???@<A>???C;
@T6XXI:00133:00760
GGGAGAAGGGAGAAAGGAAGCTAGTCACTCGAGATCTGCGGCTTTGGACAGTCCCTCTGTACTGTGAAGCCACAGAT
GTCGAGTCGACTAGCTTCCTTTCTCCCTTCTCCC
+
```

```
CB<AA><??:??>>9>;><?@???@@7969990>99<AAA?ABB>B@AAACCCC>@A<===A<=@@=?AA>@ABAAA
<@<<<<=<<<:=;;4424244-4;;;-424434,
@T6XXI:00133:00764
GGGGAATTCTAATACGACTCACTATAGGGAGAGGGAGAATAGTCAACAAATCGTTTGGGGCGACTTCTCCTTCCTTT
CTCCCTTCTCCC
+
===6><A?CBBACBAA??B><A<97111.7>>BF>A@@?A<<<@><@@@<BBBBC>?@?8>>@@@@??<;A><;??<
?A@A<@>??@A;
@T6XXI:00133:00773
GGGGAATTCTAATACGACTCACTATAGGGAGAGGGAGAATAGTTTCCTACAACTGATCGG
+
CDCCC@B?BDC@B>>>BBBB9>@>>111.7<<@BAB??@A@@?@<A>=443)*******.
@T6XXI:00133:00780
GGGAGAAGGGAGAAAGGAAGGAGAACGCGCACCCGAACCAACGGTGATCTATTCTCCCTCTCCCTATAGTGAGTCGT
ATTAGAATTCCCC
+
CE@CCB@BB>BBBB>B>?>>@BBB@BBBBBCBB===8;9<DDN@BCCCC<<;=@988/888??<ABBBCDC@@=BBB
BC@BA@>CEDCC9
@T6XXI:00133:00783
GGGGAATTCTAATACGACTCACTATAGGGAGAGGGAGAATAGTCAACAAATCGTTTGGGGCGACTTCTCCTTCCTTT
CTCCCTTCTCCC
+
@@@8A?B?CCB@B???BBBB?AB77111.6===A>A@@?B;;;AB>???<AB???8???5?ABBE>ABA>@>A>@A=
ABBB:B=@@@B:
@T6XXI:00133:00786
GGGGAATTCTAATACGACTCACTATAGGGAGAGGGAGAATAGCTACGTTTAACCGATCGTGTTGTTCTCCTTCCTTT
CTCCCTTCTCCC
+
944,424:;CCACCCCBBBBBB@?=766.7@>@C@@==>AA@@BB@?@C>AAB<??A?===A>@A>???>@@A9<=9
===DAC<>?AC;
@T6XXI:00133:00791
AATACGACTCACTATAGGGAGAGGGAGAATAGTCACCATCCTTCCCCCGGCAACTTGCACTTCCTTTCTCCCTTCTC
CC
+
C@BBBBBBBBBBA;:33308@??B?DCB@BBBBDBA>>8:7:=?BBC2C@AA>AB?BAAFC@B6::::@?B<E?AAC
D@
@T6XXI:00133:00794
GGGGAATTCTAATACGACTCACTATAGGGAGAGGGAGAATAGTCAACAAATCGTTTGGGGCGACTTCTCCTTCCTTT
CTCCCTTCTCCC
+
999/42929ABACDBBBBBBC@BA?9995:??CC>CAA?A==@<=>A@?7A:::B<AAB9BAACC?BB@>?>?>AD<
@@?A<A>@@@@<
@T6XXI:00133:00795
GGGAGAAGGGAGAAAGGAAGCTAGTCGACTCGACATCTGTGGCTTCACAGTACAGAGGACTGTCCAAAGCCGCAG
+
@@<>@>5880;?>@9A>B9<;DBBBBBDCAB@::;:77?=:/4957AABC@=;?A7858@@@AA>BB<AB>@@<=
@T6XXI:00133:00796
GGGGAATTCTAATACGACTCACTATAGGAGAGGGAGAATAGCCTCTTCACATTCTCTCATCCGCTTATCTTCCTTTC
TCCCTTCTCC
+
CDKBC=?AADC@CCBBBABAB@;;96957@>>@>A??>A???<BAB@C@??C?CAA@@@@?=???<??==8=88848
/..,/46//,
@T6XXI:00134:00752
GGGAGAAGGGAGAAAGGAAGGAGAAGTCGCCCCAAACGATTTGTTGACTATTCTCCCTCTCCCTATAGTGAGTCGTA
TTAGAATTCCCC
+
```

```
AC?BBB@CB>>BBB?B?B?B?ABC@CCEA@>>.8:6:CCDC=BB?B??<8858@CC?ABBBB=A@<<;<@@@?<==?
?>AAA>A;>==1
@T6XXI:00134:00754
GGGAGAAGGGAGAAAGGAAGGAGAACGCGCACCCGAACCAACGGTGATCTATTCTCCCTCTCCCTATAGTGAGTCGT
ATTAGAATTCCCC
+
CC?CCCACB?BAAA?B@B@B@BB?>ABBBBBBB>BC@C@CACC@CCCBA@AB@B>>><@CBBC?BC@CCC@BBACCA
BB@A==;BDCCC9
@T6XXI:00134:00770
GGGGAATTCTAATACGACTCACTATAGGAGAGGGAGAATAGTCAACAAATCGTTGGGGCG
+
:::5;;=<>=>58/6/..4..17866998???@9?6849.----0123376660177,16
@T6XXI:00134:00773
CTATCACTCGAGATCTGCGGCTTTGGACAGTCCCTCTGTACTGTGAAGCCACAGATGTCGAGTCGACTAGCTTCCTT
TCTCCCTTCTCCC
+
BBBA9::EDCCCCBBBBAB?BBB<A>@B>>7::.7@>??A@=9==A?AC@BBBCABBABCDDC;:;BBC:<8?@>CB
?CAAC?A;==?HA
@T6XXI:00134:00788
GGGGAATTCTAATACGACTCACTATAGGGAGAGGGAGAATAGTTCTCCCATGACTCGTGTTACCGTGGCCTTCCTTT
CTCCCTTCTCCC
+
CCC9C@E@DBB@BBBBBBB<?C?77111.7==>@>BAA>BA@<;<A==;@@@?@@@CCBB@@@>@@@<?<>;=;=;7
:44418=88:C<
@T6XXI:00134:00792
GGGAGAAGGGAGAAAGGAAGGAGAAGTCGCCCCCAAAACGATTTGTTGACTATTCTCCCTCTCCCTATAGTGAGTCG
TATTCAGAATTCCCC
+
CC@BA=899999990820,.6...,.34.....*...*1666989@>????==8=<;<7=AAACA>>?@@=:?<<;9
666,//77275==64
@T6XXI:00134:00795
GGGAGAAGGGAGAAAGGAAGAGCAGGGCGCCAGACTCGATCCGACGACTATTCTCCCTCTCCCTATAGTGAGTCGTA
TTAGAATTCCCCTAGCTTCCTTTCTCCCTTCTCCC
+
9>1944294-444;-
42377;;BCDF@AAD?BCCA;;<BBBACCCCED;::>AA=?:899AA<AAABAAAA?@@B@@A@@??<A@CCD9BB8
:686366,616668434443
@T6XXI:00135:00769
GGGAGAAGGGAGAAAGGAAGGAGAAGTCGCCCCAAACGATTTGTTGACTATTCTCCCTCTCCCTATAGTGAGTCGTA
TTAGAATTCCCC
+
CCABBB@DB?B??ABB@C>>>ABB@@?CBBBB9CC>BBBCC<A<ABB@==A<?:==;;:?<<<A<@@<@A<A@@@<=
=?CA@C?38870
@T6XXI:00135:00784
AGTCACTCGAGATCTGCGGCTTTGGACAGTCCCTCTGTACTGTGAAGCCACAGATGTCGAGTCGACTAGCTTCCTTC
TCCCTTCTCCC
+
CCCBABBB998?CCCB@@<99909=?ABCCCC<@><7::99BBBC>;;8:CBA??>>>=78887:;<>=:<:<,0,0
;;;6:6:>?=;
@T6XXI:00135:00789
GGGGAATTCTAATACGACTCACTATAGGGAGAGGGAGAATAGGGCCGACGTCGCATGCTCCCGGCCGCCATGGCCGG
CCGCGGGAATTCGATTGGGGAATTCTAATACGACTCACTATAGGAGAGGGAGAATAGTCAACAAATCGTTTGGGGCG
ACTTCTCCTTCCTTTCTCCCT
+
CBC7C=@=ABBBCDA@@CAA??@AAABC>????A9?9998665+,+,,+003477@??<<>;>;=:??<99;-
/,/78789CC=A=B?A@@?>ACC4@=@?AAA>?788=<;<@A:::/./98885;;;8;@@=AA?CBB>BCEAB?AA
A6><<;:7::9788:76616///,/
```

```
@T6XXI:00136:00758
GGGAGAAGGGAGAAAGGAAGCTAGTGACTCGACATCTGTGGCTTCACAGTACAGAGGGACTGTCCAAAGCCGCAGAT
CTCGAGTCGACTAG
+
BE@DDB@BA:=;94-
;=?<@CCBBC@@CCAC=?@BBBBBC@BC@CA>?BBB;:843317=<>?@>CC<??=?AAACAA??@@A@@@@@?AA
@T6XXI:00136:00760
GGGGAATTCTAATACGACTCACTATAGGGAGAGGGAGAGTAGCCGACAATTCGACTCCCCGGCAATGTTCTTCCTTT
CTCCCTTCTCCC
+
449/99@>BBD?BCBB????>AB>9111.6<<BC>CAAAA8;;@CCAA?C?BBCB@AAA8?@@A>=>@>?>>A>A;:
=A==;=55:AA?
@T6XXI:00136:00765
GGGAGAAGGGAGAAAGGAAGCTAGTCACTCGACATCTGTGGCTTCACAGTACAGAGGGACTGTCCAAAGCCGCAGAT
CTCGAGTCGACTAG
+
CC>BA?<=@=;<<C@C?B?BC>==A@;::?=773?<9@BC@AB?BBCB?BC==<33317>?@@@?BB<@@>@AA@?:
;====<=588????
@T6XXI:00136:00774
CTAGTCGACTCGAGATCTGCGGCTTTGGACAGTCCCTCTGTACTGTGAAGCCACAGATGTCGAGTCGACTAGCTTCC
TTTCCTCCCTTCTCCC
+
=7-,--
,,//7;@?AEBAA@C@@@@<@@A???7775:??CBABC@?8838A>A@@BDEDBBBBCBBBB;;:://65447+.,/
7728.87533
@T6XXI:00136:00777
CTATCGACTCGAGATCTGCGGCTTTGGACAGTCCCTCTGTACTGGGGCCACAGATGTCGAGTCGACTAGCTTCCTTT
CTCCCTTCTCCC
+
D>;;111>;;ADCCCBBBAA?ABB?B@CBAB9994:BBCCDB?=;:5><??@@@===DA@?@==<==A88<?BACC=
BBCCBC@CACB>
@T6XXI:00136:00791
GCTATCACTCGAGATCTGCGGGCTTTGGACAGTCCCTCTGGTACTGTGAAGCC
+
444444:////1//66/4333,317-81719<>CA;>;/.,..--1144531,
@T6XXI:00136:00793
GGGAGAAGGGAGAAAGGAAGGGAATAGCAGCAGTCGGCAACGTGAAAACTATTCTCCCCTCTCCCTATAGTGAGTCG
TATTAAGAACTTCCCC
+
CC>BBB>AB>A=;;8;-
0,0407,..,......5757:>97971111,3977011111,1114946444859;8//.,+0*+*+23377===5
@T6XXI:00137:00762
GGGGAATTCTAATACGACTCACTATAGGAGAGGGAGAATAGCCACCAACTATTGTTCGTCGCCGTTCTCTTCCTTTC
TCCCTTCTCCC
+
BBB7D:;;<CCBCCBBB@@AA:965..,0::>C@CAA@C:??:<><=<;;:?9;;?????AC?AA88<7837=@A<A
CAA;?/34881
@T6XXI:00137:00763
GGGAGAAGGGAGAAAGGAAGCTAGTCACTCGACATCTGTGGCTTCACAGTACAGAGGGACTGTCCAAAGCCGCAGAT
CTCGAGTCGACTAG
+
CC@CCC@CC:?444-96848?>=<??:7::@::7@?BBBB>:;9:BBBCC?;89333-
4748BB=?@<@@=@AAAAAA???@@A@@@AAA@
@T6XXI:00137:00783
GGGAGAAGGGAGAAAGGAAGGAGACACGGTCACAAACATGAATGTGACTATTCTCCCTCTCCCTATA
+
9@8<;A>A@:999901::,0,0..7668;88>99919@@>?=?435///3533-00+0334436556
```

```
@T6XXI:00137:00784
GGGAGAAGGGAGAAAGGAAGGAGAAGTCGCCCCAAACGATTTGTTGACTATTCTCCCTCTCCCTATAGTGAGTCGTA
TTAGAATTCCC
+
BB@CEB@DD>EAAB?C?B?A?ACC@@@CAAAA8@B>@CCCC>??>??B?==;<<<=:::?=B;=:;?<?A???????
?>DAA?A>@@<
@T6XXI:00137:00798
GGGAGAAGGGAGAAAGGAAGGATAGGCCATGGAGTGTGTCGCATGGCTATTCTCCCTCTCCCTATAGTGAGTCGTAT
TAGAATTCCCC
+
CH>BDA89809@@@:@<A=?=:A::,0,.;<;>CCBDCCC??===;=ACB??98809@@AC@CCBAAACCBBBA::7
<>11042889/
@T6XXI:00138:00750
GGGAGAAGGGAGAAAGGAAGCTAGTCACTCGACATCTGTGGCTTCACAGTACAGAGGGACTGTCCAAAGCCGCAGAT
CTCGAGTCGACTAG
+
@BBCDC>>=5=844-
9584;BA>B@6;:??=774:96@AB?BB@CC?>??@=<;88848<<;;=;<=;<A?A=<<<<<?=====;;<=AA@
@T6XXI:00138:00755
GGGAGAAGGGAGAAAGGAAGCTAGTCACTACATCTGTGGCTTCACAGTACAGAGGGACTGTCCAAAGCCGCAGATCT
CGAGTCGACATAGCTCCCTTTCTCCC
+
49144424>=99<H@C?B?@@@?:77,,,,,,,.0444201011110433355564666:8888=;<=<<AA@@@88
8><<<@@@@@?@43300+04243333
@T6XXI:00138:00770
GGGGAGAAGGGAGAAAGGAAGCTAGTCACTCGACATCTGTGGCTTCACAGTACAGAGGACTGTCCAAAGCCGCAGAT
CTCGAGTCGACTAGCTTCCTTTCTCCCTTCTCCC
+
999,444:;@:=@@><>=?;??877@00/....42./7?:<=@C@DABBABB>>>8969DB??@388-
4;9788B@8888==;=87777838:4::=>:?ACC;@588==7
@T6XXI:00138:00777
GGGAGAAGGAGA
+
11-1//,/,,**
@T6XXI:00138:00783
GGGGAATTCTAATACGACTCACTATAGGAGAGGGAGAATAGCTCCCGAGACTATTGGCCATAGATGTACTTCCTTTC
TCCCTTCTCCC
+
CCC9A>B?BBC@CCBBB???B:997..-
0>=BK>CBA@D:::=??>CBB98?==<<;?=@AA@ABB@?AC?B?AC>CAAA;::03441
@T6XXI:00138:00796
GGGGAATTCTAATACGACTCACTATAGGGAGAGGGAGAATAGACATCTACCAATCGCACGTCTGTTCACCTTCCTTT
CTCCCTTCTCCC
+
9=>4>=@@DCC@CCCCAAAB?AD==66656::CCAB?@>A====AAA>==3738=?=??A<<<==;<999=>A>@A;
@??@<?>AAAA<
@T6XXI:00138:00799
GGGAGAAGGGAGAAAGGAAGGAGAACGCGCACCCGAACCAACGGTGATCTATTCTCCCTCTCCCTATAGTGAGTCGT
ATTAGAATTCCCC
+
CDDCCB===:@@@B@B@B?B@BB?=ABBBBBBC>BB?A?GDCCBCBBBBCBC?>:995:BBCC>CCBBBBB@?@BA>
?@@AA@>CABCC:
@T6XXI:00139:00750
AGTCACTCGAGATCTGCGGCTTTGGACAGTCCCTCTGTACTGTGAAGCCACAGATGTCGAGTCGACTAGCTTCCTTT
CTCCCTTCTCCC
+
```

```
AADDBBCBCCAAABBBCB?BCG@C@CCBCBBB?@AAA??BCCBBBCCC?BBBBC@>>;;;==89///-
.47>B?BC>DCCE=?9<<>>7
@T6XXI:00139:00768
GGGGAATTCTAATACGACTCACTATAGGAGAGGGAGAATAGCGACAAATTGATGG
+
BB@:C@C??@?@@AABBBB@@:<;<00,//0==8?9:88@88388=4355.,,0*
@T6XXI:00139:00770
GGGGAATTCTAATACGACTCACTATAGGGAGAGGGAGAATAGCTAAAACATTATCCCCGTCGCTGTTCTCTTCCTTT
CTCCCTTCTCCC
+
CCD:C?A>9<;=@BABCC@@@BA?=677.777=A@BA@?AA@@????5?@><>????4?@A==@CG@ACBB>?=AC<
??DC<A?AA?A<
@T6XXI:00139:00776
GGGAGAAGGGAGAAAGGAAGGAGAAGTCGCCCCAAACGATTTGTTGACTATTCTCCCTCTCCCTATAGTGAGTCGTA
TTAGAATTCCCC
+
CKDCCBADB?CDBB>B?A?A?ABB?@BCAA;;7@B>BCBCD>??9???=AC?=9==;=:?<=;;:??AAA<AA@@@@
@>A@>:;2444-
@T6XXI:00139:00784
GGGGAATTCTAATACGACTCACTATAGGGAGAGGGAGAATAGTCAACAAATCGTTTGGGGCGACTTCCTCCTTCC
+
BBB8B>=7;9=;==BBABBBBB<<:?;;1//555+.--,--00002000+1666>;::8232,,,*,*,000033
@T6XXI:00139:00792
GGGGAATTCTAATACGACTCACTATAGGGAGAGGGAGAATAGTCAACAAATCGTTTGGGGCGACTTCTCCTTCCTTT
CTCCCTTCTCCC
+
BAA7A>A@CCB@CBBBABBAA>;971165:@@CE<A@@?A<??@@<<<::AA9?98???5?CBCC@AA?>@>@>AA<
@@AA<A<?@AA<
@T6XXI:00140:00756
GGGGAATTCTAATACGACTCACTATAGGAGAGGGAGAATAGTCAACAAATCGTTTGGGGCGACTTCTCCTTCCTTTC
TCCCTTCTCCC
+
994,424248899=BBBB@@?;:<<00,000?A<BAA;==@AA@<8881:B@AB?BEL9BBCBD8::A696:@CH@@
@@@<C>@@@@<
@T6XXI:00140:00765
GGGAGAAGGGAGAAAGGAAGGTGAACAGAGCGCAAACGAGTACGTCTCTATTCTCCCTCTCCCTATAGTGAGTCGTA
TTAGAATTCCCC
+
CCAB@@644-
9999094848<>>?:=@BCCGBBBB>AAABBBA?@@@@>?B<>>>@<@ABB@;;;000:::7>?BABB@C?A=B@BB
C9
@T6XXI:00140:00767
GGGAGAAGGGAGAAAGGAAGGGGAGAAGGGAGCAAAG
+
BE=<3?>>B;113GB@?@;A??.800,/:25//,202
@T6XXI:00140:00770
GGGAGAAGGGAGAAAGGAAGGGGAGAAGGGAGAAAGGAAGCTAGTCGACTCGA
+
CC@???BCC=9@<E?B@A@CCC9BCC@A=91766,7:=:?>7==@0..,++--
@T6XXI:00140:00778
GGGGAATTCTAATACGACTCACTATAGGGAGAGGGAGAATAGTCAACAAATCGTTTGGGGCGACTTCTCCTTCCTTT
CTCCCTTCTCCC
+
BAA6@=@@BBD@CCBBB@@@>@;77111.7>>AB>BAA>A=@@??<<<<7?CAAA9???5??AAB?ABA=@>??AA<
A???:>;=>>?<
@T6XXI:00140:00782
GAGATCTGCGGCTTTGGACAGTCCCTCTGTACTGTGAAGCCACAGATGTCGAGTCGACTACGTCCTTT
```

```
+
GBCBBBBABC@CCCAC@CBCCCCB<BB??>>@B@@CC>B>>@@@>>ACABCABBCBB=;1111-***)
@T6XXI:00140:00785
CTAGTCGACCGAGATCTGCGGCTTTGGACAGTCCCTCTGTACTGTGAAGCCACAGATGTCGAGTCGACTAGCTTCCT
TTCTCCCTTCTCCCCTT
+
C=9999491,////8:;>>><98709@?BCDDCB=CCCC??==>CCC??C?BBCCCCCBBBAAABBBBABB<;;<AC
C?CBBB<?B?++**)*)
@T6XXI:00141:00750
CTATCGACTCGAGATCTGCGGCTTTGGACAGTCCCTCTGTACTGTGAAGCCACAGATGTCGAGTCGACTAGCTTCCT
TTCTCCCTTCTCCC
+
B=77//118:ACA@ABBBAA?BCB?B>BBCCAAB>ABCA><<==@@@=AC@BB>>>>;?CCCBBBC@@@FBCB<<;;
J@A?>A<@>@AAA=
@T6XXI:00141:00752
GGGGAATTCTAATACGACTCACTATAGGGAGAGGGAGAATAGTCAACAAATCGTTTGGGGCGACTTCTCCTTCCTTT
CTCCCTTCTCCC
+
9>>6@>AACCC@CCC?B?B?B@B?966659??CC>B@@?A;@@AA;<@@;?BAAC=@@@7@CAAC@CB><@?B>A@:
??AB>B:<<AA<
@T6XXI:00141:00753
GGGGAATTCTAATACGACTCACTATAGGGAGAGGGAGAATAGCCTAGCTTTGTACTACCGGCTGTTCGCCTTCCTTT
CTCCCTTCTCCC
+
BA?9B=?BACCCBB?BBBCB?AA?>611.6<<=><@>><?@@A>BBCCAA<CAA???=>?>@@==<=:;:<;<;<=;
====;=;8::=;
@T6XXI:00141:00760
GGGAGAAGGGAGAAAGGAAGCTAGTCACTCGACATCTGTGGCTTCACAGTACAGAGGGACTGTCCAAAGCCGCAGAT
CTCGAGTCGACTAG
+
49-9996990488858,.,...743........-8<>>@CCBEBBAABBC@=:;444-
4:::=?>AA;@C9<<<<66////.5;<?AA>??
@T6XXI:00141:00796
GGGAGAAGGGAGAAAGGAAGCTAGTCGACTCGACATCTGTGGCTTCACAGTACAGAGGGACTGTCCAAAGCCGCAAA
C
+
B>:A<959A:>999147>;@DABBC:::@>=<=711116642368?:::AA===;44818=;;;;344,4;43333+
-
@T6XXI:00142:00753
GGGGAATTCTAATACGACTCACTATAGGGAGAGGGAGAATAGTTTTTCCCTTCACTAACCGCGAACTGCACTTCCTT
TCTCCCTTCTCCC
+
;>>5>198==@BB<<<AAAAA@@?>777.7====9?@;9;:44444,44,423=?AB?B?AAA>9==@@BB?37477
.79:94:7:444+
@T6XXI:00142:00754
GGGAGAAGGGAGAAAGGAAGGAGAAGTCGCCCCAAACGAGTTTGTTGAC
+
9>:999599-444858977<;77376673333,372611111,117111
@T6XXI:00142:00772
GGGGAATTCTAATACGACTCACTATAGGGAGAGGGAGAATAGTCAACAAATCGTTTGGGGCGACTTCTCCTTCCTTT
CTCCCTTCTCCC
+
@?=6@=>=@CCABBBBCBBBBB@==616.777>A=?==>AA@?@A?@AA<CB?@A=@@@7@AABC@AB?>@=?>@@<
@AAA=B>A???;
@T6XXI:00142:00776
TCTAATACGACTCACTATAGGAGAGGGAGAATAGTCAACAAATCGTTTGGGGTGACTTCTCCTTCCTTTCTCCCTTC
TCCC
```

```
+
@CCCACBBBBBBBBBB>>7607?7776:@B?B@?@CC@C@?;<<<==:<<;4;<<==;<::;?>=<=A<<?<=;@>?
??A=
@T6XXI:00142:00787
CTAGTCACTCGAGATCTGCGGCTTTGGACAGTCCCTCTGTACTGTGAAGCCACAGATGTCGAGTCGACTAGCTTCCT
TTCTCCCTTCTCCC
+
B;444=449BBBABBBAA@@=@BC>B=<?==67:5:BBCCBBA<<@C;=?@BBBAAA>>>@@CAAA@@@B@AA?A>A
C>B@@C=B>@@@@<
@T6XXI:00142:00799
GGGAGAAGGGAGAAAGGAAGGAGAAGTCGCCCCCAAAACGATTTGTTGACTATTCTCCCTCTCCCTATAGTGAGTCG
TATTAGAATTCCCC
+
99-99>599-
9999042343773311=C88888+333,11111,1=:?A?=<?9;47714;;>@<@@@A@??@=<<<@??==<;565
;;<1
@T6XXI:00143:00753
GGGAGAAGGGAGAAAGGAAGGGTATGTGGCAGGGGAGTCTGGTCTGGCTATTCTCCCTCTCCCTATAGTGAGTCGTA
TTAGAACTTCCCC
+
CD@:;;@CHABBBE>A?@@BB?BCB@B?>?8?AA5?@@??A=@?7737>??6::AA::;88814==?=?=@@@?=78
8.//;9;;9AAA6
@T6XXI:00143:00765
GGGGAATTCTAATACGACTCACTATAGGGAGAGGGAGAATAGCTTCACGTTTTCCTGCGGTGCCCTGTTCTTCCTTT
CTCCCTTCTCCC
+
999-
4<A?CBB@CBBBBBB?@@;76166.777CC>B@@>AA==????=;;;;.;;=@@@;?<884<AA>==>@>@@;:===
;=<<<==:
@T6XXI:00143:00768
GGGAGAAGGGAGAAAGGAAGGAGAAGTCGCCCCAAACGATTTGTTGACTATTCTCCCTCTCCCTATAGTGAGTCGTA
TTAGAATTCCCC
+
CCCDCCBCD=BBBC?C@C=@=@CC@CBBBCCC5;;8;CABC?CB?B??=@@>@ABA=B==8=AB<<=@B@>>>878<
=><<=::2.//+
@T6XXI:00143:00769
GAATTCTAATACGACTCACTATAGGGAGAGGGAGAATAGCTCCCAAACCACGTTACGTGCGACCCACTTCCTTTCTC
CCTTCTCCC
+
CB8;;<BBBCBBBBAA@<>;77166.7>>AC@B@@>BB??ABC>CB?B>AABB>AABBBCABCC@B;;79;>?<ACD
C?B<>777.
@T6XXI:00143:00776
GGGGAATTCTAATACGACTCACTATAGGGAGAGGGAGAATAGTCAACAAATCGTTTGGGGCGACTTCTCCTTCCTTT
CTCCCTTCTCCC
+
BBA7A@B@CCB@CBAA?>>>BCB97111.6<<BDA?<<>AB@@@@>@@@;BA???8?886?BBCC?CCA;=;=?BA<
A===7=>@AAA<
@T6XXI:00143:00784
GGGGAATTCTAATACGACTCACTATAGGGAGAGGGAGAATAGATGACGCGCGGCCGCATCTCTATTCTTCTTCCTTT
CTCCCTTCCTCCC
+
BBA7A=@=@CC@CCBAAABAA<@A=166.7??BC>B@@>@=<<?<B?<<B??=@@AACCBBBBBB?BD?BB=?=??9
;>@<6<,0,000,
@T6XXI:00143:00787
GGGAGAAGGGAGAAAGGAAGAACAGGATGGGTCCAGTTAATGGAGAACTATTCTCCCTCTCCCTATAGTGAGTCGTA
TTAGAATTCCCC
+
```

```
BB>CCB@BCACBBB=B>A@BA?AB??CBCC;?<?BBB@B@BB????>?BBB@AA??>BBBCC>AA?@@AA@??AAA?
?>AAA=;2444-
@T6XXI:00143:00789
GGGGAATTCTAATACGACTCACTATAGGGAGAGGGAGAATAGTCAACAAATCGTTTGGGGCGACTTCTCCTTCCTTT
CTCCCTTCTCCC
+
@@>4>9A>BCD@CCAAAAAB>@A??666-
6;;;<9???=@AAA@?;<<<7?B@@A<BAB7BBCBC?BBC;=;==??:??>?4=894881
@T6XXI:00144:00762
GGGGAATTCTAATACGACTCACTATAGGGAGAGGGAGAATAGTCAACAAATCGTTTGGGGCGACTTCTCCTTCCTTT
CTCCCTTCTCCC
+
DDD9B?B@CBC@;11>11.07777?777,7=>ACB??B=440003/0443::667,111,1==9>?CAA@@><;>@9
;444-34839=6
@T6XXI:00144:00765
GGGAGAAGGGAGAAAGGAAGGAGAAGTCGCCCCAAACGATTTGTTGACTATTCTCCCTCTCCCTATAGTGAGTCGTA
TTAGAATTCCCCC
+
EGBCBC@BD@AFAG@C@C@C?@@@@CB?8888.8?:A@AAB>??=?BBBC@>@?@@7788>>:<=<=@@@@??===@
@>A6523/-///+
@T6XXI:00144:00782
GGGAGAAGGGAGAAAGGAAGCTAGTCACTCGACATCTGTGGCTTCACAGTACAGAGGGACTGTCCAAAGCCGCAGAT
CTCGAGTCGACTAG
+
BB?CCC>>=5948809594;B::;D?:::?>77:???>AA>?@=@BCCCCA?>855556<??>>37717=;=AAA==
=AA????899AAD@
@T6XXI:00144:00783
GGGAGAAGGGAGAAAGGAAGCTAGTCACTCGACATCTGTGGCTTCACAGTACAGAGGGACTGTCCAAAGCCGCAGAT
CTCGAGTCGACTAG
+
DCADCD@?=:=944-
9;=;?B???@A?=???:::???ABD;;<@CCCBBBCCC:434139=<==>@C=AB>B>88;====:::=:??AA==
@T6XXI:00144:00796
GGGAGAAGGGAGAAAGGAAGAGCGCATGACGACAATTTTGTGGGAGCTATTCTCCCTCTCCCTATAGTGAGTCGTAT
TAGAATTCCCC
+
BB>AAA=@@:>AAB=BBCBCBBCCCBAAACCCCBABCD9BBBB>:::CCC@CAA?9<777<9?@@BAAAA???==<;
=;8;8=8=@A7
@T6XXI:00145:00761
GGGAGAAGGGAGAAAGGAAGGAGAAGTCGCCCCAAACGATTTGTTGACTATTCTCCCTCTCCCTATAGTGAGTCGTA
TTAGAATTCCCC
+
DEDDEC?AA=ACCC>B@B@B@AAA>?=BBBBB8BBABB@@@;>??BAA@B<>;;@C=A=?<=;<@<??@?@@@???A
A>B@@=?3444,
@T6XXI:00145:00793
GGGAGAAGGGAGAAAGGAAGCTAGTCGACTGACATCTGTGGCTTCACAGTACAGAGGACTGTCCAAAGCCGCAGATC
TCGAGTCGACTAGATCACCGATCTGTACA
+
4909=@ABD;<;A@9?;><@?88:?:16766;;77666789?B?AAGCBCB;;;//-
/;::;C?@A<@@=A=>=@@AAA@@@8888:;888<;977++++***...
@T6XXI:00145:00794
GGGAGAAGGGAGAAAGGAAGTGCAGTTCGCGGTTAGTGAAGGGAAAAACTATTCTCCCTCTCCCTATAGTGAGTCGT
ATTAGAATTCCCC
+
GDCCCC@BB>CA??=B>B>ABCDCCB?BBBB@B?AAABB>=98????0@CCC@CCCC>B?BCC;B:@?AAAAA@A@@
??<?@>:>8;<<4
@T6XXI:00146:00762
```

```
GGGAGAAGGGAGAAAGGAAGGGAATAGCAGCAGTCGACAACGTGAAACTATTCTCCCTCTCCCTATAGTGAGTCGTA
TTAGAATTCCCC
+
EDACCB;==<@AAA>CBC@CC?B@B??BBBBBBB?BBCC@BAA???9@BCC@CB?@>@????=?@AAA=====588>
>;=?@;=5<;;1
@T6XXI:00146:00766
CTATCACTCGAGATCTGCGGCTTTGGACAGTCCCTCTGTACTGTGAAGCCACAGATGTCGAGTCGACTAGCTTCCTT
TCTCCCTTCTCCC
+
DCDC<:::CBBBBCBA@AB@BBBAB?AABA===;?@@@==?B@B=@=<@=ACCCCAAAAC@@???BCCCCBB?B?CE
?AAAB9<9<??A<
@T6XXI:00146:00774
GGGGAATCAATACGACTCAACTATAGGGAGACGGGAGAAATAGGTCAAACCAAATCGTTTGGGGGCGACCTTCCTC
+
>@B6>.21:7799444300;;;00/67700/,,2*,,,,*,0601004442663>>>>B?BBB>/77..,.443,,
@T6XXI:00147:00752
GGGGAATTCTAATACGACTCACTATAGGAGAGGGAGAATAGTCAACAAATCGTTTGGGGCGACTTCTCCTTCCTTTC
TCCCTTCTCCC
+
DDD:D@DBCCB@CCBBBBBBB:695..,0;;?C>CB@>B;=C@<;;@A<B<967-116-
7>>@B?????A>>=@?:@@@@;A88:AC<
@T6XXI:00147:00769
CTAGTCGACTCGAGATCTGCGGCTTTGGACAGTCCCTCTGTACTGTGAAGCCACAGATGTCGAGACTAGCTTCCTTT
CTCCCTTCTCCC
+
FCCCCCCEAEBCDBBBBAAAA@9??<B<AABB???8???CEBBA@BBA88;@CCCCCCDCBAAB:;<B<;:?A=@A<
??AC@E=??CCC
@T6XXI:00147:00770
GGGAGAAGGGAGAAAGGAAGGGAACAAACCATGCTGAGAAGCCATGGCTATTCTCCCTCTCCCTATAGTGAGTCGTA
TTAGAATTCCCC
+
CDDCCC@BB>BBBB>B=@=@B?B@CCC>B@CA@?<8811,11::/,/=AB?CDDB<B?BBB>BBCCB;AAAC@@@?
?>?AB?B;@@@6
@T6XXI:00148:00756
GGGAGAAGGGAGAAAGGAAGCTAGTCACTCGACATCTGTGGCTTCACAGTACAGAGGGACTGTCCAAAGCCGCAGAT
CTCGAGTCGACTAG
+
DD?BDC@CD=AAEC@B=@=@B>;?B9;7:@?779::7BCE@AA?BC@???AA?655505;<?@A?CC:??=A?@@==
===<<<<488@@@@
@T6XXI:00148:00760
GGGAGAAGGGAGAAAGGAAGTAGAATAGCACGTTGAAGATGTCGGGACTCATTCTCCCTCTCCCTATAGTGAGTCGT
ATTAGAATTCCCC
+
CEBBBC@CC=BA@=:@=><@@@?B?BBCEBBCCCCA@D994444;-//////:::BC:99444-
49??@BBBBBBB=?773768?@CCC6
@T6XXI:00148:00765
GGGGAATTCTAATACGACTCACTATAGGAGAGGGAGAATAGTCCTCCCGTATGCATTAGCCGAATGCCCTTCCTTTC
TCCCTTCTCCC
+
CCH:C?B@BCB@BABA@BBBB:664..,0<:=?>?ED@@<<=><AAA<=====?@@>AAA>==;=AAA<A?C58888
@@C7=9=??=7
@T6XXI:00148:00769
GGGGAATTCTAATACGACTCACTATAGGAGAGGGAGAATAGTTCTGGACTGTACTCTGGGGCTGTCTCCTTCCTTTC
TCCCTTCTCCC
+
499-969699459>@9;<BBC?>>>00.0;>??=A??><AAA<??@6;8>>7788>1667-
777:ABC@B@C@CC=BBACBC@????9
```

```
@T6XXI:00148:00776
GGGGAATTCTAATACGACTCACTATAGGGAGAGGAGAATAGTTTA
+
AAC6>>B@ACCAB???@@???CB9;111.7???@C00,/////+-
@T6XXI:00148:00778
GGGAGAAGGGAGAAAGGAAGGGGAGAAGGGAGAAAGGAAGCTAGTCACTCGACAGTCGAT
+
GDACCB@BC>BCBB>B@C=???9@BB@CB9=161,7374:CA?>CA74;0,,+***++++
@T6XXI:00148:00780
GGGGAATTCTAATACGACTCACTATAGGAGAGGGAGAATAGTCAACAAATCGTTTGGGGCGACTTCTCCTTCCTTTC
TCCCTTCTCCC
+
GBB9BCD@CCC@B@??AA??B:996..,0::?C>CBB@A;9<><;44419=;;;-667-
7?BBC@CBB?B?B>AC>A???<?;==@C=
@T6XXI:00149:00753
GGGAGAAGGGAGAAAGGAAAGAGAAATCACGCGGGCCAACGTATGCGCTATTCTCCCTCTCCCTATAGTGAGTCGTA
TTAGAACTTCCCC
+
//+///,/65679;7;244-
49333,39777111,1;?=?B<<99=AA=BBA998?699...+......161004000/000/088488-
@T6XXI:00149:00790
GGGAGAAGGGAGAAAGGAAGGAGAACGCGCACCCGAACCAACGGTGATCTATTCTCCCTCTCCCTATAGTGAGTCGT
ATTAGAATTCCCC
+
DD@BCB@CC@AA@C@C@C?@>@BB?BABBBBBB>BC@C@E?BCACBABCA@><>9991888?A>BCCC????:::BB
BC@CCB>B?BCEB
@T6XXI:00149:00795
GGGAGAAGGGAGAAAGGAAGCTAGTCACTCGACATCTGTGGCTTCACAGTACAGAGGGACTGTCCAAAGCCGCAGAT
CTCGAGTCGACTAG
+
IH?AAC?BBABBBB=?=?<@?>>>>77711=7766669AA>BE@BBCCBBB@=9330-
4;;=@@?CC>BB>@@@A@<8>>AAAA????AAA
@T6XXI:00100:00801
GGGAGAAGGGAGAAAGGAAGCTAGTCGACTCGACATCTGTGGCTTCACAGTACAGAGGACTGTCCAAAGCCGCAGAT
CTCG
+
49149?599-
4499692424333377/........3335?A;=?@CCA@???BB@@@=>=<;;<9=CAAA>@??=<<?@@A
@T6XXI:00100:00815
CTAGTCACTCGAGATCTGCGGCTTTGGACAGTCCCTCTGTACTGTGAAGCCACAGATGTCGAGTCGACTAGCTTCCT
TTCCTC
+
4;9449444:99899@;;<C@BBDAA=@BDCCCCADBC;::BC?8<99:>899@@@??????==?>99733..33,.
.+.,.4
@T6XXI:00100:00836
GGAGAAGGGAGAAAGGAAGCGAGTCGACTCGACATCTGTGGCTTCACAGTACAGAGGGACTGTCCAAAGCCGCAGAT
CTCGAGTCCGACTAAG
+
HAFCC@CB=ABBC<B=@>@888>7937<<7?BB@BB;;<L@CC>BACBBBC===676.7???@@>CC>CC@C@?@AA
A??<???=::==8838
@T6XXI:00100:00838
GTCGACTCGAGATCTGCGGCTTTGGACAGTCCCTCTGTACTGTGAAGCCACAGATGTACGTAG
+
CC<D<CCCBBBBEDCCCC@CCDACBFDAB@BB>BBBBBBBBC???@BB@BB:8877,,,,,,0
@T6XXI:00100:00847
GGGAGAAGGGAGAAAGGAAGGAGAAGTCGCCCCAAACGATTTGTTGACTATTCTCCCTCTCCCTATAGTGAGTCGTA
TTAGAATTCCCC
```

```
+
DDB<<<DCCB<=<DAD?B>B>;;;=@@CCCCB8CC>BA@45+..,.554>@>>AAA:887;?6<<@?;:0//::8;A
@>?<44456881
@T6XXI:00101:00801
GGGAGAAGGGAGAAAGGAAGCTAGTCGACTGACATC
+
IC>AAA>@@<>>?A1989<@>7004.......4644
@T6XXI:00101:00817
GGGGAATTCTAATACGACTCACTATAGGGAGAGGGAGAATAGCACTGCTGTTGTTGCAGTTGACCTCTTCTTCCTTT
CTCCCTTCTCCC
+
;44,9696<@=@CBBBCCCCCCB??861.7??AC>BCC@C@>449ABC<BBAAA=ACCCC=AAC?@<=:=@>@ACD?
@???:<:<;<<7
@T6XXI:00101:00818
GGGAGAAGGGAGAAAGGAAGGGGAGAAGGGAGAAAGGAAGCTAGTCACTCGACAGTCTGTG
+
EE?BBB@CDBBBBD>B@B@BAA8><<@CB;B961,74737?;89BB11/4...---13.30
@T6XXI:00101:00837
GTCGACTCGAGATCTGCGGCTTTGGACAGTCCCTCTGTACTGTGAAAGC
+
DA:::BAA@@AA944=9959@959899BCAAA>;;;B@@77><7--*,,
@T6XXI:00101:00839
GGGAGAAGGGAGAAAGGAAGCTAGTCGACTCGACATCTGTGGCTTCACAGTACAGAGGGACTGTCCAAAGCCGCAGA
TCTCGAGTCGACT
+
99149@<@@0949919?A@BBBB??=77116133166678@>@B<><>877>774000+0000038:=:==5544<:
777A8<;<;=;90
@T6XXI:00101:00840
GGGAGAAGGGAGAAAGGAAGAACAATGTTCGTTTGTGCGGCGAGAACTATTCTCCCTCTCCCTATAGTGAGTCGTAT
TAGAATTCCCC
+
CHBFCCACB?@BBB>B@B@BB?@<>?BB@CCBC@B@@@C@CCCCC@AAAC@C@??9=>=@@;?=:6;:::;;==::@
>@AA?B?AA@5
@T6XXI:00101:00841
GGGAGAAGGGAGAAAGGAAGCACACCAGTTTAGGGGTATCGGACTGGCTATTCTCCCTCTCCCTATAGTGAGTCGTA
TTAGAATTCCCC
+
ED@CBC@CI>B@@=9>;>=>?>?>>;;777-116626,,,,*,,000383478444-
4:8>@<AAA@<<<??@@@@AA?AAB>@>@AA6
@T6XXI:00101:00842
GGGGAATTCTAATACGACTCACTATAGGGAGAGGGAGAATAGTCAACAAATCGTTTGGGGCGACTTCTCCTTCCTTT
CTCCCTTCTCCC
+
944,424:9?A?AAB;<<C<<<DDEAAA9::7>>7<??:8>?:382000+33166266?5@AA=?=>7757>?;@C9
=<9=;C59.545
@T6XXI:00101:00845
GGGAGAAAGGGGAGAAAGGAAGCTAGTCACACTCT
+
44-4444-444,449;-42474333.47...3.1.
@T6XXI:00101:00846
GGGAGAAGGGAGAAAGGAAGTGCAGTTCGCGGTTAGTGAAGGGAAAAACTATTCTCCCTCTCCCTATAGTGAGTCGT
ATTAGAATTCCCC
+
DDBCCC@CCBBCCB?CAB>@@BDCBD?BCBB@C@CBCCB>BB;????2ABCC@BBBB>BBBDE>BAA@AACB???@@
@@>@@A@B38880
@T6XXI:00102:00812
GGGAGAAGGGAGAAAGGAAGGAGAAGTCGCCCAAA
```

```
+
BA099?<>><@ACC8;595924??77./0--+,2*
@T6XXI:00102:00814
GGGGAATTCTAATACGACTCACTATAGGGAGAGGGAGAATAGCCCTCCTTGTCCGGCGTCCATCCCCCTTCCTTTCT
CCCTTCTCCC
+
FFF:D?B@BDC@CBCCBCCBBCB>?796.7?>?A>ABB=?>600-773168???A>@@@C>A?><<8.848244-
4;;;7>:=???:
@T6XXI:00102:00824
GGGGAATTCTAATACGACTCACTATAGGGAGAGGGAGAATAGTCCTCCCAACGATTTAGCTGTTTTGCACTTCCTTT
CTCCCTTCTCCC
+
DCB4B:;:<A;@<;<BB@?@AA>>>8776:AACC>BBCA??A?A<??A=C@@@ACC>BBCB>>=>6?@@BC@C@CC@
CAAA=A?BCCB>
@T6XXI:00102:00832
GGGGAATTCTAATACGACTCACTATAGGGAGAGGGAGAATAGTCAACAAATCGTTTGGGGCGACTTCTCCTTCCTTT
CTCCCTTCTCCC
+
CDD:C?A?ABC@BBB?BBBBBB>99311.7===?<?EC@C?><<<>@@@<BCCCC>@@@9BCA@??@CC>@??>==;
=@A>;A>@@@@<
@T6XXI:00102:00833
GGGAGAAGGGAGAAAGGAAGCTAGTCGACTCGAGATCTGCGGCTTTGGACAGTCCCTCTGTACTGTGAAGCCACAGA
TGTCGAGTCGACTAGCCTTCCTTTCTCCCTTCTCCC
+
CC>AA@=@B<@ABB>A=@=@C:7:A9;:?AB?@?@AB?AAA?ABB>B?BBCCBBB>B@>@<<<8568<888>BAAA@
@>>>AABA@@<<;@7727:=:=?:@@@A<A<??@A;
@T6XXI:00102:00838
GGGAGAAGGGAGAAAGGAAGCTAGTCACTCGACATCTGTGGCTTCACAGTACAGAGGGACTGTCCAAAGCCGCAGAT
CTCGAGTCGACTAG
+
CCBCBB>@@:BBBB>C?B>BC<;?>;=77177768<:CCC=?@BCCC???B?=700017==?AB=??;?C=?>A;;=
:?????@A@??AA@
@T6XXI:00102:00844
GGGGAATTCTAATACGACTCACTATAGGAGAGGGAGAATAGCTCAACACTTTCCCCCGCAACGCTATCCTTCCTTTC
TCCCTTCTCCC
+
AAA6A=@>ACDBBBB@BABBB:9990/-
0>?BL>BAB>?BB@BCC?AABBB?BBBB1@@A<??@@==;7:7:244+399;4=9=9@A<
@T6XXI:00102:00847
GGGGAATTCTAATACGACTCACTATAGGGAGAGGGAGAATAGTCAACAAATCGTTTGGGGCGACTTCTCCTTCCTTT
CTCCCTTCTCCC
+
;::49>B?CBC@BBBBB@@@BBCBA@AA<79:CCBCCC@C??<AA>@@@<BBAAB9???5??BBC@CBB?A;=8;;7
<?=A4>9;444-
@T6XXI:00102:00849
GGGGAATTCTAATACGACTCACTATAGGGAGG
+
999/4:C@BBA@>9999939<937..,,+,,*
@T6XXI:00103:00820
GGGGAATTCTAATACGACTCACTATAGGGAGAGGGAGAATAGTCAACAAATCGTTTGGGGCGACTTCTCCTTCCTTT
CTCCCTTCTCCC
+
DE20@+1,/66::44994??;;><;;<=-7617?8??D:8;C@<;8;663>>==?8999-
7666>;A?@>?>@>AA<A?>>7>488@A<
@T6XXI:00103:00821
GGGAGAAGGGAGAAAGGAAGGAGAAGTCGCCCCA
+
```

```
44-4499=>>CDCC>>;>999/..6..10,22*.
@T6XXI:00103:00832
GGGGAATTCTAATACGACTCACTATAGGGAGAGGGAGAATAGTTACCAGCTTGCACGAGTCTCCCTGGTCTCTTCCT
TTCTCCCTTCTCCC
+
EA?7A598<BC@BBBBBBB@AA<99311.7??AC>BCC>>>>9;AA?A??::>>C@@@=8<>AA<AA>AA878;;7:
;6<;;;:;794881
@T6XXI:00103:00835
GGGGAATTCTAATACGACTCACTATAGGAGAGGGAGAATAGTAGA
+
ICB17+/,//6,/4444433333777<:?<=>>-71736.,++**
@T6XXI:00103:00841
GGGGAATTCTAATACGACTCACTATAGGGAGAGGGAGAATAGTCAACAAATCGTTTGGGGCGACTTCTCCTTCCTTT
CTCCCTTCTCCC
+
CCC:D@A@ACC@BAAA??<??E?99311.7====<?BBACBB@A=<=<<;ACA@?77777:CCCCACCC=A>A@C=:
;AAC;@>=@A=;
@T6XXI:00104:00812
GGGGAATTCTAATACGACTCACTATAGGGAGAGGGAGAATAGTAAACAAATCGTTTGGGGCGACTTCTCCTTCCTTT
CTCCCTTCCTCC
+
AA?4><@=ACCBB???BBBBBBBBB<<<88?9BC>BCC??;>===;===<A===@;@@@5@@A@@>@AA>A>@9==9
=@AC<A>A>==;
@T6XXI:00104:00817
GGGGAATTCTAATACGACTCACTATAGGGAGAGGGAGAATAGCTACATGTCCCTGGCATCGCTATCTCCTTCCTTTC
TCCCTTCTCC
+
:>:58198<BC@CBB@@@>>?BBBB><866=666.777:=??;::689885=430/437877388377;999178,6
111,171110
@T6XXI:00104:00822
GCTAGGTCGAACTCGAGATCATGCGGCTTTGGAACAGTTCCCTCTGTACTGTGAAGCCACAGATGGACTGTCAC
+
9////,////,444444499944442333,.3444...,.4+..6111696163/04;<;9;61.,.......,
@T6XXI:00104:00827
GGGGAATTCTAATACGACTCACTATAGGGAGAGGGAGAATAGTCAACAAATCGTTTGGGGCGACTTCTCCTTCCTTT
CTCCCTTCTCCC
+
FCC9@698;A@@CCCBBBBBBBBCB><:867;??;?BBAFCCA@@=?@A<BCCCC>CCC7BBDBB?BBB=?>=>@A:
@777,3/44481
@T6XXI:00104:00834
AGTCACTCGAGATCTGCGGCTTTGGACAGTCCCTCTGTACTGTGAAGCCACAGATGTCGAGTCGACTAGTCCTTCCT
T
+
ECCCBCCBBBBBCCCCBC@AEB?BCBBCBBCB=BCDBBBBBBBBA@??>;;??CCBABB@@@@@@=:+++,*,*,*-
,
@T6XXI:00104:00837
GGGAGAAGGGAGAAAGGAAGGAGAAGTCGCCCCAAACGATTTGTTGACTATTCTCCCTCTCCCTATAGTGAGTCGTA
TTAGAATTCCCC
+
DC?CBBBCC>@A?B=B?C?B@BCC@@9>?BBB8BB<BBAAA=AA?CC?>??><=<<;::?>?;@???AAA??===??
?<?@@>@<???5
@T6XXI:00104:00844
TCTGCGGCTTTGGACAGTCCCTCTGTACTGTGAAGCCACAGATGTCGAGTCGACTAGCTTCCTTTCTCCCTTCTCCC
+
?CBBBE@ECC@DBEBCC@?@>@CCBCBCCCCBE?BC?A<;<AAA?BAABB==<CCBBDBBBADC???=?.747777.
@T6XXI:00105:00808
```

```
GGGGAATTCTAATACGACTCACTATAGGGAGAGGGAGAATAGTCAACAAATCGTTTGGGGCGACTTCTCCTTCCTTT
CTCCCTTCTCCC
+
AAA6C>B?BCC@C???BBBBBA@99311.777>AABCC@C>>9:=;=AA;CBBBA>@CA8B>>>C@BBB=?;<:@@;
AA@A<@=@===9
@T6XXI:00105:00812
GGGAGAAGGGAGAAAGGAAGCTAGTCGACTCGACATCTGTGGCTTCACAGTACAGAGGGACTGTCCAAAGCCGCAGA
TCTCG
+
AA:?AA;>>:>99919<@5;>AEBCABA?=A;::::;:BBC?BB@CC??>>@A@=44413;>?@A>A@<@A?AB??<
=:??@
@T6XXI:00105:00835
GGGAGAAGGGAGAAAGGAAGG
+
DEBCCC@A=:=9*,)*00)*)
@T6XXI:00105:00842
GGGGAATTCTAATACGACTCACTATAGGGAGAGGGAGAATAGTCAACAAATCGTTTGGGGCGACTTCTCCTTCCTTT
CTCCCTTCTCCC
+
999-
49@<AAA=ACCCCCCBBB>9<777.7?@BC?CAA=AA@AAA>???<BCCCC=AAA7ACABB>6962957?AA<?@CA
=B<??BA<
@T6XXI:00106:00800
GGGAGAAGGGAGAAAGGAAGGTAGTGCAGTTCGCGGTTAGTGAAGGGAAAACTATTCTCCCTCTCCCTATAGTGAGT
CGTATTAGAATTCCCC
+
BD?BBA;=>19944-
4247878;;766711011117?=:=63758@<@@A6;8447448@;@@>=A;@;;///8779;;<>?9<;949/444
+
@T6XXI:00106:00807
GGGAGAAGGGAGAAAGGAAGGATATGCCGCAGTGGGAGATTAAACGGCTATTCTCCCCTCCTCCATATGTAG
+
DFBCCB@CCCBBBC>B@B@B?@@>::;8;BBBBAB>BB@B@CC>CC<;=BDCCCCCC9@>71-+-,,,,,,,
@T6XXI:00106:00817
GGGGAATTCTAATACGACTCACTATAGGGAGAGGGAGAATAGTGCTAAACCATTGACCCGCAACTATTTTCCTTCCT
TTCTCCCTTCTCCC
+
FFF:C>?=@BB>BCBBBC?BBBB>;366.777AC?CCB@CCC?A?@BB9;7:B?AABC>AAA?AA@A?=2=374747
7,1111,04446A8
@T6XXI:00106:00823
GGGAGAAGGGAGAAAGGAAGTGCAGTTCGCGGTTAGTGAAGGGAAAAACTATTCTCCCTCTCCCTATAGTGAGTCGT
ATTAGAACTTCCCC
+
491949;>9-
449;9@=B?B@ABDH@:>>11,1,1=.77><<28?==/>??>;?>==677711,1?@=>??=443333399==9865
553.
@T6XXI:00106:00828
CTATCGACTCGAGATCTGCGGCTTTGGACAGTCCCTCTGTACTGTGAAGCCACAGATGTCGAGTCGACTAGCTTCCT
TTCTCCCTTCTCCCTTCCTTTCTCCCT
+
C9949999=;=?ABBAA@@@=?@@<A@BCECCBB>BCC>>>>>?CCC<<@@CCCCBBCBC::::CCBBAA;;8>?>B
E=@=BC>C=??AA::9;2//,////,/
@T6XXI:00106:00834
GGGAGAAGGGAGAAAGGAAGCTAGTCGACTCGACATCTGTGGCTTCACAGTACAGAGGGACTGTCCAAAGCCGCAGA
TCTCGAGTCGACTAG
+
```

CDBCDD@CHABBCB:@=@=@@?<:@0..7/...36677?A99?@=@CAAAAB@>;433-
4;;;=?<?A<@?>?AA@AAA@@@@AAA@<?@@?
@T6XXI:00106:00836
GGGAGAAGGGAGAAAGGAAGTGCAGTTCGCGTATTGAGG
+
GA?AAC@CDACCDD?B@B@CCA=<3<:;;11/**),,,*
@T6XXI:00106:00839
GCTAGTACACTCGAGATCTGCGGCTTTGGACAGTCCCTCTGTACTGTGAAGCCACAGATGTCGAGTCGTAC
+
4/6///////494999BCBAAB?877,31178<=@@>@9AADC@==633/0=888??;777;;6636,,,,
@T6XXI:00107:00806
AGGGAGAAGGGAGAAAGGAAGGGGAGAAGGGAGAAAGGAAGCTAGTCGACTCACTCTGTGGCTTCACAGTACAGAGG
ACTGTCCAAAGCCGCAGACTGTCGAGT
+
<;;8;@A?AA8<<AA<@<?5888.888688/7>??:A>A>BB@@A>7777448448333313494443773..4..,
/54554/11,16311043000000111
@T6XXI:00107:00817
CTATCGATCGAGATCTGCGGCTTTGGACAGTCCCTCTGTACTGTGAAGCCACAGATGTCGAGTCGACTAGCTTCCTT
TCTCCCTTCTCCC
+
EDCBDCCDCECBACCBB?B>?BB>B=?@CCCCB>CCC?B?@AACBB>BC@CCCDCBBBCCCCACBCCEEDDB?B?BB
>ABBC>C@CBBB>
@T6XXI:00107:00819
GGGGAATTCTAATACGACTCACTATAGGGAGAGGGAGAATAGTCAACAAATCGTTTGGGGCGACTTCTCCTT
+
?@B2>5959@;<@AAABBCBA@@BA???6=====9?AB?DCC@@@B===9@B>>?.777.1@:00,0//,0,
@T6XXI:00107:00832
CTAGTCACTCGGATCTGCGGCTTTGGACAGTCCCTCTGTACTGTGAAGCCACAGATGTCGAGTCGACTAGCTTCCTT
TCTCCCTTCTCCC
+
?>9:;;BDCCC?BB9;;<A<@@A<A>AAABAAA>@@?@BB:::BBC?AB?BBBB@::766988537610 46?=@;=?
-:66999>=;<<1
@T6XXI:00107:00837
GGGAGAAGGGAGAAAGGAAGGAGACACGGTCGAACAAACATGGAGTGTGACTATTCTCCCTCATCCTAT
+
44-49;99=699880969689?3344/,*,,,,*,,200/1114A;;996@@66269776711110-2-
@T6XXI:00107:00841
GGGGGAAATTCTAATACGACTCACTATAGGGAGAGGGAGGAAATAGTTTTCCTTTCACTAACCGCGA
+
9999+44-/76//,/668;?933737..55+//..50..,11,1113==4:5220-....,.33111
@T6XXI:00108:00806
GGGAGAAGGGAGAAAGGAAGCTAGTCGACTCGACATCTGTGGCTTCACAGTACAGAGGGACTGTCCAAAGCCGCAGA
TCTCGAGTCACAT
+
DD?@@?5990999918<><AC@@@C???=777887?????@=?B@CAABBBAAAA;;;7<<;?AA?@@<AA;==@@?
66=;7//*+****
@T6XXI:00108:00838
GGGGAATTCTAATACGACTCACTATAGGGAGAGGGAGAATAGCCACCAACTATTGTTCGTCGCCGTTCTCTTCCTTT
CTCCCTTCTCCC
+
CDB9B8;9;BB@CCBBBCCBBBB;9316.777BE@BBBBC?A?>@@>A<??@@>?A>@@@@??<?A?@@8737:=>:
@@BB?A;==AC?
@T6XXI:00108:00846
GGGGAATTCTAATACGACTCACTATAGGGAGAGGGAGAATAGCTCCCTGGTATTTATCAATCTACCAAACTCTTCCT
TTCTCCCTTCTCCC
+

```
BBA4@<>=@BC@CBBBB?@>BB?99311.7>>?C>DCC>?>;8??C>CC@C@?@;@<<=;=<<<<?==;<<@@<>?A
A:===A>C9<<AA<
@T6XXI:00109:00800
GGGAGAAGGGAGAAAGGAAGAAAGCTATAGGACTTGCCGATGGGAGATACGGTAGCTATTCTCCCTCTCCCTATAGT
GAGTCGTATTAGAATTCCCC
+
EA>BB>>BCABDCC>B@B@BAA=@BBBCCC@DBB@BB@@@BBBAA@?AADC@C7::CCD@@@@A<AA@@=<<??<=9
??AA@AAA@68:@>BBACC6
@T6XXI:00109:00801
AATTCTAATACGACTCACTATAGGGAGAGGGAGAATAGTCAACAAATCGTTTGGGGCGACTTCTCCTTCCTTTCTCC
CTTCTCCC
+
DBD@DCC@AEBBBBA@<ACBB99753;6??=?CC@CAAAAA?@@@;CBBB>:8885?@AAC?BAA>A>B?BB>B@@@
;??AAA?;
@T6XXI:00109:00802
GGGGAATTCTAATACGACTCACTATAGGAGAGGGAGAATAGCAAACGTTCCC
+
CDI99+;;<BB@C@@?BB?B>797000,/7.11,1670111044+0110111
@T6XXI:00109:00822
GGGAGAAGGGAGAAAGGAAGCTAGTCACTCGACATCTGTGGCTTCACAGTACAGAGGGACTGTCCAAAGCCGCAGAT
CTCGAGTCGACTAG
+
491449<B@094=>:=8>4;7>A@=7........6499:::8B>@<<?:;:???477.4:;?@A?@C???>?>=>@>
===:<9;838====
@T6XXI:00109:00828
GGGGAATTCTAATACGACTCACTATAGGGAGAGGGAGAATAGTCAACAAATCGTTTGGGGCGACTTCTCCTTCCTTT
CTCCCTTCCTCCC
+
BBB7A>B>CBEACAA;;<CCCAA??877.77777-7=A?A@A>302353077111,111,3::9:-
0.53331,//,4.--+-,/,//40
@T6XXI:00109:00841
GGGAGAAGGGAGAAAGGAAGGAGAACATGACCCGGCGCGATTGGTGTCTATTCTCCCTCTCCCTATAGTGAGTCGTA
TTAGAATTCCCCA
+
/1,/19594-///7+/,/565/..6499994;-4244477713139189;;7;9B?:9>::;-
:@>?B???<;<<=??=?AA>A;ABA5@
@T6XXI:00109:00847
GGGAGAAGGGAGAAAGGAAGGAGAAGTCGCCCAAC
+
EEBCCC@BB>AA@==B=@=@?BBBA>;8,,,+-+*
@T6XXI:00110:00801
GGGAGAAGGGAGAAAGGAAGGAGAACAAGTAGCGGACCATCGCGAGACTATTCTCCCTCTCCCTATAGTGAGTCGTA
TTAGAATTCCCC
+
99-
4@@BNCAA=?C>B=?=?>ABD@DDADB@A@C@CC@CDBBBBBB?@@BB@BBBB<AAAA@>>>??@@@BBA@@===>@
A>=@8;<;0
@T6XXI:00110:00830
GGGAGAAGGGAGAAAGGAAGTGCAGTTCGCGGTTAGTGAAGGGAAAACTATTCTCCCTCTCCCTATAGTGAGTCGTA
TTAGAACTTCCCC
+
HIFCDC=??>>@@B8;;<CCCBDC<<8;>>>;>;>????<AB0777.7887?BBAE>AC???66:68?B??>?@888
89<@BCHC>A@@4
@T6XXI:00110:00836
GGGGAATTCTAATACGACTCACTATAGGGAGAGGGAGAATAGCTACGTTTAACGACCGTGCTGTTCTCCTTCCTTTC
TCCCTTCTCCC
+
```

```
999/42489AA@CCCBBBAAA?;79366.777>@>BCC@C??:ABCCCC::8:<CC@B@ACCCC?AAA;;7934418
@?@;@;:4871
@T6XXI:00110:00844
GGGGAATTCTAATACGACTCACTATAGGGAGAGGAGAATAGTCAACAAATCGTTTGGGGCGACTTCTCCTTCCTTTC
TCCCTTCTCC
+
EDB:A7989449?CEECCC;;<CBC877-0/0/,...447;<??;==?1;8==?-
766,3771126==;@37300+34449;74-.,
@T6XXI:00110:00846
GGGGAATTCTAATACGACTCACTATAGGGAGAGGGAGAATAGATACTCGTTGTGGCCTTTCTATTCTACCTTCCTTT
CTCCCTTCTCCC
+
=A?6ACA?BDC@BBBBB@>>?BCBB7695:=?CD>CBCAC??<;<<AAA@>@@@;?>@C>BA??>????<?<?=?A=
BECC>C@C:::8
@T6XXI:00110:00849
GGGAGAAGGGAGAAAGGAAGGGCAAGAATAAACAACCGAGTAATTATCTATTCTCCTCTCCTATAGTGAGTCATATA
TGAG
+
A95>9969@:@@AA<?<><A@<@C@CC?BBD>BB?BDBBBBB@C@??<<<<8<;;78://-
.444<<=>;>84/11++++*
@T6XXI:00111:00802
GGGGAATTCTAATACGACTCACTATAGGGAGAGGGAGAATAGCCTTCGCGTTTTGGCTCCCATGTTCCATCTTCCTT
TCTCCCTTCTCCC
+
;99/<CB@CDDBB999BCCCBBB9<3196:??CE>BBB@A?:85?:?@BCBBC9B>?@CA<A@==>C@=4382849;
7==DDAC8<<AA<
@T6XXI:00111:00832
GGGAGAGAATTTGGTAATGGGGTTAGTTCGAGAGCAGTACACAAGGCAAGGGTCCCTTATATCTCTCTCCCTATAGT
GAGTCGTATTCAGAATTCCCC
+
CE?AAABB@BB=B@BB<:777-
:@CCC@CCCBBABBBBBBBBC@B=;;7;B>BCDAC@CCCCDCC@@@CC>BCCBBBCBAA?=778.//;;99=???4
@T6XXI:00111:00833
GGGAGAAGGGAGAAAGGAAGCGCGGTGTTGTGGGAAATAGGAGTTGACTATTCTCCCTCTCCCTATAGTGAGTCGTA
TTAGAATTCCCC
+
AB?ABB>BB?BCGE>C@C;<<CDGICCC?B???9?C>CCB@AAA@AA@888=@@A@<@DA??;?AA@=;::==<<@?
?<?@A>A>@A@5
@T6XXI:00112:00802
GGGAGAAGGGAGAAAGGAAGGAGAAGTCGCCCCAAACGATTTGTTGACTATTCTCCCTCTCCCTATAGTGAGTCGTA
TTAGAATTCCCC
+
CD@DCC@CC=ABBCCB@B9:;:BB?BBBBABC8BB?BCCCC>BB?AAABAA?@@@@<A===B=AA?@AAAA===@@A
A?AAA>A=???5
@T6XXI:00112:00807
GGGGAATTCTAATACGACTCACTATAGGAGAGGGAGAATAGTCAACAAATCGTTTGGGGCGACTTCTCCTTCCTTTC
TCCCTTCTCCC
+
AAA6CCBADBC@CCBBBBBBB:996..,0;;>A>ACD@BBBBA=<:?:4ACA@@>???7BAA====CC?B@@<@?:?
>>?:;>=AAA=
@T6XXI:00112:00818
GGGGAATTCTAATACGACTCACTATAGGAGAGGGAGAATAGTTTGTACATCGC
+
DIE7@69694424449?>@?@@@?;;:,0.7777:7:5300--+--.38<97/
@T6XXI:00112:00832
GGGAGAAGGGAGAAAGGAAGCTAGTCGACTCAACATCTGTGGCTTCACAGTACAGAGGACTGTCCAAAGCCGCAG
+
```

=A<ACA?BD?@@BB<@=@?BCBAAA:77=>78683967CCC>:;7;>77:?<9:<00,-1---3/-32113...2
@T6XXI:00112:00835
GGGAGAAGGGAGAAAGGAAGGAGAACGCGCGACCCGAACCAACGGTGATCTATTCTCCCTCCTCCCTTATAGTGAGT
CGTATTAGAATTCCCC
+
491449899-4444-
49=@B>BBC@A>>>211;:59=8924249:4//8....44...+16367>1626971710001666<:=<;:A@ABC
9
@T6XXI:00112:00844
GGGGAATTCTAATACGACTCACTATAGGAGAGGGAGAATAGTCAACAAATCGTTTGGGGCGACTTCTCCTTCCTTTC
TCCCTTCTCCC
+
DDD:C?@?ACCBCCCCCABBB>;;900,0<077,1;7ACBB@AD<??A<C@@@C>CEC7BAAAA=??B7:7:?>;:>
;<<6<:;8885
@T6XXI:00112:00845
GGGAGAAGGGAGAAAGGAAGCTAGTCGACTCGACATCTGTGGCTTCACAGTACAGAGGACTGTCCAAAGCCGCAGAT
CTCG
+
CDBAAA@CC>CBBB:@=@6;;C@@BCCC@<????????BCC?><<<CCCCBC?74..-
/7;;;A=@@<@C???=A<<<=<:
@T6XXI:00113:00802
GGGGAATTCTAATACGACTCACTATAGGAGAGGGAGAATAGTCAACAAATCGTTTGGGGCGACTTCTCCTTCCTTTC
TCCCTTCTCCC
+
CCA6;246999:@<<<DCCBBBCBA;:37=77:7:BBBCBCAA=99=@<BBA@B5:77.8=A@B;???<?:;8;<7=
@@@;@:==AAA
@T6XXI:00113:00812
GGGGAATTCTAATACGACTCACTATAGGAGAGGGAGAATAGCTAAAACATTATTCCCGTCGCTGTTCTCTTCCTTTC
TCCCTTCTCCC
+
999/9?B?CBEACBBB@@ABC:9=90.,0:0776:BC?BBC?AAAA6ABB@B@>AC>B?>>=EDC?BABC@C@CB<B
BCC?D==@BC>
@T6XXI:00113:00819
GGGAGAAGGGAGAAAGGAAGGAAACACGCGTGACGAATAGGTAGAAACTATTCTCCCTCTCCCTATAGTGAGTCGTA
TTAGAATTCCCC
+
EI?CCB@CC>BBCEAC?A>B@BB9?B?B>>ABCCBB@BCC@CCBBB?BDCC@C@@@<?BBBB>BCCCCCC@@@BCBB
B@CCC?A>@CC8
@T6XXI:00113:00820
GGGGAATTCTAATACGACTCACTATAGGGAGAGGGAGAATAGCTCCACACCCTCCATCACGCACATGTACTTCCTTT
CTCCCTTCTCCC
+
===6?<>@A@B@BCCBBA??BCB?7311.7=?CJ@CBB>A?@=@@??????;@=;<;:?AA<==@==<;;;8<8<@;
@AAA<A<????:
@T6XXI:00113:00823
GGGAGAAGGGAGAAAGGAAGGAGAAGTCGCCCCAAACGATTTGTTGACTATTCTCCCTCTCCCTATAGTGAGTCGTA
TTAGAATTCCCC
+
DEBCCC@CD@AAAD@DDCAC@?@?>;:<BBCC8BBAABCDC>BB?BBAAACA??==;=<??@<AAA@@@@AAAAAA?
?>?DH@A>@A@5
@T6XXI:00113:00841
GGGAGAAGGGAGAAAGGAAGGAGAACAGAATGCCACCCAGGTCTAAGCTATTCTCCCTCTCCCTATAGTGAGTCGTA
TTAGAATTCCCC
+
DC?BBB@CC>BBBB?B?B>BABAA?BC;98<BC@CCB?CC@CCBB@AAABC@A@>?>BACCC>?998<===<<<???
A>A@D>@=@@@5
@T6XXI:00114:00826

GGGAGAAGGGAGAAAGGAAGAGCGTGGGTGCTGCATCGGGGAGTTAGCTATTCTCCCCCTCCCTATAGTGAGTCGTA
TTAGAATTCCCC
+
:B=@9>:<@<@CDDACAD=AAAB<<<C@ECCCC@;@@ABB7BCC?BAA<;99>88888,37727111888?@@@@@@
@<8=?565;;;1
@T6XXI:00114:00832
CTAGTCACTCGAGATCTGCGGCTTTGGACAGTCCCTCTGTACTGTGAAGCCACAGATGTCGAGTCGACTAGCTTCCT
TTCCTCCCTTCTCCC
+
<11////44;449==A;;;A>@@@<@=@AAB@??9=7777=76:9>?<=>;>>>333>@@?@@?BB@@@@70/6555
7-/./::6:,7111,
@T6XXI:00114:00838
GGGAGAAGGGAGAAAGGAAGCTAGTCACTCAACATCTGTGGCTTCACAGTACAGAGGGACTGTCCAAAGCCGCAGAT
CTCGAGTCGACTAG
+
CC>ABB===5@;44-
95949B??@BA???77471311;9927;?ACCCCBCBAA?::8?@>>?A3:947?:??AAAA?===<<@@889AAA
@T6XXI:00114:00841
GGGAGAGGAG
+
//+1///,,,
@T6XXI:00114:00846
GGGGAATTCTAATACGACTCACTATAGGGAGAGGGAGAAATAGTCAACAAATCGTTTGGGGCGACTTCTCCTTCCTT
TCTCCCTTCTCCC
+
IKH:C>?<=9954?BACBBBBB??>877-
7<=6626116,996388234507911116>>8C?AA@>6662106;::43004,4/005=4
@T6XXI:00115:00815
CTATCGACTCGAGATCTGCGGCTTTGGACAGTCCCTCTGTACTGTGAAGCCACGATGTCGAGTCGACT
+
/0,,,,,,//44448>1111,.4636.00033668888>6663...4429649>====88733/,,,,
@T6XXI:00115:00820
GGGAGAAGGGAGAAAGGAAGGAGAAGTCGCCCAAACGATTGTTG
+
DEBCCB@CB:=;94-9;>;@>?8868743.4+..+.6..,..,.
@T6XXI:00115:00822
GGGAGAAGGGAGAAAGGAAGCTAGTCGACTGACTCTGATGGCTTCACAGTACAGAGGGACTGTCCAAAGCCG
+
/1,//4294-444;-4242477333117611377311111017011666600000344+000000/00+0443
@T6XXI:00115:00840
GGGAGAAGGGAGAAAGGAAGACAGTCCATCTGTGGCTTCACAGTACAGAGGGACTGTCCAAAGCCGCAGATCTCGAG
TCGACTAG
+
CCBC?=644-
9>==9>595;BBBBAA>A@@@ECC?BB?BB??>;?@CC9999=AAA@<<==5::<<;;;=====<9==CCCDCCC
@T6XXI:00116:00803
GGGGAATTCTAATACGACTCACTATAGGAGAGG
+
CCC:D?B@CBB=A<:<F;::;10::00,.3.11
@T6XXI:00116:00807
GGGAGAAGGGAGAAAGGAAGGAGATAGAAGCAGCAATGGTCAGACATCTATTCTCCCTCTCCCTATAGTGAGTCGTA
TAG
+
CF?BBA@CC>BCBB?C?B?B@CBBCB?>;>>ABBB@AA?ABBBAAAABBAA?ABAA<BBCCC>BA;?9./..,,,,+
+**
@T6XXI:00116:00812

GGGGAATTCTAATACGACTCACTATAGGGAGAGGGAGAATAGTCAACAAATCGTTTGGGGCGACTTCTCCTTCCTTT
CTCCCTTCTCCC
+
999,424:BBC@C?@?BBBCBBBB9311.6=>AB>BAB@AABA??<?@A<CDCC>46664:??BC?AAC=?=@>?D=
@@@C;@<>AAC;
@T6XXI:00116:00816
GGGGAATTCTAATACGACTCACTATAGGAGAGGAGAATAG
+
88202*1,//67;8=@;:79B?>>B9647?77467131.,
@T6XXI:00116:00818
GGGAGAAGGGAGAAAGGAAGCTAGTCACTCGACATCTGTGGCTTCACAGTACAGAGGGACTGTCCAAAGCCGCAGAT
CTCGAGTCGACTAG
+
AA??BG@BB>BCCEAC@C?BABB>8,,,,,,22256<==@?FCAD???;::BCCCCC898=988244-
4;2<<848AA?@@A88777:353
@T6XXI:00116:00832
GGGAGAAGGGAGAAAGGAAGGGGAGAAGGGAGAAAGGAAGGGGAGAAGGGAGAAAAGGAAGCTAGTCGACTCGACAT
CTGTGGCTTCACAGTACAGAGGGACTGTCCAAAGCCGCAGATCTCGAGTCGACTAGCTTCCTTTCTCCCTTCTCCC
+
FD?A@A>AA>BABC>F>@@BBB9BAA?AC>AA?@;B=A>ABB59??:<=9=4444+49444;7:;6110/...3422
5<;=;844/400046::7CA@?<>?==4823;:36:;7307933333339=;;;>@>9793?88666B6<07.--+
@T6XXI:00116:00844
GGGAGAAGGGAGAAAGGAAGGATGAGGCAGCGTCGACCGAACGACAGACTATTCTCCCTCTCCCTATAGTGAGTCGT
ATTAGAACTTCCCC
+
=?:@DBACC>BBBC@>;>:<,10...36444;;>6;:AFB@C@<2211/6//,007?:>:<8>;87:7>>8:77<::
:@855>110;=;7-
@T6XXI:00117:00813
GGGAGAAGGGAGAAAGGAAGTGCAGTTCGCGGTTAGTGAAGGGAAAAACTATTCTCCCTCTCCCTATAGTGA
+
HC?BCC?B=6>444-95969434;ABDBBCCBG@B?>87177,1111+3937599::9>0//:,..;693-6
@T6XXI:00117:00826
GGAGAAGGGAGAAAGGAAGCTAGTCACTCGACATCTGTGGCTTCACAGTACAGAGGGACTGTCCAAAGCCGCAGATC
TCGAGTCGACT
+
BECCA@CC?AB=95=;=<@@ACCCCCC???:::::6@AB?AB@BAABB>@@CA:<<;::8==A><@;@B<?>?@==<
@:@?@@@@AA<
@T6XXI:00117:00831
GGGGAATTCTAATACGACTCACTATAGGGAGAGGGAGAATAGCAAACGTTTCCCCGACGTCGCTTTCTCCTTCCTTT
CTCCCTTCTCCC
+
::449?B@CCCAC@B@BBBBBCB><3115:?<CE@???@CBB@@?;?CCC>BBB8BBBCABBCCC>B::47=?444-
43444=8<8AA<
@T6XXI:00118:00802
GGGGAATTCTAATACGACTCACTATAGGGAGAGGGAGAATAGTCTCCAGATCTCCCTTTGGCACGTACTCTTCCTTT
CTCCCTTCTCCC
+
CEB8B:;69995988994;;BA?>7777.777?B>???:;>7400327714::BB=BCAA8;:::;???<=@C?BC>
A>>A<A>A==<7
@T6XXI:00118:00813
GGGAGAAGGGAGAAAGGAAGCTAGTCGACTCGACATCTGTGGCTTCACAGTACAGAGGGACTGTCCAAAGCCGCAGA
TCTCGAGTCGACTAG
+
DDBCCB?A@:@=995>584;A>::=/00;;;;3;:AAACCC?@@ADCCAA?@AA=<<<;==>===;<<;:@>@=<??
=<@@9==A===AA??
@T6XXI:00118:00816

```
GGGAGAAGGGAGAAAGGAAGCTAGTCGACTCGACATCTGTGGCTTCACAGTACAGAGGGACTGTCCAAAGCCGCAGA
TCTCGAGTCGACTAG
+
CC?CBB===5=944-
95949>>::;/000099086787ABD=?@@BBB???AA?;44313<9>?@;==9=A>A@<?==<@@?:?AAAAAA<;
@T6XXI:00118:00826
GGGGAATTCTAATACGACTCACTATAGGGAGAGGGAGAATAGTTTTCCCTTCACTAACCGCGAACTGCACTTCCTTT
CTCCCTTCTCCC
+
BBB4AC?<=?BABBFB@BBC;;;C?777.7>>@C?AAA@CBB???5==7>58;DCB@BCBB??;??;;??><?:??9
?BBC>C?A?@@:
@T6XXI:00118:00829
GGGGAATTCTAATACGACTCACTATAGGGAGAGGGAGAATAGTCAACAAATCGTTTGGGGCGACTTCTCCTTCCTTT
CTCCCTTCTCCC
+
DEE:KAA>@BB>BCCCBCB?BCB?;799.6?>@BBBBC@ABA?@@>@AB;BBBBB>BB>8>C@C@?AAA=?;<:??<
@@@CAC@?@@@<
@T6XXI:00118:00834
GGGGAATTCTAATACGACTCACTATAGGGAGAGGGAGAATAGTTTTCGACCCCATTACACTTTTTCTCTTCCTTTCT
CCCTTCTCCC
+
AAA9ABCACAA?ACCCDCB?@@A@>>?=6==7776;@@@DAA>CC6;;4443+4?=?>=79999-
94>:<?;>?:?@@DAC?BBBE>
@T6XXI:00118:00839
GGGGAATTCTAATACGACTCACTATAGGGAGAGGGAGAATAGTCTTGCGCTTTCTAGCCCAACGTTCTCCTTCCTTT
CTCCCTTCTCCC
+
?B?/9695994?CCCCC9;;A@>>?877.7==>@<:::@CCCA@@>@A@AAA7<;?77718=@AB?@@C<?;?;==7
<=<A=B388881
@T6XXI:00118:00848
GGGAGAAGGGAGAAAGGAAGCTAGTCGACTCGACATCTGTGGCTTCACAGTACAGAGGGACTGTCCAAAGCCG
+
CE?BCC@CB>BAAB=AAH?B@B>@??CCAA@@CBABACCBB?@@=@CCB@@@;74000,3;;>?@=@B<A@=@
@T6XXI:00119:00800
CTATCGACTCGAGATCTGCGGCTTTGGACAGTCCCTCTGTACTGTGAAGCCACAGATGACGAGTCGACTAGCTTCCT
TTCTCCCTTCTCCC
+
==A?999@A;9A9;;@AA;;:;BB<B<<<CCCDC@A@??C;::@???88<@CDBDB<;<BAA@@<99>>4437;<;@
B5667726366666
@T6XXI:00119:00813
GGGAGAAGGGAGAAAGGAAGCGTAGTTCGCGT
+
BCBCCC==95=844-1;8+--,,,,,*.7611
@T6XXI:00119:00819
GGGGAATTCTAATACGACTCACTATAGGGAGAGGGAGAATAGTTTTCCCTTCA
+
999,4:;:@9989@@>9;;@??>?88:?<AABED=B::6761000+04+0/38
@T6XXI:00119:00823
GGGGAATTCTAATACGACTCACTATAGGGAGAGGGAGAATAGTCAACAAATCGTTTGGGGCGACTCTCCTTCCTTTC
TCCCTTCTCCC
+
AAA6@ACBCBC@CBBBBBCBA@>>;39:5:??CC@C??=@CDA==:773,8?>>>.777.8=7716;;8>=A?AA<A
;=@9>0/0>>9
@T6XXI:00119:00832
GGGAGAAGGGAGAAAGGAAGGAGAAGTCGCCCCAAACGATTTGTTGACTATTCTCCCTCTCCCTATAGTGAGTCGTA
TTAGAATTCCCC
+
```

```
CD>CCC@BB>BBCC?B?A?B@BAA?ACB;;??5?A<BBCC@;@C@C==?AB@@AAC>@?=?B=A<??@@AAAA===A
A>@AB::2444-
@T6XXI:00119:00842
CTAGTCACTCGAGATCTGCGGCTTTGGACAGTCCCTCTGTACTGTGTAGCCACAGATGTCGAGTCGACTAGCTTCCT
TTCTCCCTTCTCCC
+
;//////44CBBBBBBBBBB<<?B;?>@@B<>?C>@@@@<<?>@CC@9<=;=====;@@==A:8@BBCBBBBB?B?B
C>BBCC=C5::AB<
@T6XXI:00120:00809
GGGGAATTCTAATACGACTCACTATAGGAGAGGGAGAATAGATCACGACCCTTCTACCACGATTGAGCTTCCTTTCT
CCCTTCTCCC
+
CFE:C8;9;BB@CABABCBBA9;;;0/,0<07:8:CC@BBBA<<B?BCCC>B;77//,0::<B?@@@<929599194
>>:>=@BAC?
@T6XXI:00120:00817
GGGAGAAGGGAGAAAGGAAGCTAGTCGACTCGACATCTGTGGCTTCACAGTACAGAGGGACTGTCCAAAGCCGCAGA
TCTCGAGTCGACTAG
+
CKBCCB==95>>>>9>5959>800>/00<0000;:::7BDD?AA?BDC>AAE?74333,7>?ABA>@@<@?<?????
???@@A@@@@@@AAA
@T6XXI:00120:00828
GGGGAATTCTAATACGACTCACTATAGGGAGAGGGAGAATAGTCAACAAATCGTTTGGGGCGACTTCTCCTTCCTTT
CTCCCTTCTCCC
+
DCC7B8;8<99::@@<<<AACB???876.7>==<9?AA>BCF???@??@:@@?8>999=5779>?=<<A=?>?@@C9
==AA:?9<<==:
@T6XXI:00120:00830
GGGAGAAGGGAGAAAGGAAGCTAGTCGACTCGAGATCTGCGGCTTTGGACAGTCCCTCTGTACTGTGAAGCCACAGA
TGTCGAGTGACTAGCTTCCTTTCTCCTCTCCC
+
44-
49><>@:?>@@>AAC@CED<><CCBBBB;<:::B;;;C@CCB?BBCBBBCCC8::?;<<><<787>48389:;;;87
8===>>=>><787;;78828//,/---/+
@T6XXI:00120:00841
CTAGTCACTCGAGATCTGCGGCTTTGGACAGTCCCTCTGTACTGTGAAGCCACAGATGTCGAGTCGACTAGCCTTCC
TTTCTCCCTTCTCCC
+
C?:;;@===CABBABCBBA?<;BB>B<>>BBB@@;@CBA@???@@@@>;A?BBBBAAAAAABA?ABCCBBCB?B@CA
CC?@@AC>C>>AA@<
@T6XXI:00120:00843
GGGAGAAGGGAGAAAGGAAGGAGAAGTCGCCCCAAACGATTTGTTGACTATTCTCCCTCTCCCTATAGTGAGTCGTA
TTAGAATTCCC
+
EJDE=<<=C>BABB>CACBC?BBC@C@A;;;;.;?9A;<?<8?A>77:67734;;@6;;;;;4769///6669/./8
87468..--32
@T6XXI:00120:00844
GGGGAATTCTAATACGACTCACTATAGGGAGAGGGAGAATAGTCAACAAATCGTTTGGGGCGACTTCTCCTTCCTTT
CTCCCTTCTCCC
+
GCB9B?C?BBB@CBBAAA??>BB99311.7==?@?BBBACBB?@C?@@@<BB???<A??5AAAAC>?@A5:5:>??<
?CCA>A=@@@@;
@T6XXI:00120:00849
GGGGAATTCTAATACGACTCACTATAGGGAGAGGGAGAATAGGCCGACGTC
+
CCB7B=@=@CC@CBBBABBBBEB?A???6199AA>B>=:?/.,-0./---0
@T6XXI:00121:00801
```

```
GGGAGAAGGGAGAAAGGAAGCTAGTCACTCGACATCTGTGGCTTCACAGTACAGAGGGACTGTCCAAAGCCGCAGAT
CTCGAGTCGACTAG
+
CFBCBB===5=844-
95949B@?@B8;::::0/.44577==AA?ACCC??BCC?;;<7<=?@AA>AA<@@>@A=====><==@@AAA@@@@
@T6XXI:00121:00818
GGGAGAAGGGAGAAAGGAAGGAGAAGTCGCCCCAAACGATTTGTTGACTATTCTCCCTCTCCCTATAGTGAGTCGTA
TTAGAATTCCCC
+
ADAFEC@DC<A<9909<@<>;>>>7@?@BCCC9BB>BAA@@;8?::7166;:;;==7;=<;;69487899A;;;://
+0++-0100000
@T6XXI:00121:00821
GGGAGAAGGGAGAAAGGAAGCTAGTCACTCGACATCTGTGGCTTCACAGTACAGAGGGACTGTCCAAAGCCGCAGAT
CTCGAGTCGACTAG
+
DD@CBB894-
998809;>;@B;;;A??<??>7:9???AAB=@@@CBBBBCCBB>5;;6;;;>>>:==7<<9=AAAAA???<<<=<<=
===<
@T6XXI:00121:00824
GGGAGAAGGGAGAAAGGAAGCTAGTCACTCGACATCTGTGGCTTCACAGTACAGAGGGACTGTCCAAAGCCGCAGAT
CTCGAGTCGACTAG
+
DD?AAA@CC?BBB?==696:BBB??97711;;663117160:;?CCDBBBBA?=377549;@AA>AC@AB699AA@@
@@A=<<@?888;;8
@T6XXI:00121:00845
CTAGTCACTCGAGATCTGCGGCTTTGGACAGTCCCTCTGTACTGTGAAGCCACAGATGTCGAGTCGACTAGCTTCCT
TTCTCCCTTCTCCC
+
B8///1/44BBBCDCB??@C@CCB?B88;AB@?@=@EECC>>>??C@?;B>BCBBB?@ABCC@@BBABBCEBB?B@C
C>??@C=A>?@BE?
@T6XXI:00122:00809
GGGGAATTCTAATACGACTCACTATAGGAGAGGGAGAATAGTCTTGCAATTTCCTGCGGTGCATTCTCCTTCCTTTC
TCCCTTCTCCC
+
DKD8B79649984;9@@@?AAA@>>873777774:AA@BBC==<<?@B@CC>A=ABDB@DDCBB>@@@6;9;@CB9;
;??6=:=777.
@T6XXI:00122:00811
GGGGAATTCTAATACGACTCACTATAGGGAGAGGGAGAATAGAGACGTATTCGTTTGCGCTCTGTTCACCTTCCTTT
CTCCCTTCTCCC
+
A?B7A=@=@BC@CBBAA><<?CB?<311.7==BC?EBB?B@=9=AAA@??>A<<<;<<<<@@<<=<==@=?>@<?<<
?CCC;<;:?:=;
@T6XXI:00122:00814
GGGAGAAGGGAGAAAGGAAGAAGAGCGCACAGCGGGAGGACCATTGGCTATTCTCCCTCTCCCTATAGTGAGTCGTA
TTAGAATTCCCC
+
CC@CBB@CC>BBBB>B@BABB?AA8<<CCDCA@AA98?:770160126ACD?AABC>@<99=9==A??:==????<<
<:==A>A;@@@5
@T6XXI:00122:00824
GGGAGAAGGGAGAAAGGAAGCTAGTCACTCGACATCTGTGGCTTCACAGTACAGAGGGACTGTCCAAAGCCGCAGAT
CTCGAGTCGACTAG
+
DDBCCB@BE>BBBB?B896;A@AAB6::??=77:::9@@A<?@@CCCBAAAB@7000-
4:;:=?>AA<???AB@A???@AABBAA>??@AA
@T6XXI:00122:00829
GGGAGAAGGGAGAAAGGAAGAATATGCAGGAGACGAACGACGCCGAACTATTCTCCCTCTCCCTATAGTGAGTCGTA
TGATTCC
```

```
+
CD@CCD?CB>BBBB>A@A@BB@B@@@BBD@>>>BB@?@A@@@?>??>>>==<=55548AAAA<@A@;;;B<<<@A?=
?@@@=?>
@T6XXI:00122:00835
GGGAGAAGGGAGAAAGGAAGCTAGTCACTCGACATCGTGTGGCTTCACAGTACAGAGGACTGTCCAAAGCCGCAGAT
CTCGAGTCGACTAG
+
490444244-449=-
429987>?776611....36...3333.3138733;00/7//,38830020;055865388;;;9964-------0
@T6XXI:00122:00836
GTCGACTCGAGATCTGCGGCTTTGGACAGTCCCTCTGTACTGTGAAGCCACAGATGTCGAGTCGACTAGCTTCCTTT
CTCCCTTCTCCC
+
CC;::?@AAAACECCBBF?@EE@F8;<CCCCC@CCC>@@DBBBBABCB@CCCBCCCE@@<BBBAAABB::;@C@CC=
BBCE=?=@ABA<
@T6XXI:00122:00837
GGGAGAAGGGAGAAAGGAAGGAGAACATGACCCGGCGCGATTGGTGTCTATTCTCCCTCTCCCTATAGTGAGTCGTA
TTAGAACTTCCCCA
+
;:64AAADCBCBBC?D?B@C@;<;BECAABJD@DCCBB;;<=?@BB?@=;?:?<A?977111,1111159;;86400
0/003:575443*-
@T6XXI:00122:00843
GGGAGAAGGGAGAAAGGAAGCTAGTCACTCGACATCTGTGGCTTCACAGTACAGAGGACTGTCCAA
+
CBACAA894-999909595;B@@ABBBBAAA;;:???@AB>?@@CCCBBBCCC?-/,///---,--
@T6XXI:00123:00805
GGGAGAAGGGAGAAAGGAAGAGAAGCCCCAGGGGAAAGACAGGAAGCTATTCTCCCTCTCCCTATAGTGAGTCGTAT
TAGAATTCCCC
+
FIBCBA@AA>===A>B@B@BCAA?ABBB9CCCC9CC>CCCCC@C>???<===AAC;?@@AB;@<<<==A@@;????@
>@@?=;3444-
@T6XXI:00123:00820
GGGGAATTCTAATACGACTCACTATAGGGAGAGGGAGAATAGATCACCGTTGGTTCGGGTGCGCGTTCTCCTTCCTT
TCTCCCTTCTTCCTTTCTCCCTTCTCCC
+
DEB9C@C@BBC@B@?@AB???BB?<76659@@CC>CBB>>@?<<=B@>?A??<<<A==:@:;:BBA@BBC@B?C?BB
>BBBD>C?CA>A>AA<@@@A7=48444-
@T6XXI:00123:00822
CTAGTCACTCGAGATCTGCGGCTTTG
+
;C944;44444999881,,00,,,*,
@T6XXI:00123:00828
GGGAGAAGGGAGAAAGGAAGCTAGTCGACTCGACATCTGTGGCTTCACAGTACAGAGGGACTGTCCAAAGCCGCAGA
TCTCGAGTCGACTAG
+
CH?BBDDDC>CBA@=B=>=@C?;;>/00;::<0811117699?ABDCCABBAA@>;;;6;;;8888;;7==;=@888
=???988@@88877;
@T6XXI:00123:00836
GGGGAATTCTAATACGACTCACTATAGGGAGAGGAGAATAGTAGACC
+
70818?AACCCACCCBBBBB<BB99711.7?>>>B;;5700,,++0*
@T6XXI:00124:00811
GGGGAATTCTAATACGACTCACTATAGGGAGAGGGAGAATAGCTCCCGCAGTTTGACCATGTTCATCTCTTCTTTCT
CCCTTCTCCC
+
999299A>AAB99<B?>=;4;001....+..11661668<AA>ABC611=777.7=>37104/--
0++*3)**3,,+5555/..>>1
```

```
@T6XXI:00124:00817
GGGAGAAGGGAGAAAGGAAGGAGAAGTCGCCCCAAACGATTTGTTGACTATTCTCCCTCTCCCTATAGTGAGTCGTA
TTAGAATTCCCC
+
DEECCB@BE>AAAC?B@B?A?BCD@;9=??BB8>B>BCC@@=@B@CBCAAA>=<<<;=>AAA<A??;=<<<<<<=<<
??@<<8<8:9;4
@T6XXI:00124:00822
GGGGAATTCTAATACGACTCACTATAGGAGAGGGAGAATAGACACGCTTAGTGTTTATGTTTCGTCTCCTTCCTTTC
TCCCTTCTCCC
+
CCB7B=@=@CC@CBBBA@BBB:9990.,0<;>@>AFC@A@?<;<<<==<A?A?<<<@@@AA<AA???@?@=@9<<6;
==?:@>AA@C<
@T6XXI:00124:00826
GGGAGAAGGGAGAAAGGAAGAGAATGTCCGGGTTGAAAAACACATGACTATTCTCCCTCTCCCTATAGTGAGTCGTA
TTAGAATTCCCC
+
FDBDDC@BB>BBBCCB=@=@B>>>AB@A<?@:?=@BCCC2CBBBBBB>>?>;:;;;:;:::=;====<<<==<??==
=>@@@=@<>==1
@T6XXI:00124:00828
GGGAGAAGGGAGAAAGGAAAGCTAGTCGACTCGACATCTGTGGGCTTACAGTACAGAGGGACTGTCCAAAAGCCGCA
GATCTCGAGTCGACTAG
+
ED?@@?5990999914244-
473337711677/00........+139978:7:;C@BBC=<?C??>;:>>100,...168;968?????CA??A
@T6XXI:00124:00835
CCTCTGTACTGTGAAGCCACAGATGTCGAGTCGACTAGCTTCCTTTCTCCCTTCTCCC
+
EBDCCCCBBBCBBB?BB@CBBBBC@@??A@BBCBBCC@@@?B?BB>BBBD>B?BBCC>
@T6XXI:00125:00804
GGGGAATTCTAATACGACTCACTATAGGGAGAGGGAGAATAGT
+
CCB8A51,11/-/6:99:77>>AA88:B:7777;,16=7700+
@T6XXI:00125:00820
GGGAGAAGGGAGAAAGGAAGCTAGTCGACTCGACATCTGTGGCTTCACAGTACAGAGGACTGTCCCAAAGCCGCAGA
TCTCGA
+
CB@@?=6996<CAC@C?B:<;A??@:;:A??=?:;;::B?A=:<9;CCAAACCC>7327;==@@@<@A=@@=@AAAA
<<?@@@
@T6XXI:00125:00824
GGGAGAAGGGAGAAAGGAAAGCTAGTCACTCGACATCTGTGGCTTCACAGTACAGAGGGACTGTCCAAAGCCGCAGA
TCTCGAGTCGACTAGCTTCCTTTCTCCCTTCTCCC
+
EB?A;;79C>>99919599-4<7746.........47776826:>DABBB@@BB:8::-
4::=<>88818;6///968830000000:<=<:<C>A:=<6;;;=7=;=>;;:
@T6XXI:00125:00827
GGGAGAAGGGAGAAAGGAAGGAGAAGTCGCCCCAAACGATTTGTTGACTATTCTCCCTCTCCCTATAGTGAGTCGTA
TTAGAATTCCCC
+
CDBCCC@CC>BB??=B@C@B@CA@>@>@B>??8??;>BB??9>>>?;?;>?>=99949;;;;:9999=====<:::<
@<AAA>@9;;;.
@T6XXI:00125:00846
GGGGAATTCTA
+
DDD7D=<:;11
@T6XXI:00126:00812
GTCGACTCGAGATCTGCGGCTTTGGACAGTCCCTCTGTACTGTGAAGCCACAGATGGACTGTCCTTCCTTCTCCCTT
CTCCC
```

```
+
DCDD@@@CA;>>CCBCCCACFFBC@BCBB;;;7;@@ABCAACCCH=<;?9967///777.//63333,.,.---+-
000030
@T6XXI:00126:00822
CTAGTCGACTCGAGATCTGCGGCTTTGGACAGTCCCTCTGTACTGTGAAGCCACAGATGTCGAGTCGACTAGCTTCC
TTTCTCCCTTCTCCC
+
:,,,,,,,//8:?@BBBBBAA=@BB:@=?>>?87;6:BABBA<CAEBB?@C@CDCBBBBB@@@CEBB@@A@@BBBC@
CC>BBBB>B@BBCC?
@T6XXI:00126:00824
GGGAGAAGGGAGAAAAGGAAGCTAGTCACTCGACATCTGTGGCTTCACAGTACAGAGGGACTGTCCAAAGCCGCAGA
TCTCGAGTCGACTACGCTTCCTTTCTCCCTTCTCCC
+
GB:@?>;>@199999/9542433339>0.......3522,.,..60..???BB@?844-
4499=?>AA<AB:@???@@AAAC<==@>////./66467886333505+***.)
@T6XXI:00126:00827
CTAGTCGACTCGAGAATCTGC
+
///49;996:;111/,/,*,,
@T6XXI:00126:00830
AGTCACTCGAGATCTGCGGCTTTGGACAGTCTGTACTGTGAAGCCACAGATGTCGAGTCGACTAGCTTCCTTTCTCC
CTTCTCCC
+
ECCBBCCCBCCBBBBBBB??=A;B@@B?A>BBBB@@AAAAA=@@@BCCABGABEBBCCCCBCDDCEB?B@BB>AABB
?CC@@A@=
@T6XXI:00126:00836
GGGAGAAGGGAGAAAGGAAGGAGAAGTCGCCCCAAACGATTTGTTGACTATTCTCCCTCTCCCTATAGTGAGTCGTA
TTAGAATTCCCC
+
CFAHCC@BA>BCCC@C?B?B@CAA@AA@??>>2??:@>???<?DBCBBCCC????C<@ACAA<@@A@A===AB899?
?=?AB7:2444-
@T6XXI:00126:00837
CGAGATCTGCGGCTTTGGACAGTCCCTCTGTACTGTGAAGCCACAGATGTCGAGCCGCT
+
<:9====@CBB?DBD@DCBB@;<:C<@@B=000333663;84<;6<<?74977,,*,,,
@T6XXI:00127:00812
GGGGAATTCTAATACGACTCACTATAGGGAGAGGAGAATAGT
+
111+/5>;>AA>BBBBCCCCBBBB?>??9?:::>B0010,+)
@T6XXI:00127:00826
GGGGAATTCTAATTACGACTCACTATAGGGAGAGGGAGAATAGTCAACAAATCGTTTGGGGCGACTTCTCCCTTCCT
TTCTCCCTTCTCCC
+
BCB4@51-1//,/,/6/44@A?;;99777.77777-16669<>:>94330+336688:>>-
7676601111,1744<<;<===;<88866:
@T6XXI:00127:00835
GGGAGAAGGGAGAAAGGAAGGAGAAGTCGCCCCAAACGATTTGTTGACTATTCTCCCTCTCCCTATAGTGAGTCGTA
TTAGAATTCCCC
+
CDBBAA@CC>>;;4-9<?<?>AAA?ABA><<<-
88/8???B>::8;CBBCB>?9;=;<:<<=;@<@@A@AAA@?????@AA@9;/444-
@T6XXI:00127:00840
GGGAGAAGGGAGAAAGGAAGTGCAGTTCGCGGTTAGTGAAGGGAAAACTATTCTCCCTCTCCCTATAGTGAGTCGTA
TTAGAATTCCCC
+
BBEAAA?BC>B9998;@B@CCCCCCB@B??B@B@ABBBC@CB>AAA7AACC@BB>@;???889????AAAAC@===A
A>@@B?A:AAA1
```

```
@T6XXI:00127:00841
GGGAGAAGGGAGAAAGGAAGCTAGTCCTCGACATCTGTGGCTTCACAGTACAGAGGGACTGTCCAAAGCCGCAGATC
TCGAGTCGACTAG
+
FI?C?=244-
9999185944@?<<0.,0/.//4444;BB?BA@CCBBBCA@>999919999@@@CC@BB?BBCC@@<;:BCAAABBC
ECC
@T6XXI:00128:00804
GGGGAATTCTAATACGACTCACTATAGGAGAGGGAGAATAGCAAACGTTTCCCCGACGTCGCTTTCTCCTTCCTTTC
TCCCTTCTCCC
+
FEF:C@BABBB@CBBB;;<BB@?@@??:=>777.7==:???;>C;988@>@AE9:;7700/0,00,,,*.55534-
..8:468;;@@7
@T6XXI:00128:00809
GGGAGAAGGGAGAAAGGAAGTGCAGTTCGCGGTTAGTGAAGGGAAAACTATTCTCCCTCTCCCTATAGTGAGTCGTA
TTAGAATTCCCC
+
CC@CCDBDDBBBA=:@796;CBBCBB?BCBB@C;;:@@@?BB>ACC8CAAAABCCC>A????9?CDAAAA@?;4444
428?@>=<==C5
@T6XXI:00128:00812
GGGGAATTCTAATACGACTCACTATAGGAGAGGGAGAATAGCGACTTTATCCCACTCGCGCTGTGCCTTCCTTCTCC
CTTCTCCC
+
EDD9B?B=<;?@BBCCCBBBC>;;000,0;:<;2;;<;?CA???@A=?===@;?AA===@77;B::467@3747<<=
.734444,
@T6XXI:00128:00816
GGGAGAAGGGAGAAAGGAAGGAGAATGCGGTACCGGTTACGTCTTGGCTATTCTCCCTCTCCCTATAGTGAGTCGTA
TTAGAATTCCCC
+
FCBBBBACB>BBBC>B@B@C@BBB@A@BB=@@C@CCC@DBBBBC@C??@@<?;?<=;88<<<7<CC=<<::@AC?<<
;8<;=;>>AA@5
@T6XXI:00128:00820
GGGGAATTCTAATACGACTCACTATAGGGAGAGGGAAGAATAGCCTTCTCTCCATCTCTCACGTGTCATCTTCCTTT
CTCCTTCT
+
BBA4A695444298@@AAB:;8?>3361,.0772*,*,,222.-,.,7<7160:67>>?7:8:6;:<6?<:24344-
///,-,--
@T6XXI:00128:00821
GGGAGAAGGGAGAAAGGAAGGAGAAGTCGCCCAACAACGTATT
+
DCDCCDACC=BBB?:@=@=ADDCC@CABA>>9790,*,,,***
@T6XXI:00128:00825
GGGAGAAGGGAGAAAGGAAGCTAGTCGACTCGACATCTGTGGCTTCACAGTACGAGGACG
+
DDBCBB:94-89880;>@=@@B@@@9::???=;;;;;:@BB?BGBCCBA:::/0097,*)
@T6XXI:00128:00828
GGGAGAAGGGAGAAAGGAAGGATAAATAGCAGCGGGATAGGTAACAGCTATTCTCCCTCTCCTATAGTGAGTCGTAT
TAGAATTCCCC
+
EDBBAA@CC>?@@C=?=B?B?BBCC>AAAC@>B=C>BC?@=?@@CCCCCCC@CCCC>CCBB@CCCCCBCBBB?????
<<@A>A>@AB6
@T6XXI:00128:00849
GGGGAATTCTAATACGACTCACTATAGGGAGAGGGAGAATAGTCAACAAAATCGTTTGGGGCGACTTCTCCTTCCTT
TC
+
AAA6?<A>@@A@B;;;@;;;CB??B;:65:??BE=?@F;::B>=:94444+3966;66662<6769:7//,/,/412
+-
```

```
@T6XXI:00129:00811
GGGGAATTCTAATACGACTCACTATAGGGAGAGGGAGAATAGCCATTCCCTATCTCCGGCCCAGCACTACTTCCTTT
CTCCCTTCTCCC
+
DCI:D?A>ABC?BCBBBCCCA@>>>767.7=777-996@C@A=89;27818?@>@A;=;=A<A@@@ABA??;;/03-
448:4:7::;=7
@T6XXI:00129:00824
CTAGTCACTCGAGATCTGCGGCTTTGGACAGTCCCTCTGTACTGTGAAGCCACAGATGTCGAGTCGACTAGCTTCCT
TTCTCCCTTCTCCC
+
B1//188==CECCABAAAAA=BBB<B>A?>>7775:CCCCBBB@A@@?@DBCCCBBBBBBBBCCCBCCCC@@@?C@B
C>BBBD>B?@BAA<
@T6XXI:00129:00826
GGGGAATTCTAATACGACTCACTATAGGAGAGGGAGAATAGCGCAATTTGGACATGTGGCCGCTTCTCCTTCCTTTC
TCCCTTCTCCC
+
CHDAD::=<AA@B:;:CBBBB>=;;0.,0:0777:CCCD??<AAC=??<>@;<ABBCB?B?@;873447:8;34418
=@C@A>A???<
@T6XXI:00129:00833
GGGAGAAGGGAGAAAGGAAGCTAGTCGACTCGACATCTGTGGCTTCACAGTACAGAGGACTGTCCAAAGCCGCAGAT
CTCGAGTCGAC
+
CCBCCE?BA:>@=9:@8;6;A88;A:::BBA7;;::91>@B8?@>AAAAAAB@@<73/4;;:<=?AD@CC<@@????
898AA@AAAAA
@T6XXI:00129:00844
GGGGAATTCTAATACGACTCACTATAGGGAGAGGGAGAATAGCTACGTTTAACCGATCGTGTTGTTCTCCTTCCTTT
CTCCCTTCTCCC
+
DED:C@C@BCC@BCDGAA@BBAAB?>??9;::>7:7665///24430/.+.,101116463323;568<;<;??CC<
@??>7=94/-22
@T6XXI:00130:00801
GGGAGAAGGGAGAAAAG
+
AA:?99599191---*,
@T6XXI:00130:00807
GGGGAATTCTAATACGACTCACTATAGGAGAGGGAGAATAGCCAAAATTATTGACCCGGCCGCATTCTCTTCCTTTC
TCCCTTCTCCC
+
999/929249;ACCCBBBBBC=<;;00,.7/11,177::77888=C6A=AA4;776,10101<30/3;==<@?A@;@
@@C<A?=<<@5
@T6XXI:00130:00815
GGGAGAAGGGAGAAAGGAAGGAGAAGTCGCCCCAAACGATTTGTTGACTATTCTCCCTCTCCCTATAGTGAGTCGTA
TTAGAATTCCCC
+
CCAA<<;<C>CCCCACAD@B?ACD@CCB>>>>6<B=?A:9:5:A>CC>AB>;::;;:55;ACAA9==????AAAA==
=>AA@>;3444,
@T6XXI:00130:00828
GGGAGAAGGGAGAAAGGAAGAGAACGGCGACGAACAATAGTTGGTGGCTATTCTCCCTCTCCCTAT
+
CE?AAA>AA?@>>@8;8;@CCDC=ADBCCDAA@>A@=@@@@?C;:1;=;;=,...5033366267;
@T6XXI:00130:00839
GGGGAATTCTAATACGACTCACTATAGGGAGAGGAGAATAGTACCGTTGTGC
+
ECB6@=@>@CCBBAABBBBBBBBB<761-6=773;==:=77448234477/,
@T6XXI:00130:00847
GGGAGAAGGGAGAAAGGAAGTGCAGTTCGCGGTTAGTGAAGGGAAAAACTATTCTCC
+
```

```
BB>CCB?CDCCCCE>A=@>@A@><;=:;;4424243<B?;<9,1111+.40.,.433
@T6XXI:00131:00810
GGGAGAAGGGAGAAAGGAAGTGCAGTTCGCGGTTAGTGAAGGGAAAACTATTCTCCCTCTCCCTATAGTGAGTCGTA
TTAGAATTCCCC
+
CC@CA?=@C>BBCC@C@C@BCBCBBB@BBAA@B@BBB:9;:C>BEC6@@BB@CBC?9A>BAA=@=?=??<<<>==78
8:=AA>=;@@A5
@T6XXI:00131:00818
GGGAGAAGGGAGAAAGGAAGAGAAGCAGATCGTGAACTAATTCACGCTATTCTCCCTCTCCCTATAGTGAGTC
+
:D8::D@CC>BB@@<A>A>BABA@DCA;;;????A?BCC8:8:AAABBCA;==A=:4;033+-----411380
@T6XXI:00131:00836
CTAGTCGACTCGAGATCTGCGGCTTTGGACAGTCCCTCTGTACTGTGAAGCCACAGATGTCAGTCGACTAGCTTCCT
TTCTCCCTTCTCCC
+
A=:<;<99@CBBBBBCCAAAC@CCC>A=?>>?87:5;BBCCCBBACBB>=A@CCB@@@?888>79;@@@BAB@?A>@
@;CCCD>B?AA>=7
@T6XXI:00131:00837
GGGAGAAGGGAGAAAGGAAGGAGAAGTCGCCCCAAACGATTTGTTGACTATTCTCCCTCCTCCCTATAGTGAGTCGT
ATTCAGAATTCCCC
+
CC?BC@@CCA???B?B?B?B@BBB@BAB?>>>7AA<ACCCC>BB@CAAAC>=57443--
,///+./03?<@@A@><<<988834/0/300+
@T6XXI:00131:00838
GGGGAATTCTAATACGACTCACTATAGGGAGAGGGAGAATAGCCTCCTGATTCCCAGCACCGTCACACCCTTCCTTT
CTCCCTTCTCCC
+
CCC:D@B?ACC@BBBBAAAABB>99311.7==AD@CAA>AAA<:A@?CBBB@CC@CCCCC@CCCCCB@@09594991
9@?A>B?B@@@<
@T6XXI:00131:00839
GGGGAATTCTAATACGACTCACTATAGGGAGAGGGAGAATAGTCAACAAATCGTTTGGGGCGACTTCTCCTTCCTTT
CTCCCTTCTCCC
+
999-
4:C@HCC@CBBABBBBBB?9?311.777>><ABBCCBB@AA>@@@;AAACC>CBB7@?;??367=4747;:;:=???
:?5<8???
@T6XXI:00131:00842
GGGAGAAGGGAGAAAGGAAGGAGAAGTCGCCCCAAACGATTTGTTGACTATTCTCCCTCTCCCTATAGTGAGTCGTA
TTAGAATTCCCC
+
CDECCB@BCBBBBD?B?B?B@CAB>AA?337:4;B>CJCCC;BB@CB;??@@<<<=;<<?AA<A<@==<<<;;;@@<
A>@B@C@9;;<4
@T6XXI:00131:00846
GGGAGAAGGGAGAAAGGAAGCTAGTCGACTCGACATCTGTGGCTTCACAGTACAGAGGACTGTCCAAAG
+
FICCCC?B@:@@@@=B@A>CB99;ABA???C:;<@??BC:;8:C@AABC@@?>>;//,/8766?9<<7<
@T6XXI:00131:00847
GGGGAATTCTAATACGACTCACTATAGGGAGAGGGAGAATAGCTACGTTTAACCGATCGTGTTGTTCTCCTTCCTTT
CTCCCTTCTCCC
+
EEB9B>@@ACE@BB@AABBBBBB>>361-
6<<@A>BCC@B?B<BCCACA;?>A?BCC?;;;=<==:==@>A>B>A@;===C<@>@AAA<
@T6XXI:00132:00817
GGGGAATTCTAATACGACTCACTATAGGAGAGGGAGAATAGAAACGCCCTTCCCTGCTGCCCACATATTCTTCCTTC
TCCCTTCTCCC
+
```

444,4:B@ACC=@@@BCA;;;@>??873667;626663;<103+055=;=5861037334;::=<786,//,/,/01
11448543344
@T6XXI:00132:00821
GGGGAATTCTAATACGACTCACTATAGGGAGAGGGAGAATAGCAAACGTTTCCCCGACGTCGCTTTCTCCTTCCTTT
CTCCCTTCTCCC
+
EEE:CCC@BBC?BBBDECBBBBBB@@?A?ABCCEAB@@=?@>78818AAB>CCC9CCCCBBA???<?BB>B?B??A<
@>?=1424444-
@T6XXI:00132:00824
CTATCACTCGAGATCTGCGGCTTTGGACAGTCCCTCTGTACTGTGAAGCCACAGATGTCGAGTCGACTAGCTTC
+
9::9444449::=><;@B=?@C=@=@DAAACB:700000///9?=89@=>>B<;:A9<31/0///.4..78>>
@T6XXI:00132:00831
GGGGAATTCTAATACGACTCACTATAGGGAGAGGGAGAATAGTCCGCCAATTCTGCGCGTTAGCATTCTCCTTCCTT
TCTCCCTTCTCCC
+
DCC9C>A>ACC?BBB@?@CCBID><311.6><=?<?DD@DCC==;=A>A?A@CCCBBA;;>>>?BB?AA>@C>B=??
=BECE>C=A<;;6
@T6XXI:00132:00835
CTAAGTCGACTCGAGATCTGCGGCTTTGGACAGTCC
+
8,-*,0,*,,,,///7:==9949433506....,,,
@T6XXI:00132:00836
GGGAGAAGGGAGAAAGGAAGGGGAGAAGGGCTAGTCGACTCGACATCTGTGGCTTCACAGTACAGAGGGACTGTCCA
AAGCCGCAGATCTCGAG
+
CC?BBA?BC?A@@C<@=?<===4>>>;>>8ABCDCCBBCCCC:9<AAAAAC@CC@CCBC??DB::44419@@?>?59
9-8@<@ABA@A;;;@@C
@T6XXI:00132:00845
CTCGGCGAGATCTGCGGCTTTGGACAGTCCCTCTGTACTGTGAAGCCACAGATGTCGAGTCGAGCATCATCATGTAC
+
CDCA?ACGC<<<CBAA?EEE@F@DDDCBBB9<;@CCBBBCBAB?@@49888:796;877;B7..3.........35-
@T6XXI:00132:00849
GGGGAATTCTAATACGACTCACTATAGGGAGAGGGAGAATAGTCAACAAATCGTTTGGGGCGACTTCTCCTTCCTTT
CTCCCTTCTCCC
+
BCC9B>B@AAA:;<BBCCBBBB>?9367.7>>?BA@?@?CCCA??:?AA<CB>;;-666-
8??::;;FF@C@A?AA:??DC=B>???A<
@T6XXI:00133:00809
CTAGTCGACTCGAGATCTGCGGCTTTGGACAGTCCCTCTGTACTGTGAAGCCACAGATGTCGAGTTCCG
+
?11//////1:=@?CDD<;<D@CCC@D@C?87776-117=>:;:?66770954444;>77773110498
@T6XXI:00133:00812
GGGGAATTCTAATACGACTCACTATAGGAGAGGGAGAATAGTGACGTGTTGTGCGGCGCTCACTGTCTCCTTCCTTT
CTCCCTTCTCCC
+
BDNAE<<?<ED@CBCBAA?BBBBBC:727>7776:A@<AECAAA=@?<@>@@ABA?===:;@@A=77887:7;;==7
844;7;8448<6
@T6XXI:00133:00820
GGGGAATTCTAATACGACTCACTATAGGAGAGGGAGAATAGCCATTTCTCCAACACCTTCGCGCGCCACTTCCTTTC
TCCCTTCTCCC
+
CED9C>A@ACCACCBCB?@@C=;;=0/,0>0775:@@>BFB@;?<<:=A><@@BB??A>A?::=@@=A??=???D=A
AAC:?<??AD=
@T6XXI:00133:00824
GGGGAATTCTAATACGACTCACTATAGGAGAGGGAGAATAGTCAACAAATCGTTTGGGGCGACTTCTCCTTCCTTTC
TCCCTTCTCCC

```
+
CFFCC8:;;BB@EBBBBAAAB966;0/,0><?@BBCC>@??@AB><<<7@???C>BBB9C:::C<??B=?==@CD<@
@B@;?388<<7
@T6XXI:00133:00836
GGGGAATTCTAATACGACTCACTATAGGGAGAGGGAGAATAGTCAACAAATCGTTTGGGGCGACTTCTCCTTCCTTT
CTCCCTTCCTCCA
+
444,4987;11,/::ACCCCD?>>8877,766@7;?::5;@?:::8344-
8?===.777.8>???@CBB@C>=;=CEA;?939,*)**)*
@T6XXI:00133:00843
GGGGAATTCTAATACGACTCACTATAGGGAGAGGGAGAATAGCTACGTCTAACCGATCGTGTTGTTCTCCTTCCTTT
CTCCCTTCTCCC
+
999/9;>@BCC@BBBBBBBBAA;?;799.7?>@B=BCC>@??<?@BCCAAA@C@BBBBBCCA?AA>@8848<@=@B;
@ABB==8:??A;
@T6XXI:00133:00844
GGGAGAAGGGAGAAAGGAAGCTAGTCACTCGACATCTGTGGTCACAGTACAGAGGGACTGTCCAAAGCCGCAGATCT
CGAGTCGACTAG
+
DD@BCC@B>:>844-
9;>;>BAAAA>:;;CABCD@?@CEC?BBBCAABBA=<9987<A8=;9/43,4=4=A=99<:?AAAA??????@@
@T6XXI:00134:00808
GGGGAATTCTAATACGACTCACTATAGGGAGAGGGAGAATAGTCAACAAATCGTTTGGGGCGACTTCTCCTTCCTTT
CTCCCTTCTCC
+
A?B2=5929>9>@ABBCCBAADB<9716.7>777.7=B@CCB@<=888A:A?===.777-0:<//,/44,.,.,34-
40442654789
@T6XXI:00134:00810
GGGAGAAGGGAGAAAGGAAGCTAGTCACTCGACATCTGTGGCTTCACAGTACAGAGGGACTGTCCAAAGCCGCAGAT
CTCGAGTCGACTACGCTTCCTTTCTCCCTTCCTCCC
+
IB<@<96<A<B@FLAG?B@CDC<<<676111111116997995:;=??CDBBCCBA88814;;;:=244-
44/4787?:864436664334...42413333342322,-,--33
@T6XXI:00134:00820
GGGAGAAGGGAGAAAGGAAGCTAGTCACTCGAGATCTGCGGCTTTGGACAGTCCCTCTGTACTGTGAAGCCACAGAT
GTCGAGTCGACTAGCTTCCTTTCTCCCTTCCTCCC
+
991444249-
4899142824A?@@B??:::AABBDAAACC>@@C@C@BB>>>>>://0;::;@???C@??<?@@???@@AA=9=A@@
<888@BAA?AC<@@?=:4/0/344-
@T6XXI:00134:00824
GGGAGAAGGGAGAAAGGAAGGAGAAGTCGCCCCAAACGATTTGTTGACTATTCTCCCTCTCCCTATAGTGAGTCGTA
TTAGAATTCCCC
+
CCBCCD@CH>A??>:@=B?BACCC?CAB><;<6@B=BCB@><@C@CB>???A====;=:?<=;A=??@AAAA@@@>>
>>BBA?C=@AA5
@T6XXI:00134:00828
GGGAGAAGGGAGAAAGGAAGCTAGTCACTCGACATCTGTGGCTTCACAGTACAGAGGGACTGTCCAAAGCCGCAGAT
CTCGAGTCGACTAGCTTCCTTTCTCCCTTCTCCC
+
AA<@DA>AA:?98809<?<ABB?<0))((((,,,07?@@88<>@@C??@CCB?78718??@AB?CC<?@>@@?<<<
<<=AAAAA@A???@AA<?<?@;@???:=:====7
@T6XXI:00134:00844
GGGGAATTCTAATACGACTCACTATAGGGAGAGGGAGAATAGTTTTCCCCTTCACTAACCGCGAACTGCACTTCCTT
TCTCCCTT
+
```

999/9548449>BBCCCBAAA@>>>877-
77717999?<=77444+000+0/039>@<<8977747;6/0/5555777/6.4033
@T6XXI:00134:00847
GGGAGAAGGGAGAAAGGAAGGGAATAGCAGCAGTCGGCAACGTGAAACTATTCTCCCTCTCCCTATAGTGAGTCGTA
TTAGAATTCCCC
+
CBECCB@EE>ABBC>B@B@BB?C@BBABABBA?B;:5;A=@ABBBB>BCDC@?AAC>:::AB<@899??AAAA===?
?<??@?96:::.
@T6XXI:00134:00848
GGGAGAAAGGAAGCTAGTCGACTCGACATCTGTGGCTTCACAGTACAGAGGGACTGTCCAAAGCCGCAGATCTGGTC
+
44-99991959598@@@ABBBCCCC@?????A??;?C=?=A@@@@@@A<<<7<?A@A@>@A=8868??8888//,-1
@T6XXI:00134:00849
TCTGCGGCTTTGGACAGTCCCTCTGTACTGTGAAGCCACAGATGTCGAGTCGACTAGCTTCCTTTCTCCCTTCTCCC
+
@DDCBB@BB@;@?BBCC99:9;BCCCCB@?AAC@BBACEGCBBBBAABCBBBCCCCCCC?B?BD>AAAC>D?BBCD<
@T6XXI:00135:00808
GGGGAATTCTAATACGACTCACTATAGGGAGAGGGAGAATAGCCAATGGTCCTCCCGCTGTGCGCTCTTCTTCCTTT
CTCCCTTCTCCC
+
;<9/9>@<A@@=@ABBBBA@DBB7;311.7>>@B@CBJ?BAA>2738A>AA?@@<;<<@@@@A@@@9848@?@<@@8
98AH<A>?@@A<
@T6XXI:00135:00815
CTATCACTCGAGATCTGCGGCTTTGGACAGTCCCTCTGTACTGTGAAGCCACAGATGTCGAGTCGACTAGCTTCCTT
TCTCCCTTCCTCCC
+
CCCBA:::CBBCCDBBBBB@AAA>A=<@@@???9?BCCEBBB>B@=;<A?BCBBBCBB@@?BBBBBAECBBB>@?@C
>C>=<6<,0,0661
@T6XXI:00135:00826
GGGAGAAGGGAGAAAGGAAGAATAAAGGGGACGTTGCAGCAGCTCAGGCTATTCTCCCTCTCCCTATAGTGAGTCGT
ATTAGAATTCCCC
+
DBE@@@@AA:==AB>B?B@BB@BCC>CAA8ABBB@BBCBBBDBBBBB@BBBB=?9995:CBED>BBAA??@ACA??8
88>@@?=?9<<<4
@T6XXI:00135:00846
GGGAGAAGGGAGAAAGGAAGGAGAAGTCGCCCCAAACGATTTGTTGACTATTCTCCCTCTCCCTATAGTGAGTCGTA
TTAGAATTCCCC
+
CDFCCCCCC>AABC>B@B@C@BCA@ABBA@AA8BD>CCC??9>A@CAA@BB@====;589@C?B@?????<=<;<;;
?>A@A>@:<<=5
@T6XXI:00135:00848
CTAGTCACTCGAGATCTGCGGCTTTGGACAGTCCCTCTGTACTGTGAAGCCACAGATGTCGAGTCGACTAGCTTCCT
TTCTCCCTTCTCCC
+
>:111=8=?CDCCACBBBAA>@BB>B@BBCC@??8?CCAAABC@CBC@AC?BBBBBB8;;BCCABBCECB;<8=@=@
C@CBDC>C;;<@A7
@T6XXI:00136:00802
GGGAGAAGGGAGAAAGGAAGTGCAGTTCGCGGTTAGTGAAGGGAAAAACTATTCTCCCTCTCCCTATAGTGAGTCGT
ATTAGAATTCCCC
+
AB?@D@@CC?CA??<@>B>BCCCDCC@CBBB@B?B??BB@??;>>??2<<??>??>?>=?@??;?BCCC>?<AA<<<
;;8:;;98<AAA6
@T6XXI:00136:00809
GGGGAATTCTAATACGACTCACTATAGGAGAGGGAGAATAGCCGCTATTCATTCAATGCCACCTGTTCTTCCTTTCT
CCCTTCTCCC
+

```
CDG4A:;8<CCACBBC??@CC>;=;0.,0<0775:??@CBCC<AA>AA@CCC@CC>?;<>B@BB>@?=736367,7=
<<8=?A@@A=
@T6XXI:00136:00810
GGGAGAAGGGAGAAAGGAAGCTAGTCACTCGACATCTGTGGCTTCACAGTACAGAGGGACTGTCCAAAGCCGCAGAT
CTCGAGTCGACTAG
+
FICCCBA@=69844-
9594;????@A????A:::???BBC?BB@CCC>>>@C@>444148;AAA<??;?@<@A=<;><<<:::AA;;:@=A
@T6XXI:00136:00819
GGGAGAAGGGAGAAAGGAAGCTAGTCACTCGACATCTGTGGCTTCACAGTACAGAGGGACTGTCCAAAGCCGCAGAT
CTCGAAGTCGACTACG
+
IIDCBB?BC@BCCCAC?B?BA@>?@771111116;>9?BB:;<@CBAA??7777477,33004=566366556588;
:66/.,-113001.--
@T6XXI:00136:00841
GGGAGAAGGGAGAAAGGAAGCTAGTCGACTCGACACTGTGGCTTCACAGTACAGAGGACTGTCAAGC
+
DDFDCB844-999909=@>BCC@B@BCBB;::@>71166631757;?BCBBBF;655,,+++++*--
@T6XXI:00136:00842
GGGAGAAGGGAGAAAGGAAGGAGACAGTGAGCGCCGCACAACACGTCACTATTCTCCCTCTCCCTATAGTGAGTCGT
ATTAGAATTCCCC
+
CB:>9989@:A999185944=9339==ABBBDBB?BBBBC@CECBAACCCBC@BABB<<;;BC<A=<<@@CCBBBCD
CA>ABBANACBB9
@T6XXI:00136:00847
GGGAGAAGGGAGAAAGGAAGCTACTCGACATCTGTGGCTTCACAGTACAGAGGGACTGTCCAAAGCCGCAGATCTCG
AGTCGACTAG
+
CDBBCB@BE>BBBB>B@BABB?AAACB99;BBBCCEBCC>@@B@@CDCBB99989BABB?>AA?AA@B@A@?@@@@A
BAABBBBB??
@T6XXI:00137:00803
GGGAGAAGGGAGAAAGGAAGCTAGTCACTCGACATCTGTGGCTTCACAGTACAGAGGGACTGTCCAAAGCCGCAGAT
CTCGAGTCGACTAG
+
CCBAA?>AA9@>>>:>5949?>>?>?:77<>776A<9@@@>?@>@CCCCBAAA:47=:?<==A@?AB:AA?=8:<==
::<?===@@BAABA
@T6XXI:00137:00805
GGGGAATTCTAATACGACTCACTATAGGGAGAGGGAGAATAGCCATGTGCCGGTACCTGTCGCGTTCTCCTTCCTTC
TCCCTTCTCC
+
CDE9C@C@BAC@BCAAAABCC@@@C@??=?BBCC=??BA???A;=<@?843887889==A@@@@@377:7646,/,,
5.....**,+
@T6XXI:00137:00807
GGGGAATTCTAATACGACTCACTATAGGGAGAGGGAGAATAGCTACGTTTAACCGATCGTGTTGTTCTCCTTC
+
A==29?B=AAB==8;@99377<777<<88?B==?9::7>B??=BCCCDB>B@C@CBB>;;//,-444333333
@T6XXI:00137:00812
GGGGAATTCTAATACGACTCACTATAGGGAGAGGGAGAATAGTCACATTCATGTCTGTGACCGTGTCTCCTTCCTTT
CTCCCTTCTCCC
+
AAA6@@C@CCC@C??=AABB?C;99311.7==BC>BAA@C>>;<<;:?@A>?@<?@CBBB?@BCCCCBB?BBC?BC=
@@BB>B>??BC>
@T6XXI:00137:00817
GGGGAATTCTAATACGACTCACTATAGGAGAGGGAGAATAGCACTTACATGTGAAAGATTCGCTGTTCTCTTCCT
+
999/9249994;=@?C<<;A@@@A;87=000;<,0.5557B@@==:;>9:40300+044/34443;94300/0/.
@T6XXI:00137:00818
```

```
GGGAGAAGGGAGAAAGGAAGGAGAAGTCGCCCCAAACGATTTGTTGACTATTCTCCCTCTCCCTATAGTGAGTCGTA
TTAGAATTCCCC
+
DDBCDCACC>@@@C>B@C?C@B@BBBBCCCCC8AA<ACCCC>CE?BCBABB@AAAA<<<<BB;<<<<AAA<<:?><<
;=?>>9;/444-
@T6XXI:00137:00819
GGGAGAAGGGAGAAAGGAAGGAGAATCAACACGCTCGGTTTACACGTAGCTATTCTCCCTCTCCCTATAGTGTCT
+
DC?CBB>BCACBCC@C?B?BACAABAB@;:::??>6?;@@<A@??CC<;9@BC?CCCC>BBCCCA7700*(((((
@T6XXI:00137:00831
GGGAGAAGGGAGAAAGGAAGAGAATAAACGGATTACAAATTGTAGCGCTATTCTCCCTCTCCCTATAGTGAGTCGTA
TAGAATTCCCC
+
CB<AAA=ABABBBC@C@CBBBBABCFK<ACAA@=@???6AD@9;;A@@@4824877233377266666769996663
0044/0/033.
@T6XXI:00137:00833
GGGAGAAGGGAGAAAGGAAGTGCAGTTCGCGGTTAGTGAAGGGAAAAACTATTCTCCCTCTCCCTATAGTGAGTCGT
ATTAGAATTCCCC
+
HE?DBB@CC>CBAA=B?C9;;CCCCB@BACB@B>?;>??@BB=>B>>1>>??>??>?;BBCCC>B?BBB?AAAAAA@
@A>@A@>@=@@@5
@T6XXI:00137:00839
GGGGAATTCTAATACGACTCACTATAGGGAGAGGGAGAATAGCCTAGAAGTCTCCCGTTGTCCGCTATTCTTCCTTT
CACCCTTCTCCC
+
EBB7B7<8<B?@DBABABAAAAB99361.7>>>@>BCC?CBBA>BC??=?<96=?7=;9=8:7:CBCCDBB>B?A;:
>>@A<@=?>>>7
@T6XXI:00137:00846
GGGGAATTCTAATACGACTCACTATAGGAGAGGGAGAATAGTCAACAAATCGTTTGGG
+
?AA6C@C@CBBABBBBB@?BB:666..,/77=;>@?@AC>>9==<<@@<883330000
@T6XXI:00138:00815
GGGGAATTCTAATACGACTCACTATAGGGAGAGGGAGAATAGTCAACAAATCGTTTGGGGCGACTTCTCCTTCCTTT
CTCCCTTCTCCC
+
CCFFD?A??@A?BCCCC@??BBB>B761-
1;<<?<?BB?B@C@@A>=999@B99>466:5;@BCC?BBB?B@A>@?;<ACC;A=?@AA<
@T6XXI:00138:00830
GGGGAATTCTAATACGACTCACTATAGGAGAGGGAGAATAGTCAACAAATCGTTTGGGGCGACTTCTCCTTCCTTTC
TCCCTTCTCCC
+
AA=6??DBBBBACCCBABBCB:6=;0/,0<<>B>BCC>@BCA@?=???;ACBBCBBBB9B?@??:;<<375:>?@;A
AAA<A>A???<
@T6XXI:00138:00831
GGGGAATTCTAATACGACTCACTATAGGAGAGGGAGAATAGTCAACAAAATCGTTTTGGGGG
+
CDE9A?A@CCCACCCBBBBBA:6=4./-0>=BC>CCC@CDCA====?AB187,,,,*,,,,,
@T6XXI:00138:00842
CTCGAGATCTGCGGCTTTGGACAGTCCTCTGTACTGTGTAGCCACAGATGTCGAGTCGACTAGCTTCCTTTCTCCCT
TCTCCC
+
?AAABBBBBCHBB=A@C>C@CBCBCDAAAAC<<;ACCACBBB>AAAAAABBAAB???8>>>77;;>B?BG@BCCD@C
=????9
@T6XXI:00138:00847
GGGAGAAGGGAGAAAGGAAGCTAGTCGACTCGACATCTGTGGCTTCACAGTACAGAGGGACTGTCCAAAGCCGCAGA
TCTCGAGTCGACTAG
+
```

```
BD@DCB?=969988-
9698<CA<>B/00::9;=@AB@@CCC?AA@?B@?@A?>9633017==?AA>AB=@@<??AAA=;@<?<@A@AAA@@@
@T6XXI:00138:00848
GGGGAATTCTAATACGACTCACTATAGGGAGAGGGAGAATAGACTTTGGTGTTACCGAACAGCTATTCCCTTCCTTT
CTCCCTTCTCCC
+
CCD9B>B?BCFCC@?@AA???DB99311.7>>?C>CBC@CA<8><=;=;99?===;:;;<===<;<<<=4656333-
4>@@;A>???CA
@T6XXI:00139:00803
GGGGAATTCTAATACGACTCACTATAGGGAGAGGGAGAATAGTGACGTGTTGTGCGGCGCTCACTGTCTCCTTCCTT
TCTCCCTTCTCCC
+
DIF9B?A@BBBBCBBBBBBBBB>99311.7==?C@@??@CC?;AA==;;<<:::<<;=<<<A@<@@?><<9<<@=AA
;@@BC:?=;@A>;
@T6XXI:00139:00816
GGGGAATTCTAATACGACTCACTATAGGAGAGGGAGAATAGTCAACAAACTCGTTTGGGGCGACTTCTCCTTCCTTT
CTCCCTTCTCCC
+
CDIBG?A@ACC@CBBBBBBBB:9990/-0;:?C>ECC>B?BAAB>?454-/////,.--
*1766686662654;<<;;??C?C899AB<
@T6XXI:00139:00831
GACTCGAGATCTGCGGCTTTGGACAGTCCCTCTGTACTGTGAAGCCACAGATGTCGAGTCGACTAGCTTCCTTTCTC
CCTTCTCC
+
:4444444=9<9:;;8444-
4899@==9=:?=?=>::498:>;?@?ABC;99999?:6877...5.1424233,39992<:8998
@T6XXI:00139:00835
GGGAGAAGGGAGAAAGGAAGCTAGTCACTCGACATCTGTGGCTTCACAGTACAGAGGGACTGTCCAAAGCCGCAGAT
CTCGAGTCGACTAGCTTCCTTTCTCCCTTCTCCC
+
A959;<:<A:><<919699;@?>?<900........67>>8:;9;BC?@?CCB?77718>>@A@>@C=AA=@@AAAA
===@<<;@<<899@@B>A=AA>AAAA;@588>>:
@T6XXI:00139:00842
CTATCGACTCGAGATCTGCGGCTTTGGACAGTCCCTCTGTACTGTGAAGCCACAGATGTCGAGTCGACTAGCTTCCT
TTCTCCCTTCTCCC
+
@:77/////8:BBDCC;;<BBECD@C@@@BCCCC>CCCBBAAA=??C@AC@CCCCBB>@@ACC:=<CCCC@@?;?=?
C@CCCC=B??@=C<
@T6XXI:00140:00802
GTCGACTCGAGATCTGCGGCTTTGGACAGTCCCTCTGTACTGTGAAGCCACAGATGTCGAGTCGACTAGCTTCCTTT
CTCCCTTCTCCC
+
BB=:;CCCBBECCCCCCCBDCC?C?ABCBBCC=BBCCCECBAAAB@@@<@DCBBBCC<<8CDCC@BBB??@>A?BB>
BBBB>C;:;CCA
@T6XXI:00140:00819
GGGGAATTCTAATACGACTCACTATAGGGAGAGGGAGAATAGCACGACTTTTTCGGCATCGCTATAATCTCCTTCCT
TTCTCCCTTCTCCC
+
CBC9C@C@CCDDC@@@CCCBBB@?9316.7>>BC@CBB>@@B@A@B??8888/??>B@A@A<=<??=778=:<@C>A
@<@AAA<=98==@:
@T6XXI:00140:00827
GGGAGAAGGGAGAAAGGAAGCTAGTCACTCGACATCTGTGGTCACTCAC
+
CC>AAA>A>:>944-9;>;@B??@CBBCCBB:::BC>;:7+,,,,,++0
@T6XXI:00140:00828
GGGAGAAGGGAGAAAGGAAGGAGAAGTCGCCCCAAACGATTTGTTGACTATTCTCCCTCTCCCTATAGTGAGTCGTA
TTAGAATT
```

```
+
CFCDCCACC>BCDD>A>A?B?BCC@BCBBBBB5995;BBCB=BA=?@???B:9:CC<??@@A<??A@@AAA779;0/
,63+-,-,
@T6XXI:00140:00833
GGGAGAAGGGAGAAAGGAAGGAGAACGCGCACCCGAACACCA
+
BEEBB>@BC?CBDB>C@C?A?BBB@BBBBC:;;611,1,*)*
@T6XXI:00140:00837
GGGGAATTCTAATACGACTCACTATAGGAGAGGGAGAATAGCTACGTTTAACCGATCGTGTTGTTCTCCTTCCTTTC
TCCCTTCTCCC
+
999,48989AB?BDCCCCCBA9;;;0/,000?=;C??=?BB?BBBBBD>B@B?CBBB?@AA>AC?@@?>=9;/33-
433306;=ACC;
@T6XXI:00140:00843
CTATCACTCGAGATCTGCGGCTTTGGACAGTCCCTCTGTACTGTGAAGCCACAGATGTCGAGTCGACTAGCTTCCTT
TCTCCCTTCTCCC
+
AADB9::====BBCEBBBC?BBC>CACCABAAA=ACCBBBB@BB:837@=@=:;;?;;;B??B@@;;:;3768<<<@
;A>B?5:5:97A;
@T6XXI:00141:00804
GGGAGAAAGGAAGCTAGTCGACTCGACATCTGTGGCTTCACAGTACAGAGGGACTGTCCAAAGCCGCAGATCTCG
+
DD?A@==:@?A=AACAA@CCCBAAA89:AAACCC@??>?=====><<833313=??@@>AA<@@>@@=<<<8<<=
@T6XXI:00141:00808
GGGAGAAGGGAGAAAGGA
+
8B8:19699-44//+/,/
@T6XXI:00141:00809
GGGAGAAGGGAGAAAGGAAGGAGAAGTCGCCCCAAACGATTTGTTGACTATTCTCCCTCTCCCTATAGTGAGTCGTA
TTAGAATTCCCC
+
CC@AAA@CC@AABD?B@B?B?BBB@B@BBBBB7>><@CBBB>BA?CB@@@@><===;::?A=;=<<<<==<<<:?>@
B>C@@<=3444,
@T6XXI:00141:00810
GGGGAATTCTAATACGACTCACTATAGGAGAGGGAGAATAGTCAACAAATCGTTTGGGCGACTTCTCCTTCC
+
CE>1<,1,///77449@@@@A==;;00,09/76,177:?<>@@@;;:;377111,11,363344433,.,-,
@T6XXI:00141:00814
CTATCACTCGAGATCTGCGGCTTTGGACAGTCCCTCTGTACTGTGAAGCCACAGATGTCGAGTCGACTAGCCTTCCT
TTCTCCCTTCTCCC
+
AAB@?=BB999@A@>AB998;?@:@7;;;;:AB<@BAB:;;CCCCC>@C@;:;@B???BA@779@10....,.5557
:6;666,1779:<9
@T6XXI:00141:00815
GGGAGAAGGGAGAAAGGAAGGAGAATGCACCGCAGGAAATTGCAAGACTATTCTCCCTCTCCCTATAGTGAGTCGTA
TTAGAATTCCCC
+
CB?BBA?AB>BCBD>C@C@B@B@@;<?BBB>;;;B@CC>B@CBC@C@@BAA?@@@?9?5;;;6<?????CCAAAAA@
@>@@A>@?@BA5
@T6XXI:00141:00822
CTAGTCACTCGAGATCTGCGGCTTTGGACAGTCCCTCTGTACTGTGAAGCCACAGATGTCGAGTCGACTAGCTTCCT
TCCT
+
C:4999444444998@;;;B=??B<C?ACCCCCCB;::BA77:@71664>9==?BBAA??88:79>>@:989755,,
*,*,
@T6XXI:00141:00830
```

```
GGGGAATTCTAATACGACTCACTATAGGGAGAGGGAGAATAGTTTTTTCCCTTCACTAACCGCGAACTGCACTTCCT
TTCTCCCTTCTCCC
+
@??4?<@=ACEACDCB@?@BCA?<>877.7===@<:;;BCCCAACCC-
??<?;=@A??:=47777477;7::C@C@BB:@:77.7<>>==7
@T6XXI:00141:00842
GGGAGAAGGGAGAAAGGAAGGGAGTAGCAGCAGTCGGCAACGTGAAAACTATTCTCCCTCTCCCTATAGTGAGTTCG
TATTACAATTCCCC
+
BC?CBA=@@:>@>>:@<?<AA<BABAA<<<<<@B?@=@A<9733878,1779<==?>716767-7>:4422--
+*+***+0++--1,4@;,
@T6XXI:00142:00800
GGGGAATTCTAATACGACTCACTATAGGAGAGGGAGAATAGTCAACAAATCGTTTGGGGCGACTTCTCCTTCCTTTC
TCCCTTCTCCC
+
CCD:F@C@CCB@BA@>BBBB@:996..,0<:==9?AA?CCB@AA;=AA<B><=C;A@A7BBCCC@CCC>A>A?@@<@
AAA;A:==AA<
@T6XXI:00142:00807
GGGGAATTCTAATACGACTCACTATAGGGAGAGGGAGAATAGATCACCGTTGGTTCGGGTGCGCGTTCTCCTTCCTT
TCTCCCTTCTCCC
+
CCC9C?A@BCC@BA@ABBBBBB<99311.7??BC@BBB?@@?9=ACB>AA5:5::@?B@C<;;CDD@BAAAA>A?AB
?ACCC>CA<??B<
@T6XXI:00142:00810
GGGGAATTCTAATACGACTCACTATAGGGAGAGGGAGAATAGTTCAACGTTTATCCGCATGAACATCTCCTTCCTTC
TCCCTTCTCCC
+
EAA9B>A?BDD@CBBBBAAABB>99311.7?>>A>BAA?CCCA>AA>A@AA<A@@?@??@@C@A?85::7474,-
,/666064838:6
@T6XXI:00142:00816
GGGAGAAGGGAGAAAGGAAGGAGAAGTCGCCCCAAACGATTTGTTGACTATTCTCCCTCTCCCTATAGTGAGTCGTA
TTAGAATTCCCC
+
BCBCCB?BC>BCCDAB@B>@>AAA?BCCCCCC9CC>CCCCC>BB=AACCCC>??<=;<?@@A<AA@==@@@@@@@?@
@?AAA>A>@@=5
@T6XXI:00142:00826
GGGGAATTCTAATACGACTCACTATAGGGAGAGG
+
999,42424442444444497737337.525...3
@T6XXI:00142:00829
GGGGAATTCTAATACGACTCACTATAGGGAGAGGGAGAATAGTTTTTCCCTTCACTAACCGCGAACTGCACTTCCTT
TCTCCCTTCTCCC
+
;<9/9<B@CCC@CBAAA?<<BB>97366.7??BE>CCC>?BB=<::.=?:?>@CCCC@@@==?B?BAA88:CDC>AA
<????9A?BB@B<
@T6XXI:00142:00835
CTATCGACTCGAGATCTGCGGCTTTGGACAGTCCCTCTGTACTGTGAAGCCACAGATGTCGAGTCGACTAGCTTCCT
TTCTCCCTTCTCCC
+
CB>>1///66499CCCCCCC@AAA=A@;<>>877.7===?CCA:;:EACC?BBCCBB@@@@@??BBBBB@??73<37
7-76676=:71384
@T6XXI:00142:00847
CTATCGACTCGAGATCTGCGGCTTTGGACAGTCCCCTCTGTACTGTGAAGCCACAGATGTCGAGTCGACTAGCTTCC
TTTCTCCCTTCTCCC
+
A<:6/////89=>??CDBBBBBBCAC?@>??8777.7??BBBCBA@@B682-
1<>110;783711?>>>@73310101;999??5:5:>982
```

```
@T6XXI:00142:00848
GGGGAATTCTAATACGACTCACTATAGGGAGAGGGAGAATAGTCAACAAATCGTTTGGGGCGACTTCTCCTTCCTTT
CTCCCTTCTCCC
+
:99/9297<CBABA@BBCCACDB9;311.7>>>A=BCC@CCB??@>@@<;CCB??466:4:@@CC?BBB=?=??AB<
A@BA=A?===C<
@T6XXI:00143:00801
GGGGAATTCTAATACGACTCACTATAGGGAGAGGGAGAATAGCTCCCAACGGTAAACTGCTACCGAACCCTTCCTTT
CTCCCTTCA
+
AAB9ACB?@AAACCBBBBBBBBB>;367.7==BB@A@?=?;=;766,776101?@9;<B@=;;744/3849794830
3.-.+-2--
@T6XXI:00143:00818
GGGAGAAGGGAGAAAGGAAGCTAGTCACTCGACATCTGTGGCTTCACAGTACAGAGGGACTGTCAAG
+
CEBBBB@BB:?B@=:@=?;?B?<>>;:77:?:::ABBBBD?BB@CC@@@?@A@@:<<8330****)*
@T6XXI:00143:00830
CTAGTCACTCGAGATCTGCGGCTTTGGACAGTCCCTCTGTACTGTGAAGCCACAGATGTCGAGTCGACTAGCTTCCT
TTCTCCCTTCTCCC
+
B?:;;B9::CBBABBCCCCC@BCB<><?B?>???9?BCCBBBCABBB>=A@BCBBBA@??@A??ABBBBA;;7<>=A
C>BBBB=B8::@@:
@T6XXI:00143:00844
GGGAGAAGGGAGAAAGGAAGCTAGTCACTCGACATCTGTGGCTTCACAGTACAGAGGGACTGTCCAAAGCCGCAGAT
CTCGAGTCGACTAGCTTCCTTTCTCCCTTCTCCC
+
IE?CEAAABA@98809596<BA>?>67777611137>71629?=?BCBBABCCC=??9;;;;<=>AA=AC?A===@@
899@==<A???ABAAA>@>@@<@@@C<A:==<<7
@T6XXI:00144:00803
GGGAGAAGGGAGAAAGGAAGGAGAAGTCGCCCCAAACGATTTGTTGACTATTCTCCCTCTCCCTATAGTGAGTCGTA
TTA
+
CD@CCD@CC?B??C@B?A>A?BCB@B@BAA@A6>A<ABBBB>CC@CBBBBB??><<5<;<@A<AAA???A=<;??>>
=;?
@T6XXI:00144:00810
GGGAGAAGGAGAAAGGAAGAGAATACGCAATCCAGGCAGGAAAAAGCTATTCTCCCT
+
/1,-,,*,*,,/6+/,4244337;AB@@?@;;537773311111+1993311111,1
@T6XXI:00144:00817
GGGGAATTCTAATACGACTCACTATAGGAGAGGGAGAATAGACCAATTTTCCTATTCCCGCACAGTTACAGCCTTCC
TTTCTCCCTTCTCCC
+
444,42454A=@CCBBBBBCB@<<@8747=;=B7:;C?AAC577:7899+/,.-
644447<<@@=<8440002094246+4000+0/0-233
@T6XXI:00144:00818
GGGGAATTCTAATACGACTCACTATAGGGAGAGGGAGAATAGTCAACAAATCGTTTGGGGCGACTTCTCCTTCCTTT
CTCCCTTCTCCC
+
CFC:C?@?ACH@CCCBBBCBBB>99311.7>>>@>ACC@CBBA@@?@@@;CB9=@-666-
8??7747==:=;><=>699AA<@38788-
@T6XXI:00144:00827
GGGAGAAGGGAGAAAGGAAGCTAGTCACTCGACATCTGTGGCTTCACAGTACAGAGGGACTGTCCAAAGCCGCAGAT
CTCGAGTCGACTAGCTTCCTTTCTCCCTTCTCCC
+
BA5=9959;:@899142424>7736<;0......45:9=@:<?<??7::?=??=777,333044/771795:<==43
...333598;;8>?>=;=<=;:=844-43489=:
@T6XXI:00144:00830
```

```
GGGAGAAGGGAGAAAGGAAGAAGAGCGCACAGCGGGAGGACCATTGGCTATTCTCCCTCTCCCTATAGTGAGTCGTA
TTAGAATTCCCC
+
DEBAAA@CC>BBBC=B?A@BBACA8<<DCCCB???;?@>?@>@@>>?>@@A>@@AA;<:??A=A=A>>8ABA@????
?=??@>A?AAA5
@T6XXI:00144:00840
GGGAGAAGGGAGAAAGGAAGGAGAAGTCGCCCCAAACGATTTGTTGACTATTCTCCCTCTCCCTATAGTGAGTCGTA
TTAGAATTCCCC
+
CD?BD@@BB?>>BC?D>@=@@BBB@CBBA?>;5;;<@BCCC;A??BCAAAC?A<<<;A==AA<A=><@@AA?<?@@?
=<?<;8;59:<4
@T6XXI:00144:00842
GGGGAATTCTAATACGACTCACTATAGGAGAGGGAGAATAGACTCTAACTATTTATCCCGCAGCATACTCTTCCTTT
CTCCCTTCTCCC
+
AEC7B8<8<A@ACCCBBBBBB:88;0/,0?:=?<?BC@CFB?????C@BB???<?CCD>BB@B@BBGDCCC?AAAC<
??AA<>;>?@@;
@T6XXI:00144:00843
GGGAGAAGGGAGAAAGGAAAGCTAGTCGACTCGAGATCTGCGGCTTTGGACAGTCCCTCTGTACTGTGAAGCCACAG
ATGTCGAGTCGACTAGCTTCCTTTCTCCCTTCTCCC
+
CDBB<964;-4444-4244-
493..90./;;;=>>AAC?;<<@BCE>C=?@BB??C>1//::///;=AKDAB@C===??985C@@@BA@@====<@B
;??7876706,/.233
@T6XXI:00145:00800
GGGGAATTCTAATACGACTCACTATAGGAGAGGGAGAATAGTCAACAAATCGTT
+
C:808,1,//677@BBCBBC<;;D?>>3770;;,0./,..3135:5::1:7117
@T6XXI:00145:00804
GGGGAATTCTAATACGACTCACTATAGGGAGAGGGAGAATAGTTCTATTGTTGTCACCCCCGCGCTATCCTTCCTTT
CTCCCTTCTCCC
+
??=6@CB?CBC@CBBB@@>>BCB99311.7==>><@BB?BCCA>A?;?>==8;;;44443+4;:=>CCC=@<>477.
76>>6=:=644,
@T6XXI:00145:00810
GGGGAATTCTAATACGACTCACTATAGGGAGAGGGAGAATAGTCAACAAATCGTTTGGGGCGACTTCTCCTTCCTTT
CTCCCTTCTCCC
+
CEIFD?@>?CJBBBAABBBBBB>99311.7>>BK<??BACA@=:;;?@@<CBA??-
99948@BBC@CDC;;;;@A?:A877188;<BB<
@T6XXI:00145:00814
GTCGACTCGAGATCTGCGGCTTTGGACAGTCCCTCTGTACTGTGAAGCCACAGATGTCGAGTCGACTAGCTTCCTTT
CTCCCTTCTCCC
+
B@;:9=BBBBBDCCBCCEBGGCBEADCCBCCB@CCBBDCCCCCCB?BB@BBBBBCCCBBBBBCBABBB??@=>;<>6
:;BF?C>AACD@
@T6XXI:00145:00820
GGGGAATTCTAATACGACTCACTATAGGAGAGGGAGAATAGATCACCGTTGGTTCGGGTGCGCGTTCTCCTTCCTTT
CTCCCTTCTCCC
+
DBB9B;<<<CC@AA@BCB>@@>;;;00,0::??>;::<AA@??ACA=AB;?:?:@<<6<123???ICBB@C@C?BC?
CBAA<A798=:5
@T6XXI:00145:00823
GGGAGAAGGGAGAAAGGAAGCTAGTCACTCGACATCTGTGGCTTCACAGTACAGAGGGACTGTCCAAAGCCGCAGAT
CTCGAGTCGACTAG
+
```

```
DBBAAA@CB?B@==:@7:6;BA@@C?>:;;<00/955>7D>AA@CAA?>?@CC@<<<;88<<AB>@@;@B>A@@?<=
=@@???AA@??AAA
@T6XXI:00145:00826
GGGGAATTCTAATACGACTCACTATAGGGAGAGGGAGAATAGTCAACAAATCGTTTGGGGCGACTTCTCCTTCCTTT
CTCCCTTCTCCC
+
NHE9BCABCCCBCCBA@@>>>B?99311.7>>BC>B@B>BBC9BA>ABB:CBBBC>@A@7BBA==;:?C49463331
7@@A<A>A===7
@T6XXI:00145:00832
GGGGAATTCTAATACGACTCACTATAGGGAGAGGGAGAATAGTGTCTCAGTTTCGCGCAGAACTTTCTCCTTCCTTT
CTCCCTTCTCCC
+
=AA6A?B?BBB?ABBBB?<?BB?97311.7??BE>CBB?@@@<;;;=B@?>><A=====999<<955<?;=;@=@@<
@AA@;@>===:;
@T6XXI:00145:00835
GGGAGAAGGGAGAAAGGAAGGCATCGGTGACGCGCTGTAACTAGGGAGCTATTCTCCCTCTCCCTATAGTGAGTCGT
ATTAGAATTCCCC
+
DB@B@@@CC?CDDB==9?69:;CCCDACBB???AC@@=<:8????8???AAB@CCCC>AA???9?CAA@?@AAAAA<
<@>A@@=B@CCB9
@T6XXI:00146:00803
GGGAGAAGGGAGAAAGGAAGCTAGTCACTCGAGATCTGCGGCTTTGGACAGTCCCTCTGTACTGTGAAGCCACAGAT
GTCGAGTCGACTAGCTTCCTTTCTCCCTTCTCCC
+
CF?CC@799199@A<B@CACBA??>::718>==@B?:??B?@B@>@@CCCCCCC>CC@:::=A===A>>>><=>><;
:??====8:<=85777347994::;;4:243:=7
@T6XXI:00146:00812
GGGGAATTCTAATACGACTCACTATAGGGAGAGGGAGAATAGATCACCGTTGGTTCGGGTGCGCGTTCTCCTTCCTT
TCTCCCTTCTCCC
+
999/9<A@DCC@AAAABBBB>A;99311.7==>A>CBB@B;?<<=@?>AC>A=?>B@B>B;<:CDCGC?@=@?A?AD
ACBBB>B?B@AA;
@T6XXI:00146:00815
GGGAGAAGGGAGAAAGGAAGAATATTGCGCGTTTAACGGGGTGATGGCTATTCTCCCTCTCCCTATAGTGAGTCGTA
TTAGAATTCCCC
+
AB?BCB?BB=AB==:B=>=AD?BAB?CBDBAAA>A?A>?>8CCE888:BBA?BCBB>@@@@=;==<<;;;<BB@@?@
A?A??;=8<;;0
@T6XXI:00146:00840
GGGGAATTCTAATACGACTCACTATAGGGAGAGGGAGAATAGCCGCTTCTCCTTTGGCTCTCAAATATTCTTCCTTT
CTCCCTTCACCC
+
B??6A>A>BCC@BBBBAABB?BC@@76659::BC>BCC@C?@A@=<@=@@@>@A<A>A???AAA<AAA=AA@A>AA9
=<??6<9<BCC>
@T6XXI:00146:00846
GGAGAAGGGAGAAAGGAAGCTAGTTCGACTCGACATCCTCGTGGCTTCACAAGTACAGAGGGACTGTCCAAAGCCGC
AGATCCTTCGAGTCCGA
+
424442495449@=;24243339;77771111....44.....53601...,-
22244333+0033332885;=:=;;7///,.,.+.+++*++
@T6XXI:00147:00813
GGGAGAAGGGAGAAAGGAAGGAGAAGTCGCCCCAAACGATTTGTTGACTATTCTCCCTCTCCCTATAGTGAGTCGTA
TTAGAATTCCCC
+
CCCDDC@CB=B@@A=B@B@B@BBB@BBBAA@@6<<<@CNC@;?@@@<<?BB@B@@?;?@AAB==:??====:==A=<
<;@@@<=8;;;0
@T6XXI:00147:00822
```

```
GCTATCACTCGAGATCTGCGGCTTTGGACAGTCCCTCTGTACTGTGAAGCCACAGATGTCGAGTCGACTAGCTTCCT
TTCTCCCTTCTCCC
+
///8::9:4999<<;@AB;;:<AB>B:>;CBDCCABBC?@@AAA?A?;<B=@ABABACCABBB@AACCBF><:?A?A
D8::@@9?;?AAA:
@T6XXI:00147:00823
GGGAGAAGGGAGAAAGGAAGCTAGTCGACTCGACATCTGTGGCTTCACAGTACAGAGGGACTGTCCAAAGCCGCAGA
TCTCGAGTCGACTAG
+
DDBCDC:99-
9==95@8;4;A?00000//..3.:3899?AA:<<?BBCBBBCBCB=;<;<<<?A@>A@<@A?@@@A<====???@??
??A=<
@T6XXI:00147:00840
GGGGAATTCTAATACGACTCACTATAGGGAGAGGGAGAATAGTCCGCCAATTCTGCGCGTTAGCATTCTCCTTCCTT
TCTCCCTTCTCCC
+
CCC9CAC@CBC@BAAAABB>BB>99311.7??BC>????CCA??;==8;:??ABBBBAAC?CCCAA?CCC>C?B?BC
>CBCC>B?BB@@<
@T6XXI:00147:00841
GGGGAATTCTAATACGACTCACTATAGGGAGAGGGAGAATAGTCAACAAATCGTTTGGGGCGACTTCTCCTTCCTTT
CTCCCTTCTCCC
+
AAA6@?C@CCEBCCCBBA>>BD?97366.7==?C>BAA@B@>>;;8;;;6?A===-
777.8;::C@AABAA?ABCB=@@AA<@89:BA=
@T6XXI:00147:00847
CTATCGACTCGAGATCTGCGGCTTTGGACAGTCCCTCTGTACTGTGAAGCCACAGATGTCGAGTCGACTAGCTTCCT
TTCCTCCCTTCCTCCCCATCCCTA
+
B;;8////689==ABBBDBC@CDD>C;:;>>87:6;BCCCBCCCC??;=B?BCCCCBCBBCDBBFCBBBBA>;:<,0
0,0,0<<6:;A8;/?50-+++*+*
@T6XXI:00148:00835
GGGAGAAGGGAGAAAGGAAGCTAGTCGACTCGACCATCTGTGGCTTCACAGTACAGAGGGACTGTCCAAAGCCGCAG
ATCTCGAGTCGACTAG
+
CB>@9959914///+/,/,/4.339770000000,....36>=@AABBBBCCBB>@888-4;;:9:244-
4A9@@@@@ADD@>;<<4447<=>
@T6XXI:00148:00847
CTATCGACTCGAGATCTGCGGCTTTGGACAGTCCCTCTGTACTGTGAAGCCACAGATGTCGAGTCGACTAGCTTCCT
TTCTCCCTTCTCCC
+
D:::1///1:;BCBCBBBBB@AAA@AA?;>@:::4:=BABCCB<??C?@CACCCCBA>@@@@AABCCAA@@@?=A?A
C?BCCB<A:??@>7
@T6XXI:00149:00809
GGGAGAAGGGAGAAAAGGAAGCTAGTCACTCGACATCTGTGGCTTCACAGTACAGAGGGACTGTCCAAAGCCGCAGA
TCTCGAGTCGACTAGCTTCCTTTCTCCCTTCTCCC
+
ED=B:9249594999,424247999>87776111177668927;>AA@????==>===6999;=??@A;@@>@@AAB
C@??@<<<;=;<<:666,/,/7588;;57-73644
@T6XXI:00149:00810
CTAGTCACTCGAGATCTGCGGCTTTGGACAGTCCCTCTGTACTGTGAAGCCACAGATGTCGAGTCGACTAGCTTCCT
TTCTCCCTTCTCCC
+
81/////44;=?>BGCCCCC?AAB>B?A@>>766-
7@@BCCCB>BABD8<8<9>>>;<;8@?=?@@998>8812626?;?>>A9>37611-
@T6XXI:00149:00813
GGGGAATTCTAATACGACTCACTATAGGGAGAGGGAGAATAGTCAACAAATCGTTTGGGGCGACTTCTCCTTCCTTT
CTCCCTTCTCCC
```

```
+
?A?4?6;:<CC@CAAA@BBBBBB99311.7==AD@DCC>B>>>==;;?@<A@99786685?A?BC@BCC@C@C?AA<
A???;?:==AA<
@T6XXI:00149:00818
GGGAGAAGGGAGAAAGGAAGCGATAC
+
DD@CCC@CB=C@@@<;;<,1...4,,
@T6XXI:00149:00819
GGGGAATTCTAATACGACTCACTATAGGAGAGGGAGAATAGCCTGCAGGTCGAGTGTAGGACTGTTCCCTTCCTTTC
TCCCTTCCTCCC
+
EDDFGCC@CCC@CCCB@@@CC:==90.,0::?@>CCC@C@@@?BCCBB?BBCCCC@===>?>>??<@@;@>A>AA<A
:9722,-,/862
@T6XXI:00149:00825
CTAGTCACTCGAGATCTGCGGCTTTGGACAGTCCCTCTGTACTGTGAAGCCACAGATGTCGAGTCGACTAGCTTCCT
TTCTCCCTTCTCCC
+
>:1//7/44CBBBBBCCCCCBCBA>A?AABB@??=@BBCC=>BCCB?>?B@CCBABCBCCCC@?@ACCCBBBA?B?C
C?CBBC>C>?@BC>
@T6XXI:00149:00827
GGGAGAAGGGAGAAAGGAAGCTAGTCACTCGAGATCTGCGGCTTTGGACAGTCCCTCTGTACTGTGAAGCCACAGAT
GTCGAGTCGACTAGCTTCCTTTCTCCCTTCTCTC
+
?A>?BC@A@1999919<@>BDCAA??::98?@@AA<6:;A>??@<@ACC?@???9?BC<<=<A==<@@?C@A==888
?>?>?@@<==@>@=>AA@<?DAA???7>9;;@@?
@T6XXI:00100:00858
GGGGAATTCTAATACGACTCACTATAGGGAGAGGGAGAATAGTAACTCCTTCTCCAACGCGTTGACCACCTTCCTTT
CTCCCTTCTCCC
+
?AA6A@B?BCCACB?@BBBB>BB??311.7??AB>BBB>@@@<;8:==<=8::@?=;<<<==<=999<?=@<@>@C;
<<==:<;<@@@;
@T6XXI:00100:00868
GGGAGAAGGGAGAAAGGAAGGAGAACGCGCACCCGAACCAACGGTGATCTATTCTCCCTCTCCCTATAGTGAGTCGT
ATTAGAATTCCCA
+
BB=C9;:9>:?BCCDCAC>A>;83799444449-89>B@DBBBEA444944494444-
44439577796661.......,.4704249-4
@T6XXI:00100:00888
GGGAGAAGGGAGAAAGGAAGAATATGGTTGCGCTAATGGGAAGATGACTATTCTCCCTCTCCCTATAGTGAGTCGTA
TTAGAACTTCCC
+
AA:>9959A?ABBC@C@CBCC@BCCC@C=AACCCC?AAAAA>BFCBB:::A???=C<AAAAA<???@AAACAA=:8/
/,.140-3/00+
@T6XXI:00100:00889
GGGAGAAGGGAGAAAGAGGAGGACGCGCACCCGAACCAACGGTGATCTATTCTCCCTCTCCTATAGTGAGTCGTATT
AGAATTCCC
+
8856/9549-1,22*,,,*,02,44994444-
449:9::442444:;:=B>@>>;73.00,....111888:<9?@>71139244-
@T6XXI:00101:00852
GGGAGAAGGGAGAAAGGAAGGAGAAGTCGCCCCAAACGATTTGTTGACTATTCTCCCTCTCCCTATAGTGAGTCGTA
TTAGAATTCCCC
+
ADBCCA@EC>BBBC>A>A>A?BBC@CCBBBBB6BB<BCAA@;BB?C?>ACCAA;;;:88>==;====<<<=<<<=<<
??C@A>@9<;<4
@T6XXI:00101:00855
```

```
GGGGAATTCTAATACGACTCACTATAGGAGAGGGAGAATAGTCAACAAATCGTTTGGGGCGACTTCTCCTTCCTTTC
TCCCTTCTCCC
+
AAA9B>B@CDC@BB?@AA???:9990/-
0@<>B=BCC>@@?<=<;===7@CCCC<A?A6@?BCC@CBBCC@C@AA;?@DB<A9<<AA<
@T6XXI:00101:00856
GGGGAATTCTAATACGACTCACTATAGGGAGAGGGAGAATAGCTAAACCAAATCCCCTACCAGCAGACCTTCCTTTC
TCCCTTCTCCC
+
AAA6@CB@CDC@BA@ABBBBBBB99311.7??AC>BCC>???<@@@;A=7726777,0102004::;;7978/00+0
444365;;::4
@T6XXI:00101:00865
GGGGAATTCTAATACGACTCACTATAGGGAGAGGGAGAATAGTCAACAAATCGTTTGGGC
+
,--*,*/,///7884;;9<;;;>73316,71666166;B@BA?@A20042331662662.
@T6XXI:00101:00874
CTATCGACTCGAGATCTGCGGCTTTGGACAGTCCCTCTGTACTGTGAAGCCACAGATGTCGAGACGACTAGCTTCCT
TTCTCCCTTCTCCC
+
CBA=A=;4999499=@;<;BACDC>C?CCCC@??<??@???A??=??::A>@>>@@880:;;0//667377;8<9><
?;7116-7:=744,
@T6XXI:00101:00880
CTCGAGATCTGCGGCTTTGGACAGTCCCTCTGTACTGTGAAGCCACAGATGTCGAGTCGACTAGCTTCCTTTCTCCC
TTCTCCC
+
DCDCCBAACCCCC@BAA>B@BBBAAAA?BCBBBBBBAAAC@@B@AABACB>@;;;CCBBBBBB@@A?C@DC>BBCC?
C?BBBB>
@T6XXI:00101:00882
CTAGTCGACTCGAGATCTGCGGCTTTGGACAGTCCCTCTGTACTGTGAAGCCACAGATGTCGAGTCGACTAGCTTCC
TTTCTCCCTTCTCCC
+
C//111///1:CCCCCCCCBB@BDD>C?CBBC@??9?BBBBBCCABAC@=@?AABBBCCABBBDBBCBBBBBDC?A?
AE?CDDF>A?ABCCB
@T6XXI:00101:00899
GGGGAATTCTAATACGACTCACTATAGGGAGAGGGAGAATAGCTTGGCTTTGCCCCACAACATGTTCTCCTTCCTTT
CTCCCTTCTCCC
+
999/4299>AA?BGA@@CB;8;>>88682<?<??<??;9:77+,+,*,020.111,11101773558:;994;2..-
-33323,--322
@T6XXI:00102:00850
GGGGAATTCTAATACGACTCACTATAGGGAGGGGAGAATAGGCGACACACAAATCCCACGGCCTATTACTTCCTTTC
TCCCTTCTCCC
+
;<;4=CCADDD?BBBBBCCB>AABB766,17=?6BCC@CBC@AA@AA@@?><5?>>7>?=8;9@BB>A@@58487,6
666264:6CC>
@T6XXI:00102:00861
GGGAGAAGGGAGAAAGGAAGGAGAAGTGCCCCAAACGATTTGTTGACTATTCTCCCTCTCCCTATAGTGAGTCGTAT
TAGAATTCCCC
+
9=5>AB6991999@:A>A=@:;::910/77:6:::::BBB<AA=AAA?@@@CAACA@???C<989@??===ACA===
>A<<;<8>>>4
@T6XXI:00102:00864
CTAGTCACTCGAGATCTGCGGCTTTGGACAGTCCCTCTGTACTGTGAAGCCACAGATGTGAGTCGACTAGCTTCCTT
TCTCCCTTCTCCC
+
>1//11/4:CBBDCCDCCBB?BCC<>=@BCCCCC>BBB<A>@@ABBB@AC@CB@;;--
,,2..4=:;;;331010176119@9?=@@=?9
```

```
@T6XXI:00102:00870
GGGGAATTCTAATACGACTCACTATAGGGAGAGGGAGAATAGCCAGGTAGTTAACGCAATCGGCTTTTTCTCTTCCT
TTCTCCCTTCTCCC
+
CCB9B?A@ABCABA@>@BBB@AAB@97778CCCC=BB>>>>?<;<<;<=AA>=;<<:8:<==999;;;0;?>?<?>@
@;@AAB;?<?AAC;
@T6XXI:00102:00877
GGGAGAAGGGAGAAAGGAAGGAGAAGTCGCCCCAAACGATTTGTTGACTATTCTCCCTCTCCCTATAGTGAGTCGTA
TTAGAATTCCCC
+
BB@CCB@BB>BBBB>B@C@B@BBB@BAB>;<<-
786;BBCE<@??ABBBCB>AAAA=>;;<CAC???AA?888=@@AA>@@@=>5;<;1
@T6XXI:00102:00879
GGGAGAAGGGAGAAAGGAAGGGAATAGCAGCAGTCGGCAACGTGAAACTATTCTCCCTCTCCCTATAGTGAGTCGTA
TTAGAATTCCCC
+
CB@??=@BB@BBBB>B@A@BB?A@??BBBB@@ACBB?BC@CBB@@A>ACAA??@@C=@B?@?<?A@==<ABAA????
?<??A>A?AAA6
@T6XXI:00102:00882
GGGAGAAGGGAGAAAGGAAGCTAGTCGACTCGACATCTGTGGCTTCACAGTACAGAGGGACTGTCCAAAGCCGCAGA
TCTCGAG
+
CCBCBB?@=:@===:@?A=@?A@@@ACCCBBB@@??@@BBC@CC@>>>>>>@AC=99999<<==<<==;<<>A<;<<
<<??AAA
@T6XXI:00102:00885
GGGGAATTCTAATACGACTCACTATAGGGAGAGGAGAATAGTAGAC
+
---*1:@@CBD@C???BBCBBCB?;769.6>>>=A00.000/4000
@T6XXI:00102:00892
GGGGAATTCTAATACGACTCACTATAGGGAGAGGGAGAATAGTCAACAAATCGTTTGGGGCGACTTCTCCTTCCTTT
CTCCCTTCTCCC
+
:::5<?BBCBB?BBBBBB?B>BB99311.7>>AB>BBC>?>?=A=><<=;?B977,1164:@BCB@DDC::::@C=<
A@@?<@=?@A@;
@T6XXI:00102:00898
GGGGAATTCTAATACGACTCACTATAGGGAGAGGGAGAATAGCTACGTTTAACCGAT
+
CFC:B>A899A<499=999BB>997377-7111626689=<63663222*,*,*,,,
@T6XXI:00103:00858
GGGAGAAGGGAGAAAGGAAGGAGAAGTGTCGGGGTCAGAAAACGCTAACTATTCTCCCTCTCCCTATAGTGAGTCGT
ATTAGAATTCCCC
+
DCBBAAACC=?BCB>B@B@B@B??>B@AA?=<=7<BBBBBB:B@@AB@BBAA?ABDB>AACAA<A@@@CCCBBB???
=>;>?@<?8<<<4
@T6XXI:00103:00861
GGGGAGAAGGGAGAAAGGAAGCTAGTCGACTCGACTCTGTGGCCTTCAC
+
//1*/69244-4444-4242477<BBCCB::7781..,,,2*,*,*.33
@T6XXI:00103:00865
GGGAGAAGGGAGAAAGGAAGAAAGGAAGGAGAAGTCGCCCCAAACGATTTGTTGACTATTCTCCCTCTCCCTATAGT
GAGTCGTATTAGAATTCCCC
+
DD>CCB@BB?@@@A=@@B@ABB>?@B>ABBEC@CBCBBBB7BD?BCCCC>CB@BAA>BB@BBCC<@@???:?AAAC@
CCBB??<<<:<AA>=<;AA1
@T6XXI:00103:00876
GGGAGAAGGGAGAAAGGAAGGAGAAGTCGCCCCAAACGATTTGTTGACTATTCTCCCTCTCCCTATAGTGAGTCGTA
TTAGAATTCCCC
```

```
+
DLBCCC@CCCCHCC@C@CAC@;;<IBBBCCLC8B@=@BBBC;BB?BBCBBB?@@AA<A===A<@????@?@>@>>88
8969444/034-
@T6XXI:00103:00886
GGGGAATTCTAATACGACTCACTATAGGGAGAGGGAGAATAGTCAACAAATCGTTGGGGCGACTTCTCCTTCCTTTC
TCCCTTCC
+
BBD8B>@>@BBAB???@ABBB><<>366.7?>>@AABB@BBAAAA>B@>6>:00,.3304368:788@>>78344-
4///,/,/,
@T6XXI:00103:00896
CTATCGATCGAGATCTGCGGCTTTGGACAGTCCCTCTGTACTGTGAAGCCACAGATGTCGAGTCGACTAGCTTCCTT
TCCTCCCTTCTCCC
+
==A49449944449===>A>A>A<A59888778/17=>?>?AA@??:?@@BBECCAABABBCBDHCA@@C1110:<<
,1,0::8:-:/577
@T6XXI:00104:00854
GGGGAATTCTAATACGACTCACTATAGGGAGAGGGAGAATAGTCAACAAATCGTTTGGGGCGACTTCTCCTTCCTTT
CTCCCTTCTCCC
+
CCD:D>@>ACCBB??=BBBBA@;79316.7?>>A=@@D@CCCA@@>@A@;BBA9;88685??ABC@CBB?B?B@AA<
A@AA<A<??AA<
@T6XXI:00104:00871
GGGAGAAGGGAGAAAGGAAGCTAGTCACTCGACATCTGTGGCTTCACAGTACAGAGGGACTGTCCAAAGCCGCAGAT
CTCGAGTCGACTAG
+
BB?BDD@A=69844-
9594;CA?@@AA@???AAA;;;DCC?@B@CCC;;?@A?@46647;;;@@9==9=DBCAA@===AAA=@@@@@A@@@
@T6XXI:00104:00882
GGGAGAAGGGAGAAAGGAAGCTAGTCACTCGACATCTGTGGCTTCACAGTACAGAGGGACTGTCCAAAGCCGCAGAT
CTCGAGTCGACTAG
+
DDACCB=>><@BBC<B8;7;B???B8>:;::00/;66>>C?BF@BB;>>>@A??44417>9@AA>??;@A>A@<<<<
<<<<<;=<<<===<
@T6XXI:00104:00886
GGGGAATTCTAATACGACTCACTATAGGGAGAGGGAGAATAGCTCCACTCCAGTTTGAGGTTACTTGCCCTTCCTTT
CTCCCTTCTCCC
+
:::19?@?CAB?BCBBBA???BB?;7665:@@DD>CBB;???=??>?@??>>===;:66;=?CB@?@@>86:8/34+
493;;A<??@C<
@T6XXI:00104:00890
AGTCACTCGAGATCTGCGGCTTTGGACAGTCCCTCTGTACTGTGAAGCCACAGATGTCGAGTCGACTAGCTTCCTTT
CTCCCTTCTCCC
+
GBBCBBBABACCDA<=<B@;;;<<@CCCCCCC@CCBBCD??ACEE;::@ECBBBBA?A??@@@?AAA?=?@@B@FDB
BBBB>B@C@@@<
@T6XXI:00105:00875
GGGAGAAGGGAGAAAGGAAGTGCAGTTCGCGGTA
+
CC?CCBA@=:====:@@C@CCCDB;;786,-+-)
@T6XXI:00105:00877
GGGAGAAGGGAGAAAGGAAGCTAGTCACTCGACATTCGTGGTGCTTCACAGTACAGAGGGACTGTCCAAAGCCGCAG
ATCTCGAGTCGACTAG
+
HKBAA@@DH@CB==6859<>?>???:::BBC??;:,....,..4444<<<?@CF?A433-
4;;;;=/43+00/0588<@@@A==>@?@>?><;
@T6XXI:00106:00850
```

```
GGGAGAAGGGAGAAAGGAAGCTAGTCACTCGACATCTGTGGCTTCACAGTACAGAGGGACTGTCCAAAGCCGCAGAT
CTCGAGTCGACTAGCTTCCTTTCTCCCTTCTCCC
+
BC994424969499142424:399693.......44111731313;??AAA@@=;::4:<<<>?>@A<@@>AAAA==
=ACDA==<>==444::76788,//46,.,+0000
@T6XXI:00106:00852
AGATCTGCGGCTTTGGACAGTCCCTCTGTACTGTGAAGCCACAGATGTCGAGTCGACTACTCCTTCTTCCT
+
<499=?B@A>BBB>BA<<9==99-373444;9799<867373116666;@;999@:::7/,*)*)*,*,*,
@T6XXI:00106:00872
GGGAGAAGGGGAGAAAGGAAGCTAGTCACTCGACATCTGTGGCTTCACAGTACAGAGGACGTGTCCAAAGCCGCAGA
TCTCGAGTCGACTAG
+
?B:>>>6999,8899048=488??>::00....,,,,...55..-7888?B@@@@//,////.--
0113398:6533344==?=AA@===AA
@T6XXI:00106:00876
GGGGAATTCTAATACGACTCACTATAGGGAGAGGGGAGAATAGTTCGTCGTGACAT
+
8@@5@:><?>=599>;;;CBBAA??877.777766,16636110/04300001144
@T6XXI:00106:00879
GGGGAATCTAATACGACTCACTATAGGAGAGGGAGAATAGTCAACAAATCGTTTGG
+
944,4244//5///822,,,,,,,,3466177,1777;<<85A:733+.6...+,0
@T6XXI:00106:00897
GGGGAATTCTAATACGACTCACTATAGGAGAGGGAGAATAGTCAACAAATCGTTTGGGGCGACTTCTCCTTCCTTTC
TCCCTTCTCC
+
:>;1=-9249:<?<<<BBCAA@@@A87417111,177>B==9@A>@AA<CCCCC<AAA5@>>==4776:;04244-
4;;>:>27373
@T6XXI:00106:00899
GGGAGAAGGGAGAAAGGAAGCTAGTCGACTCGACATCTGTGGCTTCACAGTACAGAGGGACTGTCCAAAGCCGCAGA
TCTCGAGTCGGCTA
+
DC@??=@BB?=@BB=A2459>?>@CBAACCED>??AAACAA>AB?ACCCCB@@@<;::;==<<<C;9=9=A=???AB
AA?>@AA=6;8<=>
@T6XXI:00107:00864
GGGAGAAGGGAGAAAGGAAGGACAGTCCATCTGTGGCTTCACAGTACAGAGGGACTGTCCAAAGCCGCAGATCTCGA
G
+
BD?BB=>AA=BBBC=B==;?7:;?BBC@BCBC>>>>>?>?????=?@@@<<=<=AA@@A?AC:?:;A@<<<<<=@@@
@
@T6XXI:00107:00885
GGGAGAAGGGAGAAAGGAAGAGAACAACAGAGGCAACAAAAGACCTGCTATTCTCCCTCTCCCTATAGTGAGTCGTA
TTAGAATTCCCC
+
ED?CBB@BB?AABC>C?B?BCCC@CCACBABB@>?>ACCC:BBB@CCCD@@=@?AB<???AB>BBCBCAAAAA=888
<9<@A>=:AAC5
@T6XXI:00107:00895
GGGGAATTCTAATACGACTCACTATAGGGAGAGGGAGAATAGTTTCACGTTGCCGACTGCTGCTATTCCCTTCCTTT
CTCCCTTCTCC
+
GB@7A=A>ACDCB>B@@BBBBBCAA><:88????9?CC>AACCC;@@@?@=@A>===AB@@AA@@@?AB?A>@>@A:
9,,,+,+,++*
@T6XXI:00108:00851
GGGAGAAGGGAGAAAGGAAGGAGAAGTCGCCCCAAACGATTTGTTGACTATTCTCCCTCTCCCTATAGTGAGTCGTA
TTAGAATTCCCC
+
```

AC?AEB?CCACHDD@C@C@C@CCCB;:9888848D=B?ABA8:;@CBB@@?@ACDDAAB??=6;<878<=A;;;?00
/9434/0/033.
@T6XXI:00108:00855
GGGAGAAGGGAGAAAGGAAGGAAACTGCGCGCGCAAATCAGCTGGGAGCTATTCTCCCTCTCCCTATAGTGAGTCGT
ATTAGAATTCCCC
+
FFBCCB?BC?BDBB=BBB@C@BB<@@CCCCABBBCC>CBBBCCBC@CFCCDE?BBBB?BBBCE>CC@@A@ABCBECC
BB?B??=A@EED:
@T6XXI:00108:00856
GGGAGAAGGGAGAAAGGAAGCTAGTCACTCGACATCTGTGGCTTCACAGTACAGAGGACTGTCCAAAGCCGCAGATC
TCGAGTCGACTAG
+
CB?;99;>@:@?@@<@@B>B@@?>>?7617;667;;99AA:?@=@??????CCA??<??@AAD>AA<??>@@@?@AA
B===@A999@@@?
@T6XXI:00108:00857
GCTATCACTCGAGATCTGCGGGCTTTGGACAGTCCCTCTGTACCTGTGAAGCCACAGAGTGTCGAGTCGACTAGCTT
CCTTTCTCCCTTCTCCC
+
4:4944:=ABBBCGA@@@999-833,3137>A@CC>AA:::?989=BB@;;@59-
,,,,,,,0.11144443;777=>=>C>@@@CCC?AA@@;
@T6XXI:00108:00860
GGGAGAAGGGAGAAAGGAAGCTAGTCGACTCGACATCTGTGGCTTCACAGTACAGAGGGACTGTCCAAAGCCGCAGA
TCTCGAGTCGACTAG
+
BA69>?6991999918;>;>>9?<<11111177676661106>7?BCCCCB>=9444-
4;;:4838?:>?>?<<;<;<8;;;7446686:<
@T6XXI:00108:00868
GGGAGAAGGGAGAAAGGAAGGGGAGAAGGGAGAAAGGAAGCTAGTCG
+
BB?BCC@CC>BBBB>B@A@AAA:BBD?B;99877-701363...5/.
@T6XXI:00108:00870
GGGAGAAGGGAGAAAGGAAGTTGAATAGCACGCCTGAGGATAGCAGTCTATTCTCCCTCTCCCTATAGTGAGTCGTA
TTAGAATTCCCC
+
EFBCAA@BC>@@AB>B@?=BB@BB@BBB?>?CC@CBAA@BCB@A@AACECA>?7777:BCCC>BA?888AC?:=A??
@?@>>=B?A==4
@T6XXI:00108:00878
GGGAGAAGGGAGAAAGGAAGGAGAAGTCGCCCCAAACGATTTGTTGACTATTCTCCCTCTCCCTATAGTGAGTCGTA
TTAGAATTCCCC
+
99144=<@@098@A:?=A>B?BBD@CAB?><<-
88/8?A=@=BB@BBBCCC?@@AA<A@@@A889@CAA@@@@???@@=@AC=>8<<;0
@T6XXI:00108:00884
GGGGAATTCTAATACGACTCACTATAGGGAGAA
+
FIN9<<<B?498;@@C@<;;;88>>800+0.,*
@T6XXI:00108:00892
GGGAGAAGGGAGAAAGGAAGGGGAGAAGGGCTAGTCGACTCGACAT
+
99149?<>?0999914242488287977;:94499//,,,,,*00*
@T6XXI:00108:00893
GGGGAATTCTAATACGACTCACTATAGGGAGAGGGAGAATAGTTTGTCGTTTTACCCCGCAGCTCTGTTCTTCCTTT
CTCCCTTCTCCC
+
EDC9C?A?ACC@BABBBB?>?B?99311.7>>?@@BCC?C??<=;:::@@BD6AAAA6AAAAACBCCC@AA?A?CC>
>>@B>C>===A<
@T6XXI:00108:00894

```
GGGAGAAGGGAGAAAGGAAGCTAGTCGACTCGACATCTGTGGCTTCACAGTACAGAGGGACTGTCCAAAGCCGCAGA
TCTCGAGTCGATTAGCTCCTTCTCCCTTCTCC
+
CC<@@>699099;;;B>A=?>>>B@:66;11677667166==>B?AAB@@???=;44418<<?CH?AA<??:==>??
?<<=<<<<==<;=;/,,++,+++++*+02034
@T6XXI:00109:00859
GGGGAATTCTAATACGACTCACTATAGGAGAGGGAGAATAGTCAACAAATCGTTTGGGGCGACTTCTCCTTCCTTTC
TCCCTTCT
+
EBB7BCB?@@B@CBBAA@BBB@::?10,...61,11799>C<==;=AA9@?@@?8A??5@??<@@CCC@C=@444-
4/-222,,*
@T6XXI:00109:00860
GGGAGAAGGGAGAAAGGAAGGAGAAGTCGCCCCAAACGATTTGTTGACTATTCTCCCTCTCCCTATAGTGAGTCGTA
+
::>:::@DG@@@@BADIC?B?BA@<>=97877-73,33319888>99996;:8034,0004::45665512....+3
@T6XXI:00109:00867
GGGGAATTCTAATACGACTCACTATAGGGAGAGGGAGAATAGTCAACAAATCGTTTGGGGCGACTTCTCCTTCCTTT
CTCCCTTCTCCC
+
:::1<?CFCAA@A>>>>A?BBBB?;7665:BBCC@BAA=A@BAAA>@@@<BDC@?;:::6??BBC@CCC?B?B?AB=
???A:A;;?AA;
@T6XXI:00109:00871
GGGAGAAGGGAGAAAGGAAGGAGAATGCACCCAGCTAG
+
CE@BCB@EC?A@@B>B@;;;;;99,0./...+.76879
@T6XXI:00110:00855
GGGAGAAGGGAGAAAAGGAAGAGAACAGGCACGCATGTAAATGGTAAC
+
CB?@>>599199999/95824;335;@@<BBBE=999444-4424/,/
@T6XXI:00110:00873
GGGAGAAGGGAGAAAGGAAGGAGAAGTCGCCCCAAACGATTTGTTGACTATTCTCCCTCTCCTCATAGTGAGTCGTA
TTCAGAATTCCCC
+
CEBHEC@CH<;;B@19:;<??>:;435=00..*.40...75-/7566686600012011--,---
1166:??<<5334-./5779,444,
@T6XXI:00110:00882
CTATCGACTCGAGATCTGCGGCTTTGGACAGTCCCTCTGTACTGTGAAGCCACAGATGTCGAGTCGACTAGCTTCCT
TTCTCCCTTCTCCC
+
@866/////8?CBBBBBB@?=@DC>B<>@BB::::CCEB=AA?CCB>?C@CDCCCCBBABBBBCDCCCC@@?@C@C
B>BBBC>C?BBBC>
@T6XXI:00110:00883
CTAGTCACTCGAGATCTGCGGCTTTGGACAGTCCCTCTGTACGTGA
+
:1///1/4:CCCDCCBBBBB@BB?=?;<BCCB@@?;5****(((,,
@T6XXI:00110:00884
GGGGAATTCTAATACGACTCACTATAGGGAGAGGGAGAATAGTCAACAAATCGTTTGGGGCGACTTCTCCTTCCTTT
CTCCCTTCTCCC
+
EED9?7;;<BB?BBCCCBBAA;<<?777.7>>>><ABA?BBB@AC>???<BC>B>;@?B9BBBBB?BBC?C>?@AC>
A===9=<??CC<
@T6XXI:00110:00886
GGGGAATTCTAATACGACTCACTATAGGGAGAGGGAGAATAGTCTTGCAATTTCCTGCGGTGCATTCTCCTTCCTTT
CTCCCTTCTCCC
+
?BB4@<==@AA?BBBBCCBBCCB?>761.77777.7?<;?CCA>88;8;:@A>AABBAAAACDCC@CCB@BBD?BA=
?@>99776166-
```

```
@T6XXI:00110:00898
GGGAGAAGGGAGAAAGGAAGGAGAAGTCGCCCCAAACGATTTGTTGGACTATTCTTCCCTCTCCC
+
991444244-444;58;=<=<A>B@?983777,33,37767,1101011665:75543+-----+
@T6XXI:00111:00851
GGGAGAAGGGAGAAAGGAAGATCATGGGAGGTACGCATATGTGTGTGCTATTCTCCCTCTCCCTATAGTGAGTCGTA
TTAGAATTCCCC
+
49-
4@@@GC>B@@@9>>@=BBCCCBBB>BB?AABBBBBCCCCCCBB@AACC<;8A@>@B???<;@@@==<?>>68<;5:=
899=?@AC8
@T6XXI:00111:00853
GGGGAATTCTAATACGACTCACTATAGGGAGAGGAGAATAGCAG
+
>986@8@>@CC@C???BBBBABBCCBBB:::/.,0,,00+-.43
@T6XXI:00111:00861
GGGAGAAGGGAGAAAGGAAGCTAGTCACTCGACATCTGTGGCTTCACAGTACAGAGGGACTGTCCAAAGCCGCAGAT
CTCGAGTCGACTAGCTTCCTTTCTCCCTTCCTCCC
+
BA?ADC@CC>B@==9>595988870,,,,,,,00,,.7110171716;;;?<=>444,499;8?>?C;==:==;;>@
77777;5>??@987?@9=;=C>;;;816,/,-140
@T6XXI:00111:00863
CTATCGACTCGAGATCTGCGGCTTTGGACAGTCCCTCTGTACTGTGAAGCCACAGATGTCGAGTCGACTAGCCTTCC
TTTCCTCCCTTCTCCC
+
D:77///11:@CCCBB?@@C@@@@>@@CCCCAAA<AACB>@BB?AAD?@C@CCBBBDCBBCBA@AB;10..,*,222
26,/+0:5+.,11662
@T6XXI:00111:00868
GGGAGAAGGGAGAAAGGAAGAGAATAGGCACGCATGTAAAATGGTAACTATTCTCCCTCTCCCTATAGTGAGTCGTA
TTAGAATTCCC
+
CC?CCB@CB>B?==:@==<@CBB?BBB@@<ACCBBBAAAA8AA?@C@CB?B@CCCC?CC???<<<<AAAAAA=<===
@?AA@?C=?9;
@T6XXI:00111:00869
GGGAGAAGGGAGAAAGGAAGCTAGTCGACTCGACATCCTGTGGCTTCACAGTACAGAGGGACTGTCCAAAGCCGCAG
ATCTCGAATCGACTAGCTTCCTTTCTCCCTTCCTCCC
+
B@:@9959A09899149:9;???:37>>7..4.0...,.4222..,.337??1613344,338=?=<=A=8958@?@
@AA?=<@;>888;?<333/3/34,39440;37/394+
@T6XXI:00111:00870
CTATCGACTCGAGATCTGCGGCTTTGGACAGTCCCTCTGTACTGTGAAGCCACAGATGTCAGTCGACTAGCTTCCTT
TCTCCCTTCTCCC
+
DAA@CBBBBA@?AAB<<<ACCCCB=B?CCCC@??8????=BAAAABC@@B@CDCBB@?::;@@ABCC?@<;927:??
<?CCC<?:=744-
@T6XXI:00111:00879
GGGAGAAGGGAGAAAGGAAGTGCAGTTCGCGGTTAGTGAAGGGGAAAAA
+
?B>BEC@CB<>>995678691./86:5884424<;:000,...*.....
@T6XXI:00111:00887
GGGAGAAGGGAGAAAGGAAGCTAGTCACTCGACATCTGTGGCTTCACAGTACAGAGGGACTGTCCAAAGCCGCAGAT
CTCGAGTCGACTAG
+
DD?AAA@CC?BB@@:===;?B@@@@6;7<<=7718::>BC?BB@BB>99>A@>9300,388;?A>AA<@@>@?AA==
:;:;<;==:::===
@T6XXI:00111:00888
```

```
GGGAGAAGGGAGAAAGGAAGAGAACGGCGACGAACAATAGTTGGTGGCTATTCTCCCTCTCCCTATAGTGAGTCGTA
TTAGAATTCCCC
+
IDFCCC=?@>ACCEAC?B?BCBB=AABCCBCCC?BB@CCBB>A>;848@>><A@@@<???CF>BBCA@@@@AA@?==
=>@@@<@38880
@T6XXI:00112:00860
GGGAGAAGGGAGAAAGGAAGGAGAAGTCGCCCCAAACGATTTGTTGACTATTCTCCCTCTCCCTATAGTGAGTCGTA
TTAGAATTCCCC
+
CH@BCAACCABBBB>C@CAB@BBB@BCC>;??5??:@CCCC<AA@CB>AB@@>===;@<@AC<A<????@===@@@A
A>AAA>A38880
@T6XXI:00112:00875
GGGGAATTCTAATACGACTCACTATAGGGAGAGGAGAATATAGTCGCAGATT
+
=>=5@?A?BBC@CBBEBBCBDCAA?><?6;=773:??:=>0++++****+,*
@T6XXI:00112:00883
CTAGTCACTCGAGATCTGCGGCTTTGGACAGTCCCTCTGTACTGTGAAGCCACAGATGTCGAGTCGACTAGCTTCCT
TTCTCCCTTCTCCC
+
B?:<;A9::CDDCBCBBBBB@A??<A>;@AB@??=?CCD?>>?@@??>:A@BBBBBBABBBBCCCCBBACCCB<?=?
B>BDCC>B=?AAAA
@T6XXI:00113:00859
GGGGAATTCTAATACGACTCACTATAGGGAGAGGGAGAATAGTCAACAAATCGTTTGGGGCGACTTCTCCTTCCTTT
CTCCCTTCTCCC
+
CBB9A=@>@BBCCCC@BBBB?A?;?761.7>>@A@CCC>C??>A;:330+8?<<=9?@@6BAAAB>?@A>@>A?@@<
@AAA<A>??@@<
@T6XXI:00113:00863
CTAGTCGACTCGAGATCTGCGGCTTTGGACAGTCCCTCTGTACTGTGAAGCCACAGATGTCGAGTCGACTAGCTTCC
TTTCTCCCTTCTCCC
+
B2,,,-
,,/68@CCBDDBBBB?BCC@C?CCCBB??;@C@@@>>>@BBC?@B????BBBBAACCCC@@@CBB@??=?;@A>BDB
C>B:;;CD>
@T6XXI:00113:00865
GGGGAATTCTAATACGACTCACTATAGGAGAGGGAG
+
8B717+1,///,/488;:333333331011177,17
@T6XXI:00113:00873
GGGGAATTCTAATACGACTCACTATAGGGAGAGGGAGAATAGTCAACAAATCGTTTGGGGCGACTTCTCCTTCCTTT
CTCCCTTCTCCC
+
BBB6C@B>BBB@BBB@@@A@ACBAA76659??CC>A@@>C@@=8656;;4??==>.777.8>>@C?AAC?C?B@CC9
==>>7=8<<@B:
@T6XXI:00113:00883
GGGGAGAAGGGAGAAAGGAAGCTAGTCGACTCGACATCTGTGGCTTCACAGTACAGAGGACTGTCCAAAGCCGCAGA
TCTCGAGTCGACTAG
+
CCC:B@B=>=:@@996@786;C>;;@/0000;;08::96@@A=AA@BCBC@?A774--
,/666:@?AA<@@>@@AAA==AA?;?@AAA@@@A
@T6XXI:00113:00887
GGGGAATTCTAATACGACTCACTATAGGAGAGGGAGAATAGTCAACAAATCGTTTGGGGCGACTTCTCCTTCCTTTC
TCCCTTCTCCC
+
AAA8A>B?BDDBCBCBB@>BB:999..,0;;?B>CBB>?>?<A=<<<;:A???=496948>@AA?A@@:?;?;=>:@
AAA<A=@@@A:
@T6XXI:00113:00888
```

```
GGGGAATTCTAATACGACTCACTATAGGGAGAGGGAGAATAGCTCCCTACTCTTTGTACAGCGGCAAAGGCTTCCTT
TCTCCCTTCTCCC
+
99<4;?BACBB@BBBBA@@CCCAAA;76.7<<@B>BBBCCB>:>>>9=0//72944:=><;<=;<;<9=>AA=@=@C
7=<AC1738;;<7
@T6XXI:00113:00899
GGGAGAAGGGAGAAAGGAAGCTAGTCACTCGAGATCTGCGGCTTTGGACAGTCCCTCTGTACTGTGAAGCCACAGAT
GTCGAGTCGACTAGCTTCCTTTCTCCCTTCTCC
+
CC?A@@=>B:>99909A?>BAA@B@?@?@DEGCBCCC@BB@?BB>@=><?;>>>;>>8::==<<<<@><<;<<<<<<
<<=<<<@<@====;;>>=<==;==<<;<=<?C<
@T6XXI:00114:00857
GGGGAATTCTAATACGACTCACTATAGGGAGAGGGAGAATAGTCAACAAATCGTTTGGGGCGACTTCTCCTTCCTTT
CTCCCTTCTCCC
+
?AA6@?B@BBBECCCBBA???BBBA976-
1=66:7:BB@BAA?BB=@AA<CCBB>>BAB9CBBBC@CCF=?<<;>?;AAAD>D689AA<
@T6XXI:00114:00861
GGGAGAAGGGAGAAAGGAAGCTAGTCACTCGACATCTGTGGCTTCACAGTACAGAGGGACTGTCCAAAGCCGCAGAT
CTCGAGTCGACTAG
+
DD@BBC@@;:499909;?<ACBA??677777776B???BC?CB@CBBBBBCCBB7::68?;;;?/44-
30/05=AAA@=====?@@@@@@<
@T6XXI:00114:00863
GGGGAATTCTAATACGACTCACTATAGGAGAGGGAGAATAGTCAACAAATCGTTTGGGGCGACTTCTCCTTCCTTTC
TCCCTTCTCCC
+
DFB08,1,/667/4444477;64::00,/93679<A>@B?853320444;7117,111,3777:;===-/-
/5892::?=4999;555
@T6XXI:00114:00868
GGGGAATTC
+
444,421,/
@T6XXI:00114:00880
GGGAGAAGGGAGAAAGGAAGAACAGGGCACCGCAGGAAAACGTGAAGCTATTCTCCCTCTCCCTATAGTGAGTCGTA
ATTAGAATTCCCC
+
<:-:B@@BC@CB@@=A>>>@@=>@9>/97;:=?BB@CCC7@;99>8=444424444-
44///+/...4.33161111010110232335.
@T6XXI:00114:00885
GGGAGAAGGGAGAAAGGAAGGAGACACGGTCACAAACATGAATGTGTACTATT
+
EC?;49599-44//+/,/,/,/4./444249999@:88=>=73333......,
@T6XXI:00114:00897
GGGAGAAGGGAGAAAGGAAGTGCAGTTCGCGGTTAGTGAAGGGAAAAACTATTCTCCCTCTCCCTATAGTGAGTCGT
ATTAGAATTCCCC
+
:;8=AA@BCABAAA>A@D@CCBCCAA?B@@@8;:<BBCJBCCACCCC2CCBB::<CC>?@??B?@AA>>?====><<
66=A<=84/344-
@T6XXI:00115:00853
GGGGAATTCTAATACGACTCACTATAGGGAGAGGAGAATATA
+
111+/7A=;;9?@@@?<<CCAAC;;;B?77700,.0/,.20+
@T6XXI:00115:00865
GGGAGAAGGGAGAAAGGAAGCTAGTCGACTCGACATCTGTGGCTTCACAGTACAGAGGGACTGTCCAAAGCCGCAGA
TCTCGAGTCGACTAG
+
```

```
CC=;94299199991959948???>?777777<>>;?87::B@DC?BCCC??A>74000-
4;;;;=8;;7<=<@@889A@AA@@@?@@@788A
@T6XXI:00115:00881
GGGAGAAGGGAGAAAGGAAGGAGAACAGAATGCCACCAAGGTCTAAGCTATTCTCCCCCTCCCTATAGTGAGTCGTA
TTAGAATTCCCC
+
CC?CCF@BA=A@===B?B?B@CCA?BC;96;A><AB=A?AECCCC@CBBB?<>71111+7?B>DCBA@@A;<<=<<=
<;A<97:3444,
@T6XXI:00115:00894
GGGAGAAGGGAGAAAGGAAGCTAGTCACTCGACATCTGTGGCTTCACAGTACAGAGGGACTGTCCAAAGCCGCAGAT
CTCGAGTCGACTAG
+
CC?BDC@CC>B@==:@==;@AAABB8??;:77;:@<=BBB?BB?B??>?@@@@?36657;?AAC<??;?C>A@:;<=
<<<===AAA==:::
@T6XXI:00115:00897
GGGGAATTCTAATACGACTCACTATAGGGAGAGGGAGAATAGTCCTGTCTTTTGCCGATCGTCTATTCTCTTCCTTC
TCC
+
A??6A79;<CC@B?A?BBBB?BCBB9775:CCCD;AAA>??>;;:===@<<<5AB@@???A??===;;57554,-
,/,,,
@T6XXI:00115:00899
GGGAGAAGGGAGAAAGGAAGCTAGTCACTCGAGATCTGCGGCTTTGGACAGTCCCTCTGTACTGTGAAGCCACAGAT
GTCGAGTCGACTAGCTTCCTTTCTCCCTTCTCCC
+
AB:A<<89B:A111,1,1,/44749>1//:>>6>>@@BCC@CCC>B?CABBBBB>BBAAAA<;;;;>@==;?@@BA?
??AA???=<<888??B=?<?A?AA><;<;?@@@;
@T6XXI:00116:00852
GGGGAATTCTAATACGACTCACTATAGGAGAGGGAGAATAGTCGACAAATCGCAAAACTCCCTACATACTTCCTTTC
TCCCTTCTCCC
+
FDD:C>@>ACC@BAAAAA???:9990.,0::AB>BCCAB;;9;=;9==;B?;66994:BCCACCCCCCBB@C@BC@C
CCC>C@@@@C>
@T6XXI:00116:00865
GGGGAATTCTAATACGACTCACTATAGGAGAGGGAGAATAGC
+
::E6A;:;99488<;111000?999873777//+.336.//.
@T6XXI:00116:00868
GGGGAATTCTAATACGACTCACTATAGGGAGAGGGAGAATAGTTTTGGTGTCTCGATCCCCACCATTCTCTTCCTTT
CTCCCTTCTCCC
+
DBB7ACB?BBB=AACBBBB@@CCBC97756??BC>?@@@BCCAAA6A<??@@AA???AAA5@@<>=;=8789>3885
8@?A:?<>8>>7
@T6XXI:00116:00876
GGGAGAAGGGAGAAAGGAAGTGCAGTTCGCGGTTAGTGGAGGGAAAACTATTCTCCCTCTCCCTATAGTGAGTCGTA
TTAGAATTCCCC
+
CB?BBB@BB?AAAC>B@B@B@@ABBB@???B@B==;77688A6?<?7DCCLEDC@@;BBBCC;?;;??<<<===A:?
?>@AA=?8;;;0
@T6XXI:00116:00883
GGGGAATTCTAATACGACTCACTATAGGAGAGGGAGAATAGTTCTGGACTGTACTCGTGGGGCTGTCTCCT
+
CEE:B?B@CDDBCBBBBB@@@;;:01.,.436688999><<?85552337766440..45505/0333311
@T6XXI:00116:00885
GGGGAATTCTAATACGACTCACTATAGGAGAGGGAGAATAGTACAACTCCCTACGAATCGACCGCATCCTTCCTTTC
TCCCTTCTCCC
+
```

```
<><5=BCACAB@BCBBBB<<@:9940/,0::?C>CCC@B@@==<<:<??C;@@@??<?A===>@?===897979:6<
AAA<@=@@AC?
@T6XXI:00116:00890
CTGCGGCTTTGGACAGTCCCTCTGTACTGTGAAGCCACAGATGTCGAGTCGACTAGCTTCCTTTCTCCCTTCTCCC
+
DED@@>@CG@C@CBCCCBB=BBBAAAB@@CBC??B@BBBBBBBBBBAABBBBBBC<<<@C@EE>AAAC>C>A???9
@T6XXI:00116:00898
GGGAGAAGGGAGAAAGGAAGAGAACGGCGACGAACAATAGTTGGTGCTATTCTCCCCCTCCCTATAGTGAGTCGTAT
TAGAATTCCCCC
+
:E>A@A699199991959=B@@@?CC@CB<<<B@@A=A@=>;=-
21096667.1111+111,19696:;;;8836=44430/0/0499.
@T6XXI:00117:00855
GGGGAATTCTAATACGACTCACTATAGGGAGAGGGAGAATAGCTACGTCACTTGCAATGCTCTCCAATTCTTCCTTT
CTCCCTTCTCC
+
BCB9B?C?CBC@CCCAAA???C;99311.7>>BD>DBB;?@@<=>BCECB@?;?AA?A??B@???A>B>CB;=316.
70//,/,///,
@T6XXI:00117:00857
GGGGAATTCTAATACGACTCACTATAGGAGAGGGAGAATGGCAAACGTTTCCCCGACGTCGCTTTCTCCTTCCTTTC
TCCCTTCTCCC
+
CHH9C@A@ACDABBB@A?<<?:9990/-0>>BC>CDC>====?>;>>>>=>>?8@>>@@?B<<:88?90634/44-
4444-8:;>?@7
@T6XXI:00117:00858
GGGAGAAGGGAGAAAGGAAGCTAGTCGGACTCGACATCTGTGGCTTCACAGTACAGAGGACTGTCCAAAGCCGCAGA
TCTCGAGTCGAC
+
BE?AAC@CC>BCCC@C@B@CCBCCA<?=>7;;=00000/;>B?BC@C;;;FC?:;9//,/;::;@>AA8::>=<<AA
???AAAA===<@
@T6XXI:00117:00864
GGGGAATTCTAATACGACTCACTATAGGGAGAGGGAGAATAGTCCCGATATATACGTCGTGGGCTCTTCGCCTTCCT
TTCTCCCTTCTCCC
+
CBB9BAB?BDD@CBB?BBBBBB<99311.7?===<?AB?BBB@<<;?=C=<<?;ABCA@A??=?CB=>@CC?C==;<
C>>@@A;CACCCA=
@T6XXI:00117:00865
GGGGAATTCTAATACGACTCACTATAGGGAGAGGGAGAATAGTTCTGTGCATTTCTCCCAGCACACCGTCCTTCCTT
CTCCCTTCTCCC
+
6<<0:,924::9:@@@@@A????>;????9?@@A@:@??;86==:7335556<@;@@<=;<A===<C=@<<882:/0/
0000+3448@A6
@T6XXI:00117:00891
GGGGAATTCTAATACGACTCACTATAGGGAGAGGGAGAATAGCTTGCACGCTCGCTCCCTGCATTTGTACTTCCTTT
CTCCCTTCTCCC
+
BBB9BBC@BBB@B>>A??BB?CB?<3165:AACE>@@@>??@=@>????BBBBBBBBB=AAABCC@AAAAA?A@ECC
DCCD=A>=@@@<
@T6XXI:00117:00896
GGGGAATTCTAATACGACTCACTATAGGGAGAAGGGGAGA
+
B??4?<77///,/768=BBCB???;87=6=00,,77*,,,
@T6XXI:00118:00850
GGGAGAAGGGAGAAAGGAAGGAGAAGTCGCCCCAAACGATTTGTTGACTATTCTCCCTCTCCCTATAGTGAGTCGTA
CATTCA
+
```

==:=AB@BDACAAA<@?B>A>@@@?BCBCBCC9CB>DBCCA;9:211116330000+,++0000.34<88=DA;88/
.++*+.
@T6XXI:00118:00854
GGGAGAAGGGAGAAAGGAAGCTAGTCACTCGAGATCTGCGGCTTGGACAGTCCCTCTGTACTGTGAAGCCACAGATG
TCGAGTCGACTAGCCTTCCTTTCTCCCTTCTCCC
+
BB?ABDABCABCCCAC?B>BB;88:00/..35777/764;900,.,..16666,110<>?@@CA@C@<;<>;;8983
03366<<<=98988/0>9586,/---+-,--6:4
@T6XXI:00118:00879
GGGAGAAGGGAGAAAGGAAGAAAAGTGCCCTGGAGAACAGTCGATAGCTATTCTCCCTCTCCCTATAGTGAGTCGTA
TTAGAATTCCCC
+
CDB?88,//+1,22*,*,*////*.6499=AB?CBD?EHCEBBBBAA?BBC@C@??=?CACC>BBBBBB==>=<?<?
A>=AB<A<???4
@T6XXI:00119:00850
GGGGAATTCTAATACGACTCACTATAGGGAGAGGGAGAATAGACCTTCGTTTTTCCGAATTCCACTGTTCTTCCTTT
CTCCCTTCTCCC
+
DDB8B?@?BBB@CBBBBBBBA@;<9377.777FCCCCC?B<?6</424;;;:=.84=@;?;???<?@A859?@>?<3
59994:7938;4
@T6XXI:00119:00853
GGGGAATTCTAATACGACTCACTATAGGAGAGGGAGAATAGATCACCCGTTGGTTCGGGTGCGCGTTCTCCTTCCTT
TCTCCCTTCTCCC
+
944,49@A<<<??;;<BA@@CCB@A:7377777-
766=AC;44:877,1601018=@A>CB;:;BA=@::887785663555-/-/4330
@T6XXI:00119:00854
GGGAGAAGGGAGAAAGG
+
CCBC?>644-91--+-+
@T6XXI:00119:00864
GGGGAATTCTAATACGACTCACTATAGGGAGAGGGAGAATAGTCAACAAATCTTTCG
+
DDC7C=@=?887/1/6667<<;???877.77777,76636@@:9320--+.435+..
@T6XXI:00119:00867
GGGAGAAGGGAGAAAGGAAGGAGAAAAGGCGTAAGCAAACATTACGGCTATTCTCCCTCTCCCTATAGTGAGTCGTA
TTAGAATTCCCC
+
CD?BCB@CB>?>>><@@B@B@A:::95;@CCCB@CBDD?>;8781112=ADBIDCDADDBBB>BBBBBBBBCCBBBB
C?BBD@D:===5
@T6XXI:00119:00872
GGGAGAAGGGAGAAAGGAAGAGAATAGCGATTGGGTTGGTTTTATATCTATTCTCCCCTCTC
+
B;:=<<89;:>899149?48;??:;A?>:00,0;6;95596991:91::;44344444-4000
@T6XXI:00119:00876
ATCTGCGGCTTTGGACAGTCCCTCTGTACTGTGAAGCCACAGATGTCGAGTCGACTAGCTTCCTTTCTCCCTTCTCC
C
+
DBBBBBC@BBC@B?A@ABCCCBBBBCC?B>6;7:,/0-
0?=@C@AB@?:<<CC>;;://0,/,/8,/333,34==?A<
@T6XXI:00119:00877
GGGGAATTCTAATACGACTCACTATAGGGAGAGGGAGAATAGCTAAAACATTATTCCCGTCGCTGTTCTCTTCCTTT
CTCCCTTCTCCC
+
999/9:CBBBBABDBBCBBBBC?99311.7==>@>BCC?B?836777.7>>=BB@BD>A@@??BCC?BAAA@C@CC=
@?BB<A=??CC>
@T6XXI:00119:00881

GGGGAATTCTAATACGACTCACTATAGGAGAGGGACGAATATCACAAATCGTTGGGCGATCT
+
CDD:C@DCCCB?BEB@AA@?;;8??8:8/=5==10/.4331300044+31160..+...45.
@T6XXI:00119:00892
GGGAGAAGGGAGAAAGGAAGAAGAATAGAGATGCGGCCGCGTGTCATCTATTCTCCCTCTCCCTATAGTGAGTCGTA
TTAGAATTCCCC
+
EHBCBA?AC@BAABBB>A>AB@BB@AAA?A@778A?A?CCB???ACCCBBB?BCCC=@@:99-
7AB?ABCCBBCCAA?>A@@=??BAA6
@T6XXI:00119:00894
GGGAGAAGGGAGAAAGGAAGGAGAAGTCGCCCCAAACGATTTGTTGACTATTCTCCCTCTCCTATAGTGAGTCGTAT
TCAGAA
+
94-4:;:DC=ABAC>C@C@D@BCD>A?@8888,37677><?;=989666?A<;;99-300-,-----
2226655633235335
@T6XXI:00119:00897
GGGAGAAGGGAGAAAGGAAGCTAGTCACTCGACATCTGTGGCTTCACAGTACAGAGGACTGTCCAAAGC
+
885;111/8588?=:=;=<>C@?:0*********,3966606318117?A=97://,.11415556452
@T6XXI:00120:00858
GGGAGAAGGGAGAAAGGAAGGAGCACTAGACGGCATTTCTCGCCAATCTATTCTCCCTCTCCCTATAGTGAGTC
+
CF899B699-4449-/,/68,/./88/49=A9888A>5=@@==;<799>@@>=<<?866611,0-----13379
@T6XXI:00120:00859
GGGGAATTCTAATACGACTCACTATAGGGAGAGGGAGAATAGCCTAACGTTTTGCACAGTTGGTATCTCCTTCCTTT
CCCCTTCTCCC
+
DED:HCD?ABBB999A@@AB@@?;;<>A:=??@A<ABB?AA?</7:<::7?>8B;8/639,/,-
5<7760101000+088+0/04494
@T6XXI:00120:00868
CTAGTCGATTCGAGATCTGCGGCTTTGGACAGTCCCTCTGTACTGTGAAGCCACAGATGTCGAGTCGACTAGCTTCC
TTTCTCCCTTCTCCC
+
@@1//<81929??@BCCCCAA?ADD?C@DD?>888/8?8::BBB:;:C=?A?ADGEECC@=<CB;<>AAABAB@AB>
AA<@<<<7?:@=>A:
@T6XXI:00120:00871
CTAGTCGACTCGAGATCTGCGGCTTTGGACAGTCCCTCTGTACTGTGAAGCCACAGATGTCGAGTCGATAGCTTCCT
TTCTCCCTTCTCCC
+
C>111111=CCCCCCCABBBB@BBB<B?A@?AAAB>BEE@>99?>BB@=?A@BCBBBB?AAAA@?BBBCBBBB>BAA
C>CCCD<B>AACC>
@T6XXI:00120:00873
CTATCACTCGAGATCTGCGGCTTTGGACAGTCCCTCTGTACTGTGAAGCCACAGATGTCGAGTCGACTAGCTTCCTT
TCTCCCTTCTCCC
+
CDCA;:::BBBCBEHBBAA?BBC?B=>???7::5:BBC@??C?CDA=<A?ACBFCBBBBBCGBBBCBCCDCC=?=?B
?AABECC=??AD<
@T6XXI:00120:00881
CTAGTCGACTCGAGATCTGCGGCTTTGGACAGTCCCTCTGTACTGTGAAGCCACAGATGGACTGTCTTCCTTTCTCC
CTTCTCCC
+
DDCBBBCCCEDBCBBBAAADB@??>=@@BCBBBCB>BCBB??BC>BBC>=AACCCFBCC@CCCDCCCB@=BFA;>:C
?DB???C>
@T6XXI:00120:00886
GGGGAATTCTAATACGACTCACTATAGGGAGAGGGAGAATAGTCACATTCATGTTTGTGGCACGTGTCTCCTTCCTT
TCTCCCTTCTCCC
+

BBB9B?B?CCC@CB@>BBBBBBB>>3115:@@DD>AAA>?>?<<<::>@CAC>6332,,+,,,2367@7A=@;>;;;
4:8==7==>@?@<
@T6XXI:00120:00892
GGGAGAAGGGAGAAAGGAAGGGAATAGCAGCAGTCGGCAACGTGAAACTATTCTCCCTCTCCCTATAGTGAGTCGTA
TTAGAATTCCCC
+
CC?B?=<==<>>>?7;7;>CCCB?BCDCBBCCCCCC?BB?BCCAAB>ABIC@BBBC=AAAAA<A@???AAACA???@
@>AAA>@<>>=4
@T6XXI:00120:00899
GGGGAATTCTAATACGACTCACTATAGGGAGAGGGAGAATAGTTCTGACGGATTGCTCTGACTATTCACCTTCCTTT
CTCCCTTCTCCC
+
A?A8A>B@BCC@BBBDBCCBBB9?9367.7?>>A>BBC?BCCA??@@ABCGDC8:;AAA@B@@::6:>?=?4737=.
716;6<;=9771
@T6XXI:00121:00850
GGGGAATTCTAATACGACTCACTATAGGAGAGGGAGAATAGTCAACAAATCGTTTGGGGACGACTCTTCCTTCCTTT
CTCCCTTCTCCC
+
=?@4??A?BBB@CBCABBBB>:9990/-
0@;>A@BDJ@@<A>AA=ACC=C>:67,166.71=*****)*)*0035533<==;<8=7:86
@T6XXI:00121:00868
GGGAGAAGGGAGAAAGGAAGGAGAAGTCGCCCCAAACGATTTGTTGACTATTCTCCCTCTCCCTATAGTGAGTCGTA
TTAGAATTCCCC
+
DECCCB694-9>>>9@=A?BECCC@CCB7777-
:A=BDCBB=>>;>>>>?C@<@==;???AB=A???AAAAAAAA???=@AA;>2444-
@T6XXI:00121:00879
GGGGAATTCTAATACGACTCACTATAGGGAGAGGGAGAATAGACATATAACCGATCATGTGTCTGATCTCCTTCCTT
TCTCCCTTCTCCC
+
499/9<A@BDF@BB???BBBBBB97711.7??AB>BCC@@@<:?=B===9=8===??AAA@@===<A@@@>A?C9==
7699<:;<?AAA<
@T6XXI:00121:00882
GGGGAATTCTAATACGACTCACTATAGGGAGAGGGAGAATAGTCAACAAATCGTTTGGGGCGACTTCTCCTTCCTTT
CTCCCTTCTCCC
+
CDF:G?A?ABC@B@@BAA>>BBBB@76656??BC>BCC?AAB<A=<9999>GAAA>???7ABCA??>CD5:578;86
<==@;@>AAAA<
@T6XXI:00121:00884
GGGGAATTCTAATACGACTCACTATAGGGAGAGGGAGAATAGTCTTGTTTCATTCCCCCTTTGACTATGCTTCCTTT
CTCCCTTCTCCC
+
CCD:E;;8<@@=ACBBCBBAAB?99361.7>??@>BCC@CBB;;?9<=A<ACC@BBBB2AA<AACB@BB;:68;<=.
77<<6=477=?6
@T6XXI:00121:00890
GGGGAATTCTAATACGACTCACTATAGGGAGAGGGAGAATAGTTCCCTTCTAACCCGTACGCAACAATGCTTCCTTT
CTCCCTTCTCCC
+
C;;2:6<:<BA?DDCCCBBCCDCB@>?A<ABBCC>C?=;@?@A=AC5834484880861;;@@??A?@::7:?::=7
===A<A487886
@T6XXI:00121:00893
GGGGAATTCTAATACGACTCACTATAGGGAGAGGGAGAATAGCCTACGACCTCACCGCAGGGCTGTTCTCTTCCTTT
CTCCCTTCTCCC
+
CDC9C?C?CBC@CCBCAABBBC?99311.7>>AE>CCC@A=?9>CCBCCC;?@==89>;;;7;??@=@???==:?@;
@A@A<@8;777-
@T6XXI:00121:00894

```
GGGGAATTCTAATACGACTCACTATAGGGAGAGGGAGAATAGTCCTCACCCGTCTGGGCCACATATCCCCTTCCTTT
CTCCCTTCCTCCC
+
@??4><@>@AA?@@@BBBBBCC?>>877.7=<>?;BBB?BBB@A=??DAC>F@????9?@???BB@@@@4=897:;4
:44422,/,,551
@T6XXI:00121:00897
GGGGAATTCTAATACGACTCACTATAGGGAGAGGGAGAATAGTGACGTGTTGTGCGGCGCTCACTGTCTCCTTCCTT
TCTCCCTTCTCCC
+
BEB9B=@?ACFBCA@BBBB>;AB??366.7=<BC>AAA@C@@<888:===;<<<<<;=:8:CA=<<=<?:8>:>8<=
<<AAC=A<@@A=;
@T6XXI:00122:00850
GGGAGAAGGGAGAAAGGAAGAGAAAGCGGGCGCGTGAATGCCTTTGCTATTCTCCCTCTCCCTATAGTGAGTCGTAT
TAGAATCCCC
+
=A<?@A5991988C09297843376<>AA<??@@@??@C@?>=A=@@C??5:6668:?6?=;??:<==C<777///-
2224/----*
@T6XXI:00122:00853
TCTAATACGACTCACTATAAGGGAGAGGGAGAATAGTCAACAAAATCGTTTGGGGCGACTTCTCCTTCCTTCTCCTT
CTC
+
24498999;BC@@?@10..,..+.4766266736=>>7706111,177>?>:86.000332;=;9;382478443/,
/--
@T6XXI:00122:00866
GGGGAATTCTAATACGACTCACTATAGGAGAGGGAGAATAGCCGTTATTTTAACTCCCGCGGCCGGTTCTTCCTTTC
TCCCTTCTCCC
+
DED8A>A@BBBAAAACBBACC=;;700,/9369;@@B@C<:427>;>BHC6A>==<B:848797:;><?@>@=@@7<
<<>:@=@;;?9
@T6XXI:00122:00868
GGGAGAAGGGAGAAAGGAAGCTAGTCGACTCGGACATCTGTGGCTTCACAGTACAGAGGGACTGTCCAAAGCCGCAG
ATCTCGAGTCGACTAG
+
BB?==?@CCBBCCA6;8:<@B>=>>/11@@?B@>@@;::BBB@BC?CCC@<<?@;833317>A;<=>@@<@A?A=8=
@A@AA===A@@@AAAA
@T6XXI:00122:00885
GGGGAATTCTAATACGACTCACTATAGGGAGAGGGAGAATAGCTACGTTT
+
C?=299A=@A@?@@@A@BAA@?>?8877.777A?<@:0,/<0-.....33
@T6XXI:00122:00886
GGGAGAAAGGGAGAAAGGAAGCTAGTCGACTCGAGATCTGCGGCTTTGGACAGTCCCTCTGTACTGTGAGCCACAGA
TGTCGAGTCGACTAGCTTCCTTTCTCCCTTCTCCC
+
44-4444-44-
4///+/,/,/4...40./6777::@@@@CBA=?=?9=;??AACKE@BBAAA===A@@B?C?AAAA>===DCAA@@@A
@@@===>@=@C<?;;;,/,/-/.+
@T6XXI:00122:00889
GGGAGAAGGGAGAAAGGAAGGATAGGGACAAGTAGTGCGAAGGCAACACTATTCTCCCTCTCCCTATAGTGAGTCGT
ATTAGAATTCCCC
+
CDBBBC>BB>BBBD>B@B@B@BBCCC><@@@CCABBBAAB?B?BB?CDCC??:=444-
4:;>@:@@@===???>==>;;89:;79/444-
@T6XXI:00122:00890
GGGGAATTCTAATACGACTCACTATAGGGAGAGGGAGAATAGTTTGCTTCGCCCCACAACATGTTCTCCTTCCTTTC
TCCCTTCTCC
+
```

```
IEC9C>A=@BB?BBBCCBB@@@CB@::7.7>777.7A?<@A?<=7=AA<?AA@@4???9;<=6=;????@>@3//./
:9;6=.0+00
@T6XXI:00122:00893
GGGGAATTCTAATACGACTCACTATAGGGAGAGGGAGAATAGTCACCAAACCTCCCATGGTGCTATTGCCTTCCTTT
CTCCCTTCTCCC
+
ECB9B8;;<CC@BCBBBCCBBC@<@76659??CC>CBB>A;;<===9=<48933318==<@@ACCB>::8;?BCDD>
CBBB>B?A:974
@T6XXI:00122:00896
GGGGAATTCTAATACGACTCACTATAGGGAGAGGGAGAATAGTCAACAAATCGTTTGGGGCGACTTCTCCTTCCTTT
CTCCCTTCTCCC
+
?@?4@@B?CDD@CBBBBBBB@C@?;311.7<<>@>BCC@C@B==<;@AA<CA997-116-
8>>??<?BB?B?C@AA<@BCC;?9<<??7
@T6XXI:00122:00897
GGGGAATTCTAATACGACTCACTATAGGGAGAGGGAGAATAGTCACGATCT
+
4;A6C?B@=99<9444;437777833166?116;961.,...---------
@T6XXI:00123:00862
GGGGAATTCTAATACGACTCACTATAGGAGAGGGAGAATAGTTTCCTAGTCTC
+
CFEAD<<<=BA@CECDCBBBB000>10,.//>A;AAC?A::4:42,-------
@T6XXI:00123:00867
GGGAGAAGGGAGAAAGGAAGAAGCATGTTGCACGGGCGATGTACAAACTATTCTCCCTCTCCCTATAGTGAGTCGTA
TTAGAATTCCCC
+
EEBCAA?A@:>9;;7<AC@CB?BCCCBB?BBBBCC?CDDHBA@AAA>ADCCCCA@A>@CDCC=B9AACCA@C===??
?=@@?<=;===1
@T6XXI:00123:00869
GGGAGAAGGGAGAAAGGAAGGAGCATAACGACCAGATATACCTGCACGCTATTCTCCCTCTCCCTATAGTGAGTCGT
ATTAGAATTCCCC
+
CE@CCC@CB>BCBD>B@B@BAB<@@@@@AAAA@BBBBBBBA@DCCBDBCBCC@C>>=:449AA<ACAABBCCCB@@?
<;866444/4440
@T6XXI:00123:00874
GGGAGAAGGGAGAAAGGAAGCTAGTCACTCGAGATCTGCGGCTTTGGACAGTCCCTCTGTACTGTGAAGCCACAGAT
GTCGAGTCGACTAGCTTCCTTTCTCCCTTCTCCC
+
IC?GAA>AD?A@@@<@=>=@@???B?AAAAABBECCBBBC@CBC>@@?>BBBBB=>?@:?<====AC?BC?A9=;=;
6;<<=9==A@?>?<39378884;88;6;9;:<<7
@T6XXI:00123:00882
GGGGAATTCTAATACGACTCACTATAGGAGAGGGAGAATAGATCACCGTTGGTTCGGGTGCGCGTTCTCCTTCCTTT
CTCCCTTCTCCC
+
AAB6A@A>BBCB@@@BBAA>B:666..,0<:==<?BB@DC:6<ACCACB6747:>@@<A<;<CCBCA@@><AB<AA:
@???9?;?=>?:
@T6XXI:00123:00888
GGGAGAAGGGAGAAAGGAAGGATACGTTGGATGCTGACTAATGGGAGCTATTCTCCCTCTCCCTATAGTGAGTCGTA
TTAGAATTCCCC
+
EKBCCB@CC>BBBB>CDB@B@BBBBBB@B@BCBBABBCCC@CBB>BBB@@@@CCCC???:8858AA@==?@A@A@AB
A?A@@AB@BBB9
@T6XXI:00123:00895
GGGAGAAGGGAGAAAGGAAGCTAGTCGACTCGACATCTGTGGCTTCACAGTACAGAGGGACTGTCCAAAGCCGCAGA
TCTCGAGTCGACTAG
+
```

```
CCFCCD?A@:=944-95849?:8-
,,,,2225097==?BAC>BB>?<<<?@CAA<99999=====9984<?=@A<<=<<<<<<<<=8::<<<<
@T6XXI:00123:00897
GGGGAATTCTAATACGACTCACTATAGGGAGAGGGGAGAATAGTCAACGAAATCGTTGGGGC
+
DCC9A>@6998248>=>98888377316,661777,1177778044843/7+.-..,---*.
@T6XXI:00124:00850
CTATCACTCGAGATCTGCGGCTTTGGACAGTCCCTCTGTACTGTGAAGCCACAGATGTCGAGTCGACTAGCTTCCTT
TCTCCCTTCTCCCTTCCTTCTCCTCTCC
+
ECBB;::ABB@BACCBBBC@BBB<@<@@BBAAA=ABAABCCCCCCA=?BACCCCCCBBB@:::@@@@;;7763;377
.7<<>:A@CCAD7=8<3/,/67,/.551
@T6XXI:00124:00858
GGGGAATTCTAATACGACTCACTATAGGGAGAGGGAGAATAGTCTCCCTATTGAGCTTATTTGCGTTTTCTTCCTTT
CTCCCTTCTCCC
+
CCD:C@@>?BC@BAA>>>???BBBB97641=;?A>ACC@CBC@A<<<7;BB@CCEBB?DCD@CCBBBB9CC@D===7
<@>>:?:<;<>7
@T6XXI:00124:00863
CTAGTCACTCGAGATCTGCGGCTTTGGACAGTCCCTCTGTACTGTGAAGCCACAGATGTCGAGTCGACTAGCTTCCT
TTCTCCCTTCTCCC
+
:6/////4:DDCCBHBBBBB@BBB@AAA>>>7788:CCB@999???>96=@ABBBB>>>BBBA@BAABCDCBB?B@B
C>BBCD>C?BB@A<
@T6XXI:00124:00867
GGGAGAAGGGAGAAAGGAAGCTAGTCACTCGACATCTGTGGCTTCACAGTACAGAGGGACTGTCCAAAGCCGCAGAT
CTCGAGTCGACTAG
+
FIBDCB@BB?B;996;=?<@GBBBC8?====776?88@AA;A@?AABBCCCC>9366439>>@A>@A<AC>@@<?@=
<A@>:?@@@@???=
@T6XXI:00124:00876
GGGGAATTCTAATACGACTCACTATAGGGAGAGGGAGAATAGTCAACAAATCGTTTGGGGCGACTTCTCCTTCCTTT
CTCCCTTCTCCC
+
CCC:CBF@AAB;@9;;99?:000;00..+.459>;==@A@A85459335473177,111,3177>788>;777000+
004444/00353
@T6XXI:00124:00888
GGGGAATTCTAATACGACTCACTATAGGGAGAGGGAGAATAGTCAACAAATCGTTTGGGGCGACTTCTCCTTCCTTT
CTCCCTTCTCCC
+
DCD9C9:<;CC@CBAAAA??BB>97311.7>>BC>CBB>B??=<999ACBBB761,7;=2@??@C?CBC?B>AAAA<
AAAA<?>ABAA;
@T6XXI:00124:00893
GGGAGAAGGGAGAAAGGAAAGGAGAAGTCGCCCCAAACGATTTGTTGACT
+
CEBABDBA@:<111-/,//+/,/..697;3333,33,3776;;7443...
@T6XXI:00125:00851
GGGGAATTCTAATACGACTCACTATAGGGAGAGGGAGAATAGTCAACAAATCGTTTGGGGCGACTTCTCCTTCCTTT
CTCCCTTCTCCC
+
999/92929A?<>@<<<CBBCC;;;>==6=?=?A<???<?BF8899<?C<BBJDB6:::.8===?<????A>A?>>7
==@A<A;?<<<7
@T6XXI:00125:00861
CTAGTCGACTCGAGATCTGCGGCTTTGGACAGTCCCTCTGTACTGTGAAGCCACAGATGTCGAGCGACTAGCTTCCT
TTCTCCCTTCTCCC
+
```

```
>:111111ABCNCCBCA@BBB@BBB>B<@@BB@??=?B@CA?@?@BBC=?A@CBCBB@@ABCCBBBCCBCBBB?B?B
C>BBBC>C@CCCC>
@T6XXI:00125:00865
GGGAGAAGGGAGAAAGGAAG
+
FIB@@=599199991/,/,,
@T6XXI:00125:00869
GGGGAATTCTAATACGACTCACTATAGGGAGAGGGAGAATAGTTTTTTCCCCTTCACTAACCGCGAACTGCACTTCC
TTTCCTCCCTTCTCCC
+
999/4:B?CACCLE<><BC::;A@@><=6=777@6??=371144430(000+0437366999999;36633...,.,
..+.,...+136366+
@T6XXI:00125:00881
CTATCACTCGAGATCTGCGGCTTTGGACAGTCCCTCTGTACTGTGAAGCCACAGATGTCGAGTCGACTAGCCTTCCT
TTCTCCCTTCTCCC
+
C===1111;;:;;;DBCBB?BBC?C@B@??777.7??BCB;<;??@??B@BCCC;;;BAAA@@BC>;?;11,0,0,0
?9>ADC>C>??<=7
@T6XXI:00125:00897
GGGGAATTCTAATACGACTCACTATAGGAGAGGGAGAATAGTCAACAAATCGTTTGGGGTGCT
+
BCB9C=A?ACFACBB?BA@@?:;66./-0>>BC>BBB>>>>===<9=<787722*,,,*,,,2
@T6XXI:00126:00860
CTAGTCACTCGAGATCTGCGGCTTTGGACAGTCCCTCTGTACTGTGAAGCCACAGATGTCGAGTCGACTAGCTTCCT
TTCTCCCTTCTCCC
+
B6/////44CCCBBCABBBB?AAB?B>?A?????;?CCCC@??<??C>?B@CCCBAABBBBB@@?BBBBC@@@@K@B
B>BBCC>C?CBCB>
@T6XXI:00126:00897
GGGAGAAGGGAGAAAGGAAGGAGAAGTCGCCCCAAACGATTTGTTGACTATTCTCCCTCTCCCTATAGTGAGTCGTA
TTAGAATTCCCC
+
DDBAAA@CCB:;9C>B@C?B?BCC@B?BBBB?8>><BBB@C>?@>???CCC?=<<=;??AAA<=<<<A==A@=====
<>@<:792444-
@T6XXI:00127:00857
GGGGGGAATTCTAATACGACTCACTATAGGGAGAGGGAGAATAGTCAACAAATCGTTTGGGGCGACTTCTCCTTCCT
TTCTCCCTTCTCCC
+
99999*4:BAJBC@CBABBCDBBA@99311.7==>BADEC?C==:A=<<<;;AA?@A=@@@7BBCAC@CBB>B@A@A
A<<@@<;@>@@AA<
@T6XXI:00127:00865
GGGAGAAGGGAGAAAGGAAGGAGAAGTCGCCCCAAACGATTTGTTGACTATTCTCCCTCTCCCTATAGTGAGTCGTA
TTAGAATTCCCC
+
CC?BBBBBB@@BBB>B@A?A@BBB@B@BBBBB8BB<>BB??9??>@@@?B?A====588?==;=;;9<<=<<<9<==
C>A==9<;==>5
@T6XXI:00127:00885
GGGGAATTCTAATACGACTCACTATAGGGAGAGGAGAATAGTA
+
8880<:A>BBCCCB@AAA?>>BCB?<8866;6737::9:,,,,
@T6XXI:00127:00890
GGGAGAAGGGAGAAAGGAAGAGAAAGTCTTTGCGTGGAAATCAACGACTATTCTCCCTCCTCCCTATAGTGAGTCGT
ATTAGAATT
+
IFBDCBADGBD:<C<A8;59@88807699><>>??>BCC899C@@@@@@@@?:7==6/0,---+-33@==58-----
--445=;55
@T6XXI:00127:00893
```

GGGAGAAGGGAGAAAGGAAGCTAGTCGACTCGACATCTGTGGCTTCACAGTACAGAGGACTGTCCAAAGCCGCAGAT
CTCGAG
+
AA<?B;2990999A:@=@=@@CCC@@@CA>>>11111/;7>-:797177711..-..,-
1223A=@@<@A:==A@A@???999
@T6XXI:00127:00894
GGGAGAAGGGAGAAAGGAAGATGCGCGATCTGTTGTCATACCCGGGAGCTATTCTCCCTCTCCCTATAGTGAGTCGT
ATTAGAATTCCCC
+
FD@BBB?CB@BBBB>C@B@@@?ADCDBBBBBBC@BBBBCBBB<BB@BB?<BB@DDBB?BBBCCABCCBA@ACCDCCC
BB?BDC@E@CDD:
@T6XXI:00128:00852
GGGAGAAGGGAGAAAGGAAGGAGAACAACACGATCGGTTAAACGTAGCTATTCTCCCTCTCCCTATAGTGAGTCGTA
TTAGAATTCCCC
+
?B>AAB>AA<<99@1969;>;>>@=AHCAABHC@@A=@=@C>BBBBBBCAACBACE?::;ED@CBBAB?A@@?7887
838?@;?=?AA6
@T6XXI:00128:00861
GGGGAATTCTAATACGACTCACTATAGGGAGAGGGAGAATAGTTTCCCTTCACTTCACGCGACTGACTTCCTTTCCT
CCTCTCC
+
999/48=8>@@?BGDCDBBCCCB@:877.7@ACE?DCB=;6734-44+42071101116.....-
11.33333+.,..33363/
@T6XXI:00128:00876
GGGAGAAGGGAGAAAGGAAGCTAGTCACTCGACATCTGTGGCTTCACAGTACAGAGGGACTGTCCAAAGCCGCAGAT
CTCGAGTCGACTAG
+
4<:9>>?BA:<84;:=<=<;9777>61111111177667<;<B@CAC@@@@>>>444-
4;;=?A>AA<@@;@AAAB@@@A@???888888@
@T6XXI:00128:00895
GGGAGAAGGGAGAAAGGAAGCTAGTCGGCTCGACATCTGTGGCTTCACAGTACAGAGGGACTGTCCAAAGCCGCAGA
TCTCGAG
+
BFBCCB@A=6=999-
;=B7<;A;;;BB@C@??::;BBBCBB??@=@CBCCCA@??<<>;<<====:<<:<=;====:::::<<<
@T6XXI:00129:00853
GGGAGAAGGGAGAAAGGAAGTGCAGTTCGCGGTTAGTGAAGGGAAAAAACTATTCTCCCTCTCCCTATAGTGAGTCG
TATTAGAACTTCCCC
+
EIBCA@>@C?ABA@=B>B?BCCCCCC@BBAA==<=;AAB?BCACCCCC-
CBBC@EA>>8???@@<ABBBD@@A@@A8899?A@@688;===5
@T6XXI:00129:00854
GGGGAATTCTAATACGACTCACTATAGGAGAGGGAGAATAGCACTCCCCATT
+
EED9CCC@CCC>BABBBCCBA:99;0/,0><BB?CCC>;;;.--1111.12,
@T6XXI:00129:00855
GGGAGAAGGGAGAAAGGAAGCTAGTCGACTCGACATCTGTGGCTTCACAGTACAGAGGGACTGTCCAAAGCCGCAGA
TCTCGAGTCGACT
+
DFBB==8=>:>999195959>>:::/00;==>3<:B>9>@@::;7;@@BBBBCCA@AA<AA@?44244-
44344433343;343?;7778
@T6XXI:00129:00859
GGGAGAAGGGAGAAAGGAAGTGCAGTTCGCGGGTTAGTGAAGGGAAAACTATTCTCCCTCTCCCTATATG
+
:?6@=8,11,/1/8+/,/,497949969999>-4243733133,111,33339997;176667,66160+
@T6XXI:00129:00862

```
GGGGAATTCTAATACGACTCACTATAGGGAGAGGGAGAATAGTTCAGCCTTCTAGGTCGTCCTGTTCTACTTCCTTT
CTCCCTTCTCCC
+
EBC9C@C@BBC@A@@@??>??CB?>311.677>@<AAD?CC@@>?@AA?A888@C@ACCCC?@@@@A@BAA<?<?C<
A@@A<A?BA@A<
@T6XXI:00129:00867
GGGGAATTCTAATACGACTCACTATAGGAGAGGGAGAATAGTCAACAAATCGTTTGGGGCGACTTCTCCTTCCTTTC
TCCCTTCTCCCACTCACC
+
B?:2:296;==<=BCCBB@@@=;;000,.4477>A778::739;9444-
8?761,111,3@68<=?CC=A=@AAC;@;;;-424:::744444//
@T6XXI:00129:00874
GGGAGAAGGGAGAAAGGAAGCTAGTCGACTCGACATCTGTGGCTTCACAGTACAGAGGGACTGTCCAAAGCCGCAGA
TCTCGAGTCGACTAG
+
DDBC?=;==9>111,1,1-/4....0..33448<<888CBB<:;9;B??@CCB@@734-
4748?A<??<?B?@AAA@@@@@@@BA@AA=<<8
@T6XXI:00129:00888
GGGGAATTCTAATACGACTCACTATAGGAGAGGGAGAATAGTGACGTGTTGTGCGGCGCTCACTGTCTCCTTCCTTT
CTCCCTTCTCCC
+
;99/9696<A>?BA@?BBBBB:9990/-
0>>CE<A@B;;?>9;;===<<;<<<==<=9====<<>====9<=A:?<<<@@D<@=8@@C>
@T6XXI:00129:00892
GGGAGAAGGGAGAAAGGAAGTGCAGTTCGCGGTTAGTGAAGGGAAAAACTATTCTCCCTCTCCCTAATAGTGAGTCG
TATTAGAACTTCCC
+
CC?@@@@BC<?ACCAC@C@CCBBEBC@BBAB<@=@BBBB>??07777,37>71336621..442..,.2--
0=8655434,..3222,00+
@T6XXI:00130:00851
GGGAGAAGGGAGAAAGGAAGCTAGTCGACTCGACATCTGTGGCTTCACAGTACAGAGGACTGTCCAAAGCGCAGA
+
BDDDDC@CC<=>>>195;@CCB::>//.4/..9@99???<<?BC@DDBBCDA<<8//./;9508,//,.----00
@T6XXI:00130:00858
GGGAGAAGGGAGAAAGGAAGGAGATGCCAACGCAAACACGATTGTGGCTATTCTCCCTCTCCCTATAGTGAGTCGTA
TTAGAATTCCCC
+
GBBECCBBE>AAAB>B@BABABBACAB?B?BA@>>=@C@AAB?A??=?BCC?BACC>CBCCC>CBAABCC@@A@@==
=>AAB>A9<<<1
@T6XXI:00130:00865
GGGAGAAGGGAGAAAGGAAGCTAGTCGACTCGACATCTGTGGCTTCACAGTACAGAGGGAGTGC
+
DF=>:CACB><9<A:@<@5433:7.757///44../3667<>@C?BABE@????8//62//+..
@T6XXI:00130:00871
GGGAGAAGGGAGAAAGGAAGAACAATGTTCGTTTGCGCGGCGAGAACTATTCTCCCTCTCCCTATAGTGAGTCGTAT
TAGAACTTCCCC
+
IEBBBBACC=BABB@C@B@CC@CB?ACC@BBBC?CCCA??ABBBB?AAAA>???>.777775:CCCAA@@<<::943
/0342445333.
@T6XXI:00130:00877
CTAGTCACTCGAGATCTGCGGCTTTGGACAGTCCCTCTGTACTGTGAAGCCACAGATGTCGAGTCGACTAGCTTCCT
TTCTCCCTTCTCCC
+
B8///6699BBEHCCBAB<;;;CD>B?BBBB:::5:@@AAABBBB>@;8;7;CCABAA@C@@?ABBCBBC<<8>B>A
B:>8BC@C;;:AA;
@T6XXI:00130:00881
```

```
GGGAGAAGGGAGAAAGGAAGTGCAGTTCGCGGTTAGTGAAGGGAAAACTATTCTCCCTCTCCCTATAGTGAGTCGTA
TTAGAACTTCCCC
+
KF>B<<9<B>ACHC?CAC=<<CCCCC@B@AA@C?A@@BD@EEAAAA6@AAA>A???9:::EL@BBBA@@@AA@@<<<
<9<<A9<<<>>>5
@T6XXI:00130:00896
GGGAGAAGGGAGAAAGGAAGCTAGTCACTCGACATCTGTGCGTTCACAGTACAGAGGGACTG
+
DDFCCC9;4-999909699<BB?BB776111163443./////::<<=54468555=443<4
@T6XXI:00131:00857
GGGAGAAGGGAGAAAGGAAGGAGACACGGTCACAAACATGAAT
+
99-999599-49>?7;69<B>B?;=A9;5444444-4///668
@T6XXI:00131:00858
GCTAGTCGACA
+
44////*****
@T6XXI:00131:00872
GGGGAATTCTAATACGACTCACTATAGGGAGAGGGAGAATAGAGCTCACTGATGCCTACTCACGCCCCTCCTTCTC
+
EEDFC>@?ABC@CBBBBA???BD?>361.6>>AB>CCF@C>>===A=:?=@<<<B@>;167171166,763757,,
@T6XXI:00131:00879
GGGAGAAGGGAGAAAGGAAGATTAGAAACTGGAGCATTGCAGATGACTATTCTCCCTCTCCCTATAGTGAGTCGTAT
TAGAATTCCCC
+
CCBCCB@BC?BBBB>B>@>@BB@B>>><@BB@ABBBB@BBCDBBBCCCCB?A===:?>?CC?CECC?=?CAAAD::;
?BFE@ECCCC9
@T6XXI:00131:00882
GGGAGAAGGGAGAAAAGGAAGCTAGTCACTCGAGATCTGCGGCTTTGGACAGTCCCTCTGTACTGTGAAGCCACAGA
TGTCGAGTCGACTAGCTTCCTTTCTCCCTTCTCCC
+
;<8<11-
/857/448,4242477;;B??9666667;66:<C:;AD8:::C@A???6777447;665=?B<==8<=;<<<<?===
=88887>><?@9;;@99879,/,,+000
@T6XXI:00131:00888
GGGAGAAGGGAGAAAGGAAGGAGAATGCACCGCAGGAAATTGCAAGACTATTCTCCCTCTCCCTATAGTGAGTCGTA
TTAGAATTCCCC
+
DE?CCB?CCBDCCD>B?B@B@BBA?BBCCB@B???>??;B?B>=>>@@@??>@@@B>?<???7>?<<<?????<==A
@>@AC?A?AAA6
@T6XXI:00131:00895
GGGAGAAGGGAGAAAGGAAGCTAGTCGACTCGACCATCTGTGGCTTCACAGTACAGAGGGACTGTCCAAAGCCGCAG
ATCTCGAGTCGAC
+
DD?BBB99>0999>:><@<>??:00/.,,****,*,,,,,,54<?>?=BBBBA??;444,...---
0110323356855377888;;766
@T6XXI:00131:00899
GGGAGAAGGGAGAAAGGAAGGAGAAGTCGCCCCAAACGATTTGTTGACTATTCTCCCTCTCCCTATAGTGAGTCGTA
TTAGAATTCCCC
+
CB>BCC?CC>@@AC@C@C@B?BBB?ABC@@>>8@C?CCC?C<AA?BBBBBCAAA==;88<?C=BAAA???A@@@@@@
A>AA@<>3444,
@T6XXI:00132:00851
GGGGAATTCTAATACGACTCACTATAGGGAGAGGGAGAATAGATCTTCTCGTCCGAGCTTGCTATTCACCTTCCTTT
CTCCCTTCTCCC
+
```

```
CCD:FCC@CBCABCBBB=>>BC<99311.7==?@>BCC?BBC@;::477?AAB?BBBCC@BBBBB@BBB>@>@@CC>
<<=C>A>?ABE:
@T6XXI:00132:00852
GGGGAATTCTAATACGACTCACTATAGGAGAGGGAGAATAGTCAACAAATCGTTTGGGGCGACTTCTCCTTCCTTTC
TCCCTTCTCCC
+
CHD:DBE@CAB@CBA@??>??:9990/,0@;>A>ACC>@>>;<<;<<<;BBACE;@@@7A@@A@>@AA@C@C?A@;=
AAA;@>@@@A;
@T6XXI:00132:00859
GCGGCTTTGGACAGTCCCTCTGTACTGTGAAGCCACAGATGTCGAGTCGACTAGCTTCCTTTCTCCCTTCTCCC
+
E<<;>BF?C@CAAADCD@CDBBBCCDCBBC>AA@CCCCC@7::CC@CBB@CCDA?A?A?AA@ABBB8?5::BB>
@T6XXI:00132:00861
GGGGAATTCTAATACGACTCACTATAGGGAGAGGGAGAATAGCTGTCCCGCCTACTGAGGTTACTTATTCTTCCTTT
CTCCCTTCTCCC
+
EDC9C=A>ADCBCB@ABB>>?CB99311.7==BC@EDC@C@>6??:::5:A>BDAAA<<;<><<@>==;<<<=>AA<
@AAA<=;=?::;
@T6XXI:00132:00868
GGGAGAAGGGAGAAAGGAAGCTAGTCGACTCGACATCTGTGGCTTCACAGTACAGAGGGACTGTCCAAAGCCGCAGA
TCTCGAGTCGACTAG
+
DC?D@@8;;7<BBC>C?B?BBA;97/00::::086;6177>;?@=@CCCAAAB@9300-
4;;:<?@AA<AA=@@AAAC?:::??A???AAA?
@T6XXI:00132:00875
GGGAGGAAAGGGGAGAAAAGGG
+
:A?<969D1999/91,22022*
@T6XXI:00132:00877
GGGAGAAGGGAGAAAGGAAGGAGAAGTCGCCCCAAACGATTTGTTGACTATTCTCCCTCTCCCTATAGTGAGTCGTA
TTAGAATTCCCC
+
CFBA@?@CC@?A?B=B>B>B@CCC>B;?<???8??;???>B<ABAAABCCB?<<<:4=?>??<?AAAAAAB@@?@@@
@>@AC>@;><<1
@T6XXI:00133:00860
GGGGAATTCTAATACGACTCACTATAGGGAGAGGAGAATAGTCAACAAATCGTTTGGGGCGACTTCTCCTTCCTTTC
TCCCTTCTCCC
+
;99/<?B@CBC@BB@A@BB?BBB?<761./:00./@@AC>BA==<9=97@A===-666-
:@ABC?CCC>@>=?AB=A???;?<??AA<
@T6XXI:00133:00866
GGGGAATTCTAATACGACTCACTATAGGGAGAGGGAGAATAGTCAACAAAATTCGTTTGGGGCGAACTTCTCCTCCT
TTCT
+
9@?2@?9:=4924>>@A@@@???>8811,16111,166068@<9440000+31377>9@>>6>9989;763333000
0*++
@T6XXI:00133:00873
CTATCACTCGAGATCTGCGGCTTTGGACAGTCCCTCTGTACTGTGAAGCCACAGATGTCGAGTCGACTAGCTTCCTT
TCTCCCTTCTCCC
+
B@;B9:::=99=??@@;:;>ACC@D@A>??777.7>>B@BBB8::@<@G@BBCCBBBDBDCDBCB;;;;7785788A
;C>>=:=37100+
@T6XXI:00133:00878
GGGAGAAGGGAGAAAGGAAGAGAATAGAAATTTGGGTAAATGTGTGACTATTCTCCCTCTCCCTATAGTGAGTCGTA
TTAGAACTTCCC
+
```

```
CIBCAA=AC>B@C?097959@885;>?==9=B<BB>BBC>AAACCCAB@??=??CC7::>>=7=;888837?644=7
73/884//,--+
@T6XXI:00133:00879
GGGAGAAGGGAGAAAGGAAGAAGAATAGGACGGATGTGGCACGATCGCTATTCTCCCTCTCCCTATAGTGAGTCGTA
TTAGAATTCCC
+
BB@AAA@AA?@@@B>B@A@AA@??>BBB>???>>@A?@>?@?A===::=AA=A77717??<=;==;====;??@@@@
?=AAA:B5990
@T6XXI:00133:00898
GGGAGAAGGGAGAAAGGAAGCGGCAGTCGGTGCTGAGGAGAGGATGGCTATTCTCCCTCTCCCTATAGTGAGTCGTA
TTAGAACTTCCCC
+
DE?DCBCCC>;<<B>B@C@CCC?CCBBBC@CBBBB@@?BBBB?A??;???=<=88818===?:@???AAA@A@=<<<
>;===8;979990
@T6XXI:00134:00850
CTATCGACTCGAGATCTGCGGCTTTGGACAGTCCCTCTGTACTGTGAAGCCACAGATGTCGAGTCGACTAGCTTCCT
TCTCCTTCTCC
+
@11//////7;999BB@>99688?8?<>?>>876-
116=@??@CCBB=>A=@@AABAAAA????;;;A@B??<:;9:9:00,/,06;3
@T6XXI:00134:00861
GGGAGAAGGGAGAAAGGAAGGAAGGAGAACAACACGATCGGCTAAACGTAGCTATTCTCCCTCTCCCTATAGTGAGT
CGTATTAGAATTCCCC
+
A=:@CCACC@@A?B=B=@=@@B:;<;BE@CC@BBBCCCCC?BBBC>CCCAAA7604444313<<?@<@998A=<@AA
?????;9;<3727@@6
@T6XXI:00134:00863
GGGAGAAGGGAGAAAGGAAGTGCAGTTCGCGGTTAGTGAAGGGAAAAACTATTCTCCCTCTCCCTATAGTGAGTCGT
ATTAGAATTCCCC
+
CFBBBA@CC@CCBB=B@CABACCEBB?AEBC996:;@@A>AC=BBBB2BCCC@CCCC7999::5:CBAA@ABAA@??
?A@AAA=?8;;;0
@T6XXI:00134:00873
GGGGAATTCTAATACGACTCACTATAGGGAGAGGGAGAATAGTCAACAAATCGTTTTGGGGCGACTTCTCCTTCCTT
TCTCCC
+
;;;,45424:;24444443777974...+...5537/601113344000+331111,17727661101110174/--
+-122+
@T6XXI:00134:00875
GGGAGAAGGGAGAAAGGAAGCTAGTCACTCGACATCTGTGGCTTCACAGTACAGAGGGACTGTCCAAAGCCGCAGAT
CTCGAGTCGACTAGCTTCCTTTCCTCCCTTCCTCCC
+
FFBCCB?A?:?884-
95959BA@>;700......./69?@8:<@CCCCCCBBBB@A?8>>????>AA:@A=BBA@@A=====<?@????<;<
<:<9;C:>598?:;3858<@:
@T6XXI:00134:00892
GGGGAATTCTAATACGACTCACTATAGGGAGAGGGAGAATAGTCAACAAATCGTTTGGGGCGACTTCTCCTTCCTTT
CTCCCTTCTCCC
+
=AA6A?B@CDC@BBBBBBBB>A;99311.7>>BC@AAAAC???AC><@<;BA>9988885?ABBDABBB=?=??AA<
AAAA<@=??AA=
@T6XXI:00134:00894
GGGAGAAGGGAGAAAGGAAGCTAGTCACTCGACATCTGTGGCTTCACAGTACAGAGGG
+
66561119C@@===+-*-*,247740....533...1111713131661111771000+
@T6XXI:00134:00897
```

```
GGGAGAAGGGAGAAAGGAAGCTAGTCGACTCGAGATCTGCGGCTTTGGACAGTCCCTCTGTACTGTGAAGCCACAGA
TGTCGAGTCGACTAGCTTCCTTTCTC
+
FCB@9959>:>499142844B?@?A9:;ACCACAEJLCA?;:767.7:=?::;CB>DD?898@?;<=B=<=@@A@AC
====A===A@>88..,+03,--+-34
@T6XXI:00134:00899
GGGAGAAGGGAGAAAGGAAGGTGAACAGGTAACGGAGGGTGCGTTAACTATTCTCCCTCTCCCTATAGTGAGTCGTA
TTAGAATTCCCC
+
CCBCCC@CDBA@@A=B>A@B@CBC@CBB@BC@BB>A@B<CBA??>>=@ABBABBBB=>>@CD>CBCC??<CGCCCA?
?>@AA=@3884+
@T6XXI:00135:00862
GGGGAATTCTAATACGACTCACTATAGGGAGAGGGAGAATAGTCAACAAATCGTTTGGGGCGACTTCTCCTTCCTTT
CTCCCTTCTCCC
+
944,9<@@BDG@B>>>>B?BBC?99311.7=7775:BC@DA@>??>BAC:BBAA?;CCC8AAABC@???5933:=:7
====7>9<<@@<
@T6XXI:00135:00863
GGGGAATTCTAATACGACTCACTATAGGGAGAGGGAGAATAGTCAACAAATCGTTTGGGGCGACTTCTCCTTCCTTT
CTCCCTTCTCCC
+
A??7@5;8<CC@BAAAAA?BBB?73377.7?>@C>BCB@?>>9AC?@@@<CCA?986665????E@CCC?B?B@AA=
A===9=>@AAA<
@T6XXI:00135:00884
GGGGAATTCTAATACGACTCACTATAGGAGAGGGAGAATAGTCAACAAATCGTTGGGGCGACTTCTCCTTCCTTTCC
TCC
+
/11*/,4244424;976100666433137/5::6///77//.8494--+....,.3304599?88/6:774411+-
,.11
@T6XXI:00135:00894
GGGGAATTCTAATACGACTCACTATAGGAGAGGGAGAATAGCTCA
+
:@B9B@>;11/,///88>BCCBBAA;;8;B>@:9:/.,.4.,,..
@T6XXI:00136:00855
GGGAGAAGGGAGAAAAGGAAGCTAGTCGA
+
991449244-44444,48878;74460,,
@T6XXI:00136:00865
GGGGAATTCTAATACGACTCACTATAGGAGAGGGAGAATAGCCCAACGAGTTGTTGTCCCGGCTTTATCTTCCTTTC
TCCCTTCTCCC
+
ADB6A>B>BBC?CDBCCCCBBAAAA>:;?A??A<AB@>?>744-
49>ABBB@CB77333,44;?C=@?AA@C?>?:==;;6;9<;<;:
@T6XXI:00136:00870
GGGAGAAGGGAGAAAGGAAGGAGAACGCGCACCCGAACCAACGGTGATCTATTCTCCCTCTCCCTATAGTGAGTCGT
ATTAGAATTCCCC
+
CCBDDC>?>:====:@@B@CCCA??BECCBBBB?BCBC?A?BD@CAAAB;;;?A;::8<BBCC>CCCB@?B@BCCBA
AA?CCB=BBCCC7
@T6XXI:00136:00873
GGGAGAAGGGAGAAAGGAAGTGCAGTTCGCGGTTAGTGAAGGGAAAAACTATTCTCCCTCTCCCTATAGTGAGTCGT
ATTAGAATTCCCC
+
HHBACA@CC>C@@@<@?A@BBBECGCCB9;;=@=;;<BBDBC6:;;;0;CDE@AA@B?BBCCC>???AAAA@?@><;
58;=@A><:@CC6
@T6XXI:00136:00875
```

```
GGGGAATTCTAATACGACTCACTATAGGGAGAGGGAGAATAGTCAACAAATCGTTTGGGGCGACTTCTCCTTCCTTT
CTCCCTTCTCCC
+
ABB7CAB?BDCABBBBBBBBBC>99311.7==?B>BCD?A@@<>=:;;;4>?666-
777.8=??C@CBB=?=<>@@;A???<?>A@AA<
@T6XXI:00136:00878
CTAGTCACTCGAGATCTGCGGCTTTGGACAGTCCCTCTGTACTGTGAAGCCACAGATGTCGAGTCGACTAGCTTCCT
TTCTCCCTTCTCCC
+
>://///44?ABBABCBBBA=???;B@BB???@@=@@@?>>>?@@@@>;B@BBBBB@@ABCCCCCGBBCAAB@<?=?
C>CBBB>B?BBCC>
@T6XXI:00136:00885
GGGAGAAGGAGAAAGGAAGGAGAAGTCGCCCCAAACGATTTGTT
+
CCB111-1,/666+/,49:78>?;8143333,39793311,1.,
@T6XXI:00136:00888
GGGGAATTCTAATACGACTCACTATAGGGAGAGGGAGAATAGTCAACAAAATCGTTTGGGGCGACTTCTCCTTCCTT
TCTCCCTTCCTCCC
+
FCC:C>A=<<;<@@A@;;;??A??8877.:@BCDAK;:7:>>;4434444+8<777-
111,71;6<:777;=:=443+443733,.,,,,+
@T6XXI:00136:00889
GGGGAATTCTAATACGACTCACTATAGGGAGAGGAGAATAGTTTTACC
+
1111<>B@CCC@B>>>AA???BCB>766-1=<<:?@>;>7,*......
@T6XXI:00136:00899
CTAGTCACTCGAGATCTGCGGCTTTGGACAGTCCCTCTGTACTGTGAAGCCACAGATGTCGAGTCGACTAGCTTCCT
TTCTCCCTTCTCCC
+
:/1//81::HFEBBDBCCAA=@DD>B=@????@A=ACCCCC???CBA@8<9<DBABCCBA@@=79999877?>;<;=
C<?9;@.7371884
@T6XXI:00137:00859
GGGGAATTCTAATACGACTCACTATAGGGAGAGGGAGAATAGCTCTCAAGAGTTTCGCCTCCCAATTCTCCTTCCTT
TCTCCCTTCTCCC
+
AAA6C?BABCC@BBBBB??@A@??>777.7>?BD>BAA>ABB@E?@?B?BCAA?9?;;8ABD>C@B?B::7:?B?CC
>FBCC>C<:8@C1
@T6XXI:00137:00863
GGGGAATTCTAATACGACTCACTATAGGAGAGGGAGAATAGCCACCAATATTC
+
444,42424445448884333333318>>?BC>?8;<7710/00442112,-
@T6XXI:00137:00868
GGGAGAAGGGAGAAAGGAAGGAGAATAGAATGCCACCAAGGTCTAAGCTATTCTCCCTCTCCCTATAGTGAGTCGTA
TTAGAATTCCCC
+
BB?BCB@AC>BBBC>C@F@C@BCC@CB::7:BB=@@BC@C?AABB@CCBBB=?@DE>=<<AA<AA@@@@==A<8<=?
@=?AC>A;AAA6
@T6XXI:00137:00869
GGGGAATTCTAATACGACTCACTATAGGGAGAGGGAGAATAGTTTTCCCTTCACTAACCGCGAACTGCACTTCCTTT
CTCCCTTCTCCC
+
999/98>=ACFCCBB@B@B?BBB?;316.7>>BC?BAB>ACB??@4<<7<<?BCCC@C?A>A?@@@<<@A@>>8::8
:ABC>A?@@AA<
@T6XXI:00137:00875
GGGGAATTCTAATACGACTCACTATAGGGAGAGGGAGAATAGTCAACAAATCGTTTGGGGCGACTTCTCCTTCCTTT
CTCCCTTCTCCC
+
```

```
:99/9>B?CCC@AAAAAA??@C@>7316.7==@A>AAA?C@A=AA>>863;?<>@<ABB9BBAAC@CBB?B?B>??;
=A@=;@:==AA<
@T6XXI:00137:00883
GGGAGAAAGGGGAGAAAAGGAAAGAATATGTTGAGGTAAGAGGAGCGCGAACTATTCTCCCTCCTCC
+
AC6@999-4;;244499,4244-/=547.0/,,,00,.,.45528<<=?><><7723044-00/-22
@T6XXI:00138:00876
CTATTCTCCCTCTCCCTATAGTGAGTCGTATTAGAACTTCCCC
+
BB@@<@9990;;;AD?DBBBB@A?BBB?:::;=882663677,
@T6XXI:00138:00881
GACTCGAGATCTGCGGCTTTGGACAGTCCCTCTGTACTGTGAAGCCACAGATGTCGAGTCGACTAGCTTCCTTTCTC
CCTCTCCC
+
CCDCC=<9B94444424996??BCFCCCC@DCCC@@=><<01,11;:9;=?;68:::=?@@:;7;678<<,01,000
0,0.3330
@T6XXI:00138:00882
GGGGAATTCTAATACGACTCACTATAGGAGAGGGAGAATAGTACGACA
+
ECB9A=@>ACC@BBBBBBBBB9664..,.736>;A@::7,(((((((*
@T6XXI:00138:00883
GGGAGAAGGGAGAAAGGAAGCTAGTCGACTCGACATCTGTGGCTTCACAGTACAGAGGGACTGTCCAAAGCCGCAGA
TCTCGAG
+
EEBCBB899-
8=9=:@@C8<;C@@@CCCC<<>@@AB>@ABC=<@@B??;;??@C999;486<8895664<@>@B<@::<AA<<<
@T6XXI:00138:00891
GGGAGAAGGGAGAAAGGAAGCTAGTCGACTCGACATCTGTGGCTTCACAGTACAGAGGGACTGTCCAAAGCCGCAGA
TCTCGAGTCGACTAG
+
CC?CCC>>=59444-
9595;F?:::.000//90:6888CCC?@@@CCCB??<??=;;;<<===AA;999=@>AA@@=<@@A<=@A@==<B@@
@T6XXI:00138:00892
ACTCGAGATCTGCGGCTTTGGACAGTCCCTCTGTACTGTGAAGCCACAGATGTCGAGTCGACTAGCTTCCTTTCTCC
CTTCTCCC
+
AAAAACDCCBBABC@BBB=B=?@CBCCC@CDFCCCDBAAAC@CDACCDB@@@A<<<CCD@A@BBBAAAC=@@<@ABC
<A<?8880
@T6XXI:00138:00899
GGGGAATTCTAATACGACTCACTATAGGGAGAGGGAGAATAGTCAACAAATCGTTTGGGGCGACTTCTCCTTCCTTT
CTCCCTTCTCCC
+
CFFAB;<;<@=BCBABCCBBA@>>>877.7==??:7::66BB@@A=844-
87<<>6???5?@@AAAACB@D@?<??7<9;?9;0//;;2
@T6XXI:00139:00853
GGGGAATTCTAATACGACTCACTATAGGGAGAGGGAGAATAGTCAACAAATCGTTTGGGGCGACTTCTCCTTCCTTT
CTCCCTTCTCCC
+
CCC9C@BDCBC=A@BBBCCBBB>97366.7====9?BB?BAC@@@??@@<BBBBB>BBB9BBBCC@BBB=?=????;
@A@A;@388>>:
@T6XXI:00139:00856
GGGAGAAGGGAGAAAGGAAGGAGACACGGTCACAAACATGAAATGT
+
991944244-444=-429<9997399>92444444-4:4444-373
@T6XXI:00139:00873
GGGGAATTCTAATACGACTCACTATAGGGAGAGGGAGAATAGTCAACAAATCGTTTGGGGCGACTTCTCCTTCCTTT
CTCCCTTCTCCC
```

```
+
BEHFH>A@@@A?BBBBCCCCC?>>?877,7===A>CCB?BCCA@?<<<<7@CBFC>CCC5?777737<><?:=443+
3003,0/0-332
@T6XXI:00139:00887
GGAGAAGGGAGAAAGGAAGGAGAAGTCGCCCCAAACGATTTGTTGACTATTCTCCCTCCTCCCTATAGTGAGTTCG
+
1,/77,//+4>>>:><>4;8;;87><>><<<2<?/83311,6>9=@?@>@5408;,3388==5:<887:88//,//
@T6XXI:00140:00864
GGGAGAAGGGAGAAAGGAAGGAGA
+
DDBCCD@BA=AB8A8822*,+-,0
@T6XXI:00140:00879
GGGGAATTCTAATACGACTCACTATAGGGAGAGGGAGAATAGTTCCCCCGTCCTCAGAAATAATGTTTCCTTCCTTT
CTCCCTTCTCCC
+
999/4:@?CBB@CBBBBBBBBBB<9311.7>>@A>CCC?B>A?=@CCC1@@@?@@BBAA;@??A??>1;8<>AACC@
CCBB<><??==6
@T6XXI:00140:00884
GGGAGAAGGGAGAAAGGAAGCTAGTGACTCGAGATCTGCGGCTTTGGACAGTCCCTCTGTACTGTGAAGCCACAGAT
GTCGAGTCGACTAGCTTCCTTTCTCCCTTCTCCC
+
44-
4:@;@@<@AAA<@=>>@@@@BCCABAAAACCB?;:<C=?@C>C?A???A@@:@7444::=<88C@AA>@AA====<=
=>;883303485?<;;>@688BA-42/../+
@T6XXI:00140:00886
GGGGAATTCTAATACGACTCACTATAGGAGAGGGAGAATAGCCCAAGGTCTTAGTTGCCGCGTGCGTTCTCCTTCCT
TTCTCCCTTCTCCC
+
CFF6B=@<>>?99;BBBBAA@?>>>875:BABC=@@@=>>744+0/0/0038;=>;>@=<<<;<78858//,/5843
75..7?7:740--+
@T6XXI:00140:00888
GGGAGAAGGGAGAAAGGAAGGAGACAGCCCCAGAGTACAGTCCAGAACTATTCTCCCTCTCCCTATAGTGAGTCGTA
TTAGAATTCCCC
+
DHBCCC@CB?BBBB?C@C@B>AA?AADCLGABBBBBBBBDCCC?BBB?BCCDGB???;?B@CC>?@====AAAA??<<
=<>>B;=5>==1
@T6XXI:00140:00892
GGGAGAAGGGAGAAAGGAAGGAGACAGTGAGCGCCGCACAACACGTCACTATTCTCCCTCTCCCTATAGTGAGTCGT
ATTAGAATTCCCC
+
AE>ADA89909>>>:@=@=@=@@ABGECABBBCC?@B@@@@CCCAAACCBB@=@@@@<@A@AA<ACC<<<@A@?888
>;3?87;>4844,
@T6XXI:00141:00857
CTATCACTCGAGATCTGCGGCTTTGGACAGTCCTCTGTACTGTGAAGCCACAGATGTCGAGTCGACTAGCTTCCTTT
CTCCCTTCTCCC
+
A=?A994449;9>?BBA==;787/848??B??=?BFB?@?B9AA@38<;?>?9838373666@@A>@>>><===?A<
??@@<C?BB@A:
@T6XXI:00141:00866
GGGAGAAGGGAGAAAGGAAGGAGAAGTCGCCAACCGAACTTGA
+
FFBCCC@BB=?BBCBA?B@C?9:;BCBA;:48<;,..,..,..
@T6XXI:00141:00870
GGGAGAAGGGAGAAAGGAAGGGGAGAAGGGAGAAAGGAAGCTA
+
4;:;995991899;-4242444,43313763311,17701113
@T6XXI:00141:00872
```

```
GGGGAATTCTAATACGACTCACCTATAGGAGAGGGAGAATAGTTGCCATTTTACATGCGTGCCTATTCTC
+
?;;2A59698488>>;<10...,..331016111,1770116320;4;;;;276611111601110166
@T6XXI:00141:00887
GGGAGAAGGGAGAAAGGAAGAGAATACGCAATCCAGGCAGGAAAAAGCTATTCTCCCTCTCCCTATAGTGAGTCGTA
TTAGAATTCCCC
+
CDBCCC@CCB@@@B=B@B@CB@@=@CCCCC?BB@AB?B@?>@CCC2;;;CC;????9???BCCAAA@@@A898A@@@
@=??A?A8<<<4
@T6XXI:00141:00898
GGGGAATTCTAATACGACTCACTATAGGGAGAGGGAGAATAGCTTGCAGCTCGAGCCTGTGACTGTTCTCTTCCTTC
TCCTTCTCCC
+
8:80=1969>9;@A@@CCAAB@>>>877.::BBC>C;;.0..-
5333711761166368677<<=9B8;5523/3/00423/3444+
@T6XXI:00141:00899
GGGAGAAGGGAGAAAGGAAGCTAGTCGACTCGACATCTGTGGCTTCACAGTACAGAGGGACTGTCCAAAGCCGCAGA
TCTCGAGTCGACTAG
+
DF@@==;9=9>988096959B>;;>.00::::0818:8ABEA?>?>/11@@ABC?88818>@@AA>@@:@A>A@=<<
<<<<==;:?@@AA=>
@T6XXI:00142:00853
CTAGTCACTCGAGATCTGCGGCTTTGGACAGTCCCTCTGTACTGTGAAGCCACAGATGTCGAGTCGACTAGCTTCCT
TTCTCCCTTCTCCC
+
B:1/111:?CCCCCCBBBBC@CBB>B?CBBB:9:5:CCCCCCA?BCC@=A@BDDBBBBCCCCCBCCC@@ADCC@C@D
C>BBDC>B37744-
@T6XXI:00142:00858
GGGAGAAGGGAGAAAGGAAGCTAGTCACTCGAGATCTGCGGCTTTGGACAGTCCCTCTGTACTGTGAAGCCACAGAT
GTCGAGTCGACTAGCTTCCTTTCTCC
+
BDACA=644-
999909;>;@@?B@C???@DCCBABCBCBB@@CA>@>A@?>>>>;?>::;=<==<<@?<=;==<<<<=<;::=CC@?
?????B?@>AA<@A=;
@T6XXI:00142:00868
GGGAGAAGGGAGAAAGGAAGCTAGTCGACTCGACATCCTGTGGCTTCACAGTACAGAGGGACTGTCCAAAAGCCGCA
GATCTCGAGTCGACTAG
+
EJ?A@A=>>:>9991424247333;://:<000.3..,/566;:@=@?=>=>0006///,444484,.--*-2,---
.4033=3305555:<??
@T6XXI:00142:00872
GGGAGAAGGGAGAAAGGAAGGAGAAGTCGCCCCAAACGATTTGTTGACTATTCTCCCTCTCCCTATAGTGAGTCGTA
TTAGAATTCCCC
+
DDBC?=@CCBBBBBB=B>BDB@BBB@B@B<<<<7>C>CGB?>9?@@?=99>?@=<<<;99=AA;<?@@A=9====<<@
<;A@=:;3444,
@T6XXI:00142:00880
GGGGAATTCTAATACGACTCACTATAGGGAGAGGGAGAAATAGCTCTAATCCTCCGCCCGCG
+
:::,:24:;>>24488443???@B<<;D@B;<:@9>777,17101699777700/000+000
@T6XXI:00143:00864
GGGAGAAGGGAGAAAGGAAGGGAACAGTCCTACACTCGACCTGCAGGCTATTCTCCCTCTCCCTATAGTGAGTCGTA
TTAGAATTCCCC
+
GEDBCB@CC>BBBB=B@B@BA<B@BBBBB>BABCCCCBBB?@@@::8:BCBCCBBB=@@ACC@BBBCCCCCCB???A
A?ACA>A<AAA5
@T6XXI:00144:00852
```

```
GGGAGAAGGGAGAAAGGAAGTGCAGTTCGCGGTTAGTGAAGGGAAAAACTATTCTCCCTCTCCCTATAGTGAGTCGT
ATTAGAATTCCCC
+
DHEB??=@@<@AAA>B=@=ABBCCCC@BABB?B?ABBBB>;9,3778,9@AB@CBBB>AAABB>BAAB?C==AAA@?
==;=AC?D>@A@5
@T6XXI:00144:00887
GGGGAATTCTAATACGACTCACTATAGGAGAGGGAGAATAGTCTTGCAATTTCCTGCGGTGCATTCTCCTTCCTTTC
TCCCTTCTCCC
+
BCC:C@B?ABB@CCCCCCCCB>=66..,09:>=<@??ACBC???<>;?<AB>CDBBBC@BBBCB@?;;58586880;
?@@<A?:::B9
@T6XXI:00144:00895
GGGAGAAGGGAGAAAGGAAGTGGGTCGCACGTAACGTGATTTGGGAGCTATTCTCCCTCTCCCTATAGTGAGTCGTA
TTAGAACTTCCCCA
+
FFBC:;8;@:?9991;;?;@BA@<BCCCAA@@@7<;;BBCC?BD@A?B>=<8<9?A<;;:>B<>>>>9767661116
63334/113666,1
@T6XXI:00144:00898
GGGAGAAGGGAGAAAGGAAGGAAGGGAGAAAGGAAGCTAGCGACTCGACATCTGTGGCTTCACAGTACAGAGGACTG
TCCAAAGCCGCAGATCTCGAGTCGACTAG
+
CCBDCC@CC=?@@A:><><>495;B?CCCC?C=@>???CDCDCC@@@A@DDA@A?B>775::4:=B==:://,/446
475<99=C=A??===@@@@988>==:<;<
@T6XXI:00145:00850
GGGGAATTCTAATACGACTCACTATAGGAGAGGGAGAATAGTTTTTCCCTTCACTAACCGCGAACTGCACTTCCTTT
CTCCCTTCTCCC
+
C??6?EBACECBCDCCCBB@B;>>@8737=7775;@@?ACC@ABB084-
49;@@BC?@=::::6:A@AECC@F=?=6=777-7:===@:
@T6XXI:00145:00857
GGGAGAAGGGAGAAAGGAAGCTAGTCACTCGACATCTGTGGCTTCACAGTACAGAGGGACTGTCCAAAGCCGCAGAT
CTCGAGTCGACTAG
+
CC@BBA?A@:A@@=:@=?<@C@?@@B???@A;::::9@BC?AC@CC????@@>966647>AAA>>AA:AA=@?<<<=
;;:89=AA?:<><A
@T6XXI:00145:00859
GGGAGAAGGGAGAAAGGAAGAACATGCGGTGTCAGGAGAGTGATGAGCTATTCTCCCTCTCCCTATAGTGAGTCGTA
TTAGAATTCCCC
+
CCFBBB@CC?CBBB=BBB@BB@BBBBCC>ABBBCC@CBCB>>??@@===A@?B??@<989AC;@@@@AAAA???@@A
A:=@D?D=AA@6
@T6XXI:00145:00860
GGAGAAGGAGAAAGAG
+
954442424///+///
@T6XXI:00145:00861
GGGGAATTCTAATACGACTCACTATAGGGAGAGGGAGAATAGTGACGTGTTGTGCGGCGCTCACTGTCTCCTTCCTT
TCTCCCTTCTCCC
+
999,49BBEBC@CA>>>BBBBBBB;7665:??CE>BBB>BA@@?==AAA;:6::<<:<<<<==<==<<??<??B>AA
;;@@B<A><@A@<
@T6XXI:00145:00867
GGGGAATTCT
+
444,42/,//
@T6XXI:00145:00879
```

GGGAGAAGGGAGAAAGGAAGTCGACATCTGTGGCTTCACAGTACAGAGGGACTGTCCAAAGCCGCAGATCTCGAGTC
GACTA
+
EFCAA?@BC>:<<C>C?C?@@AB;:<BBBBBB@CC@CB>>>;?ABCBBB>B@@@AA?AB<AC;@@B@AAAAA<==?<
;;CBB
@T6XXI:00145:00887
GGGGAATTCTAATACGACTCACTATAGGGAGAGGGAGAATAGTCAACAAATCGTTTGGGGCGACTTCTCCTTCCTTT
CTCCCTTCTCCC
+
999/929<ABC@BBB?ABAAABB>93165:?>CE@A@@>C???AA;<<<:>B9<<;>>>9CA?AB?AAC?A?ADAA<
@@AA<A>A@AA;
@T6XXI:00145:00889
GGGAGAAGGGAGAAAGGAAGCTAGTCGACTCAACATCTGTGGCTTCACAGTACAGAGGGACTGTCCAAAGCCGCAGA
TCTCGAGTCGACTA
+
DEBA@=6440999909;><??@::=3/,22,,*..4435:@>>@@C<<;BBCCCC:::7:==?@@>CC;=<699@@A
ABB@?<<873734;
@T6XXI:00145:00899
GGGAGAAGGGAGAAAGGAAGCTAGTCGACTCGACATCTGTGGCTTCACAGTACAGAGGACTGTCCAAAGCCGCAGAT
CTCGAGT
+
CDBAA?==99>98809<>69;C@B@CCC@:;:@@@???@A@??@=@CCCCB@?CC8427@>>?@>@A=AA<===@@C
<?@@A@A
@T6XXI:00146:00856
CTAGTCACTCGAGATCTGCGGCTTTGGACAGTCCCTCTGTACTGTAAAGCCACAGATGTCGAGTCGACTAGCTTCCT
TTCTCCCTCCTCCC
+
111////44;;4444449487333,313333...+...333377111,0;244866...4..17;444444887;<>
;:>4440..,,,,*
@T6XXI:00146:00859
GGGAGAAGGGAGAAAGGAAGGAGAAGTCGCCCCAAACGATTTGTCGACTATTCTCCCTC
+
9:6@?@=@A<>>>>0959287><<77698777-39:<>?9;816111111100444-3;
@T6XXI:00146:00861
GGGGAATTCTAATACGACTCACTATAGGGAGAGGGAGAATAGCC
+
499,42499@A79<BA;;;;;CC<<1?9>000/,07750,,+*
@T6XXI:00146:00867
CTAGTCACTCGAGATCTGCGGCTTTGGACAGTCCCCTCTGTACTGTGAAGCCACAGATGTCGAGTCGACTAGCTTC
+
;11////44:;::==;;9>>=88803137773111,16111;99:979:5@;;988:77773711///8....344
@T6XXI:00146:00874
GGGGAATTCTAATACGACTCACTATAGGGAGAGGGAGAATAGTCAACAAATCGTTTGGGGCGACTTCTCCTTCCTTT
CTCCCTTCTCCC
+
BBB9BCCABBB@CAAA??<??BB?<311.7@@CL>BAA;?AAA<<;?<:4>?666-666-
8>>@B?BBC@B?B@AA:>>AC;A689AB<
@T6XXI:00146:00889
GGGGAATTCTAATACGACTCACTATAGGGAGAGGGAGAATAGCTACGTTTAACCGATCGTGTTGTTCTCCTTCCTTT
CTCCCTTCTCCC
+
BBB9B?CBDCB@BBBBBBBBBCCC<;??9???BC?BCC@B??;?BCCDD=@>@?AAABB@@@?AA>@??<?>@=?@<
@@@A<A=@<<;6
@T6XXI:00146:00898
GGGGAATTCTAATACGACTCACTATAGGAGAGGGAGAATAGAGACGTATTCGTTTGCGCTCTGTTCACCTTCCTTTC
TCCCTTCTCCC
+

```
CEE9C?CBDCB?BCCCB?@>B9;;=00-
0<:BD<??CAC?@=??AA@==9=<?@<???A@???>;:74288<=@C<@@AC<A:===A<
@T6XXI:00147:00860
GGGGAATTCTAATACGACTCACTATAGGGAGAGGGAGAATAGATCACCGTTGGTCGGTGGTGCG
+
999/4:ABEBB?BCCBBA??BB>97316.7A?BD@AAA>@C<6693107=:=>=--+--+,*0*
@T6XXI:00147:00869
GGGAGAAGGGAGAAAGGAAGGAGAAGTCGCCCCAAACGATTTGTTGACTATTCTCCCTCTCCCTATAGTGAGTCGTA
TTAGAATTCCCC
+
CDEAA?@BD@BCBB?DDC?A?BBB@AAB@@@A8@C>CCBBB>BC?CB=?CC@=9994<??@A<@@@@A===:==A<<
<;AAB?B3844,
@T6XXI:00147:00879
GGGGAATTCTAATACGACTCACTATAGGAGAGGGAGAATAGCTAAACGCTGTTTGCAACCCTCTTACTCTTCCTTTC
TCCCTTCCTCCC
+
BBB6A@@>ABBBDBBCCB?>>>9=60/-
0;;BC>CCC>CC@=:<?;?BCCC>>;?>>=AC>CC>;>>>A=;=;<>;<:8424,-,///,
@T6XXI:00147:00884
GGGGAATTCTAATACGACTCACTATAGGAGAGGGAGAATAGACACACGTTATGTCTACTCTTGCTGCCCCTTCCTTT
CTCCCTTCTCCC
+
DFC:H?A>ACC@CBCCCBBB>AA@@:63777?A?BCB@CBB?@A@@@CC????A???BBBJBBBA??8;2<;>477.
7:;;:@588?A<
@T6XXI:00147:00895
GGGAGAAGGGAGAAAAGGA
+
CEBA94299194999,42,
@T6XXI:00147:00896
GGGGAATTCTAATACGACTCACTATAGGAGAGGGAGAATAGTCTTGCAATTTCCTGCGGTGCATTCTCCTTCCTTTC
TCCCTTCTCCC
+
CFF:C@C@ABA@CBB@?ABBB:996..,0;;?C>CBB?B?B?A=?==@>BB>BCCCBB@CCABB@CBB>A>A?CC?C
CCC@@>?ABC<
@T6XXI:00147:00897
ATACGACTCACTATAGGGAGAGGGAGAATAGTCAACAAATCGTTTGGGGCGACTTCTCCTTCCTTTCTCCCTTCTCC
C
+
AAABCBBBCBC@@9433/8@@BE@BA?BBA>@DFA@@@>@BBBB89991;>@@C?AEB@B@C;??;???@>B?AABE
>
@T6XXI:00148:00850
GGGAGAAGGGAGAAAGGAAGGAGAAGTCGCCCCAAACGATTTGTTGACTATTCTCCCCTCTCCCTATAGTGAGTCGT
ATTAGAATTCCCC
+
DDDFDC@CC>?@@>:A>B>B>BBB?BCBBBBB8BB67>877,113661777443333+49444,038;;??<=<788
;<9<???A;@@@5
@T6XXI:00148:00851
GGGAGAAGGGAGAAAAGGAAGCAT
+
CCB@>=599184899,42/,/...
@T6XXI:00148:00856
GGGAGAAAGGGAGAAAGGAAGCTAGTCAGCGAGAT
+
B>:9499-4;:9=BC?A?A@>>90******,,22.
@T6XXI:00148:00863
GGGGAATTCTAATACGACTCACTATAGGGAGAGGGAGAATAGTTTTTCCCTTCACTAACCGCGAACTGCACTTCCTT
TCTCCCTTCTCCC
```

```
+
999/429@DCC@BBB?BBCBBC?99316.7>>BE>BAB>@CA;=>;,44-
88;@DDD@C?BBB@??B?ACBB?C@BB>AAAC>@?AA?B<
@T6XXI:00148:00867
GGGGAATTCTAATACGACTCACTATAGGGAGAGGGAGAATAGTTTTTCCCTTCACTAACCGCGAACTGCACTTCCTT
TCTCCCTTCTCCC
+
BA;6==A?BCCBCBBBAABBBB99<777-
7<<;;2961=@@A====1=85@9=@CBB?B?@??A@::8>?==<>477.7?BB>C@A?;;4
@T6XXI:00148:00878
GGGGAATTCTAATACGACTCACTATAGGGAGAGGGAGAATAGCCATGCCTCCAGCTGCTTCTCCGCAATCTTCCTTT
CTCCCTTCTCCC
+
CCC:C@B@BBC@C?B?BBCBBB;99311.7>===<???@FBBB?@<??<???AA;;66699?=:;99;?==;=;<<;
====3523444-
@T6XXI:00148:00884
GGGAGAAGGGAGAAAGGAAGGAGAAGTCGCCCAACAACGTATTGAT
+
GC>@A@>AA>A@AC<=:<@@6;:7;;;AA::8>=>6,.....,,..
@T6XXI:00148:00892
GGGAGAAGGGAGAAAGGAAGGTGAACAGAGCGCAAACGAACACGTCTCTATTCTCCCTCTCCCTATAGTGAGTCGTA
TTAGAATTCCCC
+
FD?CB=@BB?ABBD?B=?=??CBC@CCCDBBB9998;BB@CCCEC@BBBDC@C@?=:9===><@CCCBABACBCC@@
@@BCB?CACCC9
@T6XXI:00149:00850
GGGGAATTCTAATACGACTCACTATAGGAGAGGGAGAATAGTCAACAAATCGTTTGGGGCGACTTCTCCTTCCTTTC
TCCCTTCTCCC
+
AAA8A?C@CCCACCBBBA@AB:696..,0;;?C>CBB>@@@<<?>=<53<?611,667-
8?@@B@BAA?AACAAB<A@AA<A;==@@;
@T6XXI:00149:00864
GGGGAATTCTAATACGACTCACTATAGGAGAGGGAGAATAGTCAACAAATCGTTTGGGGCGACTTCTCCTTCCTTTC
TCCCTTCTCCC
+
?=A8A@GBCBB@CBB?BBBB?:996..,0::=?=@DC@C;;@==;8=84@BB??5:66-
:89:B>AAC@B?BBAA<A@??;@;==@C=
@T6XXI:00149:00880
GGGGAATTCTAATACGACTCACTATAGGAGAGGGAGAATAGTCAACAAATCGTTTGGGGCGACTTCTCCTTCCTTTC
TCCCTT
+
944,424:;BCBAAACDBBCC;9970/,0><?B;??A7;7?6;;8<@>:A@777./5500..3:5;6@=966330+0
11101,
@T6XXI:00149:00884
GGGGAATTCTAATACGACTCACTATAGGAGAGGGAGAATAGTCAACAAATTGTTTGGGCGACTTCTCCTTCCTTTCT
CCCTTCTCCCGGGATTCTAATACGACTCACTATAGGAGAGGAGAATAGATCACGAACCTTACCTGCATC
+
??A6ACCACCC@CAAABBBB?:999..,0:0775:CB>B?9844/466,0,..5+..+373444433,.,/,./,/6
996<=;;;;6:8,/-2..-,0446568;999AABB;==44344423;>99<:55320/0200/004400
@T6XXI:00100:00916
GGGGAATTCTAATACGACTCACTATAGGGAGAGGGAGAATAGTCAACAAATCGTTTGGGGCGACTTCTCCTTCCTTT
CTCCCTTCTCCC
+
DIFCG?@?ADDBB@BAAA???CB>9311.777==<?A@?BBA?AA>@@@:BA??@9???5?AAAF@CCB?B>B?AC:
?@BA=A8<<AA<
@T6XXI:00100:00917
```

```
GGGGAATTCTAATACGACTCACTATAGGGAGAGGGAGAATAGTCAACAAATCGTTTGGGGCGACTTCTCCTTCCTTT
CTCCCTTCTCCC
+
DEE:C?B@BDC@BBABBBBBBBB><766.6@=?A=ACC@@?;9AA=ABA>BBBC@;AAA7AB@@?>ACC?A=?<==;
;?AA<A=?@AA;
@T6XXI:00100:00922
GGGGAATTCTAATACGACTCACTATAGGGAGGGAG
+
CCC9A;=;==::==999=;?;9777766,6..+..
@T6XXI:00100:00927
GGGGAATTCTAATACGACTCACTATAGGGAGAGGGAGAATAGCACAAATGAATTCGGG
+
999/;:CACBB@CBBBBBCCCCCBA799.6=;<?9?@@=AAA<5344+--,00*,,,*
@T6XXI:00100:00929
GGGAGAAGGGAGAAAGGAAGGAGAACAACACGATCGGTTAAACGTAGCTATTCTCCCTCTCCCTATAGTGAGTCGTA
TTAGAATCCCC
+
FFBA@@@CC>BBCC>B>@=?@BCC@BB>FBBGCCDC@C@BBAFBA@ACBBB?BBCC>BAACC>@=ABBB=@@A?===
A?AA@?@AAA6
@T6XXI:00100:00934
GGGAGAAGGGAGAAAGGAAGAACAAACGGTCGCTGGGAATCGAGACGCTATTCTCCCTCTCCCTATAGTGAGTCGTA
TTAGAATTCCCC
+
ECBCCC@CCBBAAD>B@B@BA>AAA<BD@CCBAABF>C@BBBCCCBBAACCBCECD@BCCCC>BBBCCCCC@@@CAA
A@CDE@B6::7-
@T6XXI:00100:00935
GGGAGAAGGGAGAAAGGAAGGAGAGATACGGGCTAACTGGTGCGCATCTATTCTCCCTCTCCCTATAGTGAGTCGTA
TTAGAATTCCCC
+
EDBCCB>?>:=====B>B?B@CDBB?BBBBC>@CC@BBC@BAABBBBBB@?=?CBC>BBBBB?@@BBBBBCCBBBAA
?>BBA?C?BBB9
@T6XXI:00100:00943
GGGGAATTCTAATACGACTCACTATAGGGAGAGGGAGAATAGCTACCGTCCGTTCCCTGCTATGCTGTTCTTCCTTT
CTCCCTTCTCCC
+
9:4-9:CACCC@CCCB??ACCCA@<:995:BBCC>B@?;?B?>ABB?AA@=>=340+09?BBAAAA@@=AA=@384-
4:::4;<779@;
@T6XXI:00100:00948
GGGGAATTCTAATACGACTCACTATAGGGAGAGGGAGAATAGCCGCAACCTTACCAACACGAGCATTTTCTTCCTTT
CTCCCTTCTCCC
+
CDC:C@B@ADN@BA@AABDBBB;77311.7==>@>BCE?C@@=;@@C?B?B@CC=?==CCBBAAABDC9@@=?=??<
???C>C?C@@?;
@T6XXI:00101:00901
GGGGAATTCTAATACGACGTCACTAATAGGGAGAGGGAGAATGAGTCAACAAATCGTTTGGGGCGACTTCTCCTTCC
TTTCTCCCTTCTCCC
+
449/421,///,////444.644//,/...+3374;9::>:0./7566<==A6:97796?;<6<BBDD?@?<3737:
=??BA?@7==?<<<7
@T6XXI:00101:00909
GGGGAATTCTAATACGACTCACTATAGGGAGAGGGAGAATAGTCAACAAATCGTTTTGGGGCGACTTCTCCTTCCTT
TCTCCCTTCTCC
+
BBB6><@?AAA?;;;;<@?@BB@?>877.7?==?9?A><???>@C?@@?4>>7777,7>>2ACCCC?BB@?@=?A?@
:@;<;498;443
@T6XXI:00101:00928
```

```
GGGGAATTCTAATACGACTCACTATAGGAGAGGGAGAATAGCGCTACAATTTGTAATCCGTTTATTCTCTTCCTTTC
TCCCTTCTCCC
+
EED9A6;9;CB@BBBBBBABB9;6;0/,000>?>BBB?BCC<@@=@?B>??<A@@=??<?BB>BC@AA==;==@@<@
@@C<A>@@@?7
@T6XXI:00101:00929
GGGAGAAGGGAGAAAGGAAGGAGAAGTCGCCCCAAACGATTTGTTGACTATTCTCCCTCTCCCTATAGTGAGTCGTA
TTAGAATTCCCC
+
CEFCCD?BB=BBBB>B@C@A@A?B>?>=;;??8??;?BB>B=>?=>=BBCC?====;=9<<?:=<<=@@A@@@?878
<<?;;79/444-
@T6XXI:00101:00935
GGGAGAAGGGAGAAAGGAAGCTAGCGACTCGACATCTGTGGCTTCACAGTACAGAGGGACTGTCCAAAGCCGCAGAT
CTCGAGTCGACTAG
+
CC>CCD=??>@DBC?B>A>ABBBBBB?AAC@99:AAABCC@BB?AB@@@@C@?;66647<9>=?9;;6;=:>?@@AA
???=99<<@=:A==
@T6XXI:00101:00936
GGGGAATTCTAATACGACTCACTATAGGGAGAGAATTGGTAATGGGTCTAGGCAGGGGAGTTGGATACGGGGGCCTT
ATATCTCTCTCCCTATAGTGAGTCGTATTAGAATTCCCC
+
DIE:CCB>;487994CAABB@@@@CCCG@100:<,0,.5..,77>6>=78353777758110177733.....*-,-
344688;=<>744+066::／/./1111-0/356:=;===4
@T6XXI:00101:00938
GGGAGAAGGGAGAAAGGAAGCTAGTCACTCGACATCTGTGGCTTCACAGTACAGAGGGACTGTCCAAAGCCGCAGAT
CTCGAGTCGACTAG
+
DEBCB@694-
9999096959B>>?B600:::00/=66>@D=?@?BB@??@@@@<6::8688<==<=<;?A?A@<<=========<<<
<<<=
@T6XXI:00102:00913
GGGAGAAGGGAGAAAGGAAGTGCAGTTCGCGGTTAGTGAAGGGAAAAACTATTCTCCCTCTCCCTATAGTGAGTCGT
ATTAGAATTCCCC
+
EE?CCB@BA:A>??>@@C@B@?BCCC@CB@B@CAAAA@A<@?6;;??0?BDE@BBAC>BBBCC>BAA@?CAAAA@@5
89>@AA>C?AAA5
@T6XXI:00102:00920
GGGGAATTCTAATACGACTCACTATAGGGAGAGGGAGAATAGCCACAATCGTGTTGCGTTGGCATCTCCTTCCTTTC
TCCCTTCTCCC
+
BBB7CACBCCC@B??B?CBA@AB<<311.7@@CE>CBB@@@=688<<8;;74/../>>A<@?@BCCCC@CGE?AAAA
CCC>D@D:875
@T6XXI:00102:00926
GGGGAATTCTAATACGACTCACTATAGGGAGAGGGAGAATAGTCAACAAATCGTTTGGGGCGACTTCTCCTTCCTTT
CTCCCTTCTCCC
+
;<<4<@CBC??@BBBBBBBBBB>99311.7@@BE>AAA>?@7544,/-
2297<<@;>@@7AABDCABCB@C@C@AA<@@AC<A889A@;
@T6XXI:00102:00940
GGGAGAAGGGAGAAAGGAAGCTAGTCGACTCGACATCTGTGGCTTCACAGTACAGAGGGACTGTCCAAAGCCGCCAG
ATCTCGAGTCGACTAG
+
CC?ABB?BB=AB@>=B=@<@C<79;/00;::;>??BBAAAD>AB@CBAA?AA=74000,7=;?AC<::68;9//./5
5//0>@@AAAA@@@@A
@T6XXI:00103:00900
GGGAGAAGGGAGAAAGGAAGGAGAAGCGACGTCGGGGAAACGTTTGCTATTCTCCCTCTCCCTATAGTGAGTCGTAT
TAGAATTCCCC
```

```
+
CD>BBC?CC@AA?B>B@A@B@B?@>@BBBBB93333-
9A?BBDCBBDCBB?ACDC>BBCCC?CBBBBB?CCCBAA??;?=>;>;?@@5
@T6XXI:00103:00916
GGGAGAAGGGAGAAAGGAAGTGCAGTTCGCGGTCAGTGAAGGGAAAAACTATTCTCCCTCTCCCTATAGTGAGTCGT
ATTAGAATTCCCC
+
FFBB?=@AA?A@BC>B?BDBBACBBB@A===ABBBBBBB@BB=ABAA2BCBB@AAAA;BBBBB<????;?@@CC=99
??<?AA>AB@A@5
@T6XXI:00103:00917
GGGGAATTCTAATACGACTCACTATAGGGAGAGGGAGAATAGTCCGCCAATTCTGCGCGTTAGCATTCTCCTTCCTT
TCTCCCTTCTCCC
+
<<9/9<@=@CA?AB:;<BABA@>><877-
7>>BD>BBB?B??@A?===B@B@@BBCCBBB?CCCBB@BBAAC?B>BC<BBCC>B:;733-
@T6XXI:00103:00920
GGGGAATTCTAATACGACTCACTATAGGGAGAGGGAGAATAGCTACAACCTTGACCGCACTGCTACTATCTTCCTTT
CTCCCTTCTCCC
+
EAA9B@B@CBB@BBB@A?BBBBB><31159>?BC>CCC>;??=????>@>A>AAC?B@??????BB@AAAAAA?AA<
@@??;>;;???;
@T6XXI:00103:00926
GGGGAATTCTAATACGACTCACTATAGGGAGAGGGAGAATAGTCACATTCATGCTTGTGACCGTGTCTCCTTCCTTT
CTCCCTTCTCCC
+
AAA6ACB?BCD@BBABBBBBBCB>>311.7==BC@CBB>B?@<<<<<B@B?@?BB;;;??>>999>BCC@C=?;?@<
@CCE>A?@AAA<
@T6XXI:00103:00930
GGGAGAAGGGAGAAAGGAAGCTAGTCGACTCGACATCTGTGGCTTCACAGTACAGAGGGACTGTCCAAAGCCGCAGA
TCTCGAGTCGACTAG
+
FE?CCC=?>:@=99-
95;<AAB8887111...........459??A?ACCDDCC899A<@===BA<??:>>7;;<<<?@@A???@AA@@@AA
@T6XXI:00104:00904
GGGGAATTCTAATACGACTCACTATAGGGAGAGGGAGAATAGTTCTGGACTGTACTCTGGGGCTGTCTCCTTCCTTT
CTCCCTTCTCCC
+
999/48>;=>98=@@DDCC>>=;;0000+07377686626;8559<@>CBBCCC@@@B=?@6A?@@CBB?B?B?A?/
7777.7;7777-
@T6XXI:00104:00920
GGGGAATTCTAATACGACTCACTATAGGGAGAGGGGAGAAATAGTTCCCTGT
+
BA@4=8?599499=9<<BA@?@;:::6>9???A??2>777,6..1013+--1
@T6XXI:00104:00922
GGGAGAAGGGAGAAAGGAAGCTAGTCGACTCGAACATCTGTGGCTTCACAGTACAGAGGACTGTCCAAAGCCGCAGA
+
::7<DD5;;79>>>:?<@9;;AABB@??>;77;,0///.5333677;ABBAABCC?7427?===?9==7<A<<==?@
@T6XXI:00104:00929
CTAGTCGACTCGATGA
+
B<1////*,,,,****
@T6XXI:00104:00932
GGGAGAAGGGAGAAAGGAAGAGAATAACAGTGCACCGAATGCGCAAAACTATTCTCCCTCTCCCTATAGTGAGTCGT
ATTAGAACTT
+
AA?ADCBCG>>99919595;CCC?CB=@@@@@@@C?CB@CAA@9999/9>9954877277766,1111111644...
445033,..,
```

```
@T6XXI:00104:00936
GGGAGAAGGGAGAAAGGAAGAATATCGCCGCGTGGAAGAGATGTTGCTATTCTCCCTCTCCCTATAGTGAGTCGTAT
TAGAATTCCCC
+
BB?BCB@BB?BCBB>C@B@BB=>??BB?@??@@@>A@BCCCC::8;?BBC@CBDD>AAAAA<AA@@@@@@988=?>>
;=>@8;55<=1
@T6XXI:00104:00946
GGGAGAAGGGAGAAAGGAAGGGGAGAAGGGAGAAAGGAAGCTAGTCGACTCGACATCTGTGGCTTCACAGTACAGAG
GGACTGTCCAAAGCCGCAGA
+
FDDCHH@AA=@@@B=B@B69:91;?A<AA<@;;50;:=-
01?>>>@BBB=B9638888<AA>@A=@@AB@@=<;>77717?>A@@>AA<@@>@A<<<
@T6XXI:00105:00905
CTAGTCACTCGAGATCTGCGGCTTTGGACAGTCCCTCTGTACTGTGAAGCCACACATGTCGAGTCGACTAGCTTCCT
TTCTCCCTTCTCCC
+
=;1////::DCFCBC??ABB@BAA=A@BBBACB@=@CA??>?>@@??57;@CCCCBBBAAAB>@BBC@@@CCD=@=?
B>BBCC>B@BBAA<
@T6XXI:00105:00927
GGGGAATTCTAATACGACTCACTATAGGGAGAGGGAGAATAGTTACCATTTTACATGCGTGCCTATTCTCTTCCTTT
CTCCCTTCTCC
+
EBA6@5469>9>9949997<78883311,1111799?>;?>0/,//,-
244477755...444499<=<:844/00+00444;:511,
@T6XXI:00105:00941
GGGGAATTCTAATACGACTCACTATAGGGAGAGGAGATAGTAGAC
+
---*-8A?CDC@CB@A@BBB>BBBB97757?7;:=//,,,++--2
@T6XXI:00106:00902
GGGGAATTCTAATACGACTCACTATAGGGAGAGGGAGAATAGTCAACAAATCGTTTGGGGCGACTTCTCCTTCCTTT
CTCCCTTCCTCCC
+
IEIBGBCACCC?@@<;;9;??;;:BACCAAA>><6:00,.774<<;???7AA=>>.777.8==773777=>==4371
322222,-,/./+
@T6XXI:00106:00904
GGGGAATTCTAATACGACTCACTATAGGAGAGGGAGAATAGTCAACAAATCGTTTGGGGCGACTTCTCCTTCCTTTC
TCCCTTCTC
+
999,9595944244999@?@;;8;;8737>777.7>@>A?BC==;=AA<AAAAA9@@@487???<?A@<>;>444,4
03448/.++
@T6XXI:00106:00910
GGGAGAAGGGAGAAAGGAAGGAGAAGTCGCCCCAAACGATTTGTTGACTATTCTCCCTCTCCCTATAGTGAGTCGTA
TTAGAATTC
+
CE?EEC@CB>@AAD>B?B?B?BBA@?>?8888.39697;8986958A@@CL@B88818;;;A5883?999;843-
///..4333,-
@T6XXI:00107:00900
CTATCGACTGAGATCTGCGGCTTTGGACAGTCCCTCTGTACTGTGAAGCCACAGATGTCGAGTCGTACTAGCTTCCT
TTCCTCCC
+
68:;9444449==:=CBBB>BBB<B<<;DCCCC<?;00/:::01160042444=:;<<;>>>011///.6.,,2433
3+.,.55+
@T6XXI:00107:00904
GGAGAGTCTGACAGGCTTTGGACAGTCCCT
+
1,111////64444899;-424499444-4
@T6XXI:00107:00913
```

```
GGGGAATTCTAATACGACTCACTATAGGAGAGGGAGAATAGTCCAGCGCCCGTTGAAACGTAATTATTCTTCCTTTC
TCCCTTCTCCC
+
?B?4>2959A=>DBBBCDCC:00=;00,/::>@>CCC@CD;99>?AA===7<A<???<BCCC?B?BE@AA:=333+0
344-47:444-
@T6XXI:00108:00906
GGGGAATTCTAATACGACTCACTATAGGGAGAGAGATATAAGGGAGATCTTTGGTTTTCTGTGCACGTCACATGCCC
CATTACCAAATTCTCTCCC
+
A??7AAC@D@?=AB@@@BCBBCCA???C>@?;;;;>??==;==;=99@CCC>C?B?@8CC@A<>>?BCBBBBCBBBB
6@B6::8;E>E@BA?;77.
@T6XXI:00108:00908
GGGGAATTCTAATACGACTCACTATAGGAGAGGGAGAATAGCCGCACCAGCGTTGACAATTTATTCTTCTTCCTTTC
TCCCTTCTCCC
+
CCC:C>@>?BCAAA@@@A??BCBBB762:@@@C>CCC>BB>=<==?==?ABCC@CAAA?BB=BA>AA>@A>@>@B<@
@A>;A=?:<=;
@T6XXI:00108:00914
GGGAGAAGGGAGAAAGGAAGCTAGTCGACTCGACATCTGTGGCTTCACAGTACAGAGGGACTGTCCAAAGCCGCAGA
TCTCGGACATCTGTGGCTTCACAGTACAGACGGATGTCCAAAGCCGCAGATCTCGAAGTGCTAG
+
CC@BBA?A@:@@==:@=?=@BBBCCBCDCAAA:::ABBBCC@BG?B???BACBB?33317>>?@??CC;>@=@B<<@
=?:5537784;;;44/34/38=>?<49::///77733323;4:<:<=<<:<966//,////334
@T6XXI:00108:00921
GGGAGAAGGGAGAAAGGAAGCTAGTCACTCGACATCTGTGGCTTCACAGTACAGAGGGACTGTCCAAAGCCGCAGAT
CTCGAGTCGACTAGCTTCCTTTCTCCCTTCTCCC
+
IICAAA?BB<CCBB>B?A>A?88-
8,,,,,,,,,.9<999>:;9;A@BBBAAAB?@@788@@CB@CH;?A=??<<<A<<=A@??A?=<=<<@@>A>@C<A@
@A183/:;92
@T6XXI:00108:00934
GGGAGAAGGGAGAAAAGGGAAAGGTCATAGCTACGACTCGA
+
A?9>99599149999,88-44-42433333397744400,,
@T6XXI:00108:00937
GGGAGAAGGGAGAAAGGAAGGAGAAGTCGCCCCAAACGATTTGTTGACTATTCTCCCTCTCCCTATAGTGAGTCGTA
TTCAGAATTC
+
DDBCBB?BB>?B@@;C8:;<CCCC@CCC@@@@6@B>CCA@@=@C@CBBBAA>>===:=834=4??@???@@@@@=<<
7,////,-,+
@T6XXI:00108:00940
GGGAGAAGGGAGAAAGGAAGGAGAAGTCGCCCCAAACGATTTGTTGACTATTCTCCCTCTCCTATAGTG
+
BB?CCC@CB>BBBCAB?C?B@BBB>;6977;>8AD>BBBA@887=9;8::<;86432----,-222440
@T6XXI:00108:00941
GGGGAATTCTAATACGACTCACTATAGGGAGAGGGAGAATAGTCAACAAATCGTTTGGGGCGACTTCTCCTTCCTTT
CTCCCTTCTCCC
+
499-99@@CBCACCBBBBBBBB<97311.7>>CL?BBB>?@?=<::<<;5?D;<<-
777.8>@AC@CBC@@==?BC>A?@?;@<??AB=
@T6XXI:00109:00902
GGGAGAAGGGAGAAAGGAAGGAGAAGTCGCCCCAAACGATTTGTTGACTATTCCTCCTCCTCCTAT
+
CE?BCB?BC>BBBA=B@C@C@BBB;<;@BBDB7AA=ACCCC<B>A?99?BC?=;<=8=>93*)*)(
@T6XXI:00109:00913
GGGGAATTCTAATACGACTCACTATAGGAGAGGGAGAATAGTCTCTCGTCCGGCGTCAGTGCTATTCTCTTCCTTTC
TCCCTTCTCCC
```

+
499/95959A9;=@BABBC;<;B??8747=>?B>AAA=?@A??A@A???@>B8:9BBAAAA@@@@===::24344-
4;;;4:34444-
@T6XXI:00109:00927
GGGAGAAGGGAGAAAGGAAGCTAGTCACTCGAGATCTGCGGCTTTGGACAGTCCCTCTGTACTGTGAAGCCACAGAT
GTCGAGTCGACTAGCTTCCTTTCTCCCTTCTCC
+
DDBBB=@BC?B9996;>@>BBBAAB7::ABLAAACABB?B==?C=@?BCCCCCC>A>:??@CA=@<@===<=8=@@@
@@???AA@@;;??A?A>?=?B:;;;=7=;??A?
@T6XXI:00109:00934
GGGAGAAGGGAGAAAGGAAGCTAGTCGACTCGACATCTGTGGCTTCACAGTACAGAGGGACTGTCCAAAGCC
+
FDB@A@>AA<AABB>B@C@CCAAAA=??BCBAACBCC@?@C:;;@CCCBCCB@>;744-4;;;;>344-4;8
@T6XXI:00109:00936
GGGAGAAGGGAGAAAGGAAGCTAGTCACTCGACATCTGTGGCTTCACAGTACAGAGGGACGTGTCCAAAGCCGCAGA
TCTCGAGTCGACTAGCTTCCTTTCTCCCTTCTCCC
+
AA<@A@=@B:?<<;89AA><;<@>8,,,,,,,,,,58:<?<AB@;::A::7:77///,////..39;;9;A>@@@@@
A<<;<<<887@888:AABC?AA<@888149444>:
@T6XXI:00109:00949
GGGGAATTCTAATACGACTCACTATAGGGAGAGGGAGAATAGTGCACCATTCTCCTTTACGTAACGCCGTCTTCCTT
TCTCCCTTCTCCC
+
A@@4?<@=ADDABB???BBBAAABBA??>?BA@?;??>99A?==ABB>>@@DCC?@@>CCCCC?CB<;@@??9<:=C
???CC<@=@8771
@T6XXI:00110:00908
GGGAGAAGGGAGAAAGGAAGAACCGGCCGCGGGAGTTAAAATAACGCTATTCTCCCTCTCCCTATAGTGAGTCGTAT
TAGAACTTCCCC
+
DIBCCC@CD@BCBB>B>@>AA>A=A=@<>???:=??4777.0<:000=@@6<;BB>?@99989@?A@A@BBC@>>==
476>:>>>@@@7
@T6XXI:00110:00909
GGGGAATTCTAATACGACTCACTATAGGAGAGGGAGAATAGCAAACGTTTCCCCGACGTCGCTTTCTCCCTTCCTTT
CT
+
B9?4>6969@@<499=BBBBBBCCC:737>777.767;>::5785///663366///=00/;9:60//50344,--
+-0
@T6XXI:00110:00926
GGGAGAGGAGA
+
//+////,,*,
@T6XXI:00110:00934
GGGAGAAGGGAGAAAGGAAGCTAGTCGACTCGAGATCTGCGGCTTTGGACAGTCCCTCTGTACTGTGAAGCCACAGA
TGTCGAGTCGACTAGCTTCCTTTCTCCCTTCTCCC
+
EB@B<959@:@999195954??;;@/00=@>>B@CCBBBBC>BBB=B>>>;????;??=::;==:;<@@=<=?AAA<
???AAA:??A<>>A99=<?>AA<A@@@:?8;;=>7
@T6XXI:00111:00907
CTAGTCACTCGAGATCTGCGGCTTTGGACAGTCCCTCTGTACTGTGAAGCCACAGATGTCGAGTCGACTAGCTTCCT
TTCTCCCTTCTCCC
+
681//6/44??AABBBBB;:6;AA<B<;<DDCCB=BCCCA<AB@CCC@@CACCCBBABBCDCC@AABBCDC@@=?@C
D>?@BC>C?BAAA<
@T6XXI:00111:00910
GGGAGAAGGGAGAAAGGAAAGCTATCGACTCGACATCTGTGGCTTCACAGTACAGAGGGACTGTCCAAAGCCGCAGA
TCTCGAGTCGACTAG
+

```
AA<@BB?CD@CCCB:C54419?@@BCB;::A?771111666267@?<<B?<611>366,000004233356/34;<=
@AAAAA?==888;<<
@T6XXI:00111:00919
GGGGAATTCTAATACGACTCACTATAGGGAGAGGGAGAATAGTTCCGAGTTTTTCCCGATTCGTTGTATTCTTCCTT
TCTCCCTTCTCCC
+
:::2:292999<ABDCDBBB:88>8866,666==997:=A@?<8979;44443+00+066365346=@@;>@<?>AB
<@@@@5731;==8
@T6XXI:00111:00921
GGGGAATTCTAATACGACTCACTATAGGGAGAGGGAGAATAGTCAACAAATCGTTTGGGGCGACTTCTCCTTCCTTT
CTCCCTTCCTCCC
+
9;</9<BACCC@CBB?BBBBBCB>7311.6>77:7:CC@CAB?AA=??H?BDBBB>BEE8BBBBE<;<B?BAB?A@;
A@>=9=3737;A7
@T6XXI:00111:00932
GGGGAATTCTAATACGACTCACTATAGGGAGAGGGAGAATAGCCAATAACTCTTTGGCCCGTGTTTCTCCTTCCTTT
CTCCCTTCTCCC
+
0@@0@?B?BBC?CDBBBBBBBBBB79:<6;=7776:??=??;43473323333306367992222+//55555-
04+0044+044444-
@T6XXI:00111:00937
GGGGAATTCTAATACGACTCACTATAGGGAGAGGGAGAATAGCGTTGAACTTTCCTGTCCCCACGTATTCTTCCTTT
CTCCCTTCTCCC
+
CCC:C@C@BBB@AAAAAA???BB99311.7>>AD?BBB>@@C<::;?C@B@?;CAB>7877,7?BABB@CC@C?@@<
@AAA;A?=@@=;
@T6XXI:00111:00941
GGGAGAAGGGAGAAAGGAAGGAGAACAACACGATCGGTTAAACGTAGCTATTCTCCCTCTCCCTATAGTGAGTCGTA
TTAGAATTCCCG
+
:E?=?=599144995888997478 79@;=@>;994428548587>BBBBBB88:CC=@A:61,17166<=<747787
3:=;898:==65
@T6XXI:00112:00903
GGGAGAAGGAGAAAGGA
+
490///,/,///7+/,4
@T6XXI:00112:00924
GGGGAATTCTAATACGACTCACTATAGGGAGAGGGAGAATAGATCACCGTTGGTTCGGGTGCGCGTTCTCCTTCCTT
TCTCCCTTCTCCC
+
==92=>B?BBBBCCCCCAAABAA<>877.77777-
66699BB<==@?=??5:7:>CBCBC=<;@@><A>><<BA>?A>ACCC@D?A@>=7
@T6XXI:00112:00926
GGGGAATTCTAATACGACTCACTATAGGGAGAGGGAGAATAGTTTGGACGTCTCCCAGTCCTCGTTACGCTTCCTTT
CTCCCTTCTCCC
+
??=6@?C@CCCABB?@BBB>>BB??76657BBCC>CCC;>>?==:;;@@A@???C<@@BB>?@@A>??<><:B588-
49996<9<@DC:
@T6XXI:00112:00929
GGGGAATTCTAATACGACTCACTATAGGGAGAGGGAGAATAGTCAACAAATCGTTTGGGGCGACTTCTCCTTCCTTT
CTCCCTTCTCCC
+
AAA6C?B?BCC@BBB@?A?>BBB?;311.7==AC?CDD?B@CA=;;=<87@@??C9???5??AAAD@BB>A><;<=;
A@AA<A689CC;
@T6XXI:00112:00931
CTATCACTCGAGATCTGCGGCTTTGGACAGTCCCTCTGTACTGTGAAGCCACAGATGTCGAGTCGACTTAG
+
```

```
C===;:::CCCCBCBAABB@??A=A?AB?>7::::CEC??B@44.-,.1:<988;;;??A:7711,-,*,,
@T6XXI:00112:00937
CTAGTCACTCGAGATCTGCGGCTTTGGACAGTCCCTCTGTACTGTGAAGCCACAGATGTCGAGTCGACTAGCTTCCT
TTCTCCCTTCTCCC
+
=8///6/44BBBCCCBBBBB=>B?<B;BBBB9777:@?A@>=B>AAA?;B?BCCBCBBCCCBBBBBBCBCCAA?B?B
C>BABC>B?AAAC=
@T6XXI:00113:00907
GGGGAATTCTAATACGACTCACTATAGGAGAGGGAGAATAGCCAAATCCCTTCTTCCAGGTGGCTCTCCTTCCTTCT
CCCTTCTCCCTATTAGTCATTCC
+
944,4292;=@>ABBGDDBBB:88:00,/.,,,*,00*...1,--+0443:;<=<C>AA><<8<85333301,-,--
222346555094/,.14//-011
@T6XXI:00113:00911
GGGAGAAGGGAGAAAGGAAGGAGATATAGACACGCCAATAGAAACGCGCTATTCTCCCTCTCCCTATAGTGAGTCGT
ATTAGAATTCCCC
+
?E?;B@694-
9@<<8<;>,1,/:9;4877=>714;24248433,39997:;;==>>>;::;BA:?????ABBA???:::?A@@=B=>
==4
@T6XXI:00113:00927
GGGGAATTCTAATACGACTCACTATAGGGAGAGGGAGAATAGCCGCACCAGCGTTGACAATTTATTCTCCTTCCTTT
CTCCCTTCTCCC
+
BB@4A=@=@CC@CBBBBAABBBBB?711.7>>?A>BFF@CC?<8;8;7:==116:;7168??9?A>@@A<?<?@CC?
B?A?<?=@@AD=
@T6XXI:00113:00935
GGGGAATTTCTAATACGACTCACTATAGGGAGAGGGAGAATAGTCAACAAATCGTTTGGGGCGACTTCTCCTTCCTT
TCTCCCTTCTCCC
+
AAA6@>BB@BBBBCAAABBBBBC?99311.7==>@>CCC@C?;<==<<?<<AC7=7-111-
8>ABC@CCC?BAB@@@;@@@A<A=@@AA<
@T6XXI:00113:00937
GGGAGAAGGGAGAAAGGAAGGAGACAGTGAGCGCCGCACAACACGTCACTATTCTTCCTTTCTCCCTTCTCCC
+
GA:?>@59919>>><B>CCE=@@CDCCBBBBCBB;;;C@?=?@:::ABBBBFBCC?C?CC?CCCC>A?BC@@=
@T6XXI:00113:00944
GGGGAATTCTAATACGACTCACTATAGGGAGAGGGAGAATAGTCCTCGTCTGA
+
BCC9B@CABBB@BBBBBBBBB@7<<777.7==>>9???:=></,++0+++,*
@T6XXI:00114:00906
CTAGTCACTCGAGATCTGCGGCTTTGGACAGTCCCCTCTGTACTGTGAAGCCACAGATGTCGAGTCGACTAGCTTCC
TTTCTCCCTTC
+
B<9448///1888:;BCDBA@??>8>;77773111,16666:<@A<9B78<?@@>@@:33<7766984879776363
6817/5535,,
@T6XXI:00114:00910
GGGAGAAGGGAGAAAGGAAGGACAGTCCATCTGTGGCTTCACAGTACAGCA
+
B?>A<<;;BAA@@@<@=@<=<=733342337311888>>77?/0...4.--
@T6XXI:00114:00917
GGGAGAAGGGAGAAAGGAAGCTAGTCGACTCGAGATCTGCGGCTTTGGACAGTCCCTCTGTACTGTGAAGCCACAGA
TGTCGAGTCGACTAGCTTCCTTTCTCCCTTCTCCC
+
B?:@ABABC>A@@>:>6969@>???777777@B@BBA??>>;777-
7;>>ABAAA=AB???AAA???B?AA=??@AAAA888>??899@?@???>:;AA9<<AB>C68:AA<
@T6XXI:00114:00927
```

```
GGGGAATTCTAATACGACTCACTATAGGGAGAGGGAGAATAGTCAACAAATCGTTTGGGGCGACTTCTCCTTCCTTT
CTCCCTTCTCCC
+
BEA6A797<BB@CCCCBBBBBB>99311.777?@<ABB@C=@=@A?<==9@CCCC>BBB8BCCCC=@@D=?<==@@;
?ACC=A<@@@@<
@T6XXI:00114:00930
CTAGTCACTCGAGATCTGCGGCTTTGGACAGTCCCTCTGTACTGTGAAGCCACAGATGTCGAGTCGACTAGCTTCCT
TTCTCCCTTCTCCC
+
=7/////44CBCBABBCBBB?ADD@B?BBBC:::8:BBCC@B@@CCC?@CABABBBCDDBBBA?@@B@@?;;7;=;=
E>CBBC@D;;;CC>
@T6XXI:00114:00947
GGGAGAAGGGAGAAAGGAAGCTAGTCACTCGACATCTGTGGCTTCACAGTACAGAGGGACTGTCCAAAGCCGCAGAT
CTCGAGTCGACTAGCTTCCTTTCTCCCTTCTCCC
+
DA=C><<<A:@999095959B>>>:00/.......59;@@8;<@@?BB;::@>>4:758;>@@A>@A<@A>AA???>
<<<????>??@999AA>A?AA:AACD@A?==<@;
@T6XXI:00115:00906
GGGAGAAGGGAGAAAGGAAGCTAGTCACTCGACCATCCTGTGGCTTCACAGTACAGAGGGACTGTCCAAAGCCGCAG
ATCTCGAGTCGACTAG
+
CC@CBB>==69==@<@?A>@@@@??7000/000,...,.55>A@B@BAAAABAB??877.4448AA>@@<?@<<<<<
?@@A@A@@ABAA===<
@T6XXI:00115:00907
GGGAATCTAT
+
//+/,/////
@T6XXI:00115:00920
GGGAGAAGGGAGAAAGGAAGAAGATAGAAGGACGTTACATGTCACAACTATTCCTCCCTCTCCCTATAGTGAGTCGT
ATTAGAATTCCCC
+
6@8<<@69918944-
42424;7;>?@@B>B8<<?C@LCCCACBAAA>><;600/0464:==<<4<======??8;;:44/489=9;>>A6
@T6XXI:00115:00941
CTAGTCGACTCGAGATCTGCGGCTTTGGACAGTCCCTCTGTACTGTGAAGCCACAGATGTCGAGTCGACTAGCTTCC
TTTCTCCCTTCTCCC
+
B7--,-
,,//7;BCBCBBB996<BB>C<;7>@:;;8:CCCCAABBBAB>;@=BAAAA;;;??@?@ABBBBAA@;>B4::5:??
?:A477??9
@T6XXI:00115:00942
GGGGAATTCTAATACGACTCACTATAGGGAGAGGGAGAATAGTTAACGCACCCTCGTTACCTGTTCACCTTCCTTTC
TCCCTTCTCC
+
CBB9B?A@CCDDCBBABBBBB?<<@BAA>888CC>BBB@CA@<:<>AAA>>:://1@=@B?AED=A??=??A?AKAB
BBC>C@CAC>
@T6XXI:00116:00900
GAGAAGGGAGAAAGGAAGGAGAAGTCGCCCCAAACGATTTGTTGACTATTCTCCCTCTCCCTATAGTGAGTCGTATT
AGAATTCCCC
+
AABB=@@<@B=?=B?B?B@@??@CB?@@@@6@C>CDC@C<AA@CB>>@@><<<=;<;@<=;=<<<<<<===;@<@@<
??=8;2444-
@T6XXI:00116:00904
GGGGAATTCTAATACGACGTCACTATAGGGAGACGGGAGAATATCACATTCATGTTTGTGACCGTGTCTCCTTCCTT
TCTCCCTTCTCCC
+
```

```
AB@2D6954995948111109=>>CBCDC>>10..4+...5..67699BABAA@@@:877770161166;548=7::
49///,/,-1110
@T6XXI:00116:00913
GGGAGAAGGGAGAAAAGGAAGCTAGTCACTCGACATCTGTGGCTTCACAGTACAGAGGGACTGTCCAAAGCCGCAGA
TCTCGAGTCGACTAGCTTCCTTTCTCCCTTCTCCC
+
AC?AAA>@@?@9999/95924;;9>>?77111166;<?@AA?BB@BAAAAABBB@>AA=??AAAA?AA<@@=@@AAA
A888???>AA@@@@@@??<?<?@<@@@@<@599?@<
@T6XXI:00116:00931
GGGGAATTCTAATACGACTCACTATAGGGAGAGGGAGAATAGTCAACAAATCGTTTGGGGCGACTTCTCCTTCCTTT
CTCCCTTCTCCC
+
DJKBD<:;;BG?BBBBBBBBBB>99311.7>>BG>BCD>AC=9<>;==>-
88???9?CC9ECCBD?BBB@C@A>@@7=9;;6;.//?@9
@T6XXI:00116:00935
GGGGAATTCTAATACGACTCACTATAGGGAGAGGGAGAATAGCCGCACCAGCGTTGACAGTTTATTCTCCTTCCTTT
CTCCCTTCTCCC
+
DBB7B=A=@CC@BB???BBBBE>99311.7>>BE<BBB>>??=:<?BB=ABBAA>A<?????<?A>A@@=@=@=;;:
;AAC;?=??@B>
@T6XXI:00116:00938
CTAGTCACTCGAGATCTGCGGCTTTGGACAGTCCCTCTGTACTGTGAAGCCACAGATGTCGAGTCGACTAGCTTCCT
TTCTCCCTTCTCCC
+
>:///1/44CBCC@CBBBBB=>BB<?;<<BBB?@;?@>>>@@@ABCC><B?BBCBB@@ACCCBBCBCBBBBBCBC@C
C<BBCC>C>AACC>
@T6XXI:00116:00945
GGGGAATTCTAATACGACTCACTATAGGGAGAGGGAGAATAGTTACCATTTACATGCGTGCCTATTCTCTTCCTTTC
TCCCTTCTCCC
+
444,42424=;<ACBAA@CBB;;<AAEE>BBBBB>BBB:?::87::4:46133688:=@A@>A??=?==<>@<??:?
?=?:?:<>??:
@T6XXI:00116:00949
GGGAGAAGGGAGAAAGGAAGCTAGTCGACTCGACATCTGTGGCTTCACAGTACAGAGGGACTGTCCAAAGCCGCAGA
TCTCGAGTCGACTAG
+
CCBDDCAA@:=@>>9>5849??;;@/00:0//081166>BB;@@@@@@BBCBB@>34417==@AA>@@<AB>@@@@@
AAAA???AA??=?9<
@T6XXI:00117:00905
GGGGAATTCTAATACGACTCACTATAGGGAGAGGAGAATAGTACGACC
+
111+/,424>><ABBBBBDC@@@BBAAD>:;:@=A0/,00/------,
@T6XXI:00117:00908
GGGGAATTCTAATACGACTCACTATAGGGAGAGGGAGAATAGTTAACGCACCCTCGTTACCTGTTCACCTTCCTTTC
TCCCTTCTCCC
+
CFD:C?B?:<@BCCCBAA???DB>9311.777>BCEBCAC@?=;=;=A@>>::/1?;?B?BDB>A??:=:=:??<?
BED>C>A?AA<
@T6XXI:00117:00927
GGGGAATTCTAATACGACTCACTATAGGAGAGGGAGAATAGCTAATCCCTGACCTATGTCACCGCGTTCTTCCTTTC
TCCCTTCTCCC
+
999,49B@CCC@B>?>BBBBB:9960/,0>;>@>BCC?B??<@@@@@@<BBAA@BCEB@<AA@DCCC>?53328A=A
BAC<?<?@AA;
@T6XXI:00117:00930
GGGAGAAGGGAGAAAGGAAGGAGAAGCACCCAAGGCCAAGCATCAGGCTATTCTCCCTCTCCCTATAGTGAGTCGTA
TTAGAATTCCCC
```

```
+
DDBB>929909@AB@B@C@C>@@B?BBBBC?C=@=??B?BEBAA;<:<DCD@CCBC>BBBBB=CBCCBCBBBC@???
??A@A?@/8880
@T6XXI:00117:00931
CTAGTCGACTCGAGATCTGCGGCTTT
+
C@499944444:99=@B<;17+-,,*
@T6XXI:00117:00941
GGGAGAAGGGAGAAAAGGAAGCTAGTCACTCGACATCTGTGGCTTCACAGTACAGAGGGACTGTCCAAAGCCGCAGA
TCTCGAGTCGACTAGCTTCCTTTCTCCCTTCTCCC
+
995999969?:>9999,42424;779977111111166;;?7;;@DC?@@C@??C@A@AAA???A>@@;@C>@???@
A889<<@?=99<AAAAA=@>AA:;444-434444-
@T6XXI:00117:00945
GGGGAATTCTAATACGACTCACTATAGGGAGAGGGAGAATAGTCAACAAATCGTTTGGGGCGACTTCTCCTTCCTTT
CTCCCTTCTCCC
+
CCF:C?B?BCC@BBA?BBBBBB;99311.7???B>CBB>??>;A;<=AA<BCB??;<<<6<??@@>@CC?B?B?=<;
==<=;A?@AAA;
@T6XXI:00117:00946
GGGAGAAGGGAGAAAGGAAGGAGACACTCGCTCTTGACACAGAGGAACTATTCTCCCTCTCCCTATAGTGAGTCGTA
TTAGAATTCCCC
+
DDBCDC>BB>BB@C:@=@>B>BDBCCCDCCCC<<<@DDCCBB@A@CA@@@B@BBC?86>67613649=@97888488
B;A@?8;<===6
@T6XXI:00118:00904
GGGGAATTCTAATACGACTCACTATAGGGAGAGGGAGAATAGTCAACAAATCGTTTGGGGTGACTTCTCCTTCCTTT
CTCCCTTCTCCC
+
9996=>CDCCC?BBBBCBBBBBB?A7165:?@CD@AAA?BBB@@@>88818?==?.77;4:???CBB??=?<?<?C?
A@@C<@;=>ACA
@T6XXI:00118:00909
GGGGAATTCTAATACGACTCACTATAGGGAGAGGGAGAATAGAGACGTATTGTTTGCGCTCTGTTACCTTCCTTTCT
CCCTTCTCCC
+
KDE9D@C?CBF?BCCCBBCB@@?C;877-
777==166877=973534368;<?C<A@=<???8733688585544 0000+04334=;
@T6XXI:00118:00920
GGGAGAAGGGAGAAAGGAAGGCTAG
+
DDBCA<<<C?@111-/,/,/96696
@T6XXI:00118:00938
GGGAGAAGGGAGAAAGGAAGCTCAATCGTGGGTAGAAGGGTCGTGATCTATTCTCCCTCTCCCTATAGTGAGTCGTA
TTAGAACTTCCCC
+
FE?CCD@CCACCCE@C?A@BB???@B@@??>8A>>?>>C>B?>?AA==<=<9877317??@A<AAA@@@AA??=<=?
?<;;?000;;991
@T6XXI:00119:00902
GGGAGAAGGGAGAAAGGAAGGAGAAGTCGCCCCAAACGATTTGTTGACTATTCTCCCTCTCCCTATAGTGAGTCGTA
TTAGAATTCCCC
+
CDBCCB@ECBDBBC>C@B@B?ABB@B;?BBDD8AA@CBBCC>B?@CBABB@?<===::?@@A;=====;<<===@??
?>CA@<?38880
@T6XXI:00119:00903
GGGAGAAGGGAGAAAGGAAGTAGTCGACTCGACATCTGTGGCTTCACAGTACAGAGGGACTGTCCAAAGCCGCAGAT
CTCGAGTCGA
+
```

BB?AAA@BB>ABBBBA@B@BBAAC:;;BBBA;;<<<>ABC@BB@BCBBBBHCBB@??<>BABCB@BB<BC@B>==??
??>@BBBAA>
@T6XXI:00119:00908
GGGGAATTCTAATACGACTCACTATAGGGAGAGGGAAGAATAGCCTAACGTTTTGCACAGTTGGTATCTCCTTCCTT
TCTCCCTTCTCCC
+
AAA9B@BB999644994>8:8>>>8877,/39::+/,.555563376361111,11133368>56>@:::;??A>@C
898<A9;24344,
@T6XXI:00119:00915
CTATCACTCGAGATCTGCGGCTTTGGACAGTCCCTCTGTACTGTGAAGCCACAGATGTCGAGTCGACTAGCTTCCTT
TCTCCCTTCTCCC
+
DEEBBAAAC??ABDCCC@@=@EC>D>ABBAAAA<??A???BCAAAD>@CABCCBB@@BBCBB;@<ABB?<;62946C
>C@EE>A>??@@7
@T6XXI:00119:00932
GGGAGAAGGGAGAAAGGAAGCAGTCCATCTGTGGCTTCACAGTACGAGGGACTGTCCAAAGCCGCAGATCTCGAGTC
GACTAG
+
CEA@@?@BC@CCDC@C;;:;B>A?><@@??BBC?BB>?6655+,,,,,,*+38B@@>AA9=@@CCCBCCB@@@@@AC
CCCCCB
@T6XXI:00119:00948
GGGGAATTCTAATACGACTCACTATAGGGAGAGGGAGAATAGCTGTAATCTGAACAACTCCCGGCTGATCTTCCTTT
CTCCCTTCTCCC
+
CDD9DCC@CCC@C?@?BCCCDA@99316.7>>BC@BCB>C?@=C@??=A???@;?;;A<==7<:@AA=9==<=9983
58333;88;==:
@T6XXI:00120:00903
GGGAGAAGGGAGAAAGGAAGGAGAAGTCGCCCCAAACGATTTGTTGACTATTCTCCCTCTCCCTATAGTGAGTCGTA
TTCAGAATTCCCCCC
+
CDDCCA>BA>BBBD@C@B@C=@@B>AABCCCC9CC?CA@?<8:?>BBB???:<;;>;@A@=C<@?<;;44033332-
-,--1103777575+
@T6XXI:00120:00909
GGGAGAAGGGAGAAAGGAAGGGGAG
+
4A6::?@BA>9911-1;<;889*,-
@T6XXI:00120:00921
GGGGAATTCTAATACGACTCACTATAGGGAGAGGGAGAATAGTCAACAAATCGTTTGGGGCGACTTCTCCTTCCTTT
CTCCCTTCTCCC
+
999/959:<BC?BBBBCBCCBB?97766.7=>?C>CBB?B??<@A>??;;BBBAC;???6?@A??>@BC@B@A>AC>
C===9=>@AAA=
@T6XXI:00121:00909
GGGAGAAGGGAGAAAGGAAGCTAGTCGACTCGACATCTGTGGCTTCACAGTACAGAGGACTGTCCAAGCCGCAGATC
TCGGTCGACTATCTTCCTTTCTCCCTTCTCCC
+
A=:=?A@CB:@<<<8;>A>BBA;;;:16<61133111176988?<>B????:::???@AAAAAA>A688<?==:://
/6646///:<33;<:=:==78677,/,/./.+
@T6XXI:00121:00910
GGGGAATTCTAATACGACTCACTATAGGGAGAGGGAGAATAGACTCCCCGCGTGGTTTGTGATCGTCCCCTTCCTTT
CTCCCTTCTCCC
+
CBA7A=@=@BCDCCCBBA??BA<99361.7>>?A@BCCDAA@?8;5;8.:;>?@>@C=@@>B??@BBAA6A;=;=A=
AAAA<A;>>AC>
@T6XXI:00121:00911
GGGGAATTCTAATACGACTCACTATAGGGAGAGGAGAATATACGGACC
+

```
>>>68>EACBB@BCBBABBBBBBBBBB@C=A@>;>9::5770++*)*.)
@T6XXI:00121:00915
GGGAGAAGGGAGAAAGGAAGAATAGAGTGGGGCGTGTCGGGTTGGTAACTATTCTCCCTCTCCCTATAGTGAGTCGT
ATTAGAATTCCCC
+
DD?C?=BCC>BBBB>B@B@BB?@AAAABB??7A?@?@???=?@C@BC@CBCBAABBB@BB@?><@B@@BCDCCBABB
BB?B@A<?;>>>1
@T6XXI:00121:00938
GGGAGAAGGGAGAAAGGAAGGAGAAGTCGCCCCAAACGATTTGTTGACTATTCTCCCTCTCCCTATAGTGAGTCGTA
TTAGAATTCCCC
+
IDBCBB@AA?BBBC>B@B@BABBB?B?BBBBB8??;BBBCC>BB>BB@??C?=<<=;=<?==<@=<<=<<<<<<<;?
@>A@@>A>@@@5
@T6XXI:00121:00939
GGGAGAAGGGAGAAAGGAAGCTAGTCACTCGACATCTGTGGCTTCACAGTACAGAGGGACTGTCCAAAGCCGCAGAT
CTCGAGTCGACTAG
+
DBBDCB:94-
99880;>?>BB@@AA6:77=>776677?CD?AB@CEBA>>?<9>33318@?@BC<??<?A>A@<<<===<;;;==<<
<=;9
@T6XXI:00121:00948
GGGGAATTCTAATACGACTCACTATAGGAGAGGGAGAATAGTCACTTCATCTGGTCGCGA
+
=6606,/,/8200/////.33973331016111,111771103000233485520.44..
@T6XXI:00122:00902
GGGAGAAGGGAGAAAGGAAGCTAGTCACTCGACATCTGTGGCTTCACAGTACAGAGGGACTGTCCAAAGCCGCAGAT
CTCGAGTCGACTAGCTTCCTTTCTCCCTTCTCCC
+
FEBC<;89@:>999195959B;88?7,,,,,,,,,,/9?A88;@DCBBBBCBBB<;;6;;;<?@?AA;@@>A===A@
>=>88888@A@@@ACC6868;7;444-4248=A<
@T6XXI:00122:00903
GGGAGAAGGGAGAAAGGAAGGAGAAGTCGCCACAAACGATTTGTTGACTATTCTCCCTCTCCCTATAGTGAGTCGTA
TTAGAATTCCCC
+
FEBCCCC@><@CCB?CBB?B@BAB@CCBBB>A??@<???DE>CC?C:<<EGBICEE@CECBC?BBBCCCCCCFAB;;
;?BBC>@38880
@T6XXI:00122:00926
GGGGAATTCTAATACGACTCACTATAGGGAGAGGGAGAATAGTTTTTCCCTTCACTAACCGCGAACTGCACTTCCTT
TCTCCCTTCTCCC
+
AA?8@>A?BCCCCB@>AA???BB??761.6>>AB>BBB?@BB@A==.A<;<:;@B@A@@????=;:?<8=BB?C?BB
>BBBC>@?@@=;5
@T6XXI:00122:00932
GGGAGAAGGAGAAAGGAGG
+
CCB@99592/,,,*,***0
@T6XXI:00122:00941
CTATCACTCGAGATCTGCGGCTTTGGACAGTCCCTCTGTACTGTGAAGCCACAGATGTCGAGTCGACTAGCTTCCTT
TCTCCCTTCTCCC
+
B9949444;==>?@@AA>>;>>>8>;?AA8777.7>>AABDBAAA@=@A@@AABABC@@@BAA??@883331;;>@C
>===C>C?BCBA=
@T6XXI:00122:00942
GGGAGAAGGGAGAAAGGAAGGAGAAGTCGCCCCAAACGATTTGTTGACTATTCTCCCTCTCCCTATAGTGAGTCGTA
TTAGAATTCCCC
+
```

BB@FDC?DB=?>>><A?B?B@BBB?C@B<<<<6@A>BCB@@>@A@BCC?>>;:===:;:@AA<=<???A@<===A@@
@>@AA?B9<<<4
@T6XXI:00123:00908
CTATCACTCGAGATCTGCGGCTTTGGACAGTCCCTCTGTACTGTGAAGCCACAGATGTCGAGTCGACTAGCTATTCT
CCCTCTCCCTATAGTGAGTCGTATTAGAATTCC
+
@AB?9::=BBBBBCCAABDBCCD?C?@??>777.7???BBBCC??;37<8;@@?;;;:A>??B<;BB;::6>A5:7
?>666167-7=760333338:<==;?:34;,0,
@T6XXI:00123:00918
GGGGAATTCTAATACGACTCACTATAGGAGAGGAGAATAGCCCAATGTTCTACCTCTATCTCAGTCCCTTCCTTTCT
CCCTTCTCCC
+
CII9H?BB<;989CBABBC;87773310111101770677 4;4?:::>;>>AB?B?@@A887875384626564444
44,3/3484:
@T6XXI:00123:00924
GGGAGAAGGGAGAAAGGAAGGAGAAGCCGCCCCAAACGATTTGTTGACTATTCTCCCTCTCCCTATAGTGAGTCGTA
TTAGAATTCCCCC
+
94-4999@A=AA@?7;8;?B?BBCBCC?@??<2>A:@@BCA>AA?ABBB?>;>444-
383444:>????@?889==<88:00/0/---.+
@T6XXI:00124:00909
CTATCGACTCGAGATCTGCGGCTTTGGACAGTCCCTCTGTACTGTGAAGCCACAGATGTCGAGTCGACTAGCTTCCT
TTCTCCCTTCTCCC
+
C:7;1////89==?ACCCCC@CCB>B?@A@B:9:5:CCBBBBB?AAC>=A@CECBBBBBCCCCC?@ABBCCCC@C?B
B>BBCCCF?BA?>7
@T6XXI:00124:00929
GGGAGAAGGGAGAAAGGAAGCTAGTCACGTCGACATCTGTGGCTTCGACAGTACAGAGGGA
+
490944244-//68+/,/684?@;7><611113371116617 7?5976771....,+++*+
@T6XXI:00124:00930
GGGGAATTCTAATACGACTCACTATAGGGAGAGGGAGAATAGTTTTCCCTTCACTAACCGCGAACTGCGCTTCCTTT
CTCCCTTCTCCC
+
EFD:EDCDCCDEDEBBCDDBBBCCBCCE@AAACC>CBCAAA?:99-
00+0237;?B?BACBBC?CCB@999@C>A@@A@@A@A>BAAD;
@T6XXI:00124:00937
GGGAGAAGGGAGAAAGGAAGGAGAAGTCGCCCCAAACGATTTGTTGACTATTTCTCCCTCTCCCTATAGTGAGTCGT
ATTTCAGA
+
990444244-444=5=<??A?BBDAD<<<CCC7AA<73311,.4,..54247///77574:<=;<<<>;555---2-
---+--33
@T6XXI:00125:00915
GGGAGAAGGGAGAAAGGAAGGAGAAGTCGCCCCAAACGATTTGTTGACTATTCTCCCTCTCCCTATAGTGAGTCGTA
TTAGAATTCCCC
+
DDBAAA@BCBBCBB=?>?>A?AAC@AABCCCC7AA=BCCCC>BB?BBCCBB@AAAC;<===@;@@AAAAAA@@??=<
<<>==:=8<:<1
@T6XXI:00125:00917
GGGGAATTCTAATACGACTCACTATAGGGAGAGGGAGAATAGTTTTTCCCTTCACTAACCGCGAACTGCACTTCCTT
TCTCCCTTCTCCC
+
DBA6B=A=@@@;>@BCC@B@AB???876.7>777-
766;?713444,00+048@BB@?BA???A;::8>@@A;?;?@:>@@@:?47444-
@T6XXI:00126:00902
GGGGAATTCTAATACGACTCACTATAGGGAGAGGGAGAATAGTCTTGCAATTTCCTGCGGTCGACTACT
+

```
IKH:C?A@AAA@DBBB@;8888>>7317,16667;???>@??884::4749@195991/5///..,,,.
@T6XXI:00126:00907
GGGAGAAGGGAGAAAGGAAGGAGAAGTCGCCCCAAACGATTTGTTGACTATTCTCCCTCTCCCTATAGTGAGTCGTA
TTAGAATTCCCC
+
CCABCB@CE>CBCC>B?B?B@BAA????3377-
88/8?ABB;>?>@@@?@B?>A><4<<>>>:????AAAAA?<<<AA>BA?;:2444-
@T6XXI:00126:00915
GGGAGAAGGGAGAAAGGAAGCTAGTCACTCGACATCTGTGGCTTCACAGTACAGAGGGACTGTCCAAAGCCGCAGAT
CTCGAGTCGACTAG
+
DF?<9959919>>>:>599;CAAAB@:761=6617667666681:;@?@:::>>:==6<;<<@@8<<7<@=@CA@@@
===@ABB===@AC@
@T6XXI:00126:00946
GGGAGAAGGGAGAAAGGAAGGAGAATGCACCACAGGAAATTGCAAGACTATTCTCCCTCTCCCTATAGTGAGTCGTA
TTAGAATTCCCC
+
CF?BCC?BB=BBCC>B?@?A@BAA?AABBB@BB>?>@@;B>@AA?BCCCC>>????;==A@@;=<>>=<<<?<@@??
?>?AA?C=@@?5
@T6XXI:00127:00904
GGGAGAAGGGAGAAAGGAAGGAGAAGTCGCCCCAAACGATTTGTTGACTATTCTCCCTCTCCCTATAGTGAGTCGTA
TTAGAATTCCCC
+
CD?FBBACC@===BAC@C998:BB?CBCCCEG8@?=@CBBB<AC@CC>>@A@<8664<>@@A<AAA@@@?;?<<==?
?>AAC>A>?@>1
@T6XXI:00127:00916
GGGGAATTCTAATACGACTCACTATAGGGAGAGGGAGAATAGTCAACAAATCGTTTGGGGCGACTTCTCCTTCCTTT
CTCCCTTCTCCC
+
9;<4;>CABBBBCBBBBCCCAA@99316.7>>CE=AAB>C?><><>4741:>==>9?<?5?@AAC?AAB8:88>A?<
A???:>?@?@@<
@T6XXI:00127:00929
GGGGAATTCTAATACGACTCACTATAGGGAGAGGGAGAATAGTCTTGTATTTTGTGCATCACTTCTCTCCTTCCTTT
CTCCCTTCTCCC
+
:::4<?B@BBB@BBCBBA???BBBD><<;<?ACE>AAC?B>?<<=<::::==5===A>>>CCC>>>=@C=?;=333,
0444,478444-
@T6XXI:00127:00934
GGGGAATTCTAATACGACTCACTATAGGGAGAGGGAGAATAGTCAACTAATCTCCTCACCTCGGCGTGCCCTTCCTT
TCCTCCCTTCTCCCATCACCGACCTA
+
DCC8A548<99@B;<<BBAACA@??877-
777711699@C?@=ABA@@@?A@?=;=86446./,.453475554011+-,..73;99337+/33..--37,44
@T6XXI:00127:00947
GGGAGAAGGGAGAAAGGAAGGGGAGAAGGGAGAAAGGAAGCTAGTCGACTCGAGATCTGCGGCTT
+
?:6:<969A1948914242444,49977;:;83:6:,/,.5.,,3.....55859<6640-,--,
@T6XXI:00128:00903
GGGAGAAGGGAGAAAGGAAGGGGAGAAGGGAGAAAGGAAGCTAGTCGACTCGACATCTGTGGCTTCACAGTACAGAG
GGACTGTCCAAAGCCGCAGATCTCGAG
+
EC?AA@?AA<@@@@<@<@;>@@4>>@=@@<@???8?;@5::B@??BBBB=??@@@AAA;;;:;;88<<====<6:43
317???@?>AA<AA>@===A????==>
@T6XXI:00128:00905
GGGAGAAGGGAGAAAGGAAGCTAGTCACTCGACATCTGTGGCTTCACAGTACAGAGGGACTGTCCAAAGCCGCAGAT
CTCGAGTCGACTAG
+
```

CCBBC=694-
9988096959A888B?==777779:76BCE??@@CCB>>>C@>;46647==@AA=@@:@A@A@8<8==<<88=@@@@
AAAA
@T6XXI:00128:00922
GGGGAATTCTAATACGACTCACTATAGGGAGAGGGAGAATAGTTCTCGCCGCACAAACGAACATTGTTCTTCCTTTC
TCCCTTCTCCC
+
944,49@>=<>BG@ABB;;;CA??>876.777?C>AAB@CC@=;=<<<@<??A@?64=8838<=?@C?AA:B>BA:?
@@A<A?AAA@<
@T6XXI:00128:00933
GGGGAATTCTAATACGACTCACTATAGGAGAGGGAGAATAGTCAACAAATCGTTTGGGGCGACTTCTCCTTCCTTTC
TCCCTTCTCCC
+
?=92>69694429;;BBBCCC@<@A:73:::@@=BBBABBCAAB>@@@187;??8<??8C?@?==<<@;?==>@C@A
AAA;?;;344,
@T6XXI:00128:00937
GGGGAATTCTAATACGACTCACTATAGGGAGAGGAGAATAGAGTC
+
CAA6A>B@B@@9;;@?@@<<AAB?A?BB:;;00,.7720,/++00
@T6XXI:00128:00946
GGGAGAGAATTTGGTAATGGGCGGCAGGGAGAAAGATCGAACCCTTATATCTCTCTCCCTATAGTGAGTCGTATTCA
GAATTCCCC
+
AA<C?>?A>AC@DACC?@@@<@B@CCCF>@ABCACCCA@@59819<B9;979?;6111,16111;80--2-
++0*++557=;===5
@T6XXI:00128:00949
GGGAGAAGGGAGAAAGGAAGGTGATCGTAGAGCGTACGAGGATTGGGCTATTCTCCCTCTCCCTATAGTGAGTCGTA
TTAGAATTCCCC
+
7=6119699-4449-4242474333377;:6??<<<;;//,-1,--
+0300/0044244355356<????BAA@A@@@>@@A=B?BCC:
@T6XXI:00129:00901
GGGAGAAGGGAGAAAGGAAAGCTAGTCACTCGACATCCT
+
::6949544-444=-424858733360,,**0*****),
@T6XXI:00129:00907
CTAGTCACTCGAGATCTGCGGCTTTGGACAGTCCCTCTGTACTGTGAAGCCACAGATGTCGAGTCGACTAGCTTCCT
TTCTCCCTTCTCCC
+
>11//814:CCB@AACCBBB@CCC>C@CCBB@??;?BBBBBCC?@@B@AGACCBBBBBBA@@??AA@@B???88?:?
@9>;=>6=476=>:
@T6XXI:00129:00909
GGGGAATTCTAATA
+
444,/,/7///7/,
@T6XXI:00129:00912
GGGGAATTCTAATACGACTCACTATAGGAGAGGGAGAATAGTCCCATCATTCCCAATCTTGACATTTTCTTCCTTTC
TCCCTTCTCCC
+
CFC:C@B@BBB@BB@A@@BBB:9990/-
0>>BC>DDC?@??<<=;A==<=>==;><;=<<=559@@@4?=;>8;;6;==@;@>@AAA<
@T6XXI:00129:00925
GGGAGAAGGGAGAAAGGAAGGAGAATCGACACACGAGCGCCTACGTGATCTATTCTCCCTCTCCCTATAGTGAGTCG
TATTAGAATTCCCC
+
DIFECC@B@69988-
9;>;@BCAA@CCCBCC@=@CCCDCC@AAADCCCBCDA>113D@><?;;;:<ACELBBBAA@@:::?BBB@C@CCC9

```
@T6XXI:00129:00941
GGGAGAAGGGAGAAAGGAAAGTG
+
44-444244-////+/,//+/80
@T6XXI:00129:00946
GGGGAATTCTAATACGACTCACTATAGGGAGAGGGAGAATAGTCAACAAATCGTTTGGGGCGACTTCTCCTTCCTTT
CTCCCTTCTCCC
+
CBB9B>A?ABC@BBB@??>>BC?97311.7==?A>BCC@B>>9:=<===;BA>9988885;ACBB?BBB>A=?;<=:
;AAA=A<??@@;
@T6XXI:00130:00909
GGGGAATTCTAATACGACTCACTATAGGGAGAGGGAGAATAGAGACGTATTCGTTTGCGCTCTGTTCACCTTCCTTT
CTCCCTTCTCCC
+
AAA6C=@=@CC=@@ABACECCBA??877,7=7@E>:88::8B<<<A883@7:48818BC@BB@8738==8:9;2471
34;@7?=??HC@
@T6XXI:00130:00915
GGGAGAAGGGAGAAAGGAAGGAGACAGTGAGCGCCGCACAACACGTCACTATTCTCCCTCTCCCTATAGTGAGTCGT
ATTAGAATTCCCC
+
AA=BAA=@A:>89918<@<@7;9;=AABBAAAADFCCDGB=??@@::;A=99<====:??>CC>:<:A@BCE@@@BB
BA7617042444,
@T6XXI:00130:00916
GGGGAATTCTAATACGACTCACTATAGGGAGAGGGAGAATAGTGACGTGTTGTGCGGCGCTCACTGTCTCCTTCCTT
TCTCCCTTCTCCC
+
=??4?59699;<>9;;A@BCCCBB>877.777BC>CCC@DBB@?>?>8888;98;=8;;=?@@@@@@@@@<>;>=@@
<@AAA1738<<@:
@T6XXI:00130:00921
GGGAGAAGGGAGAAAGGAAGACCGCGAAGGCGCCGGGGAGTTATCGGCTATTCTCCCTCTCCCTATAGTGAGTCGTA
TTAGAATTCCCC
+
FDE==<AEC@B<;;7;>A>CDC>@@::47;><<:===2?AA>AA:869AAC@BBBC>?;?AA<@?@@@???AAA@==
<<;<;8;5<@@5
@T6XXI:00130:00923
GGGAGAAGGGAGAAAGGAAGGAGAAGTCGCCCCAAACGATTTGTTGACTATTCTCCCTCTCCCTATAGTGAGTCGTA
TTAGAATTCCCC
+
CDBBBB>BC@<@<CAC=A?A@A@CICDCBBCC7@@<@B?@A8::=A???@>30330+03000+0444:@@???@AB=
=9<:43443050
@T6XXI:00130:00924
GGGAGAAGGGAGAAAGGAAGCTAGTCGACTCGAGATCTGCGGCTTTGGACAGTCCCTCTGTACTGTGAAGCCACAGA
TGTCGAGTCGACTAGCTTCCTTTCTCCCTTCTCC
+
DC?C@=;@@:@99809<@>BDB>>>/1000>>B@CCCCCA?;<?<9??@CCBBCB>BB@A@@@A???D?@A?AA@@?
878<>?>?AAA@@A=<;;:88443444-424442
@T6XXI:00130:00930
GGGAGAAGGGAGAAAGGAAGCTAGTCGACTCGACATCTGTGGCTTCACAGTACAGAGGACTGTCCAAAGCCGCAGAT
CTCGAGCGA
+
DD?CGB=@@9>@@@<A?B;<<CECCCDBB???AAA:;:ABB=@@@ECC@BBBAA<;;8;==>?A>@C?DB<?@A==<
788898@AA
@T6XXI:00130:00932
GGGAGAAGGGAGAAAGGAAGGCAATAGCACCATGGGAGGTTTGGTGACTATTCTCCCTCTCCCTATAGTGAGTCGTA
ATTAGAACTTCCCC
+
```

```
>>>;CCBCD:<9991959449?B@?@@AAA=@?BB=7957;:9566=;;<<576999666972661333--33-
+++*+*-2223656<9.
@T6XXI:00130:00933
GGGAGAAGGGAGAAAGGAAGCTAGTCACTCGACATCTGTGGCTTCACAGTACAGAGGGACTGTCCAAAGCCGCAGAT
CTCGAGTCGACTAG
+
DDBCCB>==6:844-
9<><>@@?????=??????@@?BDC?CC@CCBC@CB?;706154;?AA@?AB=@?<?B==<<<A=<<?A???@A@@
@T6XXI:00130:00938
GGGGAATTCTAATACGACTCACTATAGGGAGAGGAGAATAGTA
+
?BB9B=?=@BC?BDCCCCBAAA@?;796./:0/,/:<:</1-/
@T6XXI:00130:00943
GGGAGAAGGGAGAAAGGAAGTGCGGTTCGCGGTTAGTGAAGGGAAAAACTATTCTCCCCCTCCCTATAGTGAGTCGT
ATTAGAATTCCCC
+
CBBCCA?BCBB?>=<@=@=@CCCC@C@BABBAB=?>ABB<>A<@>@@1BBCC>B78777+777.:AABC@CA@@=??
CC>A@?;;2444,
@T6XXI:00130:00946
GGGGAATTCTAATACGACTCACTATAGGAGAGGGAGAATAGCTGTCTATTCCATTCATCGCTCATTCTCTTCCTTTC
TCCCTTCTCCC
+
4441:@CDBBBADAAABBBBB;::700,...779<?<>@F;8;CC@@@C?B?@@=?CCC@?=A>A>A???>@:==9=
AAA<A?7884-
@T6XXI:00131:00910
GGGGAATTCTAATACGACTCACTATAGGGAGAGGGAGAATAGTTTTCCCTTCACTAACCGCGAACTGCACTTCCTTT
CTCCCTTCTCCC
+
EBB9B>A?BCC@BBBBBBBB@A<99311.7==BC>CCC?C@B==<5;;:<>=AACC?B>87:A>AA?@AAA@E?BB>
AAAA<A=AA@A<
@T6XXI:00131:00932
GGGGAATTCTAATACGACTCACTATAGGAGAGGGAGAATAGCAAACGTTTCCCCGACGTCGCTTTCTCCTTCCTTTC
TCCCTTCTCCC
+
A??6A>C@AA@@CCDCCBAAB=;;000,../69;>7>;>/0/940366767::66A??7611;;6<76369?>BD?=
==<:>:6?881
@T6XXI:00131:00938
GGGAGAAGGGAGAAAAGGAAGGAGAAGTCGCCCCAAACGATTTGTTGACTATTCTCCCTCTCCCTATAGTGAGTCGT
ATTAGAATTCCCC
+
DICDAA644-99999-
9=@7@=:<:@A@A@CCC9CC>CCCBB<:95:??7?@;==@B@???<A;AA@==<@<;<?@899>@AA?A>BAA5
@T6XXI:00131:00945
GGGGAATTCTAATACGACTCACTATAGGGAGAGGGAGAATAGGGCCGACGTCGCATGCTCCCGGCCGCCATGGCCGG
CCGCGGGAATTCGATTGGGGAATTCTAATACGACTCACTATAGGAGAGGGAGAATAGTCAACAAATCGTTTGGGGCG
ACTTCTCCTTCCTTTCTCCCTTCTCCC
+
CFD:B8;9;CC?CCBBBBBBBBBBBB@C?<:8888???ACCCC=?;777888?=<?>A===;<99888=:99,0,-
,-,-
4544<8856>>8:=AA6A;<9<<;8<@@>@@??????@??;>====7???<?AAA@@=??B:>>777.7==27::AB
>@@@?C@A>@@;@B@@;@=@874-
@T6XXI:00132:00903
GGGAGAAGGGAGAAAAGGAAGGACGGTGTGCTGGGAGAAATGCACAGAACTATTCTCCCTCTCCCATAGTGAGTCGT
ATTAGAACTTCCCC
+
FDBCEEDCA:>9999-
959442433777766333357>?B=CCCBBAABBCCC@;>978/37767999@@:::@ABCCAA>???4;<3776-
```

```
@T6XXI:00132:00904
GGGGAATTCTAATACGACTCACTATAGGGAGAGGGAGAATAGATCACCGTTGGTTCGGGTGCGCGTTCTCCTTCCTT
TCTCCCTTCTCCC
+
;;9/9;@=CCHACC@@@BAAA@>>>877-
7<==<6<9==FBA==B8637=:<:A>B>>??310=;;,:1666O626?9>::7.73738A:
@T6XXI:00132:00910
GGGGAATTCTAATACGACTCACTATAGGGAGAGGGAGAATAGCTCCCTGCTTTCTCGCACTGAACGTCACTTCCTTT
CTCCCTTCTCCC
+
DCA4B5469>==CCCCBBA@B;>>>877-7><<;8?:;@CCB=>9:69/.--+-
112:8;99949379;;677011,16==9?:=777,
@T6XXI:00132:00911
CTCGAGATCTGCGGCTTTGGACAGTCCCTCTGTACTGTGAACCACAGATGGAC
+
EBB<<;BBBBBDD?BBG<@=>@C=BBD8::==99=9=>>A@8:80,,,,,***
@T6XXI:00132:00928
GGGGAATTCTAATACGACTCACTATAGGAGAGGGAGAATAGTCAACAAAATCGTTTGGGGCGACTTCTCCTTCCTTT
CTCCCTTCCTCCC
+
CCC:C?A@ACC@CCCCC<;;D>==;00,0<<>@>B>B??>6..-7/---
*396;;6===2>=77:;@=?;?>@=>@:@88824,/,/442
@T6XXI:00132:00931
GTCGACTCGAGATCTGGCGGCTTTGGACAGTCCCTCTGTACTGGTGAAGCCACAGATGTCGAGTCGACTAGCTTCCT
TTCTCCCTTCTCCC
+
4;=B=@@?>B:866/1,/77:CEBDACECB>>=:==10-
00,,*../6/423777666133:0..555;CACC@C@BA97777.737111,
@T6XXI:00132:00932
GGGAGAAGGGAGAAAGGAAGAAGCGTGCTGGAGATTGGAGATGATCGTGACTATTCTCCCTCCTCCTATAGTGACGT
CTATTAGAATTCCCC
+
BD6?>?644-444;-
424@996:AC@@@942494424244444444484./////,..99777137119B==000.00...10177770111,
@T6XXI:00132:00934
GGGAGAAGGGAGAAAGGAAGATTACGCTGGGAATGCAAAGTTGGGAGCTATTCTCCCTCTCCCTATAGTGAGTCGTA
TTAGAATTCCCC
+
CB@@@@@AA@?BBB?B@B@BBB>?AAABAC?B?AABBB>BB@BB>BABCCB@BBBB=BCCCC>B@>B>BBABAAB?=
=?AAA?H:<<<4
@T6XXI:00132:00937
GGGGAATTCTAATACGACTCACTATAGGGAGAGGGAGAATAGTCAACAAATCGTTTGGGGCGACTTCTCCTTCCTTT
CTCCCTTCTCCC
+
EDC9C>@=ACC@CABABCAAAA@99311.77777-
7???BBB@AA=??A:B@?@A;?>>6><<>B?BBB=?;;8;<7=@BD>C588AA<
@T6XXI:00132:00943
TCGAGATCTGCGGCTTTGGACAGTCCCTCTGTACTGTGAACACAGCATGT
+
@BF@@AAA@@@A??>?;?=>BBB?====CBBB>B?@C??21//,,++,,,
@T6XXI:00133:00900
GGGGAATTCTAATACGACTCACTATAGGGAGGGGAGAATAGACATTTGTGTCCGACCTGCCCGAGTAACTTCCTTTC
TCCCTTCTCCC
+
499/9692499;?@A@BBAABB?@?877.7777.1=7;;@@;<777.7>>=;8744799::49444;237=;78958
899,/,----+
@T6XXI:00133:00908
```

GGGAGAAGGGAGAAAGGAAGCTAGTCACTCGACATCTGTGGCTTCACAGTACGAGGAGGACGTCCT
+
B?>AA@=@B<<<DGBCBCAA@@BBBB?856900/0//7:::@A?A:::@??>?>..**)*****),
@T6XXI:00133:00909
GGGAGAGAATTTGGTAATGGGTGGCGGCTCACAGTCACCCAAACCAGTACGTCCCCTTATATCTCTCTCCCTATAGT
GAGTCGTATTAGAATTCCCC
+
CEBCCDBB?BC?B@:;;;88,/-
588*//899CBBBBCD@CC=B7::A??8=AA=4<<CCA==911116:8;>BBCB@@@>@888?<??A;@9<;;5
@T6XXI:00133:00920
GGGGAATTCTAATACGACTCACTATAGGGAGAGGGAGAATAGCTCAGCTTTTGTTGCATGTG
+
88000)/,///,14?;;;AAA?B>;7::.7>777.7179=@@?AA>@7444+1.,.-----1
@T6XXI:00133:00925
GGGGAATTCTAATTACGACTCACTATAGGGTAGAGGGAGAATAGTTTCCGTCTCTCCCTTGCACAGCAGCCAACCTT
CCTT
+
>B<1<,,*,,,*,*/6/449777;;;7672111111,16633445=;=:?8846:44+0/0339=<=;352.,-
3000*+*
@T6XXI:00133:00931
GGGAGAGGAGAAAGAGGAGACAGTGA
+
490444424444-///5/..////44
@T6XXI:00133:00935
GGGAGAAGGGAGAAAGGAAGTAGAGGAGGATAGAAGACAAACGTTTGCTATTCTCCCTCTCCCTATAGTGAGTCGTA
TTAGAATTCCCC
+
DDBCCCACC>BBBB>B@BABBA>>@>AA@BBBCB?AAA??<?AAC:??ACCBCCCC?@@@@@<@CCCCCCBCBBDCE
A@ABA@EBEBCA
@T6XXI:00133:00937
GGGAGAAGGGAGAAAGGAAGGAGAAGTCGCCCCAAACGATTTGTTGACTATTCTCCCCCCTCCCTATAGTGAGTCGT
ATTAGAATTCCCC
+
A;594959>:>9991969694937137@8888.77277;<?;?B=?77133,-------(-
33247:../4/./5333856=:9;/344,
@T6XXI:00133:00944
CTAGTCACTCGAGATCTGCGGCTTTGGACAGTCCCTCTGTACTGTGAAGCCACAGATGTCGAGTCGACTAGCTTCCT
TTCTCCCTTCTCCC
+
B8//1=1::DCCCBCBBBBB?BBB>B>A@BB<::8??A>BCCCACCD@=@>ACCCD@@@BBA??@@@@?B;;7>A?B
B>BCEC@DA@@@@;
@T6XXI:00133:00948
CTAGTCACTCGAGATCTGCGGCTTTGGACAGTCCCTCTGTACTGTGAAGCCACAGATGTCGAGTCGACTAGCTTCCT
TTCTCCCTTCTCCC
+
>>/111/4:BBDCBCBBB??=>CC>B>AABB9788:CCE@????@@@>:A?ABBBBA>?@CCBB@@@B@@@AA?B@E
C?BCDFBC=@@@A;
@T6XXI:00133:00949
GGGAGAAGGGAGAAAGGAAGGAGACAGTGAGCGCCGCACAACACGTCACTATTCTCCCTCTCCCTATAGTGAGTCGT
ATTAGAATTCCCC
+
EBBB<;:<C?BBBG@DACBB?BA:;<@B@CBCAA=@BC@@=?@@@?@ABEBBA@;;;89>@CC=AACBABC@@?>>>
>=8?66082444,
@T6XXI:00134:00910
GGGGAATTCTAATACGACTCACTATAGGAGAGGGAGAATA
+
:BD1:842999644::8=@??::;;10,.3.11,1..,.4

```
@T6XXI:00134:00918
GGGAGAAGGGAGAAAGGAAGAATAAGACGGTATCAGGGTGTTGTTGCTATTCTCCCCTCTCCCTATAGTGAGTCCGT
ATTAAGAAACTTCCCC
+
BC=::9>A@1<9@B?B@CACC@BB;??8713333444-/88777,//669244444,44444-
433390,,,,,*,,24473000+177>@A7
@T6XXI:00134:00921
GGGAGAGGAGAAAGGAAGGAGAAGAGCTGCGCACATTGATGACAATAGCTAATTCTTCCCGT
+
11,,,,,*,,/8+/,42499;;;73.344.......,1111149137731913130,0;6;/
@T6XXI:00134:00927
GGGAGAAGGGAGAAAGGAAGAACTAGGAGTAGAAGTCGATATGACAGCTATTCTCCCTCTCCCTATAGTGAGTCGTA
TTAGAATTCCCC
+
DEBAAA@CC>BBBB>C@BABB?<<<?>?>>>>>;??@@??<<<<@@BBBBA>A==<;@<A==<=;??<?<=?::A=?
:88?=>A=???5
@T6XXI:00134:00933
GGGGAATTCTAATACGACTCACTATAGGAGAGGGAGAATAGTTTTTTCCCTTCACTAACCGCGAACTGCACTTCCTT
TCTCCCTTCTCCC
+
EBB9A>B?BCCABBBBBBBBB:9990/-
0>>BC>BBC@CA?55388*:;7>9=@CCC@C>B=@B?ABA=??A@C?BD>C???=?@AB@A<
@T6XXI:00134:00938
GGGGAATTCTAATACGACTCACTATAGGAGAGGGAGAATAGCTTGCAGCTCGAGCCTGTGACTGTTCTCTTCCTTTC
TCCCTTCTCCC
+
EDC9B?A?BBC@CBBBBBAAB9666./-
```
[truncated: 312,430 more chars]
