# Supplementary material for: MRP1-CD28 bi-specific oligonucleotide aptamers: target costimulation to drug-resistant melanoma cancer stem cells
Source: Oncotarget. 2016 Mar 15;7(17):23182–96. doi: 10.18632/oncotarget.8095 (PMC5029619; doi:10.18632/oncotarget.8095)
Supplement: Supplementary file 4 [file oncotarget-07-23182-s004.pdf]

CLUSTAL 2.1 multiple sequence alignment

```
FC88X_00034_00263      ------------------TTTTTCCCTTCACT-AA---CCGCGA-AC-TGCA--
------
FC88X_00031_00353      ------------------TTTTTCCCTTCACT-AA---CCGCGA-AC-TGCA--
------
FC88X_00027_00395      ------------------TTTTTCCCTTCACT-AA---CCGCGA-AC-TGCA--
------
FC88X_00014_00349      ------------------TTTTTCCCTTCACT-AA---CCGCGA-AC-TGCA--
------
FC88X_00014_00335      ------------------TTTTTCCCTTCACT-AA---CCGCGA-AC-TGCA--
------
FC88X_00040_00407      ------------------TTTTTCCCTTCACT-AA---CCGCGA-AC-TGCA--
------
FC88X_00020_00420      ------------------TTTTTCCCTTCACT-AA---CCGCGA-AC-TGCA--
------
FC88X_00016_00418      ------------------TTTTTCCCTTCACT-AA---CCGCGA-AC-TGCA--
------
FC88X_00013_00439      ------------------TTTTTCCCTTCACT-AA---CCGCGA-AC-TGCA--
------
FC88X_00011_00407      ------------------TTTTTCCCTTCACT-AA---CCGCGA-AC-TGCA--
------
FC88X_00044_00407      ------------------TTTTTCCCTTCACT-AA---CCGCGA-AC-TGCA--
------
FC88X_00037_00343      ------------------TTTTTCCCTTCACT-AA---CCGCGA-AC-TGCA--
------
FC88X_00044_00306      ------------------TTTTTCCCTTCACT-AA---CCGCGA-AC-TGCA--
------
FC88X_00024_00349      ------------------TTTTTCCCTTCACT-AA---CCGCGA-AC-TGCA--
------
FC88X_00089_00017      ------------------TTTTTCCCTTCACT-AA---CCGCGA-AC-TGCA--
------
FC88X_00081_00020      ------------------TTTTTCCCTTCACT-AA---CCGCGA-AC-TGCA--
------
FC88X_00074_00032      ------------------TTTTTCCCTTCACT-AA---CCGCGA-AC-TGCA--
------
FC88X_00021_00751      ------------------TTTTTCCCTTCACT-AA---CCGCGA-AC-TGCA--
------
FC88X_00048_00725      ------------------TTTTTCCCTTCACT-AA---CCGCGA-AC-TGCA--
------
FC88X_00079_00065      ------------------TTTTTCCCTTCACT-AA---CCGCGA-AC-TGCA--
------
FC88X_00074_00051      ------------------TTTTTCCCTTCACT-AA---CCGCGA-AC-TGCA--
------
FC88X_00062_00284      ------------------TTTTTCCCTTCACT-AA---CCGCGA-AC-TGCA--
------
FC88X_00058_00294      ------------------TTTTTCCCTTCACT-AA---CCGCGA-AC-TGCA--
------
FC88X_00013_00456      ------------------TTTTTCCCTTCACT-AA---CCGCGA-AC-TGCA--
------
FC88X_00016_00476      ------------------TTTTTCCCTTCACT-AA---CCGCGA-AC-TGCA--
------
FC88X_00018_00511      ------------------TTTTTCCCTTCACT-AA---CCGCGA-AC-TGCA--
------
FC88X_00079_00209      ------------------TTTTTCCCTTCACT-AA---CCGCGA-AC-TGCA--
------
```

FC88X_00121_00499 ------------------TTTTTCCCTTCACT-AA---CCGCGA-AC-TGCA--
------
FC88X_00092_00153 ------------------TTTTTCCCTTCACT-AA---CCGCGA-AC-TGCA--
------
FC88X_00051_00179 ------------------TTTTTCCCTTCACT-AA---CCGCGA-AC-TGCA--
------
FC88X_00122_00567 ------------------TTTTTCCCTTCACT-AA---CCGCGA-AC-TGCA--
------
FC88X_00112_00582 ------------------TTTTTCCCTTCACT-AA---CCGCGA-AC-TGCA--
------
FC88X_00112_00579 ------------------TTTTTCCCTTCACT-AA---CCGCGA-AC-TGCA--
------
FC88X_00043_00688 ------------------TTTTTCCCTTCACT-AA---CCGCGA-AC-TGCA--
------
FC88X_00038_00654 ------------------TTTTTCCCTTCACT-AA---CCGCGA-AC-TGCA--
------
FC88X_00037_00697 ------------------TTTTTCCCTTCACT-AA---CCGCGA-AC-TGCA--
------
FC88X_00024_00735 ------------------TTTTTCCCTTCACT-AA---CCGCGA-AC-TGCA--
------
FC88X_00114_00540 ------------------TTTTTCCCTTCACT-AA---CCGCGA-AC-TGCA--
------
FC88X_00852_00286 ------------------TTTTTCCCTTCACT-AA---CCGCGA-AC-TGCA--
------
FC88X_00883_00360 ------------------TTTTTCCCTTCACT-AA---CCGCGA-AC-TGCA--
------
FC88X_00155_00588 ------------------TTTTTCCCTTCACT-AA---CCGCGA-AC-TGCA--
------
FC88X_00199_00545 ------------------TTTTTCCCTTCACT-AA---CCGCGA-AC-TGCA--
------
FC88X_00151_00479 ------------------TTTTTCCCTTCACT-AA---CCGCGA-AC-TGCA--
------
FC88X_00121_00372 ------------------TTTTTCCCTTCACT-AA---CCGCGA-AC-TGCA--
------
FC88X_00149_00314 ------------------TTTTTCCCTTCACT-AA---CCGCGA-AC-TGCA--
------
FC88X_00114_00313 ------------------TTTTTCCCTTCACT-AA---CCGCGA-AC-TGCA--
------
FC88X_00178_00524 ------------------TTTTTCCCTTCACT-AA---CCGCGA-AC-TGCA--
------
FC88X_00176_00527 ------------------TTTTTCCCTTCACT-AA---CCGCGA-AC-TGCA--
------
FC88X_00158_00326 ------------------TTTTTCCCTTCACT-AA---CCGCGA-AC-TGCA--
------
FC88X_00195_00163 ------------------TTTTTCCCTTCACT-AA---CCGCGA-AC-TGCA--
------
FC88X_00175_00659 ------------------TTTTTCCCTTCACT-AA---CCGCGA-AC-TGCA--
------
FC88X_00164_00696 ------------------TTTTTCCCTTCACT-AA---CCGCGA-AC-TGCA--
------
FC88X_00159_00677 ------------------TTTTTCCCTTCACT-AA---CCGCGA-AC-TGCA--
------
FC88X_00109_00710 ------------------TTTTTCCCTTCACT-AA---CCGCGA-AC-TGCA--
------
FC88X_00121_00298 ------------------TTTTTCCCTTCACT-AA---CCGCGA-AC-TGCA--
------

```
FC88X_00139_00210       ------------------TTTTTCCCTTCACT-AA---CCGCGA-AC-TGCA--
------
FC88X_00400_01136       -------------------TTTTCCCTTCACT-AA---CCGCGA-AC-TGCA--
------
FC88X_00123_00245       ------------------TTTTTCCCTTCACT-AA---CCGCGA-AC-TGCA--
------
FC88X_00737_01226       ------------------TTTTTCCCTTCACT-AA---CCGCGA-AC-TGCA--
------
FC88X_00819_00193       ------------------TTTTTCCCTTCACT-AA---CCGCGA-AC-TGCA--
------
FC88X_00806_00136       ------------------TTTTTCCCTTCACT-AA---CCGCGA-AC-TGCA--
------
FC88X_00805_00094       ------------------TTTTTCCCTTCACT-AA---CCGCGA-AC-TGCA--
------
FC88X_00770_01244       ------------------TTTTTCCCTTCACT-AA---CCGCGA-AC-TGCA--
------
FC88X_00770_01061       ------------------TTTTTCCCTTCACT-AA---CCGCGA-AC-TGCA--
------
FC88X_00768_01055       ------------------TTTTTCCCTTCACT-AA---CCGCGA-AC-TGCA--
------
FC88X_00777_00833       ------------------TTTTTCCCTTCACT-AA---CCGCGA-AC-TGCA--
------
FC88X_00781_00726       ------------------TTTTTCCCTTCACT-AA---CCGCGA-AC-TGCA--
------
FC88X_00126_00936       ------------------TTTTTCCCTTCACT-AA---CCGCGA-AC-TGCA--
------
FC88X_00134_00879       ------------------TTTTTCCCTTCACT-AA---CCGCGA-AC-TGCA--
------
FC88X_00132_00889       ------------------TTTTTCCCTTCACT-AA---CCGCGA-AC-TGCA--
------
FC88X_00128_00895       ------------------TTTTTCCCTTCACT-AA---CCGCGA-AC-TGCA--
------
FC88X_00126_00893       ------------------TTTTTCCCTTCACT-AA---CCGCGA-AC-TGCA--
------
FC88X_00191_00086       ------------------TTTTTCCCTTCACT-AA---CCGCGA-AC-TGCA--
------
FC88X_00183_00083       ------------------TTTTTCCCTTCACT-AA---CCGCGA-AC-TGCA--
------
FC88X_00175_00068       ------------------TTTTTCCCTTCACT-AA---CCGCGA-AC-TGCA--
------
FC88X_00164_00089       ------------------TTTTTCCCTTCACT-AA---CCGCGA-AC-TGCA--
------
FC88X_00162_00057       ------------------TTTTTCCCTTCACT-AA---CCGCGA-AC-TGCA--
------
FC88X_00155_00095       ------------------TTTTTCCCTTCACT-AA---CCGCGA-AC-TGCA--
------
FC88X_00134_00974       ------------------TTTTTCCCTTCACT-AA---CCGCGA-AC-TGCA--
------
FC88X_00103_00887       ------------------TTTTTCCCTTCACT-AA---CCGCGA-AC-TGCA--
------
FC88X_01083_00789       ------------------TTTTTCCCTTCACT-AA---CCGCGA-AC-TGCA--
------
FC88X_01096_00739       ------------------TTTTTCCCTTCACT-AA---CCGCGA-AC-TGCA--
------
FC88X_01082_00701       ------------------TTTTTCCCTTCACT-AA---CCGCGA-AC-TGCA--
------
```

FC88X_01063_00722 ------------------TTTTTCCCTTCACT-AA---CCGCGA-AC-TGCA--
------
FC88X_01092_00658 ------------------TTTTTCCCTTCACT-AA---CCGCGA-AC-TGCA--
------
FC88X_00120_00706 ------------------TTTTTCCCTTCACT-AA---CCGCGA-AC-TGCA--
------
FC88X_00118_00935 ------------------TTTTTCCCTTCACT-AA---CCGCGA-AC-TGCA--
------
FC88X_00035_00651 ------------------TTTTTCCCTTCACT-AA---CCGCGA-AC-TGCA--
------
FC88X_00144_00827 ------------------TTTTTCCCTTCACT-AA---CCGCGA-AC-TGCA--
------
FC88X_00137_00844 ------------------TTTTTCCCTTCACT-AA---CCGCGA-AC-TGCA--
------
FC88X_00103_00817 ------------------TTTTTCCCTTCACT-AA---CCGCGA-AC-TGCA--
------
FC88X_00009_00685 ------------------TTTTTCCCTTCACT-AA---CCGCGA-AC-TGCA--
------
FC88X_00125_00694 ------------------TTTTTCCCTTCACT-AA---CCGCGA-AC-TGCA--
------
FC88X_00127_00183 ------------------TTTTTCCCTTCACT-AA---CCGCGA-AC-TGCA--
------
FC88X_00125_00165 ------------------TTTTTCCCTTCACT-AA---CCGCGA-AC-TGCA--
------
FC88X_00110_00150 ------------------TTTTTCCCTTCACT-AA---CCGCGA-AC-TGCA--
------
FC88X_00146_00752 ------------------TTTTTCCCTTCACT-AA---CCGCGA-AC-TGCA--
------
FC88X_00144_00788 ------------------TTTTTCCCTTCACT-AA---CCGCGA-AC-TGCA--
------
FC88X_00144_00761 ------------------TTTTTCCCTTCACT-AA---CCGCGA-AC-TGCA--
------
FC88X_00133_00760 ------------------TTTTTCCCTTCACT-AA---CCGCGA-AC-TGCA--
------
FC88X_00848_00259 ------------------TTTTTCCCTTCACT-AA---CCGCGA-AC-TGCA--
------
FC88X_00830_00293 ------------------TTTTTCCCTTCACT-AA---CCGCGA-AC-TGCA--
------
FC88X_00807_00201 ------------------TTTTTCCCTTCACT-AA---CCGCGA-AC-TGCA--
------
FC88X_00843_00153 ------------------TTTTTCCCTTCACT-AA---CCGCGA-AC-TGCA--
------
FC88X_00841_00184 ------------------TTTTTCCCTTCACT-AA---CCGCGA-AC-TGCA--
------
FC88X_00840_00154 ------------------TTTTTCCCTTCACT-AA---CCGCGA-AC-TGCA--
------
FC88X_00798_00477 ------------------TTTTTCCCTTCACT-AA---CCGCGA-AC-TGCA--
------
FC88X_00778_00463 ------------------TTTTTCCCTTCACT-AA---CCGCGA-AC-TGCA--
------
FC88X_00779_00295 ------------------TTTTTCCCTTCACT-AA---CCGCGA-AC-TGCA--
------
FC88X_00777_00264 ------------------TTTTTCCCTTCACT-AA---CCGCGA-AC-TGCA--
------
FC88X_00770_00257 ------------------TTTTTCCCTTCACT-AA---CCGCGA-AC-TGCA--
------

FC88X_00763_00298 ------------------TTTTTCCCTTCACT-AA---CCGCGA-AC-TGCA--
------
FC88X_00755_00289 ------------------TTTTTCCCTTCACT-AA---CCGCGA-AC-TGCA--
------
FC88X_00896_00322 ------------------TTTTTCCCTTCACT-AA---CCGCGA-AC-TGCA--
------
FC88X_00868_00318 ------------------TTTTTCCCTTCACT-AA---CCGCGA-AC-TGCA--
------
FC88X_00884_00261 ------------------TTTTTCCCTTCACT-AA---CCGCGA-AC-TGCA--
------
FC88X_00896_00183 ------------------TTTTTCCCTTCACT-AA---CCGCGA-AC-TGCA--
------
FC88X_00835_00865 ------------------TTTTTCCCTTCACT-AA---CCGCGA-AC-TGCA--
------
FC88X_00891_00402 ------------------TTTTTCCCTTCACT-AA---CCGCGA-AC-TGCA--
------
FC88X_00875_00435 ------------------TTTTTCCCTTCACT-AA---CCGCGA-AC-TGCA--
------
FC88X_00866_00409 ------------------TTTTTCCCTTCACT-AA---CCGCGA-AC-TGCA--
------
FC88X_01127_00890 ------------------TTTTTCCCTTCACT-AA---CCGCGA-AC-TGCA--
------
FC88X_01142_00817 ------------------TTTTTCCCTTCACT-AA---CCGCGA-AC-TGCA--
------
FC88X_01100_00939 ------------------TTTTTCCCTTCACT-AA---CCGCGA-AC-TGCA--
------
FC88X_00860_00195 ------------------TTTTTCCCTTCACT-AA---CCGCGA-AC-TGCA--
------
FC88X_00851_00164 ------------------TTTTTCCCTTCACT-AA---CCGCGA-AC-TGCA--
------
FC88X_00850_00151 ------------------TTTTTCCCTTCACT-AA---CCGCGA-AC-TGCA--
------
FC88X_00842_01257 ------------------TTTTTCCCTTCACT-AA---CCGCGA-AC-TGCA--
------
FC88X_00847_01210 ------------------TTTTTCCCTTCACT-AA---CCGCGA-AC-TGCA--
------
FC88X_00834_01246 ------------------TTTTTCCCTTCACT-AA---CCGCGA-AC-TGCA--
------
FC88X_00834_01207 ------------------TTTTTCCCTTCACT-AA---CCGCGA-AC-TGCA--
------
FC88X_00807_01211 ------------------TTTTTCCCTTCACT-AA---CCGCGA-AC-TGCA--
------
FC88X_00805_01243 ------------------TTTTTCCCTTCACT-AA---CCGCGA-AC-TGCA--
------
FC88X_00816_01150 ------------------TTTTTCCCTTCACT-AA---CCGCGA-AC-TGCA--
------
FC88X_00815_01161 ------------------TTTTTCCCTTCACT-AA---CCGCGA-AC-TGCA--
------
FC88X_00813_01150 ------------------TTTTTCCCTTCACT-AA---CCGCGA-AC-TGCA--
------
FC88X_00824_00908 ------------------TTTTTCCCTTCACT-AA---CCGCGA-AC-TGCA--
------
FC88X_00804_00938 ------------------TTTTTCCCTTCACT-AA---CCGCGA-AC-TGCA--
------
FC88X_00014_00260 ------------------TTTTTCCCTTCACT-AA---CCGCGA-AC-TGCA--
------

FC88X_00014_00254 ------------------TTTTTCCCTTCACT-AA---CCGCGA-AC-TGCA--
------
FC88X_00010_00260 ------------------TTTTTCCCTTCACT-AA---CCGCGA-AC-TGCA--
------
FC88X_00780_00098 ------------------TTTTTCCCTTCACT-AA---CCGCGA-AC-TGCA--
------
FC88X_00730_01201 ------------------TTTTTCCCTTCACT-AA---CCGCGA-AC-TGCA--
------
FC88X_00719_01221 ------------------TTTTTCCCTTCACT-AA---CCGCGA-AC-TGCA--
------
FC88X_00767_00193 ------------------TTTTTCCCTTCACT-AA---CCGCGA-AC-TGCA--
------
FC88X_00763_00175 ------------------TTTTTCCCTTCACT-AA---CCGCGA-AC-TGCA--
------
FC88X_00796_00113 ------------------TTTTTCCCTTCACT-AA---CCGCGA-AC-TGCA--
------
FC88X_00792_00136 ------------------TTTTTCCCTTCACT-AA---CCGCGA-AC-TGCA--
------
FC88X_00775_00104 ------------------TTTTTCCCTTCACT-AA---CCGCGA-AC-TGCA--
------
FC88X_00793_00214 ------------------TTTTTCCCTTCACT-AA---CCGCGA-AC-TGCA--
------
FC88X_00775_00961 ------------------TTTTTCCCTTCACT-AA---CCGCGA-AC-TGCA--
------
FC88X_00144_00594 ------------------TTTTTCCCTTCACT-AA---CCGCGA-AC-TGCA--
------
FC88X_00138_00569 ------------------TTTTTCCCTTCACT-AA---CCGCGA-AC-TGCA--
------
FC88X_00856_00299 ------------------TTTTTCCCTTCACT-AA---CCGCGA-AC-TGCA--
------
FC88X_00895_00608 ------------------TTTTTCCCTTCACT-AA---CCGCGA-AC-TGCA--
------
FC88X_00887_00638 ------------------TTTTTCCCTTCACT-AA---CCGCGA-AC-TGCA--
------
FC88X_00887_00623 ------------------TTTTTCCCTTCACT-AA---CCGCGA-AC-TGCA--
------
FC88X_00881_00601 ------------------TTTTTCCCTTCACT-AA---CCGCGA-AC-TGCA--
------
FC88X_00889_00561 ------------------TTTTTCCCTTCACT-AA---CCGCGA-AC-TGCA--
------
FC88X_00877_00586 ------------------TTTTTCCCTTCACT-AA---CCGCGA-AC-TGCA--
------
FC88X_00891_00509 ------------------TTTTTCCCTTCACT-AA---CCGCGA-AC-TGCA--
------
FC88X_00884_00517 ------------------TTTTTCCCTTCACT-AA---CCGCGA-AC-TGCA--
------
FC88X_00802_00863 ------------------TTTTTCCCTTCACT-AA---CCGCGA-AC-TGCA--
------
FC88X_00807_00779 ------------------TTTTTCCCTTCACT-AA---CCGCGA-AC-TGCA--
------
FC88X_00839_00404 ------------------TTTTTCCCTTCACT-AA---CCGCGA-AC-TGCA--
------
FC88X_00835_00328 ------------------TTTTTCCCTTCACT-AA---CCGCGA-AC-TGCA--
------
FC88X_00833_00322 ------------------TTTTTCCCTTCACT-AA---CCGCGA-AC-TGCA--
------

```
FC88X_00802_01151      ------------------TTTTTCCCTTCACT-AA---CCGCGA-AC-TGCA--
------
FC88X_00818_01104      ------------------TTTTTCCCTTCACT-AA---CCGCGA-AC-TGCA--
------
FC88X_00805_01051      ------------------TTTTTCCCTTCACT-AA---CCGCGA-AC-TGCA--
------
FC88X_00826_01004      ------------------TTTTTCCCTTCACT-AA---CCGCGA-AC-TGCA--
------
FC88X_00806_01043      ------------------TTTTTCCCTTCACT-AA---CCGCGA-AC-TGCA--
------
FC88X_00819_00981      ------------------TTTTTCCCTTCACT-AA---CCGCGA-AC-TGCA--
------
FC88X_00813_00953      ------------------TTTTTCCCTTCACT-AA---CCGCGA-AC-TGCA--
------
FC88X_00485_00996      -------------------TTTTCCCTTCACT-AA---CCGCGA-AC-TGCA--
------
FC88X_00488_00693      -------------------TTTTCCCTTCACT-AA---CCGCGA-AC-TGCA--
------
FC88X_00462_00568      -------------------TTTTCCCTTCACT-AA---CCGCGA-AC-TGCA--
------
FC88X_00460_00585      -------------------TTTTCCCTTCACT-AA---CCGCGA-AC-TGCA--
------
FC88X_00488_00548      -------------------TTTTCCCTTCACT-AA---CCGCGA-AC-TGCA--
------
FC88X_00480_00465      -------------------TTTTCCCTTCACT-AA---CCGCGA-AC-TGCA--
------
FC88X_00459_00427      -------------------TTTTCCCTTCACT-AA---CCGCGA-AC-TGCA--
------
FC88X_00460_00302      -------------------TTTTCCCTTCACT-AA---CCGCGA-AC-TGCA--
------
FC88X_00455_00251      -------------------TTTTCCCTTCACT-AA---CCGCGA-AC-TGCA--
------
FC88X_00482_00077      -------------------TTTTCCCTTCACT-AA---CCGCGA-AC-TGCA--
------
FC88X_00475_00093      -------------------TTTTCCCTTCACT-AA---CCGCGA-AC-TGCA--
------
FC88X_00472_00070      -------------------TTTTCCCTTCACT-AA---CCGCGA-AC-TGCA--
------
FC88X_00471_00076      -------------------TTTTCCCTTCACT-AA---CCGCGA-AC-TGCA--
------
FC88X_00457_00051      -------------------TTTTCCCTTCACT-AA---CCGCGA-AC-TGCA--
------
FC88X_00456_00087      -------------------TTTTCCCTTCACT-AA---CCGCGA-AC-TGCA--
------
FC88X_00437_01131      -------------------TTTTCCCTTCACT-AA---CCGCGA-AC-TGCA--
------
FC88X_00709_01249      ------------------TTTTTCCCTTCACT-AA---CCGCGA-AC-TGCA--
------
FC88X_00719_01153      ------------------TTTTTCCCTTCACT-AA---CCGCGA-AC-TGCA--
------
FC88X_01126_00506      ------------------TTTTTCCCTTCACT-AA---CCGCGA-AC-TGCA--
------
FC88X_01109_00529      ------------------TTTTTCCCTTCACT-AA---CCGCGA-AC-TGCA--
------
FC88X_01102_00432      ------------------TTTTTCCCTTCACT-AA---CCGCGA-AC-TGCA--
------
```

```
FC88X_01053_01209      ------------------TTTTTCCCTTCACT-AA---CCGCGA-AC-TGCA--
------
FC88X_01075_01153      ------------------TTTTTCCCTTCACT-AA---CCGCGA-AC-TGCA--
------
FC88X_01055_01168      ------------------TTTTTCCCTTCACT-AA---CCGCGA-AC-TGCA--
------
FC88X_01052_01182      ------------------TTTTTCCCTTCACT-AA---CCGCGA-AC-TGCA--
------
FC88X_01079_01114      ------------------TTTTTCCCTTCACT-AA---CCGCGA-AC-TGCA--
------
FC88X_01074_01125      ------------------TTTTTCCCTTCACT-AA---CCGCGA-AC-TGCA--
------
FC88X_01073_01118      ------------------TTTTTCCCTTCACT-AA---CCGCGA-AC-TGCA--
------
FC88X_01069_01141      ------------------TTTTTCCCTTCACT-AA---CCGCGA-AC-TGCA--
------
FC88X_01065_01140      ------------------TTTTTCCCTTCACT-AA---CCGCGA-AC-TGCA--
------
FC88X_01053_01107      ------------------TTTTTCCCTTCACT-AA---CCGCGA-AC-TGCA--
------
FC88X_01074_01061      ------------------TTTTTCCCTTCACT-AA---CCGCGA-AC-TGCA--
------
FC88X_01067_01058      ------------------TTTTTCCCTTCACT-AA---CCGCGA-AC-TGCA--
------
FC88X_01053_01099      ------------------TTTTTCCCTTCACT-AA---CCGCGA-AC-TGCA--
------
FC88X_01057_01032      ------------------TTTTTCCCTTCACT-AA---CCGCGA-AC-TGCA--
------
FC88X_01056_01039      ------------------TTTTTCCCTTCACT-AA---CCGCGA-AC-TGCA--
------
FC88X_01075_00956      ------------------TTTTTCCCTTCACT-AA---CCGCGA-AC-TGCA--
------
FC88X_00703_01062      ------------------TTTTTCCCTTCACT-AA---CCGCGA-AC-TGCA--
------
FC88X_00027_00452      ------------------TTTTTCCCTTCACT-AA---CCGCGA-AC-TGCA--
------
FC88X_01086_00936      ------------------TTTTTCCCTTCACT-AA---CCGCGA-AC-TGCA--
------
FC88X_01086_00865      ------------------TTTTTCCCTTCACT-AA---CCGCGA-AC-TGCA--
------
FC88X_01095_00831      ------------------TTTTTCCCTTCACT-AA---CCGCGA-AC-TGCA--
------
FC88X_01087_00605      ------------------TTTTTCCCTTCACT-AA---CCGCGA-AC-TGCA--
------
FC88X_00968_00698      ------------------TTTTTCCCTTCACT-AA---CCGCGA-AC-TGCA--
------
FC88X_00956_00678      ------------------TTTTTCCCTTCACT-AA---CCGCGA-AC-TGCA--
------
FC88X_00957_00334      ------------------TTTTTCCCTTCACT-AA---CCGCGA-AC-TGCA--
------
FC88X_00998_00274      ------------------TTTTTCCCTTCACT-AA---CCGCGA-AC-TGCA--
------
FC88X_00953_00282      ------------------TTTTTCCCTTCACT-AA---CCGCGA-AC-TGCA--
------
FC88X_00741_00763      ------------------TTTTTCCCTTCACT-AA---CCGCGA-AC-TGCA--
------
```

FC88X_00748_00358 ------------------TTTTTCCCTTCACT-AA---CCGCGA-AC-TGCA--
------
FC88X_00084_00406 ------------------TTTTTCCCTTCACT-AA---CCGCGA-AC-TGCA--
------
FC88X_00888_00803 ------------------TTTTTCCCTTCACT-AA---CCGCGA-AC-TGCA--
------
FC88X_00868_00772 ------------------TTTTTCCCTTCACT-AA---CCGCGA-AC-TGCA--
------
FC88X_00853_00761 ------------------TTTTTCCCTTCACT-AA---CCGCGA-AC-TGCA--
------
FC88X_00992_00671 ------------------TTTTTCCCTTCACT-AA---CCGCGA-AC-TGCA--
------
FC88X_00979_00671 ------------------TTTTTCCCTTCACT-AA---CCGCGA-AC-TGCA--
------
FC88X_01109_00813 ------------------TTTTTCCCTTCACT-AA---CCGCGA-AC-TGCA--
------
FC88X_01120_00784 ------------------TTTTTCCCTTCACT-AA---CCGCGA-AC-TGCA--
------
FC88X_01105_00779 ------------------TTTTTCCCTTCACT-AA---CCGCGA-AC-TGCA--
------
FC88X_01100_00781 ------------------TTTTTCCCTTCACT-AA---CCGCGA-AC-TGCA--
------
FC88X_01130_00749 ------------------TTTTTCCCTTCACT-AA---CCGCGA-AC-TGCA--
------
FC88X_01103_00730 ------------------TTTTTCCCTTCACT-AA---CCGCGA-AC-TGCA--
------
FC88X_01100_00709 ------------------TTTTTCCCTTCACT-AA---CCGCGA-AC-TGCA--
------
FC88X_01108_00678 ------------------TTTTTCCCTTCACT-AA---CCGCGA-AC-TGCA--
------
FC88X_01128_00600 ------------------TTTTTCCCTTCACT-AA---CCGCGA-AC-TGCA--
------
FC88X_01112_00586 ------------------TTTTTCCCTTCACT-AA---CCGCGA-AC-TGCA--
------
FC88X_01112_00584 ------------------TTTTTCCCTTCACT-AA---CCGCGA-AC-TGCA--
------
FC88X_01107_01023 ------------------TTTTTCCCTTCACT-AA---CCGCGA-AC-TGCA--
------
FC88X_01121_00960 ------------------TTTTTCCCTTCACT-AA---CCGCGA-AC-TGCA--
------
FC88X_01106_00958 ------------------TTTTTCCCTTCACT-AA---CCGCGA-AC-TGCA--
------
FC88X_00877_00999 ------------------TTTTTCCCTTCACT-AA---CCGCGA-AC-TGCA--
------
FC88X_00871_00978 ------------------TTTTTCCCTTCACT-AA---CCGCGA-AC-TGCA--
------
FC88X_00870_00988 ------------------TTTTTCCCTTCACT-AA---CCGCGA-AC-TGCA--
------
FC88X_00867_00986 ------------------TTTTTCCCTTCACT-AA---CCGCGA-AC-TGCA--
------
FC88X_00864_00994 ------------------TTTTTCCCTTCACT-AA---CCGCGA-AC-TGCA--
------
FC88X_00843_00752 ------------------TTTTTCCCTTCACT-AA---CCGCGA-AC-TGCA--
------
FC88X_00005_00253 ------------------TTTTTCCCTTCACT-AA---CCGCGA-AC-TGCA--
------

```
FC88X_00049_00217      ------------------TTTTTCCCTTCACT-AA---CCGCGA-AC-TGCA--
------
FC88X_00046_00240      ------------------TTTTTCCCTTCACT-AA---CCGCGA-AC-TGCA--
------
FC88X_01038_01181      ------------------TTTTTCCCTTCACT-AA---CCGCGA-AC-TGCA--
------
FC88X_01030_01190      ------------------TTTTTCCCTTCACT-AA---CCGCGA-AC-TGCA--
------
FC88X_01026_01177      ------------------TTTTTCCCTTCACT-AA---CCGCGA-AC-TGCA--
------
FC88X_01017_01168      ------------------TTTTTCCCTTCACT-AA---CCGCGA-AC-TGCA--
------
FC88X_00976_00486      ------------------TTTTTCCCTTCACT-AA---CCGCGA-AC-TGCA--
------
FC88X_00972_00458      ------------------TTTTTCCCTTCACT-AA---CCGCGA-AC-TGCA--
------
FC88X_00007_00292      ------------------TTTTTCCCTTCACT-AA---CCGCGA-AC-TGCA--
------
FC88X_01030_01079      ------------------TTTTTCCCTTCACT-AA---CCGCGA-AC-TGCA--
------
FC88X_01025_01095      ------------------TTTTTCCCTTCACT-AA---CCGCGA-AC-TGCA--
------
FC88X_00178_00984      ------------------TTTTTCCCTTCACT-AA---CCGCGA-AC-TGCA--
------
FC88X_00709_01164      ------------------TTTTTCCCTTCACT-AA---CCGCGA-AC-TGCA--
------
FC88X_00729_01135      ------------------TTTTTCCCTTCACT-AA---CCGCGA-AC-TGCA--
------
FC88X_01002_00845      ------------------TTTTTCCCTTCACT-AA---CCGCGA-AC-TGCA--
------
FC88X_01001_00628      ------------------TTTTTCCCTTCACT-AA---CCGCGA-AC-TGCA--
------
FC88X_01001_00564      ------------------TTTTTCCCTTCACT-AA---CCGCGA-AC-TGCA--
------
FC88X_00939_01025      ------------------TTTTTCCCTTCACT-AA---CCGCGA-AC-TGCA--
------
FC88X_01000_00985      ------------------TTTTTCCCTTCACT-AA---CCGCGA-AC-TGCA--
------
FC88X_01040_00937      ------------------TTTTTCCCTTCACT-AA---CCGCGA-AC-TGCA--
------
FC88X_01019_00924      ------------------TTTTTCCCTTCACT-AA---CCGCGA-AC-TGCA--
------
FC88X_01047_00854      ------------------TTTTTCCCTTCACT-AA---CCGCGA-AC-TGCA--
------
FC88X_01027_00871      ------------------TTTTTCCCTTCACT-AA---CCGCGA-AC-TGCA--
------
FC88X_01017_00877      ------------------TTTTTCCCTTCACT-AA---CCGCGA-AC-TGCA--
------
FC88X_01023_00812      ------------------TTTTTCCCTTCACT-AA---CCGCGA-AC-TGCA--
------
FC88X_01018_00813      ------------------TTTTTCCCTTCACT-AA---CCGCGA-AC-TGCA--
------
FC88X_00946_00915      ------------------TTTTTCCCTTCACT-AA---CCGCGA-AC-TGCA--
------
FC88X_00862_01144      ------------------TTTTTCCCTTCACT-AA---CCGCGA-AC-TGCA--
------
```

FC88X_00874_01063 -------------------TTTTTCCCTTCACT-AA---CCGCGA-AC-TGCA--
------
FC88X_00889_01023 -------------------TTTTTCCCTTCACT-AA---CCGCGA-AC-TGCA--
------
FC88X_00851_01008 -------------------TTTTTCCCTTCACT-AA---CCGCGA-AC-TGCA--
------
FC88X_00990_00511 -------------------TTTTTCCCTTCACT-AA---CCGCGA-AC-TGCA--
------
FC88X_00978_00515 -------------------TTTTTCCCTTCACT-AA---CCGCGA-AC-TGCA--
------
FC88X_00966_00521 -------------------TTTTTCCCTTCACT-AA---CCGCGA-AC-TGCA--
------
FC88X_00042_00479 -------------------TTTTTCCCTTCACT-AA---CCGCGA-AC-TGCA--
------
FC88X_00040_00488 -------------------TTTTTCCCTTCACT-AA---CCGCGA-AC-TGCA--
------
FC88X_00112_00106 -------------------TTTTTCCCTTCACT-AA---CCGCGA-AC-TGCA--
------
FC88X_00976_00376 -------------------TTTTTCCCTTCACT-AA---CCGCGA-AC-TGCA--
------
FC88X_00976_00368 -------------------TTTTTCCCTTCACT-AA---CCGCGA-AC-TGCA--
------
FC88X_00964_00367 -------------------TTTTTCCCTTCACT-AA---CCGCGA-AC-TGCA--
------
FC88X_00959_00396 -------------------TTTTTCCCTTCACT-AA---CCGCGA-AC-TGCA--
------
FC88X_00956_00390 -------------------TTTTTCCCTTCACT-AA---CCGCGA-AC-TGCA--
------
FC88X_00959_00237 -------------------TTTTTCCCTTCACT-AA---CCGCGA-AC-TGCA--
------
FC88X_00926_01250 -------------------TTTTTCCCTTCACT-AA---CCGCGA-AC-TGCA--
------
FC88X_00911_01226 -------------------TTTTTCCCTTCACT-AA---CCGCGA-AC-TGCA--
------
FC88X_00941_01166 -------------------TTTTTCCCTTCACT-AA---CCGCGA-AC-TGCA--
------
FC88X_00931_01120 -------------------TTTTTCCCTTCACT-AA---CCGCGA-AC-TGCA--
------
FC88X_00920_01123 -------------------TTTTTCCCTTCACT-AA---CCGCGA-AC-TGCA--
------
FC88X_00748_00895 -------------------TTTTTCCCTTCACT-AA---CCGCGA-AC-TGCA--
------
FC88X_00738_00334 -------------------TTTTTCCCTTCACT-AA---CCGCGA-AC-TGCA--
------
FC88X_00719_00322 -------------------TTTTTCCCTTCACT-AA---CCGCGA-AC-TGCA--
------
FC88X_00715_00323 -------------------TTTTTCCCTTCACT-AA---CCGCGA-AC-TGCA--
------
FC88X_00171_00953 -------------------TTTTTCCCTTCACT-AA---CCGCGA-AC-TGCA--
------
FC88X_00151_00979 -------------------TTTTTCCCTTCACT-AA---CCGCGA-AC-TGCA--
------
FC88X_00199_00918 -------------------TTTTTCCCTTCACT-AA---CCGCGA-AC-TGCA--
------
FC88X_00192_00948 -------------------TTTTTCCCTTCACT-AA---CCGCGA-AC-TGCA--
------

FC88X_00172_00902 ------------------TTTTTCCCTTCACT-AA---CCGCGA-AC-TGCA--
------
FC88X_00167_00916 ------------------TTTTTCCCTTCACT-AA---CCGCGA-AC-TGCA--
------
FC88X_00953_01017 ------------------TTTTTCCCTTCACT-AA---CCGCGA-AC-TGCA--
------
FC88X_00953_00956 ------------------TTTTTCCCTTCACT-AA---CCGCGA-AC-TGCA--
------
FC88X_01027_00514 ------------------TTTTTCCCTTCACT-AA---CCGCGA-AC-TGCA--
------
FC88X_01025_00522 ------------------TTTTTCCCTTCACT-AA---CCGCGA-AC-TGCA--
------
FC88X_01040_00456 ------------------TTTTTCCCTTCACT-AA---CCGCGA-AC-TGCA--
------
FC88X_01024_00455 ------------------TTTTTCCCTTCACT-AA---CCGCGA-AC-TGCA--
------
FC88X_01011_00466 ------------------TTTTTCCCTTCACT-AA---CCGCGA-AC-TGCA--
------
FC88X_00981_00804 ------------------TTTTTCCCTTCACT-AA---CCGCGA-AC-TGCA--
------
FC88X_00967_01205 ------------------TTTTTCCCTTCACT-AA---CCGCGA-AC-TGCA--
------
FC88X_00957_01241 ------------------TTTTTCCCTTCACT-AA---CCGCGA-AC-TGCA--
------
FC88X_00981_01160 ------------------TTTTTCCCTTCACT-AA---CCGCGA-AC-TGCA--
------
FC88X_00057_00503 ------------------TTTTTCCCTTCACT-AA---CCGCGA-AC-TGCA--
------
FC88X_00945_00866 ------------------TTTTTCCCTTCACT-AA---CCGCGA-AC-TGCA--
------
FC88X_00926_00876 ------------------TTTTTCCCTTCACT-AA---CCGCGA-AC-TGCA--
------
FC88X_00905_00852 ------------------TTTTTCCCTTCACT-AA---CCGCGA-AC-TGCA--
------
FC88X_00939_00816 ------------------TTTTTCCCTTCACT-AA---CCGCGA-AC-TGCA--
------
FC88X_00050_00644 ------------------TTTTTCCCTTCACT-AA---CCGCGA-AC-TGCA--
------
FC88X_00091_00579 ------------------TTTTTCCCTTCACT-AA---CCGCGA-AC-TGCA--
------
FC88X_01027_00429 ------------------TTTTTCCCTTCACT-AA---CCGCGA-AC-TGCA--
------
FC88X_01025_00352 ------------------TTTTTCCCTTCACT-AA---CCGCGA-AC-TGCA--
------
FC88X_01044_00324 ------------------TTTTTCCCTTCACT-AA---CCGCGA-AC-TGCA--
------
FC88X_01029_00315 ------------------TTTTTCCCTTCACT-AA---CCGCGA-AC-TGCA--
------
FC88X_01012_00342 ------------------TTTTTCCCTTCACT-AA---CCGCGA-AC-TGCA--
------
FC88X_01011_00305 ------------------TTTTTCCCTTCACT-AA---CCGCGA-AC-TGCA--
------
FC88X_00973_01257 ------------------TTTTTCCCTTCACT-AA---CCGCGA-AC-TGCA--
------
FC88X_00642_01071 -------------------TTTTCCCTTCACT-AA---CCGCGA-AC-TGCA--
------

```
FC88X_00620_01082       -------------------TTTTCCCTTCACT-AA---CCGCGA-AC-TGCA--
------
FC88X_00624_00941       -------------------TTTTCCCTTCACT-AA---CCGCGA-AC-TGCA--
------
FC88X_00620_00834       -------------------TTTTCCCTTCACT-AA---CCGCGA-AC-TGCA--
------
FC88X_00609_00707       -------------------TTTTCCCTTCACT-AA---CCGCGA-AC-TGCA--
------
FC88X_00620_00425       -------------------TTTTCCCTTCACT-AA---CCGCGA-AC-TGCA--
------
FC88X_00197_00838       ------------------TTTTTCCCTTCACT-AA---CCGCGA-AC-TGCA--
------
FC88X_00099_00625       ------------------TTTTTCCCTTCACT-AA---CCGCGA-AC-TGCA--
------
FC88X_00288_01000       -------------------TTTTCCCTTCACT-AA---CCGCGA-AC-TGCA--
------
FC88X_00254_01039       -------------------TTTTCCCTTCACT-AA---CCGCGA-AC-TGCA--
------
FC88X_00279_00976       -------------------TTTTCCCTTCACT-AA---CCGCGA-AC-TGCA--
------
FC88X_01093_00560       ------------------TTTTTCCCTTCACT-AA---CCGCGA-AC-TGCA--
------
FC88X_01072_00575       ------------------TTTTTCCCTTCACT-AA---CCGCGA-AC-TGCA--
------
FC88X_01092_00538       ------------------TTTTTCCCTTCACT-AA---CCGCGA-AC-TGCA--
------
FC88X_01096_00456       ------------------TTTTTCCCTTCACT-AA---CCGCGA-AC-TGCA--
------
FC88X_00152_00853       ------------------TTTTTCCCTTCACT-AA---CCGCGA-AC-TGCA--
------
FC88X_00068_00602       ------------------TTTTTCCCTTCACT-AA---CCGCGA-AC-TGCA--
------
FC88X_00618_00256       -------------------TTTTCCCTTCACT-AA---CCGCGA-AC-TGCA--
------
FC88X_00606_00262       -------------------TTTTCCCTTCACT-AA---CCGCGA-AC-TGCA--
------
FC88X_00643_00088       -------------------TTTTCCCTTCACT-AA---CCGCGA-AC-TGCA--
------
FC88X_00274_00835       -------------------TTTTCCCTTCACT-AA---CCGCGA-AC-TGCA--
------
FC88X_00058_00658       ------------------TTTTTCCCTTCACT-AA---CCGCGA-AC-TGCA--
------
FC88X_00546_01002       -------------------TTTTCCCTTCACT-AA---CCGCGA-AC-TGCA--
------
FC88X_00544_00955       -------------------TTTTCCCTTCACT-AA---CCGCGA-AC-TGCA--
------
FC88X_00521_00952       -------------------TTTTCCCTTCACT-AA---CCGCGA-AC-TGCA--
------
FC88X_00523_00269       -------------------TTTTCCCTTCACT-AA---CCGCGA-AC-TGCA--
------
FC88X_00530_00122       -------------------TTTTCCCTTCACT-AA---CCGCGA-AC-TGCA--
------
FC88X_00521_00064       -------------------TTTTCCCTTCACT-AA---CCGCGA-AC-TGCA--
------
FC88X_00471_01215       -------------------TTTTCCCTTCACT-AA---CCGCGA-AC-TGCA--
------
```

```
FC88X_00463_01209      -------------------TTTTCCCTTCACT-AA---CCGCGA-AC-TGCA--
------
FC88X_00453_01171      -------------------TTTTCCCTTCACT-AA---CCGCGA-AC-TGCA--
------
FC88X_00476_01126      -------------------TTTTCCCTTCACT-AA---CCGCGA-AC-TGCA--
------
FC88X_00461_00014      -------------------TTTTCCCTTCACT-AA---CCGCGA-AC-TGCA--
------
FC88X_00408_00718      -------------------TTTTCCCTTCACT-AA---CCGCGA-AC-TGCA--
------
FC88X_00422_00607      -------------------TTTTCCCTTCACT-AA---CCGCGA-AC-TGCA--
------
FC88X_00401_00365      -------------------TTTTCCCTTCACT-AA---CCGCGA-AC-TGCA--
------
FC88X_00449_00153      -------------------TTTTCCCTTCACT-AA---CCGCGA-AC-TGCA--
------
FC88X_00190_00820      ------------------TTTTTCCCTTCACT-AA---CCGCGA-AC-TGCA--
------
FC88X_00272_01105      -------------------TTTTCCCTTCACT-AA---CCGCGA-AC-TGCA--
------
FC88X_00174_00891      ------------------TTTTTCCCTTCACT-AA---CCGCGA-AC-TGCA--
------
FC88X_00625_01188      ------------------TTTTTCCCTTCACT-AA---CCGCGA-AC-TGCA--
------
FC88X_00647_01145      ------------------TTTTTCCCTTCACT-AA---CCGCGA-AC-TGCA--
------
FC88X_00622_01128      ------------------TTTTTCCCTTCACT-AA---CCGCGA-AC-TGCA--
------
FC88X_00617_01137      ------------------TTTTTCCCTTCACT-AA---CCGCGA-AC-TGCA--
------
FC88X_00601_01116      ------------------TTTTTCCCTTCACT-AA---CCGCGA-AC-TGCA--
------
FC88X_00600_01135      ------------------TTTTTCCCTTCACT-AA---CCGCGA-AC-TGCA--
------
FC88X_00619_01021      ------------------TTTTTCCCTTCACT-AA---CCGCGA-AC-TGCA--
------
FC88X_00072_00557      ------------------TTTTTCCCTTCACT-AA---CCGCGA-AC-TGCA--
------
FC88X_00114_00134      ------------------TTTTTCCCTTCACT-AA---CCGCGA-AC-TGCA--
------
FC88X_00080_00568      ------------------TTTTTCCCTTCACT-AA---CCGCGA-AC-TGCA--
------
FC88X_00890_01170      ------------------TTTTTCCCTTCACT-AA---CCGCGA-AC-TGCA--
------
FC88X_00878_01176      ------------------TTTTTCCCTTCACT-AA---CCGCGA-AC-TGCA--
------
FC88X_00979_00632      ------------------TTTTTCCCTTCACT-AA---CCGCGA-AC-TGCA--
------
FC88X_00089_00259      ------------------TTTTTCCCTTCACT-AA---CCGCGA-AC-TGCA--
------
FC88X_00561_01165      ------------------TTTTTCCCTTCACT-AA---CCGCGA-AC-TGCA--
------
FC88X_00865_00441      -------------------TTTTCCCTTCACT-AA---CCGCGA-AC-TGCA--
------
FC88X_00890_00390      -------------------TTTTCCCTTCACT-AA---CCGCGA-AC-TGCA--
------
```

FC88X_00879_00371 -------------------TTTTCCCTTCACT-AA---CCGCGA-AC-TGCA--
------
FC88X_00677_00735 ------------------TTTTTCCCTTCACT-AA---CCGCGA-AC-TGCA--
------
FC88X_00668_00745 ------------------TTTTTCCCTTCACT-AA---CCGCGA-AC-TGCA--
------
FC88X_00665_00747 ------------------TTTTTCCCTTCACT-AA---CCGCGA-AC-TGCA--
------
FC88X_00661_00557 ------------------TTTTTCCCTTCACT-AA---CCGCGA-AC-TGCA--
------
FC88X_00025_00430 ------------------TTTTTCCCTTCACT-AA---CCGCGA-AC-TGCA--
------
FC88X_00004_00273 ------------------TTTTTCCCTTCACT-AA---CCGCGA-AC-TGCA--
------
FC88X_00075_00102 ------------------TTTTTCCCTTCACT-AA---CCGCGA-AC-TGCA--
------
FC88X_00075_00101 ------------------TTTTTCCCTTCACT-AA---CCGCGA-AC-TGCA--
------
FC88X_00937_00456 ------------------TTTTTCCCTTCACT-AA---CCGCGA-AC-TGCA--
------
FC88X_00943_00426 ------------------TTTTTCCCTTCACT-AA---CCGCGA-AC-TGCA--
------
FC88X_00931_00417 ------------------TTTTTCCCTTCACT-AA---CCGCGA-AC-TGCA--
------
FC88X_00941_00266 ------------------TTTTTCCCTTCACT-AA---CCGCGA-AC-TGCA--
------
FC88X_00926_00252 ------------------TTTTTCCCTTCACT-AA---CCGCGA-AC-TGCA--
------
FC88X_00907_00262 ------------------TTTTTCCCTTCACT-AA---CCGCGA-AC-TGCA--
------
FC88X_00919_00174 ------------------TTTTTCCCTTCACT-AA---CCGCGA-AC-TGCA--
------
FC88X_00904_00181 ------------------TTTTTCCCTTCACT-AA---CCGCGA-AC-TGCA--
------
FC88X_00892_01256 ------------------TTTTTCCCTTCACT-AA---CCGCGA-AC-TGCA--
------
FC88X_00644_01161 ------------------TTTTTCCCTTCACT-AA---CCGCGA-AC-TGCA--
------
FC88X_00015_00201 ------------------TTTTTCCCTTCACT-AA---CCGCGA-AC-TGCA--
------
FC88X_00016_00198 ------------------TTTTTCCCTTCACT-AA---CCGCGA-AC-TGCA--
------
FC88X_00007_00181 ------------------TTTTTCCCTTCACT-AA---CCGCGA-AC-TGCA--
------
FC88X_00031_00147 ------------------TTTTTCCCTTCACT-AA---CCGCGA-AC-TGCA--
------
FC88X_00012_00057 ------------------TTTTTCCCTTCACT-AA---CCGCGA-AC-TGCA--
------
FC88X_00010_00099 ------------------TTTTTCCCTTCACT-AA---CCGCGA-AC-TGCA--
------
FC88X_00007_00080 ------------------TTTTTCCCTTCACT-AA---CCGCGA-AC-TGCA--
------
FC88X_00100_00070 ------------------TTTTTCCCTTCACT-AA---CCGCGA-AC-TGCA--
------
FC88X_00123_00038 ------------------TTTTTCCCTTCACT-AA---CCGCGA-AC-TGCA--
------

FC88X_00113_00048 ------------------TTTTTCCCTTCACT-AA---CCGCGA-AC-TGCA--
------
FC88X_00990_00729 ------------------TTTTTCCCTTCACT-AA---CCGCGA-AC-TGCA--
------
FC88X_00989_00704 ------------------TTTTTCCCTTCACT-AA---CCGCGA-AC-TGCA--
------
FC88X_00091_00805 ------------------TTTTTCCCTTCACT-AA---CCGCGA-AC-TGCA--
------
FC88X_00072_00818 ------------------TTTTTCCCTTCACT-AA---CCGCGA-AC-TGCA--
------
FC88X_01121_00603 ------------------TTTTTCCCTTCACT-AA---CCGCGA-AC-TGCA--
------
FC88X_01105_00621 ------------------TTTTTCCCTTCACT-AA---CCGCGA-AC-TGCA--
------
FC88X_01137_01210 ------------------TTTTTCCCTTCACT-AA---CCGCGA-AC-TGCA--
------
FC88X_01138_01156 ------------------TTTTTCCCTTCACT-AA---CCGCGA-AC-TGCA--
------
FC88X_01126_01144 ------------------TTTTTCCCTTCACT-AA---CCGCGA-AC-TGCA--
------
FC88X_01109_01132 ------------------TTTTTCCCTTCACT-AA---CCGCGA-AC-TGCA--
------
FC88X_01113_01090 ------------------TTTTTCCCTTCACT-AA---CCGCGA-AC-TGCA--
------
FC88X_00935_00757 ------------------TTTTTCCCTTCACT-AA---CCGCGA-AC-TGCA--
------
FC88X_00932_00765 ------------------TTTTTCCCTTCACT-AA---CCGCGA-AC-TGCA--
------
FC88X_00911_00788 ------------------TTTTTCCCTTCACT-AA---CCGCGA-AC-TGCA--
------
FC88X_00938_00726 ------------------TTTTTCCCTTCACT-AA---CCGCGA-AC-TGCA--
------
FC88X_00911_00732 ------------------TTTTTCCCTTCACT-AA---CCGCGA-AC-TGCA--
------
FC88X_00905_00737 ------------------TTTTTCCCTTCACT-AA---CCGCGA-AC-TGCA--
------
FC88X_00931_00618 ------------------TTTTTCCCTTCACT-AA---CCGCGA-AC-TGCA--
------
FC88X_00916_00604 ------------------TTTTTCCCTTCACT-AA---CCGCGA-AC-TGCA--
------
FC88X_00926_00577 ------------------TTTTTCCCTTCACT-AA---CCGCGA-AC-TGCA--
------
FC88X_00947_00516 ------------------TTTTTCCCTTCACT-AA---CCGCGA-AC-TGCA--
------
FC88X_00857_01259 ------------------TTTTTCCCTTCACT-AA---CCGCGA-AC-TGCA--
------
FC88X_00880_01219 ------------------TTTTTCCCTTCACT-AA---CCGCGA-AC-TGCA--
------
FC88X_00082_00851 ------------------TTTTTCCCTTCACT-AA---CCGCGA-AC-TGCA--
------
FC88X_00674_01166 -------------------TTTTCCCTTCACT-AA---CCGCGA-AC-TGCA--
------
FC88X_00112_00791 -------------------TTTTCCCTTCACT-AA---CCGCGA-AC-TGCA--
------
FC88X_00270_00037 -------------------TTTTCCCTTCACT-AA---CCGCGA-AC-TGCA--
------

FC88X_00211_01039 -------------------TTTTCCCTTCACT-AA---CCGCGA-AC-TGCA--
------
FC88X_00224_00955 -------------------TTTTCCCTTCACT-AA---CCGCGA-AC-TGCA--
------
FC88X_00241_00925 -------------------TTTTCCCTTCACT-AA---CCGCGA-AC-TGCA--
------
FC88X_00241_00915 -------------------TTTTCCCTTCACT-AA---CCGCGA-AC-TGCA--
------
FC88X_00225_00930 -------------------TTTTCCCTTCACT-AA---CCGCGA-AC-TGCA--
------
FC88X_00241_00899 -------------------TTTTCCCTTCACT-AA---CCGCGA-AC-TGCA--
------
FC88X_00229_00566 -------------------TTTTCCCTTCACT-AA---CCGCGA-AC-TGCA--
------
FC88X_00226_00531 -------------------TTTTCCCTTCACT-AA---CCGCGA-AC-TGCA--
------
FC88X_00200_00496 -------------------TTTTCCCTTCACT-AA---CCGCGA-AC-TGCA--
------
FC88X_00233_00393 -------------------TTTTCCCTTCACT-AA---CCGCGA-AC-TGCA--
------
FC88X_00119_00092 ------------------TTTTTCCCTTCACT-AA---CCGCGA-AC-TGCA--
------
FC88X_00933_00394 ------------------TTTTTCCCTTCACT-AA---CCGCGA-AC-TGCA--
------
FC88X_00906_00388 ------------------TTTTTCCCTTCACT-AA---CCGCGA-AC-TGCA--
------
FC88X_00592_01206 -------------------TTTTCCCTTCACT-AA---CCGCGA-AC-TGCA--
------
FC88X_00573_01113 -------------------TTTTCCCTTCACT-AA---CCGCGA-AC-TGCA--
------
FC88X_00597_01002 -------------------TTTTCCCTTCACT-AA---CCGCGA-AC-TGCA--
------
FC88X_00074_00782 ------------------TTTTTCCCTTCACT-AA---CCGCGA-AC-TGCA--
------
FC88X_00597_00924 -------------------TTTTCCCTTCACT-AA---CCGCGA-AC-TGCA--
------
FC88X_00556_00820 -------------------TTTTCCCTTCACT-AA---CCGCGA-AC-TGCA--
------
FC88X_00571_00709 -------------------TTTTCCCTTCACT-AA---CCGCGA-AC-TGCA--
------
FC88X_00597_00662 -------------------TTTTCCCTTCACT-AA---CCGCGA-AC-TGCA--
------
FC88X_00567_00256 -------------------TTTTCCCTTCACT-AA---CCGCGA-AC-TGCA--
------
FC88X_00550_00201 -------------------TTTTCCCTTCACT-AA---CCGCGA-AC-TGCA--
------
FC88X_00579_00170 -------------------TTTTCCCTTCACT-AA---CCGCGA-AC-TGCA--
------
FC88X_00581_00103 -------------------TTTTCCCTTCACT-AA---CCGCGA-AC-TGCA--
------
FC88X_00583_00055 -------------------TTTTCCCTTCACT-AA---CCGCGA-AC-TGCA--
------
FC88X_00571_00063 -------------------TTTTCCCTTCACT-AA---CCGCGA-AC-TGCA--
------
FC88X_00524_01251 -------------------TTTTCCCTTCACT-AA---CCGCGA-AC-TGCA--
------

```
FC88X_00259_00591       -------------------TTTTCCCTTCACT-AA---CCGCGA-AC-TGCA--
------
FC88X_00516_01226       -------------------TTTTCCCTTCACT-AA---CCGCGA-AC-TGCA--
------
FC88X_00511_01230       -------------------TTTTCCCTTCACT-AA---CCGCGA-AC-TGCA--
------
FC88X_00503_01216       -------------------TTTTCCCTTCACT-AA---CCGCGA-AC-TGCA--
------
FC88X_00543_01180       -------------------TTTTCCCTTCACT-AA---CCGCGA-AC-TGCA--
------
FC88X_00521_01128       -------------------TTTTCCCTTCACT-AA---CCGCGA-AC-TGCA--
------
FC88X_00546_01093       -------------------TTTTCCCTTCACT-AA---CCGCGA-AC-TGCA--
------
FC88X_00050_00822       ------------------TTTTTCCCTTCACT-AA---CCGCGA-AC-TGCA--
------
FC88X_00071_00145       ------------------TTTTTCCCTTCACT-AA---CCGCGA-AC-TGCA--
------
FC88X_00052_00770       ------------------TTTTTCCCTTCACT-AA---CCGCGA-AC-TGCA--
------
FC88X_00068_00746       ------------------TTTTTCCCTTCACT-AA---CCGCGA-AC-TGCA--
------
FC88X_00653_00079       ------------------TTTTTCCCTTCACT-AA---CCGCGA-AC-TGCA--
------
FC88X_00628_01251       ------------------TTTTTCCCTTCACT-AA---CCGCGA-AC-TGCA--
------
FC88X_01037_00789       ------------------TTTTTCCCTTCACT-AA---CCGCGA-AC-TGCA--
------
FC88X_01010_00775       ------------------TTTTTCCCTTCACT-AA---CCGCGA-AC-TGCA--
------
FC88X_01032_00723       ------------------TTTTTCCCTTCACT-AA---CCGCGA-AC-TGCA--
------
FC88X_00857_01165       ------------------TTTTTCCCTTCACT-AA---CCGCGA-AC-TGCA--
------
FC88X_00633_00730       ------------------TTTTTCCCTTCACT-AA---CCGCGA-AC-TGCA--
------
FC88X_00589_01164       ------------------TTTTTCCCTTCACT-AA---CCGCGA-AC-TGCA--
------
FC88X_00896_00728       -------------------TTTTCCCTTCACT-AA---CCGCGA-AC-TGCA--
------
FC88X_00877_00742       -------------------TTTTCCCTTCACT-AA---CCGCGA-AC-TGCA--
------
FC88X_00890_00324       -------------------TTTTCCCTTCACT-AA---CCGCGA-AC-TGCA--
------
FC88X_00876_00250       -------------------TTTTCCCTTCACT-AA---CCGCGA-AC-TGCA--
------
FC88X_00819_01216       -------------------TTTTCCCTTCACT-AA---CCGCGA-AC-TGCA--
------
FC88X_00828_01142       -------------------TTTTCCCTTCACT-AA---CCGCGA-AC-TGCA--
------
FC88X_00825_01139       -------------------TTTTCCCTTCACT-AA---CCGCGA-AC-TGCA--
------
FC88X_00807_01145       -------------------TTTTCCCTTCACT-AA---CCGCGA-AC-TGCA--
------
FC88X_00807_01110       -------------------TTTTCCCTTCACT-AA---CCGCGA-AC-TGCA--
------
```

FC88X_00803_01145 -------------------TTTTCCCTTCACT-AA---CCGCGA-AC-TGCA--
------
FC88X_00842_00984 -------------------TTTTCCCTTCACT-AA---CCGCGA-AC-TGCA--
------
FC88X_00674_00908 ------------------TTTTTCCCTTCACT-AA---CCGCGA-AC-TGCA--
------
FC88X_00672_00921 ------------------TTTTTCCCTTCACT-AA---CCGCGA-AC-TGCA--
------
FC88X_00669_00941 ------------------TTTTTCCCTTCACT-AA---CCGCGA-AC-TGCA--
------
FC88X_00694_00887 ------------------TTTTTCCCTTCACT-AA---CCGCGA-AC-TGCA--
------
FC88X_00693_00860 ------------------TTTTTCCCTTCACT-AA---CCGCGA-AC-TGCA--
------
FC88X_00683_00889 ------------------TTTTTCCCTTCACT-AA---CCGCGA-AC-TGCA--
------
FC88X_00690_00500 ------------------TTTTTCCCTTCACT-AA---CCGCGA-AC-TGCA--
------
FC88X_00654_00528 ------------------TTTTTCCCTTCACT-AA---CCGCGA-AC-TGCA--
------
FC88X_00685_00451 ------------------TTTTTCCCTTCACT-AA---CCGCGA-AC-TGCA--
------
FC88X_00666_00458 ------------------TTTTTCCCTTCACT-AA---CCGCGA-AC-TGCA--
------
FC88X_00665_00465 ------------------TTTTTCCCTTCACT-AA---CCGCGA-AC-TGCA--
------
FC88X_00687_00431 ------------------TTTTTCCCTTCACT-AA---CCGCGA-AC-TGCA--
------
FC88X_00674_00422 ------------------TTTTTCCCTTCACT-AA---CCGCGA-AC-TGCA--
------
FC88X_00676_00347 ------------------TTTTTCCCTTCACT-AA---CCGCGA-AC-TGCA--
------
FC88X_00655_00339 ------------------TTTTTCCCTTCACT-AA---CCGCGA-AC-TGCA--
------
FC88X_00672_00259 ------------------TTTTTCCCTTCACT-AA---CCGCGA-AC-TGCA--
------
FC88X_00668_00295 ------------------TTTTTCCCTTCACT-AA---CCGCGA-AC-TGCA--
------
FC88X_00613_01267 ------------------TTTTTCCCTTCACT-AA---CCGCGA-AC-TGCA--
------
FC88X_00635_00602 ------------------TTTTTCCCTTCACT-AA---CCGCGA-AC-TGCA--
------
FC88X_00646_01183 -------------------TTTTCCCTTCACT-AA---CCGCGA-AC-TGCA--
------
FC88X_00632_01161 -------------------TTTTCCCTTCACT-AA---CCGCGA-AC-TGCA--
------
FC88X_00606_01183 -------------------TTTTCCCTTCACT-AA---CCGCGA-AC-TGCA--
------
FC88X_00604_01187 -------------------TTTTCCCTTCACT-AA---CCGCGA-AC-TGCA--
------
FC88X_00630_01109 -------------------TTTTCCCTTCACT-AA---CCGCGA-AC-TGCA--
------
FC88X_00142_00964 -------------------TTTTCCCTTCACT-AA---CCGCGA-AC-TGCA--
------
FC88X_00126_00852 -------------------TTTTCCCTTCACT-AA---CCGCGA-AC-TGCA--
------

FC88X_00244_00027 -------------------TTTTCCCTTCACT-AA---CCGCGA-AC-TGCA--
------
FC88X_00959_00935 -------------------TTTTCCCTTCACT-AA---CCGCGA-AC-TGCA--
------
FC88X_00984_00883 -------------------TTTTCCCTTCACT-AA---CCGCGA-AC-TGCA--
------
FC88X_00979_00856 -------------------TTTTCCCTTCACT-AA---CCGCGA-AC-TGCA--
------
FC88X_00954_00897 -------------------TTTTCCCTTCACT-AA---CCGCGA-AC-TGCA--
------
FC88X_00886_00855 -------------------TTTTCCCTTCACT-AA---CCGCGA-AC-TGCA--
------
FC88X_00856_00872 -------------------TTTTCCCTTCACT-AA---CCGCGA-AC-TGCA--
------
FC88X_00874_00822 -------------------TTTTCCCTTCACT-AA---CCGCGA-AC-TGCA--
------
FC88X_00873_00824 -------------------TTTTCCCTTCACT-AA---CCGCGA-AC-TGCA--
------
FC88X_01026_01240 ------------------TTTTTCCCTTCACT-AA---CCGCGA-AC-TGCA--
------
FC88X_01010_01212 ------------------TTTTTCCCTTCACT-AA---CCGCGA-AC-TGCA--
------
FC88X_01077_00470 ------------------TTTTTCCCTTCACT-AA---CCGCGA-AC-TGCA--
------
FC88X_01082_00396 ------------------TTTTTCCCTTCACT-AA---CCGCGA-AC-TGCA--
------
FC88X_01082_00392 ------------------TTTTTCCCTTCACT-AA---CCGCGA-AC-TGCA--
------
FC88X_00667_00200 ------------------TTTTTCCCTTCACT-AA---CCGCGA-AC-TGCA--
------
FC88X_00694_00170 ------------------TTTTTCCCTTCACT-AA---CCGCGA-AC-TGCA--
------
FC88X_00675_01233 ------------------TTTTTCCCTTCACT-AA---CCGCGA-AC-TGCA--
------
FC88X_00692_01167 ------------------TTTTTCCCTTCACT-AA---CCGCGA-AC-TGCA--
------
FC88X_00689_01162 ------------------TTTTTCCCTTCACT-AA---CCGCGA-AC-TGCA--
------
FC88X_00419_00020 -------------------TTTTCCCTTCACT-AA---CCGCGA-AC-TGCA--
------
FC88X_00390_01141 -------------------TTTTCCCTTCACT-AA---CCGCGA-AC-TGCA--
------
FC88X_00395_00608 -------------------TTTTCCCTTCACT-AA---CCGCGA-AC-TGCA--
------
FC88X_00396_00590 -------------------TTTTCCCTTCACT-AA---CCGCGA-AC-TGCA--
------
FC88X_00392_00578 -------------------TTTTCCCTTCACT-AA---CCGCGA-AC-TGCA--
------
FC88X_00368_00471 -------------------TTTTCCCTTCACT-AA---CCGCGA-AC-TGCA--
------
FC88X_00378_00291 -------------------TTTTCCCTTCACT-AA---CCGCGA-AC-TGCA--
------
FC88X_00352_00200 -------------------TTTTCCCTTCACT-AA---CCGCGA-AC-TGCA--
------
FC88X_00371_00186 -------------------TTTTCCCTTCACT-AA---CCGCGA-AC-TGCA--
------

FC88X_00399_00040 -------------------TTTTCCCTTCACT-AA---CCGCGA-AC-TGCA--
------
FC88X_00338_01124 -------------------TTTTCCCTTCACT-AA---CCGCGA-AC-TGCA--
------
FC88X_00336_01095 -------------------TTTTCCCTTCACT-AA---CCGCGA-AC-TGCA--
------
FC88X_00308_01002 -------------------TTTTCCCTTCACT-AA---CCGCGA-AC-TGCA--
------
FC88X_00319_00996 -------------------TTTTCCCTTCACT-AA---CCGCGA-AC-TGCA--
------
FC88X_00338_00932 -------------------TTTTCCCTTCACT-AA---CCGCGA-AC-TGCA--
------
FC88X_00340_00816 -------------------TTTTCCCTTCACT-AA---CCGCGA-AC-TGCA--
------
FC88X_00680_00096 ------------------TTTTTCCCTTCACT-AA---CCGCGA-AC-TGCA--
------
FC88X_00637_00900 ------------------TTTTTCCCTTCACT-AA---CCGCGA-AC-TGCA--
------
FC88X_00620_00313 ------------------TTTTTCCCTTCACT-AA---CCGCGA-AC-TGCA--
------
FC88X_00637_00281 ------------------TTTTTCCCTTCACT-AA---CCGCGA-AC-TGCA--
------
FC88X_00359_00952 -------------------TTTTCCCTTCACT-AA---CCGCGA-AC-TGCA--
------
FC88X_00571_00965 ------------------TTTTTCCCTTCACT-AA---CCGCGA-AC-TGCA--
------
FC88X_00569_00965 ------------------TTTTTCCCTTCACT-AA---CCGCGA-AC-TGCA--
------
FC88X_00899_01025 -------------------TTTTCCCTTCACT-AA---CCGCGA-AC-TGCA--
------
FC88X_00888_00967 -------------------TTTTCCCTTCACT-AA---CCGCGA-AC-TGCA--
------
FC88X_00883_00975 -------------------TTTTCCCTTCACT-AA---CCGCGA-AC-TGCA--
------
FC88X_00888_00942 -------------------TTTTCCCTTCACT-AA---CCGCGA-AC-TGCA--
------
FC88X_00875_00939 -------------------TTTTCCCTTCACT-AA---CCGCGA-AC-TGCA--
------
FC88X_00695_01026 ------------------TTTTTCCCTTCACT-AA---CCGCGA-AC-TGCA--
------
FC88X_00016_00409 ------------------TTTTTCCCTTCACT-AA---CCGCGA-AC-TGCA--
------
FC88X_00042_00392 ------------------TTTTTCCCTTCACT-AA---CCGCGA-AC-TGCA--
------
FC88X_00016_00382 ------------------TTTTTCCCTTCACT-AA---CCGCGA-AC-TGCA--
------
FC88X_00043_00300 ------------------TTTTTCCCTTCACT-AA---CCGCGA-AC-TGCA--
------
FC88X_00030_00308 ------------------TTTTTCCCTTCACT-AA---CCGCGA-AC-TGCA--
------
FC88X_00022_00301 ------------------TTTTTCCCTTCACT-AA---CCGCGA-AC-TGCA--
------
FC88X_00012_00305 ------------------TTTTTCCCTTCACT-AA---CCGCGA-AC-TGCA--
------
FC88X_00005_00321 ------------------TTTTTCCCTTCACT-AA---CCGCGA-AC-TGCA--
------

FC88X_00048_00296 ------------------TTTTTCCCTTCACT-AA---CCGCGA-AC-TGCA--
------
FC88X_00044_00293 ------------------TTTTTCCCTTCACT-AA---CCGCGA-AC-TGCA--
------
FC88X_00313_01028 -------------------TTTTCCCTTCACT-AA---CCGCGA-AC-TGCA--
------
FC88X_00717_00091 ------------------TTTTTCCCTTCACT-AA---CCGCGA-AC-TGCA--
------
FC88X_00715_00097 ------------------TTTTTCCCTTCACT-AA---CCGCGA-AC-TGCA--
------
FC88X_00047_00173 ------------------TTTTTCCCTTCACT-AA---CCGCGA-AC-TGCA--
------
FC88X_00126_00150 -------------------TTTTCCCTTCACT-AA---CCGCGA-AC-TGCA--
------
FC88X_00115_00151 -------------------TTTTCCCTTCACT-AA---CCGCGA-AC-TGCA--
------
FC88X_00726_01234 -------------------TTTTCCCTTCACT-AA---CCGCGA-AC-TGCA--
------
FC88X_00716_01172 -------------------TTTTCCCTTCACT-AA---CCGCGA-AC-TGCA--
------
FC88X_00628_00276 ------------------TTTTTCCCTTCACT-AA---CCGCGA-AC-TGCA--
------
FC88X_00696_00405 -------------------TTTTCCCTTCACT-AA---CCGCGA-AC-TGCA--
------
FC88X_00698_00337 -------------------TTTTCCCTTCACT-AA---CCGCGA-AC-TGCA--
------
FC88X_00141_00741 -------------------TTTTCCCTTCACT-AA---CCGCGA-AC-TGCA--
------
FC88X_00108_00707 -------------------TTTTCCCTTCACT-AA---CCGCGA-AC-TGCA--
------
FC88X_00582_00524 ------------------TTTTTCCCTTCACT-AA---CCGCGA-AC-TGCA--
------
FC88X_00564_00479 ------------------TTTTTCCCTTCACT-AA---CCGCGA-AC-TGCA--
------
FC88X_00592_00414 ------------------TTTTTCCCTTCACT-AA---CCGCGA-AC-TGCA--
------
FC88X_00594_00228 ------------------TTTTTCCCTTCACT-AA---CCGCGA-AC-TGCA--
------
FC88X_00587_00178 ------------------TTTTTCCCTTCACT-AA---CCGCGA-AC-TGCA--
------
FC88X_00594_00135 ------------------TTTTTCCCTTCACT-AA---CCGCGA-AC-TGCA--
------
FC88X_00955_01171 -------------------TTTTCCCTTCACT-AA---CCGCGA-AC-TGCA--
------
FC88X_00589_01037 ------------------TTTTTCCCTTCACT-AA---CCGCGA-AC-TGCA--
------
FC88X_00805_00817 -------------------TTTTCCCTTCACT-AA---CCGCGA-AC-TGCA--
------
FC88X_00802_00807 -------------------TTTTCCCTTCACT-AA---CCGCGA-AC-TGCA--
------
FC88X_00842_00762 -------------------TTTTCCCTTCACT-AA---CCGCGA-AC-TGCA--
------
FC88X_00820_00786 -------------------TTTTCCCTTCACT-AA---CCGCGA-AC-TGCA--
------
FC88X_00820_00778 -------------------TTTTCCCTTCACT-AA---CCGCGA-AC-TGCA--
------

```
FC88X_00809_00679       -------------------TTTTCCCTTCACT-AA---CCGCGA-AC-TGCA--
------
FC88X_00800_00554       -------------------TTTTCCCTTCACT-AA---CCGCGA-AC-TGCA--
------
FC88X_00910_00731       -------------------TTTTCCCTTCACT-AA---CCGCGA-AC-TGCA--
------
FC88X_00945_00665       -------------------TTTTCCCTTCACT-AA---CCGCGA-AC-TGCA--
------
FC88X_00930_00603       -------------------TTTTCCCTTCACT-AA---CCGCGA-AC-TGCA--
------
FC88X_00925_00627       -------------------TTTTCCCTTCACT-AA---CCGCGA-AC-TGCA--
------
FC88X_00551_01144       -------------------TTTTCCCTTCACT-AA---CCGCGA-AC-TGCA--
------
FC88X_01039_00448       -------------------TTTTCCCTTCACT-AA---CCGCGA-AC-TGCA--
------
FC88X_01027_00330       -------------------TTTTCCCTTCACT-AA---CCGCGA-AC-TGCA--
------
FC88X_00922_00554       -------------------TTTTCCCTTCACT-AA---CCGCGA-AC-TGCA--
------
FC88X_00942_00503       -------------------TTTTCCCTTCACT-AA---CCGCGA-AC-TGCA--
------
FC88X_00499_00162       ------------------TTTTTCCCTTCACT-AA---CCGCGA-AC-TGCA--
------
FC88X_00871_01222       -------------------TTTTCCCTTCACT-AA---CCGCGA-AC-TGCA--
------
FC88X_00965_01259       -------------------TTTTCCCTTCACT-AA---CCGCGA-AC-TGCA--
------
FC88X_00996_01228       -------------------TTTTCCCTTCACT-AA---CCGCGA-AC-TGCA--
------
FC88X_00979_01174       -------------------TTTTCCCTTCACT-AA---CCGCGA-AC-TGCA--
------
FC88X_00341_01128       -------------------TTTTCCCTTCACT-AA---CCGCGA-AC-TGCA--
------
FC88X_00694_01212       ------------------TTTTTCCCTTCACT-AA---CCGCGA-AC-TGCA--
------
FC88X_00928_01096       -------------------TTTTCCCTTCACT-AA---CCGCGA-AC-TGCA--
------
FC88X_00490_00318       ------------------TTTTTCCCTTCACT-AA---CCGCGA-AC-TGCA--
------
FC88X_00486_00260       ------------------TTTTTCCCTTCACT-AA---CCGCGA-AC-TGCA--
------
FC88X_00372_00042       -------------------TTTTCCCTTCACT-AA---CCGCGA-AC-TGCA--
------
FC88X_00345_01153       -------------------TTTTCCCTTCACT-AA---CCGCGA-AC-TGCA--
------
FC88X_00482_00120       ------------------TTTTTCCCTTCACT-AA---CCGCGA-AC-TGCA--
------
FC88X_00454_00136       ------------------TTTTTCCCTTCACT-AA---CCGCGA-AC-TGCA--
------
FC88X_00470_00031       ------------------TTTTTCCCTTCACT-AA---CCGCGA-AC-TGCA--
------
FC88X_00459_00021       ------------------TTTTTCCCTTCACT-AA---CCGCGA-AC-TGCA--
------
FC88X_00456_00044       ------------------TTTTTCCCTTCACT-AA---CCGCGA-AC-TGCA--
------
```

FC88X_00179_00958 -------------------TTTTCCCTTCACT-AA---CCGCGA-AC-TGCA--
------
FC88X_00168_00987 -------------------TTTTCCCTTCACT-AA---CCGCGA-AC-TGCA--
------
FC88X_01143_01186 -------------------TTTTCCCTTCACT-AA---CCGCGA-AC-TGCA--
------
FC88X_01140_01183 -------------------TTTTCCCTTCACT-AA---CCGCGA-AC-TGCA--
------
FC88X_01100_01150 -------------------TTTTCCCTTCACT-AA---CCGCGA-AC-TGCA--
------
FC88X_01137_01083 -------------------TTTTCCCTTCACT-AA---CCGCGA-AC-TGCA--
------
FC88X_00181_01040 ------------------TTTTTCCCTTCACT-AA---CCGCGA-AC-TGCA--
------
FC88X_00302_01115 -------------------TTTTCCCTTCACT-AA---CCGCGA-AC-TGCA--
------
FC88X_00133_00628 -------------------TTTTCCCTTCACT-AA---CCGCGA-AC-TGCA--
------
FC88X_00356_01005 -------------------TTTTCCCTTCACT-AA---CCGCGA-AC-TGCA--
------
FC88X_00598_00588 ------------------TTTTTCCCTTCACT-AA---CCGCGA-AC-TGCA--
------
FC88X_00043_00178 -------------------TTTTCCCTTCACT-AA---CCGCGA-AC-TGCA--
------
FC88X_01007_00925 -------------------TTTTCCCTTCACT-AA---CCGCGA-AC-TGCA--
------
FC88X_01016_00807 -------------------TTTTCCCTTCACT-AA---CCGCGA-AC-TGCA--
------
FC88X_01034_00730 -------------------TTTTCCCTTCACT-AA---CCGCGA-AC-TGCA--
------
FC88X_01010_00743 -------------------TTTTCCCTTCACT-AA---CCGCGA-AC-TGCA--
------
FC88X_00988_00765 -------------------TTTTCCCTTCACT-AA---CCGCGA-AC-TGCA--
------
FC88X_00966_00790 -------------------TTTTCCCTTCACT-AA---CCGCGA-AC-TGCA--
------
FC88X_00972_00388 -------------------TTTTCCCTTCACT-AA---CCGCGA-AC-TGCA--
------
FC88X_00922_01208 -------------------TTTTCCCTTCACT-AA---CCGCGA-AC-TGCA--
------
FC88X_00948_01150 -------------------TTTTCCCTTCACT-AA---CCGCGA-AC-TGCA--
------
FC88X_00944_01164 -------------------TTTTCCCTTCACT-AA---CCGCGA-AC-TGCA--
------
FC88X_00937_01182 -------------------TTTTCCCTTCACT-AA---CCGCGA-AC-TGCA--
------
FC88X_00927_01197 -------------------TTTTCCCTTCACT-AA---CCGCGA-AC-TGCA--
------
FC88X_00938_01141 -------------------TTTTCCCTTCACT-AA---CCGCGA-AC-TGCA--
------
FC88X_00595_00645 ------------------TTTTTCCCTTCACT-AA---CCGCGA-AC-TGCA--
------
FC88X_00911_00685 -------------------TTTTCCCTTCACT-AA---CCGCGA-AC-TGCA--
------
FC88X_00903_00583 -------------------TTTTCCCTTCACT-AA---CCGCGA-AC-TGCA--
------

FC88X_00945_00525 -------------------TTTTCCCTTCACT-AA---CCGCGA-AC-TGCA--
------
FC88X_00903_00439 -------------------TTTTCCCTTCACT-AA---CCGCGA-AC-TGCA--
------
FC88X_00942_00355 -------------------TTTTCCCTTCACT-AA---CCGCGA-AC-TGCA--
------
FC88X_00929_00393 -------------------TTTTCCCTTCACT-AA---CCGCGA-AC-TGCA--
------
FC88X_00924_00394 -------------------TTTTCCCTTCACT-AA---CCGCGA-AC-TGCA--
------
FC88X_00900_00389 -------------------TTTTCCCTTCACT-AA---CCGCGA-AC-TGCA--
------
FC88X_00939_00300 -------------------TTTTCCCTTCACT-AA---CCGCGA-AC-TGCA--
------
FC88X_00915_00343 -------------------TTTTCCCTTCACT-AA---CCGCGA-AC-TGCA--
------
FC88X_00942_00248 -------------------TTTTCCCTTCACT-AA---CCGCGA-AC-TGCA--
------
FC88X_00915_00235 -------------------TTTTCCCTTCACT-AA---CCGCGA-AC-TGCA--
------
FC88X_00901_00197 -------------------TTTTCCCTTCACT-AA---CCGCGA-AC-TGCA--
------
FC88X_00891_01259 -------------------TTTTCCCTTCACT-AA---CCGCGA-AC-TGCA--
------
FC88X_00865_01253 -------------------TTTTCCCTTCACT-AA---CCGCGA-AC-TGCA--
------
FC88X_00876_01229 -------------------TTTTCCCTTCACT-AA---CCGCGA-AC-TGCA--
------
FC88X_00869_01241 -------------------TTTTCCCTTCACT-AA---CCGCGA-AC-TGCA--
------
FC88X_00857_01203 -------------------TTTTCCCTTCACT-AA---CCGCGA-AC-TGCA--
------
FC88X_00889_01194 -------------------TTTTCCCTTCACT-AA---CCGCGA-AC-TGCA--
------
FC88X_00472_01188 ------------------TTTTTCCCTTCACT-AA---CCGCGA-AC-TGCA--
------
FC88X_00471_01188 ------------------TTTTTCCCTTCACT-AA---CCGCGA-AC-TGCA--
------
FC88X_00497_01127 ------------------TTTTTCCCTTCACT-AA---CCGCGA-AC-TGCA--
------
FC88X_00213_00009 ------------------TTTTTCCCTTCACT-AA---CCGCGA-AC-TGCA--
------
FC88X_00199_01068 ------------------TTTTTCCCTTCACT-AA---CCGCGA-AC-TGCA--
------
FC88X_00197_01061 ------------------TTTTTCCCTTCACT-AA---CCGCGA-AC-TGCA--
------
FC88X_00938_00910 -------------------TTTTCCCTTCACT-AA---CCGCGA-AC-TGCA--
------
FC88X_00918_00787 -------------------TTTTCCCTTCACT-AA---CCGCGA-AC-TGCA--
------
FC88X_00915_00715 -------------------TTTTCCCTTCACT-AA---CCGCGA-AC-TGCA--
------
FC88X_00913_00721 -------------------TTTTCCCTTCACT-AA---CCGCGA-AC-TGCA--
------
FC88X_00042_00031 -------------------TTTTCCCTTCACT-AA---CCGCGA-AC-TGCA--
------

FC88X_00529_00116 ------------------TTTTTCCCTTCACT-AA---CCGCGA-AC-TGCA--
------
FC88X_00526_00078 ------------------TTTTTCCCTTCACT-AA---CCGCGA-AC-TGCA--
------
FC88X_00500_00052 ------------------TTTTTCCCTTCACT-AA---CCGCGA-AC-TGCA--
------
FC88X_00537_00036 ------------------TTTTTCCCTTCACT-AA---CCGCGA-AC-TGCA--
------
FC88X_00506_00027 ------------------TTTTTCCCTTCACT-AA---CCGCGA-AC-TGCA--
------
FC88X_00494_01222 ------------------TTTTTCCCTTCACT-AA---CCGCGA-AC-TGCA--
------
FC88X_00483_01137 ------------------TTTTTCCCTTCACT-AA---CCGCGA-AC-TGCA--
------
FC88X_00469_01146 ------------------TTTTTCCCTTCACT-AA---CCGCGA-AC-TGCA--
------
FC88X_00817_00820 -------------------TTTTCCCTTCACT-AA---CCGCGA-AC-TGCA--
------
FC88X_00800_00803 -------------------TTTTCCCTTCACT-AA---CCGCGA-AC-TGCA--
------
FC88X_00812_00763 -------------------TTTTCCCTTCACT-AA---CCGCGA-AC-TGCA--
------
FC88X_00833_00433 -------------------TTTTCCCTTCACT-AA---CCGCGA-AC-TGCA--
------
FC88X_00805_00401 -------------------TTTTCCCTTCACT-AA---CCGCGA-AC-TGCA--
------
FC88X_00838_00328 -------------------TTTTCCCTTCACT-AA---CCGCGA-AC-TGCA--
------
FC88X_00834_00339 -------------------TTTTCCCTTCACT-AA---CCGCGA-AC-TGCA--
------
FC88X_00842_00291 -------------------TTTTCCCTTCACT-AA---CCGCGA-AC-TGCA--
------
FC88X_00800_00250 -------------------TTTTCCCTTCACT-AA---CCGCGA-AC-TGCA--
------
FC88X_00826_00226 -------------------TTTTCCCTTCACT-AA---CCGCGA-AC-TGCA--
------
FC88X_00815_00193 -------------------TTTTCCCTTCACT-AA---CCGCGA-AC-TGCA--
------
FC88X_00806_00152 -------------------TTTTCCCTTCACT-AA---CCGCGA-AC-TGCA--
------
FC88X_00763_01253 -------------------TTTTCCCTTCACT-AA---CCGCGA-AC-TGCA--
------
FC88X_00770_01127 -------------------TTTTCCCTTCACT-AA---CCGCGA-AC-TGCA--
------
FC88X_00765_01124 -------------------TTTTCCCTTCACT-AA---CCGCGA-AC-TGCA--
------
FC88X_00757_01148 -------------------TTTTCCCTTCACT-AA---CCGCGA-AC-TGCA--
------
FC88X_00770_01014 -------------------TTTTCCCTTCACT-AA---CCGCGA-AC-TGCA--
------
FC88X_00753_00959 -------------------TTTTCCCTTCACT-AA---CCGCGA-AC-TGCA--
------
FC88X_00796_00824 -------------------TTTTCCCTTCACT-AA---CCGCGA-AC-TGCA--
------
FC88X_00777_00765 -------------------TTTTCCCTTCACT-AA---CCGCGA-AC-TGCA--
------

```
FC88X_00796_00584       -------------------TTTTCCCTTCACT-AA---CCGCGA-AC-TGCA--
------
FC88X_00761_00403       -------------------TTTTCCCTTCACT-AA---CCGCGA-AC-TGCA--
------
FC88X_00761_00290       -------------------TTTTCCCTTCACT-AA---CCGCGA-AC-TGCA--
------
FC88X_00759_00215       -------------------TTTTCCCTTCACT-AA---CCGCGA-AC-TGCA--
------
FC88X_00782_00178       -------------------TTTTCCCTTCACT-AA---CCGCGA-AC-TGCA--
------
FC88X_00019_00584       ------------------TTTTTCCCTTCACT-AA---CCGCGA-AC-TGCA--
------
FC88X_00387_00626       -------------------TTTTCCCTTCACT-AA---CCGCGA-AC-TGCA--
------
FC88X_00176_00907       -------------------TTTTCCCTTCACT-AA---CCGCGA-AC-TGCA--
------
FC88X_01127_01000       -------------------TTTTCCCTTCACT-AA---CCGCGA-AC-TGCA--
------
FC88X_01101_00985       -------------------TTTTCCCTTCACT-AA---CCGCGA-AC-TGCA--
------
FC88X_01134_00919       -------------------TTTTCCCTTCACT-AA---CCGCGA-AC-TGCA--
------
FC88X_01016_00568       -------------------TTTTCCCTTCACT-AA---CCGCGA-AC-TGCA--
------
FC88X_01040_00508       -------------------TTTTCCCTTCACT-AA---CCGCGA-AC-TGCA--
------
FC88X_00637_00125       ------------------TTTTTCCCTTCACT-AA---CCGCGA-AC-TGCA--
------
FC88X_00634_00127       ------------------TTTTTCCCTTCACT-AA---CCGCGA-AC-TGCA--
------
FC88X_00620_00127       ------------------TTTTTCCCTTCACT-AA---CCGCGA-AC-TGCA--
------
FC88X_00610_00133       ------------------TTTTTCCCTTCACT-AA---CCGCGA-AC-TGCA--
------
FC88X_00628_00081       ------------------TTTTTCCCTTCACT-AA---CCGCGA-AC-TGCA--
------
FC88X_00587_01252       ------------------TTTTTCCCTTCACT-AA---CCGCGA-AC-TGCA--
------
FC88X_00493_00711       ------------------TTTTTCCCTTCACT-AA---CCGCGA-AC-TGCA--
------
FC88X_00454_00731       ------------------TTTTTCCCTTCACT-AA---CCGCGA-AC-TGCA--
------
FC88X_00475_00639       ------------------TTTTTCCCTTCACT-AA---CCGCGA-AC-TGCA--
------
FC88X_00479_00553       ------------------TTTTTCCCTTCACT-AA---CCGCGA-AC-TGCA--
------
FC88X_00483_00514       ------------------TTTTTCCCTTCACT-AA---CCGCGA-AC-TGCA--
------
FC88X_00125_00631       -------------------TTTTCCCTTCACT-AA---CCGCGA-AC-TGCA--
------
FC88X_00457_01114       ------------------TTTTTCCCTTCACT-AA---CCGCGA-AC-TGCA--
------
FC88X_00430_01202       ------------------TTTTTCCCTTCACT-AA---CCGCGA-AC-TGCA--
------
FC88X_00427_01223       ------------------TTTTTCCCTTCACT-AA---CCGCGA-AC-TGCA--
------
```

```
FC88X_00432_01198       ------------------TTTTTCCCTTCACT-AA---CCGCGA-AC-TGCA--
------
FC88X_00019_00180       ------------------TTTTTCCCTTCACT-AA---CCGCGA-AC-TGCA--
------
FC88X_00109_00454       -------------------TTTTCCCTTCACT-AA---CCGCGA-AC-TGCA--
------
FC88X_00804_01101       -------------------TTTTCCCTTCACT-AA---CCGCGA-AC-TGCA--
------
FC88X_00838_00973       -------------------TTTTCCCTTCACT-AA---CCGCGA-AC-TGCA--
------
FC88X_00127_00138       -------------------TTTTCCCTTCACT-AA---CCGCGA-AC-TGCA--
------
FC88X_00116_00129       -------------------TTTTCCCTTCACT-AA---CCGCGA-AC-TGCA--
------
FC88X_00147_00098       -------------------TTTTCCCTTCACT-AA---CCGCGA-AC-TGCA--
------
FC88X_00147_00065       -------------------TTTTCCCTTCACT-AA---CCGCGA-AC-TGCA--
------
FC88X_00692_00167       -------------------TTTTCCCTTCACT-AA---CCGCGA-AC-TGCA--
------
FC88X_00621_01245       -------------------TTTTCCCTTCACT-AA---CCGCGA-AC-TGCA--
------
FC88X_00163_00408       -------------------TTTTCCCTTCACT-AA---CCGCGA-AC-TGCA--
------
FC88X_00195_00294       -------------------TTTTCCCTTCACT-AA---CCGCGA-AC-TGCA--
------
FC88X_00163_00184       -------------------TTTTCCCTTCACT-AA---CCGCGA-AC-TGCA--
------
FC88X_00162_00106       -------------------TTTTCCCTTCACT-AA---CCGCGA-AC-TGCA--
------
FC88X_00178_00037       -------------------TTTTCCCTTCACT-AA---CCGCGA-AC-TGCA--
------
FC88X_00169_00021       -------------------TTTTCCCTTCACT-AA---CCGCGA-AC-TGCA--
------
FC88X_00161_00011       -------------------TTTTCCCTTCACT-AA---CCGCGA-AC-TGCA--
------
FC88X_00213_00052       -------------------TTTTCCCTTCACT-AA---CCGCGA-AC-TGCA--
------
FC88X_00203_00074       -------------------TTTTCCCTTCACT-AA---CCGCGA-AC-TGCA--
------
FC88X_01060_00642       ------------------TTTTTCCCTTCACT-AA---CCGCGA-AC-TGCA--
------
FC88X_00195_01061       -------------------TTTTCCCTTCACT-AA---CCGCGA-AC-TGCA--
------
FC88X_00197_01007       -------------------TTTTCCCTTCACT-AA---CCGCGA-AC-TGCA--
------
FC88X_00222_00497       -------------------TTTTCCCTTCACT-AA---CCGCGA-AC-TGCA--
------
FC88X_00848_00342       -------------------TTTTCCCTTCACT-AA---CCGCGA-AC-TGCA--
------
FC88X_00843_00279       -------------------TTTTCCCTTCACT-AA---CCGCGA-AC-TGCA--
------
FC88X_00529_00955       ------------------TTTTTCCCTTCACT-AA---CCGCGA-AC-TGCA--
------
FC88X_00549_00938       ------------------TTTTTCCCTTCACT-AA---CCGCGA-AC-TGCA--
------
```

FC88X_00527_00775 ------------------TTTTTCCCTTCACT-AA---CCGCGA-AC-TGCA--
------
FC88X_00519_00674 ------------------TTTTTCCCTTCACT-AA---CCGCGA-AC-TGCA--
------
FC88X_00513_00593 ------------------TTTTTCCCTTCACT-AA---CCGCGA-AC-TGCA--
------
FC88X_00545_00517 ------------------TTTTTCCCTTCACT-AA---CCGCGA-AC-TGCA--
------
FC88X_00513_00450 ------------------TTTTTCCCTTCACT-AA---CCGCGA-AC-TGCA--
------
FC88X_00527_00304 ------------------TTTTTCCCTTCACT-AA---CCGCGA-AC-TGCA--
------
FC88X_00511_00280 ------------------TTTTTCCCTTCACT-AA---CCGCGA-AC-TGCA--
------
FC88X_00515_00105 ------------------TTTTTCCCTTCACT-AA---CCGCGA-AC-TGCA--
------
FC88X_00107_00733 -------------------TTTTCCCTTCACT-AA---CCGCGA-AC-TGCA--
------
FC88X_00866_00897 -------------------TTTTCCCTTCACT-AA---CCGCGA-AC-TGCA--
------
FC88X_00863_00865 -------------------TTTTCCCTTCACT-AA---CCGCGA-AC-TGCA--
------
FC88X_00864_00843 -------------------TTTTCCCTTCACT-AA---CCGCGA-AC-TGCA--
------
FC88X_00863_00796 -------------------TTTTCCCTTCACT-AA---CCGCGA-AC-TGCA--
------
FC88X_00871_00747 -------------------TTTTCCCTTCACT-AA---CCGCGA-AC-TGCA--
------
FC88X_00858_00714 -------------------TTTTCCCTTCACT-AA---CCGCGA-AC-TGCA--
------
FC88X_00893_00677 -------------------TTTTCCCTTCACT-AA---CCGCGA-AC-TGCA--
------
FC88X_00508_01191 ------------------TTTTTCCCTTCACT-AA---CCGCGA-AC-TGCA--
------
FC88X_00549_01137 ------------------TTTTTCCCTTCACT-AA---CCGCGA-AC-TGCA--
------
FC88X_00545_01129 ------------------TTTTTCCCTTCACT-AA---CCGCGA-AC-TGCA--
------
FC88X_00536_01130 ------------------TTTTTCCCTTCACT-AA---CCGCGA-AC-TGCA--
------
FC88X_00533_01141 ------------------TTTTTCCCTTCACT-AA---CCGCGA-AC-TGCA--
------
FC88X_00570_00084 ------------------TTTTTCCCTTCACT-AA---CCGCGA-AC-TGCA--
------
FC88X_00569_00087 ------------------TTTTTCCCTTCACT-AA---CCGCGA-AC-TGCA--
------
FC88X_00547_01251 ------------------TTTTTCCCTTCACT-AA---CCGCGA-AC-TGCA--
------
FC88X_00524_01213 ------------------TTTTTCCCTTCACT-AA---CCGCGA-AC-TGCA--
------
FC88X_00869_00675 -------------------TTTTCCCTTCACT-AA---CCGCGA-AC-TGCA--
------
FC88X_00895_00635 -------------------TTTTCCCTTCACT-AA---CCGCGA-AC-TGCA--
------
FC88X_00882_00574 -------------------TTTTCCCTTCACT-AA---CCGCGA-AC-TGCA--
------

FC88X_00880_00551 -------------------TTTTCCCTTCACT-AA---CCGCGA-AC-TGCA--
------
FC88X_00890_00493 -------------------TTTTCCCTTCACT-AA---CCGCGA-AC-TGCA--
------
FC88X_00885_00473 -------------------TTTTCCCTTCACT-AA---CCGCGA-AC-TGCA--
------
FC88X_00875_00465 -------------------TTTTCCCTTCACT-AA---CCGCGA-AC-TGCA--
------
FC88X_00877_00400 -------------------TTTTCCCTTCACT-AA---CCGCGA-AC-TGCA--
------
FC88X_00884_00342 -------------------TTTTCCCTTCACT-AA---CCGCGA-AC-TGCA--
------
FC88X_00877_00264 -------------------TTTTCCCTTCACT-AA---CCGCGA-AC-TGCA--
------
FC88X_00868_00228 -------------------TTTTCCCTTCACT-AA---CCGCGA-AC-TGCA--
------
FC88X_00890_00172 -------------------TTTTCCCTTCACT-AA---CCGCGA-AC-TGCA--
------
FC88X_00881_00150 -------------------TTTTCCCTTCACT-AA---CCGCGA-AC-TGCA--
------
FC88X_00868_00177 -------------------TTTTCCCTTCACT-AA---CCGCGA-AC-TGCA--
------
FC88X_00858_00183 -------------------TTTTCCCTTCACT-AA---CCGCGA-AC-TGCA--
------
FC88X_00838_01223 -------------------TTTTCCCTTCACT-AA---CCGCGA-AC-TGCA--
------
FC88X_00487_00903 ------------------TTTTTCCCTTCACT-AA---CCGCGA-AC-TGCA--
------
FC88X_00211_00655 -------------------TTTTCCCTTCACT-AA---CCGCGA-AC-TGCA--
------
FC88X_00111_00554 -------------------TTTTCCCTTCACT-AA---CCGCGA-AC-TGCA--
------
FC88X_00115_00816 -------------------TTTTCCCTTCACT-AA---CCGCGA-AC-TGCA--
------
FC88X_00169_00073 -------------------TTTTCCCTTCACT-AA---CCGCGA-AC-TGCA--
------
FC88X_00169_00060 -------------------TTTTCCCTTCACT-AA---CCGCGA-AC-TGCA--
------
FC88X_00238_00073 -------------------TTTTCCCTTCACT-AA---CCGCGA-AC-TGCA--
------
FC88X_00046_00606 ------------------TTTTTCCCTTCACT-AA---CCGCGA-AC-TGCA--
------
FC88X_00133_00576 -------------------TTTTCCCTTCACT-AA---CCGCGA-AC-TGCA--
------
FC88X_00143_00924 -------------------TTTTCCCTTCACT-AA---CCGCGA-AC-TGCA--
------
FC88X_00132_00934 -------------------TTTTCCCTTCACT-AA---CCGCGA-AC-TGCA--
------
FC88X_00150_00263 -------------------TTTTCCCTTCACT-AA---CCGCGA-AC-TGCA--
------
FC88X_00224_00872 -------------------TTTTCCCTTCACT-AA---CCGCGA-AC-TGCA--
------
FC88X_00073_00858 ------------------TTTTTCCCTTCACT-AA---CCGCGA-AC-TGCA--
------
FC88X_00997_00371 -------------------TTTTCCCTTCACT-AA---CCGCGA-AC-TGCA--
------

FC88X_00959_00280 -------------------TTTTCCCTTCACT-AA---CCGCGA-AC-TGCA--
------
FC88X_00983_00242 -------------------TTTTCCCTTCACT-AA---CCGCGA-AC-TGCA--
------
FC88X_00162_00024 -------------------TTTTCCCTTCACT-AA---CCGCGA-AC-TGCA--
------
FC88X_00162_00023 -------------------TTTTCCCTTCACT-AA---CCGCGA-AC-TGCA--
------
FC88X_00165_00415 -------------------TTTTCCCTTCACT-AA---CCGCGA-AC-TGCA--
------
FC88X_00856_01139 -------------------TTTTCCCTTCACT-AA---CCGCGA-AC-TGCA--
------
FC88X_00183_00783 -------------------TTTTCCCTTCACT-AA---CCGCGA-AC-TGCA--
------
FC88X_00151_00608 -------------------TTTTCCCTTCACT-AA---CCGCGA-AC-TGCA--
------
FC88X_01109_01156 ------------------TTTTTCCCTTCACT-AA---CCGCGA-AC-TGCA--
------
FC88X_01121_01188 ------------------TTTTTCCCTTCACT-AA---CCGCGA-AC-TGCA--
------
FC88X_01139_01137 ------------------TTTTTCCCTTCACT-AA---CCGCGA-AC-TGCA--
------
FC88X_01100_01158 ------------------TTTTTCCCTTCACT-AA---CCGCGA-AC-TGCA--
------
FC88X_01134_01051 ------------------TTTTTCCCTTCACT-AA---CCGCGA-AC-TGCA--
------
FC88X_01142_01056 ------------------TTTTTCCCTTCACT-AA---CCGCGA-AC-TGCA--
------
FC88X_01133_01005 ------------------TTTTTCCCTTCACT-AA---CCGCGA-AC-TGCA--
------
FC88X_01121_01060 ------------------TTTTTCCCTTCACT-AA---CCGCGA-AC-TGCA--
------
FC88X_01142_00954 ------------------TTTTTCCCTTCACT-AA---CCGCGA-AC-TGCA--
------
FC88X_01108_01032 ------------------TTTTTCCCTTCACT-AA---CCGCGA-AC-TGCA--
------
FC88X_01119_00971 -------------------TTTTCCCTTCACT-AA---CCGCGA-AC-TGCA--
------
FC88X_01119_00978 -------------------TTTTCCCTTCACT-AA---CCGCGA-AC-TGCA--
------
FC88X_01102_00994 ------------------TTTTTCCCTTCACT-AA---CCGCGA-AC-TGCA--
------
FC88X_01118_00960 ------------------TTTTTCCCTTCACT-AA---CCGCGA-AC-TGCA--
------
FC88X_01121_00907 ------------------TTTTTCCCTTCACT-AA---CCGCGA-AC-TGCA--
------
FC88X_01122_00901 ------------------TTTTTCCCTTCACT-AA---CCGCGA-AC-TGCA--
------
FC88X_01144_00883 -------------------TTTTCCCTTCACT-AA---CCGCGA-AC-TGCA--
------
FC88X_01144_00898 -------------------TTTTCCCTTCACT-AA---CCGCGA-AC-TGCA--
------
FC88X_01107_00849 ------------------TTTTTCCCTTCACT-AA---CCGCGA-AC-TGCA--
------
FC88X_01141_00806 ------------------TTTTTCCCTTCACT-AA---CCGCGA-AC-TGCA--
------

FC88X_01116_00782 ------------------TTTTTCCCTTCACT-AA---CCGCGA-AC-TGCA--
------
FC88X_01147_00756 ------------------TTTTTCCCTTCACT-AA---CCGCGA-AC-TGCA--
------
FC88X_01147_00687 ------------------TTTTTCCCTTCACT-AA---CCGCGA-AC-TGCA--
------
FC88X_01102_00742 ------------------TTTTTCCCTTCACT-AA---CCGCGA-AC-TGCA--
------
FC88X_01105_00684 ------------------TTTTTCCCTTCACT-AA---CCGCGA-AC-TGCA--
------
FC88X_01146_00683 ------------------TTTTTCCCTTCACT-AA---CCGCGA-AC-TGCA--
------
FC88X_01132_00589 ------------------TTTTTCCCTTCACT-AA---CCGCGA-AC-TGCA--
------
FC88X_01105_00785 ------------------TTTTTCCCTTCACT-AA---CCGCGA-AC-TGCA--
------
FC88X_01110_00591 ------------------TTTTTCCCTTCACT-AA---CCGCGA-AC-TGCA--
------
FC88X_01131_00630 ------------------TTTTTCCCTTCACT-AA---CCGCGA-AC-TGCA--
------
FC88X_01053_01239 ------------------TTTTTCCCTTCACT-AA---CCGCGA-AC-TGCA--
------
FC88X_01057_01222 ------------------TTTTTCCCTTCACT-AA---CCGCGA-AC-TGCA--
------
FC88X_01073_01165 ------------------TTTTTCCCTTCACT-AA---CCGCGA-AC-TGCA--
------
FC88X_01102_00438 ------------------TTTTTCCCTTCACT-AA---CCGCGA-AC-TGCA--
------
FC88X_01071_01067 ------------------TTTTTCCCTTCACT-AA---CCGCGA-AC-TGCA--
------
FC88X_01081_01100 ------------------TTTTTCCCTTCACT-AA---CCGCGA-AC-TGCA--
------
FC88X_01077_00987 ------------------TTTTTCCCTTCACT-AA---CCGCGA-AC-TGCA--
------
FC88X_01069_01076 ------------------TTTTTCCCTTCACT-AA---CCGCGA-AC-TGCA--
------
FC88X_01053_00960 ------------------TTTTTCCCTTCACT-AA---CCGCGA-AC-TGCA--
------
FC88X_01063_00994 ------------------TTTTTCCCTTCACT-AA---CCGCGA-AC-TGCA--
------
FC88X_01082_00881 ------------------TTTTTCCCTTCACT-AA---CCGCGA-AC-TGCA--
------
FC88X_01097_00851 ------------------TTTTTCCCTTCACT-AA---CCGCGA-AC-TGCA--
------
FC88X_01085_00814 ------------------TTTTTCCCTTCACT-AA---CCGCGA-AC-TGCA--
------
FC88X_01075_00884 ------------------TTTTTCCCTTCACT-AA---CCGCGA-AC-TGCA--
------
FC88X_01094_00787 ------------------TTTTTCCCTTCACT-AA---CCGCGA-AC-TGCA--
------
FC88X_01063_00823 ------------------TTTTTCCCTTCACT-AA---CCGCGA-AC-TGCA--
------
FC88X_01066_00760 ------------------TTTTTCCCTTCACT-AA---CCGCGA-AC-TGCA--
------
FC88X_01090_00778 ------------------TTTTTCCCTTCACT-AA---CCGCGA-AC-TGCA--
------

FC88X_01052_00778 ------------------TTTTTCCCTTCACT-AA---CCGCGA-AC-TGCA--
------
FC88X_01058_00798 ------------------TTTTTCCCTTCACT-AA---CCGCGA-AC-TGCA--
------
FC88X_01067_00651 ------------------TTTTTCCCTTCACT-AA---CCGCGA-AC-TGCA--
------
FC88X_01090_00652 ------------------TTTTTCCCTTCACT-AA---CCGCGA-AC-TGCA--
------
FC88X_01099_00549 ------------------TTTTTCCCTTCACT-AA---CCGCGA-AC-TGCA--
------
FC88X_01093_00593 ------------------TTTTTCCCTTCACT-AA---CCGCGA-AC-TGCA--
------
FC88X_01095_00514 ------------------TTTTTCCCTTCACT-AA---CCGCGA-AC-TGCA--
------
FC88X_01096_00512 ------------------TTTTTCCCTTCACT-AA---CCGCGA-AC-TGCA--
------
FC88X_01012_01271 ------------------TTTTTCCCTTCACT-AA---CCGCGA-AC-TGCA--
------
FC88X_01054_00435 ------------------TTTTTCCCTTCACT-AA---CCGCGA-AC-TGCA--
------
FC88X_01009_01176 ------------------TTTTTCCCTTCACT-AA---CCGCGA-AC-TGCA--
------
FC88X_01023_01166 ------------------TTTTTCCCTTCACT-AA---CCGCGA-AC-TGCA--
------
FC88X_01049_01129 ------------------TTTTTCCCTTCACT-AA---CCGCGA-AC-TGCA--
------
FC88X_01009_01171 ------------------TTTTTCCCTTCACT-AA---CCGCGA-AC-TGCA--
------
FC88X_01022_01094 ------------------TTTTTCCCTTCACT-AA---CCGCGA-AC-TGCA--
------
FC88X_01030_01086 ------------------TTTTTCCCTTCACT-AA---CCGCGA-AC-TGCA--
------
FC88X_01020_01046 ------------------TTTTTCCCTTCACT-AA---CCGCGA-AC-TGCA--
------
FC88X_01000_01098 ------------------TTTTTCCCTTCACT-AA---CCGCGA-AC-TGCA--
------
FC88X_01047_00986 ------------------TTTTTCCCTTCACT-AA---CCGCGA-AC-TGCA--
------
FC88X_01013_01003 ------------------TTTTTCCCTTCACT-AA---CCGCGA-AC-TGCA--
------
FC88X_01041_00933 -------------------TTTTCCCTTCACT-AA---CCGCGA-AC-TGCA--
------
FC88X_01022_01014 -------------------TTTTCCCTTCACT-AA---CCGCGA-AC-TGCA--
------
FC88X_01044_00856 ------------------TTTTTCCCTTCACT-AA---CCGCGA-AC-TGCA--
------
FC88X_01038_00950 ------------------TTTTTCCCTTCACT-AA---CCGCGA-AC-TGCA--
------
FC88X_01041_00886 ------------------TTTTTCCCTTCACT-AA---CCGCGA-AC-TGCA--
------
FC88X_01018_00912 ------------------TTTTTCCCTTCACT-AA---CCGCGA-AC-TGCA--
------
FC88X_01032_00878 -------------------TTTTCCCTTCACT-AA---CCGCGA-AC-TGCA--
------
FC88X_01000_00929 -------------------TTTTCCCTTCACT-AA---CCGCGA-AC-TGCA--
------

FC88X_01017_00898 ------------------TTTTTCCCTTCACT-AA---CCGCGA-AC-TGCA--
------
FC88X_01029_00866 ------------------TTTTTCCCTTCACT-AA---CCGCGA-AC-TGCA--
------
FC88X_01023_00848 ------------------TTTTTCCCTTCACT-AA---CCGCGA-AC-TGCA--
------
FC88X_01026_00816 ------------------TTTTTCCCTTCACT-AA---CCGCGA-AC-TGCA--
------
FC88X_01009_00758 -------------------TTTTCCCTTCACT-AA---CCGCGA-AC-TGCA--
------
FC88X_01021_00822 -------------------TTTTCCCTTCACT-AA---CCGCGA-AC-TGCA--
------
FC88X_01019_00711 ------------------TTTTTCCCTTCACT-AA---CCGCGA-AC-TGCA--
------
FC88X_01007_00795 ------------------TTTTTCCCTTCACT-AA---CCGCGA-AC-TGCA--
------
FC88X_01005_00718 ------------------TTTTTCCCTTCACT-AA---CCGCGA-AC-TGCA--
------
FC88X_01005_00738 ------------------TTTTTCCCTTCACT-AA---CCGCGA-AC-TGCA--
------
FC88X_01022_00636 ------------------TTTTTCCCTTCACT-AA---CCGCGA-AC-TGCA--
------
FC88X_01007_00655 ------------------TTTTTCCCTTCACT-AA---CCGCGA-AC-TGCA--
------
FC88X_01041_00594 ------------------TTTTTCCCTTCACT-AA---CCGCGA-AC-TGCA--
------
FC88X_01046_00581 ------------------TTTTTCCCTTCACT-AA---CCGCGA-AC-TGCA--
------
FC88X_01016_00598 ------------------TTTTTCCCTTCACT-AA---CCGCGA-AC-TGCA--
------
FC88X_01040_00575 ------------------TTTTTCCCTTCACT-AA---CCGCGA-AC-TGCA--
------
FC88X_01004_00502 -------------------TTTTCCCTTCACT-AA---CCGCGA-AC-TGCA--
------
FC88X_01025_00590 -------------------TTTTCCCTTCACT-AA---CCGCGA-AC-TGCA--
------
FC88X_01000_00473 ------------------TTTTTCCCTTCACT-AA---CCGCGA-AC-TGCA--
------
FC88X_01019_00451 ------------------TTTTTCCCTTCACT-AA---CCGCGA-AC-TGCA--
------
FC88X_01014_00407 -------------------TTTTCCCTTCACT-AA---CCGCGA-AC-TGCA--
------
FC88X_01019_00431 -------------------TTTTCCCTTCACT-AA---CCGCGA-AC-TGCA--
------
FC88X_01006_00405 -------------------TTTTCCCTTCACT-AA---CCGCGA-AC-TGCA--
------
FC88X_01010_00401 -------------------TTTTCCCTTCACT-AA---CCGCGA-AC-TGCA--
------
FC88X_01006_00363 ------------------TTTTTCCCTTCACT-AA---CCGCGA-AC-TGCA--
------
FC88X_01009_00448 ------------------TTTTTCCCTTCACT-AA---CCGCGA-AC-TGCA--
------
FC88X_00998_01257 ------------------TTTTTCCCTTCACT-AA---CCGCGA-AC-TGCA--
------
FC88X_01027_00303 ------------------TTTTTCCCTTCACT-AA---CCGCGA-AC-TGCA--
------

FC88X_00995_01169 ------------------TTTTTCCCTTCACT-AA---CCGCGA-AC-TGCA--
------
FC88X_00951_01204 ------------------TTTTTCCCTTCACT-AA---CCGCGA-AC-TGCA--
------
FC88X_00993_01028 ------------------TTTTTCCCTTCACT-AA---CCGCGA-AC-TGCA--
------
FC88X_00967_01147 ------------------TTTTTCCCTTCACT-AA---CCGCGA-AC-TGCA--
------
FC88X_00978_01032 ------------------TTTTTCCCTTCACT-AA---CCGCGA-AC-TGCA--
------
FC88X_01047_00930 ------------------TTTTTCCCTTCACT-AA---CCGCGA-AC-TGCA--
------
FC88X_00987_00870 ------------------TTTTTCCCTTCACT-AA---CCGCGA-AC-TGCA--
------
FC88X_00992_00918 ------------------TTTTTCCCTTCACT-AA---CCGCGA-AC-TGCA--
------
FC88X_00950_00895 ------------------TTTTTCCCTTCACT-AA---CCGCGA-AC-TGCA--
------
FC88X_00972_00867 ------------------TTTTTCCCTTCACT-AA---CCGCGA-AC-TGCA--
------
FC88X_00974_00789 ------------------TTTTTCCCTTCACT-AA---CCGCGA-AC-TGCA--
------
FC88X_00990_00807 ------------------TTTTTCCCTTCACT-AA---CCGCGA-AC-TGCA--
------
FC88X_00999_00635 ------------------TTTTTCCCTTCACT-AA---CCGCGA-AC-TGCA--
------
FC88X_00950_00676 ------------------TTTTTCCCTTCACT-AA---CCGCGA-AC-TGCA--
------
FC88X_00966_00646 ------------------TTTTTCCCTTCACT-AA---CCGCGA-AC-TGCA--
------
FC88X_00988_00708 ------------------TTTTTCCCTTCACT-AA---CCGCGA-AC-TGCA--
------
FC88X_00960_00558 ------------------TTTTTCCCTTCACT-AA---CCGCGA-AC-TGCA--
------
FC88X_00967_00573 ------------------TTTTTCCCTTCACT-AA---CCGCGA-AC-TGCA--
------
FC88X_00985_00540 ------------------TTTTTCCCTTCACT-AA---CCGCGA-AC-TGCA--
------
FC88X_00996_00688 ------------------TTTTTCCCTTCACT-AA---CCGCGA-AC-TGCA--
------
FC88X_00968_00428 -------------------TTTTCCCTTCACT-AA---CCGCGA-AC-TGCA--
------
FC88X_00989_00403 -------------------TTTTCCCTTCACT-AA---CCGCGA-AC-TGCA--
------
FC88X_00990_00397 ------------------TTTTTCCCTTCACT-AA---CCGCGA-AC-TGCA--
------
FC88X_00994_00443 ------------------TTTTTCCCTTCACT-AA---CCGCGA-AC-TGCA--
------
FC88X_00954_00383 -------------------TTTTCCCTTCACT-AA---CCGCGA-AC-TGCA--
------
FC88X_00962_00425 -------------------TTTTCCCTTCACT-AA---CCGCGA-AC-TGCA--
------
FC88X_00973_00348 ------------------TTTTTCCCTTCACT-AA---CCGCGA-AC-TGCA--
------
FC88X_00996_00349 ------------------TTTTTCCCTTCACT-AA---CCGCGA-AC-TGCA--
------

FC88X_00982_00255 ------------------TTTTTCCCTTCACT-AA---CCGCGA-AC-TGCA--
------
FC88X_00992_00288 ------------------TTTTTCCCTTCACT-AA---CCGCGA-AC-TGCA--
------
FC88X_00979_00268 -------------------TTTTCCCTTCACT-AA---CCGCGA-AC-TGCA--
------
FC88X_00968_00307 -------------------TTTTCCCTTCACT-AA---CCGCGA-AC-TGCA--
------
FC88X_00984_00247 -------------------TTTTCCCTTCACT-AA---CCGCGA-AC-TGCA--
------
FC88X_00953_00288 -------------------TTTTCCCTTCACT-AA---CCGCGA-AC-TGCA--
------
FC88X_00978_00233 ------------------TTTTTCCCTTCACT-AA---CCGCGA-AC-TGCA--
------
FC88X_00987_00246 ------------------TTTTTCCCTTCACT-AA---CCGCGA-AC-TGCA--
------
FC88X_00945_01271 ------------------TTTTTCCCTTCACT-AA---CCGCGA-AC-TGCA--
------
FC88X_00946_01260 ------------------TTTTTCCCTTCACT-AA---CCGCGA-AC-TGCA--
------
FC88X_00916_01227 -------------------TTTTCCCTTCACT-AA---CCGCGA-AC-TGCA--
------
FC88X_00925_01246 -------------------TTTTCCCTTCACT-AA---CCGCGA-AC-TGCA--
------
FC88X_00903_01138 -------------------TTTTCCCTTCACT-AA---CCGCGA-AC-TGCA--
------
FC88X_01028_00722 -------------------TTTTCCCTTCACT-AA---CCGCGA-AC-TGCA--
------
FC88X_00917_01017 -------------------TTTTCCCTTCACT-AA---CCGCGA-AC-TGCA--
------
FC88X_00900_01138 -------------------TTTTCCCTTCACT-AA---CCGCGA-AC-TGCA--
------
FC88X_00910_00924 -------------------TTTTCCCTTCACT-AA---CCGCGA-AC-TGCA--
------
FC88X_00936_00948 -------------------TTTTCCCTTCACT-AA---CCGCGA-AC-TGCA--
------
FC88X_00940_00886 -------------------TTTTCCCTTCACT-AA---CCGCGA-AC-TGCA--
------
FC88X_00942_00873 -------------------TTTTCCCTTCACT-AA---CCGCGA-AC-TGCA--
------
FC88X_00930_00898 -------------------TTTTCCCTTCACT-AA---CCGCGA-AC-TGCA--
------
FC88X_00932_00875 -------------------TTTTCCCTTCACT-AA---CCGCGA-AC-TGCA--
------
FC88X_00907_00797 -------------------TTTTCCCTTCACT-AA---CCGCGA-AC-TGCA--
------
FC88X_00921_00793 -------------------TTTTCCCTTCACT-AA---CCGCGA-AC-TGCA--
------
FC88X_00908_00599 ------------------TTTTTCCCTTCACT-AA---CCGCGA-AC-TGCA--
------
FC88X_00927_00596 ------------------TTTTTCCCTTCACT-AA---CCGCGA-AC-TGCA--
------
FC88X_00916_00522 -------------------TTTTCCCTTCACT-AA---CCGCGA-AC-TGCA--
------
FC88X_00921_00549 -------------------TTTTCCCTTCACT-AA---CCGCGA-AC-TGCA--
------

```
FC88X_00936_00430       -------------------TTTTCCCTTCACT-AA---CCGCGA-AC-TGCA--
------
FC88X_00941_00466       -------------------TTTTCCCTTCACT-AA---CCGCGA-AC-TGCA--
------
FC88X_00903_00360       -------------------TTTTCCCTTCACT-AA---CCGCGA-AC-TGCA--
------
FC88X_00932_00428       -------------------TTTTCCCTTCACT-AA---CCGCGA-AC-TGCA--
------
FC88X_00903_00284       ------------------TTTTTCCCTTCACT-AA---CCGCGA-AC-TGCA--
------
FC88X_00928_00391       ------------------TTTTTCCCTTCACT-AA---CCGCGA-AC-TGCA--
------
FC88X_00906_00200       ------------------TTTTTCCCTTCACT-AA---CCGCGA-AC-TGCA--
------
FC88X_00902_00294       ------------------TTTTTCCCTTCACT-AA---CCGCGA-AC-TGCA--
------
FC88X_00904_00159       ------------------TTTTTCCCTTCACT-AA---CCGCGA-AC-TGCA--
------
FC88X_00905_00239       ------------------TTTTTCCCTTCACT-AA---CCGCGA-AC-TGCA--
------
FC88X_00879_01256       ------------------TTTTTCCCTTCACT-AA---CCGCGA-AC-TGCA--
------
FC88X_01109_00932       ------------------TTTTTCCCTTCACT-AA---CCGCGA-AC-TGCA--
------
FC88X_00866_01018       -------------------TTTTCCCTTCACT-AA---CCGCGA-AC-TGCA--
------
FC88X_00942_00313       -------------------TTTTCCCTTCACT-AA---CCGCGA-AC-TGCA--
------
FC88X_00713_00662       -------------------TTTTCCCTTCACT-AA---CCGCGA-AC-TGCA--
------
FC88X_00729_00876       -------------------TTTTCCCTTCACT-AA---CCGCGA-AC-TGCA--
------
FC88X_00736_00462       -------------------TTTTCCCTTCACT-AA---CCGCGA-AC-TGCA--
------
FC88X_00725_00569       -------------------TTTTCCCTTCACT-AA---CCGCGA-AC-TGCA--
------
FC88X_00716_00429       -------------------TTTTCCCTTCACT-AA---CCGCGA-AC-TGCA--
------
FC88X_00722_00480       -------------------TTTTCCCTTCACT-AA---CCGCGA-AC-TGCA--
------
FC88X_00665_01215       ------------------TTTTTCCCTTCACT-AA---CCGCGA-AC-TGCA--
------
FC88X_01080_00641       ------------------TTTTTCCCTTCACT-AA---CCGCGA-AC-TGCA--
------
FC88X_00689_01152       ------------------TTTTTCCCTTCACT-AA---CCGCGA-AC-TGCA--
------
FC88X_00692_01159       ------------------TTTTTCCCTTCACT-AA---CCGCGA-AC-TGCA--
------
FC88X_00690_01048       ------------------TTTTTCCCTTCACT-AA---CCGCGA-AC-TGCA--
------
FC88X_01142_00840       ------------------TTTTTCCCTTCACT-AA---CCGCGA-AC-TGCA--
------
FC88X_00660_01030       -------------------TTTTCCCTTCACT-AA---CCGCGA-AC-TGCA--
------
FC88X_00676_01230       -------------------TTTTCCCTTCACT-AA---CCGCGA-AC-TGCA--
------
```

FC88X_00692_00974 -------------------TTTTCCCTTCACT-AA---CCGCGA-AC-TGCA--
------
FC88X_00706_00132 -------------------TTTTCCCTTCACT-AA---CCGCGA-AC-TGCA--
------
FC88X_00695_00623 ------------------TTTTTCCCTTCACT-AA---CCGCGA-AC-TGCA--
------
FC88X_00665_00847 ------------------TTTTTCCCTTCACT-AA---CCGCGA-AC-TGCA--
------
FC88X_00685_00493 ------------------TTTTTCCCTTCACT-AA---CCGCGA-AC-TGCA--
------
FC88X_00678_00588 ------------------TTTTTCCCTTCACT-AA---CCGCGA-AC-TGCA--
------
FC88X_00672_00152 ------------------TTTTTCCCTTCACT-AA---CCGCGA-AC-TGCA--
------
FC88X_00656_00241 ------------------TTTTTCCCTTCACT-AA---CCGCGA-AC-TGCA--
------
FC88X_00665_00071 ------------------TTTTTCCCTTCACT-AA---CCGCGA-AC-TGCA--
------
FC88X_00651_00139 ------------------TTTTTCCCTTCACT-AA---CCGCGA-AC-TGCA--
------
FC88X_00619_01265 ------------------TTTTTCCCTTCACT-AA---CCGCGA-AC-TGCA--
------
FC88X_00701_00127 ------------------TTTTTCCCTTCACT-AA---CCGCGA-AC-TGCA--
------
FC88X_00620_01137 ------------------TTTTTCCCTTCACT-AA---CCGCGA-AC-TGCA--
------
FC88X_00628_01209 ------------------TTTTTCCCTTCACT-AA---CCGCGA-AC-TGCA--
------
FC88X_00600_01024 ------------------TTTTTCCCTTCACT-AA---CCGCGA-AC-TGCA--
------
FC88X_00602_01143 ------------------TTTTTCCCTTCACT-AA---CCGCGA-AC-TGCA--
------
FC88X_00612_00920 ------------------TTTTTCCCTTCACT-AA---CCGCGA-AC-TGCA--
------
FC88X_00968_01016 ------------------TTTTTCCCTTCACT-AA---CCGCGA-AC-TGCA--
------
FC88X_00627_00503 ------------------TTTTTCCCTTCACT-AA---CCGCGA-AC-TGCA--
------
FC88X_00630_00840 ------------------TTTTTCCCTTCACT-AA---CCGCGA-AC-TGCA--
------
FC88X_00610_00434 ------------------TTTTTCCCTTCACT-AA---CCGCGA-AC-TGCA--
------
FC88X_00642_00410 ------------------TTTTTCCCTTCACT-AA---CCGCGA-AC-TGCA--
------
FC88X_00646_00345 ------------------TTTTTCCCTTCACT-AA---CCGCGA-AC-TGCA--
------
FC88X_00607_00444 ------------------TTTTTCCCTTCACT-AA---CCGCGA-AC-TGCA--
------
FC88X_00635_00111 ------------------TTTTTCCCTTCACT-AA---CCGCGA-AC-TGCA--
------
FC88X_00690_01108 ------------------TTTTTCCCTTCACT-AA---CCGCGA-AC-TGCA--
------
FC88X_00551_01239 ------------------TTTTTCCCTTCACT-AA---CCGCGA-AC-TGCA--
------
FC88X_00560_01219 ------------------TTTTTCCCTTCACT-AA---CCGCGA-AC-TGCA--
------

FC88X_00585_01166 ------------------TTTTTCCCTTCACT-AA---CCGCGA-AC-TGCA--
------
FC88X_00588_01166 ------------------TTTTTCCCTTCACT-AA---CCGCGA-AC-TGCA--
------
FC88X_00559_01124 ------------------TTTTTCCCTTCACT-AA---CCGCGA-AC-TGCA--
------
FC88X_00584_01174 ------------------TTTTTCCCTTCACT-AA---CCGCGA-AC-TGCA--
------
FC88X_00582_00988 ------------------TTTTTCCCTTCACT-AA---CCGCGA-AC-TGCA--
------
FC88X_00592_00964 ------------------TTTTTCCCTTCACT-AA---CCGCGA-AC-TGCA--
------
FC88X_00575_00870 ------------------TTTTTCCCTTCACT-AA---CCGCGA-AC-TGCA--
------
FC88X_00568_00934 ------------------TTTTTCCCTTCACT-AA---CCGCGA-AC-TGCA--
------
FC88X_00599_00752 ------------------TTTTTCCCTTCACT-AA---CCGCGA-AC-TGCA--
------
FC88X_00589_00823 ------------------TTTTTCCCTTCACT-AA---CCGCGA-AC-TGCA--
------
FC88X_00578_00563 ------------------TTTTTCCCTTCACT-AA---CCGCGA-AC-TGCA--
------
FC88X_00565_00683 ------------------TTTTTCCCTTCACT-AA---CCGCGA-AC-TGCA--
------
FC88X_00575_00175 ------------------TTTTTCCCTTCACT-AA---CCGCGA-AC-TGCA--
------
FC88X_00596_01202 ------------------TTTTTCCCTTCACT-AA---CCGCGA-AC-TGCA--
------
FC88X_00582_00076 ------------------TTTTTCCCTTCACT-AA---CCGCGA-AC-TGCA--
------
FC88X_00567_00111 ------------------TTTTTCCCTTCACT-AA---CCGCGA-AC-TGCA--
------
FC88X_00533_01241 ------------------TTTTTCCCTTCACT-AA---CCGCGA-AC-TGCA--
------
FC88X_00562_00083 ------------------TTTTTCCCTTCACT-AA---CCGCGA-AC-TGCA--
------
FC88X_00516_01106 ------------------TTTTTCCCTTCACT-AA---CCGCGA-AC-TGCA--
------
FC88X_00511_01217 ------------------TTTTTCCCTTCACT-AA---CCGCGA-AC-TGCA--
------
FC88X_00523_01007 ------------------TTTTTCCCTTCACT-AA---CCGCGA-AC-TGCA--
------
FC88X_00544_01000 ------------------TTTTTCCCTTCACT-AA---CCGCGA-AC-TGCA--
------
FC88X_00539_00997 ------------------TTTTTCCCTTCACT-AA---CCGCGA-AC-TGCA--
------
FC88X_00512_01040 ------------------TTTTTCCCTTCACT-AA---CCGCGA-AC-TGCA--
------
FC88X_00514_00965 ------------------TTTTTCCCTTCACT-AA---CCGCGA-AC-TGCA--
------
FC88X_00524_00990 ------------------TTTTTCCCTTCACT-AA---CCGCGA-AC-TGCA--
------
FC88X_00547_00830 ------------------TTTTTCCCTTCACT-AA---CCGCGA-AC-TGCA--
------
FC88X_00510_00976 ------------------TTTTTCCCTTCACT-AA---CCGCGA-AC-TGCA--
------

FC88X_00540_00622 ------------------TTTTTCCCTTCACT-AA---CCGCGA-AC-TGCA--
------
FC88X_00547_00735 ------------------TTTTTCCCTTCACT-AA---CCGCGA-AC-TGCA--
------
FC88X_00548_00046 ------------------TTTTTCCCTTCACT-AA---CCGCGA-AC-TGCA--
------
FC88X_00534_00109 ------------------TTTTTCCCTTCACT-AA---CCGCGA-AC-TGCA--
------
FC88X_00500_00026 ------------------TTTTTCCCTTCACT-AA---CCGCGA-AC-TGCA--
------
FC88X_00513_00020 ------------------TTTTTCCCTTCACT-AA---CCGCGA-AC-TGCA--
------
FC88X_00469_01227 ------------------TTTTTCCCTTCACT-AA---CCGCGA-AC-TGCA--
------
FC88X_00495_01244 ------------------TTTTTCCCTTCACT-AA---CCGCGA-AC-TGCA--
------
FC88X_00499_01195 ------------------TTTTTCCCTTCACT-AA---CCGCGA-AC-TGCA--
------
FC88X_00466_01219 ------------------TTTTTCCCTTCACT-AA---CCGCGA-AC-TGCA--
------
FC88X_00486_01136 ------------------TTTTTCCCTTCACT-AA---CCGCGA-AC-TGCA--
------
FC88X_00472_01179 ------------------TTTTTCCCTTCACT-AA---CCGCGA-AC-TGCA--
------
FC88X_00484_01071 ------------------TTTTTCCCTTCACT-AA---CCGCGA-AC-TGCA--
------
FC88X_00568_00198 ------------------TTTTTCCCTTCACT-AA---CCGCGA-AC-TGCA--
------
FC88X_00452_00964 ------------------TTTTTCCCTTCACT-AA---CCGCGA-AC-TGCA--
------
FC88X_00468_01000 ------------------TTTTTCCCTTCACT-AA---CCGCGA-AC-TGCA--
------
FC88X_00459_00776 ------------------TTTTTCCCTTCACT-AA---CCGCGA-AC-TGCA--
------
FC88X_00488_00898 ------------------TTTTTCCCTTCACT-AA---CCGCGA-AC-TGCA--
------
FC88X_00494_00678 ------------------TTTTTCCCTTCACT-AA---CCGCGA-AC-TGCA--
------
FC88X_00499_00736 ------------------TTTTTCCCTTCACT-AA---CCGCGA-AC-TGCA--
------
FC88X_00473_00287 ------------------TTTTTCCCTTCACT-AA---CCGCGA-AC-TGCA--
------
FC88X_00465_00522 ------------------TTTTTCCCTTCACT-AA---CCGCGA-AC-TGCA--
------
FC88X_00480_00105 ------------------TTTTTCCCTTCACT-AA---CCGCGA-AC-TGCA--
------
FC88X_00483_00136 ------------------TTTTTCCCTTCACT-AA---CCGCGA-AC-TGCA--
------
FC88X_00483_00067 ------------------TTTTTCCCTTCACT-AA---CCGCGA-AC-TGCA--
------
FC88X_00498_00089 ------------------TTTTTCCCTTCACT-AA---CCGCGA-AC-TGCA--
------
FC88X_00462_00058 ------------------TTTTTCCCTTCACT-AA---CCGCGA-AC-TGCA--
------
FC88X_00476_00055 ------------------TTTTTCCCTTCACT-AA---CCGCGA-AC-TGCA--
------

FC88X_00432_01229 ------------------TTTTTCCCTTCACT-AA---CCGCGA-AC-TGCA--
------
FC88X_00448_01222 ------------------TTTTTCCCTTCACT-AA---CCGCGA-AC-TGCA--
------
FC88X_00420_01185 ------------------TTTTTCCCTTCACT-AA---CCGCGA-AC-TGCA--
------
FC88X_00497_00029 ------------------TTTTTCCCTTCACT-AA---CCGCGA-AC-TGCA--
------
FC88X_00436_01075 ------------------TTTTTCCCTTCACT-AA---CCGCGA-AC-TGCA--
------
FC88X_00424_01200 ------------------TTTTTCCCTTCACT-AA---CCGCGA-AC-TGCA--
------
FC88X_00418_01071 ------------------TTTTTCCCTTCACT-AA---CCGCGA-AC-TGCA--
------
FC88X_00417_01192 ------------------TTTTTCCCTTCACT-AA---CCGCGA-AC-TGCA--
------
FC88X_00440_00116 ------------------TTTTTCCCTTCACT-AA---CCGCGA-AC-TGCA--
------
FC88X_00400_00291 ------------------TTTTTCCCTTCACT-AA---CCGCGA-AC-TGCA--
------
FC88X_00360_01168 ------------------TTTTTCCCTTCACT-AA---CCGCGA-AC-TGCA--
------
FC88X_00431_00049 ------------------TTTTTCCCTTCACT-AA---CCGCGA-AC-TGCA--
------
FC88X_00369_01143 ------------------TTTTTCCCTTCACT-AA---CCGCGA-AC-TGCA--
------
FC88X_00352_01162 ------------------TTTTTCCCTTCACT-AA---CCGCGA-AC-TGCA--
------
FC88X_00375_01079 ------------------TTTTTCCCTTCACT-AA---CCGCGA-AC-TGCA--
------
FC88X_00394_01055 ------------------TTTTTCCCTTCACT-AA---CCGCGA-AC-TGCA--
------
FC88X_00387_00868 ------------------TTTTTCCCTTCACT-AA---CCGCGA-AC-TGCA--
------
FC88X_00367_00978 ------------------TTTTTCCCTTCACT-AA---CCGCGA-AC-TGCA--
------
FC88X_00384_00656 ------------------TTTTTCCCTTCACT-AA---CCGCGA-AC-TGCA--
------
FC88X_00395_01049 ------------------TTTTTCCCTTCACT-AA---CCGCGA-AC-TGCA--
------
FC88X_00399_00193 ------------------TTTTTCCCTTCACT-AA---CCGCGA-AC-TGCA--
------
FC88X_00393_00533 ------------------TTTTTCCCTTCACT-AA---CCGCGA-AC-TGCA--
------
FC88X_00318_01144 ------------------TTTTTCCCTTCACT-AA---CCGCGA-AC-TGCA--
------
FC88X_00348_01159 ------------------TTTTTCCCTTCACT-AA---CCGCGA-AC-TGCA--
------
FC88X_00346_01085 ------------------TTTTTCCCTTCACT-AA---CCGCGA-AC-TGCA--
------
FC88X_00349_01067 ------------------TTTTTCCCTTCACT-AA---CCGCGA-AC-TGCA--
------
FC88X_00329_01039 ------------------TTTTTCCCTTCACT-AA---CCGCGA-AC-TGCA--
------
FC88X_00303_01082 ------------------TTTTTCCCTTCACT-AA---CCGCGA-AC-TGCA--
------

FC88X_00348_00911 ------------------TTTTTCCCTTCACT-AA---CCGCGA-AC-TGCA--
------
FC88X_00340_00985 ------------------TTTTTCCCTTCACT-AA---CCGCGA-AC-TGCA--
------
FC88X_00323_00655 ------------------TTTTTCCCTTCACT-AA---CCGCGA-AC-TGCA--
------
FC88X_00330_00793 ------------------TTTTTCCCTTCACT-AA---CCGCGA-AC-TGCA--
------
FC88X_00303_00411 ------------------TTTTTCCCTTCACT-AA---CCGCGA-AC-TGCA--
------
FC88X_00341_00553 ------------------TTTTTCCCTTCACT-AA---CCGCGA-AC-TGCA--
------
FC88X_00304_00188 ------------------TTTTTCCCTTCACT-AA---CCGCGA-AC-TGCA--
------
FC88X_00311_00328 ------------------TTTTTCCCTTCACT-AA---CCGCGA-AC-TGCA--
------
FC88X_00326_00036 ------------------TTTTTCCCTTCACT-AA---CCGCGA-AC-TGCA--
------
FC88X_00327_00017 ------------------TTTTTCCCTTCACT-AA---CCGCGA-AC-TGCA--
------
FC88X_00307_00048 ------------------TTTTTCCCTTCACT-AA---CCGCGA-AC-TGCA--
------
FC88X_00989_00490 ------------------TTTTTCCCTTCACT-AA---CCGCGA-AC-TGCA--
------
FC88X_00275_01089 ------------------TTTTTCCCTTCACT-AA---CCGCGA-AC-TGCA--
------
FC88X_00294_01089 ------------------TTTTTCCCTTCACT-AA---CCGCGA-AC-TGCA--
------
FC88X_00299_01025 ------------------TTTTTCCCTTCACT-AA---CCGCGA-AC-TGCA--
------
FC88X_00252_01055 ------------------TTTTTCCCTTCACT-AA---CCGCGA-AC-TGCA--
------
FC88X_00262_00990 ------------------TTTTTCCCTTCACT-AA---CCGCGA-AC-TGCA--
------
FC88X_00266_00984 ------------------TTTTTCCCTTCACT-AA---CCGCGA-AC-TGCA--
------
FC88X_00268_00940 ------------------TTTTTCCCTTCACT-AA---CCGCGA-AC-TGCA--
------
FC88X_00271_00933 ------------------TTTTTCCCTTCACT-AA---CCGCGA-AC-TGCA--
------
FC88X_00269_00611 ------------------TTTTTCCCTTCACT-AA---CCGCGA-AC-TGCA--
------
FC88X_00250_00922 ------------------TTTTTCCCTTCACT-AA---CCGCGA-AC-TGCA--
------
FC88X_00277_00427 ------------------TTTTTCCCTTCACT-AA---CCGCGA-AC-TGCA--
------
FC88X_00280_00560 ------------------TTTTTCCCTTCACT-AA---CCGCGA-AC-TGCA--
------
FC88X_00296_00198 ------------------TTTTTCCCTTCACT-AA---CCGCGA-AC-TGCA--
------
FC88X_00299_00296 ------------------TTTTTCCCTTCACT-AA---CCGCGA-AC-TGCA--
------
FC88X_00280_00060 ------------------TTTTTCCCTTCACT-AA---CCGCGA-AC-TGCA--
------
FC88X_00280_00144 ------------------TTTTTCCCTTCACT-AA---CCGCGA-AC-TGCA--
------

FC88X_00299_00033 ------------------TTTTTCCCTTCACT-AA---CCGCGA-AC-TGCA--
------
FC88X_00259_00058 ------------------TTTTTCCCTTCACT-AA---CCGCGA-AC-TGCA--
------
FC88X_00259_00044 ------------------TTTTTCCCTTCACT-AA---CCGCGA-AC-TGCA--
------
FC88X_00358_01007 ------------------TTTTTCCCTTCACT-AA---CCGCGA-AC-TGCA--
------
FC88X_00217_00976 ------------------TTTTTCCCTTCACT-AA---CCGCGA-AC-TGCA--
------
FC88X_00233_01014 ------------------TTTTTCCCTTCACT-AA---CCGCGA-AC-TGCA--
------
FC88X_00244_00900 ------------------TTTTTCCCTTCACT-AA---CCGCGA-AC-TGCA--
------
FC88X_00205_00956 ------------------TTTTTCCCTTCACT-AA---CCGCGA-AC-TGCA--
------
FC88X_00226_00774 ------------------TTTTTCCCTTCACT-AA---CCGCGA-AC-TGCA--
------
FC88X_00227_00790 ------------------TTTTTCCCTTCACT-AA---CCGCGA-AC-TGCA--
------
FC88X_00203_00691 ------------------TTTTTCCCTTCACT-AA---CCGCGA-AC-TGCA--
------
FC88X_00231_01101 ------------------TTTTTCCCTTCACT-AA---CCGCGA-AC-TGCA--
------
FC88X_00230_00426 ------------------TTTTTCCCTTCACT-AA---CCGCGA-AC-TGCA--
------
FC88X_00209_00799 ------------------TTTTTCCCTTCACT-AA---CCGCGA-AC-TGCA--
------
FC88X_00225_00212 ------------------TTTTTCCCTTCACT-AA---CCGCGA-AC-TGCA--
------
FC88X_00244_00208 ------------------TTTTTCCCTTCACT-AA---CCGCGA-AC-TGCA--
------
FC88X_00239_00037 ------------------TTTTTCCCTTCACT-AA---CCGCGA-AC-TGCA--
------
FC88X_00243_00017 ------------------TTTTTCCCTTCACT-AA---CCGCGA-AC-TGCA--
------
FC88X_00233_00031 ------------------TTTTTCCCTTCACT-AA---CCGCGA-AC-TGCA--
------
FC88X_00238_00049 ------------------TTTTTCCCTTCACT-AA---CCGCGA-AC-TGCA--
------
FC88X_00176_00987 -------------------TTTTCCCTTCACT-AA---CCGCGA-AC-TGCA--
------
FC88X_00157_01021 -------------------TTTTCCCTTCACT-AA---CCGCGA-AC-TGCA--
------
FC88X_00158_00995 ------------------TTTTTCCCTTCACT-AA---CCGCGA-AC-TGCA--
------
FC88X_00229_00033 ------------------TTTTTCCCTTCACT-AA---CCGCGA-AC-TGCA--
------
FC88X_00189_00913 ------------------TTTTTCCCTTCACT-AA---CCGCGA-AC-TGCA--
------
FC88X_00199_00941 ------------------TTTTTCCCTTCACT-AA---CCGCGA-AC-TGCA--
------
FC88X_00165_00932 -------------------TTTTCCCTTCACT-AA---CCGCGA-AC-TGCA--
------
FC88X_00153_00960 -------------------TTTTCCCTTCACT-AA---CCGCGA-AC-TGCA--
------

FC88X_00195_00865 -------------------TTTTCCCTTCACT-AA---CCGCGA-AC-TGCA--
------
FC88X_00199_00887 -------------------TTTTCCCTTCACT-AA---CCGCGA-AC-TGCA--
------
FC88X_00174_00806 -------------------TTTTCCCTTCACT-AA---CCGCGA-AC-TGCA--
------
FC88X_00168_00879 -------------------TTTTCCCTTCACT-AA---CCGCGA-AC-TGCA--
------
FC88X_00174_00801 ------------------TTTTTCCCTTCACT-AA---CCGCGA-AC-TGCA--
------
FC88X_00166_00869 ------------------TTTTTCCCTTCACT-AA---CCGCGA-AC-TGCA--
------
FC88X_00152_00794 ------------------TTTTTCCCTTCACT-AA---CCGCGA-AC-TGCA--
------
FC88X_00155_00778 ------------------TTTTTCCCTTCACT-AA---CCGCGA-AC-TGCA--
------
FC88X_00156_00575 ------------------TTTTTCCCTTCACT-AA---CCGCGA-AC-TGCA--
------
FC88X_00173_00794 ------------------TTTTTCCCTTCACT-AA---CCGCGA-AC-TGCA--
------
FC88X_00161_00533 ------------------TTTTTCCCTTCACT-AA---CCGCGA-AC-TGCA--
------
FC88X_00209_00301 ------------------TTTTTCCCTTCACT-AA---CCGCGA-AC-TGCA--
------
FC88X_00174_00209 ------------------TTTTTCCCTTCACT-AA---CCGCGA-AC-TGCA--
------
FC88X_00163_00463 ------------------TTTTTCCCTTCACT-AA---CCGCGA-AC-TGCA--
------
FC88X_00196_00125 ------------------TTTTTCCCTTCACT-AA---CCGCGA-AC-TGCA--
------
FC88X_00197_00134 ------------------TTTTTCCCTTCACT-AA---CCGCGA-AC-TGCA--
------
FC88X_00190_00062 ------------------TTTTTCCCTTCACT-AA---CCGCGA-AC-TGCA--
------
FC88X_00194_00142 ------------------TTTTTCCCTTCACT-AA---CCGCGA-AC-TGCA--
------
FC88X_00173_00053 ------------------TTTTTCCCTTCACT-AA---CCGCGA-AC-TGCA--
------
FC88X_00182_00087 ------------------TTTTTCCCTTCACT-AA---CCGCGA-AC-TGCA--
------
FC88X_00166_00099 ------------------TTTTTCCCTTCACT-AA---CCGCGA-AC-TGCA--
------
FC88X_00160_00348 ------------------TTTTTCCCTTCACT-AA---CCGCGA-AC-TGCA--
------
FC88X_00154_00083 ------------------TTTTTCCCTTCACT-AA---CCGCGA-AC-TGCA--
------
FC88X_00163_00063 ------------------TTTTTCCCTTCACT-AA---CCGCGA-AC-TGCA--
------
FC88X_00166_00048 ------------------TTTTTCCCTTCACT-AA---CCGCGA-AC-TGCA--
------
FC88X_00191_00020 ------------------TTTTTCCCTTCACT-AA---CCGCGA-AC-TGCA--
------
FC88X_00130_00889 ------------------TTTTTCCCTTCACT-AA---CCGCGA-AC-TGCA--
------
FC88X_00135_00884 ------------------TTTTTCCCTTCACT-AA---CCGCGA-AC-TGCA--
------

FC88X_00114_00836 ------------------TTTTTCCCTTCACT-AA---CCGCGA-AC-TGCA--
------
FC88X_00684_01123 -------------------TTTTCCCTTCACT-AA---CCGCGA-AC-TGCA--
------
FC88X_00132_00795 ------------------TTTTTCCCTTCACT-AA---CCGCGA-AC-TGCA--
------
FC88X_00667_01095 -------------------TTTTCCCTTCACT-AA---CCGCGA-AC-TGCA--
------
FC88X_00116_00718 ------------------TTTTTCCCTTCACT-AA---CCGCGA-AC-TGCA--
------
FC88X_00130_00727 ------------------TTTTTCCCTTCACT-AA---CCGCGA-AC-TGCA--
------
FC88X_00142_00669 ------------------TTTTTCCCTTCACT-AA---CCGCGA-AC-TGCA--
------
FC88X_00114_00744 ------------------TTTTTCCCTTCACT-AA---CCGCGA-AC-TGCA--
------
FC88X_00120_00685 ------------------TTTTTCCCTTCACT-AA---CCGCGA-AC-TGCA--
------
FC88X_00140_00699 ------------------TTTTTCCCTTCACT-AA---CCGCGA-AC-TGCA--
------
FC88X_00144_00560 ------------------TTTTTCCCTTCACT-AA---CCGCGA-AC-TGCA--
------
FC88X_00375_00056 ------------------TTTTTCCCTTCACT-AA---CCGCGA-AC-TGCA--
------
FC88X_00101_00591 -------------------TTTTCCCTTCACT-AA---CCGCGA-AC-TGCA--
------
FC88X_00108_00551 -------------------TTTTCCCTTCACT-AA---CCGCGA-AC-TGCA--
------
FC88X_00136_00536 ------------------TTTTTCCCTTCACT-AA---CCGCGA-AC-TGCA--
------
FC88X_00267_01000 ------------------TTTTTCCCTTCACT-AA---CCGCGA-AC-TGCA--
------
FC88X_00148_00456 ------------------TTTTTCCCTTCACT-AA---CCGCGA-AC-TGCA--
------
FC88X_00132_00546 ------------------TTTTTCCCTTCACT-AA---CCGCGA-AC-TGCA--
------
FC88X_00130_00417 -------------------TTTTCCCTTCACT-AA---CCGCGA-AC-TGCA--
------
FC88X_00121_00540 -------------------TTTTCCCTTCACT-AA---CCGCGA-AC-TGCA--
------
FC88X_00118_00420 ------------------TTTTTCCCTTCACT-AA---CCGCGA-AC-TGCA--
------
FC88X_00133_00410 ------------------TTTTTCCCTTCACT-AA---CCGCGA-AC-TGCA--
------
FC88X_00113_00412 ------------------TTTTTCCCTTCACT-AA---CCGCGA-AC-TGCA--
------
FC88X_00113_00482 ------------------TTTTTCCCTTCACT-AA---CCGCGA-AC-TGCA--
------
FC88X_00114_00250 ------------------TTTTTCCCTTCACT-AA---CCGCGA-AC-TGCA--
------
FC88X_00100_00397 ------------------TTTTTCCCTTCACT-AA---CCGCGA-AC-TGCA--
------
FC88X_00139_00219 ------------------TTTTTCCCTTCACT-AA---CCGCGA-AC-TGCA--
------
FC88X_00261_00983 ------------------TTTTTCCCTTCACT-AA---CCGCGA-AC-TGCA--
------

```
FC88X_00111_00237        ------------------TTTTTCCCTTCACT-AA---CCGCGA-AC-TGCA--
------
FC88X_00103_00340        ------------------TTTTTCCCTTCACT-AA---CCGCGA-AC-TGCA--
------
FC88X_00119_00180        ------------------TTTTTCCCTTCACT-AA---CCGCGA-AC-TGCA--
------
FC88X_00148_00192        ------------------TTTTTCCCTTCACT-AA---CCGCGA-AC-TGCA--
------
FC88X_00110_00130        ------------------TTTTTCCCTTCACT-AA---CCGCGA-AC-TGCA--
------
FC88X_00138_00213        ------------------TTTTTCCCTTCACT-AA---CCGCGA-AC-TGCA--
------
FC88X_00123_00063        ------------------TTTTTCCCTTCACT-AA---CCGCGA-AC-TGCA--
------
FC88X_00135_00078        ------------------TTTTTCCCTTCACT-AA---CCGCGA-AC-TGCA--
------
FC88X_00111_00067        -------------------TTTTCCCTTCACT-AA---CCGCGA-AC-TGCA--
------
FC88X_00731_01036        -------------------TTTTCCCTTCACT-AA---CCGCGA-AC-TGCA--
------
FC88X_00065_00776        ------------------TTTTTCCCTTCACT-AA---CCGCGA-AC-TGCA--
------
FC88X_00087_00755        ------------------TTTTTCCCTTCACT-AA---CCGCGA-AC-TGCA--
------
FC88X_00065_00654        ------------------TTTTTCCCTTCACT-AA---CCGCGA-AC-TGCA--
------
FC88X_00061_00708        ------------------TTTTTCCCTTCACT-AA---CCGCGA-AC-TGCA--
------
FC88X_00013_00511        -------------------TTTTCCCTTCACT-AA---CCGCGA-AC-TGCA--
------
FC88X_00116_00752        ------------------TTTTTCCCTTCACT-AA---CCGCGA-AC-TGCA--
------
FC88X_00908_00538        -----------------TTTTTTCCCTTCACT-AA---CCGCGA-AC-TGCA--
------
FC88X_01013_00727        -----------------TTTTTTCCCTTCACT-AA---CCGCGA-ACGTGCAA-
------
FC88X_00635_01061        -----------------TTTTTTCCCTTCACT-AA---CCGCGA-AC-TGCA--
------
FC88X_01099_00495        -----------------TTTTTTCCCTTCACT-AA---CCGCGA-AC-TGCA--
------
FC88X_00102_00470        -----------------TTTTTTCCCTTCACT-AA---CCGCGA-AC-TGCA--
------
FC88X_00037_00698        -----------------TTTTTTCCCTTCACT-AA---CCGCGA-AC-TGCA--
------
FC88X_00725_00973        -----------------TTTTTTCCCTTCACT-AA---CCGCGA-AC-TGCA--
------
FC88X_01002_00811        -----------------TTTTTTCCCTTCACT-AA---CCGCGA-AC-TGCA--
------
FC88X_01022_00342        -----------------TTTTTTCCCTTCACT-AA---CCGCGA-AC-TGCA--
------
FC88X_00557_00358        -----------------TTTTTTCCCTTCACT-AA---CCGCGA-AC-TGCA--
------
FC88X_00915_00906        -----------------TTTTTTCCCTTCACT-AA---CCGCGA-AC-TGCA--
------
FC88X_00945_00746        -----------------TTTTTTCCCTTCACT-AA---CCGCGA-AC-TGCA--
------
```

FC88X_00091_00200 -----------------TTTTTTCCCTTCACT-AA---CCGCGA-AC-TGCA--
------
FC88X_00028_00757 -------------------TTTTCCCTTCACT-AA---CCGCGA-AC-TGCA--
------
FC88X_00625_00829 -------------------TTTTCCCTTCACT-AA---CCGCGA-AC-TGCA--
------
FC88X_00892_01164 ------------------TTTTTCCCTTCACT-AA---CCGCGA-AC-TGCA--
------
FC88X_00918_00777 ------------------TTTTTCCCTTCACT-AA---CCGCGA-AC-TGCA--
------
FC88X_00713_00412 ------------------TTTTTCCCTTCACT-AA---CCGCGA-AC-TGCA--
------
FC88X_00931_00498 ------------------TTTTTCCCTTCACT-AA---CCGCGA-AC-TGCA--
------
FC88X_00268_00685 ------------------TTTTTCCCTTCACT-AA---CCGCGA-AC-TGCA--
------
FC88X_00445_00070 ------------------TTTTTCCCTTCACT-AA---CCGCGA-AC-TGCA--
------
FC88X_00035_00511 ------------------TTTTTCCCTTCACT-AA---CCGCGA-AC-TGCA--
------
FC88X_00400_00905 ------------------TTTTTCCCTTCACT-AA---CCGCGA-AC-TGCA--
------
FC88X_00045_00614 -------------------TTTTCCCTTCACT-AA---CCGCGA-AC-TGCA--
------
FC88X_00962_00487 -------------------TTTTCCCTTCACT-AA---CCGCGA-AC-TGCA--
------
FC88X_00073_00304 -------------------TTTTCCCTTCACT-AA---CCGCGA-AC-TGCA--
------
FC88X_00056_00444 -------------------TTTTCCCTTCACT-AA---CCGCGA-AC-TGCA--
------
FC88X_00089_00890 -------------------TTTTCCCTTCACT-AA---CCGCGA-AC-TGCA--
------
FC88X_00042_00649 -------------------TTTTCCCTTCACT-AA---CCGCGA-AC-TGCA--
------
FC88X_00318_00004 -------------------TTTTCCCTTCACT-AA---CCGCGA-AC-TGCA--
------
FC88X_00701_01113 -------------------TTTTCCCTTCACT-AA---CCGCGA-AC-TGCA--
------
FC88X_00413_01166 -------------------TTTTCCCTTCACT-AA---CCGCGA-AC-TGCA--
------
FC88X_00093_00912 -------------------TTTTCCCTTCACT-AA---CCGCGA-AC-TGCA--
------
FC88X_00460_01163 -------------------TTTTCCCTTCACT-AA---CCGCGA-AC-TGCA--
------
FC88X_00462_01186 -------------------TTTTCCCTTCACT-AA---CCGCGA-AC-TGCA--
------
FC88X_00504_01040 -------------------TTTTCCCTTCACT-AA---CCGCGA-AC-TGCA--
------
FC88X_00515_01112 -------------------TTTTCCCTTCACT-AA---CCGCGA-AC-TGCA--
------
FC88X_00585_01152 -------------------TTTTCCCTTCACT-AA---CCGCGA-AC-TGCA--
------
FC88X_00675_01262 -------------------TTTTCCCTTCACT-AA---CCGCGA-AC-TGCA--
------
FC88X_00747_00175 -------------------TTTTCCCTTCACT-AA---CCGCGA-AC-TGCA--
------

FC88X_00702_00812 -------------------TTTTCCCTTCACT-AA---CCGCGA-AC-TGCA--
------
FC88X_00758_00776 -------------------TTTTCCCTTCACT-AA---CCGCGA-AC-TGCA--
------
FC88X_00791_00847 -------------------TTTTCCCTTCACT-AA---CCGCGA-AC-TGCA--
------
FC88X_01008_01175 -------------------TTTTCCCTTCACT-AA---CCGCGA-AC-TGCA--
------
FC88X_01049_01203 -------------------TTTTCCCTTCACT-AA---CCGCGA-AC-TGCA--
------
FC88X_01062_01202 -------------------TTTTCCCTTCACT-AA---CCGCGA-AC-TGCA--
------
FC88X_01069_01234 -------------------TTTTCCCTTCACT-AA---CCGCGA-AC-TGCA--
------
FC88X_00017_00200 -------------------TTTTCCCTTCACT-AA---CCGCGA-AC-TGCA--
------
FC88X_00026_00224 -------------------TTTTCCCTTCACT-AA---CCGCGA-AC-TGCA--
------
FC88X_00035_00520 ------------------TTTTTCCCTTCACT-AA---CCGCGA-AC-TGCA--
------
FC88X_00178_00794 -------------------TTTTCCCTTCACT-AA---CCGCGA-AC-TGCA--
------
FC88X_00103_00131 -------------------TTTTCCCTTCACT-AA---CCGCGA-AC-TGCA--
------
FC88X_00113_00082 ------------------TTTTTCCCTTCACT-AA---CCGCGA-AC-TGCA--
------
FC88X_00219_00951 ------------------TTTTTCCCTTCACT-AA---CCGCGA-AC-TGCA--
------
FC88X_00333_00584 -------------------TTTTCCCTTCACT-AA---CCGCGA-AC-TGCA--
------
FC88X_00391_00752 ------------------TTTTTCCCTTCACT-AA---CCGCGA-AC-TGCA--
------
FC88X_01147_01195 -------------------TTTTCCCTTCACT-AA---CCGCGA-AC-TGCA--
------
FC88X_00090_00565 ------------------TTTTTCCCTTCACT-AA---CCGCGA-AC-TGCA--
------
FC88X_00103_00035 ------------------TTTTTCCCTTCACT-AA---CCGCGA-AC-TGCA--
------
FC88X_00068_00563 ------------------TTTTTCCCTTCACT-AA---CCGCGA-AC-TGCA--
------
FC88X_00077_00559 ------------------TTTTTCCCTTCACT-AA---CCGCGA-AC-TGCA--
------
FC88X_00091_00459 ------------------TTTTTCCCTTCACT-AA---CCGCGA-AC-TGCA--
------
FC88X_00093_00456 ------------------TTTTTCCCTTCACT-AA---CCGCGA-AC-TGCA--
------
FC88X_00079_00476 ------------------TTTTTCCCTTCACT-AA---CCGCGA-AC-TGCA--
------
FC88X_00086_00462 ------------------TTTTTCCCTTCACT-AA---CCGCGA-AC-TGCA--
------
FC88X_00069_00490 ------------------TTTTTCCCTTCACT-AA---CCGCGA-AC-TGCA--
------
FC88X_00071_00494 ------------------TTTTTCCCTTCACT-AA---CCGCGA-AC-TGCA--
------
FC88X_00077_00330 ------------------TTTTTCCCTTCACT-AA---CCGCGA-AC-TGCA--
------

```
FC88X_00086_00375      ------------------TTTTTCCCTTCACT-AA---CCGCGA-AC-TGCA--
------
FC88X_00072_00209      -------------------TTTTCCCTTCACT-AA---CCGCGA-AC-TGCA--
------
FC88X_00093_00420      ------------------TTTTTCCCTTCACT-AA---CCGCGA-AC-TGCA--
------
FC88X_00053_00249      ------------------TTTTTCCCTTCACT-AA---CCGCGA-AC-TGCA--
------
FC88X_00062_00205      ------------------TTTTTCCCTTCACT-AA---CCGCGA-AC-TGCA--
------
FC88X_00007_00108      -------------------TTTTCCCTTCACT-AA---CCGCGA-AC-TGCA--
------
FC88X_00333_00057      ------------------TTTTTCCCTTCACT-AA---CCGCGA-AC-TGCA--
------
FC88X_00018_00563      -------------------TTTTCCCTTCACT-AA---CCGCGA-AC-TGCA--
------
FC88X_00386_01176      -------------------TTTTCCCTTCACT-AA---CCGCGA-AC-TGCA--
------
FC88X_01101_00689      -----------------TTTTTTCCCTTCACTCAA---CCGCGA-AC-TGCA--
------
FC88X_00893_00743      -----------------TTTTTTCCCTTCACT-AA---CCGCGA-AC-TGCA--
------
FC88X_01120_00584      -----------------TTTTTTCCCTTCACT-AA---CCGCGA-AC-TGCA--
------
FC88X_00506_00577      -----------------TTTTTTCCCTTCACT-AA---CCGCGA-AC-TGCA--
------
FC88X_00651_00506      -----------------TTTTTTCCCTTCACT-AA---CCGCGA-AC-TGCA--
------
FC88X_00145_00884      -----------------TTTTTTCCCTTCACT-AA---CCGCGA-AC-TGCA--
------
FC88X_01077_01190      -----------------TTTTTTCCCTTCACT-AA---CCGCGA-AC-TGCA--
------
FC88X_00248_01011      -----------------TTTTTTCCCTTCACT-AA---CCGCGA-AC-TGCA--
------
FC88X_00488_01127      -----------------TTTTTTCCCTTCACT-AA---CCGCGA-AC-TGCA--
------
FC88X_00979_00812      -----------------TTTTTTCCCTTCACT-AA---CCGCGA-AC-TGCA--
------
FC88X_00374_00998      -----------------TTTTTTCCCTTCACT-AA---CCGCGA-AC-TGCA--
------
FC88X_00817_01173      -----------------TTTTTTCCCTTCACT-AA---CCGCGA-AC-TGCA--
------
FC88X_00975_01198      -------------------TTTTCCCTTCACT-AA---CCGCGA-AC-TGCA--
------
FC88X_00567_00965      -------------------TTTTCCCTTCACT-AA---CCGCGA-AC-TGCA--
------
FC88X_00882_01001      ------------------TTTTTCCCTTCACT-AA---CCGCGA-AC-TGCA--
------
FC88X_00938_00613      ------------------TTTTTCCCTTCACT-AA---CCGCGA-AC-TGCA--
------
FC88X_00725_00294      ------------------TTTTTCCCTTCACT-AA---CCGCGA-AC-TGCA--
------
FC88X_00938_00353      ------------------TTTTTCCCTTCACT-AA---CCGCGA-AC-TGCA--
------
FC88X_00605_01074      ------------------TTTTTCCCTTCACT-AA---CCGCGA-AC-TGCA--
------
```

FC88X_00135_00048 ------------------TTTTTCCCTTCACT-AA---CCGCGA-AC-TGCA--
------
FC88X_00084_00808 ------------------TTTTTCCCTTCACT-AA---CCGCGA-AC-TGCA--
------
FC88X_00120_00939 -------------------TTTTCCCTTCACT-AA---CCGCGA-AC-TGCA--
------
FC88X_00097_00188 -------------------TTTTCCCTTCACT-AA---CCGCGA-AC-TGCA--
------
FC88X_00050_00687 -------------------TTTTCCCTTCACT-AA---CCGCGA-AC-TGCA--
------
FC88X_00059_00689 -------------------TTTTCCCTTCACT-AA---CCGCGA-AC-TGCA--
------
FC88X_00102_00136 -------------------TTTTCCCTTCACT-AA---CCGCGA-AC-TGCA--
------
FC88X_00278_00780 -------------------TTTTCCCTTCACT-AA---CCGCGA-AC-TGCA--
------
FC88X_00251_00927 -------------------TTTTCCCTTCACT-AA---CCGCGA-AC-TGCA--
------
FC88X_00411_00154 -------------------TTTTCCCTTCACT-AA---CCGCGA-AC-TGCA--
------
FC88X_00403_00292 -------------------TTTTCCCTTCACT-AA---CCGCGA-AC-TGCA--
------
FC88X_00493_00203 -------------------TTTTCCCTTCACT-AA---CCGCGA-AC-TGCA--
------
FC88X_00469_00662 -------------------TTTTCCCTTCACT-AA---CCGCGA-AC-TGCA--
------
FC88X_00534_00633 -------------------TTTTCCCTTCACT-AA---CCGCGA-AC-TGCA--
------
FC88X_00503_00702 -------------------TTTTCCCTTCACT-AA---CCGCGA-AC-TGCA--
------
FC88X_00594_00231 -------------------TTTTCCCTTCACT-AA---CCGCGA-AC-TGCA--
------
FC88X_00595_00210 -------------------TTTTCCCTTCACT-AA---CCGCGA-AC-TGCA--
------
FC88X_00680_00884 -------------------TTTTCCCTTCACT-AA---CCGCGA-AC-TGCA--
------
FC88X_00679_00918 -------------------TTTTCCCTTCACT-AA---CCGCGA-AC-TGCA--
------
FC88X_00766_00250 -------------------TTTTCCCTTCACT-AA---CCGCGA-AC-TGCA--
------
FC88X_00793_00358 -------------------TTTTCCCTTCACT-AA---CCGCGA-AC-TGCA--
------
FC88X_00823_00199 -------------------TTTTCCCTTCACT-AA---CCGCGA-AC-TGCA--
------
FC88X_00838_00242 -------------------TTTTCCCTTCACT-AA---CCGCGA-AC-TGCA--
------
FC88X_01078_00971 -------------------TTTTCCCTTCACT-AA---CCGCGA-AC-TGCA--
------
FC88X_01078_01015 -------------------TTTTCCCTTCACT-AA---CCGCGA-AC-TGCA--
------
FC88X_01105_00802 -------------------TTTTCCCTTCACT-AA---CCGCGA-AC-TGCA--
------
FC88X_01134_00871 -------------------TTTTCCCTTCACT-AA---CCGCGA-AC-TGCA--
------
FC88X_00060_00046 -------------------TTTTCCCTTCACT-AA---CCGCGA-AC-TGCA--
------

```
FC88X_00067_00069       -------------------TTTTCCCTTCACT-AA---CCGCGA-AC-TGCA--
------
FC88X_00067_00380       -------------------TTTTCCCTTCACT-AA---CCGCGA-AC-TGCA--
------
FC88X_00042_00762       ------------------TTTTTCCCTTCACT-AA---CCGCGA-AC-TGCA--
------
FC88X_00113_00341       -------------------TTTTCCCTTCACT-AA---CCGCGA-AC-TGCA--
------
FC88X_00300_00522       ------------------TTTTTCCCTTCACT-AA---CCGCGA-AC-TGCA--
------
FC88X_00266_00210       ------------------TTTTTCCCTTCACT-AA---CCGCGA-AC-TGCA--
------
FC88X_00674_00157       -------------------TTTTCCCTTCACT-AA---CCGCGA-AC-TGCA--
------
FC88X_00331_01149       ------------------TTTTTCCCTTCACT-AA---CCGCGA-AC-TGCA--
------
FC88X_00096_00708       -------------------TTTTCCCTTCACT-AA---CCGCGA-AC-TGCA--
------
FC88X_00086_00118       ------------------TTTTTCCCTTCACT-AA---CCGCGA-AC-TGCA--
------
FC88X_00936_00533       -------------------TTTTCCCTTCACT-AA---CCGCGA-AC-TGCA--
------
FC88X_00076_00145       -------------------TTTTCCCTTCACT-AA---CCGCGA-AC-TGCA--
------
FC88X_00134_00931       ------------------TTTTTCCCTTCACT-AA---CCGCGA-AC-TGCA--
------
FC88X_00070_00139       -------------------TTTTCCCTTCACT-AA---CCGCGA-AC-TGCA--
------
FC88X_00136_00645       ------------------TTTTTCCCTTCACT-AA---CCGCGA-AC-TGCA--
------
FC88X_00015_00520       -------------------TTTTCCCTTCACT-AA---CCGCGA-AC-TGCA--
------
FC88X_00082_00610       ------------------TTTTTCCCTTCACT-AA---CCGCGA-AC-TGCA--
------
FC88X_00645_01213       -------------------TTTTCCCTTCACT-AA---CCGCGA-AC-TGCA--
------
FC88X_00275_00918       ------------------TTTTTCCCTTCACT-AA---CCGCGA-AC-TGCA--
------
FC88X_00049_00019       ------------------TTTTTCCCTTCA-CTAA---CCGCGA-A-CTGCA--
------
FC88X_01057_00716       ------------------TTTTTCCCTTCA-CTAA---CCGCGA-A-CTGCA--
------
FC88X_00548_01133       -------------------TTTTCCCTTCA-CTAAC---CGCGA-G-CTGCA--
------
FC88X_01101_00978       ------------------TTTTTCCCTTCA-CTAAC---CGCGA-G-CTGCA--
------
FC88X_01027_00578       -------------------TTTTCCCTTCA-CTAAC---CGCGA-G-CTGCA--
------
FC88X_00635_01159       ------------------CTTTTCTCCCCG-GTGAC---CGATA-C-CTGCT--
------
FC88X_00096_00395       ---------------CTACTTTTCAAGCCG--TGAC---TGCTA-T-A------
------
FC88X_00821_00311       ----------------CTCCCTTCTGACCG--GAAT---CGGCA-T-GTG----
------
FC88X_01010_00716       ------------------TTTCTCCCTTCA-CTAAC---CGCGA-A-CTGCA--
------
```

FC88X_00873_00774 -----------------TTTTATCCCTTCA-CTAAC---CGCGA-A-CTGCA--
------
FC88X_00580_00692 -----------------TTTTTTCCCTTCA-CTAAC---CGCGA-A-
CTGCACTCCTTCT
FC88X_00033_00115 -----------------TTTTTTCCCTTCA-CTAAC---CGCGA-A-CTGCA--
------
FC88X_01062_00830 -----------------TTTTTTCCCTTCA-CTAAC---CGCGA-A-CTGCA--
------
FC88X_00641_01148 -----------------TTTTTTCCCTTCA-CTAAC---CGCGA-A-CTGCA--
------
FC88X_01020_00593 -----------------TTTTTTCCCTTCA-CTAAC---CGCGA-A-CTGCA--
------
FC88X_01117_00751 -----------------TTTTTTCCCTTCA-CTAAC---CGCGA-A-CTGCA--
------
FC88X_00032_00526 -----------------TTTTTTCCCTTCA-CTAAC---CGCGA-A-
CTGCACC------
FC88X_00183_00034 -----------------TTTTTTCCCTTCA-CTAAC---CGCGA-A-
CTGCACCT-----
FC88X_00137_00507 -----------GGGGAAGTTTTTCCCTTCATCTAAC---TGCGA-A-CTGCA--
------
FC88X_00973_00503 ------------------TTTTTCCCTTCA-CTAAC---TGCGA-A-CTGCA--
------
FC88X_00538_01074 ------------------TTTTTCCCTTCA-CTAAC---TGCGA-A-CTGCA--
------
FC88X_00036_00672 -------------------TTTTCCCTTCA-CTAAC---TGCGA-A-CTGCA--
------
FC88X_00714_00942 -------------------TTTTCCCTTCA-CTAACG--TGCGA-A-CTGCA--
------
FC88X_00089_00080 ------------------TTTTTCCCTTCA-CTAAC---TGCGA-A-CTGCA--
------
FC88X_01110_00676 ------------------TTTTTCCCTTCA-CTAAC---TGCGA-A-
CTGCACTTCCTTT
FC88X_00727_00941 ------------------TTTTTCCCTTCA-CTAAC---TGCGA-A-CTGCA--
------
FC88X_00797_00958 -------------------TCTTCCCTTCA-CTAAC---AGCGA-A-CTGCA--
------
FC88X_00067_00454 -------------------TTTTCCCTTCA-CTAAC---AGCGA-A-CTGCA--
------
FC88X_00075_00623 -------------------TTTTCCCTTCA-CTAAC---AGCGA-A-CTGCA--
------
FC88X_00978_00895 -------------------TTTTCCCTTCA-CTAAC---AGCGA-A-CTGCA--
------
FC88X_01095_01134 -------------------TTTTCCCTTCA-CTAAC---AGCGA-A-CTGCA--
------
FC88X_00179_00129 ------------------TTTTTCCCTTCA-CTAACG--GC-GA-A-CTGCA--
------
FC88X_00372_01144 ------------------TTTTTCCCTTCA-CTAACG--GC-GA-A-CTGCA--
------
FC88X_00993_00713 ------------------TTTTTCCCTTCA-CTAACG--GC-GA-A-CTGCA--
------
FC88X_00685_01030 ------------------TTTTTCCCTTCA-CTAACC-----GA-A-CTGCA--
------
FC88X_00935_00638 -----------------TTTTTTCCCTTCA-CTAACG--CCGAA-A-CTGCA--
------
FC88X_00790_00217 -----------------TTTTTTCCCTTCA-CTAACG--C--GA-A-CTGCA--
------

FC88X_00875_00137 -----------------TTTTTTCCCTTCA-CTAAC---CGCGA-A-CTGCA--
------
FC88X_00883_01033 -----------------TTTTTTCCCTTCA-CTAAC---CGCGA-A-CTGCA--
------
FC88X_00415_00952 -----------------TTTTTTCCCTTCA-CTAAC---CGCGA-A-CTGCAC-
------
FC88X_00422_00985 -----------------TTTTTTCCCTTCA-CTAAC---CGCGA-A-CTGCAC-
------
FC88X_00864_00678 -----------------TTTTTTCCCTTCA-CTAAC---CGCGA-A-CTGCA--
------
FC88X_00958_00587 -----------------TTTTTTCCCTTCA-CTAAC---CGCGA-A-CTGCA--
------
FC88X_01036_00780 -----------------TTTTTTCCCTTCA-CTAAC---CGCGA-A-CTGCA--
------
FC88X_01074_00908 -----------------TTTTTTCCCTTCA-CTAAC---CGCGA-A-CTGCA--
------
FC88X_01063_00488 -----------------TTTTTTCCCTTCA-CTAAC---CGCGA-A-CTGCA--
------
FC88X_00489_01171 -----------------TTTTTTCCCTTCA-CTAAC---CGCGA-A-CTGCA--
------
FC88X_00309_01131 -----------------TTTTTTCCCTTCA-CTAAC---CGCGA-A-CTGCA--
------
FC88X_00099_00127 -----------------TTTTTTCCCTTCA-CTAAC---CGCGA-A-CTGCA--
------
FC88X_00234_01048 -----------------TTTTTTCCCTTCA-CTAAC---CGCGA-A-CTGCA--
------
FC88X_00129_00890 -----------------TTTTTTCCCTTCA-CTAAC---CGCGA-A-CTGCA--
------
FC88X_01046_00706 -----------------TTTTTTCCCTTCA-CTAAC---CGCGA-A-CTGCA--
------
FC88X_00024_00086 -----------------TTTTTTCCCTTCA-CTAAC---CGCGA-A-CTGCA--
------
FC88X_01117_01209 ------------------TTTTTCCCTTCA--TAAC---CGCGA-A-CTGCA--
------
FC88X_00529_00974 ----------------TTTTTTTCCCTTCA--TAAC---CGCGA-A-CTGCA--
------
FC88X_00936_00880 ------------GTGATTTTTTTCCCTTCA-CTAAC---CGCGA-A-CTGCA--
------
FC88X_00071_00392 ----------------TTTTTTTCCCTTCA-CTAAC---CGCGA-A-CTGCA--
------
FC88X_00601_00093 ----------------TTTTTTTCCCTTCA-CTAAC---CGCGA-A-CTGCA--
------
FC88X_00380_00284 ----------------TTTTTTTCCCTTCA-CTAAC---CGCGA-A-CTGCA--
------
FC88X_01045_00708 ----------------TTTTTTTCCCTTCA-CTAAC---CGCGA-A-CTGCA--
------
FC88X_00819_00170 ----------------TTTTTTTCCCTTCA-CTAAC---CGCGA-A-CTGCA--
------
FC88X_00770_00110 ----------------TTTTTTTCCCTTCA-CTAAC---CGCGA-A-CTGCA--
------
FC88X_00967_00873 ----------------TTTTTTTCCCTTCA-CTAAC---CGCGA-A-CTGCA--
------
FC88X_00126_00784 ---------------TTTTTTTTCCCTTCA-CTAAC---CGCGA-A-CTGCA--
------
FC88X_00653_00114 ---------------TTTTTTTTCCCTTCA-CTAAC---CGCGA-A-CTGCA--
------

FC88X_00907_01182 ----------------TTTTTTTCCCTTCA-CTAAC---CGCGA-A-CTGCA--
------
FC88X_00911_00817 ----------------TTTTTTTCCCTTCA-CTAAC---CGCGA-A-CTGCA--
------
FC88X_00763_00199 -----------------TTTTTTCCCTTCA-CTAAC---CGCGA-A-CTGCA--
------
FC88X_00652_00798 -----------------TTTTTTCCCTTCA-CTAAC---CGCGA-A-CTGCA--
------
FC88X_00139_00233 -----------------TTTTTTCCCTTCA-CTAAC---CGCGA-A-CTGCA--
------
FC88X_00088_00390 -----------------TTTTTTCCCTTCA-CTAAC---CGCGA-A-CTGCA--
------
FC88X_00048_00291 -----------------TTTTTTCCCTTCA-CTAAC---CGCGA-A-CTGCA--
------
FC88X_00144_00964 -----------------TTTTTTCCCTTCA-CTAAC---CGCGA-A-CTGCA--
------
FC88X_00653_01260 -----------------TTTTTTCCCTTCA-CTAAC---CGCGA-A-CTGCA--
------
FC88X_00747_01054 -----------------TTTTTTCCCTTCA-CTAAC---CGCGA-A-CTGCA--
------
FC88X_00598_01165 -----------------TTTTTTCCCTTCA-CTAAC---CGCGA-A-CTGCA--
------
FC88X_01004_00912 -----------------TTTTTTCCCTTCA-CTAAC---CGCGA-A-CTGCA--
------
FC88X_01012_01025 -----------------TTTTTTCCCTTCA-CTAAC---CGCGA-A-CTGCA--
------
FC88X_00990_01027 -----------------TTTTTTCCCTTCA-CTAAC---CGCGA-A-CTGCA--
------
FC88X_00690_01028 ----------------TTTTTTTCCCTTCA-CTAAC---CGCGA-A-
CTGCACTTCCTTT
FC88X_00970_00453 ----------------TTTTTTTCCCTTCA-CTAAC---CGCGA-A-CTGCA--
------
FC88X_00906_01186 -----------------TTTTTTCCCTTCA-CTAAC---CGCGA-A-CTGCA--
------
FC88X_01022_00580 -----------------TTTTTTCCCTTCA-CTAAC---CGCGA-A-CTGCA--
------
FC88X_00050_00488 -----------------TTTTTTCCCTTCA-CTAAC---CGCGA-A-CTGCA--
------
FC88X_00227_00918 -----------------TTTTTTCCCTTCA-CTAAC---CGCGA-A-CTGCA--
------
FC88X_00732_00297 -----------------TTTTTTCCCTTCA-CTAAC---CGCGA-A-CTGCA--
------
FC88X_01081_00652 -----------------TTTTTTCCCTTCA-CTAAC---CGCGA-A-CTGCA--
------
FC88X_00635_00694 -----------------TTTTTTCCCTTCA-CTAAC---CGCGA-A-CTGCA--
------
FC88X_00523_01184 -----------------TTTTTTCCCTTCA-CTAAC---CGCGA-A-CTGCA--
------
FC88X_00187_00944 -----------------TTTTTTCCCTTCA-CTAAC---CGCGA-A-CTGCA--
------
FC88X_00849_00201 -----------------TTTTTTCCCTTCA-CTAAC---CGCGA-A-CTGCA--
------
FC88X_00410_01219 -----------------TTTTTTCCCTTCA-CTAAC---CGCGA-A-CTGCA--
------
FC88X_00904_00774 -----------------TTTTTTCCCTTCA-CTAAC---CGCGA-A-CTGCA--
------

FC88X_00807_01198 -----------------TTTTTTCCCTTCA-CTAAC---CGCGA-A-CTGCA--
------
FC88X_00850_00806 ------------------TTTTTCCCTTCA-CTAAC---CGCGA-A-CTGCA--
------
FC88X_00861_00811 ------------------TTTTTCCCTTCA-CTAAC---CGCGA-A-CTGCA--
------
FC88X_00850_00874 ------------------TTTTTCCCTTCA-CTAAC---CGCGA-A-CTGCA--
------
FC88X_00059_00135 -----------GGGGAATTTTTCCCTT-CA-CTAA---CCGCGA-A-CTGCA--
------
FC88X_00738_00502 -----------GGGGAATTTTTCCCTT-CA-CTAA---CCGCGA-A-CTGCA--
------
FC88X_00886_01259 -----------GGGGAATTTTTCCCTT-CA-CTAA---CCGCGA-A-CTGCA--
------
FC88X_00149_00722 ------------GGGAATTTTTCCCTT-CA-CTAA---CCGCGA-A-CTGCA--
------
FC88X_00992_01104 ----------GGGGAAGTTTTTCCCTT-CA-CTAA---CCGCGA-A-CTGCA--
------
FC88X_00441_00206 ----------GGGGAAGTTTTTCCCTT-CA-CTAA---CCGCGA-A-CTGCA--
------
FC88X_00507_00171 ----------GGGGAAGTTTTTCCCTT-CA-CTAA---CCGCGA-A-CTGCA--
------
FC88X_00541_00245 ----------GGGGAAGTTTTTCCCTT-CA-CTAA---CCGCGA-A-CTGCA--
------
FC88X_00164_00130 ----------GGGGAAGTTTTTCCCTT-CA-CTAA---CCGCGA-A-CTGCA--
------
FC88X_00093_00910 ----------GGGGAAGTTTTTCCCTT-CA-CTAA---CCGCGA-A-CTGCA--
------
FC88X_00110_00464 ----------GGGGAAGTTTTTCCCTTACA-CTAA---CCGCGA-A-CTGCAC-
------
FC88X_00910_00730 ----------GGGGAAGTTTTTCCCTT-CA-CTAA---CCGCGA-A-CTGCAC-
------
FC88X_00740_01143 ----------------GTTTTTCCCTT-CA-CTAA---CCGCGA-A-CTGCA--
------
FC88X_01023_00708 ----------------GTTTTTCCCTT-CA-CTAA---CCGCGA-A-CTGCA--
------
FC88X_01082_00902 ----------------GTTTTTCCCTT-CA-CTAA---CCGCGA-A-CTGCA--
------
FC88X_01143_00811 ----------------GTTTTTCCCTT-CA-CTAA---CCGCGA-A-CTGCA--
------
FC88X_01033_00981 ----------------GTTTTTCCCTT-CA-CTAA---CCGCGA-A-CTGCA--
------
FC88X_00814_00122 -------------TAAGTTTTTCCCTT-CA-CTAA---CCGCGA-A-CTGCA--
------
FC88X_00477_00172 ----------------GTTTTTCCCTT-CA-CTAA---CCGCGA-A-CTGCA--
------
FC88X_00305_00359 ----------------GTTTTTCCCTT-CA-CTAA---CCGCGA-A-CTGCA--
------
FC88X_01069_00412 ----------------GTTTTTCCCTT-CA-CTAA---CCGCGA-A-CTGCA--
------
FC88X_01120_00594 ----------------GTTTTTCCCTT-CA-CTAA---CCGCGA-A-CTGCA--
------
FC88X_00947_01159 ----------------GTTTTTCCCTT-CA-CTAA---CCGCGA-A-CTGCA--
------
FC88X_00877_01156 ----------------GTTTTTCCCTT-CA-CTAA---CCGCGA-A-CTGCA--
------

FC88X_00878_01092 ----------------GTTTTTCCCTT-CA-CTAA---CCGCGA-A-CTGCA--
------
FC88X_01133_01170 ----------------GTTTTTCCCTT-CA-CTAA---CCGCGA-A-CTGCA--
------
FC88X_00650_01075 ---------------GGTTTTTCCCTT-CA-CTAA---CCGCGA-A-CTGCA--
------
FC88X_01127_00526 ----------------GTTTTTCCCTT-CA-CTAA---CCGCGA-A-CTGCA--
------
FC88X_00926_01015 ----------------GTTTTTCCCTT-CA-CTAA---CCGCGA-A-CTGCA--
------
FC88X_00227_00042 ----------------GTTTTTCCCTT-CA-CTAA---CCGCGA-A-CTGCA--
------
FC88X_00239_00051 ----------------GTTTTTCCCTT-CA-CTAA---CCGCGA-A-CTGCA--
------
FC88X_00073_00222 ----------------GTTTTTCCCTT-CA-CTAA---CCGCGA-A-CTGCA--
------
FC88X_00946_00304 ----------------GTTTTTCCCTT-CA-CTAA---CCGCGA-A-CTGCA--
------
FC88X_00973_01011 ----------------GTTTTTCCCTT-CA-CTAA---CCGCGA-A-CTGCA--
------
FC88X_00975_00764 ----------------GTTTTTCCCTT-CA-CTAA---CCGCGA-A-CTGCA--
------
FC88X_01077_00659 ----------------GTTTTTCCCTT-CA-CTAA---CCGCGA-A-CTGCA--
------
FC88X_00662_01059 ----------------GTTTTTCCCTT-CA-CTAA---CCGCGA-A-CTGCA--
------
FC88X_00995_00773 ----------------GTTTTTCCCTT-CA-CTAA---CCGCGA-A-CTGCA--
------
FC88X_01037_01041 ----------------GTTTTTCCCTT-CA-CTAA---CCGCGA-A-CTGCA--
------
FC88X_00913_00744 ----------------GTTTTTCCCTT-CA-CTAA---CCGCGA-A-CTGCA--
------
FC88X_00203_00951 ----------------GTTTTTCCCTT-CA-CTAA---CCGCGA-A-CTGCA--
------
FC88X_00698_01131 ----------------GTTTTTCCCTT-CA-CTAA---CCGCGA-A-CTGCA--
------
FC88X_00732_01032 ----------------GTTTTTCCCTT-CA-CTAA---CCGCGA-A-CTGCA--
------
FC88X_00978_00592 ----------------GTTTTTCCCTT-CA-CTAA---CCGCGA-A-CTGCA--
------
FC88X_00799_00147 ----------------GTTTTTCCCTT-CA-CTAA---CCGCGA-A-CTGCA--
------
FC88X_00307_01037 ----------------GTTTTTCCCTT-CA-CTAA---CCGCGA-A-CTGCA--
------
FC88X_00072_00313 ----------------GTTTTTCCCTT-CA-CTAA---CCGCGA-A-CTGCA--
------
FC88X_00797_00904 ----------------GTTTTTCCCTT-CA-CTAA---CCGCGA-A-CTGCA--
------
FC88X_00192_00823 ----------------GTTTTTCCCTT-CA-CTAA---CCGCGA-A-CTGCA--
------
FC88X_00412_01145 ----------------GTTTTTCCCTT-CA-CTAA---CCGCGA-A-CTGCA--
------
FC88X_00459_01229 ----------------GTTTTTCCCTT-CA-CTAA---CCGCGA-A-CTGCA--
------
FC88X_00578_00971 ----------------GTTTTTCCCTT-CA-CTAA---CCGCGA-A-CTGCA--
------

```
FC88X_00127_00753      ----------------GTTTTTCCCTT-CA-CTAA---CCGCGA-A-CTGCA--
------
FC88X_00839_00892      ----------------GTTTTTCCCTT-CA-CTAA---CCGCGA-A-CTGCA--
------
FC88X_00863_00293      ----------------GTTTTTCCCTT-CA-CTAA---CCGCGA-A-CTGCA--
------
FC88X_00874_00348      ----------------GTTTTTCCCTT-CA-CTAA---CCGCGA-A-CTGCA--
------
FC88X_00403_01000      ----------------GTTTTTCCCTT-CA-CTAA---CCGCGA-A-CTGCA--
------
FC88X_00406_01031      ----------------GTTTTTCCCTT-CA-CTAA---CCGCGA-A-CTGCA--
------
FC88X_00566_00210      ----------------GTTTTTCCCTT-CA-CTAA---CCGCGA-A-CTGCA--
------
FC88X_00397_01105      ----------------GTTTTTCCCTT-CA-CTAA---CCGCGA-A-CTGCA--
------
FC88X_00039_00777      ----------------GTTTTTCCCTT-CA-CTAA---CCGCGA-A-CTGCA--
------
FC88X_00078_00623      ----------------GTTTTTCCCTT-CA-CTAA---CCGCGA-A-CTGCA--
------
FC88X_00580_01219      ----------------GTTTTTCCCTT-CA-CTAA---CCGCGA-A-CTGCA--
------
FC88X_00157_00826      ----------------GTTTTTCCCTT-CA-CTAA---CCGCGA-A-CTGCA--
------
FC88X_00024_00256      ----------------GTTTTTCCCTT-CA-CTAA---CCGCGA-A-CTGCA--
------
FC88X_01012_00481      ----------------GTTTTTCCCTT-CA-CTAA---CCGCGA-A-CTGCA--
------
FC88X_00989_00901      ------------TTAAACATTCTCTTGACT-CTAC---CGCGCG----------
------
FC88X_00026_00408      ------------TTAAACATTCTCTTGACT-CCAC---CGCGCG----------
------
FC88X_00509_01069      -------------TGTTTCTGTTCTCTCCC-CGCG---CGCGCA----------
------
FC88X_00769_00145      ---------------------TTCTCTTCT-CAAC---CGCGCA-G--
TGTGCT------
FC88X_00946_00419      ---------------------TTCTCTTCT-CAAC---CGCGCA-G-
GTGTGCT------
FC88X_00582_01128      ------------------TTTTCCCTTCAC-TAAC---CGCGAG-T-GCA----
------
FC88X_00278_00720      -----------------TTTTTCCCTTCAC-TAAC---CGCGAC-T-G------
------
FC88X_00126_00453      ------------------TTTTTCCCTTCA-CCAC-----ACCG-A-
CTACGCGA-----
FC88X_00584_00964      -----------------TTTTTTCCCTTCA-CCAT---CGACCG-A-CTAC---
------
FC88X_00969_00827      -------------------TTTTCCCTTCA-CAAT---C-ACCG-A-CTAGCA-
------
FC88X_00962_00943      -------------------TTTTCCCTTCA-CAAT---C-AC-G-A-CTA----
------
FC88X_00554_00106      -----------------TTTTTCCCTTCAC-TAAC---CG--------------
------
FC88X_00049_00028      ------------------TTTTTCCCTTCA-CTAA---CCGTA--C-TAGCA--
------
FC88X_00512_00136      ------------------TTTTTCCCTTCA-CTAA---CCGCAA-C-TAGCA--
------
```

```
FC88X_00375_01139      ------------------TTTTTCCCTTCA-CTAA---CCGTGA-C--
TACGCA------
FC88X_00664_01155      ------------------TTTTTCCCTTCA-CTAA---CCGTGA-C-GTACG--
------
FC88X_01119_01198      -----------------TTTTTCCCTTTCG-CTAA---C---------------
------
FC88X_00920_01242      ----------------TTTTTTCCCTTACA-CTAA---C---------------
------
FC88X_00024_00767      -----------------TTTTTTCCCTTCA-CTAC---G---------------
------
FC88X_01141_00623      ---------------GTTTTTTCCCTTTCA-CTA--------------------
------
FC88X_00159_00053      -----------------TTTTTTCCCTTCA-CTAA---ACCGCC-G--------
------
FC88X_00360_00739      -----------------TTTTTTCCCTTCA-CTAA---CCGCGA----------
------
FC88X_00624_00246      ------------------TTTTTCCCTTCA-CTAA---CC--------------
------
FC88X_00880_00901      ------------------TTTTTCCCTTCA-CTAA---CCGCGA-A-CCTGA--
------
FC88X_00596_01139      ------------------TTTTTCCCTTCA-CTAA---CCG-------------
------
FC88X_00600_00085      -----------------GTTTTTCCCTTCA-CTAA---CCGCGG-A-CTGCA--
------
FC88X_00023_00186      ------------------TTTTTCCCTTCA-CTAA---CCGCGT-A-CTGCA--
------
FC88X_00359_01177      ------------------TTTTTCCCTTCA-CTAA---CCGCGC-A-CTGCA--
------
FC88X_00839_00369      ------------------TTTTTCCCTTCA-CTAA---CCGCGA-A-CTGCA--
------
FC88X_00056_00851      -----------------TTTTTCCCTTCAC-C-AA---CCGCGA-A-CTGCA--
------
FC88X_00362_01166      ----------------GTTTTTCCCTTCAC-C-AA---CCGCGA-A-CTGCA--
------
FC88X_01000_00283      -----------------TTTTTCCCTTCAC-C-AA---CCGCGA-A-CTGCA--
------
FC88X_00654_00154      -----------------TTTTTCCCTTCAC-C-AA---CCGCGA-A-CTGCA--
------
FC88X_01030_01202      ---------------------CCCCTTGAC-C-TA---CC-CGA-C-
CCGCATGCA----
FC88X_00345_01091      -----------------TTTTTCCCTTCAC-C-AA---CCGCGA-A-CCGCA--
------
FC88X_00564_01074      ----------------TTTCTTCCCTTCAC-C-AA---CCGCGA-A-CTGCA--
------
FC88X_00842_00222      -----------------TTTTTCCCTTCAC-C-AA---CCGCGA-A-CTGCA--
------
FC88X_01023_00779      -----------------TTTTTCCCTTCAC-C-AA---CCGCGA-A-CTGCA--
------
FC88X_00957_00772      -----------------TTTTTCCCTTCAC-C-AA---CCGCGA-A-CTGCA--
------
FC88X_00792_00433      -----------------TTTTTCCCTTCA--C-AA---CCGCGA---CTGCA--
------
FC88X_01078_01130      -----------------TTTTTCCCTTCAT-C-AA---CCGCGA-A-CTGCA--
------
FC88X_00144_00457      -----------------TTTTTCCCTTCA--C-AA---CCGCGA-A-CTGCA--
------
```

```
FC88X_00138_00748      -----------------TTTTTCCCTTCAC-G-AA---CCGCGA-A-CTGCA--
------
FC88X_00475_01064      -----------------TTTTTCCCTTCAC-G-AA---CCGCGA-A-CTGCA--
------
FC88X_00256_00963      -----------------TTTTTCCCTTCAC-G-AA---CCGCGA-A-CTGCA--
------
FC88X_00361_00015      -----------------TTTTTCCCTTCAC-G-AA---CCGCGA-A-CTGCA--
------
FC88X_00289_01098      -----------------TTTTTCCCTTCAC---AA---CCGCGA-A-CTGCA--
------
FC88X_00595_00754      -----------------TTTTTCCCTTCAC---AA---CCGCGA-A-CTGCA--
------
FC88X_00284_00993      ------------------TTTTCCCTTCAC-AACC---GA-CGA-A-CTGC---
------
FC88X_00698_00282      -----------------TTTTTCCCTTCAC-TGAA---CC-CGA-A-
CTACTCA------
FC88X_00609_01183      ------------------TTTTCCCTTCAC-TGAA---CCGCGA-A-CTGCA--
------
FC88X_00590_01081      -----------------TTTTTCCCTTCAC-TGAA---CCGCGA-A-CTGCA--
------
FC88X_00192_00071      -----------------TTTTTCCCTTCAC-TGAA---CCGCGA-A-CTGCA--
------
FC88X_00847_00882      -----------------TTTTTCCCTTCAC-T-AA---CCGCGA-A-CTGCA--
------
FC88X_00829_00361      -----------------TTTTTCCCTTCAC-T-AA---CCGCGA-A-CTGCA--
------
FC88X_00816_00604      ------------------TTTTTCCCTTCA-CTAA---CCGCGA-A-CTGCA--
------
FC88X_00915_00665      ----------------GTTTTTTCCCGTCA-CTAA---CCGCGA-A-CTGCA--
------
FC88X_00269_00997      -------------------TTTTTCCCTCA-CTAA---CCGCGA-A-CTGCA--
------
FC88X_00412_00960      -------------------TTTTTCCCTCA-CTAA---CCGCGA-A-CTGCA--
------
FC88X_00461_00334      -------------------TTTTTCCCTCA-CTAA---CCGCGA-A-CTGCA--
------
FC88X_00413_00023      ----------------GTTTTTTCCCTTCA-CTAA---CCGCGA-
AACTGCACTTCCT--
FC88X_00344_00864      ------------------TTTTTCCCTTCA-CTAA---CCGCGA-AACTGCA--
------
FC88X_00367_00988      ------------------TTTTTCCCTTCA-CTAA---CCGC-A-AACTGCA--
------
FC88X_00281_01072      ------------------TTTTTCCCTTCA-CTAA---CCGC-A-AACTGCA--
------
FC88X_00105_00475      -------------------TTTTCCCTTCA-CTAA---CCGC-A-AACTGCAC-
------
FC88X_00981_00653      -------------------TTTTCCCTTCA-CTAA---CCGC-A-AACTGCA--
------
FC88X_00384_00959      -----------------TTTTTCCCTTTCA-CTAA---CCGCGA-AACTGCAC-
------
FC88X_01074_00392      -------------------TTTTCCCTTCA-CTAA---CCGCGA-AACTGCA--
------
FC88X_00692_01065      ------------------TTTTTCCCTTCA-CTAA---CCGCGA-AACTGCAC-
------
FC88X_00423_00521      ------------------TTTTTCCCTTCA-CTAA---CCGCGA-AACTGCA--
------
```

```
FC88X_00804_00915       ------------------TTTTTCCCTTCA-CTAA---CCGCGA-A-CTGCA--
------
FC88X_00826_00449       ------------------TTTTTCCCTTCA-CTAA---CCGCGA-A-CTGCA--
------
FC88X_00837_00794       ------------------TTTTTCCCTTCA-CTAA---CCGCGA-A-CTGCA--
------
FC88X_00726_00936       ------------------TTTTTCCCTTCA-CTAA---CCGCGA-A-CTGCA--
------
FC88X_00030_00332       ------------------TTTTTCCCTTCA-CT-A---CCG-CG-AACTGCA--
------
FC88X_00516_00787       ------------------TTTTTCCCTTCA-CTCA---CCGACG-AACTGCA--
------
FC88X_00918_01207       ------------------TTTTTCCCTTCA-CT-A---CCG-CG-AACTGCA--
------
FC88X_00414_00054       ------------------TTTTTCCCTTCA-CTAT---CCG-CG-AACTGCA--
------
FC88X_00517_00058       ------------------TTTTTCCCTTCA-CTA----CCG-CG-AACTGCA--
------
FC88X_00930_00620       ------------------TTTTTCCCTTCA-CTA----CCG-CG-AACTGCA--
------
FC88X_00637_00287       ------------------TTTTTCCCTTCA-CTAG---CCG-CG-AACTGCA--
------
FC88X_00988_00389       ------------------TTTTTCCCTTCA-CTAG---CCG-CG-AACTGCA--
------
FC88X_01083_00689       ------------------TTTTTCCCTTCA-CTAG---CCG-CG-AACTGCA--
------
FC88X_00677_00166       ------------------TTTTTCCCTTCA-CTAG---CCG-CG-AACTGCA--
------
FC88X_00849_00962       ------------------TTTTTCCCTTCA-CTAG---CCG-CG-AACTGCA--
------
FC88X_00088_00076       ------------------TTTTTCCCTTCA-CTAG---CCG-CG-AACTGCA--
------
FC88X_00070_00370       -------------------TTTTCCCTTCA-CTAG---CCG-CG-AACTGCA--
------
FC88X_00469_00810       -------------------TTTTCCCTTCA-CTAG---CCG-CG-AACTGCA--
------
FC88X_00873_00770       -------------------TTTTCCCTTCA-CTAG---CCG-CG-AACTGCA--
------
FC88X_00563_00073       -----------------CGTTTTCCCTTCA-CTAC---CCG-CG-AACTGCAC-
------
FC88X_01074_00543       ------------------TTTTTCCCTTCA-CTAC---C-G-CG-AACTGCAC-
------
FC88X_00445_00073       ------------------TTTTTCCCTTCA-CTAA---CCG-CG-AACTGCAC-
------
FC88X_01143_01120       ------------------TTTTTCCCTTCA-CTAA---CCG-CG-AACTGCAC-
------
FC88X_00147_00771       --------------------TCTCTCCTCA-ATAG---C-GCGA-A-
TTGCGTA------
FC88X_00207_00595       -------------------TTCTCCCTTCA-CTAA---CCGCGA-
ACTTGCACTTCC---
FC88X_00877_01166       -------------------TTTTCCCTTCA-CTAA---CAGCGA-A-TTGCA--
------
FC88X_00672_00153       ------------------TTTTTCCCTTCA-CTAA---CCGCGA-A-TTGCA--
------
FC88X_00151_00688       ------------------TTTTTCCCTTCA-CTAA---CCGCGA-A-
CTGCACTTCCTTT
```

FC88X_00343_00499 ------------------TTTTTCCCTTCA-CTAA---CCGCGA-A-
CTGCACTTCCTTT
FC88X_00343_01076 ------------------TTTTTCCCTTCA-CTAA---CCGCGA-A-
CTGCACTTCCTTT
FC88X_00634_01160 ------------------TTTTTCCCTTCA-CTAA---CCGCGA-A-
CTGCACT------
FC88X_00336_00177 ------------------TTTTTCCCTTCA-CTAA---CCGCGA-A-CTGCAC-
------
FC88X_00193_00042 ------------------TTTTTCCCTTCA-CTAA---CCG-CG-AACTGCAC-
------
FC88X_00123_00666 ------------------TTTTTCCCTTCA-CTAA---CCG-CG-
AACTGCACAC-----
FC88X_00120_00443 ------------------TTTTTCCCTTCA-CTAA---CCG-CG-AACTGCAC-
------
FC88X_00889_00459 ------------------TTTTTCCCTTCA-CTAA---CCG-CG-AACTGCAC-
------
FC88X_00722_01008 ------------------TTTTTCCCTTCA-CTAA---CCG-CG-AACTGCA--
------
FC88X_00050_00280 ------------------TTTTTCCCTTCA-CTAA---CCG-CG-
AACTGCACC------
FC88X_00449_00056 ------------------TTTTTCCCTTCA-CTAA---CCG-CG-
AACTGCACC------
FC88X_00087_00903 ------------------TTTTTCCCTTCA-CTAA---CCG-CG-
AACTGCACC------
FC88X_00290_00773 ------------------TTTTTCCCTTCA-CTAA---CCG-CG-
AACTGCACC------
FC88X_00116_00688 ------------------TTTTTCCCTTCA-CTAA---CCG-CG-
AACTGCACC------
FC88X_00652_00211 ------------------TTTTTCCCTTCA-CTAA---CCG-CG-
AACTGCACC------
FC88X_00947_01139 ------------------TTTTTCCCTTCA-CTAA---CCG-CG-
AACTGCACC------
FC88X_01096_01137 ------------------TTTTTCCCTTCA-CTAA---CCG-CG-
AACTGCACC------
FC88X_00846_01271 -----------------GTTTTTCCCTTCA-CTAA---CCG-CG-
AACTGCACC------
FC88X_01013_01193 ------------------TTTTTCCCTTCA-CTAA---CCG-CG-AACTGCAC-
------
FC88X_00825_00352 ------------------TTTTTCCCTTCA-CTAA---CCG-CG-AACTGCA--
------
FC88X_00705_01273 ------------------TTTTTCCCTTCA-CTAA---CCG-CG-AACTGCA--
------
FC88X_00825_00124 ------------------TTTTTCCCTTCA-CTAA---CCG-CG-AACTGCA--
------
FC88X_00762_00284 ------------------TTTTTCCCTTCA-CTAA---CCGCGA-A-CTGCA--
------
FC88X_00020_00692 ------------------TTTTTCCCTTCA-CTAA---CCC-CG-AACTGCA--
------
FC88X_00463_01239 ------------------TTTTTCCCTTCA-CTAA---CCC-CG-AACTGCA--
------
FC88X_00670_00945 ----------TGGGGAAGTTTTTCCCTTCA-CTAA---CCCGCG-AACTGCA--
------
FC88X_00618_01137 ------------------TTTTTCCCTTCA-CTAA---CCCGCG-AACTGCA--
------
FC88X_00207_00155 ------------------TTTTTCCCTTCA-CTAA---CCCGCG-AACTGCA--
------

FC88X_00732_01017 ------------------TTTTTCCCTTCA-CTAA---CCG-CG-AACTGCA--
------
FC88X_00847_00725 ------------------TTTTTCCCTTCA-CTAA---CCG-CG-AACTGCA--
------
FC88X_00835_00110 ------------------TTTTTCCCTTCA-CTAA---CCG-CG-AACTGCA--
------
FC88X_00776_00303 ------------------TTTTTCCCTTCA-CTAA---CCG-CG-AACTGCA--
------
FC88X_00766_00828 ------------------TTTTTCCCTTCA-CTAA---CCGCGA-A-CTGCA--
------
FC88X_00799_00891 ------------------TTTTTCCCTTCA-CTAA---CCGCGA-A-CTGCA--
------
FC88X_00117_00620 ------------------TTTTTCCCTTCA-CTAA-----CGCG-AACTGCA--
------
FC88X_00380_00958 ------------------TTTTTCCCTTCA-CTAA---A-CGCG-AACTGCA--
------
FC88X_00579_01004 ------------------TTTTTCCCTTCA-CTAA---ACCGCG-AACTGCA--
------
FC88X_00384_00196 ------------------TTTTTCCCTTCA-CTAA---ACCGCG-AACTGCA--
------
FC88X_01022_00319 ------------------TTTTTCCCTTCA-CTAA---ACCGCG-AACTGCA--
------
FC88X_00792_01025 ------------------TTTTTCCCTTCA-CTAA---ACCGCG-AACTGCA--
------
FC88X_00542_01096 ------------------TTTTTCCCTTCA-CTAA---ACCGCG-AACTGCA--
------
FC88X_00712_01010 ------------------TTTTTCCCTTCA-CTAA---ACCGCG-AACTGCA--
------
FC88X_00650_01091 ------------------TTTTTCCCTTCA-CTAA---ACCGCG-AACTGCA--
------
FC88X_00627_00131 ------------------TTTTTCCCTTCA-CTAA---ACCGCG-AACTGCA--
------
FC88X_00747_00679 ------------------TTTTTCCCTTCA-CTAA---ACCGCG-AACTGCA--
------
FC88X_00437_00030 ----------------TTTTTTTCCCTTCA-CTAA---ACCGCG-AACTGCA--
------
FC88X_00573_00903 -----------------TTTTTTCCCTTCA-CTAA---ACCGCG-AACTGCA--
------
FC88X_00819_01028 ------------------TTTTTCCCTTCA-CTAA----CCGCG-AACTGCA--
------
FC88X_00751_00227 ------------------TTTTTCCCTTCA-CTAA----CCGCG-AACTGCA--
------
FC88X_00754_00480 ------------------TTTTTCCCTTCA-CTAA----CCGCG-AACTGCA--
------
FC88X_00795_00851 ------------------TTTTTCCCTTCA-CTAA----CCGCG-AACTGCA--
------
FC88X_00833_01133 ------------------TTTTTCCCTTCA-CTAA---CCGCGA-A-CTGCA--
------
FC88X_00804_01077 ------------------TTTTTCCCTTCA-CTAA---CCGCGA-A-CTGCA--
------
FC88X_00848_01214 ------------------TTTTTCCCTTCA-CTAA---CCGCGA-A-CTGCA--
------
FC88X_00778_00232 -----------------GTTTTTCCCTTCA-CTAA---TCGCGA-A-CTGCA--
------
FC88X_00694_00940 -----------GGGGAAGTTTTTCCCTTCA-CTTAA-TCCGCGA-A-CTGCAC-
------

FC88X_00887_00705 ------------------TTTTTCCCTTCA-CTAA---TCGCGA-A-CTGCA--
------
FC88X_00306_00060 ------------------TTTTTCCCTTCA-CTAA---TCGCGA-A-CTGCA--
------
FC88X_00638_00210 ------------------TTTTTCCCTTCA-CTAA---TCGCGA-A-CTGCA--
------
FC88X_00893_00595 ------------------TTTTTCCCTTCA-CTAA---TCGCGA-A-CTGCA--
------
FC88X_00039_00751 ------------------TTTTTCCCTTCA-CTAA---TCGCGA-A-CTGCA--
------
FC88X_01107_00926 -----------------TTTTTTCCCTTCA-CTAA---TCGCGA-A-CTGCA--
------
FC88X_00591_00379 ------------------TTTTTCCCTTCA-CTAA---TCGCGA-A-CTGCA--
------
FC88X_00858_01099 ------------------TTTTTCCCTTCA-CTAA---TCGCGA-A-CTGCA--
------
FC88X_01000_00568 -------------------TTTTCCCTTCA-CTAA---TCGCGA-T-CTGCA--
------
FC88X_01080_00556 -------------------TTTTCCCTTCA-CTAA---TCGCGA-T-CTGCA--
------
FC88X_00321_01021 -----------------TTTTTTCCCTTCA-CTAA--TCCGCGA-A-CTGCA--
------
FC88X_00789_00212 ------------------TTTTTCCCTTCA-CTAA---CCGCGA-A-CTGCA--
------
FC88X_00776_00828 ------------------TTTTTCCCTTCA-CTAA---CCGCGA-A-CTGCA--
------
FC88X_00826_01132 ------------------TTTTTCCCTTCA-CTAA---CCGCGA-A-CTGCA--
------
FC88X_00800_01191 ------------------TTTTTCCCTTCA-CTAA---CCGCGA-A-CTGCA--
------
FC88X_00818_01094 ------------------TTTTTCCCTTCA-CTAA---CCGCGA-A-CTGCA--
------
FC88X_00790_01144 ------------------TTTTTCCCTTCA-CTAA---CCGCGA-A-CTGCA--
------
FC88X_00782_01155 ------------------TTTTTCCCTTCA-CTAA---CCGCGA-A-CTGCA--
------
FC88X_00775_01221 ------------------TTTTTCCCTTCA-CTAA---CCGCGA-A-CTGCA--
------
FC88X_00878_00308 ------------------TTTTTCCCTTCA-CTAA---CCGCGA-A-CTGCA--
------
FC88X_00022_00390 -------------------TTTCCCCTTCA-CTAA---CCGCGA-A-CTGCA--
------
FC88X_00577_01209 ------------------GTTTCCCCTTCA-CTAA---CCGCGA-A-CTGCA--
------
FC88X_00164_00405 -------------------TTTCCCCTTCA-CTAA---CCGCGA-A-CTGCA--
------
FC88X_00919_00636 -------------------TTTCCCCTTCA-CTAA---CCGCGA-A-CTGCA--
------
FC88X_00070_00091 ----------------TCTTTTCCCCTTCA-CTAA---CCGCGA-A-CTGCA--
------
FC88X_00489_00749 -------------------TTTCCCCTTCA-CTAA---CCGCGA-A-CTGCA--
------
FC88X_00058_00513 -------------------TTTCCCCTTCA-CTAA---CCGCGA-A-CTGCA--
------
FC88X_00908_01047 -------------------TTTCCCCTTCA-CTAA---CCGCGA-A-CTGCA--
------

FC88X_01031_01043 -----------------GTTTTCCCCTTCA-CTAA---CCGCGA-A-CTGCA--
------
FC88X_00613_00188 ------------------TTTTCCCCTTCA-CTAA---CCGCGA-A-CTGCA--
------
FC88X_00715_00898 ------------------TTTTCCCCTTCA-CTAA---CCGCGA-A-CTGCA--
------
FC88X_00187_00817 ------------------TTTTCCCCTTCA-CTAA---CCGCGA-A-CTGCA--
------
FC88X_00240_00126 ------------------TTTTCCCCTTCA-CTAA---CCGCGA-A-CTGCA--
------
FC88X_00382_00997 ------------------TTTTCCCCTTCA-CTAA---CCGCGA-A-CTGCA--
------
FC88X_00601_00720 ------------------TTTTCCCCTTCA-CTAA---CCGCGA-A-CTGCA--
------
FC88X_00111_00936 ------------------TTTTCCCCTTCA-CTAA---CCGCGA-A-CTGCA--
------
FC88X_00012_00641 ------------------TTTTCCCCTTCA-CTAA---CCGCGA-A-CTGCA--
------
FC88X_00120_00151 ------------------TTTTCCCCTTCA-CTAA---CCGCGA-A-CTGCA--
------
FC88X_00869_00265 ------------------TTTTCCCCTTCA-CTAA---CCGCGA-A-CTGCA--
------
FC88X_00878_01144 ------------------TTTTCCCCTTCA-CTAA---CCGCGA-A-CTGCA--
------
FC88X_00912_00626 ------------------TTTTCCCCTTCA-CTAA---CCGCGA-A-CTGCA--
------
FC88X_00933_00724 ------------------TTTTCCCCTTCA-CTAA---CCGCGA-A-CTGCA--
------
FC88X_01060_00865 -------------------TTTTCCCCTCA-CTAA---CCGCGA-A-CTGCA--
------
FC88X_00201_00132 -------------------TTTTCCCCTCA-CTAA---CCGCGA-A-CTGCA--
------
FC88X_00414_00963 ----------------TTTTTTCCCCTTCA-CTAA---CCGCGA-A-CTGCA--
------
FC88X_01046_00335 ----------------TTTTTTCCCCTTCA-CTAA---CCGCGA-A-CTGCA--
------
FC88X_01075_00489 ----------------TTTTTTCCCCTTCA-CTAA---CCGCGA-A-CTGCA--
------
FC88X_00915_00681 ----------------TTTTTTCCCCTTCA-CTAA---CCGCGA-A-CTGCA--
------
FC88X_00936_00660 -----------------TTTTTCCCCTTCA-CTAA---CCGCGA-A-CTGCA--
------
FC88X_00096_00200 -----------------TTTTTCCCCTTCA-CTAA---CCGCGA-A-CTGCA--
------
FC88X_00275_01031 -----------------TTTTTCCCCTTCA-CTAA---CCGCGA-A-CTGCA--
------
FC88X_00445_00908 -----------------TTTTTCCCCTTCA-CTAA---CCGCGA-A-CTGCA--
------
FC88X_00169_00818 ----------------GTTTTTCCCCTTCA-CTAA---CCGCGA-A-CTGCA--
------
FC88X_01085_00409 ---------------GGTTTTTCCCCTTCA-CTAA---CCGCGA-A-CTGCA--
------
FC88X_00714_01232 -----------------TTTTTCCCCTTCA-CTAA---CCGCGA-A-CTGCA--
------
FC88X_00945_00667 -----------------TTTTTCCCCTTCA-CTAA---CCGCGA-A-CTGCA--
------

```
FC88X_00150_00718       -----------------TTTTTCCCCTTCA-CTAA---CCGCGA-A-CTGCA--
------
FC88X_00255_01060       -----------------TTTTTCCCCTTCA-CTAA---CCGCGA-A-CTGCA--
------
FC88X_00429_01030       -----------------TTTTTCCCCTTCA-CTAA---CCGCGA-A-CTGCA--
------
FC88X_00510_01091       ------------------TTTTTCCCCTCA-CTAA---CCGCGA-A-CTGCA--
------
FC88X_00463_00737       ------------------TTTTTCCCCTCA-CTAA---CCGCGA-A-CTGCA--
------
FC88X_00485_00064       ------------------TTTTTCCCCTCA--TAA---CCGCGA-A-CTGCA--
------
FC88X_00935_00673       ----------------TTTTTCCCCCTTCA-CTAA---CCGCGA-A-CTGCA--
------
FC88X_00753_00250       ------------------TTTTTCCCCTCA-CTAA---CCGCGA-A-CTGCA--
------
FC88X_00914_00384       ------------------TTTTTCCCCTCA-CTAA---CCGCGA-A-CTGCA--
------
FC88X_01135_00938       ------------------TTTTTCCCCTCA-CTAA---CCGCGA-A-CTGCA--
------
FC88X_00829_00778       ------------------TTTTTCCCCTCA-CTAT---CCGCGA-A-CTGCA--
------
FC88X_01010_00511       -----------GGGGAACTTTTTCCCCTCA-CTAA---CCGCGA-A-CTGCA--
------
FC88X_01117_01177       ------------------TTTTTCCCCTCA-CTAA---CCGCGA-A-CTGCA--
------
FC88X_00639_00203       ------------------TTTTTCCCCTCA-CTAA---CCGCGA-A-CTGCA--
------
FC88X_00754_01230       ------------------TTTTTCCCCTCA-CTAA---CCGCGA-A-CTGCA--
------
FC88X_00897_00679       ------------------TTTTTCCCCTCA-CTAA---CCGCGA-A-CTGCA--
------
FC88X_01024_00718       ------------------TTTTTCCCCTCA-CTAA---CCGCGA-A-CTGCA--
------
FC88X_01046_01237       ------------------TTTTTCCCCTCA-CTAA---CCGCGA-A-CTGCA--
------
FC88X_00023_00574       ------------------TTTTTCCCCTCA-CTAA---CCGCGA-A-CTGCA--
------
FC88X_00034_00803       -------------------TTTTCCCCTCA-CTAA---CCGCGA-A-CTGCA--
------
FC88X_00802_00963       -------------------TTTTCCCCTCA-CTAA---CCGCGA-A-CTGCA--
------
FC88X_01110_00536       -------------------TTTTCCCCTCA-CTAA---CCGCGA-A-CTGCA--
------
FC88X_00112_00496       ---------------TTTTTTCCCCTTTCA-CTAA---CCGCGA-A-CTGCA--
------
FC88X_01075_00765       -----------------TTTTTTCCCTTCA-CTAA---CCGCGA-A-CTGCA--
------
FC88X_00604_01222       -----------------TTTTTTCCCTTCA-CTAA---CCGCGA-A-CTGCA--
------
FC88X_00690_01015       -----------------TTTTTTCCCTTCA-CTAA---CCGCGA-A-CTGCA--
------
FC88X_00495_01179       -----------------TTTTTTCCCTTCA-CTAA---CCGCGA-A-CTGCA--
------
FC88X_00754_01158       ------------------TTTTTCCCTTCA-CTAA---CCGCGA-A-CTGCA--
------
```

```
FC88X_00782_01186       ------------------TTTTTCCCTTCA-CTAA---CCGCGA-A-CTGCA--
------
FC88X_00850_00326       ------------------TTTTTCCCTTCA-CTAA---CCGCGA-A-CTGCA--
------
FC88X_00893_00385       ------------------TTTTTCCCTTCA-CTAA---CCGCGA-A-CTGCA--
------
FC88X_00861_00437       ------------------TTTTTCCCTTCA-CTAA---CCGCGA-A-CTGCA--
------
FC88X_00879_00404       ------------------TTTTTCCCTTCA-CTAA---CCGCGA-A-CTGCA--
------
FC88X_00889_00471       ------------------TTTTTCCCTTCA-CTAA---CCGCGA-A-CTGCA--
------
FC88X_00884_00599       ------------------TTTTTCCCTTCA-CTAA---CCGCGA-A-CTGCA--
------
FC88X_00898_00568       ------------------TTTTTCCCTTCA-CTAA---CCGCGA-A-CTGCA--
------
FC88X_00879_00601       ------------------TTTTTCCCTTCA-CTAA---CCGCGA-A-CTGCA--
------
FC88X_00880_00622       ------------------TTTTTCCCTTCA-CTAA---CCGCGA-A-CTGCA--
------
FC88X_00873_00674       ------------------TTTTTCCCTTCA-CTAA---CCGCGA-A-CTGCA--
------
FC88X_00896_00671       ------------------TTTTTCCCTTCA-CTAA---CCGCGA-A-CTGCA--
------
FC88X_00898_00691       ------------------TTTTTCCCTTCA-CTAA---CCGCGA-A-CTGCA--
------
FC88X_00865_00725       ------------------TTTTTCCCTTCA-CTAA---CCGCGA-A-CTGCA--
------
FC88X_00898_00744       ------------------TTTTTCCCTTCA-CTAA---CCGCGA-A-CTGCA--
------
FC88X_00868_00793       ------------------TTTTTCCCTTCA-CTAA---CCGCGA-A-CTGCA--
------
FC88X_00869_00764       ------------------TTTTTCCCTTCA-CTAA---CCGCGA-A-CTGCA--
------
FC88X_00852_00840       ------------------TTTTTCCCTTCA-CTAA---CCGCGA-A-CTGCA--
------
FC88X_00884_00814       ------------------TTTTTCCCTTCA-CTAA---CCGCGA-A-CTGCA--
------
FC88X_00865_00857       ------------------TTTTTCCCTTCA-CTAA---CCGCGA-A-CTGCA--
------
FC88X_00867_00909       ------------------TTTTTCCCTTCA-CTAA---CCGCGA-A-CTGCA--
------
FC88X_00882_01143       ------------------TTTTTCCCTTCA-CTAA---CCGCGA-A-CTGCA--
------
FC88X_00948_00872       ------------------TTTTTCCCTTCA-CTAA---CCGCGA-A-CTGCA--
------
FC88X_00939_00729       ------------------TTTTTCCCTTCA-CTAA---CCGCGA-A-CTGCA--
------
FC88X_00886_01215       ------------------TTTTTCCCTTCA-CTAA---CCGCGA-A-CTGCA--
------
FC88X_00749_00257       ------------------TTTTTCCCTTCA-CTAA---CCGCGA-A-CTGCA--
------
FC88X_00061_00101       ---------------GTTTTTTTCCCTTCA-CTAA---CCGCGA-A-CTGCA--
------
FC88X_00744_01024       ---------------GTTTTTTTCCCTTCA-CTAA---CCGCGA-A-CTGCA--
------
```

FC88X_00901_00600 ----------------TTTTTTTCCCTTCA-CTAA---CCGCGA-A-CTGCA--
------
FC88X_00819_00177 -----------------TTTTTTCCCTTCA-CTAA---CCGCGA-A-CTGCA--
------
FC88X_01107_00847 -----------------TTTTTTCCCTTCA-CTAA---CCGCGA-A-CTGCA--
------
FC88X_00385_00045 -----------------TTTTTTCCCTTCA-CTAA---CCGCGA-A-CTGCA--
------
FC88X_00239_01014 -----------------TTTTTTCCCTTCA-CTAA---CCGCGA-A-CTGCA--
------
FC88X_01099_00812 -----------------TTTTTTCCCTTCA-CTAA---CCGCGA-A-CTGCA--
------
FC88X_00788_00987 -----------------TTTTTTCCCTTCA-CTAA---CCGCGA-A-CTGCA--
------
FC88X_01039_00866 -----------------TTTTTTCCCTTCA-CTAA---CCGCGA-A-CTGCA--
------
FC88X_00539_00504 -----------------TTTTTTCCCTTCA-CTAA---CCGCGA-A-CTGCA--
------
FC88X_00831_00225 -----------------TTTTTTCCCTTCA-CTAA---CCGCGA-A-CTGCA--
------
FC88X_00926_01274 -----------------TTTTTTCCCTTCA-CTAA---CCGCGA-A-CTGCA--
------
FC88X_00838_00797 -----------------TTTTTTCCCTTCA-CTAA---CCGCGA-A-CTGCA--
------
FC88X_00964_01118 -------------------TTTTCCCTTCA-CTAA---CCGCGA-A-CTGCA--
------
FC88X_00646_00387 -------------------TTTTCCCTTCA-CTAA---CCGCGA-A-CTGCA--
------
FC88X_00857_00933 ------------------TTTTTCCCTTCA-CTAA---CCGCGA-A-CTGCA--
------
FC88X_00937_00885 ------------------TTTTTCCCTTCA-CTAA---CCGCGA-A-CTGCA--
------
FC88X_00932_00839 ------------------TTTTTCCCTTCA-CTAA---CCGCGA-A-CTGCA--
------
FC88X_00605_01168 ------------------TTTTTCCCTTCA-CTAA---CCGCGA-A-CTGCA--
------
FC88X_00929_00492 ------------------TTTTTCCCTTCA-CTAA---CCGCGA-A-CTGCA--
------
FC88X_00674_00803 ------------------TTTTTCCCTTCA-CTAA---CCGCGA-A-CTGCA--
------
FC88X_00246_01099 ------------------TTTTTCCCTTCA-CTAA---CCGCGA-A-CTGCA--
------
FC88X_00056_00804 ------------------TTTTTCCCTTCA-CTAA---CCGCGA-A-CTGCA--
------
FC88X_00218_00128 ------------------TTTTTCCCTTCA-CTAA---CCGCGA-A-CTGCA--
------
FC88X_00442_01095 ------------------TTTTTCCCTTCA-CTAA---CCGCGA-A-CTGCA--
------
FC88X_00472_01078 ------------------TTTTTCCCTTCA-CTAA---CCGCGA-A-CTGCA--
------
FC88X_00404_00767 ------------------TTTTTCCCTTCA-CTAA---CCGCGA-A-CTGCA--
------
FC88X_00012_00249 ------------------TTTTTCCCTTCA-CTAA---CCGCGA-A-CTGCA--
------
FC88X_00423_00694 ------------------TTTTTCCCTTCA-CTAA---CCGCGA-A-CTGCA--
------

FC88X_00022_00508 ------------------TTTTTCCCTTCA-CTAA---CCGCGA-A-CTGCA--
------
FC88X_00029_00543 ------------------TTTTTCCCTTCA-CTAA---CCGCGA-A-CTGCA--
------
FC88X_00048_00553 ------------------TTTTTCCCTTCA-CTAA---CCGCGA-A-CTGCA--
------
FC88X_00374_00117 ------------------TTTTTCCCTTCA-CTAA---CCGCGA-A-CTGCA--
------
FC88X_00224_00010 ------------------TTTTTCCCTTCA-CTAA---CCGCGA-A-CTGCA--
------
FC88X_00210_00065 ------------------TTTTTCCCTTCA-CTAA---CCGCGA-A-CTGCA--
------
FC88X_00232_00159 ------------------TTTTTCCCTTCA-CTAA---CCGCGA-A-CTGCA--
------
FC88X_00462_01044 ------------------TTTTTCCCTTCA-CTAA---CCGCGA-A-CTGCA--
------
FC88X_00201_00661 ------------------TTTTTCCCTTCA-CTAA---CCGCGA-A-CTGCA--
------
FC88X_00239_00748 ------------------TTTTTCCCTTCA-CTAA---CCGCGA-A-CTGCA--
------
FC88X_00238_01110 ------------------TTTTTCCCTTCA-CTAA---CCGCGA-A-CTGCA--
------
FC88X_00266_00035 ------------------TTTTTCCCTTCA-CTAA---CCGCGA-A-CTGCA--
------
FC88X_00298_00058 ------------------TTTTTCCCTTCA-CTAA---CCGCGA-A-CTGCA--
------
FC88X_00214_00504 ------------------TTTTTCCCTTCA-CTAA---CCGCGA-A-CTGCA--
------
FC88X_00277_00515 ------------------TTTTTCCCTTCA-CTAA---CCGCGA-A-CTGCA--
------
FC88X_00269_00729 ------------------TTTTTCCCTTCA-CTAA---CCGCGA-A-CTGCA--
------
FC88X_00296_00516 ------------------TTTTTCCCTTCA-CTAA---CCGCGA-A-CTGCA--
------
FC88X_00112_00296 ------------------TTTTTCCCTTCA-CTAA---CCGCGA-A-CTGCA--
------
FC88X_00280_00952 ------------------TTTTTCCCTTCA-CTAA---CCGCGA-A-CTGCA--
------
FC88X_00308_01037 ------------------TTTTTCCCTTCA-CTAA---CCGCGA-A-CTGCA--
------
FC88X_00264_01130 ------------------TTTTTCCCTTCA-CTAA---CCGCGA-A-CTGCA--
------
FC88X_00425_00884 ------------------TTTTTCCCTTCA-CTAA---CCGCGA-A-CTGCA--
------
FC88X_00314_00056 ------------------TTTTTCCCTTCA-CTAA---CCGCGA-A-CTGCA--
------
FC88X_00160_00946 ------------------TTTTTCCCTTCA-CTAA---CCGCGA-A-CTGCA--
------
FC88X_00337_00055 ------------------TTTTTCCCTTCA-CTAA---CCGCGA-A-CTGCA--
------
FC88X_00346_00148 ------------------TTTTTCCCTTCA-CTAA---CCGCGA-A-CTGCA--
------
FC88X_00331_00622 ------------------TTTTTCCCTTCA-CTAA---CCGCGA-A-CTGCA--
------
FC88X_00335_00610 ------------------TTTTTCCCTTCA-CTAA---CCGCGA-A-CTGCA--
------

FC88X_00339_00620 ------------------TTTTTCCCTTCA-CTAA---CCGCGA-A-CTGCA--
------
FC88X_00054_00328 ------------------TTTTTCCCTTCA-CTAA---CCGCGA-A-CTGCA--
------
FC88X_00322_01022 ------------------TTTTTCCCTTCA-CTAA---CCGCGA-A-CTGCA--
------
FC88X_00327_01051 ------------------TTTTTCCCTTCA-CTAA---CCGCGA-A-CTGCA--
------
FC88X_00347_01075 ------------------TTTTTCCCTTCA-CTAA---CCGCGA-A-CTGCA--
------
FC88X_00316_01117 ------------------TTTTTCCCTTCA-CTAA---CCGCGA-A-CTGCA--
------
FC88X_00326_01169 ------------------TTTTTCCCTTCA-CTAA---CCGCGA-A-CTGCA--
------
FC88X_00418_00380 ------------------TTTTTCCCTTCA-CTAA---CCGCGA-A-CTGCA--
------
FC88X_00348_01158 ------------------TTTTTCCCTTCA-CTAA---CCGCGA-A-CTGCA--
------
FC88X_00417_00164 ------------------TTTTTCCCTTCA-CTAA---CCGCGA-A-CTGCA--
------
FC88X_00366_00183 ------------------TTTTTCCCTTCA-CTAA---CCGCGA-A-CTGCA--
------
FC88X_00398_00247 ------------------TTTTTCCCTTCA-CTAA---CCGCGA-A-CTGCA--
------
FC88X_00392_00397 ------------------TTTTTCCCTTCA-CTAA---CCGCGA-A-CTGCA--
------
FC88X_00395_00603 ------------------TTTTTCCCTTCA-CTAA---CCGCGA-A-CTGCA--
------
FC88X_00387_00948 ------------------TTTTTCCCTTCA-CTAA---CCGCGA-A-CTGCA--
------
FC88X_00364_01141 ------------------TTTTTCCCTTCA-CTAA---CCGCGA-A-CTGCA--
------
FC88X_00388_00962 ------------------TTTTTCCCTTCA-CTAA---CCGCGA-A-CTGCA--
------
FC88X_00017_00744 ------------------TTTTTCCCTTCA-CTAA---CCGCGA-A-CTGCA--
------
FC88X_00379_01028 ------------------TTTTTCCCTTCA-CTAA---CCGCGA-A-CTGCA--
------
FC88X_00366_01088 ------------------TTTTTCCCTTCA-CTAA---CCGCGA-A-CTGCA--
------
FC88X_00371_01143 ------------------TTTTTCCCTTCA-CTAA---CCGCGA-A-CTGCA--
------
FC88X_00392_01156 ------------------TTTTTCCCTTCA-CTAA---CCGCGA-A-CTGCA--
------
FC88X_00352_01182 ------------------TTTTTCCCTTCA-CTAA---CCGCGA-A-CTGCA--
------
FC88X_00404_00063 ------------------TTTTTCCCTTCA-CTAA---CCGCGA-A-CTGCA--
------
FC88X_00421_00058 ------------------TTTTTCCCTTCA-CTAA---CCGCGA-A-CTGCA--
------
FC88X_00430_00080 ------------------TTTTTCCCTTCA-CTAA---CCGCGA-A-CTGCA--
------
FC88X_00431_00061 ------------------TTTTTCCCTTCA-CTAA---CCGCGA-A-CTGCA--
------
FC88X_00436_00132 ------------------TTTTTCCCTTCA-CTAA---CCGCGA-A-CTGCA--
------

FC88X_00439_00144 ------------------TTTTTCCCTTCA-CTAA---CCGCGA-A-CTGCA--
------
FC88X_00440_00112 ------------------TTTTTCCCTTCA-CTAA---CCGCGA-A-CTGCA--
------
FC88X_00445_00629 ------------------TTTTTCCCTTCA-CTAA---CCGCGA-A-CTGCA--
------
FC88X_00407_00797 ------------------TTTTTCCCTTCA-CTAA---CCGCGA-A-CTGCA--
------
FC88X_00441_00850 ------------------TTTTTCCCTTCA-CTAA---CCGCGA-A-CTGCA--
------
FC88X_00064_00868 ------------------TTTTTCCCTTCA-CTAA---CCGCGA-A-CTGCA--
------
FC88X_00419_01123 ------------------TTTTTCCCTTCA-CTAA---CCGCGA-A-CTGCA--
------
FC88X_00091_00164 ------------------TTTTTCCCTTCA-CTAA---CCGCGA-A-CTGCA--
------
FC88X_00432_01140 ------------------TTTTTCCCTTCA-CTAA---CCGCGA-A-CTGCA--
------
FC88X_00401_01157 ------------------TTTTTCCCTTCA-CTAA---CCGCGA-A-CTGCA--
------
FC88X_00458_01035 ------------------TTTTTCCCTTCA-CTAA---CCGCGA-A-CTGCA--
------
FC88X_00200_00117 ------------------TTTTTCCCTTCA-CTAA---CCGCGA-A-CTGCA--
------
FC88X_00034_00090 ------------------TTTTTCCCTTCA-CTAAC--C-GCGA-A-CTGCA--
------
FC88X_00393_01086 -------------------TTTTCCCTTCA-CTAAC--C-GCGA-A-CTGCA--
------
FC88X_00038_00522 ----------------GTTTTTTCCCTTCA-CTAAC--C-GCGA-A-CTGCA--
------
FC88X_01130_00705 ----------------GTTTTTTCCCTTCA-CTAAC--CCGCGA-A-CTGCA--
------
FC88X_01129_00887 ----------------GTTTTTTCCCTTCA-CTAAC--C-GCGA-A-CTGCA--
------
FC88X_00115_00464 ----------------GTTTTTTCCCTTCA-CTAAC--C-GCGA-A-CTGCA--
------
FC88X_00586_01029 ----------------GTTTTTTCCCTTCA-CTAAC--C-GCGA-A-CTGCA--
------
FC88X_00679_00949 ----------------GTTTTTTCCCTTCA-CTAAC--C-GCGA-A-CTGCA--
------
FC88X_01016_00525 ----------------GTTTTTTCCCTTCA-CTAAC--C-GCGA-A-CTGCA--
------
FC88X_00200_00098 ----------------GTTTTTTCCCTTCA-CTAAC--C-GCGA-A-CTGCA--
------
FC88X_00990_00319 ----------------GTTTTTTCCCTTCA-CTAAC--C-GCGA-A-CTGCA--
------
FC88X_00461_01239 ----------------GTTTTTTCCCTTCA-CTAAA--CCGCGA-A-CTGCA--
------
FC88X_01054_00678 ----------------GTTTTTTCCCTTCA-CTAA---CCGCGA-A-CTGCA--
------
FC88X_01085_01077 ----------------GTTTTTTCCCTTCA-CTAA---CCGCGA-A-CTGCA--
------
FC88X_00699_01126 ----------------GTTTTTTCCCTTCA-CTAA---CCGCGA-A-CTGCA--
------
FC88X_00768_00946 ----------------GTTTTTTCCCTTCA-CTAA---CCGCGA-A-CTGCA--
------

FC88X_00792_01206 ----------------GTTTTTTCCCTTCA-CTAA---CCGCGA-A-CTGCA--
------
FC88X_00983_01053 ----------------GTTTTTTCCCTTCA-CTAA---CCGCGA-A-CTGCA--
------
FC88X_00456_00099 ----------------GTTTTTTCCCTTCA-CTAA---CCGCGA-A-CTGCA--
------
FC88X_01026_00491 -----------------TTTTTTCCCTTCA-CTAAC--C-GCGA-A-CTGCA--
------
FC88X_00992_00961 -----------------TTTTTTCCCTTCA-CTAAC--C-GCGA-A-CTGCA--
------
FC88X_00131_00228 -----------------TTTTTTCCCTTCA-CTAAC--C-GCGA-A-CTGCA--
------
FC88X_00072_00656 -----------------TTTTTTCCCTTCA-CTAAC--C-GCGA-A-CTGCA--
------
FC88X_00171_00736 -----------------TTTTTTCCCTTCA-CTAAC--C-GCGA-A-CTGCA--
------
FC88X_01065_01024 -----------------TTTTTTCCCTTCA-CTAAC--C-GCGA-A-CTGCA--
------
FC88X_00247_00902 -----------------TTTTTTCCCTTCA-CTAAC--C-GCGA-A-CTGCA--
------
FC88X_00168_00767 -----------------TTTTTTCCCTTCA-CTAAC--C-GCGA-A-CTGCA--
------
FC88X_00080_00887 -----------------TTTTTTCCCTTCA-CTAAC--C-GCGA-A-CTGCA--
------
FC88X_00421_01207 -----------------TTTTTTCCCTTCA-CTAAC--C-GCGA-A-CTGCA--
------
FC88X_00838_01050 -----------------TTTTTTCCCTTCA-CTAAC--C-GCGA-A-CTGCA--
------
FC88X_00994_01147 -------------------TTTTCCCTTCA-CTAAC--C-GCGA-A-CTGCA--
------
FC88X_00564_00969 -------------------TTTTCCCTTCA-CTAAC--C-GCGA-A-CTGCA--
------
FC88X_00864_01014 ------------------TTTTTCCCTTCA-CTAAC--C-GCGA-A-CTGCA--
------
FC88X_00934_00998 ------------------TTTTTCCCTTCA-CTAAC--C-GCGA-A-CTGCA--
------
FC88X_00718_00299 ------------------TTTTTCCCTTCA-CTAAC--C-GCGA-A-CTGCA--
------
FC88X_00612_01235 ------------------TTTTTCCCTTCA-CTAAC--C-GCGA-A-CTGCA--
------
FC88X_01050_00788 ------------------TTTTTCCCTTCA-CTAAC--C-GCGA-A-CTGCA--
------
FC88X_00584_01080 ------------------TTTTTCCCTTCA-CTAAC--C-GCGA-A-CTGCA--
------
FC88X_01040_01117 -------------------TTTTCCCTTCA-CTAAC--C-GCGA-A-CTGCA--
------
FC88X_00459_01060 ------------------TTTTTCCCTTCA-CTAAC--C-GCGA-A-CTGCA--
------
FC88X_00030_00589 ------------------TTTTTCCCTTCA-CTAAC--C-GCGA-A-CTGCA--
------
FC88X_00094_00164 -------------------TTTTCCCTTCA-CTAAC--C-GCGA-A-CTGCA--
------
FC88X_00061_00232 -------------------TTTTCCCTTCA-CTAAC--C-GCGA-A-CTGCA--
------
FC88X_00063_00544 -------------------TTTTCCCTTCA-CTAAC--C-GCGA-A-CTGCA--
------

FC88X_00051_00445 -------------------TTTTCCCTTCA-CTAAC--C-GCGA-A-CTGCA--
------
FC88X_00231_01024 -------------------TTTTCCCTTCA-CTAAC--C-GCGA-A-CTGCA--
------
FC88X_00257_00030 -------------------TTTTCCCTTCA-CTAAC--C-GCGA-A-CTGCA--
------
FC88X_00424_00142 -------------------TTTTCCCTTCA-CTAAC--C-GCGA-A-CTGCA--
------
FC88X_00432_00115 -------------------TTTTCCCTTCA-CTAAC--C-GCGA-A-CTGCA--
------
FC88X_00438_01172 -------------------TTTTCCCTTCA-CTAAC--C-GCGA-A-CTGCA--
------
FC88X_01133_00877 -------------------TTTTCCCTTCA-CTAAC--C-GCGA-A-CTGCA--
------
FC88X_00502_00101 -------------------TTTTCCCTTCA-CTAAC--C-GCGA-A-CTGCA--
------
FC88X_00521_00177 -------------------TTTTCCCTTCA-CTAAC--C-GCGA-A-CTGCA--
------
FC88X_00515_01204 -------------------TTTTCCCTTCA-CTAAC--C-GCGA-A-CTGCA--
------
FC88X_00588_00059 -------------------TTTTCCCTTCA-CTAAC--C-GCGA-A-CTGCA--
------
FC88X_00676_00627 -------------------TTTTCCCTTCA-CTAAC--C-GCGA-A-CTGCA--
------
FC88X_00662_00782 -------------------TTTTCCCTTCA-CTAAC--C-GCGA-A-CTGCA--
------
FC88X_00768_00116 -------------------TTTTCCCTTCA-CTAAC--C-GCGA-A-CTGCA--
------
FC88X_00787_00184 -------------------TTTTCCCTTCA-CTAAC--C-GCGA-A-CTGCA--
------
FC88X_00763_01015 -------------------TTTTCCCTTCA-CTAAC--C-GCGA-A-CTGCA--
------
FC88X_00767_01013 -------------------TTTTCCCTTCA-CTAAC--C-GCGA-A-CTGCA--
------
FC88X_01067_00667 -------------------TTTTCCCTTCA-CTAAC--C-GCGA-A-CTGCA--
------
FC88X_01089_00734 -------------------TTTTCCCTTCA-CTAAC--C-GCGA-A-CTGCA--
------
FC88X_01135_00686 -------------------TTTTCCCTTCA-CTAAC--C-GCGA-A-CTGCA--
------
FC88X_01138_00787 -------------------TTTTCCCTTCA-CTAAC--C-GCGA-A-CTGCA--
------
FC88X_00012_00472 -------------------TTTTCCCTTCA-CTAAC--C-GCGA-A-CTGCA--
------
FC88X_00026_00495 -------------------TTTTCCCTTCA-CTAAC--C-GCGA-A-CTGCA--
------
FC88X_00050_00141 -------------------TTTTCCCTTCA-CTAAC--C-GCGA-A-CTGCA--
------
FC88X_00085_00020 ------------------TTTTTCCCTTCA-CTAAC--C-GCGA-A-CTGCA--
------
FC88X_00107_00137 -------------------TTTTCCCTTCA-CTAAC--C-GCGA-A-CTGCA--
------
FC88X_00076_00230 ------------------TTTTTCCCTTCA-CTAAC--C-GCGA-A-CTGCA--
------
FC88X_00233_00988 ------------------TTTTTCCCTTCA-CTAAC--C-GCGA-A-CTGCA--
------

```
FC88X_00310_00218       -------------------TTTTCCCTTCA-CTAAC--C-GCGA-A-CTGCA--
------
FC88X_00022_00707       ------------------TTTTTCCCTTCA-CTAA---CCGCGA-A-CTGCA--
------
FC88X_00040_00739       ------------------TTTTTCCCTTCA-CTAA---CCGCGA-A-CTGCA--
------
FC88X_00062_00122       ------------------TTTTTCCCTTCA-CTAA---CCGCGA-A-CTGCA--
------
FC88X_00415_01028       ------------------TTTTTCCCTTCA-CTAA---CCGCGA-A-CTGCA--
------
FC88X_00606_01017       -------------------TTTTCCCTTCACT-AA---CCGCGA-AC-TGCA--
------
FC88X_00065_00043       ------------------TTTTTCCCTTCACT-AA---CCGCGA-AC-TGCA--
------
FC88X_00629_00953       -------------------TTTTCCCTTCACT-AA---CCGCGA-AC-TGCA--
------
FC88X_00062_00311       ------------------TTTTTCCCTTCACT-AA---CCGCGA-AC-TGCA--
------
FC88X_00630_00180       -------------------TTTTCCCTTCACT-AA---CCGCGA-AC-TGCA--
------
FC88X_00554_01024       ------------------TTTTTCCCTTCACT-AA---CCGCGA-AC-TGCA--
------
FC88X_00551_00109       ------------------TTTTTCCCTTCACT-AA---CCGCGA-AC-TGCA--
------
FC88X_00164_01004       -------------------TTTTCCCTTCACT-AA---CCGCGA-AC-TGCA--
------
FC88X_00418_00082       ------------------TTTTTCCCTTCACT-AA---CCGCGA-AC-TGCA--
------
FC88X_00183_00153       -------------------TTTTCCCTTCACT-AA---CCGCGA-AC-TGCA--
------
FC88X_00327_00603       ------------------TTTTTCCCTTCACT-AA---CCGCGA-AC-TGCA--
------
FC88X_00591_01109       -------------------TTTTCCCTTCACT-AA---CCGCGA-AC-TGCA--
------
FC88X_00340_00478       ------------------TTTTTCCCTTCACT-AA---CCGCGA-AC-TGCA--
------
FC88X_00860_00229       -------------------TTTTCCCTTCACT-AA---CCGCGA-AC-TGCA--
------
FC88X_00067_00790       -------------------TTTTCCCTTCACT-AA---CCGCGA-AC-TGCA--
------
FC88X_00095_00783       -------------------TTTTCCCTTCACT-AA---CCGCGA-AC-TGCA--
------
FC88X_00837_00950       -----------------TTTTTTCCCTTCACT-AA---CCGCGA-A-CTGCA--
------
FC88X_00888_00615       -----------------TTTTTTCCCTTCACT-AA---CCGCGA-AACTGCA--
------
FC88X_01097_01047       -----------------TTTTTTCCCTTCACT-AA---CCGCGA-A-CTGCA--
------
FC88X_00461_01074       -----------------TTTTTTCCCTTCACT-AA---CCGCGA-A-CTGCA--
------
FC88X_00083_00186       -----------------TTTTTTCCCTTCACT-AA---CCGCGA-A-CTGCA--
------
FC88X_00090_00619       -----------------TTTTTTCCCTTCACT-AA---CCGCGA-A-CTGCA--
------
FC88X_01062_01031       -----------------TTTTTTCCCTTCACT-AA---CCGCGA-A-CTGCA--
------
```

FC88X_00557_01122 -----------------TTTTTTCCCTTCACT-AA---CCGCGA-A-CTGCA--
------
FC88X_00963_01177 -----------------TTTTTTCCCTTCACT-AA---CCGCGA-A-CTGCA--
------
FC88X_00794_01267 -----------------TTTTTTCCCTTCACT-AA---CCGCGA-A-CTGCA--
------
FC88X_00982_01026 -----------------TTTTTTCCCTTCACT-AA---CCGCGA-A-CTGCA--
------
FC88X_00861_00433 -----------------TTTTTTCCCTTCACT-AA---CCGCGA-A-CTGCA--
------
FC88X_00997_01086 -------------------TTTTCCCTTCACT-AA---CCGCGA-A-CTGCA--
------
FC88X_00641_00361 -------------------TTTTCCCTTCACT-AA---CCGCGA-A-CTGCA--
------
FC88X_00886_00893 ------------------TTTTTCCCTTCACT-AA---CCGCGA-A-CTGCA--
------
FC88X_00895_01192 ------------------TTTTTCCCTTCACT-AA---CCGCGA-A-CTGCA--
------
FC88X_00930_00781 ------------------TTTTTCCCTTCACT-AA---CCGCGA-A-CTGCA--
------
FC88X_00725_00451 ------------------TTTTTCCCTTCACT-AA---CCGCGA-A-CTGCA--
------
FC88X_00929_00576 ------------------TTTTTCCCTTCACT-AA---CCGCGA-A-CTGCA--
------
FC88X_00029_00126 -------------------TTTTCCCTTCACT-AA---CCGCGA-A-CTGCA--
------
FC88X_00216_00253 ------------------TTTTTCCCTTCACT-AA---CCGCGA-A-CTGCA--
------
FC88X_00116_00575 -------------------TTTTCCCTTCACT-AA---CCGCGA-A-CTGCA--
------
FC88X_00410_00922 ------------------TTTTTCCCTTCACT-AA---CCGCGA-A-CTGCA--
------
FC88X_00087_00086 -------------------TTTTCCCTTCACT-AA---CCGCGA-A-CTGCA--
------
FC88X_00098_00744 -------------------TTTTCCCTTCACT-AA---CCGCGA-A-CTGCA--
------
FC88X_00080_00371 -------------------TTTTCCCTTCACT-AA---CCGCGA-A-CTGCA--
------
FC88X_00697_00972 -------------------TTTTCCCTTCACT-AA---CCGCGA-A-CTGCA--
------
FC88X_00143_00024 -------------------TTTTCCCTTCACT-AA---CCGCGA-A-CTGCA--
------
FC88X_00690_00542 -------------------TTTTCCCTTCACT-AA---CCGCGA-A-CTGCA--
------
FC88X_00390_01150 -------------------TTTTCCCTTCACT-AA---CCGCGA-A-CTGCA--
------
FC88X_00390_01166 -------------------TTTTCCCTTCACT-AA---CCGCGA-A-CTGCA--
------
FC88X_00430_01184 -------------------TTTTCCCTTCACT-AA---CCGCGA-A-CTGCA--
------
FC88X_00590_00435 -------------------TTTTCCCTTCACT-AA---CCGCGA-A-CTGCA--
------
FC88X_00507_00048 -------------------TTTTCCCTTCACT-AA---CCGCGA-A-CTGCA--
------
FC88X_00517_00037 -------------------TTTTCCCTTCACT-AA---CCGCGA-A-CTGCA--
------

```
FC88X_00506_01158      -------------------TTTTCCCTTCACT-AA---CCGCGA-A-CTGCA--
------
FC88X_01119_00991      -------------------TTTTCCCTTCACT-AA---CCGCGA-A-CTGCA--
------
FC88X_00610_01124      -------------------TTTTCCCTTCACT-AA---CCGCGA-A-CTGCA--
------
FC88X_00614_01135      -------------------TTTTCCCTTCACT-AA---CCGCGA-A-CTGCA--
------
FC88X_00735_01178      -------------------TTTTCCCTTCACT-AA---CCGCGA-A-CTGCA--
------
FC88X_00020_00441      -------------------TTTTCCCTTCACT-AA---CCGCGA-A-CTGCA--
------
FC88X_00769_00987      -------------------TTTTCCCTTCACT-AA---CCGCGA-A-CTGCA--
------
FC88X_00773_00992      -------------------TTTTCCCTTCACT-AA---CCGCGA-A-CTGCA--
------
FC88X_01076_00466      -------------------TTTTCCCTTCACT-AA---CCGCGA-A-CTGCA--
------
FC88X_01063_00557      -------------------TTTTCCCTTCACT-AA---CCGCGA-A-CTGCA--
------
FC88X_01055_01093      -------------------TTTTCCCTTCACT-AA---CCGCGA-A-CTGCA--
------
FC88X_01080_01084      -------------------TTTTCCCTTCACT-AA---CCGCGA-A-CTGCA--
------
FC88X_00024_00087      -------------------TTTTCCCTTCACT-AA---CCGCGA-A-CTGCA--
------
FC88X_00015_00153      -------------------TTTTCCCTTCACT-AA---CCGCGA-A-CTGCA--
------
FC88X_00009_00220      ------------------TTTTTCCCTTCACT-AA---CCGCGA-A-CTGCA--
------
FC88X_00029_00420      -------------------TTTTCCCTTCACT-AA---CCGCGA-A-CTGCA--
------
FC88X_00029_00539      -------------------TTTTCCCTTCACT-AA---CCGCGA-A-CTGCA--
------
FC88X_00110_00930      ------------------TTTTTCCCTTCACT-AA---CCGCGA-A-CTGCA--
------
FC88X_00127_00013      -------------------TTTTCCCTTCACT-AA---CCGCGA-A-CTGCA--
------
FC88X_00099_00215      ------------------TTTTTCCCTTCACT-AA---CCGCGA-A-CTGCA--
------
FC88X_00234_00899      ------------------TTTTTCCCTTCACT-AA---CCGCGA-A-CTGCA--
------
FC88X_00287_00856      -------------------TTTTCCCTTCACT-AA---CCGCGA-A-CTGCA--
------
FC88X_00079_00647      -------------------TTTTCCCTTCACT-AA---CCGCGA-AC-TGCA--
------
FC88X_00097_00649      -------------------TTTTCCCTTCACT-AA---CCGCGA-AC-TGCA--
------
FC88X_00088_00556      -------------------TTTTCCCTTCACT-AA---CCGCGA-AC-TGCA--
------
FC88X_00067_00616      -------------------TTTTCCCTTCACT-AA---CCGCGA-AC-TGCA--
------
FC88X_00072_00512      -------------------TTTTCCCTTCACT-AA---CCGCGA-AC-TGCA--
------
FC88X_00093_00540      -------------------TTTTCCCTTCACT-AA---CCGCGA-AC-TGCA--
------
```

```
FC88X_00069_00423       -------------------TTTTCCCTTCACT-AA---CCGCGA-AC-TGCA--
------
FC88X_00098_00488       -------------------TTTTCCCTTCACT-AA---CCGCGA-AC-TGCA--
------
FC88X_00099_00158       -------------------TTTTCCCTTCACT-AA---CCGCGA-AC-TGCA--
------
FC88X_00052_00218       -------------------TTTTCCCTTCACT-AA---CCGCGA-AC-TGCA--
------
FC88X_00050_00126       -------------------TTTTCCCTTCACT-AA---CCGCGA-AC-TGCA--
------
FC88X_00058_00126       -------------------TTTTCCCTTCACT-AA---CCGCGA-AC-TGCA--
------
FC88X_00031_00305       -------------------TTTTCCCTTCACT-AA---CCGCGA-AC-TGCA--
------
FC88X_00015_00393       -------------------TTTTCCCTTCACT-AA---CCGCGA-AC-TGCA--
------
FC88X_00014_00287       -------------------TTTTCCCTTCACT-AA---CCGCGA-AC-TGCA--
------
FC88X_00045_00286       -------------------TTTTCCCTTCACT-AA---CCGCGA-AC-TGCA--
------
FC88X_00037_00201       -------------------TTTTCCCTTCACT-AA---CCGCGA-AC-TGCA--
------
FC88X_00049_00211       -------------------TTTTCCCTTCACT-AA---CCGCGA-AC-TGCA--
------
FC88X_01145_00892       -------------------TTTTCCCTTCACT-AA---CCGCGA-AC-TGCA--
------
FC88X_01103_01143       -------------------TTTTCCCTTCACT-AA---CCGCGA-AC-TGCA--
------
FC88X_01107_00674       -------------------TTTTCCCTTCACT-AA---CCGCGA-AC-TGCA--
------
FC88X_01115_00691       -------------------TTTTCCCTTCACT-AA---CCGCGA-AC-TGCA--
------
FC88X_01054_00935       -------------------TTTTCCCTTCACT-AA---CCGCGA-AC-TGCA--
------
FC88X_01053_00958       -------------------TTTTCCCTTCACT-AA---CCGCGA-AC-TGCA--
------
FC88X_01071_00417       -------------------TTTTCCCTTCACT-AA---CCGCGA-AC-TGCA--
------
FC88X_01078_00403       -------------------TTTTCCCTTCACT-AA---CCGCGA-AC-TGCA--
------
FC88X_00796_01073       -------------------TTTTCCCTTCACT-AA---CCGCGA-AC-TGCA--
------
FC88X_00763_01145       -------------------TTTTCCCTTCACT-AA---CCGCGA-AC-TGCA--
------
FC88X_00775_00924       -------------------TTTTCCCTTCACT-AA---CCGCGA-AC-TGCA--
------
FC88X_00766_00966       -------------------TTTTCCCTTCACT-AA---CCGCGA-AC-TGCA--
------
FC88X_00790_00195       -------------------TTTTCCCTTCACT-AA---CCGCGA-AC-TGCA--
------
FC88X_00913_00990       -------------------TTTTCCCTTCACT-AA---CCGCGA-AC-TGCA--
------
FC88X_00661_01131       -------------------TTTTCCCTTCACT-AA---CCGCGA-AC-TGCA--
------
FC88X_00682_01220       -------------------TTTTCCCTTCACT-AA---CCGCGA-AC-TGCA--
------
```

```
FC88X_00632_01183       -------------------TTTTCCCTTCACT-AA---CCGCGA-AC-TGCA--
------
FC88X_00681_00301       -------------------TTTTCCCTTCACT-AA---CCGCGA-AC-TGCA--
------
FC88X_00066_00339       ----------------ATTTTTTCCCTTCACT-AA---CCGCGA-AC-TGCA--
------
FC88X_00792_00121       -----------------TTTTTTCCCTTCACT-AA---CCGCGA-AC-TGCA--
------
FC88X_01002_00436       -----------------TTTTTTCCCTTCACT-AA---CCGCGA-AC-TGCA--
------
FC88X_00047_00574       -----------------TTTTTTCCCTTCACT-AA---CCGCGA-AC-TGCA--
------
FC88X_00672_00845       -----------------TTTTTTCCCTTCACT-AA---CCGCGA-AC-TGCA--
------
FC88X_00694_01166       -----------------TTTTTTCCCTTCACT-AA---CCGCGA-AC-TGCA--
------
FC88X_00152_00028       -----------------TTTTTTCCCTTCACT-AA---CCGCGA-AC-TGCA--
------
FC88X_01005_00929       -----------------TTTTTTCCCTTCACT-AA---CCGCGA-AC-TGCA--
------
FC88X_00990_00380       -----------------TTTTTTCCCTTCACT-AA---CCGCGA-AC-TGCA--
------
FC88X_00498_00486       -----------------TTTTTTCCCTTCACT-AA---CCGCGA-AC-TGCA--
------
FC88X_00943_00724       -----------------TTTTTTCCCTTCACT-AA---CCGCGA-AC-TGCA--
------
FC88X_00878_00172       -----------------TTTTTTCCCTTCACT-AA---CCGCGA-AC-TGCA--
------
FC88X_01029_01202       -------------------TTTTCCCTTCACT-AA---CCGCGA-AC-TGCA--
------
FC88X_00570_00989       -------------------TTTTCCCTTCACT-AA---CCGCGA-AC-TGCA--
------
FC88X_00870_01112       ------------------TTTTTCCCTTCACT-AA---CCGCGA-AC-TGCA--
------
FC88X_00926_00715       ------------------TTTTTCCCTTCACT-AA---CCGCGA-AC-TGCA--
------
FC88X_00661_00458       ------------------TTTTTCCCTTCACT-AA---CCGCGA-AC-TGCA--
------
FC88X_00918_00447       ------------------TTTTTCCCTTCACT-AA---CCGCGA-AC-TGCA--
------
FC88X_00996_01275       ------------------TTTTTCCCTTCACT-AA---CCGCGA-AC-TGCA--
------
FC88X_00023_00502       -------------------TTTTCCCTTCACT-AA---CCGCGA-AC-TGCA--
------
FC88X_00435_01077       ------------------TTTTTCCCTTCACT-AA---CCGCGA-AC-TGCA--
------
FC88X_00612_00083       ------------------TTTTTCCCTTCACT-AA---CCGCGA-AC-TGCA--
------
FC88X_00035_00605       -------------------TTTTCCCTTCACT-AA---CCGCGA-AC-TGCA--
------
FC88X_00499_00445       -------------------TTTTCCCTTCACT-AA---CCGCGA-AC-TGCA--
------
FC88X_00080_00275       -------------------TTTTCCCTTCACT-AA---CCGCGA-AC-TGCA--
------
FC88X_00073_00473       -------------------TTTTCCCTTCACT-AA---CCGCGA-AC-TGCA--
------
```

FC88X_00098_00764 -------------------TTTTCCCTTCACT-AA---CCGCGA-AC-TGCA--
------
FC88X_00089_00896 -------------------TTTTCCCTTCACT-AA---CCGCGA-AC-TGCA--
------
FC88X_00251_01030 -------------------TTTTCCCTTCACT-AA---CCGCGA-AC-TGCA--
------
FC88X_00470_01001 -------------------TTTTCCCTTCACT-AA---CCGCGA-AC-TGCA--
------
FC88X_00415_00818 -------------------TTTTCCCTTCACT-AA---CCGCGA-AC-TGCA--
------
FC88X_01031_01164 -------------------TTTTCCCTTCACT-AA---CCGCGA-AC-TGCA--
------
FC88X_00499_00966 -------------------TTTTCCCTTCACT-AA---CCGCGA-AC-TGCA--
------
FC88X_00953_00969 -------------------TTTTCCCTTCACT-AA---CCGCGA-AC-TGCA--
------
FC88X_00529_00731 -------------------TTTTCCCTTCACT-AA---CCGCGA-AC-TGCA--
------
FC88X_00533_00750 -------------------TTTTCCCTTCACT-AA---CCGCGA-AC-TGCA--
------
FC88X_00557_00813 -------------------TTTTCCCTTCACT-AA---CCGCGA-AC-TGCA--
------
FC88X_00050_00560 -------------------TTTTCCCTTCACT-AA---CCGCGA-AC-TGCA--
------
FC88X_00557_01118 -------------------TTTTCCCTTCACT-AA---CCGCGA-AC-TGCA--
------
FC88X_00565_01108 -------------------TTTTCCCTTCACT-AA---CCGCGA-AC-TGCA--
------
FC88X_00597_00088 -------------------TTTTCCCTTCACT-AA---CCGCGA-AC-TGCA--
------
FC88X_00591_00165 -------------------TTTTCCCTTCACT-AA---CCGCGA-AC-TGCA--
------
FC88X_00540_00871 -------------------TTTTCCCTTCACT-AA---CCGCGA-AC-TGCA--
------
FC88X_00522_00974 -------------------TTTTCCCTTCACT-AA---CCGCGA-AC-TGCA--
------
FC88X_00535_00205 -------------------TTTTCCCTTCACT-AA---CCGCGA-AC-TGCA--
------
FC88X_00547_00584 -------------------TTTTCCCTTCACT-AA---CCGCGA-AC-TGCA--
------
FC88X_00477_01049 -------------------TTTTCCCTTCACT-AA---CCGCGA-AC-TGCA--
------
FC88X_00468_01065 -------------------TTTTCCCTTCACT-AA---CCGCGA-AC-TGCA--
------
FC88X_00467_00014 -------------------TTTTCCCTTCACT-AA---CCGCGA-AC-TGCA--
------
FC88X_00470_00176 -------------------TTTTCCCTTCACT-AA---CCGCGA-AC-TGCA--
------
FC88X_00419_01131 -------------------TTTTCCCTTCACT-AA---CCGCGA-AC-TGCA--
------
FC88X_00431_01131 -------------------TTTTCCCTTCACT-AA---CCGCGA-AC-TGCA--
------
FC88X_00442_00059 -------------------TTTTCCCTTCACT-AA---CCGCGA-AC-TGCA--
------
FC88X_00422_00123 -------------------TTTTCCCTTCACT-AA---CCGCGA-AC-TGCA--
------

```
FC88X_00263_01127      -------------------TTTTCCCTTCACT-AA---CCGCGA-AC-TGCA--
------
FC88X_00030_00558      -------------------TTTTCCCTTCACT-AA---CCGCGA-AC-TGCA--
------
FC88X_00128_00322      -------------------TTTTCCCTTCACT-AA---CCGCGA-AC-TGCA--
------
FC88X_00116_00373      -------------------TTTTCCCTTCACT-AA---CCGCGA-AC-TGCA--
------
FC88X_00070_00879      -------------------TTTTCCCTTCACT-AA---CCGCGA-AC-TGCA--
------
FC88X_00106_00209      -------------------TTTTCCCTTCACT-AA---CCGCGA-AC-TGCA--
------
FC88X_00051_00472      -------------------TTTTCCCTTCACT-AA---CCGCGA-AC-TGCA--
------
FC88X_00573_00649      -------------------TTTTCCCTTCACT-AA---CCGCGA-AC-TGCA--
------
FC88X_00090_00239      -------------------TTTTCCCTTCACT-AA---CCGCGA-AC-TGCA--
------
FC88X_00089_00329      -------------------TTTTCCCTTCACT-AA---CCGCGA-AC-TGCA--
------
FC88X_00078_00026      -------------------TTTTCCCTTCACT-AA---CCGCGA-AC-TGCA--
------
FC88X_00090_00016      -------------------TTTTCCCTTCACT-AA---CCGCGA-AC-TGCA--
------
FC88X_00033_00526      -------------------TTTTCCCTTCACT-AA---CCGCGA-AC-TGCA--
------
FC88X_00938_00903      -------------------TTTTCCCTTCACT-AA---CCGCGA-AC-TGCA--
------
FC88X_00765_00618      -------------GTGATTTTTTCCCTTCACT-AA---CCGCGA-AC-
TGCACC------
FC88X_00888_00922      -----------------TTTTTTCCCTTCACT-AA---CCGCGA-AC-
TGCACC------
FC88X_00885_00730      -----------------TTTTTTCCCTTCACT-AA---CCGCGA-AC-TGCA--
------
FC88X_01075_00855      -----------------TTTTTTCCCTTCACT-AA---CCGCGA-AC-TGCA--
------
FC88X_00660_00379      -----------------TTTTTTCCCTTCACT-AA---CCGCGA-AC-TGCA--
------
FC88X_00144_00617      -----------------TTTTTTCCCTTCACT-AA---CCGCGA-AC-TGCA--
------
FC88X_00188_00634      -----------------TTTTTTCCCTTCACT-AA---CCGCGA-AC-TGCA--
------
FC88X_00746_00850      -----------------TTTTTTCCCTTCACT-AA---CCGCGA-AC-TGCA--
------
FC88X_00644_00493      -----------------TTTTTTCCCTTCACT-AA---CCGCGA-AC-TGCA--
------
FC88X_01019_00519      -----------------TTTTTTCCCTTCACT-AA---CCGCGA-AC-TGCA--
------
FC88X_00835_00177      -----------------TTTTTTCCCTTCACT-AA---CCGCGA-AC-TGCA--
------
FC88X_00302_00167      -----------------TTTTTTCCCTTCACT-AA---CCGCGA-AC-TGCA--
------
FC88X_00883_00949      -----------------TTTTTTCCCTTCACT-AA---CCGCGA-AC-TGCA--
------
FC88X_00985_01130      -------------------TTTTCCCTTCACT-AA---CCGCGA-AC-TGCA--
------
```

FC88X_00712_00111 -------------------TTTTCCCTTCACT-AA---CCGCGA-AC-TGCA--
------
FC88X_00859_00973 ------------------TTTTTCCCTTCACT-AA---CCGCGA-AC-TGCA--
------
FC88X_00922_00921 ------------------TTTTTCCCTTCACT-AA---CCGCGA-AC-TGCA--
------
FC88X_00894_01247 ------------------TTTTTCCCTTCACT-AA---CCGCGA-AC-TGCA--
------
FC88X_00608_01239 ------------------TTTTTCCCTTCACT-AA---CCGCGA-AC-TGCA--
------
FC88X_00914_00646 ------------------TTTTTCCCTTCACT-AA---CCGCGA-AC-TGCA--
------
FC88X_00098_00625 -------------------TTTTCCCTTCACT-AA---CCGCGA-AC-TGCA--
------
FC88X_01021_00600 ------------------TTTTTCCCTTCACT-AA---CCGCGA-AC-TGCA--
------
FC88X_00132_00988 -------------------TTTTCCCTTCACT-AA---CCGCGA-AC-TGCA--
------
FC88X_00020_00481 -------------------TTTTCCCTTCACT-AA---CCGCGA-AC-TGCA--
------
FC88X_00407_00405 -------------------TTTTCCCTTCACT-AA---CCGCGA-AC-TGCA--
------
FC88X_00185_00795 -------------------TTTTCCCTTCACT-AA---CCGCGA-AC-TGCA--
------
FC88X_00220_01038 -------------------TTTTCCCTTCACT-AA---CCGCGA-AC-TGCA--
------
FC88X_00895_01195 -------------------TTTTCCCTTCACT-AA---CCGCGA-AC-TGCA--
------
FC88X_00045_00748 -------------------TTTTCCCTTCACT-AA---CCGCGA-AC-TGCA--
------
FC88X_00144_00239 -------------------TTTTCCCTTCACT-AA---CCGCGA-AC-TGCA--
------
FC88X_00668_01184 -------------------TTTTCCCTTCACT-AA---CCGCGA-AC-TGCA--
------
FC88X_00635_00468 -------------------TTTTCCCTTCACT-AA---CCGCGA-AC-TGCA--
------
FC88X_00606_00272 -------------------TTTTCCCTTCACT-AA---CCGCGA-AC-TGCA--
------
FC88X_00643_00230 -------------------TTTTCCCTTCACT-AA---CCGCGA-AC-TGCA--
------
FC88X_00635_00235 -------------------TTTTCCCTTCACT-AA---CCGCGA-AC-TGCA--
------
FC88X_00634_00089 -------------------TTTTCCCTTCACT-AA---CCGCGA-AC-TGCA--
------
FC88X_00624_00094 -------------------TTTTCCCTTCACT-AA---CCGCGA-AC-TGCA--
------
FC88X_00591_01207 -------------------TTTTCCCTTCACT-AA---CCGCGA-AC-TGCA--
------
FC88X_00579_01205 -------------------TTTTCCCTTCACT-AA---CCGCGA-AC-TGCA--
------
FC88X_00733_00991 -------------------TTTTCCCTTCACT-AA---CCGCGA-AC-TGCA--
------
FC88X_00604_00753 -------------------TTTTCCCTTCACT-AA---CCGCGA-AC-TGCA--
------
FC88X_00729_00072 -------------------TTTTCCCTTCACT-AA---CCGCGA-AC-TGCA--
------

FC88X_01013_00294 -------------------TTTTCCCTTCACT-AA---CCGCGA-AC-TGCA--
------
FC88X_00961_01056 -------------------TTTTCCCTTCACT-AA---CCGCGA-AC-TGCA--
------
FC88X_00980_01042 -------------------TTTTCCCTTCACT-AA---CCGCGA-AC-TGCA--
------
FC88X_00991_00888 -------------------TTTTCCCTTCACT-AA---CCGCGA-AC-TGCA--
------
FC88X_00967_00860 -------------------TTTTCCCTTCACT-AA---CCGCGA-AC-TGCA--
------
FC88X_00963_00883 -------------------TTTTCCCTTCACT-AA---CCGCGA-AC-TGCA--
------
FC88X_00977_00812 -------------------TTTTCCCTTCACT-AA---CCGCGA-AC-TGCA--
------
FC88X_01142_00831 -------------------TTTTCCCTTCACT-AA---CCGCGA-AC-TGCA--
------
FC88X_01117_00841 -------------------TTTTCCCTTCACT-AA---CCGCGA-AC-TGCA--
------
FC88X_01128_00719 -------------------TTTTCCCTTCACT-AA---CCGCGA-AC-TGCA--
------
FC88X_01143_00668 -------------------TTTTCCCTTCACT-AA---CCGCGA-AC-TGCA--
------
FC88X_01134_00656 -------------------TTTTCCCTTCACT-AA---CCGCGA-AC-TGCA--
------
FC88X_01130_00659 -------------------TTTTCCCTTCACT-AA---CCGCGA-AC-TGCA--
------
FC88X_01113_00686 -------------------TTTTCCCTTCACT-AA---CCGCGA-AC-TGCA--
------
FC88X_01137_00565 -------------------TTTTCCCTTCACT-AA---CCGCGA-AC-TGCA--
------
FC88X_01097_01193 -------------------TTTTCCCTTCACT-AA---CCGCGA-AC-TGCA--
------
FC88X_01084_01190 -------------------TTTTCCCTTCACT-AA---CCGCGA-AC-TGCA--
------
FC88X_01081_01160 -------------------TTTTCCCTTCACT-AA---CCGCGA-AC-TGCA--
------
FC88X_00297_00703 -------------GTGATTTTT-CCCTTCACT-AA---CCGCGA-AC-TGCA--
------
FC88X_00986_01132 -------------GTGATTTTT-CCCTTCACT-AA---CCGCGA-AC-TGCA--
------
FC88X_01122_01127 ------------GGTGATTTTTTCCCTTCACT-AA---CCGCGA-AC-TGCA--
------
FC88X_00162_00662 -------------GTGATTTTTTCCCTTCACT-AA---CCGCGA-AC-TGCA--
------
FC88X_00708_01217 ------------------TTTTTCCCTTCACT-AA---CCGCGA-AC-TGCA--
------
FC88X_00780_00181 ------------------TTTTTCCCTTCACT-AA---CCGCGA-AC-TGCA--
------
FC88X_00992_00693 -------------------TTTTCCCTTCACT-AA---CCGCGA-AC-TGCA--
------
FC88X_00954_00831 -------------------TTTTCCCTTCACT-AA---CCGCGA-AC-TGCA--
------
FC88X_01086_01060 -------------------TTTTCCCTTCACT-AA---CCGCGA-AC-TGCA--
------
FC88X_01058_01137 -------------------TTTTCCCTTCACT-AA---CCGCGA-AC-TGCA--
------

```
FC88X_00963_00809	-------------------TTTTCCCTTCACT-AA---CCGCGA-AC-TGCA--
------
FC88X_00927_01014	-------------------TTTTCCCTTCACT-AA---CCGCG--AACTGCAT-
------
FC88X_00399_00076	-------------------TTTTCCCTTCACT-AA---CCGCG--AACTGCAT-
------
FC88X_00620_01186	------------------TTTTTCCCTTCACT-AA---CCGCG--AACTGCAT-
------
FC88X_00721_01072	------------------TTTTTCCCTTCACT-AA---CCGCG--AACTGCAT-
------
FC88X_00501_00164	------------------TTTTTCCCTTCACT-AA---CCGCG--AACTGCAT-
------
FC88X_00505_00563	------------------TTTTTCCCTTCACT-AA---CCGCG--AACTGCAT-
------
FC88X_00300_00063	------------------TTTTTCCCTTCACT-AA---CCGCG--AACTGCAT-
------
FC88X_00918_00820	-----------------TTTTTTCCCTTCACT-AA---CCGCG--AACTGCAT-
------
FC88X_01074_00837	-------------------TTTTCCCTTCACT-AA---CCGCG--AACTGCA--
------
FC88X_00022_00317	-------------------TTTTCCCTTCACT-AA---CCGCG--AACTGCA--
------
FC88X_00951_00682	-------------------TTTTCCCTTCACT-AA---CCGCG--AACTGCA--
------
FC88X_00991_00676	-------------------TTTTCCCTTCACT-AA---CCGCG--AACTGCA--
------
FC88X_00032_00663	------------------TTTTTCCCTTCACT-AA---CCGCG--AACCGCA--
------
FC88X_00038_00755	------------------TTTTTCCCTTCACT-AA---CCGCG--AACCGCA--
------
FC88X_00854_00952	------------------TTTTTCCCTTCACT-AA---CCGCG--AACCGCA--
------
FC88X_01035_01028	------------------TTTTTCCCTTCACT-AA---CCGCG--AACCGCA--
------
FC88X_00090_00322	------------------TTTTTCCCTTCACT-AA---CCGCG--AACCGCA--
------
FC88X_00477_01174	-------------------TTTTCCCTTCACT-AA---CCGCG--AACCGCA--
------
FC88X_00993_00762	-------------------TTTTCCCTTCACT-AA---CCGCG--AACCGCA--
------
FC88X_00189_00794	------------------TTTTTCCCTTCACT-AA---CCGCG--AACTGCGA-
------
FC88X_00299_01081	-------------------TTTTCCCTTCACT-AA---CCGCG--AACTGCG--
------
FC88X_00315_00341	------------------TTTTTCCCTTCACT-AA---CCGCG--AACTGCGC-
------
FC88X_00868_00770	------------------TTTTTCCCTTCACT-AG---CCGCG--
AACTGCTCA------
FC88X_01011_01205	------------------TTTTTCCCTTCACT-AA---CCGCG--
AACTGCTCA------
FC88X_00993_00780	------------------TTTTTCCCTTCACT-AA---CCGCG--AACTGCT--
------
FC88X_00598_00805	------------------TTTTTCCCTTCACT-AA---CCGCG--AACTG----
------
FC88X_00648_00102	--------------------TTTCCCTTCACT-AA---CCGCG--AACTGCCA-
------
```

FC88X_00908_01146 -------------------TTTTCCCTTCACTTAA---CCGCGT-AACTGCCA-
------
FC88X_00372_01196 ------------------TTTTTCCCTTCACT-AA---CCGCG--AACTGCC--
------
FC88X_00060_00036 -------------------TTTTCCCTTCACT-AA---CCGCG--AACTGA-
CTTCCTTT
FC88X_00346_01091 -------------------TTTTCCCTTCACT-AA---CCGCG--AACTTC-
CTTTCTCC
FC88X_00416_01001 -------------------TTTTCCCTTCACT-AA---CCGCG--
AACTGCACTTCCTTT
FC88X_01033_00340 -------------------TTTTCCCTTCACT-AA---CCGCG--
AACTGCACTTCCTTT
FC88X_00810_00950 -------------------TTTTCCCTTCACT-AA---CCGCG--AACT-----
------
FC88X_00226_00917 ------------------CTTAGCCTT-CCGT-CA---CCGCG--GACCTCCC-
------
FC88X_00269_01129 ------------------CTTAGCCTT-CCGT-CA---CCGCG--GACCTCCC-
------
FC88X_00546_01060 ------------------CTTAGCCTT-CCGT-CA---CCGCG--GACCTCCC-
------
FC88X_00187_01061 ------------------CTTAGCCTTTCCGT-CA---CCGCG--GAC------
------
FC88X_00259_00927 ------------------CTTTCCCTT--CGT-CC---CCGCG--CACACTGG-
------
FC88X_00065_00587 ------------------CTTTTTCCT-ATTC-CT---CCGCG--AAGTCTT--
------
FC88X_00099_00911 ------------------TTTTTCCCTTCACT-AA---CCGCG--AACTCGCA-
------
FC88X_00794_01056 -------------------TTTTCCCTTCACT-AA---CCGCG--AACTCGCA-
------
FC88X_00141_00760 ------------------TTTTTCCCTTCACT-AA---CCGCG--
AACTGGCAATTTT--
FC88X_00195_00815 -------------------TTTTCCCTTCACT-AA---CCGCG--AACTGTCA-
------
FC88X_00927_00564 -------------------TTTTCCCTTCACT-AA---CCGCG--AACTGTCA-
------
FC88X_00871_00895 ------------------TTTTTCCCTTCACT-AA---CCGCG--AACTGTCA-
------
FC88X_00092_00459 ------------------TTTTTCCCTTCACT-AA---CCGCG--AAC------
------
FC88X_01124_00530 ----------------TTTTTTTCCCTTCACT-AA---CCGCG--AA-------
------
FC88X_00684_01260 ----------------TCTTTTCCCATCCCGT-AT---CTCCG--AATT-----
------
FC88X_00471_00765 ------------GTGATTTTTTTCCCTTCACT-AA---CCGCG--AACTGCG--
------
FC88X_00197_00531 -----------------TTTTTTCCCTTCACT-AA---CCGCG--AACTG----
------
FC88X_01030_01253 -------------------TTTTCCCTTCACT-AA---CCGCG--AAC------
------
FC88X_00239_00963 ------------------TTTTTCCCTTCACT-AA---CCGCG--AACTGAA--
------
FC88X_00713_00398 -------------------TTTTCCCTTCACT-AA---CCGCG--AACTGCA--
------
FC88X_00986_00651 -------------------TTTTCCCTTCACT-AA---CCGCG--AACTGCA--
------

```
FC88X_00953_00831       -------------------TTTTCCCTTCACT-AA---CCGCG--AACTGCA--
------
FC88X_00931_01080       ------------------TTTTTCCCTTCAC-TAA---CC-ACG-AACTGCA--
------
FC88X_01126_00811       ------------------TTTTTCCCTTCAC-TAA---CC-ACG-AACTGCA--
------
FC88X_00029_00450       -------------------TTTTCCCTTCAC-TAA---CC-ACG-AACTGCA--
------
FC88X_01008_01206       -------------------TTTTCCCTTCAC-TAA---CC-ACG-AACTGCA--
------
FC88X_01103_00526       ------------------TTTTTCCCTTCACATAA---CCGACG-
AACTGCAAC------
FC88X_00912_00809       ------------------TTTTTCCCTTCAC-TAA---CCGACG-AACTGCA--
------
FC88X_00762_01115       ------------------TTTTTCCCTTCAC-TAA---CCGACG-AACTGCA--
------
FC88X_01050_00849       ------------------TTTTTCCCTTCAC-TAA---CCGACG-AACTGCA--
------
FC88X_00173_00309       ------------------TTTTTCCCTTCAC-TAA---CCGACG-AACTGCA--
------
FC88X_00766_00880       ------------------TTTTTCCCTTCAC-TAA---CCGACG-AACTGCA--
------
FC88X_00817_00334       ------------------TTTTTCCCTTCAC-TAA---CC-GCG-AACTGCA--
------
FC88X_00743_01155       ------------------TTTTTCCCTTCAC-TAA---CC-GCG-AACTGCA--
------
FC88X_00753_00981       ------------------TTTTTCCCTTCAC-TAA---CC-GCG-AACTGCA--
------
FC88X_00759_00256       ------------------TTTTTCCCTTCAC-TAA---CC-GCG-AACTGCA--
------
FC88X_00454_00123       ---------------GGATTTTTCCCTTCAC-TAA---CCGC-G-AACTGCA--
------
FC88X_00715_01264       --------------GGGATTTTTCCCTTCAC-TAA---CCGC-G-AACTGCA--
------
FC88X_00765_00129       ------------------TTTTTCCCTTCAC-TAA---CCGC-G-AACTGCA--
------
FC88X_00723_01072       ------------------TTTTTCCCTTCAC-TAA---CCGC-G-AACTGCA--
------
FC88X_00800_00841       ------------------TTTTTCCCTTCAC-TAA---CCGC-G-AACTGCA--
------
FC88X_01060_00407       -------------------TTTTCCCTTCAC-TAA---CCGC-G-AACTGCA--
------
FC88X_00367_00043       -----------------GTTTTTCCCTTCAC-TAA---CCGCCG-AACTGCA--
------
FC88X_00533_00142       ------------------TTTTTCCCTTCAC-TAA---CCGCCG-AACTGCA--
------
FC88X_00912_00916       ------------------TTTTTCCCTTCAC-TAA---CCGCCG-AACTGCA--
------
FC88X_00824_00919       ------------------TTTTTCCCTTCAC-TAA---CCGCCG-AACTGCA--
------
FC88X_00919_00556       ------------------TTTTTCCCTTCAC-TAA---CCGCCG-AACTGCA--
------
FC88X_01008_01199       ------------------TTTTTCCCTTCAC-TAA---CCGCCG-AACTGCA--
------
FC88X_00841_00285       ------------------TTTTTCCCTTCAC-TAA---CCGC-G-AACTGCA--
------
```

FC88X_00704_01117 ------------------TTTTTCCCTTCAC-TAA---CCGC-G-AACTGCA--
------
FC88X_00835_00814 ------------------TTTTTCCCTTCAC-TAA---CCGC-G-AACTGCA--
------
FC88X_01095_00439 -------------------TTTTCCCTTCAC-TAA---CCGC-G-AACTGCA--
------
FC88X_00751_00841 ------------------TTTTTCCCTTCAC-TAA---CCGC-G-AACTGCA--
------
FC88X_00734_00263 -----------------TTCTTTCCACTGACA-CA---CCGCAA-CT-TTCC--
------
FC88X_00925_00819 -----------------TTCTTTCCACTGACA-CA---CCGCAA-CT-TT----
------
FC88X_00754_00277 -----------------TTCTTTCCACTGACA-CA---CCGCAA-CT-TT----
------
FC88X_00912_00999 -----------------TTCTTTCC--CAGAA-CC---CCGCGA-TT-TACAC-
------
FC88X_00086_00609 -----------GGGGAATTTTTTCCCTTCACA-AA---CCGCGA-AC-TGCA--
------
FC88X_00471_00007 -----------------TTTTTTCCCTTCACA-AA---CCGCGA-AC-TGCA--
------
FC88X_00552_01231 ------------------TTTTTCCCTTCACA-AA---CCGCGA-AC-TGCA--
------
FC88X_00512_00968 -----------------GTTTTTTCCCTTCACA-AA---CCGCGA-AC-TGCA--
------
FC88X_00244_00944 ------------------TTTTTCCCTTCACA-AA---CCGCGA-AC-TGCA--
------
FC88X_00151_00187 ------------------TTTTTCCCTTCACA-AA---CCGCGA-AC-TGCA--
------
FC88X_01065_01192 ------------------TTTTTCCCTTCACA-AA---CCGCGA-AC-TGCA--
------
FC88X_00169_00987 -------------------TTTTCCCTTCACA-AA---CCGCGA-AC-TGCA--
------
FC88X_00995_00656 -------------------TTTTCCCTTCACA-AA---CCGCGA-AC-
TGCACC------
FC88X_00755_00686 -------------------TTTTCCCTTCACA-AA---CCGCGA-AC-TGCA--
------
FC88X_00389_01111 -------------------TTTTCCCTTCACA-AA---CCGCGA-AC-TGCA--
------
FC88X_01030_00782 -------------------TTTTCCCTTCACA--A---CCGCGA-AC-TGCA--
------
FC88X_00300_00959 ------------------TTTTTCCCTTCACATAA---CCGCGA-AC-TGCA--
------
FC88X_01096_00934 -------------------TTTTCCCTTCACT-AA---CCGCGA-AC-TGCA--
------
FC88X_00018_00659 -------------------TTTTCCCTTCACT-AA---CCGCGA-AC-TGCA--
------
FC88X_01027_01268 -------------------TTTTCCCTTCACT-AA---CCGCGA-AC-TGCA--
------
FC88X_00994_00677 -------------------TTTTCCCTTCACT-AA---CCGCGA-AC-TGCA--
------
FC88X_00980_00722 -------------------TTTTCCCTTCACTAA----CCGCGA-AC-TGCA--
------
FC88X_00975_00299 ---------TCCTTCTCCTTTTTCCCTTCACTAA----CCGCGA-AC-TGCA--
------
FC88X_00166_00459 -----------------CTTTTTCCCTTCACTAA----CCGCGA-AC-TGCA--
------

```
FC88X_00793_01257      -----------------CTTTTTCCCTTCACTAA----CCGCGA-AC-TGCA--
------
FC88X_00819_00394      ------------------TTTTTCCCTTCACTAA----CCGCGA-AC-TGCA--
------
FC88X_00705_01141      ------------------TTTTTCCCTTCACTAA----CCGCGA-AC-TGCA--
------
FC88X_00764_00913      ------------------TTTTTCCCTTCACTAA----CCGCGA-AC-TGCA--
------
FC88X_00966_00585      -------------------TTTTCCCTTCACTAA----CCGCGA-AC-TGCA--
------
FC88X_00838_01022      -----------------TCTTTTCCCTTCACT-AAC--CG-CGA-AC-TGCA--
------
FC88X_01019_00703      -----------------TCTTTTCCCTTCACT-AAC--CG-CGA-AC-TGCA--
------
FC88X_01078_00747      -----------------TCTTTTCCCTTCACT-AAC--CG-CGA-AC-TGCA--
------
FC88X_00771_01236      ------------------CTTTTCCCTTCACT-AAC--CG-CGA-AC-TGCA--
------
FC88X_01027_00851      ------------------CTTTTCCCTTCACT-AAC--CG-CGA-AC-TGCA--
------
FC88X_00974_01258      ------------------CTTTTCCCTTCACT-AAC--CG-CGA-AC-TGCA--
------
FC88X_00050_00794      ------------------CTTTTCCCTTCACT-AAC--CG-CGA-AC-TGCA--
------
FC88X_00030_00148      -------------------TTTTCCCTTCACT-AAC--CG-CGA-AC-TGCA--
------
FC88X_00145_00804      ------------GGGGAATTTTCCC-TTCACT-AAC--CG-CGA-AC-TGCA--
------
FC88X_00509_00633      -----------GGGGAACTTTTCCC-TTCACT-AAC--CG-CGA-AC-TGCA--
------
FC88X_00197_00139      ----------------AGTTTTCC--TTCACT-AAC--CG-CGA-AC-TGCA--
------
FC88X_00494_00212      -----------GGGGAAGTTTTCCCCTTCACT-AAC--CG-CGA-AC-TGCA--
------
FC88X_01139_00933      -----------GGGGAAGTTTTCCC-TTCACT-AAC--CG-CGA-AC-TGCA--
------
FC88X_00643_00912      -----------GGGGAAGTTTTCCC-TTCACT-AAC--CG-CGA-AC-TGCAC-
------
FC88X_00202_00851      ------------------TTTTCCC--TCACT-AAC--CG-CGA-AC-TGCA--
------
FC88X_00817_00139      -----------GGGGAAGTTTTTCCCTTCACT-GAC--CG-CGA-AC-TGCA--
------
FC88X_01027_00700      -----------------GTTTTTCCCTTCACT-GAC--CG-CGA-AC-TGCA--
------
FC88X_01008_00683      ------------------TTTTTCCCTTCACT-GAC--CG-CGA-AC-TGCA--
------
FC88X_00571_01109      ------------------TTTTTCCCTTCACT-GAC--CG-CGA-AC-TGCA--
------
FC88X_00939_00655      ------------------TTTTTCCCTTCACT-GAC--CG-CGA-AC-TGCA--
------
FC88X_00225_00018      -------------------TTTTCCCTTCACT-GAC--CG-CGA-AC-TGCA--
------
FC88X_00463_00112      -----------------TTTTTTCCCTTCACT-GAC--CG-CGA-AC-TGCA--
------
FC88X_00370_00032      -----------------TTTTTTCCCTTCACT-GAC--CG-CGA-AC-TGCA--
------
```

```
FC88X_00674_00933      -----------------TTTTTTCCCTTCACT-GAC--CG-CGA-AC-TGCA--
------
FC88X_00746_00660      ------------------TTTTTCCCTTCACT-GAC--CG-CGA-AC-TGCA--
------
FC88X_00261_01082      ------------------TTTTTCCCTTCACT-GAC--CG-CGA-AC-TGCA--
------
FC88X_00454_01102      -------------------TTTTCCCTTCACT-GAC--CG-CGA-AC-TGCA--
------
FC88X_00528_01134      -------------------TTTTCCCTTCACT-GAC--CG-CGA-AC-TGCA--
------
FC88X_00731_00342      -------------------TTTTCCCTTCACT-GAC--CG-CGA-AC-TGCA--
------
FC88X_01005_00523      -------------------TTTTCCCTTCACT-GAC--CG-CGA-AC-TGCA--
------
FC88X_00453_00103      -------------------TTTTCCCTTCACT-GAC--CG-CGA-AC-TGCA--
------
FC88X_00706_00412      -------------------TTTTCCCTTCACTAGAC--CG-CGA-AC-TGCA--
------
FC88X_00525_00117      ------------------GTTTTCCCTTCACT-AAC--CG-CGA-AC-
TGCACC------
FC88X_00809_01170      -------------------TTTTCCCTTCACT-AAC--CG-CGA-AC-
TGCACC------
FC88X_00769_01035      -------------------TTTTCCCTTCACT-AAC--CG-CGA-AC-
TGCACC------
FC88X_00723_00058      -------------------TTTTCCCTTCACT-AAC--CG-CGA-AC-
TGCACC------
FC88X_00777_00148      -------------------TTTTCCCTTCACT-AAC--CG-CGA-AC-
TGCACC------
FC88X_00912_00825      -------------------TTTTCCCTTCACT-AAC--CG-CGA-AC-
TGCACC------
FC88X_00147_00340      -------------------TTTTCCCTTCACT-AAC--CG-CGA-AC-
TGCACC------
FC88X_00785_00310      -------------------TTTTCCCTTCACT-AAC--CG-CGA-AC-TGCAC-
------
FC88X_00994_00489      -------------------TTTTCCCTTCACT-AAC--CG-CGA-AC-
TGCACTC-----
FC88X_00598_00127      -------------------TTTTCCCTTCACT-AAC--CG-CGA-AC-
TGCACTTCCTTT
FC88X_00616_01063      -------------------TTTTCCCTTCA-T-AAC--CG-CGA-AC-
TGCACTTCCCT-
FC88X_00994_01168      -------------------TTTTCCCTTCACT-AAC--CG-CGA-AC-
TGCACTTCCTTT
FC88X_00488_00658      -------------------TTTTCCCTTCACT-AAC--CG-CGA-AC-
TGCACTTCCTTT
FC88X_00028_00093      -------------------TTTTCCCTTCACT-AAC--CG-CGA-AC-
TGCACT------
FC88X_00845_00119      -------------------TTTTCCCTTCACT-AAC--CG-CGA-AC-
TGCACTTCCT--
FC88X_00421_00994      -------------------TTTTCCCTTCACT-AAC--CG-CGA-AC-TGCAC-
------
FC88X_00914_00448      -------------------TTTTCCCTTCACT-AAC--CGGCGA-AC-TGCAC-
------
FC88X_01106_00504      -------------------TTTTCCCTTCACT-AAC--CG-CGA-AC-TGCAC-
------
FC88X_00826_00314      --------------------TTTTCCCTCACT-AAC--CG-CGA-AC-TGCA--
------
```

FC88X_00821_00871 -------------------TTTTCCCTTCACT-AAC--CGCGTA--C-TGCA--
------
FC88X_00592_00923 -----------------TTTACCCTCTCAACTGAAC--CGCGTA-AC-
TGCACT------
FC88X_00955_01091 -------------------TTTTCCCTTCACT-AAC--CGCGTA-AC-TGCAC-
------
FC88X_01052_00668 -------------------TTTTCCCTTCACT-AAC--CGC-TA-AC-TGCA--
------
FC88X_00042_00104 -------------------TTTTCCCTTCACT-AAC--CG-CGA-AC-TGCA--
------
FC88X_01053_00606 -------------------TTTTCCCTTCACT-AAC--CG-CGA-AC-TGCA--
------
FC88X_00734_01040 -----------------TTTTTTCCCTCCACT-AAC--CG-CGA-AC-TGCA--
------
FC88X_01091_00761 ------------------TTTTTCCCTCCACT-AAC--CG-CGA-AC-TGCA--
------
FC88X_01080_00761 -------------------TTTTCCCTCCACT-AAC--CG-CGA-AC-TGCA--
------
FC88X_00649_00415 ----------------TTTTACCTTTCCTACT-AAC--CG-CGA-AC-TGCA--
------
FC88X_00841_00225 ------------------TTTTCCCTCCTACT-AAC--CG-CGA-AC-TGCA--
------
FC88X_01071_00541 -------------------TTTTCCCTTCACT-AAC--CG-CGA-AC-TGCA--
------
FC88X_00642_00235 -------------------TTTTCCCTTCACTAA----CCGCGA-AC-TGTA--
------
FC88X_01066_00842 ------------------TTTTTCCCTTCACTAA----CCGCGA-AC-TGTA--
------
FC88X_01021_00732 -------------------TTTTCCCTTCACTAA----CCGCGA-AC-TGTA--
------
FC88X_00061_00305 -------------------TTTTCCCTTCACTAA----CCGCGA-AC-TGTA--
------
FC88X_00061_00672 -------------------TTTTCCCTTCACTAA----CCGCGA-AC-TGTA--
------
FC88X_00432_00016 -------------------TTTTCCCTTCACTAA----CCGCGA-AC-TGTA--
------
FC88X_00904_00633 -------------------TTTTCCCTTCACTAA----CCGCGA-AC-TGTA--
------
FC88X_00034_00042 -------------------TTCTCCCTT-ACTAA----CCGTGA-AC-TGTA--
------
FC88X_00877_00787 -------------------TTCTCCCTTCACTAAAA--CCGCGA-AC-TGCA--
------
FC88X_00784_00203 -------------------TTTTCCCTTCACTAAC---CGTCGA-AC-TGCA--
------
FC88X_00219_00899 -----------------TTTTTTCCCTTCACTAAC---CGT-GA-AC-TGCA--
------
FC88X_01095_01143 ------------------TTTTTCCCTTCACTAAC---CGT-GA-AC-TGCA--
------
FC88X_00889_00365 ------------------TTTTTCCCTTCACTAAC---CGT-GA-AC-TGCA--
------
FC88X_00768_01275 ----------------TTTTTTTCCCTTCACTAAC---CGTCGA-AC-TGCA--
------
FC88X_00086_00704 ------------------TTTTTCCCTTCACTAAC---C-TCGA-AC-TGCA--
------
FC88X_01120_00730 ------------------TTTTTCCCTTCACTAAC---CGTCGA-AC-TGCA--
------

FC88X_00009_00161 -------------------TTTTCCCTTCACTAA----CCGCGA-AC-TGCA--
------
FC88X_00041_00360 -------------------CTTTCCCTTCACTAAC---CG-CGA-AC-TGCA--
------
FC88X_00630_00105 -------------------CTTTCCCTTCACTAAC---CGTCGA-AC-TGCA--
------
FC88X_00076_00459 -------------------CTTTCCCTTCACTAAC---CG-CGA-AC-TGCA--
------
FC88X_00432_00372 ------------------TCTTTCCCTTCACTAAC---C-GCGA-AC-TGCA--
------
FC88X_00504_00706 ------------------TCTTTCCCTTCACTAAC---CCGCGA-ACCTGCA--
------
FC88X_00913_00860 ------------------TCTTTCCCTTCACTAAC---C-GCGA-AC-TGCA--
------
FC88X_01031_00494 ------------------TCTTTCCCTTCACTAAC---C-GCGA-AC-TGCA--
------
FC88X_00951_00878 ------------------TCTTTCCCTTCACTAAC---C-GCGA-AC-TGCAC-
------
FC88X_01014_01030 ------------------TCTTTCCCTTCACTAAC---C-GCGA-AC-TGCA--
------
FC88X_00747_01118 ------------------TCTTTCCCTTCACTAAC---C-GCGA-AC-TGCA--
------
FC88X_00977_00594 -----------------GTCTTTCCCTTCACTAAC---C-GCGA-AC-TGCA--
------
FC88X_00268_01005 ------------------TCTTTCCCTTCACTAAC---C-GCGA-AC-TGCA--
------
FC88X_00577_01231 -------------------GTTTCCCTTCACTAAC---C-GCGA-AC-TGCA--
------
FC88X_00266_00988 ------------------CGTTTCCCTTCACTAAC---C-GCGA-AC-TGCA--
------
FC88X_00772_00522 --------------------TTTCCCTTCACTAAC---C-GCGA-AC-TGCA--
------
FC88X_00656_01240 --------------------TTTCCCTTCACTAAC---C-GCGA-AC-TGCA--
------
FC88X_00047_00530 ----------------GTTTTCCCCCTTCACTAAC---CG-CGA-AC-TGCA--
------
FC88X_00996_01027 ------------------GTTTTCCCTTCACTA-C---CG-CGA-AC-TGCA--
------
FC88X_00069_00357 ------------------GTTTTCCCTTCACTAAC---CG-CGA-AC-TGCA--
------
FC88X_00131_00783 ------------------GTTTTCCCTTCACTAAC---CG-CGA-AC-TGCA--
------
FC88X_00176_00829 ------------------GTTTTCCCTTCACTAAC---CGTCGA-AC-TGCA--
------
FC88X_00161_00826 ------------------GTTTTCCCTTCACTAAC---CG-CGA-AC-TGCA--
------
FC88X_00290_01001 ------------------GTTTTCCCTTCACTAAC---CG-CGA-AC-TGCA--
------
FC88X_00380_01193 ------------------GTTTTCCCTTCACTAAC---CG-CGA-AC-TGCA--
------
FC88X_00416_01104 ------------------GTTTTCCCTTCACTAAC---CG-CGA-AC-TGCA--
------
FC88X_00436_01140 ------------------GTTTTCCCTTCACTAAC---CG-CGA-AC-TGCA--
------
FC88X_00532_01116 ------------------GTTTTCCCTTCACTAAC---CG-CGA-AC-TGCA--
------

FC88X_01008_01044 ------------------GTTTTCCCTTCACTAAC---CG-CGA-AC-TGCA--
------
FC88X_01051_01268 ------------------GTTTTCCCTTCACTAAC---CG-CGA-AC-TGCA--
------
FC88X_01003_00593 ------------------GTTTTCCCTTCACTAAC---CG-CGA-AC-TGCA--
------
FC88X_00723_01007 ------------------GTTTTCCCTTCACTAAC---CG-CGA-AC-TGCA--
------
FC88X_00797_00886 ------------------GTTTTCCCTTCACTAAC---CG-CGA-AC-TGCA--
------
FC88X_00783_01211 ------------------GTTTTCCCTTCACTAAC---CG-CGA-AC-TGCA--
------
FC88X_00823_00846 ------------------GTTTTCCCTTCACTAAC---CG-CGA-AC-TGCA--
------
FC88X_00933_00431 ------------------GTTTTCCCTTCACTAAC---CG-CGA-AC-TGCA--
------
FC88X_00912_00777 ------------------GTTTTCCCTTCACTAAC---CG-CGA-AC-TGCA--
------
FC88X_00993_00759 ------------------GTTTTCCCTTCACTAAC---CG-CGA-AC-TGCA--
------
FC88X_00983_01045 ------------------GTTTTCCCTTCACTAAC---CG-CGA-AC-TGCA--
------
FC88X_00984_01051 ------------------GTTTTCCCTTCACTAAC---CG-CGA-AC-TGCA--
------
FC88X_01072_00732 ------------------GTTTTCCCTTCACTAAC---CG-CGA-AC-TGCA--
------
FC88X_01138_00553 ------------------GTTTTCCCTTCACTAAC---CG-CGA-AC-TGCA--
------
FC88X_01130_00623 ------------------GTTTTCCCTTCACTAAC---CG-CGA-AC-TGCA--
------
FC88X_01138_00630 ------------------GTTTTCCCTTCACTAAC---CG-CGA-AC-TGCA--
------
FC88X_01115_00848 ------------------GTTTTCCCTTCACTAAC---CG-CGA-AC-TGCA--
------
FC88X_01112_01184 ------------------GTTTTCCCTTCACTAAC---CG-CGA-AC-TGCA--
------
FC88X_01120_01177 ------------------GTTTTCCCTTCACTAAC---CG-CGA-AC-TGCA--
------
FC88X_01146_01208 ------------------GTTTTCCCTTCACTAAC---CG-CGA-AC-TGCA--
------
FC88X_01041_00775 ------------------GTTTTCCCTTCACTAAC---CG-CGA-AC-TGCA--
------
FC88X_00021_00641 ------------------TTTTCCCGTTCACTAAC---CGCCGA-AC-TGCA--
------
FC88X_00461_01079 --------------------TTTCCCTTCACTAAC---CGCCGA-AC-TGCA--
------
FC88X_00205_00933 -------------------TTTTCCCTTCACTAAC---CGCCGA-AC-TGCA--
------
FC88X_00146_00509 ------------------GTTTTCCCTTCACTAAC---CGCCGA-AC-TGCA--
------
FC88X_00710_00118 --------------------TTTCCCTTCACTAAC---CG-CGA-AC-TGCA--
------
FC88X_00249_00944 -----------------TATTTTCCCTTCACTAAC---CG-CGA-AC-TGCA--
------
FC88X_00648_00447 -----------------TATTTTCCCTTCACTAAC---CG-CGA-AC-TGCA--
------

```
FC88X_00326_01060       ------------GGGGAAATTTTCCCTTCACTAAC---CG-CGA-AC-TGCA--
------
FC88X_00380_00055       ------------------ATTTTCCCTTCACTAAC---CG-CGA-AC-TGCA--
------
FC88X_00460_01124       --------------GGGGATTTTCCCTTCACTAAC---CG-CGA-AC-TGCA--
------
FC88X_00101_00562       --------------GGGGATTTTCCCTTCACTAAC---CG-CGA-AC-TGCA--
------
FC88X_00780_00151       ---------------GGGATTTTCCCTTCACTAAC---CG-CGA-AC-TGCA--
------
FC88X_00339_00584       ------------------ATTTTCCCTTCACTAAC---CG-CGA-AC-TGCA--
------
FC88X_00635_00278       --------------------TTTCCCTTCACTAAC---CG-CGA-AC-TGCA--
------
FC88X_00630_01223       -------------------TTTTCCCTTCACTAAC---CCGCGA-AC-TGCA--
------
FC88X_00991_01063       ------------------TTTTCCCTTCTACTAAC---CCGCGA-AC-TGCA--
------
FC88X_00867_00144       -------------------TTTTCCCTTCACTAAC---CCGCGA-AC-TGCA--
------
FC88X_00259_00935       --------------------TTTCCCTTCACTAAC---C-GCGA-AC-TGCA--
------
FC88X_00043_00352       -------------------TTTTCCCTTCACTAAC---CG-CGA-AC-TGCA--
------
FC88X_01065_00626       -------------------TTTTCCCTTCACTAAC---CG-CGA-AC-TGCA--
------
FC88X_01085_00709       -------------------TTTTCCCTTCACTAAC---CG-CGA-AC-TGCA--
------
FC88X_01110_00512       -------------------TTTTCCCTTCACTAAC---CG-CGA-AC-TGCA--
------
FC88X_01075_00781       -------------------CTTTTCCTTCACTAAC---CGTCGA-AC-TGCAT-
------
FC88X_00727_00261       ---------------------TTTCCTTCACTAAC---CG-CGA-AC-TGCA--
------
FC88X_00030_00051       ------------------TTTCCTTCCT-ACTAAC---CG-CGA-AC-TGCA--
------
FC88X_01033_01215       ------------------TTTCCTTCCT-ACTAAC---CG-CGA-AC-TGCAC-
------
FC88X_00061_00592       ------------------TTTCCTTCCT-ACTAAC---CG-CGA-AC-TGCA--
------
FC88X_00938_01188       ------------------TTTCCTTCCT-ACTAAC---CG-CGA-AC-TGCA--
------
FC88X_00012_00170       -----------------TTTACTTTCCTCACTAAC---CG-CGA-AC-TGCA--
------
FC88X_00018_00697       ------------------TTACTT-CCTCACTAAC---CG-CGA-AC-TGCA--
------
FC88X_00702_00928       ------------------TTTCCTTCCTCACTAAC---CG-CGA-AC-TGTA--
------
FC88X_00113_00492       ------------------TTTTCCTCCT-ACTAAC---CG-CGA-AC-TGCA--
------
FC88X_00878_00348       -------------------TTTCCTCCT-ACTAAC---CG-CGA-AC-TGCA--
------
FC88X_00107_00087       -----------------TTTTCCTTCCT-ACTAAC---CG-CGA-AC-TGCA--
------
FC88X_01093_00416       ----------------TTTTTCCTTCCT-ACTAAC---CG-CGA-AC-TGCA--
------
```

FC88X_00100_00454 --------------------TTTTCCTTCACTAAC---CG-CGA-AC-TGCA--
------
FC88X_00151_00507 --------------------TTTTCCTTCACTAAC---CGCG-A-AC-TGCA--
------
FC88X_00209_00873 ------------------GTTTTTCCTTCACTAAC---CGCGGA-AC-TGCA--
------
FC88X_00498_00318 ------------------TTTTTCCTTTCACTAAC---CG-CGA-AC-TGCA--
------
FC88X_01060_00410 -------GCAGTCGGTGATTTTTTCCTTCACTAAC---CG-CGA-AC-TGCA--
------
FC88X_01134_00854 -----------------GTTTTTTCCTTCACTAAC---CG-CGA-AC-TGCA--
------
FC88X_01082_00729 -------------------TTTTCCCTTCACTAAC---CG-CGA-AC-TGCA--
------
FC88X_01054_01257 -------------------TTTTCCCTTCACTAAC---CG-CGA-AC-TGCA--
------
FC88X_00055_00216 -------------------TTTTCCCTTCACCAA----CCGCG--AACTGCA--
------
FC88X_00066_00374 ------------------CTTTTCCCTTCACCAA----CCGCG--AACTGCA--
------
FC88X_01096_00677 -------------------TTTTCCCTTCACCAA----CCGCG--AACTGTA--
------
FC88X_00777_00143 -------------------TTTTCCCTTCACCAA----CCGCG--
AACTGCACTTCCTTT
FC88X_00103_00674 -------------------TTTTCCCTTCACCAA----CCGCG--AACTGCA--
------
FC88X_00493_01134 ------------------GTTTTCCCTTCACCAA----CCGCG--AACTGCA--
------
FC88X_00506_00918 -------------------TTTTCCCTTCACCAA----CCGCG--AACTGCA--
------
FC88X_00720_01190 -------------------TTTTCCCTTCACCAA----CCGCG--AACTGCA--
------
FC88X_00938_00460 -------------------TTTTCCCTTCACCAA----CCGCG--AACTGCA--
------
FC88X_00935_01247 -------------------TTTTCCCTTCACCAA----CCGCG--AACTGCA--
------
FC88X_00840_00430 --------------------CCTTCCCTCACCGA-----CACGT-AACTGCA--
------
FC88X_00896_00520 --------------------CCTTCCCTCACCGA-----CACGT-AACTGCA--
------
FC88X_00994_00290 ---------------------TTTCCTTCACCTGAA--CCGCGT-
AACTGCACTTTC---
FC88X_00468_00960 -------------------TTTTTCCTTCACCAA----CCGCGA-ATCTGCA--
------
FC88X_00911_00634 -----------------GTTTTTCCCTTCACTCAA---CCGCG--AACTGCA--
------
FC88X_00024_00537 -----------------GTTTTTCCCTTCACTCAA---CCGCG--AACTGCA--
------
FC88X_01071_00648 -------------------TTTTCCCTTCACTAA----CCGCG--AACTGCA--
------
FC88X_01052_00845 -------------------TTTTCCCTTCACTAA----CCGCG--AACTGCA--
------
FC88X_00048_00257 -------------------TTTTCCCTTCACTAA----CCGCG--AACTGCA--
------
FC88X_00588_01257 -------------------TTTTCCCTTCACTAAC---CG-CGA-ACCTGCA--
------

FC88X_00764_00228 -------------------TTTTCCCTTCACTAAC---CG-CGA-ACCTGCA--
------
FC88X_00922_00646 -------------------TTTTCCCTTCACTAAC---CG-CGA-ACCTGCA--
------
FC88X_01032_00699 -------------------TTTTCCCTTCACTAAC---CG-CGA-ACCTGCA--
------
FC88X_00431_00616 ------------------TTTTTCCCTTCACTAAC---CG-CGA-ACCTGCA--
------
FC88X_00930_00527 ------------------TTTTTCCCTTCACTAAC---CG-CGA-ACCTGCA--
------
FC88X_01017_00440 ------------------TTTTTCCCTTCACTAAC---CG-CGA-ACCTGCA--
------
FC88X_01093_00474 -------------------TTTTCCCTTCACTAAC---CG-CGA-AC-TGCA--
------
FC88X_01085_00765 -------------------TTTTCCCTTCACTAAC---CG-CGA-AC-TGCA--
------
FC88X_00023_00179 -------------------TTTTCCCTTCACTAAC---CG-CGA-AC-TGCA--
------
FC88X_00034_00174 -------------------TTTTCCCTTCACTAAC---CG-CGA-AC-TGCA--
------
FC88X_00046_00133 -----------------TTTTTTCCCTTCG-CTAAC--CG-CGA-AC-TGCA--
------
FC88X_00963_01269 ------------------TTTTTCCCTTCG-CTAAC--CG-CGA-AC-TGCA--
------
FC88X_00676_01008 ------------------TTTTTCCCTTCG-CTAAC--CG-CGA-AC-TGCA--
------
FC88X_00553_00960 ------------------TTTTTCCCTTCG-CTAAC--CG-CGA-AC-TGCA--
------
FC88X_00684_01174 ------------------TTTTTCCCTTCG-CTAAC--CG-CGA-AC-TGCA--
------
FC88X_00018_00055 ------------------TTTTTCCCTTCG-CTAAC--CG-CGA-AC-TGCA--
------
FC88X_00004_00530 -------------------TTTTCCCTTCG-CTAAC--CG-CGA-AC-TGCA--
------
FC88X_01102_00734 -------------------TTTTCCCTTCG-CTAAC--CG-CGA-AC-TGCA--
------
FC88X_00788_01078 ------------------TTTTCCCTTCGA-CTAAC--CG-CGA-AC-
TACACT------
FC88X_00926_00892 -------------------CTCCCTTGTCG-ATAAC--CG-TGG-AC-TCCA--
------
FC88X_01059_00653 ------------------TTTTTCCCTTCC-CTAAC--CG-CGA-AC-TGCA--
------
FC88X_01084_00760 -------------------TTTTCCCTTCA-CTAAC--CG-CGA-AC-TGCA--
------
FC88X_01115_00534 -------------------TTTTCCCTTCA-CTAAC--CG-CGA-AC-TGCA--
------
FC88X_00074_00814 --------------------TTTCTCATCT-TTCAG--CA-CGG-AA-
CGGCTG------
FC88X_00976_00615 ---------------------CTCTCTTCA-TTTAC--CA-CG-----------
------
FC88X_00754_00840 -------------------TTTTCCCTTCA-TTAAC--CA-CGA-AC-TGCA--
------
FC88X_00692_00599 -------------------TTTTCCCTTCA-TTAAC--CG-CGA-AC-CTGCA-
------
FC88X_00815_01062 -------------------TTTTCCCTTCA-TTAAC--CG-CGA-AC--TGCA-
------

FC88X_00730_01242 -----------------TTTTTTCCCTTCA-TTAAC--CG-CGA-AC-TGCA--
------
FC88X_00857_00800 ------------------TTTTTCCCTTCA-TTAAC--CG-CGA-AC-TGCA--
------
FC88X_00928_00404 -----------------TTTTTCCCTTCCA-TTAAC--CG-CGA-AC-TGCA--
------
FC88X_00575_00350 ------------------TTTTTCCCTTCA-TTAAC--CG-CGA-AC-TGCA--
------
FC88X_00152_00396 ------------------TTTTTCCCTTCACTTAAC--CG-CGA-AC-TGCA--
------
FC88X_00868_01271 -------------------TTTTCCCTTCACTTAAC--CG-CGA-AC-TGCA--
------
FC88X_00626_00178 ------------------TTTTTCCCTTCACTTAAC--CG-CGA-AC-TGCA--
------
FC88X_01134_00730 ------------------TTTTTCCCTTCACTTAAC--CG-CGA-AC-TGCA--
------
FC88X_01060_00704 -------------------TTTTCCCTTCA-CTAAC--CG-CGA-AC-TGCA--
------
FC88X_01097_00819 -------------------TTTTCCCTTCA-CTAAC--CG-CGA-AC-TGCA--
------
FC88X_00008_00283 -------------------TTTTCCCTTCA-CTAAC--CG-CGA-AC-TGCA--
------
FC88X_00746_00297 -------------------TTTTCCCTTCA-CTAAC--CG-CGA-AC-TGCA--
------
FC88X_00581_00215 ------------------TTTTTCCCTTCACTAAC---CG-CGA-AC-TGCAG-
------
FC88X_01094_00742 -------------------TTTTCCCTTCACTAAC---CG-CGA-AC-TGCA--
------
FC88X_00784_00095 ------------------TTTTTCCCTTCACTAAC---CG-CGA-AC-TGCA--
------
FC88X_00049_00171 -------------------TTTTCCCTTCACTAAC---CG-CGA-AC-TGCA--
------
FC88X_00961_00688 -------------------TTTTCCCTTCACTAAC---CG-CGA-AC-TGCA--
------
FC88X_00981_00667 -------------------TTTTCCCTTCACT-AA---CCGCGA-AC-TGCA--
------
FC88X_00025_00714 -------------------TTTTCCCTTCACT-AA---CCGCGA-AC-TGCA--
------
FC88X_00049_00287 -------------------TTTTCCCTTCACT-AA---CCGCGA-AC-TGCA--
------
FC88X_01095_00742 -------------------TTTTCCCTTCACT-AA---CCGCGA-AC-TGCA--
------
FC88X_00522_00185 ------------------TTTTTCCCTTCACT-AA---CCGCGA-AC-TGCAA-
------
FC88X_00774_01188 ------------------TTTTTCCCTTCACT-AA---CCGCGA-AC-TGCAA-
------
FC88X_00811_00327 ------------------TTTTTCCCTTCACT-AA---CCGCGA-AC-
TGCAACC-----
FC88X_00007_00164 ----------------CGATTCGTCCCTTGTG-TA---ACGCAC-TG-CCC---
------
FC88X_00955_00581 --------------------TTACTCCCTCTA-CG---TTGCGC-TA-CTGCA-
------
FC88X_00646_00181 -------------------TTTCCTCCCTACT-TG---TCGCGT-AC-TGAC--
------
FC88X_00218_00037 -------------------TCTCCCCTTTGCT-AA---TCTTTG-CG-TACT--
------

```
FC88X_00431_00952       ------------------TTCTTCCCTTCACT-AA---CCGCGA-AC-TGCAC-
------
FC88X_00938_00457       ------------------TTCTTCCCTTCACT-AA---CCGCGA-AC-TGCA--
------
FC88X_01134_00857       ------------------TTCTTCCCTTCACT-AA---CCGCGA-AC-TGCA--
------
FC88X_00758_00879       ------------------TTCTTCCCTTCACT-AA---CCGCGA-AC-TGCA--
------
FC88X_01004_00889       ------------------TTCTTCCCTTCACT-AA---CCGCGA-AC-TGCA--
------
FC88X_00807_01137       -------------------TCTTCCCTTCACT-AA---CCGCGA-AC-TGCA--
------
FC88X_00068_00713       -------------------TCTTCCCTTCACT-AA---CCGCGA-AC-TGCA--
------
FC88X_00629_00099       -------------------TCTTCCCTTCACT-AA---CCGCGA-ACCTGCA--
------
FC88X_00778_01244       -------------------TCTTCCCTTCACT-AA---CCGCGA-AC-TGCA--
------
FC88X_00953_00296       -------------------TCTTCCCTTCACT-AA---CCGCGA-AC-TGCA--
------
FC88X_00926_00676       -------------------TCTTCCCTTCACT-AA---CCGCGA-AC-TGCA--
------
FC88X_00445_00481       -------------------TCTTCCCTTCACT-AA---CCGCGA-AC-TGCA--
------
FC88X_00005_00070       -------------------TCTTCCCTTCACT-AA---CCGCGA-AC-TGCA--
------
FC88X_00430_00440       -------------------TCTTCCCTTCACT-AA---CCGCGA-AC-TGCA--
------
FC88X_00837_01245       -------------------TCTTCCCTTCACT-AA---CCGCGA-AC-TGCA--
------
FC88X_00998_00497       -------------------TCTTCCCTTCACT-AA---CCGCGA-AC-TGCA--
------
FC88X_00575_00426       --------------------TTGCCCATTATCCAAAAGC-GCAC-TTCC-----
------
FC88X_00840_00827       -----------------------CCCATTCTCTACAAACTGCAA-TTTTTCGC-
------
FC88X_00495_01228       -----------------------CCTCCACTCAACCAAATGCAG-CCTCCCA--
------
FC88X_00554_01153       -----------------------TCCTACAACACA--ACAGCGG-
GCTTGTTCT------
FC88X_01137_00988       -----------------------TCCTACA-CACA--ACAGCGG--
CTTGTTCT------
FC88X_00741_00949       ----------------------GTCCTGCA-CACA--ACAGCAG-
GCTTGTTCT------
FC88X_01094_01012       -----------------------TCCTACG-CACA--ACAGCGG-
GCTTGTTCT------
FC88X_00929_00650       ---------------------GGTCCTACA-CACA--ACAGCGG-
GCTTGTTCT------
FC88X_00492_00974       ----------------------GTCCTACA-CACA--ACAGCGG-
GCTTGTTCT------
FC88X_00774_00278       ----------------------GTCCTACA-CACA--ACAGCGG-
GCTTGTTCT------
FC88X_00741_00925       -----------------------TCCTACA-CACA--ACAGCGG-
GCTTGTTCT------
FC88X_00718_01130       -----------------------TCCTACA-CACA--ACAGCGG-
GCTTGTTCT------
```

FC88X_01119_00878 -----------------------TCCTACA-CACA--ACAGCGG-
GCTTGTTCT------
FC88X_00188_00848 -----------------------TCCTACA-CACA--ACAGCGG-
GCTTGTTCT------
FC88X_00252_00940 -----------------------TCCTACA-CACA--ACAGCGG-
GCTTGTTCT------
FC88X_00580_01247 -----------------------TCCTACA-CACA--ACAGCGG-
GCTTGTTCT------
FC88X_00864_01024 -----------------------TCCTACA-CACA--ACAGCGG-
GCTTGTTCT------
FC88X_00858_01067 -----------------------TCCTACA-CACA--ACAGCGG-
GCTTGTTCT------
FC88X_00892_01191 -----------------------TCCTACA-CACA--ACAGCGG-
GCTTGTTCT------
FC88X_01077_00770 -----------------------TCCTACA-CACA--ACAGCGG-
GCTTGTTCT------
FC88X_00514_01125 -----------------------TCCTACA-CACA--ACAGCGG-
GCTTGTCCT------
FC88X_01038_00992 ------------------------CCTGTG-CACAT-ACCGTGA-
GCTAATCTC------
FC88X_00940_01232 ------------------------CCCATCACAAA--CCCGCGA-
GCGCTTACT------
FC88X_00300_00264 -----------------------GCCCATCACAAA--CCCGCGA-GC-------
------
FC88X_00553_00132 ---------------------TATTCCACTACACA--ACGGCCG-GCTGTT---
------
FC88X_00670_01123 ------------------CCTGGCGCTTCTCTTTCCCATCACTA-A--------
------
FC88X_00089_00524 -------------------ATCGCTTCTCCTTTTCTGACCACTA-TC-------
------
FC88X_00487_01246 -----------------GCTCGTCGCTTGACTATTCTACCCCTA-ACTT-----
------
FC88X_01036_00566 -----------------CCATGGCACTCCTCTACCCGCTAACTT-T--------
------
FC88X_00961_00529 ---------------------CTCCCTGGTCTCTACTCCCGCTA-TGCT-----
------
FC88X_00546_01230 -------------------ACCGTTCGCAACCCTCGAATGGCTA-GTT------
------
FC88X_00872_00697 ------------------ACTCCCGATATAAAGCGC-AAACCTC-AT-------
------
FC88X_01123_00522 -----------------TACTCCCGTTGCAGACAGCCAAACCTT----------
------
FC88X_01135_00664 -------------------CTCCCCAAAGTAAGCCC-AGATCTG-AGC------
------
FC88X_00452_00303 -------------------CCGAGGATCAGGAACACCCAAACTG-AG-------
------
FC88X_00063_00637 ------------------------CTGTAAGCTAACACTTGCTC-TATTCAC--
------
FC88X_00074_00672 -----------------------GCTGTAAGCTAACACTTGCTC-TATTCAC--
------
FC88X_00177_00099 ------------------------CTGTAAGCTAACACTTGCTC-TATTCAC--
------
FC88X_00202_01014 ------------------------CTGTAAGCTAACACTTGCTC-TATTCAC--
------
FC88X_00868_01020 ------------------------CTGTAAGCTAACACTTGCTC-TATTCAC--
------

```
FC88X_00905_00216      ------------------------CTGTAAGCTAACACTTGCTC-TATTCAC--
------
FC88X_00948_00854      ------------------------CTGTAAGCTAACACTTGCTC-TATTCAC--
------
FC88X_00305_01135      ------------------------CTGTAAGCTAACACTTGCTC-TATTCAC--
------
FC88X_00971_01073      ------------------------CTGTAAGCTAACACTTGCTC-TATTCAC--
------
FC88X_01073_00659      ------------------------CTGTAAGCTAACACTTGCTC-TATTCAC--
------
FC88X_00089_00120      -------------------CCTCACTGTCA-CCAACACT-GATC-TGTT-----
------
FC88X_00099_00101      -------------------CCTCACTGTCA-CCAACACT-GATC-TGTT-----
------
FC88X_00470_01180      -------------------CCTCACTGTCA-CCAACACT-GATC-TGTT-----
------
FC88X_00578_01151      -------------------CCTCACTGTCA-CCAACACT-GATC-TGTT-----
------
FC88X_00090_00743      -------------------CCTCACTGTCA-CTAACACT-GATC-TGTT-----
------
FC88X_00033_00246      -----------------CATTATGCTTTCT-CCAACACCGAATC-
TTTTCAATCT-----
FC88X_00403_01134      -------------GAGAATAGTTATCATTTATCTATTCTTGTCA-CAGCGC---
------
FC88X_01017_00438      --------------------GTTATCATTTATCTATTCT-GTCA-CAGCG----
------
FC88X_00904_00776      ---------------------TTATCATTTATCTATTCT-GCCA-CAGCG----
------
FC88X_00881_00633      ---------------------TTATCATTTATCTATTCC-GTCA-CAGCG----
------
FC88X_00100_00053      --------------------TGCATCATTCTCCTTTACGTAACG-CCGT-----
------
FC88X_00935_00365      --------------------TGCATCATTCTCCTTTACGTAACG-CCGT-----
------
FC88X_00510_01121      --------------------TGCATCATTCTCCTTTACGTAGCG-CCGT-----
------
FC88X_01024_00388      ---------------------ACATTGTCTTTCTCCAATCGCCA-ACAG-----
------
FC88X_00157_01024      --------------------TTCTCCTTCACCAATGACCAAGCG-TCA------
------
FC88X_00881_00702      --------------------TTCTCCTTCACCAATGACCAAGCG-TCA------
------
FC88X_00151_00922      --------------------TTCTCCTTCACCAATGACCAAGCG-TCA------
------
FC88X_00530_00203      --------------------TTCTCCTTCACCAATGACCAAGCG-TCA------
------
FC88X_00582_00820      --------------------TTCTCCTTCACCAATGACCAAGCG-TCA------
------
FC88X_00653_01232      --------------------TTCTCCTTCACCAATGACCAAGCG-TCA------
------
FC88X_00848_00256      --------------------TTCTCCTTCACCAATGACCAAGCG-TCA------
------
FC88X_00172_00139      --------------------TTCTCCTTCACCAATGAC-AAGCG-TCA------
------
FC88X_00447_00113      --------------------TTCTCCTTCACCAACGACCAAGCG-TCA------
------
```

FC88X_00127_00109 ------------------CTTTTCCCTTGACAAACTACTCAGCA-T--------
------
FC88X_00141_00566 -----------------GCTTTTCCCTTGACAAACTACTCAACG-TC-------
------
FC88X_00949_00951 ------------------TCTTCTCCTTTGCCAATCGCGCGCTT-CG-------
------
FC88X_00772_01039 ------------------TCTTCTCCTTTGCCAATCGCGCGCTT-CG-------
------
FC88X_01100_00956 --------------------CTTCTCTTCCCCAAATGCGCATTT-ATT------
------
FC88X_00118_00483 ---------------TTTTTCCCTTCACTAAC---CGCGTAGCA-TCT------
------
FC88X_00352_01011 --------------------CCTTGCACTAACTCCCACCTAGCG-TC-------
------
FC88X_00130_00719 -------------------TTCCCCTTTCTTGACGCGCTAACGT-GTT------
------
FC88X_01134_00837 -------------------TTCCCCTTTCTTGACGCGCTAACGT-GTT------
------
FC88X_00228_00031 -------------------TTCCCCCTTCTTGACGCGCTAACGT-GTT------
------
FC88X_00068_00588 ---------------ACACTTCCCCGAAACTACCGCGCTAAT------------
------
FC88X_00501_01231 -------------------CTTTTCGATTCTCCTAACCAAACCT-GTC------
------
FC88X_00987_00605 -------------------GGTCCTTCTTCACTTTACCTAACCT-GTT------
------
FC88X_00622_00929 --------------------TTTTCCCTTCACTTAAAC----------------
------
FC88X_00876_00360 --------------------ATTTCCCTCCTACTACACGCAGCT-CGC------
------
FC88X_00486_00162 --------------------ATTTTCCTCCTACTACACGCAGCT-CGC------
------
FC88X_00970_00680 ------------------GTTTTCGACTCCTTATA-ACGAACCT-AAT------
------
FC88X_00891_00741 -------------------TTTTCGACTCCTTATA-ACGAACCT-AAT------
------
FC88X_00339_00223 --------------------ATTTTCCTCCTACTA-ACG---------------
------
FC88X_00986_01223 -------------------TTTTTCCTCCCACTTGACCGCGACT-CGTTCTA--
------
FC88X_00847_01055 -------------------TTTTTCTCATTCCTAACCCCCTGGT-CGT------
------
FC88X_01058_01181 --------------------TTCTCTCAACTCTTACACGCTAGC-TGT------
------
FC88X_00803_00180 -----------------CCACTTCCCTACTGTCGCC-G-CACTG-TT-------
------
FC88X_00203_00834 -----------------CCACTTCCCTACTGTCGCCCG-CACTG-TT-------
------
FC88X_00046_00145 --------------GTGCCACTTCCC--CTGC--TCTG-CACTG-TT-------
------
FC88X_00121_00867 --------------------CTTCCCGTCTCTC-CACG-TACTA-TTAT-----
------
FC88X_00105_00583 ---------------------TTCCCTTCCAACCCGTA-CGCAA-CAATG----
------
FC88X_00805_00957 ---------------------TTCCCTTCTAACCCGTA-CGCAA-CAATG----
------

FC88X_00394_00613 ---------------------TTCCCTTCTAACCCGTA-CGCAA-CGATGCC--
------
FC88X_00905_00669 --------------------TTCCCCTTCTAACCCGTA-CGCAA-CAATG----
------
FC88X_01143_00899 --------------------ATTCCCTCTAACCCCGCAACATGC-CAA------
------
FC88X_00426_01171 ----------------------CCGCTTTCCCCGCTAACCACTG-T--------
------
FC88X_00589_00647 ----------------------CTACTCCTCTCTATCACCACAA-CTGCT----
------
FC88X_00385_00037 -------------------TTTTACTGTCTCCCACGGCTAACTC-CG-------
------
FC88X_01077_00889 -------------------TTTTACTGTCTCCCACG-CTAACTC-CG-------
------
FC88X_00424_00989 -------------------TTTTACTGTCTCCCACGGCTAACTC-TG-------
------
FC88X_00455_01199 ------------------TTTTAACTGTCTCCCACGGCTAACT-----------
------
FC88X_00843_00347 -------------------TTACATCTCCCATCACGGCGAACTA-CT-------
------
FC88X_00870_00444 -------------------TTACATCTCCCATCACGGCGAACTA-CT-------
------
FC88X_00601_00941 -------------------TTACGAGCTCCCTGACGTCTAACTA-CT-------
------
FC88X_00544_00082 ---------------------TTCTCGTCAGTTGTTGCTAACTA-TTCC-----
------
FC88X_00095_00787 ---------------------TTCTCGTCAGTTGTTGCTAGCTA-TTCC-----
------
FC88X_00561_00116 ---------------------TTCTCGTCAGTTGTTGCTAACTA-TTCC-----
------
FC88X_00642_00933 ---------------------TTCTCGTCAGTTGTTGCTAACTA-TTCC-----
------
FC88X_01025_01119 ---------------------TTCTCGTCAGTTGTTGCTAACTA-TTCC-----
------
FC88X_00195_00050 ---------------------TTCTCGTCAGTTGTTGCTAACTA-TTCC-----
------
FC88X_00625_00094 ---------------------TTCTCGTCAGTTGTTGCTAACTA-TTCC-----
------
FC88X_00880_00885 ---------------------TTCTCGTCAGTTGTTGCTAACTA-TTCC-----
------
FC88X_00140_00656 ---------------------TTCTCGTCAGTTGTTGCCAACTA-TTCC-----
------
FC88X_00462_01235 ------------------GTCCACCCGTCAATAGTCGCCAGCTT-TT-------
------
FC88X_00613_00698 -------------------TCCACCCGTCAATAGTCGCCAGCTT-TT-------
------
FC88X_00109_00962 ------------------CCTATCCTTATCGTAAATTGCACTTG-TC-------
------
FC88X_00190_00354 -------------------CTTCGTTCTCTGCAGATTGCAGCTA-TC-------
------
FC88X_00172_00886 -------------------TTGTCTCCGCACTGGCTTTGAACTA-TC-------
------
FC88X_00542_00634 -------------------CTTACCCTTTTCTCATCCCGATGTC-GT-------
------
FC88X_01057_00645 -------------------TTTTCCCGTTAGTTTCCCCGATGTT-TT-------
------

FC88X_00603_00202 ----------------TTTTTTACTCCCCATTATTCGCGTGCC-----------
------
FC88X_01120_00721 -----------------TTTTTACTCCCCATTATTCGCATGCC-----------
------
FC88X_00954_01045 ------------------TTATAC-CCTCACTATTCTCGTTGTC-TG-------
------
FC88X_01039_00804 ------------------TTATAC-CCTCACTATTCTCGTTGTC-TG-------
------
FC88X_00185_00571 ----------------TTTCCATCACTTTACGCGTACCGGTATC----------
------
FC88X_00913_01023 -------------------TTCGCTCATAATTAGCGTCAACTCA-TT-------
------
FC88X_01091_00942 -------------------TTCGCTCATAATTAACGTCAACTCA-TT-------
------
FC88X_01002_01039 -------------GGGGGATTCTCTCGTTTCTAACAGTCACCTA-TC-------
------
FC88X_00968_00852 ---------------------------TTATTACCATTCACT------------
------
FC88X_00029_00234 ------------------TTTCTTCCCTGATTAAAG-GCGGCGT-GTCC-----
------
FC88X_00955_00736 ------------------TTTCTTCCCTGATTAAAG-GCGGCGT-GTCC-----
------
FC88X_00119_00570 ------------------TTTCTTCCCTGATTAAAG-GCGGCGT-GTCC-----
------
FC88X_00186_00585 ------------------TTTCTTCCCTGATTAAAG-GCGGCGT-GTCC-----
------
FC88X_00466_00043 ------------------TTTCTTCCCTGATTAAAG-GCGGCGT-GTCC-----
------
FC88X_00616_00373 ------------------TTTCTTCCCTGATTAAAG-GCGGCGT-GTCC-----
------
FC88X_00897_01254 ------------------TTTCTTCCCTGATTAAAG-GCGGCGT-GTCC-----
------
FC88X_00914_00709 ------------------TTTCTTCCCTGATTAAAG-GCGGCGT-GTCC-----
------
FC88X_00965_00702 -----------------TTTTCTTCCCTGATTAAAG-GCGGCGT-GTCC-----
------
FC88X_00821_00198 -------------------TTTCTCCCTGATTAAAG-GCGGCGT-GCCC-----
------
FC88X_00090_00463 -------------------AATTTGAGTACCTGTCT-ACGGCTC-GCC------
------
FC88X_00923_00391 -------------------CTTCTGAGTTAATGACT-CCGAGCT-CTC------
------
FC88X_00868_00882 ----------------------TCACGTAACAGACT-GCGACT-----------
------
FC88X_00256_00966 ----------------TTCCCAAATCCCTGACTGCCTGACACG-----------
------
FC88X_00704_00957 ----------------TTCCCAAATCCCTGACTGCCTGACACG-----------
------
FC88X_00856_00265 ------------------CCCAAACACTGCCTTCACTAACATCC-A--------
------
FC88X_01017_00847 -----------------TCCCCAAATTTGTCTTATCTAACCAC-----------
------
FC88X_00408_00275 ------------------TTCTTCCTCCTTCGTATCCGCCTTGT-G--------
------
FC88X_00920_00842 ------------------CTCTTCCTCTTACG--ATCGCTTTGT-CGT------
------

FC88X_00860_00768 ------------------TTCTCCCTTCTCTTATTGCGTTGTGT-G--------
------
FC88X_01083_00653 ------------------TTCTCCCTTCTCTTATTGCGTTGTGT-G--------
------
FC88X_01068_00621 -----------------CCAAACTCCCTTCACAAAACAGCCTGT----------
------
FC88X_00116_00231 -----------------CCAAACTCCCTTCACAAAACAGCCTGT----------
------
FC88X_00776_00995 ------------------TTAACTCCCTTCTTTAACCGACTTGT----------
------
FC88X_00632_00165 -----------------TCCTCGTTTTCTCCTCACGAAACCATC-TGTT-----
------
FC88X_00513_01138 -----------------TCCTCGTTT-CTCCTCACGAAACCATC-TGTT-----
------
FC88X_00284_00940 -------------------CTCATCC-CTCCTCACACCGC-ATG-TGTT-----
------
FC88X_00031_00275 ----------------------ACCTTTCTCTCTCACAACCATG----------
------
FC88X_00300_01016 ----------------------TTTCTCCCGTT-CACGACGTGA-TCCTCGT--
------
FC88X_00747_01146 ----------------------TTTCTCCCGTT-CACGACGTGA-TCCTCGT--
------
FC88X_00730_00973 ------------------------TCTCCCATTACCCTACGCGA--CCTCGTT-
------
FC88X_00758_00966 ----------------TCTCTTATTCTCT--CTACAAGTCGTGG-T--------
------
FC88X_00216_00134 ----------------TCTCTTATTCTCT--CTACAAGTCGTGG-T--------
------
FC88X_00279_00994 ----------------TCTCTTATTCTCT--CTACAAGTCGTGG-T--------
------
FC88X_00852_00835 ----------------TCTCTTATTCTCT--CTACAAGTCGTGG-TCTTC----
------
FC88X_01094_00563 --------GGGGAAGTTCTATAGTTCCCTGCTCACAATTCGC------------
------
FC88X_00380_00025 ------------------GAATCCTATTCTTCCACGGTCCGCGC-GCC------
------
FC88X_00576_01219 ----------------TCAACTCCTATTTCCCACGACACCGCGC----------
------
FC88X_00975_00806 ----------------------CTTTTTTGCTCGAACCCCGATC-CATGAC---
------
FC88X_00845_00920 ------------------------CCCTTCCACACATCGCGCTT-GAGTCCC--
------
FC88X_01033_00493 ------------------------TCCCTTCGTAAACCCCGCTT-GTCATGCG-
------
FC88X_00821_01136 ------------------------TTTTCCCTCCAACCGCTCCC-GTCTCAT--
------
FC88X_00402_01137 ---------------------TTCCCATCACATTCACGACATTA-TTTT-----
------
FC88X_00563_00129 ---------------------TTCCCATCACATTCACGACATT-----------
------
FC88X_00216_00015 ---------------------TTCCCATCACATTCGCGACATTA-TTTT-----
------
FC88X_00675_00991 ------------------------CCATCAACTCG-AGGCGTCG-TTTTC----
------
FC88X_00661_00100 ------------------------ACACCAACTCTCACGCGTCA-TTCTAAT--
------

FC88X_00040_00505 -------------------TTCTCCCTTCAACTGT--CGCGTCA-TTGT-----
------
FC88X_01088_00725 ---------------------CCTTCCCTGCATGTATCGCGACG-TTGT-----
------
FC88X_00842_00891 ----------------CTTTCGTCCCTACACTCGCGAGACATT-----------
------
FC88X_01100_00482 -----------------------CTCCCTTAGTAACGTCGTTCG-ACTAAC---
------
FC88X_00940_00583 -----------------------CTCCCTTAGTAACGTCATTCG-ACTAAC---
------
FC88X_00029_00128 -----------------------CTCCCTGACGCGCGACCTTCG-AATGAC---
------
FC88X_00527_01102 -------------------TTACCTCCTCTGCCTACGAACGCTG-TT-------
------
FC88X_00857_00749 -------------------TTACCTCCTCTGCCTACGAACGCTG-TT-------
------
FC88X_00855_01026 -------------------TTACCTCCTCTGCCTACGAACGCTG-TT-------
------
FC88X_00984_00816 -------------------TTACCTCCTCTGCCTACGAACGCTG-TT-------
------
FC88X_01119_00662 -------------------TTACCTCCTCTGCCTACGAACGCTG-TT-------
------
FC88X_00030_00287 -------------------TTACCTCCTCTGCCTACGAACGCTG-TT-------
------
FC88X_00124_00076 -------------------TTACCTCCTCTGCCTACGAACGCTG-TT-------
------
FC88X_00229_01029 -------------------TTACCTCCTCTGCCTACGAACGCTG-TT-------
------
FC88X_01074_00454 -------------------TTACCTCCTCTGCCTACGAACGCTG-TT-------
------
FC88X_00514_00169 -------------------CTACCTCCTCTGCCTACGAACGCTG-TT-------
------
FC88X_00191_00889 -------------------TTACCTCCTCTGCCTACGAACGCTC-GTT------
------
FC88X_00956_00732 -------------------CCGTCGGATCTTCCTACGCGCGTTC-TG-------
------
FC88X_00024_00344 ------------------CTTTGCACTTTGACCAGCCGGCGTCT-C--------
------
FC88X_00409_00032 ------------------CTTTGCACTTTGACCAGCCGGCGTCT-C--------
------
FC88X_00827_00547 ------------------CTTTGCACTTTGACCAGCCGGCGTCT-C--------
------
FC88X_00960_01087 -------------------ATTACACCTTCTCGATCGGACGCCG-CAC------
------
FC88X_00095_00070 ------------------TCCTCTACTGTCTCGAGTGTGTCTCT-T--------
------
FC88X_00393_01087 ------------------TCACTCAACTTCTCGAGC-TGGCTCT-CC-------
------
FC88X_00121_00817 --------------------CCTCACTATCTTCAACCTCCGACC-CAT------
------
FC88X_00668_00959 ------------------ACGCACACTAGCCGATATCTCCGTAT-T--------
------
FC88X_01049_01227 ------------------ACGCACACTAGCCGATATCTCCGTAT-T--------
------
FC88X_00915_00377 ------------------CCCTTCACCACTCCCTAACGCAGTAT-C--------
------

FC88X_00926_00936 ------------------GTATACACCAA-CCCTTGCGCGCTAT-C--------
------
FC88X_01079_00855 -------------------TCGACAACTCCCAAA--CGACGCAT-GCA------
------
FC88X_00614_00179 -------------------TCGACAACTTCCAAAA-CGACGCAT-GCA------
------
FC88X_00127_00367 -----------------GTTCACCAACTCCCGATTACGACGCAT-C--------
------
FC88X_00509_00426 ------------------TTCACCAACTCCCGATTACGACGCAT-C--------
------
FC88X_00068_00474 ------------------ATCTCCTTACTCCGGACACGACGCGC-G--------
------
FC88X_01058_01182 ------------------TCGTCAACCCTCGCCATCCAGCGTAC-A--------
------
FC88X_00529_01188 ------------------CTCTTCGACCATCCGCA-CGATCTGT-TC-------
------
FC88X_00996_00323 ------------------CCCTTCAACCATTAACGTCGAACTGT-T--------
------
FC88X_01097_00987 -----------------------CTATCTCTCCTCCT---ACTG-TGCCTTA--
------
FC88X_00193_00079 -----------------------CTATCCGTTCTCCTGCAACCG-TGCCTA---
------
FC88X_00026_00081 -----------------------CTATCCCTTCGGCC-TAACCG-TGCTTTA--
------
FC88X_00718_01059 -----------------------CTCCCTTCTTGCCTAACAAGG-CATGAC---
------
FC88X_00757_00174 -----------------------CTCCCTTCTTGCCTAACAAGG-CATGAC---
------
FC88X_00797_00799 -----------------------CTCCCTTCTTGCCTAACAAGG-CATGAC---
------
FC88X_00615_01204 -----------------------TTCTCGCTTCTCTTCACAGTG-CTTTAC---
------
FC88X_00190_00889 -------------------------CTTCCTTTTCCTCACCTGC-TTATTCAC-
------
FC88X_00426_00054 -------------------------CTTCCTTTTCCTCACCTGC-TTATTCAC-
------
FC88X_00730_01272 -------------------------CTTCCTTTTCCTTACCTGC-TTATTCAC-
------
FC88X_00924_00947 ------------------------CGTTTCCCTTCTTACCGTGC-TTTTCT---
------
FC88X_01131_00538 ------------------------CGCTTCCCTTCTTACCGTGT-TTTTTTCT-
------
FC88X_00927_00918 -----------------------TTATCCCTTGTCCACTCGCGC-CTTACA---
------
FC88X_00567_00050 --------------------CACATGATTCTCTTCCTCC-GCGT-ACA------
------
FC88X_00881_00356 --------------------CACATGATTCTTTTCCTCCCGCGT-ACA------
------
FC88X_00073_00832 --------------------CTCATTTCTCTATACCTCCCGCGT-GGA------
------
FC88X_00253_00947 --------------------CTCGACTCCCTTTACTGCCGCGTT-GCAC-----
------
FC88X_00968_01268 ----------------------CCATTCCCTTCTCCC--CTCAG-CACTGCG--
------
FC88X_01079_00691 ----------------------CCATTCCCTATCTCCGGCCCAG-CACTA----
------

```
FC88X_01069_00863       -----------------GGGTCCCAATACCTGCATCTC-CCGAG-TACT-----
------
FC88X_00726_01258       ----------------------ACGTGCCCTTCTCCTGCCTACC-CGCGTA---
------
FC88X_00133_00236       -----------------------CCTTCCCTTCTGTGTGCGCGT-ATTAAC---
------
FC88X_00068_00589       -----------------------CCTTCCCTTCGGTGTGCGCGT-ATTAAC---
------
FC88X_00484_01009       -----------------------CTTCCATCCCTGTGCTGCCCG-CATAAC---
------
FC88X_01014_00441       ---------------------CTCTTCTCTCCATGTGTT-CCCG-AATGAC---
------
FC88X_00459_00080       -----------------------CACCTCGTTCTGTTGACTCGC-TATTTA---
------
FC88X_01045_00341       -----------------------CATCTCGTTCTGTTGACTCGC-TATTTA---
------
FC88X_01071_01158       --------------------------TTCGTTCTGTTTATGAAT-
CGTCTATTCT-----
FC88X_00409_01208       -----------------TTAACTTCCCTCGTGCTGTTGAATCGC----------
------
FC88X_00710_00948       --------------------TTCTCCCTTTTTTTGCATTTGTGC-GATG-----
------
FC88X_00229_01006       ---------------------TTCTCCCTTTTTTGCATTTGTGC-GATG-----
------
FC88X_00934_00679       -----------------------CTTCCTTTTCAGTTGTAACGC-GATATT---
------
FC88X_01124_00539       -------------------------CTTCCTTTTGCTCTCGCGC-GATGTCGC-
------
FC88X_01014_01144       ---------------------CTTCCTCTTTACTGCACGCGTGT-GGT------
------
FC88X_00059_00270       -------------------TTCCTCTTTTTACCTGCACTTGAGT-GC-------
------
FC88X_00814_00944       ---------------------GCCGCCGTT--TCAATGACGTAC-TATTCGC--
------
FC88X_00042_00262       ----------------------CCGCCGTT--TCAATGACGTAC-TATTCGC--
------
FC88X_00345_01005       ----------------------CCGCCGTT--TCAATGACGTAC-TATTCGC--
------
FC88X_00412_00054       ----------------------CCGCCGTT--TCAATGACGTAC-TATTCGC--
------
FC88X_00488_01183       ----------------------CCGCCGTT--TCAATGACGTAC-TATTCGC--
------
FC88X_00936_01196       ----------------------CCGCCGTT--TCAATGACGTAC-TATTCGC--
------
FC88X_01046_00470       ----------------------CCGCCGTT--TCAATGACGTAC-TATTCGC--
------
FC88X_00479_00395       ----------------------CCGCCGTT--TCAATGACGTAC-TATTCGC--
------
FC88X_00588_00105       ----------------------CCGCCGTT--TCAATGACGTAC-TATTCGC--
------
FC88X_00780_00826       ----------------------CCGCCGTT--TCAATGACGTAC-TATTCGC--
------
FC88X_01038_00497       ----------------------CCGCCGTT--TCAATGACGTAC-TATTCGC--
------
FC88X_00069_00274       ----------------------CCGCCGTT--TCAATGACGTAC-TATTCG---
------
```

FC88X_00450_00371 ----------------------CCGCCGTT--TCAATGACGTAC-TATTCAC--
------
FC88X_00479_00166 ----------------------CCGCCGCT--TCAATGACGTAC-TATTCGC--
------
FC88X_00796_00193 --------------------TCTCGACGCC-TTGAAAGCCGTAT-TGTT-----
------
FC88X_00630_00451 --------------------TCTCGACACC-TTGAAAGCCGTAC-TGTT-----
------
FC88X_00914_00476 ---------------------CTCGGCACCATTGATCGTACTAC-TGTT-----
------
FC88X_00955_00772 -------------------CAATCGGCACT--TCACGAACCGAC-
TACTCTTCCTTTT--
FC88X_01105_00855 ---------------------TTCGCTCTTCAGTGAAGCC-GAC-GTCTC----
------
FC88X_00706_00281 ---------------------TTCGCTCTTTAGTGAAGCCCGAC-GTCTC----
------
FC88X_00293_00833 ---------------------TTCGCTCTTCAGTGAAGCC-GAC-GTCTC----
------
FC88X_01051_01107 ---------------------TTCGCTCTTCAGTGAAGCC-GAC-GTCTC----
------
FC88X_00007_00301 ----------------------CACCATTCCTTCCCTATCGCAC-TATT-----
------
FC88X_00157_00939 ---------------------CCACTATTCCTTCCCTAGCGCAC-TATT-----
------
FC88X_00479_01134 --------------------CTTCGCACTATG-ACCTTT-GCAC-TATTC----
------
FC88X_00603_01199 --------------------CTTCGCACTATG-ACCTTT-GCAC-TATTC----
------
FC88X_00036_00256 --------------------CTTCGCACTATG-ACCTTT-GCAC-TATTC----
------
FC88X_01050_00481 --------------------CTTCGCACTATG-ACCTTT-GCAC-TATTC----
------
FC88X_00720_01255 --------------------CCTCGTACTGTGCGACCGT-GAAC-TATT-----
------
FC88X_00713_00109 ---------------------ACCGTACTTTCAACCTCGAACAC-TATT-----
------
FC88X_00128_00116 ---------------------ACCGTACTTTCAACCTCGAGCAC-TATT-----
------
FC88X_00257_00508 ---------------------CTCGTGTCTTTCCCTCTGCGAAC-TAGT-----
------
FC88X_00400_01095 ---------------------CCCTTTTGCCTCCAATATCGCAC-TGTT-----
------
FC88X_00948_00890 --------------------TCAGTCGCTTTTCCCACCTCTTAC-TGTT-----
------
FC88X_00126_00690 -------------GGGGAAGCTACCAACTAAACTC--ACCGCAG-CTTTCT---
------
FC88X_00248_00072 --------------------CTACCAACTAAACTC--ACCGCAG-CTTTCT---
------
FC88X_00833_01034 --------------------CTACCAACTAAACTC--ACCGCAG-CTTTCT---
------
FC88X_00900_00412 --------------------CTACCAACTAAACTC--ACCGCAG-CTTTCT---
------
FC88X_01078_00998 --------------------CTACCAACTAAACTC--ACCGCAG-CTTTCT---
------
FC88X_00659_00378 --------------------CTACCAACTAAACTC--ACCGCAG-CTTTCT---
------

FC88X_00777_01163 --------------------CTACCAACTAAACTC--ACCGCAG-CTTTCT---
------
FC88X_01012_00779 --------------------CTACCAACTAAACTC--ACCGCAG-
CTTTCTCTT------
FC88X_00499_01103 --------------------CTACCAACTAAACTC--ACCGTAG-CTTTCT---
------
FC88X_01116_00598 --------------------CTACCAACTAAACTC--ACCGCAG-CTTTTCT--
------
FC88X_00604_00179 ------------------------CTACTAAACTCTTACAGGAG-CTCTCTC--
------
FC88X_00110_00688 -----------------------CCATTTCATCTCCCACCGTGA-GCTATT---
------
FC88X_00923_00841 -----------------------CCATTTCATCTCCCACCGTGA-GCTATT---
------
FC88X_00940_00777 -----------------------CCATCTCATCTCCCACCGTGA-GCTATT---
------
FC88X_00642_00804 --------------------CTCTCAGCATCAGCT---CCGTGG-ACTATCC--
------
FC88X_00905_00869 --------------------CTCCCATCTCAGCCA---CCGCGG-ACTGTC---
------
FC88X_00739_01141 ---------------------TCTCATCCCTTTCA---CCGCTA-ACCATCCC-
------
FC88X_00196_00615 ----------------------CTCCATTAAATC-CACGGCTAT-ATGTCTC--
------
FC88X_00806_00303 ----------------------CTCCCTTACCTCACGTGGTGAT-ATGTC----
------
FC88X_01004_00970 --------------------CTTGCCATTATCTCCCGGCGACAT-TTG------
------
FC88X_00260_00988 --------------------CTTGCCATTATCTCCCGGCGACAT-TTG------
------
FC88X_00965_00440 --------------------CTTGCCATTATCTCCCGGCGACAT-TTG------
------
FC88X_01117_00922 --------------------CTTGCCATTATCTCCCGGCGACAT-TTG------
------
FC88X_00656_00170 --------------------TTTAACATTAACTCCCCCGCATCT-ATT------
------
FC88X_00880_00197 --------------------TTTAACATTAACTCCCCCGCATCT-ATT------
------
FC88X_00891_00164 --------------------TTTAACATTACTCCCCGCA---------------
------
FC88X_00295_00847 --------------------TTCTACATTATTCACCGCCAATTT-GGC------
------
FC88X_00583_01220 --------------------TTCTACATTATTCACCGCCAATTT-GGC------
------
FC88X_00848_01096 --------------------TTCTACATTATTCACCGCCAATTT-GGC------
------
FC88X_00216_00103 -------------------TCTTCTCATGTTTCATCGCCAATTT-CGC------
------
FC88X_00012_00180 --------------------CTCCACTCCAGTTTGAGGCTACTT-GCC------
------
FC88X_01080_00655 --------------------CTCCACTCCAGTTTGAGGTTACTT-GCC------
------
FC88X_00908_00486 -------------------CCTGCTCCTCAGTGTCATG-TACCT-GCC------
------
FC88X_00356_01097 -----------------CACCTCCATCCCTCTCTCACG--ACTT-GC-------
------

FC88X_00865_00882 -------------------GCTCCTCCCTTTTCGACGGCAACTT-GCA------
------
FC88X_00099_00479 ------------------CTTGGCTATCTGCTAAA----TGCGT-ATTCT----
------
FC88X_00112_00397 ------------------CTTGGCTATCTGCTAAA----TGCGT-ATTCTCTT-
------
FC88X_00906_00315 ------------------CTTGGCTATCTGCTAAA----TGCGT-ATTCT----
------
FC88X_00997_00785 ------------------CTTGGCTATCTGCTAAA----TGCGT-ATTCT----
------
FC88X_00277_00935 ------------------CTTGGCTATCTGCTAAA----TGCGT-ATTCT----
------
FC88X_00662_00783 ------------------CTTGGCTATCTGCTAAA----TGCGT-ATTCT----
------
FC88X_00924_00402 ------------------CTTGGCTATCTGCTAAA----TGCGT-ATTCT----
------
FC88X_00920_01016 ------------------CTTGGCTATCTGCTAAA----TGCGT-ATTCT----
------
FC88X_00955_01012 ------------------CTTGGCTATCTGCTAAA----TGCGT-ATTCT----
------
FC88X_00951_01262 ------------------CTTGGCTATCTGCTAAA----TGCGT-ATTCT----
------
FC88X_00203_00873 ------------------CTCGGCTATCTGCTAAA----TGCGT-ATTCT----
------
FC88X_00910_00993 ------------------CTTGGCTATCTGCTAAA----TGCGT-
ATTCTCTTCC-----
FC88X_00503_00654 ------------------CTTGGCTATTTGCTAAA----TGCGT-ATTCT----
------
FC88X_00142_00757 ------------------CTTGGTTATCTGCTAAA----TGCGT-ATTCT----
------
FC88X_00576_00295 ------------CCTCATGTTCTGTAGCGTCCC-C----GTCAT----------
------
FC88X_00765_00946 -------------------TTCGCTAGCATGC-------GTCAT-CC-------
------
FC88X_01113_00642 ------------------TCATGCTGTCTTCCC-C----GGCAC-GATTCG---
------
FC88X_00944_00918 -------------------TTCGCTATCTTCCAGC----GGCAT-GTTCCC---
------
FC88X_00167_00892 -------------------TTCGCTATCTTCCAGC----GGCAT-GTTCCC---
------
FC88X_00988_00830 -------------------TTCGCTATCTTCCAGC----GGCAT-GTTCCC---
------
FC88X_00723_00066 ----------------------TTAC-TAACTA-CCAGGCGCAA-CTGTTCT--
------
FC88X_01005_00721 ---------------GGGGAATTTAC-TAACTA-CTAGGCGCAA-CTGTTCT--
------
FC88X_00018_00145 ----------------------TTAC-TAACTA-CTAGGCGCAA-CTGTTCT--
------
FC88X_00050_00135 ----------------------TTAC-TAACTA-CTAGGCGCAA-CTGTTCT--
------
FC88X_01079_01271 ----------------------TTAC-TAACTA-CTAGGCGCAAACTGTTCT--
------
FC88X_00086_00509 ----------------------TTAC-TAACTA-CTAGGCGCAA-CTGTTCT--
------
FC88X_00168_00935 ----------------------TTAC-TAACTA-CTAGGCGCAA-CTGTTCT--
------

FC88X_00176_00190 ----------------------TTAC-TAACTA-CTAGGCGCAA-CTGTTCT--
------
FC88X_00850_00338 ----------------------TTAC-TAACTA-CTAGGCGCAA-CTGTTCTA-
------
FC88X_00292_01131 ----------------------TTAC-TAACTA-CTAGGCGCAA-CTGTTCT--
------
FC88X_00487_00035 ----------------------TTAC-TAACTA-CTAGGCGCAA-CTGTTCT--
------
FC88X_00613_00279 ----------------------TTAC-TAACTA-CTAGGCGCAA-CTGTTCT--
------
FC88X_00629_01215 ----------------------TTAC-TAACTA-CTAGGCGCAA-CTGTTCT--
------
FC88X_00774_00291 ----------------------TTAC-TAACTA-CTAGGCGCAA-CTGTTCT--
------
FC88X_00864_01230 ----------------------TTAC-TAACTA-CTAGGCGCAA-CTGTTCT--
------
FC88X_00979_01000 ----------------------TTAC-TAACTA-CTAGGCGCAA-CTGTTCT--
------
FC88X_01027_00296 ----------------------TTAC-TAACTA-CTAGGCGCAA-CTGTTCT--
------
FC88X_01061_00591 ----------------------TTAC-TAACTA-CTAGGCGCAA-CTGTTCT--
------
FC88X_00165_00170 ----------------------TTAC-TAACTA-CTAGGCGCAA-CTGTTCT--
------
FC88X_00478_01120 ----------------------TTAC-TAACTA-CTAGGCGCAA-CTGTTCT--
------
FC88X_00580_00689 ----------------------TTAC-TAACTA-CTAGGCGCAA-CTGTTCT--
------
FC88X_00719_01125 ----------------------TTAC-TAACTA-CTAGGCGCAA-CTGTTCT--
------
FC88X_00789_01221 ----------------------TTAC-TAACTA-CTAGGCGCAA-CTGTTCT--
------
FC88X_00934_00444 ----------------------TTAC-TAACTA-CTAGGCGCAA-CTGTTCT--
------
FC88X_00938_00521 ----------------------TTAC-TAACTA-CTAGGCGCAA-CTGTTCT--
------
FC88X_00900_00979 ----------------------TTAC-TAACTA-CTAGGCGCAA-CTGTTCT--
------
FC88X_00923_01247 ----------------------TTAC-TAACTA-CTAGGCGCAA-CTGTTCT--
------
FC88X_00963_00493 ----------------------TTAC-TAACTA-CTAGGCGCAA-CTGTTCT--
------
FC88X_00985_00726 ----------------------TTAC-TAACTA-CTAGGCGCAA-CTGTTCT--
------
FC88X_00964_00891 ----------------------TTAC-TAACTA-CTAGGCGCAA-CTGTTCT--
------
FC88X_01021_00480 ----------------------TTAC-TAACTA-CTAGGCGCAA-CTGTTCT--
------
FC88X_01050_00382 ----------------------TTAC-TAACTA-CTAGGCGCAA-CTGTTCT--
------
FC88X_00736_00999 ---------------------GTTAC-TAACTA-CTAGGCGCAA-CTGTTCTC-
------
FC88X_00965_00650 ----------------------TCAC-TAACTA-CTAGGCGCAA-CTGTTCT--
------
FC88X_00880_00383 ----------------------TTAC-TAACTA-CTAGGCGCAA-
CTGTTCTCC------

FC88X_00896_00608 ----------------------TTAC-TAACTA-CTAGGCGCAA-
CTGTTCTCC------
FC88X_00854_00265 ----------------------TTAC-TAACTA-CTAGGCGTGA-CTGTTCT--
------
FC88X_00969_01270 ----------------------TTAC-TAACTA-CTAGGCGCGA-CTGTTCT--
------
FC88X_00850_00643 ----------------------TTAC-TAGCTA-CTAGGCCGCAACTGTTCT--
------
FC88X_00799_01060 ----------------------TTAC-TAACAA-CTAGGCGCAA-CTGTTCT--
------
FC88X_01089_00887 ----------------------TTAC-TAACTA-CTAGGCGCAA-
CTGTTCTCTTCCTTT
FC88X_00835_00351 ----------------------TTAC-TAACTA-CTAGGCGCAA-CTGTTCC--
------
FC88X_00528_01103 ----------------------TTAC-TAACTA-CTAGGCGCAA-CTGTCCT--
------
FC88X_01018_00556 ----------------------TTAC-TAACTA-CTAGGTGCAA-CTGTTCT--
------
FC88X_01029_00918 ----------------------TTACGTAACTAGCCAGGTCGCAACTGT-----
------
FC88X_00131_00469 ----------------------TTAC-TAACCA-CTAGGCGCAA-CTGTTCT--
------
FC88X_01118_01038 ----------------------TCAC-TAACCA-CTAGGCGCAA-CTGTTCT--
------
FC88X_00965_00735 ----------------------CTAC-TGACTA-CTAGGCGCAA-CTGTTCT--
------
FC88X_00027_00414 ------------------------CTATAACCCCGTAGTTGCAA--TGTTCTC-
------
FC88X_00089_00846 ------------------------CTATAACCCCGTAGTTGCAA--TGTTCTC-
------
FC88X_00418_00136 ------------------------CTATAACCCCGTAGTTGCAA--TGTTCTC-
------
FC88X_00840_00787 ------------------------CTATAACCCCGTAGTTGCAA--TGTTCTC-
------
FC88X_00891_00492 ------------------------CTATAACCCCGTAGTTGCAA--TGTTCTC-
------
FC88X_00724_01151 ------------------------CTATAACCCCGTAGTTGCAA--TGTTCTC-
------
FC88X_00949_00967 ------------------------CTATAACCCCGTAGTTGCAA--TGTTCTC-
------
FC88X_01072_00912 ------------------------CTATAGCCCCGTAGTTGCAA--TGTTCTC-
------
FC88X_00630_01049 ------------------------CTATAACCCCGTAGCTGCAA--
TGTCCTCCCATC--
FC88X_00092_00447 ------------------------CCAAGACGTAACCGGTACCG-CA-TTCTC-
------
FC88X_00459_01183 ------------------------CCAAGACGTAACCGGTACCG-CAATTCTC-
------
FC88X_00278_00046 ------------------------CCAAGACGTAACCGGTACCG-CA-TTCTC-
------
FC88X_00562_00213 ------------------------CCAAGACGTAACCGGTATC--GCATTCTC-
------
FC88X_00055_00605 ------------------------CCAAGACGTAACCGGTACCC-GCATTCTC-
------
FC88X_00323_00049 -----------------------CCAAGAACGTAACCGGTACC--GCTTTCTC-
------

```
FC88X_00081_00616       ------------------------CCAAGACGTAACCGGTACC--GCATTCTC-
------
FC88X_00532_01084       ------------------------CCAAGACGTAACCGGTACC--GCATTCTC-
------
FC88X_00761_00795       ------------------------CCAAGACGTAACCGGTACC--GCATTCTC-
------
FC88X_00773_01176       ------------------------CCAAGACGTAACCGGTACC--GCATTCTC-
------
FC88X_00896_00827       ------------------------CCAAGACGTAACCGGTACC--GCATTCTC-
------
FC88X_01050_00348       ------------------------CCAAGACGTAACCGGTACC--GCATTCTC-
------
FC88X_01133_00903       ------------------------CCAAGACGTAACCGGTACC--GCATTCTC-
------
FC88X_00067_00409       ------------------------CCAAGACGTAACCGGTACC--GCATTCTC-
------
FC88X_00088_00847       ------------------------CCAAGACGTAACCGGTACC--GCATTCTC-
------
FC88X_00149_00077       ------------------------CCAAGACGTAACCGGTACC--GCATTCTC-
------
FC88X_00104_00906       ------------------------CCAAGACGTAACCGGTACC--GCATTCTC-
------
FC88X_00164_00050       ------------------------CCAAGACGTAACCGGTACC--GCATTCTC-
------
FC88X_00166_00659       ------------------------CCAAGACGTAACCGGTACC--GCATTCTC-
------
FC88X_00318_00042       ------------------------CCAAGACGTAACCGGTACC--GCATTCTC-
------
FC88X_00614_01156       ------------------------CCAAGACGTAACCGGTACC--GCATTCTC-
------
FC88X_00649_01258       ------------------------CCAAGACGTAACCGGTACC--GCATTCTC-
------
FC88X_00711_00394       ------------------------CCAAGACGTAACCGGTACC--GCATTCTC-
------
FC88X_00790_00201       ------------------------CCAAGACGTAACCGGTACC--GCATTCTC-
------
FC88X_01125_00945       ------------------------CCAAGACGTAACCGGTACC--GCATTCTC-
------
FC88X_00616_00126       ------------------------CCAAGACGTAACCGGTACC--GCATTCT--
------
FC88X_00985_00869       ------------------------CCAAGACGCAACCGGTACC--GCATTCTC-
------
FC88X_01042_00842       ------------------------CCAAGACGCAACCGGTACC--GCATTCTC-
------
FC88X_00925_00297       ------------------------CCAAGGCGTAACCGGTACC--GCATTCTC-
------
FC88X_01059_00488       ------------------------CCAAAACGTAACCGGTACC--GCATTCTC-
------
FC88X_00728_01141       ---------------------------CCACGAGCTCC---CCG-
GCAGTACCGTTAT--
FC88X_00121_00581       ---------------------------CCACGAGCTCC---CCG-
GCAGTACCGTTAT--
FC88X_00277_01109       ---------------------------CCACAAGCTATGAACCT-
GCAGTACCGC-----
FC88X_01071_01152       -----------------------TTCGCAACGCGCTCC----CG-
GCATCACAGT-----
```

```
FC88X_00451_00004       -------------CTAACAACCGACCGCATGCTTCTTCTC--------------
------
FC88X_00759_01160       -------------CTAACAACCGACCGCATGCTTCTTCTC--------------
------
FC88X_00077_00793       -------------CTAACAACCGACCGCATGCTTCTTCTCCTTCCTTT------
------
FC88X_00025_00494       ------------GCTAATAACCATCCGCTAAGCTACTCCC--------------
------
FC88X_00277_00014       -------------CTACGATCGATGCACTACCCTCGTCCC--------------
------
FC88X_00075_00768       --------------------CTTTTCGCTATTTCTACCCCAGGATGT-------
------
FC88X_00130_00878       --------------------CTTTTCGCTATTTCTACCCCAGGATGT-------
------
FC88X_01090_00681       --------------------CTTTTCGCTATTTCTACCCCAGGATGT-------
------
FC88X_00977_01082       --------------------CTTTTCGCTATTTCTACCCCAGGATGT-------
------
FC88X_00826_00932       --------------------CTTTTCGCTATTTCTACCCCAGGATGA-------
------
FC88X_01078_00940       -------------------TCTTTTCGCTACTTCTACCCCAGGATGT-------
------
FC88X_00337_00274       --------------------TTTTTCGCTA-TTCTACCCCAGGATGT-------
------
FC88X_01087_00650       --------------------TTTTCCGCTA-GACTACTCCCGCATCAT------
------
FC88X_01118_00712       --------------------TTTTCCGCTA-GACTACTCCCGCATCAT------
------
FC88X_00075_00297       -------ATACCAGCGCTATCCTCTC-CCGAACCT-------------------
------
FC88X_00204_00931       -------CCACCCGAGCTTTTCTCCTGCCGAACGC-------------------
------
FC88X_00975_01207       ---------CTACACGCTTTCCTCTCCACGAACAAT------------------
------
FC88X_00781_00186       ----------------CGATATTATCCCCAAACAATGCGCTTCGA---------
------
FC88X_01048_01119       ----------------CGTTATTATCCCCAAACAATGCGCTTCGA---------
------
FC88X_00871_00261       ----------------CGTTATTATCCCCAAACAA-------------------
------
FC88X_00945_00767       ---------------ACAATATTATCGCCCCACAAACTGCTT------------
------
FC88X_00030_00270       ------------------ATCGTCTATTCTCTTAA-CCACGCGTCAG-------
------
FC88X_00278_00420       ------------------ATCGTCTATTCTCTTAA-CCACGCGTCAG-------
------
FC88X_00571_01114       ------------------ATCGTCTATTCTCTTAA-CCACGCGCCAG-------
------
FC88X_00135_00665       -------------------TCGAATA--CTCCCAG-TACCAGCGCTAAA-----
------
FC88X_00946_00325       -------------------ACGAATATTCTCTCAA-TCCCGACGC---------
------
FC88X_00183_00471       ----------------CTTCCTTCTCTTCACATG--CAACGTGCA---------
------
FC88X_00347_00832       ----------------CTTCCTTCTCTTCACATG--CAACGTGCA---------
------
```

FC88X_00996_00693 ----------------CTTCCTTCTCTTCACATG--CAACGTGCA---------
------
FC88X_01062_01157 ----------------CTTCCTTCTCTTCACATG--CAACGTGCA---------
------
FC88X_00144_00149 ----------------CTTCCTTCTCTTCACATG--CAACGTGCA---------
------
FC88X_00129_00548 ----------------CTTCCTTCTCTTCACATG--CAACGTGCA---------
------
FC88X_00216_00970 ----------------CTTCCTTCTCTTCACATG--CAACGTGCA---------
------
FC88X_00477_01000 ----------------CTTCCTTCTCTTCACATG--CAACGTGCA---------
------
FC88X_00603_00139 ----------------CTTCCTTCTCTTCACATG--CAACGTGCA---------
------
FC88X_00666_01218 ----------------CTTCCTTCTCTTCACATG--CAACGTGCA---------
------
FC88X_00850_01016 ----------------CTTCCTTCTCTTCACATG--CAACGTGCA---------
------
FC88X_00926_00659 ----------------CTTCCTTCTCTTCACATG--CAACGTGCA---------
------
FC88X_01021_00496 ----------------CTTCCTTCTCTTCACATG--CAACGTGCA---------
------
FC88X_01084_00676 ----------------CTTCCTTCTCTTCACATG--CAACGTGCA---------
------
FC88X_00530_00950 ---------------CTTACCTTCTCTTCACATG--CAACGTGCA---------
------
FC88X_01144_00761 -----------------TTCCTTCGGTTCCGAGAT-CAAAGTGCT---------
------
FC88X_00023_00666 -----------------CTTTCTCTTCTGTCATAC-CAACGTGCC---------
------
FC88X_00022_00278 ----------------ATATCTTCTGTTCTCATAT-CCGCGTGCA---------
------
FC88X_00280_00945 ------------------TCTTTCCCTTTGGACAT-TGACGTCTC---------
------
FC88X_00805_00318 -----------------ATCTCCCTTGATCGACAT-TGACGTGTC---------
------
FC88X_00950_00369 -----------------CTATTCTCCTTTCGTCAT-CCACGTCGC---------
------
FC88X_00870_01269 -----------------TCTCCTTTTCCCCGACTC-TGGCGTGTC---------
------
FC88X_01059_01090 -----------------TTTCCTCTTCTCCGATCC-TGGCGGGTC---------
------
FC88X_00609_01147 -----------------ATTTCTCCTTTTCACCAC-CGGCGTGTCC--------
------
FC88X_00775_01270 -------------------TCGGTTACTCCACCG--CGCTATCCTCGT------
------
FC88X_00891_00295 -------------------ACCTATTCTCCACTGT-CGCTATCTTCG-------
------
FC88X_00162_00193 -------------------ACATATACTCC-CTAG-CACTATCGTAAC------
------
FC88X_01119_00738 ------------------CATATATTCACCACGAT-CCACGTACAC--------
------
FC88X_00171_00165 ----------------TCAACTTATTTTCCCTTG--CACTATCTCCC-------
------
FC88X_00773_01163 ----------------TCAACTTATCTTCCCTTG--CACTATCTC---------
------

FC88X_00930_00400 -----------------ACGTTACCAA-TCCCGAATACTGTCGTT---------
------
FC88X_01092_00791 -----------------ACGTTACCAA-TCCCGAATACTGTCGTT---------
------
FC88X_01128_01145 -----------------ACGTTACCAA-TCCCGAATACTGTCGTT---------
------
FC88X_00105_00127 -----------------ACGTTACCGA-TCCCGAATACTGTTGTT---------
------
FC88X_00275_00081 -----------------ACGTTACCAAATCCCGAATACTGTCGCCT--------
------
FC88X_00871_00966 ------------TGTACACGTCACCTC--CCGCAATATTGT-------------
------
FC88X_00961_00666 ------------TGTACACGTCACCTC--CCGCAATATTGT-------------
------
FC88X_01114_00518 ------------ATCCCCACTCCTCAATCGCGCGATATT---------------
------
FC88X_00905_01053 ------------TCCCCAGATAATAATTCTCGCGATGTT---------------
------
FC88X_00941_00625 ------------CTCCCACCTTACCAATCACGTAATGTT---------------
------
FC88X_00692_00143 ------------------TTCCCCATCCCGCAAAGGCTTGTTTAC---------
------
FC88X_00684_00172 -------------------TCTCCCAATGACCGCAACTTGTTTAAG--------
------
FC88X_00492_00414 -----------------CCTTCCCATTCCCCGAATTGTTGTTTC----------
------
FC88X_00201_00135 -----------CAAACACGTTTTTACGACCTG-CGCCTCTT-------------
------
FC88X_01003_00982 -----------GCAAAACGTTTTCCCGACCCGTCGTCGCTTCTC----------
------
FC88X_00655_00117 --------------CTCCGTTTTCTCCTTCCCGCGCCTCGCT------------
------
FC88X_00586_01226 --------------AGATGCATTCTCGTCCCG-CACCTCGCT------------
------
FC88X_00417_00978 -------------------TTTTCCCCTCCACGATCG-CGCTATGTC-------
------
FC88X_00765_01139 -------------------TTATCCCATCCCGGATCCTCGCTTTGT--------
------
FC88X_00587_00191 ------------------CCTAACGAACCCAACGATCACAGCTAT---------
------
FC88X_00764_00131 ------------------CCTAACGAACCCAACGATCACAGCTAT---------
------
FC88X_00281_01085 -----------GGGGAATCCTAACGAACCCAACGATCACAGCTAT---------
------
FC88X_00129_00547 -------------------CCAAACACCCCGCGGACCAGACCTATT--------
------
FC88X_01125_00645 -------------------TTAACCCATC-ACGACCTACGCCTATT--------
------
FC88X_00392_01207 ---------------TTCCCCTTCCCGAACAAAGCCCACGGT------------
------
FC88X_00048_00031 --------------ACACCCAATTCCCGAACG-CATCGTACT------------
------
FC88X_00053_00467 --------------ACCGCAATTCTCCGACCG-CCACGTACT------------
------
FC88X_00949_00819 ---------------CGGTAACTTCCCTACCGTCATCGTTCT------------
------

FC88X_00625_00924 ---------------ATACTCTCTTCTCCACCG-CAACGTAAC------------
------
FC88X_00165_00297 ---------------CCATCCCTTCTGCAACCGCACAGTCCT------------
------
FC88X_00728_00898 -----------------ATCTATTCT-CAACCACATACTCCTAAC---------
------
FC88X_00570_00670 ----------------CAAACCCTTCTCCTCGGCCCA-ATGCC-----------
------
FC88X_00098_00470 ---------------TCTATCCTTCCCCTGCGGTCCACATGCT-----------
------
FC88X_00958_00828 --------------CCCAATCCTTCTCGAACGGCACAAAT--------------
------
FC88X_00703_01114 ---------------TCCCAGCTTCGTCAACGTAACA-ATATT-----------
------
FC88X_00813_00799 ---------------TCCCAGCTTCGTCAACGTAACA-ATATT-----------
------
FC88X_00186_01022 ---------------TCCCAGCTTCGTCAACGCAACA-ATATT-----------
------
FC88X_00038_00426 --------------TTGCTAACTTCTCTACCACA-CA-CTACT-----------
------
FC88X_00746_01060 ---------------TTAAAGCTTCTTCAACGGCCCA-CTATC-----------
------
FC88X_00081_00876 --------------TCATCAGCTAGTCAGTGGCCACATTCT-------------
------
FC88X_00957_00705 --------------ACATATGCTATTCCGTGCCCAAATTTT-------------
------
FC88X_00163_00814 -----------------CTGACTAGT----CACTAGATTCGCTTTTCC------
------
FC88X_00139_00052 -----------------CTGACTAGT----CACTAGATTCGCTTTTCC------
------
FC88X_00313_01036 ---------TAGTGTGACTGACTACTGCCTCCCTCGCTTCCTTTCTCC------
------
FC88X_00545_00942 --------------CTACTTACTATTCCCTTCCGAGATTCG-------------
------
FC88X_00304_00041 --------------TTATTCGCTTGGTCACCGCTTCTATTC-------------
------
FC88X_01136_00849 --------------TTATTCGCTTGGTCACCGCTTCTATTC-------------
------
FC88X_01128_00863 --------------GTATTCGCTAATGCGTGCCCGATTCTT-------------
------
FC88X_00937_01245 ---------------TATTCACTTGCCCTACCGACTCATTCT------------
------
FC88X_00099_00142 ---------------TTTTCGCTAT-TCGATGGCCTAACGCCTG----------
------
FC88X_00509_01182 ---------------TTTTCGCTAT-TCGATGGCCTAACGCCTG----------
------
FC88X_00221_00110 ---------------TTTTCGCTAT-TCGATGGCCTAACGCCTC----------
------
FC88X_00621_00407 ------------------TCGCTACATCTCTCGACTAACGCTT-----------
------
FC88X_00637_01127 -----------------TCTCCCGCAGTAACGTGTCCTTCTGTT----------
------
FC88X_00958_00718 ----------------GTCTCCCGCAGTAACGTGTCCTTCTGTT----------
------
FC88X_00129_00959 -----------------TCTCCCGCAGTAACGTGTCCTTCTGTT----------
------

```
FC88X_00944_00553      -----------------TCTCCCGCAGTAACGTGTCCTTCTGTT----------
------
FC88X_00773_01207      -----------------TCTCCCGCAGTAACATGTCCTTCTGTT----------
------
FC88X_01143_00690      ---------------ACTTTCTCTCACTGACGCGTCCATTCT------------
------
FC88X_00926_01021      -------------TTATATTTCTCTTCCCCAGCGTCATTCC-------------
------
FC88X_00277_00956      --------------TACATTTCTCTTCTGACCCATCATCTT-------------
------
FC88X_00663_01120      -------------TGCCTTTCTCAGCCTTACTCTACCCGT--------------
------
FC88X_00859_00709      --------------ACTTTCTCTAATCCAACCCTACCTACT-------------
------
FC88X_00108_00622      --------------CTGTAGTCTAT--CCGCAGCCCAGTTCTC-----------
------
FC88X_00061_00138      --------------CTGTAGTCTAT--CCGCAGCCCAGTTCTC-----------
------
FC88X_00056_00217      --------------CTGTAGTCTAT--CCGCAGCCCAGTTCTC-----------
------
FC88X_00100_00712      --------------CTGTAGTCTAT--CCGCAGCCCAGTTCTC-----------
------
FC88X_00151_00196      --------------CTGTAGTCTAT--CCGCAGCCCAGTTCTC-----------
------
FC88X_00356_00961      --------------CTGTAGTCTAT--CCGCAGCCCAGTTCTC-----------
------
FC88X_00478_00152      --------------CTGTAGTCTAT--CCGCAGCCCAGTTCTC-----------
------
FC88X_00703_00535      --------------CTGTAGTCTAT--CCGCAGCCCAGTTCTC-----------
------
FC88X_00135_00727      --------------CTGTAGTCTAT--CCGCAGCCC------------------
------
FC88X_00048_00277      --------------CTATACTCTCT--ACGCACCGCATTCTTTT----------
------
FC88X_00574_00538      ------------AAATACTATCTAT--TCTC-GCTGCATGGC------------
------
FC88X_01004_00330      ---------TCGAAAAGACACCTCT--TCGC-CCTGCAA---------------
------
FC88X_00074_00236      -------------------ACCTCT--ATTC-GCTGCAATGGCCCTTGC-----
------
FC88X_00906_00522      --------------CTTACTTCTCT--ATCC-ACTGCATTGCAC----------
------
FC88X_00989_01026      --------------CTAACCTCTCT--ATCCCACTGCATTGCA-----------
------
FC88X_01063_00880      --------------ACTATGTCTTT--TCT-TACCGCACGCTTCC---------
------
FC88X_01062_01073      --------------ACACTTTCTAT--TCGATACCGCCTTCCC-----------
------
FC88X_00144_00848      -------------CCGTGTTTCGAT--TCACA-CCTAGTTCTC-----------
------
FC88X_00500_00054      -------------CCGTGTTTCGAT--TCACA-CCTAGTTCTC-----------
------
FC88X_00550_00102      -------------CCGTGTTTCGAT--TCACA-CCTAGTTCTC-----------
------
FC88X_00020_00235      -------------CCGTGTTTCGAT--TCACA-CCTAGTTCTC-----------
------
```

FC88X_00158_00714 -------------CCGTGTTTCGAT--TCACA-CCTAGTTCTC-----------
------
FC88X_00238_00995 -------------CCGTGTTTCGAT--TCACA-CCTAGTTCTC-----------
------
FC88X_00276_00977 -------------CCGTGTTTCGAT--TCACA-CCTAGTTCTC-----------
------
FC88X_00487_01092 -------------CCGTGTTTCGAT--TCACA-CCTAGTTCTC-----------
------
FC88X_00334_00509 -------------CCGTGTTTCGAT--TCACA-CCTAGTTCT------------
------
FC88X_00393_01104 -------------CCGTGTTTCGAT--TCACAACCTAGTTCTC-----------
------
FC88X_00531_00299 -------------TCGTGTTTCGAT--TCACA-CCTAGTTCTC-----------
------
FC88X_00993_00588 -------------CCTTGTTGTGAT--TCACTACGCTGTTCT------------
------
FC88X_01110_00651 -------------CCTTGTTGTGCTCTTCGCTA-GCTATTC-------------
------
FC88X_01124_01073 ------------TCTCAGGTCCCGTCTTTGTC-GCTATGT--------------
------
FC88X_00193_00119 ------------TCTCAGGTCCCGTCTTTGTC-GCTATGT--------------
------
FC88X_00370_01031 ------------TCTCAGGTCCCGTCTTTGTC-GCTATGT--------------
------
FC88X_00403_01159 ------------TCTCAGGTCCCGTCTTTGTC-GCTATGT--------------
------
FC88X_00709_01252 ------------TCTCAGGTCCCGTCTTTGTC-GCTATGT--------------
------
FC88X_01133_00982 ------------TCTCAGGTCCCGTCTTTGTC-GCTATGT--------------
------
FC88X_00554_00211 ------------TCTCAGGTCCCGTCTTTGTC-GCTATGT--------------
------
FC88X_00930_00948 ------------TCTCAGGTCCCGTCTTTGTC-GCTATGT--------------
------
FC88X_00235_00981 ------------TCTCAGGTCCCGTC--TGTC-GCTATGT--------------
------
FC88X_00059_00837 ------------TCTCAGGTCCCGTCTTTGCC-GCTATGT--------------
------
FC88X_00050_00129 ------------TCTCAGGTCCTGTCTTTGTC-GCTATGT--------------
------
FC88X_00173_00615 ------------TCTCAGGTCCCGTCTTTG------------------------
------
FC88X_00496_00952 -----------TCTCCAGGTCCCGTCTTTGTCCGCTATGT--------------
------
FC88X_01001_00402 -----------TTCTCAACTCCCGGATTGGTA-GCACTT---------------
------
FC88X_00137_00629 -----------------CTCCCAGCTTTTGTGCACTTTACGACT----------
------
FC88X_00502_00029 -----------------CTCCCAGCTTTTGTGCACTTTACGACT----------
------
FC88X_00292_00960 -----------------CTCCCAGCTTTTGTGCACTTTACGACT----------
------
FC88X_00557_01206 -----------------CTCCCAGCTTTTGTGCATTTTACGACT----------
------
FC88X_00272_01082 -------------CCAAACTCCCGCTTATGTGCACTAAAC--------------
------

```
FC88X_00656_00231      -------------CCAAACTCCCGCTTATGTGCACTAAAC--------------
------
FC88X_00970_00512      -----------------CTCCTGCTTTTCTCGCACTGAACGTCA----------
------
FC88X_00496_01249      -----------------CTCCCAACTTGTCCGCGCTTACGTATT----------
------
FC88X_00823_01074      -----------------CTCCCAACTTGTCCGCGCTTACGTATT----------
------
FC88X_00564_00104      -----------------CTCCCTTCTTGTCTGCACGTACGTCCGT---------
------
FC88X_00463_01137      -----------------CTCCCAACTTTTCAGTGCACGTAACGC----------
------
FC88X_00447_00117      -----------------ATTACCGCTCATGTTCCCTGTACTATT----------
------
FC88X_00095_00527      -----------------ATTACCGCTCATGTTCCCTGTACTATTCC--------
------
FC88X_00571_00071      -----------------ATTACCGCTCATGTTCCCTGTACTATT----------
------
FC88X_00486_00357      -----------------ATTACCGCTCATGTTCCCTGTACTATT----------
------
FC88X_00539_00177      -----------------ATTACCGCTCATGTTCCCTGTACTATT----------
------
FC88X_00682_00122      -----------------ATTACCGCTCATGTTCCCTGTACTATT----------
------
FC88X_00762_00932      -----------------ATTACCGCTCATGTTCCCTGTACTATT----------
------
FC88X_01030_00465      -----------------ATTACCGCTCATGTTCCCTGTACTATT----------
------
FC88X_00076_00747      -----------------ATTACCGCTCAAGTTCCCTGTACTATT----------
------
FC88X_00228_00547      -----------------ATTACCGCTCAC-TTCCCTGTACTATT----------
------
FC88X_00126_00515      -----------------ATAACCGCTCATGTTCCCTGTACTATT----------
------
FC88X_00889_00721      -----------------TTGACCGCTCTGTGCGCCTCTCCTACT----------
------
FC88X_00599_00651      ------------------ATAACACCCGGTGCCCTTGT-CTATTCG--------
------
FC88X_00625_00121      ------------------ATACCACCAGGTGCCCTTGT-CTATTCG--------
------
FC88X_00865_01205      -------------------CAAAAGCATCTTG-TCTACTCGCCCCTA-------
------
FC88X_00857_00935      -------------------AAAAAGCCTGTTGGCCTGCTCTATTCGC-------
------
FC88X_00448_01193      -----------------CTTCTCCTTTATTTTCACCCGACCTGGTG--------
------
FC88X_00701_00150      -----------------CTTCTCCTTTATTT-CATCCGACCTGGTG--------
------
FC88X_00052_00449      -----------------CTTCTCCTTGGGTT-TACCAGGCCCAACCC-------
------
FC88X_00175_00390      ----------------TCCCCAGGTTTACAATGACCGGCTCCC-----------
------
FC88X_01081_01256      -----------------------CTTTACACT--CCGGCCCGG-----------
------
FC88X_00263_00958      -------------CTATCTCCTC-TCTGTACGCACTTGAC--------------
------
```

FC88X_01075_00894 -------------TTCTCTCCTCCTCGGTATACATTCGAC--------------
------
FC88X_01083_00472 -------------CTCTTCTCCATTAGTTAACCACTCGAC--------------
------
FC88X_00006_00125 --------------CCAATCATTCTTGCGCTGACCT--CCCAT-----------
------
FC88X_00670_01250 -----------------TTCATTGTT-CGCCGACTTGACCCATT----------
------
FC88X_00552_00401 -----------------ATCGTTCTT-CGATCA-CTCCCCAGTCTT--------
------
FC88X_01011_00330 -----------------ATCGTAATTGCGATCAACTCACCCGTG----------
------
FC88X_00091_00371 --------------TTCTCCGATTTT--CAACATGTTGCTGTT-----------
------
FC88X_00028_00493 --------------TTCTCCGATTTT--CAACATGTTGCTGTT-----------
------
FC88X_00259_00958 --------------TTCTCCGATTTT--CAACATGTTGCTGTT-----------
------
FC88X_00337_01093 --------------TTCTCCGATTTT--CAACATGTTGCTGTT-----------
------
FC88X_00477_00057 --------------TTCTCCGATTTT--CAACATGTTGCTGTT-----------
------
FC88X_00954_01116 --------------TTCTCCGATTTT--CAACATGTTGCTGTT-----------
------
FC88X_01064_00467 --------------TTCTCCGATTTT--CAACATGTTGCTGTT-----------
------
FC88X_01112_00694 --------------TTCTCCGATTTT--CAACATGTTGCTGTT-----------
------
FC88X_00759_01265 --------------TTCTCCGATTTT--CAACATGTTGCTGTTTC---------
------
FC88X_00055_00751 --------------TTCTCCGATTTT--CGACATGTTGCTGTT-----------
------
FC88X_00074_00309 --------------TTCTCCGATTCT--CAACATGTTGCTGTT-----------
------
FC88X_00579_00451 --------------TTCCCCGATTTT--CAACATGTTGCTGTT-----------
------
FC88X_00827_01123 --------------TTCTCCGATTTT--CAACACGTTGCTGTT-----------
------
FC88X_00129_00755 --------------TTCTCCGACTTT--CAACATGTTGCTGTT-----------
------
FC88X_00664_00936 -------------ATTCTCCGACTTT--CAACATGTTGCTGTT-----------
------
FC88X_01074_00426 --------------TTCTCCGATTTTT-CAACATGTCGCTGTT-----------
------
FC88X_01120_00740 -------------TTTCTCCGATTTT--TAACATGTTGCTGTT-----------
------
FC88X_00769_00151 --------------TTCTCCCATTTTGCTAACATGCCACGT-------------
------
FC88X_00484_00783 ------------CTTTCTCCTACCTATGCAAACCTGCGTT--------------
------
FC88X_01129_00684 ---------------TCTCCTACTTGCG-AACCCTGCGCTAAC-----------
------
FC88X_00037_00144 --------------------TCTCTATCTATCCCTAAACTGCATTTT-------
------
FC88X_00360_01111 --------------------TCTCCACATTTCCCTCTACCGAATTTAA------
------

```
FC88X_00258_00204       ---------------------CTCCACGTTTCCCCTTACTGGACTGTA------
------
FC88X_00390_01177       ---------------------CTCCATGTTTCCCCTTACTGGACGGTA------
------
FC88X_01030_00691       ----------------TTGCGCTCTAAATTTCTCCGGACTATC-----------
------
FC88X_00975_00769       ----------------CCGCGCTCCCTATAACTACGTACTGTC-----------
------
FC88X_00049_00292       ----------------CCATTCCTTGTTTCAGTTCCCGCTGTT-----------
------
FC88X_00910_00668       ----------------CCACTCCTTGTTTCAGTTCCCGCTGTT-----------
------
FC88X_00635_00937       ----------------CCATCCCTTGTTTCAGTTCCCGCTGTT-----------
------
FC88X_00462_00270       ----------------CCAATCATTATCTCACTCCCCGCTGTT-----------
------
FC88X_00016_00532       ----------------CCAGACTTTCTATGACGCCGAGCTGTTC----------
------
FC88X_00334_00966       ----------------CCACTCTCTCTCTAGCCCGCTACTAA------------
------
FC88X_00917_00532       ----------------CCGATCTTTCTTTAGTTCCTCGCTGCT-----------
------
FC88X_00797_00236       ----------------CTATTCTATCTTTGGCCCTCTGCTGAC-----------
------
FC88X_00120_00741       --------------TTTATATCTCTCCCCGTTGCTAAGCCCAC-----------
------
FC88X_00722_01215       ----------------TTAATCTATCTCTCACCCCGTCGTGTC-----------
------
FC88X_01079_00494       ----------------ATTAACTCTATCTCACCCTGTACTGCC-----------
------
FC88X_00264_00396       ----------------TTACTTCTT-TCTTACCCTGACCTGCGGC---------
------
FC88X_01079_01223       ----------------CCACTTCCTATCTGACCCCCAACTGTC-----------
------
FC88X_00040_00179       -------------TTCTGTG-CATTTCTCCCAGCAC-ACCGTC-----------
------
FC88X_01106_00451       -------------TTCTGTGGCATTTCTCCCAGCAC-ACCGTCCC---------
------
FC88X_00083_00266       -------------TTCTGTG-CATTTCTCCCAGCAC-ACCGTC-----------
------
FC88X_00133_00328       -------------TTCTGTG-CATTTCTCCCAGCAC-ACCGTC-----------
------
FC88X_00109_00741       -------------TTCTGTG-CATTTCTCCCAGCAC-ACCGTC-----------
------
FC88X_00131_00872       -------------TTCTGTG-CATTTCTCCCAGCAC-ACCGTC-----------
------
FC88X_00185_00163       -------------TTCTGTG-CATTTCTCCCAGCAC-ACCGTC-----------
------
FC88X_00200_00929       -------------TTCTGTG-CATTTCTCCCAGCAC-ACCGTC-----------
------
FC88X_00268_00283       -------------TTCTGTG-CATTTCTCCCAGCAC-ACCGTC-----------
------
FC88X_00380_00050       -------------TTCTGTG-CATTTCTCCCAGCAC-ACCGTC-----------
------
FC88X_00594_00198       -------------TTCTGTG-CATTTCTCCCAGCAC-ACCGTC-----------
------
```

FC88X_00621_00497 -------------TTCTGTG-CATTTCTCCCAGCAC-ACCGTC-----------
------
FC88X_00656_00973 -------------TTCTGTG-CATTTCTCCCAGCAC-ACCGTC-----------
------
FC88X_00660_01003 -------------TTCTGTG-CATTTCTCCCAGCAC-ACCGTC-----------
------
FC88X_00700_01151 -------------TTCTGTG-CATTTCTCCCAGCAC-ACCGTC-----------
------
FC88X_00760_00982 -------------TTCTGTG-CATTTCTCCCAGCAC-ACCGTC-----------
------
FC88X_00765_01264 -------------TTCTGTG-CATTTCTCCCAGCAC-ACCGTC-----------
------
FC88X_00846_00131 -------------TTCTGTG-CATTTCTCCCAGCAC-ACCGTC-----------
------
FC88X_00845_00310 -------------TTCTGTG-CATTTCTCCCAGCAC-ACCGTC-----------
------
FC88X_00836_00744 -------------TTCTGTG-CATTTCTCCCAGCAC-ACCGTC-----------
------
FC88X_00885_00779 -------------TTCTGTG-CATTTCTCCCAGCAC-ACCGTC-----------
------
FC88X_00940_00818 -------------TTCTGTG-CATTTCTCCCAGCAC-ACCGTC-----------
------
FC88X_00966_00227 -------------TTCTGTG-CATTTCTCCCAGCAC-ACCGTC-----------
------
FC88X_00980_00568 -------------TTCTGTG-CATTTCTCCCAGCAC-ACCGTC-----------
------
FC88X_00997_00695 -------------TTCTGTG-CATTTCTCCCAGCAC-ACCGTC-----------
------
FC88X_01021_00867 -------------TTCTGTG-CATTTCTCCCAGCAC-ACCGTC-----------
------
FC88X_01079_01228 -------------TTCTGTG-CATTTCTCCCAGCAC-ACCGTC-----------
------
FC88X_01141_00635 -------------TTCTGTG-CATTTCTCCCAGCAC-ACCGTC-----------
------
FC88X_01127_00684 -------------TTCTGTG-CATTTCTCCCAGCAC-ACCGTC-----------
------
FC88X_01114_00948 -------------TTCTGTG-CATTTCTCCCAGCAC-ACCGTC-----------
------
FC88X_00056_00674 -------------TTCTGTG-CATTTCTCCCAGCAC-ACCGTC-----------
------
FC88X_00245_00093 -------------TTCTGTG-CATTTCTCCCAGCAC-ACCGTC-----------
------
FC88X_00210_01023 -------------TTCTGTG-CATTTCTCCCAGCAC-ACCGTC-----------
------
FC88X_00260_00042 -------------TTCTGTG-CATTTCTCCCAGCAC-ACCGTC-----------
------
FC88X_00272_00526 -------------TTCTGTG-CATTTCTCCCAGCAC-ACCGTC-----------
------
FC88X_00270_01002 -------------TTCTGTG-CATTTCTCCCAGCAC-ACCGTC-----------
------
FC88X_00378_01195 -------------TTCTGTG-CATTTCTCCCAGCAC-ACCGTC-----------
------
FC88X_00488_01124 -------------TTCTGTG-CATTTCTCCCAGCAC-ACCGTC-----------
------
FC88X_00517_01125 -------------TTCTGTG-CATTTCTCCCAGCAC-ACCGTC-----------
------

```
FC88X_00586_01230       -------------TTCTGTG-CATTTCTCCCAGCAC-ACCGTC-----------
------
FC88X_00619_01053       -------------TTCTGTG-CATTTCTCCCAGCAC-ACCGTC-----------
------
FC88X_00669_00368       -------------TTCTGTG-CATTTCTCCCAGCAC-ACCGTC-----------
------
FC88X_00747_01134       -------------TTCTGTG-CATTTCTCCCAGCAC-ACCGTC-----------
------
FC88X_00797_00968       -------------TTCTGTG-CATTTCTCCCAGCAC-ACCGTC-----------
------
FC88X_00767_01102       -------------TTCTGTG-CATTTCTCCCAGCAC-ACCGTC-----------
------
FC88X_00858_00456       -------------TTCTGTG-CATTTCTCCCAGCAC-ACCGTC-----------
------
FC88X_00938_00196       -------------TTCTGTG-CATTTCTCCCAGCAC-ACCGTC-----------
------
FC88X_00913_00358       -------------TTCTGTG-CATTTCTCCCAGCAC-ACCGTC-----------
------
FC88X_00921_00946       -------------TTCTGTG-CATTTCTCCCAGCAC-ACCGTC-----------
------
FC88X_00919_00999       -------------TTCTGTG-CATTTCTCCCAGCAC-ACCGTC-----------
------
FC88X_00949_01185       -------------TTCTGTG-CATTTCTCCCAGCAC-ACCGTC-----------
------
FC88X_00989_00761       -------------TTCTGTG-CATTTCTCCCAGCAC-ACCGTC-----------
------
FC88X_00954_00822       -------------TTCTGTG-CATTTCTCCCAGCAC-ACCGTC-----------
------
FC88X_00996_01013       -------------TTCTGTG-CATTTCTCCCAGCAC-ACCGTC-----------
------
FC88X_00999_01200       -------------TTCTGTG-CATTTCTCCCAGCAC-ACCGTC-----------
------
FC88X_01011_00567       -------------TTCTGTG-CATTTCTCCCAGCAC-ACCGTC-----------
------
FC88X_01038_00577       -------------TTCTGTG-CATTTCTCCCAGCAC-ACCGTC-----------
------
FC88X_01011_00652       -------------TTCTGTG-CATTTCTCCCAGCAC-ACCGTC-----------
------
FC88X_01013_00933       -------------TTCTGTG-CATTTCTCCCAGCAC-ACCGTC-----------
------
FC88X_01056_00712       -------------TTCTGTG-CATTTCTCCCAGCAC-ACCGTC-----------
------
FC88X_01079_00884       -------------TTCTGTG-CATTTCTCCCAGCAC-ACCGTC-----------
------
FC88X_01147_00999       -------------TTCTGTG-CATTTCTCCCAGCAC-ACCGTC-----------
------
FC88X_00768_01202       ------------GTTCTGTG-CATTTCTCCCAGCAC-ACCGTC-----------
------
FC88X_00909_00694       ------------GTTCTGTG-CATTTCTCCCAGCAC-ACCGTC-----------
------
FC88X_00756_00927       ------------GTTCTGTG-CATTTCTCCCAGCAC-ACCGTC-----------
------
FC88X_00178_00733       -------------TTCTGTG-CATTCCTCCCAGCAC-ACCGTC-----------
------
FC88X_00099_00914       --------------TCTGTG-CATTTCTCCCAGCAC-ACCGTC-----------
------
```

```
FC88X_00850_00917       -------------ATCTGTG-CATTTCTCCCAGCAC-ACCGTC-----------
------
FC88X_00175_01008       -------------TTCTGTG-CATTTCTCCCAGCAC-ACCGTCCC---------
------
FC88X_00893_01125       -------------TTCTGTGCATTTTCTCCCAGCAC-ACCGTC-----------
------
FC88X_00032_00468       -------------TTCTGTG-CATTTCTCCCAGCAC-ACCGAC-----------
------
FC88X_00219_00930       -------------TTCTGTG-CATTTCTCCCAGCAC-ATCGTC-----------
------
FC88X_00784_01139       -------------TTCTGTG-CATTTCTCCCAGCAC-ACCGTCATCACCG----
------
FC88X_00373_01165       -------------TTCTGTG-CATTTCTCCCCGCAC-ACCGTC-----------
------
FC88X_00670_00930       -------------TTCTGTG-CATTTCTCCCAGCACCACCGTC-----------
------
FC88X_00947_00944       ---------------TTGTG-CATTTCTCCCAGCAC-ACCGTC-----------
------
FC88X_00742_01069       -------------TTCCGTG-CATTTCTCCCAGCAC-ACCGTT-----------
------
FC88X_00960_00714       -------------TTCCGTG-CATTTCTCCCAGCAC-ACCGTC-----------
------
FC88X_01051_00872       ------------GTTCTGTG-CATTTCTCCCAGCAC-ACCGCC-----------
------
FC88X_00817_00987       -----------------ACATTACACTCTCCGC-ATCACCTCCTG---------
------
FC88X_01125_00883       ------------------CTTTCCCCTCTCGGCCATCACCCACC----------
------
FC88X_01083_00829       -----------------ACATACCTTCTCCGCAGTCCACCGCAT----------
------
FC88X_00065_00770       -----------------ACACACTCTTGTCTGCCTCCACTCCG-----------
------
FC88X_00033_00502       -------------CTCTGCAGCTCTCCCCACACAACTTTA--------------
------
FC88X_00099_00336       -------------CTCCGCAGCTGTGACTCCACGAACATT--------------
------
FC88X_00996_01146       -----------AACGCGGCAACTCTCTCTCCACTAATT----------------
------
FC88X_01038_01033       ------------ACATTCACTCTGCCCTCTCTTCATAAA---------------
------
FC88X_00697_00359       -------------CATT-ACACTCCTCTCACGTCA-------------------
------
FC88X_00387_01042       ------------TGCCTAACCCTTCTCTACGACCAGTACCTTCCTTTCCTT---
------
FC88X_00962_00853       ---------------CTAACTCCTTCCCGTTATGATCATTAC------------
------
FC88X_00091_00246       ----------------TTACTCTCTCTCCTCACGACAATTCCC-----------
------
FC88X_00882_00606       -----------TTCTCCGAC-ACTAGCTG-CCTGCCTATC--------------
------
FC88X_00515_01027       ------------CTTCCGACTACTCTGCC-TTCACCTATCAT------------
------
FC88X_00040_00156       ---------------CTATGTTCTCTGTC-CACACCT-----------------
------
FC88X_00800_00325       ---------------CTAT-TTCTTTGCT-TCCACCACTCTGT-----------
------
```

FC88X_00689_00883 ---------------CGAACTTCTCTGCCGCTCACCAACCTGCC----------
------
FC88X_00054_00752 ----------------CCCGAGACTCTCCAATCCCCTATTGCA-----------
------
FC88X_00753_00965 -----------------CCGATACTTCTCC-TCCACTATAGCCCA---------
------
FC88X_00181_00055 -----------------CCCGAACTTCTCC-TCAACTGACCTATT---------
------
FC88X_00786_00089 -----------------CCCGAACTTCTCC-TCAACTGACCTATT---------
------
FC88X_00783_00190 -----------------CCCGAACTTCTCC-TCAACTGACCTATT---------
------
FC88X_00355_01115 -----------------CCAAACCCTCTCA-TCCAGCGACTTATT---------
------
FC88X_00258_00808 -----------------CCAAACCTCCAC--TCCAGCGACTTATT---------
------
FC88X_00030_00210 ----------------TTCGAACCTTCCCAACTGACGTATCAC-----------
------
FC88X_00092_00908 ----------------TTTAAACCTCCTC--CTCACGTATCTCGT---------
------
FC88X_00476_01241 -----------------CCGAGCCTTCTC--CAAACGTGTCTTAAC--------
------
FC88X_00575_00138 ----------CAGTCCCTCTGTAC-TGTGAAGCCACAGATGTCGAGTC------
------
FC88X_00837_01000 --------------CCCTCTGTAC-TGTGAAGCCACAGATGTCGAGT-------
------
FC88X_01025_00464 --------------TCCTCTGTAC-TGTGAAGCCACAGATGTCG----------
------
FC88X_00819_00322 --------------CCCTCTGTAC-TGTGAAGCCACAGATGTCGAGTGAG----
------
FC88X_00770_00203 ---------ACAGTCCCTCTGTAC-TGTGAAGCCACAGATGACTAGC-------
------
FC88X_01047_01108 ----------CAGTCC-TCTGTAC-TGTGAAGCCACAGATGACTAG--------
------
FC88X_00166_00930 -------TGGACAGTCCTCTGTAC-TGTGAAGCCACAGATGGACTGT-------
------
FC88X_00858_00908 ---------------TCTCTATTCATGTGACACC-CAGATATC-----------
------
FC88X_01111_00650 ---------------CTCTTTCGTTTGCAAACCCGCATGTT-------------
------
FC88X_00480_00937 ----------------CCTATAGCTCCCCAACACAAA--TGTCCT---------
------
FC88X_01086_01193 ----------------CCGATTATTCTCCGACACCGACCTGTCCT---------
------
FC88X_00562_01254 ----------------ACTTTAACCTTTCCCAACGCTCCTTGC-----------
------
FC88X_00375_00532 ----------------CCTATAACTCCCGAACACTGGCCTTGA-----------
------
FC88X_00855_00238 -----------------GCAGCGCGTCCATAGACCGGTTTCGACT---------
------
FC88X_00920_01245 ----------------ACGAACTCCTCCA-AGACCAACCTCGA-----------
------
FC88X_00067_00337 ---------------------TTCGTACACTGACCTTTCGTAA-----------
------
FC88X_00187_00271 ---------------------TTCCTCCCAAACCAGTGTCGTGCTGTC------
------

FC88X_00292_01139 ---------------------TTCCTCCCAAACCAGTGTCGTGCTGTC------
------
FC88X_00472_00309 ---------------------TTCCTCCCAAACCAGTGTCGTGCTGT-------
------
FC88X_00607_00391 ---------------------TTTCTCCCTAACC-GTGATGTGTTATTCCG---
------
FC88X_00977_00695 ---------------------CTCTTCCCCAAACTGCCCTGTTCTTGT------
------
FC88X_00623_01077 ---------------------CTCTTCCCCAGACGGCTGTGT------------
------
FC88X_00527_01215 ----------------CCCGGTCTTCTCCCACCCTGCTGTGCA-----------
------
FC88X_00427_01036 --------------CTCCCTGGTACTTATCAATCTACCAAACT-----------
------
FC88X_00117_00391 --------------CTCCCTGGTATTTATCAATCTACCAAACT-----------
------
FC88X_00545_00081 ----------------CTGTTATTTCTCTCGACCCACCTTGAT-----------
------
FC88X_00991_00771 ----------------CTGTGTTCTTTCCCACCTAGCCCTGTT-----------
------
FC88X_00427_01030 ----------------CTGTGTTCTTTC--------------------------
------
FC88X_00022_00503 ----------------CTGTGTTCTCTCTCAACGCCAAATGGC-----------
------
FC88X_00848_00528 -GTTACAGGTAATTAACTGTGATCTCTC--------------------------
------
FC88X_00916_00512 CGATACATGTAAGTA-TTGCTCTCTCAC--------------------------
------
FC88X_00215_00059 --------------TCTGA-CTG-TTTCCAAAGCA--GCTGTTCT---------
------
FC88X_00338_01044 -------------GTCTGA-CTG-TTTCCAAAGCA--GCTGTTCT---------
------
FC88X_00569_00835 --------------TCTGA-CTG-TTTCCAAAGCA--GCTGTTCT---------
------
FC88X_00664_00226 --------------TCTGA-CTG-TTTCCAAAGCA--GCTGTTCT---------
------
FC88X_00071_00396 --------------TCTGA-CTG-TTTCCAAAGCA--GCTGTTCT---------
------
FC88X_00063_00702 --------------TCTGA-CTG-TTTCCAAAGCA--GCTGTTCT---------
------
FC88X_00071_00726 --------------TCTGA-CTG-TTTCCAAAGCA--GCTGTTCT---------
------
FC88X_00389_01199 --------------TCTGA-CTG-TTTCCAAAGCA--GCTGTTCT---------
------
FC88X_00879_00929 --------------TCTGA-CTG-TTTCCAAAGCA--GCTGTTCT---------
------
FC88X_00964_00299 --------------TCTGA-CTG-TTTCCAAAGCA--GCTGTTCT---------
------
FC88X_00012_00426 --------------TCTGA-CTG-TTTCCAAAGCA--GCTGTTCT---------
------
FC88X_00187_00951 --------------TCTGAACTGATTTCCAAAGCA--GCTGTTCCT--------
------
FC88X_00959_01229 --------------TCTGA-CTG-TTTCCAGAGCA--GCTGTTCT---------
------
FC88X_00367_00974 --------------TCTGA-CTG-TTTCCAAAGCA--GCTGCTCTC--------
------

FC88X_00270_00023 ---------------CCTGACC---TTTCCAGTGCGC-GCTGTTTT---------
------
FC88X_00512_01210 ---------------CCTGACC---TTTCCAGTGCGC-GCTGTTTT---------
------
FC88X_00556_01124 ---------------CCTGACC---TTTCCAGTGCGC-GCTGTTTT---------
------
FC88X_00661_00120 ---------------CCTGACC---TTTCCAGTGCGC-GCTGTTTT---------
------
FC88X_00912_00854 ---------------CCTGACC---TTTCCAGTGCGC-GCTGTTTT---------
------
FC88X_01040_00840 ---------------CCTGACC---TTTCCAGTGCGC-GCTGTTTT---------
------
FC88X_01057_00683 ---------------CCTGACC---TTTCCAGTGCGC-GCTGTTTT---------
------
FC88X_00942_01270 ---------------CCTGACC---TTTCCAGTGCGC-GCTGTTTTCTTCCTTT-
------
FC88X_01021_00781 ---------------CCTGA-CGTTCCCTCAGCGCGC-GCTTTA-----------
------
FC88X_01106_00900 ---------------CCTGA-CGTTCCCCCAGCGCGC-GCTTTA-----------
------
FC88X_00846_01197 ----------------CTACTGTCACGTCGACTGCGC-CCTGTT-----------
------
FC88X_00636_00367 ----------------CTACTGTCACGTCGACTGCGC-C-TGTT-----------
------
FC88X_00921_00918 ----------------CTACTGTCACGTCGACTGCGC-CCTGTT-----------
------
FC88X_00612_00093 ----------------CTACTGTCACGTCGACTGCGC-CCTGTT-----------
------
FC88X_00117_00421 ----------------CTACTGTCACGTCGACTGCGC-CCTGTC-----------
------
FC88X_01110_00755 ----------------CTACCGAGTCTTCCGCTCAGC-CGTGTT-----------
------
FC88X_00120_00093 ----------------CTACCGAGTCTTCCGCTCAGC-CGTGTT-----------
------
FC88X_00172_00725 ----------------CTATTGTTTCACCAGCTGCTC-CCTGAT-----------
------
FC88X_00961_00538 ---------------CTCCTGTTTA-ACCAGCCGTGA-CCTGTT-----------
------
FC88X_00979_00795 --------------GCTCCTGTTTA-ACCAGCCGTGA-CCTGTT-----------
------
FC88X_01061_00733 --------------GCTCCTGCTTG-GCCA-CGGTGC-CCTTTT-----------
------
FC88X_01029_00986 ---------------CTACTATTTATACCACTCGCAC-ACCAC------------
------
FC88X_00434_00133 ---------------TAACTCCTTCTTCAACGCGTTG-ACCAC------------
------
FC88X_01109_00850 ---------------CTCCTCTTTCTTCCCTGATGCC-GTTCC------------
------
FC88X_00180_00889 -------------TAGCTCCCTGCCTTCCCTGACCGT-GTCCTTCCTTT------
------
FC88X_00150_00899 --------------GCTCAAGCTTTCCGTCTTAACTGATCTC-------------
------
FC88X_01115_01041 --------------GCTCAAGCTT-CCGCTTAACCTGATCTC-------------
------
FC88X_01002_00461 ---------------CTCTGGGATCACATCAGGCTACTACTC-------------
------

FC88X_00315_00806 ---------------CTCTGGGATCACATCAGGCTACTACTC-------------
------
FC88X_00519_00159 ---------------CTCTGGGATCACATCAGGCTACTACTC-------------
------
FC88X_00739_00257 ---------------CTCTGGGATCACATCAGGCTACTACTC-------------
------
FC88X_00860_01179 ---------------CTCTGGGATCACATCAGGCTACTACTC-------------
------
FC88X_00342_01128 ---------------CTCTGGGATCACATCAGGCTACTACT--------------
------
FC88X_00625_00684 ---------------CTCTGGGATCACATCAGGCTACTACTCATCAC--------
------
FC88X_00093_00026 ---------------ATCCGGGCTCACTTCCGGCGAATTCTC-------------
------
FC88X_00229_00065 ---------------ATCCGGGCTCACTTCCGGCGAATTCTC-------------
------
FC88X_01085_00773 ---------------ATCCGGGCTCACTTCCGGCGAATTCTC-------------
------
FC88X_00575_01012 ---------------ATCCGGGCTCATTTCCGGCGAATTCTC-------------
------
FC88X_00508_00652 ------------------------CTAATCCGTCAACTTCTCCTATCTCAT----
------
FC88X_01133_00599 -------------------CTTCTCTAATCCGGCTA--TCACTTATCT-------
------
FC88X_00634_00194 ---------GGGGAAGCTCGCCCTTCTCTAAATGATTGTTCCC-----------
------
FC88X_00654_00259 --------------CTACTCCTCCTTTTACAACTC-TCGTTCCC-----------
------
FC88X_00032_00469 -----------------CTTTTCGCTTCCCTGACTATTGTTTT------------
------
FC88X_00105_00406 -----------------CTTTTCGCTTCCCTGACTATTGTTTTT-----------
------
FC88X_00141_00620 -----------------CTCTTAGCTGCTCCTTCACTTAATGTT-----------
------
FC88X_00728_01243 ---------------TTTATCCCTCATCTTCGTCGTCCGCAC-------------
------
FC88X_01131_01131 ---------------TTTATCTCTCATCTTCGTCGTCCGCAC-------------
------
FC88X_00486_01205 --------------CCTTATCCTTT-TCTTCCACTCCCGC---------------
------
FC88X_00094_00151 ---------------TTTACTCCTAATCCTTGTTCTACCCAGC------------
------
FC88X_00179_00085 -------------------CTCCTCATCCTTGCCCTTTCCACTAAC---------
------
FC88X_00041_00075 ----------------CGCGTTTCTA-CTGGCGTGTC-TATATCTC---------
------
FC88X_00237_00936 ----------------CGCGTTTCTA-CTGGCGTGTC-TATATCTC---------
------
FC88X_00048_00094 ----------------CGCGTTTCTA-TTGGCGTGTC-TATATCTC---------
------
FC88X_00948_00530 ----------------CGCGTTTCTA-TTGGCGTGTCCTATATCTC---------
------
FC88X_00163_00505 ----------------CGCGTTTCTA-TTGGCGTGTC-TATATCTC---------
------
FC88X_00294_00260 ----------------CGCGTTTCTA-TTGGCGTGTC-TATATCTC---------
------

FC88X_00597_00966 ---------------CGCGTTTCTA-TTGGCGTGTC-TATATCTC---------
------
FC88X_00665_00902 ---------------CGCGTTTCTA-TTGGCGTGTC-TATATCTC---------
------
FC88X_00871_00574 ---------------CGCGTTTCTA-TTGGCGTGTC-TATATCTC---------
------
FC88X_00892_00811 ---------------CGCGTTTCTA-TTGGCGTGTC-TATATCTC---------
------
FC88X_00954_00863 ---------------CGCGTTTCTA-TTGGCGTGTC-TATATCTC---------
------
FC88X_00968_00890 ---------------CGCGTTTCTA-TTGGCGTGTC-TATATCTC---------
------
FC88X_00995_01185 ---------------CGCGTTTCTA-TTGGCGTGTC-TATATCTC---------
------
FC88X_01008_00735 ---------------CGCGTTTCTA-TTGGCGTGTC-TATATCTC---------
------
FC88X_01090_01038 ---------------CGCGTTTCTA-TTGGCGTGTC-TATATCTC---------
------
FC88X_01108_01047 ---------------CGCGTTTCTA-TTGGCGTGTC-TATATCTC---------
------
FC88X_00013_00130 ---------------CGCGTTTCTA-TTGGCGTGTC-TATATCTC---------
------
FC88X_00055_00045 ---------------CGCGTTTCTA-TTGGCGTGTC-TATATCTC---------
------
FC88X_00092_00404 ---------------CGCGTTTCTA-TTGGCGTGTC-TATATCTC---------
------
FC88X_00092_00620 ---------------CGCGTTTCTA-TTGGCGTGTC-TATATCTC---------
------
FC88X_00110_00176 ---------------CGCGTTTCTA-TTGGCGTGTC-TATATCTC---------
------
FC88X_00121_00304 ---------------CGCGTTTCTA-TTGGCGTGTC-TATATCTC---------
------
FC88X_00311_01059 ---------------CGCGTTTCTA-TTGGCGTGTC-TATATCTC---------
------
FC88X_00385_00976 ---------------CGCGTTTCTA-TTGGCGTGTC-TATATCTC---------
------
FC88X_00427_01218 ---------------CGCGTTTCTA-TTGGCGTGTC-TATATCTC---------
------
FC88X_00572_00977 ---------------CGCGTTTCTA-TTGGCGTGTC-TATATCTC---------
------
FC88X_00642_01256 ---------------CGCGTTTCTA-TTGGCGTGTC-TATATCTC---------
------
FC88X_00665_00334 ---------------CGCGTTTCTA-TTGGCGTGTC-TATATCTC---------
------
FC88X_00693_01037 ---------------CGCGTTTCTA-TTGGCGTGTC-TATATCTC---------
------
FC88X_00778_00801 ---------------CGCGTTTCTA-TTGGCGTGTC-TATATCTC---------
------
FC88X_00827_00548 ---------------CGCGTTTCTA-TTGGCGTGTC-TATATCTC---------
------
FC88X_00868_00218 ---------------CGCGTTTCTA-TTGGCGTGTC-TATATCTC---------
------
FC88X_00895_00437 ---------------CGCGTTTCTA-TTGGCGTGTC-TATATCTC---------
------
FC88X_00871_00870 ---------------CGCGTTTCTA-TTGGCGTGTC-TATATCTC---------
------

FC88X_00904_00228 ---------------CGCGTTTCTA-TTGGCGTGTC-TATATCTC---------
------
FC88X_00917_00210 ---------------CGCGTTTCTA-TTGGCGTGTC-TATATCTC---------
------
FC88X_00940_00917 ---------------CGCGTTTCTA-TTGGCGTGTC-TATATCTC---------
------
FC88X_00914_01234 ---------------CGCGTTTCTA-TTGGCGTGTC-TATATCTC---------
------
FC88X_00975_00261 ---------------CGCGTTTCTA-TTGGCGTGTC-TATATCTC---------
------
FC88X_00971_00405 ---------------CGCGTTTCTA-TTGGCGTGTC-TATATCTC---------
------
FC88X_00973_00596 ---------------CGCGTTTCTA-TTGGCGTGTC-TATATCTC---------
------
FC88X_00969_00670 ---------------CGCGTTTCTA-TTGGCGTGTC-TATATCTC---------
------
FC88X_01013_00434 ---------------CGCGTTTCTA-TTGGCGTGTC-TATATCTC---------
------
FC88X_01020_00648 ---------------CGCGTTTCTA-TTGGCGTGTC-TATATCTC---------
------
FC88X_01045_01091 ---------------CGCGTTTCTA-TTGGCGTGTC-TATATCTC---------
------
FC88X_01069_00925 ---------------CGCGTTTCTA-TTGGCGTGTC-TATATCTC---------
------
FC88X_00786_00800 --------------GCGCGTTTCTA-TTGGCGTGTC-TATATCTC---------
------
FC88X_00131_00797 --------------GCGCGTTTCTA-TTGGCGTGTC-TATATCTC---------
------
FC88X_01021_00396 ---------------CGCGTTTCTA-TTGTCGTGTC-TATATCTC---------
------
FC88X_00984_00971 ---------------CGCGTTTCTA-TTGGCGTGTC-TATATCTCCTTCCTTT-
------
FC88X_01033_01049 ---------------CGCGTTTCTA-TTGGCGTGTC-TATATCTCCTTCCTTT-
------
FC88X_00986_00931 ---------------CGCGTTTCTA-TTAGCGTGTC-TATATCTC---------
------
FC88X_01077_00803 ---------------CGCGTTTTTA-TTGGCGTGTC-TATATCTC---------
------
FC88X_00727_01124 ---------------CGCGTTTTTA-TTGGCGTGTC-TATATCT----------
------
FC88X_00470_00147 --------------GCGCGTTTTTA-TTGGCGTGTC-TATATCTC---------
------
FC88X_00435_00079 ---------------CGCGCTTCTA-TTGGCGTGTC-TATATCTC---------
------
FC88X_00588_01015 ---------------CGCGCTTCTAATTGGCGTGTC-TATATCTC---------
------
FC88X_00116_00391 ---------------CGCGTTTCTA-TCGGCGTGTC-TATATCTC---------
------
FC88X_00688_01238 ---------------CGCGTTTCTA-TCGGCGTGTC-TATATCTC---------
------
FC88X_00096_00388 ---------------CGCGTTTCTA-TTGGCGTGTC-TATGTCTC---------
------
FC88X_00238_00927 ---------------CGCGTCT-TA-TTGGCGTGTC-TATGTCTC---------
------
FC88X_00358_01078 ---------------CGCGTCTCTA-TTGGCGTGTC-TATATCTC---------
------

FC88X_00574_00238 ---------------CGCGTTTCTA-TTGGCGTGTC-TGCATCTC---------
------
FC88X_00019_00705 --------------CGTCGTTTCTA-TTGGCGTGTC-TATATCTC---------
------
FC88X_00176_00877 ---------------CGCGTTTCCG-TTGGCGTGTC-TATATCTC---------
------
FC88X_00038_00238 --------------TCGCGTCCCTA-TTGGCGTGTC-TATATCTC---------
------
FC88X_00164_00644 ---------GGGGAACGCGTTTCTA-TTGGCGTGCC------------------
------
FC88X_00413_00116 ---------------CCCGTGATTACTGGACGCGCC-TATACC-----------
------
FC88X_00487_00328 ---------------ACCCTTATTTCTTGTCGCGCC-ACTAC------------
------
FC88X_00858_00913 ---------------CAGCTGTTTT-TT-ACGCGCCTACTATTCC---------
------
FC88X_00941_00963 ---------------CAGCTGTTTT-TT-ACGCGCCTACTATTCC---------
------
FC88X_01070_00963 ---------------CAGCTGTTTT-TT-ACGCGCCTACTATTCC---------
------
FC88X_00037_00238 ---------------CAGCTGTTTT-TT-ACGCGCCTACTATTCC---------
------
FC88X_00010_00649 ---------------CAGCTGTTTT-T--ACGCGCCTACTATTCC---------
------
FC88X_00057_00617 ---------------CAGCTGTTTT-T--ACGCGCCTACTATTCC---------
------
FC88X_00506_01210 ---------------CAGCTGTTTT-T--ACGCGCCTACTATTCC---------
------
FC88X_00832_01090 ---------------CAGCTGTTTT-T--ACGCGCCTACTATTCC---------
------
FC88X_00974_00978 ---------------CAGCTGTTTT-T--ACGCGCCTACTATTCC---------
------
FC88X_00954_01179 ---------------CAGCTGTTTT-T--ACGCGCCTACTATTCC---------
------
FC88X_01086_00719 ---------------CAGCTGTTTT-T--ACGCGCCTACTATTCC---------
------
FC88X_00033_00597 ---------------CAGCTGTTTT-T--ACGCGCCTACTATTCC---------
------
FC88X_00118_00130 ---------------CAGCTGTTTT-T--ACGCGCCTACTATTCC---------
------
FC88X_00192_00602 ---------------CAGCTGTTTT-T--ACGCGCCTACTATTCC---------
------
FC88X_00361_00074 ---------------CAGCTGTTTT-T--ACGCGCCTACTATTCC---------
------
FC88X_00430_00510 ---------------CAGCTGTTTT-T--ACGCGCCTACTATTCC---------
------
FC88X_00535_00056 ---------------CAGCTGTTTT-T--ACGCGCCTACTATTCC---------
------
FC88X_00811_00904 ---------------CAGCTGTTTT-T--ACGCGCCTACTATTCC---------
------
FC88X_00948_00616 ---------------CAGCTGTTTT-T--ACGCGCCTACTATTCC---------
------
FC88X_00974_00572 ---------------CAGCTGTTTT-T--ACGCGCCTACTATTCC---------
------
FC88X_01012_00803 ---------------CAGCTGTTTT-T--ACGCGCCTACTATTCC---------
------

FC88X_01138_01214 ---------------CAGCTGTTTT-T--ACGCGCCTACTATTCC---------
------
FC88X_00405_00119 ---------------CAGCTGTTTT-TTTACGCGCCTACTATTCC---------
------
FC88X_00967_00296 ---------------CAGCTGTTTT-TTTACGCGCCTACTATTCC---------
------
FC88X_00166_00093 ---------------CAGCTGTTTT-T--GCGCGCCTACTATTCC---------
------
FC88X_00786_00097 ---------------TAGCTGTTTT-T--ACGCGCCTACTATTCC---------
------
FC88X_01080_01058 ---------------CAGCTGCTTT-T--ACGCGCCTACTATTCC---------
------
FC88X_00876_00182 --------GGGGAATCAGCTGTTGT-T--ACGCGCCTACTATTCCC--------
------
FC88X_00314_00972 ---------------CAGCTGTTTT-T--ACGCGTCTACTATTCC---------
------
FC88X_00532_01235 --------------GCAGCTGTTTT-TT-ACGCGCTCACTATTCC---------
------
FC88X_00773_00142 ---------------CTTATGTTTC-TC-GCGTGCCTATTCGTTCCC-------
------
FC88X_01044_00468 ---------------CTTATGTTTC-CC-GCGTGCCTATTCGT-----------
------
FC88X_00114_00884 ---------------CTTATGTTTC-TC-GCGTGCCTATTCGT-----------
------
FC88X_00220_01042 ---------------CTTATGTTTC-TC-GCGTGCCTATTCGT-----------
------
FC88X_00978_00447 ---------------CTTATGTTTC-TC-GCGTGCCTATTCGT-----------
------
FC88X_01069_00402 ---------------CTTATGTTTC-TC-GCGTGCCTATTCGT-----------
------
FC88X_00516_01110 ---------------CTTATGTTTC-TC-GCGTGCCTATTCGT-----------
------
FC88X_00895_00723 ---------------CTTATGTTTC-TC-GCGTGCCTATTCGT-----------
------
FC88X_00878_00854 ---------------CTTATGTTTC-TC-GCGTGCCTATTCGT-----------
------
FC88X_00888_00934 ---------------CTTATGTTTC-TC-GCGTGCCTATTCGT-----------
------
FC88X_00922_00248 ---------------CTTATGTTTC-TC-GCGTGCCTATTCGT-----------
------
FC88X_00485_01186 ---------------CTTATGCTTC-TC-GCGTGCCTATTCGT-----------
------
FC88X_00223_00560 ---------------CATATGTTTC-TC-GCGTGCCTATTCGT-----------
------
FC88X_00317_00485 ---------------CTCATGTTTC-TC-GCGTGCCTATTCGT-----------
------
FC88X_00626_00229 --------------TCCTGTCTTTT----GCCGATCGTCTATTCT---------
------
FC88X_00142_00867 --------------TCCTGTCTTTT----GCCGATCGTCTATTCT---------
------
FC88X_00919_00501 --------------TCCTGTCTTTT----GCCGATCGTCTATTCT---------
------
FC88X_00903_00402 --------------CCCTGTATTAT----GCC-ACACTCTATTCTT--------
------
FC88X_00031_00422 ----------------CCGTGTTTT----ATGCATTCTCTCTTCATT-------
------

FC88X_00072_00338 -------------GTCTTGTATTTT----GTGCATC-ACTTTTCTC--------
------
FC88X_00924_00930 -------------GTCTTGTATTTT----GTGCATC-ACCTTTTCTC-------
------
FC88X_00761_00130 -------------GTCTTGTATTTT----GTGCATC-ACTTTTCTC--------
------
FC88X_00050_00303 --------------TCTTGTATTTT----GTGCATC-ACTTTTCTC--------
------
FC88X_00051_00561 --------------TCTTGTATTTT----GTGCATC-ACTTTTCTC--------
------
FC88X_00113_00763 --------------TCTTGTATTTT----GTGCATC-ACTTTTCTC--------
------
FC88X_00166_00696 --------------TCTTGTATTTT----GTGCATC-ACTTTTCTC--------
------
FC88X_00206_00956 --------------TCTTGTATTTT----GTGCATC-ACTTTTCTC--------
------
FC88X_00319_01172 --------------TCTTGTATTTT----GTGCATC-ACTTTTCTC--------
------
FC88X_00167_00703 --------------TCTTGTATTTT----GTGCATC-ACTTTTCTC--------
------
FC88X_00438_00645 -------------ATCTTGTATTTT----GTGCATC-ACTTTTCTC--------
------
FC88X_00253_00973 --------------TCTTGTATTTT----GTGCATC-ACTTTTCTC--------
------
FC88X_00486_00111 --------------TCTTGTATTTT----GTGCATC-ACTTTTCTC--------
------
FC88X_00547_01179 --------------TCTTGTATTTT----GTGCATC-ACTTTTCTC--------
------
FC88X_00698_00928 --------------TCTTGTATTTT----GTGCATC-ACTTTTCTC--------
------
FC88X_00701_00196 --------------TCTTGTATTTT----GTGCATC-ACTTTTCTC--------
------
FC88X_00744_00989 --------------TCTTGTATTTT----GTGCATC-ACTTTTCTC--------
------
FC88X_00240_00986 --------------TCTTGTATTTTT---GTGCATC-ACTTTTCTC--------
------
FC88X_00996_00539 --------------TCTTGTATTTTT---GTGCATC-ACTTTTCTC--------
------
FC88X_00274_01106 --------------TCTTGTATTTT----GTGCATC-ACTTTTCTC--------
------
FC88X_00494_00994 --------------TCTTGTATTTT----GTGCATC-ACTTTTCTC--------
------
FC88X_00644_00277 --------------TCTTGTATTTT----GTGCATC-ACTTTTCTC--------
------
FC88X_00694_01115 --------------TCTTGTATTTT----GTGCATC-ACTTTTCTC--------
------
FC88X_00715_00795 --------------TCTTGTATTTT----GTGCATC-ACTTTTCTC--------
------
FC88X_00780_00226 --------------TCTTGTATTTT----GTGCATC-ACTTTTCTC--------
------
FC88X_00762_00577 --------------TCTTGTATTTT----GTGCATC-ACTTTTCTC--------
------
FC88X_00949_00503 --------------TCTTGTATTTT----GTGCATC-ACTTTTCTC--------
------
FC88X_00919_00615 --------------TCTTGTATTTT----GTGCATC-ACTTTTCTC--------
------

FC88X_00955_00771 --------------TCTTGTATTTT----GTGCATC-ACTTTTCTC--------
------
FC88X_01007_00523 --------------TCTTGTATTTT----GTGCATC-ACTTTTCTC--------
------
FC88X_00007_00307 --------------TCTTGTATTTT----GTGCATC-ACTTTTCTC--------
------
FC88X_00016_00642 --------------TCTTGTATTTT----GTGCATC-ACTTTTCTC--------
------
FC88X_00051_00013 --------------TCTTGTATTTT----GTGCATC-ACTTTTCTC--------
------
FC88X_00072_00128 --------------TCTTGTATTTT----GTGCATC-ACTTTTCTC--------
------
FC88X_00067_00291 --------------TCTTGTATTTT----GTGCATC-ACTTTTCTC--------
------
FC88X_00079_00537 --------------TCTTGTATTTT----GTGCATC-ACTTTTCTC--------
------
FC88X_00130_00223 --------------TCTTGTATTTT----GTGCATC-ACTTTTCTC--------
------
FC88X_00125_00420 --------------TCTTGTATTTT----GTGCATC-ACTTTTCTC--------
------
FC88X_00116_00802 --------------TCTTGTATTTT----GTGCATC-ACTTTTCTC--------
------
FC88X_00105_00912 --------------TCTTGTATTTT----GTGCATC-ACTTTTCTC--------
------
FC88X_00229_00214 --------------TCTTGTATTTT----GTGCATC-ACTTTTCTC--------
------
FC88X_00273_00181 --------------TCTTGTATTTT----GTGCATC-ACTTTTCTC--------
------
FC88X_00292_01099 --------------TCTTGTATTTT----GTGCATC-ACTTTTCTC--------
------
FC88X_00364_00053 --------------TCTTGTATTTT----GTGCATC-ACTTTTCTC--------
------
FC88X_00466_00081 --------------TCTTGTATTTT----GTGCATC-ACTTTTCTC--------
------
FC88X_00515_00200 --------------TCTTGTATTTT----GTGCATC-ACTTTTCTC--------
------
FC88X_00541_01009 --------------TCTTGTATTTT----GTGCATC-ACTTTTCTC--------
------
FC88X_00532_01238 --------------TCTTGTATTTT----GTGCATC-ACTTTTCTC--------
------
FC88X_00568_00119 --------------TCTTGTATTTT----GTGCATC-ACTTTTCTC--------
------
FC88X_00598_00160 --------------TCTTGTATTTT----GTGCATC-ACTTTTCTC--------
------
FC88X_00743_00540 --------------TCTTGTATTTT----GTGCATC-ACTTTTCTC--------
------
FC88X_00734_00634 --------------TCTTGTATTTT----GTGCATC-ACTTTTCTC--------
------
FC88X_00815_00203 --------------TCTTGTATTTT----GTGCATC-ACTTTTCTC--------
------
FC88X_00842_00464 --------------TCTTGTATTTT----GTGCATC-ACTTTTCTC--------
------
FC88X_00840_00567 --------------TCTTGTATTTT----GTGCATC-ACTTTTCTC--------
------
FC88X_00832_01266 --------------TCTTGTATTTT----GTGCATC-ACTTTTCTC--------
------

FC88X_00933_00392 --------------TCTTGTATTTT----GTGCATC-ACTTTTCTC--------
------
FC88X_00906_00547 --------------TCTTGTATTTT----GTGCATC-ACTTTTCTC--------
------
FC88X_00906_00733 --------------TCTTGTATTTT----GTGCATC-ACTTTTCTC--------
------
FC88X_00905_01005 --------------TCTTGTATTTT----GTGCATC-ACTTTTCTC--------
------
FC88X_00938_01207 --------------TCTTGTATTTT----GTGCATC-ACTTTTCTC--------
------
FC88X_00991_00602 --------------TCTTGTATTTT----GTGCATC-ACTTTTCTC--------
------
FC88X_01050_00855 --------------TCTTGTATTTT----GTGCATC-ACTTTTCTC--------
------
FC88X_01056_01022 --------------TCTTGTATTTT----GTGCATC-ACTTTTCTC--------
------
FC88X_01126_00629 --------------TCTTGTATTTT----GTGCATC-ACTTTTCTC--------
------
FC88X_00096_00400 --------------TCTTGTATTTTTTT-GTGCATC-ACTTTTCTC--------
------
FC88X_00495_01060 --------------TCTTGTATTTT----GTGCATC-GCTTTTCTC--------
------
FC88X_00819_01231 --------------TCTTGTATTTT----GTGCATC-GCTTTTCTC--------
------
FC88X_01085_00879 --------------TCTTGTATTTT----GTGCACC-ACTTTTCTC--------
------
FC88X_00059_00846 --------------TCTTGTATTCT----GTGCATC-ACTTTTCTC--------
------
FC88X_00036_00073 --------------TCTTGTATTCT----GTGCATC-ACTTTTCTC--------
------
FC88X_00039_00635 --------------TCTTGTATTCT----GTGCATC-ACTTTTCTC--------
------
FC88X_00625_00332 --------------TCTTGTATTTT----GTGCATC-ACTTTTCTCATCACCG-
------
FC88X_00996_00712 --------------TCTTGTATTTC----GTGCATC-ACTTTTCTC--------
------
FC88X_01115_00652 --------------TCTTGTATTTC----GTGCATC-ACTTTTCTC--------
------
FC88X_00301_00295 --------------TCTTGTATCTC----GTGCATC-ACTTTTCTC--------
------
FC88X_00970_00523 --------------TCTTGTATCTT----GTGCATC-ACTTTTCTC--------
------
FC88X_00091_00603 --------------TCTTGTATTTT----GTGCATC-ACTTCTCTC--------
------
FC88X_00646_01111 --------------TCTTGTATTTT----GTGCATCTACTTGTCTC--------
------
FC88X_00560_01202 --------------TCTTGTACTTT----GTGCATC-ACTTTTCTC--------
------
FC88X_00043_00819 --------------TCTTGTACTTT----GTGCATC-ACTTTTCTC--------
------
FC88X_00579_01223 --------------TCTTGTACTTT----GTGCATC-ACTTTTCTC--------
------
FC88X_01098_00570 --------------TCTTGTACTTT----GTGCATC-ACTTTTCTC--------
------
FC88X_00483_01181 --------------TCTTGTATTTT----GTGCATC-
ACTTTTCTCCTTCCTTT------

```
FC88X_00216_00690      --------------TCTTGTATTTT----GTGCATC-
ACTTTTCTCCTTCCTTT------
FC88X_01022_00361      --------------TCTTGTATTTT----GTGCATC-
ACTTTTCTCCTTCCTTT------
FC88X_00455_01008      --------------TCTTGTATTTT----GTGCATC-
ACTTTTCTCCTTCCTTTCT----
FC88X_00317_00031      --------------TCATGTATTTT----GTGCATC-ACTTTTCTC--------
------
FC88X_00311_01097      --------------CCTTGTATTTT----GTGCATC-ACTTTTCTC--------
------
FC88X_00819_01162      --------------CCTTGTGTTTT----GTGCATC-ACTTTTCTC--------
------
FC88X_00205_00012      --------------TCTTGTGTTTT----GTGCATC-ACTTTTCTC--------
------
FC88X_00466_00056      --------------TCTTGCGTTTT----GTGCATC-ACTTTTCTC--------
------
FC88X_00670_01108      --------------TCTTGTATTTT----GTGCCATCACTCTTCTC--------
------
FC88X_00141_00201      --------------TCTTGTA-TTTT---GCGCATC-ACTTTTCTC--------
------
FC88X_01083_01196      --------------TCTTGTA-TTTT---GCGCATC-ACTTTTCTC--------
------
FC88X_00609_00256      --------------TCTTGTA-TCTT---GCGCATC-ACTTTTCTC--------
------
FC88X_00342_00989      --------------TCTTGTATTTT------GCATC-ACCTTTCTC--------
------
FC88X_00719_01084      --------------TCTTGTATTTTT---TTGCATC-ACTTTTCTC--------
------
FC88X_00368_01182      --------------TCTTGCA-TTTT---GTGCATC-ACTTTTCTC--------
------
FC88X_00627_01243      --------------TCTTGCAATTTT---GTGCATC-ACTTTTCTC--------
------
FC88X_00049_00619      --------------TCTTGCA-TTTT---GTGCATC-ACTTTTCTC--------
------
FC88X_00075_00779      --------------TCTTGAATTTTT---GTGCATC-ACTTTTCTC--------
------
FC88X_00356_00698      --------------TCTTGTATTTTC----TGCATC-ACTTCTCTC--------
------
FC88X_00791_00129      --------------TCTCGTATTTT----GTGCATC-ACTTTTTTCTC------
------
FC88X_00287_01151      --------------TCTTGTATTTTT---GTGCATCTACTTTTCGTCCC-----
------
FC88X_00609_00943      --------------TCTTGTATTTT----GTGCATC-ACTTTTCTCCC------
------
FC88X_00274_00608      --------------TCTTGTATTTTT---GTGCATC-ACTTTTCTTC-------
------
FC88X_00750_00528      --------------TCTTGTATTTT----GTGCATC-ACTTTC-----------
------
FC88X_00872_00856      --------------TCTTGTATTTT----GTGCATC-ACTTTTTCTC-------
------
FC88X_00167_00124      --------------TCTTGTATTTT----GTGCATC-ATTTTTCTC--------
------
FC88X_00957_00948      --------------TCTTGTATTTT----GTGCATC-ACTTTTTCTC-------
------
FC88X_00758_00935      --------------TCTTGTATTTT----GTGCATC-ACTTTTTCTCCTTCCT-
------
```

FC88X_00581_01002 --------------TCTTGTATTTG----TTGCATC-GCTTTTCCTC-------
------
FC88X_00575_00437 -------------TCTTAGTATTTGT----CGCATC-ACTTTTCTC--------
------
FC88X_00070_00057 --------------TTCCGAGTTTTCCCGATTCGTTGTATT-------------
------
FC88X_00083_00069 --------------TTCCGAGTTTTCCCGATTCGTTGTATT-------------
------
FC88X_00055_00373 --------------TTCCGAGTTTTCCCGATTCGTTGTATT-------------
------
FC88X_00993_00753 --------------TTCCGAGTTTTCCCGATTCGTTGTATTC------------
------
FC88X_00880_00723 -------------CTTTGGTGTTTTCGCCGTTCTATTTCT--------------
------
FC88X_00529_01222 -------------CTACTATTTTTCCCAGACTTTTTCTATT-------------
------
FC88X_00897_01212 -------------------TTTTTCCC---CTTTTTGTGT--------------
------
FC88X_00294_00980 -----------CCCGTTGGCGTTTTCACGGTTCTCTAT----------------
------
FC88X_00867_00701 -----------CCCGTTGGCGTTTTCACGGTTCTCTAT----------------
------
FC88X_01042_00994 -----------CCCGTTGGCGTTTTCACGGTTCTCTAT----------------
------
FC88X_00214_00630 ------------CTCGGGGTATCCCGTCCGCTGTTTGC----------------
------
FC88X_01137_00712 ------------TTCTCGGGTCTGTATCCTCCGTCTGTT---------------
------
FC88X_00970_00341 ------------CTTTCCGCGGGTTATCCCTTTTCTGTT---------------
------
FC88X_01004_00716 ------------CTTTCCGCGGGTTATCCCTTTTCTGTTC--------------
------
FC88X_00037_00275 ------------CTTTCCGCGGGTTATCCCTTTTCTGTTT--------------
------
FC88X_00935_00544 ----------------TTCCCAACTAATTTCGCC-TACCTGTTAC---------
------
FC88X_00332_00628 ---------------TTGCACGATTATCTCCGCC-TACCTGTT-----------
------
FC88X_01097_01106 ---------------TTCCCAGCCTCTTTCCCCG-TAACTTTT-----------
------
FC88X_00508_00366 --------------TTCTCCCATCTGTTTCCGCGGTACCTG-------------
------
FC88X_01011_00974 ---------------TTTCCCCTTCATTTGCACCGCTTTTCT------------
------
FC88X_00483_01246 ---------------TTTCCCCTTCATTTGCACCGCTTTTCT------------
------
FC88X_00744_01208 -----------TAGTTTCACCCTTCACTTTGGCCGCGTTTC-------------
------
FC88X_00056_00274 --------------GTTTGACCCTTCTAGACACCGCGTTTATTCT---------
------
FC88X_00012_00459 --------------CTGTGACCCTTGCGGA----ACGTTTATTCC---------
------
FC88X_00062_00559 --------------TTTACCCTTCTCTCCGAGTGACTTTATC------------
------
FC88X_00022_00107 --------------TACTCTCCTTACCTGACGTGACCTTGC-------------
------

FC88X_00777_00238 --------------TTATCCCTTCCCTTGACGAGACGTTGC-------------
------
FC88X_00929_01172 ---------------TAACCCTTCT-TGGGGAGTACGTTCGAA-----------
------
FC88X_00071_00393 ----------------TCATTGAGTAT-TCCTCACTGCTATTTC----------
------
FC88X_00096_00135 ----------------TCATTGAGTAT-TCCTCACTGCTATTTC----------
------
FC88X_00060_00369 ----------------TCATCAGTTAT-TCCCCAGCGCCATTTC----------
------
FC88X_01139_00931 ----------------ACTCAGTCTCT-TCCCTATCACCATTTA----------
------
FC88X_00882_01173 ----------------CTGTTCGCTCT-TCCCTTTCACCGTCATC---------
------
FC88X_00623_01046 ----------------CTATCAGCTCT-TCCCTTGCACTATCAC----------
------
FC88X_00023_00129 ----------------CTATAACAAGCGTTCCCGTCACCATCAC----------
------
FC88X_00277_01114 ---------------TTTATCACTAGTCTTTCC--CATCATCC-----------
------
FC88X_00242_00803 --------------------TTCTCTCTTTTGCACCACGGTTTTGTT-------
------
FC88X_00408_01071 --------------------TTCTCTCTTTTGCATCACGGTTTTGTT-------
------
FC88X_00266_00402 -------------------TTCCCCATTCTGACAACACG-TTTTGTT-------
------
FC88X_00460_00889 -----------------CCTTTGCCTCTCTTTCACCACAGTTAG----------
------
FC88X_00225_01013 -------------------TCGCTAGTTCTCCCATTACCACACGGT--------
------
FC88X_01086_00735 -------------------TCGCTAGTTCTCCCATTACCACACGGT--------
------
FC88X_00026_00171 -------------------TCGCTAGTTCTCCCATTACCACACGGT--------
------
FC88X_00613_01039 --------------------TTTTTCCCTTCACATCACGACG------------
------
FC88X_00994_00702 -----------------CTGTTTACCCCTTCACATCACGATGCT----------
------
FC88X_00552_01219 ---------------CTATTCCATAGTTGTCCCGACACGTTT------------
------
FC88X_00907_01023 ---------------CTATTCCATAGTTGTCCCGACACGTTT------------
------
FC88X_00163_00789 ---------------CTTTTCCCT--TCGTCCCACCACGGTTAC----------
------
FC88X_00098_00347 -----------------CTACCAGATTTCTCCCGTCACGGTGAC----------
------
FC88X_00067_00142 ----------------CCAATCGATT-TCTCCTGCCAGGCTGTC----------
------
FC88X_00478_01171 ----------------CCAATCGATT-TCTCCTGCCAGGCTGTC----------
------
FC88X_00822_00803 ----------------CCAATCGATT-TCTCCTGCCAGGCTGTC----------
------
FC88X_00322_00027 ----------------CCAATCGATT-TCTCCTGCCAGGCTGT-----------
------
FC88X_01077_01243 ----------------CCAATCGATT-TCTCCTGTCAGGCTGTC----------
------

```
FC88X_00984_00888       -------------TTGAATTTCAATTCTCTCCCGCCATGCCC------------
------
FC88X_00383_01099       --------------CTCTTCCCCATGGCTTCCCGCCGCTGT-------------
------
FC88X_00589_00427       --------------CTCTTCCCCATG-CTTCCCG--------------------
------
FC88X_00777_01158       ------------TTCTCTTCTCGATGCCTTCCCAGCCTGC--------------
------
FC88X_01002_00443       ------------CTATCTTCCAAATGCCTCCCGAAACAT---------------
------
FC88X_00399_01200       -------------TCTCGCTAATAA-GTCTCCCGTCACAAA-------------
------
FC88X_00902_00831       -----------------ACTAATAATGTCTCCCTACACGTGCC-----------
------
FC88X_00484_01122       -----------------CACCTAATTCGCTATCCCTGCCTTGTA----------
------
FC88X_00910_00316       -----------------CACCTAATTCGCTATCCCTGCCTTGTA----------
------
FC88X_00052_00682       ----------------CTAACTAATAGCTCTCACATGCCCTGTT----------
------
FC88X_00856_00939       ----------------CTAACCCATTTCCCATCACAGCCCGCT-----------
------
FC88X_00926_00428       ----------------CTAACCTATTTCCCATCACAGCCCGCT-----------
------
FC88X_00097_00720       -----------GCTTTACA--CGCTGTTC---CCCTG-GAATTA----------
------
FC88X_00560_00280       ------------CTTTACA--CGCTGTTC---CCCTG-GAATTA----------
------
FC88X_00112_00931       --------------TTACA--AACTGTTCATCCCCTGCGAATT-----------
------
FC88X_00253_00913       -------------GTTCGG--CGTCGTTCGTCTCCTGCATATT-----------
------
FC88X_00036_00365       -------------GTTCGG--CGTCGTTCGTCTCCTGCATATT-----------
------
FC88X_00138_00526       --------------TTCGG--CGTCGTTCGTCTCCTGCATATT-----------
------
FC88X_00206_00877       --------------TTCGG--CGTCGTTCGTCTCCTGCATATT-----------
------
FC88X_00754_00161       --------------TTCGG--CGTCGTTCGTCTCCTGCATATT-----------
------
FC88X_00630_00453       --------------TTCGG--CGTCGTTCGTCTCCTGCATATT-----------
------
FC88X_00976_00715       --------------TTCGG--CGTCGTTCGTCTCCTGCATATT-----------
------
FC88X_00071_00017       --------------TTCGG--CGTGGTTCGTCTCCTGCATATT-----------
------
FC88X_00644_00108       --------------TTCGG--CGTCGCTCGTCTCCTGCACATT-----------
------
FC88X_00174_00719       --------------TTCGG--CGTCGTTCTTCCCCTGCATATT-----------
------
FC88X_00042_00292       --------------CTAAA--CGTCATTCACCACCTGCTAATT-----------
------
FC88X_00842_00121       --------------TTACT--TTCCCTTCGCATCTGGCATTTTCC---------
------
FC88X_00269_00680       --------------TTACT--TTCCCTTCGCATCTGGCATTTT-----------
------
```

FC88X_00609_00715 --------------CTTTC--CGCTCCATGCACCTACAATTTTC----------------
FC88X_00539_00106 -----------TAGTTAAAATTACTGCTCTCCACCTGCA---------------------
FC88X_00540_00105 -----------TAGTTAAA-TTACTGCTCTCCACCTGCA---------------------
FC88X_00231_00016 -----------TAGCTAAA-TTACTGCTCTCCACCTGCA---------------------
FC88X_00812_01126 -------------ATCAC-GACCC-TTCTACCCACCGATTGAG-----------------
FC88X_00608_00955 -------------ATCAC-GACCC-TCCTACCCACCGATTGAG-----------------
FC88X_00016_00093 -------------ATCAC-GACCC-TTCTACCCAC-GATTGAG-----------------
FC88X_00017_00321 -------------ATCAC-GACCC-TTCTACCCAC-GATTGAG-----------------
FC88X_00059_00826 -------------ATCAC-GACCC-TTCTACCCAC-GATTGAG-----------------
FC88X_00028_00163 -------------ATCAC-GACCC-TTCTACCCAC-GATTGAG-----------------
FC88X_00437_01098 -------------ATCAC-GACCC-TTCTACCCAC-GATTGAGAAA--------------
FC88X_00079_00629 -------------ATCAC-GACCC-TTCTACCCAC-GATTGAG-----------------
FC88X_00146_00809 -------------ATCAC-GACCC-TTCTACCCAC-GATTGAG-----------------
FC88X_00467_00988 -------------ATCAC-GACCC-TTCTACCCACAA-TTGAG-----------------
FC88X_00207_00053 -------------ATCAC-GACCC-TTCTACCCACAA-TTGAG-----------------
FC88X_00210_00046 -------------ATCAC-GACCC-TTCTACC-ACAA-TTGAG-----------------
FC88X_00976_01206 ------------GATCAC-GACCC-TTCTACCACCAA-TTGAG-----------------
FC88X_00944_00625 -------------ATCAC-AACCC-TTCTACCCACAA-TTGAG-----------------
FC88X_01023_00348 ------------GATCAC-TACCC-TTCTACCCACGA-TTGAG-----------------
FC88X_01015_00650 -------------ATCAC-GACCCGTTCTACCCACGACTTGAG-----------------
FC88X_01027_01181 -------------ATCAC-GACCC--TCTACC-ACGA-TTGAG-----------------
FC88X_00742_01129 -------------ATCAC-GACAC-TTCTACCCACGA-TTGAG-----------------
FC88X_00820_00121 -------------ATCAC-GACTC-TTCTACCCACGA-TTGAG-----------------
FC88X_00532_01228 -------------ATCAC-GACCC-CTCTACCCACGA-TTGAG-----------------
FC88X_00663_00111 -------------ATCAC-GACCC-CTCTACCCACGA-TTGAG-----------------
FC88X_01037_00791 -------------ATCAC-GACCC-CTCTACCCACGA-TTGAGCTTCCTTT---------
FC88X_00021_00113 -------------ATCAC-GACCC--TCTACC-ACGA-TTGAG-----------------
FC88X_00034_00600 ------------GATCAC-GACCC-TTCTACCCACGA-TTGAG-----------------

```
FC88X_00255_01061        ------------GATCAC-GACCC-TTCTACCCACGA-TTGAG-----------
------
FC88X_00359_00004        ------------GATCAC-GACCC-TTCTACCCACGA-TTGAG-----------
------
FC88X_00364_00050        -----------GGATCAC-GACCC-TTCTACCCACGA-TTGAG-----------
------
FC88X_00738_00968        ------------GATCAC-GACCC-TTCTACCCACGA-TTGAG-----------
------
FC88X_00746_01019        ------------GATCAC-GACCC-TTCTACCCACGA-TTGAG-----------
------
FC88X_00047_00344        -------------ATCAC-GACCC-TTCTACCCACGA-TTGAG-----------
------
FC88X_00140_00048        -------------ATCAC-GACCC-TTCTACCCACGA-TTGAG-----------
------
FC88X_00149_00870        -------------ATCAC-GACCC-TTCTACCCACGA-TTGAG-----------
------
FC88X_00492_01172        ------------AATCAC-GACCC-TTCTACCCACGA-TTGAG-----------
------
FC88X_00939_00193        ------------AATCAC-GACCC-TTCTACCCACGA-TTGAG-----------
------
FC88X_01070_00752        ------------AATCAC-GACCC-TTCTACCCACGA-TTGAG-----------
------
FC88X_01079_00397        ------------AATCAC-GACCC-TTCTACCCACGA-TTGAG-----------
------
FC88X_00007_00599        -------------ATCAC-GACCC-TTCTACCCACGA-TTGAG-----------
------
FC88X_00105_00309        -------------ATCAC-GACCC-TTCTACCCACGA-TTGAG-----------
------
FC88X_00150_00105        -------------ATCAC-GACCC-TTCTACCCACGA-TTGAG-----------
------
FC88X_00243_00485        -------------ATCAC-GACCC-TTCTACCCACGA-TTGAG-----------
------
FC88X_00120_00424        -------------ATCAC-GACCC-TTCCA-CCCACGATTGAG-----------
------
FC88X_00139_00910        -------------ATCAC-GACCC-TTCCA-CCCACGATTGAG-----------
------
FC88X_00498_01152        -------------ATCAC-GACCC-TTCTAACCCACGATTGAG-----------
------
FC88X_00162_01004        -------------ATCAC-GACCC-TTCTGCCCAC-GATTGAG-----------
------
FC88X_00963_01049        -------------ATCAC-GACCC-TTCTGCCCAC-GATTGAGC----------
------
FC88X_00020_00181        -------------ATCAC-GACCC-TTCTGCCCAC-GATTGAG-----------
------
FC88X_00960_00501        -------------ATCAC-GACCC-TTCTGCCCAC-GATTGAG-----------
------
FC88X_01030_00734        -------------ATCAC-GACCC-TTCT--CCAC-GATTGAG-----------
------
FC88X_00017_00181        -------------ATCAC-GACCC-TTCT-ACCAC-GATTGAG-----------
------
FC88X_00885_00159        ------------AATCAC-GACCC-TTCT-ACCAC-GATTGAG-----------
------
FC88X_00040_00257        -------------ATCAC-GACCC-TTCT-ACCAC-GATTGAG-----------
------
FC88X_00048_00416        -------------ATCAC-GACCC-TTCT-ACCAC-GATTGAG-----------
------
```

FC88X_00090_00111 -------------ATCAC-GACCC-TTCT-ACCAC-GATTGAG-----------
------
FC88X_00180_01039 -------------ATCAC-GACCC-TTCT-ACCAC-GATTGAGCC---------
------
FC88X_00718_01131 -------------ATCAC-GACCC-TTCT-ACCAC-GATTGAGCC---------
------
FC88X_00316_00570 -------------ATCAC-GACCC-TTCT-ACCAC-GATTGAGCCC--------
------
FC88X_00374_00381 -------------ATCAC-GACCC-TTCT-ACCAC-GATTGAGC----------
------
FC88X_00416_01154 -------------ATCAC-GACCC-TTCT-ACCAC-GATTGAGC----------
------
FC88X_00045_00214 -------------ATCAC-GACCC-TTCT-ACCAC-GATTGAG-----------
------
FC88X_00029_00403 -------------ATCAC-GACCC-TTCT-ACCAC-GATTGAG-----------
------
FC88X_00020_00521 -------------ATCAC-GACCC-TTCT-ACCAC-GATTGAG-----------
------
FC88X_00054_00170 -------------ATCAC-GACCC-TTCT-ACCAC-GATTGAG-----------
------
FC88X_00051_00200 -------------ATCAC-GACCC-TTCT-ACCAC-GATTGAG-----------
------
FC88X_00067_00262 -------------ATCAC-GACCC-TTCT-ACCAC-GATTGAG-----------
------
FC88X_00090_00428 -------------ATCAC-GACCC-TTCT-ACCAC-GATTGAG-----------
------
FC88X_00065_00624 -------------ATCAC-GACCC-TTCT-ACCAC-GATTGAG-----------
------
FC88X_00070_00734 -------------ATCAC-GACCC-TTCT-ACCAC-GATTGAG-----------
------
FC88X_00095_00886 -------------ATCAC-GACCC-TTCT-ACCAC-GATTGAG-----------
------
FC88X_00127_00142 -------------ATCAC-GACCC-TTCT-ACCAC-GATTGAG-----------
------
FC88X_00146_00574 -------------ATCAC-GACCC-TTCT-ACCAC-GATTGAG-----------
------
FC88X_00148_00823 -------------ATCAC-GACCC-TTCT-ACCAC-GATTGAG-----------
------
FC88X_00146_00911 -------------ATCAC-GACCC-TTCT-ACCAC-GATTGAG-----------
------
FC88X_00161_00063 -------------ATCAC-GACCC-TTCT-ACCAC-GATTGAG-----------
------
FC88X_00165_00103 -------------ATCAC-GACCC-TTCT-ACCAC-GATTGAG-----------
------
FC88X_00170_00400 -------------ATCAC-GACCC-TTCT-ACCAC-GATTGAG-----------
------
FC88X_00164_00760 -------------ATCAC-GACCC-TTCT-ACCAC-GATTGAG-----------
------
FC88X_00166_00981 -------------ATCAC-GACCC-TTCT-ACCAC-GATTGAG-----------
------
FC88X_00154_01008 -------------ATCAC-GACCC-TTCT-ACCAC-GATTGAG-----------
------
FC88X_00218_00125 -------------ATCAC-GACCC-TTCT-ACCAC-GATTGAG-----------
------
FC88X_00221_00201 -------------ATCAC-GACCC-TTCT-ACCAC-GATTGAG-----------
------

FC88X_00216_00630 -------------ATCAC-GACCC-TTCT-ACCAC-GATTGAG-----------
------
FC88X_00229_00915 -------------ATCAC-GACCC-TTCT-ACCAC-GATTGAG-----------
------
FC88X_00242_01002 -------------ATCAC-GACCC-TTCT-ACCAC-GATTGAG-----------
------
FC88X_00256_00939 -------------ATCAC-GACCC-TTCT-ACCAC-GATTGAG-----------
------
FC88X_00253_01005 -------------ATCAC-GACCC-TTCT-ACCAC-GATTGAG-----------
------
FC88X_00280_01069 -------------ATCAC-GACCC-TTCT-ACCAC-GATTGAG-----------
------
FC88X_00291_01071 -------------ATCAC-GACCC-TTCT-ACCAC-GATTGAG-----------
------
FC88X_00329_01124 -------------ATCAC-GACCC-TTCT-ACCAC-GATTGAG-----------
------
FC88X_00391_00577 -------------ATCAC-GACCC-TTCT-ACCAC-GATTGAG-----------
------
FC88X_00375_00930 -------------ATCAC-GACCC-TTCT-ACCAC-GATTGAG-----------
------
FC88X_00389_01079 -------------ATCAC-GACCC-TTCT-ACCAC-GATTGAG-----------
------
FC88X_00401_00011 -------------ATCAC-GACCC-TTCT-ACCAC-GATTGAG-----------
------
FC88X_00407_00321 -------------ATCAC-GACCC-TTCT-ACCAC-GATTGAG-----------
------
FC88X_00404_00490 -------------ATCAC-GACCC-TTCT-ACCAC-GATTGAG-----------
------
FC88X_00400_00657 -------------ATCAC-GACCC-TTCT-ACCAC-GATTGAG-----------
------
FC88X_00441_00651 -------------ATCAC-GACCC-TTCT-ACCAC-GATTGAG-----------
------
FC88X_00427_01204 -------------ATCAC-GACCC-TTCT-ACCAC-GATTGAG-----------
------
FC88X_00499_00912 -------------ATCAC-GACCC-TTCT-ACCAC-GATTGAG-----------
------
FC88X_00472_00955 -------------ATCAC-GACCC-TTCT-ACCAC-GATTGAG-----------
------
FC88X_00461_01212 -------------ATCAC-GACCC-TTCT-ACCAC-GATTGAG-----------
------
FC88X_00517_00032 -------------ATCAC-GACCC-TTCT-ACCAC-GATTGAG-----------
------
FC88X_00505_00300 -------------ATCAC-GACCC-TTCT-ACCAC-GATTGAG-----------
------
FC88X_00510_00657 -------------ATCAC-GACCC-TTCT-ACCAC-GATTGAG-----------
------
FC88X_00527_00897 -------------ATCAC-GACCC-TTCT-ACCAC-GATTGAG-----------
------
FC88X_00541_00893 -------------ATCAC-GACCC-TTCT-ACCAC-GATTGAG-----------
------
FC88X_00508_01018 -------------ATCAC-GACCC-TTCT-ACCAC-GATTGAG-----------
------
FC88X_00526_01065 -------------ATCAC-GACCC-TTCT-ACCAC-GATTGAG-----------
------
FC88X_00585_00486 -------------ATCAC-GACCC-TTCT-ACCAC-GATTGAG-----------
------

FC88X_00595_00575 -------------ATCAC-GACCC-TTCT-ACCAC-GATTGAG-----------
------
FC88X_00589_00629 -------------ATCAC-GACCC-TTCT-ACCAC-GATTGAG-----------
------
FC88X_00560_00876 -------------ATCAC-GACCC-TTCT-ACCAC-GATTGAG-----------
------
FC88X_00597_00861 -------------ATCAC-GACCC-TTCT-ACCAC-GATTGAG-----------
------
FC88X_00568_00946 -------------ATCAC-GACCC-TTCT-ACCAC-GATTGAG-----------
------
FC88X_00557_01103 -------------ATCAC-GACCC-TTCT-ACCAC-GATTGAG-----------
------
FC88X_00596_01195 -------------ATCAC-GACCC-TTCT-ACCAC-GATTGAG-----------
------
FC88X_00562_01248 -------------ATCAC-GACCC-TTCT-ACCAC-GATTGAG-----------
------
FC88X_00625_00208 -------------ATCAC-GACCC-TTCT-ACCAC-GATTGAG-----------
------
FC88X_00635_00252 -------------ATCAC-GACCC-TTCT-ACCAC-GATTGAG-----------
------
FC88X_00637_00638 -------------ATCAC-GACCC-TTCT-ACCAC-GATTGAG-----------
------
FC88X_00622_00917 -------------ATCAC-GACCC-TTCT-ACCAC-GATTGAG-----------
------
FC88X_00607_00959 -------------ATCAC-GACCC-TTCT-ACCAC-GATTGAG-----------
------
FC88X_00616_01124 -------------ATCAC-GACCC-TTCT-ACCAC-GATTGAG-----------
------
FC88X_00611_01160 -------------ATCAC-GACCC-TTCT-ACCAC-GATTGAG-----------
------
FC88X_00647_01249 -------------ATCAC-GACCC-TTCT-ACCAC-GATTGAG-----------
------
FC88X_00655_00412 -------------ATCAC-GACCC-TTCT-ACCAC-GATTGAG-----------
------
FC88X_00693_00696 -------------ATCAC-GACCC-TTCT-ACCAC-GATTGAG-----------
------
FC88X_00671_00714 -------------ATCAC-GACCC-TTCT-ACCAC-GATTGAG-----------
------
FC88X_00693_00885 -------------ATCAC-GACCC-TTCT-ACCAC-GATTGAG-----------
------
FC88X_00688_01215 -------------ATCAC-GACCC-TTCT-ACCAC-GATTGAG-----------
------
FC88X_00720_00758 -------------ATCAC-GACCC-TTCT-ACCAC-GATTGAG-----------
------
FC88X_00732_00841 -------------ATCAC-GACCC-TTCT-ACCAC-GATTGAG-----------
------
FC88X_00733_01089 -------------ATCAC-GACCC-TTCT-ACCAC-GATTGAG-----------
------
FC88X_00736_01110 -------------ATCAC-GACCC-TTCT-ACCAC-GATTGAG-----------
------
FC88X_00721_01224 -------------ATCAC-GACCC-TTCT-ACCAC-GATTGAG-----------
------
FC88X_00778_00082 -------------ATCAC-GACCC-TTCT-ACCAC-GATTGAG-----------
------
FC88X_00778_00675 -------------ATCAC-GACCC-TTCT-ACCAC-GATTGAG-----------
------

FC88X_00787_00693 -------------ATCAC-GACCC-TTCT-ACCAC-GATTGAG-----------
------
FC88X_00786_00799 -------------ATCAC-GACCC-TTCT-ACCAC-GATTGAG-----------
------
FC88X_00765_00872 -------------ATCAC-GACCC-TTCT-ACCAC-GATTGAG-----------
------
FC88X_00774_00895 -------------ATCAC-GACCC-TTCT-ACCAC-GATTGAG-----------
------
FC88X_00754_01131 -------------ATCAC-GACCC-TTCT-ACCAC-GATTGAG-----------
------
FC88X_00795_01131 -------------ATCAC-GACCC-TTCT-ACCAC-GATTGAG-----------
------
FC88X_00831_00228 -------------ATCAC-GACCC-TTCT-ACCAC-GATTGAG-----------
------
FC88X_00809_00806 -------------ATCAC-GACCC-TTCT-ACCAC-GATTGAG-----------
------
FC88X_00819_00990 -------------ATCAC-GACCC-TTCT-ACCAC-GATTGAG-----------
------
FC88X_00807_01018 -------------ATCAC-GACCC-TTCT-ACCAC-GATTGAG-----------
------
FC88X_00835_01220 -------------ATCAC-GACCC-TTCT-ACCAC-GATTGAG-----------
------
FC88X_00867_00138 -------------ATCAC-GACCC-TTCT-ACCAC-GATTGAG-----------
------
FC88X_00857_00354 -------------ATCAC-GACCC-TTCT-ACCAC-GATTGAG-----------
------
FC88X_00895_00403 -------------ATCAC-GACCC-TTCT-ACCAC-GATTGAG-----------
------
FC88X_00897_00861 -------------ATCAC-GACCC-TTCT-ACCAC-GATTGAG-----------
------
FC88X_00899_00941 -------------ATCAC-GACCC-TTCT-ACCAC-GATTGAG-----------
------
FC88X_00898_00976 -------------ATCAC-GACCC-TTCT-ACCAC-GATTGAG-----------
------
FC88X_00872_01023 -------------ATCAC-GACCC-TTCT-ACCAC-GATTGAG-----------
------
FC88X_00897_01000 -------------ATCAC-GACCC-TTCT-ACCAC-GATTGAG-----------
------
FC88X_00883_01185 -------------ATCAC-GACCC-TTCT-ACCAC-GATTGAG-----------
------
FC88X_00855_01236 -------------ATCAC-GACCC-TTCT-ACCAC-GATTGAG-----------
------
FC88X_00863_01249 -------------ATCAC-GACCC-TTCT-ACCAC-GATTGAG-----------
------
FC88X_00931_00204 -------------ATCAC-GACCC-TTCT-ACCAC-GATTGAG-----------
------
FC88X_00900_00454 -------------ATCAC-GACCC-TTCT-ACCAC-GATTGAG-----------
------
FC88X_00934_00846 -------------ATCAC-GACCC-TTCT-ACCAC-GATTGAG-----------
------
FC88X_00941_00836 -------------ATCAC-GACCC-TTCT-ACCAC-GATTGAG-----------
------
FC88X_00920_00986 -------------ATCAC-GACCC-TTCT-ACCAC-GATTGAG-----------
------
FC88X_00922_00953 -------------ATCAC-GACCC-TTCT-ACCAC-GATTGAG-----------
------

```
FC88X_00920_01128      -------------ATCAC-GACCC-TTCT-ACCAC-GATTGAG-----------
------
FC88X_00981_00238      -------------ATCAC-GACCC-TTCT-ACCAC-GATTGAG-----------
------
FC88X_00955_00354      -------------ATCAC-GACCC-TTCT-ACCAC-GATTGAG-----------
------
FC88X_00966_00377      -------------ATCAC-GACCC-TTCT-ACCAC-GATTGAG-----------
------
FC88X_00980_00497      -------------ATCAC-GACCC-TTCT-ACCAC-GATTGAG-----------
------
FC88X_00980_00530      -------------ATCAC-GACCC-TTCT-ACCAC-GATTGAG-----------
------
FC88X_00999_00506      -------------ATCAC-GACCC-TTCT-ACCAC-GATTGAG-----------
------
FC88X_00961_01084      -------------ATCAC-GACCC-TTCT-ACCAC-GATTGAG-----------
------
FC88X_00983_01182      -------------ATCAC-GACCC-TTCT-ACCAC-GATTGAG-----------
------
FC88X_00978_01227      -------------ATCAC-GACCC-TTCT-ACCAC-GATTGAG-----------
------
FC88X_00986_01231      -------------ATCAC-GACCC-TTCT-ACCAC-GATTGAG-----------
------
FC88X_01020_00497      -------------ATCAC-GACCC-TTCT-ACCAC-GATTGAG-----------
------
FC88X_01031_00616      -------------ATCAC-GACCC-TTCT-ACCAC-GATTGAG-----------
------
FC88X_01009_00756      -------------ATCAC-GACCC-TTCT-ACCAC-GATTGAG-----------
------
FC88X_01001_00811      -------------ATCAC-GACCC-TTCT-ACCAC-GATTGAG-----------
------
FC88X_01021_00843      -------------ATCAC-GACCC-TTCT-ACCAC-GATTGAG-----------
------
FC88X_01023_00834      -------------ATCAC-GACCC-TTCT-ACCAC-GATTGAG-----------
------
FC88X_01032_00806      -------------ATCAC-GACCC-TTCT-ACCAC-GATTGAG-----------
------
FC88X_01048_00950      -------------ATCAC-GACCC-TTCT-ACCAC-GATTGAG-----------
------
FC88X_01032_01093      -------------ATCAC-GACCC-TTCT-ACCAC-GATTGAG-----------
------
FC88X_01024_01139      -------------ATCAC-GACCC-TTCT-ACCAC-GATTGAG-----------
------
FC88X_01021_01167      -------------ATCAC-GACCC-TTCT-ACCAC-GATTGAG-----------
------
FC88X_01040_01194      -------------ATCAC-GACCC-TTCT-ACCAC-GATTGAG-----------
------
FC88X_01066_00626      -------------ATCAC-GACCC-TTCT-ACCAC-GATTGAG-----------
------
FC88X_01076_00603      -------------ATCAC-GACCC-TTCT-ACCAC-GATTGAG-----------
------
FC88X_01062_00738      -------------ATCAC-GACCC-TTCT-ACCAC-GATTGAG-----------
------
FC88X_01083_00803      -------------ATCAC-GACCC-TTCT-ACCAC-GATTGAG-----------
------
FC88X_01054_00934      -------------ATCAC-GACCC-TTCT-ACCAC-GATTGAG-----------
------
```

FC88X_01084_00964 -------------ATCAC-GACCC-TTCT-ACCAC-GATTGAG-----------
------
FC88X_01091_00983 -------------ATCAC-GACCC-TTCT-ACCAC-GATTGAG-----------
------
FC88X_01083_01129 -------------ATCAC-GACCC-TTCT-ACCAC-GATTGAG-----------
------
FC88X_01070_01203 -------------ATCAC-GACCC-TTCT-ACCAC-GATTGAG-----------
------
FC88X_01077_01221 -------------ATCAC-GACCC-TTCT-ACCAC-GATTGAG-----------
------
FC88X_01108_00544 -------------ATCAC-GACCC-TTCT-ACCAC-GATTGAG-----------
------
FC88X_01126_00835 -------------ATCAC-GACCC-TTCT-ACCAC-GATTGAG-----------
------
FC88X_01118_00854 -------------ATCAC-GACCC-TTCT-ACCAC-GATTGAG-----------
------
FC88X_01120_00959 -------------ATCAC-GACCC-TTCT-ACCAC-GATTGAG-----------
------
FC88X_01120_00977 -------------ATCAC-GACCC-TTCT-ACCAC-GATTGAG-----------
------
FC88X_01113_01023 -------------ATCAC-GACCC-TTCT-ACCAC-GATTGAG-----------
------
FC88X_01101_01072 -------------ATCAC-GACCC-TTCT-ACCAC-GATTGAG-----------
------
FC88X_01118_01103 -------------ATCAC-GACCC-TTCT-ACCAC-GATTGAG-----------
------
FC88X_01118_01181 -------------ATCAC-GACCC-TTCT-ACCAC-GATTGAG-----------
------
FC88X_00769_01006 -------------ATCAC-GACCC-TTCTTACCACCGATTGAG-----------
------
FC88X_00125_00293 -------------ATCAC-GACCC-TTCT--CCAC-GATTGAG-----------
------
FC88X_00815_00951 -------------ATCAT-GACCC-TTCT-ACCAC-GATTGAG-----------
------
FC88X_00385_00067 -------------ATCAC-GACCC-TTCT-ACCACGA-TTGA------------
------
FC88X_00455_00560 -------------ATCAC-GACCC-TTCT-ACCACGA-TTGATGCTTC------
------
FC88X_00269_00409 -------------ATCAC-GACCC-TTCT-ACCACGACTTGAT-----------
------
FC88X_00425_00070 -------------ATCAC-GACCC-TTCT-ACCACGACTG--------------
------
FC88X_00960_00838 -------------ATCAC-GTCCC-TTCT-ACCAC-GATTGAG-----------
------
FC88X_00176_01017 -------------ATCAC-GACCC-TTCTACCCAC-GATTGAGC----------
------
FC88X_00134_00234 -------------ATCAC-GACCC-TTCTA--CAC-GATTGAGC----------
------
FC88X_00023_00613 -------------ATCAC-GACCC-TTCTACCCAC-GATTGAGC----------
------
FC88X_00083_00116 -------------ATCAC-GACCC-TTCTACCCAC-GATTGAGCC---------
------
FC88X_00241_00091 -------------ATCAC-GACCC-TTCTACCCAC-GATTGAGC----------
------
FC88X_00369_01135 -------------ATCAC-GACCC-TTCTACCCAC-GATTGAGC----------
------

FC88X_00126_00463   -------------ATCAC-GACCC-TTCTACCCAC-GATTGAGCCTC-------
------
FC88X_00019_00516   -------------ATCAC-GACCC-TTCTACCCAC-GATTGAG-----------
------
FC88X_00117_00141   -------------ATCAC-GACCC-TTCTACCCAC-GATTGAG-----------
------
FC88X_00170_00088   -------------ATCAC-GACCC-TTCTACCCAC-GATTGAG-----------
------
FC88X_00216_01028   -------------ATCAC-GACCC-TTCTACCCAC-GATTGAG-----------
------
FC88X_00123_00079   -------------ATCAC-GACCC-TTCTACCCAC-GATTGAG-----------
------
FC88X_00890_00156   -------------ATCAC-GACCCCTTCTACCCAC-GATTGAG-----------
------
FC88X_00110_00905   -------------ATCAC-GACCC-TTCTACCCAC-GATTGAG-----------
------
FC88X_00197_01002   -------------ATCAC-GACCC-TTCTACCCAC-GATTGAG-----------
------
FC88X_00040_00564   -------------ATCAC-GACCC-TTCTACCCAC-GATTGAG-----------
------
FC88X_00542_01030   -----------CTATCAC-GACCC-TTCTACCCAC-GATTGAG-----------
------
FC88X_00130_00369   -------------ATCAC-GACCC-TTCTACCCAC-GATTGAG-----------
------
FC88X_00151_00120   -------------ATCAC-GACCC-TTCTACCCAC-GATTGAG-----------
------
FC88X_00228_00549   -------------ATCAC-GACCC-TTCTACCCAC-GATTGAG-----------
------
FC88X_00045_00724   -------------ATCAC-GACCC-TTCTACCCAC-GATTGAG-----------
------
FC88X_00814_01195   -----GGGGGAATATCAC-GACCC-TTCTACCCAC-GATTGAG-----------
------
FC88X_00106_00414   -------------ATCAC-GACCC-TTCTACCCAC-GATTGAG-----------
------
FC88X_00158_00527   -------------ATCAC-GACCC-TTCTACCCAC-GATTGAG-----------
------
FC88X_00220_00704   -------------ATCAC-GACCC-TTCTACCCAC-GATTGAG-----------
------
FC88X_00234_01062   -------------ATCAC-GACCC-TTCTACCCAC-GATTGAG-----------
------
FC88X_00271_01073   -------------ATCAC-GACCC-TTCTACCCAC-GATTGAG-----------
------
FC88X_00383_00078   -------------ATCAC-GACCC-TTCTACCCAC-GATTGAG-----------
------
FC88X_00424_01158   -------------ATCAC-GACCC-TTCTACCCAC-GATTGAG-----------
------
FC88X_00158_00759   -------------ATCAC-GACCC-TTCTACCCAC-GATTGAG-----------
------
FC88X_00872_00651   -------------ATCAC-GACCC-TTCTACCCAC-GATTGAGCTTCCT-----
------
FC88X_00238_01021   -------------ATCAC-GACCC-TTCTACCCAC-GATTGAG-----------
------
FC88X_00270_00078   -------------ATCAC-GACCC-TTCTACCCAC-GATTGAG-----------
------
FC88X_00334_01024   -------------ATCAC-GACCC-TTCTACCCAC-GATTGAG-----------
------

FC88X_00441_00097 --------------ATCAC-GACCC-TTCTACCCAC-GATTGAG------------
------
FC88X_00475_01195 --------------ATCAC-GACCC-TTCTACCCAC-GATTGAG------------
------
FC88X_00219_00680 --------------ATCAC-GACCCTTC-TACCCAC-GATTGAG------------
------
FC88X_00303_01076 --------------ATCAC-GACCCTTCATACCCAC-GATTGAG------------
------
FC88X_00243_01104 --------------ATCAC-GACCCTTC-TACCCAC-GATTGAG------------
------
FC88X_00314_00741 --------------ATCAC-GACCCTTC-TACCCAC-GATTGAG------------
------
FC88X_00427_00086 --------------ATCAC-GACCCTTC-TACCCAC-GATTGAG------------
------
FC88X_00473_00147 --------------ATCAC-GACCCTTC-TACCCAC-GATTGAG------------
------
FC88X_00452_01225 --------------ATCAC-GACCCTTC-TACCCAC-GATTGAG------------
------
FC88X_00467_01224 --------------ATCAC-GACCC-TTCTACCCAC-GATTGAG------------
------
FC88X_00539_00139 --------------ATCAC-GACCC-TTCTACCCAC-GATTGAG------------
------
FC88X_00526_00563 --------------ATCAC-GACCC-TTCTACCCAC-GATTGAG------------
------
FC88X_00507_00695 --------------ATCAC-GACCC-TTCTACCCAC-GATTGAG------------
------
FC88X_00546_00747 --------------ATCAC-GACCC-TTCTACCCAC-GATTGAG------------
------
FC88X_00530_00947 --------------ATCAC-GACCC-TTCTACCCAC-GATTGAG------------
------
FC88X_00504_00994 --------------ATCAC-GACCC-TTCTACCCAC-GATTGAG------------
------
FC88X_00522_01086 --------------ATCAC-GACCC-TTCTACCCAC-GATTGAG------------
------
FC88X_00562_00940 --------------ATCAC-GACCC-TTCTACCCAC-GATTGAG------------
------
FC88X_00576_00951 --------------ATCAC-GACCC-TTCTACCCAC-GATTGAG------------
------
FC88X_00579_01092 --------------ATCAC-GACCC-TTCTACCCAC-GATTGAG------------
------
FC88X_00556_01139 --------------ATCAC-GACCC-TTCTACCCAC-GATTGAG------------
------
FC88X_00558_01250 --------------ATCAC-GACCC-TTCTACCCAC-GATTGAG------------
------
FC88X_00621_00976 --------------ATCAC-GACCC-TTCTACCCAC-GATTGAG------------
------
FC88X_00600_01017 --------------ATCAC-GACCC-TTCTACCCAC-GATTGAG------------
------
FC88X_00673_00104 --------------ATCAC-GACCC-TTCTACCCAC-GATTGAG------------
------
FC88X_00652_00278 --------------ATCAC-GACCC-TTCTACCCAC-GATTGAG------------
------
FC88X_00693_01217 --------------ATCAC-GACCC-TTCTACCCAC-GATTGAG------------
------
FC88X_00748_00087 --------------ATCAC-GACCC-TTCTACCCAC-GATTGAG------------
------

FC88X_00726_00249 -------------ATCAC-GACCC-TTCTACCCAC-GATTGAG-----------
------
FC88X_00728_00294 -------------ATCAC-GACCC-TTCTACCCAC-GATTGAG-----------
------
FC88X_00744_01201 -------------ATCAC-GACCC-TTCTACCCAC-GATTGAG-----------
------
FC88X_00793_00388 -------------ATCAC-GACCC-TTCTACCCAC-GATTGAG-----------
------
FC88X_00755_01003 -------------ATCAC-GACCC-TTCTACCCAC-GATTGAG-----------
------
FC88X_00814_00287 -------------ATCAC-GACCC-TTCTACCCAC-GATTGAG-----------
------
FC88X_00843_00334 -------------ATCAC-GACCC-TTCTACCCAC-GATTGAG-----------
------
FC88X_00800_00514 -------------ATCAC-GACCC-TTCTACCCAC-GATTGAG-----------
------
FC88X_00824_00872 -------------ATCAC-GACCC-TTCTACCCAC-GATTGAG-----------
------
FC88X_00806_01231 -------------ATCAC-GACCC-TTCTACCCAC-GATTGAG-----------
------
FC88X_00876_00217 -------------ATCAC-GACCC-TTCTACCCAC-GATTGAG-----------
------
FC88X_00855_00332 -------------ATCAC-GACCC-TTCTACCCAC-GATTGAG-----------
------
FC88X_00866_00535 -------------ATCAC-GACCC-TTCTACCCAC-GATTGAG-----------
------
FC88X_00881_00903 -------------ATCAC-GACCC-TTCTACCCAC-GATTGAG-----------
------
FC88X_00891_00988 -------------ATCAC-GACCC-TTCTACCCAC-GATTGAG-----------
------
FC88X_00880_01043 -------------ATCAC-GACCC-TTCTACCCAC-GATTGAG-----------
------
FC88X_00895_01219 -------------ATCAC-GACCC-TTCTACCCAC-GATTGAG-----------
------
FC88X_00858_01259 -------------ATCAC-GACCC-TTCTACCCAC-GATTGAG-----------
------
FC88X_00941_00236 -------------ATCAC-GACCC-TTCTACCCAC-GATTGAG-----------
------
FC88X_00918_00426 -------------ATCAC-GACCC-TTCTACCCAC-GATTGAG-----------
------
FC88X_00942_00452 -------------ATCAC-GACCC-TTCTACCCAC-GATTGAG-----------
------
FC88X_00942_00504 -------------ATCAC-GACCC-TTCTACCCAC-GATTGAG-----------
------
FC88X_00902_00598 -------------ATCAC-GACCC-TTCTACCCAC-GATTGAG-----------
------
FC88X_00949_00587 -------------ATCAC-GACCC-TTCTACCCAC-GATTGAG-----------
------
FC88X_00908_00704 -------------ATCAC-GACCC-TTCTACCCAC-GATTGAG-----------
------
FC88X_00989_00619 -------------ATCAC-GACCC-TTCTACCCAC-GATTGAG-----------
------
FC88X_00953_00662 -------------ATCAC-GACCC-TTCTACCCAC-GATTGAG-----------
------
FC88X_00990_00668 -------------ATCAC-GACCC-TTCTACCCAC-GATTGAG-----------
------

```
FC88X_00988_00817      -------------ATCAC-GACCC-TTCTACCCAC-GATTGAG-----------
------
FC88X_00973_00886      -------------ATCAC-GACCC-TTCTACCCAC-GATTGAG-----------
------
FC88X_00975_00893      -------------ATCAC-GACCC-TTCTACCCAC-GATTGAG-----------
------
FC88X_01015_00375      -------------ATCAC-GACCC-TTCTACCCAC-GATTGAG-----------
------
FC88X_01010_00485      -------------ATCAC-GACCC-TTCTACCCAC-GATTGAG-----------
------
FC88X_01010_00545      -------------ATCAC-GACCC-TTCTACCCAC-GATTGAG-----------
------
FC88X_01019_00557      -------------ATCAC-GACCC-TTCTACCCAC-GATTGAG-----------
------
FC88X_01020_00719      -------------ATCAC-GACCC-TTCTACCCAC-GATTGAG-----------
------
FC88X_01007_00793      -------------ATCAC-GACCC-TTCTACCCAC-GATTGAG-----------
------
FC88X_01001_00830      -------------ATCAC-GACCC-TTCTACCCAC-GATTGAG-----------
------
FC88X_01039_00872      -------------ATCAC-GACCC-TTCTACCCAC-GATTGAG-----------
------
FC88X_01024_00941      -------------ATCAC-GACCC-TTCTACCCAC-GATTGAG-----------
------
FC88X_01033_01248      -------------ATCAC-GACCC-TTCTACCCAC-GATTGAG-----------
------
FC88X_01082_00524      -------------ATCAC-GACCC-TTCTACCCAC-GATTGAG-----------
------
FC88X_01055_00550      -------------ATCAC-GACCC-TTCTACCCAC-GATTGAG-----------
------
FC88X_01094_00832      -------------ATCAC-GACCC-TTCTACCCAC-GATTGAG-----------
------
FC88X_01087_00867      -------------ATCAC-GACCC-TTCTACCCAC-GATTGAG-----------
------
FC88X_01058_00963      -------------ATCAC-GACCC-TTCTACCCAC-GATTGAG-----------
------
FC88X_01142_00893      -------------ATCAC-GACCC-TTCTACCCAC-GATTGAG-----------
------
FC88X_01146_00937      -------------ATCAC-GACCC-TTCTACCCAC-GATTGAG-----------
------
FC88X_01117_01167      -------------ATCAC-GACCC-TTCTACCCAC-GATTGAG-----------
------
FC88X_00006_00284      -------------ATCAC-GACCC-TTCTACCCAC-GATTGAG-----------
------
FC88X_00019_00363      -------------ATCAC-GACCC-TTCTACCCAC-GATTGAG-----------
------
FC88X_00033_00457      -------------ATCAC-GACCC-TTCTACCCAC-GATTGAG-----------
------
FC88X_00021_00523      -------------ATCAC-GACCC-TTCTACCCAC-GATTGAG-----------
------
FC88X_00008_00692      -------------ATCAC-GACCC-TTCTACCCAC-GATTGAG-----------
------
FC88X_00053_00039      -------------ATCAC-GACCC-TTCTACCCAC-GATTGAG-----------
------
FC88X_00079_00040      -------------ATCAC-GACCC-TTCTACCCAC-GATTGAG-----------
------
```

FC88X_00096_00100 -------------ATCAC-GACCC-TTCTACCCAC-GATTGAG-----------
------
FC88X_00051_00593 -------------ATCAC-GACCC-TTCTACCCAC-GATTGAG-----------
------
FC88X_00130_00011 -------------ATCAC-GACCC-TTCTACCCAC-GATTGAG-----------
------
FC88X_00146_00020 -------------ATCAC-GACCC-TTCTACCCAC-GATTGAG-----------
------
FC88X_00138_00068 -------------ATCAC-GACCC-TTCTACCCAC-GATTGAG-----------
------
FC88X_00105_00140 -------------ATCAC-GACCC-TTCTACCCAC-GATTGAG-----------
------
FC88X_00102_00192 -------------ATCAC-GACCC-TTCTACCCAC-GATTGAG-----------
------
FC88X_00138_00173 -------------ATCAC-GACCC-TTCTACCCAC-GATTGAG-----------
------
FC88X_00125_00496 -------------ATCAC-GACCC-TTCTACCCAC-GATTGAG-----------
------
FC88X_00119_00511 -------------ATCAC-GACCC-TTCTACCCAC-GATTGAG-----------
------
FC88X_00100_00727 -------------ATCAC-GACCC-TTCTACCCAC-GATTGAG-----------
------
FC88X_00129_00975 -------------ATCAC-GACCC-TTCTACCCAC-GATTGAG-----------
------
FC88X_00156_00036 -------------ATCAC-GACCC-TTCTACCCAC-GATTGAG-----------
------
FC88X_00163_00067 -------------ATCAC-GACCC-TTCTACCCAC-GATTGAG-----------
------
FC88X_00181_00120 -------------ATCAC-GACCC-TTCTACCCAC-GATTGAG-----------
------
FC88X_00198_00818 -------------ATCAC-GACCC-TTCTACCCAC-GATTGAG-----------
------
FC88X_00173_00888 -------------ATCAC-GACCC-TTCTACCCAC-GATTGAG-----------
------
FC88X_00175_00969 -------------ATCAC-GACCC-TTCTACCCAC-GATTGAG-----------
------
FC88X_00189_00990 -------------ATCAC-GACCC-TTCTACCCAC-GATTGAG-----------
------
FC88X_00234_00392 -------------ATCAC-GACCC-TTCTACCCAC-GATTGAG-----------
------
FC88X_00236_00890 -------------ATCAC-GACCC-TTCTACCCAC-GATTGAG-----------
------
FC88X_00279_00990 -------------ATCAC-GACCC-TTCTACCCAC-GATTGAG-----------
------
FC88X_00338_00972 -------------ATCAC-GACCC-TTCTACCCAC-GATTGAG-----------
------
FC88X_00382_00188 -------------ATCAC-GACCC-TTCTACCCAC-GATTGAG-----------
------
FC88X_00395_00981 -------------ATCAC-GACCC-TTCTACCCAC-GATTGAG-----------
------
FC88X_00351_01033 -------------ATCAC-GACCC-TTCTACCCAC-GATTGAG-----------
------
FC88X_00384_01075 -------------ATCAC-GACCC-TTCTACCCAC-GATTGAG-----------
------
FC88X_00354_01102 -------------ATCAC-GACCC-TTCTACCCAC-GATTGAG-----------
------

FC88X_00390_01201 --------------ATCAC-GACCC-TTCTACCCAC-GATTGAG-----------
------
FC88X_00413_01097 --------------ATCAC-GACCC-TTCTACCCAC-GATTGAG-----------
------
FC88X_00406_01158 --------------ATCAC-GACCC-TTCTACCCAC-GATTGAG-----------
------
FC88X_00462_00304 --------------ATCAC-GACCC-TTCTACCCAC-GATTGAG-----------
------
FC88X_00467_01029 --------------ATCAC-GACCC-TTCTACCCAC-GATTGAG-----------
------
FC88X_00497_01042 --------------ATCAC-GACCC-TTCTACCCAC-GATTGAG-----------
------
FC88X_00490_01227 --------------ATCAC-GACCC-TTCTACCCAC-GATTGAG-----------
------
FC88X_00513_00138 --------------ATCAC-GACCC-TTCTACCCAC-GATTGAG-----------
------
FC88X_00532_00165 --------------ATCAC-GACCC-TTCTACCCAC-GATTGAG-----------
------
FC88X_00542_01202 --------------ATCAC-GACCC-TTCTACCCAC-GATTGAG-----------
------
FC88X_00583_00130 --------------ATCAC-GACCC-TTCTACCCAC-GATTGAG-----------
------
FC88X_00587_00137 --------------ATCAC-GACCC-TTCTACCCAC-GATTGAG-----------
------
FC88X_00593_00579 --------------ATCAC-GACCC-TTCTACCCAC-GATTGAG-----------
------
FC88X_00558_00815 --------------ATCAC-GACCC-TTCTACCCAC-GATTGAG-----------
------
FC88X_00593_01099 --------------ATCAC-GACCC-TTCTACCCAC-GATTGAG-----------
------
FC88X_00594_01240 --------------ATCAC-GACCC-TTCTACCCAC-GATTGAG-----------
------
FC88X_00606_00067 --------------ATCAC-GACCC-TTCTACCCAC-GATTGAG-----------
------
FC88X_00606_00118 --------------ATCAC-GACCC-TTCTACCCAC-GATTGAG-----------
------
FC88X_00612_00143 --------------ATCAC-GACCC-TTCTACCCAC-GATTGAG-----------
------
FC88X_00609_00979 --------------ATCAC-GACCC-TTCTACCCAC-GATTGAG-----------
------
FC88X_00634_01270 --------------ATCAC-GACCC-TTCTACCCAC-GATTGAG-----------
------
FC88X_00695_00123 --------------ATCAC-GACCC-TTCTACCCAC-GATTGAG-----------
------
FC88X_00698_00936 --------------ATCAC-GACCC-TTCTACCCAC-GATTGAG-----------
------
FC88X_00659_00955 --------------ATCAC-GACCC-TTCTACCCAC-GATTGAG-----------
------
FC88X_00732_01004 --------------ATCAC-GACCC-TTCTACCCAC-GATTGAG-----------
------
FC88X_00702_01062 --------------ATCAC-GACCC-TTCTACCCAC-GATTGAG-----------
------
FC88X_00718_01099 --------------ATCAC-GACCC-TTCTACCCAC-GATTGAG-----------
------
FC88X_00734_01128 --------------ATCAC-GACCC-TTCTACCCAC-GATTGAG-----------
------

FC88X_00747_01222 --------------ATCAC-GACCC-TTCTACCCAC-GATTGAG------------------
FC88X_00741_01263 --------------ATCAC-GACCC-TTCTACCCAC-GATTGAG------------------
FC88X_00765_00137 --------------ATCAC-GACCC-TTCTACCCAC-GATTGAG------------------
FC88X_00792_00333 --------------ATCAC-GACCC-TTCTACCCAC-GATTGAG------------------
FC88X_00753_01249 --------------ATCAC-GACCC-TTCTACCCAC-GATTGAG------------------
FC88X_00808_00121 --------------ATCAC-GACCC-TTCTACCCAC-GATTGAG------------------
FC88X_00803_00240 --------------ATCAC-GACCC-TTCTACCCAC-GATTGAG------------------
FC88X_00821_00259 --------------ATCAC-GACCC-TTCTACCCAC-GATTGAG------------------
FC88X_00852_00226 --------------ATCAC-GACCC-TTCTACCCAC-GATTGAG------------------
FC88X_00889_00210 --------------ATCAC-GACCC-TTCTACCCAC-GATTGAG------------------
FC88X_00898_00247 --------------ATCAC-GACCC-TTCTACCCAC-GATTGAG------------------
FC88X_00887_00651 --------------ATCAC-GACCC-TTCTACCCAC-GATTGAG------------------
FC88X_00859_00770 --------------ATCAC-GACCC-TTCTACCCAC-GATTGAG------------------
FC88X_00871_00820 --------------ATCAC-GACCC-TTCTACCCAC-GATTGAG------------------
FC88X_00880_00887 --------------ATCAC-GACCC-TTCTACCCAC-GATTGAG------------------
FC88X_00887_01264 --------------ATCAC-GACCC-TTCTACCCAC-GATTGAG------------------
FC88X_00920_00220 --------------ATCAC-GACCC-TTCTACCCAC-GATTGAG------------------
FC88X_00925_00241 --------------ATCAC-GACCC-TTCTACCCAC-GATTGAG------------------
FC88X_00936_00280 --------------ATCAC-GACCC-TTCTACCCAC-GATTGAG------------------
FC88X_00901_00326 --------------ATCAC-GACCC-TTCTACCCAC-GATTGAG------------------
FC88X_00902_00366 --------------ATCAC-GACCC-TTCTACCCAC-GATTGAG------------------
FC88X_00929_00455 --------------ATCAC-GACCC-TTCTACCCAC-GATTGAG------------------
FC88X_00901_00629 --------------ATCAC-GACCC-TTCTACCCAC-GATTGAG------------------
FC88X_00918_00841 --------------ATCAC-GACCC-TTCTACCCAC-GATTGAG------------------
FC88X_00968_00243 --------------ATCAC-GACCC-TTCTACCCAC-GATTGAG------------------
FC88X_00963_00364 --------------ATCAC-GACCC-TTCTACCCAC-GATTGAG------------------
FC88X_00986_00461 --------------ATCAC-GACCC-TTCTACCCAC-GATTGAG------------------
FC88X_00989_00461 --------------ATCAC-GACCC-TTCTACCCAC-GATTGAG------------------

FC88X_00992_00464 -------------ATCAC-GACCC-TTCTACCCAC-GATTGAG-----------
------
FC88X_00998_00588 -------------ATCAC-GACCC-TTCTACCCAC-GATTGAG-----------
------
FC88X_01006_00492 -------------ATCAC-GACCC-TTCTACCCAC-GATTGAG-----------
------
FC88X_01041_00505 -------------ATCAC-GACCC-TTCTACCCAC-GATTGAG-----------
------
FC88X_01008_00979 -------------ATCAC-GACCC-TTCTACCCAC-GATTGAG-----------
------
FC88X_01087_00475 -------------ATCAC-GACCC-TTCTACCCAC-GATTGAG-----------
------
FC88X_01065_00743 -------------ATCAC-GACCC-TTCTACCCAC-GATTGAG-----------
------
FC88X_01062_00886 -------------ATCAC-GACCC-TTCTACCCAC-GATTGAG-----------
------
FC88X_01114_00538 -------------ATCAC-GACCC-TTCTACCCAC-GATTGAG-----------
------
FC88X_01117_00572 -------------ATCAC-GACCC-TTCTACCCAC-GATTGAG-----------
------
FC88X_01131_00947 -------------ATCAC-GACCC-TTCTACCCAC-GATTGAG-----------
------
FC88X_01146_01105 -------------ATCAC-GACCC-TTCTACCCAC-GATTGAG-----------
------
FC88X_01102_00836 -------------ATCAC-GACCC-TTCTACCCAC-GATTGAGCTTCCTTT---
------
FC88X_00004_00257 -------------ATCAC-GG-CCCTTCTACCCAC-GATTGAG-----------
------
FC88X_00548_00214 -------------ATCAC-GGACCCTTCTACCCAC-GATTGAG-----------
------
FC88X_00068_00277 -------------ATCAC-GACCC-TTCTACC-AC-GACTGAG-----------
------
FC88X_00221_00925 -------------ATCAC-GACCC-TTCTACC-AC-GACTGAG-----------
------
FC88X_00278_01118 -------------ATCAC-GACCC-TTCTACC-AC-GACTGAG-----------
------
FC88X_00503_00218 -------------ATCAC-GACCC-TTCTACC-AC-GACTGAG-----------
------
FC88X_00604_00595 -------------ATCAC-GACCC-TTCTACC-AC-GACTGAG-----------
------
FC88X_00653_00406 -------------ATCAC-GACCC-TTCTACC-AC-GACTGAG-----------
------
FC88X_00672_00642 -------------ATCAC-GACCC-TTCTACC-AC-GACTGAG-----------
------
FC88X_00680_00677 -------------ATCAC-GACCC-TTCTACC-AC-GACTGAG-----------
------
FC88X_00790_00107 -------------ATCAC-GACCC-TTCTACC-AC-GACTGAG-----------
------
FC88X_00769_01264 -------------ATCAC-GACCC-TTCTACC-AC-GACTGAG-----------
------
FC88X_00998_01174 -------------ATCAC-GACCC-TTCTACC-AC-GACTGAG-----------
------
FC88X_01137_01201 -------------ATCAC-GACCC-TTCTACC-AC-GACTGAG-----------
------
FC88X_00671_01059 -------------ATCAC-GACCC-TTCTACC-AC-GACTGAGCTTCCT-----
------

FC88X_00507_00068 -------------ATCAC-GACCC-TTCTACC-AC-GA-TGAG-----------
------
FC88X_00863_01247 -------------ATCAC-GACCC-TTCTACC-AC-GA-TGAG-----------
------
FC88X_00891_01123 ------------GATCAC-GACCC-TTCTGCCCAC-GACTGAG-----------
------
FC88X_00377_00325 -------------ATCAC-GACCC-TTCTACCCAC-GACTGAG-----------
------
FC88X_00538_00136 -------------ATCAC-GACCC-TTCTACCCAC-GACTGAG-----------
------
FC88X_00816_00890 -------------ATCAC-GACCC-TTCTACCCAC-GACTGAG-----------
------
FC88X_00928_00511 -------------ATCAC-GACCC-TTCTACCCAC-GACTGAG-----------
------
FC88X_00969_00669 -------------ATCAC-GACCC-TTCTACCCAC-GACTGAG-----------
------
FC88X_00041_00795 -------------ATCAC-GACCC-TTCTACCCAC-GACTGAG-----------
------
FC88X_00373_00973 -------------ATCAC-GACCC-TTCTACCCAC-GACTGAG-----------
------
FC88X_00732_01098 -------------ATCAC-GACCC-TTCTACCCAC-GACTGAG-----------
------
FC88X_00872_00594 -------------ATCAC-GACCC-TTCTACCCAC-GACTGAG-----------
------
FC88X_00100_00617 -------------GTCAC-GACCC-TTCTACCCAC-GATTGAG-----------
------
FC88X_00325_01162 -------------ATCAC-GACCC-TTCTACCCAC-GATTAAGCC---------
------
FC88X_00296_00073 -------------ATTAC-GACCC-TTCTACCCAC-GATTGAG-----------
------
FC88X_00066_00648 -------------ATCAC-GACCC-TTCTACCCGC-GATTGAG-----------
------
FC88X_00691_00427 -------------ATCAC-GACCC-TTCTACC-GC-GATTGAG-----------
------
FC88X_00613_00904 -------------ATCAC-GACCC-TTCTACCCGC-GATTGAG-----------
------
FC88X_00438_00650 -------------ACCAC-GACCC-TTCTACC-AC-GATTGAG-----------
------
FC88X_00553_00866 -------------ACCAC-GACCC-TTCTACC-AC-GATTGAG-----------
------
FC88X_00869_00993 -------------ACCAC-GACCC-TTCTACC-AC-GATTGAG-----------
------
FC88X_00071_00325 -------------ACCAC-GACCC-TTCTACCCAC-GATTGAG-----------
------
FC88X_00439_01089 -------------ACCAC-GACCC-TTCTACCCAC-GATTGAG-----------
------
FC88X_00211_00021 -------------ACCAC-GACCC-TTCTACCCAC-GATTGAG-----------
------
FC88X_00255_01039 -------------ACCAC-GACCC-TTCTACCCAC-GATTGAG-----------
------
FC88X_01019_00408 -------------ACCAC-GACCC-TTCTACCCAC-GATTGAG-----------
------
FC88X_01001_00636 -------------ACCAC-GACCC-TTCTACCCAC-GATTGAG-----------
------
FC88X_00756_01032 -------------ATCAC-GACCC-TTCTACCCAC-GACTTGAG----------
------

FC88X_01117_00990 -------------ATCAC-GACCC-TTCTACC-AC-GATTCGAG----------
------
FC88X_00970_00849 -------------ATCAC-GACCC-TTCTACCCAC-GACTTGAG----------
------
FC88X_00504_01149 -------------ATCAC-GACCC-TTCTACCCAC-GATTGACGTCCTTTCCT-
------
FC88X_00900_00202 -------------ATCAC-GACCC-TTCTACCCAC-GATTGATG----------
------
FC88X_00642_01157 -------------ATCAC-GACCC-TTCTACCCAC--ATTGAGCTCCT------
------
FC88X_01106_00976 -------------ATCAC-GACCC-TTCTACC-AT--ATTGAG-----------
------
FC88X_00829_00301 -------------ATCAC-GACCC-T-CTACC-AC--ACTGAGCT---------
------
FC88X_00589_00143 -------------ATCAC-GACCC-TTCTAACCAC-GATTGAG-----------
------
FC88X_00032_00134 -------------ATCAC-GACCC-TTCTACG-AC-CACTGATGA---------
------
FC88X_00161_00634 --------GGGGAATCAC-GACCCATTCTACCA---------------------
------
FC88X_00805_00115 -------------ATCAC-GACCC-TTCTACCACG-AACTGAG-----------
------
FC88X_00021_00448 --------------TCAC-GAGCTCTTCTTCCC-CGACCTGAC-----------
------
FC88X_01103_00565 -------------ATCACTGACCCCTTCTACCCACGATTTGACG----------
------
FC88X_00569_00122 ------------ACTCACTGAACCCTTCCTACC-CACC----------------
------
FC88X_00031_00146 ------TCCCGACATCTTCAACG--TGCTATTCTA-------------------
------
FC88X_00467_00755 ------TCCCGACATCTTCAACG--TGCTATTCTAATCACC-------------
------
FC88X_00027_00168 ------TCCCGACATCTTCAACG--TGCTATTCTA-------------------
------
FC88X_00015_00128 ------TCCCGACATCTTCAACG--TGCTATTCTA-------------------
------
FC88X_00089_00763 ------TCCCGACATCTTCAACG--TGCTATTCTA-------------------
------
FC88X_00088_00153 ------TCCCGACATCTTCAACG--TGCTATTCTA-------------------
------
FC88X_00675_01024 ------TCCCGACATCTTCAACG--TGCTATTCTAT------------------
------
FC88X_00007_00568 ------TCCCGACATCTTCAACG--TGCTATTCTA-------------------
------
FC88X_00161_00556 ------TCCCGACATCTTCAACG--TGCTATTCTA-------------------
------
FC88X_00412_00492 ------TCCCGACATCTTCAACG--TGCTATTCTA-------------------
------
FC88X_00416_01103 ------TCCCGACATCTTCAACG--TGCTATTCTA-------------------
------
FC88X_00483_01108 ------TCCCGACATCTTCAACG--TGCTATTCTA-------------------
------
FC88X_00739_00265 ------TCCCGACATCTTCAACG--TGCTATTCTA-------------------
------
FC88X_00895_00743 ------TCCCGACATCTTCAACG--TGCTATTCTA-------------------
------

FC88X_00864_00827 ------TCCCGACATCTTCAACG--TGCTATTCTA-------------------
------
FC88X_00892_01137 ------TCCCGACATCTTCAACG--TGCTATTCTA-------------------
------
FC88X_00727_01249 ------TCCCGACATCTTCAAC---TGCTATTCTA-------------------
------
FC88X_00063_00266 ------TCCTGACATCTTCAGCG--TGCTATTCTA-------------------
------
FC88X_00689_01225 ------TCCTGACATCTTCAACG--TGCTATTCTA-------------------
------
FC88X_00323_01145 -------CTCGACACTGTACGCGT-TGCTATTCTG-------------------
------
FC88X_00123_00073 -------CTCGACATCGTACGCGT-TGCTATTCTG-------------------
------
FC88X_00712_01214 ------TCCCGTCATTGACCACGC-CACTATTCT--------------------
------
FC88X_00174_00845 ------TCCCGTCATTGACCACGC-CACTATTCT--------------------
------
FC88X_00080_00852 ------TCCAGTCATTGACCACGC-CACTATTCT--------------------
------
FC88X_00165_00642 ------TCGTGGCATTTAACACAC-GGCTCTCCT--------------------
------
FC88X_00981_00649 ------TCGTGGCATTTTACACAC-GGCTCTCCT--------------------
------
FC88X_01077_00818 ------TCGCGGCATTTTACACAC-GGCTCTCCT--------------------
------
FC88X_00287_00807 ------CCGGGTTATTTCACACAC-AGCGATTCT--------------------
------
FC88X_01091_01209 -------CTGTACACTGTACA-ACCCACTATGCTC-------------------
------
FC88X_00915_00962 -------CTGTACACTGTACA-ACCCACTATGCTC-------------------
------
FC88X_00972_01116 -------CTGTACACTGTACA-ACCCGCTATGCTC-------------------
------
FC88X_00606_00273 -------TCAAATACTGTTCATACCCGCGATCCT--------------------
------
FC88X_00481_01065 -----------TTGCTCGTCACCAACACTTCCCAT--AAT--------------
------
FC88X_00575_01150 -----------TTGCGCGT-ACCTTCTCTGCCCAT--ATT--------------
------
FC88X_00900_00760 ------------TACGCAC-ACCGACTCTCCCTTG--ATTAA------------
------
FC88X_00137_00129 ------------------TCTCCCTTTAATTACACTTGATAAATA---------
------
FC88X_00461_00412 -----------ACCCACGACTCCCCGAAACTCGAATTGTTATTCT---------
------
FC88X_00725_01085 ----------------GTCCCATCAACCTCAACCAAAATCGCGT----------
------
FC88X_00808_01132 -----------------TCCTAACAATTCCCCAACAGATCGCGCC---------
------
FC88X_00154_00781 ------------------CCTTCAATTCACCTCAACAATAGCGTT---------
------
FC88X_00613_00974 ------------------CCTTCAATTCACCTCAACAATAGCGTT---------
------
FC88X_01048_00693 ------------------ACACCACATATCCCGTCCCAGCGCATT---------
------

FC88X_01095_01019 ------------------ACACCACATATCCCGTCCCAGCGCATT---------
------
FC88X_00301_00369 ------------------ACACCACATATCCCGTCCCAGCGCATT---------
------
FC88X_00018_00576 ------------------CCTCCACTTATCCCGTCA-ATCGCATGTGC------
------
FC88X_00117_00837 -------------------CTCAACAACTCACGTCAAATCGCATTT--------
------
FC88X_00037_00225 ----------------CTCACTACCC-CGAGACA-CTCCCTCCGC---------
------
FC88X_00138_00119 ---------------------TCCCC-CTAGACA-CTCTCCC------------
------
FC88X_00122_00448 -----------------TCAATCCCC-GCACACATCTCCCATCGC---------
------
FC88X_00160_00904 ---------------CTTCACTCCCAGCGATACACATCTCTC------------
------
FC88X_00925_01059 ----------------GCCCTTCTCTACCGCGCGCATCTCTCTG----------
------
FC88X_00216_00953 ----------------CTCACAAATCGCCACTCCCAGCCGCCT-----------
------
FC88X_01107_00675 ----------------CCCACATCTC-CCACTAGCAACCGTCTG----------
------
FC88X_00084_00794 --------------------CTCCTTCTTCCTTTGAGCGGCCGTTAT-------
------
FC88X_00074_00142 --------------------CCCCTTCTTCCTTTGAGCGGCCGTTAT-------
------
FC88X_00572_00262 --------------------CCCCTTCTCCTCCGGCCGGACCGTTCT-------
------
FC88X_00743_00194 --------------------ACTTCTACCCCTCAGCCCGCCCGTTAA-------
------
FC88X_00565_00793 ------------------TTGACCTTCTCCTCACAGCCGCACGTGC--------
------
FC88X_00997_00962 -------------------TCGTCTAATTTCCCACGCCGCACTCTC--------
------
FC88X_01096_01002 -------------------CCGTCAAATTTCCCACGTCCGGCACTC--------
------
FC88X_01071_01091 -------------------TCCCCAAATCTCCAACGGCCGCACTTT--------
------
FC88X_00590_00533 -------------------TCCCCAAATCTCCAACGGCCGCACTTT--------
------
FC88X_00351_00313 -----------------TCGCGTCAATTCTCTCACCCCGCACAT----------
------
FC88X_01041_01132 -----------------TCGCGTCAATTCTCTCACCCCGCACAT----------
------
FC88X_00060_00301 -----------------TCCCCAAATAGCTACCGACGACAGCGTC---------
------
FC88X_00336_00957 ---------------TCCCCAAAAATAGCTACCGACGACAGCGTC---------
------
FC88X_00484_00090 -----------------TCCCCAAATAGCTACCGACGACAGCGTC---------
------
FC88X_00485_00178 -----------------TCCCCAAATAGCTACCGACGATAGCGTC---------
------
FC88X_00557_00639 -----------------TCTCCAAATAGCTACCGACGACAGCGTC---------
------
FC88X_00037_00330 ---------------TCTCCTCTAATCGGTAC--ACGACAGCCC----------
------

```
FC88X_00095_00215      ---------------TTCACCCCTATTCCGACC-GCTACAGCC-----------
------
FC88X_00105_00505      -----------------TTCGACCCTTGAC-CGACTGTTTGCCCA---------
------
FC88X_00677_00931      -----------------TTCAACCCCTGAC-CGACTGTTTGCCCA---------
------
FC88X_00596_00374      ---------------CCTCTAACCACTGACGCGACT--TTGCC-----------
------
FC88X_00974_00902      ---------------CGACACATACACGAC-CGCG---TAGTTCTC--------
------
FC88X_00998_00941      ---------------CGACACATACACGAC-CGCG---TAGTTCTC--------
------
FC88X_00320_01058      ------------------CACCTACCCGAC-GGCTC--TAGCTCTGCC------
------
FC88X_00759_00199      -----------------TTACTCCCATAGC-CGCCGTTTAGCTCTT--------
------
FC88X_01031_00538      -------------CTCCACATCCCACGAATACCTCTGACA--------------
------
FC88X_01143_00824      ------------------------TCGTAAACCTCTGACAGC------------
------
FC88X_00303_00966      ------------------TTCCTC-TCTCAACTAAA-GCTGCGTGCT-------
------
FC88X_00994_00913      ------------------TTCCTC-TCTCAACTAAA-GCTGCGTGCT-------
------
FC88X_00136_00562      ------------------TTCCTC-TCTCAACTAAA-GCTGCGTGCT-------
------
FC88X_00658_00145      ------------------TTCCTC-TCTCAACTAAA-GCTGCGTGCT-------
------
FC88X_01121_00492      ------------------TTCCTC-TCTCAACTAAA-GCTGCGTGCT-------
------
FC88X_00549_01060      ------------------TTCCTCGTATCCACAACACGCAACTTGCTCCC----
------
FC88X_00782_00277      -------------------TCCTTCAATCAATCACA-GCAAATTTCT-------
------
FC88X_01077_00812      -------------------TCTGCTATCCCTCTACA-GCCAAGTGCT-------
------
FC88X_00073_00107      ---------------------TTCCCATAACTTGA-CACCACATCTCCC-----
------
FC88X_00168_00851      -------------------ATCTCCCACAACT--A-AACTACGTCTC-------
------
FC88X_01119_00913      ----------------CTAATCTCTCATCCCT----AGACACATCTC-------
------
FC88X_00133_00595      -------------------CTCCCGCATCACTACA-GGT-ACACCACA------
------
FC88X_00766_00223      -------------------CTCCCACATCACTACA-GGT-ACACCACA------
------
FC88X_00380_00777      -------------------CTCACTCCTCACTATA-TACCACATTTC-------
------
FC88X_00928_01139      -------------------TTCCTTCATCCCTAAA-CTCGACGAGTG-------
------
FC88X_01106_00869      -------------------TTCCTTCATCCCTAAA-CTCGACGAGTG-------
------
FC88X_00874_00547      ------------GGGGAAGTTCCTCATTCCCTAAATCTCGACGAGTG-------
------
FC88X_00146_00086      --------------------------ATCCCCAAT-
CCCGACGAGTGCAACGCC------
```

FC88X_01040_00666 -------------------TTTACTATTCGCCAAA-ATCATCGTTGC-------
------
FC88X_00873_00382 -------------------TTTACTATTCGCCAAA-ATCATCGTTGC-------
------
FC88X_00911_00447 -------------------TTCTCCAATTCCCAAA-ATTGCTGTTGC-------
------
FC88X_00753_00300 ----------------CGCTGTATTGTTTGCTATT-CCCAAAAT----------
------
FC88X_00222_00102 -------------------TGCAATAATCGCTAAT-CCCTAAACCCT-------
------
FC88X_00343_01068 ------------------ACCAAC-AATCCCCTACATCCGACTATT--------
------
FC88X_00924_00252 ------------------ACCAAC-AATCCCCTACATCCGACTATT--------
------
FC88X_00999_00410 ------------------ACCAAC-AATCCCCTACATCCGACTATT--------
------
FC88X_00124_00723 ------------------ACCAAC-
AATCCCCTACATCCGACTATTCTTCCTTTC-----
FC88X_01093_00507 ------------------TCCAAC-AATCCCCTACATCCGACTATT--------
------
FC88X_01073_00941 ------------------ACCAACCAATCCCCTACATCCGACTACTTCTT----
------
FC88X_00144_00959 -------------------CCAACATTCCCCAATCACGCAACTACT--------
------
FC88X_00311_01115 -----------------TCGTCACAAATCCCGTA-ATCACACTAT---------
------
FC88X_00428_01110 -----------------TCGTCACAAATCCCGTA-ATCACACTAT---------
------
FC88X_00983_01170 -----------------TCGTCACAAATCCCGTA-ATCACACTAT---------
------
FC88X_00740_01060 -----------------CACCCAAAATTCCTGT----TCACCTATTCTC-----
------
FC88X_00831_00238 ------------------ACCTAATATCCCTGTA-GTCCCCCTATTATCAC---
------
FC88X_00064_00493 ------------------TCTCACAATGAGC-AACTCCCGACTTACG-------
------
FC88X_01113_00700 ------------------TCTCACAATGAGC-AACTCCCGACTTACG-------
------
FC88X_00436_01145 ------------------TCTCACAATGAGC-AACTCCCGACTTACG-------
------
FC88X_00964_00630 -----------------GTCTCACAATGAGC-AACTCCCGACTTACG-------
------
FC88X_00963_00650 ------------------TCTCACAATGAGCCAACTCCCGACTTACG-------
------
FC88X_01108_01272 ------------GGGGAATCTCACAATGAGCCAACTCCCGACGTACG-------
------
FC88X_00429_00025 ----------------CCTCAAACTATGATCC-ACCCCCATGTA----------
------
FC88X_00220_01082 -----------------ATCACACAACGATCCTATTCCCATTTA----------
------
FC88X_00875_00929 -------------CCTGATCCTAAAACGAACCAACTCCCG--------------
------
FC88X_00445_01059 -------------------CTCCCAAAGTGACAAACACCGAAATACT-------
------
FC88X_00033_00493 -------------------CTCCCAAAGTGACAAACACCGAAATACT-------
------

FC88X_00046_00798 -----------AACCAAGACAAACAAACAAAAAAATGCCCTGA-----------
------
FC88X_00511_00190 --------------------TCACGACGCTCGAAATCCCAAGATATT-------
------
FC88X_00432_00711 --------------------TCACGACGCTCGAAATCCCAAGATATT-------
------
FC88X_00101_00078 --------------------TCACGACGCTCGAGATCCCAAGATATT-------
------
FC88X_01055_00959 --------------------TCACGACGCCCGGAATCCCAAGATATT-------
------
FC88X_00130_00880 -------------------CCAACGGACTAGGCAATTTCTCATCAT--------
------
FC88X_00669_00380 ------------GGGGAACCAAACGGACTAGGCAATTTCTCATCAT--------
------
FC88X_00738_00933 -------------------
CCAACGTACTAGGCAATTTCTCATCATCTTCCTTTCT----
FC88X_00950_00502 -------------------CTAACGACTCCGCAGAACTCCCAACAT--------
------
FC88X_00159_00523 ------------------TCCAACCAACGACACCAACTCCCAACAG--------
------
FC88X_01128_00526 ------------------TCCAACCAACGACAC-AACTCCCATCAG--------
------
FC88X_00027_00293 -------------CCGTATTCTCGGCCCCTC-TACACTGCT-------------
------
FC88X_01089_00779 -------------ACATTTCCTCGCCCGCTCCTACATCGC--------------
------
FC88X_01097_00539 -------------CCGTTTTCTCCCCGGCCC-TAGCCTGCG-------------
------
FC88X_00835_00136 -------------TTATCTCCTCGGCTTCGCTAAC-CTGCG-------------
------
FC88X_00933_00967 ------------GTTATCTCCTCGGCTTCGCTAAC-CTGCG-------------
------
FC88X_00864_00330 -------------TTATCTCCTCGGCTTCGCTAAC-CTGCG-------------
------
FC88X_01053_00537 -------------TTATCTCCTCGGCTTCGCTAAC-CTGCG-------------
------
FC88X_00017_00745 -------------TTATCTCCTCGGCTTCGCTAAC-CTGCG-------------
------
FC88X_00879_00167 -------------TTATCTCCTCGGCTTCGCTAAC-CTGCG-------------
------
FC88X_01090_00962 -------------TTATCTCCTCGGCTTCGCTAAC-CTGCG-------------
------
FC88X_01136_01079 -------------TTATCTCCTCGGCTTCGCTAAC-CTGCG-------------
------
FC88X_00561_01193 --------------TAACTCCTCCTCTCCACTACCACTGCG-------------
------
FC88X_00231_00074 -----------------CTGC-CCCAGCAACTGAATTCCCTGACCT--------
------
FC88X_00752_00940 -------------CTGACTGCTCCCCGTCACTAACATACCC-------------
------
FC88X_00326_00924 -------------CTATTCCTCGCCTTGCAACCCCATTGC--------------
------
FC88X_00866_00280 -------------CTACACCTC-CCTAGCCGCCACATTCCGC------------
------
FC88X_00509_00971 ---------------CTCCTGTACCTA-TAACAATAAT----------------
------

FC88X_00840_00321 ----------TTCCCCTCCTGTGCCAAATATTAATGAT----------------
------
FC88X_00018_00212 -----------ACGCGACTTCTCCGATGCCACAACATT----------------
------
FC88X_01101_00697 -----------TTGCCGCTCCTTCCCAATGACAATATTC---------------
------
FC88X_00056_00276 -------------CCGACTCCTCTCCAATGGCAACATGCC--------------
------
FC88X_00014_00329 ------------GCCACGCGCTTCACTCCACCAATCTTAT--------------
------
FC88X_00695_00953 -------------CCACAGCGTTTCTCTCACCAAGCTACT--------------
------
FC88X_00412_01192 -------------CCACACATTCTTCACCCCCGACGCTCT--------------
------
FC88X_00877_00245 -------------CAACAAGTTATTTTCCCCC-ACGCTCCT-------------
------
FC88X_00117_00052 -------------CCACACGCTTTTGTCTCCCCCTAACCT--------------
------
FC88X_00873_00822 -------------GCACTCGCACTTTCTTTCCAACCTATT--------------
------
FC88X_00280_01092 -------------CCAATCGCTACTTGCCACCA-CCTATTC-------------
------
FC88X_00319_00089 -------------CCACTCCCTTATCACTACCA-CCTAGC--------------
------
FC88X_00021_00516 -----------------CGCGTATGC-CTCCCTACGTTGACACGT---------
------
FC88X_01004_00587 -----------------CGCGTATAC-CTCCCTACGTTGACACGT---------
------
FC88X_00101_00205 -------------CCACCGCCTATTCACTCGCTACCTACAC-------------
------
FC88X_00045_00624 -------------CCCACGTATCTA-CTCCCCAACATTGAC-------------
------
FC88X_01032_00820 -------------CCCCCGCCTCTATCTCGCTAACGTAGC--------------
------
FC88X_00323_00020 -------------TTCGCGCTCCTCTTACCTCAACATATT--------------
------
FC88X_00100_00175 -------------TTCGCGCTCCTTCTACCTCAACATATT--------------
------
FC88X_00975_00935 -------------TTCGCGCTCCTCTTACCTCAACATATT--------------
------
FC88X_01007_00653 -------------TTCGCGCTCCTCTTACCTCAACATATT--------------
------
FC88X_00593_01225 -------------TCCTTCGCGCTAGGACCTCATCATACC--------------
------
FC88X_00845_01174 ----------------CCCTTCCTTATCCCTCGATAAATTGCA-----------
------
FC88X_00567_00077 ----------------CCCTTGCTCGTCA-TCACTAAATTCCT-----------
------
FC88X_01095_00901 ----------------TCGTCCCTTAGCTCCTTGGCAATCCAC-----------
------
FC88X_00891_00244 ---------------CCCGTAGCTCTTGCCTTGGAAACTGTTCT----------
------
FC88X_00009_00431 -----------------CGTACCTATCACCTCGCCAAACTCGAC----------
------
FC88X_00558_00109 ----------------CCCCATATCCGGTCCACG-CTACTCGC-----------
------

```
FC88X_00589_01074       -------------CTATCCCATCTCCAGCCCACGGCTACC--------------
------
FC88X_00354_01170       -----------GTCTATCCCCTTACCTCCTCGCCACAAC---------------
------
FC88X_00995_00898       --------------TGTCTCCCACAGCGCTACCTGCCACCT-------------
------
FC88X_00309_01124       --------------TGTCTCC-ACAGCGCTACCTGCCACCT-------------
------
FC88X_00412_00031       -------------CTATATCCCATCGCTAGCTCCACCAAC--------------
------
FC88X_00227_01038       --------------CCGCACCCCGAGACAAACC-ACGTTATT------------
------
FC88X_00059_00415       --------------CCGCAGCCCGTCCCCAACCCGTGATATT------------
------
FC88X_00503_00758       -------------CTACTTGCCCGCCACTATCC--AGATATT------------
------
FC88X_00875_01259       ------------CTCCCCAACATGCGCTCCACGTAACCAT--------------
------
FC88X_00986_00836       ------------CCCAACGCTTCGCCCCGCCCGTAAACA---------------
------
FC88X_00441_00093       --------------CCGCACATTACCTACTTC-CAGCTTATT------------
------
FC88X_00633_01257       ----------------TCACCTTACCGACCTCTCAGCCTATT------------
------
FC88X_01025_00504       -------------ACCCAACGTTCCCGACCCC-TAGCCGC--------------
------
FC88X_00577_00800       ------------TCAACAATTACCAGCCCGACCTATACT---------------
------
FC88X_00908_00569       ------------CCCAACATACCGGACTCGACCCAAGCT---------------
------
FC88X_01019_00471       ------------ATCGTTCCCTACCGCGCGACCTAAACT---------------
------
FC88X_01047_00415       ----------------TGGAC-TGCTCTCACCACA-TACCTTATT---------
------
FC88X_00781_00243       ----------------TGGAC-TGCTCTCACCACA-TACCTTATT---------
------
FC88X_00841_00148       ----------------TGGAC-TGCTCTCACCACA-TACCTTATT---------
------
FC88X_01053_00578       ----------------TGGAC-TGCTCTCACCACA-TACCTTATT---------
------
FC88X_01086_01018       ----------------TGGAC-TGCTCTCACCACA-TACCTTATT---------
------
FC88X_00105_00225       ----------------TGGGC-TGCTCTCACCACA-TACCTTATT---------
------
FC88X_00510_00066       ----------------TGGAC-TGCTCTCACCACA-TA----------------
------
FC88X_00628_01021       ----------------TGGAC-TGCTCTCACCACG-
TACCTTATTCTTCCTTCT------
FC88X_00785_00280       ---------------GTGGACCTGCTCTCACCACA-TA----------------
------
FC88X_01025_00653       ----------------TCGAC-TGCCTCCCCAACTACTATTTA-----------
------
FC88X_00595_01269       ----------------ACGTC-TGCTCTCTCGACC-TAATCTATT---------
------
FC88X_00435_00020       ------------GCCCCTGAC-CGCGCACACCACC-TATCA-------------
------
```

FC88X_00596_00089 --------------CTCCTTCATTCCC--GTCCCCGTGATACT-----------
------
FC88X_00889_00996 --------------CTCCTTCATTCCC--GTCCCCGTGACACT-----------
------
FC88X_00839_01231 --------------CCCCATCATGCCTCAGTCCCCGTTACA-------------
------
FC88X_00286_00970 ---------------ATCATCCCGTCCCCGGAACAGCAACACC-----------
------
FC88X_00932_01038 -------------TTCCCTCCGCTCCCCGGGACGCACCAT--------------
------
FC88X_00436_01035 -----------ATTTCCCATCCTGAGCCCGGCACAGCGCC--------------
------
FC88X_01049_01183 -----------ATTTCCCATCCTGAGCCCGGCACAGCGCC--------------
------
FC88X_00035_00440 ------------TCTCCGATCCTGCACCCGAAAGCGCGC---------------
------
FC88X_00020_00208 ----------------------TCTATCACGAGCCCGC-CCGAGGTTATT----
------
FC88X_00095_00336 ----------------------TCTATCACGAGCCCGC-CCGAGGTTATT----
------
FC88X_01001_00737 ----------------------TCTATCACGAGCCCGC-CCGAGGTTATT----
------
FC88X_00203_00868 ------------------------ACTTCCGATCCCCCGCCGAGTTTACTC---
------
FC88X_00429_01038 ------------------------ACCCCTCATCGCCCGCCGAGTTTCTCG---
------
FC88X_00044_00123 ------------------------ATTCCCTATCGCCCACCGT-TTTCTC----
------
FC88X_00092_00149 ---------------GGGGAAGTCCG-CCAATTCTG-CGCGTCAGC-ATTCTC-
------
FC88X_00902_01243 ----------------------TCCG-CCAATTCTG-CGCGTCAGC-ATTCTC-
------
FC88X_01100_00846 ----------------------TCCG-C-AATTCTG-CGCGTTAGC-ATTCTC-
------
FC88X_00377_01197 ----------------------TCCG-CCAATTCTGACGCGTTAGC-ATTCTC-
------
FC88X_00068_00204 ----------------------TCCG-CCAATTCTG-CGCGTTAGC-ATTCTC-
------
FC88X_00187_00160 ----------------------TCCG-CCAATTCTG-CGCGTTAGC-ATTCTC-
------
FC88X_00240_00840 ----------------------TCCG-CCAATTCTG-CGCGTTAGC-ATTCTC-
------
FC88X_00222_00956 ----------------------TCCG-CCAATTCTG-CGCGTTAGC-ATTCTC-
------
FC88X_00301_00421 ----------------------TCCG-CCAATTCTG-CGCGTTAGC-ATTCTC-
------
FC88X_00366_00784 ----------------------TCCG-CCAATTCTG-CGCGTTAGC-ATTCTC-
------
FC88X_00305_00714 ----------------------TCCG-CCAAATTCTGCGCGTTAGC-ATTCTC-
------
FC88X_00488_00959 ----------------------TCCG-CCAA-TTCTGCGCGTTAGC-ATTCTC-
------
FC88X_00566_01180 ----------------------TCCG-CCAATTCTG-CGCGTTAGC-ATTCTC-
------
FC88X_00716_00896 ----------------------TCCG-CCAATTCTG-CGCGTTAGC-ATTCTC-
------

FC88X_00755_01138 ----------------------TCCG-CCAATTCTG-CGCGTTAGC-ATTCTC-
------
FC88X_00818_00118 ----------------------TCCG-CCAATTCTG-CGCGTTAGC-ATTCTC-
------
FC88X_00873_00690 ----------------------TCCG-CCAATTCTG-CGCGTTAGC-ATTCTC-
------
FC88X_00997_00376 ----------------------TCCG-CCAATTCTG-CGCGTTAGC-ATTCTC-
------
FC88X_00960_00920 ----------------------TCCG-CCAATTCTG-CGCGTTAGC-ATTCTC-
------
FC88X_01012_00272 ----------------------TCCG-CCAATTCTG-CGCGTTAGC-ATTCTC-
------
FC88X_01013_00640 ----------------------TCCG-CCAATTCTG-CGCGTTAGC-ATTCTC-
------
FC88X_01054_00367 ----------------------TCCG-CCAATTCTG-CGCGTTAGC-ATTCTC-
------
FC88X_01106_00923 ----------------------TCCG-CCAATTCTG-CGCGTTAGC-ATTCTC-
------
FC88X_00012_00388 ----------------------TCCG-CCAATTCTG-CGCGTTAGC-ATTCTC-
------
FC88X_00045_00465 ----------------------TCCG-CCAATTCTG-CGCGTTAGC-ATTCTC-
------
FC88X_00016_00692 ----------------------TCCG-CCAATTCTG-CGCGTTAGC-ATTCTC-
------
FC88X_00094_00088 ----------------------TCCG-CCAATTCTG-CGCGTTAGC-ATTCTC-
------
FC88X_00091_00201 ----------------------TCCG-CCAATTCTG-CGCGTTAGC-ATTCTC-
------
FC88X_00065_00325 ----------------------TCCG-CCAATTCTG-CGCGTTAGC-ATTCTC-
------
FC88X_00075_00325 ----------------------TCCG-CCAATTCTG-CGCGTTAGC-ATTCTC-
------
FC88X_00081_00854 ----------------------TCCG-CCAATTCTG-CGCGTTAGC-ATTCTC-
------
FC88X_00146_00754 ----------------------TCCG-CCAATTCTG-CGCGTTAGC-ATTCTC-
------
FC88X_00198_00016 ----------------------TCCG-CCAATTCTG-CGCGTTAGC-ATTCTC-
------
FC88X_00204_00898 ----------------------TCCG-CCAATTCTG-CGCGTTAGC-ATTCTC-
------
FC88X_00256_00902 ----------------------TCCG-CCAATTCTG-CGCGTTAGC-ATTCTC-
------
FC88X_00312_00034 ----------------------TCCG-CCAATTCTG-CGCGTTAGC-ATTCTC-
------
FC88X_00320_00023 ----------------------TCCG-CCAATTCTG-CGCGTTAGC-ATTCTC-
------
FC88X_00310_01003 ----------------------TCCG-CCAATTCTG-CGCGTTAGC-ATTCTC-
------
FC88X_00360_01013 ----------------------TCCG-CCAATTCTG-CGCGTTAGC-ATTCTC-
------
FC88X_00438_00032 ----------------------TCCG-CCAATTCTG-CGCGTTAGC-ATTCTC-
------
FC88X_00419_00651 ----------------------TCCG-CCAATTCTG-CGCGTTAGC-ATTCTC-
------
FC88X_00433_00958 ----------------------TCCG-CCAATTCTG-CGCGTTAGC-ATTCTC-
------

FC88X_00491_00563 -----------------------TCCG-CCAATTCTG-CGCGTTAGC-ATTCTC-
------
FC88X_00494_00588 -----------------------TCCG-CCAATTCTG-CGCGTTAGC-ATTCTC-
------
FC88X_00580_00873 -----------------------TCCG-CCAATTCTG-CGCGTTAGC-ATTCTC-
------
FC88X_00552_01091 -----------------------TCCG-CCAATTCTG-CGCGTTAGC-ATTCTC-
------
FC88X_00591_01235 -----------------------TCCG-CCAATTCTG-CGCGTTAGC-ATTCTC-
------
FC88X_00604_00067 -----------------------TCCG-CCAATTCTG-CGCGTTAGC-ATTCTC-
------
FC88X_00621_00618 -----------------------TCCG-CCAATTCTG-CGCGTTAGC-ATTCTC-
------
FC88X_00611_00873 -----------------------TCCG-CCAATTCTG-CGCGTTAGC-ATTCTC-
------
FC88X_00682_00288 -----------------------TCCG-CCAATTCTG-CGCGTTAGC-ATTCTC-
------
FC88X_00666_00367 -----------------------TCCG-CCAATTCTG-CGCGTTAGC-ATTCTC-
------
FC88X_00686_01187 -----------------------TCCG-CCAATTCTG-CGCGTTAGC-ATTCTC-
------
FC88X_00720_01027 -----------------------TCCG-CCAATTCTG-CGCGTTAGC-ATTCTC-
------
FC88X_00775_00102 -----------------------TCCG-CCAATTCTG-CGCGTTAGC-ATTCTC-
------
FC88X_00764_01084 -----------------------TCCG-CCAATTCTG-CGCGTTAGC-ATTCTC-
------
FC88X_00776_01163 -----------------------TCCG-CCAATTCTG-CGCGTTAGC-ATTCTC-
------
FC88X_00782_01173 -----------------------TCCG-CCAATTCTG-CGCGTTAGC-ATTCTC-
------
FC88X_00787_01208 -----------------------TCCG-CCAATTCTG-CGCGTTAGC-ATTCTC-
------
FC88X_00839_00229 -----------------------TCCG-CCAATTCTG-CGCGTTAGC-ATTCTC-
------
FC88X_00835_01158 -----------------------TCCG-CCAATTCTG-CGCGTTAGC-ATTCTC-
------
FC88X_00852_00545 -----------------------TCCG-CCAATTCTG-CGCGTTAGC-ATTCTC-
------
FC88X_00860_01091 -----------------------TCCG-CCAATTCTG-CGCGTTAGC-ATTCTC-
------
FC88X_00862_01175 -----------------------TCCG-CCAATTCTG-CGCGTTAGC-ATTCTC-
------
FC88X_00909_00279 -----------------------TCCG-CCAATTCTG-CGCGTTAGC-ATTCTC-
------
FC88X_00964_00557 -----------------------TCCG-CCAATTCTG-CGCGTTAGC-ATTCTC-
------
FC88X_00966_01213 -----------------------TCCG-CCAATTCTG-CGCGTTAGC-ATTCTC-
------
FC88X_01006_00340 -----------------------TCCG-CCAATTCTG-CGCGTTAGC-ATTCTC-
------
FC88X_01014_00333 -----------------------TCCG-CCAATTCTG-CGCGTTAGC-ATTCTC-
------
FC88X_01002_00372 -----------------------TCCG-CCAATTCTG-CGCGTTAGC-ATTCTC-
------

FC88X_01086_00431 ----------------------TCCG-CCAATTCTG-CGCGTTAGC-ATTCTC-
------
FC88X_01090_01053 ----------------------TCCG-CCAATTCTG-CGCGTTAGC-ATTCTC-
------
FC88X_01120_00551 ----------------------TCCG-CCAATTCTG-CGCGTTAGC-ATTCTC-
------
FC88X_01139_01024 ----------------------TCCG-CCAATTCTG-CGCGTTAGC-ATTCTC-
------
FC88X_00435_00609 ----------------------TCCG-CCAATTCTG-CGCGTTAGC-
ATTCTCCC-----
FC88X_00728_01073 ----------------------TCCG-CCAATTCTG-CGCGTTAGC-
ATTCTCCC-----
FC88X_00978_00738 -----------------------CCG-CCAATTCTG-CGCGTTAGC-ATTCTC-
------
FC88X_00784_01259 ----------------------CCCG-CCAATTCTG-CGCGTTAGC-ATTCTC-
------
FC88X_00528_00984 ----------------------TCCG-CCAATTCTG-CGCGTTAGC--TTCTC-
------
FC88X_00728_00305 ---------------------ATCCG-CCAATTCTG-CGCGTTAGC--TTCTC-
------
FC88X_01042_00744 ---------------------GTCCG-CCAATTCTG-CGCGTTAGC-ATTCTC-
------
FC88X_00908_00682 --------------------GTCCTG-CCAATTCTG-CGCGTTAGC-ATTCTC-
------
FC88X_01066_00743 ---------------------GTCCG-CCAATTCTG-CGCGTTAGC-ATTCTC-
------
FC88X_00368_00629 ----------------------TCCG-CCAGTTCTG-CGCGTTAGC-ATTCTC-
------
FC88X_00776_01040 ----------------------TCCG-CCAATTTTG-CGCGTTAGC-ATTCTC-
------
FC88X_01132_00922 ----------------------TCCG-CCAATT--G-CGCGTTAGC-ATTCTC-
------
FC88X_00259_00567 ----------------------TACG-CCAATTCTG-CGCGTTAGC-ATTCTC-
------
FC88X_01028_01218 ----------------------TTCG-CCAATTCTG-CGCGTTAGC-ATTCTC-
------
FC88X_01103_01080 ----------------------TCCG-CCAATTCTG-CGCGTTAGCTACTCTC-
------
FC88X_01036_00661 ----------------------TCCG-CCAATTCTG-CGCGTTAGC-ACTCTC-
------
FC88X_00893_00300 ----------------------TCCG-CCAATTCTG-CGCGTTGGC-ATTCTC-
------
FC88X_00989_00264 ----------------------TCCG-CC-AATTCCGCGCGTTAGC-ATTCTC-
------
FC88X_00071_00642 ----------------------TCCG-CC-AATTCCGCGCGTTAGC-ATTCTC-
------
FC88X_00551_01102 ----------------------TCCG-CCCAATTCTGCGCGTTAGC-
ATTCCTC------
FC88X_00603_00931 ----------------------TCCG-CC-AATTCTGCGCGTTAGC-ATT----
------
FC88X_01034_00551 ----------------------TCCG-CCAATTCTCGCGCGTTAGC-AT-----
------
FC88X_00347_01029 ----------------------ACCG-CCAATTCTG-CGCGCTAGC-ATTCTC-
------
FC88X_00936_01220 ----------------------TCGGTCCAAGTTCTGCGCGTTAGC-ATTCT--
------

```
FC88X_01026_00862       ---------------------TTCCATCCAATCCCG-CAC-TTAACCATT----
------
FC88X_01049_00622       ----------------TTTCGAACCTCCACATCCCG-CGCGT------------
------
FC88X_00149_00097       --------------------TTTCCTGATCTCCTCTGCCGCC-CGCAG------
------
FC88X_00861_00804       ---------------------TTCCCAATTACGTCGGGCCCA-CGCAGCG----
------
FC88X_00877_01183       ----------------------TCCCATTCTTTTCTGCCCTAGCGCGGC-----
------
FC88X_00072_00352       ----------------------TCCAAATTTCCACTCCCCGCGCGCAGC-----
------
FC88X_00193_00141       ----------------------CCCAAATTTCCACTCTCCGCGCGCAGC-----
------
FC88X_00511_00186       -------------GGGGAGTTATCCGAACTTCTCCT-TGCGCTATT--------
------
FC88X_01072_00820       ------------------GTTATCCGAACTTCTCCT-CGCGCTATT--------
------
FC88X_00926_00813       -------------------TTATCCGCTAAACTCCT--GCTCTATTCA------
------
FC88X_00849_00899       ------------------CTTATCCCTACACTCCCG-AGCGCTGTC--------
------
FC88X_00646_00321       ------------------TCTCCTTCATAATGTCCC-CACACTGGG--------
------
FC88X_01027_00493       ------------------CCTTTATGCCACTGACCC-CACGCTGTT--------
------
FC88X_00008_00359       ------------------------ACACCAA-TCGT---C--
GGGTCATGTTCTC-----
FC88X_00896_00206       -------------------GGGGAACACCAA-TCGT-GCC--
GGGTCATGTTCTC-----
FC88X_00433_00006       -----------------------AACACCAA-TCGT-GCC--
GGGTCATGTTCTC-----
FC88X_00424_01225       ------------------------ACACCAA-TCGT-GCC--
GGGTCATGTTCTC-----
FC88X_00118_00261       ------------------------ACACCAA-TCGT-GCC--
GGGTCATGTTCTC-----
FC88X_00845_00242       ------------------------ACACCAAATCGT-GCC--
GGGTCATGTTCTC-----
FC88X_00861_00932       ------------------------ACACCAA-TCGT-
GCCCGGGGTCATGTTCTCC----
FC88X_00252_00954       ------------------------ACACCAA-TCGT-GCC--
GGGTCATGTTCTC-----
FC88X_00269_01044       ------------------------ACACCAA-TCGT-GCC--
GGGTCATGTTCTC-----
FC88X_00369_00996       ------------------------ACACCAA-TCGT-GCC--
GGGTCATGTTCTC-----
FC88X_00446_01189       ------------------------ACACCAA-TCGT-GCC--
GGGTCATGTTCTC-----
FC88X_00478_01208       ------------------------ACACCAA-TCGT-GCC--
GGGTCATGTTCTC-----
FC88X_00526_00994       ------------------------ACACCAA-TCGT-GCC--
GGGTCATGTTCTC-----
FC88X_00589_00100       ------------------------ACACCAA-TCGT-GCC--
GGGTCATGTTCTC-----
FC88X_00563_00183       ------------------------ACACCAA-TCGT-GCC--
GGGTCATGTTCTC-----
```

FC88X_00801_01146 ------------------------ACACCAA-TCGT-GCC--
GGGTCATGTTCTC-----
FC88X_00868_00293 ------------------------ACACCAA-TCGT-GCC--
GGGTCATGTTCTC-----
FC88X_00868_00997 ------------------------ACACCAA-TCGT-GCC--
GGGTCATGTTCTC-----
FC88X_00916_00192 ------------------------ACACCAA-TCGT-GCC--
GGGTCATGTTCTC-----
FC88X_00994_00322 ------------------------ACACCAA-TCGT-GCC--
GGGTCATGTTCTC-----
FC88X_00968_01253 ------------------------ACACCAA-TCGT-GCC--
GGGTCATGTTCTC-----
FC88X_01098_00755 ------------------------ACACCAA-TCGT-GCC--
GGGTCATGTTCTC-----
FC88X_01089_01144 ------------------------ACACCAA-TCGT-GCC--
GGGTCATGTTCTC-----
FC88X_01091_01130 ------------------------ACACCAA-TCGT-GCC--
GGGTCATGTTCTC-----
FC88X_01138_00948 ------------------------ACACCAA-TCGT-GCC--
GGGTCATGTTCTC-----
FC88X_01133_01031 ------------------------ACACCAA-TCGT-GCC--
GGGTCATGTTCTC-----
FC88X_00069_00177 ------------------------ACACCAA-TCGT-GCC--
GGGTCATGTTCTC-----
FC88X_00122_00034 ------------------------ACACCAA-TCGT-GCC--
GGGTCATGTTCTC-----
FC88X_00111_00564 ------------------------ACACCAA-TCGT-GCC--
GGGTCATGTTCTC-----
FC88X_00136_00629 ------------------------ACACCAA-TCGT-GCC--
GGGTCATGTTCTC-----
FC88X_00172_00624 ------------------------ACACCAA-TCGT-GCC--
GGGTCATGTTCTC-----
FC88X_00236_00949 ------------------------ACACCAA-TCGT-GCC--
GGGTCATGTTCTC-----
FC88X_00292_01114 ------------------------ACACCAA-TCGT-GCC--
GGGTCATGTTCTC-----
FC88X_00428_01173 ------------------------ACACCAA-TCGT-GCC--
GGGTCATGTTCTC-----
FC88X_00474_00081 ------------------------ACACCAA-TCGT-GCC--
GGGTCATGTTCTC-----
FC88X_00451_00325 ------------------------ACACCAA-TCGT-GCC--
GGGTCATGTTCTC-----
FC88X_00582_00248 ------------------------ACACCAA-TCGT-GCC--
GGGTCATGTTCTC-----
FC88X_00594_01151 ------------------------ACACCAA-TCGT-GCC--
GGGTCATGTTCTC-----
FC88X_00637_01112 ------------------------ACACCAA-TCGT-GCC--
GGGTCATGTTCTC-----
FC88X_00665_01180 ------------------------ACACCAA-TCGT-GCC--
GGGTCATGTTCTC-----
FC88X_00710_00097 ------------------------ACACCAA-TCGT-GCC--
GGGTCATGTTCTC-----
FC88X_00729_00073 ------------------------ACACCAA-TCGT-GCC--
GGGTCATGTTCTC-----
FC88X_00796_00854 ------------------------ACACCAA-TCGT-GCC--
GGGTCATGTTCTC-----

FC88X_00781_01027 ------------------------ACACCAA-TCGT-GCC--
GGGTCATGTTCTC-----
FC88X_00796_01051 ------------------------ACACCAA-TCGT-GCC--
GGGTCATGTTCTC-----
FC88X_00819_00754 ------------------------ACACCAA-TCGT-GCC--
GGGTCATGTTCTC-----
FC88X_00843_00791 ------------------------ACACCAA-TCGT-GCC--
GGGTCATGTTCTC-----
FC88X_00875_00878 ------------------------ACACCAA-TCGT-GCC--
GGGTCATGTTCTC-----
FC88X_00923_00752 ------------------------ACACCAA-TCGT-GCC--
GGGTCATGTTCTC-----
FC88X_00978_00574 ------------------------ACACCAA-TCGT-GCC--
GGGTCATGTTCTC-----
FC88X_00974_01124 ------------------------ACACCAA-TCGT-GCC--
GGGTCATGTTCTC-----
FC88X_01009_00552 ------------------------ACACCAA-TCGT-GCC--
GGGTCATGTTCTC-----
FC88X_01029_00952 ------------------------ACACCAA-TCGT-GCC--
GGGTCATGTTCTC-----
FC88X_01050_00514 ------------------------ACACCAA-TCGT-GCC--
GGGTCATGTTCTC-----
FC88X_01083_00669 ------------------------ACACCAA-TCGT-GCC--
GGGTCATGTTCTC-----
FC88X_01088_00667 ------------------------ACACCAA-TCGT-GCC--
GGGTCATGTTCTC-----
FC88X_01089_01186 ------------------------ACACCAA-TCGT-GCC--
GGGTCATGTTCTC-----
FC88X_01118_00629 ------------------------ACACCAA-TCGT-GCC--
GGGTCATGTTCTC-----
FC88X_01123_00613 ------------------------ACACCAA-TCGT-GCC--
GGGTCATGTTCTC-----
FC88X_01139_00633 ------------------------ACACCAA-TCGT-GCC--
GGGTCATGTTCTC-----
FC88X_01142_00879 ------------------------ACACCAA-TCGT-GCC--
GGGTCATGTTCTC-----
FC88X_01122_00979 ------------------------ACACCAA-TCGT-GCC--
GGGTCATGTTCTC-----
FC88X_00033_00152 ------------------------ACACCAA-TCGT-GCC--
GGGTCATGTTCTCCC---
FC88X_00661_01052 ------------------------ACACCAA-TCAT-GCC--
GGGTCATGTTCTC-----
FC88X_00105_00122 ------------------------ACACCAA-TCGT-GCT--
GGGTCATGTTCTC-----
FC88X_00569_01215 ------------------------ACACCAA-TCGT-GCT--
GGGTCATGTTCTC-----
FC88X_00622_00663 ------------------------ACACCAA-TCGT-GC---
GGGTCATGTTCTC-----
FC88X_01090_00919 ------------------------ACACCAA-TCGT-GCC--
GGGTCATGTTCTCCTTCC
FC88X_00268_00921 ------------------------ACACCAA-TCGT-GCC--
GGGTCATGTTCTCCTTCC
FC88X_00834_00959 ------------------------ACACCAA-TCGT-GCC--
GGGTTATGTTCTC-----
FC88X_00027_00187 ------------------------ACACCAA-TCGT-GCC--
GGGTCATGTTCTCCTTCC

```
FC88X_00281_00349       ------------------------ACACCAA-TCGT-GCC--
TGGTCATGTTCT------
FC88X_00098_00837       ------------------------ACACCAA-TCGT-GCC--
GGGTCATGCTCTC-----
FC88X_01127_00967       ------------------------ACACCAA-TCGT-GCC--
GGGTCATGCTCTCCTTCC
FC88X_00199_00958       ------------------------ACACCAA-TCGC-GCC--
GGGTCATGCTCTC-----
FC88X_00099_00350       ------------------------ACACCAA-TCGC-GTC-
CGGGTCATGTTCTCC----
FC88X_00775_00942       ------------------------ACACCAA-TCGC-G-C-
CGGGTCATGTTCTC-----
FC88X_00182_00921       ------------------------ACACCAA-TCGC-G-C-
CGGGTCATGTTCTC-----
FC88X_00735_00297       ------------------------ACACCAA-TCGC-G-C-
CGGGTCATGTTCTC-----
FC88X_00836_00388       ------------------------ACACCAA-TCGC-G-C-
CGGGTCATGTTCTC-----
FC88X_00862_01099       ------------------------ACACCAA-TCGC-G-C-
CGGGTCATGTTCTC-----
FC88X_01054_01013       ------------------GGGGAAACACCAA-TCGC-G-C-
CGGGTCATGTTCTC-----
FC88X_00200_00942       ------------------------ACACCAA-TCGC-G-C-
CGGGTCATGTTCTC-----
FC88X_00331_01068       ------------------------ACACCAA-TCGC-G-C-
CGGGTCATGTTCTC-----
FC88X_00466_01179       ------------------------ACACCAA-TCGC-G-C-
CGGGTCATGTTCTC-----
FC88X_00458_01205       ------------------------ACACCAA-TCGC-G-C-
CGGGTCATGTTCTC-----
FC88X_00496_01236       ------------------------ACACCAA-TCGC-G-C-
CGGGTCATGTTCTC-----
FC88X_00693_01260       ------------------------ACACCAA-TCGC-G-C-
CGGGTCATGTTCTC-----
FC88X_00829_00225       ------------------------ACACCAA-TCGC-G-C-
CGGGTCATGTTCTC-----
FC88X_00888_00167       ------------------------ACACCAA-TCGC-G-C-
CGGGTCATGTTCTC-----
FC88X_00962_00618       ------------------------ACACCAA-TCGC-G-C-
CGGGTCATGTTCTC-----
FC88X_01093_00727       ------------------------ACACCAA-TCGC-G-C-
CGGGTCATGTTCTC-----
FC88X_00084_00276       ------------------------ACACCAA-TCGC-G-C-
CGGGTCATGTTCTC-----
FC88X_00144_00737       ------------------------ACACCAA-TCGC-G-C-
CGGGTCATGTTCTC-----
FC88X_00349_01055       ------------------------ACACCAA-TCGC-G-C-
CGGGTCATGTTCTC-----
FC88X_00443_00213       ------------------------ACACCAA-TCGC-G-C-
CGGGTCATGTTCTC-----
FC88X_00574_00187       ------------------------ACACCAA-TCGC-G-C-
CGGGTCATGTTCTC-----
FC88X_00612_01151       ------------------------ACACCAA-TCGC-G-C-
CGGGTCATGTTCTC-----
FC88X_00910_00642       ------------------------ACACCAA-TCGC-G-C-
CGGGTCATGTTCTCCC---
```

```
FC88X_00836_00445      ------------------------ACACCAA-TCGC-G---
CGGGTCATGTTCTC-----
FC88X_01034_00992      -----------------------AACACCAA-TCGC-G-C-
CGGGTCATGTTCTCCCTTC
FC88X_00949_01061      ------------------------ACACCAA-TCGC-G---
CCGGTCATGTTCTC-----
FC88X_00093_00826      ------------------------ACGCCAA-TCGC-G-C-
CGGGTCATGTTCTC-----
FC88X_00238_00453      ------------------------ACACCAA-TCGC-GCC-GGGT-
GATGTTCTC-----
FC88X_01145_01109      ------------------------ACACCAA-TCGC-GCC-
GGGTCGATGTTCTCCTTCC
FC88X_00939_00557      ------------------------ACACCAA-TCGC-G-C-
CGGGTCATGTTCCC-----
FC88X_00450_00119      ------------------------ACACCAA-TCGC-G-C-
CGGGTCATGTTCTCCTTCC
FC88X_00434_00175      ------------------------ACACCAA-TCGC-G-C-
CGGGTCGTGTTCT------
FC88X_00962_00902      ------------------------TCACCAA-TCGC-G-C-
CGGGTCATGTTCTC-----
FC88X_00333_00397      ------------------------ACACCAA-TCGC-GC---GGGTC-------
------
FC88X_00259_00499      --------------------------TCCACTCCACCGACTCCGC-
GATACTCTC-----
FC88X_00032_00456      --------------------------ACCACTCCAT-GGCCGCGT-
GACATTCTC-----
FC88X_00140_00436      ----------------------------------
ACCAACCGCGCTGGGATTCTC-----
FC88X_00851_00962      ------------------------CCAACGACTCAC-AACCCCGGGGCTGCTC-
------
FC88X_00089_00554      -----------------------ACCAAATCTCC----
CCTGCGCGCGCCTCTT------
FC88X_00455_00199      -----------------------ACCAAATCTCC----CCTGCGCGCGCCT---
------
FC88X_00823_00934      ------------------------CCATTTCTCCAACACCTTCGCGCGCCA---
------
FC88X_00952_00702      ------------------------CCATTTCTCCAACACCTTCGCGCGCCA---
------
FC88X_00690_01040      ------------------------CTTTACTCAAAGCCCC-TGACGCGCATT--
------
FC88X_00299_01132      ------------------------CTTTACTCAAAGCCCC-TGACGCGCATT--
------
FC88X_00763_00261      ------------------------CTTTACTCAAAGCCCC-TGACGCGCATT--
------
FC88X_01098_00718      ------------------------CTTTACTCAAAGCCCC-TGACGCGCATT--
------
FC88X_00699_00972      -----------------------GCTTTACTCAAAGCCCC-TGACGCGCATT--
------
FC88X_00888_01233      ------------------------CTTTACTCAAAGCCCCCTGACGCGCATT--
------
FC88X_00427_00992      --------------------------CAAACTCAGCATCTCCGGCCCGCCATC-
------
FC88X_00210_00880      -----------------------TCTCAAAATGACTATCTGCG--CCGCCAC--
------
FC88X_01131_01126      -------------------------------TCAACGTCACTTGCGCCCAAC--
------
```

FC88X_00383_01105 --------------------------TTTAGCACGTTACCTCGGCCTATTTTC-
------
FC88X_00525_01222 --------------------------TTTAGCACGTTACCTCGGCCTATTTTC-
------
FC88X_00573_01116 --------------------------TTTAGCACGTTACCTCGGCCTATTTTC-
------
FC88X_00619_01251 --------------------------TTTAGCACGTTACCTCGGCCTATTTTC-
------
FC88X_00841_00164 --------------------------TTTAGCACGTTACCTCGGCCTATTTTC-
------
FC88X_01112_00763 --------------------------TTTAGCACGTTACCTCGGCCTATTTTC-
------
FC88X_00094_00055 --------------------------TTTAGCACGTTACCTCGGCCTATTTTC-
------
FC88X_00054_00445 --------------------------TTTAGCACGTTACCTCGGCCTATTTTC-
------
FC88X_00089_00784 --------------------------TTTAGCACGTTACCTCGGCCTATTTTC-
------
FC88X_00360_01188 --------------------------TTTAGCACGTTACCTCGGCCTATTTTC-
------
FC88X_00536_01143 --------------------------TTTAGCACGTTACCTCGGCCTATTTTC-
------
FC88X_00815_00797 --------------------------TTTAGCACGTTACCTCGGCCTATTTTC-
------
FC88X_00923_01241 --------------------------TTTAGCACGTTACCTCGGCCTATTTTC-
------
FC88X_00979_01164 --------------------------TTTAGCACGTTACCTCGGCCTATTTTC-
------
FC88X_01130_00614 --------------------------TTTAGCACGTTACCTCGGCCTATTTTC-
------
FC88X_00793_01089 -------------------------GTTTAGCACGTTACCTCGGCCTATTTTC-
------
FC88X_00913_01245 --------------------------
TTTAGCACGTTACCTCGGCCTATTTTCCTTCCTT
FC88X_00984_00744 --------------------------
TTTAGCACGTTACCTCGGCCTATTTTTC------
FC88X_00988_01094 --------------------------
TTTAGCACGTTACCTCGGCCTATTTTTC------
FC88X_01043_01125 --------------------------TTTAGCACGTTACCTCGGCCTATTCTC-
------
FC88X_00231_00120 --------------------------
TTTAGCACGTTACCTCGGCCTATCTTCTT-----
FC88X_00730_01231 ----------------------------TGGCACATAACC-CGGCCA-------
------
FC88X_01094_00861 -----------------------TCTTGCGTCACATGACC-CGGACTGTCCA--
------
FC88X_00889_00938 ------------------------CAATTCGC-TCAACTCCCGGTCCTCCATC-
------
FC88X_00978_00756 ---------------------------TACGT-
TTATATCCCGGCTATCCTCCAG-----
FC88X_00836_00961 --------------------------CCTCGCGTCAATGTCCGGGCATGCTCC-
------
FC88X_00007_00085 --------------------TCGGCGCCCAAACTTCGTACTCATTCT-------
------
FC88X_00688_00911 --------------------TCGGCGCCCAAACTTCGTACTCATTCT-------
------

```
FC88X_00746_00974      --------------------TCGGCGCCCAAACTTCGTACTCATTCT-------
------
FC88X_00869_01097      --------------------TCGGCGCT-AAACTTCGTACTCATTCT-------
------
FC88X_00208_00897      ---------------------TCGCGCCCAAACTTCGTACTCATTCT-------
------
FC88X_00536_01232      ---------------------TCGCGCCCAAACTTCGTACTCATTCT-------
------
FC88X_00108_00214      -------------------ACTGTTGCTAAA-CTTCAGTCTCGTTCT-------
------
FC88X_00240_00528      -------------------ACTGTTGCTAAA-CTTCAGTCTCGTTCT-------
------
FC88X_00415_01071      -------------------ACTGTTGCTAAA-CTTCAGTCTCGTTCT-------
------
FC88X_00598_00624      -------------------ACTGTTGCTAAA-CTTCAGTCTCGTTCT-------
------
FC88X_00578_01055      -------------------ACTGTTGCTAAA-CTTCAGTCTCGTTCT-------
------
FC88X_00607_00915      -------------------ACTGTTGCTAAA-CTTCAGTCTCGTTCT-------
------
FC88X_01126_00533      -------------------ACTGTTGCTAAA-CTTCAGTCTCGTTCT-------
------
FC88X_01125_01050      -------------------ACTGTTGCTAAA-CTTCAGTCTCGTTCT-------
------
FC88X_00012_00410      -------------------ACTGTTGCTAAA-CTTCAGTCTCGTTCT-------
------
FC88X_00177_00154      -------------------ACTGTTGCTAAA-CTTCAGTCTCGTTCT-------
------
FC88X_00615_00345      -------------------ACTGTTGCTAAA-CTTCAGTCTCGTTCT-------
------
FC88X_00799_00332      -------------------ACTGTTGCTAAA-CTTCAGTCTCGTTCT-------
------
FC88X_00795_01175      -------------------ACTGTTGCTAAA-CTTCAGTCTCGTTCT-------
------
FC88X_00970_00266      -------------------ACTGTTGCTAAA-CTTCAGTCTCGTTCT-------
------
FC88X_00988_00481      -------------------ACTGTTGCTAAA-CTTCAGTCTCGTTCT-------
------
FC88X_01026_00964      -------------------ACTGTTGCTAAA-CTTCAGTCTCGTTCT-------
------
FC88X_01086_01077      -------------------ACTGTTGCTAAA-CTTCAGTCTCGTTCT-------
------
FC88X_00413_01106      -------------------ACTGTTGCTAAAACTTCAGTCTCGTTCTCC-----
------
FC88X_00765_00973      -------------------ACTGTTGCTAAC--TTCAGTCTCGTTCT-------
------
FC88X_00222_00873      -------------------ACTGTTGCTAAA-CTTCAGTCTCGTTAT-------
------
FC88X_00147_00803      -------------------ACTGTTGCTAAA-CTTCAGTCCCGTTCT-------
------
FC88X_00005_00286      -------------------ACTGTTGCTAAA-
CTTCAGTCTCGTTCTCTTCCTTT-----
FC88X_00776_00574      -------------------ACTGTTGCTAAA-
CTTCAGTCTCGTTCTCTTCCTTT-----
FC88X_00023_00627      -------------------ACTGCTGCTAAA-CTTCAGTCTCGTTCT-------
------
```

```
FC88X_00927_00492      -------------------ACTGTTGCCAAA-CTTCAGTCTCGTTCT-------
------
FC88X_00508_00058      -------------------ACTGTCGCTAAA-CTTCAGTCTCGTTCT-------
------
FC88X_00770_01268      -------------------ACTATTGCTAAA-CTTCAGT---------------
------
FC88X_00557_00238      -----------------------TCGCCACGTC-TGCGAAATCTCCCTGAT---
------
FC88X_01138_00670      -------------------------TCCACGTCACGACTAATCTTCATAATC--
------
FC88X_00826_00326      -----------------------TCCCCACGGCACAACCTTCATCCCTTA----
------
FC88X_00200_00109      ---------------CACATCCTTCTCCACAGCACACTTCTTCTCCTTCTCCC-
------
FC88X_00160_00098      ------------------TCCAAGCCTAGCAGACTACGCTTCCCT---------
------
FC88X_01083_01208      -------------------CCAAGCACGGCAAA--ACGCTTACATATTCTTCC-
------
FC88X_00461_01195      -------------------CCAAGCGCGGCAACCTTCACCACAATT--------
------
FC88X_01046_01232      ------------------TTTAAGCACGACTACCTTGAACACACT---------
------
FC88X_01132_00539      --------------------CCAACAGCATGCCAAACTCGTAAATATT------
------
FC88X_00090_00842      -----------------------ACATCACTCCCAAC-CGTAGCTATCGA----
------
FC88X_00197_00148      ------------------CTCCCTAGCATGATCCGCCAGTACATCGT-------
------
FC88X_00636_01218      ------------------CTCCCTAGCATGATCCGC-AGTACA-CGT-------
------
FC88X_00948_00316      ---------------TTTCACCCTAGTA-GACCCGC-AACACGC----------
------
FC88X_00846_01154      --------------------CTCCCGCGTAATCCGCAACTTCGAACT-------
------
FC88X_00239_00759      --------------ACGTGAGTTGAGTAAAACCTCTAAATT-------------
------
FC88X_00358_00978      ---------------GCCTCGTAAAGCAAAACCCTGAAATATT-----------
------
FC88X_00167_00728      ---------------TTCCGAAGTTGTCTTAACCGGGACGTCC-----------
------
FC88X_00882_00899      ---------------CCAGGCACTCGTCTACACCACGTCCT-------------
------
FC88X_00115_00777      ----------------CCACTCGCTATCTGC-CACGTCCCTCTA----------
------
FC88X_00663_00559      -------------------CTGCCGATCTGCGCACGTCTCCCCTAC--------
------
FC88X_00949_00853      ---------------TCCGCAAGCT---TGATCGGCCACTCTCCA---------
------
FC88X_00989_01204      ----------------CCGCAAGCTACCTGACTGGGCACCTCTC----------
------
FC88X_00155_00753      ----------------AGTACTTCCATGTACCGCAAC--TCTCAGCTTCATTT-
------
FC88X_00827_00323      ------------------------CATGTCACGCGTCAACTCCAGCTTCTT---
------
FC88X_00939_00250      ----------------------CCCGTTCATCCGTGCAACTCCCGCAAT-----
------
```

FC88X_00859_00780 -----------------------CCGTGCA-CCGGACAACTCCATCATTCT---
------
FC88X_01005_00921 ------------------ATCACTTCTCTGCATCGTCTACTTCAC---------
------
FC88X_00865_00162 ------------------CACATTATTCAGCA-CGTGTACCTCAC---------
------
FC88X_00273_00466 ------------------------CCTATACATCGG-CAGCTCCACCTTCTC--
------
FC88X_01082_01218 ------------------------CCTCTACCCCGGGTAGCTCAAACATGT---
------
FC88X_00098_00598 --------------TTTTCCCATT--GGCGAACTGCAACTCCT-----------
------
FC88X_00464_00966 --------------TTTTCCCATT--GGCGAACTGCAACTCCT-----------
------
FC88X_00561_01159 ----------------CTCCCATG--GGTGAACCGCCAATACTGT---------
------
FC88X_00864_00361 ----------------CTCCCATG--GGTGAACCGTCAATACTGT---------
------
FC88X_00553_00133 ---------------CCCGCAACT--TGTGAACGGCAACTCCT-----------
------
FC88X_00824_01218 ----------------CCGCTACTCCTGTGTCCCCCAACGTCCT----------
------
FC88X_00018_00195 --------------------CCTGAATTCATCGGC-GGCAACTTCTC-------
------
FC88X_01140_00821 -------------------ACCTGAATTCATCGGC-GGCAACTTCTC-------
------
FC88X_00084_00410 --------------------CCTGAATTCATCGGC-GGCAACTTCTC-------
------
FC88X_00288_00225 --------------------CCTGAATTCATCGGC-GGCAACTTCTC-------
------
FC88X_00073_00051 --------------------CCTGAATTCATCGGC-GGCAACTTCTC-------
------
FC88X_00794_01205 -------------------TCCTGAATTCATCGGC-GGCAACTTCTC-------
------
FC88X_00211_01026 --------------------CCTGAATTCATCGGC-GGCAACTTCTC-------
------
FC88X_00371_00018 --------------------CCTGAATTCATCGGC-GGCAACTTCTC-------
------
FC88X_00468_01033 --------------------CCTGAATTCATCGGC-GGCAACTTCTC-------
------
FC88X_00519_00427 --------------------CCTGAATTCATCGGC-GGCAACTTCTC-------
------
FC88X_00824_00244 --------------------CCTGAATTCATCGGC-GGCAACTTCTC-------
------
FC88X_00871_01205 --------------------CCTGAATTCATCGGC-GGCAACTTCTC-------
------
FC88X_00908_00335 --------------------CCTGAATTCATCGGC-GGCAACTTCTC-------
------
FC88X_00967_00853 --------------------CCTGAATTCATCGGC-GGCAACTTCTC-------
------
FC88X_01010_00667 --------------------CCTGAATTCATCGGC-GGCAACTTCTC-------
------
FC88X_00889_01076 --------------------CCTGAATTCATCGGC-GGCAACCTCTC-------
------
FC88X_00715_00865 --------------------CCTGAATTCGTCGGC-GGCAACTTCTC-------
------

FC88X_00098_00139 -------------------CCCTGGATTCATCGGC-GGCAACTTCTC-------
------
FC88X_00737_01152 --------------------CCTGACATCATCGGC-GGCAACTTCTC-------
------
FC88X_00441_00730 --------------------ACGTGTTGTATCGGCAGGCGTCTTCTC-------
------
FC88X_00559_01043 --------------------ACGTGTTGTATCGGCAGGCGTCTTCTC-------
------
FC88X_00948_00827 ------------------CCACGAGTT-CATCAGTTGGCGACTTCT--------
------
FC88X_00578_00607 -------------------CCCGGTAAACATCCGCCCGCACGTTCTC-------
------
FC88X_01011_01122 ------------------GTCCGGGTATCATCTGCACGGAC-TTCTC-------
------
FC88X_00990_00914 --------------------CTAGTTCATGTGCGACGGCGATTTCTC-------
------
FC88X_00557_00638 --------------------CTACTACATGAACGTCGGAGCATTCAC-------
------
FC88X_00862_00312 --------------------CTACTGCATGTACATCAGTAACGTCTC-------
------
FC88X_01057_01269 -------------------GCTACTGCATGTACATCAGTAACGTCTC-------
------
FC88X_01104_00802 --------------------CTACTGCATGTACATCAGTAACGTCTC-------
------
FC88X_00247_00893 --------------------CTACTGCATGTACATCAGTAACGTCTC-------
------
FC88X_00385_00519 --------------------CTACTGCATGTACATCAGTAACGTCTC-------
------
FC88X_00767_00514 --------------------CTACTGCATGTACATCAGTAACGTCTC-------
------
FC88X_00993_01141 --------------------CTACTGCATGTACATCAGTAACGTCTC-------
------
FC88X_00040_00607 --------------------CTACTGC-
TATGTAACAATCAAGTAACCGTCCTCTCCC--
FC88X_00393_00757 --------------------CGGATTGGTATACCACATTCGCATCTC-------
------
FC88X_00137_00589 -------------------GCGGATTGGTATACCACATTCGCATCTC-------
------
FC88X_00078_00494 --------------------CGGATTGGTATACCACATTCGCATCTC-------
------
FC88X_00495_01035 --------------------CGGATTGGTATACCACATTCGCATCTC-------
------
FC88X_00433_00822 --------------------TCTATTAGTCAACCACGTAGACATCTC-------
------
FC88X_00243_00898 --------------------TCTATTAGTCAACCACGTAGATATCTC-------
------
FC88X_01102_00909 ---------------------CGCGCACTTACATAGGTAGCCATCCCA------
------
FC88X_00340_00083 --------------------TCCCGTAGTTAACAACATAGACATCCCA------
------
FC88X_00043_00263 --------------------CTTTACTAACGTTAGAAGCCGGTTCTC-------
------
FC88X_00412_01210 -------------------ACTTTACTAACGTTAGAAGCCGGTTCTC-------
------
FC88X_00305_00957 --------------------CTTTACTAACGTTAGAAGCCGGTTCTC-------
------

FC88X_00201_00886 --------------------CTTTACTAACGTTAGAAGCCGGTTCTC-------
------
FC88X_00657_00071 --------------------CTTTACTAACGTTAGAAGCCGGTTCTC-------
------
FC88X_01138_00671 --------------------CTTTACTAACGTTAGAAGCCGGTTCTC-------
------
FC88X_00859_01222 --------------------CTTTACTAACGTTAGAAGCCGGTTCTC-------
------
FC88X_01143_00926 --------------------CTTTACTAACGTTAGAAGCCGGTTCTCT------
------
FC88X_00531_00923 --------------------CTTTACTAACGTTAGAAGCCGGTTCTC-------
------
FC88X_00797_00136 --------------------CTTTACTAACGTTAGAAGCCGGTTCTC-------
------
FC88X_01112_01086 --------------------CTTTACTAACGTTAGAAGCCGGTTCTC-------
------
FC88X_00928_00756 --------------------CTTTACTAACGTTAGAAGCCGGTTCTCC------
------
FC88X_00628_00213 --------------------CTTTACTAACGTTAGAAGCCGGTTCTC-------
------
FC88X_00835_01042 --------------------CTTTACTAACGTTAGAAGCCGGTTCTC-------
------
FC88X_00096_00344 --------------------
CTTTACTAACGTTAGAAGCCGGTTCTCCTTCCTTT-----
FC88X_00762_01242 --------------------CTTTACTAACGTTGGAAGCCGGTTCTC-------
------
FC88X_00340_00383 --------------------CTTTACTAACGTTAGT-GCCGGTTCTC-------
------
FC88X_00910_00935 --------------------TTTTACTAACGCTAGAAGCCGGTTCTC-------
------
FC88X_00880_00732 --------------------CTTTACTAACGTTAGAAG----------------
------
FC88X_00452_01062 --------------------CTTTACTAACGTTAGAAGCCGGTTCCTC------
------
FC88X_00215_00731 -------------------GCTTTAACATCGGTACCAGCTTGTTCTC-------
------
FC88X_01145_01065 -------------------GCTTTAACATCGATACGAGCTTGTTCTC-------
------
FC88X_00939_01063 --------------------CTTATACATCGTTATAATCATATTGTC-------
------
FC88X_00191_00008 --------------------CCAAAATTATTGACCCGGCCGCATTCT-------
------
FC88X_00507_00327 --------------------CCAAAATTATTGACCCGGCCGCATTCT-------
------
FC88X_00085_00452 --------------------CCAAAATTATTGACCCGGCCGCATTCT-------
------
FC88X_00029_00267 --------------------CCAAAATTATTGACC-GGCCGCATTCT-------
------
FC88X_00962_00287 --------------------CCAAAATTATTGACCAGGCCGCATTCT-------
------
FC88X_00228_00907 --------------------CCAAAATTATTGACC-GGCCGCATTCT-------
------
FC88X_00013_00680 --------------------CCAAAATTATTGACC-GGCCGCAATTCT------
------
FC88X_00462_01131 --------------------CCAAAATTATTGACC-GGCCGCATTCT-------
------

```
FC88X_00408_01003      -------------------GCTAAAATTATTGACCCGGCCGCATTCT-------
------
FC88X_00643_00982      --------------------CCAAAGTTATTGACCCGGCCGCATTCT-------
------
FC88X_00409_00769      --------------------CCAAAATTATCGACC-GGCCGCATTCT-------
------
FC88X_00010_00139      -----------------CCAAAAAAATTATTGACC-GGCCGCATTCT-------
------
FC88X_00091_00380      ------------------CGTTAACATTATGAAC---GGCGCACTCCT------
------
FC88X_01058_01016      --------------------GTAACATAACGTACTAGGCC-CATTCTC------
------
FC88X_00927_01262      --------------------GTAACATAACGTACTAGGCC-CATTCTC------
------
FC88X_01125_00855      -----------------CCAGTAACTCAA--TAACGGGGC-CATTCT-------
------
FC88X_00161_00623      ------------------CCCCCAAAATGTTGACCGACCGCTACT---------
------
FC88X_01124_00561      ------------------CCACAAACATGATATACGAGCACCACG---------
------
FC88X_00659_00142      ------------------CTCCCAACAGATTCGACGAACCGTCC----------
------
FC88X_01000_00843      ------------------CCCAAGATCGAGCGGACGAGCCGGCATA--------
------
FC88X_00993_00446      -------------------CCGCAAAACATCTGCCGGCCCCCTTAT--------
------
FC88X_01137_00662      ---------------------CCGAAAGATCGTCGGACCCTTATTCAA------
------
FC88X_00033_00779      -----------------CTCCCAA-ATCACGTTACGTGCGACCCA---------
------
FC88X_00172_00077      --------------GGACTCCCAA-ATCACGTTACGTGCGACCCA---------
------
FC88X_00891_00754      -----------------CTCCCAA-ATCACGTTACGTGCGACCCA---------
------
FC88X_00089_00180      -----------------CTCCCAA-ATCACGTTACGTGCGACCCA---------
------
FC88X_00164_00150      -----------------CTCCCAA-ATCACGTTACGTGCGACCCA---------
------
FC88X_00369_01152      ----------------GCTCCCAA-ATCACGTTACGTGCGACCCA---------
------
FC88X_00670_01237      -----------------CTCCCAA-ATCACGTTACGTGCGACCCA---------
------
FC88X_01077_00439      -----------------CTCCCAA-ATCACGTTACGTGCGACCCA---------
------
FC88X_00079_00736      -----------------CTCCCAA-ATCACGTTACGTGCGACCCA---------
------
FC88X_00439_01223      -----------------CTCCCAA-ATCACGTTACGTGCGACCCA---------
------
FC88X_00464_01129      -----------------CTCCCAA-ATCACGTTACGTGCGACCCA---------
------
FC88X_00672_01272      -----------------CTCCCAA-ATCACGTTACGTGCGACCCA---------
------
FC88X_00992_00619      -----------------CTCCCAA-ATCACGTTACGTGCGACCCA---------
------
FC88X_01115_01271      -----------------CTCCCAA-ATCACGTTACGTGCGACCCA---------
------
```

FC88X_00485_01032 -----------------CTCCCAA-ATCACGTTACGTGCCACCCA---------
------
FC88X_00866_00799 ----------------GCTCCCAA-ATCACGTTACGCGCGACCCA---------
------
FC88X_00032_00087 -----------------CTCCCAA-ATCACGTTACGTGCGACCCG---------
------
FC88X_00724_01080 -----------------CTCCCAA-ATCACGCTACGTGCGACCCA---------
------
FC88X_00780_01102 -----------------CTCCCAA-ATCACGTTACGTACGA-------------
------
FC88X_01129_00912 -----------------CTCCCAA-ATCACGTTACGTGCGCCCA----------
------
FC88X_00828_01195 -------------CCTTCTCCAGA-ACCAAG-TACGTGTGAC------------
------
FC88X_00811_01254 -------------CCTTCTCCAGA-ACCAAG-TACGTGTGAC------------
------
FC88X_00954_00958 -------------CCTTCTCCAGA-ACCAAG-TACGTGTGAC------------
------
FC88X_00922_00888 ---------------------------------ACGTACG--------------
------
FC88X_01049_00579 ---------------ACTCCTTTT-ATAACACGACGTACGCTC-----------
------
FC88X_00351_00947 ------------CTCTCCTGACAA-ATGACCGAACGTACTC-------------
------
FC88X_00583_00743 ------------CTCTCCTGACAA-ATGACCGAACGTACTC-------------
------
FC88X_01120_00887 ------------CTCTCCTGACAA-ATGACCGGACGTACTC-------------
------
FC88X_00097_00295 --------------CTCCT-ACAA-ATC-CCGAACATACTGATC----------
------
FC88X_00909_00214 ---------------TCCTACCAACAACACCAAACGTACTGTT-----------
------
FC88X_00772_00217 ---------------TCCTACCAACAACACCAAACGTACTGTT-----------
------
FC88X_01118_00986 ---------------TCCTACCAACAACACCAAACGTACTGTT-----------
------
FC88X_00943_00662 ---------------CTTCTATCTCCAACAAATCACCGTGCC------------
------
FC88X_01010_00478 ---------------GCTCTACTCCAATGACATCCCGGCGCCCAAC--------
------
FC88X_00536_01060 -----------------TCTCCATTACCGACAACACCGCACCCA----------
------
FC88X_00973_00270 ----------------TTCCCTGACAACAATTACCGCACGCCT-----------
------
FC88X_00809_01007 ---------------TTTCCCTGATATCCAGAACCCGACGACTC----------
------
FC88X_00160_00094 ---------------TCTCCTTACTATCACGACCGACGTGTT------------
------
FC88X_00543_01094 -----------CTGGTCTCCTT--TACCGC-ACCAACATGTT------------
------
FC88X_00820_00858 ------------------TCGCAATC--TGATCAACGTGGACATCTC-------
------
FC88X_00853_00990 -----------GGGGAAGTCGCAATC--TGATCAACGTGGACATCTC-------
------
FC88X_00045_00267 ------------------TCGCAATC--TGATCAACGTGGACATCTC-------
------

FC88X_00049_00250 ------------------CCGCAATC--TGATCAACGTAGACATCTC-------
------
FC88X_00045_00714 ------------------CCACAATC-GTGTTT-GCGTTGGCATCTC-------
------
FC88X_00170_00764 ------------GGGAAGCCACAATC-GTGTTT-GCGTTGGCATCTC-------
------
FC88X_00078_00378 ------------------CCACAATC-GTGTTT-GCGTTGGCATCTC-------
------
FC88X_00159_00613 ------------------CCACAATC-GTGTTT-GCGTTGGCATCTC-------
------
FC88X_00437_01081 ------------------CCACAATC-GTGTTT-GCGTTGGCATCTC-------
------
FC88X_00590_00367 ------------------CCACAATC-GTGTTT-GCGTTGGCATCTC-------
------
FC88X_00779_01182 ------------------CCACAATC-GTGTTT-GCGTTGGCATCTC-------
------
FC88X_00192_00049 ------------------CCACAATC-GTGTTT-GCGTTGGCATCTC-------
------
FC88X_00819_01143 ------------------CCACAATC-GTGTTT-GCGTTGGCATCTCT------
------
FC88X_00218_00733 ------------------CCACAATC-GTGTTT-GCGTTGGCATCTC-------
------
FC88X_00521_00972 ------------------CCACAATC-GTGTTT-GCGTTGGCATCTC-------
------
FC88X_00764_00830 ------------------CCACAATC-GTGTTT-GCGTTGGCATCTC-------
------
FC88X_00852_00327 ------------------CCACAATC-GTGTTT-GCGTTGGCATCTC-------
------
FC88X_00962_00362 ------------------CCACAATC-GTGTTT-GCGTTGGCATCTC-------
------
FC88X_00965_00751 ------------------CCACAATC-GTGTTT-GCGTTGGCATCTC-------
------
FC88X_01008_01149 ------------------CCACAATC-GTGTTT-GCGTTGGCATCTC-------
------
FC88X_00119_00732 ------------------CCACAATC-GTGTTT-GCGTTGGCATCTC-------
------
FC88X_00180_00961 ------------------CCACAATC-GTGTTT-GCGTTGGCATCTC-------
------
FC88X_00210_00917 ------------------CCACAATC-GTGTTT-GCGTTGGCATCTC-------
------
FC88X_00213_00944 ------------------CCACAATC-GTGTTT-GCGTTGGCATCTC-------
------
FC88X_00208_01025 ------------------CCACAATC-GTGTTT-GCGTTGGCATCTC-------
------
FC88X_00213_01080 ------------------CCACAATC-GTGTTT-GCGTTGGCATCTC-------
------
FC88X_00359_00508 ------------------CCACAATC-GTGTTT-GCGTTGGCATCTC-------
------
FC88X_00384_01190 ------------------CCACAATC-GTGTTT-GCGTTGGCATCTC-------
------
FC88X_00601_00735 ------------------CCACAATC-GTGTTT-GCGTTGGCATCTC-------
------
FC88X_00706_00722 ------------------CCACAATC-GTGTTT-GCGTTGGCATCTC-------
------
FC88X_00826_00192 ------------------CCACAATC-GTGTTT-GCGTTGGCATCTC-------
------

```
FC88X_00811_00230       ------------------CCACAATC-GTGTTT-GCGTTGGCATCTC-------
------
FC88X_00928_00209       ------------------CCACAATC-GTGTTT-GCGTTGGCATCTC-------
------
FC88X_00957_00428       ------------------CCACAATC-GTGTTT-GCGTTGGCATCTC-------
------
FC88X_00967_00569       ------------------CCACAATC-GTGTTT-GCGTTGGCATCTC-------
------
FC88X_00970_01186       ------------------CCACAATC-GTGTTT-GCGTTGGCATCTC-------
------
FC88X_01134_00603       ------------------CCACAATC-GTGTTT-GCGTTGGCATCTC-------
------
FC88X_01112_00846       ------------------CCACAATC-GTGTTT-GCGTTGGCATCTC-------
------
FC88X_01147_01062       ------------------CCACAATC-GTGTTT-GCGTTGGCATCTC-------
------
FC88X_00198_00112       -------------------CACAATC-GTGTTT-GCGTTGGCATCTC-------
------
FC88X_00166_00822       ------------------CCACAATC-GTGTTTTGCGTTGGCATCTC-------
------
FC88X_00283_00024       ------------------CCACAATC-GTGTTC-GCGTTGGCATCTC-------
------
FC88X_00409_01218       ------------------CCACAATTCGTGTTT-GCGTTGGCATCTC-------
------
FC88X_00956_01088       ------------------CCACAATT-GTGTTT-GCGTTGGCATCTC-------
------
FC88X_00762_00879       ------------------CCACATTC-GTGTTT-GCGTTGGCATCTC-------
------
FC88X_00045_00207       ------------------CCACAATC-GTGTTT-GCGTTGGCATCCC-------
------
FC88X_00495_00018       ------------------CCACAATC-GTATTT-GCGTTGGCATCTC-------
------
FC88X_00012_00339       ------------------CCGCAATC-GTGTTT-GCGTTGGCATCTC-------
------
FC88X_00557_00135       ------------------CTGTAATG-TT-ATTCACGCGGCCATCTT-------
------
FC88X_00588_00292       ------------------CTGTAATG-TT-ATTCACGCGGCCATCTT-------
------
FC88X_01019_00544       ------------------CTGTAATG-TT-ATTCACGCGGCCATCTT-------
------
FC88X_01068_00955       ------------------CTGTAATG-TT-ATTCACGCGGCCATCTT-------
------
FC88X_01079_01141       ------------------CTGTAATG-TT-ATTCACGCGGCCATCTT-------
------
FC88X_00464_01073       ------------------CTGTAATG-TTCATTCACGCGGCCATCTTCC-----
------
FC88X_01050_01074       ------------------CTGTAACG-TT-ATTCACGCGGCCATCTT-------
------
FC88X_01054_00781       --------------------TCGAACCCTCATTTTCGCTCCCATCAC-------
------
FC88X_00450_00841       --------------------TCACTCCCTTATTTACGTTAGCATCAC-------
------
FC88X_00061_00147       ------------------ACGCGCCC--TAGTCACCGCT--CACTTCTC-----
------
FC88X_00860_00277       --------------------GCACCC--TGGTGAACGATGCCATTTCTC-----
------
```

FC88X_00478_01233 -----------------GTTGCAGCGATATCTGCACCCCCACATCTC-------
------
FC88X_00829_00751 ------------------TTGCAGCGATATCTGCACCCC-ACATCTC-------
------
FC88X_00149_00693 -----------------CCTGCAACCATATTGCCGGTC--ACGTCTC-------
------
FC88X_00366_00952 -----------------CCTGCAACCATATTGCCGGTC--ACGTCTC-------
------
FC88X_00527_00710 -----------------CCTGCAACCATATTGCCGGTC--ACGTCTC-------
------
FC88X_00614_00921 -----------------CCTGCAACCATATTGCCGGTC--ACGTCTC-------
------
FC88X_01049_00713 -----------------CCTGCAACCATATTGCCGGTC--ACGTCTC-------
------
FC88X_01108_00533 -----------------CCTGCAACCATATTGCCGGTC--ACGTCTC-------
------
FC88X_01108_00671 -----------------CCTGCAACCATATTGCCGGTC--ACGTCTC-------
------
FC88X_00675_00212 ------------------CTGCAACCATATTGCCGGTC--ACGTCTC-------
------
FC88X_00995_01019 -------------------TCCCAGC--T--ACGACTCCGGTCTCAT-------
------
FC88X_01140_01048 ------------------CTCCCATC--TCGACGGCTCACG-CTCATT------
------
FC88X_00186_00286 ------------------CTCCCGCC--CAGCTGACTGCACACTCTG-------
------
FC88X_00959_00710 -----------------TCTCCCGCC--AACCACGCACTCTCGTGT--------
------
FC88X_01041_01001 -----------------TCTCCCAAC--AAA-GTGCCCTCGCGTGTC-------
------
FC88X_00083_00883 ---------------------CTCCAGGAAACTGCACACGGCCTACT-------
------
FC88X_00957_00831 ---------------------GCACGATGAATCTCGCACCGCCTGCTA------
------
FC88X_00111_00407 --------------------TTGGCCAGAAACCGATGCCGCACTCCC-------
------
FC88X_01013_00706 ------------------------CCATAATCCCATGATGCCAGCACTGTC---
------
FC88X_01130_00761 ------------------------CCCTAGCCTGATGACGCCGTACTGCC----
------
FC88X_00011_00558 ------------------CTAATGTCTCTACCTCACGATGCTGTT---------
------
FC88X_00966_00314 ---------------GGACTAATGTCTCTACCTCACGATGCTGTT---------
------
FC88X_00206_00126 ------------------CTAATGTCTCTACCTCACGATGCTGTT---------
------
FC88X_00511_00026 ------------------CTAATGTCTCTACCTCACGATGCTGTT---------
------
FC88X_00758_00255 -----------------TCCAATGTCA-TCTCTCAACAGGCTGCT---------
------
FC88X_00200_00071 -----------------GCTCATGACTGCAAC-CACGA-ACTGTTCC-------
------
FC88X_00911_00560 -----------GGGGAAGCTCATGACTGCAAC-CACGA-ACTGTTCC-------
------
FC88X_00378_01197 ------------------CTCATGACTGCAAC-CACGA-ACTGTTCC-------
------

FC88X_00957_00671 ------------------CTCATGACTGCAAC-CACGA-ACTGTTCC-------
------
FC88X_00955_01052 --------------------TCCTACTACCACTGTAATCACTATTCT-------
------
FC88X_00082_00593 -------------------GTCCTACTACCACTGTAATCACTATTCT-------
------
FC88X_01015_00321 --------------------TTCTACTACCTTTGGTACGAATGTTCG-------
------
FC88X_00054_00143 ---------------------TCTACCACTCACGGTAAAGACGATCTC------
------
FC88X_00078_00115 ----------------------TCACTCCCAACGAAGAT-CTGCACCGTC----
------
FC88X_00165_00658 -----------------------CACCGCTAGAGAAGCTGTTGCACTATC----
------
FC88X_00493_00695 -------------------CTCCCAACTTAAACAC-GGCAAGCTGTC-------
------
FC88X_01029_00400 -------------------CTCCCAACTTAAACACCGGCAAGCTGTC-------
------
FC88X_00059_00421 ------------------TCTCCCAACATAAG-AC-AGCGAGTTGTC-------
------
FC88X_00062_00034 -----------------CACTCCCTACGAAAAAA--CGCAGCGTATC-------
------
FC88X_00125_00686 -----------------CACTCCCTACGAAAAA---CGCAGCGTATC-------
------
FC88X_00637_01171 -----------------CACTCCCTACGAAAAA---CGCAGCGTATC-------
------
FC88X_00933_01199 -----------------CACTCCCTACGAAAAGAA-CGCAGCGTAATCC-----
------
FC88X_00869_00134 -------------------CTCCCCAACGTAAACA-AGCAGCGATGCC------
------
FC88X_00268_00421 --------------------ATCTCCC-AAACTCAATGCCATGTTCGC------
------
FC88X_00831_00140 --------------------ATCTCCCCAAACTCAATGCCATGTTCGC------
------
FC88X_01087_00873 --------------------CTCCCACAACTGTAAATGCAACGTACC-------
------
FC88X_00996_00293 --------------------TTCCCACACTTCCAATGGCAAAATGCA-------
------
FC88X_00737_00941 ------------------TCGCACGACGTACTAT--TGCCACATTAT-------
------
FC88X_00795_00970 ------------------TCGCACGACGTACTAT--TGCCACATTAT-------
------
FC88X_00059_00368 ------------------TCGCACGACGTACTAT--TGCCACATTAT-------
------
FC88X_00241_00014 ------------------TCGCACGACGTACTAT--TGCCACATTAT-------
------
FC88X_00510_00961 -----------------CTCCCAATACGAACGAT--GGC-ACACTAT-------
------
FC88X_00582_01146 -----------------CTCCCAATACGAACGAT--GGC-ACACTATCC-----
------
FC88X_00443_00842 ----------------CTACTCCCCACGAACTATA-TGCCACAC----------
------
FC88X_00061_00581 ----------------CACACCCCACCCTCTGCCCGGACAACA-----------
------
FC88X_00073_00131 ----------------TCCACTCCCTACAACCTCCAGACACTGG----------
------

FC88X_00120_00464 ----------------CGCCTCGCTCTCCATCCCCTGACACAG-----------
------
FC88X_00877_01110 -----------------TTCCACGGTCTCCACTTGCAATGTCTG----------
------
FC88X_00292_01025 -----------------TTCCTCGGTATCGACCCGCGACATCTT----------
------
FC88X_00170_00894 -----------------CCTTTCCATCTCCACCCCTATTGTC------------
------
FC88X_00267_01118 -----------------TTCCAAACTCTCCACCCCTGATATCCC----------
------
FC88X_00600_00994 -------------TCCCTCGC-TCCTATCAACG---GCCACGCC----------
------
FC88X_00013_00598 -----------------TCGCGTACTCTCCACA---GCCATGTACGG-------
------
FC88X_00811_00842 -----------------TCTCTTCCTATCCACACATGCCATGCC----------
------
FC88X_00185_00779 --------------------TCTTTTCCCCGCGCATGCAAAGCTCTC-------
------
FC88X_00857_00242 ------------------GTCTTTCTCTCCACAAACGCTATGTCTC--------
------
FC88X_00778_01051 --------------------CTGTTTCTCCACTGAGACGATGTCTTGCTTTC--
------
FC88X_01100_01033 --------------------CTGATTCGCGCACCGAACGGTCTTCTC-------
------
FC88X_00302_00545 ---------------ATCTTGGTCTCCCCAACGCACGGTCGTT-----------
------
FC88X_00581_01257 ---------------ATCTTGGTCTCCCCAACGCACGGTCGTT-----------
------
FC88X_00928_00642 ---------------ATCTTGGTCTCCCCAACGCACGGTCGTT-----------
------
FC88X_00195_00886 ---------------ATCTTGGTCTCCCCAACGCACGGTCGTT-----------
------
FC88X_00501_01101 ---------------ATCTTGGTCTCCCCAACGCACGGTCGTT-----------
------
FC88X_01109_00833 ---------------ATCTTGGTCTCCCCAACGCACGGTCGTT-----------
------
FC88X_00398_00076 ---------------ATCTTG-TCTCCCCAACGCACGGTCGTT-----------
------
FC88X_00232_00064 ----------------TCGTTGTCTCCCCA--GCACCGTCATCTC---------
------
FC88X_00705_00871 ------------------CTTTTCTCCCCA-CGCATCGCCGCTTAT--------
------
FC88X_00102_00260 ------------------CTTTTCTCCCCA-CGCATCGCCGCTTAT--------
------
FC88X_01090_00793 -------------------CCAAATTCTCCATATGA-CACCCCACA--------
------
FC88X_01055_00515 ------------------CCCAAATTCTCCATATGA-CACCCCACACC------
------
FC88X_00374_00669 -------------------TCAAAATCTGCTCCTAATCACCCCAC---------
------
FC88X_00733_01134 -----------------------TTTCATAATTTGAAGCCCCTATGCACTC---
------
FC88X_00548_01167 -----------------------TTTCATAATTTGAAGCCCCTATGCACTC---
------
FC88X_00947_00755 -----------------------TTTCATAATTTGAAGCCCCTATGCACTC---
------

FC88X_01146_00939 --------------------TTCGTC-AACATTTGGCGCCTCCCTGAT------
------
FC88X_00757_01242 ---------------------TCGTCGAACGTATGATGCTCCCATGAT------
------
FC88X_00089_00173 -------------------------TCTCAACATGAGCCTCCTGATTAACT---
------
FC88X_00929_00758 --------------------GGTGTTTGAAACATAAAGCCTCTAGAT-------
------
FC88X_00567_00899 -----------------------CCCACGAACCTAGGCCCCGTATCACCAC---
------
FC88X_00726_00539 ------------------------TCACCAGATTAGGCTCCCCATGAACAC---
------
FC88X_00514_00190 ------------------------CTCCCCGATCATCGACACTGACTTAAT---
------
FC88X_00804_00984 -------------------TCCACTCCCCTGATGATC-ACACTGAACC------
------
FC88X_00909_01248 ------------------TCCTTATCTCCACATTATC-GCCCTGAT--------
------
FC88X_00415_01151 ------------------------ATCACAACAAAGACCTATCTCCAGCCG---
------
FC88X_00774_01084 ------------------------ATCACAACAAAGACCTATCTCCAGCCG---
------
FC88X_00149_00809 -------------------------TCACAA-
AAAAATCTCTCCCAACCCGGTGGCC---
FC88X_00840_00270 ---------------------------
ATAACAAAATCTTCTCTCCCCGCCAAGTA----
FC88X_00856_00898 ---------------------------ATAACAAA-
TCTTCTCTCCCCGCCAAGTA----
FC88X_00439_00988 --------------------CTGTCTCCTAACAAAAAATCTCCGTACT------
------
FC88X_00606_00920 -----------CCTTCGAGCTTGTCTCCTGGCCAATCTCCTTCCTTT-------
------
FC88X_00193_00807 --------------------TTGACTCCGATCCCAAATCGACTTTCC-------
------
FC88X_00201_00543 -------------------CTTGTCTCTGCTCACGAACTGGCCTCTT-------
------
FC88X_00208_00959 -------------------CTGGTCTC-GCTCCCAAACACCTCTTGT-------
------
FC88X_00170_00072 --------------------TTCCTGTCCCGACAAACACGCTGTATC-------
------
FC88X_00730_01235 --------------------TTCCTGTCCCGACAAACACGCTGTATC-------
------
FC88X_00032_00758 --------------------TTCATGCGCCG-CAAAGACTCTCC----------
------
FC88X_00537_00052 -----------------------CCCAGTGATCATGCACCCCAGACTATC----
------
FC88X_00234_00958 -----------------------CCCACTAA-CAGGCACCACAGACCTCTC---
------
FC88X_00072_00877 ---------------TTTTCGC-CCCCGACAACCGACGTATT------------
------
FC88X_00670_00256 --------------TTTTTCGCTCCCCGACAACCGACGTATT------------
------
FC88X_00705_01234 --------------TTTTTCGCTCCCCGACAACCGACGTATT------------
------
FC88X_00935_00737 --------------TTTTTCGCTCCCCGACAACCGACGTATT------------
------

```
FC88X_00058_00161      ---------------TTTTTCGCTCCCCGACAACCGACGTATT------------
------
FC88X_00932_01260      ---------------TTTTTCGCTCCCCGACAACCGACGTATT------------
------
FC88X_01059_00712      --------------TTTTTTCGCTCCCCGACAACCGACGTATT------------
------
FC88X_00137_00174      --------------TTTTTTCGCTCCCCGACAACCGACGTATT------------
------
FC88X_01015_01059      --------------TTTTTTCGCTCCCCGACAACCGACGTATT------------
------
FC88X_00886_00428      --------------TTTTTTTGCTCCCCGACAACCGACGTATT------------
------
FC88X_00063_00481      ------------TTTTTTTTCGCTCCCCGACAACCGACGTATT------------
------
FC88X_00665_01243      ---------------TTTTTCGCTCCCCGACGACCGACGTATT------------
------
FC88X_00005_00254      ---------------TTTTTCGCTCCCCGGCAACCGACGTATT------------
------
FC88X_00948_00562      --------------TTTTTTCGCTCCCCGACAACCGACGCATT------------
------
FC88X_00879_00738      ------------------TTCTCGCCGCACAAACGAAC--ATTGTT---------
------
FC88X_00883_00812      ------------------TTCTCGCCGCACAAACGAAC--ATTGTT---------
------
FC88X_01033_00501      ------------------TTCTCGCCGCACAAACGAAC--ATTGTT---------
------
FC88X_01094_00741      ------------------TTCTCGCCGCACAAACGAAC--ATTGTT---------
------
FC88X_00255_01089      ------------------TTCTCGCCGCACAAACGAAC--ATTGTT---------
------
FC88X_00437_01068      ------------------TTCTCGCCGCACAAACGAAC--ATTGTT---------
------
FC88X_00496_00336      ------------------TTCTCGCCGCACAAACGAAC--ATTGTT---------
------
FC88X_00460_00601      ------------------TTCTCGCCGCACAAACGAAC--ATTGTT---------
------
FC88X_00472_01234      ------------------TTCTCGCCGCACAAACGAAC--ATTGTT---------
------
FC88X_00539_00717      ------------------TTCTCGCCGCACAAACGAAC--ATTGTT---------
------
FC88X_00929_00603      ------------------TTCTCGCCGCACAAACGAAC--ATTGTT---------
------
FC88X_00946_01037      ------------------TTCTCGCCGCACAAACGAAC--ATTGTT---------
------
FC88X_00942_01233      ------------------TTCTCGCCGCACAAACGAAC--ATTGTT---------
------
FC88X_00790_00315      ------------------TTCTCGCCGCACAAACGAACC-ATTGTT---------
------
FC88X_00466_00219      ------------GGGAAGTTCTCGCCGCACAAACGAAC--ATTGTT---------
------
FC88X_00105_00243      ----------------ATTACGCTCCCAATAAACCAAAG-ATCAC----------
------
FC88X_00228_01092      -------------CACCGTACACTCCGAAAAAACGTAAT-ATT------------
------
FC88X_00077_00113      -------------------ACACTCCCT-AGAAA-CATGCGCCATTCG-------
------
```

```
FC88X_00120_00069      ------------------ACACTCCCT-AGAAA-CATGCGCCATTCG-------
------
FC88X_00067_00476      ------------------ACACTCCCT-AGAAAACATGCGCCATTTG-------
------
FC88X_00308_00991      ------------------ACACTCCCT-AGAAAACATGCGCCATTTG-------
------
FC88X_01007_00438      ------------------ACACTCCCT-AGAAAACATGCGCCATTTG-------
------
FC88X_00057_00645      ------------------ACACTCCCT-AGAAA-CATGCGCCATTTG-------
------
FC88X_00201_00890      ------------------ACACTCCCT-AGAAA-CATGCGCCATTTG-------
------
FC88X_00388_00970      ------------------ACACTCCCT-AGAAA-CATGCGCCATTTG-------
------
FC88X_00459_00031      ------------------ACACTCCCT-AGAAA-CATGCGCCATTTG-------
------
FC88X_00743_01268      ------------------ACACTCCCT-AGAAA-CATGCGCCATTTG-------
------
FC88X_00928_00975      ------------------ACACTCCCT-AGAAA-CATGCGCCATTTG-------
------
FC88X_01116_01025      ------------------ACACTCCCT-AGAAA-CATGCGCCATTTG-------
------
FC88X_00076_00048      ------------------ACACTCCCT-AGAAA-CATGCGCCATTTG-------
------
FC88X_00129_00705      ------------------ACACTCCCT-AGAAA-CATGCGCCATTTG-------
------
FC88X_00199_00382      ------------------ACACTCCCT-AGAAA-CATGCGCCATTTG-------
------
FC88X_00454_01033      ------------------ACACTCCCT-AGAAA-CATGCGCCATTTG-------
------
FC88X_00665_01261      ------------------ACACTCCCT-AGAAA-CATGCGCCATTTG-------
------
FC88X_00890_00593      ------------------ACACTCCCT-AGAAA-CATGCGCCATTTG-------
------
FC88X_00916_00205      ------------------ACACTCCCT-AGAAA-CATGCGCCATTTG-------
------
FC88X_00930_00507      ------------------ACACTCCCT-AGAAA-CATGCGCCATTTG-------
------
FC88X_00917_01225      ------------------ACACTCCCT-AGAAA-CATGCGCCATTTG-------
------
FC88X_00106_00118      -----------------GACACTCCCT-AGAAA-CATGCGCCATTTG-------
------
FC88X_00531_00478      -----------------GACACTCCCT-AGAAA-CATGCGCCATTTG-------
------
FC88X_00678_00398      ------------------ACACTCCCT-GGAAA-CATGCGCCATTTG-------
------
FC88X_00734_01265      ------------------ACACTCCCT-AGAAACATCGCGCCATTTG-------
------
FC88X_00089_00139      ------------------ACACTCCCT-AGAAA-CATGCGCCATTTTG------
------
FC88X_00344_00600      ------------------ACACTCCCTTACAAACCACGCGCGAAT---------
------
FC88X_00078_00028      --------------TTGGATACTCCCC-ACAAACCGTGCGCT------------
------
FC88X_00402_01066      --------------------CCCATCAGCGAC-CAATGCGGCATCCCT------
------
```

```
FC88X_00819_00200      --------------------CCCATCAGCGAC-CAATGCGGCATCCCT------
------
FC88X_00916_01261      ---------------------CCATGAGCTACTCCATACTCCATCCCA------
------
FC88X_00688_01216      ----------------CTCCCTCATCAACCGCGGCATACTGCT-----------
------
FC88X_00973_01194      ---------------ATTCCCCCTAGTCCCACGC--ACGTGCTA----------
------
FC88X_00977_00596      ----------------CTCCCACCTATCCAGCGCCCACCTGCT-----------
------
FC88X_01065_01084      ----------------TTCATCTGTCCCTAGCTTGAACGTGCT-----------
------
FC88X_01005_01143      ----------GGGGAATTCATCTGTCCCTAGCTTGAACGTGCTCTCTT------
------
FC88X_00584_01138      ----------------CTCACCTGTGACCAGCGCGAGACTGCT-----------
------
FC88X_00924_00832      --------------CTCCCAATTACC---AACAAGCAGTTTCAT----------
------
FC88X_01060_00565      ---------------TCCCAATACCCCTTACCAAGCACGTTTT-----------
------
FC88X_00295_00224      ----------------TCCCTTTATCCACCGCCTGTATCATCTA----------
------
FC88X_00499_00406      --------------TTCTCTCCTTTATACAGCCTGTATCAA-------------
------
FC88X_00558_00183      -------------------ATCTCCGTTCTTCCTGCACACACATAGT-------
------
FC88X_00022_00106      --------------------TCTCCGTTCGGCCCGCACCAACACATC-------
------
FC88X_00026_00327      -------------------ATTTCCCTTCGAC-TGCACAACCATATT-------
------
FC88X_00111_00908      ----------------ACTTTTCCCTTGTGACTGCGACTACCA-----------
------
FC88X_00522_01229      ----------------ACTTTTCCCTTGCGACTGCGACTATCA-----------
------
FC88X_00917_01229      -----------------------------CGATGGGACTG--------------
------
FC88X_00215_01058      -----------------TCCTTCTCTCGTCGATTGCACTGTCAG----------
------
FC88X_00141_00492      -------------------CACTCCCAGTTGAACAAGTCGACATAGC-------
------
FC88X_00726_00875      --------------------------------------CGAC-TAGC-------
------
FC88X_00323_00647      -------------------ATCATTCCCTTCGACGAAACGAC-TAGCA------
------
FC88X_00085_00211      ----------------------CCCTGGACTACACCG-CTGCAGATTATT----
------
FC88X_00984_01196      ----------------------CCCTGGACTACACCG-CTGCAGATTATT----
------
FC88X_00642_00919      -------------------GTCCCGTGCTTCACGGCGGCTCCTAATT-------
------
FC88X_00717_00803      ----------------------CCGTGTTTTATGCCA-CTCCGACTTATT----
------
FC88X_00013_00091      -------------------CTTTCCCATCAACG-GACTTATCTTGTT-------
------
FC88X_00288_01013      -------------------CTTTCCCATCAACG-GACTTATCTTGTT-------
------
```

FC88X_00976_00639 -------------------CTTTCCCATCAACG-GACTTATCTTGTT-------
------
FC88X_00567_00942 ----------------TCGCTTTACCATTAACA-GAACTATCTC----------
------
FC88X_00640_00073 ----------------TCGCTTTACCATTAACA-GAACTATCTC----------
------
FC88X_00859_00296 -------------------TTTTACCAAAAGCGTAAACTATGTCC---------
------
FC88X_00329_01153 -------------------CTAATCCCTGA-CCTATGTCACCGCGTT-------
------
FC88X_00514_00338 -------------------CTAATCCCTGA-CCTATGTCACCGCGTT-------
------
FC88X_00843_00434 -------------------CTAATCCCTGA-CCTATGTCACCGCGTT-------
------
FC88X_00953_01069 -------------------CTAATCCCTGA-CCTATGCCACCGCGTT-------
------
FC88X_01100_00434 -------------------CTAATCCCTGAGCCTATGTCATCGCGTTCTT----
------
FC88X_00884_00889 -------------------CTAATCCCCGA-CCTATGTCACCGCGTT-------
------
FC88X_00586_00188 ----------------------TTCCCTTA-CCCATGCACGCGTTTTCT-----
------
FC88X_00868_00299 ----------------------TTCCCTTA-
CCCATGCACGCGTTTTTTCTCTTCCTTT-
FC88X_00388_00983 -------------------TTAGCTATTCATCGTGTTCAGCGC-----------
------
FC88X_00944_00535 ------------------CTTAGCTATTCATCGTGTTCAGCCGCC---------
------
FC88X_00402_00998 -----------------GTTTAGCTATTCATCGCGTTCAGCCGCC---------
------
FC88X_00350_01040 --------------------ACTTTTGCTATTCCCATGAACTATCTT-------
------
FC88X_00967_00383 ------------------TACAATTTGCTATTCACC---ACTTTCTTG------
------
FC88X_00074_00872 -------------------TTTTCCCGTTGTCA-GACGAGCTATGTT-------
------
FC88X_00490_00006 -------------------TTTTCCCGTTGTCA-GACGAGCTATGTTT------
------
FC88X_00989_00896 -------------------TTTTCCCGTTGCCA-GACGAGCTATGTT-------
------
FC88X_00849_00267 -------------------CTTTTGCCTTGTAA-TGCAAGCTATCTC-------
------
FC88X_00135_00872 ------------------ACTTTGGTGTTACCC-GAACAGCTATTCC-------
------
FC88X_00083_00317 ------------------ACTTTGGTGTTACCC-GAACAGCTATTCC-------
------
FC88X_00589_00317 ------------------ACTTTGGTGTTACCC-GAACAGCTATTCC-------
------
FC88X_01081_00712 ------------------ACTTTGGTGTTACCC-GAACAGCTATTCC-------
------
FC88X_00928_00320 ------------------ACTTTGGCGTTACCC-GAACAGCTATTCC-------
------
FC88X_00623_00921 ------------------CCTTTGGCATTACC--AAACCACTATCTC-------
------
FC88X_00006_00539 --------------------CTGTACATTGTCTGTTAGAACTATTCT-------
------

FC88X_00908_00426 --------------------TTGTACATTGTCTGTTAGTACTATT---------
------
FC88X_00933_00528 --------------------TTGTACATTGTCTGTTAGAGCTATTCT-------
------
FC88X_00908_00890 --------------------ATCTACCATGACT-TGAGCGCTATTCTT------
------
FC88X_00342_00698 -------------------TTCTGCCATTATCTAT--GATCTATTCGC------
------
FC88X_00964_00242 ------------------------CCATTGTCCCTACGAACTAGTCCTGTT---
------
FC88X_00052_00653 ------------------------CCATTGTCCCTACGAACTAATCCTGTT---
------
FC88X_00029_00410 --------------------CCAACAGGTAT-TGATTGC-GCGTTCTC------
------
FC88X_00806_00554 --------------------CCAACAGGTAT-TGATTGC-GCGTTCTC------
------
FC88X_00539_00143 --------------------CCAACAGGTATCTGATTGCCGCGTTCTC------
------
FC88X_00037_00288 --------------------CCAACAGGTATCTGATTGC-GCGTTCTC------
------
FC88X_00046_00653 --------------------CCAACAGGTATCTGATTGC-GCGTTCTC------
------
FC88X_00129_00111 --------------------CCAACAGGTATCTGATTGC-GCGTTCTC------
------
FC88X_00962_00365 ---------------------CAACAGG-ATCTGATTGC-GCGTTCTC------
------
FC88X_00346_01170 --------------------CCAACAGG-ATCTGATTGC-GCGTTCTC------
------
FC88X_00875_00277 -------------------GCCAACAGGTATCTGATTGC-GCGTTCTC------
------
FC88X_00948_00325 -------------------GCCAACAGGTATCTGATTGC-GCGTTCTC------
------
FC88X_00161_00602 -------------------GCCAACAGGTATCTGATTGC-GCGTTCTC------
------
FC88X_00086_00103 --------------------CCAACAGGTATCTGATTGC-GCGTTCTC------
------
FC88X_00159_00564 --------------------CCAACAGGTATCTGATTGC-GCGTTCTC------
------
FC88X_00209_01006 --------------------CCAACAGGTATCTGATTGC-GCGTTCTC------
------
FC88X_00326_01026 --------------------CCAACAGGTATCTGATTGC-GCGTTCTC------
------
FC88X_00182_00568 --------------------CCAACAGGTATCTGATTGC-GCGTTCTC------
------
FC88X_01067_01167 --------------------CCAACAGGTATCTGATTGC-GCGTTCTCT-----
------
FC88X_00274_00407 --------------------CCAACAGGTATCTGATTGC-GCGTTCTC------
------
FC88X_00417_00091 --------------------CCAACAGGTATCTGATTGC-GCGTTCTC------
------
FC88X_00453_01000 --------------------CCAACAGGTATCTGATTGC-GCGTTCTC------
------
FC88X_00526_00686 --------------------CCAACAGGTATCTGATTGC-GCGTTCTC------
------
FC88X_00602_00274 --------------------CCAACAGGTATCTGATTGC-GCGTTCTC------
------

FC88X_00614_01005 --------------------CCAACAGGTATCTGATTGC-GCGTTCTC------
------
FC88X_00846_00286 --------------------CCAACAGGTATCTGATTGC-GCGTTCTC------
------
FC88X_00547_00145 --------------------CCAACAGGTATCTGATTGC-GCGTTCTC------
------
FC88X_00261_00086 --------------------CCAACAGGTATCTGATTGC-
GCGTTCTCCTTCCTTT----
FC88X_00576_00560 --------------------CCAACAGGTATCTGATTGC-GCGTTCTC------
------
FC88X_00634_00929 --------------------CCAACAGGTATCTGATTGC-GCGTTCTC------
------
FC88X_00788_01223 --------------------CCAACAGGTATCTGATTGC-GCGTTCTC------
------
FC88X_00892_00627 --------------------CCAACAGGTATCTGATTGC-GCGTTCTC------
------
FC88X_00921_00468 --------------------CCAACAGGTATCTGATTGC-GCGTTCTC------
------
FC88X_00942_00979 --------------------CCAACAGGTATCTGATTGC-GCGTTCTC------
------
FC88X_00952_00393 --------------------CCAACAGGTATCTGATTGC-GCGTTCTC------
------
FC88X_00981_00403 --------------------CCAACAGGTATCTGATTGC-GCGTTCTC------
------
FC88X_00986_00933 --------------------CCAACAGGTATCTGATTGC-GCGTTCTC------
------
FC88X_01018_00845 --------------------CCAACAGGTATCTGATTGC-GCGTTCTC------
------
FC88X_01061_00376 --------------------CCAACAGGTATCTGATTGC-GCGTTCTC------
------
FC88X_01050_01068 --------------------CCAACAGGTATCTGATTGC-GCGTTCTC------
------
FC88X_01082_01264 --------------------CCAACAGGTATCTGATTGC-GCGTTCTC------
------
FC88X_01142_01227 --------------------CCAACAGGTATCTGATTGC-GCGTTCTC------
------
FC88X_00027_00214 --------------------CCAACAGGTATCTGATTGC-GCGTTCTC------
------
FC88X_00006_00592 --------------------CCAACAGGTATCTGATTGC-GCGTTCTC------
------
FC88X_00031_00619 --------------------CCAACAGGTATCTGATTGC-GCGTTCTC------
------
FC88X_00093_00071 --------------------CCAACAGGTATCTGATTGC-GCGTTCTC------
------
FC88X_00118_00888 --------------------CCAACAGGTATCTGATTGC-GCGTTCTC------
------
FC88X_00163_00560 --------------------CCAACAGGTATCTGATTGC-GCGTTCTC------
------
FC88X_00157_00805 --------------------CCAACAGGTATCTGATTGC-GCGTTCTC------
------
FC88X_00233_00092 --------------------CCAACAGGTATCTGATTGC-GCGTTCTC------
------
FC88X_00245_00591 --------------------CCAACAGGTATCTGATTGC-GCGTTCTC------
------
FC88X_00226_01079 --------------------CCAACAGGTATCTGATTGC-GCGTTCTC------
------

FC88X_00275_00096 --------------------CCAACAGGTATCTGATTGC-GCGTTCTC------
------
FC88X_00290_01107 --------------------CCAACAGGTATCTGATTGC-GCGTTCTC------
------
FC88X_00339_01038 --------------------CCAACAGGTATCTGATTGC-GCGTTCTC------
------
FC88X_00399_01184 --------------------CCAACAGGTATCTGATTGC-GCGTTCTC------
------
FC88X_00429_00863 --------------------CCAACAGGTATCTGATTGC-GCGTTCTC------
------
FC88X_00400_01138 --------------------CCAACAGGTATCTGATTGC-GCGTTCTC------
------
FC88X_00548_01200 --------------------CCAACAGGTATCTGATTGC-GCGTTCTC------
------
FC88X_00595_00220 --------------------CCAACAGGTATCTGATTGC-GCGTTCTC------
------
FC88X_00574_00476 --------------------CCAACAGGTATCTGATTGC-GCGTTCTC------
------
FC88X_00558_01113 --------------------CCAACAGGTATCTGATTGC-GCGTTCTC------
------
FC88X_00582_01178 --------------------CCAACAGGTATCTGATTGC-GCGTTCTC------
------
FC88X_00618_01153 --------------------CCAACAGGTATCTGATTGC-GCGTTCTC------
------
FC88X_00607_01263 --------------------CCAACAGGTATCTGATTGC-GCGTTCTC------
------
FC88X_00733_00859 --------------------CCAACAGGTATCTGATTGC-GCGTTCTC------
------
FC88X_00837_00259 --------------------CCAACAGGTATCTGATTGC-GCGTTCTC------
------
FC88X_00840_00758 --------------------CCAACAGGTATCTGATTGC-GCGTTCTC------
------
FC88X_00860_00885 --------------------CCAACAGGTATCTGATTGC-GCGTTCTC------
------
FC88X_00936_00560 --------------------CCAACAGGTATCTGATTGC-GCGTTCTC------
------
FC88X_00992_00258 --------------------CCAACAGGTATCTGATTGC-GCGTTCTC------
------
FC88X_00972_00805 --------------------CCAACAGGTATCTGATTGC-GCGTTCTC------
------
FC88X_00976_01044 --------------------CCAACAGGTATCTGATTGC-GCGTTCTC------
------
FC88X_00980_01094 --------------------CCAACAGGTATCTGATTGC-GCGTTCTC------
------
FC88X_01010_00336 --------------------CCAACAGGTATCTGATTGC-GCGTTCTC------
------
FC88X_01015_01012 --------------------CCAACAGGTATCTGATTGC-GCGTTCTC------
------
FC88X_01077_00451 --------------------CCAACAGGTATCTGATTGC-GCGTTCTC------
------
FC88X_01075_00779 --------------------CCAACAGGTATCTGATTGC-GCGTTCTC------
------
FC88X_01080_00793 --------------------CCAACAGGTATCTGATTGC-GCGTTCTC------
------
FC88X_01147_00787 --------------------CCAACAGGTATCTGATTGC-GCGTTCTC------
------

```
FC88X_01128_00815       --------------------CCAACAGGTATCTGATTGC-GCGTTCTC------
------
FC88X_01070_00433       ----------------GGGACCAACAGGTATCTGATTGC-GCGTTCTC------
------
FC88X_00142_00505       --------------------CCAGCAGGTATCTGATTGC-GCGTTCTC------
------
FC88X_00266_01099       --------------------CCAACAGGTGTCTGATTGC-GCGTTCTC------
------
FC88X_00089_00877       --------------------CCAACAGGTGTCTGATTGC-GCGTTCTC------
------
FC88X_00840_00831       -------------------ACCAACAGGTATCTGATTGC-GCGTTCTCC-----
------
FC88X_01108_01038       -------------------ACCCACAGGTATCTGATTGC-GCGTTCTC------
------
FC88X_01073_00823       --------------------CCGACAGG-ATCTGATTGC-GCGTTCTC------
------
FC88X_00953_01148       --------------------CCAACAGGTATCTGATTGC-GCGCTCTC------
------
FC88X_01004_00551       --------------------CCAACAGGTATCTGATTGC-GCGCTCTC------
------
FC88X_00976_00306       --------------------CCAACAGGTATTTGATTGC-GCGTTCTC------
------
FC88X_00963_00961       --------------------CCAACAGGTATCCGATTGC-GCGTTCTC------
------
FC88X_01081_00985       --------------------CCAACAGGTATCCGATTGC-GCGTTCTC------
------
FC88X_00091_00906       ----------------TGATGCAACAGGTATCTGATTGC-GCGTTCTC------
------
FC88X_00011_00700       --------------------GCAACAGGTATCTGATTGC-GCGTTCTC------
------
FC88X_00784_00255       --------------------CCAACAGGTATCTGATTAC-GCGTTCTC------
------
FC88X_00280_01076       --------------------CCAACAGGTATCTGGTTGC-GCGTTCTC------
------
FC88X_00849_00326       --------------------CCAACGGGTATCTGATTGC-GCGTTCTC------
------
FC88X_00643_01033       --------------------CCAACAGGTACCTGATTGC-GCGTTCTC------
------
FC88X_00077_00606       -------------------CCAAACAGGTATCTGATTGC-GCGTTCTC------
------
FC88X_00989_01144       -------------------CCAAACAGGTATCTGATTGC-GCGTTCTC------
------
FC88X_01081_00436       --------------------CTAACAGGTATCTGATTGC-GCGTTCTC------
------
FC88X_00051_00767       --------------------CCAACAGGTATCTGAT-CGCGCGTTCTC------
------
FC88X_00329_00974       --------------------CCAACAGGTATCTGATTCGCGCGTTCTCC-----
------
FC88X_00781_00850       --------------------CCAACAGGTATCTGAT-CGCGCGTTCTC------
------
FC88X_00027_00371       --------------------CCAACAGGTATCTGAT-CGCGCGTTCTC------
------
FC88X_00517_01102       --------------------CCAACAGGTATCTGAT-CGCGCGTTCTC------
------
FC88X_00115_00583       --------------------CCAACAGGTATCTGAT-CGTGCGTTCTC------
------
```

FC88X_00314_00975 --------------------CCAACAGGTATCTGAC-TGCGCGTTCTC------
------
FC88X_00548_00084 --------------------CCAACAGGTATCTGAC-TGCGCGTTCTC------
------
FC88X_00991_00489 --------------------CCAACAGGTATCTGAC-TGCGCGTTCTC------
------
FC88X_01083_01012 --------------------CCAACAGGTATCTGAC-TGCGCGTTCTC------
------
FC88X_00465_00306 --------------------CCAACAGGTATCTGAC-TGTGCGTTCTC------
------
FC88X_00942_00291 ---------------------CAACAGGCATCTGAT-TGCGCGTTCTC------
------
FC88X_00282_01053 --------------------CCAACAGGCATCTGAT-TGCGCGTTCTC------
------
FC88X_00263_01100 --------------------CCAACAGGCATCTGAT-TGTGCGTTCTC------
------
FC88X_00964_00981 --------------------CCAACAGGTAATTGATCGC-GCGTTCTC------
------
FC88X_00730_00975 -------------------CCAAACAGGAATCTGATTG----------------
------
FC88X_00710_01261 --------------CTTTACCCAAGTGGCAGTTGACTGGGA-------------
------
FC88X_00030_00708 --------------------GCGCGATATCTGCACTTGT-CTATTCTT------
------
FC88X_00931_01200 --------------------GCGCGATATCTGCACTTGT-CTATTCTT------
------
FC88X_00524_01003 --------------------TCGCGATATCTGCACTTGT-CTATTCTT------
------
FC88X_00092_00026 --------------GGGGGACCGCGTTATTTGTTGTTGC-CTATTCTT------
------
FC88X_00666_01039 --------------------TCGTCATTTCTGTTGTTCG-CTCTACTT------
------
FC88X_00026_00391 --------------------TCGTCATTTCTGTTGTTCG-CTCTACTT------
------
FC88X_00431_00990 --------------------TCGTCATTTCTGTTGTTCG-CTCTACTT------
------
FC88X_00055_00551 --------------------TCCACCGATTGGTGTTTGT-CTATTATT------
------
FC88X_00088_00778 -------------------CCACACGATGTGCTATTTGT-CTGTAAT-------
------
FC88X_00231_00050 ---------------------------ATCACCGTTGGT-CGGTCG--------
------
FC88X_00852_00780 ----------------------CCCGAAGCACGGTT--T-CGGTAGTTGCCC--
------
FC88X_00538_00248 ------------------TCAACCGCTGTCTGGATTTGC-CTTTAT--------
------
FC88X_00747_01213 --------------------ATACGTCCTCACCAACGTTGG-TTCATT------
------
FC88X_00817_00863 -------------------CCCCAGACATCTCCGACTTTGGCTTCGTT------
------
FC88X_00308_01157 ----------------------------
TCTCCGACAGCGGTCTGGCTGCGTTCT-----
FC88X_00358_00843 --------------------ATGTCTGACCATTGCTGC-TTCTATCTC------
------
FC88X_00558_00348 --------------------ATGTCTGACCATTGCTGC-TTCTATCTC------
------

FC88X_00655_00959 --------------------ATGTCTGACCATTGCTGC-TTCTATCTC------
------
FC88X_00127_00128 --------------------ATGTCTGACCATTGCTGC-TTCTATCTC------
------
FC88X_00368_00817 --------------------ATGTCTGACCATTGCTGC-TTCTATCTC------
------
FC88X_00939_00683 --------------------ATGTCTGACCATTGCTGC-TTCTATCTC------
------
FC88X_01060_00837 --------------------ATGTCTGACCATTGCTGC-TTCTATCTC------
------
FC88X_01082_01051 --------------------ATGTCTGACCATTGCTGC-TTCTATCTC------
------
FC88X_01131_00927 --------------------ATGTCTGACCATTGCTGC-TTCTATCTC------
------
FC88X_00161_01013 -------------GGGGAATATGTCTGACCATTGCTGC-TTCTATCTC------
------
FC88X_00933_00481 --------------------ATGTCTGACAATTGCTGC-TTCTATCTC------
------
FC88X_00913_00653 --------------------ATGTCTGACCATTGCTGCCTTCTATCTC------
------
FC88X_00511_00061 --------------------ATGCCTGACCATTGCTGC-TTCTATCTC------
------
FC88X_00913_00967 --------------------ATGTCTGACCACTGCT-GCTTCTATCTC------
------
FC88X_01089_01195 --------------------ATGTCTGACCATTGCTCGCTTCTATCTC------
------
FC88X_01075_00560 --------------------ATGTCTGACCATTGCTGC-
TTCTATCTCCTTTCCTTT---
FC88X_00068_00397 --------------------ATGTCTGACCATTGTTGC-TTCTATCTC------
------
FC88X_01080_00777 -------------------TATGTTCTGACCATGCTGCCTTCTATCTCCT----
------
FC88X_00647_01064 --------------------GTTTCTGATCACTGACAG-CCCTATCTC------
------
FC88X_01100_01103 --------------------GTTTCTGATCACTGACAG-CCCTATCTC------
------
FC88X_00338_00989 --------------------GTTTCTGATCACTGACAG-CCCTATCTC------
------
FC88X_00891_00257 --------------------GTTTCTGATCACTGACAG-CCCTATCTC------
------
FC88X_00874_00566 --------------------CTTCCGGATCTCTAACGC-GGCTGTCTT------
------
FC88X_01068_00490 --------------------CTTCCGGATCTCTAACGC-GGCTATCTT------
------
FC88X_01094_00427 -------------------CCTTGCTGTTCTCTTA-GC-CCCTGTCTA------
------
FC88X_00368_01064 ---------------GTGATCTTTGCGCTCTCCG-ACGGCCGTGTGTT------
------
FC88X_00854_00892 --------------------CTTTGCGCTCTCCG-ACGGCCGTGTGTT------
------
FC88X_01000_00679 --------------------CTTACCGCTCTCTA-ACTCCCGCTTGCT------
------
FC88X_01021_00909 --------------------CTAAGCGCCCTCCAGACTCCCGATTTC-------
------
FC88X_00438_00170 -------------------CTCGATTTCAGAACGGGGACTGTCTC---------
------

FC88X_00973_00678 ------------------TCTCGATTTCAGAACGGGGACTGCCTC---------
------
FC88X_00261_01078 --------------------TTAACCCACAACAGTGGAATGCCTCTT-------
------
FC88X_00037_00155 --------------------CCTAGCTTTGTACT---ACCGGCTGTTCGC----
------
FC88X_00624_00076 --------------------CCTAGCTTTGTACT---ACCGGCTGTTCGC----
------
FC88X_00694_01238 --------------------CCTAGCTTTGTACT---ACCGGCTGTTCGC----
------
FC88X_00775_00216 --------------------CCTAGCTTTGTACT---ACCGGCTGTTCGC----
------
FC88X_00795_01212 --------------------CCTAGCTTTGTACT---ACCGGCTGTTCGC----
------
FC88X_00884_00335 --------------------CCTAGCTTTGTACT---ACCGGCTGTTCGC----
------
FC88X_00850_00893 --------------------CCTAGCTTTGTACT---ACCGGCTGTTCGC----
------
FC88X_01041_00490 --------------------CCTAGCTTTGTACT---ACCGGCTGTTCGC----
------
FC88X_01070_00470 --------------------CCTAGCCTTGTACT---ACCGGCTGTTCGC----
------
FC88X_00426_00099 --------------------GCCTCCATTGCACT-GGAACGCCTGTTCTG----
------
FC88X_00735_00508 --------------------GCCTCCATTGCACTTGGAACGCCTGTTCTG----
------
FC88X_00075_00232 --------------------GCCTCCATTGCACT-GGAACGCCTGTTCTG----
------
FC88X_00667_01258 ---------------------CTCCCGGGTGCTTATAGCCAATTTTTTT-----
------
FC88X_01075_00924 ---------------------CTCCCGGGTGCTTATAGCCAATTTTTT------
------
FC88X_00097_00822 --------------------TCTCCCAGCGTCTTGT--CCAGTTGTTGT-----
------
FC88X_00852_01209 ---------------------CTAACGCGTTCTGTAAAGTTATCTTTTC-----
------
FC88X_00172_00926 --------------------CCCAACAAC-CGGAACAGTTGGCATCTC------
------
FC88X_00563_00053 --------------------CCCAACAAC-CGGAACAGTTGGCATCTC------
------
FC88X_00836_00283 --------------------CCAGACATTATGTCACAGTGCCTCTCT-------
------
FC88X_00883_00807 --------------------CCAGACATTATGTCACAGTGCCTCTCT-------
------
FC88X_00983_00811 --------------------CCAGACATTATGTCACAGTGCCTCTCT-------
------
FC88X_00603_01241 --------------------CCAGACATTATGTCACAGTGCCTCTCT-------
------
FC88X_00856_00405 --------------------CCAGACATTATGTCACAGTGCCTCTCT-------
------
FC88X_00931_01176 --------------------
CCAGACATTATGTCACAGTGCCTCTCTCTTCCTTT-----
FC88X_00897_00614 --------------------CCCAACGTTATG-CGCAGTTTGGCTTCTC-----
------
FC88X_01076_01206 --------------------CCCAACGTTATG-CGCAGTTTGGCTTCTC-----
------

FC88X_00176_00988 ---------------------TACAGCATTTGCGG--GTACCTATAACTC----
------
FC88X_00929_00982 ---------------------TACAGCATTTGCGG--GTACCTATAACTC----
------
FC88X_00369_00263 ---------------------TACAGCATTTGCGG--GTACCTATAACTC----
------
FC88X_00552_01239 ---------------------TACAGCATTTGCGG--GTACCTATAACTC----
------
FC88X_00809_00851 ---------------------TACAGCATTTGCGG--GTACCTATAACTC----
------
FC88X_00499_00351 -----------------------CTGCACTTGCCGTAGTGCGGATATCTC----
------
FC88X_00159_00049 -----------------------CTCCCTTGGCGT--GTTGCTTTCACGTGA--
------
FC88X_01085_00579 -----------------------CTCCCTTGGCGT--GTTGCTTTCACGTGA--
------
FC88X_01003_00605 ------------------------TCTCATTACGT--GTTCCTTGAACTTATT-
------
FC88X_00021_00399 --------------------CTGACACTCTTTCGTTAGCCGTTCCTC-------
------
FC88X_00772_00967 --------------------CTGACACTCTTTCGTTAGCCGTTCCTC-------
------
FC88X_00904_01242 --------------------CTGACACTCTTTCGTTAGCCGTTCCTC-------
------
FC88X_00296_01001 --------------------CTGACACTCTTTCGTTAGCCGTTCCTC-------
------
FC88X_01118_01050 --------------------CTGACACTCTTTCGTTAGCCGTTCCTC-------
------
FC88X_00522_00105 --------------------CTGACACTCTCTCGTTAGCCGTTCCTCGG-----
------
FC88X_00879_01053 --------------------CTGACTCTCTTTCGTTAGCCGTTCCTC-------
------
FC88X_00807_00937 --------------------CTGACACTCTTTCGTTAGC---------------
------
FC88X_00953_01030 --------------------CTGGCACTCTTTCGCTAGCCGTTCCTC-------
------
FC88X_00100_00083 ----------------CTACCCGGCAATTTGCCATTAGCGTTCT----------
------
FC88X_00503_01086 ----------------CTACCCGGCAATTTGCCATTAGCGTTCT----------
------
FC88X_00039_00488 ----------------CTACCCGGCAATTTGCCATTAGCGTTCT----------
------
FC88X_00263_00550 ----------------CTACCCGGCAATTTGCCATTAGCGTTCT----------
------
FC88X_00437_00887 ----------------CTACCCGGCAATTTGCCATTAGCGTTCT----------
------
FC88X_01132_00865 ----------------CTACCCGGCAATTTGCCATTAGCGTTCT----------
------
FC88X_00540_01221 ----------------CTACCCGGCAATTTGCCATTAGCGTTCC----------
------
FC88X_00179_00673 ----------------CTACCCGGCAATCTGCCATTAGCGTTCT----------
------
FC88X_00081_00033 ----------------CTACTCCCTATCTA--CCCGCACTTTGC----------
------
FC88X_00548_00179 ----------------TTACACACTATTTTGCCCTGCACTTGG-----------
------

```
FC88X_00051_00672       ------------------TTAGCGTTTTCTGACCCC-GACATTTCTC-------
------
FC88X_00684_01246       ------------------TTAGCGTTTTCTGGCCCC-GACATTTCTC-------
------
FC88X_00987_01274       ------------------TTAGCGTTTTCTGACCCCCGACA-------------
------
FC88X_00121_00297       ------------------TTAGCGTTTTCTGACCCC-GACATTTCTC-------
------
FC88X_00695_00674       ------------------TTAGCGTTTTCTGACCCC-GACATTTCTC-------
------
FC88X_00422_01084       ------------------TTAGCGTTTTCTGACCCC-GACATTTCTC-------
------
FC88X_00621_00529       ------------------TTAGCGTTTTCTGACCCC-GACATTTCTC-------
------
FC88X_00980_00660       ------------------TTAGCGTTTTCTGACCCC-GACATTTCTC-------
------
FC88X_01055_00451       ------------------TTAGCGTTTTCTGACCCC-GACATTTCTC-------
------
FC88X_01017_00589       ------------------TTAGCGTTCTCTGACCCC-GACATTTCTC-------
------
FC88X_00064_00807       -----------------ACGTACCGTTTTCGGCACC-AACTTCTC---------
------
FC88X_00864_00308       -----------------ACGTACCGTTTTCGGCACC-AACTTCTC---------
------
FC88X_00079_00297       -----------------ACGTACCGTTTTCGGCACC-AACTTCTC---------
------
FC88X_00063_00571       -----------------ACGTACCGTTTTCGGCACC-AACTTCTC---------
------
FC88X_01044_00344       -----------------ACGTACCGTTTTCGGCACC-AACTTCTC---------
------
FC88X_01079_00547       ----------------TACGTACCGTTTTCGGCGCC-AACTTCTC---------
------
FC88X_00789_00872       -----------------CTACCCTTCTTCAGACACC-AGCTTTGA---------
------
FC88X_00495_00125       ---------------GCTACTGGCTTTTTCGACTCC-CTCTTGGA---------
------
FC88X_00070_00005       ----------------CTACTAGCTTCTCCGAC-CC-CGCTTGGC---------
------
FC88X_00252_01078       -----------------TTCTAAGCTATTTGCCACTACTCTTCT----------
------
FC88X_00309_01055       -----------------TTCTAAGCTATTTGCCACTACTCTTCT----------
------
FC88X_00677_00502       -----------------TTCTAAGCTATTTGCCACTACTCTTCT----------
------
FC88X_00378_00988       -----------------TTCTAAGCTATTTGCCACCACTCTTCT----------
------
FC88X_00061_00120       -----------------CTATATGCTATT-GCCACCGCGTCTTCGT--------
------
FC88X_01117_00727       -----------------CTATATGCTATT-GCCACCGCGTCTTCGT--------
------
FC88X_00237_00198       -----------------CTATTGGCTTCTTGCCGACGGCGTTCT----------
------
FC88X_00865_00191       ----------------GCTATTC-TTTATGCTCAATGCCCTTCTA---------
------
FC88X_00897_01242       -----------------CTTCTCCTTATTCAACACCGTGCTCGG----------
------
```

FC88X_00491_01247 -----------------CTTTCTACTTCTCCCCACCGTTATCGG----------
------
FC88X_00114_00748 -----------------CTTTATGCTTATTCCAACCGAGTTCCT----------
------
FC88X_00956_00612 ----------------CTCCTACTTAATTCGCATTATCTTGCG-----------
------
FC88X_00957_00737 ----------------CTACTCCTT--TGCGCAACAGCTTTCGC----------
------
FC88X_00375_01177 ------------------TATACGCTTTTTCTAACCGCT---------------
------
FC88X_00073_00154 -----------------------ACATTTCACCTCCCACTCCTTACTAGA----
------
FC88X_00768_01175 ---------CCCTGCCATGTCGGACATTTCATTCCCCTTCCTTT----------
------
FC88X_00110_00702 --------------------TTCGCATTTGACTTTCGCCTACTTTCT-------
------
FC88X_00580_00257 --------------------TTCGCATTTGACTTTCGCCTACTTTCT-------
------
FC88X_01041_00444 --------------------TTCGCATTTGACCTTCGCCTACCTTCT-------
------
FC88X_00243_01093 -------------------CTTAGCCATTTGCTTTGC-CTCCCTGCT-------
------
FC88X_00442_00277 -------------------CTTAGCCATTTGCTTTGC-CTCCCTGCT-------
------
FC88X_01115_00754 -------------------CTTAGCCATCTGCTTTGC-CTCCCTGCT-------
------
FC88X_00158_00716 -------------------CTTGGTAATTGGCCTTGCACTCCCCGA--------
------
FC88X_00061_00225 --------------------CGGCCAACTCGCCCTA-ACTGCTATAAT------
------
FC88X_00817_00260 --------------------CGGCCAACTTGCCCTA-ACTGCTATAAT------
------
FC88X_00881_00608 ------------------TATTGCACAATTGCCTTACACTCCT-----------
------
FC88X_01097_00838 ------------------TATTGCACAATTGCCTTACACTCCT-----------
------
FC88X_00085_00251 -----------------TTTCCAACCCTTC-GGAACCGCTTTCTC---------
------
FC88X_00884_00410 -----------------TTTCCAACCCTTC-GGAACCGCTTTCTC---------
------
FC88X_00389_01038 -----------------CTTCCAACCCTTC-GGAACCGCTTTCTC---------
------
FC88X_00155_00614 -------------------CCAAGCCATTT-
GGGACTGCTGTTTATCCTTCCTT------
FC88X_01129_00517 -------------------CCAAGCCATTT-
GGGACTGCTGTTTATCCTTCCTT------
FC88X_01108_00990 -------------------CCAAGCCATTT-GGGACTGCTGTATATC-------
------
FC88X_00263_00652 -------------------ACCAGCGCTTT-ATATCTCCTCATTCTC-------
------
FC88X_00198_00930 --------------------
CCAGCCCTTCGATAACCCCTGATTCTCCTTCCTTTCTC--
FC88X_00357_01114 -------------------TCACACTGTTT-GTGCCTCCCTTACACA-------
------
FC88X_00447_01206 -------------------TCACATTGTTT-GTGCCTCCCTTACACA-------
------

FC88X_01000_00271 --------------------TCACATTGTTT-GTGCCTCCCCTACACA-------
------
FC88X_00768_00822 ------------------TGTGACGCTGTTT-ACACCTCC-TTACCC--------
------
FC88X_00044_00021 ---------------------CCAATGACTCTTCTTCATCCTATTCTT-------
------
FC88X_01011_01081 ---------------------CCGATGACTCTTCTTCATCTTATTCTT-------
------
FC88X_00191_00435 ---------------------CTGATTTCTATTCTTCACCCTATGCAC-------
------
FC88X_00584_00156 --------------------GTTAACC-CTATTCACT-GCCTTATTGCG------
------
FC88X_01093_00604 ---------------------TTAACCACTATTCTTCAGCCTCTTTGT-------
------
FC88X_00244_00928 ---------------------CCAAACCATATTCGTCTGCCTATTTTC-------
------
FC88X_00745_00185 ---------------------CCAAACCATATTCGTCTGCCTATTTTC-------
------
FC88X_00823_00997 ---------------------CCAAACCATATTCGTCTGCCTATTTTC-------
------
FC88X_00820_01204 ---------------------CCAAACCATATTCGTCTGCCTATTTTC-------
------
FC88X_00748_00485 ----------------GTGATACAAACTATTCTTCGTTGCCAAATTCG-------
------
FC88X_00058_00201 -------------------TTGCAACCTATT--CAT-TACCTAATTCCT------
------
FC88X_00486_01123 ---------------------TTTACCTATTTGCGT-AACCTACTTCCT------
------
FC88X_00711_01159 --------------------ATTCATCTATATTCTCCACCTGCTTTGG-------
------
FC88X_01053_01232 -------------------ATTTACACTATTCTTCG-ACTGCCTTAC--------
------
FC88X_00523_00152 --------------------TTTTACCTCTACTCCGTACAGTCTTTT--------
------
FC88X_00315_00012 ------------------GATGTGAGCT-TTCATCTCCACTATTGTC--------
------
FC88X_00045_00832 -------------------ATGTGAGCT-TTCATCTCCACTATTGTC--------
------
FC88X_00494_00080 -------------------ATGTGAGCT-TTCATCTCCACTATTGTC--------
------
FC88X_00795_00972 -------------------ATGTGAGCT-TTCATCTCCACTATTGTC--------
------
FC88X_00930_00810 -------------------ATGTGAGCT-TTCATCTCCACTATTGTC--------
------
FC88X_01060_01078 -------------------ATGTGAGCT-TTCATCTCCACTATTGTC--------
------
FC88X_00090_00860 -------------------ATGTGAGCT-
TTCATCTCCACTATTCTCCTTCCTTT------
FC88X_00804_01222 -----------------CCGTTTCAGCTATTCGTCAACACTCTG-----------
------
FC88X_00885_00381 ------------------CTTTTTTGCT-ATGCTCTTCACTCTTTGT--------
------
FC88X_00894_00751 ------------------CTGTTTAGCA-GGCCCTTCCACTATTTAC--------
------
FC88X_00038_00074 -------------------CTCCCCGGGACTATTCACTGACTATCT---------
------

```
FC88X_01034_00696       -----------------TCTCCCCTGGTCTAAGCTC-GACGATCCCCCTTCC--
------
FC88X_00233_00155       --------------------TCTTGCGCGTTCCCATTGCATATTCTC-------
------
FC88X_00017_00502       --------------------TCTTGCGCGTTCCCATTGCATATTCTC-------
------
FC88X_00459_01198       --------------------TCTTGCGCGTTCCCATTGCATATTCTC-------
------
FC88X_00069_00171       --------------------TCTTGTGCGTTCCCATTGCATATTCTC-------
------
FC88X_00014_00450       --------------------TCTTTAGTTCTCCCCTGGACTAGTCTC-------
------
FC88X_00621_00135       --------------------CCTCCCGTTGTTTCGCAGTTCTACGCT-------
------
FC88X_00672_01137       --------------------CCTCCCGTTGTTTCGCAGTTCTACGCT-------
------
FC88X_00356_01152       ---------------------CTCCCGTTGTTTCGCAGTTCTACGCT-------
------
FC88X_00882_00810       --------------------CCTCCCGTTGTCTCGCAGTTCTACGCT-------
------
FC88X_00569_01165       --------------------CCTCCCGTTGTTTCGCAGCTCTACGCT-------
------
FC88X_00161_00953       --------------------TCTCGCCTTCTTTC-CTCTTCTACTCTC------
------
FC88X_00070_00214       -------------------TCTTCTCGATTATTCCCAGCACTACTTG-------
------
FC88X_00035_00298       --------------TCCCTTGTTTT--CCCACGTCAGCATGTC-----------
------
FC88X_00283_00253       --------------TCCCTTGTTTT--CCCACGTCAGCATGTC-----------
------
FC88X_00979_00638       --------------TCCCTTGTTTTT-CCCACGTCA------------------
------
FC88X_00894_01077       --------------TTCCTTGTCCTA-CCTCCCAC-GCATGTC-----------
------
FC88X_00280_00517       ------------ACATCCCCGTC----TATATCCCCATATGTTT----------
------
FC88X_00895_00434       --------------TTCCCCGTTA--TTCCGCGCCCAAATGTT-----------
------
FC88X_00995_00844       -------------TCTCCCCGCTTACGCCGACGTCCCAAT--------------
------
FC88X_01128_00892       -------------TCTCCCCGTTTACGCCGACGTCCCAAT--------------
------
FC88X_00057_00634       -------------TCTCCCTGTAT---CCCGCGTCCCGATGCA-----------
------
FC88X_00618_01234       --------------TCCTGATTTTTTTTCCACCCCAAGCTGTT-----------
------
FC88X_00952_00910       --------------TCCTGATTTTTT--CCACCCCAAGCTGTT-----------
------
FC88X_00654_00277       --------------TCCTGATTTTT---CCACCC-AAGCTGTT-----------
------
FC88X_00137_00558       --------------TCCTGATTTCTT--CCACCCCAAGCTGTT-----------
------
FC88X_00426_01098       --------------TCCTGATTTTTT--CCACCC-AAGCTGTTCTTCC------
------
FC88X_00563_00243       --------------TCCTGATTTTTTT-CCACCC-AAGCTGTT-----------
------
```

FC88X_00582_00461 ---------------TCCTGATTTTTT--CCACCCCGAGCTGTT-----------
------
FC88X_00066_00585 ---------------TCCTGATTTTCT--CCACCCCAAGCTGTT-----------
------
FC88X_00662_01148 ----------------CCAGAGGTTTAA-CTCCCCACGACTGTT-----------
------
FC88X_00274_00457 ---------------CTCTTGCTATCAT-CCACTTCCACGTGTT-----------
------
FC88X_01041_01044 ----------------CCTCTTTCATTC-CCAATGGCACGTGTT-----------
------
FC88X_00959_01195 ---------------TTCTTTCTTATTC-TCCACA-CAGGTGTT-----------
------
FC88X_00561_01191 ------------------TTCTTTTTTC-CTCTCCACCGATGTTTA---------
------
FC88X_00064_00389 ----------------TGATCGAATTTTATCCCGCGACACATG------------
------
FC88X_00603_00102 ----------------TGATCGAATTTTATCCCGCGACACATG------------
------
FC88X_00047_00284 ----------------TGATCGAATTTTATCCCGCGACACATG------------
------
FC88X_00060_00395 ----------------TCATAGATTAACACCTCCCAACACATA------------
------
FC88X_00068_00438 ----------------TTTGTCGTTTT-ACCCCGCAGCTCTGTT-----------
------
FC88X_00433_00122 ----------------TTTGTCGTTTT-ACCCCGCAGCTCTGTT-----------
------
FC88X_00576_00257 ----------------TTTGTCGTTTT-ACCCCGCAGCTCTGTT-----------
------
FC88X_00588_00956 ----------------TTTGTCGTTTT-ACCCCGCAGCTCTGTT-----------
------
FC88X_00998_00736 ----------------TTTGTCGTTTTTGCCCCGCAGCCCTGTT-----------
------
FC88X_00014_00379 ----------------TCTGTCGTTTTACTCCCGCAGCACACT------------
------
FC88X_00636_00626 ----------------TCTGTCGTTTTACTCCCGCAGCACACT------------
------
FC88X_00700_00132 ----------------TCTGTCGTTTTACTCCCGCAGCACACT------------
------
FC88X_00739_01205 ----------------TCTGTCGTTTTACTCCCGCAGCACACT------------
------
FC88X_00185_01024 ----------------CTTCTCAGTTTCAACCCGCACCGCATT------------
------
FC88X_00735_00328 ------------CCGTCTTTTCAGCTTC--CCATCACCGTATT------------
------
FC88X_00309_01077 --------------GACCTTCGTTTT--TCCGAATTCCACTGTT-----------
------
FC88X_00899_01173 -----------------CTTGGTTTTGTTCCGCCTCGCATTGTT-----------
------
FC88X_00447_00371 ----------------TCCTCGGTTTT--CACCATCACAATATT-----------
------
FC88X_00557_01209 ----------------TCCTCGGTTTTT-CACGATCACAATATT-----------
------
FC88X_00412_00169 ----------------TCCTCGGTTTTTTCACCATCACAATATT-----------
------
FC88X_00982_00616 ----------------TCCTCGGTTTTT-CACCATCACAATATT-----------
------

```
FC88X_00627_01245      -------------TTCCGCTTTGTCGTCTATTTCCGTATC--------------
------
FC88X_00267_00960      -------------TTCCGCTTTGTCGTCTATTCCCGTATC--------------
------
FC88X_01068_00855      --------------TTCGCTTGGTCTTCCACTCCCGTAAATA------------
------
FC88X_01000_01099      ----------------CTTGTTGTTCTACTTCCCCAACGTTAT-----------
------
FC88X_00873_00199      ---------------TCGTGTTGTCTTCCTTCCACGTAATGTT-----------
------
FC88X_00099_00543      ----------------CTTTTTGTTCTTCGCCTTGGTAATGCC-----------
------
FC88X_00109_00337      ----------------CTTTTTGTTCTTCGCCTTGGTAATGCC-----------
------
FC88X_00273_00936      ----------------CTTTTTCTTATTCTTCTCGGCAATGTT-----------
------
FC88X_00301_00474      ---------------ACTTTTCGTTGTCTACG--CCCCATT-------------
------
FC88X_00768_00963      ----------------CTTTCCGTTGTTTTCGTGCCCCATGTT-----------
------
FC88X_00188_00477      -------------TCTCGTCTTATTCACATTCCCGCTAAT--------------
------
FC88X_00933_00576      -------------TCTCGTCTTATTCACATTCCCGCTAAT--------------
------
FC88X_00593_01219      -------------TCTCGTTCTCTTCTACCTGCCCCCGGT--------------
------
FC88X_00984_01267      -------------TCTCTTCTTCTTATACCTGTCCCCGGT--------------
------
FC88X_00092_00106      ----------------TTCTTCTTTTCGCCACTCCACGTTATT-----------
------
FC88X_00986_01089      ---------------TTCTTCTTTTTCGCCACTCCACGTTATT-----------
------
FC88X_00953_00653      ----------------CTTCTTCTTTACCGACACCACATTTT------------
------
FC88X_00622_01216      ----------------CACTTTACTCCACTGCACATCATGATA-----------
------
FC88X_00693_00155      ----------------ACTTCTCCTACACTGCACCTCACCATA-----------
------
FC88X_00968_01111      ----------------TCTCCTGATCTTCCACCTCACCATGTG-----------
------
FC88X_00029_00293      ----------------TCTCCTGATCTTCCACCTCACCATGTG-----------
------
FC88X_01033_00709      ----------------TCTTCCCGTATACCACATTACAATGTG-----------
------
FC88X_01117_01154      -----------------CTTCTCCTGTTCTGCTTGACTGCGTGC----------
------
FC88X_01084_00855      -----------------CTTTTATTCTTCTGCTCGTGCGTGTC-----------
------
FC88X_00267_00588      ----------------TCTTCTAGTAAACCGCTTCTCCGTGCC-----------
------
FC88X_00333_00403      ------------------CTTTCCTTCGCGTGGCAACAGTTA------------
------
FC88X_01054_01175      ------------------CTTTCCTTCGCGCGGCAACAGTTATTTC--------
------
FC88X_00156_00995      --------------CCCCCTTTTCTCCTCTCTGCAACATGTA------------
------
```

FC88X_00024_00081 -----------TTTCATCTTTTCCTCCCC-GAGCATCAT---------------
------
FC88X_00027_00733 ----------------TCTTTTCATCCCCTGATCAACATTGTTCTT--------
------
FC88X_00855_01240 ----------------TCTTTCCCTCACC-GTACAACTCT--------------
------
FC88X_01115_00824 ----------------TCTTTCCCTCACC-GTACAACACTTTTC----------
------
FC88X_00505_00645 ----------------TTTTCCTGTTCCCTTCACTACAATTGT-----------
------
FC88X_00524_00155 ----------------TTTTCCCTCTTACACTGCAACGATTGT-----------
------
FC88X_00573_00118 --------------TCTCCTGTTCCTCACATTGCA-CTCGTG------------
------
FC88X_00844_00715 ----------------TCCTCAACCTTACACTGCAGCGCGTGC-----------
------
FC88X_00544_00712 ----------------CTTTTCGCTGTTCACCATCGTGGTTAC-----------
------
FC88X_00324_01012 ----------------CTTTTCGCTGTTCACCATCGTGGTTAC-----------
------
FC88X_00466_01163 ----------------CTTTTCGCTGTTCACCATCGTGGTTAC-----------
------
FC88X_00197_00856 ---------------GTTCTTCGCTGTTCACTCCCATCATGGT-----------
------
FC88X_00490_00077 ------------------CTTCCCTTTTCCCCAACATG----------------
------
FC88X_01119_00816 -------------------TTCCCTTTTCTCCATCCTAACCTTGT---------
------
FC88X_00577_00912 ----------------CTACCCTTCTTTCCACAG-ACACCTTTT----------
------
FC88X_00912_00988 ----------------CCATCACCTATTTTCCAC-AGACCTTTT----------
------
FC88X_00516_00733 ---------------CTCCCCTTTTACTCACTGC---ACCTTTT----------
------
FC88X_00798_00211 --------------TCTCCCCATTTTTTCAGCGCCAAACCATTTC---------
------
FC88X_01094_00673 ---------------CTTCCCTTTTCCCCACAGC--AACCAATG----------
------
FC88X_00031_00542 ----------GGGGGAATTTATGC-TCTAT-CTCGCTGCGAACCTTT-------
------
FC88X_00037_00162 ------------------TTATGC-TCTAT-CTCGCTGCGAACCTTT-------
------
FC88X_00143_00023 ----------------ACTTATTC-TCTAT-CTCTCTGTAATCC----------
------
FC88X_00972_01174 ------------------CCATCC-CCTATCCTCTCTGCAGTCGA---------
------
FC88X_00501_00064 -----------------TCTATCCACCTATGCACTCC-CAGTCGC---------
------
FC88X_00679_00254 -----------------CCTCACGCTATGA-TTT--TCCTGTCACGC-------
------
FC88X_00854_00834 -----------------CCTAACGCTATAC-TGTAATGCTGTCTC---------
------
FC88X_00370_00690 -----------------CCTATCCCTTTGC-TTTCCTACTGTCTT---------
------
FC88X_00025_00683 -----------------TCCTTCTCTCTCCA-ACTTTACGTTATA---------
------

```
FC88X_01043_01017       -----------------TCCTTCTCTCTCCA-ACTTTACGTTATACTTCCTT--
------
FC88X_01028_00339       -----------------TCTATCTCTCTCCTTACTCCACGTA------------
------
FC88X_01009_00685       -----------------TCTATCTCTCTCCTCACTCCACGTGTT----------
------
FC88X_00354_00574       --------------AGTAACATTCTATCTCGTACCTCAGGCC------------
------
FC88X_00449_00095       --------------CCTTTCAACCT-CCTCACCGTTCGGGCC------------
------
FC88X_00785_00808       -------------------TTTTCTACTCCCTACCTTACGCCAAT---------
------
FC88X_00094_00574       --------------CCACATTCTCATTCCCTCGCCTTTTGC-------------
------
FC88X_00450_00008       --------------CCACATTCTCATTCCCTCGCCTTTTTC-------------
------
FC88X_00047_00812       --------------CCACGTTCTCGATACCAACGCTTTTCT-------------
------
FC88X_01004_00480       -----------------CTTTCTCGTCCCCACGCTTCTTGCACA----------
------
FC88X_00059_00601       -------------------TTTCCTTTCCCTCGCGCTGCTCACCGC--------
------
FC88X_00681_00992       ---------------CCTGTTCCTATTCCCTCGCGCTTCCCAT-----------
------
FC88X_00111_00030       -----------------AATTTCTATTCTCTCGTGT-GATGCTTGT--------
------
FC88X_01058_01000       ------------------ATTTCTATTCTCTCGTGT-GATGCTTGT--------
------
FC88X_01075_01246       ------------------ATTTCTATTCTCTCGTGT-GATGCTTGT--------
------
FC88X_00879_00680       ------------------
ATTTCTATTCTCTCGTGTCGATGCTTGTCCTTCCTT------
FC88X_00890_00301       -------------------ATTCTATTCTCTCGTGT-GATGCTTGT--------
------
FC88X_00847_00293       ----------------AATTTTCTATTCTCTCGTGT-GATGCTTGT--------
------
FC88X_00431_00126       -----------------GCTTTCTATCCACTCTCTT-GACACTTG---------
------
FC88X_00055_00064       ---------------TTCGCTGTTCCTATCAATCCACCTGTT------------
------
FC88X_00056_00765       ---------------TTCTCCAATCTTCTTATTGCGCCTGTT------------
------
FC88X_00283_00783       ---------------ACCACTATTCTATTCCCTTCGCCTGTC------------
------
FC88X_00236_00006       ------------------ACTATATTCCTACTCCCCGCAGCTACA---------
------
FC88X_00533_00386       ------------------ACTATATTCCTACTCCCCGCAGCTACA---------
------
FC88X_00173_00066       -----------------CGCACTACTTTCCACCCCCGCTGTTCC----------
------
FC88X_00647_00961       -----------------ACCAATTTTCTATTCCCGCACAGTTACAG--------
------
FC88X_00733_00538       -----------------ACCAATTTTCTATTCCCGCACAGTTACAG--------
------
FC88X_00718_01212       -----------------ACCAATTTTCTATTCCCGCACAGTTACAG--------
------
```

```
FC88X_00842_00201       -----------------ACCAATTTTCTATTCCCGCACAGTTACAG--------
------
FC88X_00134_00801       -----------------ACCAATTTTCTATTCCCGCGTAGTTACAG--------
------
FC88X_01030_01177       -----------------ACCAATTTTCTATTCCCGCGCAGTTACAG--------
------
FC88X_00277_00078       -----------------ACCAATTCTCTATTCCCGCACAGTTACAG--------
------
FC88X_00970_00812       ------------CTAACGACTATTCACTATTGCCCTGCGCTT------------
------
FC88X_00874_00756       ------------CTAACGACTATTCTCTATTGCCCTGCGCTT------------
------
FC88X_00458_00095       ------------CTAACGACTATTCCCTATTGCCCTGCGCTT------------
------
FC88X_01042_01049       ---------------CTTACGAATTCCCAACGTCCCGCGCGC------------
------
FC88X_00730_01050       ------------CACTTTACTATTC-CTCCTTGTCGCGAC--------------
------
FC88X_01084_00407       ------------TACTTCACTATTC-AGACTGCCTGGCGC--------------
------
FC88X_00108_00511       ------------------CACCGTCTCTACGACTCCTG--TCCTCCC-------
------
FC88X_00832_01137       --------------------TCTTCTCCACTACGCCTGGGTACTCCC-------
------
FC88X_01025_00639       ----------------------TTGTCTACTAGCCT-GCCTCCGCGTCCC----
------
FC88X_01013_01040       ---------------------CAACTCTATTCGCCTAGCCTCTCCGTTCT----
------
FC88X_00485_00534       -----------------CCCTAGTCTCTACTCATCCTGCGTTA-----------
------
FC88X_00070_00628       ----------------CCGCTAGT-TCTCTACAGCCTGCTTACTT---------
------
FC88X_00941_00391       -------------------CATGCGACCCTACACGCTGCTCAACTCCC------
------
FC88X_00079_00145       ----------------------CCGACTCTTCGC-CTGCT-AATTCCCGCA---
------
FC88X_00650_01038       -----------------AACTATTCCCTTCTCATC-CACTGTGA----------
------
FC88X_00965_00244       -----------------AACTATTCCCTTCTCATC-CACTGTGA----------
------
FC88X_00967_00786       -----------------AACTATTCCCTTCTCATC-CACTGTGA----------
------
FC88X_00488_01106       -----------------AACTATTCCCTTCTCATC-CACTGTGA----------
------
FC88X_00176_00885       -----------------AACTATTCCCTTCTCATC--ACTGTGA----------
------
FC88X_00736_00843       -----------------AACTATTCCCTTCTCATCACCTTGTGTACT-------
------
FC88X_00156_00163       ---------------CCACTATTCGCCTGCTCATCCCGAGATC-----------
------
FC88X_00931_00975       ---------------CCAACTCTTGGCCACGCATCCCGGTAT------------
------
FC88X_00657_01101       -------------------CGTAATACTTCTCCCTACGCTTCCACTC-------
------
FC88X_00943_01177       -------------------CGTAATACTTTTCCCTACGCTTCCACT--------
------
```

FC88X_00451_00133 ----------------TAACGTAATA-TTCTCTCTGCCAGTCC-----------
------
FC88X_01000_00423 ----------------TGCTAGC-ATAATCCCCTGCCTATTCTT----------
------
FC88X_01080_00549 ----------------TGCTAGC-ATAATCCCCTGCCTATTCTT----------
------
FC88X_00124_00889 ----------------TGCTAGC-ATAATCCCATGCCTATTCTT----------
------
FC88X_00883_00415 ----------------TGCTAGC-ATAATCCCCTGCCTACTCTT----------
------
FC88X_00179_00825 ---------------CTTATAGCTGTA-TCCCCTCTTACCGCCT----------
------
FC88X_00978_00816 ---------------CCACTAGCTGTACTCCCCTGTCTCTGCC-----------
------
FC88X_00710_01090 -----------TCCTGTGTTAGCGCTGT---CCCTTGTTTT-------------
------
FC88X_00150_00080 -----------TCCTGTGTTAGCGCTGT---CCCTTGTTTT-------------
------
FC88X_01061_00488 -----------TCCTGTGTTAGCGCTGT---CCCTTGTTTT-------------
------
FC88X_00945_01169 ------CATGCAGCTATGTTTGCTCTATTTACCCTTCCTTT-------------
------
FC88X_00951_00936 -----------CCGTGTAATAGCGGGTC---TCCTCTTGTT-------------
------
FC88X_01134_00721 ----------TCCTGGTAATATTCAGTT---CCTTTGTTCCC------------
------
FC88X_00918_00488 ---------------CTGTATACTCA-CTGCAGTTTATCTCTC-----------
------
FC88X_01071_01071 ---------------CTGTATACTCA-CTGCAGTTTATCTCTCC----------
------
FC88X_00248_00077 ---------------CTGTATACTCA-CTGCAGTTTATCTCTC-----------
------
FC88X_00347_00639 ---------------CTGTATACTCA-CTGCAGTTTATCTCTC-----------
------
FC88X_00660_00913 ---------------CTGTATACTCA-CTGCAGT--------------------
------
FC88X_00311_00004 ---------------CTGCAAACTCCTCTCCAGTATCTTACT------------
------
FC88X_01013_01213 ---------------CTGTGCACTCACATCCGGCTTTGTCTC------------
------
FC88X_00153_00614 ---------------CTGTGCACTCACATCCGGCTTTGTCTCT-----------
------
FC88X_00657_00383 ---------------CTGTGCACTCACATCCGGCTTTGTCTC------------
------
FC88X_00246_00033 ---------------CTGTGCACTCACATCCGGCTTTGTCTC------------
------
FC88X_01012_00533 ---------------CTGTGCACTCACATCCGGCTTTGTCTCC-----------
------
FC88X_00881_00614 ---------------CTGTGCACTCACATCCGGCTTTGTCTC------------
------
FC88X_00375_00718 ---------------CTGTGCACTCACATCCGGCTTTGTCTCATCAC-------
------
FC88X_01067_00652 ---------------CTGT-TATCTCCATTCCGCAATTTTTCT-----------
------
FC88X_00911_00545 -----------TCCATCCAGCATACTGC-CCTGCCTTATT--------------
------

FC88X_00984_00382 -----------TCCATCCAGCATACTGC-CCTGCCTTATT--------------
------
FC88X_00903_00785 ---------------TCGTTCCCTCGACACTCGGCCCACCCGC-----------
------
FC88X_00957_00358 -----------TCCTTCCTCTCTACTGTACTTGGCTTACCT-------------
------
FC88X_00557_00666 ----------TTCCATCCGCTGCCGTTCTATCGTCCC-----------------
------
FC88X_00663_00546 ----------TCCTTCCCTGCGTCGTCCTAACATCTC-----------------
------
FC88X_00978_00690 --------CCGGCTTCCCTCCAGTGGTTCTCTATA-------------------
------
FC88X_01014_01217 -----------TCTTTCCTCCAGTGCTTGTATGTCCC-----------------
------
FC88X_00137_00482 ---------------TTTTCCACTGTTCCCTCGCGTTCTTAT------------
------
FC88X_00158_00949 ---------------TTTTCCACTGTTCCCTTGCGTTCTTAC------------
------
FC88X_00550_00753 ---------------ATCTCCAAACTTTCCTCCCGT-CTTACA-----------
------
FC88X_00031_00212 --------------CCCTACCAGCGTTCGTGC----------------------
------
FC88X_00110_00395 ---------------CCCTCCAGCGTTTGTGCGCGTATTCTC------------
------
FC88X_01122_00872 --------------CCATCCCGGCTTTCATCCCGTCTTCGC-------------
------
FC88X_00575_01095 ---------------TCCTCCACTATTTCCCCC-TACGACCGT-----------
------
FC88X_00995_01023 ---------------TCCCCCACTATTTCCTCT-TACGACCGT-----------
------
FC88X_00670_00928 ----------------CCTCCACACTTTCCTCGGTCCGACTGTT----------
------
FC88X_00843_00927 -----------------CTCCTCAGTCTTTCCTCTTGCTATGTT----------
------
FC88X_00997_00544 ----------GGGGACTCTCCATCACTCTTTCCGTGGCTATA------------
------
FC88X_00145_00019 -----------------CCTTACGCTTTCCTTCGCTCCCATAAC----------
------
FC88X_00324_00300 -----------------CCACAAGCTTTCTTCTATCATATTACC----------
------
FC88X_00495_00809 ----------------TCCCCTGCTATTCTCAATGACCTTTGT-----------
------
FC88X_00616_01236 ----------------TCCCCTGCTATTCTCAATGACCTTTGT-----------
------
FC88X_00998_01148 ----------------CCTCCGCTTACTCAACCTGTCCTTTGTTC---------
------
FC88X_00374_00033 ----------------CCCTGCATTCTCTCAACCGTTCTTCGT-----------
------
FC88X_00487_00262 ---------------GTGTACCCTCCTTCTGC-CAACTCTTGCT----------
------
FC88X_00543_00637 ------------------TCCCCGCATTTTTCACAACTCTTTCA----------
------
FC88X_00818_00558 -------------------CTGCTCCCTTACTTCTGTTTACCCT----------
------
FC88X_00879_01197 --------------CCTGTCTATTCCCT--CTACTGTTTTCCC-----------
------

```
FC88X_00861_00163       ---------------CTAATCTGCTTCCT-ATCCTCGCTTACC------------
------
FC88X_00076_00310       ----------------CGTCTCTTCCTTTCCTCCCTTCTCCC-------------
------
FC88X_00695_01036       ----------------------CTTCCTTTCTCCCTTTTCCC-------------
------
FC88X_00279_01121       -CACAGATGTCGAGTC--GCT-TCCTTTTCTCCCTTCTCCC-------------
------
FC88X_01030_00648       --ACAGATGTCGAGTTCGACTGTTCCTTTCTCCCTTCTCCC-------------
------
FC88X_00714_01173       ------TCGA-T-CGACTAGCTTTCCTTTCTCCCTTCTCCC-------------
------
FC88X_00970_01260       ---ATGTCGAGT-CGACTAGCTTTCCTTTCTCCCTTCTCCC-------------
------
FC88X_00785_01182       ----------------ACTAGCTTTCCTTTCTCCCTTCTCCC-------------
------
FC88X_01106_01174       -AGATGTCGA-G-TGACTAGCTTTCCTTTCTCCCTTCTCCC-------------
------
FC88X_00798_01239       ---ATGTCGAGT-CGACTAGCTTCCTTTTCTCCCTTCTCCC-------------
------
FC88X_01069_00962       ----TGTCGAGTTCGACTAGCTTC--TTTCTCCCTTCTCCC-------------
------
FC88X_00786_00101       ------------GTCGACTAGCTTCCTTTCGTCCCTTCTCCCCCC----------
------
FC88X_01136_01052       --------------CGACTAGCTTCCTTTC-TCCTTCGTCCC-------------
------
FC88X_01056_00997       ----------------------TTTCCTTTCTCCCTTCTCCCT------------
------
FC88X_01082_00764       ---CGCAACCCCTGCCTGTGTCTTCCTTTCTCCCCTTCTCCC------------
------
FC88X_00743_00880       --------------------------------TCCCTTCTCCCCC-----------
------
FC88X_00312_00498       ------------------------CTCTTTCTCCCTTCTCCCCC-----------
------
FC88X_00659_00165       -------------TTCCTTCCTTCCCCTTTCTCCCTTCTCCC-------------
------
FC88X_00679_01042       -----------------CCTTCTCCCCTTTCTTTCTCTCCC--------------
------
FC88X_00392_00079       --------------------CCCCCCCTTTCTCCCTTCTCCCT------------
------
FC88X_00360_01135       -----------------TCCTTCTCCCTTCCTTTCTCCTTCTCCC----------
------
FC88X_00964_00715       --------------TCTCCCTTCTCCCTTCCTTTCTCCTCTCCC-----------
------
FC88X_01124_00649       -------------------CTTCTCCCCTTCTCTCCCTTCTC-------------
------
FC88X_00143_00529       ------------------CTTTCCCCTCCTCTCCTATCCCATTTA----------
------
FC88X_01132_00762       -------------------CCCCCTCCTGTATTCCTTCCCACTTAC---------
------
FC88X_00153_00933       ---------------AGCTTAGATCTTGGC-TCACGTTTTCTC------------
------
FC88X_00988_00926       ---------------AGCTTAGATCTTGGC-TCACGTTTCTCTC-----------
------
FC88X_00415_00809       ---------------AGCTTAGATCTTGGC-TCACGTTTTCTC------------
------
```

FC88X_01026_00858   --------------AGCTTAGATCTTGGC-TCACGTTTTCTC------------
------
FC88X_01115_00782   --------------AGCTTAGATCTTGGC-TCACGTTTTCTC------------
------
FC88X_00069_00129   --------------AGCTTAGATCTTGGC-TCACGTTTTCTC------------
------
FC88X_00224_00945   --------------AGCTTAGATCTTGGC-TCACGTTTTCTC------------
------
FC88X_00278_00560   --------------AGCTTAGATCTTGGC-TCACGTTTTCTC------------
------
FC88X_00405_00113   --------------AGCTTAGATCTTGGC-TCACGTTTTCTC------------
------
FC88X_00510_01015   --------------AGCTTAGATCTTGGC-TCACGTTTTCTC------------
------
FC88X_00884_00349   --------------AGCTTAGATCTTGGC-TCACGTTTTCTC------------
------
FC88X_00923_00417   --------------AGCTTAGATCTTGGC-TCACGTTTTCTC------------
------
FC88X_01096_00978   --------------AGCTTAGATCTTGGC-TCACGTTTTCTC------------
------
FC88X_01122_00694   --------------AGCTTAGATCTTGGC-TCACGTTTTCTC------------
------
FC88X_01140_01084   --------------AGCTTAGATCTTGGC-TCACGTTTTCTC------------
------
FC88X_00991_00592   --------------AGCTTAGATCTTGGCCTCACGTTTTCTC------------
------
FC88X_00572_00201   --------------AGCCTAGATCTTGGC-TCACGTTTTCTC------------
------
FC88X_00834_00382   --------------AGCTTAGATCTTGGC-TCACGCTTTCTCCC----------
------
FC88X_00604_01043   --------------AGCTTGGATCTTGGC-TCACGTTTTCTC------------
------
FC88X_01093_01221   --------------AGCTTAGATCCTGGC-TCACGTTTTCTC------------
------
FC88X_00112_00435   --------------AGCTTAGGTCTCGGC-TCACGTTTTCTC------------
------
FC88X_00066_00061   --------------AGCTCAGATCTTGGC-TCACGTTTTCTCCTTCC-------
------
FC88X_00981_01067   ----------------CCAAGAGCTCGGCCACGGCTACTTTCCCTTCCT-----
------
FC88X_00430_00988   -------------GCAATGAGTTCTAACCCTGCCGTTCTCCCTTCCTTT-----
------
FC88X_00476_00175   -----------------------CTTACTCTCCCTTTCTCCCTTC---------
------
FC88X_00357_01141   ------------CGCTACGCTCTTTTTTTCCCTTGTATA---------------
------
FC88X_01049_00509   -------------CCGGCTAACTTTTTCCATCCTGTATTCT-------------
------
FC88X_00633_01240   -----------------TTTCATCTTTTTCTCTCCTCCATAGTTAC--------
------
FC88X_01074_00829   ----------------TCGACCTCTTTTTCACCCTTCGATAATT----------
------
FC88X_00011_00424   -------------CACGA-CTTTTTCGGCATCGCTATAATCTC-----------
------
FC88X_00161_00718   -------------CACGA-CTTTTTCGGCATCGCTATAATCT------------
------

```
FC88X_00091_00791       -------------CACGA-CTTTTTCGGCATCGCTATAATCTC-----------
------
FC88X_00056_00854       -------------CACGA-CTTTTTCGGCATCGCTATAATCTC-----------
------
FC88X_00218_00007       -------------CACGA-CTTTTTCGGCATCGCTATAATCTC-----------
------
FC88X_00402_00894       -------------CACGA-CTTTTTCGGCATCGCTATAATCTC-----------
------
FC88X_00636_01036       -------------CACGA-CTTTTTCGGCATCGCTATAATCTC-----------
------
FC88X_00787_00675       -------------CACGA-CTTTTTCGGCATCGCTATAATCTC-----------
------
FC88X_00092_00188       -------------CACGA-CTTTTTCGGCATCGCTATAATCTC-----------
------
FC88X_00103_00726       -------------CACGA-CTTTTTCGGCATCGCTATAATCTC-----------
------
FC88X_00181_00808       -------------CACGA-CTTTTTCGGCATCGCTATAATCTC-----------
------
FC88X_00201_00136       -------------CACGA-CTTTTTCGGCATCGCTATAATCTC-----------
------
FC88X_00224_00904       -------------CACGA-CTTTTTCGGCATCGCTATAATCTC-----------
------
FC88X_00400_01011       -------------CACGA-CTTTTTCGGCATCGCTATAATCTC-----------
------
FC88X_00486_01197       -------------CACGA-CTTTTTCGGCATCGCTATAATCTC-----------
------
FC88X_00506_01240       -------------CACGA-CTTTTTCGGCATCGCTATAATCTC-----------
------
FC88X_00683_01078       -------------CACGA-CTTTTTCGGCATCGCTATAATCTC-----------
------
FC88X_00794_01226       -------------CACGA-CTTTTTCGGCATCGCTATAATCTC-----------
------
FC88X_00895_00247       -------------CACGA-CTTTTTCGGCATCGCTATAATCTC-----------
------
FC88X_00962_00733       -------------CACGA-CTTTTTCGGCATCGCTATAATCTC-----------
------
FC88X_01079_00638       -------------CACGA-CTTTTTCGGCATCGCTATAATCTC-----------
------
FC88X_01098_00653       -------------CACGA-CTTTTTCGGCATCGCTATAATCTC-----------
------
FC88X_01116_01159       -------------CACGA-CTTTTTCGGCATCGCTATAATCTC-----------
------
FC88X_00596_01235       ------------GCACGA-CTTTTTCGGCATCGCTATAATCTC-----------
------
FC88X_00205_00830       -------------CACGA-CTTTTTCGGCATTGCTATAATCTC-----------
------
FC88X_00217_00980       -------------CACGA-CTTTTTCGGGATCGCTATAATCTC-----------
------
FC88X_00116_00815       -------------CACGA-CCTTTTCGGCATCGCTATAATCTC-----------
------
FC88X_00076_00167       -------------CACGAATTTTTTCGGCATCGCTATAACCTC-----------
------
FC88X_00936_00757       -------------CACGA-TTTTTTCGGCATCGCTATAATCTC-----------
------
FC88X_00023_00667       -----------CTCAACACTTTTCCCCGCAACGCTATC----------------
------
```

FC88X_00860_00873 -----------CTCAACACTTTTCCCCGCAACGCTATC----------------
------
FC88X_00883_01242 -----------CTCAACACTTTTCCCCGCAACGCTATC----------------
------
FC88X_00983_00461 -----------CCCAACACTTTTCCC-GCAACGCTATC----------------
------
FC88X_00918_01133 -----------GCCATCAATCTATTCCGCCTCACTATC----------------
------
FC88X_00089_00254 --------------ACCAACATGTTTTTC-TCCCCACTGTC-------------
------
FC88X_00800_00796 --------CGTAACACGATCACGTTTTTC-ACGCCACA----------------
------
FC88X_00163_00892 -------------CTACAACCTGTTCGCTCTCCCAATGCG--------------
------
FC88X_01139_00705 -------------CTACCTCCTGT--ACTCCCGCAATGTGACC-----------
------
FC88X_01065_00820 -------------CTCCCTGTTTCTCGCACCGATGATGCA--------------
------
FC88X_00890_00841 -------------CTCCCTGTTTCTCGCACCGATGATGCA--------------
------
FC88X_00990_00810 ----------------TCTCCTTCTCGTCACCAATATGCGCAT-----------
------
FC88X_00971_00449 -------------CTTTCTCCTCACTGTTGCTAAAATATC--------------
------
FC88X_00442_00642 -------------CTTGTCCCTCACCATTGTGAAAATCACCC------------
------
FC88X_00200_00439 -------------CTTCTTTCCTTCCCCAATGAGAATAAA--------------
------
FC88X_00047_00126 -----------------ATAATGCTTTTCTCGTTAT-CGTACTCTC--------
------
FC88X_00829_00792 -----------------ATAATGCTTTTCTCGTTAT-CGTACTCTC--------
------
FC88X_00658_01176 ------------------------ACTACACGTTATGCCTACTCT---------
------
FC88X_00699_00166 ----------------CAAAACGTTCTTCCCCATTTGCCTACT-----------
------
FC88X_00335_01126 ----------------CAAAACGTTCTTCCCCATTTGCCTACT-----------
------
FC88X_00775_00818 ----------------CAAAACGTTCTTCCCCATTTGCCTACT-----------
------
FC88X_00777_01137 ----------------CAAAACGTTCTTCCCCATTTGCCTACT-----------
------
FC88X_01027_00537 ----------------CAAAACGTTCTTCCCCATTTGCCTACT-----------
------
FC88X_00199_00146 ---------GGGGAAGCAAAACGTTCTTCCCCATTTGCCTACTC----------
------
FC88X_01132_00943 ------------------AAACGTTCTTCCCCATTTGCCTACT-----------
------
FC88X_01065_00971 ----------------CAAAACGCTCTTCCCCATTTGCCTACT-----------
------
FC88X_00902_00988 ----------------CAAAACGTTCTTCCCCATCTGCCTACT-----------
------
FC88X_01012_01028 ----------------CACATTGCTTTTCCCCTTTTCCCTATA-----------
------
FC88X_00505_01167 -----------------------CCTTTCCCTCTTTGGC--CTCACACTATC--
------

FC88X_00841_01232 ------------------------CATTATCACTTTGTCA-CTGACGCTTTCT-
------
FC88X_00103_00191 -----------------------GCATTCTCGCTTTGCCTACTCACCCTG----
------
FC88X_00877_00761 ---------------------TATCACGCCCCGTATAGTTACTTTCTC------
------
FC88X_00893_00998 ---------------------TATCACGCCCCGTATAGTTACTTTCTC------
------
FC88X_00497_00552 ---------------------TATCACGCCCCGTATAGTTACTTTCTC------
------
FC88X_00164_00820 -------------GGGGGAATTATCACGCCCCGTATAGTTACTTTCTC------
------
FC88X_01140_00637 -------------------CTAATCTCTCTCTGTA-AGTCATTTCTC-------
------
FC88X_00886_00745 -------------------------TCTCTCCGTGTCCTTACTCTAATTGTC--
------
FC88X_01067_00955 ------------------------CTCTCTCCTTTACGTTA-TCTATCTGC---
------
FC88X_00575_00986 ----------------------------
CCAATCACTCTCCTTCTCCGTCATCCGC----
FC88X_00736_01159 ----------------------------CCCATTATTGTCCTTCTCCC------
------
FC88X_01116_00925 -----------------------TCTTCCCACCCACAATCGCTCTTTCTCC---
------
FC88X_00657_01107 ----------------------TCCTTCCCGTCTACCGCTCCACTGACC-----
------
FC88X_00891_00712 ---------------------TTCCCTACCCTTTATCGCTACAGTGTC------
------
FC88X_00021_00326 -----------------TTTTGTACTATTTCTCT-GCGTTGTTGC---------
------
FC88X_00720_01243 ----------------GTTTTGTACTATTTCTCT-GCGTTGTTGC---------
------
FC88X_00090_00320 -----------------TTTTGTACTATTTCTCT-GCGTTGTTGC---------
------
FC88X_00829_01010 -----------------TTTTGTACTATTTCTCT-GCGTTGTTGC---------
------
FC88X_00625_01023 -----------------TTTTGTACTATTTCTCT-GCGTTGTTGC---------
------
FC88X_00903_00966 -----------------TTTTGTACTATTTCTCT-GCGTTGTTGC---------
------
FC88X_01047_00581 -----------------TTTTGTACTATTTCTCT-ACGTTGTTGC---------
------
FC88X_00912_00923 -----------------TTTTGTACTATTTCTCT-GCGCTGTTGC---------
------
FC88X_00987_01215 ---------------TCCCAAG-ATGATTTCAATCTCGCTGTT-----------
------
FC88X_00506_00059 ---------------TCCCAAG-ATGATTTCAATCTCGCTGTT-----------
------
FC88X_00970_00685 ---------------TCCCAAG-ATGATTTCAATCTCGCTGTT-----------
------
FC88X_00122_00074 ---------------TCCCAAG-ATGATTTCAATCTCGCTGTTCTTCCTTT---
------
FC88X_00066_00121 ------------ATATCCCATGTACGATGTCTGT-TCGCT--------------
------
FC88X_00572_00684 ------------ATATCCCATGTACGATGTCTGT-TCGCT--------------
------

```
FC88X_00029_00576       -----------------CCAATTGTTTTCCTTCTTTCAGTTCAT----------
------
FC88X_00115_00303       ----------------CTCAATTCTCTTTCT-CTGACAATTCTT----------
------
FC88X_01091_00708       ---------------TTGTCCATTTCTCTACT--CTAGCTATTT----------
------
FC88X_01131_00687       ---------------TTGTCCATTTCTCTACT--CTAGCTATTT----------
------
FC88X_00089_00778       ---------------TTGTCCATTTCTCTACT--CTAGCTATTT----------
------
FC88X_00824_00906       ---------------TTGTCCATTTCTCTACT--CTAGCTATTT----------
------
FC88X_00704_00080       ------------------CCAATTGCCCTCCTTCCTAACTAATT----------
------
FC88X_00372_01014       --------------------ACATGTCTCCTTCTCAAGCTTTGTTTT-------
------
FC88X_00925_00559       -------------------CACTTCTTACCTCCTCACGCTGTTGTT--------
------
FC88X_01005_00515       ----------------TGTCACTGTTTCCCTTCTTGCTCTGTT-----------
------
FC88X_01046_01064       ------------------CCATTTTTCCTGATGCTCAGCTGTTTT---------
------
FC88X_00634_01023       ------------------CCATTTTTCCTGATGCTCAGCTGTTTT---------
------
FC88X_00909_00754       ------------------CCATTTTTCCTGATGCTCAGCTGTTTT---------
------
FC88X_00072_00858       ------------------CCGTTTTTCCCGATGCTCAGCTGTTTT---------
------
FC88X_00612_00415       -----------------CCATTTTTTCCTGATGCTCAGCTGTTTT---------
------
FC88X_00787_00818       ---------------CTTTTGTCTCTCCTGACTCAC-GCTGTT-----------
------
FC88X_00112_00614       ------------------CCGATACTTCTCCTATATCGCGTTGCC---------
------
FC88X_00911_00195       --------------TTTTCGGTTATTCTGCCTATA---CGTTGCC---------
------
FC88X_00057_00031       ------------------CCGTACTTTTCCCTGTATCGAG--------------
------
FC88X_00131_00765       ------------------CCGTACTTTTCCCTGTATCGAG--------------
------
FC88X_00608_01230       ------------------CCGTACTTTTCCCTGTATCGAG--------------
------
FC88X_00852_01018       ------------------CCAATATCTTGCCCTAATCGAGCTCTT---------
------
FC88X_00370_01114       ------------CGACCATGTCTTCTCCCGTCATATCTC---------------
------
FC88X_00925_00292       ------------CGACCATGTCTTCTCCCGTCATATCTC---------------
------
FC88X_00969_00387       ---------------CCATGAAATTTCCCG-CATCTCGGTGTT-----------
------
FC88X_00188_00982       -----------TTGACAATTCTCTGCTCCCG-ATATCGA---------------
------
FC88X_00879_00341       ----------------TTCTCCATTCTCCTGCATATCGATAGG-----------
------
FC88X_00531_01208       --------------CTATCAACTATCCTCCACTTATCGAAT-------------
------
```

```
FC88X_00213_01070      ----------------CTCACA-GTCTGCTAAATCCTTTTTCAT----------
------
FC88X_00957_00645      ---------------CCTCACACGCTTGCTA--TCCATTTTCAC----------
------
FC88X_00402_01086      ------------------CTCACGTCTGCTCT---CCTTCCAACCTCA------
------
FC88X_00855_00779      ---------CCCGAAACTCCCGTGACTGCACTAAACCTTCCTTTCTC-------
------
FC88X_00506_01016      ----------------CCTCCACGTTCGCTCTCTGCCAAATTCT----------
------
FC88X_00102_00466      ----------------TTCCCAACCTCATTA---CTCCCTGTGTATT-------
------
FC88X_00407_01083      ----------------TTCCCAACCTCGTTA---CTCCCTGCGTATT-------
------
FC88X_00272_00727      -----------------ACCGAACTTCGGTAATCCTCCCTGTGC----------
------
FC88X_00226_00954      ----------------CCCCCAACCTAGTTGTCT--CCCTGTGCTT--------
------
FC88X_00735_00462      -------------------CCAACTTAGTTCTCCACCCCTGTGCTCCC------
------
FC88X_01108_00865      ---------------------CACCACGTTGACTCCCCGTG-TGATAGT-----
------
FC88X_00919_00892      ---------------------CACCACGTTGACTCCCCGTGGTGATAGT-----
------
FC88X_00437_01139      ----------------------ACCACATT-TCTCTCCCTGCACAACACG----
------
FC88X_00946_00254      --------------------CCATCACATAATCTCTCCCTGTTGAAC-------
------
FC88X_00759_00124      ---------------------CAACTCGCTGTTCCCTGACGATGAAAAC-----
------
FC88X_01072_00930      ------------------CTCCTACTC-CTGTATCCGCGCGATGAT--------
------
FC88X_00752_01004      -------------------TCAACTTCTCTCTTCATGGCTGATGCC--------
------
FC88X_00680_00611      -------------------CCAACTTCTCTCTTCATG-CTGATGCC--------
------
FC88X_00839_00975      -----------------CTTCAAATTCCCTCTCCGCG--TGATGCGC-------
------
FC88X_00206_01003      ----------------TTTTCGACCCCC-ATTACACTGCTTTTCT---------
------
FC88X_00520_00454      ----------------TTTTCGACCCCC-ATTACACTGCTTTTCT---------
------
FC88X_00553_00833      ----------------TTTTCGACCCCC-ATTACACTGCTTTTCT---------
------
FC88X_01006_01096      ----------------TTTTCGACCCCC-ATTACACTGCTTTTCT---------
------
FC88X_01086_01075      ----------------TTTTCGACCCCC-ATTACACTGCTTTTCT---------
------
FC88X_01028_00303      ----------------TTTTCGACCCCC-ATTACACTGCTTTTCT---------
------
FC88X_00062_00120      ----------------TTTTCGACCCCC-ATTACACTGCTTTTCT---------
------
FC88X_00152_00659      ----------------TTTTCGACCCC--ATTACACTGCTTTTCT---------
------
FC88X_00025_00174      ----------------TTTTCGACCCCC-ATTACACTGCTCTTCT---------
------
```

```
FC88X_00375_01175     ----------------TTTTCGACCCCC-ATTACATTGCTTTTCT---------
------
FC88X_00063_00808     ----------------TTTTCGACCCCC-ATTACGCTGCTTTTCT---------
------
FC88X_00639_00970     ----------------TTTTCGACCCCC-ATCACACTGCTTTTCT---------
------
FC88X_00425_00951     ----------------TTTTCGACCCCCCATTACACTGCTTTTTCTC-------
------
FC88X_00827_01030     ----------------TTTTCGACCCCC-ACTACGCTGCTTTTCT---------
------
FC88X_00572_01114     ------------------CTCAACCCCC---CGTACTGTTTTCTCCAG------
------
FC88X_00827_00741     -------------GGGAAGTTTACCCCCGTACTCCCCGATTTCTCA--------
------
FC88X_00843_01254     --------------------TGACGCTCCAACTCACCGCTACTTCTC-------
------
FC88X_00681_01255     ---------------CTTCTTGCCCACT--ACCGCTACAGTTTTT---------
------
FC88X_00678_00941     ---------------TTCTTCGAGTACT--CCCTTCTGATTTTT----------
------
FC88X_00150_00947     ---------------CCTCTGG-CCGAG--CCCCGCTAATCTGTT---------
------
FC88X_00779_00239     ---------------CCACTGGTCCGAC--TCCCCCTTCATTGTT---------
------
FC88X_00050_00479     ------------------TTTTACCGAC--TCCCGC-ACTAGTT----------
------
FC88X_00063_00745     ---------------TCCTTTCAGCGAC--CCCGACTACTTTTT----------
------
FC88X_00063_00342     ------------------CCCCACGCTCGACCACAC-TGATATATT--------
------
FC88X_00787_00599     ------------------CACCACGCTCGACCACAC-TGATATATT--------
------
FC88X_00935_00847     ------------------CTCGGCCGACCCCAACAC-TAACATATT--------
------
FC88X_00820_00979     -------------------TTTACGCTAATCCCAACGTATCATATT--------
------
FC88X_00345_01149     ---------------------AGTCAGCGACCCTTCCTACCA------------
------
FC88X_00551_00190     ---------------------CGTTCTCAGCTAATCCCGCCATACCAT------
------
FC88X_01003_00811     -----------------------TTCTCGTCTACTCCCATCCTAACATCC----
------
FC88X_00984_00325     ----------------CTAAAAAAACTCCCCGACACGCAATTTCC---------
------
FC88X_01116_00597     ----------------CTGACAGATATCACCTACA-GCATTTTC----------
------
FC88X_00616_00792     ----------------CTGTAAAACCACCCTCACC-GCAGTATT----------
------
FC88X_00113_00506     ----------------CTTGGTACTCCCGCACACA-GCAATTTT----------
------
FC88X_00977_00975     ----------------CTTGGTACTCCCGCACACA-GCAATGTT----------
------
FC88X_00170_00865     -----------------CAAACTCCCGCTCATCCC-GCAATGGC----------
------
FC88X_00102_00064     -------------------CCCTACTTCCGCCATAGACAGGTTCCC--------
------
```

FC88X_00781_00348 -------------------ACCTATCTCCTGTAATCCACAATTTCCCTTCC----
------
FC88X_00630_00982 -------------------CTAACTCCTCGACTACCGCAATTTTGC--------
------
FC88X_00076_00589 ----------------------TTCTCGACTGACCATCGAGCCGTTCTT-----
------
FC88X_00181_00913 ----------------------TCCTCGGCTGACCAACGGCCCGATTTT-----
------
FC88X_00217_00244 -------------------TACTCTGCTACTAGCCCAAGCACTTTT--------
------
FC88X_00873_00244 -------------------TACTCTGCTACTAGCCCAAGCACTTTT--------
------
FC88X_00806_01205 ----------------------TCCACTACTACCCGACGGCCACTTTGTT----
------
FC88X_00899_01267 ----------------------TCCACTACTACCCGACGGCCACTTTGTT----
------
FC88X_00156_00876 --------------------TCTCTACTACAACAACAGGCCGATTTT-------
------
FC88X_00598_00940 --------------CTCAATTCATCTCCACTTTCCGACGCGC------------
------
FC88X_00817_01267 ---------------TTCTTCTTCTTCCACTGTCCAACGC--------------
------
FC88X_00161_00878 --------------TCCCTGCATTT-CG----ACTC-TCCAACGTT--------
------
FC88X_00573_00287 --------------TCCCTGCATTT-CG----ACTC-TCCAACGTT--------
------
FC88X_01059_00744 ---------------CCTTGCAATA-CGGGCAACTC-CCCAACA----------
------
FC88X_00509_00091 ----------------CCGACAATT-CGA-CTCCCCCGGCAATGTT--------
------
FC88X_00928_00549 ----------------CCGACAATT-CGA-CTCCCCCGGCAATGTT--------
------
FC88X_00704_01027 ----------------CCGACAATT-CGA-CTCCCC-GGCAATGTT--------
------
FC88X_00839_00789 ----------------CCGACAATT-CGA-CTCCCC-GGCAATGTT--------
------
FC88X_00924_00595 ----------------CCGACAATT-CGA-CTCCCC-GGCAATGTT--------
------
FC88X_00935_01090 ----------------CCGACAATT-CGA-CTCCCC-GGCAATGTT--------
------
FC88X_01070_00544 ----------------CCGACAATT-CGA-CTCCCC-GGCAATGTT--------
------
FC88X_00034_00523 ----------------CCGACAATT-CGA-CTCCCC-GGCAATGTT--------
------
FC88X_00065_00206 ----------------CCGACAATT-CGA-CTCCCC-GGCAATGTT--------
------
FC88X_00082_00365 ----------------CCGACAATT-CGA-CTCCCC-GGCAATGTT--------
------
FC88X_00080_00545 ----------------CCGACAATT-CGA-CTCCCC-GGCAATGTT--------
------
FC88X_00128_00302 ----------------CCGACAATT-CGA-CTCCCC-GGCAATGTT--------
------
FC88X_00109_00869 ----------------CCGACAATT-CGA-CTCCCC-GGCAATGTT--------
------
FC88X_00230_00906 ----------------CCGACAATT-CGA-CTCCCC-GGCAATGTT--------
------

FC88X_00299_00533 ----------------CCGACAATT-CGA-CTCCCC-GGCAATGTT--------
------
FC88X_00413_00080 ----------------CCGACAATT-CGA-CTCCCC-GGCAATGTT--------
------
FC88X_00450_01111 ----------------CCGACAATT-CGA-CTCCCC-GGCAATGTT--------
------
FC88X_00624_00339 ----------------CCGACAATT-CGA-CTCCCC-GGCAATGTT--------
------
FC88X_00768_01038 ----------------CCGACAATT-CGA-CTCCCC-GGCAATGTT--------
------
FC88X_00755_01089 ----------------CCGACAATT-CGA-CTCCCC-GGCAATGTT--------
------
FC88X_00846_00791 ----------------CCGACAATT-CGA-CTCCCC-GGCAATGTT--------
------
FC88X_00867_00828 ----------------CCGACAATT-CGA-CTCCCC-GGCAATGTT--------
------
FC88X_00900_00287 ----------------CCGACAATT-CGA-CTCCCC-GGCAATGTT--------
------
FC88X_00937_00472 ----------------CCGACAATT-CGA-CTCCCC-GGCAATGTT--------
------
FC88X_00902_01215 ----------------CCGACAATT-CGA-CTCCCC-GGCAATGTT--------
------
FC88X_00924_01251 ----------------CCGACAATT-CGA-CTCCCC-GGCAATGTT--------
------
FC88X_01064_00945 ----------------CCGACAATT-CGA-CTCCCC-GGCAATGTT--------
------
FC88X_00447_01163 ----------------CCGACAATT-CGA-CTCCCC-
GGCAATGTTCTTCCTTT------
FC88X_01085_00758 ----------------CCGACAATT-CGA--CTCCCCGGCACT-----------
------
FC88X_01008_00640 ----------------CCGACAATTTCGAACTCCCCCGGCAATCGTTCTTC---
------
FC88X_00667_00523 -------------CCTGTTGCAACA-CGA-CTCCCC-GGCACT-----------
------
FC88X_00802_00895 -------------CCTGTTGCAACA-CGA-CTCCCC-GGCACT-----------
------
FC88X_00250_00481 -------------CCTGTTGCAACA-CGA-CTCCCC-GGCACT-----------
------
FC88X_00916_00443 -------------CCTGTTGCAACA-CGA-CTCCCC-GGCACT-----------
------
FC88X_00417_00047 -------------CCTGCTGCAACA-CGA-CTCCCC-GGCACT-----------
------
FC88X_00084_00879 ---------------------CACCCACCGAACTCCCGTGACTATCCC------
------
FC88X_00055_00792 --------------------CCACCCTCCCA-TTCCAGTGATGATCCA------
------
FC88X_00926_00527 ---------------------TCTCCTTTGAACCAGCGACACCATGTC------
------
FC88X_00050_00264 ---------------------TCTCCTTTGAACCAGCGACACCATGTC------
------
FC88X_00491_00401 --------------------CTATCCTCCGAATCC-TGCGAACATGTC------
------
FC88X_00166_00794 -------------------CCGATCCTCCAACTCCCTGGGAATAT---------
------
FC88X_00155_00184 -----------------TTCACCCGATCCTATGTACGACACTTAC---------
------

FC88X_01124_01113 -----------------TTTACCCATTCCT-TGCGACACGTTTGC---------------
FC88X_01123_00931 ------------------TCTCCCCATCT-ACCACGTTTCCGTGTC--------------
FC88X_00189_00421 -------------------CTCCCCACCTGACCACCTAGGCGTTTC--------------
FC88X_00847_00315 ------------------CCCCATCTTCCGACCACGTGTTCGTATT--------------
FC88X_00525_00499 --------------------AGCTAGCTGCGACCAGTGTCCTCCT---------------
FC88X_01030_01152 ---------------------CTCAGCTGCCTAGCGAGCCTTTGTCCC------------
FC88X_00552_01012 -------------------CTCGTACTCCAGAGACCTGCCCATATT--------------
FC88X_00870_01201 -------------------CTCGTACTCCAGAGACCTGCC-ATATT--------------
FC88X_00893_00853 -------------------CTCGTACCTCCCAAGTCTTGCCGTATT--------------
FC88X_00355_01118 -------------------CCGGTACCTCCCGAACCGTGGCTTCTT--------------
FC88X_00191_00853 -------------CTGGGGACCTCCCATACCCCATATCCCTTCCTTT-------------
FC88X_00645_01124 -----------------TCACATCTCATCCCGCACCTTGCTTTT----------------
FC88X_01075_00404 -----------------ACATCTGCTCCCCGTCACTTGGCGTTAT---------------
FC88X_00070_00094 -----------------ACACCTGCTCCCCGTCACTTGGCGTTAT---------------
FC88X_00812_00186 ---------------CTGATCCCGGTCCCTGCTA--TAGCGTTA----------------
FC88X_00481_00081 -------------CCACCGACTATGCTCC-AGTAACTTGCC-------------------
FC88X_01011_00886 --------------TTCCGACACGGCTCCCACTAACTTAAA-------------------
FC88X_00069_00087 -------------AACGAGGACCTCCTCCCGCCACGTTACCC------------------
FC88X_00923_00624 ---------------TACGACCTCCCTTCCACCAGCGTTCC-------------------
FC88X_00959_00863 -------------CCATAGAACCTCCTCCCGCAACATTCC--------------------
FC88X_00599_00996 ---------------TCGGACCTCCCTCCCGTCAACAGCC--------------------
FC88X_00189_00998 ------------------CTCAACCCCC-CGTTACTACCGAATCAT--------------
FC88X_00017_00704 -----------------CTACAACACCCGCGGGACTCCCGAAAC----------------
FC88X_00740_00066 ----------------TCATCAGCTCCCGTCGTACGCCCGACC-----------------
FC88X_00538_01255 ------------------CCCAGCCCGCGTGGTACTGCCCATAGT---------------
FC88X_00039_00374 -------------------ACATCGCCTCGTCTCCCGCACCGTGTAC-------------
FC88X_00497_00993 -------------------ACAACGCGCATGACTCCCCACGGTCCT--------------
FC88X_00079_00313 ----------------CAGTTAACTCCCCTCCACGACGATGGC-----------------

FC88X_00083_00808 -------------------TCAAATCCCTTGCG-GAGGATGGCGTGG-------
------
FC88X_00377_01168 -----------------CCTCTTCCAACCCCCAG-ATGATGGCA----------
------
FC88X_00976_01185 -------------------TCAACAAACCTCCAGCATGATTGGACC--------
------
FC88X_00240_00081 ---------------ACTCCCAATTACTGTCTGACCAAGCTA------------
------
FC88X_01089_00661 ---------------ACTCCCAATTACTGTCTGACCAAGCTAC-----------
------
FC88X_00467_01221 ----------------CTCCCAGATGTCTTTCGACAAATCACC-----------
------
FC88X_00797_01062 -------------CAACTCCCTATCGATGGCTAACCACAC--------------
------
FC88X_00911_01230 ---------TCGACAACTCCCCGATGCTTTCCAAAC------------------
------
FC88X_00414_00078 ------------CTAACTCCCGAAACCTTTCACAAGCCA---------------
------
FC88X_01140_00900 ------------CCAACGCCCATTCGCTTTGACTATCGC---------------
------
FC88X_00478_00271 ----------------TTCGACATTCCCCGACTCCGTGCTATC-----------
------
FC88X_00810_00162 ----------------TTCGACATTCCCCGACTCCGTGCTATC-----------
------
FC88X_01125_00764 ----------------TTCGACATTCCCCGACTCCGTGCTATC-----------
------
FC88X_00335_00690 --------------CATTCGTGATTCCCCAACTCGGCGTTCTA-----------
------
FC88X_00042_00342 ----------------CTGGAGATCTCCCACCTCGGCCTTAT------------
------
FC88X_00065_00059 -----------------CTGTAATTTCCCCAGACAGCGCCGTAGT---------
------
FC88X_00357_00652 -----------------CTGTAATTTCCCCAGACAGCGCCGTAGT---------
------
FC88X_00380_01152 -----------------CTGTAATTTCCCCAGACAGCGCCGTAGT---------
------
FC88X_00695_01253 -----------------CTGTAATTTCCCCAGACAGCGCCGTAGT---------
------
FC88X_00066_00883 -----------------CTGTAATTTCCCCAGACAGCGCCGTAGT---------
------
FC88X_00169_00886 -----------------CTGTAATTTCCCCAGACAGCGCCGTAGT---------
------
FC88X_00407_00822 -----------------CTGTAATTTCCCCAGACAGCGCCGTAGT---------
------
FC88X_00671_00216 -----------------CTGTAATTTCCCCAGACAGCGCCGTAGT---------
------
FC88X_00680_01087 -----------------CTGTAATTTCCCCAGACAGCGCCGTAGT---------
------
FC88X_00915_01192 -----------------CTGTAATTTCCCCAGACAGCGCCGTAGT---------
------
FC88X_01046_00573 -----------------CTGTAATTTCCCCAGACAGCGCCGTAGT---------
------
FC88X_01142_00618 -----------------CTGTAATTTCCCCAGACAGCGCCGTAGT---------
------
FC88X_00100_00054 -----------------CTGTAATTTCCCCAGACAGCGCCGTAGTG--------
------

FC88X_00988_01179 ------------------CTTCGTTTTATTTCCCACCGCACCGGT---------
------
FC88X_00086_00082 ---------------CCGTTTAATCTCCCACTGCGGCA-TATC-----------
------
FC88X_00676_00082 ---------------CCGTTTAATCTCCCACTGCGGCA-TATCCC---------
------
FC88X_00052_00644 ---------------CCGTTTAATCTCCCACTGCGGCA-TATC-----------
------
FC88X_00171_00039 ---------------CCGTTTAATCTCCCACTGCGGCA-TATC-----------
------
FC88X_00151_00071 ---------------CCGTTTAATCTCCCACTGCGGCA-TATC-----------
------
FC88X_00278_00377 ---------------CCGTTTAATCTCCCACTGCGGCA-TATC-----------
------
FC88X_00278_00975 ---------------CCGTTTAATCTCCCACTGCGGCA-TATC-----------
------
FC88X_00457_00174 ---------------CCGTTTAATCTCCCACTGCGGCA-TATC-----------
------
FC88X_00564_00661 ---------------CCGTTTAATCTCCCACTGCGGCA-TATC-----------
------
FC88X_00593_01235 ---------------CCGTTTAATCTCCCACTGCGGCA-TATC-----------
------
FC88X_00614_00313 ---------------CCGTTTAATCTCCCACTGCGGCA-TATC-----------
------
FC88X_00616_00976 ---------------CCGTTTAATCTCCCACTGCGGCA-TATC-----------
------
FC88X_00714_01059 ---------------CCGTTTAATCTCCCACTGCGGCA-TATC-----------
------
FC88X_00946_00738 ---------------CCGTTTAATCTCCCACTGCGGCA-TATC-----------
------
FC88X_00946_01222 ---------------CCGTTTAATCTCCCACTGCGGCA-TATC-----------
------
FC88X_00950_00238 ---------------CCGTTTAATCTCCCACTGCGGCA-TATC-----------
------
FC88X_00977_00232 ---------------CCGTTTAATCTCCCACTGCGGCA-TATC-----------
------
FC88X_00981_01053 ---------------CCGTTTAATCTCCCACTGCGGCA-TATC-----------
------
FC88X_01008_00711 ---------------CCGTTTAATCTCCCACTGCGGCA-TATC-----------
------
FC88X_01102_00795 ---------------CCGTTTAATCTCCCACTGCGGCA-TATC-----------
------
FC88X_01147_00758 ---------------CCGTTTAATCTCCCACTGCGGCA-TATC-----------
------
FC88X_00051_00203 ---------------CCGTTTAATCTCCCACTGCGGCA-TATC-----------
------
FC88X_00108_00553 ---------------CCGTTTAATCTCCCACTGCGGCA-TATC-----------
------
FC88X_00116_00924 ---------------CCGTTTAATCTCCCACTGCGGCA-TATC-----------
------
FC88X_00168_00040 ---------------CCGTTTAATCTCCCACTGCGGCA-TATC-----------
------
FC88X_00161_00655 ---------------CCGTTTAATCTCCCACTGCGGCA-TATC-----------
------
FC88X_00337_00585 ---------------CCGTTTAATCTCCCACTGCGGCA-TATC-----------
------

```
FC88X_00337_01025      ---------------CCGTTTAATCTCCCACTGCGGCA-TATC-----------
------
FC88X_00389_00496      ---------------CCGTTTAATCTCCCACTGCGGCA-TATC-----------
------
FC88X_00382_01152      ---------------CCGTTTAATCTCCCACTGCGGCA-TATC-----------
------
FC88X_00448_01080      ---------------CCGTTTAATCTCCCACTGCGGCA-TATC-----------
------
FC88X_00542_01119      ---------------CCGTTTAATCTCCCACTGCGGCA-TATC-----------
------
FC88X_00502_01178      ---------------CCGTTTAATCTCCCACTGCGGCA-TATC-----------
------
FC88X_00597_01138      ---------------CCGTTTAATCTCCCACTGCGGCA-TATC-----------
------
FC88X_00618_00961      ---------------CCGTTTAATCTCCCACTGCGGCA-TATC-----------
------
FC88X_00691_00361      ---------------CCGTTTAATCTCCCACTGCGGCA-TATC-----------
------
FC88X_00739_00247      ---------------CCGTTTAATCTCCCACTGCGGCA-TATC-----------
------
FC88X_00710_01091      ---------------CCGTTTAATCTCCCACTGCGGCA-TATC-----------
------
FC88X_00769_01016      ---------------CCGTTTAATCTCCCACTGCGGCA-TATC-----------
------
FC88X_00819_00242      ---------------CCGTTTAATCTCCCACTGCGGCA-TATC-----------
------
FC88X_00876_01234      ---------------CCGTTTAATCTCCCACTGCGGCA-TATC-----------
------
FC88X_00901_00711      ---------------CCGTTTAATCTCCCACTGCGGCA-TATC-----------
------
FC88X_00964_00980      ---------------CCGTTTAATCTCCCACTGCGGCA-TATC-----------
------
FC88X_01000_00461      ---------------CCGTTTAATCTCCCACTGCGGCA-TATC-----------
------
FC88X_01020_00736      ---------------CCGTTTAATCTCCCACTGCGGCA-TATC-----------
------
FC88X_01048_00813      ---------------CCGTTTAATCTCCCACTGCGGCA-TATC-----------
------
FC88X_01006_01029      ---------------CCGTTTAATCTCCCACTGCGGCA-TATC-----------
------
FC88X_01093_01011      ---------------CCGTTTAATCTCCCACTGCGGCA-TATC-----------
------
FC88X_01118_00518      ---------------CCGTTTAATCTCCCACTGCGGCA-TATC-----------
------
FC88X_00102_00129      --------GGGGAAGCCGTTTAATCTCCCACTGCGGCA-TATC-----------
------
FC88X_00006_00596      ---------------CCGTTTAATCTCCCACTACGGCA-TATC-----------
------
FC88X_00935_00414      ---------------CCGTTTAATCTCCCACTGCGGCACTATCT----------
------
FC88X_00811_01027      ---------------CCGTTTAATCTCCCGCTGCGGCA-TATC-----------
------
FC88X_00961_00646      ---------------CCGTTCAATCTCCCACTGCGGCA-TATC-----------
------
FC88X_00650_00153      ---------------CCGTTTAATCTCCCACTGCGGCA-TATAC----------
------
```

FC88X_00521_00050 ---------------CCGTTTAAT-TCCCACTGCGGC--CATAT----------
------
FC88X_01037_00325 ---------------CCGTTTAATCTCCCACTGCGGCA-TATTCCTTCCTTTC-
------
FC88X_01042_01238 ---------------CCGTTTAATCTCCCACTGCGGCA-TAT-CCTTCCTTTC-
------
FC88X_00518_00180 ---------------CCGTTTAATCTCCCACTGCGGCA-TATCCTTCCTTTCT-
------
FC88X_00571_01009 ---------------CCGTTTAATCCCCCACTGCGGCCATATC-----------
------
FC88X_01058_00353 ---------------CCGTTTAATCTCCCACTGCGGCG-TATC-----------
------
FC88X_00141_00886 --------------CCGTTTTAATCTCCCACTGCGGCA-TATC-----------
------
FC88X_00776_00116 --------------CCGTTTTAATCTCCCACTGCGGCA-TATC-----------
------
FC88X_00478_00031 ---------------TCGAATACTCCCTGAAATCGACCTTAC------------
------
FC88X_00906_00486 --------------ATCAAACACCCCTT-ACATCG-CCTTATT-----------
------
FC88X_00181_00760 ---------------TCAATCAATTCTC-CAATCGGCCGTATA-----------
------
FC88X_00703_01207 --------------ATCCATCTACTCCCCCAATCGGCTGAC-------------
------
FC88X_00943_00602 -----------TCATTTCATCACCTTCCCCATCAGTCTGA--------------
------
FC88X_00552_01038 ----------------GGAAAAAATTTCCCTAAATCCCAGCACATTGC-------
------
FC88X_01063_01023 ----------------TCCCAAAATCTCCAGAACCCAATTGCT-----------
------
FC88X_01074_00864 ---------------CCGAAAACTTTCCCAAATCGCTATTGC------------
------
FC88X_01032_00491 ---------------CCACATAATTTCCCAAACCGGTCTCCC------------
------
FC88X_00034_00375 -------------TCACCCATTCTCCATAGATGCCATCGTCTT-----------
------
FC88X_00398_00852 -------------ATTCTCATTCTAAATGATTATCGTCTC--------------
------
FC88X_00694_00466 ---------------CTTCTCCATTGGTGTAAGCCAGATTCCT-----------
------
FC88X_00653_01000 ---------------CTTCTCCATTGGTGTAAGCCAGATTCCT-----------
------
FC88X_01119_00867 ---------------CTTCTCCATTGGTGTAAGCCAGATTCCT-----------
------
FC88X_01071_00754 ---------------CTTCTCCATTGGTGTAAGCCAGATTCCC-----------
------
FC88X_00562_01169 ---------------CTTCTCCATTGGTGCAAGCCAGATTCCT-----------
------
FC88X_00173_00797 ---------------CCTTCTCCTTGTTACAA-TCATATTCGT-----------
------
FC88X_00249_01113 ----------------CTTCCCATTCCCGGAAATGACATTACT-----------
------
FC88X_00785_00954 ---------------CTTTCAATA-CTCCAAACTCACGTTATT-----------
------
FC88X_00547_01198 ----------------CTTCAATATCTCCAAAATCATGCTTAT-----------
------

FC88X_00082_00308 ----------------CTGTCTTCTACGAAGCTGTTCTATATT-----------
------
FC88X_00521_00510 ----------------CTGCCTCTTCGGATACACTTGTATATT-----------
------
FC88X_00598_00739 ----------------CTATCCGCTTCACAAACCTGTTTTATT-----------
------
FC88X_00129_00644 -------------------TGCTCAGCCCAACTGCTGCGAATTCTC--------
------
FC88X_00118_00871 -------------------TGCTCAGCCCAACTGCTGCGAATTCTC--------
------
FC88X_00534_00236 ------------------TTACTCA-CCCAAC-GCTTCAAAGCCTATT------
------
FC88X_00524_00095 ------------------CTACTCA-CCCAAC-GCTTCAAAGCCTATT------
------
FC88X_00797_00862 ------------------CTACTCA-CCCAAC-GCTTCAAA-------------
------
FC88X_00363_00364 ------------------CCACTCAACCC---ACTTCAGCGCTATTTC------
------
FC88X_00456_01143 ---------------------
CTCAACCCTGGACTTCGGCGCTGTCTCCTTCCTTT----
FC88X_00438_00705 ------------------CTCCTCATCCGGATTCGCG-GCTTTATC--------
------
FC88X_00834_00901 ------------------CTACTTAGCCCCCCTCGCATGCCTTAT---------
------
FC88X_00349_00669 ---------------ACGCATCACAGT--AACGGTT-CTAATTCT---------
------
FC88X_00300_01160 ---------------ACGCATCACAGT--AACGGTTTCTAATTCT---------
------
FC88X_00337_01136 ---------------ACGCATCACAGT--AACGGTT-CTAATTCT---------
------
FC88X_00648_01004 ---------------ACGCATCACAGT--AACGGTT-CTAATTCT---------
------
FC88X_00722_01250 ---------------ACGCATCACAGT--AACGGTT-CTAATTCT---------
------
FC88X_00171_00837 ---------------ACGCATCACAG---AACGGTT-CTAATTCT---------
------
FC88X_00830_00195 ---------------ACGCATCACATGTAAACGGTT------------------
------
FC88X_00009_00499 --------------CGCACATCCCAAT---ACCGAT--AAATTCTCT-------
------
FC88X_00446_01181 --------------CGCACATCCCAAT---ACCGAT--AAATTCTC--------
------
FC88X_00986_01018 -------------CCGCACACCGCACC---CCTGCT--ATATTCT---------
------
FC88X_00249_00947 ---------------ACATCTACCA----ATCGCACGTCTGTTCAC--------
------
FC88X_00360_00135 ---------------ACATCTACCA----ATCGCACGTCTGTTCAC--------
------
FC88X_00891_00368 ---------------ACATCTACCA----ATCGCACGTCTGTTCAC--------
------
FC88X_00950_00823 ---------------ACATCTACCA----ATCGCACGTCTGTTCAC--------
------
FC88X_00672_00175 ---------------ACATCTACCA----ATCGCACGTCTGTTCA---------
------
FC88X_00463_01039 --------------GACATCGACCA----ATCGCACGTCTGTTCAC--------
------

```
FC88X_01011_00402       ----------GGGGAACATCTACCA----ACCGCACGTCTGTTCAC--------
------
FC88X_00836_01263       ---------------ACATCTGCTA----ATCGCACGTCTGTTCAC--------
------
FC88X_00644_00668       ---------------ACATTTACCA----ATCGCACG-----------------
------
FC88X_01120_01174       ------------CGCAAACCTATTC---TAATGCACTCTCGT------------
------
FC88X_00447_01027       ------------CGCAAACCTATTC---TAATGCACTCTCGT------------
------
FC88X_01032_00863       ------------CGCAAACCTATTC---TAATGCACTCTCGT------------
------
FC88X_00570_00614       ------------CTGACAGCTATTCGC-TAACGCACTCTCT-------------
------
FC88X_00634_01102       -------------TCACACCTTCTC-C-AAAAGCACTTTTAT------------
------
FC88X_00392_00628       -------------------TTCCCCCATCT-ATGATT-GCAGTCTGCG------
------
FC88X_00607_01216       ----------------GAGTTCCCCCATCTGATGATTAGCAGTCTGCGCC----
------
FC88X_00739_00258       -------------------TTCCCCCATCT-ATGATT-GCAGTCTGCG------
------
FC88X_01091_00704       -------------------TTCCCCCATCT-ATGATT-GCAGTCTGCG------
------
FC88X_00899_00937       -------------------CTCCCACATTTACAGATC-TCAGACTCCT------
------
FC88X_00304_00761       -------------------TTACCACATACACCG-TT-GTAGTCTCCT------
------
FC88X_00536_01217       ----------------TCTCGTCACCGTATATGGAACAGCATT-----------
------
FC88X_01079_01077       -----------------
CTCTTGCCGGCATATAAAGTAGCAGTCCTTTCCCTTTT-----
FC88X_00807_01200       -------------------CTTCAACATTCATAGAGTCAGCATTTC--------
------
FC88X_00120_00406       -------------------CTCCTACATCGTTAGAGTTCATTTCTC--------
------
FC88X_00112_00722       -------------------TCTCTCGCTCGATAGCATAGACTTTCGC-------
------
FC88X_00900_01197       -------------------TCTGCCGCTAGCAAGCATGGACTTTCC--------
------
FC88X_00158_00939       -------------------TCCCTCACTATGTAGCATGGTCGTCCC--------
------
FC88X_00107_00404       -----------------TCCCTGCCCCAATGGAGGATGTC--------------
------
FC88X_00713_01212       ------------------TCATTGCCTCACATCAATTACCGTTCT---------
------
FC88X_00871_01249       ------------------TCATTGCCTCACATCAATTACCGTTCT---------
------
FC88X_00119_00223       ------------------TCATTGCCTCACATCAATTACCGTTCT---------
------
FC88X_00839_01147       ------------------TTTATGCTTCCACGCAAATAACATTCT---------
------
FC88X_00936_00286       ------------------TTTATGCTTCCACGCAAATAACATTCT---------
------
FC88X_00868_00886       ------------------TTTATGCTTCCGCGCAAATAACGTTCTCTTCCTTT-
------
```

```
FC88X_00880_01032       -------------------CCTTGCCGCCACGCTATTAGCCTTTA---------
------
FC88X_00130_00743       ----------------CCGACTGACCGCTCGTTCCTACTTATT-----------
------
FC88X_00835_01041       ----------------CCACACAACTCTCGAATTGCTATTATC-----------
------
FC88X_00864_00849       -----------------TCAATGTCTGCACTATTCCAATTATAT----------
------
FC88X_00337_01044       -----------------TCAATGTCTGCACTATTCCAATTATAC----------
------
FC88X_00854_00766       ----------------------GTCTGGACT--TCCAGTTATCGC---------
------
FC88X_00467_00229       ----------------TCAAATCTTATTCTCTCGCGATATCCC-----------
------
FC88X_00397_01101       ----------------TCAAATGCTGACCTGCTCCCGAATCCT-----------
------
FC88X_00490_00027       -------------CTTATGATTAGTTC---CTCCCCGTACTTT-----------
------
FC88X_00604_00363       --------------CTATAATTCGTACGCACTCCCCAAACT-------------
------
FC88X_01078_01087       -----------------TCTTCTGCTATCTCCCCACGAACATTTA---------
------
FC88X_00326_00321       -----------------TCTTCTGCTATCTCCCCACGAACATTTA---------
------
FC88X_01123_00936       ------------------CTTTTGCTCACTCTTCCCGAACGTTTA---------
------
FC88X_00633_01192       ------------------CTTTCTCTTTCTCGCCACGATAGTAAT---------
------
FC88X_01105_00632       -------------------CCTTACTATTTCCTCGCGATCGATAT---------
------
FC88X_00932_00759       ------------------CCCTGCTATTTTCGCAACGATAATC-----------
------
FC88X_00631_00350       --------------------CTCGTACCGCATCGATATTCATTATTCC------
------
FC88X_00643_01203       --------------------CTCGTACCGCATCGATATTCATTATT--------
------
FC88X_01110_00764       --------------------CTCGTACCGCATCGATATTCATTATT--------
------
FC88X_00719_00968       -------------------TTCCCTAATTCCGCACGTTACCTTATT--------
------
FC88X_00655_00225       -------------TCTCTCATC-CACGAGTTTCAGCATATT-------------
------
FC88X_00974_00511       -------------TCTCTCATC-CACGAGTTTCAGCATATT-------------
------
FC88X_00154_00705       -------------TCTTTCATC-CACGAGTTTCAGCATATT-------------
------
FC88X_00290_00697       ---------------TCTGATCTCATTAATCTCCGCAGATTTT-----------
------
FC88X_00871_01261       ---------------TCTGATCTCATTAATCTCCGCAGATGTT-----------
------
FC88X_00364_00369       ------------------TCTCTCATTCCTCCGCCCGAAATTTCTC--------
------
FC88X_00603_00385       ------------------TTAGCTCTTATCCTCCGCGAACTTTCT---------
------
FC88X_00195_00996       -----------------CTAGCTCCCAATTATCAGCGAAATACGC---------
------
```

FC88X_00026_00056 --------------------CTAGCA-TTATTCCGCTAGG-TGCTTCTC-----
------
FC88X_00023_00638 --------------------CTAGCA-TTATTCCGCTAGGGTGCTTCTC-----
------
FC88X_00028_00168 --------------------CTAGCA-TTATTCCGCTAGG-TGCTTCTC-----
------
FC88X_00127_00761 --------------------CTAGCA-TTATTCCGCTAGG-TGCTTCTC-----
------
FC88X_00700_01149 --------------------CTAGCA-TTATTCCGCTAGG-TGCTTCTC-----
------
FC88X_00077_00531 --------------------CTAGCA-TTATTCCGCTAGG-TGCTTCTC-----
------
FC88X_01002_01170 --------------------CTAGCACTTATTCCGCTAGG-TGCTTCTC-----
------
FC88X_00573_00431 --------------------CTAGCA-TTATTCCGCTAGG-TGCTTCTC-----
------
FC88X_00876_00788 --------------------CTAGCA-TTATTCCGCTAGG-TGCTTCTC-----
------
FC88X_01044_00798 --------------------CTAGCA-TTATTCCGCTAGG-TGCTTCTC-----
------
FC88X_01114_01095 --------------------CTAGCA-TTATTCCGCTAGG-TGCTTCTC-----
------
FC88X_00235_00093 --------------------CTAGCGATTATTCCGCTAGG-TGCTTCTC-----
------
FC88X_00475_00081 --------------------CTAGC-ATTATTCCGCTAGG-TGCTTCTC-----
------
FC88X_00818_00098 --------------------CTAGC-ATTATTCCGCTAGG-TGCTTCTC-----
------
FC88X_00909_01227 --------------------CTAGC-ATTATTCCGCTAGG-TGCTTCTC-----
------
FC88X_01050_00837 --------------------CTAGC-ATTATTCCGCTAGG-TGCTTCTC-----
------
FC88X_00099_00157 --------------------CTAGCA-TTATTCCGCTAGG-TGCTTCTC-----
------
FC88X_00050_00495 --------------------CTAGCA-TTATTCCGCTAGG-TGCTTCTC-----
------
FC88X_00109_00276 --------------------CTAGCA-TTATTCCGCTAGG-TGCTTCTC-----
------
FC88X_00151_00617 --------------------CTAGCA-TTATTCCGCTAGG-TGCTTCTC-----
------
FC88X_00199_00703 --------------------CTAGCA-TTATTCCGCTAGG-TGCTTCTC-----
------
FC88X_00250_00969 --------------------CTAGCA-TTATTCCGCTAGG-TGCTTCTC-----
------
FC88X_00254_01069 --------------------CTAGCA-TTATTCCGCTAGG-TGCTTCTC-----
------
FC88X_00357_00594 --------------------CTAGCA-TTATTCCGCTAGG-TGCTTCTC-----
------
FC88X_00462_01031 --------------------CTAGCA-TTATTCCGCTAGG-TGCTTCTC-----
------
FC88X_00675_00413 --------------------CTAGCA-TTATTCCGCTAGG-TGCTTCTC-----
------
FC88X_00800_00208 --------------------CTAGCA-TTATTCCGCTAGG-TGCTTCTC-----
------
FC88X_00802_00213 --------------------CTAGCA-TTATTCCGCTAGG-TGCTTCTC-----
------

FC88X_00837_00867 --------------------CTAGCA-TTATTCCGCTAGG-TGCTTCTC-----
------
FC88X_00822_01076 --------------------CTAGCA-TTATTCCGCTAGG-TGCTTCTC-----
------
FC88X_00936_00325 --------------------CTAGCA-TTATTCCGCTAGG-TGCTTCTC-----
------
FC88X_00929_01086 --------------------CTAGCA-TTATTCCGCTAGG-TGCTTCTC-----
------
FC88X_00951_01221 --------------------CTAGCA-TTATTCCGCTAGG-TGCTTCTC-----
------
FC88X_01023_01016 --------------------CTAGCA-TTATTCCGCTAGG-TGCTTCTC-----
------
FC88X_01100_01086 --------------------CTAGCA-TTATTCCGCTAGG-TGCTTCTC-----
------
FC88X_00274_00926 --------------------CTAGCA-TTATTCCGCTAGG-TGCTTCTCCC---
------
FC88X_00825_01038 --------------------CTAGCA-TTATTCCGCTAGG-TGCTTCCC-----
------
FC88X_00197_00985 --------------------CTAGCA-TTATTCCGCTAGG-
TGCTTCTCCTTCCTTT---
FC88X_00890_00228 --------------------CTAGCA-TTATTCCGCTAGG-TGCCTCTC-----
------
FC88X_00181_00722 --------------------CTAGCA-TTACTCCGCTAGG-TGCTTCTC-----
------
FC88X_00783_01232 ---------------------CAGCA-TTATTCCGCTAGG-TGCTTCTC-----
------
FC88X_00915_00571 -----------------------TCATTAATTCTCCCCAGGTGATTC-------
------
FC88X_00284_00081 --------------------TTTATGACTATTC-GCCAGGTGACGACC------
------
FC88X_00958_00892 --------------------TTTATGACTATTC-GCCAGGTGACGACC------
------
FC88X_00716_00717 ---------------CTAATATGACC-TTCTCCGGCTGA-CTGTA---------
------
FC88X_00930_00779 ---------------CTAATATGACC-TTCTCCGGCTGA-CTGTA---------
------
FC88X_00781_00183 --------------------ATGATC-CTCGCCGGATGATCTGTTCC-------
------
FC88X_00972_00658 -------------------GATGATC-CTCGCCGGATGATCTGTTCCCC-----
------
FC88X_00072_00751 --------------------ATGATC-CTCGCCGGATGATCTGTTCC-------
------
FC88X_00346_00528 --------------------ATGATC-CTCGCCGGATGATCTGTTCC-------
------
FC88X_00616_01151 --------------------ATGATC-CTCGCCGGATGATCTGTTCC-------
------
FC88X_01096_00833 --------------------ATGATC-CTCGCCGGATGATCTGTTCC-------
------
FC88X_01085_01081 --------------------ATGATC-CTCGCCGGATGATCTGTTCC-------
------
FC88X_01069_00620 -------------TCTTTTCATGACCGCTCTCCCGATGACT-------------
------
FC88X_00214_00016 -------------------CACG-TTGATAACAAGC-GCGACTATTCCA-----
------
FC88X_00233_00963 -------------------CACG-TTGATAACAAGC-GCGACTATTCCA-----
------

FC88X_00021_00623 -------------------CACG-TTGATAACAAGC-GCGACTATTCC------
------
FC88X_00059_00210 -------------------CACG-TTGATAACAAGC-GCGACTATTCC------
------
FC88X_00185_00854 -------------------CACG-TTGATAACAAGC-GCGACTATTCC------
------
FC88X_00323_00355 -------------------CACG-TTGATAACAAGC-GCGACTATTCC------
------
FC88X_00446_00945 -------------------CACG-TTGATAACAAGC-GCGACTATTCC------
------
FC88X_00421_01026 -------------------CACG-TTGATAACAAGC-GCGACTATTCC------
------
FC88X_00463_00382 -------------------CACG-TTGATAACAAGC-GCGACTATTCC------
------
FC88X_00502_01201 -------------------CACG-TTGATAACAAGC-GCGACTATTCC------
------
FC88X_00771_00101 -------------------CACG-TTGATAACAAGC-GCGACTATTCC------
------
FC88X_00822_00894 -------------------CACG-TTGATAACAAGC-GCGACTATTCC------
------
FC88X_01042_00360 -------------------CACG-TTGATAACAAGC-GCGACTATTCC------
------
FC88X_01045_00559 -------------------CACG-TTGATAACAAGC-GCGACTATTCC------
------
FC88X_01023_00776 -------------------CACG-TTGATAACAAGC-GCGACTATTCC------
------
FC88X_00127_00536 -------------------CACG-TTGATAACAAGC-GCGACTATTCC------
------
FC88X_00138_00727 -------------------CACG-TTGATAACAAGC-GCGACTATTCC------
------
FC88X_00231_00630 -------------------CACG-TTGATAACAAGC-GCGACTATTCC------
------
FC88X_00212_00877 -------------------CACG-TTGATAACAAGC-GCGACTATTCC------
------
FC88X_00362_00456 -------------------CACG-TTGATAACAAGC-GCGACTATTCC------
------
FC88X_00567_00213 -------------------CACG-TTGATAACAAGC-GCGACTATTCC------
------
FC88X_00636_01072 -------------------CACG-TTGATAACAAGC-GCGACTATTCC------
------
FC88X_00842_00269 -------------------CACG-TTGATAACAAGC-GCGACTATTCC------
------
FC88X_00823_00894 -------------------CACG-TTGATAACAAGC-GCGACTATTCC------
------
FC88X_00931_00358 -------------------CACG-TTGATAACAAGC-GCGACTATTCC------
------
FC88X_00937_01086 -------------------CACG-TTGATAACAAGC-GCGACTATTCC------
------
FC88X_01033_00783 -------------------CACG-TTGATAACAAGC-GCGACTATTCC------
------
FC88X_01038_01013 -------------------CACG-TTGATAACAAGC-GCGACTATTCC------
------
FC88X_01054_00522 -------------------CACG-TTGATAACAAGC-GCGACTATTCC------
------
FC88X_01121_00786 -------------------CACG-TTGATAACAAGC-GCGACTATTCC------
------

```
FC88X_01102_01048       --------------------CACG-TTGATAACAAGC-GCGACTATTCC------
------
FC88X_01122_01085       --------------------CACG-TTGATAACAAGC-GCGACTATTCC------
------
FC88X_00153_00824       --------------------CACG-TTGATAACAAGC-GCGACTATTCCCC----
------
FC88X_00159_00897       --------------------CACG-TTGATAACAAGC-GCGACTATTCCCC----
------
FC88X_00506_00054       --------------------CACG-TTGATAACAAGC-GCGACTATTCCCC----
------
FC88X_00118_00008       --------------------CACG-TTGATAACAAGC-GCGACTACTCCT-----
------
FC88X_00758_00785       --------------------CACG-TTGATAACAAGC-GCGACT-----------
------
FC88X_00604_00932       --------------------CACG-TTGATAACAAGC-GCG--------------
------
FC88X_00605_00246       --------------GGGGAACACG-TTGATAACAGGC-GCGACTATTCC------
------
FC88X_00738_00906       --------------------CACG-TTGATAATAAGC-GCGACTATTCC------
------
FC88X_01114_00735       --------------------CACG-TTGACAACAAGC-GCGACTATTCC------
------
FC88X_01089_01253       --------------------CACGGTTGATAACAAGC-
GCGACTATTCCCTTCCTT-----
FC88X_00407_01040       ------------------TCTGCAGTTG-TAGTATTC-CCCATTATT--------
------
FC88X_00232_01068       -------------------CCGCCGTTGATAATAGCT-GCGATTATTC-------
------
FC88X_00097_00085       ---------------------CCGTGTGACAATCAGCCGCCGTTATTC-------
------
FC88X_00728_01057       ---------------------CCGTGTGACAATCAGCCGCCGTTATTC-------
------
FC88X_00142_00115       ---------------------CCGTGTGACAATCAGCCGCCGTTATTC-------
------
FC88X_00541_00215       ---------------------CCGTGTGACAATCAGCCGCCGTTATTC-------
------
FC88X_00121_00087       ---------------------CCGTGTGACAATCAGCCGC-GTTATTC-------
------
FC88X_00437_00039       ---------------------CCGTGTGACAATCAGC-GCGGTTATTC-------
------
FC88X_00545_00282       ----------------------CTTGTGACTAT-GACCGCCGTTATTCT------
------
FC88X_00837_01110       ----------------------CTTGTGACTAT-GACCGCCGTTATTCT------
------
FC88X_00095_00877       ----------------------CGTATGATTATGCTGCCGCTATTTGCC------
------
FC88X_01074_00825       ----------------------CCTATGACTATCAGCGGCCTATTCGC-------
------
FC88X_00032_00800       ----------------------TCGTTGGTTAATCCACGGACCGTTCTC------
------
FC88X_00149_00590       ----------------------TCGTTGGTTAATCTACGGACCGTTCTC------
------
FC88X_00353_01089       ----------------------CCGTCCGTTAATCGGTCGAAGCTTCTC------
------
FC88X_00031_00424       --------------------CCCGGTGGATAGACCATC--TTTATTCTC------
------
```

```
FC88X_00476_00800       -------------------CCCGGTGGATAGACCGTC--TTTATTCTC------
------
FC88X_01146_00861       ------------------CCCCGGTGGATGGACCATCG-TTTATTCTC------
------
FC88X_00654_01197       -------------------GTCGGTTGCTCGATTAACGGTCTATTCT-------
------
FC88X_00813_00155       --------------------TCGGTTGCTCGATTAACGGTCTATTCT-------
------
FC88X_00171_00828       --------------------TCGGTTGCTCGATTAACGGTCTACTCT-------
------
FC88X_00144_00089       --------------------ATCGTAGC-TTATC-TCGATGCATTCTC------
------
FC88X_00575_00118       --------------------ATCGTAGCCTTATC-CCGACGCATTCTC------
------
FC88X_00687_00507       --------------------CTCGT-GCCTTGAC-CCGCTGCGTATTCT-----
------
FC88X_00027_00222       -----------------------AGCCCTTCTCCCC------------------
------
FC88X_00477_00959       ----------TTTGCTCTTCTCCAGCGCTTCTCCCCC-----------------
------
FC88X_00772_00133       --------------GGGGAAGTCAACCCTTCTCTCGCGCGGCGATTGT------
------
FC88X_00970_00360       --------------------GTCAACCCTTCTCTCGCGCGGCGATTGT------
------
FC88X_00062_00188       ---------------------TCAACCCTTCTCTCGCGCGGCGATTGT------
------
FC88X_00258_01055       ---------------------CCAACCTTACTCCCGCG-AGCGACTGTT-----
------
FC88X_00513_01056       ---------------------CCAACCTTACTCCCGCG-AGCGACTGTT-----
------
FC88X_00826_00332       ---------------------CCAACCTTACTCCCGCG-AGCGACTGTT-----
------
FC88X_00027_00176       ---------------------CCAACCTTACTCCCGCG-AGCGACTGTT-----
------
FC88X_00944_00766       ---------------------CCAACCTTACTCCCGCG-AGCGACTGTT-----
------
FC88X_00261_00900       ---------------------CCAACCTTACTCC-GCG-AGCGACTGTT-----
------
FC88X_00897_00585       -----------------------CATCCTTCTCTCCAG-CACGTCTATTCA---
------
FC88X_01114_01042       -----------------------CACCCTTCTCACGCG-CGCATCCAATAA---
------
FC88X_00777_00887       --------------------CCGAGTCCCTCACCCGT--GTGCACTGTC-----
------
FC88X_00120_00008       --------------------CCGAGTCCCTCACCCGT--GTGCACTGTC-----
------
FC88X_00096_00158       ---------------------CCAGCCCTTCTTCCGTA-GCACACTGTC-----
------
FC88X_00171_00074       ------------------CCGACATCCCTTCGCCCGT--GAGTATTA-------
------
FC88X_00936_01219       ------------------CCGACATCCCTTCGCCCGT--GAGCATTA-------
------
FC88X_00646_00084       --------------------------CCTTGTCCCGAG-TGATCTAACGAACC-
------
FC88X_01002_00858       --------------------CTTAACCCTTTCCCCGTGCTAACGTAAT------
------
```

FC88X_00902_00891 -----------------CCGAGCACCCTTTTCCCGCGAACTAGC-----------
------
FC88X_00366_01175 -----------------CCGAGCACCCTTTTCCCGCGAACTAGC-----------
------
FC88X_00120_00097 ----------------------CCTGCCCCTTTCCCGAACTAGCGTTAGC-----
------
FC88X_00765_01219 --------------------TTCTAGCTCTTTCCGTGCGCACACTTT--------
------
FC88X_00936_00773 --------------------TTCTAGCTCTTTCCGTGCGCACACTTT--------
------
FC88X_00067_00117 --------------------TTCTAGCTCTTTCCGTGCGCACACTTT--------
------
FC88X_00167_00054 --------------------TTCTAGCTCTTTCCGTGCGCACACTTT--------
------
FC88X_00574_00242 --------------------TTCTAGCTCTTTCCGTGCGCACACTTT--------
------
FC88X_01017_01219 --------------------TTCTAGCTCTTTCCGTGCGCACACTTT--------
------
FC88X_01128_01188 --------------------TTCTAGCTCTTTCCGTGCGCACACTTT--------
------
FC88X_00778_00457 --------------------TTCTAGCTCTTTCCGCGCGCACACTTT--------
------
FC88X_00522_00965 --------------------TTCTAGCTCTTTCCATGCGCACACTTT--------
------
FC88X_00376_00763 ---------------------TCGAGCCCTTTGCCCCGCGACACTGTT-------
------
FC88X_00396_00075 -------------------TTGCTTGCTATTTTCC-TTCGGACGGAC--------
------
FC88X_00467_00971 -------------------TTGCTTGCTATTTTCC-TTCGGACGGAC--------
------
FC88X_01005_00872 -------------------TTGCTTGCTATTTTCC-TTCGGACGGAC--------
------
FC88X_00873_00525 -------------------TTGCTTGCTATTTTCC-TTCGGACGGAC--------
------
FC88X_00906_00657 -------------------TTGCTTGCTATTTTCC-TTCGGACGGAC--------
------
FC88X_00972_01046 -------------------TTGCTTGCTATTTTCC-TTCGGACGGAC--------
------
FC88X_01056_00853 -------------------TTGCTTGCTATTTTCC-TTCGGACGGAC--------
------
FC88X_00149_00750 -------------------TTGCTTGCTATTTTCCCTTCGGACGGAC--------
------
FC88X_00442_00027 -------------------TCACGGTATTTCAGCCCTTGCGACGCT---------
------
FC88X_00788_00860 -------------------TCACGGTATTTTAGCCCTTGCGACGCT---------
------
FC88X_01077_00800 -------------------TCACGGTATTTTAGCCCTTGTGACGCT---------
------
FC88X_00762_00859 -------------------TCACGTAATTTC--TCCTCGCGACGTCCGC------
------
FC88X_00897_00834 -------------------TTCCAGCCC--TTGCCCTACCGACGTCAT-------
------
FC88X_01101_00903 ---------------------CCAGCCTATTTCCACTCCCGACGTGAC-------
------
FC88X_00108_00111 ------------------CAACCAACTTA--TAA-CTCCCGTCGCACTC------
------

FC88X_00788_00112 -----------------CAACCAACCTA--TAAACTCCCGTCGCACTC------
------
FC88X_00111_00345 ------------------AGCCAACCTA--TCCACTCCCGACGACCT-------
------
FC88X_00568_01077 -------------------CTGTGACTTAGATGCCG-CCAGCTTCTC-------
------
FC88X_00774_01103 -------------------CTGTGACTTAGATGCCG-CCAGCTTCTC-------
------
FC88X_00994_00808 -------------------CTGTGACTTAGATGCCG-CCAGCTTCTC-------
------
FC88X_00036_00166 -------------------CTGTGACTTAGATGCCG-CCAGCTTCTC-------
------
FC88X_00304_01044 -------------------CTGTGACTTAGATGCCG-CCAGCTTCTC-------
------
FC88X_00956_00356 -------------------CTGTGACTTAGATGCCG-CCAGCTTCTC-------
------
FC88X_00983_00398 -------------------CTGTGACTTAGATGCCG-CCAGCTTCTC-------
------
FC88X_01108_00616 -------------------CTGTGACTTAGATGCCG-CCAGCTTCCC-------
------
FC88X_00032_00731 -------------------CTGTGACCTAGATGCCG-CCAGCTTCTC-------
------
FC88X_00224_01068 -------------------CTGTGAATTTGATCCCGGCCGGCTGCTC-------
------
FC88X_00812_00842 -------------------CTGTGAATTTGATCCCGGCCGGCTGCT--------
------
FC88X_00186_00984 -------------------CCGTGAATTAGTTCACGATCTGCTTCT--------
------
FC88X_00532_00146 -------------------CCGTGAATTAGTTCACGATCTGCTTCT--------
------
FC88X_00965_00895 -------------------CCGTGAATTAGTTCACGATCTGCTTCT--------
------
FC88X_00978_00560 ----------------GGGGACTAAGTAATTCCCCAA-CTTCAGCTCGA-----
------
FC88X_01070_00834 ----------------------CACGTAATTCCCGACTCTTCTCCACGA-----
------
FC88X_00395_01002 ----------------------------
GTTTCACGACCTTCTGCACAAACCTCAC----
FC88X_00100_00535 ------------------CACACCTTTGAGTTGTTCCCGCGTATT---------
------
FC88X_00646_01038 ------------------CACACCTTTGAGTTGTTCCCGCGTATT---------
------
FC88X_00055_00015 ------------------CATACCTTTGAGTTGTTCCCGCGTATT---------
------
FC88X_00180_00417 ------------------CAGACCGCTGA-TTGCTCGCG---------------
------
FC88X_00397_01149 ---------------TTCCCTGCGTTTTTGACTAAGGCTCCC------------
------
FC88X_01076_00826 ---------------TTTCCAGGCCTTTGGTTGTCTGCTCCC------------
------
FC88X_00326_00941 -------------------CTACGTTTCAG-TGACTGCTCCCGCCAGACC----
------
FC88X_00556_00604 ------------------CGTAATGTTT-GCTTACGCCTTTTCTC---------
------
FC88X_00651_01231 ------------------CGTAATGTTT-GCTTACGCCTTTTCTCCTTCCTTT-
------

```
FC88X_00449_00051      -----------------CCGTAATATTT-GCTTACGCCTTTTCTCCTTCCTTT-
------
FC88X_00434_01064      -----------------CCGTAGTGTTTTGCTTACGCCTTTTTCTCC-------
------
FC88X_00883_01090      -----------------CTGTGTCTTGT-GCCGACCGCCTATCTC---------
------
FC88X_00422_00046      -----------------CTGTATTTTA--GCCTACGGCC-ATCTCTC-------
------
FC88X_00564_00086      ----------------TTCCTGATTTTGTGCCTCCGACTATT------------
------
FC88X_00745_01150      -----------------TCGTGATTT-GTTTCGCCGACCCCTGCG---------
------
FC88X_00208_00037      ------------------CGCGCGAACCTTTAATCCAGCTTTCTC---------
------
FC88X_00534_00233      ------------------CGCGCGAACCTTTAA-CCAGCTTTCTC---------
------
FC88X_00039_00503      -------------------ACGTGAACCTTTAT-CCGTCTGTTTCTT-------
------
FC88X_00097_00119      -------------------ACGTGAACCTTTAT-CCGTCTGTTTCTT-------
------
FC88X_00564_00490      -------------------CTGCGGTTCTTTAA-CCAACTGATTCTC-------
------
FC88X_00810_01187      -------------------CTGCGGTTCTTTAA-CCGACTGATTCTC-------
------
FC88X_00786_01007      ---------------------CTGGTTCTTTAT-CCAACTGATTCTC-------
------
FC88X_00822_00247      ------------------TCTCCGGATCTTTAC-CGAGATTACTCT--------
------
FC88X_01094_00468      -------------------CTCCGAACTGATCC-GAATGAATCTCCC-------
------
FC88X_00883_01017      -----------------------CCGTTTTCTCTCCGGAATAATGTTCTC----
------
FC88X_01090_00660      -----------------------CTTTTCTTCCTCCAGCTGAGTCTTCTC----
------
FC88X_00995_01098      -----------------GCTGCACATTACCT--CCCATTTGAATATT-------
------
FC88X_00350_01147      ----------------------TCTTTACCTGACCGAACTGAACATTCTC----
------
FC88X_00436_00493      ------------------GCTGTCTATTTAACTCCCGCCAAATGCGCTT-----
------
FC88X_00943_00297      ----------------------CTCACTCTTTACCGGCATTATGCTCTT-----
------
FC88X_00159_00044      --------------------TCTCTTTACCTTCTGCCACAGCTTGTT-------
------
FC88X_00964_00311      --------------------TCTATAGACCTT-TGCCGAAACCTGTT-------
------
FC88X_00812_00860      --------------------GTTCATTTTCCCTTCCAGCAGCCTTTT-------
------
FC88X_00975_01098      --------------------ATTCAATCCCGACTCCAGCAGCCTCTT-------
------
FC88X_00066_00137      -----------------TTCCGGCCTTCTCCGACCAAGCGGTAGT---------
------
FC88X_01003_00633      ------------------TCATGCCTTCTCCATTCATACAGTATA---------
------
FC88X_00517_01185      -----------------TCCCTGT-GTTCTCCCCACAGCGGTTAA---------
------
```

FC88X_01110_00905 -----------------TCCCTGT-GTTCTCCCCACAGCGGTTAA---------
------
FC88X_00163_00844 -----------------TCCCTGT-GTTCTCCCCACAGCGGTTAA---------
------
FC88X_00269_00744 -----------------TCCCTGT-GTTCTCCCCACAGCGGTTAA---------
------
FC88X_00782_00820 -----------------TCCCTGTTGTTCTCCCCACAGCGGTTA----------
------
FC88X_00391_00015 ------------------CCATGT-TTTCTCCTACAAGCGGCTCGT--------
------
FC88X_00416_00307 -----------------------GCTTATAATAGATGCCTGCGATCCCTGA---
------
FC88X_00637_00789 -----------------------CCTCATACCCGATTCCTCCCACCTGAA----
------
FC88X_00928_01028 --------------------TGTACGCTTGCCTGGCTTCACCGACCT-------
------
FC88X_00892_01247 ---------------------TTGTTCTTGCTAT-TACCGACTATTCCT-----
------
FC88X_01000_00883 --------------------GTTGTTCTTGCTAT-TACCGACTATTCCT-----
------
FC88X_00107_00491 ----------------TGCTGTTCTCCATGCTACGTACCTACTCTTCCTTT---
------
FC88X_00593_01222 ----------------TGCTGTTCTCCATGCTACGTACCTACT-----------
------
FC88X_00975_00852 ------------------ACGTGCTCCCTGCTAC--ACCGACTTTCC-------
------
FC88X_00942_00444 ---------------TTGCCGAGTTTTCCCCTGCGACTTGACCTTCCTTT----
------
FC88X_00974_00544 -----------------GCCGAGATTTTCTGCCTGCAACGACCTT---------
------
FC88X_00121_00092 -------------------CCAAGTTCACTCCCTGCGGCGACATCTT-------
------
FC88X_00244_01028 -------------------CCAAGTTCACTCCCTGCGGCGACATCTT-------
------
FC88X_00426_00581 -------------------CCAAGTTCACTCCCTGCGGCGACATCTT-------
------
FC88X_00811_00148 -------------------CCAAGTTCACTCCCTGCGGCGACATCTT-------
------
FC88X_00898_00769 -------------------CCAAGTTCACTCCCTGCGGCGACATCTT-------
------
FC88X_00039_00367 -------------------CCAAGTTCACTCCCTGCGGCGACATCTT-------
------
FC88X_00511_00642 -------------------CCAAGTTCACTCCCTGCGGCGACATCTT-------
------
FC88X_00657_00083 -------------------CCAAGTTCACTCCCTGCGGCGACATCTT-------
------
FC88X_00855_01015 -------------------CCAAGTTCACTCCCTGCGGCGACATCTT-------
------
FC88X_01072_00659 ------------------GCCAAGTTCACTCCCTGCGGCGACATCTT-------
------
FC88X_00729_00741 -------------------CCAAGTTCACTCCCTGCGGCGACAT----------
------
FC88X_00967_00478 -------------------CCAAGCTCACTCCCTGCGGCGACATCTT-------
------
FC88X_00139_00733 -------------------CCAAGCTCACTCCCTGCGGCGACATCTT-------
------

FC88X_01091_00940 --------------------CCAAGTTCACTCCCTGCGGCGACATCTC-------
------
FC88X_00955_00785 --------------------CCAAGTTCACTCCCTGCGGCGACAC----------
------
FC88X_00140_00972 ------------------CTCCCAGCTCAGTCTTCGCGGCGTCGC----------
------
FC88X_00506_00114 ------------------CTCCCAGTTCAGTCTTCGCGGCGTCGC----------
------
FC88X_00934_00711 ------------------CTCCCAACTTCTCTCCTCCGGTGTCAC----------
------
FC88X_00531_01255 ------------------CTCCCAGTTCAATCCCTCCGCGGGTACC---------
------
FC88X_00630_00931 ----------------------CCAATTACTCCCTACAGCAGCGACATT------
------
FC88X_00632_01153 ----------------------CCAATTACTCCCTACAGCAGCGACATT------
------
FC88X_00816_01035 ----------------------CCAATTACTCCCTACAGCAGCGACATT------
------
FC88X_00885_00891 ----------------------CCAATTACTCCCTACAGCAGCGACATT------
------
FC88X_00953_00452 ----------------------CCAATTACTCCCTACAGCAGCGACATT------
------
FC88X_01136_00869 ----------------------CCAACTACTCCCTACAGCAGCGACATT------
------
FC88X_01077_00627 ----------------------CCAATTACTTCCTACAGCAGCGACATT------
------
FC88X_00586_00313 ----------------------CCACTTACTTCCCGCAGCTGACACTTA------
------
FC88X_00418_00685 ---------------------CGCACACTTTCCCTGCAGA--CAGCTACT-----
------
FC88X_01113_00849 ---------------------CTCACACTTTCCCTGCAGA--CAGCTACT-----
------
FC88X_00159_00779 ---------------------CTGCAGTCCTTCCCGCTCC--GAGCTACT-----
------
FC88X_00320_00767 ---------------------CTGACAAATTCCCCCCTGC-GCTACTTCTC----
------
FC88X_01143_00892 ---------------------CCGACAACTTCCCCGTTGC-G-TCCTTCT-----
------
FC88X_00927_00708 ---------------------CCGACGCCTTTCCCGCTGC-T-AGCTACT-----
------
FC88X_00807_00165 ----------------------AGACATCTATTCC-CTGC-GTCGCTACCGC---
------
FC88X_00579_00067 ----------------------CCCTTTATTCCCCGTTGCGAGCGCTTGC-----
------
FC88X_00858_00749 ------------------------CTTTGTTCCCTTCCGC-AGCGCTATCTC---
------
FC88X_00025_00477 -------------------TTTTGCCCTTTACCCCATGTGTGTTTT---------
------
FC88X_00827_00840 -------------------TTTTGCCCTTTACCCCATGTGTGTTTT---------
------
FC88X_00955_01267 -------------------TTTTGCCCTTTACCCCGTGTGTGTTTC---------
------
FC88X_00407_00968 ----------------GCGTTTCTTCGTCTACTCCGTCTATCC------------
------
FC88X_00698_00229 ----------------GCGTTTCTTCGTCTACTCCGTCTATCC------------
------

FC88X_00319_01168 ---------------ACGTTTTTCGTCTTACCCCTTCGTGTC------------
------
FC88X_00048_00601 --------------TCCTGTCTTCTCCCTGTGGAACGTTTG-------------
------
FC88X_00455_00512 ----------------CTGTCTTCTCC-TGTGGTGCTCTTGTCGT---------
------
FC88X_01043_00579 ----------------CTGTTTCCCCTGTGCTACCCGTTTGTC-----------
------
FC88X_00453_00292 -------------------ACCTCCCCTAGTTATCGCTTCGTTGCG--------
------
FC88X_00074_00720 ------------------CTCCCCAATTACTTGTTGCATCGTTGC---------
------
FC88X_00845_00865 ---------------------TCCCCTTGTTCATTGCGTCATCGCCCGCTT---
------
FC88X_00224_00892 -------------------TTCTCCCCTTGTTATCTTTTCGTTAAC--------
------
FC88X_00884_00404 ------------------ACACTCCCTACGTTACTCGTTTATTCG---------
------
FC88X_00349_00618 ------------------ACACTCCCTATGTTACTCGTTTATTCG---------
------
FC88X_00973_00669 ----------------CCACGCTCCCTACGATA-TCGTCTTTTA----------
------
FC88X_00589_01101 ----------GGGGAACTCGAATTCCTGTGACTCGTTGCTGTG-----------
------
FC88X_00050_00428 ----------------CCAAATTCCCTGTGCCCCGTTGTTCTT-----------
------
FC88X_00115_00802 ----------------CCGCATTCCCTTCGATACCTTGGTGTT-----------
------
FC88X_00216_00404 ---------------------CCACGCACGTC-ACGACGCCAA-ATTCTC----
------
FC88X_00621_00921 ---------------------CCACGCACGTC-ACGACGCCAA-ATTCTC----
------
FC88X_01089_00853 ---------------------CCACGCACGTC-ACGACGCCAA-ATTCTC----
------
FC88X_00876_01172 ---------------------CCACGCACGTC-ACGACGCCAA-ATTCTC----
------
FC88X_01049_00719 ---------------------CCACGCACGTC-ACGACGCCAA-ATTCTC----
------
FC88X_00559_00211 ---------------------CCACGCACGTC-ACGACGCCAA-ATTCTC----
------
FC88X_00346_01004 ---------------------CCACGCACGTC-ACGACGCCAA-ATTCTC----
------
FC88X_00427_01037 ---------------------CCACGCACGTC-ACGACGCCAA-ATTCTC----
------
FC88X_00944_01035 ---------------------CCACGCACGTC-ACGACGCCAA-ATTCTCC---
------
FC88X_00439_00119 ---------------------CCACGCACGTC-ACGACGCCAA-
ATTCTCCTTCCTTT--
FC88X_00009_00233 ----------------------CCCGCATGTCGACAATGCCAGCATTCT-----
------
FC88X_00097_00209 --------------------GCCGCAAT--TCACTGACGCCAA-CCTCTC----
------
FC88X_00416_00659 ----------------------CGCAATAATCGCTAAT-CCAA-CCTCTCTT--
------
FC88X_00029_00207 ------------------TCACCAAA-CCTCCCATGGTGCTATTGC--------
------

```
FC88X_00956_00978      -----------------GTCACCAAA-CCTCCCATGGTGCTATTGC--------
------
FC88X_00051_00401      ------------------TCACCAAA-CCTCCCATGGTGCTATTGC--------
------
FC88X_00095_00581      ------------------TCACCAAA-CCTCCCATGGTGCTATTGC--------
------
FC88X_00251_00142      ------------------TCACCAAA-CCTCCCATGGTGCTATTGC--------
------
FC88X_00697_00661      ------------------TCACCAAA-CCTCCCATGGTGCTATTGC--------
------
FC88X_00883_00306      ------------------TCACCAAA-CCTCCCATGGTGCTATTGC--------
------
FC88X_01100_00691      ------------------TCACCAAA-CCTCCCATGGTGCTATTGC--------
------
FC88X_01130_01033      ------------------TCACCAAA-CCTCCCATGGTGCTATTGC--------
------
FC88X_00025_00141      ------------------TCACCAAA-CCTCCCATGGTGCTATTGC--------
------
FC88X_00030_00260      ------------------TCACCAAA-CCTCCCATGGTGCTATTGC--------
------
FC88X_00054_00148      ------------------TCACCAAA-CCTCCCATGGTGCTATTGC--------
------
FC88X_00071_00314      ------------------TCACCAAA-CCTCCCATGGTGCTATTGC--------
------
FC88X_00119_00728      ------------------TCACCAAA-CCTCCCATGGTGCTATTGC--------
------
FC88X_00209_01050      ------------------TCACCAAA-CCTCCCATGGTGCTATTGC--------
------
FC88X_00349_00180      ------------------TCACCAAA-CCTCCCATGGTGCTATTGC--------
------
FC88X_00442_01055      ------------------TCACCAAA-CCTCCCATGGTGCTATTGC--------
------
FC88X_00706_00067      ------------------TCACCAAA-CCTCCCATGGTGCTATTGC--------
------
FC88X_00857_00696      ------------------TCACCAAA-CCTCCCATGGTGCTATTGC--------
------
FC88X_00910_00847      ------------------TCACCAAA-CCTCCCATGGTGCTATTGC--------
------
FC88X_01068_00820      ------------------TCACCAAA-CCTCCCATGGTGCTATTGC--------
------
FC88X_01077_00907      ------------------TCACCAAA-CCTCCCATGGTGCTATTGC--------
------
FC88X_00155_00783      ------------------TCACCAAA-CCTCC-ATGGTGCTATTGC--------
------
FC88X_00463_01194      ------------------TCACCAAA-CCTCGCATGGTGCTATTGC--------
------
FC88X_00454_01212      ------------------TCACCAAA-CCTCCCATG-TGCTATTGC--------
------
FC88X_00769_01066      ------------------TCACCAA--CCTCCCATGGTGCTATTGC--------
------
FC88X_00912_00457      ------------------TCACCAAA-CCTCCCATGGTGCTATTTC--------
------
FC88X_00566_00222      ------------------TCACCAAA-CCTCCCATGGTGCCACTGC--------
------
FC88X_00658_00129      ----------------CCTCACCAAA-AATCTCCCGATGACTGC----------
------
```

```
FC88X_00874_01239      ----------------TCTCATCAACTAATCTGCCCATGTCT------------
------
FC88X_00283_01053      ----------TTTGTCGCTATTCACA-GCTCCCTTGGTC---------------
------
FC88X_00923_01266      ----------TTTGTCTCTCTTCACA-CGCCCCATTGT----------------
------
FC88X_00573_00408      ----------CTTATCACTAAGCACA-CCTCCCATTGAC---------------
------
FC88X_00643_01063      --------------------CTCACATTTCCCTCCTATCTATTCGTC-------
------
FC88X_00291_01087      -------------------TCTCGCATTACTCCAACTCCTCGTCAT--------
------
FC88X_00883_00419      -------------------TCACACATTTACCCAAATTTC-TATTCT-------
------
FC88X_00492_01200      -------------------TCACACATTTACCCAAATTTC-TATTCT-------
------
FC88X_00922_00430      -------------------TCACACATTTACCCAAATTTC-TATTCT-------
------
FC88X_01139_00652      ------------------GTCACACATTTACCCAAATTTCCTATTCT-------
------
FC88X_00674_00298      -------------------TCACACATTTACC-AAATTTC-TATTCT-------
------
FC88X_00709_00548      -------------------TCACACATTTACCCAAACTTC-TATTCT-------
------
FC88X_00879_01086      -------------------TCAAACCTTCACTCCCATTCC-TCTTCT-------
------
FC88X_01044_00762      -------------------CCACAATTCTCACCACGCTTC-TATGCG-------
------
FC88X_01090_00747      -------------------CCACAATTCTCACCACGCTTC-TATGCG-------
------
FC88X_00348_01171      -----------------CTCCGCAATTTACCCCGT-TGTC-TATTC--------
------
FC88X_00696_00089      -----------------ACTGCGGCTTTT-CGCCAACCTACTCTTCTTCCTTT-
------
FC88X_00142_00818      -----------------ACTGCTGCTTTT-CGCCAGCCTACTCTT---------
------
FC88X_00524_01024      ------------------AGCCGGCTATT-CCCCAGCTAATCTTTTCTTTT---
------
FC88X_01105_01041      ----------------TTATGCGGCATTCACCCCAAACTTCTC-----------
------
FC88X_00098_00760      -------------------TCCTATTGCTCCCTGCGCCCCAATGCA--------
------
FC88X_00457_01228      -------------------TCCCATTGCTCCCTGCGCCCCAATGCA--------
------
FC88X_00178_00853      ---------------------CCATTCTTCCTGCTGCCCCGATGTATC------
------
FC88X_00524_00049      ------------------CTCCCATTGTCCCTGCAGCCAC-ATGTA--------
------
FC88X_00934_00358      -------------------TCCCATTCTCTTTGACGCCACAGTCCC--------
------
FC88X_00923_01047      ------------------------TTCATTTCTATGCGCCAATTCCCCGAT---
------
FC88X_00506_01007      ------------------CTCCCGCAGTGCACGATTCTTGTATT----------
------
FC88X_01018_00522      -----------------CCTCCCGCAGTGCACGATTCTTGTATT----------
------
```

FC88X_00069_00610 -------------------CCCTGCGATTCACATCCTTGCTATTTC--------
------
FC88X_00716_01022 -------------------ATTCGCGATTCCCTGCACTGGACTATT--------
------
FC88X_00022_00258 -------------------ATTCGCGATTCCCTGCACTGGACTATT--------
------
FC88X_00495_00142 -------------------ATTCGCGATTCCCTGCACTGGACTATT--------
------
FC88X_01081_00392 -------------------ATTCGCGATTCCCTGCACTGGACTATT--------
------
FC88X_00616_00437 --------------------ATCCCTCTTTCTTGATTTGGAACTATG-------
------
FC88X_00274_01100 ------------------CTCCCCGCATTCGCTGATATTCAACTA---------
------
FC88X_00999_00469 -------------------CTCCCCGATTTCCTGACATTGACCCCAT-------
------
FC88X_00915_01250 -------------------TCCCACGATCTCCGACACTCCCGTATG--------
------
FC88X_00076_00737 -------------------CTCCATTATTTCCGGGAATCGCGTTTT--------
------
FC88X_01080_00639 ------------------CTCCCACGACTTCCCAAAATCGGCCTAC--------
------
FC88X_00214_00035 ------------------CTCCCACAGTT--CGGAATCTCTCCTACTT------
------
FC88X_00243_01098 --------------------TTCACAGA-TGCGTAATCAGGAGCTCCT------
------
FC88X_00498_00671 --------------------TTCACAGA-TGCGTAATCAGGAGCTCCT------
------
FC88X_00767_00290 ---------------------CCACAGTCTGCGTTCTGGGGCTCTCCT------
------
FC88X_00958_01203 ---------------------CCACAAT-TCCCTTCTCAGGACCGCCTT-----
------
FC88X_00499_00993 --------------------TCTCCATTCTGTGGACCCACTATTCG--------
------
FC88X_00831_00106 -------------------CTATCTATTCT-CGGGACCGTCATTCGC-------
------
FC88X_00085_00109 -------------------CTTTCCATTATGCGGATCCCGGCTACT--------
------
FC88X_00776_01128 -------------------CTTTTCATTATGCGGATCCCGGCTACT--------
------
FC88X_00787_00910 -------------------CCTTTTATTCTCTGGACCCCCGATAAC--------
------
FC88X_00417_00400 -------------------CGTCTCGATTCCCAGCG-ACCGTTTGTT-------
------
FC88X_00556_00801 -------------------CGTCTCGATTCCCAGCG-ACCGTTTGTT-------
------
FC88X_00789_00874 -------------------CGTCTCGATTCCCAGCG-ACCGTTTGTT-------
------
FC88X_00954_00419 -------------------CGTCTCGATTCCCAGCG-ACCGTTTGTT-------
------
FC88X_00432_01111 -------------------CGTCTCGATTCCCAGCG-ACCGTTTGTT-------
------
FC88X_00596_00061 -------------------CGTCTCGATTCCCAGCG-ACCGTTTGTT-------
------
FC88X_00817_00358 -------------------CGTCTCGATTCCCAGCG-ACCGTTTGTT-------
------

FC88X_00966_00972 -------------------CGTCTCGATTCCCAGCG-ACCGTTTGTT-------
------
FC88X_01065_01200 -------------------CGTCTCGATTCCCAGCG-ACCGTTTGTT-------
------
FC88X_01122_00676 -------------------CGTCTCGATTCCCAGCG-ACCGTTTGTT-------
------
FC88X_00930_00712 -------------------CGTCTCGATTCCCAGCG-ACCGGTTGTT-------
------
FC88X_01092_00772 -------------------CATCTCGATTCCCAGCG-ACCGTTTGTT-------
------
FC88X_00568_00497 ------------------CCATATC-ATTCCCAGCATACCGTTTCT--------
------
FC88X_00416_01043 ---------------TTCGCGATCCGAATCCCAGCTA-CTGTT-----------
------
FC88X_00023_00675 ---------------TTCGCGATC-GAATCCCAGCTA-CTGTT-----------
------
FC88X_00502_00164 ---------------TTCGCGATCCGAATCCCAGCTA-CTGTT-----------
------
FC88X_01010_01245 ---------------TTCGCGATCCGAATCCCAGCTA-CTGTT-----------
------
FC88X_01130_01155 ---------------TTCGCGATCCGAATCCCAGCTA-CTGTT-----------
------
FC88X_00525_00442 ---------------TTCGCGATCCGAATCCCAGCTA-CTGTT-----------
------
FC88X_00968_01019 ---------------TTCGCGATCCGAATCCCAGCTA-CTGTT-----------
------
FC88X_00724_00451 ---------------TTCGCGATCCGAGTCCCAGCTAACTGTT-----------
------
FC88X_00424_00672 ---------------------TCCTGATTCCCAGCAAACGGCTCGAC-------
------
FC88X_00814_00973 --------------------TCCCTGATTCCCAGCAAACGGCTCGAC-------
------
FC88X_00678_01260 ------------------TCTGCCAATTTCCCTATGCCCGATATT---------
------
FC88X_01130_00665 ------------------TCTGCCAATTTCCCTATGCCCGATATTCTT------
------
FC88X_00607_01044 ------------------TCCGCCAATTTCCCTATGCCCGATATT---------
------
FC88X_00674_00916 -------------------CTGCCAGCTTCCCTATTCCCTATTGAT--------
------
FC88X_00813_00381 -------------------CTGCATGATTCCCTGCCCCCGTATTGAC-------
------
FC88X_01036_00854 -------------------CTGCATGATTCCCTGCCCCCGTATTGAC-------
------
FC88X_00154_00821 -----------CTTTCCGTTATCCCCTTTTGCCATTCT----------------
------
FC88X_00937_00737 -----------CTTTCCGTTATCCCCTTTTGCCATTCT----------------
------
FC88X_00434_01081 -----------ATTTGCTCTATCTC-TTTTCCCATTGTT---------------
------
FC88X_00614_00065 ----------------CCTTATCCC-TTTACCCATTGAACAAT-----------
------
FC88X_00183_00166 ---------------CTCTCAACCCACTAA-GCATCGCATTTT-----------
------
FC88X_00645_01185 ---------------CTTTCAACCCACTAA-GCATCGCATTTTT----------
------

FC88X_00598_01246 ------------------TTATCCCATTATCGCATCGAGTTTCAC---------
------
FC88X_00226_00890 ------------------TCGGCCATTTGACCGATTCTC-TCACAT--------
------
FC88X_01024_00667 --------------------ATCGGCTCGATCGATTGCCATCACATT-------
------
FC88X_01098_00928 -----------------GCTAACCACGATCTCCTTCAATAACACA---------
------
FC88X_00427_00064 -----------------CCTAACCCTTCGACTCATTCATATCAC----------
------
FC88X_00013_00532 --------------------TTCGCGACTCCGTGTCCTTGACTTTTT-------
------
FC88X_00081_00308 --------------------TTCGCGACTCCGTGTCCTTGACTTTTT-------
------
FC88X_00210_00124 -------------------CTTCGC-ACTCCGTCACCGTCAGTTATT-------
------
FC88X_00743_01048 -------------------CTTCGC-ACTCCGTCACCGTCGGTTATT-------
------
FC88X_00128_00582 ---------------------TTACCACTACGTAACGTTCACTACCTC------
------
FC88X_00918_00814 ---------------------------CTACGTACCAGTCACCTCC--------
------
FC88X_00171_00883 ---------------CTAACTACGAAACCGCGCGCTGCCCCT------------
------
FC88X_00105_00931 -------------ATCTAACTTAGACACCGCTCCCTGGCTC-------------
------
FC88X_00415_01136 -------------CATGCAACAAAA--CTGCGTAGCTGTCCC------------
------
FC88X_00856_00971 -------------CATGCAACAAAA--CTGCGCAGCTGTCCC------------
------
FC88X_00465_00013 ----------------CCGATAAAA--CTTCGCAGTCGTCCCATT---------
------
FC88X_00663_01259 ----------------CCGATAAAAAACTTCGCAGTCGTCCCATT---------
------
FC88X_00797_00960 ----------------CCGATAAAA--CTTCGCAGTCGTCCCATT---------
------
FC88X_01101_00896 ----------------CCGATAAAA--CTTCGCAGTCGTCCCATT---------
------
FC88X_00021_00357 ----------------CCGATAAAA--CTTCGCAGTCGTCCCATT---------
------
FC88X_00621_01169 ----------------CCGATAAAA--CTTCGCAGTCGTCCCATT---------
------
FC88X_00904_01076 ----------------CCGATAAAA--CTTCGCAGTCGTCCCATT---------
------
FC88X_00956_00810 ----------------CCGATAAAA--CTTCGCAGTCGTCCCATTC--------
------
FC88X_00905_00929 ----------------CCGATAAAA--CTTCGCAGTCGTCCCATTCC-------
------
FC88X_00958_00264 ----------------CCGATAAAA--CTTCGCAGTGGTCCCATT---------
------
FC88X_00880_00204 ----------------CCGATAAAA--CTTCGCAGTGGTCCCATT---------
------
FC88X_01103_01185 ------------------GCCACAAACTCTGGCACTTCTCCTTGCG--------
------
FC88X_01140_00675 ------------CCATTGGTAACAAAC-CTGGCGCTCACCTTCCT---------
------

FC88X_00706_00613 ----------------CTATTGGCATCCCTAGCACTCCCCGGTT----------
------
FC88X_00926_00842 -------------------TTCTGCCACTTGGCAT-CGCTGATCGCTC------
------
FC88X_00988_00863 -------------------TTCTGCCACTTGGCAT-CGCTGATCGCTC------
------
FC88X_00667_00065 -------------------TTCTGCCACTTGGCAT-CGCTGATCGCT-------
------
FC88X_00126_00950 -------------------TTCCTCAACTTGCCTCGCGATGATCGC--------
------
FC88X_00249_00288 -------------------CTCCCAGACCGACCGCATAGCCATGAC--------
------
FC88X_01133_00610 --------------------TTCCTGACC--CCTCGTAGCGTTGATCGC-----
------
FC88X_00881_00704 -------------------CTACATG-TCCGCTGG-CATCGCTATCTC------
------
FC88X_01114_00553 -------------------CTACATG-TCCGCTGG-CATCGCTATCTC------
------
FC88X_00613_00917 -------------------CTACATG-TCCGCTGG-CATCGCTATCTC------
------
FC88X_00008_00445 -------------------CTACATG-TCCGCTGG-CATCGCTATCTC------
------
FC88X_00045_00537 -------------------CTACATG-TCCGCTGG-CATCGCTATCTC------
------
FC88X_00940_00724 -------------------CTACATG-TCCGCTGG-CATCGCTATCTCACCC--
------
FC88X_00226_00017 -------------------CTACATG-TCCGCTGG-CATCGTTATCTC------
------
FC88X_00924_00395 -------------------CTACATG-TCCGCTGG-CATCGCTATTTC------
------
FC88X_00448_00459 -------------------CTACATG-TCCGTTGG-CATCGCTATCTC------
------
FC88X_01034_00917 -------------------CTACATGCTCCACGGA-CTTTGCTATCT-------
------
FC88X_00910_00798 -------------------CTACACGCTCCACGGA-CTTTGCTATCT-------
------
FC88X_00948_00832 -----------------TTTGCCACTGATTGGCAT-C---GCTATCAC------
------
FC88X_00364_01187 -----------------TTTGCCACTGGTTGGCAT-C---GCTATCAC------
------
FC88X_00937_00497 --------------------CCCACTCCTTGGCAC-TTGCGCTCTCAC------
------
FC88X_00986_00719 --------------------ACCAAAACTTGGCGC-A---GCTCCCAC------
------
FC88X_00817_01128 ---------------------CCGCTGCCTGGCCA-TCACTCTCATTCT-----
------
FC88X_00980_01163 ---------------------ATGCCGCCTGGCCG-TCCCGATCTTATT-----
------
FC88X_00404_00022 -----------------CGACACGAATGTCTTGCGCTTTTCCTC----------
------
FC88X_00317_01002 -----------------CGACACGAATGTCTTGCGCTTTCCTC-----------
------
FC88X_00462_00448 -----------------CGACACGAATGTCTTGCGCTTTCCTC-----------
------
FC88X_00731_01100 -----------------CGACACGAATGTCTTGCGCTTTCCTC-----------
------

FC88X_01010_00512 -----------------CGACACGAATGTCTTGCGCTTTCCTC-----------------
FC88X_00468_00099 -----------------CGACACGAATGTCCTGCGCTTTCCTC-----------------
FC88X_00192_00966 ---------------CACGAAACGA-TATTTTGCTATTTTCAC-----------------
FC88X_00608_01185 -------------------ACACGAAA-CTCGATGAATTCCTATTCT-------------
FC88X_01081_00892 -------------------ACACGAAAACTCGATGAATTCCTATTCT-------------
FC88X_00757_01236 -------------------ACACGAAA-CTCGATGAATTCCTATTCT-------------
FC88X_00754_01275 -------------------ACACGAAA-CTCGATGAATTCCTATTCT-------------
FC88X_00602_00958 -------------------ACACGAAA-CTCGATGAATTCCTATTCT-------------
FC88X_01083_00929 -------------------ACACGTCC-CTCGTGAGTTCTCTATCC--------------
FC88X_00683_00078 ----------------CTGACACTAACTCAAGCTCTATTCTC------------------
FC88X_00780_01018 ----------------CTGACACTAACTCAAACTCTATTCTC------------------
FC88X_00721_00937 --------------ATATAAAACCAACCCAATCGCTATTCT-------------------
FC88X_00860_00868 --------------------ATATCACACATACCTCGCTCT-------------------
FC88X_00026_00169 ---------------------CTCCCAT---TGTTACGTTCCATCACGTT----------
FC88X_00089_00482 --------------------CCTCCCAT---TGTTACGTTCCATCACGTT----------
FC88X_00918_00672 --------------------CCTCCCAT---TGTTACGTTCCATCACGCT----------
FC88X_00106_00942 --------------------CTCCACAG---TGTTGCACGTTTCCATCCC----------
FC88X_01054_01035 ------------CCTATGAATCTCCCAA---TTTG-CGCTTGC-----------------
FC88X_01111_00908 --------------------TCCCAAGT---TTTGACGTTTGCT----------------
FC88X_00119_00524 -----------CCTAGT-GATCTCCCAC-AACGTAACGTT--------------------
FC88X_00991_00861 -----------CCTAGTAGATCTCCCACCAACGTAACGTT--------------------
FC88X_00759_01151 -----------CCTAGT-GATCTCCCAC-AACGTAACGTT--------------------
FC88X_01093_00879 -----------CCTAGT-GATCTCCCAC-AACGTAACGTT--------------------
FC88X_00207_00900 -----------CCTAGT-GATCTCCCAC-AACGTAACGTT--------------------
FC88X_01040_00470 -----------CCTAGT-GATCTCCCAC-AACGTAACGTT--------------------
FC88X_01091_00917 -----------CCTAGT-GATCTCCCAC-AACGTAACGTT--------------------
FC88X_01056_00958 -----------CCTAGT-GATCTCCCAC-AACGTAACGTT--------------------
FC88X_01139_01044 -----------CCTAGT-GATCTCCCAC-AACGTAACGTT--------------------

FC88X_00401_00461 -----------CCTGGT-GATCTCCCAC-AACGTAACGTT--------------
------
FC88X_00832_00802 -----------CCTAGT-GATCTCCCAC-AGCGTAACGTT--------------
------
FC88X_00137_00750 ------------CTAGTCGATCTCCCAC-AACGTAACGTTC-------------
------
FC88X_00543_00582 -----------CTCAGC-GATCTCCCA---ACGCAACGTCCT------------
------
FC88X_00954_01061 -----------CTCAGC-GATCTCCCA---ACGCAACGTCCT------------
------
FC88X_00216_00999 ------------TTACCAATTCTCCCAC--GGT-AACGTTCA------------
------
FC88X_01070_01182 ------------TTACCAATTCTCCCAC--GGT-AACGTTCA------------
------
FC88X_00278_00734 ------------TTACCAATTCTCCCAC--GGT-AACGTTCA------------
------
FC88X_00577_00350 ------------TTACCAATTCTCCCAC--GGT-AACGTTCA------------
------
FC88X_00857_01099 ------------TTACCAATTCTCCCAC--GGT-AACGTTCA------------
------
FC88X_01116_00584 ------------TTACCAATTCTCCCAC--GGT-AACGTTCA------------
------
FC88X_01108_00754 ------------TTACCAATTCTCCCAC--GGT-AACGCTCA------------
------
FC88X_00122_00429 -----------GTTACCAAT-CTCCCAC--GGTCAACGTTCA------------
------
FC88X_00443_01196 ------------TCCAGAGACCTCCCAC---TGTGACG-TGCA-----------
------
FC88X_00039_00160 ------------CGCATGAACACCCCAC----GTGACGCTGCA-----------
------
FC88X_00696_01219 -----------------CCATTGTTCT-TACATTGTCGCGTTCAC---------
------
FC88X_01110_00979 -----------------CCATGCCTCT-TACTTTCCCGCGCTTTT---------
------
FC88X_00085_00542 ----------------GCCATCTCTCTCTACATAGCCGCACGTT----------
------
FC88X_00966_00278 ------------------CTTCCCTCGCTATCACCGTGCGCCGTGC--------
------
FC88X_00342_00409 ------------------CTTCCTTCTCTGTTTCAGTGCCGCGTT---------
------
FC88X_00081_00380 -------------------TCCCACTATTTCAACCGCGCCGCACGT--------
------
FC88X_00697_00742 --------------------CTCCCTATTT-AACTGTGCCGCACATTG------
------
FC88X_00070_00116 ------------------CCTCCCGTTATGTAGCACCAA--ATTGTT-------
------
FC88X_00544_01063 ------------------CCTCCCACTATAGAAACCCAAGAATTGT--------
------
FC88X_00476_00516 -------------------CTCCCACGGTAATATAGTACCGATCAT--------
------
FC88X_00769_00989 -------------------CTCCCACGGTAATATAGTACCGATCAT--------
------
FC88X_00981_01156 --------------------TCCCACGTTACATTACCGCGCATTCGC-------
------
FC88X_00074_00175 ---------------TTTACTCCTCTACTTTGCCGTCTTGTT------------
------

FC88X_01098_00807 ----------------CTTTACCTACGACTCTCCCATCCTGTA------------
------
FC88X_00328_00338 ----------------CCCCTACCCTCCTCTTGCCGTCATGTT------------
------
FC88X_00403_01033 -----------------TCAAAATCT-AACTTCCCGTCGCCTTATT---------
------
FC88X_00838_01222 -----------------TCACGAACT-ATCTTGCCGTCACTTTCT----------
------
FC88X_00750_00427 ----------------------CTCT-CACTTGACGTTGCGTT------------
------
FC88X_01085_01131 --------------------
CTCTCTACTCCTCACGTTGTTTTCCTCTTCCTTTC------
FC88X_00144_00068 -----------------CTATTCCCTG-GTTTGCAAACG-CGTGTT---------
------
FC88X_00102_00822 -----------------CTATTCCCTG-GTTTGCAAACG-CGTGTT---------
------
FC88X_00419_00436 -----------------CTATTCCCTG-GTTTGCAAACG-CGTGTT---------
------
FC88X_00975_00248 -----------------CTATTCCCTG-GTTTGCAAACG-CGTGTT---------
------
FC88X_01031_01093 -----------------CTATTCCCTG-GTTTGCAAACG-CGTGTT---------
------
FC88X_00520_00141 -----------------CTATTCCCTG-GTTTGCAAACG-CGTGTT---------
------
FC88X_00588_00159 -----------------CTATTCCCTG-GTTTGCAAACG-CGTGTT---------
------
FC88X_00556_01246 -----------------CTATTCCCTG-GTTTGCAAACG-CGTGTT---------
------
FC88X_01029_00997 -----------------TTATTCCCTGAGTTTGCAAACG-CGTGTT---------
------
FC88X_01088_01037 ---------------------CCCCTG-ATTTCCCCACG-CGCGTTATT------
------
FC88X_00983_00672 -------------------CATCCCAGCTCTCCTAGGCA-CGTTCTT--------
------
FC88X_00756_00987 ---------------------TCCCTACTCTCC-AGACG-CGTGCTAGC------
------
FC88X_00566_01163 -------------------TTTCCCTGATCTCCTAACTGGCATGTTT--------
------
FC88X_00734_00953 -------------------TTTCCCTGATCTCCTAACTGGCATGTTT--------
------
FC88X_00951_01082 -------------------TTTCCCTGATCTCCTAACTGGCATGTTT--------
------
FC88X_01073_00856 -------------------TTTCCCTGATCTCCTAACTGGCATGTTT--------
------
FC88X_01084_00713 -------------------TTTCCCTGATCTCCTAACTGGCATGTTTT-------
------
FC88X_00409_00756 -------------------TTTCCCTGACCTCCTAACTGGCATGTTT--------
------
FC88X_00658_01054 -------------------TTTCCCTGATCTCCTAACTGTCATGTTT--------
------
FC88X_00389_01196 ----------------------CCCTGACCTCCTTATTCCCATGCTACA------
------
FC88X_00246_01025 ----------------------TCTTGAACTCCCTAGTCTAGCGCCT--------
------
FC88X_00565_00242 -----------------CAGTACCCTGACCTCCCAACGCT-GCGC----------
------

```
FC88X_00157_00820       --------------------CTCCCACTTTTCCGGATTGACTATGCC-------
------
FC88X_01084_00835       --------------------CTCCCACTTTTCCGGATTGACTATGCC-------
------
FC88X_00522_01201       -------------------ACTCCCACTGTCCCG-ACTCACCATTCC-------
------
FC88X_00410_00787       -------------CCGTTAGCGCTCCCATTTCCCGAATGAC-------------
------
FC88X_00518_00179       -------------CCGGTGCACTCCCCTTGCCCGCATTAA--------------
------
FC88X_00798_01006       -------------CAATAATCTCCCCAGTCTCCGCATGGT--------------
------
FC88X_01020_00323       -------------CAATAATCTCCCCAGTCTCCGCATGGT--------------
------
FC88X_00539_00061       -------------CAATAATCTCCCCAGTCTCCGCATGGT--------------
------
FC88X_01020_00476       -------------CAATAATCTCCCCAGTCTCCGCATGGT--------------
------
FC88X_00997_00587       -------------CCGTAGTCTGCCCCTTCTCCTCATGTT--------------
------
FC88X_00119_00501       --------------------CTGGCACTTCTCCTACAGTCCGGTGTT-------
------
FC88X_00115_00578       -------------------CCTGGCCCTTCCCC-ACTGTACGATACA-------
------
FC88X_00197_00407       -------------------TCTCGCCATTCCCT-TCGCCCCGGTAAA-------
------
FC88X_00914_00975       -------------------CCTTGCCCTGTCCC-GTAGACCGCTAAAC------
------
FC88X_00005_00633       ------------------TTTCTAGCTATTCGCCACCTTGGAGGT---------
------
FC88X_00365_01089       ------------------TTTCTAGCTATTCGCCACCTTGGAGGT---------
------
FC88X_00124_00694       -------------------CTATAGCT-TTCCCTGCGTTGGCGTTCCCC-----
------
FC88X_00789_00718       ------------------CTCCCATCACCGGGTTGCCAACCTGACT--------
------
FC88X_00089_00881       ------------------CTCCCAATTCCCCTGCGTTGACATAGT---------
------
FC88X_00131_00089       -----------------TTCTCCCAGTTTGAGTTGTCAACGCGT----------
------
FC88X_00885_00569       ----------------GTTCTCCCAGTTTGAGTTGTCAACGCGT----------
------
FC88X_00191_00863       -----------------TTCTCCCAGTTTGAGTTGTCAACGCGT----------
------
FC88X_00422_00029       -----------------TTCTCCCAGTTTGAGTTGTCAACGCGT----------
------
FC88X_00668_01057       -----------------TTCTCCCAGTTTGAGTTGTCAACGCGT----------
------
FC88X_00109_00606       -----------------TTCTCCCAGTTTGAGTTGTCAACGCGT----------
------
FC88X_00150_00799       -----------------TTCTCCCAGTTTGAGTTGTCAACGCGT----------
------
FC88X_01010_00271       -----------------TTCTCCCAGTTTGAGTTGTCAACGCGT----------
------
FC88X_00741_00336       ----------------ACTATCCCTTTTTTTGTTATCAACTGACA---------
------
```

FC88X_00899_00395 -------------------CTCCCAA--CGATTCAACACCACCAGTGGT-----
------
FC88X_01002_00422 -------------------CTCCCAA--CGATTCAACACCACCAGTGGT-----
------
FC88X_00397_01147 -----------------GTCTCCCAATACGGTGCCATAACACCTG---------
------
FC88X_00035_00346 -----------------TTCTCCCATTGCTTGTCATCACCTGCA----------
------
FC88X_00318_00673 -----------------TTCTCCCATTGCTTGTCATCACCTGCA----------
------
FC88X_00664_01156 -----------------TTCTCCCATTGCTTGTCATCACCTGCA----------
------
FC88X_01077_00804 -----------------CTATCGTCACTTGACATGGTGC-TTCT----------
------
FC88X_00685_00065 ----------------ACTATCGTCACTTGACATGGTGC-TTCTC---------
------
FC88X_00733_00916 -----------------CTATCGTCACTTGACATGGTGC-TTCTC---------
------
FC88X_00752_01034 -----------------CTATCGTCACTTGACATGGTGC-TTCTC---------
------
FC88X_00994_00829 -----------------CTATCGTCACTTGACATGGTGC-TTCTC---------
------
FC88X_00044_00445 -----------------CTATCGTCACTTGACATGGTGC-TTCTC---------
------
FC88X_00720_01145 ----------------GCTATCGTCACTTGACATGGTGC-TTCTC---------
------
FC88X_01090_00875 ----------------GCTATCGTCACTTGACATGGTGC-TTCTC---------
------
FC88X_00611_00944 ----------------GCTATCGTCACTTGACATGGTGC-TTCTC---------
------
FC88X_00928_00239 -----------------CTATCGTCACTTGACATGGTGC-TTCTC---------
------
FC88X_00981_01167 -----------------CTATCGTCACTTGACATGGTGC-TTCTC---------
------
FC88X_00025_00470 -----------------CTATCGTCACTTGACATGGTGC-TTCTC---------
------
FC88X_00130_00989 -----------------CTATCGTCACTTGACATGGTGC-TTCTC---------
------
FC88X_00139_00960 -----------------CTATCGTCACTTGACATGGTGC-TTCTC---------
------
FC88X_00394_00070 -----------------CTATCGTCACTTGACATGGTGC-TTCTC---------
------
FC88X_00386_00998 -----------------CTATCGTCACTTGACATGGTGC-TTCTC---------
------
FC88X_00461_01166 -----------------CTATCGTCACTTGACATGGTGC-TTCTC---------
------
FC88X_00625_00678 -----------------CTATCGTCACTTGACATGGTGC-TTCTC---------
------
FC88X_00614_01034 -----------------CTATCGTCACTTGACATGGTGC-TTCTC---------
------
FC88X_00600_01171 -----------------CTATCGTCACTTGACATGGTGC-TTCTC---------
------
FC88X_00732_01076 -----------------CTATCGTCACTTGACATGGTGC-TTCTC---------
------
FC88X_00860_00232 -----------------CTATCGTCACTTGACATGGTGC-TTCTC---------
------

```
FC88X_00877_00238      -----------------CTATCGTCACTTGACATGGTGC-TTCTC---------
------
FC88X_00851_00966      -----------------CTATCGTCACTTGACATGGTGC-TTCTC---------
------
FC88X_00861_00966      -----------------CTATCGTCACTTGACATGGTGC-TTCTC---------
------
FC88X_00919_00436      -----------------CTATCGTCACTTGACATGGTGC-TTCTC---------
------
FC88X_00901_00687      -----------------CTATCGTCACTTGACATGGTGC-TTCTC---------
------
FC88X_00959_01254      -----------------CTATCGTCACTTGACATGGTGC-TTCTC---------
------
FC88X_01074_00470      -----------------CTATCGTCACTTGACATGGTGC-TTCTC---------
------
FC88X_01125_00614      -----------------CTATCGTCACTTGACATGGTGC-TTCTC---------
------
FC88X_01123_00793      -----------------CTATCGTCACTTGACATGGTGC-TTCTC---------
------
FC88X_00040_00098      -----------------CTATCGTCACTTGACATGGTGC-TTCTC---------
------
FC88X_00128_00628      -----------------CTATCGTCACTTGACATGGTGC-TTCTC---------
------
FC88X_00118_00718      -----------------CTATCGTCACTTGACATGGTGC-TTCTC---------
------
FC88X_00193_00045      -----------------CTATCGTCACTTGACATGGTGC-TTCTC---------
------
FC88X_00194_00143      -----------------CTATCGTCACTTGACATGGTGC-TTCTC---------
------
FC88X_00194_01052      -----------------CTATCGTCACTTGACATGGTGC-TTCTC---------
------
FC88X_00232_00969      -----------------CTATCGTCACTTGACATGGTGC-TTCTC---------
------
FC88X_00271_00833      -----------------CTATCGTCACTTGACATGGTGC-TTCTC---------
------
FC88X_00255_01064      -----------------CTATCGTCACTTGACATGGTGC-TTCTC---------
------
FC88X_00341_00822      -----------------CTATCGTCACTTGACATGGTGC-TTCTC---------
------
FC88X_00362_01056      -----------------CTATCGTCACTTGACATGGTGC-TTCTC---------
------
FC88X_00352_01176      -----------------CTATCGTCACTTGACATGGTGC-TTCTC---------
------
FC88X_00515_00302      -----------------CTATCGTCACTTGACATGGTGC-TTCTC---------
------
FC88X_00522_00631      -----------------CTATCGTCACTTGACATGGTGC-TTCTC---------
------
FC88X_00511_00651      -----------------CTATCGTCACTTGACATGGTGCGTTCTC---------
------
FC88X_00216_00301      -----------------CTATTGTCACTTGACATGGTGC-CTCTC---------
------
FC88X_00957_00964      -------------------CTTGTCACTTGACATGATGC-TTCTC---------
------
FC88X_00115_00645      ---------------TCGCACCATCTTTAGAT-C-GCCTTTTCT----------
------
FC88X_00165_00923      ---------------TCGCACCATCTTTAGAT-C-GCCTTTTCT----------
------
```

FC88X_00877_00743 ---------------TCGCATCATCTTTAGAT-C-GCCTTTTCT----------
------
FC88X_00125_00968 ----------------TGCACCATTATTTGAT-TTGCCGCTTCT----------
------
FC88X_00400_00468 ----------GGGGACCTGACTGTCACTAGTC-TG-CGCTTTCT----------
------
FC88X_00684_01263 ---------------CCTGACTGTCACTAGTC-TG-CGCTTTCT----------
------
FC88X_01001_00740 ---------------CCTGACTGTCACTAGTC-TG-CGCTTTCT----------
------
FC88X_01083_00850 ---------------CCTGACTGTCACTAGTC-TG-CGCTTTCT----------
------
FC88X_00383_00481 ---------------CCTGACTGTCACTAGTC-TG-CGCTTTCT----------
------
FC88X_00366_00602 ---------------CCTGACTGTCACTAGTC-TG-CGCTTTCT----------
------
FC88X_00004_00519 ---------------CCTGACTGTCACTAGTC-TG-CGCTTTCT----------
------
FC88X_00036_00808 ---------------CCTGACTGTCACTAGTC-TG-CGCTTTCT----------
------
FC88X_00080_00172 ---------------CCTGACTGTCACTAGTC-TG-CGCTTTCT----------
------
FC88X_00510_00645 ---------------CCTGACTGTCACTAGTC-TG-CGCTTTCT----------
------
FC88X_00140_00222 ---------------CCTGACTGTTACTAGTC-TG-CGCTTTCT----------
------
FC88X_00482_00131 ---------------CCTGACTGTTACTAGTC-TG-CGCTTTCT----------
------
FC88X_00921_00226 ---------------CCTGACTGTCACTAGTT-TG-CGCTTTCT----------
------
FC88X_00173_00852 ---------------CCTGACTGTCACTAGTC-TG-CGCGTTCT----------
------
FC88X_00135_00721 ---------------CCTAAC-GTAACTTGTT-TGGCGCCTTCTC---------
------
FC88X_00083_00495 -------------ACAAGAC-AGTCAGTTGTCTTC-TATTCC------------
------
FC88X_00793_00964 -------------ACAAGAC-AGTCAGTTGTCTTC-TATTCC------------
------
FC88X_00907_00396 -------------ACAAGAC-AGTCAGTTGTCTTC-TATTCC------------
------
FC88X_00149_00082 -------------ACAAGAC-AGTCAGTTGTCTTC-TATTCC------------
------
FC88X_00786_01214 -------------ACAAGAC-AGTCAGTTGTCTTC-TATTCC------------
------
FC88X_00845_01206 -------------ACAAGAC-AGTCAGTTGTCTTC-TATTCCCTTCCTTT----
------
FC88X_01082_00800 ------GGGGAAGACAAGACCAGTCAGTTGTCTTCCTACTTCCCT---------
------
FC88X_00791_00323 -----------------CACCAGTCATTTTGCAGC-TATTTCGC----------
------
FC88X_00710_00691 ----------------CCACCAGTCATTTTGCAGC-TATTTCGC----------
------
FC88X_00559_00942 -----------------CACCAGTCATTT-GCAGC-TATTTCGC----------
------
FC88X_00652_01204 ----------------CCACCGGTCATTTTGCAGC-TATTTCGC----------
------

FC88X_00895_01017 ---------------CGCACTAGTCATCA-GTTGT-TTCTCCCC----------
------
FC88X_00658_00126 ---------------TGTACCGTTATCTCGTCTTTGCCTGTT------------
------
FC88X_00801_01214 ---------GGGGATTGTACCGTTATCTCGTCTT-GCCTGTT------------
------
FC88X_00970_00504 ---------------TGTACCAGTTACCAGGTGTGTCCCATT------------
------
FC88X_00966_00957 ---------------TGTACCAGTTACCAGGTGTGTCCCATT------------
------
FC88X_00926_01227 -------------CTAGACACTTT-ACTGTGC--GCTGTTCGC-----------
------
FC88X_01032_00524 -------------CTTGTCATTTTGACTTAGC-TGCTGTTCCTC----------
------
FC88X_00084_00520 -------------------CTTTCCCTTCTCTTGATTGCGCGTAGT--------
------
FC88X_00949_01000 -------------------CTTTCCCTTCTCTTGATTGCGCGTAGT--------
------
FC88X_00945_01173 -------------------CTTTCCCTTCTCTTGATTGCGCGTAGT--------
------
FC88X_00049_00830 -------------------CTCTCCCTTCTCTTGATTGCGCGTAGT--------
------
FC88X_01004_00576 -------------------CTTTTCCCTTCCTTGTTCCCGC-TAGT--------
------
FC88X_00575_01036 -------------------TCTGTCCCTTCTTTGTTTGCGCGTTTT--------
------
FC88X_00990_00577 -------------------TCTGTCCCTTCTTTGTTTGCGCGTTTTT-------
------
FC88X_00131_00881 -------------------TCTGTCCCTTCTTTGTTTGCGCGTTTT--------
------
FC88X_00199_01027 -------------------TCTGTCCCTTCTTTGTTTGCGCGTTTT--------
------
FC88X_00409_01163 -------------------TCTGTCCCTTCTTTGTTTGCGCGTTTT--------
------
FC88X_00567_00239 -------------------TCTGTCCCTTCTTTGTTTGCGCGTTTT--------
------
FC88X_01021_00469 -------------------TCTGTCCCTTCTTTGTTTGCGCGTTTT--------
------
FC88X_01084_00562 -------------------TCTGTCCCTTCTTTGTTTGCGCGTTTTTTT-----
------
FC88X_00970_01214 -------------------TCTGTCCCTTCTTTGTTTGCGCG------------
------
FC88X_00408_01099 ------------------GTCTGTCCCTCCTTTGTTTGCGCGTTTT--------
------
FC88X_00118_00456 -------------------TCTGTCCCTTCTTTGTTTGCGCTGTTTTCTTCCT-
------
FC88X_00121_00897 -------------------TCTGTCCCTTCTTTTGTTTGGCGTTTT--------
------
FC88X_01064_00714 ------------------ATCTGCTTCTCCTTATGTTTTGGTTTTT--------
------
FC88X_00954_00683 --------------------TCAAATCCTGTTCTTTTGCGTGTTCGC-------
------
FC88X_00950_00858 --------------------TCAAATCCTGTTCTTTTGCGTGTTCGC-------
------
FC88X_00925_00516 ------------------GTCAAAATCCTGTTCTTTTGCGTGTTCGC-------
------

FC88X_00985_00928 ---------------------
TCAACTCTGATTCTTTGCGTGTTCGCTCTTCACCC----
FC88X_00090_00303 -------------------CCTGTCGCTTAT-CTGATTTGTT-GTTCT------
------
FC88X_01035_01012 -------------------CCTGTCGCTTATTCTGATTTGTTCGTTCT------
------
FC88X_00082_00621 -------------------CCTGTCGCTTAT-CTGATTTGTT-GTTCT------
------
FC88X_00444_00863 -------------------CCTGTCGCTTAT-CTGATTTGTT-GTTCT------
------
FC88X_00608_00511 -------------------CCTGTCGCTTAT-CTGATTTGTT-GTTCT------
------
FC88X_00865_00320 -------------------TTTGCCTGTCGG-CCATCTAGTTTTCTC-------
------
FC88X_00679_01059 -------------------TTTGCCTGTCGG-CCATCTAGTTTTCTC-------
------
FC88X_00686_00359 -------------------TTTGCCTGTCGG-CCATCTAGTTTTTTC-------
------
FC88X_00572_00989 -------------------TTTGCCGTTTTG-CGCATCTGTTGTCTC-------
------
FC88X_00075_00720 -------------------CCTTTCGTTTT-CCGTTTCTGCGCGTTT-------
------
FC88X_00188_00045 -------------------CCTTTCGTTTT-CCGTTTCTGCGCGTTT-------
------
FC88X_00454_01198 -------------------CCTTTCGTTTT-CCGTTTCTGCGCGTTT-------
------
FC88X_00694_00981 -------------------CCTTTCGTTTT-CCGTTTCTGCGCGTTT-------
------
FC88X_00169_00983 -------------------CCTTTCGTTTT-CCGTTTCTGCGCGTTT-------
------
FC88X_00693_01105 -------------------CCTTTCGTTTT-CCGTTTCTGCGCGTTT-------
------
FC88X_01076_00663 -------------------CCTTTCGTTTTTCCGTTTCTGCGCGTTT-------
------
FC88X_00827_01072 ------------------GCCTTTCGTTTT-CCATTTCTGCGCGTTT-------
------
FC88X_00759_00732 -------------------CCTTTCGTTTT-CCGCTTCTGCGCGTTT-------
------
FC88X_00624_00663 -------------------CCTTCCGCATTTTCTGTCTTCTGTTTCTTCCTT--
------
FC88X_00631_01005 -------------------CCTTTCGC-TTGTCCTTCTTCTGTTTAC-------
------
FC88X_00270_00026 ---------------------CTTCTCTCTTCTGCATCGTTATTGGCA------
------
FC88X_00373_00065 -----------------------CTTTTCGCTTTCTTCATTTTTTGCCAT----
------
FC88X_00897_00645 --------------------CCTTGCCTTCACCGTTTGTGTGTTTTAC------
------
FC88X_01052_01096 --------------------CCTTGCCTTCACCGTTTGGGTGTGTTAC------
------
FC88X_00774_00158 --------------------CCATGGCTTCTCAGCATG-GTTTGTTCC------
------
FC88X_01055_00721 --------------------CCATGGCTTCTCAGCATG-GTTTGTTCC------
------
FC88X_00048_00572 --------------------CCTTGTCTTCTCTGTCTTGCTACTGTT-------
------

FC88X_00630_00121 --------------------CCTTGTCTTCTCTGTCTTGCTACTGTT-------
------
FC88X_00910_00516 --------------------CCTTGTCTTCCCTGTCTTGCTACTGTT-------
------
FC88X_00452_00115 --------------------CCTTGTCTTCTCTGTCTTGCTTGT----------
------
FC88X_01139_00900 -------------CTCGCGCTTTTC-CATCCCTGGTTTTATT------------
------
FC88X_00119_00076 -------------CTCGTGCTTTTC-CATCCCTGGTTTTATT------------
------
FC88X_00127_00772 ------------CTTTACGCTTTTCACATCCCTTGTTTTG--------------
------
FC88X_00432_00794 ------------CCGGGTGCTGTTCATATC-CGGTTTCCT--------------
------
FC88X_00926_00512 ------------CCGGGTGCTGTTCATATC-CGGTTTCCT--------------
------
FC88X_00874_00803 ------------TCTTTTGCTGTTCTGATCACTGTTTCC---------------
------
FC88X_00109_00824 ------------------------CACGCGCCTTTTT-CGTTCTCCTCATTTT-
------
FC88X_00802_01158 ------------------------CGCGTGCCTTTTT-CGTTCTCCTCATTTT-
------
FC88X_00106_00525 -----------------------CCACGTGCGCTGTA-CATTC-CCTCATCTT-
------
FC88X_00187_00145 ----------------CTACTACTCAAGTGCGCGTTT-CGTTCTC---------
------
FC88X_00468_00697 ----------------CTACTACTCAAGTGCGCGTTT-CGTTCTC---------
------
FC88X_00082_00207 ----------------CTACTACTCAAGTGCGCGTTT-CGTTCTC---------
------
FC88X_00078_00530 ----------------CTACTACTCAAGTGCGCGTTT-CGTTCTC---------
------
FC88X_00164_00856 ----------------CTACTACTCAAGTGCGCGTTT-CGTTCTC---------
------
FC88X_00316_00079 ----------------CTACTACTCAAGTGCGCGTTT-CGTTCTC---------
------
FC88X_00424_01027 ----------------CTACTACTCAAGTGCGCGTTT-CGTTCTC---------
------
FC88X_00674_01115 ----------------CTACTACTCAAGTGCGCGTTT-CGTTCTC---------
------
FC88X_00729_00457 ----------------CTACTACTCAAGTGCGCGTTT-CGTTCTC---------
------
FC88X_00829_00928 ----------------CTACTACTCAAGTGCGCGTTT-CGTTCTC---------
------
FC88X_00926_00637 ----------------CTACTACTCAAGTGCGCGTTT-CGTTCTC---------
------
FC88X_00043_00103 ---------------GCTACTACTCAAGTGCGCGTTT-CGTTCTC---------
------
FC88X_01039_00507 ----------------CTACTACTCAAGTGCGCGTTT-CGTTCCC---------
------
FC88X_00754_00881 ----------------CCACTACTCAAGTGCGCGTTT-CGTTCTC---------
------
FC88X_00904_00303 ----------------CTACTACTCAAGTGCGCGTTTTCGTTCTC---------
------
FC88X_01059_00710 ----------------CTACTACTCAAGTGCGCGTTTTCGTTCTC---------
------

FC88X_00157_00725 ----------------TTACGACTCAAGTGCGCGTTT-CGTTCTC---------
------
FC88X_00555_01201 ----------------CCGACTCCCAA-CGCGCGTTT-CGTTCTC---------
------
FC88X_01089_00666 ----------------CCGACTCCCAA-CGCGCGTTT-CGTTCTC---------
------
FC88X_00087_00471 -----------------------CCTAGCGC-TGTTT-CGTCCCCCATA-----
------
FC88X_00293_00873 -----------------------CCTAGCGC-TGTTC-CGTTCC----------
------
FC88X_00382_01073 -------------------CCTCGCCC-TGCGTTTTTT-CCCAGTTTC------
------
FC88X_00033_00653 -------------------CCTCGCCC-TGCGTTTTTTTCCCAGTTTC------
------
FC88X_00547_01131 -------------------CCTCGCCC-TGCGTTTTTT-CCCAGTTTC------
------
FC88X_01102_00561 -------------------CCTCGCCC-TGCGTTTTTT-CCCAGTTTC------
------
FC88X_00159_01022 -------------------CCTCGCCC-TGCGTTTTTT-CCCAGTTTC------
------
FC88X_00416_01028 ------------------GCCTCGCCCCTGCGTTTTTT-CCCAGTTTC------
------
FC88X_00954_00326 -------------------CCTCGCCC-TGCGTTTTTT-CCCAGTTTC------
------
FC88X_00210_00004 -------------------CCTCGCCC-TGCGTTTTTC--CCAGTTTC------
------
FC88X_00427_00005 -------------------CCTCGCCC-TGCGTTTTTC--CCAGTTTC------
------
FC88X_00549_01104 -------------------CCTCGCCC-TGCGTTTTTC--CCAGTTTC------
------
FC88X_00750_00324 -------------------CCTCGCCC-TGCGTTTTTC--CCAGTTTC------
------
FC88X_01138_01063 -------------------CCTCGCCC-TGCGTTTTTC--CCAGTTTC------
------
FC88X_01133_01153 -------------------CCTCGCCC-TGCGTTTTTC--CCAGTTTC------
------
FC88X_01007_00370 -------------------CCTCGCCC-TGGGTTTTTC--CCAGTTTC------
------
FC88X_00599_01167 -------------------CCTTGCCC-TGCGTTTTTTTCCCAGGTTC------
------
FC88X_00378_01086 -------------------CCTCACCC-TGCGTTTTTC--CCAG-----------
------
FC88X_00922_00897 -------------------CCACGCCC-TGCGTTCTTC--CCAGTTTC------
------
FC88X_00440_00074 -------------------CCTTGCCC-
TGCTGTTTTTCCCATGTTCCTTCCTT------
FC88X_00899_01170 -------------------CCTTGCCC-TGCACTTTTT-TTCAGTTTC------
------
FC88X_00948_00882 --------------------CGTTCATTTGTCGTTCACCTGCTGTTC-------
------
FC88X_01050_00797 --------------------CGTTCATTTGTCGTTTACCTGCTGTTC-------
------
FC88X_01029_00713 -------------------GTCTTCATTTGACCTGC-CCTGCTGTTTC------
------
FC88X_00498_00964 ----------------------CTCAAATGTCCTTTCCGTGCTGTTTTT-----
------

FC88X_00194_00112 --------------------TCATCTTTGTCACT--CTGGCTACTTCT------
------
FC88X_00554_00203 --------------------TCTCCTTTTTCACT--CTGGCTACTTCT------
------
FC88X_00753_00182 ---------------------CTTCTTTATCCGTTGCTTGCTACTTCT------
------
FC88X_00101_00708 ---------------------CCAATCCTTTTCCCGTGCGCTACTTCT------
------
FC88X_00605_00925 -----------------TCCTTGTCTCAT----TTGCGCGTTACTACT------
------
FC88X_01026_01171 -----------------TCCTTGTCTCAT----TTGCGCGTTACTACTC-----
------
FC88X_00788_00850 -----------------TCCTTGTCTCAT----TTGTGCGTTACTACT------
------
FC88X_00984_00603 -----------------TCCTTGTCTCAT----TTGCGCGCTACTT--------
------
FC88X_00168_00869 -----------------CCCTTGACTATTC--ATTTTGCGATACTGCC------
------
FC88X_00419_01205 ------------------CTCCGACGTTTTAGCCTTCGTGTTATTC--------
------
FC88X_00917_00881 ------------------CCTTGACCTTTT--CCTTCGTGCTATGCT-------
------
FC88X_00695_00528 -----------------CCTTGACCAGTTTTTCTGTTATTCTATT---------
------
FC88X_01076_00793 ------------------CTTTACCCTAGCTCTTCTTGTTTTACT---------
------
FC88X_00947_00591 -----------------CTACTAACCGTTAGATGGGCATTTCTC----------
------
FC88X_00901_01020 -----------------CTACTAACCGTTAGATGGGCATTTCTC----------
------
FC88X_01127_01032 -----------------CTACTAACCGTTAGATGGGCATTTCTC----------
------
FC88X_00690_00387 -----------------CTACTAACCGTTAGATGGGCATTTCTC----------
------
FC88X_00668_01009 -----------------CTACTAACCGTTAGATGGGCATTTCTC----------
------
FC88X_00671_01078 -----------------CTACTAACCGTTAGATGGGCATTTCTC----------
------
FC88X_00835_00331 -----------------CTACTAACCGTTAGATGGGCATTTCTC----------
------
FC88X_00893_00641 -----------------CTACTAACCGTTAGATGGGCATTTCTC----------
------
FC88X_00860_00815 -----------------CTACTAACCGTTAGATGGGCATTTCTC----------
------
FC88X_00936_00545 -----------------CTACTAACCGTTAGATGGGCATTTCTC----------
------
FC88X_00693_00448 -----------------CTACTAACCGTTAGATGGGCATTTCTCCTTCCTTT--
------
FC88X_00103_00553 -----------------CCACTAACCGTTAGATGGGCGTTTCTC----------
------
FC88X_01030_00844 -----------------CTACTAACCGTTAGATGGGCATTTTCTC---------
------
FC88X_00634_00626 ---------------CCGTACTACTACACGTATGGACTTTCCCTTCCTTT----
------
FC88X_00447_00456 ---------------CCGTACTACTACACGTATGGGCTTTCC------------
------

```
FC88X_01118_00580      --------------------GCTATGCACTAATGGCCGTTTTTCCCTAT-----
------
FC88X_00789_00097      ---------------------CTATGCACTAATGGCCGTTTT-CCTTAT-----
------
FC88X_00705_01022      ------------------------TTCACAAATGTGAATTTCCCAAGCCGA---
------
FC88X_00744_00969      ---------------------TCCTACACTGATGGACATGTTCTCGCA------
------
FC88X_00066_00048      ---------------------TCCTACACTGATGGACATGTTCTCGCA------
------
FC88X_00361_01106      ---------------------CTACAATCTGTTGAGCTTTTTCTGTCCA-----
------
FC88X_00679_00229      ---------------------CTACAATCTGTTGAGCTTTTTCTCTCCA-----
------
FC88X_00392_01104      ------------------CTCAGCTGTATTTGGCGTTATTATCTC---------
------
FC88X_00386_01152      ------------------CTCAGCTGTATTTGGCGTTATTATCTC---------
------
FC88X_00118_00263      ------------------CTCAGCTGTATTTGGCGTAATTATCTC---------
------
FC88X_00519_01122      -----------------CTATAATCTCGTCGATTGTCGTATTCT----------
------
FC88X_00643_01125      -----------------CTATAATCTCGTCGATTGTCGTATTCT----------
------
FC88X_00585_00078      -----------------CTATAATCTCGTCGATTGTCGTATTCT----------
------
FC88X_00023_00301      -----------------CTATAATCTCGTCGATTGTCGTATTCT----------
------
FC88X_00209_00069      -----------------CTATAATCTCGTCGATTGTCGTATTCT----------
------
FC88X_00326_01054      -----------------CTATAATCTCGTCGATTGTCGTATTCTCC--------
------
FC88X_00730_01027      -----------------CTATAATCTCGTCGATTGCCGTATTCT----------
------
FC88X_00414_01028      --------------CCAATGAGCTCTCGGTGGTCGTTATTCTCT----------
------
FC88X_00870_00698      -------------------CCCCGGCATTGACTCA-GCTATCGTCTC-------
------
FC88X_00908_01229      -------------------CCCCGGCATTGACTCA-GCTATCGTCTC-------
------
FC88X_00975_00966      -----------------CCACCCAGTTGTGACTCGCGCTATTGTC---------
------
FC88X_00014_00322      -----------------CCACCCAGTTGTGACTCGCGCTATTGTC---------
------
FC88X_00107_00067      -----------------CCACCCAGTTGTGACTCGCGCTATTGTC---------
------
FC88X_00421_01105      -----------------CCACCCAGTTGTGACTCGCGCTATTGTC---------
------
FC88X_00716_01212      -----------------CCACCCAGTTGTGACTCTCGCTATTGTC---------
------
FC88X_00201_00964      ----------------CCTACCCTGGTATCATTTGAGCCATTTT----------
------
FC88X_00388_00819      ----------------CCTACCCTGGTATCATTTGAGCCATTTT----------
------
FC88X_00637_00116      ----------------CCTACCCTGGTATCATTTGAGCCATTTT----------
------
```

FC88X_00235_01011 ----------------CCTACCCAGGTATCATTTGAGCCATTTTC---------
------
FC88X_00103_00024 -----------------CTACTC-GCTATATTCTGCCCTATTTTC---------
------
FC88X_00950_00794 ------------------TCCCCTTTTATGTTTAGACATATTGCT---------
------
FC88X_01071_00971 ----------------TCTCCCGGTCTCTGTTTAGGCGTATC------------
------
FC88X_01036_00494 ----------------CACACCGGTGTTTATTTCGTCCTATTT-----------
------
FC88X_01055_00494 ----------------CACACCGGTGTTTATTTCGTCCTATTT-----------
------
FC88X_00708_00164 ----------------------CTGCTATTACTGGCGCTTTTCTCTTTA-----
------
FC88X_01095_00869 ----------------------CTGCTATTACTGGCGCTTTTCTCTTTA-----
------
FC88X_00114_00419 ----------------------
CTGCTATTACTGGCGCTTTTCTCTTTACTTCTT-----
FC88X_01123_00555 ----------------------CTGCTCAGACTTATGCGCTATCCTCGC-----
------
FC88X_00475_00987 ----------------TCTTAATTTCCTCAGCTTTACGCTATT-----------
------
FC88X_00460_00454 ------------ACGTATTTACTGTGTTCAGTTTTAC-CTATTC----------
------
FC88X_00962_00718 -----------------TTGTGTTGTCCCAGCATTGC-CTATATT---------
------
FC88X_00921_00434 -----------------CTGACTTTGCTTTCTCCTGGAAATCCC----------
------
FC88X_00995_01173 -----------------CTATGTCTCTCCGTTCGCTTAAATCTC----------
------
FC88X_01093_00491 ------------------CTTCCCGATCTTTCG--ACATGATATTCT-------
------
FC88X_00017_00289 ------------------CTTCCCGATCTTTCG--ACATGATATTCT-------
------
FC88X_00647_00464 ------------------CTTCCCGATCTTTCG--ACATGATATTC--------
------
FC88X_00107_00665 ------------------CCTCCCAATTGTTCGTGACATGATCTT---------
------
FC88X_01017_01197 ------------------TTCCCTGATTATCCGACAGTTCATATT---------
------
FC88X_00027_00534 -----------------TCTTCCCTTATCTTATCGACATGCTCG----------
------
FC88X_00471_00882 ------------------CTGCCCGTCTATTCTTATCAATTTTCT---------
------
FC88X_00068_00313 ----------------TCCCGACTTTCCTATCCCGAGATTTTCTTCCTTTCT--
------
FC88X_00176_00049 -----------------CTTTCCCGCCTCTCACTAACGATTTTCT---------
------
FC88X_00677_00262 ------------------TTCCCGGGTATCC-CGATCGACCTTCTT--------
------
FC88X_01017_00934 ------------------TTCCCGTATCCTTACTATGCACCTTCT---------
------
FC88X_00140_00728 ---------------CTTCTTCCCCGCTGTTCTCGCCCGTTTTTCTT-------
------
FC88X_00160_00646 -------------------TCTTCCCCTTCTCGCACCGATTCTCTG--------
------

FC88X_00107_00472 ---------------------CTAGCTCATCGCTGCACATGTTTTCCT------
------
FC88X_01093_00753 ---------------------ATCGCTCTTCTCTGTTTATGTTTTCTT------
------
FC88X_00470_01130 ------------------CTCTTACATCGTCGCTGTTCGTTTTC----------
------
FC88X_00126_00132 -------------------CCAGCCACCGCCGCTGCTTCTGTTTTC--------
------
FC88X_00413_01190 -----------------ATCCCTGGTCCGCGGGTGCTCG-TGTATT--------
------
FC88X_00425_00109 -----------------TTCCCTGGCCCGCGGGTGCTC--TGTATT--------
------
FC88X_00593_00265 -----------------CTCCCGGATCCGCGCTTTCTCTTTGTA----------
------
FC88X_00507_00534 ---------------------CCTGAGCATCGCTTCTCGTTATTAACC------
------
FC88X_00127_00549 ------------------ATGGCTACCTGTCGGCCTCATCGTTCT---------
------
FC88X_00254_00642 ------------------CTAACCGTCAGCCGCGGTTGTCGTTCTC--------
------
FC88X_00135_00964 ----------------CTTGCATCCCTTCTCCATGTCATGGTT-----------
------
FC88X_01001_01224 ----------------CTTGCATCCCTTCTCCATGTCATGGTT-----------
------
FC88X_00792_00298 -------------CATCTGGCATCCCTTCCCGTTATCGA---------------
------
FC88X_01065_00372 -----------------TCGCTTCCCTGCTCCGACTGATCGTGAC---------
------
FC88X_00816_01136 ------------------TGCAACCCTG-TCCCACTTATCGTGCCC--------
------
FC88X_00168_00779 -------------------CCTTCCCATCGGA-CGTCTGGTCGCATT-------
------
FC88X_00426_00048 -------------------CCTTCCCATCGGA-CGTCTGGTCGCATT-------
------
FC88X_00427_00014 -------------------CCTTCCCATCGGAACGTCTG---------------
------
FC88X_00613_00835 ---------------CCCTCGTTCCCGTGGTA--GTCTGGGCCC----------
------
FC88X_00860_00752 -----------------TTCCTTCTCGTTGTA-TGTGTGCCATGC---------
------
FC88X_01104_00480 ----------------TTCCCTTCCCGTCAGA-TG-GCGCCCTGCT--------
------
FC88X_01013_00866 ----------------TTACCTTCTCAC-GTA-TGTTGGCATTTT---------
------
FC88X_00985_00328 ----------------TTACCTTCTCAC-GTA-TGTTGGCATTTTTT-------
------
FC88X_01052_00997 ----------------TTACCTTCTCAC-GTA-TGTTGGCATTTT---------
------
FC88X_01102_00581 ----------------TTACCTTCTCAC-GTA-TGTTGGCATTTT---------
------
FC88X_00159_00023 ----------------TTACCTTCTCAC-GTA-TGTTGGCATTTT---------
------
FC88X_00320_00163 ----------------TTACCTTCTCAC-GTA-TGTTGGCATTTT---------
------
FC88X_00817_00833 ----------------TTACCTTCTCAC-GTA-TGTTGGCATTTT---------
------

FC88X_00886_00607 ----------------TTACCTTCTCAC-GTA-TGTTGGCATTTT---------
------
FC88X_00976_00544 ----------------TTACCTTCTCAC-GTA-TGTTGGCATTTT---------
------
FC88X_01137_00774 ----------------TTACCTTCTCAC-GTA-TGTTGGCATTTT---------
------
FC88X_00670_01128 ---------------GTTACCTTCTCAC-GTA-TGTTGGCATTTT---------
------
FC88X_01023_01095 ----------------CTACCTTCTCAC-GTA-TGTTGGCATTTT---------
------
FC88X_01013_01203 ----------------TTACCTTCTCAC-GTG-TGTTGGCATTTTT--------
------
FC88X_00690_01135 ----------------TTACCTTCTCGCAGTA-TGTTGGCAT------------
------
FC88X_00204_00910 ----------------CTCCCTTCTCTGTGT--TGTCGGCATAGGT--------
------
FC88X_00073_00316 -----------------ATCATGCTAACGGTG-CGTCATCTCTTTT--------
------
FC88X_00044_00455 -----------------ATCATGCTAACGGTG-CGTCATCTCTTTT--------
------
FC88X_00465_00644 -----------------ATCATGCTAACGGTG-CGTCATCTCTTTT--------
------
FC88X_00607_00340 -----------------ATCATGCTAACGGTG-CGTCATCTCTTTT--------
------
FC88X_00883_00810 -----------------ATCATGCTAACGGTG-CGTCATCTCTTTT--------
------
FC88X_00915_01120 -----------------ATCATGCTAACGGTG-CGTCATCTCTTTT--------
------
FC88X_00902_00182 -----------------ATCATGCTAACGGTG-CGTCATCTCTTTTTT------
------
FC88X_00188_00139 -----------------ATCATGCTAACGGTG-CGTCATCTCTTTCTT------
------
FC88X_00162_00806 ------------------TCTAGCTAACTATGGCGTTCACTTTTTT--------
------
FC88X_00130_00656 ---------------------ATCAACCCAAGGCAATA-GTTATCTCTT-----
------
FC88X_00418_01217 ---------------------ATCAACCCAAGGCAATA-GTTATCTCTT-----
------
FC88X_00466_01197 -------------------CTATCAACCGTCGCCATTACGTTATT---------
------
FC88X_00904_00781 -------------------CTATCAACCGTCGCCATTACGTTATT---------
------
FC88X_00563_01105 ---------------------------CCATT-TGGCATCCCTTCCGT------
------
FC88X_00935_00440 ---------------------------
CCATTGTGTTATCTCCTTCCTTCTTTA------
FC88X_00447_01012 ---------------------------CCATT-
CGCTATCTCTCTCGTGGTTAC------
FC88X_00707_00312 ------------------CCTAACGTTCCCTCGCAGTATGGTATT---------
------
FC88X_00091_00526 ------------------CCTAACGTTCCCTCGCAGTATGGTATT---------
------
FC88X_01011_00569 ------------------CCTAACGTTCCCTCGCAGTATGGTATT---------
------
FC88X_01142_00800 ------------------CCTAACGTTCCCTCGCAGTATGGTATT---------
------

FC88X_00316_00639   ------------------TCTAACGTTCCCTCGCAGTATGGTATT---------
------
FC88X_00664_01061   --------------GCTTTCTCACGTTGC--CGCAGTTGTTTATT---------
------
FC88X_00735_01161   ----------------CCGCTATCTTTCC--CGTAGTAACTTATT---------
------
FC88X_00095_00304   -----------------CACTTACATGTGAA-GATTCGCTGTTCT---------
------
FC88X_00290_00857   ----------------GCACTTACATGTGAA-GATTCGCTGTTCT---------
------
FC88X_00051_00636   -----------------CACTTACATGTGAA-GATTCGCTGTTCT---------
------
FC88X_00140_00589   -----------------CACTTACATGTGAA-GATTCGCTGTTCT---------
------
FC88X_00541_00074   -----------------CACTTACATGTGAA-GATTCGCTGTTCT---------
------
FC88X_00587_00393   -----------------CACTTACATGTGAA-GATTCGCTGTTCT---------
------
FC88X_00703_01110   -----------------CACTTACATGTGAA-GATTCGCTGTTCT---------
------
FC88X_00058_00103   -----------------CACTTACATGTGAA-GATTCGCTGTTCT---------
------
FC88X_00098_00326   -----------------CACTTACATGTGAA-GATTCGCTGTTCT---------
------
FC88X_00098_00552   -----------------CACTTACATGTGAA-GATTCGCTGTTCT---------
------
FC88X_00118_00013   -----------------CACTTACATGTGAA-GATTCGCTGTTCT---------
------
FC88X_00136_00060   -----------------CACTTACATGTGAA-GATTCGCTGTTCT---------
------
FC88X_00211_00055   -----------------CACTTACATGTGAA-GATTCGCTGTTCT---------
------
FC88X_00270_00498   -----------------CACTTACATGTGAA-GATTCGCTGTTCT---------
------
FC88X_00426_00042   -----------------CACTTACATGTGAA-GATTCGCTGTTCT---------
------
FC88X_00441_00733   -----------------CACTTACATGTGAA-GATTCGCTGTTCT---------
------
FC88X_00415_01166   -----------------CACTTACATGTGAA-GATTCGCTGTTCT---------
------
FC88X_00573_00647   -----------------CACTTACATGTGAA-GATTCGCTGTTCT---------
------
FC88X_00557_00881   -----------------CACTTACATGTGAA-GATTCGCTGTTCT---------
------
FC88X_00662_00533   -----------------CACTTACATGTGAA-GATTCGCTGTTCT---------
------
FC88X_00768_00204   -----------------CACTTACATGTGAA-GATTCGCTGTTCT---------
------
FC88X_00942_00974   -----------------CACTTACATGTGAA-GATTCGCTGTTCT---------
------
FC88X_00998_00623   -----------------CACTTACATGTGAA-GATTCGCTGTTCT---------
------
FC88X_01051_00699   -----------------CACTTACATGTGAA-GATTCGCTGTTCT---------
------
FC88X_01096_01061   -----------------CACTTACATGTGAA-GATTCGCTGTTCT---------
------

```
FC88X_01101_00795      -----------------CACTTACATGTGAA-GATTCGCTGTTCT---------
------
FC88X_01115_00902      -----------------CACTTACATGTGAA-GATTCGCTGTTCT---------
------
FC88X_00566_00151      -----------------CACTTACATGTGAA-GATTCGCTGTTCTC--------
------
FC88X_00515_01171      -----------------CACTTACATGTGAA-GATTCGCTGTTCTC--------
------
FC88X_00763_01242      -----------------CACTTACATGTGAA-GATTGGCTGTTCT---------
------
FC88X_00058_00847      -----------------TACTTACATGTGAA-GATTCGCTGTTCT---------
------
FC88X_00521_00746      -----------------CACTTACATTTGAA-GATTCGCTGTTCT---------
------
FC88X_01100_00693      -----------------CACTTACATGTGGA-GATTCGCTGTTCT---------
------
FC88X_00384_00847      -----------------CACTTACATGTGAA-GGTTCGCTGTTCT---------
------
FC88X_00583_00093      -----------------CACTTACATGTGAA-GATTC-----------------
------
FC88X_00363_00062      -----------------CACTCACATGTGAA-GATTCGCTGTTCTCTTCCTTT-
------
FC88X_00553_00143      -----------------CACTTACACGTGAA-GATTCGCTGTTCT---------
------
FC88X_00928_00767      -----------------CGCTTACACATGAA-GATTCGCTGTTCT---------
------
FC88X_00855_01154      -------------------CTAACATCTAAACGTTTCGCTATTAAC--------
------
FC88X_01044_00639      ------------------CCCTATCATCGTTGGATCTTCCCTTTTT--------
------
FC88X_00394_00661      -----------------CTTCTCTCA-CGTTACATCTGACGTTTT---------
------
FC88X_00152_01003      --------------------TTCTCTCCTATCATATTTGCGGTTTTCC------
------
FC88X_00294_01034      --------------------TTCTCTCCTATCATATTTGCGGTTTTCC------
------
FC88X_00410_00072      -----------------CTTTGTTATCTCACCCAATTTCCGTTTTTC-------
------
FC88X_00842_01108      -----------------CCCTTGTCTCTAACACGTTTGGCGCTT----------
------
FC88X_01106_01094      -----------------CCCTTGTCTCTAACACGTTTGGCGCTT----------
------
FC88X_00951_01179      -----------------
CCCTTGTCTCTAACACGTTTGGCGCTTCTTCCTTTCTCC----
FC88X_00340_01137      --------------CCGTTCTTATCTCGACA-CGTTTTGTATT-----------
------
FC88X_00999_00812      --------------CCGTTCTTATCTCGACA-CGTTTTGTATT-----------
------
FC88X_01023_00525      --------------CCGTTCTTATCTCGACA-CGTTTTGTATT-----------
------
FC88X_01031_01061      ------------------TCTTATCCCTCGT-ACTTTTTTGTTTT---------
------
FC88X_00118_00592      ---------------TCGTCCCATCTCTAAC-GTTTCAGTATT-----------
------
FC88X_00165_00716      ------------------TCCTCCTTTCGCTGACGCTATTTCTT----------
------
```

```
FC88X_00901_00829      ------------------TATACGCCTTTCTAACGCTACTATCTT---------
------
FC88X_00431_01096      ------------------TTTTCTCCTGG--AGCGAGGCTATTTCAC-------
------
FC88X_00470_00840      ------------------TTTTCTCCTGG--AGCGAGGCTATTTCAC-------
------
FC88X_00861_00774      ------------------TTTTCTCCTGG--AGCGAGGCTATTTCAC-------
------
FC88X_00938_01218      ------------------TTTTCTCCTGG--AGCGAGGCTATTTCAC-------
------
FC88X_01091_00887      -----------------GTCTTCTCCTGCCTATCGATGCTATGTT---------
------
FC88X_00278_00548      ------------------TCTCTACTTCGACAGCTCTAATCTGGCC--------
------
FC88X_00304_01070      ------------------CTCTTCTCTCGTTAGCTGCGGTATGGC---------
------
FC88X_00982_00314      -------------------CTCCTCTCCCTCAGCGCTATTGTGTCCC-------
------
FC88X_00023_00342      --------------ATCACGTAGGCGTTCGTGTGTCGATTCTC-----------
------
FC88X_00206_00036      --------------ATCACGTAGGCGTTCGTGTGTC-ATTCTC-----------
------
FC88X_00132_00125      --------------ATCACGTAGGCGTTCGTGTGTCGATTCTC-----------
------
FC88X_00304_00429      --------------ATCACGTAGGCGTTCGTGTGTCGATTCTC-----------
------
FC88X_00430_00085      --------------ATCACGTAGGCGTTCGTGTGTCGATTCTC-----------
------
FC88X_00420_00584      --------------ATCACGTAGGCGTTCGTGTGTCGATTCTC-----------
------
FC88X_00485_01030      --------------ATCACGTAGGCGTTCGTGTGTCGATTCTC-----------
------
FC88X_00586_00178      --------------ATCACGTAGGCGTTCGTGTGTCGATTCTC-----------
------
FC88X_00897_00691      --------------ATCACGTAGGCGTTCGTGTGTCGATTCTC-----------
------
FC88X_00892_01121      --------------ATCACGTAGGCGTTCGTGTGTCGATTCTC-----------
------
FC88X_01083_00772      --------------ATCACGTAGGCGTTCGTGTGTCGATTCTC-----------
------
FC88X_01130_00602      --------------ATCACGTAGGCGTTCGTGTGTCGATTCTC-----------
------
FC88X_01147_01060      --------------ATCACGTAGGCGTTCGTGTGTCGATTCTC-----------
------
FC88X_00771_00985      --------------ATCACGTAGGCGTTCGTGTGTCGATTCTC-----------
------
FC88X_00948_00977      -------------GATCACGTAGGCGTTCGTGTGTCGATTCTC-----------
------
FC88X_00689_01159      --------------ATCACGTAGGCGTTCGTGTGTCGACTCTC-----------
------
FC88X_01141_00602      -------------AATCACGTAGGCGTTCGTGTGTCGATTCTC-----------
------
FC88X_00944_01153      --------------ATCACGTAGGCGTTCGTGTATCGATTCTC-----------
------
FC88X_01097_00793      --------------ATCACGTAG-CGTTCGTGTGTCGATTCT------------
------
```

```
FC88X_01026_00952       --------------ATCACGTAGGCGCTCGTGTGTCGATTCTC-----------
------
FC88X_00784_00120       --------------ATCACGTAG-CGTTCGTGTGTCGATTCTC-----------
------
FC88X_00767_00717       --------------ATCACGTAG-CGTTCGTGTGTCGATTCTC-----------
------
FC88X_00802_01262       --------------ATCACGTAG-CGTTCGTGTGTCGATTCTC-----------
------
FC88X_00938_00862       --------------ATCACGTAG-CGTTCGTGTGTCGATTCTC-----------
------
FC88X_00909_01024       --------------ATCACGTAG-CGTTCGTGTGTCGATTCTC-----------
------
FC88X_00974_01193       --------------ATCACGTAG-CGTTCGTGTGTCGATTCTC-----------
------
FC88X_01006_00584       --------------ATCACGTAG-CGTTCGTGTGTCGATTCTC-----------
------
FC88X_01030_00781       --------------ATCACGTAG-CGTTCGTGTGTCGATTCTC-----------
------
FC88X_01098_00861       --------------ATCACGTAG-CGTTCGTGTGTCGATTCTC-----------
------
FC88X_01120_01216       --------------ATCACGTAG-CGTTCGTGTGTCGATTCTC-----------
------
FC88X_00721_00719       --------------ATCACGTAG-CGTTCGTGTGTCGATTCTC-----------
------
FC88X_00586_00080       --------------ATCACGTAG-CGTTCGTGTGTCGATTCTC-----------
------
FC88X_00238_00038       --------------ATCACGTAG-CGTTCGTGTGTCGATTCTC-----------
------
FC88X_00479_00683       --------------ATCACGTAG-CGTTCGTGTGTCGATTCTC-----------
------
FC88X_00464_01079       --------------ATCACGTAG-CGTTCGTGTGTCGATTCTC-----------
------
FC88X_00080_00509       --------------ATCACGTAG-CGTTCGTGTGTCGATTCTC-----------
------
FC88X_01059_00465       --------------ATCACGTAGGCGTTCGTGTGTCGATTCTCCTTCCTTT---
------
FC88X_01143_00949       --------------ATCACGTAGGCGTTCGTGTGTCGATTCTCCTTCCTTT---
------
FC88X_00812_00112       --------------ATCACGTAG-CGTTCGTGTGTCGATTCTCC----------
------
FC88X_00792_01042       --------------ATCACGTAG-CGTTCGTGTGTCGATTCTCTCCC-------
------
FC88X_00875_00807       ----------------AACATTG-CTTTCGTGACCGGTCTATTC----------
------
FC88X_00995_00273       ----------------AACATTG-CTTTCGTGACCGGTCTATTC----------
------
FC88X_00986_00355       ----------------AACATTG-CTTTCGTGACCGGTCTATTC----------
------
FC88X_00039_00242       ----------------AACATTG-CCTTCGTGACCAGTCTATTC----------
------
FC88X_00455_00941       --------------CTTCCGTGA--GTGTTC-CGTCTATTGTC-----------
------
FC88X_00089_00618       --------------CTTCCGTGA--GTGTTC-CGTCTATTGTC-----------
------
FC88X_00030_00408       ------------TTATACCGTGT--GCTGAT-CTGTTATTCTC-----------
------
```

```
FC88X_00834_00907      ------------TTATACCGTGT--GCTGAT-CTGTTATTCTC-----------
------
FC88X_00525_00211      ------------TTATACCGTGT--GCTGAT-CTGTTATTCTC-----------
------
FC88X_00985_00996      ----------CCTTTCACCGTGT--CGTGTT-TGATTATT--------------
------
FC88X_01133_00747      -------------CTTACCGTTC--GTTCTCCTGATTATTCT------------
------
FC88X_00520_00786      -------------CCGAACTTGGGTTTT-CAGTGCTATTCG-------------
------
FC88X_01047_01224      -------------CCGAACTTGGGTTTT-CAGTGCTATTCG-------------
------
FC88X_00797_00171      -------------CCGAACTTGGGTTTT-CAGTGCTATTCG-------------
------
FC88X_00084_00176      -------------CCGAACTTGGGTTTT-CAGTGCTATTCG-------------
------
FC88X_00126_00019      -------------CCGAACTTGGGTTTT-CAGTGCTATTCG-------------
------
FC88X_00323_00010      -------------CCGAACTTGGGTTTT-CAGTGCTATTCG-------------
------
FC88X_00402_01184      -------------CCGAACTTGGGTTTT-CAGTGCTATTCG-------------
------
FC88X_00980_00951      -------------CCGAACTTGGGTTTTTCAGTGCTATTCG-------------
------
FC88X_00115_00309      -------------CCGAACTTGGGTTTTTCAGTGCTATTCG-------------
------
FC88X_01085_00751      -------------CCGAACTTGGGTTTC--AGTGCTATTCG-------------
------
FC88X_00691_00958      -------------CCGAACGTGGGTTTT-CAGTGCTATTCG-------------
------
FC88X_00196_00683      -----------------CTCCCGCGTGTACAGATCTATCTCCGT----------
------
FC88X_01007_01172      --------------------------CTACAG-TCTATTT--------------
------
FC88X_00556_01255      --------TTCTTCCGTGTC-TATTTGCCCAATATT------------------
------
FC88X_01132_01077      ---------CCATGTGTTCCGTGCTATCCCAACAAT------------------
------
FC88X_00130_00364      ---------------CCAAGTATTCCGTGACATGCTATTCGT------------
------
FC88X_00084_00368      ---------------CCAAGTATTCCGTGACATGCTATTCGT------------
------
FC88X_00117_00107      ---------------CCAAGTATTCCGTGACATGCTATTCGT------------
------
FC88X_00157_00748      ---------------CCAAGTATTCCGTGACATGCTATTCGT------------
------
FC88X_00841_00145      ---------------CCAAGTATTCCGTGACATGCTATTCGT------------
------
FC88X_00155_00943      ---------------CCAAGTGCTCCGTGACATGCTATTCGT------------
------
FC88X_00741_00311      ---------------CCAAGTATTCCGGTGACCAT-------------------
------
FC88X_00948_00951      ----------CTCAACAC-GTGCTCCCATCCATCCTATT---------------
------
FC88X_00071_00056      -----------TCAACTCCGTGTGTACATC--TCCTATT---------------
------
```

FC88X_00092_00779 ------------CTCCTCGTGGTCCGTTACTATCCTAGC---------------
------
FC88X_00086_00269 ---------------ACGTTTCGTTTACGGCATCCTATTCTT------------
------
FC88X_00621_01215 ---------------ACGTTTCGTATACGGCATCCTATTCTT------------
------
FC88X_00244_00939 ---------------ACGTTTCGTTTACGGCATCCTATTCTT------------
------
FC88X_00446_00124 ---------------ACGTTTCGTTTACGGCATCCTATTCTT------------
------
FC88X_00558_01246 ---------------ACGTTTCGTTTACGGCATCCTATTCTT------------
------
FC88X_00678_00230 ---------------ACGTTTCGTTTACGGCATCCTATTCTT------------
------
FC88X_00677_01170 ---------------ACGTTTCGTTTACGGCATCCTATTCTT------------
------
FC88X_00758_00877 ---------------ACGTTTCGTTTACGGCATCCTATTCTT------------
------
FC88X_00846_01114 ---------------ACGTTTCGTTTACGGCATCCTATTCTT------------
------
FC88X_00934_00479 ---------------ACGTTTCGTTTACGGCATCCTATTCTT------------
------
FC88X_01059_00601 ---------------ACGTTTCGTTTACGGCATCCTATTCTT------------
------
FC88X_00813_00883 ---------------ACGTTTCGTTTACGGCATCCTATTCTTCT----------
------
FC88X_00891_01226 ---------------ACGTCTCGTTTACGGCATCCTATTCTT------------
------
FC88X_00785_01002 ---------------ACGTTTCGTTTACGGC---CTATTCTT------------
------
FC88X_00159_00870 ---------------ACGCTTCGTTTACGGCATCCTATTCTT------------
------
FC88X_00391_01090 ---------------ACGTTTCGTTTACGGCATCCTATCCT-------------
------
FC88X_00101_00557 ---------------ACGTTCCGTTTACGGCATCCTATTCTTTCCC--------
------
FC88X_00912_00299 ---------------ACGTTCCGTTTACGGCACCCTATTCTT------------
------
FC88X_00119_00543 -----------CTTCACGTTCCCCTT-TACAGTCCTATT---------------
------
FC88X_00604_01107 ---------------GCCGCAGGTTAGCACTCCCGGTCCGTT------------
------
FC88X_00977_00462 ------------AATCCCGTTGGTGCTCACTTCCCGTCTATT------------
------
FC88X_00508_00246 --------------------GGGTTCTCACTTACGTCC----------------
------
FC88X_00883_00711 ---------------TCCATTCCTGTTCGTCACTTCGCTATT------------
------
FC88X_00864_00860 ---------------TCCATTCCCGTTCGTCACTTCGCTATT------------
------
FC88X_00928_00302 ---------------ACCATCCCTGTACGGCTCTCACCTATT------------
------
FC88X_01143_01239 ---------------ACCATCCCTGTACGGCTCTCACCTATT------------
------
FC88X_00244_01117 ---------------ACCATCCCTGTACGGCTCTCACCTATT------------
------

```
FC88X_00079_00715      -------------TACACTTAGCCCTCTCGT-TACTATTCAC------------
------
FC88X_01062_00646      -------------TACACTTAGCCCTCTCGT-TACTATTCAC------------
------
FC88X_01019_01156      -------------TACACTTAGCCCTCTCGT-CACTATTCAC------------
------
FC88X_00620_00339      ---------------TTGTGGGCCCTGTCCTGCAGTATTCAC------------
------
FC88X_00422_01146      -------------CCACATCGCCCCTGT--CCTACTATTGTC------------
------
FC88X_00884_00403      -------------CCACGTTGTCCCTCTAACTTACTATTC--------------
------
FC88X_00051_00010      --------------CTGACA--AATCTCCCTTAGCT-ACTTCGA----------
------
FC88X_01037_00431      --------------CTGACA--AATCTCCCTTAGCT-ACTTCGA----------
------
FC88X_00026_00600      --------------CTGACA--AATCTCCCTTAGCC-ACTTCGA----------
------
FC88X_00989_00462      ----------------TCAA--AATCTCCCCTGACA-AGTTC------------
------
FC88X_00074_00397      ----------TCCCCAAACC-----TCCCGTCGACA-ATTACGCT---------
------
FC88X_00549_00090      -----------CCCCAAGCT--CGTTCCCGCCAACA-ATTT-------------
------
FC88X_01021_01236      ------------ACCGAACT-CCTTCTCCGC-GACA-ATTTG------------
------
FC88X_00882_00399      ------------ACCGAACT-CCTTCTCCGC-GACA-ATTTG------------
------
FC88X_00861_00993      ------------ACCGAACT-CCTTCTCCGC-GACA-ATTTG------------
------
FC88X_00939_01096      ------------ACCGAACT-CCTTCTCCGC-GACA-ATTTTGCTT--------
------
FC88X_00442_00141      ------------ACCGAACT-CCTTCTCCGC-GACG-ATTTG------------
------
FC88X_00841_00316      ------------ACCGAACT-CCTTCTCCGC-GAC--ATTTG------------
------
FC88X_00539_01190      -----------CACCAATCTACCTTGCCCACTGACA-AC---------------
------
FC88X_00130_00926      -------------------------CTCCCGGAACA-ATATTGTGCTGCCAA--
------
FC88X_00068_00717      ------------------------CCTCCCGGAACA-ATATTGTGCTGCCAA--
------
FC88X_00539_01199      -----------------------TCCTCCCTGTCCATATAGCGCGCTACA----
------
FC88X_00929_00450      ----------------TTGACACTTCTCCCGTGACA-ATGTAGC----------
------
FC88X_00313_00946      -------------CCTTTGTCCCATTACCGTGAACGTATT--------------
------
FC88X_00867_01082      -------------CCTTTGTCCCATTACCGTGAACGTATT--------------
------
FC88X_00200_00844      --------------CTATGGCGCCCTCCCGTGAACGTATT--------------
------
FC88X_00862_00167      -------------------CTCCCATGCCTCCATCGTATTGTCGAC--------
------
FC88X_00953_00443      -----------------------CCTCCCATCAGCGTAACGTCCACGACA----
------
```

```
FC88X_00608_00205      ---------------CTTTGTTAATTCCCGCCAATCTATTCAC-----------
------
FC88X_01069_01027      ---------------CTTTGTTAATTCCCGCCAATCTATTCAC-----------
------
FC88X_01024_00376      ---------------CTTTGTCAATTCCCGCCAATCTATTCAC-----------
------
FC88X_00797_01227      ---------------GTTTGTCATTCTCCCGTGTCTACTTCAC-----------
------
FC88X_00025_00766      -----------CTCCGTCATTGTCGTCTAGCTGGTCTT----------------
------
FC88X_01074_00691      ------------------------GTCGATCTAG--------------------
------
FC88X_00227_00547      -------------CATGACATATCGTTCGACTAGTTCTCCTTCCTTT-------
------
FC88X_00593_01027      --------------------------TCGACTAG--------------------
------
FC88X_00931_00790      ------------TCTGGACGTTGTATCGAGCTAGCTTCTCCTTCCTTT------
------
FC88X_01046_00727      ------------TCTGGACGTTGTGTCGAGCTAGCTTCTCC-------------
------
FC88X_00566_00932      ------------TCTGCACGTTGCTCCA--CTAGTTCTCCC-------------
------
FC88X_00499_00029      -------------TTCCTAACTCCGTCGTACTAGTCTATT--------------
------
FC88X_00510_01184      -------------TTCCTAACTCCGTCGTACTAGTCTATT--------------
------
FC88X_00961_00821      -------------TTCCTAACTCCGTCGTACTAGTCTATT--------------
------
FC88X_00155_00835      -------------TTCCTAACTCCGTCGTACTAGTCTACT--------------
------
FC88X_00883_01234      ------------------------GTCG-ACTAG--------------------
------
FC88X_01008_00506      -----------GCTCCCTTTCTCCCTTCTCCCAGTCGACTAG------------
------
FC88X_00791_01036      -----------------------GATCGT-CGAGTCGACTAG------------
------
FC88X_00879_00720      ----------------------AGATCGTACGAGTCGACTAG------------
------
FC88X_01073_01055      ----------------------CGATCGTCTGAGTCGACTAG------------
------
FC88X_00499_01199      ---------------------------------GTCGACTAG------------
------
FC88X_00520_01019      -----------------TCGAAGCACAGATGGAGTCGACTAG------------
------
FC88X_00678_00556      --------------------------------AGTCGACTAG------------
------
FC88X_00892_00942      -----------------------------ATGAGTCGACTA-------------
------
FC88X_00933_00319      -----------------GTTCGGTGAAGAACGAGTCGCCTATCG----------
------
FC88X_00079_00845      ----------------CTTGTCATTTGATCAATGTCTACATCTC----------
------
FC88X_00217_00997      ----------------CTTGTCATTTGATCAATGTCTACATCTC----------
------
FC88X_00500_01027      ----------------CTTGTCATTTGATCAATGTCTACATCTC----------
------
```

```
FC88X_00849_00288      ----------------CTTGTCATTTGATCAATGTCTACATCTC----------
------
FC88X_01125_00995      ---------GGGGAAGCTTGTCATTTGATCAATGTCTACATCTC----------
------
FC88X_00590_01222      ---------------GCTTGTCATCTGATCAATGTCTACATCTC----------
------
FC88X_00882_00275      ---------------ACTTGTCATTTGATCAATGATTCTACCA-----------
------
FC88X_01031_00455      ---------------TTTTCCCTTCTGAT-AAAGTCTACTCGC-----------
------
FC88X_01000_00536      -----------------TTTTCTTCTGAT-AAAGTCTATTCGC-----------
------
FC88X_00981_00672      ---------------ACTTCTCCTTCTTTGAACGTATACCTA------------
------
FC88X_00071_00788      -------------------TCTCCTCTGGGAACGTTTGCCTGTC----------
------
FC88X_00046_00434      --------------------CCTTCCCTTAGTCTGTGATTATGTTTC-------
------
FC88X_00392_00992      --------------------CCTTCCCTTAGTCTGTGATTATGTTTCCC-----
------
FC88X_00706_00131      --------------------CCTGCCCTTAGTCTGTGATTATG-----------
------
FC88X_01141_01006      --------------------CCTGCCCTTAGTCTGTGATTATGTTTC-------
------
FC88X_00437_01213      --------------------CCTTCCCTCAGTCTGTGATTATGTT---------
------
FC88X_00960_00979      --------------------
CCTTCCCTTAGTCTGTGACTATGTTTTCTTCCTTTCT---
FC88X_00225_01073      --------------------TTCCCCTCCAGTCTTTGGCCATGTCCA-------
------
FC88X_00090_00551      -----------------------TTCCCCACAATGCGACCATGTCTTAGC----
------
FC88X_00896_00356      ----------------------CTCCTCCTCAATCC--CCATGTTCTAGATT--
------
FC88X_00078_00155      ------------------CAGTCTCCGCGGAGTCGTGCCCATGTT---------
------
FC88X_00792_00280      ------------------CAGTCTCCGCGGAGTCGTGCCCATGTT---------
------
FC88X_00897_00791      ------------------CAGTCTCCGCGGAGTCGTGCCCATGTT---------
------
FC88X_00050_00820      -----------TCCTTCTCCCCCAACGCTATGTCGCG-TCAATCTAGT------
------
FC88X_00320_00041      --------------------CCCAACGCTATGTCGCG-TCAATCTAGT------
------
FC88X_01091_00757      -------------------TCCCGTCTTTATGTCGCGCTCAATTTAT-------
------
FC88X_00287_01001      ---------------------CTGTCTCTGAGTCGGG-CCAAATTATTCT----
------
FC88X_00680_00920      ---------------------CTGTCTCTGAGTCGGG-CCAA-TTATTCT----
------
FC88X_00068_00614      ---------------------CTGTCTCTGAGTCGGG-CCAA-TTATTCT----
------
FC88X_00134_00253      ---------------------CTGTCTCTGAGTCGGG-CCAA-TTATTCT----
------
FC88X_01123_00639      ---------------------CTGTCTCTGAGTCGGG-CCAA-TTATT------
------
```

```
FC88X_00654_01162      ---------------------CTGTCTCTGAGTTGGG-CCAA-TTATTCT----
------
FC88X_00054_00125      --------------------CCTGTCTCCGTGTTGCG-CCGATGTATT------
------
FC88X_00135_00498      -------------CCTAAGTAATGTACCTGCGCC--GTGTT-------------
------
FC88X_00174_00037      --------------CCAATCATTGTACCCTCGCC--GTGTTATT----------
------
FC88X_00626_01043      ---------------ACTTCAGTGTCCCTGCTCCC-GTGTCCT-----------
------
FC88X_00825_00923      ------------------ACATTTGCCCTTCGCCATGTGTACTAT---------
------
FC88X_00952_00698      --------------------TTCATCCCTTCGTCGAAATCCCCGTGTG------
------
FC88X_01096_00637      --------------------TTCATCCCTTCGTCGAAATCCCCGTGTG------
------
FC88X_01055_01100      -------------------TTACATCCCTCCTGCAATGCACCTGTT--------
------
FC88X_00328_01131      -------------------CTCCACCCCTCAGCGAGTGCTCGTGTT--------
------
FC88X_00137_00509      -----------------ATCCTCCAACATTCGACGTGTC-TATTCG--------
------
FC88X_01066_00487      -----------------ATCCTCCAACATTCGACGTGTC-TATTCG--------
------
FC88X_01102_00648      --------------------CCTCGAGCTTCGCAGTGTCCTATTCGC-------
------
FC88X_00131_00104      -------------------CCTCGACCCTTCGCCGTT-----------------
------
FC88X_00742_00967      ------------------TCCATG--TCTCTGCCCATTGGTTATTCT-------
------
FC88X_00849_01211      ------------------TCCATT--TCTCTGCCCATTCGTTATTCT-------
------
FC88X_00805_00809      -------------------CCATGG-TCTGTTCCCCTTCGGTTTCTC-------
------
FC88X_00220_01026      ------------------CCCTTGACTCTTCGCCCTGTCGGTATACTT------
------
FC88X_00032_00226      ----------------CTTAGAGTCTCTCGGCGGACGGCTATTC----------
------
FC88X_01114_01116      ----------------CTTAGAGTCTCTCGGCGGACGGCTATTC----------
------
FC88X_00033_00202      ----------------CTTAGAGTCTCTCGGCGGACGGCTATTC----------
------
FC88X_00024_00715      ----------------CTTAGAGTCTCTCGGCGGACGGCTATTC----------
------
FC88X_00663_00261      ----------------CTTAGAGTCTCTCGGTGTACGGCTATTC----------
------
FC88X_00129_00144      ----------------CCTTTTGTCCCTGGGTGGTCGGCGTCT-----------
------
FC88X_00963_00763      -----------------CTATCGGCTCTCCGTTGTCAGCTTCTC----------
------
FC88X_00493_00978      ------------------ATACGTGCTCTGCCCCACGTCTATCTC---------
------
FC88X_00512_01120      ------------------ATACGTGCTCTGCCCCACGTCTATCTC---------
------
FC88X_00259_00072      ------------------ATACGTGCTCTGCCCCACGTCTATCTCCTTCCTTT-
------
```

```
FC88X_00975_00532       ------------------ATACGTGCTCTGCCCCACGTCTACCTC---------
------
FC88X_00367_01155       ---------------TGCTCACGTGTTCTGTCCCA-GTCTATT-----------
------
FC88X_00687_01164       ---------------ACAATCCGTCATCGGCGCCT-GTCTAGC-----------
------
FC88X_00126_00081       ------------------CAGGCTACAATGCTGAGTGGTCGGCCTCTT------
------
FC88X_00721_00201       ------------------------ACTATGCT---CGGTCGGCCCT--------
------
FC88X_00067_00207       ----------------CCCTACGATCTTTTGCAGACGTCACCC-----------
------
FC88X_00069_00733       -----------------CCTACGAATTATCATTAGCAGCCTGTC----------
------
FC88X_00313_01136       ----------------CCTTCCGATCATCAGCGGTCTGCATTC-----------
------
FC88X_00188_00009       ----------------CCCCATCATCTTCCGAATGAGCCTGTT-----------
------
FC88X_00970_01167       ----------------CCCCATCATCTTCCGAATGAGCCTGTT-----------
------
FC88X_00892_00668       ----------------CCCCATCATCGCCTGTCTCAACCTATT-----------
------
FC88X_00961_00329       ----------------CAACTCCATCTTCTCTACGAACCTGCC-----------
------
FC88X_00324_00740       ------------------TCACCATCT-CTCTA-GATTCTGCCCGTT-------
------
FC88X_00946_00874       ----------------TCCTCCCATAATCTGCAGTAACCTGCT-----------
------
FC88X_01092_00420       ----------------TCCTCCCATAATCTGCAGTAACCTGCT-----------
------
FC88X_00965_01157       ----------------CCTCCTCGTATCCGCATGTTTCCTGCG-----------
------
FC88X_01059_00793       ----------------TCCTCTTACCACCGCTAGTTACCTTCG-----------
------
FC88X_01108_00500       ----------------CTTCTTGCTCTTCTTCTGTAACATCAC-----------
------
FC88X_00707_00272       ---------------TCTTCTTA-TACTCTCCTGTCACTGCAC-----------
------
FC88X_00160_00506       ----------------CCTCCCGATCATCTCCGTTCACATTTT-----------
------
FC88X_00210_00940       ----------------TCCTTCCGTCGTATCCCTGGACCTCTT-----------
------
FC88X_00052_00631       -----------------TGCAAA-CCATTGACCCGCA-A-CTATTTT-------
------
FC88X_01126_00803       -----------------TGCAAA-CCATTGACCCGCA-A-CTATTTTCCC----
------
FC88X_00024_00091       -----------------TGCAAA-CCATTGACCCGCA-A-CTATTTTC------
------
FC88X_00440_00070       -----------------TGCAAA-CCATTGACCCGCA-A-CTATTTTC------
------
FC88X_00553_01158       -----------------TGCAAA-CCATTGACCCGCA-A-CTATTTTC------
------
FC88X_00645_00189       -----------------TGCAAA-CCATTGACCCGCA-A-CTATTTTC------
------
FC88X_00822_00249       -----------------TGCAAA-CCATTGACCCGCA-A-CTATTTTC------
------
```

```
FC88X_00048_00142       -----------------TGCAAA-CCATTGACCCGCA-A-CTATTTTC------
------
FC88X_00055_00032       -----------------TGCAAA-CCATTGACCCGCA-A-CTATTTTC------
------
FC88X_00070_00325       -----------------TGCAAA-CCATTGACCCGCA-A-CTATTTTC------
------
FC88X_00542_00656       -----------------TGCAAA-CCATTGACCCGCA-A-CTATTTTC------
------
FC88X_00634_00922       -----------------TGCAAA-CCATTGACCCGCA-A-CTATTTTC------
------
FC88X_00681_01169       -----------------TGCAAA-CCATTGACCCGCA-A-CTATTTTC------
------
FC88X_00841_01198       -----------------TGCAAA-CCATTGACCCGCA-A-CTATTTTC------
------
FC88X_00893_00669       -----------------TGCAAA-CCATTGACCCGCA-A-CTATTTTC------
------
FC88X_01014_00463       -----------------TGCAAA-CCATTGACCCGCA-A-CTATTTTC------
------
FC88X_01139_00720       -----------------TGCAAA-CCATTGACCCGCA-A-CTATTTTC------
------
FC88X_00013_00174       -----------------TGCAAA-CCATTGACCTGCA-A-CTATTTTC------
------
FC88X_00477_00983       -----------------TGCAAA-CCATTGACCTGCA-A-CTATTTTC------
------
FC88X_00792_01202       -----------------TGCAAA-CCATTGACCCGTA-A-CTATTTTC------
------
FC88X_00418_01190       -----------------TGCAGA-CCATTGACCCGCA-A-CTATTTTC------
------
FC88X_00726_01260       -----------------TACAAA-CCATTGACCCGCA-A-CTATTTTC------
------
FC88X_00568_01045       -----------------TGCAAA-CCATTGACCCGCG-A-CTAT----------
------
FC88X_00907_01133       -----------------TGCAAA-CCACTGACCCGCA-A-CTATTTTC------
------
FC88X_00279_01085       -----------------TGCAAAACCATTGACCCGCA-AACTATTTTTC-----
------
FC88X_00902_00493       -----------------TGCAAAACCATTGACCCGCA-A-CTATTTTC------
------
FC88X_01039_00367       -------------------------CATTGAGCTCGG-ACCTTTTTTCGCCCG-
------
FC88X_00719_01167       -------------------TTTGGCT--G-TCCCACA-ACATGTTCTC------
------
FC88X_00885_01042       -------------------TTTGGCTT-G-TCCCACA-ACATGTTCTC------
------
FC88X_00111_00862       ------------------TTTTGGCTTTG-TCCCACA-ACATGTTCTC------
------
FC88X_00068_00654       -------------------TTTGGCTTTG-TCCCACA-ACATGTTCTC------
------
FC88X_00126_00109       -------------------TTTGGCTTTG-TCCCACA-ACATGTTCTC------
------
FC88X_00100_00249       -------------------TTTGGCTTTG-TCCCACA-ACATGTTCTC------
------
FC88X_00127_00743       -------------------TTTGGCTTTG-TCCCACA-ACATGTTCTC------
------
FC88X_00190_00022       -------------------TTTGGCTTTG-TCCCACA-ACATGTTCTC------
------
```

```
FC88X_00286_00679       -------------------TTTGGCTTTG-TCCCACA-ACATGTTCTC------
------
FC88X_00315_00975       -------------------TTTGGCTTTG-TCCCACA-ACATGTTCTC------
------
FC88X_00492_00103       -------------------TTTGGCTTTG-TCCCACA-ACATGTTCTC------
------
FC88X_00729_00289       -------------------TTTGGCTTTG-TCCCACA-ACATGTTCTC------
------
FC88X_01133_00946       -------------------TTTGGCTTTG-TCCCACA-ACATGTTCTCCC----
------
FC88X_00200_00452       -------------------TTTGGCTTTG-TCCCACA-ACATGTTCTCCC----
------
FC88X_00516_01235       -------------------TTTGGCTTTG-TCCCACA-ACATGTTCTC------
------
FC88X_00782_00526       -------------------TTTGGCTTTG-TCCCACA-ACATGTTCTC------
------
FC88X_00800_00302       -------------------TTTGGCTTTG-TCCCACA-ACATGTTCTC------
------
FC88X_00888_00153       -------------------TTTGGCTTTG-TCCCACA-ACATGTTCTC------
------
FC88X_00878_00673       -------------------TTTGGCTTTG-TCCCACA-ACATGTTCTC------
------
FC88X_00928_00669       -------------------TTTGGCTTTG-TCCCACA-ACATGTTCTC------
------
FC88X_01048_00625       -------------------TTTGGCTTTG-TCCCACA-ACATGTTCTC------
------
FC88X_01137_00875       -------------------TTTGGCTTTG-TCCCACA-ACATGTTCTC------
------
FC88X_00016_00169       -------------------TTTGGCTTTG-TCCCACA-ACATGTTCTC------
------
FC88X_00047_00459       -------------------TTTGGCTTTG-TCCCACA-ACATGTTCTC------
------
FC88X_00043_00553       -------------------TTTGGCTTTG-TCCCACA-ACATGTTCTC------
------
FC88X_00040_00735       -------------------TTTGGCTTTG-TCCCACA-ACATGTTCTC------
------
FC88X_00142_00081       -------------------TTTGGCTTTG-TCCCACA-ACATGTTCTC------
------
FC88X_00118_00395       -------------------TTTGGCTTTG-TCCCACA-ACATGTTCTC------
------
FC88X_00191_00031       -------------------TTTGGCTTTG-TCCCACA-ACATGTTCTC------
------
FC88X_00159_00636       -------------------TTTGGCTTTG-TCCCACA-ACATGTTCTC------
------
FC88X_00238_00482       -------------------TTTGGCTTTG-TCCCACA-ACATGTTCTC------
------
FC88X_00290_01035       -------------------TTTGGCTTTG-TCCCACA-ACATGTTCTC------
------
FC88X_00339_00806       -------------------TTTGGCTTTG-TCCCACA-ACATGTTCTC------
------
FC88X_00370_01027       -------------------TTTGGCTTTG-TCCCACA-ACATGTTCTC------
------
FC88X_00444_01182       -------------------TTTGGCTTTG-TCCCACA-ACATGTTCTC------
------
FC88X_00477_00061       -------------------TTTGGCTTTG-TCCCACA-ACATGTTCTC------
------
```

FC88X_00495_01135 -------------------TTTGGCTTTG-TCCCACA-ACATGTTCTC------
------
FC88X_00694_00958 -------------------TTTGGCTTTG-TCCCACA-ACATGTTCTC------
------
FC88X_00787_01053 -------------------TTTGGCTTTG-TCCCACA-ACATGTTCTC------
------
FC88X_00826_01206 -------------------TTTGGCTTTG-TCCCACA-ACATGTTCTC------
------
FC88X_00882_00412 -------------------TTTGGCTTTG-TCCCACA-ACATGTTCTC------
------
FC88X_00859_00745 -------------------TTTGGCTTTG-TCCCACA-ACATGTTCTC------
------
FC88X_00860_00993 -------------------TTTGGCTTTG-TCCCACA-ACATGTTCTC------
------
FC88X_00940_00372 -------------------TTTGGCTTTG-TCCCACA-ACATGTTCTC------
------
FC88X_00942_01148 -------------------TTTGGCTTTG-TCCCACA-ACATGTTCTC------
------
FC88X_01013_00763 -------------------TTTGGCTTTG-TCCCACA-ACATGTTCTC------
------
FC88X_01035_00780 -------------------TTTGGCTTTG-TCCCACA-ACATGTTCTC------
------
FC88X_01053_00480 -------------------TTTGGCTTTG-TCCCACA-ACATGTTCTC------
------
FC88X_01061_00807 -------------------TTTGGCTTTG-TCCCACA-ACATGTTCTC------
------
FC88X_01082_01237 -------------------TTTGGCTTTG-TCCCACA-ACATGTTCTC------
------
FC88X_01097_01203 -------------------TTTGGCTTTG-TCCCACA-ACATGTTCTC------
------
FC88X_01127_00713 -------------------TTTGGCTTTG-TCCCACA-ACATGTTCTC------
------
FC88X_01133_00862 -------------------TTTGGCTTTG-TCCCACA-ACATGTTCTC------
------
FC88X_00775_00945 -----------------GTTTTGGCTTTG-TCCCACA-ACATGTTCTC------
------
FC88X_00867_00696 -------------------TCTGGCTTTG-TCCCACA-ACATGTTCTC------
------
FC88X_00535_01121 -------------------TTTGGCTTTG-CCCCACA-ACATGTTCTC------
------
FC88X_00963_00535 -------------------TTTGGCTTTG-CCCCACA-ACATGTTCTC------
------
FC88X_00943_01272 -------------------TTTGGCTTTGTCCCCACA-ACATGTTCTCC-----
------
FC88X_00024_00759 -------------------TTTGGCTTTG-TCCCACG-ACATGTTCTC------
------
FC88X_00203_01033 -------------------TTTGGCTTTG-TCCCACA-ACGTGTTCTC------
------
FC88X_00026_00359 -------------------TTTGGCTTTG-TCCCACA-ACGTGTTCTC------
------
FC88X_00434_01074 -------------------TTTGGCTTTG-TCCCACA-ACACGTTCTC------
------
FC88X_00906_01128 -------------------TCTGGCTTTG-TCCCACA-ACACGTTCTC------
------
FC88X_00141_00532 --------------------GCGGCTTTTTGCCCCCACGTAAGTTCTC------
------

FC88X_00070_00720 --------------------ACGGTTATGCCCCTGCACCGATGTTCT-------
------
FC88X_00948_00463 --------------------ACGGTTATGACCCTGCACCGATGTTCTCC-----
------
FC88X_00074_00268 -------------------CCAAACTATTTGTCCCACCACCCATTTC-------
------
FC88X_00902_01116 -----------------ATGCACAATCTTTG-CCCGCCACATATT---------
------
FC88X_00323_00994 --------------------CTGCTCGATCCCTCTGCCCGGCACCA--------
------
FC88X_00941_00551 ------------------CGCTGCTCTATCTCTCT---CGACACCGGA------
------
FC88X_00485_01182 -------------------TCTGTTCTT--CTCCCCACGCACCTTGGT------
------
FC88X_00154_00955 -------------------TCTGTTCTT--CTCCC-ACGCACCTTGGT------
------
FC88X_01003_00919 -------------------CCTGTTCCCAATCTCCGCAACACCTTT--------
------
FC88X_00534_00983 ---------------TCCGCCAAGTC-TTCTCCCCGGAACTCAT----------
------
FC88X_00120_00775 ---------------TCCGCCAAGTC-TTCTCCCCGGAACTCAT----------
------
FC88X_00251_00656 --------------TATTGCTATGTC-TTCTCCGCGGATCTT------------
------
FC88X_00077_00887 -------------CCTCAGCTAAGCTGTTCTCGCCGTATC--------------
------
FC88X_00089_00909 ----------------TTACCAAGCTTTTC-TCCGGACATCAACA---------
------
FC88X_01011_01022 ----------------TTACCAAGCTTTTC-TCCGGACATCAACA---------
------
FC88X_00641_00837 ------------------ACGAGCTTTATCATCCCGCCGTCGACA---------
------
FC88X_00476_00915 ------------------TCCGAGCATTTC-CCCGCCCATGGCTAT--------
------
FC88X_00806_00928 ------------------TCCGAGCATTTC-CCCGCCCATGGCTAT--------
------
FC88X_00522_01197 -----------GGGGAAGCCCAGCATCT-TTCCCCGCTGACGTGTT--------
------
FC88X_00501_01105 -------------------CCATGATCTATTCTCCGGCGACGTGCC--------
------
FC88X_00912_01108 ---------------------ACGATCCTTCTCCGCACGACGTTTTGC------
------
FC88X_00876_01151 -------------------TTACGGTCTTTGCGCCGCAGACGTTCT--------
------
FC88X_01104_00875 ----------------CTACCAAGGCTCTATTCCCGACATCAT-----------
------
FC88X_00937_00931 ----------------CTCACAGTGCTTTTTTCCCCACATCCT-----------
------
FC88X_00206_00398 --------------------CTGTATCGCG-TCCAACACAGCGTTCTC------
------
FC88X_00628_00444 --------------------CTGTATCGCG-TCCAACACAGCGTTCTC------
------
FC88X_00682_01060 --------------------CTGTATCGCG-TCCAACACAGCGTTCTC------
------
FC88X_00788_00155 --------------------CTGTATCGCG-TCCAACACAGCGTTCTC------
------

FC88X_00049_00450 --------------------CTGTATCGCG-TCCAACACAGCGTTCTC------
------
FC88X_00584_00112 --------------------CTGTATCGCG-CCCAACACAGCGTTCTC------
------
FC88X_00847_01191 ------------------TGCTAC-TCCCGCCCTGACACCGCTTAGTCC-----
------
FC88X_01043_01215 --------------------CTACATCCCGGCCCGCGACATCGTACT-------
------
FC88X_00294_00422 ----------------------CCAACTGCTCCCCTGCCCAATCGTGGC-----
------
FC88X_01062_01172 ----------------------CCAACGCTCCCGCCATCGTGTTCTC-------
------
FC88X_01065_01206 -------------------CCCCCAACTC-ACCAGCCGCATCGTCCC-------
------
FC88X_00880_01251 -----------------------CAACTCTAACCCGCGCATCTCCCACT-----
------
FC88X_01090_00957 --------------------CCAGCTACTCC-CCGGCGC-CCTTGTTCT-----
------
FC88X_00497_00084 --------------------
CCACCACCTCCGTCGTCGCGCCTTGTTCTTCCTTTCTC--
FC88X_01050_01055 --------------------ATATCACCTCCCCCGCAGC--GCTGTTCT-----
------
FC88X_00600_01162 --------------------CCGATCTTCCCTCAC-AACGCCTGTTCT------
------
FC88X_00790_00190 ----------------ACCACCTATCTTCTCTCCCGAACGCCTCTTCCTTTC--
------
FC88X_00933_00644 --------------------CCTACCTATCTCCCCCAACGCTGGATC-------
------
FC88X_00236_00065 ---------------------TAACCCGTTCTCCCCGTCGACGCTGCG------
------
FC88X_00755_01160 --------------------TTTAAACCTTACTCCCACCGACGCTCA-------
------
FC88X_00084_00543 ---------------------CCGCCGATTCTCCCACGGCATTGTCAC------
------
FC88X_01049_01248 ---------------------CCACCGATTCTCCCACGGCATTGTCAC------
------
FC88X_00916_00864 ---------------------TTTCAGGTTCTCCCCCGGTGCATTGGC------
------
FC88X_00638_01127 --------------------CTTGCCGATCGTCCGCACGCATTTCTCT------
------
FC88X_00829_01113 --------------------CTTGCCGATCGTCCGCACGCATTTCTCT------
------
FC88X_01132_01102 --------------------CTTGCCGATCGTCCGCACGCATTTCTCT------
------
FC88X_00238_00225 --------------------CTTGCCGATCGTCCGCACGCATTTCTCT------
------
FC88X_00357_01068 --------------------CTTGCCGATCGTCCGCACGCATTTCTCT------
------
FC88X_01131_01138 --------------------CTTGCCGATCGTCCGCACGCATTTCTCT------
------
FC88X_00364_01120 --------------------CTTGCCGATCGTCCACACGCATTTCTCT------
------
FC88X_00497_01174 --------------------CTTGCCTATCTTCCCCGCCTGCATTGC-------
------
FC88X_00040_00601 -------------------TCTTACACATCCTTCTCACCTGCATTGC-------
------

FC88X_00615_00880   ------------------TCTTCTCAACTCCGCATTGTACCTTGT---------
------
FC88X_00880_00870   ----------------GTTATCTTCTCCCCGGCATTG-ACATTGT---------
------
FC88X_00919_00670   --------------------CTCGGAAACCTCCCACCG-GGCATTGT-------
------
FC88X_00997_01013   -------------------GCTCGGAAACCTCCCACCG-GGCATTGT-------
------
FC88X_00440_01069   --------------------CTCGGAAACCTCCCACCG-GGCATTGT-------
------
FC88X_00598_01061   --------------------CTCGGAAACCTCCCACCG-GGCATTGT-------
------
FC88X_00781_00861   --------------------CTCGGAAACCTCCCACCG-GGCATTGT-------
------
FC88X_00950_00777   --------------------CTCGGAAACCTCCCACCG-GGCATTGT-------
------
FC88X_00494_00204   --------------------CTCGGAAACCTCCCACCG-GGCATTGTCC-----
------
FC88X_00683_00635   --------------------CTCGGAAACCTCCCACCG-GGCATTGT-------
------
FC88X_00950_00518   --------------------CTCGGAAACCTCCCACCG-GGCATTGT-------
------
FC88X_01074_00406   --------------------CTCGGAAACCTCCCACCG-GGCATTGT-------
------
FC88X_00793_01169   --------------------CTCGGAA-CCTCCCACCG-GGCATTGT-------
------
FC88X_01100_01114   --------------------CTCGGAGACCTCCCACCG-G-CATTGT-------
------
FC88X_00371_00989   --------------------CTCGGAAACCTCCCACCG-
GGCATTGTCTTCCTTTCT---
FC88X_00661_01239   --------------------CTCGGAAACCTCCCACCG-G-TATTGT-------
------
FC88X_00943_00509   -------------------CCGGCGACTTC-CCGACTTCGGCATTGA-------
------
FC88X_00832_01230   -------------------CCAGTGACTCCTCCCGCTGCCGCATT---------
------
FC88X_00300_01119   -----------------------ACTTCTCCACCGCCACATTATCGTTAT----
------
FC88X_00953_01010   ----------------------ACCTTCTCAGCCGGCATAACGTT---------
------
FC88X_00947_00335   ---------------------CTACCTTCCCTCCGCCAAGGCATTACT------
------
FC88X_00889_00967   --------------------CTCTCTACTCCCC-AGGGTTGACGCGTT------
------
FC88X_00241_00948   --------------------CTCTCTACTCCC--
AGGGTTGACGCGTTCTTCTTTCTCCC
FC88X_01125_01176   ---------------------TCTCCACCCCGACAGAGTTA-------------
------
FC88X_00086_00081   ----------------------TTCCGTCCCCGCAGCGTTGCGCCTTT------
------
FC88X_00370_01161   --------------------CCGCCATTTGTTC-ATTGGTGCCCTATC------
------
FC88X_00118_00124   --------------------CCGCCATTTGTTC-ATTGGTGCCCTATC------
------
FC88X_00288_00607   --------------------CCGCCATTTGTTC-ATTGGTGCCCTATC------
------

```
FC88X_00456_00998       --------------------CCGCCATTTGTTC-ATTGGTGCCCTATC------
------
FC88X_00911_00278       --------------------CCGCCATTTGTTC-ATTGGTGCCCTATC------
------
FC88X_00979_01019       --------------------CCGCCATTTGTTC-ATTGGTGCCCTATC------
------
FC88X_00057_00762       --------------------CCGCCATTTGTTC-ATTGGTGCCCTATCCC----
------
FC88X_00097_00520       --------------------CCGCCACTTGTTC-ATTGGTGCCCTATC------
------
FC88X_01141_00706       --------------------CCGCCAGTAGT---ATTGGCCCACTATCTC----
------
FC88X_00032_00165       --------------------CCGCCAGTAGT---ATTGGCCCACTATCTC----
------
FC88X_01088_01043       --------------------CCGCCAGTAGT---ATTGGCCCACTATCTC----
------
FC88X_00384_00566       --------------------CCGCCATATGTTT-ATTGCAGTTATCTC------
------
FC88X_00098_00871       --------------------CCGCCATATGTTT-ATTGCAGTTATCTC------
------
FC88X_00156_00736       --------------------CCGCCATATGTTT-ATTGCAGTTATCTC------
------
FC88X_00416_00634       --------------------CCGCCATATGTTT-ATTGCAGTTATCTC------
------
FC88X_00781_00135       --------------------CCGCCATATGTTT-ATTGCAGTTATCTC------
------
FC88X_01140_00687       --------------------CCGCCATATGTTT-ATCGCAGTTATCTC------
------
FC88X_00871_00892       ---------------------GACAATACGTCT-TTCCCAGTCAGGTCAT----
------
FC88X_00019_00241       --------------------CCACCAGATGTGT-TACCCAGCGATGTT------
------
FC88X_00827_00301       ---------------------CTTAGTACTATT-GTCCCAGAAAGCTCC-----
------
FC88X_00942_00898       ----------------------GCCATACTTTT-TGCTCTGAAACCTCCA----
------
FC88X_00752_00502       --------------------CCAAAAACTCTTC-CCCAGCAGGCTGAT------
------
FC88X_00765_00722       ---------------GTGATCCAAAAACTCTTC-CCCAGCAGGCTGAT------
------
FC88X_00870_01186       --------------------CCAAAAACTCTTC-CCCAGCAGGCTGAT------
------
FC88X_00380_01123       --------------------CCAAAAACTCTTC-CC-AGCAGGCTGAT------
------
FC88X_00460_00640       --------------------CCAAAAACTCTTC-CCCAGCAGGCTGATCC----
------
FC88X_00837_01236       -------------------CCAAAAAACTCTTC-CCCAGCAGGCTGAT------
------
FC88X_00870_00819       -------------------CCAAAAAACTCTTC-CCCAGCAGGCTGAT------
------
FC88X_00040_00503       -------------------CCAAAAAACTCTTC-CCCAGCAGGCTGAT------
------
FC88X_00427_01205       -------------------CCAAAAAACTCTTC-CCCAGCAGGCTGAT------
------
FC88X_00252_01087       --------------------CCAAATTCTCTCC-GCCGGCAAGTTGTT------
------
```

```
FC88X_00321_01101       --------------------CCAAATTCTCTCC-GCCGGCAAGTTGTT------
------
FC88X_00443_00971       --------------------CCAAATTCTCTCC-GCCGGCAAGTTGTT------
------
FC88X_00424_00707       --------------------CCAAGTTCTCTCC-GCCGGCAAGTTGTT------
------
FC88X_00653_00193       --------------------CCAAATTCTCTCC-GCCGGCAAGTTGTC------
------
FC88X_00676_01066       ----------------CTCCCAACTATGTCTCC-GCAAGCCTGC----------
------
FC88X_00980_00282       ----------------CTCCCAAAT-CGTCTTC-GCCAGAATGCT---------
------
FC88X_00734_00339       ---------------TTCCCCCATCGTTTGTCC-GCATGCTTGC----------
------
FC88X_00426_01048       --------------------CCATAGTGTGTAC-TCCCGCAAGCTAGT------
------
FC88X_00590_00412       -------------------TGCGTGCGTTTT-C-CCCAGCCGTGTGTC------
------
FC88X_01120_00679       -------------------TGCGTGCGTTTTTC-CCCAGCCGTGTGTC------
------
FC88X_00063_00579       -------------------TGCGTGCGTTTT-C-CCCAGCCGTGTGTC------
------
FC88X_00593_00763       -------------------TGCGTGCGTTTT-C-CCCAGCCGTGTGTC------
------
FC88X_01091_00893       --------------------CCGCGCGCTGTTC-CACCCCCGGATGAC------
------
FC88X_00691_00134       --------------GGGGAAGCACACGGTTTTC-TCCCACCGGCTGGCC-----
------
FC88X_00845_01167       --------------------GCACACGGTTTTC-TCCCACCGGCTGGCC-----
------
FC88X_00799_00087       ---------------------CACACGGTTTTC-TCCCACCGGCTGGCC-----
------
FC88X_00797_00224       ---------------------CACACGGTTTTC-TCCCACCGGCTGGCC-----
------
FC88X_00815_01093       ---------------------CACACGGTTTTC-TCCCACCGGCTGGCC-----
------
FC88X_00808_01173       ---------------------CACACGGTTTTC-TCCCACCGGCTGGCC-----
------
FC88X_00866_00576       ---------------------CACACGGTTTTC-TCCCACCGGCTGGCC-----
------
FC88X_00986_00766       ---------------------CACACGGTTTTC-TCCCACCGGCTGGCC-----
------
FC88X_00381_00720       ---------------------CACACGGTTTTC-TCCCACCGGCTGGCC-----
------
FC88X_00902_01203       ---------------------CACACGGTTTTC-TCCCACCGGCTGGCC-----
------
FC88X_00958_00634       ---------------------CACACGGTTTTC-TCCCACCGGCTGGCC-----
------
FC88X_01003_00529       ---------------------CACACGGTTTTC-TCCCACCGGCTGGCC-----
------
FC88X_01006_00722       ---------------------CACACGGTTTTC-TCCCACCGGCTGGCC-----
------
FC88X_01035_00891       ---------------------CACACGGTTTTC-TCCCACCGGCTGGCC-----
------
FC88X_00183_00836       ---------------GGGGAACACACGGTTTTC-TCCCACCGGCTGGCC-----
------
```

FC88X_00795_01168 ---------------------CACACGGTTTTC-TCCCGCCGGCTGGCC-----
------
FC88X_00334_00931 -----------------------TACGCTTTTC-TCAGACCAGCTTGCCTT---
------
FC88X_00953_00513 --------------------TCATGTGTTTTTT--CAACCCGGACATTCT----
------
FC88X_00264_00653 --------------------TCATGTGTTTTTT--CAACCCGGACATTCT----
------
FC88X_00496_00866 --------------------TCATGTGTTTTT---CAACCCGGACATTCT----
------
FC88X_00066_00757 --------------------TCATGTGTTTTT---CAACCCGGACAATTCT---
------
FC88X_00201_00819 --------------------TCATGTGTTTTT---CAACCCGGACATTCT----
------
FC88X_00724_00880 --------------------TCATGTGTTTTT---CAACCCGGACATTCTC---
------
FC88X_01011_00467 --------------------TCATGTGTTTTT---CAACCCGGGCATTCT----
------
FC88X_00456_01048 -------------------GTCATGTGTTTTT---CAGCCCGGACATTCT----
------
FC88X_00137_00807 -------------------GTCATGTGTTTAT---CAACCTGGACATTCTCTC-
------
FC88X_00565_01210 -------------------TCTATGTGTTTTT---CAACCCGGA----------
------
FC88X_00654_00605 --------------------TCTTGGGTTTT----CTAGCCCAACGTTCTC---
------
FC88X_01027_00767 --------------------TCTTGCGTTTTTC--CTAGCCCAACGTTCTC---
------
FC88X_00562_00807 --------------------TCTTGCGTTTT----CTAGCCCAACGTTCTC---
------
FC88X_00819_00198 --------------------TCTTGCGTTTT----CTAGCCCAACGTTCTC---
------
FC88X_00613_01246 --------------------TCTTGCGTTTT----CTAGCCCAACGTTCTC---
------
FC88X_00807_01275 -------------------GTCTCGCGTTTT----CTAGCCCAACGTTCTC---
------
FC88X_00092_00471 --------------------TCTTGCGTTTT----CTAGCCCAACGTTCTC---
------
FC88X_01076_00463 --------------------TCTTGCGTTTT----CTAGCCCGACGTTCTC---
------
FC88X_00050_00512 --------------------TCTTGCGTTTT----CTAGCCCGACGTTCTC---
------
FC88X_00585_01256 --------------------TCTTGCGTTTT----CTAGCCCGACGTTCTC---
------
FC88X_01105_00506 ---------------------CTTGCTATTTTA--CCACACCGACGTTC-----
------
FC88X_00184_01058 --------------------TCTTGCATTTTTA--CCCCCGCGAGCTTCT----
------
FC88X_00206_00989 --------------------TCGAGCGTTTTT---CTCCCACGACGTATA----
------
FC88X_00419_00995 -------------------GTCGAGCGTTTTT---CTCCCACGACGTATA----
------
FC88X_00701_01026 --------------------TCGAGCGTTTTT---CTCCCACGACGTATA----
------
FC88X_00885_00795 --------------------TCGAGCGTTTTT---CTCCCACGACGTATA----
------

FC88X_00539_00756 --------------------TCGAGCGTTTTT---CTCCCACGACGTATA----
------
FC88X_00592_01168 --------------------TCGAGCGTTTTT---CTCCCACGACGTATA----
------
FC88X_00711_00879 --------------------TCGAGCGTTTTT---CTCCCACGACGTATA----
------
FC88X_00728_01003 --------------------TCGAGCGTTTTT---CTCCCACGACGTATA----
------
FC88X_00798_01184 --------------------TCGAGCGTTTTT---CTCCCACGACGTATA----
------
FC88X_00781_01273 --------------------TCGAGCGTTTTT---CTCCCACGACGTATA----
------
FC88X_01042_00878 --------------------TCGAGCGTTTTT---CTCCCACGACGTATA----
------
FC88X_00447_01196 --------------------TCGAGCGTTTTTT--CTCCCACGACGTATA----
------
FC88X_00896_00262 --------------------TCGAGCGTTTTTT--CTCCCACGACGTATA----
------
FC88X_00913_00691 -------------------
TCCGAGCGTTTTTTTTCTCCCACGACGTATATCCC------
FC88X_00865_00330 --------------------TCGAGCGTTTTTTC-ATCCCAC------------
------
FC88X_01107_00702 --------------------TCGGGCGTTTTT---CTCCCACGACGTATA----
------
FC88X_01136_01055 -----------------------AGTCTTCCT---CACCCAAG-
CGTATAACGC------
FC88X_00149_00194 --------------------ACTGGCCAT-AGCAGCCATACCGTTCTC------
------
FC88X_00325_00013 --------------------ACTGGCCAT-AGCAGCCATACCGTTCTC------
------
FC88X_00590_01234 --------------------ACTGGCCAT-AGCAGCCATACCGTTCTC------
------
FC88X_00202_01039 --------------------ACTGGCCAT-AGCAGTCATACCGTTCTC------
------
FC88X_00906_00431 -----------CTCATTGCTATTTACCATCAGCAGTCTT-CCTTTCT-------
------
FC88X_00588_01197 -------------------------CTATTTCCTCCCAGACGGTCATCTATT--
------
FC88X_00724_00055 -------------------------CTATTTCCTCCCAGACGGTCATCTATT--
------
FC88X_00795_01236 -------------------------CTATTTCCACCCAGACGGTCATCTACT--
------
FC88X_00452_01182 -----------------------CCCTATCTTCCCCATCCGAGTCATCCG----
------
FC88X_00598_00783 ---------------------CACTTATATTCAACCACCC-CGTCATGAC----
------
FC88X_00118_00840 ------------------CCACATTTAT-CCCAACGACCT-CATCTT-------
------
FC88X_00954_00538 -------------------TCAACTTTTCTTCACTCGCCCGCGTCAT-------
------
FC88X_00550_01049 -------------------TACTAGTATCTACAGTCTCCC-CGTCAC-------
------
FC88X_00029_00578 -------------------TACTAGTATCTACAGCCTCCC-CGTCAC-------
------
FC88X_00917_00770 --------------------GCTAACATTTCGCGTCTCCC-CGGCATCC-----
------

FC88X_00573_01240 ------------------GTTGTCGTCTCTTCCGCTCCCA-CGGCAC-------
------
FC88X_00333_01028 -------------------TTGTAGTGTATTCTCCCGCGG-CGTGGT-------
------
FC88X_00020_00718 --------------------ACGTCTCCTATCT-G-CCACTGG-CTTACC----
------
FC88X_00288_00098 --------------------TCATCTCCTCTTTTG-CCGCTCG-TCTGCC----
------
FC88X_00148_00939 --------------------ACATCTTCTCTCCTG-CATGCCG-CGTCA-----
------
FC88X_00957_00746 --------------------ACATCTTCTCTCCTG-CATGCCG-CGTCA-----
------
FC88X_00512_00316 --------------------ACATTTTCTCTCCTT-C-TGCCA-CTTGGGC---
------
FC88X_00124_00273 --------------------ACTAACTATCTCCCAATCTGCCA-TGTG------
------
FC88X_00138_00555 --------------------ACTAACTATCTCCCAATCTGCCACTGTG------
------
FC88X_00135_00917 ---------------------CTAATTATCTCCCA-TCGGCCG-CGTGCC----
------
FC88X_00467_01046 ---------------------CTAATTATCTCCCA-TCGGCCG-CGTGCC----
------
FC88X_00482_01174 ---------------------CTAATTATCTCCCA-TCGGCCG-CGTGCC----
------
FC88X_00596_00339 ---------------------CTAATTATCTCCCA-TCGGCCG-CGTGCC----
------
FC88X_00991_00723 ---------------------CTAATTATCTCCCA-TCGGCCG-CGTGCC----
------
FC88X_00131_00971 ---------------------CTAATTATCTCCCA-TCGGCCG-CGTGCC----
------
FC88X_00593_01062 ---------------------CTAATTATCTCCCA-TCGGCCG-CGTGCC----
------
FC88X_00820_00918 ---------------------CTAATTATCTCCCA-TCGGCCG-CGTGCC----
------
FC88X_00844_00962 ---------------------CTAATTATCTCCCA-TCGGCCG-CGTGCC----
------
FC88X_00123_00677 ---------------------CTAATTACCTCCCA-TCGGCCG-CGTGCC----
------
FC88X_00741_00393 -------------------GGCTAATTATCTCCCA-TCGGCCG-CGTGCC----
------
FC88X_00896_01209 ---------------------CTAATTATCTCCCA-TCGGCCG-CGTGC-----
------
FC88X_00671_01256 ------------------TCACGATC-ATCTCCAA-TCTCCAG-CACGCTT---
------
FC88X_01012_00305 ------------------TCACGATC-ATCTCCAA-TCTCCAG-CACGCTT---
------
FC88X_01012_00327 ------------------TCACGATC-ATCTCCAA-TCTCCAG-CACGCTT---
------
FC88X_00128_00270 ------------------TCACGATC-ATCTCCAA-TCTCCAG-CACGCTC---
------
FC88X_00968_00569 ------------------TCACGATC-ATCTCCAA-TCTCCAG-CACGTTT---
------
FC88X_00076_00268 --------------------ACGATCTATCTCCCG-CCGTCAG-CACGC-----
------
FC88X_00611_00992 --------------------ATAAACTATTTT---CCCACGGCGAGTGTTG---
------

```
FC88X_00859_00205      --------------------ATAAACTATTTT---CCCACGGCGAGTGTTG---
------
FC88X_00468_01001      --------------------ATAAACTATTTT---CCCACGGCGAGTGTTG---
------
FC88X_00677_00467      --------------------ATAAACTATTTT---CCCACGGCGAGTGTTG---
------
FC88X_01034_01216      --------------------ATAAACTATTTT---CCCACGGCGAGTGTTG---
------
FC88X_00997_01033      --------------------ATAAACTATTTTTTCCCCACGGCGAGTGTTG---
------
FC88X_01059_00791      ---------------------TCAACTAATCTC-CTCACCTCGGCGTGCC----
------
FC88X_00854_00726      ---------------------TCAACCATTTTC-CACCTCCGCGAGCGC-----
------
FC88X_00834_00191      ------------------------ACTATTTCTCCTCCGCTGCGACGTCAC---
------
FC88X_00969_00788      ------------------GGGGAAACTATTTCTCCTCCGCTGCGA---------
------
FC88X_00401_00256      -------------------ACCGTTCTATTTTCTCCCAGCCAGTA---------
------
FC88X_00796_00306      -------------------ACCATTC--TTTTTCCCCGGCAGTTAAAT------
------
FC88X_00417_01211      --------------------CCCTTCGATTTTTTCCCTCCAGTGTACG------
------
FC88X_00496_01187      --------------------TCTCGTCTTCTCTCACCC-CAGTGTGTG------
------
FC88X_00800_00842      --------------------TCGTCTATTCTCCTGCGC-CAGTATGTG------
------
FC88X_00691_00716      --------------------ACTACAATCCTTCTCCGC-CAGTGTCCG------
------
FC88X_00761_00163      ------------------GACGCACAACTCTCCTTCTCGCAGTGGCCCTCCC--
------
FC88X_00130_00992      -----------------------TTCTATGTTCTCCCGGAGGCGCCCGTC----
------
FC88X_00912_00633      ----------------------GTGCCATGTATCTCCCCTGGGCCAGAT-----
------
FC88X_00698_01078      -----------------------GCTTGTTTCTTCCCCTGAGC-GTGATGGC--
------
FC88X_01049_00725      ------------------------CTTGTTTCTTCCCCTGAGCCGTGATGGC--
------
FC88X_00100_00510      ---------------------CCCGCAATTTCTCTCCCC--GCAGCTTGA----
------
FC88X_01025_00992      ---------------------CTGACATTCTCTCATCCCACGCAGCGCGA----
------
FC88X_00921_00821      ---------------------TGTGCTATTTCCTCCCACAGCTATCGA------
------
FC88X_00902_00889      ---------------------CCTGCTATTCCTCTTCGCCAGTATCGT------
------
FC88X_00205_00439      -----------------CCTAGAAG---TCTCCCATTGTCCGCTATT-------
------
FC88X_00767_00314      -----------------CCTAGAAG---TCTCCCATTGTCCGCTATT-------
------
FC88X_01005_00319      -----------------CCTAGAAG---TCTCCCATTGTCCGCTATT-------
------
FC88X_00490_01119      -----------------CCTAGAAG---TCTCCCATTGTCCGCTATTC------
------
```

FC88X_00041_00076 ------------------CTAGAAG---TCTCCCATTGTCCGCTATT-------
------
FC88X_00197_00994 --------------------ACATC---TCTCAGCTTCACCGTCTATTTT----
------
FC88X_00156_00034 --------------------ACATC---TCTCAGCTTCACCGTCTATTTT----
------
FC88X_01103_00838 --------------------ACATC---TCTCAGCTTCACCGTCTATTTT----
------
FC88X_01083_00796 -----------------CCGACAAG---TCTC--CTGCTCCGTAATTTT-----
------
FC88X_00011_00420 ------------------CTACAT----GCCCACTTTTACCGCTATTCA-----
------
FC88X_01129_00962 -----------------CCTACATCT--ATCCCACTCAACCGCTAT--------
------
FC88X_00025_00336 --------------------CCAACA--TCTCTTCCACGCGGCGA-TATT----
------
FC88X_00811_00951 --------------------CCAACA--TCTCTTCCACGCGGCGAATATT----
------
FC88X_01022_00778 --------------------CCAACA--TCTCTTCCACGCGGCGA-TATT----
------
FC88X_01030_00783 --------------------CCAACA--TCTCTTCCACGCGGCGA-TATT----
------
FC88X_00200_00911 ---------------------CAACA--TCTCTTCCACGCGGCGA-TATT----
------
FC88X_00809_01260 ---------------------CAACA--TCTCTTCCACGCGGCGA-TATT----
------
FC88X_00265_00784 ---------------------CAACA--TCTCT-CCACGCGGCGA-TATT----
------
FC88X_01037_01039 --------------------CCAACA--TCCCTTCCACGCGGCGA-
TATTATCAC-----
FC88X_00148_00784 --------------------CCAAAA--TCCCTTCCGCGCCGGAA-TACA----
------
FC88X_00536_00097 -------------------CCTAAAC--TCTCCTACGCAC-GCTATT-------
------
FC88X_01023_00939 -------------------CCTAAAC--CCTCCTACGCACCGCTATT-------
------
FC88X_00592_00272 --------------------CCAAGT--CCCTCTGCGCAC-GCTATTAGT----
------
FC88X_00647_00911 -------------------ACCACGA--CCCT-TCTACCC-ACGATTAG-----
------
FC88X_00106_00246 ------------------GTTCAAC---GTTTATCCGCATGAACATCTC-----
------
FC88X_00781_00306 ------------------GTTCAAC---GTTTATCCGCATGAACATCTC-----
------
FC88X_01066_01269 -----------------GGTTCAAC---GTTTATCCGCATGAACATCTC-----
------
FC88X_00045_00351 -------------------TTCAAC---GTTTATCCGCATGAACATCTC-----
------
FC88X_00177_01037 -------------------TTCAAC---GTTTATCCGCATGAACATCTC-----
------
FC88X_00201_00795 -------------------TTCAAC---GTTTATCCGCATGAACATCTC-----
------
FC88X_00203_00946 -------------------TTCAAC---GTTTATCCGCATGAACATCTC-----
------
FC88X_00293_01091 -------------------TTCAAC---GTTTATCCGCATGAACATCTC-----
------

FC88X_00575_01207 -------------------TTCAAC---GTTTATCCGCATGAACATCTC-----
------
FC88X_00689_00194 -------------------TTCAAC---GTTTATCCGCATGAACATCTC-----
------
FC88X_00742_01004 -------------------TTCAAC---GTTTATCCGCATGAACATCTC-----
------
FC88X_00772_00357 -------------------TTCAAC---GTTTATCCGCATGAACATCTC-----
------
FC88X_00920_00520 -------------------TTCAAC---GTTTATCCGCATGAACATCTC-----
------
FC88X_00006_00058 -------------------TTCAAC---GTTTATCCGCATGAACATCTC-----
------
FC88X_00051_00174 -------------------TTCAAC---GTTTATCCGCATGAACATCTC-----
------
FC88X_00133_00190 -------------------TTCAAC---GTTTATCCGCATGAACATCTC-----
------
FC88X_00119_00273 -------------------TTCAAC---GTTTATCCGCATGAACATCTC-----
------
FC88X_00152_00152 -------------------TTCAAC---GTTTATCCGCATGAACATCTC-----
------
FC88X_00278_01074 -------------------TTCAAC---GTTTATCCGCATGAACATCTC-----
------
FC88X_00323_00586 -------------------TTCAAC---GTTTATCCGCATGAACATCTC-----
------
FC88X_00349_01016 -------------------TTCAAC---GTTTATCCGCATGAACATCTC-----
------
FC88X_00671_00127 -------------------TTCAAC---GTTTATCCGCATGAACATCTC-----
------
FC88X_00767_00877 -------------------TTCAAC---GTTTATCCGCATGAACATCTC-----
------
FC88X_00828_00556 -------------------TTCAAC---GTTTATCCGCATGAACATCTC-----
------
FC88X_00929_00553 -------------------TTCAAC---GTTTATCCGCATGAACATCTC-----
------
FC88X_00999_00548 -------------------TTCAAC---GTTTATCCGCATGAACATCTC-----
------
FC88X_01003_01216 -------------------TTCAAC---
GTTTATCCGCATGAACATCTCCTTCCTTT---
FC88X_00276_00937 -------------------TTCAAC---GTCTATCCGCATGAACATCTC-----
------
FC88X_00836_00174 -------------------TTCAAC---GTCTATCCGCATGAACATCTC-----
------
FC88X_00103_00613 -------------------TTCAAC---GCTTATCCGCATGAACATCTC-----
------
FC88X_00849_01066 -------------------TTCAAC---
GTTTATCCGCATGAACATCTCCTTCCTTTCTC
FC88X_00928_00639 -------------------TTCAAC---GTTTATCCGCATGA------------
------
FC88X_00628_00180 -------------------TTCAAC---GTTTATCCGCACGAACATCTC-----
------
FC88X_00372_00014 ----------------CCCTTCAACCCTGTATATCCGCATGCTC----------
------
FC88X_00596_01091 ----------------CCCTTCAACC-
TGTATATCCGCATGCTCCTTCCTTTCTC-----
FC88X_00855_01210 -----------------CCTCAAACGTCTTCTCCGCACTGCA------------
------

FC88X_00270_01012 ------------------CTCCAACGTTCTTTATACGCAGTGCCGCT-------
------
FC88X_00037_00612 ------------------------CTACCTTTTACCATCTTC------------
------
FC88X_00489_00058 ----------------------CCCAACCTTTTAACGATTTCAGCTGCT-----
------
FC88X_00428_00824 -----------------CCAACTCCTTCTTCATACCAGATTCTG----------
------
FC88X_00439_01217 --------------------ACTACTATTTCCTACCACATTCTTTCA-------
------
FC88X_00767_00695 -------------------TTTTGCAACTGCTA--TATCTCACCACGAT-----
------
FC88X_00988_00430 -------------GGGGAATTTTGCAACTGCTA--TATCTCACCACGAT-----
------
FC88X_01011_00298 ---------------------TTACAACTTCTAACCTTCTCTCTACGG------
------
FC88X_00100_00216 -------------------TTCTCCTACCTC---TACCACGTGTCTTC------
------
FC88X_00182_01040 ------------------CTTCTCCAATCCTCCATACAACGTGCC---------
------
FC88X_00495_00167 ---------------------CTACAACCTCCCTTCAAGCGTATTCAT------
------
FC88X_00914_00495 ---------------------CTACAAACTCCACTACATCAAGCACAT------
------
FC88X_00008_00568 --------------------CCACGTATCTTGCCCCTGGCTGTTCGC-------
------
FC88X_00372_01089 --------------------CCACGTATCTTGCCCCTGGCTGTTCGCT------
------
FC88X_00284_01129 --------------------CCACGTATCTTGCCCCTGGCTGTTCGC-------
------
FC88X_00320_00691 --------------------CCACGTATCTTGCCCCTGGCTGTTCGC-------
------
FC88X_00640_00453 --------------------CCACGTATCTTGCCCCTGGCTGTTCGC-------
------
FC88X_01060_00970 --------------------CCACGTATCTTGCCCCTGGCTGTTCGC-------
------
FC88X_00480_00177 ---------------------CACATGTCTCTCTACCATTGCCTGATT------
------
FC88X_00882_00641 ---------------------CACATGTTTCTCTACCATTGCCTGATT------
------
FC88X_00440_01223 --------------------CCATATGCTCGTCCCCCATTGCCTGTT-------
------
FC88X_00902_00538 -------------------TCCACGTATTCCTTCTCCATTGCCCTA--------
------
FC88X_00459_01101 -----------------GTGCAACCGCATCCTCCCTTGTCTTT-----------
------
FC88X_01143_00626 ----------------CCGACAACCGTTTCTCCCCTGGCTGCA-----------
------
FC88X_00794_00282 --------------------CAACAACACCTGCATA--TTCCCTCTGTC-----
------
FC88X_00515_00028 --------------------CAACAACACCTGCATA--TTCCCTCTGTC-----
------
FC88X_00746_00231 ----------------------ACCACATCTCCATGGGTTCAATCTGTT-----
------
FC88X_00791_00300 ---------------TGACACAACCACACCTGCCCGTATTCCT-----------
------

```
FC88X_01027_01201      ----------------------CCAGTAATT-CCGCGACTGT-AATCTCTT---
------
FC88X_00994_01088      ----------------------CCAGTAATT-CTGCGACTGG-AATCTCTT---
------
FC88X_00067_00097      --------------------CCCCAGCATTT-CTGCGACTGT-TATCGC-----
------
FC88X_00552_01138      ----------------------TCAATAATT-CTACGCCCGT-GACCTATC---
------
FC88X_00423_00063      ----------------------ACACCTATT-CCCCGGCCGT-TATCAATG---
------
FC88X_00828_00806      ----------------------TCATCGACT-CGCCGCCCGCATATTAATC---
------
FC88X_01012_00668      ---------------TCTCACGTGACTAATC-CCT-GTCTATA-----------
------
FC88X_00169_00815      ----------------GTCAC-TCACTATTC-CCTCGTCTGTTAAC--------
------
FC88X_00063_00131      -------------------TTCACACACATT-TAC-AGGGGCTGTTCT------
------
FC88X_00455_00505      -------------------TTCACACACATT-TAC-AGGGGCTGTTCTT-----
------
FC88X_00122_00268      -------------------TTCACACACATT-TAC-AGGGGCTGTTCT------
------
FC88X_00132_00510      -------------------TTCACACACATT-TAC-AGGGGCTGTTCT------
------
FC88X_00125_00582      -------------------TTCACACACATT-TAC-AGGGGCTGTTCT------
------
FC88X_00118_00694      -------------------TTCACACACATT-TAC-AGGGGCTGTTCT------
------
FC88X_00173_00414      -------------------TTCACACACATT-TAC-AGGGGCTGTTCT------
------
FC88X_00206_00023      -------------------TTCACACACATT-TAC-AGGGGCTGTTCT------
------
FC88X_00200_00871      -------------------TTCACACACATT-TAC-AGGGGCTGTTCT------
------
FC88X_00525_01146      -------------------TTCACACACATT-TAC-AGGGGCTGTTCT------
------
FC88X_00590_00266      -------------------TTCACACACATT-TAC-AGGGGCTGTTCT------
------
FC88X_00555_01019      -------------------TTCACACACATT-TAC-AGGGGCTGTTCT------
------
FC88X_00684_01137      -------------------TTCACACACATT-TAC-AGGGGCTGTTCT------
------
FC88X_00835_00649      -------------------TTCACACACATT-TAC-AGGGGCTGTTCT------
------
FC88X_00800_00736      -------------------TTCACACACATT-TAC-AGGGGCTGTTCT------
------
FC88X_00951_00760      -------------------TTCACACACATT-TAC-AGGGGCTGTTCT------
------
FC88X_01059_00410      -------------------TTCACACACATT-TAC-AGGGGCTGTTCT------
------
FC88X_01092_00815      -------------------TTCACACACATT-TAC-AGGGGCTGTTCT------
------
FC88X_00014_00078      -------------------TTCACACACATT-TAC-AGGGGCTGTTCT------
------
FC88X_00113_00209      -------------------TTCACACACATT-TAC-AGGGGCTGTTCT------
------
```

```
FC88X_00274_00871       -------------------TTCACACACATT-TAC-AGGGGCTGTTCT------
------
FC88X_00286_01080       -------------------TTCACACACATT-TAC-AGGGGCTGTTCT------
------
FC88X_00306_00119       -------------------TTCACACACATT-TAC-AGGGGCTGTTCT------
------
FC88X_00435_00041       -------------------TTCACACACATT-TAC-AGGGGCTGTTCT------
------
FC88X_00408_01194       -------------------TTCACACACATT-TAC-AGGGGCTGTTCT------
------
FC88X_00483_00488       -------------------TTCACACACATT-TAC-AGGGGCTGTTCT------
------
FC88X_00526_01031       -------------------TTCACACACATT-TAC-AGGGGCTGTTCT------
------
FC88X_00570_01122       -------------------TTCACACACATT-TAC-AGGGGCTGTTCT------
------
FC88X_00593_01147       -------------------TTCACACACATT-TAC-AGGGGCTGTTCT------
------
FC88X_00611_00219       -------------------TTCACACACATT-TAC-AGGGGCTGTTCT------
------
FC88X_00612_00214       -------------------TTCACACACATT-TAC-AGGGGCTGTTCT------
------
FC88X_00665_01211       -------------------TTCACACACATT-TAC-AGGGGCTGTTCT------
------
FC88X_00685_01274       -------------------TTCACACACATT-TAC-AGGGGCTGTTCT------
------
FC88X_00720_01211       -------------------TTCACACACATT-TAC-AGGGGCTGTTCT------
------
FC88X_00839_00717       -------------------TTCACACACATT-TAC-AGGGGCTGTTCT------
------
FC88X_00826_01141       -------------------TTCACACACATT-TAC-AGGGGCTGTTCT------
------
FC88X_00857_00821       -------------------TTCACACACATT-TAC-AGGGGCTGTTCT------
------
FC88X_00851_00932       -------------------TTCACACACATT-TAC-AGGGGCTGTTCT------
------
FC88X_00931_01175       -------------------TTCACACACATT-TAC-AGGGGCTGTTCT------
------
FC88X_01037_00490       -------------------TTCACACACATT-TAC-AGGGGCTGTTCT------
------
FC88X_01000_01016       -------------------TTCACACACATT-TAC-AGGGGCTGTTCT------
------
FC88X_01012_01089       -------------------TTCACACACATT-TAC-AGGGGCTGTTCT------
------
FC88X_01071_00460       -------------------TTCACACACATT-TAC-AGGGGCTGTTCT------
------
FC88X_01087_01020       -------------------TTCACACACATT-TAC-AGGGGCTGTTCT------
------
FC88X_01103_00470       -------------------TTCACACACATT-TAC-AGGGGCTGTTCT------
------
FC88X_01147_01017       -------------------TTCACACACATT-TAC-AGGGGCTGTTCT------
------
FC88X_00950_01022       -------------GGGAAGTTCACACACATT-TACTAGGGGCTGTTCT------
------
FC88X_00601_01221       -------------------TTCACACACATT-TAC-AGG-GCTGTTCT------
------
```

FC88X_01109_00569 -------------------TTCACACACATT-TAC-AGGAGCTGTTCT------
------
FC88X_00130_00801 -------------------TTCACACACATT-TGC-AGGGGCTGTTCT------
------
FC88X_01007_00956 -------------------TTCACACACATT-TGC-AGGGGCTGTTCT------
------
FC88X_00170_00832 -------------------TTCACACACATT-TAC-AGGGGCTGCTCT------
------
FC88X_00908_00852 -------------------TTCATGCACATT-TAC-AGGGGCTGTTCT------
------
FC88X_00391_00958 -------------------TTCACGCACATT-TAC-AGGGGCTGTTCT------
------
FC88X_00237_00278 -------------------TTCACACACGCT-TAC-AGGGGCTGTTCT------
------
FC88X_00345_00844 -------------------TTCACACACGTT-TAC-AGGGGCTGTTCT------
------
FC88X_00064_00767 -------------------TTCACACACATT-TAC-GGGGGCTGTTCT------
------
FC88X_00450_01205 -------------------TTCACACACATT-TAC-GGGGGCTGTTCT------
------
FC88X_00559_01119 -------------------TTCACACACATT-TTT-AGGGGCTGTTCT------
------
FC88X_00914_01187 ------------------GTTCACACACATT-TAC--AGGGC------------
------
FC88X_00041_00156 -------------------TTCCCACTTTTG-CTGCGCGTGAGTCGC-------
------
FC88X_00742_01249 --------------------TCTTCCCCTTT-TCCCGCGTTAGGCGCT------
------
FC88X_01108_00846 -----------------TCTCCCCGCACGTT-TTCAGCGTTTGAT----------
------
FC88X_00592_01190 -------------------CTAAAAACATTA-TTCCCGTCGCTGTTCT------
------
FC88X_00749_00320 -------------------CTAAAAACATTA-TTCCCGTCGCTGTTCT------
------
FC88X_00097_00134 -------------------CTAAAAACATTA-TTCCCGTCGCTGTTCT------
------
FC88X_00379_00990 -------------------CTAAAAACATTA-TTCCCGTCGCTGTTCT------
------
FC88X_00037_00526 -------------------CTAAAAACATTA-TCCCCGTCGCTGTTCT------
------
FC88X_00061_00828 ------------------CTAAAAAACATTA-TTCCCGTCGCTGTTCT------
------
FC88X_00106_00426 -------------------CTAAAAC-ATTA-TTCCCGTCGCTGTTCT------
------
FC88X_00150_00624 -------------------CTAAAAC-ATTA-TTCCCGTCGCTGTTCT------
------
FC88X_00474_01078 -------------------CTAAAAC-ATTA-TTCCCGTCGCTGTTCT------
------
FC88X_00536_00164 -------------------CTAAAAC-ATTA-TTCCCGTCGCTGTTCT------
------
FC88X_00866_00303 -------------------CTAAAAC-ATTA-TTCCCGTCGCTGTTCT------
------
FC88X_00864_00997 -------------------CTAAAAC-ATTA-TTCCCGTCGCTGTTCT------
------
FC88X_00931_00593 -------------------CTAAAAC-ATTA-TTCCCGTCGCTGTTCT------
------

FC88X_01081_01087 -------------------CTAAAAC-ATTA-TTCCCGTCGCTGTTCT------
------
FC88X_00040_00485 -------------------CTAAAAC-ATTA-TTCCCGTCGCTGTTCT------
------
FC88X_00136_00271 -------------------CTAAAAC-ATTA-TTCCCGTCGCTGTTCT------
------
FC88X_00142_00927 -------------------CTAAAAC-ATTA-TTCCCGTCGCTGTTCT------
------
FC88X_00225_00584 -------------------CTAAAAC-ATTA-TTCCCGTCGCTGTTCT------
------
FC88X_00271_00100 -------------------CTAAAAC-ATTA-TTCCCGTCGCTGTTCT------
------
FC88X_00453_00062 -------------------CTAAAAC-ATTA-TTCCCGTCGCTGTTCT------
------
FC88X_00459_00907 -------------------CTAAAAC-ATTA-TTCCCGTCGCTGTTCT------
------
FC88X_00992_01191 -------------------CTAAAAC-ATTA-TTCCCGTCGCTGTTCT------
------
FC88X_01041_00690 -------------------CTAAAAC-ATTA-TTCCCGTCGCTGTTCT------
------
FC88X_01080_01193 -------------------CTAAAAC-ATTA-TTCCCGTCGCTGTTCT------
------
FC88X_00610_00981 -------------------CTAAAAC-ATTA-TTCCCGTCGCTGTTCTCC----
------
FC88X_00687_00824 -------------------CTAAAACGATTAGTTCCCGTCGCTGTTCTCT----
------
FC88X_00712_00093 -------------------CTAAAAC-ATCA--TCCCGTCGCTGTTCT------
------
FC88X_00932_00217 -------------------CTAAAAC-ATTA-TCCCCGTCGCTGTTCT------
------
FC88X_01062_00958 -------------------CTAAAAC-ATTA-TCCCCGTCGCTGTTCT------
------
FC88X_00853_00724 -------------------CTAAAAC-ATTA-TTCCCGTCGCTGTTCTCTTC--
------
FC88X_00144_00637 -------------------CTAAAAC-ATTA-TTCCCGCCGCTGTTCT------
------
FC88X_00655_00977 -------------------CTAAAAC-ATTA-TTCCCGCCGCTGTTCT------
------
FC88X_00845_00194 -------------------CTAAAGC-ATTA-TTCCCGACGCTGTTCT------
------
FC88X_00711_00254 --------------------CTAAAC-ACTA--TTCCGTCG-------------
------
FC88X_00928_01000 ------------------CCAATAGC--CCAATCCCCGTCGCATGTC-------
------
FC88X_00457_01046 ----------------------TAAC--ACATTTTCCGTTGC-TGTCTCCC---
------
FC88X_00311_00006 --------------------CCGCAATTGA---CGTCATCCCT-GGTTACT---
------
FC88X_00039_00041 --------------------CCGCAATTGA---CGTCATCCCT-GGTTACT---
------
FC88X_00797_00091 --------------------CCGCAATTGA---CGTCATCCCT-GGTTACT---
------
FC88X_00721_01209 --------------------CCGCAATTGA---CGTCATCCCT-GGTTACTC--
------
FC88X_00328_00584 --------------------CCGCAATTGA---CGTCATCCCC-GGTTACT---
------

FC88X_00375_00080 --------------------CCACAATTCC---TCTCATTTCG-TGTTACT---
------
FC88X_00071_00769 --------------------CCAAACTTTT---CCTGGTCCCC-GGCTACT---
------
FC88X_00208_00137 -------------------GCCAAACTTTT---CCTGGTCCCC-GGCTACT---
------
FC88X_00149_00342 --------------------CCAAACTTTT---CCTGGTCCCC-GGCTACT---
------
FC88X_00935_00236 --------------------CCAAACTTTT---CCTGGTCCCC-GGCTACT---
------
FC88X_01135_00931 -------------------CCAAAACTTTT---CCTGGTCCCCCGGCTACT---
------
FC88X_00777_00853 --------------------CCAAACTTTT---CCCGGCCCCC-GGCTACT---
------
FC88X_00476_00133 -------------------CCAAAACTTTTT-CCCTGGTCCCC-
GGCTACTCTTCCT---
FC88X_01024_00484 -------------------CCCACAAATTC----TTCCTTCCC-GGCTACT---
------
FC88X_00942_01003 --------------------ACAAACACTCT-CTCCTGTCCGTCTGCT------
------
FC88X_00930_00284 --------------------ACAAACACTCT-CTCCTGTCCGTCTGCT------
------
FC88X_01001_00774 --------------------ACAAACACTCT-CTCCTGTCCGTCTCCT------
------
FC88X_00620_01195 -------------------TTACAACAACTT-CTCCTGTCCCTTTCT-------
------
FC88X_01125_00956 --------------------ACAAACCTTAT-CCCGTTCCGTTGTGCA------
------
FC88X_00520_00028 ----------------------CGACAATCT-CCCCCGTTCCGCCGTGTA----
------
FC88X_00784_01109 ---------------------ACAACAAT-T-CTACCGT-CCGCTATGTAC---
------
FC88X_00327_01064 -------------------GGCCGGCATCTATCCCGTGTCGGTTCGTG------
------
FC88X_00611_00938 ---------------------CCGGCATCTATCCCGTGTCGGTTCGTG------
------
FC88X_00997_00900 ---------------------CCGGCATCTATCCCGTGTCGGTTCGTG------
------
FC88X_00131_00110 ---------------------CCGGCATCTATCCCGTGTCGGTTCGTG------
------
FC88X_00150_00580 ---------------------CCGGCATCTATCCCGTGTCGGTTCGTG------
------
FC88X_00283_00204 ---------------------CCGGCATCTATCCCGTGTCGGTTCGTG------
------
FC88X_00845_00188 ---------------------CCGGCATCTATCCCGTGTCGGTTCGTG------
------
FC88X_00944_00489 ---------------------CCGGCATCTATCCCGTGTCGGTTCGTG------
------
FC88X_00478_00059 ---------------------CCGGCATCTATCCCGTGTCGGTTCGTG------
------
FC88X_00502_01019 ---------------------CCGGCATCTATCC-GTGTCGGT-----------
------
FC88X_00026_00156 -------------------ATCCAGCATCTTCTCCGTTCCCGTGCA--------
------
FC88X_00016_00337 -------------------ATCAATCAT-TTCTCCGTACGCCT-----------
------

```
FC88X_00156_00307        -------------------CTCTACATCACCGAGTAATATATTCTC--------
------
FC88X_00521_00882        -------------------CTCTACATCACCGAGTAATATATTCTC--------
------
FC88X_00170_01037        -------------------TTCTAGCTCCTCGTCTAGTATATTCGC--------
------
FC88X_00977_00848        -------------------TTCTAGTTCCTCGTCTAGTATATTCGC--------
------
FC88X_00045_00288        -------------------TTCTAGTCCCTCGTCTAGTATATTCGC--------
------
FC88X_00623_00089        ---------------------CCCATTCTCTT--CAGTCCACAAGTAACA----
------
FC88X_01142_00860        ----------------------CCATTCTCTTTCCTGTTCGTTAGTAAC-----
------
FC88X_00406_00475        -------------------ATCTCACTACCCTGC--TCCTGTACGCAC------
------
FC88X_00585_00575        --------------------TCTCAG-ACTCTGCAGTGCTGTTCCCAC------
------
FC88X_00915_00438        ------------------ACTCTCCGTCCCCGGC---CCCGAACGCTAC-----
------
FC88X_01050_00972        -----------------GACTCTCAGTACTCCGC---TCCGCACGCCT------
------
FC88X_00567_01256        ---------------------CGCCGCGCTCCGTT-CCCAGTATCGTATT----
------
FC88X_00811_00099        ------------------TTTCTCCGC-CTCCGAC-ACCTATAACCTCTTC---
------
FC88X_00804_00936        ------------------TTACTCTGT-CTCCAGC-ACGCGCGTATT-------
------
FC88X_00480_00182        ----------------------TTCGCGGCTGTCCGTCCCCCAACTACT-----
------
FC88X_00286_00103        ----------------------TTCGCGGCTGTCCGTCCCCCAACTACT-----
------
FC88X_00158_00774        --------------------CATTCGCTTATATTCGTCCC--AGCTACT-----
------
FC88X_00949_00542        --------------CTCTTTCCTTCGTGTCTGCGCGTGCCC-------------
------
FC88X_00989_01038        --------------TTTACTCCCTGACTACGGTGCGTGCCC-------------
------
FC88X_00857_00285        --------------------GCTCCCTGGCAGCCGGCATCTCTGCGTA------
------
FC88X_00112_00385        -----------------------CCGTTACGTCCCTGGCCCCGGCATATG----
------
FC88X_00323_00293        --------------------CGCTCATTTG-TCCCTTGT--CGCGTTGTA----
------
FC88X_00747_01124        --------------------CGCTCATTTG-TCCCTTGT--CGCGTTGTA----
------
FC88X_00720_00564        -----------------------CCATTCGCTCCCTTGTACCGCCTTCTA----
------
FC88X_00897_00749        -----------------------CCCTTGCCTCCCTGGTACCAACCACAC----
------
FC88X_00723_00986        -----------------------CCCTTTACTTTCCTGTGCCACCATCAC----
------
FC88X_00472_00081        ----------------------ATTAGGTATTTTCAAGTCCTAGCCTGCTC---
------
FC88X_00858_01000        -----------------------CCGTTTAAGTTCTTGCCCCTGCTCATT----
------
```

FC88X_01043_00959 ----------------------ACCTTTCTATTCGTGCCCTGCCACCTC----
------
FC88X_01019_00467 -----------------GTTTCTCGTTAGGCGCCCTGTGACTGTT--------
------
FC88X_00397_00019 -------------------TTTCTGTTAGGCGCCCTGTGACTGTT--------
------
FC88X_00188_01015 ------------------TTTCTCGTTAGGCGCCCTGTGACTGTT--------
------
FC88X_00358_00794 ------------------TTTCTCGTTAGGCGCCCTGTGACTGTT--------
------
FC88X_00652_00896 ------------------TTTCTCGTTAGGCGCCCTGTGACTGTT--------
------
FC88X_00667_01007 ------------------TTTCTCGTTAGGCGCCCTGTGACTGTT--------
------
FC88X_00910_00326 ------------------TTTCTCGTTAGGCGCCCTGTGACTGTT--------
------
FC88X_01033_00781 ------------------TTTCTCGTTAGGCGCCCTGTGACTGTT--------
------
FC88X_00048_00150 ------------------TTTCTCGTTAGGCGCCCTGTGACTGTT--------
------
FC88X_00327_00985 ------------------TTTCTCGTTAGGCGCCCTGTGACTGTT--------
------
FC88X_00398_00554 ------------------TTTCTCGTTAGGCGCCCTGTGACTGTT--------
------
FC88X_00638_01042 ------------------TTTCTCGTTAGGCGCCCTGTGACTGTT--------
------
FC88X_00839_00224 ------------------TTTCTCGTTAGGCGCCCTGTGACTGTT--------
------
FC88X_00874_00878 ------------------TTTCTCGTTAGGCGCCCTGTGACTGTT--------
------
FC88X_00602_00910 --------------GGGGATTTCTCGTTAGGCGCCCTGTGACTGTT--------
------
FC88X_00159_00591 ------------------TTTCTCGTTAGGCGCC-TGTGACTGTT--------
------
FC88X_00569_00510 ------------------TTTCTCGTTAGGCGCC-TGTGACTGTT--------
------
FC88X_00610_00597 ------------------TTTCTCGTTAGGCGCC-TGTGACTGTT--------
------
FC88X_00735_00805 ------------------TTTCTCGTTAGGCGCC-TGTGACTGTT--------
------
FC88X_00901_00466 ------------------
TTTCTCGTTAGGCGCCCTGTGACTGTTCTTCTTTCT-----
FC88X_00446_01138 ------------------TTCCTCGTTAGGCGCCCTGTGACTGTT--------
------
FC88X_00260_01097 ------------------TTTCTCGTTAGGCGCCCCGTGACTGTT--------
------
FC88X_00875_00854 ------------------TCTCTCGTTAGGCGCCCTGTGACTGTT--------
------
FC88X_00891_01244 ------------------TTTCTCGTTAGGCGCCCTGTTACTGTT--------
------
FC88X_00509_00304 ------------------TTTCTCGTTAGGCGCC-TGTGACCGTT--------
------
FC88X_00935_00232 ------------------CTTCTATCGTTGTCGCCCGTCACTATT--------
------
FC88X_00394_01164 ---------------TCTCTTCTCGTTCGGCATCACGGTGTC-----------
------

FC88X_00855_00170 -----------------CTATCTTCCCGCGCCATCACCCTATC-----------
------
FC88X_00409_00516 ------------ACCCTAACTATTTATCCCGCAGCATACT--------------
------
FC88X_00474_00978 ------------TCTACTATTTTTCCTCCCTCAGCATAT---------------
------
FC88X_00110_00566 -------------TTCACTCTTTTCTCCCCATCACATATT--------------
------
FC88X_00519_00233 -----------------ATCAAGCTTCTAGATCTCTCAC-CGTCTC--------
------
FC88X_00146_00064 -------------------CTAGCTGCTAGATCCCTCGCACGTCCT--------
------
FC88X_00140_00527 --------------------TACTTCTACGTTCGCTCGCCCATCAT--------
------
FC88X_00216_00335 --------------------CATAGCCTCACGTGGTTCACTGTTTTC-------
------
FC88X_00622_00888 --------------------CATAGCCTCACGTGGTTCACTGTTTTC-------
------
FC88X_01049_01026 --------------------CATAGCCTCACGTGGTTCACTGTTTTC-------
------
FC88X_01129_00627 --------------------CATAGCCTCACGTGGTTCACTGTTTTC-------
------
FC88X_00046_00443 --------------------CATAGCCTCACGTG-TTCACTGTTTTC-------
------
FC88X_00575_01015 --------------------CATAGCCTCACGTG-TTCACTGTTTTC-------
------
FC88X_00659_00294 --------------------CATAGCCTCACGTG-TTCACTGTTTTC-------
------
FC88X_00813_00920 --------------------CATAGCCTCACGTG-TTCACTGTTTTC-------
------
FC88X_00515_00211 --------------------CATAGCCTCACGTGGTTCACTGTTCTC-------
------
FC88X_00226_00894 --------------------CATAGCCTCACGTGGTTCACTATTTTC-------
------
FC88X_01098_01093 -------------------GCATAGCCTCACGTG-TTCGCTGTTTTC-------
------
FC88X_01066_00430 ----------------------TAGCC---------------------------
------
FC88X_00524_00508 ------------------CTCACGATTTTT-CTGAGCC-TCCCTGGG-------
------
FC88X_00898_01058 ------------------CTCACGATTTT--CTGAGCC-TCCCTGGG-------
------
FC88X_00189_00837 ------------------CTCACGATTTTTTCTGAGCCCTCCCTGGG-------
------
FC88X_00451_01126 ----------------------CTAATTT--CTTAGCC-TCACTGGGCTTATT-
------
FC88X_00788_00695 --------CCATCCCTACGTCACAATTCAT-TTTAC------------------
------
FC88X_00114_00491 --------CCAAACCTGCTCCACTATTGAA-TTTAT------------------
------
FC88X_01093_00838 ---------------ACCTCCGCTATTTATTCTTACCCTCTA------------
------
FC88X_01043_00875 ---------------ACCTTCACTGTCTAT-CTTACCTACT-------------
------
FC88X_01024_00498 ------------------TTATCCGTTCA--TCGGCTCCCTGGTTTA-------
------

FC88X_00579_00898   ------------------TTATCCGTTTAATCTCGCTCCCTGTTATCTTC----
------
FC88X_00141_00572   --------------CTCATTGTCCGTACC--TGGCCCCACTGTT----------
------
FC88X_00107_00954   -------------------TCATGCGTTTTAGACCTCACCCCTGTT--------
------
FC88X_00067_00290   -------------------TCATGCGTTTTAGACCTCACC--------------
------
FC88X_00878_00543   -------------------TCATGCACTCTA-ACCTCTCCGCGGATC-------
------
FC88X_01030_00587   -------------------TTATACACTCACCCGCT--CGGACTTATT------
------
FC88X_00910_00370   --------------GGGGATTATACACTCACCCGCT--CGGACTTATT------
------
FC88X_00353_01188   -------------------TTATACACTCACCCGCT--CGGACTTATT------
------
FC88X_00930_00857   -------------------TTATACACTCACCCGCT--CGGACTTATT------
------
FC88X_00981_00418   -------------------TTATACACTCACCCGCT--CGGACTTATT------
------
FC88X_00981_01195   -------------------TTATACACTCACCCGCT--CGGACTTATT------
------
FC88X_01074_01014   -------------------TTATACACTCACCCGCT--CGGACTTATT------
------
FC88X_00246_00127   -------------------TTCTAAACTCGCCTCCA--CGGACCTATC------
------
FC88X_00623_00097   -------------------
TTTTAAACTCCCCGGCAGTCGCATTTTCTTCCTTT------
FC88X_00981_00675   --------------------TCTATACTACCCCTCA--CGCATTATTCT-----
------
FC88X_00429_01014   -------------------TTATCCACTCCCCGCATGCCGTATTGTA-------
------
FC88X_00013_00529   -------------------CTTCGTTCTTA-CTGGCGGGCTTATCTC-------
------
FC88X_00667_01129   -------------------CTTCGTTCTTAGCTGGCGGGCTTATCTC-------
------
FC88X_00415_01178   -------------------CTTCGTTCTTA-CTGGCGGGCTTATCTC-------
------
FC88X_00895_00663   -------------------CTTCGTTCTTA-CTGGCGGGCTTATCTC-------
------
FC88X_00951_00423   -------------------CTTCGTTCTTA-CTGGCGGGCTTATCTC-------
------
FC88X_01085_00622   -------------------CTTCGTTCTTA-CTGGCGGGCTTATCTC-------
------
FC88X_01098_00867   -------------------CTTCGTTCTTA-CTGGCGGGCTTATCTC-------
------
FC88X_00294_00978   -------------------CTTCGTTCTTA-CTGGCGGGCTTATCTC-------
------
FC88X_00345_01112   -------------------CTTCGTTCTTA-CTGGCGGGCTTATCTC-------
------
FC88X_00463_00102   -------------------CTTCGTTCTTA-CTGGCGGGCTTATCTC-------
------
FC88X_00459_00458   -------------------CTTCGTTCTTA-CTGGCGGGCTTATCTC-------
------
FC88X_00459_01091   -------------------CTTCGTTCTTA-CTGGCGGGCTTATCTC-------
------

FC88X_00508_01146 -------------------CTTCGTTCTTA-CTGGCGGGCTTATCTC-------
------
FC88X_00804_00293 -------------------CTTCGTTCTTA-CTGGCGGGCTTATCTC-------
------
FC88X_00925_01021 -------------------CTTCGTTCTTA-CTGGCGGGCTTATCTC-------
------
FC88X_00996_00396 -------------------CTTCGTTCTTA-CTGGCGGGCTTATCTC-------
------
FC88X_00959_00525 -------------------CTTCGTTCTTA-CTGGCGGGCTTATCTC-------
------
FC88X_01046_00872 -------------------CTTCGTTCTTA-CTGGCGGGCTTATCTC-------
------
FC88X_01001_01086 -------------------CTTCGTTCTTA-CTGGCGGGCTTATCTC-------
------
FC88X_01121_01096 -------------------CTTCGTTCTTA-CTGGCGGGCTTATCTC-------
------
FC88X_00094_00078 -------------------CTTCGTTCTTT---GGCGGGCTTATCTC-------
------
FC88X_00023_00279 -------------------CTTCGTGCTTA-CTGGCGGGCTTATCTC-------
------
FC88X_00872_00134 -------------------CTCCGTTCTTA-CTGGCGGGCTTATCTC-------
------
FC88X_00465_01152 -------------------CTTCGTTCCTA-
CTGGCGGGCTTATCTCCTTCCTTT-----
FC88X_01086_00925 ------------------GCTTCCTGTCACACTGCCGGTCCTATAT--------
------
FC88X_00539_00216 -------------------TCTTTCTTGCACCTGCTGGACTTTCTC--------
------
FC88X_00873_00851 -------------------TCTTTCTTGCACCTGCTGGACTTTCTC--------
------
FC88X_00085_00431 -------------------TCTTACTTGCACCTGCTG-----------------
------
FC88X_00035_00690 --------------------CTTTTTCCTGCCTGGATTGCGTATTCT-------
------
FC88X_00289_00550 --------------------CTTTTTCCTGCCTGGATTGCGTATTCT-------
------
FC88X_00484_00785 --------------------CTTTTTCCTGCCTGGATTGCGTATTCT-------
------
FC88X_01078_00815 --------------------CTTTTTCCTGCCTGGATTGCGTATTCT-------
------
FC88X_01138_00686 --------------------CTTTTTCCTGCCTGGATTGCGTATTCT-------
------
FC88X_00015_00261 --------------------CTTTTTCCTGCCTGGATTGCGTATTCT-------
------
FC88X_00059_00085 --------------------CTTTTTCCTGCCTGGATTGCGTATTCT-------
------
FC88X_00537_00121 --------------------CTTTTTCCTGCCTGGATTGCGTATTCT-------
------
FC88X_00537_00955 --------------------CTTTTTCCTGCCTGGATTGCGTATTCT-------
------
FC88X_01012_00489 --------------------CTTTTTCCTGCCTGGATTGCGTATTCT-------
------
FC88X_01105_00686 --------------------CTTTTTCCTGCCTGGATTGCGTATTCT-------
------
FC88X_00897_00763 --------------------CCTTTTCCTGCCTGGACTGCGTATTCT-------
------

FC88X_00070_00811 ---------------------CTTTTCCTGCCTGGATTGCGTATTCT-------
------
FC88X_00704_00413 -------------------CTTTCTTCCTGCCTGGATTGCGTATTCT-------
------
FC88X_00255_00149 -------------------CTTTTTTCCTGCCTGGATTGCGTATTCT-------
------
FC88X_00179_00847 ------------------TACTTTTTCTTGCCTGTCGTATTCTATC--------
------
FC88X_00525_00222 --------------------CTATGCTCCTTACTGACTGCCTATTCT-------
------
FC88X_00141_00703 -----------TCATTTGTTCTCCCTAGTGACTGCCTT----------------
------
FC88X_01126_01079 -----------TCGTTTTTGATTCTCCCTGACCGCCT-----------------
------
FC88X_00360_01033 ------------CTTAGAT-CTTTCCACCGCCCGGCTTTT--------------
------
FC88X_00976_00491 -----------GCTTAGAT-CTTTCCACCGCCCGGCTTTTC-------------
------
FC88X_00896_00243 ------------CTTAGAT-CTTTCCACCGCCCGGCTTTTTC------------
------
FC88X_00900_00973 -------------TTAGGTACTTGCTCCCGCCTGACTGAA--------------
------
FC88X_00213_00034 -------------------GCTACACAATCGCTCATTCTCGACTACT-------
------
FC88X_00585_00126 --------------------CTACACAATCGCTCATTCTCG-CTACT-------
------
FC88X_00655_00263 --------------------CTACACAATCGCTCGTTCTCG-CTACT-------
------
FC88X_00587_01039 --------------------CTTCCCACTTTCCTACTAC-CTCCTGAA------
------
FC88X_00735_00475 --------------------CTGCCCAATTTGCTGCTTCTCTACTGTT------
------
FC88X_00443_00132 --------------------TTGCCCA--ATCTGGCTTTCCTCGACTACT----
------
FC88X_00770_00346 --------------------TTGCCCGTTATCTTGCTCCCTTCTATT-------
------
FC88X_00024_00658 --------------------ACGATGCTTTACTCATCGCTCTGTCTC-------
------
FC88X_00320_00600 --------------------TCGATGCTTTACTCATCGCTCTGTCTC-------
------
FC88X_00107_00274 --------------------CCGATGCTTTACTCATCGCTCTGTCTC-------
------
FC88X_00640_00863 --------------------CCGATGCTTTACTCATCGCTCTGCCTC-------
------
FC88X_00088_00767 --------------------CTGATGATAAACTCA-CGCCCTGGCTATG-----
------
FC88X_00183_00244 --------------------CTGATGATAAACTCA-CGCCCTGGCTATG-----
------
FC88X_01091_00669 --------------------CTGATGATAAACTCA-CGCCCTGGCTATG-----
------
FC88X_00278_00015 --------------------CAGATGATAAACTCA-CGCCCTGGCTATG-----
------
FC88X_00308_01015 --------------------ACGTGGCGTCACCGACTGCTCTATCTC-------
------
FC88X_00583_00779 --------------------ACGTGGCGTCACCGACTGCTCTATCTC-------
------

```
FC88X_00871_00328      --------------------ACGTGGCGTCACCGACTGCTCTATCTC-------
------
FC88X_01141_00841      --------------------ACGTGGCGTCACCGACTGCTCTATCTC-------
------
FC88X_00593_01176      --------------------ACGTGGCGTCACCGACTGCTCTATCTC-------
------
FC88X_00159_00815      --------------------
ATGTGGCGTCACCGACTGCTCTATCTCCTTCCTTT-----
FC88X_00133_00821      --------------------CTGTTGCTTCTCCGCCCGCGCTGTGTC-------
------
FC88X_00065_00406      -----------------------CCACGTAATCAT-CGCTCCCGGCGTATC---
------
FC88X_00489_01035      -----------------------CCACGTAATCAT-CGCTCCCGGCGTATC---
------
FC88X_00795_00092      -----------------------CCACGTAATCAT-CGCTCCCGGCGTATC---
------
FC88X_00629_00290      -----------------------CCACGTAATCAT-CGCTCCCGGCGTACT---
------
FC88X_00081_00873      ---------------------CTATGTGTAATCATGCGTTCCCGGCCTCC----
------
FC88X_00129_00358      -------------------CCGTGTGTTGAGTCATGGCCTCCCGGT--------
------
FC88X_00428_00584      ---------------------TTGCACACTCCTTC-GGCCCCGACTGAC-----
------
FC88X_00431_00820      ---------------------CTGCACATTATCTC-GCTCCCGACGGACC----
------
FC88X_00109_00053      ---------------------CATATTGCTATCAC-AGCTCCTGTCGGC-----
------
FC88X_00628_00162      ----------------------CCGGCACTATCAT-
CGCCCCCTGTCTATCCTTCCTTTC
FC88X_00846_00957      ----------------------CTGGCACTATCAT-CGCCCCCTGTCTATC---
------
FC88X_01014_00328      -----------------------GCGCACTATCGC-GCTTCCTCTCATATC---
------
FC88X_00688_01263      -----------------------CCGTGATATCTTCTC-CGGCACGTTTCTC--
------
FC88X_00949_00718      -----------------------CCGTGATATCTTCTC-CGGCACGTTTCTC--
------
FC88X_00909_01245      -----------------------CCGTGATATCTTCTC-CGGCACGTTTCTC--
------
FC88X_01085_00957      ------------------------CGTGATATCTTCTC-CGGCACGTTTCTC--
------
FC88X_00011_00272      ------------------------CGTGATATCTTCTC-CGGCACGTTTCTC--
------
FC88X_00037_00049      -----------------------CTGTGATATCTTCTC-CGGCACGTTTCTC--
------
FC88X_00478_00389      ------------------------TTCGATATTTTTCGACGGTACTCTCTCC--
------
FC88X_00215_00853      ------------------------
CTTAGATTTTTGTCTCAGGCTACTTTCCCTTCCTTT
FC88X_00739_01059      ------------------------CTTAGATTTT-GTCTCAGGCTACTTTCC--
------
FC88X_00037_00472      ---------------------CCCCATCGATTCTT--GCTGCCACAGTGC----
------
FC88X_00989_00772      -----------------------CTATCAATTCTTTTACTGCCAGATTTC----
------
```

FC88X_00944_00735 ----------------------GCCATCGACGATTTTGTTGGCAACTCTC----
------
FC88X_00143_00975 -----------------------CTTTCGTCTATGCTCTTTCCA-ACCGTC---
------
FC88X_00555_01249 -----------------------
CTCCCATCTATTTTTTTTTCACACCGACTTCGT----
FC88X_01140_00568 -----------------------CTCCTTGATCTT-TCTCCCGCCGTTCGC---
------
FC88X_01113_01117 -----------------------CTCCTTGATCTT-TCTCCCGCCGTTCGC---
------
FC88X_00078_00438 -----------------------CTCTTTGTTATTCTCTTCCGCCGTTTG----
------
FC88X_00346_01139 -----------------------CTCTTTGTTATTCTCTTCCGTCGTTTG----
------
FC88X_00268_00022 -----------------------CTATCATAAACTCTGACCCAGCATTCTC---
------
FC88X_01005_01227 -----------------------CTATCGTAAACTCTGACCCAGCATTCT----
------
FC88X_01123_00762 ----------------------CCTATGGTTATTTCT--CCCAGCGTTCGA---
------
FC88X_00040_00364 ----------------------CTTTGCCGTTTCTGCCATTGC-TGTTCC----
------
FC88X_01046_01032 --------------------TTGCCAGTTTCCTACGGCATTTC-TGTTCT----
------
FC88X_00459_01178 -----------------------CTTGCCATTTTATTGCCTGC-AGTTCTC---
------
FC88X_00118_00543 ---------------------GTCCTTTGGTGTTT-GCCCGAC-TGTTCTC---
------
FC88X_00656_01178 ----------------------TCCTTTGGTGTTT-GCCCGAC-TGTTCTC---
------
FC88X_00304_00107 ----------------------TCCTTTGGTGTTT-GCCCGAC-TGTTCTC---
------
FC88X_00904_00410 ----------------------TCCTTTGGTGTTT-GCCCGAC-TGTTCTC---
------
FC88X_00270_00966 ----------------------TCCTTTGGTGTTTTGCCCGAC-TGTTCTC---
------
FC88X_00669_00293 ----------------------TCCTTTGGTGTTT-GCCCGGC-TGTTCTC---
------
FC88X_00623_00644 ------------------------TGTTTGTGTTGTTCACGGCATGTTCTC---
------
FC88X_00772_00928 ------------------------TGTTTGTGTTGTTCACGGCATGTTCTC---
------
FC88X_01052_00823 ------------------------TGTTTGTGTTGTTCACGGCATGTTCTC---
------
FC88X_01013_00569 ----------------------CCGATTATTTTTGTG-GCGGCACGTTCTC---
------
FC88X_01039_00553 ----------------------CCAATTCATGTTGTACGCTGCA-GTTCT----
------
FC88X_00220_00312 ----------------GTGATTTCCATATT-TATCCACGG-CGCTGTTCT----
------
FC88X_00768_01214 ---------------------TTCCATATT-TATCCACGG-CGCTGTTCT----
------
FC88X_00272_00674 ---------------------TTCCATATT-TATCCACAG-CGCTGTTCT----
------
FC88X_00519_01161 ---------------------TTCCATATT-TATCCACAG-CGCTGTTCT----
------

FC88X_00993_00320 ---------------------TTCCATATT-TATCCACAG-CGCTGTTCT----
------
FC88X_00960_01052 ---------------------TTCCATATT-TATCCACAG-CGCTGTTCT----
------
FC88X_00535_00551 ---------------------TTCCATATT-TATCCACAG-CGCTGTTCT----
------
FC88X_00569_01130 ---------------------TTCCATATT-TATCCACAG-CGCTGTTCT----
------
FC88X_00634_00963 ---------------------TTCCATATT-TATCCACAG-CGCTGTTCT----
------
FC88X_01040_00495 ---------------------TTCCATATT-TATCCACAG-CGCTGTTCT----
------
FC88X_01147_00671 ---------------------TTCCATATT-TATCCACAG-CGCTGTTCT----
------
FC88X_00761_00698 ---------------------TTCCATATT-TATCCAC-G-CGCTGTTCT----
------
FC88X_00791_00757 ---------------------TGCCATATT-TATCCACAG-CGCTGTTCT----
------
FC88X_01122_00996 ---------------------TGCCATGAT-T-TTTACAG-
CCCTGCTCTCCTTCCTTT-
FC88X_00307_01021 ---------------------GTCCAAGT--TTTCGACAGGCACTATTCC----
------
FC88X_00821_00281 ---------------------GTCCAAGT--TTTCGACAGGCACTATTCC----
------
FC88X_01046_01136 ---------------------ATCCAAGT--TTTCGACAGGCACTATTCC----
------
FC88X_00485_00199 ---------------------CTCCCAGTGGTATGGACAGACCTAATC------
------
FC88X_01055_00432 ------------------------GCTGTTGTGCTCCAGCTGCCTGTTCTG---
------
FC88X_00603_00120 ------------------------GCTGTTGTGCTCCAGCTGCCTGTTCTG---
------
FC88X_00559_00331 ------------------------GCTGTTGTGTTCCAGCTGCCTGTTCTG---
------
FC88X_00747_00906 ------------------------GCTGTTGTGTCCCAGCTGCCTGTTCTG---
------
FC88X_00326_00215 -----------------------GGCCGTTGTGTTCCAGCTGCCTGTTCTG---
------
FC88X_00387_00971 ----------------------TTGCAGTTTTTTTC--ACAGCCCCCTCTA---
------
FC88X_00659_00223 ----------------------CCGTCAGTTTTTGCAAGCGCCCGGTTCT----
------
FC88X_00814_01108 --------------------CACCGT-AGTTTGTCTATGTGCCCTATT------
------
FC88X_00106_00851 --------------------TCGTTATTTT-AACTCCCGCGGCCGGTT------
------
FC88X_00624_00223 --------------------TCGTTATTTT-AACTCCCGCGGCCGGTT------
------
FC88X_00536_00488 ---------------------CGTTATTTT-AACTCCCGCGGCCGGTT------
------
FC88X_00324_01131 --------------------CCGTTATTTT-AACTCCCGCGGCCGGTT------
------
FC88X_00584_01122 -------------------GCCGTTATTTT-AACTCCCGCGGCCGGTT------
------
FC88X_00041_00139 --------------------CCGTTATTTT-AACTCCCGCGGCCGGTT------
------

```
FC88X_00075_00113       --------------------CCGTTATTTT-AACTCCCGCGGCCGGTT------
------
FC88X_00079_00154       --------------------CCGTTATTTT-AACTCCCGCGGCCGGTT------
------
FC88X_00104_00574       --------------------CCGTTATTTT-AACTCCCGCGGCCGGTT------
------
FC88X_00162_00359       --------------------CCGTTATTTT-AACTCCCGCGGCCGGTT------
------
FC88X_00184_00989       --------------------CCGTTATTTT-AACTCCCGCGGCCGGTT------
------
FC88X_00397_00386       --------------------CCGTTATTTT-AACTCCCGCGGCCGGTT------
------
FC88X_00494_01108       --------------------CCGTTATTTT-AACTCCCGCGGCCGGTT------
------
FC88X_00522_00387       --------------------CCGTTATTTT-AACTCCCGCGGCCGGTT------
------
FC88X_00787_00306       --------------------CCGTTATTTT-AACTCCCGCGGCCGGTT------
------
FC88X_00788_01019       --------------------CCGTTATTTT-AACTCCCGCGGCCGGTT------
------
FC88X_01062_00852       --------------------CCGTTATTTT-AACTCCCGCGGCCGGTT------
------
FC88X_01089_00863       --------------------CCGTTATTTT-AACTCCCGCGGCCGGTT------
------
FC88X_01125_00522       --------------------CCGTTATTTT-AACTCCCGCGGCCGGTT------
------
FC88X_00462_01032       --------------------CCGTTATTTT-AACTCCCGCGGCCGGTTCC----
------
FC88X_00949_00924       ---------------------CGTTATTTT-AACTCCCGCGGCCGGTT------
------
FC88X_00928_01042       --------------------CCGTTATTTTTAACTCCCGCGGCCGGTT------
------
FC88X_01028_00720       --------------------CCGTTATTTT-AACTCCCGCGGCCGATT------
------
FC88X_00059_00239       --------------------CCGTTATTTT-GACTCCCGCGGCCGGTT------
------
FC88X_00920_00965       ---------------------CGTTATTTC-AACTCCCGCGGCCGGTT------
------
FC88X_00601_00208       --------------------CCGTTATTTTTAACTCCCGC--------------
------
FC88X_00554_01023       ----------------GCTTTCGTTATTTACGACTCCCGCAGATTC--------
------
FC88X_00991_01231       -----------------CTTTCGTTATTTACGACTCCCGCAGATTC--------
------
FC88X_00108_00616       -----------------TTTCGCCTATTTTC--CTCCCGCTGTTTA--------
------
FC88X_00563_00683       -----------------CTACAAAATTGTTTTCCTCTCCGTTTTT---------
------
FC88X_00644_00417       -----------------CTACAAG-TTGTTTAGCTCCCGTATTCT---------
------
FC88X_00162_00839       --------------------CCACTTGTTTTG--ACTCCC----
AGGTCTTCTC------
FC88X_00905_01204       --------------------CCACTTGTTTTG--ACTCCC----
AGGTCTTCTC------
FC88X_00150_00051       --------------------CCGGTTGTTATGTTACTCCCGAAAAGCTCTTCT-
------
```

FC88X_00958_00233 -------------------TCCTTTCGTTTTAGCTATTCTTTTATGTC------
------
FC88X_00106_00074 -------------------CCCGTTTGTTTTTCCCAGCTATTCTAAC-------
------
FC88X_00247_01091 ---------------------GCTCATGTTTT-GTGACCCG-CCCGTTCTC---
------
FC88X_00889_00739 ---------------------GCTCATGTTTT-GTGACCCG-CCCGTTCTC---
------
FC88X_00069_00030 ---------------------GCTCATGTTACAGTGCTCCGGCCCGTT------
------
FC88X_00399_00049 ---------------------TTACATGCTTTGCCCCTCAGTTCTGTT------
------
FC88X_00335_00976 ----------------------CTCACGTTCCTTTGCGCCGTCTTATT------
------
FC88X_00572_00176 -----------------------TCTCAGTCCCTTTCCCCGCCGTCGTTGT---
------
FC88X_00029_00400 --------------------------TGTTACTTTTAACCGCGTCGTTCTT---
------
FC88X_00652_00080 ----------------------
CCCATGTTCCTTTACCCGTCTTTCCTCTTCCTTTCTC-
FC88X_00389_00062 ----------------------CATTTGCTTCAGCCGCCCGTGC-GTTATT---
------
FC88X_01094_00679 ----------------------CATTTGCTTCAGCCGCCCGTGC-GTTATT---
------
FC88X_00114_00439 ----------------------CATTTGCTTCAGCCGCCCGTGC-GTTATT---
------
FC88X_00129_00943 ----------------------CATTTGCTTCAGCCGCCCGTGC-GTTATT---
------
FC88X_00431_00082 ----------------------CATTTGCTTCAGCCGCCCGTGC-GTTATT---
------
FC88X_00873_00733 ----------------------CATTTGCTTCAGCCGCCCGTGC-GTTATT---
------
FC88X_00014_00405 ----------------------CATTTGCTTCAGCCGCCCGCGC-GTTATT---
------
FC88X_01119_00934 ------------------------TTTACCTTTGTCCCACGTGCCGTTGTT---
------
FC88X_00087_00373 -------------------TCACCTGTTGTT--CAGCGCCTAGTTCTT------
------
FC88X_00186_00013 -------------------TCACCTGTTGTT--CAGCGCCTAGTTCTT------
------
FC88X_00406_00632 -----------------CTTGACCTGAGGTTA-CAGCGCC--GTTCT-------
------
FC88X_00841_00338 -------------------CGACCTGATTTT--CAGTGCCGTGCTTCT------
------
FC88X_00954_00600 -------------------CGACCTGATTTT--CAGTGCCGTGCTTCT------
------
FC88X_01132_01210 -------------------CGACCTGATTTT--CAGTGCCGTGCTTCT------
------
FC88X_00104_00414 -------------------CGACCTGATTTT--CAGTGCCGTGCTTCT------
------
FC88X_00339_01143 -------------------CGACCTGATTTT--CAGTGCCGTGCTTCT------
------
FC88X_01038_00757 -------------------CGACCTGATTTT--CAGTGCCGTGCTTCT------
------
FC88X_00891_00679 --------------------ACCGTGATTTTT-CAGTCCCGGCTCTC-------
------

FC88X_00414_01220 --------------------ACCGTGATTTTT-CAGTCCCGGGCTCTC------
------
FC88X_01028_00961 --------------------ACCGTGATTTTTTCAGTCCCGGGCTCTC------
------
FC88X_01050_00509 --------------------ACCGCGATTTTC-AGTCCCCGGGCTCTC------
------
FC88X_00598_00960 ---------------------TCTTTACTTTTGC---GCCACAGACTTCTC---
------
FC88X_00734_00065 ---------------------TCTTTACTTTTGC---GCCACGGACTTCTC---
------
FC88X_00024_00515 ---------------------CTTTGACTCTTTCT--GCCACTGATTTCTC---
------
FC88X_00913_00496 ----------------------CTATATTTTCGCT--CCCACAGTTCTCAC---
------
FC88X_00078_00189 ---------------------TTTAAATTTCTCCGG-CTCACAGTTCTT-----
------
FC88X_00241_00928 --------------------------
CTGTTTGCAATCCCACAGTTCTCCTACT------
FC88X_00890_00925 --------------------------
CTGTTTGCAATCCCACAGTTCTCCTACT------
FC88X_00944_00863 -----------------------------TTTGCA--
CCCACCGGCCTCTCACTTTT---
FC88X_00021_00095 ------------------TGACGT-GTTG-TG---CGGC-GCTCAC-TG-
TCTC------
FC88X_01113_00870 ------------------TGACGT-GTTGATG---CGGC-GCTCAC-TG-
TCTC------
FC88X_00024_00499 ------------------TGACGT-GTTG-TG---CGGC-GCTCAC-TG-
TCTC------
FC88X_00047_00588 ------------------TGACGT-GTTG-TG---CGGC-GCTCAC-TG-
TCTC------
FC88X_00009_00122 ------------------TGACGT-GTTG-TG---CGGC-GCTCAC-TG-
TCTC------
FC88X_00947_01010 ------------------TGACGT-GTTG-TG---CGGC-GCTCACCTG-
TCTC------
FC88X_00041_00463 ------------------TGACGT-GTTG-TG---CGGC-GCTCAC-TG-
TCTC------
FC88X_00055_00186 ------------------TGACGT-GTTG-TG---CGGC-GCTCAC-TG-
TCTC------
FC88X_00024_00178 ------------------TGACGT-GTTG-TG---CGGC-GCTC-ACTG-
TCTC------
FC88X_00031_00727 ------------------TGACGTTGTTG-TG---CGGC-GCTC-ACTG-
TCTC------
FC88X_00030_00549 ------------------TGACGT-GTTG-TG---CGGC-GCTC-ACTG-
TCTC------
FC88X_00039_00679 ------------------TGACGT-GTTG-TG---CGGC-GCTC-ACTG-
TCTC------
FC88X_00036_00428 ------------------TGACGT-GTTG-TG---CGGC-GCTC-ACTG-
TCTC------
FC88X_00254_00028 ------------------TGACGT-GTTG-TG---CGGC-GCTCTACTG-
TCTC------
FC88X_00041_00513 ------------------TGACGT-GTTG-TG---CGGC-GCTC-ACTG-
TCTC------
FC88X_00067_00196 ------------------TGACGT-GTTG-TG---CGGC-GCTC-ACTG-
TCTC------
FC88X_00064_00879 ------------------TGACGT-GTTG-TG---CGGC-GCTC-ACTG-
TCTC------

FC88X_00088_00907 ------------------TGACGT-GTTG-TG---CGGC-GCTC-ACTG-
TCTC------
FC88X_00093_00356 -----------------GTGACGT-GTTG-TG---CGGC-GCTC-ACTG-
TCTC------
FC88X_00126_00781 -----------------GTGACGT-GTTG-TG---CGGC-GCTC-ACTG-
TCTC------
FC88X_00496_00770 -----------------GTGACGT-GTTG-TG---CGGC-GCTC-ACTG-
TCTC------
FC88X_00526_01061 -----------------GTGACGT-GTTG-TG---CGGC-GCTC-ACTG-
TCTC------
FC88X_00849_00772 -----------------GTGACGT-GTTG-TG---CGGC-GCTC-ACTG-
TCTC------
FC88X_00856_00246 -----------------GTGACGT-GTTG-TG---CGGC-GCTC-ACTG-
TCTC------
FC88X_00996_01080 -----------------GTGACGT-GTTG-TG---CGGC-GCTC-ACTG-
TCTC------
FC88X_01073_00942 -----------------GTGACGT-GTTG-TG---CGGC-GCTC-ACTG-
TCTC------
FC88X_01131_00652 -----------------GTGACGT-GTTG-TG---CGGC-GCTC-ACTG-
TCTC------
FC88X_00075_00806 ------------------TGACGT-GTTG-TG---CGGC-GCTC-ACTG-
TCTC------
FC88X_00082_00874 ------------------TGACGT-GTTG-TG---CGGC-GCTC-ACTG-
TCTC------
FC88X_00123_00008 ------------------TGACGT-GTTG-TG---CGGC-GCTC-ACTG-
TCTC------
FC88X_00105_00054 ------------------TGACGT-GTTG-TG---CGGC-GCTC-ACTG-
TCTC------
FC88X_00101_00141 ------------------TGACGT-GTTG-TG---CGGC-GCTC-ACTG-
TCTC------
FC88X_00122_00111 ------------------TGACGT-GTTG-TG---CGGC-GCTC-ACTG-
TCTC------
FC88X_00101_00482 ------------------TGACGT-GTTG-TG---CGGC-GCTC-ACTG-
TCTC------
FC88X_00142_00740 ------------------TGACGT-GTTG-TG---CGGC-GCTC-ACTG-
TCTC------
FC88X_00158_00182 ------------------TGACGT-GTTG-TG---CGGC-GCTC-ACTG-
TCTC------
FC88X_00177_00326 ------------------TGACGT-GTTG-TG---CGGC-GCTC-ACTG-
TCTC------
FC88X_00174_00608 ------------------TGACGT-GTTG-TG---CGGC-GCTC-ACTG-
TCTC------
FC88X_00159_00756 ------------------TGACGT-GTTG-TG---CGGC-GCTC-ACTG-
TCTC------
FC88X_00162_00835 ------------------TGACGT-GTTG-TG---CGGC-GCTC-ACTG-
TCTC------
FC88X_00155_00869 ------------------TGACGT-GTTG-TG---CGGC-GCTC-ACTG-
TCTC------
FC88X_00163_00947 ------------------TGACGT-GTTG-TG---CGGC-GCTC-ACTG-
TCTC------
FC88X_00221_00463 ------------------TGACGT-GTTG-TG---CGGC-GCTC-ACTG-
TCTC------
FC88X_00218_00623 ------------------TGACGT-GTTG-TG---CGGC-GCTC-ACTG-
TCTC------
FC88X_00250_00011 ------------------TGACGT-GTTG-TG---CGGC-GCTC-ACTG-
TCTC------

FC88X_00291_00683 ------------------TGACGT-GTTG-TG---CGGC-GCTC-ACTG-
TCTC------
FC88X_00293_00923 ------------------TGACGT-GTTG-TG---CGGC-GCTC-ACTG-
TCTC------
FC88X_00324_00340 ------------------TGACGT-GTTG-TG---CGGC-GCTC-ACTG-
TCTC------
FC88X_00302_01007 ------------------TGACGT-GTTG-TG---CGGC-GCTC-ACTG-
TCTC------
FC88X_00333_01070 ------------------TGACGT-GTTG-TG---CGGC-GCTC-ACTG-
TCTC------
FC88X_00319_01153 ------------------TGACGT-GTTG-TG---CGGC-GCTC-ACTG-
TCTC------
FC88X_00366_00312 ------------------TGACGT-GTTG-TG---CGGC-GCTC-ACTG-
TCTC------
FC88X_00357_00480 ------------------TGACGT-GTTG-TG---CGGC-GCTC-ACTG-
TCTC------
FC88X_00379_00512 ------------------TGACGT-GTTG-TG---CGGC-GCTC-ACTG-
TCTC------
FC88X_00416_00070 ------------------TGACGT-GTTG-TG---CGGC-GCTC-ACTG-
TCTC------
FC88X_00424_00096 ------------------TGACGT-GTTG-TG---CGGC-GCTC-ACTG-
TCTC------
FC88X_00404_00863 ------------------TGACGT-GTTG-TG---CGGC-GCTC-ACTG-
TCTC------
FC88X_00431_01084 ------------------TGACGT-GTTG-TG---CGGC-GCTC-ACTG-
TCTC------
FC88X_00438_01137 ------------------TGACGT-GTTG-TG---CGGC-GCTC-ACTG-
TCTC------
FC88X_00457_00081 ------------------TGACGT-GTTG-TG---CGGC-GCTC-ACTG-
TCTC------
FC88X_00458_00947 ------------------TGACGT-GTTG-TG---CGGC-GCTC-ACTG-
TCTC------
FC88X_00478_00939 ------------------TGACGT-GTTG-TG---CGGC-GCTC-ACTG-
TCTC------
FC88X_00457_00996 ------------------TGACGT-GTTG-TG---CGGC-GCTC-ACTG-
TCTC------
FC88X_00498_01179 ------------------TGACGT-GTTG-TG---CGGC-GCTC-ACTG-
TCTC------
FC88X_00525_00840 ------------------TGACGT-GTTG-TG---CGGC-GCTC-ACTG-
TCTC------
FC88X_00538_01062 ------------------TGACGT-GTTG-TG---CGGC-GCTC-ACTG-
TCTC------
FC88X_00594_00065 ------------------TGACGT-GTTG-TG---CGGC-GCTC-ACTG-
TCTC------
FC88X_00595_00140 ------------------TGACGT-GTTG-TG---CGGC-GCTC-ACTG-
TCTC------
FC88X_00599_00218 ------------------TGACGT-GTTG-TG---CGGC-GCTC-ACTG-
TCTC------
FC88X_00571_00523 ------------------TGACGT-GTTG-TG---CGGC-GCTC-ACTG-
TCTC------
FC88X_00579_00667 ------------------TGACGT-GTTG-TG---CGGC-GCTC-ACTG-
TCTC------
FC88X_00592_00748 ------------------TGACGT-GTTG-TG---CGGC-GCTC-ACTG-
TCTC------
FC88X_00599_01012 ------------------TGACGT-GTTG-TG---CGGC-GCTC-ACTG-
TCTC------

```
FC88X_00556_01264       ------------------TGACGT-GTTG-TG---CGGC-GCTC-ACTG-
TCTC------
FC88X_00616_00079       ------------------TGACGT-GTTG-TG---CGGC-GCTC-ACTG-
TCTC------
FC88X_00641_00205       ------------------TGACGT-GTTG-TG---CGGC-GCTC-ACTG-
TCTC------
FC88X_00643_00970       ------------------TGACGT-GTTG-TG---CGGC-GCTC-ACTG-
TCTC------
FC88X_00689_00124       ------------------TGACGT-GTTG-TG---CGGC-GCTC-ACTG-
TCTC------
FC88X_00676_00211       ------------------TGACGT-GTTG-TG---CGGC-GCTC-ACTG-
TCTC------
FC88X_00652_00298       ------------------TGACGT-GTTG-TG---CGGC-GCTC-ACTG-
TCTC------
FC88X_00655_00347       ------------------TGACGT-GTTG-TG---CGGC-GCTC-ACTG-
TCTC------
FC88X_00679_00760       ------------------TGACGT-GTTG-TG---CGGC-GCTC-ACTG-
TCTC------
FC88X_00677_00837       ------------------TGACGT-GTTG-TG---CGGC-GCTC-ACTG-
TCTC------
FC88X_00691_00941       ------------------TGACGT-GTTG-TG---CGGC-GCTC-ACTG-
TCTC------
FC88X_00743_00444       ------------------TGACGT-GTTG-TG---CGGC-GCTC-ACTG-
TCTC------
FC88X_00703_00735       ------------------TGACGT-GTTG-TG---CGGC-GCTC-ACTG-
TCTC------
FC88X_00739_00777       ------------------TGACGT-GTTG-TG---CGGC-GCTC-ACTG-
TCTC------
FC88X_00737_00876       ------------------TGACGT-GTTG-TG---CGGC-GCTC-ACTG-
TCTC------
FC88X_00754_00205       ------------------TGACGT-GTTG-TG---CGGC-GCTC-ACTG-
TCTC------
FC88X_00793_00262       ------------------TGACGT-GTTG-TG---CGGC-GCTC-ACTG-
TCTC------
FC88X_00757_00544       ------------------TGACGT-GTTG-TG---CGGC-GCTC-ACTG-
TCTC------
FC88X_00776_00819       ------------------TGACGT-GTTG-TG---CGGC-GCTC-ACTG-
TCTC------
FC88X_00793_01014       ------------------TGACGT-GTTG-TG---CGGC-GCTC-ACTG-
TCTC------
FC88X_00805_00240       ------------------TGACGT-GTTG-TG---CGGC-GCTC-ACTG-
TCTC------
FC88X_00818_00845       ------------------TGACGT-GTTG-TG---CGGC-GCTC-ACTG-
TCTC------
FC88X_00849_01035       ------------------TGACGT-GTTG-TG---CGGC-GCTC-ACTG-
TCTC------
FC88X_00813_01190       ------------------TGACGT-GTTG-TG---CGGC-GCTC-ACTG-
TCTC------
FC88X_00835_01227       ------------------TGACGT-GTTG-TG---CGGC-GCTC-ACTG-
TCTC------
FC88X_00878_00259       ------------------TGACGT-GTTG-TG---CGGC-GCTC-ACTG-
TCTC------
FC88X_00874_00645       ------------------TGACGT-GTTG-TG---CGGC-GCTC-ACTG-
TCTC------
FC88X_00864_00818       ------------------TGACGT-GTTG-TG---CGGC-GCTC-ACTG-
TCTC------
```

FC88X_00870_00815 -------------------TGACGT-GTTG-TG---CGGC-GCTC-ACTG-
TCTC------
FC88X_00910_00923 -------------------TGACGT-GTTG-TG---CGGC-GCTC-ACTG-
TCTC------
FC88X_00920_00901 -------------------TGACGT-GTTG-TG---CGGC-GCTC-ACTG-
TCTC------
FC88X_00917_00999 -------------------TGACGT-GTTG-TG---CGGC-GCTC-ACTG-
TCTC------
FC88X_00968_00576 -------------------TGACGT-GTTG-TG---CGGC-GCTC-ACTG-
TCTC------
FC88X_00975_00556 -------------------TGACGT-GTTG-TG---CGGC-GCTC-ACTG-
TCTC------
FC88X_00995_00556 -------------------TGACGT-GTTG-TG---CGGC-GCTC-ACTG-
TCTC------
FC88X_00979_00621 -------------------TGACGT-GTTG-TG---CGGC-GCTC-ACTG-
TCTC------
FC88X_00971_00781 -------------------TGACGT-GTTG-TG---CGGC-GCTC-ACTG-
TCTC------
FC88X_00978_00860 -------------------TGACGT-GTTG-TG---CGGC-GCTC-ACTG-
TCTC------
FC88X_00983_01232 -------------------TGACGT-GTTG-TG---CGGC-GCTC-ACTG-
TCTC------
FC88X_01024_00485 -------------------TGACGT-GTTG-TG---CGGC-GCTC-ACTG-
TCTC------
FC88X_01032_00486 -------------------TGACGT-GTTG-TG---CGGC-GCTC-ACTG-
TCTC------
FC88X_01021_00534 -------------------TGACGT-GTTG-TG---CGGC-GCTC-ACTG-
TCTC------
FC88X_01022_00644 -------------------TGACGT-GTTG-TG---CGGC-GCTC-ACTG-
TCTC------
FC88X_01046_00704 -------------------TGACGT-GTTG-TG---CGGC-GCTC-ACTG-
TCTC------
FC88X_01002_00884 -------------------TGACGT-GTTG-TG---CGGC-GCTC-ACTG-
TCTC------
FC88X_01088_00588 -------------------TGACGT-GTTG-TG---CGGC-GCTC-ACTG-
TCTC------
FC88X_01093_00568 -------------------TGACGT-GTTG-TG---CGGC-GCTC-ACTG-
TCTC------
FC88X_01089_00731 -------------------TGACGT-GTTG-TG---CGGC-GCTC-ACTG-
TCTC------
FC88X_01082_00795 -------------------TGACGT-GTTG-TG---CGGC-GCTC-ACTG-
TCTC------
FC88X_01082_01202 -------------------TGACGT-GTTG-TG---CGGC-GCTC-ACTG-
TCTC------
FC88X_01090_01261 -------------------TGACGT-GTTG-TG---CGGC-GCTC-ACTG-
TCTC------
FC88X_01111_00461 -------------------TGACGT-GTTG-TG---CGGC-GCTC-ACTG-
TCTC------
FC88X_01116_00766 -------------------TGACGT-GTTG-TG---CGGC-GCTC-ACTG-
TCTC------
FC88X_01147_00861 -------------------TGACGT-GTTG-TG---CGGC-GCTC-ACTG-
TCTC------
FC88X_01103_01103 -------------------TGACGT-GTTG-TG---CGGC-GCTC-ACTG-
TCTC------
FC88X_01138_01135 -------------------TGACGT-GTTG-TG---CGGC-GCTC-ACTG-
TCTC------

FC88X_01120_01161 ------------------TGACGT-GTTG-TG---CGGC-GCTC-ACTG-
TCTC------
FC88X_00027_00102 ------------------TGACGT-GTTG-TG---CGGC-GCTC-ACTG-
TCTC------
FC88X_00014_00192 ------------------TGACGT-GTTG-TG---CGGC-GCTC-ACTG-
TCTC------
FC88X_00029_00242 ------------------TGACGT-GTTG-TG---CGGC-GCTC-ACTG-
TCTC------
FC88X_00041_00399 ------------------TGACGT-GTTG-TG---CGGC-GCTC-ACTG-
TCTC------
FC88X_00010_00468 ------------------TGACGT-GTTG-TG---CGGC-GCTC-ACTG-
TCTC------
FC88X_00025_00505 ------------------TGACGT-GTTG-TG---CGGC-GCTC-ACTG-
TCTC------
FC88X_00014_00558 ------------------TGACGT-GTTG-TG---CGGC-GCTC-ACTG-
TCTC------
FC88X_00067_00043 ------------------TGACGT-GTTG-TG---CGGC-GCTC-ACTG-
TCTC------
FC88X_00070_00126 ------------------TGACGT-GTTG-TG---CGGC-GCTC-ACTG-
TCTC------
FC88X_00066_00219 ------------------TGACGT-GTTG-TG---CGGC-GCTC-ACTG-
TCTC------
FC88X_00082_00247 ------------------TGACGT-GTTG-TG---CGGC-GCTC-ACTG-
TCTC------
FC88X_00056_00373 ------------------TGACGT-GTTG-TG---CGGC-GCTC-ACTG-
TCTC------
FC88X_00065_00385 ------------------TGACGT-GTTG-TG---CGGC-GCTC-ACTG-
TCTC------
FC88X_00084_00469 ------------------TGACGT-GTTG-TG---CGGC-GCTC-ACTG-
TCTC------
FC88X_00084_00541 ------------------TGACGT-GTTG-TG---CGGC-GCTC-ACTG-
TCTC------
FC88X_00065_00610 ------------------TGACGT-GTTG-TG---CGGC-GCTC-ACTG-
TCTC------
FC88X_00084_00751 ------------------TGACGT-GTTG-TG---CGGC-GCTC-ACTG-
TCTC------
FC88X_00094_00757 ------------------TGACGT-GTTG-TG---CGGC-GCTC-ACTG-
TCTC------
FC88X_00071_00893 ------------------TGACGT-GTTG-TG---CGGC-GCTC-ACTG-
TCTC------
FC88X_00095_00914 ------------------TGACGT-GTTG-TG---CGGC-GCTC-ACTG-
TCTC------
FC88X_00133_00038 ------------------TGACGT-GTTG-TG---CGGC-GCTC-ACTG-
TCTC------
FC88X_00134_00350 ------------------TGACGT-GTTG-TG---CGGC-GCTC-ACTG-
TCTC------
FC88X_00142_00423 ------------------TGACGT-GTTG-TG---CGGC-GCTC-ACTG-
TCTC------
FC88X_00126_00499 ------------------TGACGT-GTTG-TG---CGGC-GCTC-ACTG-
TCTC------
FC88X_00131_00497 ------------------TGACGT-GTTG-TG---CGGC-GCTC-ACTG-
TCTC------
FC88X_00129_00741 ------------------TGACGT-GTTG-TG---CGGC-GCTC-ACTG-
TCTC------
FC88X_00149_01019 ------------------TGACGT-GTTG-TG---CGGC-GCTC-ACTG-
TCTC------

FC88X_00185_00051 ------------------TGACGT-GTTG-TG---CGGC-GCTC-ACTG-
TCTC------
FC88X_00170_00107 ------------------TGACGT-GTTG-TG---CGGC-GCTC-ACTG-
TCTC------
FC88X_00179_00126 ------------------TGACGT-GTTG-TG---CGGC-GCTC-ACTG-
TCTC------
FC88X_00185_00386 ------------------TGACGT-GTTG-TG---CGGC-GCTC-ACTG-
TCTC------
FC88X_00164_00430 ------------------TGACGT-GTTG-TG---CGGC-GCTC-ACTG-
TCTC------
FC88X_00162_00765 ------------------TGACGT-GTTG-TG---CGGC-GCTC-ACTG-
TCTC------
FC88X_00169_00802 ------------------TGACGT-GTTG-TG---CGGC-GCTC-ACTG-
TCTC------
FC88X_00176_00804 ------------------TGACGT-GTTG-TG---CGGC-GCTC-ACTG-
TCTC------
FC88X_00170_00873 ------------------TGACGT-GTTG-TG---CGGC-GCTC-ACTG-
TCTC------
FC88X_00175_01022 ------------------TGACGT-GTTG-TG---CGGC-GCTC-ACTG-
TCTC------
FC88X_00188_01055 ------------------TGACGT-GTTG-TG---CGGC-GCTC-ACTG-
TCTC------
FC88X_00202_00046 ------------------TGACGT-GTTG-TG---CGGC-GCTC-ACTG-
TCTC------
FC88X_00208_00063 ------------------TGACGT-GTTG-TG---CGGC-GCTC-ACTG-
TCTC------
FC88X_00205_00145 ------------------TGACGT-GTTG-TG---CGGC-GCTC-ACTG-
TCTC------
FC88X_00228_00790 ------------------TGACGT-GTTG-TG---CGGC-GCTC-ACTG-
TCTC------
FC88X_00205_00914 ------------------TGACGT-GTTG-TG---CGGC-GCTC-ACTG-
TCTC------
FC88X_00240_00977 ------------------TGACGT-GTTG-TG---CGGC-GCTC-ACTG-
TCTC------
FC88X_00272_00948 ------------------TGACGT-GTTG-TG---CGGC-GCTC-ACTG-
TCTC------
FC88X_00281_01077 ------------------TGACGT-GTTG-TG---CGGC-GCTC-ACTG-
TCTC------
FC88X_00324_00026 ------------------TGACGT-GTTG-TG---CGGC-GCTC-ACTG-
TCTC------
FC88X_00319_00106 ------------------TGACGT-GTTG-TG---CGGC-GCTC-ACTG-
TCTC------
FC88X_00346_00394 ------------------TGACGT-GTTG-TG---CGGC-GCTC-ACTG-
TCTC------
FC88X_00334_00689 ------------------TGACGT-GTTG-TG---CGGC-GCTC-ACTG-
TCTC------
FC88X_00332_00821 ------------------TGACGT-GTTG-TG---CGGC-GCTC-ACTG-
TCTC------
FC88X_00346_01180 ------------------TGACGT-GTTG-TG---CGGC-GCTC-ACTG-
TCTC------
FC88X_00351_00991 ------------------TGACGT-GTTG-TG---CGGC-GCTC-ACTG-
TCTC------
FC88X_00424_00688 ------------------TGACGT-GTTG-TG---CGGC-GCTC-ACTG-
TCTC------
FC88X_00433_00947 ------------------TGACGT-GTTG-TG---CGGC-GCTC-ACTG-
TCTC------

FC88X_00431_01028 ------------------TGACGT-GTTG-TG---CGGC-GCTC-ACTG-
TCTC------
FC88X_00441_01113 ------------------TGACGT-GTTG-TG---CGGC-GCTC-ACTG-
TCTC------
FC88X_00436_01186 ------------------TGACGT-GTTG-TG---CGGC-GCTC-ACTG-
TCTC------
FC88X_00426_01206 ------------------TGACGT-GTTG-TG---CGGC-GCTC-ACTG-
TCTC------
FC88X_00486_00030 ------------------TGACGT-GTTG-TG---CGGC-GCTC-ACTG-
TCTC------
FC88X_00494_00166 ------------------TGACGT-GTTG-TG---CGGC-GCTC-ACTG-
TCTC------
FC88X_00480_00943 ------------------TGACGT-GTTG-TG---CGGC-GCTC-ACTG-
TCTC------
FC88X_00490_01002 ------------------TGACGT-GTTG-TG---CGGC-GCTC-ACTG-
TCTC------
FC88X_00493_01017 ------------------TGACGT-GTTG-TG---CGGC-GCTC-ACTG-
TCTC------
FC88X_00484_01123 ------------------TGACGT-GTTG-TG---CGGC-GCTC-ACTG-
TCTC------
FC88X_00459_01182 ------------------TGACGT-GTTG-TG---CGGC-GCTC-ACTG-
TCTC------
FC88X_00542_00132 ------------------TGACGT-GTTG-TG---CGGC-GCTC-ACTG-
TCTC------
FC88X_00511_00253 ------------------TGACGT-GTTG-TG---CGGC-GCTC-ACTG-
TCTC------
FC88X_00507_00954 ------------------TGACGT-GTTG-TG---CGGC-GCTC-ACTG-
TCTC------
FC88X_00515_00960 ------------------TGACGT-GTTG-TG---CGGC-GCTC-ACTG-
TCTC------
FC88X_00509_01062 ------------------TGACGT-GTTG-TG---CGGC-GCTC-ACTG-
TCTC------
FC88X_00534_01174 ------------------TGACGT-GTTG-TG---CGGC-GCTC-ACTG-
TCTC------
FC88X_00505_01204 ------------------TGACGT-GTTG-TG---CGGC-GCTC-ACTG-
TCTC------
FC88X_00556_00038 ------------------TGACGT-GTTG-TG---CGGC-GCTC-ACTG-
TCTC------
FC88X_00576_00252 ------------------TGACGT-GTTG-TG---CGGC-GCTC-ACTG-
TCTC------
FC88X_00566_00615 ------------------TGACGT-GTTG-TG---CGGC-GCTC-ACTG-
TCTC------
FC88X_00554_00670 ------------------TGACGT-GTTG-TG---CGGC-GCTC-ACTG-
TCTC------
FC88X_00560_01095 ------------------TGACGT-GTTG-TG---CGGC-GCTC-ACTG-
TCTC------
FC88X_00585_01227 ------------------TGACGT-GTTG-TG---CGGC-GCTC-ACTG-
TCTC------
FC88X_00643_00235 ------------------TGACGT-GTTG-TG---CGGC-GCTC-ACTG-
TCTC------
FC88X_00645_00269 ------------------TGACGT-GTTG-TG---CGGC-GCTC-ACTG-
TCTC------
FC88X_00626_00791 ------------------TGACGT-GTTG-TG---CGGC-GCTC-ACTG-
TCTC------
FC88X_00612_00925 ------------------TGACGT-GTTG-TG---CGGC-GCTC-ACTG-
TCTC------

FC88X_00644_00954 ------------------TGACGT-GTTG-TG---CGGC-GCTC-ACTG-
TCTC------
FC88X_00611_01029 ------------------TGACGT-GTTG-TG---CGGC-GCTC-ACTG-
TCTC------
FC88X_00687_00238 ------------------TGACGT-GTTG-TG---CGGC-GCTC-ACTG-
TCTC------
FC88X_00678_00329 ------------------TGACGT-GTTG-TG---CGGC-GCTC-ACTG-
TCTC------
FC88X_00696_00398 ------------------TGACGT-GTTG-TG---CGGC-GCTC-ACTG-
TCTC------
FC88X_00653_00609 ------------------TGACGT-GTTG-TG---CGGC-GCTC-ACTG-
TCTC------
FC88X_00679_00938 ------------------TGACGT-GTTG-TG---CGGC-GCTC-ACTG-
TCTC------
FC88X_00661_00991 ------------------TGACGT-GTTG-TG---CGGC-GCTC-ACTG-
TCTC------
FC88X_00654_01062 ------------------TGACGT-GTTG-TG---CGGC-GCTC-ACTG-
TCTC------
FC88X_00651_01176 ------------------TGACGT-GTTG-TG---CGGC-GCTC-ACTG-
TCTC------
FC88X_00665_01150 ------------------TGACGT-GTTG-TG---CGGC-GCTC-ACTG-
TCTC------
FC88X_00711_00867 ------------------TGACGT-GTTG-TG---CGGC-GCTC-ACTG-
TCTC------
FC88X_00712_00920 ------------------TGACGT-GTTG-TG---CGGC-GCTC-ACTG-
TCTC------
FC88X_00744_00920 ------------------TGACGT-GTTG-TG---CGGC-GCTC-ACTG-
TCTC------
FC88X_00734_00999 ------------------TGACGT-GTTG-TG---CGGC-GCTC-ACTG-
TCTC------
FC88X_00723_01109 ------------------TGACGT-GTTG-TG---CGGC-GCTC-ACTG-
TCTC------
FC88X_00783_00118 ------------------TGACGT-GTTG-TG---CGGC-GCTC-ACTG-
TCTC------
FC88X_00790_00259 ------------------TGACGT-GTTG-TG---CGGC-GCTC-ACTG-
TCTC------
FC88X_00773_00346 ------------------TGACGT-GTTG-TG---CGGC-GCTC-ACTG-
TCTC------
FC88X_00798_00325 ------------------TGACGT-GTTG-TG---CGGC-GCTC-ACTG-
TCTC------
FC88X_00752_00734 ------------------TGACGT-GTTG-TG---CGGC-GCTC-ACTG-
TCTC------
FC88X_00783_00889 ------------------TGACGT-GTTG-TG---CGGC-GCTC-ACTG-
TCTC------
FC88X_00782_01270 ------------------TGACGT-GTTG-TG---CGGC-GCTC-ACTG-
TCTC------
FC88X_00824_00309 ------------------TGACGT-GTTG-TG---CGGC-GCTC-ACTG-
TCTC------
FC88X_00812_00686 ------------------TGACGT-GTTG-TG---CGGC-GCTC-ACTG-
TCTC------
FC88X_00814_00763 ------------------TGACGT-GTTG-TG---CGGC-GCTC-ACTG-
TCTC------
FC88X_00833_00858 ------------------TGACGT-GTTG-TG---CGGC-GCTC-ACTG-
TCTC------
FC88X_00800_01249 ------------------TGACGT-GTTG-TG---CGGC-GCTC-ACTG-
TCTC------

FC88X_00861_00187 ------------------TGACGT-GTTG-TG---CGGC-GCTC-ACTG-
TCTC------
FC88X_00895_00216 ------------------TGACGT-GTTG-TG---CGGC-GCTC-ACTG-
TCTC------
FC88X_00860_00272 ------------------TGACGT-GTTG-TG---CGGC-GCTC-ACTG-
TCTC------
FC88X_00889_00265 ------------------TGACGT-GTTG-TG---CGGC-GCTC-ACTG-
TCTC------
FC88X_00882_00344 ------------------TGACGT-GTTG-TG---CGGC-GCTC-ACTG-
TCTC------
FC88X_00893_00803 ------------------TGACGT-GTTG-TG---CGGC-GCTC-ACTG-
TCTC------
FC88X_00863_00911 ------------------TGACGT-GTTG-TG---CGGC-GCTC-ACTG-
TCTC------
FC88X_00898_01105 ------------------TGACGT-GTTG-TG---CGGC-GCTC-ACTG-
TCTC------
FC88X_00900_00178 ------------------TGACGT-GTTG-TG---CGGC-GCTC-ACTG-
TCTC------
FC88X_00939_00308 ------------------TGACGT-GTTG-TG---CGGC-GCTC-ACTG-
TCTC------
FC88X_00905_00502 ------------------TGACGT-GTTG-TG---CGGC-GCTC-ACTG-
TCTC------
FC88X_00943_00548 ------------------TGACGT-GTTG-TG---CGGC-GCTC-ACTG-
TCTC------
FC88X_00914_00645 ------------------TGACGT-GTTG-TG---CGGC-GCTC-ACTG-
TCTC------
FC88X_00943_00631 ------------------TGACGT-GTTG-TG---CGGC-GCTC-ACTG-
TCTC------
FC88X_00903_00678 ------------------TGACGT-GTTG-TG---CGGC-GCTC-ACTG-
TCTC------
FC88X_00929_00689 ------------------TGACGT-GTTG-TG---CGGC-GCTC-ACTG-
TCTC------
FC88X_00904_00706 ------------------TGACGT-GTTG-TG---CGGC-GCTC-ACTG-
TCTC------
FC88X_00922_00740 ------------------TGACGT-GTTG-TG---CGGC-GCTC-ACTG-
TCTC------
FC88X_00915_00888 ------------------TGACGT-GTTG-TG---CGGC-GCTC-ACTG-
TCTC------
FC88X_00913_00922 ------------------TGACGT-GTTG-TG---CGGC-GCTC-ACTG-
TCTC------
FC88X_00922_01184 ------------------TGACGT-GTTG-TG---CGGC-GCTC-ACTG-
TCTC------
FC88X_00939_01174 ------------------TGACGT-GTTG-TG---CGGC-GCTC-ACTG-
TCTC------
FC88X_00942_01168 ------------------TGACGT-GTTG-TG---CGGC-GCTC-ACTG-
TCTC------
FC88X_00980_00304 ------------------TGACGT-GTTG-TG---CGGC-GCTC-ACTG-
TCTC------
FC88X_00991_00333 ------------------TGACGT-GTTG-TG---CGGC-GCTC-ACTG-
TCTC------
FC88X_00969_00504 ------------------TGACGT-GTTG-TG---CGGC-GCTC-ACTG-
TCTC------
FC88X_00979_00527 ------------------TGACGT-GTTG-TG---CGGC-GCTC-ACTG-
TCTC------
FC88X_00982_00548 ------------------TGACGT-GTTG-TG---CGGC-GCTC-ACTG-
TCTC------

FC88X_00984_00875 ------------------TGACGT-GTTG-TG---CGGC-GCTC-ACTG-TCTC------
FC88X_00989_00874 ------------------TGACGT-GTTG-TG---CGGC-GCTC-ACTG-TCTC------
FC88X_00970_01011 ------------------TGACGT-GTTG-TG---CGGC-GCTC-ACTG-TCTC------
FC88X_00961_01143 ------------------TGACGT-GTTG-TG---CGGC-GCTC-ACTG-TCTC------
FC88X_00999_01191 ------------------TGACGT-GTTG-TG---CGGC-GCTC-ACTG-TCTC------
FC88X_00964_01254 ------------------TGACGT-GTTG-TG---CGGC-GCTC-ACTG-TCTC------
FC88X_01028_00315 ------------------TGACGT-GTTG-TG---CGGC-GCTC-ACTG-TCTC------
FC88X_01035_00337 ------------------TGACGT-GTTG-TG---CGGC-GCTC-ACTG-TCTC------
FC88X_01042_00339 ------------------TGACGT-GTTG-TG---CGGC-GCTC-ACTG-TCTC------
FC88X_01000_00624 ------------------TGACGT-GTTG-TG---CGGC-GCTC-ACTG-TCTC------
FC88X_01017_00664 ------------------TGACGT-GTTG-TG---CGGC-GCTC-ACTG-TCTC------
FC88X_01017_00683 ------------------TGACGT-GTTG-TG---CGGC-GCTC-ACTG-TCTC------
FC88X_01023_00733 ------------------TGACGT-GTTG-TG---CGGC-GCTC-ACTG-TCTC------
FC88X_01046_00837 ------------------TGACGT-GTTG-TG---CGGC-GCTC-ACTG-TCTC------
FC88X_01046_00886 ------------------TGACGT-GTTG-TG---CGGC-GCTC-ACTG-TCTC------
FC88X_01049_00917 ------------------TGACGT-GTTG-TG---CGGC-GCTC-ACTG-TCTC------
FC88X_01015_00979 ------------------TGACGT-GTTG-TG---CGGC-GCTC-ACTG-TCTC------
FC88X_01037_01005 ------------------TGACGT-GTTG-TG---CGGC-GCTC-ACTG-TCTC------
FC88X_01045_01012 ------------------TGACGT-GTTG-TG---CGGC-GCTC-ACTG-TCTC------
FC88X_01011_01060 ------------------TGACGT-GTTG-TG---CGGC-GCTC-ACTG-TCTC------
FC88X_01049_01218 ------------------TGACGT-GTTG-TG---CGGC-GCTC-ACTG-TCTC------
FC88X_01047_01269 ------------------TGACGT-GTTG-TG---CGGC-GCTC-ACTG-TCTC------
FC88X_01090_00634 ------------------TGACGT-GTTG-TG---CGGC-GCTC-ACTG-TCTC------
FC88X_01098_00795 ------------------TGACGT-GTTG-TG---CGGC-GCTC-ACTG-TCTC------
FC88X_01099_00755 ------------------TGACGT-GTTG-TG---CGGC-GCTC-ACTG-TCTC------
FC88X_01051_00853 ------------------TGACGT-GTTG-TG---CGGC-GCTC-ACTG-TCTC------
FC88X_01055_00873 ------------------TGACGT-GTTG-TG---CGGC-GCTC-ACTG-TCTC------
FC88X_01064_00871 ------------------TGACGT-GTTG-TG---CGGC-GCTC-ACTG-TCTC------

FC88X_01081_00943 ------------------TGACGT-GTTG-TG---CGGC-GCTC-ACTG-
TCTC------
FC88X_01066_00972 ------------------TGACGT-GTTG-TG---CGGC-GCTC-ACTG-
TCTC------
FC88X_01050_01045 ------------------TGACGT-GTTG-TG---CGGC-GCTC-ACTG-
TCTC------
FC88X_01062_01125 ------------------TGACGT-GTTG-TG---CGGC-GCTC-ACTG-
TCTC------
FC88X_01123_00620 ------------------TGACGT-GTTG-TG---CGGC-GCTC-ACTG-
TCTC------
FC88X_01147_00637 ------------------TGACGT-GTTG-TG---CGGC-GCTC-ACTG-
TCTC------
FC88X_01129_00652 ------------------TGACGT-GTTG-TG---CGGC-GCTC-ACTG-
TCTC------
FC88X_01143_00661 ------------------TGACGT-GTTG-TG---CGGC-GCTC-ACTG-
TCTC------
FC88X_01103_00714 ------------------TGACGT-GTTG-TG---CGGC-GCTC-ACTG-
TCTC------
FC88X_01118_00805 ------------------TGACGT-GTTG-TG---CGGC-GCTC-ACTG-
TCTC------
FC88X_01131_01012 ------------------TGACGT-GTTG-TG---CGGC-GCTC-ACTG-
TCTC------
FC88X_00345_00067 ------------------TGACGT-GTTG-TG---CGGC-GCTC-ACTG-
TCTCT-----
FC88X_00552_00217 ------------------TGACGT-GTTG-TG---CGGC-GATC-ACTG-
TCTC------
FC88X_00056_00378 ------------------TGACGT-GTTG-TG---CGGC---TC-ACTG-
TCTC------
FC88X_00524_00903 ------------------TGACGT-GTTG-TG---CGGC-GCTCACTGA-
TCTCC-----
FC88X_00080_00513 ------------------TGATGT-GTTG-TG---CGGC-GCTC-ACTG-
TCTC------
FC88X_00745_00869 ------------------TGACGT-GTTG-TG---CGGC-GCTC-ACTG-
TCTCCC----
FC88X_00973_00616 ------------------TGACGT-GTTG-TG---CGGC-GCTC-ACTG-
TCTCCC----
FC88X_00557_01264 ------------------TGACGT-GTTG-TG---CGGC-GCTC-ACTG-
TCTCCC----
FC88X_00786_00267 ------------------TGACGT-GTTG-TG---CGGC-GCTC-ACTG-
TCTCCC----
FC88X_00912_01077 ------------------TGACGT-GTTG-TG---CGGC-GCTC-ACTG-
TCTCC-----
FC88X_00965_00996 ------------------TGACGT-GTTG-TG---CGGC-GCTC-ACTG-
TCTCC-----
FC88X_00898_01070 ------------------TGACGT-GTTG-TG---CGGC-GCTCTACTG-
CTCTCC----
FC88X_00910_00334 -----------------GTGACGT-GCTG-TG---CGGC-GCTC-ACTG-
TCTC------
FC88X_00332_00085 ------------------TGACGT-GCTG-TG---CGGC-GCTC-ACTG-
TCTC------
FC88X_00526_00191 ------------------TGACGT-GCTG-TG---CGGC-GCTC-ACTG-
TCTC------
FC88X_00944_00879 ------------------TGACGT-GCTG-TG---CGGC-GCTC-ACTG-
TCTC------
FC88X_00955_01106 ------------------TGACGT-GCTG-TG---CGGC-GCTC-ACTG-
TCTC------

```
FC88X_00947_00201       ------------------TGACGT-G-TG-TG---CGGC-GCTC-ACTG-
TCTC------
FC88X_00017_00584       ------------------TGACGT-GTTG-TG---CGGC-GCTC-ACT--
GTCT------
FC88X_00133_00454       ------------------TGACGT-GTTG-TG---CGGC-GCTC-ACTC-
GTCT------
FC88X_00575_00395       ------------------TGACGT-GTTG-TG---CGGC-GCTC-ACT------
------
FC88X_00900_00805       -------------GGGGATGACGT-GTTG-TG---CGGC-GCTC-ACTG-
TCTC------
FC88X_01073_01011       ------------------TGACGC-GTTG-TG---CGGC-GCTC-ACTG-
TCTC------
FC88X_00067_00765       ------------------TGGCGT-GTTG-TG---CGGC-GCTC-ACTG-
TCTC------
FC88X_00389_00024       ------------------TGGCGT-GTTG-TG---CGGC-GCTC-ACTG-
TCTC------
FC88X_00872_01068       ------------------TGGCGT-GTTG-TG---CGGC-GCTC-ACTG-
TCTC------
FC88X_00549_01059       ------------------TGGCGT-GTTG-TG---CGGC-GCTC-ACTG-
TCTC------
FC88X_00080_00126       ------------------TGACGTG--TTGTG---CGGC-GCTC-GCTG-
TCTC------
FC88X_00766_01030       ------------------TGACGTGGTTTGTG---CGGC-GCTC-ACTG-
TCTCCTTCCT
FC88X_00835_00819       ------------------TGACGT-GTTG-TG---CGGC-GCTC-ACTG-
TTTC------
FC88X_01027_00760       ------------------TGACGT-GTTG-TG---CGGC-GCTC-ACTG-
TTTC------
FC88X_01042_00813       ------------------TGACGT-GTTG-TG---CGGC-GCTC-ACTG-
TATC------
FC88X_00301_01023       ------------------TGACGT-GTCG-TG---CGGC-GCTC-ACTG-
TCTC------
FC88X_00945_01210       ------------------TGACGT-GTCG-TG---CGGC-GCTC-ACTG-
TCTC------
FC88X_01071_00980       ------------------TGACGT-GTCG-TG---CGGC-GCTC-ACTG-
TCTC------
FC88X_01093_00984       ------------------TGACGT-GTCG-TG---CGGC-GCTC-ACTG-
TCTC------
FC88X_00978_00865       ------------------TGACGT-GTCG-TG---CGGC-GCCC-ACTG-
TCTC------
FC88X_00782_00142       ------------------TGACGT-GTTG-TG---CGGC-GCCC-ACTG-
TCTC------
FC88X_00119_00669       -------------GGGGACGACGT-GTTG-TG---CGGC-GCTC-ACTG-
TCTC------
FC88X_00606_01273       ------------------CGACGT-GTTG-TG---CGGC-GCTC-ACTG-
TCTC------
FC88X_00013_00656       -----------------TGGACGT-GTTG-TG---CGGC-GCTC-ACTG-
TCTC------
FC88X_00928_00309       ------------------TGACGT-GTTG-TG---CGAC-GCTC-ACTG-
TCTC------
FC88X_01061_00380       ------------------TGACGT-ATT-GTG---CGGC-GCTC-ACTG-
TCTC------
FC88X_00941_00764       -----------------ATGACGT-ATT-GTG---CGGC-GCTC-ACTG-
TCTC------
FC88X_00918_01205       ------------------TGACGT-ATT-GTG---CGGC-GCTC-ACTG-
TCTC------
```

FC88X_01004_00848 ------------------TGACGT-ATT-GTG---CGGC-GCTC-ACTG-
TCTC------
FC88X_01080_00925 ------------------TGACGT-TTTTGTG---CGGC-GCTC-ACTG-
TCTC------
FC88X_00975_00320 ------------------TGACGT-GTTT-TG---CGGC-GCTC-ACTG-
TCTCC-----
FC88X_01127_01040 ------------------TGACGT-GTTG-TG---CGGC-GCTC-ACTG-
TCTCCCTTCT
FC88X_01023_00346 ------------------TGACGT-GTGG-TG---CGGC-GT-C-ACTG-
TCTC------
FC88X_01011_00943 ------------------TGACGT-GTTG-TG---CGGC-GTTC-ACTG-
TCTC------
FC88X_01097_00635 ------------------TGACGT-GTTG-TG---TGGC-GCTC-ACTG-
TCTC------
FC88X_00064_00732 ------------------TGACGT-GTTG-TG---TGGC-GCTC-ACTG-
TCTC------
FC88X_00302_00394 ------------------TGACGT-GTTG-TG---TGGC-GCTC-ACTG-
TCTC------
FC88X_00555_00971 ------------------TGACGT-GTTG-TG---TGGC-GCTC-ACTG-
TCTC------
FC88X_01006_00920 ------------------TGACGT-GTTG-TG---TGGC-GCTC-ACTG-
TCTC------
FC88X_00201_01051 ------------------TGACGT-GTTG-TG---TGGC-GCTC-ACTG-
CTCTC-----
FC88X_00261_00905 ------------------TGACGT-GTTG-CG---CGGC-GCTC-ACTG-
TCTC------
FC88X_00215_01087 ------------------TGACGT-GTTG-CG---CGGC-GCTC-ACTG-
TCTC------
FC88X_00491_01209 ------------------TGACGT-GTTG-TCGGTCGGC-GTTC-ACTG-
TCTC------
FC88X_00640_00673 ------------------TGACGT-GTTG-TG---CGGC-ACTC-ACTG-
TCTC------
FC88X_00867_01227 ------------------TGACGT-GTTG-TG---CGGC-ACTC-ACTG-
TCTC------
FC88X_00289_00122 ------------------TGACGT-GTTG-TG---CGGC-ACTC-ACCG-
TCTC------
FC88X_00377_00993 ------------------TGACGT-GTTG-TG---CGGC-GCTC-
ACTGGTCTCCTTCCT
FC88X_00489_00267 ------------------TGACGT-GTTG-TG---CGGC-GCTC-ACTG-
TCTCCTTCC-
FC88X_00658_01107 ------------------TGACGT-GTTG-TG---CGGC-GCTC-ACTG-
TCTCCTTCCT
FC88X_00934_00654 ------------------TGACGT-GTTG-TG---CGGC-GCTC-ACTG-
TCTCCTTCCT
FC88X_00878_00823 ------------------TGACGT-GTTG-TG---CGGC-GCTC-ACTG-
TCTCCTTCCT
FC88X_01093_01233 ------------------TGACGT-GTTG-TG---CGGC-GCTC-ACTG-
TCTCCTTCCT
FC88X_00970_01015 ------------------TGACGT-GTTG-TG----GGC-GCTC-ACTG-
TCTCCTTCCT
FC88X_01079_01046 ------------------TGACCT-GTTG-TG---CGGC-GCTC-ACTG-
TCTC------
FC88X_00055_00455 ------------------TGACGCG-TTG-TG---CAGC-GCTC-ACTG-
TCTC------
FC88X_00712_01106 ------------------TGACGTGATTG-TG---CAGC-GCTC-ACTG-
TCTC------

FC88X_00427_00515 ------------GGGGAATTGACGT-GTCG-TG---TGGC-GCTC-ACTG-TCTCC-----
FC88X_00408_00100 --------------------TGACGT-GTTG-TG---CGGC-GCTC-ACTGTCGTCC-----
FC88X_00163_00046 --------------------TGACGT-GTTG-TG---CGGC-GC------------------
FC88X_00576_00163 --------------------TGACGT-GTTG-TG---CGGC-GCTC-ACTGTCCTC------
FC88X_01099_00802 --------------------TGACGT-GTTG-TG---CGGC-GCTC-ACTGTCCTC------
FC88X_00839_01259 --------------------TGACGT-GTTG-TG---CGGC-GCTC-ACTGTCCTC------
FC88X_00158_00151 --------------------TGACGT-GTTGGTG---CGGC-GCTC-ACTGTCCTCCC----
FC88X_00191_00880 --------------------TGACGT-GTTG-TG---CGGC-GCTC----------------
FC88X_00638_01192 ------------GGGGAAGTGACGT-GTTG-TG---CGGC-GCTC-ACTGTC---------
FC88X_00979_00273 -----------------TGGACGTGA-GTTG-TG---CGGC-GCTC-ACTGT----------
FC88X_00079_00148 --------------------TGACGT-GTTGTCG---CGGC-G-------------------
FC88X_00041_00691 --------------------TGACGT-GCTG-TG---CGGCTGCTC-AC-------------
FC88X_00180_00798 -------------------TCGACGT-GCTG-TG---CGGC-GCTC-ACTCGT---------
FC88X_00537_01250 --------------------TGACGTGTTTGCGG---CGGC---------------------
FC88X_00663_00306 --------------------CCACTT-GTTG-TG---CAGC-TCTCCCTTGGT---------
FC88X_00430_00619 --------------------TTGCGT-GTTG-TG---CTACTGTCT-CCTGCC---------
FC88X_00936_01246 --------------------TTGCGT-GTTG-TG---CTACTGTCT-CCTGCC---------
FC88X_00743_01241 --------------------TTGCGT-GTTG-TG---CTACTGT-T-CCTGCC---------
FC88X_00855_00194 --------------------TGACGT-GTTG-TG---GCGC-GCCT-ACTGCTCCTTCCTTC
FC88X_00420_00018 -------------------TTCCACGATTCCCTG---TGTTGGCCT-ACT------------
FC88X_00382_00068 ---------------------CCACAATTTCGTG----GTTTGGCT-ACTC-----------
FC88X_00754_00302 -----------------CTGTGCCATATTCGGTG---CGTCTGGTC-TC-------------
FC88X_01066_00355 -----------------CTGTGCCATATTCGGTG---CGTCTGGTC-TC-------------
FC88X_00010_00098 -----------------CTGTGCCATATTCGGTG---TGTCTGGTC-TC-------------
FC88X_00009_00675 -----------------CTGCGCCATATTCGGTG---TGTCTGGTC-TC-------------
FC88X_01080_00787 -----------------CTGCGCCATATTCGGTG---TGTCTGGTC-TC-------------
FC88X_00341_01077 -----------------CTTCCACAGATTTTGTG---TG-CTGGTC-TC-------------
FC88X_01105_00662 -----------------ACCCTTCATTTGTGCTG---TG-CTGCTC-AC-------------

FC88X_00791_01255 -----------------ACTCTATTTGTGCTG---TG-CTGCTC-AC-------
------
FC88X_00493_00607 ---------------CCCTTCGATGTGTGTC----TG-TTCCTC-AAC------
------
FC88X_00150_00763 ---------------------CGTGCCTG-TA----TACGGTAC-
CCTGTTCGC------
FC88X_00255_00098 ---------------------CGTGCCTG-TA----TACGGTAC-
CCTGTTCGC------
FC88X_00717_00563 ---------------------CGTGCCTG-TA----TACGGTAC-
CCTGTTCGC------
FC88X_00789_00306 ---------------------CGTGCCTG-TA----TACGGTAC-
CCTGTTCGC------
FC88X_00915_01066 ---------------------CGTGCCTG-TA----TACGGTAC-
CCTGTTCGC------
FC88X_01074_00575 ---------------------CGTGCCTG-TA----TACGGTAC-
CCTGTTCGC------
FC88X_00098_00227 ---------------------CGTGCCTG-TA----TACGGTAC-
CCTGTTCGC------
FC88X_00094_00387 ---------------------CGTGCCTG-TA----TACGGTAC-
CCTGTTCGC------
FC88X_00085_00563 ---------------------CGTGCCTG-TA----TACGGTAC-
CCTGTTCGC------
FC88X_00130_00698 ---------------------CGTGCCTG-TA----TACGGTAC-
CCTGTTCGC------
FC88X_00264_00958 ---------------------CGTGCCTG-TA----TACGGTAC-
CCTGTTCGC------
FC88X_00395_01099 ---------------------CGTGCCTG-TA----TACGGTAC-
CCTGTTCGC------
FC88X_00683_01123 ---------------------CGTGCCTG-TA----TACGGTAC-
CCTGTTCGC------
FC88X_00738_01000 ---------------------CGTGCCTG-TA----TACGGTAC-
CCTGTTCGC------
FC88X_00764_01156 ---------------------CGTGCCTG-TA----TACGGTAC-
CCTGTTCGC------
FC88X_00908_00179 ---------------------CGTGCCTG-TA----TACGGTAC-
CCTGTTCGC------
FC88X_00904_00401 ---------------------CGTGCCTG-TA----TACGGTAC-
CCTGTTCGC------
FC88X_00911_00994 ---------------------CGTGCCTG-TA----TACGGTAC-
CCTGTTCGC------
FC88X_01000_00535 ---------------------CGTGCCTG-TA----TACGGTAC-
CCTGTTCGC------
FC88X_01010_00758 ---------------------CGTGCCTG-TA----TACGGTAC-
CCTGTTCGC------
FC88X_01078_01077 ---------------------CGTGCCTG-TA----TACGGTAC-
CCTGTTCGC------
FC88X_01132_00575 ---------------------CGTGCCTG-TA----TACGGTAC-
CCTGTTCGC------
FC88X_00247_00820 ---------------------CGTGCCTG-TA----TACGGTAC-C-
TGTTCGC------
FC88X_00805_01087 ---------------------CGTGCCTG-CA----TACGGTAC-
CCTGTTCGC------
FC88X_00574_00283 ---------------------CGTGCCTG-TA----TACGGTAC-
CCTGTTCGCCTTCCT
FC88X_00797_00086 ---------------------CGTGCCTG-TA----TACGGTAC-
CCTGCTCGC------

FC88X_00982_00945 ---------------------CGTGC-TG-TA----TACGGTGC-
CCTGTTCGC------
FC88X_00556_01060 ---------------------CGTTACGT-TA----TGTTGTGC-G--------
------
FC88X_01043_00889 ---------------------CGTTACGT-TA----TGTTGTGC-
GCCTTTTTTT-----
FC88X_01014_00554 ---------------------CGTTACGT-CA----TGTTGTGC-
GCCTTTTTT------
FC88X_01095_00920 ---------------------CGTTACGT-TA----CGTTGTGC-
GCCTTTTTC------
FC88X_01068_00532 ---------------------CGTGATGC-TA----TATTGTGC-
CCCTGTTCT------
FC88X_00034_00428 ------------------TTACGGCACAT-TC----TGCAGTCT-CCTGTT---
------
FC88X_00111_00926 ------------------TTACGGCACAT-TC----TGCAGTCT-CCTGTT---
------
FC88X_00571_01002 ------------------TTACGGCACAT-TC----TGCAGTCT-CCTGTT---
------
FC88X_00881_00626 ------------------TTACGGCACAT-TC----TGCAGTCT-CCTGTT---
------
FC88X_00947_00644 ------------------TTACGGCACAT-TC----TGCAGTCT-CCTGTT---
------
FC88X_01022_00469 ------------------TTACGGCACAT-TC----TGCAGTCT-CCTGTT---
------
FC88X_00782_01177 -------------------TACGGCACAT-TC----TGCAGTCT-CCTGTT---
------
FC88X_00816_00199 ------------------TTACGGCACAT-TC----TGCAGTCT-CCTGCT---
------
FC88X_00710_00083 ------------------TCACGGCACAT-TC----TGCAGTCT-CCTGTT---
------
FC88X_00055_00215 ------------------TTACGGCACAT-TC----TGCGGTCT-CCTGTTA--
------
FC88X_00242_00395 ------------------CCATGGCAC-C-TA----TGCTGTGT-ACTGTTCC-
------
FC88X_00815_00254 ------------------CCATGGCAC-C-TA----TGCTGTGT-ACTGTTCC-
------
FC88X_00966_00912 -------------------CATGGCAC-C-TA----TGCTGTGT-ACTGTTCC-
------
FC88X_01053_00353 ------------------CATGGGCAC-C-TA----TGCTGTGT-ACTGTTCC-
------
FC88X_00027_00727 ----------------------ACGCCATTTTGATCGGCTC-CC--CTGATT--
------
FC88X_00812_00330 ----------------------TCCCCGCTTTGAGCGTTTCTCC--CTGAT---
------
FC88X_00206_00095 ----------------------CTAACTATTG--TTGGGCATCC--
TTGCTGTT------
FC88X_00974_00307 ----------------------CTAACTATTG--TTGGGCATCC--
TTGCTGTT------
FC88X_01016_00683 ----------------------CTAACTATTG--TTGGGCATCC--
TTGCTGTT------
FC88X_00443_00328 ----------------------CTAACTATTG--TTGGGC-TCC--
TTGCTGTT------
FC88X_01025_00592 ----------------------CTGACTATTTCTTTCGGCTCCC--CTGCA---
------
FC88X_00661_00074 -------------------------TCCTTTTGTTTGCGGCCCC--
CTACTCTT------

FC88X_00679_01223 ------------------------ATCCATTTTTTTGTGGTCTC--
CTCCTGTT------
FC88X_00030_00317 ----------------------CAACTTATGT--CTGTTCCCGGCTTTCCC---
------
FC88X_00178_00983 ---------------------CCAACTTACGT--CTGTTCCCGGCTTTCCC---
------
FC88X_00091_00759 ---------------------TCAACCAA-ATTACGGT-GCCTGTTCGAA----
------
FC88X_00969_00761 ---------------------TCAACTTACATCACGCTTGCCTGTTTTC-----
------
FC88X_00256_01000 -----------------------GCTCAGGATTATCATCCGCGCTGTCCTC---
------
FC88X_00973_00516 ------------------------CTCAGGATTATCATCCGCGCTGTCCTC---
------
FC88X_00073_00208 ------------------------CTCAGGATTATCATCCACGCTGTCCTC---
------
FC88X_00077_00411 --------------------------CAGAATTACGTCTTACTCTGCTGTCCC-
------
FC88X_00932_01186 --------------------------CAGAATTACGTCTTACTCTGCTGTCCC-
------
FC88X_00193_00039 ------------------------CTACATACTATGTTCCCAGCTGTTCGT---
------
FC88X_01103_00546 ------------------------CTACATACTATGTTCTCAGCTGTTCGT---
------
FC88X_00908_00456 ------------------------CTACATACTATGTAC-CAGCTGTTCGTCC-
------
FC88X_01060_00908 ------------------------CTCCGTACGATCTCCCCTGATGTTTGT---
------
FC88X_01119_01213 ------------------------CTCCGTACGATCTCCCCTGATGTTTGT---
------
FC88X_00109_00561 -----------------------CCTCCAACCGATTTAACACG-TGCTCCC---
------
FC88X_00626_01132 ------------------------TTGCAAACTATCTCTCATGCTGCTCCC---
------
FC88X_00020_00389 -------------------------CTCCTAC--ATGTTGATCTTTTGTGTC--
------
FC88X_00057_00685 -------------------------CTCCCACTGATGTCTAACTATTTTGTC--
------
FC88X_00538_00337 ----------------------CCACAGGCCCTGGTGTGTGTATATTCC-----
------
FC88X_00227_01101 ----------------------CCACAGACTCTGTTATCTTTTGATTGTT----
------
FC88X_00082_00117 ---------------------------
TTATCTGTTGTGTGACTTTCTCCCGGC------
FC88X_00494_01186 ---------------------------TTATCTGTTGTGTGA------------
------
FC88X_00087_00340 --------------------TCTAGTTGGTTTTATTGCT-GCTATTCTC-----
------
FC88X_00149_00487 --------------------TCTAGTTGGTTTTATTGCT-GCTATTCTC-----
------
FC88X_00316_01114 --------------------TCTAGTTGGTTTTATTGCT-GCTATTCTC-----
------
FC88X_00603_00268 --------------------TCTAGTTGGTTTTATTGCT-GCTATTCTC-----
------
FC88X_00859_00270 --------------------TCTAGTTGGTTTTATTGCT-GCTATTCTC-----
------

FC88X_01002_01264 --------------------TCTAGTTGGTTTTATTGCT-GCTATTCTC-----
------
FC88X_00038_00344 --------------------TCTAGTTGGTTTTATTGCT-GCTATTCTC-----
------
FC88X_00117_00065 --------------------TCTAGTTGGTTTTATTGCT-GCTATTCTC-----
------
FC88X_00231_00983 --------------------TCTAGTTGGTTTTATTGCT-GCTATTCTC-----
------
FC88X_00342_00921 --------------------TCTAGTTGGTTTTATTGCT-GCTATTCTC-----
------
FC88X_00454_00057 --------------------TCTAGTTGGTTTTATTGCT-GCTATTCTC-----
------
FC88X_00743_01091 --------------------TCTAGTTGGTTTTATTGCT-GCTATTCTC-----
------
FC88X_00820_00820 --------------------TCTAGTTGGTTTTATTGCT-GCTATTCTC-----
------
FC88X_00869_00685 --------------------TCTAGTTGGTTTTATTGCT-GCTATTCTC-----
------
FC88X_00879_00925 --------------------TCTAGTTGGTTTTATTGCT-GCTATTCTC-----
------
FC88X_00933_00310 --------------------TCTAGTTGGTTTTATTGCT-GCTATTCTC-----
------
FC88X_00286_01019 ---------------------TTAGTTGGTTTTATTGCT-GCTATTCTC-----
------
FC88X_00796_00921 --------------------TCTAGTTGGTTTTATTGCT-GCTGTTCTC-----
------
FC88X_00016_00480 --------------------TCTAGCTGGTTTTACTGCT-GCTATTCGC-----
------
FC88X_00028_00122 ---------------------CTTGTTGTTTTAGGCCTG-CGTATTCGT-----
------
FC88X_00308_00729 ----------------------CGTTGTTATCTATCGCT-GCGTCTCGTC----
------
FC88X_00885_00886 ----------------------CGTTGTTATCTATCGCT-GCGTCTCGTC----
------
FC88X_01033_00979 ---------------------ACTCTTTTATCCCTCGTTTGCGTCTCGC-----
------
FC88X_00477_01119 ------------------------AGTCAATATATCGTGCATGCTCGTCTC---
------
FC88X_00692_01244 ------------------------AGTCAATATATCGTGCAGGCTCGTCTC---
------
FC88X_00337_00986 -----------------------
AGGTCAATATATCGTGCAGGCTCGTCTCCTTCCTTT-
FC88X_00110_00201 -------------------------CCGAATT-ATCTTGTGCTGTCATCTCCT-
------
FC88X_00456_01125 -------------------------CCGAATTTATCTTGTGCTGTCATCTCCT-
------
FC88X_00055_00848 --------------------------CTGTTTTATCTC-TGCTGTCTGATCTC-
------
FC88X_00071_00612 ------------------TGACTAGCTGG-TCTA-CGTT-GCTCTCTC------
------
FC88X_00166_00586 ------------------TGACTAGCTGG-TCTA-CGTT-GCTCTCTC------
------
FC88X_00488_01204 ------------------TGACTAGCTGG-TCTA-CGTT-GCTCTCTC------
------
FC88X_00613_00170 ------------------TGACTAGCTGG-TCTA-CGTT-GCTCTCTC------
------

```
FC88X_00629_01108      ------------------TGACTAGCTGG-TCTA-CGTT-GCTCTCTC------
------
FC88X_00622_01207      ------------------TGACTAGCTGG-TCTA-CGTT-GCTCTCTC------
------
FC88X_00677_00676      ------------------TGACTAGCTGG-TCTA-CGTT-GCTCTCTC------
------
FC88X_00654_00920      ------------------TGACTAGCTGG-TCTA-CGTT-GCTCTCTC------
------
FC88X_00720_00092      ------------------TGACTAGCTGG-TCTA-CGTT-GCTCTCTC------
------
FC88X_01014_00773      ------------------TGACTAGCTGG-TCTA-CGTT-GCTCTCTC------
------
FC88X_01109_01171      ------------------TGACTAGCTGG-TCTA-CGTT-GCTCTCTC------
------
FC88X_00019_00087      ------------------TGACTAGCTGG-TCTA-CGTT-GCTCTCTC------
------
FC88X_00014_00580      ------------------TGACTAGCTGG-TCTA-CGTT-GCTCTCTC------
------
FC88X_00163_00462      ------------------TGACTAGCTGG-TCTA-CGTT-GCTCTCTC------
------
FC88X_00163_00670      ------------------TGACTAGCTGG-TCTA-CGTT-GCTCTCTC------
------
FC88X_00254_00018      ------------------TGACTAGCTGG-TCTA-CGTT-GCTCTCTC------
------
FC88X_00371_00997      ------------------TGACTAGCTGG-TCTA-CGTT-GCTCTCTC------
------
FC88X_00506_00431      ------------------TGACTAGCTGG-TCTA-CGTT-GCTCTCTC------
------
FC88X_00846_00303      ------------------TGACTAGCTGG-TCTA-CGTT-GCTCTCTC------
------
FC88X_00814_01031      ------------------TGACTAGCTGG-TCTA-CGTT-GCTCTCTC------
------
FC88X_00811_01148      ------------------TGACTAGCTGG-TCTA-CGTT-GCTCTCTC------
------
FC88X_00880_00279      ------------------TGACTAGCTGG-TCTA-CGTT-GCTCTCTC------
------
FC88X_00905_00806      ------------------TGACTAGCTGG-TCTA-CGTT-GCTCTCTC------
------
FC88X_00988_00255      ------------------TGACTAGCTGG-TCTA-CGTT-GCTCTCTC------
------
FC88X_01094_00655      ------------------TGACTAGCTGG-TCTA-CGTT-GCTCTCTC------
------
FC88X_01057_01124      ------------------TGACTAGCTGG-TCTA-CGTT-GCTCTCTC------
------
FC88X_01115_00772      ------------------TGACTAGCTGG-TCTA-CGTT-GCTCTCTC------
------
FC88X_01141_00757      ------------------TGACTAGCTGG-TCTA-CGTT-GCTCTCTC------
------
FC88X_01063_01044      ------------------TGACTAGCTGG-TCTA-CGTT-GCTCTCTCT-----
------
FC88X_00905_00554      ------------------TGACTAGCTGG-TCTA-CGTT-
GCTCTCTCCTTCCTTT----
FC88X_00508_00231      ------------------TGACTAGCTGG-TCTA-CGTT-GCT-----------
------
FC88X_00768_01092      ------------------TGACTAGCTGG-CCTA-CGTT-GCTCTCTC------
------
```

FC88X_00950_00851 ------------------TGACTAGCTGG-TCTA-CGCT-GCTCTCTC------
------
FC88X_00145_00907 ------------------TAACTAGCTGG-TCTA-CGTT-GCTCTCTC------
------
FC88X_00072_00537 ------------------TGACTAGCTGG-TCTA-CGTC-GCTCTCTC------
------
FC88X_00700_00425 ------------------TGACTAGCTGG-TCTA-TGTT-GCTGAG--------
------
FC88X_00989_00757 -----------------GACACACGTTATGTCTACTCTT-GCTGCCC-------
------
FC88X_00613_01148 ------------------CCACATGCTGT-TCTACTCCT-GCTTCCC-------
------
FC88X_00844_01090 ------------------TCACATGCTGT-TTTAGCGTT-GCTCCCCCCTTC--
------
FC88X_00514_01074 ------------------TCACA-TTCATGTTTGTCGACCG-TGTCTC------
------
FC88X_01011_00430 ------------------TCACA-TTCATGTTTGT-GACCG-TGTCT-------
------
FC88X_01084_00880 ------------------TCACA-TTCATGTTTGT-GACCG-TGTCT-------
------
FC88X_00067_00164 ------------------TCACA-TTCATGTTTGT-GACCG-TGTCTC------
------
FC88X_00072_00426 ------------------TCACA-TTCATGTTTGT-GACCG-TGTCTC------
------
FC88X_00413_00324 ---------------GGATCACA-TTCATGTTTGT-GACCG-TGTCTC------
------
FC88X_00415_01212 -----------------ATCACA-TTCATGTTTGT-GACCG-TGTCTC------
------
FC88X_00091_00225 ------------------TCACA-TTCATGTTTGT-GACCG-TGTCTC------
------
FC88X_00138_00081 ------------------TCACA-TTCATGTTTGT-GACCG-TGTCTC------
------
FC88X_00159_00645 ------------------TCACA-TTCATGTTTGT-GACCG-TGTCTC------
------
FC88X_00204_01064 ------------------TCACA-TTCATGTTTGT-GACCG-TGTCTC------
------
FC88X_00312_01003 ------------------TCACA-TTCATGTTTGT-GACCG-TGTCTC------
------
FC88X_00582_00153 -----------------GTCACA-TTCATGTTTGT-GACCG-TGTCTC------
------
FC88X_00802_01109 -----------------GTCACA-TTCATGTTTGT-GACCG-TGTCTC------
------
FC88X_00871_00739 -----------------GTCACA-TTCATGTTTGT-GACCG-TGTCTC------
------
FC88X_00932_00349 -----------------GTCACA-TTCATGTTTGT-GACCG-TGTCTC------
------
FC88X_01128_00633 -----------------GTCACA-TTCATGTTTGT-GACCG-TGTCTC------
------
FC88X_00529_00677 -----------------GTCACA-TTCATGTTTGT-GACCG-TGTCTC------
------
FC88X_00432_00143 -----------GGGGAAGTCACA-TTCATGTTTGT-GACCG-TGTCTC------
------
FC88X_00113_00894 ------------------TCACA-TTCATGTTTGT-GACCG-TGTCTC------
------
FC88X_00227_01045 ------------------TCACA-TTCATGTTTGT-GACCG-TGTCTC------
------

FC88X_00253_00420 ------------------TCACA-TTCATGTTTGT-GACCG-TGTCTC------
------
FC88X_00443_01214 ------------------TCACA-TTCATGTTTGT-GACCG-TGTCTC------
------
FC88X_00506_00068 ------------------TCACA-TTCATGTTTGT-GACCG-TGTCTC------
------
FC88X_00564_01143 ------------------TCACA-TTCATGTTTGT-GACCG-TGTCTC------
------
FC88X_00666_00708 ------------------TCACA-TTCATGTTTGT-GACCG-TGTCTC------
------
FC88X_00315_00820 ------------------TCACA-TTCATGTTTGT-GACCG-TGTCTC------
------
FC88X_00939_00200 ------------------TCACA-TTCATGTTTGT-GACCG-TGTCTCC-----
------
FC88X_00462_00975 ------------------TCACA-TTCATGTTTGT-GACCG-TGTCTC------
------
FC88X_00515_01032 ------------------TCACA-TTCATGTTTGT-GACCG-TGTCTC------
------
FC88X_00594_01182 ------------------TCACA-TTCATGTTTGT-GACCG-TGTCTC------
------
FC88X_00657_01075 ------------------TCACA-TTCATGTTTGT-GACCG-TGTCTC------
------
FC88X_00727_00780 ------------------TCACA-TTCATGTTTGT-GACCG-TGTCTC------
------
FC88X_00712_00871 ------------------TCACA-TTCATGTTTGT-GACCG-TGTCTC------
------
FC88X_00703_00979 ------------------TCACA-TTCATGTTTGT-GACCG-TGTCTC------
------
FC88X_00842_00263 ------------------TCACA-TTCATGTTTGT-GACCG-TGTCTC------
------
FC88X_00807_01273 ------------------TCACA-TTCATGTTTGT-GACCG-TGTCTC------
------
FC88X_00879_00280 ------------------TCACA-TTCATGTTTGT-GACCG-TGTCTC------
------
FC88X_00858_00699 ------------------TCACA-TTCATGTTTGT-GACCG-TGTCTC------
------
FC88X_00942_00296 ------------------TCACA-TTCATGTTTGT-GACCG-TGTCTC------
------
FC88X_00901_00650 ------------------TCACA-TTCATGTTTGT-GACCG-TGTCTC------
------
FC88X_00902_01149 ------------------TCACA-TTCATGTTTGT-GACCG-TGTCTC------
------
FC88X_00906_01265 ------------------TCACA-TTCATGTTTGT-GACCG-TGTCTC------
------
FC88X_00970_00954 ------------------TCACA-TTCATGTTTGT-GACCG-TGTCTC------
------
FC88X_01021_00356 ------------------TCACA-TTCATGTTTGT-GACCG-TGTCTC------
------
FC88X_01060_00360 ------------------TCACA-TTCATGTTTGT-GACCG-TGTCTC------
------
FC88X_01091_00639 ------------------TCACA-TTCATGTTTGT-GACCG-TGTCTC------
------
FC88X_01123_00715 ------------------TCACA-TTCATGTTTGT-GACCG-TGTCTC------
------
FC88X_01136_01087 ------------------TCACA-TTCATGTTTGT-GACCG-TGTCTC------
------

FC88X_00030_00157 ------------------TCACA-TTCATGTTTGT-GACCG-TGTCTC------
------
FC88X_00049_00400 ------------------TCACA-TTCATGTTTGT-GACCG-TGTCTC------
------
FC88X_00028_00479 ------------------TCACA-TTCATGTTTGT-GACCG-TGTCTC------
------
FC88X_00030_00490 ------------------TCACA-TTCATGTTTGT-GACCG-TGTCTC------
------
FC88X_00005_00516 ------------------TCACA-TTCATGTTTGT-GACCG-TGTCTC------
------
FC88X_00006_00510 ------------------TCACA-TTCATGTTTGT-GACCG-TGTCTC------
------
FC88X_00070_00007 ------------------TCACA-TTCATGTTTGT-GACCG-TGTCTC------
------
FC88X_00060_00103 ------------------TCACA-TTCATGTTTGT-GACCG-TGTCTC------
------
FC88X_00092_00185 ------------------TCACA-TTCATGTTTGT-GACCG-TGTCTC------
------
FC88X_00095_00439 ------------------TCACA-TTCATGTTTGT-GACCG-TGTCTC------
------
FC88X_00078_00473 ------------------TCACA-TTCATGTTTGT-GACCG-TGTCTC------
------
FC88X_00070_00515 ------------------TCACA-TTCATGTTTGT-GACCG-TGTCTC------
------
FC88X_00074_00503 ------------------TCACA-TTCATGTTTGT-GACCG-TGTCTC------
------
FC88X_00078_00524 ------------------TCACA-TTCATGTTTGT-GACCG-TGTCTC------
------
FC88X_00116_00441 ------------------TCACA-TTCATGTTTGT-GACCG-TGTCTC------
------
FC88X_00104_00662 ------------------TCACA-TTCATGTTTGT-GACCG-TGTCTC------
------
FC88X_00147_00837 ------------------TCACA-TTCATGTTTGT-GACCG-TGTCTC------
------
FC88X_00155_00759 ------------------TCACA-TTCATGTTTGT-GACCG-TGTCTC------
------
FC88X_00183_00791 ------------------TCACA-TTCATGTTTGT-GACCG-TGTCTC------
------
FC88X_00188_00808 ------------------TCACA-TTCATGTTTGT-GACCG-TGTCTC------
------
FC88X_00226_00008 ------------------TCACA-TTCATGTTTGT-GACCG-TGTCTC------
------
FC88X_00233_00014 ------------------TCACA-TTCATGTTTGT-GACCG-TGTCTC------
------
FC88X_00233_00045 ------------------TCACA-TTCATGTTTGT-GACCG-TGTCTC------
------
FC88X_00217_00079 ------------------TCACA-TTCATGTTTGT-GACCG-TGTCTC------
------
FC88X_00243_00119 ------------------TCACA-TTCATGTTTGT-GACCG-TGTCTC------
------
FC88X_00229_00562 ------------------TCACA-TTCATGTTTGT-GACCG-TGTCTC------
------
FC88X_00259_00896 ------------------TCACA-TTCATGTTTGT-GACCG-TGTCTC------
------
FC88X_00266_01089 ------------------TCACA-TTCATGTTTGT-GACCG-TGTCTC------
------

FC88X_00270_01111 ------------------TCACA-TTCATGTTTGT-GACCG-TGTCTC------
------
FC88X_00297_01111 ------------------TCACA-TTCATGTTTGT-GACCG-TGTCTC------
------
FC88X_00327_00996 ------------------TCACA-TTCATGTTTGT-GACCG-TGTCTC------
------
FC88X_00330_01054 ------------------TCACA-TTCATGTTTGT-GACCG-TGTCTC------
------
FC88X_00338_01071 ------------------TCACA-TTCATGTTTGT-GACCG-TGTCTC------
------
FC88X_00355_00023 ------------------TCACA-TTCATGTTTGT-GACCG-TGTCTC------
------
FC88X_00356_00500 ------------------TCACA-TTCATGTTTGT-GACCG-TGTCTC------
------
FC88X_00391_00976 ------------------TCACA-TTCATGTTTGT-GACCG-TGTCTC------
------
FC88X_00398_01208 ------------------TCACA-TTCATGTTTGT-GACCG-TGTCTC------
------
FC88X_00442_00040 ------------------TCACA-TTCATGTTTGT-GACCG-TGTCTC------
------
FC88X_00406_00110 ------------------TCACA-TTCATGTTTGT-GACCG-TGTCTC------
------
FC88X_00438_00588 ------------------TCACA-TTCATGTTTGT-GACCG-TGTCTC------
------
FC88X_00401_01194 ------------------TCACA-TTCATGTTTGT-GACCG-TGTCTC------
------
FC88X_00429_01219 ------------------TCACA-TTCATGTTTGT-GACCG-TGTCTC------
------
FC88X_00492_00030 ------------------TCACA-TTCATGTTTGT-GACCG-TGTCTC------
------
FC88X_00474_00570 ------------------TCACA-TTCATGTTTGT-GACCG-TGTCTC------
------
FC88X_00497_00756 ------------------TCACA-TTCATGTTTGT-GACCG-TGTCTC------
------
FC88X_00491_01146 ------------------TCACA-TTCATGTTTGT-GACCG-TGTCTC------
------
FC88X_00466_01238 ------------------TCACA-TTCATGTTTGT-GACCG-TGTCTC------
------
FC88X_00485_01225 ------------------TCACA-TTCATGTTTGT-GACCG-TGTCTC------
------
FC88X_00509_00088 ------------------TCACA-TTCATGTTTGT-GACCG-TGTCTC------
------
FC88X_00530_00192 ------------------TCACA-TTCATGTTTGT-GACCG-TGTCTC------
------
FC88X_00546_00299 ------------------TCACA-TTCATGTTTGT-GACCG-TGTCTC------
------
FC88X_00510_00501 ------------------TCACA-TTCATGTTTGT-GACCG-TGTCTC------
------
FC88X_00509_01135 ------------------TCACA-TTCATGTTTGT-GACCG-TGTCTC------
------
FC88X_00560_00131 ------------------TCACA-TTCATGTTTGT-GACCG-TGTCTC------
------
FC88X_00592_01117 ------------------TCACA-TTCATGTTTGT-GACCG-TGTCTC------
------
FC88X_00604_00137 ------------------TCACA-TTCATGTTTGT-GACCG-TGTCTC------
------

```
FC88X_00640_01266      ------------------TCACA-TTCATGTTTGT-GACCG-TGTCTC------
------
FC88X_00698_00094      ------------------TCACA-TTCATGTTTGT-GACCG-TGTCTC------
------
FC88X_00690_00137      ------------------TCACA-TTCATGTTTGT-GACCG-TGTCTC------
------
FC88X_00740_00998      ------------------TCACA-TTCATGTTTGT-GACCG-TGTCTC------
------
FC88X_00701_01262      ------------------TCACA-TTCATGTTTGT-GACCG-TGTCTC------
------
FC88X_00742_01267      ------------------TCACA-TTCATGTTTGT-GACCG-TGTCTC------
------
FC88X_00793_00099      ------------------TCACA-TTCATGTTTGT-GACCG-TGTCTC------
------
FC88X_00769_00195      ------------------TCACA-TTCATGTTTGT-GACCG-TGTCTC------
------
FC88X_00769_00822      ------------------TCACA-TTCATGTTTGT-GACCG-TGTCTC------
------
FC88X_00783_00936      ------------------TCACA-TTCATGTTTGT-GACCG-TGTCTC------
------
FC88X_00760_01123      ------------------TCACA-TTCATGTTTGT-GACCG-TGTCTC------
------
FC88X_00795_01219      ------------------TCACA-TTCATGTTTGT-GACCG-TGTCTC------
------
FC88X_00831_00762      ------------------TCACA-TTCATGTTTGT-GACCG-TGTCTC------
------
FC88X_00845_00968      ------------------TCACA-TTCATGTTTGT-GACCG-TGTCTC------
------
FC88X_00828_01170      ------------------TCACA-TTCATGTTTGT-GACCG-TGTCTC------
------
FC88X_00897_00655      ------------------TCACA-TTCATGTTTGT-GACCG-TGTCTC------
------
FC88X_00871_00783      ------------------TCACA-TTCATGTTTGT-GACCG-TGTCTC------
------
FC88X_00884_00813      ------------------TCACA-TTCATGTTTGT-GACCG-TGTCTC------
------
FC88X_00895_01080      ------------------TCACA-TTCATGTTTGT-GACCG-TGTCTC------
------
FC88X_00884_01175      ------------------TCACA-TTCATGTTTGT-GACCG-TGTCTC------
------
FC88X_00858_01203      ------------------TCACA-TTCATGTTTGT-GACCG-TGTCTC------
------
FC88X_00924_00240      ------------------TCACA-TTCATGTTTGT-GACCG-TGTCTC------
------
FC88X_00927_00356      ------------------TCACA-TTCATGTTTGT-GACCG-TGTCTC------
------
FC88X_00932_00367      ------------------TCACA-TTCATGTTTGT-GACCG-TGTCTC------
------
FC88X_00935_00428      ------------------TCACA-TTCATGTTTGT-GACCG-TGTCTC------
------
FC88X_00907_00742      ------------------TCACA-TTCATGTTTGT-GACCG-TGTCTC------
------
FC88X_00922_00816      ------------------TCACA-TTCATGTTTGT-GACCG-TGTCTC------
------
FC88X_00924_00830      ------------------TCACA-TTCATGTTTGT-GACCG-TGTCTC------
------
```

FC88X_00940_01199 ------------------TCACA-TTCATGTTTGT-GACCG-TGTCTC------
------
FC88X_00987_00293 ------------------TCACA-TTCATGTTTGT-GACCG-TGTCTC------
------
FC88X_00993_00419 ------------------TCACA-TTCATGTTTGT-GACCG-TGTCTC------
------
FC88X_00999_00516 ------------------TCACA-TTCATGTTTGT-GACCG-TGTCTC------
------
FC88X_00982_00770 ------------------TCACA-TTCATGTTTGT-GACCG-TGTCTC------
------
FC88X_00988_00762 ------------------TCACA-TTCATGTTTGT-GACCG-TGTCTC------
------
FC88X_00967_00822 ------------------TCACA-TTCATGTTTGT-GACCG-TGTCTC------
------
FC88X_00973_00800 ------------------TCACA-TTCATGTTTGT-GACCG-TGTCTC------
------
FC88X_00999_00938 ------------------TCACA-TTCATGTTTGT-GACCG-TGTCTC------
------
FC88X_00985_01001 ------------------TCACA-TTCATGTTTGT-GACCG-TGTCTC------
------
FC88X_00954_01064 ------------------TCACA-TTCATGTTTGT-GACCG-TGTCTC------
------
FC88X_00953_01207 ------------------TCACA-TTCATGTTTGT-GACCG-TGTCTC------
------
FC88X_00989_01227 ------------------TCACA-TTCATGTTTGT-GACCG-TGTCTC------
------
FC88X_01040_00430 ------------------TCACA-TTCATGTTTGT-GACCG-TGTCTC------
------
FC88X_01021_00661 ------------------TCACA-TTCATGTTTGT-GACCG-TGTCTC------
------
FC88X_01029_00811 ------------------TCACA-TTCATGTTTGT-GACCG-TGTCTC------
------
FC88X_01018_00863 ------------------TCACA-TTCATGTTTGT-GACCG-TGTCTC------
------
FC88X_01016_00993 ------------------TCACA-TTCATGTTTGT-GACCG-TGTCTC------
------
FC88X_01035_01015 ------------------TCACA-TTCATGTTTGT-GACCG-TGTCTC------
------
FC88X_01004_01105 ------------------TCACA-TTCATGTTTGT-GACCG-TGTCTC------
------
FC88X_01010_01104 ------------------TCACA-TTCATGTTTGT-GACCG-TGTCTC------
------
FC88X_01034_01134 ------------------TCACA-TTCATGTTTGT-GACCG-TGTCTC------
------
FC88X_01038_01230 ------------------TCACA-TTCATGTTTGT-GACCG-TGTCTC------
------
FC88X_01076_00724 ------------------TCACA-TTCATGTTTGT-GACCG-TGTCTC------
------
FC88X_01085_00753 ------------------TCACA-TTCATGTTTGT-GACCG-TGTCTC------
------
FC88X_01062_00946 ------------------TCACA-TTCATGTTTGT-GACCG-TGTCTC------
------
FC88X_01059_00968 ------------------TCACA-TTCATGTTTGT-GACCG-TGTCTC------
------
FC88X_01081_00958 ------------------TCACA-TTCATGTTTGT-GACCG-TGTCTC------
------

FC88X_01120_00505 ------------------TCACA-TTCATGTTTGT-GACCG-TGTCTC------
------
FC88X_01116_00606 ------------------TCACA-TTCATGTTTGT-GACCG-TGTCTC------
------
FC88X_01126_00672 ------------------TCACA-TTCATGTTTGT-GACCG-TGTCTC------
------
FC88X_01114_00875 ------------------TCACA-TTCATGTTTGT-GACCG-TGTCTC------
------
FC88X_01117_00867 ------------------TCACA-TTCATGTTTGT-GACCG-TGTCTC------
------
FC88X_01113_00919 ------------------TCACA-TTCATGTTTGT-GACCG-TGTCTC------
------
FC88X_01131_00911 ------------------TCACA-TTCATGTTTGT-GACCG-TGTCTC------
------
FC88X_01109_01124 ------------------TCACA-TTCATGTTTGT-GACCG-TGTCTC------
------
FC88X_01112_01144 ------------------TCACA-TTCATGTTTGT-GACCG-TGTCTC------
------
FC88X_01142_01131 ------------------TCACA-TTCATGTTTGT-GACCG-TGTCTC------
------
FC88X_01116_01150 ------------------TCACA-TTCATGTTTGT-GACCG-TGTCTC------
------
FC88X_00463_00048 ------------------TCACA-TTCATGTTTTGTGACCG-TGTCTC------
------
FC88X_01072_00784 ------------------TCACA-TTCATGTTTTGTGACCG-TGTCTC------
------
FC88X_00065_00322 ------------------TCACA-TTCATGTTTTGTGACCG-TGTCTC------
------
FC88X_00438_01192 ------------------TCACA-TTCATGTTTTGTGACCG-TGTCTC------
------
FC88X_00567_00106 ------------------TCACA-TTCATGTTTTGTGACCG-TGTCTC------
------
FC88X_00822_00240 ------------------TCACA-TTCATGTTTTGTGACCG-TGTCTC------
------
FC88X_00550_01141 ------------------TCACA-TTCATGTTTGT-GACCG-TGTCCC------
------
FC88X_00906_00540 ------------------TCACA-TTCATGTTTGT-GACCG-TGTC--------
------
FC88X_00442_00519 ------------------TCACA-TTCATGTTTGT-GACCG-TGTCTCCC----
------
FC88X_00487_01224 ------------------TCACA-TTCATGTTTGT-GACCG-TGTCTCCC----
------
FC88X_00371_01070 ------------------TCACA-TTCATGCTTGT-GACCG-TGTCTC------
------
FC88X_00875_00563 ------------------TCACA-TTCATGCTTGT-GACCG-TGTCTC------
------
FC88X_00929_00389 ------------------TCACA-TTCATGCTTGT-GACCG-TGTCTC------
------
FC88X_01092_01171 ------------------TCACA-TTCATGCTTGT-GACCG-TGTCTC------
------
FC88X_00497_00134 ------------------TCACA-TTCATGTTTGT-GACCG-TATCTC------
------
FC88X_00923_00376 ------------------TCACAATTCATGTTTGT-GACCG-TGTCTC------
------
FC88X_00275_00481 ------------------TCACA-TTCATGTTCGT-GACCG-TGTCTC------
------

FC88X_00284_00547 ------------------TCACA-TTCATGTTCGT-GACCG-TGTCTC------
------
FC88X_00789_01228 ------------------TCACA-TTCATGTTGGT-GACCG-TGTCTC------
------
FC88X_01142_00663 ------------------TCACA-TTCGTGTTTGT-GACCG-TGTCTC------
------
FC88X_00575_00229 ------------------TCACATTCA-CGTTTGT-GACCG-TGTCTC------
------
FC88X_00909_00209 -----------GGGGAAGTCACATTCATCGTTTGT-GACCG-TGTCTC------
------
FC88X_00925_00970 -----------------GTCACATTCA-TGTTTGT-GACTG-TGTCTC------
------
FC88X_00795_01259 -----------------GTCACATTCA-TGTTTGT-GATCG-TGTCTC------
------
FC88X_00192_00375 ------------------TCACA-TTTATGTTTGT-GACCG-TGTCTC------
------
FC88X_00450_00394 ------------------TCACA-TTTATGTTTGT-GACCG-TGTCTC------
------
FC88X_00803_00309 -----------GGGGAAGTCACA-TTCATGTTTGT-GACCGTCGTCTC------
------
FC88X_00897_00340 ------------------TCACA-TTCATGTTTGT-GACCGTCGTCTC------
------
FC88X_00482_01122 ------------------TCACA-TTCATGTTTGT-GACCG-CGTCTC------
------
FC88X_00581_01059 ------------------TCACA-TTCATGTTTGT-GACCG-CGTCTC------
------
FC88X_01075_00537 ------------------CCACG-TTCATGTTTGT-GACCG-TGTCTC------
------
FC88X_01056_00756 ------------------TCACG-TTCATGTTTGT-GACCG-TGTCTC------
------
FC88X_00455_00894 ------------------TCACA-TTCATGTTTGT-GACCG-
TGTCTCTTTCCTCTCC--
FC88X_00893_00590 ------------------TCACA-CTCATGTTTGT-GACCG-TGTCTC------
------
FC88X_00989_00349 -----------------GTCACA-CTCATGTTTGT-GACCG-TGTCTC------
------
FC88X_00263_00064 ------------------TCACA-CTCATGTTTGTTGACCG-
TGTCTCCTTCCTTT----
FC88X_00204_00144 ------------------TCACA-TTCATGTTTGT-GACCG-
TGTCTCCTTCCTTT----
FC88X_01072_00404 ------------------TCACA-TTCATGTTTGT-GACCG-
TGTCTCCTTCCTTT----
FC88X_01142_01062 ------------------TCACA-TTCATGTTTGT-GACCG-
TGTCTCCTTCCTTT----
FC88X_00899_00290 ------------------TCACA-TTC-TGTTTGT-GACCG-
TGTCTCCTTCCTT-----
FC88X_00817_01121 ------------------TCACA-TTCATGTTTGT-GACCG-
TGTCTTCCTTCCTTT---
FC88X_00816_00969 ------------------TCACA-TTCATGTTT-GTGGCCG-TGTCTC------
------
FC88X_00070_00410 ------------------TCACA-TTCATGTTT-GTGGCCG-TGTCTC------
------
FC88X_00483_00216 ------------------TCACA-TTCATGTTT-GTGGCCG-TGTCTC------
------
FC88X_00933_00419 ------------------TCACA-TTCATGTTT-GTGGCCG-TGTCTC------
------

FC88X_00970_01081 ------------------TCACA-TTCATGTTT-GTGGCCG-TGTCTC------
------
FC88X_00769_01209 ------------------TCACA-TTCATGTTT-GTGGCCA-TGTCTC------
------
FC88X_00476_01197 -----------------GTCACA-TTCATGTGT-GTGGCCG-TGTCTC------
------
FC88X_00910_00404 ------------------TCACA-TTCATGTTT-GTGGCCG-TGTCTTC-----
------
FC88X_00921_00556 -----------------GTCGCA-TTCATGTTTTGTGGCCG-TGTCTC------
------
FC88X_00765_01106 ------------------TCGCA-TTCATGTTT-GTGACCG-TGTCTC------
------
FC88X_00598_00923 ------------------TCACA-TTCATGTTTGT-GACCGTTGCTC-------
------
FC88X_00044_00211 ------------------TCACA-TTCATGTTTGT-G-ACG-T-----------
------
FC88X_00977_00276 ------------------TCACA-TTCATGTTTGT-GCACG-TGTCTC------
------
FC88X_00308_00332 ------------------TCACA-TTCATGTTTGT-GACCG-TGTCCTC-----
------
FC88X_00423_00711 ------------------TCACA-TTCATGTCTGT-GACCG-TGTCTCTCC---
------
FC88X_00813_00331 ------------------TCACA-TTCATGTCTGT-GACCG-CGTCTC------
------
FC88X_00208_00013 ------------------TCACA-TTCATGTTTTGTGGCCA-------------
------
FC88X_00576_00087 -----------------TCCACA-TTC-TGTTTTGTGACCG-
TGTCTCCTTCCTTTTCT-
FC88X_00553_01084 ------------------TCACTATTCATGTTC---TAGTGCTA----------
------
FC88X_00847_00256 ------------------TCACTATCAAGTTTTGT-TAGTGCCTCCT-------
------
FC88X_00632_00171 ---------------CGTGCC-GTTTTGGGT---CGTCCTGTTTGT--------
------
FC88X_00590_00087 ----------------TTGCCAGTCTTGTGTG--CGCGCTGTTCT---------
------
FC88X_00047_00330 ------------------TTCAGCCTTATAGG-TCGTCCTGTTCTA--------
------
FC88X_00004_00419 ------------------TTCAGCCTTATAGG-TCGTCCTGTTCTA--------
------
FC88X_00076_00336 ------------------TTCAGCCTTATAGG-TCGTCCTGTTCTA--------
------
FC88X_00147_00668 ------------------TTCAGCCTTATAGG-TCGTCCTGTTCTA--------
------
FC88X_00160_00801 ------------------TTCAGCCTTATAGG-TCGTCCTGTTCTA--------
------
FC88X_00173_00961 ------------------TTCAGCCTTATAGG-TCGTCCTGTTCTA--------
------
FC88X_00231_00974 ------------------TTCAGCCTTATAGG-TCGTCCTGTTCTA--------
------
FC88X_00307_00005 ------------------TTCAGCCTTATAGG-TCGTCCTGTTCTA--------
------
FC88X_00313_01092 ------------------TTCAGCCTTATAGG-TCGTCCTGTTCTA--------
------
FC88X_00383_00639 ------------------TTCAGCCTTATAGG-TCGTCCTGTTCTA--------
------

```
FC88X_00442_01194       ------------------TTCAGCCTTATAGG-TCGTCCTGTTCTA--------
------
FC88X_00475_00032       ------------------TTCAGCCTTATAGG-TCGTCCTGTTCTA--------
------
FC88X_00477_00166       ------------------TTCAGCCTTATAGG-TCGTCCTGTTCTA--------
------
FC88X_00636_01101       ------------------TTCAGCCTTATAGG-TCGTCCTGTTCTA--------
------
FC88X_00686_01035       ------------------TTCAGCCTTATAGG-TCGTCCTGTTCTA--------
------
FC88X_00745_01239       ------------------TTCAGCCTTATAGG-TCGTCCTGTTCTA--------
------
FC88X_00780_00126       ------------------TTCAGCCTTATAGG-TCGTCCTGTTCTA--------
------
FC88X_00819_00747       ------------------TTCAGCCTTATAGG-TCGTCCTGTTCTA--------
------
FC88X_00842_00886       ------------------TTCAGCCTTATAGG-TCGTCCTGTTCTA--------
------
FC88X_00836_01266       ------------------TTCAGCCTTATAGG-TCGTCCTGTTCTA--------
------
FC88X_00893_01213       ------------------TTCAGCCTTATAGG-TCGTCCTGTTCTA--------
------
FC88X_00918_00909       ------------------TTCAGCCTTATAGG-TCGTCCTGTTCTA--------
------
FC88X_01112_00835       ------------------TTCAGCCTTATAGG-TCGTCCTGTTCTA--------
------
FC88X_01141_01027       ------------------TTCAGCCTTATAGG-TCGTCCTGTTCTA--------
------
FC88X_00992_00537       ------------------TTCAGCCTTATAG--TCGTCCTGTTCTA--------
------
FC88X_00916_01005       ------------------TTCAGCCTTATAGG-TCGTCCTGTCCTA--------
------
FC88X_01077_00891       ------------------TTCAGCCTTATAGG-TCGTCCTGTTCTC--------
------
FC88X_00854_00945       -----------------GTTCAGCCTAGTAGGATCGTCCTGTTCT---------
------
FC88X_00596_00197       --------------------TGCTCTC-TAGTTCGTGCCTGTTCGTC-------
------
FC88X_00935_00238       --------------------TGCGCTCACAGTTGGCGTCCTTTTTTC-------
------
FC88X_00672_00701       ------------------------CCTACACCCTGATCGTGCTATTTTATC---
------
FC88X_00073_00006       ------------------------CCTACACCCTGATCGTGCTATTTTATC---
------
FC88X_00879_00374       ---------------------CCGTCTACTCCCTGA-CGTGT--TTTTATC---
------
FC88X_01121_00787       -----------------CCACTGCATCATCTCCTT--CGCGCTTTT--------
------
FC88X_00772_01233       -----------------CCACTGCATCATCTCCTT--CGCGCTTTT--------
------
FC88X_01088_00844       -----------------CCACTGCATCATCTCCTT--CGCGCTTTT--------
------
FC88X_01026_00363       -----------------CCACTGCATCATCTCCTT--CGTGCTTTT--------
------
FC88X_01030_00990       ---------------------TGTCTCATCTCCCTGTCGTGCATTTCT------
------
```

FC88X_00720_01002 --------------------TCTTGTTCTGTGCATT-CCCTACTTATT------
------
FC88X_00307_00187 --------------------TCTTGTTCTGTGCATT-CCCTACTTATT------
------
FC88X_00073_00734 --------------------CTTTCTTGTTCGTGCC-TTTACCCTATT------
------
FC88X_00307_00888 ---------------------CGTCTCGTTGGCGTGTCTTCTCCTACT------
------
FC88X_00992_00565 --------------------CGTTCTCTTTCGCGTG-CTTGACCTACT------
------
FC88X_00019_00509 ------------------CTCAGCTT--TTGTT-GCATGTGATTTCTC------
------
FC88X_00741_00971 -----------------GCTCAGCTT--TTGTT-GCATGTGATTTCTC------
------
FC88X_00140_00587 ------------------CTCAGCTT--TTGTT-GCATGTGATTTCTC------
------
FC88X_00249_00925 ------------------CTCAGCTT--TTGTT-GCATGTGATTTCTC------
------
FC88X_00503_01184 ------------------CTCAGCTT--TTGTT-GCATGTGATTTCTC------
------
FC88X_00755_01068 ------------------CTCAGCTT--TTGTT-GCATGTGATTTCTC------
------
FC88X_00924_00659 ------------------CTCAGCTT--TTGTT-GCATGTGATTTCTC------
------
FC88X_01049_01197 ------------------CTCAGCTT--TTGTT-GCATGTGATTTCTC------
------
FC88X_01096_00612 ------------------CTCAGCTT--TTGTT-GCATGTGATTTCTC------
------
FC88X_00021_00252 ------------------CTCAGCTT--TTGTT-GCATGTGATTTCTC------
------
FC88X_00087_00635 ------------------CTCAGCTT--TTGTT-GCATGTGATTTCTC------
------
FC88X_00125_00625 ------------------CTCAGCTT--TTGTT-GCATGTGATTTCTC------
------
FC88X_00152_00657 ------------------CTCAGCTT--TTGTT-GCATGTGATTTCTC------
------
FC88X_00320_00038 ------------------CTCAGCTT--TTGTT-GCATGTGATTTCTC------
------
FC88X_00408_01060 ------------------CTCAGCTT--TTGTT-GCATGTGATTTCTC------
------
FC88X_00647_01110 ------------------CTCAGCTT--TTGTT-GCATGTGATTTCTC------
------
FC88X_00940_00585 ------------------CTCAGCTT--TTGTT-GCATGTGATTTCTC------
------
FC88X_00945_00694 ------------------CTCAGCTT--TTGTT-GCATGTGATTTCTC------
------
FC88X_00966_00903 ------------------CTCAGCTT--TTGTT-GCATGTGATTTCTC------
------
FC88X_00970_00999 ------------------CTCAGCTT--TTGTT-GCATGTGATTTCTC------
------
FC88X_00972_01251 ------------------CTCAGCTT--TTGTT-GCATGTGATTTCTC------
------
FC88X_01037_00522 ------------------CTCAGCTT--TTGTT-GCATGTGATTTCTC------
------
FC88X_01030_00753 ------------------CTCAGCTT--TTGTT-GCATGTGATTTCTC------
------

FC88X_01145_00875 ------------------CTCAGCTT--TTGTT-GCATGTGATTTCTC------
------
FC88X_01038_00733 -----------GGGGAAGCTCAGCTT--TTGTTTGCATGTGATTTCTC------
------
FC88X_00618_00688 ------------------CTCAGCTT--TTGCT-GCATGTGATTTCTC------
------
FC88X_00137_00524 ------------------CTCAGCTT--TTGTT-GCATGTGATTTCTCC-----
------
FC88X_00949_00719 ------------------CTCAGCTC--TTGTT-GCATGTGATTTCTC------
------
FC88X_01080_00969 ------------------CTCAGCTC--TTGTT-GCATGTGATTTCTC------
------
FC88X_00123_00454 ------------------CTCAGCTT--TTTGTTGCATGTGATTTCTCC-----
------
FC88X_00914_01072 ------------------CTCAGCTT--TTTGTTGCATGTGATT----------
------
FC88X_00043_00404 ------------------CTCAGCTT--TTGTT-GCATGTGGTTTCTC------
------
FC88X_00953_00454 ------------------CTCAGCTT--TTGTT-GCACGTGATTTCTC------
------
FC88X_00398_00015 ------------------CTCAGCTT--TTGTT-
GCATGTGATTTCTCCTTCCTTT----
FC88X_00302_00044 ------------------CTCAGCTT--TCGTT-GCATGTGATTTCTC------
------
FC88X_00369_00829 ------------------CTCAGCTT--TCGTT-GCATGTGATTTCTC------
------
FC88X_00857_01214 ------------------CTCAGCTT--TTGTT-GCATGTGACTTCTC------
------
FC88X_01101_00468 ------------------CTCAGCTT--TTGTT-GCATGTGATTCTC-------
------
FC88X_00694_00226 ------------------CACTGCTG--TTGTT-GCAGTTGACCTCTT------
------
FC88X_01093_00862 ------------------CACTGCTG--TTGTT-GCAGTTGACCTCTT------
------
FC88X_00611_00971 ------------------CACTGCTG--TTGTT-GCAGTTGACCTCTT------
------
FC88X_00708_01231 ------------------CACTGCTG--TTGTT-GCAGTTGACCTCTT------
------
FC88X_00822_00102 ------------------CACTGCTG--TTGTT-GCAGTTGACCTCTT------
------
FC88X_00172_00013 ------------GGGGAACACTGCTG--TTGTT-GCAGTTGACCTCTTCTTCC-
------
FC88X_00880_00408 ------------------CGCTTCTG--TTGTT-GCAGTTGACCTCTT------
------
FC88X_00125_00265 -------------------CCAGCAG--TTGTCTGCCGTTGAGTTCTC------
------
FC88X_00849_00332 -------------------CCAGCAG--TTGTCTGCCGTTGAGTTCTC------
------
FC88X_00806_01227 -------------------CCAGCAG--TTGTCTGCCGTTGAGTTCTC------
------
FC88X_00064_00424 -------------------CCAGCAG--TTGTCTGCCGTTGAGTTCTC------
------
FC88X_00158_00895 -------------------CCAGCAG--TTGTCTGCCGTTGAGTTCTC------
------
FC88X_00500_00595 -------------------CCAGCAG--TTGTCTGCCGTTGAGTTCTC------
------

FC88X_00778_01140 -------------------CCAGCAG--TTGTCTGCCGTTGAGTTCTC------
------
FC88X_00825_00937 -------------------CCAGCAG--TTGTCTGCCGTTGAGTTCTC------
------
FC88X_00865_00501 -------------------CCAGCAG--TTGTCTGCCGTTGAGTTCTC------
------
FC88X_01026_00978 -------------------CCAGCAG--TTGTCTGCCGTTGAGTTCTC------
------
FC88X_00525_00160 --------------------CAGCAG--TTGTCTGCCGTTGAGTTCTC------
------
FC88X_01093_00570 --------------------CAGCAG--TTGTCTGCCGTTGAGTTCTC------
------
FC88X_00986_00543 -------------------CCAGCAG--TTGTCTGCCGTTGAATTCTC------
------
FC88X_00150_00326 -------------------CCAGCAG--
TTGTCTGCCGTTGAGTTCTCCTTCTTTCTCC-
FC88X_00160_01028 -------------------CCAGCAACTTTGTCTGCCGTTGAGTTCTCC-----
------
FC88X_00338_00247 -------------------CCAGCA--TTTGTCTGCCGTTGAGTTCTC------
------
FC88X_00583_01212 -------------------CCAGCAG--
TTGTGCGCCGTTGAGTTCTCCTTCCTTT----
FC88X_00140_00565 -------------------CCACCTG---TTTTTGTGGTTGAGCTATCTC----
------
FC88X_00035_00086 -------------------CCACCTG---TTTTTGTGGTTGAGCTATCTC----
------
FC88X_00921_00332 -------------------CCACCTG---TTTTTGTGGTTGAGCTATCTC----
------
FC88X_00118_00131 -------------------CCACCTG---TTTT-GTGGTTGAGCTATCTC----
------
FC88X_00152_00932 -------------------CCACCTG---TTTT-GTGGTTGAGCTATCTC----
------
FC88X_00200_01053 -------------------CCACCTG---TTTT-GTGGTTGAGCTATCTC----
------
FC88X_00362_01028 -------------------CCACCTG---TTTT-GTGGTTGAGCTATCTC----
------
FC88X_00497_00544 -------------------CCACCTG---TTTT-GTGGTTGAGCTATCTC----
------
FC88X_00543_01004 -------------------CCACCTG---TTTT-GTGGTTGAGCTATCTC----
------
FC88X_00594_00993 -------------------CCACCTG---TTTT-GTGGTTGAGCTATCTC----
------
FC88X_00560_01191 -------------------CCACCTG---TTTT-GTGGTTGAGCTATCTC----
------
FC88X_00822_00124 -------------------CCACCTG---TTTT-GTGGTTGAGCTATCTC----
------
FC88X_00959_00753 -------------------CCACCTG---TTTT-GTGGTTGAGCTATCTC----
------
FC88X_01012_00692 -------------------CCACCTG---TTTT-GTGGTTGAGCTATCTC----
------
FC88X_01033_01176 -------------------CCACCTG---TTTT-GTGGTTGAGCTATCTC----
------
FC88X_01138_00644 -------------------CCACCTG---TTTT-GTGGTTGAGCTATCTC----
------
FC88X_01146_00741 -------------------CCACCTG---TTTT-GTGGTTGAGCTATCTC----
------

```
FC88X_00098_00319      -------------------CCACCTG---TTTT-GTGGTTGAGCTATCTC----
------
FC88X_00081_00372      -------------------CCACCTG---TTTT-GTGGTTGAGCTATCTC----
------
FC88X_00135_00800      -------------------CCACCTG---TTTT-GTGGTTGAGCTATCTC----
------
FC88X_00186_01039      -------------------CCACCTG---TTTT-GTGGTTGAGCTATCTC----
------
FC88X_00263_01072      -------------------CCACCTG---TTTT-GTGGTTGAGCTATCTC----
------
FC88X_00322_00502      -------------------CCACCTG---TTTT-GTGGTTGAGCTATCTC----
------
FC88X_00353_00634      -------------------CCACCTG---TTTT-GTGGTTGAGCTATCTC----
------
FC88X_00423_01191      -------------------CCACCTG---TTTT-GTGGTTGAGCTATCTC----
------
FC88X_00529_00101      -------------------CCACCTG---TTTT-GTGGTTGAGCTATCTC----
------
FC88X_00537_00143      -------------------CCACCTG---TTTT-GTGGTTGAGCTATCTC----
------
FC88X_00551_00844      -------------------CCACCTG---TTTT-GTGGTTGAGCTATCTC----
------
FC88X_00602_01148      -------------------CCACCTG---TTTT-GTGGTTGAGCTATCTC----
------
FC88X_00780_00862      -------------------CCACCTG---TTTT-GTGGTTGAGCTATCTC----
------
FC88X_00750_00977      -------------------CCACCTG---TTTT-GTGGTTGAGCTATCTC----
------
FC88X_00814_00261      -------------------CCACCTG---TTTT-GTGGTTGAGCTATCTC----
------
FC88X_00931_00759      -------------------CCACCTG---TTTT-GTGGTTGAGCTATCTC----
------
FC88X_00961_01096      -------------------CCACCTG---TTTT-GTGGTTGAGCTATCTC----
------
FC88X_01006_00974      -------------------CCACCTG---TTTT-GTGGTTGAGCTATCTC----
------
FC88X_01130_00788      -------------------CCACCTG---TTTT-GTGGTTGAGCTATCTC----
------
FC88X_01135_01176      -------------------CCACCTG---TTTT-GTGGTTGAGCTATCTC----
------
FC88X_00583_00142      ------------------GCCACCTG---TTTT-GTGGTTGAGCTATCTC----
------
FC88X_00995_01269      ------------------GCCACCTG---TTTT-GTGGTTGAGCTATCTC----
------
FC88X_00601_01237      -------------------CCACCTG---CTTT-GTGGTTGAGCTATCTC----
------
FC88X_00981_00966      -------------------CCACCTG---CTTT-GTGGTTGAGCTATCTC----
------
FC88X_00009_00245      -------------------CCACCTG---TTTT-GTGGTTGAGCTATCTCCC--
------
FC88X_00796_00928      -------------------CCACCTG---TTTT-GTGGTTGAGCTATCTCCC--
------
FC88X_00067_00016      -------------------CGACCTG---TTTT-GTGGTTGAGCTATCTC----
------
FC88X_00864_01177      -------------------CCACCTG---TTTT-GTGGTTGAGCTATCTT----
------
```

FC88X_00980_00636 --------------------CCACCTG---TTTT-
GTGGTTGAGCTATCTCCTTCCTTT--
FC88X_00192_00987 --------------------CCACCTA---TTTT-GTGGTTGAGCTATCTC----
------
FC88X_00637_01134 --------------------CCACCTG---TTTT-GTGGTTGAGCTGTCTC----
------
FC88X_00269_00940 ---------------------CACCTG---TTTTCGTGGTTGAGCTATCTC----
------
FC88X_01095_00619 --------------------TCACCTG---TTTT-GTGGTTGAGCTATCTC----
------
FC88X_00616_00661 ---------------------CACCTG---TCTT-GTGGTTGAGCTATCTC----
------
FC88X_00043_00058 --------------------CCACCTG---TTT--
GTGGTTGAGCTATCTCCTTCCTTCTC
FC88X_00937_00676 --------------------CCACCCT---GTTTTGTGGTTGAGCTATCTC----
------
FC88X_00712_01251 --------------------CCACATC---TTTT--CGGGTGCTATGTCCTC---
------
FC88X_01049_00487 --------------------CCAGCTT---GTTT-GCAGTGATGCCTTCTC----
------
FC88X_00032_00793 --------------------CCAGCTT---GTTT-GCAGTGATGCCTTCTC----
------
FC88X_00109_00231 --------------------CCAGCTT---GTTT-GCAGTGATGCCTTCTC----
------
FC88X_00126_00643 --------------------CCAGCTT---GTTT-GCAGTGATGCCTTCTC----
------
FC88X_01100_01047 --------------------CCAGCTT---GTTT-
GCAGTGATGCCTTCTCCTTCCTTTC-
FC88X_00468_00984 --------------------CCAGCTT---GTTT-GCAGTGATGCTTTCTC----
------
FC88X_00283_00960 --------------------CCAGCTT---GTTT-GCAGTGGTGCCTTCTC----
------
FC88X_00596_00185 --------------------CCAGCTT---GTTT-GCAGTGTTGCCTTCTC----
------
FC88X_00042_00538 --------------------CCAGCTT---GTTT-GCAGCGATGCCTTCTC----
------
FC88X_00921_00343 --------------------ACCCCAC---GTTG-TTTGTGATGCTTTCTC----
------
FC88X_00138_00710 --------------------CTAGCATATTTGTT--TTGTGGGCCTTCT------
------
FC88X_00375_01199 --------------------CTAGCATACTTGTT--TTGTGGGCCTTCT------
------
FC88X_00686_01144 --------------------CCATCATATGTGGT--TATTTATCCTTACGC----
------
FC88X_00627_01240 ----------------------CACTCGTATGTTGAAACTCCCTGTAAC------
------
FC88X_01094_01187 ----------------------CACCCGTATGTTGAAACTCCCTGTAAC------
------
FC88X_00836_00855 ------------------ACTACGTTTATGTTGTCATTCTTCCTGTAAGCCTGG-
------
FC88X_00931_00802 -----------------CACCAAGCACGTGTATTGTCATCCCTC-----------
------
FC88X_00954_01076 --------------------GCCATTGCATAATGTGTG-CAGCTCTTCTC-----
------
FC88X_00086_00520 --------------------GCCATTGCATAATGTGTG-CAGCTCTTCTC-----
------

```
FC88X_00071_00093      --------------------CCATTGCATAATGTGTG-CAGCTCTTCTC-----
------
FC88X_00161_00568      --------------------CCATTGCATAATGTGTG-CAGCTCTTCTC-----
------
FC88X_00430_00112      --------------------CCATTGCATAATGTGTG-CAGCTCTTCTC-----
------
FC88X_00694_01029      --------------------CCATTGCATAATGTGTG-CAGCTCTTCTC-----
------
FC88X_00655_01114      --------------------CCATTGCATAATGTGTG-CAGCTCTTCTC-----
------
FC88X_00740_00221      --------------------CCATTGCATAATGTGTG-CAGCTCTTCTC-----
------
FC88X_00902_00239      --------------------CCATTGCATAATGTGTG-CAGCTCTTCTC-----
------
FC88X_00946_00663      --------------------CCATTGCATAATGTGTG-CAGCTCTTCTC-----
------
FC88X_00018_00097      --------------------CCATTGCATAATGTGTG-CAGCTCTTCTC-----
------
FC88X_00052_00202      --------------------CCATTGCATAATGTGTG-CAGCTCTTCTC-----
------
FC88X_00053_00754      --------------------CCATTGCATAATGTGTG-CAGCTCTTCTC-----
------
FC88X_00175_00600      --------------------CCATTGCATAATGTGTG-CAGCTCTTCTC-----
------
FC88X_00229_00097      --------------------CCATTGCATAATGTGTG-CAGCTCTTCTC-----
------
FC88X_00220_00488      --------------------CCATTGCATAATGTGTG-CAGCTCTTCTC-----
------
FC88X_00232_01012      --------------------CCATTGCATAATGTGTG-CAGCTCTTCTC-----
------
FC88X_00347_00563      --------------------CCATTGCATAATGTGTG-CAGCTCTTCTC-----
------
FC88X_00351_01170      --------------------CCATTGCATAATGTGTG-CAGCTCTTCTC-----
------
FC88X_00370_01167      --------------------CCATTGCATAATGTGTG-CAGCTCTTCTC-----
------
FC88X_00447_01160      --------------------CCATTGCATAATGTGTG-CAGCTCTTCTC-----
------
FC88X_00404_01213      --------------------CCATTGCATAATGTGTG-CAGCTCTTCTC-----
------
FC88X_00469_00689      --------------------CCATTGCATAATGTGTG-CAGCTCTTCTC-----
------
FC88X_00586_01106      --------------------CCATTGCATAATGTGTG-CAGCTCTTCTC-----
------
FC88X_00600_00173      --------------------CCATTGCATAATGTGTG-CAGCTCTTCTC-----
------
FC88X_00601_01160      --------------------CCATTGCATAATGTGTG-CAGCTCTTCTC-----
------
FC88X_00723_00384      --------------------CCATTGCATAATGTGTG-CAGCTCTTCTC-----
------
FC88X_00701_00994      --------------------CCATTGCATAATGTGTG-CAGCTCTTCTC-----
------
FC88X_00795_01183      --------------------CCATTGCATAATGTGTG-CAGCTCTTCTC-----
------
FC88X_00859_00968      --------------------CCATTGCATAATGTGTG-CAGCTCTTCTC-----
------
```

FC88X_00891_01059 --------------------CCATTGCATAATGTGTG-CAGCTCTTCTC-----
------
FC88X_00926_00183 --------------------CCATTGCATAATGTGTG-CAGCTCTTCTC-----
------
FC88X_00928_01196 --------------------CCATTGCATAATGTGTG-CAGCTCTTCTC-----
------
FC88X_00963_00404 --------------------CCATTGCATAATGTGTG-CAGCTCTTCTC-----
------
FC88X_00977_01036 --------------------CCATTGCATAATGTGTG-CAGCTCTTCTC-----
------
FC88X_01067_00896 --------------------CCATTGCATAATGTGTG-CAGCTCTTCTC-----
------
FC88X_01077_01077 --------------------CCATTGCATAATGTGTG-CAGCTCTTCTC-----
------
FC88X_00443_00098 --------------------CCATTGCATAATGTGTG-CAGCTCTTCTCCC---
------
FC88X_00490_01143 --------------------CCATTGCATAATGTGTG-CAGCTCTTCTCCC---
------
FC88X_00675_00188 --------------------CTATTGCATAATGTGTG-CAGCTCTTCTC-----
------
FC88X_00308_00306 --------------------CCATTGCATAATGTGTG-CAGCTCTTCTT-----
------
FC88X_00911_01019 --------------------CCATTGCATAATGTGTG-CAGCTCTTCCC-----
------
FC88X_00880_01036 --------------------CCATTGCGTAATGTGTG-CAGCTCTTCTC-----
------
FC88X_00858_01082 -------------------GCCATCGCATAATGTGTG-CAGCTCTTCTC-----
------
FC88X_00780_01239 --------------------CCATCGCATAATGTGTG-CAGCTCTTCTC-----
------
FC88X_00105_00457 --------------------CCATCGCATAATGTGTG-CAGCTCTTCTC-----
------
FC88X_01077_00673 --------------------CCATCGCATAATGTGTG-
CAGCTCTTCTCCTTCCTTT---
FC88X_00997_01129 --------------------CCATTGCATAATGTGTG-TAGCGCTTCTC-----
------
FC88X_01118_01207 ---------------------CATCGAATATTATGT--CCGCTTTTCT------
------
FC88X_00458_00792 ------------------------TTGTCTC-ATTGTGTCAGCTTATCTCTC---
------
FC88X_00723_01009 -----------------------GTTGTCTC-ATTGTGTCAGCTTATCTCTC---
------
FC88X_00619_00541 --------------------TCTTTGTCTCTAATGCGGCAGCTGTTCTCT----
------
FC88X_00435_00141 ---------------------TGTGGTGCAATTGT-GCCG-CCTATCTGC----
------
FC88X_00535_01241 -----------------------TTGGTGCTTTTGTAGCCATCCTATCTC-----
------
FC88X_00611_01140 ---------------------GCGTGTACTTTTGTCGCCACCTCATCTC-----
------
FC88X_00672_00424 -----------------------AGTGGTACTTTGTCGCCACCTCATCTC-----
------
FC88X_00073_00108 --------------------TCATGTGTTCAATCATCTCGGCTTTCT-------
------
FC88X_00177_00121 --------------------TCATGTGTTCAATCATCTCGGCTTTCT-------
------

```
FC88X_00865_00733      --------------------TCATGTGTTCAATCATCTCGGCTTTCT-------
------
FC88X_00925_00529      --------------------TCATGTGTTCAATCATCTCGGCTTTCT-------
------
FC88X_00928_00484      --------------------TCATGTGTTCAATCATCTCG-CTTTCT-------
------
FC88X_00568_00699      ---------------------CTCGGTGTCATTTGTATCCTCCCGCTTT-----
------
FC88X_00210_00979      ------------------CGGCACGTTTTTGTTTTTCCCGCCTTACT-------
------
FC88X_00649_01083      ------------------CGGCACGTTTTATTTTTTCCCGCCTTACT-------
------
FC88X_00792_00174      ---------------------TGTCTCAGTTTCGCGCAGA-ACTTTCTC-----
------
FC88X_01032_00874      ---------------------TGTCTCAGTTTCGCGCAGA-ACTTTCTC-----
------
FC88X_00008_00458      ---------------------TGTCTCAGTTTCGCGCAGA-ACTTTCTC-----
------
FC88X_00055_00190      ---------------------TGTCTCAGTTTCGCGCAGA-ACTTTCTC-----
------
FC88X_00442_00822      ---------------------TGTCTCAGTTTCGCGCAGA-ACTTTCTC-----
------
FC88X_01086_00760      ---------------------TGTCTCAGTTTCGCGCAGA-ACTTTCTC-----
------
FC88X_01128_00528      ---------------------TGTCTCAGTTTCGCGCAGA-ACTTTCTC-----
------
FC88X_00194_00815      ---------------------TGTCTCAGTTTCGCGCAGA-ACTTTCTC-----
------
FC88X_00482_00322      ---------------------TGTCTCAGTTTCGTGCAGA-ACTTTCTC-----
------
FC88X_00189_00902      ---------------------CGTCTCAGTTTCGCGCAGA-ACTTTCTC-----
------
FC88X_00040_00112      ---------------------TGTCTCAGTTTCGCGCAGA-GCTTTCTC-----
------
FC88X_00045_00712      ---------------------CATCGCCCTTTGGAGCACG-CCGTTCTC-----
------
FC88X_00866_00933      ---------------------CATCGCCCTTTGGAGCACGGCCGTTCTC-----
------
FC88X_00085_00487      ---------------------CATCGCCCTTTGGAGCACG-CCGTTCTC-----
------
FC88X_00586_01241      --------------------TCTTAGCTGATTGGGGCA-A-GCATTCTC-----
------
FC88X_00677_00906      ---------------------TCTTGCTACTTGCAGCATA-CCGTTCTC-----
------
FC88X_00388_01143      ---------------------TCTCTCAATTTGCAGTTCA-GCTTTCTC-----
------
FC88X_00895_00295      ---------------------TCTCTCAATTTGCAGTTCA-GCTTTCTC-----
------
FC88X_00871_00327      ---------------------TCTCTCAATTTGCAGTTCA-GCTTTCTC-----
------
FC88X_00144_00425      ---------------------TCTCTCAATTTGCAGTTCA-GCTTTCTC-----
------
FC88X_00035_00653      ----------------------CTCAATGTTCACGGCGCATGCTTTCTC-----
------
FC88X_00268_00019      -----------------------TTCCTGAATCTGGGGCAGCTATTTTCC----
------
```

FC88X_00602_00105   ----------------------GTGCCTGTA-CAGGTGCACTGTTTTTCTC---
------
FC88X_00102_00911   -----------------------CTCGTGCTCCTCGTGCAGTCTTTCTC-----
------
FC88X_01006_00761   ----------------------CCTCCTGCTCA-CGTGCAACAGTCTTTT----
------
FC88X_00594_00425   ----------------------CTGGTAAAGTCAAATGCACGCGTTCTC-----
------
FC88X_00975_00437   ----------------------
CTGGTAAAGTCAAATGCACGCGTTCTCCTTCCTTT---
FC88X_00688_01086   ----------------------CTCGTAAAGTCAAATGCACGCGTTCTC-----
------
FC88X_00058_00846   ---------------------CCTGTTAATGTCGCAT-CATGCTTCTCC-----
------
FC88X_00099_00478   ---------------------CTTGTAGTT-TTGCAC-AGTATTCTACC-----
------
FC88X_00504_00171   -----------------CTCAGGAGTCGTTCTTGCGC-TGTTTTCTCCCC----
------
FC88X_00364_00080   --------------------CCGGGGCATTATTGCAC-GAGTTATCTC------
------
FC88X_00511_00158   --------------------CCGGGGCATTATTGCAC-GAGTTATCTC------
------
FC88X_00526_01019   --------------------CCGGGGCATTATTGCAC-GAGTTATCTC------
------
FC88X_00908_00457   --------------------CCGGGGCATTATTGCAC-GAGTTATCTC------
------
FC88X_00908_00891   --------------------CCGGGGCATTATTGCAC-GAGTTATCTC------
------
FC88X_00525_01193   --------------------CCGGGGCATTATTGCAC-GAGTTATCTC------
------
FC88X_00753_01202   --------------------CCGGGGCATTATTGCAC-GAGTTATCTC------
------
FC88X_00818_01061   --------------------CCGGGGCATTATTGCAC-GAGTTATCTC------
------
FC88X_01016_01062   --------------------CCGGGGCATTATTGCAC-GAGTTATCTC------
------
FC88X_01071_00542   --------------------CCGGGGCATTATTGCAC-GAGTTATCTC------
------
FC88X_01079_00510   --------------------CCGGGGCATTATTGCAC-GAGTTATCTC------
------
FC88X_00972_00718   -------------------CCGGTGGCATTATTGCAC-GAGTTATCTC------
------
FC88X_00426_00945   ---------------------CGGGGCATTATTGCAC-GAGTTATCTC------
------
FC88X_00599_00659   --------------------CCGGGGCATTATTGCAC-GAGTTATCTCATC---
------
FC88X_00178_01050   --------------------CCAGGGCATTATTGCAC-GAGTTATCTC------
------
FC88X_00095_00506   ---------------CGCGCTACTCTTCTTCCA-C---GTGTTATC--------
------
FC88X_00894_00668   -----------------CGCAACTGGTCTTGGAGC---GTGTTA----------
------
FC88X_00740_01236   -------------------CTACTGGTTATTCAGT---GAGTTATGTC------
------
FC88X_00949_00771   -------------------CTACTAGTGCTGCAGTACTGAGTTCTCC-------
------

```
FC88X_00381_00766      --------------------GCATTTCGTAATGAGA-GCCTATTTGTC------
------
FC88X_00787_00866      --------------------GCATTTCGTAATGAGAAGCCTATTTGTC------
------
FC88X_00056_00319      ------------------------CCTGTAATTGCAGCCTGTTTTCTCCGC---
------
FC88X_00514_01066      ------------------------CCTGTAATTGCAGCCTGTTTTCTCCGC---
------
FC88X_00825_00278      ------------------------CCTGTAATTGCAGCCTGTTTTCTCCGC---
------
FC88X_00463_00051      ------------------------CCTGTAATTGCAGCCT-TTTTCTCCGC---
------
FC88X_01020_00803      -------------------------CTGTAATTGCAGCCTGTTTTCTCCGCC--
------
FC88X_00894_00837      ---------------------CCTAACGTTTTTGCACAGTTGGTATCTC-----
------
FC88X_00606_01269      ---------------------CCTAACGTTTT-GCACAGTTGGTATCTC-----
------
FC88X_00947_00727      ---------------------CCTAACGTTTT-GCACAGTTGGTATCTC-----
------
FC88X_00960_01128      ---------------------CCTAACGTTTT-GCACAGTTGGTATCTC-----
------
FC88X_01058_01127      ---------------------CCTAACGTTTT-GCACAGTTGGTATCTC-----
------
FC88X_01104_01070      ---------------------CCTAACGTTTT-GCACAGTTGGTATCTC-----
------
FC88X_00437_01202      ---------------------CCTAACGTTTT-GCACAGTTGGTATCTC-----
------
FC88X_00535_01078      ---------------------CCTAACGTTTT-GCACAGTTGGTATCTC-----
------
FC88X_00101_00119      ---------------------CCTAACGTTTT-GCACAGTTGGTATCTC-----
------
FC88X_00395_01144      ---------------------CCTAACGTTTT-GCACAGTTGGTATCTC-----
------
FC88X_00132_00229      ---------------------CCTAACGTTTT-GCACAGTTGGTATCTC-----
------
FC88X_00434_01191      ---------------------CCTAACGTTTT-GCACAGTTGGTATCTC-----
------
FC88X_00596_00819      ---------------------CCTAACGTTTT-GCACAGTTGGTATCTC-----
------
FC88X_00757_00259      ---------------------CCTAACGTTTT-GCACAGTTGGTATCTC-----
------
FC88X_00883_01215      ---------------------CCTAACGTTTT-GCACAGTTGGTATCTC-----
------
FC88X_00914_01039      ---------------------CCTAACGTTTT-GCACAGTTGGTATCTC-----
------
FC88X_00045_00097      ---------------------CCTAACGTTTT-GCACAGTTGGTATCTC-----
------
FC88X_00807_00880      ---------------------CCTAACGTGTT-GCACAGTTGGTATCTC-----
------
FC88X_00934_00666      ---------------------CCTAACTTTTG--TGCAGTTGGTATCTC-----
------
FC88X_00489_01230      ---------------------CCTAACGTTTT-GCGCAGTTGGTATGCC-----
------
FC88X_00829_00268      ---------------------CCTAACGTTTTG---------------------
------
```

```
FC88X_01066_01031      ---------------------ACTAACGTTTG--TGCAGGTCGTAGCTCC----
------
FC88X_00455_00032      --------------------GCGTTATGTTTT-ATGAACTGCGTATCTC-----
------
FC88X_00485_00973      ---------------------CGTTATGTTTT-ATGAACTGCGTATCTC-----
------
FC88X_00822_00869      ---------------------CGTTATGTTTT-ATGAACTGCGTATCTC-----
------
FC88X_01123_00773      ---------------------CGTTATGTTTT-
ATGAACTGCGTATCTCCTTCCTTT---
FC88X_00096_00152      ---------------------CGCTATGTTTT-ATGAACTGCGTATCTC-----
------
FC88X_00528_01069      ----------------------CGATCACTTTTTTGTAACATGGCTCTTCT---
------
FC88X_00989_00544      ----------------------CCATTAGTTACGTGCAACAGGGCTCTC-----
------
FC88X_00570_00927      -----------------------TCCTGTTTTCGTCTAACGGGCCGTGTC----
------
FC88X_01029_00716      -----------------------TCTTGTTTT-ATGTAACTGGCTGTATCT---
------
FC88X_00667_00290      ---------------------ACCCGAAGTTCTTGGAGCTGGGGTGCCT-----
------
FC88X_00860_00698      ----------------------CCCGATGTTGTCCGCCATTGGGTGCCC-----
------
FC88X_00912_00511      ----------------GGGACCAATAACTCTTTGGCCCGTGTTTCTC-------
------
FC88X_01006_00420      --------------------CCAATAACTCTTTGGCCCGTGTTTCTC-------
------
FC88X_00028_00226      --------------------CCAATAACTCTTTGGCCCGTGTTTCTC-------
------
FC88X_00065_00744      --------------------CCAATAACTCTTTGGCCCGTGTTTCTC-------
------
FC88X_00380_00285      --------------------CCAATAACTCTTTGGCCCGTGTTTCTC-------
------
FC88X_00178_00162      -----------------GGGCCAATAACTCTTTGGCCCGTGTTTCTC-------
------
FC88X_00893_01217      --------------------CCAATAACTCTTTGGCCCGTGTTTCTC-------
------
FC88X_01052_00718      --------------------CCAATAACTCTTTGGCCCGTGTTTCTC-------
------
FC88X_00049_00477      --------------------CCAATAACTCTTTGGCCCGTGTTTCTC-------
------
FC88X_00232_00747      --------------------CCAATAACTCTTTGGCCCGTGTTTCTC-------
------
FC88X_00053_00699      --------------------CCAATAACTCTTTGGCCCGTGTTTCTC-------
------
FC88X_00160_00779      --------------------CCAATAACTCTTTGGCCCGTGTTTCTC-------
------
FC88X_00360_01187      --------------------CCAATAACTCTTTGGCCCGTGTTTCTC-------
------
FC88X_00498_01243      --------------------CCAATAACTCTTTGGCCCGTGTTTCTC-------
------
FC88X_00598_01176      --------------------CCAATAACTCTTTGGCCCGTGTTTCTC-------
------
FC88X_00582_01237      --------------------CCAATAACTCTTTGGCCCGTGTTTCTC-------
------
```

```
FC88X_00660_00457      --------------------CCAATAACTCTTTGGCCCGTGTTTCTC-------
------
FC88X_00732_00289      --------------------CCAATAACTCTTTGGCCCGTGTTTCTC-------
------
FC88X_00747_00961      --------------------CCAATAACTCTTTGGCCCGTGTTTCTC-------
------
FC88X_00717_01102      --------------------CCAATAACTCTTTGGCCCGTGTTTCTC-------
------
FC88X_00862_01147      --------------------CCAATAACTCTTTGGCCCGTGTTTCTCCC-----
------
FC88X_00463_00520      --------------------CCAATAACTCTTTGGCCCATGTTTCTC-------
------
FC88X_00522_00215      --------------------
CCAATAACTCTTTGGCCCGTGTTTCTCCTTCCTTT-----
FC88X_00779_00827      --------------------CCAATAACTCTTTGGCCCGCGTGTCTC-------
------
FC88X_00983_00874      --------------------CCAATAACTCTTTGGCCCGCGTTTCTC-------
------
FC88X_00138_00319      --------------------CCAATAACTCTTTGGCCCGTGTTTTCTC------
------
FC88X_00608_00505      -------------------ACCAATC-CCCTTTGGCAGTCTTTTCTC-------
------
FC88X_00417_00880      ---------------------CCATTGGCTCCTTCGCCGCGTTTTCTT------
------
FC88X_00479_00653      -----------------CCAGCCATTCCTCCTTGCGGCGTATT-----------
------
FC88X_00164_00063      --------------------CCCATCTCCT---TGCAGCGTTGGCATTGAC---
------
FC88X_00581_00271      -----------------GACCCGATCTCCT---GGGCGTGGCATTGTT------
------
FC88X_00463_01177      ----------------------AATCTCCT-TCGTCCGTGTCGTCGTTGAC---
------
FC88X_01145_01274      ---------------------CTATCTCCTATTCGCCGTGGCCGCGTA------
------
FC88X_00121_00377      ---------------------TCTTCTCTCTTTCGCGGCATGCATAA-------
------
FC88X_00321_01164      -------------------ATCGTTCTCTCTTTCCCCGATGCCATG--------
------
FC88X_00350_01026      --------------------TTCATTTTCTGTCTCCCTGGGCATTGTT------
------
FC88X_00357_00967      --------------------TTCATCTCTCTTTCCCCGCGGCATAGTT------
------
FC88X_00146_00923      -----------------CTATCTCCTCTGCATACGCGGTATTAC----------
------
FC88X_00645_00662      -----------------CCATCGGCTCTCC-TACGCGGTCATACC---------
------
FC88X_01089_00771      --------------------TCTTTTCTCCCCTTGCGGTAACACGCG-------
------
FC88X_00578_00104      -----------------------CTCTCTCCTTTGCGGTAATACCGTACG----
------
FC88X_00559_01188      -----------------------CTCTCTCCTTTGCGGTAATACCGTACG----
------
FC88X_01102_01012      -----------------------TTCTCCTCTTGGCGTAATGACCGTTTT----
------
FC88X_01134_01087      -----------------------CTCTCCTCTTGGCGTAATGACCGCTTTTT--
------
```

```
FC88X_00048_00267      ------------------CGTCATTTTTTCT-CCCTCGGCGCCTT---------
------
FC88X_01101_00685      ------------TCTTATTCTCACTTTTTCTGTCCTCGGGGCC-----------
------
FC88X_00435_00402      -----------------------CTTCTTTTCTCCCGAGGGCCTTTACT-----
------
FC88X_00576_01120      --------------ACCATGCAGGTTTATCTCCCGACGAGG-------------
------
FC88X_00435_01221      ------------------CTCAGGTTT--CCTCCCACGGGGCTTCTT-------
------
FC88X_00275_00931      -----------------AACCTGATCTGTACTCCCCGGGCGTGTA---------
------
FC88X_00460_01172      ------------------ACCTGATCTGTACTCCCCGGGCGTGTA---------
------
FC88X_00509_00156      ------------------ACCTGATCTGTACTCCCCGGGCGTGTA---------
------
FC88X_00547_01190      ------------------ACCTGATCTGTACTCCCCGGGCGTGTA---------
------
FC88X_00611_00683      ------------------ACCTGATCTGTACTCCCCGGGCGTGTA---------
------
FC88X_00633_00765      ------------------ACCTGATCTGTACTCCCCGGGCGTGTA---------
------
FC88X_00628_00967      ------------------ACCTGATCTGTACTCCCCGGGCGTGTA---------
------
FC88X_00810_00219      ------------------ACCTGATCTGTACTCCCCGGGCGTGTA---------
------
FC88X_00860_00140      ------------------ACCTGATCTGTACTCCCCGGGCGTGTA---------
------
FC88X_01013_00986      ------------------ACCTGATCTGTACTCCCCGGGCGTGTA---------
------
FC88X_00405_01162      -------------------CCGGATTTATCTCCCCGAGGCTTGCT---------
------
FC88X_00871_00912      ------------------CCCTGTTCTCTCTCTCTGCGGTGTCGG---------
------
FC88X_00880_00993      ----------------TCCGCTGTTCTCTCTCCCAAGGGTGTC-----------
------
FC88X_00960_00367      --------------------CCGTTCATTTTCTCCCGCGGCCGTGAG-------
------
FC88X_00357_00924      --------------------CCGTTCATTTTCTCCCGCGGCCGTGAG-------
------
FC88X_00950_00951      --------------------CCGTTCATTTTCTCCCGCGGCCGTGAG-------
------
FC88X_01137_01064      --------------------CCGTTCATTTTCTCCCGCGGCCGTGAG-------
------
FC88X_00121_00905      --------------------CCGTTC--TATCTCCCGTGGCGGCGTGCC-----
------
FC88X_00542_00063      ----------------------TCTCCTTGTCTCCTGAGTCGGTGTGCC-----
------
FC88X_01129_00606      ----------------------GCTCCTGTTATCCAGTGTCCATGTGAC-----
------
FC88X_00845_01091      --------------------CTGATGGTTA-CTCGTCGGCTCCTTGCG------
------
FC88X_00614_00987      --------------------CTCATGTCTATCTCCACCGGTCCCTGC-------
------
FC88X_00076_00509      ---------------------CCATGCCTTTCTCCACGGCACCTATGT------
------
```

FC88X_00815_01006 ---------------------CCATGCCTTTCTCCACGGCACCTATGT------
------
FC88X_00214_01087 ---------------------CCATCGTCTTCTCCACAGGCCCTTATT------
------
FC88X_00742_00237 ----------------------ACTGTTGTTGTCCCGATGTCGTTATCTC----
------
FC88X_01099_00722 ---------------------ACATGTTGTTGTCCCCTGGCCGTT---------
------
FC88X_00050_00357 ------------------TTCCAACTG---TTTCTCCCCGGTGGTATC------
------
FC88X_00995_00798 -----------------GTTCCAACTG---TTTCTCCCCGGTGGTATC------
------
FC88X_00015_00318 ------------------TTCCAACTG---TTTCTCCCCGGTGGTATC------
------
FC88X_00014_00454 ------------------TTCCAACTG---TTTCTCCCCGGTGGTATC------
------
FC88X_00194_00118 ------------------TTCCAACTG---TTTCTCCCCGGTGGTATC------
------
FC88X_01120_00704 ------------------TTCCAACTG---TTTCTCCCCGGTGGTATC------
------
FC88X_00058_00239 ------------------TTCCAACTG---TTCCTCCCCGGTGGTATC------
------
FC88X_00712_01023 -----------GGGGAAGTTCCAACTG---CTTCTCCCCGTTGGTATC------
------
FC88X_00707_00148 ------------------TTCCAACTCGATTTTCTCCCCGGTGGTACTCCCG--
------
FC88X_00255_01004 ------------------TTGCACGTT---TGTCTCCCGTGTCGTATT------
------
FC88X_00704_00192 ------------------TTGCACGTT---TGTCTCCCGTGTCGTATT------
------
FC88X_00243_01057 ------------------TTGCACGTT---TGTCTCCCGTGTCGTATT------
------
FC88X_00974_00377 ------------------TTGCACGTT---TGTCTCCCGTGTCGTATT------
------
FC88X_01065_01260 ------------------TTGCACGTT---TGTCTCCCGTGTCGTATT------
------
FC88X_00102_00104 ------------------TTGCACGTT---TATCTCCCGTGTCGTATT------
------
FC88X_00896_01086 ----------------GCTTGCTCGTT---ATTTTCCCCCGCGTTGTT------
------
FC88X_01132_01170 -----------------CTTGCTCGTT---ATTTTCCCCCGCGTTGTT------
------
FC88X_00366_01080 -----------------CTTGCTCGTT---ATTTTCCCCCGCGTTGTT------
------
FC88X_00196_00006 -----------------CTATGAACTGTTATGTCTCCCTGGAGT----------
------
FC88X_00422_00413 -----------------CTATGAACTGTTATGTCTCCCTGGAGT----------
------
FC88X_00934_00346 -----------------CTATCAACTCCT-TCTCTCTTTTGCGTC---------
------
FC88X_00388_00054 -----------------CTATATCCCGTTTTCTCCCCTGCTTGT----------
------
FC88X_00503_01019 ---------------------CTACCCGGTTTGGGCCTGTGCCGTTCT------
------
FC88X_00883_00678 ---------------------CTACCCGGTTTGGGCCTGTGCCGTTCT------
------

FC88X_00895_00537 ---------------------CTACC-GGTTTGGGCCTGTGCCGTTCT------
------
FC88X_00949_00339 ---------------------CTACTCGTTATTCGCCCCTGCCGTCC-------
------
FC88X_00149_00920 ---------------------CGCCTTTGTGGCCGGCTGCGCGTTCTT------
------
FC88X_00915_00854 ---------------------CGCCTTTGTGGCCGGCTGCGCGTTCTT------
------
FC88X_01047_00988 ---------------------CGCCTTTGTGGCCGGTTGCGCGTTCTT------
------
FC88X_00683_00087 ---------------------TGCCTTTGCGTCTCG--GCTCGTTCTC------
------
FC88X_00561_00984 ---------------------CCTTCGCGTTTTGGCTCCCATGTTCCATCC---
------
FC88X_01113_00678 -----------------------TTCGCCTTTATGC-CGCGTGACCCATT----
------
FC88X_00232_00374 -----------------------TCCGCGTTTATGCGCTCCCTGCTTATA----
------
FC88X_01080_00650 ---------------------CCTTCGCGTTTTTG-GCTCCATGC---------
------
FC88X_01046_01251 --------------------GCCGTCTCGTTTG-GCGCTCCCTTCTTGTT----
------
FC88X_00095_00661 --------------------TCAGTGCATTC-ACAGGGCTTGGTTCTT------
------
FC88X_00872_01019 --------------------TCAGTGCATTC-ACAGGGCTTGGTTCTT------
------
FC88X_00544_01162 --------------GGGGGATCAGTGCATTC-ACAGGGCTTGGTTCT-------
------
FC88X_01089_00607 ---------------------TCATGCGTTCGACTCCGCTGGCTTCCC------
------
FC88X_00353_01159 --------------------CTCAGTAATTCGCT-CAGCTTCAATCTC------
------
FC88X_01032_00635 --------------------CTCAGTAATTCGCT-CAGCTTCAATCTC------
------
FC88X_01129_00844 --------------------CTCAGTAATTCGCT-CAGCTTCAATCTC------
------
FC88X_00826_00881 ---------------------CTTGTCATTCGCGGCTTGAGCAATCTC------
------
FC88X_01002_01052 ---------------------CTTGTCATTCGCGGCTTGAGCAATCTC------
------
FC88X_00072_00788 --------------------CCAATGCCTTGG-CCCGTGCTTATTCT-------
------
FC88X_00842_01201 --------------------CCAATGCCTTGGGCCCGTGCTTATTCT-------
------
FC88X_00398_01056 --------------------CCAATGCCTTGGGCCCGCGCTTATTCT-------
------
FC88X_00957_00648 --------------------CCATCGTGTTCGCCCCGCGATGAGTCT-------
------
FC88X_00827_00318 --------------------TCCATGCGTTGC-
CGTGTGCTGTATCTACCTTCC------
FC88X_00814_01148 --------------------TCCATGCGTTGC--GTGTGCTGTATCTAC-----
------
FC88X_00628_00152 ------------------TCTCCGTGCGTTC----TTCGCTGGATCTGTC----
------
FC88X_00234_00933 --------------------TCACAGCGACTGTTC-TATGATGGCATC------
------

```
FC88X_00050_00241      --------------------TCCCAACGTTTGCTCCTAGCATGGAGTC------
------
FC88X_00811_00190      ----------------------CTTTAGTCTCCTCCCAGCATAGTTCTC-----
------
FC88X_00814_00817      ----------------------CTTTAGTCTCCTCCCAGCATAGTTCT------
------
FC88X_00459_00987      ----------------CTATGGCTTTAGC--CCTCCCAGCGTGTTC--------
------
FC88X_01132_00626      ------------------TCTCCCCATGGATCTCGCCTGCGCAAC---------
------
FC88X_00561_00596      ------------------TCTCCCCATGGATTTCGCCTGCGCAACCTTCCTT--
------
FC88X_00447_00999      ------------------CTCCTCCTTTCATGTTGTCTCGGCAAC---------
------
FC88X_00140_00225      ----------------CACTCCGCGT-TTCTGTTGTATGTCAAT----------
------
FC88X_00886_00602      ----------------CACTCCGCGT-TTCCGTTGTATGTTAAT----------
------
FC88X_00200_00027      -----------------CTCCCACGTCTCCTGTTGCATGTACAA----------
------
FC88X_01106_00842      --------------------CCACGTCTTCTCTGGCATCTCCAACAC-------
------
FC88X_00458_00038      -----------------------CTCCGT--GTTATTT-CGCTCCGTGTTACT-
------
FC88X_00293_00668      -----------------------CTCCGT--GTTATTT-CGCTCCGTGTTACT-
------
FC88X_00358_00388      --------------------TGCCTCCGTCAGTCCATT-CGCTCCATT------
------
FC88X_00552_00743      ------------------------TTTGTACATCGCCCGTGCAACATGCTT---
------
FC88X_00885_00582      ------------------------TTTGTACATCGCCCGTGCAACATGCTT---
------
FC88X_00906_00384      ------------------------TTTGTACATCGCCCGTGCAACATGCTT---
------
FC88X_00234_00115      ------------------------TTTGTACATCGCCCGTGCAACATGCTT---
------
FC88X_00421_00514      ------------------------TTTGTACATCGCCCGTGCAACATGCTT---
------
FC88X_00835_01240      ------------------------TTTGTACATCGCCCGTGCAACATGCTT---
------
FC88X_00890_00393      ------------------------TTTGTACATCGCCCGTGCAACATGCTT---
------
FC88X_00902_00759      ------------------------TTTGTACATCGCCCGTGCAACATGCTT---
------
FC88X_00937_01045      ------------------------TTTGTACATCGCCCGTGCAACATGCTT---
------
FC88X_00968_00285      ------------------------TTTGTACATCGCCCGTGCAACATGCTT---
------
FC88X_00667_01079      ------------------------TTTGTACATCGCCCGTGCAACATG------
------
FC88X_00882_00453      ------------------------TTTGTACATCGCCCGTGCAACATGTTT---
------
FC88X_01011_01178      ------------------------TTTGTACATCGCCCGTGCAACATG------
------
FC88X_01028_00618      -----------------------TTTTGTACATCGCCCGTGCAACATG------
------
```

FC88X_00592_01172 ------------------------TTTGTACATCGCCCGTGCAACACG------
------
FC88X_00363_00411 ---------------------TCAAATGTCTGTCGACCGTTTAATGTT------
------
FC88X_00927_00922 -----------------CCAATCAATTGTGCTGTGCCCGTGTAACC--------
------
FC88X_00089_00573 ------------------CCCCACTATCTCCTTCGCCCGAGTCCT---------
------
FC88X_00910_00936 -------------------CCAACTGT-TCTTTCGCCCCA--------------
------
FC88X_00461_01141 ----------------------TTCTGATCTGTTCTTCACGCTCCTTCC-----
------
FC88X_00986_00809 --------------------CCTCCTCACCTGTTCCGC-CGTCCTACCC-----
------
FC88X_01089_01190 ----------------------ATGTTAGCTCTCCTCCCCACGCATTCC-----
------
FC88X_00090_00931 ----------------------ATCATTGATCTCCTCCGCTCTAGTTCC-----
------
FC88X_00751_00709 ----------------------ATGATCTGTC-TATTCCACCTGATTCCC----
------
FC88X_00788_01228 --------------------TGATGATTTCTCGTGCGCCACC--ATTCC-----
------
FC88X_00662_01132 -------------------GCGACTCGTTCAGTTGCCTC--CCTTTTGCT----
------
FC88X_00781_00248 --------------------CGACTCGTTCAGTTGCCTC--CCTTTTGCT----
------
FC88X_00156_00874 --------------------CGACTCGTTCAGTTGCCTC--CCTTTTGCT----
------
FC88X_00207_00054 --------------------CGACTCGTTCAGTTGCCTC--CCTTTTGCT----
------
FC88X_00376_00552 --------------------CGACTCGTTCAGTTGCCTC--CCTTTTGCT----
------
FC88X_00391_01005 --------------------CGACTCGTTCAGTTGCCTC--CCTTTTGCT----
------
FC88X_00431_00397 --------------------CGACTCGTTCAGTTGCCTC--CCTTTTGCT----
------
FC88X_00461_00535 --------------------CGACTCGTTCAGTTGCCTC--CCTTTTGCT----
------
FC88X_00613_01110 --------------------CGACTCGTTCAGTTGCCTC--CCTTTTGCT----
------
FC88X_00661_00143 --------------------CGACTCGTTCAGTTGCCTC--CCTTTTGCT----
------
FC88X_00707_00085 --------------------CGACTCGTTCAGTTGCCTC--CCTTTTGCT----
------
FC88X_00818_01161 --------------------CGACTCGTTCAGTTGCCTC--CCTTTTGCT----
------
FC88X_00939_00482 --------------------CGACTCGTTCAGTTGCCTC--CCTTTTGCT----
------
FC88X_00939_00535 --------------------CGACTCGTTCAGTTGCCTC--CCTTTTGCT----
------
FC88X_01091_00478 -----------------CGGCAGACTGTTAAATCGCGTCACCCTCTTCCT----
------
FC88X_00405_01071 --------------------CCACTCAATCGGTGCCACC--CGTCTCCC-----
------
FC88X_00979_01049 --------------------CCAATTGATCTGTGTCCCC--CCCTCTGTT----
------

FC88X_01053_01082 --------------------CTGACGAAT-CTCCCGAGTCAGACTGGG------
------
FC88X_00604_00673 --------------------CTGACGAAT-CTCCCGAGTCAGACTGGG------
------
FC88X_00796_00971 --------------------CTGACGAAT-CTCCCGAGTCAGACTGGG------
------
FC88X_00897_00201 --------------------CTGACGAAT-CTCCCGAGTCAGACTGGG------
------
FC88X_00860_00872 --------------------CTGACGAAT-CTCCCGAGTCAGACTGGG------
------
FC88X_01031_00848 --------------------CTGACGAAT-CTCCCGAGTCAGACTGGG------
------
FC88X_00064_00445 ----------------------AACGAGT-GTCT-GAGT--GACTGG-------
------
FC88X_00249_00616 --------------------CTAACGATTATTCTCCAGGCACACACT-------
------
FC88X_00924_00496 --------------------CTAACGATTATTCTCCAGGCACACACT-------
------
FC88X_00928_00823 --------------------CTAACGATTATTCTCCAGGCACACACT-------
------
FC88X_01015_01129 ---------------GGGGACTAACGATTATTCTCCAGGCACACACT-------
------
FC88X_00472_01066 -------------------TCTAACGAAT-CTCTCCAGT---------------
------
FC88X_00753_00584 --------------------TCGCTGAATCTCCGACGTCATGACTGT-------
------
FC88X_00927_00411 -------------------GTCGCTGAATCTCCGACGTCATGACTGT-------
------
FC88X_00867_00269 --------------------TCGCTGAATCTCCGACGTCATGACTGT-------
------
FC88X_00625_00123 --------------------TCGCTGAATCTCCGACGTCATGACTGT-------
------
FC88X_01072_00842 --------------------TCGCTGAATCTCCGACGTCATGACTGT-------
------
FC88X_01131_00977 --------------------TCGCTGAATCTCCGACGTCATGACTGT-------
------
FC88X_00958_00777 -------------------GTCGTTGA--TTTCCACGCAAAGACTTTCT-----
------
FC88X_00907_00738 --------------------TCGTTGA--TTTCCACGCAAAGACTTTCT-----
------
FC88X_00544_00559 --------------------GCCATGGATCTCCCGCACTGGCTCGCA-------
------
FC88X_00583_01265 --------------------GCCATGGATCTCCCGCACTGGCTCGCA-------
------
FC88X_00500_00810 ----------------TCCATGGATATATCTCCCGCCCTGTCT-----------
------
FC88X_00988_01093 -----------------CCACGTATGTAAATACCGCACTAGTT-----------
------
FC88X_00788_00177 ----------------CCCAAAAACGTACATCCCGCGCTCATT-----------
------
FC88X_01131_00958 ------------------CATGAACATCAATACCGCGCGTGCCTC---------
------
FC88X_00743_00727 -----------------TTCTCCACGTATTT-CTCTCTCGTACT----------
------
FC88X_01072_00898 ------------------TCTTGACGTACTCACTCCCTCGGAAAT---------
------

FC88X_00074_00886 ------------------CTCCCAAGTAAATTTATCGCACGTTAT---------
------
FC88X_00420_00052 ------------------CTCCCAAGTAAATTTATCGCACGTTAT---------
------
FC88X_00949_00896 ------------------CTCCCAAGTAAATTTATCGCACGTTAT---------
------
FC88X_00091_00281 ------------------CTCCCAAGTAAATTTATCGCACGTTATT--------
------
FC88X_00704_00999 ------------------CTCCCAAGTAAATTTATCGCACGTTAC---------
------
FC88X_00361_01102 ------------------CTCCCAAGTAAATTTATCGCGCGTTAT---------
------
FC88X_00717_01230 ------------------CCGCAAAGTCAATCT--CGCTGGTTACTC-------
------
FC88X_00756_00868 ------------------CTCCCAACGACATTCG-CTCACGTTAT---------
------
FC88X_00971_00669 -----------------ACTCCCGACCG-ACACG-CTAAAATTACA--------
------
FC88X_00993_00464 -----------------CTCCCGAAGATTCAT-CGCACGCATTTA---------
------
FC88X_00525_00623 -----------------CTCCCGAAGATTCAT-CGCACGCATTTA---------
------
FC88X_00403_00753 ----------------ACGCACGAAAATTGACATGCTCCCATT-----------
------
FC88X_00991_00906 ----------------ACGCACGAAAATTGACATGCTCCCATT-----------
------
FC88X_01129_00979 ----------------ACGCACGAAAATTGACATGCTCCCATT-----------
------
FC88X_00574_00647 -------------------ACCGAACGTTTT-CAACACGCGTCATCA-------
------
FC88X_00627_00160 -------------------ACCGAACGTTTTTCAACACGCGTCATCA-------
------
FC88X_00937_00462 --------------------CCGAAAGTTGACCGACACGCGTACAGAA------
------
FC88X_00866_00676 ---------------------TCCCGAATTTTATCCGCGCGTCTGTCTC-----
------
FC88X_00007_00424 ---------------------TCCCGAATTT-ATCCGCGCGTCTGTCTC-----
------
FC88X_00065_00211 ---------------------TCCCGAATTT-ATCCGCGCGTCTGTCTC-----
------
FC88X_00060_00264 ---------------------TCCCGAATTT-ATCCGCGCGTCTGTCTC-----
------
FC88X_00590_01094 ---------------------TCCCGAATTT-ATCCGCGCGTCTGTCTC-----
------
FC88X_00848_00920 ---------------------TCCCGAATTT-ATCCGCGCGTCTGTCTC-----
------
FC88X_00303_01010 ---------------------TCCCGAATTT-ATCCGCGCGTCTGTCTCC----
------
FC88X_00561_00186 ---------------------TCCCGAATTT-ATCCGCGCGTCTGTCTC-----
------
FC88X_00741_00265 ---------------------TCCCGAATTT-ATCCGCGCGTCTGTCTC-----
------
FC88X_00887_01076 ---------------------TCCCGAATTT-ATCCGCGCGTCTGTCTC-----
------
FC88X_00922_00878 ---------------------TCCCGAATTT-ATCCGCGCGTCTGTCTC-----
------

FC88X_00935_01121 ---------------------TCCCGAATTT-ATCCGCGCGTCTGTCTC-----
------
FC88X_01058_01255 ---------------------TCCCGAATTT-ATCCGCGCGTCTGTCTC-----
------
FC88X_01135_00904 ---------------------TCCCGAATTT-ATCCGCGCGTCTGTCTC-----
------
FC88X_00061_00491 ---------------------TCCCGAATTT-ATCCGCGCGTCTGTCTC-----
------
FC88X_00456_01213 ---------------------TCCCGAATTT-ATCCGCGCGTCTGTCTC-----
------
FC88X_00505_00843 ---------------------TCCCGAATTT-ATCCGCGCGTCTGTCTC-----
------
FC88X_00669_01179 ---------------------TCCCGAATTT-ATC-GCGCGTCTGTCTC-----
------
FC88X_00807_01109 ---------------------TCCCGAATTT-ATCTGCGCGTCTGTCTC-----
------
FC88X_00716_01187 ---------------------TCTCGAATTT-ATCCGCGCGTCTGTCTC-----
------
FC88X_00675_01182 ---------------------TCCCGAATTT-ATCCGCGCGTC-----------
------
FC88X_00960_00527 ---------------------TCCCGAATTT-ATCCGCGCGTCTGCCTC-----
------
FC88X_01142_00852 ---------------------TCCCGAATTT-ATTTGCGCGTCTGTCTC-----
------
FC88X_00408_01179 --------------------CTCACGAACCT--TTCGCGTGTCTCTATC-----
------
FC88X_00679_01274 --------------------CTCACGAACTT--TTCGCGTGTCTCTATC-----
------
FC88X_00283_00944 --------------------CTCACGAACCT--TTTGCGTGTCTCTATC-----
------
FC88X_00994_00627 --------------------CTCACGAACCT--TTTGCGTGTCTCTATC-----
------
FC88X_00896_00424 --------------------TCTCCCGATAT--TTCGCGCATCCATCGCA----
------
FC88X_01086_00415 --------------------CTCCCGGATTT--TTGGCACATGCGCCGC-----
------
FC88X_00986_00300 --------------------CTCCGGTATTT--CTG-CGCG-------------
------
FC88X_01036_00552 --------------------CTCCCGGATAT--TTGTCGCGCACCTGAC-----
------
FC88X_00015_00399 -------------------CCCAACGAGTTGTTGTCCCGGCTTTAT--------
------
FC88X_01142_01033 -------------------CCCAACGAGTTGTTGTCCCGGCTTTAT--------
------
FC88X_01024_00341 -------------------CACGACTA-TCTTCTCCCTGGATTTATA-------
------
FC88X_00157_00446 --------------------CCGAA--TATTTCTCCCTGGGATCGTCTC-----
------
FC88X_00776_00622 --------------------CCGAA--TATTTCTCCCTGGGATCGTCTC-----
------
FC88X_00987_00895 -------------------CCCGAAACTATTTACCGCCCCGATCAT--------
------
FC88X_00267_00999 --------------------CTACCGTCCGTTCCCT--GCTATGCTGTT-----
------
FC88X_00219_00639 --------------------CTACCGTCCGTTCCCT--GCTATGCTGTT-----
------

```
FC88X_00398_00567       --------------------CTACCGTCCGTTCCCT--GCTATGCTGTT-----
------
FC88X_00502_00545       --------------------CTACCGTCCGTTCCCT--GCTATGCTGTT-----
------
FC88X_00943_00862       --------------------CTACCGTCCGTTCCCT--GCTATGCTGTT-----
------
FC88X_00953_01149       --------------------CTACCGTCCGTTCCCT--GCTATGCTGTT-----
------
FC88X_00750_00847       --------------------CTACCGTCCGTTCCCC--GCTATGCTGTT-----
------
FC88X_00849_00256       --------------------CTACCGTCCGTCCCCCT-GCTATGCTGTTC----
------
FC88X_00308_00569       --------------------CTACCGTCCGTTCCCT--
GCTATGCTGTTCTTCCTTTCTC
FC88X_00876_01007       --------------------CTACCGTCCGTTCCCT--GCTATGTCGTT-----
------
FC88X_01062_00922       --------------------TCACCGGCCGCCCCGC--CCTGTCATGTT-----
------
FC88X_00803_00409       ---------------------TCCTGGATCCCCG-T--CCCATGATGTTGTT--
------
FC88X_00809_00799       --------------------TTTCTGGGTTCCCGCT--CCTATGATATT-----
------
FC88X_00939_00911       -----------------------CCTTTCGTTTCCCT-GCTTTCGTATTTT---
------
FC88X_00938_00369       -----------------------CCATGCGTCTCCCC-GTTTTGTAATGTT---
------
FC88X_00857_00195       ---------------------GGCTGCAGATTTGTGGCCCCCCTATGTCCC---
------
FC88X_01079_01195       ----------------------CCTGCAAATCGTCGGCCCCGTGATATC-----
------
FC88X_00124_00799       ----------------------CCTGTGAACATTCTTCGCCGTGCTCTG-----
------
FC88X_00917_00918       ----------------------CCTGTGAACATTCTTCGCCGTGCTCTG-----
------
FC88X_01067_00933       ----------------------GCCGATTACTGGCTGACCCGCAATTTCTC---
------
FC88X_00123_00107       -----------------------CCTATTAC-GACTCGGCCCCAAGCTCTC---
------
FC88X_00034_00798       -------------CTTACTCCTAGCTC-GACTTGCCATTA--------------
------
FC88X_00957_01271       -------------CCTTCTCCTTGCTG-AACACTCCATCAA-------------
------
FC88X_00607_01239       -----------------CTCCCAGCG--TACTCTCAGG----------------
------
FC88X_00839_01152       -----------------CTCCCAGCGG-TACTCTCAGG----------------
------
FC88X_01049_00908       ---------------TCCTCCCAGCTCGTAAACTCAAGCGCC------------
------
FC88X_00866_00377       ---------------------CAACGTTTGCT-CCCAGATCCCCGTGTA-----
------
FC88X_01116_01075       ---------------------CAACGTTTGCT-CCCAGATCCCCGTGTA-----
------
FC88X_00561_01183       ---------------------CAACGTTTGCT-CCCAGATCCTCGTGTA-----
------
FC88X_00125_00570       --------------------GCAACGGTTGCT-CCCAGATTCCCGTGTA-----
------
```

FC88X_00837_00779   ---------------------CAGCAGTTTAT-CCCAGCTCGTCGCGTCC----
------
FC88X_00972_00723   ---------------------CAGCAGTTTAT-CCCAGCTCGTCGCGTCC----
------
FC88X_00819_00786   ---------------------CAGCAGTTTAT-CCCAGCTCGTCGCGTCC----
------
FC88X_00322_00997   ---------------------CGGCAGTTTAT-CCCAGCTCGTCGCGTCC----
------
FC88X_00613_00947   ---------------------CAGCAGTTTAT-CCCAGCTCGTCGCGTTT----
------
FC88X_00653_01153   --------------------ACGGCAGTTCG--CCCACCTCGTCATGTT-----
------
FC88X_00678_01168   ----------------GTTACCAATGTTTGC--TCCCTGTAACGGC--------
------
FC88X_00012_00428   -----------------TTACCAATGTTTGC--TCCCTGTAACGGC--------
------
FC88X_00107_00378   -----------------TTACCAATGTTTGC--TCCCTGTAACGGC--------
------
FC88X_00317_00833   -----------------TTACCAATGTTTGC--TCCCTGTAACGGC--------
------
FC88X_00770_00140   -----------------TTACCAATGTTTGC--TCCCTGTAACGGC--------
------
FC88X_00750_00936   -----------------TTACTAATGTTTGC--TCCCTGTAACGGC--------
------
FC88X_00638_01102   -----------------TTACCAATACT-AC--TCCCCG-AACGACGC------
------
FC88X_00348_01102   --------------------ACCATACTTATCGTCCCAATGGTCGCG-------
------
FC88X_00979_00791   ---------------------CCATACTTGC--TCCCAGTTGGCG---------
------
FC88X_00049_00297   -------------------CTCCGAGATACCATGGTGGCAACGTGCC-------
------
FC88X_00083_00125   -------------------CTCCGAGATACCATGGTGGCAACGTGCC-------
------
FC88X_00860_00260   -------------------CTCCGAGATACCATGGTG-CAACGTGCC-------
------
FC88X_00686_01016   ------------------TCTCCGATCT-CCTTGCTGGTAAC-TGCC-------
------
FC88X_00067_00862   -------------------CTCCGCACTGCCTTGGTGG-GCCTCC---
ATCTTCCTTT--
FC88X_00881_00367   -----------------------
TTAATGCCTTAGTGGCGCCTCCCACATCTTCCTTT--
FC88X_00170_00094   --------------------TCCGCGATGTCGTTCTTGGACCATCCTC------
------
FC88X_00519_00258   --------------------TCTCCCATGGCCTTGCGGGACCATCCT-------
------
FC88X_00573_01238   --------------------CTCGCAATTCGATTGCACCAGCCTCTT-------
------
FC88X_00716_00062   --------------------CTCGAAATTCCGTCCAAGCATGCTCTT-------
------
FC88X_00885_00948   --------------------CTCGTAGTTTTCTCCCACCGTCCTTGG-------
------
FC88X_00681_00466   --------------------TGACCTTG--TTCGGC-ACAC-CGTGTTCTC---
------
FC88X_00928_00924   --------------------TGACCTTG--TTCGGC-ACAC-CGTGTTCTC---
------

FC88X_00064_00691 --------------------TGACCTTG--TTCGGC-ACAC-CGTGTTCTC---
------
FC88X_00933_01040 --------------------TGACCTTG--TTCGGC-ACAC-CGTGTTCTC---
------
FC88X_00810_01094 -------------------GTGACCTTG--TTCGGC-ACAC-CGCGTTCTC---
------
FC88X_00995_00901 --------------------TGACCTTG--TTCGGC-ACAC-CGCGTTCTC---
------
FC88X_00082_00805 --------------------TGACCTTG--TTCGGC-ACAC-CGCGTTCTC---
------
FC88X_00536_01067 --------------------TGACCTTG--TTCGGC-ACAC-CGCGTTCTC---
------
FC88X_00622_01234 --------------------TGACCTTG--TTCGGCCACAC-CGCGTTCTC---
------
FC88X_00964_01262 --------------------TGACCTTG--TTCGGC-ACAC-CGCGTTCTC---
------
FC88X_00987_00669 --------------------TGACCTTG--TTCGGC-ACAC-TGCGTTCTC---
------
FC88X_00017_00396 --------------------TGACCCTG--TTCGGC-ACAC-CGCGTTCTC---
------
FC88X_00684_01040 --------------------TGACCTTG--CTCGGC-ACAC-CTCGTTCTC---
------
FC88X_00134_00492 ----------------------GCTTAGACCTTGGTGGCAC-TCTGTTCTC---
------
FC88X_00745_01248 ----------------------GCTTGGACCTTGGTGGCAT-TCTGTTCTC---
------
FC88X_00154_00648 ----------------GGGGAAGCTTAGACCTTGGTGGCAT-TCTGTTTTCC--
------
FC88X_00219_01054 -----------------------CTTAGACCTTGGTGGCAT-TCTGTTCTC---
------
FC88X_00431_00758 ----------------------GCTTAGACCTTGGTGGCAT-TCTGTTCTC---
------
FC88X_00820_00946 -----------------------CTTAGACCTTGGTGGCAT-TCTGTTCTC---
------
FC88X_00895_00902 -----------------------CTTAGACCTTGGTGGCAT-TCTGTTCTC---
------
FC88X_00023_00617 -----------------------CTTAGACCTTGGTGGCAT-TCTGTTCTC---
------
FC88X_00366_01053 ----------------------ACTTAGACCTTGGTGGCAT-TCTGTTCTC---
------
FC88X_00677_01204 -----------------------CTTAGACCTTGGTGGCAT-TCTGTTCTC---
------
FC88X_00881_00720 -----------------------CTTAGACCTTGGTGGCAT-TCTGTTCTC---
------
FC88X_01083_00504 -----------------------CTTAGACCTTGGTGGCAT-TCTGTTCTC---
------
FC88X_00061_00723 -----------------------CTTAGACCTTGGTGGCAT-TCTGTTCTC---
------
FC88X_00421_00988 -----------------------CTTAGACCTTGGTGGCAT-TCTGTTCTC---
------
FC88X_00747_01132 -----------------------CTTAGACCTTGGTGGCAT-TCTGTTCTC---
------
FC88X_01032_00449 -----------------------CTTAGACCTTGGTGGCAT-TCTGTTCTCCC-
------
FC88X_00869_00822 -----------------------CTTAGACCTTGGTGGCAT-TCTGTTCTC---
------

FC88X_01123_01088 ------------------------CTTAGACCTTGGTGGCAT-TCTGTTCTC---
------
FC88X_00378_01062 ------------------------CTTAGACCTTGGTGGCAT-TCTGTTCTC---
------
FC88X_00452_01214 ------------------------CTTAGACCTTGGTGGCAT-TCTGTTCTC---
------
FC88X_00512_00343 ------------------------CTTAGACCTTGGTGGCAT-TCTGTTCTC---
------
FC88X_00580_00296 ------------------------CTTAGACCTTGGTGGCAT-TCTGTTCTC---
------
FC88X_00564_01018 ------------------------CTTAGACCTTGGTGGCAT-TCTGTTCTC---
------
FC88X_00948_01101 ------------------------CTTAGACCTTGGTGGCAT-TCTGTTCTC---
------
FC88X_00986_00245 ------------------------CTTAGACCTTGGTGGCAT-TCTGTTCTC---
------
FC88X_00976_00271 ------------------------CTTAGACCTTGGTGGCAT-TCTGTTCTC---
------
FC88X_01084_00709 ------------------------CTTAGACCTTGGTGGCAT-TCTGTTCTC---
------
FC88X_00730_00205 ------------------------CTTAGACCTTGGTGGCAT-
TCTGTTCTCCTC------
FC88X_00657_00111 ------------------------CTTAGACCTTGGTGGCAT-TCTGTTCTCT--
------
FC88X_00598_00054 ------------------------CTTAGACCTTGATGGCAT-TCTGTTCTC---
------
FC88X_00224_00332 ------------------------CTTAGACCTTGGTGGCAT-
TCTGTTCTCCTTCC----
FC88X_00310_01038 ------------------------CTTAGACCTTGGTGGCAT-
TCTGTTCTCCTTCCTTT-
FC88X_00867_00871 ------------------------CTTAGACCTTGGTGGCAT-
TCTGTTCTCCTTCCTTTC
FC88X_00175_00808 ------------------------TTTAGACCTTGGTGGCAT-TCTGTTCTC---
------
FC88X_00564_01015 ------------------------CTTAGACCCTGGTGGCAT-TCTGTTCTC---
------
FC88X_00177_00992 ------------------------CTTAGACCTCGGTGGCAT-TCTGTTCTC---
------
FC88X_01095_00809 ------------------------CTTAGACCTCGGTGGCAT-TCTGTTCTC---
------
FC88X_00061_00716 -----------------------CTTCAGACCTTGGCGGCAC-TCTGTTCTC---
------
FC88X_00419_00041 ------------------------TGCGTACCTTAGACCAAC-ACTTTTCTT---
------
FC88X_00795_00734 ------------------------TGCGTACCTTAGACCAAC-ACTTTTCTT---
------
FC88X_01107_00876 ------------------------TGCGTACCTTAGACCAAC-ACTTTTCTT---
------
FC88X_00609_01177 -------------------------GTGTAGCTCCGGGCTAC-CCTTGTTCTC--
------
FC88X_00113_00916 -------------------------TTGTACCTT-GGTGTAC-ACTTCTCTCCA-
------
FC88X_00150_00792 ---------------------TCCCTTCGTCTTGAGTGTCAC-TTTTCT------
------
FC88X_00760_00913 ---------------------TCCCTTCGTCTTGAGTGTCAC-TTTTCT------
------

```
FC88X_00155_00390      --------------------TCCCTTCGTCTTGAGTGTCAC-TTTTCT------
------
FC88X_00183_00814      --------------------TCCCTTCGTCTTGAGTGTCAC-TTTTCT------
------
FC88X_00192_01032      --------------------TCCCTTCGTCTTGAGTGTCAC-TTTTCT------
------
FC88X_00220_00881      --------------------TCCCTTCGTCTTGAGTGTCAC-TTTTCT------
------
FC88X_00409_00679      --------------------TCCCTTCGTCTTGAGTGTCAC-TTTTCT------
------
FC88X_00718_00482      --------------------TCCCTTCGTCTTGAGTGTCAC-TTTTCT------
------
FC88X_00618_00151      -------------------GTCCCATCGTCTTGAGTGTCAC-TTTTCT------
------
FC88X_00206_00026      --------------------TCCCCTCGTCTTGAGTGTCAC-TTTTCT------
------
FC88X_00580_00426      --------------------TCCCTTCGTCTTGAGTGTCAC-TTTTCC------
------
FC88X_00114_00484      -------------------CTTCCTTCGATCTT-GTCTCAC-TATTCT------
------
FC88X_00859_01225      -------------------CTTCCTTCGATCTT-GTCTCAC-TATTCT------
------
FC88X_00929_00510      --------------------TGCCATCATGTTGTGACACTT-ATTTCT------
------
FC88X_00914_01167      --------------------TGCCATCATGTTGTGACACTT-ATTTCT------
------
FC88X_00959_00554      --------------------TGCCATCATGTTGTGACACTT-ATTTCT------
------
FC88X_00295_01039      --------------------TGCCATCATGTTGTGACACTT-ACTTCT------
------
FC88X_00815_00194      --------------------TTCCATCATCTTAGACCGCGA-CACTTT------
------
FC88X_00526_01195      ----------------------CCACAAACCTTGGTGTGGA-CCCTTGTT----
------
FC88X_00914_00539      --------------------CTCCTCCATCCTGTGTGTCGA-C--TTGTT----
------
FC88X_00408_01124      --------------------AGCATCCATCGTTCGTGTTCG-CTATTC------
------
FC88X_00744_00902      ---------------------GCAACCGTCGTGTCGCCTTG-TTCTCTG-----
------
FC88X_00729_00293      -----------------------ACCACCCATCTGTGGCAC-CATGTTCTC---
------
FC88X_00894_00919      -----------------------ACCACCCATCTGTGGCAC-CATGTTCTC---
------
FC88X_00082_00229      -----------------------ACCACCCATCTGTGGCAC-CATGTTCTC---
------
FC88X_00750_01067      -----------------------ACCACCCATCTGTGGCAC-CATGTTCTC---
------
FC88X_00864_00911      -----------------------ACCACCCATCTGTGGCAC-CATGTTCTC---
------
FC88X_00922_00272      -----------------------ACCACCCATCTGTGGCAC-CATGTTCTC---
------
FC88X_00945_00600      -----------------------ACCACCCATCTGTGGCAC-CATGTTCTC---
------
FC88X_01117_00494      -----------------------ACCACCCATCTGTGGCAC-CATGTTCTC---
------
```

FC88X_00844_01033 ------------------------ACACCCATCTGTGGCAC-CATGTTCTC---
------
FC88X_01024_01007 -----------------------ACCACCAATCTGTGGCAC-CATGTTCTC---
------
FC88X_00147_00692 -----------------------TCCCGCAATCTGTGGCAC-TA-GTTCTT---
------
FC88X_00067_00475 -----------------------TTCGTCAATCAATGGCGTAC-TGTTCTT---
------
FC88X_00963_00427 -----------------------TCCGTCAATCAATGGCGTACGTGTTCTT---
------
FC88X_01072_00785 -----------------------TTTATGCACCTATTGCCTCACTGTTCTC---
------
FC88X_00810_00114 -------------------------CTTGCAACTAGTGGTTCAAGGTTCTC---
------
FC88X_00744_01134 -----------------------
ACCGTGATACTGATTCACTGGTCCTCGCCTTCCT---
FC88X_01111_01125 -----------------------
ACCGTGATACTGATTCACTGGTCCTCGCCTCCC----
FC88X_01065_00716 -----------------------TCCTTCATTCT-ATTCCCTCGTGTTCGC---
------
FC88X_00918_00364 -----------------CAAACGTTTCCCC---GACGT-CGCATTTCTC-----
------
FC88X_00619_01046 -----------------CAAACGTTTCCCC---GACGT-CGCTTTCT-------
------
FC88X_00632_01019 -----------------CAAACGTTTCCCC---GACGTTCGCCTTTCTCC----
------
FC88X_00010_00063 -----------------CAAACGTTTCCCC---GACGT-CGCTTTCTC------
------
FC88X_00019_00102 -----------------CAAACGTTTCCCC---GACGT-CGCTTTCTC------
------
FC88X_00028_00339 -----------------CAAACGTTTCCCC---GACGT-CGCTTTCTC------
------
FC88X_00221_00049 -----------------CAAACGTTTCCCC---GACGT-CGCTTTCTCC-----
------
FC88X_00527_00498 -----------------CAAACGTTTCCCC---GACGT-CGCTTTCTCC-----
------
FC88X_00070_00804 -----------------CAAACGTTTCCCC---GACGT-CGCTTTCTCC-----
------
FC88X_00474_00965 -----------------CAAACGTTTCCCC---GACGT-CGCTTTCTCC-----
------
FC88X_00473_01178 -----------------CAAACGTTTCCCC---GACGT-CGCTTTCTCC-----
------
FC88X_00561_01117 -----------------CAAACGTTTCCCC---GACGT-CGCTTTCTCC-----
------
FC88X_00852_00172 -----------------CAAACGTTTCCCC---GACGT-CGCTTTCTCC-----
------
FC88X_01028_01013 -----------------CAAACGTTTCCCC---GACGT-CGCTTTCTCC-----
------
FC88X_00013_00623 -----------------CAAACGTTTCCCC---GACGT-CGCTTTCTC------
------
FC88X_00048_00805 -----------------CAAACGTTTCCCC---GACGT-CGCTTTCTC------
------
FC88X_00091_00896 -----------------CAAACGTTTCCCC---GACGT-CGCTTTCTC------
------
FC88X_00484_00122 ----------GGGGAAGCAAACGTTTCCCC---GACGT-CGCCTTTCTC-----
------

FC88X_00546_01127 ----------------GCAAACGTTTCCCC---GACGT-CGCTTTCTC------
------
FC88X_00615_01094 ----------------GCAAACGTTTCCCC---GACGT-CGCTTTCTC------
------
FC88X_00701_01215 ----------------GCAAACGTTTCCCC---GACGT-CGCTTTCTC------
------
FC88X_00812_00874 ----------------GCAAACGTTTCCCC---GACGT-CGCTTTCTC------
------
FC88X_00927_00890 ----------------GCAAACGTTTCCCC---GACGT-CGCTTTCTC------
------
FC88X_00010_00276 -----------------CAAACGTTTCCCC---GACGT-CGCTTTCTC------
------
FC88X_00048_00566 -----------------CAAACGTTTCCCC---GACGT-CGCTTTCTC------
------
FC88X_00085_00205 -----------------CAAACGTTTCCCC---GACGT-CGCTTTCTC------
------
FC88X_00143_00445 -----------------CAAACGTTTCCCC---GACGT-CGCTTTCTC------
------
FC88X_00113_00450 -----------------CAAACGTTTCCCC---GACGT-CGCTTTCTC------
------
FC88X_00146_00578 -----------------CAAACGTTTCCCC---GACGT-CGCTTTCTC------
------
FC88X_00107_00913 -----------------CAAACGTTTCCCC---GACGT-CGCTTTCTC------
------
FC88X_00156_00422 -----------------CAAACGTTTCCCC---GACGT-CGCTTTCTC------
------
FC88X_00193_00886 -----------------CAAACGTTTCCCC---GACGT-CGCTTTCTC------
------
FC88X_00162_00936 -----------------CAAACGTTTCCCC---GACGT-CGCTTTCTC------
------
FC88X_00161_00984 -----------------CAAACGTTTCCCC---GACGT-CGCTTTCTC------
------
FC88X_00182_01022 -----------------CAAACGTTTCCCC---GACGT-CGCTTTCTC------
------
FC88X_00188_01052 -----------------CAAACGTTTCCCC---GACGT-CGCTTTCTC------
------
FC88X_00220_00017 -----------------CAAACGTTTCCCC---GACGT-CGCTTTCTC------
------
FC88X_00215_00092 -----------------CAAACGTTTCCCC---GACGT-CGCTTTCTC------
------
FC88X_00228_00095 -----------------CAAACGTTTCCCC---GACGT-CGCTTTCTC------
------
FC88X_00235_00829 -----------------CAAACGTTTCCCC---GACGT-CGCTTTCTC------
------
FC88X_00222_01040 -----------------CAAACGTTTCCCC---GACGT-CGCTTTCTC------
------
FC88X_00238_01027 -----------------CAAACGTTTCCCC---GACGT-CGCTTTCTC------
------
FC88X_00284_01083 -----------------CAAACGTTTCCCC---GACGT-CGCTTTCTC------
------
FC88X_00339_01134 -----------------CAAACGTTTCCCC---GACGT-CGCTTTCTC------
------
FC88X_00386_00010 -----------------CAAACGTTTCCCC---GACGT-CGCTTTCTC------
------
FC88X_00376_00384 -----------------CAAACGTTTCCCC---GACGT-CGCTTTCTC------
------

FC88X_00389_00913 -----------------CAAACGTTTCCCC---GACGT-CGCTTTCTC------
------
FC88X_00352_01026 -----------------CAAACGTTTCCCC---GACGT-CGCTTTCTC------
------
FC88X_00409_00451 -----------------CAAACGTTTCCCC---GACGT-CGCTTTCTC------
------
FC88X_00440_01027 -----------------CAAACGTTTCCCC---GACGT-CGCTTTCTC------
------
FC88X_00479_00030 -----------------CAAACGTTTCCCC---GACGT-CGCTTTCTC------
------
FC88X_00493_00385 -----------------CAAACGTTTCCCC---GACGT-CGCTTTCTC------
------
FC88X_00484_01111 -----------------CAAACGTTTCCCC---GACGT-CGCTTTCTC------
------
FC88X_00474_01172 -----------------CAAACGTTTCCCC---GACGT-CGCTTTCTC------
------
FC88X_00522_00130 -----------------CAAACGTTTCCCC---GACGT-CGCTTTCTC------
------
FC88X_00501_00198 -----------------CAAACGTTTCCCC---GACGT-CGCTTTCTC------
------
FC88X_00539_00718 -----------------CAAACGTTTCCCC---GACGT-CGCTTTCTC------
------
FC88X_00529_01007 -----------------CAAACGTTTCCCC---GACGT-CGCTTTCTC------
------
FC88X_00537_01024 -----------------CAAACGTTTCCCC---GACGT-CGCTTTCTC------
------
FC88X_00544_01124 -----------------CAAACGTTTCCCC---GACGT-CGCTTTCTC------
------
FC88X_00549_01172 -----------------CAAACGTTTCCCC---GACGT-CGCTTTCTC------
------
FC88X_00552_00066 -----------------CAAACGTTTCCCC---GACGT-CGCTTTCTC------
------
FC88X_00568_01080 -----------------CAAACGTTTCCCC---GACGT-CGCTTTCTC------
------
FC88X_00563_01139 -----------------CAAACGTTTCCCC---GACGT-CGCTTTCTC------
------
FC88X_00641_00193 -----------------CAAACGTTTCCCC---GACGT-CGCTTTCTC------
------
FC88X_00601_00287 -----------------CAAACGTTTCCCC---GACGT-CGCTTTCTC------
------
FC88X_00612_00648 -----------------CAAACGTTTCCCC---GACGT-CGCTTTCTC------
------
FC88X_00647_00931 -----------------CAAACGTTTCCCC---GACGT-CGCTTTCTC------
------
FC88X_00618_01217 -----------------CAAACGTTTCCCC---GACGT-CGCTTTCTC------
------
FC88X_00615_01272 -----------------CAAACGTTTCCCC---GACGT-CGCTTTCTC------
------
FC88X_00769_00312 -----------------CAAACGTTTCCCC---GACGT-CGCTTTCTC------
------
FC88X_00765_01048 -----------------CAAACGTTTCCCC---GACGT-CGCTTTCTC------
------
FC88X_00777_01175 -----------------CAAACGTTTCCCC---GACGT-CGCTTTCTC------
------
FC88X_00763_01232 -----------------CAAACGTTTCCCC---GACGT-CGCTTTCTC------
------

```
FC88X_00767_01236       -----------------CAAACGTTTCCCC---GACGT-CGCTTTCTC------
------
FC88X_00798_01251       -----------------CAAACGTTTCCCC---GACGT-CGCTTTCTC------
------
FC88X_00830_00769       -----------------CAAACGTTTCCCC---GACGT-CGCTTTCTC------
------
FC88X_00818_00854       -----------------CAAACGTTTCCCC---GACGT-CGCTTTCTC------
------
FC88X_00896_00198       -----------------CAAACGTTTCCCC---GACGT-CGCTTTCTC------
------
FC88X_00882_00325       -----------------CAAACGTTTCCCC---GACGT-CGCTTTCTC------
------
FC88X_00875_00525       -----------------CAAACGTTTCCCC---GACGT-CGCTTTCTC------
------
FC88X_00882_00566       -----------------CAAACGTTTCCCC---GACGT-CGCTTTCTC------
------
FC88X_00891_00793       -----------------CAAACGTTTCCCC---GACGT-CGCTTTCTC------
------
FC88X_00857_00833       -----------------CAAACGTTTCCCC---GACGT-CGCTTTCTC------
------
FC88X_00890_01009       -----------------CAAACGTTTCCCC---GACGT-CGCTTTCTC------
------
FC88X_00933_00186       -----------------CAAACGTTTCCCC---GACGT-CGCTTTCTC------
------
FC88X_00941_00373       -----------------CAAACGTTTCCCC---GACGT-CGCTTTCTC------
------
FC88X_00932_00571       -----------------CAAACGTTTCCCC---GACGT-CGCTTTCTC------
------
FC88X_00910_00611       -----------------CAAACGTTTCCCC---GACGT-CGCTTTCTC------
------
FC88X_00916_00806       -----------------CAAACGTTTCCCC---GACGT-CGCTTTCTC------
------
FC88X_00914_01199       -----------------CAAACGTTTCCCC---GACGT-CGCTTTCTC------
------
FC88X_00993_00561       -----------------CAAACGTTTCCCC---GACGT-CGCTTTCTC------
------
FC88X_00996_00911       -----------------CAAACGTTTCCCC---GACGT-CGCTTTCTC------
------
FC88X_00962_01111       -----------------CAAACGTTTCCCC---GACGT-CGCTTTCTC------
------
FC88X_01033_00664       -----------------CAAACGTTTCCCC---GACGT-CGCTTTCTC------
------
FC88X_01073_00404       -----------------CAAACGTTTCCCC---GACGT-CGCTTTCTC------
------
FC88X_01087_00633       -----------------CAAACGTTTCCCC---GACGT-CGCTTTCTC------
------
FC88X_01098_00965       -----------------CAAACGTTTCCCC---GACGT-CGCTTTCTC------
------
FC88X_01089_01167       -----------------CAAACGTTTCCCC---GACGT-CGCTTTCTC------
------
FC88X_01127_00703       -----------------CAAACGTTTCCCC---GACGT-CGCTTTCTC------
------
FC88X_01101_00792       -----------------CAAACGTTTCCCC---GACGT-CGCTTTCTC------
------
FC88X_00020_00315       -----------------CAAACGTTTCCCC---GACGT-CGCTTTCTC------
------
```

FC88X_00005_00357 -----------------CAAACGTTTCCCC---GACGT-CGCTTTCTC------
------
FC88X_00006_00383 -----------------CAAACGTTTCCCC---GACGT-CGCTTTCTC------
------
FC88X_00040_00472 -----------------CAAACGTTTCCCC---GACGT-CGCTTTCTC------
------
FC88X_00061_00032 -----------------CAAACGTTTCCCC---GACGT-CGCTTTCTC------
------
FC88X_00068_00062 -----------------CAAACGTTTCCCC---GACGT-CGCTTTCTC------
------
FC88X_00081_00104 -----------------CAAACGTTTCCCC---GACGT-CGCTTTCTC------
------
FC88X_00070_00345 -----------------CAAACGTTTCCCC---GACGT-CGCTTTCTC------
------
FC88X_00098_00362 -----------------CAAACGTTTCCCC---GACGT-CGCTTTCTC------
------
FC88X_00092_00474 -----------------CAAACGTTTCCCC---GACGT-CGCTTTCTC------
------
FC88X_00097_00475 -----------------CAAACGTTTCCCC---GACGT-CGCTTTCTC------
------
FC88X_00121_00065 -----------------CAAACGTTTCCCC---GACGT-CGCTTTCTC------
------
FC88X_00138_00140 -----------------CAAACGTTTCCCC---GACGT-CGCTTTCTC------
------
FC88X_00125_00224 -----------------CAAACGTTTCCCC---GACGT-CGCTTTCTC------
------
FC88X_00105_00432 -----------------CAAACGTTTCCCC---GACGT-CGCTTTCTC------
------
FC88X_00104_00689 -----------------CAAACGTTTCCCC---GACGT-CGCTTTCTC------
------
FC88X_00138_00696 -----------------CAAACGTTTCCCC---GACGT-CGCTTTCTC------
------
FC88X_00135_00759 -----------------CAAACGTTTCCCC---GACGT-CGCTTTCTC------
------
FC88X_00149_00795 -----------------CAAACGTTTCCCC---GACGT-CGCTTTCTC------
------
FC88X_00104_00885 -----------------CAAACGTTTCCCC---GACGT-CGCTTTCTC------
------
FC88X_00110_00877 -----------------CAAACGTTTCCCC---GACGT-CGCTTTCTC------
------
FC88X_00136_00925 -----------------CAAACGTTTCCCC---GACGT-CGCTTTCTC------
------
FC88X_00143_00984 -----------------CAAACGTTTCCCC---GACGT-CGCTTTCTC------
------
FC88X_00163_00043 -----------------CAAACGTTTCCCC---GACGT-CGCTTTCTC------
------
FC88X_00174_00075 -----------------CAAACGTTTCCCC---GACGT-CGCTTTCTC------
------
FC88X_00222_00095 -----------------CAAACGTTTCCCC---GACGT-CGCTTTCTC------
------
FC88X_00204_00107 -----------------CAAACGTTTCCCC---GACGT-CGCTTTCTC------
------
FC88X_00208_00115 -----------------CAAACGTTTCCCC---GACGT-CGCTTTCTC------
------
FC88X_00237_00650 -----------------CAAACGTTTCCCC---GACGT-CGCTTTCTC------
------

FC88X_00242_00747 -----------------CAAACGTTTCCCC---GACGT-CGCTTTCTC------
------
FC88X_00240_00965 -----------------CAAACGTTTCCCC---GACGT-CGCTTTCTC------
------
FC88X_00240_01077 -----------------CAAACGTTTCCCC---GACGT-CGCTTTCTC------
------
FC88X_00276_00565 -----------------CAAACGTTTCCCC---GACGT-CGCTTTCTC------
------
FC88X_00258_00749 -----------------CAAACGTTTCCCC---GACGT-CGCTTTCTC------
------
FC88X_00251_00972 -----------------CAAACGTTTCCCC---GACGT-CGCTTTCTC------
------
FC88X_00301_00665 -----------------CAAACGTTTCCCC---GACGT-CGCTTTCTC------
------
FC88X_00370_00596 -----------------CAAACGTTTCCCC---GACGT-CGCTTTCTC------
------
FC88X_00385_00619 -----------------CAAACGTTTCCCC---GACGT-CGCTTTCTC------
------
FC88X_00392_01212 -----------------CAAACGTTTCCCC---GACGT-CGCTTTCTC------
------
FC88X_00432_00017 -----------------CAAACGTTTCCCC---GACGT-CGCTTTCTC------
------
FC88X_00448_00468 -----------------CAAACGTTTCCCC---GACGT-CGCTTTCTC------
------
FC88X_00440_00674 -----------------CAAACGTTTCCCC---GACGT-CGCTTTCTC------
------
FC88X_00417_00998 -----------------CAAACGTTTCCCC---GACGT-CGCTTTCTC------
------
FC88X_00437_01054 -----------------CAAACGTTTCCCC---GACGT-CGCTTTCTC------
------
FC88X_00429_01103 -----------------CAAACGTTTCCCC---GACGT-CGCTTTCTC------
------
FC88X_00428_01211 -----------------CAAACGTTTCCCC---GACGT-CGCTTTCTC------
------
FC88X_00470_00077 -----------------CAAACGTTTCCCC---GACGT-CGCTTTCTC------
------
FC88X_00470_00386 -----------------CAAACGTTTCCCC---GACGT-CGCTTTCTC------
------
FC88X_00468_01099 -----------------CAAACGTTTCCCC---GACGT-CGCTTTCTC------
------
FC88X_00462_01135 -----------------CAAACGTTTCCCC---GACGT-CGCTTTCTC------
------
FC88X_00480_01109 -----------------CAAACGTTTCCCC---GACGT-CGCTTTCTC------
------
FC88X_00456_01167 -----------------CAAACGTTTCCCC---GACGT-CGCTTTCTC------
------
FC88X_00544_01080 -----------------CAAACGTTTCCCC---GACGT-CGCTTTCTC------
------
FC88X_00536_01124 -----------------CAAACGTTTCCCC---GACGT-CGCTTTCTC------
------
FC88X_00501_01151 -----------------CAAACGTTTCCCC---GACGT-CGCTTTCTC------
------
FC88X_00559_00772 -----------------CAAACGTTTCCCC---GACGT-CGCTTTCTC------
------
FC88X_00571_01183 -----------------CAAACGTTTCCCC---GACGT-CGCTTTCTC------
------

FC88X_00611_00123 ------------------CAAACGTTTCCCC---GACGT-CGCTTTCTC------
------
FC88X_00637_00871 ------------------CAAACGTTTCCCC---GACGT-CGCTTTCTC------
------
FC88X_00609_01158 ------------------CAAACGTTTCCCC---GACGT-CGCTTTCTC------
------
FC88X_00629_01193 ------------------CAAACGTTTCCCC---GACGT-CGCTTTCTC------
------
FC88X_00654_00237 ------------------CAAACGTTTCCCC---GACGT-CGCTTTCTC------
------
FC88X_00691_00364 ------------------CAAACGTTTCCCC---GACGT-CGCTTTCTC------
------
FC88X_00660_00897 ------------------CAAACGTTTCCCC---GACGT-CGCTTTCTC------
------
FC88X_00691_00948 ------------------CAAACGTTTCCCC---GACGT-CGCTTTCTC------
------
FC88X_00662_01251 ------------------CAAACGTTTCCCC---GACGT-CGCTTTCTC------
------
FC88X_00732_00059 ------------------CAAACGTTTCCCC---GACGT-CGCTTTCTC------
------
FC88X_00744_00915 ------------------CAAACGTTTCCCC---GACGT-CGCTTTCTC------
------
FC88X_00732_00959 ------------------CAAACGTTTCCCC---GACGT-CGCTTTCTC------
------
FC88X_00740_01158 ------------------CAAACGTTTCCCC---GACGT-CGCTTTCTC------
------
FC88X_00789_00100 ------------------CAAACGTTTCCCC---GACGT-CGCTTTCTC------
------
FC88X_00779_00219 ------------------CAAACGTTTCCCC---GACGT-CGCTTTCTC------
------
FC88X_00768_00260 ------------------CAAACGTTTCCCC---GACGT-CGCTTTCTC------
------
FC88X_00756_00860 ------------------CAAACGTTTCCCC---GACGT-CGCTTTCTC------
------
FC88X_00756_01250 ------------------CAAACGTTTCCCC---GACGT-CGCTTTCTC------
------
FC88X_00838_00243 ------------------CAAACGTTTCCCC---GACGT-CGCTTTCTC------
------
FC88X_00803_00250 ------------------CAAACGTTTCCCC---GACGT-CGCTTTCTC------
------
FC88X_00817_00672 ------------------CAAACGTTTCCCC---GACGT-CGCTTTCTC------
------
FC88X_00873_00648 ------------------CAAACGTTTCCCC---GACGT-CGCTTTCTC------
------
FC88X_00850_00793 ------------------CAAACGTTTCCCC---GACGT-CGCTTTCTC------
------
FC88X_00896_00832 ------------------CAAACGTTTCCCC---GACGT-CGCTTTCTC------
------
FC88X_00896_00845 ------------------CAAACGTTTCCCC---GACGT-CGCTTTCTC------
------
FC88X_00874_00971 ------------------CAAACGTTTCCCC---GACGT-CGCTTTCTC------
------
FC88X_00898_01121 ------------------CAAACGTTTCCCC---GACGT-CGCTTTCTC------
------
FC88X_00913_00224 ------------------CAAACGTTTCCCC---GACGT-CGCTTTCTC------
------

FC88X_00922_00381 -----------------CAAACGTTTCCCC---GACGT-CGCTTTCTC------
------
FC88X_00901_00583 -----------------CAAACGTTTCCCC---GACGT-CGCTTTCTC------
------
FC88X_00916_00559 -----------------CAAACGTTTCCCC---GACGT-CGCTTTCTC------
------
FC88X_00931_00578 -----------------CAAACGTTTCCCC---GACGT-CGCTTTCTC------
------
FC88X_00904_00647 -----------------CAAACGTTTCCCC---GACGT-CGCTTTCTC------
------
FC88X_00907_00745 -----------------CAAACGTTTCCCC---GACGT-CGCTTTCTC------
------
FC88X_00945_00768 -----------------CAAACGTTTCCCC---GACGT-CGCTTTCTC------
------
FC88X_00919_00939 -----------------CAAACGTTTCCCC---GACGT-CGCTTTCTC------
------
FC88X_00937_00984 -----------------CAAACGTTTCCCC---GACGT-CGCTTTCTC------
------
FC88X_00929_01205 -----------------CAAACGTTTCCCC---GACGT-CGCTTTCTC------
------
FC88X_00958_00225 -----------------CAAACGTTTCCCC---GACGT-CGCTTTCTC------
------
FC88X_00967_00539 -----------------CAAACGTTTCCCC---GACGT-CGCTTTCTC------
------
FC88X_00968_00524 -----------------CAAACGTTTCCCC---GACGT-CGCTTTCTC------
------
FC88X_00985_00548 -----------------CAAACGTTTCCCC---GACGT-CGCTTTCTC------
------
FC88X_00961_00557 -----------------CAAACGTTTCCCC---GACGT-CGCTTTCTC------
------
FC88X_00965_00621 -----------------CAAACGTTTCCCC---GACGT-CGCTTTCTC------
------
FC88X_00974_00781 -----------------CAAACGTTTCCCC---GACGT-CGCTTTCTC------
------
FC88X_00982_00847 -----------------CAAACGTTTCCCC---GACGT-CGCTTTCTC------
------
FC88X_00959_01028 -----------------CAAACGTTTCCCC---GACGT-CGCTTTCTC------
------
FC88X_00989_01183 -----------------CAAACGTTTCCCC---GACGT-CGCTTTCTC------
------
FC88X_00966_01207 -----------------CAAACGTTTCCCC---GACGT-CGCTTTCTC------
------
FC88X_01009_00433 -----------------CAAACGTTTCCCC---GACGT-CGCTTTCTC------
------
FC88X_01047_00409 -----------------CAAACGTTTCCCC---GACGT-CGCTTTCTC------
------
FC88X_01045_00460 -----------------CAAACGTTTCCCC---GACGT-CGCTTTCTC------
------
FC88X_01000_00546 -----------------CAAACGTTTCCCC---GACGT-CGCTTTCTC------
------
FC88X_01013_01035 -----------------CAAACGTTTCCCC---GACGT-CGCTTTCTC------
------
FC88X_01046_01234 -----------------CAAACGTTTCCCC---GACGT-CGCTTTCTC------
------
FC88X_01006_01274 -----------------CAAACGTTTCCCC---GACGT-CGCTTTCTC------
------

FC88X_01037_01256 -----------------CAAACGTTTCCCC---GACGT-CGCTTTCTC------
------
FC88X_01078_00643 -----------------CAAACGTTTCCCC---GACGT-CGCTTTCTC------
------
FC88X_01098_00629 -----------------CAAACGTTTCCCC---GACGT-CGCTTTCTC------
------
FC88X_01072_00828 -----------------CAAACGTTTCCCC---GACGT-CGCTTTCTC------
------
FC88X_01051_01027 -----------------CAAACGTTTCCCC---GACGT-CGCTTTCTC------
------
FC88X_01103_00435 -----------------CAAACGTTTCCCC---GACGT-CGCTTTCTC------
------
FC88X_01103_00510 -----------------CAAACGTTTCCCC---GACGT-CGCTTTCTC------
------
FC88X_01120_00748 -----------------CAAACGTTTCCCC---GACGT-CGCTTTCTC------
------
FC88X_01104_00858 -----------------CAAACGTTTCCCC---GACGT-CGCTTTCTC------
------
FC88X_01139_00902 -----------------CAAACGTTTCCCC---GACGT-CGCTTTCTC------
------
FC88X_01134_00954 -----------------CAAACGTTTCCCC---GACGT-CGCTTTCTC------
------
FC88X_01138_00956 -----------------CAAACGTTTCCCC---GACGT-CGCTTTCTC------
------
FC88X_01119_01028 -----------------CAAACGTTTCCCC---GACGT-CGCTTTCTC------
------
FC88X_01139_01007 -----------------CAAACGTTTCCCC---GACGT-CGCTTTCTC------
------
FC88X_00426_00231 ----------------ACAAACGTTTCCCC---GACGT-CGCTTTCTC------
------
FC88X_00128_00693 -----------------CAAACGTTTCCC----GACGT-CGCTTTCTC------
------
FC88X_00328_01078 -----------------CAAACGTTTCCCA---GACGT-CGCTTTCTC------
------
FC88X_00644_00365 -----------------CAAACGTTTCCCC---GACGT-CGCTTTCTCCC----
------
FC88X_01110_00883 -----------------CAAACGTTTCCCC---GACGT-CGCTTTCTCCC----
------
FC88X_00115_00707 -----------------CAAACGTTTCCCC---GACGT-CGCTTTCTCCC----
------
FC88X_01136_01166 -----------------CAAACGTTTCCCC---GACG-CCGCTTTCTC------
------
FC88X_00145_01001 -----------------CAAACGTTTCCCC---GACGTCCGCTTTCTC------
------
FC88X_00050_00620 -----------------CAAACGTCTCCCC---GACGT-CGCTTTCTC------
------
FC88X_01015_01027 -----------------CAAACGTCTCCCC---GACGT-CGCTTTCTC------
------
FC88X_00957_00510 -----------------CAAACGTTTTCCCC--GACGT-CGCCTTTCTC-----
------
FC88X_00807_00493 -----------------CAAACGTTTTCCC---GACGT-CGCTTTCTC------
------
FC88X_00101_00725 -----------------CAAACGTTTTCCCC--GACGT-CGCTTTCTC------
------
FC88X_00609_01141 -----------------CAAACGTTTTCCCC--GACGT-CGCTTTCTC------
------

FC88X_01015_00454 -----------------CAAACGTTTTCCCC--GACGT-CGCTTTCTC------
------
FC88X_01026_01186 -----------------CAAACGTTTTCCCC--GACGT-CGCTTTCTC------
------
FC88X_01137_00880 -----------------CAAACGTTTTCCCC--GACGT-CGCTTTCTC------
------
FC88X_00060_00128 ----------------GCAAACGTTTTCCCC--GACGT-CGCTTTCTC------
------
FC88X_00969_00940 -----------------CAAACGCTTCCCC---GACGT-CGCTTTCTC------
------
FC88X_01139_00864 -----------------CAAACGCTTCCCC---GACGT--GCTTTCTC------
------
FC88X_00042_00196 ----------------ACAAACGTTTTCCCC--GACGT-CGCTTTCTC------
------
FC88X_00394_01032 ----------------CAAAACGTTTCCCC---GACGT-CGCTTTCTC------
------
FC88X_01130_01109 ----------------CAAAACGTTTCCCC---GACGT-CGCTTTCTC------
------
FC88X_00094_00495 ----------------CAAAACGTTTCCCC---GACGT-CGCTTTCTC------
------
FC88X_00312_01016 ----------------CAAAACGTTTCCCC---GACGT-CGCTTTCTC------
------
FC88X_00367_01151 ----------------CAAAACGTTTCCCC---GACGT-CGCTTTCTC------
------
FC88X_00815_00167 ----------------CAAAACGTTTCCCC---GACGT-CGCTTTCTC------
------
FC88X_01080_01262 ----------------CAAAACGTTTCCCC---GACGT-CGCTTTCTCC-----
------
FC88X_00895_00982 -----------------TAAACGTTTCCCC---GACGT-CGCTTTCTC------
------
FC88X_00916_01008 -----------------CAAACGTTCCCCC---GACGT-CGCTTTCTC------
------
FC88X_00971_00625 -----------------CAAACGTTCCCCC---GACGT-CGCTTTCTC------
------
FC88X_00537_00948 ----------------CAAAACGTTCCCCC---GACGT-CGCTTTCTC------
------
FC88X_00623_01026 -----------------CAAACGTTACCCC---GACGT-CGCTTTCTC------
------
FC88X_00178_00867 ----------------GCAAACGTTTCCCC---GCCGT-CGCTTTCTC------
------
FC88X_00093_00596 -----------------CAAACGTTTCCCC---GGCGT-CGCTTTCTC------
------
FC88X_00104_00218 -----------------CAAACGTTTCCCC---AACGT-CGCTTTCTC------
------
FC88X_00118_00073 -----------------CAAACGTTTCCCC---AACGT-CGCTTTCTCC-----
------
FC88X_00579_00950 -----------------CAAACGTTTCCCC---GACGT-
CGCTTTCTCCTTCCTTT----
FC88X_00865_00765 -----------------CAAACGTTTCCCC---GACGT-CGCTTTCTCCTTCC-
------
FC88X_00640_01121 -----------------CAAACGTTTCCCC---GACGT-
CGCTTTCTCCTTCCTTT----
FC88X_00733_00942 -----------------CAAACGTTTCCCC---GACGT-
CGCTTTCTCCTTCCTTT----
FC88X_00089_00385 -----------------CAAACGTTTCCCC---GACGT-
CGCTTTCTCCTTCCTTT----

FC88X_00367_01180 -----------------CAAACGTTTCCCC---GACGT-
CGCTTTCTCCTTCCTTT----
FC88X_01128_00844 -----------------CAAACGTTTCCCC---GACGT-
CGCTTTCTCCTTCCTTT----
FC88X_00079_00910 -----------------CAAACGTTTCC-C---GACGT-CACTTTCTC------
------
FC88X_00697_01237 -----------------CAAACGTTTCCTC---GACGT-CGCTTTCTC------
------
FC88X_00786_00577 -----------------CAAACGTTTCTCC---GACGT-CGCTTTCTC------
------
FC88X_00213_00900 -----------------CAAACGTTTCTCC---GACGT-CGCTTTCTC------
------
FC88X_00648_00948 -----------------CAAACGTTTCTCC---GACGT-CGCTTTCTC------
------
FC88X_00205_00082 ----------------GCAAACGTTTCTTC---GACGT-CGCTTTCTC------
------
FC88X_00339_01041 -----------------CAAACGTTTCTCC---GACGC-CGCTTTCTC------
------
FC88X_00137_00476 -----------------CAAACGTTTCCCC---GACGT-CGCTTTTCTC-----
------
FC88X_00945_00270 ----------------GCAAACGTTTCCCC---GACGT-CGCTTTTCTC-----
------
FC88X_01071_00703 -----------------CAAACGTTTCCCC---GACGT-CGCTTTTCTC-----
------
FC88X_00864_00774 -----------------CAAACGTTTCCCC---GACGT-CGCTTTTCTC-----
------
FC88X_00469_00090 -----------------CAAACGTTTCCCC---GACGT-CGTTTTCTC------
------
FC88X_00203_00134 -----------------CAAACGTTTCCCC---GACGT-CG-------------
------
FC88X_00514_00981 -----------------CAAACGTTTCCCC---GACGT-CGCTTCCCC------
------
FC88X_00809_00816 -----------------CAAACGTTTCCCC---GACGT-CGCTT----------
------
FC88X_00155_00158 -----------------CAAACGTTTCCCC---GACGT-CGCTTTCCC------
------
FC88X_01034_00726 -----------------CAAACGTTTCCCC---GACGT-CGCTTTCCCC-----
------
FC88X_00504_01048 -----------------CAAACGTTTCCCC---GACGT-CGCTTTCCC------
------
FC88X_00549_01243 ----------------GCAAACGTTTCCCC---GACGT-CGCTTTTTC------
------
FC88X_01012_00811 -----------------CAAACGTTTCCCC---GACGT-CGCTTTTTC------
------
FC88X_00805_00315 -----------------CAAACGTTTCCCC---GACGT-CG-------------
------
FC88X_00384_01189 -----------------CAAACGTTTCCCC---GACGT-CGCCTTCTC------
------
FC88X_00654_01138 -----------------CAAACGTTTCCCC---GACGT-CGCCTTCTC------
------
FC88X_00917_00438 -----------------CAAACGTTTCCCC---GACGT-CGCCTTCTC------
------
FC88X_01022_00482 -----------------CAAACGTTTCCCC---GACGT-CGCCTTCTC------
------
FC88X_01004_00524 -----------------CAAACGTTTCCCC---GACGT-CGCCTTCTC------
------

FC88X_01059_00689   ----------------GCAAACGTTTCCCC---GACGT-CGCTGTCTC------
------
FC88X_00190_01037   -----------------CAAACGTTTCCCC---GACGT-CGCT-TCTC------
------
FC88X_00920_01230   -----------------CAAACGTTTCCCC---GACGT-CGCT-TCTC------
------
FC88X_00491_00156   -----------------CAAACGTTTCCCC---GACGT-CGCTCTCTC------
------
FC88X_00889_00569   -----------------CAAACGTTTCCCC---GACGT-CGCTCTCTC------
------
FC88X_01086_00743   ----------GGGGAAGCAAACGTTTCCCC---GACGT-CGCTCTCTC------
------
FC88X_01120_01064   -----------------CAAACGTTTCCCC---GACGT-CGCTTCTC-------
------
FC88X_00090_00425   -----------------CAAACGTTTCCCC---GACGT-CGCATTCTC------
------
FC88X_00488_00356   -----------------CAAACGTTTCCCC---GACGT-CGCGTTCTC------
------
FC88X_01005_00941   -----------------CAAACGTTTCCCC---GACGT-CGCGTTCTC------
------
FC88X_01116_00599   -----------------CAAACGTTTCCCC---GACGT-CGCGTTCTC------
------
FC88X_00976_00529   -----------------CAAACGTTTCCCC---GACGT-
CGCTTCTCCTCCTTCCTTCCT
FC88X_00751_01186   ----------------CAAAACGTTTCCCC---
GACGTCGCTTTTCTCCTTCCTTTC---
FC88X_00841_00314   -----------------CTAACCGTTTCCCG--CACGTCCGCGTTTCTCC----
------
FC88X_00061_00362   -----------------CAAACGTTTCCGCA-
CGCCGCTTTCTCCTTCCTTCTC------
FC88X_00630_00088   -----------------CAAACGTTTCCACG-
CCGCTTCTCCTTCCTTCTCCTTC-----
FC88X_00874_00385   ---------------------TAATCGCGCG-TCGTTTCTACTTTTCTC-----
------
FC88X_00247_00943   ---------------------TAATCGCGCG-TCGTTTCTACTTTTCTC-----
------
FC88X_00399_00342   -----------------------CTGCGCGC-TGT--TCTACTTCTTGATTCC-
------
FC88X_00884_00989   -----------------------CTGCACCC-TTTCCTCTGCTACTCTGAT---
------
FC88X_00463_01220   -----------------------CTGCACGC-GGCTTTCATCTCCTTTAAC---
------
FC88X_00126_00818   ----------------CTTTACACTGCTTTG-CTCTCTATTTAC----------
------
FC88X_01025_01055   ----------------CTTCACATCGGCCGT-GCATCTCTTTTC----------
------
FC88X_00887_00146   --------------------ACACAGGACCG-ACCCCTAGCTATTTTC------
------
FC88X_00478_00734   ----------------GCTTACACAG---CA-GTGCCTAGATTTTCTC------
------
FC88X_01001_01146   -------------------CCCGCGCTTCTT-CTGATTAAATTTATT-------
------
FC88X_01046_01048   -------------------CCCGGACTTCAC-CTGCTTGAAAATACT-------
------
FC88X_00239_00987   -------------------ACCGCCGTTCTC-CTATTTTAAAG-----------
------

FC88X_00920_00377 -------------GGCTACCACGCTCTACTC-CTAGTTAAC-------------
------
FC88X_00796_00901 ---------------GATGA-CACGCGGCCG----CATCTCTATTCTT------
------
FC88X_01013_00573 ----------------ATGA-CACGCGGCCG----CATCTCTATTCTT------
------
FC88X_00064_00191 ----------------ATGA-CACGCGGCCG----CATCTCTATTCTT------
------
FC88X_00123_00579 ----------------ATGA-CACGCGGCCG----CATCTCTATTCTT------
------
FC88X_00161_00875 ----------------ATGA-CACGCGGCCG----CATCTCTATTCTT------
------
FC88X_00594_00140 ----------------ATGA-CACGCGGCCG----CATCTCTATTCTT------
------
FC88X_00551_00330 ----------------ATGA-CACGCGGCCG----CATCTCTATTCTT------
------
FC88X_00703_00394 ----------------ATGA-CACGCGGCCG----CATCTCTATTCTT------
------
FC88X_00844_00976 ----------------ATGA-CACGCGGCCG----CATCTCTATTCTT------
------
FC88X_00822_01178 ----------------ATGA-CACGCGGCCG----CATCTCTATTCTT------
------
FC88X_01033_00323 ----------------ATGA-CACGCGGCCG----CATCTCTATTCTT------
------
FC88X_01016_00644 ----------------ATGA-CACGCGGCCG----CATCTCTATTCTT------
------
FC88X_01032_00995 ----------------ATGA-CACGCGGCCG----CATCTCTATTCTT------
------
FC88X_01051_00517 ----------------ATGA-CACGCGGCCG----CATCTCTATTCTT------
------
FC88X_00289_00974 ----------------ATGA-CACGCGGCCG----CATCTCTATTCTT------
------
FC88X_00299_01092 ----------------ATGA-CACGCGGCCG----CATCTCTATTCTT------
------
FC88X_00340_00422 ----------------ATGA-CACGCGGCCG----CATCTCTATTCTT------
------
FC88X_00315_01056 ----------------ATGA-CACGCGGCCG----CATCTCTATTCTT------
------
FC88X_00477_00637 ----------------ATGA-CACGCGGCCG----CATCTCTATTCTT------
------
FC88X_00631_00982 ----------------ATGA-CACGCGGCCG----CATCTCTATTCTT------
------
FC88X_00766_00999 ----------------ATGA-CACGCGGCCG----CATCTCTATTCTT------
------
FC88X_00813_00121 ----------------ATGA-CACGCGGCCG----CATCTCTATTCTT------
------
FC88X_00878_00645 ----------------ATGA-CACGCGGCCG----CATCTCTATTCTT------
------
FC88X_00904_00393 ----------------ATGA-CACGCGGCCG----CATCTCTATTCTT------
------
FC88X_00300_00319 ----------------ATGA-CACGCGGCC-----CAT-TCTATTCTT------
------
FC88X_00055_00415 ----------------ATGA-CACGCGGCCG----CATCTCTATTCCT------
------
FC88X_00763_00908 ----------------ATGA-CACGCGGTCCG--CCATCTCTATTCTTCC----
------

```
FC88X_00654_00168       ----------------ATGAACACGCGGCCG----CATCTCTACTTCTT-----
------
FC88X_00987_01003       -------------CCGGTAGTTTCGCGGAC-----CATAGCTCTT---------
------
FC88X_00694_00453       -------------CCGGTAGTTTCGCGGAC-----CATAGCTCTT---------
------
FC88X_01107_00947       -------------CCGGTAGTTTCGCGGAC-----CATAGCTCTT---------
------
FC88X_01124_01260       -------------CCGGTAGTTTCGCGGAC-----CATAGCTCTTC--------
------
FC88X_00964_00707       -------------CCAGTA-TCTCGCGGTC-----TCCATCTGTT---------
------
FC88X_00162_00848       -----------------CTCTTGCGTAT-GTGTTGTCTAATTCTC---------
------
FC88X_00228_01028       -----------------CTCTTGCGTAT-GTGTTGTCTAATTCTC---------
------
FC88X_00642_01241       -----------------CTCTTGCGTAT-GTGTTGTCTAATTCTC---------
------
FC88X_01007_00338       -----------------CTCTTGCGTAT-GTGTTGTCTAATTCTC---------
------
FC88X_01023_00312       -----------------CTCTTGCGTAT-GTGTTGTCTAATTCTC---------
------
FC88X_01077_01199       -----------------CTCTTGCGTAT-GTGTTGTCTAATTCTC---------
------
FC88X_00085_00265       -----------------CTCTTGCGTAT-GTGTTGTCTAATTCTC---------
------
FC88X_00192_00392       -----------------CTCTTGCGTAT-GTGTTGTCTAATTCTC---------
------
FC88X_00385_00987       -----------------CTCTTGCGTAT-GTGTTGTCTAATTCTC---------
------
FC88X_00472_01000       -----------------CTCTTGCGTAT-GTGTTGTCTAATTCTC---------
------
FC88X_00488_01210       -----------------CTCTTGCGTAT-GTGTTGTCTAATTCTC---------
------
FC88X_00840_00531       -----------------CTCTTGCGTAT-GTGTTGTCTAATTCTC---------
------
FC88X_00835_00987       -----------------CTCTTGCGTAT-GTGTTGTCTAATTCTC---------
------
FC88X_01039_01125       -----------------CTCTTGCGTAT-GTGTTGTCTAATTCTC---------
------
FC88X_00029_00211       -----------------CTCTTGCGTAT-GTGTTGTCTAATTCTC---------
------
FC88X_00635_00186       -----------------CTCTTGCGTAT-GTGTTGTCTAATTCT----------
------
FC88X_00516_01234       -----------------CTCCTGCGTAT-GTGTTGTCTAATTCTC---------
------
FC88X_00389_00117       -----------------CTCTCGCGTAT-GTGTTGTCTAATTCTC---------
------
FC88X_00762_01126       -----------------CTCTCGCGTAT-GTGTTGTCTAATTCTC---------
------
FC88X_00461_01219       -------------------CTTGCGCATCGAGCTGTCCAACTCCTT--------
------
FC88X_00338_01144       ----------------TTCGTTGCCGAC--TGCTG-CTATTCCCC---------
------
FC88X_01078_00864       ---------------TTTCGTTGCCGAC--TGCTG-CTATTCT-----------
------
```

```
FC88X_00280_00944       ----------------AACGGTGCTCAC-GTGTTGTCTATTCTT----------
------
FC88X_00669_00796       -----------------ACCTCCCTTAGTC-ATTTTCCACTTTTT---------
------
FC88X_00782_00146       -----------------ACGTTTCTTCGTCTGTTGCCCACTTAT----------
------
FC88X_00521_00042       -----------------GCCCTACGTAGCCAGTTCTCCAATTTT----------
------
FC88X_00147_00087       --------------------CCCCGGTCT-TGTCGACGGTTATTCGTA------
------
FC88X_00151_00211       --------------------CCCCGGTCT-TGTCGACGGTTATTCGTA------
------
FC88X_00490_00042       --------------------CCCCGGCCT-TGTCGACGGTTATTCGTA------
------
FC88X_00478_01218       --------------------TCCCTGTCT-TCTCAACGTTTTCGTATG------
------
FC88X_00254_00911       -------------TTCGACTCCC-TGTCG-TATCGA-CATTTTC----------
------
FC88X_00588_01156       -------------TTCGACTCCCCTGTCG-TATCGA-CATTTTC----------
------
FC88X_01143_00804       -------------TTCGACTCCC-TGTCG-TATCGAACATTTTC----------
------
FC88X_00605_01212       -------------CTCGACTCCC-TGTCG-TATCGA-CATTTTC----------
------
FC88X_00472_01235       -------------TTCGACCTCCCTGTCG-TATCGA-CATTTTT----------
------
FC88X_00019_00197       --------------CCTAACCCACGGCCT-TATCGACTTATT------------
------
FC88X_00158_00938       --------------ACTATCCCTTGGCCGATACCCACTTTT-------------
------
FC88X_00138_00652       -----------------CTTGCGCCTATAGTGTCTAGGCCTCCT----------
------
FC88X_01099_00793       ---------------TCTTTGCACTTAT-GTTTCTCGTCCTCT-----------
------
FC88X_00888_01194       --------------TTACTACTCCTGTGTCTGACGCGTCCT-------------
------
FC88X_01106_00816       ------------------CTCCTGCGTGTTTGACGCCTCCTTTT----------
------
FC88X_00898_00517       -----------------CTATCCCTTTGAGTTTCGAGTCCTTGAC---------
------
FC88X_00492_01012       -----------------GCCACGCCTTGTGTGTCGCCTCCACTT----------
------
FC88X_00057_00482       ----------------ATCCACACCCT---TGGCCTCTCCACTTAC--------
------
FC88X_00006_00496       -----------------TGGGTCACTTGTAGGTGCCACTGTTTTC---------
------
FC88X_00822_00780       -----------------TGGGTCACTTGTAGGTGCCACTGTTTTC---------
------
FC88X_00828_00848       -----------------TGGGTCACTTGTAGGTGCCACTGTTTTC---------
------
FC88X_01044_01192       -----------------TGGGTCACTTGTAGGTGCCACTGTTTTC---------
------
FC88X_00072_00439       -----------------TGGGTCACTTGTAGGTGCCACTGTTTTC---------
------
FC88X_00109_00361       -----------------TGGGTCACTTGTAGGTGCCACTGTTTTC---------
------
```

FC88X_00498_01122 -----------------TGGGTCACTTGTAGGTGCCACTGTTTTC---------
------
FC88X_00537_00242 -----------------TGGGTCACTTGTAGGTGCCACTGTTTTC---------
------
FC88X_00536_01204 -----------------TGGGTCACTTGTAGGTGCCACTGTTTTC---------
------
FC88X_00981_00299 -----------------TGGGTCACTTGTAGGTGCCACTGTTTTC---------
------
FC88X_01085_00493 -----------------TGGGTCACTTGTAGGTGCCACTGTTTTC---------
------
FC88X_00047_00773 ---------GGGGGAATTGGGTCACTTGTAGGTGCCACTGTTTTC---------
------
FC88X_00192_00561 -----------------TGGGTCACTTGTAGGTGC-ACTGTTTTC---------
------
FC88X_00667_01250 -----------------TGGGTCACTTGTAGGTGC-ACTGTTTTC---------
------
FC88X_00050_00092 -----------------TGGGCCACTTGTAGGTGCCACTGTTTTC---------
------
FC88X_00267_01111 -----------------TGGGCCACTTGTAGGTGCCACTGTTTTC---------
------
FC88X_00066_00379 -----------------TGGGTCACTTGTAGGTGCCGCTGTTTTC---------
------
FC88X_00671_00909 -----------------TGGGTCACTTGTAGGTGCCGCTGTTTTC---------
------
FC88X_00752_00873 -----------------TGGGTCACTTGTAGGTGCCGCTGTTTTC---------
------
FC88X_00092_00661 -----------------TGGGTCACTTATAGGTGCCACTGTTTTC---------
------
FC88X_01126_01171 -----------------TGGGTCACTTATAGGTGCCACTGTTTTCCTTCCTTT-
------
FC88X_01035_00632 -----------------
TGGGTCACTTGTAGGTGCCACTGTTTTTCCTTCCTTT------
FC88X_01020_00701 -----------------TGGGTCACTTGTAGGTGCCACTGTTTTTC--------
------
FC88X_00060_00470 -----------------CAGGTCTTTTGTTGCCTCTGTTGTTCT----------
------
FC88X_00114_00871 -----------------CAGGTCTTTTGTTGCCTCTGTTGTTCT----------
------
FC88X_00723_00593 -----------------CAGGTCTTTTGTTGCCTCTGTTGTTCT----------
------
FC88X_00362_00485 -----------------CAGGTCTTTTGTTGCCTCTGTTGTTCTCTTCCTTT--
------
FC88X_00102_00131 --------------------TCCATCCCTTTGTTGTTCTGTGCTCTT-------
------
FC88X_00449_00432 -----------------ATCTCTTTCTTTTTACCCCACTGTTCTCTT-------
------
FC88X_01123_00870 ----------------------TTCCTTCTTGTTGC-CTGTTTTCTTGT-----
------
FC88X_00454_01031 --------------------ACTGCTCGTTGCCTGTTT-ATTCTTATC------
------
FC88X_00962_00357 --------------------ACTGCTCGTTGCCTGTTTTATTCTTATC------
------
FC88X_00177_00682 --------------------ACTGCTCGTTGCCTGTTT-ATTCTTATC------
------
FC88X_00870_00205 -----------------TCCACTACTCGGAGC-TGTTCACTTATT---------
------

FC88X_00670_00285 -------------ACCGCTGGCTCCCTAC--CTGGT-T-GTTCT----------
------
FC88X_01124_00557 ----------------CCTGGCACCTTACGCCTGGTGT-ATTCT----------
------
FC88X_00137_00068 -------------CAACCTGGGAAACTAGGCTTTGT-T-TTTCT----------
------
FC88X_00250_00194 ---------------ATCTAACG-TTTACGCCTGGTTT-ATTCT----------
------
FC88X_00610_00416 ---------------ATCTAACG-TTTACGCCTGGTTT-ATTCT----------
------
FC88X_00006_00626 ---------------ATCTAACG-TTTACGCCTGGTTT-ATTCT----------
------
FC88X_00033_00681 ---------------ATCTAACG-TTTACGCCTGGTTT-ATTCT----------
------
FC88X_00137_00079 ---------------ATCTAACG-TTTACGCCTGGTTT-ATTCT----------
------
FC88X_00372_01159 ---------------ATCTAACG-TTTACGCCTGGTTT-ATTCT----------
------
FC88X_00942_01218 ---------------ATCTAACG-TTTACGCCTGGTTT-ATTCT----------
------
FC88X_01027_00594 ---------------ATCTAACG-TTTACGCCTGGTTT-ATTCT----------
------
FC88X_01004_00984 ---------------ATCTAACG-TTTACGCCTGGTTT-ATTCT----------
------
FC88X_00066_00819 ---------------ATCTAACG-TTTACGCCTAGTTT-ATTCT----------
------
FC88X_00992_01008 ---------------ATCTAACG-TTTACGCCTGGTTTTATTCTCTTC------
------
FC88X_00413_00070 ---------------ATCTAACG-TTTGCGCCTGGTTT-ATTCT----------
------
FC88X_00067_00808 -------------CTCTCTAAC--TCTCCGCATGGTTT--TTCT----------
------
FC88X_00626_01214 ----------------ACTAGTG-ACTCCCCGTGGTTTTTTTTATC--------
------
FC88X_00314_00287 -----------------CCAACGGCTCGCTCATGGCTGGTTCTC----------
------
FC88X_00444_00482 -----------------CCAACGGCTCGCTCATGGCTGGTTCTC----------
------
FC88X_00950_00556 -----------------CCAACGGCTCGCTCATGGCTGGTTCTC----------
------
FC88X_00923_01264 -----------------CCAACGGCTCGCTCATGGCTGGTTCTC----------
------
FC88X_01075_00705 -----------------CCAACGGCTCGCTCATGGCTGGTTCTC----------
------
FC88X_00089_00045 -----------------CCAACGGTT----CATGGCTGGTTCTC----------
------
FC88X_00610_01246 ----------------CCAATCGATC-GATGCATGGTAGTTCTC----------
------
FC88X_00942_00351 ----------------ACTATCGCTCCGTTGCATAGTA-TTCTC----------
------
FC88X_00473_00445 -------------------ATCCCGCTATCCTTGGCTATTTCTCCT--------
------
FC88X_01029_00944 -------------------ATCCCGCTATCCTTGGCTATTTCTCCT--------
------
FC88X_00539_01036 --------------------TCCCGCGTTCTTTGCTGGTCATTTCTC-------
------

FC88X_00094_00121 ---------------GCCTGGCCTTTTTGTACCGGTTATTACTC----------
------
FC88X_00388_01115 ----------------CCTGGCCTTTTTG-ACCGGTTATTACTC----------
------
FC88X_00484_00967 ----------------CCTGGCCTTTTTG-ACCGGTTATTACTC----------
------
FC88X_00606_01247 ----------------CCTGGC-TTTTTG-ACCGGTTATTACTC----------
------
FC88X_00151_00632 ----------------CCTGGCCTTTTTG-ACCGATTATTACTC----------
------
FC88X_00918_00760 ----------------GCTTACTTTTCCCGGTTGCCT-GTTTTC----------
------
FC88X_00601_01271 ----------------GCTTACTTTTTCCGGTTGCCTTGTTTTCC---------
------
FC88X_00179_01049 ----------------GCTTGCCTTTTCCTGCCCATTTATCCC-----------
------
FC88X_00086_00139 -----------------TTCAACCATATCAGG--GCCTGCTATTCC--------
------
FC88X_01125_00845 -----------------TTCAACCATATCAGG--GCCTGCTATTCC--------
------
FC88X_00036_00601 -----------------TTCAACCATATCAGG--GCCTGCTATTCC--------
------
FC88X_00155_00624 ------------GGGGATTCAACCATATCAGG--GCCAGCTATTCC--------
------
FC88X_00938_00549 -----------------CTCAACCATATCAGG--GC-TGCTATTCC--------
------
FC88X_00922_00539 ----------GGGGAAGCTTAGGCATATCACCCTGCCTTCTATTCCT-------
------
FC88X_00124_00667 --------------CCAATGCGTCAA-TCAGACGGCCATCTCCTTCTTTCTCC-
------
FC88X_00458_00055 ------------------TTAGTCAT-TC-GACGGCCGCCTATTCTT-------
------
FC88X_00600_01015 ------------------------CTGTCATATCGACTTCTACTCCTAGTT---
------
FC88X_01035_00439 ------------------------CTGTCATATCGACTTCTACTCCTAGTT---
------
FC88X_00206_00103 ------------------------CTGTCATATCGACTTCTACTCCTAGTT---
------
FC88X_00599_01231 ------------------------CTGTCATATCGACTTCTACTCCTAGTT---
------
FC88X_00325_00968 -----------------------------ATACCGACTTCTATTCTC-------
------
FC88X_00875_00906 ---------------------------
TCATGTCGTCTCCTTTTCTTCACCCGT------
FC88X_00567_01229 --------------------ACTGCTATCCTCAGGCGTGCTATTCAA-------
------
FC88X_00758_01009 --------------------ACTGCTATCCTCAGGCGTGCTATTCAA-------
------
FC88X_01055_00732 --------------------ACTGCTATCCTCAGGCGTGCTATTCAA-------
------
FC88X_01137_00877 --------------------ACTGCTATCCTCAGGCGTGCTATTCAA-------
------
FC88X_00431_00065 --------------------ACTGCTATCCTCAGGCGTGCTATTCAA-------
------
FC88X_00546_01164 --------------------ACTGCTATCCTCAGGCGTGCTATTCAA-------
------

FC88X_00668_00099 --------------------ACTGCTATCCTCAGGCGTGCTATTCAA-------
------
FC88X_00728_01043 --------------------ACTGCTATCCTCAGGCGTGCTATTCAA-------
------
FC88X_00793_00806 --------------------ACTGCTATCCTCAGGCGTGCTATTCAA-------
------
FC88X_00895_00615 --------------------ACTGCTATCCTCAGGCGTGCTATTCAA-------
------
FC88X_00895_00652 --------------------ACTGCTATCCTCAGGCGTGCTATTCAA-------
------
FC88X_01122_00646 --------------------ACTGCTATCCTCAGGCGTGCTATTCAA-------
------
FC88X_00099_00656 --------------------ACTGCTATC-TCAGGCGTGCTATTCAA-------
------
FC88X_00402_01176 --------------------ACTGCTATCCCCGGGCGTGCTATTCAA-------
------
FC88X_00937_00908 --------------------ACTGTTATGCCGTGGCCTTCTATTTTC-------
------
FC88X_00808_01172 --------------------ACTGTTATGCCGTCGCCTTCTATTTTC-------
------
FC88X_01141_01114 --------------------ACTGGTATGCCGTCGCCTTCTATTTTC-------
------
FC88X_00621_00219 --------------------ACTATGCTCGGTGGCCTAT-TATTCTC-------
------
FC88X_00017_00204 -----------------GGGACTATGCTCGGTGGCCTAT-TATTCTC-------
------
FC88X_00891_00844 --------------------ACTATGCTCGGTGGCCTAT-TATTCTC-------
------
FC88X_00968_00602 --------------------ACTATGCTCGGTGGCCTAT-TATTCTC-------
------
FC88X_00995_01168 --------------------ACTATGCTCGGTGGCCTAT-TATTCTC-------
------
FC88X_00587_01212 --------------------ACTATGCTCGGTGGCCTAT-TATTCTC-------
------
FC88X_00994_00816 --------------------ACTATGCTCGGTGGCCTAT-TATTATTCTC----
------
FC88X_00065_00765 ---------------------ATACTCGTTGTGGCCTTTCTATTCTAC------
------
FC88X_00500_01127 ---------------------ATACTCGTTGTGGCCTTTCTATTCTAC------
------
FC88X_00073_00212 --------------------TCACGCAGTCTAC-GTTCCGTCT-ATTCT-----
------
FC88X_00089_00281 --------------------TCACGCAGTCTAC-GTTCCGTCT-ATTCT-----
------
FC88X_00999_00685 -------------------GTCACGCAGTCTAC-GTTTCGTCT-ATTCT-----
------
FC88X_00423_01126 --------------------TCGCGCAGTCTAC-GTTCCGTCT-ATTCT-----
------
FC88X_00786_00245 -------------------TCACCGCAGTCTACCGTTCCGTCT-ATTCTCCT--
------
FC88X_00644_00969 --------------------CCGCGTCTTCTCC--TTCCGTGTCATTCT-----
------
FC88X_00805_00795 --------------------CCGCGTCTTCTCC--TTCCGTGTCATTCT-----
------
FC88X_00083_00349 -----------------------CTGCGGCTTATGTTCCGTGGCCTTCTC----
------

```
FC88X_00577_01142      -----------------------CTGCGGCTTATGTTCCGTGGCCTTCTC----
------
FC88X_00799_00396      ----------------------
GCTGCGGCTTATGTTCCGTGGCCTTCTCCTTCCTT---
FC88X_00995_00727      -----------------------CTGCGGCTTATGTTCCGTGACCTTCTC----
------
FC88X_00868_00695      ----------------------TCTCGGGCTACTTGTCCGTCACTTCTC-----
------
FC88X_00813_00139      ----------------------TCTCGGGCTACTTGTCCGTCACTTCTC-----
------
FC88X_00924_00986      ---------------------TCTGTCCCTCACTTCTCCGACATTGTT------
------
FC88X_00818_01076      --------------------------CCGTTACTTCTTCGACAT----------
------
FC88X_00210_00591      --------------------CTTCCCGTACTCCTGTTGCGACATTGG-------
------
FC88X_00768_00155      --------------------TTTCCCGGTCTCCTTCTGCAGCATTGC-------
------
FC88X_00126_00633      ------------------------TTCTCCATTTGTCCCG-TTCTCCTTCTC--
------
FC88X_01143_00910      -----------------------CTTCACCATTTTCCGAGATTCTCCTAC----
------
FC88X_00125_00671      ------------------TGTGATTCCACCTTCTTTCACTATTCT---------
------
FC88X_00817_01071      --------------------CTCCGACACCTTTTTTCAGGATATTCTC------
------
FC88X_00478_00169      --------------------TGCCAAACCCTTCTG---CCACATGTCCT-----
------
FC88X_00518_01252      ---------------------CCCGACCCTGTCTGTGACCACATTGCC------
------
FC88X_00290_00962      -----------------------CCATTGCGCATGTGCCGTCTTCCTGTT----
------
FC88X_00794_00814      -----------------------CCATTGCGCATGTGCCGTCTTCCTGTT----
------
FC88X_00830_01096      -----------------------CCATTGCGCATGTGCCGTCTTCCTGTT----
------
FC88X_01009_00461      ----------------------CCCATTGCGC-TGCCGAATCTCCCTGTA----
------
FC88X_00872_00708      --------------TTTTCACCCCTTCTGCAGTTGCCGTAACTTCCTTTTCT--
------
FC88X_01121_00527      --------------------CCCCTTCTGCAGCAGACGTGTTATCCT-------
------
FC88X_00822_01143      ---------------CCAAGTTCCTTCTCCATGGCAATTACCCTTCCTTTC---
------
FC88X_00877_00809      ----------------------CCTTCTACATTCCGGT--CCCTTCTTTAT---
------
FC88X_00086_00409      ------------------CGATCGTGCCACATCCGTCCTATTCTT---------
------
FC88X_00826_00136      ------------------CGATCGTGCCACATCCGTCCTATTCTT---------
------
FC88X_00811_00171      ------------------CGATCGTGCCACATCCGTCCTATTCTT---------
------
FC88X_00004_00644      ------------------CGATCGTGCCACATCCGTCCTATTCTT---------
------
FC88X_00539_01250      ------------------CGATCGTGCCACATCCGTCCTATTCTT---------
------
```

```
FC88X_00586_00191      ------------------CGATCGTGCCACATCCGTCCTATTCTT---------
------
FC88X_00659_00302      ------------------CGATCGTGCCACATCCGTCCTATTCTT---------
------
FC88X_00935_00573      ------------------CGATCGTGCCACATCCGTCCTATTCTT---------
------
FC88X_01048_00561      ------------------CGATCGTGCCACATCCGTCCTATTCTT---------
------
FC88X_00945_01134      ------------------CGATCGTGCCACATCCGTCCTATTCTTCC-------
------
FC88X_00950_00808      ------------------CGATCGTGCCACGTCCGTCCTATTCTT---------
------
FC88X_00686_00464      -----------------GCGATCGTGCCACATCCGTCCTATTCTTCTTCCTTT-
------
FC88X_00056_00591      ---------------------TCTTGCACCTTCCCTCGGATTCTTCTTT-----
------
FC88X_00454_01000      ------------------------TCTCCCGTCCATCGCAACTCCCTTGTG---
------
FC88X_00982_00560      ----------------CACAAACGTGTCCCGTCCCGCGCAACCTTCCTT-----
------
FC88X_01029_00528      ------------------ATCTGATTACGCAACATCGCCCCTGTT---------
------
FC88X_00939_00277      --------------------CTGGTTACGCCATCGCACCAGTCTTCTC------
------
FC88X_00035_00371      ------------------CTATGCAACCGCTCTTG--TTATTCTCTT-------
------
FC88X_00882_00711      ------------------CGATGCCTCTGCTTTCGAATTATTCTCCTTCCTTT-
------
FC88X_01001_01117      --------------------CCGTTGCATCTTTTGCCCCCGCTTTCT-------
------
FC88X_01068_00889      --------------------CCGTTGCATCTTTTGCCCCCGCTTTCT-------
------
FC88X_00130_00581      --------------------CCGTTGCATCTTTTGCCCCCGCTTTCT-------
------
FC88X_00518_00156      --------------------CCGTTGCATCTTTTGCCCCCGCTTTCT-------
------
FC88X_00685_00972      --------------------CCGTTGCATCTTTTGCCCCCGCTTTCT-------
------
FC88X_01077_00506      --------------------CCGTTGCATCTTTTGCCCCCGCTTTCT-------
------
FC88X_00047_00456      --------------------CCGTTGCATCTTTTGCGCCCGCTTTCT-------
------
FC88X_01081_00835      --------------------CTGTCGCATC---TGCACTCGAATTCTGTT----
------
FC88X_00545_01074      ----------------------CATGCATCTTTTCC-GCATCATTTTGTT----
------
FC88X_00842_00228      ----------------------CATGCATCTTTTCC-GCATCATTTTGTT----
------
FC88X_00970_00936      ----------------------ATTGCATCTATTCTTACTTTGTTTCGT-----
------
FC88X_00320_01082      --------------------------TCTCATTGACATCCCTTCTTCATTTG--
------
FC88X_00942_00243      ------------------------CTTCTCATTGA-
GCCTCCTCTCTGCTCATT------
FC88X_00043_00759      -----------------GTTTCGCGTCTTCAGCGTTTCGATTCTC---------
------
```

FC88X_00667_00082 ------------------TTTCGCGTCTTCAGCGTTTCGATTCTC---------
------
FC88X_00904_01237 ------------------TTTCGCGTCTTCAGCCTTTCGATTCTC---------
------
FC88X_00923_00238 ------------------TTTCGCGTCCTCAGCCTTTCGATTCTC---------
------
FC88X_00566_00466 -----------------CTTTGCCGTATCCCGCATTATCGCTTT----------
------
FC88X_00068_00442 -----------------TCTTACCGTCCTCCGCA-CCTAGCTGTT---------
------
FC88X_00780_00154 -----------------CCTTACCGTCCTCCGCA-CCTAGCTGTT---------
------
FC88X_00116_00304 -----------------CCTTGACGTCTCCCGCATCCTTGATATT---------
------
FC88X_00105_00430 --------------TTTCGTTGCCGCCTCCCGCAGCGTGTC-------------
------
FC88X_00866_00772 -----------CCACGTCGTAGCCGTC-CCCGCATCATT---------------
------
FC88X_00254_00250 ---------------ATCAGAACCTTTCT--GCATCCCACTATT----------
------
FC88X_00924_00565 ---------------ATCAGAACCTTTCT--GCATCCCACTATT----------
------
FC88X_00056_00328 ---------------ATCAGAACCTTTCT--GCATCCCACTATT----------
------
FC88X_00161_00134 ---------------ATCAGAACCTTTCT--GCATCCCACTATT----------
------
FC88X_00224_00604 ---------------ATCAGAACCTTTCT--GCATCCCACTATT----------
------
FC88X_00206_00905 ---------------ATCAGAACCTTTCT--GCATCCCACTATT----------
------
FC88X_00941_00342 ---------------ATCAGAACCTTTCT--GCATCCCACTATT----------
------
FC88X_01078_01187 ---------------ATCAGAACCTTTCT--GCATCCCACTATT----------
------
FC88X_01144_01036 ---------------ATCAGAACCTTTCT--GCATCCCACTATT----------
------
FC88X_01017_00303 ---------------ATCAGAACCTTTCT--GCATCCCGCTATT----------
------
FC88X_00078_00296 ---------------ATCAGAACCTTTCT--GCATCC-ACTATTGTCTCCC---
------
FC88X_00379_00953 ---------------ATCAGAACCTTTCT--GCATCCCTCTCATT---------
------
FC88X_00936_00676 ----------------TCTGATCCTTGCC-AGCCTCCCGTTTTTC---------
------
FC88X_01045_00574 ---------------CTCGGACCATTGCCGAATCTCCCATTAT-----------
------
FC88X_00096_00719 -----------------CCAAATCCCTTTCATCATGCAAATGTT----------
------
FC88X_00923_00652 -----------------CCAAATCCCTTTCATCACGCAAATGTT----------
------
FC88X_00956_00955 ----------------TCAAAACCATTCTCCGCATCCAGTTGTT----------
------
FC88X_00667_00279 ------------------CCAAGAGTTCTCCTCATCCCGCGGTTC---------
------
FC88X_00901_00492 ------------------CCAAGAGTTCTCCTCATCCCGCGGTTC---------
------

FC88X_00251_00816 -----------------CCAAAACGTTATTGGCATCCCGCTGTG----------
------
FC88X_01089_00702 -----------------TTCTTACGTTATTCCCGTCCTGCTGTT----------
------
FC88X_00580_00213 -----------------CTACCTCAATCCCGTCATCGCGCTGTT----------
------
FC88X_00934_00584 -----------------CTACCTCAATCCCGTCATCGCGCTGTT----------
------
FC88X_00685_01234 ----------------TCCTCAACCTTTATCTCAATGTGCTGTT----------
------
FC88X_00330_01137 ----------------TTCTCAACCTTTATCTCAATGTGCTGTT----------
------
FC88X_00797_00616 ----------------TCCTCAACCTTCATCTCAACGTGCTGTT----------
------
FC88X_00130_00920 -------------------CTTGATGTCCTGAGGACCTTGCTTCTC--------
------
FC88X_00195_01053 -------------------CTTGATGTCCTGAGGACCTTGCTTCTC--------
------
FC88X_00735_00921 -------------------CTTGATGTCCTGAGGACCTTGCTTCTC--------
------
FC88X_00062_00828 -------------------CTTGATGTCCTGAGGACCTTGCTTCTC--------
------
FC88X_00698_00148 -------------------CTTGATGTCCTGAGGACCTTGCTTCTC--------
------
FC88X_00689_00669 -------------------CTTGATGTCCTGAGGACCTTGCTTCTC--------
------
FC88X_00699_01138 -------------------CTTGATGTCCTGAGGACCTTGCTTCTC--------
------
FC88X_00916_01212 -------------------CTTGATGTCCTGAGGACCTTGCTTCTC--------
------
FC88X_00999_00774 -------------------CTTGATGTCCTGAGGACCTTGCTTCTC--------
------
FC88X_01046_00717 -------------------CTTGATGTCCTGAGGACCTTGCTTCTC--------
------
FC88X_00156_00670 -------------------
CTTGATGTCCTGAGGACCTTGCTTCTCCTTCCTTT------
FC88X_00288_01061 ---------------------TCATGTCCTAA----CTTGTCTCTCATTATT--
------
FC88X_00562_00178 -----------------TTCATGACGTACCTATTCTCTCATCAC----------
------
FC88X_00820_00987 --------------------GCATTCCTGTACATGTCTAGCTTCTC--------
------
FC88X_00808_00094 --------------------GCATTCCTGTACATGTCTAGCTTCTC--------
------
FC88X_00220_00081 -------------------TCCTTCCCGATGCATCTCTACTTTTCT--------
------
FC88X_01118_00754 -----------------CTATTCCCGTTGTTATGCTTTT-CTTCGC--------
------
FC88X_00158_00549 -----------GGGGAAGTGTTACCTTTGTGATGCTCCTACTTCGCTCA-----
------
FC88X_00835_00187 --------------------CCCTAATATGGCCGACGTTCCTCTACT-------
------
FC88X_00944_00528 -------------------ATCCTACTAATGG-GACACTCCTGAACT-------
------
FC88X_00546_01117 --------------------ATATCGCACTCCCGACCTTACTGTTCT-------
------

```
FC88X_00813_01184       ----------------------TTCGCA-TGCCGACGTTCCTCCTCTTAT----
------
FC88X_00429_00013       --------------ACCGCCGTGCCACCGACGTGACTCCCC-------------
------
FC88X_00692_00213       ---------------ACTCGGTGCTAGCCGACTGTTCCTCCT------------
------
FC88X_00322_00015       -------------------
CCCGCTGCTAGCCTTTACCTCCCTGAACTTCCTTT------
FC88X_00845_00208       ------------------CCGCGCTACC-GCCTGTTCCTTCCTGGA--------
------
FC88X_00954_00821       ------------------ACGTGCGAACGGTCTGCATCTCCCTTCT--------
------
FC88X_01048_00487       -------------------CCCGTGCTTACTCTGTCTCTCCATGAT--------
------
FC88X_01093_00421       -----------------ACTGGAACTCCCCCACGTCTATGCTCG----------
------
FC88X_00992_00966       -----------------ACTGGAACTCCCCCACGTCTATGCTCG----------
------
FC88X_01105_00994       -----------------ACTGGAACTCTTCCACGTCTATGCTCG----------
------
FC88X_00757_00759       ------------------CTTGAACGCCTCC-CTTATTTGCTTTGCC-------
------
FC88X_00870_00881       ------------------ACGTCACGTATCTGCCTCCCTC-CTTAT--------
------
FC88X_01140_00889       -------------------CCGCACGTATCGGAACCTCCCACTTATT-------
------
FC88X_01008_00898       --------------------CCACGAGACCTGTGGACCTCCCTGCTG-------
------
FC88X_00894_00612       -------------------TTTACGTGTGTTGTGAACCTCCCTGGT--------
------
FC88X_00110_00381       -------------------TTGACGTATCTACCGTCTCTGCGTATT--------
------
FC88X_00404_01056       ------------------TTTGACGTATCCCCTGTCCCTGCCTGCT--------
------
FC88X_00023_00076       -------------------TTCACC-GATGTGTGACCTTCG--CCTGTT-----
------
FC88X_00205_00091       -------------------TTCACCCGATGTGTGACCTTCG--CCTGTT-----
------
FC88X_00101_00897       -------------------TTCACC-GATGTGTGACCTTCG--CCTGTT-----
------
FC88X_00433_00014       -------------------TTCACC-GATGTGTGACCTTCG--CCTGTT-----
------
FC88X_00519_01215       -------------------TTCACC-GATGTGTGACCTTCG--CCTGTT-----
------
FC88X_00250_00874       -------------------TTCACC-GATGTGTGACCTTCG--CCTGTT-----
------
FC88X_00075_00472       ------------------ATTCACC-GATGTGTGACCTTCG--CCTGTT-----
------
FC88X_00512_00438       -------------------TTCACC-GATGTGTGACCTTCG--CCTGTT-----
------
FC88X_00841_01264       -------------------TTCACC-GATGTGTGACCTTCG--CCTGTT-----
------
FC88X_00521_00745       -------------------TTCACC-GATGTGTGACCTTCG--CCTGTT-----
------
FC88X_01046_00949       -------------------TTCACC-GATGTGTGACCTTCG--CCTGTT-----
------
```

FC88X_01011_00967 -------------------TTCACC-GATGTGTGACCTTCG--CCTGTT-----
------
FC88X_00843_01155 -------------------TTCACCGATGGTGTGACCTTCGGCCCTGTT-----
------
FC88X_00341_00596 -------------------TTCACC-GACGTGTGACCTTCG--CCTGTT-----
------
FC88X_00898_00217 ------------TCAATCATTCACCTGAGGTACGTTCGCC--------------
------
FC88X_00821_00849 --------------------CTGGCATCGATGTGCCTTCGACTTCTC-------
------
FC88X_00657_01061 --------------------CTGGCATCGATGTGCCTTCGACTTCTC-------
------
FC88X_00940_00845 -----------------GGCGCAACTTTG---TGACGTCCACTTCTC-------
------
FC88X_00729_00361 -----------------GGCGCAACTTCG---TGACGTCCACTTCTC-------
------
FC88X_00589_00258 ----------------GGCTGCAACTTTG---AGACGTCCTCTTCTC-------
------
FC88X_00982_00793 ------------------CTTCAATTTCGATATGACGTCCACGTC---------
------
FC88X_00806_01240 --------------------TTATTTCATCTCTCGTCTCCATTTTTT-------
------
FC88X_00155_00878 ---------------------TCTTTGATCTCCGACTCTTGCACTTTT------
------
FC88X_00607_00642 ---------------------CAATTACTTTGTGTCCCTTTTGCCTTTT-----
------
FC88X_01083_00583 ------------------CATCAATAACACGCTGTGCTGTCCGCCTTCC-----
------
FC88X_00355_00292 --------------------CCAATAACGTGGC---CTCTCCGACTTTAC----
------
FC88X_01036_00512 ---------------------CTTCATTGGTGGCAAAGTCGGCTTTCT------
------
FC88X_00724_01217 ---------------------CTTCATTGGTGGCAAAGTCGGCTTTCT------
------
FC88X_01094_00445 --------------------CCTACTTTGATGGCCCAGTCTCCTTAC-------
------
FC88X_00106_00556 --------------CTTGTAACTATCATGATGGCACGGCGACTTCCT-------
------
FC88X_00991_00398 ----------------CTTAACTTCTATGTTGGC-CGGCT--------------
------
FC88X_00032_00182 --------------------------ACTTTGACCAAGTCGACTTGCCCTGTT-
------
FC88X_01021_00887 ----------------------------GTTGAC-
ATGTCGCCTTCTCCCGTTTACG---
FC88X_00787_01171 -------------------TTCGTCGACTTTT--TGTCCCGCATTCTT------
------
FC88X_01104_01087 -------------------TTCGTCGACCTTT--TGTCCCGTATTCTT------
------
FC88X_00594_01235 ---------------------CGTTGAACTTTCCTGTCCCCACGTATT------
------
FC88X_00072_00054 ------------------CCACACGTTATTCCCATTCTTGGCTGTC--------
------
FC88X_00268_00779 ------------------CCACACGTTATTCCCATTCTTGGCTGTC--------
------
FC88X_00755_00514 ------------------CCACACGTTATTCCCATTCTTGGCTGTC--------
------

FC88X_00954_00357 ------------------CCACACGTTATTCCCATTCTTGGCTGTC--------
------
FC88X_01145_00992 ------------------CCACACGTTACTCCCATTCTTGGCTGTCC-------
------
FC88X_00254_00926 ------------------CCACACGTTATTCCCACTCTTGGCTGTC--------
------
FC88X_00326_01144 ------------------CCGCACGTTATTCCCATTCTTGGCTGTC--------
------
FC88X_00151_00787 ------------------CCTCACGCTTTCCGCACG-TTCGCTGTC--------
------
FC88X_00915_00750 ------------------CCCCACGCTGTTCTTTC--TTCGCTTTCT-------
------
FC88X_00566_00057 ---------------------CCACATTCTC-CCATGTACGC-GTGTTAT----
------
FC88X_00979_00966 --------------------GTCACATTCTC-CCATGTACGC-GTGTTAT----
------
FC88X_00893_00369 ------------------------CCTTCTCACCATATACACCGTTTTACG---
------
FC88X_00161_00815 --------------------------ACTCCCGCATGTGGATTATGCTCCGCA-
------
FC88X_00773_00262 ---------------------------
CTCCACCTTCTGGGCTATGTACTCCGA------
FC88X_00856_01060 ---------------------------
CTCCCGCAGTTTGACCATGTTCATCCT------
FC88X_00910_00655 ---------------------------CTCCCCCGTTTGAATGTTGTGTGA---
------
FC88X_00065_00290 --------------------CCACCAGCTCCCATCTAC--ACGGTCCA------
------
FC88X_01081_00934 --------------------CCGCCAGTTCTCCCCTGTCTGCGGTGC-------
------
FC88X_00360_01095 ------------------CCTCACTTCATCCCGCGTCTGCTCAAT---------
------
FC88X_00520_00725 -------------------CTCACATCTTCTCG-GTCTACC-------------
------
FC88X_00049_00608 -------------------TTCCCTATGACCCG-GTTTACAA-ACTCG------
------
FC88X_00678_00074 --------------------TCACTTTGTCCCGAGTGCACAATACTC-------
------
FC88X_00639_00957 -----------------ACTTCCCTTTATCCCCGT--TGCAGTACA--------
------
FC88X_00911_00485 -----------------ACTTCCCTTTATCCCCGT--TGCAGTACA--------
------
FC88X_00825_01047 ---------------------CCCTTCATCGCGTTACTGGACCATAGT------
------
FC88X_00862_00791 ----------------------------TCGCATT--TGCACCGTA--------
------
FC88X_00100_00182 -------------------CTACGCATTTTGCGTGTTT-CACTATCT-------
------
FC88X_00467_00060 -------------------CTACGCATTTTGCGTGTTT-CACTATCT-------
------
FC88X_00271_01120 -------------------CTACGCATTTTGCGTGTTT-CACTATCT-------
------
FC88X_00890_00853 -------------------CTACGCATTTTGCGTGTTT-CACTATCT-------
------
FC88X_00073_00557 ------------------GCTACGCATTTTGCGTGTTTTCACTATCT-------
------

FC88X_00624_01020 -------------------CTACGCATCTTGCGTGTTT-CACTATCT-------
------
FC88X_00478_00817 -------------------CTACGCATTCCGCGTGTTT-CACTATCTC------
------
FC88X_00101_00418 -------------------TCACGCATTT-GCCCGTTT-GACTTTCAC------
------
FC88X_00336_01172 -------------------TCACGCATTT-GCTCGTTT-GACTTTCAC------
------
FC88X_00476_00566 -------------------TCACGCATCT-GC-CGTTT-GACTTTCAC------
------
FC88X_00564_00067 ------------------TCAACGCATTT-GCCCGGTT-GACTTTCA-------
------
FC88X_00160_00142 ------------------CCAATGCATTT-CACGTATA-G-CTGTCCT------
------
FC88X_00279_00874 -------------------CAACGCCTTGGCTCGCATT-GACTGTCT-------
------
FC88X_00087_00574 ---------------------ACGC-TTGATCGGCGTT-G-CTGTCTTCTC---
------
FC88X_00497_00950 ---------------------ACGC-TTGATCGGCGTT-G-CTGTCTTCTC---
------
FC88X_00512_01241 ---------------------ACGC-TTGATCGGCGTT-G-CTGTCTTCTC---
------
FC88X_01014_00755 ---------------------ACGC-TTGATCGGCGTT-G-CTGTCTTCTC---
------
FC88X_00814_00438 ------------------TGAACGCGTTGCGCGGCGCT---CCACTGTCTC---
------
FC88X_00063_00196 ------------------ACTTCTCATCACC-TCTAGCGCATTATA--------
------
FC88X_00824_00207 ------------------ACTTCTCATCACC-TCTAGCGCATTATA--------
------
FC88X_00962_00960 ------------------ACTTGGCA-CACG-TCACGCTCTTGATT--------
------
FC88X_00713_00433 -------------------CCTACGATTAGGACCCGAGGCGTTCTTA-------
------
FC88X_00862_00824 -------------------CCTACGATTAGGACCCGAGGCGTTCTTA-------
------
FC88X_00884_00399 ------------------CCCTTTGATCAGG-TCAACGGCTTTCTT--------
------
FC88X_00745_01221 ------------------CCCTTTGATCAGG-TCAACGGCTTTCTT--------
------
FC88X_00907_00228 ------------------CCCTTTGATCAGG-TCAACGGCTTTCTT--------
------
FC88X_01146_00802 ------------------CCCTTTGATCAGG-TCAACGGCTTTCTT--------
------
FC88X_00531_00961 ------------------CCCTTTGATCAGG-
TCAACGGCTTTCTTCTTCCTTT------
FC88X_00129_00204 ---------------------CCTGTAC-GTTTGT-ACACGTTATTCGCA----
------
FC88X_01061_01272 ---------------------CCTGTAC-GTTTGT-ACACGTTATTCGCAC---
------
FC88X_00754_00462 ---------------------CCTGTAC-GTTTGT-ACACGTTATTCGCA----
------
FC88X_00165_00123 ---------------------CCTGTAC-GTTTGT-ACACGTTATTCGCA----
------
FC88X_00292_01152 ---------------------CCTGTAC-GTTTGT-ACACGTTATTCGCA----
------

FC88X_00466_01137 ---------------------CCTGTAC-GTTTGT-ACACGTTATTCGCA----
------
FC88X_00456_01158 ---------------------CCTGTAC-GTTTGT-ACACGTTATTCGCA----
------
FC88X_00739_01075 ---------------------CCTGTAC-GTTTGT-ACACGTTATTCGCA----
------
FC88X_00994_00675 ---------------------CCTGTAC-GTTTGT-ACACGTTATTCGCA----
------
FC88X_00139_00057 ---------------------CCTGTAC-GTCTGT-ACACGTTATTCGCA----
------
FC88X_00016_00729 ---------------------CTCATGT-ACTTGTCACACGGGATTCTC-----
------
FC88X_00855_01054 ---------------------CTCATGT-ACTTGTCACACGGGATTCTC-----
------
FC88X_00567_00474 ---------------------
CCTGTTTTACCTTTAACTCGTTATTCTCCTTCCTTT---
FC88X_00904_00429 ---------------------CCTGTTT-
ACCTTTAACTCGTTATTCTCCTTCCTTT---
FC88X_00573_01135 ---------------------CCTGTTT-ACCTTTAACTCGTTATTCTT-----
------
FC88X_01046_00411 ---------------------CCTGTTT-ACCTTTAACTCGTTATTCTT-----
------
FC88X_00048_00312 ---------------------CCTGTTT-ACCTTTAACTCGTTATTCTT-----
------
FC88X_00043_00402 ---------------------CCTGTTT-ACCTTTAACTCGTTATTCTT-----
------
FC88X_00628_00985 ----------------------CTGTTT-ACCTTTAACTCGTTATTCTT-----
------
FC88X_01089_00430 --------------------GCCTGTTT-ACCTCTAACTCGTTATTCTT-----
------
FC88X_00351_00384 ---------------------CCCATTGCTTGTTCACACTGTTATTCT------
------
FC88X_01011_00601 -----------------------CCTTGCTGGTTCATCGCGTTTTTTCT-----
------
FC88X_00172_00916 ----------------GGGGACCCTTGGTTCTCTAACGTCGTTATTCT------
------
FC88X_00334_00343 ---------------------CCCTTGGTTCTCTAACGTCGTTATTCT------
------
FC88X_00030_00180 ---------------------CCCTTGATCCTTTAACA-CGTTATTGCT-----
------
FC88X_00841_00183 ----------------CTGTACGATGGATACTGCAGCCTTCT------------
------
FC88X_01086_00665 ----------------CTGTACGATGGATACTGCAGCCTTCT------------
------
FC88X_00096_00131 ----------------CTGTACGATGGATACTGCAGCCTTCT------------
------
FC88X_00647_01136 ----------------CTGTACGATGGATACTGCAGCCTTCT------------
------
FC88X_00678_00494 ----------------CTGTACGATGGATACTGCAGCCTTCT------------
------
FC88X_00818_00225 ----------------CTGTACGATGGATACTGCAGCCTTCT------------
------
FC88X_00834_00842 ----------------CTGTACGATGGATACTGCAGCCTTCT------------
------
FC88X_00651_01079 ----------------CTGTACGATGGATACTGCAGCCTTCT------------
------

```
FC88X_00014_00082      ---------------CCTGCACAGTTGATCATGCACCTTCTT------------
------
FC88X_00681_01219      --------------CTCGGCATAGT--TCATTGCGACCTCTT------------
------
FC88X_00173_00005      --------------CAATGCACAGTA-TCATTGCGCCCTTTCAC----------
------
FC88X_00186_00810      -------------------CCCAGGCATCAGTGCACCGTTGTTCTC--------
------
FC88X_00700_01014      -------------------CCCAGGCATCAGTGCACCGTTGTTCTC--------
------
FC88X_00655_00336      --------------------CCAGGCATCAGTGCACCGTTGTTCTC--------
------
FC88X_00527_00735      -------------------CCCAAGCATCAGTGCACCGTTGTTCTC--------
------
FC88X_00529_00973      -------------------CCCAAGCATCAGTGCACCGTTGTTCTC--------
------
FC88X_00788_01208      -------------------CCCAAGCATCAGTGCACCGTTGTTCTC--------
------
FC88X_00840_00890      -------------------CCCAAGCATCAGTGCACCGTTGTTCTC--------
------
FC88X_00892_00783      -------------------CCCAAGCATCAGTGCACCGTTGTTCTC--------
------
FC88X_00922_00884      -------------------CCCAAGCATCAGTGCACCGTTGTTCTC--------
------
FC88X_01123_00578      -------------------CCCAAGCATCAGTGCACCGTTGTTCTC--------
------
FC88X_00267_00924      -------------------CCCAAGCATCAGTGCACCGTTGTTCTC--------
------
FC88X_00456_01236      -------------------CCCAAGCATCAGTGCACCGTTGTTCTC--------
------
FC88X_00808_01040      -------------------CCCAAGCATCAGTGCACCGTTGTTCTC--------
------
FC88X_00875_01150      -------------------CCCAAGCATCAGTGCACCGTTGTTCTC--------
------
FC88X_00613_01020      -------------------CCCAAGCATCGGTGCACCGTTGTTCTC--------
------
FC88X_00721_00915      -------------------CCCGAGCATCAGTGCACCGTTGTTCTC--------
------
FC88X_00803_00222      -------------------CCTAAGCATCAGTGCACCGTTGTTCTC--------
------
FC88X_00441_01094      -------------------CCCAAGCATCAGTGCACCGTTGTTCCC--------
------
FC88X_00315_00761      ---------------------CTAGCA-CATTGCACCGCTCTTCCCCAA-----
------
FC88X_00997_01199      ---------------------CTAGCA-CATTGCACCGCTCTTCCCAA------
------
FC88X_00047_00580      ----------------TTTCAAAA--GCCAGTGCGACGTTCTCCC---------
------
FC88X_00481_00024      ----------------TTTAAAAATCGTCCCTGCCCCGTTGTCCT---------
------
FC88X_00137_00626      -----------------TTCACCGCTGTTCA-TCCCCACCGTCGC---------
------
FC88X_01014_00816      -----------------CTAACCACTGTGCA-TCACCGCTATCAC---------
------
FC88X_00172_00246      -----------------CTAACCAATTTCCA-TCGGCACCGTATT---------
------
```

FC88X_00350_01163 -----------------CTAACCAATTTCCA-TCGGCACCGTATT---------
------
FC88X_00559_00201 -----------------CTAACCAATTTCCA-TCGGCACCGTATT---------
------
FC88X_00770_01032 -----------------CTAACCAATTTCCA-TCGGCACCGTATT---------
------
FC88X_01117_00643 -----------------CTAACCAATTTCCA-TCGGCACCGTATT---------
------
FC88X_00882_00840 -----------------CTAACCAATTTTCA-TCGGCACCGTATT---------
------
FC88X_01007_01039 ----------------GCTAACCAATTCCCA-TCGGCACCGTACT---------
------
FC88X_00563_01243 ------------------TAACCACTATTC---CTGCACCGTCTCATC------
------
FC88X_00325_00967 -------------------CACCAACTTTGCCACGTTCCCAGACCT--------
------
FC88X_00023_00640 -----------------TTTACCAATGTTA--ACACTCCCGGTCCT--------
------
FC88X_00771_00210 --------------------ACCACCGTTCA-CCGCTCCCGTTATTCA------
------
FC88X_00048_00364 --------------GGGGAAACCACCGTTCA-CCGCTCCCGTTATTCT------
------
FC88X_00840_00770 --------------------ACCACCGTTTA-CCGCTCCCG--------------
------
FC88X_00062_00136 -----------------CCACGGTCCCTCTATCTG-GCACGTTCTC--------
------
FC88X_01039_00737 -----------------CCACGGTCCCTCTATCTG-
GCACGTTCTCCTTCCTTT------
FC88X_00533_00147 -------------------ACTATCCCTATACCAGAGCATGTTCTC--------
------
FC88X_00590_01057 -----------------CCATAGTACCAGAACCTG-GGTCGTTCT---------
------
FC88X_01083_00938 -----------------CCATAGTACCAGAACCTG-GGTCGTTCT---------
------
FC88X_00132_00508 ----------------CCCATAGTACCAGAACTTG-GGTCGTTCT---------
------
FC88X_00840_00891 -----------------CCAT-GTGCCGGTACCTG-TCGCGTTCTC--------
------
FC88X_00322_00613 -----------------CCAT-GTGCCGGTACCTG-TCGCGTTCTC--------
------
FC88X_00937_00969 -----------------CCAT-GTGCCGGTACCTG-TCGCGTTCTC--------
------
FC88X_00720_01217 -----------------CCAT-GTGCCGGTACCTG-TCGCGTTCTC--------
------
FC88X_00889_00441 -----------------CCATGTCGCGTCTGGCCC--CACGTTCTC--------
------
FC88X_01037_01257 ----------------GCCATGTCGCGTCTGGCCC--CACGTTCTC--------
------
FC88X_00959_01184 -----------------CCATGTCGCGTCTGGCCC--CACGTTCTC--------
------
FC88X_01101_01045 -----------------CCATGTCGCGTCTGGCCC--CACGTTCTC--------
------
FC88X_00144_00967 -----------------CCATGTCGCGTCTGGCCC--CACGTTCTC--------
------
FC88X_00176_00176 -----------------CCATGTCGCGTCTGGCCC--CACGTTCTC--------
------

```
FC88X_00410_00388      -----------------CCATGTCGCGTCTGGCCC--CACGTTCTC--------
------
FC88X_01137_01082      -----------------CCGCGTCGTTTCTGGATT--CGCATTCTC--------
------
FC88X_00404_00504      -----------------CCGCGTCGTTTCTGGATT--CGCATTCTC--------
------
FC88X_00829_00925      -----------------CCGCGTCGTTTCTGGATT--CGCATTCTC--------
------
FC88X_00069_00831      -----------------CCGCGTCGTTTCTGGACT--CGCATTCTC--------
------
FC88X_00870_00170      -----------------CCGTATCCTGTCGGTCTG--CACATTCTC--------
------
FC88X_00126_00702      -----------------CCGTATCCTGTCGGTCTG--CACATTCTC--------
------
FC88X_00940_01071      ----------------CTCCTGTCCCTGCACAGTT--CTCATGCA---------
------
FC88X_00032_00283      ----------------CTTCCCGATCTACCCC---AGCACACGTTGC-------
------
FC88X_00820_00130      ---------------TCTCCGCCTTCTACCCCGTTAGCACACCTT---------
------
FC88X_00696_00183      ----------------CCTCCCGCGTTCCCCC---AGCGCACGTTT--------
------
FC88X_00914_01195      -------------------------ATCCCCC---CGGGCTCGTTT--------
------
FC88X_00190_01059      -----------------TCACTGGATTCTCC-CGCAGTAC-GTTTAT-------
------
FC88X_00739_01022      -----------------TCACTGGATTCTCC-CGCAGTAC-GTTTAT-------
------
FC88X_00721_01234      --------------------CCGGCTTCTCGACACCGTACTGTTTCT-------
------
FC88X_00897_01238      --------------------CCGGCTTCTCGACACCGTACTGTTTCT-------
------
FC88X_01076_01215      ---------------------CCCCGGCCTCCGACAGTACATGTAAAT------
------
FC88X_00249_00295      --------------------TTCCCAACCCTGCGCAGTACAAGTATC-------
------
FC88X_00082_00512      -----------------TTACCGCATTTTCCCTGCCGTTCGCAT----------
------
FC88X_00404_00995      ----------------GTTACCGCATTTTCCCTGCCGTTCGCAT----------
------
FC88X_00719_00988      -----------------TTACCGCATTTTCCCTGCCGTTCGCAT----------
------
FC88X_01136_00954      -----------------TTACCGCATTTTCCCTGCCGTTCGCAT----------
------
FC88X_01015_00712      -----------------TTACCGCATTTCCCCTGCCGTTCGCAT----------
------
FC88X_00946_01195      --------------------CCGC-TGATCCGCCGCTGTAC-ATATCTCC----
------
FC88X_00938_00445      --------------------CCGCGTGATCCCTGCCTGTTCGATATT-------
------
FC88X_00121_00106      -----------------CCGATGATTGATTGGCCCCGCGCGTACTCC-------
------
FC88X_00022_00493      -----------------CCGATGATTGATTGGCCCCGTGCGTAC----------
------
FC88X_00096_00195      ------------------CGATAATTGATTGGCCCCGCGCGTAC----------
------
```

FC88X_00498_01213 ---------------TCTCAATGATTTAT-TCCCCCGGCGC-ACG---------
------
FC88X_00719_01105 ---------------TCTCAATGATTTAT-TCCCCCGGCGC-ACG---------
------
FC88X_00862_00929 ---------------TCTCAATGATTTAT-TCCCCCGGCGC-ACG---------
------
FC88X_00744_00335 ---------------TCTCAATGATTTAT-TCCCCTGGCGC-ACG---------
------
FC88X_01128_00594 ---------------TCTCAATGATTTAT-TCCCCCGGCGCCACG---------
------
FC88X_01104_01121 -----------------TCAATGACTATTGTCCGCCGGCGCCAC----------
------
FC88X_00288_00941 ------------------CTCTACT-GTT-TTCCCAGGCGTGTCGTT-------
------
FC88X_00816_01259 ------------------CTCTACT-GTT-TTCCCAGGCGTGTCGTT-------
------
FC88X_01108_00750 ------------------CTCTACT-GTT-TTCCCAGGCGTGTCGTT-------
------
FC88X_00912_00372 ------------------CTCTACTCGTT-TTCCCAGGCGTGTCGTT-------
------
FC88X_00608_00416 -------------------TCTATTGATTATTCCTGTGCCTCTCGT--------
------
FC88X_00727_01117 -------------------TCTATTGATTATTCCTGTGCCTCTCGT--------
------
FC88X_00114_00092 --------------------CTGCGTGTCCGATCGCGTCTGTATCTC-------
------
FC88X_00646_01064 --------------------CTGCGTGTCCGATCGCGTCTGTATCTCC------
------
FC88X_00888_00986 --------------------CTGCGTGTCCGATCGCGTCTGTATCTC-------
------
FC88X_00857_01029 --------------------CTGCGTGTCCGATCGCGTCTGTATCTC-------
------
FC88X_00853_01119 --------------------CTGCGTGTCCGATCGCGTCTGTATCTC-------
------
FC88X_00908_00217 --------------------CTGCGTGTCCGATCGCGTCTGTATCTC-------
------
FC88X_01064_00374 --------------------CTGCGTGTCCGATCGCGTCTGTATCTC-------
------
FC88X_01078_00934 --------------------CTGCGTGTCCGATCGCGTCTGTATCTC-------
------
FC88X_00022_00174 --------------------CTGCGTGTCCGATCGCGTCTGTATCTC-------
------
FC88X_00022_00250 --------------------CTGCGTGTCCGATCGCGTCTGTATCTC-------
------
FC88X_00057_00057 --------------------CTGCGTGTCCGATCGCGTCTGTATCTC-------
------
FC88X_00052_00207 --------------------CTGCGTGTCCGATCGCGTCTGTATCTC-------
------
FC88X_00093_00395 --------------------CTGCGTGTCCGATCGCGTCTGTATCTC-------
------
FC88X_00125_00121 --------------------CTGCGTGTCCGATCGCGTCTGTATCTC-------
------
FC88X_00454_01075 --------------------CTGCGTGTCCGATCGCGTCTGTATCTC-------
------
FC88X_00533_01170 --------------------CTGCGTGTCCGATCGCGTCTGTATCTC-------
------

FC88X_00646_01192 ---------------------CTGCGTGTCCGATCGCGTCTGTATCTC-------
------
FC88X_00794_00876 ---------------------CTGCGTGTCCGATCGCGTCTGTATCTC-------
------
FC88X_00843_00156 ---------------------CTGCGTGTCCGATCGCGTCTGTATCTC-------
------
FC88X_00874_00870 ---------------------CTGCGTGTCCGATCGCGTCTGTATCTC-------
------
FC88X_01078_01252 ---------------------CTGCGTGTCCGATCGCGTCTGTATCTC-------
------
FC88X_00074_00228 ---------------------CTGCGTGTCCGATCGCGTCTGTATCTCCC-----
------
FC88X_00415_00626 ---------------------CTGCGTGTCCGATCGCGCCTGTATCTC-------
------
FC88X_00520_00219 ---------------------TTGCGTGTCCGATCGCGTCTGTATCTC-------
------
FC88X_00157_00665 ---------------------CTGCGTGTCCGGTCGCGTCTGTATCTC-------
------
FC88X_00437_00019 ---------------------CTGCGTGTCCGATCGCGTCTGTGTCTC-------
------
FC88X_00351_00431 ---------------------CTGCGTGTCTGATCGCGTCTGTATCTC-------
------
FC88X_00760_00814 ---------------------
CTGCGTGTCCGATCGCGTCTGTATCTCCTTCCTTCTCTC-
FC88X_00794_00842 ---------------------CTGCGTGTCCGATCGCGTCTGTATCCC-------
------
FC88X_00509_01007 ---------------------CTGCGTGTCCGATCGCGTGTG-ATCT--------
------
FC88X_01027_00312 -----------------CACTTTACTTGTCCG-TCGCGTCTGTT-----------
------
FC88X_00829_00995 -----------------CACTTTACTTGTCCA-TCGCGTCTGTT-----------
------
FC88X_00113_00743 ---------------------TTGCCTGCTGTCC-ACGCCGCTGTTCT-------
------
FC88X_01007_00817 ---------------------TTGCCTGCTGCCCCACGCCGCTGTTCT-------
------
FC88X_01133_01014 ---------------------TTCTCTGCTTCTCCACTCGCGTGTCCT-------
------
FC88X_00559_01174 --------------------TTAGCCTGTCCCCG-GCCTTGTGTTCTC-------
------
FC88X_00593_00261 ---------------------TCGCCTCCCCTCGCGCAATGTGTTGTT-------
------
FC88X_01081_00755 -------------------CCCCGCACGTCGCTT-GCCCAGAGTTGTT-------
------
FC88X_00440_00308 -------------------CCCCGCACGTCGCTT-GCCCAGAGTTGTT-------
------
FC88X_00933_00411 -------------------CCCCGCACGTCGCTT-GCCCAGAGTTGTT-------
------
FC88X_00968_00650 -------------------CCCCGCACGTCGCTT-GCCCAGAGTTGTT-------
------
FC88X_00012_00391 -------------------CCTTGACCCCGGCCC--TCGCGCGCTGTT-------
------
FC88X_00995_00308 ------------------ACCACGACCACGTGTC--TCGGCTGTTACC-------
------
FC88X_00020_00384 --------------------CCTAGGCCTATCCGCCCGC-GTTGTTCT-------
------

FC88X_00965_01093 -------------------CCTAGGCCTATCCGCCCGCCGTTGTTCT-------
------
FC88X_00071_00112 -------------------CCTAGGCCTATCCGTCCGC-GTTGTTCT-------
------
FC88X_00454_00835 -------------------CCTAGGCCTATCCG-CCGC-GTTGTTCT-------
------
FC88X_00006_00199 -------------------CCTAGGCCTACCCGCCCGC-GTTGTTCT-------
------
FC88X_00324_01123 -------------------CCTAGGCCTATCCGCCCGC-
GTTGTTCTCTTCCTTTCTCC-
FC88X_00013_00126 --------------------CTAGGATCCGTCACGATTTGCTCTTCT-------
------
FC88X_00055_00859 -------------------TGTTACCGCGATGTACTGACACTTCTC--------
------
FC88X_00104_00422 -------------------TGTTACCGCGATGTACTGACACTTCTC--------
------
FC88X_00283_00528 -------------------TGTTACCGCGATGTACTGACACTTCTC--------
------
FC88X_00284_01102 -------------------TGTTACCGCGATGTACTGACACTTCTC--------
------
FC88X_00891_01034 -------------------TGTTACCGCGATGTACTGACACTTCTC--------
------
FC88X_00959_00411 -------------------TGTTACCGCGATGTACTGACACTTCTC--------
------
FC88X_01120_01150 -------------------TGTTACCGCGATGTACTGACACTTCTC--------
------
FC88X_01078_00782 -------------------TGTTACCGCGATGTATTGACACTTCTC--------
------
FC88X_00519_01247 ---------------------GCGTCGCGCTGTACGCACCTGTCTCATT-----
------
FC88X_00806_00880 ----------------------CCTCGCGCCGTTCGGCACTGCCTTGTT-----
------
FC88X_00189_00951 ------------------CACGGATCCTGCTCTCGCCGTGTTTCT---------
------
FC88X_00620_00410 ------------------CACGGATCCTGCTCTCGCCGTGTTTCT---------
------
FC88X_00461_00017 ------------------CACGGATCCTGCTCTCGCCGTGTTTTTCT-------
------
FC88X_00673_01186 -----------------CCACGGATCCCGAT-TCACTGCGCTTTTCT-------
------
FC88X_01073_00730 -----------------CCACGGATCCCGAT-TTACTGCGCTTTTCT-------
------
FC88X_00518_00514 -------------------TACGGACGT-CTCACCGTGCTGCTCTT--------
------
FC88X_00165_00731 ------------------GTACGGACGT-CTCACCGTGCTGCTCTT--------
------
FC88X_00449_01168 -------------------TACGGACGT-CTCACCGTGCTGCTCTT--------
------
FC88X_00604_01180 -------------------TACGGACGT-CTCACCGTGCTGCTCTT--------
------
FC88X_00796_01108 -------------------TACGGACGT-CTCACCGTGCTGCTCTT--------
------
FC88X_00576_01202 -------------------TACGGACGT-CTCACCGTGCTGCTCTTC-------
------
FC88X_00474_00985 -------------------TACGGACGT-CTCACCGTGCTGCTCTT--------
------

FC88X_00771_01165 -------------------TACGGACGT-CTCACCGTGCTGCTCTT--------
------
FC88X_00883_00291 -------------------TACGGACGT-CTCACCGTGCTGCTCTT--------
------
FC88X_01009_00533 -------------------TACGGACGT-CTCACCGTGCTGCTCTT--------
------
FC88X_01016_00962 -------------------TACGGACGT-CTCACCGTGCTGCTCTT--------
------
FC88X_00023_00539 -------------------TACGGACGC-CTCACCGTGCTGCTCTT--------
------
FC88X_00493_01212 -------------------TACGGACGT-CTCACCGTACTGCTCTT--------
------
FC88X_00276_00930 -------------------TACGGACGT-CTCACCGTGCT--------------
------
FC88X_00814_00934 -------------------TACGGACCT-CCCACCGTGCTGCTCTT--------
------
FC88X_00840_00936 -----------------CCAATGGTCCT-CCCGCTGTGC-GCTCTT--------
------
FC88X_00896_01242 -----------------CCAATGGTCCT-CCCGCTGTGC-GCTCTT--------
------
FC88X_00031_00634 -----------------CCAATGGTCCT-CCCGCTGTGC-GCTCTT--------
------
FC88X_00556_01229 -----------------CCAATGGTCCT-CCCGCTGTGC-GCTCTT--------
------
FC88X_00833_01212 -----------------CCAATGGTCCT-CCCGCTGTGC-GCTCTT--------
------
FC88X_01084_00403 -----------------CCAATGGTCCT-CCCGCTGTGC-GCTCTT--------
------
FC88X_00105_00395 -----------------CAAATGGTCCT-CCCGCTGTGC-GCTCTT--------
------
FC88X_00666_00087 -----------------CCAATGGTCCT-TCCGCTGTGC-GCTCTT--------
------
FC88X_00483_00026 -----------------CCAATGGTCCTACCCGCTGTGC-GCTCCTTCTTC---
------
FC88X_00811_00824 -----------------CCAATGGTCCT-CCCGCTGTGC-GCTCCT--------
------
FC88X_00114_00495 -------------------CATGATTTCTCCGT-TCTGC-GCTTGTC-------
------
FC88X_00635_00238 -------------------CATGAGTTCTCCGT-TCTGC-GCTTGTC-------
------
FC88X_00208_00124 ------------------ACATGTGATCTCCGCACTTGC-GCTCTT--------
------
FC88X_00290_00974 ------------------ACATGTGATCTCCGCACTTGC-GCTCTT--------
------
FC88X_00325_00942 ------------------ACATGTGATCTCCGCACTTGC-GCTCTT--------
------
FC88X_00627_00943 ------------------ACATGTGATCTCCGCACTTGC-GCTCTT--------
------
FC88X_00882_00192 ------------------ACATGTGATCTCCGCACTTGC-GCTCTT--------
------
FC88X_00895_00775 ------------------ACATGTGATCTCCGCACTTGC-GCTCCT--------
------
FC88X_00209_00127 ------------------ACATGCGATCTCCGCACTTGC-GCTCTT--------
------
FC88X_00229_01025 --------------------TTCACCGATCTGCCCC-TGTGGACTGCACT----
------

```
FC88X_00585_01028       --------------------TTCACCGATCTGCCCCCTGTGGACTGCA------
------
FC88X_00362_00662       -----------------ATCTAG-CCCGACTGGCCCGTCTGTTCT---------
------
FC88X_00422_01162       -----------------ATCTAG-CCCGACTGGCCCGTCTGTTCT---------
------
FC88X_00848_00251       -----------------ATCTAG-CCCGACTGGCCCGTCTGTTCT---------
------
FC88X_00956_00909       -----------------ATCTAG-CCCGACTGGCCCGTCTGTTCT---------
------
FC88X_00687_01118       -----------------ATCTAG-CCCGACTGGCCCGTCTGTTCT---------
------
FC88X_00992_01144       -----------------ATCTAG-CCCGACTGGCCTGTCTGTTCT---------
------
FC88X_01147_01058       ---------------CCATCTGATCCCTAGTGGCCTCGTTGTT-----------
------
FC88X_00091_00027       -------------------ACGAGGTGTCCTCCCCACCTTGTACTA--------
------
FC88X_00094_00418       -------------------ACGAGGTGTCCTCCCCACCTTGTACTA--------
------
FC88X_00923_00594       -------------------ACGAGGTGTCCTCCCCACCTTGTACTA--------
------
FC88X_00051_00712       -------------------ACGAGGTGTCCTCCC-ACCTTGTACTA--------
------
FC88X_00076_00823       -------------------ACGAGGTGTCCTCCCAACCTTGTACTA--------
------
FC88X_00278_01000       -------------------CTCCG--ATCTTGGCCACTCTGTACACGCC-----
------
FC88X_00643_01001       -------------------CTCCGTCAACCTGGCCA---TGTACAAGCC-----
------
FC88X_00185_00345       ----------------TCCCTATCCATGAGAAA-GCG--TGTACCC--------
------
FC88X_01140_00848       ------------------CATATCGATCCGATACGCGC-TGTACCC--------
------
FC88X_00470_00179       ----------------CCTATCCCGCTCTACCCCTCCGTTGTAC----------
------
FC88X_00836_01067       ------------------TTTCCCGATCTCCTACACACTTGTACC---------
------
FC88X_00086_00248       ------------------TTTTCCGACTAATGGAATTACTGATTCT--------
------
FC88X_00789_00207       ------------------TTTTCCGACTAATGGAATTACTGATTCT--------
------
FC88X_00066_00570       ------------------TTTTCCGACCAATGGAATTACTGATTCT--------
------
FC88X_00666_00902       -----------------TTTTACCGGCTAATGGAATTACTGATTCT--------
------
FC88X_00592_01175       --------------------TTCTCCCGAATTGAATGAGTGCGTCAT-------
------
FC88X_00095_00829       --------------------TTCTCCCGAATTGAATGAGTGCGTCAT-------
------
FC88X_00120_00329       --------------------TTCTCCCGAATTGAATGAGTGCGTCAT-------
------
FC88X_00833_00780       ---------------------TCTCCCAACTTGAGTCACTGAGTCCC-------
------
FC88X_01004_01195       ---------------------TCTCCCAACTTGAGTCACTGAGTCCC-------
------
```

```
FC88X_00272_00765      -----------------------CTCCCATCTTTGCGACTGCGTA-TTGGC---
------
FC88X_01033_00544      -----------------------CTCCCATCTTTGCGACTGCGTA-TTGGC---
------
FC88X_00082_00031      -----------------------CTCCCATCTTTGCGGCTGCGTACTTGGC---
------
FC88X_00863_00656      ----------------------TCGTCCGATTTTCCGCCTAAGCGTATT-----
------
FC88X_00667_01225      -----------------------CCACAGAT-GACCCGTTGTGCTGCTGAT---
------
FC88X_00610_00707      ----------------------CCAACAAATCGACCCATTTAGCTACT------
------
FC88X_00373_00822      --------------------------TAATTCGACCCATTTTGCTGACTATGC-
------
FC88X_00452_00136      ---------------------TTTATCTATTTTACGCATTTCCCTGT-------
------
FC88X_00649_00185      -------------------GTCATAACTACGTTAGAC-TTGCCCTGTA------
------
FC88X_00906_00619      ---------------------TTATCCTACCTTCAACGTTTACATGTT------
------
FC88X_00929_00244      ---------------------CTATCCCCCATTTAATTTGCAC-TGTT------
------
FC88X_00576_01164      --------------------GTCTGCCTAAATTCAGCATCTCATATT-------
------
FC88X_00707_00243      ---------------------CCAGCCTACTTTCATCAT---------------
------
FC88X_00642_01112      --------------CCTAGAGTTCACCGACTTTACGCAT---------------
------
FC88X_00116_00146      -----------------------CTCCCAACTAGACTAACGTTT-GTATA----
------
FC88X_01028_00922      ----------------------CTCCCAAACTAGACTAACGTTTTGTATA----
------
FC88X_00153_00840      -----------------------CTCCCAACTAGACTAACGTTT-GTATA----
------
FC88X_00206_00067      -------------------------CCCGACTCTCC--
TCGTTTTCCACGTCTC------
FC88X_00057_00138      -------------------------CCCGACTTTAC--TCGTTTTGGGAT----
------
FC88X_00944_01163      ------------------TCCTGACCCCGACTAACCGATCGTTTT---------
------
FC88X_00842_00590      -------------------TTACCACCTTGTCCGA-ATCAACTCATT-------
------
FC88X_00963_00901      -------------------TTACCGCCTGGCCCGCTATACACTCAT--------
------
FC88X_00051_00771      --------------------ATCCCCCTTTTGCGGTGTAAGCTTATT-------
------
FC88X_00541_01229      --------------------ATCCCCCTTTTGCGGTGTAAGCTTATT-------
------
FC88X_00815_00829      ------------------GTTCACGCCATTTCCGGTCTAA-CTCATT-------
------
FC88X_00113_00720      ----------------------TTCTCCGACACCCTGTAAACAGTTTTTTG---
------
FC88X_00429_01152      ----------------------TTCTCCGACACCCTGTAA-CAGTTTTTTG---
------
FC88X_01074_00734      --------------------CCTTCCCTTACGCTGTGTACTCATTTT-------
------
```

FC88X_00634_00283 ------------------------TCCTTTCCCTGAGAAACTTATGTTGCG---
------
FC88X_00028_00224 ---------------TCAGCGCATAT---GCCCGTTTACAATTCTC--------
------
FC88X_00685_00478 ---------------TCAGCGCATAT---GCCCGTTTACAATTCTCCC------
------
FC88X_00110_00422 ---------------TCAGCGCATAT---GCCCGTTTACAATTCTC--------
------
FC88X_00541_01166 ---------------TCAGCGCATAT---GCCCGTTTACAATTCTC--------
------
FC88X_00568_01218 ---------------TCAGCGCATAT---GCCCGTTTACAATTCTC--------
------
FC88X_00750_00651 ---------------TCAGCGCATAT---GCCCGTTTACAATTCTC--------
------
FC88X_00983_00514 ---------------TCAGCGCATAT---GCCCGTTTACAATTCTC--------
------
FC88X_00042_00270 ---------------TCAGCGCATAT---GCCCGTTTACAATTCTC--------
------
FC88X_00106_00923 ---------------TCAGCGCATAT---GCCCGTTTACAATTCTC--------
------
FC88X_00746_01228 --------------ATCAGCGCATAT---GCCCGTTTACAATTCTC--------
------
FC88X_00893_00461 ---------------TCAGCGCATAT---GCCCGTTTACAATTCTC--------
------
FC88X_01071_00523 ---------------TCAGCGCATAT---GCCCGTTTACAATTCTC--------
------
FC88X_00146_00769 ---------------TCAGCGCATAT---GCCCGTTTACAATTCTC--------
------
FC88X_00346_00544 ---------------TCAGCGCATAT---GCCCGTTTACAATTCTC--------
------
FC88X_00376_00463 ---------------TCAGCGCATAT---GCCCGTTTACAATTCTC--------
------
FC88X_00412_00297 ---------------TCAGCGCATAT---GCCCGTTTACAATTCTC--------
------
FC88X_00400_01156 ---------------TCAGCGCATAT---GCCCGTTTACAATTCTC--------
------
FC88X_00688_00250 ---------------TCAGCGCATAT---GCCCGTTTACAATTCTC--------
------
FC88X_00652_01152 ---------------TCAGCGCATAT---GCCCGTTTACAATTCTC--------
------
FC88X_00664_01255 ---------------TCAGCGCATAT---GCCCGTTTACAATTCTC--------
------
FC88X_00751_01009 ---------------TCAGCGCATAT---GCCCGTTTACAATTCTC--------
------
FC88X_00878_00370 ---------------TCAGCGCATAT---GCCCGTTTACAATTCTC--------
------
FC88X_00867_00588 ---------------TCAGCGCATAT---GCCCGTTTACAATTCTC--------
------
FC88X_00947_00276 ---------------TCAGCGCATAT---GCCCGTTTACAATTCTC--------
------
FC88X_01006_00369 ---------------TCAGCGCATAT---GCCCGTTTACAATTCTC--------
------
FC88X_01083_00452 ---------------TCAGCGCATAT---GCCCGTTTACAATTCTC--------
------
FC88X_01069_00735 ---------------TCAGCGCATAT---GCCCGTTTACAATTCTC--------
------

FC88X_01079_00997 ---------------TCAGCGCATAT---GCCCGTTTACAATTCTC--------
------
FC88X_00989_01039 --------------GTCAGCGCATAT---GCCCGTTTACAATTCTC--------
------
FC88X_00920_00456 ---------------TCAGCGCATAT---GCCCGTATACAATTCTC--------
------
FC88X_00686_00330 ---------------TCAGCGCATAT---GCTCGTTTACAATTCTC--------
------
FC88X_00045_00454 ---------------CCAGCGCATAT---GCCCGTTTACAATTCTC--------
------
FC88X_00930_00543 ---------------TCAGCGCATAT---GCCCGTTTACAGTTCTCC-------
------
FC88X_00942_00214 ---------------TCAGCGCATGT---GCCCGTTTACAATTCTC--------
------
FC88X_00860_01037 ---------------TCAGCGCATAT---TCCCGTTTACAATTCTC--------
------
FC88X_00089_00779 ----------------TGACGCATACTAGGGCCGTTCACACCTCTTCCTTT---
------
FC88X_00730_00939 ----------------TGACGCATACTAGGGCCGTTCACACCT-----------
------
FC88X_00081_00687 -----------------------TGA---GTCGTATTAGAATTCCCC-------
------
FC88X_00862_01067 ----------------------GTGA---GCGTCTTACCGATTTCACCTTTC--
------
FC88X_00992_01068 ---------------------TGTTATGCGCTTATCGTTGACACCTCC------
------
FC88X_01002_00993 ---------------------TGTTATGCGCTTATCGTTGACACCTCCC-----
------
FC88X_00076_00840 ---------------GGGGGATGTTATGCGCTTATCGTTGACACCTCC------
------
FC88X_00079_00196 ---------------------CTCTATGCGCT--TCGTTCCCACTTACC-----
------
FC88X_00464_00983 ----------------TCAAGG-TACCGTGCCTATGATTACTCCT---------
------
FC88X_00833_00940 ----------------TCAAGG-TACCGTGCCTATGATTACTCCT---------
------
FC88X_01001_00653 ----------------TCAAGCCTTCCGCGCCTA--ATTACTGTT---------
------
FC88X_00130_00301 -----------------------TTTCGACCCCACTTACATCGTGC--------
------
FC88X_00770_00482 -----------------TCAACCCTTCGACCACACCTAAATCCT----------
------
FC88X_00467_00169 ----------------------CTCCCCGGTCTTTCGAACTGCATTACT-----
------
FC88X_00997_00583 ------------------ACAACTCCCCGGCCTC----GCTGCATCAC------
------
FC88X_00144_00070 ----------------------ACCCACCGTTTATCGATGCATCTCATT-----
------
FC88X_00223_00988 --------------------GGACCCATGGCTTAC--GTGCGTGTCATTATT--
------
FC88X_00426_01038 -----------------TTACCATTTTA-ACATGC-GTGCCT-ATTCT------
------
FC88X_00939_01228 -----------------TTACCATTTTA-ACATGC-GTGCCT-ATTCT------
------
FC88X_00020_00225 -----------------TTACCATTTTA--CATGC-GTGCCT-ATTCT------
------

```
FC88X_00073_00475      -----------------TTACCATTTTA--CATGC-GTGCCT-ATTCT------
------
FC88X_00098_00478      -----------------TTACCATTTTA--CATGC-GTGCCT-ATTCT------
------
FC88X_00238_00091      -----------------TTACCATTTTA--CATGC-GTGCCT-ATTCT------
------
FC88X_00206_01075      -----------------TTACCATTTTA--CATGC-GTGCCT-ATTCT------
------
FC88X_00383_00278      -----------------TTACCATTTTA--CATGC-GTGCCT-ATTCT------
------
FC88X_00232_00936      -----------------TTACCATTTTA--CATGC-GTGCCT-ATTCT------
------
FC88X_01140_00880      -----------------TTACCATTTTA--CATGC-GTGCCT-ATTCTT-----
------
FC88X_00314_01127      -----------------TTACCATTTTA--CATGC-GTGCCT-ATTCT------
------
FC88X_00476_00982      -----------------TTACCATTTTA--CATGC-GTGCCT-ATTCT------
------
FC88X_00621_01219      -----------------TTACCATTTTA--CATGC-GTGCCT-ATTCT------
------
FC88X_00663_00843      -----------------TTACCATTTTA--CATGC-GTGCCT-ATTCT------
------
FC88X_00338_00853      -----------------TTACCATTTT--ACATGC-GTGCCT-ATTCT------
------
FC88X_01115_00621      -----------------TTACCATTTTTACATGC-GTGCCT-ATTCT------
------
FC88X_00409_00028      -----------------TTACCATTTT--ACATGC-GTGCCT-ATTCT------
------
FC88X_00566_01230      -----------------TTACCATTTT--ACATGC-GTGCCT-ATTCT------
------
FC88X_00674_00593      -----------------TTACCATTTT--ACATGC-GTGCCT-ATTCT------
------
FC88X_00737_00892      -----------------TTACCATTTT--ACATGC-GTGCCT-ATTCT------
------
FC88X_00848_00134      -----------------TTACCATTTTA--CATGC-GTGCCT-ATTCT------
------
FC88X_00899_00494      -----------------TTACCATTTTA--CATGC-GTGCCT-ATTCT------
------
FC88X_00865_00994      -----------------TTACCATTTTA--CATGC-GTGCCT-ATTCT------
------
FC88X_00927_00226      -----------------TTACCATTTTA--CATGC-GTGCCT-ATTCT------
------
FC88X_00902_00461      -----------------TTACCATTTTA--CATGC-GTGCCT-ATTCT------
------
FC88X_00949_01203      -----------------TTACCATTTTA--CATGC-GTGCCT-ATTCT------
------
FC88X_00983_00843      -----------------TTACCATTTTA--CATGC-GTGCCT-ATTCT------
------
FC88X_01048_00826      -----------------TTACCATTTTA--CATGC-GTGCCT-ATTCT------
------
FC88X_01141_00999      -----------------TTACCATTTTA--CATGC-GTGCCT-ATTCT------
------
FC88X_00031_00420      -----------------TTACCATTTTA--CATGC-GTGCCT-ATTCT------
------
FC88X_00030_00758      -----------------TTACCATTTTA--CATGC-GTGCCT-ATTCT------
------
```

FC88X_00079_00693 ------------------TTACCATTTTA--CATGC-GTGCCT-ATTCT------
------
FC88X_00079_00814 ------------------TTACCATTTTA--CATGC-GTGCCT-ATTCT------
------
FC88X_00225_00123 ------------------TTACCATTTTA--CATGC-GTGCCT-ATTCT------
------
FC88X_00325_00941 ------------------TTACCATTTTA--CATGC-GTGCCT-ATTCT------
------
FC88X_00396_00302 ------------------TTACCATTTTA--CATGC-GTGCCT-ATTCT------
------
FC88X_00436_00121 ------------------TTACCATTTTA--CATGC-GTGCCT-ATTCT------
------
FC88X_00408_00801 ------------------TTACCATTTTA--CATGC-GTGCCT-ATTCT------
------
FC88X_00467_01188 ------------------TTACCATTTTA--CATGC-GTGCCT-ATTCT------
------
FC88X_00536_00106 ------------------TTACCATTTTA--CATGC-GTGCCT-ATTCT------
------
FC88X_00518_01070 ------------------TTACCATTTTA--CATGC-GTGCCT-ATTCT------
------
FC88X_00559_00210 ------------------TTACCATTTTA--CATGC-GTGCCT-ATTCT------
------
FC88X_00618_00226 ------------------TTACCATTTTA--CATGC-GTGCCT-ATTCT------
------
FC88X_00665_00553 ------------------TTACCATTTTA--CATGC-GTGCCT-ATTCT------
------
FC88X_00696_01003 ------------------TTACCATTTTA--CATGC-GTGCCT-ATTCT------
------
FC88X_00709_00967 ------------------TTACCATTTTA--CATGC-GTGCCT-ATTCT------
------
FC88X_00799_00238 ------------------TTACCATTTTA--CATGC-GTGCCT-ATTCT------
------
FC88X_00862_00207 ------------------TTACCATTTTA--CATGC-GTGCCT-ATTCT------
------
FC88X_00865_01105 ------------------TTACCATTTTA--CATGC-GTGCCT-ATTCT------
------
FC88X_00927_00677 ------------------TTACCATTTTA--CATGC-GTGCCT-ATTCT------
------
FC88X_00927_00911 ------------------TTACCATTTTA--CATGC-GTGCCT-ATTCT------
------
FC88X_00990_00552 ------------------TTACCATTTTA--CATGC-GTGCCT-ATTCT------
------
FC88X_00992_00742 ------------------TTACCATTTTA--CATGC-GTGCCT-ATTCT------
------
FC88X_01088_00861 ------------------TTACCATTTTA--CATGC-GTGCCT-ATTCT------
------
FC88X_01116_00737 ------------------TTACCATTTTA--CATGC-GTGCCT-ATTCT------
------
FC88X_01103_01139 ------------------TTACCATTTTA--CATGC-GTGCCT-ATTCT------
------
FC88X_00967_01222 ------------------TTACCATTTA---CATGC-GTGCCT-ATTCT------
------
FC88X_00735_01268 ------------------TTACCATCTTA--CATGC-GTGCCT-ATTCT------
------
FC88X_00807_01266 ------------------TTACCAT-TTA--CATGC-GTGCCT-ATTCT------
------

FC88X_00483_00032 ----------------GTTACCATTTTA--CATGTCGTGCCTCATTCT------
------
FC88X_00058_00548 -----------------TTACCATTTTA--CATGT-GTGCCT-ATTCT------
------
FC88X_00964_00772 -----------------TTACCATTTTA--CATGC-GTGCCT-ATTCC------
------
FC88X_01087_00719 -----------------TTGCCATTTTA--CATGC-GTGCCT-ATTCT------
------
FC88X_00198_00254 -----------------TTACCATTTTA--CATGC-GTGCCT-
ATTCTCTTCCTTT----
FC88X_00962_00723 -----------------TTACCATTTTA--CATGC-GTGTCT-ATTCT------
------
FC88X_01131_01045 -----------------TTACCATTTTA--CATGC-GTGCCT-GTTCT------
------
FC88X_00560_00636 -----------------TTACCATTTTA--CATGC-GTGCCT-GTTCT------
------
FC88X_01082_00812 -----------------TTACCATTTTA--CATGC-GTGCCT-ACTCT------
------
FC88X_00318_00937 -----------------TTACCATTTTA--CATGC-GCGCCT-ATTCT------
------
FC88X_01043_00592 -----------------TTACCATTTTA--CATGC-GTGCGT-ATTCTC-----
------
FC88X_00034_00278 -----------------TTACCATTCTA--CATGC-GTGCCT-ATTCT------
------
FC88X_00122_00955 -----------------TTACCATTCTA--CATGC-GTGCCT-ATTCT------
------
FC88X_00936_00831 -----------------TTACCATTCTA--CATGC-GTGCCT-ATTCT------
------
FC88X_00781_00197 -----------------TTACCACTCTA--CATGC-GTGCCT-ATTCT------
------
FC88X_00134_00637 -----------------TTACCATTTTA--CGTGC-GTGCCT-ATTCT------
------
FC88X_00469_01241 -----------------TTACCATTT-A--CGTGC-GTGCCT-ATTCT------
------
FC88X_00838_00329 -----------------TTACCACTTTA--CATGC-GTGCCC-ATTCT------
------
FC88X_00738_01104 -----------------TTACCACTTTA--CATGC-GTGCCT-
ATTCTCTTCCTTTCTC-
FC88X_01006_00292 -----------------CTACCGTTTTA--CATGC-GTGCCT-ATTCT------
------
FC88X_00865_01230 -----------------TTACCATTTTA--CACTGTCTGCCT-ATTCT------
------
FC88X_01019_00860 -------------------TTGATATTT--CACGTTCTGCCC-CTGCTC-----
------
FC88X_00096_00861 -----------------TCGGGATATTT--CAC-TCTCGCCC-ATTCT------
------
FC88X_00572_00490 -----------------CTAACACTTCA-A-ATTCCCAGATT-ATT--------
------
FC88X_00875_00770 -----------------TTTACCCTTGA-ACATTCCGTGACT-ATC--------
------
FC88X_00038_00266 -----------------CCGACA-TACTTA-GTTTGCCGCAT-TCTC-------
------
FC88X_00149_00687 -----------------CCGACA-TACTTA-GTTTGCCGCAT-TCTC-------
------
FC88X_00761_01095 -----------------CCGACA-TACTTA-GTTTGCCGCAT-TCTC-------
------

FC88X_00916_00254 -----------------CCGACA-TACTTA-GTTTGCCGCAT-TCTC-------
------
FC88X_00931_01124 -----------------CCGACA-TACTTA-GTTTGCCGCAT-TCTC-------
------
FC88X_00951_00623 -----------------CCGACA-TACTTA-GTTTGCCGCAT-TCTC-------
------
FC88X_01049_00969 -----------------CCGACA-TACTTA-GTTTGCCGCAT-TCTC-------
------
FC88X_01120_01171 -----------------CCGACA-TACTTA-GTTTGCCGCAT-TCTC-------
------
FC88X_00012_00479 ------------------CGACA-TACTTA-GTTTGCCGCAT-TCTC-------
------
FC88X_00192_00870 ------------------CGACA-TACTTA-GTTTGCCGCAT-TCTC-------
------
FC88X_00275_00543 ------------------CGACA-TACTTA-GTTTGCCGCAT-TCTC-------
------
FC88X_00669_00177 ------------------CGACA-TACTTA-GTTTGCCGCAT-TCTC-------
------
FC88X_00887_00649 ------------------CGACA-TACTTA-GTTTGCCGCAT-TCTC-------
------
FC88X_00970_00897 ------------------CGACA-TACTTA-GTTTGCCGCAT-TCTC-------
------
FC88X_01140_00828 ------------------CGACA-TACTTA-GTTTGCCGCAT-TCTC-------
------
FC88X_00376_01126 -----------------CCGACAATACTTA-GTTTGCCGCAT-TCTCT------
------
FC88X_00878_00653 ------------------CGACA-TACTTA-GTTTGCCACAT-TCTC-------
------
FC88X_00111_00720 -----------------CCGACA-TACTTA-GTTTGCCGCAC-TCTC-------
------
FC88X_00866_00382 -----------------CCGACA-TACTTA-GTTTGCCGCAC-TCTC-------
------
FC88X_00118_00062 -----------------CCGACA-TACTTA-GGTTGCCGCAT-TCTC-------
------
FC88X_00919_01179 -----------------CCGACA-CACTTA-GTTTGCCATTC-TC---------
------
FC88X_00971_00798 -----------------CCGACA-CACTCA-GTTTGCCGCAT-TCTC-------
------
FC88X_00847_01083 ----------------GCCGTACATACTTACGTTTGCCGCAT-TCTCC------
------
FC88X_00980_01272 ---------------GTTCAACACTTT-CA-GTTTTCCGGTT-ATTCT------
------
FC88X_01100_00984 -----------------TCAACACTTT-CA-GTTTTC-GGTT-ATTCT------
------
FC88X_00036_00665 -----------------TCAACACTTT-CA-GTTTCC-GGTT-ATTCT------
------
FC88X_00235_00392 -----------------TCAACACTTT-CA-GTTTCC-GGTT-ATTCT------
------
FC88X_00147_00638 -----------------TCAACACTTT-CA-GTTTCC-GGTT-ATTCT------
------
FC88X_00073_00340 -----------------TCAACACTTT-CA-GTTTCC-GGTT-ATTCT------
------
FC88X_01071_00745 ----------------GTCAACACTTT-CA-GTTTCC-GGTT-ATTCT------
------
FC88X_00691_00756 -----------------TCAACACTTT-CA-GTTTCC-GGTT-ATTCT------
------

FC88X_00688_01264 -----------------TCAACACTTT-CA-GTTTCC-GGTT-ATTCT------
------
FC88X_00981_00340 -----------------TCAACACTTT-CA-GTTTCC-GGTT-ATTCT------
------
FC88X_00116_00033 -----------------TCAACACTTT-CA-GTTTCC-GGTT-ATTCT------
------
FC88X_00842_01142 ----------------ATCAACACTTT-CA-GTTTCC-GGTT-ATTCT------
------
FC88X_00841_00767 -----------------TCAACACTTT-CA-GTTTCC-GGTT-ATTCT------
------
FC88X_00773_00926 -----------------TCAACACTTT-CA-GTTTCC-GGTT-ATTCT------
------
FC88X_01074_00462 -----------------TCAACACTTT-CA-GTTTCC-GGTT-ATTCT------
------
FC88X_01120_00489 -----------------TCAACACTTT-CA-GTTTCC-GGTT-ATTCT------
------
FC88X_01143_01141 -----------------TCAACACTTT-CA-GTTTCC-GGTT-ATTCT------
------
FC88X_00111_00951 -----------------TCAACACTTT-CA-GTTTCC-GGTT-ATTCTC-----
------
FC88X_00989_00820 -----------------TCAACACTCT-CA-GTTTCC-GGTT-ATTCT------
------
FC88X_00921_00699 -----------------TCAACACTCT-CA-GTTTCC-GGTT-ATTCT------
------
FC88X_00645_01094 -----------------TCAACACTTT-CA-GTTTCC-GGTT-ATTCTATCAC-
------
FC88X_00793_00366 -----------------TCAACACTTT-CA-GTTTCC-AGTT-ATTCT------
------
FC88X_00207_00943 -----------------TCAACACTTT-TC-AGTTTCCGGTT-ATTCT------
------
FC88X_00031_00207 -----------------TCAACACTTT-CA-GCTTCC-GGTT-ATTCT------
------
FC88X_00676_00437 -----------------TCGACACTTTTCA-GTTTCC-GGTT-ATTCT------
------
FC88X_00942_00658 -----------------TCGACACTTT-CA-GTTTCC-GGTT-ATTCT------
------
FC88X_01015_00746 -----------------TCGACACTTT-CA-GTTTCC-GGTT-ATTCT------
------
FC88X_00810_01009 -----------------TCAACACTTT-CA-GTTTCC-GGCT-ATTCT------
------
FC88X_00317_01026 ----------------CCAAGTACTTT-GACGCTCCC-GGTT-ATC--------
------
FC88X_00633_00846 ----------------CCAGGTACTTT-GACGCTCCC-GGTT-ATC--------
------
FC88X_00743_01010 ----------------CCAAGCACTTT-GACGCTCCC-GGTT-ATC--------
------
FC88X_00799_00782 ----------------CCAAGTACTTT-GACGCTCCC-GGCT-ATC--------
------
FC88X_00948_00221 ----------------CCAAATACTTT-GACGCTCCC-GGTT-ATC--------
------
FC88X_00319_01148 ----------------CTACATACGATAGACGCTCCC-GGTT-AC---------
------
FC88X_00322_00943 ----------------CTACATACGATAGACGCTCCC-GGTT-AC---------
------
FC88X_00965_01091 ----------------CTACATACGATAGACGCTCCC-GGTT-AC---------
------

FC88X_00533_00824 ----------------CTACATACGAT-GACGCTCCC-GGTT-AC---------
------
FC88X_00821_01023 --------------CTCTAAACACTAGCACCTCCCA--GATT-C----------
------
FC88X_00306_01078 ---------------TTTTAACGCCTAGACCGCCCAC-GATT-T----------
------
FC88X_00030_00245 ----------------CGCTACAATTT----GTAATCCGTTT-ATTCT------
------
FC88X_00042_00443 ----------------CGCTACAATTT----GTAATCCGTTT-ATTCT------
------
FC88X_00077_00512 ----------------CGCTACAATTT----GTAATCCGTTT-ATTCT------
------
FC88X_00514_00224 ----------------CGCTACAATTT----GTAATCCGTTT-ATTCT------
------
FC88X_00550_00253 ----------------CGCTACAATTT----GTAATCCGTTT-ATTCT------
------
FC88X_00591_00546 ----------------CGCTACAATTT----GTAATCCGTTT-ATTCT------
------
FC88X_00647_00320 ----------------CGCTACAATTT----GTAATCCGTTT-ATTCT------
------
FC88X_00697_00197 ----------------CGCTACAATTT----GTAATCCGTTT-ATTCT------
------
FC88X_00911_00641 ----------------CGCTACAATTT----GTAATCCGTTT-ATTCT------
------
FC88X_00956_00670 ----------------CGCTACAATTT----GTAATCCGTTT-ATTCT------
------
FC88X_00954_00728 ----------------CGCTACAATTT----GTAATCCGTTT-ATTCT------
------
FC88X_00996_00988 ----------------CGCTACAATTT----GTAATCCGTTT-ATTCT------
------
FC88X_01017_00360 ----------------CGCTACAATTT----GTAATCCGTTT-ATTCT------
------
FC88X_01055_00972 ----------------CGCTACAATTT----GTAATCCGTTT-ATTCT------
------
FC88X_00009_00290 ----------------CGCTACAATTT----GTAATCCGTTT-ATTCT------
------
FC88X_00063_00215 ----------------CGCTACAATTT----GTAATCCGTTT-ATTCT------
------
FC88X_00076_00557 ----------------CGCTACAATTT----GTAATCCGTTT-ATTCT------
------
FC88X_00062_00629 ----------------CGCTACAATTT----GTAATCCGTTT-ATTCT------
------
FC88X_00172_00035 ----------------CGCTACAATTT----GTAATCCGTTT-ATTCT------
------
FC88X_00352_01057 ----------------CGCTACAATTT----GTAATCCGTTT-ATTCT------
------
FC88X_00405_01100 ----------------CGCTACAATTT----GTAATCCGTTT-ATTCT------
------
FC88X_00502_00160 ----------------CGCTACAATTT----GTAATCCGTTT-ATTCT------
------
FC88X_00595_00965 ----------------CGCTACAATTT----GTAATCCGTTT-ATTCT------
------
FC88X_00709_00110 ----------------CGCTACAATTT----GTAATCCGTTT-ATTCT------
------
FC88X_00763_00215 ----------------CGCTACAATTT----GTAATCCGTTT-ATTCT------
------

FC88X_00784_00375 ----------------CGCTACAATTT----GTAATCCGTTT-ATTCT------
------
FC88X_00779_00973 ----------------CGCTACAATTT----GTAATCCGTTT-ATTCT------
------
FC88X_00855_00694 ----------------CGCTACAATTT----GTAATCCGTTT-ATTCT------
------
FC88X_01003_00456 ----------------CGCTACAATTT----GTAATCCGTTT-ATTCT------
------
FC88X_01039_01024 ----------------CGCTACAATTT----GTAATCCGTTT-ATTCT------
------
FC88X_01086_00572 ----------------CGCTACAATTT----GTAATCCGTTT-ATTCT------
------
FC88X_00166_00699 ----------------CGCTACAATTT----GTAATCCGCTT-ATTCT------
------
FC88X_00948_00739 ----------------CGCTACAATTT----GTAATCCGCTTTATTCT------
------
FC88X_00143_00698 ----------------CGCTACAATTTT---GTAATCCGTTT-ATTCTCC----
------
FC88X_01126_00927 ----------------CGCTACAATTTT---GTAATCCGTTT-ATTCTCC----
------
FC88X_00576_01044 ----------------CGCTACAATTT----GTAATCCGGTTTATT--------
------
FC88X_00572_00656 ----------------CGCTACAATTC----GTAATCCGTTT-ATTCT------
------
FC88X_00100_00836 ----------------CGTTACAATTT----GTAATCCGTTT-
ATTCTCTTCCTTT----
FC88X_01068_00963 ----------------CGTTACAATTT----GTAATCCGTTT-ATTCT------
------
FC88X_00512_01174 -------------------TCGTACAAT---GTGATGCCGCAAATTCTC-----
------
FC88X_00990_00505 -------------------TCGTACAAT---GTGATGCCGCAAATTCTCC----
------
FC88X_00145_00228 ------------------CTCGTTCGTC---GTTATTCC-CAAATTCTC-----
------
FC88X_01091_00871 --------------CTCATTGCA-ATAC---GCAGTGC-GTTGTTC--------
------
FC88X_00069_00104 --------------CTCATTGCA-ATAC---GCAGTGC-GTTGTTC--------
------
FC88X_00797_00865 --------------CTCATTGCA-ATAC---GCAGTGC-GTTGTTC--------
------
FC88X_00850_00754 --------------CTCATTGCA-ATAC---GCAGTGCCGTTGCT---------
------
FC88X_00638_00068 --------------CTCATTGCA-ATAC---GCCAG------------------
------
FC88X_00056_00117 -----------CCACACATTGCATAGAC---GCAGATTTAG-------------
------
FC88X_00598_01230 --------------------------GCCTAAGTCTTC-
CCCAAATGTTCTCAC------
FC88X_00017_00683 -------------------GCCACCGTCCTATGTCTTCACCCAAAT--------
------
FC88X_00469_00142 ------------------CTCCACCGACCCAAATTTTCTCCCGCA---------
------
FC88X_00823_00323 -----------------CTATCTT-GACGCAAATGTTCCCC-TATT--------
------
FC88X_01033_00468 -----------------CTATCTT-GACGCAAATGTTCCCCCTATT--------
------

FC88X_01031_01209   -----------------CTATCTT-GACGCAAATGTTCCCC-TATT--------
------
FC88X_00597_01101   -----------------TTATCCT-GACGCAAATTGTTCCCCTATT--------
------
FC88X_00535_00339   -----------TGCACACTATCTTCGACGCAACTTCTCAA--------------
------
FC88X_00660_00461   ------------------------CCACATAATTCTCCACCCCACGTTGCC---
------
FC88X_00361_01045   ------------------------CCACGTAATTCTTTACGCCTA---------
------
FC88X_00475_00746   --------------------------
CAATAAGTCGTCAGCCTATGTTTTTCCTTCTT--
FC88X_00760_00189   ----------------------------ACCATACATTGACGTACGTTTT----
------
FC88X_00151_00829   ----------------------ACAAACGTCATACATCTACTCCCTTTT-----
------
FC88X_00152_00349   --------------------CTGCTACCTGTGCCGTGCAAGCATTCT-------
------
FC88X_00183_00872   --------------------CTACCACCTATTCAGC---AACATTCCCAC----
------
FC88X_00647_00933   -----------------------CTACCCATTCTGC---CATATTTGCCTGCA-
------
FC88X_00732_01057   -----------------------CTATCCGCTCTCCAAACATTTTCGCCCT---
------
FC88X_00157_00540   -----------------CTCATTCTACCAATTTGGCCTGC-TGTT---------
------
FC88X_01119_01091   -----------------CTCATTCTACCAATTTGGCCTGC-TGTT---------
------
FC88X_00113_00880   -----------------CTCATTCTACCAATTTGGCCTGC-TGTT---------
------
FC88X_00192_00104   -----------------CTCATTCTACCAATTTGGCCTGC-TGTT---------
------
FC88X_00229_00054   -----------------CTCATTCTACCAATTTGGCCTGC-TGTT---------
------
FC88X_00974_00537   -----------------CTCATTCTACCAATTTGGCCTGC-TGTT---------
------
FC88X_00643_00829   --------------------TTTCTACCTATTCGTCACGCGTGTTTCC------
------
FC88X_00801_00255   --------------------TTTCTACCTATTCGTCACGCGTGTTTCCC-----
------
FC88X_00912_00975   --------------------TTTCTACCTATTCGTCACGCGTGTTTC-------
------
FC88X_00958_00625   --------------------TTTCTACCTATTCGTCACGCGTGTTTC-------
------
FC88X_01009_00527   --------------------TTTCTACCTATTCGTCACGCGTGTTTC-------
------
FC88X_00853_00517   --------------------TTTCTACCTATTCGTCACGCGTGTTTC-------
------
FC88X_00103_00094   --------------------TTTCTACCTATTCGTCACGCGTGTTTC-------
------
FC88X_00181_00050   --------------------TTTCTACCTATTCGTCACGCGTGTTTC-------
------
FC88X_00390_01030   --------------------TTTCTACCTATTCGTCACGCGTGTTTC-------
------
FC88X_00393_01107   --------------------TTTCTACCTATTCGTCACGCGTGTTTC-------
------

FC88X_00659_00553 --------------------TTTCTACCTATTCGTCACGCGTGTTTC-------
------
FC88X_00799_00932 --------------------TTTCTACCTATTCGTCACGCGTGTTTC-------
------
FC88X_00534_00678 --------------------TTTCAACCTATTCGTCACGCGTGTTTC-------
------
FC88X_00566_01138 --------------TCAATCATCCTCCCACGTTGCACTGTG-------------
------
FC88X_00856_01150 ---------------TACTCTTCATACCTC-TTGC-CTGTT-------------
------
FC88X_01000_01221 --------------------TCCAATCCTCTACCGTGGCCAGCTGTT-------
------
FC88X_00153_00750 ------------------CTTTCCAACCTATGCCGC--CCTGCTGTT-------
------
FC88X_00034_00161 ----------------------TCAACCTATTCCTTGCCCTACTCTTCG-----
------
FC88X_00502_01020 -----------------TTGTCTA-ACTTGACCTATCGCCCCGCT---------
------
FC88X_00784_01150 --------------------TCTATACTTGAGACCCTACGCCCCTATT------
------
FC88X_00076_00793 ------------------TCACGTGATACATAGACCCCAACCATT---------
------
FC88X_00911_00228 -------------------TGCGTCACACTATACCTCCTACCGTT---------
------
FC88X_00135_00086 --------------------TTGTCACTACAACCCTTCGAACCTTTT-------
------
FC88X_00925_00433 --------------------TTGACACC-TAGCTCCCCTCACGTTTTTAC----
------
FC88X_00638_00227 ---------------------CTACATTTCGTCCTATTTTCCCGCTTC------
------
FC88X_00828_01084 ---------------------ATACATTGATACTACTATTCCCCGCGC------
------
FC88X_00880_00669 ---------------------CTATACATTAGCTCCTTTTTCCCGTCCT-----
------
FC88X_00669_01260 ---------------------CCAAACATT-GCCCTTCGCCCGATTCTT-----
------
FC88X_00974_00308 ---------------------CCAAACATTAGGCTTTTGGCCGCTTCC------
------
FC88X_00045_00320 ----------------TTTCAC-GTTGCCGACTGCTGC-TATTCC---------
------
FC88X_00392_01109 ----------------TTTCAC-GTTGCCGACTGCTGC-TATTCCC--------
------
FC88X_00056_00585 ----------------TTTCAC-GTTGCCGACTGCTGC-TATTCC---------
------
FC88X_00069_00635 ----------------TTTCAC-GTTGCCGACTGCTGC-TATTCC---------
------
FC88X_00078_00745 ----------------TTTCAC-GTTGCCGACTGCTGC-TATTCC---------
------
FC88X_00123_00165 ----------------TTTCAC-GTTGCCGACTGCTGC-TATTCC---------
------
FC88X_00117_00569 ----------------TTTCAC-GTTGCCGACTGCTGC-TATTCC---------
------
FC88X_00109_00826 ----------------TTTCAC-GTTGCCGACTGCTGC-TATTCC---------
------
FC88X_00138_00881 ----------------TTTCAC-GTTGCCGACTGCTGC-TATTCC---------
------

```
FC88X_00189_00834       ----------------TTTCAC-GTTGCCGACTGCTGC-TATTCC---------
------
FC88X_00183_01039       ----------------TTTCAC-GTTGCCGACTGCTGC-TATTCC---------
------
FC88X_00231_00042       ----------------TTTCAC-GTTGCCGACTGCTGC-TATTCC---------
------
FC88X_00200_00138       ----------------TTTCAC-GTTGCCGACTGCTGC-TATTCC---------
------
FC88X_00215_00247       ----------------TTTCAC-GTTGCCGACTGCTGC-TATTCC---------
------
FC88X_00203_00596       ----------------TTTCAC-GTTGCCGACTGCTGC-TATTCC---------
------
FC88X_00227_00897       ----------------TTTCAC-GTTGCCGACTGCTGC-TATTCC---------
------
FC88X_00203_01008       ----------------TTTCAC-GTTGCCGACTGCTGC-TATTCC---------
------
FC88X_00284_00918       ----------------TTTCAC-GTTGCCGACTGCTGC-TATTCC---------
------
FC88X_00336_00966       ----------------TTTCAC-GTTGCCGACTGCTGC-TATTCC---------
------
FC88X_00367_00248       ----------------TTTCAC-GTTGCCGACTGCTGC-TATTCC---------
------
FC88X_00392_00250       ----------------TTTCAC-GTTGCCGACTGCTGC-TATTCC---------
------
FC88X_00409_00297       ----------------TTTCAC-GTTGCCGACTGCTGC-TATTCC---------
------
FC88X_00506_00023       ----------------TTTCAC-GTTGCCGACTGCTGC-TATTCC---------
------
FC88X_00547_00197       ----------------TTTCAC-GTTGCCGACTGCTGC-TATTCC---------
------
FC88X_00541_00450       ----------------TTTCAC-GTTGCCGACTGCTGC-TATTCC---------
------
FC88X_00530_00791       ----------------TTTCAC-GTTGCCGACTGCTGC-TATTCC---------
------
FC88X_00515_01106       ----------------TTTCAC-GTTGCCGACTGCTGC-TATTCC---------
------
FC88X_00521_01168       ----------------TTTCAC-GTTGCCGACTGCTGC-TATTCC---------
------
FC88X_00598_00084       ----------------TTTCAC-GTTGCCGACTGCTGC-TATTCC---------
------
FC88X_00661_01101       ----------------TTTCAC-GTTGCCGACTGCTGC-TATTCC---------
------
FC88X_00698_01248       ----------------TTTCAC-GTTGCCGACTGCTGC-TATTCC---------
------
FC88X_00720_00296       ----------------TTTCAC-GTTGCCGACTGCTGC-TATTCC---------
------
FC88X_00711_00918       ----------------TTTCAC-GTTGCCGACTGCTGC-TATTCC---------
------
FC88X_00718_01267       ----------------TTTCAC-GTTGCCGACTGCTGC-TATTCC---------
------
FC88X_00796_00087       ----------------TTTCAC-GTTGCCGACTGCTGC-TATTCC---------
------
FC88X_00772_00290       ----------------TTTCAC-GTTGCCGACTGCTGC-TATTCC---------
------
FC88X_00789_00990       ----------------TTTCAC-GTTGCCGACTGCTGC-TATTCC---------
------
```

```
FC88X_00839_00115        ----------------TTTCAC-GTTGCCGACTGCTGC-TATTCC---------
------
FC88X_00839_00194        ----------------TTTCAC-GTTGCCGACTGCTGC-TATTCC---------
------
FC88X_00805_00210        ----------------TTTCAC-GTTGCCGACTGCTGC-TATTCC---------
------
FC88X_00815_00884        ----------------TTTCAC-GTTGCCGACTGCTGC-TATTCC---------
------
FC88X_00870_00157        ----------------TTTCAC-GTTGCCGACTGCTGC-TATTCC---------
------
FC88X_00852_00318        ----------------TTTCAC-GTTGCCGACTGCTGC-TATTCC---------
------
FC88X_00861_00536        ----------------TTTCAC-GTTGCCGACTGCTGC-TATTCC---------
------
FC88X_00856_00695        ----------------TTTCAC-GTTGCCGACTGCTGC-TATTCC---------
------
FC88X_00882_00969        ----------------TTTCAC-GTTGCCGACTGCTGC-TATTCC---------
------
FC88X_00865_01086        ----------------TTTCAC-GTTGCCGACTGCTGC-TATTCC---------
------
FC88X_00862_01217        ----------------TTTCAC-GTTGCCGACTGCTGC-TATTCC---------
------
FC88X_00928_00269        ----------------TTTCAC-GTTGCCGACTGCTGC-TATTCC---------
------
FC88X_00933_00256        ----------------TTTCAC-GTTGCCGACTGCTGC-TATTCC---------
------
FC88X_00940_00692        ----------------TTTCAC-GTTGCCGACTGCTGC-TATTCC---------
------
FC88X_00912_00827        ----------------TTTCAC-GTTGCCGACTGCTGC-TATTCC---------
------
FC88X_00953_00309        ----------------TTTCAC-GTTGCCGACTGCTGC-TATTCC---------
------
FC88X_00976_00523        ----------------TTTCAC-GTTGCCGACTGCTGC-TATTCC---------
------
FC88X_00984_01078        ----------------TTTCAC-GTTGCCGACTGCTGC-TATTCC---------
------
FC88X_00952_01197        ----------------TTTCAC-GTTGCCGACTGCTGC-TATTCC---------
------
FC88X_00991_01195        ----------------TTTCAC-GTTGCCGACTGCTGC-TATTCC---------
------
FC88X_01003_00408        ----------------TTTCAC-GTTGCCGACTGCTGC-TATTCC---------
------
FC88X_01006_00999        ----------------TTTCAC-GTTGCCGACTGCTGC-TATTCC---------
------
FC88X_01044_00978        ----------------TTTCAC-GTTGCCGACTGCTGC-TATTCC---------
------
FC88X_01043_01171        ----------------TTTCAC-GTTGCCGACTGCTGC-TATTCC---------
------
FC88X_01077_00381        ----------------TTTCAC-GTTGCCGACTGCTGC-TATTCC---------
------
FC88X_01099_00783        ----------------TTTCAC-GTTGCCGACTGCTGC-TATTCC---------
------
FC88X_01084_00850        ----------------TTTCAC-GTTGCCGACTGCTGC-TATTCC---------
------
FC88X_01077_00989        ----------------TTTCAC-GTTGCCGACTGCTGC-TATTCC---------
------
```

```
FC88X_01120_00575      ----------------TTTCAC-GTTGCCGACTGCTGC-TATTCC---------
------
FC88X_01115_00729      ----------------TTTCAC-GTTGCCGACTGCTGC-TATTCC---------
------
FC88X_01138_00846      ----------------TTTCAC-GTTGCCGACTGCTGC-TATTCC---------
------
FC88X_01133_00853      ----------------TTTCAC-GTTGCCGACTGCTGC-TATTCC---------
------
FC88X_01125_01101      ----------------TTTCAC-GTTGCCGACTGCTGC-TATTCC---------
------
FC88X_00083_00422      ----------------TTTCAC-GTTGCCGACTGCTGC-TATTCC---------
------
FC88X_00080_00624      ----------------TTTCAC-GTTGCCGACTGCTGC-TATTCC---------
------
FC88X_00053_00680      ----------------TTTCAC-GTTGCCGACTGCTGC-TATTCC---------
------
FC88X_00142_00161      ----------------TTTCAC-GTTGCCGACTGCTGC-TATTCC---------
------
FC88X_00148_00197      ----------------TTTCAC-GTTGCCGACTGCTGC-TATTCC---------
------
FC88X_00163_00151      ----------------TTTCAC-GTTGCCGACTGCTGC-TATTCC---------
------
FC88X_00166_00684      ----------------TTTCAC-GTTGCCGACTGCTGC-TATTCC---------
------
FC88X_00159_01009      ----------------TTTCAC-GTTGCCGACTGCTGC-TATTCC---------
------
FC88X_00237_00386      ----------------TTTCAC-GTTGCCGACTGCTGC-TATTCC---------
------
FC88X_00222_01060      ----------------TTTCAC-GTTGCCGACTGCTGC-TATTCC---------
------
FC88X_00381_01099      ----------------TTTCAC-GTTGCCGACTGCTGC-TATTCC---------
------
FC88X_00431_01104      ----------------TTTCAC-GTTGCCGACTGCTGC-TATTCC---------
------
FC88X_00501_01104      ----------------TTTCAC-GTTGCCGACTGCTGC-TATTCC---------
------
FC88X_00560_01138      ----------------TTTCAC-GTTGCCGACTGCTGC-TATTCC---------
------
FC88X_00583_01156      ----------------TTTCAC-GTTGCCGACTGCTGC-TATTCC---------
------
FC88X_00623_01213      ----------------TTTCAC-GTTGCCGACTGCTGC-TATTCC---------
------
FC88X_00724_00963      ----------------TTTCAC-GTTGCCGACTGCTGC-TATTCC---------
------
FC88X_00792_00312      ----------------TTTCAC-GTTGCCGACTGCTGC-TATTCC---------
------
FC88X_00803_00228      ----------------TTTCAC-GTTGCCGACTGCTGC-TATTCC---------
------
FC88X_00842_00975      ----------------TTTCAC-GTTGCCGACTGCTGC-TATTCC---------
------
FC88X_00822_01112      ----------------TTTCAC-GTTGCCGACTGCTGC-TATTCC---------
------
FC88X_00807_01184      ----------------TTTCAC-GTTGCCGACTGCTGC-TATTCC---------
------
FC88X_00888_00324      ----------------TTTCAC-GTTGCCGACTGCTGC-TATTCC---------
------
```

```
FC88X_00879_00959       ----------------TTTCAC-GTTGCCGACTGCTGC-TATTCC---------
------
FC88X_00875_01015       ----------------TTTCAC-GTTGCCGACTGCTGC-TATTCC---------
------
FC88X_00868_01111       ----------------TTTCAC-GTTGCCGACTGCTGC-TATTCC---------
------
FC88X_00913_00225       ----------------TTTCAC-GTTGCCGACTGCTGC-TATTCC---------
------
FC88X_00924_00390       ----------------TTTCAC-GTTGCCGACTGCTGC-TATTCC---------
------
FC88X_00914_00856       ----------------TTTCAC-GTTGCCGACTGCTGC-TATTCC---------
------
FC88X_00961_00367       ----------------TTTCAC-GTTGCCGACTGCTGC-TATTCC---------
------
FC88X_00968_00872       ----------------TTTCAC-GTTGCCGACTGCTGC-TATTCC---------
------
FC88X_00975_01088       ----------------TTTCAC-GTTGCCGACTGCTGC-TATTCC---------
------
FC88X_00983_01161       ----------------TTTCAC-GTTGCCGACTGCTGC-TATTCC---------
------
FC88X_01011_00924       ----------------TTTCAC-GTTGCCGACTGCTGC-TATTCC---------
------
FC88X_01045_01223       ----------------TTTCAC-GTTGCCGACTGCTGC-TATTCC---------
------
FC88X_01084_00794       ----------------TTTCAC-GTTGCCGACTGCTGC-TATTCC---------
------
FC88X_01059_00940       ----------------TTTCAC-GTTGCCGACTGCTGC-TATTCC---------
------
FC88X_01078_01033       ----------------TTTCAC-GTTGCCGACTGCTGC-TATTCC---------
------
FC88X_01055_01218       ----------------TTTCAC-GTTGCCGACTGCTGC-TATTCC---------
------
FC88X_01114_00705       ----------------TTTCAC-GTTGCCGACTGCTGC-TATTCC---------
------
FC88X_00446_01187       ---------------GTTTCAC-GTTGCCGACTGCTGC-TATTCC---------
------
FC88X_00878_00849       ---------------GTTTCAC-GTTGCCGACTGCTGC-TATTCC---------
------
FC88X_00897_00809       ---------------GTTTCAC-GTTGCCGACTGCTGC-TATTCC---------
------
FC88X_01070_00388       ---------------GTTTCAC-GTTGCCGACTGCTGC-TATTCC---------
------
FC88X_01074_00947       ---------------GTTTCAC-GTTGCCGACTGCTGC-TATTCC---------
------
FC88X_01101_00482       ---------------GTTTCAC-GTTGCCGACTGCTGC-TATTCC---------
------
FC88X_00188_00525       ---------GGGGAAGTTTCAC-GTTGCCGACTGCTGC-TATTCC---------
------
FC88X_00180_00996       ----------------TTTCAC-GTTGCCGACTGT-GC-TATTCC---------
------
FC88X_00942_00637       ---------------GTTTCAC-GTTGCCGACTGTTGC-TATTCC---------
------
FC88X_00603_00755       ----------------TTTCAC-GTTGCCGGCTGCTGC-TATTCC---------
------
FC88X_00652_01075       ---------------GTTTCAC-GTTGCCGGCTGCTGC-TATTCC---------
------
```

FC88X_00652_00334   ----------------TTTCAC-GTTGCCGTCTGCTGC-TATTCC---------
------
FC88X_00725_00861   ----------------TTTCAC-GTTGCCGACTGCTGC-TATTC----------
------
FC88X_00990_00544   ----------------TTTCAC-GTTGCCGACTGCTGC-TATTCCCC-------
------
FC88X_00029_00556   ----------------TTTCAC-GTTGCCGACTGCTGC-TATTCT---------
------
FC88X_00407_00762   ----------------TTTCAC-GTTGCCGACTGCTGCCTATTCC---------
------
FC88X_00924_00436   ----------------CTTCAC-GTTGCCGACTGCTGC-TATTCC---------
------
FC88X_00399_00551   ----------------TTTCAC-GTTTCCGACTGCTGC-TATTCC---------
------
FC88X_00192_00897   ----------------TCTCAC-GTTGCCGACTGCTGC-TATTCC---------
------
FC88X_00515_00423   ----------------TCTCAC-GTTGCCGACTGCTGC-TATTCC---------
------
FC88X_00091_00466   ----------------TTTCAC-GTTGCCGATTGCTGC-TATTCC---------
------
FC88X_00799_00513   ----------------TTTCAC-GTTGCCGACTGCTGC-
TATTCCCTTCCTTTC------
FC88X_00943_00338   ----------------TTTCAC-GTTGCCGACTGCTGC-TGTTCC---------
------
FC88X_01026_00796   ---------------TTTTCAC-GTTGCCGACTGCTGC-TATTCC---------
------
FC88X_00386_01092   ----------------TTTCGC-GTTGCCGACTGCTGC-TATTCC---------
------
FC88X_00816_00797   ----------------TTTCGC-GTTGCCGACTGCTGC-TATTCC---------
------
FC88X_00842_01041   -----------GTGATTTTCAC-GTTGCCGACTGCTGC-TATTCC---------
------
FC88X_00787_00539   ----------------TTTCACCGTTGCCGACTGCT-ACTATTCC---------
------
FC88X_00850_00235   ----------------TTTCAC-GTTGCCGACTGCT-ACTATTCC---------
------
FC88X_00674_00062   ----------------TTTCAC-GTTGCCGACGGCT-ACTATTCC---------
------
FC88X_00716_01072   ----------------TTTCAC-GTTGCCGACTGCC-GCTATTCC---------
------
FC88X_01087_00767   ---------GGGGAAGTTTCAC-GTTGTCGACTGCCTGCTATTCC---------
------
FC88X_00109_00249   ----------------TTTCAT-GTTGCCGACTGCT-GCTATTCC---------
------
FC88X_00272_00109   ----------------TTTCAT-GTTGCCGACTGCT-GCTATTCC---------
------
FC88X_00749_00196   ----------------TTTCA--GTTGCCGACTGCT-GCTATTCC---------
------
FC88X_00916_00587   ----------------TTTCAC--GTGCCGACTGCTGC-TATTCC---------
------
FC88X_01069_00638   ----------------TTTCAC-GTCGCCGACTGCTGC-TATTCC---------
------
FC88X_00978_00458   ----------------TTTCAC-GTTGCTGACTGCTGC-TATTCC---------
------
FC88X_00795_00407   ----------------TTTCAC-GTTACCGACTGCTGC-TATTCC---------
------

```
FC88X_00725_01035       ----------------TTTCACGTT-GCCGACTGC-------------------
------
FC88X_00957_00971       ----------------TTTCACGTTTGCCGACTGCT------------------
------
FC88X_00684_00273       ----------------TTTCACGTT-GCCGA-----------------------
------
FC88X_00925_00495       ----------------TTTCACGTT-GCCGA-----------------------
------
FC88X_00551_01218       -----------------TCGGCTATTACACGGTGCCAC-TGTCTC---------
------
FC88X_00681_01201       -----------------TCGGCTATTACACGGTGCCAC-TGTCTC---------
------
FC88X_01034_00842       -----------------TCGGCTATTACACGGTGCCAC-TGTCTC---------
------
FC88X_00043_00021       -----------------TCGGCTATTACACGGTGCCAC-TGTCTC---------
------
FC88X_00091_00839       -----------------TCGGCTATTACACGGTGCCAC-TGTCTC---------
------
FC88X_00133_00082       -----------------TCGGCTATTACACGGTGCCAC-TGTCTC---------
------
FC88X_00504_00891       -----------------TCGGCTATTACACGGTGCCAC-TGTCTC---------
------
FC88X_00899_00876       -----------------TCGGCTATTACACGGTGCCAC-TGTCTC---------
------
FC88X_01040_01023       ---------------CTACGGTGTTTACTCGTTG---C-TGTCTC---------
------
FC88X_00268_01106       ------TCACCGGACATTCTAC--GTTCCGAAATCCTTCCTTTCCC--------
------
FC88X_00562_00237       ----------------TTCTAC--TATCCGACAGTCTCCCTTTGCC--------
------
FC88X_00543_01148       ----------------TCCTACCTTCTCCACTAGTGCCATACG-----------
------
FC88X_00614_01104       ----------------TCCTACCTTCTCCACTAGTGCCATATG-----------
------
FC88X_00066_00870       ------------------CTACCGACTCCC----TGCCATAC------------
------
FC88X_00972_00665       ----------------CCGCGGACTGGCTACCGTTCCCCTACT-----------
------
FC88X_00926_01197       ----------------CCTTCATTGGGCTACCATTCCACTGCT-----------
------
FC88X_00096_00352       -------------GGTTTGGACG-TCTCCCAGTCCTCGTTACG-----------
------
FC88X_00826_01154       --------------GTTTGGACG-TCTCCCAGTCCTCGTTACG-----------
------
FC88X_01022_00748       ---------------TTTGGACG-TCTCCCAGTCCTCGTTACG-----------
------
FC88X_00245_01079       ---------------TTTGGACG-TCTCCCAGTCCTCGTTACG-----------
------
FC88X_00507_00983       ---------------TTTGGACG-TCTCCCAGTCCTCGTTACG-----------
------
FC88X_00619_00269       ---------------TTTGGACG-TCTCCCAGTCCTCGTTACG-----------
------
FC88X_00272_00878       ---------------TTTGGACG-TCTCCCAGTCCTCGTTACG-----------
------
FC88X_00248_00388       ---------------TTTGGACGGTCTCCCAGTCCTCGTTACGC----------
------
```

FC88X_00419_00982 ---------------TTTGGACG-TCTCCCAGTCCTCGTTACG-----------
------
FC88X_00129_00092 ---------------TTTGGACG-TCTCCCAGTCCTCGTTACG-----------
------
FC88X_00470_01131 ---------------TTTGGACG-TCTCCCAGTCCTCGTTACG-----------
------
FC88X_00598_01007 ---------------TTTGGACG-TCTCCCAGTCCTCGTTACG-----------
------
FC88X_00749_01223 ---------------TTTGGACG-TCTCCCAGTCCTCGTTACG-----------
------
FC88X_00767_00198 ---------------TTTGGACG-TCTCCCAGTCCTCGTTACG-----------
------
FC88X_00896_00174 ---------------TTTGGACG-TCTCCCAGTCCTCGTTACG-----------
------
FC88X_00949_00716 ---------------TTTGGACG-TCTCCCAGTCCTCGTTACG-----------
------
FC88X_01038_00781 ---------------TTTGGACG-TCTCCCAGTCCTCGTTACG-----------
------
FC88X_00907_00480 ---------------TTTGGACG-TCTCCTAGTCCTCGTTACG-----------
------
FC88X_01106_00863 ---------------TTTGGACG-TCTCCCAATCCTCGTTACG-----------
------
FC88X_00455_01213 ---------CTCTCGTTTCATCG-TCTCCCAGTCCTCGTTACG-----------
------
FC88X_00069_00624 --------CTCATTGAT----CG-TCTCCTACGTGCTCTT--------------
------
FC88X_00996_00578 --------CTCATTGAT----CG-TCTCCTACGTGCTCTT--------------
------
FC88X_00852_00518 --------CTCATGGCT-AAACG-TCTCCAAAGTGTT-----------------
------
FC88X_01002_00676 --------CTCATGGCTTAAACG-TCTCCAAAGTGTT-----------------
------
FC88X_00996_01173 --------CTCATGGCT-AAACG-TCTCCAAAGTGTT-----------------
------
FC88X_00719_01219 --------CTCATGGCT-AAACG-TCTCCAAAGTGTT-----------------
------
FC88X_00748_01261 --------CTCATGGCT-AAACG-TCTCCAAAGTGTT-----------------
------
FC88X_00473_01186 --------CCAATTCCCATGACCGCCTCCCAGTGC-------------------
------
FC88X_00052_00522 --------CACTTCTCTATAAATGTCGCCACGTGGC------------------
------
FC88X_00512_00220 -------------GCGTCGATCTGA---CCACGTCCCACTATT-----------
------
FC88X_00605_00095 -------------GCGTCGATCTGA---CCACGTCCCACTATT-----------
------
FC88X_00193_00804 -------------GCGTCGATCTGA---CCGCGTCCCACTATT-----------
------
FC88X_00049_00093 -------------GCGTCGATCTGA---CCGCGTCCCACTGTT-----------
------
FC88X_00792_00732 -------------------ATCTGAACTCCAAGTTCCGCTATTTTC--------
------
FC88X_00966_01143 ------------------GATCTGAACTCCAAGTTCCGCTATTTTC--------
------
FC88X_01091_01010 -------------------ATCTGAACTCCAAGTTCCGCTATTTTC--------
------

```
FC88X_01134_00755       -------------------ATCTGAACTCCAAGTTCCGCTATTTTC--------
------
FC88X_00018_00309       -------------------ATCTGAACTCCAAGTTCCGCTATTTTC--------
------
FC88X_00127_00094       -------------------ATCTGAACTCCAAGTTCCGCTATTTTC--------
------
FC88X_00293_00242       -------------------ATCTGAACTCCAAGTTCCGCTATTTTC--------
------
FC88X_00480_00053       -------------------ATCTGAACTCCAAGTTCCGCTATTTTC--------
------
FC88X_00720_00941       -------------------ATCTGAACTCCAAGTTCCGCTATTTTC--------
------
FC88X_00130_00008       -------------------ATCTGAACTCCAAGTTCCGCTATTTTC--------
------
FC88X_01046_00931       -------------------ATCTGAACTCCAAGTTCCGCTATTTTCT-------
------
FC88X_00208_00855       -------------------ATCTGAACTCCAAGTTCCGCTATTTTC--------
------
FC88X_00253_00435       -------------------ATCTGAACTCCAAGTTCCGCTATTTTC--------
------
FC88X_00546_00684       -------------------ATCTGAACTCCAAGTTCCGCTATTTTC--------
------
FC88X_00852_00351       -------------------ATCTGAACTCCAAGTTCCGCTATTTTC--------
------
FC88X_01012_00680       -------------------ATCTGAACTCCAAGTTCCGCTATTTTC--------
------
FC88X_01048_00879       -------------------ATCTGAACTCCAAGTTCCGCTATTTTC--------
------
FC88X_00903_00248       -------------------ATCTGAACTCCAAGTTCCGCTATTTTCC-------
------
FC88X_00440_00065       -------------------ATCTGAACTCCAAGTTCCGCTATTTTC--------
------
FC88X_00520_01192       -------------------ATCTGAACTCCAAGTTCCGCTATTTTC--------
------
FC88X_00884_01094       -------------------ATCTGAACTCCAAGTTCCGCTATTTTC--------
------
FC88X_01002_00828       -------------------ATCTGAACTCCAAGTTCCGCTATTTTC--------
------
FC88X_00854_01240       -------------------ATCTGAACCCCAAGTTCCGCTATTTTC--------
------
FC88X_01088_00651       -------------------ATCTGAACTCCAAGTTCCGCTATCTTC--------
------
FC88X_00995_00460       -------------------ATCTGAACTCCAAGTTCCGCTATTTTTC-------
------
FC88X_00979_00561       -------------------ATCTGAACTCCAAGTTCCGCTATTTTTC-------
------
FC88X_00974_00732       -------------------ATCTGAACTCCAAGTTCCGCTATTTTTC-------
------
FC88X_01021_00476       -------------------ATCTGAACTCCGAGTTCCGCTGTTTTC--------
------
FC88X_00675_00978       -----------GGGGAAGATTCTGAACTCCAAGTTCCGCTATTTC---------
------
FC88X_00649_00633       ----------------CCCGACGCTGCCCAACGTTTCCCTGTT-----------
------
FC88X_00674_00183       ----------------CCCGACGCTGCCCAACGTTTCCCTGTT-----------
------
```

FC88X_00684_01053 ----------------CCCGACGCTGCCCAACGTTTCCCTGTT-----------
------
FC88X_00851_00960 ----------------CCCGACGCTGCCCAACGTTTCCCTGTT-----------
------
FC88X_00828_01066 ----------------CCCGGGACTGACCCGCGTCTCCCTGTT-----------
------
FC88X_00254_01081 ----------------ATCGTAGCTGTTTCT-ATGTGTCCATTCCC--------
------
FC88X_00911_00961 ----------------ATCGTAGCTGTTTCT-ATGTGTCCATTCC---------
------
FC88X_00032_00074 ----------------ACCGTAGCTGTTTCT-ATGTGTCCATTCC---------
------
FC88X_00490_00986 --------------TGCCCACAGCTGTACTA-TTG---CCATTCC---------
------
FC88X_00011_00243 ---------------CACAACAGC-ATGTT--CTACTCCCATTTA---------
------
FC88X_00507_00218 ---------------CACAACAGCCATGTTTCCTACTCCCATTTA---------
------
FC88X_01000_01004 ---------------CACAACAGC-ATGTT--CTACTCCCATTTA---------
------
FC88X_01100_00853 ---------------CACAACAGC-ATGTT--CTACTCCCATTTA---------
------
FC88X_00880_01268 ---------------CACAGCAGC-ATGTT--CTACTCCCATTTA---------
------
FC88X_00170_00740 ---------------CACAACAGC-ATGTT--
CTACTCCCATTTACTTCCTTTCTCC---
FC88X_00067_00817 --------------TTCCTCCAGC--TTTT--CTATTCCCGTTTA---------
------
FC88X_00149_00057 ----------------TCCACACT-ATGACC-CAATGACCGTTCAC--------
------
FC88X_00125_00358 -----------------CCACAAC-ATGTTG-CA-TGGCCGTTCAC--------
------
FC88X_01022_01274 -----------------TCACACG-ATTTCT-CCATTGCCCTTAGC--------
------
FC88X_00251_00519 ----------------------TTACAGCAGCACCGCTATTCTCCAATC-----
------
FC88X_00917_01093 -----------------GCCGTCTACGGCAGTAGCTGCATATTCTT--------
------
FC88X_00889_00310 -----------------ACCGTTCATTCCAGCAACTAGTTTATC----------
------
FC88X_00186_00128 ----------------CTCCCTTTACTCCAGC--CTAGCTTTTCT---------
------
FC88X_00335_01140 -------------------CTAGCAGCGTCTGGCTTTTCTGTCCA---------
------
FC88X_00473_01205 ---------------------------------------TGTCCA---------
------
FC88X_00756_00890 ------------------ACTCGCTTTGTCCAG-CTTGTTTCTTCT--------
------
FC88X_00189_00864 ------------------ACTCGCTTTGTCCAG-CTTGTTTCTTCT--------
------
FC88X_00795_01004 ------------------ACTCGCTTTGTCCAG-CTTGTTTCTTCT--------
------
FC88X_00070_00575 ----------------TCTCCCTTGCTGCCCAGGCGTGTTGCT-----------
------
FC88X_00503_01254 ----------------TCTCCCTTGCTGTCCAGGCGTGTTGCT-----------
------

```
FC88X_00004_00459       --------------CTCCCACTGTG---TTCATTACACGTATT-----------
------
FC88X_00134_00108       --------------CTCCCACTGTG---TTCATTACACGTATT-----------
------
FC88X_01059_00783       --------------CTCCCACTGTGG--TTCATTACACGTACT-----------
------
FC88X_00928_00835       -------------TCTCCCTATGTGCAGTTCGTGAAACGC--------------
------
FC88X_00864_00606       ------------------CTGT-TACTCCC-ATGTCACGCACATCGT-------
------
FC88X_00295_00635       ------------------CTGT-TACTCCC-ATGTCACGCACATCGT-------
------
FC88X_01006_01049       ------------------CTGT-TACTCCC-ATGTCACGCACATCGT-------
------
FC88X_01023_01182       ------------------CTGT-TACTCCC-ATGTCACGCACATCGT-------
------
FC88X_00465_00112       ------------------CTGT-TACTCCCCATGTCACGCACATCGTCT-----
------
FC88X_00580_01200       ------------------CTGTGTACTCTACATTTCAC----------------
------
FC88X_00788_01097       ------------------CTGTGTACTCTACATTTCACGCTTCTC---------
------
FC88X_00127_00813       ---------------CTCCCGCATGGTCTACGATTTATTTTCC-----------
------
FC88X_01086_00831       ---------------CTCCCGCATGGTCTACGATTTATTTTCC-----------
------
FC88X_00175_00066       ---------------CTCCCACATG---TGCAACTTATTGACCAC---------
------
FC88X_01120_00555       -----------------CCGGCATG---TCCGGTTCATTCACCGCAAC------
------
FC88X_00046_00248       -------------------CTTCTCCAGTTTCCCACTCGCCAAGGCGC------
------
FC88X_00833_00931       ------------------GTTCCTCCAGTTTCCCTCTCGCCAAGGCGC------
------
FC88X_00033_00360       ---------------------TCTCCCGCTTTGCGCCAAAGGCCTCCT------
------
FC88X_00857_00144       ----------------------CTCCAGCTCTGTCGCAACGGCCTACT------
------
FC88X_00984_01060       ---------------------TCCCGAACTTAGCGACG-GACCTCCCGG-----
------
FC88X_00690_01005       ---------------------TCCCGAACTTAGCGACG-GACCTCCCGG-----
------
FC88X_01040_00831       --------------------CTCCCGAG--TACCGACGTGATCTCTCTA-----
------
FC88X_00114_00276       -------------ACCCTCATTCCCGCA--TAGCCACAGGCGCT----------
------
FC88X_00530_01134       --------------------ATCCCGCA--TAGGGACAGTCGCCCATCCT----
------
FC88X_00297_00939       -----------------------ACTTACTTTTTG-AGTCACCGTCCCAAA---
------
FC88X_00022_00139       --------------------CTCTCTTGCTTTTGGCAGTCCCCGCGCC------
------
FC88X_00307_00864       ---------------------TCTCCCAATTGCCAGCAACGCGCCCCA------
------
FC88X_00877_00225       ----------------------CTCCCGCTGTTGACAAACGTCCCTCAC-----
------
```

```
FC88X_00041_00265      -----------------TCTTCCCA-TATTTTGCACCGCTATTCG---------
------
FC88X_01133_01194      ----------------GTCTTCCCA-TATTTTGCACCGCTATTCG---------
------
FC88X_00918_00487      -----------------TCTTCCCA-TATTTTGCACCGCTATTCG---------
------
FC88X_00479_01058      -----------------TCTTCCCAATATTTTGCACCGCTATTCG---------
------
FC88X_00567_01083      -----------------TCTTCCCA-TATTTTGCACCGCTATTCG---------
------
FC88X_00515_01004      -----------------TCTTCCCA-TATTTTGCACCGCTATTCG---------
------
FC88X_00844_00907      -----------------TCTTCCCA-TATTTCGCACCGCTATTCG---------
------
FC88X_00239_00031      -----------------TACTTCCC-TATTTTGGACC-TTATTCTC--------
------
FC88X_00863_00991      -----------------TCCTTCCG--TCTTTGTAACCGTATTCAC--------
------
FC88X_01057_01144      ------------------TCTCCCG--T-TTTGTAACCCTACTAACCT------
------
FC88X_00806_00900      ---------------TCTCTTCCTCCGTATAGTCACCGTTAT------------
------
FC88X_00953_00305      ---------------TCTCTTCCCTCGTATAGTCACCGTTAT------------
------
FC88X_00183_00995      ---------------TATTTCTCTCATGTGCGTCGCTGTTAC------------
------
FC88X_01098_00687      ---------------TATTTCTCTCATGTGCGTCGCTGTTAC------------
------
FC88X_00096_00145      ---------------TTTTCACTCCCTTTGAGACACTGTCAC------------
------
FC88X_00164_00019      -------------------TCTCCCACGGTAGGCATCGCTGCGTC---------
------
FC88X_00421_01224      -------------------TCTCCCACGGTAGGCATCGCTGCGTC---------
------
FC88X_00056_00152      -------------------TCTCCCACGGTAGGCATCGCTGCGGTC--------
------
FC88X_00850_00706      -------------------TCTCCCACGGTAGGCATCGCTGCGGTCC-------
------
FC88X_00950_00437      ---------------------TTCCCCCGTATGCATCGTTGCACTCCC------
------
FC88X_00313_00579      ---------------CTTATC-CCCGTGTGACCCACCGTTATT-----------
------
FC88X_00911_00424      ---------------CTTATC-CCCGTGTGACCCACCGTTATT-----------
------
FC88X_00477_00096      ---------------CTCATCTCCCGTTTACGGCAACGTTGT------------
------
FC88X_00013_00105      --------------TCCCAGCTA---TATTGCGCGTAGGCTCTT----------
------
FC88X_00058_00730      --------------TCCCAGCTA---TATTGCGCGTAGGCTCTT----------
------
FC88X_00174_00177      --------------TCCCAGCTA---TATTGCGCGTAGGCTCTT----------
------
FC88X_00064_00090      --------------TCCCAGCTA---TATTGCGCGTAGGCTCTT----------
------
FC88X_00079_00486      --------------TCCCAGCTA---TATTGCGCGTAGGCTCTT----------
------
```

FC88X_00232_00085 --------------TCCCAGCTA---TATTGCGCGTAGGCTCTT----------
------
FC88X_00538_00035 --------------TCCCAGCTA---TATTGCGCGTAGGCTCTT----------
------
FC88X_00907_01262 --------------TCCCAGCTA---TATTGCGCGTAGGCTCTT----------
------
FC88X_01070_00793 --------------TCCCAGCTA---TATTGCGCGTAGGCTCTT----------
------
FC88X_00576_00965 ---------------ACCAACAA---TCTTCCGCCTAGGCTATTA---------
------
FC88X_00072_00797 ---------------ACCAACAA---TCTTCCGCCTAGGCTATTA---------
------
FC88X_00588_00162 ----------------CCAACTAC-CTCTTCCACTGTGGCTGTTT---------
------
FC88X_00081_00865 -------------CTCACAACTC---TCCTCTGCGGGGGCTAT-----------
------
FC88X_00930_01158 -------------CTCACAACTC---TCCTCTGCGGGGGCTAT-----------
------
FC88X_01105_00523 -------------TTTACAACTCGTCTCCTCTGCCTGGC---------------
------
FC88X_00348_00877 -------------GGGGGACATTATCTCCTGTACATTGCGCTTTGC--------
------
FC88X_00892_01275 -------------------CATTATCTCCTGTACATTGCGCTTTGC--------
------
FC88X_00745_00382 -------------------CATCATCTCCTGTACATTGCGCTTTGC--------
------
FC88X_00867_01261 ------------------CCCCAACATCTCCGTCTTCGCGCTATG---------
------
FC88X_00049_00815 ------------------ACCAACTTTCTCGCCCGTTGCCTTATC---------
------
FC88X_00911_00236 ------------------ACCGACCTTCTC-CTCGTGACGCTATCTT-------
------
FC88X_00641_00770 ------------------CTCACCACTATCCTGCATCCCGGCTTA---------
------
FC88X_01025_00941 -----------------GTCCTCACCTATCCCGCGTGGCTTTATC---------
------
FC88X_00135_00995 ----------------TCTCCCGAC-TTTCCGCCATGGTGGTACT---------
------
FC88X_00574_00918 ----------------TCTCCCGAC-TTTCCGCCATGGTGGTACT---------
------
FC88X_00840_01217 ----------------TCTCCCGAC-TTTCCGCCATGGTGGTACT---------
------
FC88X_00911_01217 ----------------TCTCCCGAC-TTTCCGCCATGGTGGTACT---------
------
FC88X_00355_01030 -----------------CTCCCATC-TTTTCACCCTGGCCGTACC---------
------
FC88X_00530_00616 -----------------CTCCCACCACTTTCGCCACGGGCAAACC---------
------
FC88X_01079_01080 ------------------TCCCAACTTCTCGCACACCGGCATAAC---------
------
FC88X_00166_00104 ------------GGGGAAGGCCAAGTTT-CCCACGCTTCGCC-CTGTG------
------
FC88X_01008_01095 ------------GGGGAAGGCCAAGTTT-CCCACGCTTCGCC-CTGTG------
------
FC88X_00679_00787 -------------------GCCAAGTTT-CCCACGCTTCGCC-CTGTG------
------

FC88X_00899_00215 -------------------GCCAAGTTT-CCCACGCTTCGCC-CTGTG------
------
FC88X_00852_01073 -------------------GCCAAGTTT-CCCACGCTTCGCC-CTGTG------
------
FC88X_01074_00502 -------------------GCCAAGTTT-CCCACGCTTCGCC-CTGTG------
------
FC88X_01076_01258 -------------------GCCAAGTTT-CCCACGCTTCGCC-CTGTG------
------
FC88X_00787_01257 -------------------GCCAAGTTTTCCCACGCCTCGCC-CTGTG------
------
FC88X_00593_00960 -------------------TCCGCGCTC-CTCACGCTTCATC-TTGTT------
------
FC88X_00392_01070 -------------------TCCGCGCTC-CTCACGCTTCACCCTTGTT------
------
FC88X_00946_00629 -------------------TCCGCGCTC-CTCACGCTTCGTC-TTATT------
------
FC88X_00496_00692 ------------------ACCCACG--TGTTAGCAGCCTCTTATT---------
------
FC88X_00754_00330 ------------------ACCCACG--TGTTAGCAGCCTCTTATT---------
------
FC88X_00640_01044 ----------------GCTCCCACGATTGTCCCCACCGTCTTTTT---------
------
FC88X_00874_00788 ------------------ACCCCGTTTCTCCAGCCCCGCCTTATT---------
------
FC88X_00112_00439 -----------------ACTCCAAGTCTTTCGCTCCCACCTGTC----------
------
FC88X_00883_00643 ------------------CTACAAGT---TTGCTACCGCCTGAC----------
------
FC88X_01125_00631 -----------------CCACCAATTTTTTCGCTCCGGCCTGTTT---------
------
FC88X_00328_01159 -----------------CCACCAATTTTT-CGCTCCGGCCTGTTT---------
------
FC88X_00290_00376 -----------------CCACCAATTTTT-CGCCCCGGC-TGTTT---------
------
FC88X_01082_00587 -----------------CCACCAAATTTGCCACTCCCGGTTGTTCA--------
------
FC88X_00483_01235 ----------------CTCTCCACATTTTGGCTTCTC-CGTTGA----------
------
FC88X_00608_01067 -----------------TCCCCAGATTTTAACCTCCCGCGTTTA----------
------
FC88X_00340_01097 -----------------TCCACAACTTTTTCCCAGCTCCTTACA----------
------
FC88X_01115_00996 -----------------TCCACAACTTTT-CCCAGCTCCTTACA----------
------
FC88X_00558_00439 ----------------TCCCCCACTATTTCGCCTGCACCTTGT-----------
------
FC88X_00017_00680 --------------------TCAACAGCTTGACCATCATCTCCATTAC------
------
FC88X_00130_00838 --------------------TCAACAGCTTGACCATCATCTCCATTAC------
------
FC88X_00868_00132 --------------------TCAACAGCTTGACCATCATCTCCATTAC------
------
FC88X_00852_01126 --------------------TCAACAGCTTGACCATCATCTCCATTAC------
------
FC88X_00153_00747 ---------------------TTTCATCTTGACCTTC-TCACCGTTCCG-----
------

```
FC88X_00625_00226       --------------------CTAACTGCTTCCTCAGCTTCC-AACTATT-----
------
FC88X_00925_00812       -------------------ACTAACTGCTTCCTCAGCTTCC-AACTATT-----
------
FC88X_00997_00390       ------------------------CTTTTTCCTCAGCTTCCCAACCATTT----
------
FC88X_00228_00319       ----------------CCTGCAAATGTCTCCCC---TAGCTGAT----------
------
FC88X_00068_00641       ----------------GCTACAGACTTCTCCCCGGCTAGCTGGT----------
------
FC88X_00550_01198       ----------------CTTGTAGTCATATCTCCCG-CAGCTGTT----------
------
FC88X_00826_00289       ---------------TCTTGT-GCCTTAGCCCCCG-TTGCTGTT----------
------
FC88X_00524_00449       -------------------CTAAACATCTTGCCCAGTTTTT--ACATT------
------
FC88X_00909_00885       -------------------CTAAACATCTTGCCCAGTTTTT-CACATT------
------
FC88X_00743_00221       -------------------CTAAACATCTTGCCCAGTTTT--CACATT------
------
FC88X_00708_01090       -------------------CTAAACATCTTGCCCAGTTTT--CACATT------
------
FC88X_00728_00845       -------------------CTAAACATCTTGCCCAGTTTTTTCACATT------
------
FC88X_00696_00214       -------------------CTAAACATCCTGCCCAGTTTT--CACATT------
------
FC88X_00111_00042       -------------------CTAAACATCTTGCCCAGTTTT--CACGTT------
------
FC88X_00496_01235       ------------------CCTGCACATCTTCCCTTGCAGCTTTCCC--------
------
FC88X_00677_00102       ------------------CCTGCACATCTTCCCTTGCAGCTTTCCC--------
------
FC88X_00460_00084       ------------------
CCTGCACATCTTCCCTTGCAGCTTTCCCCTTCCTTT------
FC88X_00225_01072       ----------------TCTGCAGA----TCTGACATGACTTTTTCTC-------
------
FC88X_00403_01012       ----------------TCTGCAGA----TCTGACATGACTTTTTCTC-------
------
FC88X_00621_00185       ----------------TCTGCAGA----TCTGACATGACTTTTTCTC-------
------
FC88X_00676_00822       ----------------TCTGCAGA----TCTGACATGACTTTTTCTC-------
------
FC88X_00796_01114       ----------------TCTGCAGA----TCTGACATGACTTTTTCTC-------
------
FC88X_00805_01262       ----------------TCTGCAGA----TCTGACATGACTTTTTCTC-------
------
FC88X_00926_00388       ----------------TCTGCAGA----TCTGACATGACTTTTTCTC-------
------
FC88X_00976_00305       ----------------TCTGCAGA----TCTGACATGACTTTTTCTC-------
------
FC88X_01082_01078       ----------------TCTGCAGA----TCTGACATGACTTTTTCTC-------
------
FC88X_00659_00146       ----------------TCTGCAGA----TCTGACATGACTTTTTCTC-------
------
FC88X_00738_01137       ----------------TCTGCAGA----TCTGACATGACTTTTTCTC-------
------
```

FC88X_00746_01211 ----------------TCTGCAGA----TCTGACATGACTTTTTCTC-------
------
FC88X_00808_00770 ----------------TCTGCAGA----TCTGACATGACTTTTTCTC-------
------
FC88X_00808_00788 ----------------TCTGCAGA----TCTGACATGACTTTTTCTC-------
------
FC88X_00882_01189 ----------------TCTGCAGA----TCTGACATGACTTTTTCTC-------
------
FC88X_00916_00792 ----------------TCTGCAGA----TCTGACATGACTTTTTCTC-------
------
FC88X_00934_00784 ----------------TCTGCAGA----TCTGACATGACTTTTTCTC-------
------
FC88X_00995_01107 ----------------TCTGCAGA----TCTGACATGACTTTTTCTC-------
------
FC88X_01044_00321 ----------------TCTGCAGA----TCTGACATGACTTTTTCTC-------
------
FC88X_01016_00480 ----------------TCTGCAGA----TCTGACATGACTTTTTCTC-------
------
FC88X_01082_00403 ----------------TCTGCAGA----TCTGACATGACTTTTTCTC-------
------
FC88X_01074_00625 ----------------TCTGCAGA----TCTGACATGACTTTTTCTC-------
------
FC88X_00055_00127 ----------------TCTGCAGA----TCTGACATGACTTTTTCTC-------
------
FC88X_00166_00467 ----------------TCTGCAGA----TCTGACATGACTTTTTCTC-------
------
FC88X_00239_00462 ----------------TCTGCAGA----TCTGACATGACTTTTTCTC-------
------
FC88X_00091_00242 ----------------TCTGCAGA----TCTGACATGACTTTTTCTC-------
------
FC88X_00093_00636 ----------------TCTGCAGA----TCTGACATGACTTTTTCTC-------
------
FC88X_00086_00788 ----------------TCTGCAGA----TCTGACATGACTTTTTCTC-------
------
FC88X_00405_00098 ----------------TCTGCAGA----TCTGACATGACTTTTTCTC-------
------
FC88X_00445_00312 ----------------TCTGCAGA----TCTGACATGACTTTTTCTC-------
------
FC88X_00435_01182 ----------------TCTGCAGA----TCTGACATGACTTTTTCTC-------
------
FC88X_00580_00186 ----------------TCTGCAGA----TCTGACATGACTTTTTCTC-------
------
FC88X_00050_00692 ----------------TCTGCAGA----TCTGACATGACTTCTTCTC-------
------
FC88X_00742_00286 ---------------GTCTGCAGA----TCTGACATGACTTTTCTC--------
------
FC88X_00942_00624 ----------------TCTGCAGA----TCTGACATGACTCTTTCTC-------
------
FC88X_00430_00676 ----------------TCTGCAGA----TCTGACATGACCTTTTCTC-------
------
FC88X_00924_00311 ----------------TCTGCAGA----TCCGACATGACATTTTCTC-------
------
FC88X_00063_00805 ----------------TCTGCAGA----TCTGACATGACTTTCTC---------
------
FC88X_01127_01112 ----------------TCTGCAGA----
TCTGACATGACTTTCTCCTTCCTTTC------

FC88X_00582_01182 ----------------TCTGCAGA----
TCTGACATGACTTTTTCTCCTTCCTTT-----
FC88X_00762_00884 ----------------TCTGCAGA-----TTGACATGACTTTTTTCTC------
------
FC88X_00950_00385 ----------------TCTGCAAGA---TCTGACATGACTTTTTTCTC------
------
FC88X_00991_01143 ----------------TCTGCAGA----TCTGACATGACTTTTTTCTC------
------
FC88X_00865_00839 ----------------TCTGCAGA----TCTGACATGACTTTTTTCTC------
------
FC88X_00354_01004 ----------------TCTGCAGA----TCTGACATGACTTTTTTCTC------
------
FC88X_00961_00582 ----------------TCTGCAGA----TCTGACATGACTTTTTTCTC------
------
FC88X_00066_00802 ----------------TCTGCAGA----TCTGACATGACTTTTTTCTC------
------
FC88X_00269_01037 ----------------TCTGCAGA----TCTGACATGACTTTTTTCTC------
------
FC88X_00453_00960 ----------------TCTGCAGA----TCTGACATGACTTTTTTTTCTC----
------
FC88X_00924_00324 ----------------TCTGCATA----TCTGTGGCTTTTCTCGCGCT------
------
FC88X_00323_01036 -----------------CTGCCGAT-TGTCTCACGTGGCTCTTCT---------
------
FC88X_00515_01045 -----------------CTGCCGAT-TGTCTCACGTGGCTCTTCT---------
------
FC88X_00571_01259 -----------------CTGCCGAT-TGTCTCACGTGGCTCTTCT---------
------
FC88X_00685_00438 -----------------CTGCCGAT-TGTCTCACGTGGCTCTTCT---------
------
FC88X_00867_00263 -----------------CTGCCGAT-TGTCTCACGTGGCTCTTCT---------
------
FC88X_00976_00890 -----------------CTGCCGAT-TGTCTCACGTGGCTCTTCT---------
------
FC88X_00167_00605 -----------------CTGCCGAT-TGTCTCACGTG-CTCTTCT---------
------
FC88X_00829_00819 -----------------CTGCTGAT-TGTCTCACGTGGCTCTTCT---------
------
FC88X_00988_00315 -----------------CTGCCGAT-TGTCTCACGTGGCTACTCATCT------
------
FC88X_00955_00392 ----------------CCTACTGATCTGCCTCACTTCGCTACT-----------
------
FC88X_00238_01045 ---------------TTCTCCAGATCT--CTCACAGCATAGCCAACA-------
------
FC88X_01099_01238 ---------------TTCTCCAGATCT--CTCACAGCATAGCCAACA-------
------
FC88X_00637_01113 ---------------TTCTCCAGATCT--CTCACAGCATAGCCAACA-------
------
FC88X_00931_00327 ---------------TTCTCCAGATCT--CTCACAGCATAGCCAACA-------
------
FC88X_00848_01124 ----------------TCTCCTGACCTGGCTAACTGCTCTGCC-----------
------
FC88X_00116_00913 ----------------TCTCCTGACCTGGCTAACTGCTCTGCC-----------
------
FC88X_00075_00873 -----------------CTCGAAAATCGTCGTCGGCTAGCTCC-----------
------

```
FC88X_00420_00135       ------------------ACGAACATCCTCCTGAGCTGTCTATCT---------
------
FC88X_00626_00534       -----------------CGCGAGCATTGTTTGCGGTTGGCTATTC---------
------
FC88X_00799_00320       -----------------CGCGAGCATTGTTTGCGGTTGGCTATTC---------
------
FC88X_00357_00019       -----------------CGCGAGCATTGTTTGCGGTTGGCTATTC---------
------
FC88X_00940_00853       -----------------CGCGAGCATTGTTTGCGGTTGGCTATTC---------
------
FC88X_00715_01219       -----------------CGCGAGCATTGTCTGCGGTTGGCTATTC---------
------
FC88X_00400_01018       --------GTCGGTGATCGCGAGCATTGTTTGCGGTTGGCTATTC---------
------
FC88X_00150_00375       -----------------CGCGAGCATTGTTTGCGGCTGGCTATTC---------
------
FC88X_01138_00620       ----------------CCGCGA-CATT-TTCCCAGCTGGCCAGCT---------
------
FC88X_00125_00773       --------------TCCCCCAAGTCTCCCGAGTCTGGCTGCT------------
------
FC88X_01127_00531       ---------------TTCTCAATTCCCCGCAGTCTAGCTTCT------------
------
FC88X_00163_00746       ---------------TCTCCAAATCTCCGTCGTTTCGCTGAC------------
------
FC88X_00383_00069       ------------CCTCCCACAAAA-TTGTC-GTCATGCGCT-------------
------
FC88X_01054_01274       ------------CCTCCCACAAAAATTGTCCGTCATGCGCT-------------
------
FC88X_00980_00369       ------------CCTCCCACAAAA-TTGTC-GTCATGCGCT-------------
------
FC88X_00964_01256       ------------CCTCCCACAAAA-TTGTC-GTCATGCGCT-------------
------
FC88X_01045_01087       ------------CCTCCCACAAAA-TTGTC-GTCATGCGCT-------------
------
FC88X_01085_00537       ------------CCTCCCACAAAA-TTGTC-GTCATGCGCT-------------
------
FC88X_00350_01140       -------------CTCCCACAAAA-TTGTC-GTCATGCGCT-------------
------
FC88X_00236_00809       --------------CCGCACAATTTCCGTC-GTGACGCTGTC------------
------
FC88X_00061_00624       ------------------CCGCTAATTGGT-ATCATGTCGCTTTCT--------
------
FC88X_00897_00670       ------------------ACGCGAATTCTTCGTCGTGTCCGTTTCATCA-----
------
FC88X_00200_01049       ------------------GCACAGGTCAATTGCCGCCTATGTCTC---------
------
FC88X_00966_00564       ------------------GCACAGGTCAATTGCCGCCTATGTCTC---------
------
FC88X_00061_00186       ------------------GCACAGGTCAATTGCCGCCTATGTCTC---------
------
FC88X_00095_00308       ------------------GCACAGGTCAATCGCCGCCTATGTCTC---------
------
FC88X_00177_00827       ------------------ATCTTGCCCAGCCTATGCATCTTTCTC---------
------
FC88X_00992_00395       ------------------ATCTTGCCCAGCCTATGCATCTTTCTC---------
------
```

FC88X_00990_00800 ------------------ATCTTGCCCAGCCTATGCATCTTTCTC---------
------
FC88X_00872_00309 ------------------ATCTTGCCCAGCCTATGCATCTTTTTTCTC------
------
FC88X_00532_01062 --------------------GATCCCAGCTCTATGGCTTCCTCTTTCT------
------
FC88X_00625_00817 ----------------TCTCCCAATGTTTTGAATGCCGCGCTCT----------
------
FC88X_00982_00449 ----------------TTTCCCAATGTTTTGGATGCCGCGCTCT----------
------
FC88X_00681_00479 -----------------CTCCCAAC---TTGGCTACCGCGGCCTCTG-------
------
FC88X_01083_00943 ------------------TCACAACATCGACAGCTCCCTATCTAA---------
------
FC88X_00014_00443 ------------------CCACCACATCGGCCTC-CCATATCTAATCC------
------
FC88X_00789_01096 -------------------CTTGGCCATCACAGCCTCCCATCTTTT--------
------
FC88X_00928_00899 -------------------CTTGACCATCACAGCCTCCCATCTTTT--------
------
FC88X_00076_00315 ------------CTCAGTGGGTCCTCTCTGTGTGTTCTC---------------
------
FC88X_00456_00154 ------------CTCAGTGGGTCCTCTCTGTGTGTTCTC---------------
------
FC88X_00176_00092 ------------CTCAGTGGGTCCTCTCTGTGTGTTCTC---------------
------
FC88X_01083_01027 ------------CTCAGTGG-TCCTCTCTGTGTGTTCTCCTTCCTTT-------
------
FC88X_00621_01259 ------------CTTAGTGTATCCCCTCTGAGGTTCCT----------------
------
FC88X_00516_01130 ------------------GCGTCCCCTTTGTGGAT-TCACCTTCCT--------
------
FC88X_00807_01223 ----------------------TCCATGTGTGGATGTCCCCTGCCTCTT-----
------
FC88X_00337_01116 ------------------CTCTTCCCTGTGAGGATTCCTCTTCTC---------
------
FC88X_01064_00372 ------------------CTCTTCCCTGCGCGGAAGCCTCGTCTC---------
------
FC88X_00878_01139 --------------------TTGAGCCCCATGGGTTCGCCACTTCTC-------
------
FC88X_01028_00524 --------------------TTTAACGCTGTGTGTACGCCATTCTC--------
------
FC88X_00463_01106 -------------CCATCCTCGCAAA---GGGTCTCCGCAATTCT---------
------
FC88X_00890_00499 -------------CCATCCTCGCAAAAAAGGGTCTCCGCAATTCT---------
------
FC88X_00227_00561 ------------------CTCACCGCTATGTGTCTCCATCATTAT---------
------
FC88X_00120_00574 -------------------CCTGTATCCCACTGTATC-ACCCTTTC--------
------
FC88X_00029_00194 -------------------CCTGTGGCTGACTTCCTCCAACCTTTC--------
------
FC88X_00619_00584 ---------GGGTATGTTCAAAGGAAACCCCTATGTCTTCCTTTCTC-------
------
FC88X_00700_01011 -----------------TCACTGTATACCCGTAGTTGTCTAACT----------
------

```
FC88X_00134_00610      -----------------CCGGTGCACAACGCGACTCCCTGAAAT----------
------
FC88X_01062_00423      ------------------CCGGGCACTCTGCGATATCTCCCTTTT---------
------
FC88X_00986_00850      -----------------ACGCTGACCCCT-CGATTTCTCCATTTC---------
------
FC88X_00101_00157      -----------------CCGCTGCCCCTCACGATCTCGCCCTGTA---------
------
FC88X_00359_01092      ------------------------------CTTCTCCCCC--------------
------
FC88X_01006_01272      ----------------CCAGCGGCCCTCTGCTTCTCCCCCTTGCA---------
------
FC88X_00802_00102      -----------------TTGCCACTCTCTGCATCTCTCTCCATGCC--------
------
FC88X_00730_01265      ----------------ATTGCCACTCT--GCAGTTCTCCCCTTTA---------
------
FC88X_00012_00192      ----------------TTCTTACC-TCTTCTCCTCGGCTTTTAT----------
------
FC88X_00933_00271      -----------------TCTTACCATCATCTCTTCCGACTTGC-----------
------
FC88X_00131_00915      -----------------TTCCCACCT-TTCTCCACCGCCGTTCGG---------
------
FC88X_00433_01109      -----------------TCCCAACCTCTTCTCCTTCGATGTGCG----------
------
FC88X_00887_00725      ----------------ATTCTAACCC-TTCTCCACCGGCATGCC----------
------
FC88X_00594_00203      -------------------TTAGCTTCTTAATCCAGCCACATTCTT--------
------
FC88X_00884_00693      -----------------TTTTGGTGTCTCGATCCC-CACCATTCT---------
------
FC88X_00551_00051      -----------------ACTTAGAGTCTTACTCCTCGGCCACACC---------
------
FC88X_01052_01258      ----------------ATTCTGACTCGTTCTCCTTGCCTTAC------------
------
FC88X_00140_00856      ----------------ATTCTGACTCGTTCTCCTTGCCTTACCC----------
------
FC88X_00705_00457      ----------------CATTCGACTC-TTATCCCCGTCGTATT-----------
------
FC88X_00034_00465      ------------------TCCCACTAATTA--TTCTGAGGGCGTCTC-------
------
FC88X_00817_00179      ----------------TTTCCCTCTATCTTACTTCCGAATGCG-----------
------
FC88X_00059_00496      ----------------ACTTCCCCTCAGCTTCTACTGACTGTT-----------
------
FC88X_00519_01074      ----------------ACTTCCCCTCAGCTTCTACTGACTGTT-----------
------
FC88X_00975_00300      ----------------ACTTCCCCTCAGCTTCTACTGACTGTT-----------
------
FC88X_00400_00509      ---------------GGGGACCCCTCGCTATTTTACTGCTGTTACGC-------
------
FC88X_01050_01202      --------------------CCCCTCGCTATTTTACTGCTGTTACGC-------
------
FC88X_01077_00544      ------------------CCTTCCACTCGATTATATTGCTATTCA---------
------
FC88X_00940_00390      ------------------CCCTGCCCTCGTTTATTCTGCTCTTAC---------
------
```

```
FC88X_00282_00101      --------------CCGTTCGCTCGATGC--CCTTGTGCTCTT-----------
------
FC88X_00343_00433      --------------CCGTTCGCTCGATGC--CCTTGTGCTCTT-----------
------
FC88X_00944_00228      --------------CCGTTCGCTCGATGC--CCTTGTGCTCTT-----------
------
FC88X_00024_00476      --------------CCGAACTCTCGCCCA--CTTGGTGCTATT-----------
------
FC88X_01077_00449      --------------CCGAACTCTCGCCCGACCTTGGTGCTATTC----------
------
FC88X_01066_01210      -----------------CCACCTCATCTC-CCGCTTGGTTCTCCA---------
------
FC88X_00271_01080      --------------TTACCATCTCA-CTC-CCGCTATGCTCTA-----------
------
FC88X_00683_00511      ---------GGGGACTATCCCCTCA-TTC-CCTTTATGCTCTT-----------
------
FC88X_00979_01046      -------------GCCCATACCCCCCTTTTGCTCCCGAGTC-------------
------
FC88X_01031_00312      -----------------CCAGCCCCCTTTT-CTCCTGAATCGTCACC-------
------
FC88X_00988_01181      ----------------CTTGTCCCCCTACTCCATTATGCTCAC-----------
------
FC88X_01128_00793      -----------------TTTTCCAACTATTCTATTGCACTATAAGT--------
------
FC88X_01135_00946      ------------------TTTCCAACTATTCTATTGCACTATAAGT--------
------
FC88X_00131_00451      --------------------TCCATAGATTCTATACTCCTATA-----------
------
FC88X_00632_00066      ----------------CTTACCCATCTCTTCCATTCCACTATT-----------
------
FC88X_00045_00549      --------------ACAGTCTGT--ACATCAAACTCTATTCTC-----------
------
FC88X_00909_00997      --------------ACAGTCTGT--ACATCAAACTCTATTCTC-----------
------
FC88X_01126_00607      --------------ACAGTCTGT--ACATCAAACTCTATTCTC-----------
------
FC88X_00019_00093      --------------ACAGTCTGT--ACATCAAACTCTATTCTC-----------
------
FC88X_00031_00247      --------------ACAGTCTGT--ACATCAAACTCTATTCTC-----------
------
FC88X_00800_00920      --------------ACAGTCTGTT-ACATCAAACTCTATTCTC-----------
------
FC88X_01094_00918      ---------------TTTCATGT--TCATC-AACTCTCCACTCCC---------
------
FC88X_01117_00668      -------------GCCAACCTAT--TCATCGAACTCGCTACTC-----------
------
FC88X_00191_00929      ------------------CTTATTCAT-CCACACACTACTATTTTC--------
------
FC88X_01063_00807      ---------------CGTGTTATAGATGCCACACACTACTCC------------
------
FC88X_00070_00756      ------------------ACTTTACATCTTCATCTCCCGGCTACC---------
------
FC88X_01108_00716      ------------------ACTTTACATCTTCATCTCC--
GCTACCTCTTCTCCTCTCC--
FC88X_00530_01184      ------------------ACTTTTCCTATTCATCTCCACGCTACTT--------
------
```

FC88X_00127_00694 ---------------CCGCCAACATGGCA---ACCCCACGCTACT---------
------
FC88X_00589_00247 ---------------CCGCCAACATGGCA---ACCCCACGCTACT---------
------
FC88X_00603_01102 ---------------CCGCCAACATGGCA---ACCCCACGCTACT---------
------
FC88X_00692_00481 ------------------CCAACTTGGCTTATACACCACCCGACT---------
------
FC88X_00641_01201 --------------------TTTACGCCTCCATGACAAT--AACTATTA-----
------
FC88X_00601_01233 --------------------TTTACGCCTCCATGACAAT--ACCTATTA-----
------
FC88X_00383_00553 -----------------------ACTCTTCCACTACAATTCAACTATTCT----
------
FC88X_00863_00758 ------------------CGTTTATGCTTC-ATCTCCAC--AACTATCA-----
------
FC88X_00482_01229 ------------------TTCAGTACTCCCAACTTTGATATTCTCCTCCT----
------
FC88X_01074_01011 ------------------TTCAGTACTCCCAACTTTGATATTCTC---------
------
FC88X_00671_00920 ------------------TTCAGCACTCCCAACTTTGATATTCTC---------
------
FC88X_00720_00969 -------------------TCACCACTCCCT-TTGAACTATTCTTGT-------
------
FC88X_00655_01180 --------------------CATTACTCCCCTTAGTATTCAGCTACT-------
------
FC88X_00945_01175 ----------------------CTGCTCCCTATTTGTATCTGTCTATA------
------
FC88X_00958_00709 ---------------------AACACTGCTCCCCTAAATATGTCTATT------
------
FC88X_00805_00940 ------------------TTTTCCTCAATCTAC-GCAGCTGTCA----------
------
FC88X_01093_00656 -------------------CTCCCTTACTCAACTACAGCTGTCAGTT-------
------
FC88X_00649_00421 ------------------TTTCCTGATCTCCGCAGTAAGTCTCTTCCT------
------
FC88X_01145_00631 -----------------TTCCTCACTTCTCCACAGTA-GTCTCCG---------
------
FC88X_00164_00370 ----------------TTTCCTCATCT-CTCACACTAA--CTATTC--------
------
FC88X_00656_00205 ------------------CCCTCATTTGCGCACATGAATTCTATTCC-------
------
FC88X_00085_00269 -----------------CCCCGTGATTAC-GAATTTAGCTGTTCT---------
------
FC88X_00498_01038 ----------------GCCCCGTGATTAC-GAATTTAGCTGTTCT---------
------
FC88X_00187_00180 -----------------CCCCGTGATTAC-GAATTTAGCTGTTCT---------
------
FC88X_00401_00758 -----------------CCCCGTGATTAC-GAATTTAGCTGTTCT---------
------
FC88X_00503_00005 -----------------CCCCGTGATTAC-GAATTTAGCTGTTCT---------
------
FC88X_00998_01186 -----------------CCCCGTGATTAC-GAATTTAGCTGTTCT---------
------
FC88X_00968_00375 -----------------CCCCGTGATTAC-GAATTTAGCTGTTCT---------
------

FC88X_01105_00749 -----------------CCCCGTGATTAC-GAATTTAGCTGTTCT---------
------
FC88X_00585_01234 -----------------CCCCGTGATTACCGAATTTAGCTGTTCT---------
------
FC88X_00302_00925 -----------------CCGCGTGATTAC-GAATTTAGCTGTTCT---------
------
FC88X_01060_00976 -----------------CCCCGTGATTAC-GAACTTAGCTGTTCT---------
------
FC88X_00348_01133 -------AGTCGGTGATCTCCGTCATATT-TAACTTACCAGTCCT---------
------
FC88X_00529_01167 ----------------ATCTTTCTTCTATTCAGCT--GCCCGTAT---------
------
FC88X_01009_00815 -----------------CTGCTTTCTTATTCAGCTCTACCCGTAT---------
------
FC88X_00143_00015 ----------------CCACTCCGCTTACAGTTGCTCACTATT-----------
------
FC88X_00019_00457 ----------------CCACTCTGCTTACAGTTGCTCACTATT-----------
------
FC88X_00721_00963 ---------------ACCACTCATCTAACTCCT-CACACTATA-----------
------
FC88X_00919_00508 ----------------CTCCTTATATTCTTC--ACCG-TCATGTT---------
------
FC88X_00883_00203 -------------------CTTAGACTCTTCTGACCGCTCATTTTC--------
------
FC88X_00839_00929 ----------------CTCCTTAGTCTCTATTCGCCACCATCTTCC--------
------
FC88X_00914_00990 ----------------CTCCTCAGA-TCTATCGCCGA-----------------
------
FC88X_00520_00980 ---------------TCCTCTCA-TCTTCCGCGGCGAACTGTT-----------
------
FC88X_00811_01065 ---------------TCCTCTCA-TCTTCCGCGGCGAACTGTT-----------
------
FC88X_01072_00454 ---------------TCCTCTCA-TCTTCCGCGGCGAACTGTT-----------
------
FC88X_00282_01128 ---------------TCACGTAT-TCCGCTGCATCGAACTTTT-----------
------
FC88X_00099_00454 ----------------CTCCTAA-TGTTCCACGGCGACGTCC------------
------
FC88X_00330_01160 ----------------CTCCTAACTGTTCCGTGGCAACCCAAT-----------
------
FC88X_00905_00762 ---------------TCTCCTGA-CATTCAACGGCAATCCGAT-----------
------
FC88X_00033_00346 ---------------TTGCTGACTCTAATTCTGTGACTTTCT------------
------
FC88X_00048_00329 ---------------TTGCTGACTCTAATTCTGTGACTTTCT------------
------
FC88X_00051_00166 ---------------TTGCTGACTCTAATTCTGTGACTTTCT------------
------
FC88X_00248_01018 ---------------TTGCTGACTCTAATTCTGTGACTTTCT------------
------
FC88X_00481_00143 ---------------TTGCTGACTCTAATTCTGTGACTTTCT------------
------
FC88X_00631_00810 ---------------TTGCTGACTCTAATTCTGTGACTTTCT------------
------
FC88X_00903_00735 ---------------TTGCTGACTCTAATTCTGTGACTTTCT------------
------

```
FC88X_00604_00171      ---------------TTGCTGACTCTAATTCTGTAACTTTCT------------
------
FC88X_00910_00612      ---------------TTGCTGACTCTAATTCTGCGACTTTCT------------
------
FC88X_00478_00704      --------------CTAGTCGACTCGAGATCTGCGG------------------
------
FC88X_00738_00082      --------------CTAGTCGACTCGAGATCTGCGG------------------
------
FC88X_00241_00708      --------------CTAGTCGACTCGAGATCTGCGC------------------
------
FC88X_00874_01096      -----------AAGCTAGTCGACTCGACATCTGTGGCTTCCTTTCTCCC-----
------
FC88X_01075_00738      --------------TCATCTGATGCGGAATCATTGACTTCTC------------
------
FC88X_01024_00971      --------------ACGTCATCTGCGGACTCACTTCTC----------------
------
FC88X_00280_00085      ----------------TTCTGACG-GATTGCTCTGACT--ATTCAC--------
------
FC88X_00873_00718      ----------------TTCTGACG-GATTGCTCTGACTC-ATTCAC--------
------
FC88X_00551_00866      ----------------TTCTGACG-GATTGCTCTGACT--ATTCAC--------
------
FC88X_00891_01160      ----------------TTCTGACG-GATTGCTCTGACT--ATTCAC--------
------
FC88X_00048_00497      ----------------TTCTGACG-GATTGCTCTGACT--ATTCAC--------
------
FC88X_00380_01096      ----------------TTCTGACG-GATTGCTCTGACT--ATTCAC--------
------
FC88X_00873_01033      ---------------GTTCTGACG-GATTGCTCTGACT--ATTCAC--------
------
FC88X_00714_01242      ----------------TTCTGACG-GATTGCTCTGACT--ATTCAC--------
------
FC88X_00921_00710      ----------------TTCTGACG-GATTGCTCTGACT--ATTCAC--------
------
FC88X_00078_00070      ----------------TTCTGACG-GATTGCTCTGACT--ATTCAC--------
------
FC88X_00134_00576      ----------------TTCTGACG-GATTGCTCTGACT--ATTCAC--------
------
FC88X_00182_00773      ----------------TTCTGACG-GATTGCTCTGACT--ATTCAC--------
------
FC88X_00437_00007      ----------------TTCTGACG-GATTGCTCTGACT--ATTCAC--------
------
FC88X_00846_00824      ----------------TTCTGACG-GATTGCTCTGACT--ATTCAC--------
------
FC88X_00815_00939      ----------------TTCTGACG-GATTGCTCTGACT--ATTCAC--------
------
FC88X_00898_01099      ----------------TTCTGACG-GATTGCTCTGACT--ATTCAC--------
------
FC88X_00861_01128      ----------------TTCTGACG-GATTGCTCTGACT--ATTCAC--------
------
FC88X_01038_01103      ----------------TTCTGACG-GATTGCTCTGACT--ATTCAC--------
------
FC88X_00066_00267      ---------GGGGAAGTTCTGACG-GATTGCTCTGACTC-ATTCAC--------
------
FC88X_00487_00825      ---------GGGGAATTTCTGACG-GATTGCTCTGACT--ATTCAC--------
------
```

FC88X_00087_00352 -----------------TTCTGACG-GATTGCTCTGACT--ATTCACC-------
------
FC88X_00961_00665 -----------------CTCTGACG-GATTGCTCTGACT--ATTCAC--------
------
FC88X_00188_00110 -----------------TTCTGACA-GATTGCTCTGACT--ATTCAC--------
------
FC88X_00066_00884 -----------------TTCCGACG-GATTGCTCTGACT--ATTCAC--------
------
FC88X_00578_01224 -----------------TTCCGACG-GATTGCTCTGACT--ATTCAC--------
------
FC88X_00140_00171 -----------------TTCTGACG-GATTGCTCTGACT--
ATTCACCTTCCTTT------
FC88X_00465_00363 -----------------TTCTGACG-GATTGCTCTGACT--ATTCAT--------
------
FC88X_00997_01146 -----------------TTCTGACG-GATTGCTCTGACT--ATTCATC-------
------
FC88X_00408_01049 -----------------TTCTGACG-GATTGCTCTGACT--ACTCAC--------
------
FC88X_00917_00571 ----------------CTTCTGGCT-CTTCGCAGTGACT--ATTC----------
------
FC88X_00034_00773 -------------------CTCCCA-AACCTCTTTCAGTGACTTCAC--------
------
FC88X_01009_01075 -------------------CTCCCT-GATCTCTGTAACTTTACAGACCC------
------
FC88X_00189_00926 ---------------CATCATTGCT-TCTT-CGGTGCCTATACT-----------
------
FC88X_00857_01210 ---------------CATTATTGCT-TCTT-CGGTGCCTATACT-----------
------
FC88X_00112_00720 ------------TTCAATCATCGCC-GCTT-TGACTATTCT--------------
------
FC88X_00057_00535 -------------------ATGACG-TCTT-CGCTGTGTCTATTTACC-------
------
FC88X_01099_01092 -------------------ATGACG-TCTT-CGCTGTGTCTATTTACC-------
------
FC88X_00158_00054 -------------------ATGACG-TCCT-CGCTGTGTCTATTTGCC-------
------
FC88X_00708_01000 ----------------TTCCTCACC-TCCT-CGCCGCGACTAAT-----------
------
FC88X_00432_00085 ------------------CCGATCGTTTTTT--GAAGTCGAGTTCTC--------
------
FC88X_00795_00138 ------------------CCGATCGTTTTTTTTGAAGTCGAGTTCTC--------
------
FC88X_00912_00806 ------------------CCGATCGTTTTTT--GAAGTCGAGTTCTC--------
------
FC88X_00296_01047 ------------------CCGATCGTTTTTT--GAAGTCGAGTTCTC--------
------
FC88X_00646_00942 ------------------CCGATCGTTTTTT--GAAGTCGAGTTCTC--------
------
FC88X_01102_00820 ------------------CCGATCGTTTTTTT-GAAGTCGAGTTCTC--------
------
FC88X_01070_00628 ------------------CTGATCGTTTGAT---ATCTTGCGTTCTC--------
------
FC88X_00576_00663 ----------------------CCGTTTGGTT--ATCTCCATTTCTCTTGT----
------
FC88X_00763_00137 ----------------------CCGTTTGGTT--ATCTCCATTTCTCTTGT----
------

FC88X_00436_00406 -----------------TCCTTTGATCACTT-CAAAT-CTGTTCTC--------
------
FC88X_00980_00326 -----------------TCCTTTGATCACTT-CAAAT-CTGTTCTC--------
------
FC88X_01080_00948 ------------------TCTCTGATTGCTT-CATTTTCTATTCTC--------
------
FC88X_00952_01269 ---------------CTTGGGCCAAGGCCTAAGACTATT---TCTT--------
------
FC88X_01098_00667 ---------------CTTGGGCCAAG-CCTAAGACTATT---TCTT--------
------
FC88X_00923_00560 ---------------CTTGGGCCAAG-CCTAAGACTATT---TCTT--------
------
FC88X_00991_00562 ---------------CCTGGGCCGCC-TGGAATTCTATT---TCTT--------
------
FC88X_00515_01244 ---------------CTCCTTGTTCGCCTCAACTATTTG---TCC---------
------
FC88X_00228_00955 ---------------CTCGTATGT-GTGGGCATTCTATCCGGTCTCCC------
------
FC88X_00928_00867 ----------------CCGCATGTTGTCCGCAGCCTAT---GTCTC--------
------
FC88X_00954_00873 -----------------CAGGCCTTGGGCTCTTCCAATC---TCTCT-------
------
FC88X_00061_00722 ------------------CCGTCAACAAATCTGGG--TCTATTCTG--------
------
FC88X_00781_01022 -----------------GCCGTCAACAAATCTGGG--TCTATTCTG--------
------
FC88X_00889_01030 ------------------CCGTCAACAAATCTGGG--TCTATTCTG--------
------
FC88X_00915_01181 -------------------CGTCAACAAATCTGGG--TCTATTCTG--------
------
FC88X_00876_00776 ------------------CCGTCAACAAATCTGGGA-TCTATTCTG--------
------
FC88X_00543_00929 ------------------CCGTCAACAAATCTGGG--TCTATTCTG--------
------
FC88X_00918_00932 ------------------CCGTCAACAAATCTGGG--TCTATTCTG--------
------
FC88X_00988_00599 ------------------CCGTCAACAAATCTGGG--TCTATTCTG--------
------
FC88X_01006_00890 ------------------CCGTCAACAAATCTGGG--TCTATTCTG--------
------
FC88X_00092_00813 ------------------CCGTCAACAAATCTGGGG-TCTATTCTG--------
------
FC88X_00127_00783 ------------------CCGTCAACAAATCTGGGG-TCTATTCTG--------
------
FC88X_00890_00816 ------------------CCGTCAACAAATCTGGGG-TCTATTCTG--------
------
FC88X_00019_00176 ------------------CCGTCAACAAATCTGGGG-TCTATTCTG--------
------
FC88X_00007_00498 ------------------CCGTCAACAAATCTGGGG-TCTATTCTG--------
------
FC88X_00045_00805 ------------------CCGTCAACAAATCTGGGG-TCTATTCTG--------
------
FC88X_00188_00036 ------------------CCGTCAACAAATCTGGGG-TCTATTCTG--------
------
FC88X_00321_00014 ------------------CCGTCAACAAATCTGGGG-TCTATTCTG--------
------

FC88X_00532_01154 -------------------CCGTCAACAAATCTGGGG-TCTATTCTG--------
------
FC88X_00581_00952 -------------------CCGTCAACAAATCTGGGG-TCTATTCTG--------
------
FC88X_00724_01023 -------------------CCGTCAACAAATCTGGGG-TCTATTCTG--------
------
FC88X_00853_00931 -------------------CCGTCAACAAATCTGGGG-TCTATTCTG--------
------
FC88X_00945_00616 -------------------CCGTCAACAAATCTGGGG-TCTATTCTG--------
------
FC88X_00970_00722 -------------------CCGTCAACAAATCTGGGG-TCTATTCTG--------
------
FC88X_00985_00779 -------------------CCGTCAACAAATCTGGGG-TCTATTCTG--------
------
FC88X_01027_00902 -------------------CCGTCAACAAATCTGGGG-TCTATTCTG--------
------
FC88X_01034_01076 -------------------CCGTCAACAAATCTGGGG-TCTATTCTG--------
------
FC88X_01061_00918 -------------------CCGTCAACAAATCTGGGG-TCTATTCTG--------
------
FC88X_00079_00810 --------------------CGTCAACAAATCTGGGG-TCTATTCTGCC------
------
FC88X_00141_00939 --------------------CGTCAACAAATCTGGGG-TCTATTCTG--------
------
FC88X_00921_00803 --------------------CGTCAACAAATCTGGGG-TCTATTCTG--------
------
FC88X_00278_00067 -------------------TCGTCAACAAATCTGGGG-TCTATTCTG--------
------
FC88X_00833_00502 --------------------CGTGAACAAATCTGGG--TCTATTCTG--------
------
FC88X_00381_00051 -------------------CCGTCAACAAATCTGGGG-TCTACTCTG--------
------
FC88X_00059_00526 -------------------CCGTCAACAAGTCTGGGG-TCTATTCTG--------
------
FC88X_00143_00887 -------------------CCGTCAACAAATCTGGG--TCTATTCTC--------
------
FC88X_00134_00115 -------------------CCGTCAACAAACCTGGG--TCTATTCTG--------
------
FC88X_00483_01187 -------------------CCGTCAACAAACCTGGGG-TCTATTCTG--------
------
FC88X_00135_00939 -------------------CCGTCAACAAATCTGGGG-CCTATTCTG--------
------
FC88X_00567_00102 -------------------CCGTCAACAAATCTGGGG-CCTATTCTG--------
------
FC88X_00366_01123 -------------------CCGTCAACAAATTTGGGG-CCTATTCTG--------
------
FC88X_00816_00432 -------------------CCGTCAACAAATCTGGGT-CTATTTCTG--------
------
FC88X_01068_01121 --------------------CGTCAACAATCCTGGG--TCTATTCTCG-------
------
FC88X_00554_00346 -----------------GTACCTCAGTAAA-CGAAGT-TCTATTCT---------
------
FC88X_00084_00697 -----------------GTACCTCAGTAAA-CGAAGT-TCTAATTCT--------
------
FC88X_00086_00695 -----------------GTACCTCAGTAAA-CGAAGT-TCTATTCT---------
------

FC88X_00130_00813 ----------------GTACCTCAGTAAA-CGAAGT-TCTATTCT---------
------
FC88X_01116_00520 ----------------GTACCTCACCAAA-CGAAGT-TCTATTCT---------
------
FC88X_00565_00183 -----------------AGCGTAAACACGTTCGCTT-CTAATCTT---------
------
FC88X_00882_00505 -----------------AGCGTAAACACGTTCGCTT-CTAATCTT---------
------
FC88X_00918_00871 -----------------AGCGTAAACACGTTCGCTT-CTAATCTT---------
------
FC88X_01014_00527 -----------------AGCGTAAACACGTTCGCTT-CTAATCTT---------
------
FC88X_00557_00171 -----------------AGCGTAAACATGTTCGCTT-CTAATCTT---------
------
FC88X_00979_00392 -----------------CCAGTACATTGGCAACTGG-GCTGTTCC---------
------
FC88X_00208_00847 -----------------CCAGTACATTGGCAACTGG-GCTGTTCC---------
------
FC88X_00227_00976 -----------------CCGGTACATTGGCAACTGG-GCTGTTCC---------
------
FC88X_01098_00925 --------------TTAACCGTACATAGGTAATCG---CTGTTCAC--------
------
FC88X_00793_00231 ------------------TTATATGCCGGTCTGCAG-TCTATTCTC--------
------
FC88X_00433_00081 ------------------TTATATGCCGGTCTGCAG-TCTATTCTC--------
------
FC88X_00980_00789 ------------------TTATATGCCGGTCTGCAG-TCTATTCTT--------
------
FC88X_00924_00979 ---------------CTATTAGACACAGGTCTCC---TCTATTCTT--------
------
FC88X_00569_00740 -------------TCGAACCGTAAACCGGTCACTAGCTCTCTTCCTTT------
------
FC88X_00102_00450 --------------TCAGCTATTTTAAACCTTTTCA-ACTATTCT---------
------
FC88X_01035_00505 --------------TCAGCTATTTAA---CCTTTCA-ACTATTCT---------
------
FC88X_01048_01094 --------------TCAGCTATTTAA---CCTTTCA-ACTATTCT---------
------
FC88X_00368_01113 --------------TCAGCTATTTAA---CCTTTCA-ACTATTCT---------
------
FC88X_00425_01128 --------------TCAGCTATTTAA---CCTTTCA-ACTATTCT---------
------
FC88X_00415_01181 --------------TCAGCTATTTAA---CCTTTCA-ACTATTCT---------
------
FC88X_00863_00938 --------------TCAGCTATTTAA---CCTTTCA-ACTATTCT---------
------
FC88X_01053_00814 --------------TCAGCTATTTAA---CCTTTCA-ACTATTCT---------
------
FC88X_01103_00882 --------------TCAGCTATTTAA---CCTTTCA-ACTATTCTCTTCCTTT-
------
FC88X_00057_00281 --------------TCGGCTATTTAA--CCTTTCAA-ACTATTCT---------
------
FC88X_01081_00658 ---------------CCATGGTATTGGTCTTTTCTA-TCTATT-----------
------
FC88X_00097_00866 --------------------TTCTAC---CCTTGGA-ACCGCTCCCTTGTT---
------

FC88X_00350_01113 --------------------TTCTAC---CCTTGGA-ACCGCTCCCTTGTT---
------
FC88X_01111_00956 --------------------CCCTTC---CCTTTGC-ACTGCTCGCTTGTT---
------
FC88X_00021_00660 ----------------------TTTA---CCATTAG-ACTGCTCTCTTCACA--
------
FC88X_00139_00978 --------------TTATCCGTT-CGGAACTATTTA-CCTTCT-----------
------
FC88X_00686_01027 ----------------CTCCGTCGCGTTACTACTAA-TCTTTTTC---------
------
FC88X_00791_00112 -------------AGGGCCTCTT-TGACATTACTCC-TCTTCGTT---------
------
FC88X_00954_00587 -----------TTCGGGTCTATT-T----TGACTCC-TGTTCTT----------
------
FC88X_00405_00425 -------ATTTTCGCTATCTGTC-CTGTCTGGTTCT-CCTTCCTTTC-------
------
FC88X_00686_00936 -----------TCG-TGGCCGTT-CTGCTCCGTTCC-TTTT-------------
------
FC88X_00020_00161 ----------ATCCTGGACTATT-TGGCCTTCTCGC-CCTTCCTT---------
------
FC88X_00029_00463 ------------------ATCTTCTC-GTCCGAGCTTGCTATTCAC--------
------
FC88X_00943_01079 ------------------ATCTTCTC-GTCCGAGCTTGCTATTCAC--------
------
FC88X_00035_00724 ------------------ATCTTCTC-GTCCGAGCTTGCTATTCAC--------
------
FC88X_00897_00158 ------------------ATCTTCTC-GTCCGAGCTTGCTATTCAC--------
------
FC88X_00959_00463 ------------------ATCTTCTC-GTCCGAGCTTGCTATTCAC--------
------
FC88X_01094_00736 ------------------ATCTTCTC-GTCCGAGCTTGCTATTCAC--------
------
FC88X_00050_00368 ------------------ATCTTCCC-GTCCGAGCTTGCTATTCAC--------
------
FC88X_01027_00521 ----------------TCGTCTTCTCCGTCCGATTGCGATTTCTG---------
------
FC88X_00187_00925 ------------------TCCCGTCCTATTTC-GCCGCATCTTCAT--------
------
FC88X_00155_00667 ------------------TCCAATCCTATCTCCGCCTTATAATCAG--------
------
FC88X_00890_00786 -----------------------TGCTATTCC-G--------------------
------
FC88X_00060_00190 ------------------CTACGTGCTATTCC-GCCGTCTGGTATCCC------
------
FC88X_00175_00134 -----------------CTCAGATTTTCTACCCAACTCCTATT-----------
------
FC88X_00729_01070 ---------------CTCTCAGATTT-CGACACGA-TGCTATTC----------
------
FC88X_00356_01116 ------------------TTATTTTCTATTCCGACTCACTATTCG---------
------
FC88X_00522_01115 -------------------CACCGTCACTCCGACACCGA-TGTATT--------
------
FC88X_01111_01059 ------------------ACACCGTCACTCCGACACCGA-TGTATT--------
------
FC88X_00983_00877 ------------------CCACCGTCACTCCGACACCGA-TGTATT--------
------

FC88X_00265_00972 ------------------TCACCCGCACTCCGACATCGC-TATTCC--------------
FC88X_00502_01244 ------------------CCACCACAACTCCGACACTGC-TATTGC--------------
FC88X_00919_00634 ------------------CCACCACAACTCCGACACTGC-TATTGC--------------
FC88X_01144_00678 ------------------CCACCACAACTCCGACACTGC-TATTGC--------------
FC88X_00013_00504 ------------------CCGCCACAACTCCGACACTGCGTATTGC--------------
FC88X_00576_01182 -----------------CCCGCCTCTACGTCAACA-TGC-TATTGT--------------
FC88X_00433_00015 ----------------GGGGAATTCTAATACGACTCACTATAGGAGAGAGAT--------
FC88X_00878_00191 ----------------GGGGAATTCTAATACGACTCACTATAGGAGA-------------
FC88X_00334_00765 ----------------GGGGAATTCTAATACGACTCACTATAGGGAGAG-----------
FC88X_00418_00942 ---------------GGGGGAATTCTAATACGACTCA-----------------------
FC88X_00087_00214 ----------------GGGGAATTCTAATACGACTCACTATTCTCCCTC-----------
FC88X_00722_00066 ------------------TCAATCGGAATACGACTC------------------------
FC88X_00391_01101 ------------------TCCGTCCGAAACCGACGCGCACTTCGC---------------
FC88X_00587_00065 ----------------AGTCCGTCCGAAACCGACGCGCACTTCGC---------------
FC88X_00097_00542 ------------------TCCGTCCGAAACCGACGCGCATTTCGCCTTCCT---------
FC88X_00909_01216 -----------------GTCTGTACCACTCGGA-CGCCTATTTTTC--------------
FC88X_00088_00358 -----------------GTCTGTACCACTCGGA-CGCCTATTTTTC--------------
FC88X_00444_01041 ----------------TAACGGCACCACTCCAC-GCTCTATATT----------------
FC88X_00803_00782 -----------------TACCGTACGTTACTACACCACTCTATT----------------
FC88X_00920_00490 ---------------CCAACCGTGCGACTCAAC-CGACTATAC-----------------
FC88X_00487_01147 -------------------TTATGCCACCAGCCCAGTCTATTTCTC--------------
FC88X_00071_00825 -------------------TTATGCCACCAGCCCAGTCTATTTCTC--------------
FC88X_00478_01213 -------------------TTATGCCACCAGCCCAGTCTATTTCTT--------------
FC88X_00741_01192 -------------------TTATGTCACCAGCCCAGTCTATTTCTC--------------
FC88X_01004_00629 -------------------CTATCCCACCAGCCCAGTCTA--------------------
FC88X_00953_00811 -----------------ATCTCTGGCGACACCCCTTTCTATTTTCCTTC-----------
FC88X_00853_01266 -----------------ATCTCTGGCGACAGCCCTTTCTATTTTC---------------
FC88X_00667_00724 -------------------TTAATCTGACCCGACAGC-TACTTGTT--------------

FC88X_00334_00227 -------------------TTAATCTGACCCGACAGCCTACTTGTT--------
------
FC88X_00910_01174 -------------------TTAATCTGACCCGACAGCCTACTTGTTATCAC---
------
FC88X_00916_00768 -------------------CTAATCTGACCCGACAGCCTACTTGTT--------
------
FC88X_00389_00403 ------------------------TCGACACGTCGTTCTACTT-----------
------
FC88X_00801_00157 --------------------ACCGTTGACACGAAGT-CTATTTCTA--------
------
FC88X_00772_01125 -------------------CCATCTTCTTGATCAATTGCTATTCTT--------
------
FC88X_00400_01100 -------------------CCATCTTCTTGATCAATTGCTATTCTT--------
------
FC88X_00158_00415 -------------------CCATCTTCTTGATCAACTGCTATTCTT--------
------
FC88X_00355_01150 ------------TCTACACCGATCTTCTTGATCAATCGCTATTCTT--------
------
FC88X_00429_01172 ------------------CCAATCTCTCGTGGTCATTGCGCTTCT---------
------
FC88X_00548_00988 -----------------GCCAATCTCTCGTGGTCATTGCGCTTCT---------
------
FC88X_00583_00138 ------------------CCAATCTCTCGTGGTCATTGCGCTTCT---------
------
FC88X_00765_00823 ------------------CCAATCTCTCGTGGTCATTGCGCTTCT---------
------
FC88X_00961_01002 ------------------CCAATCTCTCGTGGTCATTGCGCTTCT---------
------
FC88X_00057_00737 ------------------CCAATCTCTCGTGGTCATTGCGCTTCT---------
------
FC88X_00112_00204 ------------------CCAATCTCTCGTGGTCATTGCGCTTCT---------
------
FC88X_00370_00565 ------------------CCAATCTCTCGTGGTCATTGCGCTTCT---------
------
FC88X_00472_00970 ------------------CCAATCTCTCGTGGTCATTGCGCTTCT---------
------
FC88X_00609_00992 ------------------CCAATCTCTCGTGGTCATTGCGCTTCT---------
------
FC88X_00796_00888 ------------------CCAATCTCTCGTGGTCATTGCGCTTCT---------
------
FC88X_00918_01145 ------------------CCAATCTCTCGTGGTCATTGCGCTTCT---------
------
FC88X_00994_00618 ------------------CCAATCTCTCGTGGTCATTGCGCTTCT---------
------
FC88X_00982_00726 ------------------CCAATCTCTCGTGGTCATTGCGCTTCT---------
------
FC88X_00994_00868 ------------------CCAATCTCTCGTGGTCATTGCGCTTCT---------
------
FC88X_01033_00554 ------------------CCAATCTCTCGTGGTCATTGCGCTTCT---------
------
FC88X_01013_00783 ------------------CCAATCTCTCGTGGTCATTGCGCTTCT---------
------
FC88X_01028_01201 ------------------CCAATCTCTCGTGGTCATTGCGCTTCT---------
------
FC88X_01016_01267 ------------------CCAATCTCTCGTGGTCATTGCGCTTCT---------
------

```
FC88X_00825_00161      ------------------CCAATCTCTCGTGGTCATTGTGCTTCT---------
------
FC88X_00100_00871      ------------------CCAATCTCTCGTGGTCATTGCGCTTCTCTT------
------
FC88X_00213_00023      ------------------CCAATCTCTCGAGGTCATTGCGCTTCT---------
------
FC88X_00954_00484      ------------------CCAATCTCTCGTGGTCATTGCGCTTCTCTTCCTTT-
------
FC88X_00626_00147      ------------------TCAATCTCTCGTGGTCATTGCGCTTCT---------
------
FC88X_00991_00415      ------------------CCAATCTCTCGTGGTCGTTGCGCTTCT---------
------
FC88X_00457_01231      ------------------CCAATCTCTCGTGGTCATTGCGCTTCC---------
------
FC88X_00295_01147      ------------------CCAATCTCTCGTGGTCATTGCTATTCTT--------
------
FC88X_00161_00611      -------------------TCCGCTCG-CTGATCATGACAACACTATT------
------
FC88X_00048_00422      -------------------TCCGCTCG-CTGATCATGACAACACTATT------
------
FC88X_00088_00105      -------------------TCCGCTCG-CTGATCATGACAACACTATT------
------
FC88X_00829_00972      -------------------TCCGCTCG-CTGATCATGACAACACTATT------
------
FC88X_00595_01254      --------------------TTGCTCGTCTGATCGGGTCAACTCTGA-------
------
FC88X_00987_00554      ---------------------TACCCG-ATCATCACATCGATATTGTT------
------
FC88X_01097_00689      --------------------CCACCCG-CTCGTCTCATCGCAATTCTT------
------
FC88X_00702_00166      -----------------TTATACTACTCCGCTCTACAAAGAATA----------
------
FC88X_01080_00899      ----------------------TTACAACGTTC-ACACAGACTATTTTCT----
------
FC88X_01018_00392      -------------------CTATCTCATCGGTCAATACTATTTCTC--------
------
FC88X_00021_00755      -------------------CTATCTCATCGGTCAATACTATTTCTC--------
------
FC88X_00085_00893      -------------------CTATCTCATCGGTCAATACTATTTCTC--------
------
FC88X_00577_01200      -------------------CTATCTCATCGGTCAATACTATTTCTC--------
------
FC88X_00854_01183      -------------------CTATCTCATCGGTCAATACTATTTCTC--------
------
FC88X_00715_00053      -------------------CTATCTCATCGGTCAATACTATTT-----------
------
FC88X_01049_00910      -------------------CTCTCACCTCGTTGAATTTGACTAATT--------
------
FC88X_00150_00620      -------------------TCACGGCTTAAAATTACAGTGGCATTCT-------
------
FC88X_00717_00541      -------------------TCACGGCTTAAAATTACAGTGGCATTCT-------
------
FC88X_00265_00120      -------------------TCACGGCTTAAAATTACAGTGGCATTTCT------
------
FC88X_00014_00345      ------------------CCCAACGGTAAAAATCAACTCGGCATTTT-------
------
```

```
FC88X_00209_00790      -------------------CCACGGGATTT-ATGGTCTTGGCATTCT-------
------
FC88X_00421_00503      -------------------CCACGGGATTT-ATGGTCTTGGCATTCT-------
------
FC88X_00638_01253      -------------------CCACGGGATTT-ATGGTCTTGGCATTCT-------
------
FC88X_00175_00206      ------------------TCCGCGGGAT---ATCATTTGGGCTTTCTC------
------
FC88X_00961_00641      --------------------CTCATCCCTCAATTTTCTGGGCCATCT-------
------
FC88X_00106_00185      --------------------CTCATCCCTCGATTTTCTGGGCCATCT-------
------
FC88X_00096_00683      --------------------CTCACCACTCGATTTCGTTGGCATTCT-------
------
FC88X_00200_00705      -------------------CCACTCATTCGATGAATTT-GCCATTCT-------
------
FC88X_00683_01010      -------------------CCACTCATTCGATGAATTT-GCCATTCT-------
------
FC88X_00282_00325      -------------------CCACTCATTCGATGAATTT-GCCATTCT-------
------
FC88X_00710_01245      -------------------CCACTCATTCGATGAATTT-GCCATTCT-------
------
FC88X_00074_00794      ------------GGGGAAGCCACTCATTCGATGAATTT-GCCATTCT-------
------
FC88X_00332_00522      -------------------CCACTCATTCGATGAATTTTGCCATTCT-------
------
FC88X_00962_00697      -------------------CAACTCATTCGATGAATTT-GCCATTCT-------
------
FC88X_00733_00241      -------------------CTACTACTTCGCTATCTCT-GCCATTTG-------
------
FC88X_00857_00924      -------------------TTAGCGATCATGGCGTAAACAATTCTC--------
------
FC88X_00927_00454      -------------------TTAGCGATCATGGCGTAAACAATTCTC--------
------
FC88X_00145_00244      -------------------TTAGCGATCATGGCGTAAACAATTCTC--------
------
FC88X_00254_00964      -------------------TTAGCGATCATGGCGTAAACAATTCTC--------
------
FC88X_00255_01015      -------------------TTAGCGATCATGGCGTAAACAATTCTC--------
------
FC88X_00323_00480      -------------------TTAGCGATCATGGCGTAAACAATTCTC--------
------
FC88X_00303_01052      -------------------TTAGCGATCATGGCGTAAACAATTCTC--------
------
FC88X_00376_01064      -------------------TTAGCGATCATGGCGTAAACAATTCTC--------
------
FC88X_00401_00653      -------------------TTAGCGATCATGGCGTAAACAATTCTC--------
------
FC88X_00442_01185      -------------------TTAGCGATCATGGCGTAAACAATTCTC--------
------
FC88X_00592_01264      -------------------TTAGCGATCATGGCGTAAACAATTCTC--------
------
FC88X_00655_01030      -------------------TTAGCGATCATGGCGTAAACAATTCTC--------
------
FC88X_00766_00893      -------------------TTAGCGATCATGGCGTAAACAATTCTC--------
------
```

```
FC88X_01023_00991      -------------------TTAGCGATCATGGCGTAAACAATTCTC--------
------
FC88X_01080_01114      -------------------TTAGCGATCATGGCGTAAACAATTCTC--------
------
FC88X_00160_00877      -------------------TTAGCGATCATGGCGTAAACAATTCTC--------
------
FC88X_00331_00243      -------------------TTAGCGATCATGGCGTAAACAATTCTC--------
------
FC88X_00549_00116      ----------------GAGTTAGCGATCATGGCGTAAACAATTCTC--------
------
FC88X_00380_00995      -------------------TTAGCGATCATGGCGTAAACAATTCT---------
------
FC88X_00827_00982      -------------------TTAGCGATCATGGCGAAAACAATTCTC--------
------
FC88X_00896_00801      -------------------TTAGCGATCCTGGCGTAAACAATTCTC--------
------
FC88X_01046_00463      -------------------TTAGCGATCGTGGCGTAAACAATTCTC--------
------
FC88X_00024_00554      -------------------TTAGCGATCATGGCGTGAACAATTCTC--------
------
FC88X_00186_01000      -------------------TTAGCGCTCCT--TCTACACCATTCTCAC------
------
FC88X_00072_00078      -------------------TTAACGCACCCTCCGTTACCTGTTCAC--------
------
FC88X_00493_00983      -------------------TTAACGCACCCTCCGTTACCTGTTCAC--------
------
FC88X_00454_01068      -------------------TTAACGCACCCTCCGTTACCTGTTCAC--------
------
FC88X_00851_00216      -------------------TTAACGCACCCTCCGTTACCTGTTCAC--------
------
FC88X_00876_01022      -------------------TTAACGCACCCTCCGTTACCTGTTCAC--------
------
FC88X_01010_00942      -------------------TTAACGCACCCTCCGTTACCTGTTCAC--------
------
FC88X_00196_00292      -------------------TTAACGCACC-TCCGTTACCTGTTCAC--------
------
FC88X_00079_00158      -------------------TTAACGCATCCTCCGTTACCTGTTCAC--------
------
FC88X_00058_00246      -------------------TTAACGCACCCTC-GTTACCTGTTCAC--------
------
FC88X_00675_00447      -------------------TTAACGCACCCTC-GTTACCTGTTCAC--------
------
FC88X_00713_00369      -------------------TTAACGCACCCTC-GTTACCTGTTCAC--------
------
FC88X_00837_00158      -------------------TTAACGCACCCTC-GTTACCTGTTCAC--------
------
FC88X_00930_00718      -------------------TTAACGCACCCTC-GTTACCTGTTCAC--------
------
FC88X_01014_00305      -------------------TTAACGCACCCTC-GTTACCTGTTCAC--------
------
FC88X_01027_01086      -------------------TTAACGCACCCTC-GTTACCTGTTCAC--------
------
FC88X_01100_00721      -------------------TTAACGCACCCTC-GTTACCTGTTCAC--------
------
FC88X_00250_00330      --------------------TAACGCACCCTC-GTTACCTGTTCAC--------
------
```

FC88X_00531_01203 -------------------TTAACGCGCCCTCCGTTACCTGTTCAC--------
------
FC88X_00590_00104 -------------------TTAACGCACCTTCCGTTACCTGTTCAC--------
------
FC88X_00072_00264 -------------------CTAACGAGCCTCCCGTTATTCATTATT--------
------
FC88X_00464_00699 -------------------CTAAGGCACCTCCAACGTCATTTTATT--------
------
FC88X_00042_00706 ------------------TTACCTGCAATCGGACGTCTATCTTCTC--------
------
FC88X_00567_00188 ------------------TTACCTGCAATCGGACGTCTATCTTCTC--------
------
FC88X_00096_00322 ------------------TCACCTGCAATCGGACGTCTATCTT-----------
------
FC88X_00684_00458 ----------TTCGTAACTCACCTGC--TCCGACATCGTCTCTT----------
------
FC88X_00277_00018 ------------------CTTCCGGCCTTCCGAAGTT-ACATTCTC--------
------
FC88X_00716_01214 ------------------CTTCCGGCCTTCCGAAGTT-ACATTCTCCTT-----
------
FC88X_00902_00793 -------------------CTCCAGCTATCCTACGTTGACATTCT---------
------
FC88X_00218_00535 ------------------CGATCGATCCTCGATCATCT----------------
------
FC88X_00643_00087 ------------------TCACCGCTCCTTCGACGTCATCTTGTT---------
------
FC88X_00165_00028 --------------------TCATCCTTTCGATCGC--GTTTCATTTAGC----
------
FC88X_00427_01096 -----------------------
CCCCTTCGATCGGTAGTTTTTTTTACCAAATA-----
FC88X_00934_00707 -------------------GTTCCACCTTCGATCGTTATCATTTTGTC------
------
FC88X_00030_00555 --------------------TTCCACCTTCGATCGTTATCATTTTGTC------
------
FC88X_01086_00479 -------------------------CCTCCGATC-TTTTGATATTGT-------
------
FC88X_00473_00126 -------------------ACTGCATCTACGAA-CGGTTTATTTTC--------
------
FC88X_01096_00869 ------------------CCTCGCATGTTCGAA-AGTTAGTTTCTC--------
------
FC88X_00060_00208 -----------------CCTCCGCATGTTCGA--AAGTTAGTTCTC--------
------
FC88X_00140_00721 -----------------CCTCCGCATGTTCGA--AAGTTAGTTCTC--------
------
FC88X_00628_01215 -----------------CCTCCGCATGTTCGA--AAGTTAGTTCTC--------
------
FC88X_00976_01253 -----------------CCTCCGCATGTTCGA--AAGTTAGTTCTC--------
------
FC88X_00776_00943 -----------------GCTCCGCATGTTCGA--AAGCTAGTTCTC--------
------
FC88X_01043_00767 -------------------TCCACGTGTATGACCAAGCTAGTTCTC--------
------
FC88X_00188_00014 -----------------TTTCGACGCACGATTCTAGCACTATTCC---------
------
FC88X_00665_01159 ------------------CCCACTCCACGCATC-ATTACTATTCC---------
------

FC88X_00962_00230 ------------------TTCAATCGACCCTTCTATTACTGTTCT---------
------

FC88X_00879_00861 ------------------ACCAGCACTCCA---TGTTGTCATGTTCTC------
------

FC88X_00903_01037 ------------------ACCAGCACTCCA---TGTTGTCATGTTCTC------
------

FC88X_01039_00459 ------------------ACCAGCACTCCA---TGTTGTCATGTTCTC------
------

FC88X_00051_00348 ------------------ACCAGCACTCCA---TGTTGTCATGTTCTC------
------

FC88X_00476_01167 ------------------ACCAGCACTCCA---TGTTGTCATGTTCTC------
------

FC88X_00948_00932 ------------------ACCAGCACTCCA---TGTTGTCATGTTCTC------
------

FC88X_00982_00485 ------------------ACCAGCACTCCA---TGTTGTCATGTTCTC------
------

FC88X_01027_00984 ------------------ACCAGCACTCCA---TGTTGTCATGTTCTC------
------

FC88X_00327_00006 ------------------ACCAGCACTCCA---TGTTGTCATGTTCTC------
------

FC88X_00537_01224 ------------------ACCAGCACTCCA---TGTTGTCATGTTCTC------
------

FC88X_00719_00372 ------------------ACCAGCACTCCA---TGTTGTCATGTTCTC------
------

FC88X_00975_00912 ------------------TCCCGCACCACA---TGTTGTTATAGTCTC------
------

FC88X_00152_01007 ----------------TCTTCAGCCCAACA---TTT-GTCCTATTCT-------
------

FC88X_00530_00378 -----------GGGGATCTTCAGCCCAACA---TTT-GTCCTATTCT-------
------

FC88X_00205_01058 ----------------TCTTCAGCCCAACA---TTT-GTCCTATTCT-------
------

FC88X_00012_00541 ----------------TCTTCAGCCCAACA---TTT-GTCCTATTCT-------
------

FC88X_00695_01264 ----------------TCTTCAGCCCAACA---TTT-GTCCTATTCT-------
------

FC88X_00567_00262 -------------ATGGCTTCAGCACACCA---TTTTATTCTA-----------
------

FC88X_00821_00244 -------------ATGGCTTCAGCACACCA---TTTTATTCTA-----------
------

FC88X_00151_00720 -------------ATGGCTTCAGCACACCA---TTTTATTCTA-----------
------

FC88X_00963_00734 -------------ATGGCTTCAGCACACCA---TTTTATTCTA-----------
------

FC88X_00890_00487 -------------------CCAGCCCTTAA--CTTTTGTTATAGTTTC------
------

FC88X_00697_01000 ------------------GCCAGCCCTTTAACTTTTTGTTATAGTTTCC-----
------

FC88X_00720_00912 -------------------CCAGCCCTTAG--CTTTTGTTATAGTTTC------
------

FC88X_01098_00453 -------------------CCAGCCCCTAA--CTTTTGTTATTGTTTC------
------

FC88X_01015_01010 ----------------TTACGAGCATATCT--AATTCACTATTCC---------
------

FC88X_00462_00019 ----------------TTACGAGCATATCT--AATTCACTATTCC---------
------

FC88X_00845_00302   ----------------TTACGAGCATATCT--AATTCACTATTCC---------
------
FC88X_00927_00683   ---------------TTACCGAGCATATCT--AATTCACTATTCC---------
------
FC88X_00323_00573   ---------------TTACCAGACACATAA--TTTTTTCTATT-----------
------
FC88X_00524_00105   ----------------ACTCTTCTTCTCGAACTTTTGGCTACT-----------
------
FC88X_00018_00421   ----------------ACTCTTCTTCTCGGACTTTTGGCTACT-----------
------
FC88X_00821_01274   ----------------CCACTCTTCCCCTGACATGTTGTTACT-----------
------
FC88X_00191_00062   ----------------CTGCTTCCTCCCTGACGTTTAGTTATT-----------
------
FC88X_00988_00446   ----------------CTGCTTCCTCTCTGACGCTTAGTTATT-----------
------
FC88X_00897_00447   ----------------CTGCAAGTTCCCAGACTTCTAGTTATTC----------
------
FC88X_01081_00806   ----------------CTGCAAGTTCCCAGACTTCTAGTTATTC----------
------
FC88X_01111_00756   ----------------CTGCAAGTTCCCAGACTTCTAGTTATTC----------
------
FC88X_00691_01240   -------------------ACCCGACCCGAACTGTTAGCTACTATT--------
------
FC88X_00130_00658   -------------------ACCCGACCCGAACTGTTAGCTACTATT--------
------
FC88X_00071_00384   -------------------ACCGGACCCGAACTGTTAGCTACTATT--------
------
FC88X_00063_00756   ------------------TCTGTCCCCTCACTTTATCGCTATTCTTCC------
------
FC88X_00777_01088   ------------------TCTGTCCCCTCACTTTATCGCTATTCTTC-------
------
FC88X_00009_00074   ------------------TCTGACCCCTCACTTTATCGCTATTCTT--------
------
FC88X_00770_01203   -----------------------CCCCGAACCTGATCCCGGCTAGTTCTT----
------
FC88X_00030_00316   ---------------------CTCCCTGATTCTTATCTCTATCGTCAT------
------
FC88X_00725_01260   -----------------------CCCCGAGCCTTCTCCTATCAGTCACAA----
------
FC88X_00106_00325   -------------------CAATCGTTTCCTCCC-AACGTGTCTCTC-------
------
FC88X_00914_00915   -------------------CAATCGTTTCCTCCC-AACGTGTCTCTC-------
------
FC88X_01094_00873   -------------------CAATCGTTTCCTCCC-AACGTGTCTCTC-------
------
FC88X_01058_01161   -------------------CAATCGTTTCCTCCC-AACGTGTCTCTC-------
------
FC88X_00470_00129   -------------------CAATCGTTTCCTCC--AACGTGTCTCTC-------
------
FC88X_00028_00494   -------------------CAATCGTTTCCTCC--AACGTGTCTCTC-------
------
FC88X_00901_00784   -------------------CAATCGTTTCCTCCT-AACGTGTCTCTC-------
------
FC88X_00779_00895   -------------------CAATCGTTTCCTCC--AACGTGTCTCTC-------
------

FC88X_00873_00666 -------------------CAATCGTTTCCTCC--AACGTGTCTCTC-------
------
FC88X_00140_00976 -------------------CAATCGTTTCCTCCC-AACCTGTCTCTC-------
------
FC88X_00929_00433 -------------------CAAACGTTTCCTCC--AACGTGTCTCTC-------
------
FC88X_00056_00507 -------------------CAATCGTTTCCTCC--AACGTG-------------
------
FC88X_00347_00050 -------------------CAATCGTTTCCTCC--AACGTG-------------
------
FC88X_00587_00983 --------------------ACTCTTTTCCCTTACAACTTGATTATT-------
------
FC88X_00552_00330 ----------------TTGTAACCATTTCCCTGCCAACGTATCT----------
------
FC88X_00939_01054 ----------------TTGTAACCATTTCCCTGCCAACGTATCT----------
------
FC88X_00030_00524 ----------------TTGTAACCATTTCCCTGCCAACGTATCT----------
------
FC88X_00071_00339 ----------------TTGTAACCATTTCCCTGCCAACGTATCT----------
------
FC88X_01027_00792 ----------------TTGTAACCATTTCCCTGCCAACGTATCT----------
------
FC88X_00701_00352 ----------------------TCATTTCTCT-CCAAC-TATC-----------
------
FC88X_00894_00601 -----------------CTGAGTGGCCAATGCGACCAACTATCTC---------
------
FC88X_01063_00608 -----------------CTGAGTGGCCAATGCGACCAACTATCTC---------
------
FC88X_00415_01003 -----------------CTGAGTGGCCAGTGCGACCAACTATCTC---------
------
FC88X_00783_00310 -----------------CTAAG-ACCCGTGTCGCTCAACTATCTC---------
------
FC88X_00872_01193 -----------------CTAAG-ACCCGTGTCGCTCAACTATCTC---------
------
FC88X_00606_00058 ------------------CTTCTCGCTATTTCGTACAACTATGTC---------
------
FC88X_00905_00515 ------------------CTTCTCACTATTTCGTACAACTATGTC---------
------
FC88X_00091_00798 ---------------------CTCGCTACTGTCGTCAACCATGATGAT------
------
FC88X_00107_00302 --------------------CCAGTCGATATC-CCTAAAACTGGTGTG------
------
FC88X_00215_00229 --------------------CCAGTCGATATC-CCTAAAACTGGTGTG------
------
FC88X_00429_01042 --------------------CCAGTCGATATC-CCTAAAACTGGTGTG------
------
FC88X_00897_00979 --------------------CCAGTCGATATC-CCTAAAACTGGTGTG------
------
FC88X_01102_01018 --------------------CCAGTCGATATC-CCTAAAACTGGTGTG------
------
FC88X_01123_01068 --------------------CCAGTCGATATC-CCTAAAACTGGTGTG------
------
FC88X_00864_00362 --------------------CCAGTCGATATC-CCTAAAACTGGTGTGC-----
------
FC88X_00296_00212 -------------GGGGAATCCAGTCGATATC-CCTAAAACTGGTGTG------
------

```
FC88X_00051_00730       -------------------------CAATATCTCCTCAGACTGGCAAGCCCG--
------
FC88X_01138_01127       ------------------------TCCTCAAA-CCGAT-TGCAATGTATT----
------
FC88X_00567_01241       --------------------CGGCTCCGCATC-CCGATACACAATGTA------
------
FC88X_00704_01144       --------------------CTCGGCTCGATT-TCTCTGCAGAATGTT------
------
FC88X_00695_00170       ---------------TTATCCCGAGTACTACTG--CATACATT-----------
------
FC88X_00991_00783       ---------------TTATCCCGAGTACTACTG--CATACATT-----------
------
FC88X_01147_00853       -----------------CTCCCGAG-ACTATTGGCCATAGATGTACC-------
------
FC88X_00086_00098       ------------------CCTCGCGCGCTATTC--CAAACATTTGC--------
------
FC88X_00895_00435       -----------CCAGACTCCCAG-GCCGTACTA-CTACA---------------
------
FC88X_01066_00512       -----------CCAGACTCCCAGAGCCGTACTA-CTACA---------------
------
FC88X_00275_00833       -----------CCAACCTCCC---GCCTCACCA-CAACATTGC-----------
------
FC88X_00064_00313       ---------------TCTCCCGCCGTACTAGCA-CTAATTGCT-----------
------
FC88X_00234_00761       ---------------TCTCCCGCCGTACTAGCA-CTAATTGCT-----------
------
FC88X_00755_00172       ---------------TCTCCCGCCGTACTAGCA-CTAATTGCT-----------
------
FC88X_00441_01080       -----------------TCCCTGACCATTGGTTTGCAAACTTCT----------
------
FC88X_00598_01266       -----------------TCTCCGTCCATACTTTGTCGAATTTCA----------
------
FC88X_00982_00717       ----------------GTGACATGCTTTTCGTATTCACTTTTC-----------
------
FC88X_00065_00514       -----------------TGACATGCTTTTCGTATTCACTTTTC-----------
------
FC88X_01099_00863       -----------------TGACATGCTTTTCGTATTCACTTTTC-----------
------
FC88X_00072_00646       -----------------TGACATGCTTTTCGTATTCACTTTTTC----------
------
FC88X_00953_00965       ------------------CCCATGGTTTTCCTATGTA-CTGTTCGTC-------
------
FC88X_00914_00760       ------------------CCCATGGTTTTCCTATGTA-CTGTTCGT--------
------
FC88X_00058_00849       ------------------CCCATGGTTTTCCTATGTAACTGTTCGT--------
------
FC88X_01103_00485       ------------------TCCATGGTTTTCCTATGTACTGTTTCGT--------
------
FC88X_00059_00168       -------------------CCATGACATCCCTTACGCCAATTTATT--------
------
FC88X_01060_00658       -----------------CTCAATGATTTTCCTA--GACGCCTTATT--------
------
FC88X_00187_00184       -------------------CCACGCTCTGCCTA-ACAGCCGTCTGTT-------
------
FC88X_00407_01019       -----------------CCCCACTCTATTCCTATACAGCGTTCTT---------
------
```

FC88X_00027_00079 ------------------CGAC-GTGTTCA-TTT-CGCTGTA-TTCTC------
------
FC88X_00596_01070 ------------------CGAC-GTGTTCA-TTTTCGCTGTA-
TTCTCCTTCCTTT----
FC88X_00041_00252 ------------------CGAC-GTGTTCA-TTT-CGCTGTA-TTCTC------
------
FC88X_00021_00593 ------------------CGAC-GTGTTCA-TTT-CGCTGTA-TTCTC------
------
FC88X_00151_00032 ------------------CGAC-GTGTTCA-TTT-CGCTGTA-TTCTC------
------
FC88X_00165_00811 ------------------CGAC-GTGTTCA-TTT-CGCTGTA-TTCTC------
------
FC88X_00290_00627 ------------------CGAC-GTGTTCA-TTT-CGCTGTA-TTCTC------
------
FC88X_00532_01177 ------------------CGAC-GTGTTCA-TTT-CGCTGTA-TTCTC------
------
FC88X_00555_01207 ------------------CGAC-GTGTTCA-TTT-CGCTGTA-TTCTC------
------
FC88X_00618_00607 ------------------CGAC-GTGTTCA-TTT-CGCTGTA-TTCTC------
------
FC88X_00958_01142 -----------GGGGAAGCGAC-GTGTTCA-TTT-CGCTGTA-TTCTC------
------
FC88X_01076_00525 -----------GGGGAAGCGAC-GTGTTCA-TTT-CGCTGTA-TTCTC------
------
FC88X_00125_00076 ------------------CGAC-GTGTTCA-TTT-CGCTGTA-TTCTC------
------
FC88X_00154_00706 ------------------CGAC-GTGTTCA-TTT-CGCTGTA-TTCTC------
------
FC88X_00243_00160 ------------------CGAC-GTGTTCA-TTT-CGCTGTA-TTCTC------
------
FC88X_00474_00701 ------------------CGAC-GTGTTCA-TTT-CGCTGTA-TTCTC------
------
FC88X_00598_00733 ------------------CGAC-GTGTTCA-TTT-CGCTGTA-TTCTC------
------
FC88X_00638_00466 ------------------CGAC-GTGTTCA-TTT-CGCTGTA-TTCTC------
------
FC88X_00636_01050 ------------------CGAC-GTGTTCA-TTT-CGCTGTA-TTCTC------
------
FC88X_00675_00482 ------------------CGAC-GTGTTCA-TTT-CGCTGTA-TTCTC------
------
FC88X_00890_00912 ------------------CGAC-GTGTTCA-TTT-CGCTGTA-TTCTC------
------
FC88X_00901_00739 ------------------CGAC-GTGTTCA-TTT-CGCTGTA-TTCTC------
------
FC88X_00926_01081 ------------------CGAC-GTGTTCA-TTT-CGCTGTA-TTCTC------
------
FC88X_00992_00714 ------------------CGAC-GTGTTCA-TTT-CGCTGTA-TTCTC------
------
FC88X_00970_00851 ------------------CGAC-GTGTTCA-TTT-CGCTGTA-TTCTC------
------
FC88X_00969_00931 ------------------CGAC-GTGTTCA-TTT-CGCTGTA-TTCTC------
------
FC88X_00548_00931 ------------GGGGACCGAC-GTGTTCA-TTT-CGCTGTA-TTCTC------
------
FC88X_00298_00005 -----------------CCGAC-GTGTTCA-TTT-CGCTGTA-TTCTC------
------

```
FC88X_00330_01157      -----------------CCGAC-GTGTTCA-TTT-CGCTGTA-TTCTC------
------
FC88X_00372_01016      -----------------CCGAC-GTGTTCA-TTT-CGCTGTA-TTCTC------
------
FC88X_00514_00191      -----------------CCGAC-GTGTTCA-TTT-CGCTGTA-TTCTC------
------
FC88X_00044_00272      ------------------CGAC-GTGTTCA-TTT-CG-TGTA-TTCTC------
------
FC88X_01012_01128      -----------------CCGAC-GTGTTCA-TTT-CG-TGTA-TTCTC------
------
FC88X_00327_00044      ---------------AGCCGAC-GTGTTCA-TTT-CGCTGTA-TTCTC------
------
FC88X_00636_00221      -----------------CCGAC-GTGTTCA-TTT-CGCTGTA-TTCTC------
------
FC88X_00063_00081      -----------------CCGAC-GTGTTCA-TTT-CGCTGTA-TTCTC------
------
FC88X_00109_00400      -----------------CCGAC-GTGTTCA-TTT-CGCTGTA-TTCTC------
------
FC88X_00201_00928      -----------------CCGAC-GTGTTCA-TTT-CGCTGTA-TTCTC------
------
FC88X_00292_01111      -----------------CCGAC-GTGTTCA-TTT-CGCTGTA-TTCTC------
------
FC88X_00294_01040      -----------------CCGAC-GTGTTCA-TGT-CGCTGTA-TTCTC------
------
FC88X_00391_00695      -----------------CCGAC-GTGTTCA-T-T-CGCTGTA-TTCTC------
------
FC88X_00346_01152      -----------------CCGACCGTGTTCA-TTT-CGCTGTA-TTCTC------
------
FC88X_00098_00732      -----------------CCGAC-GTGTTCA-TTT-CGCTGTA-TTCTC------
------
FC88X_00135_00497      -----------------CCGAC-GTGTTCA-TTT-CGCTGTA-TTCTC------
------
FC88X_00268_00775      -----------------CCGAC-GTGTTCA-TTT-CGCTGTA-TTCTC------
------
FC88X_00398_00894      -----------------CCGAC-GTGTTCA-TTT-CGCTGTA-TTCTC------
------
FC88X_00546_00115      -----------------CCGAC-GTGTTCA-TTT-CGCTGTA-TTCTC------
------
FC88X_00518_01233      -----------------CCGAC-GTGTTCA-TTT-CGCTGTA-TTCTC------
------
FC88X_00557_00113      -----------------CCGAC-GTGTTCA-TTT-CGCTGTA-TTCTC------
------
FC88X_00592_00184      -----------------CCGAC-GTGTTCA-TTT-CGCTGTA-TTCTC------
------
FC88X_00625_00328      -----------------CCGAC-GTGTTCA-TTT-CGCTGTA-TTCTC------
------
FC88X_00744_00836      -----------------CCGAC-GTGTTCA-TTT-CGCTGTA-TTCTC------
------
FC88X_00751_00166      -----------------CCGAC-GTGTTCA-TTT-CGCTGTA-TTCTC------
------
FC88X_00819_00854      -----------------CCGAC-GTGTTCA-TTT-CGCTGTA-TTCTC------
------
FC88X_00838_00912      -----------------CCGAC-GTGTTCA-TTT-CGCTGTA-TTCTC------
------
FC88X_00865_00215      -----------------CCGAC-GTGTTCA-TTT-CGCTGTA-TTCTC------
------
```

FC88X_00854_00324 -----------------CCGAC-GTGTTCA-TTT-CGCTGTA-TTCTC------
------
FC88X_00899_00595 -----------------CCGAC-GTGTTCA-TTT-CGCTGTA-TTCTC------
------
FC88X_00859_00819 -----------------CCGAC-GTGTTCA-TTT-CGCTGTA-TTCTC------
------
FC88X_00929_00373 -----------------CCGAC-GTGTTCA-TTT-CGCTGTA-TTCTC------
------
FC88X_00949_00481 -----------------CCGAC-GTGTTCA-TTT-CGCTGTA-TTCTC------
------
FC88X_00940_00959 -----------------CCGAC-GTGTTCA-TTT-CGCTGTA-TTCTC------
------
FC88X_00963_00632 -----------------CCGAC-GTGTTCA-TTT-CGCTGTA-TTCTC------
------
FC88X_00950_00696 -----------------CCGAC-GTGTTCA-TTT-CGCTGTA-TTCTC------
------
FC88X_00989_00659 -----------------CCGAC-GTGTTCA-TTT-CGCTGTA-TTCTC------
------
FC88X_00952_01046 -----------------CCGAC-GTGTTCA-TTT-CGCTGTA-TTCTC------
------
FC88X_01026_01131 -----------------CCGAC-GTGTTCA-TTT-CGCTGTA-TTCTC------
------
FC88X_01027_01160 -----------------CCGAC-GTGTTCA-TTT-CGCTGTA-TTCTC------
------
FC88X_01122_00505 -----------------CCGAC-GTGTTCA-TTT-CGCTGTA-TTCTC------
------
FC88X_01105_00956 -----------------CCGAC-GTGTTCA-TTT-CGCTGTA-TTCTC------
------
FC88X_00431_00981 -----------------CCGAC-GTGTTCA-TTT-CGCTGTA-TTCTCCC----
------
FC88X_00345_00188 -----------------CCGAC-GTGTTCA-TTT-CGCTGTAATTCTC------
------
FC88X_00202_01025 -----------------CCGAT-GTGTTCA-TTT-CGCTGTA-TTCTC------
------
FC88X_00102_00271 -----------------CCGAT-GTGTTCA-TTT-CGCTGTA-TTCTC------
------
FC88X_00704_00596 -----------------CCGAT-GTGTTCA-TTT-CGCTGTA-TTCTC------
------
FC88X_01015_00334 -----------------CCGAC-GCGTTCA-TTT-CGCTGTA-TTCTC------
------
FC88X_00732_00257 -----------------CCGAC-GCGTTCA-TTT-CGCTGTA-TTCTC------
------
FC88X_00104_00739 -----------------CTGAC-GTGTTCA-TTT-CGCTGTA-TTCTC------
------
FC88X_00848_00341 -----------------CCGTC-GTGTTCA-TTT-CGCTGTA-TTCTC------
------
FC88X_00952_00691 ------------------CGAC-GTGTTCA-TTT-CGCTGTA-
TTCTCCTTCCTTT----
FC88X_01027_00504 -----------------CCGAC-GTGTT-A-TTT-CGCTGTA-
TTCTCCTTCCTTT----
FC88X_00854_00778 -----------------CCGAC-GTGTTCA-TTT-CGGTGTA-
TTCTCCTTCCT------
FC88X_00142_00143 -----------------CCGAC-GTGTTCA-TTT-CGCTGTG-TTCTC------
------
FC88X_00973_00739 ------------------CGAC-GTGTTCG-TTTTCGCTGTA-TTCTC------
------

```
FC88X_01056_01185      -----------------CCGAC-GTGTTCG-TTT-CGCTGTA-TTCTC------
------
FC88X_01012_00782      ------------------CGAC-GTGTTCA-TTT-CGCTTTA-TTCTC------
------
FC88X_00116_00134      -----------------CCGAC-GTGTTCA-TTT-CGCCGTA-TTCTC------
------
FC88X_00938_01068      -----------------TCGAC-GTGTTCA-TTT-CGCCGTA-TTCTC------
------
FC88X_00308_00986      -----------------CCGAC-GTATTCA-TTT-CGCTGTA-TTCTC------
------
FC88X_00958_00502      -----------------CCGAC-GTTTTCA-TTT-CGCTGTA-TTCTC------
------
FC88X_00026_00314      -----------------CCGAC-GTGTTCA-TCT-CACTGTA-TTCTC------
------
FC88X_00613_01256      -----------------CCGGC-GTGTTCA-CTT-CGCTGTA-TTCTC------
------
FC88X_00995_00396      -----------------CCGAC-GTGTTCA-TTT-CGCTGTACTCCTC------
------
FC88X_00986_00422      -----------------CCGAC-GTGCTCA-TTT-CGC----------------
------
FC88X_00814_00807      ----------------CCCAAC-GTGTTCA-TTT-CGTCTGTA------------
------
FC88X_00216_00018      -----------------TCCAG-TGTTTAA-TTTTCTTGGCTTTTCT-------
------
FC88X_00671_00571      -----------------TCCAG-TGTTTAA-TTTTCTTGGCTTTTCT-------
------
FC88X_00074_00185      -----------------TCCAG-TGTTTAA-TTT-CTTGGCTTTTCT-------
------
FC88X_00113_00254      -----------------TCCAG-TGTTTAA-TTT-CTTGGCTTTTCT-------
------
FC88X_00218_00890      -----------------TCCAG-TGTTTAA-TTT-CTTGGCTTTTCT-------
------
FC88X_00431_00934      -----------------TCCAG-TGTTTAA-TTT-CTTGGCTTTTCT-------
------
FC88X_00894_00788      -----------------TCCAG-TGTTTAA-TTT-CTTGGCTTTTCT-------
------
FC88X_01017_01252      -----------------TCCAG-TGTTTAA-TTT-CTTGGCTTTTCT-------
------
FC88X_00118_00531      -----------------TCCAG-TGTTTAA-TTT-CTTGGCTTTTCT-------
------
FC88X_00940_00916      ----------------GTCCAG-TGTTTAA-TTT-CTTGGCTTTTCT-------
------
FC88X_00377_00728      -----------------TCCAG-TGTTTAA-TTT-CTTGGCTTTTCT-------
------
FC88X_00478_00504      -----------------TCCAG-TGTTTAA-TTT-CTTGGCTTTTCT-------
------
FC88X_00913_00543      -----------------TCCAG-TGTTTAA-TTT-CTTGGCTTTTCT-------
------
FC88X_00133_00933      -----------------TCCAG-TGTTTAA-TTT-CTTGGCTTTTCT-------
------
FC88X_00175_00879      -----------------TCCAG-TGTTTAA-TTT-CTTGGCTTTTCT-------
------
FC88X_00373_00038      -----------------TCCAG-TGTTTAA-TTT-CTTGGCTTTTCT-------
------
FC88X_00621_00413      -----------------TCCAG-TGTTTAA-TTT-CTTGGCTTTTCT-------
------
```

FC88X_00710_00050 -----------------TCCAG-TGTTTAA-TTT-CTTGGCTTTTCT-------
------
FC88X_00755_01037 -----------------TCCAG-TGTTTAA-TTT-CTTGGCTTTTCT-------
------
FC88X_00888_00739 -----------------TCCAG-TGTTTAA-TTT-CTTGGCTTTTCT-------
------
FC88X_00871_00989 -----------------TCCAG-TGTTTAA-TTT-CTTGGCTTTTCT-------
------
FC88X_00917_00243 -----------------TCCAG-TGTTTAA-TTT-CTTGGCTTTTCT-------
------
FC88X_00967_00584 -----------------TCCAG-TGTTTAA-TTT-CTTGGCTTTTCT-------
------
FC88X_01010_00273 -----------------TCCAG-TGTTTAA-TTT-CTTGGCTTTTCT-------
------
FC88X_01136_01229 -----------------TCCAG-TGTTTAA-TTT-CTTGGCTTTTCTCTT----
------
FC88X_01090_01229 -----------------TCCAG-TGTTTAA-TTT-CATGGCTTTTCT-------
------
FC88X_00831_00692 -----------------TCCAG-TCTTTAA-TTT-CTTGGCTTTTCT-------
------
FC88X_01010_00915 -----------------CCCAG-TGTTTAA-TTTTCTTGGCTTTTCT-------
------
FC88X_00963_00853 -----------------TCCAG-CGTTTAA-TTT-CTTGGCTTTTCT-------
------
FC88X_00389_01098 -----------------TCCAG-TGCTTAA-TTT-CTTGGCTTTTCT-------
------
FC88X_00230_00300 -----------------TCCAG-TGTTTAA-TTC-CCTGGCTTTTCT-------
------
FC88X_01126_01182 ---------GGGGGAATTCCAG-TGTTTAA-TTT-CTTGGCTTTTTCT------
------
FC88X_00151_00759 -----------------TCCAG-TGTTTAA-CTT-CTTGGCCTTTCT-------
------
FC88X_00728_00297 ---------------TCCTACG-TGTTTAA-TTTCCTTGGCTTTTCTC------
------
FC88X_00596_01213 ------------------CTAC-GTTTTTA-GTTACGTTGCATTTCTC------
------
FC88X_00191_00524 ------------------CTAC-GTTTT-A-GTTACGTTGCATTTCTC------
------
FC88X_00850_01214 ------------------CTAC-GTTTT-A-GTTACATTGCATTTCTC------
------
FC88X_00916_00830 ------------------CTAC-GTTTT-A-GTTACATTGCATTTCTC------
------
FC88X_00886_00670 ------------------CTAC-GTTTT-A-GTTACATTGCATTTCTC------
------
FC88X_00937_01115 ------------------CTAC-GTTTT-A-GTTACATTGCATTTCTC------
------
FC88X_01057_01052 ------------------CTAC-GTTTT-A-GTTACATTGCATTTCTC------
------
FC88X_00092_00164 ------------------CTAC-GTTTT-A-GTTACATTGCATTTCTC------
------
FC88X_00627_00604 ------------------CTAC-GTTTT-A-GTTACATTGCATTTCTC------
------
FC88X_00853_00138 ------------------CTAC-GTTTT-A-GTTACATTGCATTTCTC------
------
FC88X_00871_00191 ------------------CTAC-GTTTT-A-GTTACATTGCATTTCTC------
------

```
FC88X_00581_01110       ------------------CTAC-GTCTT-A-GTTACATTGCATTTCTC------
------
FC88X_00684_00346       ------------------CTAC-GTTTT-A-GTTACATTGCATCTCTC------
------
FC88X_01084_00568       ------------------CTAC-GTTTT-G-GTTACATTGCATTTCTC------
------
FC88X_00710_01098       ------------------CTAC-GTTTT-A-GTTACATTGCATTTTCTC-----
------
FC88X_00951_00742       ------------------CAAC-GTTTT-A-GTTACACTGCATTTCTC------
------
FC88X_00059_00464       -----------------TCTAC-GACTTCA-CTC-CATTCATTTGCT-------
------
FC88X_00673_00389       ----------------TTCAAC-GA-TTAA-TTG-CGTTCCACTGCT-------
------
FC88X_00004_00168       ------------------AGACGT-ATTCA-TTTGCGC-TCTGTTCAC------
------
FC88X_00960_00551       ------------------AGACGT-ATTCAGTTTGCGC-TCTGTTCAC------
------
FC88X_01096_01158       ------------------AGACGT-ATTCA-TTTGCGC-TCTGTTCAC------
------
FC88X_00581_00954       ------------------AGACGT-ATTCA-TTTGCGC-TCTGTTCAC------
------
FC88X_00797_00242       ------------------AGACGT-ATTCA-TTTGCGC-TCTGTTCAC------
------
FC88X_00461_00078       ------------------AGACGT-ATTCATTTGCGC-TCTGTTCAC------
------
FC88X_00625_01010       ------------------AGACGT-ATTCC-TTTGCGC-TCTGTTCAC------
------
FC88X_00082_00427       ------------------AGACGT-ATTCGTT-CGCGC-TCTGTTCAC------
------
FC88X_00540_00440       ------------------AGACGT-ATTCGTT-CGCGC-TCTGTTCAC------
------
FC88X_00767_01088       ------------------AGACGT-ATTCGTT-CGCGC-TCTGTTCAC------
------
FC88X_01099_00723       ------------------AGACGT-ATTCGTTTCGCGC-TCTGTTCAC------
------
FC88X_00017_00357       ------------------AGACGT-ATTCG-TTTGCGC-TCTGTTCAC------
------
FC88X_01080_01028       ------------------AGACGT-ATTCGGTTTGCGC-TCTGTTCAC------
------
FC88X_00038_00453       ------------------AGACGT-ATTCG-TTTGCGC-TCTGTTCAC------
------
FC88X_00107_00468       ------------------AGACGT-ATTCG-TTTGCGC-TCTGTTCAC------
------
FC88X_00031_00372       ------------------AGACGT-ATTCGTTT-GCGC-TCTGTTCAC------
------
FC88X_01094_00793       ------------------AGACGT-ATTCGTTTTGCGC-TCTGTTCAC------
------
FC88X_00023_00767       ------------------AGACGT-ATTCGTTT-GCGC-TCTGTTCAC------
------
FC88X_00121_00812       ------------------AGACGT-ATTCGTTT-GCGC-TCTGTTCAC------
------
FC88X_00187_00633       ------------------AGACGT-ATTCG-TTTGCGC-TCTGTTCAC------
------
FC88X_00599_01270       -----------------GAGACGT-ATTCG-TTTGCGC-TCTGTTCAC------
------
```

FC88X_00967_00810 ---------------GAGAGACGT-ATTCG-TTTGCGC-TCTGTTCAC------
------
FC88X_00934_01110 -----------------GAGACGT-ATTCG-TTTGCGC-TCTGTTCAC------
------
FC88X_00625_01147 -----------------GAGACGT-ATTCG-TTTGCGC-TCTGTTCAC------
------
FC88X_00940_00570 ----------------GGAGACGT-ATTCG-TTTGCGC-TCTGTTCAC------
------
FC88X_00995_00726 -----------------GAGACGT-ATTCG-TTTGCGC-TCTGTTCAC------
------
FC88X_00072_00056 ------------------AGACGT-ATTCG-TTTGCGC-TCTGTTCAC------
------
FC88X_00133_00945 ------------------AGACGT-ATTCG-TTTGCGC-TCTGTTCAC------
------
FC88X_00199_00823 ------------------AGACGT-ATTCG-TTTGCGC-TCTGTTCAC------
------
FC88X_00217_01037 ------------------AGACGT-ATTCG-TTTGCGC-TCTGTTCAC------
------
FC88X_00839_00616 ------------GGGGGAAGACGT-ATTCG-TTTGCGC-TCTGTTCAC------
------
FC88X_00827_00663 -----------------AAGACGT-ATTCG-TTTGCGC-TCTGTTCAC------
------
FC88X_00064_00175 ------------------AGACGT-ATTCG-TTTGCGC-TCTGTTCAC------
------
FC88X_00145_00980 ------------------AGACGT-ATTCG-TTTGCGC-TCTGTTCAC------
------
FC88X_00181_00894 ------------------AGACGT-ATTCG-TTTGCGC-TCTGTTCAC------
------
FC88X_00272_00005 ------------------AGACGT-ATTCG-TTTGCGC-TCTGTTCAC------
------
FC88X_00264_00999 ------------------AGACGT-ATTCG-TTTGCGC-TCTGTTCAC------
------
FC88X_00860_00143 -----------------GAGACGT-AATTCGTTTGCGC-TCTGTTCACCC----
------
FC88X_00869_00274 ------------------AGACGT-AATTCGTTTGCGC-TCTGTTCAC------
------
FC88X_00105_00404 ------------------AGACGT-A-TTCGTTTGCGC-TCTGTTCAC------
------
FC88X_00150_00632 ------------------AGACGT-A-TTCGTTTGCGC-TCTGTTCAC------
------
FC88X_00213_00517 ------------------AGACGT-A-TTCGTTTGCGC-TCTGTTCAC------
------
FC88X_00282_00473 ------------------AGACGT-A-TTCGTTTGCGC-TCTGTTCAC------
------
FC88X_00293_00973 ------------------AGACGT-ATTCG-TTTGCGC-TCTGTTCAC------
------
FC88X_00311_01078 ------------------AGACGT-ATTCG-TTTGCGC-TCTGTTCAC------
------
FC88X_00415_01063 ------------------AGACGT-ATTCG-TTTGCGC-TCTGTTCAC------
------
FC88X_00748_00959 ------------------AGACGT-ATTCG-TTTGCGC-TCTGTTCACC-----
------
FC88X_00437_00964 ------------------AGACGT-ATTCG-TTTGCGC-TCTGTTCACC-----
------
FC88X_00970_00425 ------------------AGACGT-ATTCG-TTTGCGC-TCTGTTCACC-----
------

FC88X_00284_00683 ------------------AGACGT-ATTCG-TTTGCGC-TCTGTTCAC------
------
FC88X_00310_00937 ------------------AGACGT-ATTCG-TTTGCGC-TCTGTTCAC------
------
FC88X_00327_01113 ------------------AGACGT-ATTCG-TTTGCGC-TCTGTTCAC------
------
FC88X_00419_01139 ------------------AGACGT-ATTCG-TTTGCGC-TCTGTTCAC------
------
FC88X_00471_00086 ------------------AGACGT-ATTCG-TTTGCGC-TCTGTTCAC------
------
FC88X_00522_00078 ------------------AGACGT-ATTCG-TTTGCGC-TCTGTTCAC------
------
FC88X_00545_00052 ------------------AGACGT-ATTCG-TTTGCGC-TCTGTTCAC------
------
FC88X_00516_00449 ------------------AGACGT-ATTCG-TTTGCGC-TCTGTTCAC------
------
FC88X_00502_01024 ------------------AGACGT-ATTCG-TTTGCGC-TCTGTTCAC------
------
FC88X_00546_01157 ------------------AGACGT-ATTCG-TTTGCGC-TCTGTTCAC------
------
FC88X_00596_00968 ------------------AGACGT-ATTCG-TTTGCGC-TCTGTTCAC------
------
FC88X_00569_01078 ------------------AGACGT-ATTCG-TTTGCGC-TCTGTTCAC------
------
FC88X_00566_01188 ------------------AGACGT-ATTCG-TTTGCGC-TCTGTTCAC------
------
FC88X_00609_00279 ------------------AGACGT-ATTCG-TTTGCGC-TCTGTTCAC------
------
FC88X_00607_00917 ------------------AGACGT-ATTCG-TTTGCGC-TCTGTTCAC------
------
FC88X_00645_00937 ------------------AGACGT-ATTCG-TTTGCGC-TCTGTTCAC------
------
FC88X_00634_01051 ------------------AGACGT-ATTCG-TTTGCGC-TCTGTTCAC------
------
FC88X_00662_00163 ------------------AGACGT-ATTCG-TTTGCGC-TCTGTTCAC------
------
FC88X_00688_00230 ------------------AGACGT-ATTCG-TTTGCGC-TCTGTTCAC------
------
FC88X_00663_00914 ------------------AGACGT-ATTCG-TTTGCGC-TCTGTTCAC------
------
FC88X_00677_01016 ------------------AGACGT-ATTCG-TTTGCGC-TCTGTTCAC------
------
FC88X_00719_00939 ------------------AGACGT-ATTCG-TTTGCGC-TCTGTTCAC------
------
FC88X_00715_00951 ------------------AGACGT-ATTCG-TTTGCGC-TCTGTTCAC------
------
FC88X_00725_01001 ------------------AGACGT-ATTCG-TTTGCGC-TCTGTTCAC------
------
FC88X_00789_00811 ------------------AGACGT-ATTCG-TTTGCGC-TCTGTTCAC------
------
FC88X_00799_00882 ------------------AGACGT-ATTCG-TTTGCGC-TCTGTTCAC------
------
FC88X_00795_00974 ------------------AGACGT-ATTCG-TTTGCGC-TCTGTTCAC------
------
FC88X_00751_01149 ------------------AGACGT-ATTCG-TTTGCGC-TCTGTTCAC------
------

FC88X_00824_01202 ------------------AGACGT-ATTCG-TTTGCGC-TCTGTTCAC------
------
FC88X_00886_00503 ------------------AGACGT-ATTCG-TTTGCGC-TCTGTTCAC------
------
FC88X_00865_00874 ------------------AGACGT-ATTCG-TTTGCGC-TCTGTTCAC------
------
FC88X_00861_00941 ------------------AGACGT-ATTCG-TTTGCGC-TCTGTTCAC------
------
FC88X_00899_01175 ------------------AGACGT-ATTCG-TTTGCGC-TCTGTTCAC------
------
FC88X_00850_01235 ------------------AGACGT-ATTCG-TTTGCGC-TCTGTTCAC------
------
FC88X_00937_00244 ------------------AGACGT-ATTCG-TTTGCGC-TCTGTTCAC------
------
FC88X_00917_00358 ------------------AGACGT-ATTCG-TTTGCGC-TCTGTTCAC------
------
FC88X_00901_00407 ------------------AGACGT-ATTCG-TTTGCGC-TCTGTTCAC------
------
FC88X_00926_00438 ------------------AGACGT-ATTCG-TTTGCGC-TCTGTTCAC------
------
FC88X_00927_00434 ------------------AGACGT-ATTCG-TTTGCGC-TCTGTTCAC------
------
FC88X_00933_00408 ------------------AGACGT-ATTCG-TTTGCGC-TCTGTTCAC------
------
FC88X_00919_00565 ------------------AGACGT-ATTCG-TTTGCGC-TCTGTTCAC------
------
FC88X_00925_00618 ------------------AGACGT-ATTCG-TTTGCGC-TCTGTTCAC------
------
FC88X_00944_00730 ------------------AGACGT-ATTCG-TTTGCGC-TCTGTTCAC------
------
FC88X_00923_01029 ------------------AGACGT-ATTCG-TTTGCGC-TCTGTTCAC------
------
FC88X_00916_01176 ------------------AGACGT-ATTCG-TTTGCGC-TCTGTTCAC------
------
FC88X_00952_00486 ------------------AGACGT-ATTCG-TTTGCGC-TCTGTTCAC------
------
FC88X_00954_00634 ------------------AGACGT-ATTCG-TTTGCGC-TCTGTTCAC------
------
FC88X_01022_00304 ------------------AGACGT-ATTCG-TTTGCGC-TCTGTTCAC------
------
FC88X_01047_00615 ------------------AGACGT-ATTCG-TTTGCGC-TCTGTTCAC------
------
FC88X_01027_00692 ------------------AGACGT-ATTCG-TTTGCGC-TCTGTTCAC------
------
FC88X_01048_01073 ------------------AGACGT-ATTCG-TTTGCGC-TCTGTTCAC------
------
FC88X_01015_01231 ------------------AGACGT-ATTCG-TTTGCGC-TCTGTTCAC------
------
FC88X_01096_00567 ------------------AGACGT-ATTCG-TTTGCGC-TCTGTTCAC------
------
FC88X_01087_00606 ------------------AGACGT-ATTCG-TTTGCGC-TCTGTTCAC------
------
FC88X_01097_00675 ------------------AGACGT-ATTCG-TTTGCGC-TCTGTTCAC------
------
FC88X_01057_01038 ------------------AGACGT-ATTCG-TTTGCGC-TCTGTTCAC------
------

FC88X_01133_01064 ------------------AGACGT-ATTCG-TTTGCGC-TCTGTTCAC------
------
FC88X_00036_00189 ------------------AGACGT-ATTCG-TTTGCGC-TCTGTTCAC------
------
FC88X_00044_00175 ------------------AGACGT-ATTCG-TTTGCGC-TCTGTTCAC------
------
FC88X_00038_00221 ------------------AGACGT-ATTCG-TTTGCGC-TCTGTTCAC------
------
FC88X_00048_00223 ------------------AGACGT-ATTCG-TTTGCGC-TCTGTTCAC------
------
FC88X_00009_00347 ------------------AGACGT-ATTCG-TTTGCGC-TCTGTTCAC------
------
FC88X_00017_00416 ------------------AGACGT-ATTCG-TTTGCGC-TCTGTTCAC------
------
FC88X_00026_00491 ------------------AGACGT-ATTCG-TTTGCGC-TCTGTTCAC------
------
FC88X_00071_00082 ------------------AGACGT-ATTCG-TTTGCGC-TCTGTTCAC------
------
FC88X_00072_00061 ------------------AGACGT-ATTCG-TTTGCGC-TCTGTTCAC------
------
FC88X_00094_00132 ------------------AGACGT-ATTCG-TTTGCGC-TCTGTTCAC------
------
FC88X_00059_00184 ------------------AGACGT-ATTCG-TTTGCGC-TCTGTTCAC------
------
FC88X_00084_00187 ------------------AGACGT-ATTCG-TTTGCGC-TCTGTTCAC------
------
FC88X_00068_00205 ------------------AGACGT-ATTCG-TTTGCGC-TCTGTTCAC------
------
FC88X_00085_00237 ------------------AGACGT-ATTCG-TTTGCGC-TCTGTTCAC------
------
FC88X_00079_00331 ------------------AGACGT-ATTCG-TTTGCGC-TCTGTTCAC------
------
FC88X_00096_00349 ------------------AGACGT-ATTCG-TTTGCGC-TCTGTTCAC------
------
FC88X_00082_00449 ------------------AGACGT-ATTCG-TTTGCGC-TCTGTTCAC------
------
FC88X_00086_00549 ------------------AGACGT-ATTCG-TTTGCGC-TCTGTTCAC------
------
FC88X_00085_00617 ------------------AGACGT-ATTCG-TTTGCGC-TCTGTTCAC------
------
FC88X_00095_00665 ------------------AGACGT-ATTCG-TTTGCGC-TCTGTTCAC------
------
FC88X_00070_00709 ------------------AGACGT-ATTCG-TTTGCGC-TCTGTTCAC------
------
FC88X_00050_00780 ------------------AGACGT-ATTCG-TTTGCGC-TCTGTTCAC------
------
FC88X_00069_00825 ------------------AGACGT-ATTCG-TTTGCGC-TCTGTTCAC------
------
FC88X_00116_00010 ------------------AGACGT-ATTCG-TTTGCGC-TCTGTTCAC------
------
FC88X_00116_00233 ------------------AGACGT-ATTCG-TTTGCGC-TCTGTTCAC------
------
FC88X_00140_00206 ------------------AGACGT-ATTCG-TTTGCGC-TCTGTTCAC------
------
FC88X_00107_00476 ------------------AGACGT-ATTCG-TTTGCGC-TCTGTTCAC------
------

FC88X_00138_00544 ------------------AGACGT-ATTCG-TTTGCGC-TCTGTTCAC------
------
FC88X_00116_00801 ------------------AGACGT-ATTCG-TTTGCGC-TCTGTTCAC------
------
FC88X_00105_00866 ------------------AGACGT-ATTCG-TTTGCGC-TCTGTTCAC------
------
FC88X_00130_00872 ------------------AGACGT-ATTCG-TTTGCGC-TCTGTTCAC------
------
FC88X_00115_00949 ------------------AGACGT-ATTCG-TTTGCGC-TCTGTTCAC------
------
FC88X_00176_00014 ------------------AGACGT-ATTCG-TTTGCGC-TCTGTTCAC------
------
FC88X_00166_00124 ------------------AGACGT-ATTCG-TTTGCGC-TCTGTTCAC------
------
FC88X_00161_00230 ------------------AGACGT-ATTCG-TTTGCGC-TCTGTTCAC------
------
FC88X_00170_00203 ------------------AGACGT-ATTCG-TTTGCGC-TCTGTTCAC------
------
FC88X_00170_00783 ------------------AGACGT-ATTCG-TTTGCGC-TCTGTTCAC------
------
FC88X_00165_00835 ------------------AGACGT-ATTCG-TTTGCGC-TCTGTTCAC------
------
FC88X_00246_00021 ------------------AGACGT-ATTCG-TTTGCGC-TCTGTTCAC------
------
FC88X_00217_00125 ------------------AGACGT-ATTCG-TTTGCGC-TCTGTTCAC------
------
FC88X_00235_00269 ------------------AGACGT-ATTCG-TTTGCGC-TCTGTTCAC------
------
FC88X_00203_00942 ------------------AGACGT-ATTCG-TTTGCGC-TCTGTTCAC------
------
FC88X_00254_00676 ------------------AGACGT-ATTCG-TTTGCGC-TCTGTTCAC------
------
FC88X_00299_00742 ------------------AGACGT-ATTCG-TTTGCGC-TCTGTTCAC------
------
FC88X_00299_00983 ------------------AGACGT-ATTCG-TTTGCGC-TCTGTTCAC------
------
FC88X_00282_01024 ------------------AGACGT-ATTCG-TTTGCGC-TCTGTTCAC------
------
FC88X_00270_01124 ------------------AGACGT-ATTCG-TTTGCGC-TCTGTTCAC------
------
FC88X_00280_01124 ------------------AGACGT-ATTCG-TTTGCGC-TCTGTTCAC------
------
FC88X_00305_00047 ------------------AGACGT-ATTCG-TTTGCGC-TCTGTTCAC------
------
FC88X_00313_00944 ------------------AGACGT-ATTCG-TTTGCGC-TCTGTTCAC------
------
FC88X_00330_01055 ------------------AGACGT-ATTCG-TTTGCGC-TCTGTTCAC------
------
FC88X_00340_01124 ------------------AGACGT-ATTCG-TTTGCGC-TCTGTTCAC------
------
FC88X_00380_00023 ------------------AGACGT-ATTCG-TTTGCGC-TCTGTTCAC------
------
FC88X_00351_00592 ------------------AGACGT-ATTCG-TTTGCGC-TCTGTTCAC------
------
FC88X_00393_00837 ------------------AGACGT-ATTCG-TTTGCGC-TCTGTTCAC------
------

FC88X_00389_00922 ------------------AGACGT-ATTCG-TTTGCGC-TCTGTTCAC------
------
FC88X_00356_01078 ------------------AGACGT-ATTCG-TTTGCGC-TCTGTTCAC------
------
FC88X_00382_01099 ------------------AGACGT-ATTCG-TTTGCGC-TCTGTTCAC------
------
FC88X_00445_01119 ------------------AGACGT-ATTCG-TTTGCGC-TCTGTTCAC------
------
FC88X_00433_01162 ------------------AGACGT-ATTCG-TTTGCGC-TCTGTTCAC------
------
FC88X_00479_01013 ------------------AGACGT-ATTCG-TTTGCGC-TCTGTTCAC------
------
FC88X_00501_00486 ------------------AGACGT-ATTCG-TTTGCGC-TCTGTTCAC------
------
FC88X_00504_00949 ------------------AGACGT-ATTCG-TTTGCGC-TCTGTTCAC------
------
FC88X_00549_01002 ------------------AGACGT-ATTCG-TTTGCGC-TCTGTTCAC------
------
FC88X_00526_01062 ------------------AGACGT-ATTCG-TTTGCGC-TCTGTTCAC------
------
FC88X_00520_01152 ------------------AGACGT-ATTCG-TTTGCGC-TCTGTTCAC------
------
FC88X_00522_01188 ------------------AGACGT-ATTCG-TTTGCGC-TCTGTTCAC------
------
FC88X_00520_01208 ------------------AGACGT-ATTCG-TTTGCGC-TCTGTTCAC------
------
FC88X_00562_00100 ------------------AGACGT-ATTCG-TTTGCGC-TCTGTTCAC------
------
FC88X_00579_00114 ------------------AGACGT-ATTCG-TTTGCGC-TCTGTTCAC------
------
FC88X_00584_00123 ------------------AGACGT-ATTCG-TTTGCGC-TCTGTTCAC------
------
FC88X_00590_00149 ------------------AGACGT-ATTCG-TTTGCGC-TCTGTTCAC------
------
FC88X_00551_00483 ------------------AGACGT-ATTCG-TTTGCGC-TCTGTTCAC------
------
FC88X_00583_00465 ------------------AGACGT-ATTCG-TTTGCGC-TCTGTTCAC------
------
FC88X_00559_00604 ------------------AGACGT-ATTCG-TTTGCGC-TCTGTTCAC------
------
FC88X_00589_01089 ------------------AGACGT-ATTCG-TTTGCGC-TCTGTTCAC------
------
FC88X_00592_01090 ------------------AGACGT-ATTCG-TTTGCGC-TCTGTTCAC------
------
FC88X_00583_01225 ------------------AGACGT-ATTCG-TTTGCGC-TCTGTTCAC------
------
FC88X_00635_00140 ------------------AGACGT-ATTCG-TTTGCGC-TCTGTTCAC------
------
FC88X_00622_00921 ------------------AGACGT-ATTCG-TTTGCGC-TCTGTTCAC------
------
FC88X_00638_01122 ------------------AGACGT-ATTCG-TTTGCGC-TCTGTTCAC------
------
FC88X_00652_00224 ------------------AGACGT-ATTCG-TTTGCGC-TCTGTTCAC------
------
FC88X_00651_00268 ------------------AGACGT-ATTCG-TTTGCGC-TCTGTTCAC------
------

FC88X_00695_00613 -------------------AGACGT-ATTCG-TTTGCGC-TCTGTTCAC------
------
FC88X_00686_01086 -------------------AGACGT-ATTCG-TTTGCGC-TCTGTTCAC------
------
FC88X_00655_01105 -------------------AGACGT-ATTCG-TTTGCGC-TCTGTTCAC------
------
FC88X_00721_00057 -------------------AGACGT-ATTCG-TTTGCGC-TCTGTTCAC------
------
FC88X_00729_00058 -------------------AGACGT-ATTCG-TTTGCGC-TCTGTTCAC------
------
FC88X_00707_00270 -------------------AGACGT-ATTCG-TTTGCGC-TCTGTTCAC------
------
FC88X_00726_00284 -------------------AGACGT-ATTCG-TTTGCGC-TCTGTTCAC------
------
FC88X_00734_00979 -------------------AGACGT-ATTCG-TTTGCGC-TCTGTTCAC------
------
FC88X_00700_01244 -------------------AGACGT-ATTCG-TTTGCGC-TCTGTTCAC------
------
FC88X_00781_00093 -------------------AGACGT-ATTCG-TTTGCGC-TCTGTTCAC------
------
FC88X_00756_00207 -------------------AGACGT-ATTCG-TTTGCGC-TCTGTTCAC------
------
FC88X_00761_00213 -------------------AGACGT-ATTCG-TTTGCGC-TCTGTTCAC------
------
FC88X_00791_00425 -------------------AGACGT-ATTCG-TTTGCGC-TCTGTTCAC------
------
FC88X_00786_00772 -------------------AGACGT-ATTCG-TTTGCGC-TCTGTTCAC------
------
FC88X_00768_00895 -------------------AGACGT-ATTCG-TTTGCGC-TCTGTTCAC------
------
FC88X_00786_01101 -------------------AGACGT-ATTCG-TTTGCGC-TCTGTTCAC------
------
FC88X_00772_01151 -------------------AGACGT-ATTCG-TTTGCGC-TCTGTTCAC------
------
FC88X_00839_00124 -------------------AGACGT-ATTCG-TTTGCGC-TCTGTTCAC------
------
FC88X_00805_00262 -------------------AGACGT-ATTCG-TTTGCGC-TCTGTTCAC------
------
FC88X_00840_00330 -------------------AGACGT-ATTCG-TTTGCGC-TCTGTTCAC------
------
FC88X_00815_00374 -------------------AGACGT-ATTCG-TTTGCGC-TCTGTTCAC------
------
FC88X_00804_00437 -------------------AGACGT-ATTCG-TTTGCGC-TCTGTTCAC------
------
FC88X_00800_00972 -------------------AGACGT-ATTCG-TTTGCGC-TCTGTTCAC------
------
FC88X_00830_01229 -------------------AGACGT-ATTCG-TTTGCGC-TCTGTTCAC------
------
FC88X_00880_00174 -------------------AGACGT-ATTCG-TTTGCGC-TCTGTTCAC------
------
FC88X_00892_00240 -------------------AGACGT-ATTCG-TTTGCGC-TCTGTTCAC------
------
FC88X_00883_00359 -------------------AGACGT-ATTCG-TTTGCGC-TCTGTTCAC------
------
FC88X_00857_00672 -------------------AGACGT-ATTCG-TTTGCGC-TCTGTTCAC------
------

```
FC88X_00893_00679       ------------------AGACGT-ATTCG-TTTGCGC-TCTGTTCAC------
------
FC88X_00865_00811       ------------------AGACGT-ATTCG-TTTGCGC-TCTGTTCAC------
------
FC88X_00860_01096       ------------------AGACGT-ATTCG-TTTGCGC-TCTGTTCAC------
------
FC88X_00881_01251       ------------------AGACGT-ATTCG-TTTGCGC-TCTGTTCAC------
------
FC88X_00909_00212       ------------------AGACGT-ATTCG-TTTGCGC-TCTGTTCAC------
------
FC88X_00932_00214       ------------------AGACGT-ATTCG-TTTGCGC-TCTGTTCAC------
------
FC88X_00932_00322       ------------------AGACGT-ATTCG-TTTGCGC-TCTGTTCAC------
------
FC88X_00942_00431       ------------------AGACGT-ATTCG-TTTGCGC-TCTGTTCAC------
------
FC88X_00944_00417       ------------------AGACGT-ATTCG-TTTGCGC-TCTGTTCAC------
------
FC88X_00943_00716       ------------------AGACGT-ATTCG-TTTGCGC-TCTGTTCAC------
------
FC88X_00913_00781       ------------------AGACGT-ATTCG-TTTGCGC-TCTGTTCAC------
------
FC88X_00929_00860       ------------------AGACGT-ATTCG-TTTGCGC-TCTGTTCAC------
------
FC88X_00903_00934       ------------------AGACGT-ATTCG-TTTGCGC-TCTGTTCAC------
------
FC88X_00944_01011       ------------------AGACGT-ATTCG-TTTGCGC-TCTGTTCAC------
------
FC88X_00935_01061       ------------------AGACGT-ATTCG-TTTGCGC-TCTGTTCAC------
------
FC88X_00998_00415       ------------------AGACGT-ATTCG-TTTGCGC-TCTGTTCAC------
------
FC88X_00994_00490       ------------------AGACGT-ATTCG-TTTGCGC-TCTGTTCAC------
------
FC88X_00971_00550       ------------------AGACGT-ATTCG-TTTGCGC-TCTGTTCAC------
------
FC88X_00985_00644       ------------------AGACGT-ATTCG-TTTGCGC-TCTGTTCAC------
------
FC88X_00957_00835       ------------------AGACGT-ATTCG-TTTGCGC-TCTGTTCAC------
------
FC88X_00961_00833       ------------------AGACGT-ATTCG-TTTGCGC-TCTGTTCAC------
------
FC88X_00951_01140       ------------------AGACGT-ATTCG-TTTGCGC-TCTGTTCAC------
------
FC88X_01020_00470       ------------------AGACGT-ATTCG-TTTGCGC-TCTGTTCAC------
------
FC88X_01049_00513       ------------------AGACGT-ATTCG-TTTGCGC-TCTGTTCAC------
------
FC88X_01013_00621       ------------------AGACGT-ATTCG-TTTGCGC-TCTGTTCAC------
------
FC88X_01032_00719       ------------------AGACGT-ATTCG-TTTGCGC-TCTGTTCAC------
------
FC88X_01037_00744       ------------------AGACGT-ATTCG-TTTGCGC-TCTGTTCAC------
------
FC88X_01015_00758       ------------------AGACGT-ATTCG-TTTGCGC-TCTGTTCAC------
------
```

FC88X_01048_00804	-------------------AGACGT-ATTCG-TTTGCGC-TCTGTTCAC------
------
FC88X_01022_00920	-------------------AGACGT-ATTCG-TTTGCGC-TCTGTTCAC------
------
FC88X_01046_00900	-------------------AGACGT-ATTCG-TTTGCGC-TCTGTTCAC------
------
FC88X_01000_01160	-------------------AGACGT-ATTCG-TTTGCGC-TCTGTTCAC------
------
FC88X_01028_01217	-------------------AGACGT-ATTCG-TTTGCGC-TCTGTTCAC------
------
FC88X_01096_00439	-------------------AGACGT-ATTCG-TTTGCGC-TCTGTTCAC------
------
FC88X_01081_00480	-------------------AGACGT-ATTCG-TTTGCGC-TCTGTTCAC------
------
FC88X_01088_00454	-------------------AGACGT-ATTCG-TTTGCGC-TCTGTTCAC------
------
FC88X_01093_00644	-------------------AGACGT-ATTCG-TTTGCGC-TCTGTTCAC------
------
FC88X_01082_00784	-------------------AGACGT-ATTCG-TTTGCGC-TCTGTTCAC------
------
FC88X_01057_00864	-------------------AGACGT-ATTCG-TTTGCGC-TCTGTTCAC------
------
FC88X_01070_01217	-------------------AGACGT-ATTCG-TTTGCGC-TCTGTTCAC------
------
FC88X_01103_00545	-------------------AGACGT-ATTCG-TTTGCGC-TCTGTTCAC------
------
FC88X_01103_00635	-------------------AGACGT-ATTCG-TTTGCGC-TCTGTTCAC------
------
FC88X_01144_00707	-------------------AGACGT-ATTCG-TTTGCGC-TCTGTTCAC------
------
FC88X_01100_00902	-------------------AGACGT-ATTCG-TTTGCGC-TCTGTTCAC------
------
FC88X_01120_00914	-------------------AGACGT-ATTCG-TTTGCGC-TCTGTTCAC------
------
FC88X_01120_01105	-------------------AGACGT-ATTCG-TTTGCGC-TCTGTTCAC------
------
FC88X_01127_01137	-------------------AGACGT-ATTCG-TTTGCGC-TCTGTTCAC------
------
FC88X_00973_00271	-------------------AGACGT-AATTCGTTTGCGCCTCTGTTCAC------
------
FC88X_00221_00949	-------------------AGATGT-ATTCG-TTTGCGC-TCTGTTCAC------
------
FC88X_00841_00269	-------------------AGACGT-ATTCG-TTTGCGC-TCTGTTCACCC----
------
FC88X_00934_00585	-------------------AGACGT-ATTCG-TTTGCGC-TCTGTTCACCC----
------
FC88X_00977_01178	-------------------AGACGT-ATTCG-TTTGCGC-TCTGTTCACCC----
------
FC88X_00701_00745	-------------------AGACGT-ATTCG-TTTGCGC-TCTGTTCAAC-----
------
FC88X_00474_00566	-------------------AGACAT-ATTCG-TTTGCGC-TCTGTTCAC------
------
FC88X_00066_00385	-------------------AGACGT-ATTCG-TCTGCGC-TCTGTTCAC------
------
FC88X_00236_00461	-------------------AGACGT-A-TTCGTTTGCGC-
TCTGTTCACCTTCCTTTC---

FC88X_00526_00386 -------------------AGACGT-A-TTCGTTTGCGC-
TCTGTTCACCTTCCTTTC---
FC88X_00933_01214 -------------------AGACGT-A-TTCGTTTGCGC-
TCTGTTCACCTTCCTT-----
FC88X_00905_00889 -------------------AGACGT-A-TTCGTTTGCGC-
TCTGTTCACCTTCCTTT----
FC88X_00707_00110 -------------------AGACGT-AATTCGTTTGCGC-
TCTGTTCACCTTCCTTTCTC-
FC88X_00137_00563 -------------------AGACGT-ATTCG-TTTGC-GCTCTGTTCGC------
------
FC88X_00191_00892 -------------------AGACGT-ATTCG-TTTGC-GCTCTGTTCGC------
------
FC88X_00598_01063 -------------------AGACGT-ATTCG-TTTGC-GCTCTGTTCGC------
------
FC88X_00747_01206 -------------------AGACGT-ATTCG-TTTGC-GCTCTGTTCGC------
------
FC88X_00933_00569 -------------------AGACGT-ATTCG-TTTGC-GCTCTGTTCGC------
------
FC88X_00942_00832 -------------------AGACGT-ATTCG-TTTGC-GCTCTGTTCGC------
------
FC88X_00183_00762 -------------------AGACGT-ATTCG-TTTGCTGCTCTGTTCCC------
------
FC88X_00010_00305 -------------------AGACGT-ATTCG-TTTGC-GCTCTGTCCAC------
------
FC88X_00382_01106 -------------------AGACGT-ATTCG-TTTGC-GCTCTGTCCAC------
------
FC88X_01026_00763 -------------------AGACGT-ATTCG-TTTGCGC-
TCTGTTCACCTTCCTTTCTCC
FC88X_00872_00953 -------------------AGACGT-GTTCG-TTTGCGC-TCTGTTCAC------
------
FC88X_00239_00436 -------------------AGGCGT--TTCG-TTTGCGC-TCTGTTCAC------
------
FC88X_00118_00201 -------------------AGACGT-ATTCG-TTTGCGC-TCCGTTCAC------
------
FC88X_00754_00898 -------------------AGACGT-ATTCG-TTTGCGC-TCTGCTCAC------
------
FC88X_00702_01045 -------------------AGACGT-ATTCG-TTTGCGC-TCTGCTCAC------
------
FC88X_00982_00664 -------------------AGACGT-ATTCG-TTTGCGC-TCTGCTCAC------
------
FC88X_01115_00614 -------------------AGACGT-ATTCG-TTTGCGC-TCTGCTCAC------
------
FC88X_00130_00088 -------------------AGACGT-ATTTG-TTTGCGC-TCTGCTCAC------
------
FC88X_00064_00590 -------------------AGACGT-A-CTCGTTTGCGC-TCTGTTCAC------
------
FC88X_00897_00310 ------------------AAGACGT-AACTCGTTTGCGC-TCTGTTCAC------
------
FC88X_00329_01046 -------------------AGACGT-A-CTCGTTTGCGC-TCTGTTCAC------
------
FC88X_00495_00088 -------------------AGACGT-A-CTCGTTTGCGC-TCTGTTCAC------
------
FC88X_00455_00914 -------------------AGACGT-A-CTCGTTTGCGC-TCTGTTCAC------
------
FC88X_00140_00312 -------------------AGACGT-A-TCCGTTTGCGC-TCTGTTCAC------
------

FC88X_00855_01099 ------------------AGACGT-A-TCCGTTTGCGC-TCTGTTCAC------
------
FC88X_00230_00469 ------------------AGACGT-A--CCGTTTGCGC-TCTGTTCAC------
------
FC88X_01111_00803 ------------------AGACGT-A-CCCGTTTGCGC-TCTGTTCAC------
------
FC88X_00835_01175 ------------------AGACGT-A-TTCGCTTGCGC-TCTGTTCAC------
------
FC88X_00913_00828 ------------------AGACGT-A-TTCGCTTGCGC-TCTGTTCAC------
------
FC88X_00141_00676 -----------------GAGACGT-A-TTCGATTGCGC-TCTGTTCAC------
------
FC88X_01124_00925 -----------------GCGACGT-A-TTCGTTTGCGC-TCTGTTCAC------
------
FC88X_00942_01239 ------------------TGACGT-A-TTCGTTTGCGC-TCTGTTCACCC----
------
FC88X_00307_00469 ------------------AGACGT-ATTCG-TTTGCG-TCCTGTTCAC------
------
FC88X_01007_00587 ------------------GGACGT-ATTCG-TTTGCG-CCCTGTTCAC------
------
FC88X_00095_00582 ---------------TACGTACGT-ATTCG-TTTGCGACTCTGTTCAC------
------
FC88X_00027_00583 ------------------AGACGT-ATTCG-TTTGCG-CCCTGTTCTC------
------
FC88X_00035_00193 ----------------AGACGTAT-ATTCGTTTTGTGC-TCTGTTCAC------
------
FC88X_00018_00709 ------------------AGACGT-ATTCGTTGT-CGC-TCTGTTCAC------
------
FC88X_00849_00801 ------------------AGACGT-ATTCGTTTGCCGC-TCTGTT---------
------
FC88X_00712_00234 -----------------GAGACGT-AATTCGTTTGCG-----------------
------
FC88X_00445_01078 ----------------TTAGACGTCATTCG-
GTTGCACTGTTGTTCTCCTTCCTT-----
FC88X_00568_00138 ------------------AGACGT-ATTCG--TTGCGCT---------------
------
FC88X_00032_00428 -----------------TTTGCGCATT-CGGT-GCACTG-TTATTCTC------
------
FC88X_00980_00792 -----------------TTTGCGCATT-CGGT-GCACTG-T-ATTCTC------
------
FC88X_00359_00015 -----------------TTTGCGCATT-CGGT-GCACTG-TTATTCTC------
------
FC88X_01075_00950 -----------------TTTGCGCATT-CGGT-GCACTG-TTATTCTCC-----
------
FC88X_01049_00632 -----------------TTTGCGCATT-CGGTCGCACTG-TTATTCT-------
------
FC88X_00136_00451 -----------GGGGAATTTGCGCATT-CGGTCGCACTG-TTATTCT-------
------
FC88X_00048_00590 -----------------TTTGCGCATT-CGGT-GCACTG-TTATTCT-------
------
FC88X_00008_00652 -----------------TTTGCGCATT-CGGT-GCACTG-TTATTCT-------
------
FC88X_00093_00122 -----------------TTTGCGCATT-CGGT-GCACTG-TTATTCT-------
------
FC88X_00149_00294 -----------------TTTGCGCATT-CGGGTGCACTGGTTATTCT-------
------

FC88X_01004_00698 -----------------TTTGCGCATT---GGTGCACTG-TTATTCT-------
------
FC88X_00690_00962 -----------------TTTGCGCATT-T-GGTGCACTG-TTATTCT-------
------
FC88X_00086_00121 -----------------TTTGCGCATT-C-GGTGCACTG-TTATTCT-------
------
FC88X_00288_00056 -----------------TTTGCGCATT-CGGT-GCACTG-TTATTCT-------
------
FC88X_00913_01040 ----------------GTTTGCGCATT-CGGT-GCACTG-TTATTCT-------
------
FC88X_01123_00989 ----------------GTTTGCGCATT-CGGT-GCACTG-TTATTCT-------
------
FC88X_00510_00088 ----------GGGGAAGTTTGCGCATT-CGGT-GCACTG-TTATTCT-------
------
FC88X_00177_00566 -----------------TTTGCGCATT-CGGT-GCACTG-TTATTCT-------
------
FC88X_00399_00443 -----------------TTTGCGCATT-CGGT-GCACTG-TTATTCT-------
------
FC88X_00502_00021 -----------------TTTGCGCATT-CGGT-GCACTG-TTATTCT-------
------
FC88X_00107_00732 -----------------TTTGCGCATT-CGGT-GCACTG-TTATTCT-------
------
FC88X_00679_01064 ------------GGGGATTTGCGCATT-CGGT-GCACTG-TTATTCT-------
------
FC88X_00258_00538 -----------------TTTGCGCATT-CGGT-GCACTG-TTATTCT-------
------
FC88X_00495_00986 -----------------TTTGCGCATT-CGGT-GCACTG-TTATTCT-------
------
FC88X_00594_01220 -----------------TTTGCGCATT-CGGT-GCACTG-TTATTCT-------
------
FC88X_00051_00475 ----------------TTTTGCGCATT-CGGT-GCACTG-TTATTCT-------
------
FC88X_01020_01009 ----------------TTTTGCGCATT-CGGT-GCACTG-TTATTCT-------
------
FC88X_00232_01085 -----------------TTTGCGCATT-CGGT-GCACTG-TTATTCT-------
------
FC88X_00441_01008 -----------------TTTGCGCATT-CGGT-GCACTG-TTATTCT-------
------
FC88X_00555_00948 -----------------TTTGCGCATT-CGGT-GCACTG-TTATTCT-------
------
FC88X_00757_00132 -----------------TTTGCGCATT-CGGT-GCACTG-TTATTCT-------
------
FC88X_00885_00507 -----------------TTTGCGCATT-CGGT-GCACTG-TTATTCT-------
------
FC88X_00939_00975 -----------------TTTGCGCATT-CGGT-GCACTG-TTATTCTT------
------
FC88X_01131_00779 -----------------TTTGCGCATT-CGGT-GCACTG-TTATTCTT------
------
FC88X_00874_00937 -----------------TTTGCGCATT-CGGT-GCACTG-TTATTCTT------
------
FC88X_00430_00776 -----------------TTTGCGCATT-CGGT-GCACTG-TTATTCT-------
------
FC88X_00511_00076 -----------------TTTGCGCATT-CGGT-GCACTG-TTATTCT-------
------
FC88X_00596_01265 -----------------TTTGCGCATT-CGGT-GCACTG-TTATTCT-------
------

FC88X_00818_00345 -----------------TTTGCGCATT-CGGT-GCACTG-TTATTCT-------
------
FC88X_00875_00780 -----------------TTTGCGCATT-CGGT-GCACTG-TTATTCT-------
------
FC88X_00936_00592 -----------------TTTGCGCATT-CGGT-GCACTG-TTATTCT-------
------
FC88X_00940_00656 -----------------TTTGCGCATT-CGGT-GCACTG-TTATTCT-------
------
FC88X_00925_01121 -----------------TTTGCGCATT-CGGT-GCACTG-TTATTCT-------
------
FC88X_00951_00659 -----------------TTTGCGCATT-CGGT-GCACTG-TTATTCT-------
------
FC88X_00982_01118 -----------------TTTGCGCATT-CGGT-GCACTG-TTATTCT-------
------
FC88X_01043_00751 -----------------TTTGCGCATT-CGGT-GCACTG-TTATTCT-------
------
FC88X_01036_01144 -----------------TTTGCGCATT-CGGT-GCACTG-TTATTCT-------
------
FC88X_01038_01154 -----------------TTTGCGCATT-CGGT-GCACTG-TTATTCT-------
------
FC88X_01113_00591 -----------------TTTGCGCATT-CGGT-GCACTG-TTATTCT-------
------
FC88X_01130_00769 -----------------TTTGCGCATT-CGGT-GCACTG-TTATTCT-------
------
FC88X_00027_00295 -----------------TTTGCGCATT-CGGT-GCACTG-TTATTCT-------
------
FC88X_00035_00474 -----------------TTTGCGCATT-CGGT-GCACTG-TTATTCT-------
------
FC88X_00043_00582 -----------------TTTGCGCATT-CGGT-GCACTG-TTATTCT-------
------
FC88X_00072_00063 -----------------TTTGCGCATT-CGGT-GCACTG-TTATTCT-------
------
FC88X_00056_00101 -----------------TTTGCGCATT-CGGT-GCACTG-TTATTCT-------
------
FC88X_00076_00311 -----------------TTTGCGCATT-CGGT-GCACTG-TTATTCT-------
------
FC88X_00086_00508 -----------------TTTGCGCATT-CGGT-GCACTG-TTATTCT-------
------
FC88X_00093_00700 -----------------TTTGCGCATT-CGGT-GCACTG-TTATTCT-------
------
FC88X_00113_00368 -----------------TTTGCGCATT-CGGT-GCACTG-TTATTCT-------
------
FC88X_00103_00478 -----------------TTTGCGCATT-CGGT-GCACTG-TTATTCT-------
------
FC88X_00120_00453 -----------------TTTGCGCATT-CGGT-GCACTG-TTATTCT-------
------
FC88X_00131_00586 -----------------TTTGCGCATT-CGGT-GCACTG-TTATTCT-------
------
FC88X_00135_00554 -----------------TTTGCGCATT-CGGT-GCACTG-TTATTCT-------
------
FC88X_00121_00744 -----------------TTTGCGCATT-CGGT-GCACTG-TTATTCT-------
------
FC88X_00109_00806 -----------------TTTGCGCATT-CGGT-GCACTG-TTATTCT-------
------
FC88X_00190_00791 -----------------TTTGCGCATT-CGGT-GCACTG-TTATTCT-------
------

FC88X_00210_00012 -----------------TTTGCGCATT-CGGT-GCACTG-TTATTCT-------
------
FC88X_00266_01128 -----------------TTTGCGCATT-CGGT-GCACTG-TTATTCT-------
------
FC88X_00328_00624 -----------------TTTGCGCATT-CGGT-GCACTG-TTATTCT-------
------
FC88X_00314_00961 -----------------TTTGCGCATT-CGGT-GCACTG-TTATTCT-------
------
FC88X_00307_01076 -----------------TTTGCGCATT-CGGT-GCACTG-TTATTCT-------
------
FC88X_00393_01135 -----------------TTTGCGCATT-CGGT-GCACTG-TTATTCT-------
------
FC88X_00429_01221 -----------------TTTGCGCATT-CGGT-GCACTG-TTATTCT-------
------
FC88X_00460_00122 -----------------TTTGCGCATT-CGGT-GCACTG-TTATTCT-------
------
FC88X_00529_01202 -----------------TTTGCGCATT-CGGT-GCACTG-TTATTCT-------
------
FC88X_00570_00934 -----------------TTTGCGCATT-CGGT-GCACTG-TTATTCT-------
------
FC88X_00598_01194 -----------------TTTGCGCATT-CGGT-GCACTG-TTATTCT-------
------
FC88X_00633_00881 -----------------TTTGCGCATT-CGGT-GCACTG-TTATTCT-------
------
FC88X_00620_01101 -----------------TTTGCGCATT-CGGT-GCACTG-TTATTCT-------
------
FC88X_00629_01115 -----------------TTTGCGCATT-CGGT-GCACTG-TTATTCT-------
------
FC88X_00617_01173 -----------------TTTGCGCATT-CGGT-GCACTG-TTATTCT-------
------
FC88X_00670_00212 -----------------TTTGCGCATT-CGGT-GCACTG-TTATTCT-------
------
FC88X_00695_01151 -----------------TTTGCGCATT-CGGT-GCACTG-TTATTCT-------
------
FC88X_00730_00077 -----------------TTTGCGCATT-CGGT-GCACTG-TTATTCT-------
------
FC88X_00744_00081 -----------------TTTGCGCATT-CGGT-GCACTG-TTATTCT-------
------
FC88X_00710_00416 -----------------TTTGCGCATT-CGGT-GCACTG-TTATTCT-------
------
FC88X_00706_01019 -----------------TTTGCGCATT-CGGT-GCACTG-TTATTCT-------
------
FC88X_00729_01210 -----------------TTTGCGCATT-CGGT-GCACTG-TTATTCT-------
------
FC88X_00743_01252 -----------------TTTGCGCATT-CGGT-GCACTG-TTATTCT-------
------
FC88X_00766_00150 -----------------TTTGCGCATT-CGGT-GCACTG-TTATTCT-------
------
FC88X_00776_01069 -----------------TTTGCGCATT-CGGT-GCACTG-TTATTCT-------
------
FC88X_00754_01227 -----------------TTTGCGCATT-CGGT-GCACTG-TTATTCT-------
------
FC88X_00825_00764 -----------------TTTGCGCATT-CGGT-GCACTG-TTATTCT-------
------
FC88X_00854_01104 -----------------TTTGCGCATT-CGGT-GCACTG-TTATTCT-------
------

```
FC88X_00863_01219      -----------------TTTGCGCATT-CGGT-GCACTG-TTATTCT-------
------
FC88X_00931_00249      -----------------TTTGCGCATT-CGGT-GCACTG-TTATTCT-------
------
FC88X_00926_00316      -----------------TTTGCGCATT-CGGT-GCACTG-TTATTCT-------
------
FC88X_00900_00740      -----------------TTTGCGCATT-CGGT-GCACTG-TTATTCT-------
------
FC88X_00918_00852      -----------------TTTGCGCATT-CGGT-GCACTG-TTATTCT-------
------
FC88X_00937_00988      -----------------TTTGCGCATT-CGGT-GCACTG-TTATTCT-------
------
FC88X_00928_01181      -----------------TTTGCGCATT-CGGT-GCACTG-TTATTCT-------
------
FC88X_00974_01123      -----------------TTTGCGCATT-CGGT-GCACTG-TTATTCT-------
------
FC88X_01038_00325      -----------------TTTGCGCATT-CGGT-GCACTG-TTATTCT-------
------
FC88X_01039_00542      -----------------TTTGCGCATT-CGGT-GCACTG-TTATTCT-------
------
FC88X_01030_00606      -----------------TTTGCGCATT-CGGT-GCACTG-TTATTCT-------
------
FC88X_01020_01055      -----------------TTTGCGCATT-CGGT-GCACTG-TTATTCT-------
------
FC88X_01059_00484      -----------------TTTGCGCATT-CGGT-GCACTG-TTATTCT-------
------
FC88X_01099_00842      -----------------TTTGCGCATT-CGGT-GCACTG-TTATTCT-------
------
FC88X_01055_00880      -----------------TTTGCGCATT-CGGT-GCACTG-TTATTCT-------
------
FC88X_01050_01047      -----------------TTTGCGCATT-CGGT-GCACTG-TTATTCT-------
------
FC88X_01108_00780      -----------------TTTGCGCATT-CGGT-GCACTG-TTATTCT-------
------
FC88X_01130_01090      -----------------TTTGCGCATT-CGGT-GCACTG-TTATTCT-------
------
FC88X_01114_01154      -----------------TTTGCGCATT-CGGT-GCACTG-TTATTCT-------
------
FC88X_00227_00132      -----------------TTTGCGCATT-CGGT-GCACAG-TTATTCT-------
------
FC88X_00526_00605      -----------------TTTGCGCATT-CGGT-GCAC-G-TTATTCTCT-----
------
FC88X_00814_00864      -----------------TTTGCGCATT-CGGT-GCACTG-
TTATTCTCTTCCTTT-----
FC88X_00084_00325      -----------------TTTGCGCATT-CGGT-GCACTG-TCATTCT-------
------
FC88X_00603_01099      -----------------TTTGCGCATT-CGGT-GCACTG-TCATTCT-------
------
FC88X_01144_00593      ---------------GGTTTGTGCATT-CGGT-GCACTG-TTATTCT-------
------
FC88X_00971_00756      -----------------TTTGCGCATT-CGGT-GCACTA-TTATTCT-------
------
FC88X_00814_00244      -----------------TTTGCGCATT-CGGT-GCACTA-TTATTCT-------
------
FC88X_00824_00333      -----------------TTTGCGCATT-CGGTGC-ACTG-TTATTCC-------
------
```

FC88X_00332_01123 ------------------TTTGCGCATT-CGGTGCCATTG-TTATTC--------
------
FC88X_00872_01266 ------------------TTTGCGCATT-CGGT-GCACTG-CTATTCT-------
------
FC88X_00386_00249 ------------------TTTGCGCATT-CGGT-GCACTG-TTACTCT-------
------
FC88X_00607_01175 ------------------TTTGCGCATT-CGGT-GCACTG-TTACTCT-------
------
FC88X_00726_00974 ------------------TTTGCGCATT-CGGT-GCACTG-TTACTCTACTAG--
------
FC88X_00918_01191 ------------------TTTGCGCATT-CTGT-GCACTG-TTATTCT-------
------
FC88X_00618_00974 ------------------TTTGCGCACT-CGGTGC-ACTG-TTATTCT-------
------
FC88X_00854_00705 ------------------TTTGCGCACT-CGGTGC-ACTG-TTATTCT-------
------
FC88X_00230_00120 ------------------TTTGCGCACTTCGGTGCCACTG-TTATTCT-------
------
FC88X_00816_00820 ------------------TTTTCGCACT-CGGTGC-ACTG-TTATTCT-------
------
FC88X_01033_01003 -----------------TTTTGCGCGCT-CGGTGC-ACTG-TTATTCT-------
------
FC88X_00415_00984 -----------------TTCTGCGCATT-CGGTGC-ACTG-TTATTCT-------
------
FC88X_00935_01250 -----------------TTTTGCGCATT-CGGCGC-ACTG-TTATTCT-------
------
FC88X_00981_01182 ------------------TTTGCGCGTT-CGGT-GCACTG-
TTATTCTCTTCCTTT-----
FC88X_00143_00933 -----------------CATGGCGCATC-CGGTTGGCCTC--TGTT---------
------
FC88X_01068_00368 ----------------CCATGGCGCATC-CGGTTGACCCC--TGTT---------
------
FC88X_00168_01009 ----------GGGGAATTTGGCGCATT-CGGTTG--CAC--TGTT---------
------
FC88X_00136_00772 ------------------TTGACGCCTTT-GGT-GACC-ACTGA-TCTC------
------
FC88X_01072_00763 ------------------TTGACGCCTTTTGGT-GACC-
ACTGAATCTCCTTCCTTT----
FC88X_00202_00932 ------------------TTGACGCCTTT-GGT-GACC-ACTGA-TCTC------
------
FC88X_00350_00793 ------------------TTGACGCCTTT-GGT-GACC-ACTGA-TCTC------
------
FC88X_00791_00914 ------------------TTGACGCCTTT-GGT-GACC-ACTGA-TCTC------
------
FC88X_00674_00921 ------------------TTGACGCCTTT-GGT-GACC-ACTGA-TCTC------
------
FC88X_00959_00832 ------------------TTGACGCCTTT-GGT-GACCCACTGA-TCTCC-----
------
FC88X_00983_00387 ------------------TTGACGCCTTT-GGT-GACC-ACTGA-TCTC------
------
FC88X_00983_00860 ------------------TTGACGCCTTT-GGT-GACC-ACTGA-TCTC------
------
FC88X_01002_00787 ------------------TTGACGCCTTT-GGT-GACC-ACTGA-TCTC------
------
FC88X_00143_00734 ------------------TTGACGCCTTT-GGT-GACC-ACTGA-TCTC------
------

```
FC88X_00539_00560      -----------------TTGACGCCTTT-GGT-GACC-ACTGA-TCTC------
------
FC88X_00905_00489      -----------------TTGACGCCTTT-GGT-GACC-ACTGA-TCTC------
------
FC88X_00933_00521      -----------------TTGACGCCTTT-GGT-GACC-ACTGA-TCTC------
------
FC88X_00931_00883      -----------------TTGACGCCTTT-GGT-GACC-ACTGA-TCTC------
------
FC88X_01143_00952      -----------------TTGACGCCTTT-GGT-GACC-ACTGA-TCTC------
------
FC88X_01034_00681      -----------------TTGACGCCCTT-GGT-GACC-ACTGA-TCTC------
------
FC88X_00880_00789      -----------------TTGACGCCTTA-GGT-GACC-ACTGA-TCTC------
------
FC88X_00119_00870      -----------------TTGACGCCTTT-GGT-GACC-GCTGA-TCTC------
------
FC88X_01080_00546      -----------------TTGACGCCTTT-GGT-GACC-GCTGA-TCTC------
------
FC88X_00211_00758      ------------------TTTCTCCGTTAT-CGTAGATCGCTGTTCT-------
------
FC88X_00102_00196      ------------------TTTCTCCGTTAT-CGTAGATCGCTGTTCTA------
------
FC88X_00439_01134      ------------------TTTCTCCGTTAT-CGTAGATCGCTGTTCT-------
------
FC88X_00531_00738      ------------------TTTCTCCGTTAT-CGTAGATCGCTGTTCT-------
------
FC88X_00296_00934      ------------------TTTCTCCGTTAT-CGTAGATCGCTGTTCT-------
------
FC88X_00345_01038      ------------------TTTCTCCGTTAT-CGTAGATCGCTGTTCT-------
------
FC88X_00523_01048      ------------------TTTCTCCGTTAT-CGTAGATCGCTGTTCT-------
------
FC88X_00834_01057      ------------------TTTCTCCGTTAT-CGTAGATCGCTGTTCT-------
------
FC88X_00940_00429      ------------------TTTCTCCGTTAT-CGTAGATCGCTGTTCT-------
------
FC88X_00913_01131      ------------------TTTCTCCGTTAT-CGTAGATCGCTGTTCT-------
------
FC88X_01094_00847      ------------------TTTCTCCGTTAT--GTAGATCGCTGTTCT-------
------
FC88X_00062_00303      ------------------TTTCTCGGTTAT-CGTAGATCGCTGTTCT-------
------
FC88X_00312_00963      ------------------TTCCTCCGTTAT-CGTAGATCGCTGTTCT-------
------
FC88X_00981_00378      ---------------------CTACGTTAT-CGTAGATCGCTGTTCT-------
------
FC88X_00632_01264      ------------------TTTCTCCGTTAT-CGTAGACCGCTGTTCT-------
------
FC88X_00364_00882      --------------------ACTCCTTTATTCGAGGACCTCTGTTCCG------
------
FC88X_00479_01158      -------------CCTGAGACCACTGC-AGATGACT-TCTTCTTCCTTTCC---
------
FC88X_00421_01146      --------------ATCAAAGCACTGTTATATGAGTACCCTCTTCCTTT-----
------
FC88X_00850_00956      --------------------TCTTCATACTTTGTA-AGCGCTATCTT-------
------
```

FC88X_01067_00656 -------------------TCCTTCATACTTTGTA-AGCGCTATCTT-------
------
FC88X_00348_01073 ------------------CTTCTTCATATGTTTTACAGCGCTATC---------
------
FC88X_00906_01258 ----------------------TCTACTC-CTTTATTACGTTGCAATTGC----
------
FC88X_00928_00528 ----------------------TCTACTCTCTTTGCCACGCCATCATTGC----
------
FC88X_00186_00143 --------------------CCACCAAAAACGTGGCACCCCGATCCA-------
------
FC88X_00974_00289 --------------------CCACTAATCCCTTGTTACCACGATATT-------
------
FC88X_00925_01134 -----------------------TCCACTTCCGTAGTGCGCATTGTTCTC----
------
FC88X_00701_00501 -----------------------TCCACTTCCGTAGTGCGCATTGTTCTC----
------
FC88X_00160_00886 -----------------------TCCACTTACGTAGTGCGCATTGTTCTC----
------
FC88X_00505_00899 ----------------------TCTCCCCTTTGTAGAACGCAATGTAAG-----
------
FC88X_00779_00201 -----------------------TTTCCTTCCT-ACTACGCCGCAC---------
------
FC88X_01080_00758 ----------------------TTTCCCGACCTGAGTACAACGCACTAT-----
------
FC88X_00044_00185 -----------------------------TTGCTCCATG-TGCA-
GCCCCATTA------
FC88X_00713_00913 --------------------------CTGTCTCCCCATG-
TGCACGCCCCATTA------
FC88X_00091_00573 ----------------------CTCGCTATTTCTCCGTGGTGCCCAGCCC----
------
FC88X_00369_00479 ----------------------TTCGCAAATTGCTCCCTGTGGCGACCA-----
------
FC88X_00980_00783 -------------------CCATACGCTATTTTCTCA-TGGGGCGAC-------
------
FC88X_00047_00457 ------------------------CGCTATTGCTCCCATGGCTTGCATGTT---
------
FC88X_00866_00188 ------------------------CGCTATTGCTCCCATGGCTTGCATGTT---
------
FC88X_00120_00607 -------------------ACAGGCGCTATTCTTCCCAGGAGTTAT--------
------
FC88X_00207_01072 -------------------ACAGGCGCTATTCTTCCCAGGAGTTAT--------
------
FC88X_00293_00568 -------------------ACAGGCGCTATTCTTCCCAGGAGTTAT--------
------
FC88X_00576_00256 -------------------ACAGGCGCTATTCTTCCCAGGAGTTAT--------
------
FC88X_00775_00805 -------------------ACAGGCGCTATTCTTCCCAGGAGTTAT--------
------
FC88X_00826_00565 -------------------ACAGGCGCTATTCTTCCCAGGAGTTAT--------
------
FC88X_00941_00936 -------------------ACAGGCGCTATTCTTCCCAGGAGTTAT--------
------
FC88X_00954_00962 -------------------ACAGGCGCTATTCTTCCCAGGAGTTAT--------
------
FC88X_00091_00856 -------------------ACAGGCGCTATTCTTCCCAGGAGTTAT--------
------

FC88X_00215_00029 -------------------ACAGGCGCTATTCTTCCCAGGAGTTAT--------
------
FC88X_00087_00411 -------------------TCAGGCGCTATTCTTCCCAGGAGTTAT--------
------
FC88X_00755_00158 -------------------TCTGTCCCTATTCTTCCCTGTGGTTTG--------
------
FC88X_00734_00507 -------------------CTATCGTGTCCACTCCCTATGCCTTGC--------
------
FC88X_00946_00737 -------------------TTCTCTCTCTTGTCTCCCATGTGTTGC--------
------
FC88X_00457_00542 ----------------------CTTGCTTTTTCCCAGCTGTGACATGTT-----
------
FC88X_00035_00368 ----------------------CTTGCTTTT-CCCAGCTGTGACATGTT-----
------
FC88X_00806_01206 ----------------------CCTGCTTTTTCCCAGCTGTGCCATGTT-----
------
FC88X_00984_00874 ---------------------CTCATCCTTTTCCCTACTG-GGCATGTT-----
------
FC88X_01058_00697 ---------------------CTCATCCTTTTCCCTACTG-GGCATGTT-----
------
FC88X_00812_00361 ----------------GGGGAACCGACTCCTTTGTCGCACTCCCATGTT-----
------
FC88X_01036_00637 ----------------------CCGACTCCTTTGTCGCACTCCCATGTT-----
------
FC88X_01025_01160 ----------------------CAACTTCTTCTGCTGCTTTCCCATGTT-----
------
FC88X_00976_00402 ----------------------TTCTCCTTTCGCTTGATTTCTCATGTT-----
------
FC88X_00032_00749 --------------------TTTCCTGTTCTTCCCCGTGGTGTTCTG-------
------
FC88X_00555_00604 ----------------------TTTCCTCTTTCCCCGTAGGTATCTGTT-----
------
FC88X_00430_01075 ----------------------TTCCTGTTTGCACCGTGTAGGTCTCCC-----
------
FC88X_00479_00097 --------------------TCTTCCCTCTTGTACCATGTA-------------
------
FC88X_00578_00434 ---------------------CTTTCCCTTGTCCCCATGTGGTATCAC------
------
FC88X_00897_01018 ----------------------TTGCTCCCTTCCAGGTTGTGTCGTCTA-----
------
FC88X_01133_00754 ---------------------CTTATTGCTCTCCTGGT-GTGTCATCTT-----
------
FC88X_00071_00118 --------------------TGTCACAATTCTAC------
TCCCGGATACTAAT------
FC88X_00783_00961 ---------------------ATAACAATTCTGCGC----TCCCGGATCCCG--
------
FC88X_00472_01131 -----------------------CGCAATTCTTCCCTTACTCCCGGAGACC---
------
FC88X_00638_01213 ---------------------TCCCGAATTCTTTTG---CTCCCCTATACAGC-
------
FC88X_00097_00382 ----------------------CTTCACGTTTTCCTGCGGTGCCCTGTT-----
------
FC88X_00086_00455 ----------------------CTTCACGTTTTCCTGCGGTGCCCTGTT-----
------
FC88X_00571_01252 ----------------------CTTCACGTTTTCCTGCGGTGCCCTGTT-----
------

FC88X_00710_01126 ----------------------CTTCACGTTTTCCTGTGGTGCCCTGTT-----
------
FC88X_00293_00980 ----------------------CTACCCCTTTTCCACTGATGCCCTGCGC----
------
FC88X_00749_00960 ----------------------CTCACGCCTTCTCGGTGTTGCCCTATA-----
------
FC88X_00138_00313 ---------------------ACTCACTCTTGCTCTAT-TTGTCCTCTA-----
------
FC88X_00440_01081 -------------------TCCCGTATTCTGTGGTTGCCTTATCCT--------
------
FC88X_00921_01077 ---------------------TCCCGTCTTGCGGTCGACCTATGCTCT------
------
FC88X_00077_00093 ----------------------ACACGTACTCCCTTG-TGT-CGCTGTAGC---
------
FC88X_00251_01120 ----------------------TCCCTTACTCCTGTGATGTTCTCTGTA-----
------
FC88X_00180_01044 -------------------------TCTCCTCTTCTTTTGC-TG-TATAGCG--
------
FC88X_01028_00563 -----------------------CCGACTCCCTTCTTCCGC-
CGATACAGCGCC------
FC88X_00111_00147 ------------------------TTTTCTCCTAGTTTGCTGCGTCTTTGC---
------
FC88X_00782_01026 ------------------------TTTTCTCCTAGTTTGCTGCGTCTTTGC---
------
FC88X_00078_00659 ------------------------TTTTCTCCTAGTTTGCTGCGTCTTTGC---
------
FC88X_00856_00796 ------------------------TTTTCTCCTAGTTTGCTGCGTCTTTGC---
------
FC88X_00990_01115 ------------------------TTTTCTCCTAGTTTGCTGCGTCTTTGC---
------
FC88X_00042_00619 --------------------------TTCTCCTAGTTTGCTGCGTCTTTGC---
------
FC88X_00106_00896 ------------------------TCTTCTCCTAGTTTGCTGCGTCTTTGC---
------
FC88X_01078_00978 ------------------------TTTTCTCCTAGTTTGCTGCGCCTTTGC---
------
FC88X_00710_01211 -------------------CTGTTTTTCTCTCCCACTTGCTGCTCTTTTTC---
------
FC88X_00320_00537 ------------------GCCGTGTTCTCCCAACGT--GCTGGTATA-------
------
FC88X_00101_00583 --------------------TTTTCTCCCTTCACGTTCGCTGGTCTC-------
------
FC88X_00492_00160 -----------------TCCCATCA-CATTCTCCTTTTAGTTTCG---------
------
FC88X_00519_00164 -----------------TCCCATCA-TATTCTCCTTTTAGTTTCG---------
------
FC88X_00482_01184 ------------------CCAAACACTATTCTGCGCTTAGTTAGC---------
------
FC88X_00637_00966 ------------------CCAAACACTATTCTGCGCTTAGTTAGC---------
------
FC88X_00624_01169 ------------------CCAAACACTATTCTGCGCTTAGTTAGC---------
------
FC88X_01139_00821 ----------------GTCCGAACA-TATTCTGCGTCTTATTCTC---------
------
FC88X_00524_01204 ----------------------CTATTGTCTCCCAAATACTGCTGTCTT-----
------

FC88X_00962_01173 ---------------------CTATTGTCTCCCAAATACTGCTGTCTT-----
------
FC88X_01019_00480 ---------------------CTATTGTCTCCCAAATACTGCTGTCTT-----
------
FC88X_00507_00988 ---------------------CTATTGTCTCCCAAATACTGCTGT--------
------
FC88X_00041_00735 ---------------------CAATTATTTTTCATATAGTGCCGGTCTT----
------
FC88X_00120_00202 --------------------CTGTTACCTATCCCGCTGCT-ATTTATC-----
------
FC88X_00331_01131 --------------------CTGTTACCTATCCCGCTGCT-ATTTATC-----
------
FC88X_01071_00497 --------------------CTGTTACCTATCCCGCTGCT-ATTTATC-----
------
FC88X_01138_01160 --------------------CTGTTACCTATCCCGCTGCT-ATTTATC-----
------
FC88X_00628_01233 --------------------CTATTATCTATCCCGCTGCT-ATTTATC-----
------
FC88X_00927_00776 --------------------CTATTTTCCTACCCGTTCTTGATTTATC-----
------
FC88X_00174_01049 -----------------CTATTACCCTCTGCCCCGTCATCGATTTTT------
------
FC88X_00921_00519 --------------------CTATTCCCTTTCCCGTTGTGATTTTGC------
------
FC88X_00197_00700 --------------------CTGTTTTCTCTCCTACTGCTGCTTTTT------
------
FC88X_00187_00893 --------------------CTGTTTTCTCTCCTACTGCTGCTTTTT------
------
FC88X_01060_00906 --------------------CTGTTTTCTCTCCTACTGCTGCTTTTT------
------
FC88X_00970_00609 --------------------
CTGTTTTCTCTCCTACTGCTGCTTTTTCTTCCTTTCTCC
FC88X_00112_00869 --------------------CTTATTTCTCTTCTGCGGTTGCTAGTT------
------
FC88X_00743_01143 -------------TACTACCTTGGTCTACT--CTCCGATTTC-----------
------
FC88X_01033_00919 -------------TACTACCTTGGTCTACT--CTCCGATTTC-----------
------
FC88X_01017_01037 -----------------ACCTTC-TCTACTTGCTTCGGTTTCTG---------
------
FC88X_00026_00464 -------------------CTTCGATTATTC-TC-CGGTTGCTTCGTCC----
------
FC88X_00893_01126 -------------------CTTCGTTGCTGT-TCTCGGTTGCTAC--------
------
FC88X_00086_00033 --------------------CTCGTTATTCTTCTCCTTTTGGACT-GTC----
------
FC88X_00477_00950 --------------------CTCGTTATTCTTCTCCTTTTGGACT-GTC----
------
FC88X_01000_00438 --------------------CTCGTTATTCTTCTCCTTTTGGACT-GTC----
------
FC88X_00435_00060 --------------------CTCGTTATTCTTCTCCCTTTGGACTTGTC----
------
FC88X_00197_00862 ---------------------TTCTATTCTTCCTCCTCGTGGGCTTTT-----
------
FC88X_00873_00867 --------------------CTGGGTAGTCCTCTCC-TTTGCTTCTT------
------

FC88X_00960_00740 ----------------------TCTATAGTCCTCTCCATCTGCGTTTTTC----
------
FC88X_00179_00152 -------------------CACTATTCTC-TTTCGCACTTTGTTCGC-------
------
FC88X_00156_00223 -------------------CACTATTCTC-TTTCGCACTTTGTTCGC-------
------
FC88X_00661_01206 -------------------CACTATTCTC-TTTCGCACTTTGTTCGC-------
------
FC88X_00784_00178 -------------------CACTATTCTC-TTTCGCACTTTGTTCGC-------
------
FC88X_00821_00846 -------------------CACTATTCTC-TTTCGCACTTTGTTCGC-------
------
FC88X_01048_01193 -------------------CACTATTCTC-TTTCGCACTTTGTTCGC-------
------
FC88X_01079_00386 -------------------CACTATTCTC-TTTCGCACTTTGTTCGC-------
------
FC88X_00885_00854 -------------------CACTATTCTC-TTTCGCACTT-GTTCGC-------
------
FC88X_00981_00511 -------------------CACTATTCTC-TTTCGCACTTTGTTCG--------
------
FC88X_00834_00364 -------------------TACTATTCTC-TTTCGCACTTTGTTCGC-------
------
FC88X_00855_01242 ----------------TCAAACTATTCTTACCTAGCCCTTTGT-----------
------
FC88X_00464_01169 -------------------CACTATTCTT-TCTAACCGTTTATGCCCA------
------
FC88X_00721_00488 ------------------CCCCATACTACACTCCCTCCGCTGCGT---------
------
FC88X_01053_01024 ------------------CCCCTATTCATTCCCCCGTTGCCTCGA---------
------
FC88X_00796_00157 -------------------CCCAACATTCGCTCCTCAGGTTGCTAT--------
------
FC88X_00145_00066 -------------------CCCAACATTCGCTCCTCAGGTTGCTAT--------
------
FC88X_00996_00579 -------------------CCCAACATTCGCTCCTCAGGTTGCTAT--------
------
FC88X_00012_00413 -------------------CCCACTATTC-TTTCTCACTTTGCGATT-------
------
FC88X_00146_00458 -----------------ACCCTTATTCTCT--CACGCGATTGTCGC--------
------
FC88X_00856_00210 ------------------ACCTTATTCTCT--CACGCGATTGTCGC--------
------
FC88X_00154_00191 ----------------CTCCCTTATTTGTCGCCGTGCGATTGT-----------
------
FC88X_00813_00758 ----------------CTCCCTTATTTGTCGCTGTGCGATTGT-----------
------
FC88X_00060_00836 --------------CTTCCCCTTATTTTCGTTCACGCTGTT-------------
------
FC88X_01047_00760 ---------------TTCCCCTATATTCTCCTTACGCGTTTT------------
------
FC88X_00486_00092 -------------------TTCTCCATATTCTTTCGCCGTCGCGGC--------
------
FC88X_00953_00962 -------------------TTCTCCATATTCTTTCGCCGTCGCGGC--------
------
FC88X_00392_00041 -------------------TTCTCCGTATTCTTTCGCCGTCGCGGC--------
------

FC88X_00194_00810 ------------------TCTTTCCCTATTCTTCCGCCA-CGTGGT--------
------
FC88X_00230_00882 ------------------TCTTTCCCTATTCTTCCGCCA-CGTGGT--------
------
FC88X_01043_00545 ------------------TCTTTCCCTATTCTTC-GCCA-CGTGGT--------
------
FC88X_00494_00236 ------------------TTTTCCGCTATTCTTCCGCGT-CGTGTC--------
------
FC88X_00575_00415 ---------------------TTCTCTATTCTGTGGCTCTTGGTTTTT------
------
FC88X_00888_00715 -------------------CTTTCACTATTCTCT--CCCTTGGTATA-------
------
FC88X_00835_00867 -------------------CTATCCCTGATCTGTC-GCCTTGTTTTA-------
------
FC88X_00004_00213 ----------------------TTTGTGTGTCTGTCTCATCGCTCTAGT-----
------
FC88X_00683_00664 ----------------------ACTATCTGTCTGTGTCTCCGGTTTCGT-----
------
FC88X_00413_01180 --------------------ACATCAGCTCTATCTTCTCTTGTTTCT-------
------
FC88X_00998_00442 --------------------ACATCGGCTCTATCTTCTCTCGTTTCT-------
------
FC88X_00375_01133 --------------------ACATCAGCGTGTCTCTCCTTGGTTTACC------
------
FC88X_00091_00106 --------------------CCATTGTCTCCGCTCCGTTACGTTTGT-------
------
FC88X_01012_00388 --------------------CCATTGTCTCCGCTCCGTTACGTTTGT-------
------
FC88X_00412_01118 --------------------CCATTGTCTCCGCTCCGTTACGCTTGT-------
------
FC88X_00141_00597 ----------------------TTTGTCTGTGTTTCTCCCTCCCGGTTG-----
------
FC88X_00019_00422 ------------------------TTTCCCTATTTCTCCGACCAAGTTTCA---
------
FC88X_00801_01192 ------------------------TTACTCTCTTTTTCCGCCC--GTTTCA---
------
FC88X_00201_00102 -------------------------TTTATCTTTTTCCTGTCCCAGTTTCAC--
------
FC88X_00052_00803 ----------------------TTACACTGTCTCCTATTCCGGATTTCTC----
------
FC88X_00730_00956 ----------------------TTACACTGTCTCCTATCCCGGATTTCTC----
------
FC88X_00994_01207 ----------------------TTAAACTGTCTCCTATCCCGGATTTCTC----
------
FC88X_00475_00169 ----------------------TTACACTGTCTCCTATCCCGGATTTTCTC---
------
FC88X_00603_01014 ---------------------CTTCCTGTTTCTCCT-CCCCGGATTAC------
------
FC88X_00989_00812 --------------------CTATTTGCTGTTCCTCTTCCC-CATGGA------
------
FC88X_00250_00019 --------------------CTTATAGCTGTTCCTTGTCCCGCATGT-------
------
FC88X_00131_00549 -----------------------CTAGCTGTTCCCTTTTCCCGATGTATC----
------
FC88X_00161_00649 ----------------CTTGTCTCT--CTTTCCCCTATCTCTCGTC--------
------

FC88X_00274_01040 ----------------CTAGTCTTTAGCTTTCCCTTATCCCTG-----------
------

FC88X_00637_01193 -----------------------CCTTCTCCTGTCTACTACG-GTCGTTGC---
------

FC88X_00911_00611 ---------------------ACCTTCTTTATGTCTATTCCGCGTCGTC-----
------

FC88X_00468_00233 -------------------------ACCTCCTGTCTATTGACCCGCGTTGCC--
------

FC88X_00829_01106 --------------------GCTTTCTCTTATCCATAC-CCGTCTTGTT-----
------

FC88X_00667_00358 ---------------------TTCTCCGTTATTCGTAGATCGTCTCGTTTCT--
------

FC88X_00557_01133 -------------------------TCCTACCTCTTTCTCCTGACCCGTGCC--
------

FC88X_00797_01069 -------------------------TCTTACCTATTCTGTTGTCCACGTGCT--
------

FC88X_00576_00961 -------------------CTCCTATCTTTTCACATGTGTCGTTTTCCTTCCT-
------

FC88X_00695_00333 -------------------------CCCTCCTATTTGTCACGTTCTCGTTTG--
------

FC88X_00016_00221 -------------------ACGTGCGATCAATCGTCGTGT-GTTCTC-------
------

FC88X_00865_00133 -------------------CCGTGCGTTGATTCACGGTGTCGTCCTC-------
------

FC88X_00510_00799 ---------------------CTGCGACGTCTAACGTTCTGGGCTCCC------
------

FC88X_00630_00725 --------------------CCCGAGAATTCTTAACATCCCGGCTCCA------
------

FC88X_00797_00946 ---------------------CCGATAATTTTCGG-ATCCCA------------
------

FC88X_00411_00900 --------------------CATCTGT-AGTTCGGCCTCCCATTAGT-------
------

FC88X_00783_01240 -------------------CCATCTGT-AGTTCGGCCTCCCATTAGTC------
------

FC88X_00070_00229 --------------------CATCTGT-AGTTTGGCCTCCCATTAGT-------
------

FC88X_00513_01250 -------------------TCATTTGT-TTTCTGACTCCCGATCGGT-------
------

FC88X_01013_00502 -------------------TCATTTGT-TTTCTGACTCCCGATCGGT-------
------

FC88X_00498_00211 --------------------CATCTGTCTTTCTCATCTTCCGTTAGT-------
------

FC88X_00329_01100 ----------------------ACCACAACTCTATCTCTACCTCCCTTG-----
------

FC88X_00488_00226 -----------------------CCGCGCTCTATTCGTCTCCTCCCTTCA----
------

FC88X_00461_01126 --------------TCCTGCACTTGGTGACGCAATTCTACTCTTCCTTTCTCC-
------

FC88X_00998_00770 ------------CTTGCTGCATTTGGTCTCTCA---CTATCCTTCCTT------
------

FC88X_00969_01034 ------------------GTTTACTCTTGTTCTATTCTCGGCTCTG--------
------

FC88X_00190_00864 ----------------CTGTTAACTCTTTATGT-TTCTGCTCTC----------
------

FC88X_01126_00910 -------------------ACTACTCTTTTTAGTCACCACTCTCCA--------
------

```
FC88X_00990_01185      -------------------CCAACTGCTCTCT--CTCTAGGTTCTCTT------
------
FC88X_01089_00747      -------------------CCTCCTACTCTCTGACTCTCCGGTCTC--------
------
FC88X_00072_00437      ------------------ACACGCTTAGTGTTTATGTTTCGTCTC---------
------
FC88X_00861_01135      ------------------CCGAGTTTCTCCCTTAGTATTTGTTTC---------
------
FC88X_00906_01073      -------------------TCTTCTCACT-TGTAGTTTCTG-CTTCGC------
------
FC88X_01126_00793      -----------------------CTTACTATCTCGTCTCTGGCGTCGCC-----
------
FC88X_00203_00923      ------------------CTGGTTTT-GTTTCCAGTGTCTCCATCGT-------
------
FC88X_00904_00653      ------------------CTGGTTTT-GTTTCCAGTGTCTCCATCGT-------
------
FC88X_01083_00700      ------------------CTGGTTTTTGTTTCCAGTGTCTCCATCGT-------
------
FC88X_00088_00926      ------------------CTGGTTTT-GCTTCCAGTGTCTCCATCGT-------
------
FC88X_00083_00063      ------------TTCAAATATGTTT-GGCGTCCAAT-TCTC-------------
------
FC88X_00306_01059      ------------TTCAAATATGTTT-GGCGTCCAAT-TCTC-------------
------
FC88X_00699_00790      ------------TTCAAATATGTTT-GGCGTCCAAT-TCTC-------------
------
FC88X_00896_00585      ------------TTCAAATATGTTT-GGCGTCCAAT-TCTC-------------
------
FC88X_01132_00529      ------------TTCAAATATGTTT-GGCGTCCAAT-TCTC-------------
------
FC88X_01032_00883      ------------TTCAAATATGTTTTGGCGTCCAAT-TCTC-------------
------
FC88X_00295_00053      ------------TTCAAATATGTTT-GGCGCCCAAT-TCTC-------------
------
FC88X_01093_00719      ------------TTCAAATATGTTT-GGCGCCCAAT-TCTC-------------
------
FC88X_00679_00430      ------------TTCAAATATGTTT-GGCGTCCAAT-TCTCCTTCCCTT-----
------
FC88X_00114_00142      ------------ATCAAATATGTTT-GGCGTCCAAT-TCTCCTTCCTTT-----
------
FC88X_00828_01193      ---------------------GTTACCGTGTCA-CTATCT--------------
------
FC88X_01041_00896      -----------------TACCGTATCCGTGTCATCTATCTTCTCA---------
------
FC88X_00399_01135      ------------------ACGCTTTTCGTGTCT-CTTTATCCTCAT--------
------
FC88X_00012_00714      ----------------------TTGTTCCCTTTTCCTCGCCGTGATTGT-----
------
FC88X_00066_00538      ----------------------TTGTTCCCTTTTCCTCGCCGTGATTGT-----
------
FC88X_00077_00533      ----------------------TTGTTCCCTTTTCCTCGCCGTGTTTGC-----
------
FC88X_00182_00022      ----------------------TCGTTGCATTATTCTCGCC-------------
------
FC88X_01004_00607      ----------------------TCGTTGTATTATTCTCGCCGGGGTCTT-----
------
```

FC88X_00444_01024 ------------------TTTATTGTTCCCTGATTCCCGCCGTAC---------
------
FC88X_01084_00592 ------------------TTTATTGTTCCCTGATTCCCGCCGTAG---------
------
FC88X_01074_00417 -----------------------TACCATATTCT-TCGCC-GGCCAACTCAA--
------
FC88X_00669_00748 ----------------------CCACCAGATTCCCTCGCCTGGCCTTCT-----
------
FC88X_00487_01222 ----------------------TTACCAGCTTCT-CCGCCTGT-----------
------
FC88X_00892_01235 ----------------------TTACCAGCTTCT-C-GCCTGTGATTGTCT---
------
FC88X_00255_00073 --------------------TGTTTCCTTCTCCT-CCGCCTGTATCAC------
------
FC88X_00168_00130 --------------------CTTGCTTGCTTCTTCCTCCCGGGTACC-------
------
FC88X_00802_01142 --------------------CTCCCTTGTTACTTCTTCCCGGCTGGA-------
------
FC88X_01057_01044 -------------------------TTACATCTTAGCCGGCGTTCCTCTATT--
------
FC88X_00580_01165 -------------------------TTACATCTTAGCCGGCGTTCCTCTATT--
------
FC88X_00622_00989 -------------------------TTACATCTTAGCCGGCGTTCCTCTATT--
------
FC88X_01142_00822 -------------------------TTACATCTTAGCCGGCGTTCCTC------
------
FC88X_00018_00560 -------------------------TTACACCTTAGCCGGCGTTCCTCTATT--
------
FC88X_00009_00605 -------------------------TTACTACTTCTCCGGCGTACGACTTTT--
------
FC88X_00931_00274 --------------------TCGGCATAACTTTTTCCCGACGTACCT-------
------
FC88X_01015_00601 --------------------CCTTATTTCCCGTACCTCCTGGCTATC-------
------
FC88X_00037_00428 --------------------CCTTATTTCCCGTACCTCCTGGCTATC-------
------
FC88X_00385_00080 ------------------TTCCCTTATTCCCGAAGCTC--GGCTGGC-------
------
FC88X_00073_00447 ------------------TTCCTCCTTCCCCGTACGACT--GCTGGC-------
------
FC88X_00472_01056 -------------------TCTTCTTCTACCTCGGCTCCACGGTGCC-------
------
FC88X_00771_00856 -------------------TCTACTTCTCCCACAAGTCCA-GGTGCT-------
------
FC88X_00987_00740 --------------------CCTTCTTACCTACGACGCCGGGCTGCT-------
------
FC88X_00126_00501 -----------------------CTTCTGCGGAATCATTCCGTTCTCCCCA---
------
FC88X_00875_01028 --------------------------------------TTCCTTCTCCC-----
------
FC88X_00114_00506 -------------------TTTGCTTCTGGGTCTGCATCCTGTTCTC-------
------
FC88X_01023_00577 -------------------TTTGCTTCTGGGTCTGCATCCTGTTCTC-------
------
FC88X_00172_00854 ----------CTTCCAGTCTTCTCATCTGAGCTTTACTTCCTTTCTCC------
------

FC88X_00535_00486 ---------------------CAACCGTTTTGGATTCTCCCGTGATTCT-----
------
FC88X_00987_00527 ---------------------GTCCTGCGTTTTGTGCTCCTGTGAAAC------
------
FC88X_00980_00444 ------------------------TGTCTTTTTGTTCTCCCCTGCATCTAT---
------
FC88X_00395_01187 --------------------CCAAGTTACTTCTTGTCTCCCGTCCAT-------
------
FC88X_00812_00901 --------------------CCAAGTTATTTCTTGTCTCCCGTCCAT-------
------
FC88X_00459_00162 --------------------CCCAGCTTCTTCCT-TCCCCCGTGCATGTC----
------
FC88X_00034_00693 ------------------TTAACCTCCCTTTGCCAGCGTGCTATC---------
------
FC88X_00471_00463 ------------------TTAACCTCCCTTTGCCAGCGTGCTATC---------
------
FC88X_01055_00444 ------------------TTAACCTCCCTTTGCCAGCGTGCTACC---------
------
FC88X_00686_00297 ------------------CTAACCCATCCCTCTCGACAGTCTGCT---------
------
FC88X_00995_00266 -------------TGCTTCTAAACCTCCCATTTAGCCAGAATGTAGCATTAT--
------
FC88X_00966_01246 -------------TACGTCTATACTCCC--TTTGACCTGAAC------------
------
FC88X_00237_00116 ------------------TTACCTG-CTATTCTACCCCTTTTCGAC--------
------
FC88X_00926_00396 -------------------TACCTGGCTATTCCATCCCCTTTTCAA--------
------
FC88X_00533_00830 -----------------CTTACGTGCGTTC-TTCTTCCTCTTATA---------
------
FC88X_00181_00487 -----------------CTTACGTGCGTTCCTTCTTCCTCTTATA---------
------
FC88X_00180_00888 -----------------CTTACGTGCGTTC-TTCTTCCTCTTATA---------
------
FC88X_00224_00871 --------------------TTACGTTATTGCTATTCCTCTTTTACC-------
------
FC88X_01067_00709 ------------------CGATCCGCTGTTCCTAATCC-CTTTTAC--------
------
FC88X_01101_01071 -------------------TTCTCGGC-CATGCATCCCCCGTTTATT-------
------
FC88X_00955_00408 -------------------CTATCGGCTCAAACATCCCACTTTTCT--------
------
FC88X_00892_00335 -----------------AACTTTCGTC-TATTCATCCCCTTTGCG---------
------
FC88X_00742_00377 -------------------------CTATCTAACGCTCCCCTTTCGAC------
------
FC88X_00968_01001 ------------------CTGTTCGCTATCTATTACCTCCCTTTCG--------
------
FC88X_00351_01085 ------------------CCAATCGCTATTTACATTCCCTTTTCCG--------
------
FC88X_00925_00421 -----------------TTCCTCCCTGGTCTGTTCGCTACTCAC----------
------
FC88X_00270_00058 -----------------CTAAACTCCCGTTTGCATGCTGCTCAC----------
------
FC88X_00248_00978 ----------------------CCTTGATCTGCTTGCTATTGACCTCTT-----
------

```
FC88X_00943_00396      ----------------------CCTTTCTTTGCTGCCCATTGACCTGTT-----
------
FC88X_00710_00672      -------------------CAACAGTATTTTTT-ACACGTGCGTCTC-------
------
FC88X_00775_00244      ------------------GCAACAGTATTTTTT-ACACGTGCGTCTC-------
------
FC88X_00735_01156      -------------------CAACAGTATTTTTT-ACACGTGCGTCTC-------
------
FC88X_00750_01116      -------------------CAACAGTATTTTTT-ACACGTGCGTCTC-------
------
FC88X_00131_00237      -------------------CAACAGTATTTTTT-ACACGTGCGTCTC-------
------
FC88X_00183_00469      -------------------CAACAGTATTTTTT-ACACGTGCGTCTC-------
------
FC88X_00408_00710      -------------------CAACAGTATTTTTT-ACACGTGCGTCTC-------
------
FC88X_00428_01039      -------------------CAACAGTATTTTTT-ACACGTGCGTCTC-------
------
FC88X_00642_00069      -------------------CAACAGTATTTTTT-ACACGTGCGTCTC-------
------
FC88X_00430_01112      -------------------CAACAGTATTTTTT-ACACGTGCGTCTCT------
------
FC88X_01136_00887      -------------------CAACAGTATTTTTTTACACGTGCGTCTC-------
------
FC88X_00523_01087      -------------------CAACAGTATTTTTTTACACGTGCGTCTC-------
------
FC88X_00996_00734      -------------------CAACAGTATTTTTTTACACGTGCGTCTC-------
------
FC88X_00942_00687      -------------------CAACAGTATTTTTTTACACGCGCGTCTC-------
------
FC88X_01003_01257      -------------------CAACAGTATTCTTT-ACACGTGCGTCTC-------
------
FC88X_00115_00082      -------------------CAACAGTAATTTTT-ACACGTGCGCCTC-------
------
FC88X_00028_00734      ---------------------ACACAACAGTTCTACTCCCGCGTCGCC------
------
FC88X_00645_01156      ------------------TCAACAATTCTATTTCCCCAACCTCATT--------
------
FC88X_01084_00698      ------------------TCAACAGTTCTATTTCCCCAATCTCATC--------
------
FC88X_00255_00907      --------------------ACCAGTTCT-TTTCTCACATCCCATTACC-----
------
FC88X_01080_00485      --------------------CCAACTGCTTTCTGACCTGCCCATCCC-------
------
FC88X_00553_00472      --------------------TCAACATCTGTTTGCCACCTGCATCCC-------
------
FC88X_01104_00484      --------------------TCTACTTCTCTTTGACACATGCGTGTC-------
------
FC88X_00212_00901      --------------------ACCGGATCTATTCGCTCTGCACCTGCG-------
------
FC88X_00098_00354      ----------------------CTCTGCTCCTTA--CTCCCTGACCCTGCC---
------
FC88X_01109_00536      ------------------TTCTCTCAACTCTTAAACCTCCCTGGCCCTGAAA--
------
FC88X_00981_01010      -------------------GACAATTTCTGCTCCGACTCCTTGATCT-------
------
```

FC88X_00985_01243 ------------------------ATTTCTCCTCTGTCTCTTTGCTCTTGC----
------
FC88X_01115_00835 --------------------CTCTAATTCTGCACCGTCTCCTGTAC---------
------
FC88X_00170_00153 ---------------------TCTCCTTATCACCCGGCTCCTGGACAAT------
------
FC88X_00423_01207 -----------------------TCTCGTATTAACACCGCTGCGA-CCTGTC---
------
FC88X_00083_00090 -----------------------TCTCGTATTAACACCGCTGCGAACCTGTC---
------
FC88X_01075_01006 -----------------------TCTCGTATTAACACCGCTGCGA-CCTGTC---
------
FC88X_00272_00033 --------------------CACTCTCGTGTTGA-ACCGCTCCTACTT-------
------
FC88X_01070_00746 -----------------------CTCTCTCTTTACACCTCTAC------------
------
FC88X_00051_00153 -----------------------CCGCTACTTATTCAATG---
CCCACCTGCTCC------
FC88X_00828_01115 --------------------------CTATTTACTCGATAATTCCCGCCTGAT--
------
FC88X_00317_01006 -------------------------ATTATTCTTTATCTACT-CCTATCTGCG--
------
FC88X_00845_00156 --------------------------CTATTCTTTGGCTACTTCCTCGCTGCT--
------
FC88X_01012_00479 ---------------------CACGCCTATTCTATGGTTACTGCTTTC-------
------
FC88X_00872_00944 ------------------TCACGCACTATTCTTATCCTTACTCAA----------
------
FC88X_00597_01169 ----------------------TATTCTCTCCTTCTCATA---------------
------
FC88X_00922_00257 -------------------TCGTCCCTTATGCTGCTCCTATTTCAT---------
------
FC88X_00069_00823 -----------------CTCGTGGTTATGC-TGCTCGTTATTCTC----------
------
FC88X_00125_00898 -----------------TTCGTGGTTATGC-TGCTCGTTATTCTC----------
------
FC88X_00991_00367 -----------------TTCGTCGTTGCGGGTGCTCGTTATTCT-----------
------
FC88X_00919_00652 -----------------------GTTATGTGCGCTATTTATTCTCCTTGGT----
------
FC88X_00445_01043 ------------------------CCGTGTGCGCTAATCTCTCTTTCTTGC----
------
FC88X_00678_01125 ------------------------CCGTGTGCGCTAATTTCTCTTTCTTGC----
------
FC88X_00645_00653 -------------------------CGTGCTCGTTATTCAATCTTTCCTACT---
------
FC88X_00957_01163 --------------------------CACTAGCCTTATAACTCCAACTTTAC---
------
FC88X_00902_00703 ----------------------------CTACCCTTTTCACTCGATC--------
------
FC88X_01090_00695 ------------------------CAGTATACGCTTGTTACTCTATTTATC----
------
FC88X_00334_01171 ------------------------CAGTATACGCTTGTTACTCTATTTATC----
------
FC88X_00025_00229 ------------------------CAGTACACGCTTGTTACTCTATTTATC----
------

FC88X_00548_01019 -----------------------CAGTATACGCTTGTTACTCTAATTGATC---
------
FC88X_00184_00518 --------------------CTATTGTCAGCTAA-CCTTATCCCACCT------
------
FC88X_01036_00458 --------------------CTATTGTCAGCTAAACCTTATCCCA---------
------
FC88X_00209_01065 ---------------CTTACCAATTTGCAACGTAACGTATTCT-----------
------
FC88X_00427_00872 -------------------CCAATTGGCAAACAAACCTGACATTCT--------
------
FC88X_00069_00102 ------------------CCAGGTAGTTAACGCAATC-GGCTTTTTCT------
------
FC88X_00898_00338 ------------------CCAGGTAGTTAACGCAATCCGGCTTTTTCT------
------
FC88X_00092_00528 ------------------CCAGGTAGTTAACGCAATC-GGCTTTTTCT------
------
FC88X_00205_00852 ------------------CCAGGTAGTTAACGCAATC-GGCTTTTTCT------
------
FC88X_00563_00381 ------------------CCAGGTAGTTAACGCAATC-GGCTTTTTCT------
------
FC88X_00623_00911 ------------------CCAGGTAGTTAACGCAATC-GGCTTTTTCT------
------
FC88X_00651_01242 ------------------CCAGGTAGTTAACGCAATC-GGCTTTTTCT------
------
FC88X_00761_00310 ------------------CCAGGTAGTTAACGCAATC-GGCTTTTTCT------
------
FC88X_00893_00475 ------------------CCAGGTAGTTAACGCAATC-GGCTTTTTCT------
------
FC88X_00884_00775 ------------------CCAGGTAGTTAACGCAATC-GGCTTTTTCT------
------
FC88X_00861_01139 ------------------CCAGGTAGTTAACGCAATC-GGCTTTTTCT------
------
FC88X_00906_00307 ------------------CCAGGTAGTTAACGCAATC-GGCTTTTTCT------
------
FC88X_01057_00531 ------------------CCAGGTAGTTAACGCAATC-GGCTTTTTCT------
------
FC88X_00388_01187 ------------------CCAGGTTGTTAACGCAATC-GGCTTTTTCT------
------
FC88X_00288_01048 ------------------CCAGGCAGTTAACGCAATC-GGCTTTTTCT------
------
FC88X_00050_00075 ------------------CCAGGTAGTTAACGCAATG-GGCTTTTTCTC-----
------
FC88X_01018_00419 ------------------CCAGGTAGTTAACGCAATCGGCTTTTTTCT------
------
FC88X_01000_00936 ------------------CCAGGTAGTTAACGCAATCGGCTTTTTTCT------
------
FC88X_00234_01008 ------------------CCAGGTAGTTAATGCAATCGGCTTTTTTCT------
------
FC88X_00254_00891 --------------------------TCAACGAATCGGCTATTCTTCTATCCA-
------
FC88X_00577_00117 --------------------------TCAACGAATCGGCTATTCTTCTATCCA-
------
FC88X_00315_00801 ------------------CCAATTCGACTCCCGAATTCCTTTATT---------
------
FC88X_00844_00138 ------------------CCAATTCGACTCCCGAATTCCTTTATT---------
------

FC88X_00966_00922 ------------------CCAATTCGACTCCCGAATTCCTTTATT---------
------
FC88X_01054_00832 ------------------CCAATTCGACTCCCGAATTCCTTTATT---------
------
FC88X_00880_00904 ------------------CCAATTCGACTCCGGAATTCCTTTATT---------
------
FC88X_00795_01235 ------------------CCAATTCAACTCCCGAATTCCTTTATT---------
------
FC88X_01087_00752 ---------------CCTCCACTTCAACTCTCCTAA-CCATTATT---------
------
FC88X_00858_01003 ----------------------TCCTTCACTCCCAATTTATTAATCCT------
------
FC88X_01109_00967 ---------------------TTTCACCTTTCCCAATACCTTGGTCCT------
------
FC88X_00910_00357 ----------------TGGCAAAACGAAACC-ATCATTCTCATT----------
------
FC88X_01143_01024 -----------------ATCAAACCGCTCCCTATGATTCTTATA----------
------
FC88X_00095_00585 ------------------GCGTACTCATCTCCTCAATCTTGTTATT--------
------
FC88X_00514_01119 -------------------CGTACTCATCTCCTCAATCTTGTTATT--------
------
FC88X_00115_00755 -------------------CGCTACTATCTCCATCCATTTGTTTT---------
------
FC88X_00640_00153 -------------------CTTTCTCGATTCCTCATTATTTTAACC--------
------
FC88X_00201_00894 ------------------CCACCAACTATCCTCATTCCTCTTTAT---------
------
FC88X_00747_00704 -----------------TTCACCA-CTATC-TCATGCCCCGTTCTT--------
------
FC88X_00812_00172 -----------------TTCTCCAGCAAATCTCCGCCCTTTTTTT---------
------
FC88X_00891_00887 -----------------TTTTCCAGTGTCTCGCCCCCTATTCTT----------
------
FC88X_00348_00216 ------------------TCGCTAACTATTTCTCCACTGTACATGTCC------
------
FC88X_01036_00935 --------------------TCTACCTACT-CTCCACTATTCTTGTCGC-----
------
FC88X_00011_00567 ---------------------CTAGCGTTTCCCCCGCAATCTATTTGTC-----
------
FC88X_00173_00806 ---------------------CTAGCGTTTCCCCCGCAATCTATTTGTC-----
------
FC88X_00970_00367 ---------------------CTAGCGTCTCCCCCGCAGTCTATTTGTC-----
------
FC88X_00103_00800 ---------------------CCAACTTCTCTCCCCATGTCTATGTGT------
------
FC88X_00880_00975 ----------------------CCACTTTCTCTTCAAAGCTATTTTGTC-----
------
FC88X_00450_01192 -------------------TCACGACTTCTCTCTTACCAATATTTT--------
------
FC88X_00970_01182 ------------------TTCCCACGTTCGTCTGCACCTCTATTTT--------
------
FC88X_00157_00945 ------------------TTCCCACGTTCGTCTGCACCTCCATTTTT-------
------
FC88X_00043_00465 -------------------CCACGTCCTCGGCAGCACCGCAATATT--------
------

FC88X_01079_00751 -------------------TCACATCCTCGCCAGCTCTCAATTATT--------
------
FC88X_01116_00872 --------------------TCCGACTTCGCCCTCACATCTATTACT-------
------
FC88X_00236_00886 -----------------TTCTGGGCTAATTCCC--ACAGACTTCTT--------
------
FC88X_00067_00146 -----------------TTCTGGGCTAATTCCC--ACAGACTTCTT--------
------
FC88X_00376_00205 ------------------TTCTGGCTAATTCCC--ACAGACTTCTT--------
------
FC88X_00820_01073 -----------------TCTCGGGTCCTTCCGA--TCAGACTTCTCCC------
------
FC88X_00968_00690 -----------------TCTCGGGTCCTTCCGA--TCAGACTTCTCCATCAG--
------
FC88X_00952_01025 ---------------CTCCTCGTTGTACTTCGA--TGAAACTTCT---------
------
FC88X_00164_00826 ----------------TTCCTGTTGTCCTTCGAGCTGCTACTTCTTCCTTTC--
------
FC88X_00029_00340 --------------------ACTGCTAATATCCCACCAA-CTGCTATT------
------
FC88X_00851_00863 --------------------ACTGCTAATATCCCACCAA-CTGCTATT------
------
FC88X_00999_00277 --------------------ACTGCTAATATCCCACCAA-CTGCTGTT------
------
FC88X_00476_00005 ---------------------TTCCTAACTCTCCATCATTCTGCTATT------
------
FC88X_00533_00029 ---------------------TTCCTAACTCTCCATCATTCTGCTATT------
------
FC88X_00917_01048 ---------------------TTCCTAACT-TCCATCAT--TGCTATT------
------
FC88X_00301_00365 --------------------ACTGTCCATGTTCT-CCTATCAAATTCT------
------
FC88X_00482_01237 ---------------------TTGTCCTCTTCCCATCTATCTTATGCTC-----
------
FC88X_00972_00664 ------------------TGCACTTCCATGCTACTTTTCCTAAAT---------
------
FC88X_00732_00450 ---------------------TCTGCTCCTCTCAACTTTTTAAATCCT------
------
FC88X_00937_00261 --------------------CTCGTGGTCATGTCATTCAGCTATTCA-------
------
FC88X_01115_00528 ---------------------TCTTGTCGTCTTCGTCAAGCTAATGCC------
------
FC88X_00901_00444 ---------------------TTGCCACCTTCTCAACCCTGTAATTCC------
------
FC88X_00314_00243 --------------------------TGTACTGCCCTTCTCCTGACACATTCG-
------
FC88X_00643_00257 ----------------------CGGCTGCCCTAAATTCCCCTGAGCACATA---
------
FC88X_00313_00038 --------------------TCTAC-TGATCCAC--ATTCCCCGACTGTT----
------
FC88X_00705_00046 --------------------TCTAC-TGA-CCAC--ATTCCC-GAC--------
------
FC88X_00689_01216 ------------------TCTCCACATCTATCACCAATTCCCCAA---------
------
FC88X_01109_00720 -----------------CTCTCCAC-TGTATTAC--ATTTCCAAATG-------
------

```
FC88X_00369_01106      --------------------CTCCCACTGACTACACATGTCATTTCC-------
------
FC88X_00905_00422      -------------------GCTCCCACTGACTACACATGTCATTTCC-------
------
FC88X_00699_00802      --------------------CTCCCACTGACTACACATGTCATTTCC-------
------
FC88X_00338_00202      --------------------CTCCCACTGACTACACATGTCATTTCC-------
------
FC88X_00914_00675      --------------------CTCCCACTGACTACACATGTCATTTCC-------
------
FC88X_01024_01166      --------------------CTCCCACTGACTACACATGTCATTTCC-------
------
FC88X_01116_01155      --------------------CTCCCACTGACTACACATGTCATTTCC-------
------
FC88X_00533_00992      ---------------------CTCCACTGACTACACATGTCATTTCC-------
------
FC88X_00061_00255      -----------------------CCAGTG-CAACTCATGCCTTTGCCGATT---
------
FC88X_01137_00598      -----------------------CCAGTG-CGACTCATGCCTTTGCCGATT---
------
FC88X_00747_00258      -------------------TCTGATTCTCACAACACCTGCCCTTGC--------
------
FC88X_01018_00466      -------------------TCTCTTTCTGACCATTCCTGCGTGTGC--------
------
FC88X_00371_00450      -----------------CTCTTTCTTCTCATTGCACCTG-CCTATT--------
------
FC88X_00699_01028      -------------------CCTGCTTCTCCATACTCCTGGCCTGTT--------
------
FC88X_00785_00164      ------------------TTCTACTCACTAC-ACCTTTGCTATTCT--------
------
FC88X_00933_01052      ------------------TTCTACTCTAAACTACTCGTTCCATTCT--------
------
FC88X_00876_00586      ----------------TCCACTACTGATCTC-CTATTCACTATT----------
------
FC88X_00570_01119      ----------------TCACCTACTTTCTCC-ATCCATGCTATT----------
------
FC88X_00521_01083      -----------------CCCTTCTCTCTCTTGACTCGCGGTATGC---------
------
FC88X_00698_00591      -----------------CCCTTCTCTCTCTTGACTCGCGGTATGC---------
------
FC88X_01024_01072      ------------------CCGTTCTCGTCCTGACTC-CGGTGTACT--------
------
FC88X_00330_00108      -------------------CTTCTCGGTCTAGACCCCAGGTTTCCT--------
------
FC88X_00455_01214      -----------------TCCCAATTTCTCCTG-CACCCG-TTTACC--------
------
FC88X_00671_00478      -----------------TCCCAATTCCTCCTG-CACCCG-TTTACC--------
------
FC88X_00597_00288      ---------------------ATTCTCTCCCGACACCTGGTTTACCCA------
------
FC88X_00766_01057      ---------------------TCTCTCCTTCTGCGAT-ATTCTACTCGC-----
------
FC88X_01011_00599      ------------------CTATCTCCTCTCTGGCGCG-ACTGTACT--------
------
FC88X_00055_00014      ---------------------TCTCTCCTGTGAC-CT-ATTGTACCTCCG----
------
```

```
FC88X_00055_00156        --------------------TTCCTATCTATTCTACGTGCTGTTTTCCC-----
------
FC88X_00149_00593        --------------------TTCTTCTCT-CTGCACG-GCTGTTTGCC------
------
FC88X_00673_00147        --------------------CTCGCTTCTCTACTGTG-CCTGTTTTAC------
------
FC88X_00365_01086        ---------------------CTTCTCTTTCCTTTCCACAGTTGTGGA------
------
FC88X_00481_00614        ---------------------CTTCCGTTTCTTCCACAC---------------
------
FC88X_00384_00112        -----------------CGTAAATCCCTTTCTC-CACACCCGTGTG--------
------
FC88X_01133_00750        -----------------CGTAAATCCCTTTCTC-CACACCCGTGTG--------
------
FC88X_00145_00056        ------------------GTTAATCCCTTTTTAGTACAGCCGTGCC--------
------
FC88X_00594_00802        -------------GGGGATCAAATCTCCTTTCATCACGGCCGTGGT--------
------
FC88X_00902_00948        ---------------AGATTATCTCTCCTGT--TCCCGGCCAATT---------
------
FC88X_00050_00798        ----------------------CTCTCTGCTTTTCTCCGGCCGGGTCAT-----
------
FC88X_00757_01007        ---------------------TCTCCCCTTTGTTGGCCGATTGG-TCAT-----
------
FC88X_00414_01187        ---------------------TCTCCCTATTTT-GCGCGACAAGTTGCA-----
------
FC88X_00460_01001        ---------------------TCTCCCTATTTT-GCGCGACAAGTTGCA-----
------
FC88X_00545_01207        ---------------------TCTCCCTATTTT-GCGCGACAAGTTGCA-----
------
FC88X_00782_00620        ---------------------TCTCCCTATTTT-GCGCGACAAGTTGCA-----
------
FC88X_00764_00951        ---------------------TCTCCCTATTTT-GCGCGACAAGTTGCA-----
------
FC88X_01079_00431        ---------------------TCTCCCTATTTT-GCGCGACAAGTTGCA-----
------
FC88X_01111_01119        ---------------------TCTCCCTATTTT-GCGCGACAAGTTGCA-----
------
FC88X_00048_00693        ---------------------TCTCCCTATTTT-GCGCGACAAGTTGCA-----
------
FC88X_00108_00095        ---------------------TCTCCCTATTTT-GCGCGACAAGTTGCA-----
------
FC88X_00142_00660        ---------------------TCTCCCTATTTT-GCGCGACAAGTTGCA-----
------
FC88X_00145_00676        ---------------------TCTCCCTATTTT-GCGCGACAAGTTGCA-----
------
FC88X_00163_00652        ---------------------TCTCCCTATTTT-GCGCGACAAGTTGCA-----
------
FC88X_00342_01037        ---------------------TCTCCCTATTTT-GCGCGACAAGTTGCA-----
------
FC88X_00327_01172        ---------------------TCTCCCTATTTT-GCGCGACAAGTTGCA-----
------
FC88X_00678_01072        ---------------------TCTCCCTATTTT-GCGCGACAAGTTGCA-----
------
FC88X_00748_01220        ---------------------TCTCCCTATTTT-GCGCGACAAGTTGCA-----
------
```

FC88X_00834_00245 ---------------------TCTCCCTATTTT-GCGCGACAAGTTGCA-----
------
FC88X_00913_00599 ---------------------TCTCCCTATTTT-GCGCGACAAGTTGCA-----
------
FC88X_00963_01006 ---------------------TCTCCCTATTTT-GCGCGACAAGTTGCA-----
------
FC88X_01042_00906 ---------------------TCTCCCTATTTT-GCGCGACAAGTTGCA-----
------
FC88X_00469_00988 ---------------------TCTCCCTATTT--GCGCGACAAGTTGCA-----
------
FC88X_00654_00440 ---------------------TCTCCCTATTTTTGCGCGACAAGTTGCA-----
------
FC88X_01075_01122 ---------------------TCTCCCTATTCT-GCGCGACAAGTTGCA-----
------
FC88X_00461_00008 ---------------------TCTCCCCATTTT-GCGCGACAAGTTGCA-----
------
FC88X_00539_00569 ---------------------TCTCCCTATTTT-GCGTGACAAGTTGCACC---
------
FC88X_01019_00934 ---------------------TCTCCCTATTTT-GCGCGACAAGCTGCA-----
------
FC88X_00377_01118 ---------------------TCTCCCTATTTT-GCGCGACAA-----------
------
FC88X_00075_00580 -------------------ACTCTCCCTGTATT---GCACAAGCTTGTT-----
------
FC88X_00478_01192 ----------------------TCTCCCGCTTA-TCGACACGC-TTGTTATC--
------
FC88X_01089_00667 ---------------------TCTCCTCACTTA-TTGACCCCCGTTGTT-----
------
FC88X_00042_00581 ---------------------TCTTTCGTTGCCCCGATTGACGTCAC-------
------
FC88X_00822_00449 ---------------------TCTTTCGTTGCCCCGATTGACGTCAC-------
------
FC88X_00106_00436 ------------------TCATGTTTCATTACACCGGTGGCCGTCT--------
------
FC88X_01102_00794 -----------------CTCATGCTCCGATCGACCCCGGCCTATT---------
------
FC88X_01077_00683 -----------------CTCATGCTCCGATCAACCC-GGCCTATT---------
------
FC88X_00384_01101 -----------------CTCATGGCTCCTATGACCTTTGCTTCTT---------
------
FC88X_01124_00910 -----------------CTCAAGACTCCTTTCCCCTTGGGCGTA----------
------
FC88X_00225_00387 ---------------------CTCCTTTCTCCTCTGACGGCGGTTGCC------
------
FC88X_01094_00876 ---------------------CTCCTTTCTCCTCTGACGGCGGTTGCC------
------
FC88X_00415_01020 --------------------
TTCCTCTATTCCTCTTATCGCGCTTGGCTTCCTTTC----
FC88X_00929_00621 --------------------TTCCTCACTCTCTCGTA-CGCGCTTGAC------
------
FC88X_01088_01093 --------------------CACTCCCATTGGCCGTTTCCCGGTTGA-------
------
FC88X_01131_01080 ---------------------CTCCTCTTCCAGTGGTCGGCGGTTGAC------
------
FC88X_00369_01177 ---------------------TCTTATCTTCTCCCTTGACAGTCTGCT------
------

FC88X_00041_00100 ---------------------TCTTATACTCCCCGCTT-CAGTTTGTT------
------
FC88X_00786_00179 --------------------TTTCATCATTCTCCGCCCTTCACTTTC-------
------
FC88X_00688_01006 --------------------TTTCTCTTTCGTTCCCCCCTTGGCTCGGG-----
------
FC88X_00562_01263 --------------------TCTTTCTTTCCTCCGCTCTTGACTATC-------
------
FC88X_00469_01097 -----------------------TCAATTCATCGTCATTGCTATCGCTC-----
------
FC88X_00952_00785 -----------------------CTACCCTATTCACGTTTGCATCGCTTT----
------
FC88X_00203_00068 -----------------------CTGTCTATTCCATTCATCGCTCATTCT----
------
FC88X_00786_00976 -----------------------CTGTCTATTCCATTCATCGCTCATTCT----
------
FC88X_00872_00169 --------------------TCATCAACTTTTTGTTCGTTTACTCTT-------
------
FC88X_00875_00628 ----------------------ATCCACCGTTCGTTCGGTGTCTCT--------
------
FC88X_00591_01226 -------------------------GTCTTTTCCTCCTTTCACTCTTTGTGCA-
------
FC88X_00008_00324 ------------------ACCCTCGATCCATTTCTCTCATACCAA---------
------
FC88X_00483_01126 -------------------ACCTCAGTTTATTTCCTCCTGCCCACT--------
------
FC88X_00159_00961 -------------------TCCAGGACTATTTTCATGCTCTTCACTC-------
------
FC88X_00858_01227 -------------------CCCTGAGCTATTTTGCACATGATCATT--------
------
FC88X_00983_00619 --------------------ACACACCGTGTTTGTTTCTCTCCAACCT------
------
FC88X_01037_00320 --------------------GTCCCACCTATTTGCATCTCTTCAATC-------
------
FC88X_00198_00074 -------------------TGACCCTATTTTCTCCTGTTCGC-GTAC-------
------
FC88X_00552_00933 --------------------ATCCCTCTTTTCTCAAGCTCCCTGTACA------
------
FC88X_00012_00464 -------------------CTCCAGACTTTTCTCC-ACTGCGTGTGC-------
------
FC88X_00874_00775 -------------------CTCCAGACTTTTCTCC-ACTGCGTGTGC-------
------
FC88X_01118_00880 -------------------ATCAGATCTTGTCTCCCGCTGCGTATT--------
------
FC88X_00599_01240 ---------------------TTCCTCACTCTTTGCAATGCTCTTGCG------
------
FC88X_01095_01129 ---------------------TTCCTCACTCTTTGCAATGCCCTTGCG------
------
FC88X_00822_01238 ----------------------TTTTCTCCTATTTGAATGC-CATGCGCTG---
------
FC88X_00466_01036 ---------------------CTTGTCTCTCCTCCCTATGCCAGTGCG------
------
FC88X_00372_00671 ---------------------CCCAGTTCTCTCTCCTTGGTGCATCCT------
------
FC88X_00595_00146 ---------------------CCCAGTTCTCTCTCCTTGGTGCATCCT------
------

FC88X_01123_00895 ---------------------CCCAGTTCTCTCTCCTTGGTGCATCCT------
------
FC88X_00530_01234 ---------------------CCCAGTTCTCTCTCCTTGGTGCATCCT------
------
FC88X_00934_01260 ---------------------CCCAGTTCTCTCTCCTTGGTGCATCCT------
------
FC88X_00620_01261 ---------------------CCCATTTCTCTCCACCTGTACAATCCT------
------
FC88X_01011_01198 ---------------------CCAGCTTTTTCCCTCTTTGTACGTATT------
------
FC88X_00839_00987 ---------------------CCAGCTTTTCCCTCCTTTGTACATATT------
------
FC88X_00435_00833 -------------------TCACAACGTTTTT-CCCCGTATGTACATT------
------
FC88X_00928_00288 -------------------TCAAAACGTTTTTTCCCCGTATGTACATT------
------
FC88X_00008_00276 ------------------CTAACCTGTTAGTTTCCTTCTCCGTTTC--------
------
FC88X_00026_00452 ------------------CTAACCTGTTAGTTTCCTTCTCCGTTTC--------
------
FC88X_00089_00022 ------------------CTAACA-GTTCTTTTGCTCCACCGCTAT--------
------
FC88X_00143_00674 -------------------TCCAAAGCTATTATCCTCCGACCGGC---------
------
FC88X_00333_01104 -------------------TCCAAAGCTATTACTCTCCGACCGGCCC-------
------
FC88X_00081_00610 -------------------CACACAGTTGTTCTCCTCC-ACCGATCC-------
------
FC88X_00860_00882 -------------------TCACACAGTCTCTTGTGCTCCCTTTTG--------
------
FC88X_00987_00739 -------------------TCAAACACTTTTCTACCCCAACTTTTT--------
------
FC88X_00043_00035 ------------------ATTGGCGA-GAAATGCCGTC-TAGTGCTC-------
------
FC88X_00048_00205 ------------------ATTGGCGA-GAAATGCCGTC-TAGTGCTC-------
------
FC88X_00056_00204 ------------------ATTGGCGA-GAAATGCCGTC-TAGTGCTC-------
------
FC88X_00355_01108 ------------------ATTGGCGA-GAAATGCCGTC-TAGTGCTC-------
------
FC88X_00405_00617 ------------------ATTGGCGA-GAAATGCCGTC-TAGTGCTC-------
------
FC88X_00575_00840 ------------------ATTGGCGA-GAAATGCCGTC-TAGTGCTC-------
------
FC88X_00785_00327 ------------------ATTGGCGA-GAAATGCCGTC-TAGTGCTC-------
------
FC88X_00806_00103 ------------------ATTGGCGA-GAAATGCCGTC-TAGTGCTC-------
------
FC88X_00812_00824 ------------------ATTGGCGA-GAAATGCCGTC-TAGTGCTC-------
------
FC88X_00891_00611 ------------------ATTGGCGA-GAAATGCCGTC-TAGTGCTC-------
------
FC88X_00891_00922 ------------------ATTGGCGA-GAAATGCCGTC-TAGTGCTC-------
------
FC88X_00934_00588 ------------------ATTGGCGA-GAAATGCCGTC-TAGTGCTC-------
------

FC88X_00952_00841 ------------------ATTGGCGA-GAAATGCCGTC-TAGTGCTC-------
------
FC88X_00024_00313 ------------------ATTGGCGA-GAAATGCCGTC-TAGTGCTC-------
------
FC88X_00048_00517 ------------------ATTGGCGA-GAAATGCCGTC-TAGTGCTC-------
------
FC88X_00036_00551 ------------------ATTGGCGA-GAAATGCCGTC-TAGTGCTC-------
------
FC88X_00141_00658 ------------------ATTGGCGA-GAAATGCCGTC-TAGTGCTC-------
------
FC88X_00214_00057 ------------------ATTGGCGA-GAAATGCCGTC-TAGTGCTC-------
------
FC88X_00236_01012 ------------------ATTGGCGA-GAAATGCCGTC-TAGTGCTC-------
------
FC88X_00288_00195 ------------------ATTGGCGA-GAAATGCCGTC-TAGTGCTC-------
------
FC88X_00319_00536 ------------------ATTGGCGA-GAAATGCCGTC-TAGTGCTC-------
------
FC88X_00324_00949 ------------------ATTGGCGA-GAAATGCCGTC-TAGTGCTC-------
------
FC88X_00311_01130 ------------------ATTGGCGA-GAAATGCCGTC-TAGTGCTC-------
------
FC88X_00471_00107 ------------------ATTGGCGA-GAAATGCCGTC-TAGTGCTC-------
------
FC88X_00456_01199 ------------------ATTGGCGA-GAAATGCCGTC-TAGTGCTC-------
------
FC88X_00514_01105 ------------------ATTGGCGA-GAAATGCCGTC-TAGTGCTC-------
------
FC88X_00614_00123 ------------------ATTGGCGA-GAAATGCCGTC-TAGTGCTC-------
------
FC88X_00692_00055 ------------------ATTGGCGA-GAAATGCCGTC-TAGTGCTC-------
------
FC88X_00720_00405 ------------------ATTGGCGA-GAAATGCCGTC-TAGTGCTC-------
------
FC88X_00714_00993 ------------------ATTGGCGA-GAAATGCCGTC-TAGTGCTC-------
------
FC88X_00737_01259 ------------------ATTGGCGA-GAAATGCCGTC-TAGTGCTC-------
------
FC88X_00770_01216 ------------------ATTGGCGA-GAAATGCCGTC-TAGTGCTC-------
------
FC88X_00892_00706 ------------------ATTGGCGA-GAAATGCCGTC-TAGTGCTC-------
------
FC88X_00950_00966 ------------------ATTGGCGA-GAAATGCCGTC-TAGTGCTC-------
------
FC88X_00967_01032 ------------------ATTGGCGA-GAAATGCCGTC-TAGTGCTC-------
------
FC88X_00961_01058 ------------------ATTGGCGA-GAAATGCCGTC-TAGTGCTC-------
------
FC88X_01010_00543 ------------------ATTGGCGA-GAAATGCCGTC-TAGTGCTC-------
------
FC88X_01087_00531 ------------------ATTGGCGA-GAAATGCCGTC-TAGTGCTC-------
------
FC88X_01073_01008 ------------------ATTGGCGA-GAAATGCCGTC-TAGTGCTC-------
------
FC88X_01142_00610 ------------------ATTGGCGA-GAAATGCCGTC-TAGTGCTC-------
------

FC88X_01111_00723 -------------------ATTGGCGA-GAAATGCCGTC-TAGTGCTC-------
------
FC88X_01049_00332 ------------------GATTGGCGA-GAAATGCCGTC-TAGTGCTCC------
------
FC88X_00752_01083 -------------------ATTGGCGA-GAAATGCCGTC-TAGTGC---------
------
FC88X_00157_00023 -------------------ATTGGCGA-AAAATGCCGTC-TAGTGCTC-------
------
FC88X_00954_00984 -------------------ATTGGCGAGAAAATGCCGTC-TAGTGCTC-------
------
FC88X_00143_00773 -------------------ATTGGCGA-GAAATGCCGTC-TAGTGCTCTCATTC-
------
FC88X_00770_00214 -------------------ATTGGCGA-GAAATGCCGTCCTAGT-----------
------
FC88X_00803_01115 -------------------ATTGGCGA-GAAATGCCGTC-TAGTACT--------
------
FC88X_00188_00086 -------------------ATTGGCG--AGAATGCCGTC-TAGTGCTC-------
------
FC88X_00911_01248 -------------------ATTGGCGGAAGAATGCCGTC-TAGTGCTC-------
------
FC88X_01017_00930 -------------------ATTGGCGA-GAAATGCCCGTCTAGT-----------
------
FC88X_00399_00265 ------------------ATTCGGCGA-GAAATGCCGTC-
TAGTGCCTCCTTCCT------
FC88X_00447_01005 ------------------GATTGGCGA-GAAATGCCGTA----------------
------
FC88X_00194_00145 ------------------CACGGCTCGAAA-GTGCAGTC--AGTTCTC-------
------
FC88X_00629_00630 ---------------GGACACGGCTCGAAA-GTGCAGTC--AGTTCTC-------
------
FC88X_00869_00946 ------------------CACGGCTCGAAA-GTGCAGTC--AGTTCTC-------
------
FC88X_01027_01229 ------------------CACGGCTCGAAA-GTGCAGTC--AGTTCTC-------
------
FC88X_00250_00090 ------------------CACGGCTCGAAA-GTGCAGTC--AGTTCTC-------
------
FC88X_00945_00844 ------------------CACGGCTCGAAA-GTGCAGTC--AGTTCTC-------
------
FC88X_00415_00977 ------------------CACGGCTCGAAA-GTGCAGTC--AGTTCTC-------
------
FC88X_00809_01237 -----------------GCACGGCTCGAAA-GTGCAGTC--AGTTCTC-------
------
FC88X_00956_01239 ------------------CACGGCTCGAAA-GTGCAGTC--AGTTCTC-------
------
FC88X_00087_00422 ------------------CACGGCTCGAAA-GTGCAGTC--AGTTCTC-------
------
FC88X_00657_01070 ------------------CACGGCTCGAAA-GTGCAGTC--AGTTCTC-------
------
FC88X_01022_00918 ------------------CACGGCTCGAAA-GTGCAGTC--AGTTCTC-------
------
FC88X_01010_01069 ------------------CACGGCTCGAAA-GTGCAGTC--AGTTCTC-------
------
FC88X_01140_00570 ------------------CACGGCTCGAAA-GTGCAGTC--AGTTCTC-------
------
FC88X_01135_00882 ------------------CACGGCTCGAAA-GTGCAGTC--AGTTCTC-------
------

FC88X_01135_01198 -----------------CACGGCTCGAAA-GTGCAGTC--AGTTCTCC------
------
FC88X_00922_00303 -----------------CACGGCTCGAAA-GTGCAGTC--AGTTCTCCACCC--
------
FC88X_00096_00722 -----------------CACGGCTCGAAAAGTGCAGTC--AGTTCTC-------
------
FC88X_00778_01116 -----------------TACGGCTCGAAA-GTGCAGTC--AGTTCTC-------
------
FC88X_01062_00500 -----------------TACGGCTCGAAA-GTGCAGTC--AGTTCTC-------
------
FC88X_00359_00449 -----------------CACGGCTCGAAA-GTGCAGTG--AGTTCTC-------
------
FC88X_00559_00704 ----------------GCACGGCTCGAAA-GTGCAGTC--
AGCTCTCCTTCCTTT-----
FC88X_00386_01165 ------------GCTGCCACGTTTGGACAATTGCAGTT--CTC-----------
------
FC88X_00952_00593 -------------CTGCCACGTTTGGACAATTG-AGTT--CTC-----------
------
FC88X_00597_01078 -------------CTGCCACGTTTGGACAATTGCAGTT--CTC-----------
------
FC88X_00687_01162 -------------CTGCCACGTTTGGACAATTGCAGTT--CTC-----------
------
FC88X_00974_00920 -------------CTGCCACGTTTGGACAATTGCAGTT--CTC-----------
------
FC88X_00217_00963 -------------CTGCCACGTTTGGACAATTGCAGTT--CTC-----------
------
FC88X_00761_00067 -------------CTGCCACGTTTGGACAATTGCAGTT--CTC-----------
------
FC88X_00935_00317 -------------CTGCCACGTTTGGACAATTGCAGTT--CTC-----------
------
FC88X_01073_00414 -------------CTGCCACGTTTGGACAATTGCAGTT--CTC-----------
------
FC88X_00525_01177 -------------CTGCCATGTTTGGACAATTGCAGTT--CTCT----------
------
FC88X_00640_00915 -------------CGGCCACGTTTGGACAATTGCAGTT--CTC-----------
------
FC88X_00392_01201 -------------CAGCCACGTTTGGACAATTGCAGTT--CTC-----------
------
FC88X_00456_00063 -------------TTGCCACGTTTGGACAATTGCAGTT--CTC-----------
------
FC88X_00936_01212 -------------TTGCCACGTTTGGACAATTGCAGTT--CTC-----------
------
FC88X_00595_00557 -------------TTGCCACGTTTGGACAATTGCAGTT--CTC-----------
------
FC88X_00128_00449 -------------CTGTCACGTTTGGACAATTGCAGTT--CTC-----------
------
FC88X_00827_01079 -------------CTGTCACGTTTGGACAATTGCAGTT--CTC-----------
------
FC88X_01080_00424 -------------CTGTCACGTTTGGACAATTGCAGTT--CTC-----------
------
FC88X_00242_00578 -------------CTGTCACATTTGGACAATTGCAGTT--CTCCTTCCTTT---
------
FC88X_00772_01261 -------------CTGCCACATTTGGACAATTGCAGTT--CTC-----------
------
FC88X_00677_01197 -------------CTGCCACGTTTGGACAACTGCATTT--CTC-----------
------

FC88X_00432_01098 --------------CTGCACGTTTGGACAATTGCAGTC----------------
------
FC88X_00636_00077 -------------------CCGTGCGTGGCGCAATGG-CTCGTCTC--------
------
FC88X_00112_00275 -------------------CCGTGCGTGGCGCAATGGGCTCGTCTC--------
------
FC88X_01102_00912 ----------------TCCCCGTGCCAGGCACGTCAAACTCGT-----------
------
FC88X_00841_01038 ----------------CCACCCCGTTCGCAGCGTTAGGCTCGT-----------
------
FC88X_01067_01274 ---------------GGCCCCGGTCGAGGGGTAGGTTAATAAT-----------
------
FC88X_00110_00340 ----------------CCCCAGTGCGAGTACCGCCGAGTCCTT-----------
------
FC88X_00744_01094 ---------------CTCCAAGTGCTAGATTCAGCAGACTC-------------
------
FC88X_00985_01169 ---------------CACGCTAGTCGAGGTGCCCCGTTGTTTT-----------
------
FC88X_01006_00601 ---------------CACGCTAGTCGAGGTGCCCCGTTGTTTTT----------
------
FC88X_01094_00992 ---------------CACGCTAGTCGAGGTGCCCCGTTGTTTT-----------
------
FC88X_01065_00734 ---------------CACGCTAGTCGAGGCGCCCCGTTGTTTT-----------
------
FC88X_00788_00946 ---------------CACGCTAGTCGAGGTGCCCCGCTGTTTT-----------
------
FC88X_00188_00796 -----------------CGTTAGT-GAGAACCCGCGCTATGTTAC---------
------
FC88X_00754_00973 -----------------CTCCAGGGCGCACACGCAGCTAATTCT----------
------
FC88X_01029_01225 -----------------CTCCAGGGCGCACACGCAGCTAATTCT----------
------
FC88X_00144_00931 -----------------CTCCAGGGCGCACGCGCAGCTAATTCTCTT-------
------
FC88X_00750_00517 ------------------GTCCAGGCGAGCTGGCACTGTATTCTC---------
------
FC88X_00390_00062 -------------------ACGGCGAAAGCGCACTAGTGGGGAGAC--------
------
FC88X_00057_00650 -------------------TTGTCAGCTGGCGCTCACACTCATTCT--------
------
FC88X_00594_00681 -------------------TTGTCAGCTGGCGCTCACACTCATTCT--------
------
FC88X_00114_00616 -------------------TTGTCAGCTGGCGCTCACACTCATTCT--------
------
FC88X_00246_00994 -------------------TTGTCAGCTGGCGCTCACACTCATTCT--------
------
FC88X_00614_00195 -------------------TTGTCAGCTGGCGCTCACACTCATTCT--------
------
FC88X_00804_00944 ------------------GTTGTCAGCTGGCGCTCACACTCATTCT--------
------
FC88X_00208_00859 -------------------TCTGCTGCGGTCAGGCACTCCATTCCC--------
------
FC88X_00666_00649 -------------------TCTGCTGCGGTCAGGCACTCCATTCCC--------
------
FC88X_01074_01104 -------------------TCTGCTGCGGTCAGGCACTCCATTCCC--------
------

FC88X_00640_00667 -------------------CTGTCAGCGATCTGGCACTGATGCTCCT-------
------
FC88X_00753_00285 ----------------------CCGCTAGTTAGGCAC-TGGTTCGCTGTC----
------
FC88X_00849_01242 ----------------------CCGCTAGTTAGGCAC-TGGTTCGCTGTC----
------
FC88X_00965_00964 ------------------CTCTCCGCTA-TTACGCACGTGATTCGC--------
------
FC88X_00345_01000 -----------------CGTGCGCG-AGTCGTGCCCTGTTCCCGT---------
------
FC88X_01067_00524 ----------------CGGTGCGCG-AGACGTGCCCCTG---------------
------
FC88X_00908_00884 -----------TCCGCGATTTTGC---GGCGTTCCCAACTA-------------
------
FC88X_00965_01267 --------------GCGCATTTGCCTGAGCGTGCCCGACTT-------------
------
FC88X_00087_00005 ----------------TCGTCGG----ATCGAGTCTGGCGCCCTGCT-------
------
FC88X_00389_00275 ----------------TCGTCGG----ATCGAGTCTGGCGCCCTGCT-------
------
FC88X_00470_00479 ----------------TCGTCGG----ATCGAGTCTGGCGCCCTGCT-------
------
FC88X_00462_00969 ----------------TCGTCGG----ATCGAGTCTGGCGCCCTGCT-------
------
FC88X_00680_00123 ----------------TCTTCGGTT--ATCGAGCCAGGCTATTCC---------
------
FC88X_00332_01128 ------------------CTCCCTTTTATCCAGTTGCGGCATCTC---------
------
FC88X_01136_01060 -------------------------TTATTTAGTAGCGATCTC-----------
------
FC88X_00646_01100 -------------TTCCC--TCATC-GACTCAGTTGACATCCT-----------
------
FC88X_01032_00918 -------------TTCCCAATTATCAGATGCAGTAGCTATCCC-----------
------
FC88X_00683_01040 ---------------------CCTCACATTAGTTGACGCGCTCCCCAT------
------
FC88X_00089_00109 ---------------------CCTCACATTAGTTGACGCGCTCCCCAT------
------
FC88X_00818_01089 ---------------------CCTCACATTAGTTGACGCGCTCCCCAT------
------
FC88X_00783_01029 ---------------------CCTCACATTAGTTGTCGCGCTCCCCAT------
------
FC88X_01119_01206 ------------------------CCCATTGAGTTGCGCTATCCCCGTGAT---
------
FC88X_01026_01021 ------------------------CCCATTGAGTTGCGCTATCCCCGTGAT---
------
FC88X_00471_01034 --------------------------------G---------------------
------
FC88X_00899_00201 ----------------CCTGTACTTGTGA-ATGGGCCACGTCTC----------
------
FC88X_00992_00341 ----------------CCTGTACTTGTGA-ATGGGCCACGTCTC----------
------
FC88X_01093_00587 ----------------CCTGTACTTGTGA-ATGGGCCACGTCTC----------
------
FC88X_00027_00280 ----------------CCTGTACTTGTGA-ATGGGCCACGTCTC----------
------

FC88X_00282_00639 ----------------CCTGTACTTGTGA-ATGGGCCACGTCTC----------
------
FC88X_00622_01255 ----------------CCTGTACTTGTGA-ATGGGCCACGTCTC----------
------
FC88X_00064_00792 ----------------CCTGTACTTGTGA-ATGGGCCACGTCTC----------
------
FC88X_00017_00586 ----------------CCTGTACTTGTGA-ATGGGCCATGTCTC----------
------
FC88X_00429_01203 ----------------CCTGTACTTGTGA-ATGGGCCATGTCTC----------
------
FC88X_01070_00376 ---------------ACATGCACTCTTGT-ATGC-CCATATCTC----------
------
FC88X_00098_00504 ---------------TTATTCTCTCTTGT-AAGGGCATTGTTG-----------
------
FC88X_00081_00828 ---------------TTATTCTCTCTTGT-AAGGGCATTGTTG-----------
------
FC88X_00449_00884 ---------------TTATTCTCTCTTGT-AAGGGCATTGTTG-----------
------
FC88X_00469_01130 ---------------TTATTCTCTCTTGT-AAGGGCATTGTTG-----------
------
FC88X_00790_00103 ---------------TTATTCTCTCTTGT-AAGGGCATTGTTG-----------
------
FC88X_00837_01257 ---------------TTATTCTCTCTTGT-AAGGGCATTGTTG-----------
------
FC88X_00890_00455 ---------------TTATTCTCTCTTGT-AAGGGCATTGTTG-----------
------
FC88X_00089_00147 ---------------TTATTCTCTCTTGT-AAGGGCATTGTTG-----------
------
FC88X_00079_00415 ---------------TTATTCTCTCTTGT-AAGGGCATTGTTG-----------
------
FC88X_00104_00128 ---------------TTATTCTCTCTTGT-AAGGGCATTGTTG-----------
------
FC88X_00114_00678 ---------------TTATTCTCTCTTGT-AAGGGCATTGTTG-----------
------
FC88X_00511_00321 ---------------TTATTCTCTCTTGT-AAGGGCATTGTTG-----------
------
FC88X_00630_00278 ---------------TTATTCTCTCTTGT-AAGGGCATTGTTG-----------
------
FC88X_00643_01185 ---------------TTATTCTCTCTTGT-AAGGGCATTGTTG-----------
------
FC88X_00809_00951 ---------------TTATTCTCTCTTGT-AAGGGCATTGTTG-----------
------
FC88X_00886_00982 ---------------TTATTCTCTCTTGT-AAGGGCATTGTTG-----------
------
FC88X_00914_00784 ---------------TTATTCTCTCTTGT-AAGGGCATTGTTG-----------
------
FC88X_01124_00962 ---------------TTATTCTCTCTTGT-AAGGGCATTGTTG-----------
------
FC88X_00781_00893 --------------GTTATTCTCTCTTGT-AAGGGCATTGTTG-----------
------
FC88X_00302_00040 ---------------TTATTCTCTCTTGATAAGGGCATTGTTGT----------
------
FC88X_00749_00498 ---------------TTATTCTCTCTTGA-AAGGGCATTGTTG-----------
------
FC88X_00794_00946 ---------------TTATTCTCTCTTGT-AAGGGCATGGTTG-----------
------

```
FC88X_00417_00554      ------------CTATTTCCTCTCTGAATGCTGGCCACC---------------
------
FC88X_00702_01230      -----------CCTACAACCTCCCTGA-TG-TGGCCACTC--------------
------
FC88X_00594_00090      -----------------AACCTCCTGGATG-CGGGGACTAGACTC---------
------
FC88X_00266_01098      ------------------AACCTCTGGATG-CGGGGACTAGACTC---------
------
FC88X_00869_00904      ------------------TCTCGCGATGGTCCGCTACTTGTTCT----------
------
FC88X_00875_00860      ------------------TCTCGCGATGGTCCGCTACTTGTTCTCA--------
------
FC88X_00081_00062      ------------------TCTCGCGATGGTCCGCTACTTGTTCTC---------
------
FC88X_00005_00106      ------------------TCTCGCGATGGTCCGCTACTTGTTCTC---------
------
FC88X_00215_00941      ------------------TCTCGCGATGGTC-GCTACTTGTTCTC---------
------
FC88X_00926_00679      -----------------GTCTCGCGATGGTCCGCTACTTGTTCTC---------
------
FC88X_00262_01100      ------------------TCTCGCGATGGTCCGCTACTTGTTCTC---------
------
FC88X_00005_00391      ------------------TCTCGCGATGGTCCGCTACTTGTTCTC---------
------
FC88X_00049_00700      ------------------TCTCGCGATGGTCCGCTACTTGTTCTC---------
------
FC88X_00079_00285      ------------------TCTCGCGATGGTCCGCTACTTGTTCTC---------
------
FC88X_00181_00574      ------------------TCTCGCGATGGTCCGCTACTTGTTCTC---------
------
FC88X_00215_00068      ------------------TCTCGCGATGGTCCGCTACTTGTTCTC---------
------
FC88X_00222_00122      ------------------TCTCGCGATGGTCCGCTACTTGTTCTC---------
------
FC88X_00222_00669      ------------------TCTCGCGATGGTCCGCTACTTGTTCTC---------
------
FC88X_00243_01102      ------------------TCTCGCGATGGTCCGCTACTTGTTCTC---------
------
FC88X_00285_01105      ------------------TCTCGCGATGGTCCGCTACTTGTTCTC---------
------
FC88X_00641_00222      ------------------TCTCGCGATGGTCCGCTACTTGTTCTC---------
------
FC88X_00744_01055      ------------------TCTCGCGATGGTCCGCTACTTGTTCTC---------
------
FC88X_00816_00889      ------------------TCTCGCGATGGTCCGCTACTTGTTCTC---------
------
FC88X_00878_00709      ------------------TCTCGCGATGGTCCGCTACTTGTTCTC---------
------
FC88X_01046_00923      ------------------TCTCGCGATGGTCCGCTACTTGTTCTC---------
------
FC88X_01047_00371      ------------------TCTCGCGATGGGCCGCTACTTGTTCTC---------
------
FC88X_00711_00941      ------------------TCTCGCGATGCTCCGCTACTTGTTCTC---------
------
FC88X_00632_00761      ------------------TCTCGCGATGGTCCGCTACTTGTTCTCCTTCCTTT-
------
```

```
FC88X_00461_00751       ------------------TCTCGCGATGGTTCGCTACTTGTTCTC---------
------
FC88X_00600_01265       ------------------TCTCGCGACAGTCCGCTACTTGTTCTC---------
------
FC88X_00120_00906       ------------------CTCCGCGATGGTAACATGCTTATTCTC---------
------
FC88X_01098_00974       ------------------ATCCGCGATGGTAACATGCTTATTCTC---------
------
FC88X_00091_00471       ------------------TCCTGCGCTATGCGGCCACTTGTTCTC---------
------
FC88X_00100_00217       ------------------TCCTGCGCTATGCGGCCACTTGTTCTC---------
------
FC88X_00484_01135       -------------------TTGGCGCTCGTGGGACGCCTATT-TTCTC------
------
FC88X_01077_00376       -------------------TTGGCGCTCGTAAGACGCCTATT-TTCTC------
------
FC88X_01093_00963       ---------------------TTCGCTTGTAGATAGCCTATTATTCTC------
------
FC88X_00237_00575       ------------------TTTCGCGCTTGCGAGCCCCTAGTTATTCTT------
------
FC88X_00025_00717       -------------ACATACTTTACGAGATT-GCCTATGCTC-------------
------
FC88X_00349_00654       -------------ACATACTTTACGAGATT-GCCTATGCTC-------------
------
FC88X_00551_00196       -------------ACATACTTTACGAGATT-GCCTATGCTC-------------
------
FC88X_01000_00861       -------------ACATACTTTACGAGATT-GCCTATGCTC-------------
------
FC88X_01086_00779       -------------ACATACTTTACGAGATT-GCCTATGCTC-------------
------
FC88X_00091_00435       -------------ACATACTTTACGAGATT-GCCTATGCTC-------------
------
FC88X_00101_00437       -------------ACATACTTTACGAGATT-GCCTATGCTC-------------
------
FC88X_00156_00664       -------------ACATACTTTACGAGATT-GCCTATGCTC-------------
------
FC88X_00258_00599       -------------ACATACTTTACGAGATT-GCCTATGCTC-------------
------
FC88X_00333_00030       -------------ACATACTTTACGAGATT-GCCTATGCTC-------------
------
FC88X_00319_01144       -------------ACATACTTTACGAGATT-GCCTATGCTC-------------
------
FC88X_00384_00546       -------------ACATACTTTACGAGATT-GCCTATGCTC-------------
------
FC88X_00653_00274       -------------ACATACTTTACGAGATT-GCCTATGCTC-------------
------
FC88X_00681_00605       -------------ACATACTTTACGAGATT-GCCTATGCTC-------------
------
FC88X_00831_00278       -------------ACATACTTTACGAGATT-GCCTATGCTC-------------
------
FC88X_01035_00924       -------------ACATACTTTACGAGATT-GCCTATGCTC-------------
------
FC88X_01000_01203       -------------ACATACTTTACGAGATT-GCCTATGCTC-------------
------
FC88X_01082_00643       -------------ACATACTTTACGAGATT-GCCTATGCTC-------------
------
```

FC88X_00084_00603 -------------ACATACTTTACGAGATT-GCCTATGCTCCTTCC--------
------
FC88X_00108_00236 -------------GCATACTTTACGAGATT-GCCTATGCTC-------------
------
FC88X_00747_00080 -------------ACATACTTTACGAGATTCGCCT-------------------
------
FC88X_00955_00457 -----------CCGCAGACTTTACGCGCTTGCCCTATA----------------
------
FC88X_00074_00606 ----------CCCACACCCTTTTCGCACCA-GCCTATA----------------
------
FC88X_00819_00209 ----------CCGTTACCCTTTGCGCGCTACTCCTAT-----------------
------
FC88X_00929_00948 ----------CCGATACTAATTGCTC-CAACTCGCCG-----------------
------
FC88X_00089_00517 --------------------TCTCCCCGTTAGCCTCCGCGCGATGTG-------
------
FC88X_00792_01045 ---------------GGGGATCTCCCCGTTAGCCTCCGCGCGATGTG-------
------
FC88X_00271_00860 --------------------TCTCCCCGTTAGCCTCCGCGCGATGTG-------
------
FC88X_00762_00319 --------------------TCTCCCCGTTAGCCTCCGCGCGATGTG-------
------
FC88X_00991_01002 --------------------TCTCCCCGTTAGCCTCCGCGCGATGTG-------
------
FC88X_01096_00747 --------------------TCTCCCCGTTAGCCTCCGCGCGATGTG-------
------
FC88X_00049_00174 --------------------TCTCCCCGTTAGCCTCCGCGCGATGTG-------
------
FC88X_00201_00731 --------------------TCTCCCCGTTAGCCTCCGCGCGATGTG-------
------
FC88X_01050_00835 --------------------TCTCCCCGTTAGCCTCCGCGCGATGTG-------
------
FC88X_00191_00958 --------------------TCTCCCCGTTAGCTTCCGCGCGATGTG-------
------
FC88X_00690_00733 --------------------TCTCCCTGCTTG--TCCGTGCTATGTACC-----
------
FC88X_00080_00870 --------------------TTCTCCCGTTTGTTA-CGCGCCCGTAGT------
------
FC88X_00482_00711 ---------------------TCACACATGTGTGAGCTGGCCCGTAAT------
------
FC88X_00527_01045 ----------------------CTGCCGATAGTGT-CGCGTCCGCTGTGCC---
------
FC88X_00698_00814 ---------------------CCTCCCGTTTGAG--CGCGCCCGCGAGTT----
------
FC88X_01026_00798 ---------------------TCTCCCAATCGTTCGCGTCAGCCC-GCG-----
------
FC88X_00466_00770 ---------------------TCTCCCAATCGTTCGCGTCAGCCC-GCG-----
------
FC88X_00548_00954 ---------------------TCTCCCAATCGTTCGCGTCAGCCC-GCG-----
------
FC88X_00798_01213 ---------------------TCTCCCAATCGTTCGCGTCAGCCC-GCG-----
------
FC88X_00868_00283 ---------------------TCTCCCAATAGTTCGCGTCAGCCC-GCG-----
------
FC88X_00594_01265 ---------------------CCTCCCTGT-GTACGCGCTAACCCCGCG-----
------

FC88X_00077_00292 ----------------CACCTCCCGGCATATCACACG-CACACT----------
------
FC88X_00518_01108 ----------------CACCTCCCGGCATATCACACG-CACACT----------
------
FC88X_01123_00631 ------------------CTCCCAGATATGATACACGGCACACTC---------
------
FC88X_00720_00497 ------------------CTCTCGGATACA-CACGGGGCGCTCCCTA-------
------
FC88X_00999_00855 ------------------CTCTCGGATACA-CACGGGGCGCTCCCTA-------
------
FC88X_00552_00235 ------------------CTCTCGGATACA-CACGGGGCGCTCCCTA-------
------
FC88X_00873_00915 ------------------CTCTCGGATACA-CACGGGGCGCTCCCTA-------
------
FC88X_00478_00163 ------------------CACGCGACTACAAAACGCTGGTATCTC---------
------
FC88X_00859_00733 ------------------CACGCGACTACAAAACGCTGGTATCTC---------
------
FC88X_01094_00868 ------------------CACGCGACTACAAAACGCTGGTATCTC---------
------
FC88X_01123_00967 ------------------CACGCGACTACAAAACGCTGGTATCTC---------
------
FC88X_00005_00064 ------------------CACGCGACTACAAAACGCTGGTATCTC---------
------
FC88X_00380_00047 ------------------CACGCGACTACAAAACGCTGGTATCTC---------
------
FC88X_01010_00727 ------------------CACGCGACTACAAAACGCTGGTATCTGC--------
------
FC88X_00109_00705 ------------------CACGCGACTACAAAACGCTGGTATCCC---------
------
FC88X_00756_00220 ------------------TTCCCCAGTATAAAACCGGC-TATCGCC--------
------
FC88X_00035_00510 ------------------TTCCCCAGTATAAAACCGGCCTATCGCC--------
------
FC88X_00228_00120 ------------------TTCCCCAGTATAAAACCGGT-TATCGCC--------
------
FC88X_00303_00061 ----------------CTCTCGAGATTACAAATCGGGGCTCTC-----------
------
FC88X_00993_00465 ----------------CTCTCGAGATTACAAATCGGGGCTCTC-----------
------
FC88X_00962_00766 ----------------CTCTCGAGATTACAAATCGGGGCTCTC-----------
------
FC88X_00075_00286 ----------------CTCTCGAGATTACAAAACGGGGCTCTC-----------
------
FC88X_00718_01150 ----------------CTCTCGAGATTACGAATCGGGGCTCTC-----------
------
FC88X_00184_00041 ----------------CTCTCCAGATTACAAATCGGGGCTCTC-----------
------
FC88X_00148_00592 ---------------------TATCTTGGCACGCGCGGTCAGCTCCCA------
------
FC88X_00370_01172 ---------------------TATCTTA-CACGCGCGGTCAGCTCCCA------
------
FC88X_00992_00666 --------------------CCGATCCGTCATACGCGTCGAGCTTCCT------
------
FC88X_01041_00322 --------------------TTGCGTCATGACCCGTGCCAACTTCCA-------
------

FC88X_00594_00078 ---------------TCCAGCGCTGT-GTCTCCCGAGGATATTC----------------
FC88X_00775_00827 ---------------TCCAGCGCTGT-GTCTCCCGAGGATATTC----------------
FC88X_00807_00226 ---------------TCCAGCGCTGT-GTCTCCCGAGGATATTC----------------
FC88X_00626_00119 ---------------TCCAGCGCTGT-GTCTCCCGAGGATATTC----------------
FC88X_00734_01124 ---------------TCCAGCGCTGT-GTCTCCCGAGGATATTC----------------
FC88X_00649_00441 ---------------TCCAGCGCTGT-GCCTCCCGAGGATATTC----------------
FC88X_01105_01001 ---------------TCCAGCGCCGT-GTCTCCCGAGGATATTC----------------
FC88X_00994_00488 ---------------TCCAGCGCTGTCGTCTCCCGGGGATATTC----------------
FC88X_00683_00125 ---------------TCCACAGCCGT-CCACCCCTCGGATATT-----------------
FC88X_01051_00814 --------------------ACCTGTTCACCCCGCGCGCTATTCGAC-------------
FC88X_00909_00873 --------------------ACCCGTTCACCCCGCGCGCTATTCGAC-------------
FC88X_00690_01217 -----------------CTATAGCGTTCTACTCCCTGCATATGTT---------------
FC88X_00923_00310 -----------------CTATAGCGT-CTACTCCCTGCATATGTA---------------
FC88X_00582_00613 --------------TTCATACAGCGCGCT-CTCCCATCATAA------------------
FC88X_00080_00563 ------------------TTTTCTGTCA-TACGCAGGGCTATTCTT--------------
FC88X_00206_00912 ------------------TTTTCTGTCA-TACGCAGGGCTATTCTT--------------
FC88X_00357_00839 ------------------TTTTCTGTCA-TACGCAGGGCTATTCTT--------------
FC88X_00772_00837 ------------------TTTTCTGTCA-TACGCAGGGCTATTCTT--------------
FC88X_00926_00780 ------------------TTTTCTGTCA-TACGCAGGGCTATTCTT--------------
FC88X_00184_00941 ------------------TTTTCTGTCA-TACGCAGGGCTATTCTT--------------
FC88X_00415_01082 ------------------TTTTCTGTCA-TACGCAGGGCTATTCTT--------------
FC88X_00406_01160 ------------------TTTTCTGTCA-TACGCAGGGCTATTCTT--------------
FC88X_00934_00885 ------------------TTTTCTGTCA-TACGCAGGGCTATTCTT--------------
FC88X_01128_01099 ----------------GTTTTTCTGTCA-TACGCAGGGCTATTCTT--------------
FC88X_00033_00134 ------------------TCTTCTGTCA-TACGCAGGGCTATTCTT--------------
FC88X_00639_00926 ------------------TTTTCTGTCA-TACGCAGGACTATTCTT--------------
FC88X_00109_00912 -----------------TTTTTCTGTCA-
TACGCAGGGCTATTCTTCTTCCTTT------
FC88X_00848_01236 -----------------TCTTTCTGTCA-TACGCAGGGCTATTCTT--------------

FC88X_00162_00073 ------------------TTTTCTGTCAATACGCAGGGCTATTCTTCC------
------
FC88X_00695_01092 ------------------TTTTCTGCCA-TACGCAGGGCTATTCTT--------
------
FC88X_01119_01043 -------------------TTTCTCTGCATTCGGCGGGCTATCCTT--------
------
FC88X_00151_00835 ------------------CTAGCCAATG-TCCAAATTGGAGCTCTC--------
------
FC88X_00690_01254 ------------------CTAGCCAATG-TCCAAATTGGAGCTCTC--------
------
FC88X_00137_00667 ------------------CTAGCCAATG-TCCAAATTGGAGCTCTC--------
------
FC88X_00498_00005 ------------------CTAGCCAATG-TCCAAATTGGAGCTCTC--------
------
FC88X_00022_00394 ------------------TCGTCTAGCA-TCTGCAGATGGGCTCTCTTCCTTT-
------
FC88X_00138_00940 -------------------CTTCTAGCA-TTGTCAGAGCTGTCCTC--------
------
FC88X_00350_01037 -------------------CTTCTAGCA-TTGTCAGAGCTGTCCTC--------
------
FC88X_00514_01045 -------------------CTTCTAGCA-TTGTCAGAGCTGTCCTC--------
------
FC88X_00872_00971 -------------------CTTCTAGCA-TTGTCAGAGCTGTCCTC--------
------
FC88X_00986_00978 -------------------CTTCTAGCA-TTGTCAGAGCTGTCCTC--------
------
FC88X_01097_00576 -------------------CTTCTAGCA-TTGTCAGAGCTGTCCTC--------
------
FC88X_00016_00552 -------------------CTTCTAGCA-TTGTCAGAGCTGTCCTC--------
------
FC88X_00079_00566 -------------------CTTCTAGCA-TTGTCAGAGCTGTCCTC--------
------
FC88X_00884_00845 -------------------CTTCTAGCA-TTGTCAGAGCTGTCCTC--------
------
FC88X_00902_00689 -------------------CTTCTAGCA-TTGTCAGAGCTGTCCTC--------
------
FC88X_01018_00630 -------------------CTTCTAGCA-TTGTCAGAGCTGTCCTC--------
------
FC88X_00480_00154 -------------------CTTCTAGCA-TTGTCAG-GCTGTCCTC--------
------
FC88X_00285_01091 -------------------CCTCTAGCA-TTGTCAGAGCTGTCCTC--------
------
FC88X_00321_00957 -------------------CCTCTAGCA-TTGTCAGAGCTGTCCTC--------
------
FC88X_00225_00882 --------------------CTCTAGCA-TTGTTAGAGCTGTCCTC--------
------
FC88X_00653_01271 -------------------CTTCTAGCA-TTGTTAGAGCTGTCCTC--------
------
FC88X_01038_00514 ----------------------CTAGC---------------------------
------
FC88X_00098_00112 ---------------------TACAGCA-TCGAGCCAGCT-GTCTAAACTC---
------
FC88X_00781_00131 -----------------------ATACT-TCGAACAAGCGAGTCTATCCTC---
------
FC88X_00477_00066 -------------------TTTACTGCA-ATGCAGCAACT-GTCCTAGCC----
------

```
FC88X_00856_00338      ---------------------TCCCGAA-ATCAAACAAATCGTGCTATC-----
------
FC88X_00032_00085      ----------------CCAAACCATCTCCAGGCTATTCGTGTA-----------
------
FC88X_00472_00035      -----------------CCAACCACTTTGCAGCTGTTCGTTTTA----------
------
FC88X_00255_00769      -----------------CCAATACCTATTTACCCGCTCCATTTT----------
------
FC88X_00788_01001      -----------------CCAAACCCAATCCAGCTCC------------------
------
FC88X_00752_01120      -----------------CCAATACATCTCCAGC-TCCGGCAACTA---------
------
FC88X_01049_01094      -----------------CCAATAAATATTCAGCCTCCCGCAGTC----------
------
FC88X_00343_00736      -----------------CCACCAATTCTCCGACTCGCACATTTT----------
------
FC88X_00343_01108      -----------------CCACCAATTCTCCGACTCGCACATTTT----------
------
FC88X_00061_00720      -----------------CCACCAACTCTCCGACTCGCACATTTT----------
------
FC88X_01122_01065      -----------------CCACCAATTCTCCGACTCGCACGTTTT----------
------
FC88X_00457_00768      -----------------CCACCAATTCTCCGACCCGCACATTTT----------
------
FC88X_01126_00762      -----------------CCACCAAGTCACTCCCACC------------------
------
FC88X_00473_00456      -----------------CCAACCAAACTATTGCTCCCACGTGCTCT--------
------
FC88X_00874_00676      -----------------CCAACCAAACTATTGCTCCCACGTGCT----------
------
FC88X_00095_00520      -----------------CCAACCAAACTATTGCTCCCACGTG------------
------
FC88X_00549_00088      -----------------CCAACCAATCCAGCTCCCGCACATAGC----------
------
FC88X_00478_01093      -----------------CCAATAGCTCCCGGGCCAACTGTT-------------
------
FC88X_00106_00955      -----------------CCAATAGCTCCCGGGCCAACTGTT-------------
------
FC88X_01121_00862      -------------CCAACCAATA-CTCCCAATCAACCTGTT-------------
------
FC88X_00085_00084      -------------CCGACCAA--GCTCCCAATCCAACGGCAA------------
------
FC88X_00103_00557      ---------------TTACACCCTA-ACCTCACCGATGCTATT-----------
------
FC88X_00410_00468      ---------------TTACACACTATACCTCACCGACGTCCC------------
------
FC88X_01020_01155      -------------ACTTACACCAAA-ACCTCCCAGTCCATT-------------
------
FC88X_00126_00630      -------------------CGCAAATCTCCGACACCT-GACACATCT-------
------
FC88X_00753_01255      --------------------GTCATATCCCGACTCCTAGACGCATATT------
------
FC88X_01062_00850      ---------------------CCAAATTCCGAAACTCATCGACGC---------
------
FC88X_00443_01071      -----------------TTACCCAACTCCCGACACGACTATGCC----------
------
```

FC88X_00635_00271 -----------------TTACCCT-CTTCCGACAACAC-ATGCCA---------
------
FC88X_00380_01115 --------------------CCGACACCTC--CCCGACTGCATCTTATT-----
------
FC88X_00423_00083 --------------------CCGACTCCTCGTCCCGACT-CATCATTCCC----
------
FC88X_00775_01254 -----------------------ACACCTC--CCAGACTGTACATTTTTGCC--
------
FC88X_00983_00618 -----------------------ACACCTC--CCAGACTGTACATTTCGCC---
------
FC88X_00402_00057 --------------------CTAAAAACTC--CCGCAC-TGATCACTGAAC---
------
FC88X_00609_01189 ---------------------CTAAAACTC--CCGCACCTGACCATTGAA----
------
FC88X_00864_00247 -------------------CTCCACAACTC---CCGCCTGAACATTCC------
------
FC88X_00157_00035 ----------------TTGCTCTGTAAATC---ACGAC--AACACTCC------
------
FC88X_00159_00805 ------------------TCCCACA-ACTCATCAACTACACA------------
------
FC88X_00471_01220 -----------------CTCCCACATACGAAACACTTACACACA----------
------
FC88X_01065_01168 -----------------CTCTCAAACCCTCAACCTTACAGTA------------
------
FC88X_00296_00168 ----------------CTAAACTTTTCCCAACTGCTCCCTTAC-----------
------
FC88X_01030_01139 ---------------CATAAGCTGATCCGGACAACTGACTCC------------
------
FC88X_00110_00943 -------------------GAGACAACCTCC----CACCGCTCCAATTCTC---
------
FC88X_00987_00318 --------------------AGACAACCTCC----CACCGCTCCAATTCTC---
------
FC88X_00959_01023 --------------------CCACAACCTTC----CCCGCTGCATATTCGCCC-
------
FC88X_01143_01179 -------------GGGGAAGTCACAACCTTC----GACGGTACGACTTCCC---
------
FC88X_00743_00204 ------------------GTGCAAAACCTCT---CGACGATACCTGTT------
------
FC88X_00006_00574 ------------------GTGCAAAACCTCT---CGACGATACCTGTT------
------
FC88X_00114_00596 ------------------GTGCAAAACCTCT---CGACGATACCTGTT------
------
FC88X_00231_01015 ------------------GTGCAAAACCTCT---CGACGATACCTGTT------
------
FC88X_00676_01012 ------------------GTGCAAAACCTCT---CGACGATACCTGTT------
------
FC88X_00763_01165 ------------------GTGCAAAACCTCT---CGACGATACCTGTT------
------
FC88X_00984_01023 --------------------GCAAATCCTTCTTCCCACGATGCCTGTA------
------
FC88X_00835_00387 ------------------------CTTATTCCTCCCACCCTACGTGTTGTT---
------
FC88X_00060_00443 ---------------------------
CTACCTCCCACGCTACATTTGTCTGAA------
FC88X_00908_00701 --------------------TCATCATTCTTGACGTATGACATTCTT-------
------

FC88X_00931_00887 --------------------TCATCATTCTTGACGTATGACATTCTT-------
------
FC88X_00046_00376 --------------------TCATCATTCTTGACGTATGACATTCTT-------
------
FC88X_00060_00712 --------------------TCATCATTCTTGACGTATGACATTCTT-------
------
FC88X_00756_00204 --------------------TCATCATTCTTGACGTATGACATTCTT-------
------
FC88X_00933_00414 --------------------TCATCATTCTTGACGTATGACATTCTT-------
------
FC88X_01080_00398 --------------------TCATCATTCTTGACGTATGACATTCTT-------
------
FC88X_00338_01105 --------------------TCATCATTCTCGACGTATGACATTCTT-------
------
FC88X_00334_01064 -----------------ATAACACCCTTCT-AACGTCTGAATTTC---------
------
FC88X_00214_00974 ---------------ACATGCTCCCATTACTCCTGTCAAACAC-----------
------
FC88X_00718_01269 ---------------CCACAGCCTCCCTACGCC-GCAAAACATT----------
------
FC88X_00330_01102 -------------------CTGCAAAACCTCCCGCGCTAGCATAAC--------
------
FC88X_00790_00906 -------------------CTGCAAAACCTCCCGCGCTAGCATAAC--------
------
FC88X_00748_00651 -------------------CTGCGAAACCTCCCGCGCTAGCATAA---------
------
FC88X_00368_00352 -------------------CTCCGACACATCCCTCACCAGCACACTCC------
------
FC88X_00559_01021 -------------------CTCCGACACATCCCTCACCAGCACACT--------
------
FC88X_00835_00892 ------------------CTATTCATCCCGCGCGCCGCAGCACAC---------
------
FC88X_01036_01269 -----------------GCTATTCATCCCGCGCGCCGCAGCACAC---------
------
FC88X_00870_01011 ------------------CTATCCAAAACGCTC-CCGTAAAACAGT--------
------
FC88X_00562_01035 --------------TTGTTCATTCTCCTCACGCGCCCTTTC-------------
------
FC88X_00521_00815 --------------TTGTTCACTATTCAAGAGCCCGCTCAT-------------
------
FC88X_00456_00057 --------------ATAATTATTCACCCAAGATCCCGTTTCG------------
------
FC88X_01008_00883 -------------------CTCCCAACTCAGTTCCGGTAAATTCGG--------
------
FC88X_01126_00521 -------------------CTCCCAACTCAGTTCCGGTAAATTCGG--------
------
FC88X_00468_00123 ---------------TTCATTCCCAA---AGTTCCCGCAAATTT----------
------
FC88X_00501_00352 ---------------CACATTCCTACCCAAGATCCCACGACA------------
------
FC88X_00049_00664 -----------------ATAGCGT---CCTCCCAAAACCAGTCTGTT-------
------
FC88X_00104_00456 -----------------ATAGCGT---CCTCCCAAAACCAGTCTGTT-------
------
FC88X_00180_00170 -----------------ATAGCGT---CCTCCCAAAACCAGTCTGTT-------
------

```
FC88X_00417_01207      -----------------ATAGCGT---CCTCCCAAAACCAGTCTGTT-------
------
FC88X_00670_00691      -----------------ATAGCGT---CCTCCCAAAACCAGTCTGTT-------
------
FC88X_00926_01259      -----------------ATAGCGT---CCTCCCAAAACCAGTCTGTT-------
------
FC88X_00186_00902      -----------------ATAGCGT---CCTCCCAAAACCAGTCTGTT-------
------
FC88X_00231_01034      -----------------ATAGCGT---CCTCCCAAAACCAGTCTGTT-------
------
FC88X_00872_00810      -----------------ATAGCGT---CCTCCCAAAACCAGTCTGTT-------
------
FC88X_01047_00492      -----------------ATAGCGT---CCTCCCAAAACCAGTCTGTT-------
------
FC88X_01106_01105      -----------------ATAGCGT---CCTCCCAAAACCAGTCTGTT-------
------
FC88X_00307_00179      -----------------ATAGCGT---CATCCCAAAACCAGTCTGTT-------
------
FC88X_00065_00260      -----------------ATAGCGC---CCTCCCAAA-CCAGTCTGTT-------
------
FC88X_00229_00881      ------TTTTGCGAATTATAGCGCTATCCTCCCA--------------------
------
FC88X_00766_00193      -----------------ATAGCGC-ATCCTCCCAAAC-----------------
------
FC88X_00112_00782      -----------------ATAGCGT-CCTCCCCAAAAACCA--------------
------
FC88X_00873_00847      --------------CTCATAGC-C-CTCCCGCGCAAACCATTA-----------
------
FC88X_01025_00391      -------------------------AACCTCCCAACCTTAGTTCGTACTAAT--
------
FC88X_00175_01024      -------------------------AACCTCCCAACCTTAGTGCGTACTAAT--
------
FC88X_00987_00486      --------------------CAGCTAATCCTCCCGACCTAATGCGTA-------
------
FC88X_00983_01222      -------------------CTAGCTCATACTCCCAGGCTAACGCGA--------
------
FC88X_00038_00572      -------------------TCAATAACTCCG-CCACACCATACTGTT-------
------
FC88X_01093_01125      -------------------TCAATAACTCCG-CCACACCATACTGTT-------
------
FC88X_00916_00968      -------------------TCAATGACTCCG-CCACACCATACTGTT-------
------
FC88X_00497_00058      --------------------CTATGTCTACGGCCACTCCATAGTGTT-------
------
FC88X_00901_00177      ------------------ACCTGTATTC-CC-GCACACCGACGTATT-------
------
FC88X_00996_00859      ------------------ACCTGTATTC-CC-GCACACCGACGTATT-------
------
FC88X_00145_00685      ------------------ACCTGTATTC-CC-GCACACCGACGTATT-------
------
FC88X_00465_01209      ------------------ACCTGTATTC-CC-GCACACCGACGTATT-------
------
FC88X_00499_00778      ------------------ACCTGTATTC-CA-GCACACCGACGTATT-------
------
FC88X_00499_01155      -----------------GTCCTGAAATCGCC-GGCCACCACCATATT-------
------
```

FC88X_00641_00967 ----------------TAGACGATACTCAATTTCTCCCCACCGG----------
------
FC88X_00011_00448 ------------CAATCAGACGA-AATGACTTGCCCCACA--------------
------
FC88X_00112_00010 ------------------GCTCCAATTCCCCGCCACGTCCCTATA---------
------
FC88X_01126_00685 ------------------GCGCTAATCACAGGCCACTCCCACTAAT--------
------
FC88X_00377_00292 --------------------CCTCACGCTCGGCAGCATCAGACTATTC------
------
FC88X_01021_00679 --------------------TCTAACGCTCCCCA--ATCACATTATTAC-----
------
FC88X_00944_00751 --------------------ACTAACTCCCGTCC--ACTACACTATTCC-----
------
FC88X_00169_00059 --------------------CTGTCACTCCTGCCAAACCCAACTATT-------
------
FC88X_00863_00835 --------------------TTGTCCGACCGGCCAATCCCTACTATT-------
------
FC88X_01039_01230 ---------------------TTCACTCCCGGCCGCTCCCAACTAATC------
------
FC88X_00327_00383 -------------------TCAACTACTCTCT-CACGCCGCGAAACC-------
------
FC88X_00378_00983 -------------------TCAACTACTCTCT-CACGCCGCGAAACC-------
------
FC88X_00786_00955 -------------------TCAACTACTCTCT-CACGCCGCGAAACC-------
------
FC88X_00857_01228 -------------------TCAACACTACCTC-TTCCCCGCGTAACC-------
------
FC88X_00038_00758 -------------------TTAACGATTCCCTGCACCCTACGTAAC--------
------
FC88X_00204_00012 -------------------
TTAACGACTCCCTGCACCCTACGTAACCTTCCTTTCTCC--
FC88X_00356_01120 --------------------ATAAGCCCCACTCTGCGCCACAATATT-------
------
FC88X_00018_00440 -----------------ACGATAAACTCC-CTCAGC-CGACGATAA--------
------
FC88X_00419_01018 --------------------CTACAGCCTCTTCTGACTGACGTTCTC-------
------
FC88X_00052_00536 --------------------CCACAGCCTCCCCGGCCCGACAATCTT-------
------
FC88X_00914_01060 -------------------CACCATACTCCCTCGTACCGACCTACG--------
------
FC88X_00788_00256 ---------------------CCAAACTCCCGCCTGCCGAC-------------
------
FC88X_00580_00069 ------------------CCTCAATACTA-CCACATTATAACAAT---------
------
FC88X_00668_00225 -------------------CTAAAAACTAGCTACACTCTAACCCACGTC-----
------
FC88X_00918_00848 ---------------CTCCTCAATAACTCCCTACCACATACC------------
------
FC88X_00904_00546 ----------------------ACACCTCACCAGTCCTAACCCAAACCCT----
------
FC88X_01103_00819 ------------------TCTCTCGTCCCAACTACCACAACCTAAT--------
------
FC88X_00065_00507 -------------------TTCACAACTAGCCCGACATCACGCCCT--------
------

FC88X_01079_00631 -------------------CCCTAGACCCGACCG-CATCACGA-----------
------
FC88X_01002_00458 ----------------------TGACTCTATCGCTCACGACTCCAATCC-----
------
FC88X_01107_00466 ------------------
ATCAATATTCTATTGCCCGACGATCCCTTCCTTTCCTTC---
FC88X_00737_01211 -------------------TCACTGAC-CAACAGTCTCCCAAGTAAC-------
------
FC88X_00046_00417 -------------------TCACTGAC-CAACAGTCTCCCAAGTAAC-------
------
FC88X_00579_00074 --------------------CATTGACTCAATCGCATCCCAGTAAACT------
------
FC88X_01071_01196 -----------------------TACTCTGACAATCGCACTCCCTGTATT----
------
FC88X_01033_00819 -------------------TCTTTGACTCGACAGTCTCA-TCCCTCTA------
------
FC88X_00816_00217 ---------------------CCAGAACTATTACTCGCCCCTCACAA-------
------
FC88X_00164_00014 --------------------ACCAGCACTATTAGGCACTCCCAACTA-------
------
FC88X_01076_00719 -------------------GCTTAGACCTAATA-GCACTCCCCTTTAC------
------
FC88X_00647_01099 ---------------------CTGGGACCAATA-TCATCCCCTCCTGCT-----
------
FC88X_00575_00984 -------------------TGGACACATCAA-GGTCATCCACTTTCT-------
------
FC88X_01134_00581 ------------------GTGGACACATCAA-GGTCATCCACTTTCT-------
------
FC88X_00619_00130 -------------------TGGACACACCAAAGGTCACTCC-------------
------
FC88X_00620_00914 -----------------CTTGGACAGACCAAGATGCTTGCACT-----------
------
FC88X_00848_00414 --------------------CGACGTACCAGATGTCTTCCAC------------
------
FC88X_00816_00916 --------------------CGCAAAAACTGCCCGCGCCACATTCCT-------
------
FC88X_00968_00390 --------------CAGTCACGCTAAAAATGAATGCCCTCCCTTCCT-------
------
FC88X_00433_01173 --------------------CTCGAAATCAAAGCTGACCTCCACAAC-------
------
FC88X_00825_01125 -------------------TCTCCAAATCAGTGTCACCCCACGACAC-------
------
FC88X_00098_00028 --------------------ATTGCAAACCAAATGCATCCTCCCAG--------
------
FC88X_00451_01026 --------------------ATTGCAAACCAAATGCATCCTCCC----------
------
FC88X_01067_00400 --------------------TGTGCACACCATAGACACTTTCGTCCC-------
------
FC88X_00305_00590 ---------------CAACCCCTAGAGTCTGAGTGCATTCTC------------
------
FC88X_00962_00534 ---------------CAACTCCTAGAGTCTGAGTGCATTCTC------------
------
FC88X_00127_00572 --------------CCACCTCCAAGACT---ATTGCATGCCCAA----------
------
FC88X_00443_00054 ------------TCGAAACTCCTATCCC---ATTACATTCTCC-----------
------

```
FC88X_01082_00982      --------------------TCTAGCTCCCAGTCAAACGATGTTCAT-------
------
FC88X_00295_00593      --------------------TCTAGCTCCCAGTCAAACGATGTTCAT-------
------
FC88X_00897_00843      --------------------TCTAGCTCCCCGTCAAGCGATGTTCAT-------
------
FC88X_00522_01205      -------------------CGCTAACATCCCTTCAAACAACATTTA--------
------
FC88X_00993_00620      ------------------------CCATCCCATCATACGAATCCA-TTTACT--
------
FC88X_00780_00844      ----------------------CCCGACCCCGTGAGACAAAGACAGTTTACC--
------
FC88X_00936_01070      ---------------------TTATCTGCACGCCCAACAACCGCTATT------
------
FC88X_01031_00976      -------------------CCGTATCTTCGTGCC-AATAAC-ACTATT------
------
FC88X_00024_00159      -------------------CATTGTACTCTGAACCTGGCGCTATTA--------
------
FC88X_00077_00425      -------------------CATTGTACTCTGAACCTGGCGCTATTA--------
------
FC88X_00144_00056      -------------------CATTGTACTCTGAACCTGGCGCTATTA--------
------
FC88X_00570_00928      -------------------CATTGTACTCTGAACCTGGCGCTATTA--------
------
FC88X_00761_00187      -------------------CATTGTACTCTGAACCTGGCGCTATTA--------
------
FC88X_00084_00853      ----------------ACTCACTGTACTCT----CTGACGATATTTT-------
------
FC88X_00086_00115      ----------------CATCATTGGCCTCC----CGGACGATATTC--------
------
FC88X_00291_01136      -------------------CACCGGGCTCTCGTAC--GTTTGT-----------
------
FC88X_00913_00546      ------------------ACACTGCGCTCCCACACTAGTTTGTTTT--------
------
FC88X_00011_00512      ----------------CCAAGATGTGATCC-AACATGTGCTTCTC---------
------
FC88X_00602_01167      ----------------CCAAGATGTGATCC-AACATGTGCTTCTC---------
------
FC88X_00466_00992      ------------------GCGATGAGCTCCTAACGCCTGTTTCTC---------
------
FC88X_00025_00303      ------------------CCAATGTGCTCCCAACACTGGTCAAAT---------
------
FC88X_00775_01213      ------------------CCAATGTGCTCCCAACACTGGTCAAAT---------
------
FC88X_00931_00917      ------------------CCAATGTGCTCCCAACACTGGTCAAAT---------
------
FC88X_00906_00330      ----------------CCACCATCTGCGCCAAACCGTGTTATT-----------
------
FC88X_00149_00664      ----------------TCTCCTTCTTCCCC--ACTGTGTTGTGTG---------
------
FC88X_00503_00243      ----------------ACTAATCTTGGCCAAGACGGTATTCTC-----------
------
FC88X_00930_00473      -------------TCTGCTTATATTCTCCACAACGGTATT--------------
------
FC88X_00073_00117      ----------------CTCCCATCCGTGATATGAGCGGACACAT----------
------
```

FC88X_00151_00638 ----------------CTCCCATCCGTGATATGAGCGGACACAT----------
------

FC88X_00882_00410 ----------------CTCCCATCCGTGATATGAGCGGACACAT----------
------

FC88X_01075_00688 -----------------TTCCCTCAGTGGTGGGACGGACCCTGTT---------
------

FC88X_00916_00395 ----------------CTCCCACC--TTCCGACGCAGCACGGCACT--------
------

FC88X_00910_00643 ----------------CTCCCAGCAGTAGCGAGGCCGAACGA------------
------

FC88X_00080_00307 ----------------GCCTGCATCGTGCACAGCCGAGTTCTC-----------
------

FC88X_00058_00564 ----------------CACTGCCAATTTCACAGCCAAGTTCTCC----------
------

FC88X_00616_00117 --------------GAGACTGA-TTGGACACAGACTATTCCTC-----------
------

FC88X_00604_00631 -----------------ACTGAATTGGACACAGACTATTCCTC-----------
------

FC88X_00613_01043 ----------------ACCTGAATTGGACACAGACTATTCCTC-----------
------

FC88X_00723_00342 --------------ACGACTG--TGGCCCACAGCATAATTCTC-----------
------

FC88X_01065_00380 --------------ACGACTG--TGGCCCACAGCATAATTCTC-----------
------

FC88X_00107_00086 --------------ACGACTG--TGGTCCACAGCATAATTCTC-----------
------

FC88X_00238_00052 --------------ACGACTG--TGGTCCACAGCATAATTCTC-----------
------

FC88X_00634_00219 --------------ACGACTG--TGGTCCACAGCATAATTCTCC----------
------

FC88X_00650_00283 --------------ACGACTG--TGGTCCACAGCACAATTCTC-----------
------

FC88X_00904_00367 -------------------CCTGACACCGACGAGTTGGGCCATCCT--------
------

FC88X_00470_00956 -------------------CCTGACACCGACGAGTTGGGCTATCCT--------
------

FC88X_00888_01257 ------------------CCATGTGACACACTCCATGGGCTATC----------
------

FC88X_00821_01266 --------------CATGTGAAGCCACAGAGAGT--GACTA-------------
------

FC88X_00818_00972 ------------------TTAAGCCGCAGCGAGTACGACTATCCTC--------
------

FC88X_00165_00707 ------------------CCCATTGATACAACA--CAGCTGGTTCTC-------
------

FC88X_00841_00808 -----------------GCCCATTGATACAACA--CAGCTGGTTCTC-------
------

FC88X_00729_01132 ------------------CCCATTGATACAACA--CAGCTGGTTCTC-------
------

FC88X_00581_00091 ------------------CCCATTGATACAACA--CAGCTGGTTCTC-------
------

FC88X_00796_00795 ------------------CCCATTGATACAACA--CAGCTGGTTCTC-------
------

FC88X_00972_00423 ------------------CCCATTGATACAACA--CAGCTGGTTCTC-------
------

FC88X_00039_00702 ------------------CACATTGATACAACA--CAGCTGGTTCTC-------
------

FC88X_00802_01143 ------------------CTCATGGATACAACA--CAGCTGGTTCTC-------
------
FC88X_00141_00733 ------------------ACCCTGGATACGAAA--CAGTGCATTCTC-------
------
FC88X_00100_00874 ------------------ACCCTGGATACGAAA--CAGTGCATTCTC-------
------
FC88X_00212_00816 ------------------ACCCTGGATACGAAA--CAGTGCATTCTC-------
------
FC88X_00639_01024 ------------------ACCCTGGATACGAAA--CAGTGCATTCTC-------
------
FC88X_00871_00776 ------------------ACCCTGGATACGAAA--CAGTGCATTCTC-------
------
FC88X_00949_00256 ------------------ACCCTGGATACGAAA--CAGTGCATTCTC-------
------
FC88X_00980_00848 ------------------ACCCTGGATACGAAA--CAGTGCATTCTC-------
------
FC88X_01044_00362 ------------------ACCCTGGATACGAAA--CAGTGCATTCTC-------
------
FC88X_01087_00406 ------------------ACCCTGGATACGAAA--CAGTGCATTCTC-------
------
FC88X_01106_00588 ------------------ACCCTGGATACGAAA--CAGTGCATTCTC-------
------
FC88X_00010_00103 ------------------ACCCTGGATACGAAA--CAGTGCATTCTC-------
------
FC88X_00015_00615 ------------------ACCCTGGATACGAAA--CAGTGCATTCTC-------
------
FC88X_00079_00089 ------------------ACCCTGGATACGAAA--CAGTGCATTCTC-------
------
FC88X_00919_00697 ------------------ACCCTGGATACGAAA--CAGTGCATTCTC-------
------
FC88X_00877_00653 ------------------ACCCTGGATACGAAAA-
CAGTGCATTCTCCTTCCTTT-----
FC88X_00044_00804 ------------------ACCCTGGATACGAAA--
CAGTGCATTCTCCTTCCTTT-----
FC88X_00032_00621 -------------------ACCTGGATACGAAA--CAGTGCATTCTC-------
------
FC88X_01047_01250 -------------------ACCTGGATACGAAA--CAGTGCATTCTCC------
------
FC88X_00152_00635 -------------------ACCTGGATACGAAA--CAGTGCATTCTC-------
------
FC88X_00220_00927 -------------------ACCTGGATACGAAA--CAGTGCATTCTC-------
------
FC88X_00539_00070 -------------------ACCTGGATACGAAA--CAGTGCATTCTC-------
------
FC88X_00762_00363 -------------------ACCTGGATACGAAA--CAGTGCATTCTC-------
------
FC88X_00807_00168 -------------------ACCTGGATACGAAA--CAGTGCATTCTC-------
------
FC88X_00871_00375 -------------------ACCTGGATACGAAA--CAGTGCATTCTC-------
------
FC88X_00935_01200 -------------------ACCTGGATACGAAA--CAGTGCATTCTC-------
------
FC88X_00999_00858 -------------------ACCTGGATACGAAA--CAGTGCATTCTC-------
------
FC88X_01033_00486 -------------------ACCTGGATACGAAA--CAGTGCATTCTC-------
------

FC88X_01053_00461 -------------------ACCTGGATACGAAA--CAGTGCATTCTC-------
------
FC88X_00546_01043 ------------------ACCCTGGATACGAAA--CAGCGCATTCTC-------
------
FC88X_00028_00316 -----------GGGGAACCCAGCATC-ACCAAC--CAGACCAACCT--------
------
FC88X_00814_00314 ------------------CCGGCTTCTACCAAA--CTGCGCATTCT--------
------
FC88X_00667_01151 ------------------------ACAACAAACC-TTGTGCAC-TCCCGTCGC-
------
FC88X_01069_00919 ---------------------ACAACACAGCACT-TAGAGCACCTCCTGTC---
------
FC88X_00497_00425 ----------------------CTACAACAATGC-GCATTCCCCTCCTAT----
------
FC88X_00911_00977 ------------------CACCAACC-ACTGTACAGAGGCCATTCTC-------
------
FC88X_00901_00793 ------------------CACCAAC--ACTGTACAGAGGCCATTCTC-------
------
FC88X_01118_00903 ------------------CACCAACC-ACTGTACAGAGGCCATTCTC-------
------
FC88X_00801_00434 ------------------CACCAAC--
ACTGTACAGAGGCCATTCTCCTTCCTTT-----
FC88X_00962_00642 -----------------CCACGAACCTACCCCACGGCGGACATT----------
------
FC88X_00106_00638 ------------------TTACCC-CCTACCGTAATCCACCATACT--------
------
FC88X_01003_00921 ------------------TTACCC-AATAGC-TATTCGGCCGTATT--------
------
FC88X_00773_00856 ------------------CTACCG-AAA--CCCTATTCCCCGTGTCAC------
------
FC88X_00135_00649 ---------------ACCCTAGCG-AAAGGCCACATTCTCCGA-----------
------
FC88X_00168_00023 ------------------CTGTTA-ACTAAGCAGCCCCACCGTATT--------
------
FC88X_00885_00176 ------------------CTGTTA-ACTAAGCAGCCCCACCGTATT--------
------
FC88X_00868_00207 ------------------CTGTTA-ACTAAGCAGCCCCACCGTATT--------
------
FC88X_00110_00922 ------------------CTGTTA-ACTAAGCAGCCCCACCGTATT--------
------
FC88X_00983_00537 ------------------CTGTTA-ACTAAGCAGCCCCACCGTATT--------
------
FC88X_01114_00566 ------------------CTGTTA-ACTAAGCAGCCCCACCGTATT--------
------
FC88X_00924_00568 ------------------CTGTTATACTCCGCCGTCACACACC-----------
------
FC88X_00481_00043 ----------------TACAGCATCTCG--CCCCAGGTACCATTTT--------
------
FC88X_00929_00992 ----------------TACAGCATCTCG--CCCCAGGTACCATTTT--------
------
FC88X_00070_00339 ------------------GTGCATCCCGATCCTCCTGCGCCATGTT--------
------
FC88X_00975_00566 ------------------GTGTTTGACTAGCCACCGGCACCTTCT---------
------
FC88X_00009_00288 -----------------TCTCATGAAAACAAAACATGGCTATACCTC-------
------

```
FC88X_00431_01093      -----------------TCTCATGAAAACAAA-CATGGCTATACCTC-------
------
FC88X_00042_00195      -----------------TCTCATGAAAACAAA-CATGGCTATACCTC-------
------
FC88X_00129_00922      -----------------TCTCATGAAA-CAAA-CATGGCTATACCTC-------
------
FC88X_00922_00991      -----------------TCTCATGAAA-CAAA-CATGGCTATACCTC-------
------
FC88X_00998_00873      -----------------TCTCATGAAA-CAAA-CATGGCTATACCTC-------
------
FC88X_00040_00476      -----------------TCTCATGAAA-CAAA-CATGGCTATACCTC-------
------
FC88X_00564_00201      -----------------TCTCATGAAA-CAAA-CATGGCTATACCTC-------
------
FC88X_00576_01095      -----------------TCTCATGAAA-CAAA-CATGGCTATACCTC-------
------
FC88X_00883_00790      -----------------TCTCATGAAA-CAAA-CATGGCTATACCTC-------
------
FC88X_00956_00427      -----------------TCTCATGAAA-CAAA-CATGGCTATACCTC-------
------
FC88X_00995_00453      -----------------TCTCATGAAA-CAAA-CATGGCTATACCTC-------
------
FC88X_00971_00729      -----------------TCTCATGAAA-CAAA-CATGGCTATACCTC-------
------
FC88X_00369_00072      -----------------TCTCATGAAA-CAAA-CATGGCTACACCTC-------
------
FC88X_00319_00012      -----------------TCTCATGAAAACAAA-CATGGCTACACCTC-------
------
FC88X_01123_01136      -----------------TCTCATGAAA-CAAG-CATGGCTATACCTC-------
------
FC88X_00876_00993      -----------------TCTCATGGAA-CAAA-CATGGCTATACCTCCTTCC--
------
FC88X_00901_01136      -----------------TCTCATGAAA-CAAA-CACGGCTATATCTC-------
------
FC88X_00371_01012      ----------------------CGAAAACACA-CACG-CTATCACTCACA----
------
FC88X_00667_00929      ----------------------CAAAACCGCA-TACGGCTAGCACTCATT----
------
FC88X_00578_00129      ---------------------GTCAAACAATA--GACGCTCTACTCCCAAT---
------
FC88X_00107_00238      ----------------------TCAAACAATA--GACGCTCTACTCCCAAT---
------
FC88X_00645_01126      ----------------------TCAAACAATA--GACGCTCTACTCCCAAT---
------
FC88X_00952_00970      --------------CTCTCCATTGAAAAAAAA--AACACTACGTCTCCCCC---
------
FC88X_00730_00291      ------------------ACCTCCAAAAAAAAACTCCCTATTGCATCAA-----
------
FC88X_01100_01064      ------------------TCTTCCACAACAGA---CCTTATTACCCCAA-----
------
FC88X_00153_00208      ---------------------CGCACATACACAAAA-CACTGGCTACTC-----
------
FC88X_00075_00121      ---------------------CGCACATACGCAAAAACACTGGCTACTC-----
------
FC88X_00396_00444      --------------------TTCCGCAC-TACCCAACCTCAGGCTATT------
------
```

FC88X_00141_00675 --------------------TTCCGCAC-TACCCAACCTCAGGCTATT------
------
FC88X_00426_01227 ---------------------GCGACACACAAA--TCCCACGGCCTATTA----
------
FC88X_00981_00398 ---------------------GCGACACACAAAA-TCCCACGGCCTATTA----
------
FC88X_00142_00239 ---------------------GCGACACACAAA--TCCCACGGCCTATTA----
------
FC88X_00059_00088 ----------------------CTACAATCTATCAACTCCCCGGCCTGGC----
------
FC88X_00321_00158 ----------------------CTACACACTATCTTCTCTCGGCCTCCG-----
------
FC88X_01066_00916 -------------------CCGCTAAATACCAACACCCCGGGCCCT--------
------
FC88X_00571_01224 ----------------TCCCGCAAAACAACCA--CTCCCGATACA---------
------
FC88X_00635_01233 ------------------CCTCCAAACTACCAGCCTCCCG--------------
------
FC88X_00657_00435 --------------------CCACAAACACAACCCTCCTGGGGGTGGT------
------
FC88X_00221_00911 ---------------------CTGCTAATCAACAATCCCTGCACCTGT------
------
FC88X_00764_00952 ---------------------CTGCGAATCAACAATCCCGGCACCTGT------
------
FC88X_00898_01002 --------------------CCTGTCACTAAAAAATAGCGCCTCCTTCTC----
------
FC88X_00073_00501 ----------------TCTCGACATATCCCAA--TTGCCACTGTT---------
------
FC88X_00224_01070 ----------------TCTCGACATATCCCAA--TTGCCACTGTT---------
------
FC88X_00758_01014 ----------------TCTCGACATATCCCAA--TTGCCACTGTT---------
------
FC88X_00900_01071 ----------------TCTCGACATATCCCAA--TTGCCACTGTT---------
------
FC88X_00171_00739 ----------------TCTCGACATATCCCCAATTTGCCACTGTT---------
------
FC88X_01095_00635 ------------------GCAACGTCTTCCCGA-CTAGCACTATTCC-------
------
FC88X_00926_00337 ------------------ACAACGTCTTCCCGA-CTAGCACTATTCC-------
------
FC88X_01045_00492 -------------------CAACATCT-CCCGA-TT-GCACTGTTATTCT----
------
FC88X_00193_00605 -----------------------CCATCGCCAAAGTAGCACTCCCATTATC---
------
FC88X_00864_00249 -----------------------CCATCGCCAAAGTAGCACTCCCATTATC---
------
FC88X_00855_00226 ---------------------------TCCCCAAATAGCTCAACC---------
------
FC88X_00546_00199 -------------------TCGCGAATCGTCCCACTCCAACAACAC--------
------
FC88X_01010_00670 -------------------TCGCGAATCGTCCCACTCCAACAACAC--------
------
FC88X_00823_00972 ------------------CGCGAAAGTCCCAACACTCCAAGTAGC---------
------
FC88X_00823_00804 ---------------ATACCCGCAA-TAGCACCGCTTCTAATT-----------
------

```
FC88X_01134_00915      ---------------ATACCCGCAA-TAGCACCGCTTCTAATT-----------
------
FC88X_00363_01182      ------------------CCCGCAACTATCCCTACTCCTAATGCT---------
------
FC88X_00483_00400      -------------------CTAGCGATTCGTACAGCAGCTCCCTGT--------
------
FC88X_01038_00887      -------------------CTAGCGATTCGTACAGCAGCTCCCTGT--------
------
FC88X_00436_01103      ------------------ACCAGCGATT--TTCCTCAGCACTCTTTT-------
------
FC88X_01033_00856      ----------------------CTATCCCCACG-GTAGCTCCCTGTTGAC----
------
FC88X_00351_00622      ---------------------ACTGTCCCGACACGTAGCTCCCATTTA------
------
FC88X_00502_01109      ----------------------TTCAGTACAGAAACAGCTCCCTACAT------
------
FC88X_00692_00111      -------------------CGACAACTAGA--CGACATTTCCCTATT-------
------
FC88X_00483_01244      -------------------CGACAAATAGC--CGCCACGTCCCTCACA------
------
FC88X_00625_01064      ------------------TCGACAAATTAA-CCAC-GGCTCCCTTTT-------
------
FC88X_00751_00290      ------------------TCGACAAATTAAACCACCGGCTCCCTTTT-------
------
FC88X_00918_00691      ------------------TCGACAAATTAA-CCAC-GGCTCCCTTTT-------
------
FC88X_00950_00540      ------------------TCGACAAATTAA-CCAC-GGCTCCCTTTT-------
------
FC88X_00896_00992      ------------------TCGACAAATTAA--CAC-GGCTC-------------
------
FC88X_01093_00581      ------------------TCGACAAATTAA-CCAC-GGCCCCCTTTT-------
------
FC88X_00852_00792      ------------------TCGACGAATTAA-CCGC-GGCTCCCTTTT-------
------
FC88X_00029_00721      ----------------CGCGGACAATCGCC-AA--TATCTCCCGGGA-------
------
FC88X_00617_00215      ----------------------CTATCGCC-TAATCAACTCCCGCCTCGT----
------
FC88X_00332_00792      ------------------TCGACAAATCGC--AT--AACTCCCTACATA-----
------
FC88X_01102_00908      ------------------TCGACAAATCGC-CAA--AACTCCCTACATA-----
------
FC88X_00928_00638      ------------------TCGACAAACCGC--AA--AACTCCCTACATA-----
------
FC88X_00801_00888      ------------------CGCATAACTGCC-CAA--CACTCCCGTATT------
------
FC88X_00950_00564      -----------------GCGCATAACTGCC-CAA--CACTCCCGTCATT-----
------
FC88X_00899_00199      -----------------TACCACAACGGCC-ACA--TCTCCCAAGCA-------
------
FC88X_00069_00120      ------------------TTCGCCAA-TATCCC-GCGGCGTGTTCTC-------
------
FC88X_00104_00330      -----------------GTTCGCCAA-TATCCC-GCGGCGTGTTCTC-------
------
FC88X_00114_00135      ------------------TTCGCCAA-TATCCC-GCGGCGTGTTCTC-------
------
```

```
FC88X_00147_00651      ------------------TTCGCCAA-TATCCC-GCGGCGTGTTCTC-------
------
FC88X_00391_00041      ------------------TTCGCCAA-TATCCC-GCGGCGTGTTCTC-------
------
FC88X_00422_00110      ------------------TTCGCCAA-TATCCC-GCGGCGTGTTCTC-------
------
FC88X_00473_01055      ------------------TTCGCCAA-TATCCC-GCGGCGTGTTCTC-------
------
FC88X_00556_01188      ------------------TTCGCCAA-TATCCC-GCGGCGTGTTCTC-------
------
FC88X_00678_01182      ------------------TTCGCCAA-TATCCC-GCGGCGTGTTCTC-------
------
FC88X_00802_01190      ------------------TTCGCCAA-TATCCC-GCGGCGTGTTCTC-------
------
FC88X_00915_00822      ------------------TTCGCCAA-TATCCC-GCGGCGTGTTCTC-------
------
FC88X_01079_01006      ------------------TTCGCCAA-TATCCC-GCGGCGTGTTCTC-------
------
FC88X_01110_00902      ------------------TTCGCCAA-TATCCC-GCGGCGTGTTCTC-------
------
FC88X_01111_00925      ------------------TTCGCCAA-TATCCC-GCGGCGTGTTCTC-------
------
FC88X_00099_00005      ------------------TTCGCCAA-TATCCC-GCGGCGTGTTCTC-------
------
FC88X_00088_00784      ------------------TTCGCCAA-TATCCC-GCGGCGTGTTCTC-------
------
FC88X_00170_00931      ------------------TTCGCCAA-TATCCC-GCGGCGTGTTCTC-------
------
FC88X_00174_00942      ------------------TTCGCCAA-TATCCC-GCGGCGTGTTCTC-------
------
FC88X_00215_01001      ------------------TTCGCCAA-TATCCC-GCGGCGTGTTCTC-------
------
FC88X_00362_00318      ------------------TTCGCCAA-TATCCC-GCGGCGTGTTCTC-------
------
FC88X_00377_01091      ------------------TTCGCCAA-TATCCC-GCGGCGTGTTCTC-------
------
FC88X_00546_01175      ------------------TTCGCCAA-TATCCC-GCGGCGTGTTCTC-------
------
FC88X_00589_00111      ------------------TTCGCCAA-TATCCC-GCGGCGTGTTCTC-------
------
FC88X_00584_01185      ------------------TTCGCCAA-TATCCC-GCGGCGTGTTCTC-------
------
FC88X_00617_00676      ------------------TTCGCCAA-TATCCC-GCGGCGTGTTCTC-------
------
FC88X_00636_01166      ------------------TTCGCCAA-TATCCC-GCGGCGTGTTCTC-------
------
FC88X_00643_01176      ------------------TTCGCCAA-TATCCC-GCGGCGTGTTCTC-------
------
FC88X_00650_00200      ------------------TTCGCCAA-TATCCC-GCGGCGTGTTCTC-------
------
FC88X_00684_00455      ------------------TTCGCCAA-TATCCC-GCGGCGTGTTCTC-------
------
FC88X_00680_01041      ------------------TTCGCCAA-TATCCC-GCGGCGTGTTCTC-------
------
FC88X_00730_00068      ------------------TTCGCCAA-TATCCC-GCGGCGTGTTCTC-------
------
```

FC88X_00753_00209 ------------------TTCGCCAA-TATCCC-GCGGCGTGTTCTC-------
------
FC88X_00797_00429 ------------------TTCGCCAA-TATCCC-GCGGCGTGTTCTC-------
------
FC88X_00780_01048 ------------------TTCGCCAA-TATCCC-GCGGCGTGTTCTC-------
------
FC88X_00754_01058 ------------------TTCGCCAA-TATCCC-GCGGCGTGTTCTC-------
------
FC88X_00853_01055 ------------------TTCGCCAA-TATCCC-GCGGCGTGTTCTC-------
------
FC88X_00914_00434 ------------------TTCGCCAA-TATCCC-GCGGCGTGTTCTC-------
------
FC88X_00928_00435 ------------------TTCGCCAA-TATCCC-GCGGCGTGTTCTC-------
------
FC88X_00932_00549 ------------------TTCGCCAA-TATCCC-GCGGCGTGTTCTC-------
------
FC88X_00982_00239 ------------------TTCGCCAA-TATCCC-GCGGCGTGTTCTC-------
------
FC88X_00991_00809 ------------------TTCGCCAA-TATCCC-GCGGCGTGTTCTC-------
------
FC88X_00957_01018 ------------------TTCGCCAA-TATCCC-GCGGCGTGTTCTC-------
------
FC88X_01019_00559 ------------------TTCGCCAA-TATCCC-GCGGCGTGTTCTC-------
------
FC88X_01071_00739 ------------------TTCGCCAA-TATCCC-GCGGCGTGTTCTC-------
------
FC88X_01071_01133 ------------------TTCGCCAA-TATCCC-GCGGCGTGTTCTC-------
------
FC88X_01115_00654 ------------------TTCGCCAA-TATCCC-GCGGCGTGTTCTC-------
------
FC88X_01147_00681 ------------------TTCGCCAA-TATCCC-GCGGCGTGTTCTC-------
------
FC88X_00896_00607 ------------------TTCGCCAA-TATCCC-GCGGCGTGTCCTC-------
------
FC88X_00551_01025 ------------------TTCGCCAA-TATCCC-GCGGCATGTTCTC-------
------
FC88X_00656_00160 ------------------TTCGCCAA-TATCCCCGCGGCGTGTTCTC-------
------
FC88X_00619_00408 ------------------TTCGCCAA-TACCCC-GCGGCGTGTTCTCCC-----
------
FC88X_00201_01045 ------------------TTCGCAAA-TATCCC-GCGGCGTGTTCTC-------
------
FC88X_00878_00700 ------------------TTCGCGAA-TATCCC-GCGGCGTGTTCTC-------
------
FC88X_00686_00149 ------------------TTCGCCAA-TATCTC-GCGGCGTGTTCTC-------
------
FC88X_00727_01256 ------------------TTCGCCAA-TATCTC-GCGGCGTGTTCTC-------
------
FC88X_00767_00462 ------------------TTCGCCAA-TATCTC-GCGGCGTGTTCTC-------
------
FC88X_00042_00461 ------------------TTCGCCAA-TATACC-GCGGCGTGTTCTC-------
------
FC88X_00846_00316 ------------------TTCGCCAA-TATTCC-GCGGCGTGTTCTCC------
------
FC88X_00370_01111 --------------------TGCCAA-TATCCC-GCGGCGTGTTCTC-------
------

FC88X_00947_00732 -------------------CTCGCCAA-TATCCC-GCGGCGTGTTCTC-------
------
FC88X_00623_01193 -------------------TTCGCCAA-TATCCC-
GCGGCGTGTTCTCCTTCCTTT-----
FC88X_00220_01079 -------------------TTCGCCAA-TATCCC-GCGGCGTGTCTCCT------
------
FC88X_00885_01076 -------------------TTCGCCAAATATCCCCGCGGCGT-------------
------
FC88X_00950_00605 -------------------TTCGTTCAATATCCC-GCGGCGTGTTCTCC------
------
FC88X_00377_00132 --------------------CAGAGAAATAGTCCCCCTGC-TGATCTC-------
------
FC88X_00771_00995 --------------------CAGAGAAATAGTCCCCCTGC-TGATCTC-------
------
FC88X_00984_00361 --------------------CAGAGAAATAGTCCCCCTGC-TGATCTC-------
------
FC88X_00784_01050 ------------------ACCTGT-AA-T-CCCGACCGGCTCTATCTC-------
------
FC88X_00881_00376 ------------------ACCTGT-AA-T-CCCGACCGGCTCTATCTC-------
------
FC88X_00043_00715 ------------------ACCTGT-AA-T-CCCGACCGGCTCTATCTC-------
------
FC88X_00589_01181 ------------------ACCTGT-AA-T-CCCGACCGGCTCTATCTC-------
------
FC88X_00788_01046 -------------------CCTGTCAA-TACCTCACCGGGTTGTTCT--------
------
FC88X_00377_00054 -----------------------CGAAACACTCACTCGCTGTTTTTTCTC-----
------
FC88X_00329_01143 -----------------------CGAAACACTCACTCGCTGTTTTTTTCTC----
------
FC88X_00464_01229 ---------------------CCAAAAACACTC-CCCGCTGTTGGTTACT-----
------
FC88X_00758_00653 --------------------CCGGTAGACCCTC-CGCGCTGATCTCTC-------
------
FC88X_00087_00106 --------------------CCGGTAGACCCTC-CGCGCTGATCTCTC-------
------
FC88X_00114_00412 -----------------------CCTTACCCTC-AGTCGCGTCATCTTTCA----
------
FC88X_00288_01050 --------------------CCAGTA--TCCC-CCCAGCCCTGATTTATT-----
------
FC88X_01000_01033 ---------------------CACTG--TCCCTCCCAGCTGATGATTATT-----
------
FC88X_00408_00872 --------------------CCAGT---CCCTTCCCAGCTGTTTTTTCTC-----
------
FC88X_00432_00033 --------------------CCAGTCA-TTCCCAACGGCTGCCCGCCT-------
------
FC88X_01028_00943 --------------------CCAGTCA-TTCCCAACGGCTGCCCGCCT-------
------
FC88X_00871_00913 ---------------------CAGTAA-TCTCCCA-GGCTGCTTTCTAGT-----
------
FC88X_00447_00018 --------------------CCGTTAC-TTAGGCCCCGTCGGCTGCTC-------
------
FC88X_00649_00725 -------------------CCCGGTGC-TTCCCTCCCGTCAGCTGCT--------
------
FC88X_00924_00941 -------------------CCCGGTAG-TTTCCCCGCACCTGCTTTTA-------
------

FC88X_00121_00208 ----------------------CTATACACTGAGATCCCGCC-CGACGCA----
------
FC88X_01111_01130 ---------------------CTAAAACTTTTAGCTCCCGTCGCGTCGC-----
------
FC88X_00444_00084 --------------------TACAAATCCCTTAGTTCCCGATCGCCT-------
------
FC88X_00884_00283 --------------------TACAAATCCCTTAGTTCCCGATCACCT-------
------
FC88X_00559_00206 ----------------------CAAATCAATAAGCTGCCATCCCCGAAT-----
------
FC88X_00768_01099 ------------------CCAATGAAAAACTCCATACGCGCTACTC--------
------
FC88X_00895_00967 ------------------CCAATGAAAA--CTCATACGCGCTACTC--------
------
FC88X_00529_00172 ------------------CCAATGAAAAAACTCCAATACGC-------------
------
FC88X_01073_00448 ------------------CCAATAAAAT--CCAATACGCTATATCCCG------
------
FC88X_00429_00657 --------------------ACGAAATTCCGATTAGCTCCTCCTATT-------
------
FC88X_00896_01018 -------------------TACTAAACTCC--CTAGCGCTTCATTTCA------
------
FC88X_00913_00421 -----------------TCACGCTAACTCCCGGCTATTTTCATTTGCTA-----
------
FC88X_00303_00771 ----------------------CTAACTCCCGGCTATTT-CATTTGCTA-----
------
FC88X_01086_00702 ----------------------CTAACTCCCGGCTATTT-CATTTGCTA-----
------
FC88X_00399_00485 ----------------------CTAACTCCCGGCTATTC--ATTG---------
------
FC88X_00038_00280 --------------CCGACTCGTTAGCTCCCG-CGATTC--ATT----------
------
FC88X_01140_01139 ------------------CCCTTTAACTCCCGTCCACTC--ATT----------
------
FC88X_00095_00461 --------------------ACCTAA-TCCCGCCTACTGGTAATTCTC------
------
FC88X_00618_01227 --------------------ACCTAA-TCCCGCCTACTGGTAATTCTC------
------
FC88X_00135_00617 ---------------------CCTAACTCCCGGCGTCACGAAAATCTC------
------
FC88X_00080_00345 ---------------------ATGTTACCGTAA-CAGCGACTCCATTCT-----
------
FC88X_00149_00612 ---------------------ATGTTACCGTAA-
CAGCGACTCTATTCTCTTCCTTT---
FC88X_01076_01000 ----------------------TCCCGTAATAATCAACGCCTCTATTGTC----
------
FC88X_00075_00114 --------------------CTCCTGACCGTAC-AAACAATGCCTTTT------
------
FC88X_00605_00375 -----------------------CTCAACGTA--AAGCGACGCC----------
------
FC88X_00650_00257 --------------GGGGAAGATCAAGTCT-AAAGTACGCCCCGCTATT-----
------
FC88X_01110_00896 ---------------------ATCAAGTCT-AAAGTACGCCCCGCTATT-----
------
FC88X_00044_00491 ---------------------ATCAAGTCT-AAAGTACGCCCCGCTATT-----
------

FC88X_00445_00007 ---------------------ATCAAGTCT-AAAGTACGCCCCGCTATT-----
------
FC88X_00689_00928 ---------------------ATCAAGTCT-AAAGTACGCCCCGCTATT-----
------
FC88X_00757_01152 ---------------------ATCAAGTCT-AAAGTACGCCCCGCTATT-----
------
FC88X_00421_01188 ---------------------ATCAAGTCT-AAAGTACGCCCCGC---------
------
FC88X_00246_01033 ---------------------ATCAAGTCTCAAAGTACGCCTCGCTATT-----
------
FC88X_00996_01241 -------------------------------
AAAATAGGCCCCGCAAACTCAAACCGA--
FC88X_00248_00392 ----------------------------
CTCAAAGCTGCTCCCTGTTGACATGAC-----
FC88X_01007_01140 ----------------------------
CCCGATGCTATTCACTTTAGAGACAAC-----
FC88X_00344_01086 ----------------------------
TCCAAAGAAACCCCGACTATTCACAAA-----
FC88X_00649_00551 ----------------------------
TCCAAAGAAACCCCGACTATTCACAAA-----
FC88X_00054_00759 ------------------------ACGCAAAACTACCCGCGGCTATTTCTT---
------
FC88X_00032_00750 ------------------------ACGCAAAACTACCCGCGGCTATTTCTT---
------
FC88X_00985_00612 -------------------GGGGTACGCAAAACTACCCGCGGCTATTTCTT---
------
FC88X_00417_00070 --------------------TTGGAGGCATTGCTACCC-CGACTTTTCT-----
------
FC88X_00660_01226 --------------------TTGGAGGCATTGCTACCC-CGACTTTTCT-----
------
FC88X_00602_01263 --------------------TTGGAGGCATTGCTACCC-CGACTTTTCT-----
------
FC88X_00979_00486 --------------------TTGGAGGCATTGCTACCC-CGACTTTTCT-----
------
FC88X_00118_00936 --------------------TCATTTCTATCGACTCCT-CTGCTATTGC-----
------
FC88X_01059_00690 --------------------CTACTCCTATCG--CACC-CGGCTAATGCC----
------
FC88X_00480_01127 -------------------CCGACACATATCG--AACC-CGGCCGTTCC-----
------
FC88X_00593_00979 -------------------CCGACACATATCG--AACC-CGGCCGTTCC-----
------
FC88X_00575_00114 --------------------CTACACAATCGA--CTCC-CGGCTTTTTCAC---
------
FC88X_00155_00853 ------------------TCCTCAATTCTACCACACCCGTCGTGT---------
------
FC88X_00492_00795 -----------------CTCCACAATTTC-TAATGCCCGACCTGTT--------
------
FC88X_00597_00983 ----------------CCCCCACAAAGAC-AACCACCGCC-CTGC---------
------
FC88X_00999_00564 -------------------TCACAAATTCTAACCACCCCTGCTGTT--------
------
FC88X_00166_01002 -------------------CCGCAACTCCTCCCACCCGTGGCTAAT--------
------
FC88X_01087_00865 -------------------CCGCAACTCCTCCCACCCGTGGCTAAT--------
------

```
FC88X_00322_01016      -----------------GTCCCCAAATCTACCCAGC-GCGGCTAAT--------
------
FC88X_00178_01019      ---------------------ACCACTTCCAACGGATCAC-GTATTATT-----
------
FC88X_00684_00375      ---------------------ACCACTTCCAACGGATCAC-GTATTATT-----
------
FC88X_01019_00989      ---------------------ACCACTTCCAACGGATCAC-GTATTATT-----
------
FC88X_00371_01154      ---------------------ACCACTTCCAACGGATCAC-GTATTATT-----
------
FC88X_00504_01067      ---------------------ACCACTTCCAACGGATCAC-GTATTATT-----
------
FC88X_00746_00868      ---------------------ACCACTTCCAACGGATCAC-GTATTATT-----
------
FC88X_00831_01209      ---------------------ACCACTTCCAACGGATCAC-GTATTATT-----
------
FC88X_00979_01172      ---------------------ACCACTTCCAACGGATCAC-GTATTATT-----
------
FC88X_00961_01256      ---------------------ACCACTTCCAACGGATCAC-GTATTATT-----
------
FC88X_01126_00961      ---------------------ACCACTTCCAACGGATCACCGTATTATT-----
------
FC88X_00203_00415      ---------------------ACCACTTCCAACGGACCAC-GTATTATT-----
------
FC88X_00470_00435      ---------------------ACCACTTCCAACGGACCAC-GTATTATT-----
------
FC88X_00456_00147      ----------------------CCACTTCTCACCGCACTG-GTAATATT-----
------
FC88X_00215_00457      ---------------------CCCACTTCTCACCGCACTG-GTAATATT-----
------
FC88X_00226_00143      ---------------------CATAAAGACGAAACCCGTCGCTCTCTT------
------
FC88X_00902_00402      --------------------CCGTCGAAACACGACC-GACGCTGTCCT------
------
FC88X_00231_01033      --------------------TCCGAGCTTATACGAAATCGGCGTCTC-------
------
FC88X_00987_00851      --------------------TCCGAGCTTATACGAAATCGGCGTCTC-------
------
FC88X_01005_00765      --------------------TCCGAGCTTATACGAAATCGGCGTCTC-------
------
FC88X_00268_00988      ---------------------TCGAGCTTATACGAAATCGGCGTCTC-------
------
FC88X_00938_01239      -------------------CTCCTAGCTCA-ACAACACCACCGTCAC-------
------
FC88X_00928_00827      -------------------CTTGCAGAACTAA-GACCGCGGCTTTCC-------
------
FC88X_00233_00957      -------------------CTTGCAGAACTAA-GACCGCGGCTTTCC-------
------
FC88X_01066_00965      -------------------CTTGCAGAACTAA-GACCGCGGCTTTCC-------
------
FC88X_01088_01263      -------------------CTTGCAGAACTAA-GAC-GCGGCTTTC--------
------
FC88X_00912_00210      -------------------CTTGCAGAACTAA-GACCGCAGCTTTCC-------
------
FC88X_00073_00802      -------------------CTTGCAGAACTAA-GACCGCGGCTTCCC-------
------
```

FC88X_00736_01072 -------------------CTTGCAGAACTAA-GACCGCGGCTTCC--------
------
FC88X_00379_00107 -------------------CTTGCAGAACTAA-
GACCGCGGCTTCCCTTCCTTCTCC---
FC88X_00777_00226 --------------------TTGCCTAGCTAACGACCGCAGCCTCCTC------
------
FC88X_00205_00879 --------------------TTATAAACTCCGCTGCTCTAAACTGTT-------
------
FC88X_00209_00024 --------------------TTATAAACTCCGCTGCTCTAAACTGTT-------
------
FC88X_00785_00166 --------------------TTATAAACTCCGCTGCTCTAAACTGTT-------
------
FC88X_00998_00485 --------------------TTATAAACTCCGCTGCTCTAAACTGTT-------
------
FC88X_00924_00389 --------------------TTATAAACTCCGCCGCTCTAAACTGTT-------
------
FC88X_00274_01086 ---------------TTGTCTTCTAATCCCTCCCGCGCTAAA------------
------
FC88X_00013_00370 ---------------CCAACGTCTAACCTGAGCCGCGCTAAA------------
------
FC88X_00686_00669 ---------------CTTGGTTGTAATCCCCGCCGCTGTAGT------------
------
FC88X_00907_01180 ---------------CTTGGTCGTAATCCCCGCCGCTGTAGT------------
------
FC88X_00958_00488 ---------------CCTTTCGTTTAATCACGCCGCTGTATT------------
------
FC88X_00022_00206 ------------------CATAACGTTATTCAGACCCACCCGGTA---------
------
FC88X_00409_01189 ---------------TGATATCACTTTTTCTCAGACCACCCGG-----------
------
FC88X_00974_00649 ------------------TCGCACTGACTCTCAACCAGGCGGATA---------
------
FC88X_00093_00559 -----------------TCCATACCGTCTC-ATACCCTCAATATT---------
------
FC88X_00881_01050 ------------------CCATACCTTATCCAGACCGCCGATATT---------
------
FC88X_00020_00679 --------------CTAATCTCGCGC-TTTCCCACAACCCAC------------
------
FC88X_00059_00086 --------------------TCGCGCATTTTCCACACCGC--------------
------
FC88X_00181_00554 --------------------TCGCGTAATCT-GACCGACACCCTGTCCT-----
------
FC88X_00779_00989 --------------------TCGCGTAATCT-GGCCGACACCCTGTCCT-----
------
FC88X_00422_01089 --------------------TCGCGTCATATCGACAGCCCCCCTGTT-------
------
FC88X_00641_00924 -------------------CCCGTCAACCTTTGACTGCTTGTCCCA--------
------
FC88X_00319_00691 -------------------CCCGTCAACTTTTGACTGCTTGCCCCA--------
------
FC88X_00815_00837 -------------------GCCGTCTAATCCAGACCGCTTGTCCCGT-------
------
FC88X_01102_00862 --------------------CCGTCTAATCCAGACCGCTTGTCCCGT-------
------
FC88X_00828_00449 -------------------TTACCGCCTGTCTCCCCAGCGCATTTT--------
------

FC88X_00257_00916 -------------------TTACCGCCTGTCTCCCCAGCGCATTTT--------
------
FC88X_00022_00260 -------------------CTACCGCCTGTCTCCCCAGCGCATTTT--------
------
FC88X_01140_01242 -------------------ACACCGCTTGCTCCCAGCGACTATTT---------
------
FC88X_00270_00418 -------------------CCACGGGCTCTCCGCAACATCATTTTTCTT-----
------
FC88X_01082_00768 -------------------CCACGGGCTCTCCGCAACATCATTTTT--------
------
FC88X_00536_00513 --------------------CTCCGGCTTTCCCCAAAACACATTTTC-------
------
FC88X_00175_00906 --------------------TTACTTTCTCCACAACAGCATATTCCCT------
------
FC88X_00521_01246 --------------------TTACTTTCTCCACAACAGCATATTCCC-------
------
FC88X_01118_00553 --------------------CTGTCACCACCAGCCATGCATATTCTC-------
------
FC88X_00035_00576 -------------------TCCCTGTTTCTA--CAATCAGCTATTCGC------
------
FC88X_00859_01028 -------------------TCCCTGTTTCTA--CAATCAGCTATTCGCC-----
------
FC88X_00562_00115 -------------------TCCCTGTTTCTA--CAATCAGCTATTCGC------
------
FC88X_01002_00819 -------------------TCCCTGTTTCTA--CAATCAGCTATTCGC------
------
FC88X_01096_01190 -------------------TCCCTGTTTCTA--CAATCAGCTATTCGC------
------
FC88X_00143_00810 -------------------TCCCTGTTTCTAA-CAATCAGCTATTCGCCC----
------
FC88X_00259_00062 -------------------TCCCTGTTTTTCCACAATCAGCTATTCGC------
------
FC88X_00458_00976 -------------------TCACTCTATTTCT-CGATCC-CTATTGGC------
------
FC88X_01093_01203 -------------------TCACTCTATTTCT-CGATCC-CTATTGGC------
------
FC88X_00241_00075 --------------GCTAATTCCTCTTTCCCA--A-CGAGCTATC---------
------
FC88X_01087_01263 ---------------CCAATTCCTCTTTCCCA--A-CGAGCTATC---------
------
FC88X_00387_00722 ----------------TTAATCCTGTCTCCCA--A-CTAGCTATGT--------
------
FC88X_00614_00108 ---------------TTAAGTCCTGTCTCCCA--AGCTAGCTATGTCTTCCCT-
------
FC88X_00550_00321 ------------------ATTATTCCTAACCCACAGCCCGCTATT---------
------
FC88X_00763_01030 ------------------ATTATTCCTAACCCACAGCCCGCTATT---------
------
FC88X_00968_00892 -----------GGGGAATATTATTCCTAACCCACAGCCCGCTATT---------
------
FC88X_00671_01130 ------------------ATTATTCCTAACC-ACAGCCCGCTATTC--------
------
FC88X_00848_00904 ------------------ATTATTCCTAACCCACAGCT-GCTATT---------
------
FC88X_01014_00306 ------------------CTTAACCCTATGCCCCAGCTTTCTAGCCT-------
------

FC88X_01061_00746 -------------------CTAACGCT---CCGCAGCCATCTATCTTAC-----
------
FC88X_00666_00822 ------------------CTTCAAGCTATCCCA--CAGCACTGTTTT-------
------
FC88X_00959_01129 ------------------CTTCAAGCTATCCCA--CAGCACTGTTTT-------
------
FC88X_01133_00625 ------------------CTTCAAGCTATCCCA--CAGCACTGTTTT-------
------
FC88X_00270_00749 ------------------CTTCAAGCTATCCCA--CAGCACTGTTTT-------
------
FC88X_00950_00721 ------------------CTTCAAGCTATCCCA--CAGCACTGTTTT-------
------
FC88X_01139_00751 ------------------CTTCAAGCTATCCCA--CAGCACTGTTTT-------
------
FC88X_01129_00910 ------------------CTTCAAGCTATCCCA--CAGCACTGTTTT-------
------
FC88X_00272_00942 ------------------CTTCAAGCTATCCCA--CAGCACTGTTTTT------
------
FC88X_01063_00837 ------------------CTTCAAGCTATCCCAA-CAGCACTGTTTT-------
------
FC88X_00971_00295 ------------------CTTCAAGCTATACCA--CAGCACTGTTTT-------
------
FC88X_00205_00048 ------------------CTTCAAGCTATCCCA--CAGCACTGGTTTT------
------
FC88X_00081_00903 ------------------CTTCAAGCTATCCCA--CAGCACTGTTCT-------
------
FC88X_00708_00605 -------------------TTCATCCTTCCCCAGCCAGCGCGGTTA--------
------
FC88X_00507_01059 ------------------CTATGCGATCACCAAGCCAGCCCGTT----------
------
FC88X_00112_00585 -------------------CCTGTGAGTTCTCCGCCAGCCCGTTTAT-------
------
FC88X_00692_00476 ----------------------CCGTGATCTCAACCAGACTCACCCTGCTCC--
------
FC88X_00762_00230 ----------------------CCGTGATCTCAACCAGACTCACCCTGCT----
------
FC88X_00843_00882 ----------------------ATGCGATCTC--CCAGCCTCA-----------
------
FC88X_00164_00591 -------------------CCACTATTTTCCATAACCCGATTGCT---------
------
FC88X_00782_00808 -----------------TTCCTCCCTTTTCCATCACCTCATCGTG---------
------
FC88X_00529_00460 -----------------CTTTCCCTTTTTCGCAACCATCGCTGTT---------
------
FC88X_00641_00237 -----------------CTTTCCCTTTTTCGCAACCATCGCTGTT---------
------
FC88X_00023_00619 ----------------CTTTCCCTTTTTTCGCAACCATCGCTGTT---------
------
FC88X_00126_00200 ---------------CTTTCCCTTTTCTTCACAACCATCGCTGTT---------
------
FC88X_00169_00849 ----------------TGCCTTCCTATTTCCACACCAT-GCTGTT---------
------
FC88X_00869_00928 -----------------CCATCCCTTATCCACCACCAC-GCTGCC---------
------
FC88X_00875_00760 ------------------TCTTCCTTCTTCCGTACCGCCATGGT----------
------

```
FC88X_01037_00504      ------------------ACTTCCCTTCTCCG--CCGCCACGGTGTT-------
------
FC88X_00667_01214      ------------------ACCCCCTTTCCCTTACACCCGGCTGCAG--------
------
FC88X_00899_00643      ----------------CTGGGCGCTTTCCCC--CAACCGGCTTCT---------
------
FC88X_00148_00573      --------------------CCAGTTTCTGGC-CATCCCGACTTGTAT------
------
FC88X_01083_00418      -------------TTCCCATCCAGTTTCGGCTACAACCCGCCTC----------
------
FC88X_00665_00111      ------------------TCCACCCTTTTTCCGC-ACCTGCCACCCGC------
------
FC88X_00830_00518      -----------------GTGTGCCTTTTTGCAGCCACCCGCCT-----------
------
FC88X_00367_00489      -----------------CTCCCACGTCTCTCTCCACGA-CGGAATT--------
------
FC88X_01061_00541      -----------------CTCCCACGTCTCTCTCCACGA-CGGAATT--------
------
FC88X_00022_00613      -----------------CTCCCACGTCTCTCTCCACGA-CGGAATT--------
------
FC88X_00493_00996      -----------------CTCCCACGTCTCTCTCCACGA-CGGAATT--------
------
FC88X_00975_00503      -----------------CTCCCACGTCTCTCTCCACGA-CGGAATT--------
------
FC88X_00566_01234      ------------------CCCAGCGGCACTCTCGAATATCGGCATT--------
------
FC88X_00078_00174      -----------------CTATCCCATCTCTGC-CACCAAGCTTAT---------
------
FC88X_00205_00130      -----------------CTATCCCATCTCTGC-CACCAAGCTTAT---------
------
FC88X_00868_01030      ------------------TTTCCCACCGTTCCTCTCCAACATTAT---------
------
FC88X_00956_01256      -----------------ATCCACTCTCGCCCAGTGCCACTCTAGT---------
------
FC88X_01099_00865      -----------------ATCCACTCTCGCCCAGTGCCACTCTAGT---------
------
FC88X_00474_01202      -----------------ATTCCACATCTCCCAACACCACGCTAGT---------
------
FC88X_00723_01156      ------------------CCCGCAATCGGCCTTCTCCACAATAGT---------
------
FC88X_00054_00331      -----------------TCTCCCAGTCTGGATTCTC-ACATTTCC---------
------
FC88X_00768_00245      -----------------TCTCCCAGTCTGGATTCTC-ACATTTCC---------
------
FC88X_00961_00386      -------------------TCCCCGACTAGCTTCTCCAGATTTCT---------
------
FC88X_00995_01027      -----------------CCGCCCCCTCTACGCATTCCAGATGTC----------
------
FC88X_00482_01179      -------------------TCCTTGATTCTGTACAGCATGACTGCT--------
------
FC88X_00534_00135      -------------------TCCTTGACTCTGTACAGCATGACTGCT--------
------
FC88X_00929_00566      ---------------------TTTGTCTCCGTCTCGCATCACACCTTG------
------
FC88X_00066_00758      -------------------TCCCCGACTCCGATCGCAATGATATGTT-------
------
```

FC88X_00365_00870 -----------------ACACTCCGTTTTCGATCGCAA-GATACA---------
------
FC88X_00731_01212 -----------------ACTCCCCGTCTCCTACCACATGATCAC----------
------
FC88X_00219_00344 ------------CTGCAACTCCCGACT--CGACACCAACCA-------------
------
FC88X_00665_00363 ------------CTGCAACTCCCGACT--CGACACCAACCA-------------
------
FC88X_00846_01092 ------------CTGCAACTCCCGACT--CGACACCAACCA-------------
------
FC88X_00819_00805 ---------------TAACTCCCTGTCTGCGATACTAACGAC------------
------
FC88X_01080_01140 ---------------TAACTCCCTGTCTGCGATACTAACGAC------------
------
FC88X_00994_01067 ----------------TCCTACTCTCCT-CCACATCAACGCTG-----------
------
FC88X_01005_00777 -----------------ACTCTCCGTCT-TCATCCCAACACTGTC---------
------
FC88X_00339_00546 ------------------TCTCCCAGTCATGATCGCCACCGCTAT---------
------
FC88X_00502_00067 ------------------TCTCCCAGTCATGATCGCCACCGCTAT---------
------
FC88X_00137_00124 -----------------ATCTCCCACGAATTCTCCGCACCGCTT----------
------
FC88X_00911_00363 -------------------CTCCCAGAAATACGCAGTGCGACTCAT--------
------
FC88X_00417_01104 -------------------CTCCCAGAAATACGCAGTGCGACTCAT--------
------
FC88X_00429_01193 -------------------CTCCCAGAAATACGCAGTGCGACTCAT--------
------
FC88X_00928_01244 -------------------CTCCAAGTAATTC-CATCGCGACTCATT-------
------
FC88X_00850_00993 ------------------ACTCCCACCAGCATGAACTGCGAATCC---------
------
FC88X_00759_00432 ------------------ACTCCCACCAGCATGAACTGCGAATCC---------
------
FC88X_00887_00808 ------------------ACTCCCACCAGCATGAACTGCGAATCC---------
------
FC88X_00988_00327 ------------------ACTCCCACCAGCATGAACTGCGAATCC---------
------
FC88X_00604_01093 ------------------ACTCCCACCAGCATGAACTGCGGATCC---------
------
FC88X_00568_00042 ------------CCGTCAGCTCCCAC--GCATCAA-TGCGAA------------
------
FC88X_00829_00914 ------------------TTTCCTAC--G-ATCAA-TCCG--------------
------
FC88X_00105_00149 -------------------CCCGCAAT-TC-TCCAACA-CACTCGTCCT-----
------
FC88X_00460_01173 ------------------TCCTGCACTCTC-TCCAACATCCCTCGT--------
------
FC88X_00834_00844 ------------------TCACGCGTCATCCTCCTGCACCTCTTCC--------
------
FC88X_00785_01178 ------------------CTCCACAC-TCTCCATCACGC-ACATGTA-------
------
FC88X_01078_01107 -------------------CCCACACGTCTCCAGCATGCCACCTGTT-------
------

FC88X_00828_00319 --------------------CTGCACGCTC--TACAGTTCTCCAATTAT-----
------
FC88X_01094_00835 --------------------CTGCACGCTCACCACACTTCACCTGTT-------
------
FC88X_00889_01232 -------------------CCTGCATCTCCCCTATTCTTCACCTCGCC------
------
FC88X_00944_01124 --------------------CTGCAGCCTCCCCAACTCGCAACC----------
------
FC88X_00148_00877 ------------------CCGCGCGCACATCTCCTCCAACATAAA---------
------
FC88X_00037_00608 -----------------CTACCGCACCTCTCGCCTCCGACATAAC---------
------
FC88X_00987_01197 ------------------CCCCACGCCATTCACCTCCAATATGTT---------
------
FC88X_00750_00873 -------------------GCTCCCCAATTATCGTCGCACATGTCAT-------
------
FC88X_00159_00598 -----------------CTGCTCGCCC-TTCTCCGCGTACAAGTC---------
------
FC88X_00498_00205 --------------------TTCGCCACTTATCCACCAACAT------------
------
FC88X_00580_00072 --------------------CCGCTAACTCCTAAACCCCTACGTCAC-------
------
FC88X_00204_00880 ------------------TCCCACTAAATCCCT--CCTCGACGTCAC-------
------
FC88X_00988_00692 -------------------TTTACAACT--CCCATCCTCAACATCAT-------
------
FC88X_00992_01023 -------------------TTTACAACTAACCCATCCTCAACATCAT-------
------
FC88X_01127_00710 -----------------CGCATCTTTCTCCTACAACACCTATCC----------
------
FC88X_00687_00209 -----------------CGCGCCTTTCTCCTACAACATCTATCC----------
------
FC88X_00058_00326 ------------------CCTCCTATCTCCTG-CATCTCTCCTCGA--------
------
FC88X_00158_00790 ------------------GCTCCCAACTCGTAACATACCTGTTCGA--------
------
FC88X_00126_00698 ------------------TCCCAACTTCTCCACAACG-CCACCTCGC-------
------
FC88X_00487_01245 ------------------TCCCAACTTCTCCACAACG-C-ACCTCGC-------
------
FC88X_00135_00657 --------------------CCAACCTCTCCGCGGCGTCCACCTTGTC------
------
FC88X_00834_01007 -----------------TTCCCAACTCCTCTATG---TCCACGTATC-------
------
FC88X_00906_01018 -----------------TTCCCAACTCCTCTACG---TCCACGTGTC-------
------
FC88X_00620_00120 --------------------CCAAAGTCTCTATGGCCTCCACGTTTA-------
------
FC88X_00159_01010 ------------------CTCCCTGTTATGCCAAGTCCTCGGTTT---------
------
FC88X_00859_01114 ------------------CCCCAGTCTCTGCAAAGTCCACTGTTA---------
------
FC88X_01133_01097 -----------------ACTCCCAATTCTCCACAGTACCCCAGC----------
------
FC88X_00224_00750 --------------------CCATTCCCTGCTCACCGGCATCCTCTT-------
------

```
FC88X_00774_00977      --------------------CCATAACCCG-TCAGCGTCATCGTAAC-------
------
FC88X_00004_00190      -----------------CTCCCATGACTCCGAGATCACACTCAT----------
------
FC88X_01080_00638      -------------------CTCCCGACTC-GACAGTATCCTCATTTG-------
------
FC88X_00684_00461      ---------------------CCCTACCCTACAAC-GTG-TCAAAGTTCA----
------
FC88X_00761_01023      -------------------CTCCCCACACGTCAACAGTC-TCATTGT-------
------
FC88X_00801_00136      -------------------CTCCCAACTTGTTCATGATCAGCATTGT-------
------
FC88X_01107_00957      --------------------TTAAAACTTGTTCATGATCAGCATCGC-------
------
FC88X_00087_00231      -------------------CTCCCAACATGTTT-TCAACATCATCGTA------
------
FC88X_00404_00462      ------------------ACTCCCTATATCC--AAATGCGACTTACG-------
------
FC88X_01027_00716      ----------------CCAATCCCTATGTTC--A--TGCAACTTACT-------
------
FC88X_00702_00222      ------------------TCTCCCTAGGTCT-ATAGCGCAATTTCC--------
------
FC88X_00873_01183      ------------------TCTCCCTAGGTCT-ATAGCGCAATTTCT--------
------
FC88X_01025_00852      ------------------TCTCCCTAGGTCTGATAGCGCAATTTCTCTTCC---
------
FC88X_00077_00031      ------------------TCTCCCTAGGTCT-ACAGCGAATTTCC---------
------
FC88X_00780_00269      -----------------GTCTCCCTAGGTCT-CTAGTGCAATTTCCTCTTC---
------
FC88X_00802_01226      ----------------TTTTCCAACATGTC----ATCCCAATTTTCT-------
------
FC88X_00039_00105      ------------------TCACCATATGTCC-GAAACCCACTTTGCT-------
------
FC88X_00233_01047      -------------CATCATCCCAAAATGTTC---ATTCCACTT-----------
------
FC88X_01129_00565      -------------------TCCCAATC-CCTATGAAACCATCGTTAT-------
------
FC88X_01115_00973      -------------------TCCCAATC-CCTATGAAACCATCATTAT-------
------
FC88X_00837_00297      ------------------CTCCCTATT-AGTAT-ACACCAACCTTAT-------
------
FC88X_00048_00097      -----------------ACTCCCGATGACCTAATCCAACAACAC----------
------
FC88X_00894_00433      -------------------CCCCGATT-CCTATAACAACAACCCAAC-------
------
FC88X_00225_00893      ------------------CTCCCATTAGTCAGC--ATCCAACGTATC-------
------
FC88X_01086_00426      ------------------CTCCCAATTGACACG--TTCCAACCTAAC-------
------
FC88X_00242_01016      --------------ATCACTCCCATTTACCA-----TCAAACATAC--------
------
FC88X_00023_00254      -------------------CTCCCTAATCTAAGGTCGTTGCTATCT--------
------
FC88X_00766_01254      -------------------CCGATAAATCTCTGG-CGTGGCTATCTA-------
------
```

FC88X_00668_00704 -------------------TCCCCAAATCCCTCG-AGCCGCTATCGC-------
------
FC88X_00993_00893 -------------------TTCCCGAATCCCAAG-ACTCGCTATCTC-------
------
FC88X_00349_00573 -------------------CTCCCGACTCTCACCAGGCCAGGTGCC--------
------
FC88X_00810_00183 -------------------TCCCCGACGATCCTGAGGCCAGCAGCC--------
------
FC88X_01072_00679 -------------------CTCCCAACC-TCGCTACGAGATGTACAC-------
------
FC88X_00754_00155 -------------------CTCCCAATAATCGTAAGCCGATGAACA--------
------
FC88X_00552_01260 -------------------TCTCCCAAACTGACGCGTAAGCTAGAA--------
------
FC88X_00667_00910 -------------------TCTCCCAAACTGACGCGTAAGCTAGAA--------
------
FC88X_00056_00206 -------------------CTCCCAAAAGTGGCTCGCGCTCTAGAAC-------
------
FC88X_00276_00922 ------------------GTTCCCAATGATCCTAACCGGACTAGTT--------
------
FC88X_00634_00092 -------------------CTCCCCATAGTCA-ATCGTGACTAGATAT------
------
FC88X_00098_00422 -------------------CTCCCAGCTGACTTGCGCGCGCAGTTTC-------
------
FC88X_00237_00118 -------------------CTCCCAGCTGACTTGCGCGCGCAG-----------
------
FC88X_00726_01059 -------------------CTCCCAGCTGACTTGCGCGCGCAGTTTC-------
------
FC88X_00763_00965 -------------------CTCCCAGCTGACTTGCGCGCGCAGTTTC-------
------
FC88X_00361_01158 -------------------CTCCCAGCTGACTTGCGCGCGCAGTTTC-------
------
FC88X_00888_00333 ------------------GCTCCCAGCTGACTTGCGCGCGCAGTTTC-------
------
FC88X_00049_00326 -------------------CTCCCAGCTGACTTGCGCGCGCAGTTTTC------
------
FC88X_00734_00255 ------------------TCTCCCAA-TCACCTGCGCGCGCATGAT--------
------
FC88X_00057_00722 -----------------GTCTCCCAA-TCACCTGCGCGCGCATGAT--------
------
FC88X_00805_00449 ------------------TCTCCCAA-TCACCTGCGCGCGCATGAT--------
------
FC88X_00786_01149 ------------------TCTCCCAA-TCACCTACGCGCGCATGAT--------
------
FC88X_00633_01121 -----------------TTCTCCCAGTCGTATCTTGCGCGTGCC----------
------
FC88X_01054_00974 -----------------TTCTCCCAGTCGTATCTTGCGCGTGCC----------
------
FC88X_00341_01100 -----------------TTCTCCCAGTCGTATCTTGCGCGTGCG----------
------
FC88X_00993_00882 ------------------TCTCCC-GTTGCACCGCGCGTGCGTTTC--------
------
FC88X_00574_01166 -------------------CTCCCAATTGGATGACTCGCGCGTGCC--------
------
FC88X_00971_00688 ------------------GCTCCCAATTGGATGACTCGCGCGTGCC--------
------

```
FC88X_00807_00789       -------------------CTCCCAATTGGATGACTCGCGCGTGCC--------
------
FC88X_00973_00373       -------------------CTCCCAATTGGATGACTCGCGCGTGCC--------
------
FC88X_00013_00283       -------------------CTCCCAATTGGATGACTCGCGCGTGCC--------
------
FC88X_00046_00465       -------------------CTCCCAATTGGATGACTCGCGCGTGCC--------
------
FC88X_00073_00188       -------------------CTCCCAATTGGATGACTCGCGCGTGCC--------
------
FC88X_00091_00176       -------------------CTCCCAATTGGATGACTCGCGCGTGCC--------
------
FC88X_00051_00237       -------------------CTCCCAATTGGATGACTCGCGCGTGCC--------
------
FC88X_00226_00973       -------------------CTCCCAATTGGATGACTCGCGCGTGCC--------
------
FC88X_00411_01130       -------------------CTCCCAATTGGATGACTCGCGCGTGCC--------
------
FC88X_00616_01194       -------------------CTCCCAATTGGATGACTCGCGCGTGCC--------
------
FC88X_00796_00364       -------------------CTCCCAATTGGATGACTCGCGCGTGCC--------
------
FC88X_00928_00576       -------------------CTCCCAATTGGATGACTCGCGCGTGCC--------
------
FC88X_00120_00150       ------------------CTTCCCTAT--GATGACTCGCGCGTAC---------
------
FC88X_00509_01141       --------------------CTCCCGTTGACCGACTCCTGCGCACA--------
------
FC88X_00549_01246       --------------GCCGTGGTCCCCTTTCCCGGCATACCTGTC----------
------
FC88X_00829_00911       -----------------CCGTCACATTACTCCCGCGGACCTGCC----------
------
FC88X_00219_00735       -----------------TCGTCCCATTCTTGCCATGCGTCTATC----------
------
FC88X_01043_00788       ---------------AGTCGTGACCTTCC--CCACGCAGCTATC----------
------
FC88X_00120_00576       --------------------TTCCGATGAACTCG-CGGCACATATCTC------
------
FC88X_00141_00105       -------------------GTCCGCATGAGCTAGTCGGTAACTTTCTC------
------
FC88X_00534_00998       ------------------CTCCCAAATGAGTCTA-CGGTCACTCAC--------
------
FC88X_01095_00500       -------------------CCCCCGATGATATCC-CGGCACATCACC-------
------
FC88X_00484_00977       ---------------CTCCCTGACGACTAACTCGATGACATT------------
------
FC88X_00838_01216       ---------------CTCCCTGACGACTAACTCGATGACATT------------
------
FC88X_00839_00445       ---------------CTCCCTGACGACTAACTCGATGACATT------------
------
FC88X_00005_00268       ---------------CTCCCTGACGACTAACCCGATGACATT------------
------
FC88X_01004_00637       -----------------CCCAGACGA--GACACGATGC----------------
------
FC88X_00898_01255       -------------CTTCCCTCGACGAC--ACCCAATGAAC--------------
------
```

```
FC88X_00285_00068      ----------------------TTAGCTAACACGACGATACTCATTCTC-----
------
FC88X_00404_00086      ----------------------TTAGCTAACACGACGATACTCATTCTC-----
------
FC88X_00584_00249      ----------------------TTAGCTAACACGACGATACTCATTCTC-----
------
FC88X_00610_00074      ----------------------TTAGCTAACACGACGATACTCATTCTC-----
------
FC88X_00653_00811      ----------------------TTAGCTAACACGACGATACTCATTCTC-----
------
FC88X_00750_00065      ----------------------TTAGCTAACACGACGATACTCATTCTC-----
------
FC88X_00769_00209      ----------------------TTAGCTAACACGACGATACTCATTCTC-----
------
FC88X_01123_00933      ----------------------TTAGCTAACACGACGATACTCATTCTC-----
------
FC88X_00406_00069      ----------------------TTAGCTAACACGACGATATTCATTCTC-----
------
FC88X_00582_00205      ----------------------TTAGCGAACACGACGATACTCATTCTC-----
------
FC88X_00956_00469      --------------GGGGAAGTTAGACTAACACGACGATACTCA----------
------
FC88X_01044_00871      -------------------------TCTACCAGGCTA-ACACACTCCCTTGCT-
------
FC88X_00644_00943      --------------------------
CTAACACGCTATACAATCTCCACTACTCC-----
FC88X_00287_00945      --------------------CCGTGTTTACCACGTTATCTATGCTCT-------
------
FC88X_00613_00284      ----------------GACACCGTATCTCCTACGTTAGC-ATGATC--------
------
FC88X_00636_00312      ------------------GACGTGGGTGAGCACATTAGTCATGCT---------
------
FC88X_00978_01254      -------------------------GTACGCACACTAGAAAAGCTCCATCAA--
------
FC88X_00869_00582      -----------------------------ACACAAAACGCTATTCTATCAT---
------
FC88X_01067_00475      ------------------------CCTAAGCACATAAGCTATCTCCAACATC--
------
FC88X_00013_00060      -----------GTGAAGCCA--TCAGA--TGT-CGAGTC--GACTAG-------
------
FC88X_00427_01006      -----------GTGAAGCCA--TCAGA--TGT-CGAGTC--GACTAG-------
------
FC88X_00274_00987      ----------TGTGAAGCCA--TCAGA--TGT-CGAGTC--GACTAG-------
------
FC88X_00903_01096      --------------AAGCCA--TCAGA--TGT-CGAGTC--GACTA--------
------
FC88X_00378_01140      --------------AAGCCAGCTCAGA--TGT-CGAGTC--GACTAG-------
------
FC88X_00858_00898      --------------AAGCCA--TCAGA--TGT-CGAGTC--GACTAG-------
------
FC88X_00685_01219      ---------------AGCCA--TCAGA--TGT-CGAGTC--GACTAG-------
------
FC88X_00142_00785      -------------GAAGCCA--TCAGA--TGT-CGAGT---GACTAG-------
------
FC88X_00846_00734      ------------GGAAGCCA--TCAGA-CTGT-CGAGT---GACTAG-------
------
```

```
FC88X_00018_00626       ---------------AGCCA--TCAGA--TGT-CGAGT---GACTAG-------
------
FC88X_00079_00324       ------GGTCTGTGAAGCCAGCTCAGA--TGT-CGAGT---GACTAG-------
------
FC88X_00953_01115       ---------CTGTGAAGCCA--TCAGA--TGT-CGAGT---GACTAG-------
------
FC88X_01117_00924       ---------------AGCCA--TCAGA--TGT-CGAGT---GACTAG-------
------
FC88X_00392_00943       ---------------------ATCAGA--TGT-CGAGT---GACTAG-------
------
FC88X_00081_00792       ------------------CGCATCAGA--TGT-CGAGTC--GACTAG-------
------
FC88X_00695_00103       --------------------CATCAGA--TGT-CGAGTC--GACTAG-------
------
FC88X_00880_00982       --------------------CATCAGA--TGT-CGAGTC--GACTAG-------
------
FC88X_00987_00600       --------------------CATCAGA--TGT-CGAGTC--GACTAG-------
------
FC88X_00838_00731       ---------------------ATCAGA--TGT-CGAGTC--GACTAG-------
------
FC88X_00633_00495       -----------------GCCACTCAGA--TGT-CGAGTC--GACTAG-------
------
FC88X_00727_00104       -----------------GCCA-TCAGA--TGT-CGAGTC--GACTAG-------
------
FC88X_00742_00276       -----------------GCCA-TCAGA--TGT-CGAGTC--GACTAG-------
------
FC88X_00884_01011       -----------------GCCA-TCAGA--TGT-CGAGTC--GACTAG-------
------
FC88X_00291_01033       ---------------------ATCAGA--TGT-CGAGTC--GACTAG-------
------
FC88X_00812_01272       ---------------------ATCAGA--TGT-CGAGTC--GACTAG-------
------
FC88X_00895_00600       ---------------------ATCAGA--TGT-CGAGTC--GACTAG-------
------
FC88X_00708_00139       ---------------------ATCAGA--TGT-CGAGTC--GACTAGC------
------
FC88X_00924_00755       ---------------------ATCAGA--TGT-CGAGTC--GACTAG-------
------
FC88X_00371_00008       ----------------------TCAGA--TGT-CGAGTC--GACTAG-------
------
FC88X_00602_01264       ----------------------TCAGA--TGT-CGAGTC--GACTAG-------
------
FC88X_00327_00121       ----------------------TCAGA--TGTACGAGTC--GACTAG-------
------
FC88X_01133_00638       ---------------------CTCAGA--TGTACGAGTC--GACTAG-------
------
FC88X_00342_00380       ----------------------TCAGA--TGT-CGAGTC--GACTAG-------
------
FC88X_00460_01198       ----------------------TCAGA--TGT-CGAGTC--GACTAG-------
------
FC88X_00718_00300       ----------------------TCAGA--TGT-CGAGTC--GACTAG-------
------
FC88X_00999_00308       ----------------------TCAGA--TGT-CGAGTC--GACTAG-------
------
FC88X_01067_00732       ----------------------TCAGA--TGT-CGAGTC--GACTAG-------
------
```

```
FC88X_01097_00799      ----------------------TCAGA--TGT-CGAGTC--GACTAG-------
------
FC88X_01115_00788      ----------------------TCAGA--TGT-CGAGTC--GACTAG-------
------
FC88X_01123_00804      ----------------------TCAGA--TGT-CGAGTC--GACTAG-------
------
FC88X_00819_00224      ----------------------TCAGA--TGTATGAGTC--GACTAG-------
------
FC88X_00823_00265      ------------TGAAGCCAGCTCAGA-CTGT-CGAGT---GACTAG-------
------
FC88X_00865_00299      -------------GAAGCCAGCTCAGA--TGT-CGAGT---GACTAG-------
------
FC88X_00051_00114      ---------------AGCCAGCTCAGA--TGT-CGAGT---GACTAG-------
------
FC88X_00026_00199      -----------------CCAGCTCAGA--TGT-CGAGT---GACTAG-------
------
FC88X_00988_00748      -----------------CCAGCTCAGA--TGT-CGAGT---GACTAG-------
------
FC88X_00103_00303      --------------ACGCCAGCTCAGA--TGT-CGAGT---GACTAG-------
------
FC88X_00136_00751      -----------------CCAGCTCAGA--TGT-CGAGT---GACTAG-------
------
FC88X_00084_00080      ------------------CAGCTCAGA--TGT-CGAGT---GACTAG-------
------
FC88X_00852_00292      ------------------CAGCTCAGA--TGT-CGAGT---GACTAG-------
------
FC88X_00086_00466      ------------------CAGCTCAGA--TGT-CGAGT---GACTAG-------
------
FC88X_00591_00354      -----------------CCAGCTCAGA--TGT-CGAGT---GACTAGCC-----
------
FC88X_00050_00654      ------------------CAGCTCAGA--TGT-CGAGT---GACTAG-------
------
FC88X_00895_00832      ------------------CAGCTCAGA-CTGT-CGAGT---GACTAG-------
------
FC88X_00370_01155      -------------------AGCTCAGA--TGT-CGAGT---GACTAG-------
------
FC88X_00184_00813      -----------------CCAGCTCAGA--TGT-CGAGTC--GACTAG-------
------
FC88X_01095_00985      -----------------CCAGCTCAGA--TGT-CGAGTC--GACTAG-------
------
FC88X_00986_01215      -------------------AGCTCAGA--TGT-CGAGTC--GACTAG-------
------
FC88X_00108_00224      -------------------AGCTCAGA--TGT-CGAGT---GACTAG-------
------
FC88X_00433_00121      -------------------AGCTCAGA--TGT-CGAGT---GACTAG-------
------
FC88X_00035_00569      --------------------GCTCAGA--TGT-CGAGT---GACTAG-------
------
FC88X_00900_00418      --------------------GCTCAGA--TGT-CGAGT---GACTAG-------
------
FC88X_00099_00125      --------------------GCTCAGA-CTGT-CGAGT---GACTAG-------
------
FC88X_00892_00670      -------------------AGCTCAGA-CTGT-CGAGT---GACTAG-------
------
FC88X_00453_01221      --------------------GCTCAGA--TGT-CGAGT---GACTAG-------
------
```

FC88X_00923_00549 --------------------GCTCAGA--TGT-CGAGT---GACTAG-------
------
FC88X_00975_00666 --------------------GCTCAGA--TGT-CGAGT---GACTAG-------
------
FC88X_00061_00507 ---------------------CTCAGA--TGT-CGAGT---GACTAG-------
------
FC88X_00401_00112 ---------------------CTCAGA--TGT-CGAGT---GACTAG-------
------
FC88X_00100_00377 -------------------CACTCAGA-TGT--CGAGTC--GACTAG-------
------
FC88X_00330_00953 -----------TGGAAGTCCACTCAGA-TGTA-CGAGTC--GACTAG-------
------
FC88X_00279_01033 -------------------CACTCAGA-TGT--CGAGT---GACTAG-------
------
FC88X_01038_00501 -------------------CACTCAGA-TGT--CGAGT---GACTAG-------
------
FC88X_00469_00091 -------------------CACTCAGA--TGT-CGAGT---GACTAG-------
------
FC88X_00515_00849 ------------------CCACTCAGA-CTGT-CGAGT---GACTAG-------
------
FC88X_00013_00254 --------------------ACTCAGA-TGT--CGAGT---GACTAG-------
------
FC88X_00395_01209 --------------------ACTCAGA-TGT--CGAGT---GACTAG-------
------
FC88X_00029_00259 ---------------------CTCAGA-TGT--CGAGT---GACTAG-------
------
FC88X_00164_00145 ---------------------CTCAGA-TGT--CGAGT---GACTAG-------
------
FC88X_00591_00766 ---------------------CTCAGA--TGT-CGAGT---GACTAG-------
------
FC88X_00611_00640 ---------------------CTCAGA--TGT-CGAGT---GACTAG-------
------
FC88X_00674_01226 ---------------------CTCAGA--TGT-CGAGT---GACTAG-------
------
FC88X_00816_00854 ---------------------CTCAGA--TGT-CGAGT---GACTAG-------
------
FC88X_00888_00440 ---------------------CTCAGA--TGT-CGAGT---GACTAG-------
------
FC88X_00914_00360 ---------------------CTCAGA--TGT-CGAGT---GACTAG-------
------
FC88X_00936_00845 ---------------------CTCAGA--TGT-CGAGT---GACTAG-------
------
FC88X_01077_00640 ---------------------CTCAGA--TGT-CGAGT---GACTAG-------
------
FC88X_00763_00863 ----------------------TCAGA-TCGT-CGAGT---GACTAG-------
------
FC88X_00982_01249 ----------------------TCAGA--TGT-CGAGT---GACTAGCC-----
------
FC88X_01123_00940 ---------------------TCCAGA--TGT-CGAGT---GACTAG-------
------
FC88X_00482_01167 -------------------CAGTCAGA-CTGT-CGAGT---GACTAG-------
------
FC88X_00529_00114 -------------------CAGTCAGA-CTGT-CGAGT---GACTAGC------
------
FC88X_00529_00189 ---------------------GTCAGA-CTGT-CGAGT---GACTAG-------
------

FC88X_00030_00295 ----------------------TCAGA-CTGT-CGAGT---GACTAG-------
------
FC88X_00024_00359 ----------------------TCAGA-CTGT-CGAGT---GACTAG-------
------
FC88X_00137_00854 ----------------------TCAGA-CTGT-CGAGT---GACTAG-------
------
FC88X_00046_00390 -----------------AGCCATCAGA-CTGT-CGAGT---GACTAG-------
------
FC88X_00024_00525 -----------------AGCCATCAGA-CTGT-CGAGT---GACTAG-------
------
FC88X_00380_00748 -----------------AGCCATCAGA-CTGT-CGAGT---GACTAG-------
------
FC88X_00897_00913 -----------------AGCCATCAGA-CTGT-CGAGT---GACTAG-------
------
FC88X_00987_00921 -----------------AGCCATCAGA-CTGT-CGAGT---GACTAG-------
------
FC88X_00952_00377 ------------------GCCATCAGA-CTGT-CGAGT---GACTAG-------
------
FC88X_01001_00415 ------------------GCCATCAGA-CTGT-CGAGT---GACTAG-------
------
FC88X_01073_01200 ------GTACTAGTGAACGCCATCAGA-CTGT-CGAGT---GACTAG-------
------
FC88X_00451_01037 -------------------CCATCAGA-CTGT-CGAGT---GACTAG-------
------
FC88X_00827_00810 -------------------CCATCAGA-CTGT-CGAGT---GACTAG-------
------
FC88X_00889_00852 -------------------CCATCAGA-CTGT-CGAGT---GACTAG-------
------
FC88X_00019_00520 -------------------CCATCAGA-CTGT-CGAGT---GACTAG-------
------
FC88X_01116_00763 -----------------GTCCATCAGA-CTGT-CGAGT---GACTAG-------
------
FC88X_00661_00907 -------------------CCATCAGA-CTGT-CGAGT---GACTAG-------
------
FC88X_00860_00896 -------------------CCATCAGA-CTGT-CGAGT---GACTAG-------
------
FC88X_00985_00742 -------------------CCATCAGA-CTGT-CGAGT---GACTAG-------
------
FC88X_01074_00850 -------------------CCATCAGA-CTGT-CGAGT---GACTAG-------
------
FC88X_00425_00028 --------------------CATCAGA-CTGT-CGAGT---GACTAG-------
------
FC88X_00715_01256 --------------------CATCAGA-CTGT-CGAGT---GACTAG-------
------
FC88X_00786_00198 --------------------CATCAGA-CTGT-CGAGT---GACTAG-------
------
FC88X_00778_01015 --------------------CATCAGA-CTGT-CGAGT---GACTAG-------
------
FC88X_00094_00382 ---------------------ATCAGA-CTGT-CGAGT---GACTAG-------
------
FC88X_00217_00748 ---------------------ATCAGA-CTGT-CGAGT---GACTAG-------
------
FC88X_00756_00299 ---------------------ATCAGA-CTGT-CGAGT---GACTAG-------
------
FC88X_00818_00949 ---------------------ATCAGA-CTGT-CGAGT---GACTAG-------
------

FC88X_00904_00469 ---------------------ATCAGA-CTGT-CGAGT---GACTAG-------
------
FC88X_00152_00607 ---------------------ATCAGA-CTGT-CGAGT---GACTAGC------
------
FC88X_00405_00021 ---------------------ATCAGA-CTGT-CGAGT---GACTAG-------
------
FC88X_00834_00143 ---------------------ATCAGA-CTGT-CGAGT---GACTAG-------
------
FC88X_00895_01208 ---------------------ATCAGA-CTGT-CGAGT---GACTAG-------
------
FC88X_00936_00493 ---------------------ATCAGA-CTGT-CGAGT---GACTAG-------
------
FC88X_01024_00909 ---------------------ATCAGA-CTGT-CGAGT---GACTAG-------
------
FC88X_01052_00704 ---------------------ATCAGA-CTGT-CGAGT---GACTAG-------
------
FC88X_01075_01091 ---------------------ATCAGA-CTGT-CGAGT---GACTAG-------
------
FC88X_00046_00391 ----------------------TCAGA-CTGT-CGAGT---GACTAG-------
------
FC88X_00503_01034 ----------------------TCAGA-CTGT-CGAGT---GACTAG-------
------
FC88X_00621_00145 ----------------------TCAGA-CTGT-CGAGT---GACTAG-------
------
FC88X_00725_00705 ----------------------TCAGA-CTGT-CGAGT---GACTAG-------
------
FC88X_00768_00273 ----------------------TCAGA-CTGT-CGAGT---GACTAG-------
------
FC88X_00773_01273 ----------------------TCAGA-CTGT-CGAGT---GACTAG-------
------
FC88X_00810_00890 ----------------------TCAGA-CTGT-CGAGT---GACTAG-------
------
FC88X_00904_00352 ----------------------TCAGA-CTGT-CGAGT---GACTAG-------
------
FC88X_01057_00735 ----------------------TCAGA-CTGT-CGAGT---GACTAG-------
------
FC88X_01080_01059 ----------------------TCAGA-CTGT-CGAGT---GACTAG-------
------
FC88X_01096_00515 ------------------CCGA-TCAG-CTGT-CGAGT---GACTAG-------
------
FC88X_01075_00967 -----------------ACCGACTCAG-CTGT-CGAGT---GACTAG-------
------
FC88X_00478_01145 --------------------CACTCAG-CTGT-CGAGT---GACTAG-------
------
FC88X_00931_00872 ---------------------ACTCAG-CTGT-CGAGT---GACTAG-------
------
FC88X_00725_01259 --------------------CACTCAG-CTGT-CGAGT---GACTAG-------
------
FC88X_00295_00793 ----------------------CTCAG-CTGT-CGAGT---GACTAG-------
------
FC88X_00008_00282 -----------CTGTGAAGCCATCAGA-CTGT-CGAGT---GACTAG-------
------
FC88X_00964_00407 -------------GTGAAGCCATCAGA-CTGT-CGAGT---GACTAG-------
------
FC88X_00252_01079 --------------TGAAGCCA-CAGA-CTGT-CGAGT---GACTAG-------
------

```
FC88X_00629_01139      --------------TGAAGCCA-CAGA-CTGT-CGAGT---GACTAG-------
------
FC88X_00918_00531      ----------ACTGTGAAGCCATCAGA-CTGT-CGAGT---GACTAG-------
------
FC88X_00274_01140      ----------ACTGTGAAGCCA-CAGA-CTGT-CGAGT---GACTAG-------
------
FC88X_00964_00569      ---------TACTGTGAAGCCATCAGA-CTGT-CGAGT---GACTAG-------
------
FC88X_00907_00594      ----------------AAGCCA-CAGA-CTGT-CGAGT---GACTAG-------
------
FC88X_00165_00943      -----------------AGCCA-CAG--CTGT-CGAGT---GACTAG-------
------
FC88X_00053_00475      -----------------AGCCA-CAGA-CTGT-CGAGT---GACTAG-------
------
FC88X_00405_01190      -----------------AGCCA-CAGA-CTGT-CGAGT---GACTAG-------
------
FC88X_00790_00252      -----------------AGCCA-CAGA-CTGT-CGAGT---GACTAG-------
------
FC88X_00632_00310      -----------TAGTGA-GCCA-CAGA--TGT-CGAGT---GACTAG-------
------
FC88X_01046_01271      -------GTACTAGTGAAGCCA-CAGA--TGT-CGAGTC--GACTAG-------
------
FC88X_01105_01026      ----TTGGTACTAGTGAAGCCATCAGA--TGT-CGAGT---GACTAG-------
------
FC88X_00144_00052      -----------------AGCCA-CAGA--TGT-CGAGT---GACTAG-------
------
FC88X_00041_00409      ----------------GAGCCA-CAGA--TGT-CGAGT---GACTAG-------
------
FC88X_00007_00450      ---------ATATCGTGAGCCA-CAGA--TGT-CGAGTC--GACTAG-------
------
FC88X_01108_00649      -----------------AGCCA-CAGA--TGT-AGAGT---GACTAG-------
------
FC88X_00452_00080      --------------------CA-CAGA--TGT-CGAGT---GACTAG-------
------
FC88X_00090_00402      --------------------CA-CAGA--TGT-CGAG---TGACTAG-------
------
FC88X_00966_01197      ----CTCTGTACTGTGAAGCCA-CAGA--TGT-CGATGTTCGACTAG-------
------
FC88X_00967_00812      --------------------CA-CAGA--TGT-CGAG---TGACTAG-------
------
FC88X_00072_00793      -----------TGTGAAGTCCA-CAGA-CTGT-CGAG---TGACTAG-------
------
FC88X_00402_00092      -------------------CCA-CAGA--TGT-CGAG---TGACTAG-------
------
FC88X_00299_01155      -------------------CCA-CAGA-CTGT-CGAG---TGACTAG-------
------
FC88X_00028_00260      -------------------CCA-CAG--ATGT-CGAGTC--GACTAG-------
------
FC88X_00873_00725      ------------TGAATGACCA-CAG--ATGT-CGAGTC--GACTAG-------
------
FC88X_00734_01153      ---CACTCCTCTGTATGAGCCA-CAG--ATGT-CGAGTC--GACTAG-------
------
FC88X_00746_00385      --------------------CA-CAG--ATGT-CGAGTC--GACTAG-------
------
FC88X_00781_00434      ----------------GAAGCA-CAGG-ATGT-CGAGTC--GACTAG-------
------
```

FC88X_00913_00960 --------------TGAAGCCA-CAGG-ATGT-CGAGTC--GACTAG-------
------
FC88X_00375_00066 -------------------CCA-CAGG-ATGT-CGAGTC--GACTAG-------
------
FC88X_00865_01117 -------------------CCA-CAGG-ATGT-CGAGTC--GACTAG-------
------
FC88X_00694_00938 --------------------CA-CAG--ATGT-CGAGTC--GACTAG-------
------
FC88X_00861_01186 -------------------CCA-CAG--ATGT-CGAGTC--GACTAG-------
------
FC88X_00941_00516 -----------GTGATGAACCA-CAG--ATGT-CGAGTC--GACTAG-------
------
FC88X_00831_01187 --------------------CA-CAG--ATGT-CGAGTC--GACTAG-------
------
FC88X_01088_00633 --------------------CA-CAGG-ATGT-CGAGTC--GACTAGT------
------
FC88X_00459_00036 --------------------CA-CAG--ATGT-CGAGT---GACTAG-------
------
FC88X_00319_00784 ------------------GCCA-CAGA-CTGT-CGAGT---GACTAG-------
------
FC88X_00915_00313 ------------------GCCA-CAGA-CTGT-CGAGT---GACTAGCC-----
------
FC88X_00361_01069 ------------------GCCA-CAGA-CTGT-CGAGT---GACTAG-------
------
FC88X_00511_00198 ------------------GCCAGCAGA-CTGT-CGAGT---GACTAG-------
------
FC88X_00902_00557 ------------------GCCA-CAGA-CTGT-CGAGT---GACTAG-------
------
FC88X_00115_00479 ----------------------ACAGA-CTGT-CGAGT---GACTAG-------
------
FC88X_00030_00668 -------------------CCA-CAGA--TGT-CGAGT---GACTAGC------
------
FC88X_00094_00537 ---------------------A-CAGA--TGT-CGAGT---GACTAGC------
------
FC88X_00057_00820 --------------------CA-CAG--CTGT-CGAGT---GACTAG-------
------
FC88X_00622_01051 ------------------GCCAT-AG--CTGT-CGAGT---GACTAG-------
------
FC88X_01040_00750 -------------------CCATCAG--CTGT-CGAGT---GACTAG-------
------
FC88X_00796_01274 ----------------AAGCCGACAG--GTGT-CGAGT---GACTAG-------
------
FC88X_01115_00493 -----------------------------TGT-CGAGT---GACTAG-------
------
FC88X_00092_00091 ----------CGTGTGAAGCCACAGAA-TGGT-CGAGTCC-GACTAGC------
------
FC88X_00976_00692 -------------------CCACAGA--TGGT-CGAGTC--GACTAG-------
------
FC88X_00761_01127 ----------------------------TGGT-CGAGTC--GACTAG-------
------
FC88X_01055_00996 ------------------------------GT-CGAGT---GACTAG-------
------
FC88X_00144_00530 --------------------CCGAGAG-ATGTACGAGT---GACTAG-------
------
FC88X_00925_00711 ----------------------CAGAG-ATGT-CGAGT---GACTAG-------
------

FC88X_00437_01231 --------GTACTGTGAAGCCACTCGA--TGT-CGAGT---GACTAG-------
------
FC88X_00542_01000 -------------------CCACTCGA--TGT-CGAGT---GACTAG-------
------
FC88X_00894_00778 ------------------GCCACTCGA-CTGT-CGAGT---GACTAG-------
------
FC88X_00055_00307 ---------------------ACTCGA--TGT-CGAGT---GACTAG-------
------
FC88X_00354_01134 ---------------------ACTCGA--TGT-CGAGT---GACTAG-------
------
FC88X_01038_00470 -------------------CCAGTCGA-CTGT-CGAGT---GACTAG-------
------
FC88X_00393_00013 --------------------AGCTCGA--TGT-CGAGT---GACTAG-------
------
FC88X_01001_01023 ------------------CATGCTCGA--TGT-CGAGT---GACTAG-------
------
FC88X_00085_00349 -------------------------GA--TGT-CGAGT---GACTAG-------
------
FC88X_00658_00104 ---------------------CATCGA--TGT-CGAGT---GACTAG-------
------
FC88X_01088_01051 --------------------GCATCGA--TGT-CGAGTC--GACTAG-------
------
FC88X_00028_00140 --------GACTGTCCAAAGCCGCAGA--TCT-CGAGTC--GACTAG-------
------
FC88X_01100_00718 -----------TGTCCAAAGCCGCAGA--TCT-CGAGT---GACTAG-------
------
FC88X_00780_01186 -------GGACTGTCCAAAGCCGCAGA--TCT-CGAGTC--GACTAG-------
------
FC88X_00681_00123 ----------------AAAGCCGCAGA--TCT-CGAGT---GACTAG-------
------
FC88X_00854_00765 ----------------AAAGCCGCAGA--TCT-CGAGT---GACTAG-------
------
FC88X_00616_00872 ---------------------CGCAGA--TCT-CGAGT---GACTAG-------
------
FC88X_00994_01196 ---------------GCAAGCCGCAGA--TCT-CGAGTC--GACTAG-------
------
FC88X_01116_01002 ---------------------CGCAGA--TCT-CGAGTC--GACTAG-------
------
FC88X_00438_01222 -------------TCCAAAGCCGCAGA--TCT-CGAGTC--GACTTAG------
------
FC88X_00158_00994 --------------------CCGCAGA--TCT-CGAGTC--
GACTAGCCCTTCTCCC---
FC88X_00473_00579 ---------------------AGCAGA--TCT-CGAGTC--GACTAG-------
------
FC88X_00119_00099 --------------------GCACAGA--TCT-CGAGT---GACTAG-------
------
FC88X_00036_00509 -----------------------CAGA-CTGT-CGAGT---GACTAGC------
------
FC88X_00374_00028 -----------------------CAGA-CTGTACGAGT---GACTAG-------
------
FC88X_00941_01241 ---------------------CACAGA--TATTCGAGTC--GACTAG-------
------
FC88X_00998_00925 ---------------------GACAGA-GTATGCGAGTC--GACTAG-------
------
FC88X_00178_00707 ----------------------TCAGA-TGTTC-GAGT---GACTAG-------
------

```
FC88X_00265_00899       -----------------------CAGA-TGTTCTGAGT---GACTAG-------
------
FC88X_00460_00966       ----------------------CACGA-CTGT-CGAGT---GACTAG-------
------
FC88X_00726_01215       -----------------------TAGA-CTGTTCGAGT---GACTAG-------
------
FC88X_00034_00057       -------------GTGAAGCC-ACAGA--TG-TCGA-GTC-GACTAG-------
------
FC88X_00098_00027       -------------GTGAAGCC-ACAGA--TG-TCGA-GTC-GACTAG-------
------
FC88X_00163_01014       -------------GTGAAGCC-ACAGA--TG-TCGA-GTC-GACTAG-------
------
FC88X_00842_01029       -------------GTGAAGCC-ACAGA--TG-TCGA-GTC-GACTAG-------
------
FC88X_00960_00578       -------------GTGAAGCC-ACAGA--TG-TCGA-GTC-GACTAG-------
------
FC88X_01117_00900       -------------GTGAAGCC-ACAGA--TG-TCGA-GTC-GACTAG-------
------
FC88X_00848_00232       --------------TGAAGCC-ACAGA--TG-TCGA-GTC-GACTAG-------
------
FC88X_00368_00021       -----------CTGTGAAGCC-ACAGA--TG-TCGA-GTC-GACTAG-------
------
FC88X_00777_01015       -----------CTGTGAAGCC-ACAGA--TG-TCGA-GTC-GACTAG-------
------
FC88X_00892_00785       -----------CTGTGAAGCC-ACAGA--TG-TCGA-GTC-GACTAG-------
------
FC88X_00537_00207       ------------TGTGAAGCCGACAGGATAG-TCGACGTC-GACTAG-------
------
FC88X_00816_01059       ------------TGTGAAGCC-ACAG---AG-TCGA-GTC-GACTAG-------
------
FC88X_00755_00883       ------------TGTGAAGCC-ACAGA--TG-TCGA-GTC-GACTAG-------
------
FC88X_01143_01159       ------------TGTGAAGCC-ACAGA--TG-TCGA-GTC-GACTAG-------
------
FC88X_00052_00559       -------------GTGAAGCC-ACAGA--TG-TCGA--GT-GACTAG-------
------
FC88X_00050_00113       ------------TGTGAAGCC-ACAGA--TG-TCGA--GT-GACTAG-------
------
FC88X_00444_00057       ------------TGTGAAGCC-ACAGA--TG-TCGA--GT-GACTAG-------
------
FC88X_00778_00186       -----------CTGTGAAGCC-ACAGA--TG-TCGA--GT-GACTAG-------
------
FC88X_01066_00558       -------------GTGAAGCC-ACAGA--TG-TCGA--GT-GACTAG-------
------
FC88X_00655_00968       -----------CTGTGAAGCC-ACAGA--TG-TCGA-GTC-GACTAGC------
------
FC88X_00855_00315       -------GTCTCTGTGAAGCC-ACAGA--TG-TCGA-GTC-GACTAG-------
------
FC88X_00873_00713       -----------CTGTGAAGCC-ACAGA--TG-TCGA-GTC-GACTGG-------
------
FC88X_00216_00544       -----------CTGTGAAGCC-ACAGA--TG-TTGA-GTC-GACTAG-------
------
FC88X_00483_00064       -------GTACCTGTGAAGCC-ACAGA--TG-TCGA-GTC-GACTAG-------
------
FC88X_00037_00074       ------TGTAACTGTGAAGCC-ACAGA--TG-TCGA-GTC-GACTAG-------
------
```

```
FC88X_00969_00947      ---------------------CACAGA--TG-TCGA-GTC-GACTAG-------
------
FC88X_00860_00774      ----------------------ACAGA--TG-TCGA-GTC-GACTAG-------
------
FC88X_00196_00676      ----------GTCTGGAA-GCCACAG--ATG-TCGA-GTC-GACTAG-------
------
FC88X_00786_00172      ------------CTGGAA-GCCACAG--ATG-TCGA-GTC-GACTAG-------
------
FC88X_00017_00345      -----------------A-GCCACAG--ATG-TCGA-GTC-GACTAG-------
------
FC88X_00018_00591      -----------------A-GCCACAG--ATG-TCGA-GTC-GACTAG-------
------
FC88X_00897_01078      ----------------AA-GCCACAGG-ATG-TCGA-GTC-GACTAG-------
------
FC88X_00656_00934      ------------GTGGAA-GCCACAG--ATG-TCGA-GTC-GACTAG-------
------
FC88X_00759_00213      ----------TAGTGGAA-GCCACAG--ATG-TCGA-GTC-GACTAG-------
------
FC88X_00531_01000      ----------------AA-GCCACAG--ATG-TCGA-GTC-GACTAG-------
------
FC88X_01062_00645      -------------TGGAA-GCCACAG--ATG-TCGATGTC-GACTAG-------
------
FC88X_00934_01008      -----------------A-GCCACAG---TG-TCGA-GTC-GACTAG-------
------
FC88X_00683_00583      --------GTCGTATGAA-GCCACAG--ATG-TCGA-GTC-GACTAG-------
------
FC88X_00840_00319      -----------------A-GCCACAG--ATG-TCGA-GTC-GACTAG-------
------
FC88X_00155_00767      -----------------A-GCCACAG--ATG-TCGA-GT--GACTAG-------
------
FC88X_00374_00608      --------TTACTGTGAA-GCCACAG--ATG-TCGAG-TC-GACTAG-------
------
FC88X_01105_00477      -----------------A-GCCACAG--ATG-TCGAG-T--GACTAG-------
------
FC88X_00723_01017      ----------ACTGTGAA-GCCACAG--ATG-TCGAG-TC-GACTAG-------
------
FC88X_01087_00663      ----------ACTGTGAA-GCCACAG--ATG-TCGAG-T--GACTAG-------
------
FC88X_00854_00349      --------GTACTGTGAA-GCCACAG--ATG-TCGAG-TC-GACTAG-------
------
FC88X_00924_00435      --------GTACTGTGAA-GCCACAG--ATG-TCGAG-TC-GACTAG-------
------
FC88X_01050_01225      --------GTACTGTGAA-GCCACAG--ATG-TCGAG-TC-GACTAG-------
------
FC88X_00851_01257      ------TGGTACTGTGAA-GCCACAG--ATG-TCGAG-TC-GACTAG-------
------
FC88X_00812_01244      ----CTTGGTACTGTGAA-GCCACAG--ATG-TCGAG-TC-GACTAG-------
------
FC88X_01068_00376      ------------TGTGAA-GCCACAG--ATG-TCGGAGTC-GACTAG-------
------
FC88X_01065_00938      ----------------AA-GCCACAG--ATG-TCGG--TC-GACTAG-------
------
FC88X_00895_01263      --------GTACTGTGAA-GCCACAG--ATG-TCGAGTCG-GACTAG-------
------
FC88X_00391_00964      ---TCTCCGTACTGTGAA-GCCACAG--ATG-TCGA-GTC-GACTAG-------
------
```

FC88X_00456_01034 -------------CTGAT-GCCACAG--ATG-TCGA-GTC-GACTAG-------
------
FC88X_00659_00691 -------------------GCCACAG--ATG-TCGA-GTC-GACTAG-------
------
FC88X_00021_00512 -------------------GCCACAG--ATG-TCGA-GT--GACTAG-------
------
FC88X_00673_01128 -------------------GCCACAG--ATG-TCGA-GTC-GACTAG-------
------
FC88X_00842_00236 ---------CTGTGGAAC-GCCACAG--ATG-TCGA-GTC-GACTAG-------
------
FC88X_00260_00981 ---CTCTGTAC-TGTGAA-GCCACAG--ATG-TCGAGTC--GACTAG-------
------
FC88X_00999_01120 ----TCTGTAC-TGTGAA-GCCACAG--ATG-TCGAGTC--GACTAG-------
------
FC88X_00578_00976 ---CTCTGTAC-TGTGAA-GCCACAG--ATG-TCGAGTC--GACTAG-------
------
FC88X_00639_00286 ---CTCTGTAC-TGTGAA-GCCACAG--ATG-TCGAGTC--GACTAG-------
------
FC88X_00050_00669 --CCTCTGTAC-TGTGAA-GCCACAG--ATG-TCGAGTC--GACTAG-------
------
FC88X_00128_00869 --CCTCTGTAC-TGTGAA-GCCACAG--ATG-TCGAGTCC-GACTAG-------
------
FC88X_00348_01105 --CCTCTGTAC-TGTGAA-GCCACAG--ATG-TCGAGT---GACTAG-------
------
FC88X_00879_00343 -CCCTCTGTAC-TGTGAA-GCCACAG--ATG-TCGAGTC--GACTAG-------
------
FC88X_01077_00378 -CCCTCTGTAC-TGTGAA-GCCACAG--ATG-TCGAGTC--GACTAG-------
------
FC88X_00181_00076 -----CTGTACGTGTGAA-GCCACAG--ATG-TCGAGTC--GACTAG-------
------
FC88X_00846_00897 -----CTGTAC-TGTGAA-GCCACAG--ATG-TCGAG-----------------
------
FC88X_00464_01138 -----CTGTAC-TGTGAA-GCCACAG--ATG-TCGAGTC--GACTAG-------
------
FC88X_00822_00895 -----CTGTAC-TGTGAA-GCCACAG--ATG-TCGAGTC--GACTAG-------
------
FC88X_00301_00989 ------TGTAC-TGTGAA-GCCACAG--ATG-TCGAGTC--GACTAG-------
------
FC88X_00795_01098 ------TGTAC-TGTGAA-GCCACAG--ATG-TCGAGT---GACTAG-------
------
FC88X_00921_00184 ------TGTAC-TGTGAA-GCCACAG--ATG-TCGAGTC--GACTAG-------
------
FC88X_00704_00868 -----CTGTAC-TGTGAAAGCCACAG--ATG-TCGAGTC--GACTAG-------
------
FC88X_00955_00343 ----GCTGTAC-TGTGAA-GCCACAG--ATG-TCGAGTC--GACTAG-------
------
FC88X_00413_01221 ----CCTGTAC-TGTGAA-GCCACAG--ATG-TCGAGTC--GACTAG-------
------
FC88X_00364_00096 -----CTGTAC-TGTGAA-GCCACAG--ATG-TCGAGT---GACTAGC------
------
FC88X_00716_01226 -----------------A-GCCACAG--ATG-TCGAGT---GACTAG-------
------
FC88X_00422_01225 --------TAC-TGTGAA-GCCACAG--ATGGTCGAGTC--GACTAG-------
------
FC88X_00933_01113 ----TCTGTAC-TGTGAA-GCCACAG--ATGGTCGAGTC--GACTAG-------
------

```
FC88X_00761_01143       ---------------------CACAG--ATG-TCGAGTC--GACTAG-------
------
FC88X_00817_00302       ----------------------ACAG--ATG-TCGAGTC--GACTAG-------
------
FC88X_00406_00465       ---TCCCTCTGTACTGTGAAGCACAG--ATG-TCGA-GTC-GACTAG-------
------
FC88X_00587_01000       ------------------AAGCACAG--ATG-TCGA-GTC-GACTAG-------
------
FC88X_00836_00120       -------------------AGCACAG--ATG-TCGA-GTC-GACTAG-------
------
FC88X_00316_01156       ----------------------ACAG--ATG-TCGA-GTC-GACTAG-------
------
FC88X_01020_00459       ----------------------ACAG--ATG-TCGA-GTC-GACTAG-------
------
FC88X_00373_00048       ---------------------CACAG--ATG-TCGA-GTC-GACTAGC------
------
FC88X_00057_00112       ----------------------ACAG--ATG-TCGAGTC--GACTAG-------
------
FC88X_00861_01217       ---------------ATGTCGAACAG--ATG-TCGAGTC--GACTAG-------
------
FC88X_00382_00054       ----------------------ACAG--ATG-TCGAGTC--GACTAG-------
------
FC88X_00253_00786       ----------------------ACAG--ATG-TCGAGTCC-GACTAG-------
------
FC88X_00763_00242       ---------------------AACAG--ATG-TCGAGTCC-GACTAG-------
------
FC88X_00974_00579       -----------------------CAG--ATG-TCGAGTC--GACTAG-------
------
FC88X_00532_00340       -----------------------CAG--ATGTACGA-GTC-GACTAG-------
------
FC88X_00733_00088       ----------------------CCAG--ATG-TCGA-GTC-GACTAG-------
------
FC88X_00805_00407       ---------------------------GATG-TCGA--TC-GACTAG-------
------
FC88X_00534_00937       --------TACTAGTGGAAGCCACTCG-ATG-TCGA-GTC-GACTAG-------
------
FC88X_01082_00737       ---------------------AGCCAG-ATG-TCGA-GTC-GACTAG-------
------
FC88X_00499_01179       ----------------------------ATG-TCGA-GTC-GACTAG-------
------
FC88X_00005_00592       --------------------------GCATG-TCGA-GTC-GACTAG-------
------
FC88X_00412_00121       -----------------------------TG-TCGA-GTC-GACTAG-------
------
FC88X_00211_00997       ----------ACTGCTGAAGCCACAGA--TG-TCGAGGTC-GACTAAGCC----
------
FC88X_00558_01022       ---------------------CACAG---TA-TGTCGGTC-GACTA--------
------
FC88X_00525_01022       ----------------CATGTCACAGA--TG-TCGAG-TC-GACTAC-------
------
FC88X_00147_00558       ------TGTACTGGTGAAGCCA-CAGAT--G-TCGAGT-C-GACTG--------
------
FC88X_00855_00517       -----------------AGCCA-CAGAT--G-TCGAGT-C-GACAG--------
------
FC88X_00913_00893       -----------------AGCCATCAGAT--G-TCGAGT-C-GACTG--------
------
```

FC88X_00154_00077 -----CTGGTACTGTGAAGCCA-CAGAT--G-TCGAGTTC-GACTA--------
------
FC88X_01092_00502 ----------------------ACAGATTCG-TCGAGT-C-GACTG--------
------
FC88X_01144_00746 -----------------------CAG--ATG-TCGACGTC-GACTCAG------
------
FC88X_00839_00823 ---------------------GCCACAGATG-TCGA-GTC-GATAG--------
------
FC88X_01009_00427 --------------------CCACTCAGATG-TCGA-GTC-GATAG--------
------
FC88X_01139_00839 ----------------GAAGCCACACGATGC-TCGA-GTC-GGCTAG-------
------
FC88X_00774_00150 --------TGTACTGTGAAGCCACAG--ATG-TCGAGT-G-ACCTAG-------
------
FC88X_01117_00613 --------------------CCACAG--ATG-TCGAGT-G-ACCTAG-------
------
FC88X_00729_00242 ----------------------ACAG--ATG-TCGAGT-G-ACCTAG-------
------
FC88X_00905_00882 ----------------------ACAG--ATG-TCGAGT-G-ACCTAG-------
------
FC88X_00350_01099 --------GTACTGTGGAAGCCACAG--ATG-TCGAGTCG-ACCTA--------
------
FC88X_00872_00958 -------CTGTACTAGTGAGCCACAG--ATG-TCGAGT-G-AT-----------
------
FC88X_01069_00720 -----------------AAGCACAGG--ATG-TCGAG-----------------
------
FC88X_00827_01048 -------------------CTCATGTACTGTGACGACAGTGGAGGAG-------
------
FC88X_00836_01133 ----------------------ATGT-TCGAGTCGATAG----------------
------
FC88X_00430_01051 --------GGGGACCATCGTCTTTGAT-GCGATTGCGCT--CCT----------
------
FC88X_00898_00319 -------------CTATTGTCTATAA--GC-ATCGCGCT--GTTC---------
------
FC88X_00946_00642 -----------------TGTGTACAATGACGATGGCGCA--CGTTG--------
------
FC88X_00916_00741 -----------------TGTGTACAATGACGATGGCGCA--CGTTG--------
------
FC88X_00879_00304 ------------------ATGTATCATCATCATCGC-CC--CGTTAGC------
------
FC88X_00072_00014 ----------------------CCAAAACTAAT-GACTGGCGAGTCCTCTC---
------
FC88X_00337_01035 ----------------------CCAAAACTAAT-GACTGGCGAGTCCTCTC---
------
FC88X_00999_00695 -----------------------CAAGACCAATCAATTCGTCGAGCCTCTC---
------
FC88X_00144_00912 ----------------------TAGCAATAAAATGATTAGGCGATTCTC-----
------
FC88X_00491_00598 ----------------------TAGCAATAAAATGATTAGGCGATTCTC-----
------
FC88X_00748_00977 ----------------------TAGCAATAAAATG-TTAGGCGATTCTC-----
------
FC88X_00551_00738 ----------------ATCGTGTTTAAATCATTTGATCTGTCT-----------
------
FC88X_00616_01051 ------------------CGTGCAGAAAAAATAAGATCTGGTCGTTATGCTC--
------

FC88X_00708_01115 -----------------------CTCCCAGTTCAATATTTCGGCCCGCCGT---
------
FC88X_01098_01004 -----------------------CTCCGATGACAAT-TGTCGACTCGAATT---
------
FC88X_00035_00408 ----------------------TGGCCAATTACAAG-AGTTGTCCCTCCT----
------
FC88X_00011_00485 ---------------------CCCAATTACAGATCATGGTCGCTTCTC------
------
FC88X_01032_00693 ---------------------CCCAATTACAGATCATGGTCGCTTCTC------
------
FC88X_00139_00124 ---------------------CCCAATTACAGATCATGGTCGCTTCTC------
------
FC88X_00970_01096 ---------------------CCCAATTACAGATCATGGTCGCTTCTC------
------
FC88X_00923_00858 ---------------------TCCAATTACAGATCATGGTCGCTTCTC------
------
FC88X_00853_00686 ---------------------CCCAATCACAGATCATGGTCG------------
------
FC88X_00444_00972 -------------------GTTCCCAATCCCGATGATGCTACAATCCT------
------
FC88X_00945_00299 ---------------------CCCCATCCCGGATCACAAAATACTTAC------
------
FC88X_00041_00778 --------------------CTCCCA-AATATACCATGATCAATTGCC------
------
FC88X_00283_00955 ------------------CCCTCCCACAATAGACCATCATCCGT----------
------
FC88X_00652_01208 -------------------TCTCCCAATTATAGCTATTATCCGAAC--------
------
FC88X_01033_00936 -----------------CCCCCACCCAGACGCAGGAGAATTATA----------
------
FC88X_00142_00218 ---------------ATCCCCATCCCAGTCACAGTATA-TTAT-----------
------
FC88X_00015_00150 ------------------TCTCCCAATATCGCTCTACAGTTATT----------
------
FC88X_00146_00034 -------------------CTCCCAA-GTCGGTC-GCAGTTGTGTGTCT-----
------
FC88X_00086_00202 ---------------------TCACACTCCCGATGATCGATTCCTCTA------
------
FC88X_00488_00515 ---------------------TCACACTCCCGATGATCGAT-------------
------
FC88X_00884_00268 --------------------CTCAAACGTGCGATGAATGAGCCTTCT-------
------
FC88X_01044_00865 --------------------CTCAAACGTGCGATGAATGAGCCTTCT-------
------
FC88X_00176_00598 --------------------CTCAAACGTGCGGTGAATGAGCCTTCT-------
------
FC88X_00683_00067 ----------------TACTCCCAAGTTCGAAA-GTCCGTACAC----------
------
FC88X_01063_01140 ----------------TTCTCCCAAATCGATCATGCCCGTACA-----------
------
FC88X_00109_00017 ---------------------TCGCACTGTCGTCGATGTG-TGCCTGTC-----
------
FC88X_00353_00371 --------------------GTCGCACTGTCGTCGATGTG-TGCCTGTC-----
------
FC88X_00123_00942 ---------------------TCGCACTGTCGTCGATGTG-TGCCTGTC-----
------

```
FC88X_00235_00943      ---------------------TCGCACTGTCGTCGATGTG-TGCCTGTC-----
------
FC88X_00259_00998      ---------------------TCGCACTGTCGTCGATGTG-TGCCTGTC-----
------
FC88X_01135_01063      ---------------------TCGCACTGTCGTCGATGTG-TGCCTGTC-----
------
FC88X_00286_00660      ---------------------TCGCACTGTCGTCGATGTG-TGCCTGTC-----
------
FC88X_00369_00344      ---------------------TCGCACTGTCGTCGATGTG-TGCCTGTC-----
------
FC88X_00745_00862      ---------------------TCGCACTGTCGTCGATGTG-TGCCTGTC-----
------
FC88X_00896_00590      ---------------------TCGCACTGTCGTCGATGTG-TGCCTGTC-----
------
FC88X_00989_00776      ---------------------TCGCACTGTCGTCGATGTG-TGCCTGTCC----
------
FC88X_00124_00596      ---------------------TCGCACTGTCGTCGATGTG-
TGCCTGTCCTTTC------
FC88X_00722_01224      ---------------------TCATCGCATGGCCGCTGTGATACCTATC-----
------
FC88X_00880_01253      ----------------------TCTCCTGATGTCCGTTGC-TGTATGCCC----
------
FC88X_00901_00847      ----------------------CCTTCTGATGTCCGTATG-TGTAACCCC----
------
FC88X_00597_00561      --------------------CTCCCCATCGATCCAA-GTGCCGACGAA------
------
FC88X_00130_00193      ---------------------CTCCTCTCAATCAAATGTGTAGACTAT------
------
FC88X_00603_00094      ---------------------TCTCTCCGAAACATACCTCCATAGAATT-----
------
FC88X_00714_00111      ---------------------CCACTCCCTATCAGACGTATACAGAAT------
------
FC88X_00707_00152      ------------------ACTACTCCTTCGATAATACGTCTGTT----------
------
FC88X_00116_00489      --------------ACTACCGCCTAAGTAAGC---TCTGTTCGC----------
------
FC88X_00307_01161      --------------ACTACCGCCTAAGTAAGC---TCTGTTCGC----------
------
FC88X_00984_00257      --------------ACTACCGCCTAAGTAAGC---TCTGTCCGC----------
------
FC88X_00712_00129      -------------------CTCCAAAGTAAGCCGTTCTGTCCA-----------
------
FC88X_00266_00016      ---------------CTTACTCCTGACTAAATAGCTGTTTTC------------
------
FC88X_01056_01097      ---------------CTTACTCCTGACTAAGTAGCTGTTTTC------------
------
FC88X_00934_01116      -----------------CTGGCCCGACTAAGATGGCGCTTTTCT----------
------
FC88X_00957_00751      --------------CTACCAATTCAAGCTACTCTCCGGGGC-------------
------
FC88X_01070_00876      -----------------CCAATTTAAATCGCTATTCACTGTTGCC---------
------
FC88X_00887_01206      ---------------------ACTAAACCAATGTCTAGCCTCCCTGTG------
------
FC88X_00951_00838      ---------------------TTGAAAATTAATTCAACCGTCCCTGTG------
------
```

FC88X_00941_01143 --------------------TTTAAAGCTATTGTCTACGTTCCCTCTT------
------
FC88X_00019_00218 -------------------CTCCCGACCGT-TTACCGGGCATTTCCT-------
------
FC88X_00060_00642 -------------------CTCCCAACTATGTCAATGCGCATTTCC--------
------
FC88X_00668_00406 -------------------CTCCCGACCAA-TTGAAATCAATTTCCT-------
------
FC88X_00840_00215 ------------------GCTCCCGAGCTAGTATCTGCGATATCCC--------
------
FC88X_01049_00575 -------------------CTCCCTACCAAGTTCCTGCGGTGCCCT--------
------
FC88X_00144_00223 ------------------CATCCCGAACAACACTGTGCGCTCCCC---------
------
FC88X_01095_00691 ------------------TGTCAGGATCAATTTTCTGGCACCCCT---------
------
FC88X_00029_00184 ----------------------ATCACGTCTCCCTGACTACACCATCTC-----
------
FC88X_00642_01098 ----------------------ATCACGTCTCCCTGACTACACCATCTC-----
------
FC88X_00505_00135 ---------------------TTTGCCGGATCCGTGGC-AGACCTTCTCCC---
------
FC88X_00523_00156 ---------------------TTTGCCG-ATCCGTGGCTAGACCTTCTC-----
------
FC88X_01112_00685 ---------------------TTTGCCG-ATCTGTGGCTAGACCTTCTC-----
------
FC88X_00976_00963 ---------------------TTTGCCG-ATCTGTGGCTAGACCTTCTC-----
------
FC88X_00798_00121 --------------------------CAACACCCTGT-
CCTACATCTCCATACT------
FC88X_00928_00701 -------------------------CCAACCCCTTGTATCTCCATCCTAAT---
------
FC88X_00603_00952 -----------------------CCCTTGCTCCACCTACTTACACATCCC----
------
FC88X_00298_00950 -------------------------
CCTACCCATTGGATATTCTTGACCACACTC-----
FC88X_00578_01196 ------------------------CCCTGGCCATTCCATCTTCC-GACAACAA-
------
FC88X_00214_00534 --------------------CTTGATCATCCTCGGCGGCATATTCTA-------
------
FC88X_00662_00484 --------------------CTTGATCATC-TCGGCGGCATATTCTA-------
------
FC88X_00105_00271 ---------------------CTGATCATC-TCGGCGGCATATTCTA-------
------
FC88X_00089_00075 ----------------CCCTTCTGAACATC-TCGTTGGCGTATC----------
------
FC88X_00193_01042 --------------------ATCCAACATCTCCTGGATGGCGTCTCC-------
------
FC88X_00316_01080 --------------------ATCCAACATCTCCTGGATGGCGTCTCC-------
------
FC88X_00072_00109 -----------------TCAATCCACCATCTCCTGCAGCGTATC----------
------
FC88X_01080_00413 ------------------ATATCTGACATCCCCA--GGTACG-TTCG-------
------
FC88X_00569_00345 ---------------------------ATCACCT--GGTTCGGTTCG-------
------

FC88X_00834_01202 ------------------CCGTAGGAGATCTCCAT-GGCACGTTTA--------
------
FC88X_00530_01091 ------------------CTCTTCAATATCACCAT-GGCA-ACTATT-------
------
FC88X_01051_01149 -------------------TCATCACCTTCTCCAT-GGCATACTAAT-------
------
FC88X_00953_00369 ----------------------CCACTATCTCCCT-GGCA-ACCAATGTTGT--
------
FC88X_01094_00860 ----------------------CCACTATCTCCCT-GGCA-ACCAATGTTGT--
------
FC88X_00132_00072 ---------------------TCCACCA-CTCCCT-GACA-CCTAATGCT----
------
FC88X_00087_00481 --------------------CTACATCCGTGTAGCACACAAGTCTC--------
------
FC88X_00015_00193 ------------------TCTGCCATCCGTG-GGTACACTCGTCTC--------
------
FC88X_00695_00132 ---------------------TACACCAGACCAGTGCACAAGCTTCTC------
------
FC88X_00655_01021 ---------------------TACACCAGACCAGTGCACAAGCTTCTC------
------
FC88X_01078_01145 ---------------------TACACCAGACCAGTGCACAAGCTTCTC------
------
FC88X_00142_00777 ---------------------TACACCAGACCAGTGCACAAGCTTCTC------
------
FC88X_00263_00363 ---------------------TACACCAGACCAGTGCACAAGCTTCTC------
------
FC88X_01121_00762 ---------------------TACACCAGACCAGTGCACAAGCTTCTC------
------
FC88X_00049_00713 ---------------------TACACCAGACCAGTGCACAAGTTTCTC------
------
FC88X_00958_00702 -----------------------CTACACACTGTCTCGAAAGCACATATTT---
------
FC88X_00519_00760 -----------------------CTACACACTGTCTCGAAAGCACATATT----
------
FC88X_00850_01248 ---------------------ATCTCCAGACGTGTCCGCAAG------------
------
FC88X_00287_00918 -----------------------CCGCCACATCGGACGCACATGTCTCCC----
------
FC88X_00407_00102 -----------------------CCGCCACATCGGACGCACATGTCTCCC----
------
FC88X_00835_01275 --------------------TCCCCACATCTACGGTCGCA-ATGTCTCCA----
------
FC88X_00729_00952 ---------------------TTCAACCGTCTCTGGCGCAACCATATT------
------
FC88X_01097_00869 ---------------------TTCAACCGTCTCTGGCGCAACCATATT------
------
FC88X_00033_00441 ---------------------TTCAACCGTCTCTGGCGCAACCATATT------
------
FC88X_00155_00642 ---------------------TTCAACCGTCTCTGGCGCAACCATATT------
------
FC88X_00795_00814 ---------------------TTCAACCGTCTCTGGCGCAACCATATT------
------
FC88X_00883_00611 ---------------------TTCAACCGTCTCTGGCGCAACCATATT------
------
FC88X_00891_01067 ---------------------TTCAACCGTCTCTGGCGCAACCATATT------
------

FC88X_00946_01223 ---------------------TTCAACCGTCTCTGGCGCAACCATATT------
------
FC88X_00749_01030 ---------------------TTCAACCGTCTCTGGCGCAACCATGTTC-----
------
FC88X_00876_01008 ---------------------TTCAACCGTCTCCGGCGCAACCATATT------
------
FC88X_00122_00595 -------------------------ACAACCTCTGTGGCA-CCATGTTTCTTT-
------
FC88X_01103_01152 ---------------CTCCAACTCTACGCTCCC-GTGTCAACCT----------
------
FC88X_00449_00760 ---------------CTCCAACTCTACGCTCC--GTGTCAACCT----------
------
FC88X_00318_01138 ----------------CTCGACTCTCCGACCCC-AGTGCAACCT----------
------
FC88X_00705_01169 ------------------TTACTCAACGTCTCCCGGAGCAATGAT---------
------
FC88X_00011_00355 ----------------------TCAACGTCTCC-GTGTCAATGACATCCT----
------
FC88X_00115_00281 --------------------CGCCAAATTGCGATGCGCCACACTCCT-------
------
FC88X_00978_00243 --------------------CGCTAAATTGCGATGCGTCACACTCCT-------
------
FC88X_00239_00908 -------------------CTGCTAAAA--TGAGGCTGCACACTTCTC------
------
FC88X_00900_00269 -------------------CTGCTAAAAA-TGAGGCTGCACACTTCTC------
------
FC88X_00811_00180 -------------------CTGCTAAAA--TGAGGCTGCACACTTCTC------
------
FC88X_01124_00674 -------------------CTGCTAAAA--TGAGGCTGCACACTTCTC------
------
FC88X_00024_00066 -------------------CTGCTAAAA--TGAGGCTGCACACTTCTC------
------
FC88X_00348_00484 -------------------CTGCTAAAA--TGAGGCTGCACACTTCTC------
------
FC88X_00612_00251 -------------------CTGCTAAAA--TGAGGCTGCACACTTCTC------
------
FC88X_00700_00900 -------------------CTGCTAAAA--TGAGGCTGCACACTTCTC------
------
FC88X_00788_00203 -------------------CTGCTAAAA--TGAGGCTGCACACTTCTC------
------
FC88X_00846_01147 -------------------CTGCTAAAA--TGAGGCTGCACACTTCTC------
------
FC88X_00992_00873 -------------------CTGCTAAAA--TGAGGCTGCACACTTCTC------
------
FC88X_01030_00598 -------------------CTGCTAAAA--TGAGGCTGCACACTTCTC------
------
FC88X_01106_00805 -------------------CTGCTAAAA--TGAGGCTGCACACTTCTC------
------
FC88X_00968_01053 -------------------CTGCTAAAA--TGAGGCTACACACTTCTC------
------
FC88X_00083_00811 -------------------CTGCTAAAA--
TGAGGCTGCACACTTCTCCTTCCTTT----
FC88X_00873_00973 -------------------CTGCTAAAA--TGAGGCCGCACACTTCTC------
------
FC88X_00684_01265 ------------------CCTGCCCAACTGTA--GCGG-ATTCTTCTC------
------

```
FC88X_01043_01169       ------------------CCTGCCCAACTGTA--GCGG-ATTCTTCTC------
------
FC88X_00035_00564       ------------------CCTGACCAACTGTA--GCGG-ATTCTTCTC------
------
FC88X_00739_00254       --------------------TGTCCAACTCTATGGCTC-ACACTTCT-------
------
FC88X_00717_01151       --------------------TGTCCAATTGTATGGCTC-ACACTTCT-------
------
FC88X_00694_01260       ---------------------ACAAATGCTGTCCACACTCCCGATGAA------
------
FC88X_01004_00740       ----------------------CCAACATGTTGCCACCTCCAGATTATT-----
------
FC88X_00426_01132       ------------------CTTACCAACATATGGT-TGCTACTCTGA--------
------
FC88X_00847_00252       ------------------CTTACCAACATATGGT-TGCTACTCTCA--------
------
FC88X_01086_00868       --------------------TCCCCATATATGCCGTACTACTCTCAC-------
------
FC88X_00134_00862       -------------------TGCCACGCTCCAGCTTGACCCATTGTT--------
------
FC88X_00750_01160       ------------------CCGCCA-GCTAAACTTTCTGCCATTGTT--------
------
FC88X_00389_00620       -------------------CTCGCACCCTCTTCCTGCCCATGTATT--------
------
FC88X_00650_00510       -------------------CTCGCACCCTCTTCCTGCCCATGTATT--------
------
FC88X_00538_01205       -------------GGGGATCTCCCACCCTATTCCCTCTGAGGTATT--------
------
FC88X_00344_00586       ----------------TTACTGGAACTCTCCTCTAGTGCCATT-----------
------
FC88X_00803_00329       ------------------GGGGAAGCTCAACTCTACTGCCAATGTC--------
------
FC88X_00583_01233       ----------------------CATGCGATTGTTACCGCCCGTTTTCTC-----
------
FC88X_00092_00322       ----------------------CATGCGATTGTTACCGCCCGTTTTCTC-----
------
FC88X_00494_00053       ----------------------CATGCGATTGTTACCGCCCGTTTTCTC-----
------
FC88X_00524_01247       ----------------------CATGCGATTGT-ACCGCCTGTTTTCTC-----
------
FC88X_00069_00342       ----------------------CATGCGATTGTCACCGCCCGTTTTCTC-----
------
FC88X_00576_01062       ----------------------CATGCGATTGTTACCGCCCGTTTTTCTC----
------
FC88X_00464_01199       ----------------------CATGCTATTGGCACTCCGTATTTCTC------
------
FC88X_00310_00049       ---------------------CCATGCCATGGTTACTACCCATTTTCC------
------
FC88X_00737_00690       --------------------GCCCTTCGATTGGCACTGCCTACATTATC-----
------
FC88X_00870_00372       ---------------------CCACCCTTCTGCCAGTGT-TACATTAAC-----
------
FC88X_00123_00199       -------------------CGCGTTACCAGTGCCACTCCCCAAGTT--------
------
FC88X_00320_00977       -------------------CGCGTTACCAGTGCCACTCCCCAAGTT--------
------
```

```
FC88X_00996_00296     -------------------CGCGTTACCAGTGCCACTCCCCAAGTT--------
------
FC88X_00957_00970     -------------------CGCGTTACCAGTGCCACTCCCCAAGTT--------
------
FC88X_00458_00057     -------------------CGCGTTACCAGTGCCACTCCCCAAGTTC-------
------
FC88X_00471_01212     -------------------CGCGTTACCAGTGCCACTCTCCAAGTT--------
------
FC88X_00541_00234     --------------------TTCACCCCTTTTCAACTGTACAATTTT-------
------
FC88X_00686_00925     -------------------TTTCACCCAATGATACCTC--CAATTTTA------
------
FC88X_00342_00363     ---------------------CCTGTGCCTCGCCCTCTTCGGAATATT------
------
FC88X_01063_00440     ---------------------CCTCTGGCTGACCACTTCCCACTAGTT------
------
FC88X_00880_00905     ---------------------CCATCGGTTTCAAGCCTCGCAGTTGTT------
------
FC88X_00082_00166     ---------------------CCATCGGTTTCAAGCCTCGCAGTTGTT------
------
FC88X_01021_01101     ---------------------CCATCGGTTTCAAGCCTCGCAGTTGTT------
------
FC88X_01052_00696     ---------------------CCATCGGTTTCAAGCCTCGCAGTTGTT------
------
FC88X_00988_00313     ---------------------CTATGGCTCACAGTCCCAGCAGTTATTC-----
------
FC88X_00596_01216     ---------------------CTATGGCTTACAGTCCCAGCAGTTATTCC----
------
FC88X_00564_00488     ---------------------CGCTAGTCTTAGCACTCCCACGTTGTA------
------
FC88X_01033_00633     -------------------TTCTCCAGTCTCACCAACCC--AGTTGT-------
------
FC88X_00990_01246     ---------------------CGCTGTCTTAGCCACCCACCCCACGTT------
------
FC88X_00536_01262     ----------------------CCTGGCAGTGCGCACTCCTGGTTACCT-----
------
FC88X_00970_00716     -----------------GGGGACCTGGCAGTGCGCACTCCTGGTTACCT-----
------
FC88X_00441_01057     ---------------GGGGAATCCTGGCAGTGCGCACTCCTGGTTACCT-----
------
FC88X_00666_01243     ----------------------CTTGGCACGCCTCCCTGCTGGCTGAA------
------
FC88X_00693_01257     ------------------CTGTTCAATTGGCACAGTCTCCGTGTT---------
------
FC88X_00923_00502     --------------TTCCCTGTTCTGGCA-TGCAGTCTCCGT------------
------
FC88X_01003_00543     ------------------CTGTTTTGGCT---AAGTCTCCGAGGTACA------
------
FC88X_00774_00104     ------------------CTGTTTTGGCT---AAGTCTCCGAGGTACA------
------
FC88X_00971_00824     ------------------CTGTTTTGGCT---AAGTCTCCGAGGTACA------
------
FC88X_00905_00913     ----------------TTCCCATCTTCTGCCGCACTCCCCGGTG----------
------
FC88X_01130_01078     -------------------TCCTCTTCTGG-GCGCTCCCCGTTTCTT-------
------
```

```
FC88X_00985_01112       -------------------CCATCTTCTGGCCGACTCCCCTATAA---------
------
FC88X_00822_00161       -------------------TGTCCAGCTACTCCGAACCCCGATACTC-------
------
FC88X_00171_00775       ---------------------------TCTCCACT-
TGATGATGTTTCAAATTTT-----
FC88X_00856_00800       ---------------------------TCTCCACT-
TGATGATGTTTCAAATTTT-----
FC88X_00049_00601       ---------------------------TCTCCACT-
TGATGATGTTTCAAAATTTT----
FC88X_00151_00700       -------------------TCACGTGGTCGCCGCTCTCATGCTGTT--------
------
FC88X_00668_01088       ------------------CTCATTTAGCCTCCGCA-TCATGATGTT--------
------
FC88X_00255_00668       -----------------------ACACTC-CCTACTTGCATGCGCTTTACG---
------
FC88X_00992_00702       -----------------------CCACTC-CCTACTTGCATGCGCTTCACG---
------
FC88X_00401_00305       -----------------------ACACTCTCCTTCTAGCTGACGATTTACG---
------
FC88X_00637_00317       -------------------------CCTCTGTATGCTCCGCGTGATTATGTT--
------
FC88X_00413_01152       -------------------------ATTCTCTCTATCTCGCCTGACGTTATG--
------
FC88X_00101_00494       -----------------------CCACTCCCTATATCGCGCATGATTAGC----
------
FC88X_00136_00841       -----------------GGGGAACCACACTCTCTATAGCTGCTGATCCGC----
------
FC88X_00291_01062       -------------------CCCACTAAACTCCCGAGATGGCGTACT--------
------
FC88X_00739_00669       -------------------CCCACTAAACTCCCGAGATGGCGTACT--------
------
FC88X_00151_01016       --------------------CCACTAAACTCCCGAGATGGCGTACT--------
------
FC88X_00294_00243       -------------GGGGAAGCCACTAA-CTCCCGAGATGGCGTACT--------
------
FC88X_00210_00873       -------------------CCCCATAAACTCCCGATGCGGCATGAC--------
------
FC88X_00696_01242       -------------------CCCCATAAACTCCCGATGCGGCATGAC--------
------
FC88X_00999_00476       -------------------CCCCATAAACTCCCGATGCGGCATGAC--------
------
FC88X_01014_01248       ------------------CCACTAGACTCCCACAATGCTCCTTGA---------
------
FC88X_00238_00956       ---------------------CCACACACTCCCTGGCTTACGCATCCC------
------
FC88X_00035_00627       ---------------------CCACACACTCCCTGGCTTACGCATCCC------
------
FC88X_00970_00330       ---------------------CCACAAACTCCCTATCATATCGATCCCC-----
------
FC88X_01061_01035       ---------------------CCACAAACTCCCTATCATATCGATCTC------
------
FC88X_00244_01091       ---------------------CCAGGCACTTCCTACTTGGCTCATCTC------
------
FC88X_01117_00589       ---------------------CCAGGCACTTCCTACTTGGTTCATCTC------
------
```

FC88X_00647_01129 ---------------------CCAGACAAT--CTCCCTAGGTCACCTTAT----
------
FC88X_00903_01063 ---------------------CCAGACAAT--CTCCCTAGGTCACCTCCC----
------
FC88X_00467_00998 ---------------------
ACAAACACTTACTCGATGTCAACTCTCCTTCCTTT----
FC88X_01003_00403 ---------------------ACAAACACTTACTCGATGTCAACTCTC------
------
FC88X_01093_00473 -------------------------ACAAACACTCTCGGTCATCTCTCACACT-
------
FC88X_01062_00610 ---------------------CTACAGCCTCGTAAATAGACCTCCCC-------
------
FC88X_00212_00028 ---------------------CTACCCCCTTGGTTATAGCCTCCCGA-------
------
FC88X_00275_01072 ------------------------CTCTCTCCTTGATACCCTTCCCGTGGT---
------
FC88X_00055_00188 ----------------------------
CTCCTAGCTTAGCTTCCCCGGTGTGTA-----
FC88X_00161_00275 ---------------------------CCTCCTAGATGA-
CATCCCGCGTGATTATGTT-
FC88X_00795_00234 ----------------------------
GCCCTAGAAAAAACTCCCCGGGGTAATGCT--
FC88X_00615_01213 ---------------TTCTCATCCTGCTCGAGTTAGGAA--CTATTC-------
------
FC88X_00750_00924 ---------------------CCTGACCCTAGATAGAAATGCTATTCT------
------
FC88X_00065_00853 ---------------------TCCCTGTCCGTGTTGAC-TAGTTAGCGC-----
------
FC88X_00228_00367 ----------------------CTCCCTGCTTCTTGACGTAGTAATCGA-----
------
FC88X_00993_00731 --------------------GTCACATTCCCTTCTGCCTAGGTAGCC-------
------
FC88X_00017_00384 -----------------------ACCAACTCGTCTCCCGTATGTTTAGCG----
------
FC88X_00922_00765 -----------------------ACCAACTCGTCTCCCGCATGTTTAGCG----
------
FC88X_00647_00912 ---------------------CTCCCAGCTCGATCACGCGATGTTTAT------
------
FC88X_00907_01244 ---------------------CTCCCAGCTCGATCACGCGATGTTTAT------
------
FC88X_00282_00080 ---------------------CTCCCAGCTCGATCACGCGATGTTTAT------
------
FC88X_00551_01234 ---------------------CTCCCAGCTCGATCACGCGATGTTTAT------
------
FC88X_00761_00357 ---------------------CTCCCAGCTCGATCACGCGATGTTTAT------
------
FC88X_00045_00569 ---------------------CTCCCAGCTCGATCACGCGATGTTTAT------
------
FC88X_00350_01030 ---------------------CTCCCAGCTCGATCGTGCGATGTTTAT------
------
FC88X_00775_01225 ---------------------CTCCCGACTAGTTAGTTTTAACGACAA------
------
FC88X_00845_00200 ---------------------CTCCATTCTCGCTTGTGTTAACGTCGT------
------
FC88X_00176_00195 -------------------CTCTCCATTCTCCGAT-TGTGACAACAA-------
------

```
FC88X_00283_00738          -------------------CTCTCCATTCTCCGAT-TGTGACAACAA-------
------
FC88X_00152_00854          ---------------------CCCCATGCTCCAATACGTTACAACAAT------
------
FC88X_00444_01139          ---------------------TGCCATCTCCCGAATCGTTACGATAAA------
------
FC88X_00529_00803          ------------------ACAATTCATCCCCGGCGCCGCTATTCT---------
------
FC88X_00658_00530          ------------------ACCAGTCATCCCCTG-ATAGCTATTCTT--------
------
FC88X_00739_01120          ----------------------TTCCTCCCCTACGTTACGCTTTTCTTTC----
------
FC88X_01041_01093          -------------------------CTCCACCATTTGGCGA-CGTTGTTCGTC-
------
FC88X_00859_00847          -------------------------CTCCACCATTTGGCGA-CGTTGTTCGTC-
------
FC88X_00709_00874          --------------------------TCCACGACTCGACAG-TGT---------
------
FC88X_00735_00688          -------------------------CTCCAAGGCATTACAAGCTTTGTCTACT-
------
FC88X_00914_00764          --------------------------TCCAAGACTCCCCAG--
TTTGGCTATTCC-----
FC88X_00159_00160          --------------------CCATCCCAGCGGC-GTAGTCAGCGTCTT------
------
FC88X_00932_00585          --------------------CCTTTCCAGCTGTTGTGTTCAGCGTTT-------
------
FC88X_00981_00840          --------------------CCCATCCCTCTGC-TTCGTTAGCGTCGC------
------
FC88X_00065_00837          ---------------------TCTCCCATTCGTCACAATTAACGTGCC------
------
FC88X_00112_00949          ---------------------TCTCCCATTCGTCACAATTAACGTGCC------
------
FC88X_00478_00564          ---------------------TCTCCCATTCGTCACAATTAACGTGCC------
------
FC88X_01064_01057          ---------------------TCTCCCATTCGTCACAATTAACGTGCC------
------
FC88X_01128_00697          ---------------------TCTCCCATTCGTCACAATTAACGTGCC------
------
FC88X_00924_00685          --------------------TCTCCCTATTCATCTCCAGCAGCGTGAC------
------
FC88X_00875_00360          --------------------GCTCCCCATCGTGCGCCATTAGAGTGGC------
------
FC88X_00165_00941          ---------------------CTCCCTTTTCAACGACGTCGGCCATTA------
------
FC88X_00822_00714          ---------------------CTCCCAGTTCCTC--CGACGGCCAG--------
------
FC88X_00236_00305          ------------------CTCCTCTATTTTCAGCTACGCAGTCGGC--------
------
FC88X_00854_01133          ---------------------CTCTCATTTCCCCGACGTCAGCCGTGT------
------
FC88X_01030_00928          ---------------------CTCCCAGTTTATCTGACTCACCCGCGT------
------
FC88X_00396_01181          ---------------------CTCCCACTGGTAAGTCGTCAGATTCTC------
------
FC88X_00093_00098          ---------------------CTCCCACTGGTAAGCCGTCAGATTCTC------
------
```

```
FC88X_00439_00700      ---------------------CTCCCACCTCGGCCGTCACAGATTGTC------
------
FC88X_00960_00276      ---------------------CTCCCACTTATGAGTCGTACTGACCGT------
------
FC88X_01023_00639      ---------------------CTCCCACTTATGAGTCGTCCTGACCGT------
------
FC88X_00565_00057      ---------------------CCCCCACGTAT---TCGTCCTCGA---------
------
FC88X_00966_00580      ---------------------CTCCCAGGTCTA--TCGTCCTCACACTCT----
------
FC88X_00676_00075      ---------------------ACTCTTAGTCTGTTCTGCCACGTTCGGT-----
------
FC88X_00780_00167      ---------------------ACTACTGCTCTTTTCTCCCAGGTACGG------
------
FC88X_00830_00992      -----------------------CGTTACTCTGTTCTCCCAAGGCATTAG----
------
FC88X_00514_01133      ---------------------ATTCCCTATTCTTTCAACCAAGTCGCA------
------
FC88X_00713_00078      -----------------------TCCACCTATTCTAGCCCATGTCACAGCG---
------
FC88X_00752_00287      ---------------------CCTCACAGCTTTGTCACCCAGCTCCC-------
------
FC88X_00738_01243      ---------------------TCCTCCCACTTCCTCACTCAGTCTCCC------
------
FC88X_00902_01229      -----------------TTATTCTTCCAAC-CTTCCTCTCCCTTCC--------
------
FC88X_00401_01079      --------------------TTCTCCCAATGCTTTGTCTCAGGTCCC-------
------
FC88X_00887_00797      ---------------------CTCTCACTTCTCCGAACTTGGTCTCC-------
------
FC88X_01135_00705      ---------------------TCTCGACTCCCACGGACTCAGTGTCCC------
------
FC88X_00037_00688      ---------------------ATGTCCTCTTGGCAGCATTTATATTCT------
------
FC88X_00986_00328      -------------------ACATGACCTAT---CAACATCTACATTCTC-----
------
FC88X_00346_01133      -------------------ACTGCTGTAGTTGACTCCATCTTGCTTCTAATT--
------
FC88X_00441_01086      -------------------ACTGCTGTAGTTGACTCCATCTTGCTTCTAATT--
------
FC88X_00787_00918      --------------------ATGATG-AATTTGCGACATCTAGCTTCC------
------
FC88X_00802_00837      ----------------------ATGCG-CTCTGTAGCAACTAACATCTCC----
------
FC88X_00539_00146      ----------------------ATGCG-CTCTGTAGCAACTAACATCTCC----
------
FC88X_00066_00017      ---------------------CCTGCGACTCTGCAACAGCTATCATC-------
------
FC88X_00207_01008      -------------------CACGCTCCTGATC--AACAATTACATATT------
------
FC88X_00919_00945      -------------------CACACTCCTGATC--AACAATTACATATT------
------
FC88X_01110_00483      ----------------------CCTCCCGATTTTAGCATTTAAATATTGC----
------
FC88X_00422_00044      ---------------CTCCCAATTATCTCACTTCAATAGTCAGACCTCTTTA--
------
```

FC88X_00415_01187 ------------------------CTCTCGACTTAGCAATAGTAACTTCTC---
------
FC88X_00137_00554 ---------------------CCAAGTCTCCCA-CTTCAGAAGCAATCT-----
------
FC88X_00120_00059 --------------------TCTGGCACTAATC-AGTCAGCAATA---------
------
FC88X_00800_01136 -------------------CACTCACTTCTG---AGTAATCTATTTCTT-----
------
FC88X_00943_00715 -------------------CACTCACTTCTG---AGTAATCTATTTCTT-----
------
FC88X_00207_00014 -------------------CACTCACTTCTG---AGTAATCTATTTCTT-----
------
FC88X_00596_00096 -------------------CACTCACTTCTG---AGTAATCTATTTCTT-----
------
FC88X_00820_00843 -------------------CACTCACTTCTG---AGTAATCTATTTCTT-----
------
FC88X_00437_01024 ------------------CTACCGACTTCTGCC-ACTGATCTATCTC-------
------
FC88X_00399_01000 ------------------TTACTCAGTCCTCCGCAGCAGCTTTTT---------
------
FC88X_00492_01025 ---------------------CCCACACCTCCGCAGCAATTT------------
------
FC88X_00877_00307 ----------------ACAGTTCCACTCTTCAG-ACAGCTCTTTC---------
------
FC88X_00911_00744 ------------------TCTCCCACTCGTCAGCAGCACTCTTTC---------
------
FC88X_00139_00963 -------------------ACCTCACTCCTCAGGAGCCCTTTATT---------
------
FC88X_00061_00730 --------------AACCCTTCACGACA-TCCCAGTCAGCTGTT----------
------
FC88X_00365_00013 ---------------ACCCTTCACGACA-TCCCAGTCAGCTATT----------
------
FC88X_00775_00944 ----------------ACCTTCACGACA-
TCCCAGTCAGCTATTCTTCCTTTCT------
FC88X_00992_00574 ------------------CTACACAACACTCCCAGACAGATCAAT---------
------
FC88X_00182_00798 ------------------CTTCACATCCTCTACTGCTGTTCTGTC---------
------
FC88X_00981_00735 ------------------CTACACACACTCCTGCAGCTATTTGTC---------
------
FC88X_00809_01138 -------------------CTCGAGTA-TCCCTCATGTGC--AGCAATT-----
------
FC88X_00573_01099 -------------------CTCGAGTACTCCCTCATGTGC--AGCAATT-----
------
FC88X_00057_00739 -------------------CTCGAGTA-TCCCTCATGTGC--AGCAATT-----
------
FC88X_00335_01042 -------------------CTCGAGTA-TCCCTCATGTGC--AGCAATT-----
------
FC88X_00412_01073 -------------------CTCGAGTA-TCCCTCATGTGC--AGCAATT-----
------
FC88X_00408_01146 -------------------CTCGAGTA-TCCCTCATGTGC--AGCAATT-----
------
FC88X_01057_00380 -------------------CTCGAGTA-TCCCTCATGTGC--AGCAATT-----
------
FC88X_00110_00515 ------------------------CTTATCGCACAAGTCCCAAGCCATT-----
------

FC88X_00457_01156   ---------------------TCATCGTAACCGTATGAGCT-CCCATTCA----
------
FC88X_00908_00904   ---------------------TCATCGTAACCGTATGAGCT-CCCATTCA----
------
FC88X_00920_00390   ---------------------TCATCGTAACCGTATGAGCT-CCCATCCA----
------
FC88X_00476_00707   ---------------------TCATCGTAACCGCATGAGCT-CCCATTCA----
------
FC88X_00909_00587   ---------------------CCATCGCACCGGTGTCAGTC-ACATATT-----
------
FC88X_00584_01258   ---------------------CCATCGCACCGGTGTCAGTC-ACATATT-----
------
FC88X_00876_00584   ---------------------CCATCGCACCGGTGTCAGTC-ACATATT-----
------
FC88X_00136_00066   ---------------------CTATCGCACCGGTGTCAGTC-ACATATT-----
------
FC88X_00621_00961   -------------------CCCGTTCAACGCCACAGCATTTTGCTATC------
------
FC88X_01146_00745   ------------------GCCCGTTCAATGCCACAGCATTTTGCTATC------
------
FC88X_01017_00998   -------------------
CCCGTTCAATGTCACAGCATTTTGCTATCCTTCCTTT----
FC88X_00557_01217   -----------------------CTTCAAGTCACACCCCATAGGTATT------
------
FC88X_00870_00737   ---------------------CACCAGTGTCCTCACCACCACGCTATC------
------
FC88X_00094_00583   ---------------------CACCAGTGTCCTCACCACCATGCTATC------
------
FC88X_01078_00450   -------------------GACACCGGTCCCATCAGCCACA-ATTATT------
------
FC88X_00897_00220   --------------------ACACCAGTCCCATCAGCCACA-ATTATT------
------
FC88X_00139_00531   --------------------TCTCCCAGT-ATCTAGCACGACGCTAAC------
------
FC88X_01027_00746   -------------------GTCTCCCAGT-ATCTAGCACGACGCTAAC------
------
FC88X_00115_00628   --------------------TCTCCCAGT-ATCTAGCACGACGCTAAC------
------
FC88X_00859_00662   --------------------TCTCCCAGT-ATCTAGCACGACGCTAAC------
------
FC88X_00968_01027   ------------------GGTCTCCCAAT-CAACGGCACGCCGGTAAC------
------
FC88X_00169_00781   --------------------TCTCGAACTCAGCGATCAACAGGCTATC------
------
FC88X_01087_00922   --------------------TCTCGAACTCAGCGATCAACAGGCTATC------
------
FC88X_01105_01078   --------------------TCTCGAACTCAGCGATCAACAGGCTATC------
------
FC88X_00077_00820   ---------------------CCCGCATTTAGCAGCAAGATGGCTCTC------
------
FC88X_00390_00845   --------------------CCACG-GAT-CTCCCACGTGTGTCTACA------
------
FC88X_00320_00712   --------------------CCACGTGAT-CTCCCTAGCTCGGCTACG------
------
FC88X_01101_00657   ---------------------AGCACGAT-GCTCTCCCTTGGTCTCCAC-----
------

FC88X_00115_00230 --------------------CCACGTGCT-ATACCTAACCAATGTTTC------
------
FC88X_00982_00461 --------------------TCTCCCGGT-CTCTACCACCATTGTTTC------
------
FC88X_00020_00275 --------------------CCGCACCAGCGTT-GACAATTTATTCTC------
------
FC88X_00197_01041 -------------------GCCGCACCAGCGTT-GACAATTTATTCTC------
------
FC88X_00059_00764 --------------------CCGCACCAGCGTT-GACAATTTATTCTC------
------
FC88X_00272_00150 --------------------CCGCACCAGCGTT-GACAATTTATTCTC------
------
FC88X_00286_00474 --------------------CCGCACCAGCGTT-GACAATTTATTCTC------
------
FC88X_00331_00826 --------------------CCGCACCAGCGTT-GACAATTTATTCTC------
------
FC88X_00491_00435 --------------------CCGCACCAGCGTT-GACAATTTATTCTC------
------
FC88X_00471_01054 --------------------CCGCACCAGCGTT-GACAATTTATTCTC------
------
FC88X_00534_00108 --------------------CCGCACCAGCGTT-GACAATTTATTCTC------
------
FC88X_00554_00587 --------------------CCGCACCAGCGTT-GACAATTTATTCTC------
------
FC88X_00770_00470 --------------------CCGCACCAGCGTT-GACAATTTATTCTC------
------
FC88X_00762_00850 --------------------CCGCACCAGCGTT-GACAATTTATTCTC------
------
FC88X_00968_00315 --------------------CCGCACCAGCGTT-GACAATTTATTCTC------
------
FC88X_00999_00535 --------------------CCGCACCAGCGTT-GACAATTTATTCTC------
------
FC88X_01101_00724 --------------------CCGCACCAGCGTT-GACAATTTATTCTC------
------
FC88X_00004_00579 --------------------CCGCACCAGCGTT-GACAATTTATTCTC------
------
FC88X_00022_00662 --------------------CCGCACCAGCGTT-GACAATTTATTCTC------
------
FC88X_00085_00579 --------------------CCGCACCAGCGTT-GACAATTTATTCTC------
------
FC88X_00107_00632 --------------------CCGCACCAGCGTT-GACAATTTATTCTC------
------
FC88X_00195_00533 --------------------CCGCACCAGCGTT-GACAATTTATTCTC------
------
FC88X_00290_01084 --------------------CCGCACCAGCGTT-GACAATTTATTCTC------
------
FC88X_00403_00743 --------------------CCGCACCAGCGTT-GACAATTTATTCTC------
------
FC88X_00494_00174 --------------------CCGCACCAGCGTT-GACAATTTATTCTC------
------
FC88X_00460_01080 --------------------CCGCACCAGCGTT-GACAATTTATTCTC------
------
FC88X_00535_00558 --------------------CCGCACCAGCGTT-GACAATTTATTCTC------
------
FC88X_00626_01079 --------------------CCGCACCAGCGTT-GACAATTTATTCTC------
------

FC88X_00650_01007 --------------------CCGCACCAGCGTT-GACAATTTATTCTC------
------
FC88X_00947_00577 --------------------CCGCACCAGCGTT-GACAATTTATTCTC------
------
FC88X_00916_00903 --------------------CCGCACCAGCGTT-GACAATTTATTCTC------
------
FC88X_00941_00938 --------------------CCGCACCAGCGTT-GACAATTTATTCTC------
------
FC88X_00986_01065 --------------------CCGCACCAGCGTT-GACAATTTATTCTC------
------
FC88X_01043_00531 --------------------CCGCACCAGCGTT-GACAATTTATTCTC------
------
FC88X_01079_00716 --------------------CCGCACCAGCGTT-GACAATTTATTCTC------
------
FC88X_01084_00833 --------------------CCGCACCAGCGTT-GACAATTTATTCTC------
------
FC88X_01118_00875 --------------------CCGCACCAGCGTT-GACAATTTATTCTC------
------
FC88X_00417_01132 --------------------CCGCACCAGCGTTCGACAATTTATTCTC------
------
FC88X_00289_01126 --------------------CCGCACCAGCGTT-GACAATATATTCTC------
------
FC88X_00976_01006 -------------GGGGAATCCGCACCAGCGTTTGACAATTTATTCTC------
------
FC88X_00668_00474 ---------------GGGGACCGCACCAGCGTT-GACAACTTATTCTC------
------
FC88X_00872_01099 --------------------CCGCACCAGCGTTGAC-AGTTTATTCTC------
------
FC88X_00039_00691 --------------------CCGCACCAGCGTTGACCAATTTATTCTC------
------
FC88X_00419_00488 --------------GACTAGCCGCACCAGCGTTGAC-AATTTATTCTC------
------
FC88X_00774_00311 --------------------CCGCACCAGCGTTGGC-AATTTATTCTC------
------
FC88X_00982_00576 --------------------CCGCACCAGCGTTGGC-AATTTATTCTC------
------
FC88X_00248_01030 -------------------CCAGCACCAGCGTTCGACAATTTATTCTTC-----
------
FC88X_00638_01236 --------------------CCACGCAGGTGTC-GCCAACTCATTCTC------
------
FC88X_00747_00970 --------------------CCACGCAGGTGTC-GCCAACTCATTCTC------
------
FC88X_00148_00032 --------------------CCACGCAGGTGTC-
GCCAACTTATTCTCCTTCCTT-----
FC88X_00238_00896 ---------------------CTAGCAGCTCTC-GC--ACTCCTTCTACT----
------
FC88X_00935_01119 -----------------CTTTCCTCCCGGTGAC-GTTAAATTATT---------
------
FC88X_00440_00108 -----------------CTTTCGCCCCGGTGTT-CTCGAATTATT---------
------
FC88X_00005_00640 -----------------CTTCCGACCCGGTTAT-CGCAAATTGTT---------
------
FC88X_00865_00737 -----------------CTTCCGACCCGGTTAT-CGCAAATTGTT---------
------
FC88X_00593_01072 -----------------------CTCCCGCGTGTGACGATTTAAA-CGTATC--
------

FC88X_00324_00484 -----------------------CTCCCGCGTGTGACGATTTAAA-CGTATC--
------
FC88X_00078_00017 -----------------------CTCCCTGATATGACAAATTAA--CGTAAC--
------
FC88X_00999_00750 -----------------------CTCCCGGGTATGACAACAGATCGCGCAT---
------
FC88X_00179_00613 -----------------------CTCCCGGGTATGACAACAGATCGCGCATCC-
------
FC88X_00599_01226 -----------------------CTCCCGGGTATGGCAACAGATCGCGCAT---
------
FC88X_00014_00093 ------------------TCCATCAATG---CCCCGATGCCAATTCCC------
------
FC88X_00859_00266 ------------------CTTTCCATTA---TGACGCGACCTATTCAC------
------
FC88X_00255_01027 ---------------ACACGACATGAAT---CGACGCTCCCAAATA--------
------
FC88X_00572_00132 ---------------ACACGACATGAAT---CGACGCTCCCAAATA--------
------
FC88X_00066_00279 ---------------ACACGACATGAAT---CGACGCTCCCAAATA--------
------
FC88X_00539_00976 ---------------ACACGACATGAAT---CGACGCTCCCAAATA--------
------
FC88X_00913_00270 ---------------ACACGACATGAAT---CGACGCTCCCAAATA--------
------
FC88X_00366_00954 ---------GGGGGAACACGACATGAAT---CGACGCTCCCAAATA--------
------
FC88X_00051_00567 ---------------ATACGACATGAAT---CGACGCTCCCAAATACC------
------
FC88X_00408_01106 --------------------CCTTGAAC---GCCCGCCGCCAAATC--------
------
FC88X_00653_00567 ---------------CCATGACCTATA----CCCCGACACCATTTT--------
------
FC88X_00087_00747 ---------------CCATGACCTCGA----CGGTGCCAGTCTTTT--------
------
FC88X_00787_00290 -----------------CCTCCCTATA----ACCCGCTGCCATGTGAA------
------
FC88X_00795_00490 ---------------ACCCGACCTATA----CGCTGGCCCTGTT----------
------
FC88X_00487_01189 -----------------CCGATTAGTCT--CCGTCGGGCCATTGTC--------
------
FC88X_00823_01023 -----------------CCGATTAGTCT--CCGTCGGGCCATTGTC--------
------
FC88X_00951_00636 -----------------CCGATTAGTCT--CCGTCGGGCCACTGTC--------
------
FC88X_00801_00192 -----------------CCGATTAGTCT--CCGTCGGGCCATTGTT--------
------
FC88X_01107_01109 -----------------CCGATTAATGT--GCGTCGGACCCTTATA--------
------
FC88X_00039_00249 -----------------TCGATTTATAC--GTCGTCGGCCATTTCCC-------
------
FC88X_00323_01043 --------------TCCCGAATTAATCG--GC------TCCGGCATAAT-----
------
FC88X_00688_01224 --------------TCCCGAATTAATCG--GC------TCCGGCACAAT-----
------
FC88X_00501_00998 --------------TCCCGAATTAATCG--GCGGGGGGTCCCGCATAA------
------

FC88X_00684_01171 ---------------CTCTGTTCTATG---TGGACGCGCCTTTCT---------
------
FC88X_00799_00828 ---------------CTCTGTTCTATG---TGGACGCGCCTTTCT---------
------
FC88X_00845_01067 ---------------CTCTGTTCTATG---TGGACGCGCCTTTCT---------
------
FC88X_00769_00845 ---------------CTCTGTTCTATGGGGTGGACGCGCCTATCT---------
------
FC88X_00978_01145 ---------------CTGCGATCTATG---CGGCCCGGCCTATCTTC-------
------
FC88X_00048_00208 --------------CTCCGCATTTATT---TGAACCGGCCTATC----------
------
FC88X_00699_00207 -------------CGTCAGCATGTACA---T--ACTGGCCCATCTC--------
------
FC88X_00597_00435 -------------CGCACC-ATCAATTTTTGTCGT-GCC-GTTCTC--------
------
FC88X_00588_01123 -------------CGCACC-ATCAATTTTTGTCGT-GCC-GTTCTC--------
------
FC88X_00006_00142 -------------CGCACC-ATCAATTTT-GTCGT-GCC-GTTCTC--------
------
FC88X_00161_00855 -------------CGCACC-ATCAATTTT-GTCGT-GCC-GTTCTC--------
------
FC88X_00150_00108 -------------CGCACC-ATCAATTTT-GTCGT-GCC-GTTCTC--------
------
FC88X_00879_00800 -------------CGCACCGATCAATTTT-GTCGT-GCC-GTTCTC--------
------
FC88X_00189_01000 -------------CGCACC-ATCAATTTT-GTCGT-GCC-GTTCTC--------
------
FC88X_00246_00912 -------------CGCACC-ATCAATTTT-GTCGT-GCC-GTTCTC--------
------
FC88X_00358_00752 -------------CGCACC-ATCAATTTT-GTCGT-GCC-GTTCTC--------
------
FC88X_00243_00034 -------------CGCACC-ATCAATTTT-GTCGT-GCC-GTTCTC--------
------
FC88X_00028_00352 ------------GCGCACC-ATCAATTTT-GTCGT-GCCCGTTCTC--------
------
FC88X_00275_00044 -------------CGCACC-ATCAATTTT-GTCGT-GCC-GTTCTC--------
------
FC88X_00407_00662 -------------CGCACC-ATCAATTTT-GTCGT-GCC-GTTCTC--------
------
FC88X_00650_00277 -------------CGCACC-ATCAATTTT-GTCGT-GCC-GTTCTC--------
------
FC88X_00765_00911 -------------CGCACC-ATCAATTTT-GTCGT-GCC-GTTCTC--------
------
FC88X_00302_00766 -------------CGCACC-ATCAATTTT-GTCGT-GCC-GTTCTC--------
------
FC88X_00713_00887 -------------CGCACC-ATCAATTTT-GTCGT-GCC-GTTCTCCC------
------
FC88X_00610_01185 -------------CGCACC-ATCAATTTT-GTCGT-GCC-GTTCTC--------
------
FC88X_00651_00568 -------------CGCACC-ATCAATTTT-GTCGT-GCC-GTTCTC--------
------
FC88X_00906_00489 -------------CGCACC-ATCAATTTT-GTCGT-GCC-GTTCTC--------
------
FC88X_00029_00042 -------------CGCACC-ATCAATTTT-GTCGT-GCC-GTTCTC--------
------

FC88X_00018_00484 --------------CGCACC-ATCAATTTT-GTCGT-GCC-GTTCTC--------
------
FC88X_00005_00582 --------------CGCACC-ATCAATTTT-GTCGT-GCC-GTTCTC--------
------
FC88X_00049_00695 --------------CGCACC-ATCAATTTT-GTCGT-GCC-GTTCTC--------
------
FC88X_00050_00031 --------------CGCACC-ATCAATTTT-GTCGT-GCC-GTTCTC--------
------
FC88X_00099_00045 --------------CGCACC-ATCAATTTT-GTCGT-GCC-GTTCTC--------
------
FC88X_00080_00793 --------------CGCACC-ATCAATTTT-GTCGT-GCC-GTTCTC--------
------
FC88X_00262_00072 --------------CGCACC-ATCAATTTT-GTCGT-GCC-GTTCTC--------
------
FC88X_00353_00052 --------------CGCACC-ATCAATTTT-GTCGT-GCC-GTTCTC--------
------
FC88X_00364_01002 --------------CGCACC-ATCAATTTT-GTCGT-GCC-GTTCTC--------
------
FC88X_00364_01090 --------------CGCACC-ATCAATTTT-GTCGT-GCC-GTTCTC--------
------
FC88X_00433_01058 --------------CGCACC-ATCAATTTT-GTCGT-GCC-GTTCTC--------
------
FC88X_00461_01143 --------------CGCACC-ATCAATTTT-GTCGT-GCC-GTTCTC--------
------
FC88X_00518_00640 --------------CGCACC-ATCAATTTT-GTCGT-GCC-GTTCTC--------
------
FC88X_00584_00532 --------------CGCACC-ATCAATTTT-GTCGT-GCC-GTTCTC--------
------
FC88X_00615_01162 --------------CGCACC-ATCAATTTT-GTCGT-GCC-GTTCTC--------
------
FC88X_00651_00426 --------------CGCACC-ATCAATTTT-GTCGT-GCC-GTTCTC--------
------
FC88X_00906_00741 --------------CGCACC-ATCAATTTT-GTCGT-GCC-GTTCTC--------
------
FC88X_00941_00898 --------------CGCACC-ATCAATTTT-GTCGT-GCC-GTTCTC--------
------
FC88X_01096_00789 --------------CGCACC-ATCAATTTT-GTCGT-GCC-GTTCTC--------
------
FC88X_01104_00629 --------------CGCACC-ATCAATTTT-GTCGT-GCC-GTTCTC--------
------
FC88X_00671_00986 -------------GCGCACC-ATCAATTTT-GTCGT-GCC-GTTCTC--------
------
FC88X_00121_00364 --------------CGCACC-ATCAATTT--GTCGT-GCC-GTTCTC--------
------
FC88X_00633_00180 --------------CGCACC-ATCAATTT--GTCGT-GCC-GTTCTC--------
------
FC88X_00100_00687 --------------CGCACC-ATCAATCTT-GTCGT-GCC-GTTCTC--------
------
FC88X_00462_00021 --------------CGCACC-ATCAACTT--GTCGT-GCC-GTTCTC--------
------
FC88X_00117_00648 --------------CGCACC-ATCAATCTT-GTCGT-GCC-
GTTCTCCTTCCTTT------
FC88X_00400_00062 --------------CGCACC-ATCAATTTTGTC-GT-GCC-
GTTCTCCTTCCTTT------
FC88X_01075_00852 --------------CGCACC-ATCAATTTTGTC-GT-GCC-
GTTCTCCTTCCTTT------

FC88X_00822_00398 -------------CGCGCC-ATCAATTTTGTC-GT-GCC-GTTCTC--------
------
FC88X_00627_00681 -------------CGCACC-GTCAATTTTGTC-GT-GCC-GTTCTC--------
------
FC88X_00545_00133 -------------CGCACC-ATCAGTTTTGTC-GT-GCC-GTTCTC--------
------
FC88X_01099_00521 -------------CGCACC-ATCAATTTTGTCCGT-GCCCGTTCTC--------
------
FC88X_00016_00141 --------------CCACCAA-CTACT-GTT-CGTCGCC-GTTCT---------
------
FC88X_00909_00307 --------------CCACCAA-CTACT-GTT-CGTCGCC-GTTCT---------
------
FC88X_01140_00854 --------------CCACCAA-CTACT-GTT-CGTCGCC-GTTCT---------
------
FC88X_00127_00461 --------GGGGAACCACCAA-CTATT-GTT-CGTCGCC-GTTCT---------
------
FC88X_00914_00690 --------------CCGCCAA-CTATT-GTT-CGTCGCC-GTTCT---------
------
FC88X_00859_01257 --------------CCACCAA-CTGTT-GTT-CGTCGCC-GTTCT---------
------
FC88X_00163_00055 --------------CCACCAA-CTGTT-GTT-CGTCGCC-GTTCT---------
------
FC88X_00612_01019 --------------CCACCAA-CTATT-GTT-CGTCGCCCGTTCT---------
------
FC88X_00852_00140 --------------CCACCAA-CTATT-GTT-CGTCGCCCGTTCT---------
------
FC88X_00555_00939 -------------GCCACCAA-CTATT-GTT-CGTCGCC-GTTCT---------
------
FC88X_00687_01074 -------------GCCACCAA-CTATT-GTT-CGTCGCC-GTTCT---------
------
FC88X_00778_00949 -------------GCCACCAA-CTATT-GTT-CGTCGCC-GTTCT---------
------
FC88X_00821_01100 -------------GCCACCAA-CTATT-GTT-CGTCGCC-GTTCT---------
------
FC88X_01121_00523 -------------GCCACCAA-CTATT-GTT-CGTCGCC-GTTCT---------
------
FC88X_01142_00949 -------------GCCACCAA-CTATT-GTT-CGTCGCC-GTTCT---------
------
FC88X_00317_01125 --------------CCACCAA-CTATT-GTT-CGTCGCC-GTTCT---------
------
FC88X_00364_01196 --------------CCACCAA-CTATT-GTT-CGTCGCC-GTTCT---------
------
FC88X_01012_00620 ---------------CACCAA-CTATT-GTT-CGTCGCC-GTTCT---------
------
FC88X_00101_00284 --------------CCACCAAACTATT-GTT-CGTCGCC-GTTCT---------
------
FC88X_00309_01082 --------------CCACCAA-CTATT-GTT-CGTCGCC-GTTCT---------
------
FC88X_00376_00644 --------------CCACCAA-CTATT-GTT-CGTCGCC-GTTCT---------
------
FC88X_00447_01189 --------------CCACCAA-CTATT-GTT-CGTCGCC-GTTCT---------
------
FC88X_00402_01201 --------------CCACCAA-CTATT-GTT-CGTCGCC-GTTCT---------
------
FC88X_00462_00041 --------------CCACCAA-CTATT-GTT-CGTCGCC-GTTCT---------
------

FC88X_00467_00595 --------------CCACCAA-CTATT-GTT-CGTCGCC-GTTCT---------
------
FC88X_00512_00117 --------------CCACCAA-CTATT-GTT-CGTCGCC-GTTCT---------
------
FC88X_00543_00965 --------------CCACCAA-CTATT-GTT-CGTCGCC-GTTCT---------
------
FC88X_00511_01241 --------------CCACCAA-CTATT-GTT-CGTCGCC-GTTCT---------
------
FC88X_00602_00174 --------------CCACCAA-CTATT-GTT-CGTCGCC-GTTCT---------
------
FC88X_00607_00320 --------------CCACCAA-CTATT-GTT-CGTCGCC-GTTCT---------
------
FC88X_00647_01014 --------------CCACCAA-CTATT-GTT-CGTCGCC-GTTCT---------
------
FC88X_00674_01200 --------------CCACCAA-CTATT-GTT-CGTCGCC-GTTCT---------
------
FC88X_00731_00256 --------------CCACCAA-CTATT-GTT-CGTCGCC-GTTCT---------
------
FC88X_00737_01029 --------------CCACCAA-CTATT-GTT-CGTCGCC-GTTCT---------
------
FC88X_00729_01090 --------------CCACCAA-CTATT-GTT-CGTCGCC-GTTCT---------
------
FC88X_00710_01154 --------------CCACCAA-CTATT-GTT-CGTCGCC-GTTCT---------
------
FC88X_00770_00268 --------------CCACCAA-CTATT-GTT-CGTCGCC-GTTCT---------
------
FC88X_00778_00431 --------------CCACCAA-CTATT-GTT-CGTCGCC-GTTCT---------
------
FC88X_00793_01175 --------------CCACCAA-CTATT-GTT-CGTCGCC-GTTCT---------
------
FC88X_00805_00813 --------------CCACCAA-CTATT-GTT-CGTCGCC-GTTCT---------
------
FC88X_00846_01121 --------------CCACCAA-CTATT-GTT-CGTCGCC-GTTCT---------
------
FC88X_00891_00787 --------------CCACCAA-CTATT-GTT-CGTCGCC-GTTCT---------
------
FC88X_00886_00803 --------------CCACCAA-CTATT-GTT-CGTCGCC-GTTCT---------
------
FC88X_00939_00756 --------------CCACCAA-CTATT-GTT-CGTCGCC-GTTCT---------
------
FC88X_00903_00833 --------------CCACCAA-CTATT-GTT-CGTCGCC-GTTCT---------
------
FC88X_00912_00948 --------------CCACCAA-CTATT-GTT-CGTCGCC-GTTCT---------
------
FC88X_00924_01006 --------------CCACCAA-CTATT-GTT-CGTCGCC-GTTCT---------
------
FC88X_00943_01014 --------------CCACCAA-CTATT-GTT-CGTCGCC-GTTCT---------
------
FC88X_00979_00439 --------------CCACCAA-CTATT-GTT-CGTCGCC-GTTCT---------
------
FC88X_00978_01077 --------------CCACCAA-CTATT-GTT-CGTCGCC-GTTCT---------
------
FC88X_01044_00605 --------------CCACCAA-CTATT-GTT-CGTCGCC-GTTCT---------
------
FC88X_01007_01149 --------------CCACCAA-CTATT-GTT-CGTCGCC-GTTCT---------
------

FC88X_01014_01129 ---------------CCACCAA-CTATT-GTT-CGTCGCC-GTTCT---------
------
FC88X_01109_00459 ---------------CCACCAA-CTATT-GTT-CGTCGCC-GTTCT---------
------
FC88X_01117_00685 ---------------CCACCAA-CTATT-GTT-CGTCGCC-GTTCT---------
------
FC88X_01115_00833 ---------------CCACCAA-CTATT-GTT-CGTCGCC-GTTCT---------
------
FC88X_01130_00811 ---------------CCACCAA-CTATT-GTT-CGTCGCC-GTTCT---------
------
FC88X_01115_01149 ---------------CCACCAA-CTATT-GTT-CGTCGCC-GTTCT---------
------
FC88X_00043_00624 ---------------CCACCAA-CTATT-GTT-CGTCGCC-GTTCT---------
------
FC88X_00093_00080 ---------------CCACCAA-CTATT-GTT-CGTCGCC-GTTCT---------
------
FC88X_00082_00217 ---------------CCACCAA-CTATT-GTT-CGTCGCC-GTTCT---------
------
FC88X_00091_00550 ---------------CCACCAA-CTATT-GTT-CGTCGCC-GTTCT---------
------
FC88X_00142_00073 ---------------CCACCAA-CTATT-GTT-CGTCGCC-GTTCT---------
------
FC88X_00129_00178 ---------------CCACCAA-CTATT-GTT-CGTCGCC-GTTCT---------
------
FC88X_00122_00262 ---------------CCACCAA-CTATT-GTT-CGTCGCC-GTTCT---------
------
FC88X_00103_00586 ---------------CCACCAA-CTATT-GTT-CGTCGCC-GTTCT---------
------
FC88X_00129_00960 ---------------CCACCAA-CTATT-GTT-CGTCGCC-GTTCT---------
------
FC88X_00163_00739 ---------------CCACCAA-CTATT-GTT-CGTCGCC-GTTCT---------
------
FC88X_00189_00912 ---------------CCACCAA-CTATT-GTT-CGTCGCC-GTTCT---------
------
FC88X_00255_00956 ---------------CCACCAA-CTATT-GTT-CGTCGCC-GTTCT---------
------
FC88X_00255_00961 ---------------CCACCAA-CTATT-GTT-CGTCGCC-GTTCT---------
------
FC88X_00364_00030 ---------------CCACCAA-CTATT-GTT-CGTCGCC-GTTCT---------
------
FC88X_00354_01064 ---------------CCACCAA-CTATT-GTT-CGTCGCC-GTTCT---------
------
FC88X_00442_00355 ---------------CCACCAA-CTATT-GTT-CGTCGCC-GTTCT---------
------
FC88X_00436_00418 ---------------CCACCAA-CTATT-GTT-CGTCGCC-GTTCT---------
------
FC88X_00586_01247 ---------------CCACCAA-CTATT-GTT-CGTCGCC-GTTCT---------
------
FC88X_00607_01228 ---------------CCACCAA-CTATT-GTT-CGTCGCC-GTTCT---------
------
FC88X_00652_00735 ---------------CCACCAA-CTATT-GTT-CGTCGCC-GTTCT---------
------
FC88X_00698_01215 ---------------CCACCAA-CTATT-GTT-CGTCGCC-GTTCT---------
------
FC88X_00675_01257 ---------------CCACCAA-CTATT-GTT-CGTCGCC-GTTCT---------
------

FC88X_00721_01153 ---------------CCACCAA-CTATT-GTT-CGTCGCC-GTTCT---------------
FC88X_00771_00184 ---------------CCACCAA-CTATT-GTT-CGTCGCC-GTTCT---------------
FC88X_00776_00841 ---------------CCACCAA-CTATT-GTT-CGTCGCC-GTTCT---------------
FC88X_00767_01134 ---------------CCACCAA-CTATT-GTT-CGTCGCC-GTTCT---------------
FC88X_00834_00297 ---------------CCACCAA-CTATT-GTT-CGTCGCC-GTTCT---------------
FC88X_00803_01068 ---------------CCACCAA-CTATT-GTT-CGTCGCC-GTTCT---------------
FC88X_00918_00828 ---------------CCACCAA-CTATT-GTT-CGTCGCC-GTTCT---------------
FC88X_00938_00808 ---------------CCACCAA-CTATT-GTT-CGTCGCC-GTTCT---------------
FC88X_00978_00682 ---------------CCACCAA-CTATT-GTT-CGTCGCC-GTTCT---------------
FC88X_01011_00429 ---------------CCACCAA-CTATT-GTT-CGTCGCC-GTTCT---------------
FC88X_01017_00420 ---------------CCACCAA-CTATT-GTT-CGTCGCC-GTTCT---------------
FC88X_01033_00892 ---------------CCACCAA-CTATT-GTT-CGTCGCC-GTTCT---------------
FC88X_01127_00611 ---------------CCACCAA-CTATT-GTT-CGTCGCC-GTTCT---------------
FC88X_01126_01029 ---------------CCACCAA-CTATT-GTT-CGTCGCC-GTTCT---------------
FC88X_00010_00198 ---------------CCACCAA-CTATT-GTT-CGTCGCC-GTTCT---------------
FC88X_00013_00163 ---------------CCACCAA-CTATT-GTT-CGTCGCC-GTTCT---------------
FC88X_00027_00718 ---------------CCACCAA-CTATT-GTT-CGTCGCC-GTTCT---------------
FC88X_00088_00098 ---------------CCACCAA-CTATT-GTT-CGTCGCC-GTTCT---------------
FC88X_00093_00803 ---------------CCACCAA-CTATT-GTT-CGTCGCC-GTTCT---------------
FC88X_00112_00524 ---------------CCACCAA-CTATT-GTT-CGTCGCC-GTTCT---------------
FC88X_00114_00574 ---------------CCACCAA-CTATT-GTT-CGTCGCC-GTTCT---------------
FC88X_00276_00072 ---------------CCACCAA-CTATT-GTT-CGTCGCC-GTTCT---------------
FC88X_00254_00466 ---------------CCACCAA-CTATT-GTT-CGTCGCC-GTTCT---------------
FC88X_00277_00932 ---------------CCACCAA-CTATT-GTT-CGTCGCC-GTTCT---------------
FC88X_00286_00336 ---------------CCACCAA-CTATT-GTTTCGTCGCC-GTTCT---------------
FC88X_00127_00088 ---------------CCACCAA-CTATT-GTT-CGTCGCC-GTTCTTCCTTT---------
FC88X_00844_00324 ---------------CCACCAA-CTATT-GTT-CGTCGCC-GTTTT---------------
FC88X_00142_00926 ---------------CCACCAA-CTATT-GTT-CGTCGCC-GTTCTATCAC----------

```
FC88X_01117_01162       --------------CCACCAA-CTATT-GTT-CGTCGTC-GTTCT---------
------
FC88X_00799_00182       --------------CCACCAACCA-TT-GTT-CGTCGCC-GTTCT---------
------
FC88X_00888_00856       --------------CCACCAACCA-TT-GTT-CGTCGCC-GTTCTC--------
------
FC88X_00394_00756       --------------CCACCAACTA-TT-GTT-CGTCGCC-GTTCTC--------
------
FC88X_00866_01223       --------------CCACCAACTACTT-GTT-CGTCGCC-GTTCTC--------
------
FC88X_00041_00703       --------------CCACCAACTA-TT-GTT-CGTCGCC-GTTCTC--------
------
FC88X_00283_00986       -------------GCCACCAA-CTATT-GTT-CGTCGCC-
GTTCTCTTCCTTTCT-----
FC88X_00010_00449       --------------CCACCAA-CTATT-GTT-CGTCGCC-GTTCTCTTCCTTT-
------
FC88X_00516_00024       --------------CCACCAA-CTATT-GTT-CGTCGCC-ATTCT---------
------
FC88X_00873_01259       --------------CCACCAA-CTATT-GTTCGTT-GCC-GTTCT---------
------
FC88X_00468_00064       --------------CCACCAA-CTATT-GTTCGTTCGCC-GTTCT---------
------
FC88X_01104_00930       --------------CCACCAA-CTATT-GTTCGTT-GCC-GTTCT---------
------
FC88X_00195_00982       --------------CCACCAAACTATTTGTTCGTCCGCC-GTTCT---------
------
FC88X_00893_00366       --------------CCACCAA-CTATT-GTTCG-CCGCC-GTTCT---------
------
FC88X_00063_00644       --------------CCACCAA-CTATT-GTTCGTC-GCC-GTCCT---------
------
FC88X_00437_01179       -------------CCCACCAA-CTATT-GTT-CGTCGCCGTTCCT---------
------
FC88X_00938_00847       --------------CCACCAA-CTATT-GTT-CGTCGCCGTTCCT---------
------
FC88X_00770_01157       --------------CCACCAA-CTATT-GTT-CGTCGCCGTTACTCTTC-----
------
FC88X_00522_00041       --------------CCACCAA-CTATT-GTT-CGT-GCC-GCTTTCTCTTCC--
------
FC88X_00601_00264       --------------CCACCAA-CTATT-GTT-CGTCGCC-GCTCTCTTCC----
------
FC88X_00606_00076       --------------CCACCAA-CTAT--GTTCCGTCGCC-GTTCT---------
------
FC88X_00549_01088       -------------CCCACCAA-CTATT-GTT-CGTCGGCCCGGTT---------
------
FC88X_00832_00274       --------------CCACCAA-CTATT-GAT-CGTCCGC---------------
------
FC88X_01046_00791       -------------ACACTCAATCACTTGC----GTCGTC---------------
------
FC88X_01114_00624       -------------ACACTCAATCACTTGC----GTCGTCCTACTC---------
------
FC88X_01070_01224       -----------------TCCAACACTTGCTG--GTCGTCCGCTATC--------
------
FC88X_00034_00774       ------------------CCAACACCTTGTC--GGCGGCCTCTCGTA-------
------
FC88X_00721_00928       -------------GCTACTCTCCAACCGTTG--GTCGTCCTCTCGTTCTTCT--
------
```

FC88X_00094_00502 ------------------TCAACACTTTT-ACGTTAGGCGACTCTC--------
------
FC88X_00710_01147 -----------------GTCAACACTTTTTACGTTAGGCGACTCTC--------
------
FC88X_00871_01154 ------------------TCAACACTTTT-ACGTTAGGCGACTCTC--------
------
FC88X_00071_00885 ------------------TCAACACTTTT-ACGTTAGGCGACTCTC--------
------
FC88X_01093_00532 ------------------TCAACACTTTT-ACGTTAGGCGACTCTC--------
------
FC88X_00225_00880 ------------------TCAACACTTTT-ACGTTAGGCGACTCTC--------
------
FC88X_00695_00960 -----------GGGGAATTCAACACTTTTTACGTTAGGCGACTCTC--------
------
FC88X_00071_00716 ------------------TCAACACTTTT-ACGTTAGGCGACTCTC--------
------
FC88X_00822_00889 ------------------TCAACACTTTT-ACGTTAGGCGACTCTC--------
------
FC88X_01043_00837 ------------------TCAACACTTTT-ACGTTAGGCGACTCTC--------
------
FC88X_00109_00025 ------------------TCAACACTTTT-ACGTTAGGCGACTCTCC-------
------
FC88X_00354_00055 ------------------TCAACACTTTT-ACGTTAGGCGACTCTC--------
------
FC88X_01088_00784 ------------------TCAACACTTTT-ACGTTAGGCGACTCTC--------
------
FC88X_00076_00108 ------------------TCAACACTTTT--AGTTAGGCGACTCTC--------
------
FC88X_00620_01142 -----------------GTCAACACTTTT-ACGTTAGGCAACTCTC--------
------
FC88X_00987_00492 ------------------TCAACGCTTTT-ACGTTAGGCGACTCTC--------
------
FC88X_01007_00498 ------------------TCAACACATTT-ACGTTAGGCGACTCTC--------
------
FC88X_00093_00804 ------------------TCAACACTTTT-ACGTTAGGCGATTCTC--------
------
FC88X_00820_00805 ------------------TCAACACTTTT-ACGTTAGGCAGTACTC--------
------
FC88X_00526_00959 ------------------TCAACAATTTT-AAGCGCGGCTGTTCTT--------
------
FC88X_00387_00057 ------------------TCAACAA-TTTATCTGCGTC-GGCTTTCT-------
------
FC88X_00652_00184 ------------------TCAACAA-TTTATCTGCGTC-GGCTTTCT-------
------
FC88X_00781_00423 ------------------TCAACAA-TTTATCTGCGTC-GGCTTTCT-------
------
FC88X_00805_01021 ------------------TCAACAA-TTTATCTGCGTC-GGCTTTCT-------
------
FC88X_00984_00267 ------------------TCAACAA-TTTATCTGCGTC-GGCTTTCT-------
------
FC88X_01080_00862 ------------------TCAACAA-TTTATCTGCGTC-GGCTTTCT-------
------
FC88X_00072_00714 ------------------TCAACAA-TTTATCTGCGTC-GGCTTTCT-------
------
FC88X_00147_01011 ------------------TCAACAA-TTTATCTGCGTC-GGCTTTCT-------
------

```
FC88X_00268_01074      ------------------TCAACAA-TTTATCTGCGTC-GGCTTTCT-------
------
FC88X_00591_01188      ------------------TCAACAA-TTTATCTGCGTC-GGCTTTCT-------
------
FC88X_00563_01237      ------------------TCAACAA-TTTATCTGCGTC-GGCTTTCT-------
------
FC88X_00620_00084      ------------------TCAACAA-TTTATCTGCGTC-GGCTTTCT-------
------
FC88X_00769_00920      ------------------TCAACAA-TTTATCTGCGTC-GGCTTTCT-------
------
FC88X_00843_00952      ------------------TCAACAA-TTTATCTGCGTC-GGCTTTCT-------
------
FC88X_00992_01040      ------------------TCAACAA-TTTATCTGCGTC-GGCTTTCT-------
------
FC88X_01128_01068      ------------------TCAACAA-TTTATCTGCGTC-GGCTTTCT-------
------
FC88X_00144_00205      ------------------TCAACAA-TTTATCTGCGTC-GGCTTTCT-------
------
FC88X_00177_00491      ------------------TCAACAA-TTTATCTGCGTC-GGCTTTCT-------
------
FC88X_00498_01209      ------------------TCAACAAATTTATCTGCGTC-GGCTTTCC-------
------
FC88X_00389_01004      ------------------TCAACAA-TTTATCTGCGTC-GGCTTTCC-------
------
FC88X_01053_00830      ------------------TCAACAA-TTTATCTGCGTC-GGCTTT---------
------
FC88X_00858_00987      ------------------TCAACAA-TTTATCTGTGTC-GGCTTTCT-------
------
FC88X_00483_01045      ------------------TCAACAA-TTTGTCTGCGTCCGGCTTTCTCTTC---
------
FC88X_00929_00727      -------------------CCACAATTTTGTCAGCGCC-GGCGTTTA-------
------
FC88X_01051_00778      -----------------ATCAACAACCTTCTCCGTGCC-TTCTTT---------
------
FC88X_00276_00961      -------------------CCAGAATCTGTTCTGAGTTC-GTTCTCT-------
------
FC88X_01065_00702      -------------------CCAGAATCTGTTCTGAGTTC-GTTCTCT-------
------
FC88X_00033_00695      ------------------TCCACAATG-GTTCGGACTCCCGCTCTC--------
------
FC88X_00608_00973      ------------------TCCACAATG-GTTCGGACTCC-GCTCC---------
------
FC88X_00450_01170      ------------------TCCACAATG-GTTCGGACTTCCGTTCTC--------
------
FC88X_00061_00183      -------------------ATCAATGCTGTCCAGCGTGACGTTCTC--------
------
FC88X_00197_00016      -------------------ATCAATGCTGTCCAGCGTGACGTTCTC--------
------
FC88X_01044_00345      ---------------GCGGATCAATTCGGATCGG-GTG-CGTTCTC--------
------
FC88X_00176_00630      -----------------TCAACAAAT-CCGTTTGGGG-CGAC-TTCTCC-----
------
FC88X_00457_01173      -----------------TCAACAAAT-CCGTTTGGGG-CGAC-TTCTCC-----
------
FC88X_00040_00129      -----------------TCAACAAAT-C-GTTTGGGG-CGAC-TTCTC------
------
```

FC88X_00015_00266 -----------------TCAACAAAT-C-GTTTGGGG-CGAC-TTCTC------
------
FC88X_00042_00595 -----------------TCAACAAAT-C-GTTTGGGG-CGAC-TTCTC------
------
FC88X_00023_00158 -----------------TCAACAAAT-C-GTTTGGGG-CGAC-TTCTC------
------
FC88X_00054_00654 -----------------TCAACAAAT-C-GTTTGGGG-CGACATTCTC------
------
FC88X_00024_00372 -----------------TCAACAAAT-C-GTTTGGGG-CGAC-TTCTC------
------
FC88X_00094_00014 -----------------TCAACAAAT-C-GTTTGGGG-CGAC-TTCTC------
------
FC88X_00310_00520 -----------------TCAACAAAT-CGTTTTGGGG-CGAC-TTCTC------
------
FC88X_01032_00531 -----------------TCAACAAAT-CGTTTTGGGG-CGAC-TTCTC------
------
FC88X_00269_01075 -----------------TCAACAAAT-CGTTTTGGGG-CGAC-TTCTC------
------
FC88X_00974_00482 -----------------TCAACAAAT-CGTTTTGGGG-CGAC-TTCTC------
------
FC88X_00993_00890 -----------------TCAACAAAT-CGTTTTGGGG-CGAC-TTCTC------
------
FC88X_01083_00990 -----------------TCAACAAAT-CGTTTTGGGG-CGAC-TTCTC------
------
FC88X_00035_00181 -----------------TCAACAAAT-CGTTT-GGGG-CGAC-TTCTC------
------
FC88X_00030_00442 -----------------TCAACAAAT-CGTTT-GGGG-CGAC-TTCTC------
------
FC88X_00058_00056 -----------------TCAACAAAT-CGTTT-GGGG-CGAC-TTCTC------
------
FC88X_00318_00290 -----------GGGGAATCAACAAAT-C-GTTTGGGG-CGAC-TTCTC------
------
FC88X_01055_00398 -----------GGGGAATCAACAAAT-C-GTTTGGGG-CGAC-TTCTC------
------
FC88X_01045_00915 ------------GGGAATCAACAAAT-C-GTTTGGGG-CGAC-TTCTC------
------
FC88X_00039_00346 -----------------TCAACAAAT-C-GTTTGGGG-CGAC-TTCTC------
------
FC88X_00042_00674 -----------------TCAACAAAT-C-GTTTGGGG-CGAC-TTCTC------
------
FC88X_00098_00429 -----------------TCAACAAAT-C-GTTTGGGG-CGAC-TTCTC------
------
FC88X_00088_00884 -----------------TCAACAAAT-C-GTTTGGGG-CGAC-TTCTC------
------
FC88X_00018_00644 ----------------GTCAACAAAT-C-GTTTGGGG-CGAC-TTCTC------
------
FC88X_00360_01072 -------------GGGGTCAACAAAT-C-GTTTGGGG-CGAC-TTCTC------
------
FC88X_00121_00093 ----------------GTCAACAAAT-C-GTTTGGGG-CGAC-TTCTC------
------
FC88X_00209_01061 ----------------GTCAACAAAT-C-GTTTGGGG-CGAC-TTCTC------
------
FC88X_00412_01023 ----------------GTCAACAAAT-C-GTTTGGGG-CGAC-TTCTC------
------
FC88X_00578_00614 ----------------GTCAACAAAT-C-GTTTGGGG-CGAC-TTCTC------
------

```
FC88X_00847_00755      ----------------GTCAACAAAT-C-GTTTGGGG-CGAC-TTCTC------
------
FC88X_00823_00932      ----------------GTCAACAAAT-C-GTTTGGGG-CGAC-TTCTC------
------
FC88X_01042_00726      ----------------GTCAACAAAT-C-GTTTGGGG-CGAC-TTCTC------
------
FC88X_01120_00942      ----------------GTCAACAAAT-C-GTTTGGGG-CGAC-TTCTC------
------
FC88X_01141_01084      ----------------GTCAACAAAT-C-GTTTGGGG-CGAC-TTCTC------
------
FC88X_00149_00828      ----------------GTCAACAAAT-C-GTTTGGGG-CGAC-TTCTC------
------
FC88X_00203_01020      ----------------GTCAACAAAT-C-GTTTGGGG-CGAC-TTCTC------
------
FC88X_00418_00118      ----------------GTCAACAAAT-C-GTTTGGGG-CGAC-TTCTC------
------
FC88X_00586_00596      ----------------GTCAACAAAT-C-GTTTGGGG-CGAC-TTCTC------
------
FC88X_00598_01233      ----------------GTCAACAAAT-C-GTTTGGGG-CGAC-TTCTC------
------
FC88X_00733_00894      ----------------GTCAACAAAT-C-GTTTGGGG-CGAC-TTCTC------
------
FC88X_00875_00712      ----------------GTCAACAAAT-C-GTTTGGGG-CGAC-TTCTC------
------
FC88X_00920_00408      ----------------GTCAACAAAT-C-GTTTGGGG-CGAC-TTCTC------
------
FC88X_01138_00575      ----------------GTCAACAAAT-C-GTTTGGGG-CGAC-TTCTC------
------
FC88X_00999_01075      ----------------GTCAACAAAT-C-GTTTGGGG-CGAC-TTCTCT-----
------
FC88X_00011_00467      -----------------TCAACAAAT-C-GTTTGGGG-CGAC-TTCTC------
------
FC88X_00066_00214      -----------------TCAACAAAT-C-GTTTGGGG-CGAC-TTCTC------
------
FC88X_00060_00751      -----------------TCAACAAAT-C-GTTTGGGG-CGAC-TTCTC------
------
FC88X_00119_00108      -----------------TCAACAAAT-C-GTTTGGGG-CGAC-TTCTC------
------
FC88X_00171_00085      -----------------TCAACAAAT-C-GTTTGGGG-CGAC-TTCTCT-----
------
FC88X_00444_00954      -----------------TCAACAAAT-C-GTTTGGGG-CGAC-TTCTCT-----
------
FC88X_01010_01055      -----------------TCAACAAAT-C-GTTTGGGG-CGAC-TTCTCT-----
------
FC88X_00060_00498      -----------------TCAACAAAT-C-GTTTGGGG-CGAC-TTCTC------
------
FC88X_00146_00095      -----------------TCAACAAAT-C-GTTTGGGG-CGAC-TTCTC------
------
FC88X_00126_00123      -----------------TCAACAAAT-C-GTTTGGGG-CGAC-TTCTC------
------
FC88X_00130_00434      -----------------TCAACAAAT-C-GTTTGGGG-CGAC-TTCTC------
------
FC88X_00108_00932      -----------------TCAACAAAT-C-GTTTGGGG-CGAC-TTCTC------
------
FC88X_00088_00417      -----------------TCAACAAAT-CGTTTGGGGG-CGAC-TTCTC------
------
```

```
FC88X_01122_00554       -----------------TCAACAAAT-CGTTTGGGGG-CGAC-TTCT-------
------
FC88X_00907_00245       -----------------TCAACAAAT-CGTTTGGGGG-CGAC-TTCTC------
------
FC88X_00677_00918       -----------------TCAACAAAT-CGTT-GGGGG-CGAC-TTCTC------
------
FC88X_00834_01095       -----------GGGGAATCAACAAAT-CGTTTGGGGG-CGAC-TTCTC------
------
FC88X_00680_00790       -----------------TCAACAAAT-CGTTTGGGGG-CGAC-TTCTC------
------
FC88X_00878_00455       -----------------TCAACAAAT-CGTTTGGGGG-CGAC-TTCTC------
------
FC88X_01082_00523       -----------------TCAACAAAT-CGTTTGGGGG-CGAC-TTCTC------
------
FC88X_00055_00314       -----------------TCAACAAAT-CGTTTGGGG--CGAC-TTCTC------
------
FC88X_00103_00008       -----------------TCAACAAAT-CGTTTGGGG--CGAC-TTCTC------
------
FC88X_00119_00123       -----------------TCAACAAAT-CGTTTGGGG--CGAC-TTCTC------
------
FC88X_00101_00265       -----------------TCAACAAAT-CGTTTGGGG--CGAC-TTCTC------
------
FC88X_00100_00853       -----------------TCAACAAAT-CGTTTGGGG--CGAC-TTCTC------
------
FC88X_00124_00937       -----------------TCAACAAAT-C-GTTTGGGG-CGAC-TTCTC------
------
FC88X_00171_00160       -----------------TCAACAAAT-C-GTTTGGGG-CGAC-TTCTC------
------
FC88X_00165_00311       -----------------TCAACAAAT-C-GTTTGGGG-CGAC-TTCTC------
------
FC88X_00163_00910       -----------------TCAACAAAT-C-GTTTGGGG-CGAC-TTCTC------
------
FC88X_00166_00974       -----------------TCAACAAAT-C-GTTTGGGG-CGAC-TTCTC------
------
FC88X_00167_00950       -----------------TCAACAAAT-C-GTTTGGGG-CGAC-TTCTC------
------
FC88X_00178_00966       -----------------TCAACAAAT-C-GTTTGGGG-CGAC-TTCTC------
------
FC88X_00205_00005       -----------------TCAACAAAT-C-GTTTGGGG-CGAC-TTCTC------
------
FC88X_00200_00580       -----------------TCAACAAAT-C-GTTTGGGG-CGAC-TTCTC------
------
FC88X_00216_00640       -----------------TCAACAAAT-C-GTTTGGGG-CGAC-TTCTC------
------
FC88X_00200_00839       -----------------TCAACAAAT-C-GTTTGGGG-CGAC-TTCTC------
------
FC88X_00239_00960       -----------------TCAACAAAT-C-GTTTGGGG-CGAC-TTCTC------
------
FC88X_00240_01092       -----------------TCAACAAAT-C-GTTTGGGG-CGAC-TTCTC------
------
FC88X_00275_00337       -----------------TCAACAAAT-C-GTTTGGGG-CGAC-TTCTC------
------
FC88X_00310_00154       -----------------TCAACAAAT-C-GTTTGGGG-CGAC-TTCTC------
------
FC88X_00313_00555       -----------------TCAACAAAT-C-GTTTGGGG-CGAC-TTCTC------
------
```

FC88X_00315_00953 -----------------TCAACAAAT-C-GTTTGGGG-CGAC-TTCTC------
------
FC88X_00309_01010 -----------------TCAACAAAT-C-GTTTGGGG-CGAC-TTCTC------
------
FC88X_00353_00100 -----------------TCAACAAAT-C-GTTTGGGG-CGAC-TTCTC------
------
FC88X_00382_00190 -----------------TCAACAAAT-C-GTTTGGGG-CGAC-TTCTC------
------
FC88X_00353_01069 -----------------TCAACAAAT-C-GTTTGGGG-CGAC-TTCTC------
------
FC88X_00356_01093 -----------------TCAACAAAT-C-GTTTGGGG-CGAC-TTCTC------
------
FC88X_00383_01072 -----------------TCAACAAAT-C-GTTTGGGG-CGAC-TTCTC------
------
FC88X_00447_00038 -----------------TCAACAAAT-C-GTTTGGGG-CGAC-TTCTC------
------
FC88X_00404_00773 -----------------TCAACAAAT-C-GTTTGGGG-CGAC-TTCTC------
------
FC88X_00418_01000 -----------------TCAACAAAT-C-GTTTGGGG-CGAC-TTCTC------
------
FC88X_00408_01184 -----------------TCAACAAAT-C-GTTTGGGG-CGAC-TTCTC------
------
FC88X_00413_01194 -----------------TCAACAAAT-C-GTTTGGGG-CGAC-TTCTC------
------
FC88X_00458_01062 -----------------TCAACAAAT-C-GTTTGGGG-CGAC-TTCTC------
------
FC88X_00476_01143 -----------------TCAACAAAT-C-GTTTGGGG-CGAC-TTCTC------
------
FC88X_00530_00209 -----------------TCAACAAAT-C-GTTTGGGG-CGAC-TTCTC------
------
FC88X_00545_00493 -----------------TCAACAAAT-C-GTTTGGGG-CGAC-TTCTC------
------
FC88X_00543_00665 -----------------TCAACAAAT-C-GTTTGGGG-CGAC-TTCTC------
------
FC88X_00520_00715 -----------------TCAACAAAT-C-GTTTGGGG-CGAC-TTCTC------
------
FC88X_00505_00983 -----------------TCAACAAAT-C-GTTTGGGG-CGAC-TTCTC------
------
FC88X_00546_00988 -----------------TCAACAAAT-C-GTTTGGGG-CGAC-TTCTC------
------
FC88X_00501_01002 -----------------TCAACAAAT-C-GTTTGGGG-CGAC-TTCTC------
------
FC88X_00537_01078 -----------------TCAACAAAT-C-GTTTGGGG-CGAC-TTCTC------
------
FC88X_00529_01162 -----------------TCAACAAAT-C-GTTTGGGG-CGAC-TTCTC------
------
FC88X_00505_01211 -----------------TCAACAAAT-C-GTTTGGGG-CGAC-TTCTC------
------
FC88X_00543_01245 -----------------TCAACAAAT-C-GTTTGGGG-CGAC-TTCTC------
------
FC88X_00562_00282 -----------------TCAACAAAT-C-GTTTGGGG-CGAC-TTCTC------
------
FC88X_00559_00446 -----------------TCAACAAAT-C-GTTTGGGG-CGAC-TTCTC------
------
FC88X_00589_00550 -----------------TCAACAAAT-C-GTTTGGGG-CGAC-TTCTC------
------

FC88X_00594_00869 -----------------TCAACAAAT-C-GTTTGGGG-CGAC-TTCTC------
------
FC88X_00562_01129 -----------------TCAACAAAT-C-GTTTGGGG-CGAC-TTCTC------
------
FC88X_00584_01136 -----------------TCAACAAAT-C-GTTTGGGG-CGAC-TTCTC------
------
FC88X_00551_01225 -----------------TCAACAAAT-C-GTTTGGGG-CGAC-TTCTC------
------
FC88X_00571_01202 -----------------TCAACAAAT-C-GTTTGGGG-CGAC-TTCTC------
------
FC88X_00584_01255 -----------------TCAACAAAT-C-GTTTGGGG-CGAC-TTCTC------
------
FC88X_00645_00315 -----------------TCAACAAAT-C-GTTTGGGG-CGAC-TTCTC------
------
FC88X_00628_00704 -----------------TCAACAAAT-C-GTTTGGGG-CGAC-TTCTC------
------
FC88X_00614_01167 -----------------TCAACAAAT-C-GTTTGGGG-CGAC-TTCTC------
------
FC88X_00681_00168 -----------------TCAACAAAT-C-GTTTGGGG-CGAC-TTCTC------
------
FC88X_00689_00223 -----------------TCAACAAAT-C-GTTTGGGG-CGAC-TTCTC------
------
FC88X_00653_00294 -----------------TCAACAAAT-C-GTTTGGGG-CGAC-TTCTC------
------
FC88X_00655_00293 -----------------TCAACAAAT-C-GTTTGGGG-CGAC-TTCTC------
------
FC88X_00654_00824 -----------------TCAACAAAT-C-GTTTGGGG-CGAC-TTCTC------
------
FC88X_00680_00942 -----------------TCAACAAAT-C-GTTTGGGG-CGAC-TTCTC------
------
FC88X_00671_00954 -----------------TCAACAAAT-C-GTTTGGGG-CGAC-TTCTC------
------
FC88X_00654_01012 -----------------TCAACAAAT-C-GTTTGGGG-CGAC-TTCTC------
------
FC88X_00661_01033 -----------------TCAACAAAT-C-GTTTGGGG-CGAC-TTCTC------
------
FC88X_00652_01067 -----------------TCAACAAAT-C-GTTTGGGG-CGAC-TTCTC------
------
FC88X_00682_01143 -----------------TCAACAAAT-C-GTTTGGGG-CGAC-TTCTC------
------
FC88X_00710_00292 -----------------TCAACAAAT-C-GTTTGGGG-CGAC-TTCTC------
------
FC88X_00747_00267 -----------------TCAACAAAT-C-GTTTGGGG-CGAC-TTCTC------
------
FC88X_00705_00305 -----------------TCAACAAAT-C-GTTTGGGG-CGAC-TTCTC------
------
FC88X_00730_01208 -----------------TCAACAAAT-C-GTTTGGGG-CGAC-TTCTC------
------
FC88X_00789_00246 -----------------TCAACAAAT-C-GTTTGGGG-CGAC-TTCTC------
------
FC88X_00793_00242 -----------------TCAACAAAT-C-GTTTGGGG-CGAC-TTCTC------
------
FC88X_00766_00278 -----------------TCAACAAAT-C-GTTTGGGG-CGAC-TTCTC------
------
FC88X_00781_00392 -----------------TCAACAAAT-C-GTTTGGGG-CGAC-TTCTC------
------

FC88X_00796_00474 ------------------TCAACAAAT-C-GTTTGGGG-CGAC-TTCTC------
------
FC88X_00765_01047 ------------------TCAACAAAT-C-GTTTGGGG-CGAC-TTCTC------
------
FC88X_00817_00174 ------------------TCAACAAAT-C-GTTTGGGG-CGAC-TTCTC------
------
FC88X_00849_00266 ------------------TCAACAAAT-C-GTTTGGGG-CGAC-TTCTC------
------
FC88X_00807_00327 ------------------TCAACAAAT-C-GTTTGGGG-CGAC-TTCTC------
------
FC88X_00808_00800 ------------------TCAACAAAT-C-GTTTGGGG-CGAC-TTCTC------
------
FC88X_00812_01001 ------------------TCAACAAAT-C-GTTTGGGG-CGAC-TTCTC------
------
FC88X_00817_01196 ------------------TCAACAAAT-C-GTTTGGGG-CGAC-TTCTC------
------
FC88X_00810_01208 ------------------TCAACAAAT-C-GTTTGGGG-CGAC-TTCTC------
------
FC88X_00897_00160 ------------------TCAACAAAT-C-GTTTGGGG-CGAC-TTCTC------
------
FC88X_00893_00259 ------------------TCAACAAAT-C-GTTTGGGG-CGAC-TTCTC------
------
FC88X_00885_00363 ------------------TCAACAAAT-C-GTTTGGGG-CGAC-TTCTC------
------
FC88X_00872_00813 ------------------TCAACAAAT-C-GTTTGGGG-CGAC-TTCTC------
------
FC88X_00878_00898 ------------------TCAACAAAT-C-GTTTGGGG-CGAC-TTCTC------
------
FC88X_00875_01039 ------------------TCAACAAAT-C-GTTTGGGG-CGAC-TTCTC------
------
FC88X_00891_01142 ------------------TCAACAAAT-C-GTTTGGGG-CGAC-TTCTC------
------
FC88X_00893_01114 ------------------TCAACAAAT-C-GTTTGGGG-CGAC-TTCTC------
------
FC88X_00938_00382 ------------------TCAACAAAT-C-GTTTGGGG-CGAC-TTCTC------
------
FC88X_00938_00410 ------------------TCAACAAAT-C-GTTTGGGG-CGAC-TTCTC------
------
FC88X_00914_00656 ------------------TCAACAAAT-C-GTTTGGGG-CGAC-TTCTC------
------
FC88X_00934_00757 ------------------TCAACAAAT-C-GTTTGGGG-CGAC-TTCTC------
------
FC88X_00905_00853 ------------------TCAACAAAT-C-GTTTGGGG-CGAC-TTCTC------
------
FC88X_00928_00854 ------------------TCAACAAAT-C-GTTTGGGG-CGAC-TTCTC------
------
FC88X_00946_00860 ------------------TCAACAAAT-C-GTTTGGGG-CGAC-TTCTC------
------
FC88X_00915_00933 ------------------TCAACAAAT-C-GTTTGGGG-CGAC-TTCTC------
------
FC88X_00930_01210 ------------------TCAACAAAT-C-GTTTGGGG-CGAC-TTCTC------
------
FC88X_00901_01270 ------------------TCAACAAAT-C-GTTTGGGG-CGAC-TTCTC------
------
FC88X_00993_00268 ------------------TCAACAAAT-C-GTTTGGGG-CGAC-TTCTC------
------

FC88X_00980_00479 -----------------TCAACAAAT-C-GTTTGGGG-CGAC-TTCTC------
------
FC88X_00998_00512 -----------------TCAACAAAT-C-GTTTGGGG-CGAC-TTCTC------
------
FC88X_00965_00633 -----------------TCAACAAAT-C-GTTTGGGG-CGAC-TTCTC------
------
FC88X_00975_00674 -----------------TCAACAAAT-C-GTTTGGGG-CGAC-TTCTC------
------
FC88X_00995_00728 -----------------TCAACAAAT-C-GTTTGGGG-CGAC-TTCTC------
------
FC88X_00960_00761 -----------------TCAACAAAT-C-GTTTGGGG-CGAC-TTCTC------
------
FC88X_00990_00771 -----------------TCAACAAAT-C-GTTTGGGG-CGAC-TTCTC------
------
FC88X_00972_00872 -----------------TCAACAAAT-C-GTTTGGGG-CGAC-TTCTC------
------
FC88X_00965_00952 -----------------TCAACAAAT-C-GTTTGGGG-CGAC-TTCTC------
------
FC88X_00984_01007 -----------------TCAACAAAT-C-GTTTGGGG-CGAC-TTCTC------
------
FC88X_00971_01108 -----------------TCAACAAAT-C-GTTTGGGG-CGAC-TTCTC------
------
FC88X_00999_01175 -----------------TCAACAAAT-C-GTTTGGGG-CGAC-TTCTC------
------
FC88X_01014_00767 -----------------TCAACAAAT-C-GTTTGGGG-CGAC-TTCTC------
------
FC88X_01044_00763 -----------------TCAACAAAT-C-GTTTGGGG-CGAC-TTCTC------
------
FC88X_01007_00916 -----------------TCAACAAAT-C-GTTTGGGG-CGAC-TTCTC------
------
FC88X_01004_01014 -----------------TCAACAAAT-C-GTTTGGGG-CGAC-TTCTC------
------
FC88X_01079_00462 -----------------TCAACAAAT-C-GTTTGGGG-CGAC-TTCTC------
------
FC88X_01053_00623 -----------------TCAACAAAT-C-GTTTGGGG-CGAC-TTCTC------
------
FC88X_01093_00653 -----------------TCAACAAAT-C-GTTTGGGG-CGAC-TTCTC------
------
FC88X_01062_00701 -----------------TCAACAAAT-C-GTTTGGGG-CGAC-TTCTC------
------
FC88X_01083_00792 -----------------TCAACAAAT-C-GTTTGGGG-CGAC-TTCTC------
------
FC88X_01086_00776 -----------------TCAACAAAT-C-GTTTGGGG-CGAC-TTCTC------
------
FC88X_01069_00827 -----------------TCAACAAAT-C-GTTTGGGG-CGAC-TTCTC------
------
FC88X_01083_00815 -----------------TCAACAAAT-C-GTTTGGGG-CGAC-TTCTC------
------
FC88X_01054_00850 -----------------TCAACAAAT-C-GTTTGGGG-CGAC-TTCTC------
------
FC88X_01094_00939 -----------------TCAACAAAT-C-GTTTGGGG-CGAC-TTCTC------
------
FC88X_01050_01192 -----------------TCAACAAAT-C-GTTTGGGG-CGAC-TTCTC------
------
FC88X_01057_01198 -----------------TCAACAAAT-C-GTTTGGGG-CGAC-TTCTC------
------

FC88X_01076_01196 -----------------TCAACAAAT-C-GTTTGGGG-CGAC-TTCTC------
------
FC88X_01097_01243 -----------------TCAACAAAT-C-GTTTGGGG-CGAC-TTCTC------
------
FC88X_01085_01254 -----------------TCAACAAAT-C-GTTTGGGG-CGAC-TTCTC------
------
FC88X_01121_00543 -----------------TCAACAAAT-C-GTTTGGGG-CGAC-TTCTC------
------
FC88X_01113_00751 -----------------TCAACAAAT-C-GTTTGGGG-CGAC-TTCTC------
------
FC88X_01119_00773 -----------------TCAACAAAT-C-GTTTGGGG-CGAC-TTCTC------
------
FC88X_01143_00864 -----------------TCAACAAAT-C-GTTTGGGG-CGAC-TTCTC------
------
FC88X_01109_00986 -----------------TCAACAAAT-C-GTTTGGGG-CGAC-TTCTC------
------
FC88X_01132_00950 -----------------TCAACAAAT-C-GTTTGGGG-CGAC-TTCTC------
------
FC88X_01134_01135 -----------------TCAACAAAT-C-GTTTGGGG-CGAC-TTCTC------
------
FC88X_01116_01160 -----------------TCAACAAAT-C-GTTTGGGG-CGAC-TTCTC------
------
FC88X_00025_00063 -----------------TCAACAAAT-C-GTTTGGGG-CGAC-TTCTC------
------
FC88X_00034_00147 -----------------TCAACAAAT-C-GTTTGGGG-CGAC-TTCTC------
------
FC88X_00032_00191 -----------------TCAACAAAT-C-GTTTGGGG-CGAC-TTCTC------
------
FC88X_00049_00150 -----------------TCAACAAAT-C-GTTTGGGG-CGAC-TTCTC------
------
FC88X_00014_00280 -----------------TCAACAAAT-C-GTTTGGGG-CGAC-TTCTC------
------
FC88X_00006_00335 -----------------TCAACAAAT-C-GTTTGGGG-CGAC-TTCTC------
------
FC88X_00048_00313 -----------------TCAACAAAT-C-GTTTGGGG-CGAC-TTCTC------
------
FC88X_00030_00398 -----------------TCAACAAAT-C-GTTTGGGG-CGAC-TTCTC------
------
FC88X_00034_00414 -----------------TCAACAAAT-C-GTTTGGGG-CGAC-TTCTC------
------
FC88X_00015_00483 -----------------TCAACAAAT-C-GTTTGGGG-CGAC-TTCTC------
------
FC88X_00021_00471 -----------------TCAACAAAT-C-GTTTGGGG-CGAC-TTCTC------
------
FC88X_00027_00595 -----------------TCAACAAAT-C-GTTTGGGG-CGAC-TTCTC------
------
FC88X_00010_00606 -----------------TCAACAAAT-C-GTTTGGGG-CGAC-TTCTC------
------
FC88X_00034_00684 -----------------TCAACAAAT-C-GTTTGGGG-CGAC-TTCTC------
------
FC88X_00065_00073 -----------------TCAACAAAT-C-GTTTGGGG-CGAC-TTCTC------
------
FC88X_00072_00135 -----------------TCAACAAAT-C-GTTTGGGG-CGAC-TTCTC------
------
FC88X_00054_00184 -----------------TCAACAAAT-C-GTTTGGGG-CGAC-TTCTC------
------

FC88X_00066_00177 -----------------TCAACAAAT-C-GTTTGGGG-CGAC-TTCTC------
------
FC88X_00083_00169 -----------------TCAACAAAT-C-GTTTGGGG-CGAC-TTCTC------
------
FC88X_00057_00449 -----------------TCAACAAAT-C-GTTTGGGG-CGAC-TTCTC------
------
FC88X_00072_00411 -----------------TCAACAAAT-C-GTTTGGGG-CGAC-TTCTC------
------
FC88X_00053_00480 -----------------TCAACAAAT-C-GTTTGGGG-CGAC-TTCTC------
------
FC88X_00073_00494 -----------------TCAACAAAT-C-GTTTGGGG-CGAC-TTCTC------
------
FC88X_00085_00589 -----------------TCAACAAAT-C-GTTTGGGG-CGAC-TTCTC------
------
FC88X_00095_00692 -----------------TCAACAAAT-C-GTTTGGGG-CGAC-TTCTC------
------
FC88X_00060_00724 -----------------TCAACAAAT-C-GTTTGGGG-CGAC-TTCTC------
------
FC88X_00078_00736 -----------------TCAACAAAT-C-GTTTGGGG-CGAC-TTCTC------
------
FC88X_00051_00797 -----------------TCAACAAAT-C-GTTTGGGG-CGAC-TTCTC------
------
FC88X_00058_00753 -----------------TCAACAAAT-C-GTTTGGGG-CGAC-TTCTC------
------
FC88X_00090_00801 -----------------TCAACAAAT-C-GTTTGGGG-CGAC-TTCTC------
------
FC88X_00072_00860 -----------------TCAACAAAT-C-GTTTGGGG-CGAC-TTCTC------
------
FC88X_00095_00888 -----------------TCAACAAAT-C-GTTTGGGG-CGAC-TTCTC------
------
FC88X_00095_00896 -----------------TCAACAAAT-C-GTTTGGGG-CGAC-TTCTC------
------
FC88X_00090_00906 -----------------TCAACAAAT-C-GTTTGGGG-CGAC-TTCTC------
------
FC88X_00119_00043 -----------------TCAACAAAT-C-GTTTGGGG-CGAC-TTCTC------
------
FC88X_00124_00044 -----------------TCAACAAAT-C-GTTTGGGG-CGAC-TTCTC------
------
FC88X_00126_00099 -----------------TCAACAAAT-C-GTTTGGGG-CGAC-TTCTC------
------
FC88X_00126_00131 -----------------TCAACAAAT-C-GTTTGGGG-CGAC-TTCTC------
------
FC88X_00129_00122 -----------------TCAACAAAT-C-GTTTGGGG-CGAC-TTCTC------
------
FC88X_00110_00168 -----------------TCAACAAAT-C-GTTTGGGG-CGAC-TTCTC------
------
FC88X_00124_00174 -----------------TCAACAAAT-C-GTTTGGGG-CGAC-TTCTC------
------
FC88X_00149_00195 -----------------TCAACAAAT-C-GTTTGGGG-CGAC-TTCTC------
------
FC88X_00103_00330 -----------------TCAACAAAT-C-GTTTGGGG-CGAC-TTCTC------
------
FC88X_00107_00519 -----------------TCAACAAAT-C-GTTTGGGG-CGAC-TTCTC------
------
FC88X_00126_00557 -----------------TCAACAAAT-C-GTTTGGGG-CGAC-TTCTC------
------

FC88X_00113_00799 -----------------TCAACAAAT-C-GTTTGGGG-CGAC-TTCTC------
------
FC88X_00137_00866 -----------------TCAACAAAT-C-GTTTGGGG-CGAC-TTCTC------
------
FC88X_00144_00874 -----------------TCAACAAAT-C-GTTTGGGG-CGAC-TTCTC------
------
FC88X_00141_00989 -----------------TCAACAAAT-C-GTTTGGGG-CGAC-TTCTC------
------
FC88X_00155_00027 -----------------TCAACAAAT-C-GTTTGGGG-CGAC-TTCTC------
------
FC88X_00196_00075 -----------------TCAACAAAT-C-GTTTGGGG-CGAC-TTCTC------
------
FC88X_00152_00112 -----------------TCAACAAAT-C-GTTTGGGG-CGAC-TTCTC------
------
FC88X_00171_00488 -----------------TCAACAAAT-C-GTTTGGGG-CGAC-TTCTC------
------
FC88X_00151_00571 -----------------TCAACAAAT-C-GTTTGGGG-CGAC-TTCTC------
------
FC88X_00157_00634 -----------------TCAACAAAT-C-GTTTGGGG-CGAC-TTCTC------
------
FC88X_00168_00663 -----------------TCAACAAAT-C-GTTTGGGG-CGAC-TTCTC------
------
FC88X_00168_00764 -----------------TCAACAAAT-C-GTTTGGGG-CGAC-TTCTC------
------
FC88X_00164_00818 -----------------TCAACAAAT-C-GTTTGGGG-CGAC-TTCTC------
------
FC88X_00178_00949 -----------------TCAACAAAT-C-GTTTGGGG-CGAC-TTCTC------
------
FC88X_00193_00918 -----------------TCAACAAAT-C-GTTTGGGG-CGAC-TTCTC------
------
FC88X_00196_00930 -----------------TCAACAAAT-C-GTTTGGGG-CGAC-TTCTC------
------
FC88X_00151_00978 -----------------TCAACAAAT-C-GTTTGGGG-CGAC-TTCTC------
------
FC88X_00153_00991 -----------------TCAACAAAT-C-GTTTGGGG-CGAC-TTCTC------
------
FC88X_00174_00982 -----------------TCAACAAAT-C-GTTTGGGG-CGAC-TTCTC------
------
FC88X_00232_00040 -----------------TCAACAAAT-C-GTTTGGGG-CGAC-TTCTC------
------
FC88X_00241_00033 -----------------TCAACAAAT-C-GTTTGGGG-CGAC-TTCTC------
------
FC88X_00219_00113 -----------------TCAACAAAT-C-GTTTGGGG-CGAC-TTCTC------
------
FC88X_00202_00965 -----------------TCAACAAAT-C-GTTTGGGG-CGAC-TTCTC------
------
FC88X_00205_00960 -----------------TCAACAAAT-C-GTTTGGGG-CGAC-TTCTC------
------
FC88X_00242_01033 -----------------TCAACAAAT-C-GTTTGGGG-CGAC-TTCTC------
------
FC88X_00256_00104 -----------------TCAACAAAT-C-GTTTGGGG-CGAC-TTCTC------
------
FC88X_00252_00475 -----------------TCAACAAAT-C-GTTTGGGG-CGAC-TTCTC------
------
FC88X_00265_00511 -----------------TCAACAAAT-C-GTTTGGGG-CGAC-TTCTC------
------

```
FC88X_00295_00602      -----------------TCAACAAAT-C-GTTTGGGG-CGAC-TTCTC------
------
FC88X_00258_00849      -----------------TCAACAAAT-C-GTTTGGGG-CGAC-TTCTC------
------
FC88X_00259_01008      -----------------TCAACAAAT-C-GTTTGGGG-CGAC-TTCTC------
------
FC88X_00284_01015      -----------------TCAACAAAT-C-GTTTGGGG-CGAC-TTCTC------
------
FC88X_00286_01000      -----------------TCAACAAAT-C-GTTTGGGG-CGAC-TTCTC------
------
FC88X_00293_01090      -----------------TCAACAAAT-C-GTTTGGGG-CGAC-TTCTC------
------
FC88X_00310_00056      -----------------TCAACAAAT-C-GTTTGGGG-CGAC-TTCTC------
------
FC88X_00305_01095      -----------------TCAACAAAT-C-GTTTGGGG-CGAC-TTCTC------
------
FC88X_00327_01080      -----------------TCAACAAAT-C-GTTTGGGG-CGAC-TTCTC------
------
FC88X_00313_01108      -----------------TCAACAAAT-C-GTTTGGGG-CGAC-TTCTC------
------
FC88X_00337_01119      -----------------TCAACAAAT-C-GTTTGGGG-CGAC-TTCTC------
------
FC88X_00378_00029      -----------------TCAACAAAT-C-GTTTGGGG-CGAC-TTCTC------
------
FC88X_00395_00297      -----------------TCAACAAAT-C-GTTTGGGG-CGAC-TTCTC------
------
FC88X_00380_00415      -----------------TCAACAAAT-C-GTTTGGGG-CGAC-TTCTC------
------
FC88X_00389_00708      -----------------TCAACAAAT-C-GTTTGGGG-CGAC-TTCTC------
------
FC88X_00358_01009      -----------------TCAACAAAT-C-GTTTGGGG-CGAC-TTCTC------
------
FC88X_00385_01012      -----------------TCAACAAAT-C-GTTTGGGG-CGAC-TTCTC------
------
FC88X_00377_01158      -----------------TCAACAAAT-C-GTTTGGGG-CGAC-TTCTC------
------
FC88X_00394_01178      -----------------TCAACAAAT-C-GTTTGGGG-CGAC-TTCTC------
------
FC88X_00417_00046      -----------------TCAACAAAT-C-GTTTGGGG-CGAC-TTCTC------
------
FC88X_00431_00022      -----------------TCAACAAAT-C-GTTTGGGG-CGAC-TTCTC------
------
FC88X_00404_00056      -----------------TCAACAAAT-C-GTTTGGGG-CGAC-TTCTC------
------
FC88X_00428_00084      -----------------TCAACAAAT-C-GTTTGGGG-CGAC-TTCTC------
------
FC88X_00411_00176      -----------------TCAACAAAT-C-GTTTGGGG-CGAC-TTCTC------
------
FC88X_00420_00689      -----------------TCAACAAAT-C-GTTTGGGG-CGAC-TTCTC------
------
FC88X_00422_00834      -----------------TCAACAAAT-C-GTTTGGGG-CGAC-TTCTC------
------
FC88X_00449_00956      -----------------TCAACAAAT-C-GTTTGGGG-CGAC-TTCTC------
------
FC88X_00431_01001      -----------------TCAACAAAT-C-GTTTGGGG-CGAC-TTCTC------
------
```

FC88X_00443_01007 ------------------TCAACAAAT-C-GTTTGGGG-CGAC-TTCTC------
------
FC88X_00404_01142 ------------------TCAACAAAT-C-GTTTGGGG-CGAC-TTCTC------
------
FC88X_00431_01146 ------------------TCAACAAAT-C-GTTTGGGG-CGAC-TTCTC------
------
FC88X_00449_01130 ------------------TCAACAAAT-C-GTTTGGGG-CGAC-TTCTC------
------
FC88X_00401_01169 ------------------TCAACAAAT-C-GTTTGGGG-CGAC-TTCTC------
------
FC88X_00408_01169 ------------------TCAACAAAT-C-GTTTGGGG-CGAC-TTCTC------
------
FC88X_00442_01172 ------------------TCAACAAAT-C-GTTTGGGG-CGAC-TTCTC------
------
FC88X_00465_00023 ------------------TCAACAAAT-C-GTTTGGGG-CGAC-TTCTC------
------
FC88X_00457_00394 ------------------TCAACAAAT-C-GTTTGGGG-CGAC-TTCTC------
------
FC88X_00481_01131 ------------------TCAACAAAT-C-GTTTGGGG-CGAC-TTCTC------
------
FC88X_00496_01125 ------------------TCAACAAAT-C-GTTTGGGG-CGAC-TTCTC------
------
FC88X_00487_01229 ------------------TCAACAAAT-C-GTTTGGGG-CGAC-TTCTC------
------
FC88X_00502_00088 ------------------TCAACAAAT-C-GTTTGGGG-CGAC-TTCTC------
------
FC88X_00548_00503 ------------------TCAACAAAT-C-GTTTGGGG-CGAC-TTCTC------
------
FC88X_00538_00944 ------------------TCAACAAAT-C-GTTTGGGG-CGAC-TTCTC------
------
FC88X_00501_01095 ------------------TCAACAAAT-C-GTTTGGGG-CGAC-TTCTC------
------
FC88X_00524_01055 ------------------TCAACAAAT-C-GTTTGGGG-CGAC-TTCTC------
------
FC88X_00507_01130 ------------------TCAACAAAT-C-GTTTGGGG-CGAC-TTCTC------
------
FC88X_00542_01157 ------------------TCAACAAAT-C-GTTTGGGG-CGAC-TTCTC------
------
FC88X_00535_01247 ------------------TCAACAAAT-C-GTTTGGGG-CGAC-TTCTC------
------
FC88X_00562_00093 ------------------TCAACAAAT-C-GTTTGGGG-CGAC-TTCTC------
------
FC88X_00586_00099 ------------------TCAACAAAT-C-GTTTGGGG-CGAC-TTCTC------
------
FC88X_00594_00069 ------------------TCAACAAAT-C-GTTTGGGG-CGAC-TTCTC------
------
FC88X_00582_00245 ------------------TCAACAAAT-C-GTTTGGGG-CGAC-TTCTC------
------
FC88X_00582_00467 ------------------TCAACAAAT-C-GTTTGGGG-CGAC-TTCTC------
------
FC88X_00560_00680 ------------------TCAACAAAT-C-GTTTGGGG-CGAC-TTCTC------
------
FC88X_00592_00707 ------------------TCAACAAAT-C-GTTTGGGG-CGAC-TTCTC------
------
FC88X_00594_00768 ------------------TCAACAAAT-C-GTTTGGGG-CGAC-TTCTC------
------

```
FC88X_00554_00954      -----------------TCAACAAAT-C-GTTTGGGG-CGAC-TTCTC------
------
FC88X_00550_01063      -----------------TCAACAAAT-C-GTTTGGGG-CGAC-TTCTC------
------
FC88X_00551_01099      -----------------TCAACAAAT-C-GTTTGGGG-CGAC-TTCTC------
------
FC88X_00586_01066      -----------------TCAACAAAT-C-GTTTGGGG-CGAC-TTCTC------
------
FC88X_00588_01139      -----------------TCAACAAAT-C-GTTTGGGG-CGAC-TTCTC------
------
FC88X_00564_01191      -----------------TCAACAAAT-C-GTTTGGGG-CGAC-TTCTC------
------
FC88X_00572_01214      -----------------TCAACAAAT-C-GTTTGGGG-CGAC-TTCTC------
------
FC88X_00616_00169      -----------------TCAACAAAT-C-GTTTGGGG-CGAC-TTCTC------
------
FC88X_00645_00579      -----------------TCAACAAAT-C-GTTTGGGG-CGAC-TTCTC------
------
FC88X_00606_01089      -----------------TCAACAAAT-C-GTTTGGGG-CGAC-TTCTC------
------
FC88X_00618_01100      -----------------TCAACAAAT-C-GTTTGGGG-CGAC-TTCTC------
------
FC88X_00644_01126      -----------------TCAACAAAT-C-GTTTGGGG-CGAC-TTCTC------
------
FC88X_00648_01101      -----------------TCAACAAAT-C-GTTTGGGG-CGAC-TTCTC------
------
FC88X_00625_01241      -----------------TCAACAAAT-C-GTTTGGGG-CGAC-TTCTC------
------
FC88X_00676_00234      -----------------TCAACAAAT-C-GTTTGGGG-CGAC-TTCTC------
------
FC88X_00681_00312      -----------------TCAACAAAT-C-GTTTGGGG-CGAC-TTCTC------
------
FC88X_00672_01130      -----------------TCAACAAAT-C-GTTTGGGG-CGAC-TTCTC------
------
FC88X_00666_01189      -----------------TCAACAAAT-C-GTTTGGGG-CGAC-TTCTC------
------
FC88X_00666_01231      -----------------TCAACAAAT-C-GTTTGGGG-CGAC-TTCTC------
------
FC88X_00670_01256      -----------------TCAACAAAT-C-GTTTGGGG-CGAC-TTCTC------
------
FC88X_00672_01271      -----------------TCAACAAAT-C-GTTTGGGG-CGAC-TTCTC------
------
FC88X_00693_01270      -----------------TCAACAAAT-C-GTTTGGGG-CGAC-TTCTC------
------
FC88X_00719_00096      -----------------TCAACAAAT-C-GTTTGGGG-CGAC-TTCTC------
------
FC88X_00745_00265      -----------------TCAACAAAT-C-GTTTGGGG-CGAC-TTCTC------
------
FC88X_00738_00302      -----------------TCAACAAAT-C-GTTTGGGG-CGAC-TTCTC------
------
FC88X_00711_00527      -----------------TCAACAAAT-C-GTTTGGGG-CGAC-TTCTC------
------
FC88X_00736_01018      -----------------TCAACAAAT-C-GTTTGGGG-CGAC-TTCTC------
------
FC88X_00746_01098      -----------------TCAACAAAT-C-GTTTGGGG-CGAC-TTCTC------
------
```

FC88X_00702_01129 ------------------TCAACAAAT-C-GTTTGGGG-CGAC-TTCTC------
------
FC88X_00708_01127 ------------------TCAACAAAT-C-GTTTGGGG-CGAC-TTCTC------
------
FC88X_00710_01103 ------------------TCAACAAAT-C-GTTTGGGG-CGAC-TTCTC------
------
FC88X_00724_01154 ------------------TCAACAAAT-C-GTTTGGGG-CGAC-TTCTC------
------
FC88X_00766_00182 ------------------TCAACAAAT-C-GTTTGGGG-CGAC-TTCTC------
------
FC88X_00785_00194 ------------------TCAACAAAT-C-GTTTGGGG-CGAC-TTCTC------
------
FC88X_00783_00214 ------------------TCAACAAAT-C-GTTTGGGG-CGAC-TTCTC------
------
FC88X_00792_00240 ------------------TCAACAAAT-C-GTTTGGGG-CGAC-TTCTC------
------
FC88X_00774_00280 ------------------TCAACAAAT-C-GTTTGGGG-CGAC-TTCTC------
------
FC88X_00788_00265 ------------------TCAACAAAT-C-GTTTGGGG-CGAC-TTCTC------
------
FC88X_00777_00316 ------------------TCAACAAAT-C-GTTTGGGG-CGAC-TTCTC------
------
FC88X_00784_00305 ------------------TCAACAAAT-C-GTTTGGGG-CGAC-TTCTC------
------
FC88X_00773_00444 ------------------TCAACAAAT-C-GTTTGGGG-CGAC-TTCTC------
------
FC88X_00785_00411 ------------------TCAACAAAT-C-GTTTGGGG-CGAC-TTCTC------
------
FC88X_00753_00898 ------------------TCAACAAAT-C-GTTTGGGG-CGAC-TTCTC------
------
FC88X_00788_00896 ------------------TCAACAAAT-C-GTTTGGGG-CGAC-TTCTC------
------
FC88X_00761_00914 ------------------TCAACAAAT-C-GTTTGGGG-CGAC-TTCTC------
------
FC88X_00777_01026 ------------------TCAACAAAT-C-GTTTGGGG-CGAC-TTCTC------
------
FC88X_00779_01031 ------------------TCAACAAAT-C-GTTTGGGG-CGAC-TTCTC------
------
FC88X_00755_01103 ------------------TCAACAAAT-C-GTTTGGGG-CGAC-TTCTC------
------
FC88X_00780_01242 ------------------TCAACAAAT-C-GTTTGGGG-CGAC-TTCTC------
------
FC88X_00782_01209 ------------------TCAACAAAT-C-GTTTGGGG-CGAC-TTCTC------
------
FC88X_00789_01224 ------------------TCAACAAAT-C-GTTTGGGG-CGAC-TTCTC------
------
FC88X_00836_00137 ------------------TCAACAAAT-C-GTTTGGGG-CGAC-TTCTC------
------
FC88X_00807_00216 ------------------TCAACAAAT-C-GTTTGGGG-CGAC-TTCTC------
------
FC88X_00811_00240 ------------------TCAACAAAT-C-GTTTGGGG-CGAC-TTCTC------
------
FC88X_00822_00203 ------------------TCAACAAAT-C-GTTTGGGG-CGAC-TTCTC------
------
FC88X_00830_00207 ------------------TCAACAAAT-C-GTTTGGGG-CGAC-TTCTC------
------

FC88X_00821_00332 -----------------TCAACAAAT-C-GTTTGGGG-CGAC-TTCTC------
------
FC88X_00830_00341 -----------------TCAACAAAT-C-GTTTGGGG-CGAC-TTCTC------
------
FC88X_00813_00787 -----------------TCAACAAAT-C-GTTTGGGG-CGAC-TTCTC------
------
FC88X_00804_00840 -----------------TCAACAAAT-C-GTTTGGGG-CGAC-TTCTC------
------
FC88X_00826_00831 -----------------TCAACAAAT-C-GTTTGGGG-CGAC-TTCTC------
------
FC88X_00838_00976 -----------------TCAACAAAT-C-GTTTGGGG-CGAC-TTCTC------
------
FC88X_00804_01002 -----------------TCAACAAAT-C-GTTTGGGG-CGAC-TTCTC------
------
FC88X_00843_01055 -----------------TCAACAAAT-C-GTTTGGGG-CGAC-TTCTC------
------
FC88X_00819_01188 -----------------TCAACAAAT-C-GTTTGGGG-CGAC-TTCTC------
------
FC88X_00801_01223 -----------------TCAACAAAT-C-GTTTGGGG-CGAC-TTCTC------
------
FC88X_00898_00323 -----------------TCAACAAAT-C-GTTTGGGG-CGAC-TTCTC------
------
FC88X_00858_00366 -----------------TCAACAAAT-C-GTTTGGGG-CGAC-TTCTC------
------
FC88X_00873_00353 -----------------TCAACAAAT-C-GTTTGGGG-CGAC-TTCTC------
------
FC88X_00876_00368 -----------------TCAACAAAT-C-GTTTGGGG-CGAC-TTCTC------
------
FC88X_00881_00387 -----------------TCAACAAAT-C-GTTTGGGG-CGAC-TTCTC------
------
FC88X_00884_00393 -----------------TCAACAAAT-C-GTTTGGGG-CGAC-TTCTC------
------
FC88X_00880_00544 -----------------TCAACAAAT-C-GTTTGGGG-CGAC-TTCTC------
------
FC88X_00887_00560 -----------------TCAACAAAT-C-GTTTGGGG-CGAC-TTCTC------
------
FC88X_00863_00693 -----------------TCAACAAAT-C-GTTTGGGG-CGAC-TTCTC------
------
FC88X_00855_00723 -----------------TCAACAAAT-C-GTTTGGGG-CGAC-TTCTC------
------
FC88X_00889_00778 -----------------TCAACAAAT-C-GTTTGGGG-CGAC-TTCTC------
------
FC88X_00851_00805 -----------------TCAACAAAT-C-GTTTGGGG-CGAC-TTCTC------
------
FC88X_00852_00804 -----------------TCAACAAAT-C-GTTTGGGG-CGAC-TTCTC------
------
FC88X_00869_00807 -----------------TCAACAAAT-C-GTTTGGGG-CGAC-TTCTC------
------
FC88X_00871_00915 -----------------TCAACAAAT-C-GTTTGGGG-CGAC-TTCTC------
------
FC88X_00867_00968 -----------------TCAACAAAT-C-GTTTGGGG-CGAC-TTCTC------
------
FC88X_00867_00994 -----------------TCAACAAAT-C-GTTTGGGG-CGAC-TTCTC------
------
FC88X_00873_00999 -----------------TCAACAAAT-C-GTTTGGGG-CGAC-TTCTC------
------

FC88X_00859_01012 -----------------TCAACAAAT-C-GTTTGGGG-CGAC-TTCTC------
------
FC88X_00872_01021 -----------------TCAACAAAT-C-GTTTGGGG-CGAC-TTCTC------
------
FC88X_00878_01096 -----------------TCAACAAAT-C-GTTTGGGG-CGAC-TTCTC------
------
FC88X_00899_01068 -----------------TCAACAAAT-C-GTTTGGGG-CGAC-TTCTC------
------
FC88X_00862_01156 -----------------TCAACAAAT-C-GTTTGGGG-CGAC-TTCTC------
------
FC88X_00933_00215 -----------------TCAACAAAT-C-GTTTGGGG-CGAC-TTCTC------
------
FC88X_00939_00203 -----------------TCAACAAAT-C-GTTTGGGG-CGAC-TTCTC------
------
FC88X_00905_00383 -----------------TCAACAAAT-C-GTTTGGGG-CGAC-TTCTC------
------
FC88X_00913_00399 -----------------TCAACAAAT-C-GTTTGGGG-CGAC-TTCTC------
------
FC88X_00934_00367 -----------------TCAACAAAT-C-GTTTGGGG-CGAC-TTCTC------
------
FC88X_00913_00448 -----------------TCAACAAAT-C-GTTTGGGG-CGAC-TTCTC------
------
FC88X_00929_00444 -----------------TCAACAAAT-C-GTTTGGGG-CGAC-TTCTC------
------
FC88X_00933_00451 -----------------TCAACAAAT-C-GTTTGGGG-CGAC-TTCTC------
------
FC88X_00937_00521 -----------------TCAACAAAT-C-GTTTGGGG-CGAC-TTCTC------
------
FC88X_00918_00677 -----------------TCAACAAAT-C-GTTTGGGG-CGAC-TTCTC------
------
FC88X_00941_00655 -----------------TCAACAAAT-C-GTTTGGGG-CGAC-TTCTC------
------
FC88X_00930_00741 -----------------TCAACAAAT-C-GTTTGGGG-CGAC-TTCTC------
------
FC88X_00901_00753 -----------------TCAACAAAT-C-GTTTGGGG-CGAC-TTCTC------
------
FC88X_00944_00846 -----------------TCAACAAAT-C-GTTTGGGG-CGAC-TTCTC------
------
FC88X_00910_00909 -----------------TCAACAAAT-C-GTTTGGGG-CGAC-TTCTC------
------
FC88X_00911_01054 -----------------TCAACAAAT-C-GTTTGGGG-CGAC-TTCTC------
------
FC88X_00915_01054 -----------------TCAACAAAT-C-GTTTGGGG-CGAC-TTCTC------
------
FC88X_00940_01127 -----------------TCAACAAAT-C-GTTTGGGG-CGAC-TTCTC------
------
FC88X_00923_01263 -----------------TCAACAAAT-C-GTTTGGGG-CGAC-TTCTC------
------
FC88X_00985_00251 -----------------TCAACAAAT-C-GTTTGGGG-CGAC-TTCTC------
------
FC88X_00986_00259 -----------------TCAACAAAT-C-GTTTGGGG-CGAC-TTCTC------
------
FC88X_00980_00348 -----------------TCAACAAAT-C-GTTTGGGG-CGAC-TTCTC------
------
FC88X_00995_00364 -----------------TCAACAAAT-C-GTTTGGGG-CGAC-TTCTC------
------

FC88X_00955_00477 ------------------TCAACAAAT-C-GTTTGGGG-CGAC-TTCTC------
------
FC88X_00975_00514 ------------------TCAACAAAT-C-GTTTGGGG-CGAC-TTCTC------
------
FC88X_00989_00529 ------------------TCAACAAAT-C-GTTTGGGG-CGAC-TTCTC------
------
FC88X_00956_00609 ------------------TCAACAAAT-C-GTTTGGGG-CGAC-TTCTC------
------
FC88X_00960_00632 ------------------TCAACAAAT-C-GTTTGGGG-CGAC-TTCTC------
------
FC88X_00952_00689 ------------------TCAACAAAT-C-GTTTGGGG-CGAC-TTCTC------
------
FC88X_00955_00740 ------------------TCAACAAAT-C-GTTTGGGG-CGAC-TTCTC------
------
FC88X_00961_00756 ------------------TCAACAAAT-C-GTTTGGGG-CGAC-TTCTC------
------
FC88X_00967_00789 ------------------TCAACAAAT-C-GTTTGGGG-CGAC-TTCTC------
------
FC88X_00992_00757 ------------------TCAACAAAT-C-GTTTGGGG-CGAC-TTCTC------
------
FC88X_00959_00866 ------------------TCAACAAAT-C-GTTTGGGG-CGAC-TTCTC------
------
FC88X_00989_00891 ------------------TCAACAAAT-C-GTTTGGGG-CGAC-TTCTC------
------
FC88X_00974_00932 ------------------TCAACAAAT-C-GTTTGGGG-CGAC-TTCTC------
------
FC88X_00953_01005 ------------------TCAACAAAT-C-GTTTGGGG-CGAC-TTCTC------
------
FC88X_00952_01104 ------------------TCAACAAAT-C-GTTTGGGG-CGAC-TTCTC------
------
FC88X_00972_01145 ------------------TCAACAAAT-C-GTTTGGGG-CGAC-TTCTC------
------
FC88X_00957_01170 ------------------TCAACAAAT-C-GTTTGGGG-CGAC-TTCTC------
------
FC88X_00959_01170 ------------------TCAACAAAT-C-GTTTGGGG-CGAC-TTCTC------
------
FC88X_00971_01171 ------------------TCAACAAAT-C-GTTTGGGG-CGAC-TTCTC------
------
FC88X_00997_01203 ------------------TCAACAAAT-C-GTTTGGGG-CGAC-TTCTC------
------
FC88X_00955_01270 ------------------TCAACAAAT-C-GTTTGGGG-CGAC-TTCTC------
------
FC88X_01007_00303 ------------------TCAACAAAT-C-GTTTGGGG-CGAC-TTCTC------
------
FC88X_01021_00398 ------------------TCAACAAAT-C-GTTTGGGG-CGAC-TTCTC------
------
FC88X_01011_00433 ------------------TCAACAAAT-C-GTTTGGGG-CGAC-TTCTC------
------
FC88X_01027_00433 ------------------TCAACAAAT-C-GTTTGGGG-CGAC-TTCTC------
------
FC88X_01031_00470 ------------------TCAACAAAT-C-GTTTGGGG-CGAC-TTCTC------
------
FC88X_01043_00461 ------------------TCAACAAAT-C-GTTTGGGG-CGAC-TTCTC------
------
FC88X_01010_00516 ------------------TCAACAAAT-C-GTTTGGGG-CGAC-TTCTC------
------

FC88X_01038_00505 -----------------TCAACAAAT-C-GTTTGGGG-CGAC-TTCTC------
------
FC88X_01005_00579 -----------------TCAACAAAT-C-GTTTGGGG-CGAC-TTCTC------
------
FC88X_01013_00589 -----------------TCAACAAAT-C-GTTTGGGG-CGAC-TTCTC------
------
FC88X_01043_00582 -----------------TCAACAAAT-C-GTTTGGGG-CGAC-TTCTC------
------
FC88X_01036_00601 -----------------TCAACAAAT-C-GTTTGGGG-CGAC-TTCTC------
------
FC88X_01017_00652 -----------------TCAACAAAT-C-GTTTGGGG-CGAC-TTCTC------
------
FC88X_01037_00721 -----------------TCAACAAAT-C-GTTTGGGG-CGAC-TTCTC------
------
FC88X_01038_00770 -----------------TCAACAAAT-C-GTTTGGGG-CGAC-TTCTC------
------
FC88X_01015_00822 -----------------TCAACAAAT-C-GTTTGGGG-CGAC-TTCTC------
------
FC88X_01032_00840 -----------------TCAACAAAT-C-GTTTGGGG-CGAC-TTCTC------
------
FC88X_01026_00992 -----------------TCAACAAAT-C-GTTTGGGG-CGAC-TTCTC------
------
FC88X_01024_01083 -----------------TCAACAAAT-C-GTTTGGGG-CGAC-TTCTC------
------
FC88X_01044_01164 -----------------TCAACAAAT-C-GTTTGGGG-CGAC-TTCTC------
------
FC88X_01023_01226 -----------------TCAACAAAT-C-GTTTGGGG-CGAC-TTCTC------
------
FC88X_01015_01260 -----------------TCAACAAAT-C-GTTTGGGG-CGAC-TTCTC------
------
FC88X_01073_00436 -----------------TCAACAAAT-C-GTTTGGGG-CGAC-TTCTC------
------
FC88X_01078_00412 -----------------TCAACAAAT-C-GTTTGGGG-CGAC-TTCTC------
------
FC88X_01072_00624 -----------------TCAACAAAT-C-GTTTGGGG-CGAC-TTCTC------
------
FC88X_01088_00618 -----------------TCAACAAAT-C-GTTTGGGG-CGAC-TTCTC------
------
FC88X_01097_00640 -----------------TCAACAAAT-C-GTTTGGGG-CGAC-TTCTC------
------
FC88X_01051_00758 -----------------TCAACAAAT-C-GTTTGGGG-CGAC-TTCTC------
------
FC88X_01071_00838 -----------------TCAACAAAT-C-GTTTGGGG-CGAC-TTCTC------
------
FC88X_01098_00811 -----------------TCAACAAAT-C-GTTTGGGG-CGAC-TTCTC------
------
FC88X_01053_00857 -----------------TCAACAAAT-C-GTTTGGGG-CGAC-TTCTC------
------
FC88X_01076_00869 -----------------TCAACAAAT-C-GTTTGGGG-CGAC-TTCTC------
------
FC88X_01089_00864 -----------------TCAACAAAT-C-GTTTGGGG-CGAC-TTCTC------
------
FC88X_01093_00856 -----------------TCAACAAAT-C-GTTTGGGG-CGAC-TTCTC------
------
FC88X_01066_00918 -----------------TCAACAAAT-C-GTTTGGGG-CGAC-TTCTC------
------

FC88X_01063_00955 ------------------TCAACAAAT-C-GTTTGGGG-CGAC-TTCTC------
------
FC88X_01084_01077 ------------------TCAACAAAT-C-GTTTGGGG-CGAC-TTCTC------
------
FC88X_01089_01081 ------------------TCAACAAAT-C-GTTTGGGG-CGAC-TTCTC------
------
FC88X_01092_01125 ------------------TCAACAAAT-C-GTTTGGGG-CGAC-TTCTC------
------
FC88X_01094_01120 ------------------TCAACAAAT-C-GTTTGGGG-CGAC-TTCTC------
------
FC88X_01098_01129 ------------------TCAACAAAT-C-GTTTGGGG-CGAC-TTCTC------
------
FC88X_01109_00489 ------------------TCAACAAAT-C-GTTTGGGG-CGAC-TTCTC------
------
FC88X_01102_00566 ------------------TCAACAAAT-C-GTTTGGGG-CGAC-TTCTC------
------
FC88X_01108_00559 ------------------TCAACAAAT-C-GTTTGGGG-CGAC-TTCTC------
------
FC88X_01117_00580 ------------------TCAACAAAT-C-GTTTGGGG-CGAC-TTCTC------
------
FC88X_01125_00690 ------------------TCAACAAAT-C-GTTTGGGG-CGAC-TTCTC------
------
FC88X_01116_00745 ------------------TCAACAAAT-C-GTTTGGGG-CGAC-TTCTC------
------
FC88X_01114_00793 ------------------TCAACAAAT-C-GTTTGGGG-CGAC-TTCTC------
------
FC88X_01132_00780 ------------------TCAACAAAT-C-GTTTGGGG-CGAC-TTCTC------
------
FC88X_01126_00851 ------------------TCAACAAAT-C-GTTTGGGG-CGAC-TTCTC------
------
FC88X_01135_00878 ------------------TCAACAAAT-C-GTTTGGGG-CGAC-TTCTC------
------
FC88X_01109_00970 ------------------TCAACAAAT-C-GTTTGGGG-CGAC-TTCTC------
------
FC88X_01103_01017 ------------------TCAACAAAT-C-GTTTGGGG-CGAC-TTCTC------
------
FC88X_01120_01025 ------------------TCAACAAAT-C-GTTTGGGG-CGAC-TTCTC------
------
FC88X_01129_01011 ------------------TCAACAAAT-C-GTTTGGGG-CGAC-TTCTC------
------
FC88X_01140_01037 ------------------TCAACAAAT-C-GTTTGGGG-CGAC-TTCTC------
------
FC88X_01136_01138 ------------------TCAACAAAT-C-GTTTGGGG-CGAC-TTCTC------
------
FC88X_01102_01177 ------------------TCAACAAAT-C-GTTTGGGG-CGAC-TTCTC------
------
FC88X_00921_01187 ------------------TCAACAAAT-C-GTTTGGGG-CGAC-TTCTCC-----
------
FC88X_01145_01082 ------------------TCAACAAAT-C-GTTTGGGG-CGAC-TTCTCC-----
------
FC88X_00744_00852 ------------------TCAACAAAT-C-GTTTGGGG-CGAC-TTCTCC-----
------
FC88X_00736_01134 ------------------TCAACAAAT-C-GTTTGGGG-CGAC-TTCTCCT----
------
FC88X_00397_01211 -------------------CAACAAAT-C-GTTTGGGG-CGAC-TTCTC------
------

FC88X_00457_01083 -----------------CCAACAAAT-C-GTTTGGGG-CGAC-TTCTC------
------
FC88X_00102_00118 -----------------CCAACAAAT-C-GTTTGGGG-CGAC-TTCTC------
------
FC88X_00647_00257 -----------------CCAACAAAT-C-GTTTGGGG-CGAC-TTCTC------
------
FC88X_00916_00365 -----------------CCAACAAAT-C-GTTTGGGG-CGAC-TTCTC------
------
FC88X_01054_00715 -----------------CCAACAAAT-C-GTTTGGGG-CGAC-TTCTC------
------
FC88X_00329_00223 ------------------CAACAAAT-C-GTTTGGGG-CGAC-TTCTC------
------
FC88X_00638_00609 -----------------TCAACAAAT-G-GTTTGGGG-C-GACTTCTC------
------
FC88X_01076_00806 -----------------TCAACAAAT-A-GTTTGGGG-C-GACTTCTC------
------
FC88X_00903_00753 -----------------TCAACAAAT-T-GTTTGGGG-C-GACTTCTC------
------
FC88X_00867_01163 -----------------TCAACAAAT-T-GTTTGGGG-C-GACTTCTC------
------
FC88X_00973_00403 -----------------TCAACAAAT-T-GTTTGGGG-C-GACTTCTC------
------
FC88X_00929_00671 -----------------TCAACAAAT-TCGTTTGGGG-C-GACTTCTC------
------
FC88X_00721_00892 -----------------TCAACAAAT-C-GTTTGGGG-C-GACTTCTCCC----
------
FC88X_01070_01175 -----------------TCAACAAAT-C-GTTTGGGG-C-GACTTCTCCC----
------
FC88X_00138_00522 -----------------TCAACAAAT-C-GTTTGGGG-C-GACTTCTCCC----
------
FC88X_00318_00993 -----------------TCAACAAAT-C-GTTTGGGG-C-GACTTCT-------
------
FC88X_00562_00206 -----------------TCAACAAAT-C-GTTTGGGG-CAGACTTCT-------
------
FC88X_01135_00786 -----------------TCAACAAAT-C-ATTTGGGG-CGAC-TTCTC------
------
FC88X_00915_00579 -----------------TCAACAAAT-C-ATTTGGGG-CGAC-TTCTC------
------
FC88X_00717_00897 -----------------TCAACAAAT-C-GTTTGGGG-CGAC-CTTCTC-----
------
FC88X_00805_01192 -----------------TCAACAAAT-C-GTTTGGGG-TGAC-TTCTC------
------
FC88X_00414_01150 ----------------TTCAACAAAT-C-GTT--GGG-CGAC-TTCTC------
------
FC88X_00972_00884 ----------------TTCAACAAAT-C-GTTTGGGG-CGAC-TTCTC------
------
FC88X_00204_00062 -----------------TCAACAAAT-CG-GTTGGGG-CGAC-TTCTC------
------
FC88X_00547_00205 -----------------TCAACAAAT-CGTGTTGGGG-CGAC-TTCTC------
------
FC88X_01052_00933 -----------------TCAACAAAT-CG-ATTGGGG-CGAC-TTCTC------
------
FC88X_00493_00963 -----------------TCAACAAAT-CG-CTTGGGG-CGAC-TTCTC------
------
FC88X_01057_01071 -----------------TCAACAAAT-CG-TTTGGGG-CGAC-TTCTCTCC---
------

FC88X_00898_00423 -----------------TCAACAAAT-C-GTCTGGGG-CGAC-TTCTC------
------
FC88X_00136_00565 -----------------TCAACAAAT-CGTT-CGGGG-CGAC-TTCTC------
------
FC88X_00862_00227 -----------------TCAACAAAT-CGTTTCGGGG-CGAC-TTCTC------
------
FC88X_01108_01033 -----------------TCAACAAAT-CGTTTGT-GG-CGAC-TTCTC------
------
FC88X_00725_00348 -----------------TCAACAAAT-CGTTTGT-GG-CGAC-TTCTC------
------
FC88X_00609_00376 -----------------TCAACAAAT-CGTTTG--GG-CGAC-TTCTC------
------
FC88X_00007_00149 -----------------TCAACAAAT-CGTTTTT-GG-CGAC-TTCTC------
------
FC88X_00884_01006 -----------------TCAACAAAT-CGTTTG--GG-CGACGTTCTC------
------
FC88X_00065_00391 -----------------TCAACAAAT-CGTTTGGGTG-CGAC-TTCTCC-----
------
FC88X_00911_00741 -----------------TCAACAAAT-CGTTTGG-TG-CGAC-TTCTC------
------
FC88X_00517_01040 -----------------TCAACAAAT-CGTTTGG-GA-CGAC-TTCTC------
------
FC88X_01098_01018 -----------------TCAATAAAT-C-GTTTGGGG-CGAC-TTCTC------
------
FC88X_00948_00668 -----------------TCAGCAAAT-C-GTTTGGGG-CGAC-TTCTC------
------
FC88X_01010_00288 -----------------TCAGCAAAT-C-GTTTGGGG-CGAC-TTCTC------
------
FC88X_01034_00941 -----------------TCACCAAAT-C-GTTTGGGG-CGAC-TTCTC------
------
FC88X_00216_00110 ----------------GTCAACAAAT-C-GTTTGGGG-CGAC-TTCTCCCTT--
------
FC88X_00269_00459 -----------------TCAACAAAT-C-GTTTGGGG-CGAC-TCTC-------
------
FC88X_00786_01006 -----------------TCAACAAAT-C-GTTTGGGG-CGAC-TCTC-------
------
FC88X_00263_00753 -----------------TCAACAAAT-C-GTTTGGGG-CGAC-CTCTC------
------
FC88X_00040_00240 -----------------TCAACAAAT-C-GTTTGGGG-CGAC-CTCTC------
------
FC88X_01019_01048 -----------------TCGACAAAT-C-GTTTGGGG-CGAC-CTCTC------
------
FC88X_00584_00425 -----------------TCGACAAAT-C-GTTTGGGG-CGAC-TTCTC------
------
FC88X_01059_00903 --------------CCGTCAACAAAT-C-GTTTGGGG-CGAC-TTCTC------
------
FC88X_00578_00935 ----------------GTCAACAAAT-C-GTTTGGGG-CGA-------------
------
FC88X_00159_00714 -----------------TCAACAAAT--CGTTTGGGG-CGAC-TCCTC------
------
FC88X_00464_00071 -----------------TCAACAAAT--CGTTTGGGG-CGAC-TTCCTC-----
------
FC88X_01097_00565 -----------------TCAACAAAT--CGTTTGGGG-CGAC-TCCTC------
------
FC88X_00842_00234 -----------------TCAACAAAT--CGTTTGGGG-CGAC-TCCTC------
------

FC88X_00445_00074 -----------------TCAACAAAT--CGTTTGGGG-CGAC-
TTCCTCCTTCCT-----
FC88X_00109_00899 -----------------TCAACAAAT--CGTTTGGGG-CGAC-TTCTCCTTC--
------
FC88X_00035_00236 -----------------TCAACAAAT--CGTTTGGGG-CGAC-
TTCTCCTTCCTTT----
FC88X_00012_00531 -----------------TCAACAAAT--CGTTTGGGG-CGAC-
TTCTCCTTCCTTT----
FC88X_00350_01084 -----------------TCAACAAAT--CGTTTGGGG-CGAC-
TTCTCCTTCCTTT----
FC88X_00645_00447 -----------------TCAACAAAT--CGTTTGGGG-CGAC-
TTCTCCTTCCTTT----
FC88X_00108_00024 -----------------TCAACAAAT--CGTTTGGGG-CGAC-
TTCTCCTTCCTTT----
FC88X_00188_00358 -----------------TCAACAAAT--CGTTTGGGG-CGAC-
TTCTCCTTCCTTT----
FC88X_00532_00949 -----------------TCAACAAAT--CGTTTGGGG-CGAC-
TTCTCCTTCCTTT----
FC88X_00925_01058 -----------------TCAACAAAT--CGTTTGGGG-CGAC-
TTCTCCTTCCTTT----
FC88X_00910_01136 -----------------TCAACAAAT--CGTTTGGGG-CGAC-
TTCTCCTTCCTTT----
FC88X_01052_01261 -----------------TCAACAAAT--CGTTTGGGG-CGAC-
TTCTCCTTCCTTT----
FC88X_00007_00410 ----------------GTCAACAAAT--CGTTTGGGG-CGAC-
TTCTCCTTCCTTTC---
FC88X_00988_00573 -----------------TCAACAA-T--CGTTTGGGG-CGAC-TTCTC------
------
FC88X_00869_00278 -----------------TCAACAAGT--CGTTTGGGG-CGAC-TTCTC------
------
FC88X_00027_00637 -----------------TCAACGAAT--CGTTTGGGG-CGAC-TTCTC------
------
FC88X_00997_00600 -----------------TCAACAGAT--CGTTTGGGG-CGAC-TTCTC------
------
FC88X_00176_00784 -----------------TCAACAAAT--CGTTTGGGG-CGGC-TTCTC------
------
FC88X_00106_00297 -----------------TCAACAAAT--CGTTTGGGG-CGGC-TTCTC------
------
FC88X_00466_00133 -----------------TCAACAAAT--CGTTTGGGG-CGAA-CTTCT------
------
FC88X_00676_00408 -----------------TCAACAAAT--CGTTTGGGG-CG--------------
------
FC88X_00505_00521 -----------------TCAACAAAT--CGTTTGGGG-CGAC-TTCTCATCAC-
------
FC88X_00554_01262 -----------------TCAACAAAA-C-GTTTGGGG-CGAC-TTCTC------
------
FC88X_00627_01212 -----------------TCAACAAAA-CCGTTTGGGG-CGAC-TTCTC------
------
FC88X_01011_00450 -----------------TCAACAAAA-TCGTTTGGGGGCGAC-TTCTC------
------
FC88X_00847_00971 -----------------TCAACAAAA-TCGTTTGGGG-CGAC-TTCTC------
------
FC88X_00918_01016 -----------------TCAACAAAA-TCGTTTGGGG-CGAC-TTCTC------
------
FC88X_00815_00462 -----------------TCAACAAAA-TCGTTTGGGG-CGAC-TTCTC------
------

FC88X_00009_00506 ------------------TCAACAAAA-TCGTTTGGGG-CGAC-TTCTC------
------
FC88X_00791_00321 -----------------GTCAACAAAA-TCGTTTGGGG-CGAC-TTCTC------
------
FC88X_00905_00219 ------------------TCAACAAAA-TCGTTTGGGG-CGAC-TTCTC------
------
FC88X_01028_00968 ------------------TCAACAAAA-TCGTTTGGGG-CGAC-TTCTC------
------
FC88X_00032_00479 ------------------TCAACAAAA-TCGTTTGGGG-CGAC-TTCTC------
------
FC88X_00550_01185 ------------------TCAACAAAA-TCGTTTGGGG-CGAC-TTCTC------
------
FC88X_00081_00045 ------------------TCAACAAAAATCGTTTGGGG-CGAC-TTCTC------
------
FC88X_00928_00907 ------------------TCAACAAAAATCGTTTGGGG-CGAC-TTCTC------
------
FC88X_00063_00531 ------------------TCAACAAAAATCGTTTGGGG-CGAC-TTCTC------
------
FC88X_00764_00872 ------------------TCAACAAAA--CGTTTGGGG-CGAC-TTCTC------
------
FC88X_00572_00140 ------------------TCAACAAAC---GTTTGGGG-CGAC-TTCTC------
------
FC88X_01015_00987 -----------------GTCAACAAAC--CGTTTGGGG-CGAC-TTCTC------
------
FC88X_00325_01096 ------------------TCAACAAAC--CGTTTGGGG-CGAC-TTCTC------
------
FC88X_00909_00547 -----------------CTCAACAAAT--CGTTTGGAG-CGAC-TTCTC------
------
FC88X_00981_00617 ------------------TCAACAAAT--CGTTTGGAG-CGAC-TTATC------
------
FC88X_00693_00932 ------------------TCAACAAAT--CGTTTGGGG-CGAC-
TTCTCCTTCTCCTTCTC
FC88X_00510_00589 ------------------TCAACAAAT--CGTTCGGG--CGAC-TTCTCC-----
------
FC88X_00955_00216 ------------------TCAACAAAT--CGTTTGGGG-CGAC-
TTCTCCCTTCCTTT---
FC88X_00884_00747 ------------------TCAACAAAT--CGTTTCGGG-CG--------------
------
FC88X_00704_00170 ------------------TCAACAAAATC-GTTTGGGGGCGACG-----------
------
FC88X_00821_00812 -------------TCAACAAAAAAAAT--CGTTTGGGG-CGA-CTTCTC------
------
FC88X_00929_00325 ---------TCAACAAAAAAAAAAAT--CGTTTGGGG-CGAACTTCTC------
------
FC88X_00190_00963 ------TCAACAAAAAAAAAAAAAAT--CGTTTGGGG-CGA-CTTCTC------
------
FC88X_00303_00993 -----------------------------CGTTTGGGG-CGA-CTTCTC------
------
FC88X_00192_00872 -----------------TCAAACAAAT--CGTTTGGG--CGACTTCTC-------
------
FC88X_00854_00699 -----------------TCTAACAAAT--CGTTTGGGG-CGACGTTCTT------
------
FC88X_01057_00781 ------------------TCAACGAGT-CGTTTTGGGGTGAACGTCTCC------
------
FC88X_00117_00624 ---------------------TTCCTCTGTGTCAAGAGCGAGTGTCTC------
------

```
FC88X_00568_00231      --------------------GTTCCTCTGTGTCAAGAGCGAGTGTCTC------
------
FC88X_00839_00480      ---------------------TTCCTCTGTGTCAAGAGCGAGTGTCTC------
------
FC88X_00472_01046      ---------------------TTCCTCTGTGTCAAGAGCGAGTGTCTC------
------
FC88X_00896_01062      ---------------------TTCCTCTGTGTCAAGAGCGAGTGTCTC------
------
FC88X_00966_00731      ---------------------TTCCTCTGTGTCAAGAGCGAGTGTCTC------
------
FC88X_00159_00973      ---------------------TTCCTCCGTGTCAAGAGCGAGTGTCTC------
------
FC88X_00350_00993      ---------------------CTCCTCTGTGTCAAGAGCGAGTGTCTC------
------
FC88X_00982_01266      -------------------TTGCCCACCGTTTGAAGGCCGACT-TCTC------
------
FC88X_00553_01253      -------------------TTGCCCACCGTTTGAAGGCCGACT-TCTC------
------
FC88X_00544_00108      --------------------CCTTGGCGT-TTGTGCCACGTTATTCTCGA----
------
FC88X_00040_00269      --------------------CCTTGGCGT-TTGTGCCACGTTATTCTCGA----
------
FC88X_00142_00808      --------------------CCTTGGCGT-TTGTGCCACGTTATTCTCGA----
------
FC88X_00319_00181      --------------------CCTTGGCGT-TTGTGCCACGTTATTCTCGA----
------
FC88X_00632_00278      --------------------CCTTGGCGT-TTGTGCCACGTTATTCTCGA----
------
FC88X_00702_00898      --------------------CCTTGGCGT-TTGTGCCACGTTATTCTCGA----
------
FC88X_01031_01021      --------------------CCTTGGCGT-TTGTGCCACGTTATTCTCGA----
------
FC88X_01126_00665      --------------------CCTTGGCGT-TTGTGCCACGTTATTCTCGA----
------
FC88X_00236_00921      ---------------------CTTGGCGT-TTGTGCCACGTTATTCTCGA----
------
FC88X_00988_01207      --------------------TCTTGGCGT-TTGTGCCACGTTATTCTCGA----
------
FC88X_00300_01061      --------------------CCTTGGCGT-TTGTGCTACGTTATTCTCGA----
------
FC88X_00418_00123      --------------------CCTTTGCGT-TTGTGCCACGTTATTCTCGA----
------
FC88X_00186_00045      --------------------CCTTGGCGTCTTGTGCCACGTTATTCTCTG----
------
FC88X_00674_00917      ---------------------CTTGGCGT-TCGCGACACGTTATTCTCGA----
------
FC88X_00300_00007      ----------------------ATGCCGT-TTGGGCCACGACATTCTCTC----
------
FC88X_00054_00028      ----------------------ATGCCGT-TTGGGCCACGACATTCTCTC----
------
FC88X_00074_00350      --------------------TCTCGCCGTTTGA-
GACGTTCTCTTGAGCTTCCTTT----
FC88X_00140_00037      --------------------TCTCGCCGTTTGA-GACGTTCTCTTGAG------
------
FC88X_00597_01005      ------------------ATGCCCGCTTTGTGACGACAATCTCTT---------
------
```

FC88X_00374_00195 --------------------ACCTGGCATTTCGGAC-GACGCTCTATC------
------
FC88X_00933_00806 --------------------ACCTGGCATTTCGGAC-GACGCTCTATC------
------
FC88X_00385_00139 --------------------ACCAGCCTGGTCCGGC-CGACATCTATC------
------
FC88X_00418_01019 ---------------------CTTGGCGCGCATGCCGTCCAACATCTC------
------
FC88X_00883_00336 --------------------TCCTGACCCGCATGGC-CGCGACATCTC------
------
FC88X_00026_00213 ---------------------ACCTTTCGTGCGGGCTTCGACTATTCT------
------
FC88X_01072_00888 ---------------------GCCTTTCGTACGGGCTTCGACTATTTCT-----
------
FC88X_01095_00801 -------------------ACATCCTTCGTGCGCCGGCGTCCTATT--------
------
FC88X_00036_00208 ---------------------TTATCTCGGTCCGGCCGCAATCATATT------
------
FC88X_00119_00521 ---------------------TTATCTCGGTCCGGCCGCAATCATATT------
------
FC88X_00197_00841 ---------------------TTATCTCGGTCCGGCCGCAATCATATT------
------
FC88X_00094_00518 ----------------------TCTCTCG-TCCGGCGTCAGTGCTATTCT----
------
FC88X_00743_00918 ---------------------GTCTCTCG-TCCGGCGTCAGTGCTATTCT----
------
FC88X_00325_00851 ----------------------TCTCTCG-TCCGGCGTCAGTGCTATTCT----
------
FC88X_00879_00905 ----------------------TCTCTCG-TCCGGCGTCAGTGCTATTCT----
------
FC88X_00387_00121 ----------------------TTGTGTGGTCTCGCCCCGCTAGTTCTC-----
------
FC88X_00838_00984 ----------------------TTGTGTGGTCTCGCCCCGCTAGTTCTC-----
------
FC88X_00363_00310 --------------------ACTTGTACGGTGCCGC--AGCTAATTCTC-----
------
FC88X_00141_00112 ---------------------CCTTTCTGGTC-CGCCGCGCTAGCTTATT----
------
FC88X_00139_00895 --------------------CACATTTCTGTTTCGT-CGCGCCGTATT------
------
FC88X_00140_00971 --------------------CACATTTCTGTTTCGT-CGCGCCGTATT------
------
FC88X_00425_01127 --------------------CACATTTCTGTTTCGT-CGCGCCGTATT------
------
FC88X_00467_00079 --------------------CACATTTCTGTTTCGT-CGCGCCGTATT------
------
FC88X_01069_00494 --------------------CACATTTCTGTTTCGT-CGCGCCGTATT------
------
FC88X_00594_01047 --------------------CACATTTCTGTTTCGT-CGCGCCGTATT------
------
FC88X_00936_00706 --------------------CACATTTCTGTTTCGT-CGCGCCGTATT------
------
FC88X_00941_01263 --------------------CACATTTCTGTTTCGT-CGCGCCGTATT------
------
FC88X_00882_01241 --------------------CACATTTCTGTCTCGT-CGCGCCGTATT------
------

```
FC88X_00854_00677      --------------------CACATTTCTGTTTCGT-CGCGCCGCATT------
------
FC88X_00852_01054      --------------------CACATTTCTGTTTCGC-CGCGCCGTATT------
------
FC88X_00938_00671      -----------------ACTCACCGTTCGGTTTCGGGTGCGCCGTTT-------
------
FC88X_00944_01267      ---------------GCTACTACAGTCTTTGCGCGCGCCC--------------
------
FC88X_01032_00622      -------------------CAACAGGCATT-CGCGCGCCCGCTTTCT-------
------
FC88X_00312_01127      -------------------CAACCTTCTGT-CTCCCGCGCACATTCC-------
------
FC88X_00020_00079      --------------TCTACGTTT-AACCGA----TCGT-GTTGTTCTC------
------
FC88X_01018_00607      ----------GTGATCTACGTTT-AACCGA----TCGT-GTTGTTCTC------
------
FC88X_00032_00550      ---------------CTACGTTT-AACCGA----TCGT-GTTGTTCTC------
------
FC88X_00060_00361      ---------------CTACGTTT-AACCGA----TCGT-GTTGTTCTC------
------
FC88X_00109_00480      ---------------CTACGTTT-AACCGA----TCGT-GTTGTTCTC------
------
FC88X_00140_00351      ---------------CTACGTTTTAACCGA----TCGT-GTTGTTCTC------
------
FC88X_00321_00298      ---------------CTACGTTTTAACCGA----TCGT-GTTGTTCTC------
------
FC88X_00129_00099      ---------------CTACGTTT-AACCGA----TCG--GTTGTTCTC------
------
FC88X_00018_00130      ---------------CTACGTTT-AACCGA----TCGT-GTTGTTCTC------
------
FC88X_00014_00385      ---------------CTACGTTT-AACCGA----TCGT-GTTGTTCTC------
------
FC88X_01134_00642      ---------------CTACGTTTGAACCGA----TCGTAGTTGTTCTC------
------
FC88X_00042_00828      ---------------CTACGTTTGA-CCGA----TCGT-GTTGTTCTC------
------
FC88X_00073_00168      ---------------CTACGTTTAA-CCGA----TCGT-GTTGTTCTC------
------
FC88X_00056_00259      ---------------CTACGTTT-AACCGA----TCGT-GTTGTTCTC------
------
FC88X_00133_00078      ---------------CTACGTTT-AACCGA----TCGT-GTTGTTCTC------
------
FC88X_00105_00799      ---------------CTACGTTT-AACCGA----TCGT-GTTGTTCTC------
------
FC88X_00287_01011      ---------------CTACGTTT-AACCGAA---TCGTGTT-GTTCTC------
------
FC88X_01095_00819      ---------------CTACGTTT-AACCGAA---TCGTGTT-GTTCTC------
------
FC88X_00036_00219      ---------------CTACGTTT-AACCGA----TCGTGTT-GTTCTC------
------
FC88X_00025_00554      ---------------CTACGTTT-AACCGA----TCGTGTT-GTTCTC------
------
FC88X_00087_00349      ---------------CTACGTTT-AACCGA----TCGTGTT-GTTCTC------
------
FC88X_00125_00200      ---------------CTACGTTT-AACCGA----TCGTGTT-GTTCTC------
------
```

FC88X_00026_00514 ---------------CTACGTTT-AACCGA----TCGTGTT-GTTCTC------
------
FC88X_00178_00068 ---------------CTACGTTT-AACCGA----TCGTGTTTGTTCTC------
------
FC88X_00091_00239 ---------------CTACGTTT-AACCGA----TCGTGTT-GTTCTC------
------
FC88X_00098_00883 ---------------CTACGTTT-AACCGA----TCGTGTT-GTTCTC------
------
FC88X_00147_00630 ---------------CTACGTTT-AACCGA----TCGTGTT-GTTCTC------
------
FC88X_00110_00965 ---------------CTACGTTT-AACCGA----TCGT-GTTGTTCTC------
------
FC88X_01035_00464 --------GGGGAAGCTACGTTT-AACCGA----TCGT-GTTGTTCTC------
------
FC88X_01142_01159 ----------GGGGACTACGTTT-AACCGA----TCGT-GTTGTTCTC------
------
FC88X_01113_00502 --------GGGGAAGCTACGTTT-AACCGA----TCGT-GTTGTTCTC------
------
FC88X_00061_00211 ---------------CTACGTTT-AACCGA----TCGT-GTTGTTCTC------
------
FC88X_00093_00481 ---------------CTACGTTT-AACCGA----TCGT-GTTGTTCTC------
------
FC88X_00079_00495 --------------GCTACGTTT-AACCGA----TCGT-GTTGTTCTC------
------
FC88X_00474_00071 --------------GCTACGTTT-AACCGA----TCGT-GTTGTTCTC------
------
FC88X_00502_01011 --------------GCTACGTTT-AACCGA----TCGT-GTTGTTCTC------
------
FC88X_00857_01236 --------------GCTACGTTT-AACCGA----TCGT-GTTGTTCTC------
------
FC88X_01028_00638 --------------GCTACGTTT-AACCGA----TCGT-GTTGTTCTC------
------
FC88X_01085_00992 --------------GCTACGTTT-AACCGA----TCGT-GTTGTTCTC------
------
FC88X_00053_00565 -------------AGCTACGTTT-AACCGA----TCGT-GTTGTTCTC------
------
FC88X_00529_00683 --------GGGGAAGCTACGTTT-AACCGA----TCGT-GTTGTTCTC------
------
FC88X_00071_00209 ---------------CTACGTTT-AACCGA----TCGT-GTTGTTCTC------
------
FC88X_00098_00785 ---------------CTACGTTT-AACCGA----TCGT-GTTGTTCTC------
------
FC88X_00130_00520 ---------------CTACGTTT-AACCGA----TCGT-GTTGTTCTC------
------
FC88X_00132_00890 ---------------CTACGTTT-AACCGA----TCGT-GTTGTTCTC------
------
FC88X_00164_00652 ---------------CTACGTTT-AACCGA----TCGT-GTTGTTCTC------
------
FC88X_00159_00835 ---------------CTACGTTT-AACCGA----TCGT-GTTGTTCTC------
------
FC88X_00183_00815 ---------------CTACGTTT-AACCGA----TCGT-GTTGTTCTC------
------
FC88X_00151_00966 ---------------CTACGTTT-AACCGA----TCGT-GTTGTTCTC------
------
FC88X_00246_00537 ---------------CTACGTTT-AACCGA----TCGT-GTTGTTCTC------
------

FC88X_00235_00956 ---------------CTACGTTT-AACCGA----TCGT-GTTGTTCTC------
------
FC88X_00175_00828 ---------------CTACGTTT-AACCGA----TCGT-GTTGTTCTC------
------
FC88X_00249_00084 --------------CCTACGTTT-AACCGA----TCGT-GTTGTTCTC------
------
FC88X_00158_00855 ---------------CTACGTTT-AACCGA----TCGT-GTTGTTCTC------
------
FC88X_00189_01017 ---------------CTACGTTT-AACCGA----TCGT-GTTGTTCTC------
------
FC88X_00227_00965 ---------------CTACGTTT-AACCGA----TCGT-GTTGTTCTC------
------
FC88X_00214_01084 ---------------CTACGTTT-AACCGA----TCGT-GTTGTTCTC------
------
FC88X_00249_01096 ---------------CTACGTTT-AACCGA----TCGT-GTTGTTCTC------
------
FC88X_00266_00025 ---------------CTACGTTT-AACCGA----TCGT-GTTGTTCTC------
------
FC88X_00288_00017 ---------------CTACGTTT-AACCGA----TCGT-GTTGTTCTC------
------
FC88X_00274_00056 ---------------CTACGTTT-AACCGA----TCGT-GTTGTTCTC------
------
FC88X_00263_00166 ---------------CTACGTTT-AACCGA----TCGT-GTTGTTCTC------
------
FC88X_00261_00494 ---------------CTACGTTT-AACCGA----TCGT-GTTGTTCTC------
------
FC88X_00256_00529 ---------------CTACGTTT-AACCGA----TCGT-GTTGTTCTC------
------
FC88X_00255_00728 ---------------CTACGTTT-AACCGA----TCGT-GTTGTTCTC------
------
FC88X_00280_01059 ---------------CTACGTTT-AACCGA----TCGT-GTTGTTCTC------
------
FC88X_00258_01106 ---------------CTACGTTT-AACCGA----TCGT-GTTGTTCTC------
------
FC88X_00278_01141 ---------------CTACGTTT-AACCGA----TCGT-GTTGTTCTC------
------
FC88X_00336_00168 ---------------CTACGTTT-AACCGA----TCGT-GTTGTTCTC------
------
FC88X_00305_00479 ---------------CTACGTTT-AACCGA----TCGT-GTTGTTCTC------
------
FC88X_00348_00965 ---------------CTACGTTT-AACCGA----TCGT-GTTGTTCTC------
------
FC88X_00304_01043 ---------------CTACGTTT-AACCGA----TCGT-GTTGTTCTC------
------
FC88X_00321_01077 ---------------CTACGTTT-AACCGA----TCGT-GTTGTTCTC------
------
FC88X_00337_01175 ---------------CTACGTTT-AACCGA----TCGT-GTTGTTCTC------
------
FC88X_00370_00004 ---------------CTACGTTT-AACCGA----TCGT-GTTGTTCTC------
------
FC88X_00365_00648 ---------------CTACGTTT-AACCGA----TCGT-GTTGTTCTC------
------
FC88X_00352_00686 ---------------CTACGTTT-AACCGA----TCGT-GTTGTTCTC------
------
FC88X_00395_01032 ---------------CTACGTTT-AACCGA----TCGT-GTTGTTCTC------
------

```
FC88X_00396_01068      ---------------CTACGTTT-AACCGA----TCGT-GTTGTTCTC------
------
FC88X_00423_00247      ---------------CTACGTTT-AACCGA----TCGT-GTTGTTCTC------
------
FC88X_00407_00328      ---------------CTACGTTT-AACCGA----TCGT-GTTGTTCTC------
------
FC88X_00422_00534      ---------------CTACGTTT-AACCGA----TCGT-GTTGTTCTC------
------
FC88X_00404_00994      ---------------CTACGTTT-AACCGA----TCGT-GTTGTTCTC------
------
FC88X_00406_00994      ---------------CTACGTTT-AACCGA----TCGT-GTTGTTCTC------
------
FC88X_00435_01108      ---------------CTACGTTT-AACCGA----TCGT-GTTGTTCTC------
------
FC88X_00452_00239      ---------------CTACGTTT-AACCGA----TCGT-GTTGTTCTC------
------
FC88X_00498_00518      ---------------CTACGTTT-AACCGA----TCGT-GTTGTTCTC------
------
FC88X_00459_00627      ---------------CTACGTTT-AACCGA----TCGT-GTTGTTCTC------
------
FC88X_00479_00607      ---------------CTACGTTT-AACCGA----TCGT-GTTGTTCTC------
------
FC88X_00494_00995      ---------------CTACGTTT-AACCGA----TCGT-GTTGTTCTC------
------
FC88X_00495_01206      ---------------CTACGTTT-AACCGA----TCGT-GTTGTTCTC------
------
FC88X_00495_01239      ---------------CTACGTTT-AACCGA----TCGT-GTTGTTCTC------
------
FC88X_00542_00533      ---------------CTACGTTT-AACCGA----TCGT-GTTGTTCTC------
------
FC88X_00532_00997      ---------------CTACGTTT-AACCGA----TCGT-GTTGTTCTC------
------
FC88X_00517_01004      ---------------CTACGTTT-AACCGA----TCGT-GTTGTTCTC------
------
FC88X_00509_01059      ---------------CTACGTTT-AACCGA----TCGT-GTTGTTCTC------
------
FC88X_00534_01083      ---------------CTACGTTT-AACCGA----TCGT-GTTGTTCTC------
------
FC88X_00578_00148      ---------------CTACGTTT-AACCGA----TCGT-GTTGTTCTC------
------
FC88X_00577_00241      ---------------CTACGTTT-AACCGA----TCGT-GTTGTTCTC------
------
FC88X_00571_00390      ---------------CTACGTTT-AACCGA----TCGT-GTTGTTCTC------
------
FC88X_00562_00927      ---------------CTACGTTT-AACCGA----TCGT-GTTGTTCTC------
------
FC88X_00590_01085      ---------------CTACGTTT-AACCGA----TCGT-GTTGTTCTC------
------
FC88X_00571_01137      ---------------CTACGTTT-AACCGA----TCGT-GTTGTTCTC------
------
FC88X_00560_01254      ---------------CTACGTTT-AACCGA----TCGT-GTTGTTCTC------
------
FC88X_00611_00148      ---------------CTACGTTT-AACCGA----TCGT-GTTGTTCTC------
------
FC88X_00630_00106      ---------------CTACGTTT-AACCGA----TCGT-GTTGTTCTC------
------
```

FC88X_00612_00652 ---------------CTACGTTT-AACCGA----TCGT-GTTGTTCTC------
------
FC88X_00600_00846 ---------------CTACGTTT-AACCGA----TCGT-GTTGTTCTC------
------
FC88X_00620_00909 ---------------CTACGTTT-AACCGA----TCGT-GTTGTTCTC------
------
FC88X_00626_01140 ---------------CTACGTTT-AACCGA----TCGT-GTTGTTCTC------
------
FC88X_00632_01147 ---------------CTACGTTT-AACCGA----TCGT-GTTGTTCTC------
------
FC88X_00605_01154 ---------------CTACGTTT-AACCGA----TCGT-GTTGTTCTC------
------
FC88X_00629_01176 ---------------CTACGTTT-AACCGA----TCGT-GTTGTTCTC------
------
FC88X_00606_01254 ---------------CTACGTTT-AACCGA----TCGT-GTTGTTCTC------
------
FC88X_00606_01267 ---------------CTACGTTT-AACCGA----TCGT-GTTGTTCTC------
------
FC88X_00651_00077 ---------------CTACGTTT-AACCGA----TCGT-GTTGTTCTC------
------
FC88X_00669_00228 ---------------CTACGTTT-AACCGA----TCGT-GTTGTTCTC------
------
FC88X_00655_00697 ---------------CTACGTTT-AACCGA----TCGT-GTTGTTCTC------
------
FC88X_00671_00884 ---------------CTACGTTT-AACCGA----TCGT-GTTGTTCTC------
------
FC88X_00680_01142 ---------------CTACGTTT-AACCGA----TCGT-GTTGTTCTC------
------
FC88X_00689_01119 ---------------CTACGTTT-AACCGA----TCGT-GTTGTTCTC------
------
FC88X_00670_01174 ---------------CTACGTTT-AACCGA----TCGT-GTTGTTCTC------
------
FC88X_00660_01249 ---------------CTACGTTT-AACCGA----TCGT-GTTGTTCTC------
------
FC88X_00699_01234 ---------------CTACGTTT-AACCGA----TCGT-GTTGTTCTC------
------
FC88X_00669_01257 ---------------CTACGTTT-AACCGA----TCGT-GTTGTTCTC------
------
FC88X_00738_00671 ---------------CTACGTTT-AACCGA----TCGT-GTTGTTCTC------
------
FC88X_00731_01240 ---------------CTACGTTT-AACCGA----TCGT-GTTGTTCTC------
------
FC88X_00703_01269 ---------------CTACGTTT-AACCGA----TCGT-GTTGTTCTC------
------
FC88X_00797_00146 ---------------CTACGTTT-AACCGA----TCGT-GTTGTTCTC------
------
FC88X_00761_00284 ---------------CTACGTTT-AACCGA----TCGT-GTTGTTCTC------
------
FC88X_00769_00814 ---------------CTACGTTT-AACCGA----TCGT-GTTGTTCTC------
------
FC88X_00791_00824 ---------------CTACGTTT-AACCGA----TCGT-GTTGTTCTC------
------
FC88X_00757_01145 ---------------CTACGTTT-AACCGA----TCGT-GTTGTTCTC------
------
FC88X_00776_01135 ---------------CTACGTTT-AACCGA----TCGT-GTTGTTCTC------
------

FC88X_00823_00287 ---------------CTACGTTT-AACCGA----TCGT-GTTGTTCTC------
------
FC88X_00809_00379 ---------------CTACGTTT-AACCGA----TCGT-GTTGTTCTC------
------
FC88X_00842_00840 ---------------CTACGTTT-AACCGA----TCGT-GTTGTTCTC------
------
FC88X_00809_00917 ---------------CTACGTTT-AACCGA----TCGT-GTTGTTCTC------
------
FC88X_00837_00935 ---------------CTACGTTT-AACCGA----TCGT-GTTGTTCTC------
------
FC88X_00830_01011 ---------------CTACGTTT-AACCGA----TCGT-GTTGTTCTC------
------
FC88X_00882_00159 ---------------CTACGTTT-AACCGA----TCGT-GTTGTTCTC------
------
FC88X_00871_00298 ---------------CTACGTTT-AACCGA----TCGT-GTTGTTCTC------
------
FC88X_00891_00490 ---------------CTACGTTT-AACCGA----TCGT-GTTGTTCTC------
------
FC88X_00864_00987 ---------------CTACGTTT-AACCGA----TCGT-GTTGTTCTC------
------
FC88X_00867_01042 ---------------CTACGTTT-AACCGA----TCGT-GTTGTTCTC------
------
FC88X_00850_01058 ---------------CTACGTTT-AACCGA----TCGT-GTTGTTCTC------
------
FC88X_00865_01125 ---------------CTACGTTT-AACCGA----TCGT-GTTGTTCTC------
------
FC88X_00897_01177 ---------------CTACGTTT-AACCGA----TCGT-GTTGTTCTC------
------
FC88X_00913_00532 ---------------CTACGTTT-AACCGA----TCGT-GTTGTTCTC------
------
FC88X_00942_00527 ---------------CTACGTTT-AACCGA----TCGT-GTTGTTCTC------
------
FC88X_00949_00564 ---------------CTACGTTT-AACCGA----TCGT-GTTGTTCTC------
------
FC88X_00938_00704 ---------------CTACGTTT-AACCGA----TCGT-GTTGTTCTC------
------
FC88X_00941_00762 ---------------CTACGTTT-AACCGA----TCGT-GTTGTTCTC------
------
FC88X_00903_00800 ---------------CTACGTTT-AACCGA----TCGT-GTTGTTCTC------
------
FC88X_00906_00810 ---------------CTACGTTT-AACCGA----TCGT-GTTGTTCTC------
------
FC88X_00911_00822 ---------------CTACGTTT-AACCGA----TCGT-GTTGTTCTC------
------
FC88X_00948_00867 ---------------CTACGTTT-AACCGA----TCGT-GTTGTTCTC------
------
FC88X_00911_01175 ---------------CTACGTTT-AACCGA----TCGT-GTTGTTCTC------
------
FC88X_00920_01183 ---------------CTACGTTT-AACCGA----TCGT-GTTGTTCTC------
------
FC88X_00907_01226 ---------------CTACGTTT-AACCGA----TCGT-GTTGTTCTC------
------
FC88X_00974_00264 ---------------CTACGTTT-AACCGA----TCGT-GTTGTTCTC------
------
FC88X_00979_00547 ---------------CTACGTTT-AACCGA----TCGT-GTTGTTCTC------
------

FC88X_00953_00610 ---------------CTACGTTT-AACCGA----TCGT-GTTGTTCTC------
------
FC88X_00959_00628 ---------------CTACGTTT-AACCGA----TCGT-GTTGTTCTC------
------
FC88X_00955_00660 ---------------CTACGTTT-AACCGA----TCGT-GTTGTTCTC------
------
FC88X_00996_00657 ---------------CTACGTTT-AACCGA----TCGT-GTTGTTCTC------
------
FC88X_00960_00863 ---------------CTACGTTT-AACCGA----TCGT-GTTGTTCTC------
------
FC88X_00958_00914 ---------------CTACGTTT-AACCGA----TCGT-GTTGTTCTC------
------
FC88X_00962_00949 ---------------CTACGTTT-AACCGA----TCGT-GTTGTTCTC------
------
FC88X_00953_01061 ---------------CTACGTTT-AACCGA----TCGT-GTTGTTCTC------
------
FC88X_00955_01273 ---------------CTACGTTT-AACCGA----TCGT-GTTGTTCTC------
------
FC88X_00982_01272 ---------------CTACGTTT-AACCGA----TCGT-GTTGTTCTC------
------
FC88X_01030_00463 ---------------CTACGTTT-AACCGA----TCGT-GTTGTTCTC------
------
FC88X_01018_00751 ---------------CTACGTTT-AACCGA----TCGT-GTTGTTCTC------
------
FC88X_01027_00874 ---------------CTACGTTT-AACCGA----TCGT-GTTGTTCTC------
------
FC88X_01042_00893 ---------------CTACGTTT-AACCGA----TCGT-GTTGTTCTC------
------
FC88X_01024_01076 ---------------CTACGTTT-AACCGA----TCGT-GTTGTTCTC------
------
FC88X_01014_01135 ---------------CTACGTTT-AACCGA----TCGT-GTTGTTCTC------
------
FC88X_01024_01106 ---------------CTACGTTT-AACCGA----TCGT-GTTGTTCTC------
------
FC88X_01003_01194 ---------------CTACGTTT-AACCGA----TCGT-GTTGTTCTC------
------
FC88X_01023_01179 ---------------CTACGTTT-AACCGA----TCGT-GTTGTTCTC------
------
FC88X_01066_00526 ---------------CTACGTTT-AACCGA----TCGT-GTTGTTCTC------
------
FC88X_01073_00616 ---------------CTACGTTT-AACCGA----TCGT-GTTGTTCTC------
------
FC88X_01099_00694 ---------------CTACGTTT-AACCGA----TCGT-GTTGTTCTC------
------
FC88X_01087_01000 ---------------CTACGTTT-AACCGA----TCGT-GTTGTTCTC------
------
FC88X_01100_00606 ---------------CTACGTTT-AACCGA----TCGT-GTTGTTCTC------
------
FC88X_01106_00675 ---------------CTACGTTT-AACCGA----TCGT-GTTGTTCTC------
------
FC88X_01110_00777 ---------------CTACGTTT-AACCGA----TCGT-GTTGTTCTC------
------
FC88X_01126_00921 ---------------CTACGTTT-AACCGA----TCGT-GTTGTTCTC------
------
FC88X_01133_00925 ---------------CTACGTTT-AACCGA----TCGT-GTTGTTCTC------
------

FC88X_01114_00985 ----------------CTACGTTT-AACCGA----TCGT-GTTGTTCTC------
------
FC88X_01136_01038 ----------------CTACGTTT-AACCGA----TCGT-GTTGTTCTC------
------
FC88X_00016_00102 ----------------CTACGTTT-AACCGA----TCGT-GTTGTTCTC------
------
FC88X_00041_00113 ----------------CTACGTTT-AACCGA----TCGT-GTTGTTCTC------
------
FC88X_00020_00287 ----------------CTACGTTT-AACCGA----TCGT-GTTGTTCTC------
------
FC88X_00044_00315 ----------------CTACGTTT-AACCGA----TCGT-GTTGTTCTC------
------
FC88X_00045_00539 ----------------CTACGTTT-AACCGA----TCGT-GTTGTTCTC------
------
FC88X_00071_00135 ----------------CTACGTTT-AACCGA----TCGT-GTTGTTCTC------
------
FC88X_00079_00115 ----------------CTACGTTT-AACCGA----TCGT-GTTGTTCTC------
------
FC88X_00093_00110 ----------------CTACGTTT-AACCGA----TCGT-GTTGTTCTC------
------
FC88X_00054_00238 ----------------CTACGTTT-AACCGA----TCGT-GTTGTTCTC------
------
FC88X_00093_00241 ----------------CTACGTTT-AACCGA----TCGT-GTTGTTCTC------
------
FC88X_00080_00385 ----------------CTACGTTT-AACCGA----TCGT-GTTGTTCTC------
------
FC88X_00055_00419 ----------------CTACGTTT-AACCGA----TCGT-GTTGTTCTC------
------
FC88X_00055_00429 ----------------CTACGTTT-AACCGA----TCGT-GTTGTTCTC------
------
FC88X_00061_00436 ----------------CTACGTTT-AACCGA----TCGT-GTTGTTCTC------
------
FC88X_00087_00466 ----------------CTACGTTT-AACCGA----TCGT-GTTGTTCTC------
------
FC88X_00064_00608 ----------------CTACGTTT-AACCGA----TCGT-GTTGTTCTC------
------
FC88X_00083_00633 ----------------CTACGTTT-AACCGA----TCGT-GTTGTTCTC------
------
FC88X_00075_00787 ----------------CTACGTTT-AACCGA----TCGT-GTTGTTCTC------
------
FC88X_00062_00824 ----------------CTACGTTT-AACCGA----TCGT-GTTGTTCTC------
------
FC88X_00073_00800 ----------------CTACGTTT-AACCGA----TCGT-GTTGTTCTC------
------
FC88X_00092_00837 ----------------CTACGTTT-AACCGA----TCGT-GTTGTTCTC------
------
FC88X_00138_00058 ----------------CTACGTTT-AACCGA----TCGT-GTTGTTCTC------
------
FC88X_00108_00103 ----------------CTACGTTT-AACCGA----TCGT-GTTGTTCTC------
------
FC88X_00113_00115 ----------------CTACGTTT-AACCGA----TCGT-GTTGTTCTC------
------
FC88X_00120_00126 ----------------CTACGTTT-AACCGA----TCGT-GTTGTTCTC------
------
FC88X_00144_00113 ----------------CTACGTTT-AACCGA----TCGT-GTTGTTCTC------
------

```
FC88X_00146_00116      ---------------CTACGTTT-AACCGA----TCGT-GTTGTTCTC------
------
FC88X_00104_00201      ---------------CTACGTTT-AACCGA----TCGT-GTTGTTCTC------
------
FC88X_00113_00342      ---------------CTACGTTT-AACCGA----TCGT-GTTGTTCTC------
------
FC88X_00140_00395      ---------------CTACGTTT-AACCGA----TCGT-GTTGTTCTC------
------
FC88X_00127_00427      ---------------CTACGTTT-AACCGA----TCGT-GTTGTTCTC------
------
FC88X_00123_00501      ---------------CTACGTTT-AACCGA----TCGT-GTTGTTCTC------
------
FC88X_00130_00548      ---------------CTACGTTT-AACCGA----TCGT-GTTGTTCTC------
------
FC88X_00100_00639      ---------------CTACGTTT-AACCGA----TCGT-GTTGTTCTC------
------
FC88X_00125_00663      ---------------CTACGTTT-AACCGA----TCGT-GTTGTTCTC------
------
FC88X_00131_00696      ---------------CTACGTTT-AACCGA----TCGT-GTTGTTCTC------
------
FC88X_00146_00990      ---------------CTACGTTT-AACCGA----TCGT-GTTGTTCTC------
------
FC88X_00159_00095      ---------------CTACGTTT-AACCGA----TCGT-GTTGTTCTC------
------
FC88X_00177_00052      ---------------CTACGTTT-AACCGA----TCGT-GTTGTTCTC------
------
FC88X_00179_00141      ---------------CTACGTTT-AACCGA----TCGT-GTTGTTCTC------
------
FC88X_00187_00114      ---------------CTACGTTT-AACCGA----TCGT-GTTGTTCTC------
------
FC88X_00159_00183      ---------------CTACGTTT-AACCGA----TCGT-GTTGTTCTC------
------
FC88X_00188_00383      ---------------CTACGTTT-AACCGA----TCGT-GTTGTTCTC------
------
FC88X_00172_00568      ---------------CTACGTTT-AACCGA----TCGT-GTTGTTCTC------
------
FC88X_00153_00617      ---------------CTACGTTT-AACCGA----TCGT-GTTGTTCTC------
------
FC88X_00158_00733      ---------------CTACGTTT-AACCGA----TCGT-GTTGTTCTC------
------
FC88X_00159_00735      ---------------CTACGTTT-AACCGA----TCGT-GTTGTTCTC------
------
FC88X_00177_00786      ---------------CTACGTTT-AACCGA----TCGT-GTTGTTCTC------
------
FC88X_00179_00773      ---------------CTACGTTT-AACCGA----TCGT-GTTGTTCTC------
------
FC88X_00158_00829      ---------------CTACGTTT-AACCGA----TCGT-GTTGTTCTC------
------
FC88X_00199_00982      ---------------CTACGTTT-AACCGA----TCGT-GTTGTTCTC------
------
FC88X_00206_00124      ---------------CTACGTTT-AACCGA----TCGT-GTTGTTCTC------
------
FC88X_00233_00782      ---------------CTACGTTT-AACCGA----TCGT-GTTGTTCTC------
------
FC88X_00202_00884      ---------------CTACGTTT-AACCGA----TCGT-GTTGTTCTC------
------
```

FC88X_00217_00964 ---------------CTACGTTT-AACCGA----TCGT-GTTGTTCTC------
------
FC88X_00219_00990 ---------------CTACGTTT-AACCGA----TCGT-GTTGTTCTC------
------
FC88X_00243_00992 ---------------CTACGTTT-AACCGA----TCGT-GTTGTTCTC------
------
FC88X_00264_00171 ---------------CTACGTTT-AACCGA----TCGT-GTTGTTCTC------
------
FC88X_00292_00462 ---------------CTACGTTT-AACCGA----TCGT-GTTGTTCTC------
------
FC88X_00268_00500 ---------------CTACGTTT-AACCGA----TCGT-GTTGTTCTC------
------
FC88X_00266_00971 ---------------CTACGTTT-AACCGA----TCGT-GTTGTTCTC------
------
FC88X_00275_00991 ---------------CTACGTTT-AACCGA----TCGT-GTTGTTCTC------
------
FC88X_00281_01044 ---------------CTACGTTT-AACCGA----TCGT-GTTGTTCTC------
------
FC88X_00333_00379 ---------------CTACGTTT-AACCGA----TCGT-GTTGTTCTC------
------
FC88X_00312_00525 ---------------CTACGTTT-AACCGA----TCGT-GTTGTTCTC------
------
FC88X_00316_01075 ---------------CTACGTTT-AACCGA----TCGT-GTTGTTCTC------
------
FC88X_00334_01146 ---------------CTACGTTT-AACCGA----TCGT-GTTGTTCTC------
------
FC88X_00338_01169 ---------------CTACGTTT-AACCGA----TCGT-GTTGTTCTC------
------
FC88X_00388_00478 ---------------CTACGTTT-AACCGA----TCGT-GTTGTTCTC------
------
FC88X_00383_00579 ---------------CTACGTTT-AACCGA----TCGT-GTTGTTCTC------
------
FC88X_00380_01006 ---------------CTACGTTT-AACCGA----TCGT-GTTGTTCTC------
------
FC88X_00380_01102 ---------------CTACGTTT-AACCGA----TCGT-GTTGTTCTC------
------
FC88X_00413_00061 ---------------CTACGTTT-AACCGA----TCGT-GTTGTTCTC------
------
FC88X_00436_00196 ---------------CTACGTTT-AACCGA----TCGT-GTTGTTCTC------
------
FC88X_00428_00368 ---------------CTACGTTT-AACCGA----TCGT-GTTGTTCTC------
------
FC88X_00411_00421 ---------------CTACGTTT-AACCGA----TCGT-GTTGTTCTC------
------
FC88X_00433_00425 ---------------CTACGTTT-AACCGA----TCGT-GTTGTTCTC------
------
FC88X_00448_00408 ---------------CTACGTTT-AACCGA----TCGT-GTTGTTCTC------
------
FC88X_00425_00650 ---------------CTACGTTT-AACCGA----TCGT-GTTGTTCTC------
------
FC88X_00401_00757 ---------------CTACGTTT-AACCGA----TCGT-GTTGTTCTC------
------
FC88X_00400_00971 ---------------CTACGTTT-AACCGA----TCGT-GTTGTTCTC------
------
FC88X_00438_00998 ---------------CTACGTTT-AACCGA----TCGT-GTTGTTCTC------
------

FC88X_00442_01091 ----------------CTACGTTT-AACCGA----TCGT-GTTGTTCTC------
------
FC88X_00439_01154 ----------------CTACGTTT-AACCGA----TCGT-GTTGTTCTC------
------
FC88X_00489_00062 ----------------CTACGTTT-AACCGA----TCGT-GTTGTTCTC------
------
FC88X_00453_00134 ----------------CTACGTTT-AACCGA----TCGT-GTTGTTCTC------
------
FC88X_00483_00284 ----------------CTACGTTT-AACCGA----TCGT-GTTGTTCTC------
------
FC88X_00466_00388 ----------------CTACGTTT-AACCGA----TCGT-GTTGTTCTC------
------
FC88X_00489_01011 ----------------CTACGTTT-AACCGA----TCGT-GTTGTTCTC------
------
FC88X_00491_01199 ----------------CTACGTTT-AACCGA----TCGT-GTTGTTCTC------
------
FC88X_00510_00019 ----------------CTACGTTT-AACCGA----TCGT-GTTGTTCTC------
------
FC88X_00513_00095 ----------------CTACGTTT-AACCGA----TCGT-GTTGTTCTC------
------
FC88X_00549_00075 ----------------CTACGTTT-AACCGA----TCGT-GTTGTTCTC------
------
FC88X_00526_00171 ----------------CTACGTTT-AACCGA----TCGT-GTTGTTCTC------
------
FC88X_00506_00526 ----------------CTACGTTT-AACCGA----TCGT-GTTGTTCTC------
------
FC88X_00514_01000 ----------------CTACGTTT-AACCGA----TCGT-GTTGTTCTC------
------
FC88X_00530_01076 ----------------CTACGTTT-AACCGA----TCGT-GTTGTTCTC------
------
FC88X_00538_01109 ----------------CTACGTTT-AACCGA----TCGT-GTTGTTCTC------
------
FC88X_00537_01237 ----------------CTACGTTT-AACCGA----TCGT-GTTGTTCTC------
------
FC88X_00538_01212 ----------------CTACGTTT-AACCGA----TCGT-GTTGTTCTC------
------
FC88X_00552_00182 ----------------CTACGTTT-AACCGA----TCGT-GTTGTTCTC------
------
FC88X_00586_00155 ----------------CTACGTTT-AACCGA----TCGT-GTTGTTCTC------
------
FC88X_00590_00312 ----------------CTACGTTT-AACCGA----TCGT-GTTGTTCTC------
------
FC88X_00586_00415 ----------------CTACGTTT-AACCGA----TCGT-GTTGTTCTC------
------
FC88X_00568_00689 ----------------CTACGTTT-AACCGA----TCGT-GTTGTTCTC------
------
FC88X_00568_00797 ----------------CTACGTTT-AACCGA----TCGT-GTTGTTCTC------
------
FC88X_00572_00762 ----------------CTACGTTT-AACCGA----TCGT-GTTGTTCTC------
------
FC88X_00560_00992 ----------------CTACGTTT-AACCGA----TCGT-GTTGTTCTC------
------
FC88X_00567_00963 ----------------CTACGTTT-AACCGA----TCGT-GTTGTTCTC------
------
FC88X_00558_01090 ----------------CTACGTTT-AACCGA----TCGT-GTTGTTCTC------
------

```
FC88X_00553_01181       ---------------CTACGTTT-AACCGA----TCGT-GTTGTTCTC------
------
FC88X_00569_01161       ---------------CTACGTTT-AACCGA----TCGT-GTTGTTCTC------
------
FC88X_00551_01244       ---------------CTACGTTT-AACCGA----TCGT-GTTGTTCTC------
------
FC88X_00560_01261       ---------------CTACGTTT-AACCGA----TCGT-GTTGTTCTC------
------
FC88X_00598_01264       ---------------CTACGTTT-AACCGA----TCGT-GTTGTTCTC------
------
FC88X_00604_00059       ---------------CTACGTTT-AACCGA----TCGT-GTTGTTCTC------
------
FC88X_00614_00093       ---------------CTACGTTT-AACCGA----TCGT-GTTGTTCTC------
------
FC88X_00617_00107       ---------------CTACGTTT-AACCGA----TCGT-GTTGTTCTC------
------
FC88X_00627_00221       ---------------CTACGTTT-AACCGA----TCGT-GTTGTTCTC------
------
FC88X_00615_00282       ---------------CTACGTTT-AACCGA----TCGT-GTTGTTCTC------
------
FC88X_00645_00341       ---------------CTACGTTT-AACCGA----TCGT-GTTGTTCTC------
------
FC88X_00648_00451       ---------------CTACGTTT-AACCGA----TCGT-GTTGTTCTC------
------
FC88X_00605_00967       ---------------CTACGTTT-AACCGA----TCGT-GTTGTTCTC------
------
FC88X_00606_00970       ---------------CTACGTTT-AACCGA----TCGT-GTTGTTCTC------
------
FC88X_00625_01008       ---------------CTACGTTT-AACCGA----TCGT-GTTGTTCTC------
------
FC88X_00617_01106       ---------------CTACGTTT-AACCGA----TCGT-GTTGTTCTC------
------
FC88X_00611_01190       ---------------CTACGTTT-AACCGA----TCGT-GTTGTTCTC------
------
FC88X_00610_01210       ---------------CTACGTTT-AACCGA----TCGT-GTTGTTCTC------
------
FC88X_00632_01258       ---------------CTACGTTT-AACCGA----TCGT-GTTGTTCTC------
------
FC88X_00634_01251       ---------------CTACGTTT-AACCGA----TCGT-GTTGTTCTC------
------
FC88X_00666_00140       ---------------CTACGTTT-AACCGA----TCGT-GTTGTTCTC------
------
FC88X_00676_00246       ---------------CTACGTTT-AACCGA----TCGT-GTTGTTCTC------
------
FC88X_00670_00709       ---------------CTACGTTT-AACCGA----TCGT-GTTGTTCTC------
------
FC88X_00652_01063       ---------------CTACGTTT-AACCGA----TCGT-GTTGTTCTC------
------
FC88X_00686_01169       ---------------CTACGTTT-AACCGA----TCGT-GTTGTTCTC------
------
FC88X_00682_01243       ---------------CTACGTTT-AACCGA----TCGT-GTTGTTCTC------
------
FC88X_00650_01265       ---------------CTACGTTT-AACCGA----TCGT-GTTGTTCTC------
------
FC88X_00730_00282       ---------------CTACGTTT-AACCGA----TCGT-GTTGTTCTC------
------
```

FC88X_00736_00722 ---------------CTACGTTT-AACCGA----TCGT-GTTGTTCTC------
------
FC88X_00749_00915 ---------------CTACGTTT-AACCGA----TCGT-GTTGTTCTC------
------
FC88X_00744_00977 ---------------CTACGTTT-AACCGA----TCGT-GTTGTTCTC------
------
FC88X_00746_00986 ---------------CTACGTTT-AACCGA----TCGT-GTTGTTCTC------
------
FC88X_00740_01107 ---------------CTACGTTT-AACCGA----TCGT-GTTGTTCTC------
------
FC88X_00705_01221 ---------------CTACGTTT-AACCGA----TCGT-GTTGTTCTC------
------
FC88X_00791_00095 ---------------CTACGTTT-AACCGA----TCGT-GTTGTTCTC------
------
FC88X_00798_00183 ---------------CTACGTTT-AACCGA----TCGT-GTTGTTCTC------
------
FC88X_00791_00802 ---------------CTACGTTT-AACCGA----TCGT-GTTGTTCTC------
------
FC88X_00769_00885 ---------------CTACGTTT-AACCGA----TCGT-GTTGTTCTC------
------
FC88X_00792_00929 ---------------CTACGTTT-AACCGA----TCGT-GTTGTTCTC------
------
FC88X_00781_00961 ---------------CTACGTTT-AACCGA----TCGT-GTTGTTCTC------
------
FC88X_00768_01212 ---------------CTACGTTT-AACCGA----TCGT-GTTGTTCTC------
------
FC88X_00778_01268 ---------------CTACGTTT-AACCGA----TCGT-GTTGTTCTC------
------
FC88X_00802_00274 ---------------CTACGTTT-AACCGA----TCGT-GTTGTTCTC------
------
FC88X_00821_00300 ---------------CTACGTTT-AACCGA----TCGT-GTTGTTCTC------
------
FC88X_00835_00838 ---------------CTACGTTT-AACCGA----TCGT-GTTGTTCTC------
------
FC88X_00806_00994 ---------------CTACGTTT-AACCGA----TCGT-GTTGTTCTC------
------
FC88X_00841_01030 ---------------CTACGTTT-AACCGA----TCGT-GTTGTTCTC------
------
FC88X_00805_01069 ---------------CTACGTTT-AACCGA----TCGT-GTTGTTCTC------
------
FC88X_00876_00210 ---------------CTACGTTT-AACCGA----TCGT-GTTGTTCTC------
------
FC88X_00853_00339 ---------------CTACGTTT-AACCGA----TCGT-GTTGTTCTC------
------
FC88X_00880_00307 ---------------CTACGTTT-AACCGA----TCGT-GTTGTTCTC------
------
FC88X_00870_00700 ---------------CTACGTTT-AACCGA----TCGT-GTTGTTCTC------
------
FC88X_00883_00796 ---------------CTACGTTT-AACCGA----TCGT-GTTGTTCTC------
------
FC88X_00856_00805 ---------------CTACGTTT-AACCGA----TCGT-GTTGTTCTC------
------
FC88X_00883_00848 ---------------CTACGTTT-AACCGA----TCGT-GTTGTTCTC------
------
FC88X_00864_00888 ---------------CTACGTTT-AACCGA----TCGT-GTTGTTCTC------
------

FC88X_00863_00925 ---------------CTACGTTT-AACCGA----TCGT-GTTGTTCTC------
------
FC88X_00892_00948 ---------------CTACGTTT-AACCGA----TCGT-GTTGTTCTC------
------
FC88X_00865_00958 ---------------CTACGTTT-AACCGA----TCGT-GTTGTTCTC------
------
FC88X_00898_00966 ---------------CTACGTTT-AACCGA----TCGT-GTTGTTCTC------
------
FC88X_00868_01098 ---------------CTACGTTT-AACCGA----TCGT-GTTGTTCTC------
------
FC88X_00872_01206 ---------------CTACGTTT-AACCGA----TCGT-GTTGTTCTC------
------
FC88X_00918_00201 ---------------CTACGTTT-AACCGA----TCGT-GTTGTTCTC------
------
FC88X_00943_00249 ---------------CTACGTTT-AACCGA----TCGT-GTTGTTCTC------
------
FC88X_00914_00346 ---------------CTACGTTT-AACCGA----TCGT-GTTGTTCTC------
------
FC88X_00949_00322 ---------------CTACGTTT-AACCGA----TCGT-GTTGTTCTC------
------
FC88X_00949_00340 ---------------CTACGTTT-AACCGA----TCGT-GTTGTTCTC------
------
FC88X_00902_00441 ---------------CTACGTTT-AACCGA----TCGT-GTTGTTCTC------
------
FC88X_00916_00479 ---------------CTACGTTT-AACCGA----TCGT-GTTGTTCTC------
------
FC88X_00940_00450 ---------------CTACGTTT-AACCGA----TCGT-GTTGTTCTC------
------
FC88X_00938_00604 ---------------CTACGTTT-AACCGA----TCGT-GTTGTTCTC------
------
FC88X_00910_00762 ---------------CTACGTTT-AACCGA----TCGT-GTTGTTCTC------
------
FC88X_00918_00786 ---------------CTACGTTT-AACCGA----TCGT-GTTGTTCTC------
------
FC88X_00948_00786 ---------------CTACGTTT-AACCGA----TCGT-GTTGTTCTC------
------
FC88X_00909_00864 ---------------CTACGTTT-AACCGA----TCGT-GTTGTTCTC------
------
FC88X_00912_00872 ---------------CTACGTTT-AACCGA----TCGT-GTTGTTCTC------
------
FC88X_00912_01065 ---------------CTACGTTT-AACCGA----TCGT-GTTGTTCTC------
------
FC88X_00935_01133 ---------------CTACGTTT-AACCGA----TCGT-GTTGTTCTC------
------
FC88X_00924_01231 ---------------CTACGTTT-AACCGA----TCGT-GTTGTTCTC------
------
FC88X_00989_00442 ---------------CTACGTTT-AACCGA----TCGT-GTTGTTCTC------
------
FC88X_00962_00469 ---------------CTACGTTT-AACCGA----TCGT-GTTGTTCTC------
------
FC88X_00994_00480 ---------------CTACGTTT-AACCGA----TCGT-GTTGTTCTC------
------
FC88X_00970_00548 ---------------CTACGTTT-AACCGA----TCGT-GTTGTTCTC------
------
FC88X_00952_00715 ---------------CTACGTTT-AACCGA----TCGT-GTTGTTCTC------
------

FC88X_00989_00723 ---------------CTACGTTT-AACCGA----TCGT-GTTGTTCTC------
------
FC88X_00985_00786 ---------------CTACGTTT-AACCGA----TCGT-GTTGTTCTC------
------
FC88X_00987_00872 ---------------CTACGTTT-AACCGA----TCGT-GTTGTTCTC------
------
FC88X_00995_00982 ---------------CTACGTTT-AACCGA----TCGT-GTTGTTCTC------
------
FC88X_00950_01045 ---------------CTACGTTT-AACCGA----TCGT-GTTGTTCTC------
------
FC88X_00957_01031 ---------------CTACGTTT-AACCGA----TCGT-GTTGTTCTC------
------
FC88X_00984_01029 ---------------CTACGTTT-AACCGA----TCGT-GTTGTTCTC------
------
FC88X_00987_01052 ---------------CTACGTTT-AACCGA----TCGT-GTTGTTCTC------
------
FC88X_00987_01089 ---------------CTACGTTT-AACCGA----TCGT-GTTGTTCTC------
------
FC88X_00956_01148 ---------------CTACGTTT-AACCGA----TCGT-GTTGTTCTC------
------
FC88X_00952_01171 ---------------CTACGTTT-AACCGA----TCGT-GTTGTTCTC------
------
FC88X_00990_01216 ---------------CTACGTTT-AACCGA----TCGT-GTTGTTCTC------
------
FC88X_01006_00284 ---------------CTACGTTT-AACCGA----TCGT-GTTGTTCTC------
------
FC88X_01003_00460 ---------------CTACGTTT-AACCGA----TCGT-GTTGTTCTC------
------
FC88X_01010_00487 ---------------CTACGTTT-AACCGA----TCGT-GTTGTTCTC------
------
FC88X_01002_00539 ---------------CTACGTTT-AACCGA----TCGT-GTTGTTCTC------
------
FC88X_01000_00637 ---------------CTACGTTT-AACCGA----TCGT-GTTGTTCTC------
------
FC88X_01023_00619 ---------------CTACGTTT-AACCGA----TCGT-GTTGTTCTC------
------
FC88X_01045_00606 ---------------CTACGTTT-AACCGA----TCGT-GTTGTTCTC------
------
FC88X_01003_00662 ---------------CTACGTTT-AACCGA----TCGT-GTTGTTCTC------
------
FC88X_01036_00715 ---------------CTACGTTT-AACCGA----TCGT-GTTGTTCTC------
------
FC88X_01002_00758 ---------------CTACGTTT-AACCGA----TCGT-GTTGTTCTC------
------
FC88X_01010_00785 ---------------CTACGTTT-AACCGA----TCGT-GTTGTTCTC------
------
FC88X_01029_00798 ---------------CTACGTTT-AACCGA----TCGT-GTTGTTCTC------
------
FC88X_01035_00787 ---------------CTACGTTT-AACCGA----TCGT-GTTGTTCTC------
------
FC88X_01010_00994 ---------------CTACGTTT-AACCGA----TCGT-GTTGTTCTC------
------
FC88X_01029_00976 ---------------CTACGTTT-AACCGA----TCGT-GTTGTTCTC------
------
FC88X_01012_01091 ---------------CTACGTTT-AACCGA----TCGT-GTTGTTCTC------
------

FC88X_01008_01160 ---------------CTACGTTT-AACCGA----TCGT-GTTGTTCTC------
------
FC88X_01061_00508 ---------------CTACGTTT-AACCGA----TCGT-GTTGTTCTC------
------
FC88X_01077_00513 ---------------CTACGTTT-AACCGA----TCGT-GTTGTTCTC------
------
FC88X_01081_00505 ---------------CTACGTTT-AACCGA----TCGT-GTTGTTCTC------
------
FC88X_01051_00644 ---------------CTACGTTT-AACCGA----TCGT-GTTGTTCTC------
------
FC88X_01088_00638 ---------------CTACGTTT-AACCGA----TCGT-GTTGTTCTC------
------
FC88X_01076_00718 ---------------CTACGTTT-AACCGA----TCGT-GTTGTTCTC------
------
FC88X_01088_00795 ---------------CTACGTTT-AACCGA----TCGT-GTTGTTCTC------
------
FC88X_01060_00828 ---------------CTACGTTT-AACCGA----TCGT-GTTGTTCTC------
------
FC88X_01062_00843 ---------------CTACGTTT-AACCGA----TCGT-GTTGTTCTC------
------
FC88X_01065_00807 ---------------CTACGTTT-AACCGA----TCGT-GTTGTTCTC------
------
FC88X_01082_00827 ---------------CTACGTTT-AACCGA----TCGT-GTTGTTCTC------
------
FC88X_01089_00845 ---------------CTACGTTT-AACCGA----TCGT-GTTGTTCTC------
------
FC88X_01084_00969 ---------------CTACGTTT-AACCGA----TCGT-GTTGTTCTC------
------
FC88X_01099_00961 ---------------CTACGTTT-AACCGA----TCGT-GTTGTTCTC------
------
FC88X_01060_01024 ---------------CTACGTTT-AACCGA----TCGT-GTTGTTCTC------
------
FC88X_01090_01021 ---------------CTACGTTT-AACCGA----TCGT-GTTGTTCTC------
------
FC88X_01070_01181 ---------------CTACGTTT-AACCGA----TCGT-GTTGTTCTC------
------
FC88X_01064_01241 ---------------CTACGTTT-AACCGA----TCGT-GTTGTTCTC------
------
FC88X_01053_01250 ---------------CTACGTTT-AACCGA----TCGT-GTTGTTCTC------
------
FC88X_01112_00647 ---------------CTACGTTT-AACCGA----TCGT-GTTGTTCTC------
------
FC88X_01119_00627 ---------------CTACGTTT-AACCGA----TCGT-GTTGTTCTC------
------
FC88X_01128_00662 ---------------CTACGTTT-AACCGA----TCGT-GTTGTTCTC------
------
FC88X_01117_00761 ---------------CTACGTTT-AACCGA----TCGT-GTTGTTCTC------
------
FC88X_01108_00819 ---------------CTACGTTT-AACCGA----TCGT-GTTGTTCTC------
------
FC88X_01120_00801 ---------------CTACGTTT-AACCGA----TCGT-GTTGTTCTC------
------
FC88X_01100_01003 ---------------CTACGTTT-AACCGA----TCGT-GTTGTTCTC------
------
FC88X_01137_01044 ---------------CTACGTTT-AACCGA----TCGT-GTTGTTCTC------
------

```
FC88X_01104_01089       ---------------CTACGTTT-AACCGA----TCGT-GTTGTTCTC------
------
FC88X_01130_01059       ---------------CTACGTTT-AACCGA----TCGT-GTTGTTCTC------
------
FC88X_01124_01179       ---------------CTACGTTT-AACCGA----TCGT-GTTGTTCTC------
------
FC88X_01121_01213       ---------------CTACGTTT-AACCGA----TCGT-GTTGTTCTC------
------
FC88X_01134_01208       ---------------CTACGTTT-AACCGA----TCGT-GTTGTTCTC------
------
FC88X_01092_00560       ---------------CTACGTTT-AACCGA----TCGT-GTTGTTCTCCC----
------
FC88X_00521_00069       ---------------CTACGTTT-AACCGA----TCGT-GTTGTTCTCC-----
------
FC88X_00982_00387       ---------------CTACGTTT-AACCGA----TCGT-GTTGTCTCC------
------
FC88X_00386_00094       ---------------CTACGTTT-AACCGA----TCGT-GTTGTTCTCC-----
------
FC88X_00732_00939       ---------------CTACGTTT-AACCGA----TCGT-GTTGTTCTCC-----
------
FC88X_00416_00622       ---------------CTACGTTT-AACCGA----TCGT-GTTGTTCTCCC----
------
FC88X_00410_01146       ---------------CTACGTTT-AACCGA----TCGT-GTTGTTCTCCC----
------
FC88X_00790_00235       ---------------CTACGTTT-AACCGA----TCGT-GTTGTTCTCCC----
------
FC88X_00238_00043       ---------------CTACGTTTAAACCGA----TCGT-GTTGTTCTC------
------
FC88X_00252_01111       ---------------CTACGTTTAA--CGA----TCGT-GTTGTTCTC------
------
FC88X_00245_00918       ---------------CTACGTTTAA--CGA----TCGT-GTTGTTCTC------
------
FC88X_00903_00973       ---------------CTACGTTTAA-TCGA----TCGT-GTTGTTCTC------
------
FC88X_01013_00560       ---------------CTACGTTTAA-TCGA----TCGT-GTTGTTCTC------
------
FC88X_00540_01172       ---------------CTACGTTT-AACGGA----TCGT-GTTGTTCTC------
------
FC88X_00802_01180       ---------------CTACGTTT-AACCGA----TCGT-ATTGTTCTC------
------
FC88X_00179_00077       ---------------CTACGTTT-AACCGA----TCGTGATTGTTCTC------
------
FC88X_00575_00090       ---------------TTACGTTT-AACCGA----TCGT-GTTGTTCTC------
------
FC88X_00942_00733       ---------------TTACGTTT-AACCGA----TCGT-GTTGTTCTCC-----
------
FC88X_01136_01008       ---------------TTACGTTT-AACCGA----TCGT-GTTGTTCTC------
------
FC88X_00173_00947       ---------------TTACGTTT-AACCGA----TCGT-GTTGTTCTC------
------
FC88X_00739_01039       ---------------TTACGTTT-AACCGA----TCGT-GTTGTTCTC------
------
FC88X_00955_01098       ---------------TTACGTTT-AACCGA----TCGT-GTTGTTCTC------
------
FC88X_01019_00626       ---------------TTACGTTT-AACCGA----TCGT-GTTGTTCTC------
------
```

FC88X_01047_01163 ----------------TACGTTT-AACCGA----TCGT-GTTGTTCTC------
------
FC88X_00374_00978 ---------------CTACGTTT-AACCGA----TCGT-
GTTGTTCTCCTTCCT------
FC88X_00551_00450 ---------------CTACGTTT-AACCGA----TCGT-
GTTGTTCTCCTTCCTT-----
FC88X_00590_01179 ---------------CTACGTTT-AACCGA----TCGT-
GTTGTTCTCCTTCCTTT----
FC88X_00736_00939 ---------------CTACGTTT-AACCGA----TCGT-
GTTGTTCTCCTTCCTTT----
FC88X_01146_01042 ---------------CTACGTTT-AACCGA----TCGT-
GTTGTTCTCCTTCCTTT----
FC88X_00959_00776 ---------------CTACGTTT-AACCGA----TCGT-
GTTGTTCTCCTTCCTTT----
FC88X_01015_00455 ---------------CTACGTTT-AACCGA----TCGT-
GTTGTTCTCCTTCCTTT----
FC88X_01051_00820 ---------------CTACGTTT-AACCGA----TCGT-
GTTGTTCTCCTTCCTTT----
FC88X_00193_00651 ---------------CTACGTTT-AACCGA----TCGT-
GTTGTTCTCCTTCCTTT----
FC88X_00083_00736 --------------GCTACGTTT-AACCGA----TCGT-
GTTGTTCTCCTTCCTTT----
FC88X_00094_00638 ---------------CTACGTTT-AACCGA----TCGT-GCTGTTCTC------
------
FC88X_00282_00018 ---------------CTACGTTT-AACCGA----TCGT-GCTGTTCTC------
------
FC88X_00617_00277 ---------------CTACGTTT-AACCGA----TCGT-GCTGTTCTC------
------
FC88X_00906_00764 ---------------CTACGTTT-AACCGA----TCGT-GCTGTTCTC------
------
FC88X_00967_00473 ---------------CTACGTTT-AACCGA----TCGT-GCTGTTCTC------
------
FC88X_01030_00309 ---------------CTACGTTT-AACCGA----TCGT-GCTGTTCTC------
------
FC88X_00933_00717 ---------------CTACGTTT-AACCGA----TCGT-GCTGTTCTC------
------
FC88X_00199_00992 ---------------CTACGTTT-AACCGA----TCGT-GGTGTTCTC------
------
FC88X_00600_01132 ---------------CTACGTTT-AACCGA----TCGT-G-TGTTCTC------
------
FC88X_00341_00131 ---------------CTACGTTC-AACCGA----TCGT-GTTGTTCTC------
------
FC88X_00318_00995 ---------------CTACGTTC-AACCGA----TCGT-GTTGTTCTC------
------
FC88X_00635_00986 ---------------CTACGTTC-AACCGA----TCGT-GTTGTTCTC------
------
FC88X_00701_01220 ---------------CTACGTTC-AACCGA----TCGT-GTTGTTCTC------
------
FC88X_00619_00924 ---------------CTGCGTTT-AACCGA----TCGT-GTTGTTCTC------
------
FC88X_00944_00634 ---------------CTGCGTTT-AACCGA----TCGT-GTTGTTCTC------
------
FC88X_01087_00870 ---------------CTGCGTTT-AACCGA----TCGT-GTTGTTCTC------
------
FC88X_00997_01269 --------------GCTATGTTT-AACCGA----TCGT-GTTGTTCTC------
------

FC88X_00594_01197 ----------------CTACGTTT-AACCGA----TCG-CGTTGTTCTC------
------
FC88X_00853_00935 ---------------GCTACGTTT-AACCGA----TCG-CGTTGTTCTC------
------
FC88X_00944_00220 ----------------CTACGTTT-AACCGA----TCG-CGTTGTTCTC------
------
FC88X_00914_01038 ----------------CTACGTTT-AACCGA----TCG-CGTTGTTCTC------
------
FC88X_00969_00810 ----------------CTACGTTT-AACCGA----TCG-CGTTGTTCTC------
------
FC88X_01039_00894 ----------------CTACGTTT-AACCGA----TCG-CGTTGTTCTC------
------
FC88X_00886_00867 ----------------CTACGTTT-AACCGA----TCG-CGTTGTTCTC------
------
FC88X_00992_00498 ----------------CTACGTTT-AACCGA----TCG-CGTTGTTCTC------
------
FC88X_01029_00444 ----------------CTACGTTT-AACCGA----TCG-CGTTGTTCTC------
------
FC88X_01103_00864 ----------------CTACGTTT-AACCGA----TCG-CGTTGTTCTC------
------
FC88X_01046_00388 ----------------CTACGTTT-AACCGA----TCGTCGTTGTTCTC------
------
FC88X_00123_00845 ----------------CTACGTTT-AACCGA----TCG-CGTTGTTCT-------
------
FC88X_00492_01235 ----------------CTACGTTT-AGCCGA----TCG-TGTTGTTCTC------
------
FC88X_00619_01230 ----------------CTACGTTT-AGC-GA----TCG-TGTTGTTCTC------
------
FC88X_00613_00793 ----------------CTACGTTT-AGCCGA----TCG-CGTTGTTCTC------
------
FC88X_00923_00306 ----------------CTACGTTT-ATCCGA----TCG-TGTTGTTCTC------
------
FC88X_00038_00733 ----------------CTACGTTT-AACCGA----TGGT-GTTGTTCTC------
------
FC88X_00940_01187 ----------------CTACGTTT-AACCGA----TGGT-GTTGTTCTC------
------
FC88X_00768_00136 ----------------CTACGTTT-AACCGA----TTGT-GTTGTTCTC------
------
FC88X_00894_00657 ----------------CTACGTTT-AACCGA----TAGT-
GTTGTTCTCCTTCCTTT----
FC88X_00114_00925 ----------------CTACGTTT-AACCGA----TCGT-
GTTGTTCTCCTTCCTTTC---
FC88X_01020_00964 ----------------CTACGTTT-AACCGA----TCGT-
GTTGTTCTCCTTCCTTTC---
FC88X_00155_00896 ----------------CTACGTCT-AACCGA----TCGT-GTTGTTCTC------
------
FC88X_00288_00926 ----------------CTACGTCT-AACCGA----TCGT-GTTGTTCTC------
------
FC88X_00027_00326 ----------------CTACGTCT-AACCGA----TCGT-GTTGTTCTC------
------
FC88X_00863_01273 ----------------CTACGTCT-AACCGA----TCGT-GTTGTTCTC------
------
FC88X_00174_01032 ----------------CTACGTTT-AACCGG----TCGT-GTTGTTCTC------
------
FC88X_00608_01133 ---------------GCTACGTTT-AACCGG----TCGT-GTTGTTCTC------
------

```
FC88X_00554_01216       ---------------CTACGTTT-AACCGG----TCGT-GTTGTTCTC------
------
FC88X_00121_00603       ---------------CTACGTTT-AACCGG----TCGT-
GTTGTTCTCCTTCCTTT----
FC88X_00417_01220       ---------------CTACGGTT-AACCGC----TCGT-GTTGTTCTC------
------
FC88X_01145_00970       ---------------CTACGGTT-AACCGA----TCGT-GTTGTTCTC------
------
FC88X_00067_00034       ---------------CTACGATT-AACCGA----CCGT-GTTGTTCTCCC----
------
FC88X_00177_00513       ---------------CTACGATT-AACCGA----TCGT-GTTGTTCTC------
------
FC88X_00861_00405       ---------GGGGAACTACGCTT-AACCGA----TCGT-GTTGTTCTC------
------
FC88X_00916_01085       ---------------CTACGCTT-AACCGA----TCGT-GTTGTTCTC------
------
FC88X_01142_00666       ---------------CTACGCTT-AACCGA----TCGT-GTTGTTCTC------
------
FC88X_00856_00306       ---------------CTACGCTT-AACCGA----TCAT-GTTGTTCTC------
------
FC88X_01093_00667       ---------------CCACGCTT-AACCGA----TCGT-GTTGTTCTC------
------
FC88X_00214_00273       ---------------CTACGTTTAA-CCGA----CCGT-GTTGTTCTC------
------
FC88X_00850_00408       ---------------CTACGTTTAA-CCGA----CCGT-GTTGTTCTC------
------
FC88X_00567_01195       ---------------CTACGTTTAAACCGA----CCGG-GTTGTTCTC------
------
FC88X_00791_01041       ------------GGGCTACGTTTAA-CCGA----CCGT-GTTGTCCTC------
------
FC88X_00748_00065       ---------------CTACGTTT-AACCAA----TCGT-GTTGTTCTC------
------
FC88X_00154_00747       ---------------CTACGTTTAA-TCGA----TCGT-GT-GTTCTC------
------
FC88X_00853_00350       ---------------CTACGTTTGA-TCGA----TCGT-GTTGTTCTC------
------
FC88X_00772_01104       ---------------CTACGTTTAAATCGA----TCGT-
GTTGTTCTCCTTCCTTTCTC-
FC88X_00151_00663       ---------------CTACG-TTTAACCG-A---TCGT-GTCGTTCTC------
------
FC88X_01031_01252       ---------------CTACG-TTTAACCG-A---TCGT-GTCGTTCTCC-----
------
FC88X_00995_01025       ---------------CTACG-TTTAACCG-A---TCGT-GTCGTTCTC------
------
FC88X_00834_00208       ---------------CTACG-TTTAACCG-A---TCGT-GTCGTTCTC------
------
FC88X_00654_00952       ---------------CTACG-TTTAACCG-A---TCGT-GT-------------
------
FC88X_00078_00270       ---------------CTACG-TTTAACCG-A---TCGC-GTCTTTCTC------
------
FC88X_00184_01039       ---------------CTACG-TTTAACCG-A---TCGT-GTTGTTCTT------
------
FC88X_00267_01115       ---------------CTACG-TTTAACCG-A---TCGT-GTTGTTCTTC-----
------
FC88X_00182_00132       ---------------CTACG-TTTAACCG-A---TCGT-GTTGTTCCTC-----
------
```

FC88X_00716_00942 ---------------GCTACG-TTTAACCGTA---TCGT-GTTGTTCCTC-----
------
FC88X_00155_00820 ----------------CTACG-TTTAACCG-A---TCGT-GTTGTTCCTC-----
------
FC88X_00731_00245 ----------------CTACG-TTTAACCG-A---TCGT-GTTGTTCCTC-----
------
FC88X_00686_00244 ----------------CTACG-TTTAACCG-A---TCGT-GTTGTTCCTCC----
------
FC88X_00196_00117 ----------------CTACG-TTTAACCG-A---TCGT-GTTGTTTCTC-----
------
FC88X_01138_00579 ----------------CTACG-TTTAACCG-A---TCGT-GTTG-----------
------
FC88X_00411_01005 ----------------CTACG-TTTAACCGAT---CGTT-GTTGTT---------
------
FC88X_00983_00267 ----------------CTACG-TTTAACCG-A---TCGT-
GTTGTTCCTCCTTCCTT----
FC88X_00845_00583 ----------------CTACG-TTTAACCG-A---TCGT----------------
------
FC88X_00463_00308 ----------------CTACG-TTTAACCG-A---TCGT-GTTGCTCTC------
------
FC88X_01030_00318 ----------------CTACG-TTTAACCG-A---TCGT-GTTGCTCTC------
------
FC88X_00482_00091 ----------------CTACG-TTTAACCG-A---TCGT-GTTGCTCTC------
------
FC88X_00693_00897 ----------------CTACG-TTTAACCG-A---TCGT-GTTGCTCTC------
------
FC88X_00685_01090 ----------------CTACG-TTTAACCG-A---TCGT-GTTGCTCTC------
------
FC88X_00854_01148 ----------------CTACG-TTTAACCG-A---TCGT-GTTGCTCTC------
------
FC88X_00943_00831 ----------------CTACG-TTTAACCG-A---TCGT-GTTGCTCTC------
------
FC88X_01021_00335 ----------------CTACG-TTTAACCG-A---TCGT-GTTGCTCTC------
------
FC88X_00614_00188 ----------------CTACG-TTTAACCG-A---TCGT-GTTGTCTC-------
------
FC88X_00829_00570 ----------------CTACG-TTTAACCG-A---TCGT-GTTGTCTC-------
------
FC88X_00937_00404 ----------------CTACG-TTTAACCG-A---TCGT-G-TGTCTCG------
------
FC88X_01138_00718 ----------------CTACG-TTTAACCG-A---TCGT-GGTGTCTCC------
------
FC88X_00298_01043 ----------------CTACG-TTTAACCG-A---TCGT-G--------------
------
FC88X_00162_00118 ----------------CTACG-TTTAACCG-A---TCGT-GTT------------
------
FC88X_01145_00996 ----------------CTACG-TTTAACCG-A---TCGT-GTTGTTTTC------
------
FC88X_00527_01164 ----------------CTACG-TTTAACCG-A---TCGT-GTT------------
------
FC88X_00690_01044 ----------------CTACGGTTTAACCGCA---TCGT-GTTGT----------
------
FC88X_00726_01012 ----------------CTACG-TTTAACCG-A---TCGT-GT-------------
------
FC88X_00392_00569 ----------------CGACGTTT-AACCGA----TCGT-GTTGTTCTC------
------

FC88X_00838_00715 ----------------CAACGTTT-AACCGA----TCGT-GTTGTTCTC------
------
FC88X_00475_00355 ----------------CCACGTTT-AACCGA----TCGT-GTTGTTCTCA-----
------
FC88X_00010_00338 ----------------CCACGTTT-AAC-GA----TCGT-GTTGTTCTC------
------
FC88X_00482_01216 ----------------CCACGTTT-AACCGA----TCGT-GTTGTTCTC------
------
FC88X_00581_01052 ----------------CCACGTTT-AACCGA----TCGT-GTTGTTCTC------
------
FC88X_01014_00283 ----------------CCACGTTT-AACCGA----TCGT-GTTGTTCTC------
------
FC88X_00016_00588 ----------------CCACGTTT-AACCGA----TCGT-GTTGTTCTC------
------
FC88X_00054_00671 ----------------CCACGTTT-AACCGA----TCGT-GTTGTTCTC------
------
FC88X_00265_00020 ----------------CCACGTTT-AACCGA----TCGT-GTTGTTCTC------
------
FC88X_00280_01125 ----------------CCACGTTT-AACCGA----TCGT-GTTGTTCTC------
------
FC88X_00611_01210 ----------------CCACGTTT-AACCGA----TCGT-GTTGTTCTC------
------
FC88X_00895_00169 ----------------CCACGTTT-AACCGA----TCGT-GTTGTTCTC------
------
FC88X_00251_00070 ----------------CCACGTTT-AACCGA----TCGT-GTTGTTCCC------
------
FC88X_00322_01004 ----------------CTACGTTT-AACCGA----TCGT-GTTGTTCCCC-----
------
FC88X_00588_00365 ----------------CTACGTTT-AACCGA----TCGT-GTTGTTCCC------
------
FC88X_00181_00893 ----------------CTACGTTT-AACCGA----GCGT-GTTGTTCCC------
------
FC88X_00092_00110 ----------------TCACGTTT-AACCGA----ACGT-GTTGTTCTC------
------
FC88X_01022_00849 ----------------CCACGTTT-AACCGA----ACGT-GTTGTTCCTC-----
------
FC88X_00764_01022 ----------------GCTCGTTT-AACCGA----TCGT-GTTGTTCTC------
------
FC88X_00814_01242 ----------------CTACGTCT-AACCGA----TCGT-GCTGCTCTC------
------
FC88X_00120_00494 ------------------ACATA-TAACCGAT--CATGTGTCTGATCTC------
------
FC88X_00969_00837 ------------------ACATAATAACCGAT--CATGTGTCTGATCTC------
------
FC88X_00162_00800 ------------------ACATA-TAACCGAT--CATGTGTCTGATCTC------
------
FC88X_00205_00018 ------------------ACATA-TAACCGAT--CATGTGTCTGATCTC------
------
FC88X_00627_01268 ------------------ACATA-TAACCGAT--CATGTGTCTGATCTC------
------
FC88X_00673_00555 ------------------ACATA-TAACCGAT--CATGTGTCTGATCTC------
------
FC88X_01048_00358 ------------------ACATA-TAACCGAT--CATGTGTCTGATCTC------
------
FC88X_00628_00516 ------------------ACATA-TAACCGAT--CATGTGTCTGATCTC------
------

```
FC88X_00742_01022       -----------------ACATA-TAACCGAT--CATGTGTCTGATCTC------
------
FC88X_00988_01273       -----------------ACATA-TAACCGAT--CATGTGTCTGATCTC------
------
FC88X_01005_01149       -----------------ACATA-TAACCGAT--CATGTGTCTGATCTC------
------
FC88X_00387_00293       -----------------ACATA-TAACCGAT--C-TGTGTCTGATCTC------
------
FC88X_00088_00242       -----------------ACATA-TAACCGAT--CATGTGTCTGGTCTC------
------
FC88X_00598_01095       --------------------TTGTGACATGT--AACGTCCTTCTATCTT-----
------
FC88X_00893_00620       --------------------TTGTGACATGT--AACGTCCTTCTATCTT-----
------
FC88X_00932_00981       --------------------TTGTGACATGT--AACGTCCTTCTATCTT-----
------
FC88X_01026_00888       --------------------TTGTGACATGT--AACGTCCTTCTATCTT-----
------
FC88X_01083_00650       --------------------TTGTGACATGT--AACGTCCTTCTATCTT-----
------
FC88X_01103_00924       --------------------TTGTGACATGT--AACGTCCTTCTATCTT-----
------
FC88X_00032_00099       --------------------TTGTGACATGT--AACGTCCTTCTATCTT-----
------
FC88X_00184_00842       --------------------TTGTGACATGT--AACGTCCTTCTATCTT-----
------
FC88X_00194_00855       --------------------TTGTGACATGT--AACGTCCTTCTATCTT-----
------
FC88X_00344_00767       --------------------TTGTGACATGT--AACGTCCTTCTATCTT-----
------
FC88X_00604_01123       --------------------TTGTGACATGT--AACGTCCTTCTATCTT-----
------
FC88X_00878_00755       --------------------TTGTGACATGT--AACGTCCTTCTATCTT-----
------
FC88X_00888_00788       --------------------TTGTGACATGT--AACGTCCTTCTATCTT-----
------
FC88X_01005_00561       --------------------TTGTGACATGT--AACGTCCTTCTATCTT-----
------
FC88X_01077_00943       --------------------TTGTGACATGT--AACGTCCTTCTATCTT-----
------
FC88X_01111_01010       --------------------TTGTGACATGT--AACGTCCTTCTATCTT-----
------
FC88X_00542_01209       -------------GGGGAATTTGTGACATGT--AACGTCCTTCTATCTT-----
------
FC88X_00757_01224       --------------------TTGCGACATGT--AACGTCCTTCTATCTT-----
------
FC88X_00917_00645       --------------------TTGTGACATGT--AGCGTCCTTCTATCTT-----
------
FC88X_00075_00146       --------------------TTGTGACATGT--AACGTCCTGCTATCTT-----
------
FC88X_00436_01220       ---------------------TTGGACATGT--AACGTCCTTCTATCTT-----
------
FC88X_00099_00844       ------------------CCAACCGACATGT-----GTTCTTCTATTCTT----
------
FC88X_00295_01126       ------------------CCAACCGACATGT-----GTTCTTCTATTCTT----
------
```

FC88X_00447_00873 ------------------CCAACCGACATGT-----GTTCTTCTATTCTT----
------
FC88X_00441_00976 ------------------CCAACCGACATGT-----GTTCTTCTATTCTT----
------
FC88X_00803_01005 ------------------CCAACCGACATGT-----GTTCTTCTATTCTT----
------
FC88X_00898_00176 ------------------CCAACCGACATGT-----GTTCTTCTATTCTT----
------
FC88X_01094_00901 ------------------CCAACCGACATGT-----GTTCTTCTATTCTT----
------
FC88X_00946_00324 ------------------CCAACCGACATGT-----GTTCTTCTATTCTT----
------
FC88X_01099_01156 ------------------CCAACCGACATGT-----GTTCTTCTATTCTT----
------
FC88X_00588_00662 ------------------CCAACCGACATGT-----GTTCTTCTATTCT-----
------
FC88X_00888_00884 ------------------CCAACCGACATGT-----
GTTCTTCTATTCTTCTTCCTTT--
FC88X_00139_00929 ------------------CCAACCGACATGT-----GTTGTTCTATTCTT----
------
FC88X_00136_00077 ------------------CCAACCGACATGC-----GTTCTTCTATTCTT----
------
FC88X_00509_00838 ------------------TCACCGGACATGT--CTTGGACTTATCTC-------
------
FC88X_00694_00314 ------------------TCACCGGACATGT--CTTGGACTTATCTC-------
------
FC88X_01147_00660 ------------------TCACCGGACATGT--CTTGGACTTATCTC-------
------
FC88X_00129_00127 ------------------TCACCGGACATGT--CTTGGACTTATCTC-------
------
FC88X_00853_00247 ------------------TCACCGG--CTGT--TACGGACTGTTCTCTC-----
------
FC88X_00085_00098 ----------------CGTGCAGGTATAT----CTGGTCGTTATGCTC------
------
FC88X_00084_00221 ----------------CGTGCAGGTATAT----CTGGTCGTTATGCTC------
------
FC88X_00106_00088 ----------------CGTGCAGGTATAT----CTGGTCGTTATGCTC------
------
FC88X_00222_00277 ----------------CGTGCAGGTATAT----CTGGTCGTTATGCTC------
------
FC88X_00372_00015 ----------------CGTGCAGGTATAT----CTGGTCGTTATGCTC------
------
FC88X_00425_00277 ----------------CGTGCAGGTATAT----CTGGTCGTTATGCTC------
------
FC88X_00483_00760 ----------------CGTGCAGGTATAT----CTGGTCGTTATGCTC------
------
FC88X_00638_00918 ----------------CGTGCAGGTATAT----CTGGTCGTTATGCTC------
------
FC88X_00745_00872 ----------------CGTGCAGGTATAT----CTGGTCGTTATGCTC------
------
FC88X_00891_00875 ----------------CGTGCAGGTATAT----CTGGTCGTTATGCTC------
------
FC88X_00885_01130 ----------------CGTGCAGGTATAT----CTGGTCGTTATGCTC------
------
FC88X_00911_01274 ----------------CGTGCAGGTATAT----CTGGTCGTTATGCTC------
------

FC88X_01088_01195 ----------------CGTGCAGGTATAT----CTGGTCGTTATGCTC------
------
FC88X_01112_01108 ----------------CGTGCAGGTATAT----CTGGTCGTTATGCTC------
------
FC88X_00028_00292 ----------------CGTGCAGGTATAT----CTGGTCGTTATGCTC------
------
FC88X_00028_00597 ----------------CGTGCAGGTATAT----CTGGTCGTTATGCTC------
------
FC88X_00073_00239 ----------------CGTGCAGGTATAT----CTGGTCGTTATGCTC------
------
FC88X_00072_00310 ----------------CGTGCAGGTATAT----CTGGTCGTTATGCTC------
------
FC88X_00167_00102 ----------------CGTGCAGGTATAT----CTGGTCGTTATGCTC------
------
FC88X_00151_00727 ----------------CGTGCAGGTATAT----CTGGTCGTTATGCTC------
------
FC88X_00163_00941 ----------------CGTGCAGGTATAT----CTGGTCGTTATGCTC------
------
FC88X_00254_00512 ----------------CGTGCAGGTATAT----CTGGTCGTTATGCTC------
------
FC88X_00393_01036 ----------------CGTGCAGGTATAT----CTGGTCGTTATGCTC------
------
FC88X_00407_01117 ----------------CGTGCAGGTATAT----CTGGTCGTTATGCTC------
------
FC88X_00442_01201 ----------------CGTGCAGGTATAT----CTGGTCGTTATGCTC------
------
FC88X_00545_01170 ----------------CGTGCAGGTATAT----CTGGTCGTTATGCTC------
------
FC88X_00594_01043 ----------------CGTGCAGGTATAT----CTGGTCGTTATGCTC------
------
FC88X_00701_01033 ----------------CGTGCAGGTATAT----CTGGTCGTTATGCTC------
------
FC88X_00837_00110 ----------------CGTGCAGGTATAT----CTGGTCGTTATGCTC------
------
FC88X_00890_00339 ----------------CGTGCAGGTATAT----CTGGTCGTTATGCTC------
------
FC88X_00933_00251 ----------------CGTGCAGGTATAT----CTGGTCGTTATGCTC------
------
FC88X_00903_00416 ----------------CGTGCAGGTATAT----CTGGTCGTTATGCTC------
------
FC88X_00935_00566 ----------------CGTGCAGGTATAT----CTGGTCGTTATGCTC------
------
FC88X_00901_00673 ----------------CGTGCAGGTATAT----CTGGTCGTTATGCTC------
------
FC88X_00933_01173 ----------------CGTGCAGGTATAT----CTGGTCGTTATGCTC------
------
FC88X_00902_01211 ----------------CGTGCAGGTATAT----CTGGTCGTTATGCTC------
------
FC88X_00985_00511 ----------------CGTGCAGGTATAT----CTGGTCGTTATGCTC------
------
FC88X_00991_00631 ----------------CGTGCAGGTATAT----CTGGTCGTTATGCTC------
------
FC88X_01008_00388 ----------------CGTGCAGGTATAT----CTGGTCGTTATGCTC------
------
FC88X_01032_00844 ----------------CGTGCAGGTATAT----CTGGTCGTTATGCTC------
------

```
FC88X_01096_00545       ----------------CGTGCAGGTATAT----CTGGTCGTTATGCTC------
------
FC88X_01069_00883       ----------------CGTGCAGGTATAT----CTGGTCGTTATGCTC------
------
FC88X_01086_01090       ----------------CGTGCAGGTATAT----CTGGTCGTTATGCTC------
------
FC88X_01124_00771       ----------------CGTGCAGGTATAT----CTGGTCGTTATGCTC------
------
FC88X_01145_01175       ----------------CGTGCAGGTATAT----CTGGTCGTTATGCTC------
------
FC88X_00231_00960       ---------------GCGTGCAGGTATAT----CTGGTCGTTATGCTC------
------
FC88X_01143_00867       ---------------GCGTGCAGGTATAT----CTGGTCGTTATGCTC------
------
FC88X_00883_01013       ----------------CGTGCAGGTATATAT--CTGGTCGTTATGCTC------
------
FC88X_00498_00406       ----------------CGTGCAGGTATAT----CTGATCGTTATGCTC------
------
FC88X_00430_01020       ------------GGAATCGTGCAGGTATAT----CTGGTCGTTATGCTC------
------
FC88X_00510_00176       --------GGGGGAATCGTGCAGGTATAT----CTGGTCGTTATGCTC------
------
FC88X_00822_00342       ----------------CGTGCAGGTATGT----CTGGTCGTTATGCTC------
------
FC88X_00573_00102       ---------------ACGTGCAGGTATAT----CTGGCCGTTATGCTC------
------
FC88X_01012_00389       ----------GGGGAACGTGCAGGTATAT----CTGGTCGTTATGCTC------
------
FC88X_00546_01176       ---------------GCGTGCAGGTATATC---T-
GGTCGTTATGCTCCTTCCTTT----
FC88X_00505_00125       ----------------CGTGCAGGTATATC---T-GGT----------------
------
FC88X_00815_00375       ----------------CGTGCAGGTATATC---C-GGTCGTTATGCTC------
------
FC88X_01114_01084       ----------------CGTGCAGGTATATC---C-GGTCGTTATGCTC------
------
FC88X_00984_00705       ----------------CGTGCAGGTATATC---
CTGGTCGTTATGCTCCTTCCTTT----
FC88X_00804_00149       ----------------CGTGCAGGTATGGAT--CTGGT----------------
------
FC88X_00684_00087       ---------------ACATGTTTCGACGTGC--CTGCATCTCCT----------
------
FC88X_01093_01053       ---------------ACATGTTTCGACGTGC--CTGCATCTCCT----------
------
FC88X_00687_01243       ---------------ACATGTTTCGACGTGC--CTGCATCTCCT----------
------
FC88X_00951_00243       ---------------ACATGTTTCGACGTGC--CTGCATCTCCT----------
------
FC88X_00985_00789       ---------------ACATGTTTCGACGTGC--CTGCATCTCCT----------
------
FC88X_01081_00491       ---------------ACATGTTTCGACGTGC--CTGCATCTCCT----------
------
FC88X_01134_00828       ---------------ACATGTTTCGACGTGC--CTGCATCTCCT----------
------
FC88X_00907_00951       ---------------ACATGTTCCGACGTGC--CTGCATCTCCT----------
------
```

FC88X_00556_00702 ------------AAAATAAACATGTACATGC--CCGGATCTC------------
------
FC88X_00036_00250 -------------------TTCTGGACTGTAGTCTGGGG-CTGTCTC-------
------
FC88X_01011_00475 -------------------TTCTGGACTGTATTCTGGGG-CTGTCTC-------
------
FC88X_00070_00100 -------------------TTCTGGACTGTACTCTGGGG-CTGTCTC-------
------
FC88X_00039_00352 -------------------TTCTGGACTGTACTCTGGGGGCTGTCTC-------
------
FC88X_00108_00130 -------------------TTCTGGACTGTACTCTGGGG-CTGTCTC-------
------
FC88X_00136_00752 -------------------TTCTGGACTGTACTCTGGGG-CTGTCTC-------
------
FC88X_00224_00298 -------------------TTCTGGACTGTACTCTGGGG-CTGTCTC-------
------
FC88X_00399_00045 ------------------GTTCTGGACTGTACTCTGGGG-CTGTCTC-------
------
FC88X_00997_01274 ------------------GTTCTGGACTGTACTCTGGGG-CTGTCTC-------
------
FC88X_01142_00991 ------------------GTTCTGGACTGTACTCTGGGG-CTGTCTC-------
------
FC88X_00517_01146 ------------------GTTCTGGACTGTACTCTGGGG-CTGTCTC-------
------
FC88X_00055_00506 -------------------TTCTGGACTGTACTCTGGGG-CTGTCTC-------
------
FC88X_00130_00519 -------------------TTCTGGACTGTACTCTGGGG-CTGTCTC-------
------
FC88X_00155_00064 -------------------TTCTGGACTGTACTCTGGGG-CTGTCTC-------
------
FC88X_00341_01182 -------------------TTCTGGACTGTACTCTGGGG-CTGTCTC-------
------
FC88X_00442_00148 -------------------TTCTGGACTGTACTCTGGGG-CTGTCTC-------
------
FC88X_00480_00401 -------------------TTCTGGACTGTACTCTGGGG-CTGTCTC-------
------
FC88X_00233_00316 -------------------TTCTGGACTGTACTCTGGGG-CTGTCTC-------
------
FC88X_01053_00842 -------------------TTCTGGACTGTACTCTGGGG-CTGTCTCC------
------
FC88X_00398_01200 -------------------TTCTGGACTGTACTCTGGGG-CTGTCTC-------
------
FC88X_00401_01171 -------------------TTCTGGACTGTACTCTGGGG-CTGTCTC-------
------
FC88X_00473_00998 -------------------TTCTGGACTGTACTCTGGGG-CTGTCTC-------
------
FC88X_00473_01170 -------------------TTCTGGACTGTACTCTGGGG-CTGTCTC-------
------
FC88X_00476_01198 -------------------TTCTGGACTGTACTCTGGGG-CTGTCTC-------
------
FC88X_00456_01222 -------------------TTCTGGACTGTACTCTGGGG-CTGTCTC-------
------
FC88X_00552_00232 -------------------TTCTGGACTGTACTCTGGGG-CTGTCTC-------
------
FC88X_00605_00270 -------------------TTCTGGACTGTACTCTGGGG-CTGTCTC-------
------

FC88X_00636_00980 ------------------TTCTGGACTGTACTCTGGGG-CTGTCTC-------
------
FC88X_00678_00956 ------------------TTCTGGACTGTACTCTGGGG-CTGTCTC-------
------
FC88X_00663_01262 ------------------TTCTGGACTGTACTCTGGGG-CTGTCTC-------
------
FC88X_00737_00260 ------------------TTCTGGACTGTACTCTGGGG-CTGTCTC-------
------
FC88X_00770_01187 ------------------TTCTGGACTGTACTCTGGGG-CTGTCTC-------
------
FC88X_00905_00646 ------------------TTCTGGACTGTACTCTGGGG-CTGTCTC-------
------
FC88X_00926_00636 ------------------TTCTGGACTGTACTCTGGGG-CTGTCTC-------
------
FC88X_00988_00603 ------------------TTCTGGACTGTACTCTGGGG-CTGTCTC-------
------
FC88X_00961_00786 ------------------TTCTGGACTGTACTCTGGGG-CTGTCTC-------
------
FC88X_01016_00529 ------------------TTCTGGACTGTACTCTGGGG-CTGTCTC-------
------
FC88X_01010_00868 ------------------TTCTGGACTGTACTCTGGGG-CTGTCTC-------
------
FC88X_01056_00432 ------------------TTCTGGACTGTACTCTGGGG-CTGTCTC-------
------
FC88X_01072_00584 ------------------TTCTGGACTGTACTCTGGGG-CTGTCTC-------
------
FC88X_01099_00906 ------------------TTCTGGACTGTACTCTGGGG-CTGTCTC-------
------
FC88X_01075_01056 ------------------TTCTGGACTGTACTCTGGGG-CTGTCTC-------
------
FC88X_01118_00858 ------------------TTCTGGACTGTACTCTGGGG-CTGTCTC-------
------
FC88X_00048_00807 ------------------TTCTGGACTGTACTCTGGGG-CTGTCTC-------
------
FC88X_00082_00328 ------------------TTCTGGACTGTACTCTGGGG-CTGTCTC-------
------
FC88X_00071_00480 ------------------TTCTGGACTGTACTCTGGGG-CTGTCTC-------
------
FC88X_00062_00557 ------------------TTCTGGACTGTACTCTGGGG-CTGTCTC-------
------
FC88X_00093_00677 ------------------TTCTGGACTGTACTCTGGGG-CTGTCTC-------
------
FC88X_00087_00812 ------------------TTCTGGACTGTACTCTGGGG-CTGTCTC-------
------
FC88X_00102_00063 ------------------TTCTGGACTGTACTCTGGGG-CTGTCTC-------
------
FC88X_00113_00261 ------------------TTCTGGACTGTACTCTGGGG-CTGTCTC-------
------
FC88X_00125_00551 ------------------TTCTGGACTGTACTCTGGGG-CTGTCTC-------
------
FC88X_00237_00030 ------------------TTCTGGACTGTACTCTGGGG-CTGTCTC-------
------
FC88X_00206_01072 ------------------TTCTGGACTGTACTCTGGGG-CTGTCTC-------
------
FC88X_00286_00753 ------------------TTCTGGACTGTACTCTGGGG-CTGTCTC-------
------

FC88X_00282_01090 -------------------TTCTGGACTGTACTCTGGGG-CTGTCTC-------
------
FC88X_00364_00498 -------------------TTCTGGACTGTACTCTGGGG-CTGTCTC-------
------
FC88X_00359_01052 -------------------TTCTGGACTGTACTCTGGGG-CTGTCTC-------
------
FC88X_00419_00049 -------------------TTCTGGACTGTACTCTGGGG-CTGTCTC-------
------
FC88X_00458_00015 -------------------TTCTGGACTGTACTCTGGGG-CTGTCTC-------
------
FC88X_00459_00241 -------------------TTCTGGACTGTACTCTGGGG-CTGTCTC-------
------
FC88X_00485_01015 -------------------TTCTGGACTGTACTCTGGGG-CTGTCTC-------
------
FC88X_00464_01176 -------------------TTCTGGACTGTACTCTGGGG-CTGTCTC-------
------
FC88X_00456_01217 -------------------TTCTGGACTGTACTCTGGGG-CTGTCTC-------
------
FC88X_00471_01230 -------------------TTCTGGACTGTACTCTGGGG-CTGTCTC-------
------
FC88X_00544_01034 -------------------TTCTGGACTGTACTCTGGGG-CTGTCTC-------
------
FC88X_00588_00171 -------------------TTCTGGACTGTACTCTGGGG-CTGTCTC-------
------
FC88X_00570_01004 -------------------TTCTGGACTGTACTCTGGGG-CTGTCTC-------
------
FC88X_00644_00231 -------------------TTCTGGACTGTACTCTGGGG-CTGTCTC-------
------
FC88X_00602_01123 -------------------TTCTGGACTGTACTCTGGGG-CTGTCTC-------
------
FC88X_00617_01156 -------------------TTCTGGACTGTACTCTGGGG-CTGTCTC-------
------
FC88X_00620_01227 -------------------TTCTGGACTGTACTCTGGGG-CTGTCTC-------
------
FC88X_00655_00305 -------------------TTCTGGACTGTACTCTGGGG-CTGTCTC-------
------
FC88X_00653_00938 -------------------TTCTGGACTGTACTCTGGGG-CTGTCTC-------
------
FC88X_00689_01240 -------------------TTCTGGACTGTACTCTGGGG-CTGTCTC-------
------
FC88X_00723_00774 -------------------TTCTGGACTGTACTCTGGGG-CTGTCTC-------
------
FC88X_00713_00898 -------------------TTCTGGACTGTACTCTGGGG-CTGTCTC-------
------
FC88X_00726_01005 -------------------TTCTGGACTGTACTCTGGGG-CTGTCTC-------
------
FC88X_00725_01132 -------------------TTCTGGACTGTACTCTGGGG-CTGTCTC-------
------
FC88X_00791_01154 -------------------TTCTGGACTGTACTCTGGGG-CTGTCTC-------
------
FC88X_00829_00298 -------------------TTCTGGACTGTACTCTGGGG-CTGTCTC-------
------
FC88X_00803_00931 -------------------TTCTGGACTGTACTCTGGGG-CTGTCTC-------
------
FC88X_00894_00332 -------------------TTCTGGACTGTACTCTGGGG-CTGTCTC-------
------

FC88X_00894_00662 --------------------TTCTGGACTGTACTCTGGGG-CTGTCTC-------
------
FC88X_00893_00788 --------------------TTCTGGACTGTACTCTGGGG-CTGTCTC-------
------
FC88X_00852_00953 --------------------TTCTGGACTGTACTCTGGGG-CTGTCTC-------
------
FC88X_00895_01201 --------------------TTCTGGACTGTACTCTGGGG-CTGTCTC-------
------
FC88X_00919_00539 --------------------TTCTGGACTGTACTCTGGGG-CTGTCTC-------
------
FC88X_00929_00801 --------------------TTCTGGACTGTACTCTGGGG-CTGTCTC-------
------
FC88X_00987_00576 --------------------TTCTGGACTGTACTCTGGGG-CTGTCTC-------
------
FC88X_00981_00733 --------------------TTCTGGACTGTACTCTGGGG-CTGTCTC-------
------
FC88X_00974_00917 --------------------TTCTGGACTGTACTCTGGGG-CTGTCTC-------
------
FC88X_00964_00984 --------------------TTCTGGACTGTACTCTGGGG-CTGTCTC-------
------
FC88X_01023_00294 --------------------TTCTGGACTGTACTCTGGGG-CTGTCTC-------
------
FC88X_01007_00687 --------------------TTCTGGACTGTACTCTGGGG-CTGTCTC-------
------
FC88X_01009_00982 --------------------TTCTGGACTGTACTCTGGGG-CTGTCTC-------
------
FC88X_01044_01066 --------------------TTCTGGACTGTACTCTGGGG-CTGTCTC-------
------
FC88X_01057_00472 --------------------TTCTGGACTGTACTCTGGGG-CTGTCTC-------
------
FC88X_01080_00461 --------------------TTCTGGACTGTACTCTGGGG-CTGTCTC-------
------
FC88X_01117_00831 --------------------TTCTGGACTGTACTCTGGGG-CTGTCTC-------
------
FC88X_01112_00989 --------------------TTCTGGACTGTACTCTGGGG-CTGTCTC-------
------
FC88X_00203_00903 --------------GGGAAGTTCTGGACTGTACTCTGGGG-CTGTCTC-------
------
FC88X_00644_00264 --------------------TTCTGGACTGTACTCT-GGG-CTGTCTC-------
------
FC88X_00984_00459 --------------------TTCTGGACTGTACTCTAGGG-CTGTCTC-------
------
FC88X_00419_00095 --------------------TTCTGGACTGTACCTCTGGGGCTGTCTC-------
------
FC88X_00527_01238 --------------------TTCTGGACTGTACC-CTGGGGCTGTCTC-------
------
FC88X_00855_01060 --------------------TTCTGGACTGTACTCTGGGG-CTGTCCC-------
------
FC88X_00425_00076 --------------------TTCTGGACTGTACTCTGGGG-
CTGTCTCCTTCCTTT-----
FC88X_00907_01068 --------------------TTCTGGACTGTACTCTGGGG-
CTGTCTCCTTCCTTT-----
FC88X_00735_01122 --------------------TTCTGGACTGTACTCTGGGG-
CTGTCTCCTTCCTTT-----
FC88X_00615_00577 --------------------TTCTAGACTGTACTCTGGGG-CTGTCTC-------
------

FC88X_00668_01266 -------------------TTCTGGACCGTACTCTGGGG-CTGTCTC-------
------
FC88X_00117_00467 -------------------TTCTGGACCGTACTCTGGGG-CTGTCTC-------
------
FC88X_01119_00525 -------------------TTCTGGACTGTGCTCTGGGG-CTGTCTC-------
------
FC88X_00838_00964 -------------------TTCTGGACTGTACTCCGGGG-CTGTCTC-------
------
FC88X_00946_01217 -------------------TTCTGGACTGTACTCCGGGG-CTGTCTC-------
------
FC88X_00010_00066 -------------------TTCTGGACTCTACTCTGGGG-CTGTCTC-------
------
FC88X_01145_00635 -------------------TCCTGGACTGTACTCTGGGG-CTGTCTC-------
------
FC88X_01040_01156 -------------------TCCTGGACTGTACTCTGGGG-CTGTCTC-------
------
FC88X_00697_00306 -------------------CTCTGGACTGTACTCTGGGG-CTGTCTC-------
------
FC88X_00790_00301 -------------------CTCTGGACTGTACTCTGGGG-CTGTCTC-------
------
FC88X_00099_00371 -------------------TTCTGGACTGTACTCTGGGG-CTATCTC-------
------
FC88X_01071_01263 -------------------TTCTGGACTGTACTCTGGG----------------
------
FC88X_00052_00515 -----------------CTGCCTAGTCTGTTAGTAGGGG-CGTTCT--------
------
FC88X_00656_00184 -----------------CTACCTAGTCTGTTAGCAGGGG-CGTTCT--------
------
FC88X_00485_01070 -----------------CCTTCCTGTCTTTCCCCTGGGG-CTTCT---------
------
FC88X_00256_00633 --------------------CCTGATGCTTGGCCTTGGGTGCTTCTC-------
------
FC88X_00551_00083 --------------------CCTGATGCTTGGCCTTGGGTGCTTCTC-------
------
FC88X_00084_00499 --------------------CCTGATGCTTGGCCTTGGGTGCTTCTC-------
------
FC88X_00171_00125 --------------------CCTGATGCTTGGCCTTGGGTGCTTCTC-------
------
FC88X_00238_01074 --------------------CCTGATGCTTGGCCTTGGGTGCTTCTC-------
------
FC88X_00732_00875 --------------------CCTGATGCTTGGCCTTGGGTGCTTCTC-------
------
FC88X_01124_00648 --------------------CCTGATGCTTGGCCTTGGGTGCTTCTC-------
------
FC88X_00507_00070 --------------------CTACCTGATTCTCGTCGGGATGTTCTC-------
------
FC88X_00487_01124 --------------------CTACCTGATTCTC-TCGGGATGTTCTC-------
------
FC88X_00538_00092 --------------------TTACCTGATTCTCGTCGGGATGTTCTC-------
------
FC88X_00974_00632 --------------------TTACCTGATTCTCGTCGGGATGTTCTC-------
------
FC88X_00578_00248 --------------------CCCGCCGATTCCCGAGGTGTGGTTTTC-------
------
FC88X_00303_00959 --------------------CCTGCTGATCGATGTGGGGCTGTTCGC-------
------

FC88X_00784_00312 -----------------TTTCCCG-ATATACGGCTGCGTCTTCTC---------
------
FC88X_00769_00973 -----------------TTTCCCG-ATATACGGCTGCGTCTTCTC---------
------
FC88X_00941_00228 -----------------TTTCCCG-ATATACGGCTGCGTATTCTC---------
------
FC88X_00872_01035 -----------------TTTCCCG-ATATACGGCTGCGTTTTCTC---------
------
FC88X_00051_00379 -----------------TTACCCT-ATATTCTGCTGCATCGTCGC---------
------
FC88X_00254_00012 -------------------TCCCG-ATATA-CGTCGTGGGCTCTT-CGC-----
------
FC88X_00316_00469 -------------------TCCCG-ATATA-CGTCGTGGGCTCTT-CGC-----
------
FC88X_00545_00200 -------------------TCCCG-ATATA-CGTCGTGGGCTCTT-CGC-----
------
FC88X_00747_00957 -------------------TCCCG-ATATA-CGTCGTGGGCTCTT-CGC-----
------
FC88X_00870_00356 -------------------TCCCG-ATATA-CGTCGTGGGCTCTT-CGC-----
------
FC88X_00964_01195 -------------------TCCCG-ATATA-CGTCGTGGGCTCTT-CGC-----
------
FC88X_00054_00481 -------------------TCCCG-ATATA-CGTCGTGGGCTCTT-CGC-----
------
FC88X_00635_00993 -------------------TCCCG-ATATA-CGTCGTGGGCTCTT-CGC-----
------
FC88X_00851_00404 -------------------TCCCG-ATATA-CGTCGTGGGCTCTT-CGC-----
------
FC88X_00982_00654 -------------------TCCCG-ATATA-CGTCGTGGGCTCTT-CGC-----
------
FC88X_01083_00573 -------------------TCCCG-ATATA-CGTCGTGGGCTCTT-CGC-----
------
FC88X_01062_00828 -------------------TCCCG-ATATA-CGTCGTGGGCTCTT-CGC-----
------
FC88X_01127_00772 -------------------TCCCG-ATATA-CGTCGTGGGCTCTT-CGC-----
------
FC88X_01122_00991 -------------------TCCCG-ATATA-CGTCGTGGGCTCTT-CGC-----
------
FC88X_01073_00764 -------------------TCCCG-ATATAACGTCGTGGGCTCTT-CGC-----
------
FC88X_00803_00983 -------------------TCCCG-ATATA-CGTCGTGG-CTCTT-CGC-----
------
FC88X_00291_00667 -------------------TCCCG-ATATACGTCCGTGGGCTCTT-CGC-----
------
FC88X_00378_00091 -------------------TCCCG-ATATACGTCCGTGGGCTCTT-CGC-----
------
FC88X_00051_00560 -------------------TCCCG-ATATA-CGTCGTGGGCTCTTTCGC-----
------
FC88X_00903_01177 -----------------TCCCCGT-ATATACGGATCTGTGCTCTT---------
------
FC88X_00795_01035 -----------------TTCCTCGCAAATGCGGTTGTTTATTCT----------
------
FC88X_00997_00272 -----------------TTCCTCGCAAATGCCGTTGTTTATTCT----------
------
FC88X_00905_00476 -----------------TTCCTCGCAAATGCCGTTGTCTATTCT----------
------

FC88X_01099_00612 -----------------TTCCGCG-ATATCCCGTGGGACCTTCTCCTTCCTTT-
------
FC88X_01112_00682 -----------------TTCCGCG-ATATCCCGTGGGACCTTCTC---------
------
FC88X_00041_00389 ------------------TCTTCCAATTTTG-GG-CGGTACTGTCTC-------
------
FC88X_01024_00927 ------------------TCTTCCAATTTTG-GGGCGGTACTGTCTC-------
------
FC88X_00052_00064 ------------------TCTTCCAATTTTG-GG-CGGTACTGTCTC-------
------
FC88X_00150_00176 ------------------TCTTCCAATTTTG-GG-CGGTACTGTCTC-------
------
FC88X_00746_01216 ------------------TCTTCCAATTTTG-GG-CGGTACTGTCTC-------
------
FC88X_00890_00230 ------------------TCTTCCAATTTTG-GG-CGGTACTGTCTC-------
------
FC88X_00781_00267 -----------------GTCTTCCAATTTTG-GG-CGGTACTGTCTC-------
------
FC88X_00926_01011 -----------------GTCTTCCAATTTTG-GG-CGGTACTGTCTC-------
------
FC88X_00013_00477 ------------------TCTTCCAATTTTG-GG-CGGTACTGTCTC-------
------
FC88X_00189_00064 ------------------TCTTCCAATTTTG-GG-CGGTACTGTCTC-------
------
FC88X_00527_01091 ------------------TCTTCCAATTTTG-GG-CGGTACTGTCTC-------
------
FC88X_00813_00153 ------------------TCTTCCAATTTTG-GG-CGGTACTGTCTC-------
------
FC88X_00925_01152 ------------------TCTTCCAATTTTG-GG-CGGTACTGTCTC-------
------
FC88X_00067_00553 ------------------TCTTCCAATTTTG-GG-CGGTACTGTCTC-------
------
FC88X_00837_00328 ------------------TCTTCCAATTTTG-GG-CGGTACTGTCTCT------
------
FC88X_00254_00261 ------------------TCTTCCAATTTTG-GG-CGGTACTGTCTC-------
------
FC88X_00798_01094 ------------------TCTTCCAATTTTG-GG-CGGTACTGTCTC-------
------
FC88X_00857_00268 ------------------TCTTCCAATTTTG-GG-CGGTACTGTCTC-------
------
FC88X_01021_01004 ------------------TCTTCCAATTTTG-GG-CGGTACTGTCTC-------
------
FC88X_01035_01240 ------------------TCTTCCAATTTTG-GG-CGGTACTGTCTC-------
------
FC88X_00030_00662 ------------------TCTTCCAATTTTG-GG-CGGTACTGTCTC-------
------
FC88X_00013_00711 ------------------TCTTCCAATTTTG-GG-CGGTACTGTCTC-------
------
FC88X_00042_00800 ------------------TCTTCCAATTTTG-GG-CGGTACTGTCTC-------
------
FC88X_00058_00080 ------------------TCTTCCAATTTTG-GG-CGGTACTGTCTC-------
------
FC88X_00089_00175 ------------------TCTTCCAATTTTG-GG-CGGTACTGTCTC-------
------
FC88X_00067_00600 ------------------TCTTCCAATTTTG-GG-CGGTACTGTCTC-------
------

```
FC88X_00085_00898       ------------------TCTTCCAATTTTG-GG-CGGTACTGTCTC-------
------
FC88X_00111_00063       ------------------TCTTCCAATTTTG-GG-CGGTACTGTCTC-------
------
FC88X_00139_00589       ------------------TCTTCCAATTTTG-GG-CGGTACTGTCTC-------
------
FC88X_00128_00799       ------------------TCTTCCAATTTTG-GG-CGGTACTGTCTC-------
------
FC88X_00130_00804       ------------------TCTTCCAATTTTG-GG-CGGTACTGTCTC-------
------
FC88X_00185_00009       ------------------TCTTCCAATTTTG-GG-CGGTACTGTCTC-------
------
FC88X_00171_00072       ------------------TCTTCCAATTTTG-GG-CGGTACTGTCTC-------
------
FC88X_00179_00334       ------------------TCTTCCAATTTTG-GG-CGGTACTGTCTC-------
------
FC88X_00167_00886       ------------------TCTTCCAATTTTG-GG-CGGTACTGTCTC-------
------
FC88X_00269_00053       ------------------TCTTCCAATTTTG-GG-CGGTACTGTCTC-------
------
FC88X_00270_00072       ------------------TCTTCCAATTTTG-GG-CGGTACTGTCTC-------
------
FC88X_00320_01012       ------------------TCTTCCAATTTTG-GG-CGGTACTGTCTC-------
------
FC88X_00369_00465       ------------------TCTTCCAATTTTG-GG-CGGTACTGTCTC-------
------
FC88X_00360_00558       ------------------TCTTCCAATTTTG-GG-CGGTACTGTCTC-------
------
FC88X_00427_00437       ------------------TCTTCCAATTTTG-GG-CGGTACTGTCTC-------
------
FC88X_00416_01077       ------------------TCTTCCAATTTTG-GG-CGGTACTGTCTC-------
------
FC88X_00465_01230       ------------------TCTTCCAATTTTG-GG-CGGTACTGTCTC-------
------
FC88X_00536_00167       ------------------TCTTCCAATTTTG-GG-CGGTACTGTCTC-------
------
FC88X_00502_01072       ------------------TCTTCCAATTTTG-GG-CGGTACTGTCTC-------
------
FC88X_00588_00265       ------------------TCTTCCAATTTTG-GG-CGGTACTGTCTC-------
------
FC88X_00609_01124       ------------------TCTTCCAATTTTG-GG-CGGTACTGTCTC-------
------
FC88X_00651_00812       ------------------TCTTCCAATTTTG-GG-CGGTACTGTCTC-------
------
FC88X_00666_00921       ------------------TCTTCCAATTTTG-GG-CGGTACTGTCTC-------
------
FC88X_00653_01151       ------------------TCTTCCAATTTTG-GG-CGGTACTGTCTC-------
------
FC88X_00724_00228       ------------------TCTTCCAATTTTG-GG-CGGTACTGTCTC-------
------
FC88X_00747_00232       ------------------TCTTCCAATTTTG-GG-CGGTACTGTCTC-------
------
FC88X_00760_00121       ------------------TCTTCCAATTTTG-GG-CGGTACTGTCTC-------
------
FC88X_00846_01003       ------------------TCTTCCAATTTTG-GG-CGGTACTGTCTC-------
------
```

```
FC88X_00834_01054      ------------------TCTTCCAATTTTG-GG-CGGTACTGTCTC-------
------
FC88X_00875_00570      ------------------TCTTCCAATTTTG-GG-CGGTACTGTCTC-------
------
FC88X_00874_00682      ------------------TCTTCCAATTTTG-GG-CGGTACTGTCTC-------
------
FC88X_00860_01119      ------------------TCTTCCAATTTTG-GG-CGGTACTGTCTC-------
------
FC88X_00948_00206      ------------------TCTTCCAATTTTG-GG-CGGTACTGTCTC-------
------
FC88X_00937_00515      ------------------TCTTCCAATTTTG-GG-CGGTACTGTCTC-------
------
FC88X_00947_00778      ------------------TCTTCCAATTTTG-GG-CGGTACTGTCTC-------
------
FC88X_00918_00859      ------------------TCTTCCAATTTTG-GG-CGGTACTGTCTC-------
------
FC88X_00939_00917      ------------------TCTTCCAATTTTG-GG-CGGTACTGTCTC-------
------
FC88X_00945_01063      ------------------TCTTCCAATTTTG-GG-CGGTACTGTCTC-------
------
FC88X_00963_00336      ------------------TCTTCCAATTTTG-GG-CGGTACTGTCTC-------
------
FC88X_00991_00487      ------------------TCTTCCAATTTTG-GG-CGGTACTGTCTC-------
------
FC88X_00999_00479      ------------------TCTTCCAATTTTG-GG-CGGTACTGTCTC-------
------
FC88X_00983_00794      ------------------TCTTCCAATTTTG-GG-CGGTACTGTCTC-------
------
FC88X_00999_00894      ------------------TCTTCCAATTTTG-GG-CGGTACTGTCTC-------
------
FC88X_00999_01007      ------------------TCTTCCAATTTTG-GG-CGGTACTGTCTC-------
------
FC88X_01042_00635      ------------------TCTTCCAATTTTG-GG-CGGTACTGTCTC-------
------
FC88X_01027_01066      ------------------TCTTCCAATTTTG-GG-CGGTACTGTCTC-------
------
FC88X_01004_01191      ------------------TCTTCCAATTTTG-GG-CGGTACTGTCTC-------
------
FC88X_01052_00630      ------------------TCTTCCAATTTTG-GG-CGGTACTGTCTC-------
------
FC88X_00888_00427      ------------------TCTTCCAATTTTT-GGGCGGTACTGTCTC-------
------
FC88X_00937_01046      ------------------TCTTCCAATTTTT-GGGCGGTACTGTCTC-------
------
FC88X_00747_01030      -----------------GTCTTCCAATTTTT-GGGCGGTACTGTCTC-------
------
FC88X_00943_00451      ------------GGGGAATCTTCCAATTTTT-GGGCGGTACTGTCTC-------
------
FC88X_00347_01026      ------------------TCTTCCAATTTTA-GG-CGGTACTGTCTC-------
------
FC88X_00666_00292      ------------------TCTTCCAATTTTTTGGGCGGTACTGTCTC-------
------
FC88X_00056_00110      ------------------TCTTCCAACTTTG-GG-CGGTACTGTCTC-------
------
FC88X_00089_00759      ------------------TCTTCCAATT-TG-GG-CGGTACTATCTC-------
------
```

FC88X_00484_00773   ------------------TCTTCCAATTCTG-GG-CGGTACTGTCTC-------
------
FC88X_00049_00022   ------------------TCTTCCAATTTCG-GG-CGGTACTGTCTC-------
------
FC88X_00945_00571   ------------------TCTTCCAATTTTG-GG-
CGGTACTGTCTCCTTCCTTT-----
FC88X_00399_01092   ------------------TCTTCCAATTTTG-GG-
CGGTACTGTCTCCTTCCTTT-----
FC88X_01100_00655   ------------------TCTTCCAATTTTG-GG-
CGGTACTGTCTCCTTCCTTT-----
FC88X_00593_00683   ------------------TCTTCCGATTTTG-GG-CGGTACTGTCTC-------
------
FC88X_00949_00644   -----------------GTCTCCCAATTTTG-GG-CGGTACTGTCTC-------
------
FC88X_00056_00740   ------------------TCTTCCAGTTTT--GGGCGGTACTGTCTC-------
------
FC88X_00449_01058   ------------------TCTTCCAGTTTTT-GGGCGGTACTGTCTC-------
------
FC88X_00888_00342   ------------------TCTACCAGTTTT--GGGCGGTACTGTCTC-------
------
FC88X_00615_01040   ------------------TCTTCCAATTTTG-GG-CGGTGCTGTCTC-------
------
FC88X_00646_00772   -------------------CTTCCAATTTTG-GG-CGGTACTATCTC-------
------
FC88X_00473_00459   ------------------CCTTCCAATTTTG-GG-
CGGTACTGTCTCCTTCCTTTCT---
FC88X_00744_00799   ------------------TCCTCCAATTTTG--GGCGGTACTGTCTC-------
------
FC88X_00459_00560   ------------------TCCTCCAATTTTG--GGCGGTACTGTCTC-------
------
FC88X_00463_01018   ------------------TCCTCCAATTTTG--GGCGGTACTGTCTC-------
------
FC88X_00688_00282   ------------------TCCTCCAATTTTG--GGCGGTACTGTCTC-------
------
FC88X_01063_01139   ------------------TCCTCCAATTTTG--GGCGGTACTGTCTC-------
------
FC88X_00175_01014   ------------------TCCTCCAATTTTG--GACGGTACTGTCTC-------
------
FC88X_00870_00422   ------------------TCTTCCAATTTTG--GGCGATACTGTCTC-------
------
FC88X_00449_01056   ------------------TCTTCCAATATTG--GGCGGTACTGTC---------
------
FC88X_01067_01120   ------------------TCTTCCAATCTTG--GGCGGTACTGTCTC-------
------
FC88X_00111_00359   ------------------TCTATCAATATTG-G-CCGGTAGCTTTCT-------
------
FC88X_00645_00268   ------------------TCTATCAATATTG-G-CCGGTAGCTTTCT-------
------
FC88X_00282_00737   ------------------TCTATCAATATTG-G-CCGGTAGCTTTCT-------
------
FC88X_00309_00374   ------------------TCTATCAATATTG-G-CCGGTAGCTTTCT-------
------
FC88X_00407_00008   ------------------TCTATCAATATTG-G-CCGGTAGCTTTCT-------
------
FC88X_00546_01132   ------------------TCTATCAATATTG-G-CCGGTAGCTTTCT-------
------

FC88X_00848_00306 ------------------TCTATCAATATTG-G-CCGGTAGCTTTCT-------
------
FC88X_00822_00865 ------------------TCTATCAATATTG-G-CCGGTAGCTTTCT-------
------
FC88X_00863_00309 ------------------TCTATCAATATTG-G-CCGGTAGCTTTCT-------
------
FC88X_00890_00702 ------------------TCTATCAATATTG-G-CCGGTAGCTTTCT-------
------
FC88X_01078_00506 ------------------TCTATCAATATTG-G-CCGGTAGCTTTCT-------
------
FC88X_01144_00590 ------------------TCTATCAATATTG-G-CCGGTAGCTTTCT-------
------
FC88X_00918_01251 ------------------TCTATCGATATTG-G-CCGGTAGCTTTCT-------
------
FC88X_00847_00944 ----------------GGCCTATCAATATTG-G-CCGGTAGCTTTCT-------
------
FC88X_00976_00263 ------------------TCTACCAATATTG-GTCCGGTAGCTTTCTC------
------
FC88X_00489_00952 ------------------CCATCCGATACTT-GCAC-GTGCGTTCTC-------
------
FC88X_00546_00378 ------------------CCATCCGATATTT-GCGC-GTACGTTCTC-------
------
FC88X_00535_00080 ------------------TCATCAAATAATT-GCGCTGTTCGTTTC--------
------
FC88X_00593_00271 ------------------TCATCAAATAATT-GCGCTGTTCGTTC---------
------
FC88X_01068_00662 ----------------------CCGATTAATTGGCCGGTCTCGTATTCTC----
------
FC88X_00348_01152 ----------------------CCGAT-AATTGGCCGGTCTCGTATTCTC----
------
FC88X_00572_01015 ----------------------CCGAT-AATTGGCCGGTCTCGTATTCTC----
------
FC88X_00629_00081 ----------------------CCGAT-AATTGGCCGGTCTCGTATTCTC----
------
FC88X_00674_00150 -----------------------CGGATAATTGGCCGGTCTCGTATTCTC----
------
FC88X_00796_00895 ----------------------CCGAT-
AGTTGGCCGGTCTCGTATTCTCCTTCCTTTCT
FC88X_01037_00322 ----------------------CCAAT-AATTCGCCG----
TGTATTCTCCTAAA-----
FC88X_00145_00682 ---------------------CCCGAT-GGTCCGCCCCTCTAGCATTCT-----
------
FC88X_00670_00985 ------------------TTGCGCAAT-AGTCAGCCC----GGCATTCTC----
------
FC88X_00225_00957 --------------------GCGCAATTT-GGACATGTGGCCGCTTCTC-----
------
FC88X_00778_00289 --------------------GCGCAATTT-GGACATGTGGCCGCTTCTC-----
------
FC88X_00025_00715 ---------------------CGCAATTT-GGACATGTGGCCGCTTCTC-----
------
FC88X_00115_00512 ---------------------CGCAATTT-GGACATGTGGCCGCTTCTC-----
------
FC88X_00178_00979 ---------------------CGCAATTT-GGACATGTGGCCGCTTCTC-----
------
FC88X_00378_00121 ---------------------CGCAATTT-GGACATGTGGCCGCTTCTC-----
------

```
FC88X_00515_00014       ---------------------CGCAATTT-GGACATGTGGCCGCTTCTC-----
------
FC88X_00860_00310       ---------------------CGCAATTT-GGACATGTGGCCGCTTCTC-----
------
FC88X_00144_00700       ---------------------CGCAATTTTGGACATGTGGCCGCTTCTC-----
------
FC88X_00152_00810       ---------------------CGCAATTT-GGACATGTGGCCGCTTCTC-----
------
FC88X_00308_00964       ---------------------CGCAATTT-GGACATGTGGCCGCTTCTC-----
------
FC88X_00406_00049       ---------------------CGCAATTT-GGACATGTGGCCGCTTCTC-----
------
FC88X_00791_00301       ---------------------CGCAATTT-GGACATGTGGCCGCTTCTC-----
------
FC88X_00860_00500       ---------------------CGCAATTT-GGACATGTGGCCGCTTCTC-----
------
FC88X_00895_01250       ---------------------CGCAATTT-GGACATGTGGCCGCTTCTC-----
------
FC88X_01061_00478       ---------------------CGCAATTT-GGACATGTGGCCGCTTCTC-----
------
FC88X_01122_00679       ---------------------CGCAATTT-GGACATGTGGCCGCTTCTC-----
------
FC88X_00026_00444       ---------------------CGCAATTT-GGACATGTGGCCGCTTCTC-----
------
FC88X_00016_00463       ---------------------CGCAATTT-GGACATGTGGCCGCTTCTC-----
------
FC88X_00099_00516       ---------------------CGCAATTT-GGACATGTGGCCGCTTCTC-----
------
FC88X_00072_00772       ---------------------CGCAATTT-GGACATGTGGCCGCTTCTC-----
------
FC88X_00101_00356       ---------------------CGCAATTT-GGACATGTGGCCGCTTCTC-----
------
FC88X_00193_00053       ---------------------CGCAATTT-GGACATGTGGCCGCTTCTC-----
------
FC88X_00179_00486       ---------------------CGCAATTT-GGACATGTGGCCGCTTCTC-----
------
FC88X_00225_01093       ---------------------CGCAATTT-GGACATGTGGCCGCTTCTC-----
------
FC88X_00476_01163       ---------------------CGCAATTT-GGACATGTGGCCGCTTCTC-----
------
FC88X_00480_01152       ---------------------CGCAATTT-GGACATGTGGCCGCTTCTC-----
------
FC88X_00569_00447       ---------------------CGCAATTT-GGACATGTGGCCGCTTCTC-----
------
FC88X_00648_00210       ---------------------CGCAATTT-GGACATGTGGCCGCTTCTC-----
------
FC88X_00699_00880       ---------------------CGCAATTT-GGACATGTGGCCGCTTCTC-----
------
FC88X_00720_01131       ---------------------CGCAATTT-GGACATGTGGCCGCTTCTC-----
------
FC88X_00867_00845       ---------------------CGCAATTT-GGACATGTGGCCGCTTCTC-----
------
FC88X_00931_00840       ---------------------CGCAATTT-GGACATGTGGCCGCTTCTC-----
------
FC88X_00960_00238       ---------------------CGCAATTT-GGACATGTGGCCGCTTCTC-----
------
```

FC88X_00973_00275 ---------------------CGCAATTT-GGACATGTGGCCGCTTCTC-----
------
FC88X_00977_00405 ---------------------CGCAATTT-GGACATGTGGCCGCTTCTC-----
------
FC88X_01015_00571 ---------------------CGCAATTT-GGACATGTGGCCGCTTCTC-----
------
FC88X_01026_00764 ---------------------CGCAATTT-GGACATGTGGCCGCTTCTC-----
------
FC88X_01010_00925 ---------------------CGCAATTT-GGACATGTGGCCGCTTCTC-----
------
FC88X_01049_01170 ---------------------CGCAATTT-GGACATGTGGCCGCTTCTC-----
------
FC88X_01100_00650 ---------------------CGCAATTT-GGACATGTGGCCGCTTCTC-----
------
FC88X_01135_00921 ---------------------CGCAATTT-GGACATGTGGCCGCTTCTC-----
------
FC88X_00470_00070 ---------------------CGCAATTT-GGACATGTGGCCGCTTCTCC----
------
FC88X_00712_01223 ---------------------CGCAATTT-GGACATGTGGCCGCTTCTCCCC--
------
FC88X_01031_00534 ---------------------CGCAATTT-GGACATGTGGCCGCTTCT------
------
FC88X_01122_00819 ---------------------CGCAATTT-GGACATGTGGCCGCTTCTAC----
------
FC88X_00836_00448 ---------------------CGCAATTT-GGACATGTGGCTGCTTCTC-----
------
FC88X_00434_01002 ---------------------CGCAATTT-GGACATGTGGCTGCTTCTC-----
------
FC88X_01083_00877 ---------------------CGCAATTT-GGACATGTGGCAGCTTCTC-----
------
FC88X_00019_00370 ---------------------CGCAATTT-GGACATGTGGCGGCTTCTC-----
------
FC88X_00743_01026 ---------------------CGCAATTT-G-ACATGTGGCCGCTTCTC-----
------
FC88X_01080_01006 --------------GGGGAAGCGCAATTT-GGACATGTGGCCGCTTCTC-----
------
FC88X_00071_00069 ---------------------CGCAATTT-GGACATGTGGCCGTTTCTC-----
------
FC88X_00988_00720 ----------------GTGATCGCAATTT-GGACATGTGGCCGCTTCTC-----
------
FC88X_00013_00218 ---------------------CGCAACTT-GGACATGTGGCCGCTTCTC-----
------
FC88X_01141_00962 ---------------------CGCAACTT-GGACATGTGGCCGCTTCTC-----
------
FC88X_00634_01232 ---------------------CGCAACTT-GGACATGCGGCCGCTTCTC-----
------
FC88X_00113_00491 -----------CGCCTTCTCCCGCAATTT-GGACATGTGGCCGCTTCTC-----
------
FC88X_00964_00873 ---------------------CGCAATTT-GGACGTGTGGCCGCTTCTC-----
------
FC88X_00135_00225 ---------------------CGCAATTT-GGACACGTGGCCGGCTTCTC----
------
FC88X_01002_01162 ---------------------CGCAATTT-
GGACACGTGGCCGCTTCTCCTTCCTTTC--
FC88X_00189_00885 ---------------------CGCAATTT-
GGACATGTGGCCGCTTCTCCCTTCCTT---

FC88X_00435_01172 ---------------------CGCAATTT-GGACATGTCGCCGCTTCTCC----
------
FC88X_00614_00070 ---------------------CGCAATCT-GGACATGTGGCCGCTTCTCCC---
------
FC88X_00779_00935 --------------------GCGCAATTT-
GGACGTGTGGCCGCTTCTCTCCCCT-----
FC88X_00039_00790 ----------------------GCAATTTAGCGATGGTGGGCCTTTCTC-----
------
FC88X_00811_00849 ----------------------GCAATTTAGCGATGGTGGGCCTTTCTC-----
------
FC88X_01144_00808 ----------------------GCAATTTAGCGATGGTGGGCCTTTCTC-----
------
FC88X_00036_00330 ----------------------GCAATTTAGCGATGGTGGGCCTTTCTC-----
------
FC88X_00520_01037 ----------------------GCAATTTAGCGATGGTGGGCCTTTCTC-----
------
FC88X_00826_00272 ----------------------GCAATTTAGCGATGGTGGGCCTTTCTC-----
------
FC88X_01128_01055 ----------------------GCAATTTAGCGATGGTGGGCCTTTCTC-----
------
FC88X_00918_00648 ----------------------GCAATTTAGCGATGGTGGGCCGTTCTC-----
------
FC88X_00376_00772 ------------------TCGTGCAATTTTCGACGCGTCTCTAGTT--------
------
FC88X_00495_00391 ------------------TCGTGCAATTTTCGACGCGTCTCTAGTT--------
------
FC88X_00164_00776 ------------------TCGTGCAATTTTCGACGCGTCTCTAGTT--------
------
FC88X_00252_00388 ------------------TCGTGCAATTTTCGACGCGTCTCTAGTT--------
------
FC88X_00554_00994 ------------------TCGTGCAATTTTCGACGCGTCTCTAGTT--------
------
FC88X_00819_00246 ------------------TCGTGCAATTTTCGACGCGTCTCTAGTT--------
------
FC88X_00817_00255 ------------------TCGTGCAATTTTCGACGCGTCTCTAGTT--------
------
FC88X_00812_00853 ------------------TCGTGCAATTTTCGACGCGTCTCTAGTT--------
------
FC88X_01142_00778 ------------------TCGTGCAATTTTCGACGCGTCTCTAGTT--------
------
FC88X_01123_00671 ------------------TCGTGCAATTTTCGACGCGTCCCTAGTT--------
------
FC88X_00738_00256 ------------------TCGTGCAATTTTCGGCGCGTCCCTAGTT--------
------
FC88X_00209_00868 ---------------------ACAAATTGTCG--GCGTCATGAGTTCTC-----
------
FC88X_00630_00267 ------------------TCCACTTATAGTCG--GTGGCT--GGTTCTC-----
------
FC88X_00222_00031 -------------------AGACCATTTGTCGACGATCCTCTATCC--------
------
FC88X_00910_01246 ------------------CTGAGCACTTGTCGATGGCTGTATCCC---------
------
FC88X_00746_00962 ---------------------ATACACAT--TACGTGACACATCTCTCTT----
------
FC88X_00446_00055 --------------------GATACACAT--TACGTGACACATCTCTCTT----
------

```
FC88X_01034_00468      ---------------------ATACACAT--TACGTGACACATCTCTCTT----
------
FC88X_00295_01006      ---------------------ATGCACAT--TACGTGACACATCTCTCCT----
------
FC88X_00041_00262      ---------------------ATACACAT--CACGTGACACATCCCTTT-----
------
FC88X_00573_01239      --------------------CTTCTTCAT--CACCTGTCACAGCTTTCTC----
------
FC88X_01006_00522      --------------------CTGGTTCGT--AACCCAAAGCGATTTCTC-----
------
FC88X_00712_00676      -------------------GCCGTTCTGT--GTCACATATCGCTTTCTC-----
------
FC88X_00272_00922      -------------------TCTCACCGGT-GACGGTA-TACGCTTTCTC-----
------
FC88X_01096_00578      -------------------TCTCACCGGTTGACGGTA-TACGCTTTCTC-----
------
FC88X_00257_00995      -------------------TCTCACCGGT-GACGGTA-TACGCTTTCTC-----
------
FC88X_00568_01142      -------------------TCTCACCGGT-GACGGTA-TACGCTTTCTC-----
------
FC88X_00640_01039      -------------------TCTCACCGGT-GACGGTA-TACGCTTTCTC-----
------
FC88X_01073_01094      -------------------TCTCACCGGT-GACGGTA-TACGCTTTCTC-----
------
FC88X_00216_00135      -------------------TCTCACCGGT-GACGGTA-TACGCTTTCTC-----
------
FC88X_00293_00011      -------------------TCTCACCGGT-GACGGTA-TACGCTTTCTC-----
------
FC88X_00557_00065      -------------------TCTCACCGGT-GACGGTA-TACGCTTTCTC-----
------
FC88X_00599_00533      -------------------TCTCACCGGT-GACGGTA-TACGCTTTCTC-----
------
FC88X_00873_01150      -------------------TCTCACCGGT-GACGGTA-TACGCTTTCTC-----
------
FC88X_00901_00455      -------------------TCTCACCGGT-GACGGTA-TACGCTTTCTC-----
------
FC88X_00972_00633      -------------------TCTCACCGGT-GACGGTA-TACGCTTTCTC-----
------
FC88X_00957_00809      -------------------TCTCACCGGT-GACGGTA-TACGCTTTCTCT----
------
FC88X_01024_00475      ------------------GTCTCACCGGT-GACGGTA-TACGCTTTCTC-----
------
FC88X_00556_00666      -------------------TCTCACCGGT-GACGGTA-TACGCTTTCTCCC---
------
FC88X_00925_01181      -------------------TCTCACCGGT-AACGGTACTACGCTTTCTC-----
------
FC88X_00257_01005      ------------------TTCTTGCTGGT--ACCATATTAGACTTTCT------
------
FC88X_00504_00961      -------------------ACTTGC-GGT--AACACAGCACGTTTTCTC-----
------
FC88X_00020_00333      --------------------CCTCCTGGT--AACCTATGACGCTATCTC-----
------
FC88X_00159_00876      --------------------CCACGTGGT--CATGTCTGA-GCATTCTC-----
------
FC88X_00708_01233      -----------------------CCCAGT-GACATTACTGATCTTGCTATC---
------
```

FC88X_00943_00408 -----------------------CCCAGT-GACATTACTGATCTTGCTAT----
------
FC88X_00818_00296 -----------------------CCCAGT-GACGTTACTGATCTTGCTATC---
------
FC88X_00956_00749 ---------------------CTCCCAGT-CACGTGAC--GTCTTGCTGTA---
------
FC88X_00554_00293 ---------------------CCCACCGT--ACACTGGTGCTGATGCTAGC---
------
FC88X_00847_00319 --------------------TGTTCCAGT--GCACCTGT-CTGA-GCTAGC---
------
FC88X_00037_00477 --------------------CTTGTGCGG-GTGTGTAAGGCAATTCTC------
------
FC88X_00074_00431 --------------------CTTGTGCGG-GTGTGTAAGGCAATTCTC------
------
FC88X_00088_00843 --------------------CTTGTGCGG-GTGTGTAAGGCAATTCTC------
------
FC88X_00284_00594 --------------------CTTGTGCGG-GTGTGTAAGGCAATTCTC------
------
FC88X_00412_00990 --------------------CTTGTGCGG-GTGTGTAAGGCAATTCTC------
------
FC88X_00699_01272 --------------------CTTGTGCGG-GTGTGTAAGGCAATTCTC------
------
FC88X_00938_00423 --------------------CTTGTGCGG-GTGTGTAAGGCAATTCTC------
------
FC88X_00974_00964 --------------------CTTGTGCGG-GTGTGTAAGGCAATTCTC------
------
FC88X_01132_00750 --------------------CTTGTGCGG-GTGTGTAAGGCAATTCTC------
------
FC88X_00011_00278 --------------------CTTGTGCGG-GTGTGTAAGGCAATTCTC------
------
FC88X_00004_00654 --------------------CTTGTGCGG-GTGTGTAAGGCAATTCTC------
------
FC88X_00082_00421 --------------------CTTGTGCGG-GTGTGTAAGGCAATTCTC------
------
FC88X_00103_00494 --------------------CTTGTGCGG-GTGTGTAAGGCAATTCTC------
------
FC88X_00232_00911 --------------------CTTGTGCGG-GTGTGTAAGGCAATTCTC------
------
FC88X_00824_00845 --------------------CTTGTGCGG-GTGTGTAAGGCAATTCTC------
------
FC88X_00933_00969 --------------------CTTGTGCGG-GTGTGTAAGGCAATTCTC------
------
FC88X_01075_00768 --------------------CTTGTGCGG-GTGTGTAAGGCAATTCTC------
------
FC88X_01147_00791 --------------------CTTGTGCGG-GTGTGTAAGGCAATTCTC------
------
FC88X_01122_00947 --------------------CTTGTGCGG-GTGTGTAAGGCAATTCTC------
------
FC88X_01022_01252 --------------------CTTGCGCGG-GTGTGTAAGGCAATTCTC------
------
FC88X_00915_00497 -------------------GCTTGTACGG-GTGTGTAAGGCAATTCTC------
------
FC88X_00503_01166 --------------------CTTGTGCGG-GTGTGTAAGGCGATTCTC------
------
FC88X_00497_00192 --------------------CTTGTGCGG-
GTGTGTAAGGCAATTCTCCTTCCTTT----

FC88X_00515_00122 -------------------------TCGC-
GTGTCCCGCGAAACTCTCCCTACT------
FC88X_00516_00384 ------------------------TTTGG-GCGTGTGCCAAA--
TCTCCTTACT------
FC88X_00377_00056 --------------------CTAAGGCGT-CTCTCT--CATTGCTATTCT----
------
FC88X_00148_00803 --------------------CTAAGGCGT-CTCTCT--CATTGCTATTCT----
------
FC88X_00900_01007 --------------------CCAACACGT-CTC-CT--TATTTAAATTCTC---
------
FC88X_00780_00257 --------------------CCAGAGCCG-TTGCTT--CGTGCCTATTC-----
------
FC88X_01140_01072 --------------------CCAGCGCGT-ATGCATACCGTTCCTTTTCC----
------
FC88X_01145_01058 --------------------CCAGCGCGT-TTGCGT--TATGCGTATTCC----
------
FC88X_00221_00783 --------------------CCAGCGCGT-TTGCGT--TATGCGTATTCC----
------
FC88X_00830_00775 --------------------CCAGCGCGT-TTGCGT--TATGCGTATTCC----
------
FC88X_00641_01223 --------------------CCAGCGCGT-CTGCGT--TATGCGTATTCC----
------
FC88X_00318_00562 ---------------------TCCTGAGG-ATTCACTCCGGGCGTTCT------
------
FC88X_00962_00366 ---------------------TCCTGAGG-ATTCACTCCGGGCGTTCT------
------
FC88X_00247_00231 ----------------CATGTGCCTGAGC-GAGTCATCGGGGGG----------
------
FC88X_00031_00451 ----------------CCAGACCATTTGT-GTTT----GAGGCATCTC------
------
FC88X_00113_00038 ----------------CCAGACCATTTGT-GTTT----GAGGCATCTC------
------
FC88X_00203_00098 ----------------CCAGACCATTTGT-GTTT----GAGGCATCTC------
------
FC88X_00518_00099 ----------------CCAGACCATTTGT-GTTT----GAGGCATCTC------
------
FC88X_00593_01121 ----------------CCAGACCATTTGT-GTTT----GAGGCATCTC------
------
FC88X_00777_00229 ----------------CCAGACCATTTGT-GTTT----GAGGCATCTC------
------
FC88X_00792_00439 ----------------CCAGACCATTTGT-GTTT----GAGGCATCTC------
------
FC88X_00860_00152 ----------------CCAGACCATTTGT-GTTT----GAGGCATCTC------
------
FC88X_00934_00523 ----------------CCAGACCATTTGT-GTTT----GAGGCATCTC------
------
FC88X_00577_00176 ---------------CCCAGACCATTTGT-GTTTT---GAGGCATCTCC-----
------
FC88X_00882_00954 ----------------CCAGACCATTTGT-GTTT----
GAGGCATCTCCTTCCTTT----
FC88X_00965_00342 ----------------CCAGTCCATTTGT-GTTC----GAGGCATCTC------
------
FC88X_00097_00834 --------------------CCCAGTTGC-GTTAGCGTAAGCCATCTC------
------
FC88X_00327_00564 ----------------CCAGACGACGGAC-ATCG----GAGGCATTCT------
------

```
FC88X_00360_01045      ----------------CCACACGACTTAC-AACG----TCAGGCTTTTC-----
------
FC88X_00730_01074      ----------------CCATACACTCCAG-CTCT----GTGTCATATT------
------
FC88X_00868_00925      ----------------CCATACATTCCAG-CTCT----GTGTCATATT------
------
FC88X_00068_00316      ------------------TTACACGCCAG-CCCT----TGGTGACATACC----
------
FC88X_00011_00345      -------------------TTTCCGAATCTATGATAGGACGTCTCCC-------
------
FC88X_00999_01185      -------------------TTTCCGAATCTATGATAGGACGTCTCCC-------
------
FC88X_00118_00971      ---------------------CCCGAATCATTGTGTGGATCTCCCAGT------
------
FC88X_01103_00777      ---------------------CCGAGACTCTGTGTAGGTGCTCCCATT------
------
FC88X_00531_01151      ---------------------CCGAGACTCTGTGTAGGTGCTCCCATT------
------
FC88X_00063_00462      ---------------------CCGAGACTCTGTGTAGGTGCTCCATT-------
------
FC88X_00186_00022      ---------------------CCGATGTCTCTGTTACGTGCGGTTCTC------
------
FC88X_00651_01186      ---------------------CCGATGTCTCTGTTACGTGCGGTTCTC------
------
FC88X_00964_00328      ---------------------CCGATGTCTCTGTTACGTGCGGTTCTC------
------
FC88X_00165_00794      ---------------------CCGATGTCTCTGTTACGTGCGGTTCTC------
------
FC88X_00499_00191      ---------------------CCGATGTCTCTGTTACGTGCGGTTCTC------
------
FC88X_00677_01218      ---------------------CCGATGTCTCTGTTACGTGCGGTTCTC------
------
FC88X_00777_00233      ---------------------CCGATGTCTCTGTTACGTGCGGTTCTC------
------
FC88X_00756_01249      ---------------------CCGATGTCTCTGTTACGTGCGGTTCTC------
------
FC88X_00847_00943      ---------------------CCGATGTCTCTGTTACGTGCGGTTCTC------
------
FC88X_00924_00655      ---------------------CCGATGTCTCTGTTACGTGCGGTTCTC------
------
FC88X_00049_00738      ---------------------CCGATGCCTCTGTTACGTGCGGTTCTC------
------
FC88X_00952_01232      --------------------CCACATCTCCCTGGAAGTCACTCTTTC-------
------
FC88X_00346_01185      -------------------TCCGACATTCTCTGGAAGTCGCGCGTC--------
------
FC88X_00826_00917      ----------------------CCACCAAGTC-GTGCGCGTCTCCCATCC----
------
FC88X_00248_00968      ----------------------TCATCAACTCTGTGGCGGTCTCCCAAC-----
------
FC88X_00617_00987      ---------------------ACCAAGCATGTGTCGCATGTCTCCCTT------
------
FC88X_00694_00902      ----------------------CCGACCATTCCACGTCAGCGTGACTCC-----
------
FC88X_00890_00333      ----------------------CCGACCATTCCACGTCAGCGTGACTCC-----
------
```

FC88X_00168_00979 ----------------------CCGACCATTCCACGTCAGCGTGACTCC-----
------
FC88X_00165_00278 ---------------------ACCGAACATATGGACGTCGCGTCTCTC------
------
FC88X_00297_00725 ---------------------ACCGAACATATGGACGTCGCGTCTCTC------
------
FC88X_00338_00754 ---------------------ACCGAACATATGGACGTCGCGTCTCTC------
------
FC88X_00315_01072 ---------------------ACCGAACATATGGACGTCGCGTCTCTC------
------
FC88X_00845_01037 ---------------------ACCGAACATATGGACGTCGCGTCTCTCT-----
------
FC88X_01084_01200 ----------------------CCGAACCTTCCGTTGTCGTGCCTTTCT-----
------
FC88X_00508_00096 ----------------------CCGAACCTTC-GTTGTCGTGCCTTTCT-----
------
FC88X_00525_01046 ----------------------CCGAACCTTCCGTTGTCGTGCCTTTCT-----
------
FC88X_00932_00955 -----------------------CTAACCGTCCGGTGGCGCTTGACTTTT----
------
FC88X_01003_00275 ----------------------CTATCCTTCCGAGGGTGTCGGCTTTCT-----
------
FC88X_00965_00598 ----------------------CTATCCTTCCGAGGGTGTCGGCTTTCT-----
------
FC88X_00208_00064 ----------------------CCATCCTTCCGAGGGTGTCGGCTTTCT-----
------
FC88X_00558_00171 ----------------------CTATCCTTCCGAGAGTGTCGGCTTTCT-----
------
FC88X_00651_00433 --------------------------CGTTCCGAATGTTCGCGCTTCTCCTTG-
------
FC88X_00181_00108 ---------------------CCGTTAACCCGGATCCCTGTGCATCTA------
------
FC88X_00466_01034 ---------------------CCGTTAACCCGGATCCCTGTGCATCTA------
------
FC88X_00670_01085 ---------------------CTGTGACCCAGCCTCCCTGTGAATGTA------
------
FC88X_00546_01208 ---------------------CCTGACAATGACAGCTCTCCGAATATT------
------
FC88X_00645_00072 ---------------------CCTGACAATGACAGCTCTCCGAATATT------
------
FC88X_00849_00364 ---------------------CCTGACAATGACAGCTCTCCGAATATT------
------
FC88X_00076_00491 ---------------------CCTGACAATGACAGCTCTCCGAATATT------
------
FC88X_00385_01167 ---------------------CCTGACAATGACAGCTCTCCGAATATT------
------
FC88X_01141_00922 ---------------------CCTGGCACCCGCTGCCCTCCGAATCTCTT----
------
FC88X_00661_00386 --------------------CTAT-CCCTCTGAGAACGTGCGTGTCTC------
------
FC88X_00973_00613 --------------------CTAT-CCCTCTGAGAACGTGCGTGTCTC------
------
FC88X_00111_00491 --------------------CTAT-CCCTCTGAGAACGTGCGTGTCTC------
------
FC88X_00863_00278 --------------------CTAT-CCCTCTGAGAACGTGCGTGTCTC------
------

FC88X_01083_00868 --------------------CTATGCCCTCAGCGGATGT-CTAGTCTC------
------
FC88X_00886_00371 --------------------CGAGCAGATCGTCGA-GTCCGATCTC--------
------
FC88X_01015_00951 --------------------CTAGCACCTCGCTGAAGCCCAATCTCC-------
------
FC88X_00294_00958 --------------------TCCTGAGTCTTATCGAGCTGGCCTGTC-------
------
FC88X_00541_00120 --------------------TCCTGAGTCTTATCGAGCTGGCCTGTC-------
------
FC88X_00814_01002 --------------------TCCTGAGTCTTATCGAGCTGGCCTGTC-------
------
FC88X_01107_00509 --------------------TCCTGAGTCTTATCGAGCTGGCCTGTC-------
------
FC88X_00548_00486 -------------------CACGTGTGTCT-ACCGCACGGCCCTGTCCC-----
------
FC88X_00992_00817 --------------------TCCTGTGACTTCGGGCTTTCCTCTGTT-------
------
FC88X_00352_01191 --------------------TCCTGTGACTTCGGGCTTTCCTCTGTT-------
------
FC88X_00314_01045 --------------------TCCTGTGACCTCGGGCTTTCCTCTGTT-------
------
FC88X_00162_00028 -------------------GTCCTGTGACTTCGGGCTCTCCTCTGTT-------
------
FC88X_01119_00634 ---------------------CCTGAGACTGATGGCACTCC-CTGTGA------
------
FC88X_00952_00430 ---------------------CCTGTGACTGTTAGCAGCTCGGCTCCTC-----
------
FC88X_00036_00121 ---------------------CCTGTGACTGTTAGCAGCTCGGCTCCTC-----
------
FC88X_01028_00464 --------------------CCCTGTGACATCCAG-AGCTCGACTTGT------
------
FC88X_00991_00502 --------------------CCACTCAGTCTTCGG----ACCTTGCCTTTT---
------
FC88X_00578_01114 --------------------CCCCTGAGTCTCTGGCAGCACGTTGCC-------
------
FC88X_00025_00082 ----------------AATCA-GGCCTGCGTGGACTGGTG-CGTTCTC------
------
FC88X_00046_00204 ----------------AATCA-GGCCTGCGTGGACTGGTG-CGTTCTC------
------
FC88X_00085_00188 ----------------AATCA-GGCCTGCGTGGACTGGTG-CGTTCTC------
------
FC88X_00063_00297 ----------------AATCA-GGCCTGCGTGGACTGGTG-CGTTCTC------
------
FC88X_00056_00499 ----------------AATCA-GGCCTGCGTGGACTGGTG-CGTTCTC------
------
FC88X_00179_00384 ----------------AATCA-GGCCTGCGTGGACTGGTG-CGTTCTC------
------
FC88X_00260_01100 ----------------AATCA-GGCCTGCGTGGACTGGTG-CGTTCTC------
------
FC88X_00354_01002 ----------------AATCA-GGCCTGCGTGGACTGGTG-CGTTCTC------
------
FC88X_00787_00851 ----------------AATCA-GGCCTGCGTGGACTGGTG-CGTTCTC------
------
FC88X_00864_00298 ----------------AATCA-GGCCTGCGTGGACTGGTG-CGTTCTC------
------

FC88X_00886_01168 ----------------AATCA-GGCCTGCGTGGACTGGTG-CGTTCTC------
------
FC88X_00935_00912 ----------------AATCA-GGCCTGCGTGGACTGGTG-CGTTCTC------
------
FC88X_01071_00836 ----------------AATCA-GGCCTGCGTGGACTGGTG-CGTTCTC------
------
FC88X_01076_00873 ----------------AATCA-GGCCTGCGTGGACTGGTG-CGTTCTC------
------
FC88X_00021_00322 ----------------AATCA-GGCCTGCGTGGACTGGTG-CGTTCTC------
------
FC88X_00034_00589 ----------------AATCA-GGCCTGCGTGGACTGGTG-CGTTCTC------
------
FC88X_00073_00457 ----------------AATCA-GGCCTGCGTGGACTGGTG-CGTTCTC------
------
FC88X_00245_00091 ----------------AATCA-GGCCTGCGTGGACTGGTG-CGTTCTC------
------
FC88X_00200_00134 ----------------AATCA-GGCCTGCGTGGACTGGTG-CGTTCTC------
------
FC88X_00487_00405 ----------------AATCA-GGCCTGCGTGGACTGGTG-CGTTCTC------
------
FC88X_00535_01212 ----------------AATCA-GGCCTGCGTGGACTGGTG-CGTTCTC------
------
FC88X_00578_01163 ----------------AATCA-GGCCTGCGTGGACTGGTG-CGTTCTC------
------
FC88X_00608_00603 ----------------AATCA-GGCCTGCGTGGACTGGTG-CGTTCTC------
------
FC88X_00615_00632 ----------------AATCA-GGCCTGCGTGGACTGGTG-CGTTCTC------
------
FC88X_00668_01268 ----------------AATCA-GGCCTGCGTGGACTGGTG-CGTTCTC------
------
FC88X_00748_00239 ----------------AATCA-GGCCTGCGTGGACTGGTG-CGTTCTC------
------
FC88X_00890_00220 ----------------AATCA-GGCCTGCGTGGACTGGTG-CGTTCTC------
------
FC88X_00877_00330 ----------------AATCA-GGCCTGCGTGGACTGGTG-CGTTCTC------
------
FC88X_00874_00834 ----------------AATCA-GGCCTGCGTGGACTGGTG-CGTTCTC------
------
FC88X_00882_01094 ----------------AATCA-GGCCTGCGTGGACTGGTG-CGTTCTC------
------
FC88X_00881_01132 ----------------AATCA-GGCCTGCGTGGACTGGTG-CGTTCTC------
------
FC88X_01001_00410 ----------------AATCA-GGCCTGCGTGGACTGGTG-CGTTCTC------
------
FC88X_01028_00427 ----------------AATCA-GGCCTGCGTGGACTGGTG-CGTTCTC------
------
FC88X_01043_00415 ----------------AATCA-GGCCTGCGTGGACTGGTG-CGTTCTC------
------
FC88X_01008_00910 ----------------AATCA-GGCCTGCGTGGACTGGTG-CGTTCTC------
------
FC88X_01042_01080 ----------------AATCA-GGCCTGCGTGGACTGGTG-CGTTCTC------
------
FC88X_01013_01129 ----------------AATCA-GGCCTGCGTGGACTGGTG-CGTTCTC------
------
FC88X_01080_00393 ----------------AATCA-GGCCTGCGTGGACTGGTG-CGTTCTC------
------

FC88X_01069_00628 -----------------AATCA-GGCCTGCGTGGACTGGTG-CGTTCTC------
------
FC88X_01081_01062 -----------------AATCA-GGCCTGCGTGGACTGGTG-CGTTCTC------
------
FC88X_00127_00373 -----------------AATCA-G-CCTGCGTGGACTGGTG-CGTTCTC------
------
FC88X_00189_00181 -----------------AATCA-GGCCTGCGTGGACTGGTG-CGTTCTCCC----
------
FC88X_00835_00769 -----------------AATCA-GGCCTGCGTGGACTGGTG-CGTTCTCCC----
------
FC88X_00675_00635 -----------------AATCA-GGCCTGCGTGGACTGGTG-CGTT---------
------
FC88X_00763_01275 -----------------AATCA-GGCCTGCGTGGACTGGTGGCGTT---------
------
FC88X_00630_01175 -----------------AATCA-GGCCTGCGTGGACTGGTG-CGTTCTT------
------
FC88X_00926_00498 -----------------AATCA-GGCCTGCGTGGAATGGTG-CGTTCTC------
------
FC88X_01107_00567 -----------------AATCATGGCCTGCGTGG-CTGGTG-CGTTCTC------
------
FC88X_00623_01166 -----------------AATCA-GGCCTGAGTGGACTGGTG-CGTTCTC------
------
FC88X_00851_00180 -----------------AATCA-GGCCTGCGTGGACTGGTG-
CGTTCTCCTTCCTTT----
FC88X_00901_00556 -----------------AATCA-GGCCTGCGTGGACTGGTG-
CGTTCTCCTTCCTTT----
FC88X_01106_00835 -----------------AATCA-GGTCTGCGTGGACTGGTG-CGTTCTC------
------
FC88X_00896_00548 -----------------AATCA-GGTCTGCGTGGACTGGTG-CGTTCTC------
------
FC88X_01006_00975 -----------------AATCA-GGTCTGCGTGGACTGGTG-CGTTCTC------
------
FC88X_00454_00008 -----------------AATTCAGGCCTGCGTGGACTGGTG-CGTTCTC------
------
FC88X_00115_00653 -----------------AATCA-GGCCTGCGTGGACTGGTG-
CGTTCTCCCGTTCT-----
FC88X_00924_00699 -----------------AATCA-GACCTGCGTGGACTGGTG-CGTTCTC------
------
FC88X_00022_00581 -----------------AATCA-GGCTTACGTGGACTGGTG-CGTTCTC------
------
FC88X_00246_01056 -----------------AATCA-GGCCTGCGTGGACTGGTG-CGTCTC-------
------
FC88X_00667_00360 -----------------AATCA-GGCTTGCGTGGACTGGTG-CGCTCTC------
------
FC88X_01071_00923 -------------------CCA-AGCTCCCGCGGATTGTTT-CGTCAT-------
------
FC88X_00355_00496 -----------------AGGTTCAGTCTCCCGGGGTCTCATTTCT----------
------
FC88X_01039_00809 --------------------TCGAGCCTGCCGGGGATAGTCTCATC---------
------
FC88X_00353_00207 ---------------------GTGGCTCGCCCCAGCCGATCGCTGTCG-------
------
FC88X_00863_00935 ------------------CTGCTGGCTCTCGC--GCCGAATGCTCTC--------
------
FC88X_00842_00734 ------------------CTACTTCCAATCTGCCGTCGA--CCTGTC--------
------

FC88X_01069_00939 -------------------GCTGGCACTCTCCCGGTGAGCCCTGTC--------
------
FC88X_00036_00453 --------------------CACGCAA-TTACTTGCCGTCTG-TCCTC------
------
FC88X_00350_01007 --------------------TCTGCAA-TTACTTGCCGTCTG-TCCTC------
------
FC88X_00242_01096 -------------------TCGTGCAAGTTAGTCCCAGTCTA-CTCTC------
------
FC88X_00640_00245 --------------------GCTGCAAGTTAGTCCCAGTCTA-CTCTC------
------
FC88X_00539_00215 -------------------CCGTGCGG-TTGGTCGCGGTCGG-TTTTC------
------
FC88X_01082_01015 -------------------CCGTGCGG-TTGGTCGCGGTCGG-TTCT-------
------
FC88X_00939_00674 -------------------CTCTGCAG-TTGATGGCAGTGGC-TTCTC------
------
FC88X_00055_00742 -------------------TCTTGCAA-TTTCCTGCGGTGCAATTCTC------
------
FC88X_00359_01194 -------------------TCTTGCAA-TT-CCTGCGGTGCA-TTCTC------
------
FC88X_00014_00555 -------------------TCTTGCAA-TTTCCTGCGGTGCA-TTCTC------
------
FC88X_00024_00600 -------------------TCTTGCAA-TTTCCTGCGGTGCA-TTCTC------
------
FC88X_00243_00511 -------------------TCTTGCAA-TTTCCTGCGGTGCA-TTCTC------
------
FC88X_00103_00222 -------------------TCTTGCAA-TTTCCTGCGGTGCA-TTCTC------
------
FC88X_00851_01136 -----------GGGGGAATTCTTGCAA-TTTCCTGCGGTGCA-TTCTC------
------
FC88X_00339_00583 -------------------TCTTGCAA-TTTCCTGCGGTGCA-TTCTC------
------
FC88X_00422_00155 -------------------TCTTGCAA-TTTCCTGCGGTGCA-TTCTC------
------
FC88X_00355_00979 ------------------GTCTTGCAA-TTTCCTGCGGTGCA-TTCTC------
------
FC88X_00540_00743 ------------------GTCTTGCAA-TTTCCTGCGGTGCA-TTCTC------
------
FC88X_00603_01090 ------------------GTCTTGCAA-TTTCCTGCGGTGCA-TTCTC------
------
FC88X_00940_00272 ------------------GTCTTGCAA-TTTCCTGCGGTGCA-TTCTC------
------
FC88X_01111_01180 ------------------GTCTTGCAA-TTTCCTGCGGTGCA-TTCTC------
------
FC88X_00088_00061 ------------------GTCTTGCAA-TTTCCTGCGGTGCA-TTCTC------
------
FC88X_00295_00054 -------------------TCTTGCAA-TTTCCTGCGGTGCA-TTCTC------
------
FC88X_00389_00041 -------------------TCTTGCAA-TTTCCTGCGGTGCA-TTCTC------
------
FC88X_00449_01193 -------------------TCTTGCAA-TTTCCTGCGGTGCA-TTCTC------
------
FC88X_00463_00057 -------------------TCTTGCAA-TTTCCTGCGGTGCA-TTCTC------
------
FC88X_00472_00225 -------------------TCTTGCAA-TTTCCTGCGGTGCA-TTCTC------
------

FC88X_00457_00527 -------------------TCTTGCAA-TTTCCTGCGGTGCA-TTCTC------
------
FC88X_00451_01020 -------------------TCTTGCAA-TTTCCTGCGGTGCA-TTCTC------
------
FC88X_00496_01052 -------------------TCTTGCAA-TTTCCTGCGGTGCA-TTCTC------
------
FC88X_00520_00068 -------------------TCTTGCAA-TTTCCTGCGGTGCA-TTCTC------
------
FC88X_00513_00445 -------------------TCTTGCAA-TTTCCTGCGGTGCA-TTCTC------
------
FC88X_00546_00961 -------------------TCTTGCAA-TTTCCTGCGGTGCA-TTCTC------
------
FC88X_00520_01239 -------------------TCTTGCAA-TTTCCTGCGGTGCA-TTCTC------
------
FC88X_00618_01198 -------------------TCTTGCAA-TTTCCTGCGGTGCA-TTCTC------
------
FC88X_00675_01019 -------------------TCTTGCAA-TTTCCTGCGGTGCA-TTCTC------
------
FC88X_00747_00074 -------------------TCTTGCAA-TTTCCTGCGGTGCA-TTCTC------
------
FC88X_00758_00573 -------------------TCTTGCAA-TTTCCTGCGGTGCA-TTCTC------
------
FC88X_00896_00604 -------------------TCTTGCAA-TTTCCTGCGGTGCA-TTCTC------
------
FC88X_00861_00959 -------------------TCTTGCAA-TTTCCTGCGGTGCA-TTCTC------
------
FC88X_00959_00320 -------------------TCTTGCAA-TTTCCTGCGGTGCA-TTCTC------
------
FC88X_00956_00959 -------------------TCTTGCAA-TTTCCTGCGGTGCA-TTCTC------
------
FC88X_00984_01178 -------------------TCTTGCAA-TTTCCTGCGGTGCA-TTCTC------
------
FC88X_01144_00857 -------------------TCTTGCAA-TTTCCTGCGGTGCA-TTCTC------
------
FC88X_01106_00942 -------------------TCTTGCAA-TTTCCTGCGGTGCA-TTCTC------
------
FC88X_00007_00320 -------------------TCTTGCAA-TTTCCTGCGGTGCA-TTCTC------
------
FC88X_00020_00307 -------------------TCTTGCAA-TTTCCTGCGGTGCA-TTCTC------
------
FC88X_00070_00107 -------------------TCTTGCAA-TTTCCTGCGGTGCA-TTCTC------
------
FC88X_00086_00140 -------------------TCTTGCAA-TTTCCTGCGGTGCA-TTCTC------
------
FC88X_00098_00116 -------------------TCTTGCAA-TTTCCTGCGGTGCA-TTCTC------
------
FC88X_00075_00356 -------------------TCTTGCAA-TTTCCTGCGGTGCA-TTCTC------
------
FC88X_00051_00606 -------------------TCTTGCAA-TTTCCTGCGGTGCA-TTCTC------
------
FC88X_00096_00689 -------------------TCTTGCAA-TTTCCTGCGGTGCA-TTCTC------
------
FC88X_00091_00850 -------------------TCTTGCAA-TTTCCTGCGGTGCA-TTCTC------
------
FC88X_00114_00732 -------------------TCTTGCAA-TTTCCTGCGGTGCA-TTCTC------
------

FC88X_00236_01043 -------------------TCTTGCAA-TTTCCTGCGGTGCA-TTCTC------
------
FC88X_00258_00075 -------------------TCTTGCAA-TTTCCTGCGGTGCA-TTCTC------
------
FC88X_00262_01104 -------------------TCTTGCAA-TTTCCTGCGGTGCA-TTCTC------
------
FC88X_00288_01145 -------------------TCTTGCAA-TTTCCTGCGGTGCA-TTCTC------
------
FC88X_00347_00037 -------------------TCTTGCAA-TTTCCTGCGGTGCA-TTCTC------
------
FC88X_00302_00188 -------------------TCTTGCAA-TTTCCTGCGGTGCA-TTCTC------
------
FC88X_00342_00602 -------------------TCTTGCAA-TTTCCTGCGGTGCA-TTCTC------
------
FC88X_00433_00084 -------------------TCTTGCAA-TTTCCTGCGGTGCA-TTCTC------
------
FC88X_00430_00101 -------------------TCTTGCAA-TTTCCTGCGGTGCA-TTCTC------
------
FC88X_00406_00262 -------------------TCTTGCAA-TTTCCTGCGGTGCA-TTCTC------
------
FC88X_00422_01145 -------------------TCTTGCAA-TTTCCTGCGGTGCA-TTCTC------
------
FC88X_00409_01172 -------------------TCTTGCAA-TTTCCTGCGGTGCA-TTCTC------
------
FC88X_00480_00488 -------------------TCTTGCAA-TTTCCTGCGGTGCA-TTCTC------
------
FC88X_00452_00976 -------------------TCTTGCAA-TTTCCTGCGGTGCA-TTCTC------
------
FC88X_00493_01136 -------------------TCTTGCAA-TTTCCTGCGGTGCA-TTCTC------
------
FC88X_00490_01218 -------------------TCTTGCAA-TTTCCTGCGGTGCA-TTCTC------
------
FC88X_00537_00579 -------------------TCTTGCAA-TTTCCTGCGGTGCA-TTCTC------
------
FC88X_00580_00165 -------------------TCTTGCAA-TTTCCTGCGGTGCA-TTCTC------
------
FC88X_00590_01150 -------------------TCTTGCAA-TTTCCTGCGGTGCA-TTCTC------
------
FC88X_00646_00926 -------------------TCTTGCAA-TTTCCTGCGGTGCA-TTCTC------
------
FC88X_00674_00295 -------------------TCTTGCAA-TTTCCTGCGGTGCA-TTCTC------
------
FC88X_00669_01076 -------------------TCTTGCAA-TTTCCTGCGGTGCA-TTCTC------
------
FC88X_00725_01135 -------------------TCTTGCAA-TTTCCTGCGGTGCA-TTCTC------
------
FC88X_00725_01235 -------------------TCTTGCAA-TTTCCTGCGGTGCA-TTCTC------
------
FC88X_00785_00183 -------------------TCTTGCAA-TTTCCTGCGGTGCA-TTCTC------
------
FC88X_00766_00637 -------------------TCTTGCAA-TTTCCTGCGGTGCA-TTCTC------
------
FC88X_00750_01269 -------------------TCTTGCAA-TTTCCTGCGGTGCA-TTCTC------
------
FC88X_00840_00886 -------------------TCTTGCAA-TTTCCTGCGGTGCA-TTCTC------
------

```
FC88X_00891_00260      -------------------TCTTGCAA-TTTCCTGCGGTGCA-TTCTC------
------
FC88X_00882_00618      -------------------TCTTGCAA-TTTCCTGCGGTGCA-TTCTC------
------
FC88X_00895_00603      -------------------TCTTGCAA-TTTCCTGCGGTGCA-TTCTC------
------
FC88X_00870_00956      -------------------TCTTGCAA-TTTCCTGCGGTGCA-TTCTC------
------
FC88X_00902_00227      -------------------TCTTGCAA-TTTCCTGCGGTGCA-TTCTC------
------
FC88X_00949_00795      -------------------TCTTGCAA-TTTCCTGCGGTGCA-TTCTC------
------
FC88X_00925_00833      -------------------TCTTGCAA-TTTCCTGCGGTGCA-TTCTC------
------
FC88X_00911_01153      -------------------TCTTGCAA-TTTCCTGCGGTGCA-TTCTC------
------
FC88X_00959_00223      -------------------TCTTGCAA-TTTCCTGCGGTGCA-TTCTC------
------
FC88X_00994_00669      -------------------TCTTGCAA-TTTCCTGCGGTGCA-TTCTC------
------
FC88X_00975_01142      -------------------TCTTGCAA-TTTCCTGCGGTGCA-TTCTC------
------
FC88X_01013_01262      -------------------TCTTGCAA-TTTCCTGCGGTGCA-TTCTC------
------
FC88X_01062_00356      -------------------TCTTGCAA-TTTCCTGCGGTGCA-TTCTC------
------
FC88X_01067_00837      -------------------TCTTGCAA-TTTCCTGCGGTGCA-TTCTC------
------
FC88X_01125_00783      -------------------TCTTGCAA-TTTCCTGCGGTGCA-TTCTC------
------
FC88X_01118_00992      -------------------TCTTGCAA-TTTCCTGCGGTGCA-TTCTC------
------
FC88X_00309_01101      ------------GGGGAAGTCTTGCAA-TTTCCTGCGGTGCA-TTCTCC-----
------
FC88X_00205_00854      -------------------TCTTGCAA-TTTC-TGCGGTGCA-TTCTC------
------
FC88X_00040_00047      -------------------TCTTGCAA-TTTCCTGCGGTGCATTCCCCC-----
------
FC88X_00848_00957      -------------------TCTTGCAA-TTTCCTGCGGTGCATTCCC-------
------
FC88X_00071_00632      -------------------TCTTGCAA-TTTCCAGCGGTGCA-TTCTC------
------
FC88X_00572_01154      -------------------TCTTGCAAATTTCCTGCGGTGCA-TTCTC------
------
FC88X_00823_00801      -------------------TCTTGCAA-TTTCCTGCGGTGCA-CTCTC------
------
FC88X_01134_01043      -------------------TCTTGCAA-TTTCCTGCGGTGCA-CTCTC------
------
FC88X_00535_01186      -------------------TCTTGCAG-TTTCCTGCGGTGCA-TTCTC------
------
FC88X_00930_00617      -------------------TCTTGCAG-TTTCCTGCGGTGCA-TTCTC------
------
FC88X_00445_01136      -------------------TCTTGCAA-TTTCCTGCAGTGCA-TTCTC------
------
FC88X_00995_00467      -------------------TCTTGCAA-TTTCCTGCAGTGCA-TTCTC------
------
```

FC88X_00182_00771	------------------TCTTAGCAA-TTTCCTGC-GTGCA-TTCTC------
------
FC88X_00855_01096	-------------------TCTTGCAA-TTTCCTGC-GTGCA-TTCTC------
------
FC88X_01129_01050	-------------------TCTTGCAA-TTTCCTGCGGGGCA-TTCTC------
------
FC88X_00558_00168	-------------------TCTTGCAA-TTTCCTGCGGGGCA-TTCTC------
------
FC88X_00060_00669	-------------------TCTTGCAA-TTTCCTGCGGCGCA-TTCTC------
------
FC88X_00998_00399	-------------------TCTTGCAA-TTTCCTGCGGCGCA-TTCTC------
------
FC88X_00241_00031	-------------------CCTTGCAATTTCCTG-CGGTGCA-TTCTC------
------
FC88X_00645_00925	-------------------CCTTGCAATTTCCTG-CGGTGCA-TTCTC------
------
FC88X_01089_01177	------------------CTCTTGCAATTTCCTGGCGGTGCA-TTCTCCC----
------
FC88X_00498_00012	-------------------TCTTGCAA-TTTCCTGCGGTGCG-TTCTC------
------
FC88X_00950_00773	-------------------TCTTGCAA-TTTCCTGCGGTGCG-TTCTC------
------
FC88X_00883_01265	-------------------TCTTGCAA-TCTCCTGCGGTGCA-TTCTC------
------
FC88X_01058_01103	-------------------TCTTGCAAATTTCCTGCGGTGCA-TTCTT------
------
FC88X_00513_01211	-------------------TCTTGCAA-TTTCCCGCGGTGCA-TTCTC------
------
FC88X_00047_00587	-------------------TCTTGCAA-TTCCCTGCGGTGCA-TTCTC------
------
FC88X_00686_01065	-------------------TCTTGCAA-TTCCCTGCGGTGCA-TTCTC------
------
FC88X_00121_00214	-------------------TCTCGCAA-TTTCCTGCTGTGCA-TTCTC------
------
FC88X_00582_01194	-------------------TCTCGCAA-TTTCCTGCGGTGCA-TTCTC------
------
FC88X_00702_00900	-------------------TCCTGCAATTT-CCTGCGGTGCA-TTCTC------
------
FC88X_00883_00530	-------------------TCCTGCAATTT-CCTGCGGTGCA-TTCTC------
------
FC88X_01102_00551	-------------------TCCTGCAATTT-CCTGCGGTGCA-TTCTC------
------
FC88X_00933_01228	-------------------TCCTGCAATTTTCCTGCGGTGCA-TTCTC------
------
FC88X_01103_01136	--------------------TCTGCAATTT-CCTGCGGTGCA-TTCTC------
------
FC88X_00853_00841	-------------------TCCTGCAATTT--CTGCGGTGCA-TTCTC------
------
FC88X_00870_00744	-------------------CCAAGCAG-TTCCATGCTGTGTCTTTCTC------
------
FC88X_00067_00013	-------------------CCAAGCAG-TTCCATGCTGTGTCTTTCTC------
------
FC88X_00178_00994	-------------------CCAAGCAG-TTCCATGCTGTGTCTTTCTC------
------
FC88X_00226_01024	-------------------CCAAGCAG-TTCCATGCTGTGTCTTTCTC------
------

FC88X_00417_00021 -------------------CCAAGCAG-TTCCATGCTGTGTCTTTCTC------
------
FC88X_00661_00784 -------------------CCAAGCAG-TTCCATGCTGTGTCTTTCTC------
------
FC88X_00776_00833 -------------------CCAAGCAG-TTCCATGCTGTGTCTTTCTC------
------
FC88X_00996_00955 -------------------CCAAGCAG-TTCCATGCTGTGTCTTTCTC------
------
FC88X_00998_01185 -------------------CCAAGCAG-
TTCCATGCTGTGTCTTTCTCCTTCCTTT----
FC88X_01116_00802 -------------------CCAAGCAG-CTCCATGCTGTGTCTTTCTC------
------
FC88X_00277_00072 -------------------CCAAGCAG-CTCCATGCTGTGTCTTTCTC------
------
FC88X_00895_01245 -------------------CCAAGTAG-TTCCATGCTGTGTCTTTCTCC-----
------
FC88X_00026_00704 -------------------CCAAGCAG-TTCCATGCTGTGTCCTTCTC------
------
FC88X_00223_00071 -------------------CCGAGCAG-TTCCATGCCGTGTCTTTCTC------
------
FC88X_00057_00567 --------------------CCTTCAA-ATTTCTTGAGTGGACGTCTC------
------
FC88X_00896_00986 ---------------------CTTCAA-ATTTCTTGAGTGGACGTCTC------
------
FC88X_00193_00097 --------------------CCTTCAA-ATTTCTTGAGTGGACGTCTC------
------
FC88X_00430_01164 --------------------CCTTCAA-ATTTCTTGAGTGGACGTCTC------
------
FC88X_00861_00258 --------------------CCTGCAG-ATAGCTGCAGTGTACATCTC------
------
FC88X_00628_00739 -------------------CTCTCCAG-TTTGCTGC-GCTGACCTATC------
------
FC88X_00724_01263 -------------------CTCTCCAG-TTTGCTGC-GTTGACCTATC------
------
FC88X_00521_01064 --------------------GCTCCAA-TCTACTGGAGATTACCTATC------
------
FC88X_00080_00332 --------------------ACGTGGAACTGTTGCCAGTGTATTCTC-------
------
FC88X_00507_00538 -------------------TACGTGGAACTGTTGCCAGTGTATTCTC-------
------
FC88X_00085_00385 --------------------ACGTGGAACTGTTGCCAGTGTATTCTC-------
------
FC88X_00092_00518 --------------------ACGTGGAACTGTTGCCAGTGTATTCTC-------
------
FC88X_00216_00101 --------------------ACGTGGAACTGTTGCCAGTGTATTCTC-------
------
FC88X_00740_00891 --------------------ACGTGGAACTGTTGCCAGTGTATTCTC-------
------
FC88X_00740_00983 --------------------ACGTGGAACTGTTGCCAGTGTATTCTC-------
------
FC88X_00978_00770 --------------------ACGTGGAACTGTTGCCAGTGTATTCTC-------
------
FC88X_01060_01208 --------------------ACGTGGAACTGTTGCCAGTGTATTCTC-------
------
FC88X_01110_00802 --------------------ACGTGGAACTGTTGCCAGTGTATTCTC-------
------

FC88X_00033_00693 --------------------ACGTGGAACTGTTGCCAGTGTATTCTC-------
------
FC88X_00065_00108 --------------------ACGTGGAACTGTTGCCAGTGTATTCTC-------
------
FC88X_00138_00217 --------------------ACGTGGAACTGTTGCCAGTGTATTCTC-------
------
FC88X_00397_00970 --------------------ACGTGGAACTGTTGCCAGTGTATTCTC-------
------
FC88X_00602_00743 --------------------ACGTGGAACTGTTGCCAGTGTATTCTC-------
------
FC88X_00693_00964 --------------------ACGTGGAACTGTTGCCAGTGTATTCTC-------
------
FC88X_00880_01097 --------------------ACGTGGAACTGTTGCCAGTGTATTCTC-------
------
FC88X_01076_00860 --------------------ACGTGGAACTGTTGCCAGTGTATTCTC-------
------
FC88X_01091_00918 --------------------ACGTGGAACTGTTGCCAGTGTATTCTCCC-----
------
FC88X_00061_00357 --------------------ACGTGGAACTGTTCCCAGTGTATTCTC-------
------
FC88X_00974_00493 -------------------GACGTGGAACTGTTGCCAGCGTATTCTC-------
------
FC88X_00622_00072 --------------------ACGTGGAACTGTTGCCGGTGTATTCTC-------
------
FC88X_00970_01273 -------------------------TTACTGTC-CCGGCGTAATCTCCATTAC-
------
FC88X_00166_00076 -------------------TCTCTTACTCTGCTCCCGTTATGTTTTC-------
------
FC88X_00952_00573 ----------------------CTAACACTGGTCCCACCGTTATCGTTT-----
------
FC88X_00059_00703 -------------------CCTCTGGCCGTACTGCTCCCGTTTTCT--------
------
FC88X_00464_00949 -------------------CCTCTGGCCGTACTGCTCCCGTTTTCTCTT-----
------
FC88X_00496_01047 --------------------ACCTGGCCCGCTGATCACCGTTAT----------
------
FC88X_00484_00231 ----------------------CGCATACGTTGGCCCGCGTGATTTCT------
------
FC88X_00153_00663 ----------------GGGGAACGCATACGTTGGCCCGCGTGATTTCT------
------
FC88X_00786_00530 ----------------------CGCATACGTTGGCCCGCGTGATTTCT------
------
FC88X_00619_00997 ----------------------CGCATACGTTGGCCCGCGTGATTTCT------
------
FC88X_00879_00825 ---------------------GCGCATACGTTGGCCCGCGTGATTTCT------
------
FC88X_00069_00131 ----------------------CGCATACGTTGGCCCGCGTGATTTCT------
------
FC88X_00311_00062 ----------------------CGCATACGTTGGCCCGCGTGATTTCT------
------
FC88X_00309_00903 ----------------------CGCATACGTTGGCCCGCGTGATTTCT------
------
FC88X_00475_00919 ----------------------CGCATACGTTGGCCCGCGTGATTTCT------
------
FC88X_00993_00279 ----------------------CGCATACGTTGGCCCGCGTGATTTCT------
------

```
FC88X_00201_00688      ----------------------CGCATACGTTGGCCCGCGTGATTTCC------
------
FC88X_00327_00642      ----------------------
CGCATACGTTGGCCCGCGTGATTTCTCTTCCTTTC---
FC88X_00650_00867      ----------------------CGCATACACTGGCCCGCGTGATTTCT------
------
FC88X_00220_00874      ---------------------CCGCCTACTCTGTACAGC-TGACTTGTT-----
------
FC88X_00496_00399      ---------------------CCGCCTACTCTGTACAGC-TGACTTGTT-----
------
FC88X_00240_01035      --------------------CGTGAATTCTTTACCTGTTCTCCTTTG-------
------
FC88X_01146_00975      --------------------CGTGAATTCTTTACCTGTTCTCCTTTG-------
------
FC88X_00114_00188      --------------------CGTGAATTCTTTACCTGTTCTCCTTTG-------
------
FC88X_01080_00643      --------------------CGTGAATTCTTTACCTGTTCTCCTTTG-------
------
FC88X_01048_00508      ------TTTCATTCTCGCTCCAGGTGATATTCTTCCTTTCTCCCT---------
------
FC88X_00284_00467      ---------------------CCGCACTCCCCGTGGATTTTACCTGAT------
------
FC88X_00133_00057      ------------------TTACCGCGTATCTGAAC--GGGCTGTTCC-------
------
FC88X_00877_00317      ------------------TTACCGCGTATCTGAAC--GGGCTGTTCC-------
------
FC88X_00935_00755      ------------------TTACCGCGTATCTGAAC--GGGCTGTTCC-------
------
FC88X_00155_00519      ------------------TTACCGCGTATCTGAAC--GGGCTGTTCT-------
------
FC88X_00750_00161      --------------------CCGGCGTATCTGTACCTGGTCTGTTCA-------
------
FC88X_00873_00699      --------------------CCGGCGTATCTGTACCTGGTCTGTTCA-------
------
FC88X_00218_00941      ----------------------CGCGAATGGTAACCTGTTCTGCGGATA-----
------
FC88X_00919_00581      ------------GGGGAAGACTGGCGTATGTTAAACGCCGCTGTTCCC------
------
FC88X_00592_00083      --------------------CTGGC-TATCTGAAGCCACACCGCTGTC------
------
FC88X_00121_00762      ------------------TCCCGTAGTAGGTGACTGCGGCTTCTC---------
------
FC88X_00099_00379      ------------------TCCCGTAGTAGGTGAC--CGGCTTCTC---------
------
FC88X_00316_00042      ------------------TCCCTTCGGATGTTACGGCATCTCTGT---------
------
FC88X_00617_01072      -----------------TTCCCTTCGGATGTCACG-CATCTCTGT---------
------
FC88X_00448_00505      ------------------CCACATGGCAGCTGTCCCGCGGTTCTT---------
------
FC88X_00506_01040      ------------------CCACATGGCAGCTGTCCCGCGGTTCTTCT-------
------
FC88X_01113_00838      ------------------CCACATGGCAGCTGTCCCGCGGTTCTTCTT------
------
FC88X_00112_00495      ------------------CCACATGGTAGCTGTCCCGCGGTTCTT---------
------
```

FC88X_00209_00062 ------------------CCACATGGCAGCTGTCCCGCGGTTCCTT--------
------
FC88X_00129_00560 ------------------
CCACATGGCAGCTGTCCCGCGGTTCTTCTTCCTTTCTC----
FC88X_00949_00788 ------------------CCCCATAACACGTGACCCACTATTCTT---------
------
FC88X_00923_01161 ------------------CCCCATAACACGTGACCCACTATTCTT---------
------
FC88X_00973_01243 ------------------CCCCATAACACGTGACCCACTATTCTT---------
------
FC88X_00979_00285 --------------------CCATAGCTC-TGCCCCGC-ATTCTTGTT------
------
FC88X_00697_00147 --------------------CCGTCGCTCCTGACCCGC----------------
------
FC88X_00845_00897 ------------------CTACGTTGGGGTTAACGGCGTGGCGGTA--------
------
FC88X_00926_00416 ------------------TTACGTTGGGGTTAACGGCGTGGCGGTA--------
------
FC88X_00426_01133 ------------------CTACGTTGGGGTTAACAGCGTGGCGGTA--------
------
FC88X_00198_00962 --------------CAGACCACGTCGGCAGAATCTGCGTTA-------------
------
FC88X_01033_01081 -----------------ACTGGTACTCTCCCGACCGCATATACT----------
------
FC88X_00082_00695 -----------------ACTGGTACTCTCCCGACCGCATACATT----------
------
FC88X_00976_00434 ---------------CAACTGGCAGT-TCGCGTCTTCATATCCTTC--------
------
FC88X_00146_00183 -----------------CCTGCAACCGGCTCACTTCCTGGACGT----------
------
FC88X_00882_00387 -----------------CCTCCCGCTACCGCATCTCCTGGTC------------
------
FC88X_00995_00423 -----------------CCTCCCAACGGCATCACGTGTGTTTTG----------
------
FC88X_00778_00617 ------------------CCGTCACCAGTTGGTCATGTTGCTATTC--------
------
FC88X_00880_00584 -------------------CACCGTTGGC-GATAATGGCACTATTCC-------
------
FC88X_01082_00856 ----------------TCTCCCCGTCGGC--ATAGTCACAATATT---------
------
FC88X_00051_00182 ----------------TCTCCCTATCGAC--ATGGCAAGATTCCC---------
------
FC88X_00186_00770 -----------------CCTCCCAGTGGCCGCTGTTGCC-ATGTT---------
------
FC88X_00274_00599 -----------------CCTCCCAGTGGCCGCTGTTGCC-ATGTT---------
------
FC88X_00744_01059 -----------------CCTCCCAGTGGCCGCTGTTGCCCATGTTCC-------
------
FC88X_01047_00862 ------------------CTCCCGGTGGCCGCTGTTGCC-ATGTT---------
------
FC88X_00925_00872 -----------------CCTCCCAGTGGCTGCTGTTGCC-ATGTT---------
------
FC88X_00890_01174 -----------------TCTCCTAATAGCCACT-TGGCATATTTC---------
------
FC88X_00240_00019 --------------ATCACCG-TTGGTTCG-GGGTGCGC---GTTCTC------
------

FC88X_00745_01084 --------------ATCACCG-T-GGTTCG-GG-TGCGC---GTTCTC------
------
FC88X_00014_00112 --------------ATCACCG-TTGGTTCG-GG-TGCGC---GTTCTC------
------
FC88X_00005_00233 --------------ATCACCG-TTGGTTCG-GG-TGCGC---GTTCTC------
------
FC88X_00038_00288 --------------ATCACCG-TTGGTTCG-GG-TGCGC---GTTCTC------
------
FC88X_00039_00345 --------------ATCACCG-TTGGTTCG-GG-TGCGC---GTTCTC------
------
FC88X_00052_00822 --------------ATCACCGGTTGGTTCG-GG-TGCGC---GTTCTC------
------
FC88X_00724_01117 --------------ATCACCG-TTGGTTCG-GG-TGCGC---GTTCT-------
------
FC88X_00033_00139 --------------ATCACCG-TTGGTTCG-GG-TGCGC---GTTCTC------
------
FC88X_00034_00284 --------------ATCACCG-TTGGTTCG-GG-TGCGC---GTTCTC------
------
FC88X_00044_00255 --------------ATCACCG-TTGGTTCG-GG-TGCGC---GTTCTC------
------
FC88X_00012_00605 --------------ATCACCG-TTGGTTCG-GG-TGCGC---GTTCTC------
------
FC88X_00076_00376 --------------ATCACCG-TTGGTTCG-GG-TGCGC---GTTCTC------
------
FC88X_00082_00561 --------------ATCACCG-TTGGTTCG-GG-TGCGC---GTTCTC------
------
FC88X_00016_00226 -------------GATCACCG-TTGGTTCG-GG-TGCGC---GTTCTC------
------
FC88X_00175_00141 -------------GATCACCG-TTGGTTCG-GG-TGCGC---GTTCTC------
------
FC88X_00194_00945 -------------GATCACCG-TTGGTTCG-GG-TGCGC---GTTCTC------
------
FC88X_00227_01054 -------------GATCACCG-TTGGTTCG-GG-TGCGC---GTTCTC------
------
FC88X_00358_00966 -------------GATCACCG-TTGGTTCG-GG-TGCGC---GTTCTC------
------
FC88X_00426_00987 -------------GATCACCG-TTGGTTCG-GG-TGCGC---GTTCTC------
------
FC88X_00648_01042 -------------GATCACCG-TTGGTTCG-GG-TGCGC---GTTCTC------
------
FC88X_00744_01270 -------------GATCACCG-TTGGTTCG-GG-TGCGC---GTTCTC------
------
FC88X_00880_00661 -------------GATCACCG-TTGGTTCG-GG-TGCGC---GTTCTC------
------
FC88X_00939_01051 -------------GATCACCG-TTGGTTCG-GG-TGCGC---GTTCTC------
------
FC88X_00953_00740 -------------GATCACCG-TTGGTTCG-GG-TGCGC---GTTCTC------
------
FC88X_01032_01260 -------------GATCACCG-TTGGTTCG-GG-TGCGC---GTTCTC------
------
FC88X_01066_00776 -------------GATCACCG-TTGGTTCG-GG-TGCGC---GTTCTC------
------
FC88X_00053_00786 -------------GATCACCG-TTGGTTCG-GG-TGCGC---GTTCTC------
------
FC88X_00523_01204 -------------GATCACCG-TTGGTTCG-GG-TGCGC---GTTCTC------
------

FC88X_00660_01259 --------------GATCACCG-TTGGTTCG-GG-TGCGC---GTTCTC------------
FC88X_00741_00181 --------------GATCACCG-TTGGTTCG-GG-TGCGC---GTTCTC------------
FC88X_00915_01171 --------------GATCACCG-TTGGTTCG-GG-TGCGC---GTTCTC------------
FC88X_01101_01041 --------------GATCACCG-TTGGTTCG-GG-TGCGC---GTTCTC------------
FC88X_00040_00587 ---------------ATCACCG-TTGGTTCG-GG-TGCGC---GTTCTC------------
FC88X_00070_00305 ---------------ATCACCG-TTGGTTCG-GG-TGCGC---GTTCTC------------
FC88X_00082_00451 ---------------ATCACCG-TTGGTTCG-GG-TGCGC---GTTCTC------------
FC88X_00083_00775 ---------------ATCACCG-TTGGTTCG-GG-TGCGC---GTTCTC------------
FC88X_00068_00880 ---------------ATCACCG-TTGGTTCG-GG-TGCGC---GTTCTC------------
FC88X_00118_00135 ---------------ATCACCG-TTGGTTCG-GG-TGCGC---GTTCTC------------
FC88X_00106_00498 ---------------ATCACCG-TTGGTTCG-GG-TGCGC---GTTCTC------------
FC88X_00209_00914 ---------------ATCACCG-TTGGTTCG-GG-TGCGC---GCTTCTC-----------
FC88X_00872_01070 ---------------ATCACCG-TTGGTTCG-GG-TGCGC---GCTTCTC-----------
FC88X_01116_00870 ---------------ATCACCG-TTGGTTCG-GG-TGCGC---GCTTCTC-----------
FC88X_00096_00066 ---------------ATCACCG-TTGGTTCG-GG-TGCGC---GTTCTC------------
FC88X_00093_00431 ---------------ATCACCG-TTGGTTCG-GG-TGCGC---GTTCTC------------
FC88X_00096_00717 ---------------ATCACCG-TTGGTTCG-GG-TGCGC---GTTCTC------------
FC88X_00082_00817 ---------------ATCACCG-TTGGTTCG-GG-TGCGC---GTTCTC------------
FC88X_00117_00095 ---------------ATCACCG-TTGGTTCG-GG-TGCGC---GTTCTC------------
FC88X_00103_00247 ---------------ATCACCG-TTGGTTCG-GG-TGCGC---GTTCTC------------
FC88X_00126_00488 ---------------ATCACCG-TTGGTTCG-GG-TGCGC---GTTCTC------------
FC88X_00115_00882 ---------------ATCACCG-TTGGTTCG-GG-TGCGC---GTTCTC------------
FC88X_00108_00913 ---------------ATCACCG-TTGGTTCG-GG-TGCGC---GTTCTC------------
FC88X_00117_00938 ---------------ATCACCG-TTGGTTCG-GG-TGCGC---GTTCTC------------
FC88X_00119_00970 ---------------ATCACCG-TTGGTTCG-GG-TGCGC---GTTCTC------------
FC88X_00156_00067 ---------------ATCACCG-TTGGTTCG-GG-TGCGC---GTTCTC------------
FC88X_00179_00067 ---------------ATCACCG-TTGGTTCG-GG-TGCGC---GTTCTC------------
FC88X_00191_00096 ---------------ATCACCG-TTGGTTCG-GG-TGCGC---GTTCTC------------

FC88X_00188_00147 ---------------ATCACCG-TTGGTTCG-GG-TGCGC---GTTCTC------
------
FC88X_00151_00177 ---------------ATCACCG-TTGGTTCG-GG-TGCGC---GTTCTC------
------
FC88X_00197_00580 ---------------ATCACCG-TTGGTTCG-GG-TGCGC---GTTCTC------
------
FC88X_00169_00749 ---------------ATCACCG-TTGGTTCG-GG-TGCGC---GTTCTC------
------
FC88X_00174_00833 ---------------ATCACCG-TTGGTTCG-GG-TGCGC---GTTCTC------
------
FC88X_00187_00816 ---------------ATCACCG-TTGGTTCG-GG-TGCGC---GTTCTC------
------
FC88X_00172_00879 ---------------ATCACCG-TTGGTTCG-GG-TGCGC---GTTCTC------
------
FC88X_00198_00875 ---------------ATCACCG-TTGGTTCG-GG-TGCGC---GTTCTC------
------
FC88X_00194_00990 ---------------ATCACCG-TTGGTTCG-GG-TGCGC---GTTCTC------
------
FC88X_00209_00055 ---------------ATCACCG-TTGGTTCG-GG-TGCGC---GTTCTC------
------
FC88X_00239_00338 ---------------ATCACCG-TTGGTTCG-GG-TGCGC---GTTCTC------
------
FC88X_00247_00481 ---------------ATCACCG-TTGGTTCG-GG-TGCGC---GTTCTC------
------
FC88X_00207_00890 ---------------ATCACCG-TTGGTTCG-GG-TGCGC---GTTCTC------
------
FC88X_00208_01052 ---------------ATCACCG-TTGGTTCG-GG-TGCGC---GTTCTC------
------
FC88X_00227_01078 ---------------ATCACCG-TTGGTTCG-GG-TGCGC---GTTCTC------
------
FC88X_00280_00016 ---------------ATCACCG-TTGGTTCG-GG-TGCGC---GTTCTC------
------
FC88X_00289_00142 ---------------ATCACCG-TTGGTTCG-GG-TGCGC---GTTCTC------
------
FC88X_00263_00443 ---------------ATCACCG-TTGGTTCG-GG-TGCGC---GTTCTC------
------
FC88X_00289_00475 ---------------ATCACCG-TTGGTTCG-GG-TGCGC---GTTCTC------
------
FC88X_00250_00834 ---------------ATCACCG-TTGGTTCG-GG-TGCGC---GTTCTC------
------
FC88X_00263_01011 ---------------ATCACCG-TTGGTTCG-GG-TGCGC---GTTCTC------
------
FC88X_00284_01097 ---------------ATCACCG-TTGGTTCG-GG-TGCGC---GTTCTC------
------
FC88X_00255_01125 ---------------ATCACCG-TTGGTTCG-GG-TGCGC---GTTCTC------
------
FC88X_00345_00040 ---------------ATCACCG-TTGGTTCG-GG-TGCGC---GTTCTC------
------
FC88X_00342_00283 ---------------ATCACCG-TTGGTTCG-GG-TGCGC---GTTCTC------
------
FC88X_00301_00366 ---------------ATCACCG-TTGGTTCG-GG-TGCGC---GTTCTC------
------
FC88X_00334_00487 ---------------ATCACCG-TTGGTTCG-GG-TGCGC---GTTCTC------
------
FC88X_00343_00662 ---------------ATCACCG-TTGGTTCG-GG-TGCGC---GTTCTC------
------

```
FC88X_00329_00739      --------------ATCACCG-TTGGTTCG-GG-TGCGC---GTTCTC------
------
FC88X_00320_01069      --------------ATCACCG-TTGGTTCG-GG-TGCGC---GTTCTC------
------
FC88X_00336_01078      --------------ATCACCG-TTGGTTCG-GG-TGCGC---GTTCTC------
------
FC88X_00339_01083      --------------ATCACCG-TTGGTTCG-GG-TGCGC---GTTCTC------
------
FC88X_00319_01119      --------------ATCACCG-TTGGTTCG-GG-TGCGC---GTTCTC------
------
FC88X_00390_00082      --------------ATCACCG-TTGGTTCG-GG-TGCGC---GTTCTC------
------
FC88X_00381_00281      --------------ATCACCG-TTGGTTCG-GG-TGCGC---GTTCTC------
------
FC88X_00373_00437      --------------ATCACCG-TTGGTTCG-GG-TGCGC---GTTCTC------
------
FC88X_00380_00578      --------------ATCACCG-TTGGTTCG-GG-TGCGC---GTTCTC------
------
FC88X_00369_00621      --------------ATCACCG-TTGGTTCG-GG-TGCGC---GTTCTC------
------
FC88X_00376_00627      --------------ATCACCG-TTGGTTCG-GG-TGCGC---GTTCTC------
------
FC88X_00394_00644      --------------ATCACCG-TTGGTTCG-GG-TGCGC---GTTCTC------
------
FC88X_00383_00789      --------------ATCACCG-TTGGTTCG-GG-TGCGC---GTTCTC------
------
FC88X_00355_00819      --------------ATCACCG-TTGGTTCG-GG-TGCGC---GTTCTC------
------
FC88X_00377_00877      --------------ATCACCG-TTGGTTCG-GG-TGCGC---GTTCTC------
------
FC88X_00380_00893      --------------ATCACCG-TTGGTTCG-GG-TGCGC---GTTCTC------
------
FC88X_00388_01113      --------------ATCACCG-TTGGTTCG-GG-TGCGC---GTTCTC------
------
FC88X_00411_00016      --------------ATCACCG-TTGGTTCG-GG-TGCGC---GTTCTC------
------
FC88X_00441_00014      --------------ATCACCG-TTGGTTCG-GG-TGCGC---GTTCTC------
------
FC88X_00448_00142      --------------ATCACCG-TTGGTTCG-GG-TGCGC---GTTCTC------
------
FC88X_00411_00284      --------------ATCACCG-TTGGTTCG-GG-TGCGC---GTTCTC------
------
FC88X_00407_00319      --------------ATCACCG-TTGGTTCG-GG-TGCGC---GTTCTC------
------
FC88X_00426_00648      --------------ATCACCG-TTGGTTCG-GG-TGCGC---GTTCTC------
------
FC88X_00406_00987      --------------ATCACCG-TTGGTTCG-GG-TGCGC---GTTCTC------
------
FC88X_00420_01026      --------------ATCACCG-TTGGTTCG-GG-TGCGC---GTTCTC------
------
FC88X_00411_01194      --------------ATCACCG-TTGGTTCG-GG-TGCGC---GTTCTC------
------
FC88X_00418_01150      --------------ATCACCG-TTGGTTCG-GG-TGCGC---GTTCTC------
------
FC88X_00433_01154      --------------ATCACCG-TTGGTTCG-GG-TGCGC---GTTCTC------
------
```

FC88X_00407_01207 --------------ATCACCG-TTGGTTCG-GG-TGCGC---GTTCTC------
------
FC88X_00431_01225 --------------ATCACCG-TTGGTTCG-GG-TGCGC---GTTCTC------
------
FC88X_00465_00078 --------------ATCACCG-TTGGTTCG-GG-TGCGC---GTTCTC------
------
FC88X_00452_00859 --------------ATCACCG-TTGGTTCG-GG-TGCGC---GTTCTC------
------
FC88X_00465_00964 --------------ATCACCG-TTGGTTCG-GG-TGCGC---GTTCTC------
------
FC88X_00486_00967 --------------ATCACCG-TTGGTTCG-GG-TGCGC---GTTCTC------
------
FC88X_00470_01197 --------------ATCACCG-TTGGTTCG-GG-TGCGC---GTTCTC------
------
FC88X_00474_01242 --------------ATCACCG-TTGGTTCG-GG-TGCGC---GTTCTC------
------
FC88X_00504_00005 --------------ATCACCG-TTGGTTCG-GG-TGCGC---GTTCTC------
------
FC88X_00548_00049 --------------ATCACCG-TTGGTTCG-GG-TGCGC---GTTCTC------
------
FC88X_00519_00161 --------------ATCACCG-TTGGTTCG-GG-TGCGC---GTTCTC------
------
FC88X_00530_00196 --------------ATCACCG-TTGGTTCG-GG-TGCGC---GTTCTC------
------
FC88X_00505_00834 --------------ATCACCG-TTGGTTCG-GG-TGCGC---GTTCTC------
------
FC88X_00513_00840 --------------ATCACCG-TTGGTTCG-GG-TGCGC---GTTCTC------
------
FC88X_00517_00995 --------------ATCACCG-TTGGTTCG-GG-TGCGC---GTTCTC------
------
FC88X_00518_01001 --------------ATCACCG-TTGGTTCG-GG-TGCGC---GTTCTC------
------
FC88X_00588_00191 --------------ATCACCG-TTGGTTCG-GG-TGCGC---GTTCTC------
------
FC88X_00591_00656 --------------ATCACCG-TTGGTTCG-GG-TGCGC---GTTCTC------
------
FC88X_00591_00711 --------------ATCACCG-TTGGTTCG-GG-TGCGC---GTTCTC------
------
FC88X_00567_00833 --------------ATCACCG-TTGGTTCG-GG-TGCGC---GTTCTC------
------
FC88X_00574_00801 --------------ATCACCG-TTGGTTCG-GG-TGCGC---GTTCTC------
------
FC88X_00556_00910 --------------ATCACCG-TTGGTTCG-GG-TGCGC---GTTCTC------
------
FC88X_00597_00937 --------------ATCACCG-TTGGTTCG-GG-TGCGC---GTTCTC------
------
FC88X_00551_00975 --------------ATCACCG-TTGGTTCG-GG-TGCGC---GTTCTC------
------
FC88X_00572_00996 --------------ATCACCG-TTGGTTCG-GG-TGCGC---GTTCTC------
------
FC88X_00557_01031 --------------ATCACCG-TTGGTTCG-GG-TGCGC---GTTCTC------
------
FC88X_00567_01015 --------------ATCACCG-TTGGTTCG-GG-TGCGC---GTTCTC------
------
FC88X_00586_01174 --------------ATCACCG-TTGGTTCG-GG-TGCGC---GTTCTC------
------

FC88X_00550_01215 --------------ATCACCG-TTGGTTCG-GG-TGCGC---GTTCTC------
------
FC88X_00552_01262 --------------ATCACCG-TTGGTTCG-GG-TGCGC---GTTCTC------
------
FC88X_00569_01252 --------------ATCACCG-TTGGTTCG-GG-TGCGC---GTTCTC------
------
FC88X_00638_00149 --------------ATCACCG-TTGGTTCG-GG-TGCGC---GTTCTC------
------
FC88X_00615_00460 --------------ATCACCG-TTGGTTCG-GG-TGCGC---GTTCTC------
------
FC88X_00631_00888 --------------ATCACCG-TTGGTTCG-GG-TGCGC---GTTCTC------
------
FC88X_00635_00960 --------------ATCACCG-TTGGTTCG-GG-TGCGC---GTTCTC------
------
FC88X_00649_00976 --------------ATCACCG-TTGGTTCG-GG-TGCGC---GTTCTC------
------
FC88X_00611_01036 --------------ATCACCG-TTGGTTCG-GG-TGCGC---GTTCTC------
------
FC88X_00609_01072 --------------ATCACCG-TTGGTTCG-GG-TGCGC---GTTCTC------
------
FC88X_00610_01198 --------------ATCACCG-TTGGTTCG-GG-TGCGC---GTTCTC------
------
FC88X_00637_01239 --------------ATCACCG-TTGGTTCG-GG-TGCGC---GTTCTC------
------
FC88X_00621_01270 --------------ATCACCG-TTGGTTCG-GG-TGCGC---GTTCTC------
------
FC88X_00683_00064 --------------ATCACCG-TTGGTTCG-GG-TGCGC---GTTCTC------
------
FC88X_00651_00303 --------------ATCACCG-TTGGTTCG-GG-TGCGC---GTTCTC------
------
FC88X_00673_00617 --------------ATCACCG-TTGGTTCG-GG-TGCGC---GTTCTC------
------
FC88X_00678_00718 --------------ATCACCG-TTGGTTCG-GG-TGCGC---GTTCTC------
------
FC88X_00691_01156 --------------ATCACCG-TTGGTTCG-GG-TGCGC---GTTCTC------
------
FC88X_00665_01240 --------------ATCACCG-TTGGTTCG-GG-TGCGC---GTTCTC------
------
FC88X_00727_00055 --------------ATCACCG-TTGGTTCG-GG-TGCGC---GTTCTC------
------
FC88X_00714_00297 --------------ATCACCG-TTGGTTCG-GG-TGCGC---GTTCTC------
------
FC88X_00717_00284 --------------ATCACCG-TTGGTTCG-GG-TGCGC---GTTCTC------
------
FC88X_00738_00312 --------------ATCACCG-TTGGTTCG-GG-TGCGC---GTTCTC------
------
FC88X_00705_00793 --------------ATCACCG-TTGGTTCG-GG-TGCGC---GTTCTC------
------
FC88X_00702_00921 --------------ATCACCG-TTGGTTCG-GG-TGCGC---GTTCTC------
------
FC88X_00739_00919 --------------ATCACCG-TTGGTTCG-GG-TGCGC---GTTCTC------
------
FC88X_00704_01051 --------------ATCACCG-TTGGTTCG-GG-TGCGC---GTTCTC------
------
FC88X_00707_01055 --------------ATCACCG-TTGGTTCG-GG-TGCGC---GTTCTC------
------

FC88X_00730_01087 --------------ATCACCG-TTGGTTCG-GG-TGCGC---GTTCTC------
------
FC88X_00702_01157 --------------ATCACCG-TTGGTTCG-GG-TGCGC---GTTCTC------
------
FC88X_00716_01175 --------------ATCACCG-TTGGTTCG-GG-TGCGC---GTTCTC------
------
FC88X_00700_01222 --------------ATCACCG-TTGGTTCG-GG-TGCGC---GTTCTC------
------
FC88X_00717_01215 --------------ATCACCG-TTGGTTCG-GG-TGCGC---GTTCTC------
------
FC88X_00761_00168 --------------ATCACCG-TTGGTTCG-GG-TGCGC---GTTCTC------
------
FC88X_00770_00311 --------------ATCACCG-TTGGTTCG-GG-TGCGC---GTTCTC------
------
FC88X_00783_00313 --------------ATCACCG-TTGGTTCG-GG-TGCGC---GTTCTC------
------
FC88X_00754_00516 --------------ATCACCG-TTGGTTCG-GG-TGCGC---GTTCTC------
------
FC88X_00782_00700 --------------ATCACCG-TTGGTTCG-GG-TGCGC---GTTCTC------
------
FC88X_00764_00886 --------------ATCACCG-TTGGTTCG-GG-TGCGC---GTTCTC------
------
FC88X_00793_00876 --------------ATCACCG-TTGGTTCG-GG-TGCGC---GTTCTC------
------
FC88X_00766_00905 --------------ATCACCG-TTGGTTCG-GG-TGCGC---GTTCTC------
------
FC88X_00787_01016 --------------ATCACCG-TTGGTTCG-GG-TGCGC---GTTCTC------
------
FC88X_00756_01226 --------------ATCACCG-TTGGTTCG-GG-TGCGC---GTTCTC------
------
FC88X_00780_01203 --------------ATCACCG-TTGGTTCG-GG-TGCGC---GTTCTC------
------
FC88X_00757_01266 --------------ATCACCG-TTGGTTCG-GG-TGCGC---GTTCTC------
------
FC88X_00803_00119 --------------ATCACCG-TTGGTTCG-GG-TGCGC---GTTCTC------
------
FC88X_00848_00117 --------------ATCACCG-TTGGTTCG-GG-TGCGC---GTTCTC------
------
FC88X_00817_00232 --------------ATCACCG-TTGGTTCG-GG-TGCGC---GTTCTC------
------
FC88X_00845_00214 --------------ATCACCG-TTGGTTCG-GG-TGCGC---GTTCTC------
------
FC88X_00849_00547 --------------ATCACCG-TTGGTTCG-GG-TGCGC---GTTCTC------
------
FC88X_00829_00630 --------------ATCACCG-TTGGTTCG-GG-TGCGC---GTTCTC------
------
FC88X_00806_00884 --------------ATCACCG-TTGGTTCG-GG-TGCGC---GTTCTC------
------
FC88X_00808_00948 --------------ATCACCG-TTGGTTCG-GG-TGCGC---GTTCTC------
------
FC88X_00813_00965 --------------ATCACCG-TTGGTTCG-GG-TGCGC---GTTCTC------
------
FC88X_00835_01139 --------------ATCACCG-TTGGTTCG-GG-TGCGC---GTTCTC------
------
FC88X_00842_01112 --------------ATCACCG-TTGGTTCG-GG-TGCGC---GTTCTC------
------

FC88X_00806_01155 --------------ATCACCG-TTGGTTCG-GG-TGCGC---GTTCTC------
------
FC88X_00847_01235 --------------ATCACCG-TTGGTTCG-GG-TGCGC---GTTCTC------
------
FC88X_00806_01252 --------------ATCACCG-TTGGTTCG-GG-TGCGC---GTTCTC------
------
FC88X_00887_00283 --------------ATCACCG-TTGGTTCG-GG-TGCGC---GTTCTC------
------
FC88X_00887_00419 --------------ATCACCG-TTGGTTCG-GG-TGCGC---GTTCTC------
------
FC88X_00897_00527 --------------ATCACCG-TTGGTTCG-GG-TGCGC---GTTCTC------
------
FC88X_00886_00592 --------------ATCACCG-TTGGTTCG-GG-TGCGC---GTTCTC------
------
FC88X_00899_00566 --------------ATCACCG-TTGGTTCG-GG-TGCGC---GTTCTC------
------
FC88X_00889_00649 --------------ATCACCG-TTGGTTCG-GG-TGCGC---GTTCTC------
------
FC88X_00889_00690 --------------ATCACCG-TTGGTTCG-GG-TGCGC---GTTCTC------
------
FC88X_00874_00700 --------------ATCACCG-TTGGTTCG-GG-TGCGC---GTTCTC------
------
FC88X_00892_00792 --------------ATCACCG-TTGGTTCG-GG-TGCGC---GTTCTC------
------
FC88X_00879_00987 --------------ATCACCG-TTGGTTCG-GG-TGCGC---GTTCTC------
------
FC88X_00870_01074 --------------ATCACCG-TTGGTTCG-GG-TGCGC---GTTCTC------
------
FC88X_00871_01178 --------------ATCACCG-TTGGTTCG-GG-TGCGC---GTTCTC------
------
FC88X_00903_00245 --------------ATCACCG-TTGGTTCG-GG-TGCGC---GTTCTC------
------
FC88X_00914_00223 --------------ATCACCG-TTGGTTCG-GG-TGCGC---GTTCTC------
------
FC88X_00936_00369 --------------ATCACCG-TTGGTTCG-GG-TGCGC---GTTCTC------
------
FC88X_00927_00402 --------------ATCACCG-TTGGTTCG-GG-TGCGC---GTTCTC------
------
FC88X_00906_00459 --------------ATCACCG-TTGGTTCG-GG-TGCGC---GTTCTC------
------
FC88X_00938_00529 --------------ATCACCG-TTGGTTCG-GG-TGCGC---GTTCTC------
------
FC88X_00945_00565 --------------ATCACCG-TTGGTTCG-GG-TGCGC---GTTCTC------
------
FC88X_00929_00642 --------------ATCACCG-TTGGTTCG-GG-TGCGC---GTTCTC------
------
FC88X_00912_00897 --------------ATCACCG-TTGGTTCG-GG-TGCGC---GTTCTC------
------
FC88X_00928_00857 --------------ATCACCG-TTGGTTCG-GG-TGCGC---GTTCTC------
------
FC88X_00933_00997 --------------ATCACCG-TTGGTTCG-GG-TGCGC---GTTCTC------
------
FC88X_00941_00965 --------------ATCACCG-TTGGTTCG-GG-TGCGC---GTTCTC------
------
FC88X_00906_01077 --------------ATCACCG-TTGGTTCG-GG-TGCGC---GTTCTC------
------

FC88X_00903_01109 --------------ATCACCG-TTGGTTCG-GG-TGCGC---GTTCTC------
------
FC88X_00909_01185 --------------ATCACCG-TTGGTTCG-GG-TGCGC---GTTCTC------
------
FC88X_00939_01200 --------------ATCACCG-TTGGTTCG-GG-TGCGC---GTTCTC------
------
FC88X_00988_00246 --------------ATCACCG-TTGGTTCG-GG-TGCGC---GTTCTC------
------
FC88X_00970_00269 --------------ATCACCG-TTGGTTCG-GG-TGCGC---GTTCTC------
------
FC88X_00988_00311 --------------ATCACCG-TTGGTTCG-GG-TGCGC---GTTCTC------
------
FC88X_00988_00318 --------------ATCACCG-TTGGTTCG-GG-TGCGC---GTTCTC------
------
FC88X_00982_00390 --------------ATCACCG-TTGGTTCG-GG-TGCGC---GTTCTC------
------
FC88X_00984_00405 --------------ATCACCG-TTGGTTCG-GG-TGCGC---GTTCTC------
------
FC88X_00954_00591 --------------ATCACCG-TTGGTTCG-GG-TGCGC---GTTCTC------
------
FC88X_00970_00572 --------------ATCACCG-TTGGTTCG-GG-TGCGC---GTTCTC------
------
FC88X_00996_00559 --------------ATCACCG-TTGGTTCG-GG-TGCGC---GTTCTC------
------
FC88X_00962_00645 --------------ATCACCG-TTGGTTCG-GG-TGCGC---GTTCTC------
------
FC88X_00991_00655 --------------ATCACCG-TTGGTTCG-GG-TGCGC---GTTCTC------
------
FC88X_00961_00762 --------------ATCACCG-TTGGTTCG-GG-TGCGC---GTTCTC------
------
FC88X_00989_00765 --------------ATCACCG-TTGGTTCG-GG-TGCGC---GTTCTC------
------
FC88X_00990_00881 --------------ATCACCG-TTGGTTCG-GG-TGCGC---GTTCTC------
------
FC88X_00995_00893 --------------ATCACCG-TTGGTTCG-GG-TGCGC---GTTCTC------
------
FC88X_00965_01030 --------------ATCACCG-TTGGTTCG-GG-TGCGC---GTTCTC------
------
FC88X_00959_01082 --------------ATCACCG-TTGGTTCG-GG-TGCGC---GTTCTC------
------
FC88X_00951_01171 --------------ATCACCG-TTGGTTCG-GG-TGCGC---GTTCTC------
------
FC88X_00973_01180 --------------ATCACCG-TTGGTTCG-GG-TGCGC---GTTCTC------
------
FC88X_00973_01204 --------------ATCACCG-TTGGTTCG-GG-TGCGC---GTTCTC------
------
FC88X_01005_00465 --------------ATCACCG-TTGGTTCG-GG-TGCGC---GTTCTC------
------
FC88X_01015_00541 --------------ATCACCG-TTGGTTCG-GG-TGCGC---GTTCTC------
------
FC88X_01026_00518 --------------ATCACCG-TTGGTTCG-GG-TGCGC---GTTCTC------
------
FC88X_01007_00566 --------------ATCACCG-TTGGTTCG-GG-TGCGC---GTTCTC------
------
FC88X_01041_00595 --------------ATCACCG-TTGGTTCG-GG-TGCGC---GTTCTC------
------

FC88X_01010_00609 --------------ATCACCG-TTGGTTCG-GG-TGCGC---GTTCTC------
------
FC88X_01002_00701 --------------ATCACCG-TTGGTTCG-GG-TGCGC---GTTCTC------
------
FC88X_01042_00705 --------------ATCACCG-TTGGTTCG-GG-TGCGC---GTTCTC------
------
FC88X_01024_00829 --------------ATCACCG-TTGGTTCG-GG-TGCGC---GTTCTC------
------
FC88X_01014_00891 --------------ATCACCG-TTGGTTCG-GG-TGCGC---GTTCTC------
------
FC88X_01014_00913 --------------ATCACCG-TTGGTTCG-GG-TGCGC---GTTCTC------
------
FC88X_01014_00997 --------------ATCACCG-TTGGTTCG-GG-TGCGC---GTTCTC------
------
FC88X_01028_01136 --------------ATCACCG-TTGGTTCG-GG-TGCGC---GTTCTC------
------
FC88X_01000_01177 --------------ATCACCG-TTGGTTCG-GG-TGCGC---GTTCTC------
------
FC88X_01028_01210 --------------ATCACCG-TTGGTTCG-GG-TGCGC---GTTCTC------
------
FC88X_01083_00407 --------------ATCACCG-TTGGTTCG-GG-TGCGC---GTTCTC------
------
FC88X_01097_00493 --------------ATCACCG-TTGGTTCG-GG-TGCGC---GTTCTC------
------
FC88X_01081_00655 --------------ATCACCG-TTGGTTCG-GG-TGCGC---GTTCTC------
------
FC88X_01056_00744 --------------ATCACCG-TTGGTTCG-GG-TGCGC---GTTCTC------
------
FC88X_01097_00771 --------------ATCACCG-TTGGTTCG-GG-TGCGC---GTTCTC------
------
FC88X_01068_00903 --------------ATCACCG-TTGGTTCG-GG-TGCGC---GTTCTC------
------
FC88X_01086_00905 --------------ATCACCG-TTGGTTCG-GG-TGCGC---GTTCTC------
------
FC88X_01092_00928 --------------ATCACCG-TTGGTTCG-GG-TGCGC---GTTCTC------
------
FC88X_01074_01003 --------------ATCACCG-TTGGTTCG-GG-TGCGC---GTTCTC------
------
FC88X_01068_01092 --------------ATCACCG-TTGGTTCG-GG-TGCGC---GTTCTC------
------
FC88X_01061_01219 --------------ATCACCG-TTGGTTCG-GG-TGCGC---GTTCTC------
------
FC88X_01086_01238 --------------ATCACCG-TTGGTTCG-GG-TGCGC---GTTCTC------
------
FC88X_01105_00462 --------------ATCACCG-TTGGTTCG-GG-TGCGC---GTTCTC------
------
FC88X_01112_00585 --------------ATCACCG-TTGGTTCG-GG-TGCGC---GTTCTC------
------
FC88X_01122_00642 --------------ATCACCG-TTGGTTCG-GG-TGCGC---GTTCTC------
------
FC88X_01111_00673 --------------ATCACCG-TTGGTTCG-GG-TGCGC---GTTCTC------
------
FC88X_01133_00742 --------------ATCACCG-TTGGTTCG-GG-TGCGC---GTTCTC------
------
FC88X_01120_00757 --------------ATCACCG-TTGGTTCG-GG-TGCGC---GTTCTC------
------

FC88X_01129_00770 --------------ATCACCG-TTGGTTCG-GG-TGCGC---GTTCTC------
------
FC88X_01137_00795 --------------ATCACCG-TTGGTTCG-GG-TGCGC---GTTCTC------
------
FC88X_01106_00849 --------------ATCACCG-TTGGTTCG-GG-TGCGC---GTTCTC------
------
FC88X_01129_00802 --------------ATCACCG-TTGGTTCG-GG-TGCGC---GTTCTC------
------
FC88X_01129_00891 --------------ATCACCG-TTGGTTCG-GG-TGCGC---GTTCTC------
------
FC88X_01142_00867 --------------ATCACCG-TTGGTTCG-GG-TGCGC---GTTCTC------
------
FC88X_01107_00921 --------------ATCACCG-TTGGTTCG-GG-TGCGC---GTTCTC------
------
FC88X_01108_00954 --------------ATCACCG-TTGGTTCG-GG-TGCGC---GTTCTC------
------
FC88X_01129_00990 --------------ATCACCG-TTGGTTCG-GG-TGCGC---GTTCTC------
------
FC88X_01123_01023 --------------ATCACCG-TTGGTTCG-GG-TGCGC---GTTCTC------
------
FC88X_01100_01076 --------------ATCACCG-TTGGTTCG-GG-TGCGC---GTTCTC------
------
FC88X_01100_01078 --------------ATCACCG-TTGGTTCG-GG-TGCGC---GTTCTC------
------
FC88X_01103_01178 --------------ATCACCG-TTGGTTCG-GG-TGCGC---GTTCTC------
------
FC88X_01123_01153 --------------ATCACCG-TTGGTTCG-GG-TGCGC---GTTCTC------
------
FC88X_00010_00080 --------------ATCACCG-TTGGTTCG-GG-TGCGC---GTTCTC------
------
FC88X_00023_00097 --------------ATCACCG-TTGGTTCG-GG-TGCGC---GTTCTC------
------
FC88X_00026_00094 --------------ATCACCG-TTGGTTCG-GG-TGCGC---GTTCTC------
------
FC88X_00049_00075 --------------ATCACCG-TTGGTTCG-GG-TGCGC---GTTCTC------
------
FC88X_00032_00141 --------------ATCACCG-TTGGTTCG-GG-TGCGC---GTTCTC------
------
FC88X_00048_00122 --------------ATCACCG-TTGGTTCG-GG-TGCGC---GTTCTC------
------
FC88X_00007_00152 --------------ATCACCG-TTGGTTCG-GG-TGCGC---GTTCTC------
------
FC88X_00046_00168 --------------ATCACCG-TTGGTTCG-GG-TGCGC---GTTCTC------
------
FC88X_00041_00257 --------------ATCACCG-TTGGTTCG-GG-TGCGC---GTTCTC------
------
FC88X_00043_00268 --------------ATCACCG-TTGGTTCG-GG-TGCGC---GTTCTC------
------
FC88X_00015_00314 --------------ATCACCG-TTGGTTCG-GG-TGCGC---GTTCTC------
------
FC88X_00022_00323 --------------ATCACCG-TTGGTTCG-GG-TGCGC---GTTCTC------
------
FC88X_00046_00377 --------------ATCACCG-TTGGTTCG-GG-TGCGC---GTTCTC------
------
FC88X_00034_00490 --------------ATCACCG-TTGGTTCG-GG-TGCGC---GTTCTC------
------

FC88X_00036_00547 --------------ATCACCG-TTGGTTCG-GG-TGCGC---GTTCTC------
------
FC88X_00004_00580 --------------ATCACCG-TTGGTTCG-GG-TGCGC---GTTCTC------
------
FC88X_00009_00589 --------------ATCACCG-TTGGTTCG-GG-TGCGC---GTTCTC------
------
FC88X_00023_00569 --------------ATCACCG-TTGGTTCG-GG-TGCGC---GTTCTC------
------
FC88X_00032_00577 --------------ATCACCG-TTGGTTCG-GG-TGCGC---GTTCTC------
------
FC88X_00040_00647 --------------ATCACCG-TTGGTTCG-GG-TGCGC---GTTCTC------
------
FC88X_00015_00669 --------------ATCACCG-TTGGTTCG-GG-TGCGC---GTTCTC------
------
FC88X_00045_00656 --------------ATCACCG-TTGGTTCG-GG-TGCGC---GTTCTC------
------
FC88X_00037_00725 --------------ATCACCG-TTGGTTCG-GG-TGCGC---GTTCTC------
------
FC88X_00057_00011 --------------ATCACCG-TTGGTTCG-GG-TGCGC---GTTCTC------
------
FC88X_00070_00066 --------------ATCACCG-TTGGTTCG-GG-TGCGC---GTTCTC------
------
FC88X_00086_00078 --------------ATCACCG-TTGGTTCG-GG-TGCGC---GTTCTC------
------
FC88X_00050_00107 --------------ATCACCG-TTGGTTCG-GG-TGCGC---GTTCTC------
------
FC88X_00073_00103 --------------ATCACCG-TTGGTTCG-GG-TGCGC---GTTCTC------
------
FC88X_00073_00134 --------------ATCACCG-TTGGTTCG-GG-TGCGC---GTTCTC------
------
FC88X_00079_00106 --------------ATCACCG-TTGGTTCG-GG-TGCGC---GTTCTC------
------
FC88X_00067_00159 --------------ATCACCG-TTGGTTCG-GG-TGCGC---GTTCTC------
------
FC88X_00070_00160 --------------ATCACCG-TTGGTTCG-GG-TGCGC---GTTCTC------
------
FC88X_00090_00187 --------------ATCACCG-TTGGTTCG-GG-TGCGC---GTTCTC------
------
FC88X_00096_00156 --------------ATCACCG-TTGGTTCG-GG-TGCGC---GTTCTC------
------
FC88X_00056_00231 --------------ATCACCG-TTGGTTCG-GG-TGCGC---GTTCTC------
------
FC88X_00081_00254 --------------ATCACCG-TTGGTTCG-GG-TGCGC---GTTCTC------
------
FC88X_00082_00333 --------------ATCACCG-TTGGTTCG-GG-TGCGC---GTTCTC------
------
FC88X_00050_00369 --------------ATCACCG-TTGGTTCG-GG-TGCGC---GTTCTC------
------
FC88X_00057_00369 --------------ATCACCG-TTGGTTCG-GG-TGCGC---GTTCTC------
------
FC88X_00059_00395 --------------ATCACCG-TTGGTTCG-GG-TGCGC---GTTCTC------
------
FC88X_00088_00353 --------------ATCACCG-TTGGTTCG-GG-TGCGC---GTTCTC------
------
FC88X_00093_00359 --------------ATCACCG-TTGGTTCG-GG-TGCGC---GTTCTC------
------

FC88X_00051_00461 ---------------ATCACCG-TTGGTTCG-GG-TGCGC---GTTCTC------------
FC88X_00073_00456 ---------------ATCACCG-TTGGTTCG-GG-TGCGC---GTTCTC------------
FC88X_00062_00509 ---------------ATCACCG-TTGGTTCG-GG-TGCGC---GTTCTC------------
FC88X_00081_00527 ---------------ATCACCG-TTGGTTCG-GG-TGCGC---GTTCTC------------
FC88X_00058_00566 ---------------ATCACCG-TTGGTTCG-GG-TGCGC---GTTCTC------------
FC88X_00094_00629 ---------------ATCACCG-TTGGTTCG-GG-TGCGC---GTTCTC------------
FC88X_00054_00741 ---------------ATCACCG-TTGGTTCG-GG-TGCGC---GTTCTC------------
FC88X_00071_00725 ---------------ATCACCG-TTGGTTCG-GG-TGCGC---GTTCTC------------
FC88X_00069_00762 ---------------ATCACCG-TTGGTTCG-GG-TGCGC---GTTCTC------------
FC88X_00093_00792 ---------------ATCACCG-TTGGTTCG-GG-TGCGC---GTTCTC------------
FC88X_00051_00847 ---------------ATCACCG-TTGGTTCG-GG-TGCGC---GTTCTC------------
FC88X_00054_00805 ---------------ATCACCG-TTGGTTCG-GG-TGCGC---GTTCTC------------
FC88X_00071_00809 ---------------ATCACCG-TTGGTTCG-GG-TGCGC---GTTCTC------------
FC88X_00108_00027 ---------------ATCACCG-TTGGTTCG-GG-TGCGC---GTTCTC------------
FC88X_00119_00044 ---------------ATCACCG-TTGGTTCG-GG-TGCGC---GTTCTC------------
FC88X_00105_00065 ---------------ATCACCG-TTGGTTCG-GG-TGCGC---GTTCTC------------
FC88X_00129_00084 ---------------ATCACCG-TTGGTTCG-GG-TGCGC---GTTCTC------------
FC88X_00133_00067 ---------------ATCACCG-TTGGTTCG-GG-TGCGC---GTTCTC------------
FC88X_00148_00090 ---------------ATCACCG-TTGGTTCG-GG-TGCGC---GTTCTC------------
FC88X_00121_00161 ---------------ATCACCG-TTGGTTCG-GG-TGCGC---GTTCTC------------
FC88X_00130_00164 ---------------ATCACCG-TTGGTTCG-GG-TGCGC---GTTCTC------------
FC88X_00145_00187 ---------------ATCACCG-TTGGTTCG-GG-TGCGC---GTTCTC------------
FC88X_00119_00266 ---------------ATCACCG-TTGGTTCG-GG-TGCGC---GTTCTC------------
FC88X_00138_00250 ---------------ATCACCG-TTGGTTCG-GG-TGCGC---GTTCTC------------
FC88X_00101_00362 ---------------ATCACCG-TTGGTTCG-GG-TGCGC---GTTCTC------------
FC88X_00120_00449 ---------------ATCACCG-TTGGTTCG-GG-TGCGC---GTTCTC------------
FC88X_00144_00467 ---------------ATCACCG-TTGGTTCG-GG-TGCGC---GTTCTC------------
FC88X_00141_00509 ---------------ATCACCG-TTGGTTCG-GG-TGCGC---GTTCTC------------

```
FC88X_00137_00621       --------------ATCACCG-TTGGTTCG-GG-TGCGC---GTTCTC------
------
FC88X_00101_00792       --------------ATCACCG-TTGGTTCG-GG-TGCGC---GTTCTC------
------
FC88X_00124_00818       --------------ATCACCG-TTGGTTCG-GG-TGCGC---GTTCTC------
------
FC88X_00104_00896       --------------ATCACCG-TTGGTTCG-GG-TGCGC---GTTCTC------
------
FC88X_00146_00863       --------------ATCACCG-TTGGTTCG-GG-TGCGC---GTTCTC------
------
FC88X_00114_00929       --------------ATCACCG-TTGGTTCG-GG-TGCGC---GTTCTC------
------
FC88X_00117_00904       --------------ATCACCG-TTGGTTCG-GG-TGCGC---GTTCTC------
------
FC88X_00134_00998       --------------ATCACCG-TTGGTTCG-GG-TGCGC---GTTCTC------
------
FC88X_00186_00059       --------------ATCACCG-TTGGTTCG-GG-TGCGC---GTTCTC------
------
FC88X_00196_00053       --------------ATCACCG-TTGGTTCG-GG-TGCGC---GTTCTC------
------
FC88X_00159_00117       --------------ATCACCG-TTGGTTCG-GG-TGCGC---GTTCTC------
------
FC88X_00161_00127       --------------ATCACCG-TTGGTTCG-GG-TGCGC---GTTCTC------
------
FC88X_00172_00147       --------------ATCACCG-TTGGTTCG-GG-TGCGC---GTTCTC------
------
FC88X_00176_00121       --------------ATCACCG-TTGGTTCG-GG-TGCGC---GTTCTC------
------
FC88X_00194_00128       --------------ATCACCG-TTGGTTCG-GG-TGCGC---GTTCTC------
------
FC88X_00151_00552       --------------ATCACCG-TTGGTTCG-GG-TGCGC---GTTCTC------
------
FC88X_00159_00590       --------------ATCACCG-TTGGTTCG-GG-TGCGC---GTTCTC------
------
FC88X_00162_00588       --------------ATCACCG-TTGGTTCG-GG-TGCGC---GTTCTC------
------
FC88X_00172_00647       --------------ATCACCG-TTGGTTCG-GG-TGCGC---GTTCTC------
------
FC88X_00181_00769       --------------ATCACCG-TTGGTTCG-GG-TGCGC---GTTCTC------
------
FC88X_00172_00869       --------------ATCACCG-TTGGTTCG-GG-TGCGC---GTTCTC------
------
FC88X_00172_00938       --------------ATCACCG-TTGGTTCG-GG-TGCGC---GTTCTC------
------
FC88X_00175_00908       --------------ATCACCG-TTGGTTCG-GG-TGCGC---GTTCTC------
------
FC88X_00179_00946       --------------ATCACCG-TTGGTTCG-GG-TGCGC---GTTCTC------
------
FC88X_00192_00974       --------------ATCACCG-TTGGTTCG-GG-TGCGC---GTTCTC------
------
FC88X_00180_01047       --------------ATCACCG-TTGGTTCG-GG-TGCGC---GTTCTC------
------
FC88X_00198_01009       --------------ATCACCG-TTGGTTCG-GG-TGCGC---GTTCTC------
------
FC88X_00249_00026       --------------ATCACCG-TTGGTTCG-GG-TGCGC---GTTCTC------
------
```

FC88X_00220_00703 ---------------ATCACCG-TTGGTTCG-GG-TGCGC---GTTCTC------
------
FC88X_00231_00785 ---------------ATCACCG-TTGGTTCG-GG-TGCGC---GTTCTC------
------
FC88X_00222_00880 ---------------ATCACCG-TTGGTTCG-GG-TGCGC---GTTCTC------
------
FC88X_00230_00890 ---------------ATCACCG-TTGGTTCG-GG-TGCGC---GTTCTC------
------
FC88X_00237_00885 ---------------ATCACCG-TTGGTTCG-GG-TGCGC---GTTCTC------
------
FC88X_00238_00891 ---------------ATCACCG-TTGGTTCG-GG-TGCGC---GTTCTC------
------
FC88X_00201_00917 ---------------ATCACCG-TTGGTTCG-GG-TGCGC---GTTCTC------
------
FC88X_00206_00943 ---------------ATCACCG-TTGGTTCG-GG-TGCGC---GTTCTC------
------
FC88X_00221_00974 ---------------ATCACCG-TTGGTTCG-GG-TGCGC---GTTCTC------
------
FC88X_00212_01070 ---------------ATCACCG-TTGGTTCG-GG-TGCGC---GTTCTC------
------
FC88X_00239_01058 ---------------ATCACCG-TTGGTTCG-GG-TGCGC---GTTCTC------
------
FC88X_00252_00019 ---------------ATCACCG-TTGGTTCG-GG-TGCGC---GTTCTC------
------
FC88X_00267_00039 ---------------ATCACCG-TTGGTTCG-GG-TGCGC---GTTCTC------
------
FC88X_00294_00018 ---------------ATCACCG-TTGGTTCG-GG-TGCGC---GTTCTC------
------
FC88X_00267_00647 ---------------ATCACCG-TTGGTTCG-GG-TGCGC---GTTCTC------
------
FC88X_00255_00853 ---------------ATCACCG-TTGGTTCG-GG-TGCGC---GTTCTC------
------
FC88X_00264_00940 ---------------ATCACCG-TTGGTTCG-GG-TGCGC---GTTCTC------
------
FC88X_00273_00923 ---------------ATCACCG-TTGGTTCG-GG-TGCGC---GTTCTC------
------
FC88X_00292_00941 ---------------ATCACCG-TTGGTTCG-GG-TGCGC---GTTCTC------
------
FC88X_00258_00951 ---------------ATCACCG-TTGGTTCG-GG-TGCGC---GTTCTC------
------
FC88X_00282_01031 ---------------ATCACCG-TTGGTTCG-GG-TGCGC---GTTCTC------
------
FC88X_00288_01049 ---------------ATCACCG-TTGGTTCG-GG-TGCGC---GTTCTC------
------
FC88X_00254_01074 ---------------ATCACCG-TTGGTTCG-GG-TGCGC---GTTCTC------
------
FC88X_00285_01121 ---------------ATCACCG-TTGGTTCG-GG-TGCGC---GTTCTC------
------
FC88X_00289_01102 ---------------ATCACCG-TTGGTTCG-GG-TGCGC---GTTCTC------
------
FC88X_00292_01153 ---------------ATCACCG-TTGGTTCG-GG-TGCGC---GTTCTC------
------
FC88X_00307_00053 ---------------ATCACCG-TTGGTTCG-GG-TGCGC---GTTCTC------
------
FC88X_00332_00530 ---------------ATCACCG-TTGGTTCG-GG-TGCGC---GTTCTC------
------

FC88X_00347_00971 --------------ATCACCG-TTGGTTCG-GG-TGCGC---GTTCTC------
------
FC88X_00336_01024 --------------ATCACCG-TTGGTTCG-GG-TGCGC---GTTCTC------
------
FC88X_00341_01020 --------------ATCACCG-TTGGTTCG-GG-TGCGC---GTTCTC------
------
FC88X_00308_01092 --------------ATCACCG-TTGGTTCG-GG-TGCGC---GTTCTC------
------
FC88X_00323_01080 --------------ATCACCG-TTGGTTCG-GG-TGCGC---GTTCTC------
------
FC88X_00330_01176 --------------ATCACCG-TTGGTTCG-GG-TGCGC---GTTCTC------
------
FC88X_00393_00018 --------------ATCACCG-TTGGTTCG-GG-TGCGC---GTTCTC------
------
FC88X_00380_00070 --------------ATCACCG-TTGGTTCG-GG-TGCGC---GTTCTC------
------
FC88X_00392_00085 --------------ATCACCG-TTGGTTCG-GG-TGCGC---GTTCTC------
------
FC88X_00365_00138 --------------ATCACCG-TTGGTTCG-GG-TGCGC---GTTCTC------
------
FC88X_00361_00430 --------------ATCACCG-TTGGTTCG-GG-TGCGC---GTTCTC------
------
FC88X_00388_00448 --------------ATCACCG-TTGGTTCG-GG-TGCGC---GTTCTC------
------
FC88X_00350_00523 --------------ATCACCG-TTGGTTCG-GG-TGCGC---GTTCTC------
------
FC88X_00388_00536 --------------ATCACCG-TTGGTTCG-GG-TGCGC---GTTCTC------
------
FC88X_00388_00713 --------------ATCACCG-TTGGTTCG-GG-TGCGC---GTTCTC------
------
FC88X_00393_00728 --------------ATCACCG-TTGGTTCG-GG-TGCGC---GTTCTC------
------
FC88X_00394_00788 --------------ATCACCG-TTGGTTCG-GG-TGCGC---GTTCTC------
------
FC88X_00362_00974 --------------ATCACCG-TTGGTTCG-GG-TGCGC---GTTCTC------
------
FC88X_00370_01149 --------------ATCACCG-TTGGTTCG-GG-TGCGC---GTTCTC------
------
FC88X_00398_01149 --------------ATCACCG-TTGGTTCG-GG-TGCGC---GTTCTC------
------
FC88X_00409_00055 --------------ATCACCG-TTGGTTCG-GG-TGCGC---GTTCTC------
------
FC88X_00437_00063 --------------ATCACCG-TTGGTTCG-GG-TGCGC---GTTCTC------
------
FC88X_00447_00134 --------------ATCACCG-TTGGTTCG-GG-TGCGC---GTTCTC------
------
FC88X_00410_00500 --------------ATCACCG-TTGGTTCG-GG-TGCGC---GTTCTC------
------
FC88X_00427_00662 --------------ATCACCG-TTGGTTCG-GG-TGCGC---GTTCTC------
------
FC88X_00402_00756 --------------ATCACCG-TTGGTTCG-GG-TGCGC---GTTCTC------
------
FC88X_00428_00862 --------------ATCACCG-TTGGTTCG-GG-TGCGC---GTTCTC------
------
FC88X_00401_01030 --------------ATCACCG-TTGGTTCG-GG-TGCGC---GTTCTC------
------

FC88X_00440_01044 ---------------ATCACCG-TTGGTTCG-GG-TGCGC---GTTCTC------
------
FC88X_00417_01080 ---------------ATCACCG-TTGGTTCG-GG-TGCGC---GTTCTC------
------
FC88X_00410_01151 ---------------ATCACCG-TTGGTTCG-GG-TGCGC---GTTCTC------
------
FC88X_00453_00016 ---------------ATCACCG-TTGGTTCG-GG-TGCGC---GTTCTC------
------
FC88X_00459_00028 ---------------ATCACCG-TTGGTTCG-GG-TGCGC---GTTCTC------
------
FC88X_00460_00009 ---------------ATCACCG-TTGGTTCG-GG-TGCGC---GTTCTC------
------
FC88X_00487_00027 ---------------ATCACCG-TTGGTTCG-GG-TGCGC---GTTCTC------
------
FC88X_00495_00039 ---------------ATCACCG-TTGGTTCG-GG-TGCGC---GTTCTC------
------
FC88X_00466_00167 ---------------ATCACCG-TTGGTTCG-GG-TGCGC---GTTCTC------
------
FC88X_00476_00170 ---------------ATCACCG-TTGGTTCG-GG-TGCGC---GTTCTC------
------
FC88X_00469_00295 ---------------ATCACCG-TTGGTTCG-GG-TGCGC---GTTCTC------
------
FC88X_00491_00349 ---------------ATCACCG-TTGGTTCG-GG-TGCGC---GTTCTC------
------
FC88X_00454_00422 ---------------ATCACCG-TTGGTTCG-GG-TGCGC---GTTCTC------
------
FC88X_00492_00436 ---------------ATCACCG-TTGGTTCG-GG-TGCGC---GTTCTC------
------
FC88X_00498_00505 ---------------ATCACCG-TTGGTTCG-GG-TGCGC---GTTCTC------
------
FC88X_00494_00978 ---------------ATCACCG-TTGGTTCG-GG-TGCGC---GTTCTC------
------
FC88X_00454_01024 ---------------ATCACCG-TTGGTTCG-GG-TGCGC---GTTCTC------
------
FC88X_00492_01060 ---------------ATCACCG-TTGGTTCG-GG-TGCGC---GTTCTC------
------
FC88X_00478_01101 ---------------ATCACCG-TTGGTTCG-GG-TGCGC---GTTCTC------
------
FC88X_00452_01184 ---------------ATCACCG-TTGGTTCG-GG-TGCGC---GTTCTC------
------
FC88X_00469_01184 ---------------ATCACCG-TTGGTTCG-GG-TGCGC---GTTCTC------
------
FC88X_00471_01175 ---------------ATCACCG-TTGGTTCG-GG-TGCGC---GTTCTC------
------
FC88X_00478_01180 ---------------ATCACCG-TTGGTTCG-GG-TGCGC---GTTCTC------
------
FC88X_00457_01205 ---------------ATCACCG-TTGGTTCG-GG-TGCGC---GTTCTC------
------
FC88X_00506_00024 ---------------ATCACCG-TTGGTTCG-GG-TGCGC---GTTCTC------
------
FC88X_00529_00085 ---------------ATCACCG-TTGGTTCG-GG-TGCGC---GTTCTC------
------
FC88X_00518_00189 ---------------ATCACCG-TTGGTTCG-GG-TGCGC---GTTCTC------
------
FC88X_00548_00197 ---------------ATCACCG-TTGGTTCG-GG-TGCGC---GTTCTC------
------

FC88X_00513_00221 ---------------ATCACCG-TTGGTTCG-GG-TGCGC---GTTCTC------
------
FC88X_00537_00458 ---------------ATCACCG-TTGGTTCG-GG-TGCGC---GTTCTC------
------
FC88X_00509_00949 ---------------ATCACCG-TTGGTTCG-GG-TGCGC---GTTCTC------
------
FC88X_00538_00955 ---------------ATCACCG-TTGGTTCG-GG-TGCGC---GTTCTC------
------
FC88X_00500_01033 ---------------ATCACCG-TTGGTTCG-GG-TGCGC---GTTCTC------
------
FC88X_00529_01091 ---------------ATCACCG-TTGGTTCG-GG-TGCGC---GTTCTC------
------
FC88X_00518_01142 ---------------ATCACCG-TTGGTTCG-GG-TGCGC---GTTCTC------
------
FC88X_00535_01254 ---------------ATCACCG-TTGGTTCG-GG-TGCGC---GTTCTC------
------
FC88X_00575_00060 ---------------ATCACCG-TTGGTTCG-GG-TGCGC---GTTCTC------
------
FC88X_00580_00087 ---------------ATCACCG-TTGGTTCG-GG-TGCGC---GTTCTC------
------
FC88X_00565_00159 ---------------ATCACCG-TTGGTTCG-GG-TGCGC---GTTCTC------
------
FC88X_00565_00245 ---------------ATCACCG-TTGGTTCG-GG-TGCGC---GTTCTC------
------
FC88X_00572_00244 ---------------ATCACCG-TTGGTTCG-GG-TGCGC---GTTCTC------
------
FC88X_00576_00222 ---------------ATCACCG-TTGGTTCG-GG-TGCGC---GTTCTC------
------
FC88X_00563_00257 ---------------ATCACCG-TTGGTTCG-GG-TGCGC---GTTCTC------
------
FC88X_00581_00266 ---------------ATCACCG-TTGGTTCG-GG-TGCGC---GTTCTC------
------
FC88X_00579_00944 ---------------ATCACCG-TTGGTTCG-GG-TGCGC---GTTCTC------
------
FC88X_00558_01037 ---------------ATCACCG-TTGGTTCG-GG-TGCGC---GTTCTC------
------
FC88X_00560_01015 ---------------ATCACCG-TTGGTTCG-GG-TGCGC---GTTCTC------
------
FC88X_00573_01029 ---------------ATCACCG-TTGGTTCG-GG-TGCGC---GTTCTC------
------
FC88X_00566_01125 ---------------ATCACCG-TTGGTTCG-GG-TGCGC---GTTCTC------
------
FC88X_00582_01111 ---------------ATCACCG-TTGGTTCG-GG-TGCGC---GTTCTC------
------
FC88X_00591_01183 ---------------ATCACCG-TTGGTTCG-GG-TGCGC---GTTCTC------
------
FC88X_00598_01154 ---------------ATCACCG-TTGGTTCG-GG-TGCGC---GTTCTC------
------
FC88X_00563_01205 ---------------ATCACCG-TTGGTTCG-GG-TGCGC---GTTCTC------
------
FC88X_00592_01254 ---------------ATCACCG-TTGGTTCG-GG-TGCGC---GTTCTC------
------
FC88X_00616_00131 ---------------ATCACCG-TTGGTTCG-GG-TGCGC---GTTCTC------
------
FC88X_00620_00115 ---------------ATCACCG-TTGGTTCG-GG-TGCGC---GTTCTC------
------

FC88X_00628_00121 ---------------ATCACCG-TTGGTTCG-GG-TGCGC---GTTCTC------
------
FC88X_00642_00141 ---------------ATCACCG-TTGGTTCG-GG-TGCGC---GTTCTC------
------
FC88X_00647_00378 ---------------ATCACCG-TTGGTTCG-GG-TGCGC---GTTCTC------
------
FC88X_00642_00598 ---------------ATCACCG-TTGGTTCG-GG-TGCGC---GTTCTC------
------
FC88X_00611_00936 ---------------ATCACCG-TTGGTTCG-GG-TGCGC---GTTCTC------
------
FC88X_00626_00945 ---------------ATCACCG-TTGGTTCG-GG-TGCGC---GTTCTC------
------
FC88X_00604_01049 ---------------ATCACCG-TTGGTTCG-GG-TGCGC---GTTCTC------
------
FC88X_00624_01005 ---------------ATCACCG-TTGGTTCG-GG-TGCGC---GTTCTC------
------
FC88X_00649_01147 ---------------ATCACCG-TTGGTTCG-GG-TGCGC---GTTCTC------
------
FC88X_00618_01152 ---------------ATCACCG-TTGGTTCG-GG-TGCGC---GTTCTC------
------
FC88X_00644_01151 ---------------ATCACCG-TTGGTTCG-GG-TGCGC---GTTCTC------
------
FC88X_00621_01216 ---------------ATCACCG-TTGGTTCG-GG-TGCGC---GTTCTC------
------
FC88X_00622_01222 ---------------ATCACCG-TTGGTTCG-GG-TGCGC---GTTCTC------
------
FC88X_00631_01241 ---------------ATCACCG-TTGGTTCG-GG-TGCGC---GTTCTC------
------
FC88X_00652_00143 ---------------ATCACCG-TTGGTTCG-GG-TGCGC---GTTCTC------
------
FC88X_00690_00248 ---------------ATCACCG-TTGGTTCG-GG-TGCGC---GTTCTC------
------
FC88X_00662_00355 ---------------ATCACCG-TTGGTTCG-GG-TGCGC---GTTCTC------
------
FC88X_00675_00371 ---------------ATCACCG-TTGGTTCG-GG-TGCGC---GTTCTC------
------
FC88X_00664_00438 ---------------ATCACCG-TTGGTTCG-GG-TGCGC---GTTCTC------
------
FC88X_00685_00462 ---------------ATCACCG-TTGGTTCG-GG-TGCGC---GTTCTC------
------
FC88X_00667_00771 ---------------ATCACCG-TTGGTTCG-GG-TGCGC---GTTCTC------
------
FC88X_00690_00886 ---------------ATCACCG-TTGGTTCG-GG-TGCGC---GTTCTC------
------
FC88X_00699_00882 ---------------ATCACCG-TTGGTTCG-GG-TGCGC---GTTCTC------
------
FC88X_00674_00981 ---------------ATCACCG-TTGGTTCG-GG-TGCGC---GTTCTC------
------
FC88X_00667_01026 ---------------ATCACCG-TTGGTTCG-GG-TGCGC---GTTCTC------
------
FC88X_00679_01033 ---------------ATCACCG-TTGGTTCG-GG-TGCGC---GTTCTC------
------
FC88X_00698_01135 ---------------ATCACCG-TTGGTTCG-GG-TGCGC---GTTCTC------
------
FC88X_00687_01169 ---------------ATCACCG-TTGGTTCG-GG-TGCGC---GTTCTC------
------

FC88X_00666_01241 --------------ATCACCG-TTGGTTCG-GG-TGCGC---GTTCTC------
------
FC88X_00680_01255 --------------ATCACCG-TTGGTTCG-GG-TGCGC---GTTCTC------
------
FC88X_00732_00248 --------------ATCACCG-TTGGTTCG-GG-TGCGC---GTTCTC------
------
FC88X_00732_00259 --------------ATCACCG-TTGGTTCG-GG-TGCGC---GTTCTC------
------
FC88X_00728_00390 --------------ATCACCG-TTGGTTCG-GG-TGCGC---GTTCTC------
------
FC88X_00734_00549 --------------ATCACCG-TTGGTTCG-GG-TGCGC---GTTCTC------
------
FC88X_00749_00668 --------------ATCACCG-TTGGTTCG-GG-TGCGC---GTTCTC------
------
FC88X_00726_00849 --------------ATCACCG-TTGGTTCG-GG-TGCGC---GTTCTC------
------
FC88X_00747_00909 --------------ATCACCG-TTGGTTCG-GG-TGCGC---GTTCTC------
------
FC88X_00704_00963 --------------ATCACCG-TTGGTTCG-GG-TGCGC---GTTCTC------
------
FC88X_00714_00980 --------------ATCACCG-TTGGTTCG-GG-TGCGC---GTTCTC------
------
FC88X_00719_00977 --------------ATCACCG-TTGGTTCG-GG-TGCGC---GTTCTC------
------
FC88X_00726_01096 --------------ATCACCG-TTGGTTCG-GG-TGCGC---GTTCTC------
------
FC88X_00729_01136 --------------ATCACCG-TTGGTTCG-GG-TGCGC---GTTCTC------
------
FC88X_00707_01153 --------------ATCACCG-TTGGTTCG-GG-TGCGC---GTTCTC------
------
FC88X_00713_01161 --------------ATCACCG-TTGGTTCG-GG-TGCGC---GTTCTC------
------
FC88X_00720_01175 --------------ATCACCG-TTGGTTCG-GG-TGCGC---GTTCTC------
------
FC88X_00735_01197 --------------ATCACCG-TTGGTTCG-GG-TGCGC---GTTCTC------
------
FC88X_00700_01212 --------------ATCACCG-TTGGTTCG-GG-TGCGC---GTTCTC------
------
FC88X_00725_01270 --------------ATCACCG-TTGGTTCG-GG-TGCGC---GTTCTC------
------
FC88X_00758_00131 --------------ATCACCG-TTGGTTCG-GG-TGCGC---GTTCTC------
------
FC88X_00781_00118 --------------ATCACCG-TTGGTTCG-GG-TGCGC---GTTCTC------
------
FC88X_00774_00219 --------------ATCACCG-TTGGTTCG-GG-TGCGC---GTTCTC------
------
FC88X_00771_00264 --------------ATCACCG-TTGGTTCG-GG-TGCGC---GTTCTC------
------
FC88X_00791_00291 --------------ATCACCG-TTGGTTCG-GG-TGCGC---GTTCTC------
------
FC88X_00792_00316 --------------ATCACCG-TTGGTTCG-GG-TGCGC---GTTCTC------
------
FC88X_00756_00578 --------------ATCACCG-TTGGTTCG-GG-TGCGC---GTTCTC------
------
FC88X_00778_00854 --------------ATCACCG-TTGGTTCG-GG-TGCGC---GTTCTC------
------

FC88X_00789_00979 ---------------ATCACCG-TTGGTTCG-GG-TGCGC---GTTCTC------
------
FC88X_00775_01018 ---------------ATCACCG-TTGGTTCG-GG-TGCGC---GTTCTC------
------
FC88X_00751_01071 ---------------ATCACCG-TTGGTTCG-GG-TGCGC---GTTCTC------
------
FC88X_00798_01089 ---------------ATCACCG-TTGGTTCG-GG-TGCGC---GTTCTC------
------
FC88X_00783_01152 ---------------ATCACCG-TTGGTTCG-GG-TGCGC---GTTCTC------
------
FC88X_00770_01234 ---------------ATCACCG-TTGGTTCG-GG-TGCGC---GTTCTC------
------
FC88X_00773_01228 ---------------ATCACCG-TTGGTTCG-GG-TGCGC---GTTCTC------
------
FC88X_00773_01244 ---------------ATCACCG-TTGGTTCG-GG-TGCGC---GTTCTC------
------
FC88X_00804_00181 ---------------ATCACCG-TTGGTTCG-GG-TGCGC---GTTCTC------
------
FC88X_00829_00160 ---------------ATCACCG-TTGGTTCG-GG-TGCGC---GTTCTC------
------
FC88X_00835_00256 ---------------ATCACCG-TTGGTTCG-GG-TGCGC---GTTCTC------
------
FC88X_00849_00252 ---------------ATCACCG-TTGGTTCG-GG-TGCGC---GTTCTC------
------
FC88X_00818_00307 ---------------ATCACCG-TTGGTTCG-GG-TGCGC---GTTCTC------
------
FC88X_00820_00326 ---------------ATCACCG-TTGGTTCG-GG-TGCGC---GTTCTC------
------
FC88X_00836_00339 ---------------ATCACCG-TTGGTTCG-GG-TGCGC---GTTCTC------
------
FC88X_00828_00400 ---------------ATCACCG-TTGGTTCG-GG-TGCGC---GTTCTC------
------
FC88X_00830_00449 ---------------ATCACCG-TTGGTTCG-GG-TGCGC---GTTCTC------
------
FC88X_00839_00511 ---------------ATCACCG-TTGGTTCG-GG-TGCGC---GTTCTC------
------
FC88X_00808_00703 ---------------ATCACCG-TTGGTTCG-GG-TGCGC---GTTCTC------
------
FC88X_00809_00873 ---------------ATCACCG-TTGGTTCG-GG-TGCGC---GTTCTC------
------
FC88X_00849_00872 ---------------ATCACCG-TTGGTTCG-GG-TGCGC---GTTCTC------
------
FC88X_00845_00977 ---------------ATCACCG-TTGGTTCG-GG-TGCGC---GTTCTC------
------
FC88X_00847_01080 ---------------ATCACCG-TTGGTTCG-GG-TGCGC---GTTCTC------
------
FC88X_00843_01120 ---------------ATCACCG-TTGGTTCG-GG-TGCGC---GTTCTC------
------
FC88X_00807_01150 ---------------ATCACCG-TTGGTTCG-GG-TGCGC---GTTCTC------
------
FC88X_00826_01221 ---------------ATCACCG-TTGGTTCG-GG-TGCGC---GTTCTC------
------
FC88X_00830_01245 ---------------ATCACCG-TTGGTTCG-GG-TGCGC---GTTCTC------
------
FC88X_00811_01251 ---------------ATCACCG-TTGGTTCG-GG-TGCGC---GTTCTC------
------

```
FC88X_00816_01270       --------------ATCACCG-TTGGTTCG-GG-TGCGC---GTTCTC------
------
FC88X_00853_00173       --------------ATCACCG-TTGGTTCG-GG-TGCGC---GTTCTC------
------
FC88X_00898_00539       --------------ATCACCG-TTGGTTCG-GG-TGCGC---GTTCTC------
------
FC88X_00888_00696       --------------ATCACCG-TTGGTTCG-GG-TGCGC---GTTCTC------
------
FC88X_00892_00662       --------------ATCACCG-TTGGTTCG-GG-TGCGC---GTTCTC------
------
FC88X_00893_00819       --------------ATCACCG-TTGGTTCG-GG-TGCGC---GTTCTC------
------
FC88X_00882_00861       --------------ATCACCG-TTGGTTCG-GG-TGCGC---GTTCTC------
------
FC88X_00862_00941       --------------ATCACCG-TTGGTTCG-GG-TGCGC---GTTCTC------
------
FC88X_00890_00914       --------------ATCACCG-TTGGTTCG-GG-TGCGC---GTTCTC------
------
FC88X_00883_00954       --------------ATCACCG-TTGGTTCG-GG-TGCGC---GTTCTC------
------
FC88X_00882_01236       --------------ATCACCG-TTGGTTCG-GG-TGCGC---GTTCTC------
------
FC88X_00886_01242       --------------ATCACCG-TTGGTTCG-GG-TGCGC---GTTCTC------
------
FC88X_00906_00224       --------------ATCACCG-TTGGTTCG-GG-TGCGC---GTTCTC------
------
FC88X_00933_00240       --------------ATCACCG-TTGGTTCG-GG-TGCGC---GTTCTC------
------
FC88X_00936_00245       --------------ATCACCG-TTGGTTCG-GG-TGCGC---GTTCTC------
------
FC88X_00928_00348       --------------ATCACCG-TTGGTTCG-GG-TGCGC---GTTCTC------
------
FC88X_00945_00407       --------------ATCACCG-TTGGTTCG-GG-TGCGC---GTTCTC------
------
FC88X_00936_00549       --------------ATCACCG-TTGGTTCG-GG-TGCGC---GTTCTC------
------
FC88X_00913_00550       --------------ATCACCG-TTGGTTCG-GG-TGCGC---GTTCTC------
------
FC88X_00941_00566       --------------ATCACCG-TTGGTTCG-GG-TGCGC---GTTCTC------
------
FC88X_00944_00587       --------------ATCACCG-TTGGTTCG-GG-TGCGC---GTTCTC------
------
FC88X_00903_00673       --------------ATCACCG-TTGGTTCG-GG-TGCGC---GTTCTC------
------
FC88X_00912_00701       --------------ATCACCG-TTGGTTCG-GG-TGCGC---GTTCTC------
------
FC88X_00928_00770       --------------ATCACCG-TTGGTTCG-GG-TGCGC---GTTCTC------
------
FC88X_00938_00774       --------------ATCACCG-TTGGTTCG-GG-TGCGC---GTTCTC------
------
FC88X_00915_00820       --------------ATCACCG-TTGGTTCG-GG-TGCGC---GTTCTC------
------
FC88X_00901_00878       --------------ATCACCG-TTGGTTCG-GG-TGCGC---GTTCTC------
------
FC88X_00912_00855       --------------ATCACCG-TTGGTTCG-GG-TGCGC---GTTCTC------
------
```

FC88X_00939_00871 --------------ATCACCG-TTGGTTCG-GG-TGCGC---GTTCTC------
------
FC88X_00906_00943 --------------ATCACCG-TTGGTTCG-GG-TGCGC---GTTCTC------
------
FC88X_00912_00917 --------------ATCACCG-TTGGTTCG-GG-TGCGC---GTTCTC------
------
FC88X_00923_00943 --------------ATCACCG-TTGGTTCG-GG-TGCGC---GTTCTC------
------
FC88X_00925_00923 --------------ATCACCG-TTGGTTCG-GG-TGCGC---GTTCTC------
------
FC88X_00913_00982 --------------ATCACCG-TTGGTTCG-GG-TGCGC---GTTCTC------
------
FC88X_00914_00987 --------------ATCACCG-TTGGTTCG-GG-TGCGC---GTTCTC------
------
FC88X_00938_00956 --------------ATCACCG-TTGGTTCG-GG-TGCGC---GTTCTC------
------
FC88X_00944_00980 --------------ATCACCG-TTGGTTCG-GG-TGCGC---GTTCTC------
------
FC88X_00903_01020 --------------ATCACCG-TTGGTTCG-GG-TGCGC---GTTCTC------
------
FC88X_00907_01056 --------------ATCACCG-TTGGTTCG-GG-TGCGC---GTTCTC------
------
FC88X_00936_01067 --------------ATCACCG-TTGGTTCG-GG-TGCGC---GTTCTC------
------
FC88X_00939_01057 --------------ATCACCG-TTGGTTCG-GG-TGCGC---GTTCTC------
------
FC88X_00914_01139 --------------ATCACCG-TTGGTTCG-GG-TGCGC---GTTCTC------
------
FC88X_00924_01142 --------------ATCACCG-TTGGTTCG-GG-TGCGC---GTTCTC------
------
FC88X_00907_01223 --------------ATCACCG-TTGGTTCG-GG-TGCGC---GTTCTC------
------
FC88X_00917_01244 --------------ATCACCG-TTGGTTCG-GG-TGCGC---GTTCTC------
------
FC88X_00914_01254 --------------ATCACCG-TTGGTTCG-GG-TGCGC---GTTCTC------
------
FC88X_00995_00373 --------------ATCACCG-TTGGTTCG-GG-TGCGC---GTTCTC------
------
FC88X_00976_00433 --------------ATCACCG-TTGGTTCG-GG-TGCGC---GTTCTC------
------
FC88X_00987_00417 --------------ATCACCG-TTGGTTCG-GG-TGCGC---GTTCTC------
------
FC88X_00973_00496 --------------ATCACCG-TTGGTTCG-GG-TGCGC---GTTCTC------
------
FC88X_00992_00467 --------------ATCACCG-TTGGTTCG-GG-TGCGC---GTTCTC------
------
FC88X_00968_00546 --------------ATCACCG-TTGGTTCG-GG-TGCGC---GTTCTC------
------
FC88X_00974_00548 --------------ATCACCG-TTGGTTCG-GG-TGCGC---GTTCTC------
------
FC88X_00995_00596 --------------ATCACCG-TTGGTTCG-GG-TGCGC---GTTCTC------
------
FC88X_00962_00604 --------------ATCACCG-TTGGTTCG-GG-TGCGC---GTTCTC------
------
FC88X_00988_00614 --------------ATCACCG-TTGGTTCG-GG-TGCGC---GTTCTC------
------

FC88X_00957_00667 --------------ATCACCG-TTGGTTCG-GG-TGCGC---GTTCTC------
------
FC88X_00958_00719 --------------ATCACCG-TTGGTTCG-GG-TGCGC---GTTCTC------
------
FC88X_00968_00758 --------------ATCACCG-TTGGTTCG-GG-TGCGC---GTTCTC------
------
FC88X_00980_00750 --------------ATCACCG-TTGGTTCG-GG-TGCGC---GTTCTC------
------
FC88X_00984_00798 --------------ATCACCG-TTGGTTCG-GG-TGCGC---GTTCTC------
------
FC88X_00958_00817 --------------ATCACCG-TTGGTTCG-GG-TGCGC---GTTCTC------
------
FC88X_00993_00813 --------------ATCACCG-TTGGTTCG-GG-TGCGC---GTTCTC------
------
FC88X_00950_00894 --------------ATCACCG-TTGGTTCG-GG-TGCGC---GTTCTC------
------
FC88X_00960_00888 --------------ATCACCG-TTGGTTCG-GG-TGCGC---GTTCTC------
------
FC88X_00995_00999 --------------ATCACCG-TTGGTTCG-GG-TGCGC---GTTCTC------
------
FC88X_00950_01048 --------------ATCACCG-TTGGTTCG-GG-TGCGC---GTTCTC------
------
FC88X_00994_01115 --------------ATCACCG-TTGGTTCG-GG-TGCGC---GTTCTC------
------
FC88X_00950_01254 --------------ATCACCG-TTGGTTCG-GG-TGCGC---GTTCTC------
------
FC88X_00986_01265 --------------ATCACCG-TTGGTTCG-GG-TGCGC---GTTCTC------
------
FC88X_01008_00286 --------------ATCACCG-TTGGTTCG-GG-TGCGC---GTTCTC------
------
FC88X_01010_00291 --------------ATCACCG-TTGGTTCG-GG-TGCGC---GTTCTC------
------
FC88X_01008_00384 --------------ATCACCG-TTGGTTCG-GG-TGCGC---GTTCTC------
------
FC88X_01023_00396 --------------ATCACCG-TTGGTTCG-GG-TGCGC---GTTCTC------
------
FC88X_01040_00406 --------------ATCACCG-TTGGTTCG-GG-TGCGC---GTTCTC------
------
FC88X_01014_00461 --------------ATCACCG-TTGGTTCG-GG-TGCGC---GTTCTC------
------
FC88X_01037_00498 --------------ATCACCG-TTGGTTCG-GG-TGCGC---GTTCTC------
------
FC88X_01040_00480 --------------ATCACCG-TTGGTTCG-GG-TGCGC---GTTCTC------
------
FC88X_01019_00523 --------------ATCACCG-TTGGTTCG-GG-TGCGC---GTTCTC------
------
FC88X_01019_00549 --------------ATCACCG-TTGGTTCG-GG-TGCGC---GTTCTC------
------
FC88X_01033_00526 --------------ATCACCG-TTGGTTCG-GG-TGCGC---GTTCTC------
------
FC88X_01042_00543 --------------ATCACCG-TTGGTTCG-GG-TGCGC---GTTCTC------
------
FC88X_01003_00587 --------------ATCACCG-TTGGTTCG-GG-TGCGC---GTTCTC------
------
FC88X_01007_00563 --------------ATCACCG-TTGGTTCG-GG-TGCGC---GTTCTC------
------

FC88X_01039_00685 ---------------ATCACCG-TTGGTTCG-GG-TGCGC---GTTCTC------------
FC88X_01009_00701 ---------------ATCACCG-TTGGTTCG-GG-TGCGC---GTTCTC------------
FC88X_01009_00748 ---------------ATCACCG-TTGGTTCG-GG-TGCGC---GTTCTC------------
FC88X_01018_00726 ---------------ATCACCG-TTGGTTCG-GG-TGCGC---GTTCTC------------
FC88X_01004_00752 ---------------ATCACCG-TTGGTTCG-GG-TGCGC---GTTCTC------------
FC88X_01017_00879 ---------------ATCACCG-TTGGTTCG-GG-TGCGC---GTTCTC------------
FC88X_01001_00906 ---------------ATCACCG-TTGGTTCG-GG-TGCGC---GTTCTC------------
FC88X_01021_00949 ---------------ATCACCG-TTGGTTCG-GG-TGCGC---GTTCTC------------
FC88X_01025_00913 ---------------ATCACCG-TTGGTTCG-GG-TGCGC---GTTCTC------------
FC88X_01027_00930 ---------------ATCACCG-TTGGTTCG-GG-TGCGC---GTTCTC------------
FC88X_01036_00922 ---------------ATCACCG-TTGGTTCG-GG-TGCGC---GTTCTC------------
FC88X_01011_00952 ---------------ATCACCG-TTGGTTCG-GG-TGCGC---GTTCTC------------
FC88X_01000_01010 ---------------ATCACCG-TTGGTTCG-GG-TGCGC---GTTCTC------------
FC88X_01027_01141 ---------------ATCACCG-TTGGTTCG-GG-TGCGC---GTTCTC------------
FC88X_01012_01187 ---------------ATCACCG-TTGGTTCG-GG-TGCGC---GTTCTC------------
FC88X_01034_01175 ---------------ATCACCG-TTGGTTCG-GG-TGCGC---GTTCTC------------
FC88X_01001_01268 ---------------ATCACCG-TTGGTTCG-GG-TGCGC---GTTCTC------------
FC88X_01052_00451 ---------------ATCACCG-TTGGTTCG-GG-TGCGC---GTTCTC------------
FC88X_01054_00488 ---------------ATCACCG-TTGGTTCG-GG-TGCGC---GTTCTC------------
FC88X_01091_00463 ---------------ATCACCG-TTGGTTCG-GG-TGCGC---GTTCTC------------
FC88X_01069_00509 ---------------ATCACCG-TTGGTTCG-GG-TGCGC---GTTCTC------------
FC88X_01073_00533 ---------------ATCACCG-TTGGTTCG-GG-TGCGC---GTTCTC------------
FC88X_01072_00608 ---------------ATCACCG-TTGGTTCG-GG-TGCGC---GTTCTC------------
FC88X_01088_00621 ---------------ATCACCG-TTGGTTCG-GG-TGCGC---GTTCTC------------
FC88X_01057_00669 ---------------ATCACCG-TTGGTTCG-GG-TGCGC---GTTCTC------------
FC88X_01093_00674 ---------------ATCACCG-TTGGTTCG-GG-TGCGC---GTTCTC------------
FC88X_01083_00723 ---------------ATCACCG-TTGGTTCG-GG-TGCGC---GTTCTC------------
FC88X_01050_00789 ---------------ATCACCG-TTGGTTCG-GG-TGCGC---GTTCTC------------

```
FC88X_01070_00795      --------------ATCACCG-TTGGTTCG-GG-TGCGC---GTTCTC------
------
FC88X_01079_00776      --------------ATCACCG-TTGGTTCG-GG-TGCGC---GTTCTC------
------
FC88X_01051_00822      --------------ATCACCG-TTGGTTCG-GG-TGCGC---GTTCTC------
------
FC88X_01058_00835      --------------ATCACCG-TTGGTTCG-GG-TGCGC---GTTCTC------
------
FC88X_01079_00826      --------------ATCACCG-TTGGTTCG-GG-TGCGC---GTTCTC------
------
FC88X_01085_00818      --------------ATCACCG-TTGGTTCG-GG-TGCGC---GTTCTC------
------
FC88X_01099_00817      --------------ATCACCG-TTGGTTCG-GG-TGCGC---GTTCTC------
------
FC88X_01081_00853      --------------ATCACCG-TTGGTTCG-GG-TGCGC---GTTCTC------
------
FC88X_01084_00871      --------------ATCACCG-TTGGTTCG-GG-TGCGC---GTTCTC------
------
FC88X_01085_00866      --------------ATCACCG-TTGGTTCG-GG-TGCGC---GTTCTC------
------
FC88X_01079_00919      --------------ATCACCG-TTGGTTCG-GG-TGCGC---GTTCTC------
------
FC88X_01067_00952      --------------ATCACCG-TTGGTTCG-GG-TGCGC---GTTCTC------
------
FC88X_01097_00976      --------------ATCACCG-TTGGTTCG-GG-TGCGC---GTTCTC------
------
FC88X_01056_01057      --------------ATCACCG-TTGGTTCG-GG-TGCGC---GTTCTC------
------
FC88X_01085_01086      --------------ATCACCG-TTGGTTCG-GG-TGCGC---GTTCTC------
------
FC88X_01089_01117      --------------ATCACCG-TTGGTTCG-GG-TGCGC---GTTCTC------
------
FC88X_01090_01108      --------------ATCACCG-TTGGTTCG-GG-TGCGC---GTTCTC------
------
FC88X_01099_01129      --------------ATCACCG-TTGGTTCG-GG-TGCGC---GTTCTC------
------
FC88X_01064_01232      --------------ATCACCG-TTGGTTCG-GG-TGCGC---GTTCTC------
------
FC88X_01112_00515      --------------ATCACCG-TTGGTTCG-GG-TGCGC---GTTCTC------
------
FC88X_01131_00524      --------------ATCACCG-TTGGTTCG-GG-TGCGC---GTTCTC------
------
FC88X_01131_00532      --------------ATCACCG-TTGGTTCG-GG-TGCGC---GTTCTC------
------
FC88X_01100_00601      --------------ATCACCG-TTGGTTCG-GG-TGCGC---GTTCTC------
------
FC88X_01121_00622      --------------ATCACCG-TTGGTTCG-GG-TGCGC---GTTCTC------
------
FC88X_01104_00692      --------------ATCACCG-TTGGTTCG-GG-TGCGC---GTTCTC------
------
FC88X_01130_00670      --------------ATCACCG-TTGGTTCG-GG-TGCGC---GTTCTC------
------
FC88X_01128_00798      --------------ATCACCG-TTGGTTCG-GG-TGCGC---GTTCTC------
------
FC88X_01140_00769      --------------ATCACCG-TTGGTTCG-GG-TGCGC---GTTCTC------
------
```

FC88X_01147_00771 --------------ATCACCG-TTGGTTCG-GG-TGCGC---GTTCTC------
------
FC88X_01109_00847 --------------ATCACCG-TTGGTTCG-GG-TGCGC---GTTCTC------
------
FC88X_01116_00864 --------------ATCACCG-TTGGTTCG-GG-TGCGC---GTTCTC------
------
FC88X_01127_00878 --------------ATCACCG-TTGGTTCG-GG-TGCGC---GTTCTC------
------
FC88X_01133_00860 --------------ATCACCG-TTGGTTCG-GG-TGCGC---GTTCTC------
------
FC88X_01143_00860 --------------ATCACCG-TTGGTTCG-GG-TGCGC---GTTCTC------
------
FC88X_01133_00917 --------------ATCACCG-TTGGTTCG-GG-TGCGC---GTTCTC------
------
FC88X_01108_00971 --------------ATCACCG-TTGGTTCG-GG-TGCGC---GTTCTC------
------
FC88X_01115_00975 --------------ATCACCG-TTGGTTCG-GG-TGCGC---GTTCTC------
------
FC88X_01122_01045 --------------ATCACCG-TTGGTTCG-GG-TGCGC---GTTCTC------
------
FC88X_01110_01086 --------------ATCACCG-TTGGTTCG-GG-TGCGC---GTTCTC------
------
FC88X_01112_01126 --------------ATCACCG-TTGGTTCG-GG-TGCGC---GTTCTC------
------
FC88X_01112_01182 --------------ATCACCG-TTGGTTCG-GG-TGCGC---GTTCTC------
------
FC88X_01113_01202 --------------ATCACCG-TTGGTTCG-GG-TGCGC---GTTCTC------
------
FC88X_00964_00802 --------------ATCACCG-TTGGTTCG-GG-TGCGC---GTTCTCA-----
------
FC88X_00098_00916 --------------ATCACCG-TTGGTTCG-GG-TGTGC---GTTCTC------
------
FC88X_00760_00195 --------------ATCACCG-TTGGTTCG-GG-TGTGC---GTTCTC------
------
FC88X_00029_00453 --------------ATCACCG-TTGGTTCG-GG-TGTGC---GTTCTC------
------
FC88X_00108_00467 --------------ATCACCG-TTGGTTCG-GG-TGTGC---GTTCTC------
------
FC88X_00497_01236 --------------ATCACCG-TTGGTTCG-GG-TGTGC---GTTCTC------
------
FC88X_00873_00797 --------------ATCACCG-TTGGTTCG-GG-TGTGC---GTTCTC------
------
FC88X_00950_00441 --------------ATCACCG-TTGGTTCG-GG-TGTGC---GTTCTC------
------
FC88X_01096_00763 --------------ATCACCG-TTGGTTCG-GG-TGTGC---GTTCTC------
------
FC88X_01123_00680 --------------ATCACCG-TTGGTTCG-GG-TGTGC---GTTCTC------
------
FC88X_00174_00227 --------------ATCACCG-TTGGTTCG-GG-TG-GC---GTTCTC------
------
FC88X_00219_00082 --------------ATCACCG-TTGGTTCG-GG-TG--C---GTTCTC------
------
FC88X_00317_00008 --------------ATCACCG-TTGGTTCG-GG-TGCGC---ATTCTC------
------
FC88X_00872_00370 --------------ATCACCG-TTGGTTCG-GG-TGCGC---ATTCTC------
------

FC88X_01111_01161 --------------ATCACCG-TTGGTTCG-GG-TGCGT---TCTC--------
------
FC88X_00375_00344 -------GGGGAATATCACCG-TTGGTTCG-GG-TGCGC---GTTCTC------
------
FC88X_00405_00449 -------------TATCACCG-TTGGTTCG-GG-TGCGC---GTTCTC------
------
FC88X_01083_00951 --------GGGGAAATCACCG-TTGGTTCG-GG-TGCGC---GTTCTC------
------
FC88X_00122_00342 --------GGGGAAATCACCG-TTGGTTCG-GG-TGCGC---GTTCTC------
------
FC88X_00905_00944 --------------ATCACCG-TTGGTTCG-GG-TGCGC----TTCTC------
------
FC88X_01034_00306 -------------AATCACC-GTTGGTTCG-GG-TGCGC---GTTCTC------
------
FC88X_00288_01132 -------------AATCACC-GTTGGTTCG-GG-TGCGC---GTTCTC------
------
FC88X_00991_00329 -------------AATCACC-GTTGGTTCG-GG-TGCGC---GTTCTC------
------
FC88X_00802_00718 ------------GAATCACC-GTTGGTTCG-GG-TGCGC---GTTCTC------
------
FC88X_00744_00064 ----------GGGAATCACC-GTTGGTTCG-GG-TGCGC---GTTCTC------
------
FC88X_00313_01130 ---------GGGGAATCACC-GTTGGTTCG-GG-TGCGC---GTTCTC------
------
FC88X_00015_00072 ---------GGGGAATCACC-GTTGGTTCG-GG-TGCGC---GTTCTC------
------
FC88X_00684_00890 ---------GGGGAATCACC-GTTGGTTCG-GG-TGCGC---GTTCTC------
------
FC88X_01007_00659 --------------ATCACC-GTTG-TTCG-GG-TGCGC---GTTCTC------
------
FC88X_00248_00959 --------------ATCACCCGTTGGTTCG-GG-TGCGC---GTTCTC------
------
FC88X_00722_01204 --------------ATCACCCGTTGGTTCG-GG-TGCGC---GTTCTC------
------
FC88X_00707_00174 --------------ATCACC-GTTG-TTCG-GG-TGCGC---GTTCTC------
------
FC88X_00821_00135 --------------ATCACC-GTTGGTTCG-GG-TGCGT---GTTCTC------
------
FC88X_00837_00872 --------------ATCACC-GTTGGTTCG-GG-TGCGT---GTTCTC------
------
FC88X_00409_01160 --------------ATCACC-GTTGGTTCG-GG-TGCGT---GTTCTC------
------
FC88X_00457_00972 --------------ATCACC-GTTGGTTCG-GG-TGCGT---GTTCTC------
------
FC88X_01026_00340 --------------ATCACC-GTTGGTTCG-GG-TGCGT---GTTCTC------
------
FC88X_01039_00574 --------------ATCACC-GTTGGTTCG-GG-TGCGT---GTTCTC------
------
FC88X_01083_00825 --------------ATCACC-GTTGGTTCG-GG-TGCGT---GTTCTC------
------
FC88X_00651_00294 --------------ATCACC-GTTGGTTCG-GG-TGCGTC--GTTCTC------
------
FC88X_00035_00156 --------------ATCACC-GTTGGTTCG-GG-TGCGC---GTTCTT------
------
FC88X_00711_00117 --------------ATCACC-GTTGGTTCG-GG-TGCGC---GTTCT-------
------

FC88X_00763_01247 --------------ATCACC-GTTGGTTCG-GG-TGCGC---GTTCT-------
------
FC88X_01111_00888 --------------ATCACC-GTTGGTTCG-GG-TGCGC---GTTCTCT-----
------
FC88X_00590_00230 --------------ATCACCTGTTGGTTCG-GG-TGCGC---GTTCTC------
------
FC88X_00965_00318 --------------ATCAC-TGTTGGTTCG-GG-TGCGC---GTTCTC------
------
FC88X_00793_00267 --------------ATCACCGTTGGC-TCG-GG-TGCGC---GTTCTC------
------
FC88X_00791_00851 --------------ATCACCGTTGGCGTCG-GG-TGCGC---GTTCTC------
------
FC88X_00893_00327 --------------ATCACCGTTGGC-TCG-GG-TGCGC---GTTCTC------
------
FC88X_01015_00940 --------------ATCACCGTTGGC-TCG-GG-TGCGC---GTTCTC------
------
FC88X_00356_01084 --------------ATCACCGTTGGC-TCG-GG-TGCGC---GTTCTC------
------
FC88X_00135_00525 --------------ATCACCGTTGGC-TCG-GG-TGCGC---GTTCTC------
------
FC88X_00070_00098 --------------ATCACCGTTGGC-TCG-GG-TGCGC---GTTCTC------
------
FC88X_00727_01228 -------------GATCACCGTTGGC-TCG-GG-TGCGC---GTTCTC------
------
FC88X_00812_00947 -------------GATCACCGTTGGC-TCG-GG-TGCGC---GTTCTC------
------
FC88X_01019_00696 -------------GATCACCGTTGGC-TCG-GG-TGCGC---GTTCTC------
------
FC88X_00246_01082 --------------ATCACCGTTGGC-TCG-GG-TGCGC---GTTCTC------
------
FC88X_00491_01028 --------------ATCACCGTTGGC-TCG-GG-TGCGC---GTTCTC------
------
FC88X_00148_00651 --------------ATCACCGTTGGC-TCG-GG-TGCGC---GTTCTC------
------
FC88X_00164_00210 --------------ATCACCGTTGGC-TCG-GG-TGCGC---GTTCTC------
------
FC88X_00605_00114 --------------ATCACCGTTGGGT-CG-GG-TGCGC---GTTCTC------
------
FC88X_00887_00748 --------------ATCACCGTTGGGT-CG-GG-TGCGC---GTTCTC------
------
FC88X_00062_00375 --------------ATCACCGTTGGGTTCG-GG-TGCGC---GTTCTC------
------
FC88X_00340_01138 --------------ATCACCGTTGGTC--G-GG-TGCGC---GTTCTC------
------
FC88X_01055_00771 --------------ATCACCGTTGGTC--G-GG-TGCGC---GTTCTC------
------
FC88X_00859_00668 --------------ATCACCGTTGG-T-CG-GG-TGCGC---GTTCTC------
------
FC88X_00172_00810 --------------ATCACCGTTGGAT-CG-GG-TGCGC---GTTCTC------
------
FC88X_00104_00018 --------------ATCACCG-TTGGT-CC-GGGTGCGC---GTTCTC------
------
FC88X_00612_00915 --------------ATCACCG-TTGGT-CC-GGGTGCGC---GTTCTC------
------
FC88X_00414_01032 --------------ATCACCG-TTGGTTCC-GGGTGCGC---GTTCTC------
------

FC88X_00344_01045 --------------ATCACCG-TTGGTTCG-G-TTGCGC---GTTCTC------
------
FC88X_00653_00083 --------------ATCACCG-TTGGTTCG-GGTTGCGC---GTTCTC------
------
FC88X_00856_00975 --------------ATCACCG-TTGGTTCG-G-ATGCGC---GTTCTC------
------
FC88X_00395_01081 --------------ATCACCG-TTGGTTCG-G-ATGCGC---GTTCTC------
------
FC88X_00903_01132 --------------ATCACCG-TTGGTTCG-GGTGCTGC---GCTTCTC-----
------
FC88X_00025_00690 --------------ATCACCG-TTGCTTCG-GG-TGCGC---GTTCTC------
------
FC88X_00330_01170 --------------ATCACCG-TTGCTTCG-GG-TGCGC---GTTCTC------
------
FC88X_00376_00981 --------------ATCACCG-TTGATTCG-GG-TGCGC---GTTCTC------
------
FC88X_01126_00847 --------------ATCACCG-TTGATTCG-GG-TGCGC---GTTCTC------
------
FC88X_00458_00091 --------------ATCACCA-TTGGTTCG-GG-TGCGC---GTTCTC------
------
FC88X_01114_00641 --------------ATCACCA-TTGGTTCG-GG-TGCGC---GTTCTC------
------
FC88X_01118_00771 --------------ATCACCA-TTGGTTCG-GG-TGCGC---GTTCTC------
------
FC88X_00932_01140 --------------ATCACCT-TTGGTTCG-GG-TGCGC---GTTCTC------
------
FC88X_00979_00846 --------------ATCACCT-TTGGTTCG-GG-TGCGC---GTTCTC------
------
FC88X_01078_00920 --------------ATCACCT-TTGGTTCG-GG-TGCGC---GTTCTC------
------
FC88X_00962_00632 --------------ATCACCC-TTG-TTCG-GG-TGCGC---GTTCTC------
------
FC88X_00838_00118 --------------ATCACCGT-TGGTTCG-GGTG-CGC---GCTCTC------
------
FC88X_01002_00406 --------------ATCACCGT-TGGTTCG-GGTG-CGC---GTCTC-------
------
FC88X_00810_00295 --------------ATCACCGT-TGGTTCG-GGTG-CGC---GCTCTC------
------
FC88X_00897_00707 --------------ATCACCGT-TGGTTCG-GGTG-CGC---GCTCTC------
------
FC88X_00033_00040 --------------ATCACCGT-TGGTTCG-GGTG-CGC---GCTCTC------
------
FC88X_00163_00581 --------------ATCACCGT-TGGTTCG-GGTG-CGC---GCTCTC------
------
FC88X_00385_00990 --------------ATCACCGT-TGGTTCG-GGTG-CGC---GCTCTC------
------
FC88X_00652_01257 --------------ATCACCGT-TGGTTCG-GGTG-CGC---GCTCTC------
------
FC88X_00903_00615 --------------ATCACCGT-TGGTTCG-GGTG-CGC---GCTCTC------
------
FC88X_01000_00484 --------------ATCACCGT-TGGTTCG-GGTG-CGC---GCTCTC------
------
FC88X_00684_00078 --------------ATCACCGT-TGGTTCG-GGTG-CGC---G-----------
------
FC88X_00756_00295 --------------ATCACCGT-TGGTTCG-GGTG-CGC---GGTTC-------
------

FC88X_00731_01259 --------------ATCACCGT-TGGTTCG-GGTGGCGC---G-----------
------
FC88X_01076_00927 --------------ATCACCGT-TGGTTCG-GGTG-CGC---GTTTTC------
------
FC88X_00888_00632 --------------ATCACCGT-TGGTTCG-GG-CGCGC---GTTCTC------
------
FC88X_01098_00633 --------------ATCACCGT-TGGTTCG-GG-CGCGC---GTTCTC------
------
FC88X_01097_00833 --------------ATCACCGT-TGGTTCG-GG-CGCGC---GTTCTC------
------
FC88X_01000_00292 --------------ATCACCGT-TGGTTCG-GGTCGCGC---GTTCTC------
------
FC88X_01093_00446 --------------ATCACCGT-CGGTTCG-GG-TGCGC---GTTCTC------
------
FC88X_00146_01013 --------------ATCACCGT-CGGTTCG-GG-TGCGC---GTTCTC------
------
FC88X_00614_01142 --------------ATCACCGT-CGGTTCG-GG-TGCGC---GTTCTC------
------
FC88X_00861_01018 --------------ATCACCGT-CGGTTCG-GG-TGCGC---GTTCTC------
------
FC88X_01068_01013 --------------ATCACCGTTCGGTTCG-GG-TGCGC---GTTCTC------
------
FC88X_00913_00307 --------------ATCACCGTTCGGTTCG-GG-TGCGC---GTTCTCC-----
------
FC88X_00308_00623 --------------ATCACCG-TTG-TTCG-GG-TTCGC---GTTCTC------
------
FC88X_01136_00619 --------------ATCACCG-TTGGTTCG-GG-TACGC---GTTCTC------
------
FC88X_00632_00254 --------------ATCACCG-T-AGTTCG-GG-TGCGC---GTTCTC------
------
FC88X_00804_00133 --------------ATCACCG-TTAGTTCG-GG-TGCGC---GTTCTC------
------
FC88X_00896_00527 --------------ATCACCG-TTAGTTCG-GG-TGCGC---GTTCTC------
------
FC88X_00963_00267 --------------ATCACCG-TTAGTTCG-GG-TGCGC---GTTCTC------
------
FC88X_00808_00940 --------------ATCACCG-TTAGTTCG-GG-TGCGC---GTTCTCT-----
------
FC88X_00449_00048 --------------ATCACCG-TTGGTT-C-GGGTGCGCG--TTTCTC------
------
FC88X_00824_00241 --------------ATCACCG-TTGGTT-C-GGGTGCGCG--TTTCTC------
------
FC88X_00867_00172 --------------ATCACCG-TTGGTT-C-GGGTGCGCG--TTTCTC------
------
FC88X_00992_00555 --------------ATCACCG-TTGGTT-C-GGGTGCGCG--TTTCTC------
------
FC88X_01102_00559 --------------ATCACCG-TTGGTT-C-GGGTGCGCG--TTTCTCC-----
------
FC88X_00155_00209 --------------ATCACCG-TTGGTT-C-GGGTGCGTT--CTC---------
------
FC88X_00610_00220 --------------ATCACCG-TTGGTT-C-GGGTGCGC---GTTCTCCC----
------
FC88X_01080_00894 --------------ATCACCG-TTGGTT-C-GGGTGCGC---GTTCTCCC----
------
FC88X_00638_00491 --------------ATCACCG-TTGGTT-C-GGGTGCGC---GTTCTCCC----
------

FC88X_00683_01026 --------------ATCACCG-TTGGTT-C-GGGTGCGC---GTTCTCCC----
------
FC88X_00862_00324 --------------ATCACCG-TTGGTT-C-GGGTGCGCC--GTTCTCCC----
------
FC88X_00991_00645 --------------ATCACCG-TTGGTT-C-GGGTGCGC---GTTCTCC-----
------
FC88X_00593_01034 --------------ATCACCG-TTGGTT-C-GGGTGCGC---GTTCTCC-----
------
FC88X_00894_00402 --------------ATCACCG-TTGGTT-C-GGGTGCGC---GTTCTCC-----
------
FC88X_00105_00025 -------------GATCACCG-TTGGTTGC-GGGTGCGC---GTTCTCC-----
------
FC88X_00208_00050 --------------ATCACCG-TTGGTT-C-GGGTGCGC---GTTCTCC-----
------
FC88X_00811_01232 --------------ATCACCG-TTGGTT-C-GGGTGCGC---GTTCTCC-----
------
FC88X_00867_00655 --------------ATCACCG-TTGGTT-C-GGGTGCGC---GTTCTCCTTCC-
------
FC88X_00594_00199 --------------ATCACCG-TTGGTTCG-GG-TGCAC---GTTCTC------
------
FC88X_00535_00102 --------------ATCACCG-TTGGTTCG-GG-TGCAC---GTTCTC------
------
FC88X_00602_01141 --------------ATCACCG-TTGGTTCG-GG-TGC-C---GTTCTC------
------
FC88X_00769_00454 --------------ATCACCG-TTGGTTCG-GG-TGC-C---GTTCTC------
------
FC88X_00107_00328 --------------ATCACCG-TTGGTTCG-GG-TGCCC---GTTCTC------
------
FC88X_00366_00984 --------------ATCACCG-TTGGTTCG-GGGTGCCC---GTTCTCC-----
------
FC88X_00038_00241 --------------ATCACCG-TTGGTT---GGGTGCGC---
GTTCTCCTTCCTT-----
FC88X_01074_00967 --------------ATCACCG-TTGGTT-C-GGGTGCGC---
GTTCTCCTTCCTTT----
FC88X_00836_01185 --------------ATCACCG-TTGGTT-C-GGGTGCGC---
GTTCTCCTTCCTTT----
FC88X_00242_00890 --------------ATCACCG-TTGGTT-C-GGGTGCGC---
GTTCTCCTTCCTTT----
FC88X_00032_00645 --------------ATCACCG-TTGGTT-C-GGGTGCGC---
GTTCTCCTTCCTTT----
FC88X_00050_00184 --------------ATCACCG-TTGGTT-C-GGGTGCGC---
GTTCTCCTTCCTTT----
FC88X_00682_00174 --------------ATCACCG-TTGGTT-C-GGGTGCGC---
GTTCTCCTTCCTTT----
FC88X_00022_00147 --------------ATCACCG-TTGGT--C-GGGTGCGC---
GTTCTCCTTCCTTT----
FC88X_00080_00096 --------------ATCACCG-TTGGTT-C-GGGTGCGC---
GTTCTCCTTCCTTT----
FC88X_00074_00160 --------------ATCACCG-TTGGTT-C-GGGTGCGC---
GTTCTCCTTCCTTT----
FC88X_00272_01102 --------------ATCACCG-TTGGTT-C-GGGTGCGC---
GTTCTCCTTCCTTT----
FC88X_00614_00926 --------------ATCACCG-TTGGTT-C-GGGTGCGC---
GTTCTCCTTCCTTT----
FC88X_00725_01075 --------------ATCACCG-TTGGTT-C-GGGTGCGC---
GTTCTCCTTCCTTT----

FC88X_00786_00121 ---------------ATCACCG-TTGGTT-C-GGGTGCGC---
GTTCTCCTTCCTTT----
FC88X_00021_00122 ---------------ATCACCG-TTGGTT-C-GGGTGCGC---
GTTCTCCTTCCTTTC---
FC88X_00074_00180 ---------------ATCACCG-TTGGTT-C-GGGTGCGC---
GTTCTCCTTCCTTTC---
FC88X_01053_00849 ---------------ATCACCG-CTGGTTCG-GG-TGCGC---GTTCTC------
------
FC88X_00523_01163 ---------------ATCACCG-CTGGTTCG-GG-TGCGC---GTTCTC------
------
FC88X_00667_01105 ---------------ATCACCG-CTGGTTCG-GG-TGCGC---GTTCTC------
------
FC88X_01097_00481 ---------------ATCACCG-CTGGTTCG-GG-TGCGC---GTTCTC------
------
FC88X_00492_00995 ---------------ATCACCG-CTGGTTCG-GG-TGCAC---GTTCTC------
------
FC88X_00953_00970 ---------------ATCACCG-CTGGTTCG-GG-TGCAC---GTTCTC------
------
FC88X_00338_01039 ---------------ATCACCG-TTGGTTCG-GG-TGCGC---
GTTCTCCTTCCTTTCTC-
FC88X_00032_00721 ---------------ATCACCG-TTGGTTCT-GG-TGCGC---GTTCTC------
------
FC88X_00845_00697 ---------------ATCACCG-TTGGTTCT-GG-TGCGC---GTTCTC------
------
FC88X_00911_00394 ---------------ATCACCG-TTGGTTCT-GG-TGCGC---GTTCTC------
------
FC88X_00101_00170 ---------------ATCACCG-TTGGTTCG-GG-TGCGC---GTTCCC------
------
FC88X_00976_00880 ---------------ATCACCG-TTGGTTCT-GG-TGCGC---GTTCCC------
------
FC88X_00084_00344 ---------------ACCACCG-TTGGTTCT-GG-TGCGC---GTTCTC------
------
FC88X_00066_00616 ---------------ACCACCG-TTGGTTCG-GG-TGCGC---GTTCTC------
------
FC88X_00292_00018 ---------------ACCACCG-TTGGTTCG-GG-TGCGC---GTTCTC------
------
FC88X_00394_01079 ---------------ACCACCG-TTGGTTCG-GG-TGCGC---GTTCTC------
------
FC88X_01098_00630 ---------------ACCACCG-TTGGTTCG-GG-TGCGC---GTTCTC------
------
FC88X_00039_00500 ---------------ACCACCG-TTGGTTCG-GG-TGCGC---GTTCTC------
------
FC88X_00083_00071 ---------------ACCACCG-TTGGTTCG-GG-TGCGC---GTTCTC------
------
FC88X_01116_01037 ---------------ACCACCG-TTGGTTCG-GG-TGCGC---GTTCTC------
------
FC88X_00667_01268 ---------------ACCACCG-TTG-TTCG-GG-TGCGC---GTTCTC------
------
FC88X_00486_01185 ---------------ACCACCG-TTGTTTCG-GG-TGCGC---GTTCTC------
------
FC88X_00043_00298 ---------------ATCACCG-TTGGTTCG-AG-TGCGC---GTTCTC------
------
FC88X_00083_00047 ---------------ATCACCG-TTGGTTCG-AG-TGCGC---GTTCTC------
------
FC88X_00100_00924 ---------------ATCACCG-TTGGTTCG-AG-TGCGC---GTTCTCC-----
------

FC88X_01014_00514 --------------ATCACCG-TTGGTTCG-AG-TGCGC---
GTTCTCCTTCCTTT----
FC88X_00934_00317 --------------ATCACCG-TTGGCTCG-AG-TGCGC---GTTCTC------
------
FC88X_00069_00862 --------------ATCACCG-TTGGCTCG-GG-TGCGC---
GTTCTCCTTCCTTT----
FC88X_00050_00103 --------------ATCACCG-TTGGATCA-GG-TGCGC---GTTCTC------
------
FC88X_00055_00402 --------------ATCACCG-TTGGTTT--GGGTGCGC---GTTCTC------
------
FC88X_00526_00999 --------------ATCACCG-TTGGTTT--GGGTGCGC---GTTCTC------
------
FC88X_00220_00921 --------------ATCACCG-TTGGTT---GGGTGCGC---GTTCTC------
------
FC88X_00900_00994 --------------ATCACCG-TTGGTTTC-GGGTGCGC---GTTCTC------
------
FC88X_00822_00857 --------------ATCACCG-TCGGTTTCGGGGTGCGCCG-GTTCTCC-----
------
FC88X_00302_00425 --------------ATCACCG-TTGTTTC--GGGTGCGC---GTTCTC------
------
FC88X_00477_01207 --------------ATCACCG-TTGTTTC--GGGTGCGC---GTTCTC------
------
FC88X_00060_00367 --------------ATCACCG-TTGTTTC--GGGTGCGC---GTTCTC------
------
FC88X_00100_00869 --------------ATCACCG-TTGTTTC--GGGTGCGC---GTTCTC------
------
FC88X_00520_00425 --------------ATCACCG-TTGTTTC--GGGTGCGC---GTTCTC------
------
FC88X_00798_00428 --------------ATCACCG-TTGTTTC--GGGTGCGC---GTTCTC------
------
FC88X_00881_00696 --------------ATCACCG-TTGTTTC--GGGTGCGC---GTTCTC------
------
FC88X_01035_00506 --------------ATCACCG-TTGTTTC--GGGTGCGC---GTTCTC------
------
FC88X_00952_00517 --------------ATCACCG-TCGTTGTC-GGGTGCGC---GTTCTC------
------
FC88X_00090_00454 --------------ATCACCG-TTATGTC--GGGTGCGC---GTTCTC------
------
FC88X_01067_01063 --------------ATCACCA-TCGGTTCG-GG-TGCGC---GTTCTC------
------
FC88X_00165_00719 -------------AGTCACCGTTGGTT-CG-GG-TGCGC---GTTCTC------
------
FC88X_00117_00411 --------------GTCACCGTTGGTT-CG-GG-TGCGC---GTTCTC------
------
FC88X_00469_00046 ------------GAGTCACCGTTGGTTTCG-GG-TGCGC---
GTTCTCCTTTCC------
FC88X_00649_00969 ---------------ACACCG-TTGGTTCG-GG-TGCGC---GTTCTC------
------
FC88X_00370_00758 ---------------ACACCG-TTGGTTCG-GG-TGCGC---GTTCTC------
------
FC88X_00215_01048 -------------ATACACCG-TTGGTTCG-GG-TGAGC---GTTCTC------
------
FC88X_00926_00568 --------------TTCACCG-TTGGTTCG-GG-TGCGC---GTTCTC------
------
FC88X_00041_00197 --------------TTCACCG-TTGGTTCG-GG-TGCGC---GTTCTC------
------

FC88X_01040_00766 --------------TTCACCG-TTGGTTCG-GG-TGCGC---GTTCTC------
------
FC88X_01021_00820 ------------CATTCACCGATTGGTTCG-GG-TGCGC---GTTCTC------
------
FC88X_00068_00326 -------------ATCCACCG-TTGGTTCG-GG-TGCGC---GTTCTCC-----
------
FC88X_00558_00102 -------------ATCAACCG-TTGGTTCG-GG-TGCGC---GTTCTC------
------
FC88X_00460_00025 -------------ATTCACCG-TTGGTTCG-GG-TGCGC---GTACTCC-----
------
FC88X_00479_01180 --------------ATCACCG-TTGGTTCG-GG-TGCGC---GTCCTC------
------
FC88X_00992_00289 --------------ATCACCG-TTGGTTCG-GG-TGCGC---GTTCCTC-----
------
FC88X_00442_00291 --------------ATCACCG-TTGGTTCG-GG-TGCGC---GTCCTC------
------
FC88X_00559_00165 --------------ATCACCG-TTGGTTCG-GG-TGCGC---GTCCTC------
------
FC88X_00130_00568 ------------GACTCACCG-TTGGTTCG-GG-TGCGC---GTTCCTC-----
------
FC88X_00946_00474 --------------ATCACCG-TTGGTTCG-GC-T-CGC---GTTCTCT-----
------
FC88X_00092_00382 --------------ATCACCG-TTGGTTCGGGTGTGCGTTC-
TCCCTTCCTTTT------
FC88X_00421_00794 --------------ATCACCG-TTGGTTCGGGTGCGCGCTC-ATC---------
------
FC88X_00997_00310 -------------GATCACCG-TTGGATTCGGGGTGCG----------------
------
FC88X_00052_00628 --------------ATCACCG-TTGG-TCGGTGGTGCGCT--------------
------
FC88X_00198_00925 -------------AGTCACCG-TTGGGTTCGGGGTGCGCGT-
TTCTCCCGTTCC------
FC88X_00035_00151 --------------ATCACCG-TTGGTTCGGGTGCGTCTCT-TCTTC-------
------
FC88X_00364_01105 --------------ATCACCG-TTGGTTCGGGTGCGTCT---------------
------
FC88X_00648_00125 --------------ATCACCG-TTGGTTCGGGTGCGTGTTT-CTCC--------
------
FC88X_00983_00316 -------------ATCTACCG-TTGGTTCG-G-GTGCGCGT-CTTCTC------
------
FC88X_00133_00282 -------------ATACACCG-CTGGTTCG-GTGCGCGCTT-
CTCCTTCCTTCT------
FC88X_00521_00111 -------------ATACACCG-TTGGTTTG-GGTGGCGCGT-
TCTCCTTCCTTTC-----
FC88X_00733_00300 -------------ATACACCG-TTGGTTCG-TGTGCACCGT-TCTC--------
------
FC88X_00040_00304 ---------------TCGCCGTTT-ATTCC-GTTGGCGC---GTTCT-------
------
FC88X_00468_00683 --------------CCAGCTGTTTGATTGC-ATTGGCGT---GCA---------
------
FC88X_00060_00314 ------------------CCACGAGTTT-GCTTGCGTGGT--TTACTC------
------
FC88X_00198_00139 ------------------CCACGAGTTT-GCTTGCGTGGT--TTACTC------
------
FC88X_00177_00696 ------------------CCACGAGTTT-GCTTGCGTGGT--TTACTC------
------

```
FC88X_00310_00418       ------------------CCACGAGTTT-GCTTGCGTGGT--TTACTC------
------
FC88X_00372_00437       ------------------CCACGAGTTT-GCTTGCGTGGT--TTACTC------
------
FC88X_00860_00784       ------------------CCACGAGTTTTGCTTGCGTGGT--TTACTC------
------
FC88X_00818_00126       -----------GGGGAAGCCACGAGTTT-GCTTGCGTGGT--TTACTC------
------
FC88X_00165_00821       ------------------CCACGAGTTT-GCTCGCGTGGT--TTACTC------
------
FC88X_00393_00032       -----------------CCCAAGCGGTT-GCTT-TGTGGA--TTATTC------
------
FC88X_00495_00792       -----------------CCCAAGCGGTT-GCTT-TGTGGA--TTATTC------
------
FC88X_00816_00102       -----------------CCCAAGCGGTT-GCTT-TGTGGA--TTATTC------
------
FC88X_00894_00841       -----------------CCCAAGCGGTT-GCTC-TGTGGA--TTATTC------
------
FC88X_01019_00839       ------------------CCAATAGGTTAGACTTGGTGGC--TTTCT-------
------
FC88X_00083_00818       ------------------CCAATAGGTTAGACTTGGTGGC--TTTCT-------
------
FC88X_00800_01269       ------------------CCAATAGGTTAGACTTGGTGGC--TTTCT-------
------
FC88X_00125_00774       ------------------CCAATAGGTTAGACTTGGTGGC--------------
------
FC88X_00083_00232       ----------------CCCCAAAATGTTT--CGTGGTGGT--CTCCT-------
------
FC88X_00793_00962       ----------------CCCCAAAATGTTT--CGTGGTGGT--CTCCT-------
------
FC88X_00063_00514       ------------------TCACTGGCTT-GGTTGTCCGTT--ATTCTC------
------
FC88X_00984_01020       ------------------TCACTGGCTT-GGTTGTCCGTT--ATTCTCC-----
------
FC88X_00212_00081       ------------------TCACTGGCTT-GGTTGTCCGTT--ATTCTC------
------
FC88X_00909_00700       ------------------TCACTGGCTT-GGTTGTCCGTT--ATTCTC------
------
FC88X_00270_00697       ------------------TCACTGGCTT-GGTTGTCCGTT--ATTCTC------
------
FC88X_01009_00651       ------------------TCACTGGCTT-GGTTGTCCGTT--ATTCTC------
------
FC88X_01026_00741       ------------------TCACTGGCTT-GGTTGTCCGTT--ATTCTC------
------
FC88X_01129_00723       ------------------TCACTGGCTT-GGTTGTCCGTT--ATTCTC------
------
FC88X_01105_01051       ------------------TCACTGGCTT-GGTTGTCCGTT--ATTCTC------
------
FC88X_00080_00431       ------------------TCACTGGCTT-GGTTGTCAGTT--ATTCTC------
------
FC88X_01126_00780       ------------------TCACTGGCTT-GGTTGTCCGTT--ATTCTCCC----
------
FC88X_00055_00157       ------------------TTGCTGGCTT-GGTTGTCCGTT--ATTCTCC-----
------
FC88X_00094_00381       -----------------ACCACCAGCTT-GGTTCGTC-TT--AGTCTC------
------
```

```
FC88X_00159_00942       ---------------TTCCCATTTGTTT-GGTTGTACGGT--ATT---------
------
FC88X_00281_00400       ---------------TTCCCATTTGTTT-GGTTGTACGGT--ATT---------
------
FC88X_00468_01038       ---------------TTCCCATTTGTTT-GGTTGTACGGT--ATT---------
------
FC88X_00963_00866       ---------------TTCCCATTTGTTT-GGTTGTACGGT--ATT---------
------
FC88X_00126_00651       ---------------TTCCCACTTGTTT-GGTTGTACGGT--ATT---------
------
FC88X_00968_00318       ---------------TTCCCATTTGTTT-GGCTGTACGGT--ATT---------
------
FC88X_00235_00944       -----------------CGTGGTTGGTTAGTTCTCGTGACA-TT----------
------
FC88X_00709_00892       ----------------CCGTGGTTGGTTAGTTCTCGTGACA-TT----------
------
FC88X_00962_00798       -----------------CGTGGTTGGTTAGTTCTCGTGACA-TT----------
------
FC88X_00760_01229       -------------TCCCGGTGTTTGGTCTATTCAGTTGAG--------------
------
FC88X_01119_00964       -------------TCCCGGTGTTTGGTCTATTCAGTTGAG--------------
------
FC88X_00023_00496       -------------TCCCGGTGTTTGGTCTATTCAGTTGAG--------------
------
FC88X_00881_01183       -------------TCCCGGTGTTTGGTCTATTCAGTTGAG--------------
------
FC88X_00397_01112       -------------TCCCGGTGCTTGGTCTATTCAGTTGAG--------------
------
FC88X_00058_00804       --------------TCTGTCGTTCGGT-CGACGCTGCCTAT-C-----------
------
FC88X_00534_01123       -------------GTCTGTCGTTCGGT-CGACGCTGCCTAT-C-----------
------
FC88X_00165_00749       --------------TCTGTCGTTCGGT-CGACGCTGCCTAT-C-----------
------
FC88X_00220_00474       --------------TCTGTCGTTCGGT-CGACGCTGCCTAT-C-----------
------
FC88X_00271_00984       --------------TCTGTCGTTCGGT-CGACGCTGCCTAT-C-----------
------
FC88X_00555_00321       --------------TCTGTCGTTCGGT-CGACGCTGCCTAT-C-----------
------
FC88X_00822_00841       --------------TCTGTCGTTCGGT-CGACGCTGCCTAT-C-----------
------
FC88X_00902_00291       --------------TCTGTCGTTCGGT-CGACGCTGCCTAT-C-----------
------
FC88X_00970_00255       --------------TCTGTCGTTCGGT-CGACGCTGCCTAT-C-----------
------
FC88X_00462_00964       --------------TCTGTCGTTCGGT-CGACGCTGCCTAT-CCT---------
------
FC88X_00555_00109       --------------TCTGTCGTTCGGT-CGACGGTGCCTAT-C-----------
------
FC88X_00041_00655       --------------TCTGACGTTCGGT-CGACGCTGCCTAT-C-----------
------
FC88X_00341_01044       --------------TCTGTCGTTCGGT-CGACGCTACCTAT-C-----------
------
FC88X_00041_00269       --------------TCTGTCGTTCGGT-CGACGCTGCTAT--------------
------
```

FC88X_00690_00948 --------------TCTGTCGTTCGGT-CGACGCTG------------------
------
FC88X_00676_01052 --------------TCTGTCGTTCGGTTCGACGCTGC-----------------
------
FC88X_00546_00108 ---------------CTGTCCTCTTAGTTCACGCTGCATCT-C-----------
------
FC88X_00652_00336 -----GGGGACAATAGTACTCTTGGAT-TGGCG--GCGTCT-C-----------
------
FC88X_00729_01269 --------------TCAACCCTCCTAT-TGGTGT-ACGTCT-C-----------
------
FC88X_00184_00388 ------GGGGAAGCCAGACTTTCCTCTTCGTTGC-GTTACT-------------
------
FC88X_00424_00103 -------GGGGAACCAGACTTTCCTCTTCGTTGC-GTTACT-------------
------
FC88X_00906_00436 -------------CCAGACTTTCCTCTTCGTTGC-GTTACT-------------
------
FC88X_00053_00101 -------------CCAGACTTTCCTCTTCGTTGC-GTTACT-------------
------
FC88X_00063_00174 -------------CCAGACTTTCCTCTTCGTTGC-GTTACT-------------
------
FC88X_01032_01274 -------------CCAGACTTTCCTCTTCGTTGC-GTTACT-------------
------
FC88X_00022_00230 -------------GTCAACTTTACCCTTCGTTGCCGTCACT-------------
------
FC88X_00122_00841 -------------------TTCCCGT-TTGTTTCGATCTGC-CATTTTC-----
------
FC88X_00161_00668 -------------------TTCCCGT-TTGTTTCGATCTGC-CAT---------
------
FC88X_01119_00988 -------------------CTCCCGTGTTGGTTGCACCTGC-TATG--------
------
FC88X_00091_00117 ---------------------CCCGTGTTCGTGTCCACTGC-TTTTTGT-----
------
FC88X_01129_00624 -----------------ATCATCCGTGTGAGTCGATGCTGT-TCT---------
------
FC88X_00367_00037 -----------------CTTCGTCTTGTGGTTGATGCCTAT-TGTC--------
------
FC88X_00707_01030 -----------------CTTCGTCTTGTGGTTGATGCCTAT-TGTC--------
------
FC88X_00750_00356 -----------------CTTCGTCTTGTGGTTGATGCCTAT-TGTC--------
------
FC88X_00317_01039 -----------------CTTCGTCTTGTGGTTGATGCCTAT-TGTC--------
------
FC88X_00549_01144 -----------------CTTCGTCTTGTGGTTGATGCCTAT-TGTC--------
------
FC88X_00758_00537 -----------------CTTCGTCTTGTGGTTGATGCCTAT-TGTC--------
------
FC88X_00861_01034 -----------------CTTCGTCTTGTGGTTGATGCCTAT-TGTC--------
------
FC88X_00923_01255 -----------------CTTCGTCTTGTGGTTGATGCCTAT-TGTC--------
------
FC88X_00995_00998 -----------------CTTCGTCTTGTGGTTGATGCCTAT-TGTC--------
------
FC88X_00280_01143 ------------------CTTCTCTTGTGGTTGATGCCTAT-TGTC--------
------
FC88X_00866_00339 -----------------CTTCGTCTTGTGGTCGATGCCTAT-TGTC--------
------

```
FC88X_00182_00057        -----------------CTTCGCCTTGTGGTTGATGCCTAT-TGTC--------
------
FC88X_00976_00462        -----------------CTTCGCCTTGTGGTTGATGCCTAT-TGTC--------
------
FC88X_00031_00081        -----------------TTTCTCGTTGTGGACG-TGGCTAT-TTTCC-------
------
FC88X_00151_00090        --------------TCGCTTCCCATTTTGGTAG--GGCTAT-TCA---------
------
FC88X_00784_01274        --------------TCGCTTCCCATTTTGGTAG--GGCTAT-TCA---------
------
FC88X_00160_00885        ------------------TTCACCTTTTGGT-----GCTTT-TCCCATACT---
------
FC88X_00827_00170        ------------------TTCACCTTTTGGT-----GCTTT-TC----------
------
FC88X_00155_00776        -------------GTCACTTCCTAT-CTGGTCGCGATATTC-T-----------
------
FC88X_00315_00469        --------------TCACTTCCTAT-CTGGTCGCGATATTC-T-----------
------
FC88X_00818_01204        --------------TCACTTCCTAT-CTGGTCGCGATATTC-T-----------
------
FC88X_00885_00792        --------------TCACTTCCTAT-CTGGTCGCGATATTC-T-----------
------
FC88X_00927_00191        --------------TCACTTCCTAT-CTGGTCGCGATATTC-T-----------
------
FC88X_00937_00846        --------------TCACTTCCTAT-CTGGTCGCGATATTC-T-----------
------
FC88X_00980_00514        --------------TCACTTCCTAT-CTGGTCGCGATATTC-T-----------
------
FC88X_01026_00446        --------------TCACTTCCTAT-CTGGTCGCGATATTC-T-----------
------
FC88X_00020_00528        --------------TCACTTCCTAT-CTGGTCGCGATATTC-T-----------
------
FC88X_00706_01113        --------------TCACTTCCTAT-CTGGTCGCGATATTC-T-----------
------
FC88X_00909_00326        --------------TCACTTCCTAT-CTGGTTGCGATATTC-T-----------
------
FC88X_00909_00769        -------------TTCACTTCCTAT-CTGGTCGCGATATTC-TTCT--------
------
FC88X_01056_01259        -------------GTCACTTCCTAG-CTGGTCGCGATATTC-T-----------
------
FC88X_00684_01259        --------------TCACTCCCTAT-CTGGTCGCGATATTC-T-----------
------
FC88X_00834_00939        --------------TCACTTCCTAT-CTGGTCGCGGTATTC-T-----------
------
FC88X_00292_01086        --------------TCTATTCCTGT-TTGTTGGCGACTTTC-T-----------
------
FC88X_00855_00158        --------------TCTATGTCTCT-TTGGTCGCCTTTCTC-CT----------
------
FC88X_00386_00068        --------------CCACGTGCTGT-TTGGTCCCTTCTTTG-T-----------
------
FC88X_01058_00941        --------------CCACGTGCTGT-TTGGTCCCTTCTTTG-T-----------
------
FC88X_00446_00164        ---------------CACGTGCCCTGTTGGTCGCTTGATTC-GC----------
------
FC88X_00131_00600        -----------CGTTGACGTTGT--TTCAATGGCTATTTA--------------
------
```

```
FC88X_01060_00922       -----------CGTTGACGTTGT--TTCAATGGCTT------------------
------
FC88X_00230_01024       ------------CCTGAGGTTGTTCTTCATCGGCTTTCT---------------
------
FC88X_00857_00295       ------------CCTGAGGTTGTTCTTCATCGGCTTTCT---------------
------
FC88X_00961_00525       ------------CCTGAGGTTGTTCTTCATCGGCTTTCT---------------
------
FC88X_00976_00575       ------------CCTGAGGTTGTTCTTCATCGGCTTTCT---------------
------
FC88X_00335_00423       ------------CCTGAGGTTGTTCTTCATTGGCTTTCT---------------
------
FC88X_00655_00189       --------CTAAATCCTCATTGTTTCTCGCTAATT-------------------
------
FC88X_00700_00920       ---------------------TCATCTCCTTA-TTTCCCGA-TCCCTC------
------
FC88X_00775_00710       -------------------GCGCACCTCATTGGTTTCGCCG-GTCCTC------
------
FC88X_00770_00945       ------------------ATCATCCCTCATTGCTCCCGGCT-TACA--------
------
FC88X_00929_01245       ------------------ATCA--CCCAGTTGGTTCCGG---------------
------
FC88X_00605_00863       -----------------TTCTGCCTTGTTTACGACTTTCTC-TTA---------
------
FC88X_00142_00743       -----------------CTCTGCCTTGTTTACGACTTTCTC-TTA---------
------
FC88X_00144_00991       -----------------CTGCTTTTTTTCGCGGACACTCCC-TTATT-------
------
FC88X_00229_00091       ------------CTTCAGACCTGTGCATTGCTGACATTCTC-------------
------
FC88X_00982_01214       ------------CTGTGCATTATTGCA--GCAGACCTTCT--------------
------
FC88X_00054_00834       -------------ACCAACCTGTTTTT-GTCGGTCATTCTC-------------
------
FC88X_00320_01042       -------------ACCAACCTGTTTTTTGTCGGTCATTCTC-------------
------
FC88X_00330_01011       -------------ACCAACCTGTTTTT-GTCGGTCACTCTC-------------
------
FC88X_00302_01138       ------------AATCAGCCTGTCGTTGACGTGG--------------------
------
FC88X_00473_00004       ------------TAGCAACCTGT-GTTTACTTGTCTCCTT--------------
------
FC88X_00457_01163       -------------------CTTGCAGCTCGAGCCTGTGA--TGTTCT-------
------
FC88X_00529_01022       -------------------CTTGCAGCTCGAGCCTGTCGACTGTTCT-------
------
FC88X_00042_00598       -------------------CTTGCAGCTCGAGCCTGTGA-CTGTTCT-------
------
FC88X_00093_00031       -------------------CTTGCAGCTCGAGCCTGTGA-CTGTTCT-------
------
FC88X_00082_00355       -------------------CTTGCAGCTCGAGCCTGTGA-CTGTTCT-------
------
FC88X_00124_00962       -------------------CTTGCAGCTCGAGCCTGTGA-CTGTTCT-------
------
FC88X_00096_00684       -------------------CTTGCAGCTCGAGCCTGTGA-CTGTTCT-------
------
```

FC88X_00635_00287 -----------------AACTTGCAGCTCGAGCCTGTGA-CTGTTCT-------
------
FC88X_00304_00606 -------------------CTTGCAGCTCGAGCCTGTGA-CTGTTCT-------
------
FC88X_00633_00094 -------------------CTTGCAGCTCGAGCCTGTGA-CTGTTCT-------
------
FC88X_00650_00084 -------------------CTTGCAGCTCGAGCCTGTGA-CTGTTCT-------
------
FC88X_00467_00105 ------------------GCTTGCAGCTCGAGCCTGTGA-CTGTTCT-------
------
FC88X_00980_00784 ------------------GCTTGCAGCTCGAGCCTGTGA-CTGTTCT-------
------
FC88X_00092_00454 -------------------CTTGCAGCTCGAGCCTGTGA-CTGTTCT-------
------
FC88X_00170_00126 -------------------CTTGCAGCTCGAGCCTGTGA-CTGTTCT-------
------
FC88X_00512_01014 -------------------CTTGCAGCTCGAGCCTGTGA-CTGTTCT-------
------
FC88X_00608_01202 -------------------CTTGCAGCTCGAGCCTGTGA-CTGTTCT-------
------
FC88X_00659_00092 -------------------CTTGCAGCTCGAGCCTGTGA-CTGTTCT-------
------
FC88X_00800_01010 -------------------CTTGCAGCTCGAGCCTGTGA-CTGTTCT-------
------
FC88X_00870_00219 -------------------CTTGCAGCTCGAGCCTGTGA-CTGTTCT-------
------
FC88X_00885_00405 -------------------CTTGCAGCTCGAGCCTGTGA-CTGTTCT-------
------
FC88X_00942_00323 -------------------CTTGCAGCTCGAGCCTGTGA-CTGTTCT-------
------
FC88X_00958_00826 -------------------CTTGCAGCTCGAGCCTGTGA-CTGTTCT-------
------
FC88X_01118_00676 -------------------CTTGCAGCTCGAGCCTGTGA-CTGTTCT-------
------
FC88X_00038_00440 -------------------CTTGCAGCTCGAGCCTGTGA-CTGTTCT-------
------
FC88X_00015_00674 -------------------CTTGCAGCTCGAGCCTGTGA-CTGTTCT-------
------
FC88X_00081_00084 -------------------CTTGCAGCTCGAGCCTGTGA-CTGTTCT-------
------
FC88X_00082_00567 -------------------CTTGCAGCTCGAGCCTGTGA-CTGTTCT-------
------
FC88X_00129_00115 -------------------CTTGCAGCTCGAGCCTGTGA-CTGTTCT-------
------
FC88X_00104_00714 -------------------CTTGCAGCTCGAGCCTGTGA-CTGTTCT-------
------
FC88X_00139_00941 -------------------CTTGCAGCTCGAGCCTGTGA-CTGTTCT-------
------
FC88X_00242_00500 -------------------CTTGCAGCTCGAGCCTGTGA-CTGTTCT-------
------
FC88X_00449_00099 -------------------CTTGCAGCTCGAGCCTGTGA-CTGTTCT-------
------
FC88X_00492_00050 -------------------CTTGCAGCTCGAGCCTGTGA-CTGTTCT-------
------
FC88X_00495_00518 -------------------CTTGCAGCTCGAGCCTGTGA-CTGTTCT-------
------

FC88X_00496_00985 -------------------CTTGCAGCTCGAGCCTGTGA-CTGTTCT-------
------
FC88X_00598_00616 -------------------CTTGCAGCTCGAGCCTGTGA-CTGTTCT-------
------
FC88X_00551_01116 -------------------CTTGCAGCTCGAGCCTGTGA-CTGTTCT-------
------
FC88X_00639_01008 -------------------CTTGCAGCTCGAGCCTGTGA-CTGTTCT-------
------
FC88X_00635_01239 -------------------CTTGCAGCTCGAGCCTGTGA-CTGTTCT-------
------
FC88X_00731_01131 -------------------CTTGCAGCTCGAGCCTGTGA-CTGTTCT-------
------
FC88X_00794_00278 -------------------CTTGCAGCTCGAGCCTGTGA-CTGTTCT-------
------
FC88X_00781_01111 -------------------CTTGCAGCTCGAGCCTGTGA-CTGTTCT-------
------
FC88X_00829_00264 -------------------CTTGCAGCTCGAGCCTGTGA-CTGTTCT-------
------
FC88X_00813_00313 -------------------CTTGCAGCTCGAGCCTGTGA-CTGTTCT-------
------
FC88X_00837_00457 -------------------CTTGCAGCTCGAGCCTGTGA-CTGTTCT-------
------
FC88X_00842_00957 -------------------CTTGCAGCTCGAGCCTGTGA-CTGTTCT-------
------
FC88X_00895_00752 -------------------CTTGCAGCTCGAGCCTGTGA-CTGTTCT-------
------
FC88X_00885_00818 -------------------CTTGCAGCTCGAGCCTGTGA-CTGTTCT-------
------
FC88X_00853_00870 -------------------CTTGCAGCTCGAGCCTGTGA-CTGTTCT-------
------
FC88X_00916_00439 -------------------CTTGCAGCTCGAGCCTGTGA-CTGTTCT-------
------
FC88X_00905_00568 -------------------CTTGCAGCTCGAGCCTGTGA-CTGTTCT-------
------
FC88X_00941_00560 -------------------CTTGCAGCTCGAGCCTGTGA-CTGTTCT-------
------
FC88X_00948_00735 -------------------CTTGCAGCTCGAGCCTGTGA-CTGTTCT-------
------
FC88X_00907_01259 -------------------CTTGCAGCTCGAGCCTGTGA-CTGTTCT-------
------
FC88X_00977_00525 -------------------CTTGCAGCTCGAGCCTGTGA-CTGTTCT-------
------
FC88X_00964_00735 -------------------CTTGCAGCTCGAGCCTGTGA-CTGTTCT-------
------
FC88X_01023_00745 -------------------CTTGCAGCTCGAGCCTGTGA-CTGTTCT-------
------
FC88X_01069_00425 -------------------CTTGCAGCTCGAGCCTGTGA-CTGTTCT-------
------
FC88X_01086_00742 -------------------CTTGCAGCTCGAGCCTGTGA-CTGTTCT-------
------
FC88X_01097_00864 -------------------CTTGCAGCTCGAGCCTGTGA-CTGTTCT-------
------
FC88X_01098_01164 -------------------CTTGCAGCTCGAGCCTGTGA-CTGTTCT-------
------
FC88X_00890_00417 -------------------CTTGCAGCTCGAGCCTGTGACCTGTTCT-------
------

FC88X_00279_00952 -------------------CTTGCAGCTCGAGCCTGTGA-CTGTTCTC------
------
FC88X_00945_00614 -------------------CTTGCAGCTCGAGCCTGTGA-CTGTTCTCC-----
------
FC88X_00940_00774 -------------------CTTGCGGCTCGAGCCTGTGA-CTGTTCT-------
------
FC88X_00537_00144 ----------------TCTCTTGCAGCTCGAGCCTGTGA-CTGTTCT-------
------
FC88X_00131_00309 -------------------CCTGCAGCTCGAGCCTGTGA-CTGTTCT-------
------
FC88X_00685_00905 ------------------GCTTGCAGCTCGAGCCTGTGA-CGGTTCT-------
------
FC88X_00738_00218 ------------------GCTTGCAGCTCGAGCCTGTGA-CTGTTCC-------
------
FC88X_00039_00786 -------------------CTTACAGCTCGAGCCTGTGA-CTGTTCT-------
------
FC88X_00950_00750 -------------------CTAGCAGCTCGAGCCTGTGA-CTGTTCT-------
------
FC88X_00060_00805 --------------GGGGACTTGCAGCTCGAGCCTGTGA-CTGTTCT-------
------
FC88X_00917_00725 -------------------CTTGCAGCTGGAGCCTGTGA-CTGTTCTT------
------
FC88X_00478_01076 -------------------CTTGCAGCTCGAGCCTGTGA-CTGTTCTCTTCT--
------
FC88X_01002_00500 -------------------CTTGCAGCTCGAGCCTGTGA-
CTGTTCTCTTCCTTT-----
FC88X_01092_00856 -------------------CTTGCAGCTCGAGCCTGTGA-
CTGTTCTCTTCCTTT-----
FC88X_00381_00118 -------------------CTTGCAGCTCGAGCCTGTGA-
CTGTTCTCTTCCTTTC----
FC88X_00861_01223 -------------------CTTGCAGCTCGAGCCTGTGA-
CCGTTCTCTTCCTTTCTCC-
FC88X_00099_00112 ------------------CCTCGCAGGTCGAGTGTAGGA-CTGTTCCCC-----
------
FC88X_00825_01196 -------------------TCTGCAGGTCGAGTGTAGGA-CTGTTCC-------
------
FC88X_00891_00215 ------------------CCTTGCAGGTCGAGTGTACGA-GTGTC---------
------
FC88X_00658_01041 -----------TCAACTCCCTTTCAGATCGATCGTATT----------------
------
FC88X_00022_00454 -----------TCAACTCCCTTTCAGATCGATCGTATT----------------
------
FC88X_00397_01025 -----------TCAACTCCCTTTCAGATCGATCGTATT----------------
------
FC88X_00511_01142 ---------------CTACGTTTTAACCCGATCGT-------------------
------
FC88X_01046_01010 -----------CTAACTTGGTTGTAGCTCGACCCTCTT----------------
------
FC88X_00037_00703 ------------------TCCTGTGCACTA--TAGTTCCG-GGTTCTT------
------
FC88X_00540_01165 ------------------TCCTGTGCACTA--TAGTTCCG-GGTTCTT------
------
FC88X_00778_00130 ---------------------CTGCCCACTAGTTACTTCCTTGGTTATT------
------
FC88X_00328_01148 -------------------GCCGCTGGTCGC-CAGAACCACAGTCCT-------
------

FC88X_01086_00408 ----------------------GCTGGTCAA-TAGCTCGACGTCCTTTATT---
------
FC88X_00068_00428 --------------------CCCCACCCTTTCCCTATGCTCATTTCT-------
------
FC88X_00610_00183 ---------------------------CTCTCCGTA-GCTCATAACT-------
------
FC88X_00946_00200 -------------------GCAATTGGTTGTCCGTT-GCTTATTACT-------
------
FC88X_00477_01216 --------------------CCATTGGCTCCCCAGCTGATTGATACT-------
------
FC88X_00394_00016 --------------------ACAACGGCTCCCCCGCTTGACACTACT-------
------
FC88X_00385_01176 ---------------------CCGCAGATT-TTAATAGACGGTGCCTCC-----
------
FC88X_01016_01026 ---------------------CCTCCGATTATTAATAGTCCGAGCATC------
------
FC88X_00095_00283 --------------------TCCACTGATTGTCAGTAGCTCCTAGC--------
------
FC88X_00088_00466 -----------------CTCCCAAACTACATTTTGTGATAGCCCGTAAC-----
------
FC88X_00955_00473 ------------------------ACTACGTTT-GTCATATCT-----------
------
FC88X_01119_01071 --------------------CTACACTGGATTTAGTCGTAACCTCTT-------
------
FC88X_00031_00185 -------------------CCTACACTTC-TTTAGTGCTAAGTTCTT-------
------
FC88X_00986_00657 ------------------TCCTACAG-TTCGT-AACAACTA-
GTTCTCCTTCCTTT----
FC88X_00965_00522 ------------------TCCTACAG-TTCGT-AACAACTA-GTTCTC------
------
FC88X_00291_00048 ------------------TCCTACAG-TTCGT-AGCAACTA-GTTCTC------
------
FC88X_00945_01176 -------------------CCTACAC-TCTGTCAGTAGCTA-GCTGTC------
------
FC88X_00021_00580 --------------------TTACACCTCTGTTGATTGCTA-TATCTC------
------
FC88X_00148_00765 --------------------TTACACCTCTGTTGATTGCTA-TATCTC------
------
FC88X_00453_00372 --------------------TTACACCTCTGTTGATTGCTA-TATCTC------
------
FC88X_00998_00761 --------------------TTACACCTCTGTTGATTGCTA-TATCTC------
------
FC88X_01084_00883 --------------------TTACACCTCTGTTGATTGCTA-TATCTC------
------
FC88X_00341_00811 --------------------TTACACCTCTGCTGATTGCTA-TATCTC------
------
FC88X_00790_00689 --------------------TTACACCTCTGTTGATTTCTA-TATCTC------
------
FC88X_01035_01062 --------------------TTACACCTCTGTTGATCGCTA-TATCTC------
------
FC88X_00965_01182 --------------------TTACACCTCTGTTGGTTGCTA-TATCTC------
------
FC88X_01046_00974 --------------------TTACACCTTTGCTAATTGCTAGTATCTC------
------
FC88X_00655_00182 ---------------------CTGGCACACACACGTCA-TATCTTCTC------
------

```
FC88X_00880_00928       ---------------------CTGGCACACACACGTCAGTATCTTCTCCC----
------
FC88X_00301_00506       --------------------GCTGGCACACACACGTCAGTATCTTCTCCC----
------
FC88X_00008_00339       ---------------------CTGGCACACACACGTCAGTATCTTCTC------
------
FC88X_00008_00621       ---------------------CTGGCACACACACGTCAGTATCTTCTC------
------
FC88X_00041_00602       ---------------------CTGGCACACACACGTCAGTATCTTCTC------
------
FC88X_00444_01133       --------------------GCTGGCACACACACGTCAGTATCTTCTC------
------
FC88X_00110_00045       ---------------------CTGGCACACACACGTCAGTATCTTCTC------
------
FC88X_00131_00035       ---------------------CTGGCACACACACGTCAGTATCTTCTC------
------
FC88X_00140_00566       ---------------------CTGGCACACACACGTCAGTATCTTCTC------
------
FC88X_00128_00626       ---------------------CTGGCACACACACGTCAGTATCTTCTC------
------
FC88X_00155_01000       ---------------------CTGGCACACACACGTCAGTATCTTCTC------
------
FC88X_00216_00245       ---------------------CTGGCACACACACGTCAGTATCTTCTC------
------
FC88X_00334_01057       ---------------------CTGGCACACACACGTCAGTATCTTCTC------
------
FC88X_00372_00959       ---------------------CTGGCACACACACGTCAGTATCTTCTC------
------
FC88X_00428_00404       ---------------------CTGGCACACACACGTCAGTATCTTCTC------
------
FC88X_00490_00785       ---------------------CTGGCACACACACGTCAGTATCTTCTC------
------
FC88X_00565_00809       ---------------------CTGGCACACACACGTCAGTATCTTCTC------
------
FC88X_00601_00925       ---------------------CTGGCACACACACGTCAGTATCTTCTC------
------
FC88X_00623_00968       ---------------------CTGGCACACACACGTCAGTATCTTCTC------
------
FC88X_00648_01043       ---------------------CTGGCACACACACGTCAGTATCTTCTC------
------
FC88X_00684_00067       ---------------------CTGGCACACACACGTCAGTATCTTCTC------
------
FC88X_00791_00948       ---------------------CTGGCACACACACGTCAGTATCTTCTC------
------
FC88X_00885_00171       ---------------------CTGGCACACACACGTCAGTATCTTCTC------
------
FC88X_00895_00639       ---------------------CTGGCACACACACGTCAGTATCTTCTC------
------
FC88X_00894_00777       ---------------------CTGGCACACACACGTCAGTATCTTCTC------
------
FC88X_00922_00548       ---------------------CTGGCACACACACGTCAGTATCTTCTC------
------
FC88X_00937_01252       ---------------------CTGGCACACACACGTCAGTATCTTCTC------
------
FC88X_01039_01160       ---------------------CTGGCACACACACGTCAGTATCTTCTC------
------
```

FC88X_01093_01004 ---------------------CTGGCACACACACGTCAGTATCTTCTC------
------
FC88X_01078_01103 ---------------------CTGGCACACACACGTCAGTATCTTCTC------
------
FC88X_01145_00882 ---------------------CTGGCACACACACGTCAGTATCTTCTC------
------
FC88X_00082_00215 ---------------------CTGGCACACACACGTCAGTATCTTCTC------
------
FC88X_00101_00131 ---------------------CTGGCACACACACGTCAGTATCTTCTC------
------
FC88X_00126_00740 ---------------------CTGGCACACACACGTCAGTATCTTCTC------
------
FC88X_00102_00930 ---------------------CTGGCACACACACGTCAGTATCTTCTC------
------
FC88X_00183_00046 ---------------------CTGGCACACACACGTCAGTATCTTCTC------
------
FC88X_00215_00878 ---------------------CTGGCACACACACGTCAGTATCTTCTC------
------
FC88X_00261_00701 ---------------------CTGGCACACACACGTCAGTATCTTCTC------
------
FC88X_00460_00499 ---------------------CTGGCACACACACGTCAGTATCTTCTC------
------
FC88X_00664_00276 ---------------------CTGGCACACACACGTCAGTATCTTCTC------
------
FC88X_00650_01098 ---------------------CTGGCACACACACGTCAGTATCTTCTC------
------
FC88X_00838_00341 ---------------------CTGGCACACACACGTCAGTATCTTCTC------
------
FC88X_00840_01250 ---------------------CTGGCACACACACGTCAGTATCTTCTC------
------
FC88X_00856_00195 ---------------------CTGGCACACACACGTCAGTATCTTCTC------
------
FC88X_00893_00698 ---------------------CTGGCACACACACGTCAGTATCTTCTC------
------
FC88X_00874_00722 ---------------------CTGGCACACACACGTCAGTATCTTCTC------
------
FC88X_00859_01062 ---------------------CTGGCACACACACGTCAGTATCTTCTC------
------
FC88X_00903_00896 ---------------------CTGGCACACACACGTCAGTATCTTCTC------
------
FC88X_00666_00187 ---------------------
CTGGCACACACACGTCAGTATCTTCTCCTTCCTTT----
FC88X_00486_00096 ---------------------CTGGCACACACGCGCCAGTATCTTCTC------
------
FC88X_00919_00632 ---------------------CTGGCACACACACGCCAGTATCTTCTC------
------
FC88X_00390_00769 ---------------------CTGGCACACGCACGTCAGTATCTTCTC------
------
FC88X_00822_00313 ---------------------CTGGCATACACACGTCAGTATCTTCTC------
------
FC88X_00606_01152 ---------------------CCGGCACACACACGTCAGTATCTTCTC------
------
FC88X_00808_00315 ---------------------CCGGCACACACACGTCAGTATCTTCTC------
------
FC88X_00786_00921 ---------------------CTGGCGCACACACGTCAGTATCTTCTC------
------

FC88X_00265_01044 --------------------CTAGGCACACACACGTCAGTATCTTCTC------
------
FC88X_00124_00024 --------------------CAGTGCAAAGATCCGTCTGTCTACTCTC------
------
FC88X_00256_00089 --------------------CAGTGCAAAGATCCGTCTGTCTACTCTC------
------
FC88X_00804_00174 -------------------GCAGTGCAAAGATCCGTCTGTCTACTCTC------
------
FC88X_00568_00154 --------------------CAGTGCAAAGATCCGCCTGTCTACTCTC------
------
FC88X_00216_00910 --------------------
CAGTGCAAAGATCCGTCTGTCTACTCTCCTTCCTTT----
FC88X_00582_01024 --------------------
CAGTGCAAAGATCCGTCTGTCTACTCTCCTTCCTTT----
FC88X_00649_00171 --------------------
CAGTGCAAAGATCCGTCTGTCTACTCTCCTTCCTTT----
FC88X_00368_00663 --------------------CAGCGCAAAGATCCGTCTGTCTACTCTC------
------
FC88X_01113_00469 --------------------CAGTGCGAAGATCCGTCTGTCTACCCTC------
------
FC88X_00074_00208 -------------------TCCCAGGGG-CGA-GGACACAATATTCTC------
------
FC88X_00812_01167 -------------------TCCCAGGGG-CGA-GGACACAATATTCTC------
------
FC88X_00954_00957 -------------------TCCCAGGGG-CGA-GGACACAATATTCTC------
------
FC88X_00520_00921 -------------------TCCCAGGGG-CGA-GGACACAATATTCTC------
------
FC88X_00809_00864 -------------------TCCCAGGGG-CGA-GGACACAATATTCTC------
------
FC88X_01071_00700 -------------------TCCCAGGGG-CGA-GGACACAATATTCTC------
------
FC88X_01103_01024 -------------------TCCCAGGGG-CGA-GGACACAATATTCTC------
------
FC88X_01120_00609 ------------------TTCCCAGGTC-GCA-AGCCACAA-ATTCTC------
------
FC88X_01051_01158 -------------------TCCTAGGGTTCGA-AGCCCGTGGATTCTC------
------
FC88X_00194_00569 --------------------ACTGGTCGTCAA-GGACAGTGTGTTCTT------
------
FC88X_00509_00099 --------------------ACTGGTCGTCAA-GGACAGTGTGTTCTT------
------
FC88X_01071_00578 --------------------ACTGGTCGTCAA-GGACAGTGTGTTCTT------
------
FC88X_00139_00134 --------------------ACTGGTCGTCAAAGGACAGTGTGTTCTT------
------
FC88X_00710_00166 -------------------AACTGGTCGTCAA-GGACAGTGTGTTCTT------
------
FC88X_00988_01251 --------------------ACTGGTCGCCAA-GGACAGTGTGTTCTT------
------
FC88X_01102_00763 --------------------CCCGTAGTTCGCAAGACTGTCTGTTTTC------
------
FC88X_00806_00250 --------------------CCCCTAGATCTCT-GACTGTGTGTTACT------
------
FC88X_00034_00473 ---------------------CCATTGCATTCATGCTGGTATATCCTC------
------

FC88X_00449_00797 ---------------------CCATTGCATTCATGCTGGTATATCCTC------
------
FC88X_00755_00911 ---------------------CCATTGCATTCATGCTGGTATATCCTC------
------
FC88X_00400_00074 ---------------------CCATTGCATTCATGCTGGTATATCCTC------
------
FC88X_01084_00482 ---------------------CCATTGCATTCATGCTGGTATATCCTC------
------
FC88X_00228_00920 ---------------------CCATTGCACTCATGCTGGTATATCCTC------
------
FC88X_00057_00354 ---------------------CCAATACACGGAAGCTTGTCTGTTCTC------
------
FC88X_00579_00121 ---------------------CCAATACACGGAAGCTTGTCTGTTCTCC-----
------
FC88X_00597_01125 ---------------------CCAATACACGGAAGCTTGTCTGTTCTC------
------
FC88X_00444_00904 ----------------------CAATACACGGAAGCTTGTCTGTTCTC------
------
FC88X_00959_00926 ---------------------
CCAATACACGGAAGCTTGTCTGTTCTCCTTCCTTT----
FC88X_00617_01158 ---------------------CCAATGCACGGAAGCTCGTCTGTTCTC------
------
FC88X_00815_01221 ---------------------CCAATGCACGGAAGCTTGTCTGTTCTC------
------
FC88X_00060_00282 ---------------------TCACGTAATCCACATTTGTGTCTTCTC------
------
FC88X_00613_00961 ---------------------TCACGTAATCCACATTTGTGTCTTCTC------
------
FC88X_00869_01133 ---------------------TCACGTAATCCACATTTGTGTCTTCTC------
------
FC88X_00716_01144 ---------------------TCACGTAATCCACATTTGTGTCTTCTC------
------
FC88X_00801_00338 ---------------------TCACGTAATCCACATTTGTGTCTTCTC------
------
FC88X_00564_00967 ---------------------TCACGTAATTCACATTTGTGTCTTCTC------
------
FC88X_00255_00680 ---------------------GGACCCTAATCAGAATGGTGTCATTTCTC----
------
FC88X_00350_01024 -----------------------ACCCTAATCAGAATGGTGTCATTTCTC----
------
FC88X_00824_00158 -----------------------ACCCTAATCAGAATGGTGTCATTTCTC----
------
FC88X_00804_00864 -----------------------ACCCTAATCAGAATGGTGTCATTTCTC----
------
FC88X_01113_00773 ------------------------ACCTAATCAGAATGGTGTCATTTCTC----
------
FC88X_00284_00079 ------------------------ACCTAATCGGAATGGTGTCATTTCTC----
------
FC88X_00026_00713 -----------------CCAACGACTGGCTGAATACTCCCGCAA----------
------
FC88X_00023_00755 --------------TTCCCAACCACACGGCAGTATCTCCCG-------------
------
FC88X_00158_00922 -----------------CCAACTACTACGCGGCTTTCTCCAGTA----------
------
FC88X_00067_00599 -----------------CCACTGATTAATCGGCTGACTCGACTT----------
------

FC88X_00436_00083 -----------------CCTAGTTCTAATCAACACGTCTGTCTC----------
------
FC88X_00935_00284 -----------------CCTAGTTCTAATCAACCA-------------------
------
FC88X_01108_00793 -----------------CCTGATACTAACCAGCTTGACTATCTC----------
------
FC88X_00943_00230 -----------------CCTGATACTAACCAGCTTGACTATCTC----------
------
FC88X_00024_00300 -----------------CCTAATACTAACCAGCTTGACTATCTC----------
------
FC88X_00219_00993 ------------------GCAATAGTCATGAGACCGTCGGCTGCTC--------
------
FC88X_00911_01136 -------------------CAATAGTCATGAGACCGTCGGCTGCTC--------
------
FC88X_00868_01089 ------------------CCAATAGTCATGAGACCGTCGGCTGCTC--------
------
FC88X_00444_00230 ------------------CCAATAGTCATGAGACCGTCGGCTGCTC--------
------
FC88X_00616_01177 ------------------CCAATAGTCATGAGACCGTCGGCTGCTC--------
------
FC88X_00845_01151 ------------------CCAATAGTCATGAGACCGTCGGCTGCTC--------
------
FC88X_00926_00593 ------------------CCAATAGTCATGAGACCGTCGGCTGCTC--------
------
FC88X_00286_00780 ------------------CCAATAGTCATGAGACCGTCGGCTGCCC--------
------
FC88X_00852_00972 -----------------CCAAATAGTCATGAGACCGTCGGCTGCTC--------
------
FC88X_00899_00913 ------------------CCAATTGTATTAAAGCTGTCTGCCCTC---------
------
FC88X_00960_00358 ------------------CCAATTGTATTAAAGCTGTCTGCCCTC---------
------
FC88X_00879_00759 ---------------CTCCCCACTAGTCTTATGCCGTCTGCC------------
------
FC88X_00094_00118 ------------------CTCACATTCTAATCCCG-CTGACCGTCAC-------
------
FC88X_01112_00702 ------------------CTCGTAATAGAATCACGACTGACTGTC---------
------
FC88X_00076_00304 --------------------TTTCCTGCTGACCAT-GCATTACTCTAT------
------
FC88X_00359_01079 --------------------TTCCCAGCTGTGCA--GCGTTAGTCGACC-----
------
FC88X_00598_00173 ----------------------TGGCGCAGATGAGCGTGCTACCCTACT-----
------
FC88X_00461_00025 ----------------------TGGCGCAGATGAGCGTGCTACCCTACT-----
------
FC88X_00902_00423 ----------------------TCAAGCAAACAACCTTGCTGCCCTAAT-----
------
FC88X_00164_00827 ----------------------TCAAGCAAACAACCTTGCTGCCCTAAT-----
------
FC88X_00235_00984 ----------------------TCAAGCAAACAACCTTGCTGCCCTAAT-----
------
FC88X_00931_00604 ----------------GCCAACACTTGCTGCTCCTG--ATAATTAT--------
------
FC88X_00975_00902 -----------------CCAACACTTGCTGCTCCTG--ATAATTAT--------
------

FC88X_01020_00869 -----------------CCAACACTTGCTGCTCCTG--ATAATTAT--------
------
FC88X_00968_00981 ----------------------CTTTGCTGCCCATGACATGATTATATT-----
------
FC88X_01116_00926 ----------------------CTTTGCTGCCCATGACATGATTATATT-----
------
FC88X_00164_00083 ----------------------CTTTGCTGCCCATGACATGATTATATT-----
------
FC88X_00162_00393 ----------------------CTTTGCTGCCCATGACATGATTATATT-----
------
FC88X_00109_00915 -------------------CTAT--GACTGGTGTCTCGGGTTATTTTC------
------
FC88X_00239_00081 -------------------CTAT--GACTGGTGTCTCGGGTTATTTTC------
------
FC88X_00903_00397 -------------------CTATCAGATCACTGGTGCGCGTGATT---------
------
FC88X_00988_01266 -------------------CTATCAGATCACTGGTGCGCGTATTTTCCC-----
------
FC88X_00386_00329 --------------------TGCCTCGTGACGGTAATGAGTACGCTG-------
------
FC88X_00772_00088 -----------------------------------ATGACTAGG----------
------
FC88X_00419_00830 ----------------------TTTGACAACATGTGT-CCTGCTGCCCTC----
------
FC88X_01137_00679 ----------------------TTTGACAACATGTGT-CCTGCTGCTCTC----
------
FC88X_00103_00063 ----------------------TTTGACAACGTGTGT-CCTGCTGTTCTC----
------
FC88X_00763_00278 -----------------------TTGACTACTCGTGAGCCTACTGTTCCC----
------
FC88X_01027_00562 -----------------------TTGACTACTCGTGAGCCTACTGTTCCC----
------
FC88X_00366_01181 ----------------------CCTAACCACCTGTGATCGGGCTGATTA-----
------
FC88X_00153_00019 --------------------CCTAGTGGTG-T-ACCGCCC-AGCTGTCTCC---
------
FC88X_00120_00468 --------------------CCTAGTGGTG-T-ACCGCCC-AGCTGTCTC----
------
FC88X_00111_00585 --------------------CCTAGTGGTG-T-ACCGCCC-AGCTGTCTC----
------
FC88X_00198_00477 --------------------CCTAGTGGTG-T-ACCGCCC-AGCTGTCTC----
------
FC88X_00187_00871 --------------------CCTAGTGGTG-T-ACCGCCC-AGCTGTCTC----
------
FC88X_00564_01131 --------------------CCTAGTGGTG-T-ACCGCCC-AGCTGTCTC----
------
FC88X_00703_01019 --------------------CCTAGTGGTG-T-ACCGCCC-AGCTGTCTC----
------
FC88X_00923_00699 --------------------CCTAGTGGTG-T-ACCGCCC-AGCTGTCTC----
------
FC88X_00553_00604 -------------------TCCTAGTGGTG-T-ACCGCCC-AGCTGTCTCCT--
------
FC88X_01030_01098 --------------------CCTAGTGGTG-T-ACCGCCCCAGCTGTCTC----
------
FC88X_00112_00438 --------------------CCTAGTGGTG-T-ACCGCCC-
AGCTGTCTCCTTCC-----

FC88X_00473_00188 --------------------ACTAGTGGTG-T-ACCGCCC-AGCTGTCTC----
------
FC88X_00490_00021 ---------------------CTAGTGGTG-T-ACCGCCT-AGCTGTCTC----
------
FC88X_00588_00122 --------------------CCTAGTGGTG-T-ACCGCCA-AGCTGTCTC----
------
FC88X_00910_00961 --------------------CCTAGTGGTG-T-ACCGCCA-AGCTGTCTC----
------
FC88X_00709_00066 --------------------CTCAGTGGTGGC-ACCGGCCCAGCTGTCTC----
------
FC88X_00596_00977 --------------------CCTAGTGGTGCTGACCGCCC--------------
------
FC88X_00883_00825 ---------------------CCACAGCTTGTGGTCAACCCCCTCCTT------
------
FC88X_01002_00982 --------------------CTAACAGCCTGT--TCACCTCCCTGCATAC----
------
FC88X_00644_00073 --------------------GCACCAGCTTGTGAACTCCCGCAACCG-------
------
FC88X_00035_00594 ---------------------CACGCGGTGGTTAACGGCCCGGCTTCA------
------
FC88X_00058_00571 --------------------CCACGATGTTGTGAACTCCCCTGCCTC-------
------
FC88X_00891_00355 -------------------CATGAGCACATC-TTACTGCCGAC-TATC------
------
FC88X_00046_00374 -------------------CATGAGCACATC-TTACTGCCGAC-TATC------
------
FC88X_00898_00218 -------------------CATGAGCACATC-TTACTGCCGAC-TATCCTTCC-
------
FC88X_00227_00061 -------------------CATGAGCACATC-TTATTGCCGAC-TATC------
------
FC88X_01057_00917 -------------------CATTAGCACATC-TTGCTGCCGAC-TATC------
------
FC88X_00521_00619 -------------------CATGAACACATC-TTACTGCCGAC-TATC------
------
FC88X_00594_00801 ---------------------CCAACAGCTCAGTACTGCTGCCCTATC------
------
FC88X_00324_00132 ---------------------CCAACAGCTCAGTACTGCTGCCCTATC------
------
FC88X_00507_00054 -------------------TGCGAACACCT--GTCGTGCCCACCTATC------
------
FC88X_00494_01138 ---------------------CTATCACTACTGTAGTGGGCACTTCTC------
------
FC88X_00515_00080 --------------------GCTATCACTACTGTAGTGGCCACTTCTC------
------
FC88X_00230_00244 ---------------------CTATCACTACTGTAGTGAGCACTTCTC------
------
FC88X_00395_00205 ---------------------CTATCACTACTGTAGTGAGCACTTCTC------
------
FC88X_00406_00009 ---------------------CTATCACTACTGTAGTGAGCACTTCTC------
------
FC88X_00451_00464 ---------------------CTATCACTACTGTAGTGAGCACTTCTC------
------
FC88X_00589_00115 ---------------------CTATCACTACTGTAGTGAGCACTTCTC------
------
FC88X_00807_01228 ---------------------CTATCACTACTGTAGTGAGCACTTCTC------
------

```
FC88X_00988_01160      ---------------------CTATCACTACTGTAGTGAGCACTTCTC------
------
FC88X_00661_00930      ---------------------CTATCACTACTGTAGTGAGCACTTCTC------
------
FC88X_00795_00119      ---------------------CTATCACTACTGTAGTGAGCACTTCTC------
------
FC88X_01097_00839      ---------------------CTATCACTACTGTAGTGAGCACTTCTC------
------
FC88X_00954_00711      ---------------------TTATCACTACTGTAGTGAGCACTTCTC------
------
FC88X_00689_01166      ---------------------CTATCTCTACTGTAGTGAGCACTTCTC------
------
FC88X_00951_00461      ---------------------CTATCACTACTGTGGTGAGCAGTTCTC------
------
FC88X_00455_00882      --------------------CTTATCACTTGT-TGGTGACAACTTCTC------
------
FC88X_00897_01172      --------------------CTTATCACTTGT-TGGTGACAACTTCTC------
------
FC88X_00648_00902      ---------------------CTAACCATACGTTGGCGCCCACTTATT------
------
FC88X_00959_01248      -------------------CCCTAGCCGTAC--TGGCGTCAACATATT------
------
FC88X_00132_00116      ------------------ATTCCTGTTCTCCGG-CTG---CACCCTGCC-----
------
FC88X_00305_01069      ------------------CTCCCTGTTGTGTGG-CTG---CAACATACC-----
------
FC88X_00979_00344      -----------------GCTCCGTACTCCGTGGACTG---CACAATCC------
------
FC88X_00849_00258      ------------------CTCCGTACTCCGTGGACTG---CACAATCC------
------
FC88X_00148_00588      ------------------CTCCAAACTGTTTGGCACG---AACACGCC------
------
FC88X_01087_00717      -----------------CTCCAAAACTGTTTGGCACG---AACACGCC------
------
FC88X_00118_00057      ------------------CTCCAAACTGTTTGGCACG---AACACGCC------
------
FC88X_00172_00087      ------------------CTCCAAACTGTTTGGCACG---AACACGCC------
------
FC88X_00863_00260      ------------------CTCCAAACTGTTTGGCACG---AACACGCC------
------
FC88X_00903_00817      ------------------CTCCAAACTGTTTGGCACG---AACACGCC------
------
FC88X_00328_00644      ------------------CTCCAAACTGTTTGGCACG---AACACGCC------
------
FC88X_00415_01069      ------------------CTCCAAACTGTTTGGCACG---AACACGCCC-----
------
FC88X_00937_00484      ------------------CTCCAAACTGTTTGGCACG---AACACGCC------
------
FC88X_01067_00799      ------------------CTCCAAACTGTTTGGCACG---AACACGCC------
------
FC88X_00627_01180      ------------------CTCCCAACTGGCTGGC-CC---GGATCTCTAC----
------
FC88X_00754_00138      ---------------TAACTCCCACTGGTGTCGCGCC---GGACCT--------
------
FC88X_00763_00951      ------------------TTCCGCATTTTATGACAGC---AATATTCT------
------
```

FC88X_00825_00849 -------------------TTCCGCATTTTATGACAGC---AATATTCT------
------
FC88X_00188_00914 -------------------TTCCGCATTTTATGACAGC---AATATTCT------
------
FC88X_00832_01242 -------------------TTCCGCATTTTATGACAGC---AATATTCT------
------
FC88X_00931_00481 -------------------TTCCGCACTTTATGACAGC---AATATTCT------
------
FC88X_00082_00506 -------------------TTCCGCATTTTATGGCAGC---AATATTCT------
------
FC88X_00182_00986 -------------------TTCCGCATTTTATGACAGC---ATTATTCT------
------
FC88X_00378_00920 -------------------CTCCGCACATTATGCCCCC---AGGATATTCC----
------
FC88X_00791_01208 -------------------CTCCTCACTATTTTGCCGG---CACGTTTT------
------
FC88X_01101_00640 -------------------CTCCTCACTATTTTGCCGG---CACGTTTTT-----
------
FC88X_00854_00183 -------------------CTCCTCACTATTTTGCCGG---CACGTTTT------
------
FC88X_00846_01160 -----------------GTCTTCGCACT-CTTTGCCGC---CCGACGTT------
------
FC88X_00498_00063 -------------------------ACTAATAAGCCGC---
TAAATCGTTACCGTC-----
FC88X_01071_00765 -------------------
CTCCCAACTTTTTAGCCGCAATCATATCCTTCCTTTC-----
FC88X_00100_00411 -------------------TTCCCTGGTGT-TTGAGCCGCGATCTTT--------
------
FC88X_00270_00355 -------------------TTCCCTGGTGTATTGAGCCGCGATCTTT--------
------
FC88X_00114_00508 -------------------TTCCCTGGTGT-TTGAGCCGCGATCTTT--------
------
FC88X_01017_00569 -------------------TTCCCTGGTGT-TTGAGCCGCGATCTTT--------
------
FC88X_01054_00361 -------------------TTCCCTGGTGT-TTGAGCCGCGATCTTT--------
------
FC88X_01113_00526 -------------------TTCCCTGGTGT-TTGAGCCGCGATCTTT--------
------
FC88X_01091_00437 -------------------TTCCCTGGTGT-TTGAGCCGCGATCTTT--------
------
FC88X_01139_00846 -------------------TTCCCTGGTGT-TTGAGCCGCGATCTTT--------
------
FC88X_00841_01002 -------------------TTCCCTGGTGT-TTGAGCCGCGATCTTCTT------
------
FC88X_00017_00681 -------------------TTCCCTGGTGT-
TTTAGCCGCGATCTTTCTTCCTTT------
FC88X_00881_00189 ---------------------CCTCCCTGGATTGAGCCACGATCATACA------
------
FC88X_00922_00394 ---------------------CCTCCCTGGATTGAGCCACGATCATACA------
------
FC88X_01020_00485 -------------------TTCCCTCGTATGTT--GCTG--ATAGTGCCT-----
------
FC88X_00403_00565 ------------------ACTCCCTTGT-TGTT--GCCGCGATATTGC-------
------
FC88X_00909_00635 --------------------TCCCTAGCCTGCTG-GCCATGACTGTCT-------
------

FC88X_00521_00578 -------------------TCCCTAGCCTGCTG-GCCATGACTGTCT-------
------
FC88X_00799_01194 ---------------CCCGTCCCTAG--TGCCT-GCCATGATAGC---------
------
FC88X_00046_00073 --------------------TCCAGCGCCCGTTGAAA-CGTAATTATT------
------
FC88X_00278_00442 --------------------TCCAGCGCCCGTTGAAA-CGTAATTATT------
------
FC88X_00937_00332 --------------------TCCAGCGCCCGTTGAAA-CGTAATTATT------
------
FC88X_00977_00821 --------------------TCCAGCGCCCGTTGAAA-CGTAATTATT------
------
FC88X_00075_00027 --------------------TCCAGCGCCCGTTGAAA-CGTAATTATT------
------
FC88X_00078_00291 --------------------TCCAGCGCCCGTTGAAA-CGTAATTATT------
------
FC88X_00104_00089 --------------------TCCAGCGCCCGTTGAAA-CGTAATTATT------
------
FC88X_00371_00181 --------------------TCCAGCGCCCGTTGAAA-CGTAATTATT------
------
FC88X_00356_01160 --------------------TCCAGCGCCCGTTGAAA-CGTAATTATT------
------
FC88X_00551_00803 --------------------TCCAGCGCCCGTTGAAA-CGTAATTATT------
------
FC88X_00744_00963 --------------------TCCAGCGCCCGTTGAAA-CGTAATTATT------
------
FC88X_00985_00489 --------------------TCCAGCGCCCGTTGAAA-CGTAATTATT------
------
FC88X_01011_00756 --------------------TCCAGCGCCCGTTGAAA-CGTAATTATT------
------
FC88X_01110_00916 --------------------TCCAGCGCCCGTTGAAA-CGTAATTATT------
------
FC88X_00873_00292 --------------------TCCAGCGCCCGTTGAAAACGTAATTATTCTT---
------
FC88X_00128_00716 --------------------TCCAGCGCCCGTTGAAA-CGAAATTATT------
------
FC88X_00116_00443 --------------------TCCGACCCCCGTTGTAG-CGTAAATCTT------
------
FC88X_00719_00058 ----------------------CTACTCCCGTTTCCTGCGTACGACTACA----
------
FC88X_01066_00603 --------------------CCCTTCGCCCGTTGCATG-GTACCACCA------
------
FC88X_00401_00102 --------------------CCAAGCGCTGTGCTTTG-GACACCTATC------
------
FC88X_01013_00681 --------------------CCAAGCGCTGTGCTTTG-GACACCTATC------
------
FC88X_00899_00539 --------------------CCAAGCGCTGTGCTTTG-GACACCTATC------
------
FC88X_00989_00408 --------------------CCAAGCGCTGCGCTTTG-GACACCTATC------
------
FC88X_00932_01173 ------------------TGCCGCGCGATCCCCTGTT-GACAACTAT-------
------
FC88X_00699_01160 --------------------TCGTGCGATCGCCGAAT-CACACCTATC------
------
FC88X_00040_00174 --------------------TCGTGCGATCGCCGAAT-CACACCTATC------
------

FC88X_00878_00959 --------------------TCGTGCGATCGCCGAAT-CACACCTATC------
------

FC88X_00257_00965 --------------------ACTTCCGATCGACCGAT-GATGCCTATC------
------

FC88X_00028_00511 -------------------GCCATCACCCCGTTAAACGCGCAATATT-------
------

FC88X_00861_00385 --------------------CCATCACCCCGTTAAACGCGCAATATT-------
------

FC88X_00877_00572 --------------------CCATCACCCCGTTAAACGCGCAATATT-------
------

FC88X_00774_01021 --------------------CCATCACCC-GTTAAACGCGCAATATT-------
------

FC88X_01034_00327 --------------------CCATCACCCTGTTAAACGCGCAATATT-------
------

FC88X_01052_00634 --------------------CCATCACCCCGTTAGACGCGCAATATT-------
------

FC88X_00816_00143 --------------------CCATCACTCCGTTGAACGCGCAATATT-------
------

FC88X_00938_01093 --------------------CCGTTCGCCCTTTGACTGCAGATTATT-------
------

FC88X_00179_00449 --------------------CCGTTCTCCGGATGACAGCCCCTTATT-------
------

FC88X_00161_00144 ------------------------CTCCCCAGGTGCACGCATAATGTATCC---
------

FC88X_00435_01230 --------------------CCACCGCCCCGGCTGCA-GCAAAATGTT------
------

FC88X_00311_00023 -------------------CTCACTGCCCCGCTCCCT--CATCATGTT------
------

FC88X_00841_00228 ------------------CGCCAATGCCCAG--TCCTCCTTCGACCT-------
------

FC88X_00881_00728 ------------------CGCCAATGCCCAG--TCCTCCTTCGACCT-------
------

FC88X_00692_00241 ------------------CCCCAATGCACAGACTCCTCCATCGC----------
------

FC88X_01002_00548 -------------------CCAATTGCCCAG-CTACTTCTTTATCCTC------
------

FC88X_00650_01274 ---------------GATCCGCGTTCGCCCG-CCGT-GATGTATCCTTC-----
------

FC88X_00357_01119 ----------------ATCCGCGTTCGCCCG-CCGT-GATGTATC---------
------

FC88X_00562_01177 ----------------CTCCGC-TTGGTCCG-CCGTTGCTGTCTC---------
------

FC88X_00813_01254 ----------------CTCCGC-TTGGTCCG-CCGTTGCTGTCTC---------
------

FC88X_00111_00940 -----------------TCCTCATTTCTCCG-C-ATTGATCTATTA--------
------

FC88X_00320_01087 -----------------TCCTCATTTCTCCG-C-ATTGATCTATTA--------
------

FC88X_00015_00096 -----------------TCCCCCTTTCTCCG-CCATTGCTATATC---------
------

FC88X_00882_01273 --------------------CCATTGCCCCG-ACATTATTCTATTCTT------
------

FC88X_00772_00122 --------------------CCATTGCCCCG-GTAACATAAACCTATT------
------

FC88X_00251_00961 --------------------CCATCAACTCCCC-GCCATGCACTCACC------
------

```
FC88X_00102_00773       -----------------------TCAACTCCCCTGTCATGC-------------
------
FC88X_00975_00430       ---------------------CTTCTACCCCCGTGACATGCACATACA------
------
FC88X_00538_01159       -------------------TCGTTCCCCTGCTCGACCATGATCATTCG------
------
FC88X_00681_00171       --------------------TCTTCCCCTGCTCGAC-ATGATCATTCG------
------
FC88X_00278_00937       -------------------TTGATCCCCCG-TCGAC--AGCTCATTCC------
------
FC88X_00787_00912       -------------------TATCTCCCTTGATCGGACGCA---ATCGTG-----
------
FC88X_00183_00688       -------------------TATCTCCCTTGATCGGACGCA---ATCGTG-----
------
FC88X_00963_00618       --------------------ACCTCCCTTG-TCTGACACA---ACGATGTA---
------
FC88X_00600_01218       ----------------------CTACCTTC-TCGCTGACA-CTATCGTACC---
------
FC88X_00695_00707       ----------------------CTCCCTTG-TCGGTCACGTCTACCGTACC---
------
FC88X_01007_00595       ----------------------TGGGCTCAGGTGACTG-GTCGACGTTC-----
------
FC88X_01037_00883       ---------------------------TTAGCTGACTCCGTCGAT---------
------
FC88X_00841_00339       ----------------------CAACCTCCCTAGAC-C-GTCGATGTCCT----
------
FC88X_01012_01263       ------------------CGCACTCCTCCCGTTGACTC-GTCAATGT-------
------
FC88X_00114_00321       --------------------CTCCCTTGTAAAGTGTCGTGTCGTGAA-------
------
FC88X_00115_00603       --------------------CTCCCTTGTAAAGTGTCGTGTCGTGAA-------
------
FC88X_00434_00283       --------------------CTCCCTTGTAAAGTGTCGTGTCGTGAA-------
------
FC88X_00767_00947       --------------------CTCCCTTGTAAAGTGTCGTGTCGTGAA-------
------
FC88X_01025_00692       --------------------CTCCCTTGTAAAGTGTCGTGTCGTGAA-------
------
FC88X_01046_00942       --------------------CTCCCTTGTAAAGTGTCGTGTCGTGAA-------
------
FC88X_00133_00019       --------------------CTCCCTTGTAAAGTGTCGTGTCGTGAA-------
------
FC88X_01082_00552       --------------------CTCCCTTGTAAAGTGTCGTGTCGTGAA-------
------
FC88X_00033_00794       ----------------TCCACTCCCTAAGCAAAGTCTGTGTCGT----------
------
FC88X_01021_00825       --------------------CTCCCTGGTAAACGTCGCCGCCGTCTC-------
------
FC88X_00105_00600       --------------------CTCCCTTGTAGACACT-CCGCCGTCCAA------
------
FC88X_00281_00961       --------------------CTCCCTAGTCAAGTAGCCGCCCGTGGC-------
------
FC88X_00953_01091       ------------------CGCTCCCTAGTGAACAGCCCGTGTCTAC--------
------
FC88X_00048_00822       ------------------CCATTACTAGTAAAAGTATCCGGCGTCA--------
------
```

FC88X_01074_01203 ------------------CCATTACTAGTAAA-GTATCCGGCGTCA--------
------
FC88X_00403_00383 --------------------TTCCCTTCTAAGTCGAGTCGTTGTACTC------
------
FC88X_00549_00975 --------------------TCCCCTTCTAAGTCGAGTGGCTGTACTC------
------
FC88X_00127_00085 ------------------CCTTCCCCTACATGTCAAG--ACTGTACG-------
------
FC88X_00078_00820 ------------------CTTTCCCCCCTATGACGCGTCTCTAGC---------
------
FC88X_00176_00710 ------------------CTTTCCTCCCTATGACGCGTCTCTAGC---------
------
FC88X_00562_00079 -------------------------AGGTATGTCG-GTCGCTAGCCC-------
------
FC88X_00025_00199 -------------------GGGGAACCGGCCAATGA-ACATAGCTCGCTTTCT-
------
FC88X_01140_00767 ---------------------------
ACCCGATGAGACCCAGATCGCTCCCTA------
FC88X_00095_00153 ----------------------------
CTCGATTGAAGCTAGTCCCCTGTTTACT----
FC88X_00222_00755 ----------------------------
CTCGAACCTGGCTAACTCTCTCTTTAC-----
FC88X_00605_01268 --------------------------CTGCCCAAGGAACCCCCGTAACTCCCA-
------
FC88X_00967_00820 -----------------------ACACTCCCCTAGCGACACC--TAGCTCCC--
------
FC88X_00620_00186 -----------------------CGCCAGTACCTACCGC-CCATA-GCTCCC--
------
FC88X_00623_00125 ------------------------------AAGTACAAC-CCGCATACTCCCG-
------
FC88X_00029_00517 ------------------------------
CAGTACCCCGCAACATACCCAGCTCTT---
FC88X_00911_00875 ----------------------CGCGATGCAAGTACCGC-CGCCATACTC----
------
FC88X_00116_00090 -----------------------TCATGTGCCTCCCGACGTGACATTCGC----
------
FC88X_00161_00924 -----------------------TCATGTGCCTCCCGACGTGACATTCGC----
------
FC88X_00262_01101 -----------------------TCATGTGCCTCCCGACGTGACATTCGC----
------
FC88X_00401_01035 -----------------------TCATGTGCCTCCCGACGTGACATTCGC----
------
FC88X_00498_00633 -----------------------TCATGTGCCTCCCGACGTGACATTCGC----
------
FC88X_00621_01201 -----------------------TCATGTGCCTCCCGACGTGACATTCGC----
------
FC88X_00065_00656 -----------------------TCATGTGCCTCCCGACGTGACATTCGC----
------
FC88X_00182_00864 -----------------------TCATGTGCCTCCCGACGTGACATTCGC----
------
FC88X_00182_00988 -----------------------TCATGTGCCTCCCGACGTGACATTCGC----
------
FC88X_00195_01067 -----------------------TCATGTGCCTCCCGACGTGACATTCGC----
------
FC88X_00586_00128 -----------------------TCATGTGCCTCCCGACGTGACATTCGC----
------

FC88X_00624_01113 -----------------------TCATGTGCCTCCCGACGTGACATTCGC----
------
FC88X_00705_01091 -----------------------TCATGTGCCTCCCGACGTGACATTCGC----
------
FC88X_00822_01014 -----------------------TCATGTGCCTCCCGACGTGACATTCGC----
------
FC88X_00827_01235 -----------------------TCATGTGCCTCCCGACGTGACATTCGC----
------
FC88X_00864_01240 -----------------------TCATGTGCCTCCCGACGTGACATTCGC----
------
FC88X_01015_00616 ----------------GGGGAATTCATGTGCCTCCCGACGTGACATTCGC----
------
FC88X_00996_00567 --------------------CTAACAAGTAACTCCCCGTGC-ACATTCC-----
------
FC88X_00425_00460 ----------------------CTCCAGTACCTCGCGCC---ACATTCTC----
------
FC88X_00115_00149 -----------------------------
TCTCCCTGGAACGACATGGCACACAA-----
FC88X_00029_00627 ---------------------------TTCTCCCCAGGACGTCGCA-
CACATGCG----
FC88X_00298_00962 ------------------GTGATCTACAACTCCCGTGGCGCGACATGCC-----
------
FC88X_00596_00485 -------------------------TAACTCCCG-
GGCACCACGTTCCAAATT------
FC88X_00130_00480 ----------------------TTGTCAAATCCCGC-GCATAAC-TATTCC---
------
FC88X_00998_00779 -------------------TTGTCAAAAAATCCCGC-GCATAAC-TATTCC---
------
FC88X_00301_00338 ----------------------TTGTCAAATCCCGC-GCATAAC-TATTCC---
------
FC88X_00701_00928 ----------------------TTGTCAAATCCCGC-GCATAAC-TATTCC---
------
FC88X_00888_00415 ----------------------TTGTCAAATCCCGC-GCATAAC-TATTCC---
------
FC88X_00975_01219 ----------------------TTGTCAAATCCCGC-GCATAAC-TATTCC---
------
FC88X_00986_00655 ----------------------TTGTCAAATCCCGC-GCATAACCTATTCC---
------
FC88X_00979_00574 ----------------------TTGTCAAATCCCGC-GCATAACCTATTCC---
------
FC88X_00355_00172 ----------------------TTGTCAAATCCCGC-GCATAAC-TATTCC---
------
FC88X_00757_00273 ----------------------TTGTCAAATCCCGC-GCATAAC-TATTCC---
------
FC88X_00957_00310 ----------------------TTGTCAAATCCCGC-GCATAAC-TATTCC---
------
FC88X_00115_00101 ----------------------TTGTCAAATCCCGC-GCATAAC-TATTCC---
------
FC88X_00227_01095 ----------------------TTGTCAAATCCCGC-GCATAAC-TATTCC---
------
FC88X_00626_00092 ----------------------TTGTCAAATCCCGC-GCATAAC-TATTCC---
------
FC88X_00625_00213 ----------------------TTGTCAAATCCCGC-GCATAAC-TATTCC---
------
FC88X_00931_00213 ----------------------TTGTCAAATCCCGC-GCATAAC-TATTCC---
------

FC88X_00912_00589 ----------------------TTGTCAAATCCCGC-GCATAAC-TATTCC---
------
FC88X_00959_01020 ----------------------TTGTCAAATCCCGC-GCATAAC-TATTCC---
------
FC88X_01067_00733 ----------------------TTGTCAAATCCCGC-GCATAAC-TATTCC---
------
FC88X_01146_01162 ----------------------TTGTCAAATCCCGC-GCATAAC-TATTCC---
------
FC88X_00558_00137 ----------------------TTGTCAAATCCCGC-GCATAAC-TATTC----
------
FC88X_01109_00590 ---------------------TTGGTCAAATCCCGC-GCATAAC-TATTCC---
------
FC88X_00501_01167 ----------------------TTGTCAAATCCCGC-GTATAAC-TATTCC---
------
FC88X_00943_00196 ----------------------TTGCCAAATCCCGC-GCATAAC-TATTCC---
------
FC88X_01071_00543 ----------------------TTGTCAAATCCCGC-GCATAAC-TATTTC---
------
FC88X_00662_01221 ----------------------TTGTCAAATTCCGC-GCATAAC-TATTCC---
------
FC88X_00351_01167 ----------------------TTGTCAAACCCCGC-GCATAAC-TATTCC---
------
FC88X_00951_00581 ----------------------TTGTCAAATCCCGC-GCACGAC-TATTCC---
------
FC88X_00898_00813 ---------------------GCTTTCAACTCCCCA-ACACTAC--ATTCCT--
------
FC88X_01084_00971 -----------------------TCATGACTCCCAA-GCACCACTTTTGAC---
------
FC88X_00872_01234 ------------------------CTCGACTCCCGCTGCACCACAATAGA----
------
FC88X_00717_00118 ------------------------CCCGACAACCGACGTAACACACCTAAT---
------
FC88X_00840_00187 ----------------------------AGTACCGACGTACCACTC--------
------
FC88X_00051_00183 ----------------------TGGCTAAACCCCCGTACGTCACTCCTC-----
------
FC88X_01002_00648 -----------------------TCCTTATCCCGGACCC-ATATACCTCGC---
------
FC88X_00972_00880 ----------------------CTCCATATCCCGGTCACGACATATCTC-----
------
FC88X_00173_00751 ---------------------TCAGCCACATTGACAAA-CTGTCCTTTT-----
------
FC88X_00637_01099 ---------------------TCAGCCACATTGACAAA-CTGTCCTTTT-----
------
FC88X_00717_00975 ---------------------TCAGCCACATTGACAAAACTGTCCCTTTT----
------
FC88X_00890_00377 ---------------------ACAGCCACATTGACAAA-CTGTCCTTTT-----
------
FC88X_00046_00496 ---------------------ACAGCCACATTGACAAA-CTGTCCTTTT-----
------
FC88X_00537_00642 ---------------------ACAGCCACATTGTCAAA-CTGTCCTTTTT----
------
FC88X_00139_00563 ---------------------ACAGCCACATTGACAAA-
CTGTCCTTTTCTTCCTTT---
FC88X_00136_00067 ---------------------ACAGCCACATTGTCAAAC---------------
------

```
FC88X_00562_00236      -------------------TCACGCTCCCGGCAGCATTGTCATACA--------
------
FC88X_00683_01017      ---------------------GCGCTCCCTGCTCCATAGACATCATGTA-----
------
FC88X_00634_01177      ------------CCGCCACCTC-GGAAACTCCCCTCATTTT-------------
------
FC88X_00237_00585      ------------ACGGCACCTCTAGACATTCTCCTCACT---------------
------
FC88X_00075_00575      -------------------GTTCTCGGACAGCCCGCGTCGAATTATC-------
------
FC88X_00088_00805      --------------------TTCTCGGACAGCCCGCGTCGAATTATC-------
------
FC88X_00450_00056      ---------------------TCATGCACACTCCGCATCGAATTAATT------
------
FC88X_00902_01257      ---------------------TCATGCACACTCCGCATCGAATTAATT------
------
FC88X_01072_00516      ---------------------TCATGCACACTCCGCATCGAATTAATT------
------
FC88X_00837_00375      ---------------------TCATGCACACTCCGCACCGAATTAATT------
------
FC88X_00652_00274      --------------------TCCCGGCACATTCGACATCAACTGATT-------
------
FC88X_00372_00111      ---------------------ACACGCACATTCAGCCCCGGGTCGTTA------
------
FC88X_00140_00706      ---------------------ACACGCACATTCAGCCCCGGGTCGTTA------
------
FC88X_00433_00785      ---------------------ACACGCACATTCAGCCCCGGGTCGTTA------
------
FC88X_00011_00609      ---------------------ACACACACATTCAGCCCCGGGTCGTTAC-----
------
FC88X_00759_00930      -------------------CCGCACCCTCCTTTTGCACTGGGTACT--------
------
FC88X_00489_00431      ---------------------TTGCACCCTCCGGACGATTGACTAGTT------
------
FC88X_00451_01137      ---------------------TTGCACCCTCCGGACGATTGACTAGTT------
------
FC88X_00924_00528      ---------------------TTGCACCCTCCGGACGATTGACTAGTT------
------
FC88X_00869_00770      ---------------------TTGCACCCTCCGGACGATTGACTAGTT------
------
FC88X_00558_01243      ------------------TCATTGAACCCTAC---CGCATCACTACTTT-----
------
FC88X_00884_00879      ------------------TCATTGAACCCTAC---CGCATCACTACTTT-----
------
FC88X_00424_01127      --------------------CACAGACACCTCCCGC--TGCACTATCGA-----
------
FC88X_00189_00878      --------------------CACAGACACCTCCCGC--TGCACTATCGA-----
------
FC88X_00474_00801      --------------------CCAGAACCCCTCCCGCT-TGGACCAACC------
------
FC88X_00939_00681      -------------------CCAAAAAGCCCTCCCGAA-TGCATCATCCC-----
------
FC88X_00078_00091      --------------------TTCCCACA-GACATGCAACACTATTCTT------
------
FC88X_01104_01189      -------------------GTTCCCACA-GACATGCAACACTATTCTT------
------
```

FC88X_00081_00225 --------------------TTCCCACA-GACATGCAACACTATTCTT------
------
FC88X_00090_00313 --------------------TTCCCACA-GACATGCAACACTATTCTT------
------
FC88X_00453_01126 --------------------TTCCCACA-GACATGCAACACTATTCTT------
------
FC88X_00864_00222 --------------------TTCCCACA-GACATGCAACACTATTCTT------
------
FC88X_00870_00697 --------------------TTCCCACA-GACATGCAACACTATTCTT------
------
FC88X_00873_00670 --------------------TTCCCACA-GACATGCAACACTATTCTT------
------
FC88X_00088_00095 --------------------TTCCCACA-GACATGCAACACTATTCTT------
------
FC88X_00087_00172 --------------------TTCCCACA-GACATGCAACACTATTCTT------
------
FC88X_00185_00910 --------------------TTCCCACA-GACATGCAACACTATTCTT------
------
FC88X_00331_01180 --------------------TTCCCACA-GACATGCAACACTATTCTT------
------
FC88X_00395_01205 --------------------TTCCCACA-GACATGCAACACTATTCTT------
------
FC88X_00483_00977 --------------------TTCCCACA-GACATGCAACACTATTCTT------
------
FC88X_00717_01132 --------------------TTCCCACA-GACATGCAACACTATTCTT------
------
FC88X_00986_00791 --------------------TTCCCACA-GACATGCAACACTATTCTT------
------
FC88X_00008_00492 --------------------TTCCCACA-GACATGCAATACTATTCTT------
------
FC88X_00946_00219 --------------------TTCCCACA-GACATGCGACACTATTCTT------
------
FC88X_00889_01249 --------------------TTCCCACA-GACATGCAACACTATTCTC------
------
FC88X_00055_00459 --------------------TTCCCACA-GAAATGCAACACTATTCTT------
------
FC88X_00643_00061 -------------------TTCCCAACA-CACCTGCACGCCCACTCT-------
------
FC88X_00486_00588 -------------------TTACCAACCCGACACGCCCCACTCTATT-------
------
FC88X_01015_00807 -------------------TTACCAACCCGACACGCCCCACTCTATT-------
------
FC88X_00377_01159 -------------------TTACCAACCCGACATGCCCCACTCTATT-------
------
FC88X_01142_01093 -------------------TTACCAAC--GACACGCCC-ACTCTATCTTCC---
------
FC88X_00841_00177 -------------------------CCACTACTAGCACACCTGCCCCGATT---
------
FC88X_00543_00957 ---------------------------
ACCACAACCAGCCAGCTCCCGATTCTT------
FC88X_00323_00867 ----------------TTACCACACCTACGACGTCCATCTATTCTTCCC-----
------
FC88X_00783_00518 ----------------TTACCACACCTGCGACGTCCATCTATTCTTCCTTTC--
------
FC88X_00819_01261 ---------------------------
ACAACGACTGTCCACTCCCTTCTTACC------

```
FC88X_00023_00679      ------------------TGTGATGAG-TACAACTCACCTCGATTCTC------
------
FC88X_00872_00916      ------------------TGTGATGAGATACAACTCACCTCGATTCTC------
------
FC88X_00042_00797      ------------------TGTGATGAG-TACAACTCACCTCGATTCTC------
------
FC88X_00088_00685      ------------------TGTGATGAG-TACAACTCACCTCGATTCTC------
------
FC88X_00107_00423      ------------------TGTGATGAG-TACAACTCACCTCGATTCTC------
------
FC88X_00103_00473      ------------------TGTGATGAG-TACAACTCACCTCGATTCTC------
------
FC88X_00462_01030      ------------------TGTGATGAG-TACAACTCACCTCGATTCTC------
------
FC88X_00501_00507      ------------------TGTGATGAG-TACAACTCACCTCGATTCTC------
------
FC88X_00622_00946      ------------------TGTGATGAG-TACAACTCACCTCGATTCTC------
------
FC88X_00680_00284      ------------------TGTGATGAG-TACAACTCACCTCGATTCTC------
------
FC88X_00719_00133      ------------------TGTGATGAG-TACAACTCACCTCGATTCTC------
------
FC88X_00730_01173      ------------------TGTGATGAG-TACAACTCACCTCGATTCTC------
------
FC88X_00757_00962      ------------------TGTGATGAG-TACAACTCACCTCGATTCTC------
------
FC88X_00812_01094      ------------------TGTGATGAG-TACAACTCACCTCGATTCTC------
------
FC88X_00862_00180      ------------------TGTGATGAG-TACAACTCACCTCGATTCTC------
------
FC88X_00934_00476      ------------------TGTGATGAG-TACAACTCACCTCGATTCTC------
------
FC88X_00920_00579      ------------------TGTGATGAG-TACAACTCACCTCGATTCTC------
------
FC88X_00918_00745      ------------------TGTGATGAG-TACAACTCACCTCGATTCTC------
------
FC88X_01112_00844      ------------------TGTGATGAG-TACAACTCACCTCGATTCTC------
------
FC88X_00007_00295      ------------------TGTGATGAG-TACAACTCACCTCGATTCTC------
------
FC88X_00078_00372      ------------------TGTGATGAG-TACAACTCACCTCGATTCTC------
------
FC88X_00064_00673      ------------------TGTGATGAG-TACAACTCACCTCGATTCTC------
------
FC88X_00102_00162      ------------------TGTGATGAG-TACAACTCACCTCGATTCTC------
------
FC88X_00134_00579      ------------------TGTGATGAG-TACAACTCACCTCGATTCTC------
------
FC88X_00169_00277      ------------------TGTGATGAG-TACAACTCACCTCGATTCTC------
------
FC88X_00214_00047      ------------------TGTGATGAG-TACAACTCACCTCGATTCTC------
------
FC88X_00306_00393      ------------------TGTGATGAG-TACAACTCACCTCGATTCTC------
------
FC88X_00328_01068      ------------------TGTGATGAG-TACAACTCACCTCGATTCTC------
------
```

FC88X_00367_01001 ------------------TGTGATGAG-TACAACTCACCTCGATTCTC------
------
FC88X_00429_00014 ------------------TGTGATGAG-TACAACTCACCTCGATTCTC------
------
FC88X_00492_00988 ------------------TGTGATGAG-TACAACTCACCTCGATTCTC------
------
FC88X_00488_01101 ------------------TGTGATGAG-TACAACTCACCTCGATTCTC------
------
FC88X_00537_00958 ------------------TGTGATGAG-TACAACTCACCTCGATTCTC------
------
FC88X_00626_00252 ------------------TGTGATGAG-TACAACTCACCTCGATTCTC------
------
FC88X_00638_00853 ------------------TGTGATGAG-TACAACTCACCTCGATTCTC------
------
FC88X_00614_00916 ------------------TGTGATGAG-TACAACTCACCTCGATTCTC------
------
FC88X_00656_00824 ------------------TGTGATGAG-TACAACTCACCTCGATTCTC------
------
FC88X_00664_01200 ------------------TGTGATGAG-TACAACTCACCTCGATTCTC------
------
FC88X_00707_01108 ------------------TGTGATGAG-TACAACTCACCTCGATTCTC------
------
FC88X_00779_01015 ------------------TGTGATGAG-TACAACTCACCTCGATTCTC------
------
FC88X_00890_00750 ------------------TGTGATGAG-TACAACTCACCTCGATTCTC------
------
FC88X_00936_00273 ------------------TGTGATGAG-TACAACTCACCTCGATTCTC------
------
FC88X_01096_00782 ------------------TGTGATGAG-TACAACTCACCTCGATTCTC------
------
FC88X_01147_01053 ------------------TGTGATGAG-TACAACTCACCTCGATTCTC------
------
FC88X_00374_01182 ------------------TGTGATGAG-TACAACTCACCTCGATTCTCC-----
------
FC88X_00168_00112 ------------------TGTGATGAGTACCAACTCACCTCGATTCTC------
------
FC88X_00128_00778 ------------------TGTGATGAG-TACAACTCACCCCGATTCTC------
------
FC88X_00888_00775 ------------------TGTGATGAG-TGCAACTCACCTCGATTCTC------
------
FC88X_00675_00884 ------------------CGTGATGAG-TACAACTCACCTCGATTCTC------
------
FC88X_00278_00770 ------------------TGTGATGAG-CACAACTCACCTCGATTCTC------
------
FC88X_00592_01202 ------------------TGTGATAAG-CACAACTCAC----------------
------
FC88X_00166_00171 --------------------------ATAACAACTCCAC--
GATGCTCCCAAATC-----
FC88X_00825_01169 --------------------------ATAACAACTCCACC-
GATGCTCCCAAATC-----
FC88X_00247_00258 --------------------------ATAACAACTCCAC--GATGCTCCCA---
------
FC88X_00714_01211 ---------------------------CAACTACCCTTC--
GATCCTCCCGTATT-----
FC88X_00050_00326 --------------------CTGCACAACCTCCCACCGC-CGCTTCTC------
------

FC88X_00744_01141 --------------------CTGCACAATCTCCCACCGC-CGCTTCTC------
------
FC88X_00298_00242 ---------------------CGACAACCAACCCACGGC-CGCTTCTC------
------
FC88X_00983_00948 --------------------CCGACAACCAACCCACGGC-CGCTTCTC------
------
FC88X_00798_00978 --------------------CCGACAACCAACCCGCGGC-CGCTTCTC------
------
FC88X_00906_00738 ---------------------CGACAACCATCCCACGGC-CGCTTCTC------
------
FC88X_00999_01172 --------------------CCGACAACCAACCA--CGC-CGCT----------
------
FC88X_01009_00695 --------------------CCGACAACCAACCA--CGC-CGCTTCTC------
------
FC88X_00854_00235 -------------------GCTAACGACTCTTCTTCGGCGCGATTCT-------
------
FC88X_00575_00194 --------------------CTAACGACTCTTCTTCGGCGCGATTCT-------
------
FC88X_00823_01228 --------------------CTAACGACTCTTCTTCGGCGCGATTCT-------
------
FC88X_01066_01150 --------------------CTAACGACTCTTCTTCGGCGCGATTCT-------
------
FC88X_00961_00672 --------------------TTAACAACCTTCTGACCGCCGCATTCTA------
------
FC88X_00377_01171 -----------------CACAACCTAATGACG-CCCTGTGCCTTCT--------
------
FC88X_00886_00833 -----------------CACAACCTAATGACG-CCCTGTGCCTTCT--------
------
FC88X_00849_01113 -----------------CACAACCTAACGACG-CCCTGTGCCTTCT--------
------
FC88X_00036_00068 --------------------AACGTAACTACGACGCTGCGTCCTTCT-------
------
FC88X_00596_00159 --------------------AACGTAACTACGACGCTGCGTCCTTCT-------
------
FC88X_00847_00346 --------------------AACGTAACTACGACGCTGCGTCCTTCT-------
------
FC88X_00890_01188 --------------------AACGTAACTACGACGCTGCGTCCTTCT-------
------
FC88X_00949_00324 ------------------------GAACTACCTCACTTCGTCCG----------
------
FC88X_00712_00665 -----------------------CTAACCACCTCCCTTCGCACTTCTAAT----
------
FC88X_00638_00428 --------------------CCGCCAACTAACTCTCGACGCGCTGTC-------
------
FC88X_01056_00868 --------------------CCGCCAACTAACTCTCGACGCGCTGTC-------
------
FC88X_01072_00833 --------------------CCGCCAACTAACTCTCGACGAGCTGTC-------
------
FC88X_00863_00799 --------------------CCACAAACTATCCCGCGGCGCGCTATG-------
------
FC88X_01047_00344 --------------------CCACAAACTATCCCGCGGCGCGCTATG-------
------
FC88X_00110_00202 ------------------CAGAACCAACT-GCCCTCGTCCCGAAGT--------
------
FC88X_00257_01051 -------------GCCAGCAATAAAAAAC-TCCCCCGGCGCGAA----------
------

FC88X_00432_01031 --------------------CCCCTAACCCCGTCACCGAGCTTCCTTCGC----
------
FC88X_00219_00112 -------------TTATTAACTCCCGACACCG-CACTCAACTCCTTCTCCCT--
------
FC88X_01066_01263 ------------------GTGACTAACACTTGTCACAACGCCTCTC--------
------
FC88X_01018_01053 ------------------CTGACAAACTTCCGTCATC-CGCTTCTC--------
------
FC88X_00753_01002 --------------GCTCGCGAGTTCA-GTCCCCGCGTCTCCT-----------
------
FC88X_00818_00186 -----------------ACCTAGTGCACGTCGACCCGTCTCCCG----------
------
FC88X_00372_00780 --------------------CTACCCACGACGGCCAGCAACGCTACT-------
------
FC88X_00490_00443 ---------------CTACCCAGCTCCCGACGGTATGCAACA------------
------
FC88X_00366_01041 -------------CTTCCGACTATAACCAGCGACGCGCTCCTTCCTTT------
------
FC88X_01099_01157 --------------------CTATAAC-ATCGACTCAGCACCTCTCCT------
------
FC88X_00112_00140 ---------------------TCCTCCCAACGATTTAGCTGTTTTGCA------
------
FC88X_00464_01020 ---------------------TCCTCCCAACGATTTAGCTGTTTTGCA------
------
FC88X_00964_01217 ---------------------TCCTCCCAACGATTTAGCTGTTTTGCA------
------
FC88X_00092_00618 ---------------------TCCTCCCAACGATTTAGCTGTTTTGCA------
------
FC88X_00167_00067 ---------------------TCCTCCCAACGATTTAGCTGTTTTGCA------
------
FC88X_00111_00255 ---------------------TCCTCCCAACGATTTAGCTGCTTTGCA------
------
FC88X_01027_00807 -------------------------CCTCCCGATTGAGCTTATTTGCGTTTT--
------
FC88X_01084_00895 -------------------------CCTCCCTATTGAGCTTATTTGCGTTTT--
------
FC88X_00051_00201 ----------------CCGTGCCGCCCCCGCACTTAACTTATT----------
------
FC88X_01022_00582 ----------------CCACTTACTCCCCTGCCACCAGCTGTC-----------
------
FC88X_00811_00233 ----------------CGACTTACCTCCCTTACGTTAGCTATC-----------
------
FC88X_00696_00633 ---------------CTTATACACCTCCC-AACGCTATCTTAC-----------
------
FC88X_00459_01148 -----------------TTTGCGCCGCTCCAAGACCTACTTTAT----------
------
FC88X_00963_00265 ----------------TTGTGCGCCCTACCGATGCC-ACTTTTT----------
------
FC88X_00764_01093 ----------------TTATTCCCCGTTCCAA-ACCAGCTTTCT----------
------
FC88X_00451_01007 --------------------CCCTCCGCTCCGCC-TAACCAGGTTACCC-----
------
FC88X_00936_00396 --------------------CCACCCTTTCCGCCGCATCCAAGTTAC-------
------
FC88X_00517_01206 ---------------------CTCCCACTTGGCCTAACGCGAGTTCCC------
------

```
FC88X_00699_01262      --------------------CTCCCACATTCGATA-CGGCGAGGTTCC------
------
FC88X_00978_00395      --------------------CTCCCACATTCGATA-CGGCGACGTTCC------
------
FC88X_00264_00819      --------------------CTTACACATATGCTATCGCCGACTATCC------
------
FC88X_00696_00886      ----------------TCTCCTTACACATGTTTTA-CGCCGAAC----------
------
FC88X_01004_00598      ------------GGGGAATCCTCCCGTATGCATTA--GCCGAATGCC-------
------
FC88X_00098_00945      -----------------------CTATCTAGCCATCTC-CCTGCGCGTTAA---
------
FC88X_00437_01103      -----------------------TTATCTACCCTTCACACCTGCTCTTTG----
------
FC88X_00228_01059      -----------------CTCCGCGGTCATACGTACCTCCCTGTT----------
------
FC88X_00674_00443      --------------------CCCGGACACAGGTACCTCTCT-------------
------
FC88X_00582_00803      --------------------CGCGTTCTCATGCACCGTCTCCCTGAT-------
------
FC88X_00254_00999      -------------------ACCGCGTCTAGCACA-CTCCCGGATACA-------
------
FC88X_00302_00958      -----------------CTTCCGAGTCAAGCACGTCTCCCTAGT----------
------
FC88X_00309_00248      -----------------TTACCGGCTTGCACGAGCCTCCC-TGTTCT-------
------
FC88X_00747_00078      -----------------TTACCAGCTTGCACGAGCCTCCC-TGTTCT-------
------
FC88X_00829_00985      -----------------CCTCCTGGTCGCAGGAACAGCCC-TGTT---------
------
FC88X_00111_00102      -----------------------TTCAGCACTAGCCTCCC-TGTTCCTTCC---
------
FC88X_00158_00779      ------------------ACCACAGCGGTACTCGTATCCC-TGGTCCTCCT---
------
FC88X_00799_00807      -------------------CCACAACGG--CTAGCACCCCATGATCCA------
------
FC88X_00839_00734      -----------------TTGCTTCCCACTAGCCGTCTCCCTGAT----------
------
FC88X_00917_00444      -----------------TTGCCTCCCACTAGCCGTCTCCCTGAT----------
------
FC88X_00823_01038      --------------------CCACCAGCTGTCCCTCATCCTGATCAC-------
------
FC88X_00912_00940      --------------------CCACTAGCGTTATACCTCCCAGATCCC-------
------
FC88X_00354_00297      -----------------CCGATAACTCCCCGGCGCCTTCGCGGT----------
------
FC88X_00843_00369      -----------------CCGATAACTCCCCGGCGCCTTCGCGGT----------
------
FC88X_01018_00997      -----------------CCGATAACTCCCCGGCGCCTTCGCGGT----------
------
FC88X_00162_00061      -----------GGGGATACGTTTAATCCCCTGTTCCTGCCCT------------
------
FC88X_00799_00229      -------------------GTTAATCCCTAATTACCCGCCCGTTCGT-------
------
FC88X_00904_00235      --------------------TTGCTAACCCCATTCCAGCGCGCTCCT-------
------
```

FC88X_00756_01225   ---------------------CTATCTCCTAACCTGTAGCGAGTTCTC--------------
FC88X_00838_00353   ---------------------CTATCTCCTAACCTGTAGCGAGTTCTC--------------
FC88X_00843_01251   ---------------------CTATCTCCTAACCTGTAGCGAGTTCTC--------------
FC88X_00974_00930   ---------------------CTATCTCCTAACCTGTAGCGAGTTCTC--------------
FC88X_00437_01012   ---------------------CTATCTCCTAACCTGTAGCGAGTTCTC--------------
FC88X_00649_00143   ---------------------CTATCTCCTAACCTGTAGCGAGTTCTC--------------
FC88X_00968_00770   ---------------------CTATCTCCTAACCTGTAGCGAGTTCTC--------------
FC88X_00797_00266   ---------------------
CTATCTCCTAACCTGTAGCGAGTTCTCCTTCCTTT-----
FC88X_00973_01103   ---------------------CTATCTCCTGACCTGTAGCGAGTTCTC--------------
FC88X_00952_00323   --------------------TCTTTCCCCAAGCCTGTACGGTTCTCGCC------------
FC88X_00082_00416   ---------------------CCTTGCCCCAACCTGTACGTTCGTTCC--------------
FC88X_00879_00660   ---------------------CCTTGCCCCAACCTGTGCGTTCGTTCC--------------
FC88X_00953_00416   --------------------CTATTCTCCTAACCCGCACGACTTCCCCT------------
FC88X_01003_00673   ----------------CCTAATATTCGGCTAACCC--
ACCATTTCCCTTCCTTTC------
FC88X_00228_00658   ---------------------CTTGCACGCTCGCTCCCTGCATTTTGTA------------
FC88X_00974_00290   ---------------------CTTGCACGCTCGCTCCCTGCATTT-GTA------------
FC88X_00130_00230   -------------------CTATTTCACGCTGACCGCCCACATTTTT--------------
FC88X_00042_00558   -------------------CTACGAGACGCAC-TCCCCGCCATTTTC--------------
FC88X_00999_00786   ------------------GCTACGAGACGCACCTCCCCGCCATTTTTC-------------
FC88X_00938_00731   --------------------TACCAGACTGGGCCCCTCGATAATTTA--------------
FC88X_00582_00424   ----------------------ATAGTAGCTTCCCCTTGCTATCTTCAT------------
FC88X_00368_00874   ----------------------ATAGTAGCTTCCCCTTGCTATCTTCAT------------
FC88X_01039_01019   ----------------------ATAGTAGCTTCCCCTTGCTATCTTCAT------------
FC88X_00369_01014   ----------------------ATAGTAGCTTCCCCCTGCTATCTTCAT------------
FC88X_01019_00925   ---------------------TTATGCAGCTTCCCCTTGCTATCTTCAT------------
FC88X_00815_00942   -------------------------CTACTTCCCGCTCTCAATTTACATTCA---------
FC88X_00987_01056   -------------------------TCACTTCCC-CTCTAAATTCATTGTCCG--------
FC88X_00597_00988   -------------------------ATCTTGTCCGATCTATCTTTCCATGAA---------

```
FC88X_00837_01201       ------------------------ATCTCGTCCGATCTATCTTTCCATGAA---
------
FC88X_00047_00539       -------------------------ACTTCTCGACTCTATACTTTGA-------
------
FC88X_00095_00081       -------------------TCGTATGTCAGACCGCACTATACTTTCC-------
------
FC88X_00404_00275       --------------------TTCGTACTTGTCCGGTCGCTATCTTTC-------
------
FC88X_00340_01161       ------------------ACCTGGACTCTAA-ATCCCGGCGCTCCT--------
------
FC88X_00136_00417       ------------------ACCTGGACTCTAA-ATCCCGGCGCTCCT--------
------
FC88X_00982_01233       -------------------CCTGGACCGTCCCATCCTGCCGCTAAT--------
------
FC88X_00864_00812       ------------------------ACGTCTTGCCGATGACCCCTTATTCTC---
------
FC88X_00971_00522       ---------------------CTAACGTCT--CCGCCGACCCTTATTTC-----
------
FC88X_00963_00320       ------------------GCTGTGACGTCGTGTC--TGTCCCCTAATT------
------
FC88X_01080_00614       -------------------CTGTGACGTCGTGTC--TGTCCCCTAATT------
------
FC88X_00365_00810       ------------------CTAACCACTGTCCGGTCCCAGCATGTT---------
------
FC88X_00617_00978       ------------------CTAACCACTGTCCGGTCCCAGCATGTT---------
------
FC88X_00358_00322       ------------------CTAACCCCTAGTTAGTTCCCAGCTGTT---------
------
FC88X_01045_00365       ------------------CTTGTAACTCCCCAATCACAGCCTATC---------
------
FC88X_01081_01184       -----------------ACCTTGAACTATCC--TCCCAGGCTGTCC--------
------
FC88X_00156_00165       --------------------CTTCGAGCTAC--TCTGACCCATCATTTT-----
------
FC88X_00706_00359       ---------------------TTCGTACTAC--TCTCGCCTATCGTTTTT----
------
FC88X_00666_01229       --------------------ACCCGTACGACA-TCTTGGCTATCCT--------
------
FC88X_00930_01016       -------------------ACTTCGTACGACA-TCTCAGCTATCCT--------
------
FC88X_01013_00876       ----------------ACAATATCGTACTAAACTCCCGGCTAAC----------
------


FC88X_00034_00263       -------
FC88X_00031_00353       -------
FC88X_00027_00395       -------
FC88X_00014_00349       -------
FC88X_00014_00335       -------
FC88X_00040_00407       -------
FC88X_00020_00420       -------
FC88X_00016_00418       -------
FC88X_00013_00439       -------
FC88X_00011_00407       -------
FC88X_00044_00407       -------
FC88X_00037_00343       -------
FC88X_00044_00306       -------
```

FC88X_00024_00349	-------
FC88X_00089_00017	-------
FC88X_00081_00020	-------
FC88X_00074_00032	-------
FC88X_00021_00751	-------
FC88X_00048_00725	-------
FC88X_00079_00065	-------
FC88X_00074_00051	-------
FC88X_00062_00284	-------
FC88X_00058_00294	-------
FC88X_00013_00456	-------
FC88X_00016_00476	-------
FC88X_00018_00511	-------
FC88X_00079_00209	-------
FC88X_00121_00499	-------
FC88X_00092_00153	-------
FC88X_00051_00179	-------
FC88X_00122_00567	-------
FC88X_00112_00582	-------
FC88X_00112_00579	-------
FC88X_00043_00688	-------
FC88X_00038_00654	-------
FC88X_00037_00697	-------
FC88X_00024_00735	-------
FC88X_00114_00540	-------
FC88X_00852_00286	-------
FC88X_00883_00360	-------
FC88X_00155_00588	-------
FC88X_00199_00545	-------
FC88X_00151_00479	-------
FC88X_00121_00372	-------
FC88X_00149_00314	-------
FC88X_00114_00313	-------
FC88X_00178_00524	-------
FC88X_00176_00527	-------
FC88X_00158_00326	-------
FC88X_00195_00163	-------
FC88X_00175_00659	-------
FC88X_00164_00696	-------
FC88X_00159_00677	-------
FC88X_00109_00710	-------
FC88X_00121_00298	-------
FC88X_00139_00210	-------
FC88X_00400_01136	-------
FC88X_00123_00245	-------
FC88X_00737_01226	-------
FC88X_00819_00193	-------
FC88X_00806_00136	-------
FC88X_00805_00094	-------
FC88X_00770_01244	-------
FC88X_00770_01061	-------
FC88X_00768_01055	-------
FC88X_00777_00833	-------
FC88X_00781_00726	-------
FC88X_00126_00936	-------
FC88X_00134_00879	-------
FC88X_00132_00889	-------

FC88X_00128_00895	-------
FC88X_00126_00893	-------
FC88X_00191_00086	-------
FC88X_00183_00083	-------
FC88X_00175_00068	-------
FC88X_00164_00089	-------
FC88X_00162_00057	-------
FC88X_00155_00095	-------
FC88X_00134_00974	-------
FC88X_00103_00887	-------
FC88X_01083_00789	-------
FC88X_01096_00739	-------
FC88X_01082_00701	-------
FC88X_01063_00722	-------
FC88X_01092_00658	-------
FC88X_00120_00706	-------
FC88X_00118_00935	-------
FC88X_00035_00651	-------
FC88X_00144_00827	-------
FC88X_00137_00844	-------
FC88X_00103_00817	-------
FC88X_00009_00685	-------
FC88X_00125_00694	-------
FC88X_00127_00183	-------
FC88X_00125_00165	-------
FC88X_00110_00150	-------
FC88X_00146_00752	-------
FC88X_00144_00788	-------
FC88X_00144_00761	-------
FC88X_00133_00760	-------
FC88X_00848_00259	-------
FC88X_00830_00293	-------
FC88X_00807_00201	-------
FC88X_00843_00153	-------
FC88X_00841_00184	-------
FC88X_00840_00154	-------
FC88X_00798_00477	-------
FC88X_00778_00463	-------
FC88X_00779_00295	-------
FC88X_00777_00264	-------
FC88X_00770_00257	-------
FC88X_00763_00298	-------
FC88X_00755_00289	-------
FC88X_00896_00322	-------
FC88X_00868_00318	-------
FC88X_00884_00261	-------
FC88X_00896_00183	-------
FC88X_00835_00865	-------
FC88X_00891_00402	-------
FC88X_00875_00435	-------
FC88X_00866_00409	-------
FC88X_01127_00890	-------
FC88X_01142_00817	-------
FC88X_01100_00939	-------
FC88X_00860_00195	-------
FC88X_00851_00164	-------
FC88X_00850_00151	-------

FC88X_00842_01257	-------
FC88X_00847_01210	-------
FC88X_00834_01246	-------
FC88X_00834_01207	-------
FC88X_00807_01211	-------
FC88X_00805_01243	-------
FC88X_00816_01150	-------
FC88X_00815_01161	-------
FC88X_00813_01150	-------
FC88X_00824_00908	-------
FC88X_00804_00938	-------
FC88X_00014_00260	-------
FC88X_00014_00254	-------
FC88X_00010_00260	-------
FC88X_00780_00098	-------
FC88X_00730_01201	-------
FC88X_00719_01221	-------
FC88X_00767_00193	-------
FC88X_00763_00175	-------
FC88X_00796_00113	-------
FC88X_00792_00136	-------
FC88X_00775_00104	-------
FC88X_00793_00214	-------
FC88X_00775_00961	-------
FC88X_00144_00594	-------
FC88X_00138_00569	-------
FC88X_00856_00299	-------
FC88X_00895_00608	-------
FC88X_00887_00638	-------
FC88X_00887_00623	-------
FC88X_00881_00601	-------
FC88X_00889_00561	-------
FC88X_00877_00586	-------
FC88X_00891_00509	-------
FC88X_00884_00517	-------
FC88X_00802_00863	-------
FC88X_00807_00779	-------
FC88X_00839_00404	-------
FC88X_00835_00328	-------
FC88X_00833_00322	-------
FC88X_00802_01151	-------
FC88X_00818_01104	-------
FC88X_00805_01051	-------
FC88X_00826_01004	-------
FC88X_00806_01043	-------
FC88X_00819_00981	-------
FC88X_00813_00953	-------
FC88X_00485_00996	-------
FC88X_00488_00693	-------
FC88X_00462_00568	-------
FC88X_00460_00585	-------
FC88X_00488_00548	-------
FC88X_00480_00465	-------
FC88X_00459_00427	-------
FC88X_00460_00302	-------
FC88X_00455_00251	-------
FC88X_00482_00077	-------

FC88X_00475_00093	-------
FC88X_00472_00070	-------
FC88X_00471_00076	-------
FC88X_00457_00051	-------
FC88X_00456_00087	-------
FC88X_00437_01131	-------
FC88X_00709_01249	-------
FC88X_00719_01153	-------
FC88X_01126_00506	-------
FC88X_01109_00529	-------
FC88X_01102_00432	-------
FC88X_01053_01209	-------
FC88X_01075_01153	-------
FC88X_01055_01168	-------
FC88X_01052_01182	-------
FC88X_01079_01114	-------
FC88X_01074_01125	-------
FC88X_01073_01118	-------
FC88X_01069_01141	-------
FC88X_01065_01140	-------
FC88X_01053_01107	-------
FC88X_01074_01061	-------
FC88X_01067_01058	-------
FC88X_01053_01099	-------
FC88X_01057_01032	-------
FC88X_01056_01039	-------
FC88X_01075_00956	-------
FC88X_00703_01062	-------
FC88X_00027_00452	-------
FC88X_01086_00936	-------
FC88X_01086_00865	-------
FC88X_01095_00831	-------
FC88X_01087_00605	-------
FC88X_00968_00698	-------
FC88X_00956_00678	-------
FC88X_00957_00334	-------
FC88X_00998_00274	-------
FC88X_00953_00282	-------
FC88X_00741_00763	-------
FC88X_00748_00358	-------
FC88X_00084_00406	-------
FC88X_00888_00803	-------
FC88X_00868_00772	-------
FC88X_00853_00761	-------
FC88X_00992_00671	-------
FC88X_00979_00671	-------
FC88X_01109_00813	-------
FC88X_01120_00784	-------
FC88X_01105_00779	-------
FC88X_01100_00781	-------
FC88X_01130_00749	-------
FC88X_01103_00730	-------
FC88X_01100_00709	-------
FC88X_01108_00678	-------
FC88X_01128_00600	-------
FC88X_01112_00586	-------
FC88X_01112_00584	-------

FC88X_01107_01023	-------
FC88X_01121_00960	-------
FC88X_01106_00958	-------
FC88X_00877_00999	-------
FC88X_00871_00978	-------
FC88X_00870_00988	-------
FC88X_00867_00986	-------
FC88X_00864_00994	-------
FC88X_00843_00752	-------
FC88X_00005_00253	-------
FC88X_00049_00217	-------
FC88X_00046_00240	-------
FC88X_01038_01181	-------
FC88X_01030_01190	-------
FC88X_01026_01177	-------
FC88X_01017_01168	-------
FC88X_00976_00486	-------
FC88X_00972_00458	-------
FC88X_00007_00292	-------
FC88X_01030_01079	-------
FC88X_01025_01095	-------
FC88X_00178_00984	-------
FC88X_00709_01164	-------
FC88X_00729_01135	-------
FC88X_01002_00845	-------
FC88X_01001_00628	-------
FC88X_01001_00564	-------
FC88X_00939_01025	-------
FC88X_01000_00985	-------
FC88X_01040_00937	-------
FC88X_01019_00924	-------
FC88X_01047_00854	-------
FC88X_01027_00871	-------
FC88X_01017_00877	-------
FC88X_01023_00812	-------
FC88X_01018_00813	-------
FC88X_00946_00915	-------
FC88X_00862_01144	-------
FC88X_00874_01063	-------
FC88X_00889_01023	-------
FC88X_00851_01008	-------
FC88X_00990_00511	-------
FC88X_00978_00515	-------
FC88X_00966_00521	-------
FC88X_00042_00479	-------
FC88X_00040_00488	-------
FC88X_00112_00106	-------
FC88X_00976_00376	-------
FC88X_00976_00368	-------
FC88X_00964_00367	-------
FC88X_00959_00396	-------
FC88X_00956_00390	-------
FC88X_00959_00237	-------
FC88X_00926_01250	-------
FC88X_00911_01226	-------
FC88X_00941_01166	-------
FC88X_00931_01120	-------

FC88X_00920_01123	-------
FC88X_00748_00895	-------
FC88X_00738_00334	-------
FC88X_00719_00322	-------
FC88X_00715_00323	-------
FC88X_00171_00953	-------
FC88X_00151_00979	-------
FC88X_00199_00918	-------
FC88X_00192_00948	-------
FC88X_00172_00902	-------
FC88X_00167_00916	-------
FC88X_00953_01017	-------
FC88X_00953_00956	-------
FC88X_01027_00514	-------
FC88X_01025_00522	-------
FC88X_01040_00456	-------
FC88X_01024_00455	-------
FC88X_01011_00466	-------
FC88X_00981_00804	-------
FC88X_00967_01205	-------
FC88X_00957_01241	-------
FC88X_00981_01160	-------
FC88X_00057_00503	-------
FC88X_00945_00866	-------
FC88X_00926_00876	-------
FC88X_00905_00852	-------
FC88X_00939_00816	-------
FC88X_00050_00644	-------
FC88X_00091_00579	-------
FC88X_01027_00429	-------
FC88X_01025_00352	-------
FC88X_01044_00324	-------
FC88X_01029_00315	-------
FC88X_01012_00342	-------
FC88X_01011_00305	-------
FC88X_00973_01257	-------
FC88X_00642_01071	-------
FC88X_00620_01082	-------
FC88X_00624_00941	-------
FC88X_00620_00834	-------
FC88X_00609_00707	-------
FC88X_00620_00425	-------
FC88X_00197_00838	-------
FC88X_00099_00625	-------
FC88X_00288_01000	-------
FC88X_00254_01039	-------
FC88X_00279_00976	-------
FC88X_01093_00560	-------
FC88X_01072_00575	-------
FC88X_01092_00538	-------
FC88X_01096_00456	-------
FC88X_00152_00853	-------
FC88X_00068_00602	-------
FC88X_00618_00256	-------
FC88X_00606_00262	-------
FC88X_00643_00088	-------
FC88X_00274_00835	-------

FC88X_00058_00658	-------
FC88X_00546_01002	-------
FC88X_00544_00955	-------
FC88X_00521_00952	-------
FC88X_00523_00269	-------
FC88X_00530_00122	-------
FC88X_00521_00064	-------
FC88X_00471_01215	-------
FC88X_00463_01209	-------
FC88X_00453_01171	-------
FC88X_00476_01126	-------
FC88X_00461_00014	-------
FC88X_00408_00718	-------
FC88X_00422_00607	-------
FC88X_00401_00365	-------
FC88X_00449_00153	-------
FC88X_00190_00820	-------
FC88X_00272_01105	-------
FC88X_00174_00891	-------
FC88X_00625_01188	-------
FC88X_00647_01145	-------
FC88X_00622_01128	-------
FC88X_00617_01137	-------
FC88X_00601_01116	-------
FC88X_00600_01135	-------
FC88X_00619_01021	-------
FC88X_00072_00557	-------
FC88X_00114_00134	-------
FC88X_00080_00568	-------
FC88X_00890_01170	-------
FC88X_00878_01176	-------
FC88X_00979_00632	-------
FC88X_00089_00259	-------
FC88X_00561_01165	-------
FC88X_00865_00441	-------
FC88X_00890_00390	-------
FC88X_00879_00371	-------
FC88X_00677_00735	-------
FC88X_00668_00745	-------
FC88X_00665_00747	-------
FC88X_00661_00557	-------
FC88X_00025_00430	-------
FC88X_00004_00273	-------
FC88X_00075_00102	-------
FC88X_00075_00101	-------
FC88X_00937_00456	-------
FC88X_00943_00426	-------
FC88X_00931_00417	-------
FC88X_00941_00266	-------
FC88X_00926_00252	-------
FC88X_00907_00262	-------
FC88X_00919_00174	-------
FC88X_00904_00181	-------
FC88X_00892_01256	-------
FC88X_00644_01161	-------
FC88X_00015_00201	-------
FC88X_00016_00198	-------

FC88X_00007_00181	-------
FC88X_00031_00147	-------
FC88X_00012_00057	-------
FC88X_00010_00099	-------
FC88X_00007_00080	-------
FC88X_00100_00070	-------
FC88X_00123_00038	-------
FC88X_00113_00048	-------
FC88X_00990_00729	-------
FC88X_00989_00704	-------
FC88X_00091_00805	-------
FC88X_00072_00818	-------
FC88X_01121_00603	-------
FC88X_01105_00621	-------
FC88X_01137_01210	-------
FC88X_01138_01156	-------
FC88X_01126_01144	-------
FC88X_01109_01132	-------
FC88X_01113_01090	-------
FC88X_00935_00757	-------
FC88X_00932_00765	-------
FC88X_00911_00788	-------
FC88X_00938_00726	-------
FC88X_00911_00732	-------
FC88X_00905_00737	-------
FC88X_00931_00618	-------
FC88X_00916_00604	-------
FC88X_00926_00577	-------
FC88X_00947_00516	-------
FC88X_00857_01259	-------
FC88X_00880_01219	-------
FC88X_00082_00851	-------
FC88X_00674_01166	-------
FC88X_00112_00791	-------
FC88X_00270_00037	-------
FC88X_00211_01039	-------
FC88X_00224_00955	-------
FC88X_00241_00925	-------
FC88X_00241_00915	-------
FC88X_00225_00930	-------
FC88X_00241_00899	-------
FC88X_00229_00566	-------
FC88X_00226_00531	-------
FC88X_00200_00496	-------
FC88X_00233_00393	-------
FC88X_00119_00092	-------
FC88X_00933_00394	-------
FC88X_00906_00388	-------
FC88X_00592_01206	-------
FC88X_00573_01113	-------
FC88X_00597_01002	-------
FC88X_00074_00782	-------
FC88X_00597_00924	-------
FC88X_00556_00820	-------
FC88X_00571_00709	-------
FC88X_00597_00662	-------
FC88X_00567_00256	-------

FC88X_00550_00201	-------
FC88X_00579_00170	-------
FC88X_00581_00103	-------
FC88X_00583_00055	-------
FC88X_00571_00063	-------
FC88X_00524_01251	-------
FC88X_00259_00591	-------
FC88X_00516_01226	-------
FC88X_00511_01230	-------
FC88X_00503_01216	-------
FC88X_00543_01180	-------
FC88X_00521_01128	-------
FC88X_00546_01093	-------
FC88X_00050_00822	-------
FC88X_00071_00145	-------
FC88X_00052_00770	-------
FC88X_00068_00746	-------
FC88X_00653_00079	-------
FC88X_00628_01251	-------
FC88X_01037_00789	-------
FC88X_01010_00775	-------
FC88X_01032_00723	-------
FC88X_00857_01165	-------
FC88X_00633_00730	-------
FC88X_00589_01164	-------
FC88X_00896_00728	-------
FC88X_00877_00742	-------
FC88X_00890_00324	-------
FC88X_00876_00250	-------
FC88X_00819_01216	-------
FC88X_00828_01142	-------
FC88X_00825_01139	-------
FC88X_00807_01145	-------
FC88X_00807_01110	-------
FC88X_00803_01145	-------
FC88X_00842_00984	-------
FC88X_00674_00908	-------
FC88X_00672_00921	-------
FC88X_00669_00941	-------
FC88X_00694_00887	-------
FC88X_00693_00860	-------
FC88X_00683_00889	-------
FC88X_00690_00500	-------
FC88X_00654_00528	-------
FC88X_00685_00451	-------
FC88X_00666_00458	-------
FC88X_00665_00465	-------
FC88X_00687_00431	-------
FC88X_00674_00422	-------
FC88X_00676_00347	-------
FC88X_00655_00339	-------
FC88X_00672_00259	-------
FC88X_00668_00295	-------
FC88X_00613_01267	-------
FC88X_00635_00602	-------
FC88X_00646_01183	-------
FC88X_00632_01161	-------

FC88X_00606_01183	-------
FC88X_00604_01187	-------
FC88X_00630_01109	-------
FC88X_00142_00964	-------
FC88X_00126_00852	-------
FC88X_00244_00027	-------
FC88X_00959_00935	-------
FC88X_00984_00883	-------
FC88X_00979_00856	-------
FC88X_00954_00897	-------
FC88X_00886_00855	-------
FC88X_00856_00872	-------
FC88X_00874_00822	-------
FC88X_00873_00824	-------
FC88X_01026_01240	-------
FC88X_01010_01212	-------
FC88X_01077_00470	-------
FC88X_01082_00396	-------
FC88X_01082_00392	-------
FC88X_00667_00200	-------
FC88X_00694_00170	-------
FC88X_00675_01233	-------
FC88X_00692_01167	-------
FC88X_00689_01162	-------
FC88X_00419_00020	-------
FC88X_00390_01141	-------
FC88X_00395_00608	-------
FC88X_00396_00590	-------
FC88X_00392_00578	-------
FC88X_00368_00471	-------
FC88X_00378_00291	-------
FC88X_00352_00200	-------
FC88X_00371_00186	-------
FC88X_00399_00040	-------
FC88X_00338_01124	-------
FC88X_00336_01095	-------
FC88X_00308_01002	-------
FC88X_00319_00996	-------
FC88X_00338_00932	-------
FC88X_00340_00816	-------
FC88X_00680_00096	-------
FC88X_00637_00900	-------
FC88X_00620_00313	-------
FC88X_00637_00281	-------
FC88X_00359_00952	-------
FC88X_00571_00965	-------
FC88X_00569_00965	-------
FC88X_00899_01025	-------
FC88X_00888_00967	-------
FC88X_00883_00975	-------
FC88X_00888_00942	-------
FC88X_00875_00939	-------
FC88X_00695_01026	-------
FC88X_00016_00409	-------
FC88X_00042_00392	-------
FC88X_00016_00382	-------
FC88X_00043_00300	-------

FC88X_00030_00308	-------
FC88X_00022_00301	-------
FC88X_00012_00305	-------
FC88X_00005_00321	-------
FC88X_00048_00296	-------
FC88X_00044_00293	-------
FC88X_00313_01028	-------
FC88X_00717_00091	-------
FC88X_00715_00097	-------
FC88X_00047_00173	-------
FC88X_00126_00150	-------
FC88X_00115_00151	-------
FC88X_00726_01234	-------
FC88X_00716_01172	-------
FC88X_00628_00276	-------
FC88X_00696_00405	-------
FC88X_00698_00337	-------
FC88X_00141_00741	-------
FC88X_00108_00707	-------
FC88X_00582_00524	-------
FC88X_00564_00479	-------
FC88X_00592_00414	-------
FC88X_00594_00228	-------
FC88X_00587_00178	-------
FC88X_00594_00135	-------
FC88X_00955_01171	-------
FC88X_00589_01037	-------
FC88X_00805_00817	-------
FC88X_00802_00807	-------
FC88X_00842_00762	-------
FC88X_00820_00786	-------
FC88X_00820_00778	-------
FC88X_00809_00679	-------
FC88X_00800_00554	-------
FC88X_00910_00731	-------
FC88X_00945_00665	-------
FC88X_00930_00603	-------
FC88X_00925_00627	-------
FC88X_00551_01144	-------
FC88X_01039_00448	-------
FC88X_01027_00330	-------
FC88X_00922_00554	-------
FC88X_00942_00503	-------
FC88X_00499_00162	-------
FC88X_00871_01222	-------
FC88X_00965_01259	-------
FC88X_00996_01228	-------
FC88X_00979_01174	-------
FC88X_00341_01128	-------
FC88X_00694_01212	-------
FC88X_00928_01096	-------
FC88X_00490_00318	-------
FC88X_00486_00260	-------
FC88X_00372_00042	-------
FC88X_00345_01153	-------
FC88X_00482_00120	-------
FC88X_00454_00136	-------

FC88X_00470_00031	-------
FC88X_00459_00021	-------
FC88X_00456_00044	-------
FC88X_00179_00958	-------
FC88X_00168_00987	-------
FC88X_01143_01186	-------
FC88X_01140_01183	-------
FC88X_01100_01150	-------
FC88X_01137_01083	-------
FC88X_00181_01040	-------
FC88X_00302_01115	-------
FC88X_00133_00628	-------
FC88X_00356_01005	-------
FC88X_00598_00588	-------
FC88X_00043_00178	-------
FC88X_01007_00925	-------
FC88X_01016_00807	-------
FC88X_01034_00730	-------
FC88X_01010_00743	-------
FC88X_00988_00765	-------
FC88X_00966_00790	-------
FC88X_00972_00388	-------
FC88X_00922_01208	-------
FC88X_00948_01150	-------
FC88X_00944_01164	-------
FC88X_00937_01182	-------
FC88X_00927_01197	-------
FC88X_00938_01141	-------
FC88X_00595_00645	-------
FC88X_00911_00685	-------
FC88X_00903_00583	-------
FC88X_00945_00525	-------
FC88X_00903_00439	-------
FC88X_00942_00355	-------
FC88X_00929_00393	-------
FC88X_00924_00394	-------
FC88X_00900_00389	-------
FC88X_00939_00300	-------
FC88X_00915_00343	-------
FC88X_00942_00248	-------
FC88X_00915_00235	-------
FC88X_00901_00197	-------
FC88X_00891_01259	-------
FC88X_00865_01253	-------
FC88X_00876_01229	-------
FC88X_00869_01241	-------
FC88X_00857_01203	-------
FC88X_00889_01194	-------
FC88X_00472_01188	-------
FC88X_00471_01188	-------
FC88X_00497_01127	-------
FC88X_00213_00009	-------
FC88X_00199_01068	-------
FC88X_00197_01061	-------
FC88X_00938_00910	-------
FC88X_00918_00787	-------
FC88X_00915_00715	-------

FC88X_00913_00721	-------
FC88X_00042_00031	-------
FC88X_00529_00116	-------
FC88X_00526_00078	-------
FC88X_00500_00052	-------
FC88X_00537_00036	-------
FC88X_00506_00027	-------
FC88X_00494_01222	-------
FC88X_00483_01137	-------
FC88X_00469_01146	-------
FC88X_00817_00820	-------
FC88X_00800_00803	-------
FC88X_00812_00763	-------
FC88X_00833_00433	-------
FC88X_00805_00401	-------
FC88X_00838_00328	-------
FC88X_00834_00339	-------
FC88X_00842_00291	-------
FC88X_00800_00250	-------
FC88X_00826_00226	-------
FC88X_00815_00193	-------
FC88X_00806_00152	-------
FC88X_00763_01253	-------
FC88X_00770_01127	-------
FC88X_00765_01124	-------
FC88X_00757_01148	-------
FC88X_00770_01014	-------
FC88X_00753_00959	-------
FC88X_00796_00824	-------
FC88X_00777_00765	-------
FC88X_00796_00584	-------
FC88X_00761_00403	-------
FC88X_00761_00290	-------
FC88X_00759_00215	-------
FC88X_00782_00178	-------
FC88X_00019_00584	-------
FC88X_00387_00626	-------
FC88X_00176_00907	-------
FC88X_01127_01000	-------
FC88X_01101_00985	-------
FC88X_01134_00919	-------
FC88X_01016_00568	-------
FC88X_01040_00508	-------
FC88X_00637_00125	-------
FC88X_00634_00127	-------
FC88X_00620_00127	-------
FC88X_00610_00133	-------
FC88X_00628_00081	-------
FC88X_00587_01252	-------
FC88X_00493_00711	-------
FC88X_00454_00731	-------
FC88X_00475_00639	-------
FC88X_00479_00553	-------
FC88X_00483_00514	-------
FC88X_00125_00631	-------
FC88X_00457_01114	-------
FC88X_00430_01202	-------

FC88X_00427_01223	-------
FC88X_00432_01198	-------
FC88X_00019_00180	-------
FC88X_00109_00454	-------
FC88X_00804_01101	-------
FC88X_00838_00973	-------
FC88X_00127_00138	-------
FC88X_00116_00129	-------
FC88X_00147_00098	-------
FC88X_00147_00065	-------
FC88X_00692_00167	-------
FC88X_00621_01245	-------
FC88X_00163_00408	-------
FC88X_00195_00294	-------
FC88X_00163_00184	-------
FC88X_00162_00106	-------
FC88X_00178_00037	-------
FC88X_00169_00021	-------
FC88X_00161_00011	-------
FC88X_00213_00052	-------
FC88X_00203_00074	-------
FC88X_01060_00642	-------
FC88X_00195_01061	-------
FC88X_00197_01007	-------
FC88X_00222_00497	-------
FC88X_00848_00342	-------
FC88X_00843_00279	-------
FC88X_00529_00955	-------
FC88X_00549_00938	-------
FC88X_00527_00775	-------
FC88X_00519_00674	-------
FC88X_00513_00593	-------
FC88X_00545_00517	-------
FC88X_00513_00450	-------
FC88X_00527_00304	-------
FC88X_00511_00280	-------
FC88X_00515_00105	-------
FC88X_00107_00733	-------
FC88X_00866_00897	-------
FC88X_00863_00865	-------
FC88X_00864_00843	-------
FC88X_00863_00796	-------
FC88X_00871_00747	-------
FC88X_00858_00714	-------
FC88X_00893_00677	-------
FC88X_00508_01191	-------
FC88X_00549_01137	-------
FC88X_00545_01129	-------
FC88X_00536_01130	-------
FC88X_00533_01141	-------
FC88X_00570_00084	-------
FC88X_00569_00087	-------
FC88X_00547_01251	-------
FC88X_00524_01213	-------
FC88X_00869_00675	-------
FC88X_00895_00635	-------
FC88X_00882_00574	-------

FC88X_00880_00551	-------
FC88X_00890_00493	-------
FC88X_00885_00473	-------
FC88X_00875_00465	-------
FC88X_00877_00400	-------
FC88X_00884_00342	-------
FC88X_00877_00264	-------
FC88X_00868_00228	-------
FC88X_00890_00172	-------
FC88X_00881_00150	-------
FC88X_00868_00177	-------
FC88X_00858_00183	-------
FC88X_00838_01223	-------
FC88X_00487_00903	-------
FC88X_00211_00655	-------
FC88X_00111_00554	-------
FC88X_00115_00816	-------
FC88X_00169_00073	-------
FC88X_00169_00060	-------
FC88X_00238_00073	-------
FC88X_00046_00606	-------
FC88X_00133_00576	-------
FC88X_00143_00924	-------
FC88X_00132_00934	-------
FC88X_00150_00263	-------
FC88X_00224_00872	-------
FC88X_00073_00858	-------
FC88X_00997_00371	-------
FC88X_00959_00280	-------
FC88X_00983_00242	-------
FC88X_00162_00024	-------
FC88X_00162_00023	-------
FC88X_00165_00415	-------
FC88X_00856_01139	-------
FC88X_00183_00783	-------
FC88X_00151_00608	-------
FC88X_01109_01156	-------
FC88X_01121_01188	-------
FC88X_01139_01137	-------
FC88X_01100_01158	-------
FC88X_01134_01051	-------
FC88X_01142_01056	-------
FC88X_01133_01005	-------
FC88X_01121_01060	-------
FC88X_01142_00954	-------
FC88X_01108_01032	-------
FC88X_01119_00971	-------
FC88X_01119_00978	-------
FC88X_01102_00994	-------
FC88X_01118_00960	-------
FC88X_01121_00907	-------
FC88X_01122_00901	-------
FC88X_01144_00883	-------
FC88X_01144_00898	-------
FC88X_01107_00849	-------
FC88X_01141_00806	-------
FC88X_01116_00782	-------

FC88X_01147_00756	-------
FC88X_01147_00687	-------
FC88X_01102_00742	-------
FC88X_01105_00684	-------
FC88X_01146_00683	-------
FC88X_01132_00589	-------
FC88X_01105_00785	-------
FC88X_01110_00591	-------
FC88X_01131_00630	-------
FC88X_01053_01239	-------
FC88X_01057_01222	-------
FC88X_01073_01165	-------
FC88X_01102_00438	-------
FC88X_01071_01067	-------
FC88X_01081_01100	-------
FC88X_01077_00987	-------
FC88X_01069_01076	-------
FC88X_01053_00960	-------
FC88X_01063_00994	-------
FC88X_01082_00881	-------
FC88X_01097_00851	-------
FC88X_01085_00814	-------
FC88X_01075_00884	-------
FC88X_01094_00787	-------
FC88X_01063_00823	-------
FC88X_01066_00760	-------
FC88X_01090_00778	-------
FC88X_01052_00778	-------
FC88X_01058_00798	-------
FC88X_01067_00651	-------
FC88X_01090_00652	-------
FC88X_01099_00549	-------
FC88X_01093_00593	-------
FC88X_01095_00514	-------
FC88X_01096_00512	-------
FC88X_01012_01271	-------
FC88X_01054_00435	-------
FC88X_01009_01176	-------
FC88X_01023_01166	-------
FC88X_01049_01129	-------
FC88X_01009_01171	-------
FC88X_01022_01094	-------
FC88X_01030_01086	-------
FC88X_01020_01046	-------
FC88X_01000_01098	-------
FC88X_01047_00986	-------
FC88X_01013_01003	-------
FC88X_01041_00933	-------
FC88X_01022_01014	-------
FC88X_01044_00856	-------
FC88X_01038_00950	-------
FC88X_01041_00886	-------
FC88X_01018_00912	-------
FC88X_01032_00878	-------
FC88X_01000_00929	-------
FC88X_01017_00898	-------
FC88X_01029_00866	-------

FC88X_01023_00848	-------
FC88X_01026_00816	-------
FC88X_01009_00758	-------
FC88X_01021_00822	-------
FC88X_01019_00711	-------
FC88X_01007_00795	-------
FC88X_01005_00718	-------
FC88X_01005_00738	-------
FC88X_01022_00636	-------
FC88X_01007_00655	-------
FC88X_01041_00594	-------
FC88X_01046_00581	-------
FC88X_01016_00598	-------
FC88X_01040_00575	-------
FC88X_01004_00502	-------
FC88X_01025_00590	-------
FC88X_01000_00473	-------
FC88X_01019_00451	-------
FC88X_01014_00407	-------
FC88X_01019_00431	-------
FC88X_01006_00405	-------
FC88X_01010_00401	-------
FC88X_01006_00363	-------
FC88X_01009_00448	-------
FC88X_00998_01257	-------
FC88X_01027_00303	-------
FC88X_00995_01169	-------
FC88X_00951_01204	-------
FC88X_00993_01028	-------
FC88X_00967_01147	-------
FC88X_00978_01032	-------
FC88X_01047_00930	-------
FC88X_00987_00870	-------
FC88X_00992_00918	-------
FC88X_00950_00895	-------
FC88X_00972_00867	-------
FC88X_00974_00789	-------
FC88X_00990_00807	-------
FC88X_00999_00635	-------
FC88X_00950_00676	-------
FC88X_00966_00646	-------
FC88X_00988_00708	-------
FC88X_00960_00558	-------
FC88X_00967_00573	-------
FC88X_00985_00540	-------
FC88X_00996_00688	-------
FC88X_00968_00428	-------
FC88X_00989_00403	-------
FC88X_00990_00397	-------
FC88X_00994_00443	-------
FC88X_00954_00383	-------
FC88X_00962_00425	-------
FC88X_00973_00348	-------
FC88X_00996_00349	-------
FC88X_00982_00255	-------
FC88X_00992_00288	-------
FC88X_00979_00268	-------

FC88X_00968_00307	-------
FC88X_00984_00247	-------
FC88X_00953_00288	-------
FC88X_00978_00233	-------
FC88X_00987_00246	-------
FC88X_00945_01271	-------
FC88X_00946_01260	-------
FC88X_00916_01227	-------
FC88X_00925_01246	-------
FC88X_00903_01138	-------
FC88X_01028_00722	-------
FC88X_00917_01017	-------
FC88X_00900_01138	-------
FC88X_00910_00924	-------
FC88X_00936_00948	-------
FC88X_00940_00886	-------
FC88X_00942_00873	-------
FC88X_00930_00898	-------
FC88X_00932_00875	-------
FC88X_00907_00797	-------
FC88X_00921_00793	-------
FC88X_00908_00599	-------
FC88X_00927_00596	-------
FC88X_00916_00522	-------
FC88X_00921_00549	-------
FC88X_00936_00430	-------
FC88X_00941_00466	-------
FC88X_00903_00360	-------
FC88X_00932_00428	-------
FC88X_00903_00284	-------
FC88X_00928_00391	-------
FC88X_00906_00200	-------
FC88X_00902_00294	-------
FC88X_00904_00159	-------
FC88X_00905_00239	-------
FC88X_00879_01256	-------
FC88X_01109_00932	-------
FC88X_00866_01018	-------
FC88X_00942_00313	-------
FC88X_00713_00662	-------
FC88X_00729_00876	-------
FC88X_00736_00462	-------
FC88X_00725_00569	-------
FC88X_00716_00429	-------
FC88X_00722_00480	-------
FC88X_00665_01215	-------
FC88X_01080_00641	-------
FC88X_00689_01152	-------
FC88X_00692_01159	-------
FC88X_00690_01048	-------
FC88X_01142_00840	-------
FC88X_00660_01030	-------
FC88X_00676_01230	-------
FC88X_00692_00974	-------
FC88X_00706_00132	-------
FC88X_00695_00623	-------
FC88X_00665_00847	-------

FC88X_00685_00493	-------
FC88X_00678_00588	-------
FC88X_00672_00152	-------
FC88X_00656_00241	-------
FC88X_00665_00071	-------
FC88X_00651_00139	-------
FC88X_00619_01265	-------
FC88X_00701_00127	-------
FC88X_00620_01137	-------
FC88X_00628_01209	-------
FC88X_00600_01024	-------
FC88X_00602_01143	-------
FC88X_00612_00920	-------
FC88X_00968_01016	-------
FC88X_00627_00503	-------
FC88X_00630_00840	-------
FC88X_00610_00434	-------
FC88X_00642_00410	-------
FC88X_00646_00345	-------
FC88X_00607_00444	-------
FC88X_00635_00111	-------
FC88X_00690_01108	-------
FC88X_00551_01239	-------
FC88X_00560_01219	-------
FC88X_00585_01166	-------
FC88X_00588_01166	-------
FC88X_00559_01124	-------
FC88X_00584_01174	-------
FC88X_00582_00988	-------
FC88X_00592_00964	-------
FC88X_00575_00870	-------
FC88X_00568_00934	-------
FC88X_00599_00752	-------
FC88X_00589_00823	-------
FC88X_00578_00563	-------
FC88X_00565_00683	-------
FC88X_00575_00175	-------
FC88X_00596_01202	-------
FC88X_00582_00076	-------
FC88X_00567_00111	-------
FC88X_00533_01241	-------
FC88X_00562_00083	-------
FC88X_00516_01106	-------
FC88X_00511_01217	-------
FC88X_00523_01007	-------
FC88X_00544_01000	-------
FC88X_00539_00997	-------
FC88X_00512_01040	-------
FC88X_00514_00965	-------
FC88X_00524_00990	-------
FC88X_00547_00830	-------
FC88X_00510_00976	-------
FC88X_00540_00622	-------
FC88X_00547_00735	-------
FC88X_00548_00046	-------
FC88X_00534_00109	-------
FC88X_00500_00026	-------

FC88X_00513_00020	-------
FC88X_00469_01227	-------
FC88X_00495_01244	-------
FC88X_00499_01195	-------
FC88X_00466_01219	-------
FC88X_00486_01136	-------
FC88X_00472_01179	-------
FC88X_00484_01071	-------
FC88X_00568_00198	-------
FC88X_00452_00964	-------
FC88X_00468_01000	-------
FC88X_00459_00776	-------
FC88X_00488_00898	-------
FC88X_00494_00678	-------
FC88X_00499_00736	-------
FC88X_00473_00287	-------
FC88X_00465_00522	-------
FC88X_00480_00105	-------
FC88X_00483_00136	-------
FC88X_00483_00067	-------
FC88X_00498_00089	-------
FC88X_00462_00058	-------
FC88X_00476_00055	-------
FC88X_00432_01229	-------
FC88X_00448_01222	-------
FC88X_00420_01185	-------
FC88X_00497_00029	-------
FC88X_00436_01075	-------
FC88X_00424_01200	-------
FC88X_00418_01071	-------
FC88X_00417_01192	-------
FC88X_00440_00116	-------
FC88X_00400_00291	-------
FC88X_00360_01168	-------
FC88X_00431_00049	-------
FC88X_00369_01143	-------
FC88X_00352_01162	-------
FC88X_00375_01079	-------
FC88X_00394_01055	-------
FC88X_00387_00868	-------
FC88X_00367_00978	-------
FC88X_00384_00656	-------
FC88X_00395_01049	-------
FC88X_00399_00193	-------
FC88X_00393_00533	-------
FC88X_00318_01144	-------
FC88X_00348_01159	-------
FC88X_00346_01085	-------
FC88X_00349_01067	-------
FC88X_00329_01039	-------
FC88X_00303_01082	-------
FC88X_00348_00911	-------
FC88X_00340_00985	-------
FC88X_00323_00655	-------
FC88X_00330_00793	-------
FC88X_00303_00411	-------
FC88X_00341_00553	-------

FC88X_00304_00188	-------
FC88X_00311_00328	-------
FC88X_00326_00036	-------
FC88X_00327_00017	-------
FC88X_00307_00048	-------
FC88X_00989_00490	-------
FC88X_00275_01089	-------
FC88X_00294_01089	-------
FC88X_00299_01025	-------
FC88X_00252_01055	-------
FC88X_00262_00990	-------
FC88X_00266_00984	-------
FC88X_00268_00940	-------
FC88X_00271_00933	-------
FC88X_00269_00611	-------
FC88X_00250_00922	-------
FC88X_00277_00427	-------
FC88X_00280_00560	-------
FC88X_00296_00198	-------
FC88X_00299_00296	-------
FC88X_00280_00060	-------
FC88X_00280_00144	-------
FC88X_00299_00033	-------
FC88X_00259_00058	-------
FC88X_00259_00044	-------
FC88X_00358_01007	-------
FC88X_00217_00976	-------
FC88X_00233_01014	-------
FC88X_00244_00900	-------
FC88X_00205_00956	-------
FC88X_00226_00774	-------
FC88X_00227_00790	-------
FC88X_00203_00691	-------
FC88X_00231_01101	-------
FC88X_00230_00426	-------
FC88X_00209_00799	-------
FC88X_00225_00212	-------
FC88X_00244_00208	-------
FC88X_00239_00037	-------
FC88X_00243_00017	-------
FC88X_00233_00031	-------
FC88X_00238_00049	-------
FC88X_00176_00987	-------
FC88X_00157_01021	-------
FC88X_00158_00995	-------
FC88X_00229_00033	-------
FC88X_00189_00913	-------
FC88X_00199_00941	-------
FC88X_00165_00932	-------
FC88X_00153_00960	-------
FC88X_00195_00865	-------
FC88X_00199_00887	-------
FC88X_00174_00806	-------
FC88X_00168_00879	-------
FC88X_00174_00801	-------
FC88X_00166_00869	-------
FC88X_00152_00794	-------

FC88X_00155_00778	-------
FC88X_00156_00575	-------
FC88X_00173_00794	-------
FC88X_00161_00533	-------
FC88X_00209_00301	-------
FC88X_00174_00209	-------
FC88X_00163_00463	-------
FC88X_00196_00125	-------
FC88X_00197_00134	-------
FC88X_00190_00062	-------
FC88X_00194_00142	-------
FC88X_00173_00053	-------
FC88X_00182_00087	-------
FC88X_00166_00099	-------
FC88X_00160_00348	-------
FC88X_00154_00083	-------
FC88X_00163_00063	-------
FC88X_00166_00048	-------
FC88X_00191_00020	-------
FC88X_00130_00889	-------
FC88X_00135_00884	-------
FC88X_00114_00836	-------
FC88X_00684_01123	-------
FC88X_00132_00795	-------
FC88X_00667_01095	-------
FC88X_00116_00718	-------
FC88X_00130_00727	-------
FC88X_00142_00669	-------
FC88X_00114_00744	-------
FC88X_00120_00685	-------
FC88X_00140_00699	-------
FC88X_00144_00560	-------
FC88X_00375_00056	-------
FC88X_00101_00591	-------
FC88X_00108_00551	-------
FC88X_00136_00536	-------
FC88X_00267_01000	-------
FC88X_00148_00456	-------
FC88X_00132_00546	-------
FC88X_00130_00417	-------
FC88X_00121_00540	-------
FC88X_00118_00420	-------
FC88X_00133_00410	-------
FC88X_00113_00412	-------
FC88X_00113_00482	-------
FC88X_00114_00250	-------
FC88X_00100_00397	-------
FC88X_00139_00219	-------
FC88X_00261_00983	-------
FC88X_00111_00237	-------
FC88X_00103_00340	-------
FC88X_00119_00180	-------
FC88X_00148_00192	-------
FC88X_00110_00130	-------
FC88X_00138_00213	-------
FC88X_00123_00063	-------
FC88X_00135_00078	-------

FC88X_00111_00067	-------
FC88X_00731_01036	-------
FC88X_00065_00776	-------
FC88X_00087_00755	-------
FC88X_00065_00654	-------
FC88X_00061_00708	-------
FC88X_00013_00511	-------
FC88X_00116_00752	-------
FC88X_00908_00538	-------
FC88X_01013_00727	-------
FC88X_00635_01061	-------
FC88X_01099_00495	-------
FC88X_00102_00470	-------
FC88X_00037_00698	-------
FC88X_00725_00973	-------
FC88X_01002_00811	-------
FC88X_01022_00342	-------
FC88X_00557_00358	-------
FC88X_00915_00906	-------
FC88X_00945_00746	-------
FC88X_00091_00200	-------
FC88X_00028_00757	-------
FC88X_00625_00829	-------
FC88X_00892_01164	-------
FC88X_00918_00777	-------
FC88X_00713_00412	-------
FC88X_00931_00498	-------
FC88X_00268_00685	-------
FC88X_00445_00070	-------
FC88X_00035_00511	-------
FC88X_00400_00905	-------
FC88X_00045_00614	-------
FC88X_00962_00487	-------
FC88X_00073_00304	-------
FC88X_00056_00444	-------
FC88X_00089_00890	-------
FC88X_00042_00649	-------
FC88X_00318_00004	-------
FC88X_00701_01113	-------
FC88X_00413_01166	-------
FC88X_00093_00912	-------
FC88X_00460_01163	-------
FC88X_00462_01186	-------
FC88X_00504_01040	-------
FC88X_00515_01112	-------
FC88X_00585_01152	-------
FC88X_00675_01262	-------
FC88X_00747_00175	-------
FC88X_00702_00812	-------
FC88X_00758_00776	-------
FC88X_00791_00847	-------
FC88X_01008_01175	-------
FC88X_01049_01203	-------
FC88X_01062_01202	-------
FC88X_01069_01234	-------
FC88X_00017_00200	-------
FC88X_00026_00224	-------

FC88X_00035_00520	-------
FC88X_00178_00794	-------
FC88X_00103_00131	-------
FC88X_00113_00082	-------
FC88X_00219_00951	-------
FC88X_00333_00584	-------
FC88X_00391_00752	-------
FC88X_01147_01195	-------
FC88X_00090_00565	-------
FC88X_00103_00035	-------
FC88X_00068_00563	-------
FC88X_00077_00559	-------
FC88X_00091_00459	-------
FC88X_00093_00456	-------
FC88X_00079_00476	-------
FC88X_00086_00462	-------
FC88X_00069_00490	-------
FC88X_00071_00494	-------
FC88X_00077_00330	-------
FC88X_00086_00375	-------
FC88X_00072_00209	-------
FC88X_00093_00420	-------
FC88X_00053_00249	-------
FC88X_00062_00205	-------
FC88X_00007_00108	-------
FC88X_00333_00057	-------
FC88X_00018_00563	-------
FC88X_00386_01176	-------
FC88X_01101_00689	-------
FC88X_00893_00743	-------
FC88X_01120_00584	-------
FC88X_00506_00577	-------
FC88X_00651_00506	-------
FC88X_00145_00884	-------
FC88X_01077_01190	-------
FC88X_00248_01011	-------
FC88X_00488_01127	-------
FC88X_00979_00812	-------
FC88X_00374_00998	-------
FC88X_00817_01173	-------
FC88X_00975_01198	-------
FC88X_00567_00965	-------
FC88X_00882_01001	-------
FC88X_00938_00613	-------
FC88X_00725_00294	-------
FC88X_00938_00353	-------
FC88X_00605_01074	-------
FC88X_00135_00048	-------
FC88X_00084_00808	-------
FC88X_00120_00939	-------
FC88X_00097_00188	-------
FC88X_00050_00687	-------
FC88X_00059_00689	-------
FC88X_00102_00136	-------
FC88X_00278_00780	-------
FC88X_00251_00927	-------
FC88X_00411_00154	-------

FC88X_00403_00292	-------
FC88X_00493_00203	-------
FC88X_00469_00662	-------
FC88X_00534_00633	-------
FC88X_00503_00702	-------
FC88X_00594_00231	-------
FC88X_00595_00210	-------
FC88X_00680_00884	-------
FC88X_00679_00918	-------
FC88X_00766_00250	-------
FC88X_00793_00358	-------
FC88X_00823_00199	-------
FC88X_00838_00242	-------
FC88X_01078_00971	-------
FC88X_01078_01015	-------
FC88X_01105_00802	-------
FC88X_01134_00871	-------
FC88X_00060_00046	-------
FC88X_00067_00069	-------
FC88X_00067_00380	-------
FC88X_00042_00762	-------
FC88X_00113_00341	-------
FC88X_00300_00522	-------
FC88X_00266_00210	-------
FC88X_00674_00157	-------
FC88X_00331_01149	-------
FC88X_00096_00708	-------
FC88X_00086_00118	-------
FC88X_00936_00533	-------
FC88X_00076_00145	-------
FC88X_00134_00931	-------
FC88X_00070_00139	-------
FC88X_00136_00645	-------
FC88X_00015_00520	-------
FC88X_00082_00610	-------
FC88X_00645_01213	-------
FC88X_00275_00918	-------
FC88X_00049_00019	-------
FC88X_01057_00716	-------
FC88X_00548_01133	-------
FC88X_01101_00978	-------
FC88X_01027_00578	-------
FC88X_00635_01159	-------
FC88X_00096_00395	-------
FC88X_00821_00311	-------
FC88X_01010_00716	-------
FC88X_00873_00774	-------
FC88X_00580_00692	-------
FC88X_00033_00115	-------
FC88X_01062_00830	-------
FC88X_00641_01148	-------
FC88X_01020_00593	-------
FC88X_01117_00751	-------
FC88X_00032_00526	-------
FC88X_00183_00034	-------
FC88X_00137_00507	-------
FC88X_00973_00503	-------

```
FC88X_00538_01074       -------
FC88X_00036_00672       -------
FC88X_00714_00942       -------
FC88X_00089_00080       -------
FC88X_01110_00676       C------
FC88X_00727_00941       -------
FC88X_00797_00958       -------
FC88X_00067_00454       -------
FC88X_00075_00623       -------
FC88X_00978_00895       -------
FC88X_01095_01134       -------
FC88X_00179_00129       -------
FC88X_00372_01144       -------
FC88X_00993_00713       -------
FC88X_00685_01030       -------
FC88X_00935_00638       -------
FC88X_00790_00217       -------
FC88X_00875_00137       -------
FC88X_00883_01033       -------
FC88X_00415_00952       -------
FC88X_00422_00985       -------
FC88X_00864_00678       -------
FC88X_00958_00587       -------
FC88X_01036_00780       -------
FC88X_01074_00908       -------
FC88X_01063_00488       -------
FC88X_00489_01171       -------
FC88X_00309_01131       -------
FC88X_00099_00127       -------
FC88X_00234_01048       -------
FC88X_00129_00890       -------
FC88X_01046_00706       -------
FC88X_00024_00086       -------
FC88X_01117_01209       -------
FC88X_00529_00974       -------
FC88X_00936_00880       -------
FC88X_00071_00392       -------
FC88X_00601_00093       -------
FC88X_00380_00284       -------
FC88X_01045_00708       -------
FC88X_00819_00170       -------
FC88X_00770_00110       -------
FC88X_00967_00873       -------
FC88X_00126_00784       -------
FC88X_00653_00114       -------
FC88X_00907_01182       -------
FC88X_00911_00817       -------
FC88X_00763_00199       -------
FC88X_00652_00798       -------
FC88X_00139_00233       -------
FC88X_00088_00390       -------
FC88X_00048_00291       -------
FC88X_00144_00964       -------
FC88X_00653_01260       -------
FC88X_00747_01054       -------
FC88X_00598_01165       -------
FC88X_01004_00912       -------
```

FC88X_01012_01025	-------
FC88X_00990_01027	-------
FC88X_00690_01028	C------
FC88X_00970_00453	-------
FC88X_00906_01186	-------
FC88X_01022_00580	-------
FC88X_00050_00488	-------
FC88X_00227_00918	-------
FC88X_00732_00297	-------
FC88X_01081_00652	-------
FC88X_00635_00694	-------
FC88X_00523_01184	-------
FC88X_00187_00944	-------
FC88X_00849_00201	-------
FC88X_00410_01219	-------
FC88X_00904_00774	-------
FC88X_00807_01198	-------
FC88X_00850_00806	-------
FC88X_00861_00811	-------
FC88X_00850_00874	-------
FC88X_00059_00135	-------
FC88X_00738_00502	-------
FC88X_00886_01259	-------
FC88X_00149_00722	-------
FC88X_00992_01104	-------
FC88X_00441_00206	-------
FC88X_00507_00171	-------
FC88X_00541_00245	-------
FC88X_00164_00130	-------
FC88X_00093_00910	-------
FC88X_00110_00464	-------
FC88X_00910_00730	-------
FC88X_00740_01143	-------
FC88X_01023_00708	-------
FC88X_01082_00902	-------
FC88X_01143_00811	-------
FC88X_01033_00981	-------
FC88X_00814_00122	-------
FC88X_00477_00172	-------
FC88X_00305_00359	-------
FC88X_01069_00412	-------
FC88X_01120_00594	-------
FC88X_00947_01159	-------
FC88X_00877_01156	-------
FC88X_00878_01092	-------
FC88X_01133_01170	-------
FC88X_00650_01075	-------
FC88X_01127_00526	-------
FC88X_00926_01015	-------
FC88X_00227_00042	-------
FC88X_00239_00051	-------
FC88X_00073_00222	-------
FC88X_00946_00304	-------
FC88X_00973_01011	-------
FC88X_00975_00764	-------
FC88X_01077_00659	-------
FC88X_00662_01059	-------

FC88X_00995_00773	-------
FC88X_01037_01041	-------
FC88X_00913_00744	-------
FC88X_00203_00951	-------
FC88X_00698_01131	-------
FC88X_00732_01032	-------
FC88X_00978_00592	-------
FC88X_00799_00147	-------
FC88X_00307_01037	-------
FC88X_00072_00313	-------
FC88X_00797_00904	-------
FC88X_00192_00823	-------
FC88X_00412_01145	-------
FC88X_00459_01229	-------
FC88X_00578_00971	-------
FC88X_00127_00753	-------
FC88X_00839_00892	-------
FC88X_00863_00293	-------
FC88X_00874_00348	-------
FC88X_00403_01000	-------
FC88X_00406_01031	-------
FC88X_00566_00210	-------
FC88X_00397_01105	-------
FC88X_00039_00777	-------
FC88X_00078_00623	-------
FC88X_00580_01219	-------
FC88X_00157_00826	-------
FC88X_00024_00256	-------
FC88X_01012_00481	-------
FC88X_00989_00901	-------
FC88X_00026_00408	-------
FC88X_00509_01069	-------
FC88X_00769_00145	-------
FC88X_00946_00419	-------
FC88X_00582_01128	-------
FC88X_00278_00720	-------
FC88X_00126_00453	-------
FC88X_00584_00964	-------
FC88X_00969_00827	-------
FC88X_00962_00943	-------
FC88X_00554_00106	-------
FC88X_00049_00028	-------
FC88X_00512_00136	-------
FC88X_00375_01139	-------
FC88X_00664_01155	-------
FC88X_01119_01198	-------
FC88X_00920_01242	-------
FC88X_00024_00767	-------
FC88X_01141_00623	-------
FC88X_00159_00053	-------
FC88X_00360_00739	-------
FC88X_00624_00246	-------
FC88X_00880_00901	-------
FC88X_00596_01139	-------
FC88X_00600_00085	-------
FC88X_00023_00186	-------
FC88X_00359_01177	-------

FC88X_00839_00369	-------
FC88X_00056_00851	-------
FC88X_00362_01166	-------
FC88X_01000_00283	-------
FC88X_00654_00154	-------
FC88X_01030_01202	-------
FC88X_00345_01091	-------
FC88X_00564_01074	-------
FC88X_00842_00222	-------
FC88X_01023_00779	-------
FC88X_00957_00772	-------
FC88X_00792_00433	-------
FC88X_01078_01130	-------
FC88X_00144_00457	-------
FC88X_00138_00748	-------
FC88X_00475_01064	-------
FC88X_00256_00963	-------
FC88X_00361_00015	-------
FC88X_00289_01098	-------
FC88X_00595_00754	-------
FC88X_00284_00993	-------
FC88X_00698_00282	-------
FC88X_00609_01183	-------
FC88X_00590_01081	-------
FC88X_00192_00071	-------
FC88X_00847_00882	-------
FC88X_00829_00361	-------
FC88X_00816_00604	-------
FC88X_00915_00665	-------
FC88X_00269_00997	-------
FC88X_00412_00960	-------
FC88X_00461_00334	-------
FC88X_00413_00023	-------
FC88X_00344_00864	-------
FC88X_00367_00988	-------
FC88X_00281_01072	-------
FC88X_00105_00475	-------
FC88X_00981_00653	-------
FC88X_00384_00959	-------
FC88X_01074_00392	-------
FC88X_00692_01065	-------
FC88X_00423_00521	-------
FC88X_00804_00915	-------
FC88X_00826_00449	-------
FC88X_00837_00794	-------
FC88X_00726_00936	-------
FC88X_00030_00332	-------
FC88X_00516_00787	-------
FC88X_00918_01207	-------
FC88X_00414_00054	-------
FC88X_00517_00058	-------
FC88X_00930_00620	-------
FC88X_00637_00287	-------
FC88X_00988_00389	-------
FC88X_01083_00689	-------
FC88X_00677_00166	-------
FC88X_00849_00962	-------

FC88X_00088_00076	-------
FC88X_00070_00370	-------
FC88X_00469_00810	-------
FC88X_00873_00770	-------
FC88X_00563_00073	-------
FC88X_01074_00543	-------
FC88X_00445_00073	-------
FC88X_01143_01120	-------
FC88X_00147_00771	-------
FC88X_00207_00595	-------
FC88X_00877_01166	-------
FC88X_00672_00153	-------
FC88X_00151_00688	C------
FC88X_00343_00499	-------
FC88X_00343_01076	-------
FC88X_00634_01160	-------
FC88X_00336_00177	-------
FC88X_00193_00042	-------
FC88X_00123_00666	-------
FC88X_00120_00443	-------
FC88X_00889_00459	-------
FC88X_00722_01008	-------
FC88X_00050_00280	-------
FC88X_00449_00056	-------
FC88X_00087_00903	-------
FC88X_00290_00773	-------
FC88X_00116_00688	-------
FC88X_00652_00211	-------
FC88X_00947_01139	-------
FC88X_01096_01137	-------
FC88X_00846_01271	-------
FC88X_01013_01193	-------
FC88X_00825_00352	-------
FC88X_00705_01273	-------
FC88X_00825_00124	-------
FC88X_00762_00284	-------
FC88X_00020_00692	-------
FC88X_00463_01239	-------
FC88X_00670_00945	-------
FC88X_00618_01137	-------
FC88X_00207_00155	-------
FC88X_00732_01017	-------
FC88X_00847_00725	-------
FC88X_00835_00110	-------
FC88X_00776_00303	-------
FC88X_00766_00828	-------
FC88X_00799_00891	-------
FC88X_00117_00620	-------
FC88X_00380_00958	-------
FC88X_00579_01004	-------
FC88X_00384_00196	-------
FC88X_01022_00319	-------
FC88X_00792_01025	-------
FC88X_00542_01096	-------
FC88X_00712_01010	-------
FC88X_00650_01091	-------
FC88X_00627_00131	-------

FC88X_00747_00679	-------
FC88X_00437_00030	-------
FC88X_00573_00903	-------
FC88X_00819_01028	-------
FC88X_00751_00227	-------
FC88X_00754_00480	-------
FC88X_00795_00851	-------
FC88X_00833_01133	-------
FC88X_00804_01077	-------
FC88X_00848_01214	-------
FC88X_00778_00232	-------
FC88X_00694_00940	-------
FC88X_00887_00705	-------
FC88X_00306_00060	-------
FC88X_00638_00210	-------
FC88X_00893_00595	-------
FC88X_00039_00751	-------
FC88X_01107_00926	-------
FC88X_00591_00379	-------
FC88X_00858_01099	-------
FC88X_01000_00568	-------
FC88X_01080_00556	-------
FC88X_00321_01021	-------
FC88X_00789_00212	-------
FC88X_00776_00828	-------
FC88X_00826_01132	-------
FC88X_00800_01191	-------
FC88X_00818_01094	-------
FC88X_00790_01144	-------
FC88X_00782_01155	-------
FC88X_00775_01221	-------
FC88X_00878_00308	-------
FC88X_00022_00390	-------
FC88X_00577_01209	-------
FC88X_00164_00405	-------
FC88X_00919_00636	-------
FC88X_00070_00091	-------
FC88X_00489_00749	-------
FC88X_00058_00513	-------
FC88X_00908_01047	-------
FC88X_01031_01043	-------
FC88X_00613_00188	-------
FC88X_00715_00898	-------
FC88X_00187_00817	-------
FC88X_00240_00126	-------
FC88X_00382_00997	-------
FC88X_00601_00720	-------
FC88X_00111_00936	-------
FC88X_00012_00641	-------
FC88X_00120_00151	-------
FC88X_00869_00265	-------
FC88X_00878_01144	-------
FC88X_00912_00626	-------
FC88X_00933_00724	-------
FC88X_01060_00865	-------
FC88X_00201_00132	-------
FC88X_00414_00963	-------

FC88X_01046_00335	-------
FC88X_01075_00489	-------
FC88X_00915_00681	-------
FC88X_00936_00660	-------
FC88X_00096_00200	-------
FC88X_00275_01031	-------
FC88X_00445_00908	-------
FC88X_00169_00818	-------
FC88X_01085_00409	-------
FC88X_00714_01232	-------
FC88X_00945_00667	-------
FC88X_00150_00718	-------
FC88X_00255_01060	-------
FC88X_00429_01030	-------
FC88X_00510_01091	-------
FC88X_00463_00737	-------
FC88X_00485_00064	-------
FC88X_00935_00673	-------
FC88X_00753_00250	-------
FC88X_00914_00384	-------
FC88X_01135_00938	-------
FC88X_00829_00778	-------
FC88X_01010_00511	-------
FC88X_01117_01177	-------
FC88X_00639_00203	-------
FC88X_00754_01230	-------
FC88X_00897_00679	-------
FC88X_01024_00718	-------
FC88X_01046_01237	-------
FC88X_00023_00574	-------
FC88X_00034_00803	-------
FC88X_00802_00963	-------
FC88X_01110_00536	-------
FC88X_00112_00496	-------
FC88X_01075_00765	-------
FC88X_00604_01222	-------
FC88X_00690_01015	-------
FC88X_00495_01179	-------
FC88X_00754_01158	-------
FC88X_00782_01186	-------
FC88X_00850_00326	-------
FC88X_00893_00385	-------
FC88X_00861_00437	-------
FC88X_00879_00404	-------
FC88X_00889_00471	-------
FC88X_00884_00599	-------
FC88X_00898_00568	-------
FC88X_00879_00601	-------
FC88X_00880_00622	-------
FC88X_00873_00674	-------
FC88X_00896_00671	-------
FC88X_00898_00691	-------
FC88X_00865_00725	-------
FC88X_00898_00744	-------
FC88X_00868_00793	-------
FC88X_00869_00764	-------
FC88X_00852_00840	-------

FC88X_00884_00814	-------
FC88X_00865_00857	-------
FC88X_00867_00909	-------
FC88X_00882_01143	-------
FC88X_00948_00872	-------
FC88X_00939_00729	-------
FC88X_00886_01215	-------
FC88X_00749_00257	-------
FC88X_00061_00101	-------
FC88X_00744_01024	-------
FC88X_00901_00600	-------
FC88X_00819_00177	-------
FC88X_01107_00847	-------
FC88X_00385_00045	-------
FC88X_00239_01014	-------
FC88X_01099_00812	-------
FC88X_00788_00987	-------
FC88X_01039_00866	-------
FC88X_00539_00504	-------
FC88X_00831_00225	-------
FC88X_00926_01274	-------
FC88X_00838_00797	-------
FC88X_00964_01118	-------
FC88X_00646_00387	-------
FC88X_00857_00933	-------
FC88X_00937_00885	-------
FC88X_00932_00839	-------
FC88X_00605_01168	-------
FC88X_00929_00492	-------
FC88X_00674_00803	-------
FC88X_00246_01099	-------
FC88X_00056_00804	-------
FC88X_00218_00128	-------
FC88X_00442_01095	-------
FC88X_00472_01078	-------
FC88X_00404_00767	-------
FC88X_00012_00249	-------
FC88X_00423_00694	-------
FC88X_00022_00508	-------
FC88X_00029_00543	-------
FC88X_00048_00553	-------
FC88X_00374_00117	-------
FC88X_00224_00010	-------
FC88X_00210_00065	-------
FC88X_00232_00159	-------
FC88X_00462_01044	-------
FC88X_00201_00661	-------
FC88X_00239_00748	-------
FC88X_00238_01110	-------
FC88X_00266_00035	-------
FC88X_00298_00058	-------
FC88X_00214_00504	-------
FC88X_00277_00515	-------
FC88X_00269_00729	-------
FC88X_00296_00516	-------
FC88X_00112_00296	-------
FC88X_00280_00952	-------

FC88X_00308_01037	-------
FC88X_00264_01130	-------
FC88X_00425_00884	-------
FC88X_00314_00056	-------
FC88X_00160_00946	-------
FC88X_00337_00055	-------
FC88X_00346_00148	-------
FC88X_00331_00622	-------
FC88X_00335_00610	-------
FC88X_00339_00620	-------
FC88X_00054_00328	-------
FC88X_00322_01022	-------
FC88X_00327_01051	-------
FC88X_00347_01075	-------
FC88X_00316_01117	-------
FC88X_00326_01169	-------
FC88X_00418_00380	-------
FC88X_00348_01158	-------
FC88X_00417_00164	-------
FC88X_00366_00183	-------
FC88X_00398_00247	-------
FC88X_00392_00397	-------
FC88X_00395_00603	-------
FC88X_00387_00948	-------
FC88X_00364_01141	-------
FC88X_00388_00962	-------
FC88X_00017_00744	-------
FC88X_00379_01028	-------
FC88X_00366_01088	-------
FC88X_00371_01143	-------
FC88X_00392_01156	-------
FC88X_00352_01182	-------
FC88X_00404_00063	-------
FC88X_00421_00058	-------
FC88X_00430_00080	-------
FC88X_00431_00061	-------
FC88X_00436_00132	-------
FC88X_00439_00144	-------
FC88X_00440_00112	-------
FC88X_00445_00629	-------
FC88X_00407_00797	-------
FC88X_00441_00850	-------
FC88X_00064_00868	-------
FC88X_00419_01123	-------
FC88X_00091_00164	-------
FC88X_00432_01140	-------
FC88X_00401_01157	-------
FC88X_00458_01035	-------
FC88X_00200_00117	-------
FC88X_00034_00090	-------
FC88X_00393_01086	-------
FC88X_00038_00522	-------
FC88X_01130_00705	-------
FC88X_01129_00887	-------
FC88X_00115_00464	-------
FC88X_00586_01029	-------
FC88X_00679_00949	-------

FC88X_01016_00525	-------
FC88X_00200_00098	-------
FC88X_00990_00319	-------
FC88X_00461_01239	-------
FC88X_01054_00678	-------
FC88X_01085_01077	-------
FC88X_00699_01126	-------
FC88X_00768_00946	-------
FC88X_00792_01206	-------
FC88X_00983_01053	-------
FC88X_00456_00099	-------
FC88X_01026_00491	-------
FC88X_00992_00961	-------
FC88X_00131_00228	-------
FC88X_00072_00656	-------
FC88X_00171_00736	-------
FC88X_01065_01024	-------
FC88X_00247_00902	-------
FC88X_00168_00767	-------
FC88X_00080_00887	-------
FC88X_00421_01207	-------
FC88X_00838_01050	-------
FC88X_00994_01147	-------
FC88X_00564_00969	-------
FC88X_00864_01014	-------
FC88X_00934_00998	-------
FC88X_00718_00299	-------
FC88X_00612_01235	-------
FC88X_01050_00788	-------
FC88X_00584_01080	-------
FC88X_01040_01117	-------
FC88X_00459_01060	-------
FC88X_00030_00589	-------
FC88X_00094_00164	-------
FC88X_00061_00232	-------
FC88X_00063_00544	-------
FC88X_00051_00445	-------
FC88X_00231_01024	-------
FC88X_00257_00030	-------
FC88X_00424_00142	-------
FC88X_00432_00115	-------
FC88X_00438_01172	-------
FC88X_01133_00877	-------
FC88X_00502_00101	-------
FC88X_00521_00177	-------
FC88X_00515_01204	-------
FC88X_00588_00059	-------
FC88X_00676_00627	-------
FC88X_00662_00782	-------
FC88X_00768_00116	-------
FC88X_00787_00184	-------
FC88X_00763_01015	-------
FC88X_00767_01013	-------
FC88X_01067_00667	-------
FC88X_01089_00734	-------
FC88X_01135_00686	-------
FC88X_01138_00787	-------

FC88X_00012_00472	-------
FC88X_00026_00495	-------
FC88X_00050_00141	-------
FC88X_00085_00020	-------
FC88X_00107_00137	-------
FC88X_00076_00230	-------
FC88X_00233_00988	-------
FC88X_00310_00218	-------
FC88X_00022_00707	-------
FC88X_00040_00739	-------
FC88X_00062_00122	-------
FC88X_00415_01028	-------
FC88X_00606_01017	-------
FC88X_00065_00043	-------
FC88X_00629_00953	-------
FC88X_00062_00311	-------
FC88X_00630_00180	-------
FC88X_00554_01024	-------
FC88X_00551_00109	-------
FC88X_00164_01004	-------
FC88X_00418_00082	-------
FC88X_00183_00153	-------
FC88X_00327_00603	-------
FC88X_00591_01109	-------
FC88X_00340_00478	-------
FC88X_00860_00229	-------
FC88X_00067_00790	-------
FC88X_00095_00783	-------
FC88X_00837_00950	-------
FC88X_00888_00615	-------
FC88X_01097_01047	-------
FC88X_00461_01074	-------
FC88X_00083_00186	-------
FC88X_00090_00619	-------
FC88X_01062_01031	-------
FC88X_00557_01122	-------
FC88X_00963_01177	-------
FC88X_00794_01267	-------
FC88X_00982_01026	-------
FC88X_00861_00433	-------
FC88X_00997_01086	-------
FC88X_00641_00361	-------
FC88X_00886_00893	-------
FC88X_00895_01192	-------
FC88X_00930_00781	-------
FC88X_00725_00451	-------
FC88X_00929_00576	-------
FC88X_00029_00126	-------
FC88X_00216_00253	-------
FC88X_00116_00575	-------
FC88X_00410_00922	-------
FC88X_00087_00086	-------
FC88X_00098_00744	-------
FC88X_00080_00371	-------
FC88X_00697_00972	-------
FC88X_00143_00024	-------
FC88X_00690_00542	-------

FC88X_00390_01150	-------
FC88X_00390_01166	-------
FC88X_00430_01184	-------
FC88X_00590_00435	-------
FC88X_00507_00048	-------
FC88X_00517_00037	-------
FC88X_00506_01158	-------
FC88X_01119_00991	-------
FC88X_00610_01124	-------
FC88X_00614_01135	-------
FC88X_00735_01178	-------
FC88X_00020_00441	-------
FC88X_00769_00987	-------
FC88X_00773_00992	-------
FC88X_01076_00466	-------
FC88X_01063_00557	-------
FC88X_01055_01093	-------
FC88X_01080_01084	-------
FC88X_00024_00087	-------
FC88X_00015_00153	-------
FC88X_00009_00220	-------
FC88X_00029_00420	-------
FC88X_00029_00539	-------
FC88X_00110_00930	-------
FC88X_00127_00013	-------
FC88X_00099_00215	-------
FC88X_00234_00899	-------
FC88X_00287_00856	-------
FC88X_00079_00647	-------
FC88X_00097_00649	-------
FC88X_00088_00556	-------
FC88X_00067_00616	-------
FC88X_00072_00512	-------
FC88X_00093_00540	-------
FC88X_00069_00423	-------
FC88X_00098_00488	-------
FC88X_00099_00158	-------
FC88X_00052_00218	-------
FC88X_00050_00126	-------
FC88X_00058_00126	-------
FC88X_00031_00305	-------
FC88X_00015_00393	-------
FC88X_00014_00287	-------
FC88X_00045_00286	-------
FC88X_00037_00201	-------
FC88X_00049_00211	-------
FC88X_01145_00892	-------
FC88X_01103_01143	-------
FC88X_01107_00674	-------
FC88X_01115_00691	-------
FC88X_01054_00935	-------
FC88X_01053_00958	-------
FC88X_01071_00417	-------
FC88X_01078_00403	-------
FC88X_00796_01073	-------
FC88X_00763_01145	-------
FC88X_00775_00924	-------

FC88X_00766_00966	-------
FC88X_00790_00195	-------
FC88X_00913_00990	-------
FC88X_00661_01131	-------
FC88X_00682_01220	-------
FC88X_00632_01183	-------
FC88X_00681_00301	-------
FC88X_00066_00339	-------
FC88X_00792_00121	-------
FC88X_01002_00436	-------
FC88X_00047_00574	-------
FC88X_00672_00845	-------
FC88X_00694_01166	-------
FC88X_00152_00028	-------
FC88X_01005_00929	-------
FC88X_00990_00380	-------
FC88X_00498_00486	-------
FC88X_00943_00724	-------
FC88X_00878_00172	-------
FC88X_01029_01202	-------
FC88X_00570_00989	-------
FC88X_00870_01112	-------
FC88X_00926_00715	-------
FC88X_00661_00458	-------
FC88X_00918_00447	-------
FC88X_00996_01275	-------
FC88X_00023_00502	-------
FC88X_00435_01077	-------
FC88X_00612_00083	-------
FC88X_00035_00605	-------
FC88X_00499_00445	-------
FC88X_00080_00275	-------
FC88X_00073_00473	-------
FC88X_00098_00764	-------
FC88X_00089_00896	-------
FC88X_00251_01030	-------
FC88X_00470_01001	-------
FC88X_00415_00818	-------
FC88X_01031_01164	-------
FC88X_00499_00966	-------
FC88X_00953_00969	-------
FC88X_00529_00731	-------
FC88X_00533_00750	-------
FC88X_00557_00813	-------
FC88X_00050_00560	-------
FC88X_00557_01118	-------
FC88X_00565_01108	-------
FC88X_00597_00088	-------
FC88X_00591_00165	-------
FC88X_00540_00871	-------
FC88X_00522_00974	-------
FC88X_00535_00205	-------
FC88X_00547_00584	-------
FC88X_00477_01049	-------
FC88X_00468_01065	-------
FC88X_00467_00014	-------
FC88X_00470_00176	-------

FC88X_00419_01131	-------
FC88X_00431_01131	-------
FC88X_00442_00059	-------
FC88X_00422_00123	-------
FC88X_00263_01127	-------
FC88X_00030_00558	-------
FC88X_00128_00322	-------
FC88X_00116_00373	-------
FC88X_00070_00879	-------
FC88X_00106_00209	-------
FC88X_00051_00472	-------
FC88X_00573_00649	-------
FC88X_00090_00239	-------
FC88X_00089_00329	-------
FC88X_00078_00026	-------
FC88X_00090_00016	-------
FC88X_00033_00526	-------
FC88X_00938_00903	-------
FC88X_00765_00618	-------
FC88X_00888_00922	-------
FC88X_00885_00730	-------
FC88X_01075_00855	-------
FC88X_00660_00379	-------
FC88X_00144_00617	-------
FC88X_00188_00634	-------
FC88X_00746_00850	-------
FC88X_00644_00493	-------
FC88X_01019_00519	-------
FC88X_00835_00177	-------
FC88X_00302_00167	-------
FC88X_00883_00949	-------
FC88X_00985_01130	-------
FC88X_00712_00111	-------
FC88X_00859_00973	-------
FC88X_00922_00921	-------
FC88X_00894_01247	-------
FC88X_00608_01239	-------
FC88X_00914_00646	-------
FC88X_00098_00625	-------
FC88X_01021_00600	-------
FC88X_00132_00988	-------
FC88X_00020_00481	-------
FC88X_00407_00405	-------
FC88X_00185_00795	-------
FC88X_00220_01038	-------
FC88X_00895_01195	-------
FC88X_00045_00748	-------
FC88X_00144_00239	-------
FC88X_00668_01184	-------
FC88X_00635_00468	-------
FC88X_00606_00272	-------
FC88X_00643_00230	-------
FC88X_00635_00235	-------
FC88X_00634_00089	-------
FC88X_00624_00094	-------
FC88X_00591_01207	-------
FC88X_00579_01205	-------

FC88X_00733_00991	-------
FC88X_00604_00753	-------
FC88X_00729_00072	-------
FC88X_01013_00294	-------
FC88X_00961_01056	-------
FC88X_00980_01042	-------
FC88X_00991_00888	-------
FC88X_00967_00860	-------
FC88X_00963_00883	-------
FC88X_00977_00812	-------
FC88X_01142_00831	-------
FC88X_01117_00841	-------
FC88X_01128_00719	-------
FC88X_01143_00668	-------
FC88X_01134_00656	-------
FC88X_01130_00659	-------
FC88X_01113_00686	-------
FC88X_01137_00565	-------
FC88X_01097_01193	-------
FC88X_01084_01190	-------
FC88X_01081_01160	-------
FC88X_00297_00703	-------
FC88X_00986_01132	-------
FC88X_01122_01127	-------
FC88X_00162_00662	-------
FC88X_00708_01217	-------
FC88X_00780_00181	-------
FC88X_00992_00693	-------
FC88X_00954_00831	-------
FC88X_01086_01060	-------
FC88X_01058_01137	-------
FC88X_00963_00809	-------
FC88X_00927_01014	-------
FC88X_00399_00076	-------
FC88X_00620_01186	-------
FC88X_00721_01072	-------
FC88X_00501_00164	-------
FC88X_00505_00563	-------
FC88X_00300_00063	-------
FC88X_00918_00820	-------
FC88X_01074_00837	-------
FC88X_00022_00317	-------
FC88X_00951_00682	-------
FC88X_00991_00676	-------
FC88X_00032_00663	-------
FC88X_00038_00755	-------
FC88X_00854_00952	-------
FC88X_01035_01028	-------
FC88X_00090_00322	-------
FC88X_00477_01174	-------
FC88X_00993_00762	-------
FC88X_00189_00794	-------
FC88X_00299_01081	-------
FC88X_00315_00341	-------
FC88X_00868_00770	-------
FC88X_01011_01205	-------
FC88X_00993_00780	-------

```
FC88X_00598_00805      -------
FC88X_00648_00102      -------
FC88X_00908_01146      -------
FC88X_00372_01196      -------
FC88X_00060_00036      CTCC---
FC88X_00346_01091      -------
FC88X_00416_01001      CTCC---
FC88X_01033_00340      CT-----
FC88X_00810_00950      -------
FC88X_00226_00917      -------
FC88X_00269_01129      -------
FC88X_00546_01060      -------
FC88X_00187_01061      -------
FC88X_00259_00927      -------
FC88X_00065_00587      -------
FC88X_00099_00911      -------
FC88X_00794_01056      -------
FC88X_00141_00760      -------
FC88X_00195_00815      -------
FC88X_00927_00564      -------
FC88X_00871_00895      -------
FC88X_00092_00459      -------
FC88X_01124_00530      -------
FC88X_00684_01260      -------
FC88X_00471_00765      -------
FC88X_00197_00531      -------
FC88X_01030_01253      -------
FC88X_00239_00963      -------
FC88X_00713_00398      -------
FC88X_00986_00651      -------
FC88X_00953_00831      -------
FC88X_00931_01080      -------
FC88X_01126_00811      -------
FC88X_00029_00450      -------
FC88X_01008_01206      -------
FC88X_01103_00526      -------
FC88X_00912_00809      -------
FC88X_00762_01115      -------
FC88X_01050_00849      -------
FC88X_00173_00309      -------
FC88X_00766_00880      -------
FC88X_00817_00334      -------
FC88X_00743_01155      -------
FC88X_00753_00981      -------
FC88X_00759_00256      -------
FC88X_00454_00123      -------
FC88X_00715_01264      -------
FC88X_00765_00129      -------
FC88X_00723_01072      -------
FC88X_00800_00841      -------
FC88X_01060_00407      -------
FC88X_00367_00043      -------
FC88X_00533_00142      -------
FC88X_00912_00916      -------
FC88X_00824_00919      -------
FC88X_00919_00556      -------
FC88X_01008_01199      -------
```

FC88X_00841_00285	-------
FC88X_00704_01117	-------
FC88X_00835_00814	-------
FC88X_01095_00439	-------
FC88X_00751_00841	-------
FC88X_00734_00263	-------
FC88X_00925_00819	-------
FC88X_00754_00277	-------
FC88X_00912_00999	-------
FC88X_00086_00609	-------
FC88X_00471_00007	-------
FC88X_00552_01231	-------
FC88X_00512_00968	-------
FC88X_00244_00944	-------
FC88X_00151_00187	-------
FC88X_01065_01192	-------
FC88X_00169_00987	-------
FC88X_00995_00656	-------
FC88X_00755_00686	-------
FC88X_00389_01111	-------
FC88X_01030_00782	-------
FC88X_00300_00959	-------
FC88X_01096_00934	-------
FC88X_00018_00659	-------
FC88X_01027_01268	-------
FC88X_00994_00677	-------
FC88X_00980_00722	-------
FC88X_00975_00299	-------
FC88X_00166_00459	-------
FC88X_00793_01257	-------
FC88X_00819_00394	-------
FC88X_00705_01141	-------
FC88X_00764_00913	-------
FC88X_00966_00585	-------
FC88X_00838_01022	-------
FC88X_01019_00703	-------
FC88X_01078_00747	-------
FC88X_00771_01236	-------
FC88X_01027_00851	-------
FC88X_00974_01258	-------
FC88X_00050_00794	-------
FC88X_00030_00148	-------
FC88X_00145_00804	-------
FC88X_00509_00633	-------
FC88X_00197_00139	-------
FC88X_00494_00212	-------
FC88X_01139_00933	-------
FC88X_00643_00912	-------
FC88X_00202_00851	-------
FC88X_00817_00139	-------
FC88X_01027_00700	-------
FC88X_01008_00683	-------
FC88X_00571_01109	-------
FC88X_00939_00655	-------
FC88X_00225_00018	-------
FC88X_00463_00112	-------
FC88X_00370_00032	-------

FC88X_00674_00933	-------
FC88X_00746_00660	-------
FC88X_00261_01082	-------
FC88X_00454_01102	-------
FC88X_00528_01134	-------
FC88X_00731_00342	-------
FC88X_01005_00523	-------
FC88X_00453_00103	-------
FC88X_00706_00412	-------
FC88X_00525_00117	-------
FC88X_00809_01170	-------
FC88X_00769_01035	-------
FC88X_00723_00058	-------
FC88X_00777_00148	-------
FC88X_00912_00825	-------
FC88X_00147_00340	-------
FC88X_00785_00310	-------
FC88X_00994_00489	-------
FC88X_00598_00127	C------
FC88X_00616_01063	-------
FC88X_00994_01168	-------
FC88X_00488_00658	-------
FC88X_00028_00093	-------
FC88X_00845_00119	-------
FC88X_00421_00994	-------
FC88X_00914_00448	-------
FC88X_01106_00504	-------
FC88X_00826_00314	-------
FC88X_00821_00871	-------
FC88X_00592_00923	-------
FC88X_00955_01091	-------
FC88X_01052_00668	-------
FC88X_00042_00104	-------
FC88X_01053_00606	-------
FC88X_00734_01040	-------
FC88X_01091_00761	-------
FC88X_01080_00761	-------
FC88X_00649_00415	-------
FC88X_00841_00225	-------
FC88X_01071_00541	-------
FC88X_00642_00235	-------
FC88X_01066_00842	-------
FC88X_01021_00732	-------
FC88X_00061_00305	-------
FC88X_00061_00672	-------
FC88X_00432_00016	-------
FC88X_00904_00633	-------
FC88X_00034_00042	-------
FC88X_00877_00787	-------
FC88X_00784_00203	-------
FC88X_00219_00899	-------
FC88X_01095_01143	-------
FC88X_00889_00365	-------
FC88X_00768_01275	-------
FC88X_00086_00704	-------
FC88X_01120_00730	-------
FC88X_00009_00161	-------

FC88X_00041_00360	-------
FC88X_00630_00105	-------
FC88X_00076_00459	-------
FC88X_00432_00372	-------
FC88X_00504_00706	-------
FC88X_00913_00860	-------
FC88X_01031_00494	-------
FC88X_00951_00878	-------
FC88X_01014_01030	-------
FC88X_00747_01118	-------
FC88X_00977_00594	-------
FC88X_00268_01005	-------
FC88X_00577_01231	-------
FC88X_00266_00988	-------
FC88X_00772_00522	-------
FC88X_00656_01240	-------
FC88X_00047_00530	-------
FC88X_00996_01027	-------
FC88X_00069_00357	-------
FC88X_00131_00783	-------
FC88X_00176_00829	-------
FC88X_00161_00826	-------
FC88X_00290_01001	-------
FC88X_00380_01193	-------
FC88X_00416_01104	-------
FC88X_00436_01140	-------
FC88X_00532_01116	-------
FC88X_01008_01044	-------
FC88X_01051_01268	-------
FC88X_01003_00593	-------
FC88X_00723_01007	-------
FC88X_00797_00886	-------
FC88X_00783_01211	-------
FC88X_00823_00846	-------
FC88X_00933_00431	-------
FC88X_00912_00777	-------
FC88X_00993_00759	-------
FC88X_00983_01045	-------
FC88X_00984_01051	-------
FC88X_01072_00732	-------
FC88X_01138_00553	-------
FC88X_01130_00623	-------
FC88X_01138_00630	-------
FC88X_01115_00848	-------
FC88X_01112_01184	-------
FC88X_01120_01177	-------
FC88X_01146_01208	-------
FC88X_01041_00775	-------
FC88X_00021_00641	-------
FC88X_00461_01079	-------
FC88X_00205_00933	-------
FC88X_00146_00509	-------
FC88X_00710_00118	-------
FC88X_00249_00944	-------
FC88X_00648_00447	-------
FC88X_00326_01060	-------
FC88X_00380_00055	-------

FC88X_00460_01124	-------
FC88X_00101_00562	-------
FC88X_00780_00151	-------
FC88X_00339_00584	-------
FC88X_00635_00278	-------
FC88X_00630_01223	-------
FC88X_00991_01063	-------
FC88X_00867_00144	-------
FC88X_00259_00935	-------
FC88X_00043_00352	-------
FC88X_01065_00626	-------
FC88X_01085_00709	-------
FC88X_01110_00512	-------
FC88X_01075_00781	-------
FC88X_00727_00261	-------
FC88X_00030_00051	-------
FC88X_01033_01215	-------
FC88X_00061_00592	-------
FC88X_00938_01188	-------
FC88X_00012_00170	-------
FC88X_00018_00697	-------
FC88X_00702_00928	-------
FC88X_00113_00492	-------
FC88X_00878_00348	-------
FC88X_00107_00087	-------
FC88X_01093_00416	-------
FC88X_00100_00454	-------
FC88X_00151_00507	-------
FC88X_00209_00873	-------
FC88X_00498_00318	-------
FC88X_01060_00410	-------
FC88X_01134_00854	-------
FC88X_01082_00729	-------
FC88X_01054_01257	-------
FC88X_00055_00216	-------
FC88X_00066_00374	-------
FC88X_01096_00677	-------
FC88X_00777_00143	C------
FC88X_00103_00674	-------
FC88X_00493_01134	-------
FC88X_00506_00918	-------
FC88X_00720_01190	-------
FC88X_00938_00460	-------
FC88X_00935_01247	-------
FC88X_00840_00430	-------
FC88X_00896_00520	-------
FC88X_00994_00290	-------
FC88X_00468_00960	-------
FC88X_00911_00634	-------
FC88X_00024_00537	-------
FC88X_01071_00648	-------
FC88X_01052_00845	-------
FC88X_00048_00257	-------
FC88X_00588_01257	-------
FC88X_00764_00228	-------
FC88X_00922_00646	-------
FC88X_01032_00699	-------

FC88X_00431_00616	-------
FC88X_00930_00527	-------
FC88X_01017_00440	-------
FC88X_01093_00474	-------
FC88X_01085_00765	-------
FC88X_00023_00179	-------
FC88X_00034_00174	-------
FC88X_00046_00133	-------
FC88X_00963_01269	-------
FC88X_00676_01008	-------
FC88X_00553_00960	-------
FC88X_00684_01174	-------
FC88X_00018_00055	-------
FC88X_00004_00530	-------
FC88X_01102_00734	-------
FC88X_00788_01078	-------
FC88X_00926_00892	-------
FC88X_01059_00653	-------
FC88X_01084_00760	-------
FC88X_01115_00534	-------
FC88X_00074_00814	-------
FC88X_00976_00615	-------
FC88X_00754_00840	-------
FC88X_00692_00599	-------
FC88X_00815_01062	-------
FC88X_00730_01242	-------
FC88X_00857_00800	-------
FC88X_00928_00404	-------
FC88X_00575_00350	-------
FC88X_00152_00396	-------
FC88X_00868_01271	-------
FC88X_00626_00178	-------
FC88X_01134_00730	-------
FC88X_01060_00704	-------
FC88X_01097_00819	-------
FC88X_00008_00283	-------
FC88X_00746_00297	-------
FC88X_00581_00215	-------
FC88X_01094_00742	-------
FC88X_00784_00095	-------
FC88X_00049_00171	-------
FC88X_00961_00688	-------
FC88X_00981_00667	-------
FC88X_00025_00714	-------
FC88X_00049_00287	-------
FC88X_01095_00742	-------
FC88X_00522_00185	-------
FC88X_00774_01188	-------
FC88X_00811_00327	-------
FC88X_00007_00164	-------
FC88X_00955_00581	-------
FC88X_00646_00181	-------
FC88X_00218_00037	-------
FC88X_00431_00952	-------
FC88X_00938_00457	-------
FC88X_01134_00857	-------
FC88X_00758_00879	-------

FC88X_01004_00889	-------
FC88X_00807_01137	-------
FC88X_00068_00713	-------
FC88X_00629_00099	-------
FC88X_00778_01244	-------
FC88X_00953_00296	-------
FC88X_00926_00676	-------
FC88X_00445_00481	-------
FC88X_00005_00070	-------
FC88X_00430_00440	-------
FC88X_00837_01245	-------
FC88X_00998_00497	-------
FC88X_00575_00426	-------
FC88X_00840_00827	-------
FC88X_00495_01228	-------
FC88X_00554_01153	-------
FC88X_01137_00988	-------
FC88X_00741_00949	-------
FC88X_01094_01012	-------
FC88X_00929_00650	-------
FC88X_00492_00974	-------
FC88X_00774_00278	-------
FC88X_00741_00925	-------
FC88X_00718_01130	-------
FC88X_01119_00878	-------
FC88X_00188_00848	-------
FC88X_00252_00940	-------
FC88X_00580_01247	-------
FC88X_00864_01024	-------
FC88X_00858_01067	-------
FC88X_00892_01191	-------
FC88X_01077_00770	-------
FC88X_00514_01125	-------
FC88X_01038_00992	-------
FC88X_00940_01232	-------
FC88X_00300_00264	-------
FC88X_00553_00132	-------
FC88X_00670_01123	-------
FC88X_00089_00524	-------
FC88X_00487_01246	-------
FC88X_01036_00566	-------
FC88X_00961_00529	-------
FC88X_00546_01230	-------
FC88X_00872_00697	-------
FC88X_01123_00522	-------
FC88X_01135_00664	-------
FC88X_00452_00303	-------
FC88X_00063_00637	-------
FC88X_00074_00672	-------
FC88X_00177_00099	-------
FC88X_00202_01014	-------
FC88X_00868_01020	-------
FC88X_00905_00216	-------
FC88X_00948_00854	-------
FC88X_00305_01135	-------
FC88X_00971_01073	-------
FC88X_01073_00659	-------

FC88X_00089_00120	-------
FC88X_00099_00101	-------
FC88X_00470_01180	-------
FC88X_00578_01151	-------
FC88X_00090_00743	-------
FC88X_00033_00246	-------
FC88X_00403_01134	-------
FC88X_01017_00438	-------
FC88X_00904_00776	-------
FC88X_00881_00633	-------
FC88X_00100_00053	-------
FC88X_00935_00365	-------
FC88X_00510_01121	-------
FC88X_01024_00388	-------
FC88X_00157_01024	-------
FC88X_00881_00702	-------
FC88X_00151_00922	-------
FC88X_00530_00203	-------
FC88X_00582_00820	-------
FC88X_00653_01232	-------
FC88X_00848_00256	-------
FC88X_00172_00139	-------
FC88X_00447_00113	-------
FC88X_00127_00109	-------
FC88X_00141_00566	-------
FC88X_00949_00951	-------
FC88X_00772_01039	-------
FC88X_01100_00956	-------
FC88X_00118_00483	-------
FC88X_00352_01011	-------
FC88X_00130_00719	-------
FC88X_01134_00837	-------
FC88X_00228_00031	-------
FC88X_00068_00588	-------
FC88X_00501_01231	-------
FC88X_00987_00605	-------
FC88X_00622_00929	-------
FC88X_00876_00360	-------
FC88X_00486_00162	-------
FC88X_00970_00680	-------
FC88X_00891_00741	-------
FC88X_00339_00223	-------
FC88X_00986_01223	-------
FC88X_00847_01055	-------
FC88X_01058_01181	-------
FC88X_00803_00180	-------
FC88X_00203_00834	-------
FC88X_00046_00145	-------
FC88X_00121_00867	-------
FC88X_00105_00583	-------
FC88X_00805_00957	-------
FC88X_00394_00613	-------
FC88X_00905_00669	-------
FC88X_01143_00899	-------
FC88X_00426_01171	-------
FC88X_00589_00647	-------
FC88X_00385_00037	-------

FC88X_01077_00889	-------
FC88X_00424_00989	-------
FC88X_00455_01199	-------
FC88X_00843_00347	-------
FC88X_00870_00444	-------
FC88X_00601_00941	-------
FC88X_00544_00082	-------
FC88X_00095_00787	-------
FC88X_00561_00116	-------
FC88X_00642_00933	-------
FC88X_01025_01119	-------
FC88X_00195_00050	-------
FC88X_00625_00094	-------
FC88X_00880_00885	-------
FC88X_00140_00656	-------
FC88X_00462_01235	-------
FC88X_00613_00698	-------
FC88X_00109_00962	-------
FC88X_00190_00354	-------
FC88X_00172_00886	-------
FC88X_00542_00634	-------
FC88X_01057_00645	-------
FC88X_00603_00202	-------
FC88X_01120_00721	-------
FC88X_00954_01045	-------
FC88X_01039_00804	-------
FC88X_00185_00571	-------
FC88X_00913_01023	-------
FC88X_01091_00942	-------
FC88X_01002_01039	-------
FC88X_00968_00852	-------
FC88X_00029_00234	-------
FC88X_00955_00736	-------
FC88X_00119_00570	-------
FC88X_00186_00585	-------
FC88X_00466_00043	-------
FC88X_00616_00373	-------
FC88X_00897_01254	-------
FC88X_00914_00709	-------
FC88X_00965_00702	-------
FC88X_00821_00198	-------
FC88X_00090_00463	-------
FC88X_00923_00391	-------
FC88X_00868_00882	-------
FC88X_00256_00966	-------
FC88X_00704_00957	-------
FC88X_00856_00265	-------
FC88X_01017_00847	-------
FC88X_00408_00275	-------
FC88X_00920_00842	-------
FC88X_00860_00768	-------
FC88X_01083_00653	-------
FC88X_01068_00621	-------
FC88X_00116_00231	-------
FC88X_00776_00995	-------
FC88X_00632_00165	-------
FC88X_00513_01138	-------

FC88X_00284_00940	-------
FC88X_00031_00275	-------
FC88X_00300_01016	-------
FC88X_00747_01146	-------
FC88X_00730_00973	-------
FC88X_00758_00966	-------
FC88X_00216_00134	-------
FC88X_00279_00994	-------
FC88X_00852_00835	-------
FC88X_01094_00563	-------
FC88X_00380_00025	-------
FC88X_00576_01219	-------
FC88X_00975_00806	-------
FC88X_00845_00920	-------
FC88X_01033_00493	-------
FC88X_00821_01136	-------
FC88X_00402_01137	-------
FC88X_00563_00129	-------
FC88X_00216_00015	-------
FC88X_00675_00991	-------
FC88X_00661_00100	-------
FC88X_00040_00505	-------
FC88X_01088_00725	-------
FC88X_00842_00891	-------
FC88X_01100_00482	-------
FC88X_00940_00583	-------
FC88X_00029_00128	-------
FC88X_00527_01102	-------
FC88X_00857_00749	-------
FC88X_00855_01026	-------
FC88X_00984_00816	-------
FC88X_01119_00662	-------
FC88X_00030_00287	-------
FC88X_00124_00076	-------
FC88X_00229_01029	-------
FC88X_01074_00454	-------
FC88X_00514_00169	-------
FC88X_00191_00889	-------
FC88X_00956_00732	-------
FC88X_00024_00344	-------
FC88X_00409_00032	-------
FC88X_00827_00547	-------
FC88X_00960_01087	-------
FC88X_00095_00070	-------
FC88X_00393_01087	-------
FC88X_00121_00817	-------
FC88X_00668_00959	-------
FC88X_01049_01227	-------
FC88X_00915_00377	-------
FC88X_00926_00936	-------
FC88X_01079_00855	-------
FC88X_00614_00179	-------
FC88X_00127_00367	-------
FC88X_00509_00426	-------
FC88X_00068_00474	-------
FC88X_01058_01182	-------
FC88X_00529_01188	-------

FC88X_00996_00323	-------
FC88X_01097_00987	-------
FC88X_00193_00079	-------
FC88X_00026_00081	-------
FC88X_00718_01059	-------
FC88X_00757_00174	-------
FC88X_00797_00799	-------
FC88X_00615_01204	-------
FC88X_00190_00889	-------
FC88X_00426_00054	-------
FC88X_00730_01272	-------
FC88X_00924_00947	-------
FC88X_01131_00538	-------
FC88X_00927_00918	-------
FC88X_00567_00050	-------
FC88X_00881_00356	-------
FC88X_00073_00832	-------
FC88X_00253_00947	-------
FC88X_00968_01268	-------
FC88X_01079_00691	-------
FC88X_01069_00863	-------
FC88X_00726_01258	-------
FC88X_00133_00236	-------
FC88X_00068_00589	-------
FC88X_00484_01009	-------
FC88X_01014_00441	-------
FC88X_00459_00080	-------
FC88X_01045_00341	-------
FC88X_01071_01158	-------
FC88X_00409_01208	-------
FC88X_00710_00948	-------
FC88X_00229_01006	-------
FC88X_00934_00679	-------
FC88X_01124_00539	-------
FC88X_01014_01144	-------
FC88X_00059_00270	-------
FC88X_00814_00944	-------
FC88X_00042_00262	-------
FC88X_00345_01005	-------
FC88X_00412_00054	-------
FC88X_00488_01183	-------
FC88X_00936_01196	-------
FC88X_01046_00470	-------
FC88X_00479_00395	-------
FC88X_00588_00105	-------
FC88X_00780_00826	-------
FC88X_01038_00497	-------
FC88X_00069_00274	-------
FC88X_00450_00371	-------
FC88X_00479_00166	-------
FC88X_00796_00193	-------
FC88X_00630_00451	-------
FC88X_00914_00476	-------
FC88X_00955_00772	-------
FC88X_01105_00855	-------
FC88X_00706_00281	-------
FC88X_00293_00833	-------

FC88X_01051_01107	-------
FC88X_00007_00301	-------
FC88X_00157_00939	-------
FC88X_00479_01134	-------
FC88X_00603_01199	-------
FC88X_00036_00256	-------
FC88X_01050_00481	-------
FC88X_00720_01255	-------
FC88X_00713_00109	-------
FC88X_00128_00116	-------
FC88X_00257_00508	-------
FC88X_00400_01095	-------
FC88X_00948_00890	-------
FC88X_00126_00690	-------
FC88X_00248_00072	-------
FC88X_00833_01034	-------
FC88X_00900_00412	-------
FC88X_01078_00998	-------
FC88X_00659_00378	-------
FC88X_00777_01163	-------
FC88X_01012_00779	-------
FC88X_00499_01103	-------
FC88X_01116_00598	-------
FC88X_00604_00179	-------
FC88X_00110_00688	-------
FC88X_00923_00841	-------
FC88X_00940_00777	-------
FC88X_00642_00804	-------
FC88X_00905_00869	-------
FC88X_00739_01141	-------
FC88X_00196_00615	-------
FC88X_00806_00303	-------
FC88X_01004_00970	-------
FC88X_00260_00988	-------
FC88X_00965_00440	-------
FC88X_01117_00922	-------
FC88X_00656_00170	-------
FC88X_00880_00197	-------
FC88X_00891_00164	-------
FC88X_00295_00847	-------
FC88X_00583_01220	-------
FC88X_00848_01096	-------
FC88X_00216_00103	-------
FC88X_00012_00180	-------
FC88X_01080_00655	-------
FC88X_00908_00486	-------
FC88X_00356_01097	-------
FC88X_00865_00882	-------
FC88X_00099_00479	-------
FC88X_00112_00397	-------
FC88X_00906_00315	-------
FC88X_00997_00785	-------
FC88X_00277_00935	-------
FC88X_00662_00783	-------
FC88X_00924_00402	-------
FC88X_00920_01016	-------
FC88X_00955_01012	-------

FC88X_00951_01262	-------
FC88X_00203_00873	-------
FC88X_00910_00993	-------
FC88X_00503_00654	-------
FC88X_00142_00757	-------
FC88X_00576_00295	-------
FC88X_00765_00946	-------
FC88X_01113_00642	-------
FC88X_00944_00918	-------
FC88X_00167_00892	-------
FC88X_00988_00830	-------
FC88X_00723_00066	-------
FC88X_01005_00721	-------
FC88X_00018_00145	-------
FC88X_00050_00135	-------
FC88X_01079_01271	-------
FC88X_00086_00509	-------
FC88X_00168_00935	-------
FC88X_00176_00190	-------
FC88X_00850_00338	-------
FC88X_00292_01131	-------
FC88X_00487_00035	-------
FC88X_00613_00279	-------
FC88X_00629_01215	-------
FC88X_00774_00291	-------
FC88X_00864_01230	-------
FC88X_00979_01000	-------
FC88X_01027_00296	-------
FC88X_01061_00591	-------
FC88X_00165_00170	-------
FC88X_00478_01120	-------
FC88X_00580_00689	-------
FC88X_00719_01125	-------
FC88X_00789_01221	-------
FC88X_00934_00444	-------
FC88X_00938_00521	-------
FC88X_00900_00979	-------
FC88X_00923_01247	-------
FC88X_00963_00493	-------
FC88X_00985_00726	-------
FC88X_00964_00891	-------
FC88X_01021_00480	-------
FC88X_01050_00382	-------
FC88X_00736_00999	-------
FC88X_00965_00650	-------
FC88X_00880_00383	-------
FC88X_00896_00608	-------
FC88X_00854_00265	-------
FC88X_00969_01270	-------
FC88X_00850_00643	-------
FC88X_00799_01060	-------
FC88X_01089_00887	-------
FC88X_00835_00351	-------
FC88X_00528_01103	-------
FC88X_01018_00556	-------
FC88X_01029_00918	-------
FC88X_00131_00469	-------

FC88X_01118_01038	-------
FC88X_00965_00735	-------
FC88X_00027_00414	-------
FC88X_00089_00846	-------
FC88X_00418_00136	-------
FC88X_00840_00787	-------
FC88X_00891_00492	-------
FC88X_00724_01151	-------
FC88X_00949_00967	-------
FC88X_01072_00912	-------
FC88X_00630_01049	-------
FC88X_00092_00447	-------
FC88X_00459_01183	-------
FC88X_00278_00046	-------
FC88X_00562_00213	-------
FC88X_00055_00605	-------
FC88X_00323_00049	-------
FC88X_00081_00616	-------
FC88X_00532_01084	-------
FC88X_00761_00795	-------
FC88X_00773_01176	-------
FC88X_00896_00827	-------
FC88X_01050_00348	-------
FC88X_01133_00903	-------
FC88X_00067_00409	-------
FC88X_00088_00847	-------
FC88X_00149_00077	-------
FC88X_00104_00906	-------
FC88X_00164_00050	-------
FC88X_00166_00659	-------
FC88X_00318_00042	-------
FC88X_00614_01156	-------
FC88X_00649_01258	-------
FC88X_00711_00394	-------
FC88X_00790_00201	-------
FC88X_01125_00945	-------
FC88X_00616_00126	-------
FC88X_00985_00869	-------
FC88X_01042_00842	-------
FC88X_00925_00297	-------
FC88X_01059_00488	-------
FC88X_00728_01141	-------
FC88X_00121_00581	-------
FC88X_00277_01109	-------
FC88X_01071_01152	-------
FC88X_00451_00004	-------
FC88X_00759_01160	-------
FC88X_00077_00793	-------
FC88X_00025_00494	-------
FC88X_00277_00014	-------
FC88X_00075_00768	-------
FC88X_00130_00878	-------
FC88X_01090_00681	-------
FC88X_00977_01082	-------
FC88X_00826_00932	-------
FC88X_01078_00940	-------
FC88X_00337_00274	-------

FC88X_01087_00650	-------
FC88X_01118_00712	-------
FC88X_00075_00297	-------
FC88X_00204_00931	-------
FC88X_00975_01207	-------
FC88X_00781_00186	-------
FC88X_01048_01119	-------
FC88X_00871_00261	-------
FC88X_00945_00767	-------
FC88X_00030_00270	-------
FC88X_00278_00420	-------
FC88X_00571_01114	-------
FC88X_00135_00665	-------
FC88X_00946_00325	-------
FC88X_00183_00471	-------
FC88X_00347_00832	-------
FC88X_00996_00693	-------
FC88X_01062_01157	-------
FC88X_00144_00149	-------
FC88X_00129_00548	-------
FC88X_00216_00970	-------
FC88X_00477_01000	-------
FC88X_00603_00139	-------
FC88X_00666_01218	-------
FC88X_00850_01016	-------
FC88X_00926_00659	-------
FC88X_01021_00496	-------
FC88X_01084_00676	-------
FC88X_00530_00950	-------
FC88X_01144_00761	-------
FC88X_00023_00666	-------
FC88X_00022_00278	-------
FC88X_00280_00945	-------
FC88X_00805_00318	-------
FC88X_00950_00369	-------
FC88X_00870_01269	-------
FC88X_01059_01090	-------
FC88X_00609_01147	-------
FC88X_00775_01270	-------
FC88X_00891_00295	-------
FC88X_00162_00193	-------
FC88X_01119_00738	-------
FC88X_00171_00165	-------
FC88X_00773_01163	-------
FC88X_00930_00400	-------
FC88X_01092_00791	-------
FC88X_01128_01145	-------
FC88X_00105_00127	-------
FC88X_00275_00081	-------
FC88X_00871_00966	-------
FC88X_00961_00666	-------
FC88X_01114_00518	-------
FC88X_00905_01053	-------
FC88X_00941_00625	-------
FC88X_00692_00143	-------
FC88X_00684_00172	-------
FC88X_00492_00414	-------

FC88X_00201_00135	-------
FC88X_01003_00982	-------
FC88X_00655_00117	-------
FC88X_00586_01226	-------
FC88X_00417_00978	-------
FC88X_00765_01139	-------
FC88X_00587_00191	-------
FC88X_00764_00131	-------
FC88X_00281_01085	-------
FC88X_00129_00547	-------
FC88X_01125_00645	-------
FC88X_00392_01207	-------
FC88X_00048_00031	-------
FC88X_00053_00467	-------
FC88X_00949_00819	-------
FC88X_00625_00924	-------
FC88X_00165_00297	-------
FC88X_00728_00898	-------
FC88X_00570_00670	-------
FC88X_00098_00470	-------
FC88X_00958_00828	-------
FC88X_00703_01114	-------
FC88X_00813_00799	-------
FC88X_00186_01022	-------
FC88X_00038_00426	-------
FC88X_00746_01060	-------
FC88X_00081_00876	-------
FC88X_00957_00705	-------
FC88X_00163_00814	-------
FC88X_00139_00052	-------
FC88X_00313_01036	-------
FC88X_00545_00942	-------
FC88X_00304_00041	-------
FC88X_01136_00849	-------
FC88X_01128_00863	-------
FC88X_00937_01245	-------
FC88X_00099_00142	-------
FC88X_00509_01182	-------
FC88X_00221_00110	-------
FC88X_00621_00407	-------
FC88X_00637_01127	-------
FC88X_00958_00718	-------
FC88X_00129_00959	-------
FC88X_00944_00553	-------
FC88X_00773_01207	-------
FC88X_01143_00690	-------
FC88X_00926_01021	-------
FC88X_00277_00956	-------
FC88X_00663_01120	-------
FC88X_00859_00709	-------
FC88X_00108_00622	-------
FC88X_00061_00138	-------
FC88X_00056_00217	-------
FC88X_00100_00712	-------
FC88X_00151_00196	-------
FC88X_00356_00961	-------
FC88X_00478_00152	-------

FC88X_00703_00535	-------
FC88X_00135_00727	-------
FC88X_00048_00277	-------
FC88X_00574_00538	-------
FC88X_01004_00330	-------
FC88X_00074_00236	-------
FC88X_00906_00522	-------
FC88X_00989_01026	-------
FC88X_01063_00880	-------
FC88X_01062_01073	-------
FC88X_00144_00848	-------
FC88X_00500_00054	-------
FC88X_00550_00102	-------
FC88X_00020_00235	-------
FC88X_00158_00714	-------
FC88X_00238_00995	-------
FC88X_00276_00977	-------
FC88X_00487_01092	-------
FC88X_00334_00509	-------
FC88X_00393_01104	-------
FC88X_00531_00299	-------
FC88X_00993_00588	-------
FC88X_01110_00651	-------
FC88X_01124_01073	-------
FC88X_00193_00119	-------
FC88X_00370_01031	-------
FC88X_00403_01159	-------
FC88X_00709_01252	-------
FC88X_01133_00982	-------
FC88X_00554_00211	-------
FC88X_00930_00948	-------
FC88X_00235_00981	-------
FC88X_00059_00837	-------
FC88X_00050_00129	-------
FC88X_00173_00615	-------
FC88X_00496_00952	-------
FC88X_01001_00402	-------
FC88X_00137_00629	-------
FC88X_00502_00029	-------
FC88X_00292_00960	-------
FC88X_00557_01206	-------
FC88X_00272_01082	-------
FC88X_00656_00231	-------
FC88X_00970_00512	-------
FC88X_00496_01249	-------
FC88X_00823_01074	-------
FC88X_00564_00104	-------
FC88X_00463_01137	-------
FC88X_00447_00117	-------
FC88X_00095_00527	-------
FC88X_00571_00071	-------
FC88X_00486_00357	-------
FC88X_00539_00177	-------
FC88X_00682_00122	-------
FC88X_00762_00932	-------
FC88X_01030_00465	-------
FC88X_00076_00747	-------

FC88X_00228_00547	-------
FC88X_00126_00515	-------
FC88X_00889_00721	-------
FC88X_00599_00651	-------
FC88X_00625_00121	-------
FC88X_00865_01205	-------
FC88X_00857_00935	-------
FC88X_00448_01193	-------
FC88X_00701_00150	-------
FC88X_00052_00449	-------
FC88X_00175_00390	-------
FC88X_01081_01256	-------
FC88X_00263_00958	-------
FC88X_01075_00894	-------
FC88X_01083_00472	-------
FC88X_00006_00125	-------
FC88X_00670_01250	-------
FC88X_00552_00401	-------
FC88X_01011_00330	-------
FC88X_00091_00371	-------
FC88X_00028_00493	-------
FC88X_00259_00958	-------
FC88X_00337_01093	-------
FC88X_00477_00057	-------
FC88X_00954_01116	-------
FC88X_01064_00467	-------
FC88X_01112_00694	-------
FC88X_00759_01265	-------
FC88X_00055_00751	-------
FC88X_00074_00309	-------
FC88X_00579_00451	-------
FC88X_00827_01123	-------
FC88X_00129_00755	-------
FC88X_00664_00936	-------
FC88X_01074_00426	-------
FC88X_01120_00740	-------
FC88X_00769_00151	-------
FC88X_00484_00783	-------
FC88X_01129_00684	-------
FC88X_00037_00144	-------
FC88X_00360_01111	-------
FC88X_00258_00204	-------
FC88X_00390_01177	-------
FC88X_01030_00691	-------
FC88X_00975_00769	-------
FC88X_00049_00292	-------
FC88X_00910_00668	-------
FC88X_00635_00937	-------
FC88X_00462_00270	-------
FC88X_00016_00532	-------
FC88X_00334_00966	-------
FC88X_00917_00532	-------
FC88X_00797_00236	-------
FC88X_00120_00741	-------
FC88X_00722_01215	-------
FC88X_01079_00494	-------
FC88X_00264_00396	-------

FC88X_01079_01223	-------
FC88X_00040_00179	-------
FC88X_01106_00451	-------
FC88X_00083_00266	-------
FC88X_00133_00328	-------
FC88X_00109_00741	-------
FC88X_00131_00872	-------
FC88X_00185_00163	-------
FC88X_00200_00929	-------
FC88X_00268_00283	-------
FC88X_00380_00050	-------
FC88X_00594_00198	-------
FC88X_00621_00497	-------
FC88X_00656_00973	-------
FC88X_00660_01003	-------
FC88X_00700_01151	-------
FC88X_00760_00982	-------
FC88X_00765_01264	-------
FC88X_00846_00131	-------
FC88X_00845_00310	-------
FC88X_00836_00744	-------
FC88X_00885_00779	-------
FC88X_00940_00818	-------
FC88X_00966_00227	-------
FC88X_00980_00568	-------
FC88X_00997_00695	-------
FC88X_01021_00867	-------
FC88X_01079_01228	-------
FC88X_01141_00635	-------
FC88X_01127_00684	-------
FC88X_01114_00948	-------
FC88X_00056_00674	-------
FC88X_00245_00093	-------
FC88X_00210_01023	-------
FC88X_00260_00042	-------
FC88X_00272_00526	-------
FC88X_00270_01002	-------
FC88X_00378_01195	-------
FC88X_00488_01124	-------
FC88X_00517_01125	-------
FC88X_00586_01230	-------
FC88X_00619_01053	-------
FC88X_00669_00368	-------
FC88X_00747_01134	-------
FC88X_00797_00968	-------
FC88X_00767_01102	-------
FC88X_00858_00456	-------
FC88X_00938_00196	-------
FC88X_00913_00358	-------
FC88X_00921_00946	-------
FC88X_00919_00999	-------
FC88X_00949_01185	-------
FC88X_00989_00761	-------
FC88X_00954_00822	-------
FC88X_00996_01013	-------
FC88X_00999_01200	-------
FC88X_01011_00567	-------

FC88X_01038_00577	-------
FC88X_01011_00652	-------
FC88X_01013_00933	-------
FC88X_01056_00712	-------
FC88X_01079_00884	-------
FC88X_01147_00999	-------
FC88X_00768_01202	-------
FC88X_00909_00694	-------
FC88X_00756_00927	-------
FC88X_00178_00733	-------
FC88X_00099_00914	-------
FC88X_00850_00917	-------
FC88X_00175_01008	-------
FC88X_00893_01125	-------
FC88X_00032_00468	-------
FC88X_00219_00930	-------
FC88X_00784_01139	-------
FC88X_00373_01165	-------
FC88X_00670_00930	-------
FC88X_00947_00944	-------
FC88X_00742_01069	-------
FC88X_00960_00714	-------
FC88X_01051_00872	-------
FC88X_00817_00987	-------
FC88X_01125_00883	-------
FC88X_01083_00829	-------
FC88X_00065_00770	-------
FC88X_00033_00502	-------
FC88X_00099_00336	-------
FC88X_00996_01146	-------
FC88X_01038_01033	-------
FC88X_00697_00359	-------
FC88X_00387_01042	-------
FC88X_00962_00853	-------
FC88X_00091_00246	-------
FC88X_00882_00606	-------
FC88X_00515_01027	-------
FC88X_00040_00156	-------
FC88X_00800_00325	-------
FC88X_00689_00883	-------
FC88X_00054_00752	-------
FC88X_00753_00965	-------
FC88X_00181_00055	-------
FC88X_00786_00089	-------
FC88X_00783_00190	-------
FC88X_00355_01115	-------
FC88X_00258_00808	-------
FC88X_00030_00210	-------
FC88X_00092_00908	-------
FC88X_00476_01241	-------
FC88X_00575_00138	-------
FC88X_00837_01000	-------
FC88X_01025_00464	-------
FC88X_00819_00322	-------
FC88X_00770_00203	-------
FC88X_01047_01108	-------
FC88X_00166_00930	-------

FC88X_00858_00908	-------
FC88X_01111_00650	-------
FC88X_00480_00937	-------
FC88X_01086_01193	-------
FC88X_00562_01254	-------
FC88X_00375_00532	-------
FC88X_00855_00238	-------
FC88X_00920_01245	-------
FC88X_00067_00337	-------
FC88X_00187_00271	-------
FC88X_00292_01139	-------
FC88X_00472_00309	-------
FC88X_00607_00391	-------
FC88X_00977_00695	-------
FC88X_00623_01077	-------
FC88X_00527_01215	-------
FC88X_00427_01036	-------
FC88X_00117_00391	-------
FC88X_00545_00081	-------
FC88X_00991_00771	-------
FC88X_00427_01030	-------
FC88X_00022_00503	-------
FC88X_00848_00528	-------
FC88X_00916_00512	-------
FC88X_00215_00059	-------
FC88X_00338_01044	-------
FC88X_00569_00835	-------
FC88X_00664_00226	-------
FC88X_00071_00396	-------
FC88X_00063_00702	-------
FC88X_00071_00726	-------
FC88X_00389_01199	-------
FC88X_00879_00929	-------
FC88X_00964_00299	-------
FC88X_00012_00426	-------
FC88X_00187_00951	-------
FC88X_00959_01229	-------
FC88X_00367_00974	-------
FC88X_00270_00023	-------
FC88X_00512_01210	-------
FC88X_00556_01124	-------
FC88X_00661_00120	-------
FC88X_00912_00854	-------
FC88X_01040_00840	-------
FC88X_01057_00683	-------
FC88X_00942_01270	-------
FC88X_01021_00781	-------
FC88X_01106_00900	-------
FC88X_00846_01197	-------
FC88X_00636_00367	-------
FC88X_00921_00918	-------
FC88X_00612_00093	-------
FC88X_00117_00421	-------
FC88X_01110_00755	-------
FC88X_00120_00093	-------
FC88X_00172_00725	-------
FC88X_00961_00538	-------

FC88X_00979_00795	-------
FC88X_01061_00733	-------
FC88X_01029_00986	-------
FC88X_00434_00133	-------
FC88X_01109_00850	-------
FC88X_00180_00889	-------
FC88X_00150_00899	-------
FC88X_01115_01041	-------
FC88X_01002_00461	-------
FC88X_00315_00806	-------
FC88X_00519_00159	-------
FC88X_00739_00257	-------
FC88X_00860_01179	-------
FC88X_00342_01128	-------
FC88X_00625_00684	-------
FC88X_00093_00026	-------
FC88X_00229_00065	-------
FC88X_01085_00773	-------
FC88X_00575_01012	-------
FC88X_00508_00652	-------
FC88X_01133_00599	-------
FC88X_00634_00194	-------
FC88X_00654_00259	-------
FC88X_00032_00469	-------
FC88X_00105_00406	-------
FC88X_00141_00620	-------
FC88X_00728_01243	-------
FC88X_01131_01131	-------
FC88X_00486_01205	-------
FC88X_00094_00151	-------
FC88X_00179_00085	-------
FC88X_00041_00075	-------
FC88X_00237_00936	-------
FC88X_00048_00094	-------
FC88X_00948_00530	-------
FC88X_00163_00505	-------
FC88X_00294_00260	-------
FC88X_00597_00966	-------
FC88X_00665_00902	-------
FC88X_00871_00574	-------
FC88X_00892_00811	-------
FC88X_00954_00863	-------
FC88X_00968_00890	-------
FC88X_00995_01185	-------
FC88X_01008_00735	-------
FC88X_01090_01038	-------
FC88X_01108_01047	-------
FC88X_00013_00130	-------
FC88X_00055_00045	-------
FC88X_00092_00404	-------
FC88X_00092_00620	-------
FC88X_00110_00176	-------
FC88X_00121_00304	-------
FC88X_00311_01059	-------
FC88X_00385_00976	-------
FC88X_00427_01218	-------
FC88X_00572_00977	-------

FC88X_00642_01256	-------
FC88X_00665_00334	-------
FC88X_00693_01037	-------
FC88X_00778_00801	-------
FC88X_00827_00548	-------
FC88X_00868_00218	-------
FC88X_00895_00437	-------
FC88X_00871_00870	-------
FC88X_00904_00228	-------
FC88X_00917_00210	-------
FC88X_00940_00917	-------
FC88X_00914_01234	-------
FC88X_00975_00261	-------
FC88X_00971_00405	-------
FC88X_00973_00596	-------
FC88X_00969_00670	-------
FC88X_01013_00434	-------
FC88X_01020_00648	-------
FC88X_01045_01091	-------
FC88X_01069_00925	-------
FC88X_00786_00800	-------
FC88X_00131_00797	-------
FC88X_01021_00396	-------
FC88X_00984_00971	-------
FC88X_01033_01049	-------
FC88X_00986_00931	-------
FC88X_01077_00803	-------
FC88X_00727_01124	-------
FC88X_00470_00147	-------
FC88X_00435_00079	-------
FC88X_00588_01015	-------
FC88X_00116_00391	-------
FC88X_00688_01238	-------
FC88X_00096_00388	-------
FC88X_00238_00927	-------
FC88X_00358_01078	-------
FC88X_00574_00238	-------
FC88X_00019_00705	-------
FC88X_00176_00877	-------
FC88X_00038_00238	-------
FC88X_00164_00644	-------
FC88X_00413_00116	-------
FC88X_00487_00328	-------
FC88X_00858_00913	-------
FC88X_00941_00963	-------
FC88X_01070_00963	-------
FC88X_00037_00238	-------
FC88X_00010_00649	-------
FC88X_00057_00617	-------
FC88X_00506_01210	-------
FC88X_00832_01090	-------
FC88X_00974_00978	-------
FC88X_00954_01179	-------
FC88X_01086_00719	-------
FC88X_00033_00597	-------
FC88X_00118_00130	-------
FC88X_00192_00602	-------

FC88X_00361_00074	-------
FC88X_00430_00510	-------
FC88X_00535_00056	-------
FC88X_00811_00904	-------
FC88X_00948_00616	-------
FC88X_00974_00572	-------
FC88X_01012_00803	-------
FC88X_01138_01214	-------
FC88X_00405_00119	-------
FC88X_00967_00296	-------
FC88X_00166_00093	-------
FC88X_00786_00097	-------
FC88X_01080_01058	-------
FC88X_00876_00182	-------
FC88X_00314_00972	-------
FC88X_00532_01235	-------
FC88X_00773_00142	-------
FC88X_01044_00468	-------
FC88X_00114_00884	-------
FC88X_00220_01042	-------
FC88X_00978_00447	-------
FC88X_01069_00402	-------
FC88X_00516_01110	-------
FC88X_00895_00723	-------
FC88X_00878_00854	-------
FC88X_00888_00934	-------
FC88X_00922_00248	-------
FC88X_00485_01186	-------
FC88X_00223_00560	-------
FC88X_00317_00485	-------
FC88X_00626_00229	-------
FC88X_00142_00867	-------
FC88X_00919_00501	-------
FC88X_00903_00402	-------
FC88X_00031_00422	-------
FC88X_00072_00338	-------
FC88X_00924_00930	-------
FC88X_00761_00130	-------
FC88X_00050_00303	-------
FC88X_00051_00561	-------
FC88X_00113_00763	-------
FC88X_00166_00696	-------
FC88X_00206_00956	-------
FC88X_00319_01172	-------
FC88X_00167_00703	-------
FC88X_00438_00645	-------
FC88X_00253_00973	-------
FC88X_00486_00111	-------
FC88X_00547_01179	-------
FC88X_00698_00928	-------
FC88X_00701_00196	-------
FC88X_00744_00989	-------
FC88X_00240_00986	-------
FC88X_00996_00539	-------
FC88X_00274_01106	-------
FC88X_00494_00994	-------
FC88X_00644_00277	-------

FC88X_00694_01115	-------
FC88X_00715_00795	-------
FC88X_00780_00226	-------
FC88X_00762_00577	-------
FC88X_00949_00503	-------
FC88X_00919_00615	-------
FC88X_00955_00771	-------
FC88X_01007_00523	-------
FC88X_00007_00307	-------
FC88X_00016_00642	-------
FC88X_00051_00013	-------
FC88X_00072_00128	-------
FC88X_00067_00291	-------
FC88X_00079_00537	-------
FC88X_00130_00223	-------
FC88X_00125_00420	-------
FC88X_00116_00802	-------
FC88X_00105_00912	-------
FC88X_00229_00214	-------
FC88X_00273_00181	-------
FC88X_00292_01099	-------
FC88X_00364_00053	-------
FC88X_00466_00081	-------
FC88X_00515_00200	-------
FC88X_00541_01009	-------
FC88X_00532_01238	-------
FC88X_00568_00119	-------
FC88X_00598_00160	-------
FC88X_00743_00540	-------
FC88X_00734_00634	-------
FC88X_00815_00203	-------
FC88X_00842_00464	-------
FC88X_00840_00567	-------
FC88X_00832_01266	-------
FC88X_00933_00392	-------
FC88X_00906_00547	-------
FC88X_00906_00733	-------
FC88X_00905_01005	-------
FC88X_00938_01207	-------
FC88X_00991_00602	-------
FC88X_01050_00855	-------
FC88X_01056_01022	-------
FC88X_01126_00629	-------
FC88X_00096_00400	-------
FC88X_00495_01060	-------
FC88X_00819_01231	-------
FC88X_01085_00879	-------
FC88X_00059_00846	-------
FC88X_00036_00073	-------
FC88X_00039_00635	-------
FC88X_00625_00332	-------
FC88X_00996_00712	-------
FC88X_01115_00652	-------
FC88X_00301_00295	-------
FC88X_00970_00523	-------
FC88X_00091_00603	-------
FC88X_00646_01111	-------

FC88X_00560_01202	-------
FC88X_00043_00819	-------
FC88X_00579_01223	-------
FC88X_01098_00570	-------
FC88X_00483_01181	-------
FC88X_00216_00690	-------
FC88X_01022_00361	-------
FC88X_00455_01008	-------
FC88X_00317_00031	-------
FC88X_00311_01097	-------
FC88X_00819_01162	-------
FC88X_00205_00012	-------
FC88X_00466_00056	-------
FC88X_00670_01108	-------
FC88X_00141_00201	-------
FC88X_01083_01196	-------
FC88X_00609_00256	-------
FC88X_00342_00989	-------
FC88X_00719_01084	-------
FC88X_00368_01182	-------
FC88X_00627_01243	-------
FC88X_00049_00619	-------
FC88X_00075_00779	-------
FC88X_00356_00698	-------
FC88X_00791_00129	-------
FC88X_00287_01151	-------
FC88X_00609_00943	-------
FC88X_00274_00608	-------
FC88X_00750_00528	-------
FC88X_00872_00856	-------
FC88X_00167_00124	-------
FC88X_00957_00948	-------
FC88X_00758_00935	-------
FC88X_00581_01002	-------
FC88X_00575_00437	-------
FC88X_00070_00057	-------
FC88X_00083_00069	-------
FC88X_00055_00373	-------
FC88X_00993_00753	-------
FC88X_00880_00723	-------
FC88X_00529_01222	-------
FC88X_00897_01212	-------
FC88X_00294_00980	-------
FC88X_00867_00701	-------
FC88X_01042_00994	-------
FC88X_00214_00630	-------
FC88X_01137_00712	-------
FC88X_00970_00341	-------
FC88X_01004_00716	-------
FC88X_00037_00275	-------
FC88X_00935_00544	-------
FC88X_00332_00628	-------
FC88X_01097_01106	-------
FC88X_00508_00366	-------
FC88X_01011_00974	-------
FC88X_00483_01246	-------
FC88X_00744_01208	-------

FC88X_00056_00274	-------
FC88X_00012_00459	-------
FC88X_00062_00559	-------
FC88X_00022_00107	-------
FC88X_00777_00238	-------
FC88X_00929_01172	-------
FC88X_00071_00393	-------
FC88X_00096_00135	-------
FC88X_00060_00369	-------
FC88X_01139_00931	-------
FC88X_00882_01173	-------
FC88X_00623_01046	-------
FC88X_00023_00129	-------
FC88X_00277_01114	-------
FC88X_00242_00803	-------
FC88X_00408_01071	-------
FC88X_00266_00402	-------
FC88X_00460_00889	-------
FC88X_00225_01013	-------
FC88X_01086_00735	-------
FC88X_00026_00171	-------
FC88X_00613_01039	-------
FC88X_00994_00702	-------
FC88X_00552_01219	-------
FC88X_00907_01023	-------
FC88X_00163_00789	-------
FC88X_00098_00347	-------
FC88X_00067_00142	-------
FC88X_00478_01171	-------
FC88X_00822_00803	-------
FC88X_00322_00027	-------
FC88X_01077_01243	-------
FC88X_00984_00888	-------
FC88X_00383_01099	-------
FC88X_00589_00427	-------
FC88X_00777_01158	-------
FC88X_01002_00443	-------
FC88X_00399_01200	-------
FC88X_00902_00831	-------
FC88X_00484_01122	-------
FC88X_00910_00316	-------
FC88X_00052_00682	-------
FC88X_00856_00939	-------
FC88X_00926_00428	-------
FC88X_00097_00720	-------
FC88X_00560_00280	-------
FC88X_00112_00931	-------
FC88X_00253_00913	-------
FC88X_00036_00365	-------
FC88X_00138_00526	-------
FC88X_00206_00877	-------
FC88X_00754_00161	-------
FC88X_00630_00453	-------
FC88X_00976_00715	-------
FC88X_00071_00017	-------
FC88X_00644_00108	-------
FC88X_00174_00719	-------

FC88X_00042_00292	-------
FC88X_00842_00121	-------
FC88X_00269_00680	-------
FC88X_00609_00715	-------
FC88X_00539_00106	-------
FC88X_00540_00105	-------
FC88X_00231_00016	-------
FC88X_00812_01126	-------
FC88X_00608_00955	-------
FC88X_00016_00093	-------
FC88X_00017_00321	-------
FC88X_00059_00826	-------
FC88X_00028_00163	-------
FC88X_00437_01098	-------
FC88X_00079_00629	-------
FC88X_00146_00809	-------
FC88X_00467_00988	-------
FC88X_00207_00053	-------
FC88X_00210_00046	-------
FC88X_00976_01206	-------
FC88X_00944_00625	-------
FC88X_01023_00348	-------
FC88X_01015_00650	-------
FC88X_01027_01181	-------
FC88X_00742_01129	-------
FC88X_00820_00121	-------
FC88X_00532_01228	-------
FC88X_00663_00111	-------
FC88X_01037_00791	-------
FC88X_00021_00113	-------
FC88X_00034_00600	-------
FC88X_00255_01061	-------
FC88X_00359_00004	-------
FC88X_00364_00050	-------
FC88X_00738_00968	-------
FC88X_00746_01019	-------
FC88X_00047_00344	-------
FC88X_00140_00048	-------
FC88X_00149_00870	-------
FC88X_00492_01172	-------
FC88X_00939_00193	-------
FC88X_01070_00752	-------
FC88X_01079_00397	-------
FC88X_00007_00599	-------
FC88X_00105_00309	-------
FC88X_00150_00105	-------
FC88X_00243_00485	-------
FC88X_00120_00424	-------
FC88X_00139_00910	-------
FC88X_00498_01152	-------
FC88X_00162_01004	-------
FC88X_00963_01049	-------
FC88X_00020_00181	-------
FC88X_00960_00501	-------
FC88X_01030_00734	-------
FC88X_00017_00181	-------
FC88X_00885_00159	-------

FC88X_00040_00257	-------
FC88X_00048_00416	-------
FC88X_00090_00111	-------
FC88X_00180_01039	-------
FC88X_00718_01131	-------
FC88X_00316_00570	-------
FC88X_00374_00381	-------
FC88X_00416_01154	-------
FC88X_00045_00214	-------
FC88X_00029_00403	-------
FC88X_00020_00521	-------
FC88X_00054_00170	-------
FC88X_00051_00200	-------
FC88X_00067_00262	-------
FC88X_00090_00428	-------
FC88X_00065_00624	-------
FC88X_00070_00734	-------
FC88X_00095_00886	-------
FC88X_00127_00142	-------
FC88X_00146_00574	-------
FC88X_00148_00823	-------
FC88X_00146_00911	-------
FC88X_00161_00063	-------
FC88X_00165_00103	-------
FC88X_00170_00400	-------
FC88X_00164_00760	-------
FC88X_00166_00981	-------
FC88X_00154_01008	-------
FC88X_00218_00125	-------
FC88X_00221_00201	-------
FC88X_00216_00630	-------
FC88X_00229_00915	-------
FC88X_00242_01002	-------
FC88X_00256_00939	-------
FC88X_00253_01005	-------
FC88X_00280_01069	-------
FC88X_00291_01071	-------
FC88X_00329_01124	-------
FC88X_00391_00577	-------
FC88X_00375_00930	-------
FC88X_00389_01079	-------
FC88X_00401_00011	-------
FC88X_00407_00321	-------
FC88X_00404_00490	-------
FC88X_00400_00657	-------
FC88X_00441_00651	-------
FC88X_00427_01204	-------
FC88X_00499_00912	-------
FC88X_00472_00955	-------
FC88X_00461_01212	-------
FC88X_00517_00032	-------
FC88X_00505_00300	-------
FC88X_00510_00657	-------
FC88X_00527_00897	-------
FC88X_00541_00893	-------
FC88X_00508_01018	-------
FC88X_00526_01065	-------

FC88X_00585_00486	-------
FC88X_00595_00575	-------
FC88X_00589_00629	-------
FC88X_00560_00876	-------
FC88X_00597_00861	-------
FC88X_00568_00946	-------
FC88X_00557_01103	-------
FC88X_00596_01195	-------
FC88X_00562_01248	-------
FC88X_00625_00208	-------
FC88X_00635_00252	-------
FC88X_00637_00638	-------
FC88X_00622_00917	-------
FC88X_00607_00959	-------
FC88X_00616_01124	-------
FC88X_00611_01160	-------
FC88X_00647_01249	-------
FC88X_00655_00412	-------
FC88X_00693_00696	-------
FC88X_00671_00714	-------
FC88X_00693_00885	-------
FC88X_00688_01215	-------
FC88X_00720_00758	-------
FC88X_00732_00841	-------
FC88X_00733_01089	-------
FC88X_00736_01110	-------
FC88X_00721_01224	-------
FC88X_00778_00082	-------
FC88X_00778_00675	-------
FC88X_00787_00693	-------
FC88X_00786_00799	-------
FC88X_00765_00872	-------
FC88X_00774_00895	-------
FC88X_00754_01131	-------
FC88X_00795_01131	-------
FC88X_00831_00228	-------
FC88X_00809_00806	-------
FC88X_00819_00990	-------
FC88X_00807_01018	-------
FC88X_00835_01220	-------
FC88X_00867_00138	-------
FC88X_00857_00354	-------
FC88X_00895_00403	-------
FC88X_00897_00861	-------
FC88X_00899_00941	-------
FC88X_00898_00976	-------
FC88X_00872_01023	-------
FC88X_00897_01000	-------
FC88X_00883_01185	-------
FC88X_00855_01236	-------
FC88X_00863_01249	-------
FC88X_00931_00204	-------
FC88X_00900_00454	-------
FC88X_00934_00846	-------
FC88X_00941_00836	-------
FC88X_00920_00986	-------
FC88X_00922_00953	-------

FC88X_00920_01128	-------
FC88X_00981_00238	-------
FC88X_00955_00354	-------
FC88X_00966_00377	-------
FC88X_00980_00497	-------
FC88X_00980_00530	-------
FC88X_00999_00506	-------
FC88X_00961_01084	-------
FC88X_00983_01182	-------
FC88X_00978_01227	-------
FC88X_00986_01231	-------
FC88X_01020_00497	-------
FC88X_01031_00616	-------
FC88X_01009_00756	-------
FC88X_01001_00811	-------
FC88X_01021_00843	-------
FC88X_01023_00834	-------
FC88X_01032_00806	-------
FC88X_01048_00950	-------
FC88X_01032_01093	-------
FC88X_01024_01139	-------
FC88X_01021_01167	-------
FC88X_01040_01194	-------
FC88X_01066_00626	-------
FC88X_01076_00603	-------
FC88X_01062_00738	-------
FC88X_01083_00803	-------
FC88X_01054_00934	-------
FC88X_01084_00964	-------
FC88X_01091_00983	-------
FC88X_01083_01129	-------
FC88X_01070_01203	-------
FC88X_01077_01221	-------
FC88X_01108_00544	-------
FC88X_01126_00835	-------
FC88X_01118_00854	-------
FC88X_01120_00959	-------
FC88X_01120_00977	-------
FC88X_01113_01023	-------
FC88X_01101_01072	-------
FC88X_01118_01103	-------
FC88X_01118_01181	-------
FC88X_00769_01006	-------
FC88X_00125_00293	-------
FC88X_00815_00951	-------
FC88X_00385_00067	-------
FC88X_00455_00560	-------
FC88X_00269_00409	-------
FC88X_00425_00070	-------
FC88X_00960_00838	-------
FC88X_00176_01017	-------
FC88X_00134_00234	-------
FC88X_00023_00613	-------
FC88X_00083_00116	-------
FC88X_00241_00091	-------
FC88X_00369_01135	-------
FC88X_00126_00463	-------

FC88X_00019_00516	-------
FC88X_00117_00141	-------
FC88X_00170_00088	-------
FC88X_00216_01028	-------
FC88X_00123_00079	-------
FC88X_00890_00156	-------
FC88X_00110_00905	-------
FC88X_00197_01002	-------
FC88X_00040_00564	-------
FC88X_00542_01030	-------
FC88X_00130_00369	-------
FC88X_00151_00120	-------
FC88X_00228_00549	-------
FC88X_00045_00724	-------
FC88X_00814_01195	-------
FC88X_00106_00414	-------
FC88X_00158_00527	-------
FC88X_00220_00704	-------
FC88X_00234_01062	-------
FC88X_00271_01073	-------
FC88X_00383_00078	-------
FC88X_00424_01158	-------
FC88X_00158_00759	-------
FC88X_00872_00651	-------
FC88X_00238_01021	-------
FC88X_00270_00078	-------
FC88X_00334_01024	-------
FC88X_00441_00097	-------
FC88X_00475_01195	-------
FC88X_00219_00680	-------
FC88X_00303_01076	-------
FC88X_00243_01104	-------
FC88X_00314_00741	-------
FC88X_00427_00086	-------
FC88X_00473_00147	-------
FC88X_00452_01225	-------
FC88X_00467_01224	-------
FC88X_00539_00139	-------
FC88X_00526_00563	-------
FC88X_00507_00695	-------
FC88X_00546_00747	-------
FC88X_00530_00947	-------
FC88X_00504_00994	-------
FC88X_00522_01086	-------
FC88X_00562_00940	-------
FC88X_00576_00951	-------
FC88X_00579_01092	-------
FC88X_00556_01139	-------
FC88X_00558_01250	-------
FC88X_00621_00976	-------
FC88X_00600_01017	-------
FC88X_00673_00104	-------
FC88X_00652_00278	-------
FC88X_00693_01217	-------
FC88X_00748_00087	-------
FC88X_00726_00249	-------
FC88X_00728_00294	-------

FC88X_00744_01201	-------
FC88X_00793_00388	-------
FC88X_00755_01003	-------
FC88X_00814_00287	-------
FC88X_00843_00334	-------
FC88X_00800_00514	-------
FC88X_00824_00872	-------
FC88X_00806_01231	-------
FC88X_00876_00217	-------
FC88X_00855_00332	-------
FC88X_00866_00535	-------
FC88X_00881_00903	-------
FC88X_00891_00988	-------
FC88X_00880_01043	-------
FC88X_00895_01219	-------
FC88X_00858_01259	-------
FC88X_00941_00236	-------
FC88X_00918_00426	-------
FC88X_00942_00452	-------
FC88X_00942_00504	-------
FC88X_00902_00598	-------
FC88X_00949_00587	-------
FC88X_00908_00704	-------
FC88X_00989_00619	-------
FC88X_00953_00662	-------
FC88X_00990_00668	-------
FC88X_00988_00817	-------
FC88X_00973_00886	-------
FC88X_00975_00893	-------
FC88X_01015_00375	-------
FC88X_01010_00485	-------
FC88X_01010_00545	-------
FC88X_01019_00557	-------
FC88X_01020_00719	-------
FC88X_01007_00793	-------
FC88X_01001_00830	-------
FC88X_01039_00872	-------
FC88X_01024_00941	-------
FC88X_01033_01248	-------
FC88X_01082_00524	-------
FC88X_01055_00550	-------
FC88X_01094_00832	-------
FC88X_01087_00867	-------
FC88X_01058_00963	-------
FC88X_01142_00893	-------
FC88X_01146_00937	-------
FC88X_01117_01167	-------
FC88X_00006_00284	-------
FC88X_00019_00363	-------
FC88X_00033_00457	-------
FC88X_00021_00523	-------
FC88X_00008_00692	-------
FC88X_00053_00039	-------
FC88X_00079_00040	-------
FC88X_00096_00100	-------
FC88X_00051_00593	-------
FC88X_00130_00011	-------

FC88X_00146_00020	-------
FC88X_00138_00068	-------
FC88X_00105_00140	-------
FC88X_00102_00192	-------
FC88X_00138_00173	-------
FC88X_00125_00496	-------
FC88X_00119_00511	-------
FC88X_00100_00727	-------
FC88X_00129_00975	-------
FC88X_00156_00036	-------
FC88X_00163_00067	-------
FC88X_00181_00120	-------
FC88X_00198_00818	-------
FC88X_00173_00888	-------
FC88X_00175_00969	-------
FC88X_00189_00990	-------
FC88X_00234_00392	-------
FC88X_00236_00890	-------
FC88X_00279_00990	-------
FC88X_00338_00972	-------
FC88X_00382_00188	-------
FC88X_00395_00981	-------
FC88X_00351_01033	-------
FC88X_00384_01075	-------
FC88X_00354_01102	-------
FC88X_00390_01201	-------
FC88X_00413_01097	-------
FC88X_00406_01158	-------
FC88X_00462_00304	-------
FC88X_00467_01029	-------
FC88X_00497_01042	-------
FC88X_00490_01227	-------
FC88X_00513_00138	-------
FC88X_00532_00165	-------
FC88X_00542_01202	-------
FC88X_00583_00130	-------
FC88X_00587_00137	-------
FC88X_00593_00579	-------
FC88X_00558_00815	-------
FC88X_00593_01099	-------
FC88X_00594_01240	-------
FC88X_00606_00067	-------
FC88X_00606_00118	-------
FC88X_00612_00143	-------
FC88X_00609_00979	-------
FC88X_00634_01270	-------
FC88X_00695_00123	-------
FC88X_00698_00936	-------
FC88X_00659_00955	-------
FC88X_00732_01004	-------
FC88X_00702_01062	-------
FC88X_00718_01099	-------
FC88X_00734_01128	-------
FC88X_00747_01222	-------
FC88X_00741_01263	-------
FC88X_00765_00137	-------
FC88X_00792_00333	-------

FC88X_00753_01249	-------
FC88X_00808_00121	-------
FC88X_00803_00240	-------
FC88X_00821_00259	-------
FC88X_00852_00226	-------
FC88X_00889_00210	-------
FC88X_00898_00247	-------
FC88X_00887_00651	-------
FC88X_00859_00770	-------
FC88X_00871_00820	-------
FC88X_00880_00887	-------
FC88X_00887_01264	-------
FC88X_00920_00220	-------
FC88X_00925_00241	-------
FC88X_00936_00280	-------
FC88X_00901_00326	-------
FC88X_00902_00366	-------
FC88X_00929_00455	-------
FC88X_00901_00629	-------
FC88X_00918_00841	-------
FC88X_00968_00243	-------
FC88X_00963_00364	-------
FC88X_00986_00461	-------
FC88X_00989_00461	-------
FC88X_00992_00464	-------
FC88X_00998_00588	-------
FC88X_01006_00492	-------
FC88X_01041_00505	-------
FC88X_01008_00979	-------
FC88X_01087_00475	-------
FC88X_01065_00743	-------
FC88X_01062_00886	-------
FC88X_01114_00538	-------
FC88X_01117_00572	-------
FC88X_01131_00947	-------
FC88X_01146_01105	-------
FC88X_01102_00836	-------
FC88X_00004_00257	-------
FC88X_00548_00214	-------
FC88X_00068_00277	-------
FC88X_00221_00925	-------
FC88X_00278_01118	-------
FC88X_00503_00218	-------
FC88X_00604_00595	-------
FC88X_00653_00406	-------
FC88X_00672_00642	-------
FC88X_00680_00677	-------
FC88X_00790_00107	-------
FC88X_00769_01264	-------
FC88X_00998_01174	-------
FC88X_01137_01201	-------
FC88X_00671_01059	-------
FC88X_00507_00068	-------
FC88X_00863_01247	-------
FC88X_00891_01123	-------
FC88X_00377_00325	-------
FC88X_00538_00136	-------

FC88X_00816_00890	-------
FC88X_00928_00511	-------
FC88X_00969_00669	-------
FC88X_00041_00795	-------
FC88X_00373_00973	-------
FC88X_00732_01098	-------
FC88X_00872_00594	-------
FC88X_00100_00617	-------
FC88X_00325_01162	-------
FC88X_00296_00073	-------
FC88X_00066_00648	-------
FC88X_00691_00427	-------
FC88X_00613_00904	-------
FC88X_00438_00650	-------
FC88X_00553_00866	-------
FC88X_00869_00993	-------
FC88X_00071_00325	-------
FC88X_00439_01089	-------
FC88X_00211_00021	-------
FC88X_00255_01039	-------
FC88X_01019_00408	-------
FC88X_01001_00636	-------
FC88X_00756_01032	-------
FC88X_01117_00990	-------
FC88X_00970_00849	-------
FC88X_00504_01149	-------
FC88X_00900_00202	-------
FC88X_00642_01157	-------
FC88X_01106_00976	-------
FC88X_00829_00301	-------
FC88X_00589_00143	-------
FC88X_00032_00134	-------
FC88X_00161_00634	-------
FC88X_00805_00115	-------
FC88X_00021_00448	-------
FC88X_01103_00565	-------
FC88X_00569_00122	-------
FC88X_00031_00146	-------
FC88X_00467_00755	-------
FC88X_00027_00168	-------
FC88X_00015_00128	-------
FC88X_00089_00763	-------
FC88X_00088_00153	-------
FC88X_00675_01024	-------
FC88X_00007_00568	-------
FC88X_00161_00556	-------
FC88X_00412_00492	-------
FC88X_00416_01103	-------
FC88X_00483_01108	-------
FC88X_00739_00265	-------
FC88X_00895_00743	-------
FC88X_00864_00827	-------
FC88X_00892_01137	-------
FC88X_00727_01249	-------
FC88X_00063_00266	-------
FC88X_00689_01225	-------
FC88X_00323_01145	-------

FC88X_00123_00073	-------
FC88X_00712_01214	-------
FC88X_00174_00845	-------
FC88X_00080_00852	-------
FC88X_00165_00642	-------
FC88X_00981_00649	-------
FC88X_01077_00818	-------
FC88X_00287_00807	-------
FC88X_01091_01209	-------
FC88X_00915_00962	-------
FC88X_00972_01116	-------
FC88X_00606_00273	-------
FC88X_00481_01065	-------
FC88X_00575_01150	-------
FC88X_00900_00760	-------
FC88X_00137_00129	-------
FC88X_00461_00412	-------
FC88X_00725_01085	-------
FC88X_00808_01132	-------
FC88X_00154_00781	-------
FC88X_00613_00974	-------
FC88X_01048_00693	-------
FC88X_01095_01019	-------
FC88X_00301_00369	-------
FC88X_00018_00576	-------
FC88X_00117_00837	-------
FC88X_00037_00225	-------
FC88X_00138_00119	-------
FC88X_00122_00448	-------
FC88X_00160_00904	-------
FC88X_00925_01059	-------
FC88X_00216_00953	-------
FC88X_01107_00675	-------
FC88X_00084_00794	-------
FC88X_00074_00142	-------
FC88X_00572_00262	-------
FC88X_00743_00194	-------
FC88X_00565_00793	-------
FC88X_00997_00962	-------
FC88X_01096_01002	-------
FC88X_01071_01091	-------
FC88X_00590_00533	-------
FC88X_00351_00313	-------
FC88X_01041_01132	-------
FC88X_00060_00301	-------
FC88X_00336_00957	-------
FC88X_00484_00090	-------
FC88X_00485_00178	-------
FC88X_00557_00639	-------
FC88X_00037_00330	-------
FC88X_00095_00215	-------
FC88X_00105_00505	-------
FC88X_00677_00931	-------
FC88X_00596_00374	-------
FC88X_00974_00902	-------
FC88X_00998_00941	-------
FC88X_00320_01058	-------

FC88X_00759_00199	-------
FC88X_01031_00538	-------
FC88X_01143_00824	-------
FC88X_00303_00966	-------
FC88X_00994_00913	-------
FC88X_00136_00562	-------
FC88X_00658_00145	-------
FC88X_01121_00492	-------
FC88X_00549_01060	-------
FC88X_00782_00277	-------
FC88X_01077_00812	-------
FC88X_00073_00107	-------
FC88X_00168_00851	-------
FC88X_01119_00913	-------
FC88X_00133_00595	-------
FC88X_00766_00223	-------
FC88X_00380_00777	-------
FC88X_00928_01139	-------
FC88X_01106_00869	-------
FC88X_00874_00547	-------
FC88X_00146_00086	-------
FC88X_01040_00666	-------
FC88X_00873_00382	-------
FC88X_00911_00447	-------
FC88X_00753_00300	-------
FC88X_00222_00102	-------
FC88X_00343_01068	-------
FC88X_00924_00252	-------
FC88X_00999_00410	-------
FC88X_00124_00723	-------
FC88X_01093_00507	-------
FC88X_01073_00941	-------
FC88X_00144_00959	-------
FC88X_00311_01115	-------
FC88X_00428_01110	-------
FC88X_00983_01170	-------
FC88X_00740_01060	-------
FC88X_00831_00238	-------
FC88X_00064_00493	-------
FC88X_01113_00700	-------
FC88X_00436_01145	-------
FC88X_00964_00630	-------
FC88X_00963_00650	-------
FC88X_01108_01272	-------
FC88X_00429_00025	-------
FC88X_00220_01082	-------
FC88X_00875_00929	-------
FC88X_00445_01059	-------
FC88X_00033_00493	-------
FC88X_00046_00798	-------
FC88X_00511_00190	-------
FC88X_00432_00711	-------
FC88X_00101_00078	-------
FC88X_01055_00959	-------
FC88X_00130_00880	-------
FC88X_00669_00380	-------
FC88X_00738_00933	-------

FC88X_00950_00502	-------
FC88X_00159_00523	-------
FC88X_01128_00526	-------
FC88X_00027_00293	-------
FC88X_01089_00779	-------
FC88X_01097_00539	-------
FC88X_00835_00136	-------
FC88X_00933_00967	-------
FC88X_00864_00330	-------
FC88X_01053_00537	-------
FC88X_00017_00745	-------
FC88X_00879_00167	-------
FC88X_01090_00962	-------
FC88X_01136_01079	-------
FC88X_00561_01193	-------
FC88X_00231_00074	-------
FC88X_00752_00940	-------
FC88X_00326_00924	-------
FC88X_00866_00280	-------
FC88X_00509_00971	-------
FC88X_00840_00321	-------
FC88X_00018_00212	-------
FC88X_01101_00697	-------
FC88X_00056_00276	-------
FC88X_00014_00329	-------
FC88X_00695_00953	-------
FC88X_00412_01192	-------
FC88X_00877_00245	-------
FC88X_00117_00052	-------
FC88X_00873_00822	-------
FC88X_00280_01092	-------
FC88X_00319_00089	-------
FC88X_00021_00516	-------
FC88X_01004_00587	-------
FC88X_00101_00205	-------
FC88X_00045_00624	-------
FC88X_01032_00820	-------
FC88X_00323_00020	-------
FC88X_00100_00175	-------
FC88X_00975_00935	-------
FC88X_01007_00653	-------
FC88X_00593_01225	-------
FC88X_00845_01174	-------
FC88X_00567_00077	-------
FC88X_01095_00901	-------
FC88X_00891_00244	-------
FC88X_00009_00431	-------
FC88X_00558_00109	-------
FC88X_00589_01074	-------
FC88X_00354_01170	-------
FC88X_00995_00898	-------
FC88X_00309_01124	-------
FC88X_00412_00031	-------
FC88X_00227_01038	-------
FC88X_00059_00415	-------
FC88X_00503_00758	-------
FC88X_00875_01259	-------

FC88X_00986_00836	-------
FC88X_00441_00093	-------
FC88X_00633_01257	-------
FC88X_01025_00504	-------
FC88X_00577_00800	-------
FC88X_00908_00569	-------
FC88X_01019_00471	-------
FC88X_01047_00415	-------
FC88X_00781_00243	-------
FC88X_00841_00148	-------
FC88X_01053_00578	-------
FC88X_01086_01018	-------
FC88X_00105_00225	-------
FC88X_00510_00066	-------
FC88X_00628_01021	-------
FC88X_00785_00280	-------
FC88X_01025_00653	-------
FC88X_00595_01269	-------
FC88X_00435_00020	-------
FC88X_00596_00089	-------
FC88X_00889_00996	-------
FC88X_00839_01231	-------
FC88X_00286_00970	-------
FC88X_00932_01038	-------
FC88X_00436_01035	-------
FC88X_01049_01183	-------
FC88X_00035_00440	-------
FC88X_00020_00208	-------
FC88X_00095_00336	-------
FC88X_01001_00737	-------
FC88X_00203_00868	-------
FC88X_00429_01038	-------
FC88X_00044_00123	-------
FC88X_00092_00149	-------
FC88X_00902_01243	-------
FC88X_01100_00846	-------
FC88X_00377_01197	-------
FC88X_00068_00204	-------
FC88X_00187_00160	-------
FC88X_00240_00840	-------
FC88X_00222_00956	-------
FC88X_00301_00421	-------
FC88X_00366_00784	-------
FC88X_00305_00714	-------
FC88X_00488_00959	-------
FC88X_00566_01180	-------
FC88X_00716_00896	-------
FC88X_00755_01138	-------
FC88X_00818_00118	-------
FC88X_00873_00690	-------
FC88X_00997_00376	-------
FC88X_00960_00920	-------
FC88X_01012_00272	-------
FC88X_01013_00640	-------
FC88X_01054_00367	-------
FC88X_01106_00923	-------
FC88X_00012_00388	-------

FC88X_00045_00465	-------
FC88X_00016_00692	-------
FC88X_00094_00088	-------
FC88X_00091_00201	-------
FC88X_00065_00325	-------
FC88X_00075_00325	-------
FC88X_00081_00854	-------
FC88X_00146_00754	-------
FC88X_00198_00016	-------
FC88X_00204_00898	-------
FC88X_00256_00902	-------
FC88X_00312_00034	-------
FC88X_00320_00023	-------
FC88X_00310_01003	-------
FC88X_00360_01013	-------
FC88X_00438_00032	-------
FC88X_00419_00651	-------
FC88X_00433_00958	-------
FC88X_00491_00563	-------
FC88X_00494_00588	-------
FC88X_00580_00873	-------
FC88X_00552_01091	-------
FC88X_00591_01235	-------
FC88X_00604_00067	-------
FC88X_00621_00618	-------
FC88X_00611_00873	-------
FC88X_00682_00288	-------
FC88X_00666_00367	-------
FC88X_00686_01187	-------
FC88X_00720_01027	-------
FC88X_00775_00102	-------
FC88X_00764_01084	-------
FC88X_00776_01163	-------
FC88X_00782_01173	-------
FC88X_00787_01208	-------
FC88X_00839_00229	-------
FC88X_00835_01158	-------
FC88X_00852_00545	-------
FC88X_00860_01091	-------
FC88X_00862_01175	-------
FC88X_00909_00279	-------
FC88X_00964_00557	-------
FC88X_00966_01213	-------
FC88X_01006_00340	-------
FC88X_01014_00333	-------
FC88X_01002_00372	-------
FC88X_01086_00431	-------
FC88X_01090_01053	-------
FC88X_01120_00551	-------
FC88X_01139_01024	-------
FC88X_00435_00609	-------
FC88X_00728_01073	-------
FC88X_00978_00738	-------
FC88X_00784_01259	-------
FC88X_00528_00984	-------
FC88X_00728_00305	-------
FC88X_01042_00744	-------

FC88X_00908_00682	-------
FC88X_01066_00743	-------
FC88X_00368_00629	-------
FC88X_00776_01040	-------
FC88X_01132_00922	-------
FC88X_00259_00567	-------
FC88X_01028_01218	-------
FC88X_01103_01080	-------
FC88X_01036_00661	-------
FC88X_00893_00300	-------
FC88X_00989_00264	-------
FC88X_00071_00642	-------
FC88X_00551_01102	-------
FC88X_00603_00931	-------
FC88X_01034_00551	-------
FC88X_00347_01029	-------
FC88X_00936_01220	-------
FC88X_01026_00862	-------
FC88X_01049_00622	-------
FC88X_00149_00097	-------
FC88X_00861_00804	-------
FC88X_00877_01183	-------
FC88X_00072_00352	-------
FC88X_00193_00141	-------
FC88X_00511_00186	-------
FC88X_01072_00820	-------
FC88X_00926_00813	-------
FC88X_00849_00899	-------
FC88X_00646_00321	-------
FC88X_01027_00493	-------
FC88X_00008_00359	-------
FC88X_00896_00206	-------
FC88X_00433_00006	-------
FC88X_00424_01225	-------
FC88X_00118_00261	-------
FC88X_00845_00242	-------
FC88X_00861_00932	-------
FC88X_00252_00954	-------
FC88X_00269_01044	-------
FC88X_00369_00996	-------
FC88X_00446_01189	-------
FC88X_00478_01208	-------
FC88X_00526_00994	-------
FC88X_00589_00100	-------
FC88X_00563_00183	-------
FC88X_00801_01146	-------
FC88X_00868_00293	-------
FC88X_00868_00997	-------
FC88X_00916_00192	-------
FC88X_00994_00322	-------
FC88X_00968_01253	-------
FC88X_01098_00755	-------
FC88X_01089_01144	-------
FC88X_01091_01130	-------
FC88X_01138_00948	-------
FC88X_01133_01031	-------
FC88X_00069_00177	-------

FC88X_00122_00034	-------
FC88X_00111_00564	-------
FC88X_00136_00629	-------
FC88X_00172_00624	-------
FC88X_00236_00949	-------
FC88X_00292_01114	-------
FC88X_00428_01173	-------
FC88X_00474_00081	-------
FC88X_00451_00325	-------
FC88X_00582_00248	-------
FC88X_00594_01151	-------
FC88X_00637_01112	-------
FC88X_00665_01180	-------
FC88X_00710_00097	-------
FC88X_00729_00073	-------
FC88X_00796_00854	-------
FC88X_00781_01027	-------
FC88X_00796_01051	-------
FC88X_00819_00754	-------
FC88X_00843_00791	-------
FC88X_00875_00878	-------
FC88X_00923_00752	-------
FC88X_00978_00574	-------
FC88X_00974_01124	-------
FC88X_01009_00552	-------
FC88X_01029_00952	-------
FC88X_01050_00514	-------
FC88X_01083_00669	-------
FC88X_01088_00667	-------
FC88X_01089_01186	-------
FC88X_01118_00629	-------
FC88X_01123_00613	-------
FC88X_01139_00633	-------
FC88X_01142_00879	-------
FC88X_01122_00979	-------
FC88X_00033_00152	-------
FC88X_00661_01052	-------
FC88X_00105_00122	-------
FC88X_00569_01215	-------
FC88X_00622_00663	-------
FC88X_01090_00919	TTT----
FC88X_00268_00921	TTT----
FC88X_00834_00959	-------
FC88X_00027_00187	TTCCT--
FC88X_00281_00349	-------
FC88X_00098_00837	-------
FC88X_01127_00967	TTT----
FC88X_00199_00958	-------
FC88X_00099_00350	-------
FC88X_00775_00942	-------
FC88X_00182_00921	-------
FC88X_00735_00297	-------
FC88X_00836_00388	-------
FC88X_00862_01099	-------
FC88X_01054_01013	-------
FC88X_00200_00942	-------
FC88X_00331_01068	-------

```
FC88X_00466_01179       -------
FC88X_00458_01205       -------
FC88X_00496_01236       -------
FC88X_00693_01260       -------
FC88X_00829_00225       -------
FC88X_00888_00167       -------
FC88X_00962_00618       -------
FC88X_01093_00727       -------
FC88X_00084_00276       -------
FC88X_00144_00737       -------
FC88X_00349_01055       -------
FC88X_00443_00213       -------
FC88X_00574_00187       -------
FC88X_00612_01151       -------
FC88X_00910_00642       -------
FC88X_00836_00445       -------
FC88X_01034_00992       C------
FC88X_00949_01061       -------
FC88X_00093_00826       -------
FC88X_00238_00453       -------
FC88X_01145_01109       TTTCTT-
FC88X_00939_00557       -------
FC88X_00450_00119       TTTCTCC
FC88X_00434_00175       -------
FC88X_00962_00902       -------
FC88X_00333_00397       -------
FC88X_00259_00499       -------
FC88X_00032_00456       -------
FC88X_00140_00436       -------
FC88X_00851_00962       -------
FC88X_00089_00554       -------
FC88X_00455_00199       -------
FC88X_00823_00934       -------
FC88X_00952_00702       -------
FC88X_00690_01040       -------
FC88X_00299_01132       -------
FC88X_00763_00261       -------
FC88X_01098_00718       -------
FC88X_00699_00972       -------
FC88X_00888_01233       -------
FC88X_00427_00992       -------
FC88X_00210_00880       -------
FC88X_01131_01126       -------
FC88X_00383_01105       -------
FC88X_00525_01222       -------
FC88X_00573_01116       -------
FC88X_00619_01251       -------
FC88X_00841_00164       -------
FC88X_01112_00763       -------
FC88X_00094_00055       -------
FC88X_00054_00445       -------
FC88X_00089_00784       -------
FC88X_00360_01188       -------
FC88X_00536_01143       -------
FC88X_00815_00797       -------
FC88X_00923_01241       -------
FC88X_00979_01164       -------
```

```
FC88X_01130_00614       -------
FC88X_00793_01089       -------
FC88X_00913_01245       T------
FC88X_00984_00744       -------
FC88X_00988_01094       -------
FC88X_01043_01125       -------
FC88X_00231_00120       -------
FC88X_00730_01231       -------
FC88X_01094_00861       -------
FC88X_00889_00938       -------
FC88X_00978_00756       -------
FC88X_00836_00961       -------
FC88X_00007_00085       -------
FC88X_00688_00911       -------
FC88X_00746_00974       -------
FC88X_00869_01097       -------
FC88X_00208_00897       -------
FC88X_00536_01232       -------
FC88X_00108_00214       -------
FC88X_00240_00528       -------
FC88X_00415_01071       -------
FC88X_00598_00624       -------
FC88X_00578_01055       -------
FC88X_00607_00915       -------
FC88X_01126_00533       -------
FC88X_01125_01050       -------
FC88X_00012_00410       -------
FC88X_00177_00154       -------
FC88X_00615_00345       -------
FC88X_00799_00332       -------
FC88X_00795_01175       -------
FC88X_00970_00266       -------
FC88X_00988_00481       -------
FC88X_01026_00964       -------
FC88X_01086_01077       -------
FC88X_00413_01106       -------
FC88X_00765_00973       -------
FC88X_00222_00873       -------
FC88X_00147_00803       -------
FC88X_00005_00286       -------
FC88X_00776_00574       -------
FC88X_00023_00627       -------
FC88X_00927_00492       -------
FC88X_00508_00058       -------
FC88X_00770_01268       -------
FC88X_00557_00238       -------
FC88X_01138_00670       -------
FC88X_00826_00326       -------
FC88X_00200_00109       -------
FC88X_00160_00098       -------
FC88X_01083_01208       -------
FC88X_00461_01195       -------
FC88X_01046_01232       -------
FC88X_01132_00539       -------
FC88X_00090_00842       -------
FC88X_00197_00148       -------
FC88X_00636_01218       -------
```

FC88X_00948_00316	-------
FC88X_00846_01154	-------
FC88X_00239_00759	-------
FC88X_00358_00978	-------
FC88X_00167_00728	-------
FC88X_00882_00899	-------
FC88X_00115_00777	-------
FC88X_00663_00559	-------
FC88X_00949_00853	-------
FC88X_00989_01204	-------
FC88X_00155_00753	-------
FC88X_00827_00323	-------
FC88X_00939_00250	-------
FC88X_00859_00780	-------
FC88X_01005_00921	-------
FC88X_00865_00162	-------
FC88X_00273_00466	-------
FC88X_01082_01218	-------
FC88X_00098_00598	-------
FC88X_00464_00966	-------
FC88X_00561_01159	-------
FC88X_00864_00361	-------
FC88X_00553_00133	-------
FC88X_00824_01218	-------
FC88X_00018_00195	-------
FC88X_01140_00821	-------
FC88X_00084_00410	-------
FC88X_00288_00225	-------
FC88X_00073_00051	-------
FC88X_00794_01205	-------
FC88X_00211_01026	-------
FC88X_00371_00018	-------
FC88X_00468_01033	-------
FC88X_00519_00427	-------
FC88X_00824_00244	-------
FC88X_00871_01205	-------
FC88X_00908_00335	-------
FC88X_00967_00853	-------
FC88X_01010_00667	-------
FC88X_00889_01076	-------
FC88X_00715_00865	-------
FC88X_00098_00139	-------
FC88X_00737_01152	-------
FC88X_00441_00730	-------
FC88X_00559_01043	-------
FC88X_00948_00827	-------
FC88X_00578_00607	-------
FC88X_01011_01122	-------
FC88X_00990_00914	-------
FC88X_00557_00638	-------
FC88X_00862_00312	-------
FC88X_01057_01269	-------
FC88X_01104_00802	-------
FC88X_00247_00893	-------
FC88X_00385_00519	-------
FC88X_00767_00514	-------
FC88X_00993_01141	-------

FC88X_00040_00607	-------
FC88X_00393_00757	-------
FC88X_00137_00589	-------
FC88X_00078_00494	-------
FC88X_00495_01035	-------
FC88X_00433_00822	-------
FC88X_00243_00898	-------
FC88X_01102_00909	-------
FC88X_00340_00083	-------
FC88X_00043_00263	-------
FC88X_00412_01210	-------
FC88X_00305_00957	-------
FC88X_00201_00886	-------
FC88X_00657_00071	-------
FC88X_01138_00671	-------
FC88X_00859_01222	-------
FC88X_01143_00926	-------
FC88X_00531_00923	-------
FC88X_00797_00136	-------
FC88X_01112_01086	-------
FC88X_00928_00756	-------
FC88X_00628_00213	-------
FC88X_00835_01042	-------
FC88X_00096_00344	-------
FC88X_00762_01242	-------
FC88X_00340_00383	-------
FC88X_00910_00935	-------
FC88X_00880_00732	-------
FC88X_00452_01062	-------
FC88X_00215_00731	-------
FC88X_01145_01065	-------
FC88X_00939_01063	-------
FC88X_00191_00008	-------
FC88X_00507_00327	-------
FC88X_00085_00452	-------
FC88X_00029_00267	-------
FC88X_00962_00287	-------
FC88X_00228_00907	-------
FC88X_00013_00680	-------
FC88X_00462_01131	-------
FC88X_00408_01003	-------
FC88X_00643_00982	-------
FC88X_00409_00769	-------
FC88X_00010_00139	-------
FC88X_00091_00380	-------
FC88X_01058_01016	-------
FC88X_00927_01262	-------
FC88X_01125_00855	-------
FC88X_00161_00623	-------
FC88X_01124_00561	-------
FC88X_00659_00142	-------
FC88X_01000_00843	-------
FC88X_00993_00446	-------
FC88X_01137_00662	-------
FC88X_00033_00779	-------
FC88X_00172_00077	-------
FC88X_00891_00754	-------

FC88X_00089_00180	-------
FC88X_00164_00150	-------
FC88X_00369_01152	-------
FC88X_00670_01237	-------
FC88X_01077_00439	-------
FC88X_00079_00736	-------
FC88X_00439_01223	-------
FC88X_00464_01129	-------
FC88X_00672_01272	-------
FC88X_00992_00619	-------
FC88X_01115_01271	-------
FC88X_00485_01032	-------
FC88X_00866_00799	-------
FC88X_00032_00087	-------
FC88X_00724_01080	-------
FC88X_00780_01102	-------
FC88X_01129_00912	-------
FC88X_00828_01195	-------
FC88X_00811_01254	-------
FC88X_00954_00958	-------
FC88X_00922_00888	-------
FC88X_01049_00579	-------
FC88X_00351_00947	-------
FC88X_00583_00743	-------
FC88X_01120_00887	-------
FC88X_00097_00295	-------
FC88X_00909_00214	-------
FC88X_00772_00217	-------
FC88X_01118_00986	-------
FC88X_00943_00662	-------
FC88X_01010_00478	-------
FC88X_00536_01060	-------
FC88X_00973_00270	-------
FC88X_00809_01007	-------
FC88X_00160_00094	-------
FC88X_00543_01094	-------
FC88X_00820_00858	-------
FC88X_00853_00990	-------
FC88X_00045_00267	-------
FC88X_00049_00250	-------
FC88X_00045_00714	-------
FC88X_00170_00764	-------
FC88X_00078_00378	-------
FC88X_00159_00613	-------
FC88X_00437_01081	-------
FC88X_00590_00367	-------
FC88X_00779_01182	-------
FC88X_00192_00049	-------
FC88X_00819_01143	-------
FC88X_00218_00733	-------
FC88X_00521_00972	-------
FC88X_00764_00830	-------
FC88X_00852_00327	-------
FC88X_00962_00362	-------
FC88X_00965_00751	-------
FC88X_01008_01149	-------
FC88X_00119_00732	-------

FC88X_00180_00961	-------
FC88X_00210_00917	-------
FC88X_00213_00944	-------
FC88X_00208_01025	-------
FC88X_00213_01080	-------
FC88X_00359_00508	-------
FC88X_00384_01190	-------
FC88X_00601_00735	-------
FC88X_00706_00722	-------
FC88X_00826_00192	-------
FC88X_00811_00230	-------
FC88X_00928_00209	-------
FC88X_00957_00428	-------
FC88X_00967_00569	-------
FC88X_00970_01186	-------
FC88X_01134_00603	-------
FC88X_01112_00846	-------
FC88X_01147_01062	-------
FC88X_00198_00112	-------
FC88X_00166_00822	-------
FC88X_00283_00024	-------
FC88X_00409_01218	-------
FC88X_00956_01088	-------
FC88X_00762_00879	-------
FC88X_00045_00207	-------
FC88X_00495_00018	-------
FC88X_00012_00339	-------
FC88X_00557_00135	-------
FC88X_00588_00292	-------
FC88X_01019_00544	-------
FC88X_01068_00955	-------
FC88X_01079_01141	-------
FC88X_00464_01073	-------
FC88X_01050_01074	-------
FC88X_01054_00781	-------
FC88X_00450_00841	-------
FC88X_00061_00147	-------
FC88X_00860_00277	-------
FC88X_00478_01233	-------
FC88X_00829_00751	-------
FC88X_00149_00693	-------
FC88X_00366_00952	-------
FC88X_00527_00710	-------
FC88X_00614_00921	-------
FC88X_01049_00713	-------
FC88X_01108_00533	-------
FC88X_01108_00671	-------
FC88X_00675_00212	-------
FC88X_00995_01019	-------
FC88X_01140_01048	-------
FC88X_00186_00286	-------
FC88X_00959_00710	-------
FC88X_01041_01001	-------
FC88X_00083_00883	-------
FC88X_00957_00831	-------
FC88X_00111_00407	-------
FC88X_01013_00706	-------

FC88X_01130_00761	-------
FC88X_00011_00558	-------
FC88X_00966_00314	-------
FC88X_00206_00126	-------
FC88X_00511_00026	-------
FC88X_00758_00255	-------
FC88X_00200_00071	-------
FC88X_00911_00560	-------
FC88X_00378_01197	-------
FC88X_00957_00671	-------
FC88X_00955_01052	-------
FC88X_00082_00593	-------
FC88X_01015_00321	-------
FC88X_00054_00143	-------
FC88X_00078_00115	-------
FC88X_00165_00658	-------
FC88X_00493_00695	-------
FC88X_01029_00400	-------
FC88X_00059_00421	-------
FC88X_00062_00034	-------
FC88X_00125_00686	-------
FC88X_00637_01171	-------
FC88X_00933_01199	-------
FC88X_00869_00134	-------
FC88X_00268_00421	-------
FC88X_00831_00140	-------
FC88X_01087_00873	-------
FC88X_00996_00293	-------
FC88X_00737_00941	-------
FC88X_00795_00970	-------
FC88X_00059_00368	-------
FC88X_00241_00014	-------
FC88X_00510_00961	-------
FC88X_00582_01146	-------
FC88X_00443_00842	-------
FC88X_00061_00581	-------
FC88X_00073_00131	-------
FC88X_00120_00464	-------
FC88X_00877_01110	-------
FC88X_00292_01025	-------
FC88X_00170_00894	-------
FC88X_00267_01118	-------
FC88X_00600_00994	-------
FC88X_00013_00598	-------
FC88X_00811_00842	-------
FC88X_00185_00779	-------
FC88X_00857_00242	-------
FC88X_00778_01051	-------
FC88X_01100_01033	-------
FC88X_00302_00545	-------
FC88X_00581_01257	-------
FC88X_00928_00642	-------
FC88X_00195_00886	-------
FC88X_00501_01101	-------
FC88X_01109_00833	-------
FC88X_00398_00076	-------
FC88X_00232_00064	-------

FC88X_00705_00871	-------
FC88X_00102_00260	-------
FC88X_01090_00793	-------
FC88X_01055_00515	-------
FC88X_00374_00669	-------
FC88X_00733_01134	-------
FC88X_00548_01167	-------
FC88X_00947_00755	-------
FC88X_01146_00939	-------
FC88X_00757_01242	-------
FC88X_00089_00173	-------
FC88X_00929_00758	-------
FC88X_00567_00899	-------
FC88X_00726_00539	-------
FC88X_00514_00190	-------
FC88X_00804_00984	-------
FC88X_00909_01248	-------
FC88X_00415_01151	-------
FC88X_00774_01084	-------
FC88X_00149_00809	-------
FC88X_00840_00270	-------
FC88X_00856_00898	-------
FC88X_00439_00988	-------
FC88X_00606_00920	-------
FC88X_00193_00807	-------
FC88X_00201_00543	-------
FC88X_00208_00959	-------
FC88X_00170_00072	-------
FC88X_00730_01235	-------
FC88X_00032_00758	-------
FC88X_00537_00052	-------
FC88X_00234_00958	-------
FC88X_00072_00877	-------
FC88X_00670_00256	-------
FC88X_00705_01234	-------
FC88X_00935_00737	-------
FC88X_00058_00161	-------
FC88X_00932_01260	-------
FC88X_01059_00712	-------
FC88X_00137_00174	-------
FC88X_01015_01059	-------
FC88X_00886_00428	-------
FC88X_00063_00481	-------
FC88X_00665_01243	-------
FC88X_00005_00254	-------
FC88X_00948_00562	-------
FC88X_00879_00738	-------
FC88X_00883_00812	-------
FC88X_01033_00501	-------
FC88X_01094_00741	-------
FC88X_00255_01089	-------
FC88X_00437_01068	-------
FC88X_00496_00336	-------
FC88X_00460_00601	-------
FC88X_00472_01234	-------
FC88X_00539_00717	-------
FC88X_00929_00603	-------

FC88X_00946_01037	-------
FC88X_00942_01233	-------
FC88X_00790_00315	-------
FC88X_00466_00219	-------
FC88X_00105_00243	-------
FC88X_00228_01092	-------
FC88X_00077_00113	-------
FC88X_00120_00069	-------
FC88X_00067_00476	-------
FC88X_00308_00991	-------
FC88X_01007_00438	-------
FC88X_00057_00645	-------
FC88X_00201_00890	-------
FC88X_00388_00970	-------
FC88X_00459_00031	-------
FC88X_00743_01268	-------
FC88X_00928_00975	-------
FC88X_01116_01025	-------
FC88X_00076_00048	-------
FC88X_00129_00705	-------
FC88X_00199_00382	-------
FC88X_00454_01033	-------
FC88X_00665_01261	-------
FC88X_00890_00593	-------
FC88X_00916_00205	-------
FC88X_00930_00507	-------
FC88X_00917_01225	-------
FC88X_00106_00118	-------
FC88X_00531_00478	-------
FC88X_00678_00398	-------
FC88X_00734_01265	-------
FC88X_00089_00139	-------
FC88X_00344_00600	-------
FC88X_00078_00028	-------
FC88X_00402_01066	-------
FC88X_00819_00200	-------
FC88X_00916_01261	-------
FC88X_00688_01216	-------
FC88X_00973_01194	-------
FC88X_00977_00596	-------
FC88X_01065_01084	-------
FC88X_01005_01143	-------
FC88X_00584_01138	-------
FC88X_00924_00832	-------
FC88X_01060_00565	-------
FC88X_00295_00224	-------
FC88X_00499_00406	-------
FC88X_00558_00183	-------
FC88X_00022_00106	-------
FC88X_00026_00327	-------
FC88X_00111_00908	-------
FC88X_00522_01229	-------
FC88X_00917_01229	-------
FC88X_00215_01058	-------
FC88X_00141_00492	-------
FC88X_00726_00875	-------
FC88X_00323_00647	-------

FC88X_00085_00211	-------
FC88X_00984_01196	-------
FC88X_00642_00919	-------
FC88X_00717_00803	-------
FC88X_00013_00091	-------
FC88X_00288_01013	-------
FC88X_00976_00639	-------
FC88X_00567_00942	-------
FC88X_00640_00073	-------
FC88X_00859_00296	-------
FC88X_00329_01153	-------
FC88X_00514_00338	-------
FC88X_00843_00434	-------
FC88X_00953_01069	-------
FC88X_01100_00434	-------
FC88X_00884_00889	-------
FC88X_00586_00188	-------
FC88X_00868_00299	-------
FC88X_00388_00983	-------
FC88X_00944_00535	-------
FC88X_00402_00998	-------
FC88X_00350_01040	-------
FC88X_00967_00383	-------
FC88X_00074_00872	-------
FC88X_00490_00006	-------
FC88X_00989_00896	-------
FC88X_00849_00267	-------
FC88X_00135_00872	-------
FC88X_00083_00317	-------
FC88X_00589_00317	-------
FC88X_01081_00712	-------
FC88X_00928_00320	-------
FC88X_00623_00921	-------
FC88X_00006_00539	-------
FC88X_00908_00426	-------
FC88X_00933_00528	-------
FC88X_00908_00890	-------
FC88X_00342_00698	-------
FC88X_00964_00242	-------
FC88X_00052_00653	-------
FC88X_00029_00410	-------
FC88X_00806_00554	-------
FC88X_00539_00143	-------
FC88X_00037_00288	-------
FC88X_00046_00653	-------
FC88X_00129_00111	-------
FC88X_00962_00365	-------
FC88X_00346_01170	-------
FC88X_00875_00277	-------
FC88X_00948_00325	-------
FC88X_00161_00602	-------
FC88X_00086_00103	-------
FC88X_00159_00564	-------
FC88X_00209_01006	-------
FC88X_00326_01026	-------
FC88X_00182_00568	-------
FC88X_01067_01167	-------

FC88X_00274_00407	-------
FC88X_00417_00091	-------
FC88X_00453_01000	-------
FC88X_00526_00686	-------
FC88X_00602_00274	-------
FC88X_00614_01005	-------
FC88X_00846_00286	-------
FC88X_00547_00145	-------
FC88X_00261_00086	-------
FC88X_00576_00560	-------
FC88X_00634_00929	-------
FC88X_00788_01223	-------
FC88X_00892_00627	-------
FC88X_00921_00468	-------
FC88X_00942_00979	-------
FC88X_00952_00393	-------
FC88X_00981_00403	-------
FC88X_00986_00933	-------
FC88X_01018_00845	-------
FC88X_01061_00376	-------
FC88X_01050_01068	-------
FC88X_01082_01264	-------
FC88X_01142_01227	-------
FC88X_00027_00214	-------
FC88X_00006_00592	-------
FC88X_00031_00619	-------
FC88X_00093_00071	-------
FC88X_00118_00888	-------
FC88X_00163_00560	-------
FC88X_00157_00805	-------
FC88X_00233_00092	-------
FC88X_00245_00591	-------
FC88X_00226_01079	-------
FC88X_00275_00096	-------
FC88X_00290_01107	-------
FC88X_00339_01038	-------
FC88X_00399_01184	-------
FC88X_00429_00863	-------
FC88X_00400_01138	-------
FC88X_00548_01200	-------
FC88X_00595_00220	-------
FC88X_00574_00476	-------
FC88X_00558_01113	-------
FC88X_00582_01178	-------
FC88X_00618_01153	-------
FC88X_00607_01263	-------
FC88X_00733_00859	-------
FC88X_00837_00259	-------
FC88X_00840_00758	-------
FC88X_00860_00885	-------
FC88X_00936_00560	-------
FC88X_00992_00258	-------
FC88X_00972_00805	-------
FC88X_00976_01044	-------
FC88X_00980_01094	-------
FC88X_01010_00336	-------
FC88X_01015_01012	-------

FC88X_01077_00451	-------
FC88X_01075_00779	-------
FC88X_01080_00793	-------
FC88X_01147_00787	-------
FC88X_01128_00815	-------
FC88X_01070_00433	-------
FC88X_00142_00505	-------
FC88X_00266_01099	-------
FC88X_00089_00877	-------
FC88X_00840_00831	-------
FC88X_01108_01038	-------
FC88X_01073_00823	-------
FC88X_00953_01148	-------
FC88X_01004_00551	-------
FC88X_00976_00306	-------
FC88X_00963_00961	-------
FC88X_01081_00985	-------
FC88X_00091_00906	-------
FC88X_00011_00700	-------
FC88X_00784_00255	-------
FC88X_00280_01076	-------
FC88X_00849_00326	-------
FC88X_00643_01033	-------
FC88X_00077_00606	-------
FC88X_00989_01144	-------
FC88X_01081_00436	-------
FC88X_00051_00767	-------
FC88X_00329_00974	-------
FC88X_00781_00850	-------
FC88X_00027_00371	-------
FC88X_00517_01102	-------
FC88X_00115_00583	-------
FC88X_00314_00975	-------
FC88X_00548_00084	-------
FC88X_00991_00489	-------
FC88X_01083_01012	-------
FC88X_00465_00306	-------
FC88X_00942_00291	-------
FC88X_00282_01053	-------
FC88X_00263_01100	-------
FC88X_00964_00981	-------
FC88X_00730_00975	-------
FC88X_00710_01261	-------
FC88X_00030_00708	-------
FC88X_00931_01200	-------
FC88X_00524_01003	-------
FC88X_00092_00026	-------
FC88X_00666_01039	-------
FC88X_00026_00391	-------
FC88X_00431_00990	-------
FC88X_00055_00551	-------
FC88X_00088_00778	-------
FC88X_00231_00050	-------
FC88X_00852_00780	-------
FC88X_00538_00248	-------
FC88X_00747_01213	-------
FC88X_00817_00863	-------

FC88X_00308_01157	-------
FC88X_00358_00843	-------
FC88X_00558_00348	-------
FC88X_00655_00959	-------
FC88X_00127_00128	-------
FC88X_00368_00817	-------
FC88X_00939_00683	-------
FC88X_01060_00837	-------
FC88X_01082_01051	-------
FC88X_01131_00927	-------
FC88X_00161_01013	-------
FC88X_00933_00481	-------
FC88X_00913_00653	-------
FC88X_00511_00061	-------
FC88X_00913_00967	-------
FC88X_01089_01195	-------
FC88X_01075_00560	-------
FC88X_00068_00397	-------
FC88X_01080_00777	-------
FC88X_00647_01064	-------
FC88X_01100_01103	-------
FC88X_00338_00989	-------
FC88X_00891_00257	-------
FC88X_00874_00566	-------
FC88X_01068_00490	-------
FC88X_01094_00427	-------
FC88X_00368_01064	-------
FC88X_00854_00892	-------
FC88X_01000_00679	-------
FC88X_01021_00909	-------
FC88X_00438_00170	-------
FC88X_00973_00678	-------
FC88X_00261_01078	-------
FC88X_00037_00155	-------
FC88X_00624_00076	-------
FC88X_00694_01238	-------
FC88X_00775_00216	-------
FC88X_00795_01212	-------
FC88X_00884_00335	-------
FC88X_00850_00893	-------
FC88X_01041_00490	-------
FC88X_01070_00470	-------
FC88X_00426_00099	-------
FC88X_00735_00508	-------
FC88X_00075_00232	-------
FC88X_00667_01258	-------
FC88X_01075_00924	-------
FC88X_00097_00822	-------
FC88X_00852_01209	-------
FC88X_00172_00926	-------
FC88X_00563_00053	-------
FC88X_00836_00283	-------
FC88X_00883_00807	-------
FC88X_00983_00811	-------
FC88X_00603_01241	-------
FC88X_00856_00405	-------
FC88X_00931_01176	-------

FC88X_00897_00614	-------
FC88X_01076_01206	-------
FC88X_00176_00988	-------
FC88X_00929_00982	-------
FC88X_00369_00263	-------
FC88X_00552_01239	-------
FC88X_00809_00851	-------
FC88X_00499_00351	-------
FC88X_00159_00049	-------
FC88X_01085_00579	-------
FC88X_01003_00605	-------
FC88X_00021_00399	-------
FC88X_00772_00967	-------
FC88X_00904_01242	-------
FC88X_00296_01001	-------
FC88X_01118_01050	-------
FC88X_00522_00105	-------
FC88X_00879_01053	-------
FC88X_00807_00937	-------
FC88X_00953_01030	-------
FC88X_00100_00083	-------
FC88X_00503_01086	-------
FC88X_00039_00488	-------
FC88X_00263_00550	-------
FC88X_00437_00887	-------
FC88X_01132_00865	-------
FC88X_00540_01221	-------
FC88X_00179_00673	-------
FC88X_00081_00033	-------
FC88X_00548_00179	-------
FC88X_00051_00672	-------
FC88X_00684_01246	-------
FC88X_00987_01274	-------
FC88X_00121_00297	-------
FC88X_00695_00674	-------
FC88X_00422_01084	-------
FC88X_00621_00529	-------
FC88X_00980_00660	-------
FC88X_01055_00451	-------
FC88X_01017_00589	-------
FC88X_00064_00807	-------
FC88X_00864_00308	-------
FC88X_00079_00297	-------
FC88X_00063_00571	-------
FC88X_01044_00344	-------
FC88X_01079_00547	-------
FC88X_00789_00872	-------
FC88X_00495_00125	-------
FC88X_00070_00005	-------
FC88X_00252_01078	-------
FC88X_00309_01055	-------
FC88X_00677_00502	-------
FC88X_00378_00988	-------
FC88X_00061_00120	-------
FC88X_01117_00727	-------
FC88X_00237_00198	-------
FC88X_00865_00191	-------

FC88X_00897_01242	-------
FC88X_00491_01247	-------
FC88X_00114_00748	-------
FC88X_00956_00612	-------
FC88X_00957_00737	-------
FC88X_00375_01177	-------
FC88X_00073_00154	-------
FC88X_00768_01175	-------
FC88X_00110_00702	-------
FC88X_00580_00257	-------
FC88X_01041_00444	-------
FC88X_00243_01093	-------
FC88X_00442_00277	-------
FC88X_01115_00754	-------
FC88X_00158_00716	-------
FC88X_00061_00225	-------
FC88X_00817_00260	-------
FC88X_00881_00608	-------
FC88X_01097_00838	-------
FC88X_00085_00251	-------
FC88X_00884_00410	-------
FC88X_00389_01038	-------
FC88X_00155_00614	-------
FC88X_01129_00517	-------
FC88X_01108_00990	-------
FC88X_00263_00652	-------
FC88X_00198_00930	-------
FC88X_00357_01114	-------
FC88X_00447_01206	-------
FC88X_01000_00271	-------
FC88X_00768_00822	-------
FC88X_00044_00021	-------
FC88X_01011_01081	-------
FC88X_00191_00435	-------
FC88X_00584_00156	-------
FC88X_01093_00604	-------
FC88X_00244_00928	-------
FC88X_00745_00185	-------
FC88X_00823_00997	-------
FC88X_00820_01204	-------
FC88X_00748_00485	-------
FC88X_00058_00201	-------
FC88X_00486_01123	-------
FC88X_00711_01159	-------
FC88X_01053_01232	-------
FC88X_00523_00152	-------
FC88X_00315_00012	-------
FC88X_00045_00832	-------
FC88X_00494_00080	-------
FC88X_00795_00972	-------
FC88X_00930_00810	-------
FC88X_01060_01078	-------
FC88X_00090_00860	-------
FC88X_00804_01222	-------
FC88X_00885_00381	-------
FC88X_00894_00751	-------
FC88X_00038_00074	-------

FC88X_01034_00696	-------
FC88X_00233_00155	-------
FC88X_00017_00502	-------
FC88X_00459_01198	-------
FC88X_00069_00171	-------
FC88X_00014_00450	-------
FC88X_00621_00135	-------
FC88X_00672_01137	-------
FC88X_00356_01152	-------
FC88X_00882_00810	-------
FC88X_00569_01165	-------
FC88X_00161_00953	-------
FC88X_00070_00214	-------
FC88X_00035_00298	-------
FC88X_00283_00253	-------
FC88X_00979_00638	-------
FC88X_00894_01077	-------
FC88X_00280_00517	-------
FC88X_00895_00434	-------
FC88X_00995_00844	-------
FC88X_01128_00892	-------
FC88X_00057_00634	-------
FC88X_00618_01234	-------
FC88X_00952_00910	-------
FC88X_00654_00277	-------
FC88X_00137_00558	-------
FC88X_00426_01098	-------
FC88X_00563_00243	-------
FC88X_00582_00461	-------
FC88X_00066_00585	-------
FC88X_00662_01148	-------
FC88X_00274_00457	-------
FC88X_01041_01044	-------
FC88X_00959_01195	-------
FC88X_00561_01191	-------
FC88X_00064_00389	-------
FC88X_00603_00102	-------
FC88X_00047_00284	-------
FC88X_00060_00395	-------
FC88X_00068_00438	-------
FC88X_00433_00122	-------
FC88X_00576_00257	-------
FC88X_00588_00956	-------
FC88X_00998_00736	-------
FC88X_00014_00379	-------
FC88X_00636_00626	-------
FC88X_00700_00132	-------
FC88X_00739_01205	-------
FC88X_00185_01024	-------
FC88X_00735_00328	-------
FC88X_00309_01077	-------
FC88X_00899_01173	-------
FC88X_00447_00371	-------
FC88X_00557_01209	-------
FC88X_00412_00169	-------
FC88X_00982_00616	-------
FC88X_00627_01245	-------

FC88X_00267_00960	-------
FC88X_01068_00855	-------
FC88X_01000_01099	-------
FC88X_00873_00199	-------
FC88X_00099_00543	-------
FC88X_00109_00337	-------
FC88X_00273_00936	-------
FC88X_00301_00474	-------
FC88X_00768_00963	-------
FC88X_00188_00477	-------
FC88X_00933_00576	-------
FC88X_00593_01219	-------
FC88X_00984_01267	-------
FC88X_00092_00106	-------
FC88X_00986_01089	-------
FC88X_00953_00653	-------
FC88X_00622_01216	-------
FC88X_00693_00155	-------
FC88X_00968_01111	-------
FC88X_00029_00293	-------
FC88X_01033_00709	-------
FC88X_01117_01154	-------
FC88X_01084_00855	-------
FC88X_00267_00588	-------
FC88X_00333_00403	-------
FC88X_01054_01175	-------
FC88X_00156_00995	-------
FC88X_00024_00081	-------
FC88X_00027_00733	-------
FC88X_00855_01240	-------
FC88X_01115_00824	-------
FC88X_00505_00645	-------
FC88X_00524_00155	-------
FC88X_00573_00118	-------
FC88X_00844_00715	-------
FC88X_00544_00712	-------
FC88X_00324_01012	-------
FC88X_00466_01163	-------
FC88X_00197_00856	-------
FC88X_00490_00077	-------
FC88X_01119_00816	-------
FC88X_00577_00912	-------
FC88X_00912_00988	-------
FC88X_00516_00733	-------
FC88X_00798_00211	-------
FC88X_01094_00673	-------
FC88X_00031_00542	-------
FC88X_00037_00162	-------
FC88X_00143_00023	-------
FC88X_00972_01174	-------
FC88X_00501_00064	-------
FC88X_00679_00254	-------
FC88X_00854_00834	-------
FC88X_00370_00690	-------
FC88X_00025_00683	-------
FC88X_01043_01017	-------
FC88X_01028_00339	-------

FC88X_01009_00685	-------
FC88X_00354_00574	-------
FC88X_00449_00095	-------
FC88X_00785_00808	-------
FC88X_00094_00574	-------
FC88X_00450_00008	-------
FC88X_00047_00812	-------
FC88X_01004_00480	-------
FC88X_00059_00601	-------
FC88X_00681_00992	-------
FC88X_00111_00030	-------
FC88X_01058_01000	-------
FC88X_01075_01246	-------
FC88X_00879_00680	-------
FC88X_00890_00301	-------
FC88X_00847_00293	-------
FC88X_00431_00126	-------
FC88X_00055_00064	-------
FC88X_00056_00765	-------
FC88X_00283_00783	-------
FC88X_00236_00006	-------
FC88X_00533_00386	-------
FC88X_00173_00066	-------
FC88X_00647_00961	-------
FC88X_00733_00538	-------
FC88X_00718_01212	-------
FC88X_00842_00201	-------
FC88X_00134_00801	-------
FC88X_01030_01177	-------
FC88X_00277_00078	-------
FC88X_00970_00812	-------
FC88X_00874_00756	-------
FC88X_00458_00095	-------
FC88X_01042_01049	-------
FC88X_00730_01050	-------
FC88X_01084_00407	-------
FC88X_00108_00511	-------
FC88X_00832_01137	-------
FC88X_01025_00639	-------
FC88X_01013_01040	-------
FC88X_00485_00534	-------
FC88X_00070_00628	-------
FC88X_00941_00391	-------
FC88X_00079_00145	-------
FC88X_00650_01038	-------
FC88X_00965_00244	-------
FC88X_00967_00786	-------
FC88X_00488_01106	-------
FC88X_00176_00885	-------
FC88X_00736_00843	-------
FC88X_00156_00163	-------
FC88X_00931_00975	-------
FC88X_00657_01101	-------
FC88X_00943_01177	-------
FC88X_00451_00133	-------
FC88X_01000_00423	-------
FC88X_01080_00549	-------

FC88X_00124_00889	-------
FC88X_00883_00415	-------
FC88X_00179_00825	-------
FC88X_00978_00816	-------
FC88X_00710_01090	-------
FC88X_00150_00080	-------
FC88X_01061_00488	-------
FC88X_00945_01169	-------
FC88X_00951_00936	-------
FC88X_01134_00721	-------
FC88X_00918_00488	-------
FC88X_01071_01071	-------
FC88X_00248_00077	-------
FC88X_00347_00639	-------
FC88X_00660_00913	-------
FC88X_00311_00004	-------
FC88X_01013_01213	-------
FC88X_00153_00614	-------
FC88X_00657_00383	-------
FC88X_00246_00033	-------
FC88X_01012_00533	-------
FC88X_00881_00614	-------
FC88X_00375_00718	-------
FC88X_01067_00652	-------
FC88X_00911_00545	-------
FC88X_00984_00382	-------
FC88X_00903_00785	-------
FC88X_00957_00358	-------
FC88X_00557_00666	-------
FC88X_00663_00546	-------
FC88X_00978_00690	-------
FC88X_01014_01217	-------
FC88X_00137_00482	-------
FC88X_00158_00949	-------
FC88X_00550_00753	-------
FC88X_00031_00212	-------
FC88X_00110_00395	-------
FC88X_01122_00872	-------
FC88X_00575_01095	-------
FC88X_00995_01023	-------
FC88X_00670_00928	-------
FC88X_00843_00927	-------
FC88X_00997_00544	-------
FC88X_00145_00019	-------
FC88X_00324_00300	-------
FC88X_00495_00809	-------
FC88X_00616_01236	-------
FC88X_00998_01148	-------
FC88X_00374_00033	-------
FC88X_00487_00262	-------
FC88X_00543_00637	-------
FC88X_00818_00558	-------
FC88X_00879_01197	-------
FC88X_00861_00163	-------
FC88X_00076_00310	-------
FC88X_00695_01036	-------
FC88X_00279_01121	-------

FC88X_01030_00648	-------
FC88X_00714_01173	-------
FC88X_00970_01260	-------
FC88X_00785_01182	-------
FC88X_01106_01174	-------
FC88X_00798_01239	-------
FC88X_01069_00962	-------
FC88X_00786_00101	-------
FC88X_01136_01052	-------
FC88X_01056_00997	-------
FC88X_01082_00764	-------
FC88X_00743_00880	-------
FC88X_00312_00498	-------
FC88X_00659_00165	-------
FC88X_00679_01042	-------
FC88X_00392_00079	-------
FC88X_00360_01135	-------
FC88X_00964_00715	-------
FC88X_01124_00649	-------
FC88X_00143_00529	-------
FC88X_01132_00762	-------
FC88X_00153_00933	-------
FC88X_00988_00926	-------
FC88X_00415_00809	-------
FC88X_01026_00858	-------
FC88X_01115_00782	-------
FC88X_00069_00129	-------
FC88X_00224_00945	-------
FC88X_00278_00560	-------
FC88X_00405_00113	-------
FC88X_00510_01015	-------
FC88X_00884_00349	-------
FC88X_00923_00417	-------
FC88X_01096_00978	-------
FC88X_01122_00694	-------
FC88X_01140_01084	-------
FC88X_00991_00592	-------
FC88X_00572_00201	-------
FC88X_00834_00382	-------
FC88X_00604_01043	-------
FC88X_01093_01221	-------
FC88X_00112_00435	-------
FC88X_00066_00061	-------
FC88X_00981_01067	-------
FC88X_00430_00988	-------
FC88X_00476_00175	-------
FC88X_00357_01141	-------
FC88X_01049_00509	-------
FC88X_00633_01240	-------
FC88X_01074_00829	-------
FC88X_00011_00424	-------
FC88X_00161_00718	-------
FC88X_00091_00791	-------
FC88X_00056_00854	-------
FC88X_00218_00007	-------
FC88X_00402_00894	-------
FC88X_00636_01036	-------

FC88X_00787_00675	-------
FC88X_00092_00188	-------
FC88X_00103_00726	-------
FC88X_00181_00808	-------
FC88X_00201_00136	-------
FC88X_00224_00904	-------
FC88X_00400_01011	-------
FC88X_00486_01197	-------
FC88X_00506_01240	-------
FC88X_00683_01078	-------
FC88X_00794_01226	-------
FC88X_00895_00247	-------
FC88X_00962_00733	-------
FC88X_01079_00638	-------
FC88X_01098_00653	-------
FC88X_01116_01159	-------
FC88X_00596_01235	-------
FC88X_00205_00830	-------
FC88X_00217_00980	-------
FC88X_00116_00815	-------
FC88X_00076_00167	-------
FC88X_00936_00757	-------
FC88X_00023_00667	-------
FC88X_00860_00873	-------
FC88X_00883_01242	-------
FC88X_00983_00461	-------
FC88X_00918_01133	-------
FC88X_00089_00254	-------
FC88X_00800_00796	-------
FC88X_00163_00892	-------
FC88X_01139_00705	-------
FC88X_01065_00820	-------
FC88X_00890_00841	-------
FC88X_00990_00810	-------
FC88X_00971_00449	-------
FC88X_00442_00642	-------
FC88X_00200_00439	-------
FC88X_00047_00126	-------
FC88X_00829_00792	-------
FC88X_00658_01176	-------
FC88X_00699_00166	-------
FC88X_00335_01126	-------
FC88X_00775_00818	-------
FC88X_00777_01137	-------
FC88X_01027_00537	-------
FC88X_00199_00146	-------
FC88X_01132_00943	-------
FC88X_01065_00971	-------
FC88X_00902_00988	-------
FC88X_01012_01028	-------
FC88X_00505_01167	-------
FC88X_00841_01232	-------
FC88X_00103_00191	-------
FC88X_00877_00761	-------
FC88X_00893_00998	-------
FC88X_00497_00552	-------
FC88X_00164_00820	-------

FC88X_01140_00637	-------
FC88X_00886_00745	-------
FC88X_01067_00955	-------
FC88X_00575_00986	-------
FC88X_00736_01159	-------
FC88X_01116_00925	-------
FC88X_00657_01107	-------
FC88X_00891_00712	-------
FC88X_00021_00326	-------
FC88X_00720_01243	-------
FC88X_00090_00320	-------
FC88X_00829_01010	-------
FC88X_00625_01023	-------
FC88X_00903_00966	-------
FC88X_01047_00581	-------
FC88X_00912_00923	-------
FC88X_00987_01215	-------
FC88X_00506_00059	-------
FC88X_00970_00685	-------
FC88X_00122_00074	-------
FC88X_00066_00121	-------
FC88X_00572_00684	-------
FC88X_00029_00576	-------
FC88X_00115_00303	-------
FC88X_01091_00708	-------
FC88X_01131_00687	-------
FC88X_00089_00778	-------
FC88X_00824_00906	-------
FC88X_00704_00080	-------
FC88X_00372_01014	-------
FC88X_00925_00559	-------
FC88X_01005_00515	-------
FC88X_01046_01064	-------
FC88X_00634_01023	-------
FC88X_00909_00754	-------
FC88X_00072_00858	-------
FC88X_00612_00415	-------
FC88X_00787_00818	-------
FC88X_00112_00614	-------
FC88X_00911_00195	-------
FC88X_00057_00031	-------
FC88X_00131_00765	-------
FC88X_00608_01230	-------
FC88X_00852_01018	-------
FC88X_00370_01114	-------
FC88X_00925_00292	-------
FC88X_00969_00387	-------
FC88X_00188_00982	-------
FC88X_00879_00341	-------
FC88X_00531_01208	-------
FC88X_00213_01070	-------
FC88X_00957_00645	-------
FC88X_00402_01086	-------
FC88X_00855_00779	-------
FC88X_00506_01016	-------
FC88X_00102_00466	-------
FC88X_00407_01083	-------

FC88X_00272_00727	-------
FC88X_00226_00954	-------
FC88X_00735_00462	-------
FC88X_01108_00865	-------
FC88X_00919_00892	-------
FC88X_00437_01139	-------
FC88X_00946_00254	-------
FC88X_00759_00124	-------
FC88X_01072_00930	-------
FC88X_00752_01004	-------
FC88X_00680_00611	-------
FC88X_00839_00975	-------
FC88X_00206_01003	-------
FC88X_00520_00454	-------
FC88X_00553_00833	-------
FC88X_01006_01096	-------
FC88X_01086_01075	-------
FC88X_01028_00303	-------
FC88X_00062_00120	-------
FC88X_00152_00659	-------
FC88X_00025_00174	-------
FC88X_00375_01175	-------
FC88X_00063_00808	-------
FC88X_00639_00970	-------
FC88X_00425_00951	-------
FC88X_00827_01030	-------
FC88X_00572_01114	-------
FC88X_00827_00741	-------
FC88X_00843_01254	-------
FC88X_00681_01255	-------
FC88X_00678_00941	-------
FC88X_00150_00947	-------
FC88X_00779_00239	-------
FC88X_00050_00479	-------
FC88X_00063_00745	-------
FC88X_00063_00342	-------
FC88X_00787_00599	-------
FC88X_00935_00847	-------
FC88X_00820_00979	-------
FC88X_00345_01149	-------
FC88X_00551_00190	-------
FC88X_01003_00811	-------
FC88X_00984_00325	-------
FC88X_01116_00597	-------
FC88X_00616_00792	-------
FC88X_00113_00506	-------
FC88X_00977_00975	-------
FC88X_00170_00865	-------
FC88X_00102_00064	-------
FC88X_00781_00348	-------
FC88X_00630_00982	-------
FC88X_00076_00589	-------
FC88X_00181_00913	-------
FC88X_00217_00244	-------
FC88X_00873_00244	-------
FC88X_00806_01205	-------
FC88X_00899_01267	-------

FC88X_00156_00876	-------
FC88X_00598_00940	-------
FC88X_00817_01267	-------
FC88X_00161_00878	-------
FC88X_00573_00287	-------
FC88X_01059_00744	-------
FC88X_00509_00091	-------
FC88X_00928_00549	-------
FC88X_00704_01027	-------
FC88X_00839_00789	-------
FC88X_00924_00595	-------
FC88X_00935_01090	-------
FC88X_01070_00544	-------
FC88X_00034_00523	-------
FC88X_00065_00206	-------
FC88X_00082_00365	-------
FC88X_00080_00545	-------
FC88X_00128_00302	-------
FC88X_00109_00869	-------
FC88X_00230_00906	-------
FC88X_00299_00533	-------
FC88X_00413_00080	-------
FC88X_00450_01111	-------
FC88X_00624_00339	-------
FC88X_00768_01038	-------
FC88X_00755_01089	-------
FC88X_00846_00791	-------
FC88X_00867_00828	-------
FC88X_00900_00287	-------
FC88X_00937_00472	-------
FC88X_00902_01215	-------
FC88X_00924_01251	-------
FC88X_01064_00945	-------
FC88X_00447_01163	-------
FC88X_01085_00758	-------
FC88X_01008_00640	-------
FC88X_00667_00523	-------
FC88X_00802_00895	-------
FC88X_00250_00481	-------
FC88X_00916_00443	-------
FC88X_00417_00047	-------
FC88X_00084_00879	-------
FC88X_00055_00792	-------
FC88X_00926_00527	-------
FC88X_00050_00264	-------
FC88X_00491_00401	-------
FC88X_00166_00794	-------
FC88X_00155_00184	-------
FC88X_01124_01113	-------
FC88X_01123_00931	-------
FC88X_00189_00421	-------
FC88X_00847_00315	-------
FC88X_00525_00499	-------
FC88X_01030_01152	-------
FC88X_00552_01012	-------
FC88X_00870_01201	-------
FC88X_00893_00853	-------

FC88X_00355_01118	-------
FC88X_00191_00853	-------
FC88X_00645_01124	-------
FC88X_01075_00404	-------
FC88X_00070_00094	-------
FC88X_00812_00186	-------
FC88X_00481_00081	-------
FC88X_01011_00886	-------
FC88X_00069_00087	-------
FC88X_00923_00624	-------
FC88X_00959_00863	-------
FC88X_00599_00996	-------
FC88X_00189_00998	-------
FC88X_00017_00704	-------
FC88X_00740_00066	-------
FC88X_00538_01255	-------
FC88X_00039_00374	-------
FC88X_00497_00993	-------
FC88X_00079_00313	-------
FC88X_00083_00808	-------
FC88X_00377_01168	-------
FC88X_00976_01185	-------
FC88X_00240_00081	-------
FC88X_01089_00661	-------
FC88X_00467_01221	-------
FC88X_00797_01062	-------
FC88X_00911_01230	-------
FC88X_00414_00078	-------
FC88X_01140_00900	-------
FC88X_00478_00271	-------
FC88X_00810_00162	-------
FC88X_01125_00764	-------
FC88X_00335_00690	-------
FC88X_00042_00342	-------
FC88X_00065_00059	-------
FC88X_00357_00652	-------
FC88X_00380_01152	-------
FC88X_00695_01253	-------
FC88X_00066_00883	-------
FC88X_00169_00886	-------
FC88X_00407_00822	-------
FC88X_00671_00216	-------
FC88X_00680_01087	-------
FC88X_00915_01192	-------
FC88X_01046_00573	-------
FC88X_01142_00618	-------
FC88X_00100_00054	-------
FC88X_00988_01179	-------
FC88X_00086_00082	-------
FC88X_00676_00082	-------
FC88X_00052_00644	-------
FC88X_00171_00039	-------
FC88X_00151_00071	-------
FC88X_00278_00377	-------
FC88X_00278_00975	-------
FC88X_00457_00174	-------
FC88X_00564_00661	-------

FC88X_00593_01235	-------
FC88X_00614_00313	-------
FC88X_00616_00976	-------
FC88X_00714_01059	-------
FC88X_00946_00738	-------
FC88X_00946_01222	-------
FC88X_00950_00238	-------
FC88X_00977_00232	-------
FC88X_00981_01053	-------
FC88X_01008_00711	-------
FC88X_01102_00795	-------
FC88X_01147_00758	-------
FC88X_00051_00203	-------
FC88X_00108_00553	-------
FC88X_00116_00924	-------
FC88X_00168_00040	-------
FC88X_00161_00655	-------
FC88X_00337_00585	-------
FC88X_00337_01025	-------
FC88X_00389_00496	-------
FC88X_00382_01152	-------
FC88X_00448_01080	-------
FC88X_00542_01119	-------
FC88X_00502_01178	-------
FC88X_00597_01138	-------
FC88X_00618_00961	-------
FC88X_00691_00361	-------
FC88X_00739_00247	-------
FC88X_00710_01091	-------
FC88X_00769_01016	-------
FC88X_00819_00242	-------
FC88X_00876_01234	-------
FC88X_00901_00711	-------
FC88X_00964_00980	-------
FC88X_01000_00461	-------
FC88X_01020_00736	-------
FC88X_01048_00813	-------
FC88X_01006_01029	-------
FC88X_01093_01011	-------
FC88X_01118_00518	-------
FC88X_00102_00129	-------
FC88X_00006_00596	-------
FC88X_00935_00414	-------
FC88X_00811_01027	-------
FC88X_00961_00646	-------
FC88X_00650_00153	-------
FC88X_00521_00050	-------
FC88X_01037_00325	-------
FC88X_01042_01238	-------
FC88X_00518_00180	-------
FC88X_00571_01009	-------
FC88X_01058_00353	-------
FC88X_00141_00886	-------
FC88X_00776_00116	-------
FC88X_00478_00031	-------
FC88X_00906_00486	-------
FC88X_00181_00760	-------

FC88X_00703_01207	-------
FC88X_00943_00602	-------
FC88X_00552_01038	-------
FC88X_01063_01023	-------
FC88X_01074_00864	-------
FC88X_01032_00491	-------
FC88X_00034_00375	-------
FC88X_00398_00852	-------
FC88X_00694_00466	-------
FC88X_00653_01000	-------
FC88X_01119_00867	-------
FC88X_01071_00754	-------
FC88X_00562_01169	-------
FC88X_00173_00797	-------
FC88X_00249_01113	-------
FC88X_00785_00954	-------
FC88X_00547_01198	-------
FC88X_00082_00308	-------
FC88X_00521_00510	-------
FC88X_00598_00739	-------
FC88X_00129_00644	-------
FC88X_00118_00871	-------
FC88X_00534_00236	-------
FC88X_00524_00095	-------
FC88X_00797_00862	-------
FC88X_00363_00364	-------
FC88X_00456_01143	-------
FC88X_00438_00705	-------
FC88X_00834_00901	-------
FC88X_00349_00669	-------
FC88X_00300_01160	-------
FC88X_00337_01136	-------
FC88X_00648_01004	-------
FC88X_00722_01250	-------
FC88X_00171_00837	-------
FC88X_00830_00195	-------
FC88X_00009_00499	-------
FC88X_00446_01181	-------
FC88X_00986_01018	-------
FC88X_00249_00947	-------
FC88X_00360_00135	-------
FC88X_00891_00368	-------
FC88X_00950_00823	-------
FC88X_00672_00175	-------
FC88X_00463_01039	-------
FC88X_01011_00402	-------
FC88X_00836_01263	-------
FC88X_00644_00668	-------
FC88X_01120_01174	-------
FC88X_00447_01027	-------
FC88X_01032_00863	-------
FC88X_00570_00614	-------
FC88X_00634_01102	-------
FC88X_00392_00628	-------
FC88X_00607_01216	-------
FC88X_00739_00258	-------
FC88X_01091_00704	-------

FC88X_00899_00937	-------
FC88X_00304_00761	-------
FC88X_00536_01217	-------
FC88X_01079_01077	-------
FC88X_00807_01200	-------
FC88X_00120_00406	-------
FC88X_00112_00722	-------
FC88X_00900_01197	-------
FC88X_00158_00939	-------
FC88X_00107_00404	-------
FC88X_00713_01212	-------
FC88X_00871_01249	-------
FC88X_00119_00223	-------
FC88X_00839_01147	-------
FC88X_00936_00286	-------
FC88X_00868_00886	-------
FC88X_00880_01032	-------
FC88X_00130_00743	-------
FC88X_00835_01041	-------
FC88X_00864_00849	-------
FC88X_00337_01044	-------
FC88X_00854_00766	-------
FC88X_00467_00229	-------
FC88X_00397_01101	-------
FC88X_00490_00027	-------
FC88X_00604_00363	-------
FC88X_01078_01087	-------
FC88X_00326_00321	-------
FC88X_01123_00936	-------
FC88X_00633_01192	-------
FC88X_01105_00632	-------
FC88X_00932_00759	-------
FC88X_00631_00350	-------
FC88X_00643_01203	-------
FC88X_01110_00764	-------
FC88X_00719_00968	-------
FC88X_00655_00225	-------
FC88X_00974_00511	-------
FC88X_00154_00705	-------
FC88X_00290_00697	-------
FC88X_00871_01261	-------
FC88X_00364_00369	-------
FC88X_00603_00385	-------
FC88X_00195_00996	-------
FC88X_00026_00056	-------
FC88X_00023_00638	-------
FC88X_00028_00168	-------
FC88X_00127_00761	-------
FC88X_00700_01149	-------
FC88X_00077_00531	-------
FC88X_01002_01170	-------
FC88X_00573_00431	-------
FC88X_00876_00788	-------
FC88X_01044_00798	-------
FC88X_01114_01095	-------
FC88X_00235_00093	-------
FC88X_00475_00081	-------

FC88X_00818_00098	-------
FC88X_00909_01227	-------
FC88X_01050_00837	-------
FC88X_00099_00157	-------
FC88X_00050_00495	-------
FC88X_00109_00276	-------
FC88X_00151_00617	-------
FC88X_00199_00703	-------
FC88X_00250_00969	-------
FC88X_00254_01069	-------
FC88X_00357_00594	-------
FC88X_00462_01031	-------
FC88X_00675_00413	-------
FC88X_00800_00208	-------
FC88X_00802_00213	-------
FC88X_00837_00867	-------
FC88X_00822_01076	-------
FC88X_00936_00325	-------
FC88X_00929_01086	-------
FC88X_00951_01221	-------
FC88X_01023_01016	-------
FC88X_01100_01086	-------
FC88X_00274_00926	-------
FC88X_00825_01038	-------
FC88X_00197_00985	-------
FC88X_00890_00228	-------
FC88X_00181_00722	-------
FC88X_00783_01232	-------
FC88X_00915_00571	-------
FC88X_00284_00081	-------
FC88X_00958_00892	-------
FC88X_00716_00717	-------
FC88X_00930_00779	-------
FC88X_00781_00183	-------
FC88X_00972_00658	-------
FC88X_00072_00751	-------
FC88X_00346_00528	-------
FC88X_00616_01151	-------
FC88X_01096_00833	-------
FC88X_01085_01081	-------
FC88X_01069_00620	-------
FC88X_00214_00016	-------
FC88X_00233_00963	-------
FC88X_00021_00623	-------
FC88X_00059_00210	-------
FC88X_00185_00854	-------
FC88X_00323_00355	-------
FC88X_00446_00945	-------
FC88X_00421_01026	-------
FC88X_00463_00382	-------
FC88X_00502_01201	-------
FC88X_00771_00101	-------
FC88X_00822_00894	-------
FC88X_01042_00360	-------
FC88X_01045_00559	-------
FC88X_01023_00776	-------
FC88X_00127_00536	-------

FC88X_00138_00727	-------
FC88X_00231_00630	-------
FC88X_00212_00877	-------
FC88X_00362_00456	-------
FC88X_00567_00213	-------
FC88X_00636_01072	-------
FC88X_00842_00269	-------
FC88X_00823_00894	-------
FC88X_00931_00358	-------
FC88X_00937_01086	-------
FC88X_01033_00783	-------
FC88X_01038_01013	-------
FC88X_01054_00522	-------
FC88X_01121_00786	-------
FC88X_01102_01048	-------
FC88X_01122_01085	-------
FC88X_00153_00824	-------
FC88X_00159_00897	-------
FC88X_00506_00054	-------
FC88X_00118_00008	-------
FC88X_00758_00785	-------
FC88X_00604_00932	-------
FC88X_00605_00246	-------
FC88X_00738_00906	-------
FC88X_01114_00735	-------
FC88X_01089_01253	-------
FC88X_00407_01040	-------
FC88X_00232_01068	-------
FC88X_00097_00085	-------
FC88X_00728_01057	-------
FC88X_00142_00115	-------
FC88X_00541_00215	-------
FC88X_00121_00087	-------
FC88X_00437_00039	-------
FC88X_00545_00282	-------
FC88X_00837_01110	-------
FC88X_00095_00877	-------
FC88X_01074_00825	-------
FC88X_00032_00800	-------
FC88X_00149_00590	-------
FC88X_00353_01089	-------
FC88X_00031_00424	-------
FC88X_00476_00800	-------
FC88X_01146_00861	-------
FC88X_00654_01197	-------
FC88X_00813_00155	-------
FC88X_00171_00828	-------
FC88X_00144_00089	-------
FC88X_00575_00118	-------
FC88X_00687_00507	-------
FC88X_00027_00222	-------
FC88X_00477_00959	-------
FC88X_00772_00133	-------
FC88X_00970_00360	-------
FC88X_00062_00188	-------
FC88X_00258_01055	-------
FC88X_00513_01056	-------

FC88X_00826_00332	-------
FC88X_00027_00176	-------
FC88X_00944_00766	-------
FC88X_00261_00900	-------
FC88X_00897_00585	-------
FC88X_01114_01042	-------
FC88X_00777_00887	-------
FC88X_00120_00008	-------
FC88X_00096_00158	-------
FC88X_00171_00074	-------
FC88X_00936_01219	-------
FC88X_00646_00084	-------
FC88X_01002_00858	-------
FC88X_00902_00891	-------
FC88X_00366_01175	-------
FC88X_00120_00097	-------
FC88X_00765_01219	-------
FC88X_00936_00773	-------
FC88X_00067_00117	-------
FC88X_00167_00054	-------
FC88X_00574_00242	-------
FC88X_01017_01219	-------
FC88X_01128_01188	-------
FC88X_00778_00457	-------
FC88X_00522_00965	-------
FC88X_00376_00763	-------
FC88X_00396_00075	-------
FC88X_00467_00971	-------
FC88X_01005_00872	-------
FC88X_00873_00525	-------
FC88X_00906_00657	-------
FC88X_00972_01046	-------
FC88X_01056_00853	-------
FC88X_00149_00750	-------
FC88X_00442_00027	-------
FC88X_00788_00860	-------
FC88X_01077_00800	-------
FC88X_00762_00859	-------
FC88X_00897_00834	-------
FC88X_01101_00903	-------
FC88X_00108_00111	-------
FC88X_00788_00112	-------
FC88X_00111_00345	-------
FC88X_00568_01077	-------
FC88X_00774_01103	-------
FC88X_00994_00808	-------
FC88X_00036_00166	-------
FC88X_00304_01044	-------
FC88X_00956_00356	-------
FC88X_00983_00398	-------
FC88X_01108_00616	-------
FC88X_00032_00731	-------
FC88X_00224_01068	-------
FC88X_00812_00842	-------
FC88X_00186_00984	-------
FC88X_00532_00146	-------
FC88X_00965_00895	-------

FC88X_00978_00560	-------
FC88X_01070_00834	-------
FC88X_00395_01002	-------
FC88X_00100_00535	-------
FC88X_00646_01038	-------
FC88X_00055_00015	-------
FC88X_00180_00417	-------
FC88X_00397_01149	-------
FC88X_01076_00826	-------
FC88X_00326_00941	-------
FC88X_00556_00604	-------
FC88X_00651_01231	-------
FC88X_00449_00051	-------
FC88X_00434_01064	-------
FC88X_00883_01090	-------
FC88X_00422_00046	-------
FC88X_00564_00086	-------
FC88X_00745_01150	-------
FC88X_00208_00037	-------
FC88X_00534_00233	-------
FC88X_00039_00503	-------
FC88X_00097_00119	-------
FC88X_00564_00490	-------
FC88X_00810_01187	-------
FC88X_00786_01007	-------
FC88X_00822_00247	-------
FC88X_01094_00468	-------
FC88X_00883_01017	-------
FC88X_01090_00660	-------
FC88X_00995_01098	-------
FC88X_00350_01147	-------
FC88X_00436_00493	-------
FC88X_00943_00297	-------
FC88X_00159_00044	-------
FC88X_00964_00311	-------
FC88X_00812_00860	-------
FC88X_00975_01098	-------
FC88X_00066_00137	-------
FC88X_01003_00633	-------
FC88X_00517_01185	-------
FC88X_01110_00905	-------
FC88X_00163_00844	-------
FC88X_00269_00744	-------
FC88X_00782_00820	-------
FC88X_00391_00015	-------
FC88X_00416_00307	-------
FC88X_00637_00789	-------
FC88X_00928_01028	-------
FC88X_00892_01247	-------
FC88X_01000_00883	-------
FC88X_00107_00491	-------
FC88X_00593_01222	-------
FC88X_00975_00852	-------
FC88X_00942_00444	-------
FC88X_00974_00544	-------
FC88X_00121_00092	-------
FC88X_00244_01028	-------

FC88X_00426_00581	-------
FC88X_00811_00148	-------
FC88X_00898_00769	-------
FC88X_00039_00367	-------
FC88X_00511_00642	-------
FC88X_00657_00083	-------
FC88X_00855_01015	-------
FC88X_01072_00659	-------
FC88X_00729_00741	-------
FC88X_00967_00478	-------
FC88X_00139_00733	-------
FC88X_01091_00940	-------
FC88X_00955_00785	-------
FC88X_00140_00972	-------
FC88X_00506_00114	-------
FC88X_00934_00711	-------
FC88X_00531_01255	-------
FC88X_00630_00931	-------
FC88X_00632_01153	-------
FC88X_00816_01035	-------
FC88X_00885_00891	-------
FC88X_00953_00452	-------
FC88X_01136_00869	-------
FC88X_01077_00627	-------
FC88X_00586_00313	-------
FC88X_00418_00685	-------
FC88X_01113_00849	-------
FC88X_00159_00779	-------
FC88X_00320_00767	-------
FC88X_01143_00892	-------
FC88X_00927_00708	-------
FC88X_00807_00165	-------
FC88X_00579_00067	-------
FC88X_00858_00749	-------
FC88X_00025_00477	-------
FC88X_00827_00840	-------
FC88X_00955_01267	-------
FC88X_00407_00968	-------
FC88X_00698_00229	-------
FC88X_00319_01168	-------
FC88X_00048_00601	-------
FC88X_00455_00512	-------
FC88X_01043_00579	-------
FC88X_00453_00292	-------
FC88X_00074_00720	-------
FC88X_00845_00865	-------
FC88X_00224_00892	-------
FC88X_00884_00404	-------
FC88X_00349_00618	-------
FC88X_00973_00669	-------
FC88X_00589_01101	-------
FC88X_00050_00428	-------
FC88X_00115_00802	-------
FC88X_00216_00404	-------
FC88X_00621_00921	-------
FC88X_01089_00853	-------
FC88X_00876_01172	-------

FC88X_01049_00719	-------
FC88X_00559_00211	-------
FC88X_00346_01004	-------
FC88X_00427_01037	-------
FC88X_00944_01035	-------
FC88X_00439_00119	-------
FC88X_00009_00233	-------
FC88X_00097_00209	-------
FC88X_00416_00659	-------
FC88X_00029_00207	-------
FC88X_00956_00978	-------
FC88X_00051_00401	-------
FC88X_00095_00581	-------
FC88X_00251_00142	-------
FC88X_00697_00661	-------
FC88X_00883_00306	-------
FC88X_01100_00691	-------
FC88X_01130_01033	-------
FC88X_00025_00141	-------
FC88X_00030_00260	-------
FC88X_00054_00148	-------
FC88X_00071_00314	-------
FC88X_00119_00728	-------
FC88X_00209_01050	-------
FC88X_00349_00180	-------
FC88X_00442_01055	-------
FC88X_00706_00067	-------
FC88X_00857_00696	-------
FC88X_00910_00847	-------
FC88X_01068_00820	-------
FC88X_01077_00907	-------
FC88X_00155_00783	-------
FC88X_00463_01194	-------
FC88X_00454_01212	-------
FC88X_00769_01066	-------
FC88X_00912_00457	-------
FC88X_00566_00222	-------
FC88X_00658_00129	-------
FC88X_00874_01239	-------
FC88X_00283_01053	-------
FC88X_00923_01266	-------
FC88X_00573_00408	-------
FC88X_00643_01063	-------
FC88X_00291_01087	-------
FC88X_00883_00419	-------
FC88X_00492_01200	-------
FC88X_00922_00430	-------
FC88X_01139_00652	-------
FC88X_00674_00298	-------
FC88X_00709_00548	-------
FC88X_00879_01086	-------
FC88X_01044_00762	-------
FC88X_01090_00747	-------
FC88X_00348_01171	-------
FC88X_00696_00089	-------
FC88X_00142_00818	-------
FC88X_00524_01024	-------

FC88X_01105_01041	-------
FC88X_00098_00760	-------
FC88X_00457_01228	-------
FC88X_00178_00853	-------
FC88X_00524_00049	-------
FC88X_00934_00358	-------
FC88X_00923_01047	-------
FC88X_00506_01007	-------
FC88X_01018_00522	-------
FC88X_00069_00610	-------
FC88X_00716_01022	-------
FC88X_00022_00258	-------
FC88X_00495_00142	-------
FC88X_01081_00392	-------
FC88X_00616_00437	-------
FC88X_00274_01100	-------
FC88X_00999_00469	-------
FC88X_00915_01250	-------
FC88X_00076_00737	-------
FC88X_01080_00639	-------
FC88X_00214_00035	-------
FC88X_00243_01098	-------
FC88X_00498_00671	-------
FC88X_00767_00290	-------
FC88X_00958_01203	-------
FC88X_00499_00993	-------
FC88X_00831_00106	-------
FC88X_00085_00109	-------
FC88X_00776_01128	-------
FC88X_00787_00910	-------
FC88X_00417_00400	-------
FC88X_00556_00801	-------
FC88X_00789_00874	-------
FC88X_00954_00419	-------
FC88X_00432_01111	-------
FC88X_00596_00061	-------
FC88X_00817_00358	-------
FC88X_00966_00972	-------
FC88X_01065_01200	-------
FC88X_01122_00676	-------
FC88X_00930_00712	-------
FC88X_01092_00772	-------
FC88X_00568_00497	-------
FC88X_00416_01043	-------
FC88X_00023_00675	-------
FC88X_00502_00164	-------
FC88X_01010_01245	-------
FC88X_01130_01155	-------
FC88X_00525_00442	-------
FC88X_00968_01019	-------
FC88X_00724_00451	-------
FC88X_00424_00672	-------
FC88X_00814_00973	-------
FC88X_00678_01260	-------
FC88X_01130_00665	-------
FC88X_00607_01044	-------
FC88X_00674_00916	-------

FC88X_00813_00381	-------
FC88X_01036_00854	-------
FC88X_00154_00821	-------
FC88X_00937_00737	-------
FC88X_00434_01081	-------
FC88X_00614_00065	-------
FC88X_00183_00166	-------
FC88X_00645_01185	-------
FC88X_00598_01246	-------
FC88X_00226_00890	-------
FC88X_01024_00667	-------
FC88X_01098_00928	-------
FC88X_00427_00064	-------
FC88X_00013_00532	-------
FC88X_00081_00308	-------
FC88X_00210_00124	-------
FC88X_00743_01048	-------
FC88X_00128_00582	-------
FC88X_00918_00814	-------
FC88X_00171_00883	-------
FC88X_00105_00931	-------
FC88X_00415_01136	-------
FC88X_00856_00971	-------
FC88X_00465_00013	-------
FC88X_00663_01259	-------
FC88X_00797_00960	-------
FC88X_01101_00896	-------
FC88X_00021_00357	-------
FC88X_00621_01169	-------
FC88X_00904_01076	-------
FC88X_00956_00810	-------
FC88X_00905_00929	-------
FC88X_00958_00264	-------
FC88X_00880_00204	-------
FC88X_01103_01185	-------
FC88X_01140_00675	-------
FC88X_00706_00613	-------
FC88X_00926_00842	-------
FC88X_00988_00863	-------
FC88X_00667_00065	-------
FC88X_00126_00950	-------
FC88X_00249_00288	-------
FC88X_01133_00610	-------
FC88X_00881_00704	-------
FC88X_01114_00553	-------
FC88X_00613_00917	-------
FC88X_00008_00445	-------
FC88X_00045_00537	-------
FC88X_00940_00724	-------
FC88X_00226_00017	-------
FC88X_00924_00395	-------
FC88X_00448_00459	-------
FC88X_01034_00917	-------
FC88X_00910_00798	-------
FC88X_00948_00832	-------
FC88X_00364_01187	-------
FC88X_00937_00497	-------

FC88X_00986_00719	-------
FC88X_00817_01128	-------
FC88X_00980_01163	-------
FC88X_00404_00022	-------
FC88X_00317_01002	-------
FC88X_00462_00448	-------
FC88X_00731_01100	-------
FC88X_01010_00512	-------
FC88X_00468_00099	-------
FC88X_00192_00966	-------
FC88X_00608_01185	-------
FC88X_01081_00892	-------
FC88X_00757_01236	-------
FC88X_00754_01275	-------
FC88X_00602_00958	-------
FC88X_01083_00929	-------
FC88X_00683_00078	-------
FC88X_00780_01018	-------
FC88X_00721_00937	-------
FC88X_00860_00868	-------
FC88X_00026_00169	-------
FC88X_00089_00482	-------
FC88X_00918_00672	-------
FC88X_00106_00942	-------
FC88X_01054_01035	-------
FC88X_01111_00908	-------
FC88X_00119_00524	-------
FC88X_00991_00861	-------
FC88X_00759_01151	-------
FC88X_01093_00879	-------
FC88X_00207_00900	-------
FC88X_01040_00470	-------
FC88X_01091_00917	-------
FC88X_01056_00958	-------
FC88X_01139_01044	-------
FC88X_00401_00461	-------
FC88X_00832_00802	-------
FC88X_00137_00750	-------
FC88X_00543_00582	-------
FC88X_00954_01061	-------
FC88X_00216_00999	-------
FC88X_01070_01182	-------
FC88X_00278_00734	-------
FC88X_00577_00350	-------
FC88X_00857_01099	-------
FC88X_01116_00584	-------
FC88X_01108_00754	-------
FC88X_00122_00429	-------
FC88X_00443_01196	-------
FC88X_00039_00160	-------
FC88X_00696_01219	-------
FC88X_01110_00979	-------
FC88X_00085_00542	-------
FC88X_00966_00278	-------
FC88X_00342_00409	-------
FC88X_00081_00380	-------
FC88X_00697_00742	-------

FC88X_00070_00116	-------
FC88X_00544_01063	-------
FC88X_00476_00516	-------
FC88X_00769_00989	-------
FC88X_00981_01156	-------
FC88X_00074_00175	-------
FC88X_01098_00807	-------
FC88X_00328_00338	-------
FC88X_00403_01033	-------
FC88X_00838_01222	-------
FC88X_00750_00427	-------
FC88X_01085_01131	-------
FC88X_00144_00068	-------
FC88X_00102_00822	-------
FC88X_00419_00436	-------
FC88X_00975_00248	-------
FC88X_01031_01093	-------
FC88X_00520_00141	-------
FC88X_00588_00159	-------
FC88X_00556_01246	-------
FC88X_01029_00997	-------
FC88X_01088_01037	-------
FC88X_00983_00672	-------
FC88X_00756_00987	-------
FC88X_00566_01163	-------
FC88X_00734_00953	-------
FC88X_00951_01082	-------
FC88X_01073_00856	-------
FC88X_01084_00713	-------
FC88X_00409_00756	-------
FC88X_00658_01054	-------
FC88X_00389_01196	-------
FC88X_00246_01025	-------
FC88X_00565_00242	-------
FC88X_00157_00820	-------
FC88X_01084_00835	-------
FC88X_00522_01201	-------
FC88X_00410_00787	-------
FC88X_00518_00179	-------
FC88X_00798_01006	-------
FC88X_01020_00323	-------
FC88X_00539_00061	-------
FC88X_01020_00476	-------
FC88X_00997_00587	-------
FC88X_00119_00501	-------
FC88X_00115_00578	-------
FC88X_00197_00407	-------
FC88X_00914_00975	-------
FC88X_00005_00633	-------
FC88X_00365_01089	-------
FC88X_00124_00694	-------
FC88X_00789_00718	-------
FC88X_00089_00881	-------
FC88X_00131_00089	-------
FC88X_00885_00569	-------
FC88X_00191_00863	-------
FC88X_00422_00029	-------

FC88X_00668_01057	-------
FC88X_00109_00606	-------
FC88X_00150_00799	-------
FC88X_01010_00271	-------
FC88X_00741_00336	-------
FC88X_00899_00395	-------
FC88X_01002_00422	-------
FC88X_00397_01147	-------
FC88X_00035_00346	-------
FC88X_00318_00673	-------
FC88X_00664_01156	-------
FC88X_01077_00804	-------
FC88X_00685_00065	-------
FC88X_00733_00916	-------
FC88X_00752_01034	-------
FC88X_00994_00829	-------
FC88X_00044_00445	-------
FC88X_00720_01145	-------
FC88X_01090_00875	-------
FC88X_00611_00944	-------
FC88X_00928_00239	-------
FC88X_00981_01167	-------
FC88X_00025_00470	-------
FC88X_00130_00989	-------
FC88X_00139_00960	-------
FC88X_00394_00070	-------
FC88X_00386_00998	-------
FC88X_00461_01166	-------
FC88X_00625_00678	-------
FC88X_00614_01034	-------
FC88X_00600_01171	-------
FC88X_00732_01076	-------
FC88X_00860_00232	-------
FC88X_00877_00238	-------
FC88X_00851_00966	-------
FC88X_00861_00966	-------
FC88X_00919_00436	-------
FC88X_00901_00687	-------
FC88X_00959_01254	-------
FC88X_01074_00470	-------
FC88X_01125_00614	-------
FC88X_01123_00793	-------
FC88X_00040_00098	-------
FC88X_00128_00628	-------
FC88X_00118_00718	-------
FC88X_00193_00045	-------
FC88X_00194_00143	-------
FC88X_00194_01052	-------
FC88X_00232_00969	-------
FC88X_00271_00833	-------
FC88X_00255_01064	-------
FC88X_00341_00822	-------
FC88X_00362_01056	-------
FC88X_00352_01176	-------
FC88X_00515_00302	-------
FC88X_00522_00631	-------
FC88X_00511_00651	-------

FC88X_00216_00301	-------
FC88X_00957_00964	-------
FC88X_00115_00645	-------
FC88X_00165_00923	-------
FC88X_00877_00743	-------
FC88X_00125_00968	-------
FC88X_00400_00468	-------
FC88X_00684_01263	-------
FC88X_01001_00740	-------
FC88X_01083_00850	-------
FC88X_00383_00481	-------
FC88X_00366_00602	-------
FC88X_00004_00519	-------
FC88X_00036_00808	-------
FC88X_00080_00172	-------
FC88X_00510_00645	-------
FC88X_00140_00222	-------
FC88X_00482_00131	-------
FC88X_00921_00226	-------
FC88X_00173_00852	-------
FC88X_00135_00721	-------
FC88X_00083_00495	-------
FC88X_00793_00964	-------
FC88X_00907_00396	-------
FC88X_00149_00082	-------
FC88X_00786_01214	-------
FC88X_00845_01206	-------
FC88X_01082_00800	-------
FC88X_00791_00323	-------
FC88X_00710_00691	-------
FC88X_00559_00942	-------
FC88X_00652_01204	-------
FC88X_00895_01017	-------
FC88X_00658_00126	-------
FC88X_00801_01214	-------
FC88X_00970_00504	-------
FC88X_00966_00957	-------
FC88X_00926_01227	-------
FC88X_01032_00524	-------
FC88X_00084_00520	-------
FC88X_00949_01000	-------
FC88X_00945_01173	-------
FC88X_00049_00830	-------
FC88X_01004_00576	-------
FC88X_00575_01036	-------
FC88X_00990_00577	-------
FC88X_00131_00881	-------
FC88X_00199_01027	-------
FC88X_00409_01163	-------
FC88X_00567_00239	-------
FC88X_01021_00469	-------
FC88X_01084_00562	-------
FC88X_00970_01214	-------
FC88X_00408_01099	-------
FC88X_00118_00456	-------
FC88X_00121_00897	-------
FC88X_01064_00714	-------

FC88X_00954_00683	-------
FC88X_00950_00858	-------
FC88X_00925_00516	-------
FC88X_00985_00928	-------
FC88X_00090_00303	-------
FC88X_01035_01012	-------
FC88X_00082_00621	-------
FC88X_00444_00863	-------
FC88X_00608_00511	-------
FC88X_00865_00320	-------
FC88X_00679_01059	-------
FC88X_00686_00359	-------
FC88X_00572_00989	-------
FC88X_00075_00720	-------
FC88X_00188_00045	-------
FC88X_00454_01198	-------
FC88X_00694_00981	-------
FC88X_00169_00983	-------
FC88X_00693_01105	-------
FC88X_01076_00663	-------
FC88X_00827_01072	-------
FC88X_00759_00732	-------
FC88X_00624_00663	-------
FC88X_00631_01005	-------
FC88X_00270_00026	-------
FC88X_00373_00065	-------
FC88X_00897_00645	-------
FC88X_01052_01096	-------
FC88X_00774_00158	-------
FC88X_01055_00721	-------
FC88X_00048_00572	-------
FC88X_00630_00121	-------
FC88X_00910_00516	-------
FC88X_00452_00115	-------
FC88X_01139_00900	-------
FC88X_00119_00076	-------
FC88X_00127_00772	-------
FC88X_00432_00794	-------
FC88X_00926_00512	-------
FC88X_00874_00803	-------
FC88X_00109_00824	-------
FC88X_00802_01158	-------
FC88X_00106_00525	-------
FC88X_00187_00145	-------
FC88X_00468_00697	-------
FC88X_00082_00207	-------
FC88X_00078_00530	-------
FC88X_00164_00856	-------
FC88X_00316_00079	-------
FC88X_00424_01027	-------
FC88X_00674_01115	-------
FC88X_00729_00457	-------
FC88X_00829_00928	-------
FC88X_00926_00637	-------
FC88X_00043_00103	-------
FC88X_01039_00507	-------
FC88X_00754_00881	-------

FC88X_00904_00303	-------
FC88X_01059_00710	-------
FC88X_00157_00725	-------
FC88X_00555_01201	-------
FC88X_01089_00666	-------
FC88X_00087_00471	-------
FC88X_00293_00873	-------
FC88X_00382_01073	-------
FC88X_00033_00653	-------
FC88X_00547_01131	-------
FC88X_01102_00561	-------
FC88X_00159_01022	-------
FC88X_00416_01028	-------
FC88X_00954_00326	-------
FC88X_00210_00004	-------
FC88X_00427_00005	-------
FC88X_00549_01104	-------
FC88X_00750_00324	-------
FC88X_01138_01063	-------
FC88X_01133_01153	-------
FC88X_01007_00370	-------
FC88X_00599_01167	-------
FC88X_00378_01086	-------
FC88X_00922_00897	-------
FC88X_00440_00074	-------
FC88X_00899_01170	-------
FC88X_00948_00882	-------
FC88X_01050_00797	-------
FC88X_01029_00713	-------
FC88X_00498_00964	-------
FC88X_00194_00112	-------
FC88X_00554_00203	-------
FC88X_00753_00182	-------
FC88X_00101_00708	-------
FC88X_00605_00925	-------
FC88X_01026_01171	-------
FC88X_00788_00850	-------
FC88X_00984_00603	-------
FC88X_00168_00869	-------
FC88X_00419_01205	-------
FC88X_00917_00881	-------
FC88X_00695_00528	-------
FC88X_01076_00793	-------
FC88X_00947_00591	-------
FC88X_00901_01020	-------
FC88X_01127_01032	-------
FC88X_00690_00387	-------
FC88X_00668_01009	-------
FC88X_00671_01078	-------
FC88X_00835_00331	-------
FC88X_00893_00641	-------
FC88X_00860_00815	-------
FC88X_00936_00545	-------
FC88X_00693_00448	-------
FC88X_00103_00553	-------
FC88X_01030_00844	-------
FC88X_00634_00626	-------

FC88X_00447_00456	-------
FC88X_01118_00580	-------
FC88X_00789_00097	-------
FC88X_00705_01022	-------
FC88X_00744_00969	-------
FC88X_00066_00048	-------
FC88X_00361_01106	-------
FC88X_00679_00229	-------
FC88X_00392_01104	-------
FC88X_00386_01152	-------
FC88X_00118_00263	-------
FC88X_00519_01122	-------
FC88X_00643_01125	-------
FC88X_00585_00078	-------
FC88X_00023_00301	-------
FC88X_00209_00069	-------
FC88X_00326_01054	-------
FC88X_00730_01027	-------
FC88X_00414_01028	-------
FC88X_00870_00698	-------
FC88X_00908_01229	-------
FC88X_00975_00966	-------
FC88X_00014_00322	-------
FC88X_00107_00067	-------
FC88X_00421_01105	-------
FC88X_00716_01212	-------
FC88X_00201_00964	-------
FC88X_00388_00819	-------
FC88X_00637_00116	-------
FC88X_00235_01011	-------
FC88X_00103_00024	-------
FC88X_00950_00794	-------
FC88X_01071_00971	-------
FC88X_01036_00494	-------
FC88X_01055_00494	-------
FC88X_00708_00164	-------
FC88X_01095_00869	-------
FC88X_00114_00419	-------
FC88X_01123_00555	-------
FC88X_00475_00987	-------
FC88X_00460_00454	-------
FC88X_00962_00718	-------
FC88X_00921_00434	-------
FC88X_00995_01173	-------
FC88X_01093_00491	-------
FC88X_00017_00289	-------
FC88X_00647_00464	-------
FC88X_00107_00665	-------
FC88X_01017_01197	-------
FC88X_00027_00534	-------
FC88X_00471_00882	-------
FC88X_00068_00313	-------
FC88X_00176_00049	-------
FC88X_00677_00262	-------
FC88X_01017_00934	-------
FC88X_00140_00728	-------
FC88X_00160_00646	-------

FC88X_00107_00472	-------
FC88X_01093_00753	-------
FC88X_00470_01130	-------
FC88X_00126_00132	-------
FC88X_00413_01190	-------
FC88X_00425_00109	-------
FC88X_00593_00265	-------
FC88X_00507_00534	-------
FC88X_00127_00549	-------
FC88X_00254_00642	-------
FC88X_00135_00964	-------
FC88X_01001_01224	-------
FC88X_00792_00298	-------
FC88X_01065_00372	-------
FC88X_00816_01136	-------
FC88X_00168_00779	-------
FC88X_00426_00048	-------
FC88X_00427_00014	-------
FC88X_00613_00835	-------
FC88X_00860_00752	-------
FC88X_01104_00480	-------
FC88X_01013_00866	-------
FC88X_00985_00328	-------
FC88X_01052_00997	-------
FC88X_01102_00581	-------
FC88X_00159_00023	-------
FC88X_00320_00163	-------
FC88X_00817_00833	-------
FC88X_00886_00607	-------
FC88X_00976_00544	-------
FC88X_01137_00774	-------
FC88X_00670_01128	-------
FC88X_01023_01095	-------
FC88X_01013_01203	-------
FC88X_00690_01135	-------
FC88X_00204_00910	-------
FC88X_00073_00316	-------
FC88X_00044_00455	-------
FC88X_00465_00644	-------
FC88X_00607_00340	-------
FC88X_00883_00810	-------
FC88X_00915_01120	-------
FC88X_00902_00182	-------
FC88X_00188_00139	-------
FC88X_00162_00806	-------
FC88X_00130_00656	-------
FC88X_00418_01217	-------
FC88X_00466_01197	-------
FC88X_00904_00781	-------
FC88X_00563_01105	-------
FC88X_00935_00440	-------
FC88X_00447_01012	-------
FC88X_00707_00312	-------
FC88X_00091_00526	-------
FC88X_01011_00569	-------
FC88X_01142_00800	-------
FC88X_00316_00639	-------

FC88X_00664_01061	-------
FC88X_00735_01161	-------
FC88X_00095_00304	-------
FC88X_00290_00857	-------
FC88X_00051_00636	-------
FC88X_00140_00589	-------
FC88X_00541_00074	-------
FC88X_00587_00393	-------
FC88X_00703_01110	-------
FC88X_00058_00103	-------
FC88X_00098_00326	-------
FC88X_00098_00552	-------
FC88X_00118_00013	-------
FC88X_00136_00060	-------
FC88X_00211_00055	-------
FC88X_00270_00498	-------
FC88X_00426_00042	-------
FC88X_00441_00733	-------
FC88X_00415_01166	-------
FC88X_00573_00647	-------
FC88X_00557_00881	-------
FC88X_00662_00533	-------
FC88X_00768_00204	-------
FC88X_00942_00974	-------
FC88X_00998_00623	-------
FC88X_01051_00699	-------
FC88X_01096_01061	-------
FC88X_01101_00795	-------
FC88X_01115_00902	-------
FC88X_00566_00151	-------
FC88X_00515_01171	-------
FC88X_00763_01242	-------
FC88X_00058_00847	-------
FC88X_00521_00746	-------
FC88X_01100_00693	-------
FC88X_00384_00847	-------
FC88X_00583_00093	-------
FC88X_00363_00062	-------
FC88X_00553_00143	-------
FC88X_00928_00767	-------
FC88X_00855_01154	-------
FC88X_01044_00639	-------
FC88X_00394_00661	-------
FC88X_00152_01003	-------
FC88X_00294_01034	-------
FC88X_00410_00072	-------
FC88X_00842_01108	-------
FC88X_01106_01094	-------
FC88X_00951_01179	-------
FC88X_00340_01137	-------
FC88X_00999_00812	-------
FC88X_01023_00525	-------
FC88X_01031_01061	-------
FC88X_00118_00592	-------
FC88X_00165_00716	-------
FC88X_00901_00829	-------
FC88X_00431_01096	-------

FC88X_00470_00840	-------
FC88X_00861_00774	-------
FC88X_00938_01218	-------
FC88X_01091_00887	-------
FC88X_00278_00548	-------
FC88X_00304_01070	-------
FC88X_00982_00314	-------
FC88X_00023_00342	-------
FC88X_00206_00036	-------
FC88X_00132_00125	-------
FC88X_00304_00429	-------
FC88X_00430_00085	-------
FC88X_00420_00584	-------
FC88X_00485_01030	-------
FC88X_00586_00178	-------
FC88X_00897_00691	-------
FC88X_00892_01121	-------
FC88X_01083_00772	-------
FC88X_01130_00602	-------
FC88X_01147_01060	-------
FC88X_00771_00985	-------
FC88X_00948_00977	-------
FC88X_00689_01159	-------
FC88X_01141_00602	-------
FC88X_00944_01153	-------
FC88X_01097_00793	-------
FC88X_01026_00952	-------
FC88X_00784_00120	-------
FC88X_00767_00717	-------
FC88X_00802_01262	-------
FC88X_00938_00862	-------
FC88X_00909_01024	-------
FC88X_00974_01193	-------
FC88X_01006_00584	-------
FC88X_01030_00781	-------
FC88X_01098_00861	-------
FC88X_01120_01216	-------
FC88X_00721_00719	-------
FC88X_00586_00080	-------
FC88X_00238_00038	-------
FC88X_00479_00683	-------
FC88X_00464_01079	-------
FC88X_00080_00509	-------
FC88X_01059_00465	-------
FC88X_01143_00949	-------
FC88X_00812_00112	-------
FC88X_00792_01042	-------
FC88X_00875_00807	-------
FC88X_00995_00273	-------
FC88X_00986_00355	-------
FC88X_00039_00242	-------
FC88X_00455_00941	-------
FC88X_00089_00618	-------
FC88X_00030_00408	-------
FC88X_00834_00907	-------
FC88X_00525_00211	-------
FC88X_00985_00996	-------

FC88X_01133_00747	-------
FC88X_00520_00786	-------
FC88X_01047_01224	-------
FC88X_00797_00171	-------
FC88X_00084_00176	-------
FC88X_00126_00019	-------
FC88X_00323_00010	-------
FC88X_00402_01184	-------
FC88X_00980_00951	-------
FC88X_00115_00309	-------
FC88X_01085_00751	-------
FC88X_00691_00958	-------
FC88X_00196_00683	-------
FC88X_01007_01172	-------
FC88X_00556_01255	-------
FC88X_01132_01077	-------
FC88X_00130_00364	-------
FC88X_00084_00368	-------
FC88X_00117_00107	-------
FC88X_00157_00748	-------
FC88X_00841_00145	-------
FC88X_00155_00943	-------
FC88X_00741_00311	-------
FC88X_00948_00951	-------
FC88X_00071_00056	-------
FC88X_00092_00779	-------
FC88X_00086_00269	-------
FC88X_00621_01215	-------
FC88X_00244_00939	-------
FC88X_00446_00124	-------
FC88X_00558_01246	-------
FC88X_00678_00230	-------
FC88X_00677_01170	-------
FC88X_00758_00877	-------
FC88X_00846_01114	-------
FC88X_00934_00479	-------
FC88X_01059_00601	-------
FC88X_00813_00883	-------
FC88X_00891_01226	-------
FC88X_00785_01002	-------
FC88X_00159_00870	-------
FC88X_00391_01090	-------
FC88X_00101_00557	-------
FC88X_00912_00299	-------
FC88X_00119_00543	-------
FC88X_00604_01107	-------
FC88X_00977_00462	-------
FC88X_00508_00246	-------
FC88X_00883_00711	-------
FC88X_00864_00860	-------
FC88X_00928_00302	-------
FC88X_01143_01239	-------
FC88X_00244_01117	-------
FC88X_00079_00715	-------
FC88X_01062_00646	-------
FC88X_01019_01156	-------
FC88X_00620_00339	-------

FC88X_00422_01146	-------
FC88X_00884_00403	-------
FC88X_00051_00010	-------
FC88X_01037_00431	-------
FC88X_00026_00600	-------
FC88X_00989_00462	-------
FC88X_00074_00397	-------
FC88X_00549_00090	-------
FC88X_01021_01236	-------
FC88X_00882_00399	-------
FC88X_00861_00993	-------
FC88X_00939_01096	-------
FC88X_00442_00141	-------
FC88X_00841_00316	-------
FC88X_00539_01190	-------
FC88X_00130_00926	-------
FC88X_00068_00717	-------
FC88X_00539_01199	-------
FC88X_00929_00450	-------
FC88X_00313_00946	-------
FC88X_00867_01082	-------
FC88X_00200_00844	-------
FC88X_00862_00167	-------
FC88X_00953_00443	-------
FC88X_00608_00205	-------
FC88X_01069_01027	-------
FC88X_01024_00376	-------
FC88X_00797_01227	-------
FC88X_00025_00766	-------
FC88X_01074_00691	-------
FC88X_00227_00547	-------
FC88X_00593_01027	-------
FC88X_00931_00790	-------
FC88X_01046_00727	-------
FC88X_00566_00932	-------
FC88X_00499_00029	-------
FC88X_00510_01184	-------
FC88X_00961_00821	-------
FC88X_00155_00835	-------
FC88X_00883_01234	-------
FC88X_01008_00506	-------
FC88X_00791_01036	-------
FC88X_00879_00720	-------
FC88X_01073_01055	-------
FC88X_00499_01199	-------
FC88X_00520_01019	-------
FC88X_00678_00556	-------
FC88X_00892_00942	-------
FC88X_00933_00319	-------
FC88X_00079_00845	-------
FC88X_00217_00997	-------
FC88X_00500_01027	-------
FC88X_00849_00288	-------
FC88X_01125_00995	-------
FC88X_00590_01222	-------
FC88X_00882_00275	-------
FC88X_01031_00455	-------

FC88X_01000_00536	-------
FC88X_00981_00672	-------
FC88X_00071_00788	-------
FC88X_00046_00434	-------
FC88X_00392_00992	-------
FC88X_00706_00131	-------
FC88X_01141_01006	-------
FC88X_00437_01213	-------
FC88X_00960_00979	-------
FC88X_00225_01073	-------
FC88X_00090_00551	-------
FC88X_00896_00356	-------
FC88X_00078_00155	-------
FC88X_00792_00280	-------
FC88X_00897_00791	-------
FC88X_00050_00820	-------
FC88X_00320_00041	-------
FC88X_01091_00757	-------
FC88X_00287_01001	-------
FC88X_00680_00920	-------
FC88X_00068_00614	-------
FC88X_00134_00253	-------
FC88X_01123_00639	-------
FC88X_00654_01162	-------
FC88X_00054_00125	-------
FC88X_00135_00498	-------
FC88X_00174_00037	-------
FC88X_00626_01043	-------
FC88X_00825_00923	-------
FC88X_00952_00698	-------
FC88X_01096_00637	-------
FC88X_01055_01100	-------
FC88X_00328_01131	-------
FC88X_00137_00509	-------
FC88X_01066_00487	-------
FC88X_01102_00648	-------
FC88X_00131_00104	-------
FC88X_00742_00967	-------
FC88X_00849_01211	-------
FC88X_00805_00809	-------
FC88X_00220_01026	-------
FC88X_00032_00226	-------
FC88X_01114_01116	-------
FC88X_00033_00202	-------
FC88X_00024_00715	-------
FC88X_00663_00261	-------
FC88X_00129_00144	-------
FC88X_00963_00763	-------
FC88X_00493_00978	-------
FC88X_00512_01120	-------
FC88X_00259_00072	-------
FC88X_00975_00532	-------
FC88X_00367_01155	-------
FC88X_00687_01164	-------
FC88X_00126_00081	-------
FC88X_00721_00201	-------
FC88X_00067_00207	-------

FC88X_00069_00733	-------
FC88X_00313_01136	-------
FC88X_00188_00009	-------
FC88X_00970_01167	-------
FC88X_00892_00668	-------
FC88X_00961_00329	-------
FC88X_00324_00740	-------
FC88X_00946_00874	-------
FC88X_01092_00420	-------
FC88X_00965_01157	-------
FC88X_01059_00793	-------
FC88X_01108_00500	-------
FC88X_00707_00272	-------
FC88X_00160_00506	-------
FC88X_00210_00940	-------
FC88X_00052_00631	-------
FC88X_01126_00803	-------
FC88X_00024_00091	-------
FC88X_00440_00070	-------
FC88X_00553_01158	-------
FC88X_00645_00189	-------
FC88X_00822_00249	-------
FC88X_00048_00142	-------
FC88X_00055_00032	-------
FC88X_00070_00325	-------
FC88X_00542_00656	-------
FC88X_00634_00922	-------
FC88X_00681_01169	-------
FC88X_00841_01198	-------
FC88X_00893_00669	-------
FC88X_01014_00463	-------
FC88X_01139_00720	-------
FC88X_00013_00174	-------
FC88X_00477_00983	-------
FC88X_00792_01202	-------
FC88X_00418_01190	-------
FC88X_00726_01260	-------
FC88X_00568_01045	-------
FC88X_00907_01133	-------
FC88X_00279_01085	-------
FC88X_00902_00493	-------
FC88X_01039_00367	-------
FC88X_00719_01167	-------
FC88X_00885_01042	-------
FC88X_00111_00862	-------
FC88X_00068_00654	-------
FC88X_00126_00109	-------
FC88X_00100_00249	-------
FC88X_00127_00743	-------
FC88X_00190_00022	-------
FC88X_00286_00679	-------
FC88X_00315_00975	-------
FC88X_00492_00103	-------
FC88X_00729_00289	-------
FC88X_01133_00946	-------
FC88X_00200_00452	-------
FC88X_00516_01235	-------

FC88X_00782_00526	-------
FC88X_00800_00302	-------
FC88X_00888_00153	-------
FC88X_00878_00673	-------
FC88X_00928_00669	-------
FC88X_01048_00625	-------
FC88X_01137_00875	-------
FC88X_00016_00169	-------
FC88X_00047_00459	-------
FC88X_00043_00553	-------
FC88X_00040_00735	-------
FC88X_00142_00081	-------
FC88X_00118_00395	-------
FC88X_00191_00031	-------
FC88X_00159_00636	-------
FC88X_00238_00482	-------
FC88X_00290_01035	-------
FC88X_00339_00806	-------
FC88X_00370_01027	-------
FC88X_00444_01182	-------
FC88X_00477_00061	-------
FC88X_00495_01135	-------
FC88X_00694_00958	-------
FC88X_00787_01053	-------
FC88X_00826_01206	-------
FC88X_00882_00412	-------
FC88X_00859_00745	-------
FC88X_00860_00993	-------
FC88X_00940_00372	-------
FC88X_00942_01148	-------
FC88X_01013_00763	-------
FC88X_01035_00780	-------
FC88X_01053_00480	-------
FC88X_01061_00807	-------
FC88X_01082_01237	-------
FC88X_01097_01203	-------
FC88X_01127_00713	-------
FC88X_01133_00862	-------
FC88X_00775_00945	-------
FC88X_00867_00696	-------
FC88X_00535_01121	-------
FC88X_00963_00535	-------
FC88X_00943_01272	-------
FC88X_00024_00759	-------
FC88X_00203_01033	-------
FC88X_00026_00359	-------
FC88X_00434_01074	-------
FC88X_00906_01128	-------
FC88X_00141_00532	-------
FC88X_00070_00720	-------
FC88X_00948_00463	-------
FC88X_00074_00268	-------
FC88X_00902_01116	-------
FC88X_00323_00994	-------
FC88X_00941_00551	-------
FC88X_00485_01182	-------
FC88X_00154_00955	-------

FC88X_01003_00919	-------
FC88X_00534_00983	-------
FC88X_00120_00775	-------
FC88X_00251_00656	-------
FC88X_00077_00887	-------
FC88X_00089_00909	-------
FC88X_01011_01022	-------
FC88X_00641_00837	-------
FC88X_00476_00915	-------
FC88X_00806_00928	-------
FC88X_00522_01197	-------
FC88X_00501_01105	-------
FC88X_00912_01108	-------
FC88X_00876_01151	-------
FC88X_01104_00875	-------
FC88X_00937_00931	-------
FC88X_00206_00398	-------
FC88X_00628_00444	-------
FC88X_00682_01060	-------
FC88X_00788_00155	-------
FC88X_00049_00450	-------
FC88X_00584_00112	-------
FC88X_00847_01191	-------
FC88X_01043_01215	-------
FC88X_00294_00422	-------
FC88X_01062_01172	-------
FC88X_01065_01206	-------
FC88X_00880_01251	-------
FC88X_01090_00957	-------
FC88X_00497_00084	-------
FC88X_01050_01055	-------
FC88X_00600_01162	-------
FC88X_00790_00190	-------
FC88X_00933_00644	-------
FC88X_00236_00065	-------
FC88X_00755_01160	-------
FC88X_00084_00543	-------
FC88X_01049_01248	-------
FC88X_00916_00864	-------
FC88X_00638_01127	-------
FC88X_00829_01113	-------
FC88X_01132_01102	-------
FC88X_00238_00225	-------
FC88X_00357_01068	-------
FC88X_01131_01138	-------
FC88X_00364_01120	-------
FC88X_00497_01174	-------
FC88X_00040_00601	-------
FC88X_00615_00880	-------
FC88X_00880_00870	-------
FC88X_00919_00670	-------
FC88X_00997_01013	-------
FC88X_00440_01069	-------
FC88X_00598_01061	-------
FC88X_00781_00861	-------
FC88X_00950_00777	-------
FC88X_00494_00204	-------

FC88X_00683_00635	-------
FC88X_00950_00518	-------
FC88X_01074_00406	-------
FC88X_00793_01169	-------
FC88X_01100_01114	-------
FC88X_00371_00989	-------
FC88X_00661_01239	-------
FC88X_00943_00509	-------
FC88X_00832_01230	-------
FC88X_00300_01119	-------
FC88X_00953_01010	-------
FC88X_00947_00335	-------
FC88X_00889_00967	-------
FC88X_00241_00948	-------
FC88X_01125_01176	-------
FC88X_00086_00081	-------
FC88X_00370_01161	-------
FC88X_00118_00124	-------
FC88X_00288_00607	-------
FC88X_00456_00998	-------
FC88X_00911_00278	-------
FC88X_00979_01019	-------
FC88X_00057_00762	-------
FC88X_00097_00520	-------
FC88X_01141_00706	-------
FC88X_00032_00165	-------
FC88X_01088_01043	-------
FC88X_00384_00566	-------
FC88X_00098_00871	-------
FC88X_00156_00736	-------
FC88X_00416_00634	-------
FC88X_00781_00135	-------
FC88X_01140_00687	-------
FC88X_00871_00892	-------
FC88X_00019_00241	-------
FC88X_00827_00301	-------
FC88X_00942_00898	-------
FC88X_00752_00502	-------
FC88X_00765_00722	-------
FC88X_00870_01186	-------
FC88X_00380_01123	-------
FC88X_00460_00640	-------
FC88X_00837_01236	-------
FC88X_00870_00819	-------
FC88X_00040_00503	-------
FC88X_00427_01205	-------
FC88X_00252_01087	-------
FC88X_00321_01101	-------
FC88X_00443_00971	-------
FC88X_00424_00707	-------
FC88X_00653_00193	-------
FC88X_00676_01066	-------
FC88X_00980_00282	-------
FC88X_00734_00339	-------
FC88X_00426_01048	-------
FC88X_00590_00412	-------
FC88X_01120_00679	-------

FC88X_00063_00579	-------
FC88X_00593_00763	-------
FC88X_01091_00893	-------
FC88X_00691_00134	-------
FC88X_00845_01167	-------
FC88X_00799_00087	-------
FC88X_00797_00224	-------
FC88X_00815_01093	-------
FC88X_00808_01173	-------
FC88X_00866_00576	-------
FC88X_00986_00766	-------
FC88X_00381_00720	-------
FC88X_00902_01203	-------
FC88X_00958_00634	-------
FC88X_01003_00529	-------
FC88X_01006_00722	-------
FC88X_01035_00891	-------
FC88X_00183_00836	-------
FC88X_00795_01168	-------
FC88X_00334_00931	-------
FC88X_00953_00513	-------
FC88X_00264_00653	-------
FC88X_00496_00866	-------
FC88X_00066_00757	-------
FC88X_00201_00819	-------
FC88X_00724_00880	-------
FC88X_01011_00467	-------
FC88X_00456_01048	-------
FC88X_00137_00807	-------
FC88X_00565_01210	-------
FC88X_00654_00605	-------
FC88X_01027_00767	-------
FC88X_00562_00807	-------
FC88X_00819_00198	-------
FC88X_00613_01246	-------
FC88X_00807_01275	-------
FC88X_00092_00471	-------
FC88X_01076_00463	-------
FC88X_00050_00512	-------
FC88X_00585_01256	-------
FC88X_01105_00506	-------
FC88X_00184_01058	-------
FC88X_00206_00989	-------
FC88X_00419_00995	-------
FC88X_00701_01026	-------
FC88X_00885_00795	-------
FC88X_00539_00756	-------
FC88X_00592_01168	-------
FC88X_00711_00879	-------
FC88X_00728_01003	-------
FC88X_00798_01184	-------
FC88X_00781_01273	-------
FC88X_01042_00878	-------
FC88X_00447_01196	-------
FC88X_00896_00262	-------
FC88X_00913_00691	-------
FC88X_00865_00330	-------

FC88X_01107_00702	-------
FC88X_01136_01055	-------
FC88X_00149_00194	-------
FC88X_00325_00013	-------
FC88X_00590_01234	-------
FC88X_00202_01039	-------
FC88X_00906_00431	-------
FC88X_00588_01197	-------
FC88X_00724_00055	-------
FC88X_00795_01236	-------
FC88X_00452_01182	-------
FC88X_00598_00783	-------
FC88X_00118_00840	-------
FC88X_00954_00538	-------
FC88X_00550_01049	-------
FC88X_00029_00578	-------
FC88X_00917_00770	-------
FC88X_00573_01240	-------
FC88X_00333_01028	-------
FC88X_00020_00718	-------
FC88X_00288_00098	-------
FC88X_00148_00939	-------
FC88X_00957_00746	-------
FC88X_00512_00316	-------
FC88X_00124_00273	-------
FC88X_00138_00555	-------
FC88X_00135_00917	-------
FC88X_00467_01046	-------
FC88X_00482_01174	-------
FC88X_00596_00339	-------
FC88X_00991_00723	-------
FC88X_00131_00971	-------
FC88X_00593_01062	-------
FC88X_00820_00918	-------
FC88X_00844_00962	-------
FC88X_00123_00677	-------
FC88X_00741_00393	-------
FC88X_00896_01209	-------
FC88X_00671_01256	-------
FC88X_01012_00305	-------
FC88X_01012_00327	-------
FC88X_00128_00270	-------
FC88X_00968_00569	-------
FC88X_00076_00268	-------
FC88X_00611_00992	-------
FC88X_00859_00205	-------
FC88X_00468_01001	-------
FC88X_00677_00467	-------
FC88X_01034_01216	-------
FC88X_00997_01033	-------
FC88X_01059_00791	-------
FC88X_00854_00726	-------
FC88X_00834_00191	-------
FC88X_00969_00788	-------
FC88X_00401_00256	-------
FC88X_00796_00306	-------
FC88X_00417_01211	-------

FC88X_00496_01187	-------
FC88X_00800_00842	-------
FC88X_00691_00716	-------
FC88X_00761_00163	-------
FC88X_00130_00992	-------
FC88X_00912_00633	-------
FC88X_00698_01078	-------
FC88X_01049_00725	-------
FC88X_00100_00510	-------
FC88X_01025_00992	-------
FC88X_00921_00821	-------
FC88X_00902_00889	-------
FC88X_00205_00439	-------
FC88X_00767_00314	-------
FC88X_01005_00319	-------
FC88X_00490_01119	-------
FC88X_00041_00076	-------
FC88X_00197_00994	-------
FC88X_00156_00034	-------
FC88X_01103_00838	-------
FC88X_01083_00796	-------
FC88X_00011_00420	-------
FC88X_01129_00962	-------
FC88X_00025_00336	-------
FC88X_00811_00951	-------
FC88X_01022_00778	-------
FC88X_01030_00783	-------
FC88X_00200_00911	-------
FC88X_00809_01260	-------
FC88X_00265_00784	-------
FC88X_01037_01039	-------
FC88X_00148_00784	-------
FC88X_00536_00097	-------
FC88X_01023_00939	-------
FC88X_00592_00272	-------
FC88X_00647_00911	-------
FC88X_00106_00246	-------
FC88X_00781_00306	-------
FC88X_01066_01269	-------
FC88X_00045_00351	-------
FC88X_00177_01037	-------
FC88X_00201_00795	-------
FC88X_00203_00946	-------
FC88X_00293_01091	-------
FC88X_00575_01207	-------
FC88X_00689_00194	-------
FC88X_00742_01004	-------
FC88X_00772_00357	-------
FC88X_00920_00520	-------
FC88X_00006_00058	-------
FC88X_00051_00174	-------
FC88X_00133_00190	-------
FC88X_00119_00273	-------
FC88X_00152_00152	-------
FC88X_00278_01074	-------
FC88X_00323_00586	-------
FC88X_00349_01016	-------

FC88X_00671_00127	-------
FC88X_00767_00877	-------
FC88X_00828_00556	-------
FC88X_00929_00553	-------
FC88X_00999_00548	-------
FC88X_01003_01216	-------
FC88X_00276_00937	-------
FC88X_00836_00174	-------
FC88X_00103_00613	-------
FC88X_00849_01066	-------
FC88X_00928_00639	-------
FC88X_00628_00180	-------
FC88X_00372_00014	-------
FC88X_00596_01091	-------
FC88X_00855_01210	-------
FC88X_00270_01012	-------
FC88X_00037_00612	-------
FC88X_00489_00058	-------
FC88X_00428_00824	-------
FC88X_00439_01217	-------
FC88X_00767_00695	-------
FC88X_00988_00430	-------
FC88X_01011_00298	-------
FC88X_00100_00216	-------
FC88X_00182_01040	-------
FC88X_00495_00167	-------
FC88X_00914_00495	-------
FC88X_00008_00568	-------
FC88X_00372_01089	-------
FC88X_00284_01129	-------
FC88X_00320_00691	-------
FC88X_00640_00453	-------
FC88X_01060_00970	-------
FC88X_00480_00177	-------
FC88X_00882_00641	-------
FC88X_00440_01223	-------
FC88X_00902_00538	-------
FC88X_00459_01101	-------
FC88X_01143_00626	-------
FC88X_00794_00282	-------
FC88X_00515_00028	-------
FC88X_00746_00231	-------
FC88X_00791_00300	-------
FC88X_01027_01201	-------
FC88X_00994_01088	-------
FC88X_00067_00097	-------
FC88X_00552_01138	-------
FC88X_00423_00063	-------
FC88X_00828_00806	-------
FC88X_01012_00668	-------
FC88X_00169_00815	-------
FC88X_00063_00131	-------
FC88X_00455_00505	-------
FC88X_00122_00268	-------
FC88X_00132_00510	-------
FC88X_00125_00582	-------
FC88X_00118_00694	-------

FC88X_00173_00414	-------
FC88X_00206_00023	-------
FC88X_00200_00871	-------
FC88X_00525_01146	-------
FC88X_00590_00266	-------
FC88X_00555_01019	-------
FC88X_00684_01137	-------
FC88X_00835_00649	-------
FC88X_00800_00736	-------
FC88X_00951_00760	-------
FC88X_01059_00410	-------
FC88X_01092_00815	-------
FC88X_00014_00078	-------
FC88X_00113_00209	-------
FC88X_00274_00871	-------
FC88X_00286_01080	-------
FC88X_00306_00119	-------
FC88X_00435_00041	-------
FC88X_00408_01194	-------
FC88X_00483_00488	-------
FC88X_00526_01031	-------
FC88X_00570_01122	-------
FC88X_00593_01147	-------
FC88X_00611_00219	-------
FC88X_00612_00214	-------
FC88X_00665_01211	-------
FC88X_00685_01274	-------
FC88X_00720_01211	-------
FC88X_00839_00717	-------
FC88X_00826_01141	-------
FC88X_00857_00821	-------
FC88X_00851_00932	-------
FC88X_00931_01175	-------
FC88X_01037_00490	-------
FC88X_01000_01016	-------
FC88X_01012_01089	-------
FC88X_01071_00460	-------
FC88X_01087_01020	-------
FC88X_01103_00470	-------
FC88X_01147_01017	-------
FC88X_00950_01022	-------
FC88X_00601_01221	-------
FC88X_01109_00569	-------
FC88X_00130_00801	-------
FC88X_01007_00956	-------
FC88X_00170_00832	-------
FC88X_00908_00852	-------
FC88X_00391_00958	-------
FC88X_00237_00278	-------
FC88X_00345_00844	-------
FC88X_00064_00767	-------
FC88X_00450_01205	-------
FC88X_00559_01119	-------
FC88X_00914_01187	-------
FC88X_00041_00156	-------
FC88X_00742_01249	-------
FC88X_01108_00846	-------

FC88X_00592_01190	-------
FC88X_00749_00320	-------
FC88X_00097_00134	-------
FC88X_00379_00990	-------
FC88X_00037_00526	-------
FC88X_00061_00828	-------
FC88X_00106_00426	-------
FC88X_00150_00624	-------
FC88X_00474_01078	-------
FC88X_00536_00164	-------
FC88X_00866_00303	-------
FC88X_00864_00997	-------
FC88X_00931_00593	-------
FC88X_01081_01087	-------
FC88X_00040_00485	-------
FC88X_00136_00271	-------
FC88X_00142_00927	-------
FC88X_00225_00584	-------
FC88X_00271_00100	-------
FC88X_00453_00062	-------
FC88X_00459_00907	-------
FC88X_00992_01191	-------
FC88X_01041_00690	-------
FC88X_01080_01193	-------
FC88X_00610_00981	-------
FC88X_00687_00824	-------
FC88X_00712_00093	-------
FC88X_00932_00217	-------
FC88X_01062_00958	-------
FC88X_00853_00724	-------
FC88X_00144_00637	-------
FC88X_00655_00977	-------
FC88X_00845_00194	-------
FC88X_00711_00254	-------
FC88X_00928_01000	-------
FC88X_00457_01046	-------
FC88X_00311_00006	-------
FC88X_00039_00041	-------
FC88X_00797_00091	-------
FC88X_00721_01209	-------
FC88X_00328_00584	-------
FC88X_00375_00080	-------
FC88X_00071_00769	-------
FC88X_00208_00137	-------
FC88X_00149_00342	-------
FC88X_00935_00236	-------
FC88X_01135_00931	-------
FC88X_00777_00853	-------
FC88X_00476_00133	-------
FC88X_01024_00484	-------
FC88X_00942_01003	-------
FC88X_00930_00284	-------
FC88X_01001_00774	-------
FC88X_00620_01195	-------
FC88X_01125_00956	-------
FC88X_00520_00028	-------
FC88X_00784_01109	-------

FC88X_00327_01064	-------
FC88X_00611_00938	-------
FC88X_00997_00900	-------
FC88X_00131_00110	-------
FC88X_00150_00580	-------
FC88X_00283_00204	-------
FC88X_00845_00188	-------
FC88X_00944_00489	-------
FC88X_00478_00059	-------
FC88X_00502_01019	-------
FC88X_00026_00156	-------
FC88X_00016_00337	-------
FC88X_00156_00307	-------
FC88X_00521_00882	-------
FC88X_00170_01037	-------
FC88X_00977_00848	-------
FC88X_00045_00288	-------
FC88X_00623_00089	-------
FC88X_01142_00860	-------
FC88X_00406_00475	-------
FC88X_00585_00575	-------
FC88X_00915_00438	-------
FC88X_01050_00972	-------
FC88X_00567_01256	-------
FC88X_00811_00099	-------
FC88X_00804_00936	-------
FC88X_00480_00182	-------
FC88X_00286_00103	-------
FC88X_00158_00774	-------
FC88X_00949_00542	-------
FC88X_00989_01038	-------
FC88X_00857_00285	-------
FC88X_00112_00385	-------
FC88X_00323_00293	-------
FC88X_00747_01124	-------
FC88X_00720_00564	-------
FC88X_00897_00749	-------
FC88X_00723_00986	-------
FC88X_00472_00081	-------
FC88X_00858_01000	-------
FC88X_01043_00959	-------
FC88X_01019_00467	-------
FC88X_00397_00019	-------
FC88X_00188_01015	-------
FC88X_00358_00794	-------
FC88X_00652_00896	-------
FC88X_00667_01007	-------
FC88X_00910_00326	-------
FC88X_01033_00781	-------
FC88X_00048_00150	-------
FC88X_00327_00985	-------
FC88X_00398_00554	-------
FC88X_00638_01042	-------
FC88X_00839_00224	-------
FC88X_00874_00878	-------
FC88X_00602_00910	-------
FC88X_00159_00591	-------

FC88X_00569_00510	-------
FC88X_00610_00597	-------
FC88X_00735_00805	-------
FC88X_00901_00466	-------
FC88X_00446_01138	-------
FC88X_00260_01097	-------
FC88X_00875_00854	-------
FC88X_00891_01244	-------
FC88X_00509_00304	-------
FC88X_00935_00232	-------
FC88X_00394_01164	-------
FC88X_00855_00170	-------
FC88X_00409_00516	-------
FC88X_00474_00978	-------
FC88X_00110_00566	-------
FC88X_00519_00233	-------
FC88X_00146_00064	-------
FC88X_00140_00527	-------
FC88X_00216_00335	-------
FC88X_00622_00888	-------
FC88X_01049_01026	-------
FC88X_01129_00627	-------
FC88X_00046_00443	-------
FC88X_00575_01015	-------
FC88X_00659_00294	-------
FC88X_00813_00920	-------
FC88X_00515_00211	-------
FC88X_00226_00894	-------
FC88X_01098_01093	-------
FC88X_01066_00430	-------
FC88X_00524_00508	-------
FC88X_00898_01058	-------
FC88X_00189_00837	-------
FC88X_00451_01126	-------
FC88X_00788_00695	-------
FC88X_00114_00491	-------
FC88X_01093_00838	-------
FC88X_01043_00875	-------
FC88X_01024_00498	-------
FC88X_00579_00898	-------
FC88X_00141_00572	-------
FC88X_00107_00954	-------
FC88X_00067_00290	-------
FC88X_00878_00543	-------
FC88X_01030_00587	-------
FC88X_00910_00370	-------
FC88X_00353_01188	-------
FC88X_00930_00857	-------
FC88X_00981_00418	-------
FC88X_00981_01195	-------
FC88X_01074_01014	-------
FC88X_00246_00127	-------
FC88X_00623_00097	-------
FC88X_00981_00675	-------
FC88X_00429_01014	-------
FC88X_00013_00529	-------
FC88X_00667_01129	-------

FC88X_00415_01178	-------
FC88X_00895_00663	-------
FC88X_00951_00423	-------
FC88X_01085_00622	-------
FC88X_01098_00867	-------
FC88X_00294_00978	-------
FC88X_00345_01112	-------
FC88X_00463_00102	-------
FC88X_00459_00458	-------
FC88X_00459_01091	-------
FC88X_00508_01146	-------
FC88X_00804_00293	-------
FC88X_00925_01021	-------
FC88X_00996_00396	-------
FC88X_00959_00525	-------
FC88X_01046_00872	-------
FC88X_01001_01086	-------
FC88X_01121_01096	-------
FC88X_00094_00078	-------
FC88X_00023_00279	-------
FC88X_00872_00134	-------
FC88X_00465_01152	-------
FC88X_01086_00925	-------
FC88X_00539_00216	-------
FC88X_00873_00851	-------
FC88X_00085_00431	-------
FC88X_00035_00690	-------
FC88X_00289_00550	-------
FC88X_00484_00785	-------
FC88X_01078_00815	-------
FC88X_01138_00686	-------
FC88X_00015_00261	-------
FC88X_00059_00085	-------
FC88X_00537_00121	-------
FC88X_00537_00955	-------
FC88X_01012_00489	-------
FC88X_01105_00686	-------
FC88X_00897_00763	-------
FC88X_00070_00811	-------
FC88X_00704_00413	-------
FC88X_00255_00149	-------
FC88X_00179_00847	-------
FC88X_00525_00222	-------
FC88X_00141_00703	-------
FC88X_01126_01079	-------
FC88X_00360_01033	-------
FC88X_00976_00491	-------
FC88X_00896_00243	-------
FC88X_00900_00973	-------
FC88X_00213_00034	-------
FC88X_00585_00126	-------
FC88X_00655_00263	-------
FC88X_00587_01039	-------
FC88X_00735_00475	-------
FC88X_00443_00132	-------
FC88X_00770_00346	-------
FC88X_00024_00658	-------

```
FC88X_00320_00600       -------
FC88X_00107_00274       -------
FC88X_00640_00863       -------
FC88X_00088_00767       -------
FC88X_00183_00244       -------
FC88X_01091_00669       -------
FC88X_00278_00015       -------
FC88X_00308_01015       -------
FC88X_00583_00779       -------
FC88X_00871_00328       -------
FC88X_01141_00841       -------
FC88X_00593_01176       -------
FC88X_00159_00815       -------
FC88X_00133_00821       -------
FC88X_00065_00406       -------
FC88X_00489_01035       -------
FC88X_00795_00092       -------
FC88X_00629_00290       -------
FC88X_00081_00873       -------
FC88X_00129_00358       -------
FC88X_00428_00584       -------
FC88X_00431_00820       -------
FC88X_00109_00053       -------
FC88X_00628_00162       T------
FC88X_00846_00957       -------
FC88X_01014_00328       -------
FC88X_00688_01263       -------
FC88X_00949_00718       -------
FC88X_00909_01245       -------
FC88X_01085_00957       -------
FC88X_00011_00272       -------
FC88X_00037_00049       -------
FC88X_00478_00389       -------
FC88X_00215_00853       -------
FC88X_00739_01059       -------
FC88X_00037_00472       -------
FC88X_00989_00772       -------
FC88X_00944_00735       -------
FC88X_00143_00975       -------
FC88X_00555_01249       -------
FC88X_01140_00568       -------
FC88X_01113_01117       -------
FC88X_00078_00438       -------
FC88X_00346_01139       -------
FC88X_00268_00022       -------
FC88X_01005_01227       -------
FC88X_01123_00762       -------
FC88X_00040_00364       -------
FC88X_01046_01032       -------
FC88X_00459_01178       -------
FC88X_00118_00543       -------
FC88X_00656_01178       -------
FC88X_00304_00107       -------
FC88X_00904_00410       -------
FC88X_00270_00966       -------
FC88X_00669_00293       -------
FC88X_00623_00644       -------
```

FC88X_00772_00928	-------
FC88X_01052_00823	-------
FC88X_01013_00569	-------
FC88X_01039_00553	-------
FC88X_00220_00312	-------
FC88X_00768_01214	-------
FC88X_00272_00674	-------
FC88X_00519_01161	-------
FC88X_00993_00320	-------
FC88X_00960_01052	-------
FC88X_00535_00551	-------
FC88X_00569_01130	-------
FC88X_00634_00963	-------
FC88X_01040_00495	-------
FC88X_01147_00671	-------
FC88X_00761_00698	-------
FC88X_00791_00757	-------
FC88X_01122_00996	-------
FC88X_00307_01021	-------
FC88X_00821_00281	-------
FC88X_01046_01136	-------
FC88X_00485_00199	-------
FC88X_01055_00432	-------
FC88X_00603_00120	-------
FC88X_00559_00331	-------
FC88X_00747_00906	-------
FC88X_00326_00215	-------
FC88X_00387_00971	-------
FC88X_00659_00223	-------
FC88X_00814_01108	-------
FC88X_00106_00851	-------
FC88X_00624_00223	-------
FC88X_00536_00488	-------
FC88X_00324_01131	-------
FC88X_00584_01122	-------
FC88X_00041_00139	-------
FC88X_00075_00113	-------
FC88X_00079_00154	-------
FC88X_00104_00574	-------
FC88X_00162_00359	-------
FC88X_00184_00989	-------
FC88X_00397_00386	-------
FC88X_00494_01108	-------
FC88X_00522_00387	-------
FC88X_00787_00306	-------
FC88X_00788_01019	-------
FC88X_01062_00852	-------
FC88X_01089_00863	-------
FC88X_01125_00522	-------
FC88X_00462_01032	-------
FC88X_00949_00924	-------
FC88X_00928_01042	-------
FC88X_01028_00720	-------
FC88X_00059_00239	-------
FC88X_00920_00965	-------
FC88X_00601_00208	-------
FC88X_00554_01023	-------

FC88X_00991_01231	-------
FC88X_00108_00616	-------
FC88X_00563_00683	-------
FC88X_00644_00417	-------
FC88X_00162_00839	-------
FC88X_00905_01204	-------
FC88X_00150_00051	-------
FC88X_00958_00233	-------
FC88X_00106_00074	-------
FC88X_00247_01091	-------
FC88X_00889_00739	-------
FC88X_00069_00030	-------
FC88X_00399_00049	-------
FC88X_00335_00976	-------
FC88X_00572_00176	-------
FC88X_00029_00400	-------
FC88X_00652_00080	-------
FC88X_00389_00062	-------
FC88X_01094_00679	-------
FC88X_00114_00439	-------
FC88X_00129_00943	-------
FC88X_00431_00082	-------
FC88X_00873_00733	-------
FC88X_00014_00405	-------
FC88X_01119_00934	-------
FC88X_00087_00373	-------
FC88X_00186_00013	-------
FC88X_00406_00632	-------
FC88X_00841_00338	-------
FC88X_00954_00600	-------
FC88X_01132_01210	-------
FC88X_00104_00414	-------
FC88X_00339_01143	-------
FC88X_01038_00757	-------
FC88X_00891_00679	-------
FC88X_00414_01220	-------
FC88X_01028_00961	-------
FC88X_01050_00509	-------
FC88X_00598_00960	-------
FC88X_00734_00065	-------
FC88X_00024_00515	-------
FC88X_00913_00496	-------
FC88X_00078_00189	-------
FC88X_00241_00928	-------
FC88X_00890_00925	-------
FC88X_00944_00863	-------
FC88X_00021_00095	-------
FC88X_01113_00870	-------
FC88X_00024_00499	-------
FC88X_00047_00588	-------
FC88X_00009_00122	-------
FC88X_00947_01010	-------
FC88X_00041_00463	-------
FC88X_00055_00186	-------
FC88X_00024_00178	-------
FC88X_00031_00727	-------
FC88X_00030_00549	-------

FC88X_00039_00679	-------
FC88X_00036_00428	-------
FC88X_00254_00028	-------
FC88X_00041_00513	-------
FC88X_00067_00196	-------
FC88X_00064_00879	-------
FC88X_00088_00907	-------
FC88X_00093_00356	-------
FC88X_00126_00781	-------
FC88X_00496_00770	-------
FC88X_00526_01061	-------
FC88X_00849_00772	-------
FC88X_00856_00246	-------
FC88X_00996_01080	-------
FC88X_01073_00942	-------
FC88X_01131_00652	-------
FC88X_00075_00806	-------
FC88X_00082_00874	-------
FC88X_00123_00008	-------
FC88X_00105_00054	-------
FC88X_00101_00141	-------
FC88X_00122_00111	-------
FC88X_00101_00482	-------
FC88X_00142_00740	-------
FC88X_00158_00182	-------
FC88X_00177_00326	-------
FC88X_00174_00608	-------
FC88X_00159_00756	-------
FC88X_00162_00835	-------
FC88X_00155_00869	-------
FC88X_00163_00947	-------
FC88X_00221_00463	-------
FC88X_00218_00623	-------
FC88X_00250_00011	-------
FC88X_00291_00683	-------
FC88X_00293_00923	-------
FC88X_00324_00340	-------
FC88X_00302_01007	-------
FC88X_00333_01070	-------
FC88X_00319_01153	-------
FC88X_00366_00312	-------
FC88X_00357_00480	-------
FC88X_00379_00512	-------
FC88X_00416_00070	-------
FC88X_00424_00096	-------
FC88X_00404_00863	-------
FC88X_00431_01084	-------
FC88X_00438_01137	-------
FC88X_00457_00081	-------
FC88X_00458_00947	-------
FC88X_00478_00939	-------
FC88X_00457_00996	-------
FC88X_00498_01179	-------
FC88X_00525_00840	-------
FC88X_00538_01062	-------
FC88X_00594_00065	-------
FC88X_00595_00140	-------

FC88X_00599_00218	-------
FC88X_00571_00523	-------
FC88X_00579_00667	-------
FC88X_00592_00748	-------
FC88X_00599_01012	-------
FC88X_00556_01264	-------
FC88X_00616_00079	-------
FC88X_00641_00205	-------
FC88X_00643_00970	-------
FC88X_00689_00124	-------
FC88X_00676_00211	-------
FC88X_00652_00298	-------
FC88X_00655_00347	-------
FC88X_00679_00760	-------
FC88X_00677_00837	-------
FC88X_00691_00941	-------
FC88X_00743_00444	-------
FC88X_00703_00735	-------
FC88X_00739_00777	-------
FC88X_00737_00876	-------
FC88X_00754_00205	-------
FC88X_00793_00262	-------
FC88X_00757_00544	-------
FC88X_00776_00819	-------
FC88X_00793_01014	-------
FC88X_00805_00240	-------
FC88X_00818_00845	-------
FC88X_00849_01035	-------
FC88X_00813_01190	-------
FC88X_00835_01227	-------
FC88X_00878_00259	-------
FC88X_00874_00645	-------
FC88X_00864_00818	-------
FC88X_00870_00815	-------
FC88X_00910_00923	-------
FC88X_00920_00901	-------
FC88X_00917_00999	-------
FC88X_00968_00576	-------
FC88X_00975_00556	-------
FC88X_00995_00556	-------
FC88X_00979_00621	-------
FC88X_00971_00781	-------
FC88X_00978_00860	-------
FC88X_00983_01232	-------
FC88X_01024_00485	-------
FC88X_01032_00486	-------
FC88X_01021_00534	-------
FC88X_01022_00644	-------
FC88X_01046_00704	-------
FC88X_01002_00884	-------
FC88X_01088_00588	-------
FC88X_01093_00568	-------
FC88X_01089_00731	-------
FC88X_01082_00795	-------
FC88X_01082_01202	-------
FC88X_01090_01261	-------
FC88X_01111_00461	-------

FC88X_01116_00766	-------
FC88X_01147_00861	-------
FC88X_01103_01103	-------
FC88X_01138_01135	-------
FC88X_01120_01161	-------
FC88X_00027_00102	-------
FC88X_00014_00192	-------
FC88X_00029_00242	-------
FC88X_00041_00399	-------
FC88X_00010_00468	-------
FC88X_00025_00505	-------
FC88X_00014_00558	-------
FC88X_00067_00043	-------
FC88X_00070_00126	-------
FC88X_00066_00219	-------
FC88X_00082_00247	-------
FC88X_00056_00373	-------
FC88X_00065_00385	-------
FC88X_00084_00469	-------
FC88X_00084_00541	-------
FC88X_00065_00610	-------
FC88X_00084_00751	-------
FC88X_00094_00757	-------
FC88X_00071_00893	-------
FC88X_00095_00914	-------
FC88X_00133_00038	-------
FC88X_00134_00350	-------
FC88X_00142_00423	-------
FC88X_00126_00499	-------
FC88X_00131_00497	-------
FC88X_00129_00741	-------
FC88X_00149_01019	-------
FC88X_00185_00051	-------
FC88X_00170_00107	-------
FC88X_00179_00126	-------
FC88X_00185_00386	-------
FC88X_00164_00430	-------
FC88X_00162_00765	-------
FC88X_00169_00802	-------
FC88X_00176_00804	-------
FC88X_00170_00873	-------
FC88X_00175_01022	-------
FC88X_00188_01055	-------
FC88X_00202_00046	-------
FC88X_00208_00063	-------
FC88X_00205_00145	-------
FC88X_00228_00790	-------
FC88X_00205_00914	-------
FC88X_00240_00977	-------
FC88X_00272_00948	-------
FC88X_00281_01077	-------
FC88X_00324_00026	-------
FC88X_00319_00106	-------
FC88X_00346_00394	-------
FC88X_00334_00689	-------
FC88X_00332_00821	-------
FC88X_00346_01180	-------

FC88X_00351_00991	-------
FC88X_00424_00688	-------
FC88X_00433_00947	-------
FC88X_00431_01028	-------
FC88X_00441_01113	-------
FC88X_00436_01186	-------
FC88X_00426_01206	-------
FC88X_00486_00030	-------
FC88X_00494_00166	-------
FC88X_00480_00943	-------
FC88X_00490_01002	-------
FC88X_00493_01017	-------
FC88X_00484_01123	-------
FC88X_00459_01182	-------
FC88X_00542_00132	-------
FC88X_00511_00253	-------
FC88X_00507_00954	-------
FC88X_00515_00960	-------
FC88X_00509_01062	-------
FC88X_00534_01174	-------
FC88X_00505_01204	-------
FC88X_00556_00038	-------
FC88X_00576_00252	-------
FC88X_00566_00615	-------
FC88X_00554_00670	-------
FC88X_00560_01095	-------
FC88X_00585_01227	-------
FC88X_00643_00235	-------
FC88X_00645_00269	-------
FC88X_00626_00791	-------
FC88X_00612_00925	-------
FC88X_00644_00954	-------
FC88X_00611_01029	-------
FC88X_00687_00238	-------
FC88X_00678_00329	-------
FC88X_00696_00398	-------
FC88X_00653_00609	-------
FC88X_00679_00938	-------
FC88X_00661_00991	-------
FC88X_00654_01062	-------
FC88X_00651_01176	-------
FC88X_00665_01150	-------
FC88X_00711_00867	-------
FC88X_00712_00920	-------
FC88X_00744_00920	-------
FC88X_00734_00999	-------
FC88X_00723_01109	-------
FC88X_00783_00118	-------
FC88X_00790_00259	-------
FC88X_00773_00346	-------
FC88X_00798_00325	-------
FC88X_00752_00734	-------
FC88X_00783_00889	-------
FC88X_00782_01270	-------
FC88X_00824_00309	-------
FC88X_00812_00686	-------
FC88X_00814_00763	-------

FC88X_00833_00858	-------
FC88X_00800_01249	-------
FC88X_00861_00187	-------
FC88X_00895_00216	-------
FC88X_00860_00272	-------
FC88X_00889_00265	-------
FC88X_00882_00344	-------
FC88X_00893_00803	-------
FC88X_00863_00911	-------
FC88X_00898_01105	-------
FC88X_00900_00178	-------
FC88X_00939_00308	-------
FC88X_00905_00502	-------
FC88X_00943_00548	-------
FC88X_00914_00645	-------
FC88X_00943_00631	-------
FC88X_00903_00678	-------
FC88X_00929_00689	-------
FC88X_00904_00706	-------
FC88X_00922_00740	-------
FC88X_00915_00888	-------
FC88X_00913_00922	-------
FC88X_00922_01184	-------
FC88X_00939_01174	-------
FC88X_00942_01168	-------
FC88X_00980_00304	-------
FC88X_00991_00333	-------
FC88X_00969_00504	-------
FC88X_00979_00527	-------
FC88X_00982_00548	-------
FC88X_00984_00875	-------
FC88X_00989_00874	-------
FC88X_00970_01011	-------
FC88X_00961_01143	-------
FC88X_00999_01191	-------
FC88X_00964_01254	-------
FC88X_01028_00315	-------
FC88X_01035_00337	-------
FC88X_01042_00339	-------
FC88X_01000_00624	-------
FC88X_01017_00664	-------
FC88X_01017_00683	-------
FC88X_01023_00733	-------
FC88X_01046_00837	-------
FC88X_01046_00886	-------
FC88X_01049_00917	-------
FC88X_01015_00979	-------
FC88X_01037_01005	-------
FC88X_01045_01012	-------
FC88X_01011_01060	-------
FC88X_01049_01218	-------
FC88X_01047_01269	-------
FC88X_01090_00634	-------
FC88X_01098_00795	-------
FC88X_01099_00755	-------
FC88X_01051_00853	-------
FC88X_01055_00873	-------

FC88X_01064_00871	-------
FC88X_01081_00943	-------
FC88X_01066_00972	-------
FC88X_01050_01045	-------
FC88X_01062_01125	-------
FC88X_01123_00620	-------
FC88X_01147_00637	-------
FC88X_01129_00652	-------
FC88X_01143_00661	-------
FC88X_01103_00714	-------
FC88X_01118_00805	-------
FC88X_01131_01012	-------
FC88X_00345_00067	-------
FC88X_00552_00217	-------
FC88X_00056_00378	-------
FC88X_00524_00903	-------
FC88X_00080_00513	-------
FC88X_00745_00869	-------
FC88X_00973_00616	-------
FC88X_00557_01264	-------
FC88X_00786_00267	-------
FC88X_00912_01077	-------
FC88X_00965_00996	-------
FC88X_00898_01070	-------
FC88X_00910_00334	-------
FC88X_00332_00085	-------
FC88X_00526_00191	-------
FC88X_00944_00879	-------
FC88X_00955_01106	-------
FC88X_00947_00201	-------
FC88X_00017_00584	-------
FC88X_00133_00454	-------
FC88X_00575_00395	-------
FC88X_00900_00805	-------
FC88X_01073_01011	-------
FC88X_00067_00765	-------
FC88X_00389_00024	-------
FC88X_00872_01068	-------
FC88X_00549_01059	-------
FC88X_00080_00126	-------
FC88X_00766_01030	-------
FC88X_00835_00819	-------
FC88X_01027_00760	-------
FC88X_01042_00813	-------
FC88X_00301_01023	-------
FC88X_00945_01210	-------
FC88X_01071_00980	-------
FC88X_01093_00984	-------
FC88X_00978_00865	-------
FC88X_00782_00142	-------
FC88X_00119_00669	-------
FC88X_00606_01273	-------
FC88X_00013_00656	-------
FC88X_00928_00309	-------
FC88X_01061_00380	-------
FC88X_00941_00764	-------
FC88X_00918_01205	-------

```
FC88X_01004_00848      -------
FC88X_01080_00925      -------
FC88X_00975_00320      -------
FC88X_01127_01040      TCTC---
FC88X_01023_00346      -------
FC88X_01011_00943      -------
FC88X_01097_00635      -------
FC88X_00064_00732      -------
FC88X_00302_00394      -------
FC88X_00555_00971      -------
FC88X_01006_00920      -------
FC88X_00201_01051      -------
FC88X_00261_00905      -------
FC88X_00215_01087      -------
FC88X_00491_01209      -------
FC88X_00640_00673      -------
FC88X_00867_01227      -------
FC88X_00289_00122      -------
FC88X_00377_00993      TTT----
FC88X_00489_00267      -------
FC88X_00658_01107      TT-----
FC88X_00934_00654      TT-----
FC88X_00878_00823      TT-----
FC88X_01093_01233      TT-----
FC88X_00970_01015      TT-----
FC88X_01079_01046      -------
FC88X_00055_00455      -------
FC88X_00712_01106      -------
FC88X_00427_00515      -------
FC88X_00408_00100      -------
FC88X_00163_00046      -------
FC88X_00576_00163      -------
FC88X_01099_00802      -------
FC88X_00839_01259      -------
FC88X_00158_00151      -------
FC88X_00191_00880      -------
FC88X_00638_01192      -------
FC88X_00979_00273      -------
FC88X_00079_00148      -------
FC88X_00041_00691      -------
FC88X_00180_00798      -------
FC88X_00537_01250      -------
FC88X_00663_00306      -------
FC88X_00430_00619      -------
FC88X_00936_01246      -------
FC88X_00743_01241      -------
FC88X_00855_00194      TT-----
FC88X_00420_00018      -------
FC88X_00382_00068      -------
FC88X_00754_00302      -------
FC88X_01066_00355      -------
FC88X_00010_00098      -------
FC88X_00009_00675      -------
FC88X_01080_00787      -------
FC88X_00341_01077      -------
FC88X_01105_00662      -------
FC88X_00791_01255      -------
```

```
FC88X_00493_00607      -------
FC88X_00150_00763      -------
FC88X_00255_00098      -------
FC88X_00717_00563      -------
FC88X_00789_00306      -------
FC88X_00915_01066      -------
FC88X_01074_00575      -------
FC88X_00098_00227      -------
FC88X_00094_00387      -------
FC88X_00085_00563      -------
FC88X_00130_00698      -------
FC88X_00264_00958      -------
FC88X_00395_01099      -------
FC88X_00683_01123      -------
FC88X_00738_01000      -------
FC88X_00764_01156      -------
FC88X_00908_00179      -------
FC88X_00904_00401      -------
FC88X_00911_00994      -------
FC88X_01000_00535      -------
FC88X_01010_00758      -------
FC88X_01078_01077      -------
FC88X_01132_00575      -------
FC88X_00247_00820      -------
FC88X_00805_01087      -------
FC88X_00574_00283      TTC----
FC88X_00797_00086      -------
FC88X_00982_00945      -------
FC88X_00556_01060      -------
FC88X_01043_00889      -------
FC88X_01014_00554      -------
FC88X_01095_00920      -------
FC88X_01068_00532      -------
FC88X_00034_00428      -------
FC88X_00111_00926      -------
FC88X_00571_01002      -------
FC88X_00881_00626      -------
FC88X_00947_00644      -------
FC88X_01022_00469      -------
FC88X_00782_01177      -------
FC88X_00816_00199      -------
FC88X_00710_00083      -------
FC88X_00055_00215      -------
FC88X_00242_00395      -------
FC88X_00815_00254      -------
FC88X_00966_00912      -------
FC88X_01053_00353      -------
FC88X_00027_00727      -------
FC88X_00812_00330      -------
FC88X_00206_00095      -------
FC88X_00974_00307      -------
FC88X_01016_00683      -------
FC88X_00443_00328      -------
FC88X_01025_00592      -------
FC88X_00661_00074      -------
FC88X_00679_01223      -------
FC88X_00030_00317      -------
```

FC88X_00178_00983	-------
FC88X_00091_00759	-------
FC88X_00969_00761	-------
FC88X_00256_01000	-------
FC88X_00973_00516	-------
FC88X_00073_00208	-------
FC88X_00077_00411	-------
FC88X_00932_01186	-------
FC88X_00193_00039	-------
FC88X_01103_00546	-------
FC88X_00908_00456	-------
FC88X_01060_00908	-------
FC88X_01119_01213	-------
FC88X_00109_00561	-------
FC88X_00626_01132	-------
FC88X_00020_00389	-------
FC88X_00057_00685	-------
FC88X_00538_00337	-------
FC88X_00227_01101	-------
FC88X_00082_00117	-------
FC88X_00494_01186	-------
FC88X_00087_00340	-------
FC88X_00149_00487	-------
FC88X_00316_01114	-------
FC88X_00603_00268	-------
FC88X_00859_00270	-------
FC88X_01002_01264	-------
FC88X_00038_00344	-------
FC88X_00117_00065	-------
FC88X_00231_00983	-------
FC88X_00342_00921	-------
FC88X_00454_00057	-------
FC88X_00743_01091	-------
FC88X_00820_00820	-------
FC88X_00869_00685	-------
FC88X_00879_00925	-------
FC88X_00933_00310	-------
FC88X_00286_01019	-------
FC88X_00796_00921	-------
FC88X_00016_00480	-------
FC88X_00028_00122	-------
FC88X_00308_00729	-------
FC88X_00885_00886	-------
FC88X_01033_00979	-------
FC88X_00477_01119	-------
FC88X_00692_01244	-------
FC88X_00337_00986	-------
FC88X_00110_00201	-------
FC88X_00456_01125	-------
FC88X_00055_00848	-------
FC88X_00071_00612	-------
FC88X_00166_00586	-------
FC88X_00488_01204	-------
FC88X_00613_00170	-------
FC88X_00629_01108	-------
FC88X_00622_01207	-------
FC88X_00677_00676	-------

FC88X_00654_00920	-------
FC88X_00720_00092	-------
FC88X_01014_00773	-------
FC88X_01109_01171	-------
FC88X_00019_00087	-------
FC88X_00014_00580	-------
FC88X_00163_00462	-------
FC88X_00163_00670	-------
FC88X_00254_00018	-------
FC88X_00371_00997	-------
FC88X_00506_00431	-------
FC88X_00846_00303	-------
FC88X_00814_01031	-------
FC88X_00811_01148	-------
FC88X_00880_00279	-------
FC88X_00905_00806	-------
FC88X_00988_00255	-------
FC88X_01094_00655	-------
FC88X_01057_01124	-------
FC88X_01115_00772	-------
FC88X_01141_00757	-------
FC88X_01063_01044	-------
FC88X_00905_00554	-------
FC88X_00508_00231	-------
FC88X_00768_01092	-------
FC88X_00950_00851	-------
FC88X_00145_00907	-------
FC88X_00072_00537	-------
FC88X_00700_00425	-------
FC88X_00989_00757	-------
FC88X_00613_01148	-------
FC88X_00844_01090	-------
FC88X_00514_01074	-------
FC88X_01011_00430	-------
FC88X_01084_00880	-------
FC88X_00067_00164	-------
FC88X_00072_00426	-------
FC88X_00413_00324	-------
FC88X_00415_01212	-------
FC88X_00091_00225	-------
FC88X_00138_00081	-------
FC88X_00159_00645	-------
FC88X_00204_01064	-------
FC88X_00312_01003	-------
FC88X_00582_00153	-------
FC88X_00802_01109	-------
FC88X_00871_00739	-------
FC88X_00932_00349	-------
FC88X_01128_00633	-------
FC88X_00529_00677	-------
FC88X_00432_00143	-------
FC88X_00113_00894	-------
FC88X_00227_01045	-------
FC88X_00253_00420	-------
FC88X_00443_01214	-------
FC88X_00506_00068	-------
FC88X_00564_01143	-------

FC88X_00666_00708	-------
FC88X_00315_00820	-------
FC88X_00939_00200	-------
FC88X_00462_00975	-------
FC88X_00515_01032	-------
FC88X_00594_01182	-------
FC88X_00657_01075	-------
FC88X_00727_00780	-------
FC88X_00712_00871	-------
FC88X_00703_00979	-------
FC88X_00842_00263	-------
FC88X_00807_01273	-------
FC88X_00879_00280	-------
FC88X_00858_00699	-------
FC88X_00942_00296	-------
FC88X_00901_00650	-------
FC88X_00902_01149	-------
FC88X_00906_01265	-------
FC88X_00970_00954	-------
FC88X_01021_00356	-------
FC88X_01060_00360	-------
FC88X_01091_00639	-------
FC88X_01123_00715	-------
FC88X_01136_01087	-------
FC88X_00030_00157	-------
FC88X_00049_00400	-------
FC88X_00028_00479	-------
FC88X_00030_00490	-------
FC88X_00005_00516	-------
FC88X_00006_00510	-------
FC88X_00070_00007	-------
FC88X_00060_00103	-------
FC88X_00092_00185	-------
FC88X_00095_00439	-------
FC88X_00078_00473	-------
FC88X_00070_00515	-------
FC88X_00074_00503	-------
FC88X_00078_00524	-------
FC88X_00116_00441	-------
FC88X_00104_00662	-------
FC88X_00147_00837	-------
FC88X_00155_00759	-------
FC88X_00183_00791	-------
FC88X_00188_00808	-------
FC88X_00226_00008	-------
FC88X_00233_00014	-------
FC88X_00233_00045	-------
FC88X_00217_00079	-------
FC88X_00243_00119	-------
FC88X_00229_00562	-------
FC88X_00259_00896	-------
FC88X_00266_01089	-------
FC88X_00270_01111	-------
FC88X_00297_01111	-------
FC88X_00327_00996	-------
FC88X_00330_01054	-------
FC88X_00338_01071	-------

FC88X_00355_00023	-------
FC88X_00356_00500	-------
FC88X_00391_00976	-------
FC88X_00398_01208	-------
FC88X_00442_00040	-------
FC88X_00406_00110	-------
FC88X_00438_00588	-------
FC88X_00401_01194	-------
FC88X_00429_01219	-------
FC88X_00492_00030	-------
FC88X_00474_00570	-------
FC88X_00497_00756	-------
FC88X_00491_01146	-------
FC88X_00466_01238	-------
FC88X_00485_01225	-------
FC88X_00509_00088	-------
FC88X_00530_00192	-------
FC88X_00546_00299	-------
FC88X_00510_00501	-------
FC88X_00509_01135	-------
FC88X_00560_00131	-------
FC88X_00592_01117	-------
FC88X_00604_00137	-------
FC88X_00640_01266	-------
FC88X_00698_00094	-------
FC88X_00690_00137	-------
FC88X_00740_00998	-------
FC88X_00701_01262	-------
FC88X_00742_01267	-------
FC88X_00793_00099	-------
FC88X_00769_00195	-------
FC88X_00769_00822	-------
FC88X_00783_00936	-------
FC88X_00760_01123	-------
FC88X_00795_01219	-------
FC88X_00831_00762	-------
FC88X_00845_00968	-------
FC88X_00828_01170	-------
FC88X_00897_00655	-------
FC88X_00871_00783	-------
FC88X_00884_00813	-------
FC88X_00895_01080	-------
FC88X_00884_01175	-------
FC88X_00858_01203	-------
FC88X_00924_00240	-------
FC88X_00927_00356	-------
FC88X_00932_00367	-------
FC88X_00935_00428	-------
FC88X_00907_00742	-------
FC88X_00922_00816	-------
FC88X_00924_00830	-------
FC88X_00940_01199	-------
FC88X_00987_00293	-------
FC88X_00993_00419	-------
FC88X_00999_00516	-------
FC88X_00982_00770	-------
FC88X_00988_00762	-------

FC88X_00967_00822	-------
FC88X_00973_00800	-------
FC88X_00999_00938	-------
FC88X_00985_01001	-------
FC88X_00954_01064	-------
FC88X_00953_01207	-------
FC88X_00989_01227	-------
FC88X_01040_00430	-------
FC88X_01021_00661	-------
FC88X_01029_00811	-------
FC88X_01018_00863	-------
FC88X_01016_00993	-------
FC88X_01035_01015	-------
FC88X_01004_01105	-------
FC88X_01010_01104	-------
FC88X_01034_01134	-------
FC88X_01038_01230	-------
FC88X_01076_00724	-------
FC88X_01085_00753	-------
FC88X_01062_00946	-------
FC88X_01059_00968	-------
FC88X_01081_00958	-------
FC88X_01120_00505	-------
FC88X_01116_00606	-------
FC88X_01126_00672	-------
FC88X_01114_00875	-------
FC88X_01117_00867	-------
FC88X_01113_00919	-------
FC88X_01131_00911	-------
FC88X_01109_01124	-------
FC88X_01112_01144	-------
FC88X_01142_01131	-------
FC88X_01116_01150	-------
FC88X_00463_00048	-------
FC88X_01072_00784	-------
FC88X_00065_00322	-------
FC88X_00438_01192	-------
FC88X_00567_00106	-------
FC88X_00822_00240	-------
FC88X_00550_01141	-------
FC88X_00906_00540	-------
FC88X_00442_00519	-------
FC88X_00487_01224	-------
FC88X_00371_01070	-------
FC88X_00875_00563	-------
FC88X_00929_00389	-------
FC88X_01092_01171	-------
FC88X_00497_00134	-------
FC88X_00923_00376	-------
FC88X_00275_00481	-------
FC88X_00284_00547	-------
FC88X_00789_01228	-------
FC88X_01142_00663	-------
FC88X_00575_00229	-------
FC88X_00909_00209	-------
FC88X_00925_00970	-------
FC88X_00795_01259	-------

FC88X_00192_00375	-------
FC88X_00450_00394	-------
FC88X_00803_00309	-------
FC88X_00897_00340	-------
FC88X_00482_01122	-------
FC88X_00581_01059	-------
FC88X_01075_00537	-------
FC88X_01056_00756	-------
FC88X_00455_00894	-------
FC88X_00893_00590	-------
FC88X_00989_00349	-------
FC88X_00263_00064	-------
FC88X_00204_00144	-------
FC88X_01072_00404	-------
FC88X_01142_01062	-------
FC88X_00899_00290	-------
FC88X_00817_01121	-------
FC88X_00816_00969	-------
FC88X_00070_00410	-------
FC88X_00483_00216	-------
FC88X_00933_00419	-------
FC88X_00970_01081	-------
FC88X_00769_01209	-------
FC88X_00476_01197	-------
FC88X_00910_00404	-------
FC88X_00921_00556	-------
FC88X_00765_01106	-------
FC88X_00598_00923	-------
FC88X_00044_00211	-------
FC88X_00977_00276	-------
FC88X_00308_00332	-------
FC88X_00423_00711	-------
FC88X_00813_00331	-------
FC88X_00208_00013	-------
FC88X_00576_00087	-------
FC88X_00553_01084	-------
FC88X_00847_00256	-------
FC88X_00632_00171	-------
FC88X_00590_00087	-------
FC88X_00047_00330	-------
FC88X_00004_00419	-------
FC88X_00076_00336	-------
FC88X_00147_00668	-------
FC88X_00160_00801	-------
FC88X_00173_00961	-------
FC88X_00231_00974	-------
FC88X_00307_00005	-------
FC88X_00313_01092	-------
FC88X_00383_00639	-------
FC88X_00442_01194	-------
FC88X_00475_00032	-------
FC88X_00477_00166	-------
FC88X_00636_01101	-------
FC88X_00686_01035	-------
FC88X_00745_01239	-------
FC88X_00780_00126	-------
FC88X_00819_00747	-------

FC88X_00842_00886	-------
FC88X_00836_01266	-------
FC88X_00893_01213	-------
FC88X_00918_00909	-------
FC88X_01112_00835	-------
FC88X_01141_01027	-------
FC88X_00992_00537	-------
FC88X_00916_01005	-------
FC88X_01077_00891	-------
FC88X_00854_00945	-------
FC88X_00596_00197	-------
FC88X_00935_00238	-------
FC88X_00672_00701	-------
FC88X_00073_00006	-------
FC88X_00879_00374	-------
FC88X_01121_00787	-------
FC88X_00772_01233	-------
FC88X_01088_00844	-------
FC88X_01026_00363	-------
FC88X_01030_00990	-------
FC88X_00720_01002	-------
FC88X_00307_00187	-------
FC88X_00073_00734	-------
FC88X_00307_00888	-------
FC88X_00992_00565	-------
FC88X_00019_00509	-------
FC88X_00741_00971	-------
FC88X_00140_00587	-------
FC88X_00249_00925	-------
FC88X_00503_01184	-------
FC88X_00755_01068	-------
FC88X_00924_00659	-------
FC88X_01049_01197	-------
FC88X_01096_00612	-------
FC88X_00021_00252	-------
FC88X_00087_00635	-------
FC88X_00125_00625	-------
FC88X_00152_00657	-------
FC88X_00320_00038	-------
FC88X_00408_01060	-------
FC88X_00647_01110	-------
FC88X_00940_00585	-------
FC88X_00945_00694	-------
FC88X_00966_00903	-------
FC88X_00970_00999	-------
FC88X_00972_01251	-------
FC88X_01037_00522	-------
FC88X_01030_00753	-------
FC88X_01145_00875	-------
FC88X_01038_00733	-------
FC88X_00618_00688	-------
FC88X_00137_00524	-------
FC88X_00949_00719	-------
FC88X_01080_00969	-------
FC88X_00123_00454	-------
FC88X_00914_01072	-------
FC88X_00043_00404	-------

FC88X_00953_00454 -------
FC88X_00398_00015 -------
FC88X_00302_00044 -------
FC88X_00369_00829 -------
FC88X_00857_01214 -------
FC88X_01101_00468 -------
FC88X_00694_00226 -------
FC88X_01093_00862 -------
FC88X_00611_00971 -------
FC88X_00708_01231 -------
FC88X_00822_00102 -------
FC88X_00172_00013 -------
FC88X_00880_00408 -------
FC88X_00125_00265 -------
FC88X_00849_00332 -------
FC88X_00806_01227 -------
FC88X_00064_00424 -------
FC88X_00158_00895 -------
FC88X_00500_00595 -------
FC88X_00778_01140 -------
FC88X_00825_00937 -------
FC88X_00865_00501 -------
FC88X_01026_00978 -------
FC88X_00525_00160 -------
FC88X_01093_00570 -------
FC88X_00986_00543 -------
FC88X_00150_00326 -------
FC88X_00160_01028 -------
FC88X_00338_00247 -------
FC88X_00583_01212 -------
FC88X_00140_00565 -------
FC88X_00035_00086 -------
FC88X_00921_00332 -------
FC88X_00118_00131 -------
FC88X_00152_00932 -------
FC88X_00200_01053 -------
FC88X_00362_01028 -------
FC88X_00497_00544 -------
FC88X_00543_01004 -------
FC88X_00594_00993 -------
FC88X_00560_01191 -------
FC88X_00822_00124 -------
FC88X_00959_00753 -------
FC88X_01012_00692 -------
FC88X_01033_01176 -------
FC88X_01138_00644 -------
FC88X_01146_00741 -------
FC88X_00098_00319 -------
FC88X_00081_00372 -------
FC88X_00135_00800 -------
FC88X_00186_01039 -------
FC88X_00263_01072 -------
FC88X_00322_00502 -------
FC88X_00353_00634 -------
FC88X_00423_01191 -------
FC88X_00529_00101 -------
FC88X_00537_00143 -------

FC88X_00551_00844	-------
FC88X_00602_01148	-------
FC88X_00780_00862	-------
FC88X_00750_00977	-------
FC88X_00814_00261	-------
FC88X_00931_00759	-------
FC88X_00961_01096	-------
FC88X_01006_00974	-------
FC88X_01130_00788	-------
FC88X_01135_01176	-------
FC88X_00583_00142	-------
FC88X_00995_01269	-------
FC88X_00601_01237	-------
FC88X_00981_00966	-------
FC88X_00009_00245	-------
FC88X_00796_00928	-------
FC88X_00067_00016	-------
FC88X_00864_01177	-------
FC88X_00980_00636	-------
FC88X_00192_00987	-------
FC88X_00637_01134	-------
FC88X_00269_00940	-------
FC88X_01095_00619	-------
FC88X_00616_00661	-------
FC88X_00043_00058	-------
FC88X_00937_00676	-------
FC88X_00712_01251	-------
FC88X_01049_00487	-------
FC88X_00032_00793	-------
FC88X_00109_00231	-------
FC88X_00126_00643	-------
FC88X_01100_01047	-------
FC88X_00468_00984	-------
FC88X_00283_00960	-------
FC88X_00596_00185	-------
FC88X_00042_00538	-------
FC88X_00921_00343	-------
FC88X_00138_00710	-------
FC88X_00375_01199	-------
FC88X_00686_01144	-------
FC88X_00627_01240	-------
FC88X_01094_01187	-------
FC88X_00836_00855	-------
FC88X_00931_00802	-------
FC88X_00954_01076	-------
FC88X_00086_00520	-------
FC88X_00071_00093	-------
FC88X_00161_00568	-------
FC88X_00430_00112	-------
FC88X_00694_01029	-------
FC88X_00655_01114	-------
FC88X_00740_00221	-------
FC88X_00902_00239	-------
FC88X_00946_00663	-------
FC88X_00018_00097	-------
FC88X_00052_00202	-------
FC88X_00053_00754	-------

FC88X_00175_00600	-------
FC88X_00229_00097	-------
FC88X_00220_00488	-------
FC88X_00232_01012	-------
FC88X_00347_00563	-------
FC88X_00351_01170	-------
FC88X_00370_01167	-------
FC88X_00447_01160	-------
FC88X_00404_01213	-------
FC88X_00469_00689	-------
FC88X_00586_01106	-------
FC88X_00600_00173	-------
FC88X_00601_01160	-------
FC88X_00723_00384	-------
FC88X_00701_00994	-------
FC88X_00795_01183	-------
FC88X_00859_00968	-------
FC88X_00891_01059	-------
FC88X_00926_00183	-------
FC88X_00928_01196	-------
FC88X_00963_00404	-------
FC88X_00977_01036	-------
FC88X_01067_00896	-------
FC88X_01077_01077	-------
FC88X_00443_00098	-------
FC88X_00490_01143	-------
FC88X_00675_00188	-------
FC88X_00308_00306	-------
FC88X_00911_01019	-------
FC88X_00880_01036	-------
FC88X_00858_01082	-------
FC88X_00780_01239	-------
FC88X_00105_00457	-------
FC88X_01077_00673	-------
FC88X_00997_01129	-------
FC88X_01118_01207	-------
FC88X_00458_00792	-------
FC88X_00723_01009	-------
FC88X_00619_00541	-------
FC88X_00435_00141	-------
FC88X_00535_01241	-------
FC88X_00611_01140	-------
FC88X_00672_00424	-------
FC88X_00073_00108	-------
FC88X_00177_00121	-------
FC88X_00865_00733	-------
FC88X_00925_00529	-------
FC88X_00928_00484	-------
FC88X_00568_00699	-------
FC88X_00210_00979	-------
FC88X_00649_01083	-------
FC88X_00792_00174	-------
FC88X_01032_00874	-------
FC88X_00008_00458	-------
FC88X_00055_00190	-------
FC88X_00442_00822	-------
FC88X_01086_00760	-------

FC88X_01128_00528	-------
FC88X_00194_00815	-------
FC88X_00482_00322	-------
FC88X_00189_00902	-------
FC88X_00040_00112	-------
FC88X_00045_00712	-------
FC88X_00866_00933	-------
FC88X_00085_00487	-------
FC88X_00586_01241	-------
FC88X_00677_00906	-------
FC88X_00388_01143	-------
FC88X_00895_00295	-------
FC88X_00871_00327	-------
FC88X_00144_00425	-------
FC88X_00035_00653	-------
FC88X_00268_00019	-------
FC88X_00602_00105	-------
FC88X_00102_00911	-------
FC88X_01006_00761	-------
FC88X_00594_00425	-------
FC88X_00975_00437	-------
FC88X_00688_01086	-------
FC88X_00058_00846	-------
FC88X_00099_00478	-------
FC88X_00504_00171	-------
FC88X_00364_00080	-------
FC88X_00511_00158	-------
FC88X_00526_01019	-------
FC88X_00908_00457	-------
FC88X_00908_00891	-------
FC88X_00525_01193	-------
FC88X_00753_01202	-------
FC88X_00818_01061	-------
FC88X_01016_01062	-------
FC88X_01071_00542	-------
FC88X_01079_00510	-------
FC88X_00972_00718	-------
FC88X_00426_00945	-------
FC88X_00599_00659	-------
FC88X_00178_01050	-------
FC88X_00095_00506	-------
FC88X_00894_00668	-------
FC88X_00740_01236	-------
FC88X_00949_00771	-------
FC88X_00381_00766	-------
FC88X_00787_00866	-------
FC88X_00056_00319	-------
FC88X_00514_01066	-------
FC88X_00825_00278	-------
FC88X_00463_00051	-------
FC88X_01020_00803	-------
FC88X_00894_00837	-------
FC88X_00606_01269	-------
FC88X_00947_00727	-------
FC88X_00960_01128	-------
FC88X_01058_01127	-------
FC88X_01104_01070	-------

FC88X_00437_01202	-------
FC88X_00535_01078	-------
FC88X_00101_00119	-------
FC88X_00395_01144	-------
FC88X_00132_00229	-------
FC88X_00434_01191	-------
FC88X_00596_00819	-------
FC88X_00757_00259	-------
FC88X_00883_01215	-------
FC88X_00914_01039	-------
FC88X_00045_00097	-------
FC88X_00807_00880	-------
FC88X_00934_00666	-------
FC88X_00489_01230	-------
FC88X_00829_00268	-------
FC88X_01066_01031	-------
FC88X_00455_00032	-------
FC88X_00485_00973	-------
FC88X_00822_00869	-------
FC88X_01123_00773	-------
FC88X_00096_00152	-------
FC88X_00528_01069	-------
FC88X_00989_00544	-------
FC88X_00570_00927	-------
FC88X_01029_00716	-------
FC88X_00667_00290	-------
FC88X_00860_00698	-------
FC88X_00912_00511	-------
FC88X_01006_00420	-------
FC88X_00028_00226	-------
FC88X_00065_00744	-------
FC88X_00380_00285	-------
FC88X_00178_00162	-------
FC88X_00893_01217	-------
FC88X_01052_00718	-------
FC88X_00049_00477	-------
FC88X_00232_00747	-------
FC88X_00053_00699	-------
FC88X_00160_00779	-------
FC88X_00360_01187	-------
FC88X_00498_01243	-------
FC88X_00598_01176	-------
FC88X_00582_01237	-------
FC88X_00660_00457	-------
FC88X_00732_00289	-------
FC88X_00747_00961	-------
FC88X_00717_01102	-------
FC88X_00862_01147	-------
FC88X_00463_00520	-------
FC88X_00522_00215	-------
FC88X_00779_00827	-------
FC88X_00983_00874	-------
FC88X_00138_00319	-------
FC88X_00608_00505	-------
FC88X_00417_00880	-------
FC88X_00479_00653	-------
FC88X_00164_00063	-------

FC88X_00581_00271	-------
FC88X_00463_01177	-------
FC88X_01145_01274	-------
FC88X_00121_00377	-------
FC88X_00321_01164	-------
FC88X_00350_01026	-------
FC88X_00357_00967	-------
FC88X_00146_00923	-------
FC88X_00645_00662	-------
FC88X_01089_00771	-------
FC88X_00578_00104	-------
FC88X_00559_01188	-------
FC88X_01102_01012	-------
FC88X_01134_01087	-------
FC88X_00048_00267	-------
FC88X_01101_00685	-------
FC88X_00435_00402	-------
FC88X_00576_01120	-------
FC88X_00435_01221	-------
FC88X_00275_00931	-------
FC88X_00460_01172	-------
FC88X_00509_00156	-------
FC88X_00547_01190	-------
FC88X_00611_00683	-------
FC88X_00633_00765	-------
FC88X_00628_00967	-------
FC88X_00810_00219	-------
FC88X_00860_00140	-------
FC88X_01013_00986	-------
FC88X_00405_01162	-------
FC88X_00871_00912	-------
FC88X_00880_00993	-------
FC88X_00960_00367	-------
FC88X_00357_00924	-------
FC88X_00950_00951	-------
FC88X_01137_01064	-------
FC88X_00121_00905	-------
FC88X_00542_00063	-------
FC88X_01129_00606	-------
FC88X_00845_01091	-------
FC88X_00614_00987	-------
FC88X_00076_00509	-------
FC88X_00815_01006	-------
FC88X_00214_01087	-------
FC88X_00742_00237	-------
FC88X_01099_00722	-------
FC88X_00050_00357	-------
FC88X_00995_00798	-------
FC88X_00015_00318	-------
FC88X_00014_00454	-------
FC88X_00194_00118	-------
FC88X_01120_00704	-------
FC88X_00058_00239	-------
FC88X_00712_01023	-------
FC88X_00707_00148	-------
FC88X_00255_01004	-------
FC88X_00704_00192	-------

FC88X_00243_01057	-------
FC88X_00974_00377	-------
FC88X_01065_01260	-------
FC88X_00102_00104	-------
FC88X_00896_01086	-------
FC88X_01132_01170	-------
FC88X_00366_01080	-------
FC88X_00196_00006	-------
FC88X_00422_00413	-------
FC88X_00934_00346	-------
FC88X_00388_00054	-------
FC88X_00503_01019	-------
FC88X_00883_00678	-------
FC88X_00895_00537	-------
FC88X_00949_00339	-------
FC88X_00149_00920	-------
FC88X_00915_00854	-------
FC88X_01047_00988	-------
FC88X_00683_00087	-------
FC88X_00561_00984	-------
FC88X_01113_00678	-------
FC88X_00232_00374	-------
FC88X_01080_00650	-------
FC88X_01046_01251	-------
FC88X_00095_00661	-------
FC88X_00872_01019	-------
FC88X_00544_01162	-------
FC88X_01089_00607	-------
FC88X_00353_01159	-------
FC88X_01032_00635	-------
FC88X_01129_00844	-------
FC88X_00826_00881	-------
FC88X_01002_01052	-------
FC88X_00072_00788	-------
FC88X_00842_01201	-------
FC88X_00398_01056	-------
FC88X_00957_00648	-------
FC88X_00827_00318	-------
FC88X_00814_01148	-------
FC88X_00628_00152	-------
FC88X_00234_00933	-------
FC88X_00050_00241	-------
FC88X_00811_00190	-------
FC88X_00814_00817	-------
FC88X_00459_00987	-------
FC88X_01132_00626	-------
FC88X_00561_00596	-------
FC88X_00447_00999	-------
FC88X_00140_00225	-------
FC88X_00886_00602	-------
FC88X_00200_00027	-------
FC88X_01106_00842	-------
FC88X_00458_00038	-------
FC88X_00293_00668	-------
FC88X_00358_00388	-------
FC88X_00552_00743	-------
FC88X_00885_00582	-------

FC88X_00906_00384	-------
FC88X_00234_00115	-------
FC88X_00421_00514	-------
FC88X_00835_01240	-------
FC88X_00890_00393	-------
FC88X_00902_00759	-------
FC88X_00937_01045	-------
FC88X_00968_00285	-------
FC88X_00667_01079	-------
FC88X_00882_00453	-------
FC88X_01011_01178	-------
FC88X_01028_00618	-------
FC88X_00592_01172	-------
FC88X_00363_00411	-------
FC88X_00927_00922	-------
FC88X_00089_00573	-------
FC88X_00910_00936	-------
FC88X_00461_01141	-------
FC88X_00986_00809	-------
FC88X_01089_01190	-------
FC88X_00090_00931	-------
FC88X_00751_00709	-------
FC88X_00788_01228	-------
FC88X_00662_01132	-------
FC88X_00781_00248	-------
FC88X_00156_00874	-------
FC88X_00207_00054	-------
FC88X_00376_00552	-------
FC88X_00391_01005	-------
FC88X_00431_00397	-------
FC88X_00461_00535	-------
FC88X_00613_01110	-------
FC88X_00661_00143	-------
FC88X_00707_00085	-------
FC88X_00818_01161	-------
FC88X_00939_00482	-------
FC88X_00939_00535	-------
FC88X_01091_00478	-------
FC88X_00405_01071	-------
FC88X_00979_01049	-------
FC88X_01053_01082	-------
FC88X_00604_00673	-------
FC88X_00796_00971	-------
FC88X_00897_00201	-------
FC88X_00860_00872	-------
FC88X_01031_00848	-------
FC88X_00064_00445	-------
FC88X_00249_00616	-------
FC88X_00924_00496	-------
FC88X_00928_00823	-------
FC88X_01015_01129	-------
FC88X_00472_01066	-------
FC88X_00753_00584	-------
FC88X_00927_00411	-------
FC88X_00867_00269	-------
FC88X_00625_00123	-------
FC88X_01072_00842	-------

FC88X_01131_00977	-------
FC88X_00958_00777	-------
FC88X_00907_00738	-------
FC88X_00544_00559	-------
FC88X_00583_01265	-------
FC88X_00500_00810	-------
FC88X_00988_01093	-------
FC88X_00788_00177	-------
FC88X_01131_00958	-------
FC88X_00743_00727	-------
FC88X_01072_00898	-------
FC88X_00074_00886	-------
FC88X_00420_00052	-------
FC88X_00949_00896	-------
FC88X_00091_00281	-------
FC88X_00704_00999	-------
FC88X_00361_01102	-------
FC88X_00717_01230	-------
FC88X_00756_00868	-------
FC88X_00971_00669	-------
FC88X_00993_00464	-------
FC88X_00525_00623	-------
FC88X_00403_00753	-------
FC88X_00991_00906	-------
FC88X_01129_00979	-------
FC88X_00574_00647	-------
FC88X_00627_00160	-------
FC88X_00937_00462	-------
FC88X_00866_00676	-------
FC88X_00007_00424	-------
FC88X_00065_00211	-------
FC88X_00060_00264	-------
FC88X_00590_01094	-------
FC88X_00848_00920	-------
FC88X_00303_01010	-------
FC88X_00561_00186	-------
FC88X_00741_00265	-------
FC88X_00887_01076	-------
FC88X_00922_00878	-------
FC88X_00935_01121	-------
FC88X_01058_01255	-------
FC88X_01135_00904	-------
FC88X_00061_00491	-------
FC88X_00456_01213	-------
FC88X_00505_00843	-------
FC88X_00669_01179	-------
FC88X_00807_01109	-------
FC88X_00716_01187	-------
FC88X_00675_01182	-------
FC88X_00960_00527	-------
FC88X_01142_00852	-------
FC88X_00408_01179	-------
FC88X_00679_01274	-------
FC88X_00283_00944	-------
FC88X_00994_00627	-------
FC88X_00896_00424	-------
FC88X_01086_00415	-------

FC88X_00986_00300	-------
FC88X_01036_00552	-------
FC88X_00015_00399	-------
FC88X_01142_01033	-------
FC88X_01024_00341	-------
FC88X_00157_00446	-------
FC88X_00776_00622	-------
FC88X_00987_00895	-------
FC88X_00267_00999	-------
FC88X_00219_00639	-------
FC88X_00398_00567	-------
FC88X_00502_00545	-------
FC88X_00943_00862	-------
FC88X_00953_01149	-------
FC88X_00750_00847	-------
FC88X_00849_00256	-------
FC88X_00308_00569	C------
FC88X_00876_01007	-------
FC88X_01062_00922	-------
FC88X_00803_00409	-------
FC88X_00809_00799	-------
FC88X_00939_00911	-------
FC88X_00938_00369	-------
FC88X_00857_00195	-------
FC88X_01079_01195	-------
FC88X_00124_00799	-------
FC88X_00917_00918	-------
FC88X_01067_00933	-------
FC88X_00123_00107	-------
FC88X_00034_00798	-------
FC88X_00957_01271	-------
FC88X_00607_01239	-------
FC88X_00839_01152	-------
FC88X_01049_00908	-------
FC88X_00866_00377	-------
FC88X_01116_01075	-------
FC88X_00561_01183	-------
FC88X_00125_00570	-------
FC88X_00837_00779	-------
FC88X_00972_00723	-------
FC88X_00819_00786	-------
FC88X_00322_00997	-------
FC88X_00613_00947	-------
FC88X_00653_01153	-------
FC88X_00678_01168	-------
FC88X_00012_00428	-------
FC88X_00107_00378	-------
FC88X_00317_00833	-------
FC88X_00770_00140	-------
FC88X_00750_00936	-------
FC88X_00638_01102	-------
FC88X_00348_01102	-------
FC88X_00979_00791	-------
FC88X_00049_00297	-------
FC88X_00083_00125	-------
FC88X_00860_00260	-------
FC88X_00686_01016	-------

FC88X_00067_00862 -------
FC88X_00881_00367 -------
FC88X_00170_00094 -------
FC88X_00519_00258 -------
FC88X_00573_01238 -------
FC88X_00716_00062 -------
FC88X_00885_00948 -------
FC88X_00681_00466 -------
FC88X_00928_00924 -------
FC88X_00064_00691 -------
FC88X_00933_01040 -------
FC88X_00810_01094 -------
FC88X_00995_00901 -------
FC88X_00082_00805 -------
FC88X_00536_01067 -------
FC88X_00622_01234 -------
FC88X_00964_01262 -------
FC88X_00987_00669 -------
FC88X_00017_00396 -------
FC88X_00684_01040 -------
FC88X_00134_00492 -------
FC88X_00745_01248 -------
FC88X_00154_00648 -------
FC88X_00219_01054 -------
FC88X_00431_00758 -------
FC88X_00820_00946 -------
FC88X_00895_00902 -------
FC88X_00023_00617 -------
FC88X_00366_01053 -------
FC88X_00677_01204 -------
FC88X_00881_00720 -------
FC88X_01083_00504 -------
FC88X_00061_00723 -------
FC88X_00421_00988 -------
FC88X_00747_01132 -------
FC88X_01032_00449 -------
FC88X_00869_00822 -------
FC88X_01123_01088 -------
FC88X_00378_01062 -------
FC88X_00452_01214 -------
FC88X_00512_00343 -------
FC88X_00580_00296 -------
FC88X_00564_01018 -------
FC88X_00948_01101 -------
FC88X_00986_00245 -------
FC88X_00976_00271 -------
FC88X_01084_00709 -------
FC88X_00730_00205 -------
FC88X_00657_00111 -------
FC88X_00598_00054 -------
FC88X_00224_00332 -------
FC88X_00310_01038 -------
FC88X_00867_00871 T------
FC88X_00175_00808 -------
FC88X_00564_01015 -------
FC88X_00177_00992 -------
FC88X_01095_00809 -------

FC88X_00061_00716	-------
FC88X_00419_00041	-------
FC88X_00795_00734	-------
FC88X_01107_00876	-------
FC88X_00609_01177	-------
FC88X_00113_00916	-------
FC88X_00150_00792	-------
FC88X_00760_00913	-------
FC88X_00155_00390	-------
FC88X_00183_00814	-------
FC88X_00192_01032	-------
FC88X_00220_00881	-------
FC88X_00409_00679	-------
FC88X_00718_00482	-------
FC88X_00618_00151	-------
FC88X_00206_00026	-------
FC88X_00580_00426	-------
FC88X_00114_00484	-------
FC88X_00859_01225	-------
FC88X_00929_00510	-------
FC88X_00914_01167	-------
FC88X_00959_00554	-------
FC88X_00295_01039	-------
FC88X_00815_00194	-------
FC88X_00526_01195	-------
FC88X_00914_00539	-------
FC88X_00408_01124	-------
FC88X_00744_00902	-------
FC88X_00729_00293	-------
FC88X_00894_00919	-------
FC88X_00082_00229	-------
FC88X_00750_01067	-------
FC88X_00864_00911	-------
FC88X_00922_00272	-------
FC88X_00945_00600	-------
FC88X_01117_00494	-------
FC88X_00844_01033	-------
FC88X_01024_01007	-------
FC88X_00147_00692	-------
FC88X_00067_00475	-------
FC88X_00963_00427	-------
FC88X_01072_00785	-------
FC88X_00810_00114	-------
FC88X_00744_01134	-------
FC88X_01111_01125	-------
FC88X_01065_00716	-------
FC88X_00918_00364	-------
FC88X_00619_01046	-------
FC88X_00632_01019	-------
FC88X_00010_00063	-------
FC88X_00019_00102	-------
FC88X_00028_00339	-------
FC88X_00221_00049	-------
FC88X_00527_00498	-------
FC88X_00070_00804	-------
FC88X_00474_00965	-------
FC88X_00473_01178	-------

FC88X_00561_01117	-------
FC88X_00852_00172	-------
FC88X_01028_01013	-------
FC88X_00013_00623	-------
FC88X_00048_00805	-------
FC88X_00091_00896	-------
FC88X_00484_00122	-------
FC88X_00546_01127	-------
FC88X_00615_01094	-------
FC88X_00701_01215	-------
FC88X_00812_00874	-------
FC88X_00927_00890	-------
FC88X_00010_00276	-------
FC88X_00048_00566	-------
FC88X_00085_00205	-------
FC88X_00143_00445	-------
FC88X_00113_00450	-------
FC88X_00146_00578	-------
FC88X_00107_00913	-------
FC88X_00156_00422	-------
FC88X_00193_00886	-------
FC88X_00162_00936	-------
FC88X_00161_00984	-------
FC88X_00182_01022	-------
FC88X_00188_01052	-------
FC88X_00220_00017	-------
FC88X_00215_00092	-------
FC88X_00228_00095	-------
FC88X_00235_00829	-------
FC88X_00222_01040	-------
FC88X_00238_01027	-------
FC88X_00284_01083	-------
FC88X_00339_01134	-------
FC88X_00386_00010	-------
FC88X_00376_00384	-------
FC88X_00389_00913	-------
FC88X_00352_01026	-------
FC88X_00409_00451	-------
FC88X_00440_01027	-------
FC88X_00479_00030	-------
FC88X_00493_00385	-------
FC88X_00484_01111	-------
FC88X_00474_01172	-------
FC88X_00522_00130	-------
FC88X_00501_00198	-------
FC88X_00539_00718	-------
FC88X_00529_01007	-------
FC88X_00537_01024	-------
FC88X_00544_01124	-------
FC88X_00549_01172	-------
FC88X_00552_00066	-------
FC88X_00568_01080	-------
FC88X_00563_01139	-------
FC88X_00641_00193	-------
FC88X_00601_00287	-------
FC88X_00612_00648	-------
FC88X_00647_00931	-------

FC88X_00618_01217	-------
FC88X_00615_01272	-------
FC88X_00769_00312	-------
FC88X_00765_01048	-------
FC88X_00777_01175	-------
FC88X_00763_01232	-------
FC88X_00767_01236	-------
FC88X_00798_01251	-------
FC88X_00830_00769	-------
FC88X_00818_00854	-------
FC88X_00896_00198	-------
FC88X_00882_00325	-------
FC88X_00875_00525	-------
FC88X_00882_00566	-------
FC88X_00891_00793	-------
FC88X_00857_00833	-------
FC88X_00890_01009	-------
FC88X_00933_00186	-------
FC88X_00941_00373	-------
FC88X_00932_00571	-------
FC88X_00910_00611	-------
FC88X_00916_00806	-------
FC88X_00914_01199	-------
FC88X_00993_00561	-------
FC88X_00996_00911	-------
FC88X_00962_01111	-------
FC88X_01033_00664	-------
FC88X_01073_00404	-------
FC88X_01087_00633	-------
FC88X_01098_00965	-------
FC88X_01089_01167	-------
FC88X_01127_00703	-------
FC88X_01101_00792	-------
FC88X_00020_00315	-------
FC88X_00005_00357	-------
FC88X_00006_00383	-------
FC88X_00040_00472	-------
FC88X_00061_00032	-------
FC88X_00068_00062	-------
FC88X_00081_00104	-------
FC88X_00070_00345	-------
FC88X_00098_00362	-------
FC88X_00092_00474	-------
FC88X_00097_00475	-------
FC88X_00121_00065	-------
FC88X_00138_00140	-------
FC88X_00125_00224	-------
FC88X_00105_00432	-------
FC88X_00104_00689	-------
FC88X_00138_00696	-------
FC88X_00135_00759	-------
FC88X_00149_00795	-------
FC88X_00104_00885	-------
FC88X_00110_00877	-------
FC88X_00136_00925	-------
FC88X_00143_00984	-------
FC88X_00163_00043	-------

FC88X_00174_00075	-------
FC88X_00222_00095	-------
FC88X_00204_00107	-------
FC88X_00208_00115	-------
FC88X_00237_00650	-------
FC88X_00242_00747	-------
FC88X_00240_00965	-------
FC88X_00240_01077	-------
FC88X_00276_00565	-------
FC88X_00258_00749	-------
FC88X_00251_00972	-------
FC88X_00301_00665	-------
FC88X_00370_00596	-------
FC88X_00385_00619	-------
FC88X_00392_01212	-------
FC88X_00432_00017	-------
FC88X_00448_00468	-------
FC88X_00440_00674	-------
FC88X_00417_00998	-------
FC88X_00437_01054	-------
FC88X_00429_01103	-------
FC88X_00428_01211	-------
FC88X_00470_00077	-------
FC88X_00470_00386	-------
FC88X_00468_01099	-------
FC88X_00462_01135	-------
FC88X_00480_01109	-------
FC88X_00456_01167	-------
FC88X_00544_01080	-------
FC88X_00536_01124	-------
FC88X_00501_01151	-------
FC88X_00559_00772	-------
FC88X_00571_01183	-------
FC88X_00611_00123	-------
FC88X_00637_00871	-------
FC88X_00609_01158	-------
FC88X_00629_01193	-------
FC88X_00654_00237	-------
FC88X_00691_00364	-------
FC88X_00660_00897	-------
FC88X_00691_00948	-------
FC88X_00662_01251	-------
FC88X_00732_00059	-------
FC88X_00744_00915	-------
FC88X_00732_00959	-------
FC88X_00740_01158	-------
FC88X_00789_00100	-------
FC88X_00779_00219	-------
FC88X_00768_00260	-------
FC88X_00756_00860	-------
FC88X_00756_01250	-------
FC88X_00838_00243	-------
FC88X_00803_00250	-------
FC88X_00817_00672	-------
FC88X_00873_00648	-------
FC88X_00850_00793	-------
FC88X_00896_00832	-------

FC88X_00896_00845	-------
FC88X_00874_00971	-------
FC88X_00898_01121	-------
FC88X_00913_00224	-------
FC88X_00922_00381	-------
FC88X_00901_00583	-------
FC88X_00916_00559	-------
FC88X_00931_00578	-------
FC88X_00904_00647	-------
FC88X_00907_00745	-------
FC88X_00945_00768	-------
FC88X_00919_00939	-------
FC88X_00937_00984	-------
FC88X_00929_01205	-------
FC88X_00958_00225	-------
FC88X_00967_00539	-------
FC88X_00968_00524	-------
FC88X_00985_00548	-------
FC88X_00961_00557	-------
FC88X_00965_00621	-------
FC88X_00974_00781	-------
FC88X_00982_00847	-------
FC88X_00959_01028	-------
FC88X_00989_01183	-------
FC88X_00966_01207	-------
FC88X_01009_00433	-------
FC88X_01047_00409	-------
FC88X_01045_00460	-------
FC88X_01000_00546	-------
FC88X_01013_01035	-------
FC88X_01046_01234	-------
FC88X_01006_01274	-------
FC88X_01037_01256	-------
FC88X_01078_00643	-------
FC88X_01098_00629	-------
FC88X_01072_00828	-------
FC88X_01051_01027	-------
FC88X_01103_00435	-------
FC88X_01103_00510	-------
FC88X_01120_00748	-------
FC88X_01104_00858	-------
FC88X_01139_00902	-------
FC88X_01134_00954	-------
FC88X_01138_00956	-------
FC88X_01119_01028	-------
FC88X_01139_01007	-------
FC88X_00426_00231	-------
FC88X_00128_00693	-------
FC88X_00328_01078	-------
FC88X_00644_00365	-------
FC88X_01110_00883	-------
FC88X_00115_00707	-------
FC88X_01136_01166	-------
FC88X_00145_01001	-------
FC88X_00050_00620	-------
FC88X_01015_01027	-------
FC88X_00957_00510	-------

FC88X_00807_00493	-------
FC88X_00101_00725	-------
FC88X_00609_01141	-------
FC88X_01015_00454	-------
FC88X_01026_01186	-------
FC88X_01137_00880	-------
FC88X_00060_00128	-------
FC88X_00969_00940	-------
FC88X_01139_00864	-------
FC88X_00042_00196	-------
FC88X_00394_01032	-------
FC88X_01130_01109	-------
FC88X_00094_00495	-------
FC88X_00312_01016	-------
FC88X_00367_01151	-------
FC88X_00815_00167	-------
FC88X_01080_01262	-------
FC88X_00895_00982	-------
FC88X_00916_01008	-------
FC88X_00971_00625	-------
FC88X_00537_00948	-------
FC88X_00623_01026	-------
FC88X_00178_00867	-------
FC88X_00093_00596	-------
FC88X_00104_00218	-------
FC88X_00118_00073	-------
FC88X_00579_00950	-------
FC88X_00865_00765	-------
FC88X_00640_01121	-------
FC88X_00733_00942	-------
FC88X_00089_00385	-------
FC88X_00367_01180	-------
FC88X_01128_00844	-------
FC88X_00079_00910	-------
FC88X_00697_01237	-------
FC88X_00786_00577	-------
FC88X_00213_00900	-------
FC88X_00648_00948	-------
FC88X_00205_00082	-------
FC88X_00339_01041	-------
FC88X_00137_00476	-------
FC88X_00945_00270	-------
FC88X_01071_00703	-------
FC88X_00864_00774	-------
FC88X_00469_00090	-------
FC88X_00203_00134	-------
FC88X_00514_00981	-------
FC88X_00809_00816	-------
FC88X_00155_00158	-------
FC88X_01034_00726	-------
FC88X_00504_01048	-------
FC88X_00549_01243	-------
FC88X_01012_00811	-------
FC88X_00805_00315	-------
FC88X_00384_01189	-------
FC88X_00654_01138	-------
FC88X_00917_00438	-------

FC88X_01022_00482	-------
FC88X_01004_00524	-------
FC88X_01059_00689	-------
FC88X_00190_01037	-------
FC88X_00920_01230	-------
FC88X_00491_00156	-------
FC88X_00889_00569	-------
FC88X_01086_00743	-------
FC88X_01120_01064	-------
FC88X_00090_00425	-------
FC88X_00488_00356	-------
FC88X_01005_00941	-------
FC88X_01116_00599	-------
FC88X_00976_00529	-------
FC88X_00751_01186	-------
FC88X_00841_00314	-------
FC88X_00061_00362	-------
FC88X_00630_00088	-------
FC88X_00874_00385	-------
FC88X_00247_00943	-------
FC88X_00399_00342	-------
FC88X_00884_00989	-------
FC88X_00463_01220	-------
FC88X_00126_00818	-------
FC88X_01025_01055	-------
FC88X_00887_00146	-------
FC88X_00478_00734	-------
FC88X_01001_01146	-------
FC88X_01046_01048	-------
FC88X_00239_00987	-------
FC88X_00920_00377	-------
FC88X_00796_00901	-------
FC88X_01013_00573	-------
FC88X_00064_00191	-------
FC88X_00123_00579	-------
FC88X_00161_00875	-------
FC88X_00594_00140	-------
FC88X_00551_00330	-------
FC88X_00703_00394	-------
FC88X_00844_00976	-------
FC88X_00822_01178	-------
FC88X_01033_00323	-------
FC88X_01016_00644	-------
FC88X_01032_00995	-------
FC88X_01051_00517	-------
FC88X_00289_00974	-------
FC88X_00299_01092	-------
FC88X_00340_00422	-------
FC88X_00315_01056	-------
FC88X_00477_00637	-------
FC88X_00631_00982	-------
FC88X_00766_00999	-------
FC88X_00813_00121	-------
FC88X_00878_00645	-------
FC88X_00904_00393	-------
FC88X_00300_00319	-------
FC88X_00055_00415	-------

FC88X_00763_00908	-------
FC88X_00654_00168	-------
FC88X_00987_01003	-------
FC88X_00694_00453	-------
FC88X_01107_00947	-------
FC88X_01124_01260	-------
FC88X_00964_00707	-------
FC88X_00162_00848	-------
FC88X_00228_01028	-------
FC88X_00642_01241	-------
FC88X_01007_00338	-------
FC88X_01023_00312	-------
FC88X_01077_01199	-------
FC88X_00085_00265	-------
FC88X_00192_00392	-------
FC88X_00385_00987	-------
FC88X_00472_01000	-------
FC88X_00488_01210	-------
FC88X_00840_00531	-------
FC88X_00835_00987	-------
FC88X_01039_01125	-------
FC88X_00029_00211	-------
FC88X_00635_00186	-------
FC88X_00516_01234	-------
FC88X_00389_00117	-------
FC88X_00762_01126	-------
FC88X_00461_01219	-------
FC88X_00338_01144	-------
FC88X_01078_00864	-------
FC88X_00280_00944	-------
FC88X_00669_00796	-------
FC88X_00782_00146	-------
FC88X_00521_00042	-------
FC88X_00147_00087	-------
FC88X_00151_00211	-------
FC88X_00490_00042	-------
FC88X_00478_01218	-------
FC88X_00254_00911	-------
FC88X_00588_01156	-------
FC88X_01143_00804	-------
FC88X_00605_01212	-------
FC88X_00472_01235	-------
FC88X_00019_00197	-------
FC88X_00158_00938	-------
FC88X_00138_00652	-------
FC88X_01099_00793	-------
FC88X_00888_01194	-------
FC88X_01106_00816	-------
FC88X_00898_00517	-------
FC88X_00492_01012	-------
FC88X_00057_00482	-------
FC88X_00006_00496	-------
FC88X_00822_00780	-------
FC88X_00828_00848	-------
FC88X_01044_01192	-------
FC88X_00072_00439	-------
FC88X_00109_00361	-------

FC88X_00498_01122	-------
FC88X_00537_00242	-------
FC88X_00536_01204	-------
FC88X_00981_00299	-------
FC88X_01085_00493	-------
FC88X_00047_00773	-------
FC88X_00192_00561	-------
FC88X_00667_01250	-------
FC88X_00050_00092	-------
FC88X_00267_01111	-------
FC88X_00066_00379	-------
FC88X_00671_00909	-------
FC88X_00752_00873	-------
FC88X_00092_00661	-------
FC88X_01126_01171	-------
FC88X_01035_00632	-------
FC88X_01020_00701	-------
FC88X_00060_00470	-------
FC88X_00114_00871	-------
FC88X_00723_00593	-------
FC88X_00362_00485	-------
FC88X_00102_00131	-------
FC88X_00449_00432	-------
FC88X_01123_00870	-------
FC88X_00454_01031	-------
FC88X_00962_00357	-------
FC88X_00177_00682	-------
FC88X_00870_00205	-------
FC88X_00670_00285	-------
FC88X_01124_00557	-------
FC88X_00137_00068	-------
FC88X_00250_00194	-------
FC88X_00610_00416	-------
FC88X_00006_00626	-------
FC88X_00033_00681	-------
FC88X_00137_00079	-------
FC88X_00372_01159	-------
FC88X_00942_01218	-------
FC88X_01027_00594	-------
FC88X_01004_00984	-------
FC88X_00066_00819	-------
FC88X_00992_01008	-------
FC88X_00413_00070	-------
FC88X_00067_00808	-------
FC88X_00626_01214	-------
FC88X_00314_00287	-------
FC88X_00444_00482	-------
FC88X_00950_00556	-------
FC88X_00923_01264	-------
FC88X_01075_00705	-------
FC88X_00089_00045	-------
FC88X_00610_01246	-------
FC88X_00942_00351	-------
FC88X_00473_00445	-------
FC88X_01029_00944	-------
FC88X_00539_01036	-------
FC88X_00094_00121	-------

FC88X_00388_01115	-------
FC88X_00484_00967	-------
FC88X_00606_01247	-------
FC88X_00151_00632	-------
FC88X_00918_00760	-------
FC88X_00601_01271	-------
FC88X_00179_01049	-------
FC88X_00086_00139	-------
FC88X_01125_00845	-------
FC88X_00036_00601	-------
FC88X_00155_00624	-------
FC88X_00938_00549	-------
FC88X_00922_00539	-------
FC88X_00124_00667	-------
FC88X_00458_00055	-------
FC88X_00600_01015	-------
FC88X_01035_00439	-------
FC88X_00206_00103	-------
FC88X_00599_01231	-------
FC88X_00325_00968	-------
FC88X_00875_00906	-------
FC88X_00567_01229	-------
FC88X_00758_01009	-------
FC88X_01055_00732	-------
FC88X_01137_00877	-------
FC88X_00431_00065	-------
FC88X_00546_01164	-------
FC88X_00668_00099	-------
FC88X_00728_01043	-------
FC88X_00793_00806	-------
FC88X_00895_00615	-------
FC88X_00895_00652	-------
FC88X_01122_00646	-------
FC88X_00099_00656	-------
FC88X_00402_01176	-------
FC88X_00937_00908	-------
FC88X_00808_01172	-------
FC88X_01141_01114	-------
FC88X_00621_00219	-------
FC88X_00017_00204	-------
FC88X_00891_00844	-------
FC88X_00968_00602	-------
FC88X_00995_01168	-------
FC88X_00587_01212	-------
FC88X_00994_00816	-------
FC88X_00065_00765	-------
FC88X_00500_01127	-------
FC88X_00073_00212	-------
FC88X_00089_00281	-------
FC88X_00999_00685	-------
FC88X_00423_01126	-------
FC88X_00786_00245	-------
FC88X_00644_00969	-------
FC88X_00805_00795	-------
FC88X_00083_00349	-------
FC88X_00577_01142	-------
FC88X_00799_00396	-------

FC88X_00995_00727	-------
FC88X_00868_00695	-------
FC88X_00813_00139	-------
FC88X_00924_00986	-------
FC88X_00818_01076	-------
FC88X_00210_00591	-------
FC88X_00768_00155	-------
FC88X_00126_00633	-------
FC88X_01143_00910	-------
FC88X_00125_00671	-------
FC88X_00817_01071	-------
FC88X_00478_00169	-------
FC88X_00518_01252	-------
FC88X_00290_00962	-------
FC88X_00794_00814	-------
FC88X_00830_01096	-------
FC88X_01009_00461	-------
FC88X_00872_00708	-------
FC88X_01121_00527	-------
FC88X_00822_01143	-------
FC88X_00877_00809	-------
FC88X_00086_00409	-------
FC88X_00826_00136	-------
FC88X_00811_00171	-------
FC88X_00004_00644	-------
FC88X_00539_01250	-------
FC88X_00586_00191	-------
FC88X_00659_00302	-------
FC88X_00935_00573	-------
FC88X_01048_00561	-------
FC88X_00945_01134	-------
FC88X_00950_00808	-------
FC88X_00686_00464	-------
FC88X_00056_00591	-------
FC88X_00454_01000	-------
FC88X_00982_00560	-------
FC88X_01029_00528	-------
FC88X_00939_00277	-------
FC88X_00035_00371	-------
FC88X_00882_00711	-------
FC88X_01001_01117	-------
FC88X_01068_00889	-------
FC88X_00130_00581	-------
FC88X_00518_00156	-------
FC88X_00685_00972	-------
FC88X_01077_00506	-------
FC88X_00047_00456	-------
FC88X_01081_00835	-------
FC88X_00545_01074	-------
FC88X_00842_00228	-------
FC88X_00970_00936	-------
FC88X_00320_01082	-------
FC88X_00942_00243	-------
FC88X_00043_00759	-------
FC88X_00667_00082	-------
FC88X_00904_01237	-------
FC88X_00923_00238	-------

FC88X_00566_00466	-------
FC88X_00068_00442	-------
FC88X_00780_00154	-------
FC88X_00116_00304	-------
FC88X_00105_00430	-------
FC88X_00866_00772	-------
FC88X_00254_00250	-------
FC88X_00924_00565	-------
FC88X_00056_00328	-------
FC88X_00161_00134	-------
FC88X_00224_00604	-------
FC88X_00206_00905	-------
FC88X_00941_00342	-------
FC88X_01078_01187	-------
FC88X_01144_01036	-------
FC88X_01017_00303	-------
FC88X_00078_00296	-------
FC88X_00379_00953	-------
FC88X_00936_00676	-------
FC88X_01045_00574	-------
FC88X_00096_00719	-------
FC88X_00923_00652	-------
FC88X_00956_00955	-------
FC88X_00667_00279	-------
FC88X_00901_00492	-------
FC88X_00251_00816	-------
FC88X_01089_00702	-------
FC88X_00580_00213	-------
FC88X_00934_00584	-------
FC88X_00685_01234	-------
FC88X_00330_01137	-------
FC88X_00797_00616	-------
FC88X_00130_00920	-------
FC88X_00195_01053	-------
FC88X_00735_00921	-------
FC88X_00062_00828	-------
FC88X_00698_00148	-------
FC88X_00689_00669	-------
FC88X_00699_01138	-------
FC88X_00916_01212	-------
FC88X_00999_00774	-------
FC88X_01046_00717	-------
FC88X_00156_00670	-------
FC88X_00288_01061	-------
FC88X_00562_00178	-------
FC88X_00820_00987	-------
FC88X_00808_00094	-------
FC88X_00220_00081	-------
FC88X_01118_00754	-------
FC88X_00158_00549	-------
FC88X_00835_00187	-------
FC88X_00944_00528	-------
FC88X_00546_01117	-------
FC88X_00813_01184	-------
FC88X_00429_00013	-------
FC88X_00692_00213	-------
FC88X_00322_00015	-------

FC88X_00845_00208	-------
FC88X_00954_00821	-------
FC88X_01048_00487	-------
FC88X_01093_00421	-------
FC88X_00992_00966	-------
FC88X_01105_00994	-------
FC88X_00757_00759	-------
FC88X_00870_00881	-------
FC88X_01140_00889	-------
FC88X_01008_00898	-------
FC88X_00894_00612	-------
FC88X_00110_00381	-------
FC88X_00404_01056	-------
FC88X_00023_00076	-------
FC88X_00205_00091	-------
FC88X_00101_00897	-------
FC88X_00433_00014	-------
FC88X_00519_01215	-------
FC88X_00250_00874	-------
FC88X_00075_00472	-------
FC88X_00512_00438	-------
FC88X_00841_01264	-------
FC88X_00521_00745	-------
FC88X_01046_00949	-------
FC88X_01011_00967	-------
FC88X_00843_01155	-------
FC88X_00341_00596	-------
FC88X_00898_00217	-------
FC88X_00821_00849	-------
FC88X_00657_01061	-------
FC88X_00940_00845	-------
FC88X_00729_00361	-------
FC88X_00589_00258	-------
FC88X_00982_00793	-------
FC88X_00806_01240	-------
FC88X_00155_00878	-------
FC88X_00607_00642	-------
FC88X_01083_00583	-------
FC88X_00355_00292	-------
FC88X_01036_00512	-------
FC88X_00724_01217	-------
FC88X_01094_00445	-------
FC88X_00106_00556	-------
FC88X_00991_00398	-------
FC88X_00032_00182	-------
FC88X_01021_00887	-------
FC88X_00787_01171	-------
FC88X_01104_01087	-------
FC88X_00594_01235	-------
FC88X_00072_00054	-------
FC88X_00268_00779	-------
FC88X_00755_00514	-------
FC88X_00954_00357	-------
FC88X_01145_00992	-------
FC88X_00254_00926	-------
FC88X_00326_01144	-------
FC88X_00151_00787	-------

FC88X_00915_00750	-------
FC88X_00566_00057	-------
FC88X_00979_00966	-------
FC88X_00893_00369	-------
FC88X_00161_00815	-------
FC88X_00773_00262	-------
FC88X_00856_01060	-------
FC88X_00910_00655	-------
FC88X_00065_00290	-------
FC88X_01081_00934	-------
FC88X_00360_01095	-------
FC88X_00520_00725	-------
FC88X_00049_00608	-------
FC88X_00678_00074	-------
FC88X_00639_00957	-------
FC88X_00911_00485	-------
FC88X_00825_01047	-------
FC88X_00862_00791	-------
FC88X_00100_00182	-------
FC88X_00467_00060	-------
FC88X_00271_01120	-------
FC88X_00890_00853	-------
FC88X_00073_00557	-------
FC88X_00624_01020	-------
FC88X_00478_00817	-------
FC88X_00101_00418	-------
FC88X_00336_01172	-------
FC88X_00476_00566	-------
FC88X_00564_00067	-------
FC88X_00160_00142	-------
FC88X_00279_00874	-------
FC88X_00087_00574	-------
FC88X_00497_00950	-------
FC88X_00512_01241	-------
FC88X_01014_00755	-------
FC88X_00814_00438	-------
FC88X_00063_00196	-------
FC88X_00824_00207	-------
FC88X_00962_00960	-------
FC88X_00713_00433	-------
FC88X_00862_00824	-------
FC88X_00884_00399	-------
FC88X_00745_01221	-------
FC88X_00907_00228	-------
FC88X_01146_00802	-------
FC88X_00531_00961	-------
FC88X_00129_00204	-------
FC88X_01061_01272	-------
FC88X_00754_00462	-------
FC88X_00165_00123	-------
FC88X_00292_01152	-------
FC88X_00466_01137	-------
FC88X_00456_01158	-------
FC88X_00739_01075	-------
FC88X_00994_00675	-------
FC88X_00139_00057	-------
FC88X_00016_00729	-------

FC88X_00855_01054	-------
FC88X_00567_00474	-------
FC88X_00904_00429	-------
FC88X_00573_01135	-------
FC88X_01046_00411	-------
FC88X_00048_00312	-------
FC88X_00043_00402	-------
FC88X_00628_00985	-------
FC88X_01089_00430	-------
FC88X_00351_00384	-------
FC88X_01011_00601	-------
FC88X_00172_00916	-------
FC88X_00334_00343	-------
FC88X_00030_00180	-------
FC88X_00841_00183	-------
FC88X_01086_00665	-------
FC88X_00096_00131	-------
FC88X_00647_01136	-------
FC88X_00678_00494	-------
FC88X_00818_00225	-------
FC88X_00834_00842	-------
FC88X_00651_01079	-------
FC88X_00014_00082	-------
FC88X_00681_01219	-------
FC88X_00173_00005	-------
FC88X_00186_00810	-------
FC88X_00700_01014	-------
FC88X_00655_00336	-------
FC88X_00527_00735	-------
FC88X_00529_00973	-------
FC88X_00788_01208	-------
FC88X_00840_00890	-------
FC88X_00892_00783	-------
FC88X_00922_00884	-------
FC88X_01123_00578	-------
FC88X_00267_00924	-------
FC88X_00456_01236	-------
FC88X_00808_01040	-------
FC88X_00875_01150	-------
FC88X_00613_01020	-------
FC88X_00721_00915	-------
FC88X_00803_00222	-------
FC88X_00441_01094	-------
FC88X_00315_00761	-------
FC88X_00997_01199	-------
FC88X_00047_00580	-------
FC88X_00481_00024	-------
FC88X_00137_00626	-------
FC88X_01014_00816	-------
FC88X_00172_00246	-------
FC88X_00350_01163	-------
FC88X_00559_00201	-------
FC88X_00770_01032	-------
FC88X_01117_00643	-------
FC88X_00882_00840	-------
FC88X_01007_01039	-------
FC88X_00563_01243	-------

FC88X_00325_00967	-------
FC88X_00023_00640	-------
FC88X_00771_00210	-------
FC88X_00048_00364	-------
FC88X_00840_00770	-------
FC88X_00062_00136	-------
FC88X_01039_00737	-------
FC88X_00533_00147	-------
FC88X_00590_01057	-------
FC88X_01083_00938	-------
FC88X_00132_00508	-------
FC88X_00840_00891	-------
FC88X_00322_00613	-------
FC88X_00937_00969	-------
FC88X_00720_01217	-------
FC88X_00889_00441	-------
FC88X_01037_01257	-------
FC88X_00959_01184	-------
FC88X_01101_01045	-------
FC88X_00144_00967	-------
FC88X_00176_00176	-------
FC88X_00410_00388	-------
FC88X_01137_01082	-------
FC88X_00404_00504	-------
FC88X_00829_00925	-------
FC88X_00069_00831	-------
FC88X_00870_00170	-------
FC88X_00126_00702	-------
FC88X_00940_01071	-------
FC88X_00032_00283	-------
FC88X_00820_00130	-------
FC88X_00696_00183	-------
FC88X_00914_01195	-------
FC88X_00190_01059	-------
FC88X_00739_01022	-------
FC88X_00721_01234	-------
FC88X_00897_01238	-------
FC88X_01076_01215	-------
FC88X_00249_00295	-------
FC88X_00082_00512	-------
FC88X_00404_00995	-------
FC88X_00719_00988	-------
FC88X_01136_00954	-------
FC88X_01015_00712	-------
FC88X_00946_01195	-------
FC88X_00938_00445	-------
FC88X_00121_00106	-------
FC88X_00022_00493	-------
FC88X_00096_00195	-------
FC88X_00498_01213	-------
FC88X_00719_01105	-------
FC88X_00862_00929	-------
FC88X_00744_00335	-------
FC88X_01128_00594	-------
FC88X_01104_01121	-------
FC88X_00288_00941	-------
FC88X_00816_01259	-------

FC88X_01108_00750	-------
FC88X_00912_00372	-------
FC88X_00608_00416	-------
FC88X_00727_01117	-------
FC88X_00114_00092	-------
FC88X_00646_01064	-------
FC88X_00888_00986	-------
FC88X_00857_01029	-------
FC88X_00853_01119	-------
FC88X_00908_00217	-------
FC88X_01064_00374	-------
FC88X_01078_00934	-------
FC88X_00022_00174	-------
FC88X_00022_00250	-------
FC88X_00057_00057	-------
FC88X_00052_00207	-------
FC88X_00093_00395	-------
FC88X_00125_00121	-------
FC88X_00454_01075	-------
FC88X_00533_01170	-------
FC88X_00646_01192	-------
FC88X_00794_00876	-------
FC88X_00843_00156	-------
FC88X_00874_00870	-------
FC88X_01078_01252	-------
FC88X_00074_00228	-------
FC88X_00415_00626	-------
FC88X_00520_00219	-------
FC88X_00157_00665	-------
FC88X_00437_00019	-------
FC88X_00351_00431	-------
FC88X_00760_00814	-------
FC88X_00794_00842	-------
FC88X_00509_01007	-------
FC88X_01027_00312	-------
FC88X_00829_00995	-------
FC88X_00113_00743	-------
FC88X_01007_00817	-------
FC88X_01133_01014	-------
FC88X_00559_01174	-------
FC88X_00593_00261	-------
FC88X_01081_00755	-------
FC88X_00440_00308	-------
FC88X_00933_00411	-------
FC88X_00968_00650	-------
FC88X_00012_00391	-------
FC88X_00995_00308	-------
FC88X_00020_00384	-------
FC88X_00965_01093	-------
FC88X_00071_00112	-------
FC88X_00454_00835	-------
FC88X_00006_00199	-------
FC88X_00324_01123	-------
FC88X_00013_00126	-------
FC88X_00055_00859	-------
FC88X_00104_00422	-------
FC88X_00283_00528	-------

FC88X_00284_01102	-------
FC88X_00891_01034	-------
FC88X_00959_00411	-------
FC88X_01120_01150	-------
FC88X_01078_00782	-------
FC88X_00519_01247	-------
FC88X_00806_00880	-------
FC88X_00189_00951	-------
FC88X_00620_00410	-------
FC88X_00461_00017	-------
FC88X_00673_01186	-------
FC88X_01073_00730	-------
FC88X_00518_00514	-------
FC88X_00165_00731	-------
FC88X_00449_01168	-------
FC88X_00604_01180	-------
FC88X_00796_01108	-------
FC88X_00576_01202	-------
FC88X_00474_00985	-------
FC88X_00771_01165	-------
FC88X_00883_00291	-------
FC88X_01009_00533	-------
FC88X_01016_00962	-------
FC88X_00023_00539	-------
FC88X_00493_01212	-------
FC88X_00276_00930	-------
FC88X_00814_00934	-------
FC88X_00840_00936	-------
FC88X_00896_01242	-------
FC88X_00031_00634	-------
FC88X_00556_01229	-------
FC88X_00833_01212	-------
FC88X_01084_00403	-------
FC88X_00105_00395	-------
FC88X_00666_00087	-------
FC88X_00483_00026	-------
FC88X_00811_00824	-------
FC88X_00114_00495	-------
FC88X_00635_00238	-------
FC88X_00208_00124	-------
FC88X_00290_00974	-------
FC88X_00325_00942	-------
FC88X_00627_00943	-------
FC88X_00882_00192	-------
FC88X_00895_00775	-------
FC88X_00209_00127	-------
FC88X_00229_01025	-------
FC88X_00585_01028	-------
FC88X_00362_00662	-------
FC88X_00422_01162	-------
FC88X_00848_00251	-------
FC88X_00956_00909	-------
FC88X_00687_01118	-------
FC88X_00992_01144	-------
FC88X_01147_01058	-------
FC88X_00091_00027	-------
FC88X_00094_00418	-------

FC88X_00923_00594	-------
FC88X_00051_00712	-------
FC88X_00076_00823	-------
FC88X_00278_01000	-------
FC88X_00643_01001	-------
FC88X_00185_00345	-------
FC88X_01140_00848	-------
FC88X_00470_00179	-------
FC88X_00836_01067	-------
FC88X_00086_00248	-------
FC88X_00789_00207	-------
FC88X_00066_00570	-------
FC88X_00666_00902	-------
FC88X_00592_01175	-------
FC88X_00095_00829	-------
FC88X_00120_00329	-------
FC88X_00833_00780	-------
FC88X_01004_01195	-------
FC88X_00272_00765	-------
FC88X_01033_00544	-------
FC88X_00082_00031	-------
FC88X_00863_00656	-------
FC88X_00667_01225	-------
FC88X_00610_00707	-------
FC88X_00373_00822	-------
FC88X_00452_00136	-------
FC88X_00649_00185	-------
FC88X_00906_00619	-------
FC88X_00929_00244	-------
FC88X_00576_01164	-------
FC88X_00707_00243	-------
FC88X_00642_01112	-------
FC88X_00116_00146	-------
FC88X_01028_00922	-------
FC88X_00153_00840	-------
FC88X_00206_00067	-------
FC88X_00057_00138	-------
FC88X_00944_01163	-------
FC88X_00842_00590	-------
FC88X_00963_00901	-------
FC88X_00051_00771	-------
FC88X_00541_01229	-------
FC88X_00815_00829	-------
FC88X_00113_00720	-------
FC88X_00429_01152	-------
FC88X_01074_00734	-------
FC88X_00634_00283	-------
FC88X_00028_00224	-------
FC88X_00685_00478	-------
FC88X_00110_00422	-------
FC88X_00541_01166	-------
FC88X_00568_01218	-------
FC88X_00750_00651	-------
FC88X_00983_00514	-------
FC88X_00042_00270	-------
FC88X_00106_00923	-------
FC88X_00746_01228	-------

FC88X_00893_00461	-------
FC88X_01071_00523	-------
FC88X_00146_00769	-------
FC88X_00346_00544	-------
FC88X_00376_00463	-------
FC88X_00412_00297	-------
FC88X_00400_01156	-------
FC88X_00688_00250	-------
FC88X_00652_01152	-------
FC88X_00664_01255	-------
FC88X_00751_01009	-------
FC88X_00878_00370	-------
FC88X_00867_00588	-------
FC88X_00947_00276	-------
FC88X_01006_00369	-------
FC88X_01083_00452	-------
FC88X_01069_00735	-------
FC88X_01079_00997	-------
FC88X_00989_01039	-------
FC88X_00920_00456	-------
FC88X_00686_00330	-------
FC88X_00045_00454	-------
FC88X_00930_00543	-------
FC88X_00942_00214	-------
FC88X_00860_01037	-------
FC88X_00089_00779	-------
FC88X_00730_00939	-------
FC88X_00081_00687	-------
FC88X_00862_01067	-------
FC88X_00992_01068	-------
FC88X_01002_00993	-------
FC88X_00076_00840	-------
FC88X_00079_00196	-------
FC88X_00464_00983	-------
FC88X_00833_00940	-------
FC88X_01001_00653	-------
FC88X_00130_00301	-------
FC88X_00770_00482	-------
FC88X_00467_00169	-------
FC88X_00997_00583	-------
FC88X_00144_00070	-------
FC88X_00223_00988	-------
FC88X_00426_01038	-------
FC88X_00939_01228	-------
FC88X_00020_00225	-------
FC88X_00073_00475	-------
FC88X_00098_00478	-------
FC88X_00238_00091	-------
FC88X_00206_01075	-------
FC88X_00383_00278	-------
FC88X_00232_00936	-------
FC88X_01140_00880	-------
FC88X_00314_01127	-------
FC88X_00476_00982	-------
FC88X_00621_01219	-------
FC88X_00663_00843	-------
FC88X_00338_00853	-------

FC88X_01115_00621	-------
FC88X_00409_00028	-------
FC88X_00566_01230	-------
FC88X_00674_00593	-------
FC88X_00737_00892	-------
FC88X_00848_00134	-------
FC88X_00899_00494	-------
FC88X_00865_00994	-------
FC88X_00927_00226	-------
FC88X_00902_00461	-------
FC88X_00949_01203	-------
FC88X_00983_00843	-------
FC88X_01048_00826	-------
FC88X_01141_00999	-------
FC88X_00031_00420	-------
FC88X_00030_00758	-------
FC88X_00079_00693	-------
FC88X_00079_00814	-------
FC88X_00225_00123	-------
FC88X_00325_00941	-------
FC88X_00396_00302	-------
FC88X_00436_00121	-------
FC88X_00408_00801	-------
FC88X_00467_01188	-------
FC88X_00536_00106	-------
FC88X_00518_01070	-------
FC88X_00559_00210	-------
FC88X_00618_00226	-------
FC88X_00665_00553	-------
FC88X_00696_01003	-------
FC88X_00709_00967	-------
FC88X_00799_00238	-------
FC88X_00862_00207	-------
FC88X_00865_01105	-------
FC88X_00927_00677	-------
FC88X_00927_00911	-------
FC88X_00990_00552	-------
FC88X_00992_00742	-------
FC88X_01088_00861	-------
FC88X_01116_00737	-------
FC88X_01103_01139	-------
FC88X_00967_01222	-------
FC88X_00735_01268	-------
FC88X_00807_01266	-------
FC88X_00483_00032	-------
FC88X_00058_00548	-------
FC88X_00964_00772	-------
FC88X_01087_00719	-------
FC88X_00198_00254	-------
FC88X_00962_00723	-------
FC88X_01131_01045	-------
FC88X_00560_00636	-------
FC88X_01082_00812	-------
FC88X_00318_00937	-------
FC88X_01043_00592	-------
FC88X_00034_00278	-------
FC88X_00122_00955	-------

FC88X_00936_00831	-------
FC88X_00781_00197	-------
FC88X_00134_00637	-------
FC88X_00469_01241	-------
FC88X_00838_00329	-------
FC88X_00738_01104	-------
FC88X_01006_00292	-------
FC88X_00865_01230	-------
FC88X_01019_00860	-------
FC88X_00096_00861	-------
FC88X_00572_00490	-------
FC88X_00875_00770	-------
FC88X_00038_00266	-------
FC88X_00149_00687	-------
FC88X_00761_01095	-------
FC88X_00916_00254	-------
FC88X_00931_01124	-------
FC88X_00951_00623	-------
FC88X_01049_00969	-------
FC88X_01120_01171	-------
FC88X_00012_00479	-------
FC88X_00192_00870	-------
FC88X_00275_00543	-------
FC88X_00669_00177	-------
FC88X_00887_00649	-------
FC88X_00970_00897	-------
FC88X_01140_00828	-------
FC88X_00376_01126	-------
FC88X_00878_00653	-------
FC88X_00111_00720	-------
FC88X_00866_00382	-------
FC88X_00118_00062	-------
FC88X_00919_01179	-------
FC88X_00971_00798	-------
FC88X_00847_01083	-------
FC88X_00980_01272	-------
FC88X_01100_00984	-------
FC88X_00036_00665	-------
FC88X_00235_00392	-------
FC88X_00147_00638	-------
FC88X_00073_00340	-------
FC88X_01071_00745	-------
FC88X_00691_00756	-------
FC88X_00688_01264	-------
FC88X_00981_00340	-------
FC88X_00116_00033	-------
FC88X_00842_01142	-------
FC88X_00841_00767	-------
FC88X_00773_00926	-------
FC88X_01074_00462	-------
FC88X_01120_00489	-------
FC88X_01143_01141	-------
FC88X_00111_00951	-------
FC88X_00989_00820	-------
FC88X_00921_00699	-------
FC88X_00645_01094	-------
FC88X_00793_00366	-------

FC88X_00207_00943	-------
FC88X_00031_00207	-------
FC88X_00676_00437	-------
FC88X_00942_00658	-------
FC88X_01015_00746	-------
FC88X_00810_01009	-------
FC88X_00317_01026	-------
FC88X_00633_00846	-------
FC88X_00743_01010	-------
FC88X_00799_00782	-------
FC88X_00948_00221	-------
FC88X_00319_01148	-------
FC88X_00322_00943	-------
FC88X_00965_01091	-------
FC88X_00533_00824	-------
FC88X_00821_01023	-------
FC88X_00306_01078	-------
FC88X_00030_00245	-------
FC88X_00042_00443	-------
FC88X_00077_00512	-------
FC88X_00514_00224	-------
FC88X_00550_00253	-------
FC88X_00591_00546	-------
FC88X_00647_00320	-------
FC88X_00697_00197	-------
FC88X_00911_00641	-------
FC88X_00956_00670	-------
FC88X_00954_00728	-------
FC88X_00996_00988	-------
FC88X_01017_00360	-------
FC88X_01055_00972	-------
FC88X_00009_00290	-------
FC88X_00063_00215	-------
FC88X_00076_00557	-------
FC88X_00062_00629	-------
FC88X_00172_00035	-------
FC88X_00352_01057	-------
FC88X_00405_01100	-------
FC88X_00502_00160	-------
FC88X_00595_00965	-------
FC88X_00709_00110	-------
FC88X_00763_00215	-------
FC88X_00784_00375	-------
FC88X_00779_00973	-------
FC88X_00855_00694	-------
FC88X_01003_00456	-------
FC88X_01039_01024	-------
FC88X_01086_00572	-------
FC88X_00166_00699	-------
FC88X_00948_00739	-------
FC88X_00143_00698	-------
FC88X_01126_00927	-------
FC88X_00576_01044	-------
FC88X_00572_00656	-------
FC88X_00100_00836	-------
FC88X_01068_00963	-------
FC88X_00512_01174	-------

FC88X_00990_00505	-------
FC88X_00145_00228	-------
FC88X_01091_00871	-------
FC88X_00069_00104	-------
FC88X_00797_00865	-------
FC88X_00850_00754	-------
FC88X_00638_00068	-------
FC88X_00056_00117	-------
FC88X_00598_01230	-------
[truncated: 282,057 more chars]
